
JOURNAL INFORMATION
==============================
NLM Title Abbreviation: Innov Aging
Journal ID: Innov Aging
NLM Journal ID: 101703706
Journal ID: innovateage

Innovation in Aging

EISSN: 2399-5300
Publisher: Oxford University Press

ARTICLE INFORMATION
==============================
PMCID: PMC12755232
DOI: 10.1093/geroni/igaf122
Article ID: igaf122
Article version: 1
Subjects: Full GSA 2025 Abstract Book PDF, AcademicSubjects/SOC02600

Full GSA 2025 Abstract Book PDF

Publication date: 2025 Dec
Electronic publication date: 2025 Dec 31
Volume: 9
Issue: Suppl 2
Issue title: Program Abstracts from the GSA 2025 Annual Scientific Meeting, “Innovative Horizons in Gerontology”
Electronic Location ID: igaf122
Copyright: © The Author(s) 2025. Published by Oxford University Press on behalf of the Gerontological Society of America.
Copyright year: 2025
License: This is an Open Access article distributed under the terms of the Creative Commons Attribution License (https://creativecommons.org/licenses/by/4.0/), which permits unrestricted reuse, distribution, and reproduction in any medium, provided the original work is properly cited.
License URL: https://creativecommons.org/licenses/by/4.0/

Page count: 1380

==============================

INNOVATION IN AGING®

GSA GERONTOLOGICAL SOCIETY OF AMERICA®

Volume 9 • Issue S2

### Editorial Board - Innovation in Aging

**Michelle Putnam, PhD, MGS, FGSA, Editor-in-Chief**
University of Massachusetts Boston, USA

**Megumi Inoue, PhD, MSW, RN, FGSA, Deputy Editor**
George Mason University, USA

**Karen J. Jung, MSW, Managing Editor**
Gerontological Society of America, USA
Email: ia@geron.org

### Associate Editors

**Christine E. Bishop, PhD, FGSA**
Brandeis University, USA
**Julie Blaskewicz Boron, PhD, FGSA**
University of Nebraska, Omaha, USA
**Howard B. Degenholtz, PhD, FGSA**
University of Pittsburgh, USA
**Nancy W. Glynn, PhD, FGSA**
University of Pittsburgh, USA
**Min-Ah Lee, PhD**
Chung-Ang University, South Korea
**Amanda N. Leggett, PhD, FGSA**
Wayne State University, USA

**Lena K. Makaroun, MD, MS**
VA Pittsburgh Healthcare System, USA
University of Pittsburgh, USA
**Duy Nguyen, PhD, MSW, FGSA**
Sacred Heart University, USA
**Joshua D. Niznik, PharmD, PhD**
UNC Chapel Hill School of Medicine, USA
**Abby J. Schwartz, MGS, MSW, PhD, FGSA**
East Carolina University, USA
**Roland J. Thorpe, Jr., PhD, FGSA**
Johns Hopkins Bloomberg School of Public Health, USA

**Jennifer R. Turner, PhD**
University of Hawai'i at Hilo, USA
**Noah J. Webster, PhD, FGSA**
University of Michigan, USA
**Yong Yang, PhD**
University of Memphis, USA
**W. Quin Yow, PhD**
Singapore University of Technology & Design, Singapore

### Editorial Board

**Steven M. Albert, PhD, MS, FGSA**
University of Pittsburgh, USA
**Jose E. Cabrero Castro, MD, PhD, MS**
University of Texas at El Paso, USA
**Yuan (Sally) Cao, PhD**
The University of Hong Kong, China
**Moon Choi, PhD, FGSA**
Korea Advanced Institute of Science & Technology, South Korea
**Sara J. Czaja, PhD, FGSA**
Weill Cornell Medicine, USA
**Adam Davey, PhD, FGSA**
University of Delaware, Newark, USA
**Megan Gilligan, PhD, FGSA**
University of Missouri, USA
**Allen Glicksman, PhD, FGSA**
NewCourtland, USA
**Sean N. Halpin, PhD**
RTI International, USA
**Tracie C. Harrison, PhD, FGSA**
University of Iowa, USA
**Ellen L. Idler, PhD, FGSA**
Emory University, USA
**Giyeon Kim, PhD, FGSA**
Chung-Ang University, South Korea
**Yeonwoo Kim, PhD**
University of Texas at Arlington, USA
**TsuAnn Kuo, PhD**
Chung Shan Medical University, Taiwan

**Yeonjung Lee, PhD, MSW**
Chung-Ang University, South Korea
**Jiaming Liang, PhD**
University of Texas Health Science Center at Houston, USA
**Darren Liu, DrPH, FGSA**
West Virginia University, USA
**Hui Liu, PhD, FGSA**
Michigan State University, USA
**Xinran Liu, PhD, MS, MBBS**
Peking University, China
**Mariana López-Ortega, PhD, MPP**
National Institute of Geriatrics, National Institutes of Health, Mexico
**Meghan K. Mattos, PhD, RN**
University of Virginia, USA
**Anne Collins McLaughlin, PhD**
North Carolina State University, USA
**Hongdao Meng, MD, PhD, FGSA**
University of South Florida, USA
**David A. Nace, MD, MPH**
University of Pittsburgh, USA
**Lycia Tramujas Vasconcellos Neumann, PhD, MPA**
Alzheimer's Association, USA
**Karen E. Nielsen, PhD**
Georgia State University, USA
**Marcia Ory, PhD, FGSA**
Texas A&M, USA
**Angela (Angie) Perone, PhD, JD, MSW, MA**
University of California, Berkeley, USA

**Alison Phinney, PhD**
University of British Columbia, Canada
**Natalie D. Pope, PhD**
University of Kentucky, USA
**Xiang Qi, PhD, RN**
New York University, USA
**Karen A. Roberto, PhD, FGSA**
Virginia Tech, USA
**Laura P. Sands, PhD, FGSA**
Virginia Tech, USA
**Huei-wern Shen, PhD FGSA**
University of North Texas, USA
**Jennifer Tehan Stanley, PhD, FGSA**
The University of Akron, USA
**J. Jill Suitor, PhD, FGSA**
Purdue University, USA
**Robert Joseph Taylor, MSW, PhD**
University of Michigan, USA
**Jasmine L. Travers, PhD, RN**
New York University, USA
**Kaipeng Wang, PhD**
University of Denver, USA
**Richard Winett, PhD**
Virginia Tech, USA
**Yijin Wu, PhD**
Shandong University, China
**Yao Yao, MD**
Peking University, China
**Minzhi Ye, PhD**
University of Texas Rio Grande Valley, USA

### Former Editors-in-Chief

**Laura P. Sands, PhD, FGSA (2017-2020)**

**Steven M. Albert, PhD, MS, FGSA (2021-2024)**

### Program, Publications, and Products Committee

**Katherine Britt, BSN, MSN, PhD**
University of Iowa, USA
**Abraham Brody, FAAN, PhD, RN, FGSA, Chair**
NYU Rory Meyers College of Nursing, USA
**Pamela Cacchione, NP, PhD, RN, FGSA**
University of Pennsylvania, USA
**Runcie C.W Chidebe, BS, MS**
Miami University, USA
**Sean Curran, PhD, FGSA**
University of Southern California, USA
**Howard Degenholtz, PhD, FGSA**
University of Pittsburgh, USA
**Laura Donorfio, PhD, FAGHE**
University of Connecticut, USA

**Cal Halvorsen, MSW, PhD**
Washington University in St. Louis, USA
**Sohyun Kim, PhD, RN**
University of Texas at Arlington, USA
**Judie Lieu, BA**
Gerontological Society of America
**Karen Lyons, PhD, FGSA**
Boston College, USA
**Mark McCormick, PhD**
University of New Mexico, USA
**Megan McCutcheon, MA**
Gerontological Society of America, USA
**Chivon Mingo, PhD, FGSA**
Georgia State University, USA

**Ronica Rooks, BA, MA, PhD, FGSA**
University of Colorado, Denver; USA
**Tamar Shovali, MS, PhD, FAGHE**
Eckerd College, USA
**Alan Stevens, PhD, FGSA**
Baylor Scott & White Health, USA
**Jeffrey Stokes, PhD**
University of Massachusetts, Boston; USA
**Elizabeth Vasquez, DrPH, FGSA**
Albany University (SUNY), USA

Fellows of the Gerontological Society of America (FGSA)

### Program Abstracts from the GSA 2025 Annual Scientific Meeting "Innovative Horizons in Gerontology"

Abstracts are arranged numerically by session and in the order of presentation within each session.
Abstracts are published as received.

### ZWEIGSESSION 1000 (SYMPOSIUM)

Abstract citation ID: igaf122.001

### TRANSFORMING LGBTQ+ AGING: FROM LONELINESS TO RESILIENCE

Chair: Christine Happel
Co-Chair: Haley Sanner
Discussant: Erin Burk-Leaver

Lesbian, gay, bisexual, transgender, queer/questioning (LGBTQ+) older adults in the United States face unique and compounded health and social challenges, including loneliness and social isolation, that compromise the quality of their aging experience. This symposium employs quantitative, qualitative, and mixed methods to provide a comprehensive understanding of the challenges and resilience strategies among LGBTQ+ older adults. The first presentation, utilizing data from the 2023 Behavioral Risk Factor Surveillance System, explores the heightened risks of loneliness and adverse social determinants of health faced by LGBTQ+ older adults. The study highlights the disparities in loneliness, food insecurity, housing insecurity, and access to medical care between LGBTQ+ and non-LGBTQ+ older adults. The second session explores the unique difficulties faced by older lesbians, focusing on their experiences of loneliness and isolation. This study investigates protective factors like community and social engagement to outline possible policy and programming adaptations to support aging well. The third presentation showcases the impact of the 'Aging Positively' program for older people living with HIV, particularly long-term survivors. This evaluation explores resilience, self-compassion, survivorship, stigma, and intersectionality. The final presentation assesses the inclusion of LGBTQ+ older adults in state plans on aging. This study uses critical discourse analysis to review documents from all 50 states and the District of Columbia, aiming to create a publicly accessible report card on LGBTQ+ inclusion. Bridging together national data, qualitative narrative, program implementation and policy. These studies offer valuable insights and practical approaches to support the well-being of this diverse and resilient population.

Abstract citation ID: igaf122.002

### AGING WELL, AGING TOGETHER: EXPERIENCES OF LONELINESS AND COMMUNITY CONNECTION FOR OLDER LESBIANS

Christine Happel, Joanne Patterson, and Julia Applegate,
*The Ohio State University, Columbus, Ohio, United States*

Older lesbians in the U.S. face challenges to aging well, including health disparities and lack of culturally competent medical care. However, older lesbians are recognized as an "at-risk, yet resilient" population. Enhancing resiliency requires understanding how protective factors like community and social engagement promote aging well in the lesbian community. This cross-sectional, qualitative study was designed to: (1) understand the experiences of loneliness among older lesbians in Central, Ohio (2) examine the impact of the disappearance of lesbian social spaces; and (3) identify strategies to support aging well for lesbians. Interviews were conducted with individuals age 55 + (N = 34); who identified as lesbian/woman loving woman/gay/queer; and spoke English. Forty percent of participants were ages 70+, 25% were people of color, 53% lived alone, 85% attained at least an undergraduate degree, and 94% still drove. Interviews were analyzed using the team-based flexible coding method. Few interviewees identified as lonely, but most expressed dissatisfaction with their social relationships. Lesbian bars impacted social capital and identity exploration in interviewee's youth but not in later life. Interviewees collectively expressed pride in their lesbian identity, shared stories of past advocacy, and felt no pressure to conform. Small, mostly non-lesbian community groups, and friendships were identified by interviewees as stress-reducing supports. Few interviewees had a formal network for instrumental support beyond their partner or, in some cases, children. Lesbian-specific shared living communities; culturally congruent assisted and nursing care; accessible transportation; and a centralized community center were identified by participants as solutions for supporting aging well.

Abstract citation ID: igaf122.003

### AGING POSITIVELY: REDEFINING RESILIENCE & LEGACY WITH HIV

Haley Sanner, *Colorado Health Network, Denver, Colorado, United States*

Founded during the AIDS epidemic, Colorado Health Network (CHN) is a statewide organization providing services for people living with HIV (PLWH). The advent of antiretroviral therapy (ART) drastically increased life expectancy, thus presenting unique quality of life concerns for older PLWH (over 50% of clients), especially long-term survivors. In addition to the physical health complexities, older PLWH, especially long-term survivors, experience higher rates of depression, loneliness, and fractured support networks. Additionally, HIV is an intersectional disease that disproportionately affects people of color, LGBTQ+ people, low-income people, people experiencing homelessness, and people with disabilities. CHN established Healthy Aging Programs (HAP) to prioritize PCTI programming to improve physical and mental health outcomes for older PLWH. In collaboration with CHN Behavioral Health and Peer Leaders, HAP has developed a robust 'Aging Positively' psychosocial program to address the intersection of ageism, HIV-stigma and social isolation. The original 6-week curriculum explores themes of

© The Author(s) 2025. Published by Oxford University Press on behalf of the Gerontological Society of America.
This is an Open Access article distributed under the terms of the Creative Commons Attribution License (https://creativecommons.org/licenses/by/4.0/), which permits unrestricted reuse, distribution, and reproduction in any medium, provided the original work is properly cited.

themes of positive aging pertaining to older PLWH - including resilience, self-compassion, survivorship, stigma, and intersectionality. In collaboration with older PLWH, a follow-up offerings that commemorates the lived experiences and legacies of older PLWH by employing storytelling, creative aging and intergenerational methods. By integrating equitable approaches to program design, CHN shifts the perspective from 'If we build it, will they come?' to 'If they build it, will they use it?' to intentionally co-create programming with older participants. This session will highlight implementation science methods, psychosocial contexts, structural elements, cross-departmental collaboration, community engagement approaches and lessons learned from this innovative program.

Abstract citation ID: igaf122.004

### LGBT+ OLDER ADULTS REPORT HIGHER RATES OF LONELINESS AND OTHER ADVERSE SOCIAL DETERMINANTS OF HEALTH

Jace Flatt[1], Joel Anderson[2], Ethan Cicero[3], Jalal Uddin[1], and Erin Rook[1], *1. University of Nevada Las Vegas, Nevada, United States, 2. University of Tennessee, Knoxville, Tennessee, United States, 3. Emory University, Atlanta, Georgia, United States*

While loneliness tends to decline with age, lesbian, gay, bisexual, transgender, and additional identity (LGBT+) older adults face heightened risks of loneliness and adverse social determinants of health (SDoH). We examined data from 33 U.S. states and 2 territories from the 2023 Behavioral Risk Factor Surveillance System that included optional SDoH and sexual orientation and gender identity (SOGI) modules. The sample included adults aged 65+ (mean:73.5 years, range:65-80+) who identified as a LGBT+ (n = 2356) and non-LGBT + (n = 68,546). Loneliness was defined as feeling socially isolated from others always/usually/sometimes. Multivariable logistic regression models examined associations between loneliness and LGBT+ identity adjusting for demographic characteristics (age, race/ethnicity, education and income). Compared with non-LGBT+ older adults, LGBT+ older adults were slightly younger and unmarried/not partnered (61% vs. 46%), and reported annual incomes ≤$20,000 (12% vs. 8%), poor/fair health (25% vs. 22%), greater depression (22% vs. 15%), and greater functional impairment (10% vs. 9%). In terms of SDoH, LGBT+ older adults also reported greater food insecurity (11% vs. 7.0%) and housing insecurity (5% vs. 3%) compared with non-LGBT+ older adults. Nearly one third of LGBT+ older adults reported current loneliness compared with one fifth of non-LGBT+ older adults ($p < 0.001$). After accounting for demographics, LGBT+ older adults were 30% more likely to report loneliness (aOR:1.30; 95% CI:1.17, 1.44) than non-LGBT+ older adults. This study highlights higher rates of loneliness among LGBT+ older adults compared with non-LGBTQ+ older adults and the critical need for interventions to bolster social connectedness and support for LGBT+ older adults.

Abstract citation ID: igaf122.005

### ASSESSING LGBTQ+ LEGITIMIZATION IN STATE PLANS ON AGING: THE LGBTQ STATE PLAN ON AGING REPORT CARD (LGBTQ-SPARC)

Megan McCoy, *Northern Arizona University, Flagstaff, Arizona, United States*

The 2020 reauthorization of the Older Americans Act (OAA) coupled with a federal rulemaking process effective in March 2024 together represent a historic shift in federal aging policy that acknowledges the importance of cultivating an aging services delivery system inclusive of LGBTQ+ older adults. Yet, to date there is no publicly available analysis of the extent to which states include LGBTQ+ older adults in the planning processes that determine how aging services funded under the OAA are implemented locally. Through a critical discourse analysis lens, we conducted a document review of current state plans on aging for all 50 states and the District of Columbia (N = 51) to assess the extent to which LGBTQ+ older adults are included in the language of state plans on aging and the context of that language in relation to key domains including: definitions of "greatest social need," community engagement, data collection, and training/professional development. Findings indicate that while plans for 36 states contain LGBTQ+ inclusive terms, the context of that language and the extent to which it directly legitimizes LGBTQ+ older adults in key domains varies geographically. In addition to sharing findings with the research community, dissemination activities include creation of a publicly accessible LGBTQ State Plan on Aging Report Card (LGBTQ-SPARC) depicting level of LGBTQ+ inclusion by state, providing a snapshot of how states currently conform to best practices for LGBTQ+ inclusion in planning and serving as a baseline against which to measure LGBTQ+ inclusion in state planning processes for the future.

### SESSION 1005 (SYMPOSIUM)

Abstract citation ID: igaf122.006

### AMPLIFYING VOICES: WORKFORCE-RELATED BARRIERS AND FACILITATORS TO PERSON-CENTERED CARE IN LTSS

Chair: Philip Taylor
Co-Chair: Jennifer Morgan
Discussant: Deke Cateau

Workforce challenges in long-term care highlight the necessity of improving job quality within the sector, where workers' voices are crucial. The diverse long-term care workforce poses significant questions about how all perspectives and concerns are represented. For many workers, particularly those on atypical contracts with low pay and minimal skill requirements, the opportunity to express their views on issues affecting their working lives is limited. Nevertheless, providing a meaningful voice is essential for cultivating a culture of empowerment, enabling workers to influence matters that are important to them. Amplifying workers' voices benefits organizations by enhancing workforce engagement, reducing absenteeism, and fostering innovation. From a social justice standpoint and within contemporary understandings of work, having a voice is considered a fundamental individual right that adds meaning to work. Therefore, facilitating this voice is central to promoting fairness and transparency in long-term care organizations, ultimately benefiting society. However, critical knowledge gaps exist regarding long-term care's current capabilities to enable employee voice amidst often conflicting demands. Drawing from the perspectives of leaders and workers across various long-term care settings and international evidence, the session will contribute new empirical evidence relating to job quality and provide key insights for human resource practice and public policymakers undertaking sector reforms. Reflections from those engaged in the everyday practice of care delivery will test the applicability of the findings.

Abstract citation ID: igaf122.007

### GIVING EVERYONE A VOICE IN ACHIEVING A JUST TRANSITION IN WELSH DOMICILIARY CARE

Philip Taylor[1], and Katharina Sarter[2], *1. University of Warwick, Coventry, England, United Kingdom, 2. Warwick Institute for Employment Research, Coventry, England, United Kingdom*

Amid efforts to decarbonize society, the voices of socially disadvantaged community members, e.g. women low-paid workers, are often overlooked. Given the nature and growing significance of domiciliary care and the characteristics and size of its workforce, it represents a potentially valuable case study of involving communities in the co-design of solutions for a sector already undergoing significant transformation and now facing scrutiny as a major carbon emitter. This paper reports the preliminary findings of JUST-Systems, a UK-wide research initiative aimed at developing integrated systems approaches that position people at the center of efforts to accelerate action on decarbonization, local economies, and social justice. JUST-Systems seeks to identify bottlenecks and design better, more inclusive interventions by understanding the interactions between people, policies, and technologies. By adopting a systems approach, it may be possible to identify synergies and trade-offs between climate strategies and other priorities, such as jobs and social inclusion, focus on key obstacles and leverage points for change, and highlight what interventions can yield co-benefits. Welsh domiciliary care is one of the project's test cases. This paper explores how systems thinking can aid in identifying synergies and trade-offs between climate strategies and the sector's other policy priorities and challenges, emphasizing key barriers and leverage points for change. The paper reports on applying participatory action research methods and the Critical Systems Heuristics framework for reflective practice to reveal worker views around delivering domiciliary care through a decarbonization lens, identifying the opportunities and challenges in utilizing such techniques in facilitating worker voice.

Abstract citation ID: igaf122.008

### STRENGTHENING LONG-TERM CARE WORKPLACE CULTURE AND WORKER VOICE THROUGH THE COACH APPROACH INTERVENTION

Kezia Scales[1], Emily Dieppa Colo[2], and McKayla Brady[2], *1. PHI, New York, New York, United States, 2. Paraprofessional Healthcare Institute, New York, New York, United States*

Communication challenges in long-term care organizations can strain relationships, curtail workers' engagement and contributions, and impact care delivery and outcomes. PHI, a national organization focused on the direct care workforce in long-term care, developed the Coach Approach organizational change model to enhance communication skills among all members of the care team in long-term care settings toward a goal of building strong, respectful relationships that foster collaboration and collective problem-solving. The Coach Approach model comprises three components: executive coaching, supervisory training ("Coaching Supervision"), and frontline worker training ("Coaching Communication"). For this evaluation study, PHI contracted with a large national home care provider to adapt and deliver the Coach Approach intervention in a hybrid format to their full staff between January and August 2024. Findings from the evaluation revealed high levels of satisfaction with the training, and 100% of supervisors and 97% of frontline staff reported that they have used the skills they acquired. Findings also indicated that the Coach Approach helps care team members appreciate one another's perspectives and work together more effectively. The evaluation results demonstrate the value of implementing communication training across all staffing levels within a large, multi-site long-term care organization with implications for improving job quality and care quality for older adults and people with disabilities being served.

Abstract citation ID: igaf122.009

### MORAL DISTRESS IN MEDICAL DIRECTORS BARRIERS TO FACILITATING EVIDENCE BASED IMPROVEMENTS IN PRACTICE

Catherine MacDonald, Annie Rhodes, and Leland Waters, *Virginia Commonwealth University, Richmond, Virginia, United States*

In nursing homes, the Medical Director (MD) serves a leadership role, as such the voice of the MD cultivates the quality of resident care, and sets standards for workforce training. MDs are responsible for coordinating care, implementing and evaluating care policies, and ensuring adherence to clinical standards. MDs use their voice in clinical oversight, the influence of administrative decisions, quality assurance, and process improvement initiatives. MDs, like other nursing home staff, must navigate organizational and regulatory barriers that accompany quality resident care, and as such, MDs are at risk for compassion fatigue and moral distress arising from systemic barriers. These conflicting institutional priorities affect both residents and staff. MDs navigate these challenges personally while simultaneously being required to use their voice and authority to serve on the leadership team. This session explores the intersection of moral distress and the MD role in the nursing home. Semi-structured interviews with MDs are analyzed through dialogical narrative analysis, with an emphasis on voice, and close, annotative top-to-bottom readings of the transcripts. The exploratory, in-depth, and narrative nature of long-form interviews provides a compelling narrative method that can elicit rich and in-depth data and understanding and participant voice. While the MDs share their voices from different facilities, their joint narratives provide unified accounts of their industry. The experiences of the MDs about compassion fatigue, moral distress and the organizational barriers and regulatory barriers that they encounter will be used to provide insight on how to improve person-centered care in the nursing home.

Abstract citation ID: igaf122.010

### NAVIGATING NURSING HOME WORKFORCE CHALLENGES: LEADERS' VOICES FOR SUSTAINING PERSON-CENTERED CARE

Nahida Akter[1], Salma Akter[2], Lucy Mondol[3], Asifa Khatun[4], Liza Behrens[1], and Kimberly Van Haitsma[1], *1. The Pennsylvania State University, University Park, Pennsylvania, United States, 2. Wayne State University, Detroit, Michigan, United States, 3. Grameen Caledonian College of Nursing, Dhaka, Dhaka, Bangladesh, 4. Dhaka Nursing College, Dhaka, Dhaka, Bangladesh*

Nursing home (NH) leaders face ongoing challenges in addressing workforce shortages while ensuring federally mandated person-centered care (PCC) for residents. This study explores NH leaders' voices in managing staffing challenges while attempting to honor residents' daily care preferences-a key aspect of PCC. This qualitative descriptive study utilized semi-structured interviews (n = 14) from four NHs in Pennsylvania. NH leaders (administrators, clinical directors and supervisors) participated in in-depth individual interviews, which were then evaluated using inductive thematic coding structured according to the Nurse Staffing Framework. Our data revealed four key themes. First, confirming honoring residents' preferences is essential for PCC, with leaders emphasizing its benefits for resident well-being and staff satisfaction despite challenges such as perceived family interference and resource limitations. Second, workforce shortages pose a significant barrier to PCC, driven by negative perceptions of nursing, recruitment difficulties, and state-mandated staffing ratios, leading to staff overload and increased reliance on agency staff. Third, leaders employ various workforce management strategies, including pay adjustments, flexible scheduling, and staff recognition, to improve staff retention and maintain care quality. Finally, advocacy for policy reforms is perceived as crucial, with leaders stressing the need for regulatory changes to enhance staffing levels, workforce stability, and the overall sustainability of high-quality, resident-centered care in NHs. The paper will discuss insights into how workforce shortages impinge upon PCC, suggest effective workforce management strategies, as well as policy and regulatory reforms to strengthen both workforce stability and ensure the consistent delivery of PCC to residents in NHs.

### SESSION 1010 (PAPER)

### LONG-TERM SERVICES AND SUPPORTS: THE BEST LAID PLANS

Abstract citation ID: igaf122.011

### DEVELOPMENT AND TESTING OF A LONG-TERM CARE COST PLANNING AND EDUCATION TOOL FOR CAREGIVERS

Louise (Siyu) Gao[1], Kelly Moeller[2], and Marguerite DeLiema[3], *1. University of Minnesota, Minneapolis, Minnesota, United States, 2. University of Minnesota Twin Cities, Minneapolis, Minnesota, United States, 3. University of Minnesota School of Social Work, Minneapolis, Minnesota, United States*

An estimated 52% of Americans over 65 years old will need high levels of long-term care late in life (Favreault & Dey, 2016), but most have not planned for their long-term care needs nor saved enough to cover the high cost of long-term care (AP-NORC Center for Public Affairs Research, 2014). Medicaid provides a critical safety net for those who have exhausted their savings and require skilled care, but the complexity of Medicaid eligibility rules and covered care options create barriers to enrollment. We developed "Care Assistant," the first website to provide state-specific and personalized Medicaid enrollment timelines for older adults who need long-term services and supports (LTSS). Designed to reduce information overload for informal caregivers, the website alleviates caregiver burden by providing information on what financial resources the care recipient may qualify for. In this study, 112 caregivers of adults aged 60+ interacted with and completed tasks on Care Assistant (mean age = 43). Participants completed a Medicaid knowledge test before and after the website testing session, which was video-recorded and analyzed using rapid qualitative insights and descriptive statistics. Results indicate that using the website and watching four Medicaid explainer videos significantly improved participants' Medicaid program knowledge ($t = 13.3$, $p < .001$). Participants found it easy to enter the care recipient's financial information to determine their Medicaid eligibility (mean= 5.9/7) and the Care Cost Calculator was particularly helpful in learning about their local LTSS costs. Results indicate that a personalized, easy-to-navigate website can ease the burden of care planning.

Abstract citation ID: igaf122.012

### IMPROVING RELOCATIONS OF NURSING HOME RESIDENTS: A PARTICIPATORY DESIGN APPROACH

Annerieke Stoop[1], Charlotte Poot[2], and Monique Caljouw[2], *1. Tilburg University, Tilburg, Noord-Brabant, Netherlands, 2. Leiden University Medical Center, Leiden, Zuid-Holland, Netherlands*

Relocations of nursing home residents within and between nursing homes occur for various reasons, including changing healthcare needs or renovation of outdated real estate. These relocations can have negative effects on physical and psychological functioning and mortality of residents and burn-out in staff. To prevent this, nursing homes deploy activities to support residents and staff during the relocation process. Yet, these activities are developed without involvement of relevant stakeholders and fail to address their needs and wishes. The objective of this study was to develop a prototype that aims to improve relocation experiences of residents, their families and staff. A participatory, user-centered design approach was used, in which residents, families and staff actively participated throughout the research. Context mapping was performed to explore current practices, relocation phases, impact on residents and staff, and identify best practices. Data were gathered from previous (Appreciative Inquiry) focus groups (n = 13) with stakeholders from sixteen nursing homes, complemented by knowledge sessions (n = 2) with researchers and social designers. Insights were used to generate design concepts which were translated into prototypes addressing key areas such as equal participation, space for emotions, feeling at home, effective communication and knowledge sharing. Prototypes were evaluated and tested with residents, families and staff in three iteration rounds and led to the development of a (comprehensive) relocation toolkit. This study demonstrates how participatory design supports the development of a tailored relocation intervention in which experiences and needs of residents, families and staff are reflected and contributes to reinforcement of research–practice partnerships.

Abstract citation ID: igaf122.013

### PHYSICAL DISABILITY, SERVICE COORDINATION, & OUTCOMES IN PUBLICLY-FUNDED LONG-TERM SERVICES & SUPPORTS

Romil Parikh[1], Dana Urbanski[2], Chanee Fabius[3], Stephanie Giordano[4], Eric Jutkowitz[5], and Tetyana Shippee[5], *1. University of Minnesota School of Public Health, Minneapolis, Minnesota, United States, 2. Indiana University Bloomington, Bloomington, Indiana, United States, 3. Johns Hopkins University, Baltimore, Maryland, United States, 4. Human Services Research Institute, Cambridge, Massachusetts, United States, 5. University of Minnesota, Minneapolis, Minnesota, United States*

People living with physical disability (PD) receiving long-term services & supports (LTSS) experience greater unmet service needs. It is unclear if having a case manager for service coordination modifies PD-associated LTSS outcomes. To fill this knowledge gap, we evaluated associations of PD with emergency department (ED) visits and community integration among older

LTSS consumers with and without a case manager. Among 6,756 respondents (age, ≥65 years) from the National Core Indicators-Aging & Disability Survey (2018-2019), we evaluated three dichotomized outcomes (yes vs no): ED visits (over 12 months); and self-reported community integration indicated by both-activity (i.e. being as active in community as preferred) and enjoyment (i.e. enjoying things outside home). We used logistic regression, adjusting for consumers' sociodemographic and health-related characteristics, with random intercept for state. PD was documented in 60% of survey respondents. People living with PD were 27% more likely to report not having a case manager ($p < 0.001$). Among those without a case manager, PD was associated with significantly greater odds of ED visits (odds ratio [OR], 1.80, 95% CI, 1.48-2.19) and lower odds of community integration [activity: OR, 0.75, 95% CI, 0.62-0.90; enjoyment: OR, 0.69, 95% CI, 0.58-0.83]. These associations were attenuated among consumers with a case manager [ED visits: OR, 1.09, 95% CI, 0.94-1.25; activity: OR, 0.86, 95%CI, 0.75-1.00; enjoyment: OR, 0.85, 95% CI, 0.73-1.00]. Older LTSS consumers living with documented PD are more likely to report not having a case manager; and having a case manager might mitigate PD-associated adverse LTSS outcomes.

Abstract citation ID: igaf122.014

### WHO MAKES IT HOME: SNF TO COMMUNITY TRANSITIONS FOR TRADITIONAL MEDICARE BENEFICIARIES WITH SERIOUS MENTAL ILLNESS

Taylor Bucy[1], Carrie Henning-Smith[2], Donovan Maust[3], Tetyana Shippee[4], and Dori Cross[2], *1. University of Kansas School of Medicine, Kansas City, Kansas, United States, 2. University of Minnesota School of Public Health, Minneapolis, Minnesota, United States, 3. University of Michigan Medical School, Ann Arbor, Michigan, United States, 4. University of Minnesota, Minneapolis, Minnesota, United States*

Discharge to the community following a skilled nursing facility (SNF) stay is a key metric of high-quality care. This aligns with preferences around aging-in-place, yet significant fragmentation across service providers has made achieving this outcome challenging. This is especially true for people with serious mental illness (SMI) (i.e. schizophrenia, bipolar disorder) who represent a growing proportion of U.S. long-term care residents. We use a 4-year (2016-2019), 100% sample of traditional Medicare data to examine individual- and organization-level predictors of SNF discharge location (community vs other) for persons with SMI. Of the 118,325 unique SNF stays for people with SMI, 54% ended in discharge to the community. Patients with SMI who were discharged to the community (vs not) were significantly younger (69 vs 72 y/o), more likely to be female (61% vs 56%), and were less likely to be dual-eligible (51% vs 71%) or to have co-occurring ADRD (33% vs 51%). SMI patients discharged to the community were also significantly more likely to receive care in smaller, urban SNFs, that were more integrated, higher quality, and saw a smaller share of SMI patients overall. These findings were reinforced by our fully adjusted regression models. While many socioenvironmental factors restrict patients' ability to return home following a SNF stay, better positioning SNFs to achieve good outcomes requires significant investments along the continuum. Policymakers should consider how to leverage value-based payment programs in a way that better accounts for the resources required to coordinate care for this population, including partnership with community-based providers.

### SESSION 1015 (SYMPOSIUM)

Abstract citation ID: igaf122.015

### ADAPTING INTERVENTIONS FOR EARLY-ONSET DEMENTIA: SUPPORTING FRONTOTEMPORAL DEGENERATION CAREGIVERS

Chair: Lauren Massimo
Discussant: Esther Kane

Caregiving for a person with dementia is demanding and stressful, particularly for caregivers of persons with frontotemporal degeneration (FTD). FTD is a common cause of early-onset dementia with no known cure. Most patients with FTD remain in the community through the end of their lives, depending on informal caregivers to assist them, yet existing interventions are designed for older adults with Alzheimer's disease. In this interdisciplinary symposium, we highlight approaches taken to adapt interventions to address the specific needs of FTD caregivers. The first session will describe Living and Learning with Advancing FTD (STELLA-FTD), an intervention that tests the efficacy of the ABC (Activator, Behavior, Consequences) approach to manage behavioral symptoms. The second session describes Primary Progressive Aphasia (PPA) Tele-Savvy, designed to help caregivers address communication in persons with PPA. The third session will describe Virtual Caregiver Coach for You (ViCCY), a motivational health coaching intervention initially designed for caregivers of persons with heart failure. ViCCY utilizes a motivational coaching approach to help caregivers to build individualized resources, with the goal of improving caregiver self-care. The last session will describe the FTD Clinical Research Learning Institute (FTD CRLI), a caregiver engagement initiative that was adapted from Amyotrophic Lateral Sclerosis (ALS). The FTD CRLI, developed in partnership with the Association for Frontotemporal Degeneration, empowers those with lived experience to be advocates in the field of FTD research by educating them on the clinical research process. Together these presentations highlight innovative efforts to tailor interventions for unique challenges faced by FTD caregivers.

Abstract citation ID: igaf122.016

### SUPPORT VIA TECHNOLOGY: LIVING AND LEARNING WITH ADVANCING FRONTOTEMPORAL DEGENERATION

Allison Lindauer, and Aimee Mooney, *Oregon Health & Science University, Portland, Oregon, United States*

Support via Telehealth: Living and Learning with Advancing FTD (STELLA-FTD) is one of the few behavioral interventions in the US designed specifically for family care partners with all types of FTD (e.g., behavioral variant, primary progressive aphasia). Our NIA-funded study provides a group-based intervention that tests the efficacy of the ABC (Activator, Behavior, Consequences) approach, both in increasing care partner self-efficacy and reducing burden. The intervention teaches care partners about how to address behavioral symptoms by engaging rehabilitation specialists in their care journey. This aligns with the World Health Association's mandate that dementia care include rehabilitation support. Along with testing the STELLA-FTD intervention, we are also testing the mechanism of action. In the early 2000's Teri (2005) advanced the science of behavior analysis into the dementia field by making the ABC analytic approach accessible to care partners. This approach is well-known in the caregiving world, tested by scientists in

Thailand, Brazil, Japan, and the US. These studies have shown positive results in reducing care partner burden, but none, to our knowledge, have isolated the essential components of the ABC analytic approach, and thus, the mechanism of action is not fully understood. Because of this, it is not known what the "essential ingredient" is in ABC-based interventions. This RCT will isolate the ABC approach in the context of a telehealth-based intervention. This will allow future studies to engage the known mechanism of action, potentially increasing the effectiveness of behavioral interventions for care partners.

Abstract citation ID: igaf122.017

### PPA TELE-SAVVY: RESULTS OF AN ONLINE PILOT INTERVENTION WITH CAREGIVERS OF PERSONS WITH PRIMARY PROGRESSIVE APHASIA

Darby Morhardt, Lauren Dowden, and Kaitlyn Lucca, *Northwestern University, Evanston, Illinois, United States*

A PPA diagnosis leads to complex psychosocial issues compounded by complicated terminology and uncertain progression and pathology. Dementia caregiver interventions do not match PPA families' needs for tailored support. Project aims: (1) Identify PPA families' most pressing challenges and find ways the existing evidence-based caregiver intervention Tele-Savvy should be adapted. (2) Pilot the resulting PPA Tele-Savvy intervention with PPA care partners in 7 weekly 90 minute videoconferences using formative evaluation to address communication, cognitive and behavioral changes, and assist caregivers in achieving competence in the caregiving role. Fourteen spousal caregivers participated in Aim 1 and identified ways Tele-Savvy was helpful and ways it was not. Revisions were incorporated into the resulting PPA Tele-Savvy caregiver intervention. Fifteen spousal caregivers participated in 2 pilot interventions (n = 9, n = 6) involving seven 90-minute weekly videoconference sessions, mindfulness exercises, PPA-specific manual readings, and homework assignments. Pre-post results demonstrated trends in decreased caregiver depression, increased competency, decreased burden, increased PPA knowledge and significant increased positive dyadic interaction. Analysis of video transcriptions and evaluative focus groups revealed: increasing knowledge of PPA, learning new approaches of communication and connection, adjusting expectations, initiating care planning, and assessing self-care. PPA Tele-Savvy demonstrates the feasibility of a tailored PPA caregiver intervention and yields positive results for improving caregiver well-being, competence, knowledge, with positive effects on the dyadic relationship. Further testing in a larger trial is warranted.

Abstract citation ID: igaf122.018

### ADAPTING A SELF-CARE INTERVENTION TO MEET THE NEEDS OF CAREGIVERS OF PERSONS WITH FRONTOTEMPORAL DEGENERATION

Lauren Fisher, Barbara Riegel, Karen Hirschman, and Lauren Massimo, *University of Pennsylvania, Philadelphia, Pennsylvania, United States*

While many caregiver interventions focus on education about symptom management for a person with dementia, the Virtual Caregiver Coach for You (ViCCY) focuses explicitly on the caregiver's ability to care for their own physical and emotional health (i.e., self-care). We use motivational coaching to bring awareness to caregiving demands, stressors, and coping strategies for caregivers to build support and resources. ViCCY is a virtual health coaching intervention that partners health coaches and caregivers in 1:1 relationships to enhance self-care over 10 sessions in 6 months. ViCCY was initially developed for caregivers of persons living with heart failure. Throughout development, our study aimed to adapt this intervention for Frontotemporal Dementia (FTD) based on a review of self-care literature, parent study findings in heart failure, the investigators' clinical expertise, and focus groups to include content related to the unique symptoms of FTD and the demands in this caregiver group. The 6-month randomized controlled pilot study demonstrated significant improvement in self-care monitoring and confidence (two of three domains on the self-care inventory) for the intervention group (n = 15) compared to a control group (n = 15). We conducted focus groups after the completion of the pilot to receive feedback from participants about their experience in the intervention group. Caregivers appreciated the structured protocol content (including self-care, stress, and coping) but suggested integrating more specialized information, such as caregiver anticipatory grief. Based on the pilot study findings and focus group feedback, we have adapted the intervention protocol for the next phase of this intervention.

Abstract citation ID: igaf122.019

### THE FRONTOTEMPORAL DEGENERATION (FTD) CLINICAL RESEARCH LEARNING INSTITUTE

Shana Dodge[1], Meaghan Sharp[2], Wanda Smith[3], and Lauren Massimo[2], *1. Association for Frontotemporal Degeneration, King of Prussia, Pennsylvania, United States, 2. University of Pennsylvania, Philadelphia, Pennsylvania, United States, 3. CalHHS Alzheimer's Disease & Related Disorders Advisory Committee, Sacramento, California, United States*

Recent years have seen a marked increase in research opportunities for frontotemporal degeneration (FTD), a common cause of early-onset dementia that impacts behavior, communication, and/or motor functioning. Recruitment and retention for trials, however, remains a challenge, as do community misconceptions about FTD research. The Parkinson's and ALS fields have seen success tackling these barriers through patient engagement initiatives designed to empower people directly impacted by neurodegenerative conditions with critical information about clinical research. Utilizing a curriculum adapted from the Northeast ALS Consortium (NEALS), in 2024, the Association for Frontotemporal Degeneration (AFTD) and University of Pennsylvania Frontotemporal Degeneration Center (Penn FTD Center) piloted an FTD Clinical Research Learning Institute (CRLI). The one-day, virtual FTD CRLI received 70 applications for 12 training spots, demonstrating a community interest in research training. The FTD CRLI program included sessions on FTD drug development and clinical trials, ethics and informed consent, study design and statistics, how to read research papers, personal experiences with research, and FTD advocacy. Graduate "research ambassadors" are given opportunities to participate in initiatives that influence and improve the FTD research process and are encouraged to bring awareness of FTD research to their own communities. On a post-training survey, ambassadors reported an eagerness to connect research information to the general public and increased enthusiasm for practical advocacy guidance, AFTD and the Penn FTD Center are expanding FTD CRLI opportunities for 2025 with a focus on diversifying the pool of research ambassadors.

### SESSION 1020 (SYMPOSIUM)

Abstract citation ID: igaf122.020

### ALZHEIMER'S DISEASE AND RELATED DEMENTIAS (ADRD) IN RURAL COMMUNITIES: EXPLORING RISKS AND PATHWAYS TO PREVENTION

Chair: Mary (Kate) Clayton-Jones
Discussant: Mary (Kate) Clayton-Jones

Approximately a quarter of U.S. older adults live in rural communities. Due to limited healthcare access and supportive resources, older adults in rural communities may face an increased risk of disease. However, culturally relevant preventative strategies can help mitigate risks. In this symposium, we will discuss challenges influencing rates of cognitive decline in rural populations and approaches to reduce this disparity. Through an instrument design study, Dr. Cassidy will discuss qualitative findings regarding considerations of usability, inclusivity, and cultural relevance for measuring rural health beliefs toward Alzheimer's Disease and Related Dementias (ADRD) prevention. Dr. Williams will share ADRD risk factors in a diverse rural population around Lake Okeechobee and correlations observed between cognitive function and factors such as education, hypertension, hearing loss, and social connectedness. Dr. Chanti-Ketterl will discuss the Alzheimer's Gut Microbiome Project implemented in Indigenous communities in North Carolina, highlighting the success of a mobile clinic research approach to exploring the relationship between cognitive function and the gut microbiome. Dr. Hoffman will report on ADRD early detection and biomarker use in Nebraska and Iowa revealing educational efforts are needed to increase knowledge and use of these important diagnostic tools in rural areas. Lastly, Dr. Ferdows will present on higher ADRD mortality rates linked with restrictive state nurse practitioners and physician assistant policies. Collectively, these studies highlight the need for targeted community and multidisciplinary-centered interventions to reduce dementia risks in rural settings.

Abstract citation ID: igaf122.021

### ADAPTING A DEMENTIA PREVENTION TOOL FOR OLDER ADULTS LIVING IN RURAL COMMUNITIES: A CONTENT ANALYSIS

Jessica Cassidy, and Kathy Lee, *The University of Texas at Arlington, Arlington, Texas, United States*

Older adults living in rural communities are at heightened risk of dementia; however, preventative strategies can reduce these risks. Before dementia prevention efforts can be effectively implemented in rural communities, gaps in the literature must be addressed by developing culturally relevant tools designed to measure the influence of community characteristics on local dementia-related health beliefs and behaviors. To achieve this objective, this study developed the Rural Older Adults Dementia prevention–Health Behaviors questionnaire (ROAD-HB) by applying a lifespan developmental approach to the Health Belief Model (HBM). The questionnaire consisted of three embedded instruments and was constructed following a stage development design. To explore participant perceptions of the ROAD-HB's acceptability, usability, and content validity, cognitive interviews were conducted with 20 older adults from two rural Northeast Texas counties and themes were developed through a qualitative content analysis. There were six major themes identified which included: (1) Acceptability and positive feedback on ROAD-HB's preventative focus; (2) Suggestions to improve inclusivity of questionnaire title; (3) Usability and Clarity Challenges due to low health literacy and a lack of familiarity with dementia; (4) Challenges with response options; (5) Differences between local rural culture health beliefs and those in items stems; and (6) Needs for Greater Specificity in Item Stems to Assess Health Behavior Norms. Overall, place-based factors were observed to shape participant's dementia-related health beliefs and behaviors in multiple ways. The findings of this study may inform future dementia prevention efforts, which are urgently needed in rural communities.

Abstract citation ID: igaf122.022

### EXAMINING THE INFLUENCE OF MODIFIABLE RISK FACTORS FOR ADRD IN A RURAL COHORT

Christine Williams[1], Lisa Wiese[1], Janet Holt[1], and Juyoung Park[2], *1. Florida Atlantic University, Boca Raton, Florida, United States, 2. University of Arizona, Tucson, Arizona, United States*

Results from year one of a three-year repeated measures descriptive study involving multicultural rural residents are offered (N = 494). The region surrounding Lake Okeechobee, 2nd largest freshwater lake in the contiguous U.S., is home to diverse groups, including farmworkers, teachers, and healthcare workers who experience all 14 potentially modifiable risk factors for Alzheimer's Disease and Related Dementias (ADRD). We report on these risk factors (Livingston et al., 2023). For example, correlations between individual risk factors and cognitive function (Montreal Cognitive Assessment) scores revealed that education, $r = .16$, $p < .001$, hypertension, $r = -.12$, $p = .008$, hearing loss, $r = -.14$, $p = .002$, smoking, $r = -.13$, $p = .005$, physical performance, $r = .22$, $p < .001$, cholesterol, $r = -.10$, $p = .02$, depression, $r = -.15$, $p < .001$, loneliness, $r = -.13$, $p = .003$, and social connectedness, $r = .22$, $p < .001$, correlated with MoCA scores. Number of years lived in this area interacted with education, $p = .007$, and diabetes, $p = .038$, to predict cognitive function. Interactions with smoking, BMI, social connectedness, and loneliness were notable trends towards significance ($p < .1$). This highlights the need to examine all ADRD risk factors prior to developing community-based interventions to decrease risk, while considering that older age correlates with many other risk factors for ADRD. Future studies will focus on improving knowledge about ADRD, health behaviors, early detection of cognitive decline and management of chronic conditions related to ADRD risk.

Abstract citation ID: igaf122.023

### ASSESSING COGNITIVE AND GUT HEALTH IN NORTH CAROLINA'S RURAL INDIGENOUS POPULATION VIA MOBILE CLINIC

Marianne Chanti-Ketterl[1], Jada Brooks[2], Jennifer Jones-Locklear[3], and Heather Whitson[1], *1. Duke University, Durham, North Carolina, United States, 2. The University of North Carolina at Chapel Hill, Chapel Hill, North Carolina, United States, 3. The University of North Carolina at Pembroke, Pembroke, North Carolina, United States*

Objective: Alzheimer's disease disproportionately affects communities of color, including rural North Carolina. These communities experience environmental hazards, limited nutritious food access, and healthcare barriers. To ensure cultural relevance, we collaborated with community members and regional research partners to adapt the Alzheimer's Gut Microbiome Project protocol (AGMP). Using a mobile clinic, we investigated the relationship between cognitive function, the gut microbiome (GMB), and the metabolome in and around Robeson County, the tribal headquarters of the Lumbee Tribe. Methods: After tribal IRB approval and informed consent, participants underwent fasting glucose and cholesterol testing via finger prick and biomarker collection through venipuncture. The AGMP protocol for metabolomics and metagenomics was followed. Participants completed cognitive assessments and provided demographic, lifestyle, medical, environmental, and dietary information through structured questionnaires. A home stool collection kit with detailed instructions was provided. Results: Of the 65 individuals expressing interest, 44 enrolled, and 39 provided both plasma and stool samples. Only three participants did not return stool samples. All participants identified as Indigenous with a mean age of 72 (SD 8); most were women (77%) with a mean of 13 years of education (SD 3). The mean Healthy Eating Index score was 64 (SD 12), and the mean 3-point adjusted Montreal Cognitive Assessment Score was 21 (SD 4). GMB analyses are ongoing. Discussion: Findings of the association between cognitive function and the GMB will be presented. The high rate of biospecimen collection highlights the effectiveness of our community-centered mobile clinic research approach in a rural area.

Abstract citation ID: igaf122.024

### RURAL HEALTH DISPARITIES IN ALZHEIMER'S DISEASE AND RELATED DEMENTIAS BIOMARKER TESTING

Rashelle Hoffman[1], Elise Choquette[1], Erin Blocker[2], Blake Murphy[1], Bryant Howren[3], and Eric Vidoni[4],
*1. Creighton University, Omaha, Nebraska, United States,*
*2. Emporia State University, Emporia, Kansas, United States,*
*3. VA Office of Rural Health, Iowa City, Iowa, United States,*
*4. University of Kansas Medical Center, Kansas City, Kansas, United States*

Early diagnosis of cognitive impairment through clinical tests and Alzheimer's disease and related dementias (ADRD) biomarkers improves psychosocial care, reduces caregiver stress, and lowers healthcare costs. With the development of plasma biomarkers, ADRD testing has become more accessible for rural providers. However, it is unclear if rural providers have integrated these biomarkers into practice and whether barriers remain. At the 2024 Nebraska Rural Health and Vulnerable Population Conferences, 25 participants from 12 counties in Nebraska and Iowa completed a questionnaire on ADRD biomarker use and perceptions of an ADRD diagnosis. Most (64%) were patient-facing providers, while 36% were healthcare administrators. Although all providers reported regular cognitive screening in their practice, 75% were not ordering biomarker testing, with 68.75% expressing no interest. Among those using biomarkers (25%), blood plasma and PET imaging were the only methods reported. Most providers (62%) felt unqualified to order biomarkers. Across both administrators and providers, 39.1% reported that their organizations were not supportive, with 26.1% reporting only slight support for biomarker use. After a 30-minute educational session, participants showed greater positivity toward an ADRD diagnosis and increased confidence in discussing early detection. While cognitive screening is common in rural settings, there is limited use of biomarker testing, primarily due to a lack of knowledge about ADRD biomarker practices.

Abstract citation ID: igaf122.025

### RURAL-URBAN DISPARITIES IN ADRD MORTALITY BY NURSE PRACTITIONER AND PHYSICIAN ASSISTANT PRACTICE POLICIES

Nasim Ferdows, *Northeastern University, Boston, Massachusetts, United States*

Alzheimer's Disease and Related Dementias (ADRD) mortality has risen over the past decade, with rural areas consistently experiencing higher rates than urban areas. Limited healthcare access, particularly in states with restrictive Nurse Practitioner (NP) and Physician Assistant (PA) policies, may contribute to these disparities. Using CDC WONDER data from 2010 to 2020, we analyzed ADRD mortality across rural and urban areas and examined the association between state-level NP and PA practice policies and mortality rates. NP practice authority was categorized as either Full Practice Authority (FPA), where NPs can practice and prescribe independently, or restricted, where physician oversight is required. PA practice was classified as restrictive, moderate, or permissive based on state regulations. ADRD mortality was consistently higher in rural areas, with rural mortality rates increasing from 95.2 per 100,000 in 2010 to 123.4 per 100,000 in 2020, compared to an increase from 76.5 to 98.7 per 100,000 in urban areas. States with restrictive NP and PA policies had the highest ADRD mortality rates, particularly in rural settings, while states granting FPA to NPs and permissive practice authority to PAs had lower mortality rates. In rural areas, states with FPA had an average ADRD mortality rate of 110.3 per 100,000, compared to 126.7 per 100,000 in restrictive states. These findings suggest that expanding NP and PA scope of practice may improve healthcare access and reduce ADRD mortality, particularly in rural communities, highlighting the need for policy changes to address rural-urban disparities.

### SESSION 1025 (SYMPOSIUM)

Abstract citation ID: igaf122.026

### CHARACTERIZING END-OF-LIFE CARE AMONG OLDER ADULTS EXPERIENCING HEIGHTENED VULNERABILITY

Chair: Caroline Stephens
Co-Chair: Laura Block
Discussant: Katherine Ornstein

Access to high-quality end-of-life (EOL) care is not equally distributed. Yet, little is known about how some of our most vulnerable older adults access this care, where they die, or the social and family factors that influence their final days. This symposium includes 4 presentations focused on EOL care and place of death among highly vulnerable older adult populations. Each study employs a retrospective cohort design to examine different cohorts using data from the Utah Caregiving Population Science (Utah C-PopS) study –the only population-based, family-linked dataset in the United States. The first presentation describes the relationship between place of death and cause of death, with key differences by rurality. The second presentation identifies unrepresented nursing home residents, or those without family, and compares their sociodemographic and EOL care utilization by rural versus urban nursing home status. The third presentation examines a cohort of persons aged 55+ with serious mental illness to identify differences in place of death and the role of comorbid dementia and family availability. The last presentation examines rates of EOL care utilization in the last two years of life for decedents with a history of non-metastatic fragility fractures, focusing on the impact of dementia and family availability. Our discussant, Dr. Ornstein, will then review cross-cutting themes from these presentations, highlighting the medical, social, and caregiving determinants driving the increased need for more formalized EOL care. She will also discuss implications for better tailoring of EOL care practices and corresponding policies for these vulnerable older adults.

Abstract citation ID: igaf122.027

### UNREPRESENTED AND VULNERABLE: A RURAL/URBAN COMPARISON OF NURSING HOME DECEDENTS WITHOUT FAMILY

Caroline Stephens[1], Rachael Alexander[1], Eli Iacob[1], Rebecca Utz[1], Lauren Hunt[2], Sei Lee[2], Laura Block[3], and Katherine Ornstein[4], *1. University of Utah, Salt Lake City, Utah, United States, 2. University of California, San Francisco, San Francisco, California, United States, 3. University of Wisconsin-Madison, Madison, Wisconsin, United States, 4. Johns Hopkins University, Baltimore, Maryland, United States*

Families often play a critical role in end-of-life (EOL) care for nursing home (NH) residents, yet one in five older adults who die in a NH do not have available family living in the same state at the time of death. These potentially ‘unrepresented’ NH residents are highly vulnerable to poor EOL care, yet little is known about how rurality affects this population, with rurality also contributing to EOL care inequities. Given these individuals are typically unable to participate in survey or intervention-based research studies, their needs remain largely unknown and unmet. Using the only population-based, family-linked NH dataset in the U.S. - Utah Caregiving Population Science (Utah C-PopS) - we conducted a retrospective cohort study of 9,318 NH residents identified as not having any available family (spouses, children, siblings, parents) at the time of death and compared sociodemographic and death characteristics by rural vs urban NH status. Compared to those who died in an urban NH (n = 6,960), those who died in a rural NH (n = 2,358) were more likely to be male (38.5% vs 33.7%), White Non-Hispanic (93.4% vs 87.6%), widowed (61.0% vs 59.0%) with less than a high school education (45.9% vs 39.4%). Unrepresented rural NH residents were more likely to have multiple comorbidities (18.2% vs 15.9%) and more hospitalizations in the last 30 days of life (27.7% vs 22.9%), compared to urban NH residents. Findings provide a better understanding of rural/urban differences in EOL care for vulnerable ‘unrepresented’ NH residents and advance research on disparities in EOL care.

Abstract citation ID: igaf122.028

### CAUSE OF DEATH PREDICTING PLACE OF DEATH, BY RURALITY: EVIDENCE FROM THE UTAH POPULATION DATABASE

Attrayee Bandyopadhyay, Rebecca Utz, Caroline Stephens, and Michael Hollingshaus, *University of Utah, Salt Lake City, Utah, United States*

Place of death is a quality indicator of end-of-life (EOL) care. How cause of death affects place of death and how place of death varies across rural-urban areas is unexplored. This study uses a cohort drawn from Utah Caregiving Population Study (C-PopS): deaths from natural causes at ages 20+ in Utah between 1998 and 2016 (n = 217,222). The cohort is stratified by rural (n = 42,055), urban (n = 164,296), and frontier (n = 10,871). C-PopS is created through linkage of administrative data, vital statistics, and detailed health records at the individual level. Results show that heart disease, cancer, and other diseases (24.2%, 21.3%, 34.5%) are the top three primary causes of death. Home (37.6%) is most common place of death, followed by hospitals (30.9%) and nursing homes (26.5%). 60.5% of cancer decedents died at home versus 61.2% of dementia decedents in nursing homes. For all other causes, hospital is most common, except for chronic obstructive pulmonary disease (COPD). Home was slightly more common among rural decedents than urban (39.3% vs. 37.3%). A lower percentage of frontier decedents died in nursing homes compared to both rural and urban areas (19.7% vs. 26.9%), indicating lower utilization of nursing homes in frontier areas. This study has important implications in examining how EOL experiences may vary among the residents of rural, urban, and frontier areas of Utah. The findings from this study inform health policies and future interventions to cater to the unmet EOL care needs of the Utah population.

Abstract citation ID: igaf122.029

### PLACE OF DEATH AMONG DECEDENTS WITH SERIOUS MENTAL ILLNESS: THE ROLE OF DEMENTIA AND FAMILY AVAILABILITY

Malek Alnajar[1], Caroline Stephens[1], Eli Iacob[1], Djin Tay[1], Katherine Ornstein[2], and Laura Block[3], *1. University of Utah, Salt Lake City, Utah, United States, 2. Johns Hopkins University, Baltimore, Maryland, United States, 3. University of Wisconsin-Madison, Madison, Wisconsin, United States*

Older adults with serious mental illness (SMI), including major depression, schizophrenia, bipolar, and generalized anxiety, face complex health and social challenges (e.g., lack of family) that significantly impact their end-of-life experiences, including place of death. Yet, no study has comprehensively examined their end-of-life (EOL) experience. This retrospective cohort study compared place of death across major SMI conditions among individuals aged 55+ and described presence of comorbid dementia, family availability, and social determinants. Using data from the Utah Caregiving Population Science (Utah C-PopS) study, we analyzed 31,193 decedents with SMI. The majority were female (60.8%), non-Hispanic White (93.8%) with a mean age at death of 77.2 years (SD = 10.68). Major depression was most common (52.4%), followed by multiple SMI (21.7%), anxiety (20.7%), schizophrenia (2.7%), and bipolar (2.1%); one-third had comorbid dementia. Decedents with schizophrenia or co-occurring dementia were most likely to die in a long-term care facility (49.5% and 50.8%). Those with schizophrenia had the lowest family availability (28.3% with none) and a younger average age at death (mean=73.29 years), as did those with bipolar disorder (mean=72.67 years). Both groups were also more likely to be dual eligible and die in an urban setting. Individuals with multiple SMIs had the highest risk of multiple hospitalizations at EOL (47.2%). Findings highlight EOL challenges for older adults with SMI, especially those with schizophrenia and bipolar disorder, likely due to dementia burden and limited family support. Such insights can guide interventions to improve community support, hospice access, and family-centered care for older adults with SMI.

Abstract citation ID: igaf122.030

**END-OF-LIFE UTILIZATION AMONG DECEDENTS WITH FRAGILITY FRACTURES: THE ROLE OF DEMENTIA AND FAMILY AVAILABILITY**

Amy Cizik[1], Eli Iacob[1], Djin Tay[1], Katherine Ornstein[2], and Caroline Stephens[1], *1. University of Utah, Salt Lake City, Utah, United States, 2. Johns Hopkins University, Baltimore, Maryland, United States*

Fragility fractures (FFx) are associated with high morbidity and mortality, particularly among persons with dementia. While families play a critical role in caring for these individuals, little is known about how comorbid dementia and family availability impact their end-of-life (EOL) outcomes. This retrospective population-based cohort study examined rates of EOL care utilization (emergency room, hospital, outpatient) in the last two years of life for 20,988 decedents with a history of non-metastatic FFx, focusing on the impact of dementia and family availability. Most decedents with FFx were female (65.0%) and non-Hispanic White (94.6%), with 35.8% having dementia, 82.8% having at least one living family member, and 45.6% having sustained a hip, pelvis, or femur fracture. Mean age at death was 81.7 (SD = 11.7). Adjusted regression models showed that comorbid dementia was associated with a 10% decrease in hospitalization rates (0.899 [0.874,0.924]), a 42% decrease in outpatient visits (0.583 [0.530,0.642]), but 13% increase in emergency visits (1.134 [1.083, 1.188]), compared to no dementia. However, compared to those without family, presence of a spouse with or without child(ren) was associated with a 23% and 35% increase in outpatient visits, respectively (1.230 [1.098,1.378]); (1.351 [1.181,1.547]). Healthcare providers should consider both the presence of dementia and family availability when planning EOL care for persons with FFx. Increased use of emergency services by those with comorbid dementia suggests alternative care pathways, such as use of palliative care or more frequent home-based services, may be needed to address the increased need for emergency medical attention near EOL.

### SESSION 1030 (SYMPOSIUM)

Abstract citation ID: igaf122.031

**CO-CREATING CARER-CENTRIC SERVICE MODEL: ASSESSING CARER NEEDS AND DEVELOPING NEEDS-STRATIFIED INTERVENTIONS**

Chair: Vivian Lou

Discussant: Rebecca Utz

This symposium aims to explore innovative approaches to enhance the well-being of family carers in Hong Kong through collaborative research and tailored interventions. Utilizing participatory co-creation methods, Jockey Club Carer Space Project underscores the importance of needs-stratified service provision and carer-centric design, illustrating how collaborative research can lead to sustainable, community-driven solutions. Two interventions were developed to support carers of older adults with different levels of need. A randomized controlled trial was conducted to examine effectiveness of a low-intensity cognitive behavioral therapy (CBT)-guided group intervention. Preliminary findings suggest that the intervention may significantly improve mental health outcomes and overall quality of life for family carers of older adults. A two-phase usability evaluation was conducted to collect participants' feedback on the functionality and user experience of an eCoaching platform, leading to refinements before its launch for evaluating its effectiveness in helping family carers of older adults manage their mental health in a three-arm randomized controlled trial. Quantifying economic burden of family caregiving in Hong Kong, particularly in light of the aging population, highlights the significant monetary value of caregiving time and related out-of-pocket expenses, reiterating the urgent need for policy measures to support family carers in their critical roles. This symposium will provide a platform for discussing innovative, evidence-based interventions that address the complex needs of family carers. By fostering collaboration among researchers, practitioners, and policymakers, this symposium aims to pave the way for more effective carer-centric service model that enhance the quality of life for both carers and care recipients.

Abstract citation ID: igaf122.032

**NAVIGATING CARE: JOCKEY CLUB CARER SPACE PROJECT AND ITS IMPACT ON DEVELOPING A CARER-FRIENDLY COMMUNITY IN HONG KONG**

Vivian Lou[1], Vera Mun Yu Tang[1], Sau Man Angela Luk[2], Tarani Chandola[1], Wai Sze Chan[1], Jianchao Quan[1], and Terry Lum[1], *1. The University of Hong Kong, Hong Kong, China (People's Republic), 2. Caritas, Hong Kong, Hong Kong, China (People's Republic)*

The Jockey Club Carer Space Project aims to enhance the well-being of carers of older adults in Hong Kong through community-centered research and support. Grounded in community-engaged scholarship, the project employs participatory co-creation methods, engaging community stakeholders in all stages of research, service model development and intervention. Key findings include improved caregiver well-being, reduced stress, and increased confidence. The project has also developed innovative methodologies that prioritize community input, enhancing the relevance and effectiveness of interventions. Policy implications highlight the need for supportive measures such as flexible respite care, and carer-centric design. This initiative exemplifies the impact of collaborative, community-driven research in creating sustainable solutions for carer-friendly communities.

Abstract citation ID: igaf122.033

**A CLUSTERED RANDOMIZED CONTROLLED TRIAL OF LOW INTENSITY COGNITIVE BEHAVIORAL THERAPY ADAPTED FOR CARERS' NEEDS**

Wai Sze Chan, Tom Chun Wai Tsoi, Yu Hon Kenneth Kwok, Vera Mun Yu Tang, Tarani Chandola, Jianchao Quan, and Vivian Weiqun Lou, *The University of Hong Kong, Hong Kong, China (People's Republic)*

Carers experience elevated levels of stress, distress, and deterioration in quality of life. A low-intensity cognitive behavioral therapy (CBT)-guided group intervention is developed for the specific needs of family carers of older adults. This intervention integrates key CBT elements including behavioral activation, mindfulness, and problem-solving, with addition of lifestyle modifications including sleep hygiene, relaxation, and healthy dietary recommendations. A two-armed, multi-site, single-blind clustered randomized controlled trial is being conducted to examine the effectiveness of the intervention. Participants are adults aged 18+ who identify as a carers of a family member aged 60+, provide caregiving for 6+ hours per week, show moderate levels of need in the Carer Multidimensional Need Assessment, and are not diagnosed Alzheimer or dementia or other acute health conditions that hinder study participation and caregiving. Primary outcomes include depressive and anxiety symptoms. Secondary outcomes include carer needs, social support, quality of life, caregiver burden, and resilience. Outcomes measures are administered at baseline, post-intervention, and at 3-month follow-up. Carers in the intervention group are hypothesized to show a greater reduction in depression and anxiety symptoms and greater improvements in secondary outcomes compared to those in the control group. The trial protocol is registered on ClinicalTrials.gov with the identifier NCT0647301. This trial seeks to provide evidence for an integrated, evidence-based intervention model that can be widely disseminated to support family carers of older adults, ultimately improving their mental health and quality of life.

Abstract citation ID: igaf122.034

### USABILITY EVALUATION OF ECOACHING FOR FAMILY CARERS OF OLDER ADULTS: THINK-ALOUD AND USER ACCEPTANCE TESTING

Tom Chun Wai Tsoi, Yu Hon Kenneth Kwok, Vera Mun Yu Tang, Wai Sze Chan, Tarani Chandola, Jianchao Quan, and Vivian Weiqun Lou, *The University of Hong Kong, Hong Kong, China (People's Republic)*

Carers of older adults encounter significant barriers in using center-based support services due to caregiving demands and flexed operation hours. Online interventions may help bridge that gap by improving flexibility and accessibility for those who are unable to receive center-based support services. The eCoaching platform promotes mental health and resilience among family carers managing caregiving demands through interactive modules on cognitive behavioral therapy, self-care strategies, resilience-building, lifestyle modifications to enhance carers' emotional well-being and self-care self-efficacy. Multiple methods were applied in two phases of this formative usability evaluation. In phase one, think-aloud methodology was employed to understand carers' interactions with the platform and identify usability challenges. Six participants engaged in a 60-minute testing session, verbalizing their thoughts and impressions while navigating the eCoaching platform. Following the think-aloud sessions, semi-structured interviews were conducted to capture retrospective insights regarding participants' experiences and difficulties encountered. In phase two, usability testing assessed the platform's functionality over a one-week period. Nine participants used the eCoaching platform for approximately 30 minutes each day. Individual semi-structured interviews gathered in-depth feedback on usability and user experience, covering ease of use, content relevance, accessibility, and visual design. Participants shared their impressions, key takeaways, likelihood of future use, and suggestions for improvement, offering valuable insights for enhancing the eCoaching platform. Results from the usability study with end-users informed refinements to the eCoaching platform before its launch for evaluating its effectiveness in helping family carers of older adults manage their mental health in a three-arm randomized controlled trial in Hong Kong.

Abstract citation ID: igaf122.035

### MEASURING THE ECONOMIC BURDEN OF FAMILY CAREGIVING IN A RAPIDLY AGING EAST ASIAN POPULATION

Xingnan Yi, Xuechen Xiong, Vivian Lou, and Jianchao Quan, *The University of Hong Kong, Hong Kong, China (People's Republic)*

Hong Kong's aging population, with 19.6% age ≥65 in 2021, has increased reliance on unpaid family caregiving to meet long-term care needs. While psychological impacts are well documented, this study quantifies the direct and indirect economic costs of family caregiving. Data were collected from 2,284 adult caregivers age ≥18 who provided at least six hours of care weekly to family members age ≥60 in 2023–2024. Direct costs included caregiving time (valued using the opportunity cost method), out-of-pocket expenses (OOP), and medical costs due to caregiving-related health issues. Indirect costs included income losses from job cessation, reduced work hours, and productivity impairment due to providing caregiving tasks. Lost hours were quantified through self-reported work absences and productivity reductions, with income loss calculated by multiplying total missed hours by wage level using the opportunity cost method. The total annual economic burden of family caregiving in Hong Kong was estimated at USD 6.65 billion, or 2% of GDP. The mean annual monetary value of caregiving time was USD 6,770 per caregiver. OOP expenses, reported by 68% of caregivers, averaged USD 3,867 annually, while medical costs related to caregiving burden, incurred by 11%, averaged USD 2,350. Indirect costs included income losses from job cessation (reported by 44%) at USD 1,077, reduced work hours (56%) at USD 146, and productivity impairment (43%) leading to USD 2,454 in lost income annually per caregiver. These findings highlight the substantial economic value of unpaid caregiving and underscore the urgent need for policy actions to support family caregivers.

### SESSION 1035 (SYMPOSIUM)

Abstract citation ID: igaf122.036

### PARTNERING WITH CAREGIVERS TO OPTIMIZE HOSPITAL-BASED GERIATRIC CARE—THE TIME IS NOW

Chair: Beth Fields
Discussant: Lauren Bangerter

A growing number of family caregivers (relatives, partners, or other support persons) are providing support to older adults during hospital stays. However, many of these caregivers report feeling unrecognized and disengaged in hospital care processes. To address this gap, AARP championed the Caregiver Advise Record and Enable (CARE) Act, now law in 45 states and US territories. This law mandates that hospitals: 1) record the name of a family caregiver in the patient's healthcare record, 2) inform the family caregiver when the patient is to be discharged, and 3) provide the family caregiver with education on tasks to be performed for the patient. In response to this legislation, this symposium will present innovative approaches to enhance partnership with family caregivers in hospital settings. The information presented aims to bridge the policy and practice gap by offering healthcare clinicians, administrators, and researchers practical examples of when, where, why, and how to involve family caregivers in hospital care. The first presentation will describe the role of caregiver feedback in the adaptation of a caregiver support intervention for hospital care. The second presentation will discuss challenges and strategies for identifying family caregivers in the hospital setting. The third presentation will report findings from implementing a caregiver readiness for hospital discharge assessment. The fourth and final presentation will share a co-designed toolkit for including and supporting family caregivers at the system level.

Abstract citation ID: igaf122.037

**HOSPITAL GAMEPLAN4CARE: ITERATIVE ADAPTATION OF A CAREGIVER SUPPORT INTERVENTION FOR HOSPITAL CARE**

Molly Horstman[1], Tracy Evans[2], Timothy Zhou[2], Aanand Naik[3], Alan Stevens[4], and Mark Kunik[5], *1. Michael E. DeBakey VA Medical Center, Houston, Texas, United States, 2. Baylor College of Medicine, Houston, Texas, United States, 3. UTHealth Houston, Houston, Texas, United States, 4. Baylor Scott & White Health, Belton, Texas, United States, 5. Baylor College of Medicine, Houston, Texas, United States*

There are no evidence-based support interventions tailored to the needs of caregivers of hospitalized adults with dementia. Planned adaptation is an iterative approach that improves the fit between an intervention and a new implementation setting. Our objective is to describe the pre-implementation and post-implementation adaptation of GamePlan4Care for the hospital setting (Hospital GamePlan4Care). GamePlan4Care is an evidence-informed intervention that provides tailored education, support, and skills training to caregivers of adults with dementia in the community. Pre-implementation adaptations were guided by a literature review, semi-structured interviews with caregivers and health professionals, and a stakeholder panel that included caregivers. Post-implementation adaptations were guided by semi-structured interviews with caregiver participants who completed a single-arm pilot study of Hospital GamePlan4Care. Pre-implementation adaptations focused on intervention content: adding new content and modifying existing content to improve accessibility. Caregivers who completed the pilot study found the content helpful but faced challenges finding time to meet with the interventionist and complete the online training. Based on caregiver feedback, adaptations were made to improve the fit between the intervention and the lived experience of caregivers following discharge. Post-implementation adaptations included reducing the length of the intervention and creating greater flexibility in the timing and mode of therapeutic sessions. Caregiver feedback informed adaptions to the content and intervention delivery. Piloting the intervention enabled caregivers to provide concrete recommendations to improve the fit between the intervention and the caregiver lived experience of the hospitalization and return to home-based care.

Abstract citation ID: igaf122.038

**USING ELECTRONIC HEALTH RECORD TO IDENTIFY CAREGIVERS OF PEOPLE WITH DEMENTIA IN HOSPITAL SETTINGS**

Te-Lien Ku[1], Kayla Dillon[2], and Beth Fields[2], *1. University of Wisconsin-Madison, Madison, Wisconsin, United States, 2. University of Wisconsin–Madison, Madison, Wisconsin, United States*

Family and friend caregivers play a crucial role in supporting people living with dementia (PLWD) during hospitalization and post-discharge. Accurate identification and documentation of these caregivers are essential for effective care planning and decision-making. However, identifying caregivers in hospital settings can be challenging, and how these processes involved are less understood in the literature. Using a qualitative description approach, we examined electronic health record (EHR)-based practices during a 10-month hospital-based clinical trial to better understand the challenges and potential solutions in caregiver identification and documentation. Out of the 1,698 screened EHR charts of hospitalized PLWD, only 111 identified a caregiver. Key challenges in identifying caregivers through the EHR included the lack of structured fields in the record for documenting caregivers within the patient's care network beyond emergency contacts, as well as accompanying details (e.g., relationship to the patient, distance caregiving, power of attorney status, level of care involvement, and contact priority among multiple caregivers). Additionally, inconsistencies in documentation practices in the EHR across different hospital units further complicate efforts to standardize caregiver identification. To address these challenges, we propose several solutions to enhance caregiver identification and documentation for practice and research. These include integrating structured EHR fields that go beyond emergency contacts and embedding prompts and automated alerts to facilitate early and systematic caregiver recognition for clinicians. Refining documentation workflows and strengthening caregiver identification will ensure the most appropriate caregivers receive the necessary information and resources as needed.

Abstract citation ID: igaf122.039

**IDENTIFYING FAMILY CAREGIVERS IN HOSPITALS: OVERCOMING CHALLENGES**

Alycia Bristol, *University of Utah, Salt Lake City, Utah, United States*

During hospitalization, family caregivers (family, friends, or other support persons) play a vital role in supporting positive health outcomes for patients. Family caregivers often feel unprepared and unsure about providing care after hospital discharge. To support identification of caregivers' needs at discharge, we are adapting and testing the Family Readiness for Hospital Discharge Scale (FamRHDS). Testing of the FamRHDS scale centers on recruitment of patients and caregivers at hospital discharge. During our study, we have observed changes in recruiting caregivers post-COVID. While we successfully recruited patients and caregivers via telephone and text during COVID, we now face challenges due to non-functional or absent telephones in patient rooms and a fast-paced discharge process that often limits the time and availability of caregivers. Moreover, identification of caregivers within the electronic health record remains a challenge. This has led to decreased response rates for caregiver recruitment, contrasting with higher patient recruitment rates. To address this, we have shifted to maintaining an in-person presence within the hospital. We meet with patients to identify a preferred primary caregiver. When engaging with caregivers, we prioritize communicating our intention to understand their needs, emphasizing the practical impact and applicability of our work, rather than focusing on research for its own sake. Furthermore, we have established relationships with clinicians and administrators to support the identification of potential participants and raise awareness of our study. From our work, we have learned the importance of focusing on the utility of our research plan to better align with caregivers' needs.

Abstract citation ID: igaf122.040

**CO-DESIGNING THE ADRD SYSTEMATIC HOSPITAL INCLUSION FAMILY TOOLKIT (ASHIFT)**

Nicole Werner[1], Sydney Hoel[1], Teresa Thuemling[1], Linda Haack[2], and Beth Fields[3], *1. Vanderbilt University Medical Center, Nashville, Tennessee, United States, 2. Caregiver Partner, Nasvhille, Tennessee, United States, 3. University of Wisconsin–Madison, Madison, Wisconsin, United States*

Care partners (family or friends) of people living with Alzheimer's disease and related dementias (ADRD) are often not systematically included when the person with ADRD is hospitalized. This lack of care partner inclusion is associated with suboptimal outcomes for both ADRD patients and their care partners. Research and policy call for care partner inclusion in hospitalizations, but no tools exist to guide healthcare systems on care partner inclusion. This study aimed to co-design ASHIFT, an adaptable toolkit to be used by healthcare systems to facilitate the inclusion of care partners of hospitalized people living with ADRD. We convened two co-design groups – one of care partners of people living with ADRD who experienced a hospitalization (n = 6) and one of clinicians who care for patients living with ADRD (n = 5). We conducted five parallel co-design sessions over Zoom. Across the sessions, co-designers created ASHIFT, starting with many ideas and converging on the final prototype. ASHIFT provides guidance on how to identify, assess, and train care partners of hospitalized people living with ADRD and resources to support each action. Guidance is divided into hospitalization stages, with specific guidance for admission, hospital stay, discharge, and post-discharge. Each stage provides specific assets and protocols and links to resources. To support implementation, ASHIFT also provides an organizational readiness assessment, suggestions on prioritizing actions, and which actions can be conducted remotely. The next step is to test the feasibility and effect of ASHIFT on increasing care partner preparedness and reducing avoidable hospital readmission for patients with ADRD.

### SESSION 1040 (SYMPOSIUM)

Abstract citation ID: igaf122.041

### LEVERAGING CROSS-NATIONAL ENVIRONMENTAL DATA: LESSONS LEARNED FROM THE GATEWAY TO GLOBAL AGING DATA

Chair: Sara Adar
Co-Chair: Emma Nichols

The environments where people live, work, and socialize are known to influence health. However, these relationships can vary across studies, raising questions as to if these inconsistencies stem from differences in populations or variations in study design, data quality, and analytic approaches. Harmonizing data helps to ensure comparability across studies, offering improved insights as to how the environment affects health and why relationships may differ by location. This symposium highlights research within the Gateway to Global Aging Data (Gateway) project, an open access data sharing platform that provides harmonized longitudinal data on the exposome for nine countries around the world. Dr. Adar will first present a summary of the environmental exposome measures available to researchers while Ms. Clements will share the trends in those exposures across places and times. Dr. Ashtan and Zaninotto will then showcase how analyses can be run in individual cohorts showcasing how two measures – greenspace and temperature – are associated with altered mental and cognitive health in India and England. Dr. Zhang will then share an example of leveraging data across studies with a harmonized analysis of air pollution and cognitive function through four HCAP studies. Collectively, these talks will introduce this resource to the research community, share lessons learned from constructing harmonized exposure metrics, and highlight analyses of these exposures on outcomes of aging both within and across countries. We will also discuss how researchers can leverage similarities and differences across locations to advance science and highlight examples of research using Gateway data.

Abstract citation ID: igaf122.042

### HARMONIZED EXPOSOME DATA FOR AGING POPULATIONS WITHIN THE GATEWAY DATA SHARING PLATFORM

Sara Adar, *University of Michigan School of Public Health, Ann Arbor, Michigan, United States*

The Gateway to Global Aging Data has harmonized environmental exposome measures for seven nationally representative aging cohorts across the Americas, Europe, and Asia. Through a collaboration across studies within the Health and Retirement Study International Network, cohorts have been linked to information about air pollution (i.e., total and source-specific fine particulate matter, nitrogen dioxide, and ozone), weather (i.e., temperature and heat), and the built environment (i.e., greenspace and blue space) allowing for the examination of risk and protective factors for health. These cohorts are committed to providing data access to the scientific community, opening new opportunities for innovative research on the environmental exposome and health in aging populations. In addition to promoting data availability, the Gateway project has also conducted methodological research on topics such as spatial confounding and data security as well as developed comprehensive user guides to support studies on the exposome and aging outcomes. This presentation will showcase these powerful resources and demonstrate how researchers can leverage them to advance science.

Abstract citation ID: igaf122.043

### THE PATTERNING OF EXPOSURES AMONG AGING POPULATIONS IN EIGHT NATIONS

Nia Clements, *University of Michigan School of Public Health, Ann Arbor, Michigan, United States*

This presentation will showcase the variability in population-weighted exposome measures for Brazil, Chile, England, India, Ireland, Northern Ireland, and the United States using harmonized data from Gateway. We used the global exposure measures derived from satellite data, empirical spatiotemporal prediction models, and physics-based deterministic models to estimate exposures at population density weighted locations in each country. We then evaluated patterns within and between countries across space and time. More pronounced differences were observed between countries for several key ambient air pollutants such as particulate matter and ozone whereas the variation within countries was larger for measures of natural spaces like greenspace and nitrogen dioxide as a traffic-related air pollutant. For instance, median particulate matter levels were nearly seven times higher in India than in the United States (52.8 ug/m3 versus 7.6 ug/m3 respectively). Although temporal trends and correlations between exposures were often consistent across countries, we will highlight some unique patterns that emerged, such as how from 1990 to 2017, ozone in India increased by 0.52 ppb per year on average whereas ozone in Mexico decreased by 0.60 ppb per year on average. Furthermore, from 2017 to 2018 alone, there was a 7.6% decrease in greenspace in Chile while greenspace levels were relatively constant in other countries evaluated. The talk will conclude with implications of similarities and differences in exposures patterns for research on environmental predictors of accelerated aging, such as opportunities for change-on-change modeling and the influence of place-based confounding by different exposures.

Abstract citation ID: igaf122.044
**GREENSPACE AND DEPRESSION IN LASI**
Samer Atshan, *Center for Economic and Social Research, Los Angeles, California, United States*
Greenspaces, such as parks, gardens, and natural vegetation, are increasingly recognized as protective against mental health challenges among older adults. However, findings have primarily emerged from high-income, urbanized contexts, and evidence from low- and middle-income countries remains limited. Using data from the Longitudinal Aging Study in India-Diagnostic Assessment of Dementia (LASI-DAD), this study examines the relationship between exposure to greenspace and depressive symptoms as assessed by the CESD-10 scale. Our analysis specifically explores how this relationship varies across urban and rural contexts. Preliminary results indicate that in rural areas, respondents residing in greener environments reported lower scores on positive affect items of the CESD-10 scale, suggesting fewer experiences of positive emotions such as happiness and optimism. We explore potential explanations including socioeconomic influences in rural settings related to agricultural labor, experiences of social isolation and loneliness, and limited access to healthcare services. This study highlights the importance of contextualizing environmental determinants of mental health in aging research and demonstrates the value of harmonized environmental data in generating nuanced insights for culturally relevant mental health interventions.

Abstract citation ID: igaf122.045
**TEMPERATURE AND COGNITION IN ELSA**
Paola Zaninotto, Shaun Scholes, and Jessica Gong, *University College London, London, England, United Kingdom*
Understanding the relationship between temperature and cognitive function is crucial, especially in older populations, as extreme temperatures may exacerbate cognitive decline and affect overall health. This study explores the longitudinal association between temperatures and cognitive function in a large, nationally representative sample of individuals aged 50 and over, participants in the English Longitudinal Study of Ageing (ELSA). Using nine waves of data collected between 2002 and 2018, temperatures were linked to participants' residential addresses to assess the impact of temperature variations on cognitive performance. We also examined whether the effect of temperature on cognitive function was more pronounced in vulnerable individuals, including those with pre-existing health conditions. Our findings suggests that repeated exposure to extreme temperatures has an effect on cognitive decline, highlighting the need for tailored public health interventions for older populations.

Abstract citation ID: igaf122.046
**AIR POLLUTION AND THE BRAIN: A HARMONIZED ANALYSIS OF FOUR COHORTS WITH THE HARMONIZED COGNITIVE ASSESSMENT PROTOCOL**
Boya Zhang, *Harvard T.H. Chan School of Public Health, Boston, Massachusetts, United States*
Growing evidence suggests that air pollution may have significant impacts on aging and cognitive health. This study investigates the cross-sectional associations of long-term exposure to total and source-specific fine particulate matter (PM2.5) with harmonized cognitive function measures across four countries. We included participants who had completed the Harmonized Cognitive Assessment Protocol battery of cognitive tests from Health and Retirement Study (HRS), a US nationally representative study, and its sister studies in England, Chile, and India. We linked modeled concentrations of total PM2.5 and PM2.5 from 19 emission sources to participants' residential addresses over the 10 years preceding the cognitive assessment. To examine associations with cognitive function, we employed weighted generalized linear models adjusted for individual- and area-level confounders. The 10-year average total PM2.5 concentrations were ranging from 9.2±1.9 µg/m3 in the US to 56.5±25.9 µg/m3 in India. Although overall associations between total PM2.5 and cognitive function were modest across all countries, we identified universal and unique sources across countries. Specifically, higher wildfire-related PM2.5 was associated with poorer cognitive function in the US and India, while higher agriculture-related PM2.5 was linked to poor cognitive function in England and Chile, particularly among those living in the non-urban areas. Associations with residential PM2.5 were only observed for India. Our findings highlight the need for cross-nation studies to resolve air pollution issues from a global perspective.

### SESSION 1045 (SYMPOSIUM)

Abstract citation ID: igaf122.047
**EMOTION REGULATION, COGNITION, AND WELL-BEING ACROSS THE ADULT LIFESPAN**
Chair: Hannah Wolfe
Discussant: Derek Isaacowitz
This symposium explores the multifaceted nature of emotion regulation (ER) and its relationship with cognition and well-being across the adult lifespan. Wolfe and Isaacowitz investigated the cognitive demand and outcomes of ER tactics in younger and older adults. They found that positive-approaching was self-reported as the most demanding tactic and was linked to the highest memory accuracy; yet, there were no differences in pupil dilation across tactics. Growney & English further examined cognitive demands of ER strategies in younger adults, cognitively normal (CN) older adults, and older adults with mild cognitive impairment (MCI). They found that positive-emotion-enhancing strategies were perceived as less demanding and that younger and CN older adults, but not older adults with MCI, used high-demand strategies less frequently. Lai et al. explored whether ER abilities assessed in the lab align with success in daily life. Their results indicated minimal associations between lab and daily-life ER success across younger adults, CN older adults, and older adults with MCI, suggesting that lab-based assessments might capture different facets of ER ability. They also found age-related advantages in daily ER success, including among older adults with MCI. Finally, Britton et al. broadened this perspective by analyzing links between psychological well-being and cognitive function in older adulthood. They found that multiple components of well-being, including sense of purpose and self-acceptance, were significantly positively associated with both concurrent and future cognitive function, suggesting that psychological well-being may contribute to the cognitive resources involved in effective ER. Derek Isaacowitz will serve as a discussant.

Abstract citation ID: igaf122.048

### COGNITIVE DEMANDS AND MEMORY-RELATED OUTCOMES OF EMOTION REGULATION TACTICS AMONG YOUNGER AND OLDER ADULTS

Hannah Wolfe[1], and Derek Isaacowitz[2], *1. Stockton University, Galloway, New Jersey, United States, 2. Washington University in St. Louis, St. Louis, Missouri, United States*

Older adults "paradoxically" report high emotional wellbeing despite declines in some cognitive abilities. The selection, optimization, and compensation with emotion regulation model (SOC-ER) suggests that older adults compensate for losses in cognitive resources by instead selecting and optimizing less cognitively-demanding (but effective) emotion regulation strategies. However, empirical evidence regarding which regulation behaviors are more cognitively demanding across the lifespan is limited. This study aimed to test the SOC-ER model by directly measuring the cognitive demand and outcomes of emotion regulation tactics. Younger (ages 18-25, N = 77) and older adults (ages 65+, N = 62) viewed blocks of negative images while instructed to use one regulation tactic per block: acceptance, positive-approaching, negative-receding, and negative-approaching. Cognitive demand was assessed for each tactic via self reports and pupillometry (a physiological measure of real time cognitive effort). Incidental memory for the stimuli was also assessed; participants completed a recognition memory test for all images presented during the regulation blocks. Results showed that while acceptance was self-reported as the least mentally demanding tactic and positive-approaching as the most, $F(3,381)=3.655$, $p<.05$, there was no significant difference in pupil dilation across tactics, $F(3,351)=0.476$, $p=.699$, $\eta p2=.004$. Furthermore, positive-approaching was associated with the highest memory accuracy and negative-receding with the lowest, $F(3,408)=13.90$, $p<.001$, $\eta p2=.093$. All interactions with age group were nonsignificant. Overall, the SOC-ER model was minimally supported, suggesting that older adults' emotion regulation behaviors are not primarily driven by cognitive demand concerns.

Abstract citation ID: igaf122.049

### DO EMOTION REGULATION ABILITIES IN THE LAB AND IN DAILY LIFE MAP ONTO EACH OTHER FOR OLDER ADULTS?

Jocelyn Lai[1], Tabea Springstein[2], Claire Growney[3], and Tammy English[1], *1. Washington University in St. Louis, St. Louis, Missouri, United States, 2. University of California, Riverside, Riverside, California, United States, 3. Stanford University, Stanford, California, United States*

Despite natural decline in physical and cognitive health, older adults experience greater emotional well-being through prioritization and selective allocation of resources towards socioemotional goals. As such, older adults are theorized to have greater emotion regulation (ER) abilities. Empirical findings, however, are mixed regarding age-related strengths in ER. Laboratory-based assessments typically elicit higher-arousal negative affect, a context that older adults experience less of in daily life and may require more cognitive effort. Thus, ER success assessed in these types of experimental paradigms may not be reflective ER success as assessed in daily life for older adults with or without mild cognitive impairment (MCI) compared to younger adults. 211 adults (n = 66 younger adults; n = 87 cognitively normal [CN] older adults; n = 58 older adults with mild cognitive impairment [MCI]) completed a lab-based paradigm of ER and ecological momentary assessments of ER (7x for 9 days). Overall, ER success in the laboratory was minimally associated with ER success in daily life across all three groups. There were, however, age-related advantages in daily ER success, even among older adults with MCI. Our findings suggest that laboratory assessments may capture distinct aspects of ER ability compared to daily life assessments. Future work should test modifications to lab-based assessments to better capture how older adults engage in ER in daily life and consider other factors that may strengthen or weaken the connection between laboratory and in daily life.

Abstract citation ID: igaf122.050

### DO ALL COMPONENTS OF PSYCHOLOGICAL WELLBEING PREDICT COGNITIVE FUNCTION?

Karysa Britton[1], Gabrielle Pfund[2], and Emily Willroth[1], *1. Washington University in St. Louis, St. Louis, Missouri, United States, 2. Auburn University, Auburn, Alabama, United States*

Psychological wellbeing, specifically sense of purpose, has been consistently associated with cognitive functioning and dementia. Less is known about associations between other components of psychological wellbeing and cognitive functioning, an indicator of dementia. Given the importance of identifying modifiable risk and protective factors for dementia, the current study assessed whether components of psychological wellbeing are cross-sectionally and prospectively associated with cognitive function and cognitive change. A total of 4,026 participants from the Midlife in the United States study were included in analyses (MAge=56.18, SD = 12.38). The sample was representative of both males (45%) and females (55%), individuals primarily identified as White (93%) followed by Black or African American (4%), and about half of the participants reported that they completed an associate's degree or higher (45%). Components of wellbeing were assessed with the Ryff Psychological Wellbeing Scales (1989) and cognition was assessed with the Brief Test of Adult Cognition. All six components of wellbeing were significantly and positively associated with cognition cross-sectionally and effect sizes ranged from small to medium across components ($\beta$s=0.07-0.19) with personal growth having the largest association. Moreover, higher levels of environmental mastery, personal growth, sense of purpose, and self-acceptance significantly predicted increased levels of cognition at a nine-year follow-up when controlling for cognition at the first assessment timepoint ($\beta$ = 0.03-0.04]). These findings extend past wellbeing research by demonstrating that in addition to sense of purpose, some components of psychological wellbeing but not others are also associated with cognitive outcomes and should be assessed as possible protective factors for ADRD.

Abstract citation ID: igaf122.051

### PERCEIVED COGNITIVE DEMANDS OF EMOTION REGULATION IN YOUNG ADULTS AND COGNITIVELY DIVERSE OLDER ADULTS

Claire Growney[1], and Tammy English[2], *1. Stanford University, Stanford, California, United States, 2. Washington University in St. Louis, St. Louis, Missouri, United States*

Effective emotion regulation (ER) often requires cognitive resources, but some strategies are theorized to be more cognitively demanding than others. Individuals can maintain emotional well-being by selecting ER strategies consistent with their available resources. Selection of low-demand strategies may be especially helpful for older adults experiencing accelerated cognitive decline. In this study, young adults (age 21–34, n = 66), cognitively normal older adults (CN; age 70–83, n = 90), and older adults with mild cognitive impairment (MCI; 70–84, n = 60) reported their perceived ER demands in a laboratory task and regarding their general use of ten ER strategies. We examined group differences in perceived demands as well as links between demands and frequency of use. Group differences varied by context: in the laboratory task, older adults with MCI reported higher demands than young and CN older adults, but reports of general demand perceptions revealed young adults perceive strategies as more demanding than older adults. Strategies varied significantly in their perceived cognitive demands, with strategies focused on enhancing positive emotion (e.g., savoring) rated as less demanding than those focused on reducing negative emotion (e.g., distraction). Multilevel models revealed that regulators used strategies less that they perceived as higher in cognitive demands among young adults (b=–0.20, SE = 0.05, p<.001) and CN older adults (b=–0.26, SE = 0.05, p<.001), but not older adults with MCI (b=–0.07, SE = 0.06, p=.220). Findings suggest that older adults may often have more ER expertise, but MCI may create ER difficulties, interfering with the ability to select easier to implement strategies.

### SESSION 1050 (SYMPOSIUM)

Abstract citation ID: igaf122.052

### INNOVATIVE STRATEGIES FOR TEAM TREATMENT PLANNING AND COLLABORATIVE GOAL-SETTING IN HOME-BASED PRIMARY CARE

Chair: Tara Afonso
Co-Chair: Michelle Mlinac
Discussant: Katie Mendoza

Collaborative treatment planning with older adults and caregivers in home-based primary care (HBPC) can be challenging for the interprofessional teams who serve them. This symposium describes a multi-faceted stepped quality improvement approach to changing the process of interdiscplinary treatment planning (IDTP) within an HBPC program. This program spans four individual teamlets and has a patient census of 580. Members of the interdisciplinary team include nurses, social workers, physicians, nurse practitioners, physicians assistants, pharmacists, dieticians, rehab therapists, medical support assistants, and psychologists. The first presentation provides an overview of the strategic planning course for the team which began with a revamp of the structure and workflow of the IDTP meeting. Planful documentation improvements include the creation of an overall 'mission' for each patient to help guide the course of longitudinal care, as well as enhancing documentation of the 4M's to underscore holistic geriatric care delivery. The second presentation describes the approach to foster the spirit of motivational interviewing (MI) within the team. In order to facilitate learning and promote buy-in, a gamification strategy was employed, and results suggest both immediate and sustained learning gains. The third presentation provides a review of how the team elicited patient SMART goals, aligned them with team treatment goals, and worked towards achievement of those goals. The strategic use of gamification was again enlisted to foster a friendly competition among the four teamlets to adopt new skills in a safe learning environment. Overall this multi-year QI process has driven person-centered care improvements for patients with multicomplexity.

Abstract citation ID: igaf122.053

### EVOLVING THE MISSION: TEAM PROCESS IMPROVEMENTS IN HOME-BASED PRIMARY CARE

Tara Afonso[1], Brian Green[1], Todd Paul[2], Cynthia Knight[2], Shallee Saquing-Nwodo[2], Katie Connor[2], Melissa Meynadasy[3], and Michelle Mlinac[4], *1. VA Boston Health Care System, Boston, Massachusetts, United States, 2. Jamaica Plain VA Medical Center, Jamaica Plain, Massachusetts, United States, 3. VA Boston Healthcare System, West Roxbury, Massachusetts, United States, 4. VA Boston Healthcare System, Boston, Massachusetts, United States*

The Home-Based Primary Care (HBPC) model is designed to be an exemplar of integrated interprofessional care, such that HBPC teams can meet the complex medical and psychosocial needs of homebound older adults. Concern for non-specific or undefined goals, challenging patient encounters, and risk of staff burnout highlighted the need to overhaul how the team engaged patients in collaborative care planning. Aims of this 5-year process improvement work were to 1) Update the structure of the interdisciplinary team treatment planning (IDTP) meeting 2) Identify an overall "mission" driving the individualized longer-term care plan for each patient 3) Align current practices with Age-Friendly Healthcare, driven by the 4M's. These aims were introduced intermittently over time, as team members mastered individual components, integrated them into routine practice, and gained confidence. This work coincided with rapid geographical expansion of the team and a 39% increase in its census. Roles within the team were de-centralized: the documentation responsibility became shared across team members and meeting structure allowed for any team member to serve as the leader. The team achieved a Level 2 Age-Friendly (Committed to Care Excellence) rating by IHI, and in the first year of implementation 98% of HBPC patients (524 out of an average daily census of 533) received 4M's care.

Abstract citation ID: igaf122.054

### USE OF MOTIVATIONAL INTERVIEWING WITHIN HOME-BASED PRIMARY CARE: A GAMIFICATION APPROACH

Michelle Mlinac[1], Angelica Boeve[2], Tara Afonso[3], Cynthia Knight[4], Katie Mendoza[3], Hannah Bashian[3], Brian Green[3], and Linda DiLalla[3], *1. VA Boston Healthcare System, Boston, Massachusetts, United States, 2. Minneapolis VA, Minneapolis, Minnesota, United States, 3. VA Boston Health Care System, Boston, Massachusetts, United States, 4. Jamaica Plain VA Medical Center, Jamaica Plain, Massachusetts, United States*

Motivational interviewing (MI) is a well-established strategy for promoting behavior change, but it has been understudied for older adults with complex health conditions. As part of a holistic patient-centered approach to the care of older patients with multi-complexity, such as those served by home-based primary care (HBPC), use of MI could be instrumental in identifying patients' priorities, strengthening interdisciplinary care, and engaging patients in managing their own healthcare decisions. A multi-pronged approach to training a home-based primary care team in using MI was undertaken to train HBPC providers in practicing MI regularly during patient care. A gamification teaching strategy was utilized to train 24 members of an interprofessional HBPC in use of MI skills. A 90-minute workshop was comprised of a 30-minute didactic and a 60-minute skills-focused game, led by three MI experts within the team.

Participants were asked to complete brief pre- and post-session knowledge checks. The number of participants correctly identifying the stages of behavior change and MI microskills increased from 54% and 75% respectively before the training, to 100% on both concepts after the session. Qualitative feedback was obtained from a focus group conducted with team members after the training highlighted collaboration and feeling valued as positive impacts of using MI. Sustainability efforts following the training included having time during team meetings to share of stories about using MI with patients and building in in-home coaching sessions by MI experts within the team to demonstrate MI skills with patients and help coach staff who requested additional learning.

Abstract citation ID: igaf122.055

**MAKE IT SMART: ELICITING AND ALIGNING GOALS IN HOME-BASED PRIMARY CARE**

Pietra Bruni[1], Tara McBride Afonso[1], Brian Green[1], Todd Paul[2], Cynthia Knight[2], Cherrylyn Alejo[1], Laura Moore[1], and Michelle Mlinac[3], *1. VA Boston Health Care System, Boston, Massachusetts, United States, 2. Jamaica Plain VA Medical Center, Jamaica Plain, Massachusetts, United States, 3. VA Boston Healthcare System, Boston, Massachusetts, United States*

Home-Based Primary Care teams aim to deliver healthcare services to homebound older adults with complex medical and psychosocial issues. Both patients and providers can become overwhelmed with attempting to manage multiple issues simultaneously. Therefore, identifying outcomes that are meaningful to patients can be facilitated through identification of personalized goals. Utilizing a problem-solving approach was identified as a high competency of the interdisciplinary team. However, problem-solving can contribute to maintenance of the status quo in patient-provider interactions and to loss of enthusiasm for goal setting. As part of VA's Whole Health model, a multi-stepped quality improvement project was implemented within a home-based primary care team. First, the team implemented team goals for each patient and then once this had become routine process, they elicited patient's own SMART goals. Achievement of these goals was tracked during care planning meetings, and friendly competition between teamlets to set and meet these goals helped to drive enthusiasm and buy-in. Measurement of meeting both team and patient SMART goals was tracked every 90-days across two years, resulting in the team's ability to meet their own goals rising from 73% to 88% respectively. Obtaining patient SMART goals was built on training and coaching in motivational interviewing skills, and led to 75% of these patient goals to be met over time. Improvements in SMART goals were identified, with qualitative feedback from the team indicating an appreciation for the visibility of progress, modeling by other team members, and feeling a better connection in clinical work.

### SESSION 1055 (SYMPOSIUM)

Abstract citation ID: igaf122.056

**MENTAL HEALTH IN HISPANIC/LATINO OLDER ADULTS WITH MULTIPLE CHRONIC CONDITIONS: EVIDENCE AND RECOMMENDATIONS**

Chair: Irina Mindlis
Co-Chair: Atami De Main
Discussant: Daniel Jimenez

Among older adults with multiple chronic conditions (MCC), poor mental health increases the risk for morbidity and mortality. Hispanic/Latino older adults are more likely to sustain early onset of MCC and age in poverty, with poorer access to health care, and greater functional limitations. However, most research on mental health related to MCC has focused primarily on non-Hispanic/Latino populations. This symposium aims to highlight findings on the mental health of Hispanic/Latino older adults with MCC through a range of approaches, including in-depth interviews, community-based approaches, and national and cross-national studies. Camacho et al. address the assessment of loneliness and wellbeing narratives among Hispanic/Latino older adults with MCC and chronic pain, highlighting the importance of culturally-informed assessments. De Main et al. discuss barriers and facilitators to recruitment of this population for mental health research, in partnership with community-based organizations. Malatyali et al. present findings from the Health and Retirement Survey (HRS) on the disproportionate burden of poor psychological outcomes in Hispanic/Latino older adults with MCC. Finally, Bishop examines the comparability of depressive symptom indicators across the HRS and the Mexican Health and Aging Study, informing cross-national examinations of depressive symptoms and comorbidities among Mexican-origin older adults. Overall, these papers will provide insights and recommendations into issues of assessment, engagement, and best practices in the evaluation of mental health in Hispanic/Latino older adults with MCC. The findings will be discussed with attention to cultural and contextual factors, and highlight implications for future interventions to improve mental health outcomes in this community.

Abstract citation ID: igaf122.057

**STRATEGIES, FACILITATORS & BARRIERS IN RECRUITING HISPANIC/LATINO OLDER ADULTS IN NYC COMMUNITY ORGANIZATIONS**

Atami De Main[1], Daphne Buitron[1], and Irina Mindlis[2], *1. Weill Cornell Medicine, New York, New York, United States, 2. Rutgers University, New York, New York, United States*

The recruitment of Hispanic/Latino older adults living with multiple chronic conditions (MCC) for mental health research poses unique ethical and practical challenges that require careful consideration. This presentation aims to outline the strategies, facilitators, and barriers encountered while recruiting Hispanic/Latino older adults with MCC in senior centers across New York City for a mental health research initiative. Despite the increasing importance of involving this demographic in research, effective outreach and engagement remain challenging. We draw from our experience on a mixed-methods study designed to assess the underlying mechanisms influencing the relationship between MCC and mental health, while incorporating the perspectives of Hispanic/Latino older adults with MCC, to lay the groundwork for a community-based intervention to improve mental health in this population. Through our partnerships and collaborations with community-based organizations (CBOs), we were able to establish a shared understanding of our research goals and recruitment strategies. Key strategies to maximize participants' engagement included using culturally relevant materials, via presentations, providing bilingual resources through flyers, and obtaining referrals from CBOs. Collaboration with site directors, staff engagement, as well as offering follow-up opportunities, significantly improved participation among Hispanic/Latino older adults in our study. However, we also identified several barriers, including site logistics, the method of survey delivery, and varying levels of survey literacy. These findings highlight the necessity for tailored recruitment approaches that address the unique needs and preferences of Hispanic/Latino older adults. Overall, this research contributes to the development of best practices for engaging underrepresented populations with MCC in mental health research.

Abstract citation ID: igaf122.058

### COMPARING DEPRESSIVE SYMPTOMS IN THE HEALTH AND RETIREMENT STUDY AND MEXICAN HEALTH AND AGING STUDY

Nicholas Bishop, *University of Arizona, Tucson, Arizona, United States*

Cross-national examination of depressive symptoms and comorbid conditions can help identify mechanisms that can be leveraged to reduce the burden of depression among Mexican-origin older adults, but the comparability of depressive symptom indicators in the Health and Retirement Study (HRS) and Mexican Health and Aging Study (MHAS) is currently untested. I used the 2018 HRS (n = 1,267) and MHAS (n = 10,883), 7 CESD-based depressive symptom indicators that were asked in both studies (felt depressed, alone, sad, happy, everything an effort, restless sleep, enjoy life), and self-reported somatic chronic conditions (hypertension, diabetes, cancer, stroke, arthritis) to assess measurement invariance across depressive symptom indicators, then examined how chronic conditions were associated with depressive symptom factor scores. The largest difference in standardized factor loadings existed for enjoy life ($\Delta = 0.17$) and felt happy ($\Delta = 0.13$), but all loadings were sufficient to assume configural invariance. Approximate fit indices (e.g., comparative fit index [CFI] and McDonald's non-centrality fit index [MFI]) were used to assess measurement invariance. Across assessments of weak, strong, and strict factorial invariance, changes in CFI and MFI were negligible (max CFI $\Delta = 0.001$, max MFI $\Delta = 0.004$), suggesting strict invariance could be assumed. When predicting depressive symptom factor scores in multiple-group models adjusted for age, sex, education, and number of doctor visits in past year, arthritis demonstrated the strongest association with depressive symptoms, followed by heart attack, hypertension, and diabetes. These associations did not significantly differ across studies. These preliminary results suggest select depressive symptom indicators in the HRS and MHAS can be used to make cross-national comparisons.

Abstract citation ID: igaf122.059

### PSYCHOLOGICAL OUTCOMES OF MULTIMORBIDITY: ANXIETY, STRESS AND DEPRESSION IN HISPANIC/LATINO OLDER ADULTS

Ayse Malatyali, Tom Cidav, Dahee Kim, Janet Lopez, Veronica Decker, and Ladda Thiamwong, *University of Central Florida, Orlando, Florida, United States*

Psychological conditions are significant health concerns among older adults with multimorbidity, particularly in the Hispanic/Latino population, where the prevalence of chronic conditions such as diabetes, hypertension, and obesity are disproportionately high. However, limited evidence exists on how multimorbidity and ethnicity influence different psychological outcomes. This study examines the associations of multimorbidity and ethnicity with anxiety, perceived stress, and depressive symptoms. Using logistic regression and generalized linear models, we analyzed cross-sectional data from 2,730 participants (age ≥ 65) in the Health and Retirement Study (2020). Multimorbidity was assessed based on the presence of up to seven chronic conditions. Psychological outcomes were measured using the 5-Item Beck Anxiety Inventory, Perceived Stress Scale, and the Center for Epidemiological Studies Depression Scale. The mean age was 75.24 years. Women had significantly higher rates of anxiety, stress, and depressive symptoms than men. Hispanic/Latino older adults had 98% higher odds of elevated anxiety and 0.18-point higher depression scores than their peers, while their perceived stress scores were not significantly different. Higher multimorbidity levels were strongly associated with the psychological burden, as individuals with three or more chronic conditions had 2.72 times higher odds of anxiety, 0.79-point higher perceived stress, and 0.62-point higher depressive symptoms compared to those without chronic conditions. Functional difficulty and lower income quartiles (Q1 and Q2) were also significant predictors of poorer psychological health. These findings highlight the disproportionate burden of psychological conditions among Hispanic/Latino older adults with multimorbidity, emphasizing the need for targeted interventions to improve psychosocial well-being in aging populations.

Abstract citation ID: igaf122.060

### CRITICAL ISSUES IN THE ASSESSMENT OF LONELINESS IN OLDER LATINO ADULTS IN THE DOLORES AND SOLEDAD QUALITATIVE STUDY

David Camacho[1], Kelly Pacheco[2], María Aranda[3], M. Carrington Reid[4], and Elaine Wethington[5], *1. University of Illinois Chicago, Chicago, Illinois, United States, 2. New York University, New York, New York, United States, 3. University of Southern California, Los Angeles, California, United States, 4. Weill Cornell Medicine, New York, New York, United States, 5. Cornell University, Ithaca, New York, United States*

Despite the large number of US older Latinos, there is a dearth of literature that examines loneliness in this population. We examine loneliness assessment issues from the Dolores and Soledad Study that included loneliness and/or chronic pain illness narrative interviews with 35 Spanish-speaking Latinos (60 years+) with multiple chronic conditions. Loneliness assessment included the question "Do you feel lonely?" and the 3-item UCLA Loneliness scale. Interviewers were bilingual (English/Spanish) gerontological social workers with training in culturally sensitive clinical research, and elicitation of "sensitive data." Participants were predominantly Puerto Rican or immigrated from Dominican Republic, Ecuador, and Mexico. Most participants had lived in the US (New York or Los Angeles) for 30+ years, and overwhelmingly reported clinically significant depressive symptoms or chronic pain (86%). We identified multiple assessment challenges including participants': 1) hesitation about discussing emotional well-being; 2) limited prior discussion of loneliness with family, friends or professionals (e.g., to prevent their emotional distress); 3) linguistic and conceptual difficulties in differentiating between loneliness, social isolation, and solitude that do not align with westernized perspectives (e.g., "soledad" may indicate loneliness and/or solitude and influenced by familism and immigrant experiences); 4) ambivalence about feelings of loneliness (e.g., felt they should not feel alone); 5) non-linear descriptions that included varying dimensions (e.g., emotional /existential, chronicity, causes); and 6) loneliness narratives that were linked to traumatic life events (e.g., physical/sexual violence). Findings support a need for culturally-informed loneliness assessments and narrative methods. These approaches may enhance trust and facilitate recognition and elicitation of loneliness narratives.

### SESSION 1060 (PAPER)

### WORKFORCE WELL-BEING AND EQUITY IN LONG-TERM CARE: ADDRESSING BURNOUT, BIAS, AND INNOVATIVE CARE MODELS

Abstract citation ID: igaf122.061

### BURNOUT AMONG NURSING HOME CARE WORKERS: THE INFLUENCE OF WORK-RELATED AND PERSONAL FACTORS

Lisa Geyskens[1], Nasrin Khan[1], Anja Declercq[1], Koen Milisen[1], and Mieke Deschodt[2], *1. KU Leuven, Leuven, Vlaams-Brabant, Belgium, 2. University of Leuven, KU Leuven, Leuven, Vlaams-Brabant, Belgium*

Nursing homes face significant challenges due to increasingly complex care needs, and difficulties in recruiting and retaining care workers. High job demands and stressful work conditions contribute to burnout, exacerbating workforce shortages and potentially impacting care quality. This study describes the prevalence of burnout and its association with organizational, work environment, demographic, and intrapersonal factors among nursing home care workers. A secondary analysis was conducted using cross-sectional survey data from the Flanders Nursing Home (FLANH) project, including 1,521 care workers from 25 Flemish nursing homes. Participants included care assistants (43.7%), registered nurses (20.5%), support staff (15.4%), allied health professionals (14.8%), and team leaders (5.7%). Burnout was assessed using the short Burnout Assessment Tool (BAT-12), and associations were analyzed using a multivariable linear mixed model. Results showed that 19.0% of care workers were at risk of burnout. Higher staffing levels ($\beta$= -0.017, $p=0.0015$), perceived staffing adequacy ($\beta$= -0.040, $p=0.0125$), role clarity ($\beta$= -0.069, $p=0.0017$), skill use ($\beta$= -0.109, $p<0.0001$), and safety climate ($\beta$= -0.052, $p=0.0364$) were significantly associated with lower burnout. Conversely, greater emotional burden at work ($\beta=0.085$, $p<0.0001$) and work-life interference ($\beta=0.266$, $p<0.0001$) were related to higher burnout. Older age, and higher self-efficacy, resilience, and optimism, emerged as significant protective factors. This study confirms the importance of adequate staffing and a supportive work environment in mitigating burnout among nursing home care workers. Targeted strategies to improve the work environment and strengthen intrapersonal resources are essential for promoting staff well-being and retention.

Abstract citation ID: igaf122.062

### COLLABORATION AND AGE-FRIENDLY INTEGRATION – THE DEVELOPMENT OF THE FLOURISHCARE CENTERS OF EXCELLENCE

Barbara Gordon, Anna Faul, Pamela Yankeelov, and Sam Cotton, *University of Louisville, Louisville, Kentucky, United States*

As part of our Geriatric Workforce Enhancement Program, Kentucky developed statewide FlourishCare Centers of Excellence (FC-CEs), bringing together experts from universities, healthcare providers, community organizations, and local governments to improve how care is provided to older adults. The goal is to make sure healthcare professionals have the specialized knowledge needed to support aging adults in all areas of care. FlourishCare Centers of Excellence works closely with community partners to ensure older adults can easily access personalized services that help them stay independent, maintain their dignity, and improve their overall well-being. By connecting different systems—such as hospitals, nursing homes, senior care programs, and government services—we created a smooth and coordinated approach to healthcare. FC-CEs ensures that older adults, including those with dementia, receive continuous and well-organized age-friendly care and support, whether they are at home, in a hospital, or in a care facility. These centers are led by Area Agencies on Aging (AAAs), which serve as regional coordinators to ensure seamless collaboration between healthcare and community systems. A dedicated steering committee guides the initiative's strategic direction. This session will explore the successes of the FC-CE initiative, including the integration of age-friendly principles in primary care and the advancement of workforce development programs. Attendees will learn about effective partnership models, strategies for replication in other regions, and solutions to challenges faced during implementation. FC-CEs are setting a new standard for age- and dementia-friendly care, ensuring that older adults receive high-quality, coordinated, and compassionate services across all care settings.

Abstract citation ID: igaf122.063

### MONTESSORI IMPACTS ON LONG-TERM CARE STAFF: RESULTS OF A STEPPED-WEDGE CLUSTER RANDOMIZED TRIAL

Michelle Hilgeman[1], Amber Collins[1], Whitney Gay[1], Teddy Bishop[1], Kimberly Curyto[2], A. Lynn Snow[3], Whitney Mills[4], and Richard Kennedy[5], *1. Tuscaloosa VA Medical Center, Tuscaloosa, Alabama, United States, 2. VA Western New York Health Care System, Batavia, New York, United States, 3. The University of Alabama, Tuscaloosa, Alabama, United States, 4. Brown University, Providence, Rhode Island, United States, 5. The University of Alabama at Birmingham, Birmingham, Alabama, United States*

Person-centered care practices and staff outcomes have been associated in cross-sectional studies in long-term care settings. However, few clinical trials have examined changes in staff outcomes following introduction of a person-centered intervention delivered to residents. This study is among the first to examine the impact of Montessori approaches on staff outcomes for an intervention focused on meaningful engagement, strengths identification, empowerment, and dignity support of residents. We conducted a hybrid type 3 effectiveness-implementation study in 8 US Veterans Health Administration Community Living Centers (CLCs, VA nursing homes) using a stepped wedge cluster randomized trial design. Staff completed online surveys of perceptions of implementation success and effectiveness during pre-intervention or control time points (i.e., baseline and 1-month before baseline, $N=229$) and post-intervention (i.e., 3-months and 6-months after training, $N=218$). Changes were modeled over time using generalized estimating equations. Implementation results indicated significant changes in perceptions about whether Montessori would become/was a normal part of routine care within their CLC ($p < .001$). Staff-reported effectiveness outcomes reflected post-intervention changes of lower staff burnout scores ($p = .028$) and higher person-centered care scores ($p = .014$) on a social connectedness and individualized care and services scale. Collection of survey data from staff about their experiences expands understanding of both implementation and effectiveness outcomes in clinical trials of non-pharmacological interventions. Approaches like Montessori may have the potential to improve both person-centered care practices for residents and outcomes for the long-term care staff workforce.

Abstract citation ID: igaf122.064

### WHO USES PAID HELP? RACIAL/ETHNIC DIFFERENCES IN CAREGIVING NETWORKS

Wen-Hua Lai, Wenjing Li, Natasha Nemmers, and Amanda Leggett, *Wayne State University, Detroit, Michigan, United States*

Racial/ethnic disparities in residential care arrangements are well-documented, with Black and Hispanic older adults relying more on unpaid family caregivers and less on long-term care than Whites. However, less is known about racial/ethnic differences in using paid helpers alongside unpaid family caregivers among community-dwelling older adults. Using longitudinal data from the National Health and Aging Trends Study (2011-2019; N = 6,876), we applied survey-weighted mixed-effects logistic regression models to examine racial/ethnic differences in whether paid helpers were utilized. We assessed caregiving network size, care needs, and dementia status as covariates, adjusting for sociodemographic characteristics and chronic health conditions. Hispanic older adults had the highest prevalence of paid helpers (13.8%), slightly above Blacks and Whites (both 11%). Individuals with dementia had increased odds of paid helper use, with Blacks (possible dementia) and Hispanics (probable dementia) more likely than Whites to use paid helpers. Larger caregiving networks generally increased paid helper use, but Blacks and Hispanics with larger networks had 20-24% lower odds than Whites. Specific care needs (self-care, mobility, medication management) increased odds of paid helper use, with Blacks (self-care/mobility needs) and Hispanics (medication, self-care, mobility needs) having higher odds than Whites. Overall, Hispanics and Blacks were more likely to utilize paid help when caregiving networks were smaller or care needs greater, but reliance on paid help decreased as networks expanded. This suggests that family or friend caregivers may compensate for paid support, reflecting cultural preferences for informal care. Findings can inform targeted interventions and policies promoting equitable and culturally sensitive care coordination.

### SESSION 1065 (SYMPOSIUM)

Abstract citation ID: igaf122.065

### PATHWAYS TO LONGEVITY

Chair: Peter Martin
Discussant: Bradley Willcox

Although life expectancy has dramatically increased over the last century, reaching the age of 100 years is still somewhat rare. Past investigations have investigated correlates of longevity, but less research has addressed different pathways to longevity. The purpose of this symposium is to highlight components distinguishing different survivorship groups. A second purpose is to assess whether longevity groups show different levels of functioning and mortality risk. All four presentations conducted latent profile analyses to determine the most optimal number of longevity groups and included additional analyses to predict functioning and mortality risks among the groups. The first presentation included longevity components based on the Georgia Centenarian Study model (e.g., family longevity, environmental support, and individual characteristics) that defined latent classes predicting levels of functioning. The second presentation used a similar set of longevity predictors in the Iowa Centenarian Study as the basis for exploring within-domain and between-domain longevity groups. The Kuakini Hawaii Centenarian Study presentation addresses the relevance of two genetic factors (i.e., APOE and FOXO3) in combination with environmental factors to distinguish between longevity groups. Finally, findings from the Health and Retirement Study display distinct profiles of longevity among adults 80 years and older based on family longevity, physical health, personality, and life satisfaction. The presentations also address profile group differences in functioning and mortality risk. The four presentations provide novel information about different profile groups of long-lived individuals, underlining policy and practical implications for these exceptional survivorship groups as they show different levels of functioning.

Abstract citation ID: igaf122.066

### HOW EARLY LIFE INFLUENCES SHAPE PATHWAYS TO LONGEVITY: INSIGHTS FROM THE GEORGIA CENTENARIAN STUDY

Grace da Rosa[1], Amin Hashemzehi[2], Peter Martin[2], and Leonard Poon[3], *1. Georgia State University, Atlanta, Georgia, United States, 2. Iowa State University, Ames, Iowa, United States, 3. University of Georgia, Athens, Georgia, United States*

Understanding early life influences is crucial for uncovering pathways to health and longevity, especially in those who live to 100 and beyond. This research examines early life conditions of centenarians and their potential influence on the pathways to longevity, focusing on 239 participants aged 98 to 109 years from the Georgia Centenarian Study. A latent profile analysis (LPA) identified two distinct groups. Group 1 (n = 90, 38.58%, Early Disadvantaged Centenarians) is characterized by lower parental and centenarian education, parents with shorter life span, poorer health during childhood, and lower financial well-being in childhood. In contrast, Group 2 (n = 142, 61.42%, Early Resourceful Centenarians) had parents with higher education levels and greater longevity, participants' higher education, and greater financial well-being during childhood compared to Group 1. T-tests and Chi-Square results confirmed significant differences with more favorable education of parents and of participants in Group 2. This group also reported growing up in a financially favorably household and their fathers and mothers lived significantly longer than their counterparts in Group 1. Group 1 had a larger percentage of African American participants and participants had significantly more children when compared to Group 2. ADL functioning also differed significantly, with Group 1 exhibiting lower ADL scores. These findings highlight the importance of early-life factors in shaping pathways to longevity and quality of life in centenarians.

Abstract citation ID: igaf122.067

### EXAMINING PREDICTORS OF LONGEVITY AMONG IOWA CENTENARIANS: A LATENT PROFILE ANALYSIS

Elnaz Abaei, *Iowa State University, Ames, Iowa, United States*

Longevity is influenced by a complex interplay of personal and social factors, yet few studies have examined the interrelationship of these factors. Using data from the Iowa Centenarian

Study, we computed latent profile analysis (LPA), including 138 centenarians to identify distinct profiles based on four domains: 1. relationship variables: marital status, marriage length, marriage influence, and relationships with parents; 2. educational experiences: years of education and learning a foreign language; 3. social support: guidance, reliable alliance, reassurance of worth, social integration, attachment, and the opportunity to provide nurturance, and 4. NEO personality traits: neuroticism, extraversion, openness to experience, agreeableness, and conscientiousness. Analyses were conducted in two steps: first, within-domain solutions were identified, followed by a between-domain solution. Preliminary analysis identified marriage length, education, neuroticism, and attachment as key distinguishing factors. These variables were then used in a between-domain LPA, resulting in a two-class solution. Class 1 (Low Education, 81.2%) is characterized by moderate marriage length, lower education, higher neuroticism, and insecure attachment. Class 2 (Long-Term Bonds, 18.8%) is characterized by long-term marriages, higher education, lower neuroticism, and secure attachment. The mean group differences revealed significant group differences for marriage length, $F(1,93)=7.234$, $p=.008$, education, $F(1,130)=39.507$, $p<.001$, and attachment, $F(1,110)=106.239$, $p<.001$, while no significant effect was found for neuroticism, $F(1,105)=2.162$, $p=.144$. Applying LPA in this study offers a novel approach to identifying heterogeneous centenarians and insights into the diverse pathways to longevity. Practical implications include targeted interventions focused on relationship stability, education, and emotional well-being to promote longevity.

Abstract citation ID: igaf122.068

### IDENTIFICATION OF NOVEL CENTENARIAN PHENOTYPES FROM THE KUAKINI HONOLULU ASIA AGING STUDY: A LATENT CLASS ANALYSIS

Yeonjung (Jane) Lee[1], Gina Lee[2], Peter Martin[3], Kamal Masaki[4], and Bradley Willcox[5], *1. University of Hawai'i at Mānoa, Honolulu, Hawaii, United States, 2. University of Wisconsin–Madison, Madison, Wisconsin, United States, 3. Iowa State University, Ames, Iowa, United States, 4. Kuakini Medical Center, Honolulu, Hawaii, United States, 5. John A. Burns School of Medicine, Honolulu, Hawaii, United States*

As society ages, understanding the determinants of healthy aging and longevity has become a research priority. Most studies of longevity lack prospectively collected data. Therefore, we utilized the Kuakini Honolulu Asia Aging Study (HAAS), a decades-long, prospective epidemiologic study of aging. Based on known predictors of longevity, including education, parents' history of Alzheimer's disease, stress management, social support, family longevity, family networks, FOXO3 and APOE genotypes. We performed a latent class analysis in Mplus and identified three novel, distinct centenarian phenotypes (subgroups). One, "socially buffered" survivors (16.20% of the sample) had a relatively higher level of education and social support, despite the lowest mother's age at death. Two, the "longevity guardians" (5.63% of the sample) had the greatest number of children, the highest proportion of healthy longevity FOXO3 gene, and the highest levels of stress tolerance but the lowest levels of education. The "academic" longevity group (78.17% of the sample) had the highest mother's age at death and highest levels of education, despite the lowest number of children. In terms of functional ability, the socially buffered survivors had the highest ADL impairment level, whereas the longevity guardians had the lowest impairment level. Our study advances the understanding of different pathways to longevity, based on family longevity, social support and network, stress management, educational attainment, and genetic factors among Japanese-American male centenarians. The findings provide implications for continued investigation on lifestyle and genetic factors promoting healthy aging and offer insights into the evidence on the strengths and resilience of long-lived individuals.

Abstract citation ID: igaf122.069

### EXPLORING HETEROGENEITY OF LONGEVITY: INSIGHTS FROM THE HEALTH RETIREMENT STUDY

Yeon Ji Ryou, and Peter Martin, *Iowa State University, Ames, Iowa, United States*

Understanding the heterogeneity in longevity among oldest-old adults is crucial for identifying distinct pathways to extended life, as aging experiences vary by individuals. This study aimed to identify unique longevity profiles among adults 80 years and older using the 2020 RAND Health and Retirement Study (HRS) data. A complete sample of 5,436 participants was selected ($M = 86.09$). Latent profile analysis (LPA) was conducted using indicators of respondents' and parental longevity, physical health (chronic conditions, activities of daily living limitations), cognitive function, personality traits (neuroticism and conscientiousness), and psychological well-being (life satisfaction). Four distinct longevity profiles were identified: (1) Burdened Survivors ($n = 483$; 8.89%), experiencing chronic diseases, ADL limitations, low conscientiousness, relatively high neuroticism, and lower life satisfaction but with more prolonged survival; (2) Goldilocks survivors ($n = 1,143$; 21.03%), with moderate longevity, average well-being, and neither the best nor worst outcomes; (3) Thriving Short-Lived Survivors ($n = 3,276$; 60.27%), enjoying excellent overall well-being but with the shortest lifespan; and (4) Longevity at a Cost ($n = 534$; 9.82%), living the longest but enduring significant physical and psychological challenges. The Cox proportional hazard model subsequently assessed mortality risk across profiles. There are significant differences in mortality risk across profiles, with Thriving Short-lived Survivors exhibiting the highest mortality risk, showing a 123.4% increased risk of death compared to the reference group (Longevity at a Cost). These findings highlight the importance of understanding varied longevity trajectories to develop tailored and nuanced interventions that address physical and psychological challenges while fostering resilience and enhancing the quality of life among the oldest-old population.

### SESSION 1070 (SYMPOSIUM)

Abstract citation ID: igaf122.070

### CHRONIC PAIN IN OLDER ADULTS: PREVALENCE, RISK FACTORS, AND CONSEQUENCES

Chair: Gillian Fennell

Over 100 million Americans experience chronic pain, and older adults are disproportionately affected. This symposium presents four studies using large population-based samples of older adults from three different countries to examine the prevalence, risk factors, consequences, and management of chronic pain. Using data from two generations of Framingham Heart Study participants, Felson identified a secular increase in widespread pain prevalence measured when both cohorts were in their 70s. This rising prevalence provides strong context for Limani's examination of chronic pain's effect on successful aging in the Canadian Longitudinal Study on Aging. Limani found that chronic pain significantly lowers respondents' perceptions of their physical, psychological, and social wellbeing, with lower income and higher pain severity exacerbating these effects. Discerning safe and effective chronic pain management in older adults is a core research priority, particularly around opioid use. Milani investigated the interplay between sex, race/ethnicity, and cognitive impairment on self-reported opioid use from the Health and Retirement Study (HRS). After integrating Medicare Claims data, Milani will additionally explore parallel patterns of opioid prescription. Finally, Huang highlights the enduring health impact of early-life trauma using data from the Vietnam Health and Aging Study: early-life exposure to the Vietnam War increased the likelihood of later-life chronic pain. These studies leverage data from well-powered surveys to document trends in widespread pain prevalence, person-level consequences of chronic pain, disparities in opioid use, and the long-term health consequences of traumatic early-life experiences.

Abstract citation ID: igaf122.071

### GENDER DIFFERENCES IN OPIOID USE AMONG OLDER ADULTS WITH CHRONIC PAIN

Sadaf Milani, Yong Shan, Yong-Fang Kuo, and Mukaila Raji, *University of Texas Medical Branch, Galveston, Texas, United States*

Women and minoritized individuals often have their pain underdiagnosed and undertreated. Cognitive status adds a layer of complexity, as self-report of pain may be affected as individuals decline. Our objective was to assess self-reported prescription opioid use among older adults, while considering race/ethnicity and cognitive status. We used 2018 data from the Health and Retirement Study linked to Medicare Claims. We included participants with chronic pain and at least one year of continuous enrollment (n = 2,990), excluding those with HMO plans. Our outcome was self-reported past three-month opioid use. Our exposure was gender. Effect modifiers included race/ethnicity (non-Hispanic White, non-Hispanic Black, Hispanic) and cognitive status (no dementia, cognitive impairment no dementia, dementia). We used logistic regression to examine the association of gender with opioid use, controlling for demographic and health characteristics. We tested the interaction of gender with race/ethnicity and cognitive status. Opioid use did not differ between women and men (15.4% vs. 13.6%). There was an interaction between gender and cognitive status as well as race/ethnicity. In gender-stratified models, non-Hispanic Black and Hispanic women had lower odds of opioid use compared to non-Hispanic White women. There were no racial/ethnic differences for men. Lastly, women with dementia had lower odds of opioid use, while men with dementia had higher odds of opioid use compared to their counterparts with normal cognition. This work highlights the complexities of opioid use among older adults. The final version will incorporate data from Medicare Part-D to examine the variation of prescriptions for chronic pain treatment.

Abstract citation ID: igaf122.072

### SECULAR CHANGES IN WIDESPREAD PAIN ACROSS TWO GENERATIONS: THE FRAMINGHAM HEART STUDY

David Felson, Juan-Pablo Zertuche, Nene Ukonu, Michael LaValley, Margaret Clancy, and Tuhina Neogi, *Boston University Chobanian & Avedisian School of Medicine, Boston, Massachusetts, United States*

Ten to eleven percent of adults have widespread musculoskeletal pain (WSP) with a higher prevalence in older persons. It is unknown whether WSP prevalence has changed nor whether increased obesity or longevity especially of women could explain this change. We studied these questions in 2 generations of the community-based Framingham Heart Study cohorts. We studied the original cohort of the Heart Study in 1992-1995 and Generation2, the offspring of the original cohort and their spouses in 2019-2021, at a similar age to the original cohort. A minoritized community sample (OMNI cohort) was also assessed in 2019-2021. Participants were asked whether they had joint pain on most days and, those saying yes used a homunculus to identify sites of pain. We then determined whether a participant met the WSP definition: pain on the left and right sides of the body; pain above and below the waist, and spinal or low back pain. We tallied the prevalence of WSP in each cohort, adjusting for confounders using logistic regression to test for cohort differences. The mean age of participants, mostly women, was in the 70s (table 1). Just over 14% of both original and OMNI cohorts reported WSP but 19-20% of Generation2 had WSP. Comparing Generation2 to the Original Cohort, WSP increased in prevalence (adjOR = 1.97 (95% CI: 1.49 - 2.61, p <.001). The prevalence of WSP has risen in older persons, an increase not explained by increasing pain with age, increased longevity of women nor increasing BMI.

Abstract citation ID: igaf122.073

### RISK, RESILIENCE, AND TIMING: THE IMPACT OF WAR ON LATER-LIFE PAIN

Rui Huang[1], Yuhang Li[2], and Hanna Grol-Prokopczyk[1], *1. University at Buffalo, Buffalo, New York, United States, 2. University at Albany, Albany, New York, United States*

Life course theories predict that early-life war experiences will impact later-life health through both risk and resilience processes, which may vary by age cohorts. However, limited research has empirically examined the resilience process following war and the heterogeneous pathways across age cohorts. This study uses data on older adults from the 2018 Vietnam Health and Aging Study (N = 1,826) to explore associations between early-life war experiences and later-life moderate/severe chronic pain, highlighting the role of war-related risk or protective factors (socioeconomic status, health behaviors, social engagement, and health conditions), and testing for heterogeneity of war impacts between child (aged 6-11), adolescent (aged 12-17), and young adult cohorts (aged 18-23) during the wartime (1965). Regression models reveal that Vietnam War exposures dramatically increase the risk of later-life pain, and that war generates both risk factors (e.g., lower educational attainment, higher smoking risk, greater physical disease count, larger distress, and PTSD score) and protective factors (e.g., greater social engagement). However, KHB mediation analyses show that the impact of war on pain is primarily driven by increases in physical and mental health problems. Children were more vulnerable to the effects of wartime violence on later-life pain than older age groups. As for risk/protective factors generated by war, the child cohort tends to exhibit poorer coping under the effect of war (as indicated by a higher risk of smoking), whereas this group appears to have a better outcome in terms of a greater increase in occupational prestige and less deterioration in physical health.

Abstract citation ID: igaf122.074

**PAIN AND SUCCESSFUL AGING AMONG CANADIAN ADULTS**

Merita Limani, and Anna Zajacova, *Western University, London, Ontario, Canada*

Chronic pain, highly prevalent among older adults, is associated with worse health. However, little is known about its impact on successful aging (SA). SA is a multidimensional concept encompassing not only physical and psychological health, but also social connectedness and functional independence in later life. This study examines the impact of chronic pain on SA over time and identifies demographic and socioeconomic factors that may buffer or exacerbate their association. Data are from the Canadian Longitudinal Study on Aging (CLSA), a 3-wave panel study including 30,097 adults aged 45 and older. The primary outcome is SA, which we constructed from 6 domains with indicators collected identically at all waves. We estimate mixed-effects models of SA as a function of pain severity (no pain, mild/moderate pain, severe pain). Models control for age, gender, marital status, race/ethnicity, immigrant status, education, and income, and include appropriate interactions to test buffering. Initial results indicate that chronic pain is associated with lower SA scores, with more severe pain linked to lower baseline scores and greater declines over time. Among covariates, income is particularly salient as a moderator of the association. These findings highlight that chronic pain undermines multiple dimensions of wellbeing essential to successful aging, extending beyond physical and mental health. The significant role of income highlights socioeconomic disparities in pain experiences, suggesting that addressing these inequities could enhance successful aging trajectories.

### SESSION 1075 (SYMPOSIUM)

Abstract citation ID: igaf122.075

**SENSORY LOSS AND MOBILITY IN AGING: INTERDISCIPLINARY PERSPECTIVES ON CHALLENGES AND INTERVENTIONS**

Chair: Shu Xu
Co-Chair: Joshua Ehrlich
Discussant: Jennifer Deal

This session will discuss sensory loss and mobility in later life from gerontological, public health, and intervention perspectives. Sensory loss is highly prevalent among older adults, with over half experiencing hearing loss and more than a quarter living with visual impairment in the U.S. As the population ages, the number of older adults with sensory loss are expected to increase accordingly. Sensory health is essential for maintaining mobility, as sensory loss is linked to poor postural balance, fall-related issues, walking difficulties, reduced participation in daily activities. However, the association between sensory loss and various forms of mobility remains underexplored. Environmental factors and the potential mechanisms underlying these associations are under-investigated. This session will investigate associations between objectively measured hearing, vision, dual sensory loss and outdoor mobility limitations, along with environmental factors, such as public transit, that may help explain these associations. We will examine the longitudinal association between different types of visual function and physical performance using a nationally representative sample of U.S. older adults. We will also describe characteristics of communities with high rates of hearing difficulties and falls using community-level data. Whether treatment of sensory loss is an effective intervention for improving mobility is unknown. This session will conclude by presenting results of a randomized controlled trial investigating the effects of hearing care on daily functioning in older adults with hearing loss. Bringing together researchers from multiple disciplines, this symposium will advance understanding of sensory loss and mobility in aging, with implications for research, policy, and practice.

Abstract citation ID: igaf122.076

**LONGITUDINAL ASSOCIATION BETWEEN VISUAL FUNCTION AND PHYSICAL PERFORMANCE AMONG OLDER ADULTS: FINDINGS FROM NHATS**

Shu Xu[1], Joshua Ehrlich[1], and Mengyao Hu[2], *1. University of Michigan, Ann Arbor, Michigan, United States, 2. The University of Texas Health Science Center, Houston, Texas, United States*

Older adults with a visual impairment (VI) are more likely to experience mobility limitations; however, few studies have examined how changes in specific visual functions influence mobility trajectories. This study investigates the longitudinal association between visual function and physical performance among U.S. older adults. We analyzed three-year longitudinal data (2021-2023) from Medicare beneficiaries ≥71 years in the 2021-2023 National Health and Aging Trends Study. Visual function was assessed based on presenting binocular distance visual acuity [DVA] and contrast sensitivity [CS]. Any VI was defined as either distance VI [>0.30 logMAR] or CS impairment [<1.55 logCS]. Physical performance was measured using Short Physical Performance Battery (SPPB; range: 0–12), with higher scores indicating better physical performance. Growth curve modeling was used to analyze longitudinal associations. Among 2,707 participants, 31.3% had any VI. Older adults with any VI had lower SPPB scores than those without any VI (mean: 6.01 vs. 7.70). Growth curve models showed significant between-person effects for both DVA ($B = -3.24$, $p < .001$) and CS ($B = -2.99$, $p < .001$), as well as significant within-person effects for DVA ($B = -0.65$, $p < .01$) and CS ($B = -0.51$, $p < .01$). In conclusion, older US adults with worse overall visual function have lower physical performance, and declines in visual function over time are associated with faster declines in physical performance. Findings highlight the critical role of vision health in maintaining mobility in later life.

Abstract citation ID: igaf122.077

**EFFECTS OF HEARING INTERVENTION ON MOBILITY DISABILITY OVER 3 YEARS: FINDINGS FROM THE ACHIEVE STUDY**

Pablo Martinez-Amezcua[1], Emmanuel Garcia-Morales[2], Sarah Bessen[3], Alison Huang[4], Jennifer Deal[1], Nicholas Reed[5], Jennifer Schrack[1], and Frank Lin[1], *1. Johns Hopkins Bloomberg School of Public Health, Baltimore, Maryland, United States, 2. New York University, New York, New York, United States,*

*3. Johns Hopkins School of Medicine, Baltimore, Maryland, United States, 4. Johns Hopkins Bloomberg School of Public Health Department of Epidemiology, Baltimore, Maryland, United States, 5. New York University Grossman School of Medicine, New York, New York, United States*

Hearing loss is associated with increased functional limitations and difficulties in daily activities. However, whether treating hearing loss slows down the decline in daily life functioning remains unknown. Using data from the Aging Cognitive Health Evaluation in Elders (ACHIEVE) study, a 1:1 randomized control trial of a hearing care intervention (including the provision of hearing aids) compared to a healthy education control group, we investigated the effect hearing care vs. control on daily life functioning, assessed by three domains (functional limitations [e.g., walking ¼ mile, crouching], activities of daily living [ADLS; e.g., getting in and out of bed, dressing], and instrumental ADLS [IADLS; e.g., house chores, preparing meals]), with the Physical Ability Questionnaire, among 977 community-dwelling older adults with hearing loss recruited from the Atherosclerosis Risk in Communities Study (ARIC [n = 238]) and de novo from the community (n = 739). Over three years, there was an increase in the proportion of participants reporting functional limitations (from 65.6% to 73.3%), difficulties in ADLS (from 11.8% to 1838%), and IADLS (from 19.3% to 26.7%). The 3-year change in these proportions did not differ between the hearing intervention arm and the health education control participants. Our findings suggest that compared to a health education control, a hearing intervention does slow down the progression of functional limitations or difficulties with daily (instrumental or not) activities. Further research is warranted to explore additional factors influencing daily function in older adults with hearing loss.

Abstract citation ID: igaf122.078

### VISION AND HEARING IMPAIRMENT IN OLDER ADULTS AND OUTDOOR MOBILITY LIMITATIONS: THE ROLE OF PUBLIC TRANSIT

Philippa Clarke, Shu Xu, Nasya Tan, and Joshua Ehrlich, *University of Michigan, Ann Arbor, Michigan, United States*

Sensory loss in older adults poses a risk for mobility limitations. Yet, little research has examined how the accessibility of the surrounding built environment, as measured through access to public transportation, mitigates the risk of mobility limitations for those with vision impairment, hearing impairment, or dual sensory impairment (both vision and hearing impairment). In this paper we examined the frequency of leaving one's home among older adults (age 65+) with objectively measured vision and hearing impairment (and dual impairment) in Round 12 (2022) of the National Health and Aging Trends Study (n = 5185), and examined the modifying role of the density of public transit stops in the local neighborhood. Weighted logistic regression models (adjusted for demographics, living arrangements, and health conditions) found that older adults with hearing impairment had 30% lower odds (OR = 0.70, 95% CI = 0.52-0.93) of leaving their home (every/most days in the past week vs. never/rarely) compared to older adults with no sensory impairment. Those with both vision and hearing impairment had a 53% lower odds of leaving their home (OR = 0.47, 95% CI = 0.32-0.69). However, these associations were attenuated when older adults with hearing impairment or dual sensory impairment lived in a neighborhood with at least one public transit stop. Findings emphasize the importance of considering dual sensory impairment in older adults and the potential role of the surrounding environment for mitigating the risk of mobility restrictions with aging.

Abstract citation ID: igaf122.079

### CONTRAST SENSITIVITY IMPAIRMENT PREDICTS DECLINE IN BALANCE OVER 30 MONTHS IN COGNITIVELY NORMAL OLDER ADULTS

Atalie Thompson, Ashley Wells, Michael Miller, Haiying Chen, Paul Laurienti, and Stephen Kritchevsky, *Wake Forest University School of Medicine, Winston-Salem, North Carolina, United States*

Contrast sensitivity impairment (CSI) increases in prevalence as older adults age, and may impact different aspects of physical performance over time. We conducted a single center prospective cohort study of 192 cognitively unimpaired older adults with good visual acuity and self-reported visual function. Linear mixed models examined the difference in the association of moderate CSI (log CS < 1.55) at baseline with standing balance time(seconds), 4m gait speed(m/s), narrow walking speed(m/s) and chair pace(stands/s) over 30 months. Multivariable models adjusted for the effect of age, race, and sex on the slopes of each type of physical performance. At baseline, the mean participant age was 76.5±4.7 years, with 56.5% (N = 108) female and 9.4% (N = 18) black. CSI predicted a significantly greater decline in balance time (Beta:-4.33, 95% CI (-7.41, -1.24), p = 0.006) relative to those with non-impaired CS (Beta:-1.05, 95% CI(-2.05, -0.04), p = 0.042), with a difference in slopes of –3.28 (95% CI (-6.53, 0.03), p = 0.048). The difference in slopes remained significant with adjustment for age, sex, and race effects (Beta:-3.51, 95% CI(-6.83, -0.20), p = 0.038). Those with CSI also experienced significant declines in 4m gait speed and narrow walking speed (p < 0.05), but the difference in slopes was not significant compared to those without CS impairment (p > 0.05), and there was no significant relationship of CSI with chair pace over time. In cognitively intact older adults with good visual acuity, CSI was associated with a significantly faster decline in balance time over 30 months of follow-up. CS testing may identify older adults at risk for developing postural instability.

Abstract citation ID: igaf122.080

### CHARACTERISTICS OF COMMUNITIES WITH HIGH RATES OF HEARING DIFFICULTIES AND FALLS: FINDINGS FROM THE RI AND MA HADR

Eric Frimpong[1], Taylor Jansen[1], Josephine Boateng[2], Seokmin Kim[3], Yan-Jhu Su[4], and Elizabeth Dugan[1], *1. University of Massachusetts Boston, Boston, Massachusetts, United States, 2. University of Massachusetts Boston, Dorchester, Massachusetts, United States, 3. University of Massachusetts Boston, Quincy, Massachusetts, United States, 4. The Pennsylvania State University, University Park, Massachusetts, United States*

The National Institutes of Health (NIH) reports that one in three older adults live with hearing loss. Not only do hearing difficulties impact one's quality of life, but it also increases the risk of falls among older adults, primarily due to their impact on balance and spatial awareness. Falls are a major public health concern, a leading cause of injury-related deaths among older adults and contribute to loss of independence. This study aims to examine communities with high rates of falls and hearing loss to identify the associated risk factors among older residents. Data was drawn from the 2025 Massachusetts and Rhode Island Healthy Aging Data Reports (HADR), the American Community

Survey (2018-2022), and the Medicare Beneficiary Summary File (2020-2021). Among the total 427 communities, the average rate of 65+ adults who fell in the past year was 28% (range: 18.47-49.18%), while 12.55% (range: 0-42.31%) reported hearing difficulties. Communities with high fall rates (N=85; Mean: 32.43%) and hearing difficulties (Mean: 17.33%) reported higher rates of older adults living alone, experiencing independent living and self-care difficulties, receiving food stamps, living below the poverty line, and being dually eligible for Medicare and Medicaid. The findings further indicate that communities with high rates of falls and hearing loss are home to older residents with self-reported independent living difficulties, lower employment, socioeconomic statuses, and physical activity. These findings are essential to guide policymakers on where to implement evidence-based programming like Matter of Balance and Tai Chi.

### SESSION 1080 (SYMPOSIUM)

Abstract citation ID: igaf122.081

### THE LONG ARC OF SELF-RATED HEALTH RESEARCH

Chair: Ellen Idler
Discussant: Ted Johnson

Research with older populations that asks respondents to assess their health with a single question began with the Duke Longitudinal Studies of Normal Aging in the 1950s. Early patterns that emerged were that physicians tended to rate overall health more negatively than study subjects, and that respondent "optimism" about health increased with age. In the 1980s a series of population-based studies of older respondents found that "poor" SRH was a significant predictor of all-cause mortality, even when physical health and other important covariates were well-characterized. Throughout the 1990s and 2000s literally hundreds of studies of representative, population-based samples in countries around the world have shown nearly universally that SRH is a reliable predictor of mortality, despite many forms of the question and different response categories. Since then, researchers have focused on understanding the precursors of these ratings; finding novel covariates that "explain" the effect and/or narrow it to a "true" self-rating of health; identifying sociodemographic groups for which the effect may be modified; and exploring the phenomenon of health optimism in old age. In this symposium, Quesnel-Vallée will present current research comparing SRH with algorithm-derived diagnoses. Jylhä will report on analyses of qualitative and quantitative data for a Finnish cohort aged 90+. Ferraro will present research on SRH and dual (physical and cognitive) functionality. Idler will present analyses of SRH and mortality where causes of death are disaggregated by treatability and preventability. The discussant will situate this current research in its long history and current translational applications.

Abstract citation ID: igaf122.082

### DOES SELF-RATED HEALTH PREDICT BOTH AVOIDABLE AND UNAVOIDABLE CAUSES OF MORTALITY?

Ellen Idler[1], Emily Dore[2], and Courtney Yarbrough[3], *1. Emory University, Atlanta, Georgia, United States, 2. Harvard T.H. Chan School of Public Health, Boston, Massachusetts, United States, 3. Emory University Rollins School of Public Health, Atlanta, Georgia, United States*

Self-rated health (SRH) has been known as a predictor of all-cause mortality for decades. The mechanism by which poor SRH is so consistently associated with higher risks of mortality, however, is still unclear. One approach taken to better understand this effect has been to disaggregate mortality by cause of death. This has yielded mixed results, given the sacrifice of sample size for single causes of death, but a benefit has been suggestions that respondents with some knowledge of their risks for, or diagnoses of, such single causes, show enhanced effects of SRH. We introduce the approach of disaggregating all-cause mortality into meaningful groups of causes. Originating in health policy studies, the concepts of preventable and treatable causes of mortality have been used to assess the effectiveness of public health and medical care systems, often by comparing countries. Does SRH have differential effects on causes of mortality that are preventable or treatable, compared with unavoidable causes of death? We analyzed data from the Panel Study of Income Dynamics for 1999-2021, with SRH as a predictor of treatable, preventable (by individual behaviors or by public health measures), preventable-plus-treatable, and unavoidable causes of death (OECD). Results from fully-adjusted competing risks models showed that Poor/Fair/Good SRH compared with Very good/Excellent had significantly increased hazards for treatable (HR 1.99, 95% CI 1.20, 3.32), and for preventable-plus-treatable (HR 1.95, 95% CI 1.57, 2.42) causes of mortality, but not for preventable or unavoidable causes. The findings underscore the mechanism of knowledge of one's health.

Abstract citation ID: igaf122.083

### BRIDGING THE GAP: ACCESS TO CARE, DIAGNOSTIC CONCORDANCE, AND SELF-RATED HEALTH IN OLDER ADULTS

Amélie Quesnel-Vallée[1], Divine-Favour Ofili[1], and Isabelle Dufour[2], *1. McGill University, Montreal, Quebec, Canada, 2. Sherbrooke University, Montreal, Quebec, Canada*

Self-rated health (SRH) is a widely utilized indicator of population health, shaped by multiple domains. Prior research has established that functioning, diseases, and pain are the most influential contributors to SRH in Canada, particularly among older adults. However, the role of access to care in shaping the diagnosis domain—and by extension, its influence on SRH—remains underexplored. We use the TorSaDE cohort, a linked dataset combining five cycles of the Canadian Community Health Survey (CCHS 2007-2016) with administrative health data covering the entire population of the province of Québec, Canada, enabling comprehensive analysis of health trajectories and outcomes among community-dwelling older adults. Using these linked data, we categorize participants into three diagnostic groups: (1) self-reported diagnosis only, (2) administrative (algorithmic) diagnosis only, and (3) concordant diagnosis across both sources. We conducted these analyses for both diabetes and major neurocognitive disorders (MNCD), and we examined the differences in self-ratings of health between these groups, and across a range of sociodemographic characteristics. We found that individuals with only algorithmically detected diagnoses reported significantly better self-rated health compared to the other two groups. This pattern was consistent for both diabetes and MNCD. These findings provide strong support for the cognitive model of self-rated health, suggesting that individuals primarily base their health assessments on known, consciously acknowledged diagnoses. Furthermore, this reliance on known information appears to override other "objective" health indicators, such as the intensity of health care utilization (as algorithmic detection inherently requires significant interaction with the health care system).

Abstract citation ID: igaf122.084

### IS SELF-RATED HEALTH DIFFERENT FOR NONAGENARIANS?

Marja Jylha, Katariina Tuominen, and Linda Enroth, *Tampere University, Tampere, Pirkanmaa, Finland*

Self-rated health (SRH) is a well-known predictor of mortality. In younger old people, chronic conditions are a major determinant of SRH. Less is known about SRH among people aged 90 and older, a small but rapidly growing population group with rather short life-expectancy and high multimorbidity. This paper discusses the contents and meanings of ´health´ in self-ratings of nonagenarians, contrasting different types of data from the Vitality 90+ project in Tampere, Finland. The project includes repeated survey studies with all individuals aged 90+ in the area plus qualitative interviews and face-to-face health examinations with subgroups. In the 2023 qualitative life story interviews (N = 54), participants framed diseases as given facts in high age and discussed health in relation to the ability to do things, manage everyday life, experienced symptoms, cognitive capacity and medications. They used comparisons to age peers with poorer condition or already deceased, to construct a relatively positive view of their own health. This is consistent with our earlier analysis in the survey data, where mobility and subjective symptoms were more important direct associates of SRH than diseases. Perhaps surprisingly, SRH showed a robust, graded association with mortality also in nonagenarians. During 4.6 years, mortality rate was 43% for good & fairly good and 89% for fairly poor & poor SRH. Our findings invite discussion about the meaning of ´health´ and about the most relevant experienced hallmarks of physiological vitality in the oldest-old.

Abstract citation ID: igaf122.085

### DOES SELF-RATED HEALTH FORETELL LOSS OF DUAL FUNCTIONALITY?

Kenneth Ferraro[1], and Mallory Bell[2], *1. Purdue University, West Lafayette, Indiana, United States, 2. Purdue, Lafayette, Indiana, United States*

Self-rated health (SRH) has been described as a global measure of health because the respondent is asked to reply based on one's assessment of overall health. Although there is a rich literature examining whether SRH predicts a variety of single measures of health, it is unknown whether it also predicts a global measure of health that is defined by a combination of physical and cognitive functions. Given that most people aspire to retain their physical function and cognitive function, we investigate if SRH is a valid predictor of dual functionality (DF). We use data from the Health and Retirement Study from 2004 to 2020 to prospectively study the relationship between SRH and DF. We used Weibull accelerated failure-time (WAFT) models with age as the time metric to investigate whether SRH is associated with the loss of DF. Among respondents with DF at baseline, each higher level of SRH was significantly related to DF in fully-adjusted models. Using poor SRH as the reference group, even respondents in fair health reported a 10% later loss of DF (time ratio = 1.10, SE = .02, $p < .001$). Compared to poor SRH, respondents who rated their health as good reported a 19% later loss of DF; those who rated their health as very good reported a 28% later loss of DF; and respondents who rated their health as excellent reported an impressive 35% later loss of DF ($p < .001$). We conclude that SRH is a valuable upstream predictor of DF.

### SESSION 1085 (SYMPOSIUM)

Abstract citation ID: igaf122.086

### FROM EVIDENCE TO IMPACT: IMPLEMENTATION PATHWAYS FOR ELDER MISTREATMENT INITIATIVES

Chair: Kristin Lees Haggerty
Discussant: Tony Rosen

This symposium explores implementation science-informed approaches to elder mistreatment (EM) screening and intervention across healthcare contexts. The five presentations highlight complementary strategies with common implementation themes: adaptation of evidence-based tools for specific settings, addressing clinician barriers, and leveraging electronic health records. The first presentation demonstrates how integrating an EM protocol into emergency department electronic records increased screening rates from 2% to 85%, illustrating the power of workflow integration. The DETECT-RPC Project adapts a validated screening tool for home-based primary care, using the Theory of Planned Behavior to address clinician uncertainty about EM recognition and reporting requirements through targeted training interventions. The University of Colorado's VESPA program showcases iterative adaptation of an elder abuse consultation service in response to implementation challenges. Their theory of change—that specialized teams offload complex and morally distressing cognitive work from medical providers—drove adaptive strategies as consult volumes outpaced resources, including developing a stepped care model and prioritizing complex cases. The Montana RISE-APS project represents a novel implementation approach, embedding the RISE intervention within Adult Protective Services (APS) rather than partnering externally. Using the Consolidated Framework for Implementation Research, this adaptation identifies contextual barriers and facilitators to implementation within APS. Finally, the Veterans Health Administration demonstrates system-wide implementation of standardized EM electronic tools, highlighting how researcher-clinician partnerships facilitate national dissemination in complex healthcare environments. Together, these presentations illustrate critical implementation science principles for EM interventions: context-sensitive adaptation, addressing workforce barriers, leveraging existing systems, and creating sustainable models that respond to real-world constraints while maintaining intervention fidelity.

Abstract citation ID: igaf122.087

### THEORY DRIVEN ADAPTATION OF AN ELDER ABUSE CONSULTATION SERVICE

Elizabeth Bloemen[1], Sarah Tietz[2], Josh Bollan[2], Sarah Cox[3], and Daniel Lindberg[2], *1. University of Colorado, Aurora, Colorado, United States, 2. University of Colorado School of Medicine, Aurora, Colorado, United States, 3. University of Colorado Hospital, Aurora, Colorado, United States*

Elder abuse consultation services are a new model of care for victims of elder mistreatment who are seen in the emergency room or hospital setting. Our team, the Vulnerable Elder Services, Protection, and Advocacy Team (VESPA) at the University of Colorado was developed and implemented in 2021. Consisting of specialized social workers and geriatricians, our team provides trauma informed care focused on reducing harm for an entire family system experiencing violence. In the first year, our team saw 42 cases and has grown to over 200 cases a year, having served over 700 victims and families to date. Due to changes in clinical volume; the improved recognition of abuse; and the challenges of the COVID 19 pandemic, our team has undergone multiple adaptations to best serve the victims and providers we care for. Our theory of change is that medical providers play a critical role in the response to elder mistreatment but that they are dis-incentivized from doing so by time constraints and a lack of training. Medical consultation teams offload this work, and the accompanying moral distress, thereby improve the recognition of abuse. As our team grew, and recognition grew, our consult numbers outpaced our funding. We have made several adaptations to our workflow to accommodate this while maintaining our core theory of change. These have included: limiting our response to stranger financial abuse; focusing on inpatient versus emergency department consultations; and developing a stepped model of care.

Abstract citation ID: igaf122.088

### ADAPTING RISE INTO MONTANA ADULT PROTECTIVE SERVICES: AN EMBEDDED MODEL

David Burnes[1], Marian Liu[2], Patricia Kimball[3], Lianna Waller[4], Jody McCampbel[5], Marie-Therese Connolly[6], Geoff Rogers[7], and Stuart Lewis[8], *1. University of Toronto, Toronto, Ontario, Canada, 2. Purdue University, West Lafayette, Indiana, United States, 3. Elder Abuse Institute of Maine, Brunswick, Maine, United States, 4. Adult Protective Services, Missoula, Montana, United States, 5. Adult Protective Services, Billings, Montana, United States, 6. RISE Collaborative, Washington, District of Columbia, United States, 7. Hunter College, New York, New York, United States, 8. Dartmouth Health, Hanover, New Hampshire, United States*

Understanding effective elder abuse and self-neglect (EASN) interventions remains limited. Adult Protective Services (APS) is primarily responsible for investigating EASN reports in the U.S. However, APS generally lacks a dedicated intervention phase to address underlying case needs/risks. RISE is an evidence-based EASN intervention that works in partnership with APS to address this intervention gap. RISE integrates core modalities (motivational interviewing, restorative approaches, teaming, engagement, goal attainment scaling) and operates at Relational, Individual, Social, and Environmental levels to work with older adult victims and alleged harmers and strengthen social supports surrounding them. To date, the RISE-APS model has involved complementary partnerships where RISE operates from external community-based organizations. In partnership with Montana APS, this study sought to understand how to adapt and implement a RISE-APS "embedded" model, where RISE operates from within Montana APS as a specialized unit providing extended services for complex cases. This RISE-APS adaptation/implementation project was guided by the Consolidated Framework for Implementation Research (CFIR), containing factors across five domains (intervention, outer setting, inner setting, individuals, implementation process) representing potential internal/external implementation barriers/facilitators. Informed by CFIR and using a descriptive phenomenological qualitative approach, we conducted focus groups and individual interviews with Montana APS management (n = 4) and social service workers (5) as part of an adaptation needs assessment to identify implementation barriers/facilitators and engaged in a process to determine strategies to address/leverage them. This presentation will present an adaptation process model for the embedded RISE-APS model, identifying implementation determinants (barriers, facilitators), actions (strategies, mechanisms), and outcomes (adoption, fidelity, sustainment).

Abstract citation ID: igaf122.089

### INTEGRATING AN ELDER MISTREATMENT INTERVENTION PROTOCOL INTO THE EMERGENCY DEPARTMENT ELECTRONIC HEALTH RECORD

Jason Burnett[1], Charles Maddow[1], and Michael Cannell[2], *1. UTHealth Houston, Houston, Texas, United States, 2. TCU, Fort Worth, Texas, United States*

Experiencing mistreatment in later life significantly increases the risk of morbidity and mortality. These heightened risks are often preceded by or closely linked to emergency department visits and subsequent hospitalizations, which are more frequent among abused older adults compared to those who have not experienced mistreatment. Despite this, standardized screening for elder mistreatment (EM) remains low across clinical settings, including emergency departments—where trauma and violence-related injuries are routinely assessed and treated. As a result, emergency departments present a crucial opportunity for improving the detection of elder mistreatment and providing potentially life-saving interventions. However, a key barrier to screening in these settings is the absence of integrated EM screening and response protocols within electronic health records, which guide most routine assessments in emergency care. This presentation outlines the process of implementing an evidence-informed EM screening and response protocol in a large Level III trauma center. Findings highlight the shift in EM screening rates among adults aged 65 and older admitted to the emergency department—from approximately 2% before implementation to 85% afterward. Additionally, challenges, barriers, and strategies for successful implementation are explored.

Abstract citation ID: igaf122.090

### ADAPTING ELDER MISTREATMENT SCREENING FOR HOME-BASED PRIMARY CARE: THE DETECT-RPC PROJECT

Michael Cannell[1], Kristin Lees Haggerty[2], Jason Burnett[3], Melvin Livingston[4], and Carolyn Pickering[3], *1. TCU, Fort Worth, Texas, United States, 2. Education Development Center, Waltham, Massachusetts, United States, 3. UTHealth Houston, Houston, Texas, United States, 4. Emory University, Atlanta, Georgia, United States*

Elder mistreatment (EM) is underrecognized and underreported in home-based primary care (HBPC) settings, where clinicians often serve highly vulnerable older adults, including those with Alzheimer's Disease and Related Dementias (ADRD). The Detection of Elder Mistreatment Through Emergency Care Technicians – Revised for Primary Care (DETECT-RPC) Project adapts a previously validated EMT-based EM screening tool for HBPC clinicians. Guided by the Theory of Planned Behavior (TPB) and the Abuse Intervention Model (AIM), DETECT-RPC seeks to overcome key barriers to EM screening, including clinician uncertainty about EM recognition, concerns about mandatory reporting, and lack of training. Through an intervention mapping process, we developed the DETECT-RPC screening tool to integrate into HBPC workflows and designed a training program to enhance clinician knowledge and confidence in identifying and responding to EM. We are currently conducting a cluster randomized controlled trial (RCT) to evaluate DETECT-RPC's impact on screening and APS reporting behaviors among HBPC clinicians. This presentation will highlight findings from our formative research, the intervention development process, and early insights from clinician training and survey responses measuring attitudes and beliefs about EM reporting. Discussion will focus on implementation challenges, clinician concerns about APS involvement, and strategies for adapting EM screening tools to non-acute care settings. By addressing these barriers, DETECT-RPC aims to improve the identification and response to EM in home-based settings, providing a scalable model for EM screening beyond emergency and hospital-based care.

Abstract citation ID: igaf122.091

### DISSEMINATING AN ELDER MISTREATMENT ELECTRONIC HEALTH RECORD TOOL IN A NATIONAL HEALTH SYSTEM

Lena Makaroun[1], Colleen McQuown[2], and Luna Ragsdale[3], *1. VA Pittsburgh Healthcare System/University of Pittsburgh, Pittsburgh, Pennsylvania, United States, 2. Louis Stokes Cleveland VA Medical Center, Cleveland, Ohio, United States, 3. Durham VA Healthcare System, Durham, North Carolina, United States*

The Veterans Health Administration (VHA), the largest integrated health system in the United States, provides comprehensive, high-quality care to approximately 5 million Veterans age ≥60 years, many with a high burden of elder abuse (EA) risk factors. VHA is a leader in research and care for complex geriatric syndromes and integrating social and medical care, however recent reviews identified a need for systems support for addressing EA. Through interdisciplinary collaborations between VHA researchers and clinical leaders in geriatrics and emergency medicine, we developed a new electronic health record (EHR) tool to support standardized a clinical process for EA identification. First, we created a national evidence-based EHR EA screening template by adapting the Elder Mistreatment Screening and Response Tool (EM-SART) to the VHA system and workflow. Second, we obtained broad stakeholder feedback on the note from leaders of different clinical service lines. Finally, the note template was piloted at three sites in preparation for dissemination. The adapted EM-SART was released for national dissemination in February 2025. In this symposium, we present practical experiences, empirical data and lessons learned from stakeholder engagement, piloting, refinement, implementation and national dissemination of an adapted tool for improving EA identification and response in a national healthcare system. Highlighting how VHA has leveraged partnerships between researchers, philanthropic foundations, and clinical operations partners to advance care for the high priority, but previously under-addressed, issue of EA can help advance implementation science knowledge for other systems looking to advance clinical care and data standardization around EA in their patients.

### SESSION 1090 (PAPER)

### AGING IN THE CONTEXT OF TRAUMA AND CONFLICT

Abstract citation ID: igaf122.092

### A QUALITATIVE STUDY OF HEALTHCARE AND AGING WHILE INCARCERATED

Michelle Suh[1], Megan Benavides[2], Stephen Chen[2], Annika Bhananker[3], Sumin Yoon[4], Marc Robinson[2], Josiah Rich[1], and Anita Chary[2], *1. Brown University, Providence, Rhode Island, United States, 2. Baylor College of Medicine, Houston, Texas, United States, 3. Rice University, Houston, Texas, United States, 4. Houston, Texas, United States*

Older adults comprise the fastest growing demographic in correctional facilities in the United States. While incarcerated, prisoners not only develop chronic conditions but also experience accelerated aging. However, little is known about how older adults navigate the correctional environment, aging, and healthcare. We sought to explore experiences of aging and healthcare in incarcerated older adults. We conducted semi-structured qualitative interviews with 16 incarcerated older adults (>50 years old) from the county jail who were receiving care in a public hospital emergency department. Interviews focused on experiences of aging and healthcare while incarcerated. We used an inductive approach to code data and identify overarching themes. Two major themes emerged. First, participants reported difficulty navigating the jail environment due to aging and disabilities. Examples included using the top bunk bed due to social pressure from other inmates, difficulty reading printed instructions due to visual impairments, and a Parkinson's tremor impeding the grasp of eating utensils. Participants relied on other inmates for assistance but were vulnerable to exploitation, such as commissary fund theft. Second, participants faced numerous challenges attempting to access healthcare. Issues included confusion regarding the protocol for accessing care especially digital kiosks, interruptions in care for chronic conditions including lapses in daily medications, and correctional staff skepticism about medical concerns. Despite a constitutional right to healthcare and high rates of medical comorbidities, older prisoners experienced a challenging physical environment and difficulty accessing healthcare. Further work is needed to identify potential age-friendly modifications to the correctional environment and improved healthcare delivery.

Abstract citation ID: igaf122.093

### BEYOND THE UNDERGROUND: EMERGENCY GERIATRIC HOSPITAL RELOCATION IN CONFLICT - A HEALTHCARE INNOVATION MODEL

Sharon Ost-Mor[1], Elena Titelman[2], Eduard Zlyesov[2], and Inna Shugaev[2], *1. University of Haifa, Haifa, HaZafon, Israel, 2. Fliman Medical Center, Haifa, HaZafon, Israel*

The unprecedented emergency relocation of Israel's largest rehabilitation facility in the north, Fliman Medical Center, to an underground facility during active conflict presents a unique case study in healthcare emergency management. This research addresses a critical gap in the literature on emergency preparedness for specialized healthcare facilities during crisis situations. This qualitative single-case study examines the strategic planning and execution of relocating individuals requiring ventilator support and other complex medical care amid active conflict. Data were collected through in-depth interviews with the emergency coordinator and analysis of contemporaneous documentation. Over eleven months of security challenges, the facility successfully implemented a carefully coordinated evacuation plan, safely transferring sixty medically complex patients—along with essential medical equipment and personnel—to an underground facility at Rambam Hospital within three hours. The relocation maintained operational independence and ensured the continuity of comprehensive rehabilitation services. The successful execution of this emergency relocation protocol demonstrates the feasibility of sustaining complex medical care during crisis situations. This study offers a model for emergency preparedness in specialized healthcare facilities, providing critical insights for institutions worldwide—particularly those serving medically complex populations in conflict-prone regions. The findings present practical guidelines for the safe relocation of vulnerable patients during crises, contributing to improved healthcare resilience in emergency settings.

Abstract citation ID: igaf122.094

### EXPLORING PREDICTORS OF DISASTER RESILIENCE AMONG OLDER ADULTS: EVIDENCE FROM A NATIONALLY REPRESENTATIVE SURVEY

Gabriella Meltzer[1], Kathleen Lynch[2], and Alexis Merdjanoff[2], *1. American University, United States, 2. New York University, New York, New York, United States*

Older adults are the fastest-growing age group in the U.S. and desire to age at home. However, many reside in places increasingly exposed to climate change-fueled disasters. The Aging in Risky Environmental Areas (AREA) Study is a nationally representative survey of community-dwelling adults ages 50+ queried in November 2022 on aging, disaster exposure, and climate change. We conducted bivariate analyses and multivariate linear regression to examine the association between disaster exposure in the past 3 years and scores on the Disaster Adaptation and Resilience Scale (DARS), adjusting for sociodemographics and healthy aging indicators. Among 1,504 participants, 40.3% were exposed to $\geq 2$ climate-related disasters. Total disaster exposure ($p = 0.002$), any indirect exposure ($p < 0.001$), and any direct exposure ($p = 0.005$) were all negatively associated with total disaster resilience. Total disaster exposure was also negatively associated with DARS subscales of social resources ($p = 0.003$), distress regulation ($p < 0.001$), and optimism ($p = 0.006$). In the final model, disaster exposure was not associated with resilience. Several healthy aging variables were associated, including place attachment ($b = 3.28$, $p < 0.001$); time spent on social and leisure activities ($b = 1.23$, $p < 0.001$); loneliness ($b = -0.47$, $p < 0.001$); cognitive impairment ($b = -8.28$, $p < 0.001$); depression ($b = -5.47$, $p = 0.002$); and psychological distress ($b = -0.88$, $p < 0.001$). Results show that while disaster exposure is associated with worse resilience, healthy aging can supersede this while facing more frequent and intense climate-related disasters.

Abstract citation ID: igaf122.095

### FORGOTTEN GRIEVERS: THE UNHEARD VOICES OF GRANDPARENTS GRIEVING FOR GRANDCHILDREN FROM THE “SWORDS OF IRON” WAR

Ile Kermel-Schiffman[1], Rinat Lifshitz[2], Ayala Eliyahou[3], and Galia Segev-Rosenberg[4], *1. Ariel University, Ariel, HaMerkaz, Israel, 2. The Max Stern Academic College of Emek Yezreel, Emek Yezreel, HaZafon, Israel, 3. Bar-Ilan University, Ramat Gan, HaMerkaz, Israel, 4. Kfar Saba Municipality, Kfar Saba, HaMerkaz, Israel*

Since the Swords of Iron War began on October 7, 2023, approximately 4,000 grandparents in Israel have lost a grandchild. Research shows their bereavement differs significantly from that of parents, spouses, or siblings, yet they often remain an overlooked group. This study examines (1) grandparents’ unique bereavement experiences and (2) their needs, vulnerabilities, and resilience in coping with grief. Using a qualitative approach, 25 grandparents who lost grandchildren in military service or terrorist attacks were interviewed. Data were analyzed through an inductive five-step qualitative content analysis. Three key themes emerged: (1) “Losing a grandchild is like nothing else”-participants described deep emotional, cognitive, physical, and behavioral distress; (2) “What is my place now?”- they struggled with identity shifts and sought formal recognition as bereaved family members; and (3) “We learned to live with the pain” - resilience and coping strategies shaped their adaptation. Bereaved grandparents require tailored support and formal acknowledgment, yet they also demonstrate resilience. Professional interventions - both individual and group-based - are crucial to addressing their grief while maintaining their family roles. This study broadens discourse on grandparents’ bereavement, providing a foundation for targeted policies and interventions to enhance their well-being.

Abstract citation ID: igaf122.096

### HOLOCAUST SURVIVOR’S STORIES FOLLOWING THE OCTOBER 7TH WAR IN ISRAEL

Yarin Cohen[1], Maryah Fram[2], and Sue Levkoff[1], *1. University of South Carolina, Columbia, South Carolina, United States, 2. Mary Baldwin University, Columbia, South Carolina, United States*

Holocaust survivors (HS) have often developed narrative identities centered on Israel as both ensuring safety from atrocities and a place where Jewish communities can thrive into the future. The October 7th Hamas attack and subsequent war potentially challenged these resilience fostering narratives. This study explored how HS processed these events and subsequent antisemitism to better understand their meaning-making and support their well-being during late life. Researchers conducted 18 in-depth, semi-structured video interviews with Israeli HS(ages 81-95), analyzing transcripts through thematic and narrative approaches. Findings revealed survivors used their historical narratives as coping tools when processing October 7, drawing guidance from past coping experiences. As a coping mechanism, they shifted between multiple perspectives—viewing events as Jews, as Israelis, and within global-historical contexts. While distinguishing October 7th from the Holocaust, some noted similarities to historical pogroms, with certain images triggering personal Holocaust memories. Many expressed concerns about Israel’s existence, their descendants’ safety, diminished hopes for peace, and anxiety about not living to see crisis resolution due to their age. The resurgence of antisemitism was particularly alarming to many who noted disturbing similarities to pre-Holocaust conditions. Their accounts revealed both psychological distress and remarkable resilience. Participants’ narrative-based coping strategies revealed mechanisms for identity preservation during periods of trauma and challenge. These findings inform how support services might be tailored for older adult HS who continue to face psychological impacts of historical trauma within contexts of ongoing uncertainty, while recognizing them as resilience “experts”, preserving their testimony and deepening understanding of genocide’s psychological impacts.

Abstract citation ID: igaf122.097

### VULNERABILITY OF OLDER ADULTS IN NATURAL DISASTER SCENARIOS: A CASE STUDY OF EARTHQUAKES

Wanwan Si, and Hong Mi, *Zhejiang University, Hangzhou, Zhejiang, China (People’s Republic)*

With the increasing frequency of extreme weather events and climate change, the impact of natural disasters on older adults has become a pressing social concern worldwide. Their vulnerability is not only associated with physiological decline but also influenced by social support systems, psychological resilience, and policy protections. Grounded in vulnerability theory, this study integrates perspectives from sociology, psychology, and medicine to examine the unique risks faced by older populations in disaster contexts. Using data from the EM-DAT International Disaster Database, the WHO Mortality Database, and the China Health and Retirement Longitudinal Study (CHARLS), this study selects three major earthquake events—the 2008 Wenchuan Earthquake in China, the 2011 Great East Japan Earthquake, and the 2016 Central Italy Earthquake—to systematically analyze how older adults experience disaster-related mortality, health impacts, social support availability, and governmental response measures across different countries. Findings highlight the role of diverse social security systems in shaping vulnerability among aging populations and explore strategies to reduce disaster risks, including optimizing emergency response policies, improving access to healthcare resources, and strengthening social support networks. This study provides theoretical insights and practical guidance for the development of age-inclusive disaster management policies, contributing to enhanced emergency preparedness, resilience, and post-disaster recovery for older adults.

### SESSION 1095 (PAPER)

### CONTEXTUALIZING DEPRESSION IN AGING: GENDER, ENVIRONMENT, AND SOCIAL VULNERABILITY

Abstract citation ID: igaf122.098

#### EFFECT OF COGNITIVE FUNCTION ON DEPRESSION SYMPTOMS IN KOREAN OLDER ADULTS LIVING ALONE: MODERATING ROLE OF GENDER

Si Young Song, *Kyung Hee University, Syracuse, New York, United States*

This study examines the effect of cognitive function on depression symptoms in older adults living alone and investigates the moderating role of gender. Data were obtained from the 2023 National Survey of Older Koreans, a nationally representative survey conducted in South Korea, focusing on individuals aged 65 and older who live alone. Cognitive function was assessed using the K-MMSE-2 (Korean Mini-Mental State Examination-2) scale (range: 0–30), and depression symptoms were measured with the SGDS-15 (Short Geriatric Depression Scale-15) (range: 0–13). Gender was analyzed as a moderating variable, while age, education level, household income, life satisfaction, and social contact frequency were controlled. A moderation analysis was conducted. The findings indicate that cognitive function is negatively associated with depression symptoms in older adults living alone. Moreover, gender significantly moderates this relationship. The simple slope test revealed that higher cognitive function is linked to lower depression symptoms in both male and female older adults living alone. However, the association was stronger in males, indicating that cognitive function plays a more critical role in mitigating depression symptoms among older men living alone. These results highlight the need for cognitive health interventions to reduce depression symptoms in older adults living alone. Mental health policies should incorporate gender-specific strategies, and community-based cognitive training programs should be expanded to promote emotional well-being in this population.

Abstract citation ID: igaf122.099

#### INVESTIGATING THE IMPACT OF TRANSPORTATION AIR/NOISE POLLUTION ON MENTAL HEALTH AND IMPLICATIONS FOR HEALTHY AGING

Dialechti Tsimpida[1], Anastasia Tsakiridi[1], and Emily Oliver[2], *1. University of Southampton, Southampton, England, United Kingdom, 2. Newcastle University, Newcastle upon Tyne, United Kingdom*

Mental ill health is shaped by both social and environmental factors, yet the combined effects of air and noise pollution on mental health, particularly among older adults, remain largely underexplored. Disadvantaged communities are often disproportionately exposed to these stressors, amplifying their vulnerability. This study investigated the independent and combined effects of transportation-related air and noise pollution on depression across two Integrated Care Systems (ICS) in northern and southern England. Using data from the 2022 Quality and Outcomes Framework, we calculated depression prevalence for small areas within the Hampshire and Isle of Wight (HIOW) and North East and North Cumbria (NENC) ICS. Transportation-related nitrogen oxides and noise mapping data were used to assess transportation air and noise pollution levels, while neighborhood deprivation was measured using all domains of the English Index of Multiple Deprivation. Depression prevalence was derived from routinely collected publicly available mental health data. Generalized Structural Equation Spatial Modeling was employed to explore mediation effects between environmental stressors, socioeconomic deprivation, and depression at a small area level. In HIOW's rural areas, noise exposure indirectly affected depression, accounting for 18.64% of the total effect of crime on depression. In NENC's urban areas, older adults with higher nighttime noise exposure were more vulnerable to the mental health impacts of income deprivation. Combined air and noise pollution further exacerbated depression in older adults [indirect effect -5.0623 ($p < 0.000001$)]. These findings highlight the need for targeted, place-based public health strategies to reduce mental health inequalities and promote healthy aging in disadvantaged communities.

Abstract citation ID: igaf122.100

#### NUANCED GENDER DIFFERENCES IN EVERYDAY AGEISM AND HEALTH

Julie Ober Allen, *University of Wisconsin–Madison, Madison, Wisconsin, United States*

Qualitative research has documented substantial gender differences in perceptions, experiences, and consequences of ageism, yet survey studies have generally documented comparable amounts of self-reported ageism for men and women. This study uses the new Everyday Ageism Scale for an in-depth investigation of gender differences in amount of routine ageism experienced, types, and salience to mental and physical health in a nationally representative sample of US adults ages 50-80 (2019 National Poll on Healthy Aging, N=2044). Men and women reported comparable amounts of everyday ageism overall and two of three types (age discrimination, internalized ageism). Women reported more exposure to ageist messages suggesting aging and older adults were unattractive, undesirable, and a source of ridicule (p=.005). No gender differences were detected in associations between 1) overall amount of ageism and all examined mental and physical health outcomes, and 2) types of ageism and physical health outcomes (number of chronic health conditions, odds of fair/poor physical health). Among types of ageism, age discrimination and internalized ageism were associated with mental health outcomes at comparable levels by gender; however, more exposure to ageist messages was associated with higher odds of having depression symptoms among women (unrelated for men) and lower odds of fair/poor mental health among men (unrelated for women). While ageism may be a health risk shared by men and women, findings inform initiatives and clinical care on nuances at the intersection of ageism and gender that may contribute to differences in types of ageism experienced and potential mechanisms affecting mental health.

Abstract citation ID: igaf122.101

### SOCIOECONOMIC AND HEALTH DISPARITIES BETWEEN TRANSGENDER AND CISGENDER OLDER ADULTS IN THE SWEDISH POPULATION

J. Lucas Tilley[1], Ylva Moberg[1], Anyah Prasad[2], and Emma Essen[3], *1. Stockholm University, Stockholm, Stockholms Lan, Sweden, 2. Vanderbilt University, Boston, Massachusetts, United States, 3. Uppsala University, Uppsala, Uppsala Lan, Sweden*

Transgender older adults are an understudied group, and most current studies are based on convenient samples which can be subject to selection bias. Recruiting a sizeable representative sample of transgender older adults can be difficult and expensive as they are a very small percentage of the population and hard to reach due to stigma, privacy, and trust issues. Population data from centralized administrative and medical health records available in some countries with universal health care are an under-tapped potential to understand aging among transgender individuals. In this study, we use 2019 data from the Swedish National Patient Register to identify individuals with a diagnosis of gender incongruence and get information on their past year health (hospitalization and anti-depression/anxiety medication usage). Their corresponding sociodemographic data were obtained from Statistics Sweden. We identified 535 transwomen and 206 transmen age 50 and above and each trans person was matched, based on their birth year, with 10 cisgender men and women each. Compared to cisgender men and women, transgender individuals were more likely to live in urban areas, less likely to be married, and tend to have lower earnings and retire early. In logistic regression, after controlling for sociodemographic factors, compared to cisgender men, transgender women and men experienced significantly higher odds of hospitalization ($OR = 1.85.$, $p < 0.001$; $OR = 1.88$, $p = 0.001$, respectively) and were significantly more likely to be on anti-depression/anxiety medication ($OR = 2.23$, $p < 0.001$; $OR = 3.04.$, $p < 0.001$, respectively). These findings highlight the need for targeted interventions to elevate the disproportionate burden of poor health among transgender older adults.

Abstract citation ID: igaf122.102

### THE ROLE OF SEX AND DEPRESSION ON COGNITIVE IMPAIRMENT RISK IN A LONGITUDINAL STUDY OF OLDER ADULTS

Lauren Rutter[1], Lucas Hamilton[2], Skylar Wilson[1], Anne Krendl[1], and Brea Perry[1], *1. Indiana University Bloomington, Bloomington, Indiana, United States, 2. Augustana University, Sioux Falls, South Dakota, United States*

Depression and biological sex independently influence Alzheimer's disease and related disorders (ADRD) risk, yet their interaction is underexplored. This study examines whether depression differentially impacts impairment risk for men and women in a large, longitudinal sample. Data were obtained from the National Alzheimer's Disease Coordinating Center Uniform Data Set ($N = 26,362$, 59.3% women). Depression status was determined using the Geriatric Depression Scale ($GDS \geq 5$), and impairment was defined as progression to mild cognitive impairment or dementia. Impairment status was decided by either a consensus panel or a single physician. Cox proportional-hazards models tested the effects of depression, sex, and their interaction on impairment risk, controlling for age and APOEε4 status. Results showed significant main effects of depression, sex, age, and APOEε4 on impairment risk, and a significant interaction of depression and sex ($p<.01$). Depression increased impairment risk by 74% in men ($HR = 1.74$, $p<.001$) and 106% in women ($HR = 2.06$, $p<.001$). While men had a higher overall hazard ratios for impairment, depression disproportionately accelerated impairment risk in women. This difference may stem from biological mechanisms such as hormonal influences and neuroinflammation or psychosocial factors including caregiving roles and stress exposure. Our current work is exploring sex differences in perceived stress, and the potential mediating role of perceived stress in predicting impairment. Findings highlight the importance of considering sex differences when considering depression as a risk factor for cognitive decline. Targeted interventions addressing depression in women may be particularly effective in mitigating dementia risk and promoting cognitive resilience in aging populations.

### SESSION 1100 (SYMPOSIUM)

Abstract citation ID: igaf122.103

### UNRAVELING FINANCIAL EXPLOITATION IN OLDER ADULTS: A MULTIDIMENSIONAL APPROACH

Chair: Gali Weissberger
Co-Chair: Yoav Bergman
Discussant: Duke Han

A growing body of work has identified the multifaceted nature of the risk factors and consequences of financial exploitation (FE) in older adults, demonstrating the importance of psychosocial, cultural, and cognitive factors. While investigating each of these domains individually is crucial to understanding the phenomenon, considering them together is rarely done. The goal of this symposium is to present novel findings which integrate cognitive, cultural, and psychosocial risk factors of FE. From a cognitive perspective, Sunderaramen introduces a new online money management task, and demonstrates a relationship between auditory memory and identification of erroneous transactions, a skill necessary for avoiding credit card fraud and other forms of FE. Moreover, Hoffman demonstrates the interactive links between associative memory, quality of life, and FE vulnerability. From a sociocultural perspective, Noriega-Makarskyy elaborates upon the relationship between FE vulnerability and social relationships to show that relationship depth, and specifically a strong sense of belonging, is associated with less FE vulnerability, while having distinct social roles is not, and Weissberger explores how culture may modify associations between experienced FE and psychosocial factors. Finally, Bergman employs a longitudinal design to examine the cross-lagged association between subjective age and FE vulnerability. Together, these presentations provide a nuanced understanding of FE, emphasizing cognition, culture, age-related perceptions, and social aspects which mitigate FE risks and consequences. The discussion (led by D. Han) will integrate the implications gleaned from each individual presentation and highlight the importance of taking a multifaceted approach to evaluating FE risks and consequences.

Abstract citation ID: igaf122.104

**FINANCIAL EXPLOITATION AMONG OLDER ADULTS IN ISRAEL: EXPLORING THE ROLE OF CULTURE**

Gali Weissberger[1], and Yoav Bergman[2], *1. Bar-Ilan University, Ramat Gan, HaMerkaz, Israel, 2. Ashkelon Academic College, Ashkelon, HaDarom, Israel*

Financial exploitation (FE) of older adults results in devastating social and psychological consequences. However, few studies have considered culture as a contextual factor that may modify risk factors and consequences of FE. In this study, we examined the psychosocial correlates of experienced FE among two main cultural groups in Israel: the majority group of Jewish Israelis, and the minority group of Arab Israelis. Through convenience sampling techniques, 357 Israeli older adults (M age = 67.89, SD = 6.21, 72.3% female; 13.4% Arab) were recruited into the study. Participants self-reported whether or not they experienced FE after the age of 50, responded to demographic questions, and completed psychosocial questionnaires (loneliness, social support, and psychological distress). A MANCOVA model tested whether the association between FE and psychosocial factors varies by cultural group. There were 79 participants (22% of the sample) who self-reported an experience of FE. Results demonstrated a main effect of FE on the combined dependent variables ($F(3, 346) = 10.98$, $p < 0.001$); there was no main effect of cultural group. A FE by cultural group interaction was found ($F(3, 346) = 3.78$, $p = 0.011$). Follow-up ANCOVA models revealed that the negative effect of FE on psychosocial functioning, and specifically loneliness and psychological distress, was stronger for Israeli Arabs compared to Israeli Jews. These findings suggest that Israeli Arabs who experience FE may be in particular need of support following a FE experience, and highlight the importance of considering culture as a contextual factor that may modify risk factors and consequences of FE.

Abstract citation ID: igaf122.105

**SUBJECTIVE AGE AND FINANCIAL EXPLOITATION VULNERABILITY IN OLDER ADULTS: A CROSS-LAGGED ANALYSIS**

Yoav Bergman[1], and Gali Weissberger[2], *1. Ashkelon Academic College, Ashkelon, HaDarom, Israel, 2. Bar-Ilan University, Ramat Gan, HaMerkaz, Israel*

During the past decade, research has demonstrated the importance of positive views of the aging process and of one's own aging self for older adults' physical, psychological, and social well-being. Recently, studies have demonstrated that older adults who feel younger than their chronological age (i.e., a young subjective age) reported reduced vulnerability for financial exploitation (FEV), as well as with additional related factors such as financial self-efficacy. However, such studies were cross-sectional, and little is known regarding the causal relationship between subjective age and FEV. The current study employs a cross-lagged model which examines both constructs over a period of six months. Data was collected from 82 older adults (Mage= 68.82, SD = 5.44, range 60-86) who filled out scales assessing FEV and subjective age at baseline (T1) and six months later (T2). Results indicated that FEV at T1 predicted an older subjective age at T2, whereas the association between subjective age at T1 and FEV at T2 was not significant. The discussion highlights the important connection between personal subjective views of aging and the risk for financial exploitation in older populations and suggests theoretical and practical implications of this connection.

Abstract citation ID: igaf122.106

**COGNITIVE CORRELATES OF A NOVEL SIMULATED ONLINE MONEY MANAGEMENT CREDIT CARD TASK**

Preeti Sunderaraman[1], Madison Bouchard-Liporto[2], Silvia Chapman[3], Sandra Rizer[3], Masanao Yajima[4], and Stephanie Cosentino[3], *1. Memory and Aging Program, Butler Hospital; Brown University, Boston, Massachusetts, United States, 2. Boston University - Chobanian & Avedisian School of Medicine, Boston, Massachusetts, United States, 3. Columbia University Irving Medical Center, New York, New York, United States, 4. Boston University Department of Mathematics and Statistics, Boston, Massachusetts, United States*

About 65% of older adults experience credit card fraud accounting for loss of $246 million. As older adults are also at risk for cognitive decline, it becomes necessary to develop novel tools to assess online money management (OMM) credit card skills. This study examined the convergent and divergent validity of a novel, simulated OMM credit card task designed to replicate real-world scenarios. Data were collected from 190 participants (M age (yrs)=67.84; SD=4.26, M education (yrs)=16.65, SD=2.26, 69.5% female, 89.5% White). The OMM task comprises of: i) Online Navigation (ON); logging into a simulated credit card account to download a statement, ii) Simple Literacy (SL); clicking on answers in response to questions about the statement, and iii) Statement Monitoring (SM); identifying erroneous transactions. Metrics for each component included number of clicks and time taken to complete each task; additionally, number of correct (accuracy) was present for SL and SM. Bivariate correlations examined the association between OMM components, NIH toolbox tests and the FunStatsTM battery components of online banking and ATM. OMM metrics related to ON and SL were most strongly associated with online banking, while SM was most strongly associated with auditory memory. Compared to other OMM metrics, SL accuracy showed more associations with cognitive abilities. This study found evidence for convergent and divergent validity of the OMM task. These findings are promising and indicate that the OMM task is useful to assess various aspects of OMM credit card skills and those at risk for financial exploitation.

Abstract citation ID: igaf122.107

**FINANCIAL EXPLOITATION VULNERABILITY AND SOCIAL CONNECTEDNESS IN MIDDLE-AGED AND OLDER ADULTS WITHOUT DEMENTIA**

Daisy Noriega-Makarskyy, *University of Southern California, Los Angeles, California, United States*

Financial exploitation of older adults is an understudied but widespread phenomenon in the United States. Previous research examining the association between social functioning and financial exploitation vulnerability suggests that social embeddedness may be protective against financial exploitation. It is not clear, however, whether relationship depth and/or social network diversity (i.e., having many different social roles) drives this protective effect. This study aimed to examine the relationship between aspects of social connectedness (i.e., social network diversity and relationship depth) and financial exploitation vulnerability amongst community-dwelling adults aged 50 or older. One hundred eighteen individuals completed a laboratory visit consisting of questionnaires assessing relationship depth (i.e., Interpersonal Support Evaluation List), social network diversity (i.e., Social Network Index Total Social Roles), and financial exploitation vulnerability (i.e., Perceived Financial Vulnerability Scale; PFVS). Hierarchical linear regressions separately tested associations between financial exploitation vulnerability, relationship depth, and social network diversity. After covarying for demographics, cognitive, and psychological variables, results showed a significant negative association between financial exploitation vulnerability and relationship depth (B(SE) = -.09(.03), p = .004). Subscale analysis indicated that a strong sense of belonging was negatively associated with financial exploitation vulnerability (B(SE) = -.28(.06), p < .001). In contrast, financial exploitation vulnerability was not significantly associated with the number of distinct social roles in one's network (B(SE) = -.02(.11), p = .85), or with other relationship depth subscales. These findings suggest potential benefits of fostering close interpersonal relationships in middle and older adulthood, such as reduced vulnerability to financial exploitation.

Abstract citation ID: igaf122.108

### ASSOCIATIVE MEMORY BUFFERS THE LINK BETWEEN LOW LIFE SATISFACTION AND HIGH FINANCIAL EXPLOITATION VULNERABILITY

Yaakov Hoffman, and Gali Weissberger, *Bar-Ilan University, Ramat Gan, HaMerkaz, Israel*

Vulnerability to financial exploitation (FE) is associated with cognitive and emotional functioning. A recent MRI study demonstrated a link between the entorhinal cortex and FE vulnerability. Accordingly, the primary goal of this study was to investigate the interactive role of associative memory and life satisfaction on FE vulnerability. We focused on the false alarm memory measure; responding "yes" to a new stimulus not previously presented during a learning phase. In associative memory, the "new" stimulus is actually a re-pairing of two old stimuli that appeared during the learning phase but were not paired together. Thus a "yes" response to such stimuli (false alarm) means that participants do not distinguish well between old items and an associated pair. Older adults (N = 144, Mage 68.23±5.26, range 60-86; 63.2% female) were assessed in a cross-sectional study which included the memory test and other demographic and psychosocial variables. There was a significant main effect between lower life satisfaction and higher FE vulnerability, but no main effect of associative memory. A significant interaction showed that the link between FE vulnerability and low life satisfaction was buffered when false alarm rates for associative memory were low. Only this memory score interacted, suggesting that the ability to form and remember new associations can buffer against factors that are linked with higher financial exploitation. While these preliminary results should be further examined, they nevertheless provide an initial indication that associative learning and memory could be important protective factors against FE.

### SESSION 1105 (PAPER)

### EDUCATION, INEQUALITY, AND AGING: UNPACKING STRUCTURAL DETERMINANTS OF LATER-LIFE HEALTH AND WELL-BEING

Abstract citation ID: igaf122.109

### ASSOCIATIONS OF CHILDHOOD SOCIOECONOMIC POSITION, AFFECTIVE PROBLEMS, AND LATER-LIFE COGNITION

Anja Leist[1], Sarah-Naomi James[2], Yiwen Liu[2], Marcus Richards[2], and Anouk Geraets[1], *1. University of Luxembourg, Esch-sur-Alzette, Grevenmacher, Luxembourg, 2. University College London, London, England, United Kingdom*

Socioeconomic inequalities may act on affective and cognitive health from early life through later-life. This study tested to what extent the association of childhood socioeconomic position (SEP) with later-life cognitive ageing is explained by life-course accumulative affective problems. Data were from the MRC National Survey of Health and Development (n = 1,593; 52.6% women). Cognitive ageing was assessed through a battery of neuropsychological tests. Later-life cognitive function was assessed at age 69, and cognitive decline was measured across ages 53, 60–64 and 69. Childhood SEP was derived from overcrowding, essential household amenities, housing condition, and paternal occupation. Affective problems measured at ages 13–64 were categorized into case-level thresholds (never/once/twice or more). Multiple linear and logistic regression analyses and structural equation modeling tested associations between childhood SEP, affective problems, and later-life cognitive ageing. Low childhood SEP was associated with lower scores on later-life (age 69) verbal memory (B=-1.87[-2.48;-1.25]), letter search speed (B=-9.98[-17.57;-2.40]), letter search accuracy (B=-0.90[-1.44;-0.37]), cognitive state (B=-2.14[-2.82;-1.46]), and accelerated decline (between ages 53-69) in letter search accuracy (B = 0.82[0.17;1.46]), but slower decline in verbal memory (B=-0.27[-0.51;-0.00]) compared to high childhood SEP. Low childhood SEP was associated with increased risk of affective problems once (OR = 1.37[1.09;1.73]) and twice or more (OR = 1.32[1.01;1.71]). The association between childhood SEP and later-life letter search accuracy was partly explained (6.3%) by incidental affective problems. Although improving affective health across the life course may enhance later-life cognitive ageing, it could only marginally explain the association between childhood SEP and later-life cognitive ageing.

Abstract citation ID: igaf122.110

### EDUCATION LOANS AND CARDIOVASCULAR DISEASE RISK AMONG OLDER ADULTS

Francisco Rios Casas[1], Christy Erving[1], and Mateo Farina[2], *1. The University of Texas at Austin, Austin, Texas, United States, 2. University of Texas at Austin, Austin, Texas, United States*

Higher levels of educational attainment have been linked to substantially lower cardiovascular risk in later life – a main cause of disability and death in older adulthood. However, due to historic increases in educational debt, the benefits of education for cardiovascular health may be more limited for those that take on student loans. This study uses data from the 2022 wave of the Health and Retirement Study (HRS) to examine the association between outstanding educational debt and health among a population-based sample of US adults between 50 and 80 years of age (N = 10,857). We used logistic regression to examine the association between outstanding debt and four risk factors of cardiovascular disease: diabetes, high blood pressure, obesity, and smoking. Older adults with education debt owed on average $40,000. Moreover, results showed that those with debt were more likely to have diabetes, high blood pressure, and obesity but not more likely to be smokers. This study suggests that outstanding educational loans are increasing in prevalence among recent cohorts of older adults and will be a growing public health concern in the coming decades as more adults continue to take on debt and/or carry it into older adulthood. Additional research is needed to understand the health profiles of older adults that will increasingly reach older adulthood with this type of debt.

Abstract citation ID: igaf122.111

### EDUCATIONAL ATTAINMENT AND SOCIAL ENGAGEMENT IN LATER LIFE: A COHORT ANALYSIS

James Iveniuk[1], Alyssa Goldman[2], and Markus Schafer[3], *1. National Opinion Research Center at The University of Chicago, Scarborough, Ontario, Canada, 2. Boston College, Chestnut Hill, Massachusetts, United States, 3. Baylor University, Waco, Texas, United States*

Associations between educational attainment and social capital are well established, however, this relationship has not been explored in the context of the changing educational landscape in the United States. Since before the 1950s, rates of college attendance and college degree completion have dramatically increased, especially among women. These trends raise questions about whether the advantages of higher education for social capital have remained consistent or exhibit heterogeneity across birth cohorts and forms of social engagement. We use four rounds of U.S. population-based data from the National Social Life, Health, and Aging Project in a series of multilevel models to estimate the effects of older adults' educational attainment on six different measures of social engagement. We pay particular attention to differences across birth cohorts between 1930 and 1965. Our findings indicate that higher levels of education are associated with significantly more frequent volunteering, socializing, religious service attendance, and participating in organized groups, as well as having more friends and larger discussion networks in later life. These findings emerge when adjusting for parental education, age, gender, and race/ethnicity. Associations between education and network size, number of friends, and number of close relatives, are stronger among earlier-born compared to later-born cohorts. However, education is more weakly associated with the frequency of socializing for earlier-born cohorts. We discuss potential mechanisms that underlie these associations, including the alignment of the cohort differences with other social and institutional phenomena in the U.S. We consider the implications for efforts to address social isolation and socioeconomic health disparities.

Abstract citation ID: igaf122.112

### LONGITUDINAL ASSOCIATIONS BETWEEN SCHOOL QUALITY IN ADOLESCENCE AND COGNITIVE HEALTH IN OLDER ADULTHOOD

Joy Bohyun Jang, Lindsay Ryan, and Megan Patrick, *University of Michigan, Ann Arbor, Michigan, United States*

Studies show that residing in states with higher-resourced education systems in childhood is associated with better cognitive health in older adulthood (Walsemann et al., 2023; 2024). One limitation is that state-level measures may not accurately reflect the quality of the schools that respondents attended earlier in life. Using data from a national sample and school administrative records, we examined the association between school quality in adolescence and cognitive health in older adulthood. Longitudinal data from individuals who were high school seniors in 1978-1981 and who participated in the age 60 survey in 2020-2023 were used from the Monitoring the Future (MTF) Panel study (n = 2,090). MTF first surveys high school seniors at their schools and follows up a selective sample over the course of their lives. Cognitive health was measured by self-reported poor memory at age 60 (coded as 1=poor/fair; 0=good/very good/excellent). School quality measures (student/teacher ratio at the schools, school-district level expenditure and revenue per student) were obtained from the National Center for Education Statistics (NCES). Survey-weighted logistic regression models were used. About 10% of the analytic sample reported poor memory at age 60. Regression findings show that a higher student/teacher ratio is associated with poor memory at age 60, particularly among those who attended schools in the South and among racial minority groups (i.e., other than Whites). Our findings suggest that school quality in adolescence may play an important role in cognitive health in older adulthood. We will further explore the mechanisms of school quality (e.g., neighborhood socioeconomic status).

Abstract citation ID: igaf122.113

### QUADRATIC AGE PATTERNS IN SELF-PERCEPTIONS OF AGING: THE MODERATING ROLE OF EDUCATIONAL ATTAINMENT

Charles Johnson[1], Yanjun Dong[2], Wenxing Wei[3], Zexi Zhou[4], and Kun Wang[5], *1. The University of Alabama at Birmingham, Birmingham, Alabama, United States, 2. SUNY at Albany, Albany, New York, United States, 3. Case Western Reserve University, Cleveland, Ohio, United States, 4. The University of Texas at Austin, Austin, Texas, United States, 5. University of Alabama at Birmingham, Birmingham, Alabama, United States*

Higher education often serves as a protective factor for health and psychological well-being among older adults. Beyond providing access to socioeconomic resources, education fosters problem-solving skills, knowledge, and a sense of agency, enabling individuals to manage aging-related challenges effectively. Consequently, older adults with higher educational attainment may hold higher expectations for aging than their less-educated peers. However, as they age, functional declines and physical limitations beyond their control may emerge. According to Discrepancy Theory, dissatisfaction arises when a gap exists between expectations and reality, suggesting that highly educated older adults may experience more pronounced changes in self-perceptions of aging (SPA). Yet, this relationship remains unexplored. This study 1) examined how positive and negative SPA vary across different ages, and 2) investigated how educational attainment moderates these associations. Data from the Health and Retirement Study (HRS) included adults aged 50+ who completed the Leave-Behind Questionnaire in 2008/2010 (baseline n = 14,538) and were followed through 2012/2014 and 2016/2018. Mixed-effects models with quadratic age terms and interactions between age and education were used. Results showed quadratic trajectories for SPA: positive SPA followed an inverted U-shape, peaking around age 74, while negative SPA exhibited a U-shape, increasing with age. Educational attainment significantly moderated these patterns (Positive SPA: B = -0.01, p < .001; Negative SPA: B = 0.01, p < .001), with higher educational attainment linked to sharper changes in SPA over the lifespan. Tailored interventions should address the unique psychological needs of highly educated older adults to support positive aging perceptions.

### SESSION 1110 (PAPER)

Abstract citation ID: igaf122.114

### GAIT SPEED IN OLDER ADULTS AND COGNITIVE HEALTH

Mounting evidence supports the relationship between Gait speed and cognition. This session will add to the science on this topic of the link of gait speed to cognition.

Abstract citation ID: igaf122.115

### DEVELOPMENT AND VALIDATION OF A COGNITIVE-MOTOR ASSESSMENT USING THE THINKING IN MOTION MODEL

Shiri Embon Magal[1], Kfir Asraf[2], and Maayan Agmon[1], *1. University of Haifa, Haifa, HaZafon, Israel, 2. Max Stern Yezreel Valley College, Yezreel Valley, HaZafon, Israel*

The effect of cognitive-motor training on motor and cognitive functions across the lifespan is well established. However, these domains are typically assessed separately, limiting our ability to evaluate their cumulative impact. This study evaluates a novel cognitive-motor assessment: Thinking in Motion (TIM). Sixty-seven healthy adults (mean age 52.8 ± 6.1 years, age range 45–66, 67.37% women) underwent cognitive testing (Trail Making Test, Stroop Test) and motor assessments, including the burpees test, single-leg stance test, step test, Four Square Step Test (FSST), and Dual-task Tandem walking test (DT). The TIM assessment is based on a novel graphic language for movement representation, integrating various manipulations affecting execution speed, symbol interpretation, and spatial perception. As hypothesized, the TIM assessment was correlated with Stroop-A (Pearson's r=-0.3625, p = 0.006), Stroop-B (Pearson's r=-0.4245, p<.001), and TMT-A (Pearson's r = 0.2951, p = 0.026), indicating its effectiveness in measuring cognitive-motor integration. Additionally, the correlation with FSST (Pearson's r = 0.4077, p = 0.002), step test (Pearson's r = 0.2468, p = 0.027), single-leg stance test (Pearson's r= -0.3442, p = 0.009), DT speed (Pearson's r=-0.2636, p = 0.05), and number of burpees (Pearson's r=-0.2972, p = 0.025) supported its relevance in evaluating motor adaptability and postural control. TIM is a valid test that is associated with traditional measures of motor and cognitive function yet provides a broader view of cognitive-motor integration which can serve as a tool for early intervention as early as midlife. Further research should deepen the understanding of this assessment tool through long-term studies examining its predictive capabilities and its relationship with daily functional performance.

Abstract citation ID: igaf122.116

### FEASIBILITY OF USING DAILY HOME GAIT TESTING TO ASSESS INTERVENTION EFFECTIVENESS IN OLDER ADULTS

Pei-An Lee[1], Juhi Digvijay Salecha[1], Brad Manor[2], and On-Yee Lo[3], *1. Hebrew SeniorLife & Harvard Medical School, Boston, Massachusetts, United States, 2. Hebrew SeniorLife/Harvard Medical School, Boston, Massachusetts, United States, 3. Harvard Medical School/ Marcus, Hebrew SeniorLife, Boston, Massachusetts, United States*

Interventions focused on improving gait performance in older adults typically assess gait within single study visits at baseline and after intervention. This approach fails to account for non-trivial, within-subject daily fluctuations in gait performance. We previously validated a smartphone application (Gait App) enabling older adults to self-administer normal and 'dual-task' gait assessments in their own homes. This study examined the feasibility of deploying the Gait App to complete daily gait assessments both during and after a two-week intervention of noninvasive brain stimulation (10, once-daily stimulation sessions over two consecutive weeks) designed to improve dual-task gait in older adults. We enrolled 15 individuals at risk of falls (79±3yrs; 4 females; fear of falling via FES-I: 18-41; SPPB: 4-11). Participants were instructed to utilize the Gait App to complete a brief normal and dual-task gait assessments once a day, at least five days each week, throughout the intervention and over a subsequent 20-day follow-up. Participants successfully completed the assessment on 8.3±3.6 days (83% adherence) during the intervention phase and 11.3±13.7 days (57% adherence) throughout the follow-up period. Participants rated their experience with the Gait App on a scale from 0 (very poor) to 10 (very good), with an average score of 7.4. Six participants gave ratings of 9 or 10. These results demonstrate that the Gait App is feasible for monitoring daily gait performance under both normal and dual-task conditions in older adults at risk of falls, thereby enabling longitudinal assessment of how interventions impact mobility changes in older adults.

Abstract citation ID: igaf122.117

### GAIT SPEED AND COGNITIVE PERFORMANCE IN OLDER ADULT WOMEN: THE STUDY OF WOMEN'S HEALTH ACROSS THE NATION

Jillian Baker[1], Lauren MacConnachie[2], Alexis Reeves[3], Michelle Hood[1], Kelley Pettee Gabriel[4], Bradley Appelhans[5], Carol Derby[6], and Carrie Karvonen-Gutierrez[2], *1. University of Michigan School of Public Health, Ann Arbor, Michigan, United States, 2. University of Michigan, Ann Arbor, Michigan, United States, 3. Stanford Medicine - Stanford University, Stanford, California, United States, 4. The University of Alabama at Birmingham, Birmingham, Alabama, United States, 5. Rush University, Chicago, Illinois, United States, 6. Albert Einstein College of Medicine, Bronx, New York, United States*

Cross-sectional studies show a bi-directional association between gait speed and cognitive function; longitudinal studies are needed to determine the true causal directionality. However, longitudinal studies, particularly of older adults, are prone to selection bias due to differential loss to follow-up. This analysis leverages longitudinal data from the Study of Women's Health Across the Nation to explore relationships between gait speed and cognitive function, accounting for meaningful loss-to-follow-up. We hypothesize that gait speed predicts cognitive functioning and vice-versa. SWAN is a multi-ethnic, community-based cohort of women followed through menopause and into older adulthood. At study visits in 2012-13 (V13) and 2015-16 (V15), 1,258 participants completed: a four-meter timed walk to quantify gait speed and tests of working memory, processing speed, and verbal memory. Linear regressions predicted V15 cognitive test scores from V13 gait speed and vice-versa. Inverse probability weights for missingness due to death, stroke, poor physical function, and other missingness adjusted for differential loss-to-follow-up from baseline. In models including weights to account for differential loss-to-follow-up and adjusting for race and ethnicity, education, age, and financial strain, faster gait speeds at V13 were associated with better processing speed at V15 ($\beta = 4.19$, 95%CI:1.69,6.70) but processing speed at V13 was not statistically significantly associated with gait speed at V15 ($\beta = 0.0010$, 95%CI:-0.0002,0.0022). No statistically significant associations were observed between gait speed and working or verbal memory. These results suggest the relationship between gait speed and cognitive function is not bi-directional, but that gait speed may have an effect on future processing speed and not the reverse.

Abstract citation ID: igaf122.118

### PERCEIVED BALANCE PROBLEM ASSOCIATED WITH FALL PREVENTION BEHAVIOR AMONG HOSPITALIZED OLDER ADULTS

Hanne Dolan, Keenan Pituch, and David Coon, *Arizona State University, Phoenix, Arizona, United States*

Inpatient falls among older adults remain a relentless problem. Older adults' engagement in fall prevention is imperative, yet research indicates that up to 82% of hospitalized older adults do not perceive themselves as at risk for falling in the hospital despite being identified as such by nursing assessment. Recent studies found that older adults may prefer the term 'balance problem' as opposed to 'fall risk.' Using the Health Belief Model as its theoretical framework, the purpose of this study was to explore the interrelationships among perceived balance problem, balance confidence, fear of falling (FOF), falls history, and fall prevention behavior among hospitalized older adults. A cross-sectional analysis was conducted using survey data collected among 70 rurally hospitalized older adults (F = 57.1%, Mage = 77.8; SD = 8.7). A multivariable regression analysis found that older adults who indicated a balance problem reported greater fall prevention behavior (b = .41; SE = .16; p = .014; rsemi-partial = .23). No other variable in the model was independently associated with fall prevention behavior, including balance confidence, FOF, falls history, health factor- and socio-demographic variables. Hospitalized older adults' fall prevention behavior was positively associated with their perceived balance problem. Our findings also align with research showing that self-reported falls was associated with having a balance problem. This suggests that assessing hospitalized older adults' perceptions of their own balance would be an important addition to inpatient fall risk assessments and may be highly important in reframing the topic of fall prevention among older adults.

Abstract citation ID: igaf122.119

### TAKING THE NEXT STEP: INCREASING GAIT SPEED ASSESSMENT IN PRIMARY CARE

Lisa Barry[1], Kathryn Callahan[2], Mariana Wingood[2], Richard Fortinsky[1], Karina Berg[3], Natong Lin[1], Song Han[1], and Nicholas Pajewski[2], *1. University of Connecticut, Farmington, Connecticut, United States, 2. Wake Forest University School of Medicine, Winston-Salem, North Carolina, United States, 3. UConn Health, Farmington, Connecticut, United States*

Gait speed is a simple and robust predictor of outcomes in older persons including disability and falls. Yet, despite widespread acceptance as an important clinical indicator, it is not routinely assessed during office visits. We sought to implement gait speed assessment into Medicare Annual Wellness Visit (AWV) workflow at four clinical practices across two healthcare systems. Gait speed was assessed using a previously-validated, radio frequency identification (RFID) technology-based system. Patients complete a 4-meter walk while wearing an armband containing an RFID tag. Wall-mounted antennae identify the tag and a computer program calculates gait speed (meters per second). We assessed implementation feasibility and fidelity using a mixed methods approach informed by the Consolidated Framework for Implementation Research 2.0. Implementation strategies included: identifying clinical champions; developing a gait speed field in Epic, the electronic medical record system; training medical assistants to conduct gait speed assessments before "rooming" patients; and assessing the proportion of AWVs where the system worked as intended. Preliminary results are available from the first clinic. Over the first 6 weeks of the 12-week implementation period, we observed 35 AWVs. In the 3-week first phase, medical assistants successfully assessed gait speed and recorded results into Epic during 16/23 (70%) of AWVs. After medical assistant re-training and re-programming range adjustment to optimize tag detection, gait speed was successfully assessed in 100% of the 12 visits observed in the 3-week second phase. Our findings provide encouraging evidence indicating that gait speed assessment using RFID technology can be implemented into AWV workflow.

### SESSION 1115 (PAPER)

### NEW HORIZONS FOR OLDER WORKERS

Abstract citation ID: igaf122.120

### BRIDGE JOBS AS PATHS TO WORKING LONGER: DO GENDER AND RETIREMENT IDENTITIES MATTER?

Yun Taek Oh[1], and Phyllis Moen[2], *1. University of Nevada, Reno, Reno, Nevada, United States, 2. University of Minnesota, Minneapolis, Minnesota, United States*

The prevalence and diversification of bridge jobs loosen the conventional coupling of older workers' retirement and their identity transitions: not, partly, or completely retired. These transitions may vary by gender due to the pre-existing gendered norms and practices and the historical changes in women's work lives. While these identities may influence job attachment and timing of workforce exit, the process of retirement identification, especially by gender, is not well-documented. To assess gender differences in retirement identities and timing of workforce exit of those taking on bridge jobs, we draw on a sample of Early Boomers in the Health and Retirement Study (ages 51-56 in 2004) who are in their career jobs in ages 55-56 (n = 792), and use multinomial logistic regression and event history analysis to investigate the gender differences in predictors of retirement identities and timing of workforce exit. Our results show gender differences in retirement identification; men tend to see themselves as "partly retired" (RRR = 6.201) while women tend to see themselves as "completely retired" (RRR = 5.008). Social Security receipt increases the likelihood of identifying as "partly retired" for women (RRR = 12.953) and "completely retired" for men (RRR = 30.062). Regardless of gender, self-identifying as "completely retired" is associated with earlier workforce exit while self-identifying as "not retired" is associated with longer work lives. The deep institutionalization of retirement as concomitant with Social Security receipt shapes the identities of those occupying bridge jobs, and these identities predict older workers' workforce exits.

Abstract citation ID: igaf122.121

### EMPLOYMENT PATTERN AND DEPRESSION FOR MIDDLE-AGED AND OLDER NATIVES AND IMMIGRANTS IN THE UNITED STATES

Yingyue Jiang, *Brown University, Providence, Rhode Island, United States*

Stable full-time employment and voluntary retirement are important to the well-being of older adults, in terms of economic status and health. This study examines the employment and retirement pattern of middle-aged adults to older adults, as well as these patterns' association with depression. This study focuses on differences between natives and immigrants in the United States, using data from the 1992-1998 Health and Retirement Study (HRS) of 4,706 natives and 513 immigrants who were aged 50 to 65 at the baseline in the United States. Sequence analysis and multivariate analysis were employed to identify key employment and retirement patterns. Findings show that natives typically exhibit greater stability in full-time employment while immigrants show more variation in transition patterns. Among respondents who have full-time employment before exiting the labor market, natives are more likely to maintain their original status and exit the labor market later. Among respondents who indicated retired, there is no significant difference in the type of retirement observed between natives and immigrants. Immigrants have higher depression scores in both the first wave and last wave compared to natives. Early exit from the labor market and job instability (part-time employment and unemployment) are associated with higher levels of depression, with a greater impact observed among immigrants. The nonsignificant effect of years in the U.S. challenges the assimilation hypothesis and underscores persistent health inequalities among immigrants. The findings highlight the importance of job stability and voluntary retirement in supporting mental health and reducing social inequalities among middle-aged to older adults.

Abstract citation ID: igaf122.122

### THE PRECARIOUS IN-BETWEEN: EARLY RETIREMENT FOR THOSE NEITHER OLD NOR YOUNG ENOUGH, RICH NOR POOR ENOUGH

Crystal Kwan, *The Hong Kong Polytechnic University, Hong Kong, Hong Kong, China (People's Republic)*

Research Aim: This study explores the perspectives, lived experiences, and expectations of early retirees with lower incomes as they adjust to retirement in Hong Kong. Methods: We conducted a qualitative study with 50 early retirees (before age 65) classified as lower-income per Hong Kong's Low Income Working Family Allowance (LIFA) criteria. Over ten weeks, data was collected through semi-structured interviews lasting 45-75 minutes and supplemented by weekly photo-diary entries. A resource-based dynamic perspective was the theoretical framework, guiding the coding. Thematic analysis of both visual and textual data helped extract themes. Findings: Most participants faced involuntary retirement due to economic recession, ageism, work pressure, caregiving demands, and health issues. A major theme was the ironic transition from long work hours to sudden free time, leading to either relief and pursuit of interests or feelings of aimlessness. Financial concerns were prevalent; however, many adapted by embracing a simpler lifestyle. Ambivalence about the adequacy of their limited savings for retirement was a common sentiment. Another significant theme was the positive impact of developing healthy habits and the additional challenges faced by subgroups dealing with caregiving and housing issues. Conclusions and Implications: This study underscores the necessity for targeted policies and early intervention programs to assist this overlooked group at risk of poverty, proposing strategies to enhance their agency and overcome ambivalence to facilitate a positive adjustment to retirement.

Abstract citation ID: igaf122.123

### USING THE FORESIGHT 50+ NATIONAL PROBABILITY PANEL OF OLDER ADULTS FOR RAPID RECRUITMENT FOR QUALITATIVE RESEARCH

Ellen Bloss[1], Nell Compernolle[1], Martha Cowley[2], and Alex Chew[2], *1. NORC at the University of Chicago, Chicago, Illinois, United States, 2. National Opinion Research Center at The University of Chicago, Chicago, Illinois, United States*

Background and Objectives: The older adult population is rapidly growing. However, they remain severely underrepresented in research, in large part due to recruitment challenges. As a result, aging research is often constrained by large recruitment budgets or unrepresentative convenience samples. Here, we tested the feasibility of a novel, high quality recruitment tool for rapid qualitative research for an NIH-funded pilot study on loneliness among older adults. Methods: We leveraged the Foresight 50+("F50+"), a sub-panel of AmeriSpeak that is offered by AARP and NORC and fields monthly Omnibus surveys to a nationally representative sample of U.S. adults ages 50 and older. As part of the F50+ Omnibus survey (fielded to N~1000 panelists), we included the 3-item UCLA loneliness scale and an item assessing their interest in participating in a 60-minute focus group discussion. Results: Coupled with the F50+ Profile variables, we used the Omnibus results to identify U.S. adults who are ages 65+(N=602), reported some loneliness (N=292), were willing to participate in a group discussion (N=92), and varied across key socio-demographic characteristics (e.g., gender and race/ethnicity). The study team scheduled and successfully conducted 4 virtual focus group discussions with our target population, the compositions of which varied by gender and race/ethnicity, within 4 weeks at a reasonable cost. In general, panelists were agreeable, engaging, and communicative, with minimal attrition or invalid contact information. Discussion: The Foresight 50+ panel offers a cost-effective option for researchers and policymakers seeking high quality participants for rapid research among sub-populations of older adults.

Abstract citation ID: igaf122.124

### WORKFORCE DEVELOPMENT PROGRAMS FOR OLDER ADULTS: PARTICIPANT, PROGRAM, AND POLICY INSIGHTS

Mary Lou Ciolfi[1], Jennifer Crittenden[2], and Rachel Coleman[2], *1. UMaine Center on Aging, Orono, Maine, United States, 2. University of Maine Center on Aging, Orono, Maine, United States*

The confluence of increased longevity and critical labor shortages presents opportunities for older adult workforce development (WFD) programming that meets the needs of older adults, employers, and communities. Research on an AmeriCorps Seniors funded demonstration of seven diverse workforce development programs reveals important insights into older worker motivation, program structures and processes, external environments, and local, state, and federal policy that can inform workforce policy and programming into the future. The variety of geographic locations, labor supply, and work sector focus of the seven programs contributes to compelling questions around how to attract older workers to compensated employment, thereby decreasing critical labor shortages in regions and commercial sectors around the country. This presentation highlights early findings from the University of Maine Center on Aging's research identifying core components of successful WFD programs. Data include survey (N = 191) and focus group and interview data (N = 45) collected across the seven programs. Year One findings illuminate promising ideas related to program design and implementation, messaging, incentives and barriers, government and higher education involvement and support, and the community and demographic data needed to boost program success, worker satisfaction, and partner collaboration. Research findings highlight valuable WFD program outcomes including participant social and emotional wellness and use of a cohort model. Lessons learned include use of specific recruitment messaging and platforms, adapting training to older workers, aligning skills and employment opportunities, and securing community partnerships to create successful, regionally responsive workforce programming that strengthens individuals, organizations, communities, and employment sectors.

### SESSION 1120 (PAPER)

### PSYCHOSOCIAL AND BEHAVIORAL DIMENSIONS OF HEALTHY AGING

Abstract citation ID: igaf122.125

### BEFORE AND AFTER DAILY STRESSORS: IMPLICATIONS FOR DAILY HEALTH AND WELL-BEING IN MIDDLE-AGED AND OLDER ADULTS

MacKenzie Hughes[1], Emily Smith[2], Shevaun Neupert[3], and Ann Pearman[4], *1. National Opinion Research Center at The University of Chicago, Gig Harbor, Washington, United States, 2. Vermont Department of Health, Burlington, Vermont, United States, 3. North Carolina State University, Raleigh, North Carolina, United States, 4. MetroHealth Medical System, Cleveland, Ohio, United States*

Stressor exposure is associated with higher negative affect, worse physical health, and poorer memory performance. However, these processes have typically been evaluated from retrospective accounts of stressors that have already occurred. The goal of the current study was to examine the simultaneous contribution of retrospective daily stressors and forecasts of future stressors as they unfold within the daily lives of middle-aged and older adults. Two hundred sixty-four adults (mean age = 64.11, range 55 - 79) participated in an online daily diary study for 30 consecutive days. Each day they reported on their negative affect as well as challenges with their physical health and memory. They also reported on the stressors encountered in the previous 24 hours and rated the likelihood of experiencing a stressor within the next 24 hours. Separate multilevel models were conducted for each outcome (affect, memory, health) with retrospective stressor exposure and stressor forecasts as within-person predictors. Across the three models, increases in forecasts of future stressors were associated with increases in negative affect, health challenges, and memory issues. However, increases in daily stressor exposure were associated with increases in negative affect and health challenges, but not memory issues. In addition to discussing the significance of these findings, we will explore how understanding this interplay of past daily stressors and appraisals of future stressors may inform targeted interventions designed to support older adults' well-being.

Abstract citation ID: igaf122.126

### IMPROVING DIET, SELF-RATED HEALTH, & BMI FOR OLDER ADULTS WITH A STUDENT-LED HEALTH EDUCATION INTERVENTION

Britteny Howell[1], Amber Worthington[1], Leslie Redmond[2], and Vanessa Hiratsuka[3], *1. University of Alaska Anchorage, Anchorage, Alaska, United States, 2. University of Manitoba, Winnipeg, Manitoba, Canada, 3. Southcentral Foundation, Anchorage, Alaska, United States*

Older adults in Alaska make up the fastest growing population in the state and face some of the greatest provider shortages in the country. Research suggests that older adults may respond better to positive, hopeful information that aligns with their desires and goals for the future. Therefore, we designed and delivered a 15-week, student-led health intervention using Persuasive Hope Theory to 39 older adults to increase feelings of hope and improve health. Our research question was: Did self-efficacy, fruit and vegetable intake, physical activity, self-rated health, or BMI improve significantly as a result of the program? Dependent sample t-tests revealed that the number of servings of fruit and vegetables consumed increased significantly after the program compared to before the program ($p<.01$). BMI was also statistically significantly lower after the program compared to before the program ($p<.05$) and participants' self-rated health was significantly higher after the program compared to before the program ($p<.01$). There was no significant difference in self-efficacy or physical activity levels over time. A power analysis was conducted using G*Power version 3.1.9.7 that indicated the required sample size to achieve 80% power at a significance criterion of $\alpha = 0.5$, is $N = 27$ for a dependent sample t-test. Despite the use of a small, convenience sample, results indicate that participants were satisfied with the student-led intervention that significantly helped to decrease BMI and increase fruit and vegetable intake and self-rated health among the sample population, which can serve as a model for other hope-based, intergenerational healthy aging initiatives.

Abstract citation ID: igaf122.127

### PERSONALITY-ACTIVITY ALIGNMENT: IMPLICATIONS FOR DEMENTIA RISK REDUCTION

Joseph Svec[1], Yulri Kim[2], Jeong Eun Lee[2], and Natasha Nemmers[3], *1. St. Joseph's University, Patchogue, New York, United States, 2. Iowa State University, Ames, Iowa, United States, 3. Wayne State University, Detroit, Michigan, United States*

Certain personality types (i.e. neuroticism) are often accompanied by higher risks of cognitive decline, whereas others of the “big five” (i.e. conscientiousness) correspond with lower risks. However, such associations largely focus on independent associations of personality types and cognitive health. We extend this literature by focusing on the connections between personality types and personal behaviors, specifically the extent to which older adults engage in social and non-social activities. Utilizing a person-environmental fit perspective, this study examines not only the associations between activity associations and cognitive decline but also whether the configurations of personality-activity dimensions amplify such activity effects. Using the Health and Retirement Study (N = 3,380), we run a series of logistic regressions results predicting dementia. Preliminary findings suggest that social activities significantly correspond with reduced odds of dementia. However, the positive and significant interaction of neuroticism and social activities (OR = 1.63) indicate that social activities have increasingly reduced benefits for those with higher levels of neuroticism. Conversely, those with the lowest conscientiousness scores, social activities increase dementia risk by 461% (OR = 5.61) with the caveat that each additional unit increase in conscientiousness scores reduce the odds of social activities by 42% (OR = 0.58). In other words, conscientiousness appears to enhance the protective effects of social activities against dementia risk. The observed relationships highlight that social engagement or activities and their alignments with personality types may represent modifiable factors associated with dementia risk. Thus, future research may benefit from more nuanced understandings of activities as conditional to personal and environmental fits.

Abstract citation ID: igaf122.128

### LIFE SATISFACTION AND RISK OF DEMENTIA: EVIDENCE FROM TWO PROSPECTIVE SAMPLES

Selin Karakose[1], Martina Luchetti[2], Yannick Stephan[3], Angelina R. Sutin[2], and Antonio Terracciano[1], *1. Florida State University, Tallahassee, Florida, United States, 2. Florida State University College of Medicine, Tallahassee, Florida, United States, 3. University of Montpellier, Montpellier, Languedoc-Roussillon, France*

Life satisfaction predicts better physical and mental health outcomes. There is promising evidence that its positive contribution may extend to healthier cognitive outcomes, including incident dementia. Samples from more countries are needed to evaluate the replicability and robustness of this association. This study examines the association between life satisfaction and dementia in two large, prospective, population-based cohorts from 15 European countries. Data from the English Longitudinal Study of Ageing (ELSA: N = 6979, 56.1% female; Mean age=65.35; Follow-up mean=11.27 years; 498 incident dementia) and the Survey of Health and Ageing and Retirement in Europe (SHARE: N = 24119, 55.5% female; Mean age=64.50; Follow-up mean: 10.90 years; 1655 incident dementia) were analyzed using Cox proportional hazards regression. In both samples, higher life satisfaction was associated with about 20% lower risk of dementia, an association that remained significant accounting for demographic (age, sex, race, education, marital status, and presence of children), psychological (depression), behavioral (physical activity and smoking), and clinical (obesity, diabetes, hypertension) risk factors. The association between life satisfaction and incident dementia was not moderated by sex, education, race, marital status, presence of children, or depressive symptoms in either sample. The associations were similar when the sample was restricted to participants 65 years and older and when participants who developed dementia within 5 years were excluded. The findings suggest that life satisfaction is a potential intervention target to reduce dementia risk and support healthier cognitive aging.

### SESSION 1125 (PAPER)

### RELATIONSHIP QUALITY AND HEALTH IN LATER LIFE: INSIGHTS FROM AGING COUPLES ACROSS CONTEXTS

Abstract citation ID: igaf122.129

### A NATIONAL DYADIC STUDY OF SPOUSAL EDUCATION AND HYPERTENSION AMONG OLDER COUPLES IN THE UNITED STATES

Hui Liu, Wencheng Zhang, and Juwen Wang, *Purdue University, West Lafayette, Indiana, United States*

Hypertension is a major public health concern, particularly for older adults. Guided by the linked lives perspective, this study examines the relationship between spousal education and hypertension risk among older couples. Using data from the National Social Life, Health & Aging Project (NSHAP) Round 3 (2015-2016), we analyzed 1,214 couple dyads aged 50 and older. Hypertension was assessed using both biological and self-reported measures. Spousal education was categorized by college degree attainment. Results from the Actor Partner Interdependence Model (APIM) suggest that a wife’s college degree was associated with lower odds of hypertension for both herself and her husband, but a husband’s college degree was not associated with the risk of hypertension for either spouse. Mediation analysis shows that husband’s health behaviors, but not household income, partly explained the association between a wife’s college degree and both spouses’ hypertension risk. These findings suggest that a wife’s education has a greater impact on a couple’s hypertension risk than a husband’s education, highlighting the need for interventions targeting couples with a lower-educated wife to promote cardiovascular health among older adults.

Abstract citation ID: igaf122.130

### DAILY CLOSENESS DISCREPANCY AND MARITAL INTERACTION QUALITY IN OLDER COUPLES: THE MEDIATING EFFECT OF LONELINESS

Yan Huang, and Lynn Martire, *The Pennsylvania State University, University Park, Pennsylvania, United States*

Feelings of closeness are key to individual and relational well-being. However, individuals may desire different degrees of relationship closeness compared to the actual closeness they feel with their partners. Such a discrepancy in closeness may affect marital interaction quality, especially in the context of chronic pain. This study aimed to investigate associations of daily closeness discrepancy with same-day marital interaction quality and the potential mediating effect of same-day loneliness among older adults with chronic back pain (N = 147) and their partners. Participants reported daily actual and desired closeness, marital interaction quality, and loneliness for 30 days. Closeness discrepancy was defined by the absolute value of actual closeness minus desired closeness. Separate multilevel models were run for patients and partners, and the bootstrapping method was applied in multilevel mediation models. We found that closeness discrepancy on a given day was significantly related to decreased marital interaction quality that day in both patients and partners, and such daily associations were in part explained by feelings of loneliness that day. Specifically, on days when participants had higher closeness discrepancy, their levels of loneliness were also higher, and loneliness was in turn related to less positive and more negative marital interaction quality. These associations remained significant after controlling for depressive symptoms at baseline. Findings provide evidence on the daily dynamics of closeness discrepancy and marital interaction quality in the context of chronic pain and suggest loneliness as an indirect psychosocial pathway linking such daily associations.

Abstract citation ID: igaf122.131

### DOUBLE BURDENS OR DOUBLE RESILIENCE: A QUALITATIVE STUDY OF CHINESE OLDER COUPLES BOTH WITH MULTIMORBIDITY

Yuanyuan Jin, and Yu Zhang, *Soochow University, Suzhou, Jiangsu, China (People's Republic)*

As the population ages, the occurrence of older adults and their spouses both living with multimorbidity is becoming increasingly common. Multimorbidity not only affects the patients themselves but also imposes a heavy burden on family caregivers. When both partners experience multimorbidity, caregiving and care-receiving roles often blur. Limited research exists on the lived experiences and daily interactions of such couples. This study aimed to explore the lived experiences and daily interactions of older couples both with multimorbidity. A qualitative study was conducted in China from May 2023 to January 2025, involving 20 couples. Reflexive thematic analysis was used to analyze data. Four key themes were generated: triadic relationship, dynamic daily health management interactions, double burdens, and double resilience. In older couples where both partners have multimorbidity, roles of "patient" and "caregiver" shift based on acute illness flares, declining function, or third-party involvement. Daily health management interactions reflect independence, interdependence, or dependence, shaped by health status and preferences. Double burdens include physical tolls, financial hardship, and emotional contagion. Conversely, double resilience is reflected in the formation of a nudge effect, the generation of emotional resonance, and the encouragement of family-wide advocacy for a healthy lifestyle. This study adopts a dyadic perspective to explore the experiences and interactions within older couples where both partners experience multimorbidity. Formal or informal caregiving support from third parties, as well as the nudge effect and emotional resonance between spouses, play a pivotal role in offering essential support, helping older couples better navigate the challenges of multimorbidity.

Abstract citation ID: igaf122.132

### END-OF-LIFE PLANNING IN AGING COUPLES: A COMPARISON OF GAY, LESBIAN, AND HETEROSEXUAL MARRIAGES

Yiwen Wang, *University of Central Florida, Florida, United States*

Research on end-of-life planning within marriage has predominantly focused on heterosexual couples, overlooking the experiences of sexual minority couples. However, evidence suggests that many processes considered normative in different-sex marriages unfold differently in same-sex marriages. This study employs a minority stress perspective to examine how heterosexual, gay, and lesbian married couples engage in both informal (e.g., discussions about future care and end-of-life preferences) and formal (e.g., legal arrangements through living wills and other documents) end-of-life planning. I leverage dyadic data from the Health and Relationships Project (2015-2022) to compare the responses of men and women in same-sex and different-sex marriages. Using the Actor- Partner Interdependence Model (APIM), I assess how advance care planning are shaped by both spouses' health, past health crises, dementia worries, relationships with family members, and experiences of discrimination. Findings suggest that gay and lesbian couples are more likely than heterosexual couples to engage in both informal and formal end-of-life planning, with these disparities largely driven by health concerns and fears of discrimination. These results highlight the need to consider the rapidly changing legal and social landscape and its implications for end-of-life planning among same- and different-sex couples.

Abstract citation ID: igaf122.133

### SIMILARITIES IN PERCEIVED MARITAL RESPONSIVENESS AMONG ARAB COUPLES AFTER RETIREMENT

Reem Nashef-Hamuda, Dikla Segel-Karpas, Roi Estlein, and Yuval Palgi, *University of Haifa, Haifa, HaZafon, Israel*

This study explores the intricate relationship between perceived marital responsiveness, life satisfaction, and marital quality among retired Arab couples—a context largely understudied in non-Western collectivist societies. A total of 108 retired heterosexual couples (216 participants; husbands' mean age = 70.31 years, wives' mean age = 65.07 years, mean marital duration = 43.58 years) completed surveys assessing perceived marital responsiveness, life satisfaction, and marital quality. Using Dyadic Response Surface Analysis (DRSA), this study examined both individual and dyadic effects of perceived responsiveness on partners' life satisfaction and marital quality. Contrary to prior research, dyadic congruence in perceived responsiveness—the degree to which partners perceive responsiveness similarly—did not significantly predict life satisfaction or marital quality. Instead, individual perceptions of responsiveness emerged as a stronger predictor of both outcomes, highlighting the centrality of personal emotional experiences over dyadic similarity in shaping well-being in later life. Drawing upon an integrative Family Systems and Socioemotional Selectivity framework, these findings challenge Western-centric assumptions about marital dynamics in aging couples and underscore the cultural nuances of emotional support in Arab society. The study contributes to a more nuanced understanding of marital resilience after retirement, with implications for relationship interventions, aging policy, and cross-cultural gerontological research.

### SESSION 1130 (SYMPOSIUM)

Abstract citation ID: igaf122.135

### "DON'T STUDY US WITHOUT US": AGE INCLUSIVITY IN RESEARCH

Chair: Nina Silverstein
Co-Chair: Lauren Bowen
Discussant: Joann Montepare

The Research Domain of the Age Inclusivity Domains in Higher Education (AIDHE) model endorsed by GSA advocates for facilitating aging-related research by faculty and students with attention to age-diversity, intergenerational dynamics, and interdisciplinary collaboration; incorporating age-inclusive perspectives into research agendas and scholarly activities across disciplines; and expanding knowledge to inform applications and interventions. Whitbourne and colleagues' (2024) study that surveyed 6,831 faculty, staff, and students across 25 U.S. institutions revealed that only about 61% of age-inclusive research practices were in place across campuses. This symposium will show creative ways these practices can be expanded in higher education. Whitbourne will begin by describing the AIDHE model and what their study revealed about the prevalence and range of age-inclusive research practices. McGaffigan will present examples from the Aging PCOR Learning Collaborative to show how involving older adults as experts fosters meaningful research and outcomes. Waterhouse adds the perspective of a medical campus and will describe a workforce development program for older adults to become research specialists and be eligible for hire onto research teams; and their Research Roadshows and Living Labs that provide experiential opportunities for older adults to engage directly with research teams. Finally, Koren will consider the value of age-diversity in educational research that involves older learners, younger learners, and faculty to provide insights about the effectiveness of intergenerational classrooms. Montepare will serve as discussant and reflect on strategies for strengthening age-inclusive practices in this critical domain.

Abstract citation ID: igaf122.136

### AIDHE: A FRAMEWORK FOR ADVANCING AGE-INCLUSIVE RESEARCH PRACTICES

Susan Whitbourne, *University of Massachusetts Boston, Boston, Massachusetts, United States*

The AIDHE model views age inclusivity as incorporating seven domains that reflect all activities within institutions of higher education. Central to the mission of many universities, the domain of research includes such age-inclusive practices by research offices as stimulating new investigations on aging through workshops, small grants, featured newsletter stories, support of travel, and assistance in grant writing. The present paper will summarize the AIDHE model in the domain of research and describe the results of the national survey's findings on the presence of age-inclusive practices and awareness of these practices among the 23 participating institutions. Administrators completed the ICCS Inventory, indicating which age-inclusive research practices their offices supported and also enumerated how many age-related funding agencies supported sponsored research on their campuses. Across all institutions, such age-inclusive practices were reported in only 61%, making research the second-lowest of all domains included in the inventory. Climate survey data from faculty on items tapping awareness of age-inclusive practices revealed even lower awareness, such as in the area of stimulating research discussions on aging, with 39% acknowledging whether these discussions occur on campus. These findings will be discussed along with suggestions for strategies that institutions can embark on to support and encourage broader awareness of the need for research on aging, both in basic and applied areas.

Abstract citation ID: igaf122.137

### "I NEVER CONSIDERED THAT": THE IMPACT OF ENGAGING OLDER ADULTS IN RESEARCH AND STRATEGIES FOR MAKING IT WORK

Erin McGaffigan[1], Marc Cohen[2], Sophia Webber[1], Melissa Destrampe[1], Myrna Finn[3], Janet Sasser[4], and Linda Alloy[5], *1. Collective Insight, North Reading, Massachusetts, United States, 2. University of Massachusetts Boston, Boston, Massachusetts, United States, 3. Osher Lifelong Learning Institute, Pittsfield, Massachusetts, United States, 4. Los Angeles, California, United States, 5. Brookline, Massachusetts, United States*

Researchers often lack training and resources to meaningfully engage older adults in research. As a result, those most impacted by aging research- older adults- are often left out of the design and implementation. The Aging PCOR Learning Collaborative, a PCORI-funded project co-lead by Collective Insight and the LeadingAge LTSS Center@UMass Boston, encouraged researchers to engage older adults as partners so research agendas and scholarly activities better reflect age-inclusive perspectives. The Learning Collaborative engaged older adults and researchers to design educational tools and trainings to build researchers' engagement capacity, reaching more than 800 aging-focused researchers, PhD students, and others in the aging research community to do just that. Among these activities was an Aging Research Advisory program, which provided opportunities for older adults to inform researchers and students on their aging-focused research activities. Ten older adults participated in introductory research training. They then hosted 8 advisory sessions from Fall 2023 to Summer 2024, providing feedback to 4 researchers on their research priorities and methods. In Spring 2025, Collective Insight and the LeadingAge LTSS Center@ UMass Boston partnered with older adult research advisors to co-investigate the Aging Research Advisory program process and outcomes. In this presentation, we share our findings from this co-investigation, including the impact of advisory sessions on improving research relevance, design, and recruitment while also challenging researcher bias. We will also explore the specific strategies used to engage older adults as research advisors as well as co-researchers and co-writers when analyzing this model for older adult engagement across higher education.

Abstract citation ID: igaf122.138

### AGING AND INNOVATION: THE ROLE OF OLDER ADULTS IN RESEARCH SUCCESS

Jodi Waterhouse[1], Cari Levy[2], and Kathryn Nearing[3], *1. CU Anschutz Medical Campus, Aurora, Colorado, United States, 2. University of Colorado, Aurora, Colorado, United States, 3. University of Colorado Anschutz Medical Campus, Denver, Colorado, United States*

At CU Anschutz Medical Campus, older adults play a vital role in research. The Older Adult Research Specialists (OARS) program is a 14-week training initiative that empowers community-dwelling older adults with essential skills in healthcare navigation, communication, and research fundamentals. The first seven weeks focus on community health worker training, while the next phase dives into peer-to-peer recruitment, informed consent, and community engagement—ensuring older adults can effectively connect their peers with critical clinical trials. Beyond training, OARS participants serve on research teams, hold paid positions, and lead CU Anschutz Research Roadshows—immersive, educational events that bring research opportunities to urban, rural, and underserved communities across Colorado. These roadshows allow community members to learn about and engage in research without leaving their hometowns. Another innovative initiative, Living Labs in Senior Congregate Care Communities, transforms everyday spaces—dining rooms, gyms, and activity rooms—into active research hubs. Guided by the Academic Research Advisory Council (ARAC), residents help vet studies to align with their healthcare priorities. This model fosters meaningful collaboration between researchers and older adults, ensuring studies are both relevant and impactful. By embedding older adults in the research process, CU Anschutz bridges the gap between academia and community, enhancing research accessibility and inclusivity while empowering older adults as key contributors to scientific discovery.

Abstract citation ID: igaf122.139

### INTERGENERATIONAL CLASSROOM EXPERIENCES: THE IMPORTANCE OF OLDER LEARNERS', STUDENTS', AND FACULTY'S INSIGHTS

Chaya Koren[1], Anna Zisberg[1], Orit Hirsch-Matsioulas[1], and Sigal Naim[2], *1. University of Haifa, Haifa, HaZafon, Israel, 2. The Center for Research and Study of Aging, University of Haifa, Haifa, HaZafon, Israel*

Age-inclusive educational practices need research that involves older learners' perspectives to provide insights about issues impacting intergenerational classroom effectiveness. Accordingly, this study aimed to understand learning/teaching experiences in university intergenerational classroom courses considering older learners' (aged 65+), along with students' and faculty's perspectives. The sample included 11 courses open to older learners. Some with 80 to 150 participants, others with up to 20 participants. Some included more older learners than students. Participants included older learners (n = 11), students (n = 11), and faculty (n = 7). Semi-structured qualitative interviews (n = 29) were audio recorded and transcribed verbatim for data analysis using thematic analysis. Findings highlight the importance of including older learners' perspectives along with students' and faculty's indicating key differences in learning motivations. Older learners sought enrichment, enjoyment, and meaningful engagement without academic obligations, whereas students focused on degree completion and professional development. These contrasting motivations shaped classroom dynamics, influencing perceptions of older learners as either contributing to discussions or disrupting structured learning. Social interactions also played a meaningful role. Older learners form peer connections while perceiving students as part of the campus environment rather than social partners. While both age groups value social engagement, generational differences led to limited cross-generational exchanges mutually shared by both generations. Coinciding with Bronfenbrenner's ecosystems perspective, findings highlight the importance of including the perspectives of all three stakeholder types for receiving a broader overall perspective on intergenerational classroom experiences. Recommendations include coordinating expectations, providing a teachers' toolbox, receiving older learners' feedback, and establishing a suitable older learners-student ratio per course.

### SESSION 1135 (PAPER)

### BUILDING COMMUNITY IN COMMUNITY WITH THE COMMUNITY

Abstract citation ID: igaf122.140

### BUILDING THE FUTURE OF AGING: INVESTIGATING STRATEGIES AND INNOVATIONS IN MASTER PLANS ON AGING

Lydia Manning[1], and Carey Peerman[2], *1. Scripps Gerontology Center, Oxford, Ohio, United States, 2. Radford University, Radford, Virginia, United States*

Master Plans on Aging (MPAs) are essential frameworks designed to address the challenges that older adults face and ensure that states have a comprehensive strategy for aging-related policies, services, and programs. This presentation explores the development and implementation of MPAs across five states: Colorado, California, Texas, Massachusetts, and Minnesota, and highlights the frameworks used, the roles of political leadership, and the multi-sector collaboration required to create such plans. This qualitative, exploratory study, with 25 stakeholder interviews, utilizes a grounded theory approach to investigate the strategies and innovations that have shaped the development and implementation of Master Plans on Aging (MPAs) in the five U.S. states with plans. The analysis reveals several commonalities and differences across states, as well as critical insights into the effectiveness of these plans. Furthermore, findings provide insight into the planning and stakeholder engagement processes, integration of these plans into state-level policies, and funding mechanisms that support their implementation. The presentation will discuss key innovations in aging policy, including creating age-friendly communities, affordable housing, and economic security for older adults, as well as successful models for service delivery and evaluation metrics. Participants will gain insights into the collaborative processes that have shaped these plans and learn best practices for overcoming aging, equity, and inclusion barriers. This session will provide a comprehensive overview of how MPAs can effectively improve the quality of life for older adults and offer a roadmap for developing similar initiatives across the U.S. and beyond.

Abstract citation ID: igaf122.141

### COMMUNITY PARTNERSHIPS IN DEVELOPING MASTER PLANS FOR AGING: LESSONS LEARNED FROM SANTA PAULA, CA

Ronald Berkowsky, *California State University Channel Islands, Camarillo, California, United States*

Master plans for aging (MPAs, or multisector plans for aging) provide frameworks to coordinate services (typically at the state-level) using evidence-based data to address the needs of older residents and their caregivers. Stakeholders in elder services recognize the value of multisector collaborations, as previous research has shown that these partnerships can help reduce health care utilization and cost among older residents. While several US states have implemented an MPA (e.g., California) or are currently developing an MPA, there is a growing need to identify the aging needs of local populations and to draft more county- or city-level MPAs to address these specific needs given local constraints (e.g., policies, resources). In this presentation,

I describe the process of drafting an MPA for the city of Santa Paula, a predominantly Hispanic/Latinx city located in Ventura County, California. This community-engaged project marked a collaborative effort between the city, a faculty and students at a local 4-year university (i.e., California State University Channel Islands), stakeholder groups representing the particular needs of aging Santa Paula residents (e.g., the Santa Paula Senior Advisory Committee), and community-members. I describe the roles and responsibilities of those involved in this project, the methods (e.g., focus groups with local elders, quantitative surveys with city residents) and process of data collection, the results gleaned from the data, and the translation of these results to actionable insights which would form the basis of the Santa Paula MPA. Challenges and lessons learned will also be addressed throughout.

Abstract citation ID: igaf122.142

### COMMUNITY-BASED PARTICIPATORY RESEARCH IN THE LENS OF SOCIAL CAPITAL: A MENTAL HEALTH LITERACY PROMOTION PROJECT

Jessie Ho-Yin Yau, Wing-Yin For, Ken Tsz-Kin Ho, Stella Lai-Kuen Wong, Cheryl Hiu-Kwan Chui, and Terry Lum, *The University of Hong Kong, Hong Kong, China (People's Republic)*

Community-based participatory research (CBPR) is an approach that integrates community knowledge into evidence-based practice, enhancing intervention validity. While existing CBPR models were developed with a top-down approach in the West, their applicability in East Asian contexts and validity may be limited. CBPR principles emphasize resource and information sharing, yet current models overlook the synergy between network and resource sharing that influences collective actions and outcomes. This study aimed to address these gaps by co-creating a conceptual framework with stakeholders to understand CBPR's operational mechanism from a social capital perspective. The research focused on a mental health literacy promotion project in an East Asian community, involving nine focus group discussions with older adults (n = 21) and social workers (n = 15), followed by co-analyzing and co-interpreting the data and findings with community stakeholders. Our data suggested six critical elements, which can be broadly grouped under the three primary forms of social capital, underlie the process of CBPR: emotional meaningfulness within a group as a foundation for sustainability, characterized by collective harmony, shared values and norms, and role models (bonding social capital); co-learning and co-production of resources across the groups, stimulated by the knowledge exchange environment (bridging social capital); and breaking hierarchical gaps and fostering equal partnership, induced by resource contributions from each stakeholder and the existence of super connectors (linking social capital). These elements promote network, skills, knowledge, and resource sharing among various stakeholders for collective actions for sustainability, community capacity building, and the common good.

Abstract citation ID: igaf122.143

### DEMENTIA-FRIENDLY COMMUNITIES: THE ROLE OF BUILT ENVIRONMENT AND IMPLEMENTATION CHALLENGES IN THE U.S.

Xi Chen, Chanam Lee, and Wenjin Wang, *Texas A&M University, College Station, Texas, United States*

The growing aging population has intensified public health concerns, particularly regarding dementia. Dementia-Friendly Communities (DFC) have gained global attention as a response to the increasing number of people living with dementia (PLWD) and the associated societal care burden. However, empirical research to inform policy briefs, design guidelines, and implementation strategies remains limited. This study explores expert perspectives on key neighborhood built environment features that support aging in place for PLWD and identifies challenges in implementing DFC in the U.S. A nationwide online expert survey was conducted from January to March 2023 and a total of 51 experts specializing in environment and aging research or practice provided complete responses and were included in data analysis. Quantitative ratings indicate that "land use & accessibility" and "safety" are the two most critical neighborhood-scale built environment domains for DFC design. Open-ended responses on environmental features highlight five key themes: (1) safety first, (2) clear space for better memory support, (3) balance between easy access and stimulation, (4) comfort by design with environmental context in mind, and (5) tailored design for different stages of dementia. Experts also identified financial challenges and knowledge barriers toward dementia or DFC as two major barriers to implementation in the U.S. By identifying these design priorities and implementation challenges, this study provides valuable insights to help policymakers, researchers, and design professionals better understand critical considerations for DFC design and facilitate the development of more feasible and effective solutions to promote dementia-friendly environments and support healthy aging in place.

Abstract citation ID: igaf122.144

### ESTABLISHING A COMMUNITY COUNCIL TO ENHANCE ARAB AMERICAN ENGAGEMENT IN DEMENTIA RESEARCH

Sara Masoud[1], Howaida Werfelli[2], Caitlin Sangdahl[1], Azizeh Sowan[1], Amani Charaf[1], and Fayron Epps[1], *1. UT Health San Antonio, San Antonio, Texas, United States, 2. Salvere Public Health Consultants, San Antonio, Texas, United States*

Arab Americans face a disproportionately high risk for Alzheimer's disease and related dementias (ADRD) but remain severely underrepresented in dementia research. Systemic barriers—including race/ethnicity misclassification, institutional exclusion, and structural discrimination—contribute to this underrepresentation, limiting the development of culturally responsive interventions. An engaged approach is necessary to ensure that research aligns with the experiences and priorities of communities most impacted by dementia. Anchored in emergent engagement science frameworks and the principles of Community Based Participatory Research (CBPR), this presentation outlines steps and strategies used to establish and build capacity for co-conducting research among community members and researchers. A Community Council (CC) (N = 9) for Arab Dementia research was established. Strategies for establishing the CC included: 1) organizing a community convening to initiate action around ADRD research disparities; 2) monthly meetings and working sessions; and 3) capacity building activities (e.g., discussing basics of research, co-planning convenings outside of the research study deliverables) to strengthen the potential for a successful research partnership. Within each strategy, approaches to overcome challenges (e.g., recruiting male CC members, balancing competing priorities) encountered will be discussed. Research conducted through this community-academic partnership will contribute to building evidence around innovative engagement science frameworks, raising awareness about the research needs and experiences of the Arab American community, and toward developing and testing models of engagement scalable to other populations.

Abstract citation ID: igaf122.145

**PLACE INCLUSION FOR OLDER LGBT+ PEOPLE AND OLDER PEOPLE WITH LEARNING DISABILITIES: UK ORGANISATIONAL PERSPECTIVES**

Judith Sixsmith[1], Mei Lan Fang[2], Meiko Makita[1], Sallie Johnson[3], Laura Roe[1], and Romel Yanez[4], *1. University of Dundee, Dundee, Scotland, United Kingdom, 2. Simon Fraser University, Vancouver, British Columbia, Canada, 3. University of Hertfordshire, Hatfield, England, United Kingdom, 4. Federal University of Ceara, Fortaleza, Ceara, Brazil*

Older LGBT+ individuals and those with Learning Disabilities (LD) have lived through eras of systemic stigma, exclusion, and social devaluation. The 46-month IncludeAge project (www.IncludeAge), funded by the UK Economic and Social Research Council, aims to examine the experiences of inclusion and exclusion among these groups and identify strategies to create more inclusive communities. By exploring both lived experiences and organizational perspectives, the project seeks to develop co-created solutions that enhance inclusion in place, space, policy, and practice. Eighty semi-structured interviews were conducted with advocacy and support groups for older LGBT+ and LD communities, age-related organizations, and third spaces such as churches, shops, hospitality, and welfare services. A thematic analysis of barriers and facilitators of inclusion across organizations in Scotland, England, and Wales was undertaken. Four key themes emerged as barriers to inclusion: (1) historical distrust and past negative experiences, (2) organizational limitations and financial constraints, (3) passive inclusion and limited visibility, and (4) digital (in)accessibility. Five themes were identified as facilitators: (1) communication and social networks, (2) external oversight mechanisms, (3) inclusive symbols and language, (4) stakeholder involvement, and (5) intergenerational initiatives. Findings highlight systemic, socio-structural, and psychological challenges in fostering inclusive environments. Despite these barriers, facilitators indicate that meaningful change is achievable. Strengthening organizational strategies and fostering sustainable institutional change are critical to building more equitable communities. Addressing these issues requires targeted policy interventions, cross-sector collaboration, and long-term investment in inclusive practices to ensure older LGBT+ people and those with LD are fully recognized and supported in society.

### SESSION 1140 (SYMPOSIUM)

Abstract citation ID: igaf122.146

**SOCIAL CONNECTIONS AND COMMUNITY CONTEXTS: SHAPING END-OF-LIFE PLANS AMONG U.S. OLDER ADULTS**

Chair: Deborah Carr
Co-Chair: Lucie Kalousova
Discussant: Clifford Ross

Advance care planning (ACP) is an important step toward ensuring individuals' end-of-life (EOL) wishes are honored. Yet, many U.S. older adults do not engage in these important practices. This symposium presents four original studies that explore how relationship histories, family dynamics, social connections, and neighborhood environments affect older adults' ACP behaviors, drawing on recent data from the Health and Retirement Study (HRS). Carr and Choi examine how complex romantic partnership histories and parental status affect four subtypes of ACP (formal advance directives only. informal discussions only, both formal and informal, or neither), revealing that recently widowed individuals are the most likely to engage in ACP, whereas childless persons and those who have never married are notably less involved. Zhang and Kalousová delve into the role of complex relationship histories, demonstrating that marital disruptions and remarriage significantly impact ACP decisions, particularly around establishing living wills and assigning durable power of attorney for healthcare (DPAHC). Lian and Kalousová explore how friendship networks influence ACP, uncovering important gender differences and emphasizing the critical role of friend-based emotional support for never-married older men. Lastly, Ding, Lou, and Carr highlight how neighborhood characteristics, such as cohesion and disorder, influence ACP participation among older adults living alone, particularly enhancing ACP engagement for women living in cohesive neighborhoods. Collectively, these studies provide valuable insights into the social and relational influences on ACP, informing strategies and policies aimed at improving end-of-life preparedness across diverse groups of older adults.

Abstract citation ID: igaf122.147

**ADVANCED CARE PLANNING OF UNMARRIED AND CHILDLESS OLDER U.S. ADULTS: ARE THEY LESS PREPARED FOR THE END-OF-LIFE?**

Deborah Carr[1], and Shinae Choi[2], *1. Boston University, Boston, Massachusetts, United States, 2. The University of Alabama, Tuscaloosa, Alabama, United States*

Planning for end-of-life health care is critical for dying persons and their families they leave behind. Advance care planning (ACP), which encompasses an advance directive and informal discussions, is an important step toward the receipt of end-of-life medical care that aligns with one's wishes. ACP is associated with better psychological adjustment and family functioning among the decedent's survivors. However, it is unclear how those growing old outside of traditional marriage or who have complex childbearing histories prepare for end-of-life. We use data from the 2016-2022 waves of the Health and Retirement Study (N = 5,295) to explore how complex romantic partnership histories and parental status affect four subtypes of ACP (advance directives only [formal], informal discussions only [informal], both, or neither). We examine the extent to which these patterns differ by gender, after adjusting for socioeconomic, health, and psychosocial factors that have been found elsewhere to affect ACP. Multivariable analyses show that divorced and widowed persons, and especially recently widowed persons, are most likely to do formal ACP and both formal/informal ACP, with the magnitude of these patterns differing slightly by gender. Never-married persons are least likely to do ACP. Childless persons have especially low rates of ACP. Parents are more likely than childless persons to have informal end-of-life discussions only. Our results reveal the complex ways that family statuses affect end-of-life preparations and can inform policies and professional strategies to encourage these practices.

Abstract citation ID: igaf122.148

### THE ROLE OF RELATIONSHIP BIOGRAPHY IN ADVANCE CARE PLANNING AMONG OLDER ADULTS

Zhe Zhang, and Lucie Kalousova, *Vanderbilt University, Nashville, Tennessee, United States*

As individuals approach end-of-life, advance care planning (ACP) becomes a critical aspect of personal and familial well-being. Prior research on ACP focuses on static marital status—such as whether an individual is currently married, divorced, or widowed—as predictors of ACP behaviors. However, this approach overlooks the cumulative and dynamic nature of relationship histories, leaving a critical gap in understanding how one's full relationship trajectory shapes end-of-life decision-making. Guided by a gendered life course perspective, this study addresses this gap by examining how relationship trajectories shape three ACP dimensions: living wills, durable power of attorney for healthcare (DPAHC), and end-of-life discussions. Using data from the 2020 Health and Retirement Study (HRS), a nationally representative longitudinal survey of older Americans, we trace individuals' relationship histories and assess their associations with ACP behaviors, with attention to gender differences. Multivariable logistic regression results show that, compared to those continuously married, older adults who are partnered, widowed, or unmarried after multiple disruptions are more likely to have a living will. Similarly, the divorced, widowed, and those unmarried after two or more disruptions have higher odds of naming a DPAHC. For end-of-life discussions, only one group significantly differs from the continuously married: individuals remarried after two or more disruptions have higher odds of having these conversations. We detect modest gender differences in these patterns. This study advances research on the social determinants of ACP and life course perspectives by demonstrating that relationship histories play a crucial role in shaping individuals' preparedness for life's final chapter.

Abstract citation ID: igaf122.149

### FRIENDSHIP AND END-OF-LIFE DISCUSSIONS AMONG OLDER ADULTS: MODERATING EFFECTS OF MARITAL STATUS AND GENDER

Zheng (Noah) Lian, and Lucie Kalousova, *Vanderbilt University, Nashville, Tennessee, United States*

Sharing healthcare preferences with others reduces the likelihood of unwanted treatments at the end-of-life (EOL), a key indicator of a "good death." Spouses and other romantic partners are key facilitators of EOL planning decision-making, but little is known about the role of broader social networks, which may be particularly important for people who are single in the final years of their lives. This study investigates the association between emotional support from friends, friendship network size, and informal and formal EOL planning using data from the 2018 and 2020 waves of the Health and Retirement Study (HRS)—a nationally representative sample of U.S. older adults (N = 4,728). We use multivariable logistic regression models and evaluate how the associations vary by gender and marital status. Our findings reveal that emotional support from friends is differentially related to older adults' engagement in EOL conversations. Among married, partnered, or previously married individuals, support from friends has no statistically significant effect on the likelihood of EOL discussion. We find evidence of moderation by marital status and gender. Older men with low emotional support are 68 percent less likely to engage in EOL discussions compared to those with high levels of support. Never married older men with strong emotional support from friends are as likely to have had informal EOL conversations as their married/partnered counterparts. These findings contribute to the literature by highlighting the critical role of high-quality friendships in EOL preparations for never married men.

Abstract citation ID: igaf122.150

### LIVING ALONE AND ADVANCE CARE PLANNING: DO NEIGHBORHOOD COHESION AND DISORDER MATTER?

Kedong Ding[1], Yifan Lou[2], and Deborah Carr[3], *1. Case Western Reserve University, Cleveland, Ohio, United States, 2. Virginia Commonwealth University, Richmond, Virginia, United States, 3. Boston University, Boston, Massachusetts, United States*

More than16 million U.S. adults ages 65+ live alone. Given the centrality of coresidential family for older adults' end-of-life preparation, it is unclear how those who live alone engage in advance care planning (ACP). Neighborhood characteristics like cohesion may enhance ACP, whereas disorder may impede it. Thus, our goal is to examine the association between living alone and three dimensions of ACP, and the extent to which these associations are moderated by self-reported neighborhood social cohesion (e.g., friendliness of people, availability of help when needed) and physical disorder (e.g., vandalism/graffiti, feeling safe alone after dark). We used data from 9,296 participants aged 65+ in the Health and Retirement Study (HRS) 2016 and 2018. ACP outcomes include living will, durable power of attorney for healthcare (DPAHC), and informal discussion. Multivariable analyses were adjusted family structure (marital status and number of children), sociodemographic, and health covariates. We find that living alone significantly increased the likelihood of having a living will, DPAHC, and discussion by 38%, 36%, and 30%, respectively. Living in neighborhoods with the highest cohesion (top 25-percentile) and lowest disorder (bottom 25-percentile) significantly increased the likelihood of formal ACP (i.e., living will and DPAHC), but not for informal discussions. Additionally, living in a socially cohesive neighborhood significantly increased the positive effect of living alone on having DPAHC (OR = 1.42). Gender-specific analyses showed that these moderating effects were particularly pronounced among women. We discussed the policy and practice implications of the role of neighborhood environment in living alone and ACP.

### SESSION 1145 (SYMPOSIUM)

Abstract citation ID: igaf122.151

### BRIDGING GAPS IN NURSING HOME CARE: INNOVATIONS IN PALLIATIVE CARE AND ADVANCE CARE PLANNING

Chair: Joan Carpenter
Co-Chair: Kathleen Unroe
Discussant: Cari Levy

The 2022 National Academies report The National Imperative to Improve Nursing Home Quality: Honoring Our Commitment to Residents, Families, and Staff and expert consensus panels have identified significant gaps in the delivery of high-quality care for people living with serious illness in nursing homes (NHs). Recommendations to address these gaps include improving NH palliative care (PC) practices and developing strategies for better integration of advance care planning (ACP) into routine care. This symposium highlights research in NHs studying PC and ACP. The first presentation describes the PC knowledge, attitudes, and practices among frontline NH staff (N = 242) in 16 facilities using a validated survey measuring family communication, provider communication, planning/intervention, and bereavement, and patient vignettes. The second presentation illustrates findings of a large qualitative study using a grounded theory approach to develop an empirically derived theoretical model describing how PC is effectively implemented in NHs using interview data from PC and NH staff (N = 33). The third presentation reviews findings from a Delphi study conducted with 18 clinical experts to develop a tool measuring quality of NH-based ACP implementation to guide ACP policy and interventions to facilitate standardized procedures and enhance NH buy-in. The fourth presentation reports outcomes of an embedded pragmatic cluster randomized clinical trial designed to evaluate the implementation of an ACP Specialist Program in 132 nursing homes. The discussant, Cari Levy—a PC and NH expert—will discuss the four presentations implications for advancing PC and ACP practices in the NH setting.

Abstract citation ID: igaf122.152

### PALLIATIVE CARE KNOWLEDGE AND ATTITUDES OF NURSING HOME STAFF: BASELINE DATA FROM THE UPLIFT TRIAL

Kathleen Unroe[1], John Cagle[2], Alex Floyd[3], Wanzhu Tu[4], and Timothy Stump[5], *1. Indiana University School of Medicine, Indianapolis, Indiana, United States, 2. University of Maryland, Batimore, Maryland, United States, 3. Regenstrief Institute, Indianapolis, Indiana, United States, 4. Indiana University Indianapolis, Indianapolis, Indiana, United States, 5. Indiana University Bloomington, Bloomington, Indiana, United States*

Palliative care and hospice are known to improve quality of care within nursing homes. However, such care is underutilized in long term care settings. One known barrier is staff knowledge and attitudes. To evaluate the palliative care knowledge, attitudes, and practices among nursing home staff, we surveyed (n = 242) frontline nursing home staff in 16 facilities located in two states, n = 8 in Maryland and n = 8 in Indiana, using a validated survey instrument. The survey contains domains including family communication, provider communication, planning/intervention, and bereavement, and utilizes patient vignettes. Participating nursing home communities were eligible if they: had >40 long stay residents with cognitive impairment and did not have an active in-house palliative care program. Surveys were collected in person by research staff. Survey completion varied across facilities (from n = 6 to n = 23). Participating nursing home staff had an average age of 43 years old, 92% female, and 49% were Black/African American. The most common job categories for nursing home staff completing the survey were certified nursing assistants (44%), followed by nurses including LPNs (20%, and RNs (8%). With higher scores indicating greater knowledge/better attitudes (possible range 1-4 on each item), we found that participating staff had: M = 3.1 (SD = 0.6) on the Family Communication subscale; M = 2.9 (SD = 0.8) on the Provider Coordination subscale; M = 2.7 (SD = 0.9) on the Planning/Intervention subscale; and M = 2.4 (SD = 0.6) on the Bereavement subscale. Overall, palliative care knowledge scores for physical symptoms were higher than psychological symptoms, which were both higher than knowledge of end of life.

Abstract citation ID: igaf122.153

### NAVIGATING SERIOUS ILLNESS: THE ROLE OF PALLIATIVE CARE CONSULTATIONS IN NURSING HOMES

Elisha Oduro[1], Ruth Palan Lopez[2], Kathleen Unroe[3], Alexandra Mora[4], Moses Akwobugi[4], and Joan Carpenter[5], *1. University of Maryland Baltimore, Baltimore, Maryland, United States, 2. Massachusetts General Hospital Institute of Health Professions, Boston, Massachusetts, United States, 3. Indiana University School of Medicine, Indianapolis, Indiana, United States, 4. University of Maryland Baltimore, Baltimore, Maryland, United States, 5. University of Maryland School of Nursing, Baltimore, Maryland, United States*

Palliative care (PC) consultations in nursing homes (NHs) are recognized for enhancing the quality of life for residents living with serious illness. Despite their benefits, the accessibility of PC consultations remains variable and inconsistent across NHs. This study aimed to develop a framework to explain PC consultation implementation and utilization in NHs from the perspective of PC and NH key partners. A constructivist grounded theory approach was employed, using semi-structured and group interviews with PC and NH administrators, nursing directors, social workers, nurses, primary care providers, and PC specialist nurse practitioners. Participants (N = 33) were purposively sampled from three PC agencies with relationships in 14 NHs to capture variability in PC implementation and utilization. Data were analyzed through open, process, and theoretical coding to develop a theoretical framework. The central issue influencing PC consultations in NHs was the need to navigate resident and family uncertainty and complexity in serious illness care, especially for residents living with dementia. NH staff used PC consultations to guide residents and families during unpredictable disease trajectories, mediate family and institutional expectations by "bridging the gap", and reframe PC as integrated support rather than only for end-of-life care. PC consultations facilitated better sensemaking, improved communication, and led to more effective care planning. PC consultations serve as strategic tools for NH staff to manage the complexities of serious illness care. Understanding and supporting the practical applications of PC consultations within NHs can lead to more effective integration and utilization of these services, ultimately enhancing resident care.

Abstract citation ID: igaf122.154

### DEVELOPING AN ASSESSMENT TOOL OF NURSING HOME-BASED ADVANCE CARE PLANNING IMPLEMENTATION QUALITY

Peiyuan Zhang[1], Joan Davitt[1], Nancy Kusmaul[1], Paul Sacco[2], Kathleen Unroe[3], Kaipeng Wang[4], Fei Sun[5], and John Cagle[6], *1. University of Maryland, Baltimore County, Baltimore, Maryland, United States, 2. University of Maryland, Baltimore, Maryland, United States, 3. Indiana University School of Medicine, Indianapolis, Indiana, United States, 4. University of Denver, Denver, Colorado, United States, 5. Michigan State University, East Lansing, Michigan, United States, 6. University of Maryland, Batimore, Maryland, United States*

Advance care planning (ACP), a key mechanism to improve the quality of end-of-life care, has significant relevance in nursing homes (NHs) where nearly 30% of older Americans spend their last days, but the quality of ACP practices vary. Thus, this study aimed to develop a tool measuring quality of NH-based ACP implementation. Firstly, we conducted a systematic review of quality indicators and factors influencing NH-based ACP

implementation. Guided by Donabedian's model of quality of care, we conducted deductive thematic analysis of the selected 19 studies and identified 13 elements for successful ACP implementation. They were categorized into two major dimensions: (1) nursing home structural support for ACP and (2) standardized implementation procedures and converted into binary questions to form the drafted tool. Then a Delphi study was conducted with 18 clinical experts with an average of 23 years working with NH residents to revise the tool. Almost half of participants conducted more than 10 ACP conversations in a typical month. The first round involved 5 focus groups on their opinions about the drafted tool, resulting in the modified tool including 19 questions. It was presented back to panelists in Delphi surveys. Response options were 1=endorse, 2=would endorse with modification, and 3=not endorse. The consensus was reached after two rounds of rating (interquartile range=0, median=1, percentage of agreement>80%). To our knowledge, this is first tool examining ACP implementation quality at the NH level, which has the potential to guide ACP policy and interventions to facilitate standardized procedures and enhance NH buy-in.

Abstract citation ID: igaf122.155

### OUTCOMES OF A PRAGMATIC RANDOMIZED CLINICAL TRIAL OF AN ADVANCE CARE PLANNING PROGRAM IN NURSING HOMES

Susan Hickman[1], Timothy Stump[2], Abigail Evans[3], Wanzhu Tu[4], and Kathleen Unroe[5], *1. Indiana University, Indianapolis, Indiana, United States, 2. Indiana University Bloomington, Bloomington, Indiana, United States, 3. Regenstrief Institute, Indianapolis, Indiana, United States, 4. Indiana University, Indianapolis, Indiana, United States, 5. Indiana University School of Medicine, Indianapolis, Indiana, United States*

Advance care planning (ACP) helps prepare residents and family caregivers for communication and decision-making in advance of a crisis and helps ensure that preferences are known, documented, and honored. This is particularly important in persons with Alzheimer's disease or related dementias (ADRD), given known complications that necessitate decisions about hospitalization, resuscitation, feeding, and infection management. We conducted an embedded pragmatic cluster randomized clinical trial to evaluate the implementation of the ACP Specialist Program in 132 nursing homes between September 2021 – August 2022. Existing nursing home staff members were trained to enhance care through improved ACP procedures, standardized online staff education on ACP, and systematic ACP facilitation. Outcomes were assessed using Medicare claims and Minimum Data Set 3.0 assessments, consistent with the pragmatic design. Across the intervention (n = 64) and control (n = 64) buildings, 37% (14,498/39,114) of residents were identified as having ADRD per documented diagnosis of Alzheimer's or dementia and/or a Cognitive Function Scale score > 2 (moderate or severe cognitive impairment). In the intervention buildings, 77 ACP Specialists engaged in ACP conversations for 21% (N = 1,470) of residents with ADRD. In a mixed effects Poisson regression analysis adjusted for covariates, there were no significant differences between intervention and control sites in our primary outcome of hospital transfers (admissions and emergency department visits) over the 12-month study period (Incident Rate Ratio for intervention versus control = 0.91 (0.79, 1.05), p = ns). Implementation challenges and variable rates of implementation across intervention buildings likely contributed to the non-significant outcome.

### SESSION 1150 (PAPER)

### LONG-TERM SERVICES AND SUPPORTS: QUALITY OF CARE

Abstract citation ID: igaf122.156

### CARE OR PROFIT? UNPACKING PRIVATE EQUITY'S IMPACT ON U.S. NURSING HOMES – A SYSTEMATIC REVIEW

Gregory Orewa[1], Aizhan Karabukayeva[2], Rohit Pradhan[3], Itopa Jimoh[4], and Robert Weech-Maldonado[4], *1. The University of Texas at San Antonio, San Antonio, Texas, United States, 2. The University of Oklahoma, Norman, Oklahoma, United States, 3. Texas State University, San Marcos, Texas, United States, 4. The University of Alabama at Birmingham, Birmingham, Alabama, United States*

In the past two decades, private equity (PE) acquisitions in nursing homes have surged, driven by financial gain and market consolidation. The increasing presence of PE firms in the U.S. nursing home industry has raised concerns about its impact on care quality and financial performance. This systematic review examined the impact of PE ownership on U.S. nursing homes, focusing on care quality and financial performance using agency theory and the structure-process-outcome framework. Adhering to PRISMA guidelines, a systematic search across five databases, including PubMed, Web of Science, and ABI/Inform. The initial search yielded 343 articles. After removing duplicates, screening for title, abstract, thorough evaluation, and full-text review, 12 studies published between 2000 and 2024 met the inclusion criteria and were included in the study. Data were extracted and synthesized across quality and financial dimensions. PE ownership was linked to higher deficiencies, increased hospitalizations, and higher mortality, though some care processes improved. PE-owned facilities showed initial financial gains but faced long-term financial challenges due to high debt and lease obligations. Staffing changes suggested an increased reliance on lower-skilled staff. PE strategies may prioritize short-term profitability, at the expense of care quality. Cost-cutting measures can undermine staffing and patient outcomes, highlighting the need for stricter staffing regulations, financial transparency, and value-based reimbursement to ensure sustainable, high-quality care. Effective policy interventions are needed to safeguard residents of nursing homes and maintain equitable care standards.

Abstract citation ID: igaf122.157

### CHATO NATIONAL TRIAL: USING LEADERSHIP CLOSEOUT MEETINGS TO IMPROVE ONLINE EDUCATION IN NURSING HOMES

Carissa Coleman[1], Maria Jaros[1], Maria Roche-Dean[2], Amy Berkley[3], Frances Yang[4], Clarissa Shaw[5], Kristine Williams[6], and Maria Hein[7], *1. University of Kansas Medical Center, Kansas City, Kansas, United States, 2. Kirkhof College of Nursing, Grand rapids, Michigan, United States, 3. University of Kansas School of Nursing, Kansas City, Kansas, United States, 4. University of Kansas, University of Kansas Medical Center, Kansas, United States, 5. University of Iowa College of Nursing, Iowa City, Iowa, United States, 6. University of Kansas, Kansas City, Kansas, United States, 7. University of Iowa, Iowa City, Iowa, United States*

The Changing Talk: Online (CHATO) intervention was developed to increase staff access to person-centered dementia care communication education in a national nursing home sample. A qualitative review of meeting notes taken from the closeout meetings (N = 75) with nursing home implementation leads was performed to determine: 1) acceptability of the modules, and 2) facilitators and barriers associated implementation, to improve uptake across nursing homes. The open-ended interviews completed over the 3 year study provided clinical leaders the opportunity to give feedback on the educational content, technical aspects of the modules, implementation strategies, and what leaders liked most and least about the program. CHATO training facilitators identified: (93.3%) of implementation leads reported the content was useful, followed by the videos illustrating behavioral examples (12.0%), and the training was useful for onboarding staff (9.3%). CHATO training barriers identified: training was too long (48.0%), login issues (30.7%), navigation (29.3%), "good refresher"(18.7%), not cell phone compatible (17.3%), and low computer literacy (16.0%). Implementation facilitators identified were site specific, tailored strategies (32.0%), rewards (22.7%), onsite discussions (17.3%), mandatory training approach (14.7%), and weekly reminders (12.0%). Barriers to implementation were lack of interest (18.7%), training was too time consuming (14.7%), too many other online trainings (10.7%), and staff were too busy (10.7%). These findings will provide input for the redesign of the CHATO modules and implementation plan for the next iteration of the Changing Talk education intervention.

Abstract citation ID: igaf122.158

### IDENTIFYING DISTINCT IMPLEMENTATION PATTERNS FOR ARTIFACTS OF CULTURE CHANGE 2.0 PRACTICES IN NURSING HOMES

Frances Yang[1], Clarissa Shaw[2], Carissa Coleman[3], Yelena Perkhounkova[4], Maria Hein[4], Amy Berkley[5], Maria Roche-Dean[6], and Kristine Williams[7], *1. University of Kansas, University of Kansas Medical Center, Kansas, United States, 2. University of Iowa College of Nursing, Iowa City, Iowa, United States, 3. University of Kansas Medical Center, Kansas City, Kansas, United States, 4. University of Iowa, Iowa City, Iowa, United States, 5. University of Kansas School of Nursing, Kansas City, Kansas, United States, 6. Kirkhof College of Nursing, Grand rapids, Michigan, United States, 7. University of Kansas, Kansas City, Kansas, United States*

The Artifacts of Culture Change 2.0 (ACC) is a self-assessment tool of 134 practices allowing nursing homes to evaluate and benchmark their culture related to person-centered care. Latent profile analysis was conducted in Mplus 8.11 to identify distinct patterns of culture change implementation across 147 nursing homes from 37 states. The analysis identified mean percent implementation ($\bar{x}$%i) across the five ACC practices groupings: 1) resident directed life, 2) being well known, 3) home environment and accommodation of needs and preferences, 4) family and community, and 5) leadership and team engagement. The overall implementation of culture change practices averaged 60.7% (SD + 15.7%, Range=19.8%-99.3%). Statistical criteria supported five implementation pattern profiles (Loglikelihood H0 -2925, AIC 5918, BIC 6020, SABIC 5913). The three most frequent patterns were categorized as 'average implementers' with medium implementation across all practices (n = 46, 31%), 'low implementers' with low scores across all practices (n = 43, 29%), and 'team empowered' with medium-high leadership and team engagement scores (n = 41, 28%). Two smaller groups emerged as 'high implementers' with high scores across all practices (n = 10, 7%) and 'connection challenged' that specifically struggled with family, community, leadership, and team engagement (n = 7, 5%). Resident's being well known was the most highly implemented practice within each profile and family and community engagement was the lowest practice across the profiles. The next phase of this research will investigate whether these distinct implementation profiles are associated with resident and nursing home outcomes collected as part of our ongoing national trial on person-centered communication in nursing home care.

Abstract citation ID: igaf122.159

### MEDICAID PAYMENTS AND FIVE STAR QUALITY RATINGS IN THE NURSING HOME SECTOR: A FACILITY LEVEL ANALYSIS

Edward Miller[1], John Bowblis[2], Elizabeth Simpson[1], and Marc Cohen[1], *1. University of Massachusetts Boston, Boston, Massachusetts, United States, 2. Miami University, Oxford, Ohio, United States*

Medicaid is the primary and largest single payer for nursing home (NH) care in the United States. The Five-Star Quality Rating System rates NHs from 1 (much below average) to 5 (much above average) based on health inspections, quality measures, and staffing. Ample research on factors that contribute to NH quality exists, yet, due to data constraints, the question of how Medicaid payment levels affect the quality at individual facilities remains largely unexplored. This study examined whether Medicaid payment rates and Medicaid payment-to-cost ratios affect NH 5-star ratings. Star ratings were obtained from Care Compare historical archives, Medicaid per diem payment rates were collected directly from the states, and Medicare payment-to-cost ratios were calculated using estimated Medicaid per diem costs from the Medicare Cost Reports. The study population consisted of 9,475 freestanding NHs in 44 states in 2019. Regression analyses accounted for the clustering of NHs within states and adjusted for known covariates that are associated with NH quality (e.g., ownership status, staffing levels) and state-level fixed effects. Results indicate a positive association between Medicaid payment rates and average star ratings. There is no association between Medicaid payment-to-cost ratios and star ratings when the Medicaid payment-to-cost ratio is below 0.90 (i.e., Medicaid covers less than 90% of estimated cost to care for a Medicaid resident), but a positive association once above 0.90. Policymakers need to consider Medicaid payment as part of NH reform, as higher payment levels that cover a greater proportion of costs are associated with higher quality.

### SESSION 1155 (PAPER)

Abstract citation ID: igaf122.160

### AGING TRAJECTORIES

These research presentations provide a research perspective on the trajectories of aging.

Abstract citation ID: igaf122.161

### ACCELERATED BIOLOGICAL AGE IN MULTIPLE ORGANS IS ASSOCIATED WITH INCIDENT DEMENTIA

Fangyu Liu[1], Aditya Surapaneni[2], Jingsha Chen[3], Hamilton Se-Hwee Oh[4], Josef Coresh[2], Morgan Grams[2], and Keenan Walker[5], *1. Rutgers University, New Brunswick, New Jersey, United States, 2. New York University, New York, New York, United States, 3. Johns Hopkins Bloomberg School of Public Health, Baltimore, Maryland, United States, 4. Icahn*

*School of Medicine at Mount Sinai, New York, New York, United States, 5. National Institute on Aging, Baltimore, Maryland, United States*

Plasma-protein based biological age has been previously examined with respect to dementia risk at individual organ level. However, less is known about how accelerated aging in multiple organs may influence dementia risk. We estimated biological age in 11 organs using plasma proteomic data from the Atherosclerosis Risk in Communities (ARIC) study for participants in midlife (N = 11,595, mean age 57±6 years) and in late life (N = 4,287, mean age 75±5 years). When examined individually, we found that accelerated age in artery, brain, heart, immune, intestine, and liver during midlife (HR = 1.02-1.07, q < 0.05) was associated with higher 20-year dementia risk, after adjusting for demographic factors, BMI, eGFR, smoking status, APOE, diabetes, and hypertension. Accelerated age in all organs except kidney during late life (HR = 1.03-1.09, q < 0.05) was individually associated with higher 7-year dementia risk. When we examined the number of organs (0, 1, 2, or 3+) with abnormally old age (>1.5 SD), we observed that increasing number of abnormally old organs exhibited supra-additive associations with dementia risk in midlife and late life, albeit not statistically significant (p > 0.20). This finding suggests that the biological age of multiple systems may have synergistic effects on dementia development. Lastly, compared to participants who only had abnormally old brain age in midlife, participants with abnormally old age in both brain and the heart (HR = 2.82, p = 0.03) or both brain and muscle (HR = 2.54, p = 0.03) had higher 20-year dementia risk, suggesting a link between body and brain health. Our study provides new insights into the interactions of organ systems on dementia.

Abstract citation ID: igaf122.162

### ASSOCIATION BETWEEN LONELINESS SUBTYPES AND BRAIN VOLUME IN OLDER ADULTS WITH HIV

Moka Yoo-Jeong[1], Eran Shorer[2], Raha Dastgheyb[3], Gregory Wade[3], Kylie Alm[4], Arnold Bakker[2], Tracey Wilson[5], and Leah Rubin[4], *1. Northeastern University, Boston, Massachusetts, United States, 2. Johns Hopkins University, Baltimore, Maryland, United States, 3. Johns Hopkins School of Medicine, Baltimore, Maryland, United States, 4. Johns Hopkins University School of Medicine, Baltimore, Maryland, United States, 5. SUNY Downstate Health Sciences University, Brooklyn, New York, United States*

Loneliness is a risk factor for cognitive impairment in older adults, with previous studies noting sex differences in both loneliness and brain volume. However, it remains unclear whether there are sex differences in the types of loneliness (i.e., emotional vs. social loneliness) and their relationship to brain volumes in older adults with HIV. Fifty-eight older people with HIV (PWH) over the age of 50 (overall mean age =62, SD = 6; 59% male; 76% Black) completed the De Jong Gierveld Loneliness Scale and underwent T1-weighted structural magnetic resonance imaging of the brain. Brain volumes from the left and right hemispheres were summed to achieve total brain volumes for total cortical gray matter (GM), frontal, parietal, temporal, occipital, subcortical, and cerebellar GM as well as cerebral and cerebellar white matter (WM). A series of multivariable regression models were conducted to assess the association between loneliness subtypes and brain volumes, controlling for relevant covariates. While loneliness did not differ significantly between sexes, associations between loneliness and brain volumes were only evident in men with HIV. Specifically, greater emotional loneliness was associated with reduced brain volumes in cortical, frontal, parietal, occipital, and subcortical GMs (R's=-0.418 to -4.80, p's=0.008-0.024), and in cerebellar WM (R=-0.431, p = 0.020). Social loneliness was not associated with brain volumes in either sex. Findings suggest that emotional loneliness is associated with reduced brain volumes in older men with HIV, highlighting the need for further investigation into sex-specific patterns of loneliness to better preserve cognitive function.

Abstract citation ID: igaf122.163

### FATIGUE, HEALTH AND MORTALITY FROM AGE 70-100

Irit Stessman-Lande[1], Aliza Rozenberg[2], Jochanan Stessman[3], and Jeremy Jacobs[4], *1. Hadassah University Medical Centers; The Department of Geriatric Rehabilitation, Jerusalem, Yerushalayim, Israel, 2. Hebrew University of Jerusalem, Hadassah Medical Center, Jerusalem, Yerushalayim, Israel, 3. Hebrew University of Jerusalem, Hadassah Medical Center Faculty of Medicine, Jerusalem, Yerushalayim, Israel, 4. Hadassah Hebrew University Hospital, Jerusalem, Yerushalayim, Israel*

Fatigue is common with aging, yet poorly described. We examined the prevalence, health and functional status, and mortality associated with fatigue between ages 70-100. The Jerusalem Longitudinal Study (1990-2021) prospectively followed a community-dwelling cohort born 1920-21, at ages 70, 78, 85, 90, 95 and 100 (n = 604, 1024, 1222, 729, 508, 205 respectively). Comprehensive assessment included fatigue ("Do you feel generally tired?") and mortality data. Logistic regression models (adjusted for gender, education, self-rated health [SRH], Charlson comorbidity index [CCI], ADL, depression, chronic musculoskeletal pain), Kaplan-Meier survival curves, and Cox proportional hazards models (adjusted for gender, education, CCI) were performed. At ages 70, 78, 85, 90, 95, 100 the prevalence of fatigue was 29.4%, 52.3%, 67.9%, 77.2%, 64.9%, and 65.6%. Between age 70-100, fatigue was significantly associated with numerous cross-sectional health and functional measures, and subsequent deterioration for loneliness, SRH, ADL dependence, and physical activity. In contrast, no consistent pattern of association with current or subsequent common medical morbidities was observed. Significantly decreased survival rates were observed among fatigued vs. non-fatigued subjects at ages 70-78, 78-85, 85-90, 90-95: 69.6% vs 80.8% (p = 0.0078), 64.9% vs 73.3% (p = 0.0016), 60.6% vs 76.4% (p < 0.0001), 51.3% vs 65.0% (p = 0.0068) respectively, yet not 95-100: 21.7% vs 18.8% (p = 0.99). After adjusting for known mortality risk factors, fatigue at age 70, 85 and 90 was associated with increased mortality. Fatigue is increasingly common with advancing age, and demonstrates significant negative impact upon health and survival. The poor association with medical morbidities raises questions concerning possible pathways of action.

Abstract citation ID: igaf122.164

### INTRINSIC CAPACITY AND LONGEVITY FROM AGE 70-100

Jeremy Jacobs[1], Irit Stessman-Lande[2], Aliza Rozenberg[3], and Jochanan Stessman[4], *1. Hadassah Hebrew University Hospital, Jerusalem, Yerushalayim, Israel, 2. Hadassah University Medical Centers; The Department of Geriatric Rehabilitation, Jerusalem, Yerushalayim, Israel, 3. Hebrew University of Jerusalem, Hadassah Medical Center, Jerusalem, Yerushalayim, Israel, 4. Hebrew University of Jerusalem, Hadassah Medical Center Faculty of Medicine, Jerusalem, Yerushalayim, Israel*

Intrinsic capacity (IC) has been proposed as a useful marker of healthy aging. This study examines the prevalence of IC between ages 70-100, and its association with survival. The Jerusalem Longitudinal Study (1990-2021) prospectively followed a community-dwelling cohort born 1920-21, at ages 70, 78, 85, 90, 95 and 100 (n=604, 1024, 1222, 729, 508, 205 respectively). Comprehensive assessment was performed, and mortality data collected. Intrinsic capacity (0-100/minimum-maximum) was a composite of Cognition (Mini-Mental State Exam), Psychological (depression using the Basic Symptom Inventory), Vitality (BMI), Locomotion (chair transfer) and Sensory (Snellen and whisper test) domains. Kaplan-Meier survival curves, and Cox Proportional Hazard models were performed. The mean IC at ages 70, 78, 85, 90, 95, and 100 years consistently declined: 75.8, 74.0, 66.7, 64.5, 63.5, 51.8, and was higher in males vs. females: 75.4/72.5, 77.1/74.1, 69.0/64/8, 68.7/61.1, 64.3/62.9, 53.1/51.3. A consistent pattern of age-dependent decline was observed within each separate domain. Using various cut-off points for IC, increased IC was consistently associated with improved survival throughout follow-up, except for age 78-85. Treating IC as a continuous variable, after adjusting for gender, education and Charlson comorbidity index, the mortality Hazard Ratios at age 70, 78, 85, 90, and 95 were 0.98 (95%CI 0.95-1.0, p=0.039), 1.0 (95%CI 0.98-1.02, p=0.91), 0.98 (95%CI 0.97-0.99, p<0.01), 0.98 (95%CI 0.96-0.99, p<0.01), 0.99 (95%CI 0.98-1.0, p=0.19). Using a simple operational definition, our findings describe the consistent trajectory of age-related decline in Intrinsic Capacity between age 70-100, and support its usefulness as a marker for subsequent longevity.

Abstract citation ID: igaf122.165

### SOCIODEMOGRAPHIC DRIVERS OF ACCELERATED BIOLOGICAL AGING IN MEXICO CITY: A PROSPECTIVE ANALYSIS OF 148,110 ADULTS

Carlos Fermin-Martinez[1], Omar Yaxmehen Bello-Chavolla[1], Neftali Eduardo Antonio-Villa[2], Daniel Ramirez-Garcia[1], and Jeronimo Perezalonso Espinosa[1], *1. Instituto Nacional de Geriatria, Mexico City, Distrito Federal, Mexico, 2. National Institute of Cardiology Ignacio Chavez, Mexico City, Distrito Federal, Mexico*

AnthropoAge is a metric designed to estimate biological age using simple anthropometric measures (weight, height, waist circumference), developed and validated in NHANES and in diverse longitudinal aging studies. Here, we sought to characterize AnthropoAge as a predictor of all-cause and cause-specific mortality in the Mexico City Prospective Study (MCPS), and to explore sociodemographic determinants associated with accelerated biological aging, which could influence the aging process in Mexican population. We analyzed 148,110 MCPS participants, including sociodemographic, anthropometric, and health information, with a subset re-evaluated 15 years later. We used c-statistic from Cox models to compare the predictive accuracy for mortality of AnthropoAge and chronological age (CA). Determinants of accelerated aging were assessed with logistic models at baseline, and with mixed-effects linear models after 15 years. AnthropoAge independently increased all-cause mortality risk (HR: 1.059, 95%CI: 1.054-1.064) and showed greater predictive accuracy (c-statistic: 0.752) than CA (0.744), and for major causes of death, including cardiovascular, respiratory, and renal mortality. Accelerated aging was associated at baseline with lower education level (OR: 2.07, 95%CI: 1.98-2.18), higher income (0.82, 0.77-0.86), social development index (0.64, 0.57-0.72), sedentary lifestyle (1.67, 1.62-1.72) and number of comorbidities (1.23, 1.21-1.25). When assessing changes in age acceleration after 15 years, lower education, residence in low-development areas, sedentary lifestyle, and comorbidity burden remain as predictors. Our findings highlight the value of AnthropoAge to characterize age-related outcomes and sociodemographic drivers of the aging process in Mexicans and vulnerable populations. Its application could inform public health strategies and interventions targeting aging-related disparities in middle-income countries.

### SESSION 1160 (PAPER)

### DEMENTIA CARE INNOVATIONS: ADDRESSING INEQUITIES, RELATIONSHIPS, AND LIVED EXPERIENCE

Abstract citation ID: igaf122.166

### CHANGING HEALTHCARE PROFESSIONALS' ATTITUDES ABOUT DEMENTIA WITH LIVED EXPERIENCE STORYTELLING

Edward Cisek[1], Olivia Cohen[1], Anne Kenny[1], Amy Puca[2], Vicki Zimmer[2], and Stephani Shivers[1], *1. CaringKind, New York, New York, United States, 2. Via Evaluation, Buffalo, New York, United States*

People living with dementia (PLWD) can combat stigma by sharing their insights and experiences and giving feedback on services they receive. Via an Administrative on Community Living – Alzheimer's Disease Program Initiatives grant, CaringKind, a dementia-focused community-based organization, launched Dementia Act II (DA2), a program aiming to change attitudes and behaviors of healthcare, research, and industry professionals/students towards PLWD. Four facilitated groups of PLWD crafted scripts about their healthcare experiences over 4-6 sessions and presented in a one-hour grand rounds format. Before and after the presentations, audiences were asked to write three words describing the experience of a PLWD. Post-performance, participants were also asked to respond on a 5-point scale from 0 ("never") to 4 ("every time") regarding their past/anticipated future interactions with PLWD around 3 themes. Results revealed the most common descriptive words changed from "frustration", "memory", "confusion" and "sad" to "hope", "resilient", "creative" and "strong" (n=84). Furthermore, compared to their prior interactions, participants are significantly more likely to speak directly to the PLWD and with empathy and candor (t(62)=-6.43, p<.001); consider the PLWD's involvement in peer support and meaningful engagement (t(63)=-9.60, p<.001); and consider the connectivity of those family/friends providing care to ongoing support, education, and planning services as care needs change (t(64)=-10.45, p<.001). We conclude that by hearing about PLWD experiences in their own words, practicing professionals improve their impression of PLWD and may modify how they interact with, and plan for the support and care of, families affected by dementia.

Abstract citation ID: igaf122.167

### CRIME EXPOSURE, DEMENTIA RISKS, AND RACIAL AND ETHNIC DISPARITIES AMONG MIDDLE-AGED AND OLDER AMERICANS

Junjie He[1], Zhuoer Lin[2], Yan Zhang[3], Yang Wang[4], and Xi Chen[5], *1. Yale University, State College, Pennsylvania, United States, 2. University of Illinois Chicago, Chicago,*

*Illinois, United States, 3. Texas State University, Greenville, North Carolina, United States, 4. University of Wisconsin–Madison, Madison, Wisconsin, United States, 5. Yale University, New Haven, Connecticut, United States*

Exposure to crime, a significant social stressor, may profoundly affect cognitive functioning, particularly among racial and ethnic minorities, yet its long-term relationship with dementia risk remains underexplored. In this study, we examined the association between crime exposure and the incidence of cognitive impairment and dementia, with a focus on racial and ethnic disparities. We analyzed data from the Health and Retirement Study, linking a nationally representative sample of Americans aged 50+ (N = 34,625, or 177,238 person-waves) with local crime rates from the Uniform Crime Reporting system (1995-2016). Crime exposure was measured at the county-year level, accounting for both current and prior year exposures to assess both immediate and cumulative effects. Cognitive outcomes were determined using a validated algorithm based on cognitive test scores and proxy assessments. Using time-varying, weighted Cox proportional hazards models, we adjusted for individual and census track-level factors and accounted for state and racial and ethnic stratifications. Our findings revealed that higher local crime rates were associated with increased risks of cognitive impairment (HR 1.29 per IQR; 95% CI, 1.21-1.38) and dementia (HR 1.23 per IQR; 95% CI, 1.14-1.32), with property crimes showing stronger associations than violent crimes. Non-Hispanic Black and Hispanic individuals experienced significantly greater associations between crime exposure and cognitive outcomes compared to non-Hispanic White individuals. These results underscore the need for targeted interventions to address structural racial and ethnic inequities in neighborhood crime exposures and associated cognitive outcomes, particularly among racial and ethnic minorities.

Abstract citation ID: igaf122.168

### DEMENTIA CARE IS RELATIONAL AND COMMUNAL: LESSONS FROM INDIGENOUS COMMUNITIES IN CANADA AND THE US

Dana Ketcher[1], Elizabeth Weigler[1], Margaret Noun[1], Melissa Blind[1], Nickolas Lambrou[2], Megan Zuelsdorff[3], Carey Gleason[2], and Kristen Jacklin[1], *1. University of Minnesota Medical School, Duluth Campus, Duluth, Minnesota, United States, 2. University of Wisconsin–Madison, Madison, Wisconsin, United States, 3. University of Wisconsin Madison, Madison, Wisconsin, United States*

This paper presents findings from the Indigenous Cultural Understandings of Alzheimer's Disease and Dementia (ICARE) Project. We share our analysis of qualitative data generated in partnership with four Indigenous communities in Canada and the US. We interviewed dementia program and health care administrators (n = 20) and held sequential focus groups with local health care staff/formal caregivers who work with Indigenous older adults (18 sessions, n = 17) between 2018-2021. Topics focused on the community experience of dementia across the disease trajectory. Four qualitative analysts coded and analyzed data in partnership with PIs, community-based researchers, and senior researchers. The analysis resulted in identifying three key cultural responses communities employ to care for people living with dementia across the disease continuum. First, a relationality perspective that underscores the importance of ongoing care needs rooted in relationship-building through cultural practices such as visiting. Second, community care activates community support systems, prioritizing training opportunities and decentralizing responsibilities and rights to care away from Western institutions. Finally, care models that support and center the family; coordinating with family members across institutions and specialists were a key concern. These findings have been translated into an Indigenous dementia care framework that highlights these three domains and prioritizes strength-based strategies that communities use to mitigate the colonial erasure of Indigenous practices and worldviews. Working with community partners, we have received further feedback on the framework, identifying differences in community uses for the framework and issues that apply more broadly across the four sites for future work.

Abstract citation ID: igaf122.169

### MORTALITY AND INSTITUTIONALIZATION AFTER A POTENTIALLY PREVENTABLE HOSPITALIZATION AMONG PEOPLE WITH DEMENTIA

Elham Mahmoudi, *University of Michigan, Ann Arbor, Michigan, United States*

Potentially preventable hospitalizations (PPHs) are costly and harmful to all older adults. There is a paucity of evidence on adverse health events of PPH among patients with dementia. We examined the effect of any PPH in 2018 on mortality and long-term institutionalization in 2019 among Medicare beneficiaries with dementia. We used 20% random sample of 2018-2019 Medicare claims data to select people with dementia who continuously enrolled in 2018 and 2019 traditional Medicare with at least one hospitalization in 2018 (n = 67,110). We also identified people with at least one PPH in 2018 (20.1% of the sample). We used the average treatment effect on treated method, matching the characteristics of people with and without PPH and applying robust standard errors. Our models controlled for age, sex, race/ethnicity, frailty index, rurality, neighborhood disadvantaged index, and number of primary care providers and hospitals at the county level. The average effect of at least one PPH on mortality was 33.5% (95% CI: 32.7%-34.3%) among people with PPH compared to 28.8% (95% CI: 28.3%-29.2%) among those with no PPH. Thus, among dementia patients, a PPH increased the risk of mortality by 4.7 percentage points (PP) (95% CI: 3.8%-5.6%). Effect of any PPH on long-term institutionalization was 21.1% (95% CI: 20.3%-22.0%) among people with PPH compared to 17.9% (95% CI: 17.4%-18.3%) among those with no PPH. Having any PPH increased the risk of long-term institutionalization by 3.3 PP (95% CI: 2.3%-4.2%). PPHs increase the risks of mortality and long-term institutionalization among older adults with dementia.

Abstract citation ID: igaf122.170

### PURPOSE IN LIFE AND ITS MEASUREMENT IN OLDER ADULTS WITH COGNITIVE IMPAIRMENT USING COGNITIVE INTERVIEWING

Elise Hu[1], Maria Caceres[1], Lijuan Yin[1], Woojin Song[1], Kelsey King[2], and Naoko Muramatsu[1], *1. University of Illinois Chicago, Chicago, Illinois, United States, 2. Roosevelt University, Chicago, Illinois, United States*

Purpose in life (PiL), defined as the subjective experience of living with meaning, critically facilitates well-being in older adults with cognitive impairment (CI). The seminal PiL scale (Ryff, 1989) is the most widely used today and spans three domains: having a life purpose, engaging in that purpose, and setting purposeful goals. However, older adults with CI may interpret the scale items differently than intended. Guided by cognitive interviewing, a qualitative tool to verbalize thought processes behind response selection, this study examined how older adults with CI interpreted Ryff's 7-item PiL scale to (1) identify purposes among and (2) assess its suitability for this population. 25 participants (ages 51-90) were enrolled in a physical activity trial designed for inactive older adults with mild CI or mild dementia. During baseline interviews, research staff displayed a 6-point Likert scale (strongly disagree to strongly agree) and read aloud each item. Participants responded and were asked to discuss their answer choice. Thematic analyses revealed that most participants identified purposes related to family. One item, "I sometimes feel as if I've done all there is to do in life," misled participants because while feeling pride in completing life goals, they sensed the item's undesirable overtone. Negatively worded items (e.g., "I don't think") confused participants with poorer cognition. PiL theory and measurement should thus account for various life stages and cognition levels. Future research will apply these findings to refine PiL measurement and explore how PiL can drive behaviors for healthy living among older adults with CI.

Abstract citation ID: igaf122.171

### CHARACTERISTICS OF OLDER ADULTS SEEKING DEMENTIA PREVENTION INTERVENTION: INSIGHTS FROM THE MY HEALTHY BRAIN TRIAL

Makenna Law[1], Christine Ritchie[1], Olivia Okereke[1], Bettina Hoeppner[1], Judson Brewer[2], Stephen Bartels[1], Ana-Maria Vranceanu[1], and Ryan Mace[1], *1. Massachusetts General Hospital, Boston, Massachusetts, United States, 2. Brown University, Providence, Rhode Island, United States*

Many older adults seek dementia prevention interventions because of heightened concern about cognitive decline, yet little is known about their demographic and clinical characteristics. To address this gap, we examine the baseline presentations and characteristics of older adults with subjective cognitive decline enrolled in a NIA Stage 1B feasibility randomized clinical trial of the virtual My Healthy Brain group mindfulness-based dementia prevention program. We received 406 inquiries, screened 314, and enrolled 62 older adults using a combination of local in-person (senior centers and Boston-area community organizations) and national online (newsletters, registries) recruitment methods. Most participants self-referred (389/406, 95.8%) through flyers, community presentations, online recruitment platforms, and friends. Descriptive statistics were used to characterize the sample according to sociodemographic factors, SCD presentation, physical function, and participation motivators collected at baseline. Participants ($M = 71$, $SD = 6.98$) were majority female (81%), white (68%), non-Hispanic or Latino/a (90%) with a family history of dementia (58%). Participants endorsed SCD across multiple domains, with only 15% reporting one domain: mainly memory (69%), language (60%), and attention (47%). Major risk factors for dementia included hypertension (61%), sleep disorders (52%), hypercholesterolemia (35%), and thyroid disease (23%). Participants reported high levels of motivation for behavioral change (92% very motivated), especially regarding physical activity (86%), nutrition (73%), and mental activity (65%). These findings can guide future work by examining associations between baseline characteristics and intervention outcomes. They also emphasize the need to evaluate strategies that promote equity across demographic groups and various stages of dementia risk.

### SESSION 1165 (SYMPOSIUM)

Abstract citation ID: igaf122.172

### BRIDGING THE GAP: USING IMPLEMENTATION SCIENCE TO SUPPORT COMMUNICATION IN DEMENTIA CARE

Chair: Natalie Douglas
Discussant: Allison Lindauer

Effective dementia care relies on person-centered, evidence-based approaches that improve outcomes for people living with dementia and their care partners. A key challenge in implementing dementia care strategies is that it relies on effective communication—yet communication impairments are a core feature of dementia. Without addressing these impairments, even well-designed interventions may be difficult to sustain, adapt, or scale. Despite the critical role of communication in dementia care, speech-language pathologists (SLPs) remain underutilized in implementation efforts. This symposium explores how communication-based strategies can be successfully integrated into dementia care using implementation science principles, with lessons for researchers, clinicians, and policymakers working to enhance care delivery. First, a case study illustrates how unaddressed communication challenges create barriers to dementia care implementation. The second study maps care partner-reported distressing behaviors to speech-language pathology interventions, highlighting how communication-based strategies can be tailored to both individual and contextual needs. The third study presents a scalable, technology-based intervention designed to foster social engagement for dementia care partners, illustrating the importance of addressing communication needs beyond direct patient care. The fourth study evaluates a real-world adaptation of Dementia Collaborative Coaching, where care providers are trained in communication strategies to improve dementia care delivery in resource-limited settings. Together, these studies illustrate how applying implementation science principles to communication-based dementia care strategies can enhance care outcomes, improve intervention sustainability, and reduce care partner burden. These findings have broad implications for gerontologists, healthcare professionals, and policymakers committed to improving dementia care.

Abstract citation ID: igaf122.173

### "BUT MY SPEECH IS FINE...": EXPANDING THE SPEECH-LANGUAGE PATHOLOGIST'S ROLE IN NURSING HOME DEMENTIA CARE

Natalie Douglas, *University of Louisiana at Lafayette, Lafayette, Louisiana, United States*

Speech-language pathologists are uniquely positioned to support communication and person-centered care for people living with dementia in nursing homes. A recent feasibility study demonstrated that Dementia Collaborative Coaching can be implemented by speech-language pathologists to train certified nursing assistants in communication strategies within usual care workflows. In that study, four speech-language pathologists implemented coaching with 10 nursing assistants and 15 residents across 90 sessions, achieving 64% fidelity and significant reductions in behavioral distress, as measured by the Cohen-Mansfield Agitation Inventory, $t(14) = 10.51$, $p < .001$, $d = 2.76$. This presentation builds upon those findings by adapting Dementia Collaborative Coaching for a nursing home in southwestern Louisiana, located in one of the most socioeconomically disadvantaged regions in the United States, ranking in the 9th decile on the Area Deprivation Index. Adaptations included reducing session frequency to improve feasibility, training multiple nursing assistants per resident to ensure continuity across shifts, and incorporating culturally responsive materials tailored to the linguistic and social diversity of the region. Speech-language pathologists developed relationships with nursing assistants, modeled skilled communication strategies, and fostered a collaborative learning environment to integrate communication-focused care into daily routines. Preliminary findings suggest these adaptations improved nursing assistant engagement, consistency in strategy use, and resident interaction quality. This study highlights how speech-language pathologists can modify evidence-based interventions to fit resource-limited nursing homes while maintaining fidelity and impact.

Abstract citation ID: igaf122.174

### ADDRESSING SOCIAL COMMUNICATION NEEDS OF DEMENTIA CARERS: A FEASIBILITY TRIAL OF THE CAREENGAGE PROGRAM

Raksha Mudar[1], Lizzy Lydon[2], Wendy Rogers[3], and Minakshi Raj[4], *1. University of Illinois Urbana-Champaign, University of Illinois Urbana-Champaign, Illinois, United States, 2. Miami University, Oxford, Ohio, United States, 3. University of Illinois Urbana-Champaign, Champaign, Illinois, United States, 4. University of Illinois Urbana-Champaign, Urbana, Illinois, United States*

Family carers of persons with dementia (PwD) often experience a decline in meaningful and engaging social communication as caregiving demands increase leading to loneliness, and negative health outcomes. Traditional support groups play an important role in assisting carers with the practical challenges of caregiving. However, they often overlook carers' broader social communication needs to engage with others in ways that are not centered on caregiving. Speech-language pathologists (SLPs), as healthcare professionals, are uniquely equipped to create person-centered programs that support social communication in carers leveraging their expertise in communication and cognitive rehabilitation, and working in collaboration with interdisciplinary teams. In this study, we developed and optimized a technology-based social engagement program called CareEngage and conducted a feasibility trial using a pragmatic approach with 68 carers of PwD (mean age = 68.8 ± 6.6). Carers joined two weekly, 30-minute Zoom sessions for four weeks, engaging in casual conversations on non-caregiving topics (e.g., Gardening; Healthy Foods). They provided feedback immediately after each session and completed a post-program assessment. The results revealed significantly higher scores on the Friendship Scale ($p = .049$) and the Social Activity Questionnaire ($p = .034$) following the program. Session-level and interview data indicated strong satisfaction with social communication with an average enjoyment rating of 4.4 out of 5. These findings suggest that carefully optimized technology-based social engagement programs can support carers' social communication and health. These results inform healthcare practice and implementation science on developing scalable programs that can be integrated into routine care to improve carer support and outcomes.

Abstract citation ID: igaf122.175

### MAPPING CARE PARTNER-REPORTED DISTRESSING BEHAVIORS TO SPEECH-LANGUAGE PATHOLOGY INTERVENTIONS

Aimee Mooney, Allison Lindauer, Christina Zonker, and Heather Franklin, *Oregon Health & Science University, Portland, Oregon, United States*

Purpose: Speech and Language Pathologists play a central role in the assessment, diagnosis, and treatment of persons with dementia (PWD) and their caregivers. The World Health Organization (WHO) has directed rehabilitation as a core component of dementia care. Dementia caregivers commonly relate distressing behaviors, e.g. "withdraws during family functions" and "repetitive questions; interrupting". This study maps distressing behaviors reported by dementia caregivers to Non-Pharmacological Treatments (NPTs), inclusive of Cognitive Rehabilitation, Expressive-Receptive Language Therapy and Swallowing. Method: Distressing behaviors were identified by 176 family caregivers as part of a larger psychoeducational intervention project: Tele-STELLA, (Support via Technology: Living and Learning with Advancing dementia, NIA R01AG067546). Definitions, treatment targets and treatment components were condensed into a final catalog of seven NPTs; coding rules were established. Two researchers mapped an initial sampling of 98 behaviors onto the NPTs, achieving substantial inter-rater reliability, with an agreement of 79% and a Cohen's Kappa of 0.68 [95% CI (0.56, 0.80)]. Results: 73% of the behaviors were categorized to the NPTs of Cognitive Rehabilitation, (43), Expressive/Receptive Language Therapy/Swallowing (16) and Psychological Management (13); 2% of behaviors were coded as 'No NPT. Conclusions: By categorizing the caregiver-generated distressing behaviors into applicable NPTs, we highlight the need for referral to Speech and Language Therapy in dementia care. This analysis suggests many dementia caregiver concerns map neatly to SLT interventions focusing on cognitive, behavioral, communication/language and swallowing challenges. This provides practical information for gerontologists and other healthcare professionals when considering referrals to optimize function and reduce disability in dementia care.

Abstract citation ID: igaf122.176

### PRESERVING CONNECTION: THE VITAL WINDOW OF SPEECH THERAPY INTERVENTION IN A DEMENTIA CARE CASE STUDY

Collean Combs, *University of New Hampshire, Durham, New Hampshire, United States*

This case illustrates that delayed implementation of best practice for early and frequent opportunities to intervene in dementia care can significantly impact patients' communication abilities as well as QoL for both patients and care partners. This situation underscores the need for systematic improvements in implementation science related to education and intervention in dementia care, emphasizing the importance of interdisciplinary management that includes SLPs. A 63-year-old man diagnosed with

Alzheimer's disease went 4.5 years without a speech-language consultation, despite declining cognitive linguistic abilities noted by his neurologist. Neither he nor his care partner received guidance on interventions, leading the care partner to independently seek services, self-referring to the clinic where a comprehensive cognitive-communication evaluation was completed. The evaluation revealed, despite severe cognitive impairment and notable word finding difficulties, the patient's syntactic and grammatical structures were largely intact. Although the foundation of language was preserved, the patient lacked compensatory communication strategies during these challenges, which hindered his ability to convey his overall message effectively, resulting in frequent communication breakdowns. These findings led to a patient-centered intervention designed to equip both the patient and their care partner with tools to address communication breakdowns. This included the implementation of cognitive-communication strategies and care partner training, providing specific methods to enhance communication and reduce frustration. This comprehensive approach improved the patient's overall functional communication, resulting in a reported increase in QoL for both the patient and care partner, highlighting the value of early referrals to speech-language services in dementia care.

### SESSION 1170 (SYMPOSIUM)

Abstract citation ID: igaf122.177

### A PROGRAM FOR THE STUDY AND SUPPORT OF CAREGIVERS: AN INNOVATIVE MODEL FOR US MEDICAL SCHOOLS

Chair: Sara Czaja
Discussant: Mark Lachs

Formal (paid) and informal (unpaid) caregivers play crucial roles in supporting the health and well-being of older adults with chronic and disabling health conditions. The physical and emotional demands of caregiving are profound, yet caregivers are not systematically engaged or supported in health care settings. The Program for the Study and Support of Caregivers at Weill Cornell Medicine (WCM) is the first program of its kind within a US medical school. It convenes a multidisciplinary team of scientists, clinicians, and educators to address the most pressing topics and knowledge gaps in the field of caregiving. A unique feature of our Program is the synergistic integration between our research patient care, and educational programs, which allows us to implement and disseminate findings to our patient and caregiving populations. This symposium is organized according to the Program's three pillars: discover, care, teach. Following the first presentation, which will provide an overview of the Program's vision, structure, and unique features (Czaja), subsequent presentations will describe (1) our world class research component that aims to address pressing gaps in the research landscape by designing, adapting, and implementing evidence-based programs (Sterling), (2) innovative practice-based initiatives that will promote caregiver assessment, education, and support in clinical practice (Adelman), and (3) comprehensive educational programing utilizing didactic teaching, reflective practice, and experiential learning methods to prepare trainees to deliver high-quality, family-centered care (Phongtankuel).Our discussant Dr. Lachs, will lead a conversation about the Program's significance and potential to serve as model for the study and support of caregivers nationwide.

Abstract citation ID: igaf122.178

### A PILOT RANDOMIZED CONTROLLED TRIAL WITH HOME HEALTH AIDES CARING FOR COMMUNITY-DWELLING ADULTS WITH HEART FAILURE

Madeline Sterling[1], Cisco Espinosa[2], Sasha Vergez[3], Joanna Ringel[1], Monika Safford[1], Jonathan Tobin[4], and Margaret McDonald[5], *1. Weill Cornell Medicine, New York, New York, United States, 2. Albert Einstein College of Medicine, Bronx, New York, United States, 3. VNS Health, Bronx, New York, United States, 4. Clinical Directors Network, New York, New York, United States, 5. VNS Health, New York, New York, United States*

Home health aides and attendants (HHAs) frequently provide care to adults with heart failure (HF), but many lack training and confidence, and cannot quickly reach their nurse supervisors when they need help. In partnership with one of the largest home care agencies in New York, NY, we conducted a pilot randomized control trial (RCT) to test an education and communication-based intervention among 105 HHAs caring for HF patients. HHAs were randomized to: a) HF training (control) or b) HF training plus mHealth app that allowed HHAs to message nurses in real-time (intervention) and followed for 90 days. Main outcomes: 1) feasibility 2) acceptability and 3) effectiveness (primary outcomes: HF knowledge Caregiver Self-Efficacy (SE), secondary outcome: self-reported preventable 911calls). Data on patient outcomes (ED visits, hospitalizations) were collected. Mixed effects models were used to compare trajectory of all outcomes between and within study arms. Qualitative data was analyzed thematically. The presentation will focus on the findings of the RCT, including completion and retention rates, feasibility and acceptability of the intervention, and HHA and patient outcomes. Overall, we found that training improved HHAs' HF knowledge and caregiving SE, with the largest gains among those with lowest baseline scores. The chat feature (intervention) did not significantly improve primary outcomes but did reduce preventable 911 calls (secondary outcome). Fewer ED visits occurred among patients cared for by intervention arm HHAs. The findings can inform the design of a future large-scale trial, and how to better support and integrate HHAs providing care HF care.

Abstract citation ID: igaf122.179

### THE CENTER FOR THE STUDY AND SUPPORT OF CAREGIVERS - CLINICAL ACTIVITIES

Veerawat Phongtankuel, *Weill Cornell Medicine, New York, New York, United States*

Many family caregivers are untrained and often feel ill-prepared to take on caregiving tasks. Our clinical program focuses on developing strategies to assess, support, and educate our patients' caregivers. In our geriatric outpatient practice at the Center on Aging, we have 10,000 patient visits annually. Approximately 50% of these visits include either paid or family caregivers who accompany the patients. We will use validated caregiver assessment tools developed by research colleagues and implement them in the practice. Based on the results of these assessments, we will develop educational and support resources for family caregivers that will be available online and in person so that isolated caregivers can be reached. These programs include a curriculum covering proper techniques for lifting and transferring patients to avoid back strain, feeding patients with swallowing difficulties, and positioning patients in beds and chairs to avoid bedsores. These resources will also be customized to educate the caregiver on the care recipient's illness and specific care needs. The curriculum will also cover counseling on caregiver stress. This will include strategies for finding relief and managing stress by learning how to ask for help, involving other family members in caregiving tasks, and participating in support groups. These practices are essential for maintaining their own health and well-being. The clinical program will train caregivers wherever we render care, whether it be in the inpatient, outpatient, or home setting.

Abstract citation ID: igaf122.180

### SUPPORTING CAREGIVERS IN CLINICAL PRACTICE: FINDINGS FROM A PILOT RANDOMIZED CONTROLLED TRIAL

Ronald Adelman, *Weill Cornell Medicine, New York, New York, United States*

Caregivers commonly accompany older adults to their routine medical visits, yet they are not systematically supported in clinical practice. Caregivers report feeling ill-prepared for their role and receive little guidance from healthcare professionals. The clinical component of our Caregiver Program aims to address these issues by launching a comprehensive approach to family-centered care that incorporates caregiver assessment, training, and support. As a first step in this initiative, our multidisciplinary team developed Collaborative Healthcare Encounters with Caregivers (CHEC), a checklist-based intervention to help caregivers identify and communicate their concerns to healthcare providers. We evaluated the feasibility, acceptability, and preliminary efficacy of CHEC in a randomized controlled pilot trial of $N = 52$ patient-caregiver dyads recruited from our geriatric outpatient practice. Visits were audio-recorded and analyzed using a standardized coding procedure. Post-visit questionnaires assessed caregivers' perceptions of the checklist, efficacy in primary care interactions and knowledge of relevant resources. CHEC caregivers reported that the checklist was easy to complete (89.3%) and useful in helping the medical team to recognize their concerns (78.6%). Analyses of audio-recordings found that CHEC visits allocated a greater proportion of the visit time to discussing caregivers' concerns about the patient. As compared to usual care participants, CHEC caregivers reported higher efficacy in primary care interactions and greater knowledge of relevant resources. These findings suggest that CHEC is feasible to administer and may promote greater involvement of caregivers in patient-centered discussions. Results lay the groundwork for future activities aimed at providing comprehensive education and support to caregivers throughout our clinical programs.

### SESSION 1175 (SYMPOSIUM)

Abstract citation ID: igaf122.181

### MODIFIABLE PSYCHOSOCIAL PREDICTORS OF HEALTHY COGNITIVE AGING IN DAILY LIFE

Chair: Jeremy Hamm
Co-Chair: Eric Cerino

Healthy cognitive aging is shaped in part by modifiable (intervenable) psychosocial factors. However, little remains known about how these dynamic processes operate in daily life, and the extent to which they are implicated in day-to-day changes in cognitive functioning. This symposium addresses the role of daily beliefs and appraisals (perceived control, stressor severity), emotions (affect valence and intensity), and strategies (goal engagement) as predictors of cognitive health and functioning in daily life. Klepacz et al. investigate how, across the adult lifespan, individual differences in perceived control predict day-to-day variability in cognitive functioning, a sensitive indicator of early changes in cognition that confers increased risk of later impairment. Cerino et al. use data from the National Study of Daily Experiences to document how interpersonal stressor control is linked to memory lapses and associated irritation in daily life, especially in older adulthood. Richards et al. use ecological momentary assessment (EMA) data to identify the contextual circumstances under which stress severity predicts moment-to-moment working memory performance. Castle et al. employ EMA data to examine how affect pleasantness, unpleasantness, and intensity are associated with momentary processing speed and working memory. Finally, Hamm et al. use measurement-burst data to document how the investment of time and persistent effort into valued goals (goal engagement) is linked to daily episodic memory and executive functioning. This symposium contributes to a more comprehensive understanding of how healthy cognitive aging may be shaped by dynamic moment-to-moment and day-to-day changes in modifiable beliefs, emotions, and strategies across the adult lifespan.

Abstract citation ID: igaf122.182

### PERCEIVED CONTROL BUFFERS AGAINST VARIABILITY IN DAY-TO-DAY COGNITION IN MIDLIFE AND OLDER AGE

Laura Klepacz[1], Eric Cerino[2], Jacqueline Mogle[3], and Jeremy Hamm[1], *1. North Dakota State University, Fargo, North Dakota, United States, 2. Northern Arizona University, Flagstaff, Arizona, United States, 3. RTI Health Solutions, Durham, North Carolina, United States*

Perceived control has been consistently identified as a modifiable predictor of healthy cognitive aging. However, little is known about its link with intraindividual variability in cognitive performance, a sensitive indicator of cognitive aging that confers increased risk of later impairment. A better understanding of the association between perceived control and variability in cognitive performance is needed to elucidate protective psychosocial factors that buffer against heightened variability. Data from the Modifiable Antecedents of Memory and Behavior in Adulthood Study ($n = 217$, Mage = 53.6 years, $SD = 14.8$, range = 30-80, 65% female) was used to (a) examine associations between baseline perceived control and day-to-day variability in cognitive performance, and (b) evaluate when in the adult lifespan this relationship becomes most prominent. Multilevel heterogenous variance models revealed that greater perceived control predicted less day-to-day variability (the Level-1 residual variances) in executive functioning ($\alpha = -0.23$, $p < .001$) and processing speed ($\alpha = -0.25$, $p < .001$), but not episodic memory ($\alpha = -0.06$, $p = .32$). Age moderation models showed that higher levels of perceived control were most protective against day-to-day variability in early-to-late midlife. Specifically, perceived control had more prominent negative associations with variability in (a) episodic memory for individuals in their 40s ($\alpha = -0.27$, $p = .057$) and (b) processing speed for individuals in their 50s ($\alpha = -0.59$, $p < .0001$). Study findings suggest that perceived control may facilitate the maintenance of more stable cognitive performance, especially among individuals who are in early-to-late midlife.

Abstract citation ID: igaf122.183

### ASSOCIATIONS BETWEEN DAILY INTERPERSONAL STRESSOR CONTROL AND MEMORY LAPSES IN DAILY LIFE

Eric Cerino[1], Faith Shannon[1], Gillian Porter[1], Dakota Witzel[2], Robert Stawski[3], Jonathan Rush[4], Jacqueline Mogle[5], and David Almeida[6], *1. Northern Arizona University, Flagstaff, Arizona, United States, 2. South Dakota State University, Brookings, South Dakota, United States, 3. Utah State University, Logan, Utah, United States, 4. University of Victoria, Victoria, British Columbia, Canada, 5. RTI Health Solutions, Durham, North Carolina, United States, 6. The Pennsylvania State University, University Park, Pennsylvania, United States*

Studying modifiable psychosocial correlates of cognitive health in daily life is crucial to inform targets for dementia prevention. Perceived control is an important psychosocial resource for cognitive health. Prior work, however, focuses largely on global aspects of control, ignoring influences of more dynamic aspects of control associated with specific experiences in daily life (i.e., daily stressor control). We used data from the National Study of Daily Experiences (N = 1,236, Mage=67.54, SE = 10.34, 57.38% Female) to address this gap and examine daily associations among stressor control and memory lapses. Over eight consecutive evenings, respondents reported perceived control over interpersonal stressors they had experienced and two types of memory lapses (retrospective, prospective) as well as the impact of these lapses each day (irritation, interference). We used multilevel modeling with statistical adjustment for number of stressors, gender, education, race, and global control. On average, people with higher stressor control reported significantly lower irritation ratings from retrospective memory lapses (Est.=-0.47, SE = 0.15, p<.01). Within-person stressor control and age significantly interacted (Est.=-0.03, SE = 0.01, p=.01) such that days when stressor control was higher than usual were associated with less irritation from prospective memory lapses only among comparatively older adults (Est.=-0.66, SE = 0.31, p<.05). Results indicate that daily stressor control may serve as a psychosocial resource related to less irritation when forgetting past information and future intentions. We discuss the value of promoting perceived control over daily interpersonal stressors to protect against detrimental impacts of memory lapses and encourage healthy cognitive aging in daily life.

Abstract citation ID: igaf122.184

### DAILY COGNITIVE PERFORMANCE UNDER STRESS: CONTEXTUAL AND GENDER DIFFERENCES

Nicole Richards[1], Elizabeth Munoz[1], Anqing Zheng[2], Sally Wadsworth[2], Martin Sliwinski[3], and Chandra Reynolds[2], *1. The University of Texas at Austin, Austin, Texas, United States, 2. University of Colorado Boulder, Boulder, Colorado, United States, 3. The Pennsylvania State University, University Park, Pennsylvania, United States*

While exposure to stressors has been widely examined in the literature in relation to cognitive function, stress severity– the extent to which stressors are perceived as threatening or overwhelming– has emerged as a critical determinant of cognitive function and change. However, the momentary effects of stress severity on everyday cognitive performance remain unclear. This study examined the momentary associations between stress severity and cognitive performance using ecological momentary assessment data from the Colorado Adoption/Twin Study of Lifespan on behavioral development and cognitive aging. Participants (N = 440, Mage = 35.81, 57.7% women) completed momentary surveys and working memory tasks via smartphones up to three times daily for 14 days. Multilevel models, accounting for momentary and between-person confounders, revealed that people who tended to report greater stress severity had poorer working memory performance indexed by higher error scores (b = .009, CI[.004, .013], p < .001). At the momentary level, activities and gender moderated the momentary effect of stress severity on performance. Specifically, participants performed worse when they reported greater stress severity while "doing nothing" (b = .007, CI[.002, .012], p < .01), with this effect being more pronounced among women (b = .013, CI[.003, .022], p < .01). Conversely, for women, socializing buffered the negative impact of stress on cognitive performance (b = -.003, CI[-0.006, -0.001], p < .05). These findings highlight the importance of momentary context in understanding stress-related cognitive vulnerability and suggest that meaningful activities and social interactions may serve as protective factors, particularly for women.

Abstract citation ID: igaf122.185

### AFFECTIVE STATES AND COGNITIVE PERFORMANCE: RESULTS FROM THE EMOTIONS IN DAILY LIFE STUDY

John Castle[1], Giselle Ferguson[2], and Stacey Scott[1], *1. Stony Brook University, Stony Brook, New York, United States, 2. Union College, Schenectady, New York, United States*

Although affective states have been observed to influence cognitive performance in experimental laboratory settings, limited research has investigated this relationship in daily life. In this study, we aimed to understand the relationship between momentary affect (pleasantness, unpleasantness, and intensity) and momentary cognition (processing speed and spatial working memory) using ecological momentary assessment (EMA). For 7 days, 221 participants (Mage=47 years, SD = 15, range=18-82) recruited from an online panel of US adults completed up to 3 EMA per day. In line with laboratory findings, we hypothesized that cognitive performance would be worse at times when individuals experienced more intense, more unpleasant, and less pleasant affective states. We hypothesized age moderation of these effects, such that older age would be associated with weaker momentary affect-cognition slopes. Intraclass correlation coefficients (ICCs) indicated variance both between- and within-persons (ICC processing speed=.66, spatial memory=.58, pleasantness=.32, unpleasantness=.29, intensity=.40). We used separate, parallel multilevel models to assess the affective dimensions' association with processing speed and spatial working memory, respectively. At times when individuals rated their affect more as more intense than usual, their spatial memory performance was worse (B=.10, p=.03). Unexpectedly, pleasantness and unpleasantness were not associated with cognitive performance either between- or within-persons. Further, we did not find evidence for age as a moderator of these relationships. Thus, in a lifespan sample of adults from across the US, we found that affective intensity, rather than valence, was a key factor associated with cognition in daily life.

Abstract citation ID: igaf122.186

### THE DYNAMIC COUPLING OF GOAL ENGAGEMENT AND COGNITION IN DAILY LIFE

Jeremy Hamm[1], Jacob Shane[2], Jacqueline Mogle[3], Laura Klepacz[1], Matthew Pierce[1], and Kelly Parker[1], *1. North Dakota State University, Fargo, North Dakota, United States, 2. Brooklyn College and the Graduate Center, The City University of New York, Brooklyn, New York, New York, United States, 3. RTI Health Solutions, Durham, North Carolina, United States*

Consistent evidence shows that investing time and persistent effort toward valued goals (goal engagement) is linked to adaptive development and healthy cognitive aging. However, little is known about the dynamic nature of goal engagement in daily life and the extent to which fluctuations in this motivational resource are linked to day-to-day changes in cognitive functioning. Using 14 days of data from an ongoing measurement-burst study ($n = 217$, Mage=54±15, 65% female), we examined the extent to which day-to-day changes in goal engagement predicted corresponding shifts in daily episodic memory and executive functioning when controlling for time, age, sex, and education. We assessed daily goal engagement using the persistence in goal engagement scale (e.g., "When I encountered problems today, I didn't give up until I solved them"), daily episodic memory using a 15-word immediate recall task, and daily executive functioning using a letter fluency task. Multilevel models showed that daily, within-person increases in goal engagement predicted corresponding increases in daily executive functioning ($\gamma=.58$, $p = .013$), but not episodic memory. Between-person differences were also observed, such that middle-aged and older adults with higher goal engagement than their peers had higher average levels of episodic memory performance ($\gamma = .65$, $p = .016$), but not executive functioning. Our findings inform lifespan theories of motivation and development by (a) providing initial evidence that goal engagement exhibits meaningful, within-person fluctuations at the daily level (ICC = .46) and (b) showing that these day-to-day changes are linked to dynamic fluctuations in daily cognition.

### SESSION 1180 (SYMPOSIUM)

Abstract citation ID: igaf122.187

### EMPLOYMENT HISTORY AND LATER-LIFE HEALTH

Chair: Wenxuan Huang

Employment provides essential financial resources, institutional protection, and social networks to workers, all of which serve as key social determinants of health throughout the life course. While the link between employment and health is well-documented, limited research has taken a holistic approach to examine how employment histories shape later-life health. Recent advances in longitudinal research methods and the growing availability of life history data have enabled new efforts to address these gaps. This symposium brings together four studies investigating the impact of employment history on various health outcomes in later life across different social contexts. Using data from Health Retirement Study, Han and Dr. Moen's apply advanced causal estimation models to assess how workforce participation in the 50s contributes to cognitive health in later life in the U.S. context, highlighting both time-varying effects and subgroup heterogeneity. Dr. Fu et al.'s paper explores how the life-course patterns of work history shape the frailty trajectories among Chinese adults aged 60 and older, emphasizing the role of cumulative disadvantage in work on frailty. Dr. Han's study examines the temporal pattern of employment history using sequence analysis approach and assesses its association with late-life functional limitations, revealing the urban-rural divide in employment patterns and its health consequences in China. Dr. Huang's paper combines sequence analysis and decomposition method to understand how typical employment patterns explains the midlife health disparities between high school graduates and GED recipients. By conducting life-course-sensitive analysis, these studies collectively highlight how unequal employment histories drive later-life heath disparities.

Abstract citation ID: igaf122.188

### DECOMPOSING MIDLIFE HS-GED HEALTH DISPARITIES: THE ROLE OF EMPLOYMENT HISTORIES

Wenxuan Huang, *Johns Hopkins University, Baltimore, Maryland, United States*

Background: The health disparities between high school (HS) diploma holders and GED recipients have been well documented. However, limited research has examined how differential employment histories may contribute the observed HS-GED health disparities. Data: This study uses data from the National Longitudinal Survey of Youth 1979 (NLSY79, $n = 3,277$) to construct the employment histories (age 21-49) and examine eight health outcomes (e.g., self-rated health, functional limitations and depressive symptoms, etc.) observed in the 50+ Health Module. Methods: This study employs sequence-based analysis to classify employment histories into typical life course patterns. It then uses multivariate decomposition approach to determine the extent to which the HS-GED health disparities are attributable to differential distribution or differential return of typical employment histories. Results: This study identifies six typical employment patterns: (1) gradual withdrawal, (2) incremental integration, (3) prolonged inactive, (4) continuous full-time, (5) part-time dominated, and (6) military to civil work. The decomposition analysis suggests that both differential distribution and differential return between HS and GED groups play a role in explaining the HS-GED disparities, while the relative shares differ across health outcomes. Especially, the differential distribution of "continuous full-time" and "prolonged inactive" employment histories arise to be the main drivers of the HS-GED health disparities. These findings highlight the crucial role of employment histories in shaping HS-GED health disparities and raise the question of why similar employment histories yield discriminatory health returns between these two groups.

Abstract citation ID: igaf122.189

### CAUSAL DYNAMICS OF WORK PARTICIPATION AND COGNITIVE HEALTH IN LATER LIFE: A LIFE COURSE PERSPECTIVE

Xiaowen Han, and Phyllis Moen, *University of Minnesota, Minneapolis, Minnesota, United States*

This study investigates how continued work participation shapes cognitive functioning in later adulthood, highlighting the timing, duration, and reciprocal nature of these causal processes within a life course framework. Drawing on data from the Health and Retirement Study (HRS), a nationally representative longitudinal survey of U.S. adults aged 50 and older, we employ advanced causal estimation methods—including marginal structural models, structural nested models, and a Bayesian model comparison algorithm—to disentangle the causal pathways between work engagement and cognitive outcomes while adjusting for time-varying confounders. We measure cognitive functioning with a 27-point scale capturing memory, working memory, and processing speed, and categorize work participation as not employed, part-time, full-time, or fully retired. Preliminary findings indicate that continued work participation exerts positive effects on cognitive functioning after accounting for dynamic confounders, with these protective effects becoming more pronounced at older ages, especially for individuals in non-managerial or non-professional jobs. We also observe reciprocity, as higher cognitive functioning increases the likelihood of remaining employed. By modeling time-varying confounders and comparing multiple model specifications, we capture both overall and subgroup-specific effects, illustrating how work participation in one's 50s may influence cognitive status well into the 70s. Moving forward, we will employ a Bayesian model comparison algorithm to identify the best-fitting models across different measures of timing and duration of work participation, capturing these complex longitudinal causal dynamics. These results have important implications for public policy and organizational practices, particularly as aging societies face extended working lives and rising concerns about cognitive decline.

Abstract citation ID: igaf122.190

### WORK HISTORY AND FUNCTIONAL LIMITATIONS IN LATER LIFE UNDER CHANGED INSTITUTION IN CHINA

Chengming Han, *UTMB Health, The University of Texas Medical Branch, Galveston, Texas, United States*

Objective: This study examines the impact of work history on functional limitations in later life among individuals who experienced China's 1978 economic reform. Methods: Data were drawn from the 2011–2018 China Health and Retirement Longitudinal Study (CHARLS) for respondents born between 1934 and 1964. Sequence analysis was used to categorize work histories by type and duration. Tobit models were applied to estimate the effects of work trajectories on functional limitations in later life. Results: The economic reform facilitated transitions from farming to non-rural work, yet hukou-based urban-rural divisions shaped access to pensions, medical insurance, and housing benefits. Urban work histories were associated with lower functional limitations and a slower decline over time. Compared to urban workers with an urban hukou, self-employed individuals with an urban hukou ($b = 1.570$, $p < 0.001$), farmers with a rural hukou ($b = 1.465$, $p < 0.05$), and older migrant workers ($b = 1.662$, $p < 0.001$) exhibited higher levels of functional limitations. Conclusion: Rural hukou holders had fewer opportunities for non-rural employment, limiting their access to pensions, healthcare, and better living conditions. Those engaged in rural work experienced significantly higher functional limitations compared to individuals in other employment types.

Abstract citation ID: igaf122.191

### LIFE-COURSE PATTERNS OF WORK HISTORY AND FRAILTY TRAJECTORIES AMONG COMMUNITY-DWELLING OLDER ADULTS IN CHINA

Peipei Fu[1], Yi Wang[2], Xingzhi Wang[3], Jiao Yu[2], and Xi Chen[2], *1. Shandong University, Jinan, Shandong, China (People's Republic), 2. Yale University, New Haven, Connecticut, United States, 3. Duke University, Durham, North Carolina, United States*

Diverse work experiences in early and midlife stages are associated with physical frailty in later life. However, whether life-course patterns of work history are associated with later life frailty trajectories in China remains unknown. This study utilized data from the China Health and Retirement Longitudinal Study and included 5,133 participants aged 60 years or older. We used sequence analysis to identify the work-history patterns of women and men respectively between age 18 and 60, and conducted group-based trajectory modelling (GBTM) to classify subsequent frailty trajectories. Multinomial logistic regression analyses were then performed to determine the association between work-history patterns and later-life frailty trajectories. Our analysis identified four work-history patterns for both genders. The GBTM revealed four frailty groups for women (persistently non-frail, low increasing frail, consistently increasing frail, persistently high frail) and three frailty groups for men (persistently non-frail, low increasing frail, persistently high frail). Notably, the non-agriculture to retirement work history was significantly associated with frailty trajectories for both genders. The association was particularly strong for participants in the persistently high frail group, with women (Relative Ratio (RR) = 0.42 [95% CI, 0.18, 0.96]) and men (RR = 0.54 [95% CI, 0.30, 0.97]) showing a lower relative risk of frailty compared to participants with lifelong agricultural work histories. Among women, transitioning from agricultural to non-agricultural employed work history was associated with lower relative risks of being frail. Our study highlights the importance of the duration, sequence and timing of transitions in work history for frailty trajectories among older Chinese.

### SESSION 1185 (SYMPOSIUM)

Abstract citation ID: igaf122.192

### LONELINESS AND SOCIAL ISOLATION INTERVENTIONS: WHAT WORKS, WHAT DOESN'T, AND WHAT'S NEXT?

Chair: Kexin Yu
Co-Chair: Chuwen Zhong
Discussant: Matthew Smith

Loneliness and social isolation in older adult populations pose significant public health concerns. Despite numerous interventions, their effectiveness remains mixed, necessitating a critical examination of what works, what doesn't, and why. This symposium brings together experts to explore diverse approaches to reducing loneliness, ranging from targeted interventions to broad public health strategies. Kexin Yu will present findings from a pilot online conversation intervention designed to enhance social connectedness and cognitive health among older Chinese immigrants, highlighting the role of culturally tailored digital solutions. Kimberly Van Orden will share results from a clinical trial on a psychotherapy/coaching program aimed at reducing loneliness, which used Ecological Momentary Assessment (EMA) and passive sensing to capture sensitive changes in aspects of loneliness, offering insights into psychological approaches and personalized support. Christina Victor will critically examine the challenges of loneliness interventions, discussing why many fail and how future efforts can be more effective. Finally, Roger O'Sullivan will explore a public health approach to addressing loneliness and social isolation, emphasizing population-level strategies and policy implications. Together, these presentations will provide a comprehensive discussion of loneliness interventions, addressing both individual and systemic factors. The session will engage attendees in a dialogue about the future of research, practice, and policy in fostering meaningful social connections among older adults.

Abstract citation ID: igaf122.193

### ONLINE CONVERSATIONS FOR COGNITIVE HEALTH: A CULTURALLY ADAPTED INTERVENTION FOR OLDER CHINESE IMMIGRANTS

Kexin Yu[1], Amanda Mar[2], Sabrina Lin[3], Kevin Duff[2], Lisa Silbert[2], and Hiroko Dodge[4], *1. UT Southwestern Medical Center, Dallas, Texas, United States, 2. Oregon Health & Science University, Portland, Oregon, United States, 3. Lewis & Clark University, Portland, Oregon, United States, 4. Massachusetts General Hospital, Harvard Medical School, Boston, Massachusetts, United States*

Social isolation is a major risk factor for Alzheimer's Disease and Related Dementias (ADRD), particularly among older Chinese immigrants who face language barriers and social adjustment challenges. To address this, we developed C-CONECT (Chinese Conversation Engagement Co-design and Pilot Test), an adaptation of the I-CONECT study (ClinicalTrials.gov Identifier: NCT02871921, PI: Dodge), which previously demonstrated that user-friendly video chats could improve cognition and social satisfaction in socially isolated older adults. C-CONECT tailored this intervention to the cultural and linguistic needs of older Chinese immigrants. Participants engaged in 30-minute video conversations four times per week over six weeks with trained research staff. Outcomes included loneliness, depressive symptoms, social isolation, and cognitive function, assessed before and after the intervention. We conducted qualitative interviews to understand participant experiences more in-depth. Eight participants (mean age 76, 75% female) enrolled, with seven completing the study. Most (88%) preferred Mandarin, and the chat completion rate was 90%. After the intervention, 62% of participants reported reduced depressive symptoms. Interviews revealed that participants found the conversations warm and supportive, with memory improvement being their primary motivation for participation. High engagement and positive feedback suggest the feasibility and acceptability of C-CONECT in this population. These findings support the potential for a future randomized clinical trial to assess its impact on cognitive function. By culturally adapting evidence-based interventions, we can expand access to effective behavioral health strategies for underrepresented groups, ultimately contributing to ADRD prevention.

Abstract citation ID: igaf122.194

### ENGAGE COACHING FOR LONELY OLDER ADULTS: CHANGES IN DAILY LONELINESS AND SOCIAL ACTIVITIES

Kimberly Van Orden, *University of Rochester Medical Center, Rochester, New York, United States*

Social disconnection—low engagement, isolation, and loneliness—is associated with poor health, low quality of life, and premature mortality for older adults. However, it is not yet clear which interventions work, especially for older adults who are not homebound (with access to social activities). Identifying mechanisms that account for improvements could help identify effective strategies. This presentation describes a clinical trial testing a brief social engagement coaching program for lonely older adults. Momentary loneliness (perceived social isolation, PROMIS Social Isolation Short Form) and frequency of activities outside the home are assessed via 10 days of ecological momentary assessment (EMA) at baseline, 3-months and 6-months. Currently, 29 out of 30 planned subjects completed baseline EMA protocols; 13 have completed follow-up EMA. The sample has an average age of 72.26 years; 5 subjects are male, 19 female, and 1 intersex. The average number of activities outside the home at each survey instance was less than 1 at baseline (0.55, std 1.10) and increased to 1.25 at 3-months (std 1.74, $p = 0.000$). Loneliness severity at baseline was 9.04 (std 1.84) and decreased to 8.88 (std 1.85) at 3-months ($p = 0.000$). Presentation results will include the full sample and 6-month follow-up; effect sizes to illustrate magnitude of effects; and examination of sequential effects in mediation. Results indicate reliable reductions in loneliness and increases in activities outside the home after social engagement coaching. Results suggest that greater activities outside the home may be one mechanism accounting for reduced loneliness in older adults participating in social engagement coaching.

Abstract citation ID: igaf122.195

### IF LONELINESS IS A PUBLIC HEALTH ISSUE, THEN WHAT IS A PUBLIC HEALTH APPROACH TO LONELINESS?

Roger O'Sullivan, *The Institute of Public Health, Belfast/Dublin, Northern Ireland, United Kingdom*

The U.S. Surgeon General and the World Health Organization (WHO) have highlighted the significant impact loneliness has on the health and well-being of the population, and loneliness is increasingly being recognized as a public health issue. However, there is less discussion on how to drive a public health approach to loneliness. This presentation outlines a public health framework to address loneliness, first setting out the context and methods of public health, as well as the steps of a public health approach. By adapting the Frieden Health Impact Pyramid and its five tiers to the issue of loneliness, the types of actions required at each tier are identified to address loneliness among older people. At the base of the pyramid, actions have the widest population focus, while those further up the pyramid have a greater individual focus. Tier 1 actions address structural factors. Tier 2 highlights interventions which aim to change the context. Tier 3 focuses on protective interventions. Tier 4 provides examples of tailored interventions for individuals, and Tier 5 outlines specialized interventions. In summary, to advance a public health approach to loneliness among older people, increasing focus should be placed on: 1) preventing loneliness; 2) protecting those groups most at risk; and 3) promoting a greater understanding of the causes and consequences of loneliness. The suggested public health actions at the structural, community, and individual levels aim to address the negative costs of loneliness, not only for lonely individuals but society as a whole.

Abstract citation ID: igaf122.196

### LONELINESS AND SOCIAL ISOLATION INTERVENTIONS: A PUBLIC HEALTH ANALYSIS

Christina Victor, *Brunel University London, Guildford, England, United Kingdom*

The 1948 World Health Organisation (WHO) definition of health identifies three distinct domains of health: physical, mental and social. Until the second decade of the 21st century the concept of social health was largely neglected. This position was transformed in November 2023 when WHO declared that 'loneliness as a 'global public health concern' and created the Commission on Social Connection. These developments are the culmination of a body of work that has drawn attention to the association between loneliness/social isolation and a range of health and health service use outcomes. A public health approach to loneliness involves primary prevention, proactively preventing loneliness; secondary prevention, identifying and intervening with those at 'high risk' of loneliness whilst tertiary prevention focuses upon minimising the consequences of established loneliness. Using a public health lens, we undertook a critical evaluation of 50 loneliness interventions for community dwelling older adults identified through existing systematic reviews from 2010 onwards. Twenty interventions focused upon secondary prevention, ten on primary prevention and was unknown/not reported for the remainder. The duration of interventions was predominantly short term with 55 being completed in under 6 months. Evidence for effectiveness is limited as sample sizes are small with 16 studies having 100+ participants. Approximately half the studies reported a post intervention statistically significant decrease in loneliness scores. This does not easily translate into how score changes enhance the quality-of-life older adults. We suggest that future research should look at identifying score changes which are meaningful to individuals alongside statistical tests.

### SESSION 1190 (SYMPOSIUM)

Abstract citation ID: igaf122.197

### THE POLITICS OF PUNISHMENT: HOW POLICY SHAPES THE AGING EXPERIENCE IN PRISON AND AFTER RELEASE

Chair: Kenzie Latham-Mintus
Co-Chair: Raya Kheirbek
Discussant: Elizabeth Vásquez

As the U.S. carceral system faces an unprecedented aging crisis, policies such as compassionate release, geriatric parole, and end-of-life care have emerged as critical yet underutilized mechanisms. This symposium explores how these policies profoundly influence the experiences of aging incarcerated individuals and those navigating reentry. Presentations will examine the systemic barriers to geriatric release and reentry, the ethical complexities of medical decision-making for incapacitated older adults in prison, and the privatization of prison hospice care. Additionally, this session will explore social vulnerability post-incarceration, analyzing how aging individuals experience social networks, social integration, and loneliness after release. By incorporating policy analysis, legal archival research, and a review of existing geriatric parole programs, this session highlights the urgent need for reform, advocacy, and interdisciplinary collaboration to address the health and social inequities faced by older adults in the carceral system.

Abstract citation ID: igaf122.198

### SOCIAL VULNERABILITY POST-INCARCERATION: ANALYZING SOCIAL ISOLATION AND LONELINESS AMONG OLDER ADULTS

Kenzie Latham-Mintus[1], Elizabeth Nelson[1], Melissa Garrido[2], and Raya Kheirbek[3], *1. Indiana University Indianapolis, Indianapolis, Indiana, United States, 2. Boston University School of Public Health, Boston, Massachusetts, United States, 3. University of Maryland School of Medicine, Baltimore, Maryland, United States*

Background: This study examines how past incarceration affects social relationships in older adults. Objective: To explore the relationship between past incarceration and the quality and quantity of social relationships among older adults. Design, Setting, and Participants: Cross-sectional analysis of 13,023 Americans aged 50 and older from the Health and Retirement Study (HRS). Participants self-reported their history of incarceration. Main Outcomes and Measures: Multivariable logistic regression determined the odds of lacking close ties, low social integration, and high loneliness, while linear regression evaluated the relationship between incarceration history and the number of close ties, controlling for demographic factors. Results: Among 13,768 respondents, 992 (7.2%) reported a history of incarceration. They were more likely to be unmarried or unpartnered (aOR: 1.34, 95% CI: 1.11-1.61), rate spousal closeness lower (aOR: 0.59, 95% CI: 0.50-0.70), and have no close relationships with children (aOR: 1.42, 95% CI: 1.19-1.69), family (aOR: 1.28, 95% CI: 1.06-1.54), or friends (aOR: 1.21, 95% CI: 1.01-1.44). They also had higher odds of low participation (aOR: 1.42, 95% CI 1:18, 1.71), low cohesion (aOR: 1.35, 95% CI 1.13, 1.60), and high loneliness (aOR: 1.67, 95% 1.43, 1.94). Linear regression revealed a similar pattern of social vulnerability across all social measures. Conclusions: Older adults with a history of incarceration are at increased risk of social isolation and loneliness. Targeted interventions could improve their social integration and well-being.

Abstract citation ID: igaf122.199

### A SCOPING REVIEW OF GERIATRIC RELEASE AND THE LATER LIFE IMPACTS FOR INCARCERATED OLDER ADULTS

Samuel Van Vleet, and Ryan Steel, *Miami University, Oxford, Ohio, United States*

As the U.S. carceral system grapples with an aging prison population, policies such as compassionate release and geriatric parole are intended to provide pathways for older adults to transition out of incarceration. However, these mechanisms remain underutilized, restrictive, and fraught with systemic barriers. While these programs are designed to provide relief, little is known about the post-incarcerated impacts of reentry for the older adults that are granted this release. This scoping review seeks to address gaps in our understanding of geriatric release programs within the United States by examining both the broader challenges of reentry and the unique barriers that older adults face. Older adults navigating reentry face barriers common to the broader formerly incarcerated population, such as parole compliance, recidivism risk, transportation, and healthcare access. These challenges are heightened by aging-related issues like chronic health conditions, limited employment opportunities, and social isolation. Beyond the individual, these difficulties place additional strain on families and communities that are essential to reintegration. Findings highlight a critical gap in research on post-release outcomes for older adults and a need for stronger evaluation of geriatric release policies. As mass incarceration's long-term consequences unfold, this paper calls for urgent policy reform, targeted reintegration support, and stronger collaborations between gerontology and criminal justice advocacy. Without meaningful intervention, the aging prison population will face worsening health and social disparities, exacerbating existing inequities and straining already limited resources.

Abstract citation ID: igaf122.200

### INCAPACITATED, INCARCERATED PATIENTS: WHO SHOULD DECIDE FOR THEM?

Anurima Chattopadhyay, and Daniel Karel, *National Institutes of Health, Bethesda, Maryland, United States*

The carceral healthcare system is reckoning with a rapidly aging patient population. This increase poses significant challenges because elderly patients are much less likely to be able to make their own medical decisions, requiring a surrogate decision-maker in about 70% of cases. Currently, prison officials–such as wardens or prison guards–are involved in making medical decisions for about 50% of decisionally incapacitated, incarcerated patients. In contrast, twelve states prohibit prison officials from serving as surrogates for incarcerated patients. This discrepancy highlights the need to more thoroughly examine which parties are appropriate surrogates for incarcerated patients. We consider what qualities make an appropriate surrogate decision-maker. They would, ideally: care about the patient, not have conflicts of interest, and know the patient's interests and wishes or be otherwise trained to make surrogate decisions. We ultimately determine that prison officials do not best fit these criteria. We then explore alternative approaches that better protect both incarcerated patients and prison officials. We advocate for efforts to raise the rate of advance medical planning amongst incarcerated individuals; currently less than 1% of incarcerated individuals have filled out an advance directive. Healthcare teams should also work to involve incarcerated patients' families (when appropriate) by addressing issues with security and travel that may prevent them from serving as surrogates. Lastly, when incarcerated patients have no available surrogates, we suggest healthcare teams rely on the relevant guidelines for non-incarcerated, 'unrepresented' patients, where decision-making often requires consensus amongst multiple physicians and the involvement of an ethics committee.

Abstract citation ID: igaf122.201

### END-OF-LIFE CARE FOR THE INCARCERATED: A CASE STUDY ON PRIVATIZATION

Hannah Schwendeman, *University of Minnesota, Minneapolis, Minnesota, United States*

The U.S. prison population is rapidly aging as an unprecedented number of older adults live and die behind bars. Due to tough-on-crime policies that lengthened sentences, the population of older adults in prison has increased nearly 400% in the last 20 years. In response to this aging crisis, state administrators are building new prison hospice units, contracting with private healthcare companies, and training both staff and incarcerated people to care for older prisoners. In this paper, I ask: what logics and practices are used by prison administrators and public officials to provide end-of-life care for incarcerated people? Through legal archival analysis, I analyze how state actors are responding to the increasing need for end-of-life care for incarcerated people. Specifically, I analyze how the state of Connecticut, the first state to receive Medicaid funding for this population, privatized end-of-life care for the incarcerated and the legal challenges that ensued.

### SESSION 1195 (SYMPOSIUM)

Abstract citation ID: igaf122.202

### PROSOCIAL ENGAGEMENT AND HEALTH: THEORETICAL AND METHODOLOGICAL INNOVATION

Chair: Seoyoun Kim
Co-Chair: Cal Halvorsen
Discussant: Nancy Morrow-Howell

All individuals should have opportunities for meaningful engagement, including volunteering and informal helping, across the life course. These prosocial activities foster purpose and connection, essential for well-being in later life. However, opportunities and benefits are shaped by structural factors such as socioeconomic status, race, and pre-existing health conditions. Research on volunteering's health effects has often overlooked these complexities and lacked rigorous causal methodologies. To that end, this symposium presents cutting-edge research on the health benefits of prosocial engagement in later life. Kim examines the causal link between volunteering and cardiovascular biomarkers using g-computation with Health and Retirement Study (HRS) data (2005-2016), enhancing causal inference. Ryu investigates the relationship between volunteering and hypertension risk, stratified by insurance status, using the entropy balancing weights to mitigate selection bias and enhance estimate accuracy. Nemeth expands the discussion by analyzing formal and informal helping and their impact on functional recovery. Chen explores how helping others influences psychological well-being, employing ecological momentary assessment (EMA) to capture real-time emotional and behavioral responses. Han utilizes HRS data (1998-2020) to examine how changes in helping behaviors—both in role status and commitment—affect cognitive health over time, using an extended asymmetric fixed-effects model to address endogeneity and establish potential causal relationships. Morrow-Howell (discussant) will provide insights into the broader implications and future directions of this research. Together, these studies advance the field by integrating robust causal inference techniques, emphasizing inclusion, and leveraging innovative data collection methods to deepen our understanding of prosocial engagement and aging.

Abstract citation ID: igaf122.203

### VOLUNTEERING AND CARDIOVASCULAR BIOMARKERS: A MACHINE-LEARNING CAUSAL APPROACH IN HEALTH AND RETIREMENT STUDY

Seoyoun Kim[1], Koichiro Shiba[2], and Cal Halvorsen[3],
*1. University of Michigan, Ann Arbor, Michigan, United States,*
*2. Boston University, Boston, Massachusetts, United States,*
*3. Washington University in St. Louis, St. Louis, Missouri, United States*

Prosocial engagement, such as volunteering, has been recognized for its protective effects against cardiovascular diseases (CVD), yet its causal impact on CVD-related biomarkers remains unclear. This study employs targeted maximum likelihood estimator (TMLE), a doubly robust causal inference method, to estimate the effect of the changes in volunteer activity (2006/2008 and 2010/2012) and seven key CVD-related biomarkers (in 2014/2016) using the Health and Retirement Study (N = 17,479). The biomarkers assessed include blood glucose (HbA1c), lipid regulation (TC/HDL ratio), chronic inflammation

(CRP), kidney function (Cystatin C), blood pressure (systolic and diastolic), and BMI. Volunteering status was categorized into non-volunteers, initiators, stoppers, and sustained volunteers. TMLE results indicate that, had the entire population engaged in volunteering, we would expect improvements in CVD biomarkers compared to no volunteering at either wave. Sustained volunteering was associated with a 4.6% lower prevalence of unhealthy HbA1c levels (-0.046, p = 0.016), a 1.7% lower prevalence of an unhealthy TC/HDL ratio (-0.02, p = 0.001), an 8.1% lower prevalence of elevated CRP levels (-0.04, p = 0.0003), a 3.7% lower prevalence of unhealthy Cystatin C levels (-0.08, p < 0.001), an 8.9% and 8.7% lower prevalences of elevated diastolic and systolic blood pressure, respectively (DBP: -0.09, p<.001, SBP: -0.09, p<.001). However, a modest 1.2% increase in BMI was also observed. These findings highlight the potential of long-term volunteering as a population-level intervention to improve cardiovascular health, underscoring the need for further research to explore underlying mechanisms and long-term clinical benefits.

Abstract citation ID: igaf122.204

### AGE MODERATES ASSOCIATIONS BETWEEN HELPING AND A SENSE OF MEANINGFULNESS IN DAILY LIFE

Enna Chen, Claire Growney, and Laura Carstensen, *Stanford University, Stanford, California, United States*

The aim of the present study was to examine whether helping others in daily life is associated with meaning in life and experienced emotions, and whether age moderates the associations. A sample of 180 individuals aged 18 to 93 years participated in an experience sampling study, where they reported their emotions, experience of meaning, and whether they were engaging in helping behavior in the moment at five randomly selected times per day for seven days. Averaging across the week-long period, age was positively associated with helping frequency. Individuals who helped others more often throughout the week reported more meaning in life. Age did not moderate this association. However, within-individual analyses suggested that when participants were helping others, they experienced higher levels of meaningfulness compared to other activities; and this relationship was stronger in younger than older participants. At the between-person level, helping was also associated with more positive emotions (and unrelated to negative emotions); this effect was stronger for older than younger participants. Despite this, at a within-person level, helping others predicted a higher level of momentary negative emotions (but not momentary positive emotions) regardless of age. Findings suggest that older adults help others more than younger adults in daily life, which is consistent with a small but growing literature suggesting that prosociality increases with age. While individuals experienced more negative emotions while helping, the tendency to help is related to reporting higher mean levels of positive emotions and a greater sense of meaningfulness.

Abstract citation ID: igaf122.205

### THE EFFECTS OF VOLUNTEERING ON HYPERTENSION BY HEALTH INSURANCE STATUS AMONG U.S. ADULTS AGED 50-64

Byeongju Ryu[1], Seoyoun Kim[2], and Halvorsen Cal[1],
*1. Washington University in St. Louis, St. Louis, Missouri, United States, 2. University of Michigan, Ann Arbor, Michigan, United States*

Hypertension is a major risk factor for cardiovascular disease, the leading cause of death in the U.S. Managing blood pressure can be challenging for individuals without health insurance due to barriers to healthcare access. Emerging evidence suggests that volunteering may serve as a public health intervention that reduces hypertension. However, it remains unclear whether the relationship between volunteering and hypertension differs by health insurance status. We analyzed data from the 2012–2018 Health and Retirement Study, restricting participants to those aged 50-64 (below Medicare eligibility age; N = 4,366). To address selection bias and ensure correct temporal order, we created entropy-balance weights using participants' characteristics from 2012/2014 to balance volunteers and non-volunteers in 2014/2016. We then employed weighted Poisson regression with robust error variance to estimate the population average treatment effects of volunteering in 2014/2016 on hypertension in 2016/2018. We pooled waves because the outcome variable was measured in half the sample in consecutive waves. We ran seemingly unrelated estimations to statistically test for differences in effect sizes between insured and uninsured volunteers. Results indicate that volunteers were less likely to be hypertensive than non-volunteers in the full sample (IRR=0.91, p = 0.008). In subgroup analyses, volunteering was associated with a lower incidence of hypertension among both uninsured (IRR=0.70, p = 0.035) and insured (IRR=0.93, p = 0.035) respondents. These effects did not significantly differ (p = 0.104), suggesting that volunteers were less likely to be hypertensive regardless of insurance status. Our findings suggest that despite limited healthcare access, volunteering may be one pathway toward reducing hypertension in uninsured older adults.

Abstract citation ID: igaf122.206

### LEVERAGING ASYMMETRIC EFFECTS TO EXAMINE DOSE AND LONG-TERM EFFECTS OF HELPING BEHAVIORS ON HEALTH TRAJECTORIES

Sae Hwang Han, *The University of Texas at Austin, Austin, Texas, United States*

Despite extensive research on how (pro)social engagement influences health, methodological approaches for examining dose changes and sustained long-term engagement in relation to health outcomes using observational data remain limited. This paper introduces a methodological approach for analyzing the health benefits of helping behaviors, emphasizing two key issues: (1) dynamic changes in helper role status and time commitment and (2) the long-term effects of sustained engagement. To this end, we extend the asymmetric fixed-effects modeling approach within a multilevel framework that allows for elucidating how intraindividual changes in helping behaviors—measured as both helper role status and time commitment—shape health trajectories. The proposed approach also incorporates strategies to address multiple sources of endogeneity and ensure proper temporal ordering of key variables, strengthening confidence in potential causal relationships. We demonstrate an empirical application of this approach in examining the relationship between helping behaviors and cognitive function using longitudinal data from the U.S. Health and Retirement Study (1998–2020; N = 31,303). The study findings reveal asymmetric effects, with cognitive benefits associated with role acquisition and increased time commitment, while role withdrawal and dose reduction were linked to cognitive decline. Notably, sustained engagement in moderate levels of helping (e.g., 2–4 weekly hours)

yielded the greatest cognitive benefits, while abrupt changes, either in adopting or withdrawing from helping behaviors, were associated with less favorable outcomes. The proposed methodological approach provides a useful framework for evaluating modifiable lifestyle factors in gerontological research, offering new insights for designing interventions aimed at promoting health outcomes.

Abstract citation ID: igaf122.207

### DUAL FUNCTIONAL RECOVERY: THE CONTRASTING ROLES OF VOLUNTEERING AND HELPING BEHAVIORS

Samuel Nemeth[1], Seoyoun Kim[2], Cal Halvorsen[3], and Shawn Bauldry[1], *1. Purdue University, West Lafayette, Indiana, United States, 2. University of Michigan, Ann Arbor, Michigan, United States, 3. Washington University in St. Louis, St. Louis, Missouri, United States*

The concept of dual functionality integrates both physical and cognitive function – two important and interrelated domains for older adult health and well-being – and represents the absence of impairment in either domain. The loss of dual function presents a significant barrier to independent living; however, this loss may not be permanent. As such, guided by social integration theory, this study investigates the effect of two prosocial behaviors – formal volunteering and informal helping – on the recovery of dual function. We use nationally representative panel data from the Health and Retirement Study (2004-2016, N = 6,110), entropy balancing weights, and discrete time hazard models. After adjusting for selection and study covariates, volunteering was associated with lowered odds of recovery while helping was associated with increased odds of recovery (Odds Ratio (OR)=0.863 [95% CI: 0.754, 0.987]; OR = 1.120 [95% CI: 1.010, 1.243], respectively). Additionally, we found that age at loss of dual function moderated the effect of volunteering and helping ($p < 0.05$). Specifically, there is a significant difference in the effects of volunteering and helping when dual function is lost at younger ages. Findings underscore the complexity of recovery from impairment, the role prosocial behaviors may play in this process for older adults, and provide implications for health policy and interventions.

### SESSION 1200 (SYMPOSIUM)

Abstract citation ID: igaf122.208

### BLOOD-BASED BIOMARKERS OF AGING AND NEURODEGENERATION IN LONGITUDINAL COHORT STUDIES OF BRAIN AGING

Chair: Natascha Merten
Co-Chair: Josef Coresh
Discussant: Bryan James

Alzheimer's disease and related dementias (ADRD) have a long preclinical stage, with early pathological brain changes starting often in midlife, decades before any clinical symptoms. Identifying individuals at risk for cognitive and physical decline early will thus be most effective for intervention and prevention methods. Blood-based biomarkers are cost-effective and non-invasive and are promising for developing screening methods. To date, different blood-based biomarkers have been studied. Most of our knowledge stems from clinical study cohorts or studies enriched for patients with neurological diseases such as ADRD. Community- and population-based cohorts on the effectiveness of different biomarkers in the general population are rare. This symposium tries to fill this gap. We present work based on longitudinal data from the community-based Atherosclerosis Risk in Communities (ARIC) Study and the ACHIEVE trial as well as from the Beaver Dam Offspring Study-Neurocognitive Aging Study. We will show how different midlife and late life plasma-based biomarkers of neurodegeneration and AD are related to i) functional changes of cognition and ii) structural brain changes in cortical thickness later in life. Moreover, we will show how i) a combined risk prediction scores of plasma biomarkers of neurodegeneration and AD and ii) a blood-based biomarker of accelerated aging might help identify individuals at high risk for cognitive decline. This work will inform our understanding of the potential utility of these blood markers for a larger scale application in general population screenings for brain aging.

Abstract citation ID: igaf122.209

### CHANGE IN PLASMA BIOMARKERS OF ALZHEIMER'S DISEASE FROM MIDLIFE TO LATE-LIFE AND ASSOCIATIONS WITH COGNITIVE CHANGE

Priya Palta[1], James Pike[2], Keenan Walker[3], Kevin Sullivan[4], Bharat Thyagarajan[5], Thomas Mosley[4], and Josef Coresh[2], *1. UNC School of Medicine, Chapel Hill, North Carolina, United States, 2. New York University, New York, New York, United States, 3. National Institute on Aging, Baltimore, Maryland, United States, 4. University of Mississippi Medical Center - The MIND Center, Jackson, Mississippi, United States, 5. University of Minnesota, Minneapolis, Minnesota, United States*

Plasma biomarkers of Alzheimer's disease (AD) pathology offer a non-invasive method to assess brain changes linked to dementia and cognitive decline. However, the associations with specific cognitive domains and subsequent cognitive change in participants with or without cognitive impairment is unclear. Plasma biomarkers were assayed in ARIC participants using Quanterix SiMoA technology on samples from midlife (visit 3, 1993-95, mean age 59) and late-life (visit 5, 2011-13, mean age 77). Biomarkers included Aβ42:Aβ40 ratio, p-Tau181, neurofilament light (NfL), and GFAP. Global and domain-specific cognitive factor scores were calculated from neuropsychological tests administered across multiple visits. Linear mixed effects models estimated the association between biomarkers and late-life cognitive change, adjusting for demographics, lifestyle and cardiovascular risk factors, and APOE-ε4 status. Exploratory analyses examined effect modification of the biomarker-global cognition associations by visit 5 cognitive impairment. Among 1515 participants with plasma biomarkers (60.7% female, 25.5% identified as Black), 39% had cognitive impairment in late-life. Higher midlife NfL and GFAP were associated with faster declines in global cognition and language. NfL also predicted faster memory decline. In late-life, lower Aβ42:Aβ40 and higher p-Tau181, NfL, and GFAP were associated with faster declines in global cognition, memory, and language. Changes in NfL, p-Tau181, and GFAP from midlife to late-life were associated with declines in global cognition and language. No consistent effect modification by cognitive impairment was observed. Plasma biomarkers of AD pathology, neuronal injury, and astrogliosis show distinct associations with cognitive decline across domains when measured in midlife and late-life.

Abstract citation ID: igaf122.210

### CHANGE IN ALZHEIMER'S DISEASE BLOOD-BASED BIOMARKERS AND ASSOCIATIONS WITH CORTICAL THICKNESS

James Pike[1], Yifei Lu[2], Keenan Walker[3], Thomas Mosley[4], Rebecca Gottesman[5], Clifford Jack[6], Josef Coresh[1], and Priya Palta[7], *1. New York University, New York, New York, United States, 2. Merck, Landsdale, Pennsylvania, United States, 3. National Institute on Aging, Baltimore, Maryland, United States, 4. University of Mississippi Medical Center - The MIND Center, Jackson, Mississippi, United States, 5. National Institutes of Health, Bethesda, Maryland, United States, 6. Mayo Clinic, Rochester, Minnesota, United States, 7. University of North Carolina School of Medicine, Chapel Hill, North Carolina, United States*

Blood-based biomarkers show promise as a noninvasive method for measuring Alzheimer's disease pathology and neurodegeneration throughout the lifecourse. However, the relationship between longitudinal changes in these biomarkers and late-life brain morphology in community-dwelling populations requires further investigation. Between 2011 and 2013, 1,977 participants from the Atherosclerosis Risk in Communities Study received underwent 3 Tesla magnetic resonance imaging scans. Whole-brain cortical thickness was quantified by Freesurfer. Stored plasma samples collected in midlife (1993-95, mean age 59.0 years) and late-life (2011-13, mean age 76.8 years) from a subsample of 1,515 participants (60.7% female, 25.5% Black adults) were assayed in 2022 using Quanterix SiMoA. The assay quantified amyloid-β (Aβ)42/40, phosphorylated tau at threonine 181 (p-Tau181), neurofilament light (NfL), and glial fibrillary acidic protein (GFAP). Linear regression models estimated the association of blood-based biomarkers from midlife, late-life, and the change from midlife to late-life with cortical thickness in late-life. In models adjusted for demographics, lifestyle factors, cardiovascular factors, and the presence of APOE ε4 alleles, higher midlife measures of p-Tau181 and increases from midlife to late-life in NfL and GFAP were associated with lower cortical thickness in late-life. Concurrent measures of p-Tau181, NfL, and GFAP were associated with lower cortical thickness in late-life and exhibited a stronger association among participants ≥75 years old compared to participants <75 years old. Additional longitudinal research is needed to determine whether blood-based biomarkers predict cortical thinning over time and may serve as an early indicator of accelerated brain atrophy.

Abstract citation ID: igaf122.211

### PLASMA NEURODEGENERATION AND AD BIOMARKERS IDENTIFY POPULATIONS WITH RAPID COGNITIVE DECLINE

Josef Coresh[1], James Pike[1], Nicholas Reed[2], Priya Palta[3], Jennifer Deal[4], Kevin Sullivan[5], Bharat Thyagarajan[6], and Frank Lin[4], *1. New York University, New York, New York, United States, 2. New York University Grossman School of Medicine, New York, New York, United States, 3. University of North Carolina School of Medicine, Chapel Hill, North Carolina, United States, 4. Johns Hopkins Bloomberg School of Public Health, Baltimore, Maryland, United States, 5. University of Mississippi Medical Center - The MIND Center, Jackson, Mississippi, United States, 6. University of Minnesota, Minneapolis, Minnesota, United States*

Predicting rapid cognitive decline is valuable for patient counseling and targeting clinical trials to slow down this rate of decline. We used neurodegeneration and AD biomarkers measured in the ARIC cohort (amyloid-β 42 to amyloid-β 40 ratio, p-tau 181, NFL, and GFAP on frozen plasma collected in 2011-13 using the Quanterix SiMoA platform, N = 1825) to develop a risk score for the rate of decline in global cognition factor score and validated its performance among participants of the ACHIEVE clinical trial (same 4 biomarkers assayed using the Alamar CNS protein panel, N = 502 at the 3-year follow-up). Biomarkers were standardized within platforms using inverse-normal transformation. Cognitive decline risk scores developed in ARIC were applied to ACHIEVE for validation. Biomarker risk scores developed in ARIC validated in ACHIEVE with the upper quintile having a mean (95% CI) progression rate of -0.123 (-0.137, -0.108) in ARIC, -0.111 (-0.158, -0.065) in ACHIEVE (compared to mean of -0.012 in the total ACHIEVE validation sample). Adding age and Mini Mental State Exam did not improve the risk scores. The power of a clinical trial would change from 5% to 96% as mean annualized cognitive decline increases from -0.012 to -0.111 (selected top risk quintile; a progression SD of 0.25) in a trial of 800 participants and a 20% reduction in cognitive decline from an intervention. In conclusion, risk scores based on plasma biomarkers allow for identification of subgroups likely to experience rapid cognitive decline and may increase the power of cognitive decline clinical trials.

Abstract citation ID: igaf122.212

### ACCELERATED PHENOTYPICAL AGING IN MIDLIFE IS ASSOCIATED WITH LONG-TERM COGNITIVE DECLINE

Natascha Merten[1], Mary Ryan Baumann[1], Richard Chappell[1], Yanjun Chen[1], Lindsay Clark[1], Sterling Johnson[1], James Pankow[2], and Art Walaszek[1], *1. University of Wisconsin-Madison, Madison, Wisconsin, United States, 2. University of Minnesota, Minneapolis, Minnesota, United States*

PhenoAge, a multi-system biomarker uses easily-obtained common clinical blood tests and determines whether a person is younger or older on a biological and physiological level than expected by chronological age. Higher PhenoAge is associated with increased risk of disability, age-related morbidities and all-cause mortality. Its associations with early cognitive changes in midlife are less understood. The aim of this study was to determine whether accelerated PhenoAge in midlife was associated with 10-year cognitive changes in middle-aged to older adults. This longitudinal study is based on N = 2,630 (54% women;mean age 50years) Beaver Dam Offspring Study participants. We measured baseline blood-based clinical markers necessary for calculation of accelerated PhenoAge (PhenoAgeAccel). We tested Trail-making Test B (TMT-B) performance at baseline, 5-year and 10-year follow-up. We used linear mixed-effects model with PhenoAgeAccel as predictor and TMT-B time as outcome, adjusting for random intercepts, education and sex. Models were repeated sex-stratified. With every additional year older in PhenoAge compared to chronological age at baseline, participants performed worse on the TMT-B at baseline [overall:0.60 seconds slower, 95% Confidence Interval (0.29,0.91); women: 0.34(-0.05,0.72); men: 0.91(0.41,1.42)] and had a faster decline in TMT-B over 10-years [overall:0.94 seconds slower (0.58,1.29); women: 0.51(0.08,0.95); men: 1.48(0.90,2.07)]. Accelerated PhenoAge in midlife was associated with 10-year cognitive decline, overall and in men. Longer follow-up will be needed to determine whether PhenoAge might be predictive of cognitive impairment later in life. If confirmed, PhenoAge could become a cost-effective marker of cognitive decline and dementia and help identify at-risk individuals early and inform targeted interventions.

### SESSION 1205 (SYMPOSIUM)

Abstract citation ID: igaf122.213

### LONGITUDINAL PERSPECTIVES ON DEPRESSIVE SYMPTOMS AMONG OLDER ADULTS OR THEIR CAREGIVERS IN ASIA

Chair: Jeremy Lim-Soh
Co-Chair: Rahul Malhotra

Mental health is critical for older adults and their caregivers, both as a contributing factor to health behaviors and other health outcomes, and an important outcome in its own right. This symposium advances our understanding of older adults' or their caregivers' mental health, specifically depressive symptoms, in three ways. First, it focuses on studies that harness longitudinal data to provide a more robust and comprehensive picture of how depressive symptoms change over time, identify causal factors for depressive symptoms and the impact of depressive symptoms on healthcare use. Second, it investigates depressive symptoms in the context of aging in Asia, where expectations of support from kin and non-kin are different from some Western contexts, leading to potentially contrasting outcomes for mental health when such expectations are not met. Third, it explores various facets of social support, including social networks, family caregiving, and elder abuse, and their implications for depressive symptoms. Together, the findings call for earlier identification and management of depressive symptoms in older adults or their caregivers, and their risk factors. While social support can help to mitigate depressive symptoms, policymakers and practitioners should be sensitive to the complex and context-dependent relationship between support and depressive symptoms for older adults and their caregivers.

Abstract citation ID: igaf122.214

### DEPRESSIVE SYMPTOMS ARE ASSOCIATED WITH INCREASED HEALTHCARE UTILIZATION AMONG OLDER ADULTS IN SINGAPORE

Rahul Malhotra[1], Abhijit Visaria[1], Rakhi Vashishtha[2], Qiqi Cheng[3], and Angelique Chan[3], *1. Duke-NUS Medical School, Singapore, Singapore, 2. Centre for Behavioural and Implementation Science Interventions (BISI), Yong Loo Lin School of Medicine, National University of Singapore, Singapore, Singapore, 3. University of Manchester Manchester Institute of Education, Manchester, England, United Kingdom*

Population ageing presents a significant challenge to healthcare systems, as older adults typically have higher rates of healthcare utilization. This study investigated the impact of depressive symptoms on healthcare utilization among community-dwelling older adults in Singapore, focusing on emergency department (ED) visits, frequent ED use (≥4 visits), and inpatient hospitalizations. We analyzed data from a nationally representative longitudinal cohort with participants' survey data linked to electronic medical records to objectively measure healthcare utilization outcomes. We utilized two-part models for ED visits and hospitalizations and logistic regression for frequent ED use. We observed that higher depressive symptoms were associated with increased odds of ED visits and hospitalizations within 12 months. Each unit increase in the depressive symptoms score was linked with 5% higher odds of at least one ED visit and 6% higher odds of hospitalization. Furthermore, depressive symptoms were associated with a 13% increase in the odds of frequent ED use. This study provides robust evidence on the longitudinal association of depressive symptoms with healthcare utilization among older adults, specifically in an Asian population. Given the growing older adult population and the significant healthcare costs associated with hospitalizations and ED visits, these findings highlight the need for proactive identification and management of depressive symptoms in older adults to potentially reduce future healthcare burdens.

Abstract citation ID: igaf122.215

### NON-KIN NETWORKS MATTER MORE FOR LATER-BORN COHORTS OF UNMARRIED OLDER MEN'S DEPRESSIVE SYMPTOMS

Shannon Ang[1], Rahul Malhotra[2], and Grace Chua[1], *1. Nanyang Technological University, Singapore, Singapore, 2. Duke-NUS Medical School, Singapore, Singapore*

It is well-established that gender disparities in health are partially due to gender differences in social support network size and diversity – women are more likely to have better health outcomes because they tend to have larger and more diverse social support networks (i.e., including both kin and non-kin). Recent studies have shown cohort changes in network size and proportion of kin and non-kin members, but we know little about whether and how broad societal changes have altered the effect of social support networks on older adults' well-being. Moreover, these societal changes are likely to differ between married and unmarried individuals' well-being outcomes, since social support networks are altered through marriage. We explore cohort differences in how kin and non-kin social support networks are associated with depressive symptoms at the intersection of gender and marital status. We rely on six waves of data from two nationally representative surveys of older adults aged 60 years and above in Singapore. Results from growth curve models show that non-kin social support networks are more beneficial for unmarried men's depressive symptoms, compared to married men. Additionally, this effect is stronger for later-born cohorts of unmarried men. Our findings highlight the need to revise our understanding of the effectiveness of kin and non-kin social support networks for later-born cohorts of older adults. Policy interventions that focus on older adults' social support networks for better well-being will need to consider both non-kin and kin social support networks.

Abstract citation ID: igaf122.216

### FRACTURED CONNECTIONS: URBAN-RURAL DIVIDE IN CHINA LINKING ELDER ABUSE, SOCIAL NETWORKS, AND DEPRESSION

Shibin Yan[1], Zi Yan[2], and Xin Sun[3], *1. University of Pennsylvania, Camden, New Jersey, United States, 2. Waseda University, Tokyo, Tokyo, Japan, 3. Fudan University, Shanghai, Shanghai, China (People's Republic)*

The contextual theory of elder abuse suggests that elder abuse emerges from interactions across individual, relational, community, and societal contexts. While prior research has established the negative effects of elder abuse on older adults' physical and psychological well-being, its impact on their social dimensions—such as changes in social networks and the subsequent effects of those changes on their psychological well-being—remains underexplored. This study examines the relationship between elder abuse, social networks, and depressive symptoms using longitudinal data from 6,424 older adults (aged60 years and older) in the 2018 and 2020 China Longitudinal Ageing Social Survey. Mediation models were employed to assess whether changes in different types of social networks (contact, emotional, and instrumental) mediated the association between elder abuse experienced in 2018 and depressive symptoms in 2020. Mediation effects were estimated using Stata 17. Findings revealed that changes in social networks mediated the relationship between elder abuse and depressive symptoms, with notable urban-rural differences. Among rural older adults, elder abuse was linked to a decline in contact and emotional networks, which in turn exacerbated depressive symptoms. Conversely, among urban older adults, elder abuse was associated with an expanded instrumental network and lower depressive symptoms. These findings highlight the critical role of social networks in shaping the psychological outcomes of elder abuse victims. The divergent patterns between urban and rural older adults suggest the need for tailored social support interventions to mitigate the psychological impact of elder abuse.

Abstract citation ID: igaf122.217
### PERSISTENT AND EPISODIC DEPRESSIVE SYMPTOMS AMONG FAMILY CAREGIVERS AND NON-CAREGIVERS IN SINGAPORE
Jeremy Lim-Soh[1], Ha-Linh Quach[2], and Rahul Malhotra[1], *1. Duke-NUS Medical School, Singapore, Singapore, 2. The University of Sydney, Sydney, New South Wales, Australia*

While family caregivers, versus non-caregivers, have a higher risk of adverse psychological outcomes, less is known about the persistence of this risk over time. We assessed (a) the risk of persistent and episodic clinically relevant depressive symptoms (CRDS; 11-item CES-D score of seven or above) in family caregivers of older adults, relative to non-caregivers, and (b) among caregivers, care-recipient and caregiver characteristics associated with such depressive symptoms. Using longitudinal data (4 waves; 6-12 months apart) on 218 caregivers and 174 non-caregivers in Singapore, we classified them into three categories of CRDS over time: (1) persistent CRDS (CRDS in two or more consecutive waves), (2) episodic CRDS (CRDS in one or two non-consecutive waves), and (3) no CRDS. The prevalence of persistent and episodic CRDS was much higher among caregivers (23.4% and 23.4%, respectively) versus non-caregivers (9.2% and 16.1%, respectively). Logistic regression, adjusting for confounders, confirmed the higher risk of caregivers, versus non-caregivers, for persistent or episodic (versus no) CRDS. Among caregivers, their own chronic diseases and their care-recipient's mood, behavior, and memory impairments were associated with persistent (versus no) CRDS. Our findings highlight the substantial and enduring psychological distress that family caregivers are exposed to, underscoring the need to support their mental well-being.

### SESSION 1210 (SYMPOSIUM)

Abstract citation ID: igaf122.218
### FEELING YOUNGER, FEELING OLDER: UNPACKING THE COMPLEXITIES OF SUBJECTIVE AGE
Chair: M. Clara de Paula Couto
Co-Chair: David Weiss

Subjective age, or how young or old individuals feel compared to their chronological age, influences various developmental outcomes. This symposium explores subjective age through five studies using diverse methodologies, including longitudinal and experimental designs, to examine the determinants, consequences, and mechanisms of subjective age. Weiss et al. examine how social and temporal comparisons shape subjective aging. Their findings reveal that social comparisons lead to a relative younger subjective age, while temporal comparisons often result in more negative self-perceptions of aging and an older subjective age. de Paula Couto et al. build on the dual-process theory of developmental regulation. showing that individuals counteract societal negative attitudes towards aging by feeling younger (assimilation) or redefining old age (accommodation). Younger participants felt younger in valued domains, while older participants raised the threshold for old age. Gourley and Chasteen investigate the drawbacks of feeling younger in later adulthood from a social identity threat perspective. They find that a younger subjective age can lead to negative social evaluations from younger and middle-aged adults, thus countering its benefits. Kornadt et al. examine loneliness as a determinant of subjective age in a longitudinal study, showing that greater loneliness leads to an older subjective age trajectory, mediated by stress, highlighting its psychological toll and accelerating effects on subjective aging. Finally, Sabatini et al. explore subjective age's relation to future care planning, finding that dementia caregivers feel older and are more engaged in care preparation compared to never-caregivers, suggesting that a relative older subjective age fosters proactive future planning.

Abstract citation ID: igaf122.219
### A DUAL-PROCESS MODEL OF SUBJECTIVE AGING: THE ROLE OF TEMPORAL AND SOCIAL COMPARISONS
David Weiss[1], M.Clara de Paula Couto[2], and Klaus Rothermund[2], *1. University of Halle, Halle (Saale), Sachsen-Anhalt, Germany, 2. Friedrich-Schiller University Jena, Jena, Thuringen, Germany*

We propose and test a dual process model of subjective aging illustrating how social and temporal comparison processes shape subjective aging in opposite ways in the second half of life. First, an information-processing pathway primarily involves temporal comparisons, including reflective processing that leads to assimilation. Second, a motivational pathway involves social comparisons, including evaluative processing that results in contrast. We predicted and found across two studies – a 10-year longitudinal (Study 1, N = 2,425, 39-93 years; 55.5% women) and an experimental study (Study 2, N = 160, 50-75 years, 58% women) - that social comparisons ("me vs. them") result in more positive self-perceptions of aging and feeling relatively younger, whereas temporal comparisons ("me vs. past/future me") lead to more negative self-perceptions of aging and feeling relatively older. We discuss how these insights can enhance our understanding of the cognitive and motivational processes that shape subjective aging and can inform interventions and policies aimed at fostering positive self-perceptions of aging and promoting healthy aging.

Abstract citation ID: igaf122.220
### SUSTAINING A YOUNG SELF-PERCEPTION: FEELING YOUNGER OR REDEFINING AGE BOUNDARIES?
M.Clara de Paula Couto[1], Fiona Rupprecht[2], Klaus Rothermund[1], and Jana Nikitin[2], *1. Friedrich-Schiller University Jena, Jena, Thuringen, Germany, 2. University of Vienna, Vienna, Wien, Austria*

Our study investigates how individuals counteract societal negative attitudes towards aging by striving to maintain a youthful self-image, either by feeling younger than their actual age or by redefining what they consider "old age". Based on the dual-process theory of developmental regulation, feeling younger is the result of an assimilative strategy, while adjusting the concept of old age is an accommodative strategy. We examined these strategies across different life domains, such as family, friendships, and work, hypothesizing that people emphasize youth-preserving strategies in areas critical to their identity. We focused on whether older adults more commonly adjust their definition of old age in important life domains (accommodative response), versus younger adults preferring to feel younger in domains that are deemed important (assimilative response). Analyzing data from 768 participants aged between 30 to 80 years through multilevel analysis, our research confirmed these hypotheses. Findings reveal that individuals indeed work to maintain a youthful identity in domains they find important. Younger participants felt younger in crucial life domains, whereas older participants adjusted their old age threshold in personally relevant domains. This behavior suggests a societal tendency to value youth, with individuals using both old age perception adjustment and feelings of youthfulness to protect their self-image and avoid the stigma of being old.

Abstract citation ID: igaf122.221

### SUBJECTIVE AGE, FUTURE CARE PREPARATION, AND THEIR ASSOCIATION IN DEMENTIA CAREGIVERS AND NEVER-CAREGIVERS

Serena Sabatini[1], Emily Mroz[2], Shelbie Turner[3], Fiona Rupprecht[4], Victoria Tischler[5], and Blossom Stephan[6], *1. University of Barcelona, Barcelona, Catalonia, Spain, 2. Emory University, Atlanta, Georgia, United States, 3. Weill Cornell Medicine, New York, New York, United States, 4. University of Vienna, Vienna, Wien, Austria, 5. University of Surrey School of Psychology, Guildford, England, United Kingdom, 6. Curtin EnAble Institute, Perth, Western Australia, Australia*

Subjective age refers to the age an individual feels like, which can differ from their chronological age. Future care preparation refers to the process of sharing preferences regarding future care with health-care professionals, family, or friends. Engagement in future care preparation is important as it is related to positive psychological outcomes (i.e., lower anxiety and depression), and it limits interpersonal and economic challenges. This study examined differences in subjective age and future care preparation in dementia caregivers vs. never-caregivers in the UK, and investigated the moderating role of caregiving status in the association of subjective age with future care preparation. An online survey was conducted with 259 informal dementia caregivers (Mean age=62.83; SD = 9.24) and 1699 never-caregivers (Mean age=67.79 years; SD = 8.05 years). Unadjusted and adjusted (for age, sex, education, marital status, working status, physical and mental health) regression models were estimated. Never-caregivers felt 4% younger than dementia caregivers (p<.001). Although both dementia caregivers and never-caregivers reported low future care preparation, being a dementia caregiver was significantly associated with more future care preparation (Mean=13.65) compared to never-caregivers (Mean=12.67). In the overall sample a higher subjective age was associated with greater future care preparation (Adjusted unstandardized regression coefficient=-1.71; 95%CI: -3.01, -0.39). Caregiving status did not moderate the association of subjective age with future care preparation. Hence, a higher subjective age is related to more future care preparation both in dementia caregivers and never-caregivers. Strategies promoting future care preparation maybe more effective if they focus on sense of ageing rather than targeting caregiver status.

Abstract citation ID: igaf122.222

### YOUNG AT HEART: HOW SUBJECTIVE AGE INFLUENCES PERCEPTIONS AND EXPECTATIONS OF OLDER ADULTS

Amy Gourley, and Alison L. Chasteen, *University of Toronto, Toronto, Ontario, Canada*

A younger subjective age is often associated with positive health outcomes among adults over the age of 65. However, it is also possible that those who attempt to look or act younger than their chronological age may face backlash given this behavior is in violation of prescriptive stereotypes that serve to maintain hierarchical age group boundaries. The degree to which younger and middle-aged perceivers anticipate violations of prescriptive age stereotypes (i.e., that older adults should act their age) may predict negative evaluations of older adult targets who feel 'younger than their years.' The present work examined younger and middle-aged adults' (N = 678; Mage = 35.8, SDage = 9.56) attitudes and evaluations regarding 65-year-old targets who varied by gender (man, woman) and felt age (65, 45, or 25). Participants also reported their expectations regarding older targets' potential violation of succession-, consumption- and identity- related prescriptive age stereotypes. Expectations of prescriptive stereotype violation mediated the relationship between older targets' younger felt age and participants' ratings of targets' warmth, competence, and interaction intentions. Specifically, targets who felt younger than their chronological age were expected to violate prescriptive stereotypes, which in turn decreased ratings of targets' warmth and competence and lessened participants' willingness to interact with targets. Our findings suggest that a younger subjective age may not always benefit older adults. Specifically, consistent with a Social Identity Threat perspective, younger perceivers' concerns about preserving strict age group boundaries may explain discriminatory attitudes and evaluations of counter-stereotypical older adults.

Abstract citation ID: igaf122.223

### ASSOCIATIONS OF SUBJECTIVE AGE TRAJECTORIES WITH LONELINESS AND STRESS ACROSS ADULTHOOD

Anna Kornadt[1], Markus Wettstein[2], Anthony Lepinteur, Claus Vögele[1], and Conchita D'Ambrosio[1], *1. University of Luxembourg, Esch-sur-alzette, Grevenmacher, Luxembourg, 2. Humboldt-Universität zu Berlin, Berlin, Berlin, Germany*

Subjective age, that is the age a person feels compared to their chronological age, is indicative of a variety of aging processes. Studies that investigate multi-variate, dynamic, longitudinal relations of subjective age with potential determinants and mechanisms of these relations have rarely been employed. In the current study, we focus on loneliness as a potential subjective age determinant, as loneliness affects a variety of psychosocial and health outcomes across life and is stereotypically perceived as a feature of old age. We investigate whether loneliness is related with levels and changes in subjective age and test whether this association is mediated via self-reported stress. N = 5,594 adults aged 18 – 93 years (Mage = 50.41, SD = 15.99) who participated in a longitudinal survey comprising up to three measurement occasions over a time span of 2.5 years reported their loneliness, subjective age, and stress as well as sociodemographic and health-related covariates. We employed latent growth modeling and found that, when controlling for sociodemographic and health-related covariates, higher loneliness was related to an older subjective age cross-sectionally and a to steeper increase in subjective age over time. These relations were mediated via stress; however, the relation between stress and subjective age was no longer statistically significant when including the covariates. All associations were qualified by significant interactions with chronological age, albeit in different directions. Our findings attest to the associations between loneliness, stress and subjective aging experiences and highlight the need for an age-informed approach when planning further studies and interventions.

### SESSION 1215 (PAPER)

### ADULT PROTECTION AND ELDER ABUSE

Abstract citation ID: igaf122.224

### CASE MANAGEMENT IMPROVES ANXIETY AND DEPRESSIVE SYMPTOMS FOR APS CLIENTS AT CASE CLOSURE

Courtney Reynolds[1], Samantha Tuft[1], Farida Ejaz[1], Jessica Bibbo[1], and Traci Lee[2], *1. Benjamin Rose Institute on Aging, Cleveland, Ohio, United States, 2. Utah Department of Health & Human Services, Salt Lake City, Utah, United States*

Caretaker neglect is the second most common type of abuse in the United States. In Utah, 74% of cases of caretaker neglect investigated by Adult Protective Services (APS) result in an 'inconclusive disposition.' With federal funding, practitioners from Utah APS collaborated with researchers from Benjamin Rose to develop and evaluate a telephone-based case management intervention for APS clients and their caretakers when there was an inconclusive finding of caretaker neglect. The goal was to prevent future reports of caretaker neglect or abuse. Using a four-month, pre-post design, care coordinators interviewed study participants, developed individualized care plans and referred to services and supports, making modifications as needed. In total, 95 clients and 83 alleged perpetrators consented to participate in the study. This presentation focuses on 54 clients who completed the baseline and posttest interviews. The average age of APS clients was 61 years (range: 18– 89), 56% were male, 26% lived in a rural/frontier area, and 24% participated with their alleged perpetrator. APS clients experienced a significant pre-post reduction in depressive symptoms using the Patient Health Questionnaire-2 ($p = .007$) and anxiety symptoms using the Generalized Anxiety Disorder-2 ($p = .049$). Discussion will focus on the impact of providing case management on APS clients' improved anxiety and depressive symptoms at case closure. Implications for policy, research and practice include the value of multidisciplinary collaborations to develop interventions to prevent future caretaker neglect, and possibly other forms of abuse, neglect and exploitation.

Abstract citation ID: igaf122.225

### IMPLEMENTATION OF A DECISION SUPPORT ALGORITHM FOR MULTIDISCIPLINARY TEAMS: INITIAL CASE COMPARISONS

Zachary Gassoumis[1], and Erin Thayer[2], *1. UTHealth Houston, Houston, Texas, United States, 2. University of Southern California, Los Angeles, California, United States*

Adult maltreatment multidisciplinary teams (MDTs) bring together professionals from health, social service, and justice systems to address complex cases of abuse, neglect, and self-neglect of older adults and younger adults with disability. MDTs are a promising intervention with demonstrated system outcomes, but they experience challenges identifying cases for review. In partnership with San Francisco Adult Protective Services (APS), we developed and implemented an algorithm to identify APS cases that appear appropriate for MDT review. Once identified by the algorithm as being potentially appropriate for an MDT, cases were randomized to a control group or for recommendation to APS staff as a decision support resource. This study attempts to better understand the algorithm-selected cases by comparing the control group with all cases presented to the MDT from October 2024 onward, using Fisher's Exact and Wilcoxon Rank Sum tests. Preliminary results ($N = 58$) include that MDT and control cases had comparable reduction in risk from case opening to closure ($p=.70$), but MDT cases were significantly more likely to be closed due to receiving services from other agencies (32% vs. 10%, $p=.048$). MDT cases were somewhat, though not significantly, less likely to have clients who refuse all interventions (0% vs. 15%, $p=.063$). These results provide insight into the operation of MDTs in the presence of this decision support tool, suggesting potential targets for further analyses. This research is one piece in a move toward developing and testing practice enhancement for APS and MDTs, as a way to improve outcomes for elder mistreatment cases.

Abstract citation ID: igaf122.226

### MOTIVATIONAL INTERVIEWING VS CASE MANAGEMENT FOR ELDER ABUSE INTERVENTION: A COMPARISON STUDY

Elsie Yan[1], Jasmin He[1], Daniel W. L. Lai[2], David Burnes[3], Karl Pillemer[4], and Mark Lachs[5], *1. The Hong Kong Polytechnic University, Hong Kong, Hong Kong, China (People's Republic), 2. Hong Kong Baptist University, Hong Kong, China (People's Republic), 3. University of Toronto, Toronto, Ontario, Canada, 4. Cornell University, Ithaca, New York, United States, 5. Weill Cornell Medicine, New York, New York, United States*

Elder abuse is a serious public health issue. Considerable efforts have been made to develop interventions to address this issue. This study compared the effectiveness of a 4-session motivational interviewing (MI) program and an 8-session case management (CM) program in reducing abuse severity, promoting physical and mental health, general self-efficacy, social support, and motivation to change. Baseline data was collected from 129 older adults screening positive for elder abuse in a community survey. Assessments were repeated at the end of the intervention for the 86 cases receiving MI intervention and 43 cases receiving case management, and at 3-month follow up for 20 cases in the MI group. No demographic difference was observed between cases assigned to MI and CM at baseline. Wilcoxon signed-rank tests were conducted to examine intervention outcomes by different time points. At post-test, the MI group demonstrated significant improvement in abuse severity, psychological distress, general self-efficacy, social support and motivation to change (p<.001). The CM group also showed improvement in these areas with the exception of general self-efficacy (p<.01). Significant difference was observed between MI and CM group in their improvement in general self-efficacy. Abuse severity and psychological distress continued to drop at 3-month post-intervention (p<.05). Abuse severity and psychological distress continued to decrease between post-intervention and 3 month-follow-up (p<.05). Our findings suggest that MI is a cost-effective intervention for reducing abuse severity and risk.

Abstract citation ID: igaf122.227

### RESPONDING TO ELDER ABUSE WHEN VICTIMS OPPOSE LEGAL INTERVENTION: INSIGHTS FROM THE FRONT LINES

Catheryn Koss, *California State University, Sacramento, Sacramento, California, United States*

Elder abuse cases present unique challenges when victims are reluctant to prosecute their abusers or take other legal action, often due to familial ties. This qualitative study examines insights from over 20 professionals, including prosecutors, Adult Protective Services (APS) workers, and private attorneys, who work directly with elder abuse victims. Several key themes were identified across the in-depth interviews that highlight the complexities of balancing legal imperatives with victim-centered approaches. One theme is asynchronous time, where the legal system moves too quickly when victims need more time and too slowly when urgent action is required. Another is the importance of an ally, emphasizing the need for a trusted advocate to support victims. Participants also stressed active and open listening, which helps victims share their stories, reduce shame, and provide prosecutors with valuable context for decision-making. A tension emerged around division of labor and continuity of support—while multiple professionals must be involved, victims also need a consistent contact person for trust and stability. Keeping victims informed is also critical, yet professionals disagreed on how much influence victims should have over prosecution decisions. Some advocated for incorporating victims' concerns into decisions about legal responses, including sentencing recommendations, while others emphasized broader criminal justice priorities and the importance of protecting victims and others from harm. This presentation will explore these and other themes and discuss strategies for navigating elder abuse cases when victims resist or oppose legal action. Attendees will gain insights into improving responses and fostering victim engagement in the legal process.

Abstract citation ID: igaf122.228

### SCREENING FOR ELDER MISTREATMENT RISK IN CAREGIVERS: CLINICIAN PERSPECTIVES ON BARRIERS, FEASIBILITY, & RESPONSE

Kelly Marnfeldt[1], Eleanor Batista-Malat[2], Zachary Gassoumis[3], Bonnie Olsen[2], Laura Mosqueda[4], and Alexis Coulourides Kogan[5], *1. University of Southern California Leonard Davis School of Gerontology, Los Angeles, California, United States, 2. University of Southern California, Los Angeles, California, United States, 3. UTHealth Houston, Houston, Texas, United States, 4. University of Southern California, Pasadena, California, United States, 5. Keck Medicine of USC, Los Angeles, California, United States*

Elder mistreatment (EM) risk screening in primary care presents challenges, particularly when aimed at assessing caregivers of people living with dementia. This secondary qualitative analysis examines how primary care clinicians conceptualize elder mistreatment, the feasibility of screening caregivers, and the perceived barriers and facilitators to screening implementation. Data were drawn from five focus groups (N = 38) conducted with multidisciplinary personnel at general and geriatric primary care clinics. Using Braun and Clarke's reflexive thematic analysis, we analyzed focus group discussions to explore attitudes toward elder mistreatment screening among caregivers of patients with dementia, its practicality in primary care workflows, and related concerns. Participants viewed EM screening as feasible under certain conditions but raised concerns about time constraints, difficulty in dementia caregiver identification, and cultural and linguistic barriers. Some worried that screening might feel accusatory rather than supportive, emphasizing the need for careful framing. Respondents struggled with defining who qualifies as a caregiver, particularly when caregivers were not included in medical records or were absent during visits. While many saw screening as a potential opportunity to assess caregiver needs and connect them with resources, they noted that without clear implementation strategies and resources, feasibility remained uncertain. Effective implementation of dementia caregiver-focused EM risk screening requires clinician and site buy-in, cultural responsiveness, and framing that reassures rather than alienates caregivers. Findings highlight the importance of embedding screening within broader caregiver support efforts and ensuring clinicians feel equipped to navigate sensitive conversations.

Abstract citation ID: igaf122.229

### UTILIZING FOCUS GROUPS TO INFORM THE DEVELOPMENT OF THE FINANCIAL EMPOWERMENT SCALE

Carson De Fries[1], Riley Coyne[2], Pilar Ingle[2], Ingrid Backes[2], Briana Kohlbrenner[2], Shannon O'Gara-Standiford[2], Asia Cutforth[2], and Eric Chess[2], *1. University of Utah, Salt Lake City, Utah, United States, 2. University of Denver, Denver, Colorado, United States*

Financial fraud and exploitation (FFE) are associated with billions of dollars lost each year and have significant adverse effects on older adults. Multiple domains have been identified that impact the risk of FFE among older adults including individual behaviors, personality traits, social factors, financial literacy, and cognitive health. However, no accessible tool comprehensively assesses these factors and the ways they intersect to better identify potential risk of FFE. This study is part of a larger ongoing project to develop a scale assessing financial wellbeing among older adults and aims to: (1) determine key domains associated with risk for FFE, (2) inform the development of the Financial Empowerment Scale (FES), and (3) assess the need for and applicability of the FES from different professional viewpoints. To achieve these aims, semi-structured focus groups were conducted with experts in the field. Thirty-six individuals across six focus groups participated, representing financial institutions, academia, and aging and social services. Data were analyzed through an iterative, mixed inductive-deductive content analysis approach. Five domains were identified as core components influencing risk factors for FFE: Cognitive Health, Financial Wellbeing, Social Health, Individual Behaviors, and Health and Environment. Focus group participants also expressed the need for a scale such as the FES to support older adults and professionals in the field. Findings from this study expand on the literature surrounding FFE among older adults and support the need for FES development. Findings around domains will be applied to item generation in the development of the FES.

### SESSION 1220 (PAPER)

Abstract citation ID: igaf122.230

### BIOMARKERS FOR FRAILTY AND FUNCTIONAL DECLINE IN AGING

In this session research will be presented on biological and clinical biomarkers for frailty and functional decline in older adults.

Abstract citation ID: igaf122.231

### DEFINING FRAILTY WITH THE SWHI TOOL IN THE SHARE DATABASE: PREDICTING FALLS AND FRACTURES OCCURRENCE ACROSS WAVES

Paolo Mazzola[1], and Justin Brathwaite[2], *1. University of Milano-Bicocca, Monza, Lombardia, Italy, 2. University of Minnesota, Minneapolis, Minnesota, United States*

Introduction: Tools for assessing frailty can be used both in clinical settings and in community medicine. However, the practice of frailty assessment in community medicine is often limited by time constraints and workload reported by general practitioners. Methods: Accessing the Survey of Health, Ageing and Retirement in Europe (SHARE), we performed descriptive statistics of the study population belonging to Wave 8 interviews. Frailty was defined with 4 questions of the Simplified sWHI tool (range 0-5): 1) extent of limitation in daily activities (score 0-2); 2) frequency of feeling tired (0-1); 3) frequency of practicing activities that require a moderate amount of energy (0-1); 4) reported weight loss of > 4 kg (0-1). Subjects were frail for sWHI score>3, pre-frail for sWHI score 1 or 2, and non-frail for sWHI=0. Outcomes were hip fracture occurrence and falls occurrence in the last 6 months (evaluated at Wave 9 interviews). Results: The study population included 31,471 individuals (mean age 71.2±9.5 years): 10,242 were Non-frail, 15,435 were Pre-frail, and 5,139 were Frail. Multivariable logistic regression analyses, adjusted for age, sex, and comorbidity, showed that being pre-frail and frail increases significantly and independently the risk of hip fractures (OR 1.9 and 3.6, respectively ) and falls occurrence (OR 1.7 and 3.7, respectively) compared to being non-frail (p<.001). Conclusions: The sWHI tool is useful and quick for identifying frailty with 4 simple questions, and can be adopted by general practitioners to guide the implementation of prevention strategies against this syndrome.

Abstract citation ID: igaf122.232

### IDENTIFYING BIOMARKERS OF FRAILTY THROUGH AN INTEGRATED MULTI-OMICS ANALYSIS

John Batsis[1], Dakota Batchek[1], David Lynch[2], Laura Herring[1], Whitney Stutts[1], Danae Gross[1], and Hillary Spangler[1], *1. The University of North Carolina at Chapel Hill, Chapel Hill, North Carolina, United States, 2. University of North Carolina School of Medicine, Chapel Hill, North Carolina, United States*

Background: Older adults are vulnerable to frailty, leading to adverse outcomes. We examined baseline metabolomic/proteomic biomarkers associated with different frailty states with a goal of advancing precision medicine in a pilot study of older adults pursuing physical therapy. Methods: We conducted a prospective study of 18 adults (≥65 years) from primary care, referred for physical therapy. Frailty was classified using a modified Cumulative Deficit Index (38 variables). Baseline gait speed, grip strength and instrumental activities of daily living (ADL) score (0-100) were assessed. Plasma samples underwent untargeted metabolomics and proteomics. Proteomics used Mag-Net enrichment to detect low-abundance proteins, followed by LC-MS/MS analysis on a Thermo Neo-Orbitrap Astral. Metabolomics involved methanol extraction and CE-MS/MS analysis using a 908Devices ZipChip-Thermo Fusion Lumos. Results: The mean participant age was 77.1±5.5 (33% female). There were 12 were pre-frail and 6 frail participants. Baseline function differed between groups: gait speed was 1.23±0.26 vs. 0.98±0.21 m/s ($p = 0.01$), grip strength was 28.8±9.5 vs. 27.1±5.6kg ($p = 0.98$), and instrumental ADL scores were 100±0 vs. 66.1±22.9 ($p = 0.005$). Among 4,300 quantified proteins, ~60 differed ($p < 0.05$; log2-fold change ±0.5) with ~40 proteins elevated in frail individuals, including nucleosome-related proteins (H2B, H3C1, H2AC3, H3-4). Metabolomic analysis assessed ~275 metabolites quantifying 115. Nicotinamide was significantly downregulated in the frail group ($p < 0.05$; log2-fold <-0.5). Conclusions: This pilot study suggests that circulating nucleosomes–complexes of histones and DNA–may serve as biomarkers for aging and related conditions. Additionally, nicotinamide, a precursor of $NAD^4$, a key coenzyme in metabolism, DNA repair, and mitochondrial function, declines with age, potentially contributing to age-related conditions.

Abstract citation ID: igaf122.233

### IDENTIFYING EARLY SIGNS OF AGING: MOBILITY AND FUNCTIONAL IMPAIRMENT IN MIDDLE-AGED ADULTS

Roee Hayek[1], Shmuel Springer[1], and Rebecca Brown[2], *1. Ariel University, Ariel, HaMerkaz, Israel, 2. University of Pennsylvania, Philadelphia, Pennsylvania, United States*

Middle age (45 to 64 years) is a pivotal stage of life when early signs of mobility and functional decline appear to emerge. Despite growing evidence of these changes, no comprehensive review has assessed them. This narrative review examined studies that use self-report assessments to investigate mobility and function and their association with chronic health conditions in middle-aged adults. Data from over 200,000 participants from several countries show that the prevalence of mobility and functional impairments varies widely, from 3.7% to 69.8%, with most studies reporting rates between 15% and 25%. Chronic health conditions have a substantial influence on the prevalence of such impairments, with chronic musculoskeletal pain and arthritis having the highest population-attributable risks (PARs) at 15-38%. Multimorbidity (i.e., having two or more chronic conditions) affects 23-33% of middle-aged adults, with a PAR exceeding 40%. Conversely, hypertension has a PAR of only 3%. Self-reported assessments may underestimate mobility impairments due to ceiling effects, as they are often based on difficulties in activities of daily living (ADLs), which are relatively less demanding for middle-aged adults and primarily capture advanced limitations rather than early or moderate impairments. This analysis underscores the importance of early detection through age-specific tools, targeted measures and interventions to address modifiable risk factors, and integrated care models to ensure long-term mobility and independence.

Abstract citation ID: igaf122.234

**SELF-RATED HEALTH AS A RELIABLE PREDICTOR OF INCIDENT FRAILTY IN THE PRESENCE OF COMPETING MORTALITY**

Mallak Alzahrani[1], Megan Huisingh-Scheetz[2], Melissa Hladek[1], and Qian-Li Xue[1], *1. Johns Hopkins University, Baltimore, Maryland, United States, 2. The University of Chicago, Chicago, Illinois, United States*

Self-rated health (SRH) predicts frailty in longitudinal studies, but failing to account for competing mortality risks may bias its predictive value for frailty. Using data from 4,755 adults aged 65+ who participated in the National Health and Aging Trends Study between 2011 and 2019, we analyzed the association between SRH and incident frailty while accounting for frailty-free mortality (i.e., death before frailty onset). Included in the analysis are those who lived in the community or residential care settings, had no probable dementia, and were not frail (per the physical frailty phenotype) at baseline. Over a median follow-up of 4 years, 1,564 developed frailty (74 per 1000 person-years), and 639 died before frailty onset (30 per 1000 person-years). After adjusting for demographics, number of chronic diseases, and psychological wellbeing, worse SRH independently predicted a higher risk of both frailty and frailty-free mortality, with a stronger association for frailty. Specifically, compared to those reporting excellent health, participants rating their health as very good, good, fair, and poor respectively had 1.3, 1.8, 2.6, 3.3 times higher frailty risk (all $p<0.05$), and 1.2, 1.5, 1.4, 2.5 times higher risk of frailty-free mortality ($p=0.11$, 0.01, 0.05, $< 0.01$). The associations remained strong across age groups. These findings suggest that older adults may intuitively perceive subtle functional declines before clinical frailty emerges. The weaker link to frailty-free mortality may reflect the role of unpredictable acute health events. As a low-cost, scalable tool, SRH can help identify early frailty risk and guide targeted prevention efforts.

Abstract citation ID: igaf122.235

**SINGLE-NUCLEUS RNA SEQUENCING REVEALS MUSCLE FIBER CELL HETEROGENEITY DURING HUMAN SKELETAL MUSCLE AGING**

Li Wu[1], Caixia Gong[1], Ange Wang[2], Jing Chen[2], and Qin Zhang[2], *1. Zhejiang University School of Medicine, Hangzhou, Zhejiang, China (People's Republic), 2. The First Affiliated Hospital, Zhejiang University School of Medicine, Hangzhou, Zhejiang, China (People's Republic)*

Skeletal muscle aging is characterized by a progressive decline in muscle mass and function, yet its cellular and molecular mechanisms remain incompletely understood. In this study, we utilized single-nucleus RNA sequencing (snRNA-seq) to construct a comprehensive molecular atlas of skeletal muscle aging by analyzing vastus lateralis muscle samples from five Chinese male adults (aged 22 to 60 years) and three older adults (aged 99 to 101 years). Our analysis revealed significant muscle fiber atrophy in the older group, particularly a reduction in type IIA and IIX fibers, accompanied by a relative increase in $RUNX1^+$, $NCAM1^+$, and $SORBS2^+$ fiber subpopulations. Differential gene expression and pathway enrichment analyses identified age-related alterations in metabolic pathways, calcium signaling, HIF-1, and MAPK pathways. Cell–cell communication analysis demonstrated an enhanced interaction network in aged muscle, with increased crosstalk between mast cells and fibro-adipogenic progenitors (FAPs), suggesting a role in extracellular matrix remodeling and chronic inflammation. Notably, ADGRL signaling was uniquely activated in type I and type II fibers in the older group, while CD99, NRG, and COLLAGEN signaling pathways were specifically upregulated, highlighting their potential roles in extracellular matrix remodeling and inflammation during aging. This study provides novel insights into the cellular diversity and molecular mechanisms underlying human skeletal muscle aging, offering potential therapeutic targets to mitigate age-related muscle decline.

### SESSION 1225 (PAPER)

### COMMUNITY, CULTURE, AND CARE: PATHWAYS TO AGING EQUITY

Abstract citation ID: igaf122.236

**ADOPTING A CULTURAL LENS ON SOCIAL CAPITAL OF OLDER CHINESE AND SOUTH ASIANS IN HONG KONG: A COMPARATIVE CFA STUDY**

Codiez Z. D. Huang[1], Daniel W. L. Lai[1], Alison Y. T. Ou[1], Vincent W. P. Lee[1], Jessica J. Li[2], Shireen Surood[3], Doris S. F. Yu[4], and Gary K. K. Lau[4], *1. Hong Kong Baptist University, Kowloon, Hong Kong, China (People's Republic), 2. The Chinese University of Hong Kong, Hong Kong, China (People's Republic), 3. Island Health, Victoria, British Columbia, Canada, 4. The University of Hong Kong, Hong Kong, China (People's Republic)*

Social capital structures play an important role in addressing social and health care needs of older adults. Knowing culture provides the context for values, beliefs and behaviors, it is imperative to use an ethnically-specific approach to understand the complexity of social capital structures in a multi-national population. Ethnic diversity is a prominent characteristic among the aging population in Hong Kong, with the South Asian aged 55 increased at almost three times the rate of the local aged cohort. This study aimed to examined how social capital structures determined their care needs among these diverse group. This study conducted telephone survey to 800 Hong Kong older adults via random digital dialing. Another purposive sample of 215 South Asians were interviewed face-to-face. Confirmatory Factor Analysis revealed notable differences in social capital structures between the groups, with both models showing excellent reliability (South Asians: CFI=0.999, TLI=0.999, RMSEA=0.078; Hong Kong: CFI=0.991, TLI=0.987, RMSEA=0.077). South Asian older adults emphasized neighborhood cohesion, community interactions, and collective support, reflecting collectivist cultural values. Conversely, Hong Kong older adults focused on individual community participation, family-oriented support, and trust networks from family to neighbors, indicative of a more individualistic cultural orientation centered on personal achievements and family ties. These findings highlight the need for culturally responsive social policies. Programs for South Asian older adults should enhance neighborhood ties and collective support, while initiatives for Hong Kong older adults should strengthen familial networks and personalized social engagement. Future research should explore these distinctions to guide inclusive aging policies and community interventions.

Abstract citation ID: igaf122.237

**DYADIC ASSOCIATIONS OF DEPRESSIVE SYMPTOMS AND CARDIOVASCULAR HEALTH IN MIDDLE-AGED AND OLDER COUPLES**

Yenee Soh[1], Richard Jones[2], Izzuddin Aris[3], Ichiro Kawachi[1], and Henning Tiemeier[1], *1. Harvard T.H. Chan School of Public Health, Boston, Massachusetts, United States, 2. Brown University, Providence, Rhode Island, United States, 3. Harvard Medical School, Boston, Massachusetts, United States*

Depressive symptoms contribute to worse cardiovascular health, yet the role of spousal depressive symptoms on this association remains unknown. We examined the longitudinal association between depressive symptoms and cardiovascular health (CVH) in spousal dyads within the Health and Retirement Study (HRS), a cohort of U.S. adults aged 50+ years. Among 4,360 heterosexual spousal dyads, depressive symptoms were assessed at baseline (Wave 1) using the modified 8-item Center for Epidemiologic Studies-Depression scale. The HRS collected sociodemographic covariates at baseline (age, race and ethnicity, and education) and measures of body mass index, blood pressure, fasting glucose, total cholesterol, and smoking (Waves 2-4), which were used to calculate a CVH score (range 0-100 points; higher scores reflect better CVH) as defined by the American Heart Association. We used the Actor-Partner Interdependence Model and latent growth curve modeling to examine how both spouses' depressive symptoms were associated with subsequent CVH, adjusting for covariates. For men, their own (B=-1.27; 95%CI:[-1.65,-0.89]) and their spouse's depressive symptoms (B=-0.42; 95%CI:[-0.74,-0.11]) at baseline were associated with their own CVH at Wave 2. Similar associations were observed for women's own and their spouse's depressive symptoms on their CVH. Additionally, only husbands' depressive symptoms were associated with a faster decline in wives' CVH (B=-0.08; 95%CI:[-0.14,-0.03]) over an 8-year period (Waves 2-4). Our findings suggest both an individual's and their spouses' depressive symptoms may mutually influence long-term CVH. Addressing depressive symptoms within the spousal context may be a strategy to improve CVH and reduce cardiovascular disease risk in middle-aged and older couples.

Abstract citation ID: igaf122.238

**LESSONS FROM THE REAL WORLD: PREPARING DEMENTIA CARE PARTNERS USING REACH-TX**

Stephani Shivers[1], Dante Tipiani[1], Amy Puca[2], Vicki Zimmer[2], Alan Stevens[3], and Edward Cisek[1], *1. CaringKind, New York, New York, United States, 2. Via Evaluation, Buffalo, New York, United States, 3. Baylor Scott & White Health, Belton, Texas, United States*

Community-based organizations (CBO) are strongly positioned to translate evidence-based interventions into real world practice. CaringKind, a dementia focused CBO, examined the effects of implementing Resources for Enhancing Alzheimer's Caregiver Health-II (REACH)-TX in a project funded by the Administration on Community Living. REACH-II is a widely studied, evidence-based education/support program for dementia family care partners (CP). The REACH-TX version of the program engages a CP with a certified REACH specialist for 2-6 meetings over the course of 3-6 months. REACH-TX includes a risk assessment, tailored plan of care, and skills-training activities focusing on 9 core areas including safety, promoting emotional well-being, and managing problem behaviors. Real world adaptations included virtual delivery, elimination of in-home visits, and an internal staff master trainer. To practically evaluate the impact of REACH-TX, CaringKind chose the Preparedness for Caregiving Scale (PCGS) as the one measure to be completed by CP at baseline and program completion. Results indicated a significant increase in PCGS scores from 2.1 to 2.8 (t(64)=6.12, p<.001; possible range 0-4). Overall, 75% of CP had an increase in their mean PCGS score. Over half of the 57 CP who answered an added question about confidence in their ability to handle challenging or distressing behaviors (31, 54%) reported a higher score post intervention. This pragmatic delivery of REACH-TX by a CBO demonstrated a positive outcome on the PCGS, which aligns with the intervention and is realistic to administer. Leveraging CBOs in intervention development, translation and implementation may accelerate intervention uptake and adoption.

Abstract citation ID: igaf122.239

**OLDER ADULTS' CONTACT PATTERNS WITH FAMILY MEMBERS DURING COVID-19: EVIDENCE FROM THE NSHAP COVID STUDY**

Xiaoyu Fu[1], Merril Silverstein[2], Catherine Garcia[2], Ying Xu[2], and Tianqi Zhou[2], *1. Syracuse University, Los Angeles, California, United States, 2. Syracuse University, Syracuse, New York, United States*

The COVID-19 pandemic significantly disrupted older adults' social connectedness, prompting many to adopt digital communication technologies to maintain family ties. This study examines distinct patterns of older adults' family communication, including in-person visits, phone calls, and digital contact (email, social media, and video call), and retrospective assessments of changes in these interactions during the pandemic. Using data from the COVID sub-study of the National Social Life, Health & Aging Project (NSHAP), we applied latent class analysis to identify communication patterns among 2,048 adults aged 60 and over. Four distinct patterns emerged: (1) Moderate Contact with Decreased Frequency, characterized by moderate but declining engagement across all contact modes; (2) High Traditional Contact with Increased Frequency, marked by frequent in-person and phone contact with increases across all modes; (3) High Contact with Increased Digital Contact, defined by high engagement across all communication modes, particularly digital contact; and (4) Low Contact with Increased Frequency, indicating that although the frequency of contact has risen across all modes, overall contact levels remain low. These findings highlight the diverse ways older adults adapted their communication strategies during the pandemic, with some maintaining high levels of traditional contact, others expanding digital engagement, and some experiencing overall declines. As digital communication continues to play a crucial role in social connectedness, interventions should focus on enhancing digital literacy, increasing access to user-friendly technologies, and providing tailored support to help older adults sustain meaningful family interactions and mitigate social isolation.

Abstract citation ID: igaf122.240

**REFRAMING AGING: TWO-SPIRIT AND INDIGENOUS LGBTQ+ PERSPECTIVES ON LIVING WELL WITH HIV**

Chase Bryer, *Brown University School of Public Health, Providence, Rhode Island, United States*

This study explored the intersections of social identities and aging experiences among Indigenous Two-Spirit (2S) and

LGBTQ+ people living with HIV (PLWH). While advances in HIV treatment have increased their lifespan, these individuals still face high infection rates and health disparities due to intersectional stigma, discriminatory stress, and historical trauma. The primary researcher aimed to understand what it means to "age well" with HIV within this community, and how their cultural perspectives challenge conventional views of "successful aging". Based on 15 oral life review interviews, the primary researcher used narrative analysis to understand how people make meaning of their life experiences by closely examining their narratives. Findings suggest that Indigenous cultural practices helped participants accept their HIV diagnoses and adhere to antiretroviral treatment (ART), while Indigenous cultural generativity—passing down cultural knowledge—was an important element in aging well with HIV. Notably, participants viewed HIV as a "gift" or "messenger," which allowed them to realign their life purposes, especially through Two-Spirit activism and HIV advocacy. These insights emphasize the importance of integrating cultural perspectives into aging and HIV care for Indigenous 2S/LGBTQ+ individuals. The study provides valuable lessons for future research, policy, and healthcare practices that are strengths-based, and aimed at addressing health inequities in this community.

### SESSION 1230 (SYMPOSIUM)

Abstract citation ID: igaf122.241

### MHEALTH INNOVATION FOR AGING WITH MOBILITY DISABILITY

Chair: Elena Remillard
Discussant: Eric Levitan

The landscape of telehealth has changed drastically over the last decade, extending beyond the remote delivery of clinical services (i.e., telemedicine). Worldwide, there has been continued growth in the popularity of mHealth (mobile health) applications to support physical, mental, and social health. From virtual exercise classes to remote health monitoring systems, mHealth can utilize a variety of mobile technologies such as smart phones, laptops, and wearable devices. mHealth applications can be especially beneficial for people aging with mobility disabilities who experience transportation and accessibility barriers with in-person programs. This session will highlight innovative mHealth solutions from the Rehabilitation Engineering Research Center on Technologies to Support Aging among People with Long-Term Disabilities (RERC TechSAge). Mitzner et al. will present results from the TechSAge Tele Tai Chi clinical trial – an evidence-based tai chi program delivered via Zoom designed to be socially engaging and inclusive of older adults with mobility limitations. Rice et al. will describe the development of a falls detection and monitoring system for older adults who use wheelchairs that integrates smart watch monitoring and a user-facing app. Hsieh et al., will highlight the design a fall risk mHealth app for people with Multiple sclerosis (MS) that enables users to measure and evaluate their fall risk and engage in preventative strategies. Eric Levitan, CEO & Founder of Vivo, an online, live fitness program designed for older adults, will serve as the discussant, engaging participants in interactive discussion and offering an industry perspective on challenges implementing mHealth applications for older adults.

Abstract citation ID: igaf122.242

### FINDINGS FROM THE TECHSAGE TELE TAI CHI CLINICAL TRIAL

Tracy Mitzner[1], Elena Remillard[2], and Kara Mumma[2],
*1. Person in Design, Atlanta, Georgia, United States, 2. Georgia Institute of Technology, Atlanta, Georgia, United States*

Tai chi is a mind-body exercise that involves a series of slow gentle movements, meditation, and controlled breathing. It has been shown to have a variety of evidence-based benefits, including improvements in balance, mobility, strength, flexibility, and relaxation, as well as reductions in falls, depression, and pain. While tai chi programs have grown in popularity and availability for older adults, many adults aging with mobility disabilities experience barriers participating in these exercise classes, including lack of transportation, appropriate exercise modifications, and building accessibility. The TechSAge Tele Tai Chi intervention was designed to address these barriers with 1) an evidence-based tai chi program (Tai Chi for Arthritis) that offers modifications appropriate for our target population (e.g., seated, movement adaptations) and 2) a translation to an interactive, online class via Zoom videoconferencing. We chose Tai Chi for Arthritis because it is a Title III-D (Highest Tier) Evidence-Based Health Promotion/Disease Prevention Program, endorsed by the Administration for Community Living. The intervention also includes moderated social time before and after the lesson as this population is at increased risk for social isolation. In this session, we will present outcomes from the 8-week clinical trial conducted with small-group cohorts of community-dwelling older adults aging with mobility disabilities ($N = 60$). Significant outcomes include increases in physical activity and social connectedness and decreases in loneliness. The findings of the clinical trial provide evidence that older adults with mobility disabilities can receive physical and social benefits from a group online tai chi program.

Abstract citation ID: igaf122.243

### DEVELOPMENT AND VALIDATION OF A FALL DETECTION SYSTEM FOR OLDER ADULTS WHO USE WHEELCHAIRS

Laura Rice[1], David Peeler[2], Peter Presti[2], and Andrea Tangonan[3],
*1. University of Illinois College of Applied Health Sciences, Urbana, Illinois, United States, 2. Georgia Tech, Atlanta, Georgia, United States, 3. University of Illinois Urbana-Champaign, Urbana, Illinois, United States*

Falls are a concern for the 1 million older adults who use a wheelchairs or scooters (WC/S) in the US. ~60% of the population are affected by falls. After a fall, people who use WC/S spend an average of 9 minutes (range: 1-45 min) on the floor and 80% require assistance to recover. Remaining on the ground for an extended period of time after a fall is associated with an increased risk of future injurious falls, long term care admissions, and death. Automated fall detection devices can provide timely assistance and minimize the consequences of a long lie. Although used widely in ambulatory populations, current automated fall detection devices do not accurately detect falls from WC/S. In response, our team developed a system to detect falls among older adults who use WC/S and increase awareness of fall circumstances. A custom algorithm was developed to detect falls with high accuracy (>99%). 20 older adults, 4 care partners, and 10 clinicians and researchers provided feedback on the design of the fall detection system.

Older adults and care partners indicated a desire for such a device, preference for a watch form, ability to modify who is contacted in the event of a fall, and ability to cancel a false alarm. Clinicians and researchers emphasized the need for such a system to guide user-centered fall interventions and better connection with patients. This system has good potential to increase the safety and quality of life of older adults who use WC/S.

Abstract citation ID: igaf122.244

### MSAFE: FALLS RISK APP FOR INDIVIDUALS WITH MULTIPLE SCLEROSIS

Katherine Hsieh[1], Deborah Backus[2], and Jon Sanford[1], *1. Georgia State University, Atlanta, Georgia, United States, 2. Shepherd Center, Atlanta, Georgia, United States*

Multiple Sclerosis (MS) is a chronic, autoimmune, disease of the central nervous system with the highest prevalence in adults aged 55 years and older. MS is a dynamic condition with symptoms fluctuating daily to weekly, placing people with MS at a high risk of falls. Symptom variability makes it challenging for older adults with MS to manage their fall risk. Our research team developed MSafe, a mobile health application designed to help older adults with MS manage their fall risk. In this co-design process, we interviewed 6 MS experts to identify important factors and components to include in the app. Experts identified key risk factors to incorporate. We then collaborated with an app development team to design MSafe, which evaluates multifactorial fall risk through assessment of: 1) patient reported outcomes (e.g., demographics, sensory function, fatigue), 2) environmental hazards, 3) physical function, and 4) cognition. MSafe displays individualized results compared to clinical thresholds. To assess usability, 10 older adults with MS independently completed MSafe, followed by semi-structured interviews that were video recorded, transcribed, and analyzed thematically. Three themes emerged: 1) ease of use, 2) fall risk feedback, and 3) bilateral adaptations. Participants reported that MSafe was intuitive and easy to follow. Following the assessment, participants expressed interest in conducting repeated assessments to track changes over time. Last, those using bilateral assisted devices requested a phone holder for dynamic tasks. These findings suggest potential for independent, fall risk assessments with mobile technology and inform next iteration of MSafe prior to home evaluation.

### SESSION 1235 (PAPER)

Abstract citation ID: igaf122.245

### ENVIRONMENTAL DETERMINANTS OF HEALTH

In this session research will be presented on the impact to the environment on healthy aging

Abstract citation ID: igaf122.246

### BEYOND GREEN: HOW URBAN GREENSPACE AND EXTREME CLIMATE SHAPE HEALTH OUTCOMES IN CHINA

Linjiang Wei[1], Shujun Chai[2], Liangwen Zhang[1], Ya Fang[1], Xinyi Wang[1], Ying Han[3], and Wen Zhang[1], *1. Xiamen University, Xiamen, Fujian, China (People's Republic), 2. Renmin University of China, Beijing, Beijing, China (People's Republic), 3. Wenzhou Medical University, Wenzhou, Zhejiang, China (People's Republic)*

Against the dual backdrop of climate change and rapid urbanization, a thorough analysis of the spatiotemporal interactions between extreme climate events, urban green spaces, and health outcomes in aging populations holds significant theoretical and practical importance. This study innovatively employs the multiscale geographically and temporally weighted regression (MGTWR) model to systematically explore the mechanisms through which extreme climate exposure and greenness coverage influence the prevalence of chronic diseases and comorbidities among urban older adults (age ≥60) in China. By integrating data from two national cohort studies (CLASS and CHARLS, 2015-2020), along with multidimensional indicators such as meteorological data, urban greenness metrics, the study yields the following key findings: First, extreme climate exposure exhibits significant spatial autocorrelation, with its impact on chronic disease comorbidity prevalence showing a spatial gradient that increases from southwest to northeast (standardized $\beta > 0.24$). Second, urban greenness demonstrates dual health effects: park greenness significantly reduces chronic disease prevalence through its ecological regulation functions, but may increase short-term comorbidity risks due to factors such as behavioral changes ($\tau = 0.2$). Third, the MGTWR model ($R^2 = 0.772$) outperforms traditional methods in terms of goodness-of-fit, effectively revealing the spatiotemporal heterogeneity of health-environment relationships. Based on these findings, the study proposes policy recommendations, including establishing a climate-resilient health protection system, optimizing the precise allocation of green space health services, and improving the framework for environmental health governance. This research offers critical guidance for addressing the dual challenges of chronic disease burden and climate vulnerability in the context of rapid urbanization.

Abstract citation ID: igaf122.247

### HOW INDOOR AIR QUALITY AFFECTS SLEEP IN OLDER ADULTS: EVIDENCE FROM THE 2018 CLHLS

Cai Xu[1], and Yen-Han Lee[2], *1. Ohio University, El Paso, Texas, United States, 2. University of Central Florida, Orlando, California, United States*

Background: Sleep is essential for health and well-being, particularly among older adults. This study examines the associations between indoor air quality and sleep outcomes in older Chinese adults. Method: We analyzed cross-sectional data from the 2018 Chinese Longitudinal Healthy Longevity Survey (CLHLS). Latent class analysis (LCA) identified ventilation patterns based on seasonal indoor air quality variations. Multiple linear and logistic regression models examined associations between ventilation patterns, air purifier use, home proximity to major roads, and sleep outcomes (quality and duration). Interaction effects between rural-urban residence and ventilation patterns were also assessed. Results: Among 9,902 adults (≥65 years), three ventilation classes were identified: Moderate with Summer-Selective Ventilation (11.2%), Strong Year-Round Ventilation (70.3%), and Minimal Ventilation (18.7%). Older adults with Strong Year-Round Ventilation ($OR = 1.16$, $p < 0.01$) and Moderate with Summer-Selective Ventilation ($OR = 1.19$, $p < 0.05$) had significantly better sleep quality. After accounting for interactions, urban residents with Strong Year-Round Ventilation had significantly higher odds of good sleep quality ($OR = 1.27$, $p < 0.05$). Additionally, older adults with Strong Year-Round Ventilation slept 0.30 hours longer per day ($p < 0.001$). After interactions, urban older adults with Moderate with Summer-Selective Ventilation slept 0.46 hours longer per day ($p < 0.01$). Conclusion: Indoor air quality, particularly ventilation patterns, is associated with sleep quality and duration among older adults in China. Improving indoor air quality may be an effective strategy to promote healthier sleep, particularly for older adults in urban settings.

Abstract citation ID: igaf122.248

### PERSONAL AND NEIGHBORHOOD SOCIOECONOMIC STATUS AS DETERMINANTS OF DEPRESSION OUTCOMES AMONG OLDER ADULTS

Yao Du[1], Stephanie Ming Yin Wong[1], Dara Kiu Yi Leung[1], Wai Chi Chan[1], Gloria HoiYan Wong[2], and Terry Lum[1], *1. The University of Hong Kong, Hong Kong, China (People's Republic), 2. University of Reading, England, United Kingdom*

Background Disparities in mental health and their determinants in old age remain a critical yet understudied global issue. This study examines how individual and neighbourhood socioeconomic status (SES) interact to shape not only concurrent mental health but also treatment responses among older adults. Methods We recruited 4,381 older adults with subclinical depressive symptoms from the JC JoyAge programme, a large-scale community-based collaborative stepped-care intervention in Hong Kong, for this longitudinal analysis (2020–2023). Using multilevel modelling, we examined how individual SES (education, welfare status, housing type) and neighbourhood SES (disadvantage index) jointly affected depressive symptoms, anxiety symptoms, health-related quality of life (HRQoL), and treatment responses over time. Findings Neighbourhood disadvantage was linked to higher depressive symptoms and lower HRQoL at baseline ($p<0.01$) and moderated treatment responses ($p<0.05$). Cross-level interactions showed that individuals with low education and welfare reliance had higher depressive symptoms at baseline, while public housing residents had lower depressive symptoms, though these effects weakened in disadvantaged neighbourhoods ($p<0.05$). Regarding treatment response, individuals with lower education and welfare reliance in disadvantaged areas had higher odds of meaningful mental health improvements ($p<0.05$), whereas public housing residents had lower odds of meaningful clinical response in these areas ($p=0.07$). Interpretations Findings reveal the nuanced interplay between individual and neighbourhood SES in mental health disparities. While some low-SES subgroups show resilience in disadvantaged environments, others remain vulnerable, emphasising the need for tailored, multilevel interventions that address structural barriers while strengthening individual coping resources.

Abstract citation ID: igaf122.249

### THE ABSOLUTE AND RELATIVE CONCENTRATION-RESPONSE PATTERNS OF AIR POLLUTANTS AND THE RISK OF ALZHEIMER'S DISEASE

Igor Akushevich[1], Arseniy Yashkin[1], Larry Tupler[1], Meishuo Ouyang[2], and Julia Kravchenko[2], *1. Duke University, Durham, North Carolina, United States, 2. Duke University School of Medicine, Durham, North Carolina, United States*

Existing literature lacks information on the relationship between exposure to ambient air contaminants and Alzheimer's disease (AD) risk represented by simultaneous assessment of absolute and relative concentration-response patterns. This study focuses on quantifying PM2.5-exposure and AD-risk relationships with detailed analyses of the role of methodologic limitations and sources of bias (e.g., population heterogeneity) and identifying notable subpopulation differences and populations most vulnerable to PM2.5 exposure. Using Medicare and SEDAC data, we evaluated the effects of PM2.5 exposure on AD risk stratified by sex, race/ethnicity, and exposure level using absolute concentration-response functions based on age-adjusted rates and relative functions estimated via Cox models. The results show that chronic low-level exposures to PM2.5 were associated with higher AD risk which varied substantially across sex- and race-specific population groups. Females showed higher absolute incidence, while males exhibited steeper relative risk increases with rising PM2.5 concentrations. Native Americans, residing primarily in low-exposure areas, demonstrated the steepest rise in hazard with increasing PM2.5 concentrations; Black and Asian subpopulations exhibited the lowest relative ratios. Nonlinearities were detected in exposure-response relationships. AD risk in Hispanics was subject to notable geographic heterogeneity. This study provides a foundation for assessing the health impacts of recent U.S. air quality standards and their effects on AD risk in vulnerable populations. Future research should incorporate flexible modeling techniques (e.g., spline-based or piecewise methods) and integrate mechanistic studies to clarify the biological and social pathways linking PM2.5 to AD, explain observed disparities, and identify underlying causal factors.

Abstract citation ID: igaf122.250

### THE RELATIONSHIP BETWEEN GEOGRAPHICAL RESIDENCE AND HOSPITALIZATION OF OLDER ADULTS IN THE UNITED STATES

Hillary Spangler[1], David Lynch[2], Haiyi Chen[1], Nina Daneshvar[2], Michael Rydberg[2], Joshua Niznik[2], Feng-Chang Lin[3], and John Batsis[1], *1. The University of North Carolina at Chapel Hill, Chapel Hill, North Carolina, United States, 2. University of North Carolina School of Medicine, Chapel Hill, North Carolina, United States, 3. University of North Carolina Gillings School of Public Health, Chapel Hill, North Carolina, United States*

Introduction: Older adults (65+ years) in rural areas are at a higher risk of frailty and costly healthcare utilization due to ecological factors, such as inconsistent support for accessing healthcare. We aim to assess the relationship between frailty trajectories and inpatient hospitalizations in rural versus urban communities. Methods: We included older adults from the National Health and Aging Trend Study (2011-2020) with complete data for Fried's frailty phenotype scoring ($n=5,540$). The Office of Management and Budget's criteria defined rurality. Previously, latent growth class analysis defined frailty trajectories (improving, stable, mildly and drastically worsening). Using linked Medicare fee-for-service data, negative binomial regression and marginalized two-part model were used to determine how geographic residence modifies the relationship of inpatient hospitalizations and frailty trajectories using incident rate ratios (IRR). Results: The mean age of the sample was 75.3±6.74(SD) years, 60.9% ($n=3,373$) female, 18.3% ($n=1,013$) living in rural communities, and mean yearly hospitalization cost $1,806 (SD $5,522). Trajectory membership breakdown: 8.6% improving, 42.6% stable, 38.4% mildly and 10.4% drastically worsening. Among those living in rural communities, we observed higher IRR associated with the mild [1.81 95%CI (1.55, 2.11)] and drastic worsening [1.81 (1.21, 2.72)] trajectories. For urban communities, this was only observed for the drastically worsening trajectory [2.05 (1.44, 2.93)]. The relationship between hospitalization rate and frailty trajectories was significantly modified by urban residence (p-value 0.018). Conclusion: Urban residence may modify the relationship between hospitalization rate and worsening frailty trajectories. Future analyses examining predictors of utilization may serve as intervention targets for frailty prevention.

### SESSION 1240 (PAPER)

### FAMILY TIES AND CAREGIVING DEMANDS: INTERGENERATIONAL BONDS, BURDENS, AND BEREAVEMENT IN LATER LIFE

Abstract citation ID: igaf122.251

#### LINKED FAMILY LIVES: THE INFLUENCES OF SIBLINGS ON CHINESE ADULTS' WEALTH AND HEALTH TRAJECTORIES IN LATER LIVES

Xinyi Chen[1], and Wei-jun Jean Yeung[2], *1. National Volunteer & Philanthropy Centre, Singapore, Singapore, 2. Agency for Science, Technology and Research, Singapore, Singapore*

Siblings are lifelong companions, providing both support and rivalry, yet their influence in later life remains underexplored in family research. This study examines how sibling number and gender shape the wealth and health trajectories of older adults in China, with a focus on gender differences in these relationships. Using four waves of China Health and Retirement Longitudinal Study (CHARLS) data and employing growth curve modeling, we find that having more siblings is generally associated with greater wealth and better physical and cognitive well-being in later life. While wealth and health decline with age, having siblings slows the trajectory of physical health decline but accelerates wealth reduction over time. Gender-specific analyses reveal that having sisters slows physical health decline, with stronger benefits for men than women. While males show greater resilience to age-related memory decline, having sisters weakens this advantage, whereas having brothers enhances it. These findings highlight the protective effects of sibling relationships on the physical and cognitive health of Chinese older adults. However, greater wealth decline among those with more siblings may stem from financial transfers for aging-related healthcare needs or diluted inheritance. Men also particularly benefit from sisters' instrumental and emotional support, aligning with traditional caregiving expectations for women. As the one-child policy parent-generation enters later life in the coming decades, these findings suggest potential vulnerabilities in physical health trajectories and early-life wealth accumulation for their children, emphasizing the need for policy adaptations to support aging families.

Abstract citation ID: igaf122.252

#### LONGITUDINAL ASSOCIATIONS BETWEEN PHYSICAL PAIN AND OLDER ADULTS' INTERGENERATIONAL SOCIAL NETWORKS

Shelbie Turner[1], Carson De Fries[2], Carly Roman-Woo[3], and Shannon Jarrott[4], *1. Weill Cornell Medicine, New York, New York, United States, 2. University of Utah, Salt Lake City, Utah, United States, 3. Archstone Foundation, Long Beach, California, United States, 4. The Ohio State University, Columbus, Ohio, United States*

Older adults with chronic pain often struggle to maintain social connections and have reduced social networks. Intergenerational social relationships may be especially challenging to maintain in the presence of pain. Yet, limited scholarship characterizes the intergenerational social networks of older adults with pain, nor how pain impacts the prevalence of intergenerational social partners over time. To fill this gap, we analyzed cross-sectional and longitudinal associations between activity-limiting pain and intergenerational social networks among older adults participating in the National Health and Aging Trends Study (n = 2,348). Based on an existing evidence-based coding process, we coded the number of close social partners who were 10, 15, 20, and 25 years younger than the age of each participant. We then ran regression models to analyze whether activity-limiting pain was associated with the number of social partners in each intergenerational group (i.e., 10, 15, 20, and 25+ years younger). Models revealed that having activity-limiting pain was associated with fewer social partners 25 years younger than the participant cross-sectionally (-0.08, p = 0.02) and longitudinally two (-0.07, p = 0.02) and three (-0.08, p = 0.04) years later. There were no associations between activity-limiting pain and the number of social partners who were 10, 15, or 20 years younger than the participant. Pain appears to be particularly consequential to older adults' relationships with people multiple generations younger than them (i.e., "skipped generation relationships"). Efforts to support older adults' engagement with skipped generation social partners are necessary, and researchers should evaluate how improved intergenerational connectivity can support older adults' pain management.

Abstract citation ID: igaf122.253

#### LOSS IN LATER LIFE: PARTNERSHIP AND HEALTH AFTER THE DEATH OF AN ADULT CHILD

Rose Anderson, and Christine Mair, *University of Maryland, Baltimore County, Baltimore, Maryland, United States*

The death of a child is one of life's most devastating experiences. The resulting health implications can extend into parents' later years, yet research on how child loss is associated with older adults' physical and mental well-being remains limited. Specifically, there is a need for additional studies on the extent to which partnership buffers or exacerbates these patterns. Using data from the 2018 Health and Retirement Study (HRS) dataset (N = 15,432), we analyze how child loss is associated with psychological and physical health outcomes among partnered and unpartnered older adults. Contrary to initial hypotheses, partnership did not buffer associations between child loss and lower psychological and physical health. Furthermore, unpartnered and "kinless" (unmarried and childless) older adults who experienced child loss faced similar declines in health as their partnered counterparts. The results of this analysis suggest that the nature of child loss, the different grieving contexts between partnered and unpartnered individuals, and the need for broader emotional and social support systems may explain this pattern. These findings highlight the importance of developing grief support approaches that address heterogeneity among bereaved parents. Future studies should investigate the potential for a wide array of social relationships and their potential buffering role following the loss of an adult child.

Abstract citation ID: igaf122.254

**LOVE, PRIDE AND WORRY: GRANDPARENTS' EMOTIONS IN DAILY INTERACTIONS WITH THEIR GRANDCHILDREN**

Flavia Chereches, Yvonne Brehmer, Gabriel Olaru, and Nicola Ballhausen, *Tilburg University, Tilburg, Noord-Brabant, Netherlands*

Past research investigating the impact of grandchild contact on grandparents' well-being showed mixed results. Some studies reported benefits, while others revealed no effects or even negative consequences of grandchild contact for grandparents' well-being. However, most of this research was cross-sectional or relied on data with large time gaps between assessments. Consequently, the short-term experiences of grandparenting remain unclear. We aimed to investigate how contact with grandchildren impacts grandparents' positive and negative emotions in their daily lives, comparing this contact to interactions with others and time spent with a partner or alone. Using snippets of ambient sound recordings, we investigated if grandparents use more positive and less negative emotion-related words with grandchildren than with others. Our study involved 126 grandparents from the "Daily Experiences and Wellbeing study" followed over 5 days (6 assessments/day). Applying multilevel structural equation modeling, we found that grandchild contact was associated with stronger positive affect, specifically more love and pride, greater pleasantness, and more positive word use compared to interactions with others and being alone. However, grandparents also reported feeling sadder and more worried when being with their grandchildren, although these effects were comparatively weaker. Integrating multiple assessment methods (self-report and objective sound recordings), our findings show that while grandchild contact evokes a mix of emotions, increases in specific positive emotions within daily life are unique for interaction with grandchildren as compared to interactions with other individuals.

Abstract citation ID: igaf122.255

**UNEQUAL EXPOSURE TO KIN LOSS ACROSS THE LIFE COURSE IN CHINA**

Jingwen Liu, *Rice University, Houston, Texas, United States*

Despite extensive research on the effects of specific bereavement events (e.g., parental or spousal loss) on health and well-being, few studies systematically examine how kin loss is structured across the life course and varies by social status, particularly in non-Western contexts. Applying nonparametric life table methods to the 2014 Life History Wave of the China Health and Retirement Longitudinal Study (CHARLS), this study investigates the cumulative risks and age-specific hazards of losing a mother, father, sibling, spouse, or child, respectively, in the full sample and by gender, education, and rural-urban hukou status. Descriptive analyses reveal that by 2014, 72.7% of respondents had lost a mother, 84.3% a father, 38.3% a sibling, 12.3% a spouse, and 11.9% a child. Kaplan-Meier survival estimates show that women are 1.1 to 1.5 times more likely than men to lose a child or spouse, with spousal loss risk peaking at 2.5 times between ages 65–70. Illiterate individuals are exposed to significantly higher risks of early parental deaths before midlife (peaking at 2-3 times), spousal (peaking at 3.2 times) and child deaths (peaking at 1.5 times) throughout adulthood, and sibling deaths (peaking at 2 times) across the whole life course. Rural residents are 1.5–2.1 times more likely to experience sibling and child loss, with child loss disparities persisting from ages 25 to 75. These findings highlight kin loss as an important but often overlooked determinant of social inequality, which may compound to further disadvantage the marginalized groups.

Abstract citation ID: igaf122.256

**WHISPERS OF LOSS, ECHOES OF STRENGTH: THE EXPERIENCE OF BEDOUIN ARAB GRANDPARENTS WHO LOST A GRANDCHILD IN WAR**

Reem Nashef-Hamuda[1], and Hadass Goldblatt[2], *1. University of Haifa, Tel Aviv University, Haifa, HaZafon, Israel, 2. University of Haifa, Haifa, HaZafon, Israel*

There are 47 unrecognized Bedouin villages located in the 'open areas' of the Negev in southern Israel that lack protection from the Iron Dome missile defense system. Having no safe shelters or permanent houses, these communities are more prone to direct injury from rocket fire, resulting in a more significant number of casualties. During the war, 19 civilians from the Negev Bedouin Arab population were killed, including six children (Arava Institute, 2023). The purpose of this study is to explore in-depth the bereavement experience of Bedouin grandparents following the death of a grandchild during the war. We examined this phenomenon from an intersectionality perspective, emphasizing the overlap between minority status, advanced age, and the experience of bereavement. This study employed a qualitative narrative approach. Data collection consisted of in-depth, semi-structured interviews with 10 Bedouin grandparents who had lost grandchildren during the war. Interviews were conducted in Arabic to ensure a culturally sensitive exploration of the grandparents' experiences. Data were analyzed using thematic analysis. Two themes will be presented: (1) "When it's war and sudden, it's harsh:" The experience of traumatic loss and accompanying emotions following bereavement; (2) "God gives you the strength to endure the suffering:" Faith in God as a source of meaning and comfort. These themes illustrate the dual reality of grief and resilience in a community shaped by conflict. This study provides a deeper understanding of bereavement processes among minority groups in conflict settings, offering insights into culturally sensitive coping mechanisms.

### SESSION 1245 (PAPER)

Abstract citation ID: igaf122.257

**GLOBAL RESEARCH IN AGING**

In this session research is presented from across the globe to address important geriatric syndromes.

Abstract citation ID: igaf122.258

**CROSS-NATIONAL PAIN AND FUNCTIONAL IMPAIRMENT IN OLDER ADULTS WITH CVD: INSIGHTS FROM INDIA, CHINA, AND MEXICO**

Lu Shao[1], Hongtao Cheng[2], Huijun Wu[2], Junwen Yu[3], and Jun-e Zhang[2], *1. University of Hong Kong, Guangzhou, Guangdong, China (People's Republic), 2. Sun Yat-sen University, Guangzhou, Guangdong, China (People's Republic), 3. Fudan University, Shanghai, Shanghai, China (People's Republic)*

Chronic pain in older adults with cardiovascular disease (CVD) may exacerbate functional disability, yet cross-cultural evidence remains limited. This study examines pain-disability associations across India, China, and Mexico, providing a comparative perspective on aging and disability. We analyzed nationally representative data from three cohorts: the Longitudinal Aging Study in India (LASI, n = 11,247), China Health and Retirement Longitudinal Study (CHARLS, n = 3,733), and Mexican Health and Aging Study (MHAS, n = 5,108). Participants aged ≥60 with self-reported CVD were included. Chronic pain was assessed via self-report, and functional disability was measured using activities of daily living (ADL), instrumental ADL (IADL), and mobility scales. Cross-sectional multivariable logistic regression models examined associations, while longitudinal analyses in China and Mexico assessed 3-year incident disability risks. Pain prevalence was 43.7% in India, 36.2% in China, and 43.6% in Mexico. Adjusted models revealed significant associations between pain and ADL disability across all countries: India (OR = 1.86, 95% CI:1.70–2.04), China (OR = 2.22, 1.86–2.63), Mexico (OR = 2.32, 2.00–2.70). Pain severity showed dose-response relationships with ADL/IADL limitations. Longitudinally, baseline pain predicted higher risks of new-onset ADL disability in China (OR = 2.27, 1.71–3.01) and Mexico (OR = 1.97, 1.57–2.47), with consistent patterns for IADL disability. Chronic pain is a strong, independent predictor of functional disability in older CVD patients across diverse middle-income countries. These findings highlight the necessity of integrating pain management into cardiovascular care to promote functional independence in aging populations. Future research should explore culturally tailored interventions to mitigate the impact of pain on disability in diverse healthcare systems.

Abstract citation ID: igaf122.259

### DETERMINATION OF ULTRASOUND REFERENCE VALUES FOR DIAGNOSING LOW MUSCLE MASS IN OLDER CHINESE ADULTS

Qin Zhang[1], Shumin Li[2], and Jing Chen[1], *1. The First Affiliated Hospital, Zhejiang University School of Medicine, Hangzhou, Zhejiang, China (People's Republic), 2. The First Affiliated Hospital, School of Medicine, Zhejiang University, Hangzhou, Zhejiang, China (People's Republic)*

Ultrasound is a promising tool for sarcopenia diagnosis, yet standardized criteria are lacking. We aimed to identify the optimal muscle sites and reference values for diagnosing low muscle mass in Chinese older adults using ultrasound. The study included 1011 participants aged ≥60 years. Dual-energy X-ray absorptiometry (DXA) served as the reference standard for assessing muscle mass. Seven muscle sites were evaluated via ultrasound for muscle thickness (MT) and cross-sectional area (CSA). Our results showed that the biceps brachii CSA (AUC=0.832, 95%CI: 0.793-0.870) in males and tibialis anterior MT (AUC=0.833, 95%CI: 0.789-0.877) in females demonstrated superior predictive power for low muscle mass compared to other assessed parameters. Diagnostic thresholds were < 7.1 $cm^2$ for male biceps brachii CSA and < 2.3 cm for female tibialis anterior MT. Ultrasound achieved sensitivities, specificities, and accuracies of 73.9%, 78.6%, and 75.7% in males, and 73.6%, 76.8%, and 75.8% in females, respectively, comparable to bioelectrical impedance analysis (BIA) at 62.6%, 80.2%, and 71.5%. The inter-rater reliability was excellent for biceps brachii CSA (ICC =0.877, 95%CI: 0.677-0.941) and good for tibialis anterior MT (ICC =0.731, 95%CI: 0.610-0.817). Low muscle mass defined by ultrasound correlated significantly with poorer physical performance outcomes, with the effect size higher than DXA or BIA diagnosed low mass, suggesting better prediction power of ultrasound than DXA or BIA. In conclusion, ultrasound could be a preferable method for sarcopenia assessment, demonstrating good inter-rater reliability and significant correlation with physical performance.

Abstract citation ID: igaf122.260

### GLOBAL DISPARITIES IN COGNITIVE AND FUNCTIONAL AGING: A CROSS-SECTIONAL STUDY ACROSS COUNTRIES

You Wu[1], Jie Shen[1], Marie-Theres Huemer[2], Magdalena Opazo Breton[3], and Yuan Ma[4], *1. Harvard T.H. Chan School of Public Health, Boston, Massachusetts, United States, 2. Helmholtz Zentrum München, German Research Center for Environmental Health, Neuherberg, Bayern, Germany, 3. University of Nottingham, Nottingham, England, United Kingdom, 4. Harvard. T.H. Chan School of Public Health, Boston, Massachusetts, United States*

With rapid population aging, understanding disparities in cognitive and functional aging between individuals and across countries is crucial for global healthy longevity. Our study described cognitive and physical function among nationally representative older adults (aged 65 and older) from China, England, India, Mexico, and USA, using data from 15,430 participants (53.7% women) in the Harmonized Cognitive Assessment Protocol (HCAP) and parent cohort studies. Cognitive function was assessed using harmonized cognitive function factors. Physical function was measured by the Activities of Daily Living scale (ADLs). We further explored how individual- and country-level characteristics may explain these variances using multi-level modelling. We observed that older adults in England and USA maintained better cognitive function than their peers in China, India, and Mexico throughout later life. The average cognitive function of older adults in China, India, and Mexico was equivalent to those approximately 16-25 years older in England and USA. England and USA also had a higher declining rate in cognitive function across age groups. Similar cross-country disparities were observed for ADLs, with an increasing proportion of dependency with age in all countries. The individual-level (age, sex, education, and multimorbidity) and country-level covariates (socio-demographics and universal health coverage) jointly explained 14.5% within-country variances and 43.8% between-country variances in cognitive function, but only 2.7% and 0.3% for ADLs. Our findings showed a wide global disparity in cognitive and functional aging, while individual- and country-specific characteristics partly explained these variances. Future studies may analyze how these characteristics interact over time to influence healthy aging.

Abstract citation ID: igaf122.261

### PHYSICAL EXAMS AND HEALTHCARE UTILIZATION AND EXPENDITURES AMONG MID-AGED AND OLDER ADULTS IN CHINA

Sijiu Wang, and Tianzi Li, *Tsinghua University, Beijing, Beijing, China (People's Republic)*

As populations continue to age in China, the effectiveness and cost-efficacy of preventive health services, especially physical examinations, have become increasingly important in addressing health challenges in an aging society. Previous research on physical examinations has found inconsistent findings on healthcare utilization and expenditures. Therefore, this study aimed to examine the association between preventive physical examinations and healthcare utilization and expenditures among middle-aged and older adults in China. We used data from the China Health and Retirement Longitudinal Study from 2011 to 2018. The independent variables were whether the individual had a physical examination within two months before the interview for outpatient analyses and one year before the interview for inpatient analyses. Six outcomes were assessed: any outpatient/inpatient care use, log-transformed outpatient/inpatient out-of-pocket expenditures, and log-transformed total outpatient/inpatient expenditures. We employed propensity score weighting and fixed effects regression models, controlling for demographics and diagnosed chronic diseases. Sensitivity analyses and subgroup analyses were also conducted. Physical examinations were associated with a 46.1% higher odds of inpatient care utilization and a 49.7% higher odds of outpatient care utilization. While no significant association with inpatient expenditures was observed, outpatient expenditures increased significantly (out-of-pocket by a factor of 1.86; total by a factor of 1.72). These results showed that preventive physical examinations were significantly associated with increased short-term healthcare utilization and outpatient expenditures. It emphasized the importance of ensuring insurance coverage, especially for vulnerable populations. More research is needed to develop causal pathways and to examine the long-term impacts.

### SESSION 1250 (PAPER)

### LANGUAGE, IMMIGRATION AND GLOBAL HEALTH: THE AMERICAS

Abstract citation ID: igaf122.262

### AMPLIFYING THE VOICES OF LATINO IMMIGRANT DEMENTIA CAREGIVERS: USING PHOTOVOICE TO ASSESS RESOURCES

Mayra Sainz, *Emory University, Atlanta, Georgia, United States*

The high prevalence of dementia among Latino individuals is particularly concerning for Latino immigrants, who on average tend to be older, have longer life expectancies, and are less likely to have health insurance compared to their U.S.-born counterparts. However, the role of immigration in dementia caregiving remains largely underexplored in the literature. This study employed photovoice methods to assess the availability and accessibility of resources for Latino immigrant caregivers of individuals living with dementia. This photovoice study design was guided by the Socio-Ecological Model and consisted of four components: pre-screening interviews, an orientation, photography exercises, and a community forum. The photograph exercises included photograph led discussion employing the SHOWED/VENCER guide. Photographs and transcripts were used to develop a codebook and identifying key themes. Nine immigrant Latino dementia caregivers participated in the photovoice exercise, all of whom were women, with an average age of 55.9 years (range: 38 to 75 years) and reported having lived in the United States for an average of 18.2 years (range: 1 to 32 years). Emerging themes include (1) insufficient support between family members, (2) limited access to mental health services, (3) lack of dementia-friendly environments, (4) the importance of prioritizing sustainability, and (5) a need to increase collaboration opportunities for Latino immigrant caregivers. Research should commit to developing scalable, long-term solutions that integrate cultural factors, enhance mental health support, and expand outreach strategies. Increasing the availability and accessibility of resources for immigrant caregivers will be crucial in fostering a comprehensive caregiving environment for Latino families.

Abstract citation ID: igaf122.263

### DUAL DIAGNOSES AND HEALTH BIOMARKERS: DIABETES AND ADRD AMONG HISPANIC OLDER ADULTS ON THE US-MEXICO BORDER

Hyeran Chung[1], Alfonso Rojas-Alvarez[1], and Brian Downer[2], *1. The University of Texas at El Paso, El Paso, Texas, United States, 2. UTMB, Galveston, Texas, United States*

Hispanic older adults near the US-Mexico border face significant healthcare challenges, especially in managing chronic conditions like diabetes and Alzheimer's Disease or Related Dementias (ADRD). Having both conditions complicates disease management and increases the likelihood of poor outcomes. However, little is known about how dual diagnoses affect health biomarkers, particularly in racially and ethnically marginalized communities along the border. This study explores the associations between dual diagnoses and key biomarkers related to diabetes and ADRD to better understand the health disparities faced by this population. Using the Hispanic Established Populations for the Epidemiologic Study of the Elderly (HEPESE) and Medicare administrative data (n = 718), we examine the relationship between dual diagnoses and biomarkers such as HbA1c and cognitive outcomes. Descriptive and regression analyses compare differences in these biomarkers among those with dual diagnoses and those with a single diagnosis. Proximity to the border is considered a moderating variable based on our previous findings about caregiver and community resources impacting health outcomes. Preliminary results show that Hispanic older adults with both diabetes and ADRD near the US-Mexico border have worse biomarker profiles, including poor glycemic control (elevated HbA1c) and more severe cognitive impairment. These disparities will be more pronounced near the border, suggesting that healthcare access and structural barriers may worsen disease progression. This project highlights the compounded health challenges of Hispanic older adults with dual diagnoses in the US-Mexico border region. It provides insights into healthcare disparities that can inform future policies to improve health outcomes.

Abstract citation ID: igaf122.264

### IS LIMITED ENGLISH PROFICIENCY OR SPEAKING SPANISH ASSOCIATED WITH MEDICARE ADVANTAGE ENROLLMENT?

Lilly Estenson[1], Eric Roberts[2], and Mireille Jacobson[1], *1. University of Southern California, Los Angeles, California, United States, 2. University of Pennsylvania, Philadelphia, Pennsylvania, United States*

Medicare beneficiaries increasingly choose to receive their Medicare benefits through private Medicare Advantage (MA) plans. While prior research has identified higher MA enrollment rates among Latino, Black, and low-income beneficiaries, trends among beneficiaries who speak Spanish and/or have limited English proficiency (LEP) are less clear. Accordingly, we used nationally representative Medicare Current Beneficiary Survey data and linear probability models to estimate differences in MA enrollment by Spanish language and LEP status. We adjusted for social, health, and local MA market characteristics, year, and county fixed effects. Among approximately 30,000 respondents,

6.0% spoke Spanish, and separately, 6.1% had LEP. Spanish-speaking beneficiaries had substantially higher rates of MA enrollment (58.9%) compared to non-Spanish-speaking beneficiaries (36.2%). Among Spanish speakers, beneficiaries with LEP had higher MA enrollment than beneficiaries with English proficiency (60.9% vs 51.1%). Among non-Spanish speakers, differences in MA enrollment by LEP status were minimal (36.6% vs 36.3%). In adjusted models, neither speaking Spanish nor LEP status was associated with MA enrollment when examined in the full sample. LEP status was not associated with MA enrollment when examined among Spanish- and non-Spanish speakers separately. Our results highlight higher rates of MA enrollment among Spanish-speaking beneficiaries, especially Spanish speakers with LEP. However, after adjusting for covariates, neither speaking Spanish nor LEP appear to independently account for MA enrollment differences. Additionally, our results support prior evidence of racial/ethnic and socioeconomic MA enrollment differences. Understanding the role of social factors in choosing MA can inform MA marketing, benefit design, and quality improvement policies.

Abstract citation ID: igaf122.265

### SOCIAL DETERMINANTS OF HEALTH IN AGING POPULATIONS: A COMPARATIVE STUDY OF U.S.-BORN AND IMMIGRANT INDIVIDUALS

Man Guo[1], and Yi Wang[2], *1. University of Iowa, Iowa City, Iowa, United States, 2. University of Iowa School of Social Work, Iowa City, Iowa, United States*

Aging experiences and health outcomes are shaped by multi-dimensional social determinants of health (SDOH), yet the latent structures of SDOH and their health impacts remain underexplored, particularly among diverse older populations. Using 2016/2018 Health and Retirement Study data, this study employs multi-group confirmatory factor analysis (MG-CFA) to uncover SDOH structures and their links to ADL limitations, depression, and cognition among U.S.-Born ($N = 9{,}258$) and Immigrant adults aged 50+ ($N = 1{,}379$). MG-CFA (metric model: CFI = 0.966, RMSEA = 0.059) identified three consistent factors—Neighborhood Environment Quality (high cohesion, low disorder), Personal Resource Capacity (SES), and Family Support (child/family support)—with uniform measurement across groups. However, group-specific factor intercepts varied: U.S.-born adults reported higher SES and neighborhood cohesion, while immigrants experienced more neighborhood disorder but stronger family support, reflecting nativity-specific challenges and strengths. Regression analyses revealed that Personal Resource Capacity was robustly associated with better health outcomes across both groups, while Family Support was linked to fewer ADL limitations and depressive symptoms. Neighborhood Environment Quality showed limited direct health associations, suggesting its influence may be distal or mediated by more proximal resources. Non-significant interactions between SDOH factors and nativity status indicate that these determinants similarly benefit both groups. These findings advance gerontological theory by demonstrating uniform SDOH structures and health associations across nativity status despite baseline disparities. They also highlight the need for targeted interventions to address immigrant-specific barriers, such as education gaps and neighborhood disadvantage, while leveraging cultural strengths like family support to promote equitable aging outcomes.

Abstract citation ID: igaf122.266

### PHYSICAL ACTIVITY AND COGNITION IN OLDER ADULTS

Yurun Cai, *University of Pittsburgh, Pittsburgh, Pennsylvania, United States*

Physical activity and exercise in older adults are associated with cognition in older adults. The results of these studies provide insight into this relationship.

Abstract citation ID: igaf122.267

### PHYSICAL ACTIVITY COMPLEXITY AND COGNITIVE FUNCTION IN OLDER ADULTS

Yurun Cai, Beth Snitz, and Ann Cohen, *University of Pittsburgh, Pittsburgh, Pennsylvania, United States*

Physical activity (PA) is associated with cognitive impairment and dementia in older adults. However, most studies have focused solely on quantities or intensities (e.g., moderate to vigorous PA) which may introduce measurement error. This study aims to examine the association between a novel PA measure–PA complexity, derived from continuous accelerometer signals using multiscale entropy method–and cognitive function in older adults in the Connectomics in Brain Aging and Dementia study. Among 190 adults who completed neuropsychological tests and 7-day wrist-worn accelerometer assessment in 2016-2021, 166 (mean age=$65.3 \pm 8.9$y) had ≥3 valid days (≤10% non-wear time of the day) of accelerometer data and were included in the analysis. Confirmatory factor analysis was used to construct three factors (memory, language, and executive function) from neuropsychological tests. In the unadjusted piecewise linear regression models, every 0.1-unit higher in PA complexity was associated with a 3.2-unit ($p = 0.004$) higher fluid cognition composite score and a 2.7-unit ($p = 0.008$) higher total cognition composite score when PA complexity was ≤0.6. After adjusting for sociodemographic characteristics and total activity counts per day, every 0.1-unit higher in PA complexity was associated with a 1.8-unit ($p = 0.044$) higher total cognition composite score. Low PA complexity (≤0.6) was associated with lower factor scores in memory ($\beta$=-0.26, $p = 0.037$), language ($\beta$=-0.33, $p = 0.008$), and executive function ($\beta$=-0.37, $p = 0.004$). Our findings highlight the importance of assessing novel PA metrics which may be more sensitive than conventional metrics to identify subtle changes in activity patterns and help predict early stages of pathological progression to dementia.

Abstract citation ID: igaf122.268

### EFFECTS OF A 6-MONTH YOGA INTERVENTION ON COGNITION & ANTERIOR CINGULATE CORTEX: RESULTS FROM THE SAY EXERCISE TRIAL

Neha Gothe[1], Angeles Tepper[1], Paul Camacho[2], Stephanie Voss[3], Jessica Hayes[4], Brad Sutton[2], Jessica Damoiseaux[4], and Edward McAuley[2], *1. Northeastern University, Boston, Massachusetts, United States, 2. University of Illinois Urbana-Champaign, Urbana, Illinois, United States, 3. Mass General Brigham, Boston, Massachusetts, United States, 4. Wayne State University, Detroit, Michigan, United States*

Lifestyle behaviors such as exercise continue to demonstrate neuroprotective effects with aging. A growing body of evidence suggests the role of aerobic exercise; however, little is known about alternative exercise approaches such as yoga. Sedentary older adults ($N = 145$, Mean age = 64.2) were randomized to one of three exercise groups: Hatha yoga, aerobic exercise, stretching-toning. Participants engaged in group exercise classes 3x/week, 60mins/session for 6 months. Neurocognitive measures including the Flanker inhibition task and Sternberg working memory task were administered pre and post intervention along with a structural MRI. Linear mixed models demonstrated improved reaction time (RT) on the Flanker incongruent condition for both yoga and aerobic groups (b_yoga = -51.17, p < .001; b_aerobics = -30.38, p = .011) but no significant improvement in the stretching-toning group (b_stretch = -9.69, p = .397). RT on the Sternberg 6-digit condition was also significantly faster among yoga participants (b_yoga = -92.79, p < 0.001; b_aerobics = -6.14, p = 0.805; b_stretch = -25.39, p = 0.317) and this change significantly correlated with changes in anterior cingulate cortex (r =-.41, p=.015) only in the yoga group. There appears to be specificity in the effects observed with the aerobic group demonstrating improvements in inhibitory control whereas yoga demonstrating improvements in both inhibitory control and working memory. For yoga, faster RT on the Sternberg 6-digit condition also associated with increased volume in the anterior cingulate cortex, a brain structure known to be impacted by aerobic exercise and play critical role in executive functioning.

Abstract citation ID: igaf122.269

### LONGITUDINAL TRANSITIONS ACROSS PHYSICAL FUNCTION STATES AMONG OLDER CANCER SURVIVORS

Emilie Duchesneau, Allison Musty, Kathryn Callahan, Heidi Klepin, Amresh Hanchate, Mara Vitolins, and Nicholas Pajewski, *Wake Forest University School of Medicine, Winston-Salem, North Carolina, United States*

Older cancer survivors are at risk of functional decline. Changes in physical function in older cancer survivors are understudied. We described longitudinal transitions in physical function among older cancer survivors using Rounds 1-12 (2011-2022) of the National Health and Aging Trends Study, a nationally representative cohort of adults aged 65+ years. We included participants with a self-reported cancer history in Round 1. Physical function was assessed annually based on limitations (unable to do without assistance) in 4 self-care, 3 mobility, and 5 household activity domains and was categorized as no limitations (0), minimal limitations (1-3), or moderate-to-severe limitations (4-12). Multistate Markov models estimated 1-year transition probabilities across physical function states and death, pooling across rounds. Among 1303 cancer survivors (56.9% female, 46.3% aged 65-74, 38.3% aged 75-84, 15.4% aged 85+), 42.2% had minimal and 32.2% had moderate-to-severe limitations at baseline. Individuals without limitations had a 62% probability of remaining stable, 32% probability of developing minimal limitations, 5% probability of developing moderate-to-severe limitations, and 2% probability of dying over one year. Those with minimal limitations had a 66% probability of maintaining their status, a 10% probability of improving to no limitations, a 19% probability of progressing to moderate-to-severe limitations, and a 5% probability of dying. In those with moderate-to-severe limitations, the probabilities of remaining in the state, improving, and dying were 72%, 13%, and 14%, respectively. These results highlight the dynamic nature of physical function in older cancer survivors and suggest that transitions to greater limitations are more common than recovery.

Abstract citation ID: igaf122.270

### MODULATING BRAIN TO IMPROVE GOAL-DIRECTED PHYSICAL ACTIVITY IN OLDER ADULTS: A PILOT STUDY

On-Yee Lo[1], Levi Ask[2], Melike Kahya[3], Thomas Travison[4], Lewis Lipsitz[5], and Brad Manor[6], *1. Harvard Medical School/Marcus, Hebrew SeniorLife, Boston, Massachusetts, United States, 2. Clark University, Worcester, Massachusetts, United States, 3. High Point University, High Point, North Carolina, United States, 4. Hebrew SeniorLife & Harvard Medical School, Hebrew SeniorLife, Massachusetts, United States, 5. Hebrew Senior Life, Boston, Massachusetts, United States, 6. Hebrew SeniorLife/Harvard Medical School, Boston, Massachusetts, United States*

Insufficient physical activity in older adults remains a global health issue. Several interrelated factors contributing to inactivity are linked to the prefrontal cortex. We conducted a pilot study to assess the feasibility, acceptability, and effects of combining transcranial direct current stimulation (tDCS) and behavior counseling to improve physical activity in older adults. Inactive older adults living in subsidized housing participated in this randomized controlled trial. Baseline physical activity (daily steps) was measured with a Fitbit for two weeks. Participants then received an eight-week intervention, including ten daily sessions of tDCS or sham stimulation during the first two weeks, along with four bi-weekly behavior sessions. Functional outcomes were assessed at baseline, post-stimulation, and after the entire intervention. Step counts were measured throughout the intervention and a 12-week retention period. Twenty-eight participants completed the study. Compliance was 97%, 93% and 92% for brain stimulation, behavior sessions, and follow-up assessments, respectively. Fitbit adherence was 96% and 71% during the intervention and retention periods. The tDCS arm, compared to sham, exhibited greater increase in average daily steps (p < 0.001). Participants increased 1179 (+22%) and 550 (+15%) steps/day from baseline in the tDCS and Sham arms, respectively. Motivation (p = 0.03) and self-reported walking performance (p = 0.02) were also improved in the tDCS arm compared to Sham. Combining tDCS and personalized behavior counseling to improve physical activity was feasible, acceptable, and appeared to be effective in a cohort of inactive older adults living within subsidized housing. Larger and more definitive studies are warranted.

### SESSION 1260 (COMMUNITY ENGAGED SCHOLARSHIP SYMPOSIUM)

Abstract citation ID: igaf122.271

### COMMUNITY-ENGAGED RESEARCH AND WORKFORCE EDUCATION IN MOBILE HEALTH: TIPS FOR SUCCESSFUL REPLICATION

Chair: Lana Sargent
Discussant: Barbara Resnick

Mobile clinics have demonstrated success in addressing community needs due to their flexibility and responsiveness. Utilizing a mobile health platform to provide workforce education and integrate community-based research can lead to sustained relationship building. Community-based workforce education and research requires engaging diverse partners, capacity building for student learning outcomes, and trust building to facilitate research participation. To advance the science of community-engaged research (CEnR), we convene leading scholars who will provide insight into the inherent issues of engaging the community in research and education with strategies for overcoming obstacles. The first presentation will present how the Mobile Health and Wellness (MHWP) program was conceived and factors leading to its sustainability over 12 years of continuous growth in scope of service, education, and research along with resources for replication. The second will demonstrate how to gain the Institute for Healthcare Improvement as an Age-Friendly Health System--Committed to Care Excellence by providing reliable practice assessing and acting on the 4Ms of age-friendly care in a community-based health and wellness program. The third will present results from a mixed methods study evaluating the training programs' results hosted at MHWP based on age-friendly care with medically underserved communities. The final presentation will present outcomes from a transdisciplinary CEnR program to address food insecurity in the mobile clinic setting, uncovering the effects of diet and social risk factors for cardiovascular disease. This symposium concludes with reflections about meaningful and authentic academic-community partnerships that align with supporting and building bridges between aspiring and existing community-engaged scholars.

Abstract citation ID: igaf122.272

### INTEGRATING CARE COORDINATION, EDUCATION, AND RESEARCH IN MOBILE HEALTH AND WELLNESS PROGRAMS

Lana Sargent[3], Patricia Slattum[1], Kimberly Battle[2], Martha Purvis[3], Katherine Falls[2], Amy Salisbury[3], Jack Weisskohl[3], and Faika Zanjani[2], *1. Virginia Commonwealth University College of Health Professions, Richmond, Virginia, United States, 2. Virginia Commonwealth University, Richmond, Virginia, United States, 3. Virginia Commonwealth University School of Nursing, Richmond, Virginia, United States*

The VCU School of Nursing's Mobile Health and Wellness Program (MHWP) is an age-friendly interdisciplinary mobile clinic operating in rural and urban Virginia. MHWP serves three critical purposes for the Commonwealth of Virginia and VCU: First, MHWP decreases barriers for medically under-resourced populations by bringing care and coordination of services directly to communities. Second, MHWP increases the number of interdisciplinary healthcare professionals prepared to meet the needs of medically under-resourced communities through interdisciplinary clinical learning experiences led by VCU faculty. MHWP facilitates the training of a public-ready healthcare professional workforce. Third, we integrate transdisciplinary research. We operate in nine under-resourced areas in Virginia, including six urban low-income housing areas and two rural sites. Decreasing barriers to care through care coordination resulted in an 8.6% reduction in emergency room visits and a 9.8% reduction in hospitalizations. HRSA funding to develop a nurse-led, interprofessional mobile health clinic through which we broadened the program's reach to two additional urban sites and two distant rural communities, further increasing the program's scope to become the VCU Mobile Health and Wellness Program. In 2022, we broadened our intentional community engagement, education, and research efforts to include four additional communities. We purchased two clinical vehicles to reach these sites and provide care. MHWP is an example of how a flexible approach works best when teams are prepared to meet specific communities' social, economic, and health needs within cultural and linguistic characteristics.

Abstract citation ID: igaf122.273

### QUALITY IMPROVEMENT TO SUSTAIN AGE-FRIENDLY PRACTICE: THE MHWP MEDICATION WORKFLOW

Emily Peron[2], Patricia Slattum[1], Krista Donohe[2], Elvin Price[2], and Rachel Regal[3], *1. Virginia Commonwealth University College of Health Professions, Richmond, Virginia, United States, 2. Virginia Commonwealth University School of Pharmacy, Richmond, Virginia, United States, 3. Virginia Commonwealth University, Richmond, Virginia, United States*

The Mobile Health and Wellness Program (MHWP) gained recognition through the Institute for Healthcare Improvement as an Age-Friendly Health System--Committed to Care Excellence (Level-2) in 2020 by demonstrating reliable practice assessing and acting on the 4Ms of age-friendly care in a community-based health and wellness program. In partnership with the Virginia Geriatric Education Center Geriatrics Workforce Enhancement Program (GWEP), workflows for each of the Ms undergo a quality improvement process–Plan-Do-Study-Act (PDSA)--to better support program participants, faculty and student learners. Workflows include assessment followed by offering education, resources, and referrals for care and services. The medication workflow included medication reconciliation and screening for medication nonadherence and medication-related problems, including use of high-risk medications. Using PDSA, data collected in REDCap to document participant encounters related to the medication workflow was reviewed by the MHWP faculty. Engaging pharmacy students (McFarlane Scholars) in the process, five focus groups were conducted with MHWP participants to gather their perspectives on a medication self-management skills building approach to medication review. A revised workflow replaced screening for medication-related problems with an age-friendly focused medication review and skill building for medication self-management. The revised workflow was presented to MHWP faculty for additional feedback. The revised workflow will be implemented, including training for faculty and student learners, followed by re-assessment of data collected from case notes of participant encounters. Continuous quality improvement is a key component of sustainable age-friendly practice. Engaging students creates a rich learning opportunity for future healthcare professionals.

Abstract citation ID: igaf122.274

### ENHANCING INTERPROFESSIONAL STUDENT TRAINING IN AGE-FRIENDLY CARE: OUTCOMES MOBILE HEALTH AND WELLNESS PROGRAM

Rachel Regal[1], Kimberly Battle[1], Katherine Falls[1], Genevieve Beaird[1], Emily Peron[2], Ashley Staton[1], Jacquelin Daniel[3], and Glenda Watkins[4], *1. Virginia Commonwealth University, Richmond, Virginia, United States, 2. Virginia Commonwealth University School of Pharmacy, Richmond, Virginia, United States, 3. Virginia Commonwealth University School of Nursing, Richmond, Virginia, United States, 4. Virginia Commonwealth University College of Health Professions, Richmond, Virginia, United States*

The Mobile Health and Wellness Program (MHWP) brings health promotion and care coordination services to medically underserved communities while training interprofessional students in age-friendly care. Since MHWP was founded in 2012, over 2700 health professions students from nursing, medicine, pharmacy, and other allied health programs have trained onsite. Students engage with interprofessional team visits and faculty debriefings, online didactic modules based in 4Ms care, morning topic discussions, and orientation simulation. Student outcomes from two mixed methods studies will be presented that demonstrate pre/post semester gains across attitudes, behaviors, and competencies. First, for students placed in older adult affordable housing building sites and a community wellness center (n = 166), students reported reduced personalized aging anxiety, increased collective affinity for older adults, and fewer negative ageist behaviors (paired t-tests, p<.05). Students also reported more favorable attitudes towards work with the medically underserved and enhanced interprofessional readiness. Second, for nursing students placed at medically underserved sites including mobile vans and rural locations (n = 174), students reported confidence gains across eight competency domains of public health nursing (Wilcox Signed-Rank Test, p<.001). Qualitative themes from student focus groups highlight the strengths of the MHWP training model in facilitating positive student outcomes, including a low-stakes learning setting to build confidence, time for and value on relationship and rapport building, inclusion of social drivers for health as part of shared health promotion, and interprofessional practice based in cultural humility. Implications for student training programs in age-friendly care with medically underserved communities will be shared.

Abstract citation ID: igaf122.275

### TRANSDISCIPLINARY COMMUNITY-ENGAGED RESEARCH FOOD INSECURITY AND CARDIOVASCULAR HEALTH: MOBILE HEALTH PLATFORM

Ana Diallo[2], Katherine Falls[1], Rachel Regal[1], Jered Wendte[2], Natalie Mansion[1], Leland Waters[1], Johnathan Williams[3], and Kimberly Battle[1], *1. Virginia Commonwealth University, Richmond, Virginia, United States, 2. Virginia Commonwealth University School of Nursing, Richmond, Virginia, United States, 3. Virginia Commonwealth University Center for Translational Research, Richmond, Virginia, United States*

The Mobile Health and Wellness Program (MHWP) serves as a platform for transdisciplinary community-engaged research (CEnR), aimed at driving innovations to address complex conditions associated with aging. In this forum, we share our experience using transdisciplinary approaches to tackle challenges older adults face, with research spanning food insecurity, cardiovascular disease, pharmacogenomics, Alzheimer's research, and brain health interventions. One aspect of our research focused on food insecurity, we implemented a 6-12 week Wellness and Nutrition Education Program (PPP). The program included weekly or bi-weekly wellness sessions and provided participants with bags of fruits and vegetables. These sessions emphasized goal-setting as a tool for promoting behavior change. We conducted a pre-post descriptive analysis to assess the program's impact on nutrition, food insecurity, and health measures such as blood pressure, blood glucose, and medication adherence. Eighty-one participants enrolled and 71 (88%) completed the program. On average, participants were 66.9 years old, and the majority identified as African American (79%) and women (70%), with 54% having a high school education or less. Participants reported an average of five chronic diseases, with hypertension (75%), type 2 diabetes (38%), and hypercholesterolemia (36%) being the most common. At baseline, 53% screened positive for food insecurity, and 51% reported nutrition insecurity. By completion, 92% of participants received six bags of fruits and vegetables. The program was significantly associated with an 18% decrease in nutrition insecurity (Z= -2.4, p = 0.02). These results highlight the effectiveness of the transdisciplinary CEnR model in addressing complex health challenges through collaboration.

### SESSION 1265 (SYMPOSIUM)

Abstract citation ID: igaf122.276

### LEVERAGING NOVEL METHODS TO CHART NEW HORIZONS IN THE STUDY OF PERSONALITY, STRESS, AND COGNITIVE AGING

Chair: Eileen Graham
Co-Chair: Emorie Beck

In order to optimize health in older adulthood, it is crucial to understand how variability in modifiable psychosocial factors fluctuate both short- and long-term. Using several innovative methodological approaches, this symposium highlights how psychosocial processes (e.g., stress, personality) that unfold across multiple time scales are critical for cognitive aging. First, Beck examines individual differences in cognitive coupling (i.e. momentary, within-person associations among cognitive domains) in later life and associations with sociodemographic and risk factors. Second, Ma explores associations between momentary Big Five personality states and cognitive function levels and variability using mixed effects location scale models, with preliminary results suggesting that personality states impact cognitive variability as much as momentary cognitive function. Third, Zavala explores whether believing stress can serve as an enhancer (i.e. stress mindset) and perceived stress are associated with momentary cognitive function. Preliminary findings indicate that older adults tended to report less momentary perceived stress but that neither stress mindset nor momentary perceived stress were associated with cognitive performance. Finally, Luo will present a coordinated analysis that explores how Big Five personality traits are associated with longitudinal patterns of social stress (i.e., social asymmetry) in multiple longitudinal studies of aging. Preliminary findings suggest that individuals who are higher in neuroticism become more socially vulnerable over the lifespan, while those higher in extraversion, conscientiousness, and agreeableness become more socially resilient. We will close with a panel discussion about the utility of investigating psychosocial and cognitive processes across multiple timescales for promoting health aging.

Abstract citation ID: igaf122.277

### INDIVIDUAL DIFFERENCES IN COGNITIVE COUPLING

Emorie Beck[1], Zoë Hawks[2], and Eileen Graham[3], *1. University of California, Davis, Davis, California, United States, 2. Brooklyn Health, New York, New York, United States, 3. Northwestern University, Evanston, Illinois, United States*

Cognition can be understood in terms of both narrower cognitive domains (e.g., working memory, processing speed) and broader cognitive function (e.g., g, cognitive impairment) across the lifespan. Yet, there are at least two lingering challenges in our understanding of cognitive domains and global cognitive functioning. First, although cognitive abilities or capacities are relatively stable over time, cognitive function shows reliable and valid fluctuations in everyday life. Such fluctuations may provide insight into how modifiable risk factors of cognitive decline (e.g., exercise, cognitive activity, etc.) "get into the person" and increase their long-term risk for poor cognitive function. Second, current understandings of links between cognitive domains (i.e. the structure of cognitive functioning) is almost entirely between-person (i.e. people with good working memory also have fast processing speed), missing the possibility that cognitive domains may be differentially coupled within a person. Such within-person coupling across cognitive domains may be a key digital risk factor of long-term cognitive decline and dysfunction. Using an ecological momentary assessment of middle and older adults (N = 200, total obs.=12,431), we examine individual differences in the coupling of three cognitive domains (working memory, episodic memory, and processing speed) using continuous time structure equation models to capture both autoregressive (i.e. stability) and cross-lagged (i.e. directional cross-domain associations) among cognitive domains within- and between-persons. We will then examine whether individual differences in coupling are concurrently associated with global cognitive functioning, cognitive impairment, personality traits, and other psychosocial factors.

Abstract citation ID: igaf122.278

### PERSONALITY STATES AND COGNITIVE ABILITY AND VARIABILITY IN OLDER ADULTS

Winkie Ma[1], Adam Nissen[1], Eileen Graham[2], Zoë Hawks[3], and Emorie Beck[1], *1. University of California, Davis, Davis, California, United States, 2. Northwestern University, Evanston, Illinois, United States, 3. McLean Hospital, Boston, Massachusetts, United States*

Psychological factors, like personality, are particularly promising behavioral indicators of AD/ADRD to target as risk-modifying treatments. Personality traits are typically defined as characteristic patterns of thinking, feeling, and behaving, which are expressed in everyday life as personality states. Prior work linking personality to cognition has largely focused on how broad, between-person differences in personality traits are associated with broad differences in cognitive function and decline, ignoring personality states and the within-person processes that unfold on shorter time scales. The present study aims to extend prior work by investigating associations between Big Five states and momentary cognitive function (i.e. momentary levels of cognitive function) and variability using an ecological momentary assessment (EMA) study of middle and older adults between 45 and 74. At baseline, 200 (Mean age = 55; 50.1% female, total obs. = 12,431) participants responded to five surveys per day for 20 days capturing their cognitive function, contextual factors, and Big Five personality states. Using mixed-effects location scale models, we simultaneously predicted momentary cognitive levels (location) and cognitive variability (scale) from fluctuations in personality states. In line with prior work on personality traits, we expected that Conscientiousness and Neuroticism states would impact both cognitive levels and variability, on average, across the sample. However, we also expected individual differences in the degree to which each was associated with cognitive function and variability across people, which would signal differences in vulnerability to personality states that could be targets for intervention.

Abstract citation ID: igaf122.279

### STRESS MINDSET AND PERCEIVED STRESS: NO MOMENTARY LINKS TO COGNITIVE FUNCTIONING IN DAILY LIFE

Daisy Zavala[1], Amanda Miller[1], Martina Luchetti[2], Elizabeth Milad[1], Selin Karakose[1], Antonio Terracciano[1], and Angelina R. Sutin[2], *1. Florida State University, Tallahassee, Florida, United States, 2. Florida State University College of Medicine, Tallahassee, Florida, United States*

Evidence suggests that individuals that are unable to adapt to daily stress are more likely to experience short-term cognitive failures. Yet, less is known about how a stress mindset affects perceptions of stress and cognition in daily life, and whether a stress mindset buffers against the negative effects of perceived stress on cognition. The present study leveraged a momentary ecological assessment (EMA) study to investigate the association between stress mindset, perceived stress, and cognitive functioning among middle-aged adults (40-70 years, N = 286). We hypothesized that a stress-is-enhancing mindset would predict lower momentary perceived stress, and that higher perceived stress would be associated with worse cognitive functioning in processing speed and working memory – as measured by the Dot Memory and Symbol Search task. Stress mindset was measured at baseline, whereas perceived stress and cognition were measured across an 8-day EMA protocol (3 assessments/day). Multilevel models that adjusted for temporal and contextual factors, age, sex, education, race/ethnicity, practice, and chronic stress, revealed no significant results. That is, stress mindset did not predict lower momentary perceived stress, nor were any momentary associations observed between perceived stress and cognitive functioning. However, people who were older reported less momentary perceived stress and those who had more years of education and chronic stress reported more momentary perceived stress. These findings suggest that further work is needed as theoretical and experimental work highlighting the benefits of a stress-is-enhancing mindset, may not fully translate to experiences of stress and cognition in daily life.

Abstract citation ID: igaf122.280

### CHANGES IN SOCIAL ASYMMETRY AS PEOPLE AGE AND THE EFFECTS OF PERSONALITY AND SOCIOECONOMIC STATUS

Jing Luo[1], Kathryn Jackson[1], Emorie Beck[2], Tomiko Yoneda[2], Karina Van Bogart[1], Daniel Mroczek[1], Anthony Ong[3], and Eileen Graham[1], *1. Northwestern University, Evanston, Illinois, United States, 2. University of California, Davis, Davis, California, United States, 3. Cornell University, Ithaca, New York, United States*

Social stress related experiences are substantial public health concerns as people age. However, prior research suggests discrepancies among different aspects of social stress such as social isolation and loneliness. Social asymmetry is a concept that captures the discordance/concordance between loneliness and social isolation levels. Social asymmetry indicates how well individuals adapt to their social environment, with individuals experiencing greater loneliness than expected given their social isolation levels considered socially vulnerable and those experiencing less loneliness than expected considered socially resilient. It remains unknown how social asymmetry changes over time as people age, and how different individual and contextual factors contribute to individual differences in the change trajectory of social asymmetry. In the current project, we used data from 9 large longitudinal studies to examine changes in social asymmetry during aging and the effects of personality traits and socioeconomic status on the levels of and changes in social asymmetry. Adopting the coordinated data analysis framework allows us to synthesize the results across samples and examine the degree to which the findings are generalizable by testing different sample-level moderators. The preliminary results suggested increases in social asymmetry in late adulthood. Higher neuroticism was found to be related to higher levels of and increases in social asymmetry (social vulnerability, whereas higher extraversion, conscientiousness, and agreeableness, as well as higher SES, were related to lower levels of and decreases in social asymmetry (social resilience). The study enhances our understanding of individual differences in the dynamics of objective and subjective social stress during aging.

### SESSION 1270 (SYMPOSIUM)

Abstract citation ID: igaf122.281

### NAVIGATING POLICY SHIFTS IN HOME HEALTH: IMPLICATIONS FOR CARE, QUALITY, AND HIGH-NEED POPULATIONS

Chair: Jamie Smith
Co-Chair: Jacy Weems
Discussant: Shekinah Fashaw-Walters

This symposium focuses on the evolving landscape of home health care (HH) and the impact of key federal and state-level policies on HH delivery. The HH setting has historically been highly responsive to regulatory shifts and policy nudges. As we analyze the effects of the new payment model, the Patient-Driven Groupings Model (PDGM), state-level Certificate of Need laws, and prioritization of quality ratings, we must understand that HH patients are not a homogeneous group. HH patients have different care needs, often driven by their health, functional status, and at-home support. This symposium features four studies that explore the impact of various policies on HH care. Two presentations focus specifically on PDGM and examine how certain aspects of the model influence HH service utilization. The first presentation examines how PDGM has impacted community-entry HH use among individuals living with dementia. The second presentation investigates changes in post-acute therapy services for older adults transitioning home following hospitalization. The third presentation examines the provision of high-quality HH for individuals with dementia, raising questions about the effectiveness of current measures in capturing care quality. The fourth presentation evaluates the impact of Certificate of Need laws on HH use among persons with Alzheimer's disease and related dementias. These innovative studies will foster nuanced discussions about the complexities of the current HH environment and inform future policy recommendations.

Abstract citation ID: igaf122.282

### THE IMPACT OF CERTIFICATE OF NEED LAWS ON HOME HEALTH CARE ACCESS FOR MEDICARE BENEFICIARIES WITH DEMENTIA

Jacy Weems[1], Xiao (Joyce) Wang[2], Katherine Ornstein[3], Anna Beeber[3], Jamie Smith[4], and Momotazur Rahman[2], *1. Brown University School of Public Health, Providence, Rhode Island, United States, 2. Brown University, Providence, Rhode Island, United States, 3. Johns Hopkins University, Baltimore, Maryland, United States, 4. Widener University, Philadelphia, Pennsylvania, United States*

Certificate of Need (CON) laws aim to control state healthcare spending by regulating the establishment of healthcare facilities based on demonstrated need. While CON laws have been largely ineffective in regulating hospital and nursing homes, emerging evidence suggests they may reduce home health care use. Currently, 14 states maintain CON regulations for home health services. Persons with Alzheimer's disease and related dementias (ADRD) increasingly rely on Medicare skilled home health care, especially through community-initiated home health services, to age in place. In this study, we examined whether CON laws reduce home health use among persons with ADRD. We analyzed data from 818,878 Medicare fee-for-service beneficiaries newly diagnosed with ADRD in 2016, tracking their healthcare use over 48 months before and after diagnosis. Using an individual fixed-effects difference-in-differences model, we compared changes in utilization between states with and without home health CON laws. Before ADRD diagnosis, home health use per person per month was 5.9%. After diagnosis, utilization increased by 7.7 percentage points in states without home health CON and by 6.9 percentage points in CON states, suggesting that CON laws lead to approximately 10% lower home health use among individuals with ADRD. We did not observe a significant impact of CON laws on hospital and nursing home utilization. Given CON laws are associated with comparatively less home health utilization among individuals with ADRD, further research is needed to understand how this affects patient outcomes.

Abstract citation ID: igaf122.283

### HOME HEALTH DELIVERY FOR PERSONS WITH DEMENTIA: IMPACTS OF THE NEW MEDICARE PAYMENT SYSTEM

Julia Burgdorf[1], and Tracy Mroz[2], *1. VNS Health, New York, New York, United States, 2. University of Washington School of Medicine, Seattle, Washington, United States*

Medicare-funded home health care (HH) is a crucial resource helping Persons with Dementia (PwD) meet their care needs while aging in place. HH includes a mix of skilled nursing, rehabilitation, and personal care services delivered in the patient's home and one-third of HH patients have dementia. In 2020, Medicare implemented a new HH payment system: the "Patient Driven Groupings Model" (PDGM). PDGM includes several provisions that effectively reduce reimbursement for the types of HH most used by PwD. Using 2019 and 2021 linked Medicare HH claims, patient assessment, geographic, and HH agency data, we modelled the types and intensity of services received as a function of patient dementia status and year (i.e., pre- or post-PDGM). Our sample included older adults (65+) receiving community-initiated HH in 2019 or 2021 (n = 907,553). We modelled outcomes using multivariable logistic and negative binomial regression, adjusting for patient sociodemographic characteristics, functional and clinical status, geographic location, and HH agency characteristics. Compared to pre-PDGM, PwD post-PDGM had lower odds of receiving nursing (Adjusted Odds Ratio (aOR): 0.73; 95% Confidence Interval (CI): 0.72-0.75), occupational therapy (aOR: 0.86; 95% CI: 0.84-0.88), social work (aOR: 0.76; 95% CI: 0.73-0.78), and aide services (aOR: 0.70; 95% CI: 0.68-0.72). Findings persisted following multiple sensitivity analyses, including adjusting for county-level COVID-19 infection rates. This work presents the first analysis of how HH care delivery shifted for PwD following PDGM; findings suggest unintended consequences that may threaten HH quality and the ability to safely age in place for the high-need population of community-living PwD.

Abstract citation ID: igaf122.284

**MEDICARE SKILLED HOME HEALTH CARE QUALITY: ARE WE MEASURING WHAT MATTERS FOR PEOPLE LIVING WITH DEMENTIA?**

Jennifer Reckrey[1], Bian Liu[2], Arushi Arora[2], Christine Ritchie[3], Bruce Leff[4], Abraham Brody[5], Julia Burgdorf[6], and Katherine Ornstein[7], *1. NYU Grossman School of Medicine, New York, New York, United States, 2. Icahn School of Medicine at Mount Sinai, New York, New York, United States, 3. Massachusetts General Hospital, Boston, Massachusetts, United States, 4. Johns Hopkins University School of Medicine, Baltimore, Maryland, United States, 5. NYU Rory Meyers College of Nursing, New York, New York, United States, 6. VNS Health, New York, New York, United States, 7. Johns Hopkins University, Baltimore, Maryland, United States*

People living with dementia frequently use Medicare skilled home health care to meet their care needs at home, but little is known about variation in care quality for this population. Using 100% 2021 Medicare Claims data, we examined receipt of high-quality home health as determined by the 1) Quality of Patient Care Star Rating and 2) Patient Survey Star Rating among fee-for-service beneficiaries with dementia. Of the over 1.6 million Medicare fee-for-service beneficiaries with dementia in 2021, 34% used home health. We found significant county-level variability in utilization of high-quality home health. Within a given county, people living with dementia were consistently less likely to use high-quality home health agencies, regardless of whether quality was measured using the Quality of Patient Care Star Rating or the Patient Survey Star Rating. These findings highlight disparities in care quality received by persons living with dementia and underscore the limited utility of existing home health quality measures for this population. Specifically, quality measures focusing on functional improvement are unlikely to meaningfully reflect care quality for people living with dementia experiencing progressive functional decline as part of their disease. Additional work is needed to optimize care quality and customize quality measures to the unique care needs and experiences of people living with dementia.

Abstract citation ID: igaf122.285

**IMPACT OF THE PATIENT-DRIVEN GROUPINGS MODEL ON REHABILITATION SERVICES IN POST-ACUTE HOME HEALTH CARE**

Tracy Mroz[1], Arati Dahal[2], Rachel Prusynski[2], Debra Saliba[3], and Natalie Leland[4], *1. University of Washington School of Medicine, Seattle, Washington, United States, 2. University of Washington, Seattle, Washington, United States, 3. University of California, Los Angeles, Los Angeles, California, United States, 4. University of Pittsburgh, Pittsburgh, Pennsylvania, United States*

Home health (HH) agencies offer skilled nursing and rehabilitation (physical therapy [PT], occupational therapy [OT], and speech language pathology [SLP]) services to facilitate patients' transition back home following hospitalization. Implemented in 2020, the Patient-Driven Groupings Model (PDGM) removed previous incentives to provide rehabilitation visits, raising concerns about reduced access to these services. Using 100% Traditional Medicare HH claims and assessment data from 2018-2021, we examined changes in receipt of rehabilitation services following PDGM implementation. We modelled odds of receiving any PT, OT, and ST visits across 2,657,967 post-acute HH stays using logistic regression. We modelled number of PT, OT, and ST visits among those who received any visits using negative binomial regression. All models accounted for clustering of stays within agencies and controlled for patient sociodemographic and clinical characteristics, length of stay, agency characteristics, and county-level COVID-19 case rates as the pandemic emerged nearly simultaneously with PDGM implementation. While the adjusted odds of receiving PT, OT, and SLP services during post-acute HH stays were not significantly lower under PDGM compared to before PDGM, the number of visits received decreased significantly for PT (aIRR: 0.86; 95%CI: 0.85,0.86), OT (aIRR: 0.82; 95%CI: 0.82,0.83), and SLP (aIRR: 0.76, 95%CI: 0.75,0.77). This reduction on average equates to about one fewer visit each of PT, OT, and SLP per post-acute HH stay. Decreases in PT, OT, and SLP visits under PDGM may lead to worse rehabilitation-related outcomes for post-acute HH patients. Additional examination of the impact of reduced rehabilitation on patient outcomes is urgently needed.

### SESSION 1275 (SYMPOSIUM)

Abstract citation ID: igaf122.286

**STEP INTO MY SHOES: EMPATHY AND SHARED EMOTION IN DEMENTIA-CAREGIVING CONTEXTS**

Chair: Emily Mroz
Co-Chair: Jenna Wells
Discussant: Carolyn Clevenger

Empathic perspective-taking and shared emotion support meaningful connections between caregivers and people living with dementia (i.e., care recipients). Strong connections, in turn, can support shared decision-making and promote wellbeing for both individuals. Yet, empathy and emotional synergy are often strained by interpersonal and cognitive challenges that are common in dementia contexts. To boost investigation into how empathy and shared emotion can develop, and be nurtured, this symposium includes cutting-edge observational and translational research. First, Dr. Brown will present results demonstrating that care recipients' poor emotion recognition is associated with caregivers' increased negative affect during interactions. Dr. Wells will complement this by describing findings that behaviorally-coded shared positive emotion in interactions between dementia caregivers and care recipients is associated with better mental health in caregivers. Next, Dr. Huo will describe a mixed-methods examination of the links between perspective-taking and addressing care recipient needs in spousal care dyads. Dr. Mroz will describe a photo captioning intervention that supports dementia-caregiver perspective-taking. She will showcase approaches caregivers can take when engaging in the intervention maximize improvements in perspective-taking. Dr. Kemp will share results from a pilot trial of an improvisation training program for dementia-caregivers. She will share how improvisation training was found to support caregiver empathy. Our discussant, Dr. Clevenger, will synthesize these talks and describe future directions for promoting empathy and shared emotion in clinical and community settings. These presentations will showcase empathy and shared emotion as vital, teachable skills that can be supported through accessible behavioral interventions in dementia-care contexts and beyond.

Abstract citation ID: igaf122.287

### "ONE MORE PICTURE": CAPTION CONTENT AND EMPATHY GAINS IN A PHOTO CAPTIONING INTERVENTION FOR DEMENTIA-CAREGIVERS

Emily Mroz[1], Rarinsri Assakul[2], Jenna Wells[2], Molly Perkins[3], Kenneth Hepburn[1], and James Rilling[1], *1. Emory University, Atlanta, Georgia, United States, 2. Cornell University, Ithaca, New York, United States, 3. Emory University School of Medicine, Atlanta, Georgia, United States*

Empathic perspective-taking allows caregivers to better understand, communicate with, and respond to the needs of family members living with dementia. A photo captioning intervention can promote empathic perspective-taking by guiding caregivers to take daily photographs of their family member living with dementia and caption those photos to capture the inner voice of that care recipient. In this study, we analyzed patterns in 190 photo captions from a pilot trial of this intervention with dementia-caregivers. We used a subgroup parallel sampling design which allowed us to identify patterns in photo captions for the participants who experienced the most and least change in perspective-taking across the intervention. Descriptive content-analysis demonstrated that caregivers who experienced high perspective-taking change wrote photo captions that 1) maintained first-person, care-recipient focused language, 2) highlighted care recipients' engagement and intention, and 3) illustrated resilient living. Caregivers who experienced low perspective-taking change captioned photos by 1) offering inconsistent, incomplete phrasing, 2) providing generic, detected descriptions, and sometimes 3) adopting a negative, critical tone. Caregivers from both groups also wrote captions that 1) connected care recipients' emotions to events and 2) recorded care recipients' wonders, wants, and wishes. The ways that caregivers approach writing photo caption content may be an important behavioral mechanism influencing the impact of this intervention. Results can guide refinement of perspective-taking interventions to maximize benefits experienced by caregivers and their care recipients.

Abstract citation ID: igaf122.288

### UNDERSTANDING NEEDS OF PEOPLE WITH ALZHEIMER'S DISEASE: THE ROLE OF CAREGIVER PERSPECTIVE TAKING

Meng Huo[1], Rose Tran[1], and Emily Mroz[2], *1. University of California, Davis, Davis, California, United States, 2. Emory University, Atlanta, Georgia, United States*

Despite receiving intensive support from caregivers, people living with Alzheimer's disease (PLWD) still face unmet care needs that exacerbate their own functional declines and increase caregiver burden. Unmet needs emerge when caregivers and PLWD are misaligned about PLWD's needs. We conducted a mixed-methods study with 67 couples in which one person had mild-to-moderate Alzheimer's disease. Both spouses completed semi-structured interviews to rate PLWD's needs across 16 areas (e.g., self-care, memory, company) using the modified Camberwell Assessment of Need for the Elderly (CANE); they also discussed how they communicated about those needs. We examined caregivers' perspective taking—their ability to understand PLWD's thoughts and feelings and observed a negative association between perspective taking and spousal discrepancies in PLWD's needs. We then conducted a thematic analysis of caregivers' qualitative descriptions about communication strategies about PLWD's needs. When caregivers had high perspective taking, spouses described engaging in open, effective communication that fostered a shared understanding of caregiving expectations and caregivers felt they adopted PLWD-centered strategies that sustained or enhanced PLWD's sense of competence. When caregivers had low perspective taking, spouses described disagreeing on the care PLWD needed and some caregivers acknowledged being transparently frustrated in conversations with PLWD. Findings illustrate how and why caregivers' perspective taking can direct their efforts to understand and meet the needs of PLWD: perspective taking can facilitate couple communication and enhance caregivers' perceptions of and responses to PLWD's needs. These insights can inform interventions to improve the quality of dementia care and promote health in caregiving dyads.

Abstract citation ID: igaf122.289

### EMPATHY, YES, AND…, IMPROVING DEMENTIA CARE THROUGH IMPROV

Candace Kemp, Jennifer Morgan, Christina Cifuentes, and Mary Jones, *Georgia State University, Atlanta, Georgia, United States*

Empathy is at the heart of person-centered dementia care and has a significant impact on care interactions and relationships. Some family caregivers of persons living with dementia instinctively approach care with empathy. Yet, many have difficulty relating to their person, particularly when faced with behaviors perceived as out-of-character, unexpected, or challenging. Identifying ways to enhance caregiver empathy is critical to improving dementia care experiences. One innovative and promising avenue involves improvisational (i.e., "improv") theatre. Improv, like empathy, requires actively listening and observing, collaborating, meeting people where they are, and being in the moment, and can enhance caregivers' engagement capacity. In this paper we examine improv's potential by analyzing data collected as part of a pilot study testing, "Improving Care through Improv," an 8-hour training program designed for family and friends caring for someone with moderate dementia. We assessed the program through a no-control study involving surveys (collected at baseline, program completion, and 3-months post-baseline) and focus groups (n = 4). Forty caregivers participated in the training and completed the study. Training was associated with decreases in stress, negative responses to challenging behaviors, and reports of frustration. In open-ended survey and focus group data, most referred to a "mindset shift," including many who described enhanced capacity to be "more patient," "accept and understand," have "more grace," and "not always "correct." One explained, understanding the "need to radically accept where my person is" and "support her from there." This training demonstrates improv's ability to impact empathy and care relationships and requires additional systematic investigation.

Abstract citation ID: igaf122.290

### GREATER POSITIVITY RESONANCE IS ASSOCIATED WITH BETTER MENTAL HEALTH IN DEMENTIA CAREGIVERS

Jenna Wells[1], Suzanne Shdo[2], Claire Yee[3], Diana Heath[4], Barbara Fredrickson[5], and Robert Levenson[2], *1. Cornell University, Ithaca, New York, United States, 2. University of*

*California, Berkeley, Berkeley, California, United States, 3. Mayo Clinic, Scottsdale, Arizona, United States, 4. Arizona State University, Tempe, Arizona, United States, 5. The University of North Carolina at Chapel Hill, Chapel Hill, North Carolina, United States*

As a group, family caregivers of people with dementia (PWDs) have substantially elevated levels of depression and anxiety, compared to same-aged non-caregiving adults. However, there is striking variation in mental health among individual caregivers. Previous research on predictors of the sources of these individual differences has focused on characteristics of individual caregivers and PWDs (rather than on the caregiver-PWD relationship), self-report measures (rather than behavioral measures), and negative emotion (rather than positive emotion). We examined 185 caregiver-PWD dyads who had an unrehearsed 10-minute conversation about an area of conflict in the laboratory. Video recordings were coded for Behavioral Indicators of Positivity Resonance (BIPR), which derives from Positivity Resonance Theory and assesses dyad-level behavioral expressions of positive affect, mutual warmth, and synchrony. Caregivers reported their level of depression, anxiety, and emotional well-being and PWDs underwent a comprehensive neurological and neuropsychological evaluation. Pearson's correlations revealed that greater dyad-level BIPR was associated with caregivers' higher emotional well-being ($r(181) = .19$, $p = .008$), lower depression ($r(179) = -.17$, $p = .025$), and lower anxiety ($r(180) = -.15$, $p = .045$). Regression analyses revealed that these associations were still obtained after controlling for caregiver age and sex and PWD dementia severity, cognitive impairment, and neuropsychiatric behavior (emotional well-being ß = .18, $p = .012$; depression ß = -.15, $p = .039$; anxiety ß = -.15, $p = .044$). Future studies should determine whether interventions that increase co-experienced positive emotions among caregiver-PWD dyads help buffer caregivers from adverse mental health consequences.

Abstract citation ID: igaf122.291

### EMOTION RECOGNITION IN PEOPLE WITH DEMENTIA AND CAREGIVER AFFECT DURING DYADIC INTERACTIONS

Casey Brown[1], Yuxuan Chen[2], and Robert Levenson[3], *1. Georgetown University, Washington, District of Columbia, United States, 2. Stanford University, Stanford, California, United States, 3. University of California, Berkeley, Berkeley, California, United States*

Dementia caregivers may experience negative emotions during interactions with their care recipients, yet little research has explored the specific factors that contribute to this response in a dyadic context. Based on research demonstrating care recipient's emotion recognition impairments are linked with their caregivers' depressive symptoms, we hypothesized that impairments in care recipient's emotion recognition relates to increases in their caregiver's negative affect during interactions with their care recipient. A sample of 100 caregiver-care recipient dyads participated in the study. Caregivers reported on their care recipients' emotion recognition using a well-validated measure. Dyads engaged in a 10-minute conflict-based conversation in a laboratory setting, after which caregivers watched video recordings of their conversation and continuously rated their emotional valence using a rating dial. These ratings were used to assess changes in caregivers' affect over the course of the conversation. Caregivers whose care recipients had greater deficits in emotion recognition experienced more pronounced increases in negative affect throughout the conversation, even after accounting for caregivers' baseline affect, sex, and age, as well as care recipients' diagnosis, level of cognitive impairment, neuropsychiatric symptoms, and functional limitations. These results demonstrate an association between care recipients' emotion recognition skills and caregivers' emotional experiences. When care recipients struggle to recognize emotions, caregivers may be more likely to experience worsening negative affect during interactions, highlighting a potential target for caregiver support interventions.

### SESSION 1280 (SYMPOSIUM)

Abstract citation ID: igaf122.292

### THE CUTTING EDGE OF GERONTOLOGICAL SCHOLARSHIP: THE GERONTOLOGIST'S EDITOR'S CHOICE SYMPOSIUM

Chair: Joseph Gaugler
Discussant: Joseph Gaugler

The mission of The Gerontologist is to publish and disseminate "applied, multidisciplinary research and analysis on social issues related to human aging. It informs the broad community of disciplines and professions involved in understanding the aging process and providing care to older people." In addition, the journal encourages "cutting-edge, conceptually-oriented work that addresses inequities in health, mental health, social status, and justice in late life as well as intersections among these areas with applicability to real-world-settings." This symposium will highlight some of the recent and best examples of gerontological scholarship in The Gerontologist by highlighting select Editor's Choice articles published in the past year. John Puxty, MbCbB, FRCP(C), Sarah Webster, MHS, and colleagues will share findings from a study that developed and validated a survey tool to evaluate Age-Friendly Community Initiatives, which will help researchers, members, and policymakers better discern the extent of age-friendliness in their communities. Jennifer Vincenzo, PhD, MPH, PT, and colleagues will present an implementation study of the Stopping Elderly Accidents, Deaths, and Injuries (STEADI) Initiative across 34 outpatient rehabilitation clinics serving older adults in a large healthcare system. Dr. Raina Croff and colleagues will describe the feasibility and health effects of a pilot triadic walking program for older Black adults with mild cognitive impairment that emphasizes culturally-centered, celebratory social reminiscence. Nikki-Anne Wilson, PhD, and Kaarin Anstey, PhD, argue for increased attention on barriers to behavior changes to reduce dementia risk in order to avoid "lifestyle" stigma.

Abstract citation ID: igaf122.293

### ASSESSING AGE-FRIENDLY COMMUNITY INITIATIVES: DEVELOPING A NOVEL SURVEY TOOL FOR ASSESSMENT AND EVALUATION

John Puxty, *Queen's University, Kingston, Ontario, Canada*

Background and Objectives: Age-friendly community initiatives (AFCIs) have gained recognition as essential responses to the needs of aging populations. Despite their growing significance, there is a notable lack of effective measurement tools to assess the planning, implementation, and sustainability of AFCIs. The purpose of this study was to develop and validate a survey tool for evaluating AFCIs. Research Design and Methods: A sequential exploratory mixed-method design was used in 2 phases.

First, we identified key themes from interviews with AFCI leads to generate AFCI survey items and regional workshops. Then, we conducted a pilot of the survey and assessed its measurement properties. Results: Thematic analysis of interviews with 68 key informants from 58 AFCIs revealed 4 main themes: AFCI priorities, enablers, challenges, and benefits. These themes, combined with feedback from AFCI stakeholders at the regional workshops and an AFCI conference, informed the development and refinement of a reliable and valid AFCI survey in 2019, supported by a high Cronbach's alpha value (α _= 0.881). Steps were identified to maintain and sustain the AFCI survey over time including during and post COVID Pandemic. Discussion and Implications: The survey accommodates AFCIs' diverse demographics, governance structures, and priorities with a standardized and flexible approach for effective measurement. This research contributes to the academic understanding of AFCIs and aids community leaders and policymakers in planning, implementing, and evaluating AFCIs.

Abstract citation ID: igaf122.294

### STEADI FOR FALLS PREVENTION IN OUTPATIENT REHABILITATION: AN OUTCOMES EVALUATION USING THE RE-AIM FRAMEWORK

Jennifer Vincenzo, *University of Arkansas for Medical Sciences, Little Rock, Arkansas, United States*

We used the RE-AIM (Reach, Effectiveness, Adoption, Implementation, and Maintenance) framework to identify the implementation outcomes of the Stopping Elderly Accidents, Deaths, and Injuries (STEADI) Initiative in 34 outpatient rehabilitation clinics within a health system between 2018–2021. STEADI was used to screen and assess all older adults ≥65 years for falls risk. STEADI Implementation strategies included health system mandates, EHR revisions, email instructions, educational sessions and resources, clinical leads and champions, and chart audits. Using Electronic Health Records (EHRs) and descriptive statistics, we identified the Reach, Adoption, and Maintenance of screenings and physical assessments to identify fall risk among older adults. Reach: 76.4% (50,023) of older adults seen by a rehabilitation provider had a completed screening, and 44.1% screened at risk for falls. Adoption: Clinic-level adoption varied, with most performing screenings. Profession-level adoption was highest for physical therapists (PTs; 94.2% initiated, 80.6% completed) and lowest for speech-language pathologists (SLPs; 79.8% initiated, 55.9% completed). Reach and Adoption of functional outcomes measures (FOM): PTs completed a FOM on 59.5% of at-risk patients, occupational therapists on 11.6%, and SLPs on 7.9%. Maintenance: All measures declined 1%–10% annually between 2018 and 2021. STEADI screening and FOMs were implemented systemwide in 34 outpatient rehabilitation clinics, reaching over 50,000 older adults. Screening adoption rates varied by clinic. PTs had the highest adoption rate. All adoption rates declined over time. Future research should consider an implementation science approach with input from key partners to identify barriers and develop strategies to support all STEADI components in outpatient rehabilitation.

Abstract citation ID: igaf122.295

### WALKING AND SOCIAL REMINISCENCE IN GENTRIFYING NEIGHBORHOODS AMONG OLDER BLACK ADULTS: FEASIBILITY AND OUTCOMES

Raina Croff[1], Sophia Aron[1], Anne Wachana[1], Patrice Fuller[1], Nora Mattek[1], Juell Towns[2], and Jeffrey Kaye[1], *1. Oregon Health & Science University, Portland, Oregon, United States, 2. University of Washington, Seattle, Washington, United States*

The Sharing History through Active Reminiscence and Photo-imagery (SHARP) study uses Black history amidst the erasure of gentrification and the disparate risk of memory loss to motivate healthier aging among older Black older adults. In two pilots, fourteen triads of healthy and mildly cognitively impaired participants walked 1-mile routes 3x/week over 24 weeks in Portland's gentrifying historically Black neighborhoods. Using a group tablet, triads accessed a menu of 72 themed routes via the SHARP walking application. Along routes, GPS-linked historical images of local Black life and culture from 1940-2010 prompted conversational reminiscence. Walking narratives were recorded for a digital oral history archive. Retention was 74% and 86% for pilots, and 100% and 92%, respectively, were "extremely likely" to recommend SHARP to friends and family. Mean rank scores indicated program readiness with minor changes, effective conversational prompts, and appropriate pace and dose. Self-rated health, mood, activity levels, and energy improved, days feeling downhearted decreased, and days feeling calm/peaceful were maintained or improved. Among Cohort 2, cognitive assessment scores were maintained or improved for 67%; for MCI, 76% had mean improvement of 2.4 (p = .045). Blood pressure and weight decreased for 78% and 44%, respectively. Thirty focus groups were completed. Participants noted that the triadic structure created group accountability, motivated sustained engagement, curated friendships, and expanded social networks. Walking also increased physical and social activity outside the program, inspired healthier habits and self-care, improved awareness of cognitive decline risk and personal agency, and cultivated a deep-seated sense of community connection.

Abstract citation ID: igaf122.296

### AVOIDING STIGMA IN DEMENTIA PREVENTION DISCOURSE: UNDERSTANDING BARRIERS AND HARNESSING STRENGTHS.

Nikki-Anne Wilson, *University of New South Wales Sydney/ Neuroscience Research Australia, Kensington, New South Wales, Australia*

Recent decades have seen exponential growth in research on modifiable risk factors for dementia across the lifespan. We now have an unprecedented understanding of ways to support brain health, particularly the need for healthy eating and physical activity which are commonly referred to as "lifestyle choices". Individualistic lifestyle modifications remain the leading dementia prevention focus and their importance should not be understated. We caution against, however, a potential short-sightedness regarding the bounds of individual responsibility and the need for a broader understanding of individual and socio-economic barriers to instigating positive health behaviors. People living with dementia already experience stigma and we know that negative health outcomes do occur for people with other non-communicable "lifestyle diseases" e.g., diabetes. Moving forward, we call for balance in dementia prevention discourse capturing both the complexities of etiology and a more inclusive approach to risk-reduction which addresses health inequities. This requires both population-level interventions and multidisciplinary individual support. Further, given our broadening understanding of psychosocial factors such as depression and social isolation as contributing to dementia risk, we suggest that a greater focus on strengths-based approaches to building social and psychological wellbeing may be beneficial. Our aim is not to detract from work which has come before nor undermine the continued importance of empowering individuals to make positive health changes. Rather, we put forward the need for a more considered and inclusive approach to the investigation and dissemination of dementia risk-reduction which avoids inadvertently increasing stigma through overly simplistic representations of a complex clinical syndrome.

Abstract citation ID: igaf122.297

### THE CHANGING DEMOGRAPHY OF LATE-LIFE FAMILY CAREGIVING: A RESEARCH AGENDA TO UNDERSTAND FUTURE CARE NETWORKS

I-Fen Lin, *University of Missouri, Columbia, Missouri, United States*

Repeated claims that a dwindling supply of potential caregivers is creating a crisis in care for the U.S. aging population have not been well grounded in empirical research. Concerns about the supply of family care do not adequately recognize factors that may modify the availability and willingness of family and friends to provide care to older persons in need of assistance or the increasing heterogeneity of the older population. In this paper, we set forth a framework that places family caregiving in the context of older adults' care needs, the alternatives available to them, and the outcomes of that care. We focus on care networks, rather than individuals, and discuss the demographic and social changes that may alter the formation of care networks in the future. Last, we identify research areas to prioritize in order to better support planning efforts to care for the aging U.S. population.

### SESSION 1285 (SYMPOSIUM)

Abstract citation ID: igaf122.298

### MOTIVATIONAL THEMES FOR ENGAGING IN RANDOMIZED CONTROLLED TRIALS INCORPORATING COGNITIVE TRAINING

Chair: Jody Nicholson
Co-Chair: Maria Vander Meulen
Discussant: Walter Boot

As of March 2024, the NIH annual budget for Alzheimer's disease and related dementias (ADRD) research is set to nearly $3.8 billion, after a $100 million increase (Alzheimer's Association, 2025). This significant increase in funding for cognitive aging research is focused on identifying the causes of age-related cognitive decline and advancing methods and technologies to improve cognitive function in older adults. Whether grant- or industry-funded, clinical trial or observational, two of the most challenging aspects of research are recruitment and retention, which are integral to maintaining validity and generalizability of study results. Computerized cognitive training is one of the most promising non-pharmaceutical interventions for ADRD prevention, so understanding participants' reasons for engaging in this type of research is crucial to the next stages of implementation research. This symposium highlights research teams who have assessed participant motivation to engage in computerized cognitive training in order understand how motivations for research engagement differ between underrepresented groups (paper 1), how motivation relates to both participant enrollment and drop-out (paper 2), and how to use participant motivation to develop tailored supportive communications in randomized controlled trials to encourage adherence and retention (paper 3). The symposium presents refinement in research examining participant motivation with the studies representing novel contributions given their sample size (paper 1; n = 5,721), link to both a theoretical framework and information on participant withdrawal (paper 2), and a manner in which participant motivation can be strategically applied to increase study retention (paper 3).

Abstract citation ID: igaf122.299

### EXPLORING MOTIVATION TO PARTICIPATE IN COGNITIVE TRAINING ACROSS UNDERREPRESENTED GROUPS IN CLINICAL RESEARCH

Lizbeth Vera Murillo[1], Maria Vander Meulen[2], Angel Collie[3], Stacey Alvarez-Alvarado[2], Kat Barrett[4], Yuting Yang[5], and Jennifer O'Brien[6], *1. Duke University School of Medicine, Durham, North Carolina, United States, 2. University of Florida College of Medicine - Jacksonville, Jacksonville, Florida, United States, 3. The Pennsylvania State University, University Park, Pennsylvania, United States, 4. Clemson University, Clemson, South Carolina, United States, 5. Health Informatics Institute, Tampa, Florida, United States, 6. University of South Florida, Tampa, Florida, United States*

Challenges with participant recruitment and lack of sample diversity are common problems in clinical research. The Alzheimer's Association highlights the need for more heterogeneous samples in clinical research; underrepresented groups in clinical research can be categorized by demographic characteristics, including race/ethnicity, gender, and participant age. In particular, more effective recruitment for Latinos/Hispanics, Blacks/African Americans, males, and old-old adults (≥75 years) is needed. The goal of the current study was to explore motivations for participating in an Alzheimer's disease and related dementias (ADRD) prevention trial and to examine whether motivations differed by demographic characteristics associated with underrepresented groups in clinical research. Cognitively healthy older adults (N = 5,721, Mage= 71.5 + 5.0) enrolled in the Preventing Alzheimer's with Cognitive Training (PACT) trial completed open-ended questions assessing motivations for participation. Thematic analyses were conducted to identify motivational topics. Demographic subgroup differences were analyzed using chi-square and logistic regression analyses. Concern about brain health was the most common motivational theme, followed by direct experience with ADRD and altruism. Motivations differed by gender, race, age, and education. Results suggest that focusing on brain health concerns may be effective for recruitment messaging in ADRD prevention trials. However, demographic differences in motivations suggest recruitment materials and messaging could be tailored to align with specific motivational factors among subgroups.

Abstract citation ID: igaf122.300

### PARTICIPANT MOTIVATION TYPOLOGIES AS CORRELATES OF STUDY PARTICIPATION AND RETENTION IN COGNITIVE AGING TRIALS

Abigail Stephan, Hye Won Chai, Jody Nicholson, Christy Phillips, and Lesley Ross, *Clemson University, Clemson, South Carolina, United States*

In recruiting and retaining participants in Alzheimer's disease and related dementias (ADRD) research, participants'

motivations may explain why they participate in and withdraw from research. Drawing on Bardach and colleagues' (2020) Activator, Motivator, Outside mediator, Re-enforcer (AMOR) model of research engagement, this presentation will report on our mixed-methods study that 1) explored participants' reasons for participating and, in some cases, withdrawing from computerized cognitive training research studies and 2) connecting the provided reasons to study retention. A convenience sample of community-dwelling adults (N = 423) between 55-88 years (M = 67.45) was drawn from two randomized controlled trials involving computerized cognitive training. We used eclectic coding procedures to analyze participants' reasons for participating (shared via written responses) and, when relevant, their stated reasons for withdrawing (ascertained via chart notes made by study personnel). Chi-square tests and logistic regression were used to test the associations between reasons for participation and study retention. Primary reasons for participating in research included activating and motivating factors, while primary reasons for withdrawing included outside contextual factors and re-enforcers. Re-enforcers were particularly relevant for withdrawal during the cognitive training phase. Reasons for participation were not associated with reasons for, nor likelihood of, withdrawal. This work suggests that individuals' reasons for participating in and withdrawing from cognitive training studies are nuanced, with participants considering multiple factors in their decisions. Findings support an adapted version of the AMOR model for explaining research engagement, and further validation of a motivational research participation model is critical for identifying effective recruitment and retention efforts.

Abstract citation ID: igaf122.301

### ENHANCING ADHERENCE IN COGNITIVE ASSESSMENT AND TRAINING: INSIGHTS FROM THE APPT TRIAL

Dawn Carr[1], Walter Boot[2], Zhe He[1], Shayok Chakraborty[1], Mia Liza Lustria[1], Shenghao Zhang[2], and Michael Dieciuc[1], *1. Florida State University, Tallahassee, Florida, United States, 2. Weill Cornell Medicine, New York, New York, United States*

The Adherence Promotion with Person-centered Technology (APPT) trial addresses adherence challenges in cognitive assessment and training by leveraging smart, tailored technologies. Early detection of cognitive decline is critical for advancing scientific understanding, improving clinical trial recruitment, and enabling early intervention. However, adherence to frequent at-home testing and behavioral interventions remains a significant barrier. Designing an effective adherence support system requires understanding the motivations that drive older adults to participate in research and engage with interventions. To initially explore these motivations, we surveyed 472 older adults about their reasons for participating in research. Results identified four motivation typologies: brain health advocates, research helpers, fun seekers, and multiple motivation enthusiasts. Individual differences—including age, employment status, and cognitive difficulties—shaped motivation profiles, informing the development of tailored engagement strategies. Building on this foundation, we then pilot-tested a just-in-time adherence support system using personalized text messages to encourage engagement in a 10-day cognitive training study. Forty-three older adults received messages aligned with their stated participation motivations. Matched messages were rated as significantly more effective in promoting adherence than mismatched ones. Participants preferred messages that were personalized and formal, reinforcing the importance of message tailoring. With these insights, the first randomized controlled trial (N = 199) evaluating the APPT system for home-based cognitive training has been completed, and a second trial, examining home-based assessment, is now underway. Findings will inform future refinements of dynamic, machine-learning-driven adherence support, with broad applications for cognitive training, telehealth, and other health-related interventions.

### SESSION 1290 (SYMPOSIUM)

Abstract citation ID: igaf122.302

### INCLUSIVE HEALTHY AGING IN A GLOBAL SOCIETY: STRENGTHENING COMMUNITY AND COGNITIVE VITALITY

Chair: Su-I Hou

This symposium explores global perspectives on aging, focusing on societal perceptions, loneliness, cognitive decline prevention, and psychological well-being. Through multidisciplinary and international viewpoints, it highlights research-driven strategies for fostering inclusive communities and promoting cognitive and emotional health among older adults worldwide. Prof. Hou (University of Central Florida) will compare older adults in neighborhood lunch programs (NLP) and lifelong learning programs (LLP) in the U.S., examining how community engagement shapes perceptions of aging, financial security, and political influence. Drs. Pan and Liu (Vrije Universiteit Brussel & National Cheng Kung University) will explore social and emotional loneliness among community-dwelling older adults in Taiwan during the early pandemic. Their findings on community and individual risk factors will inform culturally adaptive intervention strategies. Drs. Chou and Hou (UCF) will analyze the role of polypharmacy in moderating the relationship between instrumental activities of daily living (IADL) and physio-cognitive decline syndromes (PCDS), emphasizing the need for medication management in supporting cognitive and physical health. Ms. Perera (FINGERS Brain Health Institute) will present updates from the MET-FINGER study, a European-led trial combining lifestyle interventions with metformin to prevent cognitive impairment. This study highlights the potential for precision-prevention strategies in reducing dementia risk globally. Ms. Chang (University of Rochester) will examine the impact of vision loss on psychological well-being among older adults in the U.S., offering insights into interventions applicable across diverse healthcare systems. Together, these studies provide global insights into aging, offering evidence-based strategies to enhance community engagement, cognitive resilience, and psychological well-being in later life. This is a collaborative symposium between the Aging Among Asians and International Comparisons of Healthy Aging Interest Groups.

Abstract citation ID: igaf122.303

### SOCIETAL VIEWS ON AGING: COMPARING NEIGHBORHOOD LUNCH AND LIFELONG LEARNING PROGRAM PARTICIPANTS

Su-I Hou, *University of Central Florida, Orlando, Florida, United States*

Purpose: Understanding societal views toward aging is crucial for fostering inclusive communities. This study examines how participation in two community programs in Florida —a neighborhood lunch program (NLP) and a university-based lifelong learning program (LLP)—influences older adults' perceptions of aging. Methods: A total of 193 older adults from the same county participated, with 57% from LLP. Significant demographic differences were observed: LLP participants were younger (70 vs. 77 years, p<.001), predominantly White (95% vs. 24%, p<.001), and more likely to have a college degree (92% vs. 31%, p<.001). Both groups had long-term community residency (NLP: 21.86 years; LLP: 18.96 years; non-significant). Results: Compared to LLP participants, NLP participants reported more positive perceptions of financial security, healthcare and caregiving support, family integration, and societal respect as they age. However, NLP participants perceived older adults as having less political influence. No significant differences were found between the groups regarding social participation opportunities or age-based discrimination. Discussion: These findings provide new insights into the characteristics and social perceptions of older adults in different community programs. While LLP participants viewed older adults as having greater political influence, NLP participants expressed stronger perceptions of financial and social support. The longer engagement and support structure of NLP may contribute to these more favorable views. Despite their higher socioeconomic status, LLP participants reported lower perceived societal support for older adults. Future research should explore factors influencing these perceptions, examine the benefits of NLP, and address the challenges faced by LLP participants.

Abstract citation ID: igaf122.304

### LONELINESS AMONG COMMUNITY-DWELLING OLDER ADULTS IN TAIWAN: EXPLORING MULTI-LEVEL RISK FACTORS

Honghui Pan[1], LiFan Liu[2], and Su-I Hou[3], *1. Vrije Universiteit Brussel, Brussels, Brussels Hoofdstedelijk Gewest, Belgium, 2. National Cheng Kung University, Tainan City, Tainan, Taiwan (Republic of China), 3. University of Central Florida, Orlando, Florida, United States*

Loneliness has emerged as a significant societal issue in many Western countries, but limited research has explored loneliness among older adults in Taiwan, a rapidly aging society in East Asia. This study aimed to examine the prevalence and risk factors of loneliness among Taiwanese older adults during the COVID-19 pandemic. We analyzed secondary data from the University Responsibility dataset at National Cheng Kung University in southern Taiwan, including 530 adults aged 65 and above. Loneliness was measured using the 6-item De Jong Gierveld loneliness scale (2006), assessing emotional and social loneliness. Hierarchical multiple linear regression was applied to examine community-level (e.g., non-age-friendly environments, low social capital, low social support) and individual-level (e.g., poor living environment perception, low social participation) risk factors. Demographic factors such as age, gender, marital status, ethnicity, education, health, and income were also included. The results showed that 59.2% of participants experienced moderate loneliness, while 19.6% reported severe loneliness. Additionally, 36.5% faced emotional loneliness, and 58.4% social loneliness. Key community-level risk factors included low social capital ($\beta$=-0.341, P = 0.024) and low social participation ($\beta$=-0.365, P = 0.035). Individual-level risk factors included negative living environment perceptions ($\beta$=-0.700, P < 0.001), poor health ($\beta$=-0.452, P < 0.001), low income ($\beta$=-0.507, P = 0.026), and being married ($\beta$=-0.391, P = 0.037). These findings underscore the need for community-based interventions to enhance social capital and participation, and for promoting positive perceptions of living environments to reduce loneliness among older Taiwanese adults.

Abstract citation ID: igaf122.305

### POLYPHARMACY MODERATES LINK BETWEEN INSTRUMENTAL DAILY ACTIVITIES AND PHYSIO-COGNITIVE DECLINE IN OLDER ADULTS

Wan-Yun Chou, and Su-I Hou, *University of Central Florida, Orlando, Florida, United States*

Introduction: Physio-cognitive decline syndrome (PCDS) increases the risk of dementia, disability, and mortality in older adults. However, little is known about the role of polypharmacy in the association between instrumental activities of daily living (IADL) and PCDS. Methods: This cross-sectional study examined the moderating effect of polypharmacy on this relationship among 183 community-dwelling adults aged 65 and older undergoing annual health screenings (March 2021–November 2022) in Taipei, Taiwan. Participants were classified into two groups: robust or PCDS. PCDS was diagnosed if a subject met a cutoff below 1.5 standard deviations of age-, sex-, and education-matched norms of the Chinese version of the Mini-Mental State Examination (CMMSE) and physical (with weakness/ and/or slowness) criteria. PCDS was identified as 21% of participants. Results showed that IADL was negatively associated with PCDS, but this relationship was moderated by polypharmacy. When polypharmacy was present, the association was more potent, and the relationship was stronger, whereas without polypharmacy, the relationship was weaker. These findings highlight the importance of managing polypharmacy alongside daily activity to protect cognitive and physical health in older adults. It is necessary to pay attention to polypharmacy and daily activity among older adults. Targeted interventions addressing both factors may help mitigate PCDS. While the convenience sampling method may limit generalizability, further research is needed to explore the impact of polypharmacy on the relationship between IADL and PCDS identification.

Abstract citation ID: igaf122.306

### COMBINING LIFESTYLE WITH METFORMIN TO REDUCE DEMENTIA RISK: UPDATES FROM MET-FINGER TRIAL

Dinithi Perera[1], Mariagnese Barbera[2], Alina Solomon[3], Jenni Lehtisalo[4], Francesca Mangialasche[5], Lefkos Middleton[6], Tiia Ngandu[7], and Miia Kivipelto[3], *1. FINGERS Brain Health Institute, Stockholm, Stockholms Lan, Sweden, 2. University of Eastern Finland, Kuopio, Pohjois-Savo, Finland, 3. Karolinska Institutet, Solna, Stockholms Lan, Sweden, 4. Finnish Institute for Health and Welfare, Helsinki, Uusimaa, Finland, 5. Division of Clinical Geriatrics, Stockholm, Stockholms Lan, Sweden, 6. Imperial College London, London, England, United Kingdom, 7. Finnish Institute for Health and Wefare, Helsinki, Uusimaa, Finland*

MET-FINGER is the first randomized controlled trial combining healthy lifestyle changes with a putative disease-modifying drug (metformin) for the prevention of cognitive impairment, in an APOEε4-enriched at-risk population of older adults. The potential benefits of metformin repurposing are based on the link between Type-II Diabetes (T2D) and Alzheimer's Disease (AD), and emerging evidence of independent neuro-protective effects. Findings will provide crucial information for innovative precision-prevention strategies aimed at cognitive impairment and dementia risk-reduction. 600 participants are randomly allocated to the FINGER 2.0 intervention (lifestyle for all + metformin, where appropriate; active arm), or regular health advice (control arm). Participants in the FINGER 2.0 group and at increased risk of T2D, are further randomised to 2000mg/day metformin, 1000mg/day metformin, or placebo (double-blind). The multimodal lifestyle intervention is an optimised version of the FINGER model. Metformin is administered as Glucophage®XR/SR 500, (Merck Healthcare KGaA, Darmstadt, Germany; 500mg oral tablets). Primary outcome is change in global cognition (combined z-score of a neuropsychological test battery). Secondary outcomes are changes in individual cognitive domains; functional ability; and risk factors for AD/dementia. The feasibility, potential lifestyle-metformin interaction, and disease-modifying effects of the lifestyle-metformin combination are exploratory outcomes. 489 eligible participants have been identified; 365 have been randomised. The main challenges experienced are linked to balancing APOEε4-carriers enrichment and the eligibility rate for metformin treatment (e.g., APOEε4-carriers were less likely to fulfil some eligibility criteria for metformin). Lessons learned, pertaining to trial design, set-up, recruitment, and delivery, for future combination-therapy studies, will be presented.

Abstract citation ID: igaf122.307

### HOW VISION LOSS AFFECTS DIFFERENT PSYCHOLOGICAL OUTCOMES AMONG OLDER ADULTS?

Ya-Han Chang[1], and Silvia Sörensen[2], *1. University of Rochester, Rochester, New York, United States, 2. University of Rochester Warner School of Education and Human Development, Rochester, New York, United States*

Background: Loneliness (Alma et al., 2011; Mick et al., 2018), anxiety and depression (Evans et al., 2007) are more prevalent among visually impaired (VI) than sighted older adults. Individuals' emotional response to vision loss (ERVL) may be important to determining these outcomes. Methods: We used baseline data from adults with macular degeneration (N = 210, Mean Age = 80.1) participating in a depression prevention intervention (Sörensen et al., 2015). ERVL was assessed using 12 items of NEI-VFQ items (RAND, 1996) focused on emotions and perceptions of living with VI ($\alpha$=.904). We used MANCOVA to test the effect of ERVL on loneliness, depression, anxiety, worries about future care, and perceived stress. Results: The multivariate effect of ERVL on the combined psychological outcomes was significant (Wilks' Lambda = .249, $p < .01$, $\eta^2 = .242$). ERVL was significantly associated with loneliness ($p<.05$, $\eta^2 = .265$), depression ($p<.05$, $\eta^2 = .290$), and perceived stress level ($p<.001$, $\eta^2 = .335$). ERVL was not associated with anxiety ($p>.05$, $\eta^2 = .224$ ) and worries about future care ($p>.05$, $\eta^2 = .257$). Conclusion: How individuals perceive and respond to challenging situations can affect psychological outcomes, supporting the idea that stress appraisal is an important aspect of coping with vision loss.

### SESSION 1295 (SYMPOSIUM)

Abstract citation ID: igaf122.308

### IN SYNC: EXPLORING DIGITAL TOOLS FOR SLEEP AND BRAIN HEALTH IN AGING RESEARCH

Chair: Raeanne Moore
Co-Chair: Yeonsu Song
Discussant: Martin Sliwinski

Understanding the interplay between sleep quality, mood, and cognitive functioning is crucial for older adults with cognitive disorders. This symposium showcases innovative research that harnesses digital health tools to elucidate these complex relationships. We will begin with an overview of new methodologies for measuring sleep and cognition in real-world settings, providing practical examples to help attendees assess the strengths and limitations of these approaches. The discussion will then shift to integrating of digital cognitive assessments and speech analytics to explore cognitive and sleep variability. Developed in collaboration with IBM and McLean Hospital/Harvard, this approach uses AI to monitor fluctuations in cognitive health, capturing the dynamic relationship between sleep and cognitive changes while offering insights into potential signal early signs of cognitive decline. This symposium with include sessions on specific patient populations, starting with dementia care dyads. We will examine daily affective dynamics via ecological momentary assessment, focusing on their impact on subsequent sleep quality for both family care partners and individuals living with dementia. We will also address middle-aged and older individuals living with schizophrenia, who face a heightened risk of dementia. Using wearable sleep trackers and mobile cognitive assessments, we will present evidence of strong correlations between improved sleep metrics—such as later wake time and longer total sleep duration—and enhanced cognitive performance, particularly in attention and memory tasks. This symposium illustrates the transformative potential of digital health tools in advancing research on sleep, mood, and cognition among aging populations, informing targeted interventions and improving brain health outcomes.

Abstract citation ID: igaf122.309

### LEVERAGING TECHNOLOGY TO DISENTANGLE THE RELATIONSHIP BETWEEN SLEEP AND COGNITION IN DAILY LIFE

Christopher Kaufmann, and Todd Manini, *University of Florida, Gainesville, Florida, United States*

There is strong interest in how poor sleep impacts cognition and neurodegenerative diseases like Alzheimer's disease. Research across basic science, clinical, and epidemiological studies highlight a strong relationship between the two; however, questions remain about causality due to concerns about reverse causation. Despite the rigor of prior studies, the bidirectional nature of this relationship necessitates a nuanced understanding on a day-to-day and night-to-night basis within community settings. Recent technological advances, such as wearables and smartphones, offer new avenues to explore these daily and weekly dynamics. While accelerometers have been used to measure sleep for nearly four decades, modern wearables can now provide valuable insights, including sleep staging and nighttime breathing rate, factors which may significantly impact daily cognition. Traditionally, cognitive assessments have been conducted in the lab using formal testing; however, the proliferation of smartphones has enabled researchers to evaluate cognition in everyday settings. These new technologies present exciting possibilities to clarify the connection between poor sleep and neurodegeneration. However, it is essential to consider the limitations of data capture for accurate interpretation of the findings. This presentation will introduce how new technologies can enhance measurement of sleep and cognition in real world contexts, addressing both their strengths and limitations. The goal is to equip audience members with insights on effectively utilizing these tools in their own research with older adults. Various applications of these technologies will be demonstrated through practical examples.

Abstract citation ID: igaf122.310

### LEVERAGING DIGITAL ASSESSMENTS AND SPEECH ANALYTICS FOR MONITORING CHANGES IN SLEEP AND COGNITION

Shifali Singh, *McLean Hospital, Boston, Massachusetts, United States*

Tracking everyday cognitive functioning, sleep, and mood, beyond a single timepoint of assessment, is essential for understanding the progression of cognitive impairment and improving early diagnosis and treatment of disorders like AD/ADRDs. In this talk, we will explore the integration of digital cognitive assessments and speech analytics, focusing on how these tools can track intraindividual and interindividual variability in sleep and cognitive performance over time. We will present our approach, developed in collaboration between IBM and McLean Hospital/Harvard Medical School, which combines traditional neuropsychological evaluations with cutting-edge speech analytics powered by AI, machine learning (ML), and large language models (LLMs). By leveraging digital cognitive assessments, we capture cognitive and sleep fluctuations through daily prompts and testing, allowing for a more dynamic understanding of cognitive health. In addition, speech data collected through open-ended prompts and summaries are analyzed using advanced ML algorithms, including Whisper from OpenAI for acoustic analysis, and chatGPT for result interpretation, to detect subtle changes in speech characteristics that correlate with cognitive and sleep changes. This talk will highlight how our tools track cognitive and sleep fluctuations over time, focusing on intraindividual variability—how an individual's sleep impacts their cognitive changes from day to day—and interindividual variability—how these changes compare across different individuals. By integrating biometric data from actigraphy monitors, such as heart rate and activity levels, we will discuss how one can further refine models to better characterize cognitive states and identify how sleep can be used to detect early signs of cognitive decline.

Abstract citation ID: igaf122.311

### FAMILY CARE PARTNERS' DAILY AFFECT AND SLEEP IN DEMENTIA CARE DYADS USING ECOLOGICAL MOMENTARY ASSESSMENT

Yeonsu Song[1], Raeanne Moore[2], Brent Mausbach[2], Jennifer Martin[3], and Mary-Lynn Brecht[1], *1. University of California, Los Angeles, Los Angeles, California, United States, 2. University of California, San Diego, San Diego, California, United States, 3. VA Greater Los Angeles Healthcare System, Los Angeles, California, United States*

Poor sleep is associated with lower positive affect and higher negative affect. Many family care partners (CP) of people living with dementia (PLWD) experience poor sleep. However, the impact of poor sleep on daily affect among CP, and the reverse relationship, remains unclear. This study examined daily variations in positive and negative affect and their relationship with poor sleep using data from an ongoing clinical trial involving dementia care dyads. A total of 72 CP completed ecological momentary assessment surveys of their positive and negative affect (three times a day) and sleep of CP and PLWD for seven days. Variables included positive (feeling energetic, happy, motivated, rested, relaxed) and negative affect (feeling anxious, depressed, helpless, hopeless, nervous, tense, worried, worthless), and 'good sleep' (no trouble falling asleep and staying asleep) on previous nights. Descriptive and mixed-effects regression models were conducted. Positive affect was higher in the morning compared to midday and evening. Negative affect was lower in the evening than the morning, and higher at midday than in the morning. The relationship between positive affect and the previous night's CP sleep differed significantly between the morning and the evening ($\beta = 0.079$, $p = 0.049$). In a lagged mixed-effect model, negative affect was significantly related to the next day's sleep for both CP and PLWD. Higher levels of negative affect were associated with a greater likelihood of poor sleep for both dyad members ($\beta = 1.435$, $p = 0.007$ for CP; $\beta = 1.398$, $p = 0.038$ for PLWD). Understanding daily affect fluctuations is essential for improving sleep among dementia care dyads.

Abstract citation ID: igaf122.312

### EXAMINING COGNITIVE FUNCTION AND SLEEP DISTURBANCES IN ADULTS WITH SCHIZOPHRENIA USING MOBILE AND WEARABLE SENSORS

Ellen Lee[1], Jerry McDonald[1], Andrea Coppola[2], and Stephanie Ibrahim[1], *1. University of California, San Diego, San Diego, California, United States, 2. VA San Diego Health Care System, San Diego, California, United States*

Due to cognitive impairment during the prodromal period/onset of schizophrenia and accelerated brain aging, older people living with schizophrenia (PLWS) have 10- to 20-fold higher prevalence of dementia, compared to the general population. While risk factors for dementia (e.g., metabolic side effects of medications and sedentary behaviors) can be difficult to treat, addressing sleep disturbances could improve or preserve cognitive functioning among PLWS. Our group has been assessing outpatient middle-aged and older PLWS with wearable sleep trackers, home sleep tests, and mobile cognitive testing to understand the complex relationships of sleep and cognition. We will present findings comparing mobile cognitive testing tasks with traditional neuropsychological battery (MATRICS). We will present associations of cognitive functioning (mobile cognitive testing and MATRICS battery) with sleep measures among PLWS and age- and sex-comparable non-psychiatric comparison (NC) participants. We will explore the relationships of sleep with mental and physical health measures. Our initial analyses (subsample of 12 PLWS and 28 NCs) found strong correlations between later mean wake-time and better scores on attention, working memory, and processing speed tasks (rs=.62-.78, p's<.05, $n = 12$), between longer total sleep time and less variable scores on working memory task (rs=.72, p=.008, $n = 12$) only in PLWS. We did not observe these relationships among the NC participants. Study recruitment and assessment is ongoing, and we will present the updated results on a larger sample at the conference.

### SESSION 1300 (SYMPOSIUM)

Abstract citation ID: igaf122.313

### INTERPROFESSIONAL COLLABORATION FOR IMPLEMENTING EVIDENCE-BASED PRACTICES N NURSING HOMES

Chair: Katherine Abbott
Discussant: Natalie Douglas

Effective long-term care (LTC) for older adults requires innovative approaches to interprofessional collaboration, team coaching, and the integration of evidence-based interventions. This symposium presents five studies exploring strategies to enhance LTC by improving communication, implementation processes, and care delivery. The first study examines care professionals' perspectives on electronic health records (EHRs) as a tool for interprofessional collaboration in Dutch nursing homes, identifying key areas for optimization. The second study explores the role of team coaching in implementing psychosocial interventions to improve sleep, mobility, and fall prevention in nursing home residents. Through qualitative analyses, it highlights facilitators and barriers to team coaching adoption. The third study investigates the characteristics and mechanisms of action of clinical site champions in a Veterans Affairs project, revealing critical traits that contribute to successful implementation of goals-of-care conversations. The fourth study details the engagement of a Community Advisory Board (CAB) in refining a hospital-to-skilled nursing facility transition intervention, emphasizing the importance of family caregiver involvement. Finally, the fifth study evaluates the impact of the Individualized Positive Psychosocial Interaction program on nursing home staff care partners, demonstrating improvements in knowledge, self-efficacy, well-being, and meaningfulness of care interactions. The Discussant, Dr. Natalie Douglas, will discuss the implications of these projects for researchers, clinicians, and policymakers seeking to enhance the quality of care for older adults in LTC settings. Together, these studies highlight the importance of interdisciplinary collaboration, implementation strategies, and person-centered interventions in improving LTC outcomes.

Abstract citation ID: igaf122.314

### TEAM COACHING TO SUPPORT NURSING HOME IMPLEMENTATION OF COMPLEX PSYCHOSOCIAL INTERVENTIONS

A. Lynn Snow[1], Megan McCullough[2], Michele Koziel[3], Lindsay Pack[3], Kelly Presser[3], Selma Stovrag[3], Alex Wong[3], and Christine Hartmann[2], *1. The University of Alabama, Tuscaloosa, Alabama, United States, 2. University of Massachusetts Lowell, Lowell, Massachusetts, United States, 3. Geriatrics and Extended Care, VA Central Office, Washington, District of Columbia, United States*

We describe the use of team coaching to support nursing home implementation across two clinical trials of a front-line centered team huddling intervention, one focused on improving sleep for people with dementia and one focused on improving mobility and reducing falls in nursing home residents. Team coaching is a sub-type of practice facilitation in which the focus is on the entity of the team as well as the individuals within the team, and their relationships, communications, processes, goals, and outcomes with their stakeholders (e.g., employees, residents, families, and corporate) as well as each other. The first trial has completed intervention in 11 nursing homes (of 24 total) and the second trial's intervention is near completion in 8 homes. Team coaching is provided by a group of coaches with nursing home and team coaching experience (including nurses, social workers, a nursing home administrator and organizational development experts). Team coaching supervision is provided by experienced coaches. Regular qualitative interviews with the teams and coaches were analyzed for relevant themes related to program implementation and the benefits and challenges of team coaching. Preliminary emergent themes from the teams include reflections on the ways in which team coaching has been helpful and emerging challenges. Preliminary emergent themes from the coaches include identification of typical "turning points" that indicate the team has begun to internalize the principles and practices of the intervention, typical "pain points" that challenge the progress of the team, and challenges and opportunities in supporting team development.

Abstract citation ID: igaf122.315

### ENSURING SUCCESS: MECHANISMS OF ACTION OF CLINICAL SITE CHAMPIONS

Joan Carpenter[1], Kate Magid[2], Andrew Murray[3], Jennifer Kononowech[4], Connie Cole[5], Leah Haverhals[6], Cari Levy[7], and Mary Ersek[3], *1. University of Maryland School of Nursing, Baltimore, Maryland, United States, 2. Eastern Colorado Healthcare System, Veterans Health Administration, Aurora, Colorado, United States, 3. Corporal Michael J. Crescenz VA Medical Center, Philadelphia, Pennsylvania, United States, 4. VA Ann Arbor Health Care System, Ann Arbor, Michigan, United States, 5. VA Eastern Colorado Health Care System, Aurora, Colorado, United States, 6. VA Eastern Colorado Healthcare System, Aurora, Colorado, United States, 7. University of Colorado, Aurora, Colorado, United States*

Clinical champions are engaged to implement evidence-based practices in health care settings. Research indicates that the presence of a champion does not ensure project success; therefore, we sought to identify the characteristics of effective clinical champions. In a Department of Veterans Affairs (VA) quality improvement project, we utilized clinical champions to implement a goals of care conversation intervention with seriously ill Veterans in VA home based primary care (HBPC) and community nursing homes (CNHs). During the five-year project, we conducted interviews (N = 99) with clinical champions and leadership at 11 HBPC programs and six VA CNH programs. Guided by the Tailored Implementation in Chronic Diseases framework and Shea's conceptual model of champion impact, we analyzed interview data to identify champion characteristics and site-level contextual factors that affected achievement of implementation outcomes. We also compared clinical champion characteristics between sites that were successful and not successful in achieving implementation outcomes. Eight HBPC programs (73%) and four CNH programs (67%) were fully successful in achieving implementation outcomes. Successful site champions were committed to serving as champions and believed in the importance of goals of care conversations and documenting Veterans' life-sustaining treatment preferences. They spent more time engaging peers, providing support, and performing implementation activities. Most successful sites reported high levels of leadership support and engagement. These findings highlight important considerations for identifying and selecting champions that may help guide other implementation programs—including champion belief and commitment to implementing a new intervention, motivation to serve as a champion, and ability to engage peers.

Abstract citation ID: igaf122.316

### IMPACT OF THE INDIVIDUALIZED POSITIVE PSYCHOSOCIAL INTERACTION (IPPI) EPCT ON STAFF CARE PARTNER OUTCOMES

Katherine Abbott[1], Allison R. Heid[2], Molly Noble[1], Kathleen Unroe[3], Amy Kotterman[4], and Kimberly Van Haitsma[5], *1. Miami University, Oxford, Ohio, United States, 2. Independent Research Consultant, Ardmore, Pennsylvania, United States, 3. Indiana University School of Medicine, Indianapolis, Indiana, United States, 4. United Church Homes, Marion, Ohio, United States, 5. The Pennsylvania State University, University Park, Pennsylvania, United States*

The Individualized Positive Psychosocial Interaction (IPPI) program is an evidence-based program that engages persons living with dementia in nursing homes in brief (i.e., 10 minute) one-to-one preference-based activities two times a week. Primary direct care providers of eligible intervention recipients (i.e., CNAs, activity professionals, nurses, and social workers) are trained to provide IPPI. This study examined the impact of program training and implementation on staff care partner outcomes of knowledge, self-efficacy, appropriateness, acceptability, satisfaction, meaningfulness of time, and well-being. Seventy staff care partners completed an online course on emotion-focused communication strategies (EFCT) with a pre-and post-training assessment of knowledge and self-efficacy. Post-training appropriateness, acceptability, and satisfaction were assessed. Care partners were then trained on IPPI delivery and implemented the program 2x a week with residents for 6-months each. Immediately following each IPPI, they rated their perception of meaningfulness of time spent. Nineteen of the care partners also completed an exit interview rating the impact of the IPPI program on their well-being. Knowledge of emotion-focused communication strategies and self-efficacy for using emotion-focused communication strategies significantly increased from pre- to post-training. Acceptability, appropriateness, and satisfaction with intervention training were also high. Following 92% (n = 3,639) of IPPIs, staff care partners indicated that they found the IPPI to be a meaningful use of their time. Mean levels of well-being associated with delivering the IPPI were also high. IPPI is a person-centered, preference-based intervention that improves outcomes for both the residents receiving the intervention and the staff care partner providing the services.

Abstract citation ID: igaf122.317

### ENGAGING A COMMUNITY ADVISORY BOARD TO INTEGRATE FAMILY CAREGIVERS IN HOSPITAL-TO-SNF TRANSITIONS

Alycia Bristol, Shinduk Lee, Brenda Luther, and Gail Towsley, *University of Utah, Salt Lake City, Utah, United States*

Transitions from hospitals to skilled nursing facilities (SNFs) are often complex and require careful planning with older adults and their family caregivers (FCGs; e.g., unpaid family or friends). Despite the numerous existing care transition interventions, few consider FCG involvement, contributing to poor communication, poor patient outcomes, increased readmission risks, and reduced care quality. Using the Integrated Research-Practice Partnerships Process Model, we formed a community advisory board (CAB) with members from hospitals (n = 2), SNF/post-acute (n = 2), and FCGs (n = 2). In three one-hour meetings, the CAB provided input on FCG integration and intervention protocols and materials to co-develop a patient- and family-centered intervention, Better Connections. A post-meeting survey gathered feedback on satisfaction and CAB structure. The CAB identified three phases for FCG involvement during hospital-SNF transitions: 1) predischarge hospital meeting; 2) planning for transfer; and 3) SNF initial care plan meeting. Key elements included improving communication, establishing points of contact, and identifying and fulfilling unmet education/transition needs. The CAB recommended developing conversation guides with questions, resources, and communication tips for FCGs and hospital/ SNF teams. Five of six surveys were completed. CAB members agreed or strongly agreed that meetings were productive, their input was valued, their time and expertise were used effectively, and their contributions influenced the direction of the project. Three CAB members found learning about the perspectives of other roles was educational and enlightening. Engaging a CAB with varied roles guided us to co-develop Better Connections, emphasizing the importance of FCG involvement and communication throughout the hospital-SNF transition process.

### SESSION 1305 (SYMPOSIUM)

Abstract citation ID: igaf122.318

### CARE OF OLDER ADULTS WITH COMPLEX NEEDS: EMERGING STRATEGIES IN THE VETERANS HEALTH ADMINISTRATION

Chair: Maresi Berry-Stoelzle

The Veterans Health Administration (VHA) is unique in its mandate and mission to provide long-term care to eligible Veterans. To meet older Veterans' increasingly complex needs, VHA has invested in a continuum of residential and community care programs, as well as support for caregiving teams that include paid and unpaid caregivers. This symposium examines promising avenues in VHA for supporting team-based, person-centered long-term care options for growing numbers of older Veterans with complex care needs, many of whom live in rural areas or face other barriers to care access. First, we explore approaches to ensuring Veterans' access to home health services. Davila et al. describe a successful pilot program to provide VHA-delivered home health (vs. contracted services) to reach high-need rural and other Veterans who may otherwise go unserved. Next, Ding et al. discuss how VHA teams strategically build trust with Veterans and families to encourage uptake of aide services among Veterans who would benefit from these services. We then consider the role of family caregivers. Ngo et al. describe the need to strengthen education and outreach to ensure family caregivers have the information needed to access VHA and community resources, while Quach et al. describe VHA primary care nurses' experiences coordinating complex care with family caregivers. Finally, Berry-Stoelzle describes how effective billing practices can enhance access to advanced care planning among rural and other hard-to-reach Veterans. Together, this research highlights innovative avenues to improve reach and quality of long-term care for those who need it most.

Abstract citation ID: igaf122.319

### FRONTLINE STAFF EXPERIENCES DELIVERING HOME HEALTH IN A PILOT VETERANS HEALTH ADMINISTRATION PROGRAM

Heather Davila[1], Mary Good[2], Tammy Walkner[2], Heather Benzel[3], Irene San Roman[2], and Aaron Seaman[4], *1. Iowa City VA Healthcare System, Iowa City, Iowa, United States, 2. Iowa City VA Health Care System, Iowa City, Iowa, United States, 3. VA Midwest Healthcare Network, St. Paul, Nebraska, United States, 4. University of Iowa, University of Iowa, Iowa, United States*

Historically, the Veterans Health Administration (VHA/VA) has provided access to skilled home health (e.g., rehabilitation, nursing) and home health aide services through contract community agencies. In 2022, VA began piloting VA-delivered home health (VA-HH) at 4 sites in the upper Midwest. Program goals were to improve access to care and care coordination. In 2024, we conducted semi-structured interviews with VA-HH nurses (n = 18) and nursing assistant/aides (n = 13) to understand motivations for joining VA-HH, resources needed to address Veterans' complex care needs, and overall job satisfaction. Most nurses (60%) and aides (85%) joined VA for their VA-HH positions. Motivations included commitment to the philosophy of home health, desire to give back to Veterans, and appreciation for flexibility and autonomy in their roles. Both groups noted the need for clear guidelines and procedures, supplies (e.g., for wound care), and technology (e.g., for in-home charting) to effectively deliver home health to Veterans with complex care needs. Whereas nurses discussed the importance of inter-team collaboration (e.g., with social work, primary care), aides focused more on collaboration within the VA-HH team. Aides described needing strong communication with nurses, plus nurse and management support related to complex care (e.g., dementia, mental health) and safety concerns. Most staff expressed high job satisfaction. Aides, particularly those with prior non-VA experience, noted being valued and included in the overall healthcare team. Moving forward, VA-HH could be expanded to serve more Veterans. Further enhancing inter- and intra-team collaboration, safety protocols, and leadership support could promote ongoing job satisfaction and high-quality care.

Abstract citation ID: igaf122.320

### ADVENTURES IN BILLING: SUSTAINABLE BILLING PRACTICES IN SERVING ADULTS WITH COMPLEX HEALTH NEEDS

Maresi Berry-Stoelzle[1], Sarah Murray[2], Tammy Walkner[1], Daniel Karr[3], and Amanda Heeren[1], *1. Iowa City VA Health Care System, Iowa City, Iowa, United States, 2. Iowa City VA Medical Center, Iowa City, Iowa, United States, 3. Department of Veterans Affairs, Iowa City, Iowa, United States*

Discussions about future medical care are first steps to life altering medical decisions. A complex medical diagnosis with accompanying therapy affects the patient and their family/support network. Communication between patients, family members, and the medical care team can lower anxiety, increase goal congruent care, and increase satisfaction in care received. This is grouped together as advance care planning (ACP). The Centers for Medicare and Medicaid Services (CMS) has added a billing code, available for all patients and licensed independent providers to have dedicated medical encounters focused on ACP. Unfortunately, integration of these encounters into routine outpatient medical care has been low. For a rural serving Veterans Health Administration (VHA) Medical Center, <1% of eligible Veterans were billed using these codes. In a more generalized study, 5.2% of eligible VHA patients had documentation of ACP in 2020. Especially rural Veterans and others with extended travel time may have challenges adding another encounter to an already busy medical care schedule. Our VHA initiative has built and tested a concise, billable telehealth ACP encounter to integrate care between VHA specialty clinics and long-term care facilities where Veterans receive care. By adhering to the CPT code guidelines and integrating practices from VHA palliative care and ethics trainings, we are able to offer Veteran-centered encounters to patients and families. Significant benefits accrue for Veterans, families, and the health care system in ensuring that patient records and Veteran/family expectations are regularly updated. We discuss offering this encounter to almost 1,000 Veterans and families.

Abstract citation ID: igaf122.321

### FAMILY CAREGIVER AND CLINICIAN PERCEPTIONS OF RESOURCE ACCESS IN RURAL AREAS

Victoria Ngo[1], Elizabeth Chamberlin[1], Elizabeth Marfeo[2], Steven Shirk[1], Bret Hicken[3], Cathy Cruise[4], Maria Venegas[5], and Lauren Moo[6], *1. VA Bedford Health Care System, Bedford, Massachusetts, United States, 2. Tufts University, Medford, Massachusetts, United States, 3. VHA Office of Rural Health, Salt Lake City, Utah, United States, 4. Veterans Health Administration, Northpoint, New York, United States, 5. Department of Veterans Affairs, Bedford, Massachusetts, United States, 6. VA Bedford Healthcare System, Bedford, Massachusetts, United States*

Family caregivers and clinicians in the Veterans Health Administration (VHA/VA) play crucial roles in supporting older Veterans, particularly in rural areas where access to resources can be challenging. A qualitative analysis of interviews with 18 rural family caregivers and 10 clinicians highlights a disconnect between these two groups regarding resource access. Thematic analysis revealed that clinicians assume that patients are being provided services through social workers, believing that these professionals effectively connect families to needed services. However, when asked, rural family caregivers report being unaware of many available VA programs and services (e.g., VA Caregiver Support Program, mileage reimbursement), leaving them feeling unsupported and unsure of where to seek help. This gap in knowledge contributes to frustration and missed opportunities for essential support services. The differing perceptions between clinicians and family caregivers highlight systemic barriers in resource navigation for older veterans. While clinicians rely on social workers as a point of contact for connecting families with community services, family caregivers in rural areas may struggle with limited awareness, inconsistent outreach, or difficulty in accessing social support altogether. This disconnect underscores the need for improved communication strategies within the VA system to ensure family caregivers are informed and empowered to access resources. Efforts such as proactive outreach, improved caregiver and clinician education, and clearer roles within care teams could bridge this gap and improve outcomes for both caregivers and the older veterans they support.

Abstract citation ID: igaf122.322

### COORDINATION OF FAMILY CAREGIVER SUPPORT SERVICES ACROSS VHA

Emma Quach[1], Ryan Cabrera[1], Victoria Ngo[1], and David Giard[2], *1. VA Bedford Health Care System, Bedford, Massachusetts, United States, 2. Department of Veterans Affairs, Jamaica Plain, Massachusetts, United States*

Veterans Affairs (VA) primary care (PC) currently treats more Veterans with dementia than any other VA setting, due to a scarcity of geriatricians and neurologists. In the course of caring for Veterans with dementia, frontline PC nurses frequently interact with family caregivers, whose support enables Veterans with dementia to live where they want—in their homes—but such support may lead to caregiver burnout, contributing to Veterans requiring institutional care. Screening for and referral to caregiver support services in PC may prevent or reduce caregiver burnout but are reportedly rare in non-VA settings. To explore caregiver screening and support by VA PC, we conducted an online focus group with 23 nurses working at a New England VA medical center. Qualitative analysis showed PC nurses do not formally conduct screening. They, however, recognized addressing caregiver burnout to be part of good patient care and were acutely concerned about the varied dilemmas facing caregivers and Veterans. For example, nurses struggle to work with family caregivers' stress related to their need to balance Veterans' independence and safety. To support distressed caregivers, nurses valued easily accessible information on VA caregiver support groups, caregiver self-care, and VA respite service eligibility criteria (services administered outside of PC) and consults to PC social work. Nurses' reflections revealed consistent coordination among these VA entities is essential to provide seamless support to family caregivers seen with Veterans in PC. Our study suggests the integral role of PC nurses in supporting family caregivers as part of providing holistic care.

Abstract citation ID: igaf122.323

### EMPOWERING VETERAN AUTONOMY AND CHOICE: THE ROLE OF TRUST IN THE UPTAKE AND ENGAGEMENT IN HOME HEALTH AID SERVICES

Margaret Ding[1], Lauren Hall[1], and Emily Franzosa[2], *1. James J Peters VA Medical Center, New York, New York, United States, 2. Bronx VA, Bronx, New York, United States*

The Veterans Health Administration's (VHA) commitment to veteran-centered care prioritizes the unique needs of veterans, offering Home Health Aid (HHA) services purchased from community providers essential for those, especially, with higher needs. Trust in the patient-provider relationship, recognition of veteran autonomy, and respect for veterans' care choices are crucial for HHA service uptake and engagement in care. To better understand how veteran-centered approaches affect use of HHA, our team conducted 60 semi-structured interviews with VA primary care providers (MD, RN, NP, Social Workers) and home health agency staff at 5 geographically diverse sites (15-20/site). Interviews were recorded, transcribed, and analyzed using inductive thematic analysis. Findings across these 5 sites highlighted how trust and autonomy impacts multiple touch points of care when connecting veterans to HHA, including 1) initial assessment (ex. encouraging disclosure of ADL needs) 2) referral and acceptance of services (ex. veterans' preferences for care and their concern for the loss of independence) 3) ongoing engagement with services (ex. veterans' voicing concerns about care quality). Additionally, VHA and agencies' strategies to build trust, such as providing information and education about available HHA services, involving family and caregivers in discussions and care, and fostering collaborative care were identified as key factors that directly influence informed clinical decision-making. Building trust at every stage of the HHA service process requires coordinated efforts across both VHA and contracted agencies, ensuring that veterans, providers, and caregivers establish strong relationships and work together to ensure high-quality care.

### SESSION 1310 (SYMPOSIUM)

Abstract citation ID: igaf122.324

### TRANSFER OF KNOWLEDGE AND ACADEMIC-PRACTICE PARTNERSHIP IN NURSING HOMES - INTERNATIONAL EXPERIENCES

Chair: Karin Wolf-Ostermann
Co-Chair: Hilde Verbeek
Discussant: Marie Boltz

Nursing homes are not only places where people in need of care are cared for, they are also increasingly seen as places of partnership between academia and practice and places of knowledge transfer. They offer practical approaches to skills development and lifelong learning for staff, promote research capacity and improve outcomes for residents. This requires continuous organizational development, the expansion of digitalization potential, the training of specialized geriatric staff and the integration of evidence-based, person-centred care into the operation of nursing homes. Academic-Practice Partnership models are therefore gaining increasingly attention as a feasible approach to achieve these goals. This symposium will describe interdisciplinary collaboration between scientists, care providers and educators in nursing homes in four countries: United States, Germany, the Netherlands, and the Austria. Experiences of stakeholders, research activities and the evaluation and implementation of digital services will be discussed. The first presentation will presentation will provide an overview of the Dutch Living Lab in Long-term care. The second presentation will describe interdisciplinary staff's experiences and the impact of Age-Friendly Care 4M Rounding with older adults living in long-term care. The third presentation will highlight the collaborative development and implementation of a practice-oriented, evidence-based toolkit for non-pharmacological pain management within the nursing home environment of the Austrian living lab. The final presentation will describe the TCALL project in Germany, with a focus on implementing innovative digital tools into daily practice. A discussion will follow addressing future directions and challenges of academic-practice partnership in nursing homes including policy implications.

Abstract citation ID: igaf122.325

### DIGITALIZATION AS A DRIVER OF INNOVATION IN LONGTERM-CARE: EXPERIENCES FROM THE GERMAN TCALL PROJECT

Karin Wolf-Ostermann[1], Emily Mena[1], Claudia Stolle-Wahl[2], Benedikt Preuss[3], and Heinz Rothgang[3], *1. University of Bremen, Bremen, Bremen, Germany, 2. City University of Applied Sciences Bremen, Bremen, Bremen, Germany, 3. Research Center on Inequality and Social Policy, University of Bremen, Bremen, Bremen, Germany*

Ensuring high-quality care is emerging as a key challenge for the future in Germany, particularly in light of demographic change and a shrinking workforce. Improving working conditions in long-term care through digital innovations seems to be a promising approach to improve quality of care and quality of work. The Transfer Cluster of Academic Teaching Nursing Homes in long-term care in Germany (TCALL) enables the implementation of digital innovations in regular operations and offers a direct transfer from academia to healthcare practice and back from healthcare practice to research and education. TCALL thus constitutes a conceptual space for innovative transfer activities and simultaneously provides a permanent physical environment for recursive innovation development, testing and implementation. Such transfer and innovation structures as regular and obligatory processes are currently lacking in Germany. For the first time, TCALL offers structures that directly contribute to the rapid and process-oriented development of the state of the art through innovative insights and products. We present the concept of TCALL, which has been implemented so far in three nursing homes in Bremen, and discuss examples of the implementation of digital solutions (e.g., an AI-based management for the prevention and documentation of falls). We identify the facilitators and barriers to implementing digital technologies in terms of structural and process-related factors, explore the implications of innovation implementation for personnel and organizational development, and share lessons learned from participatory approaches.

Abstract citation ID: igaf122.326

### HEALTHCARE PROFESSIONALS' PERSPECTIVE OF THE IMPLEMENTATION OF AGE-FRIENDLY CARE 4MS ROUNDING: A CASE STUDY

Erin Kitt-Lewis[1], and Zahra Ezzi[2], *1. Penn State, State College, Pennsylvania, United States, 2. Penn State Ross and Carol Nese College of Nursing, University Park, Pennsylvania, United States*

The Age-Friendly Care Health System (AFHS) initiative, launched by the Institute for Healthcare Improvement in collaboration with John A. Hartford Foundation, focuses on providing care that addresses the unique needs of older adults. The 4Ms Framework – What Matters, Medications, Mentation, and Mobility – provides a model to ensure that care for older adults is comprehensive and focused on their goals and priorities. AHFS 4Ms rounding process was implemented into one long-term care facility by this PI and an interdisciplinary team of nursing home staff. The purpose of this case study was to explore the perspectives of the interdisciplinary staff (e.g., physician, nurse practitioner, social worker, registered nurse, licensed practical nurse, nursing home assistant administrator) that participated in the implementation of the Age-Friendly Care 4Ms Rounding with residents. Interdisciplinary staff were interviewed using a semi-structured interview guide. Audio recorded interviews were transcribed, verified, and de-identified. Thematic analysis was conducted. Findings revealed healthcare professionals' perspective of the implementation of Age-Friendly Care 4Ms rounding. Themes include the impact of 4Ms rounding; factors that support and hinder the integration of the rounding process; and recommendation for improving and sustaining rounding for better integration into this care setting and potential future care settings. These findings will be used to refine the rounding process and create sustainable changes to procedures and practices in this nursing home. In addition, a template will be created so that additional long-term care facilities can replicate this process of implementing Age-Friendly Care 4Ms rounding.

Abstract citation ID: igaf122.327

### DEVELOPMENT OF TOOLKIT FOR NON-PHARMACOLOGICAL PAIN MANAGEMENT IN THE AUSTRIAN LIVING LAB

Daniela Schoberer, Manuela Hoedl, Eva Pock, and Doris Eglseer, *Medical University of Graz, Graz, Steiermark, Austria*

The OPINION Lab, established as a Living Lab in an Austrian nursing home, aimed to develop a practical, evidence-based toolkit for non-pharmacological pain management, addressing the high prevalence of chronic pain, especially among residents with dementia. In this Living Lab, researchers, caregivers, residents, and family members collaboratively engaged in the process of developing and testing the toolkit, ensuring that the interventions were relevant and feasible for everyday nursing practice. The study followed a systematic, participatory approach, incorporating nursing staff from the beginning to identify relevant interventions, synthesize current evidence, and assess the acceptability, feasibility, and required resources for implementation. Rapid reviews were conducted for interventions lacking sufficient evidence, using Cochrane Rapid Reviews guidelines. The toolkit includes 20 non-pharmacological interventions, addressing key aspects such as effectiveness, indications, application areas, and required resources. This method of participatory research within the OPINION Lab encouraged close collaboration between nursing researchers and caregivers, facilitating a strong connection between theory and practice. The toolkit was developed to be user-friendly, ensuring its practical applicability in the nursing home setting. It is currently being implemented and evaluated under scientific supervision. The participatory approach at the OPINION Lab has not only resulted in an evidence-based toolkit but also enhanced mutual understanding between research and practice, supporting a holistic, patient-centered approach to pain management in nursing homes.

Abstract citation ID: igaf122.328

### IT TAKES TWO TO TANGO: PARTNERSHIP BETWEEN ACADEMIA AND LONG-TERM CARE

Jan Hamers[1], Judith Urlings[2], and Hilde Verbeek[1], *1. Maastricht University, Maastricht, Limburg, Netherlands, 2. Living Lab in Ageing and Long-Term Care, Maastricht, Limburg, Netherlands*

Older people and their families, health care professionals, policy makers, and educators need to benefit from new advancements and best available evidence to improve their well-being and meet their needs. Researchers aim to have an impact in broader society with their scientific work and influence everyday care practice as well as policy and education. In the Netherlands, we have developed a partnership approach for innovation in long-term care through scientific research which we will present in this symposium. The Limburg Living Lab in Ageing and Long-Term care originated 27 years ago and has proven to be a successful partnership that drives scientific research in long-term care in co-creation with end-users, including older people and their relatives, professional caregivers, managers, policy makers and educators. Thirteen organizations collaborate structurally to improve quality of life, care and work in long-term care. It has three key characteristics, including linking pins (scientific and practice-based), interdisciplinarity and community engamenennt throughout the entire scientific process. Older people and their caregivers together set the agenda for scientific research. This is of great importance to include outcomes that matter to make an impact in practice. Furthermore, we translate results into education via hybrid teaching methods to attract early career professionals and life long learning approaches to support current staff. Using the ecosystem approach, return on investment is large as organizations profit from knowledge created by the network as a whole. Other countries have implemented our model and adapted it to their local context.

### SESSION 1315 (PAPER)

### ALZHEIMER'S DISEASE AND OTHER RELATED DEMENTIAS: CROSSING BOUNDARIES AND BREAKING BARRIERS

Abstract citation ID: igaf122.329

### ASSESSING DEMENTIA NEEDS IN ILLINOIS: A PUBLIC HEALTH-ACADEMIC PARTNERSHIP FOR SERVICE IMPROVEMENT

Julie Bobitt[1], and Jessica Link[2], *1. University of Illinois at Chicago, Chicago, Illinois, United States, 2. Illinois Department of Public Health, Springfield, Illinois, United States*

Alzheimer's Disease and Related Dementias (ADRD) pose a significant public health challenge, affecting approximately 240,000 Illinois residents over age 65. Additionally, 6.1% of adults aged 45 and older in Illinois experience subjective cognitive decline (SCD), a key early indicator of dementia. Given ADRD's progressive nature and its impact on individuals, caregivers, and healthcare systems, identifying service gaps and strengthening dementia-capable systems is critical. To address these needs, the Illinois Department of Public Health (IDPH) partnered with the University of Illinois Chicago (UIC) Center for Dissemination and Implementation Science to conduct a statewide needs assessment. This process, guided by the Needs Assessment Toolkit from the Alzheimer's Association and the Association of State and Territorial Health Officials (ASTHO), included a landscape report, a survey of 83 professionals across aging services, healthcare, and public health, and qualitative data, collected in collaboration with state and professional organizations, from over 500 formal and informal caregivers and persons living with dementia. Findings highlight dementia prevalence and underscore the need for improved early detection, care coordination, and community-based supports. Results also provide key insights into the lived experiences of informal and formal caregivers. Challenges identified include workforce shortages, gaps in caregiver support, and the need for culturally tailored dementia care. This session will share insights from the needs assessment, highlighting efforts to enhance dementia-capable services and strengthen cross-sector partnerships. Attendees will better understand how public health-academic collaborations can drive policy and inform program development to better support persons with dementia and their caregivers.

Abstract citation ID: igaf122.330

### BEYOND BURDEN: CROSS-CULTURAL PERSPECTIVES ON LAY EXPERTISE IN DEMENTIA CAREGIVING

Cameron Beckett, *University of Chicago, Chicago, Illinois, United States*

Alzheimer's disease and related dementias (ADRD) represent a 21st-century public health crisis, with global prevalence rising alongside aging populations. In the United States, individuals living with Alzheimer's are projected to double by 2060, intensifying reliance on informal caregivers—mostly family—who navigate complex medical, emotional, and logistical demands without formal training. While existing research documents effects of caregiver burden, including cardiovascular risks, social isolation, and suicidal ideation, this deficit-centric lens obscures caregivers' potential for adaptation and growth. This multi-sited qualitative study examines how caregivers in Italy and the U.S. acquire "lay expertise" managing ADRD. Drawing on 20 in-depth interviews, ethnographic observations, and grounded-theory analysis, two processes emerge: Building Lay Expertise (practical competence via trial-and-error, self-assessment, and integrating medical advice) and confronting a Constellation of Burdens (role disruption, isolation, and fatigue compounded by structural gaps). Self-efficacy—caregivers' confidence in managing tasks—buffers strain and sustains skill development. Italian caregivers often view caregiving as a seamless extension of familial obligation, whereas U.S. caregivers experience abrupt, individualized caregiving roles that exacerbate isolation. These differences yield variations in diagnosis timelines and the onset of caregiving burdens. Despite adopting coping strategies—ranging from peer support to creative adaptations—caregivers in both contexts remain at risk of burnout when respite services and community resources are insufficient or poorly tailored. This study reframes caregiving as a process of dynamic skill acquisition and a source of profound vulnerability, underscoring the need for interventions that validate evolving caregiver expertise, and contribute to broader debate on how cultural norms and institutional structures shape family caregiving.

Abstract citation ID: igaf122.331

### DISPARITIES IN ACCESS AND UTILIZATION OF SERVICES FOR OLDER ADULTS IN CONNECTICUT'S MFP PROGRAM

Asmita Aasaavari, Ellis Dillon, Kathy Kellett, Christine Bailey, Amal Alsamawi, Richard Fortinsky, Lisa Barry, and Julie Robison, *University of Connecticut, Farmington, Connecticut, United States*

Money Follows the Person (MFP) is a Medicaid program that helps older adults move from Long-Term Care institutions to the community. In this study, we examine the mechanisms that contribute to disparities for older persons living with dementia (PLWD) in access to, utilization of, and quality of the MFP program in Connecticut. This qualitative study thematically analyzes in-depth interviews and focus groups with MFP-involved professionals (n = 23), family caregivers (n = 14), and older adults (n = 24) who (1) never applied to MFP, (2) applied but never moved, (3) applied, moved, and sustained community-living, and (4) moved but were reinstitutionalized. Dominant themes include: (1) Mismatch between many PLWDs' care needs and MFP program resources: participants described how MFP budgets, services, and training were not designed to address the multiple supports many PLWDs need. (2) Importance of self/family-advocacy: Learning about the MFP program, completing the application, and arranging a move required advocacy to make calls, assemble paperwork, find housing, and have family members provide informal support. Despite Connecticut's longstanding MFP program, interviews and focus groups underscored how program procedures pose additional obstacles for PLWD and their families. Recommendations for innovative solutions include upstream planning upon nursing home admission, re-designing siloed and inflexible HCBS programs, leveraging new technologies (e.g., remote monitoring/supports), expanding assistive technology, and developing supportive housing to better integrate PLWD into communities. Identifying better solutions for community living and improving concordance between the design of MFP and HCBS programs are paramount in meeting the unique needs of PLWD who are transitioning to the community.

Abstract citation ID: igaf122.332

**UNDERSTANDING AND ADDRESSING BARRIERS TO CARE FOR LGBTQ+ OLDER ADULTS LIVING WITH SUBJECTIVE COGNITIVE PROBLEMS**

Jessica VanderWerf[1], Sarah McCrackin[2], Nadine Austin[3], and Nik Lampe[1], *1. University of South Florida, Tampa, Florida, United States, 2. University of South Florida Health, Tampa, Florida, United States, 3. Georgia State University, Atlanta, Georgia, United States*

Lesbian, gay, bisexual, transgender, and queer (LGBTQ+) older adults living with cognitive health issues face multiple, compounding barriers in accessing and utilizing quality care. These barriers are driven by structural mechanisms such as providers lacking LGBTQ+ medical education and training, limited culturally tailored resources, and the exacerbated effects of the family caregiver availability crisis due to policies restricting access to marriage and family formation. In this study, we aim to answer the following research question: "What are the unique or specific barriers to healthcare services, support, and resources for LGBTQ+ older adults living with subjective cognitive problems?" From June 2024 to February 2025, the study team conducted semi-structured interviews (N = 22) with LGBTQ+ adults aged 60+ diagnosed with mild cognitive impairment (MCI) by a licensed clinician (n = 8); or who self-reported significant cognitive health problems (n = 9); and licensed clinicians (n = 5). Data were coded using NVivo software and analyzed inductively through grounded theory analysis. Three thematic themes were analyzed: (1) lack of LGBTQ+ culturally-responsive medical education and training; (2) comorbidities, including social isolation, depression, and anxiety, that complicate barriers to care; and (3) systemic biases and facility-related issues on LGBTQ+ patients in cognitive care settings. These findings highlight the need to enhance and expand culturally tailored programs, services, support, and resources for LGBTQ+ older populations living with cognitive health issues and conditions. This study highlights the need for culturally tailored programming, improved care access, and LGBTQ+ inclusive policy initiatives to address the unique barriers faced by this medically vulnerable population.

### SESSION 1320 (SYMPOSIUM)

Abstract citation ID: igaf122.333

**ESPO AND SOCIAL RESEARCH, POLICY, AND PRACTICE SECTION SYMPOSIUM: INNOVATIVE TRANSLATIONAL RESEARCH TO IMPROVE HOME ENVIRONMENT AND ACTIVITY PERFORMANCE FOR AGING IN PLACE**

Chair: Mengzhao Yan
Co-Chair: Taylor Jansen
Discussant: Sarah Szanton

Ensuring home safety and accessibility is essential for aging in place, yet the diverse needs of older adults demand innovative and person-centered approaches. The 2025 GSA SRPP ESPO Symposium brings together a panel of established transdisciplinary gerontology researchers with training backgrounds in multiple disciplines. They will share insights from their multidimensional careers and highlight their innovative research projects that translate science and technologies into practical applications, devices, and programs to improve the home environment and activity performance for older adults. Professor Wendy Rogers of the University of Illinois Urbana-Champaign will introduce her work at the Human Factors and Aging Laboratory and the McKechnie Family LIFE Home to illuminate human-technology interaction. Professor Susan Stark of the Washington University in Saint Louis will focus on the Home Hazard Removal Program (HARP) to illustrate collaborative efforts and strategies to develop and deliver interventions. Professor Laura Gitlin of Drexel University will elucidate the promise and future of intervention science, using the Care of Older Persons in their Environment (COPE) program and similar programs as exemplars. Professor Jon Sanford of Georgia State University will present his career in evidence-based, user-centered design. Professor Sarah Szanton of Johns Hopkins University, Co-Developer of the Community Aging in Place Advancing Better Living for Elders (CAPABLE) program, will serve as the discussant and lead a discussion aimed at offering advice for emerging scholars and professionals on integrating knowledge across disciplines, driving innovations to advance gerontology, and bridging research, policy, and practice to promote person-centered approaches for aging in place.

Abstract citation ID: igaf122.334

**THE LONG AND WINDING ROAD: MY JOURNEY DESIGNING TECHNOLOGY TO SUPPORT SUCCESSFUL AGING**

Wendy Rogers, *University of Illinois Urbana-Champaign, Champaign, Illinois, United States*

In the Human Factors and Aging Laboratory, which I direct, we take a broad view of successful aging. We think about it is being able to do what you want, when you want, where you want, how you want, and with whom you want. In short, the goal is autonomy, and the quest is to determine how technology can be used as an augmentative tool. We explore technology in many forms from apps on a smart phone, websites, videochat platforms, digital home assistants, to assistive robots – social, mobile, and interactive. Our approach is to think about the context in which the technology will be used and the range of users (e.g., older adults, family members, professional caregivers). We follow a user-centered design process that engages subject matter experts and end-users throughout, designing with them not for them. My degrees are all in psychology, thus my focus is always on the human side of the human-technology interaction. I think about what the person wants to do; what their limitations and preferences are; and especially what kind of support they will need to be able to take advantage of what the technology has to offer. I combine my training in cognitive psychology, human factors, experimental research methods, and qualitative assessments to develop mixed methods approaches to the research questions. I will provide examples of our research in the McKechnie Family LIFE Home that illustrate the process of fulfilling the potential for technology to support the autonomy of older adults.

Abstract citation ID: igaf122.335

**HOME MODIFICATIONS FOR OLDER ADULTS: THE HARP PROGRAM**

Susan Stark, *Washington University in St. Louis, St. Louis, Missouri, United States*

Occupational therapy (OT) plays an important role in ensuring safe and accessible home environments for aging populations. The Home Hazard Removal Program (HARP) is an OT intervention designed to reduce falls. Initially developed through pilot studies that assessed the feasibility, HARP advanced to clinical trials. Subsequently, the program was implemented within the Long-Term Services and Supports (LTSS) network, demonstrating its scalability and effectiveness in community settings. The current phase of HARP development involves its adaptation for individuals aging with long-term disabilities, addressing the complex needs of a diverse aging population. This series of studies, progressing through the translational continuum, underscores the importance of interdisciplinary collaboration and team science in the development and dissemination of innovative OT interventions. Collaborative efforts among occupational therapists, gerontologists, public health experts, and other stakeholders have been integral to refining HARP's methodology and achieving measurable improvements in home safety outcomes. These findings provide a valuable roadmap for emerging scholars and professionals seeking to implement evidence-based strategies in gerontology and occupational therapy. This presentation delineates the evolution of HARP through pilot testing, clinical trials, and LTSS network implementation and describes barriers to implementation.

Abstract citation ID: igaf122.336

**MAKING LIFE BETTER FOR OLDER ADULTS AND FAMILIES: MY JOURNEY IN INTERVENTION SCIENCE**

Laura Gitlin, *Drexel University, Philadelphia, Pennsylvania, United States*

Intervention science is a dynamic and challenging field that addresses critical public health issues by bridging the gap between theory and practice. It focuses on designing, implementing, and evaluating novel approaches that have the potential to significantly improve the health and well-being of individuals, families, and communities. My research journey in this area has been driven by a deep commitment to understanding how behavioral, psychological, and environmental factors intersect and can be modified, even for individuals facing serious chronic illnesses. Over my 40+ year career, I have had the privilege of developing and testing numerous interventions, in collaboration with various teams, which have been shown through clinical trials to be efficacious, acceptable, feasible, and have cost-savings. Many of these interventions have been adopted worldwide and adapted for different delivery contexts. Using COPE and similar programs as exemplars, I will trace my journey through intervention science—from the initial inception of ideas to their implementation. This includes the transition from developing and testing face-to-face, home-based intensive programs to innovative, technology-driven digital solutions. While intervention science holds great promise, significant gaps remain between the generation of effective interventions and their integration into real-world settings. These knowledge-to-practice gaps continue to restrict access to evidence-based practices for many families, posing a challenge to the field and driving my career decisions. This talk will explore the personal, ethical, and professional dilemmas encountered on the journey of an intervention scientist, offering insights into how we can bridge these gaps and improve the reach and impact of interventions.

Abstract citation ID: igaf122.337

**WHAT'S AN ARCHITECT DOING IN A PLACE LIKE THIS?...MY JOURNEY FROM FORM TO FUNCTION TO FACILITATE AGING IN PLACE**

Jon Sanford, *Georgia State University, Atlanta, Georgia, United States*

My journey in evidence-based, user-centered design to promote activity performance and aging in place started long before these were clinical buzzwords. With an undergraduate degree in social psychology, I recognized early on in my architecture education that there is an inherent disconnect between architects' assumptions about human needs, preferences, and abilities and the people they design for. As luck would have it, Georgia Tech had one of the earliest programs in environment and behavior research, and funding for accessibility and falls research put me through graduate school. This was followed by my own research funding (which also created a faculty position) for the early accessibility standards. Soon after, I moved on to the new Atlanta VA Rehab R&D Center on Geriatric Rehabilitation, where I was one of 3 research architects in the VA system. At the same time, I helped start the Center on Universal Design at NC State, where I co-authored the Principles of Universal Design. I also secured funding for a Chicago aging-in-place remodeler to develop one of the earliest remote home modifications programs. Then, it was back to GA Tech at the Center for Assistive Technology and Environmental Access (CATEA), where I was a professor of industrial design and one of the three founding PIs of RERC TechSAge. The one commonality in each project along the journey has been my collaboration with occupational therapists (OTs). With this in mind, it is easy to see what an architect is doing as the Research Director in an OT program.

### SESSION 1325 (COMMUNITY ENGAGED SCHOLARSHIP SYMPOSIUM)

Abstract citation ID: igaf122.338

**ENGAGING WITH, NOT FOR: COMMUNITY-BASED RESEARCH TOWARDS INCLUSIVE THIRD PLACES FOR HEALTHY AGING**

Chair: Mei Lan Fang
Co-Chair: Rebecca White
Discussant: Ryan Woolrych

Despite increasing attention to participatory research, older adults remain underrepresented as active research partners. This symposium presents community-based participatory research (CBPR) projects that go beyond inclusion to foster authentic engagement, challenge ageist stereotypes, and empower marginalized older populations. Each study applies key CBPR principles—equity, inclusivity, empowerment, and co-creation—to innovative participatory methods such as citizen science, co-researcher models, participatory realist review and strategic bricolage, ensuring older adults play a central role in knowledge production in third places. The symposium highlights how community spaces—particularly third places—can serve as catalysts for participation, inclusion, and rights to place. Four papers will illustrate diverse approaches to authentic engagement. White presents the Collective of Older Adult Researchers (COAR) initiative, which addresses tokenistic involvement by embedding intergenerational research training and experiential learning within senior centres, positioning them as research hubs. Fang explores ways to improve collaboration between senior centres, their members, and healthcare partners to develop a community-integrated health model for older adults through strategic cross-sectoral workshops. Stone examines how urban gardening and access to green spaces affect the well-being of older adults, using a realist review co-designed with community partners. Sixsmith shares insights from RomaPlaceAge, a project co-led by Roma community members to address the health and well-being of Roma people aged 40+ in the UK. To conclude, Woolrych, Director of the Urban Institute at Heriot-Watt University, will lead a discussion on the future of participatory research and the critical role of third places in fostering meaningful engagement and social connectivity.

Abstract citation ID: igaf122.339

### INTEGRATING SENIORS CENTRES INTO PRIMARY HEALTHCARE: A COMMUNITY-ENGAGED APPROACH TO COLLABORATIVE CARE

Mei Lan Fang[1], Rebecca White[1], Anthony Kupferschmidt[2], and Michael Volker[3], *1. Simon Fraser University, Vancouver, British Columbia, Canada, 2. City of Vancouver, Vancouver, British Columbia, Canada, 3. 411 Seniors Centre Society, Vancouver, British Columbia, Canada*

This presentation highlights the vital role of seniors centres as third places in promoting older adults' well-being and social connectivity, yet their integration into primary healthcare is often overlooked. This project aims to foster collaboration between seniors centres and healthcare organizations to co-develop a community-integrated health model that enhances healthcare access, social inclusion, and well-being for older adults. Through five cross-sectoral workshops rotated across senior centre locations, we engaged representatives from four Vancouver-based seniors centres through the Alliance of Seniors Centres and four healthcare organizations—Providence Health Care, Vancouver Division of Family Practice, Vancouver Coastal Health, and Mid-Main Community Health Centre. Guided by community-engaged research principles, the workshops focused on a future research and practice agenda, long-term goals, and identifying conditions for success with measurable impact indicators toward a Theory of Change framework. A key engagement feature was the intergenerational pairing of seniors with students, fostering cross-generational learning, research inclusion, and community ties. Findings were analyzed using a thematic framework approach, and key themes were validated across stakeholder groups to ensure relevance in shaping integrated healthcare models. Preliminary themes include improved collaboration between seniors centres and healthcare providers, the role of third places in supporting healthy aging outcomes, and establishing a sustainable research agenda. A key implication involves opportunities to advance community-based research by reshaping healthcare delivery to be more inclusive, accessible, and responsive to older adults. Findings will inform policymakers, practitioners, researchers, and seniors centre staff and Board members, offering actionable strategies for integrating seniors centres into primary healthcare.

Abstract citation ID: igaf122.340

### BUILDING RESEARCH CAPACITY WITH OLDER ADULTS: THE COLLECTIVE OF OLDER ADULT RESEARCHERS (COAR) INITIATIVE

Rebecca White[1], Atiya Mahmood[1], Michael Volker[2], and Mei Lan Fang[1], *1. Simon Fraser University, Vancouver, British Columbia, Canada, 2. 411 Seniors Centre Society, Vancouver, British Columbia, Canada*

Developing the capacity of older adults to meaningfully engage in research is an increasingly recognized priority in the field of aging. However, practical frameworks for effectively fostering research capacity through collaborative partnerships with older adult community members remain limited. This paper introduces the Collective of Older Adult Researchers (COAR) project, an innovative intergenerational initiative between 411 Seniors Centre Society and Simon Fraser University (Vancouver, Canada). Designed to advance the vision of community research hubs within third places that serve older adults, this initiative prioritizes key aging-related issues while increasing older adults' direct involvement in research. Through a series of three interactive 2.5-hour workshops, participants engaged in a blend of research methodology presentations and hands-on activities conducted in community spaces. Older adults ($n = 10$) were paired with graduate students ($n = 10$) to co-develop and implement a practical research project. A reflexive analysis of the process, conducted in collaboration with older adult co-researchers, identified four key themes that offer insights for strengthening this model and informing broader implementation: (1) Engaging Community Partners in Workshop Design, (2) Fostering Intergenerational Collaboration, (3) Prioritizing Informal Collaborative Dialogue, and (4) Building Pathways for Sustainable Change. Findings highlight the potential of community-based research training to equip older adults with the knowledge and skills needed to become equitable research partners. However, further exploration is required to enhance, sustain, and scale these models, which will be investigated through forthcoming community-engaged research collaborations.

Abstract citation ID: igaf122.341

### EXPLORING SOCIAL SUSTAINABILITY THROUGH URBAN GARDENING: A PARTICIPATORY RAPID REALIST REVIEW

Elise Stone[1], TJ Roberts[2], Andrew Sixsmith[1], and Mei Lan Fang[1], *1. Simon Fraser University, Vancouver, British Columbia, Canada, 2. Pendrellis Housing Society, Vancouver, British Columbia, Canada*

Among the classic triad of sustainable development—ecology, economy, and society—social sustainability remains the least studied and critically evaluated. Conceptualizing wellbeing as a core component of social sustainability, this study explores how the social fabric of urban life, including third spaces, influences the wellbeing of older residents. Using a Participatory Rapid Realist Review (PRRR) methodology, co-designed with Pendrellis Housing Society, this research synthesizes peer-reviewed literature and integrates community-based perspectives to examine how urban gardening as a mechanism for developing engaging third places contributes to social sustainability. The PRRR approach investigates why, how, and under what circumstances urban gardening supports wellbeing. It follows a six-step process: (1) defining the review scope, (2) identifying relevant studies, (3) synthesizing data, (4) engaging stakeholders, (5) validating findings, and (6) disseminating results. Recognizing the complexity of linking urban gardening to wellbeing, this research takes a reflexive approach, embedding subjective perspectives within the analysis. Focusing on urban-dwelling older adults, gardening, and wellbeing, findings highlight the significance of small-scale social practices within third places that create vibrancy in urban life. Given the diversity of housing arrangements and motivations for gardening, understanding social sustainability at a micro scale is essential. Through community-engaged scholarship, older adults actively contributed to a co-designed Theory of Change, ensuring findings are grounded in lived experiences and applicable across the life course. Ultimately, this collaboration supported the development of an onsite community garden, ensuring older adults' voices shaped both the research process and its practical application.

Abstract citation ID: igaf122.342

### BRIDGING THE GAP: THIRD PLACES, AGEING, AND HEALTH IN UK ROMA COMMUNITIES

Judith Sixsmith[1], Ryan Woolrych[2], Aleksandar Marinov[3], and Margaret Greenfields[4], *1. University of Dundee, Dundee, Scotland, United Kingdom, 2. Heriot Watt University, Edinburgh, Scotland, United Kingdom, 3. The Urban Institute, Heriot Watt University, Edinburgh, Scotland, United Kingdom, 4. Anglia Ruskin University, School of Allied Health and Social Care, Cambridge, England, United Kingdom*

Roma people, Europe's largest ethnic minority, face systemic exclusion, discrimination, and health disparities, contributing to significantly lower life expectancy than the general population. In the UK, these inequities persist, particularly for older Roma individuals who navigate intersecting challenges related to health, housing, and social inclusion. The RomaPlaceAge Project, a research initiative co-led with Roma community members, explores the health and well-being experiences of Roma individuals aged 40+ across three case study sites - Govanhill (Scotland), Peterborough, and Luton (England). Central to this research is the role of third places - public and semi-public environments such as GP practices, outdoor spaces, community centres, and housing estates - where Roma individuals engage with services and social networks, often in the face of complex barriers including language difficulties, digital exclusion, and insecure housing. Through three knowledge café events with stakeholders (healthcare providers, NGOs, police, educators, and individuals with lived experience), and in-depth interviews with Roma aged 40+, the project uncovers how third places either support or hinder health and well-being. Preliminary findings highlight the importance of micro-interactions and relationality in shaping access to services, the inaccessibility of healthcare services, the underutilisation of green spaces, and the role of community-led initiatives in fostering inclusion. By positioning third places as critical to health and well-being, this research contributes to discourses on participatory and place-based approaches to ageing, advocating for targeted interventions that promote equity, inclusion, and ageing well in the right place.

### SESSION 1330 (SYMPOSIUM)

Abstract citation ID: igaf122.343

### AGING IN TIME: PSYCHOLOGICAL AND BEHAVIORAL ADAPTATIONS TO SHIFTING TEMPORAL HORIZONS

Chair: Yaeji Kim-Knauss
Co-Chair: Frieder Lang
Discussant: Yoav Bergman

Human aging unfolds within a dynamic temporal perspective that encompasses the past, present, and future. Lifespan development theories suggest that as individuals grow older, heightened awareness of life's finitude influences psychological adaptation, motivation, and behavioral choices, which should in turn influence their well-being. Building on these theoretical foundations, five presentations will examine various dimensions of human experience shaped by shifting temporal perspectives. First, Nicole Long Ki Fung explores the trade-offs older adults make among pro-hedonic, pro-family, and prosocial goals as they adapt to shorter perceived time horizons. Expanding on the theme of goals and preferences, Claire Growney examines how appreciation of remaining time is linked to well-being and social preferences in older adulthood. Addressing the topic of coping and resilience, JoNell Strough investigates the mediating role of different time perspective in the relationship between age and emotional well-being, particularly in response to challenging life events such as natural disasters. Yaeji Kim-Knauss further examines how older adults experiencing health declines over time cope with mortality through two self-regulation strategies, namely, death preparation and death acceptance, and their respective effects on life satisfaction. Finally, highlighting the expression of shifting time perspectives, Talia Elkarif demonstrates how creative activities serve as a medium for older adults to articulate and construct evolving views of the future. In closing, Yoav Bergman will synthesize the key findings of the five talks, emphasizing different adaptive responses to limited temporal horizons in later life and their theoretical as well as practical implications.

Abstract citation ID: igaf122.344

### DEATH ACCEPTANCE AND PREPARATION: THEIR ROLES IN LIFE SATISFACTION AMONG OLDER ADULTS PERCEIVING HEALTH DECLINES

Yaeji Kim-Knauss, and Frieder Lang, *Friedrich-Alexander-Universität Erlangen-Nürnberg, Nuremberg, Bayern, Germany*

Confronting mortality is an inevitable aspect of aging. This study distinguishes two strategies older adults may employ to cope with life's finitude: death preparation (an assimilative, instrumental approach) and death acceptance (an accommodative approach). We examined whether these strategies differently predict life satisfaction, particularly when individuals perceive declining health. Data were drawn from the longitudinal Ageing as Future online study in Germany (2012–2023), collected at two-year intervals. A total of 664 older adults (aged 60–89 at baseline) contributed 1,592 observations in total. Using linear mixed modeling, we tested how engagement in death acceptance and death preparation (i.e., having a living will and/or having designated power of attorney) predicted life satisfaction at both between- and within-individual levels, depending on subjective health. We also explored whether the interplay of these approaches provides additional protective effects under deteriorating health conditions. Results showed that, although the presence of death preparation alone was significantly associated with higher life satisfaction, it was not moderated by subjective health. In contrast, at both between- and within-individual levels, individuals who accepted death showed greater life satisfaction, especially when health perceptions were poorer. Notably, this protective effect of death acceptance was most pronounced among those without death preparation in place. Our findings indicate that, under conditions of declining health, death acceptance, conceptualized as an accommodative coping strategy, seems particularly adaptive. Therefore, fostering psychological preparedness and personal meaning in one's remaining time may be beneficial as older adults face increasing aging-related challenges.

Abstract citation ID: igaf122.345

### WHEN IMPORTANT GOALS COLLIDE: PRIORITIZATION OF PROSOCIALITY OVER EMOTION REGULATION AND FAMILY IN OLDER ADULTS

Nicole Long-ki Fung[1], Molly Lin[2], Hongmei Lin[1], and Helene Fung[1], *1. The Chinese University of Hong Kong, Hong Kong, China (People's Republic), 2. University of Zurich, Zürich, Switzerland*

The socioemotional selectivity theory (Carstensen et al., 1999) suggests that as future time perspective becomes more limited in old age, motivational priorities shift toward emotionally meaningful goals. For instance, compared with younger adults, older adults become more prosocial (Bailey et al., 2020), prefer their own family members (Fung et al., 1999), and put higher emphasis on emotional regulation (Isaacowitz et al., 2021). However, these important goals are not always compatible with one and other. It remains unclear how older adults will prioritize these important goals when they are in conflict. To address this question, we recruited with 144 older (M = 67.8, SD = 5.20, 60.4% female) and 130 younger adults (M = 24.0, SD = 4.44, 74.6% female) for two studies. Participants read hypothetical scenarios where important goals compete. In Study 1, participants decided whether they would engage in prosocial behaviors with less emotional value over pleasant activities with less prosocial value (prosocial vs. emotional regulation). Older adults prioritized being prosocial over emotional regulation ($t = 4.02$, $p < .001$), while such tendency was not observed in younger adults. In Study 2, participants decided whether they would choose to help others at the expense of their family over benefiting their family against the welfare of others (prosocial vs. profamily). Again, older adults prioritize being prosocial over benefiting family ($t = 9.14$, $p < .001$), while younger adults did not show such a preference. The findings suggest that prosocial goal may take precedence in the motivational shift in late adulthood.

Abstract citation ID: igaf122.346

### APPRECIATION OF TIME AND EMOTIONAL WELL-BEING IN ADULTHOOD

Claire Growney, Tyler Matteson, and Laura Carstensen, *Stanford University, Stanford, California, United States*

According to socioemotional selectivity theory, as individuals age and experience time as increasingly limited, appreciation for remaining time in life increases. Theoretically, this leads people to prioritize meaningful experiences and social relationships that contribute to emotional well-being. To examine this postulate, we developed the Appreciation of Remaining Time (ART) scale and established its convergent and discriminant validity. The present study examined the relationship of the ART scale with existing measures of savoring and measures of well-being. 406 participants aged 18–90 (Mage = 49.33, SD = 19.28) completed the ART scale, as well as measures of time perspective, savoring, and gratitude. Participants also completed assessments of emotional well-being and a social preference paradigm task. Analyses established discriminant validity for the ART scale from measures of time perspective, savoring, and gratitude. ART mediated associations between age and outcomes of interest. Specifically, age was associated with greater appreciation of remaining time, which in turn was associated with greater emotional well-being and stronger preferences for engaging with emotionally meaningful social partners. These findings replicate and extend prior research, suggesting that the ART scale taps a distinct construct that holds meaningful implications for socioemotional aging. Viewing time as both limited and valuable appears to contribute to greater emotional well-being at older ages.

Abstract citation ID: igaf122.347

### DON'T LOOK BACK: POST-DISASTER MENTAL HEALTH BENEFITS OF AGING THROUGH REDUCED FOCUS ON THE PAST

JoNell Strough[1], Andrew Parker[2], Esha Azhar[1], Samer Atshan[3], and Ryan Best[1], *1. West Virginia University, Morgantown, West Virginia, United States, 2. RAND Corporation, Pittsburgh, Pennsylvania, United States, 3. Center for Economic and Social Research, Los Angeles, California, United States*

Disasters negatively impact mental health (NIH, 2022) but some research found that older age was protective (Strough et al., 2023). We used data ($n = 936$) from six monthly surveys of the Understanding America Study to investigate whether the impact of disasters on depression and anxiety varied across adulthood and if so, whether temporal orientations to the past, present, or future mediated any age differences. Panelists reported whether they had experienced any disaster in the past month (e.g., hurricane, wildfire) and if so, whether they had personally experienced any negative consequence (e.g., evacuation, property loss). Older age was consistently associated with reporting fewer symptoms of depression and anxiety even among those who had experienced a disaster or negative consequence. Depression and anxiety symptoms were significantly greater among individuals who had experienced a negative consequence of a disaster versus not having experienced any disaster, or having experienced a disaster without a negative consequence. Mediation analyses tested present, past, and future temporal orientations as pathways between older age and fewer anxiety and depression symptoms, controlling for income, education, race, and gender. Older age was associated with lesser past and future temporal orientations. Greater temporal orientation toward the past (but not future or present) was significantly associated with increased depression and anxiety symptoms. Age-related reductions in anxiety and depression symptoms were partially explained by a lesser tendency to focus on the past at older ages. Findings are interpreted from the perspective of Carstensen's (2006) socio-emotional selectivity theory. Implications for policy and practice are discussed.

Abstract citation ID: igaf122.348

### THE FUTURE PERSPECTIVES OF OLDER ADULTS AS REFLECTED THROUGH DIGITAL PHOTOCOLLAGE

Talia Elkarif[1], Shoshi Keisari[1], Hod Orkibi[1], Silvia Piol[1], Giada Mola[2], Frieder Lang[3], and Ines Testoni[4], *1. University of Haifa, Haifa, HaZafon, Israel, 2. Università degli Studi di Padova DPSS Department, Padova, Veneto, Italy, 3. Friedrich-Alexander-Universität Erlangen-Nürnberg, Nuremberg, Bayern, Germany, 4. Università degli Studi di Padova FISPPA Department, Section of Applied Psychology, Padova, Veneto, Italy*

Future time perspective refers to our subjective perceptions of the future. These perspectives change across the lifespan, shaping our goals, and accordingly, our actions. Exploring future perspectives can support self-development while aging but may also evoke perspectives that are difficult to discuss. While the arts can provide a safe and creative environment for older adults to explore future time perspectives, studies in this field are scarce. The current qualitative study explored the future time perspectives of older adults as expressed through digital photocollage. This study was part of a larger project aimed at developing an online arts-based intervention for community-dwelling older adults. Twenty-four Italian and Israeli older adults aged 78–92 participated in a brief therapeutic online intervention integrating Dignity Therapy and digital photocollage. Visual and verbal data were analyzed using polytextual thematic analysis. Four themes emerged: three reflecting participants' attitudes toward the future and one reflecting the evolution of future time perspectives during the creative process. The findings indicate that despite the short-term nature of the creative online intervention, and the complex topic at hand, participants expressed multiple future perspectives. The study also highlights the potential of digital and artistic methods to facilitate both the expression and the expansion of older adults' future perspectives. These findings contribute to the growing literature on creative arts therapies in later life, demonstrating the potential of digital arts-based approaches in fostering meaningful engagement with future-oriented narratives among older adults.

### SESSION 1335 (SYMPOSIUM)

Abstract citation ID: igaf122.349

### USING DATA FOR GOOD IN NEW ENGLAND, MISSISSIPPI, AND 38 CITIES IN CHINA: BUILDING AGE-FRIENDLY MOMENTUM

Chair: Elizabeth Dugan
Discussant: James Connell

This symposium describes how researchers can collaborate with state leaders to transform data into tools that identify health inequities and support social change. The 2025 Healthy Aging Data Reports (see healthyagingdatareports.org) have been released in five New England states (Connecticut, Maine, Massachusetts, New Hampshire, and Rhode Island), Mississippi (2023), and Wyoming (2023). Related work is underway in 38 cities in China. The background, data sources, statistical methods, and findings are reviewed in Dugan's opening overview presentation. Then state leaders describe efforts and progress in Maine, Massachusetts, Mississippi, New Hampshire, and Rhode Island. James Fuccione describes the Massachusetts Healthy Aging Collaborative work with policy leaders, philanthropy, academics, service providers, and private sector leaders. James Connell describes Age-Friendly Rhode Island efforts, including developing a strategic plan, convening high-level policy meetings, creating a website, and successfully advocating for resources. Jennifer Rablais describes the New Hampshire Alliance for Healthy Aging (NHAHA) efforts to develop a strategic plan, engage stakeholders to prioritize activities, and utilize a collective impact approach. Dr. Kina White describes the inspiring progress of age-friendly Mississippi which has convened briefings, trainings, and annual Age-Friendly Summits. Jess Mauer describes the Maine Council on Aging and the Power in Aging movement in Maine that aims to eliminate ageism by 2032. Finally, Dr. ShuangShuang Wang describes the development and use of a healthy aging index in China. Dr. Wang leads a panel discussion of best practices, lessons learned, and mistakes to avoid to help those in other places seeking to create age-friendly social change.

Abstract citation ID: igaf122.350

### AGE-FRIENDLY AND DEMENTIA-FRIENDLY MASSACHUSETTS

James Fuccione, *Massachusetts Healthy Aging Collaborative, Boston, Massachusetts, United States*

The Massachusetts Healthy Aging Collaborative (MHAC), funded by Point32Health Foundation, is a statewide, cross-sector network committed to advancing inclusive healthy aging and age- and dementia friendly communities throughout the state. There are now 131 active age-friendly and 122 active dementia-friendly communities with 81 involved in both initiatives. In 2018, MA became the second US state to become age-friendly through AARP's Network of Age-Friendly States and Communities and the first with an action plan. MHAC supported the newly-rebranded Executive Office of Aging & Independence on a community engagement process to refresh this plan that will be launched with a range of partners and communities in 2025. MHAC provides an age-friendly voice as part of multiple state commissions and workgroups that promote housing, digital equity, safe pedestrian and bicycle infrastructure, falls prevention and dementia-friendly community design. MHAC has supported equity in the movement with its "Healthy Aging for All Guide" and co-leads a $3.3 million digital equity initiative that supports older adults in 74 cities and towns. MHAC uses data to inform all of it's outreach and advocacy work and helps communities to leverage local data as well.

Abstract citation ID: igaf122.351

### NEW HAMPSHIRE ALLIANCE FOR HEALTHY AGING

Jennifer Rabalais, *University of New Hampshire Institute on Disability, Durham, New Hampshire, United States*

The NH Alliance for Healthy Aging (NHAHA) is a statewide collective impact initiative of over 600 stakeholders, representing 300+ organizations (http://nhaha.info/). AHA's approach is: a) change the conversation about aging; b) change public policy to promote a strong, stable infrastructure for aging; and c) change practices across public and private sectors to improve care and support for older adults, their families, and communities. The work is guided by a shared vision for communities to advance culture, policies, and services which support older adults and their families. Strategic priority areas of NHAHA include: develop and strengthen an advocacy infrastructure to advocate on behalf of the needs of older adults; enhance family caregiver support; improve availability of quality direct care workforce; ensure transportation options are available for older adults across the state; and advance options for diverse housing to allow older adults to remain in their community of choice. . The NH Healthy Aging Data Report continues to support this state level approach by providing data for state plans and other state level initiatives as well as helping communities across the state understand the needs of older adults in their communities.

Abstract citation ID: igaf122.352

### AGE-FRIENDLY MOMENTUM IN MISSISSIPPI

Kina White, *Mississippi State Department of Health, Jackson, Mississippi, United States*

The Mississippi State Department of Health (MSDH) is advancing healthy aging through emerging public health policy and practice. While the overall state's population is declining, between 2010 and 2021, the 65+ population of adults experienced a 29.3% increase. In partnership with Trust for America's Health (TFAH) and The John A. Hartford Foundation (JAHF),

MSDH adopted the 6C's Framework for Creating an Age-Friendly Public Health Systems (AFPHS) Framework and established a systematic approach to supporting older health and well-being. These efforts resulted in the achievement of exemplar status as a recognized Age-Friendly Public Health System. To achieve AFPHS Recognition, MSDH established a multi-sectoral Advisory Committee and developed a state-wide action plan based on the AFPHS 6C's Framework using the collective impact process; developed a Healthy Aging Data Report (HADR) and county data profiles with over 120 indicators; partnered with AARP MS and the Mississippi Public Health Association on several endeavors, including the development of a champion program for older adult volunteers to encourage their communities to be age-friendly and promote intergenerational activities. At the intersection of policy and practice, HADRs were hand-delivered to mayors across all 82 counties, translating findings of chronic disease and disability indicators to advance policies that establish age-friendly communities and health systems to improve older adult health outcomes.

Abstract citation ID: igaf122.353

### MAINE COUNCIL ON AGING: EMPOWERING CHANGE AGENTS

Jess Mauer, *Maine Council on Aging, Brunswick, Maine, United States*

The Maine Council on Aging (MCOA) is a broad, multidisciplinary network of more than 135 organizations, businesses, municipalities, and community members working to ensure we can all live healthy, engaged, and secure lives as we age in our homes and community settings. The Power in Aging Project is a campaign to dismantle ageism and build an Age-Positive Maine by 2032. The Project's primary strategy is to engage audiences from different sectors in direct conversations about age- bias, the impacts of ageism, and the benefits of living and working in an age-positive culture. Last year we achieved a historic victory that will increase the financial security of more than 45,000 older Mainers. These Mainers are now eligible for the Medicare Savings Program, a federal health insurance program that pays their Medicare costs. They will save on average $7,300 every year for the rest of their lives. We also celebrate our partnership with Hannaford Supermarkets and the $1.3 million grant they gave us to increase the food security and social connection of older people in our region, prioritizing diverse older people, older people living rurally, and veterans. By completion of the project, MCOA will have helped Hannaford make over $800,000 in investments in sustainable programs. Another exciting program was a relaunched Creative Aging Program that will be facilitating and hosting conversations and using creative processes to engage older people in discussions about their experiences with aging in Maine. In all our outreach data helps inform our work.

Abstract citation ID: igaf122.354

### HEALTHY AGING INDEX REPORT: INSIGHTS FROM 38 MAJOR CHINESE CITIES

ShuangShuang Wang, *Southwest Jiaotong University, Chengdu, Sichuan, China (People's Republic)*

The construction of a Health Aging Index is pivotal for evaluating cities' readiness for an aging society. Utilizing the analytic hierarchy process, this study developed an index encompassing five areas (each score ranged 1-20): health care, living environment, transportation, social fairness and participation, and social security and finance, with 43 specific indicators. The evaluation of 38 Chinese large and medium size cities (bigger than 1 million population) revealed uneven development. Coastal cities, for instance, averaged 10.5 in health care, compared to 8.5 for some inland cities. Overall, Beijing led with a score of 58.5, while Guiyang scored 42.30, with a notably low score (2.47) in social security and finance. To foster healthy aging in China, tailored policies should be developed based on city-specific needs. Long-term care insurance systems must be strengthened to integrate medical and care services, and urban infrastructure should be enhanced to create age-friendly environments.

### SESSION 1340 (SYMPOSIUM)

Abstract citation ID: igaf122.355

### USING REAL-WORLD DATA TO STUDY DEPRESCRIBING OF POTENTIALLY INAPPROPRIATE MEDICATIONS

Chair: Joshua Lin

Deprescribing potentially inappropriate medications (PIM) in a timely fashion is pivotal for drug safety in older adults. However, high-quality evidence to inform how to deprescribe PIM is often lacking. Randomized controlled trials (RCTs) typically severely underrepresent complex and frail patients in routine care. Insurance claims data and electronic health records (EHRs) are the two most commonly used real-world data sources in deprescribing research. Using 5 highly relevant examples, we will introduce common biases and mitigation strategies when using EHR and claims data for deprescribing research. Specifically, Study 1 integrated EHR with Medicare claims data to establish a scalable natural language processing (NLP)-based framework to validate claims-based medication discontinuation definitions. Study 2 demonstrated how one could use the Veterans Affairs nursing home EHR to study deprescribing of anticholinergic medications in which misclassification due to over-the-counter anticholinergic agent use can be minimized. Study 3 illustrated how we can investigate the rates, predictors, and barriers of deprescribing antipsychotics used for behavioral and psychological symptoms in dementia using a US national commercial insurance database (Optum's Clinformatics DataMart [CDM]). The last two studies utilized both Medicare data and Optum CDM. Study 4 compared discontinuation vs. continuation of antipsychotics following hospitalization in older adults. Study 5 compared the likelihood of discontinuing chronic opioid therapy after initiating gabapentinoids vs. muscle relaxants among community-dwelling older adults with chronic back pain. With a variety of data sources and deprescribing clinical questions, we will discuss the opportunities and potential pitfalls of conducting deprescribing research using observational routine care data.

Abstract citation ID: igaf122.356

### VALIDATION OF TREATMENT DISCONTINUATION IN CLAIMS DATA USING NLP AND ELECTRONIC HEALTH RECORDS

Chun-Ting Yang[1], Kerry Ngan[1], Dae Hyun Kim[2], Jie Yang[1], Jun Liu[1], and Joshua Lin[1], *1. Brigham and Women's Hospital, Boston, Massachusetts, United States, 2. Hebrew SeniorLife, Boston, Massachusetts, United States*

Measuring medication discontinuation in claims data primarily relies on the gaps between prescription fills, but such definitions are rarely validated. This study aimed to establish a natural language processing (NLP)-based framework to validate claims-based discontinuation algorithms for commonly used medications against NLP-based reference standards from electronic health records (EHRs). We identified 35,010 patients receiving antipsychotic medications (APMs), benzodiazepines, warfarin, or direct oral anticoagulants (DOACs) from EHRs at the Mass General Brigham in 2007-2020. These EHR data were linked with 82,455 Medicare Part D claims. An NLP-aided chart review was applied to determine medication discontinuation from EHRs (reference standard). In claims data, we defined discontinuation based on a prescription gap larger than 15-90 days (claims-based algorithms). Sensitivity, specificity, and predictive values of claims-based algorithms against the reference standard were calculated. We found that the sensitivity and specificity of 90-day-gap-based algorithms were 0.49 and 0.82 for haloperidol, 0.47 and 0.86 for atypical APMs, 0.54 and 0.75 for benzodiazepines, 0.40 and 0.81 for warfarin, and 0.46 and 0.89 for DOACs, respectively. The corresponding estimates for 15-day-gap-based algorithms were 0.71 and 0.58 for haloperidol, 0.61 and 0.64 for atypical APMs, 0.75 and 0.45 for benzodiazepines, 0.64 and 0.50 for warfarin, and 0.61 and 0.66 for DOACs, respectively. Positive predictive values were primarily affected by medication discontinuation rates and less by gap lengths. In summary, the accuracy of claims-based discontinuation algorithms differs by medications and prescription gaps. This study demonstrates the scalability and utility of the NLP-based validation framework for multiple medications.

Abstract citation ID: igaf122.357

### DISCONTINUATION OF ANTICHOLINERGIC MEDICATIONS AMONG VA NURSING HOME RESIDENTS WITH AND WITHOUT DEMENTIA

Cellas Hayes, *Stanford University, Stanford, California, United States*

Anticholinesterase drugs are used commonly among older adults despite their known adverse effects on cognitive outcomes. A major challenge in studying their use is the difficulty of capturing over-the-counter anticholinergic medications, such as antihistamines, in electronic health records. In Veterans Affairs (VA) nursing homes, healthcare providers administer and track medications using a barcode medication administration system, which allows us to capture all anticholinergic medications, including over-the-counter ones. The present study used data from 45,183 VA long-term care residents (admitted between 2006 – 2019) aged 65 years and older. We captured antihistamine and non-antihistamine anticholinergic medication use, and linked with data on demographics, chronic conditions, laboratory measures, and functional status data from the minimum dataset. Residents were an average of 78.1 years old and 2.5% were women and 44.5% had dementia. The median length of stay was 9 months. Residents with dementia used antihistamine anticholinergics for a median of 6.5% (0.15–100%) weeks of their stay, compared to 10.7% (0.13–100%) in those without dementia. Additionally, 44% of residents with dementia used non-antihistamine anticholinergic medications at some point during their stay compared with 56% of those without dementia. Over 80% of residents with and without dementia discontinued their non-antihistamine anticholinergic medication before discharge or death. Anticholinergic medication use is high among nursing home residents but discontinuation is common.

Abstract citation ID: igaf122.358

### RATES, PREDICTORS, AND BARRIERS OF DEPRESCRIBING ANTIPSYCHOTICS FOR BEHAVIORAL SYMPTOMS IN DEMENTIA

Yichi Zhang[1], Chun-Ting Yang[1], James Wilkins[2], Dae Hyun Kim[3], and Joshua Lin[1], *1. Brigham and Women's Hospital, Boston, Massachusetts, United States, 2. McLean Hospital, Belmont, Massachusetts, United States, 3. Hebrew SeniorLife, Boston, Massachusetts, United States*

Antipsychotic medications (APMs) are commonly used to manage behavioral and psychological symptoms in dementia (BPSD), but it is recommended that APMs should be discontinued in a timely fashion when possible. We aimed to investigate the discontinuation rates, predictors, and barriers of discontinuing APMs for BPSD. We used Optum Clinformatics Data Mart database (01/01/2005 to 08/31/2024). Eligible patients were adults aged ≥65 years without prior psychiatric disorders taking APM continuously for ≥3 months, with a dementia diagnosis prior to the use of APM. The primary outcome was APM discontinuation, defined as an absence of refill for >30 days. We estimated cumulative incidence of discontinuation by calculating one minus the Kaplan-Meier estimate at 1, 3, 6, 12, and 24 months following the index date. Our study included 15,886 patients, with 524 (3.3%) haloperidol users (mean [SD] age, 82.94 [5.33] years; 327 women [62.4%]) and 15,362 (96.7%) atypical APM users (mean [SD] age, 82.18 [5.70] years; 9575 women [62.3%]). The cumulative incidence of discontinuation at 6 months of follow-up was 30.4% (95% CI, 29.5%-31.2%) among atypical APM users and 48.6% (95% CI, 43.0%-53.7%) among haloperidol users (P < .001 for the difference between haloperidol vs. atypical APMs). In the multivariate-adjusted Cox proportional hazard model, use of haloperidol, venous thromboembolism, Black or Asian race, and use of antiarrhythmics were associated with a significantly higher rate of APM discontinuation, and alcohol abuse or dependence, hip/pelvic fracture, use of anticoagulants, and use of dementia drug were associated with a significantly lower rate of APM discontinuation.

Abstract citation ID: igaf122.359

### HEALTH OUTCOMES OF DISCONTINUING ANTIPSYCHOTICS AFTER HOSPITALIZATION IN OLDER ADULTS

Dae Hyun Kim[1], Chun-Ting Yang[2], James Wilkins[3], Elyse DiCesare[2], Kevin Pritchard[2], Qiaoxi Chen[2], Yichi Zhang[2], and Joshua Lin[2], *1. Hebrew SeniorLife, Boston, Massachusetts, United States, 2. Brigham and Women's Hospital, Boston, Massachusetts, United States, 3. McLean Hospital, Belmont, Massachusetts, United States*

Antipsychotic medications (APMs) prescribed to manage behavioral symptoms of delirium during hospitalization are often continued after discharge. Whether discontinuing APM post-discharge is associated with better clinical outcomes is unknown. This retrospective cohort study utilized Medicare 2013-2018 and Optum Clinformatics® 2004-2024 to examine the clinical outcomes of discontinuing versus continuing APMs following hospitalization in older adults. Patients aged≥65 years without psychiatric disorders or prior APM use who filled an APM prescription within 30 days of hospital discharge were included. APM discontinuation was defined as having a dispensing gap of more than 45 days following the end of an APM

prescription. We used an incidence density sampling approach to select APM continuers, matching the type of APM prescribed, time since the first APM prescription, and intensive care unit admission as the discontinuers. Propensity score matching was applied to balance baseline covariates. Study outcomes included all-cause rehospitalization, specific rehospitalization reasons, and all-cause mortality. Hazard ratio (HR) comparing APM discontinuation versus continuation was estimated using Cox proportional hazards model. Estimates from the two databases were pooled by inverse variance weighting. A total of 13,955 propensity score-matched pairs were identified. Over the median follow-up of 180 days, APM discontinuers showed significantly lower risks of rehospitalization (HR: 0.88 [0.84-0.93]), inpatient delirium (0.87 [0.79-0.96]), fall-related emergency visits or hospitalizations (0.80 [0.69-0.93]), and all-cause mortality (0.78 [0.70-0.88]) compared with the continuers. Non-significant reductions were observed for pneumonia (0.95 [0.78-1.15]) and urinary tract infections (0.85 [0.72-1.02]). These findings suggest the importance of minimizing duration of APM use after discharge.

Abstract citation ID: igaf122.360

### ADJUVANT GABAPENTINOIDS VERSUS MUSCLE RELAXANTS TO AID OPIOID DISCONTINUATION IN OLDER ADULTS

Kevin Pritchard[1], Chun-Ting Yang[1], Xiaojuan Liu[1], James Wilkins[2], Dae Hyun Kim[3], and Joshua Lin[1], *1. Brigham and Women's Hospital, Boston, Massachusetts, United States, 2. McLean Hospital, Belmont, Massachusetts, United States, 3. Hebrew SeniorLife, Boston, Massachusetts, United States*

We compared the likelihood to discontinue chronic opioid therapy after initiating gabapentinoids vs. muscle relaxants among community-dwelling older adults with chronic back pain. We conducted a retrospective cohort study of adults ≥65 years of age in U.S. fee-for-service Medicare data (2013-2018) and Optum Clinformatics® Data Mart Database (2004-2023). We included community-dwelling individuals diagnosed with chronic back pain and opioid use lasting >90 consecutive days, who newly initiated gabapentinoids or muscle relaxants. Our primary outcome of opioid discontinuation was defined by an absence of a refill within 60 days of the supply ending. Secondary outcomes included all-cause mortality and all-cause hospitalization. We controlled for 95 covariates using propensity score 1:1 matching. During the 1-year follow-up, we used Cox proportional hazard regression to estimate hazard ratios (HR) and generalized linear models to estimate risk difference (RD). We pooled database-specific estimates with inverse-variance weighting. We matched 17,063 muscle relaxant and gabapentinoid initiators from Medicare (Mean age [SD]=74 [6] years, female=67%, White=74%) and 138,141 from Optum (Mean age [SD]=74 [7] years, female=71%, White=88%). Compared to muscle relaxant initiators, gabapentinoid initiation was not associated with the rate of discontinuing opioids (HR [95% CI], 0.97 [0.84, 1.11]; RD [95% CI] per 1,000 PYs, -55.4 [-191.3, 80.6]). However, gabapentinoid initiation was associated higher rates of death (HR [95% CI], 1.08 [1.01, 1.16]; RD per 1,000 PYs, 1.6 [0.4, 2.9] and hospitalization (HR, 1.09 [1.06, 1.11]; RD per 1,000 PYs, 33.4 [14.5, 52.4]). Compared to muscle relaxants, initiating gabapentinoids was associated with a higher likelihood of adverse events.

### SESSION 1345 (SYMPOSIUM)

Abstract citation ID: igaf122.361

### ADVANCING EARLY DEMENTIA DIAGNOSIS: INSIGHTS FROM COMMUNITY-BASED STUDIES IN KOREAN AMERICAN OLDER ADULTS

Chair: Hae-Ra Han

With the rapid growth of the aging population, disparities in dementia diagnosis and care persist, particularly among individuals with limited English proficiency (LEP), such as Korean Americans. Early detection improves quality of life, reduces healthcare costs, and enhances the benefits of emerging disease-modifying therapies. However, limited dementia literacy and language barriers often delay diagnosis and treatment. This symposium presents insights from two community-based studies on Korean American older adults—MASK-MD (Memory and Aging Study of Koreans in Maryland), an ethnic church-based cognitive assessment study, and PLAN (Preparing for health aging through dementia Literacy And Navigation), a multi-site, community-based randomized controlled trial. Discussions will cover: 1) Findings from MASK-MD which screened over 1,000 older Korean Americans in ethnic churches in Maryland, along with key insights from the PLAN trial, such as 2) unique barriers and facilitators related to recruiting and retaining community-dwelling Korean American older adults with undiagnosed dementia and their caregivers, 3) the impact of digital literacy on social isolation in the sample, and 4) findings from PLAN's outcomes analyses, focusing on its effectiveness in promoting linkage to medical services for formal dementia evaluation and care planning. This symposium aims to strengthen the evidence base on dementia prevalence and strategies for promoting early diagnosis and care planning among LEP older adults in community settings. These findings are critical for addressing health inequities through behavioral and social science research.

Abstract citation ID: igaf122.362

### FIRST FINDINGS FROM PLAN: MULTI-SITE RCT FOR KOREAN AMERICAN OLDER ADULTS WITH UNDIAGNOSED DEMENTIA AND CAREGIVERS

Hae-Ra Han[1], Nancy Perrin[1], Ji-Young Yun[2], Deborah Min[3], Simona Kwon[4] Jin hui Joo[5], and Hochang Lee[6], *1. Johns Hopkins University, Baltimore, Maryland, United States, 2. Johns Hopkins University, Wellesley, Massachusetts, United States, 3. Johns Hopkins Bloomberg School of Public Health, United States, 4. New York University, New York, New York, United States, 5. Massachusetts General Hospital, Boston, Massachusetts, United States, 6. University of Rochester, Rochester, New York, United States*

Linking individuals to medical services is critical for early dementia detection, diagnosis, and care. Racial and ethnic minoritized older adults, particularly those with limited English proficiency (LEP), face significant barriers to dementia evaluation and care. Older Korean Americans (KAs)—one of the fastest-growing aging populations in the US—are predominantly monolingual first-generation immigrants, making them especially vulnerable to dementia inequities. Community health workers (CHWs) can help bridge linguistic and health literacy gaps. We evaluated the effectiveness of PLAN (Preparing for healthy aging through dementia Literacy education And Navigation), a CHW-led intervention promoting dementia care access, in a multi-site clinical trial across the Greater Washington and New York metropolitan areas. Using a two-step eligibility screening with Mini-Mental State Exam (n = 2,698) followed by Clinical Dementia Rating (CDR, n = 393), a total of 288 dyads of KA older adults with undiagnosed dementia (CDR 1+; mean age=83, 73% women) and their caregivers (mean age=67, 69% women) were enrolled and followed for six months. Results showed a significantly higher rate of linkage to medical services in the intervention group compared to the control group (16.5% vs. 0%, X2 [1]=23.69, p<.001). No significant differences were observed in secondary outcomes, including caregiver dementia knowledge, self-efficacy, depression, and quality of life. To our knowledge, this is the first study to test the efficacy of a CHW-led, community-based transition model for dementia evaluation and care in an older LEP population. Findings highlight the effectiveness of PLAN in facilitating dementia care access for underserved immigrant communities facing significant language barriers.

Abstract citation ID: igaf122.363

### THE PREVALENCE OF DEMENTIA IN KOREAN AMERICAN OLDER ADULTS IN MARYLAND: THE MEMORY AND AGING STUDY OF KOREANS (MASK)

Hyun Kim[1], Patrick Walsh[2], Jean Guan[3], and Hochang Lee[2], *1. Columbia University, New York, New York, United States, 2. University of Rochester, Rochester, New York, United States, 3. University of Missouri School of Medicine, Springfield, Missouri, United States*

Korean American (KA) older adults are among the fastest growing ethnic groups in the U.S. older adult population. KAs face an elevated risk of dementia due to high rates of well-established risk factors (e.g., cardiovascular and lifestyle factors). However, data on the prevalence of dementia in this population remain limited. To address this gap, Memory and Aging Study of Koreans (MASK) was developed, recruiting community-representative sample of KA older adults from ethnic churches, senior centers, and business establishments of Baltimore-Washington D.C. area. A total of 1,118 KA older adults were screened by trained, bilingual community health workers based on a Korean version of the Mini-Mental State Exam during the initial survey stage, and 91 participants underwent a second-stage clinical evaluation, including the Clinical Dementia Rating scale and physician evaluation. The estimated prevalence of dementia was 7.2% (SE ± 2.3), while mild cognitive impairment (MCI) was estimated to be 27.8% (SE = 5.9). Dementia prevalence increased with age (e.g., 35.3% in those aged 80+ years vs. 2.1% in those aged 70-79 years) and differed by subtypes (4.8% Alzheimer's dementia vs. 2.4 vascular subtype). These findings provide a crucial first step in addressing dementia burden in culturally and linguistically isolated KA minority groups across the U.S.

Abstract citation ID: igaf122.364

### ADAPTING RECRUITMENT AND RETENTION FOR KOREAN AMERICAN OLDER ADULTS IN A COMMUNITY CLINICAL TRIAL DURING COVID-19

Ji-Young Yun[1], Deborah Min[2], Ji-Young Cho[3], Sara Kim[4], and Hae-Ra Han[5], *1. Johns Hopkins University, Wellesley, Massachusetts, United States, 2. Johns Hopkins Bloomberg School of Public Health, United States, 3. Korean Community Service Center of Greater Washington, Annandale, Virginia, United States, 4. Korean Community Services of Metropolitan New York, Bayside, New York, United States, 5. Johns Hopkins University, Baltimore, Maryland, United States*

Recruiting and retaining underrepresented populations, particularly those with limited English proficiency, remains challenging in clinical trials, especially during public health crises like COVID-19. The PLAN study (Preparing Healthy Aging Through Dementia Literacy Education and Navigation), a community-based clinical trial, contacted 2,997 Korean American older adults for cognitive screening between February 2021 and January 2024, enrolling 288 dyads of Korean American older adults with probable dementia and their caregiver (N = 576). The study adapted recruitment and retention strategies to evolving pandemic conditions. In the first phase (Feb–Jul 2021), strict COVID-19 protocols limited outreach to virtual methods, engaging 461 dyads. The second phase (Aug 2021–Mar 2023) combined virtual and in-person approaches, reaching 1,981 dyads as restrictions eased. The final phase (Apr 2023–Jan 2024), post-pandemic, engaged 555 dyads primarily through in-person efforts. Recruitment strategies included social media, ethnic media, and outreach at adult daycare centers, churches, and senior centers. Virtual methods, such as ethnic newspapers and KakaoTalk, were effective early on, while in-person engagement—particularly through adult daycare centers and word-of-mouth referrals—proved more successful later. Virtual recruitment yielded a 5% success rate, compared to 10% for in-person efforts. Retention strategies, including monetary compensation and consistent communication, contributed to a low 6% dropout rate. This study underscores the importance of population-tailored recruitment strategies and provides valuable insights into optimizing outreach for future community-based clinical trials.

Abstract citation ID: igaf122.365

### THE IMPACT OF DIGITAL LITERACY ON SOCIAL ISOLATION AMONG KOREAN AMERICAN OLDER ADULTS: INSIGHTS FROM THE PLAN TRIAL

Deborah Min[1], Tina Kim[2], and Hae-Ra Han[3], *1. Johns Hopkins Bloomberg School of Public Health, United States, 2. Johns Hopkins Hospital, Baltimore, Maryland, United States, 3. Johns Hopkins University, Baltimore, Maryland, United States*

Social isolation is a pressing public health concern both in the United States and globally. While growing research highlights the urgent need to address social isolation to enhance the health and well-being of older adults, the magnitude and nature of this issue among immigrant older adults—especially those with limited English proficiency—is not completely known. Immigrant older adults with limited English proficiency are particularly at risk for social isolation, which may be further exacerbated by low digital literacy and engagement. This multi-method study examines the relationship between digital literacy and social isolation using quantitative data from Korean American older adults (N = 733) screened for the PLAN trial (Preparing healthy aging through dementia Literacy education And Navigation; ClinicalTrials.gov Identifier: NCT03909347). The quantitative sample had a mean age of 77 years, with 70% female, 58% having a high school education or less, 28% reporting comfort with technology, and 37% identified as at risk for social isolation. To contextualize our understanding of these findings, we are currently conducting qualitative interviews with a subset of participants to explore the lived experiences of social isolation and the role of digital literacy among community-dwelling Korean American older adults. These combined insights will serve as a critical first step in informing culturally-responsive intervention strategies aimed at reducing social isolation and improving the well-being of immigrant older adults with limited English proficiency in the United States.

### SESSION 1350 (SYMPOSIUM)

Abstract citation ID: igaf122.366

### SOLITUDE AND LONELINESS ACROSS THE LIFESPAN

Chair: Gizem Hueluer
Co-Chair: Jana Nikitin

Solitude and loneliness are complex, multifaceted experiences that shape individuals' well-being across the lifespan. This symposium presents novel perspectives on how solitude and loneliness are shaped by media use, personal motivations, age related factors, and life transitions. Dwight Tse examines social integration and loneliness among older digital gamers, comparing them to non-gamers in a large-scale secondary data analysis of the UK Biobank. Gizem Hueluer presents findings from two experience sampling studies on media use during solitude, exploring how different forms of media consumption are related to loneliness. Jana Nikitin investigates situational solitude in an experimental study, framing it as an opportunity to pursue intrinsically motivated goals and examining its potential to reduce loneliness. Jennifer Lay examines age differences in the recall of solitude experiences. Iris Wahring analyzes loneliness during relationship transitions, conceptualizing solitude as a life situation. Together, these presentations provide key insights into how solitude intersects with media use, motivation, age-related factors, and life transitions in shaping loneliness, contributing to a deeper understanding of these interconnected phenomena across the lifespan.

Abstract citation ID: igaf122.367

### ARE OLDER GAMERS LONERS? FINDINGS FROM A COUNTRY-WIDE STUDY IN THE UNITED KINGDOM

Dwight Tse, and Emma Anderson, *University of Strathclyde, Glasgow, Scotland, United Kingdom*

The stereotypical perceptions of digital gamers as young and lonely are common. Do these perceptions apply to older gamers? In this presentation, we report findings from the UK Biobank data collected between 2006 and 2010, involving N > 70,000 participants aged 50 or older, who reported their computer gaming habits as "never" (non-gamers; 83%), "sometimes" (occasional gamers; 15%), or "often" (frequent gamers; 3%). Despite statistical significance driven by the large sample size, the effect sizes of differences in social engagement between gamers and non-gamers were mostly negligible to small, as indicated by measures of loneliness, family and friend visits, the ability to confide, and participation in leisure and social activities. Further exploratory results suggested negligible differences between older gamers and non-gamers in demographics (e.g., age, sex), sleep patterns (e.g., chronotype, insomnia), and psychological well-being (e.g., satisfaction with life, depressed mood). However, older gamers were less likely to adopt an active lifestyle and exhibited higher BMI, increased body fat, and lower self-rated physical health compared to non-gamers. These findings shed light on the distinct characteristics of older gamers and offer valuable perspectives for gerontologists and gaming industry professionals.

Abstract citation ID: igaf122.368

### DO PEOPLE FEEL LESS LONELY WHEN ALONE IF THEY USE MEDIA? FINDINGS FROM TWO EXPERIENCE SAMPLING STUDIES

Gizem Hueluer[1], Christina Ristl[2], and Jana Nikitin[2],
*1. University of Bamberg, Bamberg, Germany, 2. University of Vienna, Vienna, Wien, Austria*

Solitude is a common daily experience that can have both negative and positive impacts on well-being depending on individual and situational factors. In this study, we examined whether media use moderates the relationship between being alone and loneliness, with a particular focus on whether this effect varies by age. To do so, we used data from two experience sampling studies, which were analyzed using multilevel regression models. In both studies, participants reported higher loneliness when alone. Media use moderated this effect in both studies: In the first study (N = 103, 55% women, mean age = 47 years, SD = 18 years, range = 19 to 79 years), media use was associated with higher overall loneliness, but it was associated with lower loneliness while being alone, particularly in older adults. The findings of the second study (N = 265, 56% women, mean age = 48 years, SD = 18 years, range = 17 to 83 years) were more nuanced: While using a computer or the internet was associated with lower loneliness when alone, watching TV was linked to greater loneliness when alone. These effects were not moderated by age. Reading and listening to the radio had no significant effects. These findings suggest that while some forms of media use may buffer against loneliness when alone, others may exacerbate it, and the effects may vary based on media type and age. The results highlight the importance of considering the specific ways in which individuals engage with media when addressing loneliness.

Abstract citation ID: igaf122.369

### REFRAMING SOLITUDE AS A STRATEGY TO REDUCE LONELINESS

Jana Nikitin, *University of Vienna, Vienna, Wien, Austria*

Loneliness is a significant societal concern with profound effects on physical and mental health. Older adults can be particularly vulnerable due to life transitions, reduced social networks, and increased time spent alone. However, while solitude may become more prevalent with age, it does not inherently lead to loneliness. Learning to experience solitude positively could offer a meaningful way to enhance well-being. This study tested a brief strategy designed to reframe solitude as a positive experience, leveraging the saying-is-believing effect to reduce loneliness. Using an experimental design in an online study (N = 211 participants aged 18–89), participants were randomly assigned to one of two groups. The experimental group actively reflected on the positive opportunities solitude offers for pursuing intrinsically motivated goals, while the control group simply described their emotions during solitude. Findings revealed significantly lower levels of loneliness in the experimental group compared to the control group. Mediation analysis identified self-efficacy as a key mechanism driving this effect. Importantly, these results do not suggest that social isolation is merely an individual responsibility; rather, they highlight the potential of psychological reframing as a complementary approach in loneliness prevention. By shifting perspectives on solitude, this research provides valuable insights into fostering resilience in older adults and underscores the need for scalable, cost-effective solutions that promote well-being without overlooking the structural factors contributing to social isolation.

Abstract citation ID: igaf122.370

### SOLITUDE MISREMEMBERED: RECALLED LONELINESS AND HAPPINESS IN SOLITUDE ARE SHAPED BY AGE, CULTURE, AND LIVING ALONE

Jennifer Lay[1], Yuen Wan Ho[2], Dwight Tse[3], Helene Fung[4], and Da Jiang[5], *1. University of Exeter, Exeter, England, United Kingdom, 2. Lingnan University, Hong Kong, Hong Kong, China (People's Republic), 3. University of Strathclyde, Glasgow, Scotland, United Kingdom, 4. The Chinese University of Hong Kong, Hong Kong, China (People's Republic), 5. The Education University of Hong Kong, Hong Kong, China (People's Republic)*

Recalling past affective experiences is key to guiding behaviour – including solitary activities – in ways that maximise wellbeing. However, we do not always accurately recall such experiences. Previous research suggests older (vs. younger) adults show a positivity effect in memory, depending on whether the content is culturally meaningful. It is unclear, however, whether this age-related positivity effect applies to recalling time in solitude (no social contact), which becomes increasingly prevalent in older adulthood. An age-stratified sample of adults aged 18-81 (M age = 40.8 years, 60.8% female) in the United Kingdom (N = 214) and Hong Kong (N = 220) provided a total of 13,531 reports of their current affective experiences and social situations over a 7-day period (5 times/day) using a smartphone app. Participants then recalled how they felt over the 7-day period at times when they were in solitude. Models controlling for peak and recent affect, overall time in solitude, independent/interdependent self-construal, and demographic factors showed that whereas younger adults tended to retrospectively overestimate how lonely they felt in solitude, older adults tended to underestimate their loneliness in solitude. Moreover, whereas UK older adults tended to retrospectively overestimate their happiness in solitude, HK older adults and those who were living alone tended to underestimate happiness in solitude. Findings suggest individuals misremember their solitude experiences in ways that align with age-normative and cultural attitudes to solitude. Additionally, there is an age-related positivity effect which is shaped by culture. We discuss implications for loneliness and wellbeing in older adulthood.

Abstract citation ID: igaf122.371

### RELATIONSHIP TRANSITIONS AND LONELINESS IN MIDLIFE AND OLDER ADULTHOOD

Iris Wahring[1], Urmimala Ghose[2], NIlam Ram[3], Christiane Hoppmann[4], and Denis Gerstorf[5], *1. Humboldt Universität zu Berlin, Berlin, Berlin, Germany, 2. Humboldt University in Berlin, Humboldt University in Berlin, Berlin, Germany, 3. Stanford University, Stanford University, California, United States, 4. UBC, Vancouver, British Columbia, Canada, 5. Humboldt University Berlin, Humboldt University Berlin, Berlin, Germany*

Prior research suggests that being married or living with a partner is linked to a lower likelihood of loneliness, with some studies indicating a stronger effect for men. Moreover, because social networks and their perception change across the lifespan, the impact of relationship status on loneliness may differ between middle-aged and older men and women. We investigated how three major relationship events—separation, moving in with a partner, and marriage—affect loneliness and whether these effects vary by gender and age. We also examined instances in which individuals both moved in and married within the same timeframe. Using data from 4,768 U.S. participants in the Health and Retirement Study (HRS), we applied propensity score matching to compare two-year changes in loneliness likelihood between individuals experiencing a relationship event and matched controls whose relationship status remained stable. Results showed that moving in with a partner was associated with a reduced likelihood of feeling lonely, particularly among older men who also married their partner within the same period. In contrast, marriage among already cohabiting individuals and separation were not linked to significant changes in loneliness. These findings highlight the protective role of relationship involvement against loneliness in midlife and older adulthood and underscore the importance of considering the co-occurrence of relationship events.

### SESSION 1355 (PAPER)

### BARRIERS TO ACCESS AND AUTONOMY: HEALTH, DISABILITY, AND MOBILITY DISPARITIES IN AGING POPULATIONS

Abstract citation ID: igaf122.372

### DENTAL EXAMS AMONG ADULTS AGING WITH INTELLECTUAL AND DEVELOPMENTAL DISABILITIES IN DIFFERENT LIVING ARRANGEMENTS

Elisabeth Stam[1], Jeffrey Stokes[1], and Danielle Waldron[2], *1. University of Massachusetts Boston, Boston, Massachusetts, United States, 2. Stonehill College, North Easton, Massachusetts, United States*

Background and Objectives: Oral health is crucial for overall health and well-being across the lifespan. Receiving routine dental examinations and regular checkups with advancing age is paramount, especially for vulnerable populations such as adults with intellectual and developmental disabilities (I/DD). The dental health needs of adults with I/DD are often overlooked and barriers exist impeding their engagement with dental care services. Research Design and Methods: We analyzed 8 panel data waves (2013-2014 through 2021-2022) from the National Core Indicators In-Person Survey (NCI-IPS), which is a national survey of adults with I/DD (18+) who receive state services (N = 133,225 observations from 49 states). We performed multilevel logistic regression with models investigating whether adults with I/DD underwent dental exams and how dental services utilization trends differed by living situation. Results: Results demonstrated that adults with I/DD living in intermediate care facilities (ICF) settings are the most likely to receive a dental exam, while individuals with I/DD residing with their families are the least likely, but this divide lessened with age. Adults living with more severe I/DD have a lower likelihood of receiving a dental exam. Engaging in activities, community involvement, having friends, and having a job were significantly associated with an increased likelihood of receiving dental exams. Discussion and Implications: Findings indicate that adults with I/DD access dental care in varying degrees based on living arrangement, I/DD level, and social and community ties. Further research is necessary to understand the factors that contribute to promoting dental services utilization among this population.

Abstract citation ID: igaf122.373

**MAJOR DEPRESSIVE DISORDER IS ASSOCIATED WITH RISKY DRIVING BEHAVIOR AMONG OLDER ADULTS: A NATURALISTIC STUDY**

David Carr[1], and Ganesh Babulal[2], *1. Washington University in St. Louis, St Louis, Missouri, United States, 2. Washington University School of Medicine in St. Louis, SAINT LOUIS, Missouri, United States*

We examined the impact of Major Depressive Disorder (MDD) on naturalistic driving behavior among older drivers. We hypothesized that cases with MDD would exhibit riskier driving behaviors, visit fewer destinations, and have fewer trips compared to nondepressed controls. A prospective, longitudinal, case-control study was conducted with 395 older adults (≥65 years). The sample included 85 cases with MDD and 310 controls. Neurology, clinical, and neuropsychological tests were collected annually. Daily driving behavior was recorded using a datalogger. Linear mixed models with propensity score weighting compared driving behaviors between groups, adjusting for sociodemographic variables, and medication use. MDD subjects exhibited higher numbers of speeding events (mean = 2.34 vs. 1.67, p<.001) and more hours driven per month (mean = 24.8 vs. 21.2, p<.001) compared to controls at baseline. Longitudinal analysis showed MDD subjects had an increase in hard braking (β = 0.27, p<.01) and hard cornering events (β = 0.21, p<.05) per trip, greater distances driven from home (β = 2.45 miles, p<.01), more unique destinations visited (β = 1.85, p<.05), and higher random entropy (β = 0.13, p<.05) over time. Post-hoc analysis adjusting for antidepressants and total medication use did not alter the results. Older adults with MDD exhibit distinct and riskier driving behaviors compared to non-depressed controls, with increased hard braking, cornering, and unpredictability in driving patterns over time. Routine depression screening and tailored interventions are essential for enhancing driving safety and maintaining independence among older adults with MDD. Comprehensive care approaches addressing both mental and physical health are crucial for this vulnerable population.

Abstract citation ID: igaf122.374

**ORAL HEALTH BARRIERS AMONG COMMUNITY-DWELLING OLDER ADULTS >=65 YEARS WITH AND WITHOUT DISABILITY**

Uma Kelekar[1], Debasree Das Gupta[2], Dinh Nguyen[3], and Diep Tran[4], *1. Marymount University, Arlington, Virginia, United States, 2. Utah State University, Logan, Utah, United States, 3. Unaffiliated, Frederick, Maryland, United States, 4. Johns Hopkins University, Baltimore, Maryland, United States*

Among US older adults (>=65 years), multiple overlapping challenges, such as progressive aging, increasing comorbidities, and lack of universal dental coverage, restrict routine dental-care visits. The presence of one or more disabilities may further exacerbate access-related dental care disparities among the 65+. However, the literature on oral healthcare disparities within this population, particularly by disability status, remains limited. To address this gap, we employ 2012-2022 Medical Expenditure Panel Survey to 1) estimate dental service utilization rates among older adults with and without a disability, and 2) identify barriers associated with dental-care access among the 65+, after controlling for socio-demographics and health conditions. The main outcome, dental service utilization, was measured as a visit to a General Practitioner (GP) dentist in the last year. A secondary outcome represented barriers to dental care, measured by inability to access care/delaying care. Disability was measured as the presence of sensory, cognitive or physical limitations. In the multivariable analysis, in 2012, the odds of having seen a dentist (OR:0.68, 95% CI: 0.50,0.93) was lower among those with any disability compared to no disability. This difference between the 65+ with and without any disability was no longer significant in 2022. However, in 2022, the odds of reporting delays in seeking dental care [OR = 1.67, 95% CI: 1.13, 2.48] was higher among the 65+ reporting any disability, compared to those reporting no disability. Oral health disparities among older adults with disabilities continue to persist and should be addressed through adequate policy reform and investments in health education programs.

Abstract citation ID: igaf122.375

**THE DETERMINANTS AND TRAJECTORIES OF TRANSPORT DISADVANTAGE AMONG AMERICAN OLDER ADULTS**

Yong Yang, *The University of Memphis, Memphis, Tennessee, United States*

Background: Maintaining daily transportation becomes increasingly challenging with age due to various barriers. Transport disadvantage—defined as inadequate access to transportation via car, public transit, or other modes—remains understudied in older adulthood. Little is known about how it evolves over time, whether it accelerates or stabilizes at different aging stages, and how trajectories vary among older adults. Methods: This study examines changes in transport disadvantage from 2011 to 2024 using data from the National Health and Aging Trends Study (NHATS), a nationally representative panel study of U.S. Medicare enrollees aged 65 and older. We apply linear mixed-effects models and group-based trajectory models to assess transport disadvantage over time, identify distinct trajectories, and examine the influence of personal characteristics (e.g., demographics, health status) and neighborhood conditions (e.g., deprivation, safety, walkability, public transit access). Results: We identify three trajectories: (1) a majority with no transport restrictions, (2) a group with low but stable or increasing disadvantage, and (3) a group with moderate to high disadvantage that declines over time. Transport disadvantage generally increases with age, with a sharp decline following driving cessation. Older adults in high-deprivation, low-safety, or poor-transit neighborhoods are more likely to experience worsening transport disadvantage, with effects amplified among those with disabilities, lower socioeconomic status, or declining health. Conclusion: Addressing disparities in transport access requires targeted interventions that consider both individual vulnerabilities and neighborhood factors to support mobility and well-being in aging populations.

Abstract citation ID: igaf122.376

**WHITE MATTER HYPERINTENSITIES AND DRIVING BEHAVIOR: MITIGATING EFFECTS OF HYPERTENSION TREATMENT**

David Carr[1], Madhur Parihar[2], Jin-Moo Lee[3], Ganesh Babulal[4], Yasheng Chen[5], and Chia-Ling Phuah[2], *1. Washington University in St. Louis, St Louis, Missouri, United States, 2. Barrow Neurological Institute, Phoenix, Arizona, United States, 3. Washington University in St. Louis, St. Louis,*

*Missouri, United States, 4. Washington University School of Medicine in St. Louis, SAINT LOUIS, Missouri, United States, 5. WashU Medicine, St. Louis, Missouri, United States*

White matter hyperintensities (WMH) compromise cognitive reserve, potentially accelerating dementia onset. We investigated WMH's impact on complex cognitive performance through longitudinal analysis of naturalistic driving behavior in older adults (OA). We examined 212 cognitively intact OA (aged >65 years, CDR=0) in the DRIVES Project cohort, analyzing 16 driving metrics using in-vehicle dataloggers, and quantifying WMH using deep learning. Linear mixed-effects models, adjusted for demographics, socioeconomic status, and composite vascular risk, assessed WMH influence on longitudinal changes in driving performance. Our study analyzed 74,275 weeks of driving data (2015-2024, average follow-up of 6.1 years). Higher WMH burden correlated with decreased trip frequency, fewer near-home trips, reduced unique destinations, and lower driving entropy over time (all $p < 0.002$). Posterior WMH lesions, particularly in parietal and occipital regions, were primarily linked to driving complexity decline (beta=-0.16, $p = 0.009$, beta =-0.15, $p = 0.01$, respectively). WMH impact intensified in participants developing cognitive impairment ($n = 36$), manifesting as increased hard braking and impact events. Anti-hypertensive medication users ($n = 147$) showed less decline in driving frequency and complexity ($p = 0.03$). WMH in OA significantly impacts driving behavior, leading to latent self-regulation and reduced driving complexity. Anti-hypertensive use may mitigate WMH effects on driving behavior change over time, despite posterior WMH suggesting a prominent role for AD in driving decline, warranting further study of blood pressure treatment. WMH shows potential as a biomarker for identifying individuals at higher risk of unsafe driving and early cessation, underscoring its value in screening and intervention for road safety among aging populations.

### SESSION 1360 (SYMPOSIUM)

Abstract citation ID: igaf122.377

### ADVANCING CARE FOR OLDER ADULTS THROUGH HEALTH INFORMATION TECHNOLOGY: AN INTERDISCIPLINARY APPROACH

Chair: Zhang Zhang
Co-Chair: Nancy Schoenborn
Discussant: Thomas Cudjoe

Technology holds transformative potential to enhance healthcare delivery for older adults by improving access, efficiency, and patient-clinician communication. However, challenges persist such as heterogeneity across population groups in technology adoption, stakeholder priorities, and ethical concerns about transparency. This symposium addressed these gaps through four interdisciplinary studies, which provide insights into telehealth, patient portals, and artificial intelligence (AI) in the care of older adults. The first study examined telehealth's role in expanding preventive care access and promoting early detection of dementia via Medicare Annual Wellness Visits (AWVs). Telehealth adoption was associated with an increase in AWVs. The second study found that among older adults, patients who send the most secure messages via the patient portal are more likely to be white, have a higher comorbidity burden, and exhibit greater patient portal and healthcare use, which require equitable digital access and tailor patient portals to better support diverse needs. The third study adopted a mixed-methods approach to test ChatGPT's ability to identify nonpatient authors of dementia-related portal messages. Interviews with people with dementia and caregivers revealed conditional acceptance of AI-drafted messages, contingent on clinician review and explicit disclosure of AI involvement. The fourth study delved into stakeholder decision-making regarding AI adoption in healthcare. Interviews with older adults, caregivers, clinicians, payers, and investors revealed shared key factors in decision-making (e.g., usability, value) but divergent emphasis on cost. Our discussant will explore implications for care innovation and approaches to address the barrier to technology, including strategies to ensure inclusive implementation across diverse aging populations.

Abstract citation ID: igaf122.378

### THE EVALUATION OF TELEHEALTH'S IMPACT ON MEDICARE ANNUAL WELLNESS VISITS AND DEMENTIA DIAGNOSIS

Zhang Zhang[1], Nancy Schoenborn[2], Katherine Miller[1], Jennifer Wolff[3], and Daniel Polsky[4], *1. Johns Hopkins Bloomberg School of Public Health, Baltimore, Maryland, United States, 2. Johns Hopkins University School of Medicine, Baltimore, Maryland, United States, 3. Johns Hopkins University - School of Public Health, Baltimore, Maryland, United States, 4. Johns Hopkins University, Baltimore, Maryland, United States*

Background: Telehealth has emerged as an essential tool in modern healthcare, providing the potential for the early screening and diagnosis of mild cognitive impairment (MCI) and Alzheimer's Disease and Related Dementias (ADRD). For example, telehealth enhances access to care and streamlines cognitive assessment during Medicare Annual Wellness Visits (AWVs). Our study aims to evaluate the impact of telehealth on AWV uptake using a large population dataset. Method: We used the data from Traditional Medicare (TM) and Medicare Advantage (MA) plans. We applied a probit regression model to examine the relationship between telehealth adoption and AWV uptake among beneficiaries aged 65 and older continuously enrolled in traditional FFS plans or MA in 2020-2022. Results: Preliminary analysis using 2020 TM data revealed that 39% of beneficiaries had ever adopted at least one telehealth visit. 7% of the AWVs were delivered via telehealth among those AWV adaptors. There was a significant increase in AWV uptake among telehealth users by 10.2 percentage points to non-telehealth users in the TM plan ($p < 0.001$). We will extend this analysis to MA beneficiaries, where we anticipate a more pronounced effect. MA likely has greater capabilities to manage health expenditures and provide resources, such as internet access and incentives for preventive care. Conclusions: Our study underscores the role of telehealth in increasing AWV uptake and facilitating cognitive assessments. Telehealth holds promises for bridging the gaps in cognitive screening and enhancing early detection of MCI and ADRD, thereby supporting timely diagnosis and care planning.

Abstract citation ID: igaf122.379

### PROFILING HIGH-INTENSITY MESSAGE SENDERS AMONG OLDER ADULT PATIENT PORTAL USERS

Aleksandra Wec[1], Jenni Seale Reiff[1], and Jennifer Wolff[2], *1. Johns Hopkins Bloomberg School of Public Health, Baltimore, Maryland, United States, 2. Johns Hopkins University - School of Public Health, Baltimore, Maryland, United States*

Rising secure messaging volume in patient portals has led to increasing demand on clinician time. Little is known about patients who send the most messages. We develop a profile of high-intensity secure message senders from a sample of 16,023 older adults who sent 199,952 messages to clinicians at an academic health system over 12 months. High-intensity messaging was defined using outlier analysis of aggregate message volume during the 12-month observation period. Additional measures of high-intensity messaging included identifying concentrated periods of messaging, message length, and ratios of messages to portal logins and to healthcare encounters. We compared socio-demographic characteristics, health status, and messaging behaviors of high-intensity senders to all other message senders. Of the total sample, 1,298 patients (8.1%) were classified as high-intensity message senders; these patients accounted for 39.7% of total messages. High-intensity message senders, compared to all other message senders, were more likely to be white (80.4% vs. 72.5%, $p<0.001$), have higher comorbidity scores (2.6 vs. 1.8, $p<0.001$), to have diagnosed cancer (35.8% vs. 22.8%, $p<0.001$) or dementia (8.3% vs. 6.1%, $p<0.002$). They were also more likely to have concentrated messaging periods, send longer messages, and have greater patient portal use and higher healthcare utilization in relation to the number of messages they sent. High-intensity message senders form a small, distinct group of older adults who contribute disproportionately to message volume. Better understanding the profile of high-intensity message senders may inform targeted initiatives seeking to improve patient care while easing clinicians' inbox workload.

Abstract citation ID: igaf122.380

### EVALUATING CHATGPT IN PORTAL MESSAGING FOR DEMENTIA CARE: IDENTIFYING CARE PARTNERS AND EXPLORING ACCEPTANCE

Kelly Gleason, and Athena DeGennaro, *Johns Hopkins School of Nursing, Baltimore, Maryland, United States*

Introduction: ChatGPT, a popular large language model, is integrated in Epic's patient portal and can create draft responses that clinicians may refine and use when answering patient messages. Application of GPT in messaging related to dementia care requires a judicious approach: persons with dementia and their care partners have distinct differences from the general population (e.g., higher care partner involvement). Methods: We conducted a two-part study. We first used a de-identified set of patient portal messages sent from persons with dementia and their care partners to assess ChatGPT's capacity to identify the sender of the message. We used a set of 1,973 messages where the sender (patient or nonpatient) was manually coded by two independent reviewers as the gold standard. We then conducted interviews with persons with dementia and their care partners to explore their perceptions of the use of artificial intelligence in patient portal messaging. We used team-based qualitative rapid analysis encapsulate major findings across the summaries of participants' interviews. Results: We found that ChatGPT had acceptable performance in detecting a nonpatient author of a patient portal message (0.92 area under the receiver operating curve). In qualitative interviews ($n=13$), we found that care partners and persons with dementia accepted the use of artificial intelligence to draft patient portal messages provided two key conditions were met: clinician review before sending and clear transparency regarding artificial intelligence's involvement. Conclusion: Use of ChatGPT in patient portal messaging is acceptable to care partners and persons with dementia, and may support care partner identification.

Abstract citation ID: igaf122.381

### HOW DO STAKEHOLDERS MAKE DECISIONS ABOUT AI AND NOVEL TECHNOLOGIES FOR THE HEALTHCARE OF OLDER ADULTS?

Nancy Schoenborn[1], Zhang Zhang[2], Ashida Sato[3], and Thomas Cudjoe[4], *1. Johns Hopkins University School of Medicine, Baltimore, Maryland, United States, 2. Johns Hopkins Bloomberg School of Public Health, Baltimore, Maryland, United States, 3. University of Iowa College of Public Health Department of Community and Behavioral Health, Iowa City, Iowa, United States, 4. Johns Hopkins School of Medicine, Baltimore, Maryland, United States*

Artificial intelligence (AI) and novel technologies offer tremendous potential for improving the health of older adults, but how key stakeholders make decisions about which technology to finance and adopt is not well understood. We used qualitative semi-structured interviews to explore how older adults, care partners, clinicians, and insurance payers decide whether to adopt and how investors decide whether to finance new AI technology. Participants included 15 older adults or care partners, 15 clinicians, 8 payers, and 5 investors. Interviews were analyzed using qualitative thematic analysis. All key stakeholders considered cost, usability, and added value, but each group had different perspectives. Older adults and care partners emphasized out-of-pocket costs and the product's user-friendliness for older adults. Clinicians were concerned about both patient and health system costs and considered usability when fitting into existing workflow. Payers considered the cost and added value from a population health perspective, including the frequency and cost of the health events (e.g., falls) that the technology aims to impact, the revenue stream (e.g., insurance coverage), and the cost of implementation and maintenance. Investors considered cost in terms of return on investment and considered competing products, market demand size, and the project team in decisions. In addition to end-user cost and usability, multiple other factors influence the likelihood that new AI technologies will become available for the healthcare of older adults. Ensuring engagement to raise awareness about key decisional factors and aligning incentives to motivate impactful products for the end-users is important.

### SESSION 1365 (PAPER)

### CROSS-CULTURAL PERSPECTIVES ON AGING, CARE, AND RESILIENCE

Abstract citation ID: igaf122.382

### ADAPTATION AND VALIDATION OF THE TRADITIONAL CHINESE FAMILY CAREGIVER MEDICATION ADMINISTRATION HASSLE SCALE

Te-Lien Ku[1], Shu-Ying Wu[2], Wei-Chu Chie[3], and Yen-Ming Huang[4], *1. University of Wisconsin-Madison, Madison, Wisconsin, United States, 2. Sijhih Cathay General Hospital, New Taipei City, New Taipei, Taiwan (Republic of China), 3. National Taiwan University, Taipei, Taipei, Taiwan (Republic of China), 4. National Taiwan University, Taipei City, Taipei, Taiwan (Republic of China)*

The Family Caregiver Medication Administration Hassle Scale (FCMAHS) measures the stress family caregivers experience in managing medication for older adults. However, its applicability to Chinese-speaking caregivers in Taiwan remains uncertain. This study adapted the FCMAHS into Traditional Chinese (FCMAHS-TC). We conducted a two-phase cross-sectional design: Phase I involved bidirectional translation and expert evaluation, while Phase II used a convergent mixed-methods field test. A purposive sample of 138 family caregivers in Taiwan completed the FCMAHS-TC, and 12 participants were interviewed. Exploratory factor analysis (EFA) determined the factor structure of the scale. Validity and reliability were assessed through content validity, internal consistency, and test-retest reliability. Caregiver interviews were audio-recorded, transcribed verbatim, and analyzed deductively using content analysis to triangulate evidence for scale adaptability by exploring caregivers' medication management experiences and associated hassles. Expert evaluation confirmed excellent content validity, with an item-and scale-level content validity index of 1.0. The EFA revealed a four-factor structure explaining 59.57% of the variance, including Information Seeking and Sharing (11 items), Scheduling Logistics (7 items), Medication Filling and Prescription Management (3 items), and Safety Issues (3 items). Reliability analysis showed strong internal consistency (Cronbach's alpha = 0.94) and good test-retest reliability (intra correlation coefficient = 0.78). Qualitative findings supported the items and factor structure while highlighting Care Coordination as a distinct hassle for Taiwanese family caregivers. The FCMAHS-TC demonstrates strong psychometric properties and cultural adaptability for the Taiwanese population, helping researchers and healthcare providers better understand family caregivers' medication management challenges and design targeted interventions.

Abstract citation ID: igaf122.383

### EXCAVATING AND UNDERSTANDING THE ECCENTRIC: TEXTUAL ANALYSIS, GENDER AND AGEING IN BRITISH CINEMA

Sian Barber, and Gemma Carney, *Queen's University Belfast, Belfast, Northern Ireland, United Kingdom*

In this paper we use our combined expertise in textual film analysis and social and cultural gerontology to interrogate visualisations of age and gender in Hampstead (2017) and The Lady in the Van (2015). Building on existing work which analyses film as a narrative (Oró-Piqueras and Casado-Gual, 2020) we engage with cinematography, mise-en-scène and aesthetics, viewing film as a vivid and moving imaginary of old age. In particular, we use Barber's (2015) expertise in British cinema to link the performances of Smith (The Lady in the Van) and Keaton (Hampstead) to incarnations of ageing women which are deeply rooted in British cinema. Both films allow us to locate the main female characters in the precise analytical frame of British cinema. Both provide a visceral and visual representation of older women's precarious property ownership in two contrasting visions of London. For British cinema-goers Maggie Smith's struggle with homelessness, mental health difficulties and status as a single, childless older woman evoke the harsh realities of London's property market whilst drawing on British social realist cinema. By contrast, Keaton's 'American in London' story of widowhood, ageing and ultimate salvation through the love of Irish man Brendan Gleeson draws heavily on heritage cinema aesthetics and familiar rom-com tropes. After attending the session participants will be able to see that a filmic gaze offers a powerful means of de-constructing homogenization of old age well documented by scholars of the 'silvering screen' (Chivers, 2011).

Abstract citation ID: igaf122.384

### HEALTHCARE ACCESS AND BARRIERS AMONG OLDER ASIAN AMERICANS: CULTURAL INFLUENCES AND SYSTEMIC CHALLENGES

Shinae Choi[1], Hee Lee[1], Hyunjin Noh[1], Peiyuan Zhang[2], and Hannah Francis[1], *1. The University of Alabama, Tuscaloosa, Alabama, United States, 2. University of Maryland, Baltimore County, Baltimore, Maryland, United States*

Although nearly a quarter of the 19 million Asian immigrants in the U.S. call the South home, many still struggle to access and utilize healthcare services, revealing critical gaps in the system. This study investigated older Asian Americans' access to healthcare services, utilization patterns, and quality of healthcare services received. We interviewed 15 Asian Americans, aged 50 or older, residing in West Alabama. Individual semi-structured interviews were conducted either virtually or in-person in English between July and November 2023. Each interview lasted 60-90 minutes. All interviews were recorded, transcribed, and thematically analyzed using open coding. Our analysis revealed six main themes on older Asian Americans' healthcare services utilization: 1) dual cultural influences on healthcare service expectations, shaped by both the country of origin and the U.S., 2) hesitancy taking medication due to concerns about over-prescription, 3) long waiting and delayed care, and 4) navigating different healthcare logistics and high medical expenses; nearly all participants reported paying at check-out in their country of origin, where healthcare costs are known upfront. However, in the U.S., medical bills often arrive weeks or months later, making it difficult to anticipate and understand any out-of-pocket costs. Additional challenges addressed 5) hesitancy to ask questions during check-ups and 6) perceived discrimination from healthcare service providers. These challenges often lead to underutilization of services and unmet healthcare needs in the community. Our findings highlight critical needs for culturally tailored healthcare policies and targeted interventions to eliminate disparities, enhance accessibility, and improve health outcomes for older Asian Americans.

Abstract citation ID: igaf122.385

### SOCIOCULTURAL PERSPECTIVES OF ABUSE OF OLDER ADULTS WITHIN FAMILY CAREGIVING SETTINGS IN NIGERIA

Tochukwu Okolie[1], Patricia Agbawodikeizu[2], Prince Ekoh[2], Ngozi Chukwu[2], Emmanuel Ugwu[2], Samuel Onuh[2], Obinna Odo[3], and Kingsley Udeh[3], *1. University of Connecticut, Mansfield Center, Connecticut, United States, 2. University of Nigeria Nsukka, Nsukka, Enugu, Nigeria, 3. Miami University, Oxford, Ohio, United States*

In Nigeria, family members remain at the core of providing care to older adults. However, these intrinsic and culturally accepted responsibilities are occasionally contrasted/undermined by inadvertent abuse of the older person within family caregiving settings. Notably, what is considered abusive or not in a caregiving situation is usually culturally defined. Therefore, this study explored the perspectives of older adults about caregiving behaviors that they consider to be abusive. Data were obtained using semi-structured interviews with 16 older adults age 60 years and above in a rural community in Awgu Local Government Area (LGA), Enugu state, Nigeria. The data were analyzed thematically with the aid of NVivo12 software. Five themes emerged as caregiving behaviors that were considered abusive (i) neglect of caregiving responsibilities (ii) forced feeding and/or medication (iii) nonconsensual use of older adults' money/assets (iv) restriction of older adults' movements (v) invasion of privacy. Majority of the participants in the study verbalized a detest for these behaviors by family members, while others added that sometimes those were normal and expected caregiving behaviors. The caregiving behaviors identified by participants in this study are usually not within the typically reported types of abuse of older adults in Nigeria. However, they may constitute detrimental consequences to the health and quality of life of older adults in Nigeria. Hence, the study recommends research into these specific caregiving behaviors to understand how and if they impact older adults' overall quality of life in any way.

Abstract citation ID: igaf122.386

### STEEL BUTTERFLIES: RESILIENCE AND POSITIVE SOLITUDE AMONG OLDER WOMEN IN ISRAELI KIBBUTZIM DURING WAR

Sharon Ost-Mor[1], and Lia Ring[2], *1. University of Haifa, Haifa, HaZafon, Israel, 2. Department of Social Work, Ruppin Academic Center, Emek Hefer, Israel*

The October 7, 2023 attacks and subsequent war exposed women living in Israeli kibbutzim to collective trauma and forced evacuation, providing an opportunity to examine both psychological distress and potential growth in later life trauma exposure. This qualitative study examined the experiences of 27 women (ages 65-91) from three kibbutzim, including both evacuated and non-evacuated communities, using semi-structured interviews analyzed through thematic analysis to explore their experiences, coping mechanisms, and the role of positive solitude in trauma processing. Three primary temporal themes emerged: (1) "Wings Growing from Pain" - leveraging past experiences and personal strengths for present coping; (2) "Reconstruction Within Chaos" - creating new meaning while maintaining individual identity within the collective, particularly through positive solitary activities; and (3) "Tomorrow's Garden" - future orientation expressed through both practical return plans and messages to younger generations, reflecting hope despite disappointment in institutional responses. The findings highlight the complex interplay between collective trauma and individual coping among women in kibbutzim. Their unique integration of resilience through positive solitude within a communal living framework demonstrates how life-course experiences contribute to trauma processing. This study offers insights for developing interventions that balance individual and collective needs in supporting people during crises, while acknowledging their capacity for growth even in the face of severe trauma.

### SESSION 1370 (PAPER)

### HEALTH CARE AND NUTRITION POLICY AND PRACTICE

Abstract citation ID: igaf122.387

### TRENDS IN TREATMENT PROVISION FOR MENTAL ILLNESS AND SUBSTANCE USE IN THE UNITED STATES:1997-2024

Marilyn Sinkewicz, Philippa Clarke, and Iris Gomez-Lopez, *University of Michigan, Ann Arbor, Michigan, United States*

Economic and health shocks, such as the COVID-19 pandemic and the opioid crisis, have intensified concerns about mental health. Recently, the Surgeon General declared a public mental health emergency. For an increasingly aging population the adequate provision of mental health and substance use (MHSU) treatment is important to successfully aging in place. However, evidence to guide treatment provision has largely been limited to specialty services, select geographic areas, and discrete time periods. To inform MHSU planning, policy, and programs for older adult populations, this study uses national longitudinal neighborhood-level data from a wide range of public sources to examine the evolution of treatment provision from 1997 through 2024. We used geospatial hot spot analysis and heat maps to examine overall patterns and trends, as well as nuanced dynamics by source of treatment, demography, and geography. Findings reveal that MHSU treatment is delivered by a wide variety of providers and facilities, and that the overall density of treatment provision increases over time. Yet the distribution of treatment provision is uneven at the turn of the 21th century, and differences widen over the next 24 years. Source-specific trends differ with respect to their trajectories, geographic impact, and demographic groups affected. For example, urban neighborhoods widen their advantage over rural areas; economically affluent neighborhoods improve more than disadvantaged ones; and white neighborhoods progress more than black and Hispanic neighborhoods. The exception is psychiatric hospitals, which become concentrated in disadvantaged rural and black rural areas. Findings suggest tailoring mental health interventions by geographic/demographic area.

Abstract citation ID: igaf122.388

### DOES DUAL ELIGIBILITY DRIVE HEALTH CARE USE PATTERNS? EVIDENCE FROM MEPS USING LATENT CLASS ANALYSIS

Mohammad Usama Toseef[1], Ramin Homayouni[2], Aastha Dharia[3], and Wassim Tarraf[4], *1. Corewell Health, Farmington Hills, Michigan, United States, 2. Oakland University, Rochester, Michigan, United States, 3. Oakland University William Beaumont School of Medicine, Rochester Hills, Michigan, United States, 4. Wayne State University, Detroit, Michigan, United States*

Dually-eligible beneficiaries (or duals) are enrolled in both Medicare and Medicaid. Duals have high and complex healthcare needs, and face substantial barriers to quality healthcare access. Thus, it is important to understand how dual-eligibility status is associated with patterns of healthcare services use and the factors that drive these patterns. We used data from the Medical Expenditure Panel Survey (2008-2022) to evaluate the association of dual-eligibility status and health care use patterns for older Medicare beneficiaries (65+-years). We used Latent Class Analysis to sequentially fit and assess multiple class solutions based on 10 healthcare services (e.g. hospital use, dental visits, home healthcare, medications) and generated healthcare use patterns profiles. Subsequently, we fit survey-weighted unadjusted and adjusted multinomial logistic regression models to link dual-eligibility status to the best fitting use patterns profiles. We found that the three class solution (typical [67.8%], high [17.5%], and low [14.7%] users) provided the best statistical and substantive fit to the data. Unadjusted models showed that duals are more likely to be both high (RRR = 2.07, $p < 0.001$) and low users (RRR=1.45, $p < 0.001$) as compared to non-duals. After adjusting for sociodemographics, comorbidities, and access to usual source of care, duals remained more likely to be high users (RRR=1.7, $p < 0.001$), but no longer low users as compared to non-duals. These results highlight the need for more nuanced approaches, by stakeholders, to policies and interventions that streamline the use of health services in a way that aligns with efficient, quality and cost-effective healthcare for this complex need population.

Abstract citation ID: igaf122.389

### ROLE OF THE COMMUNITY HEALTH REPRESENTATIVES IN CARING FOR OLDER PEOPLE IN TRIBAL COMMUNITIES

Susan Chapman, and Angela "Jackie" Kaslow, *University of California, San Francisco, San Francisco, California, United States*

The Community Health Representative (CHR) Program was established 57 years ago to meet the healthcare needs of American Indian and Alaska Native (AIAN) populations. The purpose of this project was to assess whether the CHR workforce in Tribal rural and urban settings had a sufficient supply of well-trained CHRs and resources to meet the health care needs of older people. Twenty (20) individuals were identified from 10 sites in California using snowball sampling. Fourteen (14) participants including 9 CHRs, 2 nurses, 2 managers and 1 clinic director agreed to participate in a 45-60 virtual semi-structured interview. We conducted a sub-thematic analysis focused on issues in caring for older people in rural tribal settings including the unique care needs, barriers to care, and the role of CHRs in meeting those needs. Home visits stood out as an essential service for rural-living older tribal members. Transportation was a highly used and valued service provided by CHRs. Older Tribal people experience social isolation due to remote living, which is also characterized by limited information technology connectivity resulting in limited health technology literacy. Older Tribal people also demonstrate a significant need for transportation to clinical appointments and to obtain medications and medical supplies. Interviewees noted that building trusting relationships that reflect AIAN cultural standards is a key component of older tribal people and acceptance of care. Policy should address the structural gaps to AIAN older people in accessing culturally-based home care services from well-trained CHRs as well as barriers to essential services.

Abstract citation ID: igaf122.390

### SOCIOECONOMIC STATUS AND OVERALL SATISFACTION WITH THE HEALTHCARE AMONG OLDER ADULTS IN THE U.S.

Rui Huang[1], Yaqi Yuan[2], and Yulin Yang[3], *1. University at Buffalo, Buffalo, New York, United States, 2. Georgia Institute of Technology, Atlanta, Georgia, United States, 3. University of California, San Francisco, San Francisco, California, United States*

This study investigates the relationship between socioeconomic status (SES) and healthcare satisfaction among older adults, with a particular emphasis on how age moderates this relationship. Although substantial research has paid attention to health inequalities, there remains limited evidence on how SES factors such as education and wealth affect healthcare satisfaction longitudinally as individuals grow older. Guided by the Fundamental Cause Theory and Cumulative Inequality theory, this study explores whether and how education and wealth predict disparities in healthcare satisfaction over time and examines the moderating effect of age on these relationships. Data from four waves (2014, 2016, 2018, and 2020) of the Health and Retirement Study (HRS) were analyzed using multilevel logistic models with random intercepts and effects to capture within- and between-individual variabilities. The key independent variables include total wealth and educational attainment. Self-reported healthcare satisfaction is the dependent variable. Age is the moderator. Control variables include demographic characteristics, Medicare coverage, and self-rated health status. Preliminary results indicate that the association between SES and healthcare satisfaction is significantly influenced by age. Specifically, older adults with higher levels of education and greater wealth exhibit increasing satisfaction with healthcare as they age compared to their lower-income and less-educated peers, highlighting a widening gap in healthcare experiences across socioeconomic lines. Policymakers should prioritize targeted interventions to reduce healthcare disparities among aging populations by addressing socioeconomic barriers. Expanding equitable access to high-quality healthcare services, improving health literacy, and increasing financial protection for lower-income older adults may mitigate the adverse effects of socioeconomic disadvantages.

Abstract citation ID: igaf122.391

### THE GROWING UNMET NEED FOR OLDER ADULT NUTRITION PROGRAMS: EVIDENCE FROM NATIONAL DATASETS

Bernadette Wright[1], and Victoria Owens[2], *1. Meals on Wheels America, Falls Church, Virginia, United States, 2. Meals on Wheels America, Arlington, Virginia, United States*

Home-delivered meal and community dining programs, such as Meals on Wheels, provide older adults with nutritious meals and the opportunity to address social and safety needs that affect health. With federal funding at risk and just 1% of philanthropic giving going to aging issues, nutrition programs need data to demonstrate the need. We examined trends over the past 25 years (based on availability) in service reach, funding and indicators of need. Data were from the National Health and Nutrition Examination Survey, Administration on Community Living, the Current Population Survey Food Security Supplement and the Health and Retirement Study. We found a stark disconnect between declining numbers of older adults served and growing indicators of need. 4.6 million Americans aged 60 and older reported receiving community or government home-delivered meals and/or community dining in the past 12 months (August 2021- August 2023), regardless of funding source. This was fewer people than in 6 of 10 prior periods since 1999-2000. 2.2 million older adults received Older Americans Act (OAA) Title III meal services in 2021, the fewest of any year since 1999. The purchasing power of OAA meal funding declined from $626 million in 1999 inflation-adjusted to $1.175 billion to $1.059 billion in FY2024. Meanwhile, the number of adults age 60 and older with food insecurity quadrupled between 1999 and 2023 (1.8 million to 7.4 million). 56% of older adults experienced loneliness in 2022, with little change since 2006. These findings suggest funding has not kept pace with the growing need.

### SESSION 1375 (PAPER)

### HOUSING AND AGING: QUALITY, STABILITY, AND HEALTH OUTCOMES IN LATER LIFE

Abstract citation ID: igaf122.392

### ASSOCIATIONS BETWEEN HOUSING INSECURITY AND PHYSIOLOGICAL HEALTH AMONG AGING ADULTS IN THE UNITED STATES

Aarti Bhat[1], Andrew Fenelon[2], Jessica Ho, Idan Shalev[1], and David Almeida[1], *1. The Pennsylvania State University, University Park, Pennsylvania, United States, 2. University of Minnesota, Minneapolis, Minnesota, United States*

Housing insecurity (HI) is a stressor that may have implications for physiological health. While extant literature has examined relationships between HI and physiological markers, these studies are limited by their cross-sectional nature and examine only composite allostatic load scores or individual physiological markers as outcomes of interest. In contrast, this study utilizes longitudinal data from the Midlife in the United States (MIDUS) study to assess associations between HI and composite allostatic load scores, as well as individual physiological subsystem scores that comprise allostatic load; and utilizes allostatic load scores from the prior wave of MIDUS to control for health selection. The analytic sample comprised 448 adults (mean age = 64.94, 55.80% female, 15.85% Black, 6.70% other non-white race). Controlling for sociodemographic and health covariates as well as prior wave inflammatory subsystem scores, HI was associated with significantly higher levels of inflammation; however, HI was not associated with overall allostatic load nor other physiological subsystems. We examined whether perceived and objective neighborhood quality moderated the association between HI and inflammation. With increased levels of HI, lower perceived neighborhood quality and higher scores of objective neighborhood deprivation were significantly associated with higher inflammation. Among participants who reported experiencing at least one HI event, those who rented their homes experienced higher inflammation compared to those who owned their homes outright. These analyses indicate inflammation may be a main biological pathway impacted by HI in aging adults, and have implications for health-related interventions to mitigate adverse health effects of HI in this vulnerable population.

Abstract citation ID: igaf122.393

### ASSOCIATIONS BETWEEN HOUSING QUALITY AND DISABILITY AMONG MIDLIFE AND OLDER ADULTS

Kimberly Rollings, Philippa Clarke, Lu Qin, Sohel Ahmed, and HwaJung Choi, *University of Michigan, Ann Arbor, Michigan, United States*

Numerous studies have linked housing quality to a variety of health outcomes throughout the life course. However, less is known about associations between housing quality and disability among midlife and older adults. This exploratory study examined these associations using biennial 2002 to 2018 data from the U.S. Health and Retirement Study (HRS). Available HRS housing data from a subsample of 28,357 community-dwelling adults aged 50 years and older (mean age=68.1 years, SD = 11.5 years) were harmonized and combined to create a housing quality scale (average of interviewer-reported and standardized: number of rooms per household member, and ratings of cleanliness, clutter, structural quality, maintenance, and safety features). Disability measures included any limitations in activities of daily living (ADL, e.g., walking, dressing, bathing) and instrumental activities of daily living (IADL, shopping, preparing meals), and any functional limitations (FL, e.g., difficulty climbing stairs). Logistic regression models indicated that a one unit increase in housing quality score (mean 0.03, range -2.56 to 2.05) was associated with up to 15% reduced odds of disability (any ADLs: OR = 0.88, 95% CI = 0.84-0.92; 2 any IADLs: OR = 0.87, 95% CI = 0.83-0.91; any FLs: OR = 0.85, 95% CI = 0.82 to 0.88) after accounting for housing type and ownership, and demographic, socioeconomic, health, and regional characteristics. These findings highlight a need for more rigorous research on housing quality and disability among midlife and older adults, and suggest that early and persistent interventions to address housing quality may support adults aging into and with disability and aging in place.

Abstract citation ID: igaf122.394

### LONGITUDINAL ASSOCIATIONS OF HOMEOWNERSHIP STATUS WITH MORTALITY AND GERIATRIC OUTCOMES IN OLDER AMERICANS

Yi Wang, Lucero Paredes, Thomas Gill, and Robert Becher, *Yale University, New Haven, Connecticut, United States*

Homeownership is a key social determinant of health, and its absence has been associated with poor self-rated health and hospitalization among older persons. However, the associations between homeownership status and subsequent mortality and incident geriatric conditions remain understudied among community-living older Americans. Using data from the 2015 cohort of the National Health and Aging Trends Study (NHATS)—a nationally representative study of Medicare beneficiaries aged 65 years or older—we evaluated whether homeownership status is associated with mortality and the incidence of frailty, disability, and dementia over a 5-year follow-up period. Mortality was determined from linked Medicare records, while the geriatric outcomes were determined using annual NHATS surveys. Homeownership status was classified as non-homeowners (renters or those in other housing arrangements) versus homeowners. We applied time-varying Cox proportional hazards models with homeownership status as a time-dependent variable, adjusting for demographic and socioeconomic factors to estimate hazard ratios (HR) and 95% confidence intervals (CI). NHATS survey weights were used to generate nationally representative estimates. Among the 7499 participants (representing 40,728,543 older Americans) in 2015, 26.0% were non-homeowners. Over the 5-year follow-up period, being a non-homeowner was significantly associated with higher mortality (HR = 1.64, 95% CI = 1.50-1.80) and higher incidence of frailty (HR = 1.39; 95% CI = 1.14-1.69), disability (HR = 1.44; 95% CI = 1.23-1.69), and dementia (HR = 1.84, 95% CI = 1.49-2.26). Overall, these findings highlight non-homeownership as an important yet under-recognized risk factor for mortality and geriatric outcomes among community-living older Americans.

Abstract citation ID: igaf122.395

### SIZE MATTERS: AGING IN A RETIREMENT METROPOLIS

Galit Nimrod, *Ben Gurion University of the Negev, Be'er Sheva, HaDarom, Israel*

Despite the abundance of retirement communities in the U.S., none compares to The Villages in Central Florida. Home to nearly 150,000 residents, it is the world's largest retirement community and the only one that can be considered a city for older adults. Based on in-depth interviews with 40 residents and two months of participant observations, this study explored (among other things) why people move there and their perceptions of its unprecedented size. Results indicated that while some residents resent the place's growth and the problems that it causes (e.g., traffic, crowdedness), The Villages' size yields its most attractive qualities: the number and variety of leisure activities, the vast population of like-minded people, and the value for money. Residents perceive a "typical villager" as active, fun-oriented, friendly, down-to-earth, and "young." These characteristics and study participants' identification and appreciation thereof point to a strong "place identity" among the villagers. To some extent, these characteristics are associated with the primary pull factors: The active and fun-oriented attributes are related to the variety of activities, and the down-to-earth casual trait is connected to the value for money. In addition, all qualities, especially the friendliness and the "young" spirit, describe the like-mindedness of the people. As the pull factors are associated with The Villages' size, it may be argued that the villagers' collective place identity indirectly results from the city's size. It is thus suggested that retirement cities may cultivate individual and collective place identity, contributing to residents' sense of belonging and pride.

### SESSION 1380 (AWARD LECTURE)

Abstract citation ID: igaf122.396

### AGHE FACULTY AWARD PRESENTATIONS AND LECTURES

Chair: Tina Newsham

The AGHE Faculty Award Presentations and Lectures will feature an address by the 2025 Distinguished Faculty Award recipient, Kara Dassell, PhD, FGSA and an address by the 2025 Rising Star Early Career Faculty Award recipient, Angela Person, PhD, JD, MSW, MA. The Distinguished Faculty Award recognizes persons whose teaching stands out as exemplary, innovative, of impact, or any combination thereof. The Rising Star Early-Career Faculty Award acknowledges new faculty whose teaching and leadership stand out as influential and innovative.

Abstract citation ID: igaf122.397

### TEACHING AS A CATALYST: DRIVING CHANGE THROUGH EDUCATION

Kara Dassel, *University of Utah, Salt Lake City, Utah, United States*

When we hear the word "teaching," many of us picture traditional classroom instruction. However, education reaches far beyond the walls of the classroom and takes on many forms across settings and disciplines. This lecture explores the diverse and dynamic nature of teaching, highlighting how meaningful educational experiences unfold within healthcare and community-based environments, through the design and implementation of interprofessional programs, through mentoring relationships with individual students, and, of course, within the classroom itself. Drawing on examples from practice, this talk will illustrate how teaching can be a collaborative, adaptive, and deeply impactful process. Whether guiding a team in a clinical setting, shaping curriculum across professional boundaries, or mentoring a student one-on-one, educators have the power to influence learning in transformative ways. Attendees will be invited to reflect on their own teaching roles and consider how education can be leveraged to create broader, lasting impact across multiple contexts.

Abstract citation ID: igaf122.398

### TEACHING HOPE, AGENCY, AND POSSIBILITIES IN AN ERA OF RADICAL RESTRUCTURING

Angela Perone, *University of California, Berkeley, Berkeley, California, United States*

The current political, social, and economic moment has brought many new challenges for instructors, students, and the communities we serve. Emerging research suggests parallels with the COVID-19 pandemic and prior historical moments of government retrenchment and overreach. Mental health and economic needs are growing--as are health disparities that have continued to plague the United States and beyond. Higher education is being transformed before our very eyes. And not necessarily always for the better. Despite all these challenges, we as instructors have opportunities to teach our students how to navigate this difficult moment and give ourselves grace to practice (and sometimes fail) in this endeavor. This lecture will provide an overview of case examples, lessons learned, and potential models for teaching hope, agency, and possibilities to support students, colleagues, and ourselves amidst a radical restructuring in higher education and many social, health, and political systems that we study and teach about to our students.

### SESSION 1385 (PAPER)

### LONG-TERM SERVICES AND SUPPORTS: NURSING HOME CARE

Abstract citation ID: igaf122.399

### COMPANION DOG IN NURSING HOME: A STUDY ON MECHANISMS OF EFFICACY AND IMPLEMENTATION

Bobo Hi Po Lau[1], David Cheung[2], Stephanie Law[3], Miu Yee Lee[4], Luis Miguel Dos Santos[5], Joe Tsz Kin Ngai[5], and Sandrine Man-chi Chung[5], *1. Hong Kong Shue Yan University, North Point, China (People's Republic), 2. Hong Kong Seeing Eye Dogs Services, Ta Kwu Ling, Hong Kong, China (People's Republic), 3. Cultural Homes, Tsuen Wan, Hong Kong, China (People's Republic), 4. Caritas Li Ka Shing Care & Attention Home, Tuen Mun, Hong Kong, China (People's Republic), 5. Hong Kong Shue Yan University, North Point, Hong Kong, China (People's Republic)*

Research has shown that human-animal interactions help older adults cope with emotional difficulties and foster social interactions. However, seldom have studies elaborated how a resident dog is engaged in a nursing home to contribute to the well-being of the residents, as well as the contextual factors that lead to practices which benefit both humans and animals. This study employed a multi-stake holder, qualitative methodology to investigate the efficacy and implementation of a 'Companion dog' program in two nursing homes in Hong Kong. Four focus groups were held with the staff and ten individual interviews were conducted with the residents. Our analysis revealed that human-dog bonding developed spontaneously during bed-side visits and group activities, and caused better mood, higher morale, fewer disruptive behaviors, and more social interactions among the residents. Some residents showed initiative and even engaged in taking care of the dogs. While the resident dogs did not significantly increase staff workload, adequate manpower, a large premises with outdoor recreational area, commitment of the staff to animal welfare, and receptiveness of the families were all vital for the success of the program. Additionally, close collaboration with the dog trainer is pivotal for ensuring the well-being of the dogs. Despite the success of the four-month program, post-program evaluation revealed the need for the resident dogs to break before continuing their tenure. A combination of a resident dog program with intermittent visits from other therapy dogs may enhance animal well-being and the therapeutic effectiveness of the dog-assisted activities.

Abstract citation ID: igaf122.400

### SHORT AND LONG-TERM NURSING HOME USE IN OLDER MEDICAID-FUNDED HOME AND COMMUNITY-BASED SERVICE RECIPIENTS

Richard Fortinsky[1], Julie Robison[1], James Grady[2], David Steffens[1], Chae Man Lee[1], Kristin Baker[1], and Deborah Migneault[1], *1. University of Connecticut, Farmington, Connecticut, United States, 2. University of Connecticut School of Medicine, Farmington, Connecticut, United States*

Medicaid-funded home and community-based service (HCBS) programs for older adults are receiving increasing attention from policymakers and researchers. While HCBS programs are intended to delay or avoid nursing home (NH) admissions, little is known about patterns of short-term and long-term NH admissions, or about factors that confer greater risk for NH admission, among HCBS recipients. We determined predictors of both types of NH admissions in a statewide population of HCBS recipients aged >65, classifying predictors as predisposing, enabling, and need variables based on the Andersen framework of health service use. We constructed a study cohort of HCBS recipients who received a standardized clinical assessment in 2022, which provided predictor data. Medicaid and Medicare claims data provided NH admission data for 12 months after the assessment date. Multinomial regression analysis compared predictors among non-NH users, short-term, and long-term users. The study cohort (n = 6,040) was 75.4% female, with mean(sd) age=80.4(8.9); 49.5% non-Hispanic white, 29.8% Hispanic, and 14.7% non-Hispanic Black individuals; 12.3% experienced short-term and 8.7% long-term NH admissions. Compared to non-NH users, short-term NH users were more likely to be > 85 years old (relative risk ratio (RRR)=1.51) have >6 comorbidities (RRR=1.76), and less likely to be Hispanic individuals (RRR=0.49; all p < 0.001). Long-term NH users were more likely to have dementia (RRR=1.33) and severe levels of ADL disability (RRR=2.0), and less likely to be Hispanic (RRR=0.29) or non-Hispanic Black individuals (RRR=0.68; all p < 0.01). Findings will enable improved targeting opportunities of HCBS recipients at greater risk for NH admission for policy makers and providers.

Abstract citation ID: igaf122.401

### THE USE OF MENTAL HEALTH SERVICES BY DUALLY-ELIGIBLE NURSING HOME RESIDENTS

Howard Degenholtz, Michael Sharbaugh, Lingshu Xue, Paige Semple, Jie Li, and Keri Kastner, *University of Pittsburgh, Pittsburgh, Pennsylvania, United States*

Previous research on mental health service among nursing home residents has documented high rates of psychoactive medication use and increasing prevalence of people with severe mental illness (SMI). This is the first study to examine how the delivery of mental health services has changed to meet the needs of this population. We merged Medicaid and Medicare claims for mental health services visits for dually-eligible nursing home residents in Pennsylvania from 2016 to 2022 provided by mental health specialists (e.g., psychiatrists, psychologists, social workers) and non-specialists (e.g., internal medicine, family medicine, advance practitioners) with nursing home minimum data set assessments. From 2016 to 2022, the total census declined by 24% (67,229 to 51,370) residents, however the dual-eligible population dropped by 46% (45,164 to 24,291). While the prevalence of schizophrenia, bipolar disorder and depression increased, Alzheimer's Disease and Related Dementia declined. The proportion of residents with any mental health visit increased from 25% to 37%, mainly due to doubling of visits by non-mental health specialists (from 6% to 14%). Mental health visits increased faster in nursing homes with a low prevalence of SMI (< 8%), from 15.09% to 30.49% compared to nursing homes with high prevalence of SMI ( > =21%) from 16% to 15%. There has been a long-term shift in the type of people living in US nursing homes. We found important shifts in process of care for this population that raise important questions about the quality of mental health care provided by non-specialists in the nursing home setting.

Abstract citation ID: igaf122.402

### TRENDS AND CHARACTERISTICS OF NURSING HOME OBESITY RATES, 2011–2021

Hye-Young Jung[1], Hyunkyung Yun[2], and Jihoon Park[3], *1. Joan and Sanford I. Weill College of Medicine, Cornell University, New York, New York, United States, 2. Brown University, Providence, Rhode Island, United States, 3. McDaniel College, Providence, Rhode Island, United States*

Nursing home (NH) residents have historically had high rates of obesity. However, recent trends and NH factors related to them are understudied. We described national trends in obesity rates among NH residents from 2011 to 2021 and examined facility characteristics associated with them in 2021. NH facility characteristics were obtained from LTCFocus. Obesity rates were defined as the proportion of residents with body mass index ≥ 35. The sample included 16,712 NHs in 49 states. Resident obesity rates increased from 26.5% in 2011 to 30.5% in 2021, a 15% relative increase, with substantial geographic variation. All states experienced increases with Idaho exhibiting the largest increase (11.8 percentage points (pp); from 29.1% to 40.9%) and Louisiana experiencing the smallest (0.5 pp; from 28.4% to 28.9%). Compared to NHs in the lowest quartile of obesity rates, residents of those in the highest quartile were younger on average (74.9 vs. 78.0; p < 0.001), had a lower proportion of non-Hispanic White residents (19.0% vs. 30.5%; p < 0.001), and a higher share of Medicaid-covered residents (65.3% vs. 61.9%; p < 0.001). They also exhibited lower occupancy rates (72.1% vs. 77.8%; p < 0.001), were more likely to be part of a multi-facility chain (65.1% vs. 53.1%; p < 0.001), and to be in a rural area (37.5% vs. 14.2%: p < 0.001). Our findings indicate continued increases in obesity rates among NH residents with wide geographic variation, and potential disparities associated with race/ethnicity, rurality, and income. Future research is warranted to examine the relationship between NH characteristics and care quality for residents with obesity.

### SESSION 1387 (BIOLOGICAL SCIENCES INVITED SYMPOSIUM)

Abstract citation ID: igaf122.403

**TESTING THE GEROSCIENCE HYPOTHESIS**

Chair: Sara Espinoza

Aging is the most significant risk factor for many chronic diseases and geriatric syndromes. The geroscience hypothesis posits that by targeting underlying cellular and molecular mechanisms of aging, it is possible to delay age-related diseases and thereby extend healthy years of life spent free of multimorbidity, disability, and frailty (healthspan). Towards this end, and informed by scientific discoveries in the biology of aging field, translational studies are ongoing to test the geroscience hypothesis by examining potential gerotherapeutics to extend healthspan. This session focuses on recent research to test the geroscience hypothesis, including studies of rapamycin and novel agents in preclinical models and human studies, including clinical trials.

Abstract citation ID: igaf122.404

**TESTING THE GEROSCIENCE HYPOTHESIS IN LOWER URINARY TRACT SYMPTOMS OF AGING**

Iman Al-Naggar, *UConn Health, Farmington, Connecticut, United States*

Aging and the metabolic syndrome—a condition that accelerates biological aging—are both major risk factors for lower urinary tract symptoms (LUTS). LUTS, including urinary frequency, urgency, incontinence, and nocturia, substantially reduce quality of life and contribute to depression, social isolation, falls, fractures, and loss of independence in older adults. Dysfunction involving the brain-to-bladder axis presents as declines in physiological resilience, and existing therapies such as anticholinergics targeting detrusor smooth muscle provide limited benefit and are often discontinued due to side effects. Recent evidence implicates fundamental biological mechanisms of aging—chronic inflammation, mitochondrial dysfunction, and oxidative stress—in the pathogenesis of metabolic syndrome–associated LUTS. Preclinical studies in mouse models of aging and metabolic syndrome demonstrate increased bladder dysfunction, supporting these mechanisms as potential therapeutic targets. This presentation will describe the rationale and design of an ongoing pilot clinical trial testing MitoQ, a mitochondria-targeted antioxidant with gerotherapeutic properties, for ability to alleviate LUTS in older women with metabolic syndrome. By moving from animal models to human trials, this work aims to test whether targeting hallmarks of aging can modify urologic symptoms. The talk will also highlight key design considerations and practical challenges in translating the geroscience hypothesis into clinical research.

Abstract citation ID: igaf122.405

**STAGE-DEPENDENT EFFECTS OF RAPAMYCIN FOR ALZHEIMER'S DISEASE: INSIGHTS FROM PREVENTION AND TREATMENT TRIALS,**

Mitzi Gonzales[1], and Ai-Lin Ling[2], *1. Cedars-Sinai Medical Center, Los Angeles, California, United States, 2. University of Missouri, Columbia, Missouri, United States*

Rapamycin is the best-established longevity-promoting drug in animal models and has been shown to attenuate Alzheimer's disease pathology in preclinical trials. Here we compare the findings of a prevention versus therapeutic clinical trial of rapamycin for Alzheimer's disease and discuss implications for applying the geroscience hypothesis to Alzheimer's disease. Two open-label pilot studies evaluated the safety and preliminary efficacy of short-term (4-12 week) low dose (1 mg/day) rapamycin treatment. The prevention trial enrolled cognitively unimpaired middle-aged APOE4 carriers and non-carriers, whereas the therapeutic trial enrolled older adults with mild cognitive impairment or dementia. Both studies examined changes in plasma inflammatory markers. The prevention study also examined cerebral blood flow, and the therapeutic study assessed Alzheimer's disease biomarkers in cerebrospinal fluid (CSF). Baseline to post-treatment changes were evaluated using paired samples t-tests or Wilcoxon signed rank tests. The prevention study enrolled 9 APOE4 carriers (mean age 53 ± 8 years, 44% female) and 14 APOE-4 non-carriers (mean age 55 ± 6 years, 50% female). APOE4 carriers demonstrated reduced plasma cytokines and increased cerebral blood flow at post-treatment (all $p < 0.05$) that were not observed in non-carriers. In contrast, participants in the therapeutic trial displayed increased plasma cytokines and CSF phosphorylated tau 181, neurofilament light, and glial fibrillary acidic protein levels (FDR-corrected p-value<0.05). Overall, the results of the two trials suggest that rapamycin may have markedly divergent effects on inflammatory and Alzheimer's disease biomarkers when administered for prevention versus treatment, highlighting the importance of tailoring geroscience-informed interventions for the appropriate disease stage.

Abstract citation ID: igaf122.406

**TESTING THE TRANSLATIONAL POTENTIAL OF RAPAMYCIN ON HEALTHY AGING**

Adam Konopka, *University of Wisconsin-Madison*

Treatment with rapamycin, an inhibitor of the mechanistic Target Of Rapamycin Complex One (mTORC1) protein kinase, has been repeatedly demonstrated to extend lifespan and prevent or delay several age-related diseases in diverse model systems. Concerns over the risk of adverse side effects, including immunosuppression and metabolic disruptions, have cautiously limited the clinical translation of rapamycin and its analogs as a treatment for aging associated conditions. Despite potential risks, an increasing number of physically active adults are taking the mTOR inhibitor rapamycin off label with the goal of extending healthspan. A working model suggests that while inhibition of mTORC1 promotes healthy aging, many of the negative side effects of rapamycin are associated with inhibition of a second mTOR complex, mTORC2. Differences in the kinetics and molecular mechanisms by which rapamycin inhibits mTORC1 and mTORC2 suggest that a therapeutic window for rapamycin could be exploited using intermittent dosing schedules or alternative rapalogs that may enable more selective inhibition of mTORC1. However, the optimal dosing schedules and the long-term efficacy of such interventions in humans is unknown. Further, it remains unknown how different rapamycin dosing schedules impact the health benefits of exercise. Here, I will provide the rationale, experimental design, and updates for two ongoing human clinical trials aimed to determine the safety, pharmacokinetics, pharmacodynamics, and efficacy of rapamycin and rapalogs on several clinically oriented outcomes. I will also share new insights from integrated multi-omics studies that reveal the impact of different rapamycin dosing schedules on the molecular and metabolic adaptations to exercise in older mice. Results from these pre-clinical and early phase studies will help guide the design of future clinical trials to determine whether rapamycin can be used safely to inhibit mTORC1 for the treatment and prevention of age-related diseases in humans.

### SESSION 1390 (SYMPOSIUM)

Abstract citation ID: igaf122.407

### ESPO AND ACADEMY FOR GERONTOLOGY IN HIGHER EDUCATION SECTION SYMPOSIUM: GERONTOLOGICAL EDUCATION AS AN INTERDISCIPLINARY PURSUIT

Chair: Abigail Stephan
Co-Chair: Erta Cenko
Discussant: Aimee Fox

As professionals across the globe recognize the critical need to support our aging population, training opportunities that allow students to experience the inherent interdisciplinarity of gerontology and geriatrics will be in high demand. Accordingly, this session explores innovative approaches for integrating gerontological concepts within educational experiences for students and early career professionals. We highlight collaborations among gerontology and geriatrics faculty and professionals from diverse fields to enhance student learning around aging. Presenters will share examples of successful partnerships and discuss challenges and opportunities in interdisciplinary gerontological education. In the first presentation, Cenko and Rani explore technology as a tool to incorporate gerontological concepts within health science education. Newsham and colleagues will then share about an innovative interdisciplinary collaboration between faculty and students exploring end-of-life and advance care planning. The third presentation centers the work of Carpenter and Aguilo, which examines an immersive visual arts program bringing together students and older artists. Stephan and colleagues will highlight a collaboration with landscape architecture and its impact on students' perceptions of aging in our fourth presentation. Finally, Mancini will share insights from mentoring across the career spectrum. By showcasing how aging-related topics intersect with fields such as healthcare, social sciences, education, technology and engineering, and the arts, this session emphasizes the importance of interdisciplinary approaches in preparing students to address the complexities of an older society. Attendees will gain insights into practical strategies for expanding gerontological education across disciplines and fostering meaningful connections between aging studies and other fields.

Abstract citation ID: igaf122.408

### DESIGNING FOR AGE-INCLUSIVITY: A CROSS-DISCIPLINARY APPROACH TO LANDSCAPE ARCHITECTURE EDUCATION

Abigail Stephan, Thomas Schurch, and Cordelia Wayt,
*Clemson University, Clemson, South Carolina, United States*

As society's proportion of older adults increases, professionals across disciplines must be aware of the opportunities and challenges associated with an aging population. Gerontology intersects with landscape architecture to promote cognitive, physical, and psychological well-being across the lifespan by enhancing access to nature. To prepare landscape architects-in-training to consider the impact of an aging population on their profession, our team developed a semester-long community design studio for second-year undergraduate landscape architecture majors to craft design plans for a local (1) 55+ townhome community and (2) residential care facility. Students engaged in project-based, experiential learning with scaffolded support that drew on the expertise of faculty, staff, and practitioners across disciplines (e.g., landscape architecture, gerontology, horticulture, environmental psychology, non-profit leadership). We conducted a mixed methods investigation to explore students' (N = 10) experience in the studio and determine its influence on perceptions of aging, rated nature exposure importance across the lifespan, and understanding of their future career's role in meeting societal challenges. Pre- and post-semester survey results revealed no statistically significant changes in students' aging perceptions and perceived nature exposure importance. However, students' accounts from focus group discussions suggest students enjoyed engaging with older adults and this experience was meaningful in advancing their understanding of the ways in which landscape architects play a critical role in shaping more nature-rich, age-friendly environments. Taken together, our findings demonstrate that the community design studio experience was valuable for students and led to a more nuanced perspective of the interdisciplinary approach required to successfully serve an aging society.

Abstract citation ID: igaf122.409

### REFRAMING AGING THROUGH AI-GENERATED PATIENT SIMULATIONS IN PRE-MEDICAL EDUCATION

Erta Cenko, and Neha Rani, *University of Florida, Gainesville, Florida, United States*

As future healthcare providers, pre-medical students' perceptions of aging influence how they will interact with older patients. However, misconceptions and implicit biases about aging persist in medical education, often reinforcing stereotypes. This study explores the use of AI-generated videos as an innovative educational tool to help undergraduate health sciences and pre-medical students reframe their understanding of aging. The study follows a three-phase design. First, students complete a survey assessing their baseline perceptions of aging and older adults. Next, they engage with AI-generated video simulations that depict aging patients with diverse health conditions and life experiences. Afterwards, students complete a follow-up survey to measure shifts in their attitudes. In the final phase, students upload their own photos into an AI system that generates "aged" versions of themselves. These personalized avatars are then integrated into additional patient simulations, allowing students to see themselves as older individuals and reflect on their potential aging experiences. By integrating AI-driven experiential learning, this project aims to increase students' empathy and challenge ageist assumptions in medical education. Preliminary findings will explore changes in students' perspectives before and after the intervention, highlighting AI's potential to transform gerontology education in the medical sciences. This approach supports gerontological education across fields, helping create a more age-inclusive healthcare workforce.

Abstract citation ID: igaf122.410

### INTERDISCIPLINARY COLLABORATION TO TEACH ABOUT END-OF-LIFE ISSUES AND ADVANCE CARE PLANNING

Tina Newsham, Alissa Dark-Freudeman,
Elizabeth Fugate-Whitlock, and Jamy Chulak, *The University of North Carolina at Wilmington, Wilmington, North Carolina, United States*

Topics related to end-of-life (EOL) and advance care planning (ACP) can be challenging as they may activate negative assumptions and experiences for students and instructors. Further complicating teaching about these topics, they can (and, we argue, should) be addressed through multiple disciplinary lenses, and ideally in an interdisciplinary fashion. In this paper, we share an interdisciplinary effort to teach about EOL and ACP at the University of North Carolina Wilmington. Faculty in Psychology, Gerontology, Philosophy, and Respiratory Therapy collaborated to create an interdisciplinary module that was integrated into one class from each participating discipline. Students were grouped across disciplines and worked together to create content for a Virtually Inclusive Patient online module that can be used in a variety of courses. Students completed guided reflections regarding what they learned about and from students with different disciplinary perspectives and how the interdisciplinary collaboration resulted in outcomes that would otherwise have been absent in a singular-discipline effort. We highlight here the interdisciplinary nature of the collaboration between the faculty leading this effort as well as that of the students participating in this project.

Abstract citation ID: igaf122.411

### BRIDGING GERONTOLOGY & THE VISUAL ARTS: A MULTICOMPONENT PROGRAM WITH UNDERGRADUATE STUDENTS

Brian Carpenter[1], and Maria Elisa Aguilo[2], *1. Washington University, St. Louis, Missouri, United States, 2. Washington University in St. Louis, St. Louis, Missouri, United States*

This session describes a program that bridged gerontology and the visual arts by exposing undergraduate students to older artists and their creative output. The program was one component in a semester-long, introductory interdisciplinary course on aging for first-year students. The program consisted of several elements, including an introductory lecture on creativity across the lifespan; a panel discussion in class with three older professional artists; a visit to a curated museum exhibition featuring works created later in life by Picasso, Miro, Chagall, and Calder, among others; and an assignment that asked students to research a visual artist and reflect on their creativity over their career. A rigorous program evaluation documented the program's impact on the students. As examples of findings, among the 73 students who participated, their perception of the age at which creativity peaks rose following each component of the program (from 33.8 to 44.5 years old, $F(2,59) = 8.59$, $p < .001$), and their rating of the extent to which older adults were "innovative" increased, $F(2,60) = 10.82$, $p < .001$. We describe each component of the program, our perceptions of how they shifted student attitudes, and information regarding resources need to replicate the program, in whole or in part. Overall, this program is an innovative melding of gerontology and the visual arts with the potential to challenge ageist beliefs and spotlight the creative potential of older artists.

Abstract citation ID: igaf122.412

### GERONTOLOGY MENTORING ACROSS THE CAREER SPECTRUM: BUILDING AN INTERDISCIPLINARY PIPELINE FOR THE FUTURE

Todd Manini, *University of Florida, Gainesville, Florida, United States*

Mentorship is a critical component of professional development in gerontology, shaping career trajectories from student training to senior leadership. As an inherently interdisciplinary field, gerontology requires mentorship models that span diverse disciplines, professional roles, and career stages. This presentation will explore how structured and informal mentorship can foster interdisciplinary collaboration, support career transitions, and sustain a strong pipeline of gerontology professionals. Drawing from real-world examples and different mentorship philosophies, the presentation will address key strategies for effective mentoring. Special attention will be given to the role of mentorship in attracting practitioners to the field, giving opportunities to early-career professionals to gerontology, and preparing mid-career scholars to serve as mentors themselves. Additionally, the presentation will discuss best practices for mentoring in a remote or hybrid environment, particularly relevant in the post-pandemic landscape of higher education and research. The session will conclude with a discussion on institutional strategies to promote mentorship cultures, including structured programs, and cross-disciplinary collaborations. Attendees will leave with actionable insights on how to enhance mentoring efforts within their own institutions and disciplines, ensuring that the next generation of gerontologists is equipped to address the complex challenges of aging in an interdisciplinary and collaborative manner.

### SESSION 1395 (PAPER)

### ALZHEIMER'S DISEASE AND OTHER RELATED DEMENTIAS: INNOVATION AND INTEGRATION

Abstract citation ID: igaf122.413

### SUCCESSES AND CHALLENGES OF SUSTAINING COMMUNITY-BASED PROGRAMS FOR PEOPLE WHO LIVE ALONE WITH DEMENTIA

Kate Gordon[1], Yoon Chung Kim[2], Lirisha Tuladhar[3], Heather Menne[3], and Michael Lepore[4], *1. Splaine Consulting, Columbia, Maryland, United States, 2. University of Maryland School of Medicine, Baltimore, Maryland, United States, 3. Miami University, Oxford, Ohio, United States, 4. University of Massachusetts Amherst, Amherst, Massachusetts, United States*

Approximately a quarter of older adults with dementia or cognitive impairment live alone in the US. To address the needs of people living alone with dementia (PLAWD), the Administration for Community Living requires PLAWD-focused services in its Alzheimer's Disease Program Initiative (ADPI) to address service gaps and improve care delivery for PLAWD. We examined 59 ADPI grants to determine the types of services delivered, and successes and challenges faced in service delivery. Grantees provided care management and/or delivered services such as companionship and support for socialization through buddy programs, phone calls, social and wellness programming, and regular home visits. This study explores the post-grant period experiences of community-based organizations that received federal funding. The study team conducted semi-structured, online interviews with 13 of the 59 grant program directors representing multiple regions of the US. Interviews were audio/video-recorded, transcribed, and manually coded by team members using an inductive approach to identify key themes. Findings highlight the factors that impacted the long-term sustainability of PLAWD-focused services after federal funding ceased. Grantees implemented strategies to maintain and expand services including modifying services, partnering with local service providers and leveraging existing relationships. Findings demonstrate that a variety of services have been successfully sustained for PLAWD. However, common challenges are experienced in sustaining service delivery after initial grant funding ends. PLAWD often lack local care partners and need specialized services and support, including care management, care monitoring, and socialization. Different approaches used and challenges faced in sustaining grant-initiated community programs for PLAWD are discussed.

Abstract citation ID: igaf122.414

### FACILITATORS OF PERSON-CENTERED DEMENTIA CARE STRATEGIES: INSIGHTS FROM STAFF AND LEADERSHIP

Yoon Chung Kim[1], Nancy Kusmaul[2], Sarah Holmes[3], Michael Lepore[4], Jing Wang[5], Laura Davie[5], Briana Murray[4], and Kirsten Corazzini[5], *1. University of Maryland School of Medicine, Baltimore, Maryland, United States, 2. University of Maryland, Baltimore County, Baltimore, Maryland, United States, 3. University of Maryland School of Nursing, Baltimore, Maryland, United States, 4. University of Massachusetts Amherst, Amherst, Massachusetts, United States, 5. University of New Hampshire, Durham, New Hampshire, United States*

Person-centered dementia care (PCDC) strategies improve the well-being of residents living with dementia (RLWD) in long-term care (LTC). We explored facilitators to PCDC strategies in low-resource LTC settings. Semi-structured interviews occurred June 2023 - February 2024 to explore stakeholder perspectives in low-resource settings. Participating facilities were two nursing homes and two assisted livings in US Census Bureau-designated medically underserved areas. Interviews were audio recorded and transcribed using NVivo 14.0. Coauthors coded the interviews guided by the Values, Individualized approach, Perspective of PLWD, and Social environment model. Participants were 20 direct care staff and 7 administrators, with between 3 months and 27 years' experience. Facilitators of PCDC were identified at multiple levels, highlighting opportunities to strengthen supportive practices. Organizational facilitators included adequate staffing, information sharing systems, regular dementia education, positive work culture, staff empowerment, and flexibility in care decisions. Interpersonal facilitators based on the LTC setting social environment included strong teamwork, inter-staff communication, good relationships with care partners, and trust/rapport with residents. Individual facilitators were based on individual staff values associated with delivering PCDC, e.g., positive attitudes about care work and motivation for additional dementia education. Given the well-documented benefits of PCDC, identifying and cultivating these facilitators, particularly in resource-limited settings, is critical to enhancing the well-being of RLWD. Although one identified facilitator is adequate staffing, low-resource settings can support PCDC through effective data sharing, regular training, and fostering teamwork between staff, RLWD, and care partners. Future research should identify factors that foster employees' positive attitudes and motivation for additional training.

Abstract citation ID: igaf122.415

### INNOVATIVE STRATEGIES FOR EXPANDING DEMENTIA EDUCATION: LEVERAGING GRANT FUNDING AND PARTNERSHIPS FOR ENGAGEMENT

Ashley Haas, Jessica Bibbo, and Megan Huth, *Benjamin Rose Institute on Aging, Cleveland, Ohio, United States*

This session will present how the organizational grant partners successfully leveraged an Administration for Community Living (ACL) Alzheimer's Disease Programs Initiative (ADPI) grant to expand dementia education to the public. The three-year grant focused on serving urban and rural communities within the state of Ohio. A dementia education webinar series was developed to engage community members. The series delivered 24 sessions, reaching over 2,782 community members, including professionals, caregivers, and individuals living with dementia. The project saw substantial growth each year, marked by increases in both registration and attendance. The average number of registrants rose from M = 141 (SD = 40.07) in Year 1 to M = 235 (SD = 89.68) in Year 3, while average attendance increased from M = 84 (SD = 34.36) in Year 1 to M = 130 (SD = 59.97) in Year 3. Initially focused on a specific geographic area in Ohio, the project expanded its reach nationwide and internationally by employing innovative engagement strategies, reaching participants from all 50 states, Washington, DC, and five additional countries. The national reach grew significantly each year, with an average increase in national participants from M = 41 (SD = 17.21) in Year 1 to M = 85 (SD = 45.02) in Year 3. This session will highlight how the strategic use of grant funding and collaborative partnerships can enhance outreach and foster impactful engagement.

Abstract citation ID: igaf122.416

### KAER I/DD AND GUIDE: CONVERGENCE TO INCREASE ALIGNMENT OF BENEFICIARIES WITH INTELLECTUAL DISABILITIES

Matthew Janicki[1], Kathryn Service[2], Jennifer Pettis[3], and Lisa McGuire[4], *1. University of Illinois Chicago, Rockport, Maine, United States, 2. Rockport, Maine, United States, 3. Gerontological Society of America, Washington, District of Columbia, United States, 4. Gerontological Society of America, Atlanta, Georgia, United States*

Background: Adults with intellectual disabilities (ID) face significant barriers accessing dementia care, particularly in marginalized communities. To address these disparities, the National-Task-Group-on-Intellectual-Disabilities-and-Dementia-Practices (NTG) developed a grant-funded educational initiative linked to the Centers-for-Medicare-&-Medicaid's Guiding-an-Improved-Dementia-Experience (GUIDE) Model to: (1) equip care navigators and practitioners with ID-specific knowledge and skills, and (2) increase rates of accommodation of Medicare beneficiaries with ID and dementia within GUIDE Participant programs. This initiative corresponded NTG technical expertise with the Gerontological-Society-of-America's (GSA) Addressing-Brain-Health-in-Adults-with-Intellectual-and-Developmental-Disabilities: A Companion to the GSA KAER-Toolkit-for-Brain-Health (KAER ID/D), an adaptation of the KAER framework designed to improve dementia screening, diagnosis, and care planning for individuals with ID and other developmental disabilities. Method: As GUIDE participant sites launched in 2024 and 2025, the NTG created specialized training materials and technical assistance resources to enhance provider capabilities to enroll more beneficiaries with ID. Training modules covered three core areas: (1) information on ID (and related conditions) and dementia care planning, (2) caregiving histories and family dynamics in ID populations, and (3) connections with ID-focused service providers. Standardized, scalable education was delivered through learning management system training packs for GUIDE navigators and an online seminar series on dementia screening and diagnostics for practitioners. Outcomes: By integrating KAER ID/D principles, NTG ensured that GUIDE

program personnel, particularly navigators, gained essential competencies in ID and dementia care. This training initiative enhanced the capacity of GUIDE agencies to provide equitable, informed, and effective support for adults with ID, ultimately increasing participation and improving care outcomes within this underserved population.

### SESSION 1400 (SYMPOSIUM)

Abstract citation ID: igaf122.417

### INNOVATIONS TO DEMENTIA CARE: ADVANCING EQUITY, COMMUNICATION, AND PREPAREDNESS ACROSS DIVERSE CONTEXTS

Chair: Jing Wang
Co-Chair: Eunjung Ko

As dementia care evolves to meet the needs of a growing and diverse aging population, innovative strategies are required to improve accessibility, cultural responsiveness, communication, and preparedness. This symposium brings together global perspectives to explore how dementia care can be optimized across different settings and populations. The first presentation provides an updated scoping review of rural palliative dementia care, identifying challenges and strategies to improve person-centered support in resource-limited areas. The second presentation examines culturally adapted interventions for Chinese and Korean American caregivers, showcasing strategies used in the New York University Caregiver Intervention—Enhanced Support (NYUCI-ES) to enhance engagement, support, and care outcomes. The third presentation explores Augmentative and Alternative Communication (AAC) in dementia care, synthesizing research on communication aids and identifying critical gaps in integration and real-world application. The final presentation focuses on disaster preparedness among Chinese American family caregivers, analyzing how caregiving-related characteristics, cultural beliefs, and cognitive appraisals influence emergency planning. This symposium highlights the need for holistic, person-centered, and contextually relevant dementia care models. By addressing rural access barriers, cultural tailoring, communication challenges, and emergency preparedness, these studies provide valuable insights into advancing dementia care across diverse settings. Attendees will gain a deeper understanding of how interdisciplinary, community-driven, and culturally sensitive approaches can enhance dementia caregiving worldwide.

Abstract citation ID: igaf122.418

### ADVANCING RURAL PALLIATIVE DEMENTIA CARE: AN UPDATED SCOPING REVIEW

Jing Wang[1], Christine Ritchie[2], and Kirsten Corazzini[1], *1. University of New Hampshire, Durham, New Hampshire, United States, 2. Massachusetts General Hospital, Boston, Massachusetts, United States*

Palliative and end-of-life care for people living with dementia in rural areas remains a significant challenge due to geographic isolation, specialized workforce shortages, financial barriers, and gaps in culturally sensitive care models. This study updates Elliot et al.'s scoping review by assessing recent evidence on rural palliative dementia care to identify stakeholders' needs and explore emerging approaches and their impact. Following Arksey and O'Malley's scoping review framework, we conducted a systematic search of PubMed, EMBASE, CINAHL, PsycINFO, Web of Science, Cochrane Library, and ProQuest for studies published from November 2018 to the present. A total of 2,592 studies were identified, with 234 full-text articles screened and 48 studies included. Compared to the previous review, which primarily identified systemic barriers and policy gaps, this update reveals a growing focus on technology-enabled interventions such as telehealth, remote monitoring, and virtual peer support programs. However, digital literacy and infrastructure limitations hinder widespread implementation. Studies continue to highlight persistent challenges in accessing dementia-specific palliative services due to provider shortages, transportation barriers, and financial constraints. Caregiver burden remains a major concern, with rural caregivers facing increased emotional and physical strain due to limited respite care and professional support. Additionally, there is a critical need for community-driven solutions to improve rural dementia palliative care. Despite progress, critical gaps remain in integrating sustainable telehealth, expanding caregiver support, and developing community-responsive care models. Future research should prioritize leveraging community networks, home-based care models, and digital health innovations to enhance equity in rural dementia care.

Abstract citation ID: igaf122.419

### CULTURAL ADAPTATION OF A VALIDATED INTERVENTION FOR CHINESE AND KOREAN AMERICAN DEMENTIA CAREGIVERS

Eunjung Ko[1], Bei Wu[2], Yaolin Pei[3], Xiang Qi[1], I tek Leong[1], Weiyu Mao[4], Jing Wang[5], and Mary Mittelman[6], *1. New York University, New York, New York, United States, 2. NYU Shanghai, Shanghai, China (People's Republic), 3. The University of Texas at Austin, Austin, Texas, United States, 4. University of Nevada, Reno, Reno, Nevada, United States, 5. University of New Hampshire, Durham, New Hampshire, United States, 6. New York University Grossman School of Medicine, New York, New York, United States*

With the growing diversity of the U.S. population, culturally adapted interventions are essential in dementia caregiving. However, approaches tailored for East Asian cultures remain limited. Based on our ongoing randomized controlled trial of the, "New York University Caregiver Intervention—Enhanced Support (NYUCI-ES)," we identified adaptation strategies for Chinese and Korean cultures and categorized them into five elements: content, context, relationship fidelity to core elements, engagement, and cultural competence. Content adaptations included translating materials into multiple languages, and incorporating culturally relevant terminology, questionnaires, community resources, and social media support groups. For context, we delivered the counseling sessions either in person or online, arranged culturally matched staff, trained staff to provide culturally tailored counseling, and established strong partnerships with Korean and Chinese organizations. Fidelity to the original intervention was maintained through the original developers' active involvement in regular meetings that also include experts in East Asian culture, and the detailed explanation about the NYUCI-ES in participants' languages. Engagement strategies included step-by-step efforts at involving prospective participants, rapport through explanation of staff roles, multiple communications, and participant compensation. For cultural competence, we used a culturally tailored counseling approach, respecting cultural beliefs on counseling, illness, and family, used euphemistic language about dementia, and provided culturally appealing refreshments. Within the NYUCI's structured framework, we adapted content to East Asian culture. These strategies address research gaps and can guide future cultural adaptations of evidence-based interventions to the preferences and needs of members of East Asian communities to improve their physical and emotional health.

Abstract citation ID: igaf122.420

**FAMILY DEMENTIA CAREGIVERS' DISASTER PREPAREDNESS IN CHINESE AMERICAN COMMUNITIES IN NEW YORK CITY**

Weiyu Mao[1], Bei Wu[2], Yaolin Pei[3], and Zhen Cong[4],
*1. University of Nevada, Reno, Reno, Nevada, United States,*
*2. NYU Shanghai, Shanghai, China (People's Republic),*
*3. The University of Texas at Austin, Austin, Texas, United States,*
*4. Chapman University, Orange, California, United States*

Disaster preparedness is a significant public health concern. Disasters manifest in different forms and often lead to disruption of daily life. This is especially challenging for family dementia caregivers and their care recipients in the community. There remains much unknown regarding dementia caregiver disaster preparedness, especially in ethnic and racial communities. Guided by Protection Motivation Theory, with the backdrop of Hurricane Ida, this study explored the association between caregiving-related characteristics, cognitive appraisals, and disaster preparedness among caregivers in Chinese American communities. Data came from a pilot study on Chinese American family dementia caregivers in New York City between November 2021 and June 2022. Purposive sampling was used to recruit caregivers to participate in a survey (online or telephone). Current caregivers (n = 76) were included. The dependent variable was caregiver disaster preparedness, measured by preparedness for Hurricane Ida (prepared or others). From group comparisons, caregivers caring for someone with mild dementia were less likely to report disaster preparedness. Caregivers with higher levels of response efficacy, having an emergency plan in place, and with lower levels of avoidance toward death tended to report disaster preparedness. From logistic regressions, spousal caregivers were 4.46 times more likely to report disaster preparedness. Caregivers with lower levels of avoidance toward death were 1.52 times more likely to report disaster preparedness. Caregivers with higher levels of response efficacy were 2.36 times more likely to report disaster preparedness. This study offers theoretical and practical implications on how to engage and support Chinese American communities in disaster planning.

Abstract citation ID: igaf122.421

**ENHANCING COMMUNICATION IN DEMENTIA CARE: RAISING THE ROLE OF AUGMENTATIVE AND ALTERNATIVE COMMUNICATION (AAC)**

Jing Wang, Chloe Mitchell, Aubrie Woodward, and Kay Chen,
*University of New Hampshire, Durham, New Hampshire, United States*

Effective communication is fundamental to maintaining quality of life and person-centered care for individuals with dementia. However, cognitive decline progressively impairs verbal expression and comprehension, leading to social withdrawal, frustration, and increased caregiver burden. Augmentative and Alternative Communication (AAC) interventions offer promising strategies to support communication in dementia care, yet their integration into routine practice remains limited. This narrative review synthesizes current research on AAC interventions for dementia, identifies key gaps, and outlines future directions to enhance their effectiveness and implementation. We conducted a narrative review of peer-reviewed studies, reports, and guidelines on AAC in dementia care, focusing on intervention types, efficacy, and integration into care practices. Findings indicate that AAC strategies range from using low-tech solutions (e.g., memory books, visual cues, and communication boards) to high-tech system (e.g., speech-generating devices and mobile applications). While research suggests AAC can improve engagement, decision-making, and social participation, its long-term effectiveness and real-world application remain underexplored. Major challenges include the need for personalization based on disease progression, cultural considerations, and scalable caregiver training. Additionally, limited research exists on how AAC can be effectively embedded into standard dementia care workflows. Addressing these gaps is essential for optimizing AAC interventions and ensuring their accessibility and sustainability. Future research should prioritize longitudinal studies to assess sustained impact, strategies for culturally responsive adaptation, and scalable caregiver training models. Advancing AAC in dementia care has the potential to enhance person-centered communication, facilitate shared decision-making, and improve quality of life for individuals with dementia and their caregivers.

### SESSION 1405 (SYMPOSIUM)

Abstract citation ID: igaf122.422

**SOCIAL SCIENCES SECTION OF THE JOURNALS OF GERONTOLOGY SERIES B: FEATURED 2024 EDITOR'S CHOICE ARTICLES**

Chair: Jessica Kelley

Social science inquiry on age, aging, and the life course spans many topics and methodologies. This symposium highlights papers that were selected as Editor's Choice articles in the Social Sciences section of The Journals of Gerontology, Series B: Psychological Sciences and Social Sciences in 2024. These papers highlight methodological innovations, important advancements in our state of knowledge in an area, or emerging issues in the study of aging and older adults. Namkung and Sung apply an age-period-cohort analysis to tracking chronic disease prevalence among older Koreans. Reyes, employs narrative qualitative methods to explore how older African American and Latine immigrants define their civic role and relationship to their new country. Thomeer et al. employ life history data to characterize how childbearing histories impact midlife cognition. Sheftel et al. examine how transitions into loneliness following a range of specific life events may be similar or different across twenty countries. Finally, Brady et al. incorporate features of one's current work experience in impacting mental health when facing parental caregiving.

Abstract citation ID: igaf122.423

**IN THEIR WORDS: AFRICAN AMERICAN AND LATINE IMMIGRANT OLDER ADULTS (RE)DEFINE CIVIC PARTICIPATION**

Laurent Reyes, *University of California, Berkeley, Berkeley, California, United States*

Older adults' civic participation has received considerable attention. However, this literature has understudied the experiences of civic participation among minoritized ethnoracial older adults. Particularly absent from this literature is the contextualization of civic participation as it exists within cultural and historical structures of inequality that influence how these populations understand, participate, and experience civic life. A phenomenological design was used to explore civic participation through participants' experiences and unique perspectives. Thirty-four in-depth, semistructured interviews were conducted with Latine immigrant and Black older adults (ages 60+) living in New Jersey and New York City. A conceptual content analysis was used to identify how older Black and Latine immigrant adults define civic participation for themselves. This study presents 3 new definitions of civic participation, that are derived directly from participants' conceptualization and applied across the lived experiences. Definitions present civic participation as the responsibility of community belonging; as a religious/spiritual practice; and as a way of life. These definitions provide new perspectives by which to study civic participation and challenge current framing of helper and needy, altruism, the voluntary nature of participation, and the separation between social, political, and spiritual participation. Findings from this study contribute to expanding gerontology's ontological imagination of how civic participation is experienced and conceptualized among older Latine immigrants and Black adults. The expertise shared by older African Americans and Latine immigrants lends us important perspectives to develop a critical theoretical framework by which scholars can more accurately study civic participation among this diverse population.

Abstract citation ID: igaf122.424

### THE TREND OF CHRONIC DISEASES AMONG OLDER KOREANS, 2004-2020: AGE-PERIOD-COHORT ANALYSIS

Eun Ha Namkung[1], and Sung Hye Kang[2], *1. Ewha Womans University, Seoul, Republic of Korea, 2. University of California, Los Angeles, Los Angeles, California, United States*

This study aimed to examine age, period, and cohort effects contributing to the prevalence of diabetes and hypertension among older Koreans. Additionally, it sought to investigate how sociodemographic characteristics interact with period and cohort effects to influence the disease prevalence. Using the 2004–2020 data from the National Survey of Older Koreans, a nationally representative sample of older adults aged 65 or older, hierarchical age–period–cohort cross-classified random effects models (HAPC-CCREMs) were employed to estimate separate age, period, and cohort components of the recent trends in diabetes and hypertension. Sociodemographic characteristics were tested for their interactions with period and cohort effects. Significant period effects were observed, indicating a steady increase in the likelihood of being diagnosed with diabetes and hypertension over time. Age effects revealed a quadratic trend, with disease risks generally increasing with age, but the rate of increase diminishing at older ages. Cohort effects exhibited an inverted U-shaped pattern, with higher risks observed in the 1930s and early 1940s cohorts compared to earlier and later cohorts. Gender and educational attainment emerged as significant moderators. Women than men born in the early 1930s exhibited higher risks of diabetes and hypertension, whereas individuals with lower educational attainment showed a steadily increasing risk of hypertension over time. The results underscore the complex interplay of age, period, and cohort effects in shaping disease prevalence among older Koreans. Our findings highlight the importance of considering historical context and sociodemographic factors in understanding disease trends and designing targeted interventions to mitigate health disparities.

Abstract citation ID: igaf122.425

### CHILDBEARING HISTORIES AND MIDLIFE COGNITION: ACCOUNTING FOR EARLY LIFE FACTORS

Mieke Thomeer[1], Rin Reczek[2], Joseph Wolfe[1], Dee Ferguson[1], and Rui Cao[2], *1. The University of Alabama at Birmingham, Birmingham, Alabama, United States, 2. The Ohio State University, Columbus, Ohio, United States*

Childbearing histories—for example, parity and age at first birth—matter for the health outcomes of mid- and later-life women. There is growing evidence that childbearing may shape cognitive outcomes. However, previous research is not definitive due to its inability to account for selection. Selection is critical to address given that multiple factors, including socioeconomic background and cognition in adolescence, place women at risk for both specific childbearing experiences and poor cognitive functioning. We analyze the 1979 National Longitudinal Survey of Youth (NLSY79; $N = 3{,}668$), a longitudinal nationally representative data set from the United States that began collecting data during respondents' late teens and early 20s. We estimate the average treatment effects of childbearing histories on midlife memory and cognition using propensity-score matching techniques that incorporate an expansive set of early life factors. In models adjusting for early life covariates, we find that any births, high parity, and early first birth are associated with worse self-reported memory, but not cognitive functioning scores. After addressing the influence of early life factors through matching techniques, the only robust differences relate to any births and early first births with self-reported memory. Selection into specific childbearing experiences partially drives the link between childbearing and cognition at midlife, with cascading implications into later life. We suggest incorporating childbearing and early life factors into models to identify social determinants of memory and cognitive functioning.

Abstract citation ID: igaf122.426

### LIFE EVENTS AND LONELINESS TRANSITIONS AMONG MIDDLE-AGED AND OLDER ADULTS AROUND THE WORLD

Mara Sheftel[1], Rachel Margolis[2], and Ashton Verdery[3], *1. Rutgers University, New Brunswick, New Jersey, United States, 2. Western University, London, Ontario, Canada, 3. The Pennsylvania State University, University Park, Pennsylvania, United States*

Objectives: Adult loneliness is a substantial social problem and a growing point of concern for policymakers around the world. We assess whether the predictors of loneliness onset among middle-aged and older adults vary from country to country in a large array of settings across world regions. Taking a life course perspective, we focus on common life events in our focal age range, including changes in partnership, coresidence, work, and health, and we test whether changes in them have comparable prospective associations with loneliness onset in different countries. Methods: We draw on respondent-level data from a diversity of world regions surveyed in 7 harmonized cross-national studies in 20 countries, representing 47% of the global population over the age of 50. Our innovative longitudinal approach estimates prospective transition probability models that examine how each life event predicts the transition into loneliness. Results: Despite substantial variation in the prevalence of loneliness and life events across the range of countries in our sample, our results highlight consistency in the predictors of loneliness transitions. Family and household changes like divorce, coresidence, and especially widowhood are paramount predictors of loneliness transition across settings, with changes in work and health playing more minor and less universal roles. Discussion: The results demonstrate the importance that family and household connections play in determining loneliness at these ages. These findings suggest that addressing late-life loneliness may require a focus on key life events, especially those concerning changes in families and households.

Abstract citation ID: igaf122.427

**AN INTERSECTIONAL APPROACH TO UNDERSTANDING THE PSYCHOLOGICAL HEALTH EFFECTS OF COMBINING WORK AND CAREGIVING**

Samantha Brady[1], Taylor Patskanick[2], and Joseph Coughlin[2], *1. Massachusetts Institute of Technology, Cambridge, Massachusetts, United States, 2. Massachusetts Institute of Technology AgeLab, Cambridge, Massachusetts, United States*

Role theory suggests occupying simultaneous family caregiving and employment roles in midlife may exert positive and negative effects on psychological health. However, there is a lack of causal evidence examining the degree to which combinations of these roles influence psychological health at the intersection of gender and racial identity. Longitudinal data from the Health and Retirement Study (2004–2018) are used to estimate a series of individual fixed effects models examining combinations of employment status and parental caregiving situation on Center for Epidemiological Studies—Depression Scale (CES-D) depression scores among Black and White men and women aged 50–65. Subsequent models were stratified by intensity of caregiving situation and work schedule. Individual fixed effects models demonstrate combining work, and parental caregiving is associated with greater depressive symp¬toms than only working, and with lower depressive symptoms than only caregiving, suggesting that paid employment exerts a protective effect on psychological health whereas parental caregiving may be a risk factor for depressive symptoms in later life. Analyses using an intersectional lens found that combining paid work with parental caregiving exerted a protective effect on CES-D scores among White women and men regardless of participants' intensity of care situation or work schedule. This effect was not present for Black men and women. Accounting for intersectionality is imperative to research on family caregiving, work, and psychological health.

### SESSION 1410 (COMMUNITY ENGAGED SCHOLARSHIP SYMPOSIUM)

Abstract citation ID: igaf122.428

**OLDER ADULTS AND COMMUNITY-BASED ORGANIZATIONS AS RESEARCH PARTNERS: THREE METHODOLOGICAL CASE EXAMPLES**

Chair: Michelle Putnam

This session presents approaches to engaging in community-based research using case examples from three individual investigators and their work. The first example, presented by Dr. Susan Stark, describes a trajectory of starting with building community partnerships, then developing participant advisory boards, and finally creating and supporting an ongoing community-based research network of community-based organizations regularly engaged in a collaborative process supporting local research ranging from fall prevention to community participation in greater St. Louis and the Missouri state area. The second example, presented by Dr. Caitlin Coyle, describes a laddered model of building capacity among older adults starting with lower levels stakeholder engagement through to paid, trained membership in the academic research team employed to evaluate age-friendly communities in New England that reflects the needs and preferences of older community members. The third example, presented by Dr. Lydia Ogden, describes a process of building trust between the researcher and study participant in ethnographic research at a clubhouse for older adults with serious mental illness by developing and sustaining a culture of belonging within the collaborative research process that honors participant voices across the study design, including co-authorship of study findings. Shared elements across case examples are identified and discussed, with the aim of facilitating a conversation with the audience about learnings from each model and recommendations for researchers seeking to engage older adults and local communities meaningfully and in a sustained way in their research.

Abstract citation ID: igaf122.429

**ENGAGING COMMUNITY IN RESEARCH: ACCELERATING TRANSLATION OF RESEARCH INTO PRACTICE**

Susan Stark, *Washington University in St. Louis, St. Louis, Missouri, United States*

Community engaged research is an approach that embraces partnerships between researchers and their communities that encourages research designed for dissemination and implementation. This session describes the continuum of community-engaged research used by the Stark Lab in St. Louis: community partnerships, participant advisory boards, and a community-based research network and how each of these has improved the quality, applicability, and translatability of research done in the Lab related to fall intervention, home modifications, and social participation. Benefits of these approaches for the Lab include higher participant recruitment rates for studies, better cultural competence of research team members, increased university-community trust, ability to tailor and adapt interventions to meet different community needs, intensive community-engaged skill-building for students and trainees. Benefits for community partners include improved community knowledge and capacity, greater readiness to adopt interventions (e.g. fall prevention programs), reduced gaps in service delivery, improved communication between community based organizations, and increased access to evidence and training. Challenges include time, funding, and availability for university and community-based partners. However, benefits far outweigh challenges and solutions such as building funding to support these efforts into grant funding is helpful.

Abstract citation ID: igaf122.430

**FROM CONSULTATION TO COLLABORATION: A LADDER OF PARTICIPATION FOR OLDER ADULTS IN RESEARCH**

Caitlin Coyle, *University of Massachusetts Boston, Boston, Massachusetts, United States*

Assumptions about what older adults want and need can drive research and planning such that systemic ageism is propagated within systems. Representation of older adults in investigations and initiatives is needed so that policies, programs and interventions developed out of research are in alignment with the needs and preferences of older people. The aim of this article is to describe the creation of a ladder of participatory opportunities for older people to contribute to a needs assessment process. This process allows for co-researchers to objectively shape of environments and services for their peers. Data comes from 5 community needs assessment projects and the accumulation of 4 older adult research associates. Data were collected via surveys, key informant interviews, and focus groups. We describe how participatory principles were enacted, the involvement of older adults in research design, data collection, and dissemination. As well, we describe the process by which older people can move from a project-based participation to becoming a member of an academic research team. Finally, we discuss the ways in which a community inclusive multi-method strategy can optimize resource allocation, identify pathways to more effective policymaking that is matched to the needs and interests of its oldest residents, and lead to unanticipated benefits. Consistent and equivalent collaboration between researchers and older people can provide critical context and perspectives of the those challenged by their aging circumstances and distribute power to older people for the purposes of being active builders of their environments.

Abstract citation ID: igaf122.431

### BELONGING IN PRACTICE: AN ETHNOGRAPHIC JOURNEY WITH A CLUBHOUSE

Lydia Ogden, *Salem State University, Salem, Massachusetts, United States*

Older adults with serious mental illnesses (SMIs) face high rates of social isolation, loneliness, and associated health risks. However, psychosocial clubhouse membership offers protection through connection, community, and meaningful roles. From December 2023 to December 2024, I conducted a collaborative ethnographic study at a clubhouse with 35% older adult membership, spending 75 days in the field and conducting 40+ interviews. My research explores the clubhouse culture that fosters inclusion and belonging for this often-isolated population. Deeply participatory, the study prioritizes member voices through relationship-building, oral histories, and long-term engagement. Member-checking and collaborative analysis ensure alignment with lived experiences, and clubhouse members are invited as co-authors in final publications. This approach strengthens research trustworthiness while supporting local and national clubhouse policy. Findings will contribute to understanding how clubhouses sustain meaningful social connections and improve outcomes for older adults with SMIs.

### SESSION 1415 (SYMPOSIUM)

Abstract citation ID: igaf122.432

### SAME OLD, SAME OLD? HETEROGENEITY IN PSYCHOSOCIAL PROCESSES AND WELL-BEING IN MINORITY AGING

Chair: Heather Farmer
Co-Chair: Jeffrey Stokes
Discussant: Jennifer Ailshire

There are well-established racial/ethnic disparities in health across the life course, and psychosocial processes (e.g., stress, socioeconomic status) are hypothesized to drive these disparities. Scholarship suggests that unique social contexts, lived experiences, and social identities may contribute to variations in health and well-being within and between racial/ethnic groups, but these associations require more attention. This symposium showcases innovative scholarship that elucidates heterogeneity in psychosocial risk and protective factors linked to health and well-being in minority aging. Dr. Forrester will examine how race and sex influence the association between psychosocial factors (e.g., lifetime trauma, chronic stress, wealth) and accelerated aging, as well as whether psychosocial factors interact with accelerated aging to predict cognitive impairment. Dr. Farmer will use dyadic data from older Black couples to examine whether individual-level and vicarious discrimination are associated with a reduction in purpose in life for individuals and their spouses, as well as whether educational attainment moderates these associations. Dr. Nguyen will explore the influence of religious participation on cognitive trajectories in African Americans and whether this association is conditional on age. Finally, Dr. Stokes will examine racial/ethnic differences in the extent to which neighborhood social context predicts later levels of loneliness and cognitive functioning. Collectively, this work highlights heterogeneity in the psychosocial mechanisms associated with health and well-being in the context of minority aging. Future research, policy, and interventions aimed at reducing longstanding racial/ethnic disparities require theoretically-informed, interdisciplinary approaches that incorporate a focus on social context and lived experiences.

Abstract citation ID: igaf122.433

### DYADIC DISCRIMINATION AND PURPOSE IN LIFE AMONG MIDLIFE AND OLDER BLACK COUPLES

Heather Farmer[1], and Jeffrey Stokes[2], *1. University of Delaware, Newark, Delaware, United States, 2. University of Massachusetts Boston, Boston, Massachusetts, United States*

Marriage is among the most central and closest relationships in adulthood. Psychosocial risks and resources are often shared, though dyadic processes in Black couples are underexplored. Given the interdependent nature of marriage, discrimination may jeopardize resilience resources, such as purpose in life, for the individual experiencing it and vicarious discrimination could affect their partner's purpose as well. Moreover, diminished purpose in life may be "contagious" among older Black spouses, particularly those who lack structural protective factors such as educational attainment. We explore the dyadic associations between discrimination and purpose in life among 180 opposite-gender Black couples ages 50+ in the 2016-2020 waves of the Health and Retirement Study. We estimate the individual and dyadic associations between discrimination and purpose using longitudinal structural equation models, controlling for age, education, employment, and marital quality. We then included an interaction term [partner's education x purpose] to the model. Results showed that individual-level discrimination was significantly associated with lower purpose only in women ($b = -0.21$, $p<.01$), and that vicarious discrimination was not associated with purpose. We found that a spouse's purpose undermined one's own purpose only when the spouse had low educational attainment. Findings provide preliminary evidence of interpersonal mechanisms that may hinder psychological well-being among Black couples. Shared psychosocial experiences in marriage may be tied to health in Black populations, for better or worse, and deserve more scholarly attention. Therefore, considering purpose as a shared, rather than individual, resource may lead to effective interventions aimed at supporting purpose to enhance well-being.

Abstract citation ID: igaf122.434

### NEIGHBORHOOD COHESION, LONELINESS, AND COGNITIVE FUNCTION: EFFECTS OF RACE AND PLACE

Jeffrey Stokes[1], Kenzie Latham-Mintus[2], Heather Farmer[3], and Setarreh Massizhadegan[1], *1. University of Massachusetts Boston, Boston, Massachusetts, United States, 2. Indiana University Indianapolis, Indianapolis, Indiana, United States, 3. University of Delaware, Newark, Delaware, United States*

Racial/ethnic disparities in cognition and dementia are well-established. Social determinants of health researchers have identified neighborhood context as one potential contributor to these disparities, though to date most research has focused on neighborhood built environment and objective neighborhood characteristics (e.g., neighborhood SES, racial/ethnic demographics). More recently, research on "cognability" has identified neighborhood social context as an important determinant of both well-being and cognitive health among the aging population. Neighborhood social cohesion, in particular, may help to reduce loneliness and thereby improve cognitive aging among midlife and older adults. However, access to high-quality and cohesive neighborhood settings varies by race/ethnicity in the U.S., as too may the association of neighborhood social cohesion with well-being outcomes such as loneliness. This study uses three-wave longitudinal data from the 2010-2020 waves of the Health and Retirement Study (HRS; N = 7,879) to examine whether (a) neighborhood social cohesion is associated with loneliness and, thereby, cognitive functioning over an 8-year period, as well as (b) whether this mediation model varies by race/ethnicity. Results indicate that non-Hispanic White adults reported the highest neighborhood social cohesion, the lowest loneliness, and exhibited the best cognitive functioning over the study period. Moreover, neighborhood social cohesion was associated with reduced loneliness only among non-Hispanic White participants. Although loneliness was associated with greater declines to cognition over time across all racial/ethnic groups, only non-Hispanic White participants' cognitive trajectories benefited from living in socially cohesive neighborhoods, via reduced loneliness. We discuss implications of findings for health disparities research in light of the "cognability" framework.

Abstract citation ID: igaf122.435

### IMPACTS OF PSYCHOSOCIAL STRESS AND AGE ACCELERATION ON COGNITIVE IMPAIRMENT: THE HEALTH AND RETIREMENT STUDY

Sarah Forrester, *University of Massachusetts Chan Medical School, Worcester, Massachusetts, United States*

Psychosocial stress affects physiological and cognitive outcomes. This study examines race and sex differences in psychosocial stressors and biological age acceleration (BAA) and how psychosocial factors and BAA impact cognitive impairment (CI). Data came from 2,083 participants in HRS who had biological (blood pressure, BMI, pulmonary function, glucose, CRP, and cholesterol), cognitive, and psychosocial data (chronic stress, wealth, lifetime trauma, depression symptoms) available 2006 – 2016. CI was measured with the Telephone interview for Cognitive Status (TICS; score <12 = CI). BAA was measured using the KDM equation as biological age minus chronological age. Participants were 66 years on average (SD = 8) at analytic baseline, majority White (76%), and female (60%). Linear mixed effects and frailty models were adjusted for age and education and adjusted for multiple testing. Black participants had higher CI, stress, and BAA. The BAA and depression relationship was stronger for men compared to women (B = 0.75, 95% CI: 0.32 – 1.19). Each year increase in BAA was associated with a significant hazard of incident CI (HR: 1.01, 95% CI: 1.01 – 1.02) and was stronger among women than men (HR: 1.02, 95% CI: 1.01 – 1.03). BAA was was associated with a lower hazard of CI when trauma was present (HR: 0.973, 95% CI: 0.96 – 0.99). Eighty-three percent of the association between chronic stress and incident CI was accounted for by BAA. BAA may be a mechanism through which stress impacts risk of CI and important for prevention efforts among those most affected.

Abstract citation ID: igaf122.436

### RELIGIOUS PARTICIPATION AND TRAJECTORIES OF COGNITIVE DECLINE IN AFRICAN AMERICANS: THE MODERATING EFFECT OF AGE

Ann Nguyen[1], Elissa Kim[2], Weidi Qin[3], Yoonkyung Shin[1], and Lisa Barnes[4], *1. Case Western Reserve University, Cleveland, Ohio, United States, 2. Wayne State University, Detroit, Michigan, United States, 3. University of Wisconsin–Madison, Madison, Wisconsin, United States, 4. Rush University Medical Center, Chicago, Illinois, United States*

African Americans are disproportionately impacted by dementia compared to other racial groups. Religion holds special meaning and significance for African Americans and is an important psychosocial resource that can protect against cognitive decline. The salubrious effects of religion tend to be greater for older adults than younger adults. However, very few studies have examined the connection between religious participation and cognitive decline specifically in African Americans, and none have investigated age differences in this connection. The aims of this study were 1) to determine whether non-organizational religious participation is associated with cognitive status trajectories in this population and 2) to determine whether the association between non-organizational religious participation and cognitive status trajectories vary by age. Data for this study come from the Minority Aging Research Study (N = 845). Non-organizational religious participation measures included religious coping and prayer/meditation. We utilized group-based trajectory modeling to identify cognitive status trajectories based on global cognitive function scores. We identified four distinct cognitive status trajectories: very low and declining; low and declining; moderate and slow decline; and high and stable. While religious participation was not directly related to cognitive status trajectories, the interactions between the religious participation variables and age were significant. The protective effects of religious participation were strongest among old-old participants. Among the oldest-old participants, more frequent religious participation was associated with increased risk for cognitive decline. These findings indicate that the relationship between non-organizational religious participation and cognitive status trajectories is dependent on age. Implications for clinical practice and interventions will be discussed.

### SESSION 1420 (SYMPOSIUM)

Abstract citation ID: igaf122.437

### CULTIVATING COMMUNITY: BUILDING STRUCTURES OF CARING IN GERONTOLOGICAL SPACES

Chair: Nicholas DiCarlo
Co-Chair: Brittney Pond
Discussant: Díana Poncé

Research suggests that building community is important for mental health, personal and professional growth, feelings of belongingness, etc. (Brower, 2020). Scientists, researchers, and academics have made efforts to form community through various avenues, such as early career writing groups (Kent et al., 2017), informal mentorship structuring (Pegg et al., 2015), and more. In this symposium, we aim to explore two interconnecting questions: 1) how do we create structures of caring within communities during times of crisis (such as the COVID-19 pandemic, sociopolitical change, etc.) 2) what is the role of resistance in healing? The three presenters address this question using various methods, including community reflections, personal experiences, and data-driven discussions. This symposium has several practical and theoretical implications, such as 1) strategies for navigating disruptions 2) ways to cultivate community and 3) discussions regarding the meaning of community within and beyond the academy. These contributions are applicable to faculty and staff, researchers, clinicians, students, community members, participants, etc. This symposium aims to include active audience discussion and resource sharing to build community specifically at GSA.

Abstract citation ID: igaf122.438

### INTELLECTUAL FREEDOM AND CRONE PEDAGOGIES

Nicholas DiCarlo, *University of California, San Francisco, San Francisco, California, United States*

Crone pedagogies (DiCarlo, 2023 & 2004) provide grounding frameworks for collective survival for teaching-learning. Re-membering the emancipatory potential of historical memory counters colonial hegemony's ongoing assault on collective vitality and indigenous ways of knowing. Opposing the logics of extraction and exploitation, crone pedagogies invest in the collective by resisting disposability and neoliberalism's insistence on competition and scarcity. The gerontological imagination (Estes, 1992) calls us to envision bridges between frameworks that understand how ideology exploits material reality (Estes, 1979) and shapes subjectivity (Gaztambide, 2023). These bridges equip scholars to sustain engagement with complex feelings about traumatic sociopolitical realities over time, pushing past analytic frames that comfort and quell and pursuing unsettling and disillusionment that antagonize institutional complicity (Layton, 2019).

Abstract citation ID: igaf122.439

### DEFENDING LGBTQIA+ AGING RESEARCH: U.S. FEDERAL POLICIES' IMPACTS ON STUDIES OF OLDER SEXUAL AND GENDER MINORITIES

Jace Flatt[1], Joel Anderson[2], and Whitney Wharton[3],
1. University of Nevada Las Vegas, Nevada, United States,
2. University of Tennessee, Knoxville, Tennessee, United States,
3. Emory University, Atlanta, Georgia, United States

Funding supporting research about sexual and gender minority aging and Alzheimer's disease and related dementias is currently being terminated by the U.S. federal government. During the past year, research grants that included older transgender people, as well as lesbian, gay, bisexual, queer, intersex, and additional identities (LGBTQIA+), have been terminated, funding decisions rescinded, and grants pulled from scientific review. Recent termination letters from the federal government have claimed that "research programs based on gender identity are often unscientific, have little identifiable return on investment, and do nothing to enhance the health of many Americans. Many such studies ignore, rather than seriously examine, biological realities." This presentation will highlight the impact of anti-LGBTQIA+ policies on the current state of science and explore potential action steps and strategies that researchers committed to supporting and conducting research with sexual and gender minority aging populations should consider. We will highlight our efforts to advocate for this research, including talking to the media, writing to policymakers, generating visibility on social media with colleagues and peers, speaking with lawyers and legal scholars, and connecting with community members. This includes a discussion of several case studies involving research with LGBTQIA+ older adults that has been abruptly terminated by the federal government. We will also discuss ways to support our colleagues, junior faculty and early career scholars, conduct emancipatory research, and stand up against current policies to abrogate diversity, equity, inclusion, and accessibility, as well as anti-transgender ideologies in research and academia.

Abstract citation ID: igaf122.440

### BACKPACKS & BRIEFCASES, WALKERS & WHEELCHAIRS? UNIVERSITY EXPANSION & HOUSING JUSTICE FOR URBAN OLDER RENTERS

Ian Johnson, *The University of Texas at San Antonio, San Antonio, Texas, United States*

University expansion and district revitalization often exacerbate existing housing inequities, particularly for urban-dwelling older adult renters. This presentation explores the university's role in housing justice, focusing on the impacts of institutional growth on vulnerable older populations. This presentation will present a media analysis of university-driven development in downtown San Antonio and how it has prompted and responded to concerns regarding displacement, rent escalation, and diminished housing security among older residents. Findings reveal that university expansion efforts, while intended to stimulate economic growth, frequently overlook the specific needs of long-term older renters. Further, language and rhetoric used to frame district planning similarly excludes older adults as stakeholders in urban places. We will discuss the complex interplay between university planning, local policy, and the lived experiences of older adults, highlighting the challenges of maintaining age-friendly communities amidst rapid urban change. This presentation aims to present a framework for actionable strategies for universities and policymakers to adopt ethical and equitable approaches to urban redevelopment. We will offer considerations for integrating age-friendly principles into university district planning, emphasizing the importance of community engagement, affordable housing preservation, and the protection of older adults' rights to remain in their neighborhoods.

### SESSION 1425 (SYMPOSIUM)

Abstract citation ID: igaf122.441

### PRODUCTIVE AGING ACROSS NATIONS AND CULTURES

Chair: Patrick Ho Lam Lai
Discussant: Nancy Morrow-Howell

Engagement in productive activities such as employment and volunteerism is essential for well-being and societal development in later life, particularly while the aging population continues to grow across different cultures and countries. However, participation in these activities varies due to cultural, economic, social, and policy factors. This symposium examines productive aging from an international and cross-cultural perspective, highlighting disparities in engagement and well-being among older adults across various cultural and national contexts. The first presentation explores how culture-related factors such as family expectations and religion shape volunteerism among older Asian adults in the United States compared to other ethnoracial groups. The second presentation examines financial disparities among older self-employed workers in Sweden, comparing limited liability company owners, sole proprietors, and employees in terms of household income and social benefits. The third presentation analyzes gender disparities in depression among older adults from a cross-national and longitudinal perspective, considering the role of work and volunteerism while accounting for cohort and age effects. The final presentation analyzes disparities in U.S. older workers' access to employer-provided health insurance, retirement savings, and flexible work arrangements by gender, race, and ethnicity, with a particular focus on Hispanic and Black workers. By exploring these diverse perspectives, this symposium advances understanding of the contextual influences on later-life engagement. The discussant will synthesize key insights and discuss implications for policies and programs that promote equitable participation in productive aging across cultures and countries.

Abstract citation ID: igaf122.442

### CULTURAL DETERMINANTS OF FORMAL AND INFORMAL VOLUNTEERISM AMONG ASIAN OLDER ADULTS AND OTHER ETHNORACIAL GROUPS

Patrick Ho Lam Lai[1], and Christina Matz[2], *1. The University of Oklahoma, Moore, Oklahoma, United States, 2. Boston College, Chestnut Hill, Massachusetts, United States*

Background: Volunteerism is a key component of productive aging, yet little is known about the cultural influences shaping formal and informal volunteerism among older Asian Americans. This study explores the role of socioeconomic resources and culture-related factors in explaining ethnoracial disparities in formal and informal volunteerism, with a focus on older Asian Americans. Methods: Using cross-sectional pooled data from the 2004-2020 waves of the Health and Retirement Study (HRS), logistic regression models examined ethnoracial differences in formal and informal volunteerism among adults aged 50-75. The study incorporated cultural indicators (e.g., family composition, emotional support, religious behavior, and immigration status) alongside socioeconomic factors. Results: Socioeconomic resources (education and income) partially explained volunteerism differences, but cultural factors—particularly religious behavior, family dynamics, and immigration status—further clarified disparities. Differences between Asians and Whites or Blacks diminished after accounting for cultural factors, while gaps with Hispanics persisted. Conclusions: This study underscores the importance of cultural factors in understanding ethnoracial disparities in volunteerism. Findings suggest that culturally informed interventions are needed to promote productive aging among Asian Americans and other ethnoracial groups. Future research should expand on these findings with more comprehensive cultural indicators and subgroup analyses.

Abstract citation ID: igaf122.443

### HOW SELF-EMPLOYMENT INFLUENCES THREE FORMS OF INCOME IN LATER LIFE: A SWEDISH REGISTRY DATA STUDY

Cal Halvorsen[1], Melody Almroth[2], Kuan-Yu Pan[2], Alicia Nevriana[2], Jessie Gevaert[3], and Daniel Falkstedt[2], *1. Washington University in St. Louis, St. Louis, Missouri, United States, 2. Karolinska Institutet, Solna, Stockholms Lan, Sweden, 3. Vrije Universiteit Brussel, Brussel, Brussels Hoofdstedelijk Gewest, Belgium*

Research that considers the relationship between self-employment and income has generated mixed results, possibly due to selection bias and imprecise measurements of self-employment. In response, we aimed to estimate the treatment effects of self-employment on three types of income—income from work, adjusted household income, and receipt of social benefits—while controlling for selection into self-employment, specifying self-employment types, and identifying treatment effects by gender. We used data from the Swedish Work, Illness, and Labor Market Participation cohort (born 1959-1969; ~1.2 million individuals). To reduce selection bias, we created entropy balancing weights to balance the self-employed and wage/salary employment groups in 2014 on sociodemographic, work, and health factors from prior years. We included these weights in regression models to predict different types of income in 2019. Compared to wage/salary employment and controlling for multiple job holding, we estimate that self-employment in 2014 led to lower annual work income (-213,777 SEK, $p<.001$; ~$21,000) and adjusted household income (-55,831 SEK, $p<.001$; ~$5,500) in 2019, with sole proprietors and women being the most financially vulnerable. Although self-employment in 2014 was negatively related to receipt of social benefit income in 2019 (OR=.729, $p=.002$), this effect was lost once accounting for multiple job holding. These results show how self-employment's influence on income is multifaceted. Future research should investigate the motivations for pursuing self-employment past midlife and their interactions with gender and household dynamics, given that it may be a useful form of work for people needing flexibility (e.g., caregivers) and desiring more autonomy.

Abstract citation ID: igaf122.444

### THE MODERATING EFFECT OF LABOR FORCE PARTICIPATION ON THE PREVALENCE OF DEPRESSION AMONG OLDER ADULTS

Antonia Diaz-Valdes, Jose Medina, Esteban Calvo, and Nicolas Montalva, *Universidad Mayor, Santiago, Region Metropolitana, Chile*

Depression among older adults is one of the most pressing issues in public health, yetis one of the less recognized problems and is one of the leading causes of disability. This study aims to examine cross-national gender gaps in the prevalence of depression risk from 2000 to 2020, controlling for age and cohort effects, and exploring labor force status as moderator. A random-effects model with robust standard errors was conducted to examine depression cases and predict probabilities. Our analysis involved a sample of 246,000 individuals aged 50 and older from 35 countries with at least 2 waves of reported measures, spanning the years 2000-2020. We used cut-points for risk of depression based on the depression scales used in the different surveys – Health and Retirement Study and sister datasets around the world. The average predicted probability of depression was 22.00 % for males and 33.62% for females. While the gender gap in depression probability remained stable over time, both males and females exhibited an upward trend in depression probability during this period – about 3.52 and 3.79 respectively. The average predicted probability of depression varied by labor status, being 19.32% and 31.70% for working males and females respectively, and 23.00% for retired males and 34.27% for retired females. Our study highlights the importance of screening and treating depression among older adults to reduce the consequences of depression, especially among women, who are disproportionately affected independent of their labor force status

Abstract citation ID: igaf122.445

### DISPARITIES IN EMPLOYER-PROVIDED BENEFITS AMONG OLDER BLACK AND HISPANIC WORKERS

Bruna Lopez[1], and Cal Halvorsen[2], *1. Boston College, Chestnut Hill, Massachusetts, United States, 2. Washington University in St. Louis, St. Louis, Missouri, United States*

Employer-provided benefits, while important for workers across the life course, may be particularly essential to older workers due to the higher cost of private health insurance for older adults, increased attention to retirement savings, and need for flexibility for caregiving responsibilities. In response, we analyzed nearly 30,000 observations of workers between the ages of 50 and 64 using six waves of the biennial Health and Retirement Study (HRS), from 2010 to 2020 and HRS sample weights to promote the representativeness of our results. We documented trends in access to employer-provided health insurance, retirement savings programs, and the ability to reduce working hours overall and by gender, race, and ethnicity. We found that about 60% of workers had access to employer-provided health insurance, about 60% of workers had access to employer-sponsored retirement savings programs, and about 30% of workers reported the ability to reduce their work hours. Rates were relatively consistent throughout the study period. Although male and White workers are more likely to have access to these benefits than female and Black workers, as well as those in the "all other races" category, the largest differences were by ethnicity: Hispanic older workers reported access to employer-sponsored retirement savings programs, for example, at rates up to 30 percentage points less than their non-Hispanic counterparts. This disparity may be attributed to workplace culture differences. Overall, results suggest that Hispanic older workers are the least likely to have access to employer-provided benefits, potentially foreshadowing more health and financial difficulties later in life.

### SESSION 1430 (PAPER)

### NEW TECHOLOGIES FOR HEALTH AND CARING: GLOBAL PERSPECTIVES

Abstract citation ID: igaf122.446

### DEVELOPING AN IMPLEMENTATION FRAMEWORK FOR WEB-BASED PRESENCE TECHNOLOGY INTEGRATION IN LONG-TERM CARE HOMES

Anna Garnett[1], Halyna Yurkiv[1], Denise Connelly[1], Richard Booth[1], and Lorie Donelle[2], *1. Western University, London, Ontario, Canada, 2. University of South Carolina, Columbia, South Carolina, United States*

Web-based presence technologies (WPT) have been recognized for their ability to mitigate social isolation and foster a sense of aging in place among older adults within long-term care homes. Despite ongoing interest in WPT use by a range of stakeholders, a guiding framework for its sustained implementation in long-term care homes is lacking. This multi-method study sought to develop an evidence-informed framework for integration of WPT within long-term care homes. Using a qualitative descriptive approach, semi-structured interviews were conducted with 12 long-term care home leadership personnel, 22 family members, 10 staff, and seven older adults. Additionally, documents such as long term-care policies and guidelines were examined. Multi-stage data analysis included: 1) directed content analysis guided by Technology Acceptance Model to examine older adults, family members, and staff experiences; 2) conventional content analysis of documents and leaders' accounts. Findings were triangulated to develop an implementation framework highlighting factors to be considered to achieve successful WPT integration within long-term care homes. Facilitating factors included: training to enhance digital literacy of WPT end users, collaboration between long-term care homes and external companies for resource acquisition (devices, funding, human resources), and diverse applications of WPT use (i.e., social connectedness, telehealth). Potential barriers included: funding constraints and competing priorities within long-term care, lack of standardized organizational guidelines on WPT use, and usability challenges among end-users. Findings highlight the need for sustained funding opportunities, ongoing digital literacy programs for end-users, and standardized guidelines to ensure equitable and sustained integration of WPT across long-term care homes.

Abstract citation ID: igaf122.447

### DIGITAL INCLUSION DRIVING THE SILVER ECONOMY AMONG OLDER ADULTS IN CHINA: A SUPPLY-DEMAND THEORETICAL PERSPECTIVE

Chuanzheng Xu, and Jing Ye, *Wuhan University, Wuhan, Hubei, China (People's Republic)*

Background: Amid accelerating global aging, the digital divide poses systemic threats to social equity. This study applies a supply-demand theoretical framework to analyze how digital inclusion bridges supply-demand gaps in the silver economy, examining dual mechanisms of supply optimization and consumption stimulation among older adults. Methodology and Data: Using Chinese provincial panel data (2012-2022) and the

China Health and Retirement Longitudinal Study (2013, 2015, 2018, 2020), this research constructs a tripartite digital inclusion index (accessibility-utilization-benefit) using the entropy weight method as the explanatory variable. To assess its causal effects on the silver economy's supply-demand dynamics, this study integrates supply-side industrial evolution with demand-side consumption trajectories as outcome variables. Findings: (1) Digital inclusion significantly drives silver economy growth, expanding aging-related industry scale and older adults' consumption. (2) The regional business environment plays a mediating role between digital inclusion and the supply-demand expansion in the silver economy. (3) Less-developed regions demonstrate a "catch-up effect" resulting from digital inclusion improvements. Discussion: To unlock older adults' economic potential and address aging challenges equitably, digital inclusion strategies must integrate technological empowerment with institutional reforms. This requires expanding digital infrastructure in underserved regions and optimizing the business environment to foster inclusive growth in the silver economy.

Abstract citation ID: igaf122.448

### TELEHEALTH USE BY RESIDENCE TYPE AMONG OLDER ADULTS RECEIVING LONG-TERM SERVICES AND SUPPORTS

Dana Urbanski[1], Romil Parikh[2], Jack Wolf[2], Benjamin Langworthy[2], Eric Jutkowitz[3], and Tetyana Shippee[3], *1. Indiana University Bloomington, Bloomington, Indiana, United States, 2. University of Minnesota School of Public Health, Minneapolis, Minnesota, United States, 3. University of Minnesota, Minneapolis, Minnesota, United States*

Telehealth can improve access to geriatric care, particularly for vulnerable older adults facing transportation, mobility, and health-related barriers to in-person care. However, integration of telehealth into geriatric care must account for the different residence types where older adults use telehealth. This study examined the association between residence type and telehealth use among older recipients of long-term services and supports (LTSS) using cross-sectional data from the 2021-2022 National Core Indicators-Aging and Disability Adult Consumer Survey. The analytic sample included 6,925 LTSS users aged ≥ 65 years without intellectual or developmental disability: 69.2% lived in the community, 20% in residential care, and 10.9% in nursing facilities. Telehealth use was reported by 39.2% of community-dwelling respondents, 33.5% in residential care, and 20.3% in nursing facilities. Complete-case multivariable logistic regression, adjusting for sociodemographic and health-related factors with state-level random intercepts, revealed that those in residential care and nursing facilities had significantly lower adjusted odds of telehealth use than their community-dwelling counterparts (residential care: OR, 0.80 [95% CI, 0.69-0.92]; nursing facilities: OR, 0.37 [95% CI, 0.29-0.47]), with nursing home residents also having lower adjusted odds than those in residential care (OR, 0.46 [95% CI, 0.36-0.60]). These findings highlight differences in telehealth use among older LTSS users, with those in residential and nursing facilities having lower odds of telehealth use compared to community-dwelling individuals. Targeted efforts to promote telehealth adoption in institutional and congregate care settings may help bridge this gap.

Abstract citation ID: igaf122.449

### THE FUTURE OF AGING: TRANSFORMING HEALTHCARE FOR NIGERIA'S AGING POPULATION

Chinenye Aniemeke[1], Joy Demesi[1], Ebi Ofrey[2], Ajibola Meraiyebu[3], and Gregory Orewa[1], *1. The University of Texas at San Antonio, San Antonio, Texas, United States, 2. GeroCare Solutions Ltd, Lagos, Lagos, Nigeria, 3. Gerocare, Abuja, Federal Capital Territory, Nigeria*

Nigeria's healthcare crisis disproportionately impacts its growing older adult population, now exceeding 10 million. The healthcare crisis has been exacerbated by the migration of skilled healthcare professionals leaving the country. Limited geriatric care, high healthcare costs, and lack of structured home services leave many individuals with undiagnosed chronic conditions and preventable complications. GeroCare, an innovative solution, addresses this gap by offering home medical visits, teleconsultations, and caregiver support through a network of physicians. This model utilizes technology to enhance accessibility and improve care coordination. This study explores how GeroCare impacts healthcare access, quality of life, and family caregiving experiences for older adults in Nigeria, as reflected in patient testimonials. This study employs thematic analysis to identify recurring patterns from qualitative data from patient testimonials collected between 2017 and 2024 in written, audio, and video formats. Key themes included increased healthcare access, enhanced quality of life, and improved peace of mind for families. Findings indicate that GeroCare improves access to care for the aging population and relieves caregiving burdens. The study emphasizes technology-driven home healthcare models to fill systemic gaps in older adult care. It highlights the need for policy reforms to integrate telehealth into national healthcare frameworks and expand insurance coverage for home-based medical services across low-resource settings. Additionally, scaling models like GeroCare can alleviate workforce shortages by leveraging telehealth, task shifting, and remote monitoring to optimize healthcare delivery. Policymakers, healthcare providers, and stakeholders should consider an innovative model like GeroCare to improve healthcare for older adults in low-resource settings.

### SESSION 1435 (SYMPOSIUM)

Abstract citation ID: igaf122.450

### TAKE THE NEXT STEP: PRACTICAL TOOLS AND STRATEGIES FOR CLINICAL TRIALS WORK IN NURSING HOMES

Chair: Kathleen Unroe
Co-Chair: Debra Saliba
Discussant: Jerry Gurwitz

Nursing home residents have been historically excluded from clinical trials. Numerous challenges have been described to conducting research in nursing homes, including barriers to recruitment, methodological difficulties, and a need for pilot funding preparatory to larger trials. The National Institute on Aging funded the $15.6 million NEXT STEPs (Nursing home EXplanatory clinical Trials: Supporting Transformation by Enhancing Partnerships) U24 initiative to directly address these challenges. The goal of NEXT STEPs is to expand the number and quality of multi-site clinical trials in the nursing home setting.

The perspectives and participation of interdisciplinary clinical providers and researchers are critical to inform the launch and activities of this innovative network. This symposium will highlight several of the activities and resources developed to date by the NEXT STEPs Network, with a goal of providing practical tools and guidance for researchers and partners in nursing home research.

Abstract citation ID: igaf122.451

### TACKLING METHODOLOGICAL CHALLENGES IN NURSING HOME RESEARCH

Susan Hickman[1], Andrew Zullo[2], Julie Lima[3], Cathleen Colon-Emeric[4], Jennifer Stevens-Lapsley[5], Stefan Gravenstein[6], and Melissa Tran[5], *1. Indiana University, Indianapolis, Indiana, United States, 2. Brown University, Providence, Rhode Island, United States, 3. Brown University School of Public Health Department of Health Services, Policy, and Practice, Providence, Rhode Island, United States, 4. Duke University School of Medicine, Durham, North Carolina, United States, 5. University of Colorado Anschutz Medical Campus, Aurora, Colorado, United States, 6. Alpert Medical School of Brown University, Providence, Rhode Island, United States*

The NEXT STEPs Methods, Measures, and Data Core (MMDC) develops guidance for researchers conducting studies in nursing homes through consultation and best practice recommendations. To help inform Year 1 priorities, a survey administered to a convenience sample of early-stage investigators (n = 23) identified key challenges, including regulatory and institutional review board (IRB) considerations, institutional engagement and resistance, and study design concerns. Concurrent with the survey and then drawing on its responses, the MMDC synthesized best practices through literature review, regulatory analysis, and expert consensus. Key guidance focuses on: 1) research regulation and compliance, including navigating HIPAA requirements and when to execute Business Associates Agreements, Data Use Agreements, Research Services Agreements, and Federal Wide Assurances; 2) accessing and using primary and secondary data sources relevant to nursing home research; and 3) selecting appropriate clinical trial designs in the nursing setting as well as strategies to overcome barriers to research. Conducting research in nursing homes presents unique challenges related to developing partnerships, identifying data sources, and study design. Newly created tools and resources, including a data sources compendium and best practice IRB and regulatory compliance guidelines, are available from the NEXT STEPS MMDC to help investigators navigate these complexities and grow the evidence base of nursing home explanatory clinical trials.

Abstract citation ID: igaf122.452

### NEXT STEPS: EVIDENCE-BASED RECRUITMENT AND RETENTION FOR NURSING HOME CLINICAL TRIALS

Sheryl Zimmerman[1], Johanna Hickey[2], Lea Efird-Green[2], Jennifer Carnahan[3], Gail Towsley[4], Alice Bonner[5], and Jasmine Altizer[6], *1. University of North Carolina at Chapel Hill, Chapel Hill, North Carolina, United States, 2. The University of North Carolina at Chapel Hill, Chapel Hill, North Carolina, United States, 3. Indiana University Center for Aging Research, Indianapolis, Indiana, United States, 4. University of Utah, Salt Lake City, Utah, United States, 5. Institute for Healthcare Improvement, Boston, Massachusetts, United States, 6. New York University, New York, New York, United States*

The National Institute on Aging funded NEXT STEPs (Nursing home EXplanatory clinical Trials: Supporting Transformation by Enhancing Partnerships) to promote the successful conduct of nursing home clinical trials. Three of NEXT STEPs' key elements recognize the importance of the relationship between researchers and those participating in research: recruiting nursing homes and participants; cultivating engagement throughout the process; and following up and following through on communication to share results and promote sustainable implementation. NEXT STEPs' Recruitment and Retention Core focuses on these areas, a key effort being the development of a range of evidence-based best practice resources for use by those who conduct research and participate in research. To begin this effort, Core members searched the literature and conferred with experts to develop an extensive and evolving list of best practice topics – such as strategies to help researchers convey the importance of research, and participants to understand their rights as a research partner – and determine the optimal format to convey the best practices (e.g., checklists, short videos). The first document created for researchers is entitled "Words Matter," employing a "use this/not that" format (e.g., use "choose not to" rather than "noncompliant"). Feedback on materials are obtained from diverse partner workgroups before they are finalized, and their utility is evaluated as they are used in NEXT STEPs' efforts. This session will overview the topics identified important for recruitment and retention, the process of topic selection and material development, and materials developed to date; audience feedback and recommendations will be solicited.

Abstract citation ID: igaf122.453

### ENGAGEMENT OPPORTUNITIES IN THE NEXT STEPS NETWORK

Cari Levy[1], Barbara Resnick[2], Joan Carpenter[3], Sarah Berry[4], and Nicole Fowler[5], *1. University of Colorado, Aurora, Colorado, United States, 2. University of Maryland, Baltimore, Maryland, United States, 3. University of Maryland School of Nursing, Baltimore, Maryland, United States, 4. Hebrew SeniorLife & Harvard Medical School, Hebrew SeniorLife, Massachusetts, United States, 5. Indiana University, Indianapolis, Indiana, United States*

The Projects and Training Core strengthens research capacity by offering critical support to investigators through three initiatives: (1) Pilot Grants, which provide funding to both early-stage and experienced researchers to collect preliminary data essential for designing rigorous clinical trials; (2) An Experiential Learning Program, designed to introduce researchers new to nursing home research to the complexities of nursing home care. This program includes webinars, guided reflective discussions, and a hands-on, day-long visit to a nursing home to interact with staff and residents, fostering a deeper understanding of the environment; and (3) Research Studios, structured interactive sessions where researchers present their project ideas to an expert panel for constructive feedback and strategic guidance to refine study designs and methodologies. By providing these resources, training opportunities, and expert mentorship, the Projects and Training Core enhances the feasibility, quality, and impact of nursing home clinical trials. This initiative ensures that researchers are well-prepared to navigate the unique challenges of conducting research in nursing homes, ultimately advancing knowledge and improving care for residents.

Abstract citation ID: igaf122.454

### ENGAGING KEY PLAYERS IN PARTNER WORKGROUPS: A COLLABORATIVE APPROACH TO EXPLANATORY TRIALS IN NHS

Jasmine Altizer[1], Alice Bonner[2], Shih-Yin Lin[3], Sonya Jha[1], Gail Towsley[4], Jennifer Carnahan[5], and Sheryl Zimmerman[6], 1. New York University, New York, New York, United States, 2. Institute for Healthcare Improvement, Boston, Massachusetts, United States, 3. New York University Rory Meyers College of Nursing, New York, New York, United States, 4. University of Utah, Salt Lake City, Utah, United States, 5. Indiana University Center for Aging Research, Indianapolis, Indiana, United States, 6. University of North Carolina at Chapel Hill, Chapel Hill, North Carolina, United States

To support the NEXT STEPs Network's goal of creating a national infrastructure that will address systemic barriers to conducting research in nursing homes, we have established four partner workgroups (i.e., nursing home residents, care partners, nursing home clinical staff and nursing home administrators and leaders). These partner workgroups play a key role in representing essential perspectives for conducting research in nursing homes. Each workgroup consists of approximately 5-8 members and meets three times a year via Zoom. To recruit workgroup members, potential candidates were first identified through NEXT project investigator connections, national organizations, the LTCDC, and other organizations. The New York University team (part of NEXT's Recruitment and Retention Core) then sent formal invitations outlining the expectations of workgroup members, along with a demographic form to ensure diversity in gender, race and ethnicity, and geographic representation within the workgroups. Workgroup members review and contribute to best practice guidance documents and training materials, advise on pilot grants, and provide feedback on specific interventions for nursing home clinical trials. To obtain input and feedback on specific products/activities, NEXT STEPs Cores (Projects and Training; Recruitment and Retention; Methods, Measures, and Data) complete a standardized online request form, specifying how they would like to receive feedback from the workgroups (e.g., attending a virtual workgroup meeting, receiving an email summary of workgroup recommendations, cognitive interviewing style via Qualtrics, etc.). In this session, we will explore facilitators, barriers, and lessons learned during recruitment and continued engagement of the partner workgroups.

### SESSION 1440 (SYMPOSIUM)

Abstract citation ID: igaf122.455

### FEARING FORGETTING: DEMENTIA-RELATED ANXIETY AND ITS EFFECTS ON CAREGIVERS, MENTAL HEALTH, AND SUPPORT STRATEGIES

Chair: Sabine Lohmar
Discussant: Allie Peckham

Alzheimer's disease and related dementias (hereafter "dementia") are among the most feared diseases (Awang et al., 2018). With increased exposure to dementia (e.g., caregiving; media portrayals), individuals can develop dementia worry (DW) or dementia-related anxiety (DRA), which can range in strength from mild worry to severe anxiety or phobia (Kessler et al., 2012). This symposium will explore various aspects of DW/DRA, focusing on how it impacts caregivers and those at risk for dementia and its impact on psychosocial outcomes. Sabine Lohmar, M.S., will introduce the topic with results from a systematic review on the relationship between DW and depression, highlighting at-risk groups and implications for dementia treatment. Dr. Maxfield will discuss the interpersonal consequences of DRA and association with anticipated suicidal and death ideation. Dr. Lundquist will present on her work utilizing a quantitative measure of anxiety about Alzheimer's disease with a focus on implications for improving support of individuals with DRA. Dr. Baker will share findings from a series of Community Advisory Board meetings, exploring how DRA might help bereaved dementia caregivers adopt health behaviors that could reduce their own dementia risk, inspired by caregiver fears of developing dementia. This symposium explores DW and DRA through diverse methodological approaches, including systematic review, qualitative and quantitative research, community engaged inquiry, and clinical assessment to explore the impact of DRA/DW in caregivers and at-risk individuals.

Abstract citation ID: igaf122.456

### DEMENTIA WORRY AND DEPRESSION: A SYSTEMATIC REVIEW

Sabine Lohmar, *West Virginia University, Morgantown, West Virginia, United States*

Dementia worry (DW), a response ranging from mild concern to severe anxiety about developing dementia, has been increasingly recognized as a significant psychological concern (Kessler et al., 2012). It is unclear, though, whether DW is associated with psychosocial variables such as depression (e.g., Kinzer & Suhr, 2016; Spalding et al., 2024) and no systematic reviews exist on the topic. Investigating this relationship is crucial to understanding how DW develops and is maintained. This systematic review evaluates and synthesizes the current literature on the relationship between DW and depressive symptoms. A comprehensive search of five databases was conducted between September and October of 2024. Studies were included if they were peer-reviewed, available in English, and statistically examined the relation between DW and depressive symptoms. This process identified 22 studies with 19 distinct samples conducted in six countries, encompassing 8,581 total participants. Data on sample characteristics, measures, statistical analyses, and results were extracted and compiled. Each study's quality was assessed with the Appraisal Tool for Cross-Sectional Studies (AXIS). Conclusions were drawn while weighing the strengths and weaknesses of each study. Results of this review suggest that DW and depressive symptoms are positively related, but that this relationship may vary in strength based on exposure to dementia, gender, and strength of DW. Moreover, this association may be related by avoidant behaviors, potentially exacerbating distress and delaying cognitive testing. Future research should investigate how exposure to dementia influences the strength of the relationship between DW and depression.

Abstract citation ID: igaf122.457

### DEMENTIA-RELATED ANXIETY AND ITS IMPACT ON SOCIAL CONNECTEDNESS AND SUICIDE RISK

Molly Maxfield[1], Allie Peckham[1], Dara James[2], Laura Lathrop[3], and Amy Fiske[4], *1. Arizona State University, Phoenix, Arizona, United States, 2. Arizona State University, Tempe, Arizona, United States, 3. University of Denver, Denver, Colorado, United States, 4. West Virginia University, Morgantown, West Virginia, United States*

Alzheimer's disease and related dementias (ADRD) lead to declines in independence, financial and psychological burden to those diagnosed and close others, and decreased social engagement, all of which can contribute to dementia-related anxiety (DRA). DRA involves anxiety about currently or eventually having dementia, often driven by self-perceived ADRD risk, feared changes to the self and identity, and anticipated interpersonal consequences. The interpersonal theory of suicide suggests that fundamental human desires to belong, contribute, and be needed are critical to well-being, and when undermined, the feelings of exclusion and burdensomeness is so painful, suicidal ideation (SI) may result (Van Orden et al., 2010). Therefore, to the extent that DRA has an interpersonal component, it is likely associated with SI. In a mixed methods study of 50 midlife and older adults without a dementia diagnosis (Mage = 70.80, SDage = 6.02), 42.0% of the sample anticipated suicidal ideation (14.0%) or death ideation (DI; 28.0%) if they were diagnosed with dementia. Three themes emerged from statements of anticipated SI and DI: fear of becoming a burden to others, devaluation of life/loss of self with dementia, and the desire for (and anticipated thwarting of) personal control. Since most DRA research focuses on cognitively healthy participants, it is unclear whether anticipated SI and DI translates to increased suicide risk post-diagnosis. Given evidence that death by suicide is highest within 90 days of ADRD diagnosis (Schmutte et al., 2021), we examine the intersection of three late-life public health challenges: ADRD, social disconnectedness, and suicide risk.

Abstract citation ID: igaf122.458

### REDUCING ANXIETY AND RISK: VIEWS OF THE SUPPORTING DEMENTIA CAREGIVERS AFTER DEATH ADVISORY BOARD

Zachary Baker[1], Joahana Segundo[1], and Ashley Millenbah[2], *1. Arizona State University, Phoenix, Arizona, United States, 2. University of Minnesota, Minneapolis, Minnesota, United States*

The Supporting Dementia Caregivers After Death Community Advisory Board (CAB) includes members with lived experience caring for people with dementia who have since died. Some CAB members expressed a strong fear of developing dementia, with a few stating they would prefer death over putting their families through caregiving. Many believed their own dementia risk was high. Given consensus that 14 lifestyle factors could potentially prevent 45% of dementia cases, we explored whether dementia-related anxiety could be leveraged to promote risk-reducing behaviors. CAB members were interested in learning about and improving these lifestyle factors, particularly social isolation, hearing loss, depression, and air pollution. While using dementia-related anxiety as motivation was not controversial, CAB members questioned whether dementia-related anxiety alone was a strong enough driver for behavior change. Instead, they suggested that discussions about dementia risk could serve as an entry point for broader conversations on prevention. Key themes emerged for designing interventions to improve lifestyle factors: (1) Timing—interventions soon after death might make risk feel more urgent but could be overwhelming during acute experiences of grief; (2) Self-Determination—interventions should empower individuals to choose which factors to address; (3) Leveraging existing resources—utilizing current programs rather than developing new ones; and (4) Perceived acuteness of risk—older adults may feel less able to modify risk, while younger adults may see the threat as too distant. These insights highlight the need for tailored, sensitive approaches to dementia risk reduction among bereaved dementia caregivers.

Abstract citation ID: igaf122.459

### USE OF A QUESTIONNAIRE MEASURING ANXIETY ABOUT ALZHEIMER'S DISEASE AND IMPLICATIONS FOR IMPROVING CARE

Tessa Lundquist, *VA Boston Health Care System, Boston, Massachusetts, United States*

Studies show that older adults have anxiety about Alzheimer's disease (AD). There has been limited research utilizing a quantitative measure of this construct, and it is not known how anxiety about AD may impact wellbeing and health behavior. This presentation will review the use of a quantitative measure of AD anxiety and implications for improving support of older adults who worry about their memory. The AD Anxiety scale was administered to a sample of 43 older adults. The AD Anxiety scale exhibited adequate internal consistency (Cronbach's alpha = .71). Participants had a mean AD Anxiety score of 19.89 (SD = 4.16, range 7-35). Participants who reported having concerns about their memory had significantly higher AD anxiety (M = 21.25, SD = 3.55; $t(33) = 2.71$, $p < .05$) than those who reported no such concerns (M = 18.11, SD = 3.45). AD Anxiety was distinct from more general state trait anxiety. Findings suggest that AD anxiety is a distinct form of anxiety and impacts participants with varying demographics differently. Research is mixed on how dementia worry may impact health related behavior. It will be important to determine if AD anxiety may be motivating for proactive health behavior or if it may lead to avoidance. Female participants had higher AD anxiety than males. Similarly, participants with a family history of dementia had more AD anxiety than those with no family history. Older adults' demographics and previous experiences with dementia should be considered when addressing their AD anxiety for optimal health outcomes.

### SESSION 1445 (SYMPOSIUM)

Abstract citation ID: igaf122.460

### THE DAILY CONTEXT OF MENTAL AND COGNITIVE HEALTH: USING AN ECOLOGICAL MOMENTARY ASSESSMENT (EMA) APPROACH

Chair: Jee eun Kang

Mental health and cognitive performance fluctuate with daily experiences, yet most research relies on retrospective reports that do not capture real-time variations. Ecological momentary assessment (EMA) enables examination of how contextual experiences unfold in everyday life. This symposium showcases studies using EMA to investigate how psychological and cognitive processes in daily life influence cognitive experiences in aging populations and how they represent potential entry points for intervention. First, Dr. Compernolle will describe social and physical contexts that contribute to fluctuations in loneliness—an important indicator of social and mental well-being—using findings from the Chicago Health and Activity Space in Real Time study. Next, Drs. Kang and Felt will highlight the dynamic relationship between loneliness and cognitive performance in daily life in an Einstein Aging Study cohort. Kang will discuss the reciprocal association between loneliness and daily memory lapses, demonstrating how loneliness both predicts and results from memory lapses. Felt will explore bidirectional links between loneliness and objective cognitive performance, showing that higher cognitive performance predicts lower subsequent loneliness, whereas loneliness is associated with slower processing speed. Finally, Dr. Sutin will describe how fluctuations in momentary purpose in life link with cognitive performance, revealing that while greater purpose is linked to better cognitive performance, it may also temporarily tax attentional resources, leading to increased memory lapses. Together, these presentations will highlight how momentary psychological experiences—including loneliness and purpose in life—shape cognitive performance and vice versa, emphasizing the importance of real-time assessments in understanding and promoting cognitive and emotional well-being in later life.

Abstract citation ID: igaf122.461

### LONELINESS AND DAILY MEMORY LAPSES IN OLDER ADULTS: FINDINGS FROM THE EINSTEIN AGING STUDY

Jee eun Kang, Christopher Engeland, Martin Sliwinski, and Jennifer Graham-Engeland, *The Pennsylvania State University, University Park, Pennsylvania, United States*

Although loneliness is a known risk factor for cognitive decline, the degree to which it predicts subjective cognitive functioning—such as daily memory lapses—is unclear. Moreover, memory difficulties themselves may contribute to feelings of loneliness by disrupting social interactions and diminishing confidence in cognitive abilities. This study utilized ecological momentary assessment (EMA) data to examine the bidirectional daily associations between loneliness and self-reported prospective (PM) and retrospective memory (RM) lapses. A sample of 263 older adults (mean age = 77 years, 67% women, 43% Black, 31% MCI) completed 14 days of EMA, reporting momentary loneliness (5×/day) and daily memory lapses each evening. Multilevel models tested the effects of loneliness on same-day and next-day memory lapses and vice versa, controlling for study day, age, gender, education, MCI status, marital status, living arrangement, and prior-day outcomes. On days when participants felt lonelier than usual, they were more likely to forget meetings (OR = 1.07, p=.052), although this effect was marginally significant; moreover, forgetting meetings predicted higher loneliness the next day (b = 4.2, p=.003). Further, greater irritation from PM lapses was associated with higher loneliness on the same (b=.03, p=.020) and next day (b=.03, p=.04). Higher loneliness was also associated with forgetting important information (OR = 1.03, p=.049) on the same day and misplacing items the next day (OR = 1.03, p=.010). Findings highlight a potentially bidirectional relationship, suggesting that loneliness contributes to daily cognitive difficulties, which, in turn, may reinforce loneliness. Addressing memory lapses as well as loneliness may help support cognitive and emotional well-being in aging populations.

Abstract citation ID: igaf122.462

### MOMENTARY LONELINESS AMONG OLDER ADULTS: REAL-TIME CORRELATES AND POTENTIAL ENTRY POINTS FOR INTERVENTION

Nell Compernolle[1], Louise Hawkley[2], and Ellen Bloss[1], *1. NORC at the University of Chicago, Chicago, Illinois, United States, 2. National Opinion Research Center at The University of Chicago, Chicago, Illinois, United States*

Background and Objectives: Momentary loneliness remains at the forefront of investigations into the complexities of loneliness. Ecological momentary assessments (EMAs) have enabled researchers to consider the relevance of real-time social context for loneliness rather than rely on the long-documented more stable measures of social integration. Here, we summarize a series of studies that provide novel insights into who may be more likely to experience momentary loneliness, when, and implications for intervention. Methods: We use data from the Chicago Health and Activity Space in Real Time study (CHART), a population-based cluster sample of 450 adults ages 65+. A series of regression models examined associations between real-time physical and social context and momentary loneliness, as well as moderating effects of more stable social factors and socio-demographic characteristics. Subsequent focus group discussions explored the nuance to older adults' momentary loneliness experiences. Results: Findings identify being momentarily alone and/or at home as key risk factors for momentary loneliness. However, these loneliness-inducing contexts were stronger for certain sub-populations, those with large personal networks, and during the COVID-19 pandemic (versus before). Qualitative research highlights social company departures, misalignment in socializing desires, and a lack of belonging as additional real-time factors increasing risk. Discussion: Results are among the first to identify contextual effects on real-time loneliness in older adults and how these associations vary across individuals and more stable and societal-level contexts. Group discussions further inform the feasibility and utility of a just-in-time adaptive intervention to reduce momentary loneliness via key antecedents personalized to users.

Abstract citation ID: igaf122.463

### CROSS-LAGGED RELATIONS BETWEEN LONELINESS AND COGNITIVE PERFORMANCE IN DAILY LIFE AMONG OLDER ADULTS

John Felt[1], Jee eun Kang[1], Karina Van Bogart[2], Martin Sliwinski[1], Christopher Engeland[1], and Jennifer Grahama-Engeland[1], *1. The Pennsylvania State University, University Park, Pennsylvania, United States, 2. Northwestern University, Chicago, Illinois, United States*

Loneliness affects 24-33% of older adults and has been associated with a 26% increased risk for developing Alzheimer's Disease and related dementias (ADRDs). Given that ADRDs have a long preclinical period, loneliness may arise in response to earlier cognitive changes and act as a preclinical indicator. The purpose of this study was to investigate bidirectional associations between loneliness and cognitive performance in older adults. Data comes from a burst of the Einstein Aging Study, a community sample of 318 adults over the age of 70 from Bronx, NY who were dementia free at baseline. Participants completed 5 ecological momentary assessments (EMAs) per day for 14 consecutive days. Participants self-reported how lonely they felt and completed 3 brief (~45 seconds per task) cognitive tasks at each EMA (Grid Memory, Color Shapes, and Symbol Search). We used dynamic structural equation modeling (DSEM) to test the cross-lagged relations between loneliness and cognitive performance on the three tasks (~every 3-4 hours). Results indicated that higher cognitive performance at one EMA was associated with lower levels of loneliness on the following EMA for the proportion of shapes correctly identified in the Color Shape task. There was also some evidence that higher levels of loneliness were associated with slower reaction time on the Symbol Search task. Further, higher levels of loneliness at one EMA were associated with slower performance on the Color Shape task. These results will be discussed in the context of pre-clinical mechanisms and additional results on multiple burst data will be presented.

Abstract citation ID: igaf122.464

### PURPOSE IN LIFE AND COGNITION: MOMENTARY ASSOCIATIONS FROM A MICRO-LONGITUDINAL STUDY OF DAILY LIFE

Angelina R. Sutin[1], Martina Luchetti[1], and Antonio Terracciano[2], *1. Florida State University College of Medicine, Tallahassee, Florida, United States, 2. Florida State University, Tallahassee, Florida, United States*

Purpose in life, the feeling the one's life is goal-oriented and has direction, is associated with healthier cognitive outcomes in older adulthood. A better understanding of how purpose supports cognitive function in daily life is needed to inform intervention development. The present study replicates and extends recent evidence that momentary feelings of purpose help support cognitive function throughout the day. Using ecological momentary assessment methodology, participants from the Purpose and Engagement in Everyday Life study (N = 1573; Mage=66.06, range 40-93) reported on their momentary purpose and cognitive complaints and completed three short cognitive tasks three times a day for eight days (n = 32,375 momentary assessments). Consistent with the literature, between-person, momentary purpose was associated with better performance on the three cognitive tasks and reporting fewer cognitive complaints. Within person, in moments when participants felt more purposeful than their average, they had faster processing speed (b=-.05, SE=.02, p=.002) and executive functioning (b=-.16, SE=.05, p<.001) and reported fewer problems with concentration (b=-.10, SE=.01, p<.001); momentary purpose was unrelated to visual memory. Momentary purpose, however, was also associated with more reported memory lapses measured concurrently in the moment (b=.03, SE=.01, p=.002) and at the next moment (b=.04, SE=.02, p=.005). The present findings replicate the momentary association between purpose and processing speed with different measures of both constructs than used in previous research, extends the momentary association to executive functioning and concentration, and suggests that purpose may generally be beneficial, but it may tax attentional resources that temporarily results in forgetfulness in daily life.

### SESSION 1450 (SYMPOSIUM)

Abstract citation ID: igaf122.465

### POLICY SERIES: CURRENT RESEARCH, OPPORTUNITIES, AND RECOMMENDATIONS FOR DIVERSE HEALTH AND AGING

Chair: Angela Perone
Discussant: Brian Lindberg

This session will identify and analyze recent research on diverse representation in study populations, research teams, and leadership, while addressing persistent gaps in minority health aging research due to a lack of robust diversity and equity principles. It will evaluate existing research opportunities to include diverse participants and team members, exploring innovations in access to care, culturally responsive interventions, and health equity. The discussion will propose future actionable research recommendations for integrating diversity and equity into all phases of minority health aging research. This includes strategies for enhancing the recruitment and retention of diverse participants, promoting inclusive research environments, and ensuring the equitable dissemination of findings to inform public policy.

### SESSION 1455 (SYMPOSIUM)

Abstract citation ID: igaf122.466

### LONG-TERM EFFECTS OF LIFESTYLE INTERVENTIONS TARGETING DIABETES AND WEIGHT LOSS ON AGING-RELATED OUTCOMES

Chair: Peter Huckfeldt
Discussant: Kristine Ensrud

Type 2 diabetes can lead to other chronic conditions (e.g., heart disease) and physical disability, and when paired with obesity, accelerates aging processes and functional decline, threaten independence and quality of life, and increase clinical care needs and complexity. Lifestyle interventions focused on reducing weight and increasing physical activity have been shown to improve diabetes control and functional status and reduce multimorbidity. However, their long-term benefits remain incompletely understood. The Diabetes Prevention Program (DPP) and the Action for Health in Diabetes (Look AHEAD) study examined the effects of lifestyle interventions on delaying the onset of type 2 diabetes and diabetes-related cardiovascular outcomes. Between 1996 and 2021, the DPP randomized 3,234 participants (mean age, 51 yrs) who were at high risk for developing type 2 diabetes to an intensive lifestyle intervention (ILI) arm, metformin, or a control group. Look AHEAD randomized 5,145 participants (mean age, 58.7 yrs) with type 2 diabetes and overweight or obesity to an ILI or a control group between 2001 and 2004. While both interventions concluded over 10 years ago, participants' health status and health care use has been continuously monitored through a combination of surveys and linkages to Medicare data, providing ideal data for studying the long-term effects of lifestyle intervention. This symposium will present research on the long-term effects of an ILI on multimorbidity in DPP (Salive and colleagues), nursing home residence and institutional days in Look AHEAD (Huckfeldt and colleagues), and mortality in Look AHEAD (Houston and colleagues).

Abstract citation ID: igaf122.467

### EFFECT OF LIFESTYLE OR METFORMIN ON CLAIMS-BASED MULTIMORBIDITY IN THE DIABETES PREVENTION PROGRAM OUTCOMES STUDY

Marcel Salive[1], Jesse Ames[2], Ashley Tjaden[2], Jill Crandall[3], Dana Dabelea[4], Helen Hazuda[5], Brandy Heckman-Stoddard[6], and Elizabeth Venditti[7], *1. National Institutes of Health, Rockville, Maryland, United States, 2. The George Washington University Biostatistics Center, Rockville, Maryland, United States, 3. Albert Einstein College of Medicine, Bronx, New York, United States, 4. Lifecourse Epidemiology of Adiposity & Diabetes Center, Aurora, Colorado, United States, 5. UT Health San Antonio, San Antonio, Texas, United States, 6. National Cancer Institute Division of Cancer Prevention, Bethesda, Maryland, United States, 7. University of Pittsburgh, Pittsburgh, Pennsylvania, United States*

Studying how to prevent or delay not just one disease, e.g., diabetes, but multiple chronic conditions would have great potential for public health, yet few interventions have demonstrated success. We aimed to examine the impact of the lifestyle and metformin interventions, compared to placebo, on the long-term occurrence of multimorbidity. 3,234 adults at high risk of diabetes joined the DPP in 1996-1999 (mean age 51 years), were randomized to lifestyle, metformin, or placebo interventions, and followed through 2020. Consenting study participants' data were matched with CMS data. Multimorbidity was defined as presence of ≥ 2 of 15 prevalent chronic conditions, as defined in CMS' Chronic Condition Warehouse for fee-for-service claims and as adapted for Medicare Advantage encounters. Logistic regression models estimated the association between randomized treatment group and multimorbidity outcomes. Of 1,173 participants for whom Medicare data were available, 85% had ≥2 conditions (median=5). Odds of multimorbidity were lower among lifestyle participants compared to placebo (OR = 0.64; 95% CI = 0.42, 0.99) after adjustment for relevant covariates, but there were no differences between metformin and placebo participants. When restricting to high-cost dyad conditions, the results were similar (lifestyle vs. placebo: OR = 0.55; 95% CI = 0.35, 0.88). Increased odds of multimorbidity (excluding diabetes) were associated with the presence of diabetes, higher time-weighted HbA1c, and longer diabetes duration. Among a diabetes-related cohort, lifestyle intervention, not metformin, was associated with lower risk of multimorbidity >15 years after the end of original intervention. Multimorbidity was related to multiple diabetes measures.

Abstract citation ID: igaf122.468

### LONG-TERM EFFECTS OF INTENSIVE LIFESTYLE INTERVENTION ON NURSING HOME STAYS AND INSTITUTIONAL DAYS

Peter Huckfeldt[1], Ann Harada[2], Lynne Wagenknecht[3], Charles Semelka[3], Mark Woodhouse[1], Govinda Yost[4], Mark Espeland[3], and Denise Houston[3], *1. University of Minnesota, Minneapolis, Minnesota, United States, 2. Leonard D. Schaeffer Center for Health Policy & Economics, Los Angeles, California, United States, 3. Wake Forest University School of Medicine, Winston-Salem, North Carolina, United States, 4. University of Minnesota School Of Public Health, Minneapolis, Minnesota, United States*

Diabetes and obesity can accelerate aging processes and functional decline and are associated with a higher lifetime risk of nursing home (NH) admission among older adults. The Look AHEAD study randomized 5,145 participants with type 2 diabetes and overweight or obesity to an intensive lifestyle intervention (ILI) focused on weight loss and increased physical activity compared to a control group receiving diabetes support and education. The Look AHEAD ILI led to long-term improvements in mobility, physical function, and moderate/severe physical disability. These benefits could translate to lower needs for care received in a NH, hospital, or post-acute care facility. We linked 3038 consenting participants in the Look AHEAD study to Medicare data and identified short NH stays (90 or fewer days) and long NH stays (more than 90 days) in the Long Term Care Minimum Data Set in the eight years immediately following the termination of the ~10-year intervention (2013-2020). We found that 22.2% of ILI and 22.1% of controls received any NH care during this period and 1.9% of ILI and 2.9% of controls had any long NH stays. There was no significant difference between arms (p = 0.706 for any NH and p = 0.093 for long NH stays), suggesting that improvements in functional status did not translate to reduced NH admissions. In ongoing work, we are examining the association of improved mobility and physical function during and after the intervention with "healthy days at home", which are defined as days not spent in a hospital, post-acute care facility, or NH.

Abstract citation ID: igaf122.469

### LONG-TERM ASSOCIATION OF AN INTENSIVE LIFESTYLE INTERVENTION ON MORTALITY: THE LOOK AHEAD STUDY

Denise Houston[1], Joni Evans[1], Charles Semelka[1], Haiying Chen[1], Peter Huckfeldt[2], Marcel Salive[3], Mark Espeland[1], and Lynne Wagenknecht[1], *1. Wake Forest University School of Medicine, Winston-Salem, North Carolina, United States, 2. University of Minnesota, Minneapolis, Minnesota, United States, 3. National Institutes of Health, Rockville, Maryland, United States*

Although weight loss is recommended for individuals with type 2 diabetes and obesity to improve diabetes- and obesity-related risk factors, few studies have examined the effects of intentional weight loss on mortality. Look AHEAD, a randomized trial in 5,145 individuals aged 45-76 yrs with type 2 diabetes and overweight/obesity comparing an intensive lifestyle intervention (ILI) designed to achieve 7% weight loss to diabetes support and education (DSE), previously found no significant differences in all-cause mortality during 10 yrs of intervention or at 17 yrs of follow-up. Since the effects of intentional weight loss on mortality risk may take many years to emerge, we conducted a follow-up examination of all-cause mortality over 23 yrs of follow-up using proportional hazards regression. Interactions between ILI and mortality were also examined in pre-specified subgroups. A total of 800 deaths in ILI and 861 deaths in DSE were observed over 80,725 person-yrs. ILI experienced a lower incidence of all-cause mortality relative to DSE (HR [95% CI]: 0.89 [0.81, 0.98]). There were no significant interactions between treatment and age, sex, or cardiovascular disease history. However, there were significant race/ethnicity by treatment interactions (p = 0.01), with HRs (95% CIs) for participants identifying as African American 0.96 (0.75, 1.23), Caucasian 0.94 (0.84, 1.05), and Hispanic 0.54 (0.39, 0.74). In middle aged and older adults with type 2 diabetes and overweight/obesity, intentional weight loss modestly reduced all-cause mortality risk over 23 yrs of follow-up, which appeared to be largely driven by a reduction in mortality among participants of Hispanic ethnicity.

### SESSION 1460 (SYMPOSIUM)

Abstract citation ID: igaf122.470

### PROTEOMICS AND AGING: INSIGHTS FROM THE OSTEOPOROTIC FRACTURES IN MEN STUDY

Chair: Peggy Cawthon

Aging-related declines in muscle mass, frailty, and bone strength significantly impact health outcomes in older adults. This symposium presents cutting-edge research leveraging proteomics to uncover biological determinants of these age-related factors, using data from the Osteoporotic Fractures in Men (MrOS) Study. The first presentation examines proteomic determinants of muscle mass assessed using deuterated creatine dilution (D3Cr) and highlights key proteins, including novel targets like SVEP1 and brorin, that may play roles in muscle preservation. The second study explores frailty-related proteomic signatures, identifying 76 proteins associated with frailty, including markers linked to inflammation, immune response, and tissue repair. The third presentation focuses on proteomic predictors of bone loss, revealing site-specific skeletal protein associations and demonstrating that a multi-protein signature is a stronger predictor of bone loss than traditional biomarkers. Finally, the symposium concludes with an AI-driven analysis integrating proteomics and metabolomics to predict biological age, showcasing transformer-based models that outperform traditional machine learning approaches in identifying molecular markers of aging. Together, these studies advance our understanding of the molecular pathways underlying muscle loss, frailty, and bone deterioration in aging, while demonstrating the power of multi-omic and AI-based approaches in geroscience. This research paves the way for novel biomarkers and therapeutic targets to improve health outcomes in aging populations.

Abstract citation ID: igaf122.471

**PROTEOMIC DETERMINANTS OF MUSCLE SIZE IN OLDER MEN: THE OSTEOPOROTIC FRACTURES IN MEN STUDY**

Peggy Cawthon[1], Tyler Mansfield[1], Jodi Lapidus[2], William Evans[3], Daniel Evans[4], and Eric Orwoll[2], *1. California Pacific Medical Center, San Francisco, California, United States, 2. Oregon Health & Science University, Portland, Oregon, United States, 3. University of California, Berkeley, Berkeley, California, United States, 4. University of California, San Francisco, San Francisco, California, United States*

In MrOS, men with low muscle mass as determined by deuterated creatine dilution (D3Cr muscle mass) are weaker, slower, and have a higher risk of incident falls, mobility limitation, disability, fractures and death. Proteomic determinants of D3Cr muscle mass are not known. Serum proteomics were assayed using the SOMAlogic 7K panel. Of the 7166 aptamers included in analyses, 475 were significantly associated with d3Cr muscle after adjusting for age and weight (adjusted $p<0.05$). In age and weight adjusted models controlling for multiple comparisons, we found significant positive associations between D3Cr muscle mass with creatine kinase M type, and creatine kinase M type:B type heterodimer ($r>0.34$, adjusted $p<3x10^(-10)$ for both). These positive correlations are expected, as the D3Cr dilution approach relies on assessment of total body creatine pool size, thus, relative abundance of creatine kinase should be related to D3Cr muscle mass given the properties of this assay. When controlling for multiple comparisons, we also found significant negative correlations between D3Cr muscle mass and SVEP1 (protein with a role in vascular disease) and brorin (a bone morphogenic protein) ($r<-0.23$, adjusted $p<3x10^(-7)$ for all); these may represent new targets to identify or target low muscle mass. Leptin and FSTL3 were negatively correlated with D3Cr muscle mass ($r<-0.25$, adjusted $p<6x10^(-6)$ for both). Further work will identify pathways rather than specific proteins that relate to low D3Cr muscle mass, and whether these associations replicate in other populations and in women.

Abstract citation ID: igaf122.472

**PROTEOMICS OF HRPQCT BONE LOSS IN OLDER MEN**

Eric Orwoll[1], Adam Burns[1], Jack Wiendrick[1], Alicia Feryn[1], Andrew Burghardt[2], and Jodi Lapidus[1], *1. Oregon Health & Science University, Portland, Oregon, United States, 2. University of California, San Francisco, San Francisco, California, United States*

The biological basis of bone loss in older people is unclear, and current biomarkers are of limited usefulness. We measured serum protein levels (SomaLogic 7K panel) in 230 men (84+ 3 years) and determined associations with HRpQCT change (distal radius, distal tibia) over ~6 yrs, with adjustments for time between measures, age, height, weight and genomic principal components. Primary skeletal outcomes were total vBMD and failure load. Linear regression of proportional change in HRpQCT was used to assess protein associations. 16 proteins were robustly associated with skeletal change, 7 at the radius and 10 at the tibia, with only one protein associated with change at both measurement sites. In almost all cases, higher proteins levels were related to increased bone loss. Associations at the radius and tibia were concordant in direction, and associations with change in total vBMD and failure load were essentially the same. Associations with changes in DXA hip BMD were similar. FSH levels were most strongly (negatively) related to change. A 19-protein signature was much more strongly predictive of bone loss than traditional bone remodeling markers (C statistics: signature - 0.83, CTX – 0.58, P1NP – 0.54). In sum, serum protein levels were strongly associated with skeletal change, the association patterns were distinct at the radius and tibia, suggesting the biological pathways governing bone change at the two sites are unique. A protein signature may represent a powerful biomarker of bone loss.

Abstract citation ID: igaf122.473

**MULTI-OMIC AGE ASSOCIATIONS IDENTIFIED WITH ARTIFICIAL INTELLIGENCE (AI)**

Daniel Evans[1], Jiashun Zheng[1], Peggy Cawthon[2], Steven Cummings[2], and Hao Li[1], *1. University of California, San Francisco, San Francisco, California, United States, 2. California Pacific Medical Center, San Francisco, California, United States*

There is substantial interest in using Machine Learning (ML) and Artificial Intelligence (AI) approaches to integrate multi-omics data to identify molecular factors associated with age. Multi-omic AI models can be used to predict "biological age" and to identify age-associated molecular features. To-date, predictions from traditional ML methods like elastic-net have outperformed AI models like deep learning with tabular (spreadsheet-like) data. However, new developments in transformer architectures have led to AI models that can outperform traditional ML methods. In this work, a tabular foundation AI model (TabPFN) built on transformer-based in-context learning (ICL) algorithms is used to integrate proteomics and metabolomics to identify models for age in a cohort study of older men, the Osteoporotic Fractures in Men (MrOS) Study. The serum proteomics assay was the SomaLogic 7K panel, and the serum metabolomics assay was the Metabolon Discovery HD platform. In 493 male participants split into training and test partitions, TabPFN models for age adjusted for body mass index (BMI) and estimated glomerular filtration rate (eGFR) achieved a root mean square error (RMSE) in the test set of 2.75 years, which outperformed an elastic-net model RMSE in the test set of 3.1 years. Proteins and metabolites contributing to the TabPFN model for age identified by Shapley Additive exPlanations (SHAP) included Pleiotrophin, Frizzled-7, secreted frizzled-related protein 1, and N-acetylcarnosine. Multi-omic AI models outperformed traditional ML models for age and identified biologically relevant features. Future directions include replication of findings in other studies and assessment of model performance in females.

Abstract citation ID: igaf122.474

**PROTEOMIC ASSOCIATIONS AND SIGNATURES OF FRAILTY AMONG OLDER MEN**

Jodi Lapidus[1], Alicia Feryn[1], Jack Wiendrick[1], Adam Burns[1], Eric Orwoll[1], and Kristine Ensrud[2], *1. Oregon Health & Science University, Portland, Oregon, United States, 2. University of Minnesota, United States*

Aging leads to biological changes that influence frailty onset and prevalence, and these changes are likely reflected in circulating proteins. In this study, we analyzed serum proteomics (SomaLogic 7K panel) from 500 older men (average age 84 ± 3.4 years) in the MrOS study at the fourth visit in 2015. A phenotypic frailty index was computed, including several components: shrinkage (recent weight loss, low BMI), weakness (grip strength <32 kg), low energy, slowness (gait speed <0.8 m/s, use of a walking aid), and low physical activity (no walking for exercise). Participants were classified as robust (22%), intermediate (58%), or frail (20%). Our objective was to identify proteins and co-expression protein modules linked to frailty and create multi-protein signatures of frailty. Seventy-six proteins showed consistent associations with frailty (e.g., odds ratio > |1.5| or standardized effects > 0.3). Frailty-associated proteins included C/EBPβ (linked to inflammation-related diseases), ARIDA1 (involved in DNA repair and stem cell maintenance), APP (related to Alzheimer's), IL4 (a cytokine regulator in inflammation and wound healing), and AKT1 (a mediator of cellular functions linked to immune issues, proteostasis failure, and cancer). Other frailty-associated proteins were involved in tissue damage/repair (e.g., collagens, TFF3, GDF15) and immune function activation (e.g., CCL15, Cystatin C, B2M). The presentation will also highlight proteins associated with specific frailty components and cluster proteins with similar associations. This research aims to enhance understanding of how proteins and their interconnected biological networks contribute to the multifaceted aspects of frailty in older populations.

### SESSION 1465 (SYMPOSIUM)

Abstract citation ID: igaf122.475

**WHAT IS DRIVING THE NEED FOR HOME-DELIVERED MEALS? FINDINGS FROM THE DELIVER-EE STUDY**

Chair: Kali Thomas
Co-Chair: Bernadette Wright
Discussant: Julie Locher

Home-delivered meals are provided to 2.2 million older adults each year. Prior research has documented the benefit of meals for older adults' health and nutrition; however, the funding for home-delivered meals has not kept pace with the need. This symposium leverages findings from the Deliver-EE study to describe the varied reasons that older adults seek out nutrition support and their experiences after receiving home-delivered meals. Deliver-EE is a pragmatic clinical trial funded by the Patient-Centered Outcomes Research Institute and operates in partnership with Meals on Wheels America and the AARP Foundation. Deliver-EE is enrolling 2,300 older adults ages 66 years and older from waitlists at 14 Meals on Wheels programs located in California, Texas, Maryland, North Carolina, South Carolina, Tennessee, Illinois and Florida. This panel features a series of presentations focused on the pre-intervention experiences of participants, bridging their insights from quantitative administrative and baseline survey data (n = 1775) with qualitative interviews (n = 99). The first presenter highlights challenges of diet quality and food insecurity among home-delivered meal recipients; The second discusses financial strain and related coping strategies observed in this sample; The third shares findings about this sample's social isolation and loneliness; and The fourth describes the benefits and unmet needs that both caregivers and older adults report from receiving home-delivered meals. Our discussant situates the findings in the landscape of nutrition research and shares reactions to findings in the context of the current and future need for home-delivered meals.

Abstract citation ID: igaf122.476

**DIETARY RISK AMONG OLDER ADULTS: EXAMINING THE ROLE OF FOOD INSECURITY AND SOCIAL ISOLATION**

Pierre Jolin[1], Patricia Markham Risica[1], Julie Locher[2], James Jolin[3], and Kali Thomas[4], *1. Brown University, Providence, Rhode Island, United States, 2. The University of Alabama at Birmingham, Birmingham, Alabama, United States, 3. University of Cambridge Department of Public Health and Primary Care, Cambridge, England, United Kingdom, 4. Johns Hopkins University, Baltimore, Maryland, United States*

As the aging U.S. population grows, so do healthcare challenges, particularly in nutrition and dietary quality. Older adults face a higher risk of malnutrition, affecting health and quality of life. Dietary intake is influenced by health, social environments, and food access, but their combined impact remains unclear among vulnerable populations. While food security is recognized as crucial for adequate nutrition, the role of social and environmental factors in dietary quality is less understood. The Deliver-EE trials aim to explore these relationships. The purpose of our investigation is to assess the relationships between social isolation, food insecurity, and dietary risk, contributing to a deeper understanding of nutrition among older adults. With a Dietary Screening Tool (DST) threshold of ≤ 60, 93% of the sample was categorized as at risk for low dietary quality. Food insecurity in the sample was 52%, yet a high proportion of both food-insecure and food-secure individuals were at risk for low dietary quality (48% and 45% respectively). No significant association was found between social isolation and DST scores. Dietary risk does not seem to be associated with food insecurity or social isolation. These findings suggest that systemic factors, rather than ability to afford food and social connectivity, drive dietary risk. Future research should examine nutritional gaps in food assistance programs and structural determinants of dietary quality in vulnerable populations.

Abstract citation ID: igaf122.477

**THE ROLE OF FOOD INSECURITY IN RELATION TO MEALS ON WHEELS PARTICIPATION**

Laura Samuel[1], Emily Gadbois[2], Kimberly Bernard[3], Claire Wang[1], Nichole Stetten[3], Tiffany Riser[1], Christopher Liu[3], and Kali Thomas[1], *1. Johns Hopkins University, Baltimore, Maryland, United States, 2. Brown University School of Public Health, Providence, Rhode Island, United States, 3. Brown University, Providence, Rhode Island, United States*

Deliver-EE participants have a five-fold higher risk of food insecurity than typical U.S. older adults (47% vs. 9% based on

2023 national data), defined as the inability to afford a consistently balanced diet. This is notable because food insecurity is associated with adverse outcomes including health care spending and dementia risk. Based on subgroup data (n = 376), 93% of participants have financial strain where they had either "just enough" or "not enough" money to make ends meet. This study explored the reasons for enrolling in Meals on Wheels using semi-structured interviews among 31 participants experiencing food insecurity or financial strain. Preliminary findings found many enrolled because they couldn't afford food and/or transportation, as evidenced by one participant, "I just didn't have the money." Others described how financial strain in the presence of functional difficulties with shopping and/or cooking led them to seek meals, including one who said "So it's kind of hard to get out and shop. Plus, we're both low income, and we're seniors.". Notably, 37% with food insecurity had Activities in Daily Living (ADL) limitations and 72% had Instrumental ADL limitations, highlighting the co-occurrence of food insecurity and functional limitations. Participants also discussed that prepared foods are typically more expensive and less nutritious than food cooked at home; many said that they enrolled because they wanted a balanced and affordable diet that included hot food. These results suggest that Meals on Wheels meets a critical nutrition gap for food insecure older adults, especially those with functional limitations.

Abstract citation ID: igaf122.478

### CRAVING MORE THAN MEALS: SOCIAL ISOLATION AMONG OLDER ADULTS ON MEALS ON WHEELS WAITLISTS

Kimberly Bernard[1], Emily Gadbois[2], Melissa Clark[1], Em Balkan[1], Laura Dionne[1], Christopher Liu[1], and Kali Thomas[3], *1. Brown University, Providence, Rhode Island, United States, 2. Brown University School of Public Health, Providence, Rhode Island, United States, 3. Johns Hopkins University, Baltimore, Maryland, United States*

Prolonged periods of social isolation are increasingly found to have negative impacts on health and well-being. Older adults are particularly at risk as social networks tend to decrease after 65 years of age. Recent nationally representative studies have found that 1 in 4 community dwelling older adults are socially isolated. Slightly more than half of the participants in the Deliver-EE clinical trial lived alone (54%). Approximately, 20% of the total sample reported that they often felt isolated from others, this percent was similar between those who lived alone and lived with others. We examined whether those experiencing social isolation were more likely to request meal schedules that provided regular contact with a paid/volunteer driver five times per week versus an alternative (once every two weeks). We found that those who were lonely were more likely to request daily deliveries (45% versus 41%). To better understand the potential impacts of delivery contacts on socially isolated participants, we analyzed 54 qualitative client interviews for reports of interactions with delivery drivers. Clients described a range of socialization experiences from "brief and polite" to having more substantive conversations with their drivers. When probed, clients often shared that they will refrain from initiating conversation out of concern that they will put the driver off their schedule. With active encouragement, socially isolated older adults could get additional benefits from meal programs that provide daily delivery touch points.

Abstract citation ID: igaf122.479

### MEALS ON WHEELS AND FAMILY CAREGIVING: BENEFITS RECEIVED AND UNMET NEEDS

Emily Gadbois[1], Nichole Stetten[2], Laura Samuel[3], Kimberly Bernard[2], Em Balkan[2], Yiqing Qian[4], and Kali Thomas[3], *1. Brown University School of Public Health, Providence, Rhode Island, United States, 2. Brown University, Providence, Rhode Island, United States, 3. Johns Hopkins University, Baltimore, Maryland, United States, 4. Johns Hopkins University School of Nursing, Baltimore, Maryland, United States*

Long-term services and supports are frequently provided by millions of unpaid family caregivers to community-dwelling older adults. Meals on Wheels programs provide home-delivered meals that help older adults continue to live in their communities. The benefits of Meals on Wheels programs to their clients are well-documented, but impacts on caregivers of those clients are less clear. In-depth, semi-structured interviews were conducted with 85 clients and 14 caregivers from 13 Meals on Wheels programs. Through these 99 interviews, we learned about the care and assistance provided by unpaid caregivers which included food-related support (acquiring and/or paying for groceries, cooking, initiating Meals on Wheels), physical care and assistance with activities of daily living and instrumental activities of daily living, transportation, medication and healthcare management, and financial support. Although clients and caregivers reported that Meals on Wheels eased some caregiving burden, they also identified substantial unmet needs. Many clients expressed that they would benefit from additional meals, as well as financial, transportation, and hands-on support. Caregivers reported substantial burden, and suggested that they would benefit from assistance with financial and care planning, identifying formal supports, and a desire for respite care. While Meals on Wheels provide essential nutrition support to older adults, this is just one component of need and its services are not intended to address all the care needs of older adults or their caregivers. This research is responsive to the Recognize, Assist, Include, Support, and Engage (RAISE) Family Caregivers Act and the associated National Strategy to Support Family Caregivers.

### SESSION 1470 (SYMPOSIUM)

Abstract citation ID: igaf122.480

### FROM INNOVATION TO IMPLEMENTATION: TRANSFORMING GERIATRIC CARE IN THE VA SYSTEM

Chair: Caroline Madrigal
Co-Chair: Lea Kiefer
Discussant: Marianne Shaughnessy

Veterans Affairs (VA) has a long-standing commitment to providing high-quality geriatric care, driven by a history of innovation and a dedication to meeting the unique needs of older Veterans. This symposium will provide an overview of the evolution of geriatric clinical innovations in VA, highlighting organizational milestones, successful models of care, and strategies for sustainable implementation. We will review the development of VA's geriatric care infrastructure, examining the establishment of Geriatrics and Extended Care and the Geriatric Research, Education, and Clinical Centers (GRECCs), and their impact on the research—clinical practice continuum. We will showcase specific examples of innovative, evidence-based geriatric care models developed in VA, supported by VA's national GRECC office, and disseminated across the enterprise via GRECC's Mentoring Partnerships Program. We will explore the origin, evidence-base, and strategies for long-term sustainment for each program—specifically: 1) Coordinated Transitional Care (CTraC)— a post-hospital transitional care program to support Veterans at high-risk of readmission; 2) Patient Priorities Care—an Age-friendly approach to align current care plans with patients' unique health priorities; 3) STRIDE (AssiSTed EaRly MobIlity for HospitalizeD VEterans )— a supervised walking program designed to enhance older Veterans' physical function during hospitalization; 4) Gerofit— an outpatient supervised group exercise program designed to improve physical function and maintain independence in older Veterans with multimorbidity. This symposium will offer valuable insights into VA's journey in geriatric innovations, demonstrating advancements in care for older Veterans and providing a roadmap for continued progress in the field of geriatric care.

Abstract citation ID: igaf122.481

### UNDERSTANDING THE TRADITION AND IMPACT OF VA INNOVATION TO GERIATRIC CARE

Lea Kiefer[1], Caroline Madrigal[2], Caitlin Kappler[3], Katherine Hall[4], Katherine Ritchey[5], Nihar Shetty[1], Anne Wang[1], and Priya Bapna[1], *1. Michael E. DeBakey VA Medical Center, Houston, Texas, United States, 2. VA Boston Healthcare System, Boston, Massachusetts, United States, 3. Durham Veterans Affairs, Durham, North Carolina, United States, 4. Veterans Health Administration, Durham, North Carolina, United States, 5. Department of Veterans Affairs, Seattle, Washington, United States*

The Department of Veterans Affairs (VA) has been a leader in geriatric care since the aftermath of World War I, pioneering specialized programs to meet the needs of aging Veterans. In 1975, Congress established the Geriatric Research, Education, and Clinical Centers (GRECCs). There are currently 20 GRECCs located in VA medical centers across the country, and each is connected with an academic medical institution. The GRECCs are the engines of discovery and innovation, and have been testing and developing models of care nearly a decade before similar programs/initiatives emerged in Medicare. This commitment deepened in 1990 with the creation of the Geriatrics and Extended Care (GEC) service line, which expanded access to comprehensive care focused on preventing disability and promoting independence for older adults through evidence-based, patient-centric programs and services for Veterans and their families/caregivers. VA's sustained investments in geriatric care resulted in numerous clinical innovations that became standard practice, not only within the VA system but also in broader healthcare settings. Many programs predated similar private sector initiatives, exemplifying VA's proactive approach to meeting the evolving needs of the nation's aging population. VA has a rich history of pioneering advancements in geriatric care and has played a crucial role in translating research into evidence-based practices. By examining the evolution of VA's geriatric initiatives and their collaboration with community-based initiatives, we can see how VA continues to shape the future of geriatric care –leveraging research, education, and clinical innovation to meet the evolving needs of the Veteran population.

Abstract citation ID: igaf122.482

### IMPLEMENTATION OF THE COORDINATED TRANSITIONAL CARE (CTRAC) PROGRAM IN VA: OVERVIEW AND LESSONS LEARNED

Caroline Madrigal[1], and Jane Driver[2], *1. VA Boston Healthcare System, Boston, Massachusetts, United States, 2. VA Boston Healthcare System, Brockton, Massachusetts, United States*

The Coordinated Transitional Care (CTraC) program is an evidence-based, nurse-led program designed to meet VA's need for transitional care that can serve a geographically dispersed population. The goal of CTraC is to reduce negative post-hospital outcomes and improve Veteran/family empowerment during the early post-hospitalization period. The program is led by a registered nurse case manager who identifies and enrolls Veterans at high risk for readmission—the nurse participates in multidisciplinary discharge planning, visits eligible Veterans while inpatient, and completes post-discharge telehealth follow-up within 48-72 hours and weekly telehealth follow-up for 30 days. CTraC does not duplicate or replace existing care; it fills the gap between inpatient and outpatient services, and provides intensive symptom assessment, medication reconciliation, and case management. By targeting only patients at increased risk of readmission, CTraC focuses resources on Veterans who need it most. CTraC protocol's feasibility and acceptability has been demonstrated in research and by its sustainment in clinical practice for over ten years. Since its inception, the program has served over 10,000 Veterans and consistently demonstrates a significant reduction in hospital readmissions, identification and reconciliation of medication discrepancies, and care coordination to needed services/supports. All of which has translated to substantial cost savings across the 15 VA sites where it operates. Important next steps toward dissemination and sustainment will be described in this presentation including ongoing program evaluation, standardization efforts across the enterprise, identification of sites for future dissemination, and development of the program's business case to encourage long-term sustainment.

Abstract citation ID: igaf122.483

### DISSEMINATION OF PATIENT PRIORITIES CARE: STRATEGIES FOR TRANSFORMING CLINICAL CARE THROUGHOUT VHA

Katherine Ritchey[1], Lea Kiefer[2], Blythe Chandler[2], and Anne Wang[2], *1. Department of Veterans Affairs, Seattle, Washington, United States, 2. Michael E. DeBakey VA Medical Center, Houston, Texas, United States*

Patient Priorities Care (PPC) is an Age-Friendly approach that aligns healthcare with patient values and priorities, especially for persons with multiple chronic conditions (MCC). It is the preferred approach to elicit and act on 'what matters' in Age-Friendly Health Systems (AFHS). Though PPC has been shown to be an evidenced based approach that reduces unwanted care and increases the receipt of supportive care in persons with MCC, large scale health system dissemination had been lacking. Therefore, we used a multi-prong approach to promote clinical integration of PPC VHA wide: establish national VHA partnerships; create integrated electronic health record (EHR) templates and tools; standardize training resources; foster a community of practice. In 2020, the Office of Geriatrics and Extended Care designated PPC as one of four Mentored Partner Programs (MPP). This collaborative learning program provides funding and guidance to frontline staff interested in PPC integration. Additional partnerships with VA Whole Health and AFHS leadership teams have further promoted PPC through AFHS action communities and Whole Health clinical champions. Creation of PPC talent management system training videos provided low-barrier access to most health professionals with professional accreditation. Finally, development of VA EHR tools and implementation website offered frontline staff frameworks and resources for PPC integration. Through these efforts, it is now practiced in a variety of clinical settings in all 170 VHA facilities touching over 10,000 veterans monthly. Lessons learned from our experience can be applicable for other health systems interested in person-centered care transformation, especially in populations with MCC.

Abstract citation ID: igaf122.484

**ADVANCING GERIATRIC CARE: THE IMPACT AND FUTURE OF STRIDE IN THE VA**

Caitlin Kappler[1], Karen Stechuchak[2], Amy Webster[2], Ashley Choate[2], and Susan Hastings[3], *1. Durham Veterans Affairs, Durham, North Carolina, United States, 2. Durham VA Health Care System, Durham, North Carolina, United States, 3. Durham VA Healthcare System, Durham, North Carolina, United States*

STRIDE (AssiSTed EaRly MobIlity for HospitalizeD VEterans) is an evidence-based, supervised walking program designed to preserve and enhance the physical function of older Veterans, a population at high risk for hospital-associated disability. As the VA serves an aging Veteran community, ensuring access to effective geriatric care is critical for maintaining independence and quality of life. Launched in 2014, STRIDE has been successfully implemented in over 70 Veterans Affairs (VA) hospitals nationwide, proactively addressing functional decline through early intervention. The program encourages enrollment ideally within 24 hours of admission, provides supervised daily walks of up to 20 minutes, and ensures dedicated staff oversee both the walks and pre/post evaluations. Evidence demonstrates that STRIDE participants experience shorter hospital stays, reduced discharge to skilled nursing facilities, and report feeling better immediately after walking. A robust set of standardized implementation support tools has been shown to facilitate STRIDE's adoption in VA hospitals, providing a scalable solution to mitigate hospital-associated disability. Sustaining and expanding STRIDE requires ongoing investment in research and implementation support, particularly in securing dedicated staffing and fostering leadership engagement at local, regional, and national levels. By improving outcomes for aging Veterans, STRIDE strengthens the VA's capacity to integrate and sustain best practices, ensuring older Veterans receive the specialized care they need to maintain mobility, independence, and overall well-being.

Abstract citation ID: igaf122.485

**A DECADE OF INNOVATION: LESSONS FROM NATIONWIDE DISSEMINATION OF A CLINICAL EXERCISE PROGRAM FOR OLDER ADULTS**

Katherine Hall[1], Megan Pearson[2], Rebekah Harris[3], Stephen Jennings[4], Tyara Mason[5], and Kenneth Manning[2], *1. Veterans Health Administration, Durham, North Carolina, United States, 2. Durham VA Healthcare System, Durham, North Carolina, United States, 3. VA Boston Healthcare System, Boston, Massachusetts, United States, 4. Durham VA Healthcare Adminstration, Durham, North Carolina, United States, 5. Durham VA Health Care System, Durham, North Carolina, United States*

The Gerofit program, established in 1986 addresses functional decline in older Veterans through tailored exercise. Initially piloted at 4 sites in 2013, it now operates at 33 VA medical centers. This abstract traces its dissemination via VA's Geriatrics and Extended Care (GEC) Mentoring Partnerships Program and Office of Rural Health (ORH) Enterprise Wide Initiative. Expansion strategies included phased funding (GEC/ORH partnerships), virtual adaptations, standardized implementation training curriculum and toolkit development, and clinical note templates. The Gerofit National Coordinating Center (NCC) provided mentorship, monthly Community of Practice forum, and data infrastructure. Sustainability metrics tracked post-funding retention and program growth. From 2013–2025, Gerofit expanded 8-fold, serving >5,000 older adults annually. Telehealth programming (58% of current enrollees) and on-demand videos (119K views) increased rural access. Post-implementation, 94% of sites sustained programming through institutional buy-in. Key facilitators included leadership alignment with VA's Whole Health initiative, multi-disciplinary provider teams, automated workload capture, and consumer demand for the program. Barriers included staff turnover, loss of clinical space, and competing facility priorities. Gerofit's success reflects VA's commitment to scaling evidence-based geriatric care. Hybrid delivery models and NCC support enable long-term viability and continued innovation, offering a blueprint for national dissemination of clinical wellness programs for medically complex older adults.

### SESSION 1475 (SYMPOSIUM)

Abstract citation ID: igaf122.486

**SEX DIFFERENCES IN SLEEP HEALTH AND AGING**

Chair: Ashley Curtis
Co-Chair: Christopher Kaufmann
Discussant: Christopher Kaufmann

Growing evidence highlights significant sex differences in sleep patterns, disorders, and their health consequences. Given well-documented age-related changes in sleep patterns overall, there is a need to study sex differences in sleep and associated outcomes in mid-to-late life. This symposium will showcase emerging research in this field and offer insights into sex-specific sleep and circadian patterns, as well as the differential effects on cardiovascular, neurocognitive, and physical health. Paper 1 will characterize sex differences in multidimensional sleep health using data from the Midlife in the United States study (MIDUS). The Ru-SATED sleep health framework proposed by Dr. Buysee will guide examinations of cross-sectional and longitudinal sex differences in core sleep health components (e.g., regularity, timing, efficiency, duration). Paper 2 will examine sex differences in circadian patterns. Using MIDUS, we will present key sex differences in actigraphy-assessed Rest-Activity Rhythms patterns, and their impact on cardiometabolic factors (e.g., HbA1C, total cholesterol). Paper 3 will describe sex-specific patterns of association between sleep health, cardiovascular risk profiles and cognition in mid-to-late life. Paper 4 will present data from the Thinking and Living with Cancer study comparing effects of poor sleep health on physical functioning in older women with breast cancer versus controls. As Discussant, Dr. Stone will integrate findings and discuss future research directions. Overall, our symposium provides unique insight into sex differences in sleep health and their impact on physical, cardiovascular and neurocognitive functioning. This session can guide future treatments for sleep disturbances, promoting successful aging in both men and women.

Abstract citation ID: igaf122.487

**THE SLEEP-HEART-BRAIN CONNECTION: SEX-SPECIFIC MODIFIABLE RISK FACTORS AND COGNITIVE IMPACT IN MID-TO-LATE LIFE**

Ashley Curtis, Amy Costa, and Natasa Billeci, *University of South Florida, Tampa, Florida, United States*

Poor sleep and cardiovascular health are considered modifiable risk factors of cognitive decline. How these functions interact in associations with cognition, and whether there are sex differences in patterns of association remains underexplored. The present study evaluated sex-specific interactive patterns of sleep health and cardiovascular risk factors on cognition in mid-to-late life. Middle-aged and older adults (N = 270; 124 women; Mage=64.5±7.8) completed measures of sleep health [(Pittsburgh Sleep Quality Index (PSQI)], medical history and demographics (including biological sex, BMI), substance use (Alcohol Use Identification Test, smoking history), and self-reported cognition (Cognitive Failures Questionnaire). A subset of participants (n = 67) completed objective cognitive tasks (Posner Cueing Task, Sternberg Working Memory Task, Stroop Task). Moderation analyses examined sex differences in interactive associations between sleep health, cardiovascular disease (CVD) risk score (combined score including presence of hypertension, cancer, diabetes, smoking, hazardous alcohol use, overweight/obesity) and cognitive function, controlling for age. Sex moderated interactive associations between PSQI-Total, CVD risk score and endogenous attentional orienting. Specifically, in men, greater CVD risk score was associated with worse attentional orienting only in those with worst global sleep quality (PSQI-Total ~11; p=.02), and this pattern was not observed in women. Worse sleep health may exacerbate the impact of cardiovascular disease risk profile on objective spatial attention in mid-to-late life men. Sex-specific monitoring and tailoring of treatments for cardiovascular and sleep health is encouraged. Prospective studies that examine underlying mechanisms (e.g., circulating testosterone) are needed to understanding sex and sleep-specific interactive risk factors of cognitive decline.

Abstract citation ID: igaf122.488

**LONGITUDINAL AND CROSS-SECTIONAL SEX DIFFERENCES IN SLEEP HEALTH ACROSS MIDDLE AND OLDER ADULTHOOD**

Kris Calfee, and Soomi Lee, *The Pennsylvania State University, University Park, Pennsylvania, United States*

Differences in sleep between males and females have been well-documented; however, this is most often examined using a single assessment of one or limited sleep variables. The current study used the Ru-SATED sleep health framework to examine potential differences in multidimensional sleep health in a sample of 434 adults (Mage=54) from the Midlife in the United States study. A subsample of N = 166 was also used to examine change in sleep health over approximately 10 years. Sleep dimensions of regularity, timing, efficiency, and duration were measured across 7 days using Actigraphy, while satisfaction and alertness were measured by sleep diary during the same 7 days. Each dimension was dichotomized (suboptimal vs. optimal) and summed to create a composite score of sleep health. Cross-sectional analyses indicated that men had more suboptimal overall sleep health compared to women. Specifically, men had less regular sleep, lower evening alertness, lower sleep efficiency, and shorter sleep duration compared to women after controlling for sociodemographic characteristics and chronic conditions. Conversely, men were more likely to have optimal sleep timing, and there were no sex differences in sleep quality. Longitudinally, overall declines in the sleep health composite and the majority of individual domains from baseline to follow-up did not differ by sex, except that sleep duration shortened more for men. These findings indicate that men and women may experience similar age-related declines in sleep health, although men have more sleep health problems at baseline and their sleep duration becomes shorter over time.

Abstract citation ID: igaf122.489

**SEX AS A MODERATOR IN THE ASSOCIATION BETWEEN REST-ACTIVITY RHYTHMS AND CARDIOMETABOLIC PROFILES**

Chooza Moon[1], and Meina Zhang[2], *1. University of Iowa, Iowa City, Iowa, United States, 2. UT Health, United States*

Cardiometabolic profiles are the significant indicators of the development of cardiovascular disease in midlife and older adults. Rest-activity rhythm (RAR) reflects the pattern of physical activity, rest, and sleep throughout 24 hours. Although sex may be linked to both RAR and cardiometabolic profiles, limited research has examined the role of sex in the relationship between RAR and cardiometabolic profiles. We analyzed data from the Midlife in the United States Survey (MIDUS 2) (N = 440, N (female)= 266(61%), mean age male (SD)= 55.4 ( 3.5), mean age female (SD) = 53.2 (11.5) ) to examine the moderating role of sex on the relationships between RAR parameters (i.e., acrophase and R-square) and cardiometabolic profiles (i.e., HbA1C, Fasting glucose, LDL, total cholesterol, IL-6, C-Reactive Protein). RAR was measured using 7-day actigraphy. After controlling for age, we found the interaction between sex and R-square were significantly associated with HbA1C (β = -3.97, p = 0.003), fasting glucose (β =-101.90, p = 0.0038), total cholesterol (β = 121.75, p = 0.007), LDL (β = 87.94, p = 0.023), but not with IL-6 and C-Reactive Protein. However, the moderating role of acrophase and sex were not significant in any of the outcomes. Our findings also demonstrated a significant interaction effect between activity consistency and sex associated with cardiometabolic profiles. These findings suggest a potential moderation effect of sex on rest-activity rhythmicity and cardiometabolic profiles rather than on peak timing. Future studies should utilize more accurate and direct measures of cardiovascular function, along with direct circadian rhythm data, in larger prospective cohorts.

Abstract citation ID: igaf122.490

**ASSOCIATION BETWEEN SLEEP AND PHYSICAL FUNCTION IN A LONGITUDINAL TLC COHORT STUDY OF WOMEN**

Cynthia Kusters[1], Wanting Zhai[2], Jeanne Mandelblatt[2], and Judith Carroll[1], *1. University of California, Los Angeles, Los Angeles, California, United States, 2. Georgetown University, Washington, District of Columbia, United States*

Sleep disturbances, such as insomnia, are common in postmenopausal women and are believed to negatively affect physical functioning. These disturbances are more prevalent in breast cancer survivors compared to the general female population. However, limited data exists on how sleep affects physical function in older breast cancer cases. We analyzed data from the Thinking and Living with Cancer study, a multicenter, longitudinal study of women aged 60+ newly diagnosed with breast cancer and frequency-matched non-cancer controls. Participants were followed annually for up to 5-years. Sleep quality was assessed using the PSQI (score >5 indicating sleep disruption); physical functioning was measured by the SF-12 physical components score (PCS) and the Timed Up and Go (TUG) test. Linear mixed models analyzed 2,659 visits from 771 women (341 controls, 430 survivors), adjusting for age, race, comorbidities, recruitment site, and visit time. The average age of the sample was 69.5 (70.0 vs. 69.3 in survivors vs. controls). Disrupted sleep was more prevalent among survivors at baseline (43.7% vs. 31.1%;p<0.001) and persisted during follow-up. Compared to controls without sleep disruption, survivors with sleep disturbances had ~0.47 SD lower adjusted PCS scores ($p < 0.001$). Survivors with sleep disruption also had longer TUG times (~1.3 sec) than controls without sleep disturbances, especially in the first 24 months ($p = 0.002$). These findings suggest older women with breast cancer and sleep disruption are vulnerable to long-term losses in physical functioning. Clinical surveillance for sleep disruption paired with intervention could help maintain function in the growing population of older cancer survivors.

### SESSION 1480 (SYMPOSIUM)

Abstract citation ID: igaf122.491

### DAILY EXPERIENCES AND WELL-BEING IN MID- AND LATER LIFE: TESTS OF INTRA AND INTERINDIVIDUAL MODERATORS

Chair: Lydia Ong
Co-Chair: Anna Kornadt
Discussant: Shevaun Neupert

Stressful and positive experiences are common in daily life and associated with fluctuations in emotions, subjective cognition, and physiology, with implications for downstream health across mid- and later life. Daily diaries and ecological momentary assessment capture naturalistic experiences and provide insight into how these experiences covary with well-being within individuals. Importantly, these associations are shaped by how individuals interact with their environment. Thus, in this symposium, we present four studies examining intra and interindividual moderators of the links between daily experiences and well-being. First, Ong et al. examine the daily association between savoring and positive affect among adults in Switzerland (n = 108, Mage=73) and British Columbia (n = 178, Mage=47), testing whether individuals with more versus less positive views of aging differ in this relationship. In a sample of 1,125 US adults (Mage=62), Saruhanlioglu et al. investigate whether elevated positive affect buffers the relationship between daily stressors and memory lapses. Furthermore, Luchetti et al. look at the daily link between loneliness and subjective cognitive difficulties among 1,828 US adults (Mage=57), testing sociodemographic factors as moderators. Lastly, among 318 US parents (Mage=40), Pauly et al. consider whether personality moderates the associations of solitude with negative affect and diurnal cortisol slopes. Results from these studies demonstrate how daily experiences are linked with emotional, cognitive, and physiological well-being, and when or for whom associations might be buffered or strengthened. Findings will be discussed in the context of understanding how individual differences shape the impact of daily experiences on developmental trajectories across different timelines.

Abstract citation ID: igaf122.492

### SAVORING DAILY LIFE: VIEWS OF AGING AS A MODERATOR OF SAVORING AND POSITIVE AFFECT

Lydia Ong[1], Theresa Pauly[2], Anna Kornadt[3], and Nancy Sin[4], *1. The University of British Columbia, Vancouver, British Columbia, Canada, 2. Simon Fraser University, Vancouver, British Columbia, Canada, 3. University of Luxembourg, Esch-sur-Alzette, Grevenmacher, Luxembourg, 4. University of British Columbia, Vancouver, British Columbia, Canada*

Positive events are common in daily life (e.g., having a good conversation) and savoring strategies can be used to up-regulate positive emotions. Drawing from Socioemotional Selectivity Theory, individuals with relatively less positive views of aging (VoA) might attend more to positive and meaningful experiences in the present as a function of shifting time perspective. Therefore, we hypothesized that people with less positive VoA would derive more positive affect from savoring their positive experiences. This pre-registered study analyzed experience sampling data from an adult lifespan sample from Canada (n = 178 persons and 14 days, Mage=47) and an older adult sample from Switzerland (n = 108 persons and 7 days, Mage=73). VoA were measured through attitudes toward own aging and age stereotypes, while savoring and positive affect were assessed at the end of each day. Two-level multilevel models revealed that positive affect was higher on days with more-than-usual savoring (Canada sample: $b = 0.16$, 95% CI[0.03, 0.30], p=.019; Switzerland sample: $b = 0.42$, 95% CI[0.35, 0.49, p<.001). VoA moderated the link between savoring and positive affect in the Swiss sample only, such that individuals with less positive VoA had smaller upticks in positive affect associated with savoring (simple slope for -1 SD: $b = 0.34$, $SE = 0.05$, p<.001), compared to those with more positive VoA (simple slope for +1 SD: $b = 0.49$, $SE = 0.05$, p<.001). Findings suggest that older adults with more positive VoA may benefit more from savoring, but it remains unclear whether VoA hold the same importance for savoring among younger and middle-aged adults.

Abstract citation ID: igaf122.493

### DAILY STRESSORS AND MEMORY LAPSES: CONSIDERING POSITIVE AFFECT AS A STRESS BUFFER

Zeynep Saruhanlioglu[1], Yvonne Brehmer[1], Keri Pekaar[1], Dorien Kooij[1], Jacqueline Mogle[2], David Almeida[3], and Patrick Klaiber[1], *1. Tilburg University, Tilburg, Noord-Brabant, Netherlands, 2. RTI Health Solutions, Durham, North Carolina, United States, 3. The Pennsylvania State University, University Park, Pennsylvania, United States*

Memory lapses are common in adulthood and can negatively affect daily functioning. Stressor occurrence has been previously linked to same-day memory lapses, yet prior research has not distinguished between retrospective memory (RM) and prospective memory (PM) lapses or examined whether positive affect buffers these effects. RM primarily depends on retrieving stored information, whereas PM requires not only retrieval but also monitoring and execution processes, that may be more susceptible to stressors. This study investigated whether positive affect moderates the within-person relationship between daily stressor occurrence and retrospective and prospective memory lapse occurrence among middle-aged and older adults. Data were drawn from 1,125 community-dwelling adults (ages 43-90; M = 62.43) in the National Study of Daily Experiences 3. Participants completed eight consecutive daily telephone interviews assessing stressor occurrence, retrospective and prospective memory lapses, and positive affect. Results from mixed-effects logistic regression models indicated that experiencing a memory lapse was significantly more likely on stressor days than on stressor-free days. However, this association was only observed for retrospective memory lapses, not prospective ones. While positive affect did not buffer the daily stressor-memory lapse link, individuals with higher overall positive affect reported fewer memory lapses across both memory types. The findings highlight that memory retrieval processes may be more vulnerable to stressor-related disruptions, whereas individuals may employ compensatory strategies to maintain daily PM functioning. Furthermore, they underscore the need to explore modifiable factors to protect against daily memory lapses throughout adulthood.

Abstract citation ID: igaf122.494

### NO TIME FOR MYSELF: PERSONALITY MODERATES ASSOCIATIONS BETWEEN SOLITUDE AND PARENTAL WELL-BEING

Theresa Pauly, *Simon Fraser University, Vancouver, British Columbia, Canada*

Parental well-being is often challenged by the demanding nature of caregiving responsibilities, leaving little opportunity for solitude (i.e., time to oneself). While prior research highlights the benefits of solitude for overall well-being, less is known about how these effects vary based on personality. Solitude may be especially important for parents who are introverted, to help recharge, or high in neuroticism, to manage heightened emotional reactivity. This study aimed to investigate whether personality traits moderate the association between solitude and daily well-being (as indicated by negative affect and cortisol levels) among parents. Data are from 318 parents of underage children (Mage=40.06 years; 45% male, 81% White) in the Refresher Daily Diary subproject of the Midlife Development in the United States Survey. Over 8 days, participants reported daily opportunities for time to themselves, negative affect, and provided saliva samples on days 2–5 to measure cortisol levels. Data were analyzed using multilevel modeling. On days on which participants reported having had time to themselves, they reported lower levels of negative affect ($b = -0.05$, $p = .001$) and steeper wake-evening cortisol slopes ($b = -0.07$, $p = .011$). Individuals with higher levels of neuroticism showed stronger associations between time to themselves and negative affect ($b = -0.06$, $p = .007$) as well as cortisol slopes ($b = -0.09$, $p = .041$). Facilitating moments of solitude for parents, especially those high in neuroticism, could serve as an effective strategy for supporting their emotional and physiological health.

Abstract citation ID: igaf122.495

### LONELINESS AND SUBJECTIVE COGNITIVE CONCERNS IN DAILY LIFE

Martina Luchetti[1], Damaris Aschwanden[2], Antonio Terracciano[3], and Angelina R. Sutin[1], *1. Florida State University College of Medicine, Tallahassee, Florida, United States, 2. Eastern Switzerland University of Applied Sciences, Institute for Ageing Research, St. Gallen, Sankt Gallen, Switzerland, 3. Florida State University, Tallahassee, Florida, United States*

Loneliness is associated with increased risk of dementia and poor cognitive functioning in middle-aged and older adults. Limited work has examined how loneliness is associated with subjective cognitive concerns, particularly in the context of everyday life. This study used data from the National Study of Daily Experiences, part of the Midlife in the United States Study to examine the daily association between feeling lonely and self-perceptions of cognitive function. Respondents (n = 1,828; mean age = 56.56; 55.7% females) participated in an 8-day daily assessment reporting on loneliness, cognitive concerns (e.g., memory lapses), and other aspects of daily life. Multilevel linear and binary logistic regressions indicated a significant between- and within-person association between loneliness and subjective cognition. At the between-person level, participants who felt lonelier tended to report more cognitive problems. At the within-person level, on days participants felt lonely (independent of the frequency of those feelings), they also reported more trouble concentrating and were more likely to experience memory lapses. Feeling lonely was also associated with more irritation and interference associated with memory lapses. The observed associations were in general not moderated by socio-demographic factors, remained significant controlling for individual- and day-level social and contextual covariates, and when excluding individuals with depression or anxiety. Results suggest that even transitory feelings of loneliness are associated with poor perceptions of everyday cognitive function, a marker with implications for future risk of cognitive decline.

### SESSION 1485 (PAPER)

Abstract citation ID: igaf122.496

### AGE FRIENDLY CARE

This session addresses opportunities to improve Age Friendly health care using both the IHI 4 M's and Geriatric Medicine's 5 M's and nursing's gerontological competencies.

Abstract citation ID: igaf122.497

### AGE-FRIENDLY CARE PRACTICES: DOCUMENTING OPPORTUNITIES FOR IMPROVEMENT

Taylor Bucy, and Jessica Kalender-Rich, *University of Kansas School of Medicine, Kansas City, Kansas, United States*

Efforts to address the multifaceted clinical and social needs of an aging population have centered around the Age Friendly Health System (AFHS) framework of 4Ms: Medication, Mentation, Mobility and what Matters. In 2025, hospital-wide AFHS structural measures were adopted by CMS, with both cost and quality implications. This study describes early efforts to document AFHS processes across a large Midwestern academic medical center. Using electronic health record data, we analyzed 447 unique patient encounters across two hospital units between July-September 2024. Mobility was measured using the bedside mobility assessment tool (BMAT), Mentation via the confusion assessment method (CAM) at least twice daily, Medication by use of antipsychotics, sedatives, and/or anticholinergics (i.e. monitored medications), and what Matters by person-reported location of expected discharge. On average, patients were 77.9 (+/- 6.3) years, with a median length of stay of 4 days (IQR=5) and an unadjusted 30-day readmission rate of 14.5%. The geriatric consult service saw 4.7% of patients. The majority (64.9%) of patients saw no improvement between admission and discharge BMAT. Roughly two-thirds of patients were administered a CAM screening twice daily, and 83.2% of patients received a monitored medication at least once. Finally, agreement between actual and expected discharge home was approximately 80%. Systematic tracking of age-friendly care facilitates early interventions aimed at standardization and prioritizes an environment of continuous learning. Our work identifies areas for targeted improvement prior to system-wide implementation of federal requirements and prompts further investigation into how geriatric consultation services can be leveraged to fill observed gaps.

Abstract citation ID: igaf122.498

### ASSESSING WHAT COUNTS: EVALUATING AND REFINING GERIATRIC NURSING COMPETENCY ASSESSMENTS THROUGH ITEM ANALYSIS

Yosefa Birati[1], Allison P. Squires, PhD, RN, FAAN[2], Mattia Gilmartin[3], Paule-Sarah Fraiman[4], Janna Sheps[5], Ilana Peterfreund[6], and Anna Zisberg[4], *1. The Cheryl Spencer Department of Nursing Faculty of Social Welfare and Health Science, Hanaton, HaZafon, Israel, 2. New York University, New York, New York, United States, 3. New York University Meyers College of Nursing, New York, New York, United States, 4. University of Haifa, Haifa, HaZafon, Israel, 5. Bnai Zion Medical Center, Haifa, Hefa, Israel, 6. Bnai Zion Medical Center, Haifa, HaZafon, Israel*

The Geriatric Institutional Assessment Profile survey, developed as part of the Nurses Improving Care for Healthsystem Elders Program (NICHE), plays a critical role in evaluating healthcare providers' knowledge and competencies in geriatric-care. With the growing demand for geriatric services, equipping healthcare professionals with essential knowledge is vital to ensuring high-quality, person-centered care for older adults. Knowledge assessments are essential for evaluating competencies, yet their effectiveness and reliability are often under-explored. This study evaluates the psychometric properties of the Clinical Geriatric Knowledge (CGK) questionnaire and General Knowledge (GK) assessment, validating these tools beyond their original development setting, and testing their applicability and reliability in an international healthcare context. The analysis was conducted among 303 nurses across two hospitals. Participants were categorized into high-achievers and low-achievers based on performance. Using item analysis, we evaluated item difficulty (DIF-I), discrimination index (DI), distractor effectiveness (DE), and internal consistency reliability (KR-20). Results revealed that 60% of items had acceptable difficulty, and 65% of CGK items, and 60% of GK items demonstrated good/excellent discrimination. Distractor analysis showed that 25% of multiple-choice items effectively used all distractors, and 45% contained one non-functioning distractor. KR-20 scores were moderate (0.58 for GK, 0.52 for CGK), indicating acceptable reliability for assessments covering diverse content areas. These findings highlight the effectiveness of these tools in evaluating geriatric nursing competencies and revealing opportunities to optimize item design, particularly by balancing difficulty and refining distractors. This work contributes to advancing geriatric care and education, ultimately improving the quality of care for older adults.

Abstract citation ID: igaf122.499

### ENHANCING AGE-FRIENDLY CARE WITH SHARED DECISION-MAKING TOOLS AND TECHNOLOGY FOR OLDER ADULTS

Gee Kim, *FAIR Health, New York, New York, United States*

Shared decision-making (SDM) tools powered by technology may improve age-friendly care for older patients with chronic conditions by facilitating SDM with clinicians. Delivered as interactive visual aids on platforms such as iPads or desktop computers, these tools present complex medical and cost information in simplified formats, enhancing patients' and caregivers' ability to access and act upon the information. For patients with hip osteoarthritis, for example, digital SDM tools improve communication with healthcare providers, enabling patients to express their preferences and concerns and decide between nonsurgical treatments and surgery. The cost information in the SDM tools allows patients and caregivers to weigh financial implications alongside clinical factors. This session will present results from four clinical sites that have achieved Age-Friendly Health Systems recognition and are collaborating with FAIR Health to use its SDM tools at the point of care, under a grant from The John A. Hartford Foundation. Early feedback suggests that using the tools to facilitate SDM discussions can improve patients' understanding of treatment options, increase confidence in decision making and foster more productive discussions with providers. Clinicians report that SDM tools help structure conversations, enhance patient comprehension and support collaborative decision making. SDM tools that leverage technology to present treatment information may improve health literacy, reduce decisional conflict and lead to treatment plans that better reflect individual values, goals and financial considerations. Integrating such tools into clinical practice fosters personalized care, improves patient satisfaction and optimizes decision making based on "what matters" to patients, a cornerstone of age-friendly healthcare.

Abstract citation ID: igaf122.500

### NATIONAL TRENDS OF THE 5MS OF GERIATRIC MEDICINE, NHANES 2001-2018

Lily Bessette[1], Linnea Wilson[2], and Timothy Anderson[1], *1. University of Pittsburgh, Pittsburgh, Pennsylvania, United States, 2. Beth Israel Deaconess Medical Center, Boston, Massachusetts, United States*

Age-friendly care of older adults embodies the Geriatric-5Ms, a framework spanning domains: Mind, Mobility, Medications, Multi-complexity, and Matters most. How the prevalence of these 5 geriatric syndromes is changing among U.S. adults is unknown. We analyzed cross-sectional data from the National Health and Nutrition Examination Survey (NHANES) using sampling weights to generate nationally representative estimates of the 5Ms in U.S. adults from 2001-2018. The geriatric syndromes (i.e. 5Ms) were defined by self-report of depression or cognitive impairment (Mind), functional impairments (Mobility), polypharmacy (Medications), >2 chronic conditions (Multi-complexity), and self-reported poor health (Matters Most). We estimated the prevalence of each of the 5Ms and performed age-stratified and sex-adjusted logistic regression to assess temporal trends. The cohort included 47,954 U.S. adults (38% younger, age 20-39; 45% middle-aged, age 40-64; 18% older, age ≥65; 52% female; 68% White). Overall, the prevalence of having at least one geriatric syndrome increased from 62% to 69% between 2001-2018, with greatest increases in the Mobility and Multi-complexity domains.

In older adults, prevalence of Multi-complexity was highest of the 5Ms (93%), while the Medication domain increased the most over time (29% to 44%). In both middle-aged and older adults, the prevalence is increasing at similar rates in Mobility (middle-aged: 23% to 32%; older: 70% to 77%) and Multi-complexity (middle-aged: 63% to 70%; older: 85% to 93%) domains. Two-thirds of U.S. adults have at least one geriatric syndrome. The burden of the 5Ms is growing in middle-aged and older adults suggesting a need for age-friendly care across the lifespan.

Abstract citation ID: igaf122.501

**THE PACE OF CHANGE: EVALUATING THE IMPLEMENTATION OF AGE-FRIENDLY HEALTH SYSTEMS IN CONVENIENT CARE CLINICS**

Grace Armstrong[1], Sarah Ball[2], Nicholas Schiltz[1], Brant Oliver[3], Anne Pohnert[2], and Mary Dolansky[1], *1. Case Western Reserve University, Cleveland, Ohio, United States, 2. CVS Health, Woonsocket, Rhode Island, United States, 3. Dartmouth Health, Hanover, New Hampshire, United States*

The Age-Friendly Health Systems 4Ms framework (What Matters, Mobility, Medication, Mentation) was implemented in CVS MinuteClinic convenient care clinics nationwide in May 2020. Since then, a vast collection of strategies have been used to enhance provider delivery of the 4Ms. These include documentation optimization, additional time for patient's 65 years and up appointments, incorporating the 4Ms into annual provider performance reviews, and extensive education initiatives. These strategies can now be retrospectively examined to determine which had the greatest impact on the pace of organizational change. Statistical Process Control (SPC) analyses assessed performance variation across over 1,000,000 eligible visits from May 2021 to March 2025. Outcome measures included the proportion of eligible patients meeting each M of the 4M criteria, the proportion meeting all 4Ms criteria, and a composite score for 4M completion (M Score). Improvement was observed across all core measures: (1) "Matters" improved from 23.1% to 55.0%; (2) "Mobility" improved from 11.5% to 52.4%; (3) "Medication" improved from 18.7% to 50.1%; (4) "Mentation" improved from 6.4% to 47.9%; and (5) "4M" improved from 4.8% to 42.1%. "Average M Score" (Min-Max Range: 0, 4.0) performance also steadily improved from 0.61 at baseline to 2.05 in 2025. Overall, consistent performance improvements were observed from 2021 to 2025. The addition of time to the visit for patients ages 65 and up, creation and enhancement of provider Best Practice Alerts and the inclusion of 4Ms metrics in annual performance reviews were some of the strategies that significantly increased delivery of 4Ms care.

### SESSION 1490 (PAPER)

### BELONGING, ENGAGEMENT, AND VULNERABILITY: SOCIAL CONNECTIONS AND DISCONNECTION IN LATER LIFE

Abstract citation ID: igaf122.502

**AGING WITH HIV IN THE SHADOWS: HOW OLDER ASIAN ADULTS WITH HIV NAVIGATE STIGMA, ISOLATION, AND SURVIVAL**

Jen-Hao Chen[1], Cheng-Shi Shiu[2], and Wei-Ti Chen[3], *1. National Chengchi University, Taipei, Taipei, Taiwan (Republic of China), 2. National Taiwan University, Taipei, Taipei, Taiwan (Republic of China), 3. University of California, Los Angeles, Los Angeles, California, United States*

This study examines how HIV-positive Asian immigrants navigate aging, disease management, and social marginalization in the United States, drawing on 34 in-depth interviews conducted in Los Angeles between 2018-2019. Our analysis reveals that the dual fear of being "found out" as HIV-positive and/or undocumented, combined with the need for resources, compels them to develop complex survival strategies while maintaining a double layer of secrecy in everyday interactions. The heightened stigmatization of HIV within Asian American communities leads many to hide their status from family and friends, withdraw from community life, and manage their illness and survival alone. In healthcare settings, withholding critical medical information to avoid discrimination and experiences of microaggressions is common. Unfortunately, such strategies limit access to social and healthcare support from communities and families that are often available to other older adults. Moreover, legal status and limited English proficiency frequently prevent them from leaving their ethnic communities, further restricting access to broader HIV resources and support networks and exacerbating social and healthcare precarity. Maintaining these strategies, however, creates heightened stress and takes a psychological toll. Consequently, these older adults age with HIV in the shadows, confined within Asian American communities, yet traditional support networks are often absent—or even sources of rejection. These findings challenge theoretical perspectives that frame family and communities as sources of support, highlighting their potential role as structural barriers to aging. The findings also underscore the need for interventions and policy reforms to address the compounded vulnerabilities of aging Asian immigrants with HIV.

Abstract citation ID: igaf122.503

**INTERCONNECTION OF MEANING AND PURPOSE IN LIFE, SENSE OF BELONGING AND SOCIAL ENGAGEMENT IN PEOPLE LIVING WITH HIV**

Atami De Main[1], Marshall J. Glesby[1], Carrie Johnston[1], Mark Brennan Ing[2], and Eugenia L Siegler[1], *1. Weill Cornell Medicine, New York, New York, United States, 2. Hunter College, New York, New York, United States*

Despite advancements in medical treatment for HIV/AIDS, people living with HIV (PWH) still encounter health, social and structural vulnerabilities. Understanding factors that contribute to PWH's mental health and well-being become increasingly important. Psychosocial factors — meaning and purpose in life, sense of belonging and social engagement — play a protective role against negative health outcomes. Our study aimed to examine the associations among these factors, using items from the Brief Multidimensional Measure of Religiousness and Spirituality scale, in older PWH (n = 349, age range=50-84) from the Research on Older Adults with HIV (ROAH 2.0) study at the Weill Cornell Campus in New York City. Hierarchical regression analyses revealed that lower levels of loneliness, fewer depressive symptoms, lower number of chronic conditions and higher engagement in social activities and media-based activities were significantly associated with strong general sense of belonging in older PWH (R2adj=0.34, F(10, 269) = 15.61, p<.001), strong feelings of being part of a community (R2adj=0.35, F(10, 262) = 15.56, p<.001) and high meaning and purpose in life (R2adj=0.34, F(9, 281) = 17.49, p<.001). Additionally, PWH experiencing financial strain and those living alone reported lower general sense of belonging (F(6, 286) = 2.99, p<.001, feelings of being part of a community F(6, 280) = 1.91, p<.001)

and meaning and purpose in life F(5, 297) = 2.50, p = 0.03). These findings underscore the importance of psychosocial indicators in promoting the mental health and well-being of older PWH and highlight the need for interventions to enhance social and community engagement in this population.

Abstract citation ID: igaf122.504

### LONGITUDINAL STUDY OF THE IMPACT OF COMMUNITY BELONGING ON MEMORY IN OLDER ADULTS RESIDING IN LIFE PLAN COMMUNITIES

Renae Smith-Ray, Jennifer Smith, and Laurel Mertz, *Mather Institute, Evanston, Illinois, United States*

Evidence suggests that social well-being, including community belonging, is an important predictor of health. Older adults with strong community belonging (i.e., feeling of connection to one's proximal places, groups, and individuals) consistently demonstrate better health outcomes compared to those with lower belonging. There is less known about the impact of belonging on older adults who reside in Life Plan Communities (LPCs). The purpose of this study was to examine the impact of belonging on memory over three years (2018-2020) among a cohort of older adults residing in LPCs. The longitudinal cohort design entailed surveying participants annually, including measures of community belonging and self-reported memory. Participants (N = 8,070) had a mean age of 83.9 years, were 67.2% female, and an average length of LPC residence of 6 years. A linear mixed model with repeated measures was used to examine the impact of belonging on memory over time. The analysis revealed significant main effects of belonging, F(1,14446.8) = 193.4, p < 0.001, and year, F(2, 5720.6)=3.43, p = 0.032, indicating that higher belonging was associated with better memory and that memory varied across years. There was also a significant interaction between year and belonging, F(2,5681.8) = 4.7, p = 0.010, indicating that the change in memory scores over time differed by participants' belonging score. Post-hoc tests revealed that memory declined over time, but the decline was slightly moderated by belonging. These results suggest that, for older adults residing in LPCs, greater community belonging is associated with better memory over time, underscoring the importance of belonging on cognitive health.

Abstract citation ID: igaf122.505

### SOCIAL ENGAGEMENT AND SENSE OF COMMUNITY AMONG OLDER ADULTS: THE ROLE OF SOCIOECONOMIC STATUS AND RACE

Bryant Carlson, *Oregon Health & Science University - Portland State University School of Public Health, Oregon, United States*

Previous research suggests that as levels of neighborhood socioeconomic and racial heterogeneity increase, rates of social engagement and sense of community (SOC) often decrease, but the extent to which this trend persists for older adults in socioeconomically and racially diverse communities remains unclear. This study estimated the relationship of engagement in social activities with SOC and its components among Black or African American, Hispanic or Latino, and White older adults. Participants included 204 older adults aged 60 years or older. Social engagement was assessed with eight questions, and the Brief Sense of Community Scale (BSCS) was used to assess total SOC and components of SOC (e.g., need fulfillment, group membership, influence, and emotional connection). Results were analyzed using confirmatory factor analysis and hierarchical linear regression. Engagement in social activities had a positive effect on SOC, but this relationship varied by socioeconomic and racial group. Specifically, engagement in social activities had a positive relationship with SOC for White and higher socioeconomic level older adults, but the strength of this relationship attenuated considerably for Black or African American, Hispanic or Latino, and lower socioeconomic level older adults. While these findings validate the importance of engagement in social activities for increasing SOC in older adults, the effect observed for neighborhood socioeconomic and racial heterogeneity poses challenges for SOC in diverse neighborhoods and communities, reinforcing enduring tensions between community cohesion and diversity.

Abstract citation ID: igaf122.506

### THE PARADOX OF MINOR PAIN: HOW PHYSICAL PAIN SHAPES LONELINESS IN SMALL NETWORKS WITH GENDER DIFFERENCES

Haosen Sun, *University of Nevada, Reno, Reno, Nevada, United States*

Loneliness in older age is widely understood as a subjective experience stemming from the gap between expected and actual levels of social connectedness. However, how this psychological discomfort intersects with physical pain in older adults—and whether these patterns differ by gender—remains unclear. Pooling data from Wave 4 (2011) and Wave 6 (2015) of the Survey of Health, Aging, and Retirement in Europe (SHARE), which provides detailed information on social network composition and self-reported pain, we conducted linear regression analysis with a longitudinal setup to examine whether individuals experiencing pain are more likely to report higher levels of loneliness compared to those with similar network sizes but without pain, with particular focus on those in smaller networks. The analysis controlled for baseline loneliness, baseline pain, network changes, social activities, personality traits, other health factors, and key sociodemographic variables. We also explored whether this intersection varies between males and females. Among individuals with small networks (fewer than two core confidants), those reporting minor pain experienced even lower levels of loneliness than their counterparts without pain, suggesting that minor discomfort may reduce expectations or justify a smaller network. In contrast, individuals experiencing moderate or severe pain reported significantly higher levels of loneliness with smaller networks, highlighting an increased need for support when pain becomes more intense. This pattern was particularly pronounced among males but not females, suggesting potential gendered coping mechanisms. Men may place greater expectations on external support and experience greater emotional vulnerability when such support is lacking when needed.

### SESSION 1495 (PAPER)

### COGNITIVE AGING: SOCIAL DETERMINANTS ACROSS DIVERSE CONTEXTS

Abstract citation ID: igaf122.507

### CHILD LOSS AND COGNITIVE HEALTH AMONG OLDER ADULTS

Sneha Mani[1], Muqi Guo[2], Lindsay Kobayashi[3], and Alden Gross[1], *1. Johns Hopkins Bloomberg School of Public Health, Baltimore, Maryland, United States, 2. University of Michigan School of Public Health, Ann Arbor, Michigan, United States, 3. University of Michigan, Ann Arbor, Michigan, United States*

The loss of a child, a severe life adversity, is associated with long-term health risks for parents, including increased mortality, cardiovascular diseases, and mental health issues. However, its potential impact on later-life cognitive health remains understudied. We examine the association between child loss and cognitive function using data from 3,456 participants aged ≥60 from the Longitudinal Aging Study in India and 1,927 participants aged ≥55 from the Mexican Health and Aging Study. Child loss was defined by self-reported loss of at least one child aged ≥5 years or (18% in India, 10% in Mexico) or ≥ 25 years (11% in India, 6% in Mexico). Cognitive function was measured using country-specific co-calibrated cognitive factor scores using measures from the Harmonized Cognitive Assessment Protocol (HCAP). A multivariable-adjusted, country-stratified linear regression model was used to examine the relationship between child loss and cognitive function. Compared to individuals who never experienced child loss, those who lost a child aged ≥5 years and those who lost a child aged ≥25 years exhibited -0.07 SD (95% CI: -0.12 to -0.01) and -0.09 SD (95% CI: -0.16 to -0.02) lower cognitive scores in India, and -0.13 SD (95% CI: -0.24 to -0.01) and -0.06 SD (95% CI: -0.20 to 0.08) lower scores in Mexico, respectively. These findings suggest that child loss across the life course may represent a significant risk factor for poorer later-life cognitive function in older adults.

Abstract citation ID: igaf122.508

### CONFLICT EXPOSURE THROUGHOUT THE LIFE-COURSE AND MENTAL HEALTH AMONG OLDER ADULTS IN LEBANON

Carlos Mendes de Leon[1], Abla Sibai[2], Martine Elbejjani[2], Alexandra Abi Nassif[2], Mayssan Kabalan[2], Che Henry Ngwa[2], Stephen McCall[2], and Monique Chaaya[2], *1. Georgetown University, Washington, District of Columbia, United States, 2. American University of Beirut, Beirut, Beyrouth, Lebanon*

Lebanon has experienced a prolonged period of political instability and conflict, starting with the onset of the civil war in 1975. Most older adults in Lebanon have been exposed to conflict, violence and losses during most of their life. We examine the mental health associations of these conflict-related exposures, using baseline data from the Lebanon Study of Aging and HeAlth (LSAHA), a population-based survey of 3,027 adults aged > 60 years (69% participation rate). The sample was obtained using a multi-stage random sampling design in two pre-selected areas: the city of Beirut and the Zahle district (southern Beqaa Valley governate). Conflict exposures were summarized in experiences of personal bodily harm (13.3%), bodily harm to close family members (27.4%), loss of own possessions (e.g., houses, jobs, 35.5%), and loss of possessions among close family members (15.7%). After accounting for the complex sampling design, weighted linear regression models controlling for age, sex, education, and sampling area showed that each unit increase in all four conflict exposure variables was significantly (p's < .01) associated with higher levels of depressive symptoms (CES-8) and anxiety symptoms (GAD-7). The strongest association for depression was found for experiences of personal bodily harm (ß=1.36, p<.005), while the strongest association for anxiety was for loss of possessions among family members (ß=1.42, p<.001). The results suggest that the high prevalence of conflict-related exposures, whether they resulted in harm or losses to self or to family members, has a substantial adverse effect on the well-being of older adults in Lebanon.

Abstract citation ID: igaf122.509

### LINKS BETWEEN HOBBIES, ACTIVITIES, VOLUNTEERING, AND COGNITIVE FUNCTION IN MEXICAN OLDER MEN AND WOMEN

Joseph Saenz[1], Yuan Zhang[2], Erika Beidelman[3], and Yang (Claire) Yang[4], *1. Arizona State University, Phoenix, Arizona, United States, 2. Columbia University, New York, New York, United States, 3. Columbia University Mailman School of Public Health, New York, New York, United States, 4. The University of North Carolina at Chapel Hill, Chapel Hill, North Carolina, United States*

Engagement in cognitively stimulating activities throughout life may support cognitive function and help prevent decline. Social participation is linked to better cognitive outcomes in older adults in high-income countries; however, benefits may be more pronounced in populations who had historically limited access to stimulating opportunities in early-life (e.g., wide access to high-quality education). We used data from the 2015 Mexican Health and Aging Study and 2016 Mex-Cog study, including comprehensive neuropsychological tests assessing multiple cognitive domains (n = 1,968). We considered both the number of activities in two activity domains: hobbies and indoor activities, and volunteering and community activities, and individual activities within each domain. We employed linear regression models to assess relationships across cognitive domains. Hobbies and indoor activities were associated with better memory, language, visuospatial ability, executive function, and orientation. Volunteering and community activities showed no significant associations. Engagement in all individual hobbies and indoor activities, except watching television, were linked to better cognitive functions. Education-stratified analyses revealed that activities such as talking to friends, performing home maintenance, and attending sporting clubs were associated with better cognitive function among individuals with lower education but not among those with higher education. These associations were largely consistent between men and women. Findings provide nuanced evidence on the role of leisure and social activities in cognitive function among older Mexican adults, highlighting similarities and differences across education. Moving forward, we will examine health-related selection into activity participation using prior waves and use 2021 Mex-Cog data to assess changes across domains over five years.

Abstract citation ID: igaf122.510

### MENOPAUSE TIMING AND LATER-LIFE COGNITION: A COMPARATIVE STUDY OF CHINA, INDIA, AND THE UNITED STATES

Muqi Guo[1], Tsai-Chin Cho[1], Alden Gross[2], and Lindsay Kobayashi[1], *1. University of Michigan School of Public Health, Ann Arbor, Michigan, United States, 2. Johns Hopkins Bloomberg School of Public Health, Baltimore, Maryland, United States*

Women have a higher dementia prevalence than men, but midlife risk factors alone do not fully explain this disparity. Estrogen, which has neuroprotective effects, declines sharply at menopause, potentially influencing cognitive function. This study examines the association between menopause age and later-life cognition in China, India, and the U.S., where cross-national comparisons are lacking. We analyzed nationally representative data from postmenopausal women aged 45+ in the China Health and Retirement Longitudinal Study (2018, n = 7,412), the

Longitudinal Aging Study in India (2017–2019, n = 22,648), and the U.S. Health and Retirement Study (2018, n = 5,577). Menopause age was categorized as < 40, 40–44, 45–49, 50–55 (reference), and >55 years. Cognitive function was assessed as total score of harmonized immediate and delayed word recall. Linear regression models adjusted for socio-demographic factors, and interaction terms tested country-specific differences. Premature (< 40) and early menopause (40–44) were more common in India, while menopause at 50–55 was most frequent in the U.S. Across countries, menopause before 50 was linked to poorer cognition. In China, late menopause (>55) was also associated with cognitive decline. U.S. women with menopause at 45–49 showed a greater decline compared to their counterparts in China and India. Overall, earlier menopause is associated with lower cognitive function across all three countries, with Indian women facing a greater burden due to higher early menopause prevalence. Additionally, late menopause may pose cognitive risks for Chinese women. These findings highlight menopause timing as a key factor in cognitive aging across diverse economic contexts.

### SESSION 1500 (PAPER)

Abstract citation ID: igaf122.511

### EPIDEMIOLOGY OF AGING IN AT RISK POPULATIONS

In this session you will gain insights into rural health as well as substance use disorders

Abstract citation ID: igaf122.512

### AGRICULTURAL INJURIES WITH DEMENTIA: DOUBLE WHAMMY?

Kanika Arora[1], Jonathan Davis[1], Lila Basnet[1], and Julie Bobitt[2], *1. University of Iowa, Iowa City, Iowa, United States, 2. University of Illinois at Chicago, Chicago, Illinois, United States*

Older adults make up about one-third of U.S. producers and account for nearly 80% of agricultural fatalities. Moreover, agricultural workers have higher odds of developing dementia, a neurodegenerative disorder with significant safety implications due to impaired judgement, memory loss, and problems with balance, vision, and communication. Farm environments with heavy equipment and livestock further amplify these risks. Despite the fact that older farmers continue to work well into advanced ages, no prior study has examined how dementia impacts agricultural injuries. We examine mechanisms and severity of agricultural injuries with dementia among those 60 years and older using national trauma data from the Trauma Quality Programs Participant Use File (TQP-PUF) over 2017-2021. Using multivariable logistic and ordered logistic regressions, we compare agricultural injuries in individuals with dementia (group 1) to those with agricultural injuries but without dementia (group 2) and those with non-agricultural injuries but with dementia (group 3). Our findings show group 1 has a unique injury profile, combining elements of both agricultural and dementia-related trauma. Compared to group 3, group 1 was less likely to experience falls but more likely to be injured by transport and machinery-related incidents. Alternatively, compared to group 2, group 1 had higher odds of experiencing falls. Group 1 was also more likely to experience "severe" injuries (Injury Severity Score > =16) relative to group 3. Additionally, group 1 members were also more likely to require ICU/surgery at ED Discharge. These results highlight the need for targeted injury prevention programs for older adults with dementia in agriculture.

Abstract citation ID: igaf122.513

### OLFACTION TESTING AND ASSOCIATED FACTORS AMONG OLDER U.S. FARMERS

Yaqun Yuan[1], Brenda Plassman[2], Zhehui Luo[1], Jayant Pinto[3], Christine Parks[4], Dale Sandler[4], and Honglei Chen[1], *1. Michigan State University, East Lansing, Michigan, United States, 2. Duke University Medical Center, Durham, North Carolina, United States, 3. The University of Chicago, Chicago, Illinois, United States, 4. National Institute of Environmental Health Sciences, Durham, North Carolina, United States*

Poor olfaction is common in older adults and may signify adverse health conditions. We tested olfaction in 2545 older farmers from North Carolina and Iowa between 2020-2021, using the 12-item Brief Smell Identification Test (B-SIT). We compared their B-SIT score with perceived testing experience and examined factors that may associate with the test results, accounting for sampling design, study participation, and covariates. Overall, 37.8% of the farmers had good olfaction (B-SIT score 11-12), 34.2% moderate olfaction (9-10), and 28.0% poor olfaction (0-8). A higher B-SIT score correlated with perceived more odorants identified (Spearman's $\rho = 0.65$) and perceived odor strength (Spearman's $\rho = 0.54$). Older age, single marital status, North Carolina residence, and a history of asthma, acute recurrent sinus infections, and surgery of the nose or brain were associated with poor olfaction. Seasonality was associated with B-SIT results, with the highest scores in the summer (June to August) and the lowest in the Spring (March to May), with a mean difference of 0.48. Allergic or cold symptoms (e.g., running nose, sore throat, sinus pain) or farming activities (e.g., working in animal confinement areas or around wood/metal dust) in the past 24 hours were generally unrelated to poor olfaction. Although not statistically significant, those who reported loading/mixing/applying pesticides and cleaning hands or machines with gasoline in the past 24 hours had lower B-SIT scores. To our knowledge, this is the first study that characterized the olfaction testing experience in older farmers and reported factors that may associate their olfaction test results.

Abstract citation ID: igaf122.514

### FUNCTIONAL LIMITATIONS AND DISABILITY AMONG OLDER ADULTS IN NEPAL

Usha Dhakal[1], Hardik Sapkota[2], Indra Chaudhary[2], Adina Gurung[2], Emily Briceño[3], Uttam Sharma[2], Dirgha Ghimire[4], and Carlos Mendes de Leon[1], *1. Georgetown University, Washington, District of Columbia, United States, 2. Institute for Social and Environmental Research-Nepal (ISER-N), Chitwan, Bagmati, Nepal, 3. University of Michigan Medical School, Ann Arbor, Michigan, United States, 4. University of Michigan, Ann Arbor, Michigan, United States*

The burden of disability among older adults in Nepal remains unclear and understanding this can help inform healthcare services and support systems to enhance their quality of life. Therefore, we examined patterns of disability as a function of functional limitations and other key predictors of late-life disability among older adults in Chitwan, Nepal. Data for this study came from 144 adults 50 years and older using a cluster random sampling design. Nearly one-third (30%) of the participants had at least one activity of daily living (ADL) disability and more than half (52%) had at least one instrumental ADL (IADL) disability. Functional limitations were assessed using physical performance tests of gait speed, chair stand, and grip strength summarized in a composite score (0-9). We used negative binomial regression models to predict the number of ADL and IADL disabilities (0-12) and found that higher physical performance test scores were associated with lower disability after adjusting for age and gender (β=-0.12, p=0.03). This association was slightly attenuated after adjusting for education and lifetime income (β=-0.10, p=0.07) and no longer significant after additional adjustment for chronic conditions (β=-0.07, p=0.19). In separate models for ADL and IADL disability, physical performance tests scores were only predictive of IADL disability. Our findings suggest a substantial burden of disability among older adults in Chitwan, even in this relatively young older age group. We may have lacked sufficient statistical power to more firmly establish the role of functional limitations in the disability process in this population.

### SESSION 1505 (PAPER)

### FAMILY TIES IN LATER LIFE: CAREGIVING, CONNECTION, AND COPING WITH LOSS

Abstract citation ID: igaf122.515

### A SCOPING REVIEW OF DELPHI METHODOLOGIES IN AGING RESEARCH: 2018-2024

Shannon Jarrott[1], Aaron Ogletree[2], Shelbie Turner[3], and Michelle Demetres[3], *1. The Ohio State University, Columbus, Ohio, United States, 2. Virginia Tech, Blacksburg, Virginia, United States, 3. Weill Cornell Medicine, New York, New York, United States*

The Delphi method engages experts to develop, refine, or establish face validity of materials, such as clinical guidelines or assessment tools. It is a convenient, efficient means of gathering input that also mitigates power dynamics that might exist among varied stakeholders. The number of Delphi studies is growing, and researchers in aging apply the methodology across a range of topics. However, Delphi methodology standards and reporting guidelines vary, potentially limiting study rigor and reproducibility. We conducted a scoping review of Delphi studies in aging research to (a) characterize methodological practices adopted by researchers and (b) identify quality indicators for Delphi studies specific to aging research. Searching multiple databases for published literature from January 2018 through November 2024, we identified 89 eligible studies and extracted key information reflecting recommended Delphi method practices (e.g., pre-identifying consensus). Results demonstrate variation across adopted practices and highlight a need for greater alignment with quality indicators. Percentage of studies adopting the recommended practices ranged from a low of 11% (specifying a target number of expert participants) to a high of 92% (providing the final product). For example, Diamond and colleagues recommended that number of rounds be determined a priori, but this occurred in only 28% of examined studies. We propose a Delphi quality indicator, or criteria, rating form for aging research based on these findings. The criteria rating form can be used to plan and implement the Delphi method and evaluate the trustworthiness of studies and resultant products.

Abstract citation ID: igaf122.516

### DIGITAL TECHNOLOGY USE AND DEMENTIA RISK IN OLDER ADULTS: EVIDENCE FROM NHATS

Wenxuan Huang[1], Jiao Yu[2], and Roland Thorpe[3], *1. Johns Hopkins University, Baltimore, Maryland, United States, 2. Yale University, New Haven, Connecticut, United States, 3. Johns Hopkins Bloomberg School of Public Health, Baltimore, Maryland, United States*

Digital technology has become integral to older adults' lives, supporting healthcare, social, and leisure needs. While the cognitive benefits of technology use have been documented in recent research, existing studies are limited by cross-sectional designs, narrow measures of technology engagement (e.g., single domains like computer use), and small sample sizes. Furthermore, no prior research has explored how this relationship varies between older adults with and without activity limitations. Leveraging 12 years of longitudinal data from the National Health and Aging Trends Study (NHATS; n=7,027), this study investigates associations between three domains of technology use—general device use (e.g., smartphones), everyday technology use (e.g., emailing, online shopping), and digital health technology use (e.g., telehealth)—and dementia incidence among community-dwelling older adults. Analyses adjust for selection bias using inverse probability weighting and stratify by three activity limitation categories (mobility limitations, self-care limitations, household limitations). Weighted mixed effects logit model results indicate that everyday technology use related to routine activities (i.e., emailing/texting, online shopping) is associated with an approximately 80% lower odds of dementia, compared to 48% and 64% respectively for using any technology devices and any digital health technology. Stratified model results demonstrate significantly greater marginal effects for older adults with activity limitations, suggesting that they may glean more cognitive health benefits from technology use than their limitation-free counterparts. These findings underscore digital technology's potential as a scalable, modifiable intervention to reduce dementia risk, particularly for vulnerable populations with disabilities.

Abstract citation ID: igaf122.517

### EVALUATING MHEALTH APPLICATIONS FOR OLDER ADULTS WITH HYPERTENSION: SYSTEMATIC META-ANALYSIS OF CLINICAL TRIALS

Renato FerreiraLeitao Azevedo[1], Michael Varzino[2], Nidhi Patel[3], and Wendy Rogers[2], *1. University of Georgia, Athens, Georgia, United States, 2. University of Illinois Urbana-Champaign, Champaign, Illinois, United States, 3. University of Illinois Urbana-Champaign, Chicago, Illinois, United States*

Hypertension is the most prevalent chronic condition affecting older adults and mobile health (mHealth) applications have the potential to support their blood pressure (BP) management and self-care. However, no meta-analysis examines the effects of mHealth app interventions for older adults. To address this gap, we conducted a meta-analysis of randomized controlled trials (RCTs) to examine the effectiveness of mHealth apps for improving older adults' hypertension outcomes. Relevant studies were sought through EBSCO, Clinical Trials, Cochrane Library, ProQuest, PubMed/MEDLINE, ScienceDirect, Scopus, and Web of Science databases. We retrieved 1,244 papers and two reviewers completed a full screen review of 79. We excluded studies that were not RCTs, did not examine mHealth apps effects, or were not conducted with older adults, resulting in 9 papers with a total of 915 participants included in the analysis. Our results indicate that mHealth apps can be effective for older adults managing hypertension, with significant reductions in Systolic BP (Hedge's g: -1.37, 95% CI [-2.25 to -0.49]), and Diastolic BP levels (g: -079, 95% CI [-1.42 to -0.16]), as well as signs of improvement in medication adherence (g: 3.70, 95% CI [-29.82 to 37.22]). These effects are consistent with those meta-analysis involving other age groups, supporting that older adults can benefit from mHealth apps. We review each intervention, assess potential biases and the app components that may impact effectiveness. This analysis aims to guide the design of future hypertension management RCTs, and more broadly, improve the design of other mHealth tools for older adults. Pre-registration: https://doi.org/10.17605/OSF.IO/NS7TP

Abstract citation ID: igaf122.518

**IMPACT OF THE SMART TABLET EDUCATION FOR HEALTHY LIVING (STEHL) INTERVENTION: RESULTS FROM A FOCUS GROUP STUDY**

Yujun Liu[1], Elizabeth Sterner[2], Bolanle Olayeni[2], Shalini Shrikanth[3], Pamela Gampetro[4], Rhea Johnson[2], Masooma Shamsi[2], and Anitha Saravanan[2], *1. Northern Illinois University, Naperville, Illinois, United States, 2. Northern Illinois University, DeKalb, Illinois, United States, 3. Loyola University Chicago, Chicago, Illinois, United States, 4. Illinois State University, Evanston, Illinois, United States*

Background and Objectives: Older adult Americans often struggle with digital health literacy (DHL), the ability to find, evaluate, and use digital information to make informed healthcare decisions. Inadequate DHL can also hinder effective physician–patient communication. The Smart Tablet Education for Healthy Living (STEHL) intervention leverages digital technology to enhance health literacy and promote healthy behaviors among older adults. This study aims to evaluate the impact of the STEHL intervention through qualitative insights from focus groups conducted with participants. Research Design and Methods: Three focus groups (n = 13) were conducted by trained facilitators in quiet rooms at three retirement homes in Illinois. Using a semi-structured interview approach, the focus groups explored participants' experiences, perceptions, and behavioral changes resulting from the intervention. Responses from each question were imported into MAXQDA Analytics Pro 2024. Results: Thematic analysis revealed 12 themes and positive outcomes including increased confidence in using digital tools for health-related tasks, improved understanding of health information, and reported positive changes in lifestyle habits. Participants highlighted the intervention's user-friendly design and personalized content as significant facilitators, while challenges such as initial apprehension toward technology were also noted. Discussion: These findings underscore the potential of the STEHL intervention to address health literacy gaps and support older adults in achieving better health outcomes. Future research should explore scalability and long-term effects of digital health interventions like STEHL in diverse populations.

Abstract citation ID: igaf122.519

**LESSONS LEARNED IN SAMPLING COMMUNITY-DWELLING BLACK/AFRICAN AMERICAN OLDER ADULTS WITHOUT ADVOCATES**

Miriam Gofine[1], Antoinette Schoenthaler[2], and Carolyn Berry[2], *1. New York University, Brookline, Massachusetts, United States, 2. New York University, New York, New York, United States*

"Older Adults Without Advocates" (OAWA), a clinical population referred to as "elder orphans" by the American Geriatrics Society, are conceptualized as older adults lacking kin support. While multidisciplinary literatures highlight the urgent need for more research on this growing, higher-need population, identifying OAWA in community and clinical settings remains a challenge, posing barriers to research recruitment. We report lessons learned from sampling community-dwelling Black/African American (B/AA) OAWA for a qualitative study and suggest avenues for future successes in sampling this population. Using best practices for sampling hidden populations and B/AA older adults, we utilized snowball sampling of community-dwelling B/AA OAWA with limited success. Despite strong community support for the study and recruitment, including four initial sampling "seeds," engagement with >50 referrers across New York City to identify >60 potential OAWA, and successfully extending referral chains up to five degrees, we were only able to interview three eligible OAWA. We encountered multiple recruitment barriers, including: discrepancies between referrers' and OAWA candidates' perceptions of familial support; discordance between stricter clinical and looser social definitions of OAWA; gatekeepers' reluctance or inaccessibility for referrals; inability to reach OAWA candidates using provided contact information; eligible candidates' reluctance to participate; language barriers (Spanish and Haitian Creole); and lack of a validated OAWA screening tool. Future research should consider engaging trusted gatekeepers as interviewers, using a larger "seed" group and including OAWA residing in long-term care facilities. A validated screening tool tailored to diverse communities is essential for improving recruitment and success of research on community-dwelling OAWA.

### SESSION 1510 (PAPER)

Abstract citation ID: igaf122.520

**HEALTH BEHAVIORS**

During this session you will gain insights into the Impact of health behaviors on Aging.

Abstract citation ID: igaf122.521

**ADAPTIVE COPING AND CAREGIVING SELF-EFFICACY AMONG BLACK FAMILY CAREGIVERS OF PERSONS WITH DEMENTIA**

Sheria Robinson-Lane[1], Lu Qin[2], Florence Johnson[1], Ivo Dinov[1], and Bruno Giordani[2], *1. University of Michigan School of Nursing, Ann Arbor, Michigan, United States, 2. University of Michigan, Ann Arbor, Michigan, United States*

Black family caregivers of persons with dementia have an increased risk of poor health, future cognitive impairment, and premature death related to stressors associated with caregiving. Understanding the relationships between adaptive coping, caregiving self-efficacy, and health can facilitate the development of resilience-focused interventions that may improve caregiver health. We surveyed 102 self-identified Black family caregivers of persons with dementia. Survey measures included the coping and adaptation processing scale (CAPS), the revised scale for caregiving self-efficacy, and the PROMIS global health scale. Analysis was completed using regression modeling. Adjusting for age, gender, marital status, employment status, income, education, and presence of chronic disease, we found that adaptive coping was positively associated with caregiver self-efficacy in managing disruptive behaviors (B = 1.57, 95% CI [.96, 2.173], p = 0.000) and controlling upsetting thoughts (B = 1.67, 95% CI [1.13, 2.22], p = 0.000). Adaptive coping was not significantly associated with mental health (i.e., emotional distress, social role satisfaction) (B=.07, 95% CI [-.28, 0.41], p = 0.707), or physical health (i.e., physical function, pain, fatigue) (B=-0.01, 95%CI [-.27, 0.24], p = 0.926). Adaptive coping strategies associated with caregiving self-efficacy and resilience theory included experience, use of planning skills, and humor. Designing caregiver support interventions that facilitate the development of effective planning skills may significantly improve caregiver mental health.

Abstract citation ID: igaf122.522

**ASSOCIATIONS BETWEEN DIGITALLY MEASURED SLEEP QUALITY AND LATERAL STEP VARIABILITY AMONG OLDER ADULTS**

Francesca Marino[1], Minzae Kim[1], Rhoda Au[1], Phillip Hwang[2], Jesse Mez[1], Mike Alosco[1], Robert Thomas[3], and Ludy Shih[3], 1. Boston University - Chobanian & Avedisian School of Medicine, Boston, Massachusetts, United States, 2. Boston University School of Public Health, Boston, Massachusetts, United States, 3. Beth Israel Deaconess Medical Center, Boston, Massachusetts, United States

Higher step variability is associated with slower gait speed, reduced leg strength, and increased fall risk. Poor sleep quality is an emerging risk factor for worse physical function, but associations between sleep and step variability are unclear. Evidence from objective sleep measures is also lacking, as most studies rely on self-reported measures that may misclassify sleep quality. Therefore, this study aimed to evaluate associations between digitally measured sleep characteristics and lateral step variability among older adults. This study included 72 older adults (mean age: 71 years) who had ≥2 nights of objectively measured sleep (SleepImage ring) and completed a 10-meter preferred speed walk while wearing APDM sensors on their back, wrists, and feet. Lateral step variability, defined as variability of perpendicular deviations of foot placement, was categorized as medial (≤-7.5 cm), minimal (within ±7.5 cm; reference), or lateral displacement (≥7.5 cm). Demographic-adjusted multinomial logistic regression models evaluated cross-sectional associations between sleep and lateral step variability categories. We found that 1% higher sleep fragmentation was associated with 1.06-times odds of step variability ≥7.5 cm (95% CI: 1.01-1.11), while 1% higher sleep stability was linked with 0.95-times odds of variability ≥7.5 cm (95% CI: 0.91-0.99) compared to variability within ±7.5 cm. There were no associations with sleep duration, efficiency, apnea, or oxygen saturation. Better quality sleep, particularly lower fragmentation and higher stability, was associated with lower lateral step variability. Future studies should evaluate whether digital sleep measures can predict individuals at risk for falls or serve as intervention targets for fall prevention.

Abstract citation ID: igaf122.523

**PHYSICAL ACTIVITY MODERATES ASSOCIATIONS BETWEEN SLEEP AND COGNITION IN MIDDLE-AGED AND OLDER ADULTS**

Marc Kaizi-Lutu[1], Daniel Callow[2], Sanjana Subramanyan[1], Casandra Nyhuis[3], William Eaton[1], Brion Maher[1], Cynthia Munro[2], and Adam Spira[1], *1. Johns Hopkins Bloomberg School of Public Health, Baltimore, Maryland, United States, 2. Johns Hopkins School of Medicine, Baltimore, Maryland, United States, 3. Penn State College of Medicine, Hershey, Pennsylvania, United States*

Higher sleep quality and greater physical activity are linked to reduced risk of cognitive decline and dementia, but little is known about the synergistic effects of sleep and physical activity on cognition in middle-aged and older adults. We examined whether physical activity moderated the association between sleep and cognition in 341 cognitively unimpaired middle-aged and older adults (mean 67.5±7.40 years, 63.6% female, 35.5% non-white, 12.9±2.3 years of education), who participated in Wave 5 of the Baltimore Epidemiologic Catchment Area Study. Sleep and physical activity measures derived from 6.8±1.6 days of wrist actigraphy included total volume of physical activity (TVPA), total sleep time (TST), sleep onset latency (SOL), sleep efficiency (SE; % time in bed spent asleep) and wake after sleep onset (WASO; minutes awake after falling asleep). Cognitive tests assessed memory, executive function, language, attention, and psychomotor speed. After adjustment for age, sex, race, and education, TVPA was positively associated with attention and language. Lower SE, greater WASO, longer SOL, and TST < 6 hours (vs. TST 6-8 hours) were associated with poorer cognitive performance. TVPA moderated the associations between SOL and executive function (interaction $p < 0.05$), and WASO and language (interaction $p = 0.04$), such that associations between lower sleep quality and poorer cognition were weaker among participants with higher TVPA. Higher TVPA was associated with better language performance among those with 6-8 or > 8 hours of TST, but not in those with TST < 6 hours. Findings suggest that greater physical activity may attenuate the negative impact of poor sleep on cognition.

Abstract citation ID: igaf122.524

**IMPROVING HEALTHY DAILY ACTIVITY IN OLDER ADULTS WITH MULTIMORBIDITY: EFFICACY AND ACCEPTABILITY OF ACTIVEOT**

Tara Klinedinst[1], Juleen Rodakowski[2], Lee Jennings[3], Nicholas Hollman[4], Sarah Beth Bell[1], Zachary Pope[1], Zsolt Nagykaldi[1], and Darla Kendzor[1], *1. The University of Oklahoma Health Sciences Center, Oklahoma City, Oklahoma, United States, 2. University of Pittsburgh, Pittsburgh, Pennsylvania, United States, 3. University of Oklahoma Health Sciences, Oklahoma City, Oklahoma, United States, 4. The University of Oklahoma School of Community Medicine, Tulsa, Oklahoma, United States*

Nearly half of U.S. older adults have multimorbidity (≥2 chronic conditions) in addition to functional limitations that restrict engagement in health-promoting daily activity (e.g., sleep, medication-taking, physical activity). We tested a combination of behavioral activation and occupational therapy ($n = 21$) compared to brief education ($n = 20$) on performance of health-promoting daily activity, measured by the Canadian Occupational Performance Measure (COPM) at baseline, 10 weeks, and 22 weeks. "ActiveOT" was delivered by occupational therapists in 10 once-weekly sessions in participant homes. On average, the 41 participants were 73 years old, female, and identified as White. They averaged 4.0 chronic conditions ($SD = 1.3$), and 1.6 functional limitations ($SD = 1.5$). Baseline scores were not different between groups. At 10-weeks, the ActiveOT group had higher COPM scores ($M = 7.3$, $SD = 1.7$) compared to controls ($M = 4.9$, $SD = 1.8$; $t(35) = 4.32$, $p < 0.001$). This trend continued at the 22-week follow-up where the ActiveOT group had higher COPM scores ($M = 7.8$, $SD = 1.2$) than controls ($M = 5.3$, $SD = 1.8$; $t(33) = 4.81$, $p < 0.001$). Between-group COPM scores reflected large effect sizes at 10 weeks (Cohen's $d = 1.42$) and 22 weeks (Cohen's $d = 1.63$), and 89% of ActiveOT participants experienced clinically meaningful improvements (MCID=3). We conducted semi-structured follow-up interviews with ActiveOT participants ($n = 15$) to understand the acceptability and satisfaction with the intervention and study processes. ActiveOT participants reported overall satisfaction with the intervention and appreciated the flexibility of goal areas and person-centered focus. ActiveOT participants demonstrated improved performance of health-promoting daily activity and evaluated the intervention favorably. Future work will focus on implementation of ActiveOT in primary care contexts.

### SESSION 1515 (PAPER)

### IMPROVING THE CARE DELIVERY SYSTEM

Abstract citation ID: igaf122.525

### IMPLEMENTING AGE-FRIENDLY HOSPITAL INITIATIVES: THE MEITAV MODEL - A SYNERGISTIC BOTTOM-UP-TOP-DOWN APPROACH

Ayelet Dagan Yarkoni[1], Natella Belinson[2], Orit Shahar[1], Rony Schenker[1], Adar Klein Druyan[2], Ruty Ofir[2], and Anna Zisberg[3], *1. JDC, Jerusalem, Yerushalayim, Israel, 2. Ministry of Health, Jerusalem, Yerushalayim, Israel, 3. University of Haifa, Haifa, HaZafon, Israel*

The unprecedented demographic shift toward an aging population necessitates integrating age-friendly principles into hospital operations. This presentation examines MEITAV (Preventing Functional Decline of Older Adults During Hospitalization), an innovative implementation model combining leadership-driven strategies with grassroots initiatives to achieve sustainable hospital transformation. MEITAV represents a pioneering collaboration between government ministries and a civil society organization, creating a scalable framework implemented across all general hospitals in Israel. Our analysis demonstrates how strategic alignment of interdisciplinary teams, staff empowerment, and executive leadership has enhanced application of the 4Ms Framework—particularly mobility and mentation (Delirium)—while strengthening geriatric consultation services. Critical success factors include targeted professional development, environmental modifications, and evidence-based protocols. Initial implementation data shows engagement across 100 internal and surgical departments in 27 general hospitals, led by 120 multidisciplinary champions. Notably, 50% of hospitals meet mobility maintenance and improvement during hospitalization, while 76% achieve delirium screening objective for patients aged 75+. This presentation provides evidence-based insights into challenges, opportunities, and best practices from the MEITAV experience, offering valuable guidance for transforming isolated age-friendly initiatives into comprehensive institutional change, Initial clinical and economic outcomes will be presented, demonstrating MEITAV's impact on improving care for hospitalized older adults

Abstract citation ID: igaf122.526

### EXPLORING THE ROLE OF SOCIAL WORKERS IN AGE FRIENDLY HEALTH SYSTEMS: A SCOPING REVIEW

Leslie Hasche, Aprille Arena, and Jina Park, *University of Denver, Denver, Colorado, United States*

Age-friendly health systems have expanded across academic medicine, hospital systems, Center for Medicare and Medicaid payment mechanisms, and federal geriatric workforce efforts. Age friendly health systems involve a 4M Framework of (1) What Matters, (2) Medication, (3) Mentation, and (4) Mobility, with corresponding implementation tools, quality measures, and interprofessional training programs. Given that 30% of social workers are employed in hospital, health care, or long-term care settings, the specific role of social workers warrants exploration. This scoping review aimed to identify how social workers, or social services have engaged in age-friendly health care. The search, conducted in March of 2025, originally yielded 159 articles. Following duplication removal and eligibility screening, 24 articles were included. Over half of the articles were commentary or review articles describing age-friendly health systems that incorporated naming social work as a key interprofessional role or explicated how social workers may be involved in geriatric emergency room responses, assessments, caregiver support, and care management. Empirical articles included eight qualitative or mixed method studies that (1) evaluated implementation or training efforts, (2) identified unmet needs, or (3) described barriers. As part of the mixed methods studies and in two unique quantitative studies, retrospective analysis of clinical chart data was used to assess health outcomes. Social workers did offer unique contributions to promote consideration of disability-friendly care needs and of cultural adaptations, particularly for older Hawaiian populations. Geriatric social work education efforts should be updated to incorporate age-friendly health care approaches and attend to adaptations for diverse older adults.

Abstract citation ID: igaf122.527

### IMPACT OF AGE-FRIENDLINESS ON OLDER ADULTS' CONSUMPTION AND WELL-BEING: INSIGHTS FROM A HONG KONG SURVEY EXPERIMENT

Xue Bai, Mengyu Liu, Youjuan Zhang, and Shuai Zhou, *The Hong Kong Polytechnic University, Hong Kong, China (People's Republic)*

Aims This study aims to investigate how older adults in Hong Kong perceive age-friendliness, focusing on how age-friendly business/societal structures influence consumption choices and wellbeing. Methods We use the second wave data from the Panel Study on Active Ageing and Society (PAAS, N = 3000), which is the first city-wide representative longitudinal survey on active ageing and society in Hong Kong. To identify the causal effect of age-friendliness, we designed a factorial survey experiment and randomly assign respondents to 16 self-developed vignettes. Multilevel models were employed to analyse the data. Results Our findings show that age-friendly practices, in general, along with its four specific domains of physical environment, staff and personnel, marketing and information, and products and services, positively impacts the consumption willingness of adults aged≥50 years, increasing their willingness to consume by 146% (mean difference: 4.03, $t = 32.07$, $P < 0.001$). Additionally, satisfaction with AFB is positively associated with the physical (e.g. lower risk of falls, sarcopenia and frailty), mental (lower risk of depression and loneliness), and cognitive health of older adults, as well as their life satisfaction. Businesses perceived as age-friendly are more likely to motivate older adults to engage in consumption that aligns with healthy ageing goals. Conclusions Age-friendliness enhances healthy aging by shaping consumption behaviors that prioritize older adults' holistic well-being. The findings highlight actionable strategies for businesses and policymakers to design inclusive markets and age-friendly cities. By addressing older adults' expectations of the silver market, this study supports creating health-supportive environments where older adults thrive.

Abstract citation ID: igaf122.528

### IMPLEMENTATION PROFILES OF AN ONLINE COMMUNICATION EDUCATION PROGRAM IN THE CHATO NATIONAL TRIAL NURSING HOMES

Carissa Coleman[1], Frances Yang[2], Maria Hein[3], Yelena Perkhounkova[3], Clarissa Shaw[4], Maria Roche-Dean[5], Amy Berkley[6], and Kristine Williams[7], *1. University of Kansas Medical Center, Kansas City, Kansas, United States, 2. University*

*of Kansas, University of Kansas Medical Center, Kansas, United States, 3. University of Iowa, Iowa City, Iowa, United States, 4. University of Iowa College of Nursing, Iowa City, Iowa, United States, 5. Kirkhof College of Nursing, Grand rapids, Michigan, United States, 6. University of Kansas School of Nursing, Kansas City, Kansas, United States, 7. University of Kansas, Kansas City, Kansas, United States*

The Changing Talk (CHAT) communication education program effectively reduces elderspeak and subsequent behavioral challenges in residents with dementia in nursing homes . As part of the nationwide pragmatic clinical trial testing Changing Talk: Online Training (CHATO), a remote implementation design was developed to capture contextual factors, ensure fidelity, and identify effective implementation strategies. Nursing homes implementing CHATO were surveyed upon completion to determine implementation strategies consistent with Expert Recommendations for Implementing Change (ERIC). A national sample of 79 nursing homes from 37 states completed the survey. Remote implementation lasted three months, one month per phase (planning, intervention implementation, and a follow-up). Staff participation rates averaged 51.6% (SD = 31.3%), ranging from 3% to 100%. Latent class analysis in Mplus version 8.10 was used to determine the implementation profiles of nursing home strategies. The three-class model demonstrated the best fit (AIC=652.05, BIC=706.55, and Sample-Size Adjusted BIC=634.03) which classified implementation profiles into (1) self-paced, (2) champion-paced, and (3) leadership-paced. Fifty-four nursing homes (68%) belonged to the Self-Paced dominant class which demonstrated all staff participation, but low participation in discussions and recognition; 22 nursing homes (28%) belonged to the Champion-Paced dominant class which used champions and reminder emails, and three nursing homes (4%) belonged to the Leadership-Paced dominant class which had relatively the highest probability of all staff participation, reminder emails and texts, discussions, and public recognition. Future analysis of these profiles will be combined with MDS data to assess which implementation styles are linked to improved resident outcomes.

Abstract citation ID: igaf122.529

### MEASURING AND IMPROVING WHAT REALLY MATTERS: A POPULATION HEALTH SOLUTION FOR AGING WELL

James Firman, *BetterAge, Arlington, Virginia, United States*

Traditional health and social service metrics often fail to capture the full picture of what enables older adults to age well. BetterAge is pioneering a new approach—one that measures holistic health and wellbeing across 13 dimensions, identifies 17 critical barriers, and provides hyper-personalized recommendations to drive meaningful action. The session will explore how BetterAge integrates emerging technologies with trusted community networks to assess, engage, and support older adults at scale. Supported by a $2.5M SBIR Fast Track grant from the National Institute on Aging, BetterAge leverages AI-driven assessments, behavioral insights, and strategic partnerships, including USAging and state-level initiatives, to generate real-world impact. Recent pilots with community organizations and state agencies through the Age Well Innovation Network (AWIN) are beginning to demonstrate its potential to identify disparities, get people to change behaviors known to improve outcomes, and reduce social isolation. Participants will learn how this population health solution is being implemented nationwide, how it aligns with evolving policy and funding landscapes, and how organizations can leverage it to support older adults more effectively. The session will include real-world case studies, data-driven insights from 4,000+ consumer records, and a discussion on the future of measuring what truly matters for aging well.

### SESSION 1520 (BIOLOGICAL SCIENCES INVITED SYMPOSIUM)

Abstract citation ID: igaf122.530

### LATE BREAKING BIOLOGICAL SCIENCES SESSION

Chair: Sean Curran

Abstract citation ID: igaf122.531

### MECHANISMS OF SLOWED AGING IN A NEW MODEL SYSTEM FOR EXTENDED LONGEVITY

Jessica Foley[1], and Stephen Montgomery[2], *1. Tufts University, Medford, Massachusetts, United States, 2. University of Bristol, Bristol, England, United Kingdom*

Evolution has given rise to lifespans in extant species ranging from days to centuries. Given that mechanisms of aging are highly conserved, studies of long-lived lineages across the animal kingdom could shed light on mechanisms of healthy aging in humans. However, typical animal models of extended lifespan often live for decades, making them intractable for longitudinal studies. Ideal model systems would instead be organisms that are long-lived relative to their close evolutionary relatives, yet have lifespans on experimentally tractable scales. Lifespans in the Heliconius butterfly genus, reaching nearly a year, are among the longest recorded in butterflies. This represents a dramatic extension over their closest relatives in the Heliconiini tribe, which typically live only 1–2 months. While previous work has attributed this difference to a plastic response to enhanced nutrition, the degree and consistency of the extension across the genus led us to hypothesise that it reflects evolved, heritable mechanisms of longevity. We conducted detailed survival and functional senescence analyses on two representative shorter- and longer-lived species to show evidence for evolved mechanisms for slowed ageing in Heliconius. Results of transcriptomic analyses then revealed a strong upregulation of pathways related to protein-folding in aged Heliconius, suggesting that an increased investment into protoeostatic mechanisms may have been a key factor in their evolution of longevity. This finding aligns with extensive evidence that proteostasis plays a central role in aging, including in humans, and highlights the value of studying diverse organisms to advance understanding of healthy aging.

Abstract citation ID: igaf122.532

### GEROTHERAPEUTIC COMPOUND PICOLINIC ACID SUPPORTS LOCOMOTOR FUNCTION AND BONE HEALTH IN AGED ZEBRAFISH

Hassan Rammal[1], Dafna Perry[2], Daniel Rivas[1], David Karasik[2], and Gustavo Duque[1], *1. McGill University, Montreal, Quebec, Canada, 2. Azrieli Faculty of Medicine, Bar-Ilan University, Bar-Ilan, Hefa, Israel*

Objectives: This study aimed to evaluate the impact of picolinic acid (PIC), a metabolite derived from tryptophan, on age-related tissue regeneration and physical decline in zebrafish. Additionally, it examined changes in whole-body mass index (WB-BMI) as an indicator of musculoskeletal aging. Methods: Siblings born in August 2022 were randomly assigned to four groups at age 20 mo: (1) PIC in water (25 mg/kg/day), (2) PIC in water + 25 mg/kg oral gavage, (3) PIC in water + 75 mg/kg oral gavage, and (4) control (system water + gavage) for eight weeks. The treatment groups consisted of 15 fish. At week 7, swimming performance was recorded over a 30-minute period. Caudal fins were amputated for regeneration analysis. In week 8, fish were euthanized for whole-body micro-CT and for β-galactosidase (X-gal) staining to evaluate cellular senescence. The Mann-Whitney U test was used to compare WB-BMI between groups. Results: Group 2 showed the highest swimming speed (44.5 m/min), followed by Group 1 (34.7 m/min) ($p = 0.01$ and $p = 0.09$, respectively) than the control group (31.7 m/min). WB-BMI was decreasing over the duration of the experiment in all 4 groups, with Group 1 maintaining the highest BMD compared to the control (non-significant, $p > 0.5$). Regeneration in Groups 2 and 3 was more advanced than in Group 1, with stronger staining by β-galactosidase. Conclusion: PIC at moderate doses supports locomotor function and overall health in aged zebrafish. It may influence regenerative outcomes through mechanisms involving cellular aging.

Abstract citation ID: igaf122.533

### THE HOUSE CRICKET (ACHETA DOMESTICUS) AS A SCALABLE INVERTEBRATE MODEL FOR GEROTHERAPEUTIC TESTING

Gerald Liao, Jenna Klug, Swastik Singh, Sherwin Dai, Elizabeth Bae, and Warren Ladiges, *University of Washington, Seattle, Washington, United States*

The house cricket (Acheta domesticus) is a promising preclinical geroscience model due to its short lifespan, low maintenance, age-associated functional decline, and responsiveness to geroprotective drugs. Continuous dosing with rapamycin, acarbose, and phenylbutyrate extends lifespan; whether intermittent dosing offers similar benefits remains unknown. We tested 274 sex-matched crickets given 2-week intermittent dosing of each drug starting at mid-age (8-weeks), followed by behavioral testing at 10-weeks (geriatric stage). Assays included Y-maze olfactory discrimination, open-field exploration, and treadmill performance. Locomotor gaits were identified by velocity-based K-means clustering (silhouette > 0.5). A subset was monitored for post-treatment survival using Kaplan-Meier analysis. Olfactory preference was preserved by all drugs ($d = -1.82$ to $-1.28$, P's $< 0.01$), with strongest effects in rapamycin-treated individuals. Rapamycin-treated males matched or exceeded juvenile locomotor activity; phenylbutyrate reduced male activity ($d = 1.49$, $P = 0.049$) and acarbose increased walking-to-running ratios ($d = -0.75$, $P = 0.029$). Rapamycin increased central exploration and freezing ($d = -1.55$, $P < 0.0001$), while acarbose and phenylbutyrate increased peripheral freezing ($d = -0.76$, $P = 0.02$). Rapamycin and phenylbutyrate extended maximum running time ($d = -2.30$ to $-1.32$, $P < 0.0001$), with sex-specific jumping gains in rapamycin-treated females and acarbose-treated males. Post-treatment lifespan was prolonged by rapamycin ($HR = 0.42$, $P = 0.0004$) and reduced by acarbose in females ($HR = 2.92$ to 3.03, P's $< 0.05$). Intermittent rapamycin preserved survival, cognition, and locomotion, while acarbose and phenylbutyrate produced selective benefits, supporting A. domesticus as a scalable model for geroprotective drug discovery.

Abstract citation ID: igaf122.534

### A NOVEL INDUCIBLE MTDNA MUTATOR MOUSE MODEL TO STUDY MITOCHONDRIAL DYSFUNCTION WITH TEMPORAL AND SPATIAL CONTROL

Hannah Tobias-Wallingford, Sydney Bartman, Lauren Gaspar, Giuseppe Coppotelli, and Jaime Ross, *University of Rhode Island, Kingston, Rhode Island, United States*

Mitochondrial dysfunction is a hallmark of aging and numerous age-related diseases. A wealth of studies supports the accumulation of mitochondrial DNA (mtDNA) mutations as a driving factor of mitochondrial dysfunction in aging and disease. One of the best models to study the relationship between mtDNA mutations and mitochondrial dysfunction is the mtDNA mutator mouse, which expresses a proofreading-deficient version of mtDNA polymerase-gamma (PolgA), resulting in accelerated accumulation of mtDNA mutations and aging-like mitochondrial dysfunction. Despite its many contributions in mitochondrial biology and aging research, this model is limited by the whole-body accumulation of mtDNA mutations, which prevents the investigation of tissue-specific differences in mitochondrial dysfunction. To overcome this limitation, we developed a novel inducible knock-in mtDNA mutator mouse model that allows spatial and temporal control of mtDNA mutations, enabling the precise study of mitochondrial dysfunction in a tissue- and time-specific manner. Here, we report the generation and validation of this novel model through whole-body induction via Cre recombinase. Our data demonstrate that, upon induction, this model recapitulates the phenotype of the original mtDNA mutator mouse manifesting the same behavioral and biochemical alterations. This work establishes the functionality of our model and highlights its value as a powerful tool for studying the impact of mtDNA mutations on aging and age-related disorders with enhanced specificity and control.

Abstract citation ID: igaf122.535

### AGE-ASSOCIATED SPLICING EVENTS IN THE INTEGRATIVE LONGEVITY OMICS (ILO) COHORT

Payton Bock[1], Stefano Monti[1], Paola Sebastiani[2], Thomas Perls[1], Stacy Andersen[1], Eric Reed[3], and Albert Tai[4], *1. Boston University, Boston, Massachusetts, United States, 2. Tufts Medical Center, Boston, Massachusetts, United States, 3. Albert Einstein College of Medicine, New York City, New York, United States, 4. Tufts University, Boston, Massachusetts, United States*

Alternative splicing regulates transcript diversity and is increasingly recognized as a key mechanism in aging and age-related disease, yet little is known about how splicing contributes to human exceptional longevity. We analyzed RNA-seq data from the Integrative Longevity Omics (ILO) cohort, which includes centenarians (the oldest-old, age ≥100) and older adult controls (ages 60–89), to identify age-associated splicing events. Splicing was quantified with rMATS to estimate percent-spliced-in (PSI) values across five event categories, and quasi-binomial regression was used to model splicing changes with age. This analysis identified 731 significant age-associated events (false discovery rate < 0.05), the majority of which were mutually exclusive exons and skipped exons. A strong example is a skipped exon in Nuclear Transcription Factor Y subunit C (NFYC), a regulator of senescence-associated pathways. In controls, NFYC PSI trended downward with age, whereas in centenarians, PSI increased significantly with age, suggesting a reversal of midlife splicing trajectories. Residual analysis comparing observed PSI to predictions from a spline model of normal aging further suggests that centenarians deviate from normal aging patterns. This work describes age-associated splicing events in a population enriched for centenarians and provides preliminary evidence for a candidate splicing signature of exceptional longevity. Ongoing analyses will evaluate functional pathways and clinical relevance in independent cohorts.

### SESSION 2000 (SYMPOSIUM)

Abstract citation ID: igaf122.536

### RECONCILING COUNTERINTUITIVE FINDINGS AND QUANTIFYING POTENTIAL SOURCES OF BIAS IN COGNITIVE AGING RESEARCH

Chair: Emma Nichols
Co-Chair: Aleda Leis
Discussant: Lindsay Kobayashi

Epidemiologic research on cognitive aging poses methodological challenges including survival bias, measurement error, and unmeasured confounding. The application of novel methodological approaches can help quantify sources of bias and reconcile counterintuitive findings to advance our understanding of the causes and consequences of cognitive decline and dementia. This session features innovative research on cognitive aging using a wide range of methodologies, including latent growth curve modeling, inverse probability weighting, assessments of potential sources of measurement error, and simulation analysis to better understand the magnitude and role of biasing factors in cognitive aging research. First, Dr. Zachary Kunicki will estimate the rate of normative cognitive changing, addressing potential biases in prior research by using the long follow-up and narrow age range available in the Children of the Depression Age (CODA) cohort, and accounting for mode and practice effects. Second, Dr. Michelle Shardell will evaluate potential reasons for the observed inverse association between cancer and dementia by using inverse probability weights to address selective attrition and data on plasma metabolomics to assess unmeasured confounding through shared biological mechanisms. Third, Dr. Yezhen Li will discuss the potential for measurement error due to noise during cognitive testing and how this may vary by hearing impairment. Finally, Dr. Ryan Andrews will estimate the impact of pre-enrollment survival bias across different country contexts in cross-national research using simulation analysis. Dr. Lindsay Kobayashi will serve as the discussant, integrating findings to discuss the benefits of quantifying sources of bias and implications for future research in cognitive aging.

This is a collaborative symposium between the Epidemiology of Aging and Measurement, Statistics, and Research Design Interest Groups.

Epidemiology of Aging Interest Group Sponsored Symposium

Abstract citation ID: igaf122.537

### THE PACE OF COGNITIVE AGING IN OLDER ADULTS WITHOUT A NEUROCOGNITIVE DISORDER

Zachary Kunicki[1], Emma Nichols[2], Alyssa De Vito[3], Cyrus Kosar[3], Adea Rich[4], Emily Briceño[5], Alden Gross[6], and Richard Jones[3], *1. Warren Alpert Medical of Brown University, Providence, Rhode Island, United States, 2. University of Southern California, Los Angeles, California, United States, 3. Brown University, Providence, Rhode Island, United States, 4. Columbia University, New York, New York, United States, 5. University of Michigan Medical School, Ann Arbor, Michigan, United States, 6. Johns Hopkins Bloomberg School of Public Health, Baltimore, Maryland, United States*

The pace of cognitive change without a neurocognitive disorder is one of the major questions in cognitive aging. The Children of the Depression Age (CODA) cohort of the Health and Retirement Study (HRS) is ideal for studying cognitive aging because it has a long follow-up (22 years) and a narrow age range at baseline (67-74 years). We analyzed data on delayed word recall performance in HRS-CODA (N = 2,295), gathered every other year during the follow-up period. Using latent growth curve modeling and treating delayed recall as a categorical outcome, we found a quadratic model showed better fit to the data than a linear model, with no strong evidence for a practice and retest effect. When calculating the pace of normative aging, our results suggested normative (defined as the absence of a dementia diagnosis over the follow-up period) memory decline is about -0.05 standard deviations per year (SD/y), but is better characterized by age specific estimates of -0.04 SD/y, -0.10 SD/y, and -0.15 SD/y for individuals in their 70s, 80s, and 90s, respectively. These results would suggest that memory decline, in the absence of a recognized dementia and without a confounding of baseline age differences and longitudinal age changes, would be present but almost imperceptible to an individual in their eighth decade, but noticeable in their ninth and quite impairing in their tenth decade.

Abstract citation ID: igaf122.538

### THE INVERSE ASSOCIATION BETWEEN CANCER AND DEMENTIA: IS IT STILL COUNTERINTUITIVE?

Michelle Shardell[1], Alan Rathbun[1], Shanshan Yao[2], Megan Marron[3], Venkatesh Murthy[2], Anne Newman[3], and Eleanor Simonsick[4], *1. University of Maryland School of Medicine, Baltimore, Maryland, United States, 2. University of Michigan, Ann Arbor, Michigan, United States, 3. University of Pittsburgh, Pittsburgh, Pennsylvania, United States, 4. National Institute on Aging, Baltimore, Maryland, United States*

Epidemiologic studies have consistently demonstrated inverse associations between cancer and dementia. One hypothesis is that differential attrition bias leads to an apparent inverse association. Another hypothesis posits that cancer and dementia result from opposite extremes of shared biological mechanisms, where dysregulation in one direction promotes cancer risk, and dysregulation in the opposite direction promotes dementia risk. Such a shared mechanism would produce unmeasured confounding. To assess attrition bias, we used data from n = 2,153 participants in the Health, Aging and Body Composition Study who had neither a history of cancer (self-report) nor dementia (dementia medications or Modified Mini-Mental <78) at baseline to examine whether time-varying cancer history was associated with subsequent incident dementia using cause-specific discrete-time Cox models with and without inverse-probability weighting (IPW) for drop-out and death. To assess possible shared biological mechanisms, we used data from a subset of 1,674 participants with baseline plasma metabolomics characterized. We fit IPW cause-specific Cox models with and without adjustment for the top 20 metabolites jointly associated with cancer and dementia. Cancer history was inversely associated with dementia (Hazard Ratio[HR]=0.648; 95%Confidence Interval[CI]=0.441-0.951), which remained when using IPW

(HR = 0.650; 95%CI=0.440-0.960). Among participants in the metabolomics subset, cancer history was inversely associated with dementia (HR = 0.620, 95%CI=0.391-0.982), but the association was slightly attenuated and lost statistical significance after adjustment for metabolites (HR = 0.669; 95%CI=0.420-1.065). Findings suggest that the inverse association between cancer and dementia is not explained by attrition bias, but may be partially explained by shared biological mechanisms (unmeasured confounding). Multi-omic replication studies are needed.

Abstract citation ID: igaf122.539

### NOISE DURING COGNITIVE TESTS AND OLDER ADULTS' COGNITIVE PERFORMANCE: EVIDENCE FROM INDIA

Yezhen Li[1], Clarice Myers[2], Wuyang Zhang[3], Jinkook Lee[1], Nicholas Reed[4], and Emma Nichols[1], *1. University of Southern California, Los Angeles, California, United States, 2. Johns Hopkins School of Medicine Department of Otolaryngology-Head and Neck Surgery, Baltimore, Maryland, United States, 3. Johns Hopkins Bloomberg School of Public Health, Baltimore, Maryland, United States, 4. New York University Grossman School of Medicine, New York, New York, United States*

Existing evidence suggests that chronic noise exposure elevates the risk of cognitive impairment among older adults. However, noise during cognitive testing may be associated with chronic noise exposure and could impact cognitive testing, leading to bias in analyses of the chronic noise-cognition association. The risk of such biases may be larger in developing regions, where creating optimal test conditions may be more challenging. Using data from the second wave of the Longitudinal Aging Study in India - Diagnostic Assessment of Dementia (n = 3,507), we examined the associations between interviewer-rated noise during assessment and cognitive functioning. We assessed whether the associations were explained by potential mediators linked to chronic noise exposure and cognition (e.g., depression, sleep quality) and whether they were moderated by hearing abilities. High noise levels during testing were significantly associated with lower general cognitive performance scores (b = -.309; p < .001) and lower scores in several cognitive domains, including executive functioning, language, visuospatial, and memory (b = -.280, -.398, -.156, and -.254; p < .05). Associations remained after accounting for potential mediators of chronic noise-cognition associations, suggesting observed effects may be due to acute impacts of noise during assessment rather than chronic noise exposures. Moreover, the moderating effects of hearing abilities were curvilinear, with stronger associations for respondents with normal hearing range and severe hearing loss. These findings suggest that testing conditions, including noise, may factor into older adults' cognitive assessment results. Further research is needed to better understand the implications of noise during cognitive tests.

Abstract citation ID: igaf122.540

### A SIMULATION APPROACH TO EVALUATE PRE-ENROLLMENT SURVIVAL BIAS IN CROSS-NATIONAL AGING EPIDEMIOLOGY RESEARCH

Ryan Andrews[1], Kelvin Zhang[2], Alden Gross[3], Sneha Mani[3], Jennifer Weuve[4], and Lindsay Kobayashi[2], *1. Columbia University, New York, New York, United States, 2. University of Michigan, Ann Arbor, Michigan, United States, 3. Johns Hopkins Bloomberg School of Public Health, Baltimore, Maryland, United States, 4. Boston University, Boston, Massachusetts, United States*

Selection bias caused by collider stratification can distort the estimated effects of exposures on outcomes. As a specific type of selection bias, pre-enrollment survival bias is especially salient in aging research because differential mortality in older populations can yield study samples that systematically deviate from their target populations. Several cross-national comparison studies have observed heterogeneity in the estimated effect of risk factors on aging outcomes, which could be due to true effect heterogeneity or spurious differences due to bias. For example, inconsistent estimates across countries could arise from various magnitudes of pre-enrollment survival bias induced by varying life expectancies across countries. To evaluate how pre-enrollment survival bias can account for the discrepancy in effect estimation in multiple countries, we used cross-national simulations to quantify potential bias in the estimated effect of type 2 diabetes duration on dementia. We simulated country-specific cohorts of individuals born between 1927 and 1951 in the US, England, and India (N = 250,000 each). Under three causal structures for each country that represented the relations among type 2 diabetes duration, vital status in 2016, and dementia status in 2016, we compared the estimated effect among survivors with the true causal effect. We found that the effect of type 2 diabetes duration on dementia could be underestimated as much as 4.5 times the estimated standard error. Age standardization was also applied to enable comparisons of estimated effects across countries. The age-standardized estimates in the US and England did not differ substantially.

### SESSION 2005 (SYMPOSIUM)

Abstract citation ID: igaf122.541

### ESPO AND BEHAVIORAL AND SOCIAL SCIENCES SECTION SYMPOSIUM: INNOVATIVE APPROACHES TO ADVANCING ENVIRONMENTAL JUSTICE IN AGING RESEARCH

Chair: Monica Walters
Co-Chair: Eun Young Choi

Environmental justice is increasingly recognized as an important dimension of aging research, particularly in understanding later-life health disparities. Structural inequities in environmental exposures disproportionately affect racially and ethnically minoritized older adults, exacerbating disparities in cognitive, mental, and functional health. However, critical gaps remain in identifying what specific environmental determinants contribute to these inequities and the mechanisms through which they operate. This symposium brings together cutting-edge conceptual and methodological approaches to advance environmental justice in aging research. The first three presentations address neighborhood-driven disparities in cognitive aging. Dr. Esposito uses a novel sequential mixed-methods approach to critically consider how conceptualizations of "place" (e.g., neighborhood risk/protective factors) shape cognitive outcomes. Dr. Muñoz investigates the temporal effects of neighborhood exposures at macro- and micro-time scales on cognition, drawing on multiple datasets including racially and ethnically diverse populations. Dr. Abdel Magid assesses the relative contributions of different spatial social polarization domains to dementia risk, utilizing Medicare claims data. The final two presentations focus on environmental hazards and racism as structural determinants of health inequities. Ms. Holland assesses relationships between extreme heat exposure and depression risk among older Mexican Americans, leveraging data from the Hispanic Established Population for the Epidemiological Study of the Elderly. Dr. Adkins-Jackson examines how early-life and adulthood exposure to environmental racism shapes late-life functional abilities among Black residents, using the Behavioral Risk Factor Surveillance System. Together, these presentations will advance understanding of how environmental exposures contribute to health disparities, pointing to policy changes that can promote health equity.

Abstract citation ID: igaf122.542

### LIFE COURSE COGNABILITY: A MIXED METHODS STUDY OF NEIGHBORHOODS AND COGNITIVE HEALTH

Michael Esposito[1], and Jessica Finlay[2], *1. University of Minnesota, Minneapolis, Minnesota, United States, 2. University of Colorado Boulder, Boulder, Colorado, United States*

This mixed methods study extends the concept of Cognability -- a new theoretical framework for understanding how neighborhood environments shape cognitive health -- to investigate specific built and social environmental features that impact cognitive health across the adult life course. Current understanding of which neighborhood factors matter when across adulthood is limited. We will conduct a sequential mixed methods approach (QUAL -> QUANT) to identify neighborhood risk and protective factors influencing cognitive health. First, qualitative interviews with 240 adults across four metropolitan areas (San Francisco, Denver, New York, Atlanta) will investigate how individuals of different ages navigate local environments to engage in cognitive health-promoting behaviors. Second, we will utilize these insights to construct sophisticated quantitative models in a coordinated integrated data analysis of four national longitudinal cohort studies (AddHealth, ACL, REGARDS, HRS) spanning adolescence to late age, with geographic data for nearly 90,000 unique respondents. Using structural nested mean models to unravel tricky counterfactual histories, we will assess whether amenities, services, and hazards identified through our qualitative data systematically predict cognitive function trajectories nationwide. This project will advance understanding of Alzheimer's Disease and Related Dementias (AD/ADRD) by identifying modifiable environmental factors across adulthood, not just in later life. By integrating qualitative insights with sophisticated quantitative methods, we aim to develop actionable evidence to inform community interventions that support cognitive health behaviors, address geographic barriers to health outcomes, and advance much-needed 'exposome' research.

Abstract citation ID: igaf122.543

### TEMPORAL DIMENSIONS OF NEIGHBORHOOD CONTEXTS AND COGNITIVE HEALTH

Elizabeth Muñoz, *University of Texas at Austin, Austin, Texas, United States*

Neighborhood environments are critical social determinants of cognitive health, yet they are structured in ways that advantage some groups while disadvantaging others. Investigating how neighborhoods shape cognitive function in racially and ethnically minoritized individuals is essential for informing policies and programs that address environmental inequities and promote cognitive health. While previous research has begun to demonstrate distinct associations between neighborhood domains—structural, physical, and social—and cognitive function, the temporal effects of neighborhood exposures are less clear. This presentation will explore the influence of neighborhood exposures across macro- and micro-time scales. At the macro level, I will summarize research demonstrating how neighborhood exposures across the life course—from childhood to adulthood—impact cognitive function, drawing on data from the Health and Retirement Study and the Colorado Adoption/Twin Study of Lifespan behavioral development and cognitive aging. At the micro level, I will demonstrate how neighborhood exposures shape cognitive performance in real time, focusing on the effects of neighborhood violence using ecological momentary assessment data from racially and ethnically diverse adults in the Effects of Stress on Cognitive Aging, Physiology, and Emotions study. I will conclude by discussing ongoing research that takes a within-group perspective on the daily lived experiences of Latino adults in Central Texas investigating how daily exposures within distinct neighborhoods shape cognitive performance in the moment and over time. By integrating macro- and micro-temporal perspectives, this presentation will highlight the complex and dynamic ways in which neighborhood contexts shape cognitive health, informing future research, prevention, and policy efforts.

Abstract citation ID: igaf122.544

### ESTIMATING THE IMPACT OF SPATIAL SOCIAL POLARIZATION ON DEMENTIA DISPARITIES

Hoda Abdel Magid, Mengya Xu, Wei-Lin Wang, Evelyn Gonzalez, and Julie Zissimopoulos, *University of Southern California, Los Angeles, California, United States*

Using a 20% sample of Medicare claims data (2010–2019, N>4 million), we examined the association between spatial social polarization (SSP) and Alzheimer's Disease and Related Dementias (ADRD) prevalence and incidence. SSP was measured using the Index of Concentration at the Extremes (ICE) across five domains: race, income, education, language proficiency, and homeownership. Logistic regression models were used to estimate the association between neighborhood SSP and incident ADRD, adjusting for age, race, socioeconomic status, and comorbidities. Significant racial disparities were observed in ADRD prevalence and incidence in 2019, with rates notably higher among Black and Hispanic populations at 8% and 3.5%, respectively. Despite a yearly decrease in incidence rates, significant associations were found between higher risks in ADRD and neighborhood-level SSP in race, income, and education, though the association with SSP in primarily spoken language was not significant. These findings suggest that neighborhood SSP is associated with ADRD prevalence and incidence, with stronger associations observed for race, income, and education domains. Further investigation into the role of spatial social polarization in ADRD disparities may help identify potential intervention targets for reducing neighborhood-driven inequities in cognitive health.

Abstract citation ID: igaf122.545

### EXTREME HEAT AND DEPRESSION RISK AMONG OLDER MEXICAN AMERICANS

Alexandra Holland[1], and Sadaf Milani[2], *1. UTMB Health, The University of Texas Medical Branch, Galveston, Texas, United States, 2. University of Texas Medical Branch, Galveston, Texas, United States*

Both Texas and California are experiencing rising average temperatures and more frequent days of dangerously elevated temperatures. Extreme temperatures are linked to many poor health outcomes among those 65+, but less is known about the potential effects of temperature change on the risk of depression among older adults, especially for older Hispanic adults. We aimed to examine the association between extremely high temperatures and depressive symptoms among older Mexican Americans living in Texas or California, using data from Wave 5 (2005) of the Hispanic Established Population for the Epidemiological Study of the Elderly (HEPESE), linked to heat index data (gridMET and National Weather Service) and geocoded to census tract. We used a count of days with a maximum heat index of 1) ≥90 °F (extreme heat) and 2) ≥103 °F (dangerous heat). Elevated depressive symptoms were measured with the Center for Epidemiologic Studies Depression Scale. We used linear regression models to test the association between heat index and depressive symptoms, controlling for sociodemographic characteristics. Our sample (n = 1,701) was 81.6 years old on average (SD: 5.0) and 62.0% female. On average, participants had 9.5 (SD: 9.0) depressive symptoms. In fully adjusted analyses, extreme heat (β: -0.006; 95% CI: -0.016, 0.004) and dangerous heat (β: -0.007; 95% CI: -0.029, 0.014) were not associated with depressive symptoms. Future work will incorporate additional waves of data from the HEPESE to detect the potential effects of changes in temperatures over time, along with the potential effects of other variables (e.g.: SES, neighborhood characteristics).

Abstract citation ID: igaf122.546

### "WE ALL GONNA GET IT IN DUE TIME": ROLE OF EARLY LIFE ENVIRONMENTAL RACISM ON LATE-LIFE FUNCTIONAL ABILITY

Paris Adkins-Jackson[1], Cailyn Clemons[2], Muriel Taks Calle[2], and Yoshira Ornelas Van Horne[3], *1. Mailman School of Public Health, Columbia University, New York, New York, United States, 2. Columbia University Mailman School of Public Health, New York, New York, United States, 3. University of California, Los Angeles, Los Angeles, California, United States*

From the National Environmental Policy Act to the Disaster Mitigation Act, the 2000s ushered in a new wave of environmental justice policies meant to protect marginalized groups. Although emergent research on adverse childhood exposures like racism reflects its enduring impact on longevity. This study examines the associations between four measures of environmental racism occurring in early-life and adulthood on late-life functional ability (mobility, independent living, and self-care) among adults racialized as Black (65+) from the Behavioral Risk Factor Surveillance System. We used FEMA, HOLC, IPUMS, and the EPA's Water Quality Portal, Landfill Methane Outreach, and Superfund data. Exposures included U.S. counties with: (1) a greater number of natural disasters than the national median (2023); (2) fewer water quality monitoring sites than the national median (1960); (3) at least one active landfill site (1990); and (4) at least one Superfund site on the National Priority List (1990). Counties were scored a "1" for Variable 1 if it had a history of redlining (1930-40s), for Variables 2-4, if it had a higher-than-national-average proportion of residents racialized as Black. We averaged county scores to the state-level and performed logistic regressions with functional ability. Living in a state with a greater number of counties with fewer water quality monitoring sites than the national median in areas with greater residents racialized as Black is associated with lower self-care in 2015, and in 2020, lower mobility, independent living, and self-care. Environmental racism during early life disrupts the longevity of residents racialized as Black in later-life.

### SESSION 2010 (SYMPOSIUM)

Abstract citation ID: igaf122.547

### ADAPTING, IMPLEMENTING, AND EVALUATING COMMUNITY-BASED DEMENTIA EDUCATION FOR DIVERSE POPULATIONS

Chair: Jace Flatt
Discussant: Kristine Talley

In this symposium, we highlight lessons learned in the adaptation, implementation and evaluation of community-based dementia education interventions. First, Dr. Moone highlights lessons learned from the Training to Serve program aimed at improving long-term services and supports (LTSS) providers understanding of LGBTQ+ aging. Using a community-engaged approach, an existing curriculum was adapted for LTSS staff and tailored to LGBTQ+ communities impacted by dementia. The second presentation by Dr. McCarthy highlights the Dementia Friends Information Session—an hour-long session adapted to an online modality during COVID-19 to educate health sciences students on creating dementia friendly communities. This presentation will also include a focus on sustainability of the program. Next, Dr. Nkimbeng shares an educational intervention, African MaDE: Memory and Dementia Education, for African immigrants living with dementia. This presentation describes a community engagement approach used to determine the feasibility and acceptability of African MaDE and efforts to disseminate the program on a larger scale. Finally, Dr. Anderson discusses the adaptation of the Savvy Caregiver® program for LGBTQ+ caregivers of persons with dementia. This will include lessons learned from the use of digital and community-engaged research methods and user-centered design approaches to culturally adapted the program for LGBTQ+ caregivers of people with dementia. Dr. Kris Talley an expert in gerontological nursing education and interventions for managing geriatric syndromes and dementia will summarize the conclusions from each speaker's presentation and place them in the context of efforts needed to advance the adaptation, implementation, evaluation, and dissemination of community-based educational interventions for dementia.

Abstract citation ID: igaf122.548

### ADAPTATION OF A LGBTQ AGING DEMENTIA EDUCATION INTERVENTION IN SENIOR LIVING

Rajean Moone, and Morgan Wright, *University of Minnesota, Minneapolis, Minnesota, United States*

Lesbian, gay, bisexual, transgender, and queer (LGBTQ) older adults have historically faced discrimination in long-term services and supports (LTSS), leading many to fear or avoid care or conceal their identities. At the same time, they often have a greater need for LTSS due to limited family or friend caregivers. In 2008, advocates, academics, state officials, and aging service providers launched the nonprofit Training to Serve (TTS) to improve LTSS providers' understanding of LGBTQ aging. In 2024, the University of Minnesota began a randomized control trial to evaluate the effectiveness of the in-person TTS intervention, a new online version, and a control group in nursing homes and assisted living. This paper examines the community-engaged approach used to adapt the TTS curriculum for LTSS staff, including expanded content on Alzheimer's and related dementias within LGBTQ communities. The curriculum development process integrated expertise from LGBTQ aging researchers and input from older LGBTQ community members to ensure cultural relevance. Additionally, the paper explores implementation strategies in 57 senior care facilities and presents findings from the evaluation study. Understanding how to adapt culturally relevant training is essential to improving care quality for LGBTQ residents in nursing homes and assisted living.

Abstract citation ID: igaf122.549

### MODIFICATION, IMPLEMENTATION, AND EVALUATION OF A COMMUNITY-BASED DEMENTIA INFORMATION SESSION

Teresa McCarthy, *University of Minnesota, Minneapolis, Minnesota, United States*

Building empathy and understanding for those with cognitive decline is a fundamental component of Dementia Friendly communities. The Dementia Friends Information Session is one hour of content delivered by community volunteers to community members in a "train the trainer" model. The intent of the sessions is to provide face-to-face information about "what dementia is and how it affects people, and that each individual can make a difference for people touched by dementia." The content was developed in the UK and licensed in Minnesota to our Area Agency on Aging. In 2019, the University of Minnesota HRSA-funded Geriatric Work Force Enhancement Program (GWEP) successfully implemented this model to educate health science students at the University of Minnesota. When the COVID 19 pandemic precluded these in-person sessions, the University transitioned the content to on-line virtual sessions. University relations with the community owners of the content allowed the program to be quickly modified and delivered virtually across the state despite the pandemic. This paper reviews the transition of this well-established community-based program to virtual delivery due to the COVID pandemic. The outcome of the intervention was assessed for a population of health sciences students utilizing the Dementia Attitude Scale. A well-established community-based information session was adapted for academic education. The academic relationship facilitated a rapid transformation of the face-to-face sessions to virtual delivery in the community that sustained the program through the COVID pandemic.

Abstract citation ID: igaf122.550

### PARTNERING WITH THE COMMUNITY TO DEVELOP AND PILOT TEST A CULTURALLY TAILORED DEMENTIA EDUCATION PROGRAM

Manka Nkimbeng[1], Truphosa (Posa) Aswani[2], Wynfred Russell[3], Tetyana Shippee[4], and Joseph Gaugler[4], *1. University of Minnesota School of Public Health, Minneapolis, Minnesota, United States, 2. University of Washington, Seattle, Minnesota, United States, 3. Strengthening African Resilience Towards Excellence, Brooklyn Park, Minnesota, United States, 4. University of Minnesota, Minneapolis, Minnesota, United States*

The Immigrant Memory Collaborative is a community university partnership that leveraged community needs and asset assessment to develop a culturally tailored dementia education program with the African immigrant community in Minnesota. Guided by a project advisory board, key informant interviews and community conversations informed the content of African MaDE: Memory and Dementia Education, by and for the African community. This presentation will describe the community engagement approach used to pilot test African MaDE with the community and pre-post evaluation. In addition to the primary partner, the team leveraged its network to collaborate with community organizations to host education sessions for 187 unique attendees at 11 events in person and remotely. Of these, 77 participants completed pre- and post-dementia knowledge questions. Most (55) attendees were first-generation (born outside the United States). Most of these participants were neither current nor past care partners and had never provided care for a person living with dementia in a professional capacity. Preliminary findings show a statistically significant 0.9-point improvement in dementia knowledge from pre- to post-education. Qualitative comments also reveal that participants appreciate the presentation and content. African MaDE is feasible and acceptable to the African community. We are currently using a community-engaged approach to conduct large-scale dissemination of this program. In addition to describing our process, we will present lessons learned in this community-engaged education development that can inform the development of similar programs in other communities.

Abstract citation ID: igaf122.551

### DIGITAL AND COMMUNITY-ENGAGED RESEARCH METHODS TO ADAPT SAVVY CAREGIVER® FOR LGBTQIA+ CAREGIVERS

Joel Anderson[1], Jace Flatt[2], Joseph Knight[1], Brittany Klenczar-Castro[2], John Hobday[3], and Carey Sherman[3], *1. University of Tennessee, Knoxville, Tennessee, United States, 2. University of Las Vegas, Nevada, United States, 3. Savvy Systems LLC, Minneapolis, Minnesota, United States*

While caring for people with dementia affects all caregivers, LGBTQIA+ caregivers experience unique minority stressors, including discrimination and stigma, that magnify the strains of caregiving. Our research and that of others finds LGBTQIA+ caregivers have significantly poorer health than heterosexual, cisgender (not transgender) caregivers, and experience higher levels of physical and emotional stress and caregiver burden. These higher levels of stress may be related to LGBTQIA+ caregivers having less access to social and community supports. The Savvy Caregiver® program is a successful psychoeducation intervention that has been shown to relieve dementia caregivers' distress and depressive symptoms as well as enhance caregivers' confidence, knowledge, and behavioral skills, outcomes of specific importance to LGBTQIA+ caregivers. Here, we present our combination of digital and community-engaged research methods and user-centered design approaches to develop a culturally adapted version of Savvy Caregiver®. This study is innovative and has potential for immediate impact as, to our knowledge, it will be the first cultural adaptation of Savvy Caregiver® for LGBTQIA+ caregivers of people with dementia.

### SESSION 2015 (SYMPOSIUM)

Abstract citation ID: igaf122.552

### PERSON-CENTERED LONG-TERM CARE STANDARDS: RESEARCH, EDUCATION, AND TOOL DEVELOPMENT

Chair: Tara Cortes

The Mayer-Rothschild Foundation (TM-RF) was founded to further Dr. Robert N. Mayer's vision of person-centered long-term care. The Foundation has generously funded this research and scholarship. The ultimate goal of the TM-RF Designation of Excellence in Person-Centered Long-Term Care process is to establish standards and processes that can be used to recognize long-term care settings which implement best practices in person-centered care. The Designation of Excellence is the result of several years of research, testing, and evaluation by innovators and early adopters across the long-term care sector. In this symposium, the HIGN team at NYU Meyers presents their endeavor in this important work, building on prior work completed by the University of Maine during the first two years of this multi-year project. This symposium describes how qualitative and quantitative data collected from multiple sites selected for geographical and population diversity were used to validate domains and concepts that can be used to describe person-centered care. The session also describes the development of an organizational assessment tool using these domains and concepts.

Abstract citation ID: igaf122.553

### PERCEPTIONS OF PERSON-CENTERED CARE AMONG RESIDENTS AND STAFF OF LONG-TERM CARE COMMUNITIES

Elizabeth Seidel[1], Shih-Yin Lin[1], and Tara Cortes[2], *1. New York University Rory Meyers College of Nursing, New York, New York, United States, 2. New York University/HIGN, New York, New York, United States*

The convergence and divergence of perceptions of good person-centered care (PCC) practices as perceived by long-term care (LTC) residents and staff are under-explored. As part of the 2nd phase of a study to establish standards of care for PCC, 8 resident (n = 56) and 9 staff (n = 66) in-person focus groups were held at 5 LTC communities: The New Jewish Home (NY), Garden Spot Communities (PA) Bethel New Life (IL), Beatitudes Campus (AZ), and Sequoia Living (CA). Deductive content analysis was used to validate 7 PCC domains previously established by the University of Maine. All 7 PCC domains were mentioned in at least 50% of focus groups. While residents and staff agreed on certain aspects of PCC there were areas where there was a disconnect either in what was important in these domains, or the importance of the domains as demonstrated by how often these themes appeared in these focus groups. Staff Empowerment was the only domain that was discussed more often among staff groups and was a top domain from staff's perspective. Resident Care, Choice, and Empowerment and Family Engagement were essential PCC domains agreed by all resident and staff focus groups, yet there were subtle differences between residents' and staff's framing of how to best address these domains. In order to better align expectations of recipient and delivery of PCC, it is important to compare and contrast perspectives of residents and staff. These findings also highlight the importance of staff empowerment in LTC staff's ability to provide PCC.

Abstract citation ID: igaf122.554

### LONG-TERM CARE RESIDENTS' PRIORITIES IN PERSON-CENTERED CARE: A SURVEY OF FIVE COMMUNITIES

Shih-Yin Lin[1], Elizabeth Seidel[1], and Tara Cortes[2], *1. New York University Rory Meyers College of Nursing, New York, New York, United States, 2. New York University/HIGN, New York, New York, United States*

As part of a multi-year project to establish person-centered care (PCC) standards for long-term care (LTC), this study explored how LTC residents perceive the importance of various PCC best practices and examined the influence of race/ethnicity and living type on their prioritization. A cross-sectional survey was conducted in five LTC communities across the U.S., where 470 residents (68.9% female; 66% non-Hispanic White, 25% non-Hispanic Black, and 9% Hispanic) rated the importance of 77 PCC best practices using a 5-point Likert-type scale. Descriptive statistics were calculated for resident characteristics and PCC ratings, and ANOVA was used to examine associations between demographics (race/ethnicity and living type) and PCC priorities. The top 10 ranked best practices focused on 'Space and Living Environment' (4 items), 'My Health Care' (3 items), 'Communication with Staff' (2 items), and 'My Family, Friends, and Community' (1 item). Findings revealed significant differences in PCC prioritization based on race/ethnicity and living type: Of the 77 best practices, Non-Hispanic White residents rated 53 items (69%) higher ($p < 0.05$) than non-Hispanic Black residents, and 10 items (13%) higher than Hispanic residents ($p < 0.05$). Independent living and skilled nursing residents rated all seven 'Communication with Staff' best practices higher than assisted living residents ($p < 0.05$). Additionally, independent living residents placed greater importance on 'Participation in Leadership' than both assisted living residents ($p < 0.05$ for 5 of the 7 items) and skilled nursing residents ($p < 0.01$ for all 7 items). These findings underscore the need to tailor PCC approaches based on resident race/ethnicity and living type.

Abstract citation ID: igaf122.555

### DEVELOPMENT AND PILOTING OF EDUCATION AND ASSESSMENT OF PERSON-CENTERED CARE STANDARDS

Tara Cortes[1], Shih-Yin Lin[2], and Elizabeth Seidel[2], *1. New York University/HIGN, New York, New York, United States, 2. New York University Rory Meyers College of Nursing, New York, New York, United States*

To further develop a tool that assesses the use of person-centered care (PCC) practices in long-term care (LTC) communities, the preliminary standards developed by the University of Maine in Years 1 and 2 were compared against focus group and survey findings from 5 new LTC communities across the United States conducted by the HIGN team at NYU Meyers. The preliminary standards were revised by consolidating overlapping, creating new standards, and reducing the reading level. For each of the standards, person-centered best practices were transformed into corresponding items in an organizational self-assessment questionnaire, along with a description of appropriate supporting documents for corroboration. The organizational assessment items to evaluate each standard were developed iteratively during weekly team meetings, in consultation with an expert panel consisting of national advocates for aging research and long-term care. Companion educational modules and booklets were created to familiarize care communities with the 7 person-centered domains, the standards, the associated behaviors, and supporting documents including policies within each domain. Findings from this research can inform the development and implementation of best practices in LTC settings. Future research should (1) evaluate comprehension of this assessment tool among LTC staff via cognitive interviewing and (2) pilot this tool along with accompanying educational materials in a larger sample of communities across the United States to ensure generalizability. The tools developed in this project can be used to evaluate person-centeredness across long-term care settings.

### SESSION 2020 (SYMPOSIUM)

Abstract citation ID: igaf122.556

### CUMULATIVE DIS/ADVANTAGE IN A CHANGING WORLD: STRUCTURAL AND POLICY IMPACTS

Chair: Dale Dannefer
Co-Chair: Stephen Crystal

.In a rapidly-changing world, improving our understanding of late-life inequality and the structural and policy factors operating over the lifecourse to drive these outcomes is vital. Research on cumulative advantage/disadvantage processes has provided important insights on these factors, but much remains to be understood. Additional insights are emerging from new research approaches. These include comparative analyses of inequality across age groups in countries with varying retirement income systems, and longitudinal analyses of the role of structural factors including marital histories, residential segregation, and education, interacting with policy factors such as retirement income systems, in shaping disparate life-course outcomes. This symposium will include present several such analyses of the operation of social processes underlying cumulative dis/advantage. Papers will cover several fresh directions of empirical research, including comparative analyses that present comparisons across age groups and cross-national comparisons (Crystal, Zhou), and evidence on mechanisms linking education (Kim, Zhou) and family (Carr) factors to inequality in mental and physical health outcomes. The symposium will also include a fresh critical analysis and assessment of the uses and misuses of cumulative dis/advantage in the gerontological and life-course literatures and trends in emerging scholarship (Kelley). Empirical contributions will address the role of midlife segregation and healthcare access as mediators of education and cognitive impairment; the role of marital histories interacting with the US Social Security system in contributing to older adults' economic outcomes; and cross-national analyses of late-life inequality before, during, and after the COVID pandemic.

Abstract citation ID: igaf122.557

### LATE-LIFE INEQUALITY, CAD AND RETIREMENT INCOME SYSTEMS: INSIGHTS FROM CROSS-NATIONAL ANALYSIS

Stephen Crystal, *Rutgers University Institute for Health, New Brunswick, New Jersey, United States*

Prior research on cumulative advantage/disadvantage in the US has shown that income inequality is higher after age 65 than at younger ages, both in within-cohort and between-cohort analyses, a pattern thought to be related to the structure of retirement income systems in the US. There has, however, been less study of late-life inequality and its relationship to inequality among non-elderly among adults across multiple countries with differing retirement income systems, particularly in the pandemic and post-pandemic period. We used OECD data to compare Gini indices of inequality for the 65+ population and the 18-64 population for 27 developed countries from 2018-2022. Results indicate that US income inequality was persistently higher at 65+ than at 18-64, with ratios ranging from 105% to 111% across years and relatively stable over the study period. In contrast, income inequality was on average lower in the other OECD countries after age 65 than before. Late-life inequality was higher in the US in each year than in any of the comparison countries, with the US inequality index (.453) exceeding the non-US rate by 36% in 2022. Countries with the lowest late-life inequality in 2022 included Czechia, Belgium, Norway, Poland, Slovenia, the Netherlands, and France, while countries with higher late-life inequality included Portugal, Chile, Israel, Latvia, New Zealand and the US. We will discuss some of the ways in which pension systems vary between countries with higher and lower late-life inequality.

Abstract citation ID: igaf122.558

### MIDLIFE RESIDENTIAL SEGREGATION AND HEALTHCARE ACCESS AS MEDIATORS OF EDUCATION AND COGNITIVE IMPAIRMENT

Min Hee Kim, *Rutgers, New Brunswick, New Jersey, United States*

Despite increasing evidence documenting the causal effect of education in reducing the risk of Alzheimer's disease and related dementias, few studies have quantified the cumulative (dis)advantage across the life course in action. This study applies a causal mediation analysis to unpack the causal effect of education on the risk of cognitive impairment risk via upstream and downstream pathways. We leverage changes in historical measures of state-level education policies as natural experiments and estimated the effects of educational attainment on cognitive impairment in the REasons for Geographic and Racial Disparities in Stroke (REGARDS) study, a national longitudinal survey of Black and White older adults aged 45+ with up to 20 years of follow-ups (N = 21,603). We decompose total effect into direct and indirect effects by mid-life exposures as mediators. Of particular interest are residential segregation and public health infrastructure and individual-level socioeconomic, stress, and healthcare access and quality conditions. Our sample includes 80% of respondents with diagnosed cardiometabolic conditions at baseline and 13% of respondents with experienced cognitive impairment incidence in the follow-up. We will discuss methodological challenges and advances in causal mediation (i.e., addressing multiple mediators and intermediate confounding). This study, which attempts to decompose some of the "synergistic impact" of policy and institutional choices, will facilitate further discussions on policy intervention points for reducing health disparities.

Abstract citation ID: igaf122.559

### OLDER ADULTS' ECONOMIC SECURITY: DO MARITAL HISTORIES CONTRIBUTE TO CUMULATIVE (DIS) ADVANTAGES?

Deborah Carr, *Boston University, Boston, Massachusetts, United States*

Social Security benefits rules privilege married persons and penalize divorced, prematurely widowed, and lifelong single older adults. Program rules may be an engine driving cumulative

(dis)advantage, because marriage is increasingly an institution of economically privileged persons. Women are more likely than men to be divorced or prematurely widowed, increasing their vulnerability to late-life economic insecurity. We examine Social Security and household income, and poverty rates of older adults based on marital categories aligned with Social Security benefits rules: (re)married; divorced (after short vs. long marriage), widowed (before vs. after age 65), and never married. Data are from the Wisconsin Longitudinal Study, which tracked white high school graduates from ages 18 (1957) to 72 (2011). Our analytic sample includes 5,269 persons (2,498 men and 2,711 women). We used OLS and logistic regression to estimate Social Security income, household income, and poverty status at age 72, adjusted for covariates. Fully adjusted models show that married older adults have higher Social Security and household income and lower poverty rates than all unmarried categories. Divorced women, regardless of marital duration, fare worst on all outcomes. Prematurely widowed persons are worse off than those widowed at older ages. Never married men are less financially secure than all other men. Revisions to Social Security including caregiver credits for years in which a worker had no/low earnings could mitigate disparities in late-life economic security. Our results suggest that cumulative dis/advantage theories should more fully address the role of marital and family roles as engines driving late-life inequalities.

Abstract citation ID: igaf122.560

### DEPRESSION, COGNITIVE IMPAIRMENT AND EDUCATION OVER THE LIFE COURSE: COMPARING CHINA AND THE US

Nan Zhou, *Case Western Reserve University, Cleveland, Ohio, United States*

Depression and cognitive impairment frequently co-occur in later life, yet their longitudinal relationship—and how it varies by educational attainment and national context—remains underexplored. This study examines the bidirectional association between depressive symptoms and cognitive impairment among older adults in China and the United States, assessing differences across education levels. Using longitudinal data from 2011 to 2020 from the China Health and Retirement Longitudinal Study (CHARLS) and the U.S. Health and Retirement Study (HRS), this study employs a multi-group cross-lagged panel model (CLPM) to examine the relationship of depressive symptoms and cognitive impairment, its stability over the life course and its relation to education. Measurement and structural invariance across four educational attainment groups: less than high school, high school diploma, associate's degree, and bachelor's degree or higher. The study explores the hypotheses that 1) depression and cognitive impairment have a bidirectional relationship within each educational group, 2) individuals with lower educational attainment will exhibit stronger reciprocal effects between depression and cognitive impairment, and 3) that the association may be more pronounced in China, where lower education levels may be linked to steeper cognitive decline, while higher education mat serve as a protective factor. Differences in healthcare access, social policies, and cultural attitudes toward aging may further shape these cross-national patterns. Findings may contribute to understanding how education influences later-life mental health trajectories and provide insights for policies and interventions aimed at reducing disparities in cognitive aging. Educational attainment, depression, cognitive aging, cross-national comparison, Cumulative Inequality Theory

Abstract citation ID: igaf122.561

### BRIGHT SPOTS AND BLIND SPOTS: THREE DECADES OF CUMULATIVE DIS/ADVANTAGE RESEARCH AND WHAT WE'RE STILL GETTING WRONG

Jessica Kelley, *Case Western Reserve University, Cleveland, Ohio, United States*

In the canon of theories that are central to understanding the social dynamics of aging, cumulative dis/advantage is one of the most widely cited. Its introduction and early application in the field by scholars Dannefer, Crystal, and others stood in stark contrast to other common frameworks such as normative aging, stress process, person-environment fit. CDA centrally focuses on social inequality that operates at micro, meso, and macro levels of society and shapes life chances of individuals from birth to death. The structural and systemic features in the allocation of resources creates a predictable set of processes, whereby the (lack of) resources becomes a central predictor in access to future resources. Empirical evidence has affirmed increasing inequality over the life course. Despite its wide-ranging explanatory potential, CDA has too often been limited in its application by researchers. Its intuitive appeal in explaining how accumulating adversity can lead to a cascade of negative outcomes as a person ages (and vice versa for accumulating advantages) can often be the only feature of the theory that is highlighted or tested. Studies can often be testing for "chains of risk" that occur as one ages rather than the systemic inequality that perpetuates the unequal opportunity structure. Likewise, focusing on social mediators that may lessen the impact of early-life adversity looks to individual-level solutions to a structural problem. I will discuss the state of the field and offer potential new avenues to expand the application of cumulative dis/advantage theory in gerontology.

### SESSION 2025 (SYMPOSIUM)

Abstract citation ID: igaf122.562

### THE SOCIAL PRESCRIPTION: ENHANCING COGNITIVE FUNCTIONING THROUGH SOCIAL ACTIVITIES

Chair: Meng Huo
Discussant: Dawn Carr

Social activities have been linked to a variety of physical and emotional benefits in later life, but the contribution these activities can make to older adults' cognitive health remains less clear. This symposium extends the growing body of work on social activities and cognitive functioning by showcasing four studies that considered the risk of Alzheimer's disease and related dementias (ADRD). Brown examined older African Americans free of dementia at baseline, observing that their social activities led to slower cognitive declines over 10 years, regardless of the APOE genotype that independently affected cognitive changes. Han identified a robust within-person association between prosocial behaviors and slower cognitive declines, but this association was stronger among older adults at higher genetic risk for AD. Turning to older adults with mild cognitive impairment (MCI)—a common precursor of ADRD, Huo found that those who volunteered, particularly those who continuously volunteered or initiated volunteering, exhibited more positive cognitive changes. Amano further revealed the stability of older adults' engagement in social activities in the presence of MCI, highlighting the importance of providing additional resources to those with limited social engagement. These studies reveal the crucial role social activities play in slowing cognitive declines in healthy adults and those already experiencing early declines that precede pathological deterioration. Findings identify social factors underlying risk and resilience as older adults experience cognitive changes and inform tailored, targeted interventions. Our discussant, Dr. Dawn Carr, will synthesize these studies, discuss their theoretical and practical applications, and address challenges and opportunities in future research.

Abstract citation ID: igaf122.563

### VOLUNTEERING WITH MILD COGNITIVE IMPAIRMENT: IMPLICATIONS FOR SUBSEQUENT COGNITIVE CHANGES

Meng Huo[1], and Kyungmin Kim[2], *1. University of California, Davis, Davis, California, United States, 2. Seoul National University, Seoul, Republic of Korea*

Volunteering has cognitive benefits in later life and has been theorized to protect against Alzheimer's disease and related dementias (ADRD). A small but growing body of volunteering programs target people with mild cognitive impairment (MCI)—who are presumably at elevated risk for ADRD, but we know surprisingly little about who volunteers in the presence of MCI and how volunteering affects their subsequent cognitive changes. The current study sought to address these gaps in the literature. We used longitudinal data from the Health and Retirement Study (2002–2018) and identified a pooled sample of 6,930 midlife and older adults (aged 50+) who met criteria for MCI based on their cognitive scores on the Telephone Interview for Cognitive Status (TICS). We tracked these participants' sociodemographic characteristics, volunteer activities, and cognitive scores in the subsequent four years. A two-level logistic regression showed that among midlife and older adults with MCI, those who were younger, attained more years of education, had greater wealth, reported better self-rated health, had fewer functional limitations, and reported a history of volunteering were more likely to report volunteering in the presence of MCI. Volunteers with MCI—particularly those who continuously volunteered or initiated volunteering exhibited more positive cognitive changes over time. This study highlights the importance of socioeconomic resources and health in predicting volunteering with MCI and reveals lasting cognitive benefits of volunteering as midlife and older adults adjust to their cognitive impairment. Findings call for more tailored volunteering opportunities for people with MCI.

Abstract citation ID: igaf122.564

### EFFECTS OF SOCIAL ACTIVITIES AND APOE ON COGNITIVE DECLINE IN AFRICAN AMERICANS

Casey Brown[1], Neke Nsor[1], Kyle Bourassa[2], and Lisa Barnes[3], *1. Georgetown University, Washington, District of Columbia, United States, 2. Durham VA Medical Center, Durham, North Carolina, United States, 3. Rush University Medical Center, Chicago, Illinois, United States*

African Americans experience disproportionate rates of cognitive decline yet remain underrepresented in research on the biopsychosocial factors contributing to this decline. This study examines whether biological factors (APOE alleles) interact with social engagement (e.g., participation in social groups) to predict cognitive decline in African Americans. A sample of 734 African American adults from the Minority Aging Research Study (MARS), aged 65 and older and free of dementia at enrollment, completed annual cognitive assessments over 10 years. At baseline, participants underwent genetic testing and reported their engagement in social and cognitive activities. Structural equation modeling assessed the effects of APOE genotype, cognitive activities, and social activities on cognitive decline, along with their interaction effects over time. Results showed that APOE count influenced cognitive decline, such that a greater number of APOE4 alleles was associated with faster cognitive decline, while a greater number of APOE2 alleles was linked to slower cognitive decline. However, social activities did not interact with APOE count in predicting decline. Instead, APOE4 and social activities had independent, additive effects on cognitive decline. These findings underscore the role of social engagement in slowing cognitive decline in African Americans, regardless of APOE status.

Abstract citation ID: igaf122.565

### PROSOCIAL HELPING BEHAVIORS, GENETIC RISK FOR ALZHEIMER'S DISEASE, AND COGNITIVE DECLINE IN LATER LIFE

Sae Hwang Han, and Shiyang Zhang, *The University of Texas at Austin, Austin, Texas, United States*

Genetic factors play a critical role in the development of cognitive impairment and Alzheimer's disease (AD), with individuals at high genetic risk—characterized by APOE ε4 status and broader polygenic risk—often experiencing symptom onset up to a decade earlier than those at low genetic risk. This early onset is partly driven by the accelerated cognitive decline associated with high genetic susceptibility to AD, yet little is known about protective lifestyle factors that may slow this progression. This study examines whether two forms of prosocial helping behaviors— volunteering through a formal organization and informal helping provided directly to friends and neighbors—attenuate the cognitive decline linked to genetic risk. The research questions were addressed using longitudinal data from individuals aged 51 and older in the nationally representative Health and Retirement Study (HRS; 1998–2020; N = 17,972; person-wave observations = 139,777). Cognitive function was assessed using a modified version of the Telephone Interview for Cognitive Status (TICS), and genetic risk was measured separately using APOE ε4 status and polygenic risk for AD, both available in the HRS. Preliminary findings from within-between random effects models indicate a robust within-person association between engagement in helping behaviors and a slower rate of cognitive decline, with particularly strong benefits observed among individuals at high genetic risk for AD. These results highlight the potential of prosocial engagement as a cost-effective public health intervention for delaying and preventing cognitive impairment and AD, particularly in high-risk populations.

Abstract citation ID: igaf122.566

### CHANGES IN SOCIAL ENGAGEMENT PATTERNS AMONG PEOPLE WITH MILD COGNITIVE IMPAIRMENT

Takashi Amano, *Rutgers University Newark, Newark, New Jersey, United States*

Promoting engagement in social activities may be an effective intervention to maintain or improve well-being in later life. People with mild cognitive impairment may be particularly vulnerable to disengagement from social activities. Nevertheless, little is known about social engagement among individuals with this condition, leading to a lack of practical interventions.

This study aims to examine how patterns of social engagement change over time among individuals with mild cognitive impairment. Data were drawn from the Health and Retirement Study (HRS). A pooled sample was created using pairs of baseline and follow-up waves (2012–2016, 2014–2018). The final sample comprised 1,112 individuals with Cognitive Impairment No Dementia (CIND). Eight indicators were utilized to measure social engagement, including engaging in activities with grandchildren, volunteering, charity work, attending educational courses, participating in organizations, meeting up with others, having conversations, and writing or emailing. Latent transition analysis was conducted to identify transitions in patterns of social engagement. Three distinct patterns were identified at each wave: low engagement (30.6%), informal social engagement only (51.0%), and both formal and informal social engagement (18.4%). The vast majority of participants (91.3%) maintained their social engagement patterns over four years. These findings suggest that individuals with CIND can remain socially engaged over time. However, those with low engagement levels may continue to be disengaged in the absence of targeted interventions. Future research is needed to identify factors that contribute to changes in social engagement, including individual characteristics, social support networks, and external influences such as community programs.

### SESSION 2030 (PAPER)

### COORDINATING CARE IN THE CONTEXT OF CHRONIC ILLNESS AND AGING

Abstract citation ID: igaf122.567

### AGE AND AGE-RELATED NETWORK DIVERSITY AS PREDICTORS OF STRESS REDUCTION AMONG CANCER SURVIVORS AND CARE PARTNERS

Kristin Cloyes[1], Jia-Wen Guo[2], Kelly Mansfield[2], Sarah Wawrzynski[3], and Maija Reblin[4], *1. Oregon Health & Science University, Portland, Oregon, United States, 2. University of Utah College of Nursing, Salt Lake City, Utah, United States, 3. Nemours Children's Health, Wilmington, Delaware, United States, 4. University of Vermont, Burlington, Vermont, United States*

Social support, an established driver of health outcomes, reflects resources conferred by social relationships that shift across life course, circumstances, and events. For example, structural social network characteristics shape support resource access and quality. Older adults may consolidate networks, prioritizing strong emotional ties, while people with chronic illness and caregivers experience shrinking networks. Broader social context like the pandemic can intensify these effects. We performed secondary analysis of cancer survivor and care partner social network (baseline) and perceived stress data (baseline and 3 months) collected October 2020 through April 2021 (N = 64, 32 survivor/partner dyads, Mean age = 43.3, SD = 17.18). Binary logistic regression examined whether network structure (density, modularity, age diversity) predicted positive or negative changes in stress, and if this differed by age. Density and modularity were non-significant and removed to improve model stability. The simplified model, retaining age and age-related diversity, showed that participants' age was significantly associated with stress reduction (B = -0.046, p = .016; Exp(B) = 0.955); for each additional year of age, the odds of decreased stress rose by 5.1%. Age-related network diversity demonstrated a trend toward reduced stress reduction (B = -0.092, p = .132; Exp(B) = 0.912). Our prior work found that age diversity positively predicted support, yet older participants had less age-diverse networks. Current findings suggest age-diverse networks may moderate stress during stressful life events, possibly due to differences in network function like availability of resources. Future research focusing on support availability within networks over time could address this gap.

Abstract citation ID: igaf122.568

### EXPLORING CAREGIVERS' UNMET NEEDS, UNMET NEEDS DISTRESS, AND WELL-BEING: THE ROLE OF PERSONAL GAIN AND MASTERY

Emma Jackson[1], Claire Grant[2], Lauren Stratton[3], and Katherine Judge[4], *1. Cleveland State University, Westlake, Ohio, United States, 2. VA, Waltham, Massachusetts, United States, 3. Alzheimer's Association, Chicago, Illinois, United States, 4. Cleveland State University, Cleveland, Ohio, United States*

Informal dementia caregivers experience a range of negative well-being outcomes and reported unmet needs and related distress. Few studies have examined how caregiver's unmet needs and corresponding distress may contribute to well-being along with intrapersonal factors as mediators. Guided by the Stress Process Model, this paper examined the mediating role of personal gain and mastery (intrapersonal factors) in the relationship between well-being outcomes (depression and anxiety), unmet needs, and unmet needs distress using 4 parallel mediation models. Of the 440 participants (Mage = 48.94), 68.9% were female and 58.6% White. Neither personal gain nor mastery were found to be significant mediators for unmet needs and the well-being outcomes. For unmet needs distress predicting depression, the indirect effect for personal gain was significant (b = -.0290, CI [-.0633, -.0022]) while mastery was not (b = -.0549, CI [-.1233, .0033]). Similarly, for unmet needs distress predicting anxiety, personal gain was significant (b = -.0364, CI [-.0721, -.0102]) while mastery was not (b = -.0503, CI [-.1112, .0017]). Results indicated 1) intrapersonal factors did not mediate the relationship between unmet needs and well-being outcomes and 2) personal gain mediated the relationship between unmet needs distress and both well-being outcomes. These findings suggest the constructs of unmet needs and unmet needs distress function differently and for unmet needs distress, personal gain rather than mastery, was important in understanding the relationship with depression and anxiety. These findings are important for expanding our theoretical understanding of the stress process along with implications for interventions.

Abstract citation ID: igaf122.569

### NETWORKS AND CAREGIVING: UNDERSTANDING RESILIENCE, WELLBEING, AND BURDEN AMONG DEMENTIA CARE PARTNER NETWORKS

M. Aaron Guest, Allie Peckham, Sage Sadow, Keenan Pituch, Casey Davies, and Giselle Reyes, *Arizona State University, Phoenix, Arizona, United States*

Research on dementia caregiving often focuses on the care partner and care recipient dyad, overlooking the broader social networks that shape caregiver well-being, resilience, and burden. This study addresses this gap by examining how social network composition influences caregiver outcomes. Using an egocentric network approach, we conducted guided interviews with 107 unpaid caregivers of individuals living with dementia in Arizona and Nevada. Data collected included caregiver and care recipient demographics, social network satisfaction, caregiver stress resilience, well-being, and social network characteristics, including demographic similarity and perceptual affinity. Caregivers were predominantly female (87%) and white (80.1%), with networks averaging 9.6 members (SD = 3.97). Participants reported high perceptual affinity with their networks (.71) but lower demographic similarity (.52) and strong tie strength (.78). Multiple regression analyses revealed that perceptual affinity was positively associated with resilience (β = .33, SE = .11) and well-being (β = .20, SE = .10) and negatively associated with burden (β = -.39, SE = .10). Additionally, total network members (β = .19, SE = .07) and caregiver tenure (β = .19, SE = .09) were positively associated with resilience. No other associations were significant. Findings highlight the importance of broader social networks in caregiving, demonstrating that perceptual affinity—shared commonalities and interest within networks—plays a key role in enhancing resilience and well-being while reducing burden. These insights underscore the need for interventions that strengthen meaningful social connections to support caregivers more effectively and additional research to identify the exact types of perceptual affinity that improve outcomes.

Abstract citation ID: igaf122.570

### PROFESSIONAL HEALTHCARE NETWORKS FOR OLDER ADULTS LIVING WITH TYPE 2 DIABETES

Catherine Clair[1], Janiece Taylor[2], and Karin Tobin[3], *1. Johns Hopkins School of Nursing, Baltimore, Maryland, United States, 2. Johns Hopkins University, Baltimore, Maryland, United States, 3. Johns Hopkins Bloomberg School of Public Health, Baltimore, Maryland, United States*

Older adults living with Type 2 diabetes often interact with healthcare providers in outpatient and home settings. These healthcare providers represent a professional healthcare network, which can be explored using egocentric social network methods and analysis. We aimed to 1) explore network characteristics in a sample of older adults living with Type 2 diabetes and 2) identify individual- and network-level variables associated with the number of emergency department visits. We conducted a cross-sectional observational study, which included a social network inventory, with community-dwelling older adults (65+ years) living with Type 2 diabetes. We ran a negative binomial regression of emergency department utilization on number of professional alters in the network providing care for five years or more, adjusting for the number of additional chronic conditions, race, driving status, and dual eligibility for Medicare and Medicaid. The final sample included 122 individuals and was 70.5% female (n = 86) and 57.4% non-White (n = 70). The mean age of participants was 73.06 ± 6.12 years. Of the 122 participants, 121 had a professional healthcare network. In adjusted analyses, for each additional professional alter in their network providing care for five years or more, older adults had 0.91 times the incidence rate of emergency department visit (95% CI: 0.80-1.04) when controlling for other covariates of interest. Although this pilot study was not powered for significance, our findings highlight the importance of care continuity and suggest a need for future confirmatory studies to explore the impact of care continuity on the health and well-being of this population.

Abstract citation ID: igaf122.571

### RESILIENCE AND DAILY MENTAL HEALTH IN HISPANIC AND LATINO DEMENTIA CAREGIVERS: A DAILY DIARY STUDY

Frank Puga[1], Natashia Bibriescas[1], Loreli Alvarez[2], Stephanie DiFiglia[3], Andres Azuero[1], and Michael Crowe[1], *1. University of Alabama at Birmingham, Birmingham, Alabama, United States, 2. The University of Alabama at Birmingham, Birmingham, Alabama, United States, 3. Metropolitan Jewish Health System, Inc. Institute for Innovation in Palliative Care, New York, New York, United States*

Hispanic and Latino (H&L) family dementia caregivers experience high levels of stress, increasing their risk for depression and anxiety. However, caregiver resilience may mitigate these risks. We examined associations between daily caregiving stress, psychological distress, and resilience using data from the Nuestros Dias (Our Days) Study. Participants (N = 155; 2,679 daily observations) completed daily diary surveys for 21 days, reporting on caregiving stress, depression, and anxiety symptoms using the Neuropsychiatric Inventory–Questionnaire distress ratings and the PROMIS Short Form Depression and Anxiety measures adapted for daily diaries. Resilience factors (perception of self, planned future, and structured style) were measured at baseline using the Resilience Scale for Adults. Data were analyzed using multilevel logistic regression models. On days when caregivers reported higher-than-average caregiving stress, daily odds increased by 7% for depression (OR = 1.07, 95% CI: 1.05–1.09, p < .001) and 9% for anxiety (OR = 1.09, 95% CI: 1.06–1.11, p < .001). For each one-point increase in planned future resilience, daily odds decreased by 25% for depression (OR = 0.75, 95% CI: 0.69–0.83, p < .001) and 21% for anxiety (OR = 0.79, 95% CI: 0.72–0.86, p < .001). Combined effects were examined using mean predicted probabilities based on dichotomized stress and resilience levels. Low resilience/high stress probabilities were 43% and 59% for depression and anxiety, respectively. For high-resilience/high-stress caregivers, probabilities were 14% and 28%, respectively. Findings highlight the need for culturally responsive interventions that foster planned future resilience to enhance H&L caregiver well-being.

Abstract citation ID: igaf122.572

### THE SOCIAL-MEDICAL NETWORK: INTEGRATION OF PERSONAL AND PROFESSIONAL HEALTHCARE NETWORKS

Catherine Clair[1], Janiece Taylor[2], and Karin Tobin[3], *1. Johns Hopkins School of Nursing, Baltimore, Maryland, United States, 2. Johns Hopkins University, Baltimore, Maryland, United States, 3. Johns Hopkins Bloomberg School of Public Health, Baltimore, Maryland, United States*

Older adults living with Type 2 diabetes may have two healthcare networks: Personal (family, friends, neighbors) and professional (healthcare providers). The social-medical network is the larger care network comprised these subnetworks and their integration. We aimed to 1) explore the characteristics of the subnetworks and social-medical network and 2) regress emergency department utilization on social-medical network integration. We conducted a cross-sectional observational study, which included a social network inventory, with community-dwelling older adults (65+ years) living with Type 2 diabetes. We ran a negative binomial regression of emergency department utilization on social-medical network integration, adjusting for the number of additional chronic conditions, having one personal alter, and an interaction term between the primary independent variable and having one personal alter. We completed data collection with 122 individuals. The analytic sample (n = 98) was 71.4% female (n = 70) and 58.2% non-White (n = 57). The mean age of participants was 73.79 ± 6.01 years. In adjusted analyses, for each 1-unit increase in integration, older adults with two or more personal alters had 0.43 times the incidence rate of emergency department visit (95% CI: 0.07-2.62) when controlling for other covariates of interest. The direction of the association was reversed for older adults with only one personal alter (IRR: 1.76, 95% CI: 0.36-8.55). Social-medical network integration had a protective effect on emergency department visit for older adults with two or more family and friends involved in their care. Integration of family and friends with healthcare providers is critical to support the health and well-being of this population.

### SESSION 2035 (SYMPOSIUM)

Abstract citation ID: igaf122.573

### USE OF THE NIA LINKAGE ENCLAVE TO STUDY OBSTRUCTIVE SLEEP APNEA, COGNITION, AND DEMENTIA

Chair: Christopher Kaufmann
Co-Chair: Marcela Blinka
Discussant: Adam Spira

As the population ages, the number of people with Alzheimer's disease (AD) and AD-related dementias (ADRD) will rise. While treatments to slow AD/ADRD are emerging, there is still no cure, highlighting the need to leverage data to identify risk factors for dementia prevention. The National Institute on Aging (NIA) established the LINKAGE Enclave, an online cloud-based platform facilitating access to NIA-funded cohort studies linked with administrative claims from the Centers for Medicare and Medicaid Services (CMS). Before LINKAGE, researchers had to individually apply for access from CMS, obtain permission from partner studies, and establish secure data infrastructure in their own institutions. This cumbersome process created barriers to collaboration across sites, limiting cross-site partnerships, and hindering use of these data. LINKAGE has improved data access to these unique resources. Our team, including investigators from three institutions, was among the early teams to work in the system. In our ongoing project, we are using LINKAGE to analyze two NIA-funded cohort studies with linked Medicare claims. Because our team involves multiple institutions and is working with two cohort studies, accessing and eventually using these data was not simple. We learned a tremendous amount and wish to share our experience with the aging research community. In this symposium, we present our experience using LINKAGE. We will discuss lessons learned and experiences gained, offering insights to help future teams succeed in their own projects. We encourage researchers to utilize this valuable resource, and hope it fosters research promoting dementia prevention and healthy aging.

Abstract citation ID: igaf122.574

### INVESTIGATING OBSTRUCTIVE SLEEP APNEA AND ITS TREATMENT ON COGNITIVE OUTCOMES USING SURVEY AND REAL WORLD DATA

Christopher Kaufmann[1], Marcela Blinka[2], Alden Gross[3], Jennifer Albrecht[4], Emerson M. Wickwire[4], Halima Amjad[5], Atul Malhotra[6], and Adam Spira[3], *1. University of Florida, Gainesville, Florida, United States, 2. Johns Hopkins University, Center on Aging and Health, Baltimore, Maryland, United States, 3. Johns Hopkins Bloomberg School of Public Health, Baltimore, Maryland, United States, 4. University of Maryland, Baltimore, Maryland, United States, 5. Johns Hopkins University School of Medicine, Baltimore, Maryland, United States, 6. University of California, San Diego, San Diego, California, United States*

With the growing prevalence of Alzheimer's disease (AD) and AD-related dementias (ADRD), investigators have sought to identify modifiable risk factors that if addressed, can slow cognitive decline and delay AD/ADRD. Obstructive sleep apnea (OSA) affects a billion people worldwide and is linked to serious health consequences, including AD/ADRD. Treatments for OSA (e.g., continuous positive airway pressure [CPAP]), are available and highly effective when used as prescribed. Even so, there is ongoing debate about how these therapies influence cognitive outcomes, largely stemming from the limited number of studies with sufficient follow-up time to account for the extended preclinical phase during which OSA affects patients' AD/ADRD pathology. In 2022, our team was awarded an R01 from the NIA to investigate the relationship between OSA and cognitive decline and AD/ADRD onset, and whether CPAP can reduce the risk of these negative cognitive outcomes. We are using data from two NIA-funded cohort studies, the Health and Retirement Study (HRS) and the National Health and Aging Trends Study (NHATS), with linked Medicare claims accessed via the newly established NIA LINKAGE Enclave to address these questions. Ultimately, our study leverages nationally representative cohort studies of older adults linked to real-world data to generate insights for dementia prevention. In this presentation, we will a) provide an overview of OSA and its link to cognitive outcomes, b) outline our study's aims, c) describe our use of HRS and NHATS data linked with Medicare claims, and d) discuss the importance of the NIA LINKAGE Enclave in effectuating our vision.

Abstract citation ID: igaf122.575

### THE NIA LINKAGE ENCLAVE APPLICATION PROCESS

Marcela Blinka[1], Jennifer Albrecht[2], Adam Spira[3], Halima Amjad[4], Alden Gross[4], Emerson M. Wickwire[2], Atul Malhotra[5], and Christopher Kaufmann[6], *1. Johns Hopkins University, Center on Aging and Health, Baltimore, Maryland, United States, 2. University of Maryland, Baltimore, Maryland, United States, 3. Johns Hopkins Bloomberg School of Public Health, Baltimore, Maryland, United States, 4. Johns Hopkins University School of Medicine, Baltimore, Maryland, United States, 5. University of California, San Diego, San Diego, California, United States, 6. University of Florida, Gainesville, Florida, United States*

As the global population continues to age, aging-related diseases, including Alzheimer's disease and other dementias, are becoming more common, creating serious challenges for healthcare systems worldwide. Addressing these challenges requires a comprehensive understanding of the genetic, environmental, and behavioral factors that contribute to the development and progression of these conditions. The National Institute on Aging Data LINKAGE Program (LINKAGE) is a valuable resource, providing access to a wide range of data on genetic variations, biomarkers, and age-related diseases with secure linkages to administrative claims data. Researchers can apply to LINKAGE to gain access to these data, which are essential for improving our understanding of these conditions and advancing efforts in diagnosis, prevention, and treatment. In this presentation, we will share our experience in applying for access to these data through the LINKAGE program, with a particular focus on the National Health and Aging Trends Study (NHATS) and the Health and Retirement Study (HRS). We will highlight the lessons learned throughout the process and offer practical recommendations for navigating the application system more effectively. Based on our experience, we found that engaging with NHATS and HRS personnel early and often throughout the application process is critical to success. Additionally, close coordination between study partners significantly streamlines the process, ensuring smoother communication and faster access to valuable data. By sharing these insights, we aim to help other researchers expedite their own applications and make the most of the resources available through LINKAGE.

Abstract citation ID: igaf122.576

### FROM DATA TO DIAGNOSIS: BUILDING OSA COHORTS IN CLAIMS DATA LINKED TO THE NATIONAL HEALTH AND AGING TRENDS STUDY

Jennifer Albrecht[1], Adam Spira[2], Alden Gross[2], Emerson M. Wickwire[1], Halima Amjad[2], Atul Malhotra[3], Marcela Blinka[4], and Christopher Kaufmann[5], *1. University of Maryland, Baltimore, Maryland, United States, 2. Johns Hopkins Bloomberg School of Public Health, Baltimore, Maryland, United States, 3. University of California, San Diego, San Diego, California, United States, 4. Johns Hopkins University, Center on Aging and Health, Baltimore, Maryland, United States, 5. University of Florida, Gainesville, Florida, United States*

Evidence suggests obstructive sleep apnea (OSA) accelerates cognitive decline, yet data containing physician-assigned OSA diagnoses often lack granular measures of cognition and vice versa. Linking administrative claims to a longitudinal study with annual cognition measurement, such as the National Health and Aging Trends Study (NHATS), can strengthen these analyses. To determine the association between OSA and cognitive decline, we linked NHATS participants' OSA diagnostic claims data to cognitive measures from NHATS study visits. We accessed NHATS data (2011-2021) linked to Medicare administrative claims (2008-2021) through NIA LINKAGE Enclave. Because patient-reported OSA diagnosis is not available in NHATS, we identified OSA by searching for ≥2 corresponding International Classification of Disease (ICD) codes from inpatient or outpatient Medicare claims. Main outcome of our analysis was cognitive performance; thus, we excluded those with pre-existing dementia (from NHATS algorithm or claims) at baseline. Finally, we matched people with OSA to those without. We identified 977 NHATS participants with OSA based on claims but without dementia. Half received the diagnosis before entering NHATS, making assignment of index date challenging. Further, participants with OSA were younger than those without, leading to difficulty matching. We assigned date of entry into NHATS as index date, assuming OSA was present the entire time, and matched participants without OSA to those with OSA based on age at NHATS entry and sex. In summary, we successfully identified NHATS participants with OSA and linked them to their cognitive data. Methodological challenges encountered during this process will be discussed.

Abstract citation ID: igaf122.577

### HARMONIZATION OF COGNITIVE DATA ACROSS COHORT STUDIES

Alden Gross[1], Adam Spira[1], Emerson M. Wickwire[2], Jennifer Albrecht[2], Atul Malhotra[3], Halima Amjad[4], Marcela Blinka[5], and Christopher Kaufmann[6], *1. Johns Hopkins Bloomberg School of Public Health, Baltimore, Maryland, United States, 2. University of Maryland, Baltimore, Maryland, United States, 3. University of California, San Diego, San Diego, California, United States, 4. Johns Hopkins University School of Medicine, Baltimore, Maryland, United States, 5. Johns Hopkins University, Center on Aging and Health, Baltimore, Maryland, United States, 6. University of Florida, Gainesville, Florida, United States*

Harmonizing longitudinal population-based studies affords the ability to compare cognitive decline even when protocols for measuring cognition differ between cohorts. We derived a longitudinally harmonized general cognitive factor score based on cognitive tests from the Health and Retirement Study (HRS) and the National Health and Aging Trends Study (NHATS), two of the largest population-based studies with longitudinal cognitive testing in the US. Using item response theory methods supplemented by an item banking approach, we leveraged common and unique tests administered in 2012-2018 Core interviews of the two studies to derive a harmonized general cognitive factor score. We evaluated concurrent and predictive criterion validity of harmonized scores by: 1) cross-sectionally comparing scores at baseline (2012) with cognitive impairment status and demographics; and 2) longitudinally assessing associations of demographics and risk factors. Cross-sectionally, the harmonized score was lowest among dementia/probable dementia groups in both HRS (z=-1.55 SD) and in NHATS (z=-2.21 SD) compared to cognitively normal participants in HRS (z = 0.27 SD) and NHATS (z=-0.05 SD). Longitudinally, in both studies, cognitive decline is steeper for participants at older ages within participants, with less education, and those reporting hypertension, stroke, or diabetes, relative to those without. This harmonized cognitive score can be a resource for aging researchers to examine and compare rare risk factors for cognitive performance and decline across large population-based studies. It returns patterns of change across demographic and health groups consistent with prior literature.

Abstract citation ID: igaf122.578

### THE CENTER FOR HEALTH IN COGNITIVE AGING (CHECA): A RCMAR PROGRAM JOINING POPULATION SCIENCE AND HEALTH PROMOTION

Uchechi Mitchell, Julia Bauer, Debaleena Chattopadhyay, Jennifer Kwok, and Zhuoer Lin, *University of Illinois Chicago, Chicago, Illinois, United States*

The Center for Health in Cognitive Aging (CHECA) is a newly funded Resource Center for Minority Aging Research (RCMAR) at the University of Illinois Chicago. CHECA aims to build behavioral and social research infrastructure, diversify the AD/ADRD research workforce through mentorship and training of early-career Scientists, and support pilot research. The center thematically focuses on research that (1) advances our understanding of environmental, sociocultural, and biobehavioral contributors of AD/ADRD inequities; (2) develops or tests health-promoting interventions or resources; and (3) cross-fertilizes population science and health promotion to address inequities in adults with or at risk for AD/ADRD. This symposium highlights the exceptional research of four CHECA Scientists: Dr. Bauer's research identified sex differences in the effects of persistent organic pollutants on biomarkers of neurodegeneration among middle-aged and older adults in the Hispanic Community Health Study. Dr. Chattopadhyay conducted mobile technology learning workshops with older adults with cognitive difficulties and identified challenges with digital security and usability that limited engagement with digital technologies. Dr. Kwok's research used public use Census data and found racial/ethnic differences in living arrangements among older adults with cognitive difficulties that can influence access to informal caregiving. Lastly, using data from NHATS, Dr. Lin found that among older adults living with dementia, social isolation was positively associated with unmet care needs. Collectively, these projects advance AD/ADRD research across multiple levels of effect—biological, behavioral, familial, and social—and epitomize the interdisciplinary approaches needed to improve cognitive outcomes in diverse populations and address AD/ADRD inequities.

Abstract citation ID: igaf122.579

### PERSISTENT ORGANIC POLLUTANTS AND PLASMA MARKERS OF NEURODEGENERATION IN SOL-INCA AND HCHS/SOL

Julia Bauer[1], Mary Turyk[2], Victoria Persky[2], Charles DeCarli[3], Bharat Thyagarajan[4], Wassim Tarraf[5], and Hector Gonzalez[6], *1. University of Illinois Chicago, Chicago, Illinois, United States, 2. School of Public Health, University of Illinois at Chicago, Chicago, Illinois, United States, 3. University of California, Davis, Sacramento, California, United States, 4. University of Minnesota, Minneapolis, Minnesota, United States, 5. Wayne State University, Detroit, Michigan, United States, 6. University of California, San Diego, San Diego, California, United States*

There is an urgent need to identify modifiable risk factors during early stages of neurodegeneration to reduce dementia risks. Persistent organic pollutants (POPs), including DDT and other organochlorine pesticides (OCPs) and polychlorinated biphenyls (PCBs), are environmental contaminants that accumulate in human tissues and have neurotoxic potential and sex-specific effects. We examined sex-specific associations between POPs and plasma biomarkers of neurodegeneration. This study included 1,837 participants (mean age 55 years) from the Hispanic Community Health Study/Study of Latinos with serum POPs measurements at baseline (2008–2011; V1) and a 7-year follow-up (2015–2018; V2). Serum POPs (DDT, OCPs, PCBs) were quantified using gas chromatography-isotope dilution high-resolution mass spectrometry. At V2, plasma biomarkers of neurodegeneration were measured at the University of Minnesota, including AD-related proteins (amyloid beta, phosphorylated tau 181) and non-specific markers of neurodegeneration (Glial Fibrillary Acid Protein (GFAP), neurofilament light chain (NfL)). Covariate-adjusted associations between lipid-adjusted POPs and neurodegeneration biomarkers were estimated using linear regression with survey weights. Sex differences were explored using sex stratified models and sex-POP interaction p-values in main models. Sex-specific associations were observed between POPs and non-specific neurodegeneration markers. Among females, DDT was associated with higher GFAP concentrations (2nd vs. 1st tertile: beta=0.29 (95% CI: 0.07, 0.51); 3rd vs. 1st tertile: beta=0.58 (0.25, 0.91); p-values for sex*DDT interaction <0.01). Among males, OCP was associated with increased NfL (2nd vs. 1st tertile: beta=0.33 (0.17, 0.49)); p-interaction sex*OCP <0.001). Findings suggest sex differences in the association between POPs and biomarkers of neurodegeneration.

Abstract citation ID: igaf122.580

### NAVIGATING DIGITAL SECURITY AND USABILITY CHALLENGES FOR OLDER ADULTS WITH COGNITIVE CONCERNS

Debaleena Chattopadhyay, and Tasneem Mubashshira, *University of Illinois Chicago, Chicago, Illinois, United States*

Emerging technologies—including smartphone apps, smart home devices, digital health services, and wearables—offer opportunities to support older adults, particularly those at risk for Alzheimer's Disease and Alzheimer's Disease Related Dementias (AD/ADRD), in maintaining health and independence. As digital tools become integral to instrumental activities of daily living (I/ADLs)—such as medication management, transportation, and telehealth—effective use becomes increasingly critical. However, cognitive changes can limit the breadth of technology use even after initial adoption. In 2024, we conducted ten mobile technology learning workshops with community-dwelling older adults in greater Chicago (median age = 74). Sixty-five percent of participants reported subjective cognitive decline (SCD), assessed using the Cognitive Function Instrument. SCD is increasingly recognized as a potential early marker of non-normative cognitive decline and ADRD progression. A reflexive thematic analysis of workshop recordings revealed a persistent tension between security and usability. Participants expressed heightened anxiety about making mistakes or compromising personal information, leading to technology avoidance—even when they were interested. At the same time, they struggled with managing authentication credentials and resisted software updates, fearing disruptions to familiarity and ease of use. Current security measures—such as frequent password changes, multi-factor authentication, and mandatory updates—exacerbate these barriers, further limiting digital engagement. To facilitate technology use among older adults at risk for AD/ADRD, technology design must balance security with cognitive accessibility. Addressing these challenges is critical for fostering digital inclusion and supporting the functional independence of older adults with emerging cognitive concerns.

Abstract citation ID: igaf122.581

### HOUSEHOLD COMPOSITION OF OLDER U.S. ADULTS WITH COGNITIVE DIFFICULTIES BY RACE AND ETHNICITY

Jennifer Kwok, *University of Illinois Chicago, Chicago, Illinois, United States*

Older adults with cognitive difficulties may receive informal support and care from family members in their households. This study investigated how the household composition of community-dwelling older adults with cognitive difficulties varies by sociodemographic characteristics, focusing on race and ethnicity. I used the U.S. Census Bureau's American Community Survey Public Use Microdata Sample from 2008-2021. The 7.0% of community-dwelling adults aged 65 and older who are reported to have serious difficulty concentrating, remembering, or making decisions because of a physical, mental, or emotional condition were classified as having cognitive difficulties. I used logistic regression models that incorporated the survey design, individual characteristics (age, sex, education, income), and control variables (years) to understand associations between household composition and race and ethnicity in this population. Black / African American individuals had a significantly lower average predicted probability of living with spouses (31.4%; 95% confidence interval (CI): 16.7%-46.1%) than White (44.6%; CI:29.9%-59.2%), Asian / Pacific Islander (44.4%; CI:36.0%-52.9%), and Hispanic/Latino (43.4%; CI:38.5%-48.3%) individuals. Asian / Pacific Islander individuals had the highest average predicted probability of living with adult children (48.5%; CI:46.2%-50.7%), followed by Hispanic/Latino (41.3%; CI:36.6%-46.0%), Black / African American (33.5%; CI:32.0%-34.9%), and White (22.5%; CI:18.9%-26.2%) individuals. Results are similar in subpopulations of individuals who also have independent living difficulties and excluding 2020-2021 data. These findings suggest that policies that aim to help older adults with cognitive difficulties and their caregivers should consider that diverse U.S. populations likely vary in their access to and reliance on family members for informal support and care.

Abstract citation ID: igaf122.582

### HOW SOCIAL ISOLATION EXACERBATES UNMET CARE NEEDS IN OLDER ADULTS WITH DEMENTIA

Zhuoer Lin[1], and Zhiyong Lin[2], *1. University of Illinois Chicago, Chicago, Illinois, United States, 2. University of Texas at San Antonio, San Antonio, Texas, United States*

Older adults living with dementia (PLWD) have substantial care needs, yet many face significant barriers to accessing adequate support. Social isolation is particularly concerning, as it restricts access to care networks and is associated with poorer health outcomes, potentially exacerbating unmet care needs among PLWD. While social isolation is recognized as a modifiable risk factor for dementia, its impact on unmet care needs remains insufficiently explored. This study examines the association between social isolation and unmet care needs among PLWD aged 65 and older using longitudinal data from the National Health and Aging Trends Study (NHATS, 2011–2018, N = 5,690). By classifying individuals based on their levels of social isolation, care needs, and care network structures, we assess how these factors intersect. Mixed-effects negative binomial regression models reveal that socially isolated PLWD, particularly those experiencing severe isolation, have significantly higher rates of unmet care needs compared to their socially integrated counterparts. While disparities in access to supportive networks and differences in care needs contribute to this pattern, severe social isolation continues to be strongly associated with higher unmet care needs even after accounting for these factors. These findings highlight the urgent need for targeted interventions to enhance social and care networks for PLWD, especially those who are most isolated. Reducing social isolation could be key to addressing unmet care needs, improving health outcomes, and enhancing overall well-being among PLWD. Policymakers and healthcare providers should prioritize efforts to mitigate social isolation as part of a comprehensive strategy to improve dementia care.

### SESSION 2045 (SYMPOSIUM)

Abstract citation ID: igaf122.583

### TAILORED RECRUITMENT STRATEGIES TO IMPROVE RESEARCH STUDY ENGAGEMENT AND ENROLLMENT

Chair: Ruth Tappen
Discussant: Joshua Chodosh

Successful recruitment of a sufficient number of older adults is a critical but challenging step in most research studies. In this symposium, experienced researchers will share the strategies they adopted to improve the results of their recruitment efforts. Dr. Jackson will report on the addition of a social media campaign to the more traditional community-based recruitment efforts in a large study on driving and dementia. A comparison of the sociodemographic characteristics of enrolled participants recruited via social media vs. those obtained through more traditional means provides some interesting insights. Ms. Villar reports the results of a qualitative analysis of the failure of the social media campaign to attract Spanish-speaking older adults, identifying adaptations that go beyond simple English-to-Spanish translation that should improve the response. Dr. Boon found the recruitment of older individuals with cancer and older individuals with AD achievable, but not the recruitment of those with both cancer and AD, for which an entirely different strategy was needed. Finally, Dr. Morgan reports a new, carefully constructed model to improve communication with persons with dementia that facilitates both recruitment and participation in study activities. Two of the themes that run through these papers are knowing the population of interest well and the importance of tailoring recruitment strategies to that population's unique characteristics, values, and preferences. Our discussant, Dr. Chodosh, will elaborate on these and additional themes. Nursing Care of Older Adults Interest Group Sponsored Symposium

Abstract citation ID: igaf122.584

### TRADITIONAL STRATEGIES VS. SOCIAL MEDIA FOR RECRUITMENT OF OLDER ADULTS IN A LONGITUDINAL STUDY

Kelley Jackson, David Newman, Alexandru Pasarariu, KwangSoo Yang, and Ruth Tappen, *Florida Atlantic University, Boca Raton, Florida, United States*

Over time, a variety of traditional recruitment strategies were employed in a 5-year longitudinal study of cognition and driving behavior in older adults. Eligible participants were older (≥65) adult drivers residing in South Florida in possession of a valid driver's license and insurance who were willing to participate in quarterly cognitive testing and to allow sensors, including driver-facing video, forward-facing video, GPS, and telemetry to capture their driving behavior, to be installed in their personal vehicles. Initial recruitment strategies included flyer distribution (virtually and onsite), health fairs, personal referrals from existing participants, and presentations to community groups. In the fourth year of our study, a digital marketing consultant was engaged to assist the research team in launching geographically targeted, customized ads appealing to older adults meeting inclusion criteria via Facebook and Instagram platforms. Close to 800 individuals responded, indicating their interest electronically, and were contacted by research team members over the next 9 months while traditional recruitment strategies continued. Those who actually enrolled in the study during this time period via social media (n = 57) when compared to traditional strategies (n = 28) were younger t(83) =2.8, p=.003, and had higher MoCA scores t(83) =2.8, p=.003. The two groups of enrolled participants did not differ in terms of gender (p=.191), ethnicity (p=.326), race (p=.236), or years of education (p=.167). In conclusion, social media shows promise to be an effective stand-alone or adjunctive strategy for the recruitment of older adults for relatively complex research studies.

Abstract citation ID: igaf122.585

### ADAPTING SOCIAL MEDIA RECRUITMENT STRATEGIES TO THE PREFERENCES OF SPANISH-SPEAKING OLDER ADULTS

Michelle Villar[1], Monica Rosselli[2], Jinwoo Jang[2], Borko Furht[2], and Ruth Tappen[2], *1. Florida Atlantic University, Lake Worth, Florida, United States, 2. Florida Atlantic University, Boca Raton, Florida, United States*

Social media is proving to be a game changer in the recruitment of older adults for research. Given the success of our initial Facebook outreach to older adults in South Florida, we expanded it to include older Spanish-speaking adults. Neither of these initial campaigns yielded Spanish-speaking participants. In our second campaign thus far, follow-up telephone outreach yielded successful contact with 11 of 39 (28%) leads. Of those 11, 2 were excluded due to distance from the testing sites, 3 did not want further information, and 6 declined after receiving more information. Many reported difficulty fully understanding the content and intent of the posted Facebook ads. Our more traditional recruitment efforts utilized personal referrals and health fairs to foster a critical personal connection, which appeared to be lacking on the social media platform. This disconnect highlighted the need to provide a more complete description of the participant's role in the study and achieve relatability with the target audience in the ads. We concluded that translation into Spanish was insufficient to engage potential participants, and an additional step is necessary, i.e., incorporating personal experiences of Spanish-speaking participants already enrolled in the study and a voiceover that clearly articulates the study's purpose, benefits, and requirements. By fostering meaningful connections and providing appropriate messaging, researchers can attract individuals genuinely interested in participation. Ultimately, adapting recruitment strategies to reflect participants' values and preferences can increase the likelihood of successful engagement and improve the effectiveness of participant recruitment efforts.

Abstract citation ID: igaf122.586

### RECRUITING COMPLEX MEDICAL PATIENTS: LESSONS FROM A STUDY OF PAIN IN ALZHEIMER'S DISEASE AND CANCER

Jeffrey Boon[1], Michelle Crum[2], Jennifer Moon[3], Ronald Cowan[4], and Todd Monroe[1], *1. The Ohio State University, Columbus, Ohio, United States, 2. The Ohio State University College of Nursing, Columbus, Ohio, United States, 3. The Ohio State University Comprehensive Cancer Center, Columbus, Ohio, United States, 4. The University of Tennessee Health Science Center Department of Psychiatry, Memphis, Tennessee, United States*

We are conducting a study on pain differences among people with Alzheimer's disease (AD), cancer, and both AD and cancer. We encountered difficulties recruiting a hard to identify group—those with AD and cancer. We initially recruited through newspaper, social media, and public transportation advertisements as well as community health fairs and organizations with low yield. We partnered with clinicians in oncology and dementia-specialty practices but experienced challenges obtaining referrals even with our staff embedded in the clinic. Developing relationships with community centers and retirement communities with memory care facilities supported recruitment of individuals with solely AD or cancer. To reach the AD and cancer group, we finally partnered with cancer clinical trials office recruitment coordinators. With trained recruiters systematically reviewing clinic schedules and proactively approaching individuals meeting screening criteria, our rates of study participants with both AD and cancer increased (0.24/month to 2.26/month). These recruiters are trained for empathy and compassion in recruitment and informed consent. This group also has access to artificial intelligence (AI) chart search tools that are being developed; these tools have not yet been utilized as we explore the ethical and regulatory challenges of AI in recruitment. While our approach included a specialty recruitment service, this proactive clinic screening approach could be modified and replicated by teams in the future to reach difficult to recruit medical populations. We have also theorized reasons for this recruitment difficulty such as concern for comfort and caregiver burden that can be considered as part of future recruitment planning.

Abstract citation ID: igaf122.587

### IDENTIFYING AND IMPLEMENTING BEST PRACTICES FOR INCLUDING PERSONS LIVING WITH DEMENTIA IN RESEARCH

Brianna Morgan[1], Elizabeth Carpenter-Song[2], Alejandra Martinez[3], Lisa Mistler[2], Salar Khaleghzadegan[2], Wambui Onsando[3], Joshua Chodosh[4], and Paul Barr[3], *1. New York University Langone Health, New York, New York, United States, 2. Dartmouth College, Hanover, New Hampshire, United States, 3. The Dartmouth Institute for Health Policy and Clinical Practice, Lebanon, New Hampshire, United States, 4. NYU Langone, New York City, New York, United States*

Including persons living with dementia (PLwD) in research is increasingly a priority for professional organizations (e.g., Gerontological Society of America) and national research strategies (e.g., National Plan to Address Alzheimer's). However, PLwD have unique barriers to effective communication, including memory impairment, word-finding difficulty, and executive dysfunction, limiting their participation. Further, few research studies explicitly describe practices for facilitating the participation of PLwD in research, henceforth termed "inclusive practices". Our aim was to develop and pilot an inclusive practice guide that allowed PLwD to share their perspectives in a study on the barriers and enablers to interpersonal communication in triadic healthcare visits (i.e., PLwD, care partner, healthcare provider). We identified best practices for including PLwD in research from the literature, which directed our interview guide and training manual development. We then mapped the interview questions onto the Capability-Opportunity-Motivation model of Behavior (COM-B) theoretical framework to understand interpersonal communication and the component(s) to target for change. We also translated the guide into Spanish. Using purposive sampling to identify participants who could provide feedback on the interview guide, we pilot-tested the interview guide with seven participants living with dementia, five in English (in Lebanon, New Hampshire) and two in Spanish (in New York City, New York). Participants found the interview guide acceptable and provided suggestions for improvement. The final interview guide generated robust responses to the research question without causing distress. Explicitly incorporating inclusive practices for including PLwD research is an essential step in soliciting the important perspectives of PLwD.

### SESSION 2050 (SYMPOSIUM)

Abstract citation ID: igaf122.588

### BEYOND THE MEDICAL BILL: THE HIDDEN FINANCIAL, EMPLOYMENT, AND HEALTH IMPACTS OF CAREGIVING

Chair: Barbara Mendez Campos
Discussant: Christina Matz

Unpaid caregivers provide an essential yet often unrecognized economic foundation for the U.S. healthcare and long-term care systems. Valued at over billions of dollars annually, their contributions surpass Medicaid's total spending on long-term services and supports. Yet, despite their critical role, caregivers frequently face financial and employment barriers that jeopardize their own well-being. Many reduce work hours, leave the workforce, or forgo career advancement—decisions that come with steep costs, including lost wages, reduced retirement savings, and diminished Social Security benefits. These barriers disproportionately impact women, low-income caregivers, and historically underrepresented racial and ethnic groups, further widening economic and health disparities. This symposium examines the financial, employment, and health implications of caregiving across diverse populations. The first presentation explores the relationship between intergenerational financial support and planned retirement timing among middle-aged and older workers. Using experimental methods, the second presentation investigates and uncovers hiring bias toward family caregivers, finding that hiring penalties for caregivers to older adults may be even greater than hiring penalties toward parents The third presentation quantifies the opportunity cost of family caregiving, estimating lost income, retirement savings, and Social Security benefits for those reducing work hours or leaving the workforce. Using AARP's latest data and the Urban Institute's simulation model, it highlights financial disparities by age, income, and care intensity, emphasizing the need for targeted policy solutions. The final presentation examines caregiving-related job disruptions and cognitive outcomes, analyzing how workforce disruptions linked to caregiving contribute to cognitive decline, with disparities evident across gender and racial/ethnic groups. This is a collaborative symposium between the Aging Workforce and Family Caregiving Interest Groups.

Abstract citation ID: igaf122.589

### WORK, CARE, AND COGNITION: HOW JOB DISRUPTIONS SHAPE ADRD RISK ACROSS RACE, ETHNICITY, AND GENDER

Barbara Mendez Campos[1], Tsai-Chin Cho[2], Wenhua Lai[3], Jacqui Smith[4], and HwaJung Choi[4], *1. University of Tennessee Knoxville, Knoxville, Tennessee, United States, 2. University of Michigan School of Public Health, Ann Arbor, Michigan, United States, 3. Wayne State University, Detroit, Michigan, United States, 4. University of Michigan, Ann Arbor, Michigan, United States*

Continuous paid work supports cognitive health, but employment disruptions, especially among historically marginalized groups, may increase the risk of cognitive decline and Alzheimer's disease and related dementias (ADRD). While prior research has explored employment trajectories and cognitive aging, limited evidence examines how caregiving-related employment disruptions affect cognitive outcomes, especially across race/ethnicity and gender. This study investigates the association between caregiving-related job disruptions and cognitive function among older adults, with a focus on disparities. Using data from 7,769 U.S. adults aged 55 and older in the 2018 Health and Retirement Study (HRS) and Life History Mail Survey (LHMS), we classified employment histories into caregiving-related disruptions, other disruptions, or continuous employment. Sequence analysis identified employment patterns, and multivariable linear regression models assessed the impact of caregiving-related job disruptions on cognitive function, adjusting for demographic and socioeconomic covariates. Results show that women experienced significantly more caregiving-related job disruptions than men (0.54 vs. 0.04 years, $p < 0.001$), and Hispanic individuals had more disruptions than non-Hispanic Whites (0.83 vs. 0.26 years, $p = 0.010$). Caregiving-related job disruptions were significantly associated with lower cognitive scores in both females ($\beta$=-0.07, $p = 0.002$) and males ($\beta$=-0.14, $p = 0.03$), with notable disparities among non-Hispanic White and non-Hispanic Black male caregivers. Findings suggest that caregiving-related employment disruptions may contribute to cognitive decline, particularly among non-Hispanic White males and non-Hispanic Black caregivers. Targeted policies and interventions addressing these disparities are essential to reduce ADRD risk across diverse caregiving populations.

Abstract citation ID: igaf122.590

### INTERGENERATIONAL FINANCIAL SUPPORT AND PLANNED RETIREMENT TIMING: AN OVERLOOKED DIMENSION OF CAREGIVING

Chin-Yi Su, *Boston College, Chestnut Hill, Massachusetts, United States*

Researchers increasingly recognize that family caregiving responsibilities influence planned retirement decisions, yet most studies focus on hands-on caregiving, overlooking the impact of intergenerational financial support. This study examines how financial support to aging parents and children/grandchildren affects planned retirement timing among middle-aged and older workers. Using 2018 Health and Retirement Study (HRS) data, we analyzed a sample of 2,557 full-time and part-time workers aged 55-61. Ordered logistic regression was employed to assess the relationship between financial support to aging parents, financial support to children/grandchildren, and planned retirement age. Findings indicate that supporting aging parents significantly increases the likelihood of planning for early retirement, whereas financial support for children and grandchildren may delay retirement, though this effect is only marginally significant. Additionally, women, Black, and Hispanic older workers, as well as those with a working pension, are more likely to plan for earlier retirement. These findings highlight the subjectivity of retirement timing and the often-overlooked role of financial caregiving. While economic responsibility for younger generations may extend workforce participation, financial support for aging parents appears to accelerate retirement planning. This research underscores financial caregiving as a critical yet under examined factor shaping retirement decisions, particularly for the sandwiched generation. Future studies should further investigate the mechanisms linking financial caregiving and retirement behavior, considering how financial strain varies across caregiving roles and demographic groups.

Abstract citation ID: igaf122.591

**THE OPPORTUNITY COST OF FAMILY CAREGIVING: NEW DIRECTIONS FOR POLICY AND PRACTICE SOLUTIONS**

Selena Caldera, *AARP, Washington, District of Columbia, United States*

This presentation details a new study from AARP providing updated national estimates of the opportunity cost of family caregiving. The Urban Institute's Dynamic Simulation of Income Model was calibrated using the latest Caregiving in the US data on the demographics, care tasks, and economic characteristics of family caregivers. This model examines counterfactual scenarios for adults who stopped or limited labor force participation to provide care to another adult with disabilities or a health condition requiring regular assistance. The study estimates potential lost income, lost savings, and lost Social Security income for caregivers who stop working altogether and for those who reduce their work hours to accommodate their care responsibilities. Disparate outcomes for caregivers of different age groups, income levels, and levels of care intensity will be summarized. This detailed analysis is critical to understanding that there is no one-size-fits-all solution to financially support family caregivers. Instead, a range of solutions targeted to the experiences and needs of different groups of caregivers is needed. These findings are crucial for developing targeted policy and practice solutions that mitigate family caregivers' potential financial losses. In conclusion, a range of innovative policy and practice solutions to protect the financial security of family caregivers will be discussed.

Abstract citation ID: igaf122.592

**JOB SEEKING WHILE CAREGIVING: IS THERE A HIRING PENALTY FOR FAMILY CAREGIVERS?**

Samantha Brady, *Massachusetts Institute of Technology, Cambridge, Massachusetts, United States*

Whether by choice or necessity, the majority of unpaid family caregivers to adults in the U.S. are employed. Prior research highlights the importance of work for caregivers' financial stability, emotional well-being, and physical health. However, less is known about how caregiving responsibilities for an adult affect job seekers, particularly in hiring decisions. While hiring bias against parents is well-documented, the impact of adult caregiving on employment opportunities remains under-explored. Using data from an original hiring experiment of hiring managers across the US, we examine how family caregivers are perceived in the hiring process and whether they face penalties due to their caregiving roles. Results show that unpaid family caregivers are 4.4 percentage points less likely to be hired than equally qualified applicants without caregiving responsibilities and 2.7 percentage points less likely to be hired than applicants with dependent children, controlling for age, gender, education, and skill level. Further analysis reveals that while both parents and caregivers to adults are seen as less available and reliable than non-caregivers, caregivers to adults are also perceived as less committed, contributing to their lower hiring rates. While existing research documents workplace challenges for employed caregivers, our findings underscore an earlier and often overlooked barrier: the hiring process itself. These hiring penalties may limit career mobility and threaten the financial security of millions of unpaid caregivers, raising critical implications for employment policies and workplace equity.

### SESSION 2055 (SYMPOSIUM)

Abstract citation ID: igaf122.593

**COLLABORATIVE RESEARCH WITH COMMUNITY-BASED HEALTHCARE PROVIDERS: CO-PRESENTED LEARNINGS FROM PILOT STUDIES**

Chair: Kali Thomas
Discussant: Emily Gadbois

This symposium highlights five community-engaged research projects conducted by the Brown University Center for Long Term Care Quality and Innovation (Q&I). Each symposium will be co-led by a community partner and a Q&I Center researcher. In each presentation, we will describe the processes for project prioritization, collaborative outcome selection, data acquisition, co-interpretation of key findings, and recruitment and consent decisions. We will also describe site- and population-specific barriers to conducting pilot studies. Community partners include: Meridian Senior Living, which owns and operates over 50 assisted living communities (ALCs) across 21 states; Bluestone Physician Services, which provides chronic disease management to over 15,000 patients at approximately 575 ALCs in three states (Florida, Minnesota, and Wisconsin); CareLink, a Rhode Island-based healthcare network providing high-quality, evidence-based care for over 5,000 older adults each year; and PACE RI, a health plan which has provided care to over 2,000 nursing home eligible, community-dwelling adults in RI, with the goal of maintaining independence. We will transparently discuss our failures and successes and share tools that may be helpful to participants conducting community-engaged research.

Abstract citation ID: igaf122.594

**ASSESSING EMOTIONAL RESPONSES IN PEOPLE WITH DEMENTIA USING FACIAL ASSESSMENT SOFTWARE**

Ellen McCreedy[1], Kelley Hurley[2], Ann Reddy[3], and Corinne Roma[3], *1. Brown University, School of Public Health, Providence, Rhode Island, United States, 2. CareLink, East Providence, Rhode Island, United States, 3. Brown University, Providence, Rhode Island, United States*

Facial assessment software may help people living with dementia better communicate their emotional states, particularly as the disease progresses, and may help researchers evaluate the effect of promising interventions on momentary affective states. For this pilot study, we partnered with CareLink, a Rhode Island-based healthcare network providing high-quality, evidence-based care for older adults, and SOLO, facial assessment software which has been integrated into a user-friendly application that can be accessed from a tablet or smartphone (https://www.imsolo.ai/). We compared SOLO output to results from Dementia Mood Picture Test (DMPT), a validated self-report tool in which people living with dementia self-report current emotion states using pictures of faces. CareLink enrolled 12 community-dwelling older adults with mild or moderate dementia between September 2023 and April 2024. There were 48 valid comparisons of SOLO and DMPT mood states (mean 4 per participant, range 2-6). Pearson correlation coefficients were computed to assess the linear relationship between self-reported happiness, worry, anger, sadness, and "good mood" and SOLO-detected happiness, stress, anger, sadness, and well-being. After controlling for repeated observations, there were moderate positive correlations between self-reported good mood and SOLO-detected well-being, self-reported happiness and SOLO-detected happiness, and self-reported worry and SOLO-detected stress. Sadness and anger were rarely self-reported. CareLink administered both tools and will discuss their acceptability and face validity with patients, care partners and clinical staff. Facial assessment software may be helpful in discerning positive responses to behavioral interventions in people living with dementia.

Abstract citation ID: igaf122.595

### ENGAGING ASSISTED LIVING RESIDENTS IN THE DEVELOPMENT OF AN EVALUATION PLAN FOR A FUTURE TRIAL OF AN EXERCISE PROGRAM

Ann Reddy[1], Kevin Carlin[2], Olivia Taylor[3], Rosa Baier[3], and Ellen McCreedy[4], *1. Brown University, Providence, Rhode Island, United States, 2. Meridian Senior Living, Bethesda, Maryland, United States, 3. Brown University School of Public Health, Providence, Rhode Island, United States, 4. Brown University, School of Public Health, Providence, Rhode Island, United States*

Many trials are conducted without first assessing which outcomes are important to patients receiving the interventions. The goal of this work was to ask participants of an ongoing exercise program what they believed the most important impacts of the program were to better align outcomes for a future trial with participant priorities. The Rev6 program is a seated, 30-minute exercise class designed for older adults with varying cognitive abilities. The program is offered twice a week by a large assisted living community (ALC) in Rhode Island. We observed three Rev6 sessions and held a focus group with seven Rev6 participants. We also interviewed one ALC staff member responsible for delivering the Rev6 program and two corporate leaders. Three members of the research team analyzed the focus group and interview transcripts to identify themes. Themes related to physical health included staff- and participant-reported impact of the Rev6 program on balance and walking, strength, and pain. Themes related to mental health included staff- and participant-reported impact of the Rev6 program on "feeling better" and increased social connection. With input from ALC staff, researchers developed visual cue cards for each theme and met with nine program participants to vote on the most important theme to evaluate as an outcome measure for a future study. Four residents voted for balance and walking, three residents voted for feeling better, and two residents voted for pain as the most important impact of the program. This framework for evaluation codesign includes the voices of people with dementia.

Abstract citation ID: igaf122.596

### RECRUITING AND CONSENTING COMMUNITY-DWELLING ADULTS WITH DEMENTIA TO TEST SLEEP TRACKING DEVICES

Rosa Baier[1], Melissa Simonian[2], Corinne Roma[3], Ann Reddy[3], and Ellen McCreedy[4], *1. Brown University School of Public Health, Providence, Rhode Island, United States, 2. Program of All-Inclusive Care for the Elderly, Riverside, Rhode Island, United States, 3. Brown University, Providence, Rhode Island, United States, 4. Brown University, School of Public Health, Providence, Rhode Island, United States*

Poor sleep is associated with decreased physical and cognitive function, poor mood, and increased falls. Understanding sleep patterns may help physicians better recommend behavioral programs for improving sleep. Researchers partnered with the PACE Organization of Rhode Island (PACE-RI) to recruit community-dwelling older adults living with dementia to pilot test sleep-tracking devices. PACE-RI provides healthcare to adults 55+ with complex medical conditions, with a focus on maintaining their independence. Participants in this study used a wearable sleep tracking device and a sleep tracking mat for three consecutive nights. Caregivers residing with participants completed a four-question sleep diary for each of the three nights. We encountered challenges recruiting up to 15 participants in this study, with only 5 participant-caregiver dyads enrolled over a one-year period. Key barriers to recruitment included: lack of Spanish language materials / bilingual recruiters, requirement to have a cohabitating caregiver, no internet service at home, lack of trust of researchers and/or technology, and concerns about researchers entering homes. At PACE-RI, approximately 45% of their clients have dementia, but most do not have a family caregiver living with them. In an effort to establish greater trust and connection with participants, the research team visited each participant three times (at consent / equipment setup, at equipment pickup, and after personalized findings were available) and called daily. We will share our one-page participant reports that summarize each participant's sleep data using simple language and pictures. In this ongoing pilot, the follow-up visits and reports have increased snowball-based recruitment.

Abstract citation ID: igaf122.597

### BLUESTONE'S EMERGENCY DEPARTMENT EARLY RESPONSE PROGRAM: PROMOTING HIGH-QUALITY AND SAFE TRANSITIONS

Grace Wittenberg[1], Martha Etzell[2], Peter Serina[3], Nichole Stetten[1], Ann Reddy[1], and Ellen McCreedy[4], *1. Brown University, Providence, Rhode Island, United States, 2. Bluestone Physician Services, Stillwater, Minnesota, United States, 3. Salem Hospital, Salem, Massachusetts, United States, 4. Brown University, School of Public Health, Providence, Rhode Island, United States*

Almost half of all Assisted Living Community (ALC) residents transfer to the emergency department (ED) each year. For people living with dementia (PLWD), providing a medical history can be a challenge, resulting in prolonged stays and risk of delirium or hospital acquired infections. Bluestone Physician Services developed a ED early response program in which complex care managers (CCM) receive an electronic Admission, Discharge, and Transfer (ADT) notification when one of their patients registers at an ED. If the notification occurs during business hours, the CCMs call the ED using a script and follow-up with a structured fax. We conducted semi-structured interviews with 12 CCMs involved in the program to assess the feasibility and acceptability of the program. There were five themes: patients with dementia and those on hospice were especially likely to benefit from the program; strengths of the program, including increased communication between Bluestone and ED providers; weakness of the program, including a lack of awareness of the program among variable ED staff and challenges with timing the call and fax to maximize benefit. The CCMs also shared some learnings and adaptations that increased contact rate over time and individual success stories. In this session, we will also provide tips on how to integrate real-time ADT notifications into CCM workflows. Next steps include examining the ED provider, patient and caregiver perceptions of the program, quantifying the impact of the program on utilization, and analyzing how the program would perform in other health care settings.

### SESSION 2060 (SYMPOSIUM)

Abstract citation ID: igaf122.598

### ASSESSING THE SOCIETAL COSTS OF COGNITIVE AGING: EVIDENCE FROM THE US AND MEXICO

Chair: Flavia Andrade
Co-Chair: Jacqueline Angel

As the United States and Mexico undergo demographic shifts, rising longevity has intensified the burden of chronic conditions such as dementia and diabetes. These conditions pose significant policy and financial challenges, particularly as families bear most caregiving costs. This symposium advances knowledge on caregiving in the U.S. and Mexico, using diverse data sources to examine barriers and facilitators in chronic disease management. Five papers reflect collaborations between emerging scholars and senior faculty from the International Conference on Aging in the Americas. Falk analyzes BRFSS data, showing that caregivers of individuals with Alzheimer's Disease and related dementias (ADRD), especially women and those facing food insecurity, experience significantly higher stress, underscoring the need for targeted interventions. Rojas-Alvarez uses HEPESE and Medicare data to show that Hispanic older adults with dual diagnoses of diabetes and ADRD near the U.S.-Mexico border experience worse healthcare utilization and self-reported health, pointing to structural barriers. Bokum analyzes the HEPESE and ACS to examine financial strain among co-residing adult child caregivers, finding younger and late-middle-aged caregivers face disproportionate financial stress, emphasizing the need for direct compensation and expanded support. Aguila draws on MHAS and HRS sister surveys to assess informal dementia care, identifying public and family-based care gaps requiring policy action. Mudrazija and Aranda estimate that unpaid dementia caregiving costs will rise substantially by 2060, disproportionately affecting minoritized caregivers and highlighting the need for culturally tailored support. Together, these studies inform strategies to enhance caregiver support and reduce health and financial disparities.

Abstract citation ID: igaf122.599

### FINANCIAL STRAIN AMONG MEXICAN AMERICAN ADULT CHILDREN CAREGIVERS LIVING WITH AGING PARENTS

Anna Bokun[1], Flavia Andrade[2], Jacqueline Angel[3], Phillip Cantu[4], and Sunshine Rote[5], *1. The University of Texas at Austin, Boston, Massachusetts, United States, 2. University of Illinois--Urbana-Champaign, Urbana, Illinois, United States, 3. The University of Texas at Austin, Austin, Texas, United States, 4. UTMB Health, The University of Texas Medical Branch, Galveston, Texas, United States, 5. University of Louisville, Louisville, Kentucky, United States*

As more individuals provide care to aging parents, the question arises of whether adult children who provide care to and live with their parents experience increased financial strain and how this might differ if the caregiver is the head of the household. Despite the cost-sharing benefits of co-residing, adult child caregivers may face greater financial strain due to out-of-pocket costs, medical expenses, and home modifications. We use the Hispanic Established Populations for the Epidemiologic Study of the Elderly (HEPESE) data (2010–11), a survey of Mexican-Americans in five southwestern states in the United States, supplemented with nationally representative data from the American Community Survey. Our dependent variable is an indicator of caregiver financial strain developed from an index of five measures of financial difficulty in meeting financial expenses. Logistic regressions show that co-residing more than doubled the odds of financial strain (OR 2.17, 95% CI [1.22, 3.82], $p < 0.01$), especially if the caregiver is younger or late-middle aged. We find no observed association between household headship status and financial strain. This study provides empirical evidence for public policies that reduce family caregivers' financial burden through direct compensation and expanded support services.

Abstract citation ID: igaf122.600

### SOCIAL AND ECONOMIC SUPPORT FOR MEXICAN OLDER ADULTS LIVING WITH DEMENTIA IN MEXICO AND THE UNITED STATES

Emma Aguila[1], Mariana Lopez-Ortega[2], Rosa Garcia-Chanes[2], and Jacqueline Angel[3], *1. University of Southern California, Los Angeles, California, United States, 2. National Institute of Geriatrics, Ciudad de Mexico, Estado de México, Mexico, 3. The University of Texas at Austin, Austin, Texas, United States*

Dementia is one of the most common causes of disability and dependence and is a frequent reason that older people require supportive living. In Mexico and the United States, Alzheimer's and other dementias represent one of main cause of DALYS (disability-adjusted life-years) and it is estimated that there are approximately 1.6 million in Mexico and at least 6 million persons living with dementia in the U.S. However, the health systems are not prepared to adequately provide diagnosis, post-diagnosis care as well as care support at home for individuals living with dementia and their family caregivers. In addition, economic conditions of a large number of Mexican-origin older population are precarious given their working life in the informal sector or "gig economy" with limited access to employer provided benefits and social safety net policies in old age, relying primarily on family financial and in-kind support. The aim of this study is to investigate the burden of informal support in Mexico and the United States. We use the Mexican Health and Aging Study (MHAS) and the Health and Retirement Study (HRS) data sets linked to their Cognitive Aging Ancillary Study (Mex-Cog and HCAP) 2015-16. This allows for a detailed characterization of support received by individuals living with dementia and compares it with the support available and received by older adults with no impairment. The results allow for specific recommendations for further support programs and activities that focus on areas of need and are currently not covered either by public or family support.

Abstract citation ID: igaf122.601

### CURRENT AND FUTURE COSTS OF FAMILY CAREGIVING FOR OLDER AMERICANS WITH AND WITHOUT DEMENTIA

Stipica Mudrazija[1], and María Aranda[2], *1. University of Washington, Seattle, Washington, United States, 2. University of Southern California, Los Angeles, California, United States*

Family caregivers in the US provide substantial value of unpaid care to older adults, while less recognized are the employment-related costs they endure and the trajectory of these costs. We estimate the replacement cost of unpaid family caregiving to US adults aged 70 and older with and without dementia and opportunity costs of forgone earnings, lost productivity, and loss of federal income tax revenue between 2011 and 2060, using data from the National Study of Caregiving, Panel Study of Income Dynamics, and various other sources such as Census Bureau's population projections. Currently, the annual replacement cost of unpaid family care is between $96-182 billion, with 44% accounted for by dementia caregiving. By 2060, it will increase to $277-571 billion, and 53% will be for dementia caregiving. The opportunity costs of forgone earnings, productivity loss, and loss of federal income tax payments, however, will grow faster, increasing from current levels of $107 billion, $26 billion, and $7 billion to $380 billion, $102 billion, and $27 billion, respectively, in 2060. These costs will be increasingly borne by minoritized caregivers. For example, Latino caregivers will account for 48% of replacement costs and 39% of opportunity costs of caregiving for older adults with dementia in 2060, compared to their current share of only 27% and 19%, respectively. These results highlight a growing need for robust and culturally tailored support for unpaid family caregivers to support their loved ones while remaining attached to the labor force.

Abstract citation ID: igaf122.602

### CAREGIVER STRESS AND SOCIAL DETERMINANTS OF HEALTH: COMPARING ALZHEIMER'S DISEASE CAREGIVERS TO OTHER CAREGIVERS

Derek Falk[1], Elizabeth Vásquez[2], Anna Bokun[3], Christian Vazquez[1], and Sunshine Rote[4], *1. The University of Texas at Arlington, Arlington, Texas, United States, 2. University at Albany (SUNY), Albany, New York, United States, 3. The University of Texas at Austin, Boston, Massachusetts, United States, 4. University of Louisville, Louisville, Kentucky, United States*

Caregivers for individuals with Alzheimer's Disease and related dementias (ADRD) experience excessive psychological and financial burdens compared to caregivers of other conditions due to the prolonged and unpredictable nature of dementia-related care. The impact of social determinants of health (SODH) on the levels and sources of stress experienced by caregivers of individuals with ADRD as compared to caregivers of individuals with other chronic conditions is unclear. Using the 2023 Behavioral Risk Factor Surveillance System (BRFSS), including the SDOH and health equity module, we assessed ADRD caregiver burden by sociodemographic and SODH measures. Univariable and bivariable statistics described the distribution of the caregivers and compared ADRD caregivers to caregivers of other chronic conditions. Weighted and adjusted multivariable logistic regressions assessed the odds of feeling stressed among caregivers. Over 25% of caregivers assisted with ADRD. Compared to caregivers of other chronic conditions, more ADRD caregivers were aged 55 + ($P = 0.002$) and female ($P = 0.003$). Our regression models showed food insecurity or not having food last and not being able to buy more when needed (odds ratio [OR]: 3.20; confidence interval [CI]: 2.29-4.48), financial strain or not being able to pay mortgage, rent, or utility bills (OR: 1.81; CI: 1.06-2.49), caregiving for someone with ADRD (OR: 1.40; CI: 1.06-1.85), and being female (OR: 1.55; CI: 1.28-1.87) were significantly associated with caregiver stress. Social determinants of health negatively impact caregiver stress. Our findings highlight the need for targeted interventions and supportive services that mitigate the psychological and financial burden faced by ADRD caregivers.

Abstract citation ID: igaf122.603

### UNPACKING HEALTHCARE UTILIZATION FOR OLDER ADULTS WITH DIABETES AND ADRD IN THE US-MEXICO BORDER REGION

Alfonso Rojas-Alvarez[1], Hyeran Chung[1], and Brian Downer[2], *1. The University of Texas at El Paso, El Paso, Texas, United States, 2. UTMB, Galveston, Texas, United States*

Hispanic older adults living near the U.S.-Mexico border encounter considerable healthcare challenges that impact the management of diabetes and Alzheimer's Disease or Related Dementias (ADRD). Studies have found that Hispanic older adults living on the U.S.-Mexico border are disproportionately affected by financial strain, limited healthcare access, and a higher prevalence of chronic diseases. Individual diagnoses of either diabetes or ADRD already present significant health challenges for Hispanic older adults, and dual diagnoses exacerbates these challenges, increasing the complexity of medical management. Using the Medicare-linked HEPESE survey ($n = 718$) we conduct statistical analyses to explore the relationship between dual diagnoses of ADRD and diabetes, with self-reported health measures and healthcare utilization. Our key stratifying variable is proximity to the border, given our previous findings that it explains caregiver and community resources that affect outcomes for Hispanic older adults aged 80+. Preliminary results indicate that Hispanic older adults with dual diagnoses near the US-Mexico border exhibit worse measures of healthcare utilization compared to those with a single diagnosis. Similarly, we find that Hispanic older adults with dual diagnoses near the US-Mexico border exhibit worse measures of self-reported health related to their conditions, compared to those with a single diagnosis. Structural and policy-driven factors, particularly healthcare access, influence healthcare utilization and health outcomes, with proximity to the border affecting access to care. This project is significant as it highlights the unique challenges faced by older adults with dual diagnoses in border region, aiming to inform policies that address healthcare issues for vulnerable populations.

### SESSION 2065 (SYMPOSIUM)

Abstract citation ID: igaf122.604

### A ENGAGEMENT STRATEGY TO BUILD A DEMENTIA CARE INFRASTRUCTURE

Chair: Sara Murphy
Co-Chair: Jennifer Severance
Discussant: Matthew Smith

With one of the fastest growing populations in the United States, Texas is home to an increasing number of older adults living with dementia. Guided by the Center for Disease Control and Prevention's Healthy Brain Initiative (HBI) Road Map, an academic health science center partnered with community, clinical, and public health sectors to engage stakeholders, identify emerging needs, braid together resources and expertise, and develop and strengthen the state's dementia care infrastructure. This symposium examines multi-sector solutions to improve the quality of care and quality of life for people at risk or living with Alzheimer's disease and related dementias (ADRD) and their caregivers, help people remain in their homes and communities, and reduce healthcare expenditures. First, presenters introduce a Dementia Consortium of community, health, and public health representatives and people with lived experience that supports dementia-friendly training and education across primary care, long-term care, and community settings. This is followed by results from implementation of an innovative primary care model of dementia navigator services designed to support comprehensive and coordinated care for persons with dementia and to strengthen community-clinical linkages. Finally, presenters explore examples and outcomes of interventions designed to promote brain health, early detection, and diagnosis as well as caregiver health. By applying the HBI public health framework, communities can enhance local collaboration and coordination, raise public and provider awareness about dementia, and design cross-sector strategies to address the healthcare costs and challenges of care for people impacted by ADRD.

Abstract citation ID: igaf122.605

### DEMENTIA CARES: A TRAINING SERIES TO IMPROVE CAREGIVER ACCESS TO RESOURCES, EDUCATION AND SUPPORT

Sara Murphy[1], Jane Oderberg[1], Jennifer Severance[2], and Janice Knebl[1], *1. University of North Texas Health Science Center, Fort Worth, Texas, United States, 2. University of North Texas Health Science Center - Fort Worth, TX, Fort Worth, Texas, United States*

The entry point into dementia caregiving is a critical time in which a caregiver needs information, emotional and psychological support, and assistance in planning for their future role. The Dementia CARES series introduces dementia caregivers to strategies and topics to promote social support and the acquisition of new skills and knowledge. Session topics include 1) living with dementia; 2) behavioral changes; 3) strategies to form a local support network; 4) improving communication with the person living with dementia and their care team; 5) exploring resources, including evidence-based programs and clinical trials; and 6) practicing self-nurturing activities. Each session involves group discussion to learn about a dementia topic followed by sharing and problem-solving activities to practice new concepts, self-evaluation, and peer support to build caregiver confidence (self-efficacy) in carrying out tasks and addressing challenges. Designed by a licensed Master's degree-level social worker at a geriatric primary care clinic, Dementia CARES is delivered as six one-hour virtual weekly group sessions for six to ten dementia caregivers. Four virtual series of the six-session CARES program were conducted in December 2018 to July 2019. Thirty-nine dementia caregivers were recruited by the primary care clinic, with twenty-nine (74%) participants completing at least four sessions. Preliminary data from post-intervention survey responses ($n=29$) show 96.5% would strongly recommend the training to a friend or relative. Participants valued the discussion with other caregivers, learning strategies and suggestions, and sharing their experiences. Findings underscore the value and feasibility of offering structured group education and support in primary care settings.

Abstract citation ID: igaf122.606

### CROSS-SECTOR COLLABORATION TO ADVANCE DEMENTIA-FRIENDLY WORKFORCE EDUCATION IN RURAL COMMUNITIES

Jennifer Severance[1], Janice Knebl[2], Sara Murphy[2], and Sarah Ross[2], *1. University of North Texas Health Science Center - Fort Worth, TX, Fort Worth, Texas, United States, 2. University of North Texas Health Science Center, Fort Worth, Texas, United States*

Limited access to quality dementia care due to workforce challenges is a critical issue, especially in rural communities. Ninety percent of Texas counties are Rural Health areas and 93% are designated as Primary Care Health Professional Shortage Areas. To increase knowledge about dementia in the geriatric care workforce, a Health Resources and Services Administration-funded Geriatric Workforce Enhancement Program in Texas designed collaborative education and training programs aimed towards rural communities. Using a multipronged strategy to engage primary care and long-term care teams across the state, interdisciplinary faculty and community and health care partners combined critical resources to develop and enhance curricula in geriatric best practices using the Institute for Healthcare Improvement's Age-Friendly Health Systems 4Ms framework. Partners developed an infrastructure to reach rural and underserved communities using learning technologies, social media platforms, and community health educators. An advisory board of older adults and caregivers was assembled to inform curricula development. In the first implementation year, partners incorporated tele-education technologies, experiential learning, and interprofessional activities into learning activities and clinical training environments for health professions students ($n=69$), primary care residents ($n=48$), health care practitioners ($n=146$), and community educators ($n=125$). Preliminary trainee feedback demonstrates improved knowledge, confidence and attitudes in providing dementia care and connecting older adults and caregivers to needed social supports. This model of collaboration continues with a goal to further enhance training models, expand organizational capacity to respond to community needs, improve health care for older adults and their caregivers, and build bridges between community and clinical settings.

Abstract citation ID: igaf122.607

### OUTCOMES OF A BRAIN HEALTH SELF-MANAGEMENT COACHING INTERVENTION

Kush Kinariwala[1], Sarah Ross[1], Sara Murphy[1], and Matthew Smith[2], *1. University of North Texas Health Science Center, Fort Worth, Texas, United States, 2. Texas A&M University, College Station, Texas, United States*

Research suggests modifiable brain healthy lifestyle behaviors account for one-third of Alzheimer's Disease and related dementia (ADRD) diagnoses. While brain-healthy lifestyle choices (e.g., diet, exercise, sleep, social engagement) can help reduce the risk of developing ADRD, individuals often lack the knowledge and support needed to identify, implement, and maintain the necessary changes. Adults without ADRD were recruited to participate in an in-person three-month brain health coaching intervention hosted by certified health and wellness coaches in a clinical context. Brain health coaching targeted the Seven Pillars of Brain Health: nutrition, exercise, social engagement, cognitive activity, sleep, outlook and mindfulness, and general health. Over a three-month period, participants met with the health coach monthly to create a personalized plan for improving brain-healthy self-management behaviors. Data were collected from 22 participants at baseline and 3-month follow-up, which were analyzed using paired-samples t-tests and sign-rank tests. The average age of participants was 76, with 72% female and 95% White. Almost half (45%) reported subjective memory complaints. Compared to baseline, on average, participants reported significant improvements in their mindfulness (t=-1.73, P = 0.001) and nutrition (t=-4.50, P < 0.001), regardless of the chosen focus area for their person brain health goals. Findings suggest the feasibility of a brain health coaching program and the effectiveness at improving mindfulness practices, including mindful eating and implementing a brain-healthy diet. Consistent engagement with certified health and wellness coaches may assist individuals in reducing their risk for cognitive impairment by adopting and adhering to brain-healthy self-management behaviors.

Abstract citation ID: igaf122.608

### EMBEDDING DEMENTIA CARE NAVIGATION INTO PRIMARY CARE

Janice Knebl[1], Jennifer Severance[2], and Sara Murphy[1], 1. University of North Texas Health Science Center, Fort Worth, Texas, United States, 2. University of North Texas Health Science Center - Fort Worth, TX, Fort Worth, Texas, United States

People living with dementia often have multiple chronic conditions and receive fragmented care, leading to high rates of hospitalization and emergency visits. Dementia care navigators provide person-centered resources, education, and support to help caregivers understand and access their options and have been found to improve health outcomes for people living with dementia. However, the adoption of dementia care navigator services by health systems is limited due to a variety of factors, including lack of defined delivery models and the absence of a payment structure for primary care. This presentation describes a primary care clinic's experience as one of two healthcare providers on the experienced program track in Texas approved by the Centers for Medicare and Medicaid Services (CMS) to offer the Guiding an Improved Dementia Experience (GUIDE) Model designed to improve the quality of life for people living with dementia and those in their lives who give them support. The GUIDE Model pays for the coordination of services, education about dementia, and the support people living with dementia often need to remain in their homes safely and delay nursing home care as long as appropriate. The clinical team also received a grant from the state to assess emerging and unmet needs of persons with dementia and their caregivers and to strengthen community-clinical linkages through dementia resources specialists. Early results and learnings reveal challenges and opportunities of implementing dementia care navigator services and the need for comprehensive and coordinated approaches to dementia care addressing needs at the individual and community levels.

### SESSION 2070 (SYMPOSIUM)

Abstract citation ID: igaf122.609

### CHALLENGES AND OPPORTUNITIES FOR CENTERS ON AGING: PART 1 - LEADERSHIP, DIVERSITY, AND ADVOCACY

Chair: Pamela Saunders

The current national landscape poses significant challenges for the future of socio-biomedical research in the US. Politicians seek to restructure the funding priorities for essential programs that support older adults, including Medicare and organizations like NIH and the National Science Foundation. As a result, leaders at aging centers are evaluating their programs, services, structures, and sources of support. This symposium is part one of a two-part series titled "Challenges and Opportunities for Centers on Aging" and addresses issues of diversity, advocacy, mentorship, and development. The first paper will discuss recommendations and lessons learned from the startup of the new Center for Healthy Aging at Georgetown University. The second paper will explore the pivotal role of the CU Anschutz Multidisciplinary Center on Aging in leading advocacy efforts for older adults and the stakeholders who support their care. The third paper will introduce a new initiative called the Leadership Excellence and Advancement of Directors (LEAD) Program, which aims to provide mentorship, professional growth, and peer support for center directors. The fourth paper will investigate academic, professional, and policy frames of equity and social justice for the Center for the Advanced Study of Aging Services, housed in UC Berkeley's School of Social Welfare. The fifth paper will explore strategies for overcoming financial and institutional constraints through the Marymount Center for Optimal Aging's interdisciplinary strategic model. This model is designed to impact older adults' well-being while providing students with hands-on training. The symposium offers recommendations to tackle challenges and seize opportunities facing aging centers.

Abstract citation ID: igaf122.610

### STARTING A NEW AGING CENTER IN UNCERTAIN TIMES: RECOMMENDATIONS AND LESSONS LEARNED

Pamela Saunders, *Georgetown University, Washington, District of Columbia, United States*

The national landscape poses significant challenges for the future of socio-biomedical research in the US. As a result, aging centers are evaluating their programs, services, structures, and sources of support. This paper discusses the launch of a new Center for Healthy Aging at Georgetown University Medical Center (GUMC), detailing its leadership, operations, and activities. GUMC includes schools of medicine, health, and nursing. While many faculty members conducted research on aging, there was no central unit to promote collaboration and networking across schools. To start, the Dean of Research formed a working group to strategize the future of aging research, resulting in a proposal for a new center. It was subsequently submitted to and approved by the Medical Center Committee on Research. The leadership structure includes a steering committee, a director, and an associate director. Members of the steering committee were invited to serve by the Dean of Research from the original working group and were primarily researchers. Later, additional faculty members were invited to join to develop educational resources and community partnerships. The Center's operations involve monthly meetings to discuss activities and priorities. The Center hosts an annual Symposium featuring speakers from NIH, Georgetown, and local universities. Panel topics included social determinants of health, cognitive aging, and cancer in relation to aging. The Center's future goals include conducting a SWOT analysis to establish priorities, submitting a conference grant to broaden national participation, and formalizing funding sources for programs and activities. Recommendations and lessons learned will be discussed.

Abstract citation ID: igaf122.611

### DRIVING CHANGE: EMPOWERING ADVOCACY FOR OLDER ADULTS THROUGH UNIVERSITY CENTERS ON AGING

Jodi Waterhouse, *CU Anschutz Medical Campus, Aurora, Colorado, United States*

This session will explore the pivotal role of University Centers on Aging in leading advocacy efforts that significantly impact older adult issues and the stakeholders who support their care. By building community consortiums to drive advocacy, education, and legislation, the CU Anschutz Multidisciplinary Center on Aging has successfully led, identified and addressed critical gaps. This session will provide a comprehensive, step-by-step guide to developing an effective advocacy strategy. Participants will gain insights from successful advocacy campaigns offering a blueprint for other states to establish similar working groups. Additionally, the session will cover strategies for securing state funding to support older adult issues, including positioning advocacy efforts within the Legislature to ensure the successful passage of legislative bills. Attendees will leave equipped with practical strategies to engage their state's elected officials and drive support for Age-Friendly initiatives across the state and country.

Abstract citation ID: igaf122.612

**MENTORING NEW RESEARCH CENTER DIRECTORS THROUGH TIMES OF UNCERTAINTY: THE LEAD PROGRAM**

Juanita-Dawne Bacsu[1], and Harleah Buck[2], *1. Thompson Rivers University, Kamloops, British Columbia, Canada, 2. University of Iowa, Iowa City, Iowa, United States*

Mentorship is vital for new research center directors to help them build leadership skills, foster diversity, and lead successful teams, especially through times of uncertainty. However, there is limited discussion on mentorship programs to support new center directors in the field of gerontology. Collaborating with the Gerontological Society of America, we developed the Leadership Excellence and Advancement of Directors (LEAD) Program as a model to provide mentorship, professional growth, and peer support for center directors. This presentation aims to: 1) outline evolving mentorship strategies to empower new centre directors in navigating unpredictable challenges and uncertainty; and ii) identify the LEAD Program as an innovative model to provide mentorship to support new directors. The LEAD Program is a trailblazing GSA initiative that was developed in response to the growing call for mentorship from incoming directors as previous leaders transitioned to retirement. The LEAD Program is quickly evolving into a hub of mentorship by: i) addressing collective challenges and leadership needs; ii) providing peer support to engage in collective problem-solving; iii) facilitating knowledge to enhance decision-making during times of uncertainty; and iv) networking to support capacity-building among center directors. Moving forward, our next steps are to assess the LEAD Program to enhance the mentorship model for future cohorts of center directors.

Abstract citation ID: igaf122.613

**FRAMING EQUITY AND SOCIAL JUSTICE IN A NEW ERA FOR AGING CENTERS**

Angela Perone, *University of California, Berkeley, Berkeley, California, United States*

As our population ages and becomes more diverse, the work of aging centers is needed now more than ever before. Founded in 1996 through generous support from the Eugene and Rose Kleiner Family Foundation, the Center for the Advanced Study of Aging Services (CASAS) conducts research, educates emerging and established leaders, and informs policies to improve the lives of older adults and their care partners. As part of the School of Social Welfare at the University of California, Berkeley, CASAS is rooted in equity and social justice values. Social justice is explicitly named as a core value and ethical principle in the code of conduct for social workers through the National Association of Social Workers (NASW). Many of the contested research, education, and language stemming from prohibited work around DEI contrasts directly with professional standards for social work researchers, educators, and practitioners. This presentation will illuminate evolving challenges and strategies employed by CASAS as a case study to reconcile these inherent contradictions. Organizational auto-ethnographic data from this case study illustrates the diversity of academic, professional, and policy frames and approaches to equity and social justice in a turbulent social and political moment of uncertainty. It also provides insights for other aging centers, researchers, and practitioners navigating these professional and ethical tensions.

Abstract citation ID: igaf122.614

**SUSTAINING A RESEARCH CENTER ON AGING: STRATEGIES FOR GROWTH, RESILIENCE, AND IMPACT IN CHALLENGING TIMES**

Patricia Heyn, *Marymount University, Arlington, Virginia, United States*

Establishing and sustaining a research center on aging requires strategic vision, institutional support, and adaptability, especially during times of financial uncertainty, shifting institutional and federal priorities, and evolving research landscapes. The Center for Optimal Aging at Marymount University was launched with the goal of fostering interdisciplinary research, enhancing community engagement, and developing mentoring and leadership training models for a diverse aging research workforce. However, launching a new aging center among many funding challenges, administrative turnover, and shifting expectations for research productivity presents unique obstacles. This presentation will outline the critical steps, challenges, and opportunities involved in developing a new research center on aging, including: 1) Strategic planning and institutional alignment – positioning the center's mission within the broader university and external funding landscape; 2) Building research infrastructure – developing administrative and faculty support for interdisciplinary research while navigating institutional and federal constraints; 3) Sustainability and funding challenges – balancing internal and external funding mechanisms, fostering collaborations, and managing shifting priorities; and 4) Opportunities for growth – leveraging partnerships, faculty mentorship, students and early-career researcher engagement to enhance research capacity. By reflecting on lessons learned in launching and leading a research center in today's uncertain academic and political environment, this session will provide practical strategies for creating and establishing a new center while positioning the center for long-term impact and success.

### SESSION 2075 (SYMPOSIUM)

Abstract citation ID: igaf122.615

**POLICY SERIES: HEALTH AND AGING POLICY FELLOWS: BRINGING TOGETHER AGING AND POLICY**

Chair: Maureen Henry
Discussant: Priti Patel

The Health and Aging Policy Fellows is a program that has trained more than 200 people through a one-year fellowship that includes an intensive training in Washington DC and a placement in Washington, within a state or local government, or with a non-profit organization. Fellows are selected from across career stages, disciplines, and emphasis within aging. Presentations will describe how policy can be used to solve discrete structural challenges that affect older persons, such as unpredictable and non-transparent medication prices or direct care worker shortages. Another presentation will report about processes of collecting and analyzing data to create a national plan for aging. The final presentation will describe aspects of the appropriations process in Congress. Together, presentations will show a breadth of approaches to tackling aging policy challenges.

Abstract citation ID: igaf122.616

**IMPROVING ACCESS TO CARE THROUGH OUT-OF-POCKET PRICE TRANSPARENCY POLICY**

Caroline Sloan, *Duke University School of Medicine, Durham, North Carolina, United States*

Technology Policy (ASTP) in the Department of Health and Human Services (DHHS) provides an opportunity to advance federal out-of-pocket price transparency regulation. As a primary care physician, I have long been frustrated by the inability to predict the costs of the medications I send to the pharmacy, limiting my ability to choose lower-cost and equally effective treatment options for my patients. In 2019, Medicare issued a regulation requiring Medicare Part D plans to provide "timely and accurate" out-of-pocket cost estimates to prescribers, via real-time benefit tools (RTBT). RTBTs are applications embedded in the electronic health records (EHR) that automatically display out-of-pocket cost estimates for prescription medications, prior authorization requirements, and any available lower-cost alternatives. Early research has found that patients whose clinicians use RTBTs are more likely to fill their prescriptions at the pharmacy and to save money on those prescriptions. However, clinicians at a third of US healthcare organizations do not have access to RTBTs. I am working on a team at ASTP that will update the RTBT regulation to account for some of the limitations in RTBT access and functionality encountered so far. Learnings will include: 1) understanding the rule-making process; 2) learning how to synthesize and respond to public comments from a variety of stakeholders; and 3) leveraging my clinical experience as an RTBT user and my research experience evaluating the impact of RTBTs on access to medications and clinical outcomes among adults with multimorbidity.

Abstract citation ID: igaf122.617

**MAKING THE ASK: BEST STRATEGIES FOR ADVOCACY AND APPROPRIATION REQUESTS**

Tracy Fasolino, *Clemson University, Clemson, South Carolina, United States*

'Waste, fraud, and abuse' are the main narratives ringing throughout the 119th congressional corridors, regardless of political affiliation or party alignment. Research, education, and public health are posed for significant revisions in staffing and services, and the consequences will likely be felt for years. The traditional strategies for seeking federal funding and support will fail to produce the anticipated outcome given this season of governmental cuts. New approaches for 'making the ask' on behalf of advocacy groups and during appropriation season will be necessary. As a Health & Aging Policy Fellow Residential Track with Senator Tim Kaine (D-VA), Dr. Tracy Fasolino witnessed first-hand the rapid changes during these unprecedented times with the flurry of Executive Orders and the dismantling of many executive branch organizations. She gained valuable insight into the best strategies for engaging congressional staff while advocating for a particular cause or initiative. During appropriation season, she developed a keen understanding of the process and can offer some insights on how to reframe the narrative. She will share new approaches for presenting requests in a bipartisan and bicameral approach beyond the typical data analytics and storyboarding. As the new administration continues with budgetary cuts, fresh approaches and restructured narratives will be necessary.

Abstract citation ID: igaf122.618

**AGING FORWARD: BUILDING A STRONGER NATION FOR OLDER AMERICANS**

Samsun Naher, *University of Washington, Seattle, Washington, United States*

Older adults make invaluable contributions to society, not only through their cultural, economic, social, and political impact during their careers but also through caregiving, mentorship, and volunteerism after retirement. However, as they age, they face significant challenges that require thoughtful policy interventions. As a health economics and health services researcher, I am honored to be a part of the Health and Aging Policy Fellowship (HAPF) program. Through this fellowship, I have the opportunity to work with the Administration for Community Living (ACL) on the Interagency Coordinating Committee (ICC) on Healthy Aging and Age-Friendly Communities, which seeks to develop policy solutions that promote healthy aging, improve coordination at all levels of government, enhance service delivery, and eliminate barriers to independence for older adults. In collaboration with the 18 federal agencies and departments included in the ICC, my team is assessing the feasibility of the Strategic Framework for the National Plan on Aging and identifying the most crucial areas for focus at this time. This evaluation is based on feedback collected from multiple sources, including individual and organizational responses via the West Health Input Portal, Gallup polling, listening sessions with older adults in AL, AZ, PA, TX, and Washington, DC, community roundtables, and conference discussions within the aging services network. My role involves synthesizing this feedback, identifying policy gaps, and formulating recommendations to support age-friendly communities. In this presentation, I will share key findings, policy challenges, and proposed solutions to advance a more inclusive and supportive aging policy framework.

Abstract citation ID: igaf122.619

**STRENGTHENING THE DIRECT CARE WORKFORCE: POLICY AND PRACTICE SOLUTIONS FOR SUSTAINABLE AGING SERVICES**

Julie Lowenthal, *Ageways Nonprofit Senior Services, Berkley, Michigan, United States*

The direct care workforce (DCW) is essential to aging services, yet persistent workforce shortages, high turnover (40-60% annually), and low wages threaten its stability. Economic insecurity, limited career advancement, inadequate training, and transportation barriers—key social determinants of health (SDOH)—exacerbate these challenges, impacting worker retention and care quality. This paper explores workforce trends, the intersection of SDOH, and evidence-based strategies to improve workforce sustainability. Key solutions include increasing Medicaid reimbursement rates to support wage growth, expanding training and credentialing opportunities, and leveraging public-private partnerships to enhance recruitment and retention. State-level case studies demonstrate that wage increases, career ladders, and technology-driven workforce innovations lead to improved job satisfaction and retention. As the U.S. population aged 65+ surpasses 70 million by 2030, a stable direct care workforce is critical to meeting long-term care demands. This research provides policy recommendations for strengthening workforce recruitment, retention, and career development to ensure high-quality care for older adults and individuals with disabilities.

### SESSION 2080 (SYMPOSIUM)

Abstract citation ID: igaf122.620

### EXAMINING THE STRUCTURE AND UTILITY OF THE EASE: ENVIRONMENTAL AUDIT SCORING EVALUATION TOOL

Chair: Margaret Calkins
Co-Chair: Migette Kaup

The designed environment is especially important for individuals with Alzheimer's Disease and related dementias, but validated tools to assess physical environments lag behind theory and practice and omit best design practices. The Environmental Audit Scoring Evaluation tool (EASE) is a unique assessment tool for long-term care settings that is: 1) evidence-based, 2) dementia-inclusive (for both segregated and integrated living areas), and 3) specifically inclusive of household model settings. Founded on the principles of person-centered care practices, the tool specifically targets environmental characteristics that distinguish the values of residential living over institutional routines. The tool was administered in 228 living areas across US and Canada, including nursing homes, assisted living and memory care. The first paper addresses the analysis of the factorial structure of the EASE tool. The second paper examines environmental characteristics that differentiate between traditional versus person-centered care settings. The third paper explores the relationship between EASE evaluations and resident function as assessed with the MDS, identifying associations between environmental characteristics and outcomes of interest. The final paper explicates the use of the EASE as a tool to inform the process of change in seven traditional care settings seeking to become more person-centered.

Abstract citation ID: igaf122.621

### EXAMINING THE PSYCHOMETRIC PROPERTIES AND FACTOR STRUCTURE OF EASE

Adam Davey[1], Margaret Calkins[2], Migette Kaup[3], and Robert Wrublowsky[4], *1. University of Delaware, Newark, Delaware, United States, 2. IDEAS Institute, Cleveland Heights, Ohio, United States, 3. Kansas State University, Manhattan, Kansas, United States, 4. RAW Consulting, Winnipeg, Manitoba, Canada*

Initial information about psychometric properties of EASE subscales as well as for the overall instrument will be discussed. Development of the tool has included initial determination of face-validity, followed by establishing construct validity ($r = 0.88$ with EAT tool), and interrater agreement ($r = 0.97$). With the administration of the EASE tool in 228 living areas (e.g., units), parallel analysis was used to investigate the structure of the 130 item EASE instrument. 12 components were initially identified, and several additional component analyses were examined to determine the most efficient structure. Results describe the structure of the EASE instrument with regard to living area characteristics, particularly focusing on items that discriminate between different models (traditional, household and hybrid). Recognition of the dual purposes of the EASE tool as both a design planning resource and as a tool for comparative research across multiple settings, different potential structures will be discussed.

Abstract citation ID: igaf122.622

### LINKING ENVIRONMENTS AND RESIDENT FUNCTION VIA MDS

Migette Kaup[1], Adam Davey[2], Margaret Calkins[3], and Robert Wrublowsky[4], *1. Kansas State University, Manhattan, Kansas, United States, 2. University of Delaware, Newark, Delaware, United States, 3. IDEAS Institute, Cleveland Heights, Ohio, United States, 4. RAW Consulting, Winnipeg, Manitoba, Canada*

Over the past three decades, research in long-term care settings has demonstrated links between design characteristics and the experiences of residents who live in these environments. Individual studies have shown how different approaches to the design of the built environment can contribute to enhanced resident safety, function, and even care outcomes but these findings have been difficult to generalize due to the lack of a validated and comprehensive assessment tool that provides a consistent metric for the discrete environmental characteristics that may be contributing factors. In addition, existing environmental tools have limited demonstrated connections to other widely used assessment tools for care outcomes such as the Minimum Data Set (MDS) the standard for facilitating care management in nursing homes. The Environmental Audit Scoring Evaluation (EASE) tool has been shown to provide a reliable set of comprehensive scoring criteria that can measure the strengths (or weaknesses) of specific design features. Further, the EASE tool items are comprised of features that specifically support quality outcomes measures that are also of focus of the MDS. In this paper, we describe the study design to link the MDS assessments of 235 residents to the EASE assessments of their (15) distinct living areas across seven skilled care buildings.

Abstract citation ID: igaf122.623

### USING A STRUCTURED ENVIRONMENTAL ASSESSMENT TOOL OF INFORM CHANGE UNDER CONSTRAINTS: ACTION MARGUERITE

Robert Wrublowsky[1], and Migette Kaup[2], *1. RAW Consulting, Winnipeg, Manitoba, Canada, 2. Kansas State University, Manhattan, Kansas, United States*

One goal of the EASE is to assist care providers who have older, traditional buildings determine how to best utilize scarce resources when considering renovations that will have the most positive impact on residents, families and staff. Analysis of differences between settings that clearly reflect traditional design elements (long, double loaded corridors and minimal space for meaningful social engagement) and settings that have adopted some person-centered care values identify smaller scale and the presence of a functional kitchen as key factors. Both of these involve significant operational as well as environmental changes and can be met with resistance. This session describes the process of working with a care provider that had seven highly traditional living areas assessed with the EASE which resulted in identification of specific recommendations to reduce the size of living areas and include functional kitchens. The evidence-based nature of the EASE supports constructive responses to perceived barriers to adoption of changes that require operational changes. The EASE also identifies numerous low-cost interventions that can be easily adopted allowing for a phased implementation process.

Abstract citation ID: igaf122.624

### USING EASE TO IDENTIFY CHARACTERISTICS THAT DISTINGUISH TRADITIONAL VERSUS PERSON CENTERED LIVING AREAS

Maggie Calkins[1], Adam Davey[2], Migette Kaup[3], and Robert Wrublowsky[4], *1. IDEAS Institute, Cleveland Heights, Ohio, United States, 2. University of Delaware, Newark, Delaware, United States, 3. Kansas State University, Manhattan, Kansas, United States, 4. RAW Consulting, Winnipeg, Manitoba, Canada*

The Environmental Audit Screening Evaluation tool (EASE) is a unique assessment tool which was administered in 228 living areas across US and Canada. One-way analysis of variance was used to estimate eta-squared values as indicators of effect sizes between settings that were identified as being either traditional or homelike. Elimination of items with eta-square values 1.0 or less resulted in 39 items that maximally distinguished between different types of settings. These 39 items were subjected to parallel analysis which generated a 4-component solution that provided high loadings with few cross-loadings. Specifically, this subset of EASE items appeared to assess: 1) residential and familiar character (14 items) (e.g., kitchen design appears residential in style, and required institutional features (such as exhaust vents) are disguised) 2) orientation and functional supports (15 items) (e.g., doors to dangerous storage largely out of sight) 3) human scale and intuitive layout (8 items) (e.g., bedrooms close to social spaces) and 4) autonomy in daily living (8 items) (e.g., showers allow privacy and independence). The value and use of this subset of items as a tool in multi-site research will be discussed.

### SESSION 2085 (SYMPOSIUM)

Abstract citation ID: igaf122.625

### AGING VETERANS: PHYSICAL HEALTH, MENTAL WELL-BEING, AND SOCIAL CONNECTIONS

Chair: Janet Wilmoth

Although the aging veteran population has received more scholarly attention in recent years, gerontologists continue to disentangle the confounding and sometimes countervailing influence of military service on later-life outcomes. This symposium provides insights into the physical health and mental well-being of aging veterans, while also offering insight into strategies for promoting social connections among rural veterans. Serier and colleagues, using a sample of Vietnam era veterans, find post-traumatic stress disorder (PTSD) is associated with hypertension and hypertension treatment among male veterans but not female veterans. London et al. estimate the joint influence of veteran status and PTSD on cardiovascular disease (CVD) risk with data from the National Wellbeing Survey. The results demonstrate increased CVD risk for veterans and those with PTSD, with the highest risk observed among veterans with PTSD. Marini and colleagues report findings from a primary data collection focused on how daily emotions are impacted by thoughts. They find between-person and within-person associations between thinking and talking about service experiences, rumination, and negative affect. Using Veteran Affairs healthcare system data, Guneet et al. find rural veterans are more likely to have a cognitive impairment diagnosis and are more likely to receive dementia medication, even prior to a pre-dementia or dementia diagnosis. Pless Kaiser and colleagues discuss a social group intervention designed to improve social connections among older rural Veterans with PTSD. They describe implementation of the program at two sites in Montana and North Carolina then offer suggestions for program dissemination.

Abstract citation ID: igaf122.626

### POSTTRAUMATIC STRESS DISORDER AND HYPERTENSION IN VIETNAM VETERAN MEN AND WOMEN

Avron Spiro[1], Kelsey Serier[2], Hannah Burns[2], Rachel Kimerling[3], Anica Pless Kaiser[2], Kathryn Magruder[4], and Brian Smith[5], *1. Boston University - Chobanian & Avedisian School of Medicine, Waban, United States, 2. VA Boston Health Care System, Boston, Massachusetts, United States, 3. VA Palo Alto Health Care System, Palo Alto, California, United States, 4. Medical University of South Carolina, Charleston, South Carolina, United States, 5. National Center for PTSD Women's Health Sciences Division, Boston, Massachusetts, United States*

Hypertension is a risk factor for cardiovascular disease, a leading cause of death for older adults. Posttraumatic stress disorder (PTSD) may increase the likelihood of developing hypertension. However, this association has seldom been studied in older adults and infrequently among veterans. The present study used data from two cohorts of older adult Vietnam era veterans: over 4104 women (Mage= 67 years) and 5767 men Mage= 62 years). Respondents completed by telephone a structured clinical interview assessing lifetime PTSD. Information on self-reported hypertension diagnosis, age of onset, and past-year treatment, as well as on covariates was obtained by mail survey. Weighted logistic regression analyses adjusted for relevant covariates revealed an association between PTSD and higher likelihood of hypertension in male veterans (OR = 1.57; 95% CI [1.30, 1.91]). There was no association between lifetime PTSD and hypertension in female veterans (OR = 0.93; 95% CI [0.78, 1.11]). Exploratory secondary analyses suggested an association between PTSD and hypertension onset in early and middle adulthood only in men. PTSD was not associated with past-year hypertension treatment for women or men. Overall, these findings suggest that PTSD may contribute to hypertension risk in older male veterans, but no contribution was found for female veterans. Prospective research is needed to confirm these findings and further clarify the relationship between PTSD and hypertension to inform veteran clinical care.

Abstract citation ID: igaf122.627

### THE JOINT ASSOCIATION OF VETERAN AND POST-TRAUMATIC STRESS DISORDER ON RISK OF CARDIOVASCULAR DISEASES

Andrew London[1], Jacob DeBlois[2], and Kevin Heffernan[3], *1. Syracuse University, Maxwell School of Citizenship and Public Affairs, Syracuse, New York, United States, 2. University of Rhode Island, Kingston, Rhode Island, United States, 3. Columbia University, Teachers College, New York, New York, United States*

Identifying subpopulations at elevated risk of cardiovascular disease (CVD) is important for preventive purposes. We use pooled data from the 2021-2023 National Wellbeing Survey (NWS) (N = 17,257) to examine the joint influence of veteran and post-traumatic stress disorder (PTSD) statuses on high blood pressure, high cholesterol, diabetes, and heart disease diagnosis by a health care professional among working-age adults. The NWS uniquely measures PTSD among veterans and non-veterans. In well-controlled multivariable logistic regression analyses, relative to non-veterans without PTSD (henceforth the reference group), the odds of high blood pressure are significantly higher among non-veterans (adjusted odds ratio [AOR]=1.48, p<.001) and veterans with PTSD (AOR=1.63, p<.01), and post-hoc tests indicate that both of these groups also have significantly higher odds of high blood pressure than veterans without PTSD. For high cholesterol, the odds are significantly higher among non-veterans with PTSD (AOR=1.33, p<.001) and both veterans without (AOR=1.24, p<.05) and with PTSD (AOR=1.51, p<.01) relative to the reference group. For diabetes, both veterans without (AOR=1.36, p<.01) and with PTSD (1.72, p<.010) have higher odds relative to the reference group, and veterans with PTSD have significantly higher odds than non-veterans with PTSD. For heart disease, the odds are higher for non-veterans with PTSD (AOR=1.90, p<.001), and both veterans without (AOR=1.38, p<.05) and with PTSD (AOR=2.44, p<.001) relative to the reference group, and veterans with PTSD have significantly higher odds than veterans without PTSD. These results suggest that both veteran and PTSD statuses contribute to CVD risk, and that their influences are at times synergistic.

Abstract citation ID: igaf122.628

### IT'S BEEN ON MY MIND: AGING VETERANS' DAILY THOUGHTS OF MILITARY SERVICE, RUMINATION, AND NEGATIVE AFFECT

Christina Marini, Katherine Fiori, Carly Lawrence, and Amanda Emma, *Adelphi University, Garden City, New York, United States*

Aging veterans may be more likely to think about their prior military service due to age-related changes, such as bereavement and declining physical health. Such losses might trigger intrusive memories about one's prior service, which may lead to distress (Davison et al., 2016). Veterans may also experience increased distress on days when they ruminate more about their current troubles/worries (Kashdan et al., 2012). This study therefore examined the degree to which veterans a) thought/talked about their prior service and b) ruminated about their current worries, and whether either was associated with their daily negative affect. The sample included 68 veterans (M age = 74.75, SD = 5.58) who completed a baseline questionnaire and up to 7 (and at least 4) end-of-day surveys. Most were male (95.6%), white/Caucasian (95.6%), married (92.6%), retired (79.4%), and served during the Vietnam era (79.4%). More than half (58.8%) completed at least one combat/warzone tour. After controlling for age and number of combat/warzone tours, findings from a series of multi-level models indicated that veterans who on average across days a) think/talk about their service more and b) ruminate more experience higher negative affect (i.e., between-person associations). Furthermore, a significant within-person association indicated that on days when veterans ruminate more than they usually do, they experience higher negative affect. Additionally, veterans who ruminate more on average tend to think/talk more about their service. These findings suggest that aging veterans' affective well-being is tied to their active processing of both past service-related experiences and current worries.

Abstract citation ID: igaf122.629

### RURAL-URBAN DIFFERENCES IN COGNITIVE IMPAIRMENT AND MEDICATION PRESCRIBING PRE- DEMENTIA DIAGNOSIS

Guneet Jasuja[1], Heather Davila[2], Haijuan Yan[3], Mark Zocchi[3], Michelle Hilgeman[4], Hilary Mosher[5], Tammy Walkner[6], and Lauren Moo[7], *1. Center for Health Optimization and Implementation Research, VA Bedford, Boston, Massachusetts, United States, 2. Iowa City VA Healthcare System, Iowa City, Iowa, United States, 3. Center for Health Optimization and Implementation Research, VA Bedford Healthcare System, Bedford, Massachusetts, United States, 4. Tuscaloosa VA Medical Center, Tuscaloosa, Alabama, United States, 5. Minneapolis VA Medical Center, Minneapolis, Minnesota, United States, 6. Iowa City VA Health Care System, Iowa City, Iowa, United States, 7. VA Bedford Healthcare System, Bedford, Massachusetts, United States*

Documentation of a dementia diagnosis provides access to a pathway of evidence-based treatment. However, delayed diagnosis is common, particularly in rural areas with limited access to dementia specialists, often resulting in documentation of less specific cognitive impairment and prescribing of dementia medications pre-dementia diagnosis. We described patterns of rural-urban differences in cognitive impairment and dementia medication prescribing pre-dementia diagnosis in the Veterans Affairs (VA), the largest integrated healthcare system in the U.S. We identified veterans ≥at least 50 years old with two ICD-10-CM dementia diagnoses, with first diagnosis in 2022 or 2023. Documentation of diagnoses of mild cognitive impairment (MCI), amnesia/memory loss, and receipt of dementia medications were compared among rural and urban veterans in the two years preceding the first dementia diagnosis. In 2022-23, 89,482 veterans had a first dementia diagnosis (mean age: 79 years, 97% men). Of these 89,482 veterans, 67% resided in urban vs. 33% in rural areas, with 43% living in the South. More urban vs. rural veterans had documentation of MCI (20% vs. 17.5%) pre-dementia diagnosis. As expected, more rural Veterans had documentation of an amnesia/memory loss (15% vs. 13%), received a dementia medication (12% vs. 10%), and received a dementia medication without documentation of MCI or amnesia/memory loss pre-dementia diagnosis (5.1% vs. 4.2%) (p < 0.01 for all comparisons above). Particularly for rural veterans, dementia-targeting treatment often began well before dementia diagnosis. Whether these patterns reflect delays in formally classifying cognitive impairment as dementia, gaps in access to specialists or other factors should be further explored.

Abstract citation ID: igaf122.630

### DISSEMINATION AND IMPLEMENTATION OF A SOCIAL GROUP INTERVENTION FOR OLDER RURAL VETERANS WITH PTSD

Anica Pless Kaiser[1], Jennifer Daks[1], Jay Gregg[2], Aaron Stusser[3], Annika Goldman[2], Shawn Dunlap[4], Madison Strouse[5], and Jennifer Moye[1], *1. VA Boston Health Care System, Boston, Massachusetts, United States, 2. Durham VA Medical Center, Durham, North Carolina, United States, 3. Montana VA Health Care System, Fort Harrison, Montana, United States,*

*4. VA Bedford Health Care System, Bedford, Massachusetts, United States, 5. VA Bronx Healthcare System, New York, New York, United States*

Older rural Veterans confront greater social isolation and health comorbidities, and have lower access to healthcare than Veterans in urban areas. Additionally, older rural Veterans with posttraumatic stress disorder (PTSD) are at elevated risk for social isolation, loneliness, and low social support. The purpose of this VA Office of Rural Health funded project was to disseminate and evaluate an 8-9 week group intervention "Enhancing Social Function for Older Veterans with PTSD" (ESVP), delivered in-person or virtually. Dissemination proceeded in several steps. (1) We identified potentially interested clinicians via 10-minute presentations at regional VA Geriatric Mental Health Champion-led meetings. (2) We provided 60-minute trainings to six clinicians, offering co-facilitation and consultation. (3) We partnered with behavioral health clinicians serving older rural Veterans at two sites. At the Montana site, our team co-facilitated two virtual ESVP groups (N = 9 Veterans) with Veterans from Montana, South Dakota, and Massachusetts. At the North Carolina site, clinicians co-facilitated one in-person ESVP group (N = 4 Veterans). (4) Leaders provided week by week feedback during consultation calls. Across the three groups, average session attendance was high (79%), despite initial technological difficulties in the virtual groups. Veterans reported modest gains in social function and decreased depressive symptoms pre to post group and provided positive qualitative feedback and suggestions for improvement. Clinicians reported high applicability (M = 4.9/5), feasibility (M = 4.4/5), and helpfulness (M = 4.8/5) of ESVP, and provided valuable feedback to inform future efforts to disseminate ESVP to older Veterans in rural areas.

### SESSION 2090 (SYMPOSIUM)

Abstract citation ID: igaf122.631

### JOY IN LONG TERM SERVICES AND SUPPORTS CAREGIVING

Chair: Nathan Boucher
Co-Chair: Lillian Hung

Retaining and empowering staff in long-term services and supports (LTSS) is essential for sustaining quality care. Despite high demands, staff often receive low wages and limited recognition, making workplace culture, professional growth, and well-being critical factors in job satisfaction/retention. This symposium presents findings from three qualitative studies examining staff experiences in LTSS, highlighting factors fostering retention, resilience, and joy in caregiving. The first study explored retention factors among Canadian LTSS staff through focus groups, identifying three key themes: (1) professional growth and development through training and mentorship, (2) recognition and workplace culture that foster team cohesion and appreciation, and (3) relational joy in caregiving, where meaningful connections with residents and colleagues enhanced resilience. The second study analyzed essays from LTSS clinicians reflecting on moments of joy in their work. Content analysis revealed that joy stemmed from (1) building relationships through residents' unique life stories, (2) teamwork that fostered belonging, and (3) shared decision-making that supported person-centered, end-of-life care. Despite workforce shortages, clinicians found fulfillment through narrative medicine, celebrating direct care staff, and engaging in palliative care discussions. The third study examined experiences of LTSS administrators during the COVID-19 pandemic. Findings underscored the importance of communication, collaboration, and relational leadership in sustaining staff morale. Innovative communication strategies helped alleviate isolation and reinforced a sense of team belonging. Collectively, these studies in Canada and the US underscore the need for evidence-based retention strategies that prioritize professional development, workplace recognition, and relational well-being to sustain and strengthen the joy of the LTSS workforce.

Abstract citation ID: igaf122.632

### JOY AT A VANCOUVER LTSS FACILITY: IMPLICATIONS FOR STAFF RETENTION

Lillian Hung[1], Nathan Boucher[2], Peter Zhou[3], and Joey Oi Yee Wong[1], *1. University of British Columbia, Vancouver, British Columbia, Canada, 2. Duke University, Durham, North Carolina, United States, 3. The University of British Columbia, Vancouver, British Columbia, Canada*

Retaining and empowering caregivers in long-term care (LTC) is critical to sustaining quality care. Despite high demands, long term care staff often receive low wages and limited societal recognition. A positive workplace culture that fosters professional growth, recognition, and well-being is essential for caregiver retention and job satisfaction. This qualitative study explored factors that contribute to staff retention among LTC staff. Four focus groups were conducted with 24 staff, including nurses, care workers, housekeeping, recreation, and rehabilitative staff, in a Canadian LTC home. Data were analyzed thematically to identify key workplace factors influencing staff retention. Three overarching themes emerged: (1) Professional Growth and Development – Staff emphasized the importance of training programs, career advancement pathways, and mentorship in fostering motivation and fulfillment. Continuing education and specialization opportunities contributed to sustained engagement. (2) Recognition and Workplace Culture – Appreciation programs, team cohesion, and a culture of respect and dignity were central to job satisfaction. Feeling valued and included promoted a sense of belonging. (3) Relational Joy in Caregiving – Joy was described as relational and evolving, emerging from supportive environments, positive attitudes, and shared experiences with colleagues and residents. A culture that prioritizes well-being and emotional support reduced burnout and enhanced resilience. In conclusion, retention strategies should be evidence-based, drawing from best practices and ongoing evaluations to measure effectiveness and return on investment. Infusing joy into caregiving strengthens team resilience and fosters compassion, ultimately benefiting both caregivers and residents.

Abstract citation ID: igaf122.633

### WHAT BRINGS JOY TO LTC PROVIDERS: VIEW FROM THE EDITORS' DESK

Elizabeth Galik[1], and Tess Bird[2], *1. University of Maryland School of Nursing, Baltimore, Maryland, United States, 2. Elsevier Publishing, Philadelphia, Pennsylvania, United States*

Recruiting and retaining competent and caring post-acute and long-term care (PALTC) clinicians is critical for quality and safe patient care. Identification and promotion of activities that foster joy in work for PALTC clinicians is protective against burnout which has increased in severity and prevalence since the COVID-19 pandemic. Qualitative methods were used to analyze ten essays solicited by and submitted to Caring for the Ages, a publication of the Post-Acute and Long-Term Care Medical Association (PALTmed). Authors were asked to write in the first person about a work experience in PALTC that made them feel good and bring joy to their work as clinicians. Essays were analyzed using content analysis. Three themes were identified: (1) Building relationships through unique life stories; (2) Collaborating as a team to provide a sense of belonging and meaningful engagement among residents and staff; and (3) Fostering shared decision making that supports person centered care at the end of life. Disciplines that were represented included medicine, nursing and social work. Despite challenges of increasing workload and staffing shortfalls, clinicians may be able to increase joyful experiences in their clinical practice in PALTC through engagement in narrative medicine, supporting and celebrating the dedication of direct care staff, and recognizing the importance of shared medical decision making, particularly within a palliative care context.

Abstract citation ID: igaf122.634

### ADMINISTRATOR PERSPECTIVE ON JOY IN THE WORKPLACE AND WORKER RETENTION

Sandi Lane[1], and Darren Liu[2], *1. The University of North Carolina at Charlotte, Charlotte, North Carolina, United States, 2. West Virginia University, Morgantown, West Virginia, United States*

Long-term care administrators faced unprecedented challenges during the COVID-19 pandemic while striving to provide quality care and maintain staff morale. Our qualitative study examined the experiences of 21 administrators from North Carolina (n = 15) and Pennsylvania (n = 6) through semi-structured interviews. The research revealed that serving as an administrator during the pandemic was emotionally draining, stressful, and challenging. Communication and relationship-building emerged as the most valuable competencies for effective leadership. Administrators who fostered collaboration among staff and residents positively influenced workplace culture and enhanced problem-solving capabilities. The psychological well-being of staff was directly linked to support mechanisms, particularly caring relationships within the facility. Despite the difficulties, 18 of the 21 participants remained in their roles, describing their work as "a calling," while three administrators left the field. Innovative communication strategies helped combat isolation, including virtual visits with colleagues, daily voicemail updates, and social media use to reassure families. For administrators, feeling valued and part of a team proved crucial in managing the challenges of caring for residents and staff during this crisis. The study also highlighted the importance of enhancing the public image of the long-term care industry to drive meaningful change and improve experiences for both staff and administrators.

### SESSION 2095 (SYMPOSIUM)

Abstract citation ID: igaf122.635

### EMOTION REGULATION ACROSS ADULTHOOD: NEW RESEARCH DIRECTIONS

Chair: Claudia Haase
Discussant: Joseph Mikels

Emotion regulation plays an important role in late-life well-being and health. While existing research has yielded important insights, numerous questions remain open. This symposium brings together four contributions to elucidate new directions in emotion regulation research across adulthood – featuring diverse emotion regulation and coping processes (e.g., acceptance, avoidance), contexts (e.g., daily stressors, repeated social stress exposure, caregiving), and study designs (e.g., laboratory-based, survey-based, daily diary, and longitudinal approaches). Dr. Haase and colleagues will present findings from a research program on acceptance (i.e., embracing emotions and thoughts without judgment). Laboratory-based and survey-based findings show that acceptance benefits physiological and mental health and becomes increasingly important in late life. Dr. Charles and colleagues will share insights into belonging from a large-scale national study of daily experiences in midlife and later adulthood. Findings show that belonging is associated with lower negative affect and buffers the effects of daily stressors on negative affect. Dr. Luong will present insights from a laboratory-based study with younger and older adults who were repeatedly exposed to social stress. While older adults use effective emotion regulation strategies (i.e., cognitive reappraisal) more frequently, they also show greater affective reactivity to repeated social stress exposure. Gad and colleagues will share findings from a longitudinal study with adult-child caregivers of parents with memory loss. Findings show that avoidant coping predicts greater caregiver burden concurrently and longitudinally, but caregivers who experience more positive emotions relative to their negative emotions may be buffered. The symposium will conclude with a discussion from Dr. Mikels.

Abstract citation ID: igaf122.636

### NEW INSIGHTS INTO ACCEPTANCE ACROSS ADULTHOOD

Claudia Haase[1], Lillian Fu[2], Chen-Wei Yu[1], David Rompilla[3], and Michael Kisley[4], *1. Northwestern University, Evanston, Illinois, United States, 2. University of California, Irvine, Evanston, Illinois, United States, 3. Texas A&M University, College Station, Texas, United States, 4. University of Colorado Colorado Springs, Colorado Springs, Colorado, United States*

Acceptance (i.e., embracing emotions and thoughts without judging them) is an emotion regulation strategy that may become particularly important in late life. While research on other strategies (e.g., positive reappraisal) has been blossoming, we know surprisingly little about acceptance. We present findings from a research program on acceptance that combines laboratory-based and survey-based approaches. Findings from our laboratory-based research show that acceptance of negative emotions (a) is preferred over other strategies (i.e., detachment, positive reappraisal), (b) predicts greater vagal reactivity during instructed emotion regulation (for habitual acceptance), and (c) predicts greater mental health, especially when executive functioning is constrained in older adults (N = 129, age 64-83). In a new line of survey-based research, we examine age differences in acceptance, distinguishing between the acceptance and rejection of positive and negative emotions. Across three studies with age-diverse samples (N = 416, 402, 412; age range: 18-87), findings show that (a) older adults are less likely to reject positive emotions and (albeit less consistently) more likely to accept positive emotions compared to younger adults. Moreover, (b) while no consistent age effects are found for negative emotions broadly, clear age differences emerge for the acceptance and rejection of anger and sadness specifically. Older adults (65+) are less likely than younger adults (18-35) to reject anger and sadness and more likely to accept anger and sadness (with sadness showing significantly steeper age gradients than anger). Together, these findings underscore the important role that acceptance plays in late life and contribute to longstanding discussions about emotion regulation across adulthood.

Abstract citation ID: igaf122.637

**UNDER PRESSURE, BUT TOGETHER: STRESSOR REACTIVITY AND DAILY SOCIAL CONNECTION IN MIDDLE AND LATER ADULTHOOD**

Susan Charles[1], Eric Cerino[2], Jennifer Piazza[3], David Almeida[4], and Jonathan Rush[5], *1. University of California, Irvine, Irvine, California, United States, 2. Northern Arizona University, Flagstaff, Arizona, United States, 3. California State University, Fullerton, Fullerton, California, United States, 4. The Pennsylvania State University, University Park, Pennsylvania, United States, 5. University of Victoria, Victoria, British Columbia, Canada*

Social belonging has long been conceptualized and studied as a stable individual difference characteristic, shaped by temperament, personality, and childhood experiences. In this talk, we will discuss how feelings of social connection vary on a daily basis, and how these variations are related to stressor reactivity. Using eight days of daily diary data from the National Study of Daily Experiences (N = 2,022), we examine how daily social connection is related to negative affect experienced in response to a stressor in a national sample of midlife and later adulthood. Each day, participants reported whether they had experienced a daily stressor (e.g., an argument, a stressor at work, a stressor at home), and the extent to which they felt a sense of belonging across the past 24 hours. We used multilevel modelling to examine how levels of daily social belonging were related to changes in negative affect on days when people responded to stressors, after adjusting for average levels of positive affect, stressor exposure, and parental warmth. In a model examining the main effects, greater sense of belonging was related to lower levels of negative affect ( $\gamma$ =-.022, p<.001), and stressor exposure was related to higher negative affect ($\gamma$ =.127, p<.001). However, the addition of the within-person interaction indicated that negative affect was lower on stressor days when people felt a higher sense of belonging ($\gamma$ = -0.065, p<.001). Results suggest that daily connection is important for well-being and how we respond to stressors in daily life.

Abstract citation ID: igaf122.638

**HERE WE GO AGAIN: AGE DIFFERENCES IN ADAPTING EMOTION REGULATION STRATEGIES ACROSS REPEATED STRESSORS**

Gloria Luong, *Colorado State University, Fort Collins, Colorado, United States*

After decades of research on emotion regulation and aging, questions remain regarding for whom and under which conditions we observe age-related advantages or disadvantages in emotion regulation in response to stressors. The current research investigated the degree to which younger and older adults adapt their emotion regulation strategies across repeated exposures to a standardized psychosocial laboratory stressor, the Trier Social Stress Test (TSST). In the Health and Daily Experiences (HEADE) study, 244 participants (138 younger adults, 106 older adults) completed 3 administrations of the TSST across three different days over one week. Participants reported their positive and negative affect at baseline and immediately after the task, as assessments of their affect reactivity to the stressor. They also rated the degree to which they used a variety of emotion regulation strategies during the task. Results revealed that although at the first lab session we found no age differences in positive or negative affect reactivity, these age differences widened with repeated stressor exposures such that older adults showed elevated affect reactivity over younger adults. Moreover, we found that although cognitive reappraisal was an effective strategy for reducing positive and negative affect reactivity and older adults used this strategy more frequently than younger adults at the second and third lab sessions but they still showed elevated affect reactivity. These findings suggest that even though older adults may use more effective strategies more frequently than younger adults, they may do so in an effort to downregulate their high levels of affect reactivity.

Abstract citation ID: igaf122.639

**COPING STRATEGIES, RATIO OF POSITIVE TO NEGATIVE AFFECT, AND BURDEN IN ADULT CHILDREN OF PARENTS WITH MEMORY LOSS**

Dustin Gad[1], Jenna Wells[1], Joan Monin[2], and Kristie Wood[3], 1. Cornell University, Ithaca, New York, United States, 2. Yale, Yale, Connecticut, United States, 3. University of Colorado Anschutz Medical Campus, Aurora, Colorado, United States

Caring for an individual with memory loss is associated with high levels of burden, leading to adverse health effects. Caregivers use behavioral and cognitive coping strategies to manage this stress. Approach coping strategies (e.g., seeking emotional support) are generally better for caregivers' mental health than avoidant coping strategies (e.g., denial). Engaging in less adaptive coping strategies may fuel decreases in caregivers' positive emotions and increases in their negative emotions, thereby amplifying burden. We evaluated whether caregivers' coping strategies (Brief COPE) are associated with their levels of burden (cross-sectionally and longitudinally over one year; ZBI-12); and whether these associations are moderated by ratios of positive to negative affect (calculated by dividing their reported positive emotions by their negative emotions; PANAS) in adult-child caregivers (N = 150) of parents with memory loss. In a multiple regression, greater use of approach coping (b= .14, p= .031) and avoidant coping (b= .59, p< .001) were associated with higher burden. Further, greater avoidant coping at baseline was associated with greater increases in burden one year later (b= .31, p= .001), accounting for baseline burden. No significant association was found between approach coping and changes in burden. Ratios of positive to negative affect moderated the cross-sectional association between avoidant coping and burden such that caregivers who reported lower ratios of positive to negative affect reported higher levels of burden (b= .51, p= .007). Although avoidant coping strategies are often associated with poor outcomes, caregivers who experience more positive emotions relative to their negative emotions may be buffered.

### SESSION 2100 (SYMPOSIUM)

Abstract citation ID: igaf122.640

**FRAILTY IN THE DIGITAL AGE: ADVANCING FRAILTY RESEARCH WITH WEARABLE TECHNOLOGY**

Chair: Jennifer Schrack
Co-Chair: Amal Wanigatunga
Discussant: Megan Huisingh-Scheetz

Phenotypic frailty is an established construct of health and function with aging. Generally assessed in laboratory and clinical settings, phenotypic frailty is defined using traditional measures of health and function, including self-reported low physical activity, exhaustion, and weight loss, and measured weakness and slowness. To date, there is limited evidence connecting phenotypic frailty with real-world markers of health and function. To this end, digital data from wearable sensors may provide unique insights into daily behaviors and their association(s) with frailty risk or recovery. This symposium will focus on 1) pooling frailty measures across large cohort studies to aid in quantifying and defining digital measures of physical activity by frailty status, 2) assessing how these digital physical activity measures are associated with frailty risk, and 3) understanding digital measures of interstitial glucose in prefrail older adults. Dr. Etzkorn will present methodology related to pooling frailty definitions among adults aged ≥70 years participating in BLSA, ARIC, ACHIEVE, NHATS, and STURDY. Dr. Schrack will present physical activity characteristics by frailty status in each of these studies and as overall normative data. Dr. Davoudi will present the association between features of accelerometry data from NHATS and risk of frailty at 1-year follow-up. Dr. Wanigatunga will present baseline continuous glucose monitoring data from the START sedentary behavior reduction trial. Dr. Huisingh-Scheetz will offer insights as discussant. Collectively, these presentations will provide novel insights into frailty status and risk using data from wearable digital sensors and highlight their utility for monitoring frailty-related outcomes in older adults.

Abstract citation ID: igaf122.641

### ASSESSMENT OF A PRE-STATISTICAL FRAILTY HARMONIZATION IN FIVE US-BASED STUDIES OF OLDER ADULTS

Lacey Etzkorn[1], Sunan Guo[2], Anis Davoudi[2], Brian Buta[2], Qian-Li Xue[2], Karen Bandeen-Roche[3], Amal Wanigatunga[1], and Jennifer Schrack[1], *1. Johns Hopkins Bloomberg School of Public Health, Baltimore, Maryland, United States, 2. Johns Hopkins University, Baltimore, Maryland, United States, 3. Johns Hopkins Center on Aging and Health, Baltimore, Maryland, United States*

Pre-statistical harmonization—careful comparison of study-to-study inclusion criteria, protocols, instruments, and data formats—is an essential step in consistently characterizing the Frailty Phenotype across studies. We explored a pre-statistical harmonization strategy for characterizing frailty in ACHIEVE, ARIC, BLSA, NHATS, and STURDY. Ambulatory study participants aged ≥70 years without Parkinson's disease who wore an accelerometer at a recent study visit were included. Robust, prefrail, and frail phenotypes were defined as 0, 1-2, or 3-5 of: low energy, low physical activity, slow gait, weak grip, and unintentional weight loss or low body mass index, respectively. Slow gait was identified over 3-6 meters or medical preclusion. Weak grip was identified using a hand dynamometer or medical preclusion. Weight loss was self-reported (except ACHIEVE). Low physical activity was defined using questions about walking, exercise, or vigorous activities. Low energy criteria included items on "energy", "exhaustion", "tiredness". The 3,558 participants (mean age of 79.2years, 52.9% female) were 11.1% frail, 55.5% prefrail, and 33.4% robust. Study-specific sample prevalences ranged widely for slow gait (ACHIEVE=5% to NHATS=37%), weak grip (ACHIEVE, BLSA=36% to STURDY=61%), weight loss (ARIC=10% to STURDY=18%), low energy (BLSA=4% to NHATS=28%), and low activity (BLSA=13% to STURDY=31%). Study-average gait speed ranged from 1.05m/s(BLSA) to 0.87m/s(STURDY) and grip strength ranged from 28.0kg (ACHIEVE) to 24.5kg(STURDY). These results were generally not attenuated by adjustment for demographics via logistic regression. Pre-statistical harmonization did not yield a seamless frailty phenotype across these five studies. Alternative frailty operationalizations using criteria that is both objective and standardized may better support cross-study comparisons.

Abstract citation ID: igaf122.642

### OBJECTIVE PHYSICAL ACTIVITY BY FRAILTY STATUS: A CROSS-STUDY COMPARISON

Jennifer Schrack[1], Lacey Etzkorn[1], Sunan Gao[2], Brian Buta[2], Anis Davoudi[2], Qian-Li Xue[2], Karen Bandeen-Roche[3], and Amal Wanigatunga[1], *1. Johns Hopkins Bloomberg School of Public Health, Baltimore, Maryland, United States, 2. Johns Hopkins University, Baltimore, Maryland, United States, 3. Johns Hopkins Center on Aging and Health, Baltimore, Maryland, United States*

Physical activity (PA) is paramount to delaying the onset and progression of frailty. Current understanding of how PA differs by frailty status is mostly limited to select populations and/or summary measures of time spent at various intensities and fails to account for the typical amount and patterns of daily PA in which most older adults engage. We investigated differences in daily PA among 3,558 older adults aged ≥70 years (mean=79.2±5.7years, 52.9% female) from five cohorts: ARIC, BLSA, NHATS, STURDY, and ACHIEVE. PA was assessed using identical 24-hour/7-day wrist-worn accelerometry protocols and defined using measures of activity volume (min/day), fragmentation (% probability of transitioning from an active-to-sedentary state), and endurance (minutes/active bout). Frailty was defined as having ≥3 of low self-reported walking for exercise or sport activities, low grip strength, slow walking speed, exhaustion, and unintentional weight loss; having 1-2 was considered prefrail. Across cohorts, 395(11.1%) were frail, 1,975(55.5%) prefrail, and 1,188(33.4%) robust. Robust older adults averaged 406±107 active min/day, 24.2±5.8% fragmentation, and 4.4±1.1 minutes/active bout. Frail and prefrail older adults had lower activity volume [317±131 and 377±115 active minutes/day, respectively], were more fragmented [31.6±11% and 26.3±7.1%, respectively], and had lower average bout length [3.6±1.3 and 4.1±1.1 min/bout, respectively], comparatively. Time-of-day analyses indicated, relative to the robust group, frail and prefrail older adults were more active overnight (12-5am) and less active during waking hours (8am-8pm). These results represent a first step in establishing normative values for objective PA across the frailty spectrum, providing a path towards earlier detection and treatment.

Abstract citation ID: igaf122.643

### PHYSICAL ACTIVITY PATTERNS AND INCIDENT FRAILTY IN THE NATIONAL HEALTH AND AGING TRENDS STUDY

Anis Davoudi[1], Lacey Etzkorn[2], Sunan Gao[1], Brian Buta[1], Qian-Li Xue[1], Karen Bandeen-Roche[3], Amal Wanigatunga[2], and Jennifer Schrack[2], *1. Johns Hopkins University, Baltimore, Maryland, United States, 2. Johns Hopkins Bloomberg School of Public Health, Baltimore, Maryland, United States, 3. Johns*

*Hopkins Center on Aging and Health, Baltimore, Maryland, United States*

Frailty is characterized in part by slow walking speed and low self-reported physical activity (PA), but there is limited evidence linking objective amount, intensity, and patterns of daily PA to frailty incidence. A deeper understanding of the characteristics of PA most strongly associated with frailty may help illuminate new markers for prevention. We examined the association between wrist-worn accelerometry-derived patterns of daily PA and incident frailty in the National Health and Aging Trends Study (NHATS). Frailty was defined as having ≥3 criteria of self-reported exhaustion, unintentional weight loss, low self-reported physical activity, and low measured grip strength and slow walking speed; having 1-2 criteria was considered prefrail. Daily PA features included total daily activity volume (total counts, active minutes/day), intensity (most active 5-minutes (M5) counts/day), fragmentation(%), and variability (intra-daily variability(IV), inter-daily stability(IS)). Survey-weighted multivariable logistic regression models were used to study the association between each accelerometer feature and incident frailty 1-year later, adjusting for sociodemographics and clinical conditions. In 519 participants with ≥3 days of valid accelerometry data who were robust or prefrail at baseline (33.5% aged ≥80 years, 56% female), 13.6% became frail. Every 30/min higher baseline active time, 100,000 more activity counts/day, and 1000 more M5 counts/day were associated with 13%[95% CI: 4%-21%], 8%[1%-15%], and 35%[21%-46%] lower odds of incident frailty, respectively. Additionally, every 1% higher fragmentation and 0.1 higher IV were associated with 7%[4%-11%] and 18%[6%-33%] higher odds of frailty. Future studies should investigate using accelerometer measures of PA to predict frailty onset.

Abstract citation ID: igaf122.644

### EXPLORING DEVICE WEAR CRITERIA TO CHARACTERIZE GLUCOSE PATTERNS IN PREFRAIL OLDER ADULTS WITHOUT DIABETES

Amal Wanigatunga[1], Xiaowen Chen[1], Sydney Schultz[1], Elizabeth Selvin[1], Karen Bandeen-Roche[2], Eleanor Simonsick[3], and Jennifer Schrack[1], *1. Johns Hopkins Bloomberg School of Public Health, Baltimore, Maryland, United States, 2. Johns Hopkins Center on Aging and Health, Baltimore, Maryland, United States, 3. National Institute on Aging, Baltimore, Maryland, United States*

Frail at-risk individuals may experience episodes of low blood sugar that may accelerate frailty onset. Continuous glucose monitoring (CGM) provides detailed interstitial glucose information throughout the day but device wear criteria (e.g., 24-hour wear; weekend behavior differences) to accurately estimate ambulatory glucose levels in prefrail older adults living without diabetes are not well defined. Baseline data from 37 participants enrolled in the Sedentary To Active Rising to Thrive (START) trial were examined (Dec 2023–Nov 2024). Each participant wore a CGM sensor for up to 14 days. Linear regression was used to estimate differences in mean ambulatory glucose level between: 1) wear days with and without complete data collection and 2) weekdays and weekends. Among 37 START participants, 32(86%) provided CGM data (mean age:76 years, 94%women). Mean wear period was 14-days (range: 9–15) and mean CGM glucose was 106 mg/dL (range:95–113 mg/dL). Glucose levels were lower on days with missing data (97.67 mg/dL; n = 64 days) compared to days with no missing (107.64 mg/dL; n = 393 days) ($p < 0.001$). Time-of-day patterns showed that mean glucose levels were lowest around 3 AM (94.5 mg/dL, SD = 27.2) and highest around 3 PM (113.4 mg/dL, SD = 16.0). Mean glucose levels were higher by 3 mg/dL on weekends compared to weekdays ($p = 0.07$). Findings suggest that addressing incomplete data collection would improve estimates of mean ambulatory glucose levels by capturing both fasting (early morning) and postprandial (afternoon) effects on glucose levels. Findings also suggest that at least one weekend day should be measured for valid ambulatory glucose assessment.

### SESSION 2105 (PAPER)

### AGING, INEQUALITY, AND MENTAL HEALTH: GLOBAL AND STRUCTURAL INSIGHTS

Abstract citation ID: igaf122.645

### ASSOCIATION BETWEEN RESIDENTIAL SEGREGATION AND SUICIDE ACROSS THE LIFECOURSE AMONG BLACK AMERICANS

Eskira Kahsay, and Briana Mezuk, *University of Michigan, Ann Arbor, Michigan, United States*

Residential segregation is a widely researched structural determinant with negative effects on physical health. However, the neighborhood context also allows for increased social capital and fewer interpersonal experiences of discrimination, potentially benefiting the mental health of those in racially/ethnically homogeneous areas. Still, the impact of segregation on suicide remains unclear, especially among Black Americans whose suicide rates have increased in the last decade. This study investigated the association between segregation and suicide among Black Americans using the National Violent Death Reporting System harmonized to1-year American Community Survey Public Use Microdata Samples (cases N = 10,462; controls N = 1,298,572) in 29 states and D.C. between 2017-2021. In addition to single suicides (N = 6,575), undetermined intent deaths (N = 3,887) were included to account for high rates of suicide misclassification. Black ethnic density (i.e., proportion of Black residents) served as a proxy measure for county-level segregation. Weighted logistic regression models were fit, adjusting for demographics, year, county population density, and county poverty. Stratification by age (early [18-24], young [25-44], middle [45-64], older [65+] adulthood) was conducted. Ethnic density was positively associated with suicide (per 10% increase, OR = 1.13, 95% CI: 1.11-1.14). The association was strongest among middle (OR: 1.31, 95% CI: 1.29 - 1.34) and older adults (OR: 1.18, 95% CI: 1.13 - 1.23). While potentially protective against other dimensions of mental health, racial/ethnic homogeneity may be a salient contributor to suicide risk, particularly later in the life-course, potentially as as a result of accumulated stressors and systemic disadvantages.

Abstract citation ID: igaf122.646

### CROSS-NATIONAL DIFFERENCES IN ASSOCIATION BETWEEN FOOD DEPRIVATION AND DEPRESSIVE SYMPTOM IN INDIA, MEXICO AND USA

Shekhar Chauhan[1], Dawn Carr[1], T Muhammad[2], and Arun Balachandran[3], *1. Florida State University, Tallahassee, Florida, United States, 2. The Pennsylvania State University, University Park, Pennsylvania, United States, 3. Columbia University, New York City, New York, United States*

Food insecurity has been shown to erode mental health as people age, but it is not known how these effects vary across different country contexts. This study examines the association between food deprivation and depressive symptoms among older adults in India, Mexico, and the USA. We use a nationally representative dataset we harmonized using Longitudinal Aging Study in India (LASI, 2017-18), the Mexican Health and Aging Study (MHAS, 2018), and the Health and Retirement Study (HRS, 2018). Descriptive analyses show differences in rates of food deprivation (5% in the USA, 7% in India, and 12% in Mexico) and depressive symptoms (4-item CESD) (0.66 symptoms in the US, 0.95 symptoms in India, and 1.13 symptoms in Mexico, on average) by country. OLS regression shows food deprivation is predictive of higher levels of depressive symptoms for older adults in all three countries relative to their non-food deprived counterparts. Moderation tests showed certain socio-demographic factors and resources differentially influence depressive symptom consequences of food deprivation in certain countries, but not others. For instance, females have higher depressive symptoms relative to their male counterparts only in Mexico (1.86 versus 1.25 symptoms; $p < 0.001$). Having higher levels of educational attainment is protective of food deprivation only in Mexico ($p < 0.001$). Living with children is protective only in India (1.3 vs 1.61; $p < 0.001$). However, being in worse health and being widowed is associated with higher depressive symptom consequences of food deprivation in all three countries ($p < 0.001$). These findings highlight the need for targeted policy interventions.

Abstract citation ID: igaf122.647

### THE ROLE OF DEATH ANXIETY IN OLDER ADULT PTSD, DEPRESSION, AND ANXIETY DURING WARTIME

Yoav Bergman[1], Rotem Saar-Ashkenazy[1], Yifat Faran[1], Eyal Klonover[1], and Yuval Palgi[2], *1. Ashkelon Academic College, Ashkelon, HaDarom, Israel, 2. University of Haifa, Haifa, Hefa, Israel*

The outbreak of the Israel–Hamas war on October 7, 2023, has presented unprecedented challenges to older adults' mental health, including increased posttraumatic stress, anxiety, and depression. The current study examined potential war- and age-related factors associated with probable posttraumatic stress disorder (PTSD), depression, and anxiety among Israeli older adults during the ongoing war. Moreover, due to the continued threat of death, we examined whether death anxiety is an additional contributing factor to older adults' probable PTSD, depression, and anxiety. Data were collected January–March 2024 from 554 community-dwelling older adults (Mage= 73.90 years, SD = 7.35, range: 61–96 years) who completed online scales assessing sociodemographic variables, war exposure (distance from the Gaza Strip, exposure to terror attacks/blasts), and age-related constructs (assistance in daily activities [ADL], cognitive decline, physical illnesses, death anxiety). Increased ADL was associated with probable depression (B = 0.62, OR = 1.87), and anxiety (B = 0.42, OR = 1.53), and cognitive decline was associated with probable depression (B = 1.52, OR = 4.56). Older adults with high levels of death anxiety were almost 3 times as likely to meet the criteria for probable PTSD (B = 1.05, OR = 2.85) and more than 1.5 as likely to meet the criteria for probable depression (B = 0.54, OR = 1.71) and anxiety (B = 0.50, OR = 1.65). The importance of death anxiety as a potential risk factor for negative psychological outcomes among older adults during war will be discussed.

Abstract citation ID: igaf122.648

### INTEGRATING GUT MICROBIOTA AND VIOLENCE EXPOSURE METRICS TO CLASSIFY PSYCHOLOGICAL DISTRESS IN MIDDLE-AGED ADULTS

Khalil Iktilat[1], Gali Levin[2], Sondra Turjeman[2], Roy Tzemah-Shahar[3], Yoram Louzoun[2], Omry Koren[2], and Maayan Agmon[3], *1. Tel Aviv University, Tel Aviv, Tel Aviv, Israel, 2. Bar-Ilan University, Ramat Gan, HaMerkaz, Israel, 3. University of Haifa, Haifa, HaZafon, Israel*

The link between violence exposure and psychological distress is well established. The study of microbiota-gut-brain crosstalk shows that the relationships between microbiota and environmental stressors are bidirectional and play a causative role in host mental state. We explored how the microbiota in a population at the onset of aging relates to violence exposure and distress levels and determined that the microbiota and exposure to violence can be used to classify patients into high- and low-distress groups. Bioinformatics and modeling were completed, underscoring the significant impact of the microbiome on violence-distress relationships and highlighting the importance of our contribution to ongoing research. We first characterized fecal microbiota of Israeli-Muslims (n = 305) aged 50.82±6.94 (40-65yrs) exposed to increasing violence in the past decade and examined correlations with exposure to violence (Screen for Adolescent Violence Exposure (SAVE) questionnaire) and psychological distress (Kessler 6) focusing on total microbiome and a subset of taxa identified from literature searches. We found unique microbial signatures associated with increasing violence exposure and distress. While some significantly associated bacteria were previously identified, many correlations were novel such as Desulfovibrio. Furthermore, microbial profiles associated with violence and distress were largely non-overlapping, yet we could classify participants into high- and low distress categories with machine learning algorithms (area-under-the-curve=0.63). This research highlights the significant role of the microbiota-gut-brain axis in linking violence exposure to psychological distress among middle-aged Muslims in Israel. It suggests that specific gut microbiota profiles, correlated with violence exposure, can help identify individuals at higher risk for psychological distress.

Abstract citation ID: igaf122.649

### RACIAL/ETHNIC DISPARITIES IN CRIME EXPOSURE AND ASSOCIATIONS WITH MIDDLE-AGED AND OLDER AMERICANS' MENTAL HEALTH

Junjie He[1], Zhuoer Lin[2], Yan Zhang[3], Yang Wang[4], and Xi Chen[5], *1. Yale University, State College, Pennsylvania, United States, 2. University of Illinois Chicago, Chicago, Illinois, United States, 3. Texas State University, Greenville, North Carolina, United States, 4. University of Wisconsin–Madison, Madison, Wisconsin, United States, 5. Yale University, New Haven, Connecticut, United States*

While the association between crime and mental health is documented, its racial and ethnic differences and interactions with crime types on late-life mental health remain unclear. This study examined the impact of crime exposure on depressive symptoms, focusing on racial and ethnic disparities and main crime types. We linked the Health and Retirement Study (HRS), a nationally representative longitudinal data, with crime data from the Uniform Crime Reporting (UCR) system. The final analytic sample included 27,899 Americans aged 50 and older. Crime exposure was measured by local crime rates at the county-year level and depressive symptoms were measured by the CES-D scale. Employing models with individual fixed-effects and adjusting for comprehensive covariates to mitigate potential biases, we found that a one-standard-deviation increase in local crime rates was associated with a 1.03% increase in the likelihood of experiencing depressive symptoms. Compared to non-Hispanic Whites, non-Hispanic Blacks and Hispanics were both exposed to higher crime levels and exhibited more depressive symptoms. Moreover, the negative effects of crime were stronger for non-Hispanic Blacks and Hispanics than for Whites, with significantly larger effects of both violent and property crimes for non-Hispanic Blacks, and larger effects of property crimes for Hispanics. These patterns held across various robustness checks. Our findings suggest that persistent racial and ethnic disparities in late-life mental health may be driven by structural racial and ethnic inequities, in particular through differential exposure to crime and the amplified impact of crime, highlighting the importance of enhancing community safety to address health disparities.

### SESSION 2110 (SYMPOSIUM)

Abstract citation ID: igaf122.650

### STRENGTHENING SYSTEMS: ADVANCING INTEGRATED BEHAVIORAL HEALTH FOR OLDER ADULTS

Chair: Allison Gibson
Discussant: Kelly O'Malley

Implementing integrated behavioral health in aging services presents several challenges, including organizational silos, cultural differences between sectors, stigma, and a lack of trained staff who are equipped to address both aging and behavioral health needs. Workforce shortages and the complexity of older adults' health needs further complicate integration efforts. This symposium will highlight a series of strategies to overcome these challenges, including systems organizational change, improving training and care coordination, enhancing students' training in geriatric competencies and workforce development, and increasing access to appropriate resources. The first session discusses collaboration processes with aging-focused and behavioral health providers to foster cross-sector integration. The discussion highlights the importance of system change in making aging services and behavioral health more comprehensive and responsive to older adults. Our second presentation explores utilizing the 4Ms-Behavioral Health Framework to support integration efforts by examining data on behavioral health and substance use among older adults in the U.S. and Australia, emphasizing how training in different settings can lead to meaningful knowledge and behavioral change among interprofessional teams. Our third presentation prepares students to apply geriatric care competencies through the Mobile Health and Wellness Program (MHWP). Presenters will discuss how this initiative enhances workforce development, preparing future providers to integrate aging and behavioral health care effectively. Our final presentation will discuss the role of the Comprehensive Healthcare Integration (CHI) Framework, which enhances patient-centered care by embedding behavioral health consultants into a team-based model that improves health outcomes and care coordination of older clients.

Abstract citation ID: igaf122.651

### INTEGRATING AGE-FRIENDLY PRACTICES IN AGING SERVICE AND BEHAVIORAL HEALTH SYSTEMS: A SYSTEMS CHANGE INITIATIVE

Allison Gibson[1], Marla Berg-Weger[2], Julie Blanco[3], Britney Parson[4], Dana Silverblatt[4], Max Zubatsky[5], and Dimitra Tziarli[6], *1. Saint Louis University, Saint Louis University, Missouri, United States, 2. Saint Louis University - - St Louis, MO, St. Louis, Missouri, United States, 3. Illume - Behavioral Health Center of Excellence, St. Louis, Missouri, United States, 4. Behavioral Health Network of Greater St. Louis, St. Louis, Missouri, United States, 5. Saint Lewis University, Saint Louis, Missouri, United States, 6. Saint Louis University, St. Louis, Missouri, United States*

The integration of aging services and behavioral health remains a significant challenge due to long-standing silos between the two systems. To address this, our team collaborated with 12 community-based aging-focused and behavioral health organizations in St. Louis, bringing together opportunities for greater collaboration. Through a series of meetings, these organizations shared their existing practices and services, allowing us to identify concrete actions to enhance integration. Each organization sets its own goals and timeline. Many organizations set goals to train staff and volunteers, adopt new assessment tools, and adjust referral processes to ensure more comprehensive care. As part of this effort, we developed an interview guide to assess the impact of these changes and better understand the barriers and facilitators to systems change. Our interview guide captures systems of change of these organizations: key insights into how organizations are implementing new strategies, what challenges they are facing, and what support they may need to sustain these efforts. Open-ended questions encouraged reflection on the effectiveness of training programs, changes in referral workflows, and overall improvements in service coordination. These agency interviews broaden the implications of these efforts, including the potential for scalability and sustainability beyond the initial implementation. In this presentation, we will discuss the best practices of system change, inform policy discussions of comprehensive care, and ensure that aging and behavioral health services are better integrated to serve the needs of the community.

Abstract citation ID: igaf122.652

### 4MS-BEHAVIORAL HEALTH IN AUSTRALIA: AN INTERNATIONAL COMPARISON

Erin Emery-Tiburcio[1], and Laura Porter[2], *1. Rush University Medical Center, Chicago, Illinois, United States, 2. Cherokee Health Systems, Knoxville, Tennessee, United States*

The 4Ms framework of an Age-Friendly Health System (What Matters, Medication, Mentation, Mobility) was originally developed by the Institute for Healthcare Improvement with support from The John A. Hartford Foundation for hospital and ambulatory care settings. As many behavioral health providers did not clearly see a role for themselves in all 4Ms, the 4Ms-Behavioral Health framework (4Ms-BH) was adapted to clarify roles and include additional critical elements for older adult mental health and substance use. Following piloting in the US, the 4Ms-BH training was tailored for Australian audiences with language, cultural, and health system references. Individual interviews with Australian health providers indicated that the training was most needed in inpatient health settings, thus the training was adapted further to meet these needs. The training has been provided to interprofessional teams at a rural hospital in Cairns, Queensland (n = 49), and an urban hospital in Gold Coast, Queensland (n = 25). Initial qualitative data analysis indicates that these Queensland health systems are far ahead of most US health systems in their screening for and addressing delirium, obtaining basic advance directives, and incorporating pharmacy in attending to polypharmacy and high risk medications. Critical elements internationally are widespread ageism and connecting the 4Ms as a set. Discussion will focus on unique cultural elements and international similarities in 4Ms-Behavioral Health implementation.

Abstract citation ID: igaf122.653

**BEHAVIORAL HEALTH STUDENT TRAINING AND INTERVENTION IN THE COMMUNITY-BASED MOBILE HEALTH AND WELLNESS PROGRAM**

Rachel Regal[1], Leland Waters[1], and Patricia Slattum[2], *1. Virginia Commonwealth University, Richmond, Virginia, United States, 2. Virginia Commonwealth University College of Health Professions, Richmond, Virginia, United States*

The Mobile Health and Wellness Program (MHWP) trains interprofessional students on 4Ms age-friendly care as part of care coordination and health promotion weekly clinics for medically underserved older adults within low-income senior housing buildings and a community-based wellness clinic. Of the nearly 3000 visits in 2024 with 374 older adults, student teams from nursing, medicine, pharmacy, and other disciplines provided behavioral health intervention in 436 visits (15%) with over a third of participants (n = 131). Unmet mental health needs identified in 56% (41 of 73) of screens for depression, anxiety, substance use, and social connection led to further assessment, mental health education, wellness goal setting, and care referrals or coordination. Interprofessional students engage with behavioral health training as part of team debriefings, morning topic discussions, a simulation orientation with motivational interviewing skill development, morning topic discussions on a range of mental health topics, and online 4Ms evidence-based training modules including trauma-informed care. Modules conclude with knowledge check questions and self-rated confidence on three key domains: identifying risk factors, conducting screenings, and intervention. Descriptive results indicate that the majority of students report confidence gains following each module. Results of Wilcoxon signed rank analyses showed a 1-rank median confidence increase for Mentation, Social Connectedness, and Ageism Modules ($p < .001$, N = 132 to 544). Further findings and details will be presented that describe how MHWP trains students and intervenes for behavioral health needs in community-based settings with underserved older adults.

Abstract citation ID: igaf122.654

**THE COMPREHENSIVE HEALTHCARE INTEGRATION FRAMEWORK: INTEGRATING BEHAVIORAL HEALTH INTO GERIATRIC PRIMARY CARE**

Anna Faul, and Joseph D'Ambrosio, *University of Louisville, Louisville, Kentucky, United States*

Integrating behavioral health into geriatric focused primary care involves collaboration between primary care and behavioral health clinicians to provide comprehensive, patient-centered care, addressing behavioral health needs alongside medical conditions. This model improves health outcomes, enhances behavioral health access, reduces stigma, and fosters holistic care. Emphasizing team-based collaboration, enables seamless coordination of treatment plans addressing physical and behavioral health needs, and is recognized as the gold standard for patient well-being. The Comprehensive Healthcare Integration Framework advances primary care behavioral health integration by outlining 8 evidence-based domains and 3 progressive constructs to guide primary care practices toward behavioral health integration. This integration requires person-centered strategies and interprofessional teamwork. At our Optimal Aging Clinic, a geriatric focused primary care practice, we developed and implemented integration workflows based on the CHI Framework to support comprehensive health care for older adults. Based on this model, we incorporate behavioral health consultants as generalists providing brief, focused interventions and biopsychosocial assessments to enhance primary care team capacity. We use a collaborative team of providers who all function at the top of their licenses. Our model ensures efficient, evidence-based management of complex cases and unmet clinical goals addressing physical, psychological, and social needs, improving care coordination and outcomes. Evaluation data shows improved management of depression and anxiety among older adults, together with overall enhanced biopsychosocial health, fostering better healthcare engagement from older adults in their care. This session will highlight our successes, the replicability of our model, and the challenges faced in integrating behavioral health into age-friendly care.

### SESSION 2115 (SYMPOSIUM)

Abstract citation ID: igaf122.655

**NEW INTERVENTIONS FOR OLDER ADULT VICTIM-SURVIVORS OF FINANCIAL FRAUD**

Chair: Marguerite DeLiema
Discussant: Surya Kolluri

Financial fraud is a growing elder justice issue. While people of all ages are targeted, isolated older adults without a financial or social safety net experience the most significant harm. Victimization often comes with deep shame, making it hard to reach out for help or even acknowledge the consequences. There is also very little research on what works to protect older adults from fraud victimization. This symposium will bring together researchers and direct practice clinicians to discuss findings from their recent work to prevent fraud and intervene to support victim-survivors. The first presentation will showcase "Successful Aging thru Financial Empowerment" (SAFE), a no cost financial coaching and economic advocacy service dedicated to preventing financial exploitation and fraud among older adults. The program also provides financial and persuasion literacy training and scam awareness education. SAFE Director, Latoya Hall, MSW, will present research findings on a pilot study and longitudinal findings on financial fraud victims who received SAFE services. Next, Marti DeLiema, PhD, will share research on the efficacy of temporary financial account holds as a stopgap measure to protect older adults from continued financial exploitation, and also the impact these holds have on victim-survivors' financial autonomy. Last, practitioners with FightCybercrime will present data on how online peer support groups for romance fraud victim-survivors assist with emotional recovery and help build community following fraud. The presentations will be followed by a panel discussion led by Surya Kolluri (Director, TIAA Institute) on the future of fraud intervention research.

Abstract citation ID: igaf122.656
**FINANCIAL AUTONOMY VERSUS FINANCIAL SAFETY: A NECESSARY TRADEOFF?**
Marguerite DeLiema, *University of Minnesota School of Social Work, Minneapolis, Minnesota, United States*
Persistent and chronic fraud victims may not recognize the harmful consequences of responding to scams, placing them at risk of continued victimization. An adult protection law in Minnesota authorizes financial institutions to place 3-week holds on transactions if an older adult is believed to be experiencing financial abuse or fraud. Many states have recently passed similar laws, but none have systematically collected data on temporary account holds and how they impact older adults' financial safety and overall well-being. In this study, researchers partnered with the Minnesota Department of Commerce and Adult Protective Services to analyze data on elder financial exploitation cases that were referred for temporary account holds between 2022 and 2024. Researchers then interviewed 12 older adults who experienced temporary account holds, or their designated proxies, to understand the material and psychological effects of the intervention. In 2024 there were 638 investigations opened following referrals for temporary account holds (mean = 53 cases/month); double the number from 2022. Temporary account holds were implemented in 19% of the investigations, resulting in the protection of $8 million dollars. Interview participants described how the perpetrators were not able to take their money during the hold period, but expressed anger and mistrust toward their financial institutions for placing the holds. Results indicate that temporary holds can protect chronic victims against significant financial losses, but when applied too broadly to funds in an account or placed without sufficient communication, account holds can cause significant psychological distress and some material harm.

Abstract citation ID: igaf122.657
**SUCCESSFUL AGING THRU FINANCIAL EMPOWERMENT (SAFE)**
LaToya Hall, and Peter Lichtenberg, *Wayne State University, Detroit, Michigan, United States*
Current estimates of financial exploitation (FE) show between 5% and 11% of older adults are victimized yearly. The rapid growth of FE and defrauding of older people impels the creation and evaluation of intervention and prevention strategies. The Successful Aging thru Financial Empowerment (SAFE) program was created in 2017 to provide older adults education, support and assistance to address FE. The SAFE program currently focuses on providing no cost financial coaching/economic advocacy services to older adult victims of FE and the administration of FE prevention intervention. This presentation will cover two recent studies on SAFE program services and outcomes: (1) One study examined the financial coaching/economic advocacy intervention. Program records and baseline and follow-up assessments of 115 SAFE clients were used to detail program dynamics and investigate participant outcomes. Participants reported high levels of program satisfaction and significantly less stress at the six-month follow-up. These findings demonstrate empowerment-centered financial coaching interventions can successfully address FE in older adult populations. (2) The second is a pilot study examining the effect and acceptability of the newly developed FE prevention program. Intake and follow up assessment data of the first 45 participants enrolled was analyzed. Findings support the prediction that financial literacy and financial vulnerability scores after the intervention would show significant improvement from baseline scores. Participants' ratings after each session, with respect to usefulness and trustworthiness, were extremely positive. The financial exploitation prevention intervention program demonstrated acceptability and a positive effect on reducing vulnerability to financial exploitation.

Abstract citation ID: igaf122.658
**THE ROLE OF ONLINE PEER SUPPORT GROUPS IN THE RECOVERY OF ROMANCE FRAUD VICTIMS**
Jennifer Lawrence, *FightCybercrime, Boston, Massachusetts, United States*
Romance frauds lead to emotional, psychological, and financial distress that last for years after the scam concludes. This presentation examines the role of online peer support groups in helping victims recover after a scam. Peer support programs that address a victim's loneliness and shame, while also educating participants in scammer tactics and cyber security best practices can help victims rebuild their lives across multiple aspects. This holistic approach helps victims through recovery and equips them with the tools needed to respond to future scams. In 2024, a FightCybercrime post-peer support group survey of 67 participants examined the factors influencing victims' initial engagement with scammers. Loneliness emerged as the most significant factor, followed by a lack of awareness of scam sophistication. In the survey, participants noted that the peer support group helped them recognize how scammers take advantage of these vulnerabilities. Also, 87% of those surveyed stated that the peer support group provided valuable resources to address these challenges. Finally, qualitative responses described the benefits of shared experiences, emotional validation, and information on cyber security best practices to help avoid future scams. These findings emphasize the role of peer support in mitigating the impact of romance scams and preventing revictimization.

### SESSION 2120 (PAPER)

### AGING SACROSS THE LIFE COURSE: COGNITIVE TRAJECTORIES, GLOBAL TRENDS, AND SOCIAL DETERMINANTS

Abstract citation ID: igaf122.659
**COGNITION IN LATER LIFE AND LIFE COURSE SOCIOECONOMIC DETERMINANTS: LONGITUDINAL EVIDENCE FROM CHINA (2011-2020)**
Mengling Cheng, *East China University of Science and Technology, Shanghai, Shanghai, China (People's Republic)*
Evidence showed that differences in later-life cognition were associated with variations in socioeconomic status (SES) in various life course periods. However, less is understood about the effects of SES over the life course and the underlying patterns. Guided by the life course perspective, this study examined the association between SES across childhood, adulthood, and older age and later-life cognition in China, testing four life course models: critical period, sensitive period, pathway, and accumulation. We used data from the China Health and Retirement Longitudinal Study (2011-2020, 17,000 participants). We measured cognition globally and across domains. We measured SES in childhood, adulthood, and older age, alongside a cumulative SES index. We performed multilevel growth curve models to test the four life course models. Results revealed that SES throughout the life course was associated with later-life cognition, with consistent evidence in global cognition and across cognitive domains. Findings supported the sensitive period, pathway, and accumulation models, but not the critical period model. In particular, older women and rural residents were more vulnerable to the adverse effects of socioeconomic disadvantages on cognition over time. Our study highlights the importance of addressing SES disparities across the life course to mitigate its impact on cognitive aging.

Abstract citation ID: igaf122.660

### CORRELATES OF PHYSICAL ACTIVITY AMONG OLDER ADULTS OF LOW VERSUS HIGH SOCIO-ECONOMIC STATUS

Olivia Malkowski[1], Jessica Harvey[2], Nick Townsend[3], Mark Kelson[4], Charlie Foster[3], and Max Western[2], *1. Yale University, New Haven, Connecticut, United States, 2. University of Bath, Bath, England, United Kingdom, 3. University of Bristol, Bristol, England, United Kingdom, 4. University of Exeter, Exeter, England, United Kingdom*

Understanding socio-economic differences in the factors influencing physical activity among older adults is essential for developing comprehensive interventions. We aimed to quantify the associations of modifiable correlates and determinants with physical activity among UK-based older adults of low versus high socio-economic status (SES). In this systematic review and meta-analysis, we searched MEDLINE, Embase, Web of Science, CENTRAL, and Scopus from inception to December 2023, for peer-reviewed studies published in English, investigating associations between a modifiable factor as an independent variable and physical activity as a dependent variable, by SES, in community-dwelling UK older adults aged 60+ years. Random effects meta-analyses were performed separately for people of low and high SES. Risk of bias was assessed with the Mixed Methods Appraisal Tool. This study was registered with PROSPERO (CRD42022351708). Searches identified 11,472 references; seventy-seven studies met the selection criteria, of which fifty-one contributed to meta-analyses (N range = 134–29,280). Of the exposures positively associated with physical activity, physical function, social participation, and perceived general health had the largest effect sizes (standardised mean difference [SMD] range = 0.53–0.81; I-squared statistic range = 54.81–91.00%). Estimates were comparable among low- and high-SES older adults, except for built physical activity facilities, walking and cycling infrastructure, and less smoking, which were positively associated with physical activity only among low-SES individuals. Our results suggest researchers need to better understand discrepancies in the prevalence of the assessed correlates (e.g., fewer low-SES participants reported good physical function) to inform policies that reduce inequalities in older adults' physical activity levels.

Abstract citation ID: igaf122.661

### DISTINCT LONGITUDINAL TRAJECTORIES OF COGNITIVE CHANGE AMONG MIDDLE-AGED AND OLDER ADULTS IN CHINA

Ying Liu, Dayoung Lee, Stefan Schneider, and John Strauss, *University of Southern California, Los Angeles, California, United States*

Monitoring and understanding the longitudinal trajectories of cognitive change is central to dementia care and research. Prior studies that examined the cognitive change in Chinese population either assumed a single trajectory, which ignored interpersonal differences, or modeled change along measurement times rather than age. The current study leveraged a longitudinal, nationally representative survey, the China Health and Retirement Longitudinal Study (CHARLS), and identified distinct trajectories of cognitive change using the Bayesian age-based Growth Mixture Modeling (GMM) techniques. In 2011-2018, four waves of cognition data were collected on 21,242 participants aged 40 years and older, with tasks including time orientation, immediate and delayed word recall, serial's 7s, and drawing interlocking pentagons. The GMM analyses classified individuals into three subgroups, each with a distinct trajectory as age increased from 40: 65.9% had a high initial cognition with a relatively flat but slightly accelerated decline; 26.8% had a middle initial cognition yet a noticeable and steady decline; and 7.3% had a low initial cognition and a noticeable decline. Adjusting for demographics (education, gender, ethnicity, coupledness, urbanicity based on Hukou or household registration, and migration status), multinomial logistic regression analyses revealed that compared to the high-initial-cognition group, the other two groups tended to be women and have lower education and rural Hukou; the associations were particularly strong for the low-initial-cognition group. These findings provided an insightful view of different patterns in cognitive change among the middle-aged and older Chinese. They also illustrated the potential of the GMM techniques in population research of aging.

Abstract citation ID: igaf122.662

### EARLY-LIFE MULTILINGUALISM AND MIDLIFE COGNITIVE HEALTH: EVIDENCE FROM HIGH SCHOOL AND BEYOND

Yue Qin[1], Eric Grodsky[1], Chandra Muller[2], and John Warren[3], *1. University of Wisconsin–Madison, Madison, Wisconsin, United States, 2. The University of Texas at Austin, Austin, Texas, United States, 3. University of Minnesota, Minneapolis, Minnesota, United States*

Learning a second language is cognitively stimulating, as it challenges the brain to make sense of an additional set of symbols and rules of syntax. However, little is known about how the timing and context of second language learning affect adulthood cognition. Drawing on the cognitive reserve theory and data from the 1980, 1982, and 2021 waves of High School and Beyond:1980 (N ≈ 13,980), we examine whether learning a second language before school or college, or taking a second language course in high school is associated with midlife cognition. We also examine potential differences in associations by race and ethnicity. We estimate linear regression models of composite cognition scores derived from multiple cognitive performances such as word recall, phonemic fluency, and semantic fluency, controlling for individual sociodemographic attributes. We find that being multilingual before college rather than before school was significantly linked to better midlife cognition before controlling for high-school academic performances, with larger benefits for Hispanics. This is possibly because learning English promotes the adaptation of Hispanics, whose first language is often Spanish, in the US educational system; Hispanics also have more opportunities to navigate different linguistic contexts between school and home. Taking second language course(s) in high school was also associated with better midlife cognition, with no significant racial or ethnic differences in the association.

This study contributes to the growing research on the early-life factors influencing adulthood cognitive health inequality, highlighting the importance of the timing and context of second language learning in shaping cognitive outcomes.

Abstract citation ID: igaf122.663

### HISTORICAL CHANGES IN TERMINAL DECLINE IN WELL-BEING AMONG JAPANESE OLDER ADULTS

Takeshi Nakagawa[1], Gizem Hueluer[2], Denis Gerstorf[3], Jersey Liang[4], and Erika Kobayashi[5], *1. The University of Osaka, Osaka, Osaka, Japan, 2. University of Bamberg, Bamberg, Germany, 3. Humboldt University Berlin, Humboldt University Berlin, Berlin, Germany, 4. University of Michigan, Ann Arbor, Michigan, United States, 5. Tokyo Metropolitan Institute for Geriatrics and Gerontology, Itabashi City, Tokyo, Japan*

Historical changes in psychosocial functioning at the end of life have been underexplored, and conflicting scenarios have been proposed regarding secular trends in the rate of decline: While the compression of morbidity hypothesis predicts a steeper rate of decline over historical time, the concept of “manufactured survival” suggests that the rate of decline has become shallower. This pre-registered study (https://osf.io/jd8xr) examined how individuals’ changes in well-being in the last years of life and their correlates differ over historical time. Data were derived from a longitudinal study of a nationally representative sample of Japanese older adults aged 60+ years. We compared two death-year cohorts based on individuals’ year of death (earlier: 1987-1999 versus later: 2000-2012) case-matched based on age at death and sex (n = 668 per cohort). Well-being was indicated by life satisfaction and depressive symptoms. Multilevel models found death-year cohort differences in life satisfaction but not depressive symptoms. While the levels of and rates of changes in life satisfaction did not differ across death-year cohorts, the association between physical health and well-being was stronger in the later-deceased cohort. These findings suggest that terminal decline in life satisfaction accelerates in interaction with disease particularly in later cohorts, with those who more recently passed away with high disease load experiencing lower levels of life satisfaction and more rapid declines. These secular trends may be due to societal advancements in healthcare systems enabling later-born cohorts to be more likely to survive with health declines.

Abstract citation ID: igaf122.664

### THE GROWING ONE CENTURY CLUB: EVOLUTION OF THE CENTENARIAN POPULATION IN EUROPE 2011–2021

Laetitia Teixeira[1], Lia Araujo[2], and Oscar Ribeiro[3], *1. ICBAS-UP, Porto, Porto, Portugal, 2. RISE-Health, Porto, Porto, Portugal, 3. University of Aveiro, Aveiro, Aveiro, Portugal*

Longevity has been increasing in Europe, leading to changes in the distribution of centenarians across countries. This study analyzes changes in the distribution of centenarians across Europe between 2011 and 2021 based on data obtained from the CensusHub platform. Given the availability of data, 26 countries were included: Belgium, Bulgaria, Czechia, Denmark, Estonia, Ireland, Greece, Spain, France, Croatia, Italy, Cyprus, Latvia, Lithuania, Luxembourg, Hungary, Malta, Netherlands, Austria, Portugal, Slovenia, Slovakia, Finland, Sweden, Iceland and Norway. The number of centenarians per 100,000 inhabitants and the variation between 2011 and 2021 (in%) were calculated for each country, for the total population and by sex. The number of centenarians increased between 2011 and 2021. In 2011, the total number of centenarians was 57,951 (18.9 centenarians per 100,000 inhabitants) and in 2021 the number increased to 78,563 (25.2 centenarians per 100,000 inhabitants). In 2011, France, Italy, and Greece had the highest number of centenarians per 100,000 inhabitants, while in 2021, France, Italy, and Portugal led the ranking. Considering the variation (in %) in the decade, some countries revealed a negative variation: Lithuania (-35.4%), Hungary (-31.7%) and Iceland (-6.5%). The countries with the higher variations were Malta (100%), Ireland (92.2%) and Portugal (83.6%), both for total and for females. For males, higher variations were observed in Ireland (171.9%), Slovakia (108.2%) and Norway (93.1%); negative variations were observed in Lithuania, Hungary, Malta, and Cyprus. Although the overall number of centenarians increased, country-specific variations highlight the need for further research to understand underlying demographic and socio-economic factors.

### SESSION 2125 (PAPER)

Abstract citation ID: igaf122.665

### CARDIOVASCULAR AGING

In this session you will gain insights into the impact of cardiovascular aging and disease specific monitoring opportunities.

Abstract citation ID: igaf122.666

### ARTIFICIAL INTELLIGENCE IDENTIFIES WORSENING HEART FAILURE BY VOICE ANALYSIS: A PILOT STUDY

Yang Yi[1], Bochao Zhao[2], Dire Ying[1], Keyao Liu[1], Wendong Xiao[2], and Qiaoqin Wan[1], *1. Peking University, Beijing, Beijing, China (People’s Republic), 2. University of Science and Technology Beijing, Beijing, Beijing, China (People’s Republic)*

Effective out-of-hospital monitoring is essential for reducing heart failure (HF) readmission rates. Emerging evidence suggests that voice biomarkers may serve as non-invasive indicators for HF monitoring. The objectives of this study were to (1) identify HF-specific speech tasks and acoustic features; and (2) validate the reliability of mobile phone recordings in HF monitoring. Recordings were collected in three phases from 160 HF patients. Phase I focused on simplifying speech tasks based on feasibility and correlation with NT-proBNP levels, with recordings taken on the same day as NT-proBNP testing. Phase II aimed to assess the cross-device stability of acoustic features through simultaneous recordings using both professional equipment and smartphones. Phase III sought to develop machine learning models to differentiate patient statuses at admission, mid-hospitalization, and discharge, and identify speech tasks and acoustic features most relevant to HF. Out of 11 tasks, 4 were retained for the final model. The accuracy, sensitivity, specificity, and area under the curve for distinguishing admission and discharge were 0.82, 0.85, 0.78, and 0.85, respectively; for mid-hospitalization and discharge, these were 0.82, 0.79, 0.85, and 0.88. Key features included Mel-frequency cepstral coefficients, chroma, fundamental frequency, frequency-domain, energy, and glottal features. No significant acoustic differences were observed between mobile phone brands. This study established a feasible, reliable set of speech tasks, identified HF-specific acoustic features, and validated mobile phone recordings for HF monitoring, supporting the potential for remote, at-home HF patient monitoring.

Abstract citation ID: igaf122.667

### LONGITUDINAL ASSOCIATIONS BETWEEN CARDIOVASCULAR KIDNEY METABOLIC SYNDROME AND DEMENTIA IN OLDER ADULTS

Benjumin Hsu, *Harvard Medical School, Boston, Massachusetts, United States*

Aim: The American Heart Association (AHA) recently introduced the cardiovascular-kidney-metabolic (CKM) syndrome. The relationship between CKM syndrome and dementia in community-dwelling older men is unclear. Method: Community-dwelling men ages 70 years and older free of dementia from the CHAMP study (n = 1270) were assessed at baseline (2005-07). CKM syndrome was defined based on the Presidential Advisory from the AHA. Incidence dementia was identified over 17 years based on linked hospital records. The Cox proportional hazards model building for all analyses adjusted for relevant covariates. Results: In this longitudinal study of older men aged 76.8 ± 5.5 years, approximately 2% in stage 0, 2% in stage 1, 46% in stage 2, 19% in stage 3, and 32% were in stage 4. Our model demonstrated significant linear trend ($p < 0.001$) as the stages of CKM become more advanced in the risk of incident dementia. In the multivariable-adjusted model, participants with stage 4 CKM were statistically significantly associated with increased risk of developing dementia (HR: 1.76, 95%CI: 1.07-2.92) when compared to participants with no CKM or stage 0. Participants with stage 3 CKM (HR: 1.60, 95%CI: 0.96-2.66), stage 2 CKM (HR:1.33, 95%CI: 0.81-2.18) and stage 1 CKM (HR: 1.70, 95%CI: 0.87-3.31) may have increased risk of dementia when compared to stage 0 participants. Conclusion: Advanced stages of CKM syndrome is associated with dementia among community-dwelling older men. Older adults with CKM may need to be followed closely and managed in preventing dementia.

Abstract citation ID: igaf122.668

### MEDICATION REFILL ADHERENCE AND MORTALITY RISK IN THE OLDEST-OLD:

Raymond Kheirbek[1], Brock Beamer[2], Patrick McArdle[2], Nadim Ojaimi[2], Bernard Davies-Teye[3], Abree Johnson[3], Farrokh Alemi[4], and Raya Kheirbek[5], *1. Johns Hopkins University, Baltimore, Maryland, United States, 2. University of Maryland, Baltimore, Maryland, United States, 3. University of Maryland School of Pharmacy, Baltimore, Maryland, United States, 4. George Mason University, Fairfax, Virginia, United States, 5. University of Maryland School of Medicine, Baltimore, Maryland, United States*

Background: The U.S. population aged 85 and older is growing rapidly, yet little research explores how medication refill adherence affects mortality in this group. Adherence is critical for managing hypertension and reducing cardiovascular risk, but missed refills may signal broader health, cognitive, or social issues beyond non-compliance. Objective: This study examines the link between medication refill adherence and all-cause mortality in Medicare beneficiaries aged 85+ with hypertension. Design, Setting, and Participants: This retrospective cohort study analyzed Medicare claims data (2006–2021), using a 5% sample (2006–2016) and a 20% sample (2017–2021) of beneficiaries aged 85+ with confirmed hypertension. Exposure: Refill patterns for antihypertensive drugs, statins, and proton pump inhibitors (PPIs) were evaluated via prescription claims, categorizing participants into regular vs. missed refill periods. Outcome Measure: All-cause mortality was the primary outcome, with mortality risk compared between refill patterns using Cox proportional hazards models with time-varying exposure. Results: Regular antihypertensive refills were linked to a 55-66% lower mortality risk (HR: 0.336–0.451), consistent even in those aged 95 + (HR: 0.366–0.441). Similar mortality reductions were seen with statins and PPIs, suggesting refill adherence reflects overall health stability rather than antihypertensive effects alone. Conclusion: Missed refills strongly predict higher mortality in the oldest-old, likely indicating cognitive decline, frailty, or social vulnerability rather than purely pharmacologic factors. Pharmacy refill data could serve as an early-warning tool to identify at-risk older adults for targeted interventions. Further studies should investigate causal pathways and policy implications.

Abstract citation ID: igaf122.669

### SEX AND AGE DEPENDENT DIFFERENCES IN EXTRACELLULAR MATRIX AND CELL PHENOTYPES IN THE MOUSE HEART

Katarzyna Cieslik, *Houston Methodist Research Institute, Houston, Texas, United States*

Fibrosis is one of the hallmarks of aging, which can lead to increased stiffness of the heart, progressing to diastolic dysfunction and heart failure with preserved ejection fraction. While the general mechanisms leading to fibrosis have been well characterized, there is a knowledge gap in our understanding of the distinct mechanisms leading to fibrosis in a male and female aging heart. Mass spectrometry was used to analyze the proteome of decellularized hearts and sorted fibroblasts. As expected, proteome from the old hearts (both sexes) was enriched for various extracellular matrix proteins, mainly involved in collagen fibril assembly. Fibroblasts isolated from the old hearts expressed higher levels of enzymes necessary for collagen processing and cross-linking. Fibroblasts from the old male hearts (as opposed to those from the old female heart) expressed several complement components and other proteins involved in immune responses. Some of the proteins/surface receptors expressed by the fibroblasts in the old hearts are usually expressed by cancer-associated fibroblasts, suggesting pathway similarities between cancer and aging, which have not been investigated in depth in the heart. Finally, Gli1, a transcription factor that plays an important role in cancer progression, is also involved in collagen synthesis in the aging heart and targeting Gli1 expression reduces collagen expression. We also discovered sex differences in mechanisms leading to collagen synthesis and secretion corresponding to tissue stiffness. In conclusion, both sexes present proteome differences, suggesting the activation of distinct pathways that should be targeted to reduce fibrosis more effectively.

### SESSION 2130 (AWARD LECTURE)

Abstract citation ID: igaf122.670

### MAXWELL A. POLLACK AWARD FOR CONTRIBUTIONS TO HEALTHY AGING PRESENTATION AND LECTURE

Chair: Jung Kwak

The Maxwell A. Pollack Award for Contributions to Healthy Aging Lecture will feature an address by the 2024 Pollack Award recipient Edward Alan Miller, PhD, MPA, FGSA, of the University of Massachusetts Boston. This session will also include the presentation of the 2025 Maxwell A. Pollack Award to recipient John W. Rowe, MD, FGSA, of Columbia University. The Maxwell A. Pollack Award for Contributions to Healthy Aging Award recognizes instances of practice informed by research and analysis, research that has directly improved policy or practice, and distinction in bridging the worlds of research and practice.

Abstract citation ID: igaf122.671

### TAKING THE LONG VIEW: AN EVOLVING, MULTI-DIMENSIONAL ARC TO ADVANCING AGING POLICY AND PRACTICE

Edward Miller, *University of Massachusetts Boston, Boston, Massachusetts, United States*

Gerontologists influence aging policy and practice in multiple rich and varied ways during their careers. Dr. Edward Miller will draw on more than 35 years working in the field to illustrate the trajectories (often unplanned) that make a career in gerontology so gratifying. This discussion will be informed by his personal history and educational background, and his professional experience as a researcher and citizen of the profession. Dr. Miller has sought to understand the determinants and effects of federal and state policies affecting older adults, especially those in need of long-term services and supports. His research has applied a range of theories and methodological approaches to elucidating varied dimensions of the aging experience. His efforts include promoting the global dissemination of knowledge in his role as an educator and journal editor. Gerontology is a team sport. Dr. Miller has benefited enormously from the colleagueship of his mentors, students, and collaborators.

### SESSION 2135 (PAPER)

### CAREGIVING REIMAGINED: MIND-BODY APPROACHES, TECHNOLOGY, AND COLLABORATIVE CARE MODELS

Abstract citation ID: igaf122.672

### DELIVERING WOOP FOR DEMENTIA CAREGIVERS TO GROUPS VIA TECHNOLOGY: A FEASIBILITY STUDY

Donna Fedus[1], Emily Mroz[2], Joan Monin[3], Thi Vu[4], and Lauren Lewis[1], *1. Borrow My Glasses, Madison, Connecticut, United States, 2. Emory University, Atlanta, Georgia, United States, 3. Yale, Yale, Connecticut, United States, 4. Yale University, New Haven, Connecticut, United States*

WOOP for Dementia Caregivers (WFDC) is an online health technology program and platform that teaches the evidence-based strategy Wish>Outcome>Obstacle>Plan (WOOP) to caregivers of people living with dementia. WOOP helps individuals engage in self-care by identifying ways to overcome internal obstacles within their control to fulfill important, challenging wishes. This session describes an NIA-funded Small Business Technology Transfer (STTR) study, where a small business (woman-owned gerontology education and evaluation company Borrow My Glasses) collaborated with scholars at research institutions (Yale University and other academic colleagues) and community members to develop WFDC. This session reports qualitative findings from focus group discussions with 24 caregiver-participants who engaged with WFDC. Caregivers learned and practiced WOOP during three online group sessions and received support via a password-protected WFDC website. Analysis of six focus group discussions revealed factors that motivated successful implementation of WFDC: individual motivators like caregivers honoring their commitment to themselves and prioritizing wellbeing; interpersonal motivators like the benefits of group practice and unique facilitator attributes; and programmatic motivators like the fact that WOOP is a simple approachable tool, and the program provides a gentle supportive culture balanced with parameters to maintain progress. Participants also described barriers to successful implementation, including individual barriers of balancing WOOP with other responsibilities and caregiver readiness for support; interpersonal barriers of limited opportunities to maintain relationships with group members and interpersonal tensions; and programmatic barriers of inconsistencies implementing study materials and scheduling constraints. These findings suggest ways to refine WFDC and benefit others developing technology-based programs for caregivers.

Abstract citation ID: igaf122.673

### FOREST BATHING IN URBAN GREEN SPACES: IMPACT ON WELLBEING OF FAMILY CAREGIVERS OF PEOPLE LIVING WITH DEMENTIA

Jacky C. P. Choy, and Maggie S. L. Ma, *The University of Hong Kong, Hong Kong, China (People's Republic)*

Forest bathing is an emerging approach aimed at addressing anxiety, depression, and overall psychological wellbeing. Given the needs of family caregivers of people living with dementia (PLwD) and the availability of public green spaces in Hong Kong, we adapted the forest bathing intervention for family caregivers and implemented it in parks in the urban areas. This study aims to assess its acceptability, feasibility, and effectiveness in reducing caregivers' burden, depressive symptoms, and anxiety symptoms. We conducted a non-randomized controlled trial with 107 dyads of family caregivers and PLwD in the community. The experimental group ($n = 54$) participated in three sessions of the forest bathing intervention, while the control group ($n = 53$) received the same number of sessions of art activity intervention. Data on attrition, satisfaction, adherence, and changes in primary outcomes over six weeks were collected and analysed. The intervention protocol was highly acceptable and feasible, with a 17% attrition rate, 89% of participants perceiving benefits for their wellbeing, 75% adhering to the homework protocol, and accessible green spaces in the neighbourhood at no additional cost. Compared to the control group, caregivers who participated in the forest bathing intervention reported lower levels of care burden, depressive symptoms, and anxiety symptoms, after controlling for demographic factors. The findings support the forest bathing intervention as an evidence-based group activity in community caregiver support services. Further investigations are needed to explore its mechanisms of change, particularly the dyadic participation of caregivers and PLwD, as well as the homework component that encourages self-practice.

Abstract citation ID: igaf122.674

### IMPACT OF AN ADRD CAREGIVER INTERVENTION DURING THE COVID-19 PANDEMIC

Lena Thompson[1], Freda Lynn[2], Emily Killian[2], Maria Donohoe[2], Hyunkeun Cho[2], and Sato Ashida[2], *1. University of Alaska Fairbanks, Fairbanks, Alaska, United States, 2. University of Iowa, Iowa City, Iowa, United States*

The Building a Bridge (BAB) intervention is a mixed-method clinical trial connecting caregivers with programs, resources, and information to support people newly diagnosed with dementia. BAB was forced to adapt during the Covid-19 pandemic—a time when services for people living with dementia (PLWD) and their caregivers were greatly reduced. This study assesses the experiences and impact of the pandemic-altered intervention to inform best practices for interventions that address caregiver needs during times of societal crisis. Participants completed baseline (n = 81) and 3-month follow-up (n = 53) telephone surveys (e.g., Zarit Burden; knowledge; self-efficacy). Qualitative data included participant responses to open-ended questions at 3-month follow-up and interventionists' notes for 31 participants. Data were analyzed using change score regression models and rapid qualitative analysis. Statistically, intervention participants gained more knowledge about ADRD and caregiving compared to control (p=.036) and those with low knowledge at baseline gained more knowledge (p=.044). On average, self-efficacy did not statistically change and distress (e.g., burden, anxiety) increased. A sensitivity analysis confirms the robustness of results despite attrition. Participants described feeling more knowledgeable about dementia and future planning, and they consistently expressed gratitude for the interventionists' emotional support. Interventionists reported providing unplanned support outside the intervention like support with selling a home. The BAB intervention did not increase self-efficacy to use services, likely because services were unavailable during the pandemic timeframe. However, it increased participants' knowledge and connected them to social systems that allow them to activate resources when needed, building resilience to withstand challenging situations like the pandemic.

Abstract citation ID: igaf122.675

### JOURNALING AS A TOOL FOR CARE PARTNERS TO PROCESS AND STAY ENGAGED BETWEEN INTERVENTION SESSIONS

Jacqueline Eaton[1], Sarah Neller[2], Moroni Fernandez Cajavilca[1], Julene Johnson[3], and Lee Ellington[1], *1. University of Utah, Salt Lake City, Utah, United States, 2. University of Tennessee, Knoxville, Tennessee, United States, 3. University of California, San Francisco, San Francisco, California, United States*

The Enhancing Active Caregiver Training (EnACT) intervention was developed to improve how care partners of persons living with dementia (PLWD) interact during training and support groups. During the pilot randomized trial, three sessions of EnACT were delivered every two weeks to 30 care partners of PLWD. To enhance retention and engagement during the two-week interval between intervention sessions, we provided optional take-home exercises. Each exercise included two open-ended questions: one related to the previous session (e.g., exploring relationship changes; what it means to keep going) and another free-write on a topic of choice. Response rates were high with 86% of participants completing all take-home exercises; the average completion time was 13 minutes per exercise. We conducted an inductive thematic analysis of the journal entries. Two major themes were identified: 1) New Connections Through Shared Caregiving Roles and 2) Persistence through challenges. Participants experienced loneliness due to caregiving demands but found friendship through shared experiences. They faced challenges related to illness, behavioral symptoms, financial concerns, changes in temperament, and loss. Perseverance was essential; faith, gratitude, and moments of connection helped them "keep going." The take-home exercises encouraged participants to reflect and expand upon EnACT topics between sessions, likely contributing to high retention rates. Post intervention participants rated exercises positively and appreciated the opportunity to "sit down and think" and "formulating your thoughts was helpful." Optional take-home exercises reinforced EnACT and supported retention. Future efforts will explore journaling as a tool to enhance application of EnACT beyond the intervention space.

Abstract citation ID: igaf122.676

### MANAGING MEDICATIONS FOR OLDER ADULTS: CONCEPTUALIZING PRIMARY AND SECONDARY CAREGIVER COLLABORATION

Te-Lien Ku[1], Laura Block[1], James Ford[2], Shu-Ying Wu[3], and Yen-Ming Huang[4], *1. University of Wisconsin-Madison, Madison, Wisconsin, United States, 2. University of Wisconsin–Madison, Madison, Wisconsin, United States, 3. Sijhih Cathay General Hospital, New Taipei City, New Taipei, Taiwan (Republic of China), 4. National Taiwan University, Taipei City, Taipei, Taiwan (Republic of China)*

Approximately 90% of older adults take at least one prescription medication, with a third taking more than five. Family members, including primary and secondary caregivers, play critical roles in helping older adults manage medications, yet research has not explored how they collaborate on these tasks. Limited understanding of multi-caregiver collaboration on medication management hinders support effort and jeopardize patient's disease management and safety. This study used a secondary analysis of qualitative interview data with 29 family caregivers in Taiwan who self-identified as primary caregivers of an older adult and collaborated with at least one other caregiver on medication management. Thematic analysis began with a deductive approach using Look and Stone's medication management activity framework, followed by inductive analysis to identify additional themes. Two themes emerged capturing caregiver collaboration: (1) Conferring variable decision-making authority and (2) Assuming an active or passive support role. Three collaboration strategies were patterned based on the two themes: (1) Partnership—both caregivers shared decision-making authority and tasks equally; (2) Delegation—the primary caregiver retained most decision-making and task responsibility but temporarily shifted tasks to the secondary caregiver when unavailable; and (3) Directive—the primary caregiver retained full decision-making while assigned the secondary caregiver's predefined routine tasks. Collaboration shifts based on physical or cognitive task demands, caregivers' knowledge and skills in medication management, and relationship and care availability factors such as gender, kinship, marital status, co-residency, and employment. Future research should explore how caregiver satisfaction with these strategies affects medication management and the role of socio-demographic determinants.

Abstract citation ID: igaf122.677

### MIND-BODY INTERVENTION FOR STRESS AND HYPERTENSION IN BLACK FEMALE DEMENTIA FAMILY CAREGIVERS: A FEASIBILITY STUDY

Kathy Wright[1], Ingrid K. Richards Adams[2], Nathan Helsabeck[2], Karen Moss[3], Donya Nemati[4],

Christopher Nguyen[2], and Maryanna Klatt[2], *1. The Ohio State University, Powell, Ohio, United States, 2. The Ohio State University, Columbus, Ohio, United States, 3. The Ohio State University, Blacklick, Ohio, United States, 4. Ohio State University, Columbus, Ohio, United States*

Black females caring for family members living with Alzheimer's disease and related dementias (ADRD) face high risks of caregiving stress and hypertension, leading to reduced quality of life and cardiovascular disease. While some interventions address ADRD caregivers' stress and quality of life, gaps remain in targeting chronic caregiving stress and hypertension self-care. This Stage I (1R21AG077069) pilot study was conducted to determine the feasibility and acceptability of Mindfulness in Motion plus the Dietary Approaches to Stop Hypertension (MIM DASH) for Black female ADRD family caregivers. Participants (N = 28) were randomized to either MIM DASH or Alzheimer's Association Caregiver Training (attention control) for eight weekly, 1.5-hour group sessions via Zoom Communications, Inc (video and telephone access). After completion of the intervention, both groups received eight weekly calls for two months, followed by one monthly call for four months. The intervention demonstrated high feasibility, with a 93% enrollment rate and 86% and 93% retention rates at three- and nine-month follow-ups, respectively. Participants in the MIM-DASH group attended an average of 6.29 sessions compared to 4.86 for the attention control group. Usability scores for MIM-DASH participants were high (mean 50.31), and satisfaction ratings averaged 8.77 out of 10. Credibility ratings were similarly robust, with a mean score of 41.23 out of 45 for the intervention group. Fidelity was maintained with minor deviations observed. MIM DASH was feasible and acceptable for Black female caregivers. Findings support the delivery of a Stage II efficacy trial.

### SESSION 2137 (BIOLOGICAL SCIENCES INVITED SYMPOSIUM)

Abstract citation ID: igaf122.678

### PRESIDENTIAL SYMPOSIUM: INTEGRATING GEROSCIENCE ACROSS SCALE

Chair: Sean Curran

Abstract citation ID: igaf122.679

### TRANSLATING SENOLYTICS FROM MICE TO HUMANS

Sundeep Khosla, *Mayo Clinic College of Medicine and Science, Rochester, Minnesota, United States*

Extensive studies in rodents have shown that senescent cells accumulate across tissues and their clearance leads to improvements in healthspan and lifespan. Based on these studies, there is intense interest in translating these findings into humans. Based on ClinicalTrials.gov, there are currently 26 ongoing studies on "senolytics" and 32 on "fisetin". However, to date, there are only 9 published clinical trials of senolytics, only 2 of which included a control group. Collectively, the initial findings from these trials have indicated possible biological efficacy of senolytics (dasatinib + quercitin) in improving biomarkers of healthspan in humans with no significant safety concerns. However, these initial studies also highlight the potential heterogeneity of aging mechanisms in humans and argue for a more personalized approach in future senolytic trials. Specifically, it is likely going to be critical to identify aging individuals with a sufficiently high burden of senescent cells that would result in a beneficial effect from the senolytic intervention.

Abstract citation ID: igaf122.680

### TIME TO LEARN: THE ROLE OF THE CLOCK GENE PER1 IN REGULATING MEMORY ACROSS THE LIFESPAN

Janine Kwapis, *Pennsylvania State University, University Park, Pennsylvania, United States*

Many biological processes, including memory formation, are strongly influenced by the circadian system, which synchronizes animals' internal states with the external time of day. Long-term memory performance changes across the day/night cycle in both humans and rodents, yet the mechanisms that support this process are largely unknown. In this talk, I will present my lab's research suggesting that a circadian clock gene, Period1 (Per1) may serve as a molecular interface between the circadian clock and memory formation. Per1 oscillates in tandem with memory in memory-relevant brain regions, like the hippocampus, with both memory and Per1 levels peaking during the day and showing a trough at night. Interestingly, old animals have repressed hippocampal Per1 levels even during the day, suggesting that they may show impairments in memory due to this persistent "nighttime state" that limits memory across the diurnal cycle. Finally, we find that young and old female mice show unique memory oscillation patterns that depend on sex hormone expression. Together, our data suggest that Per1 may function locally in memory-relevant brain regions to exert diurnal control over memory and age-related disruption of Per1 may contribute to age-related cognitive decline.

Abstract citation ID: igaf122.682

### GEROSCIENCE MEETS MEDICAL AND SOCIAL SCIENCE: EXPLAINING DIFFERENTIAL AGING HEALTH

Eileen Crimmins, *University of Southern California, Los Angeles, California, United States*

Geroscience has hypothesized that all age-related health outcomes are related to a set of age-related biological processes that combine to produce health deterioration with age. Medical science has been developing an increasingly wider array of risk factors for age-related health outcomes. Social science has long focused on differential aging; i.e. aging that occurs at different rates for population subgroups, those exposed to adverse social conditions, or health behaviors that put them at risk. Using insights from geroscience and medical science, I will provide greater clarity to how the social world gets under the skin to produce differential rates of aging through the mediation of biological processes. This will be done through the integration of biological measures at two levels – the molecular and cellular features included in the "Hallmarks of Aging" and risk factors identified at the biochemical or clinical level – into models assessing social differences in important health outcomes linked to age.

### SESSION 2140 (PAPER)

### AGING, ETHICS AND SOCIETY

Abstract citation ID: igaf122.683

### "TREAT ME WITH RESPECT": OLDER SEXUAL MINORITY MEN'S INTERACTIONS WITH HEALTHCARE PROVIDERS

Mekiayla (Meki) Singleton[1], Jennafer Kwait[2], Deborah Konkle-Parker[3], Deanna Ware[4], Michael Plankey[5], Janet Turan[6], Katherine Wu[7], and Mackey Friedman[1], *1. Rutgers University, Newark, New Jersey, United States, 2. Health Research & Policy Institute at Whitman Walker Institute, Washington, District of Columbia, United States, 3. University of Mississippi Medical Center, Jackson, Mississippi, United States, 4. Georgetown University Medical Center, Washington, District of Columbia, United States, 5. Georgetown University, Washington, District of Columbia, United States, 6. The University of Alabama at Birmingham, Birmingham, Alabama, United States, 7. Johns Hopkins University, Baltimore, Maryland, United States*

Engagement with the healthcare system, including interactions with healthcare providers, can have a critical impact on health outcomes. For marginalized communities, experiences with healthcare providers may be more challenging to navigate. The purpose of this study was to explore older sexual minority (SM) men's experiences of their interactions with healthcare providers. Data for this study originated from the MACS/WIHS Combined Cohort Study (Stigma and Non-Communicable Syndemic Sub-Study). Participants (N = 887) were asked to use open-text responses included in a survey to describe times when they felt a) the most comfortable and b) least comfortable in their interactions with a healthcare provider. Data consisted of unstructured, free-text responses, which was analyzed using content analysis and organized into themes. The average age of participants was 60.7 (SD = 12.7) years; 69% identified as White, 10% were Hispanic; and 54% were living with HIV. Results showed that themes describing most comfortable interactions included: 1) having a provider that "listens and is attentive"; 2) "takes questions and concerns seriously"; 3) "discusses personal issues outside of health"; 4) "cares for the person as a whole". Themes describing least comfortable interactions included: 1) "experiencing bias" around sexual orientation and HIV status and 2) "negative provider demeanor/attitude". Outcomes from this work highlight that for older SM men there are specific characteristics that support feeling comfortable when interacting with a healthcare provider. These findings provide useful information that can inform and guide the implementation of policies and procedures in healthcare settings that serve a diverse older adult population.

Abstract citation ID: igaf122.684

### AGE DISPARITIES IN FREQUENCY OF EVERYDAY DISCRIMINATION AND HEIGHTENED VIGILANCE IN A NATIONAL UNITED STATES SAMPLE

Roger Wong, Karen Gonzalez, and Kiersten Crawford, *SUNY Upstate Medical University, Syracuse, New York, United States*

Discrimination and vigilance are known to be crucial psychological factors that influence how individuals interact with others in their environment, however, whether these experiences vary across the lifespan remains poorly understood. Our present study compared age differences in the frequency of discrimination and vigilance. The 2023 National Health Interview Survey administered the validated five-item Everyday Discrimination Scale (e.g. treated with disrespect) and four-item Heightened Vigilance Scale (e.g. careful about your appearance) among a nationally representative sample of 28,583 adults in the United States. We categorized respondents into four age groups: young adult (18-24 years), early middle-age (25-39 years), late middle-age (40-64 years), and older adult (65+ years). Composite scores were created separately for discrimination (range 0-20) and vigilance (range 0-16) by adding all scale items. Average discrimination and vigilance scores were highest among young adults, but gradually decreased for each subsequent age group, which were statistically significant based on ANOVA tests. Two separate negative binomial regression models indicated compared to young adults, older adults had a significantly lower frequency of discrimination by 48% (Incidence Rate Ratio [IRR]=0.52, 95% CI = 0.48-0.57, p<.001) and vigilance by 39% (IRR=0.61, 95% CI = 0.56-0.66, p<.001), after applying complex survey sampling weights and adjusting for sociodemographic, geographic, and health covariates. These findings suggest that as age increases, frequencies of exposure to discrimination and exhibiting vigilant behaviors decrease. Further research is needed to explore the underlying mechanisms behind these age-related differences and how these associations may intersect with other sociodemographic factors such as race, gender, and sexual orientation.

Abstract citation ID: igaf122.685

### PERCEPTION OF AGING AND CHANGES IN LATER LIFE: FINDINGS FROM ETHNOGRAPHIC INTERVIEWS WITH OLDER ADULTS

Lun Li[1], and Yeonjung Lee[2], *1. MacEwan University, Edmonton, Alberta, Canada, 2. University of Calgary, Calgary, Alberta, Canada*

Older adults' understanding of aging and their perception of aging-related issues (e.g., health, social life) have fundamental impacts on their preparation for later life, and the quality of life during the aging process. This study intends to explore how older adults understand aging and aging-related issues through later life experiences during and after the COVID-19 pandemic. This study is based on 21 ethnographic interviews conducted by social work students who enrolled in a gerontology course in a western Canada university between 2022 and 2024. The participants of interviews are older adults aged 70 years and older. A thematic analysis was conducted to all 21 transcripts. Four main themes were identified, including 1) Older adults normalize the aging process through the changes of family/generation structure, a shift from a productive to a recreative social life, and the narrowed networking size; 2) Older adults adjust to the deterioration of physical health and functional capability using various strategies; 3) Older adults distinguish the perception of aging from the concept of oldness; and 4) The COVID-19 pandemic and recent developments (e.g., technology advancement) contribute to older adults' challenges in navigating their later life. Findings suggest that older adults actively make changes to their different life aspects to enhance the quality of later life. It also reveals older adults' understanding of aging process, particularly their acceptance of age but not oldness. This study further emphasizes the salience of aging-friendly society without barriers to enable healthy and active aging.

Abstract citation ID: igaf122.686
### KING LEAR FIGHTING THE DEMENTORS: RETHINKING THE POLITICS OF DEMENTIA REPRESENTATION IN POPULAR CULTURE
Ulla Kriebernegg[1], and Heike Hartung[2], *1. University of Graz, Graz, Steiermark, Austria, 2. University of Potsdam, Potsdam, Brandenburg, Germany*

This paper examines how contemporary literature, film, and theater shape public perceptions of dementia, often reinforcing negative stereotypes that contribute to the othering of individuals living with the condition. Popular fiction, such as J.K. Rowling's Harry Potter series, employs dementia-adjacent metaphors—like the soul-sucking Dementors—to depict memory loss as an erasure of identity. Similarly, films like Still Alice and The Father use dementia metonymically, reducing individuals to symbols of loss. Since the 1980s, cultural representations of dementia have ranged from depictions of the disease as a "living death" to more nuanced explorations of forgetfulness. However, today, dementia is frequently framed as a crisis of aging, often through symbolic narratives that heighten societal anxieties about cognitive decline. A key example that will be analyzed is Shakespeare's King Lear, whose modern reinterpretations increasingly medicalize Lear's descent into madness, diagnosing him with Lewy Body Dementia. This reading shifts the focus from Lear's emotional and psychological turmoil to an ageist burden narrative that aligns with contemporary fears of cognitive decline, loss of autonomy, and aging as a social and economic crisis. This paper first analyzes these culturally influential examples to explore how mainstream narratives shape public discourse on dementia. It then turns to more nuanced portrayals to examine aesthetic strategies that offer alternative and more humane representations. Ultimately, we argue for a cultural politics of dementia informed by critical aging studies to challenge ageist frameworks and promote attitudinal change toward aging and dementia.

### SESSION 2145 (SYMPOSIUM)

Abstract citation ID: igaf122.687
### (UN)FORBIDDEN WORDS: DIVERSITY, EQUITY & INCLUSION IN GERONTOLOGY & GERIATRICS EDUCATION
Chair: Rona Karasik
Discussant: Mark Brennan Ing

Gerontology and geriatrics have long advocated for the consideration and inclusion of older adults in community, policy, practice and research. Systemic ageism, however, cannot be separated from the context and power dynamics inherent in structural racism, sexism, heterosexism, ableism, xenophobia and the like. Understanding aging requires consideration of the entire life course, including the social determinants that impact the lives and experiences of all older adults. So how do gerontological educators proceed as higher education grapples with directives determined to dismantle scientific inquiry in general, and exploration of diversity, equity and inclusion in particular? This symposium, sponsored by Gerontology & Geriatrics Education, reflects on current challenges and considers pathways forward as we continue to advocate for diversity, equity and inclusion in gerontological education. First, as an introduction, Karasik & Kishimoto discuss the concepts of diversity, equity and inclusion as they relate to gerontology and geriatrics education. Next, Kishimoto and Karasik consider the role of antiracist pedagogy in gerontological education and how it can be applied to teaching about health disparities throughout the life course and across generations. Third, Montepare and colleagues address the concept of age inclusivity as perceived by students/faculty/staff and its implications for educational efforts. Fourth, Flatt highlights current obstacles and ways for education, training, and activism to support sexual gender and minority aging research to move forward. Finally, discussant Brennan-Ing will engage participants in a structured discussion of next steps for defending diversity, equity, and inclusion as the essence of gerontological inquiry and education.

Abstract citation ID: igaf122.688
### IT'S ABOUT ALL OF US: DEFINING AND DEFENDING DEI IN GERONTOLOGICAL EDUCATION
Rona Karasik[1], and Kyoko Kishimoto[2], *1. Saint Cloud State University, Saint Cloud, Minnesota, United States, 2. St. Cloud State University, St. Cloud, Minnesota, United States*

This is a critical period in teaching about DEI, where the concepts of diversity, equity and inclusion are under attack, demonized, and purposefully misrepresented for political gain. Now, more than ever, students preparing to work in gerontology and geriatrics need a broad understanding of how policies and events impact groups differently over their life course. This presentation lays the groundwork for demystifying DEI -- what it is, what it does, and why DEI is essential to gerontology and geriatrics education, theory, research, policy and practice. Despite its focus on diminishing discrimination and increasing recognition of historically marginalized groups (e.g., people of color, women, older adults, sexual and gender minorities, the economically and socially disenfranchised), DEI's scope and purpose are often misunderstood and mistakenly maligned as a zero-sum game such that any effort to obtain basic rights for some necessarily means a loss for others. Rather than recognizing the mutual societal benefit of educational programs, employment opportunities, and research (both medical and social), the current attack on "all things DEI" threatens to erase the hard-won (albeit sorely incomplete) advances made through civil rights actions, the women's movement, the Americans with Disabilities and Older American's Acts, socioeconomic and healthcare programs including Social Security, Medicare and Medicaid, and the like. By defining and clarifying the concepts of diversity, equity and inclusion as they relate to gerontological education, this presentation highlights DEI's connection to people of all ages and backgrounds. Ultimately, DEI isn't about "them"; it's about "us" -- all of us.

Abstract citation ID: igaf122.689
### JOURNEYS OF A LIFETIME: APPLYING ANTIRACIST PEDAGOGY TO HEALTH DISPARITIES ACROSS THE LIFE COURSE
Kyoko Kishimoto[1], and Rona Karasik[2], *1. St. Cloud State University, St. Cloud, Minnesota, United States, 2. Saint Cloud State University, Saint Cloud, Minnesota, United States*

Pervasive health disparities among ethnic and racial groups throughout the life-course and across generations are well documented and become amplified as they intersect with factors of age and gender (Martinez, 2024; Steward et al., 2025). Despite this knowledge, racial disparities in all aspects of health continue to persist, suggesting simple acknowledgement is insufficient to address them. Rather, educators must guide the next generation of researchers and practitioners to uncover root causes of these disparities – an essential step toward effecting social change. A significant barrier to this process is a lack of historical knowledge and policy context among students and many educators (Karasik & Kishimoto, 2018; 2022). This challenge is exacerbated by current political attacks on DEI, which by design, create confusion and fear to even talk about health disparities – much less try to alleviate them. Silencing the problem, however, does not make it go away. Therefore, new and enhanced strategies are needed to engage and support students in exploring the sources and impact of these disparities. Using an antiracist pedagogical approach, this project pairs an extensive annotated timeline of key historical events and multi-media resources with health care related policies/programs in a digitized format – allowing students to follow historically accurate, fictionalized life-story vignettes to their current circumstances, while also providing opportunities to extrapolate potential future outcomes. In addition to providing an analysis of the underlying principles and inner workings of this interactive interface, this presentation offers insight into the student learning experience and strategies for next steps.

Abstract citation ID: igaf122.690

### AGE INCLUSIVITY: PERCEPTIONS BY STUDENTS, FACULTY, AND STAFF OF THE NEGLECTED DEI CATEGORY

Joann Montepare[1], Lauren Bowen[2], Susan Whitbourne[2], and Nina Silverstein[2], *1. Lasell University, Bedford, Massachusetts, United States, 2. University of Massachusetts Boston, Boston, Massachusetts, United States*

Diversity, equity, and inclusion (DEI) efforts in higher education seldom incorporate age-inclusive values, practices, and policies in intentional ways into ongoing programs. However, the need to advance age inclusivity across our colleges and universities has become a pressing issue for many reasons which call for extending attention to this neglected category. The results of a study are described in which a qualitative analysis was conducted of responses by 412 students, faculty, and staff to an open-ended question about age inclusivity. Although some responses indicated that age inclusivity was an unfamiliar concept (12%), many other responses indicated that respondents perceived age inclusivity in higher education as a valuable focus (40%). Differing views included that age inclusivity should not be a focus in higher education for various reasons including that the needs of younger students are more central to the mission of higher education or that the efforts should be "age-blind" just as other DEI efforts should be "color-blind" (26%), or that it can only work if it is enacted appropriately and thoughtfully (12%). Other comments revealed the existence of ageism on campuses, and the need to rethink how higher education refers to age-diverse students and uses language to communicate information about aging to avoid reinforcing ageist thinking. Study results offer several insights about integrating a more age-inclusive focus into broader DEI efforts including the need to raise awareness, make a clear case for the benefits, and utilize meaningful strategies with specific goals.

Abstract citation ID: igaf122.691

### NAVIGATING CHALLENGES IN SEXUAL AND GENDER MINORITY AGING RESEARCH AND EDUCATION: ADVOCACY AND COMMUNITY ACTION

Jace Flatt[1], and Ethan Cicero[2], *1. University of Nevada Las Vegas, Nevada, United States, 2. Emory University, Atlanta, Georgia, United States*

On October 16, 2016, the directors of the National Institute of Minority Health Disparities, with support from the Agency for Healthcare Research and Quality, designated sexual and gender minorities (SGMs)—including lesbian, gay, bisexual, transgender, queer, intersex, asexual, and additional identities (LGBTQIA+)—as a health disparity population for National Institutes of Health research. As of January 20, 2025, Executive Order 14168, titled "Defending Women from Gender Ideology Extremism and Restoring Biological Truth to the Federal Government," has been used to overrule this designation and terminate federally-funded research involving SGM aging research, as well as rescind funding decisions and peer review of federal research grants including SGM aging populations. In this talk, we will highlight the current state of the science and challenges and pathways forward for education, training, and activism to support LGBTQIA+ aging populations and SGM aging research. This includes supporting early-career scholars, collecting sexual orientation and gender identity data in aging research, and developing strategies for advocating for terminated and rescinded research grants. We will provide examples from our nearly five million in federal research grants that have been terminated or rescinded. We will also share lessons learned from our efforts to promote visibility, advocacy, and community-driven action to support LGBTQIA+ aging research and education.

### SESSION 2150 (SYMPOSIUM)

Abstract citation ID: igaf122.692

### ADVANCING PERSON-CENTERED DEMENTIA CARE IN LONG-TERM CARE SETTINGS: GLOBAL PERSPECTIVES FROM FIVE COUNTRIES

Chair: Jing Wang
Co-Chair: Kirsten Corazzini
Discussant: Edward Miller

As the global population ages, long-term care (LTC) systems across diverse cultural and economic contexts face mounting challenges in providing person-centered dementia care (PCDC). This symposium brings together five studies from five countries, highlighting innovative strategies, workforce-driven facilitation, quality-of-life conceptualizations, behavioral symptom management, and technology-driven interventions in LTC settings. By examining experiences across different healthcare systems, this session offers insights into strengthening dementia care through interdisciplinary, culturally responsive, and sustainable approaches. The first presentation (United States) explores resilience-based strategies that enable LTC staff to uphold PCDC despite resource limitations, identifying organizational, interpersonal, and individual strengths that foster adaptability. The second study (Canada) challenges traditional facilitation frameworks by demonstrating how frontline workers in nursing homes informally drive care innovations, filling gaps between leadership initiatives and direct caregiving. The third presentation (Netherlands) investigates how nursing home administrators conceptualize and promote quality of life (QoL) for persons with dementia, revealing tensions between individualized and community-centered approaches. The fourth study (China) examines agitation in older LTC residents with cognitive impairment, identifying key individual, family, staff, and facility-level factors that contribute to behavioral symptoms. The final presentation (Singapore) evaluates an interdisciplinary telemedicine program aimed at reducing avoidable emergency department visits among nursing home residents, demonstrating how technology can optimize acute care management. Together, these studies provide a comprehensive, cross-national perspective on sustaining and enhancing person-centered dementia care in LTC. They underscore the need for workforce support, culturally informed care strategies, innovative facilitation models, and scalable technology-enabled solutions to improve dementia care across diverse healthcare settings. Common Data Elements for International Research in Residential Long-Term Care Interest Group Sponsored Symposium

Abstract citation ID: igaf122.693

**REDEFINING FACILITATION IN NURSING HOMES: THE CRITICAL ROLE OF FRONTLINE WORKERS IN IMPLEMENTING CARE INNOVATIONS**

Jing Wang[1], Ruth Anderson[2], Yinfei Duan[3], Carole Estabrooks[3], and Anna Beeber[4], *1. University of New Hampshire, Durham, New Hampshire, United States, 2. Duke University, Durham, North Carolina, United States, 3. University of Alberta, Edmonton, Alberta, Canada, 4. Johns Hopkins University, Baltimore, Maryland, United States*

Facilitation in healthcare is traditionally conceptualized as a structured, leadership-driven process within frameworks such as Promoting Action on Research Implementation in Health Services (PARIHS) and integrated-PARIHS (i-PARIHS). However, in nursing homes, where frontline workers—particularly care aides—have the most direct interactions with residents, facilitation occurs informally and is embedded in daily caregiving. This study highlights how frontline workers function as facilitators of care innovations, despite not being formally recognized in existing facilitation frameworks. We conducted qualitative analysis of data from the Translating Research in Elder Care (TREC) program, analyzing 60 in-depth interviews and 150 pages of fieldnotes from three nursing homes. Thematic analysis using ATLAS.ti mapped out the relationships between facilitation, innovation, and implementation strategies, revealing distinct patterns of facilitation. Frontline workers played a key role in bridging gaps between leadership-driven initiatives and the realities of daily care, adapting innovations to fit practical constraints. In settings where facilitation was distributed across multiple staff levels, care aides took initiative in leading pressure ulcer prevention, falls reduction, and dementia behavior management strategies. Conversely, in facilities with hierarchical, top-down facilitation models, staff disengagement and resistance led to ineffective adoption of care innovations. This study challenges traditional paradigms by demonstrating that facilitation in nursing homes is a system-wide, collaborative process rather than solely a leadership function. Recognizing and legitimizing frontline staff as facilitators is critical to sustaining innovation, improving care quality, and fostering adaptive implementation strategies in nursing home settings.

Abstract citation ID: igaf122.694

**FACTORS OF AGITATION IN OLDER CHINESE WITH COGNITIVE IMPAIRMENT IN LONG-TERM CARE FACILITIES: A MULTILEVEL ANALYSIS**

Kaipeng Wang[1], Fei Sun[2], and Xiang Gao[3], *1. University of Denver, Denver, Colorado, United States, 2. Michigan State University, East Lansing, Michigan, United States, 3. Huazhong University of Science and Technology, Wuhan, Hubei, China (People's Republic)*

Agitation among older residents with cognitive impairment is a common challenge in long-term care facilities. However, systematic investigations into contributing factors of agitation of older Chinese residents with cognitive impairment in long-term care facilities remain limited. This study examines individual, family, staff, and facility-level factors associated with agitation in older Chinese with cognitive impairment in long-term care facilities. We collected individual and family data from 266 older adults with cognitive impairment (Mini-Mental State Examination (MMSE) score < 24) and facility staff members across 19 long-term care facilities. Data on staff and facility characteristics were ascertained from direct care workers (DCWs) and facility administrators. Results of linear mixed-effects models show that higher education levels and poorer psychological adjustment to the facility were associated with a higher level of agitation. In contrast, residents with better cognitive functioning and those who called family members at least once a week exhibited lower aggression. At the facility level, a higher mean of DCWs' perceived organizational support and the presence of at least one DCW on a permanent contract were associated with lower agitation. These results underscore the complex influence of individual, family, staff, and facility factors on agitation among older Chinese residents. Interventions aimed at strengthening older resident family connections and enhancing DCW support systems may be critical in mitigating agitation and improving the well-being of this population.

Abstract citation ID: igaf122.695

**TELEMEDICINE BRIDGE BETWEEN EDS AND NHS: REDUCING NH RESIDENTS' AVOIDABLE EMERGENCY VISITS**

Hanzhang Xu[1], Chong Yau Ong[2], Angus Jun Jie Ng[2], Jiameng Yuan[1], Gayathri Devi Nadarajan[3], Marcus Ong[4], and Jean Mui Hua Lee[2], *1. Duke University, Durham, North Carolina, United States, 2. Sengkang General Hospital, Singapore, Singapore, 3. Singapore General Hospital, Singapore, Singapore, 4. Duke-NUS Medical School, Singapore, Singapore*

Nursing Home (NH) residents have high rates of emergency department (ED) visits, with nearly half potentially avoidable. We evaluated the impact of the EAGLEcareACT (Enhancing Advance Care Planning, Geriatrics, and End-of-Life Care Acute Care Team) --an interdisciplinary telemedicine program at Sengkang General Hospital (SKH), Singapore linking ED physicians with NH staff—on reducing avoidable ED visits among NHs residents. Between 2020-2023, 7 out of the 35 NHs in the catchment area were enrolled in EAGLEcareACT. We analyzed data from all ED visits at SKH from NHs residents 2020-2023. Outcomes included hospitalization, National Early Warning Score (NEWS), and ED acuity levels. We used generalized estimating equations models, to assess associations between EAGLEcareACT enrollment and outcomes accounting for within-NH clustering. Among 6,344 ED visits from NH residents (median age 78 years, 37.8% female, 85.8% Chinese), 2,483 (39.1%) were from EAGLEcareACT partnered NHs. Patients from partnered NHs were slightly older (median 79 vs. 78 years) and more likely to be female (43.4% vs. 34.2%) than patients from non-partnered NHs. After adjustment, comparing to patients from non-partners NHs, those from partnered NHs were more likely to be hospitalized (odds ratio [OR] 1.59, 95% CI 1.10-2.29), have a critical condition as NEWS scores ≥5 (OR 1.76, 95% CI 1.27-2.45), and present with high acuity (OR 1.69, 95% CI 1.15-2.50). These findings suggest that EAGLEcareACT effectively manages non-urgent cases at NHs via telemedicine while appropriately identifying high-risk older adults requiring ED care. Further research is needed to evaluate long-term outcomes and healthcare utilization impacts.

Abstract citation ID: igaf122.696

### PERSON-CENTERED VS PERSONS-CENTERED: EXPLORING QUALITY-OF-LIFE PROMOTION AMONG ADMINISTRATORS IN NURSING HOMES

Olivia Black[1], and Jing Wang[2], *1. Boston College, Hampton, New Hampshire, United States, 2. University of New Hampshire, Durham, New Hampshire, United States*

As dementia is a progressive and incurable condition, improving QoL stands as a paramount objective in care. While there are strategies to improve QoL for persons with dementia (PWD), person-centered care is particularly prominent. However, defining QoL for PWD is complex; it is widely acknowledged to be subjective and multifaceted. There is a notable gap in research regarding how PWD themselves, as well as those who care for and support them, perceive QoL. Our study investigates general perceptions of QoL and specifically how it is understood and promoted for PWD in nursing homes, as described by nursing home administrators (i.e. executive directors, therapeutic directors). We utilized an exploratory and descriptive qualitative study design, gathering data through semi-structured interviews with thirteen administrators from a variety of nursing homes, which ranged from independent to chain-affiliated facilities that accommodate PWD. Results demonstrate a divergence in perspectives, with some interviewees stating that QoL is tied to social relationships, whereas others stating that QoL is more about preserving individual agency. Results also revealed a divergence in prioritization among administrators, with some emphasizing person-centered QoL measures while others focused on broader QoL promotion for the resident community. Additionally, interviewees highlighted variations in the conceptualization of QoL across micro-communities within the facility, particularly in facilities with separate memory care units. Moreover, some administrators expressed challenges in maintaining consistent practices of QoL promotion due to the changing needs of residents. These findings have significant implications for the conceptualization and development of person-centered QoL measures.

Abstract citation ID: igaf122.697

### RESILIENCE THROUGH STRENGTHS-BASED STRATEGIES: PERSON-CENTERED CARE IN LOW-RESOURCE LONG-TERM CARE SETTINGS

Jing Wang[1], Yoon Chung Kim[2], Alison Rataj[3], Sarah Holmes[4], Michael Lepore[5], Nancy Kusmaul[6], Laura Davie[1], and Kirsten Corazzini[1], *1. University of New Hampshire, Durham, New Hampshire, United States, 2. University of Maryland School of Medicine, Baltimore, Maryland, United States, 3. College of Health and Human Services, Center on Aging and Community Living, Durham, New Hampshire, United States, 4. University of Maryland School of Nursing, Baltimore, Maryland, United States, 5. University of Massachusetts Amherst, Amherst, Massachusetts, United States, 6. University of Maryland, Baltimore County, Baltimore, Maryland, United States*

Low-resource long-term care (LTC) settings face significant challenges in implementing person-centered dementia care (PCDC) due to staffing shortages, financial constraints, and limited organizational capacity. Care teams can utilize resilience and strengths-based strategies to maintain staff commitment and enhance resident engagement in meaningful activities despite resource limitations. This qualitative study examined how four LTC settings (two nursing homes; two assisted livings) in federally designated underserved areas navigate resource limitations while upholding PCDC principles. Using purposive sampling, 59 participants, including administrators, staff, residents, and care partners were recruited. Semi-structured interviews were conducted in person or via Zoom, lasting approximately 30 minutes, and analyzed using framework analysis. Findings reveal that resilience is embedded in strengths-based approaches, including adaptability, teamwork, and creative problem-solving, allowing formal care partners to focus on available resources rather than deficits. Organizational resilience was demonstrated through flexible scheduling, structured dementia training, and mission-driven leadership to mitigate workforce challenges. Interpersonal resilience was reflected in strong staff-resident relationships and collaborative teamwork, fostering trust and engagement. At the individual level, strengths such as optimism, coping mechanisms, and self-efficacy helped staff navigate daily complexities in providing PCDC. Residents and care partners played a key role in reinforcing strengths-based care through shared routines, communication, and maintaining meaningful engagement. Participants emphasized low-cost, high-impact interventions, such as preference books, step-by-step task guidance, and integrating familiar activities like music or reminiscence, reinforcing residents' autonomy and dignity. Future initiatives should embed strengths-based approaches into staff training, care policies, and dementia care models to enhance sustainability and resident well-being.

### SESSION 2155 (SYMPOSIUM)

Abstract citation ID: igaf122.698

### PERSPECTIVES ON SUCCESSFUL AGING ACROSS DIVERSE CULTURAL AND SOCIAL CONTEXTS

Chair: Emily Willroth
Co-Chair: Payton Rule

Promoting successful aging is a global priority (e.g., World Health Organization, 2022). However, perceptions of successful aging can differ significantly across cultural and social contexts. Understanding these diverse perspectives is crucial for promoting successful aging for all individuals. This symposium features research on perspectives of successful aging from underrepresented groups, including LGBTQ individuals, people in low- and middle-income countries, individuals with disabilities, and those from diverse racial, ethnic, and cultural backgrounds. First, Dr. Fredriksen Goldsen will present results from a longitudinal study identifying life affirming pathways to sexual and gender diverse older adults' wellbeing and good health. Dr. Ivan will discuss the impact of e-health literacy on reducing loneliness and sustaining wellbeing during the pandemic, comparing middle- and high-income countries. Rule will present findings from a study examining older adult perceptions of a new, more disability-inclusive definition of successful aging. Lewis will discuss healthy aging experiences among Black women. Lastly, Dr. Nguyen will present findings from a study investigating how individual notions of successful aging can be linked to, and co-constituted by, relational and intergenerational notions of personhood within the broader socio-economic, familial, and cultural contexts of migration. In sum, this symposium presents evidence for differences in definitions of and pathways to successful aging across cultural and social contexts.

Abstract citation ID: igaf122.699
### BEYOND DISABILITY: OLDER ADULT PERCEPTIONS OF SUCCESSFUL AGING
Payton Rule[1], Jennifer Pearlstein[2], and Emily Willroth[1], *1. Washington University in St. Louis, St. Louis, Missouri, United States, 2. WashU Medicine, St. Louis, Missouri, United States*
High physical and mental function and the absence of disability have long been included as key components of most successful aging models. However, prior work suggests these components may not be necessary for successful aging. Moreover, this definition focuses on individual abilities without acknowledging the role of inclusive environments (e.g., no stairs, large print materials) in promoting participation and successful aging. To address these limitations, we created a broader and more inclusive definition of successful aging as continued engagement in the activities one needs and wants to participate in—with or without accommodation, assistance, and environmental changes. To empirically evaluate this proposed definition, we recruited 427 older adults, with and without disabilities, to complete an online survey. Participants rated how closely their perception of successful aging aligned with three existing definitions and our proposed definition. They also answered questions regarding their physical health, wellbeing, and perceptions of their own aging. A series of linear regression analyses and paired-samples t-tests were conducted to assess older adults' perceptions of our proposed definition compared to existing definitions of successful aging. Additional analyses examined associations between aging successfully according to each definition and subjective wellbeing and subjective successful aging. Results indicated that older adults' perceptions of successful aging aligned significantly better with our proposed definition than with existing physiologically-focused definitions. Furthermore, our proposed definition was significantly associated with wellbeing and individuals' subjective sense of aging successfully. This talk will discuss implications of this disability-inclusive successful aging conceptualization for future research, clinical practice, and policy.

Abstract citation ID: igaf122.700
### HETEROGENEITY AND TRANSITIONS IN OLDER BLACK WOMEN'S HEALTHY AGING EXPERIENCES OVER TIME
Joshua Lewis, *The University of Maryland, United States*
Within group heterogeneity in healthy aging experiences among Black women over time have little been examined. Consequently, I explore the plurality of aging experiences among older Black women over time. I used 2010-2022 data from the U.S. Health and Retirement Study limited to Black women who completed the Psychosocial Leave Behind Questionnaire (N = 1049). I conducted latent transition analysis over two time points using indicators of physical health, psychological well-being, social support/strain, and social engagement to longitudinally characterize aging experiences, the patterning of membership transitions between aging experiences, and which experiences were associated with higher mortality risk. There were six latent statuses/experiences observed and one additional absorbing status representing mortality. I adopted status labels in accordance with their distinctive characteristics previously identified cross-sectionally (Lewis, Drentea, and Warner, 2025): infirm, isolated, taxed, independent, vivacious, and robust. Membership in aging statuses were predominantly stable over time, especially for unpartnered and partnered women with high indications of aging well, vivacious (74% maintained) and robust (80% maintained) respectively. Women who were isolated, indicated by fewest social ties and poor overall well-being, were most likely to transition into either another status of aging (16% -> infirm; 12% -> taxed; 14% ->independent; 9% ->vivacious) or experience mortality (12%). Using nationally representative data, I reveal significant heterogeneity in Black women's aging experiences over time. While some are more likely to experience mortality and challenging aging experiences, most older Black women are likely to maintain or transition into experiences of aging well.

Abstract citation ID: igaf122.701
### LIFE AFFIRMING PATHWAYS TO AGING AND WELL-BEING AMONG SEXUAL AND GENDER DIVERSE OLDER ADULTS
Karen Fredriksen-Goldsen, *University of Washington, Seattle, Washington, United States*
Sexual and gender diverse older adults comprise a growing and resilient, yet health disparate and underserved population. Based on the Health Equity Promotion Model, Dr. Karen Fredriksen- Goldsen, PI, presents findings from the first national, longitudinal 15-year study of this population, Aging with Pride: National Health, Aging and Sexuality/Gender Study (N = 2,450). Utilizing structural equation modeling, the findings identify life affirming pathways to sexual and gender diverse older adults' well-being and good health. Specific factors, including identity affirmation, social and psychological resources, and health promoting behaviors are associated with positive aging and health outcomes, while the adverse consequences of social exclusion, discrimination and violence are related to poor physical and mental health. The effects of marginalization on identity affirmation are conditional on identity management style, indicating that in case of heightened vulnerabilities (e.g., frailty or poor health) being open about one's identity may be associated with worse health outcomes, especially in adverse environments. Thus, strategically concealing one's sexual or gender diverse identity in hostile situations may be socially protective yet may take a toll personally and lead to social isolation. While being consistently open may be psychologically protective, it may increase vulnerability to marginalization and violence. Providers must remain aware of the historical and contemporary contexts and lived experiences of sexual and gender diverse older adults to develop tailored prevention strategies and interventions promoting well-being and good health. The lessons learned from these communities provide important insights for aging well in our increasingly diverse older adult population.

### SESSION 2160 (PAPER)

### ASSISTED LIVING

Abstract citation ID: igaf122.702
### A MULTILEVEL ANALYSIS OF DETERMINANTS OF DYING IN PLACE AMONG ASSISTED LIVING RESIDENTS
Emmanuelle Belanger[1], Xiao (Joyce) Wang[1], Melissa Clark[1], Joan Teno[1], Kali Thomas[2], and Pedro Gozalo[1], *1. Brown University, Providence, Rhode Island, United States, 2. Johns Hopkins University, Baltimore, Maryland, United States*
Many older adults residing in assisted living (AL) consider this setting their home; yet, there is limited evidence about determinants of their ability to die in place, with state regulations explaining only some variation in this outcome. We examined the likelihood of residents dying in place as a function of state regulations and AL-community factors. To do this, we combined an inventory of state regulations with a nationally-representative survey of over 2,000 AL administrators from 48 states about care processes, and claims data for 9,803 deceased fee-for-service Medicare beneficiaries in 2021-2022. Variables included state regulations related to third-party services (e.g. license explicitly allows hospice), survey variables about organizational characteristics (size, neighboring facilities, for-profit status, accepting Medicaid), care processes (staffing, quality of collaboration with hospice), resident characteristics (demographics, chronic conditions) and a geographic (HSA) indicator of intensity of end-of-life care. Multilevel models were estimated with sampling weights to obtain nationally-representative estimates. State regulations were not significantly associated with dying in place in fully adjusted models. Residents were more likely to die in place when residing in AL communities with for-profit status and those that did not accept Medicaid. Residents at communities near a nursing home were significantly less likely to die in place. In terms of care processes, dying in place was associated with administrators arranging for hospice care (rather than referring out), and reporting higher quality of collaboration with hospice providers. AL characteristics and care processes are more important determinants of dying in place than state regulations.

Abstract citation ID: igaf122.703

### A STUDY OF CAREGIVER BURDEN AMONG INFORMAL DEMENTIA CAREGIVERS IN ASSISTED LIVING

Molly Perkins[1], Candace Kemp[2], Kenneth Hepburn[3], Regina Shih[4], Regine Haardoerfer[3], Carolyn Clevenger[3], Miranda Moore[1], and Alexis Bender[3], *1. Emory University School of Medicine, Atlanta, Georgia, United States, 2. Georgia State University, Atlanta, Georgia, United States, 3. Emory University, Atlanta, Georgia, United States, 4. Emory University Rollins School of Public Health, Atlanta, Georgia, United States*

Informal caregivers, especially relatives and friends, play a critical role in caring for assisted living (AL) residents with dementia. Limited empirical evidence suggests that caregiver burden may be greater for informal caregivers in AL compared with other long-term care settings, particularly within context of caring for residents with advanced dementia. This study reports baseline findings from a 5-year NIA-funded (RF1AG069114/R01AG069114) longitudinal project designed to address this knowledge gap and identify potential interventions aimed at reducing challenges and improving care and care experiences. Data derived from baseline surveys, including qualitative open-ended questions, with 134 informal caregivers from 39 diverse AL communities in Georgia. We calculated descriptive statistics to characterize the sample and used thematic analysis to analyze the qualitative data. Most participants (mean age = 61) were female (85%), Non-Hispanic White (84%), and had some college education or higher (91%). Close to half (45%) reported mild to moderate burden on the Zarit Burden Interview (ZBI-12). More than one-third (35%) reported severe burden. Despite high levels of education, the average percentage of correct answers on the Dementia Knowledge Assessment Tool (DKAT2) was less than half (47%). In addition to lack of knowledge about dementia and the disease process, qualitative data showed that other key factors contributing to caregiver burden included limited time for self-care, staff turnover and lack of continuity of care, and inconsistent communication from AL staff and hospice. Findings have important implications for interventions aimed at improving care for AL residents with advanced dementia and reducing informal caregiver burden.

Abstract citation ID: igaf122.704

### ASSOCIATION OF REPORTED COLLABORATION QUALITY WITH HOSPICE PROVIDERS AND HOSPICE UTILIZATION IN ASSISTED LIVING

Xiao (Joyce) Wang[1], Joan Teno[1], Melissa Clark[1], Kali Thomas[2], Pedro Gozalo[1], and Emmanuelle Belanger[1], *1. Brown University, Providence, Rhode Island, United States, 2. Johns Hopkins University, Baltimore, Maryland, United States*

Hospice is crucial in meeting the increasing care needs of dying residents in assisted living (AL) and supporting dying in place. We know little about how AL-hospice collaboration relates to staying in AL at the end of life. We conducted a national survey of AL administrators in 2021-2023 about end-of-life care processes in large ALs (25+ beds). The survey contained instruments adapted from other long-term care settings, including a Likert-scale with items about AL-hospice collaboration quality (e.g., AL staff know when to expect a hospice nurse visit for all/most/some/none of the time). The sample included 9,069 AL residents who died in 2021 or 2022 in 1,501 surveyed ALs. We operationalized "staying in place with support at the end of life" as the number of days with hospice in AL in the last 90 days of life, excluding days receiving hospice in other settings. Two-part models were used to estimate the relationship between reported AL-hospice collaboration quality and days receiving hospice in AL, accounting for individual-, AL-, and market-level characteristics. Decedents spent 2.6 more days on hospice in AL (95% CI: 1.1, 4.2) if administrators reported residents were always assessed within 24 hours of hospice referrals, compared to residents in ALs where this happened none/some/most of the time. This difference represents 11% more days in place for residents who lived in ALs where administrators reported that hospice providers always respond timely to hospice referrals. The collaboration between hospice and AL staff is a modifiable factor that can improve end-of-life care experiences.

Abstract citation ID: igaf122.705

### RESIDENT- AND FACILITY-LEVEL PREDICTORS OF RESIDENT-REPORTED QUALITY OF LIFE IN ASSISTED LIVING

Tetyana Shippee[1], Romil Parikh[2], Molin Ji[1], Timothy Beebe[1], and Mark Woodhouse[1], *1. University of Minnesota, Minneapolis, Minnesota, United States, 2. University of Minnesota School of Public Health, Minneapolis, Minnesota, United States*

Ensuring high-quality care in assisted living (AL) is a growing priority, yet little is known about factors shaping resident-reported quality of life (QoL). Minnesota recently implemented statewide measurement and public reporting of QoL, providing a unique opportunity to examine predictors of resident experiences. This study evaluated resident- and facility-level factors associated with seven QoL domains: staff, environment, food, engagement, autonomy, culture, and security. We analyzed cross-sectional data from 11,684 AL residents in facilities with more than five beds who participated in the 2024 state QoL survey (90% aged ≥65 years, 66% female, 84% White). Using multivariable linear mixed regression with a facility-level random intercept, we accounted for facility-level clustering. Mean QoL scores ranged from 1.6 to 1.9 out of 2, with food and engagement rated lowest and environment highest. Older residents reported higher scores across most QoL domains (β, 0.02–0.08; $p < 0.05$). Women rated food and security lower (β, -0.04 and -0.02; $p < 0.05$), while American Indian/Alaskan Native residents reported lower scores in culture, security, staff, and environment (β, -0.05 to -0.10; $p < 0.05$). Smaller facilities (< 25 beds) had higher scores in food, staff, and autonomy (β, 0.02–0.07; $p < 0.05$). Facilities with dementia care unit licensure had higher food scores but lower autonomy, staff, and security scores (β, -0.02 to -0.03; $p < 0.05$). Findings highlight disparities in resident QoL, underscoring the need for targeted interventions and policy efforts to enhance resident well-being in AL.

### SESSION 2165 (SYMPOSIUM)

Abstract citation ID: igaf122.706

### REIMAGINING MUSIC FOR OLDER ADULTS: FRESH IDEAS AND BOLD POSSIBILITIES

Chair: Lisa Lehmberg
Co-Chair: Victor Fung
Discussant: Justine McGovern

In this interdisciplinary symposium, attendees are invited to "think outside the box" as new avenues regarding the role of music in older adult well-being and quality of life are explored via four interconnected research presentations spanning philosophy, education, social science, and health. The first presentation establishes a theoretical foundation through classic Daoist and Confucian perspectives, offering philosophical analyses into music's capacity to foster harmony, balance, and societal well-being. Building on this, the second presentation shares empirical research findings on intergenerational music-making as a means to counteract ageism, strengthen social bonds, and support lifelong music participation, with implications for multigenerational collaboration within school music programs. The third presentation extends the theme of social connection by introducing research on the use of silent disco headphones in long-term care settings, demonstrating their potential to cultivate immersive, shared musical experiences that enhance joy and engagement. Shifting toward a clinical lens, the final presentation explores group drumming as a non-pharmacological intervention to support well-being, inclusion, and participation among older adults with intellectual and developmental disabilities. A concluding discussion synthesizes these findings, highlighting key intersections and inviting attendees to contribute insights on reimagining music's role in aging. Attendees are encouraged to take the ideas and insights from this symposium back to their own context and reimagine new possibilities in using music to promote the well-being of older adults and the broader community.

Abstract citation ID: igaf122.707

### MUSIC AND AGING: PHILOSOPHICALLY SPEAKING

Victor Fung, *University of South Florida, Tampa, Florida, United States*

The presenter will explore questions about humans being part of nature through classic Daoism and about human relationships across the lifespan through classic Confucianism. Like all living beings, aging in human is inevitable and is a natural process. Classic Daoists see that all phenomena are guided by Dao, which should be void of egoistic desires. Actions should be taken to support the natural way of being and growth. Music has a natural bond with humans since the earliest recorded history. The exchange of energies, yin and yang, within the music, within the human, and between the two becomes critical in maintaining a balance. An imbalance in this exchange could cause discomfort, unhappiness, illness, hopelessness, or other physical or psychological adversities. Classic Confucians focus on relationships and interactions among humans across the entire lifespan. Especially noteworthy is the role orientation in relation to the expected behaviors. Based on this role orientation and characteristics of different life stages, everyone in all stages of life could help to promote deep harmony. Based on these philosophical analyses, findings suggest that (a) all generations need to be better educated about the aging process, (b) music continues to have a special place in support of a high quality of life and a harmonious society, and (c) everyone's understanding of the music of all living generations could promote a balanced, harmonious household, community, and society.

Abstract citation ID: igaf122.708

### COUNTERACTING AGEISM AND FOSTERING LIFELONG ENGAGEMENT VIA MULTI-GENERATIONAL SCHOOL MUSIC OPPORTUNITIES

Lisa Lehmberg, *University of Massachusetts Amherst, Amherst, Massachusetts, United States*

Research has consistently shown that ageism—a socially constructed form of discrimination—negatively impacts the self-esteem and overall well-being of older adults worldwide, including in the United States. However, participation in musical activities has been shown to counteract these effects by enhancing self-esteem, fostering social belonging, and instilling a sense of purpose in older adults. While these benefits are well-documented, a critical question remains: How can younger individuals be encouraged to experience the benefits of music participation as they age? Through the lens of the strategies to reduce ageism shared in the World Health Organization's Global Report on Ageism (2021), this presentation synthesizes research findings related to integrating older adults in U.S. school music settings, highlighting approaches for (a) fostering meaningful music connections between older adults and school music students, and (b) cultivating an early mindset of "lifelong musician in training." In particular, multi-generational school music activities are recommended as potentially valuable opportunities to promote lifelong musical participation while challenging age-related stereotypes and enhancing the well-being of older adults.

Abstract citation ID: igaf122.709

### SILENT DISCO HEADPHONE PARTIES IN CANADIAN LONG-TERM CARE HOMES

Lillian Hung, *University of British Columbia, Vancouver, British Columbia, Canada*

Silent disco headphone parties offer an innovative way to foster social connection and engagement among older adults in long-term care (LTC) by allowing residents to dance and enjoy music through wireless, multichannel headphones. Unlike traditional group activities, silent discos provide residents with the autonomy to select their preferred music while participating in a shared, joyful and immersive experience. Given the high prevalence of loneliness and social isolation among LTC residents, finding creative approaches to encourage social interaction and meaningful engagement is essential. This study examined the experiences of 180 participants—including residents, family members, and interdisciplinary staff—across three Canadian LTC homes. Data were collected using video ethnography, incorporating video recordings, conversational interviews, direct observations, and focus groups to capture diverse perspectives on the experience. Thematic analysis revealed three central themes: bridging generations, fostering social interaction, and promoting a sense of community. Participants expressed that silent disco parties created opportunities for spontaneous interactions, strengthened connections between residents and caregivers, and facilitated intergenerational engagement. Findings suggest that silent disco parties have the potential to enhance social well-being in LTC by providing an inclusive, accessible, and adaptable social activity that fosters participation and enjoyment. These events may also contribute to reducing feelings of isolation by promoting movement, shared experiences, and emotional expression through music. Future research should further investigate the long-term benefits of silent disco parties on residents' social health, emotional well-being, and overall quality of life.

Abstract citation ID: igaf122.710

### RHYTHM & RESILIENCE: DRUMMING AS A PATHWAY TO WELLNESS IN LATER LIFE

Emily Ihara, and Michelle Hand, *George Mason University, Fairfax, Virginia, United States*

Music engagement in later life fosters cognitive, emotional, and social well-being. Drumming, a uniquely accessible and embodied musical activity, holds promise as a non-pharmacological intervention for older adults, including those living with intellectual and developmental disabilities (IDD) and neurodegenerative conditions. This presentation highlights findings from Rhythm & Nature, a drumming and horticulture-based intervention designed to promote wellness and engagement among older adults with IDD. Drumming provides a multisensory experience, activating broad neural networks and facilitating bilateral stimulation, which is theorized to enhance cognitive processing, emotional regulation, and motor coordination. Group drumming fosters interpersonal synchrony, reinforcing social connections and reducing feelings of isolation. Participants in Rhythm & Nature reported increased confidence, improved mood, and eagerness for future sessions. Additionally, drumming's adaptability enables individuals with diverse abilities to participate, reinforcing agency and self-expression. This session will explore the multidimensional benefits of drumming in older populations, emphasizing its impact on cognitive health, emotional resilience, and social inclusion. Findings will be discussed within a broader framework of music-based interventions for older adults, underscoring drumming's potential to support intergenerational engagement and community-building. In reimagining music for older adults, we propose that drumming – rooted in rhythm and collective participation – offers a bold and innovative avenue for enhancing well-being in later life.

### SESSION 2170 (COMMUNITY ENGAGED SCHOLARSHIP SYMPOSIUM)

Abstract citation ID: igaf122.711

### BUILDING CAPACITY FOR COMMUNITY-ENGAGED RESEARCH: FRAMEWORKS AND METHODS FOR SUCCESSFUL AGING SCIENCE

Chair: Liza Behrens
Co-Chair: Ally Brothers
Discussant: Cal Halvorsen

Capacity building is integral to the success of community engaged research (CEnR). This is especially important for collaborating with new community and research partners. With the rapidly changing healthcare environment, researchers are often challenged to build capacity for successful research with older adults and their communities. This symposium will explore innovative frameworks and methodologies for capacity building in CEnR, focused on dementia care and healthy aging outcomes. The session will feature four presentations that highlight different approaches to fostering capacity development and community engagement in diverse settings. The first paper will present a framework for capacity development in Villages, a community-centered model for healthy aging. This research identifies key thematic categories for capacity growth, including understanding and motivation for research, cultivating partnerships, theorizing interventions, and procuring data systems. The second paper integrates CEnR and Two-Eyed Seeing, combining biomedical and indigenous knowledge frameworks to create a comprehensive dementia evaluation toolkit for Indigenous populations. The third paper shares insights on how capacity building using CEnR frameworks can drive sustainable changes in a significant public health challenge. The fourth paper highlights the barriers and challenges encountered while implementing CEnR frameworks and offers valuable lessons on what not to do in similar initiatives. The session will conclude with a moderated discussion that will engage presenters and participants in exploring strategies to advocate for community engagement in aging science, reports to funders, and plan for future capacity building work with community partners.

Abstract citation ID: igaf122.712

### ENGAGING VILLAGES AS KEY PARTNERS FOR HEALTHY AGING OUTCOMES RESEARCH: A FRAMEWORK FOR CAPACITY DEVELOPMENT

Natalie Pope, and Emily Greenfield, *Rutgers University, New Brunswick, New Jersey, United States*

There is sustained enthusiasm for Villages as a community-centered model to promote the health and well-being of people as they age in their communities. Yet there are many challenges to advancing rigorous empirical evidence on the outcomes of Villages, including their small size, grassroots nature, variable implementation, and multiple components. As part of a larger engagement project, we aimed to develop a framework for capacity development toward outcomes research with Villages. We used qualitative data from virtual summits with approximately 400 Village participants across the United States and several other countries, including Village members, volunteers, staff, and partners. Data were primarily from discussion groups that asked participants to reflect on how Villages influence healthy aging, how Villages connect with broader health systems, and conditions for high-quality research partnerships. Through iteratively coding the data, our analysis identified four thematic categories for capacity development among Villages to engage in healthy aging outcomes research: (a) understanding and motivation for research (e.g., the desire to engage in research that "proves" the benefits of Villages), (b) cultivating partnerships (e.g., with other Villages and researchers), (c) theorizing Village interventions (including Villages as an intervention, as well as interventions embedded within Villages), and (d) procuring data systems and research protocols. Findings indicate how capacity development is not only a matter of technical skills and resources, but also encompasses social capital, human motivation, and theory development. The framework can assess readiness and capacity needs for Villages and similar community-based organizations to engage in healthy aging outcomes research.

Abstract citation ID: igaf122.713

### COMMUNITY-ENGAGED RESEARCH & TWO-EYED SEEING: CREATING A CULTURALLY APPROPRIATE DEMENTIA SCREENING TOOLKIT

Melissa Blind[1], Kandyce Garcia[2], January Johnson[3], Cassandra Thomas[4], Carey Gleason[5], Tassy Parker[6], Kristen Jacklin[1], and Sheamus Cavanaugh[1], *1. University of Minnesota Medical School, Duluth Campus, Duluth, Minnesota, United States, 2. Center For Native American Health, University of New Mexico Health Sciences Center, Albuquerque, New Mexico, United States, 3. University of Minnesota Medical School Memory Keepers Medical Discovery Team, Duluth, Minnesota, United States, 4. WI Alzheimer's Disease Research Center, UW Madison School of Medicine and Public Health, Madison, Wisconsin, United States, 5. University of Wisconsin–Madison, Madison, Wisconsin, United States, 6. University of New Mexico, Albuquerque, New Mexico, United States*

In this paper, we present our framework for community-engaged research in our American Indigenous Cognitive Assessment (AMICA) project (R01AG074231). AMICA aims to develop and validate a culturally appropriate dementia screening toolkit, which includes a cognitive assessment, caregiver report, depression scale, and inventory of Activities of Daily Living. Our multi-site project involves community partners in Red Lake Nation, Minnesota; an urban Indigenous population in Albuquerque, New Mexico; and the Oneida Nation in Wisconsin. Each site includes local community-based researchers and Indigenous Knowledge Advisory Groups (IKAGs) that review and adapt dementia screening tools to be more culturally safe and relevant. In order to develop a single dementia evaluation toolkit for Indigenous populations throughout the United States, two IKAG members from each partner site were nominated to form a National IKAG to come to consensus on the adapted tools. Our methodology also includes the integration and use of Two-Eyed Seeing as an iterative framework, which incorporates both biomedical and Indigenous ways of knowing. In addition to the National IKAG, the AMICA project also includes an Assessment Expert Panel (AEP) consisting of experts in medical anthropology, geriatric medicine, neuropsychology, and other disciplines. We implement our community-engaged research framework to create a single intercultural dementia screening toolkit for Indigenous populations throughout the United States.

Abstract citation ID: igaf122.714

### "CAPACITY BUILDING IN COMMUNITY-ENGAGED RESEARCH: THE FLAGSTAFF DEMENTIA-FRIENDLY CITY INITIATIVE"

Eric Cerino[1], Michael McCarthy[1], Charlton Wilson[2], Terry Smith[1], Karin Von Kay[3], Cindy Martin[2], and Mary Gemma O'Donnell[1], *1. Northern Arizona University, Flagstaff, Arizona, United States, 2. Northern Arizona Regional Behavioral Health Authority Institute, Flagstaff, Arizona, United States, 3. Northern Arizona Alzheimer's and Dementia Alliance, Flagstaff, Arizona, United States*

Research Aims & Significance: The Flagstaff Dementia-Friendly City Initiative demonstrates how capacity building in community-engaged research can drive sustainable change. While research was not the initial goal, the initiative naturally integrated Community-Based Participatory Research (CBPR) principles, strengthening local research capacity while addressing dementia as a growing public health challenge in northern Arizona. Methods & Approach: Collaborative partnerships among Northern Arizona University (NAU), the Northern Arizona Regional Behavioral Health Authority Institute (NARBHA), the Northern Arizona Alzheimer's and Dementia Alliance (NAZADA), caregivers, people with memory challenges, healthcare providers, and local organizations formed the foundation for an inclusive framework. Using the Dementia Friendly America Community Toolkit, the initiative followed a four-phase process: Convene, Engage, Analyze, and Act, leading to the formation of a 30+ member Dementia-Friendly Community Council representing diverse sectors. Key Findings: Capacity-building strategies included establishing collaborative governance structures, conducting a community needs assessment, and expanding dementia-friendly education programs. Within a year, Flagstaff achieved Dementia-Friendly City designation, integrating research-driven frameworks into community programs, public service improvements, and caregiver respite initiatives. Implications for Research, Policy & Practice: Challenges such as sustaining engagement and aligning research priorities with community needs highlighted the necessity of distributed leadership and ongoing relationship-building. Future directions focus on broadening dementia-friendly policies, increasing community education and respite care resources, and enhancing accessibility across public and healthcare spaces. This initiative illustrates how CBPR enhances local research capacity while informing dementia-friendly policy and services, offering a model for other communities pursuing similar designations.

Abstract citation ID: igaf122.715

### LEARNING WHAT NOT TO DO IN COMMUNITY-ENGAGED NURSING RESEARCH IN RURAL SETTINGS

Lisa Wiese[2], Magdalena Tolea[1], and Janet Holt[2], *1. University of Miami Miller School of Medicine, Miami, Florida, United States, 2. Florida Atlantic University, Boca Raton, Florida, United States*

Although the rural community setting is well-suited for ADRD screening and care management due to trust established between providers and patients, barriers to diagnosis and management reflect a combination of factors. When conducting our "ORCHID" study (Optimizing Rural Community Health to Increase Dementia Detection and Care) to address these barriers, we discovered unique barriers to building capacity for community-engaged and dementia-focused research. Aims in this community and provider education intervention were 1) identify community-specific provider barriers to clinical ADRD assessments, 2) evaluate the effectiveness of pairing local nursing students with faith-based health educators to provide community education and cognitive screening, and 3) compare ADRD diagnosis and treatment outcomes between provider education and control groups. Interventions included support from regional neurology teams, adult gerontology nurse practitioner assessments/recommendations, and training office staff regarding ADRD resources for patients/caregivers. Measures included objective/subjective cognitive assessment surveys for residents, and provider dementia knowledge and confidence surveys. The sample (N = 232 residents;14 providers) was primarily non-White (71.2%), female (72.2%), and living alone (57.5%). Seventy-three (31%) of older adults (age: 65.0±10.1 yrs.) scored at risk for cognitive impairment (Mini-MoCA < 12). The intervention provider group was small (n = 4) but they did increase diagnosis and treatment by 60%, while the control group (n = 10) rates were unchanged. Other findings included determining what was unsuccessful regarding partnering nursing students with community health educators, provider training, referrals, data-gathering events, team composition, timing, and participant stipends. The ORCHID framework revealed important considerations for multidisciplinary approaches to improving ADRD detection and management in rural settings.

### SESSION 2175 (SYMPOSIUM)

Abstract citation ID: igaf122.716

### PERSPECTIVES OF FAMILY CAREGIVING ACROSS MICRO, MESO AND MACRO LEVELS

Chair: Hsueh-Fen Kao
Discussant: Patricia Heyn

Family caregivers provide essential support to older adults, addressing both their own and the care recipient's bio-psycho-social needs. While they play a vital role in promoting older adults' well-being, their own health often suffers, leading to their recognition as 'invisible patients.' As the U.S. population rapidly ages and the healthcare system increasingly shifts toward community-based settings, family caregiving demands continue to rise, especially among minoritized populations who rely heavily on family support. These challenges extend beyond individual families (micro level) to communities and societal structures (meso level) and national policies, highlighting the need for systemic solutions (macro level). This symposium presents diverse perspectives on family caregiving across multiple health disciplines, including nursing, aging research, health sciences, and counseling. It begins with a personal journey illustrating the use of digital communication tools for long-distance caregiving. Next, It shares insights from long-term women caregivers of Mexican origin, detailing their experiences and the need to refine research protocols. The symposium also highlights two interventions for Alzheimer's disease and related dementias: a web-based meaning-centered psychotherapy platform designed to enhance caregivers' sense of purpose and reduce their loneliness, and a community-based wellness-focused initiative supporting Hispanic/Latino caregivers through the 'health and wellness passport' program to support caregivers who often report subjective cognitive decline and promote their well-being. Through these discussions, we aim to enhance understanding, encourage dialogue, and promote the well-being of at-risk caregivers.

Abstract citation ID: igaf122.717

### MANAGING REMOTE CAREGIVING: ORCHESTRATING A CARE NETWORK FOR PEOPLE WITH DEMENTIA VIA DIGITAL COMMUNICATION

Patricia Heyn, *Marymount University, Arlington, Virginia, United States*

The growing reliance on digital communication tools has transformed caregiving, particularly for those managing care from a distance. This autoethnographic reflection examines the complexities of orchestrating a remote care network for a loved one, utilizing WhatsApp and other digital platforms to coordinate with healthcare providers in real time. As more adults navigate the sandwich generation—balancing professional responsibilities while caring for both children and aging parents—many assume the dual role of care manager and emotional anchor, ensuring their loved one's medical needs, well-being, and support systems remain integrated despite physical distance. This narrative explores the benefits of digital communication in caregiving, including immediate access to medical updates, medication management, and emergency decision-making, alongside the emotional and logistical burdens of long-distance caregiving. Key themes include the role of technology in coordinating multidisciplinary care teams, the challenges of remote medical advocacy, and strategies for balancing work and caregiving responsibilities. The discussion also addresses the limitations of digital caregiving, highlighting gaps in telemedicine support and the burden on long-distance caregivers. By integrating personal insights, digital health data, and multimedia documentation—including photos, videos, and shared decision-making moments—this abstract contributes to the broader discourse on technology-driven caregiving, caregiver stress, and the evolving role of digital health tools. As gerontologists, we have long studied the aging experience, yet today, we find ourselves living it firsthand—navigating the very challenges we research and advocate for, as our personal and professional worlds converge in the realities of caregiving.

Abstract citation ID: igaf122.718

### LESSONS LEARNED IN A FEASIBILITY STUDY WITH WOMEN CAREGIVERS OF MEXICAN ORIGIN

Hsueh-Fen Kao, *The University of Texas at El Paso, El Paso, Texas, United States*

With the rapidly aging Hispanic/Latino population and the traditional caregiving roles of women, it is critical to study caregiving stress in the largest yet understudied subgroup of women caregivers of Mexican origin. This feasibility study aimed to adapt a research protocol examining the impact of long-term caregiving stress on coronary heart disease (CHD) risk among women caregivers of Mexican origin, using the allostatic load model. A convenience sample of 20 women who provided family care (an average of 24 hours per week) for at least past six months to a dependent older relative aged 60 or older was recruited through community networks, home healthcare agencies, promotoras, the Alzheimer's Association local chapter, and hospital outpatient services in El Paso, Texas. Key adjustments included tailoring terminology to align with participants' preferences, managing complex data collection, and refining recruitment criteria to reflect cultural caregiving norms. COVID-related delays required further adaptations, such as proactive licensing management and alternative recruitment strategies. Inclusion and exclusion criteria were revised to better capture family caregiving dynamics, ensuring continuity despite staff turnover and rising costs due to the study's extended timeline and inflation. By addressing these challenges, the study established a foundation for future research on women caregivers of Mexican origin. This research will focus on developing preventive interventions to reduce caregiving stress and CHD risk, ultimately supporting age-in-place for the dependent older adults in family care.

Abstract citation ID: igaf122.719

**THE DEVELOPMENT AND TESTING OF RELOAD-C, A WEB-BASED PLATFORM TO REDUCE LONELINESS IN DEMENTIA CAREGIVERS**

Allison Marziliano[1], Allison Applebaum[2], Edith Burns[3], Marzena Gieniusz[1], Jessica Mongelli[1], Huma Babar[1], Liron Sinvani[1], and Michael Diefenbach[4], *1. Northwell Health, New Hyde Park, New York, United States, 2. Icahn School of Medicine at Mount Sinai, New York, New York, United States, 3. Zucker School of Medicine Hofstra-Northwell, Manhasset, New York, United States, 4. Northwell, Manhasset, New York, United States*

There is a need for evidence-based interventions to reduce loneliness in family caregivers of patients with dementia. Given the inverse relationship between finding meaning in life and loneliness, interventions to reduce loneliness may be strengthened by incorporating concepts from Meaning-Centered Psychotherapy (MCP), a psychotherapy effective in increasing meaning in life in patients with advanced cancer and their caregivers. The purpose of this presentation is to describe the development and preliminary testing of RELOAD-C (REducing LOneliness in Alzeheimer's Disease-Caregivers), a web-based platform that delivers concepts from MCP, adapted for caregivers of patients with dementia, via 6 brief videos, 7 virtual peer group meetings, and written content. During development, N = 15 dementia caregivers completed one-on-one interviews with the study PI. Input from stakeholders indicated: 1) preference for specific terminology; 2) removing MCP content that urges caregivers to engage in complex conversations with the person they care for; 3) providing an explanation of ambiguous concepts, such as "unfinished business"; and 4) refraining from using the term "intervention" given its negative connotations. Following integration of stakeholder feedback and creation of the RELOAD-C web-based intervention, usability/acceptability testing occurred with a separate 16 caregivers. Of the 16 caregivers, n = 13 (81.3%) correctly completed all 10 discreet tasks. Averages on 9/10 items of the System Usability Scale were below 2.0 (possible range 1-5), indicating favorable usability of RELOAD-C. In sum, RELOAD-C is a user-friendly intervention, informed by multiple rounds of review by a multidisciplinary research team and stakeholders from the very population it is designed to help.

Abstract citation ID: igaf122.720

**A HEALTH AND WELLNESS PASSPORT FOR HISPANIC/LATINO INFORMAL CAREGIVERS TO PERSONS WITH ADRD**

Shelly Aboagye, *Marymount University, Arlington, Virginia, United States*

The mental and functional health of Hispanic/Latino informal caregivers for older adults with cognitive impairments, such as Alzheimer's disease and related dementias (ADRD), is a growing concern. Nearly 20% of American adults serve as informal or unpaid caregivers (Jeffers et al., 2021). However, the mild cognitive impairment that caregivers of these patients experience has not been thoroughly explored. According to the Centers for Disease Control and Prevention (CDC) Behavioral Risk Factor Surveillance System (BRFSS), a cross-sectional survey on subjective cognitive decline (SCD) among caregivers over the age of 45 in 22 states, 11% of the 1,434 caregivers surveyed in Virginia reported experiencing SCD between 2015 and 2019 (Jeffers et al., 2021). Furthermore, the cognitive function of Hispanic/Latino caregivers is adversely affected by stress and caregiver burden. To address this issue, we plan to develop a pilot study aimed at bridging the gap between caregiver needs and the efforts to address those needs. This study will evaluate the effectiveness and cultural sensitivity of a customized Health and Wellness Passport (Pasaporte de la Salud) specifically designed for Hispanic/Latino caregivers. The passport will include personalized health assessments, educational materials, and wellness resources. Evaluating the Health and Wellness Passport program will help determine its effectiveness and whether it reduces caregiver burden. This initiative will provide a deeper understanding of how to support vulnerable, at-risk Hispanic/Latino caregivers and promote their mental, physical, and functional well-being. Additionally, we will assess the practicality and usefulness of the bilingual (English and Spanish) Health and Wellness Passports.

### SESSION 2180 (SYMPOSIUM)

Abstract citation ID: igaf122.721

**IMPLEMENTATION OF FUNCTION-FOCUSED CARE IN THE HOSPITAL SETTING: RESULTS OF THE FFC-AC-EIT TRIAL**

Chair: Barbara Resnick
Co-Chair: Marie Boltz

Once hospitalized, individuals living with dementia are more likely to experience negative impacts of hospitalization including a decline in physical activity, function, falls, delirium, and behavioral symptoms associated with dementia, when compared to individuals without cognitive impairment. The Function Focused Care for Acute Care Using the Evidence Integration Triangle (FFC-AC-EIT) intervention, in contrast to structured exercise or mobility programs, helps nurses engage patients in routine care activities and therefore provides a more realistic and sustainable approach to increase function and physical activity. The FFC-AC-EIT intervention was implemented by a nurse interventionist working with a unit-based interdisciplinary stakeholder team and champion using a four step approach: environment and policy assessment; education of staff, patients, and families; development of function-focused care goals; and mentoring and motivating the staff, patients, and families. A clustered randomized design was used with 12 hospitals randomized to FFC-AC-EIT versus Education Only. This symposium reports the main outcomes of the FFC-AC-EIT trial, as well as other relevant, secondary analyses that provide insights into factors to consider when implementing and evaluating FFC-AC-EIT. The first and second presenters will report the primary and secondary outcomes of the trial, respectively. The third presenter will discuss the impact of delirium upon our major outcome, physical function, in a sample of older adults with dementia. Finally, the fourth presenter will describe the association between patient engagement and the quality of staff-patient interactions.

Abstract citation ID: igaf122.722

### FUNCTION-FOCUSED CARE AND ITS ASSOCIATION WITH ADVERSE EVENTS

Marie Boltz, *Pennsylvania State University, University Park, Pennsylvania, United States*

Although the benefits of function-focused care (FFC) are well known, its implementations continues to be slow to take hold in the hospital setting, in part due to concerns for patient safety. Clinicians, patients, and families have expressed worry about the potential for falls and fall-related injuries which is associated with increased hospitalizations and mortality. This study addressed that issue by examining whether FFC was associated with increased falls, transfers to the hospital, and hospital readmission. Mixed-effects logistic regression demonstrated that, adjusting for race, age, co-morbidity, discharge location, and dementia severity, hospitalized older adults in the intervention arm had no more falls at one ( p=.512) and six (p= .668) months as compared to those in the control (education-only) arm. Findings suggest that, contrary to commonly held beliefs, FFC can be implemented without concern for increasing falls or risk of emergency room visits or hospitalizations.

Abstract citation ID: igaf122.723

### THE IMPACT OF FFC-AC-EIT UPON PATIENT OUTCOMES

Barbara Resnick[1], Boltz Marie[2], and Ashley Kuzmik[3], *1. University of Maryland, Baltimore, Maryland, United States, 2. The Pennsylvania State University, University Park, Pennsylvania, United States, 3. Alzheimer's Association, Chicago, Illinois, United States*

This study tested the impact of FFC-AC-EIT on hospitalized patients living with dementia. A total of 455 patients were recruited from 12 hospitals. The mean age of the participants was 82.5 (SD = 8.5) and the majority were women (62.6%), White older adults (65.3%), married (64.4%) and with high school or less education (67%). All had evidence of cognitive impairment with a mean SLUMS score of 7.5 (SD = 5.9) and a mean of 3.0 (SD = 2.4) comorbidities. At discharge, both FFC-AC-EIT and Education-only groups improved in function and physical activity, patients in the FFC-AC-EIT group had less increase in pain than those in the Education-only group ($\beta$ = -0.58, [95% CI] (-0.89, -0.26), $p < 0.001$), with no group differences in behavioral symptoms or delirium. At one month post-discharge there was a significant improvement in function ($\beta = 5.55$, 95% CI [0.36, 10.75], $p = 0.036$) and physical activity ($\beta = 3.07$, 95% CI [1.87, 4.28], $p < 0.001$) in the FFC-AC-EIT arm. The decrease in pain between admission and discharge indicates that addressing pain and engaging patients in FFC-AC-EIT did not result in exacerbation of pain. Exposure to information about FFC-AC-EIT, including Education-only, may help hospital staff maintain and increase function and physical activity for older patients living with dementia and help facilitate an increase in function and physical activity after discharge.

Abstract citation ID: igaf122.724

### DELIRIUM AND IMPACT ON PHYSICAL FUNCTION IN HOSPITALIZED PERSONS LIVING WITH DEMENTIA

Ashley Kuzmik, *Alzheimer's Association, Chicago, Illinois, United States*

Delirium is a common occurrence among hospitalized older adults living with dementia, and it poses significant risks to their health and wellbeing. The presence of delirium among older adults, especially those with dementia, is associated with low levels of physical function while hospitalized. The aim of this study was to investigate whether demographic characteristics (i.e., age, sex, and race) moderate any differences in delirium severity as predictors of physical function in 351 hospitalized persons living with dementia. Age and sex but not race had significant moderating effects on the relationship between delirium severity and physical function ($\beta = 2.22$; $p = 0.02$ and $\beta = 1.34$; $p = 0.04$, respectively). Notably, older adults aged ≥ 85 years with higher levels of delirium severity had lower levels of physical function compared to older adults aged 65-84 years . Males with higher levels of delirium severity had lower levels of physical function compared to females. These findings suggest that future clinical interventions, such as use of function focused care, be tailored to address the unique needs and challenges faced by older males particularly to best address delirium and optimize physical function when hospitalized.

Abstract citation ID: igaf122.725

### THE ASSOCIATION BETWEEN PATIENT ENGAGEMENT AND QUALITY OF CARE INTERACTIONS AMONG ACUTE CARE PATIENTS WITH DEMENTIA

Rachel McPherson, *University of Maryland School of Nursing, Baltimore, Maryland, United States*

Effective staff-patient interaction is critical to promote effective care delivery, which includes function-focused care to hospitalized persons with dementia. The purpose of this study was to determine whether the quality of staff-patient care interactions were associated with active patient engagement during the interaction after controlling for relevant covariates. The study was a secondary data analysis using baseline data from the Function Focused Care for Acute Care intervention study, with a total sample of 286 patients. Generalized linear mixed model indicated that there was a significant relationship between the quality of care interactions and patient engagement such that receiving positive care interactions resulted in higher odds of active patient engagement. These findings can inform future interventions and training for acute care staff to improve quality of care interactions and patient engagement.

### SESSION 2185 (PAPER)

### ALZHEIMER'S DISEASE AND OTHER RELATED DEMENTIAS: HEALTH SERVICES AND POLICY OUTCOMES

Abstract citation ID: igaf122.726

### EXPERIENCES OF OLDER ADULTS LIVING WITH DEMENTIA ELIGIBLE FOR A MONEY FOLLOWS THE PERSON PROGRAM

Julie Robison, Ellis Dillon, Chae Man Lee, Deborah Migneault, Kristin Baker, Richard Fortinsky, and Lisa Barry, *University of Connecticut, Farmington, Connecticut, United States*

We sought to identify mechanisms contributing to disparities for older persons living with dementia (PLWD) in access, utilization, and quality in Connecticut's Money Follows the Person (MFP) program. MFP enables Medicaid-insured nursing home (NH) residents to move back to the community with Home and Community-Based Services (HCBS). We constructed a retrospective cohort of Connecticut MFP-eligible Medicaid-insured individuals age 55+ who were long-term (90+ days) NH residents between 2018-2022 (N = 29,050) from population-level claims and assessment data combined with MFP administrative records. 60.3% of MFP-eligible residents had dementia (race/ethnicity composition: 80.8% White; 11.2% Black; 5.4% Hispanic; 2.6% other). Overall, 15.3% of MFP-eligible residents applied to MFP (access), 27.6% of MFP applicants moved to the community within 2 years (utilization), and 76.0% of movers sustained community living for 60+ days (quality). PLWD had significantly lower rates of applying to MFP (9.4% vs 24.1%), moving (22.7% vs 30.6%), and sustaining community living (71.9% vs 77.8%). Black (28.8%) and Hispanic (30.6%) residents had significantly higher MFP application rates than White (12.3%) residents and similar likelihood of moving and sustaining community living. Despite Connecticut's successful MFP program, rates of access, utilization and quality are significantly lower for PLWD, although disparities shrank at each step. PLWDs' lower rates of MFP applications, moves, and sustained community living may indicate a disparity and/or selection effect as may differences by race/ethnicity. Growth in PLWD and variations by race/ethnicity necessitate identifying better solutions for community-living and improving concordance between design of MFP and HCBS programs and individual needs.

Abstract citation ID: igaf122.727

### HEALTH SERVICES IMPACT OF HOSPITAL-OLDER ADULT CENTER PARTNERSHIPS FOR PEOPLE LIVING WITH DEMENTIA

Divya Bhagianadh[1], Clara Scher[2], Emily Greenfield[3], Ceara Somerville[4], Caitlin Coyle[4], and Ayse Akincigil[3], *1. University of Arkansas, Fayetteville, Arkansas, United States, 2. Weill Cornell Medicine, New York, New York, United States, 3. Rutgers University, New Brunswick, New Jersey, United States, 4. University of Massachusetts Boston, Boston, Massachusetts, United States*

There is growing enthusiasm for policies and practices that promote cross-sectoral collaboration to improve care for people aging in community with dementia. Our study aimed to expand empirical evidence in this area by examining the impact of partnerships between hospitals and older adult centers on healthcare utilization and costs among community-dwelling older adults with dementia. Older adult centers are community-based organizations that provide essential services and social connections for participants. Our study uses a novel, statewide hierarchical dataset of older adults with dementia in Massachusetts nested within the geographic catchment areas of municipally-run older adult centers that did or did not report partnerships with hospitals. Outcomes including number of hospital stays, readmissions and total Medicare payments were from 2018 - 2019 Medicare claims data. We employed a multilevel mixed-effects generalized linear model. Results suggested that individuals with dementia living in communities where older adult centers engaged in partnerships with hospitals had significantly fewer hospital stays. Among subgroups of dementia patients with multiple comorbidities and among dual-eligible older adults, these partnerships were associated with significantly lower levels of healthcare utilization and Medicare costs. In addition, people with dementia at the intersection of other minoritized identities (e.g., racialized as African American, lower socioeconomic status) were less likely to live in communities reporting hospital-older adult center partnerships. Our findings highlight the importance of continued research, policy, and practice on the interfaces across health and social care settings to improve outcomes for people with dementia, especially for those aging in place with greater precarity.

Abstract citation ID: igaf122.728

### OUT OF POCKET COSTS FOR PEOPLE WITH DEMENTIA: A COMPARISON ACROSS INSURANCE TYPES

Krista Harrison[1], Karen McKendrick[2], Lihua Li[2], Claire Ankuda[2], Lauren Hunt[1], and Melissa Aldridge[2], 1. University of California, San Francisco, San Francisco, California, United States, 2. Icahn School of Medicine at Mount Sinai, New York, New York, United States

People with dementia (PWD) comprise a significant proportion of enrollees in Traditional Medicare (TM), Medicare Advantage (MA), and Medicaid, with notable increases in MA. Although overall health differs by insurance, we know little about the types of healthcare driving out-of-pocket costs for people with dementia. We conducted a cohort study of PWD age 65+ in the Medicare Current Beneficiary Survey, 2002-2022. We categorized PWD based on 12 months of TM, MA, partial or full Medicaid dual enrollment. All analyses are survey-weighted and adjusted to 2022 dollars. Of 72,758 PWD, mean age was 75.7, 78.8% were White, 55.9% were female; 5.5% died. Mean annual out-of-pocket costs for PWD were $3,944; 31% due to outpatient costs, 20% prescriptions, 17% nursing homes, 17% assisted living and other facilities, 7% durable medical equipment, 3% inpatient costs, and 2% home health. Mean annual out-of-pocket costs were highest for PWD in TM ($4,590) compared to MA ($2,990) and Medicaid ($2,900). Despite differences in total out-of-pocket costs between TM and MA, proportional spending by healthcare type were similar, except for higher spending on prescriptions (MA 25% versus TM 20%). Among PWD with Medicaid, out-of-pocket costs for nursing homes and assisted living facilities were significantly higher (60%) compared to those TM (33%) and MA (30%). Although nursing home use declined among PWD using Medicaid, mean annual out-of-pocket costs increased from $10,950 in 2002 to $14,260 in 2022. PWD in TM spend the most in out-of-pocket costs; residential care for PWD continues to drive out-of-pocket spending across insurance types.

Abstract citation ID: igaf122.729

### WHO'S MISSING OUT? EXPLORING POTENTIAL MEDICAID ENROLLMENT GAPS AMONG PEOPLE WITH DEMENTIA

Sijiu Wang[1], Siying Yang[1], and Jing Li[2], *1. Tsinghua University, Beijing, Beijing, China (People's Republic), 2. University of Washington, Seattle, Washington, United States*

Medicaid serves as a critical safety net for older adults with Alzheimer's Disease and Related Dementias (ADRD), yet many eligible individuals remain unenrolled, exacerbating their health and financial vulnerabilities. This study analyzes data from the National Health and Aging Trends Study (2011-2023), a nationally representative panel survey of Medicare beneficiaries, to identify Medicaid-eligible but unenrolled older adults with ADRD and assess their health and economic risks. We conducted a panel data analysis using survey waves immediately before and after Medicaid enrollment to examine enrollee characteristics, including health, functioning, and financial status prior to enrollment. Two approaches were employed to identify potentially eligible but unenrolled people with ADRD: (1) defining potential eligibility based on income below 100% and 138% of the Federal Poverty Level, and (2) estimating expected Medicaid enrollment likelihood using socioeconomic and health characteristics of non-ADRD enrollees as a reference. Comparative analyses revealed that unenrolled ADRD individuals were disproportionately aged 85+, Hispanic, and residing in the West, with higher rates of functional impairment, hospitalizations, and depressive symptoms compared to their enrolled counterparts. While they exhibited relatively better financial status than Medicaid enrollees with ADRD, their economic hardship was significantly greater than that of non-ADRD individuals. Findings suggest that structural barriers, rather than eligibility criteria alone, contribute to Medicaid enrollment gaps. Targeted outreach strategies, streamlined enrollment processes, and culturally responsive interventions are needed to close these gaps and mitigate adverse health and financial outcomes among this vulnerable population.

### SESSION 2190 (SYMPOSIUM)

Abstract citation ID: igaf122.730

### SOCIAL ENGAGEMENT, ISOLATION, AND LONELINESS AMONG DIVERSE CAREGIVING POPULATIONS: INSIGHTS FROM NATIONAL DATA

Chair: Yiqing Qian
Co-Chair: Mara Rosenberg
Discussant: Christine Ritchie

Social isolation and loneliness are critical issues that shape the well-being of older adults and their family caregivers. Despite growing scientific inquiries into isolation and loneliness since the COVID-19 pandemic, these psychosocial challenges remain underexplored in family caregiving research. This symposium draws on multiple nationally representative datasets to examine how social connection, or lack thereof, influences caregiver experiences and mental health. Together, these papers explore the intersections of social engagement, isolation, and stress among diverse caregiving populations. First, Dr. Rosenberg presents data from the National Study of Caregiving (NSOC) to demonstrate the associations between caregiver participation in social activities and burden. Next, we examine the isolation and mental health of two subgroups of family caregivers for older adults. Dr. Pomeroy focuses on dementia caregivers, a subgroup of caregivers who bear high intensity and burden of care, to characterize how their social isolation and loneliness experiences differ by gender. Linking NSOC with the National Health and Aging Trends Study, Dr. Elmore focuses on understudied long-distance caregivers and examines the association between proximity and mental health. Lastly, Dr. Li presents a comparative analysis between caregivers and non-caregivers in the National Poll on Healthy Aging, demonstrating that despite the high prevalence of loneliness during the COVID-19 pandemic, caregivers are still less likely to use mental health self-care services. Overall, these papers explore caregivers' distinct social experiences and offer potential targets to improve caregiver well-being. As the discussant, Dr. Kotwal will synthesize these presentations and propose targets for research, policy, and practice.

Abstract citation ID: igaf122.731

### WHEN MISSING OUT MATTERS: ASSOCIATIONS BETWEEN SOCIAL ACTIVITIES AND CAREGIVER BURDEN IN NATIONAL DATA

Mara Rosenberg, Alexander Smith, and Ashwin Kotwal, *University of California, San Francisco, San Francisco, California, United States*

Engaging in social activities is essential for well-being, yet caregivers often face restrictions due to caregiving responsibilities. We analyzed cross-sectional data from the National Study of Caregiving Round 11 ($N = 1,619$, weighted $N = 17.9$ million) to examine the association between social restriction and burden among unpaid caregivers of adults aged 65+. Caregivers reported social activity participation (visiting friends/family, attending religious services, going out, group activities), the importance of these, and burden (emotional, physical, financial). Using weighted logistic regressions adjusting for sociodemographic covariates, we examined associations between restriction and burden. A subgroup analysis compared missing important versus unimportant activities. Average age was 65 years, 65% were female, 53% children of their care partner, 19% spouses. 43% reported any burden: 35% emotional, 17% physical, 9% financial. Caregivers kept from activities reported higher burden: when kept from going out (Emotional: 76% vs 43%; Physical: 37% v 16%; Financial: 24% v 7%), visiting family/friends (Emotional: 79% vs 38%; Physical: 41% v 14%; Financial: 15% v 6%), or group activities (Emotional: 82% vs 42%; Physical: 53% v 14%; Financial: 15% v 7%). The more missed activities, the greater any burden (37% v. 68% v. 81% for missing zero, one, or two, $p < 0.05$). Among religious activities, burden was higher if caregivers valued the missed activity (Emotional: 91% v. 47%; Physical: 64% v. 22%, $p < 0.05$). Findings underscore the need for personalized recommendations that align with caregiver preferences, while recognizing that any social interaction is beneficial. Providers should screen for burden when caregivers restrict participation and offer supportive resources.

Abstract citation ID: igaf122.732

### GENDER DIFFERENCES IN SOCIAL ISOLATION AND LONELINESS AMONG A NATIONAL SAMPLE OF DEMENTIA CAREGIVERS

Mary Louise Pomeroy[1], Yiqing Qian[2], Gilbert Gimm[3], Katherine Ornstein[4], and Thomas Cudjoe[5], *1. Johns Hopkins Bloomberg School of Public Health, Baltimore, Maryland, United States, 2. Johns Hopkins University School of Nursing, Baltimore, Maryland, United States, 3. George Mason University, Fairfax, Virginia, United States, 4. Johns Hopkins University, Baltimore, Maryland, United States, 5. Johns Hopkins School of Medicine, Baltimore, Maryland, United States*

As life expectancy rises in the U.S., more older adults and their family/friend caregivers will face the challenges of managing dementia. Social connections can play a crucial role in providing support that may attenuate these challenges. However, little is known about gender differences in social isolation and loneliness among dementia caregivers. This study used Round 12 of the National Study of Caregiving (NSOC) to examine social isolation and loneliness among a nationally-representative sample of caregivers to community-dwelling older adults with dementia in 2022. Among 753 dementia caregivers, 39% ($n = 242$) were men. Using chi-squared tests and a multi-domain social isolation typology, we found that male caregivers were more likely to be socially isolated compared to female caregivers (21.4% vs. 12.4%; $p < 0.01$). Underlying components driving isolation among men included small core discussion network size (25.4% vs. 15.4%; $p < 0.05$) and no religious service attendance (64.6% vs. 45.5%; $p < 0.001$). However, men were less likely to live alone (3.9% vs. 12.6%; $p < 0.001$) and there were no significant gender differences in participation in community-based activities (66.5% vs.

64.5%, p = 0.74). Male caregivers were less likely to report loneliness than females, though this difference was not significant (23.8% vs. 30.0%, p = 0.12). Because women are at greater risk for dementia than men, the number of male caregivers for women with dementia is expected to grow. However, male caregivers are underrepresented in dementia research. Interventions and policies to support dementia care management (e.g., the GUIDE model) should carefully consider gender differences in social connection among dementia caregivers.

Abstract citation ID: igaf122.733

### RESIDENTIAL PROXIMITY AND SYMPTOMS OF DEPRESSION AND ANXIETY AMONG ADULT CHILDREN CARING FOR AGING PARENTS

Catherine Elmore[1], Soojung Ahn[2], Malek Alnajar[1], Megan Hebdon[3], Sarah Small[4], Gregory Stoddard[5], and Katherine Ornstein[6], *1. University of Utah, Salt Lake City, Utah, United States, 2. Boston College William F. Connell School of Nursing, Chestnut Hill, Massachusetts, United States, 3. University of Utah College of Nursing, Salt Lake City, Utah, United States, 4. University of Utah Department of Economics, Salt Lake City, Utah, United States, 5. University of Utah, Study Design and Biostatistics Center, Salt Lake City, Utah, United States, 6. Johns Hopkins University, Baltimore, Maryland, United States*

Long-distance caregivers are an understudied subgroup of family caregivers. This study characterizes adult-child caregivers based on residential proximity to their older-adult parent, and explores associations of proximity with caregiver symptoms of depression and anxiety. Data from the 2021 National Health and Aging Trends Study Round 11 was linked with National Study of Caregiving IV. The sample included adult-child caregivers of Medicare beneficiaries aged 71 and older. Bivariate and multivariable Poisson regression models (stratified by education attainment) analyzed associations between proximity categories, caregiver demographics, care activities, and symptoms of depression and anxiety. The sample included 932 caregivers, representing a population of 9,171,811. In weighted analysis, 26.3% of caregivers coreside, 47.4% live 1–20 minutes away, 13.1% live 21–59 minutes away, and 13.2% live ≥1 hour away. Residential proximity to their aging parent was not a significant predictor of depression, and only marginally significant for anxiety in those with some college education. Poor/fair health predicted anxiety across all education groups; employment reduced the risk of depression for those with some college education. While adult-children provide varying types of caregiving assistance regardless of residential proximity, depression and anxiety are largely associated with caregivers' health and financial status rather than residential proximity. Findings highlight the need for policies, services and programs that support the needs of distance caregivers, given that their needs are relatively overlooked compared to proximate/local caregivers.

Abstract citation ID: igaf122.734

### CAREGIVING, LACK OF COMPANIONSHIP, AND MENTAL HEALTH SELF-CARE DURING COVID-19

Niying Li[1], Darshan Chudasama[2], Lin-Na Chou[3], Mary Louise Pomeroy[4], and Yiqing Qian[5], *1. University of Georgia, Athens, Georgia, United States, 2. University of Georgia College of Public Health, Athens, Georgia, United States, 3. University of Utah, Saratoga Springs, Utah, United States, 4. Johns Hopkins Bloomberg School of Public Health, Baltimore, Maryland, United States, 5. Johns Hopkins University School of Nursing, Baltimore, Maryland, United States*

The COVID-19 pandemic exposed public health challenges in social isolation and loneliness and urgent demands for mental health self-care services (e.g., professional services, wellness apps). Family caregivers face unique barriers to socialization and mental health self-care. Using nationally representative data from the 2021 National Poll on Healthy Aging of adults aged 50-80, we examined the association between a lack of companionship and use of mental health self-care services between March 2020 and January 2021, and explored family caregiver status as an effect modifier. The survey-weighted sample was mostly non-Hispanic White (70.9%), between ages 50-64 (59.5%), living alone (78.6%), and non-caregivers (87.5%). Compared with non-caregivers, caregivers were more likely to report lacking companionship (45.4% vs 35.9%) and using self-care services (46.1% vs 35.8%). In the full sample including both caregivers and non-caregivers, we conducted a logistic regression adjusting for socio-demographic and health characteristics, demonstrating that lacking companionship was associated with a higher average predicted probability of using self-care services (40.0%, 95%CI: 36.1%-44.0%) than not lacking companionship (35.9%, 95%CI: 32.9%-38.8%). Caregiver status moderated this association. Among caregivers, those lacking companionship (35.6%, 95%CI: 26.3%-45.0%) had a lower average predicted probability of self-care use than those not lacking companionship (45.8%, 95%CI: 37.3%-54.4%). Our work highlights that caregivers who lack companionship are also less likely to use mental health self-care services, suggesting a potential lack of time, resources, or knowledge to access mental health self-care during COVID-19. Future work should examine loneliness during caregiving and tailoring self-care services to caregivers' social and emotional needs.

### SESSION 2195 (SYMPOSIUM)

Abstract citation ID: igaf122.735

### NEW NATIONAL GERIATRIC 5MS INTERPROFESSIONAL CORE CURRICULUM OF GERIATRIC RESEARCH EDUCATION AND CLINICAL CENTERS

Chair: Kathryn Nearing
Co-Chair: Beth Hogans
Discussant: Andrea Schwartz

The Veterans Health Administration (VHA) is the largest provider of geriatric/gerontologic education in the US. Geriatric Research, Education, and Clinical Centers (GRECCs) are VHA centers of excellence focused on aging. Each GRECC has a health professions training program with a unique constellation of health professions trainees, ranging from chaplaincy to podiatry, pharmacy to audiology. In 2020, GRECC Associate Directors for Education and Evaluation (ADEEs) initiated the development of a common core curriculum to ensure this broad spectrum of trainees gained essential knowledge in best practices for caring for older adults. Organized by the Geriatric 5Ms, the interprofessional core curriculum was designed 1) for broad appropriateness across professions, 2) to optimize adoption by GRECC sites given diverse local contexts, and 3) to support accessibility and acceptability among trainees given expanding training requirements. This symposium will address the following topics:
- Need for geriatric/gerontologic training and GRECCs' history of training allied health professionals to promote Age Friendly

Health Systems (AFHS) within the VHA and beyond - Needs assessment to inform content, which engaged subject matter experts nationally through roundtable discussions and, subsequently, ADEEs in a modified e-Delphi process - Overview of the interprofessional Geriatric 5Ms core curriculum, including its unique contribution in relation to other AFHS and Geriatric 5Ms training resources - Development of modules, delivered as micro-learning segments to optimize feasibility/utilization - Robust evaluation informed by the RE-AIM framework from implementation science. We will highlight initial evaluation findings, including emerging implementation models. The symposium will conclude with a moderated panel discussion.

Abstract citation ID: igaf122.736

### GERIATRIC RESEARCH, EDUCATION AND CLINICAL CENTERS: TRAINING TOMORROW'S HEALTH CARE WORKFORCE

Marianne Shaughnessy, *Veterans Health Administration, MIDDLE RIVER, Maryland, United States*

Geriatric Research, Education and Clinical Centers (GRECC) are geriatric centers of excellence located throughout the U.S. One of the mandates of the GRECC program is to educate VA staff and trainees from their academic affiliates on evidence-based best practices in geriatric care. Over 45 years, GRECCs have educated thousands of students, residents and fellows in a range of health care professions, including, but not limited to: medicine, nursing, rehabilitation science, pharmacy, psychology, optometry, chaplaincy and social work. In fiscal year 2024, GRECCs collectively trained over 1600 medical students, residents and fellows, and over 400 Health Professions Trainees. GRECC educators introduce concepts of interdisciplinary and interprofessional team care informed by both the Geriatric 5Ms model, as an outgrowth of Age-Friendly Health Systems (AFHS), as well as the interprofessional education collaboration (IPEC) competencies. Finally, they have been a critical component of the spread of Age-Friendly Health Systems principles of care enterprise wide. Historically, each GRECC has developed and deployed its own educational curriculum. In 2022, the VA's Office of Academic Affiliations made a formal request that GRECCs standardize their basic core curriculum in geriatrics. This presentation will introduce the GRECC interprofessional 5Ms core curriculum, discuss the educational resources in GRECCs and illuminate the creative and demonstrated achievements of GRECC educators. The vision for the curriculum was to meet the needs of a variety of health professions trainees organized around the Geriatric 5 Ms, (what matters, medication, mentation, mobility and multicomplexity) with considerations for former military not available in existing geriatric training materials.

Abstract citation ID: igaf122.737

### INFORMING THE DEVELOPMENT OF AN INTERPROFESSIONAL GERIATRICS CORE CURRICULUM: STAKEHOLDER ENGAGEMENT

Katharina Echt[1], Kathryn Nearing[2], Iriana Hammel[3], Elizabeth Chapman[4], Becky Powers[5], Sumi Misra[6], and Beth Hogans[7], *1. U.S. Department of Veterans Affairs, Brookhaven, Georgia, United States, 2. University of Colorado Anschutz Medical Campus, Denver, Colorado, United States, 3. Miami VA Medical Center, Miami, Florida, United States, 4. William S. Middleton Memorial Veterans' Hospital, Madison, Wisconsin, United States, 5. The University of Texas Health Science Center at San Antonio, San Antonio, Texas, United States, 6. Vanderbilt University Medical Center, Nashville, Tennessee, United States, 7. Johns Hopkins School of Medicine, Baltimore, Maryland, United States*

Early-stage, formative solicitation of stakeholder needs and preferences can inform curriculum development that is responsive and fosters a sense of co-ownership. Clinical discipline preceptors are key collaborators in the ultimate implementation of an interprofessional core curriculum in geriatrics with their respective health professions trainees. This presentation describes the formative environmental scan and subsequent stakeholder engagement process to identify 1) core geriatric knowledge needed by interprofessional trainees, 2) gaps in geriatric education across discipline-specific programs, and 3) perceived barriers and delivery/modality preferences. The Geriatric Interprofessional Core Curriculum development team conducted an environmental scan to identify existing interprofessional core curricula that provide essential knowledge in best practices for caring for older adults. The team identified multiple relevant and validated resources and iteratively discussed strengths and limitations of each. Few were sufficiently inclusive to meaningfully engage the range of disciplines and levels of professional preparation represented in the GRECC health professions training program. The Geriatric 5M's Framework (What Matters, Mentation, Medication, Mobility, Multicomplexity) provided an interprofessional scaffold that, with preceptors' subject matter expertise, facilitated curriculum development. Accordingly, five roundtable discussions were held, each focusing on one of the 5Ms. A total of 49 interprofessional educators were engaged and asked to identify core geriatric training needs, discuss preferred curriculum modality and potential barriers to implementation. Thematic analysis of transcripts and detailed session notes yielded a set of priority content topics, as well as implementation preferences regarding usable formats and delivery, in support of the next phase of the development process.

Abstract citation ID: igaf122.738

### BUILDING A BIGGER TENT – INTERPROFESSIONAL ENGAGEMENT IN GERIATRIC CURRICULUM DEVELOPMENT

Beth Hogans[1], and Katharina Echt[2], *1. Johns Hopkins School of Medicine, Baltimore, Maryland, United States, 2. U.S. Department of Veterans Affairs, Brookhaven, Georgia, United States*

This presentation will provide an overview of curriculum development including needs assessment and collaborative creation of interprofessional learning objectives before describing a national effort building geriatric interprofessional core curriculum for Veterans Administration (VA) health professions trainees. Using modified Delphi processes and multi-level interprofessional feedback, the curriculum development focused on distilling information from the task, needs, and gap assessments and synthesizing a coordinated set of learning objectives to support module development. Following the needs assessment process which included resource appraisal and a series of national stakeholder roundtable meetings, one for each of the geriatrics 5 Ms, we developed and delivered a survey directed to the cohort of associate directors for education and evaluation at the 20 VA Geriatric Research Education and Clinical Centers of Excellence (GRECCs) nationally. The purpose of the survey was to review and finalize the recommendations of the stakeholder meetings, ensuring that topics proposed were relevant to identified education needs across the national program. Subsequently the results of the survey were discussed, and core content topics harmonized across the curriculum. Once the curriculum topics were finalized, interprofessional curriculum team members were assigned to draft learning macro and micro-objectives designed to describe the specifics of the course content. Draft learning objectives were presented to the curriculum team as a whole and underwent multiple rounds of editing for alignment of purpose, clarity and concision. National education experts provided additional peer-review feedback and learning objectives were reviewed by diverse VA education stakeholders. Final learning objectives were utilized to shape module development.

Abstract citation ID: igaf122.739

**GERIATRIC CORE CURRICULUM MODULE DEVELOPMENT**

Iriana Hammel[1], and Elizabeth Chapman[2], *1. Miami VA Medical Center, Miami, Florida, United States, 2. William S. Middleton Memorial Veterans' Hospital, Madison, Wisconsin, United States*

GRECC ADEEs formed a workgroup to develop an interprofessional geriatric core curriculum using the Geriatric 5Ms ('AFHS+1') framework. Our workgroup created six modules: an introduction and one module focused on each of the Ms: (Mobility, Mentation, Medication, What Matters Most, and Multicomplexity). We created 60-minute, web-based modules to meet accreditation requirements of a broad spectrum of health professions while remaining feasible to complete in the context of other responsibilities. To enhance curriculum integration and adoption, we divided modules into 10–15-minute micro-learning segments. This presentation provides an overview of the process and challenges encountered in developing one such module for Multicomplexity. Providing trainees from varied backgrounds with an overview of Multicomplexity is daunting, given the breadth of topics encompassed and the differing levels of clinical knowledge among the targeted professions. A modified Delphi process helped narrow the focus to six topics: balancing treatment benefits and harms, engaging interprofessional teams, addressing communication challenges, frailty, care settings, and care transitions. Module materials were revised via an iterative process using input from colleagues and informal feedback from trainees. The final web-based training segments consisted of 508-compliant audio-visual presentations together with interactive cases, knowledge checks, and selected resources that ensured access to materials designed to support deeper learning and integration of geriatric best practices into care of older Veterans. Curriculum module development was a complex but worthwhile endeavor and required support from the national ILEAD office who provided assistance with 508 compliance, accreditation, and development of web-based trainings.

Abstract citation ID: igaf122.740

**RE-AIM FRAMEWORK GUIDES COMPREHENSIVE MIXED-METHODS EVALUATION OF GRECC INTERPROFESSIONAL 5MS CORE CURRICULUM**

Kathryn Nearing[1], Huai Cheng[2], Elizabeth Chapman[3], Katharina Echt[4], Iriana Hammel[5], Beth Hogans[6], Sumi Misra[7], and Marianne Shaughnessy[8], *1. University of Colorado Anschutz Medical Campus, Denver, Colorado, United States, 2. Minneapolis VA Hospital, Minneapolis, Minnesota, United States, 3. William S. Middleton Memorial Veterans' Hospital, Madison, Wisconsin, United States, 4. U.S. Department of Veterans Affairs, Brookhaven, Georgia, United States, 5. Miami VA Medical Center, Miami, Florida, United States, 6. Johns Hopkins School of Medicine, Baltimore, Maryland, United States, 7. Vanderbilt University Medical Center, Nashville, Tennessee, United States, 8. Veterans Health Administration, MIDDLE RIVER, Maryland, United States*

The national evaluation of the interprofessional Geriatric 5Ms core curriculum is guided by the RE-AIM framework from implementation science. RE-AIM stands for reach (who accesses and completes the curriculum), effectiveness (the degree to which the curriculum increases awareness, knowledge, self-efficacy and intentions to apply new learning in practice), adoption (uptake of the curriculum by the national GRECC network), implementation (how the curriculum is integrated into other training requirements locally), and maintenance (sustained use of the core curriculum by GRECC health professions training programs). RE-AIM focuses our comprehensive evaluation at multiple levels salient to the national roll-out of a new curriculum across varied local contexts and different configurations of associated health professions training programs. RE-AIM directs us to assess the national reach of the curriculum to address current gaps/needs for geriatric/gerontologic education, while also assessing facilitators and barriers to local adoption and implementation. Key lessons learned can be applied to promote dissemination. This individual abstract session will provide an overview of the mixed-methods evaluation of the new core curriculum, including the national learning management system available to ensure all GRECC health professions trainees are assigned and complete the curriculum as part of their learning plans and receive a standardized evaluation to assess key outcomes such as satisfaction with the quality of content and delivery, gains in core competencies, and intentions to apply new learning in practice. We will conclude by sharing early evaluation findings, including emerging implementation models from the field.

### SESSION 2200 (SYMPOSIUM)

Abstract citation ID: igaf122.741

**SOCIAL DETERMINANTS OF COGNITIVE AND BRAIN AGING: NOVEL APPROACHES AND NEW FINDINGS**

Chair: Andrea Rosso
Discussant: Lisa Barnes

Social determinants of health, including socioeconomic status (SES), neighborhood conditions and discrimination, are now recognized as critical modifiable risk factors for poor cognitive and brain aging. However, there remain a number of uncertainties regarding their role, including specific pathways and important aspects of exposure. Here, we explore some of these questions by interrogating exposure definitions, employing a lifecourse perspective, exploring study design effects on findings, and testing specific mediation pathways. We incorporate data from 5 cohorts, spanning local neighborhood studies to nationally-representative samples, to explore these questions. First, Meredith Phillips assesses how subjective and objective measures of SES relate to neuroimaging markers of brain health in a middle-aged cohort to address which aspects of SES are most critical to brain health. Second, Andrea Rosso examines neighborhood deprivation measures across the lifecourse in relation to general cognitive aging in a cohort based in 2 historically Black neighborhoods to address critical periods of exposure. Third, Erica Fan assesses the relation of everyday discrimination in relation to cognitive tests across 3 cohorts, exploring how study design and race may affect results. Finally, Greta Cheng tests a mediation pathway from neighborhood SES to general cognitive function through hemoglobin A1C in the Health and Retirement Study. Together, these results advance our understanding of the role of social determinants in cognitive and brain aging with implications for study design and methods. A discussion led by Lisa Barnes will address the advances presented in these studies and consider next steps.

Abstract citation ID: igaf122.742

### LIFECOURSE NEIGHBORHOOD DISADVANTAGE AND GLOBAL COGNITION IN A COMMUNITY-BASED STUDY OF BLACK ADULTS

Andrea Rosso[1], Greta Jianjia Cheng[1], Gerald Hunter[2], Tanisha Hill-Jarrett[3], Bonnie Dastidar[4], Wendy Troxel[2], and Tamara Dubowitz[5], *1. University of Pittsburgh, Pittsburgh, Pennsylvania, United States, 2. RAND, Pittsburgh, Pennsylvania, United States, 3. University of California Memory and Aging Center, San Francisco, California, United States, 4. RAND, Santa Monica, California, United States, 5. University of Pittsburgh School of Public Health, Pittsburgh, Pennsylvania, United States*

Evidence suggests that neighborhood characteristics affect cognitive health in later life. Nearly all existing studies have assessed neighborhood context at study enrollment and lack a lifecourse perspective. For cognitive aging, early life exposures may play a critical role. We obtained lifetime residential histories from participants in the Think PHRESH study, a cohort of participants (n = 423, mean age=63, 75% female, 95% Black) from two historically disinvested, predominantly Black neighborhoods in Pittsburgh, PA. Participants were randomly sampled from a complete list of addresses in each neighborhood. Addresses were linked to historical census data to quantify neighborhood deprivation using 14 census measures across five lifecourse periods (childhood, adolescence, young adulthood, middle age, late life). Linear models estimated associations of deprivation at each lifecourse period with current Modified Mini Mental State Examination (3MS) scores, adjusted for demographic characteristics. Mean 3MS score was 89 (SD = 8). Greater deprivation in childhood and adolescence was not related to 3MS scores. Greater neighborhood deprivation during young adulthood (beta=-1.59 (95% CI: -2.72, -0.46)) and midlife (beta=-1.63 (95% CI: -3.00, -0.28)) was related to lower 3MS scores. Greater deprivation in late life was marginally related to lower 3MS scores (beta=-1.79 (95% CI: -3.65, 0.08)). These results highlight young adulthood and midlife as potential critical windows of exposure to neighborhood deprivation for later life global cognitive functioning and may suggest specific pathways by which these exposures influence dementia risk.

Abstract citation ID: igaf122.743

### OBJECTIVE AND SUBJECTIVE MEASURES OF MIDLIFE SOCIOECONOMIC STATUS AND WHITE MATTER HYPERINTENSITIES

Meredith Phillips, Peter Gianaros, Tae Kim, C. Shaaban, and Andrea Rosso, *University of Pittsburgh, Pittsburgh, Pennsylvania, United States*

The risk of cognitive impairment and dementia is greater among people with lower socioeconomic status (SES). SES is a multifaceted factor encompassing many potential pathways influencing brain structure but is often measured using single variables. Adverse brain features in midlife are associated with faster cognitive aging. We estimated the association between white matter hyperintensity (WMH) volumes and three SES measures: household income, income relative to neighborhood average, and subjective SES in a middle-aged cohort. We fit a series of linear regression models estimating the associations between SES and log-transformed WMHs controlling for age, educational attainment, race/ethnicity. We tested effect measure modification by sex using its interaction with SES measures. Our sample (N = 191) was 40% male, primarily White (84%), and highly educated (88% bachelor's degree or higher), with a mean age of 42 (SD: 9). We found no associations between total overall WMHs and household income or relative income. Women with higher subjective SES had smaller total overall WMH volume (log-transformed sex*subjective SES β: -0.26, 95% CI: -0.44 – -0.08) but there was no significant association in men. We also found that men with higher subjective SES had larger deep WMH volumes (log-transformed β: 0.55, 95% CI 0.17 – 0.92), while women with higher subjective SES had smaller deep WMH volumes (log-transformed sex* subjective SES β: -0.40, 95% CI: -0.61 – -0.18). Only subjective SES was associated with WMH. We also identified sex differences, as the association between subjective SES and deep WMHs was positive for men but negative for women.

Abstract citation ID: igaf122.744

### DISCRIMINATION AND COGNITION IN THREE COMMUNITY-BASED COHORTS: THINK PHRESH, COBRA, AND HEART-SCORE

Erica Fan[1], Sarah Royse[2], Tanisha Hill-Jarrett[3], Wendy Troxel[4], Tamara Dubowitz[1], Ann Cohen[2], and Andrea Rosso[2], *1. University of Pittsburgh School of Public Health, Pittsburgh, Pennsylvania, United States, 2. University of Pittsburgh, Pittsburgh, Pennsylvania, United States, 3. University of California Memory and Aging Center, San Francisco, California, United States, 4. RAND, Pittsburgh, Pennsylvania, United States*

Previous work indicates an association between exposure to discrimination and poorer cognition in older adults; however, structural and demographic factors may affect the strength and direction of this association. Such factors differentially impact study enrollment and thus, without a clear understanding of how study design impacts these factors, inference from population studies may be limited. As such, in three cohort studies of cognitive health, we aimed to (1) examine differences in sample characteristics, (2) assess the association between discrimination and cognition, and (3) test the modifying effect of racialized group and study type. Of these community-based cohorts (n = 402), two included neuroimaging and all included older Black adults. Discrimination was measured using 5 items from the Everyday Discrimination Scale. Cognition was assessed using the Boston Naming test, Trails A/B, FAS fluency, and animal naming. Participants enrolled in imaging studies were more educated, reported lower perceived discrimination, and performed better on cognitive testing. We found that in the full cohort, discrimination was significantly associated with worse performance on total FAS fluency and animal naming (β=-0.26, p = 0.04; β=-0.13, p = 0.03) in linear regression models adjusted for age, sex, and education. The association between discrimination and total FAS fluency differed by racialized group (p = 0.005), where discrimination was not associated with cognition in Black participants across studies, but was associated with cognition in White participants. This suggests that the associations between discrimination and cognition may vary by racialized group; however, additional work and more sensitive measures may be needed to understand these complex, multifactorial relationships.

Abstract citation ID: igaf122.745

**CUMULATIVE MIDLIFE NEIGHBORHOOD DISADVANTAGE AND LATE-LIFE COGNITIVE CHANGE: THE MEDIATING ROLE OF HEMOGLOBIN A1C**

Greta Jianjia Cheng, Jeanine Buchanich, Tiffany Gary-Webb, Christina Mair, C. Shaaban, and Andrea Rosso, *University of Pittsburgh, Pittsburgh, Pennsylvania, United States*

Neighborhood factors are recognized determinants of late-life cognitive function; mechanisms remain unclear but may include metabolic factors. Using data from the Health and Retirement Study, we examined whether hemoglobin A1C, a 3-month measure of blood glucose level, partially explains the association between cumulative midlife neighborhood disadvantage and late-life cognitive change in 4,707 older adults aged 50+. Cumulative midlife neighborhood disadvantage (continuous score) captured average exposure to tract-level disadvantage, including poverty, unemployment, % female-headed households, and % households with public assistance. A1C level was categorized as < 5.7%, 5.7%-6.5%, and > =6.5%. Cognitive function was repeatedly assessed using the Telephone Interview for Cognitive Status. Cognitive change groups (maintainers, minor decliners, major decliners) were identified based on participant-specific random slopes from a linear mixed model. Path analyses estimated coefficients and product method calculated the mediation effect. Higher neighborhood disadvantage was associated with elevated A1C (5.7%-6.5%, RRR—relative risk ratio: 1.2, 95% CI: 1.1, 1.4; > =6.5%, RRR: 1.4, 95% CI: 1.2, 1.6), and was associated with minor (RRR: 1.34, 95% CI: 1.16, 1.55) or major cognitive decline (RRR: 1.71, 95% CI: 1.43, 2.03). After controlling for A1c, neighborhood disadvantage remained associated with cognitive change, suggesting a partial mediating effect from blood glucose. Controlling the A1C level from > =6.5% to < 5.7% would potentially reduce the adverse effect of neighborhood disadvantage by 12% for minor and 16% for major cognitive decline. Midlife is a critical period of neighborhood exposure for cognitive health; targeting blood glucose could partially mitigate the adverse effect of neighborhood disadvantage on late-life cognitive decline.

### SESSION 2205 (SYMPOSIUM)

Abstract citation ID: igaf122.746

**PROJECT ECHO IN GERIATRICS EDUCATION: INNOVATIVE INTERPROFESSIONAL MODELS AND METHODS FOR THE FUTURE**

Chair: Phillip Clark

Discussant: Thomas Caprio

Future innovation in geriatrics education depends on integrating interprofessional teamwork with Age-Friendly 4M Care principles in targeted settings and with specific types of trainees. The 4M framework requires coordinated participation of multiple professions to address the multifaceted and complex healthcare needs of older adults. Project ECHO (Extension for Community Healthcare Outcomes) is custom-made for this purpose. It is a Zoom-based telemedicine model with an interprofessional hub of specialists delivering sessions to primary care and other teams in the community. Key components are a short didactic presentation, followed by a case presentation and discussion. An "all teach, all learn" educational philosophy levels professional hierarchies, with participants sharing perspectives and recommendations for a complex case in a community of practice built on mutual support and trust. Papers in this symposium are based on the extensive ECHO experience of several HRSA-funded Geriatric Workforce Enhancement Programs (GWEPs) and focus on key lessons learned from specific settings and trainees for expanding future ECHOs. First, the conceptual basis of ECHO for the development of interprofessional education (IPE) is presented. Second, the implications of lessons learned from multi-GWEP collaboration in designing Dementia-Friendly education for primary care teams are presented. Third, the challenges of fostering teamwork with front-line CNA providers are explored. Finally, methods of combining IPE and geriatrics education of students and other trainees are explored. Recommendations include developing more explicit methods for integrating 4M care and teamwork, targeting ECHOs on specific clinical teams and sites, and more effectively assessing impacts on learners and clinical practice.

Abstract citation ID: igaf122.747

**THE THEORETICAL FOUNDATIONS OF ECHO: THE KEY TO INTERPROFESSIONAL INNOVATION IN GERIATRICS EDUCATION**

Phillip Clark, *University of Rhode Island, Kingston, Rhode Island, United States*

"There is nothing so practical as a good theory," observed social psychologist Kurt Lewin. In developing innovative methods of interprofessional geriatrics education, this maxim applies to how the theoretical foundations of Project ECHO can enhance educators' ability to apply its dual concepts of "tele-mentoring" and "tele-networking" to improve geriatric care. The multiple, complex, and chronic conditions of many older adults require interprofessional and interorganizational teamwork to provide Age-Friendly care. Two of the educational theories underpinning ECHO are especially relevant to addressing this need: (1) community of practice, and (2) single- and double-loop learning. Community of practice requires an intentional commitment of professionals to an ongoing group for learning and improving care. In ECHO, it provides the structure within which educational methods are employed and supports members in coping with challenging patients and situations. The ECHO principle of "all teach, all learn" is the foundation of this community. Single-loop learning involves acquiring professional knowledge to improve care and is embodied in didactic presentations. In contrast, double-loop learning entails transforming familiar modes of practice by recognizing the limits of one's own discipline and supporting interprofessional collaboration for Age Friendly care. This outcome is based on the ECHO case presentation and discussion. Future innovations incorporating these theoretical principles are presented: (1) advantage of learning 4M care within a community of practice, (2) need for interprofessional teamwork made explicit in ECHO programs, and (3) requirement for mixed-methods evaluation of ECHOs to capture the impacts of changes in practice on patient care and outcomes.

Abstract citation ID: igaf122.748

### PROJECT ECHO DEMENTIA FOR PACE PROGRAMS: STRENGTH IN PARTNERSHIPS AND LESSONS FOR THE FUTURE

Leland Waters[1], Phillip Clark[2], Faith Helm[3], Dana Sohmer[4], and Jane Marks[5], *1. Virginia Commonwealth University, Richmond, Virginia, United States, 2. University of Rhode Island, Kingston, Rhode Island, United States, 3. Rhode Island Geriatric Education Center, Kingston, Rhode Island, United States, 4. Alzheimer's Association, Chicago, Illinois, United States, 5. Johns Hopkins University, Baltimore, Maryland, United States*

Project ECHO Dementia has been a collaborative initiative designed to enhance dementia care. Over the past five years, three Geriatrics Workforce Enhancement Programs (GWEPs) have partnered with the Alzheimer's Association National Office to implement two distinct Project ECHO series. This new series builds upon an established history of collaboration between GWEPs and regional Program of All-Inclusive Care for the Elderly (PACE) programs, which have actively participated in previous geriatrics education programs, including ECHO-based learning. Our ECHO framework follows the structured model with a dedicated interprofessional Hub team, fostering a collaborative "all teach, all learn" approach. PACE programs, already operating within an interprofessional care model, naturally align with this educational approach, leveraging team-based training to enhance dementia care. Through iterative development, we have adapted our program in response to participant feedback. Initially structured as twelve biweekly sessions, we transitioned to a more focused format of four weekly sessions based on a pre-series needs assessment. Key lessons learned include the importance of continuity of engagement among PACE learning sites and among subject matter experts to establish trust and rapport and the ability to build on previous sessions for progressive learning. We implemented a needs assessment to identify specific educational priorities for PACE programs, refining session content to enhance engagement and applicability and expanding interprofessional collaboration to strengthen dementia care training. Recommendations include maintaining continuity of subject matter experts, integrating participant feedback into future ECHO series, and exploring opportunities to scale and sustain the program for broader impact within geriatrics education.

Abstract citation ID: igaf122.749

### BENEFITS AND CHALLENGES OF ECHO INNOVATION TO REACH NEW INTERPROFESSIONAL TEAM MEMBERS AND MEET WORKFORCE NEEDS

Lauren Gleason[1], Monica Long[2], and Katherine Thompson[1], 1. UChicago Medicine, Chicago, Illinois, United States, 2. University of Chicago Medicine/SHARE Network, Glenwood, Illinois, United States

The University of Chicago GWEP has a long history of utilizing the Project ECHO model to train interprofessional teams of healthcare workers. One group not historically included in the ECHO training model is certified nursing assistants (CNAs) working in skilled nursing facilities (SNFs). This workforce is particularly vulnerable to workplace injury, burnout, and high job turnover. Using a needs assessment of CNAs to guide our program development, we created an innovative ECHO curriculum to train CNAs in SNFs, focusing on such high yield topics as empowerment, leadership, peer support, and quality improvement. Data from mixed methods assessment of barriers and facilitators to participation and learning were collected for future program quality improvement. Barriers included irregularity of participant availability due to work schedules and lack of comfort and familiarity with case-style presentations. Facilitators included ability to feel empowered in the workplace by developing additional skills and a peer community of practice. Adaptations for quality improvement included providing sample cases for discussion and tailoring topics to evolving needs of participants. Using the ECHO model to develop innovative programs may be a scalable way to meet interprofessional workforce needs as well as support vulnerable workers by developing a sense of empowerment and a community of practice. Traditional ECHO teaching strategies (e.g., case presentations) may need to be adapted based on comfort level and familiarity of the learner group.

Abstract citation ID: igaf122.750

### UTILIZING PROJECT ECHO-GERIATRICS FOR INTERPROFESSIONAL EDUCATION OF HEALTH PROFESSIONS STUDENTS AND TRAINEES

Katherine Bennett[1], Ashley McPeek[2], and Noa Brazg[3], *1. University of Washington School of Medicine, Seattle, Washington, United States, 2. University of Washington Center for Health Sciences Interprofessional Education, Research, and Practice, Seattle, Washington, United States, 3. University of Washington, Seattle, Washington, United States*

With the rapid expansion of the Project ECHO model over the past decade, there have been increasing numbers of ECHO programs that include interprofessional expert panels. Observation of interprofessional clinical practice is not readily available to most health professions students, and ECHO provides a venue for observing and participating in discussions that exemplify interprofessional teamwork. The University of Washington's Project ECHO-Geriatrics has run monthly from 2016 to the present to increase geriatrics education for family medicine residents throughout a regional residency network. It includes a robust panel of health professionals, including geriatricians, psychiatrists, nurses, pharmacists, social workers, and Area Agency on Aging staff members. Each session includes a brief didactic, followed by an interprofessional case discussion of a patient presented by a program participant. In 2022, a program was launched for UW health professions trainees to participate longitudinally in Project ECHO-Geriatrics. Students attend an introduction to Project ECHO and Age-Friendly Care and then participate in at least three monthly Project ECHO-Geriatrics Sessions that are followed by a separate debrief for students. Health professions students across ten disciplines (e.g., medicine, nursing, pharmacy, social work, dentistry, and public health) have participated thus far. Surveys of participants have consistently shown increased confidence in both interprofessional teamwork and geriatrics skills as a result of program participation. These programs at UW for trainees and students offer a framework for the growing number of ECHOs with interprofessional panels to leverage their existing program to increase geriatrics and interprofessional team skills among trainees across many health professions.

### SESSION 2210 (SYMPOSIUM)

Abstract citation ID: igaf122.751

### EMPOWERING AGING IN PLACE THROUGH COMMUNITY HEALTH INNOVATION FOR ASIAN OLDER ADULTS

Chair: Bada Kang
Co-Chair: Noriko Yamamoto-Mitani
Discussant: Eleanor McConnell

With the super-aging of South Korea and Japan, enabling older adults to age in place has become a critical priority in both countries. This symposium explores transformative approaches that leverage digital technologies and community-driven initiatives in these countries to develop scalable solutions for aging in place. The first presentation introduces a university-led community health program in Japan, designed to establish a sustainable care-delivery system. This program focuses on care-competency education, healthy living environments, and mutual support among care professionals. Using a community-based participatory research approach, it seeks to extend healthy life expectancy by actively involving citizens in their own care. The second presentation highlights an educational program aimed at enhancing end-of-life care skills for both nurses and caregivers. The program creates an engaging and collaborative learning environment for palliative care by integrating dramatic e-learning, expert-led lectures, and professional shadowing. The third presentation examines caregiver burden in dementia care using multimodal monitoring techniques, including sweat patches, actigraphy, and surveys. In identifying the biological, psychological, and social factors contributing to caregiver burden, this study emphasizes the need for tailored interventions to improve care outcomes. The final presentation investigates the feasibility of mobile-based Ecological Momentary Assessment (EMA) for measuring social connectedness among older adults. Using real-time data collected from a senior living lab cohort, this study refines assessment methodologies to better understand social connectedness, emotions, and contextual influences. This symposium provides valuable insights into innovative solutions for aging in place to inform the future direction of community-based healthcare for older adults.

Abstract citation ID: igaf122.752

### A UNIVERSITY-LED COMMUNITY PROGRAM FOR SUPER-AGED SOCIETY

Noriko Yamamoto-Mitani[1], Yuka Sumikawa[2], Hanako Numata[3], Manami Takaoka[1], Taeko Mizukami[4], Yurina Miyano[4], and Ayumi Igarashi[5], *1. The University of Tokyo, Bunkyo, Tokyo, Japan, 2. University of Tokyo, Bunkyo-City, Tokyo, Japan, 3. University of Tokyo, University of Tokyo, Tokyo, Japan, 4. Bunkyo City Social Welfare Council, Bunkyo City, Tokyo, Japan, 5. The University of Tokyo, Tokyo, Tokyo, Japan*

How to maintain a sustainable care delivery system for those who need care is an urgent issue for Japan, which has become a super-aged society and a society with a declining population. The solution to such social issues cannot be found through conventional research and surveys, but rather through community-based participatory research (CBPR), in which solutions are found together with citizens. Based on such a concept, the Global Nursing Research Center of the University of Tokyo School of Medicine is constructing a CBPR for community development aimed at extending life expectancy with well-being. This program will have: Care Competency Education Program, Room for Healthy Living, and Mutual Support for Care Professionals. In the Care Competency Education Program, nurses provide education on the ability to care for the health and safety of themselves and those around them. Room for Healthy Living will be a hub for health in the community, similar to the health centers in schools. It is a place where anyone can casually drop in and gather, and where they can easily consult with professional nurses as needed. It is a place where people with the same difficulties can get together and share information. Mutual Support for Care Professionals is a place where various professionals involved in care in the community can get together and see each other, deepen mutual understanding through case studies, etc., and learn the latest knowledge through lectures by researchers. The above initiatives are being implemented and developed based on the needs of citizens.

Abstract citation ID: igaf122.753

### A JOINT LEARNING PROGRAM FOR LICENSED NURSES AND CAREGIVERS ON END-OF-LIFE CARE IN THE COMMUNITY

Yuka Sumikawa[1], Hanako Numata[2], Akari Maeda[3], and Noriko Yamamoto-Mitani[4], *1. University of Tokyo, Bunkyo-City, Tokyo, Japan, 2. University of Tokyo, University of Tokyo, Tokyo, Japan, 3. The University of Tokyo Global Nursing Research Center, Tokyo, Tokyo, Japan, 4. The University of Tokyo, Bunkyo, Tokyo, Japan*

While hospital deaths have long been the norm in Japan, the super-aging society is also a time of multiple deaths, and many people are now dying outside of medical facilities. In particular, many people who die from diseases other than cancer often have significantly impaired ADLs by the time of death and require care. However, current training programs for caregivers do not include education on end-of-life care, and many caregivers face difficulties in providing end-of-life care. On the other hand, most nurses who work outside of hospitals have experience in end-of-life care, but most of their experience is in hospitals and they are not used to work with non-medical professionals such as formal caregivers. Therefore, we have developed an educational program on end-of-life care, in which nurses and caregivers can learn about end-of-life care together. The concept was to make the education enjoyable and to enable nurses and caregivers to learn together. The educational program was planned mainly by the researchers, incorporating the opinions of nurses and caregivers in the field. An e-learning program was created in which a drama of end-of-life care was created to enhance their empathy for the patients, and lectures by experts were added at key points so that participants could learn specific end-of-life care techniques. Some possible participants shadowed different professions than themselves to learn more about the observations and judgments of other professions. In the future, we are considering evolving the program so that more professions, such as care managers, can learn together.

Abstract citation ID: igaf122.754

### BEYOND SELF-REPORTS: LEVERAGING MULTIMODAL MONITORING TO UNDERSTAND CAREGIVER BURDEN FOR PERSONS WITH DEMENTIA

Kyung Hee Lee[1], Bomgyeol Kim[2], and Jiyoung Shin[3], *1. Yonsei University, Seodaemun-gu, Seoul-t'ukpyolsi, Republic of Korea, 2. Yonsei University College of Nursing, Seodaemun-gu,*

*Seoul-t'ukpyolsi, Republic of Korea, 3. Health Insurance Research Institute, Wonju, Kangwon-do, Republic of Korea*

Although numerous studies have examined the determinants of caregiver burden for people with dementia, the measurement of this burden has predominantly relied on self-report methods. The aim of this study was to identify the key determinants of the burden among family caregivers dealing with people with dementia using a multimodal monitoring approach. An observational cross-sectional study was conducted with 75 dyads of individuals with dementia and their caregivers. The determinants of caregiver burden were assessed within a biopsychosocial framework—encompassing biological, psychological, and social factors—using sweat patches, actigraphy, and surveys. A hierarchical multiple regression analysis was used to examine the associations. Psychological factors explained 39.3% of the variance in this burden, and an additional 17.5% increase was observed when biological factors were included, accounting for 56.8% of the variance. Higher levels of the pro-inflammatory cytokine TNF-α in persons with dementia were significantly associated with an increased caregiver burden, whereas longer total sleep time was linked to a decreased burden. Among the psychological factors, higher depressive symptoms and more severe behavioral and psychological symptoms of dementia were significantly related to an increased caregiver burden. Our findings underscore the need to integrate objective physiological measures with survey-based assessments to gain a more comprehensive understanding of caregiver burden. Furthermore, this study highlights the importance of tailored interventions that address both biological and psychological factors to alleviate caregiver burden and improve care outcomes. Reducing caregiver burden may also facilitate aging in place, enabling individuals with dementia to remain in familiar environments.

Abstract citation ID: igaf122.755

### EXPLORING MOBILE EMA AND GPS TO UNDERSTAND SOCIAL CONNECTEDNESS IN COMMUNITY-DWELLING OLDER ADULTS

JiYeon Choi[1], Bomgyeol Kim[2], Seongmi Choi[3], JaeWon Hyun, and Hun Kang[1], *1. Yonsei University, Seoul, Republic of Korea, 2. Yonsei University College of Nursing, Seoul, Republic of Korea, 3. Daegu Catholic University, Daegu, Republic of Korea*

Social connectedness is a key determinant of health and well-being in older adults. Ecological Momentary Assessment (EMA) has been widely used to study dynamic social interactions in daily life, and technological advancements continue to refine EMA for real-world applications. This presentation shares our experience of conducting a series of studies that assess social connectedness and associated psychosocial responses among community-dwelling older adults using mobile EMA and Global Positioning System (GPS) tracking. In 2023, in collaboration with the Active Senior Living Lab Support Group (Seongnam Senior Industry Innovation Center, Gyeonggi-do, South Korea), we established a cohort of older adults (n = 35, 72.2±6.7 years old) for longitudinal research on social connectedness. A pilot study assessed the feasibility of mobile EMA-based data collection by measuring social activities and emotions four times daily for two weeks (56 data points). Thirty-four participants successfully completed data collection (completion rate, 99.7%). Four group interviews with 20 pilot study participants provided feedback on EMA applicability, assessment appropriateness, and research staff interactions. Participants reported increased awareness of their social interactions, reinforced their sense of responsibility, and fostered a stronger sense of community belonging. Based on these findings, our 2025 follow-up study integrates the use of mobile EMA with GPS tracking system to further explore the spatial dynamics of social connectedness in older adults. This approach records the location-based social engagement pattern information, thereby providing contextual insights into social connectedness, loneliness, and emotions. These findings will help to refine methodologies for assessing social connectedness and contextual factors in community-dwelling older adults.

### SESSION 2215 (SYMPOSIUM)

Abstract citation ID: igaf122.756

### POLICY SERIES: IMPACTS AND OPPORTUNITIES: RESEARCH AND POLICY PRIORITIES AT NIH

Chair: Patricia D'Antonio
Discussant: Brian Lindberg

This symposium will explore the significant policy changes implemented by the current administration at the National Institutes of Health (NIH) and the National Institute on Aging (NIA), with a focus on their impact on the research community. We will examine how these shifts affect grant funding, application processes, and the overall research landscape.

### SESSION 2220 (SYMPOSIUM)

Abstract citation ID: igaf122.757

### PSYCHOSOCIAL AND COGNITIVE DIMENSIONS OF AGING AND CANCER: COMMUNICATION, RESILIENCE, AND HEALTH CHALLENGES

Chair: Sean Halpin

In this symposium, we explore the psychosocial and cognitive dimensions of aging and cancer, focusing on how humor, communication, and psychological well-being shape patient experiences across different health contexts, including cancer and other chronic conditions. We bring together diverse methodological approaches to examine the complexities of navigating health challenges as an older adult. First, Halpin will present on the intersection of cognitive load and socio-emotional adaptation theory in patient education for older adults with multiple myeloma. He will focus on how information delivery strategies affect patient understanding and emotional adaptation. Next, Hardt will discuss older adults' experiences of depression during cancer. Using qualitative methods, she describes older adults' views of depression during cancer and strategies for coping with cancer, including mental health services. Chidebe will then examine access to psychotherapy, psychological support and psycho-oncology concerns among older cancer patients, using quantitative methods to explore the psychological challenges faced by older adults with cancer and potential avenues for mental health support. Finally, Krok-Schoen will explore the relationship between physical functioning, self-efficacy, and quality of life in cancer survivors, drawing on quantitative data to assess key psychosocial factors influencing survivorship. By integrating our perspectives, we provide a broader understanding of how psychosocial adaptation, humor, and cognitive processing intersect in aging populations facing complex health challenges. Our discussion will offer practical applications for improving communication, patient education, and psychosocial support strategies for older adults. Cancer and Aging Interest Group Sponsored Symposium

Abstract citation ID: igaf122.758

### ENHANCING PATIENT EDUCATION IN MULTIPLE MYELOMA: COGNITIVE LOAD AND SOCIO-EMOTIONAL ADAPTATION

Sean Halpin, *RTI International, Decatur, Georgia, United States*

This study expands on Socio-Emotional Adaptation (SEA) Theory by integrating cognitive load concepts to examine the educational experiences of older adults with multiple myeloma (MM) undergoing autologous stem cell transplantation (ASCT). SEA Theory highlights how perceived control, adaptation, and relationships with caregivers modulate emotional responses to illness. Adding cognitive load underscores how these factors impact patients' bandwidth to process health information. Over 150 hours of ethnographic observation of nurse-patient education sessions (N = 70), supplemented by patient interviews (N = 35) and clinician interviews (N = 7), were conducted over 18 months. Findings reveal barriers, including anxiety, overwhelming information, and lack of medical expertise, alongside facilitators such as tailored education and supportive social networks. By iteratively developing video-based tools addressing these barriers, the intervention decreased cognitive overload while enhancing emotional readiness. This case underscores how cognitive and socio-emotional models inform patient-centered educational practices for older adults with life-limiting illnesses.

Abstract citation ID: igaf122.759

### AGE DIFFERENCES IN PSYCHOLOGICAL IMPACT OF CANCER AND ACCESS TO PSYCHOTHERAPY IN NIGERIA: A NATIONAL STUDY.

Runcie Chidebe[1], Sampson C. Ipiankama[2], Maria Chidi C. Onyedibe[3], Darlingtina Esiaka[4], and JohnBosco Chika Chukwuorji[5], *1. Miami University, Oxford, Ohio, United States, 2. Project PINK BLUE - Health & Psychological Trust Centre, Abuja, Federal Capital Territory, Nigeria, 3. University of Nigeria Nsukka Department of Psychology, Nsukka, Enugu, Nigeria, 4. University of Kentucky - College of Medicine, Lexington, Kentucky, United States, 5. Michigan State University, East Lansing, Michigan, United States*

The cumulative risk of developing and dying from cancer before the age of 75 years is 12% and 8.0% in Nigeria. While certain cancer types (e.g., prostate, liver, cervical, and breast) more commonly affect people ages 30+, the age of cancer diagnosis is low in Nigeria compared to many countries. Yet, the psychological impact of cancer diagnosis among younger and older adults is largely unknown. We aimed to estimate the age difference in the psychological impact of a cancer diagnosis and access to psychotherapy and psycho-oncologists among cancer patients receiving treatment (i.e., chemotherapy, radiotherapy, and immunotherapy) across all the cancer hospitals in Nigeria. Cancer patients (N = 1,374, Mage=52.15) completed the national access to psychotherapy and psycho-oncology questionnaire. We analyzed the data using multiple linear regression and Fisher's discriminant functions. The result showed that older adults (M = 53.81, SD = 14.58) were more likely than younger adults (M = 51.76, SD = 12.58) to be affected psychologically when they received the news of their cancer diagnosis, $F(1, 1317) = 4.31$, $p=.038$. However, there was no age difference in their receiving psychological services after they learned of their cancer, access to psychological support during treatment, need to see a psychologist during treatment, utilization of psychological services during treatment and knowledge of where they could receive psychological or counseling services. These findings highlight the need for targeted psychological interventions to support older adults in coping with the emotional impact of a cancer diagnosis in Nigeria.

Abstract citation ID: igaf122.760

### PHYSICAL FUNCTIONING, SELF-EFFICACY, AND QUALITY OF LIFE AMONG OLDER CANCER SURVIVORS

Jessica Krok-Schoen[1], Cara Chase[1], Colleen Spees[1], Ashley Rosko[2], Mike Beck[1], Brett Nickerson[1], Roberto Benzo[1], and Zachary Chaplow[1], *1. The Ohio State University, Columbus, Ohio, United States, 2. The Ohio State University Comprehensive Cancer Center, Columbus, Ohio, United States*

Introduction: Older cancer survivors are at heightened risk of physical limitations, which can negatively impact their quality of life and self-efficacy for healthy behaviors. The E-PROOF (E-intervention for Protein Intake and Resistance Training to Optimize Function) study is the first synchronous, online, protein-focused dietary and resistance training randomized controlled trial among older cancer survivors. Objective: This study seeks to understand the changes in and association among physical functioning, self-efficacy, and quality of life among older cancer survivors over the 12-week online diet and resistance training intervention. Methods: Eligibility criteria include adults age ≥65 years, stage I-III breast, colorectal, or prostate cancer, and completion of curative treatment. Intervention participants received online nutrition counseling sessions and online resistance training sessions. Control participants received online counseling on stretching and healthy eating. Results: In interim analysis (n = 61, median age=70), 72% of the sample are breast cancer survivors. Improvements in physical function were observed for the whole sample (mean scores=9.6 and 10.2, $p = 0.054$). Participants also reported improvements in exercise self-efficacy and dietary self-efficacy. Health-related quality of life significantly improved in physical function (75.3 to 81.7, $p = 0.03$), physical role limitations (55.7 to 73.4, $p = 0.01$), and energy (58.7 to 69.3, $p = 0.001$). No significant differences were observed between groups. Significant positive correlations were observed after the intervention among physical function, quality of life, and self-efficacy. Final sample results will be presented. Conclusions: Trial results will inform the preliminary efficacy of online diet and resistance training interventions to improve physical function and related psychological outcomes among older cancer survivors.

Abstract citation ID: igaf122.761

### THE EXPERIENCE OF DEPRESSION IN OLDER ADULTS WITH CANCER: A QUALITATIVE ANALYSIS

Madeleine Hardt[1], Jaime Gilliland[2], Lisbeth Rubi[2], Rinchen Dolma[2], Jo Anne Sirey[3], and Kelly McConnell[2], *1. Stony Brook Medicine, New York, United States, 2. Memorial Sloan Kettering Cancer Center, New York, New York, United States, 3. Weill Cornell Medical College, New York City, New York, United States*

Older adults (≥65 years) will make up 73% of U.S. cancer survivors by 2040. Over 25% of older adults with cancer (OACs)

report significant depressive symptoms, linked to worse outcomes. Yet, little is known about OACs' depression experience. This study explored OACs' perspectives via focus groups (n = 13 patients receiving or within 6 months of receiving active treatment). Five key themes emerged: (1) OACs conceptualized their depressive symptoms within the context of their cancer experience (e.g., fear of treatment); (2) Depressive symptoms impact daily life (e.g., reduced activity engagement); (3) OACs perceive their depressive symptoms as distinct from clinical depression and "not bad enough" to warrant treatment; (4) OACs prefer self-reliance or support networks over mental health services; (5) Barriers (e.g., appointment burden) limit access to mental health services. Findings highlight OACs' unique views on depression and suggest that their preference for personal coping strategies may impact mental health service engagement.

### SESSION 2225 (SYMPOSIUM)

Abstract citation ID: igaf122.762

### DIRECT CARE STAFF TRAINING STANDARDS AND EDUCATIONAL INTERVENTIONS IN LONG-TERM CARE

Chair: Katherine Kennedy
Discussant: Carissa Coleman

The training of direct care staff including personal care aides (PCAs), nursing assistants (NAs), and student nurses is critically important to delivery of high-quality care and a more stable long-term care workforce. Relevant quality training is key to ensuring optimum care and coordination for patient populations facing a spectrum of needs, such as those with dementia, mental illness, and other physical and cognitive impairments. Positive perceptions of training quality and attributes (e.g., greater hours, clinical training, facilitator, length, modality, content, impact on interprofessional practice) are associated with more positive perceptions of dementia, improved job satisfaction, and better nursing home resident health outcomes. While training contributes to job quality, current evaluations of training standards, actual training opportunities, and educational interventions or programs have been lacking in the field. A primary focus of this symposium is to describe the importance of workforce development in the post-pandemic world from a macro-policy lens focused on training consistency, rigor, and portability, to a micro-level focus on areas of opportunity for developing and implementing training (e.g., increasing job relevant training, enhancing digital literacy, microlearning). Presentations will highlight current information about training standards for PCAs, on-the-job training of nursing home staff (primarily NAs), and an innovative research-practice partnership to build a pipeline for student nurses into nursing home careers. Through examinations of training standards, training opportunities, and intervention/program delivery, we gain knowledge about potential practice gaps such as important knowledge to mitigate care challenges, knowledge translation strategies, and opportunities for program and policy development.

Abstract citation ID: igaf122.763

### A COMPREHENSIVE REVIEW OF PERSONAL CARE AIDE TRAINING STANDARDS ACROSS THE UNITED STATES

Jessica King, and Kezia Scales, *PHI, New York, New York, United States*

Personal care aides (PCAs), unlike other direct care workers, are not subject to any federal minimum training requirements. Therefore, PCA training standards vary across states. This study aimed to generate a comprehensive analysis of state-by-state PCA training standards, updating and extending a 2014 analysis. To accomplish this, we conducted a content analysis of state Medicaid manuals, waiver documents, and administrative code pertaining to home care programs and services. We assessed the requirements according to three domains: consistency (whether the training requirements were consistent across Medicaid programs); rigor (according to indicators such as named competencies and minimum training hours, among others); and portability (whether training is tied to a recognized certification and/or centrally recorded on a registry), as well as whether states had training requirements for independent providers and private-pay PCAs. We identified six "leader states" who scored highly across all domains: Washington D.C., New Jersey, New York, Oregon, Rhode Island, and Washington State. We found that 65 percent of states (33 states) had consistent training requirements across all Medicaid programs, while 14 percent (7 states) did not have any requirements. This patchwork of state training standards has implications for workforce preparedness and mobility, recruitment and retention, and care quality. The study findings can help state leaders assess the adequacy of their training standards, learn from other states, and implement changes, working toward a goal of building a stable, competent workforce to care for older adults and people with disabilities.

Abstract citation ID: igaf122.764

### NURSING ASSISTANT TRAINING IN VHA COMMUNITY LIVING CENTERS: RESOURCES AND RECOMMENDATIONS

Katherine Kennedy[1], Nathalie Mcintosh[2], Sylvia Haigh[3], David Mohr[4], Ciaran Phibbs[5], and Whitney Mills[6],
*1. Providence VA Healthcare System, Providence, Rhode Island, United States, 2. THRIVE, Providence VA Medical Center, Providence, Rhode Island, United States, 3. VA Providence Healthcare System, Providence, Rhode Island, United States, 4. Veterans Health Administration, Mason, Ohio, United States, 5. Health Economics Resource Center, VA Palo Alto Health Care System, Menlo Park, California, United States, 6. Brown University, Providence, Rhode Island, United States*

Quality training is one component of direct care job quality in long-term care based on non-VA research. This study aimed to identify patterns in nursing assistant (NA) training in Veterans Health Administration Community Living Centers (CLCs; nursing homes). We recruited 2 high-performing and 2 low-performing CLCs based on NA intent to stay from a 2022 survey and completed 6-9 interviews at each (N = 30). We employed qualitative content analysis based on Paraprofessional Healthcare Institute's (PHI) Five Pillars of Direct Care Job Quality to understand training factors impacting intent to stay. The high-performing CLCs (88% average intent to stay) provided structured and well-coordinated orientation, longer floor orientation, offered NA skills workshops, and leveraged existing relationships with clinical resource nurses (CRNs) who supplemented nurse manager coaching. Whereas the low-performing CLCs (32% average intent to stay) had less well coordinated orientations, shorter floor orientation, lacked regular skills workshops, experienced turnover in nurse educator/CRNs, and reported NA training as an area needing improvement. Recommendations included:

NA-tailored, standardized training in various topics (i.e., mental health, dementia, end-of-life, nursing) and more continuing education; synergizing NA and nurse educator/CRN relationships and directly communicating with NAs about training opportunities; and modifications needed to address barriers related to time, staffing, computer access and learning preferences. This study confirms the importance of training to NA job satisfaction in VHA CLCs. Ways to improve training and access to training (e.g., microlearning) may improve job satisfaction and retention.

Abstract citation ID: igaf122.765

**EVALUATING OUTCOMES OF COMMUNICATION EDUCATION FOR LINGUISTICALLY DIVERSE NURSING HOME STAFF**

Maria Roche-Dean[1], Maria Hein[2], Yelena Perkhounkova[2], Carissa Coleman[3], Frances Yang[4], Clarissa Shaw[5], and Kristine Williams[6], *1. Kirkhof College of Nursing, Grand rapids, Michigan, United States, 2. University of Iowa, Iowa City, Iowa, United States, 3. University of Kansas Medical Center, Kansas City, Kansas, United States, 4. University of Kansas, University of Kansas Medical Center, Kansas, United States, 5. University of Iowa College of Nursing, Iowa City, Iowa, United States, 6. University of Kansas, Kansas City, Kansas, United States*

The ethnic and linguistic diversity of staff working in healthcare has increased, along with the critical role of immigrant workers in meeting workforce demand in long-term service and support settings (LTSS). The focus of this analysis is to compare learning outcomes for nursing home staff who identified a primary language other than English (LOE) to primary English speakers participating in Changing Talk Online Training (CHATO), a pragmatic clinical trial. CHATO is a psychoeducational intervention designed to reduce elderspeak, a form of communication that sounds like baby talk, and has been linked to increased restiveness to care and need for psychoactive medications in LTSS residents with dementia. LOE staff (n = 88) had a greater gain in knowledge than the primary English speakers (n = 1586) with mean scores increasing from 48% on pre-test to 72% on post-test, compared to primary English speakers whose mean scores increased from 57% to 74% (p=.009). Both LOE and primary English-speaking staff expressed positive views regarding CHATO education content. However, LOE participants rated the intervention more difficult than primary English speakers (8.5% v 3.3%, p= .001) which may be associated with technological challenges. Preparing educational content in healthcare that also reaches LOE staff is necessary for a well-prepared workforce; modifications for learning may include digital literacy training and developing organizational policies that support these practices.

Abstract citation ID: igaf122.766

**INNOVATIVE EDUCATION TO CAREER PIPELINES IN LONG-TERM CARE: THE SCHOLARS WITH A.G. RHODES PROGRAM**

Wendy Simonds, Jennifer Morgan, and Antonius Skipper, *Georgia State University, Atlanta, Georgia, United States*

Gerontology programs often seek to enter into education-practice partnerships to support students to gain hands-on knowledge and experience that prepares them for careers serving older adults. This collaboration, known as “Scholars With A.G. Rhodes at GSU” (SWAGR at GSU), offers master’s students the opportunity to gain valuable hands-on experience in long-term care settings while completing their graduate training in Gerontology. By combining education with real-world practice, the program aims to strengthen the elder care workforce, enrich the students’ professional development, and elaborate a research-practice partnership. A.G. Rhodes has committed to helping students remain working in its centers after graduation. The SWAGR program extends the usual partnerships that foreground service learning or internships while seeking to create educational/career pipelines for international students with undergraduate nursing degrees. The program not only helps fill the growing need for skilled elder care professionals but also provides a much-needed pathway to establish nursing careers in nursing homes. Our presentation will describe the pilot program, in which nine students participated during the first year. Most SWAGRs served as “non-clinical interns” and assisted with resident engagement programs; two who had already passed their NCLEX exams did more traditional clinical internships. We will discuss the challenges and accomplishments the students experienced on site at three A.G. Rhodes centers. We will also discuss our plans to develop a program of research embedded in A.G. Rhodes to support model development, translation of research to practice and engaged scholarship.

### SESSION 2230 (SYMPOSIUM)

Abstract citation ID: igaf122.767

**SLEEP AND AGING ACROSS THE GLOBE**

Chair: Tuo Yu Chen
Co-Chair: Soomi Lee
Discussant: Orfeu Buxton

The increasing prevalence of poor sleep worldwide is a growing concern, given its detrimental effects on physical health, mental health, longevity, and overall quality of life. The relationship between sleep, aging, and health varies across different countries and cultures, highlighting the need to explore sleep in diverse contexts and establish standardized assessment methods. This symposium brings together four presentations that delve into sleep and aging among various populations, focusing on its implications for life and health expectancy, sociobehavioral and lifestyle risk factors, and methodological advancements in sleep research. The first study investigates the associations between sleep and nap duration with total life expectancy and cardiovascular disease-free life expectancy in older adults in Singapore, shedding light on sleep’s role in promoting healthy aging. The second study examines how work and family spillover—the interplay between two central social roles—relates to sleep quality over time among midlife adults in the US and Japan, highlighting cultural similarities and differences in occupational stress and sleep disruption. The third study explores lifestyle factors related to multidimensional sleep health among Japanese adults aged 40 years and older, as well as age-related variations in these associations. The last study focuses on innovative methodologies for assessing multidimensional sleep health, leveraging data from older cohorts in the US and the Netherlands. Finally, Dr. Orfeu Buxton will lead a discussion on the key findings from these various countries and methodological approaches, fostering a deeper understanding of sleep health’s role in aging and identifying future directions for global sleep research.

Abstract citation ID: igaf122.768

**ASSOCIATION OF SLEEP AND NAP DURATION WITH CARDIOVASCULAR DISEASE-FREE LIFE EXPECTANCY AMONG OLDER ADULTS**

Ruoxuan Rosalyn Li[1], Abhijit Visaria[1], Stefan Ma[2], and Rahul Malhotra[1], *1. Duke-NUS Medical School, Singapore, Singapore, 2. Singapore Ministry of Health, Singapore, Singapore*

Sleep and nap durations have been linked with many health outcomes, yet their impact on health expectancy remains understudied. We investigated the association of nocturnal sleep and daytime nap durations with total life expectancy (TLE), and life expectancy (LE) with and without cardiovascular diseases (CVD), among older adults in Singapore. Representative longitudinal data (three waves; 2009-2015) of community-dwelling Singapore residents aged ≥60 years (n = 3452) was used. We applied multistate life table methods to estimate TLE, and LE with and without CVD, by sleep duration and nap duration, adjusting for a range of socio-demographic and health variables. At ages 60, 70, and 80 years, there was no difference in TLE or LE without CVD among those with short, recommended and long sleep. However, long (versus recommended) sleep was associated with a shorter LE with CVD. At ages 60 and 70, the proportion of remaining life without CVD was higher, and with CVD was lower, among those with long (versus recommended) sleep. At ages 60, 70, and 80 years, long nap was associated with a shorter TLE and LE without CVD, versus none or short nap. LE with CVD or proportion of remaining life with and without CVD did not differ by nap duration. Our findings reflect the complex relationship between sleep and nap durations and life and health expectancy outcomes. Further research is needed to elucidate underlying mechanisms and guide recommendations for optimal nocturnal sleep and daytime nap durations in older adults.

Abstract citation ID: igaf122.769

**SHORTER BUT BETTER? A CROSS-CULTURAL ANALYSIS OF SLEEP IN JAPAN AND THE U.S. USING**

Kyoungmin Cho, and Soomi Lee, *The Pennsylvania State University, University Park, Pennsylvania, United States*

Poor sleep is an increasing global concern, yet research comparing cross-country differences in sleep quality remains limited. This study examined cross-cultural differences in sleep patterns between Japan and the United States. We analyzed two-wave data from 618 adults who participated in the Midlife in the United States (MIDUS; Wave1=2004–2006, Wave2=2013–2014, n = 535, M_age=51.6) and Midlife in Japan (MIDJA; Wave1=2008, Wave2=2012, n = 83, M_age=50.5). Sleep was commonly assessed using the Pittsburgh Sleep Quality Index, a well-validated measure assessing habitual sleep quality, sleep latency, sleep duration, habitual sleep efficiency, sleep disturbance, sleeping medication use, and daytime dysfunction (PSQI; Range=0-21; the higher score, the better). In the ANCOVA, baseline age, gender, education, and self-rated health were controlled for. At Wave1, Japanese adults showed better global PSQI scores compared to US adults (F(1,611)=20.80, p<.001, Mean_US =5.96 vs. Mean_Japan=4.08). They also exhibited shorter sleep latency, higher sleep efficiency, fewer sleep disturbances, and lower medication use. However, sleep duration, sleep quality, and daytime dysfunction did not differ significantly. At Wave2, Japanese adults continued to have better sleep (F(1,611)=14.66, p<.001, Mean_US=6.37 vs. Mean_Japan=4.83). Japanese adults reported shorter sleep latency, fewer sleep disturbances, and lower medication use. However, they slept for a shorter duration than their U.S. counterparts (F(1, 611)=17.94, p<.001, Mean_US=6.91 hrs vs. Mean_Japan=6.27 hrs). Sleep quality, sleep efficiency and daytime dysfunction did not significantly differ at Wave2. Despite shorter duration, Japanese adults reported better sleep quality than U.S. adults. Further research should explore cultural and lifestyle influences in cross-cultural differences in sleep.

Abstract citation ID: igaf122.770

**FACTORS ASSOCIATED WITH MULTIDIMENSIONAL SLEEP HEALTH IN JAPANESE ADULTS: AN AGE-STRATIFIED ANALYSIS**

Ryuji Furihata[1], Yukiko Tateyama[1], Tomonari Shimamoto[1], Nishioka Norihiro[1], Takahashi Yoshimitsu[1], Hiroshi Okada[2], Takeo Nakayama[1], and Taku Iwami[1], *1. Kyoto University, Kyoto, Kyoto, Japan, 2. Wakayama Medical University, Wakayama, Wakayama, Japan*

Objective: To investigate factors associated with multidimensional sleep health among community-dwelling adults and to assess variations by age group. Methods: A self-administered survey was conducted from December 2022 to February 2023 among Kyoto City residents aged 40 years or older. Multidimensional sleep health was measured using the RU_SATED questionnaire, which evaluates regularity, satisfaction, alertness, timing, efficiency, and duration. A score of 10 or higher defined good sleep health. Multivariable logistic regression was used to investigate associated factors. The study was approved by the Kyoto University Ethics Committee. Results: Data from 661 respondents (57.9% female, mean age 64.9 years) were analyzed. Good sleep health was observed in 45.8% of participants, including 44.5% of those younger than 75 years and 49.7% of those 75 years or older. Age group and alcohol drinking (OR = 1.64) were positively associated with good sleep health. In participants younger than 75 years, exercise for health (OR = 1.60) and alcohol drinking (OR = 1.83) were positively associated with good sleep health. In those 75 years or older, obesity (OR = 0.40) was negatively associated with good sleep health. Conclusion: Factors associated with good sleep health differ by age group. These findings may provide important evidence for community-based health promotion focused on multidimensional sleep health.

Abstract citation ID: igaf122.771

**MULTIDIMENSIONAL SLEEP HEALTH METHODS: FINDINGS FROM US AND NETHERLANDS COHORTS OF OLDER ADULTS**

Meredith Wallace, *University of Pittsburgh, Pittsburgh, Pennsylvania, United States*

Sleep health is a multidimensional construct, with dimensions including Regularity, Satisfaction, Alertness/Sleepiness, Timing, Efficiency, and Duration. Global estimates from large cohort studies suggest that approximately 30-40% of adults report ≥2 suboptimal sleep health dimensions. Moreover, poor sleep health dimensions are established risk factors for disease and disability, regardless of the presence or absence of a sleep disorder. Although current literature indicates that different sleep health dimensions are associated with different health outcomes, several methodological challenges have created hurdles to understanding which combinations of sleep dimensions are most relevant for each health outcome. In this presentation, Dr. Wallace will outline and demonstrate approaches for quantifying multidimensional sleep health and relating it to health outcomes. These methods range from the simple and clinically practical (e.g., using simple self-reported sleep health questionnaires) to the statistically complex (e.g., machine learning models that empirically derive weights to optimally combine sleep health features; or empirically deriving common within-person combinations of sleep health dimensions). Dr. Wallace will compare, contrast and demonstrate these methods using an international multi-cohort sample of older adults from the United States and the Netherlands, including data from the Osteoporotic Fractures in Men Study, the Study of Osteoporotic Fractures, the Multi-Ethnic Study of Atherosclerosis, the Rush Memory and Aging Project, Minority Aging Research Study, and the Rotterdam Study. With these data and methods, she will discuss the role of sleep health in relation to longitudinal trajectories of depression and cognitive outcomes.

### SESSION 2235 (SYMPOSIUM)

Abstract citation ID: igaf122.772

### RETHINKING SOCIAL PARTICIPATION SERVICES AND INTERVENTIONS: INNOVATIVE APPROACHES FOR DIVERSE AGING REALITIES

Chair: Mélanie Levasseur
Co-Chair: Dolores Majon-Valpuesta

As a key determinant of health, particularly in later life when the risk of social isolation and exclusion increases, social participation and connection requires effective services and interventions addressing the diverse needs, life conditions and trajectories of aging populations. This symposium aims to present innovative services and interventions designed to meet older adults' specific participation and connection needs, improve access to resources, and foster their active engagement in the community according to diverse aging realities. Drawing on theorical and empirical evidence, it will provide a forum for broader reflection and discussion on how to strengthen the participation and connection opportunities for older adults by proposing interventions that range from taking advantage of technological advances to favoring self-questioning of services. Levasseur and colleagues explored experiences of older adults interacting with one social robot having the ability to converse and promote social participation. Majón-Valpuesta and colleagues considered the strategies to update services aimed to promote social participation considering the baby boomer generation's needs. Lévesque and colleagues examined the compatibility of social prescribing by preventive occupational therapy intervention to expand older adults' access to meaningful and health-promoting activities. Lord and colleagues, identified factors associated with and counteracting epistemic injustices to innovate in health services, better supporting social participation and connection. Together, these presentations foster interdisciplinary dialogue between earlier and mid-career as well as senior researchers, bringing diverse perspectives to help shape policy, practice, and advocacy that strengthen innovation in social participation and connection, and better support the health and well-being of aging populations.

Abstract citation ID: igaf122.773

### STRATEGIES FOR UPDATING SOCIAL PARTICIPATION SERVICES: THE CASE OF BABY BOOMERS IN QUEBEC AND SPAIN

Dolores Majon-Valpuesta[1], and Melanie Levasseur[2],
*1. Université de Sherbrooke, Sherbrooke, Quebec, Canada, 2. Université de Sherbrooke Faculty of Medicine and Health Sciences, Sherbrooke, Quebec, Canada*

Services designed to promote social participation as a key factor in healthy aging are struggling to meet the expectations of a large generation of older adults, the baby boomer, which in 2021 represented more than a quarter (26%) of the population in both Quebec (Canada) and Spain. Despite the challenge of adapting services to the participation needs of this cohort, little is known about the strategies and changes required to make these services attractive to them. This presentation aims to report results of two qualitative studies exploring the strategies to update services aimed to promote social participation of baby boomer generation. These two studies were both developed in Spain and Quebec with 53 individuals and 12 group interviews, involving a total of 107 baby boomers and 52 community representatives. Three strategies were identified: 1) the consideration of intragenerational diversity from an intersectional perspective, according to criteria such as life trajectories, autonomy levels, identity factors, and daily environment; 2) the organization of services and participation beyond the age criterion, i.e., by interests, needs, common values in which a real interaction between generations is favored; 3) the promotion of services flexible where baby boomers have control over their time, i.e., services friendly to conciliation (caring for oneself, others, continuing professional life, etc.). The services will respond to meaningful participation for members of this generation according to the willingness of decision-makers and service designers to implement these strategies. Enhancing these services leads to greater motivation to participate and responsible use of community resources.

Abstract citation ID: igaf122.774

### OLDER ADULTS INTERACTING WITH SOCIAL ROBOTS: TOWARD GREATER SOCIAL PARTICIPATION AND CONNECTION

Mélanie Levasseur[1], Mariam Fdil[2], Francois Michaud[1], Marika Lussier-Therrien[3], and Dominic Létourneau[2],
*1. Université de Sherbrooke, Sherbrooke, Quebec, Canada, 2. Université de Sherbrooke Faculty of Engineering and Interdisciplinary Institute for Technological Innovation, Sherbrooke, Quebec, Canada, 3. Université de Sherbrooke Faculty of Medicine and Health Sciences, Sherbrooke, Quebec, Canada*

Some older adults require stimulation, learning, or assistance in their interactions, particularly when having disabilities. Social robots are promising to foster their participation and connection, and prevent situations of isolation. To our knowledge, Tabletop (T-Top) presents the most advanced communication and autonomous reasoning capabilities, but little is known about how older adults interact and perceived their experience with this robot. This study thus aimed to explore how older adults interact and perceive their experience with the social robot T-Top. An exploratory qualitative clinical research design was used with semi-directed interviews and observation of six older adults living in one senior residence. Older participants interacted with

T-Top twice for about 30 minutes, an observation grid was used to identify their reactions and interactions, followed by interviews with a semi-structured guide to explore their experience and satisfaction. During the interaction sessions, all older adults expressed joy and surprise and maintained interested eye contact with the robot. The majority of older adults engaged in conversation with T-Top and actively participated. Although all participants appreciated the robot's responsiveness and interactive behaviour, and, for most of them, its thinking and listening skills, some older adults were bothered by lack of perseverance and artificial appearance of T-Top. This study highlighted that social robots like T-Top should be personalized to older adults' needs, preferences, and habits to facilitate interactions. More studies are needed to evaluate how such promising interventions can be used to foster older adults' social participation and connection, and to prevent situations of isolation.

Abstract citation ID: igaf122.775

### LINKING SOCIAL PRESCRIBING AND PREVENTIVE OCCUPATIONAL THERAPY: A WAY TO ADVANCE HEALTH AND EQUITY?

Marie-Hélène Lévesque[1], Mya Fischer[2], and Mélanie Levasseur[1], *1. Université de Sherbrooke, Sherbrooke, Quebec, Canada, 2. Bishop's University, Sherbrooke, Quebec, Canada*

Presented as a response to unmet social needs and a way to foster a more equitable, sustainable, and healthy society, social prescribing (SP) faces challenges and criticisms, and little is known about its compatibility with preventive occupational therapy, particularly the Lifestyle Redesign program (LR), a landmark intervention with shared principles. To explore this compatibility this narrative umbrella review draws on seven systematic reviews supplemented by expert contributions from the National Academy of Social Prescribing. Results indicate conflicting evidence of SP on individuals, communities and healthcare systems. Despite widespread implemented in the UK and gaining traction globally, including in Canada and U.S., few peer-reviewed studies have examined its effects on older adults, and those available are of limited quality. Most studies reported positive changes in health self-management, loneliness, social isolation, and well-being, with high acceptability among participants and general practitioners. The COVID-19 pandemic highlighted both the importance of social prescribing in addressing these issues and the challenges of its implementation during crises. SP remains underutilized by occupational therapists, even though their expertise aligns with its objectives. Strengthening SP-LR linkages could advance interdisciplinary collaboration through three approaches: (1) LR as an activity within SP, (2) a navigator dedicated to LR, and (3) the occupational therapist as navigator. While not a unique solution, integrating SP within occupational therapy practice could improve access to preventive interventions, notably for older adults at risk of loneliness, social isolation, and chronic disease, and empower clinicians to address social determinants of health and drive innovation in gerontology.

Abstract citation ID: igaf122.776

### OVERCOMING THE EPISTEMIC INJUSTICES EXPERIENCED BY OLDER ADULTS IN HEALTH INNOVATIONS: HOW AND WHAT CAN THIS LEAD TO?

Marie-Michele Lord[1], Valérie Poulin[1], and Marie-Josee Drolet[2], *1. Université du Québec à Trois-Rivières, Trois-Rivières, Quebec, Canada, 2. Université du Québec à Trois-Rivières, Trois-Rivieres, Quebec, Canada*

Epistemic injustices experienced by older adults refer to the marginalization of their knowledge, experiences, and perspectives, which are often ignored or devalued in social, cultural, and political discourses and decisions. This leads to limited inclusion of their perspectives when health innovations concerning them are developed, including those designed to support their social participation. This presentation aims to discuss a research project conducted with the goal of: 1) identifying factors associated with epistemic injustices when including the knowledge of older adults in the development of health innovations, and 2) implementing actions to counter these factors to co-create, with various societal actors, including older adults and their loved ones, a health innovation that places them at the center of medical and support interactions concerning them. This project was carried out using a living lab method, which is a participatory and collaborative research approach where participants (n = 40), from local communities, were actively involved in co-constructing knowledge and practical solutions. The results highlighted actors contributing to epistemic injustices were identified, including organizational disharmony and internalized ageism. The principles and values of a philosophical research community, and steps proposed by the Occupational Justice Collaborative Framework were used to address these factors. Multiple iterative working meetings with a co-development committee and an advisory committee led to the development of an innovative technological tool that enhances the agency and strengthens older adults' social participation by fostering greater inclusion and engagement in decision-making processes that affect their lives.

### SESSION 2240 (SYMPOSIUM)

Abstract citation ID: igaf122.777

### INNOVATIONS IN GEROPSYCHOLOGY TRAINING AND WORKFORCE DEVELOPMENT: GRADUATE SCHOOL THROUGH POST-LICENSURE

Chair: Michelle Mlinac
Co-Chair: Erin Kube
Discussant: Patricia Bamonti

Following the 2021 Building Bridges Conference on geropsychology workforce development, this symposium highlights novel innovations in geropsychology training. In 2023, the specialty updated its training taxonomy, formal guidance on the recommended offerings for each level of opportunity ("exposure" through "major area of study") from graduate school through post-licensure levels of development. The first presentation reviews results from a recent survey of the landscape of geropsychology training following the COVID pandemic, highlighting issues of burnout among geropsychology trainees and faculty. The second presentation offers a new competency framework outlining the knowledge and skills recommended to provide clinical supervision to geropsychology trainees. The third presentation shares the results of a 2025 survey of geropsychologists' learning needs around behavioral sleep medicine, to create continuing professional education to bolster geropsychologists' clinical skills in this area. The fourth presentation reviews a pilot program to broaden the workforce and foster exposure to clinical practice with older adults via monthly consultation for generalist mental health providers at the masters and doctoral levels. The fifth presentation describes the process of board-certification for geropsychologists with the aim of increasing the numbers of those who deliver specialty care to the public. It also describes the development of a clinical consultation model to support a pathway for practicing psychologists who were not specialty trained in geropsychology in graduate school or fellowship to become eligible for board-certification at the post-licensure level. The innovations in this symposium may interest other geriatric clinical professions looking to meet similar training and workforce needs.

Abstract citation ID: igaf122.778

### BURNOUT IN THE GEROPSYCHOLOGY WORKFORCE: RESULTS FROM A NATIONAL SURVEY ON THE IMPACT OF COVID-19

Sophia Geisser[1], Zoe Geisser[2], Julia Boyle[3], Hannah Bashian[4], and Michelle Mlinac[3], *1. University of Alabama, Tuscaloosa, Alabama, United States, 2. SUNY Albany, Albany, New York, United States, 3. VA Boston Healthcare System, Boston, Massachusetts, United States, 4. VA Boston Health Care System, Boston, Massachusetts, United States*

A geropsychology workforce is critical as the aging population increases. Faculty burnout, exacerbated by COVID-19, poses a significant risk to the growth of the geropsychology field. Faculty and trainees that worked with older adults reported difficulty with pandemic-related mental health challenges, such as an increased demand for services and telehealth platforms and difficulty separating the home and work environment, as they are often interrelated. The Building Bridges team focused on creating training opportunities at the practicum, internship, and fellowship level to foster an interest in working with older adults. However, faculty burnout emerged as a barrier, prompting the development of a Qualtrics survey to explore this. Geropsychology faculty (n = 35) and students/trainees (n = 24) completed an online survey assessing perceptions of burnout and support during the COVID-19 pandemic. Using mixed methods (SPSS for quantitative and NVivo for qualitative analysis), findings revealed significant negative impacts on faculty and training programs, including burnout, fewer and less competent applicants, and worsened professional behaviors (i.e., reduced camaraderie and formality). Despite these challenges, some faculty noted positive student changes, such as improved work-life boundaries (31.4%) and strengthened intention to specialize in Geropsychology (16.7%). Positive adaptations aimed at enhancing training and student support during the pandemic was also highlighted. Recommendations for training programs to address COVID-19 impacts on faculty and trainee wellness and burnout will be discussed. This study emphasizes implications of pandemic-related effects and supports the development and maintenance of the geropsychology workforce.

Abstract citation ID: igaf122.779

### IDENTIFYING AND ASSESSING FOUNDATIONAL COMPETENCIES IN GEROPSYCHOLOGY SUPERVISION

Holly Malette[1], Jacqueline Gurevitch[2], and Michelle Mlinac[2], *1. VA Boston Healthcare System, Boston, MA, Massachusetts, United States, 2. VA Boston Healthcare System, Boston, Massachusetts, United States*

While previous generations of older adults were less likely to engage in mental health care, efforts to reduce mental health stigma and the aging of progressive generations will likely increase demand for these services. The geriatric mental health workforce must be equipped to meet this need. The Pikes Peak Geropsychology Knowledge and Skill Assessment Tool outlines and measures competencies for professional practice in geropsychology. These competencies do not include providing geropsychology supervision, yet supervised clinical experiences are integral for developing the knowledge, skills, and attitudes required for independent practice. The aim of this project is to define competencies for the provision of geropsychology supervision and develop a tool for their assessment. Proposed supervision knowledge and skills were generated from both literature review and professional experience. This first version of the competency tool was piloted with geropsychology trainees as part of a didactic seminar series. This second version was disseminated to psychologists involved in training geropsychology students. This group of geropsychologists provided feedback and a qualitative analysis was conducted. Respondents underscored the importance of supervisors competent in geropsychology and also highlighted supervision competencies unique to geropsychological practice (e.g. interprofessional collaboration, managing countertransference and supporting caregivers). While further assessment of the utility and acceptability of this tool is warranted, the eventual goal is to utilize it alongside the Pikes Peak tool to evaluate trainee competencies throughout their training and to guide the supervision practices of geropsychologists.

Abstract citation ID: igaf122.780

### EVALUATING BEHAVIORAL SLEEP MEDICINE KNOWLEDGE AND SKILLS IN GEROPSYCHOLOGISTS AND TRAINEES: A NEEDS ASSESSMENT

Catherine Ju[1], Amy Fiske[1], Michelle Mlinac[2], Angelica Boeve[3], and Julia Boyle[2], *1. West Virginia University, Morgantown, West Virginia, United States, 2. VA Boston Healthcare System, Boston, Massachusetts, United States, 3. Minneapolis VA, Minneapolis, Minnesota, United States*

Older adults are at greater risk for sleep difficulties which may impact their physical health, mental health, and quality of life. Despite this, research suggests that few clinical psychologists have formal sleep-related training. Given the shortage of clinicians specifically trained to work with older adults, the number of providers who feel competent in delivering behavioral treatment for older adults with sleeping problems is likely lacking. The current study aimed to identify gaps in behavioral sleep medicine knowledge and training in geropsychologists and trainees who work with older adults. Geropsychologists and trainees (N = 83) completed an online survey to evaluate gaps in knowledge and skills related to behavioral sleep medicine within geropsychology. The study found that the community had a general understanding of how sleep presents in older adults; however, they were less familiar with the assessment of sleep disorders beyond insomnia. Respondents were most knowledgeable about presentations of insomnia (94%) and sleep disordered breathing (93%) and least familiar with disorders of hypersomnolence and periodic limb movement disorder. Respondents had provided sleep hygiene education and cognitive behavioral therapy for insomnia more than anything else. Interest in learning about more interventions was distributed across multiple treatments, with brief behavioral therapy for insomnia being the highest, followed by exposure relaxation and rescription therapy. Geropsychologists are uniquely positioned to assess and treat sleep disorders in older adults, and our findings suggest the need for more comprehensive training in order to effectively treat sleep disorders in older adults.

Abstract citation ID: igaf122.781

**BUILDING BRIDGES POST-LICENSURE CONSULTATION: STRENGTHENING THE GEROPSYCHOLOGY WORKFORCE**

Erin Emery-Tiburcio[1], Erin Kube[2], Ann Steffen[3], Suzann Ogland-Hand[4], Julia Kasl-Godley[5], Michelle Mlinac[6], Janet Yang[7], and Andrew Heck[8], *1. Rush University Medical Center, Chicago, Illinois, United States, 2. VISN 19 Clinical Resource Hub, Salt Lake City, Utah, United States, 3. University of Missouri-St. Louis, St. Louis, Missouri, United States, 4. Ogland-Hand Consulting, LLC, Grand Rapids, Michigan, United States, 5. The Wright Institute, Berkely, California, United States, 6. VA Boston Healthcare System, Boston, Massachusetts, United States, 7. Heritage Clinic, Pasadena, California, United States, 8. GeroPartners, LLC, Midlothian, Virginia, United States*

U.S. Census trends indicate significantly increased numbers of older adults and thus, a greater need for medical and mental health providers equipped to address older adult needs. Since the Building Bridges Conference in March 2021, the Building Bridges Post-Licensure (BBPL) group has been working to advance clinical practice with older adults. In 2022, we conducted a needs assessment of 80 post-licensure psychologists and identified a strong desire for gero-focused case consultation. We subsequently launched two six-month pilot consultation groups, occurring monthly for 90 minutes and led separately by a board certified geropsychologist. The groups consisted of didactic teaching, case presentation, and case discussion, focusing on foundational knowledge, assessment, and intervention geropsychology competencies. Nine licensed psychologists and one licensed clinical social worker completed consultation and eight of these participants completed the post-assessment. Most agreed consultation added value to their clinical practice (62% 'strongly agree'; 38% 'agree), was responsive to their professional needs (75% 'strongly agree'; 25% 'agree'), and 88% strongly agreed they received direction with difficult cases. Qualitative accounts of skills implemented were highlighted. This presentation will describe the BBPL's efforts in developing these groups, including recruitment strategies, structure and format of monthly groups, and outcome measures. This approach may serve as a model for other disciplines interested in developing post-licensure training and educational opportunities for providers working with older adults.

Abstract citation ID: igaf122.782

**POST-LICENSURE GEROPSYCHOLOGY CONSULTATION PROGRAM: STRENGTHENING PATHWAYS TO BOARD CERTIFICATION**

Erin Kube[1], Michelle Mlinac[2], Lisa Lind[3], Dolores Gallagher Thompson[4], and Tonita Wroolie[5], *1. VISN 19 Clinical Resource Hub, Salt Lake City, Utah, United States, 2. VA Boston Healthcare System, Boston, Massachusetts, United States, 3. Deer Oaks Behavioral Health, San Antonio, Texas, United States, 4. Dept. of Psychiatry & Behavioral Sciences, Stanford Univ. School of Medicine, Los Altos, California, United States, 5. Department of Psychiatry and Behavioral Sciences, Stanford University School of Medicine, Stanford, California, United States*

There are approximately 4500 licensed psychologists in the US. Of these, 4% are board certified and only 2% of those are boarded in geropsychology. This is in contrast with the growing numbers of older adults being seen in mental health settings. The American Board of Geropsychology (ABGERO) conducted a needs assessment of psychologists, sampled from various aging-related listservs/practice settings to clarify interest in and barriers to board certification in geropsychology. Results among 20 respondents indicated gaps in education, supervised training, and general understanding of the board certification process. For practicing psychologists who developed an interest in geropsychology only after becoming licensed, a substantial barrier to board certification is lack of supervised clinical training experiences with older adults at the graduate, internship, or postdoctoral level. This presentation will describe this committee's efforts to remove these barriers and demystify the board-certification process. We will identify opportunities to increase the number of board certified specialists in geropsychology, specifically through creation of a year-long consultation program aimed at post-licensure psychologists to build competence in working with older adults. This process may serve as a model to other gerontological professions about how to build pathways for generalists to specialize in clinical aging care.

Abstract citation ID: igaf122.783

**BIOMARKERS OF FUNCTIONAL DECLINE, MORBIDITY AND MORTALITY**

Keisha Lovence, *University of Pittsburgh, Pittsburgh, Pennsylvania, United States*

Research in this session will describe clinical and biological markers for functional decline, morbidity and mortality.

Abstract citation ID: igaf122.784

**BLOOD IMMUNOMETABOLISM CORRELATES WITH EXERCISE INTOLERANCE IN OLDER ADULTS WITH HEART FAILURE**

Philip Kramer, Zhengrong Gao, Iris Leng, and Dalane Kitzman, *Wake Forest University School of Medicine, Winston-Salem, North Carolina, United States*

Heart failure with preserved ejection fraction (HFpEF) is a common and costly geriatric syndrome, causing exercise intolerance in over three million new Americans every year. Blood bioenergetic profiling has been used to assess systemic metabolic health and immunometabolism in older individuals with mobility impairments and other age-related conditions. To test whether the immunometabolism of CD15+ neutrophils and CD14- / CD61- lymphocytes correlate with exercise intolerance, as measured by 6-minute walking distance, we obtained blood from 23 individuals with HFpEF (Avg. 72 YOA, 74% female) and assessed bioenergetics using Seahorse with and without immune activation by phorbol 12-myristate 13-acetate (PMA). PMA-stimulated oxidant production in neutrophils was significantly and positively correlated with walking distance ($r = 0.47$, $p = 0.04$). However, the percent of neutrophils in the blood of these subjects tended to be lower in individuals with higher walking speeds ($r = -0.33$). Lymphocyte ATP-linked respiration tended to be higher in individuals with longer walking distances ($r = 0.37$), but lymphocyte PMA-stimulated glycolysis, which represents the ability of lymphocytes to switch fuel type upon activation, was not correlated. Older age (60-88) tended to lower walking distance ($r = -0.33$), neutrophil oxidant production ($r = -0.32$), lymphocyte ATP-linked respiration ($r = -0.21$), and significantly lower PMA-stimulated lymphocyte glycolysis ($r = -0.45$, $p = 0.03$). These data suggest neutrophil and lymphocyte immunometabolism may serve as live-cell bioenergetic biomarkers for exercise intolerance and aging. Here we report preliminary data on the first subjects in an ongoing study which will help elucidate the role of neutrophil oxidant production and immunometabolism on heart and skeletal muscle function in older patients with HFpEF.

Abstract citation ID: igaf122.785

**COGNITIVE DOMAIN IMPAIRMENTS RELATED TO WALKING DIFFICULTY: A HARMONIZED COGNITIVE ASSESSMENT PROTOCOL STUDY**

Matthew Miller, Pamela Ygrubay, Irena Cenzer, Deborah Barnes, Katherine Possin, and Kenneth Covinsky, *University of California, San Francisco, San Francisco, California, United States*

Community-dwelling older adults with cognitive impairment are at risk of disability. This study aimed to identify if adding specific cognitive domain impairments increase the odds of walking difficulty beyond single domain impairments. We analyzed data from 3,374 community-dwelling older adults (mean[SD] age: 74.1[7.6] yrs, 55.5% female, 80.5% non-Hispanic White) in the Health and Retirement Study Harmonized Cognitive Assessment Protocol, a data-source for cognitive aging research. A validated algorithm was used to classify cognitive domain impairment for executive function (EXF), memory (MEM), language fluency (LFL), visuospatial (VIS), and orientation (ORI). Difficulty walking (≤1 block) was identified using participants' self-report. We first calculated the prevalence of difficulty walking for each domain impairment. Next, we estimated age, sex, and race/ethnicity-adjusted odds of difficulty walking (aOR-[95%CI]) for each domain impairment alone, and then in the presence of another domain impairment. The prevalence of difficulty walking was highest for EXF impairment (27.2%), followed by ORI (25.5%), VIS (19.1%), MEM (14.8%), and LFL (10.4%), relative to 13.6% without any domain impairment. Compared to no impairment, the odds of difficulty walking were greater with EXF impairment (2.23 [95%CI:1.41,3.52]), but no other single domain impairments (P > 0.05). EXF impairment increased the odds of difficulty walking beyond MEM (MEM+EXF:3.65[95%CI:1.36-9.84] and LFL (LFL+EXF: 8.57[95%CI:2.26-32.56]), but no other dual-domain impairments increased the odds above referent EXF, MEM, LFL, VIS, ORI impairments alone. To enhance clinical outcomes, further research must examine the consistency of relationships of EXF impairment, alone and with other domains, across meaningful measures of older adult function.

Abstract citation ID: igaf122.786

**EVERY STEP COUNTS: REPORTED WALKING ABILITY AS A PREDICTOR OF ALL-CAUSE MORTALITY**

Keisha Lovence[1], Shanshan Yao[2], Ziling Mao[1], Elsa Strotmeyer[1], Eleanor Simonsick[3], and Anne Newman[4], *1. University of Pittsburgh, Pittsburgh, Pennsylvania, United States, 2. University of Michigan, Ann Arbor, Michigan, United States, 3. National Institute on Aging, Baltimore, Maryland, United States, 4. University of Pittsburgh, Pittsburgh, Pennsylvania, United States*

Evaluation of mortality risk with respect to physical function has focused on limitation. Here, we evaluated all-cause mortality risk in relation to increments of capacity in mobility intact older adults using the Walking Ability Index (WAI). In the Health, Aging and Body Composition study (Health ABC, N = 3075, aged 70-79), self-reported ease/difficulty walking ¼ mile and 1 mile, scored zero (worst) to nine (best) was assessed. Per study eligibility requirements, the WAI ranged from 4-9 at baseline. This analysis includes 2812 participants (57% White; 49% men; aged 73.6±2.9 years). Mortality over 15 years was ascertained from death certificates and proxy reports. Analyses used Kaplan-Meier plots, and Cox regression adjusting for demographic, lifestyle, and health factors. For baseline WAI, 673 (24%) scored 4-6, 855 (30%) scored 7-8, and 1284 (46%) scored 9. During a follow-up of 15 years, 1578 (56%) participants died. Kaplan-Meier analysis demonstrated significant association between higher WAI scores and better survival (log-rank test $p < 0.001$). A 1-point higher WAI score corresponded to a lower adjusted mortality risk (HR = 0.88, 95% CI: 0.85–0.92). A dose-response relationship was observed, with scoring 9 on the WAI associated with lowest mortality risk (0.61[0.46–0.81]) referent to scoring 4. Findings strongly indicate that attention to functional capacity provides important information about the likelihood of longevity and argues for including capacity assessments within research and health care settings involving older adults. Future work should interrogate relationships between self-reported walking ability and other aging outcomes.

Abstract citation ID: igaf122.787

**LINKING SOCIAL COHESION TO BIOLOGICAL MARKERS OF AGING: EVIDENCE FROM NHATS**

Tara Klinedinst[1], Nicholas Hollman[2], Michael Hankes[3], and Raymond Jones[3], *1. The University of Oklahoma Health Sciences Center, Oklahoma City, Oklahoma, United States, 2. The University of Oklahoma School of Community Medicine, Tulsa, Oklahoma, United States, 3. The University of Alabama at Birmingham, Birmingham, Alabama, United States*

Interest in the complex intersection between social determinants of health and biological aging markers is growing. Social cohesion, a measure of community belonging, and inflammatory biomarkers, like interleukin-6 (IL-6) and c-reactive protein (CRP), have emerged as significant factors influencing age-related chronic conditions and functional decline among older adults. With data from the National Health and Aging Trends Study (NHATS), we used logistic regression to assess the relationship between sociodemographic variables and low social cohesion, as well as multivariable linear regression to test the association between low social cohesion and IL-6 and CRP. In our sample of 4,648 older adults, the average age was 76 years old, majority female (55%), and non-Hispanic White (81%). We found that low social cohesion was associated with minority status, lower income, higher BMI, >3 chronic conditions, probable dementia, frailty, depression, and lack of walking for exercise. Additionally, low social cohesion was associated with higher levels of CRP and IL-6 after adjusting for the influence of sociodemographic variables. Understanding the link between social cohesion and inflammation is particularly relevant in aging populations, as with age, social networks often diminish, and the prevalence of inflammatory conditions increases. This research provides critical insights into the potential benefits of improved social engagement and feelings of community, and the relationship to inflammation and other health outcomes among older adults. These findings establish a target for health policy and future intervention research, namely improving health outcomes through a specific focus on improving social cohesion and community.

Abstract citation ID: igaf122.788
**METABOLIC MARKERS DEMONSTRATE THE HETEROGENEITY OF WALKING ABILITY IN COMMUNITY-DWELLING OLDER ADULTS**
Shanshan Yao[1], Ziling Mao[2], Megan Marron[2], Eleanor Simonsick[3], Venkatesh Murthy[1], Ravi Shah[4], and Anne Newman[4], *1. University of Michigan, Ann Arbor, Michigan, United States, 2. University of Pittsburgh, Pittsburgh, Pennsylvania, United States, 3. National Institute on Aging, Baltimore, Maryland, United States, 4. Vanderbilt University Medical Center, Nashville, Tennessee, United States*

Walking ability is important to life quality of older adults. A self-reported walking ability index (WAI) covering the difficulty and ease of walking captures a broader spectrum of walking ability in older persons. Using metabolomics in the Health, Aging and Body Composition study, we identified metabolites related to WAI (0–9, higher scores indicate better walking ability). Among 2,334 participants (mean age 74.6 years, 51% women, 37% Black), 27% scored 0–5, 36% scored 6–8, and 37% scored 9 at baseline (i.e. Year 2). Over 4 years, 52% maintained a stable WAI, 6% improved, while 42% declined (22% 1-2 point and 20% >2 points decline). Using probabilistic index models for cross-sectional and multinomial logistic models for longitudinal analyses, we identified 81 metabolites significantly associated with both poorer concurrent WAI and faster decline, including higher carnitine species, long-chain saturated diglycerides and triglycerides, and TCA cycle intermediates (cis-aconitic, fumaric, malic acids), and lower phospholipids levels. Eighteen additional metabolites were only associated with faster WAI decline: higher short-chain saturated triglycerides and energy metabolism markers (ATP/ADP/AMP), and lower margaric acid and glycine levels. Notably, those with improved WAI, despite poorer baseline WAI and lifestyles, showed more favorable metabolic profiles than others. Metabolites linked to inflammation, energy metabolism, and fatty acid utilization were related to mobility function. Some metabolites might be particularly important for early detection of mobility decline risk. Metabolic profiles may also help identify older individuals (i.e. with improving WAI) with greater metabolic resilience to lifestyle risk factors and health conditions.

Abstract citation ID: igaf122.789
**EARLY DETECTION OF MOBILITY DECLINE: SMARTPHONE-BASED ANALYSIS OF GAIT AND STAIR NEGOTIATION**
Roee Hayek[1], Rebecca Brown[2], Itai Gutman[1], Guy Baranes[1], and Shmuel Springer[1], *1. Ariel University, Ariel, HaMerkaz, Israel, 2. University of Pennsylvania, Philadelphia, Pennsylvania, United States*

Aging is associated with gradual mobility decline, often undetected until it affects daily activities. Although most mobility impairments are linked to old age, they can emerge as early as middle age. We examined the utility of smartphone-based accelerometry to detect early age-related mobility changes. Participants were 88 healthy adults divided into four age groups, young (20-35 years), early middle-aged (45-54 years), late middle-aged (55-65 years), and older adults (65-80 years). They completed gait assessments with and without cognitive (texting) and physical (carrying load) dual tasks and stair negotiation test. Gait measures included gait speed (m/sec), stride time variability (%), and movement similarity, measured with dynamic time warping (DTW) of anteroposterior (AP) and mediolateral (ML) acceleration signal. Dual-task cost (DTC) of the cognitive and physical tasks were calculated for each gait measure. Stair negotiation outcomes included total ascent and descent time (sec), muscle power (watts/m), and movement similarity using vertical (V) DTW. Late middle-aged adults had significantly higher cognitive DTC of stride time variability than young adults ($p = 0.006$). A strong indicator of early mobility decline was movement similarity. Young adults showed significantly lower cognitive DTC of DTW-AP compared to all other groups, early middle-age ($p = 0.035$), late middle-age ($p = 0.007$) and older adults ($p = 0.006$), as well as lower DTW-V during stair ascending ($p < 0.001$, compared to all groups). These findings highlight the utility of smartphone-based indices, particularly movement similarity, to detect subtle mobility changes in midlife, allowing for targeted interventions to promote healthy aging.

### SESSION 2250 (SYMPOSIUM)

Abstract citation ID: igaf122.790
**AGEING IN A CHANGING CLIMATE: RISKS, ADAPTATIONS, AND HEALTH IMPACTS**
Chair: Paola Zaninotto
Discussant: Liat Ayalon

Older adults are disproportionately vulnerable to climate change, yet research and policy often overlook their specific risks and adaptive strategies. This symposium examines the intersection of ageing, environmental hazards, and health, shedding light on the lived experiences and physiological impacts of climate change on older populations. We begin by exploring how older adults navigate disaster recovery in hazardous environments, using Hurricane Ian as a case study to highlight social infrastructure needs and barriers to ageing in place. Next, we examine the complex relationship between thermal perception, acceptance, and physical symptoms, offering insights into heat adaptation behaviours among older individuals. A further presentation addresses the respiratory health risks associated with domestic solid fuel use, revealing its dual impact on individual health and climate change. Lastly, we present findings on the effects of temperature on kidney function, providing further evidence of climate-related health risks for older adults. Together, these studies emphasise the urgent need for policies and interventions that enhance resilience and mitigate health risks in an ageing population. By integrating perspectives from different disciplines, this symposium offers a critical dialogue on the challenges and opportunities of ageing in a warming world.

Abstract citation ID: igaf122.791
**AGING IN PRECARIOUS PLACE: HOW OLDER ADULTS NAVIGATE DISASTER RECOVERY AND NEGOTIATE CLIMATE RISK**
Alexis Merdjanoff, *New York University, New York, New York, United States*

Community-dwelling older adults frequently age in states that are susceptible to various disasters, such as hurricanes, heat waves, wildfires, blizzards, and flooding. Despite their vulnerability, there has been little planning focused on providing community-dwelling older adults with the resources they need to prepare for and recover from climate-related disasters. This presentation will use Hurricane Ian as a case study to understand the social resources that older adults need to recover from disaster and age in precarious place. Hurricane Ian was responsible for the direct and indirect deaths of 150 Floridians and caused over $112 billion in damage, making it the costliest hurricane in Florida's history and the third-costliest in U.S. history. Analyses includes 73 in-depth interviews with older adults who were living in Southwest Florida at the time of the storm, and collected between September 2023 and November 2023. This presentation will examine three themes that have been identified using a grounded theory approach: 1) homeowners' insurance as a barrier to aging in place; 2) adult children as a key facilitator of older adult recovery; and, 3) a commitment to aging in precarious place. This study's initial themes raise questions regarding the social infrastructure needs of older adult communities that are at-risk for climate-related disasters. This presentation will conclude by addressing the types of local programming, community non-profits and public spaces that older adults need in order to age well, despite the climate crisis.

Abstract citation ID: igaf122.792

### FEELING HOT BUT ACCEPTING IT: RELATIONSHIP BETWEEN THERMAL PERCEPTION-ACCEPTANCE DISCREPANCY & PHYSICAL SYMPTOMS

Molly Lin[1], Rachel Leung[2], and Helene Fung[2], *1. University of Zurich, Zürich, Switzerland, 2. The Chinese University of Hong Kong, Hong Kong, China (People's Republic)*

The increasing severity of hot weather is considered an emerging threat to public health, especially for older adults. While research has demonstrated the negative effects of hot temperatures on older adults' physical well-being, limited research has examined whether their perceptions of the temperature provide distinct predictions of physical well-being beyond the objective temperature, let alone the discrepancies between thermal perception and thermal acceptance. A time-sampling study was conducted during the hottest months (May – September) in 2021 and 2022 in Hong Kong. A total of 321 participants (k = 9,282) aged 60 and above reported their physical symptoms, thermal perception, and thermal acceptance three times a day across ten days and wore sensors that tracked the instantaneous temperature of their immediate environment. Multilevel models showed that thermal perception, thermal acceptance, and the perception-acceptance discrepancy distinctively predicted the likelihood of experiencing physical symptoms, after controlling for the objective temperature. A larger perception-acceptance discrepancy predicted a higher likelihood of reporting physical symptoms. Factors influencing this perception-acceptance discrepancy were explored. These findings provide implications for promoting heat adaptation behaviors among older adults.

Abstract citation ID: igaf122.793

### DOMESTIC SOLID FUEL USE AND RESPIRATORY HEALTH: IMPLICATIONS FOR CLIMATE CHANGE

Shaun Scholes, Laura Horsfall, and Paola Zaninotto, *University College London, London, England, United Kingdom*

The domestic use of solid fuels such as coal and wood for heating is a significant source of air pollution, contributing to poor indoor and outdoor air quality and exacerbating climate change through the release of particulate matter and black carbon. These emissions not only accelerate global warming but also pose serious risks to human health, particularly for older adults who are more vulnerable to respiratory diseases. Despite growing concerns about air pollution and climate change, longitudinal evidence on the health impacts of domestic solid fuel use remains limited. This study explores how the use of solid fuels affects lung function trajectories among older adults, using FEV1 data from the English Longitudinal Study of Ageing (ELSA) from 2004-05 to 2012-13. Preliminary findings indicate that domestic solid fuel use was most common in both affluent households (20% in the highest wealth tertile) and fuel-poor households (19%) at wave 2. Over the 8-year study period, lung function declined more rapidly among those using solid fuels. For participants aged 70-79, FEV1 decreased on average by 0.12 litres among solid fuel users, compared to 0.07 litres among non-users. These results highlight the urgent need for public health and environmental policies promoting the transition to cleaner, more sustainable domestic energy sources. Given the dual burden of air pollution on both human health and climate change, reducing reliance on solid fuels is essential to protect older adults from respiratory decline while mitigating the broader environmental impacts of household emissions.

Abstract citation ID: igaf122.794

### EXPOSURE TO AMBIENT HEAT AND KIDNEY FUNCTION IN THE HEALTH AND RETIREMENT STUDY

Kristina Van Dang, Eun Young Choi, Eileen Crimmins, and Jennifer Ailshire, *University of Southern California, Los Angeles, California, United States*

Increased heat events are one of the defining characteristics of climate change affecting human health. The kidney's role in homeostasis makes them particularly susceptible to heat, especially in older populations in which kidney function is declining. Using data from the Health and Retirement Study (HRS) Venous Blood Study (N = 9,933), we linked respondents to census tract level daily heat index from 7 days prior to blood collection. Kidney function was assessed with estimated glomerular filtration rate based on serum creatinine (eGFRcr). The average age of our analytic sample was 69 years (SD = 10). About 18% of the sample would be classified as having chronic kidney disease based on an eGFRcr < = 60 mL/min/1.83m2. Using linear models with cluster-adjusted standard errors, and controlling for age, gender, race/ethnicity, education, and urbanicity, we found that each unit increase in prior 7-day average heat-index was associated with a decreased eGFRcr of 0.04 (95% CI: -0.06, -0.02). We also categorized heat index days into cautionary (heat index > 80, the level at which the National Weather Service reports fatigue is possible) and extreme cautionary levels (heat index>90, the level at which NWS reports sunstroke, muscle cramps, and/or heat exhaustion is possible). We found that more days at cautionary level was associated with -0.96 lower (95% CI: -2.0, 0.05) eGFRcr and more days at extreme cautionary levels was associated with -2.5 lower (95% CI: -3.7, -1.3) eGFRcr. Our findings suggest kidney function in older adults can be adversely affected by exposure to short-term high heat.

### SESSION 2255 (PAPER)

Abstract citation ID: igaf122.795

### CAREGIVING OF OLDER ADULTS: CHALLENGES AND OPPORTUNITIES

Presenters address caregiver burden challenges and strategies to improve the caregiving experience.

Abstract citation ID: igaf122.796

### ADAPTING TO CHANGE: CARE PARTNER CHALLENGES AND STRATEGIES IN MILD COGNITIVE IMPAIRMENT AND MILD DEMENTIA

Cassandra Dictus[1], Youngmin Cho[2], Ya-Ning Chan[1], Chiao-Hsin Teng[3], Jing Wang[4], Victoria Crowder[5], Ruth Anderson[1], and Bianca Shieu[6], *1. Duke University, Durham, North Carolina, United States, 2. Johns Hopkins University, Baltimore, Maryland, United States, 3. Chang Gung University, Taoyuan City, Taoyuan, Taiwan (Republic of China), 4. University of New Hampshire, Durham, New Hampshire, United States, 5. The University of North Carolina at Chapel Hill, Chapel Hill, North Carolina, United States, 6. University of Texas Health Science Center at San Antonio, San Antonio, Texas, United States*

Care partners are critical for helping people living with mild cognitive impairment (MCI) and mild dementia (MD) maintain daily routines. This study compared the experiences of care partners working to support people living with MCI and MD with their daily tasks. Using a secondary qualitative analysis, we analyzed recorded intervention coaching sessions where care partners (10 MCI care partners and 8 MD care partners) learned communication techniques to support individuals with MCI and MD in learning oral hygiene techniques. Guided by the Adaptive Leadership Framework for Chronic Illness, we applied directed content analysis to explore patterns in their caregiving experiences. Findings revealed that care partners encountered a range of adaptive challenges, from managing functional changes and communication difficulties to balancing caregiving responsibilities with personal needs. MCI care partners expressed concerns about future cognitive decline, whereas MD care partners focused on balancing efficiency with autonomy in caregiving. Strategies varied by cognitive stage: MD care partners relied on tactile cues to reinforce routines, while MCI care partners were more collaborative and less directive in their communication. Care partners in both MCI and MD groups also engaged in adaptive work by refining techniques, seeking support, and adjusting their approaches over time. These findings underscore the importance of tailoring interventions and clinical support based on the stage of cognitive decline. Programs designed for care partners should address relationship dynamics, communication strategies, access to resources, and future planning to enhance caregiving experiences and improve outcomes for both care partners and individuals with MCI and MD.

Abstract citation ID: igaf122.797

### ASSESSMENT AND MITIGATION OF CAREGIVER BURDEN IN A MOVEMENT DISORDER CLINIC

Morgan Wilhelmi, Joseph Quinn, Shannon Anderson, Rachel Lynn, and Jiayun Xu, *Oregon Health & Science University, Portland, Oregon, United States*

Finding ways to assess and mitigate caregiver burden in real life clinical settings is a challenge. Using a pre-post test design in a quality improvement project, we aimed to assess the feasibility and preliminary efficacy of a social work intervention to reduce caregiver burden. From June 2023 to May 2024, caregiver burden was assessed using the Zarit Caregiver Burden Inventory on family caregivers who accompanied patients to a movement disorders clinic visit. Caregivers who scored above 30 on the Zarit were referred to a social worker who completed a structured needs assessment and resource referrals. The Zarit was measured again 2 months post social work referral. Burden was assessed among 405 caregivers at baseline, and only 3.5% (n = 14) caregivers completed a two-week assessment. Out of the caregivers who reported higher burden (n = 106, 30.1%), 69.8% (n = 74) were contacted by social work, and 44.3% (n = 47) completed a social work visit. The most common sources of caregiver burden were: mental health (n = 25, 29.8%), and financial concerns (25.5%). Of the caregivers who completed a 2-week Zarit, 42.9% (n = 6) reported an improvement in caregiver burden. The low follow up rate highlights the challenges of measuring post-intervention scores in a real-life clinic setting. Nevertheless, results indicate caregivers were willing to engage with social workers to discuss their burden and come up with a plan to address burden. Future projects need to more systematically assess burden post-intervention and determine ways to measure engagement with community resources after social work visits occur.

Abstract citation ID: igaf122.798

### CANCER CARE PARTNERS' TRANSITIONS IN SELF-IDENTITY FROM PRE-CANCER INTO SURVIVORSHIP: A MIXED METHODS ANALYSIS

Ann Kuglin Jones[1], Lee Ellington[2], Linda Edelman[2], Lanell Bellury[3], Darryl Somayaji[4], and Kristin Cloyes[5], *1. Wings of Hope Cancer Support Center, Council Bluffs, Iowa, United States, 2. University of Utah, Salt Lake City, Utah, United States, 3. Mercer University, Macon, Georgia, United States, 4. University of Buffalo, Buffalo, New York, United States, 5. Oregon Health & Science University, Portland, Oregon, United States*

Cancer survivors' transitions from diagnosis into post-treatment life are well-described. Care partners' transition experiences, however, remain underrepresented. Our mixed methods study combined NLP and qualitative analysis to examine shifts in care partners' self-identity from pre-cancer into survivorship. We enrolled n = 18 spouses/partners (n = 9 women, n = 9 men) of post-treatment survivors. Participants completed two free listing exercises and two interviews describing "who am I" before and after cancer. Selected LIWC linguistic markers measures identity-related and affective content, and Wilcoxon signed-rank and Mann-Whitney U tests compared differences pre/post cancer and by gender. Abductive interview coding, integrating transition theory concepts and inductive analysis, identified themes. When describing their before-cancer self-identity, participants used more positive (Mdn = 96.94 vs. 54.60, p = .025) and self-focused language (Mdn = 0.50 vs. 3.50, p = .008). Post-cancer descriptions were more relational in focus with more "we" language (Mdn = 0.00 vs. 1.00, p = .009) with more negative affect, including anxiety (p = .019), and sadness (p = .043). Described changes in self-identity evoked uncertainty, role ambiguity, evolving relationship dynamics, and dissatisfaction with available identity labels. Participants rejected "caregiver' as too unidirectional and "survivor" as inapt, while also describing their experiences a life- and identity-changing transition. Care partners lack a shared, recognized vocabulary to articulate their survivorship experiences, challenging their ability to find meaning in and connect with current survivorship discourse. Health care providers can support and validate care partners' identities through person-centered communication that uses their language, acknowledges their unique experiences, and assesses their unique support needs.

Abstract citation ID: igaf122.799

### CAREGIVERS' PERSPECTIVES ON THEIR SELF-CARE PRACTICES WHILE CARING FOR OLDER ADULTS WITH CHRONIC ILLNESS

Soojung Ahn[1], Kemberlee Bonnet[2], Ashley Sellers[2], Christopher Lee[1], and Mulubrhan Mogos[2], *1. Boston College William F. Connell School of Nursing, Chestnut Hill, Massachusetts, United States, 2. Vanderbilt University, Nashville, Tennessee, United States*

The demands of family caregiving can significantly impede self-care practices, often resulting in self-neglect, health decline, diminished quality of life, and vulnerability to chronic illness. Despite these impacts, few studies have investigated the underlying factors contributing to caregivers' diminished self-care practices. This study aimed to explore caregivers' perspectives on barriers and facilitators to their self-care practices. We conducted four focus groups with caregivers primarily responsible for providing in-home care to older adults with chronic illnesses. Sessions were audio-recorded, transcribed, and analyzed using a hierarchical coding system refined by experienced qualitative researchers through an iterative inductive-deductive approach. Five major themes emerged: 1) varied definitions and perceptions of self-care practices among caregivers; 2) psychosocial barriers to self-care practices, including emotional strain, cognitive demands of caregiving, maladaptive or avoidant coping, and loss of sense of control; 3) contextual barriers, including caregiving roles, the health status of the care recipient, family dynamics, and resource availability; 4) social support and knowledge of resources as facilitators to better self-care; and 5) problem-based coping strategies developed by caregivers, such as systems for organization, to regain sense of control and alleviate stress. These findings underscore the importance of understanding caregivers' perceptions and the multi-level factors influencing their self-care practices. Future research should focus on identifying and implementing strategies to mitigate these barriers, ultimately fostering better self-care among caregivers to improve their quality of life and health outcomes.

Abstract citation ID: igaf122.800

### DRIVERS AND MODERATORS OF RURAL-URBAN DIFFERENCES IN CAREGIVING INTENSITY AND HEALTH-RELATED QUALITY OF LIFE

Steven Cohen[1], and Mary Greaney[2], *1. University of Rhode Island, Kingston, Rhode Island, United States, 2. University of Rhode Island Department of Health Studies, Kingston, Rhode Island, United States*

Rural-urban disparities are pervasive among older adults and their caregivers. Rural areas in the US have less access to services and a disproportionately high population of older adults, and are more reliant on informal, family caregivers than those in urban and suburban areas. There is a need to understand critical, rural-urban differences in caregiving intensity and caregiver health-related quality of life (HRQoL) across the US. Data were abstracted and merged from the 2022 and 2023 Behavioral Risk Factor Surveillance System (n = 31,164), a nationally representative data set. Generalized linear models were used to assess associations between rural-urban status and caregiving intensity and HRQoL, and model associations between caregiving intensity and HRQoL, adjusting for demographics and social determinants of health and accounting for complex sampling. Rural caregivers had a significantly higher likelihood of providing >20 hours/week of care (odds ratio [OR] 1.10, 95%CI 1.01-1.19), reporting fair/poor general health (OR 1.30, 95%CI 1.19-1.42) and having >5 days/month of poor physical health (OR 1.16, 95%CI 1.07-1.26). However, rural caregivers had a lower likelihood of reporting >5 days/month of poor mental health (OR 0.88, 95%CI 0.82-0.96) and providing care for someone with Alzheimer's disease/dementia (OR 0.87, 95%CI 0.77-0.98) than urban caregivers. There were no significant differences in associations between caregiving intensity and HRQoL by rural-urban status. The study identified notable rural-urban differences in caregiving intensity and aspects of HRQoL that likely impact the health and quality of life of informal caregivers, suggesting that policies and interventions to address caregiver needs must account for geographic context.

### SESSION 2260 (PAPER)

### COVID CHANGED EVERYTHING, EVERYWHERE, ALL AT ONCE

Abstract citation ID: igaf122.801

### "WE ALSO HAD A BIG HIT DURING COVID": REALITIES OF THE COVID-19 PANDEMIC ON ADULT DAY SERVICES

Kingsley Udeh[1], Molly Noble[1], Heather Menne[1], Sara McLaughlin[1], Randi Hamill[2], MaKenna McClure[2], and Laurinda Johnson[2], *1. Miami University, Oxford, Ohio, United States, 2. LeadingAge Ohio, Columbus, Ohio, United States*

Adult day service (ADS) is a model of home-and-community-based services within the long-term care services and support continuum. ADS offers a system of professionally delivered, integrated, therapeutic, social, and health-related services in a structured and secure environment that helps individuals sustain living within the community. It also offers respite to family caregivers. There is growing need to understand the realities and struggles experienced by ADS providers, particularly in the years following the COVID-19 pandemic when many ADS programs were shuttered. The current study seeks to understand factors that made it easier or more difficult for providers to navigate through/after the pandemic. Seventeen ADS providers in Ohio participated in two focus groups. Transcripts were reconciled for accuracy and coded thematically to identify facilitators and barriers to service provision. Two key themes from the analysis include the importance of partnerships and education/awareness about ADS. Transcripts were further coded for constructs included in the Outer and Inner Setting domain of the updated Consolidated Framework for Implementation Science (CFIR). Outer and Inner setting domain constructs include external and internal influences that might affect organizational performance. Both facilitators and barriers to service provision fell primarily within the CFIR's Outer setting, including critical incidence, local attitude and conditions, partnership and connections, and financing. Inner setting domain constructs included structural characteristics, relational connections, communications, and available resources. Study findings highlight the realities faced by ADS providers and highlight potential points of intervention for researchers and policy makers seeking to foster use and access to adult day services.

Abstract citation ID: igaf122.802

### CAREGIVING DURING THE COVID-19 PANDEMIC THROUGH PUBLICLY-FUNDED HOME- AND COMMUNITY-BASED SERVICES

Romil Parikh[1], Tetyana Shippee[2], Jack Wolf[1], Chanee Fabius[3], Janette Dill[1], Dana Urbanski[4], Stephanie Giordano[5], and Eric Jutkowitz[2], *1. University of Minnesota School of Public*

*Health, Minneapolis, Minnesota, United States, 2. University of Minnesota, Minneapolis, Minnesota, United States, 3. Johns Hopkins University, Baltimore, Maryland, United States, 4. Indiana University Bloomington, Bloomington, Indiana, United States, 5. Human Services Research Institute, Cambridge, Massachusetts, United States*

Workforce shortages in home- and community-based services (HCBS) were exacerbated during the COVID-19 pandemic. We evaluated changes in HCBS use and consumer-reported unmet HCBS needs for (1) personal care aide (PCA) and (2) caregiver support/respite (CS/R) in 2021-2022 (during the pandemic) versus 2018-2019 (pre-pandemic), in the US National Core Indicators- Aging & Disability Adult Consumer Survey. We included 7143 community-dwelling HCBS consumers (age, ≥65 years, from 11 states). We calculated adjusted odds ratios (aOR) and 95% confidence interval (CI) for HCBS use and consumer-reported unmet HCBS needs for PCA and CS/R using logistic regression, adjusting for sociodemographic and health-related variables (fixed effects), with random intercept for state (to account for clustering by state). For PCA, compared to 2018-2019, during 2021-2022, there was a significant increase in the odds of both PCA use (aOR, 1.24; 95% CI, 1.09, 1.40) and unmet PCA needs (aOR, 1.23; 95% CI, 1.03, 1.46), suggesting that pandemic policy efforts to mitigate service disruptions were insufficient to decrease unmet PCA needs. For CS/R, during 2021-2022, there was a significant increase in the odds of CS/R use (aOR, 1.24; 95% CI, 1.09, 1.40) and a simultaneous significant decrease in the odds of unmet CS/R needs (aOR, 0.49; 95% CI, 0.35, 0.70), suggesting that temporary pandemic policies for CS/R may have successfully abated consumers-reported unmet CS/R service needs. These cross-sectional findings call for more rigorous investigation to identify which temporary pandemic-related CS/R policies were effective; to inform disaster preparedness efforts for mitigating HCBS disruptions during future public health emergencies.

Abstract citation ID: igaf122.803

### DIFFERENCES IN COVID-19 VACCINE UPTAKE AMONG IMMIGRANTS AND NON-IMMIGRANTS IN THE UNITED STATES: EVIDENCE FROM MEPS

Mohammad Usama Toseef[1], Indrakshi Roy[2], and Nasim Ferdows[3], *1. Corewell Health, Farmington Hills, Michigan, United States, 2. Indiana University, Bloomington, Bloomington, Indiana, United States, 3. Northeastern University, Boston, Massachusetts, United States*

Background: Understanding differences in COVID-19 vaccine uptake is crucial for addressing public health inequities. Immigrants in the United States face unique structural and socioeconomic barriers that may influence vaccination rates, including access to healthcare, acculturation, and trust in medical institutions. This study examines differences in COVID-19 vaccine uptake among immigrant and non-immigrant populations, with attention to the role of socioeconomic and demographic factors. Methods: This study utilizes data from the 2021-2022 Medical Expenditure Panel Survey (MEPS), a nationally representative survey of the U.S. non-institutionalized population. The analytic sample includes 39,767 adults aged 18 and older. Immigration status is categorized as U.S.-born, foreign-born residing in the U.S. for less than 15 years, and acculturated immigrants (residing in the U.S. for 15 years or more). Logistic regression models, adjusted for sociodemographic factors, estimate the association between immigration status and COVID-19 vaccine uptake. Results: Preliminary unweighted analyses indicate higher COVID-19 vaccination rates among foreign-born individuals compared to U.S.-born populations. However, differences emerge when considering acculturation, with acculturated immigrants showing a distinct vaccination trajectory. Adjusted models will further explore the impact of socioeconomic factors, such as education and income, on vaccination likelihood. Conclusion: Findings suggest that immigration status and acculturation play significant roles in shaping COVID-19 vaccine uptake. Future research should explore barriers to vaccination among acculturated immigrants and identify targeted interventions to improve vaccine accessibility and trust among immigrant communities.

Abstract citation ID: igaf122.804

### SHELTER-IN-PLACE POLICIES, PROXIMITY, AND CHANGES IN CAREGIVING FOR OLDER ADULTS DURING THE COVID-19 PANDEMIC

Lei Chen, and Joanne Spetz, *University of California, San Francisco, San Francisco, California, United States*

During the COVID-19 pandemic, the dynamics of family support for older adults living with disabilities were disrupted due to “Shelter-in-Place (SIP)” orders. This study examined the impact of SIP policies on caregiving changes and how these changes were related to family members’ geographic proximity. This study used the National Health and Aging Trends Survey (NHATS) round 10 data, COVID-19 geographic data, and restricted COVID-19 data files. We added published data about SIP policies at the county level. The study sample was NHATS respondents who needed assistance with activities of daily living (ADL) or instrumental activities of daily living (IADL), and who did not live with a spouse (N = 512). We estimated weighted multivariate logistic regression models and estimated separately by dementia status and ADL/IADL needs. 55.2% reported no change in receiving help, 36.7% reported receiving less help, and 8.1% reported receiving more help during COVID than before. Compared to those who did not have a change in help during COVID, a person living in a place with a higher percentage of SIP days was less likely to receive more help. No significant changes were found as stratified by dementia status and ADL/IADL needs. No significant associations were found between proximity and changes in caregiving. It is important to address caregiving needs of older adults with disabilities who do not live with a spouse during public health emergencies and to examine how disruptions caused by policies impact the provision of help for people who rely on family support.

Abstract citation ID: igaf122.805

### UNDERSTANDING PANDEMIC EXPERIENCE OF OLDER SOUTH ASIANS IN CHINESE SOCIETY IN HONG KONG DURING COVID-19

Zhijing Qu[1], Daniel W. L. Lai[2], and Jiajia Zhou[1], *1. Hong Kong Baptist University, Kowloon, Hong Kong, China (People’s Republic), 2. Hong Kong Baptist University, Kowloon, China (People’s Republic)*

The COVID-19 pandemic significantly disrupted lives worldwide, yet the experiences of marginalized groups, particularly older individuals in racialized minority communities, have received limited attention. This study aims to explore the social contexts of older South Asians in Hong Kong during the pandemic. Through in-depth narrative interviews with 20 South Asians aged 65 and over, thematic analysis revealed the critical roles of social capital in bonding, bridging, and linking. Participants highlighted the importance of family and close friends in bonding social capital, providing emotional, informational, and social support. Bridging social capital was evident in interactions with neighbours and the local Chinese community, who offered support but could also be sources of discrimination. Linking social capital was demonstrated through the provision of essential resources and services by local, voluntary, and religious organizations. These findings underscore the multifaceted functions of social capital in promoting social inclusion and support for aging racialized minorities, especially during health crises. The study highlights the urgent need for targeted policies and interventions to address the unique challenges faced by older South Asians in Hong Kong. By understanding the intricate dynamics of social capital within these communities, policymakers can more effectively tailor efforts to build robust and inclusive support systems, fostering a more resilient and equitable society in the face of future pandemics or similar crises.

### SESSION 2265 (PAPER)

### END OF LIFE CARE: NEW HORIZONS FOR ADVANCE DIRECTIVES

Abstract citation ID: igaf122.806

### ENHANCING CARE TRANSITIONS WITH AN EXPLICIT LIVE DISCHARGE PROTOCOL (LDP) FROM HOSPICE

Stephanie P. Wladkowski[1], Susan Enguidanos[2], and Tracy Schroepfer[3], *1. Bowling Green State University, Bowling Green, Ohio, United States, 2. University of Southern California, Los Angeles, California, United States, 3. University of Wisconsin–Madison, Madison, Wisconsin, United States*

Implementing a live discharge protocol (LDP) from hospice care is a critical component of ensuring safe and compassionate transitions for hospice patients (and their caregivers) who no longer meet eligibility criteria. A live discharge protocol (LDP) for hospice was recently developed with aims to (1) enhance patient and caregiver experience, (2) minimize disruptions of care, and (3) support hospice providers in navigating the complexities of care transitions. Key elements of the LDP include an inventory of clinical needs, assessment of psychosocial and financial concerns, needed community referrals, and post-discharge follow-up to prevent care gaps and assess caregiving well-being post-discharge. We tested the LDP in 4 US hospice agencies for live discharges experienced due to extended prognosis (n = 30). Following each discharge, in-depth, semi-structured interviews were conducted with hospice providers to elicit their perspectives on the usability of the LDP and suggestions for improving LDP implementation. A second set of interviews (n = 19) were conducted after the post-discharge phone call to explore the clinicians' experiences with the LDP process. Thematic analysis was used. Providers noted the LDP was useful in ensuring a thorough collection of currently used equipment and supplies for improved discharge process. The need for the LDP referral section was specifically valuable for patients living at home. Finally, the follow-up phone call was found to be useful to support the hospice clinician in this care transition, and as a mechanism to remain connected to patients/caregivers for potential future re-enrollment onto hospice. Future research, policy and practice implications will be discussed.

Abstract citation ID: igaf122.807

### FEASIBILITY OF IMPLEMENTING THE ADVANCED DEMENTIA PROGNOSTIC TOOL IN NURSING HOME ASSESSMENTS

Susanny Beltran[1], Latarsha Chisholm[2], and Vittoria Figueiro[1], *1. University of Central Florida, Orlando, Florida, United States, 2. Univeresity of Central Florida, University of Central Florida, Florida, United States*

Identifying hospice eligibility for nursing home residents with advanced dementia remains inconsistent, leading to delays in appropriate end-of-life care. The Advanced Dementia Prognostic Tool (ADEPT) has the potential to standardize prognostic assessments, but its feasibility for routine nursing home integration is unclear. This study evaluated ADEPT's feasibility across four monthly assessment cycles in a skilled nursing facility. Data were collected on completion rates, documentation consistency, staff adherence to protocol, and perceived barriers and facilitators. Compliance with recommended actions based on ADEPT scores was assessed, and deviations were analyzed for justification and impact on care planning. ADEPT was successfully integrated into Minimum Data Set (MDS) evaluations, with compliance improving over time. Of the 48 assessments due, 34 were submitted in compliance. However, staff frequently omitted score documentation, citing concerns over regulatory scrutiny and potential repercussions. Uncertainty regarding score interpretation underscored the need for a clear threshold to guide decision-making. Barriers included staff training needs and regulatory apprehension, while facilitators included workflow alignment and structured interdisciplinary communication. Staff preferred using ADEPT to support goals-of-care discussions at family conferences rather than as an automatic hospice referral trigger. Findings demonstrate that ADEPT is a feasible addition to routine nursing home assessments, improving consistency in hospice eligibility identification. However, additional strategies are needed to support consistent score documentation, ensure regulatory confidence, and integrate ADEPT into structured decision-making processes. Future research should focus on best practices for incorporating ADEPT scores into clinical workflows to enhance goals-of-care discussions and promote care concordance with residents' needs and preferences.

Abstract citation ID: igaf122.808

### REVIEW OF COURT CASES ON ADVANCECARE PLANNING: IDENTIFYING IIMPLEMENTATION ISSUES AND CHALLENGES

Megumi Inoue[1], Meng-Hao Li[1], Yifan Lou[2], Nina Buckley[1], Lana El-Khatib[1], and Isabelle Davis[1], *1. George Mason University, Fairfax, Virginia, United States, 2. Virginia Commonwealth University, Richmond, Virginia, United States*

Introduction: Advance directives (ADs) are a critical component of advance care planning (ACP), formally documenting healthcare preferences to ensure an individual's end-of-life care wishes are respected and followed. However, if ADs are incomplete or improperly completed, legal disputes may arise, potentially leading to unsatisfactory or unforeseen outcomes. This study analyzed court records related to ACP to identify issues in its implementation. Design: Data for this study were extracted from the Caselaw Access Project (CAP) database, the largest publicly available dataset of legal documents. A total of 291 court reports published between 1980 and 2019 in the U.S. that contained the keywords “advance directive” or “advance care planning” were extracted for analysis. A thematic analysis was conducted to identify the key issues that led to court cases. Results: After removing unrelated cases, such as those concerning asset wills, 79 cases were included in the analysis. The major issues identified were the conflicts among stakeholders over appropriate treatments compounded by the absence of ADs or related ACP measures (n = 53), physician noncompliance with existing ADs (n = 14), and challenges to the legitimacy of ADs (n = 12). Conclusion: The findings highlight the importance of having an AD in place as a legal document to safeguard individual wishes and prevent family and/or family-physician conflicts. Our study also found that even with an AD, goal-concordant care isn’t guaranteed, leading to legal cases. This emphasizes that discussions with family and healthcare providers, ensuring awareness and understanding of ACP, are as important as, if not more than, the AD itself.

### SESSION 2270 (AWARD LECTURE)

Abstract citation ID: igaf122.809

### THE MARGRET M. AND PAUL B. BALTES AWARD LECTURE AND PRESENTATION

Chair: Jennifer Ailshire

The Margret M. and Paul B. Baltes Award Lecture will feature an address by the 2024 Baltes Award recipient, Nicole E. Werner, PhD, of Vanderbilt University. This session will also include the presentation of the 2025 Margret M. and Paul B. Baltes Award to recipient Jeremy M. Hamm, PhD, of North Dakota State University. The Margret M. and Paul B. Baltes Foundation Award in Behavioral and Social Gerontology recognizes outstanding early-career contributions in behavioral and social gerontology. The award is generously funded by the Margret M. and Paul B. Baltes Foundation.

Abstract citation ID: igaf122.810

### DESIGNING FOR TO DESIGNING WITH: CO-DESIGNING INTERVENTIONS WITH DEMENTIA CARE PARTNERS

Nicole Werner, *Vanderbilt University Medical Center, Nashville, Tennessee, United States*

Co-design as applied to the design, evaluation, and implementation of interventions for family and friend care partners of people living with dementia has great potential to address the needs and outcomes that matter most to dementia care partners. Co-design democratizes the intervention pipeline through sustained partnership in which co-designers have the decisional and generative power to identify the problem to be solved and collaboratively create the solution. However, fully implementing intervention co-design with dementia care partners can be challenging given care partners’ context and the current structures of academic research. This talk will describe one researcher’s journey from ‘designing for’ to ‘designing with’ family and friend care partners of people living with dementia, sharing approaches, strategies, and lessons learned across ten years of research.

### SESSION 2275 (PAPER)

### ENVIRONMENTAL AND PSYCHOSOCIAL RISKS ACROSS THE LIFE COURSE: A PUBLILC HEALTH PERSPECITVE

Abstract citation ID: igaf122.811

### ADOLESCENT FLOOD EXPOSURE AND COGNITIVE AGING: TRAJECTORY PATTERNS AMONG MIDDLE-AGED AND OLDER ADULTS IN CHINA

Yunchen Ruan[1], Shiqi Lin[1], and Yexin Zheng[2], *1. Fuzhou University, Fuzhou, Fujian, China (People’s Republic), 2. Fujian Normal University, Fuzhou, Fujian, China (People’s Republic)*

Objective: This study aims to investigate the relationship between adolescent flood exposure and cognitive function trajectories among middle-aged and older adults in China from a dynamic perspective. The study is intended to address the gap in research on the long-term effects of flood exposure on cognitive function. Methods: A total of 10,352 adults aged 45 and older participating in the 2011, 2013, 2015, 2018, and 2020 waves of the China Health and Retirement Longitudinal Study were analyzed. Cognitive function trajectories were analyzed using group-based trajectory modeling. The associations between trajectory memberships and adolescent flood exposure were assessed using multinomial logistic regression. Results: Three distinct cognitive function trajectories were identified: “low start–slow decline” (18.36%), “moderate start–fast decline” (44.34%), and “high start–accelerated decline” (37.20%). After adjusting for sociodemographic variables, economic status, and health conditions, adolescent flood exposure was inversely associated with unfavorable cognitive trajectories (RRR = 0.56, 95% CI: 0.42–0.75). Mediation analysis via the Karlson–Holm–Breen method indicated that socioeconomic status significantly mediated this relationship, with years of schooling showing the strongest explanatory power (29.3%). Conclusions: Adolescent flood exposure is associated with cognitive decline trajectories in middle and old age. Disaster response strategies should prioritize long-term health benefits, enhance socioeconomic policies, and implement targeted interventions for high-risk populations. Keywords: adolescent flood exposure, cognitive aging, group-based trajectory modeling, cognitive function trajectories, Chinese middle-aged and older adults

Abstract citation ID: igaf122.812

### FATAL DROWNING IN OLDER ADULTS WITH ALCOHOL, OPIOIDS, CANNABINOIDS, AND OTHER DRUG EXPOSURES

Armiel Suriaga[1], Jessica Mangila[2], Hien Do[1], Lindsey Gensoli[1], Grace Abrams[1], Karina Del Carpio[1], Alondra Gittleson[1], and Gianna Nemia[1], *1. Florida Atlantic University, Boca Raton, Florida, United States, 2. Boston University, Boston, Massachusetts, United States*

More than 4,500 people die annually from drowning in the U.S., with a 19% increase among 65+ in 2022 compared to 2019. However, research on drowning-related mortality (DRM) among older adults exposed to alcohol, opioids, cannabis, and other substances is scarce. We aimed to report the characteristics of older adults who drowned with substances, using de-identified data from the Florida Department of Law Enforcement (2020-2022). We used descriptive statistics and regression modeling in Stata 17. The medical examiners ascertained the outcome (drowning) through autopsy and toxicology results. The analysis included 46,437 decedents with substances, 1,162 (2.5%) decedents with DRM, and 265 (4.64%) were older adults. 187 (70.57%) of the 265 were males, white= 228 (86.04%), Black=28 (10.57%), and 252 (95.1%) occurred in urban counties. 177 (66.79%) used a single drug and 88 (33.21%) with polydrug exposures. 70 (26.42%) had cardiac-related conditions, 16 (6.04%) with respiratory, and 15 (5.66%) had neurocognitive-related conditions. Older adults have lower odds of drowning than the reference group (0-17), OR=.68, 95% CI, .48-.95, p = 0.03. Those with alcohol had 3.26 higher odds of drowning compared to those without alcohol, OR = 3.26, 95% CI, 2.88-3.70, p < 0.001; opioids, OR=.19, 95% CI, .16-.22, p < 0.001; cannabinoids, OR=.85, 95% CI, .73-.99, p = 0.04; benzodiazepine, OR=.70, 95% CI, .59-.81, p < 0.001, and stimulant, OR=.73, 95% CI, .61-.87, p < 0.001. Polydrug exposures were significantly associated, OR=.42, 95% CI, .38-48, p < 0.001. Our results suggest that using multiple substances such as alcohol, opioids, and cannabis increases the odds of DRM, underscoring the need for tailored interventions to safeguard older adults.

Abstract citation ID: igaf122.813

### FINE PARTICULATE MATTER AIR POLLUTION AND LUNG FUNCTION AMONG U.S. OLDER ADULTS

Sung Eun Cho, Eun Young Choi, and Jennifer Ailshire, *University of Southern California, Los Angeles, California, United States*

Although prior research has found associations between fine particulate matter air pollution (PM2.5) and lung function among older adults, the findings have come from region-specific studies and have focused on individuals with pre-existing conditions. Furthermore, it remains unclear how individual health behaviors contribute to variations in vulnerability to PM2.5 exposure. This study examines the association between outdoor PM2.5 levels and lung function in a nationally representative and diverse sample of U.S. older adults from the 2016 and 2018 Health and Retirement Study (HRS). Our study sample includes 7,119 adults aged 65 and older linked with 2015 and 2017 census tract-level annual average PM2.5 concentrations from the HRS Contextual Data Resource. Regression models examined the cross-sectional association between PM2.5 concentration quartiles and lung function, measured through the peak flow meter tests. Older adults living in areas with the second-highest PM2.5 quartile had significantly lower lung function (b = -1.95, p = 0.013) compared to those in the lowest quartile, even after adjusting for demographic, socioeconomic, and health characteristics. Living in the highest PM2.5 quartile was not associated with lower lung function (b = -0.53, p = 0.505). However, in this quartile, those who were physically active exhibited worse lung function compared to those who were physically inactive (predicted lung function: 80.91 vs. 83.49, p = 0.000). These findings highlight the negative impact of PM2.5 on older adults' lung health and suggest that physical activity, potentially indicative of increased outdoor exposure, may heighten vulnerability to air pollution in highly polluted areas.

Abstract citation ID: igaf122.814

### GROUP PSYCHOLOGICAL COUNSELING BASED GROWTH MINDSET INTERVENTION TO PROMOTE ACTIVE AGING BEHAVIORS IN OLDER PEOPLE

Qian Yang[1], and Xiaohua Xiao[2], *1. School of Public Health, Zhejiang University School of Medicine, Hangzhou, Zhejiang, China (People's Republic), 2. Zhejiang University, Hangzhou, Zhejiang, China (People's Republic)*

Misconceptions that aging leads only to decline and is uncontrollable undermine older people' autonomy and discourage active aging behaviors. Few studies focus on older people' autonomy. The growth mindset theory, which posits that abilities can be developed through effort, aligns with the active aging framework and serve as a key intervention target to enhance engagement in active aging behaviors. However, research on growth mindset interventions for older people remains scarce. This study addresses the gap in growth mindset interventions for older people by using group counseling to promote active aging behaviors. A randomized controlled trial design was employed, with an autonomous intervention group receiving four structured counseling sessions and a control group engaging in equivalent community activities. The study was conducted in six communities across eastern and western China. Growth mindset, active health behaviors, social participation, and self-security behaviors were evaluated at four timepoints: baseline (T1), post-intervention (T2), and 1- and 3-month follow-ups (T3/T4). The intervention group showed significant improvements in growth mindset and active social participation behavior at (T2/T3/T4) (B > 0.00, p < 0.00) and demonstrated greater improvement in active health behaviors at T2 compared to the control group (B = 0.12, p < 0.00). The intervention group showed improvements in active self-security behavior at T3 (B = 0.16, p < 0.00). This study is the first to validate the effectiveness of growth mindset interventions tailored to older people, providing a novel approach to enhancing active aging behaviors and addressing the challenges posed by an aging population.

Abstract citation ID: igaf122.815

### PRODUCTIVE AGING PROFILES AMONG VIETNAMESE WAR SURVIVORS: THE ROLE OF EARLY-LIFE WAR EXPOSURE AND MILITARY SERVICE

Bussarawan Teerawichitchainan[1], Sara Hamm[2], Zachary Zimmer[2], and Minh Nguyen[3], *1. National University of Singapore, Singapore, Singapore, 2. Mount Saint Vincent University, Halifax, Nova Scotia, Canada, 3. Vietnam Academy of Social Sciences, Hanoi, Ha Noi, Vietnam*

War disrupts millions of lives, particularly in low- and middle-income countries (LMICs), where armed conflicts are most frequent. While much is known about war's detrimental effects on physical and psychological health, its long-term impact on productive aging remains unclear. This study investigates how early-life war exposure affects productive aging among older Vietnamese war survivors and examines the moderating role of past military service and war trauma exposure. Data come from the Vietnam Health and Aging Study (N = 2,447 war survivors aged 60+). Latent class analysis identifies patterns of engagement across five domains: work, in-kind support, caregiving, community involvement, and self-development. Multinomial logistic regressions assess relationships between war exposure intensity, military role, and engagement patterns. Our analyses identify five patterns of later-life productive engagement: High Engagers, Altruistic, Low Engagers, Civic Developers, and Active Workers. Greater exposure intensity is associated with profiles indicative of higher levels of engagement. Military roles moderate this relationship, with formal military veterans more likely to belong to the Active Workers class under high exposure conditions, while informal military members are more likely to be classified as Low

Engagers. To promote productive aging in conflict-affected LMICs, policymakers should consider the long-term impacts of early-life war exposure and past military service. Addressing inequalities and introducing interventions earlier in the life course could enhance effectiveness.

Abstract citation ID: igaf122.816

### THE EFFECT OF INCENTIVES ON DISASTER MITIGATION BEHAVIOR: AN AGE-BASED ANALYSIS

Wenqian He[1], Jeffrey Wickliffe[1], and Zhen Cong[2], *1. The University of Alabama at Birmingham, Birmingham, Alabama, United States, 2. Chapman University, Orange, California, United States*

Background: As climate change accelerates, the frequency and severity of natural disasters are increasing. However, most individuals cannot get sufficient protective measures due to financial pressure or environmental barriers. This study aims to examine how different incentives influence people's willingness to take mitigation behavior. Methods: Data were collected from 781 tornado survivors in Texas, Alabama, and Tennessee, as part of the 'Vulnerability and Resilience to Disasters' project. Participants were randomly assigned to 12 conditions based on cost coverage ratios (25%, 50%, 75%), improvement types (storm shelters, structural reinforcement), and incentive forms (cash rebates, insurance discounts). Multivariate logistic regression models were used, with a focus on age differences (< 49 years vs. ≥50 years). Results: Higher cost coverage increased protective willingness, and participants are more willing to get structural upgrades than storm shelters. No significant interaction effects were found between incentive methods. Besides, older participants, people with minor children, people experienced stress during the last tornado, and people anticipated future tornadoes were more likely to take mitigation behavior. Stress increased younger participants' willingness, while the presence of minor children increased older participants' willingness. 75% cost coverage was effective across all groups, while 50% coverage alone worked for younger participants but need to be combined with insurance discounts to work for older participants. Conclusion: Cost coverage is important in disaster mitigation. Younger participants are more likely to be driven by stress, while older participants prefer combined incentives and prioritize family safety. Policies should consider demographic differences to enhance effectiveness and equity.

### SESSION 2277 (BIOLOGICAL SCIENCES INVITED SYMPOSIUM)

Abstract citation ID: igaf122.817

### IMMUNOLOGICAL ASPECTS OF AGING

Chair: Anthony Covarubbias

The immune system undergoes significant changes and dysregulation during aging, leading to immune cell exhaustion and senescence, impaired tissue homeostasis, age-related chronic inflammation (inflammaging), and decreased pathogen immunity. This session will focus on the immunological aspects of aging and highlight new insights into the molecular and cellular mechanisms that drive immune dysfunction and inflammaging. Dr. Anthony J. Covarrubias (UCLA) will chair this session and also discuss recent discoveries that macrophages can undergo cellular senescence, adopting pro-inflammatory phenotypes that promote chronic inflammation and fibrosis in aging and metabolic disease. Dr. Emily Goldberg (UCSF) will explore the cross-talk between metabolism and immune cells during aging, and how this impacts tissue inflammation during aging. Dr. Duygu Ucar (The Jackson Laboratory) will present systems-level and single-cell genomic approaches that reveal molecular signatures of immune aging, including how biological sex, infection history, and epigenetic remodeling shape immune responses and vaccine efficacy across aging. Dr. Seokjo Kang (Cedars-Sinai Medical Center) will conclude by examining molecular mechanisms controlling microglial aging, including sex-specific differences in microglial gene expression, metabolism, and function, and how these changes contribute to neuroinflammation and cognitive decline. Together, these talks will explore cutting-edge approaches and recent discoveries in molecular and cellular biology, metabolism, and computational fields to enhance our understanding of immune aging and its systemic effects on the aging process. The session will also showcase emerging therapeutic strategies aimed at rejuvenating the immune system and extending health span.

Abstract citation ID: igaf122.818

### MACROPHAGE SENESCENCE: A NEW AXIS IN INFLAMMAGING AND MASLD WITH IMPLICATIONS FOR SENOLYTIC THERAPY

Anthony Covarubbias, *University of California, Los Angeles, Los Angeles, California, United States*

DNA damage or other cellular stress can lead to an irreversible cell cycle arrest, known as cellular senescence in immune cells. While cellular senescence in immune cells can be beneficial in some contexts such as wound healing and development, it is now recognized as a major source of sterile inflammation in aging tissues via the secretion of inflammatory factors known as the senescence-associated secretory phenotype (SASP). Targeting senescent immune cells is therefore an emerging strategy for treating age-related diseases, however, the identity and function of specific senescent immune cell types remain unclear. Here, we identify p21$^{4}$senescent macrophages as a key source of chronic inflammation in aging and in metabolic dysfunction-associated steatotic liver disease (MASLD). To further investigate macrophage senescence, we developed an in vitro model of DNA damage-induced macrophage senescence in primary mouse and human macrophages. Using a multi-omic approach, we leveraged these biomarkers to distinguish senescent macrophages from other established polarized states. We also found that DNA damage or metabolic stress induces stable macrophage senescence marked by an elevated SASP, mitochondrial dysfunction, and interferon signaling. These senescent macrophages accumulate in aged mouse livers, particularly in Kupffer cells, and are enriched in human cirrhotic liver tissue. Using transcriptomic profiling we identified a unique transcriptomic signature to identify macrophage senescence both in vitro and in vivo for both mice and humans. We also showed that senescent macrophages can be selectively targeted for depletion using senolytics, which reduces liver steatosis and inflammation in MASLD models, highlighting macrophage senescence as a major source of inflammaging and promising therapeutic target in age-related diseases.

Abstract citation ID: igaf122.819

### NEUTROPHILS AND CHRONIC INFLAMMATION

Emily Goldberg, *University of California, San Francisco, California, United States*

Neutrophils are short-lived immune cells that are best known for their important role in antimicrobial immunity. Recently, neutrophils have been implicated in sterile inflammatory diseases like diabetes, cardiovascular disease, and cancer. We have been investigating how neutrophils might contribute to chronic inflammatory diseases, and particularly in age-related inflammation.

Abstract citation ID: igaf122.820

**HETEROGENEITY IN HUMAN IMMUNE AGING AND VACCINE RESPONSES**

Duygu Ucar, *The Jackson Laboratory for Genomic Medicine, Farmington, Connecticut, United States*

Aging increases the risk for morbidity and mortality from severe infections and reduces vaccine responses, particularly among older adults. While we know how aging affects immune cells, the heterogeneity among older adults both in terms of immune aging and immune responsiveness to vaccination are less understood. The overarching goal of my laboratory is to uncover the drivers of this heterogeneity, with the ultimate aim of improving immune resilience in older adults. We have identified transcriptomic and epigenomic signatures of immune aging in peripheral blood mononuclear cells (PBMCs) and discovered predictive genomic markers of vaccine responsiveness to pneumococcal and influenza vaccines in older adults. We recently showed that elevated frequencies of cytotoxic CD16+ NK cells in older adults are associated with poor antibody responses to T cell-dependent pneumococcal vaccination, suggesting a novel NK and T helper regulatory axis in immune aging.

Abstract citation ID: igaf122.821

**MICROGLIAL SEX DIFFERENCES IN THE AGING BRAIN**

Seokjo Kang, *Cedars-Sinai Medical Center*

Microglia, the brain's resident macrophages, are key players in both neuroprotection and neurodegeneration. Microglial heterogeneity is thought to account for at least some of the diverse functional properties of microglia, especially in the context of neurodegeneration. For instance, neuroprotective disease-associated microglia (DAM) become more abundant during healthy aging, as well as in mice and humans with neurodegenerative diseases such as Alzheimer's disease. Variation in microglial states and subsets may also underlie sex differences in neurodegenerative disease risk. Interestingly, we observed more aging-associated transcriptomic changes in hippocampal microglia from female mice than male mice. We also detected more abundant DAM in the hippocampus of old female mice than old male mice, reflecting a stronger glycolytic shift in females that is promoted by autocrine complement (C3a) signaling to support their phagocytic properties. In ongoing studies, we are evaluating whether these differences are a consequence of more damage in the aging female brain or reveal a stronger neuroprotective response. We are also probing the implications of these findings in the context of healthy aging and aging-associated neurodegenerative diseases.

### SESSION 2280 (SYMPOSIUM)

Abstract citation ID: igaf122.822

**FROM DAILY LIFE PROCESSES TO LONG-TERM HEALTH: INNOVATIONS IN MEASUREMENT-BURST DESIGNS**

Chair: Sun Ah Lee
Discussant: David Almeida

Measurement-burst designs offer a nuanced approach for examining developmental processes by capturing both short-term dynamics and long-term changes in psychosocial experiences, behaviors, and health. Despite their unique capacity to assess changes across multiple temporal intervals, these designs remain underutilized, due in part to limited refinement of their methodological application. This symposium highlights innovative research questions and modeling approaches that leverage measurement-burst data to examine developmental changes in daily or momentary experiences and their implications for health and well-being across adulthood. Lee and Almeida use two bursts of daily diary and biomarker data to investigate the mediating role of daily affect dynamics in the longitudinal and bidirectional relationship between depressive symptoms and inflammation. Rush et al. use three bursts of daily diary data to examine longitudinal changes in daily stress reactivity over 20 years and its mediating role in linking declines in functional health and life satisfaction. Chai et al. use three bursts of ecological momentary assessments to evaluate the impact of a cognitive training intervention on longitudinal changes in daily subjective sleep and age. Witzel et al. use two bursts of daily diary data to explore age differences and longitudinal changes in the daily associations between positive experiences and physical symptoms. Almeida will discuss recent innovations and opportunities in measurement-burst designs and their potential to advance research on daily experiences and health across adulthood. This symposium demonstrates how measurement-burst approaches enhance our understanding of developmental changes across different timescales and provide insights into healthy aging. Measurement, Statistics, and Research Design Interest Group Sponsored Symposium

Abstract citation ID: igaf122.823

**DAILY AFFECT DYNAMICS MEDIATE THE LONGITUDINAL AND RECIPROCAL LINK BETWEEN DEPRESSIVE SYMPTOMS AND INFLAMMATION**

Sun Ah Lee, and David Almeida, *The Pennsylvania State University, University Park, Pennsylvania, United States*

Depression is a leading mental health concern among middle-aged and older adults. Extensive research has established an intricate relationship between depression and inflammation, yet greater attention is needed to elucidate the mechanisms through which these processes unfold across multiple time scales and levels of analysis. This study evaluated the mediating role of daily affect dynamics in the bidirectional and longitudinal association between depressive symptoms and inflammation. Using data from the second and third waves of the Midlife in the United States (MIDUS) study, the sample included 563 adults who participated in both the Daily Diary and Biomarker Projects (Mage = 52.4; 57% female; 84% White). Daily affect and stress were measured over eight consecutive days. Depressive symptoms were assessed using the CESD-20. Inflammatory markers were used to create two separate indicators: composite cytokine scores (IL-6, IL-8, TNFα, IL-10) and CRP, derived from fasting blood samples. Multilevel structural equation models simultaneously tested between-person autoregressive and cross-lagged paths of the depressive symptoms-inflammation link, as well as within-person mediated paths involving daily affect dynamic indicators. Results revealed that positive affective variability and negative affective reactivity to daily stressors significantly mediated the associations between depressive symptoms and CRP. These patterns were more evident among middle-aged than older adults. Between-person autoregressive paths for depressive symptoms and inflammation were significant, whereas cross-lagged paths showed no significant associations. Findings suggest daily affective processes as potential mechanisms linking mental and physical health, underscoring the need for interventions that promote affective stability and adaptive stress responses in everyday life.

Abstract citation ID: igaf122.824

### LONG-TERM CHANGES IN DAILY STRESS REACTIVITY AS A MEDIATOR BETWEEN CHANGES IN HEALTH AND WELL-BEING ACROSS 20 YEARS

Jonathan Rush[1], Eric Cerino[2], Dakota Witzel[3], Jennifer Piazza[4], Susan Charles[5], and David Almeida[6], *1. University of Victoria, Victoria, British Columbia, Canada, 2. Northern Arizona University, Flagstaff, Arizona, United States, 3. South Dakota State University, Brookings, South Dakota, United States, 4. California State University, Fullerton, Fullerton, California, United States, 5. University of California, Irvine, Irvine, California, United States, 6. The Pennsylvania State University, University Park, Pennsylvania, United States*

The daily within-person association between stress exposure and negative affect (i.e., stress reactivity) has been shown to be predictive of adverse health and well-being outcomes. These findings typically rely on a single burst of daily assessments. Recent findings have shown that long-term changes in daily stress reactivity across adulthood are associated with changes in health outcomes. The present study extends this work by examining whether long-term changes in daily stress reactivity mediates the association between longitudinal changes in functional health and life satisfaction across 20 years. We used measurement burst data from the National Study of Daily Experiences (N=2,880) embedded within the MIDUS longitudinal study. Three measurement bursts were separated by ten years, with each containing daily measures of stress and affect across eight consecutive days, yielding 33,942 days of data across 20 years of adulthood. Functional health and life satisfaction were also measured every 10 years. Multilevel SEM were fit to simultaneously model daily within-person associations between stress and affect (i.e., stress reactivity) at Level 1; long-term changes in stress reactivity at Level 2; and the mediational effect of changes in stress reactivity on the pathway of changes in functional health predicting changes in life satisfaction at Level 3. Declines in functional health predicted declines in life satisfaction across 20 years (estimate=1.12, SE=0.37, p<.001). Importantly, changes in stress reactivity mediated this effect (indirect effect=17.51, SE=8.40, p=.02). Results suggest that the link between declines in functional health and life satisfaction may be accounted for by increases in daily stress reactivity.

Abstract citation ID: igaf122.825

### LONGITUDINAL CHANGES IN DAILY SUBJECTIVE SLEEP AND AGE FROM THE EVERYDAY FUNCTION INTERVENTION TRIAL

Hye Won Chai, Alyssa Gamaldo, and Lesley Ross, *Clemson University, Clemson, South Carolina, United States*

Some cognitive training interventions, such as Useful Field of View training (UFOVt) are effective in maintaining brain health, cognition, and everyday functioning. However, daily changes associated with training are less understood. This study examined daily changes in sleep and subjective age as outcomes of UFOVt. Data came from the Everyday Function Intervention Trial (EFIT; NCT04651582) study, a randomized clinical controlled trial designed to assess the moderators and mechanisms of UFOVt transfer effects among older adults aged between 65 to 90 years (N=94). EFIT participants completed laptop-based cognitive, health, and psychosocial surveys during baseline, post-test, and three-month follow-up as well as smartphone-based daily assessments throughout the study period. Data utilized for this study included daily assessments of subjective sleep (e.g., sleep quality, restfulness, difficulty falling asleep, difficulty staying asleep) and subjective age (e.g., "Compared to your actual age, what age do you feel right now?") collected across 14 days at baseline, post-test, and follow-up, respectively (i.e., three 14-day bursts of daily data). Multilevel analyses assessed the longitudinal changes between baseline and follow-up in person-level average and fluctuations in daily sleep and subjective age, and whether these changes differ by training. Results demonstrated that UFOVt group reported reductions in difficulty falling asleep and in feeling older than their actual age compared to the active control group. These results suggest UFOVt as a potentially useful tool for improving older adults' subjective assessment of their everyday experiences and well-being which may be protective of cognitive decline.

Abstract citation ID: igaf122.826

### USING TWO BURSTS OF MIDUS TO INFORM DAILY POSITIVE EXPERIENCE AND PHYSICAL HEALTH SYMPTOM ASSOCIATIONS

Dakota Witzel[1], Maria Kurth[2], Shelbie Turner[3], and Robert Stawski[4], *1. South Dakota State University, Brookings, South Dakota, United States, 2. The Pennsylvania State University, University Park, Pennsylvania, United States, 3. Weill Cornell Medicine, New York, New York, United States, 4. Utah State University, Logan, Utah, United States*

Positive experiences are buffers for long-term health outcomes such as mortality and daily objective health indicators such as cortisol and inflammation. However, less is known about the implications of positive experiences on proximal (i.e., same day) subjective physical health symptoms. Using two bursts of data from the National Study of Daily Experiences (N=1,327, Magewave2~56, Nobs=7,966), we examined whether the number and type of daily positive experience was associated with same day physical health symptoms (M=19), as well as 10-year changes therein. Further, we explored age-related resilience of positive experiences by considering age as a moderator. In both Wave 2 (year: 2005) and Wave 3 (year: 2015), participants completed eight days of end-of-day telephone interviews reporting on their daily experiences including daily positive experiences (interpersonal interactions, at work, and at home) and physical health symptoms (e.g., headache, muscle soreness, diarrhea). Both the number and type of positive experiences were significantly related to the number of physical health symptoms reported in a day (ps < .05). Days when adults reported either a positive interpersonal interaction, work, or home experiences was associated with more physical health symptoms compared to when no positive experience was reported. The effect of positive interpersonal interactions was statistically significant for middle aged adults (estimate=.12, p< .001) but not older adults (estimate=.01 p=.56). Discussion will include how daily positive experiences may be an indicator of an active life, thus resulting in more daily symptoms such as aches and highlight potential bidirectional effects.

### SESSION 2285 (SYMPOSIUM)

Abstract citation ID: igaf122.827

### IMPLEMENTING EVIDENCE-BASED DEMENTIA CARE INTERVENTIONS: THE CMS GUIDE MODEL AND BEYOND

Chair: David Reuben
Discussant: Christine Ritchie

There is high need for access to evidence-based dementia care interventions. Use of such services can contribute to higher quality health care, improved clinical outcomes, and cost savings. Multiple strategies have been implemented, many in response to the 2024

launch of the CMS payment demonstration project, the Guiding an Improved Dementia Experience (GUIDE) Model. For health systems participating in GUIDE, efforts are focused on supporting their successful operations or initiating new dementia care programs. The National Dementia Care Collaborative (NDCC) aims to promote dissemination of evidence-based comprehensive dementia care to both GUIDE participants and health care organizations ineligible for GUIDE. The Best Programs for Caregiving (BPC) online database is a resource dedicated to linking health systems, community-based organizations and family caregivers to evidence-based interventions proven to support family caregivers of people living with dementia. Work at Indiana University related to comprehensive dementia care has adapted related approaches to address delirium and critical illness care, and to support navigation during diagnostic processes related to mild cognitive impairment. The objectives of this session are to explain the goals and outcomes of the NDCC, BPC, and work at Indiana University to address multiple chronic conditions, and to show how these efforts can contribute to dementia care implementation. Participants in the session will learn strategies for how health systems and community-based organizations can increase access to evidence-based dementia care while integrating it with other health care and services to improve overall care quality.

Abstract citation ID: igaf122.828

### THE NATIONAL DEMENTIA CARE COLLABORATIVE: OUTCOMES FROM YEAR 1

Gary Epstein-Lubow, Kristin Haggerty, Maria Avalos, Madelyn Johnson, and Rebecca Stoeckle, *Education Development Center, Waltham, Massachusetts, United States*

The National Dementia Care Collaborative (NDCC) aims to improve access to and successful implementation of evidence-based comprehensive dementia care. NDCC provides a common learning platform for health systems and other provider organizations implementing or interested in implementing a proven model of comprehensive dementia care. Evidence-based model teams participating with NDCC include the Aging Brain Care (ABC) Program from Indiana University, the Alzheimer's and Dementia Care (ADC) Program from UCLA, the Benjamin Rose Institute's Care Consultation (BRI-CC), the Care Ecosystem from UCSF, Maximizing Independence (MIND) at Home from Johns Hopkins University, and the Integrated Memory Care (IMC) program from Emory University, This session will provide descriptions of implementation strategies that are common across these comprehensive dementia care models as well as examples of specific implementation strategies that are unique to one or more model(s). Outcomes will be described regarding the number of new implementations of an evidence-based comprehensive dementia care program in health systems and community-based organizations participating in the CMS GUIDE Model and external to it; this included 51 total new programs in 2024. New emphasis on growing the interest in dementia care models in value-based care, Medicare Advantage, and commercial health plans will also be described.

Abstract citation ID: igaf122.829

### USING BEST PROGRAMS FOR CAREGIVING TO ACCESS EVIDENCE-BASED DEMENTIA CARE

Morgan Minyo[1], David Bass[1], Rachel Cannon[1], Zoe Fete[2], Megan Huth[1], and Sara Powers[2], *1. Benjamin Rose Institute on Aging, Cleveland, Ohio, United States, 2. Benjamin Rose, Cleveland, Ohio, United States*

Best Program for Caregiving (BPC) is a free, online directory with detailed information on 45 dementia caregiving programs. This easy-to-use database has Professional and Public Versions, making BPC an invaluable resource for both providers and caregivers to access information on proven, evidence-based dementia caregiving programs that meet their needs. The Professional Version was designed for providers to use for identifying and comparing programs, and for making informed decisions on adopting implementation-ready programs. Available data include program characteristics, research outcomes, and delivery organizations' experiences. The Public Version displays more than 200 organizations verified as delivering one or more of the 45 programs on the Professional Version. By inserting a zip code, providers and family caregivers can find and enroll in locally available programs that align with each caregiver's individual preferences and care situation. Selected results from the Public Version indicate 85.0% of programs are free; 18 are offered virtually online or by phone to caregivers nationwide; 23.2% were adapted for a diverse group of caregivers such as those living in rural and other underserved communities. Common adaptations include consulting with members or experts of the diverse group (100.0%), adapting program content (92.8%), and delivering the program in the group's language (53.8%). This presentation provides an overview of programs in and findings from BPC, as well as providing a demonstration and discussion on how providers can use the database as a resource for accessing and implementing evidence-based dementia care at their organization.

Abstract citation ID: igaf122.830

### COMPREHENSIVE DEMENTIA CARE TO ADDRESS MULTIMORBIDITY ACROSS INPATIENT, RESIDENTIAL AND COMMUNITY SETTINGS

Malaz Boustani, *Indiana University, Indianapolis, Indiana, United States*

Fully addressing dementia requires attention to patients at risk for dementia. The Aging Brain Care (ABC) program has been addressing this along with its sustainability and scalability over the past 17 years. The ABC populations disproportionately suffer from dementia, comorbid medical conditions, and high social frailty. To address comorbidities, the ABC program conducts a comprehensive assessment to diagnose dementia and its severity, reviews medical conditions, assesses the burden on informal caregivers, determines the biopsychosocial needs of the dyads, and identify their goals and preferences. Then it co-develops a personalized care plan including de-prescribing inappropriate medications and doing co management of comorbid conditions in collaboration with primary care. In a clinical trial, conducted in Federally Qualified Health Centers, the ABC program reduced the behavioral and psychological symptoms related to dementia, the burden of informal caregivers, and the use of inappropriate medications. To enhance the ABC program's work with vulnerable populations, it facilitates care transition from home to hospital and subacute rehabilitation and back to home. ABC also partners with community-based organizations to identify home and community-based resources to overcome care needs and address social determinants of health. Community health workers trained and supported to deliver care across settings in accordance with the patients' and family preference have provided care to an average 400 dyads per year. Work is now underway to expand the care navigation roles to address conditions other than dementia, and to support the diagnostic process for patients at risk for dementia.

### SESSION 2290 (SYMPOSIUM)

Abstract citation ID: igaf122.831

### INNOVATIONS IN RURAL AGING: COMMUNITY-BASED APPROACHES TO SUPPORT OLDER ADULTS

Chair: Heather Fuller

Rural communities provide a unique context, posing both challenges and advantages as individuals age. The purpose of this symposium is to showcase innovative community-based approaches to assessing needs and implementing interventions for rural older adults across five distinct U.S. regions. Monaghan-Geernaert used a community-driven approach to examine modalities of caregiving for Native American elders within rural settings in South Dakota, finding that implementing Indigenous healing methods into care environments helps improve patient well-being and caregiver effectiveness. Kopera-Frye examined caregiving needs among rural New Mexico Latinx caregivers and found culturally appropriate, family-based solutions for caregiving despite gaps in access to formal support services. Liu et al. examined challenges faced by rural older Veterans with vision loss in Northern Florida/South Georgia, determining that implementing low-tech solutions by generalist healthcare providers could support aging well in rural communities and sharing initial implementation steps of a low-tech toolkit intervention to foster independent living. Roberts & Chen implemented an intergenerational Active Aging for L.I.F.E. project for promoting shared positive health perspectives among older adults and young adults in rural Oklahoma and found outcomes demonstrating effectiveness for train the trainer programming. Fuller et al. assessed the implementation and effectiveness of the community-driven Aging in Community program for fostering aging in place in rural North Dakota, finding that a responsive and flexible intervention resulted in older adults' improved independence, community connection, and quality of life. Together, these papers highlight innovative strategies for addressing the diverse needs of aging in rural communities, emphasizing the importance of community-informed approaches. Rural Aging Interest Group Sponsored Symposium

Abstract citation ID: igaf122.832

### HONORING ELDERS, HONORING CULTURE: RETHINKING RURAL HEALTHCARE FOR NATIVE COMMUNITIES

Pamela Monaghan-Geernaert, *Northern State University, Aberdeen, South Dakota, United States*

Providing culturally competent care for Native American elders in rural communities requires an understanding of their history and culture. Many Indigenous elders prefer that the care they receive focus on community, spirituality, and traditional healing practices. These modalities of care often differ from mainstream Western healthcare approaches and are furthermore, not reimbursable expenses in nursing home and other health care environments. Additionally, it is unlikely that staff have been trained to manage, promote or administer this kind of culturally responsive care. Barriers such as lack of culturally trained staff, geographic isolation, and systemic healthcare disparities make it challenging to implement culturally responsive care in rural settings. This study explores how rural communities engage with Native elders to develop care models that honor specific tribal cultural practices while addressing medical needs. Through qualitative research with Native and non-Native caregivers, this presentation highlights best practices. Findings demonstrate that community-driven solutions, staff cultural training, and integration of Indigenous healing methods improve patient well-being and caregiver effectiveness. By recognizing and respecting Native elders' preferences, healthcare providers in rural communities can create inclusive, culturally sustaining care environments that uphold dignity and cultural continuity.

Abstract citation ID: igaf122.833

### THE IMPORTANCE OF LATINX CULTURAL VALUES IN CAREGIVING IN RURAL COMMUNITIES

Karen Kopera-Frye, *New Mexico State University, Las Cruces, New Mexico, United States*

CDC reports almost 23% of adults are caregivers to family or friends in New Mexico; 10.4 % provide care to an individual with dementia. The southern Border region of New Mexico is remote and desert, with high rates of poverty and inaccessibility to health care and services. The incidence of Latinx caregivers is rising in light of the increasing population trends. Yet, available culturally appropriate support services are lacking. Familismo, a Latinx primary cultural value, refers to the importance of strong family loyalty, closeness, and getting along with and contributing to the well-being of the nuclear family, extended family, and kinship networks. This value is additionally responsible for the increased caregiving seen among many Latinx families. The family is extremely important, yet appropriate services and resources may not be available, causing distress. Seventy-six primarily female Latinx caregivers were surveyed on caregiving they provide, perceptions of preparedness, needs, positive and negative aspects of caregiving. Results indicated the top three provided aspects of caregiving were (in descending order): Food, emotional support, and transportation. Caregivers reported a high degree of preparedness, e.g., taking care of the recipient's physical needs. Needed resources included emotional support, financial assistance, and respite. Greater caregiving provision was significantly associated with greater resource needs ($r = .35$, $p <.01$). Despite multiple needs, caregiving perceptions were largely positive, highlighting the importance of the cultural value of familismo in Latinx families. Having culturally responsive service providers can decrease some of the challenges such as health disparities in rural communities.

Abstract citation ID: igaf122.834

### SUPPORTING RURAL VETERANS WITH VISION LOSS USING A LOW-TECH TOOL KIT: THE FIRST STEP

Chiung-Ju Liu[1], Kimberly Findley[2], Consuelo Kreider[1], and Hongwu Wang[1], *1. University of Florida College of Public Health and Health Professions, Gainesville, Florida, United States, 2. Malcom Randall VA Medical Center, Gainesville, Florida, United States*

Age-related vision loss can significantly impact older people's daily activities, leading to care dependency. Access to vision care services is challenging for rural older Veterans with vision loss due to long travel distances and limited capacity to drive. Low-tech solutions to enhance activity participation could be taught by VA generalist clinicians who are not vision specialists. For example, applying tactile cues to help operate a microwave. As an initial step of an implementation study, this survey aimed to understand 1) VA generalist clinicians' awareness of older Veterans' vision concerns and 2) their preferences for a tool kit that offers low-tech solutions to address these concerns. Community-based VA clinicians in the Northern Florida/South Georgia Veterans Health System were invited to complete the survey, with 39 respondents representing seven health professions. Descriptive analysis showed that 92% of respondents had served rural Veterans in the past three months, and only 59% routinely reviewed medical records for vision-related concerns. The top identified concerns were difficulty reading, driving, and falls. Respondents mostly referred the Veterans to vision care services (67%). Additionally, respondents' top-ranked features for the low-tech tool kit as "very important" were: having simple instructions with clear illustrations (77%), kit items that are intuitive to use (64%), and items that can be easily replaced (64%). Survey results suggested a need to increase VA generalist clinicians' awareness of activity challenges faced by rural older Veterans with vision loss. Results also guided the development of a low-tech tool kit to meet the clinicians' preferences.

Abstract citation ID: igaf122.835

### INTERGENERATIONAL RURAL HEALTH PROGRAMMING THROUGH ACTIVE AGING FOR L.I.F.E.

Emily Roberts, and Xuewei Chen, *Oklahoma State University, Stillwater, Oklahoma, United States*

Aging is a process in which everyone is involved, as it is a lifelong experience, not just a later life occurrence. The Active Aging for L.I.F.E. collaborative project provides a novel rural health education approach promoting positive health outcomes for intergenerational participants. The program is developed to focus on the interrelationships between Longevity, Independence, Fitness and Engagement (L.I.F.E.), bringing together L.I.F.E. Leader teams of older adults (50+) and college-age students who are trained in the four L.I.F.E. modules. The L.I.F.E. Leaders then take the program to rural high school students in four sessions, focusing on the interconnected network of personal and professional health and wellness opportunities. This presentation provides guidance and tools for the Active Aging for L.I.F.E. program and demonstrates through mixed-method findings the potential of intergenerational rural community-based active aging programming.

Abstract citation ID: igaf122.836

### AGING IN COMMUNITY: IMPLEMENTATION OF A GRASSROOTS PROGRAM TO SUPPORT RURAL AGING IN PLACE

Heather Fuller[1], Jane Strommen[1], Debarati Kole[1], Bryce Van Vleet[2], and Sharon Query[1], *1. North Dakota State University, Fargo, North Dakota, United States, 2. Eastern Mennonite University, Harrisonburg, Virginia, United States*

Though most older adults prefer to age in place, aging in rural communities can pose unique challenges such as geographic distance to resources, lack of social services, and workforce shortages. ND Aging in Community (AIC) is a grassroots program aimed at improving quality of life by increasing community-level support to help older adults age in place in two rural North Dakotan communities. A multidimensional, systematic evaluation was conducted over 4 years to assess the process and impact of program implementation. Across the first two years, three surveys were conducted with community-based steering committee members (N = 20-29) to assess strengths and areas for growth in service development and community engagement. Findings indicated growth and success over time in service implementation, the critical importance of responsiveness to community voices, and persistent challenges related to workforce and geography. During the final three years, annual surveys were conducted with AIC participants (N = 70-99) to assess satisfaction and impact. Findings indicated older adult participants reported improvement over time in confidence living at home, connection to community, and quality of life; additionally, 100% would recommend the program to others. Through an iterative process, evaluation results were used to inform program refinement. In the end, a holistic Aging in Community Framework was developed with a multi-pronged focus on housing, health and wellness, transportation, connection and growth, personal finance and community engagement. This framework provides an adaptable model for replication in other rural communities. Future directions will be discussed, including examining long-term efficacy and sustainability.

### SESSION 2295 (SYMPOSIUM)

Abstract citation ID: igaf122.837

### MARRIAGE, COHABITATION AND INTIMATE RELATIONSHIPS IN LATER LIFE

Chair: Hui Liu
Discussant: Sara Moorman

The five papers presented in this symposium utilize data from the National Social Life, Health, and Aging Project (NSHAP) to examine various types of intimate relationships in later life. Two papers focus on marital relationships. Li and Waite explore the likelihood of older adults naming their spouse or partner as a "core confidant" and how relationship quality influences this designation, finding that relationship quality significantly predicts spousal confidant status. Avent and Ivenuik examine how spousal retirement affects social networks and shared relationships, showing that retirement leads to more interconnected but smaller networks for men, while women's networks initially grow before shrinking. The other three papers examine other intimate ties. Brown and Newmyer extend research on the marital biographies of older adults by incorporating cohabitation histories, offering a more comprehensive view of union experiences among U.S. older adults. Warner and Lyons investigate how couple-level sexual expression predicts subsequent loneliness for both spouses, showing that less-than-desired sexual activity is associated with greater loneliness, particularly among couples who view sex as an important part of life. Finally, Pope and Thomas move beyond human intimate ties to examine the impact of human-animal relationships on older adults' well-being, finding that pet touch may benefit mental health of older adults, with effects varying by gender and marital satisfaction. Together, these five papers provide valuable insights into the diverse intimate ties that shape later life, including marriage, cohabitation, sexual expression, and human-animal relationships, and how these intimate ties influence the well-being of older adults. Human-Animal Interaction Interest Group Sponsored Symposium

Abstract citation ID: igaf122.838

### PARTNER RELATIONSHIP QUALITY AND INCLUSION OF INTIMATE PARTNERS AS NETWORK CONFIDANTS OF OLDER ADULTS

Yiang Li, and Linda Waite, *The University of Chicago, Chicago, Illinois, United States*

Robust social networks and high-quality intimate partnerships each promote health in later life, but little is known about how older adults integrate spouses and partners into their core discussion networks. Using data from the National Social Life, Health, and Aging Project (NSHAP), this paper examines the likelihood that older adults name their spouse or partner as a "core confidant" and how relationship quality shapes that designation. Among 1,844 respondents with an intimate partner, nearly one in four did not list that partner as a confidant. Naming a spouse as a confidant is strongly predicted by positive indicators of relationship quality, including time spent together, partner support, and relationship satisfaction. Conversely, partner strain lowers the probability of inclusion. The link between relationship support and confidant naming is most pronounced for highly educated respondents. We discuss the findings as reflecting social inequalities in the ability to integrate intimate ties into broader networks.

Abstract citation ID: igaf122.839

**GOLDEN YEARS, GOLDEN BONDS: EXPLORING THE IMPACT OF RETIREMENT ON SPOUSES' SOCIAL NETWORKS AND SHARED RELATIONSHIPS**

Elizabeth Avent, and James Ivenuik, *National Opinion Research Center at The University of Chicago, Chicago, Illinois, United States*

Research has shown that retirement influences spouses' social networks, though the extent of post-retirement network structure and overlap may differ by gender and ethnicity. Using data from the National Social Life, Health, and Aging Project (NSHAP) (N = 1870) this study examines how spousal retirement affects social networks and shared relationships. Results from generalized structural equation models show that retired men's networks neither expanded nor diminished but became more interconnected ($\beta = 0.05$, $p < .05$), even as their overall size declined. Additionally, retired men spent less time with their partners ($\beta = -0.42$, $p<.05$), while their retired partners exhibited a similar decrease ($\beta = -0.37$, $p<.05$). Retired women's networks initially increased ($\beta = 0.15$, $p < .05$) before decreasing over time ($\beta = -0.20$, $p < .05$), with reduced time spent with their network ($\beta = -10.60$, $p<.05$). Although retirement did not significantly alter overall network overlap, greater overlap was observed among Black ($\beta = 32.21$, $p<.01$) and Hispanic ($\beta = 38.63$, $p<.01$) couples when the male retired. Hispanic couples in which the female retired had even higher levels of network overlap ($\beta = 66.26$, $p<.01$). These findings suggest gender and racial/ethnic differences in how couples navigate social networks post-retirement. Understanding these dynamics can inform strategies to promote social engagement and support in later life.

Abstract citation ID: igaf122.840

**COHABITATION HISTORIES OF U.S. OLDER ADULTS**

Susan Brown, *Bowling Green State University, Bowling Green, Ohio, United States*

The rapid acceleration in unmarried cohabitation during the second half of the 20th century reshaped the family life course as cohabitation shifted from the margins to being the modal path to marriage. Today's older adults came of age during an era marked by low levels of cohabitation. But as they aged into midlife and beyond, cohabitation grew in popularity, diffusing across the population. As remarriage after divorce plummeted, a corresponding increase in cohabitation largely offset this decline. Cohabitation is now a common living arrangement among all age groups, including older adults. This diffusion process raises important questions about the levels and patterns of cohabitation experienced by today's older adults over their life courses. Drawing on three waves of data from the National Social Life, Health, and Aging Project (NSHAP), a national sample of adults aged 57-85 begun in 2005-06 and refreshed in 2015-16, we use demographic techniques to describe the cohabitation histories of older adults, tracking variation by sex and across birth cohorts (born 1920-1965). We perform a series of life table estimates of having ever cohabited by various ages as well as having ever experienced premarital cohabitation, postmarital cohabitation, and serial cohabitation. We also assess the disposition of cohabiting unions to determine whether they eventuated in marriage or separation. Our study extends the growing literature on the marital biographies of older adults by integrating cohabitation histories, providing a more comprehensive portrait of the union experiences of U.S. older adults.

Abstract citation ID: igaf122.841

**LESS THAN DESIRED: SEXUAL EXPRESSION AND SUBSEQUENT LONELINESS AMONG OLDER MARRIED COUPLES**

David Warner[1], and Heidi Lyons[2], *1. The University of Alabama at Birmingham, Birmingham, Alabama, United States, 2. Oakland University, Rochester, Michigan, United States*

Older married couples continue to engage in sexual activity—even as the frequency wanes with age—and this is associated with better physical and mental health. Sexual activity, however, is but one component of sexual expression. Indeed, we have previously shown there is considerable heterogeneity in sexual expression among older couples when behavior, desire, and attitudes are considered simultaneously, and objectively low levels of sexual activity may or may not be congruent with expectations. Some couples, for example, with infrequent behavior and ambivalent about whether sex is an important part of life still indicate that they are having sex less than desired. The implications of this incongruence for subsequent well-being are unclear. We address this gap with data on 595 heterosexual couples in long-term marriages from Rounds 2 and 3 of the National Social Life, Health, and Aging Project (NSHAP). Using latent class analysis with distal outcomes, we examined how couple-level sexual expression predicted subsequent loneliness—an indicator of unmet psychosocial need—for both spouses. We found that latent classes characterized by less than desired sexual activity (not simply low activity) were associated with higher subsequent loneliness among both wives and husbands, and this effect was strongest for those classes where couples viewed sex as important part of life. This pattern was robust to controls for physical health and marital quality. The findings add nuance to our understanding of the health consequences of sexual activity and point to the importance of evaluating behaviors in the context of exp ectations.

Abstract citation ID: igaf122.842

**DOES PET TOUCH INFLUENCE MENTAL HEALTH?**

Taylor Pope, and Patricia Thomas, *Purdue University, West Lafayette, Indiana, United States*

Older adults are often at risk of social isolation and mental health consequences. Touch between humans has established benefits in reducing feelings of anxiety, depression, and loneliness; however, touch between humans and their pets may also improve the mental health of older adults. Applying stress process theory, we examine the relationship between pet touch and self-reported mental health using data from the National Social Life, Health, and Aging Project (NSHAP), a nationally representative longitudinal, population-based household survey of older adults in the United States. Our sample includes 1,815 participants aged 57 to 85 (Mean age = 68) across two rounds of data collection (2006-2011). Using a lagged dependent variable model, we assess whether pet touch in Round 1 predicts changes in mental health by Round 2, while controlling for socio-demographic and health-related factors. Greater pet touch in Round 1 was associated with improved mental health in Round 2 (B = .022, 95% CI: .003, .041, p = .021). Next, we examined an interaction between frequency of pet touch and marital satisfaction among the married/partnered, to understand whether pet touch was more important for the mental health of those experiencing low marital satisfaction or whether these two resources combined for particularly better mental health. Pet touch was not beneficial for those with low marital satisfaction. However, individuals with higher marital satisfaction and frequent pet touch experienced better mental health in Round 2. Our findings suggest that pet touch could benefit mental health, improving the wellbeing of older adults.

### SESSION 2300 (SYMPOSIUM)

Abstract citation ID: igaf122.843

### INNOVATIONS IN HEALTHY AGING AROUND THE GLOBE

Chair: Chien-Ching Li
Co-Chair: Tung Sung Tseng
Discussant: Darren Liu

As populations continue to age, the demand for effective healthcare and social support strategies becomes increasingly urgent. This symposium presents five studies that highlight significant advancements in aging-related care and emphasize the importance of tailored interventions to address the needs of older adults in the United States, Taiwan, and Nigeria. The first study examines the impact of social function, fatigue, and smoking status on the well-being of American prostate cancer survivors, offering insights to improve patient care. The second study introduces an innovative decision-support tool designed to assist Chinese American family caregivers in navigating dementia care options effectively. The third study explores the shift to virtual healthcare during the COVID-19 pandemic and its impact on provider satisfaction, underscoring the need for enhanced virtual communication training to ensure high-quality care for older adults in the U.S. The fourth study identifies key barriers to diabetes management among older adults in Nigeria, highlighting the need for improved healthcare access and support systems. The fifth study investigates Dance Movement Therapy as a non-pharmacological intervention that enhances bodily awareness, emotional expression, and social connection, fostering engagement, resilience, and a holistic, person-centered approach to care. Overall, this symposium presents cutting-edge research addressing the complex challenges faced by older adults. It offers valuable perspectives on aging-related healthcare and social support, fostering discussions on innovative, culturally responsive interventions for diverse aging populations. International Comparisons of Healthy Aging Interest Group Sponsored Symposium

Abstract citation ID: igaf122.844

### A CULTURALLY TAILORED DECISION AID TO SUPPORT DEMENTIA CARE PLANNING AMONG CHINESE AMERICAN FAMILY CAREGIVERS

Chien-Ching Li[1], Tingfei Hu[1], Sharon Mei[2], Samantha Wu[2], and Olimpia Paun[1], *1. Rush University, Chicago, Illinois, United States, 2. University of Illinois Chicago, Chicago, Illinois, United States*

The Chinese American population, the largest Asian subgroup in the U.S., is aging rapidly. Consequently, Chinese American family caregivers of individuals with dementia face increasing challenges in healthcare planning. We have developed a culturally tailored, web-based decision aid to help caregivers make informed choices about home and long-term care. The tool provides essential information on dementia symptoms across stages, care options, costs, insurance coverage, and key decision factors. It also includes a Self-Assessment that generates a personalized summary to guide caregivers' decision-making process. In partnership with two Chinese community organizations in Chicago, we recruited a total of n = 10 Chinese American family caregivers of loved ones living with dementia or potential dementia symptoms for the study. Participants were asked to use the web-based DA tool and complete usability and acceptability surveys. Descriptive analysis was conducted to describe the usability and acceptability testing results. Results indicated that study participants were highly satisfied with the DA tool. Key feedback reported by the majority of participants includes: (1) the length and amount of information presented in the tool was just right, (2) this tool can provide sufficient information to make appropriate care decisions for loved ones with dementia., (3) various functions in this tool are well integrated, and (4) I will frequently use this tool to help me discuss dementia care plans and decisions with family and providers. Future efforts should focus on developing and implementing interventions that integrate the web-based decision-aid tool to better support Chinese American family caregivers.

Abstract citation ID: igaf122.845

### FACTORS ASSOCIATED WITH QUALITY OF LIFE AMONG OLDER PROSTATE CANCER SURVIVORS: NIH ALL OF US RESEARCH PROGRAM

Tung Sung Tseng[1], Masuma Mannan[2], Yu-Wen Chiu[3], Ya-Hsin Li[4], and Hui-Yi Lin[5], *1. Louisiana State University School of Public Health, New Orleans, Louisiana, United States, 2. Louisiana State University Health Sciences Center School of Public Health, New Orleans, Louisiana, United States, 3. Louisiana State University Health Sciences Center School of Public Health, Mew Orleans, Louisiana, United States, 4. Chung Shan Medical University, Taichung, Taichung, Taiwan (Republic of China), 5. Louisiana State University Health Sciences Center, New Orleans, Louisiana, United States*

Prostate cancer (PCa) is the first leading cause of cancer incidence and the 2nd leading cause of cancer deaths among American men in 2024. Most of the PCa patients have long-term survivorship, and the impact of the disease and quality of life (QoL) becomes a crucial aspect of well-being and overall survival. This study included 6,426 PCa patients, mean age 75 (SD = 8.4), from the NIH All of Us research program. Results showed that 558 (8.8%) PCa patients reported low QoL, 1,324 (20.9%) reported poor physical health and 534 (9.0%) reported poor mental health. The multivariable models indicate that African Americans, those not married or living with a partner, those with lower education levels, low income, current smokers, high fatigue levels, and poor social role reported lower QoL. Age (OR = 0.98, $p < 0.0001$), overweight (OR = 0.82 vs. under or normal weight, $p < 0.0001$), smoking status (OR = 1.34, $p < 0.040$ for current smokers), alcohol consumption (OR = 0.59, $p < 0.0001$ for high consumption), social function (OR = 6.64 for poor function, $p < 0.0001$), and fatigue (OR = 4.62 for high level, $p < 0.0001$) significantly impact on poor physical health. This study identified several factors (especially social function and fatigue level) that had a major impact on QoL and related health measures for PCa patients. These findings may help healthcare providers better understand PCa patients' overall well-being and help physicians and PCa patients make medical decisions accordingly. This consideration can improve PCa patient satisfaction and outcomes.

Abstract citation ID: igaf122.846

### VIRTUAL HEALTHCARE FOR HEALTHY AGING: COMMUNICATION SHIFTS & PROVIDER SATISFACTION IN THE PANDEMIC

Su-I Hou, *University of Central Florida, Orlando, Florida, United States*

Purpose: The COVID-19 pandemic significantly altered healthcare delivery, impacting provider interactions with older patients. This study examines shifts in communication methods and their effects on provider satisfaction. Methods: A survey including of 59 healthcare providers working with older patients in healthcare and long-term care settings analyzed communication modes before and during the pandemic. Open-ended questions explored provider experiences and recommendations for improving virtual interactions. Results: Before the pandemic, most communication occurred through in-office visits (41%), phone calls (10%), or a combination (32%), with high provider satisfaction (52% very satisfied, 39% satisfied). During the pandemic, over 70% of providers increased telemedicine and virtual communication, while in-office visits declined (27%), phone use increased (15%), email was introduced (5%), and mixed methods rose (44%). Provider satisfaction dropped significantly, with fewer than 25% very satisfied and 58% satisfied. Challenges included communication difficulties due to masks and face shields, as well as the need for better video systems and patient portal enrollment. While statistical significance was limited, qualitative findings emphasized provider skills and strong provider-patient relationships as key to effective care. Discussion: These findings highlight the need for further research with larger samples to assess the impact of virtual communication on provider satisfaction. The decline in satisfaction underscores the importance of training and support to optimize virtual healthcare interactions and ensure high-quality care for older clients.

Abstract citation ID: igaf122.847

### BARRIERS TO DIABETES SELF-MANAGEMENT AND FAMILY SUPPORT AMONG OLDER ADULTS IN RESOURCE-LIMITED SETTINGS IN NIGERIA

Jimmy Reyes, *Proteus Clinic, Des Moines, Iowa, United States*

Diabetes is a growing public health concern among older adults, particularly in resource-limited settings where access to healthcare is constrained. This study examined diabetes self-management and family support among older adults in Adamawa State, Nigeria, using a cross-sectional, descriptive design with 810 participants across five local government areas. Data were collected through validated surveys and collected using the Kobo Toolbox. Results indicated that older adults face significant barriers to diabetes management, including lack of health insurance (89%), high out-of-pocket expenses, and limited access to medication, with 74.5% of rural participants not taking Metformin. Dietary and lifestyle interventions remain insufficient, as only 21% of participants reported receiving guidance on low-fat diets, and 34% engaged in regular physical activity. Correlation analyses revealed significant associations between diabetes management and factors such as health insurance status ($p < .001$), adherence to prescribed medication ($p < .001$), and healthcare follow-up ($p < .001$). Regression analysis identified local government area, diabetes diagnosis duration, and healthcare access as key predictors of diabetes self-management ($R^2 = 0.221$, $p < .001$). The findings underscore the need for targeted interventions to improve diabetes care among older adults, particularly in rural communities. Strengthening healthcare access, financial support mechanisms, and culturally tailored education programs could enhance self-management and reduce diabetes-related complications in this vulnerable population.

Abstract citation ID: igaf122.848

### DANCE/MOVEMENT GROUP THERAPY FOR OLDER ADULTS WITH DEMENTIA

YinHui Hong[1], and Hao-Min Chen[2], *1. University of Taipei, Taipei, Taipei, Taiwan (Republic of China), 2. Texas A&M University, Central Texas, Killeen, Texas, United States*

Dementia significantly affects cognitive, psychosocial, and neuroendocrine functioning in older adults. Globally, 8% of individuals over 60 and 7.99% of those over 65 in Taiwan are diagnosed with dementia. This study examined the therapeutic process of older adults with dementia participating in a Dance/Movement Therapy (DMT) group at a Taiwanese hospital. The intervention aimed to enhance bodily awareness, emotional expression, and social connection through semi-structured movement activities. Participants attended 20 sessions, each lasting 1.5 hours, held twice weekly. A social constructionist and narrative inquiry approach was adopted. Using purposeful sampling, nine older adults with dementia of various stages (mean age = 71.8) and their caregivers were recruited. Data sources included video and audio recordings, progress notes, and field observations. Narrative analysis identified three key themes: (1) a shift from unconscious bodily presence to heightened awareness and support; (2) progression from rigid postures to fluid movement, and (3) transformation from isolation to social resonance and connection. Findings underscore the importance of leveraging participants' strengths, resilience, and inherent capacities, helping them rediscover abilities they may not have previously recognized. This empowerment fostered engagement, agency, and enhanced well-being. The key takeaway from this study is that DMT offers a promising non-pharmacological approach for individuals with dementia, emphasizing the importance of preserving functionality, fostering individual experiences, and enhancing relational connections. It encourages a paradigm shift in focus from diagnosis and decline to holistic care centered on people and their interactions, underscoring the transformative potential of the therapeutic process.

### SESSION 2305 (SYMPOSIUM)

Abstract citation ID: igaf122.849

### FROM EAST TO WEST: EFFECTS OF RESPITE CARE MODELS FOR LONG-TERM CARE

Chair: Hong Mi
Co-Chair: Fei Sun
Discussant: Joseph Gaugler

As populations around the world grow older, respite care has become an essential component of long-term care systems, providing temporary relief for family caregivers while supporting the well-being of individuals with care needs. This symposium examines respite care models in Eastern and Western contexts, exploring their effectiveness, policy frameworks, and cultural adaptations. By bringing together scholars, policymakers, and industry experts, the discussions aim to advance both research and practice in long-term care. A key focus will be on the health outcomes of respite care, including its effects on physical and mental well-being for both caregivers and those receiving care. Cross-cultural differences in intervention design and evaluation methods will also be explored. Another area of discussion will address the factors influencing respite care utilization, including economic conditions, policy support, and technological innovations, while identifying barriers to access and service delivery. Comparative analyses of respite care models will highlight how different long-term care insurance systems shape service provision and how cultural values influence program design and usage. Policy discussions will examine the effectiveness of government-led initiatives, incentive structures, and international best practices. Finally, the symposium will explore the role of social enterprises, digital platforms, and innovative business models in expanding and improving respite care services. By fostering interdisciplinary dialogue, this symposium aims to inform policy development and promote sustainable, person-centered respite care solutions worldwide.

Abstract citation ID: igaf122.850

### ASSOCIATES OF RESPITE CARE UTILIZATION AMONG FAMILY CAREGIVERS OF OLDER ADULTS IN THE US

Jun Chu[1], Joseph Svec[2], and Fei Sun[3], *1. University of Maryland, Baltimore County, Baltimore, Maryland, United States, 2. St. Joseph's University, Patchogue, New York, United States, 3. Michigan State University, East Lansing, Michigan, United States*

Respite care provides a relief to family caregivers by temporarily stepping in to provide care for older adults. Prior literature has shown that respite care services can improve both physical and mental health outcomes for caregivers and care recipients. Despite these benefits, respite utilization remains low, and little is known about the profiles of caregivers and care recipients who uses respite care. Using 2015, 2017 and 2021 National Health and Aging Trends Study (NHATS) and National Study of Caregiving (NSOC), we examined the prevalence and associates of respite usage among family caregivers of older adults. Our study sample represented weighted 15,329,939 caregivers, with average age around 59.14 years. 16.45% caregivers used respite care services. Findings revealed that caregivers with higher income and education levels were more likely to use respite. Those with higher emotional burdens, including feeling little joy in caregiving (6.0 percentage points more, $p<0.001$), and struggling emotionally with caregiving responsibilities (5.62 percentage points more, $p<0.001$), were more likely to use respite. Older care recipients with Medicaid coverage (5.8 percentage point more, $p<0.01$), dementia diagnosis (9.76 percentage points more, $p<0.001$) and more chronic conditions (5.23 percentage points more, $p=0.02$) were positively associated with respite usage of caregivers. Our results suggests the current respite care demand is driven by caregivers' desire to reduce caregiving burden, as well as the care recipient's Medicaid status. The results underscore the need for expanded respite care funding and policies to improve access for low-income caregivers and those with emotionally and financially challenging circumstances.

Abstract citation ID: igaf122.851

### RESPITE CARE IN ADULT DAY SERVICES: AN UPDATED SYSTEMATIC REVIEW FROM 2015 TO 2025

Yawen Li, *California State University, San Bernardino, San Bernardino, California, United States*

Adult day services provide structured activities and supervised care for individuals with developmental disabilities and chronic health conditions such as diabetes and dementia, offering respite for caregivers. Building upon previous systematic reviews, this review synthesizes global evidence from 2015 to 2025 on the impact of ADS on caregiver burden, well-being, economic implications, utilization barriers, and service quality. A systematic search of PubMed, Cochrane Library, Google Scholar, and other databases identified over 25 relevant studies, including randomized controlled trials, observational studies and qualitative research. Findings indicate that regular ADS use is associated with reduced caregiver stress, improved mood, and physiological stress reduction. On ADS days, caregivers report lower negative affect and higher positive affect, with potential protective effects on stress biomarkers. ADS participation is linked to delayed nursing home placement, translating into cost savings. However, utilization remains suboptimal due to financial constraints, limited awareness, caregiver reluctance, and service availability. Facilitators include caregiver education, culturally tailored programming, financial assistance, and high-quality staff engagement. While early Cochrane reviews found inconclusive effects due to limited randomized trial data, recent observational and longitudinal studies provide stronger evidence of ADS benefits. Cost-effectiveness analyses support ADS as a strategy to delay institutionalization and reduce health care expenses. To maximize benefits, efforts should focus on increasing awareness, affordability, and cultural accessibility, alongside continued research to enhance service quality and utilization. Expanding high-quality ADS programs, such as ADS Plus, and addressing financial and logistical barriers are essential for optimizing respite care effectiveness and supporting caregivers.

Abstract citation ID: igaf122.852

### A QUALITATIVE STUDY ON BARRIERS TO HOSPITAL AT HOME

Panpan Wang, Ying Qin, Mengting Liu, and Changqing Sun, *Zhengzhou University, Zhengzhou, Henan, China (People's Republic)*

As the process of population aging accelerates and the pressure on family caregiving continues to increase in China, building a comprehensive service system that can both meet patients' home healthcare needs and effectively alleviate the burden on family caregivers has become an urgent task. Hospital at Home, as an important model of integrated healthcare and eldercare, not only provide continuous home medical care for those with mobility impairments or chronic diseases but also help alleviate the strain on healthcare resources to some extent. However, despite the gradual promotion of this service model across various regions, its implementation is still constrained by multiple factors. Following the concept mapping methodology, this study systematically explores the main barriers to the promotion of Hospital at Home through in-depth interviews with the medical professionals of different community medical institution. The study first classifies the identified barrier factors and uses expert scoring methods to assess the priority and feasibility of addressing each factor. Next, through multidimensional scaling and hierarchical cluster analysis, a concept map is constructed to analyze the relationships between the various factors. Finally, based on this analysis, the study explores the integration mechanism of respite care, aiming to achieve a complementary and collaborative relationship between medical support and social services. The theoretical significance of this study lies in two aspects: on the one hand, it provides empirical evidence to improve the Hospital at Home system, and on the other, it offers theoretical guidance for promoting the implementation of respite care services in primary healthcare institutions.

Abstract citation ID: igaf122.853

### INTERNATIONAL EXPERIENCES IN RESPITE CARE AND INSIGHTS FOR CHINA: A SCOPING REVIEW BASED ON HEALTH SYSTEM FRAMEWORK

Yuxuan Ma[1], Ye-Fan Glavin[2], Zhen Wu[1], and Linlin Hu[1], *1. Chinese Academy of Medical Sciences & Peking Union Medical College, Dongcheng District, Beijing, China (People's Republic), 2. Case Western Reserve University, Cleveland, Ohio, United States*

Background: As China faces the rapid aging challenge, integrating global best practices into respite care systems is of great urgency. Methods: A systematic review was conducted using "Respite Care" and the four-dimension framework of Health System (service delivery, financing and payment, resource supply and regulation) across PubMed, CNKI, and government portals through February 20, 2025. From 483 records, 23 studies were selected and analyzed. Results: Analyses revealed diverse respite care models in Europe, North America, Australia. Service delivery: The U.S. NFCSP offers adult day care and emergency respite, Denmark's "All In One" flexible home visits, and employment support initiatives in the UK and Sweden. Financing and payment: Australia's RRC caps costs under federal funding, Germany splits insurance costs between employers and employees, while the U.S. relies on federal and state funding. Resource supply: In terms of workforce, Canada's PRISMA program integrates community-based respite care via case managers, while the U.S. PACE model deploys multidisciplinary teams. Regulation: The UK's Care Act and the U.S. Lifespan Respite Care Act ensure quality and accessibility. Implications: China's pilot programs in Beijing and Hangzhou suggest future priorities: scaling community respite centers with diversified services; building professional-informal caregiver networks; expanding long-term care insurance coverage through public-private financing; and establishing quality standards with third-party evaluations. Conclusion: International experiences emphasize four-dimension system synergy for respite care. China's path forward lies in adapting these insights to its familial care traditions, fostering a government-led, market-engaged, and socially supported respite care model to cope with the surging wave of population aging.

Abstract citation ID: igaf122.854

### WHAT ENABLES TRUE "RESPITE"? MEDIATION PATHWAYS OF CAREGIVER WELL-BEING AND SUPPLY-DEMAND MATCHING IN RESPITE CARE

Chen Chen[1], Xiuting Cai[2], Guoyong Ma[3], and Hong Mi[4], 1. School of Public Administration, Zhejiang University, Hangzhou, Zhejiang, China (People's Republic), 2. College of Economics and Management, Northeast Forestry University, Harbin City, Heilongjiang, China (People's Republic), 3. Northeast Forestry University, Harbin, Heilongjiang, China (People's Republic), 4. Zhejiang University, Hangzhou, Zhejiang, China (People's Republic)

The effectiveness of respite care, as an external support mechanism within family elderly care systems, not only directly impacts the multidimensional well-being of older individuals and their family caregivers but also bears significant relevance to regional social security and the long-term stability of population development. By deconstructing the core connotation of "respite"—encompassing the dimensions of "service utilization intensity" and "respite capacity"—this study empirically verifies the significant enhancement of caregiver well-being through respite effects, while controlling for heterogeneity in prior research conclusions. Notably, improvements in caregivers' well-being can synchronously optimize elderly care quality through intergenerational interaction mechanisms, thereby establishing a bidirectional virtuous cycle. Through deconstructing caregiver well-being into three dimensions—positive affect, negative emotions, and abusive tendencies—path analysis reveals that caregivers' respite capacity exerts significant effects across all three well-being indicators, with systematic variations in directional impact and magnitude. Besides, variables such as caregivers' health status, familial support pressures, intergenerational relationship quality, economic burden levels, and older adults' dependence on daily activities significantly influence well-being dimensions. Mediation effect analyses further demonstrate that intrinsic motivation mechanisms partially mediate the pathways through which familial support and parental support affect positive affect, while resource constraint mechanisms significantly mediate the transmission of economic pressures and daily activity dependence to negative emotions and abusive tendencies. Crucially, structural differences exist between the pathways of respite service utilization intensity and respite capacity: the former only significantly enhances positive affect, whereas its effects on mitigating negative emotions and abusive tendencies lack statistical significance, indicating functional limitations in current service models.

### SESSION 2310 (SYMPOSIUM)

Abstract citation ID: igaf122.855

### FROM CONCEPT TO COMPLETION: STRATEGIES FOR ENGAGING OLDER ADULTS AND COMMUNITIES IN BEHAVIORAL CLINICAL TRIALS

Chair: Ryan Mace
Discussant: Kathi Heffner

Behavioral clinical trials increasingly emphasize community engagement to enhance recruitment, retention, and intervention effectiveness, particularly for underserved populations. However, translating community partnerships into successful trials presents challenges requiring innovative approaches. This symposium aligns with the GSA 2025 theme, "Innovative Horizons in Gerontology," by showcasing strategies for integrating older adult communities into behavioral clinical trials, from initial planning to large-scale implementation. Presenter 1 will discuss the process of engaging the Massachusetts Councils on Aging (MCOA; 350+ senior centers) in the development of a comparative effectiveness trial of Cognitive Behavioral Therapy with Social Enhancement (CBT-SOUL) versus social prescribing to reduce loneliness and social isolation in low-income and minoritized older adults. Presenter 2 will examine strategies for community partnership with MCOA from planning through execution of a feasibility RCT testing My Healthy Brain, a mindfulness-based dementia prevention intervention for older adults with subjective cognitive decline, supported by an NIA K23. Presenter 3 will present community engagement strategies for the Hoping & Coping project, an NCCIH K23 and RCMAR study culturally tailoring and testing the feasibility of mindfulness-based cognitive therapy (MBCT) for Black older adults with chronic pain, depression, and early cognitive decline. Presenter 4 will discuss the transition from an NCCIH R61 feasibility trial to an R33 effectiveness trial of a mind-body walking group intervention delivered in community clinics for English- and Spanish-speaking adults with chronic pain. The Discussant will synthesize these presentations, highlighting methodological innovations in community engagement and the role of behavioral trials in advancing gerontology research. Behavioral Interventions for Older Adults Interest Group Sponsored Symposium

Abstract citation ID: igaf122.856

### UNITING COUNCILS ON AGING AND RESEARCH TEAMS: A COLLABORATIVE APPROACH TO BRAIN HEALTH AND DEMENTIA PREVENTION

Ryan Mace[1], Makenna Law[1], Elizabeth Connell[2], Madeline Noonan[2], Corinne White[3], and Ana-Maria Vranceanu[1], *1. Massachusetts General Hospital, Boston, Massachusetts, United States, 2. Massachusetts Councils on Aging, Franklin, Massachusetts, United States, 3. Age Strong Commission, Boston, Massachusetts, United States*

Older adults with subjective cognitive decline (SCD) face elevated dementia risk, yet preventive lifestyle trials frequently encounter enrollment barriers, including low awareness, limited trust, and accessibility challenges. Senior centers are ideal partners to address these barriers, given their established roles as trusted community hubs offering health promotion, social engagement, and educational programs for older adults. My Healthy Brain (MHB), an NIA K23 Stage 1b RCT, evaluated the feasibility of a mindfulness-based group intervention targeting lifestyle dementia risk factors for older adults with SCD. MHB collaborated with +350 senior centers through the Massachusetts Councils on Aging (MCOA) to enhance recruitment, diversify enrollment, and improve intervention accessibility. Engagement strategies included co-creation of trial procedures with MCOA leadership and local senior center staff through planning meetings and mentor involvement; community brain health presentations (11 events; 22 hours total); early ethics and regulatory approval from the community partner; presence at community events without enrollment pressure; and recruiting through local champions and peer ambassadors. Inclusive practices involved parallel clinical groups for individuals ineligible for trial participation and a participant "reunion" post-trial for feedback and dissemination. MCOA-affiliated channels generated over one-third (35%) of trial enrollment. All enrolled participants (N = 60) completed the intervention, demonstrating high feasibility and acceptability. The community-academic partnership enhanced enrollment diversity, improved community trust, and catalyzed new initiatives (e.g., PCORI submission to reduce loneliness in underserved older adults). This collaboration advances a concept-to-completion framework for dementia prevention trials, offering a replicable and sustainable blueprint for engaging community organizations and older adults in gerontological research.

Abstract citation ID: igaf122.857

### DEVELOPING, IMPLEMENTING AND SUSTAINING COMMUNITY ENGAGED RESEARCH WITHIN AN UNDERSERVED COMMUNITY CLINIC

Ana-Maria Vranceanu[1], Kate McDermott[2], Courtney Kilduff[2], Clara Vanderheide[2], Yoojee Kim[2], Natalia Giraldo-Santiago[2], Jonathan Greenberg[2], and Christine Ritchie[1], *1. Massachusetts General Hospital, Boston, Massachusetts, United States, 2. Massachusetts General Hospital Center for Health Outcomes and Interdisciplinary Research, Boston, Massachusetts, United States*

Chronic musculoskeletal pain (pain > 3-6 months) is prevalent, costly, challenging to treat and a national priority area. Older adults have the highest rates of chronic pain, and these are expected to increase over the next three decades. Mounting evidence suggests that older adults with chronic pain from disadvantaged populations (e.g., lower social and economic resources, racial and ethnic minorities) are at the highest risk, but underserved community clinics where these older adults seek care, lack evidence based nonpharmacological pain treatments tailored to their needs. This presentation will describe the process of co-design, testing and implementation of a mind-body activity program tailored for the unique needs of these older adults with chronic pain, through community engaged methods that included a partnership with primary care doctors, nurses, medical interpreters, leadership and patients within an underserved community clinic. We will specifically focus on: 1) strategies used to establish a successful partnership (e.g., identifying key stakeholders, building trust, and shared decision-making); 2) conducting community needs assessments (e.g., listening sessions, surveys and focus groups); 3) utilizing cultural and linguistic adaptations; 4) integrating research into the clinical workflow (e.g., minimizing burden, flexible scheduling); 5) leveraging clinic providers (e.g., nurses, social workers, psychologists); 6) ongoing feedback from stakeholders for optimization of procedures; 7) co-creating dissemination strategies (accessible results sharing at multiple time-points, celebrating milestones); 8) ensuring mutual benefit (capacity building, resource sharing); 9) maintaining long term relationships (sustainability planning through attention to intervention cost, billing and patient co-pays) and 10) ethical considerations (transparency and reciprocity).

Abstract citation ID: igaf122.858

### METHODS TO ENGAGE COMMUNITY PARTNERS FOR RESEARCH TO INCREASE SOCIAL CONNECTION AMONG AT-RISK OLDER ADULTS

Jin hui Joo[1], Ryan Mace[1], Rebekka Lee[2], Namkee Choi[3], Evan Plys[1], Nancy Donovan[4], and Joseph Locascio[1], *1. Massachusetts General Hospital, Boston, Massachusetts,*

*United States, 2. Harvard University, Cambridge, Massachusetts, United States, 3. The University of Texas at Austin, Austin, Texas, United States, 4. Harvard Medical School, Boston, Massachusetts, United States*

Social isolation and loneliness are critical public health issues in the U.S. and worldwide, with studies reporting a prevalence ranging from 22% to 61% of adults who feel lonely. Loneliness puts a person at risk for multiple medical and mental health problems such as cardiovascular disease, depression, diabetes, dementia as well as increased the risk of all cause mortality. Systematic reviews have concluded that established interventions, cognitive behavioral therapy (CBT, addressing unhelpful social cognitions and behaviors) and Social Prescribing (SP, linking to non-clinical community-based resources). We partnered with the Massachusetts Council on Aging and the Cambridge Housing Authority to develop a comparative effectiveness RCT PCORI proposal of these two interventions. In the process, we used multiple strategies to engage our community partners. These strategies included: ongoing engagement over 12 months, numerous in person and virtual listening sessions with community members ranging from older adults, organization staff and leaders, and scientific experts, attention to building capacity for research through knowledge exchange, built in processes and organizational structure to integrate fully various community partners with complementary expertise and experience, and finally ongoing assessment of engagement throughout the project. Numerous discussions and listening sessions were held with elders, leaders, and frontline staff at senior centers and public housing organizations. The result was bidirectional knowledge about CBO (community-based organization) priorities, needs, resources and processes "on the ground" as well as research principles and process that shaped the research design and established an ongoing foundation of trust and rapport.

Abstract citation ID: igaf122.859

### CULTURALLY ADAPTING MINDFULNESS BASED COGNITIVE THERAPY FOR THE CHRONIC PAIN RELATED NEEDS OF OLDER BLACK ADULTS

Tony Pham, *Massachusetts General Hospital, Boston, Massachusetts, United States*

Up to 78% of older Black individuals experience chronic musculoskeletal pain. Among older Black individuals, chronic pain co-occurs with depression and early cognitive decline, and these co-morbidities worsen physical function. Addressing chronic pain and its co-morbidities among older Black adults is a public health priority. We utilized the Community Engagement (CE) Studio Toolkit to examine (1) barriers and facilitators to participation in Mindfulness-Based Cognitive Therapy (MBCT) and (2) perceptions of MBCT skills. We facilitated facilitated CE Studio focus groups among older Black individuals with chronic pain and depression (n = 31 across 6 groups). We thematically analyzed the CE Studio focus group transcripts using a hybrid deductive–inductive approach. Based on our analyses, five themes emerged about how MBCT should be culturally adapted for the chronic pain, depression, and early cognitive decline needs of older Black individuals. CE Studio members suggested that MBCT (1) address relevant socio-cultural issues; (2) include coping strategies already accepted by the Black community; (3) reducing the access barriers to MBCT by offering it for a less expensive stipend and in local group settings; (4) focus on providers who are sensitive to the older black community's cultural, spiritual, and sociological needs (e.g., by expanding the definition of MBCT instructors to include lay instructors; and (5) abbreviating MBCT's duration from 2- to 1-hour sessions. MBCT, culturally tailored to address the unique needs of older Black adults in the community, may be an effective and efficient solution to improve physical, emotional, and cognitive outcomes among this underserved population.

### SESSION 2315 (SYMPOSIUM)

Abstract citation ID: igaf122.860

### STRENGTHENING PERSONAL CAPACITY OF OLDER ADULTS IN CULTURALLY DIVERSE CONTEXT

Chair: Daniel W. L. Lai

The critical interplay of cultural diversity, digital access, lifelong learning, and personal capacity is a key focus, particularly for older adults in multicultural settings. These complex factors across diverse landscapes demand exploration to address challenges and enhance well-being. This symposium presents five studies examining personal capacity, social resilience, mental health, digital inclusion, and community empowerment among aging populations, spotlighting South Asian elders in Hong Kong, racialized immigrants in Canada, older adults in China, vulnerable seniors in Hong Kong's digital context, and older Chinese immigrants in Canada. These studies underscore the need for culturally congruent, tech-savvy, and education-driven interventions. They highlight challenges in social connectivity, community engagement, mental health, digital literacy, and discrimination, alongside the power of intergenerational ties and learning. Critical elements like cross-cultural understanding, resilience depth, access to community, digital, and educational resources, family dynamics, and unmet care needs significantly shape older adults' lives. The research strongly supports culturally attuned, digitally inclusive, and learning-focused policies and practices. It emphasizes empowerment, resilience-building, mental health support, digital capacity enhancement, and community advocacy tailored to diverse identities and needs. These findings are vital for crafting targeted interventions, policies, and frameworks to overcome unique barriers faced by ageing individuals in culturally diverse, digitally advancing, and socially evolving contexts worldwide. International Aging and Migration Interest Group Sponsored Symposium

Abstract citation ID: igaf122.861

### ENHANCING CROSS-CULTURAL INTERGENERATIONAL CAPACITY IN SOCIAL CAPITAL

Daniel W. L. Lai[1], Hamza Saghir Aslam[2], Hazy H.Y. Lee[3], and Alice C. Wong[3], *1. Hong Kong Baptist University, Kowloon, China (People's Republic), 2. Hong Kong Baptist University Department of Sports and Health Sciences, Kowloon, China (People's Republic), 3. Hong Kong Baptist University, Kowloon, Hong Kong, China (People's Republic)*

This research presents the impactful findings of the "Enhancing Cross-Cultural Intergenerational Capacity in Social Capital"

project, which successfully connected university students with older South Asians in Hong Kong, addressing critical issues of social isolation and cultural misunderstanding. Older South Asians often encounter barriers to community engagement and resource access. This initiative aimed to enhance students' cultural competency while empowering older adults, fostering stronger community ties and promoting inclusivity. The project sought to cultivate cross-cultural communication skills among students, facilitate meaningful intergenerational interactions, and co-create culturally relevant services tailored to the unique needs of older South Asians. Through cultural competency training and collaborative co-creation workshops, students worked alongside community partners and older adults to design services that resonate with the community. Engaging over 50 participants, the project led to transformative outcomes: participants reported a 30% increase in cultural understanding and improved social connectivity, while students experienced a 40% boost in confidence in their service delivery. Qualitative feedback highlighted powerful personal stories of connection and growth, illustrating the profound impact on both students and older adults. These findings underscore the essential role of inclusive service-learning initiatives in fostering social capital and intergenerational solidarity, offering valuable insights for future community engagement practices and research.

Abstract citation ID: igaf122.862

### PROMOTING SOCIAL RESILIENCE FOR RACIALIZED OLDER IMMIGRANTS IN CANADA: STAKEHOLDER ENGAGEMENT PROJECT FINDINGS

Lun Li, *MacEwan University, Edmonton, Alberta, Canada*

The Canadian population is increasingly aging and diversifying. The proportion of racialized older immigrants among older Canadians is projected to be about 25 percent after 2030. However, Racialized older immigrants experience greater social isolation and loneliness when compared to their Canada-born counterparts. Social resilience, understood as the maintenance of positive social relationships and interactions, is essential to reduce social isolation and loneliness. Thus, this study explores the strategies to promote social resilience for racialized old immigrants in Canada. A stakeholder engagement project was conducted in September 2024, including five focus groups with Chinese, Southeast Asia, South Asia, Black and Muslim communities in a metropolitan city in western Canada. Stakeholders in each focus group included community-based racialized older immigrants, service providers to older immigrants, and scholars in immigration studies. Four main groups of strategies were identified from the focus group discussion, including 1) Empowering racialized older immigrants through various learning programs and volunteering opportunities, 2) Developing awareness of the situation of racialized older immigrants within the family, 3) Increasing community-based programs, such as intergenerational projects and neighborhood activities, to enable social interaction and participation for older immigrants, and 4) Advocate for society-level changes (e.g., inclusiveness, public fundings) to support racialized older immigrants. Findings of the project reveal the urgent need to promote social resilience for racialized older immigrants. Multiple levels of strategies were proposed and discussed, with an emphasis on resilience from the society level. The project supports the idea of "social resilience is the resilience of society.

Abstract citation ID: igaf122.863

### ENHANCING DIGITAL CAPACITY OF VULNERABLE OLDER ADULTS IN HONG KONG

Xue Bai, Youjuan Zhang, and Shuai Zhou, *The Hong Kong Polytechnic University, Hong Kong, China (People's Republic)*

The global digital revolution has reshaped society, yet older adults, particularly vulnerable ones, struggle to adapt, lagging behind due to limited access and skills. In Hong Kong, where the aging population is projected to rise from 1.45 million in 2021 to 2.74 million by 2046 (20.5% to 36.0%), and despite a leading ICT development index, a triple digital divide—access, capability, and outcomes—persists. Research shows older adults face secondary and tertiary divides, with inadequate digital literacy hampering technology use, a gap widened during the pandemic when digital reliance surged unevenly. Strengthening their digital capacity is vital to reduce loneliness, enhance memory, and boost well-being. The "Evergreen Digital Fuel Station—Fun Fun Needs You" project, funded by the Social Innovation and Entrepreneurship Development Fund and hosted by The Hong Kong Polytechnic University's Centre for Gerontology and Family Studies, addresses this through a community-based approach. This initiative promotes intergenerational inclusion via diverse training, including three-tiered, six-module courses, consultation points, and post-training activities like service and documentary production. Integrating the PERMA model, it fosters positive engagement, peer support, and lifelong learning. Evaluations reveal improved digital literacy, reduced anxiety, and enhanced mental health among participants—predominantly female retirees with moderate education—especially in the positive education group. Qualitative feedback highlights increased autonomy, shifted stereotypes, and stronger intergenerational bonds. This scalable model narrows the digital divide, empowers older adults, and fosters a cohesive, age-friendly society.

Abstract citation ID: igaf122.864

### LIFELONG LEARNING AND COMMUNITY EMPOWERMENT AMONG OLDER CHINESE IMMIGRANTS IN CANADA

Weiguo Zhang, *University of Toronto, Toronto, Ontario, Canada*

Lifelong learning is essential for encouraging social inclusion, resilience, and empowerment among older adults, especially in immigrant communities. The Chinese Age-well Research and Education (CARE) initiative tackles key challenges faced by older Chinese immigrants in Canada through several projects. The Intergenerational Dialogue on Elder Abuse project builds awareness, provides emotional support, and strengthens ties between generations to prevent older adult's mistreatment. The Intergenerational and Intercultural Dialogue to Fight Racism project teaches older Chinese immigrants about systemic racism, how it connects with ageism, and ways to respond to discrimination. CARE also supports digital literacy and storytelling, giving older adults tools to share their stories and connect with wider social issues. These efforts show how working across generations and offering focused education help older Chinese immigrants address social challenges, create strong support networks, and take part in community advocacy. Many older immigrants deal with language difficulties, cultural gaps, and struggles to recognize or handle discrimination, making accessible and culturally sensitive learning vital. Through research, education, and community collaboration, CARE uses lifelong learning as a means of social change, emphasizing the need for inclusive policies and programs to support the well-being and active involvement of older immigrant.

### SESSION 2320 (SYMPOSIUM)

Abstract citation ID: igaf122.865

### RECENT INVESTIGATIONS IN MEDICAL AID IN DYING: AWARENESS, PREFERENCES, POLICIES, AND CLINICAL PRACTICE

Chair: Samuel Nemeth
Discussant: Elissa Kozlov

Medical Aid in Dying (MAID) is an increasingly available, legal end-of-life (EOL) option for Americans who meet eligibility criteria (less than 6 months prognosis, aged 18+, have capacity to consent to end of life medical care). Data suggests that while many in the US are unaware of MAID laws, there is considerable interest in potentially pursuing MAID if diagnosed with a terminal illness in the future. However, empirical studies of MAID at the macro and micro levels remains scarce. To that end, this symposium presents a collection of new insights on MAID. Nemeth, using data from a cross-sectional online survey, examines the potential demographic, belief, and experiences predictors of preference for future MAID use. Kozlov expands this discussion by analyzing these predictors and their relationships to US and State level certainty of knowledge of MAID legality. Phillips and Kumar explore and analyze hospice polices on MAID throughout the US. In the first of two studies, Becker, using cross-sectional survey data, examines hospice clinician in-person presence during patient MAID usage. In the second study, Becker continues this line of work by examining the timing (e.g., during self-administration) of clinicians in the room during patient MAID usage. Dr. Kozlov (discussant) will provide insights into current MAID policies, implications of the present studies, and directions for future research. These studies advance our understanding of MAID in the US by using a variety of data sources and populations, highlighting the need for and importance of unbiased, rigorous, empirical MAID research. Hospice, Palliative and End-of-Life Care Interest Group Sponsored Symposium

Abstract citation ID: igaf122.866

### FACTORS ASSOCIATED WITH CONSIDERATION OF FUTURE PREFERENCE FOR MEDICAL AID IN DYING IN THE U.S.

Samuel Nemeth[1], Elizabeth Luth[2], and Elissa Kozlov[3], *1. Purdue University, West Lafayette, Indiana, United States, 2. Rutgers University, New Brunswick, New Jersey, United States, 3. Rutgers School of Public Health, Piscataway, New Jersey, United States*

Medical Aid in Dying (MAID) is a legal medical option for terminally ill patients in 10 U.S. states and Washington DC. Several other states are considering adopting MAID laws. However, it remains unclear what factors predict future preference for using MAID. Using a national convenience sample of 3,221 U.S adults and multinomial logistic regression models, we examine demographic, belief, knowledge, and experience predictors of potential future MAID usage (definitely not [reference], probably not, not sure, probably, definitely). Net of all predictors, Catholicism and Protestantism were both significantly and robustly associated with lowered odds of considering future MAID usage compared to Agnostic/Atheist individuals. For example, Catholicism was associated with 74% lower odds while Protestantism was associated with 79% lower odds of selecting "definitely" (Adjusted Odds Ratio (AOR)=0.26 [95% CI: 0.10, 0.66] and AOR=0.21 [CI: 0.08, 0.53], respectively). Additionally, individuals who believe MAID should not be legal (vs. not sure, AOR=0.48, CI: 0.25, 0.95) were less likely to select "definitely." Conversely, individuals who were older (AOR=1.01 CI: 1.00, 1.03), believe MAID is moral (vs. not sure, AOR=12.09, CI: 6.23, 23.46) and who had ever euthanized a pet (AOR=2.44 CI: 1.71, 3.48) were more likely to say they definitely would consider pursuing MAID in the future. These findings suggest that preference for considering future MAID usage varies across demographic, belief, and experience factors. While future preferences are not completely indicative of actual behaviors, this study provides insight into possible end-of-life treatment preferences.

Abstract citation ID: igaf122.867

### FACTORS ASSOCIATED WITH CERTAINTY REGARDING MEDICAL AID IN DYING LAWS IN A NATIONAL SAMPLE OF U.S. ADULTS

Elissa Kozlov[1], Samuel Nemeth[2], and Elizabeth Luth[3], *1. Rutgers School of Public Health, Piscataway, New Jersey, United States, 2. Purdue University, West Lafayette, Indiana, United States, 3. Rutgers University, New Brunswick, New Jersey, United States*

Medical Aid in Dying (MAID) is a legal medical option for terminally ill patients in a growing number of U.S. states, covering over one-fifth of the population. Using a national convenience sample of 3,221 U.S. adults in states where MAID is legal ($n = 2,159$) and not legal ($n = 1,062$), this study examines predictors of certainty regarding MAID legality (correctly identifying legality, incorrectly identifying legality, or not knowing). Controlling for sociodemographic factors and attitudes toward MAID, lower socioeconomic status was significantly associated with lower odds of correctly identifying MAID legality nationwide. Compared to financially "comfortable" individuals, those with "enough" (AOR: 0.71, $p < 0.001$) or "not enough" (AOR: 0.61, $p < 0.001$) to make ends meet had lower odds of correctly stating MAID is legal anywhere in the U.S. Similarly, those with some college (AOR: 0.75, $p < 0.01$) or a high school education or less (AOR: 0.66, $p < 0.001$) were less likely to correctly identify MAID legality. Being non-Hispanic Black (AOR: 0.62, $p < 0.05$), having a high school education or less (AOR: 0.52, $p < 0.001$), and reporting financial insecurity (AOR: 0.63, $p < 0.05$) were associated with incorrectly stating MAID was illegal in their state. In states where MAID is legal, longer duration of legality was associated with greater awareness (AOR: 1.06, $p < 0.001$). These findings suggest that certainty about MAID laws varies by socioeconomic and demographic factors, which may have implications for informed consent and patient-centered end-of-life decision-making.

Abstract citation ID: igaf122.868

### EXAMINING PUBLIC-FACING HOSPICE MEDICAL AID IN DYING PARTICIPATION POLICIES IN LEGALIZING US JURISDICTIONS

Kira Philips[1], Sanjana Kumar[2], Todd Becker[3], Elizabeth Luth[4], Veda Kota[5], Samuel Nemeth[6], Elissa Kozlov[5], and Paul Duberstein[4], *1. Brown University, Providence, Rhode Island, United States, 2. Brandeis University, Waltham, Massachusetts,*

*United States, 3. WashU Medicine, St. Louis, Missouri, United States, 4. Rutgers University, New Brunswick, New Jersey, United States, 5. Rutgers School of Public Health, Piscataway, New Jersey, United States, 6. Purdue University, West Lafayette, Indiana, United States*

Medical aid in dying (MAID) permits qualified individuals to self-administer a medication to hasten their death. Although currently legal for roughly 74 million Americans across 10 states and Washington, DC, little is known about hospice organizational policies toward participation. This study aimed to characterize the availability and content of public-facing hospice MAID participation policies across legalizing jurisdictions. We used 2024 Centers for Medicare & Medicaid Services' Hospice General Information data to identify all Medicare-certified hospices in legalizing jurisdictions. Random selection of 15.9% of California hospices, due to increased representation, and inclusion of all hospices from the remaining jurisdictions resulted in a sample of 724 hospices. We identified public-facing policy availability by searching sample hospices' websites. Policy content was independently extracted by two analysts using a researcher-constructed tool and subsequently coded as "yes," "no," or "unreported." We described policy availability and content via frequency and percentage. Results indicated that only 5.4% of hospice websites contained a public-facing MAID participation policy. Policy content across reporting hospices was highly variable. Whereas 69.2% reported on the provision of patient about local MAID laws, only 20.6% indicated having a protocol outlining patient MAID initiation. When reported, language was often vague, complicating discernment of how hospices formatively participated in MAID. For instance, 46.2% did not report whether or not employed providers could prescribe MAID medications. The lack of availability and specificity in these policies likely affects patients' informed decision making about hospice selection, which may then affect receipt of goal-concordant care. Greater organizational transparency is needed.

Abstract citation ID: igaf122.869

### INTERDISCIPLINARY HOSPICE CLINICIAN PRESENCE DURING PATIENT MEDICAL AID IN DYING SELF-ADMINISTRATION

Todd Becker[1], Denae Gerasta[2], Grant Yoder[3], Daniel Matlock[4], Elissa Kozlov[5], Stacy Fischer[6], and Karla Washington[7], *1. WashU Medicine, St. Louis, Missouri, United States, 2. University of Colorado Anschutz, Aurora, Colorado, United States, 3. University of Colorado School of Medicine, Aurora, Colorado, United States, 4. University of Colorado Anschutz Medical Campus, Aurora, Colorado, United States, 5. Rutgers School of Public Health, Piscataway, New Jersey, United States, 6. UCHealth University of Colorado Hospital (UCH), Aurora, Colorado, United States, 7. Washington University in St. Louis, St. Louis, Missouri, United States*

Many older adults using medical aid in dying (MAID) have reported a preference to have clinicians present while they self-administer medication to hasten death. However, limited data exist to characterize hospice clinician involvement during this time. This study aimed to examine correlates of hospice clinician presence during patient MAID self-administration. This secondary analysis used cross-sectional data from our earlier survey of hospice physicians, nurses, social workers, and chaplains. This convenience sample was recruited through national hospice and palliative care professional membership associations. We restricted the analytic sample to participants working for hospices servicing states where MAID is legal and permitting at least partial MAID participation (N = 100). We assessed personal, professional, organizational, and MAID-specific factors as correlates of ever having been present during patient MAID self-administration via a binary logistic regression model adjusted for small-sample bias. Results indicated that being a chaplain (vs. physician: OR = 5.29, p = .017) and working for a hospice permitting full MAID participation (vs. partial: OR = 4.84, p = .004) were each significantly associated with greater odds of being present during MAID self-administration. Findings represent the first exploration of factors associated with hospice clinician presence during patient MAID self-administration. Differences between chaplains and physicians may reflect discipline-specific skill sets corresponding to involvement at distinct points across the delivery of care. Conversely, hospices permitting full participation may be more amenable—or even encourage—clinician presence when requested. Future studies should examine how clinician roles and hospice policy shape patient perceptions of care receipt.

Abstract citation ID: igaf122.870

### MAPPING TRAJECTORIES OF HOSPICE CLINICIAN PRESENCE THROUGHOUT THE MEDICAL AID IN DYING PROCEDURE

Todd Becker[1], Karla Washington[2], Stacy Fischer[3], Elissa Kozlov[4], Daniel Matlock[5], Denae Gerasta[6], and Grant Yoder[7], *1. WashU Medicine, St. Louis, Missouri, United States, 2. Washington University in St. Louis, St. Louis, Missouri, United States, 3. UCHealth University of Colorado Hospital (UCH), Aurora, Colorado, United States, 4. Rutgers School of Public Health, Piscataway, New Jersey, United States, 5. University of Colorado Anschutz Medical Campus, Aurora, Colorado, United States, 6. University of Colorado Anschutz, Aurora, Colorado, United States, 7. University of Colorado School of Medicine, Aurora, Colorado, United States*

Many older patients have requested that a clinician be present during their medical aid in dying (MAID) procedure. Perceptions of organizational liability have led participating hospices to define the procedure as a sequence of key stages. Concerns that liability varies across stages has informed organizational restrictions regarding where in patient residences clinicians can be present. These "leave-the-room" policies have prompted dynamic—yet underexplored—patterns of clinician presence. This study aimed to map these trajectories. We used cross-sectional survey data from a convenience sample of hospice physicians, nurses, social workers, and chaplains. Eligible participants self-reported employment by a hospice that services states where MAID is legal and permits at least partial MAID participation (N = 100). Participants completed a matrix question inquiring about past presence. Rows indicated stages (during self-administration, after self-administration, after death). Columns indicated locations (in the same room, in the same residence but not the same room, never present). We consolidated responses to identify distinct trajectories, subsequently described via frequency distributions. Results revealed 13 distinct trajectories. The most common trajectories indicated never having been present (49%) and consistent presence in the room (24%). The 11 remaining trajectories reflected trends of increasing proximity to the patient (22%), increasing distance from the patient

(2%), increasing proximity followed by increasing distance (2%), and consistent presence in the residence (1%). These 13 trajectories indicate substantial heterogeneity. Trends culminating in closer proximity suggest that perceived liability during self-administration declines across subsequent stages. Future studies should explore experiences of these trajectories alongside perceived quality of care.

### SESSION 2325 (SYMPOSIUM)

Abstract citation ID: igaf122.871

### IMPROVING SOCIAL RELATIONSHIPS IN LONG-TERM CARE SETTINGS: INSIGHTS FOR INNOVATIVE STRATEGIES

Chair: E-Shien Chang
Co-Chair: Amy Roberts
Discussant: Noah Webster

Residents of long-term care (LTC) homes represent a unique population with significant needs for meaningful social connections. Building strong social connections with other residents and staff can assist residents in managing the challenges of communal living. In contrast, poor social interactions can seriously affect residents' quality of care and well-being. Despite the implications of vital social relationships for LTC residents, there has been less focus on understanding the full spectrum of social interactions, both from a resident-resident and resident-staff perspective, to guide intervention programming. In this symposium, we will discuss new insights for building meaningful social relationships across various LTC residential settings. Dr. Amy Roberts will describe existing practices, gaps, and training opportunities for improving social engagement and managing resident conflicts from the viewpoints of social services workers in U.S. nursing homes. Dr. E-Shien Chang will discuss findings from a qualitative study of nursing home clinical and non-clinical providers regarding predisposing factors and acute precipitants for race/ethnicity-related resident-to-resident aggression. Using a social network analysis approach, Dr. Rebecca Mauldin will explore negative social interactions found in social networks among residents of assisted living communities with data from a longitudinal survey. Dr. Elsie Yan will discuss the prevalence and risk factors of resident-initiated aggression towards staff and their impact on resident relationships based on a cohort study conducted in Hong Kong. Finally, Dr. Noah Webster, a policy and intervention expert in the area of social relations, will provide practice recommendations for prioritizing psycho-social needs and relation-based support tailored towards the LTC community.

Abstract citation ID: igaf122.872

### PREVENTING AND ADDRESSING INTERPERSONAL CONFLICTS AMONG RESIDENTS IN DIVERSE NURSING HOME SETTINGS

Amy Roberts[1], Lirisha Tuladhar[1], Veronica Barrios[1], Dongxia Sang[1], and E-Shien Chang[2], *1. Miami University, Oxford, Ohio, United States, 2. Weill Cornell Medicine, New York, New York, United States*

This study examines how nursing home staff can prevent and address interpersonal conflicts among residents in long-term care facilities. As these facilities increasingly serve diverse populations, understanding conflict dynamics is crucial for promoting a sense of belonging, acceptance, and respect. Interviews with twelve social services workers (with 1–25 years of experience) from nursing homes across the U.S. revealed that conflicts often stem from cultural misunderstandings and differences in social identities. Participants highlighted the importance of trauma-informed, person-centered care and suggested increased monitoring, early intervention, and environmental adjustments (e.g., changing roommates) as strategies to prevent or resolve resident-to-resident conflicts. Four facilities had formal inclusion programs with staff or resident leaders to help new residents adjust and socially integrate. While many described efforts to incorporate culturally appropriate activities, match roommates based on shared interests and preferences, and provide dementia training for staff, only one respondent reported participating in a program aimed at addressing unconscious biases among staff. Overall, respondents felt that there is limited training available for staff on how to help residents live harmoniously with others who differ from them. Factors contributing to resident conflicts included cultural differences (n = 9), cognitive impairments (n = 9), disruptive behavior (n = 7), race (n = 6), younger age (n = 5), religion (n = 5), political divisions (n = 2), gender identity (n = 1), and substance use (n = 1). The findings suggest a need for staff training on managing high-conflict situations and effectively engaging with residents from diverse backgrounds, using strategies such as active listening, motivational interviewing, and goal-setting.

Abstract citation ID: igaf122.873

### PREDISPOSING FACTORS AND ACUTE PRECIPITANTS FOR RACE/ETHNICITY-RELATED RESIDENT-TO-RESIDENT AGGRESSION

E-Shien Chang[1], Hannah Mason[1], Sara Czaja[1], Karl Pillemer[2], Mark Lachs[1], and Tony Rosen[3], *1. Weill Cornell Medicine, New York, New York, United States, 2. Cornell University, Ithaca, New York, United States, 3. Weill Cornell Medicine / New York-Presbyterian Hospital, New York, New York, United States*

Resident-to-resident aggression (RRA) is a prevalent form of mistreatment in long-term care facilities. Some episodes of RRA are motivated by racial or ethnic differences between residents, but little research has investigated this aspect of the phenomenon. Guided by a theory-informed perspective, we qualitatively explored the predisposing factors and acute precipitants of this race/ethnicity-related resident-to-resident aggression (RE-RRA), defined as RRA motivated by racial or ethnic differences or conflicts that would not have occurred between residents of the same race or ethnicity. We conducted focus groups (n = 9) and in-depth interviews (n = 12) with 81 clinical and non-clinical staff members at two nursing homes in New York. Qualitative data was coded using a thematic approach. We identified nine significant types of RE-RRA, ranging from physical violence and microaggression to racial avoidance. Consistent with previous research, we found 20 predisposing factors and 14 acute precipitants contributing to RRA overall that contributed to RE-RRA. Notably, 10 predisposing factors and 8 precipitants were uniquely drivers of RE-RRA. We synthesized these findings into a conceptual framework to illustrate how distinct factors operate across resident, staff, facility, and structural levels to cause RE-RRA. Our findings provide the preliminary foundation for developing necessary prevention and intervention programs and emphasize the need to consider distinct race/ethnicity-related dynamics in RRA prevention and intervention research. Also, this represents a critical area of focus to enhance the quality of care among racially and ethnically diverse long-term care residents.

Abstract citation ID: igaf122.874

**PREVALENCE AND RISK FACTORS OF RESIDENT TO STAFF AGGRESSION IN LONG TERM CARE FACILITIES IN HONG KONG**

Elsie Yan[1], Daniel W. L. Lai[2], Habib Chaudhury[3], Karl Pillemer[4], and Mark Lachs[5], *1. The Hong Kong Polytechnic University, Hong Kong, Hong Kong, China (People's Republic), 2. Hong Kong Baptist University, Hong Kong, China (People's Republic), 3. Simon Fraser University, Burnaby, British Columbia, Canada, 4. Cornell University, Ithaca, New York, United States, 5. Weill Cornell Medicine, New York, New York, United States*

Resident-to-staff aggression (RSA) is common in long-term care facilities. It is associated with adverse physical and psychological consequences for staff, deteriorates resident-staff relationships, and greater staff turnover intention. Drawing on a sample of 703 care workers from 70 long-term care facilities, this study sought to determine the prevalence and risk factors of RSA in Hong Kong. RSA is common in this sample: 97.6% reported verbal aggression, 10.7% physical assault, 8.5% sexual violence, 13.7% annoying behaviors. Logistic regression analyses were conducted to determine factors associated with physical assaults, sexual violence, and annoying behaviors, controlling for duration (minutes) and location (common area vs resident rooms) of RSA. Physical assaults was associated with perpetrator behavioral problems (OR = 1.08, p<.001), resident male gender (OR = 2.40, p<.05), dementia (OR = 21.87, p<.001), staff lack of experience in dementia care (OR = 17.59, p<.001), need to provide dementia care (OR = 15.89, p<.01), lack of training (OR = 10.06, p<.01, and perceived insufficient training (OR = 2.97, p<.01). Sexual violence was associated with perpetrator male gender (OR = 22.51, p<.001), staff younger age (OR=.93. p<.05) and female gender (OR=.14, p<.01). Annoying behaviors was associated with perpetrator behavioral problems (OR = 1.07, p<.001), younger age (OR=.94, p<.95), male gender (OR = 3.04, p<.01), dementia (OR = 2.31, p<.01), staff female gender (OR=.32, p<.01), lack of experience in dementia care (OR = 3.56, p<.05), needs to provide dementia care (OR = 12.31, p<.01), lack of training (OR = 11.52, p<.001), and perceived insufficient training (OR = 2.52, p<.01). Addressing resident behavioral problems and providing sufficient staff training may help prevent RSA

### SESSION 2330 (SYMPOSIUM)

Abstract citation ID: igaf122.875

**INNOVATIVE INTERGENERATIONAL INITIATIVES**

Chair: Tamar Shovali

Discussant: Lisa Borrero

This session highlights lessons learned, strategies for success, and setbacks to avoid when developing innovative and collaborative intergenerational initiatives. The first presentation examines the process and outcomes of a series of course-based intergenerational Death Cafes that brought together students, community members, and members of a university-affiliated Academy of Senior Professionals to discuss death and dying. The second presentation shares collaborative strategies for forming an interdisciplinary team to develop a 100th day of school evidence-based toolkit for pre-K-2 teachers to foster intergenerational understanding from an early age. The third presentation features the Sages and Scholars innovative program to promote age inclusivity that creates shared learning experiences between students and a university-affiliated Osher Lifelong Learning Institute. The fourth presentation explores bringing traditional students and older learners together to create, express, and learn together in a course titled Intergenerational Artistic Expression. The final presentation highlights an innovative model for intergenerational advocacy and partnership through the Intergenerational Community Health Collaborative, a program that connects residential older adults with students to discuss pressing community health issues. In addition to presenting multiple intergenerational initiatives using a variety of innovative approaches, this symposium session highlights the creativity and dynamism that intergenerational innovations can provide to foster meaningful connections. These presentations demonstrate expanding horizons to create inclusive spaces that foster contributions of people of all ages, encourage the bi-directional exchange of life experiences, and inspire innovative approaches to promoting connectedness across our increasingly diverse society. Intergenerational Learning, Research, and Community Engagement Interest Group Sponsored Symposium

Abstract citation ID: igaf122.876

**OPENING THE DIALOGUE ON DEATH: THE VALUE OF INTERGENERATIONAL DEATH CAFÉS**

Tamar Shovali, *Eckerd College, St. Petersburg, Florida, United States*

This project intentionally fostered open, honest conversations about mortality by building an intergenerational Death Café, recognizing that such spaces are not inherently intergenerational. Undergraduates in a Death and Dying course, community members, and members of our university-affiliated Academy of Senior Professionals joined a series of Death Cafés in 2023 and 2024, with an average of 43 participants ranging in age from 18 to 84 years. Attendees were surveyed following each session about their frequency of engagement in discussions about death and dying in their daily lives, their perceptions of the value of intergenerational Death Cafés, whether they learned something new about death and dying, and whether their views on death and dying changed as a result of attending. Average responses indicate consistently high value and learning across all sessions, with a 12.2% increase in participants responding affirmatively that their views on death and dying changed. Nearly half of attendees reported rarely discussing death and dying in their lives, though such conversations increased slightly from 33.3% after the first café to 58% after the third café. Qualitative results indicate that each café built upon the last, shifting from initial openness and comfort (café 1), to refining the dialogue process and balancing perspectives (café 2), to encouraging deeper personal action and longer-term comfort with the subject (café 3). Clearly, meaningful intergenerational conversations about mortality can foster fulfilling and deeply human connections. The process of preparing and planning cafés will be discussed in this presentation.

Abstract citation ID: igaf122.877

**COLLABORATING ON THE 100TH DAY OF SCHOOL: GERONTOLOGY, SOCIOLOGY, EDUCATION, AND SOCIAL WORK, OH MY!**

Cynthia Hancock[1], Tina Newsham[2], Daniel Alston[1], Lisa Borrero[3], Katherina Nikzad-Terhune[4], Sarah Tesar[1], and Elizabeth Fugate-Whitlock[2], *1. The University of North Carolina at Charlotte, Charlotte, North Carolina, United States, 2. The University of North Carolina at Wilmington,*

*Wilmington, North Carolina, United States, 3. University of Indianapolis, Indianapolis, Indiana, United States, 4. Northern Kentucky University, Highland Heights, Kentucky, United States*

Given that some celebrations of the 100th day of school involve having young children dressing up "like a 100-year-old," a gerontologist and a social worker with expertise in gerontology questioned the ageist ideas this activity could reinforce. Recognizing that children adopt beliefs about various social groups at an early age, we saw the 100th day of school as an opportunity to teach accurate and positive information about aging and older adulthood rather than perpetuate ingrained stereotypes. The small team recognized that additional expertise was needed and reached out to others, including a sociologist, other social workers with gerontology expertise, an expert in health and aging, and an elementary educator with expertise in diversity and inclusion. The interdisciplinary team has collaborated for over three years to provide pre-K-2 teachers with evidence-based educational materials related to aging (centenarians in particular). We created a toolkit about aging and ageism to give teachers options for celebrating the 100th day of school in a way that reinforces academic content while supporting age-inclusivity. In this presentation, we share how the team was formed, describe strategies we use for collaborating, and identify the benefits of working on an innovative interdisciplinary collaboration to help eliminate ageism.

Abstract citation ID: igaf122.878

### SAGES & SCHOLARS: CONNECTING OLLI MEMBERS AND ACADEMIC STUDENTS FOR INTERGENERATIONAL EXCHANGE

Tina Newsham[1], Amy Keith[2], and Jennifer Heber-Brown[2], *1. The University of North Carolina at Wilmington, Wilmington, North Carolina, United States, 2. Osher Lifelong Learning Institute, Wilmington, North Carolina, United States*

While the population is aging, higher education classrooms remain filled primarily with "traditionally-aged" students, which limits opportunities for exchange and shared learning experiences across generations. This limitation is detrimental to all students (and those who do not feel welcome or able to engage in higher ed classes) and is particularly unfortunate in gerontology classes where many students have limited connection with older adults outside their families. To foster intergenerational connection and exchange, gerontology students at the University of North Carolina Wilmington (UNCW) are partnered with members of the UNCW Osher Lifelong Learning Institute and asked to engage in a series of social activities in a program called Sages & Scholars. In this presentation, we share the history of the development of the Sages & Scholars program at UNCW and lessons learned through the first two years of implementation. We believe this program serves as a model for others seeking to implement innovative intergenerational initiatives on their campuses. Programs like Sages & Scholars foster meaningful connections across generations and contribute to age-inclusivity on college campuses.

Abstract citation ID: igaf122.879

### ESTABLISHING THE INTERGENERATIONAL COMMUNITY HEALTH COLLABORATIVE: AN INNOVATIVE PARTNERSHIP MODEL

Grace Savard[1], Maya Koffski[2], Azylen Lunak[2], and Fyzeen Ahmad[3], *1. University of Minnesota Twin Cities, Minneapolis, Minnesota, United States, 2. University of Minnesota School of Public Health, Minneapolis, Minnesota, United States, 3. University of Minnesota Department of Neuroscience, Minneapolis, Minnesota, United States*

Established in Fall 2023, the Intergenerational Community Health Collaborative (ICHC) is a partnership initiative between the University of Minnesota's Aging Studies Interdisciplinary Group—a GSA Student Chapter—and the Pillars of Prospect Park, a residential community for older adults located in Minneapolis, MN. The group meets monthly and includes over 35 members—University of Minnesota students and Pillars residents alike. Together, we explore Minnesota's most pressing societal issues through an intergenerational lens. Grounded in shared leadership between students and residents, we cultivate curiosity and engagement through discussions, hosting expert speakers, and participating in civic advocacy efforts. Members collectively brainstorm and vote on focus areas at the beginning of each semester, and a planning committee of students and residents coordinates meetings and activities throughout the academic year. Over the past two years, we have focused on healthy aging initiatives, food insecurity in the Twin Cities, bridging divides through constructive dialogue, and protection from scams and fraud. This group uniquely positions members to develop intergenerational relationships and share lived experiences while participating in practical learning. In an effort to inform other GSA Student Chapters who may be interested in enacting a program similar to ICHC, this presentation will feature lessons learned about shared intergenerational leadership, tools utilized to sustain engagement from students and residents, and specific implementation challenges to consider. Finally, we will highlight future directions and goals of ICHC, emphasizing the potential for innovative intergenerational partnership and elevated community impact.

### SESSION 2335 (SYMPOSIUM)

Abstract citation ID: igaf122.880

### LIVING ALONE WITH DEMENTIA: CHALLENGES, SUPPORT, AND PATHS FORWARD

Chair: Heather Menne
Discussant: Michael Lepore

Whether due to choice or circumstance, across the globe there are significant numbers of people living alone with dementia in the community. This symposium brings together scholars from the United States and the United Kingdom to explore the experiences of people living alone with dementia, highlighting their unmet needs, well-being, and daily challenges, while emphasizing the need for improved support, policy changes, and community responsiveness. Dr. Kate Singer highlights the increasing number of people living alone with dementia as represented in national surveys, and she presents the rates of people living alone with dementia who report feeling lonely every day or even most days of the week. Dr. Allison Gibson presents on the experiences of individuals living alone with dementia, underscoring their challenges with medication adherence, finances, emergency services, and fear of institutionalization, while also examining their social support, healthcare use, and interest in additional assistance to age in place. Mr. Matt Nelson outlines data from Older Americans Act (OAA) clients who are living alone with dementia, and he shares respondents' assessments of whether the OAA service helps them remain independent and at home. Dr. Linda Clare presents a scoping review of 150+ unique studies about people living alone with dementia; her results reveal significant unmet needs, social differences, and a lack of targeted support, while recommending policy and practice improvements in service responsiveness, community support, and research inclusivity. To conclude, Dr. Michael Lepore, who has supported policies, programs, and research on people living alone with dementia, offers discussion comments. Alzheimer's Disease and Related Dementias Interest Group Sponsored Symposium

Abstract citation ID: igaf122.881

### THE HIDDEN STRUGGLES OF PEOPLE LIVING ALONE WITH DEMENTIA: LONELINESS AND WELLBEING

Kate Singer, Matt Nelson, and Heather Menne, *Miami University, Oxford, Ohio, United States*

The percentage of people living with dementia (PLWD) who also live alone in community settings (i.e., private residences) varies across sources. Analyses of the 2015 National Health and Aging Trends Study (NHATS) found that 16% of people with probable dementia were living alone whereas more recent analyses of the 2022 Health and Retirement Study (HRS) found that 32.5% of people 65+ years old with probable dementia were living alone. Other examinations of PLWD and people living alone with dementia (PLAwD) in the United Kingdom suggest that these populations experience higher rates of loneliness compared to the general population of older adults. The present study uses the 2022 wave of the NHATS to determine how many PLAWD are present in the sample and examines the loneliness rates among PLWD and PLAwD. The data reveals 36.0% of adults 65+ with probable dementia are living alone. Amongst PLAwD in the sample, 4.3% report feeling lonely everyday and 13.1% most days of the week. Compared to PLWD, these rates are higher, as 3.7% of PLWD report feeling lonely everyday and 3.3% most days of the week. Implications of these results are two-fold. First, there is a consistent trend that there are more people with probable dementia living alone, which begs for further programs and services for this population. Second, these findings reinforce the need to investigate ways to promote wellbeing among community-dwelling PLWD who may not have access to care partners.

Abstract citation ID: igaf122.882

### AGING ALONE WITH DEMENTIA: NAVIGATING CHALLENGES AND SUPPORT NEEDS

Allison Gibson[1], Dimitra Tziarli[2], Leslie McClure[2], Max Zubatsky[3], and Marla Berg-Weger[4], *1. Saint Louis University, Saint Louis University, Missouri, United States, 2. Saint Louis University, St. Louis, Missouri, United States, 3. Saint Lewis University, Saint Louis, Missouri, United States, 4. Saint Louis University - - St Louis, MO, St. Louis, Missouri, United States*

More than one-third of older adults live alone in the U.S., and the proportion increases with age. Fueling this phenomenon is a preference for the independence and privacy that living alone affords, a desire to remain in one's private residence in later life, and long-standing sociodemographic trends. The prevalence of dementia is also steadily increasing. Aging in place, the concept of residing within one's home as one age is a common goal for most older adults, and a supportive environment can help achieve such goals. However, the onset of dementia often derails one's ability to age in place due to the necessity of caregiving for support and safety. This study explored the experience of persons living alone with dementia about their preferences about their memory concerns, their use of healthcare and social services, their informal social support, and their preferences for aging in place. Qualitative interviews with 15 individuals were identified through collaboration with an emergency medical services program. Themes from the interviews included challenges to medication adherence, difficulty managing finances, use of emergency services, and fear of being forced into institutionalization. While some participants had someone who was assisting them (i.e., nephew, neighbor, sibling), many participants reported they did not have any support. The majority of the participants also indicated an interest in getting additional support for their needs but felt they needed help to do so. The presentation will conclude with a discussion on potential interventions and policy implications to better support individuals living alone with dementia.

Abstract citation ID: igaf122.883

### PEOPLE LIVING WITH DEMENTIA LIVING INDEPENDENTLY: THE ROLE OF OLDER AMERICANS ACT SERVICES

Ian Matthew Nelson, Heather Menne, Kate Singer, and Emerson McSparran, *Miami University, Oxford, Ohio, United States*

Older Americans Act (OAA) funding provides community-based services to help individuals age 60 and over live independently in their home and communities. The National Survey of Older Americans Act Participants (NSOAAP) is a nationally representative survey of OAA program participants receiving services such as case management, homemaking, home-delivered meals, transportation, and congregate meals. We used the 2023 NSOAAP survey to examine whether the receipt of OAA services helps participants living with dementia (PLWD) remain independent and at home. The percentage of PLWD responding to the survey across the 5 programs varied between 13% home delivered meal services and 4% for those attending congregate meals. Very high percentages report that these services help them live independently regardless of dementia status (75-98%). Fewer PLWD report that transportation services assist them with remaining at home when compared to those without dementia (86% versus 93%). Finally, a significantly higher percentage of PLWD report that congregate meals help them remain home when compare to those without dementia (91% versus 75%). A smaller portion of the sample, up to 7% of OAA participants, are people living alone with dementia (PLAWD). Eighty-one to 100% of PLAWD report that the service helps them live independently. Based on these results of vulnerable OAA participants, many opportunities are available for further research on other factors contributing to participants' perceptions and ability to remain independently. In addition, policymakers can use these results to explore mechanisms that enhance supports for older adults living with dementia to live independently.

Abstract citation ID: igaf122.884

### LIVING ALONE WITH DEMENTIA IS A NEGLECTED SOURCE OF INEQUALITY: FINDINGS FROM A SCOPING REVIEW

Linda Clare[1], Anthony Martyr[1], Laura Gamble[2], Maria Caulfield[3], Catherine Charlwood[4], Claire Hulme[1], Jan Oyebode[5], and Matthew Prina[2], *1. University of Exeter, Exeter, England, United Kingdom, 2. Newcastle University, Newcastle upon Tyne, England, United Kingdom, 3. University*

*of Bradford, Bradford, England, United Kingdom, 4. University of Exeter Medical School, Exeter, England, United Kingdom, 5. Bradford University, Bradford, England, United Kingdom*

The finding that 40% of community-dwelling individuals with dementia in England live alone challenges still-prevalent assumptions about availability of informal carers. We conducted a scoping review to synthesise knowledge about their characteristics and needs and how best to support them, in consultation with people with lived experience and other stakeholders. Following PRISMA-ScR guidelines, we searched for English-language publications in seven databases without date restriction. After screening, 200 articles reporting on 161 discrete studies met inclusion criteria. Findings showed that people living alone with dementia are more likely to be female and older, with pre-existing social disadvantage, significant unmet needs, and varying levels of informal support. Although no different to those living with others in dementia symptoms or general health, people living alone are subject to inequalities in relation to diagnosis, provision of formal support, financial burden and transition to institutional care. They experience greater loneliness and isolation and more challenges in everyday life. Family members and professionals are primarily concerned with balancing risk and autonomy, but family members involved at a distance receive little support. Although people living alone have more unmet needs and different needs to those living with others, surprisingly few studies explored ways of addressing these needs or improving support. We conclude that living alone with dementia is a neglected source of inequality. We offer recommendations for policy and practice in four key areas: robust estimation of numbers, increasing service responsiveness, strengthening community support, and ensuring research is inclusive and focused on practical ways of improving support.

### SESSION 2340 (SYMPOSIUM)

Abstract citation ID: igaf122.885

### CUSTODIAL GRANDFAMILIES: SYSTEMIC INEQUITIES, CULTURAL RESILIENCE, AND TRAUMA-INFORMED INTERVENTIONS

Chair: Danielle Nadorff
Discussant: Deborah Whitley

Custodial grandparents face intersecting systemic, cultural, and psychological challenges that require culturally responsive, equity-driven interventions. This symposium integrates four studies to advance research and practice. First, a mixed-methods study (Shillingsburg) identifies custodial grandparents' preferences for trauma-informed parenting interventions (e.g., PC-Care), emphasizing relational skill-building and accessibility. Second, Littleton's analysis of National Survey of Children's Health data reveals rural-urban disparities in social determinants of health, with rural grandparents disproportionately facing barriers in neighborhood built environments. Third, a cross-cultural phenomenological study (Lee & Mendoza) compares Korean and U.S. grandparents through role theory, demonstrating how cultural norms shape caregiving experiences: Korean grandparents report role conformity and U.S. grandparents role conflict, yet both groups exhibit resilience during the pandemic. Finally, McLin's survey of ethno-racially marginalized grandparents uncovers a counterintuitive link between resilience and prospective memory failures (objective lapses in remembering tasks), where resilience directly predicts more frequent failures—a relationship potentially explained by heightened cognitive load. However, physical activity mediates this pathway, reducing failures among grandparents with strong ethnic identity resolution (commitment to one's identity). Collectively, these talks highlight: 1. The need for trauma-responsive interventions addressing systemic barriers (e.g., rural service gaps, legal advocacy); 2. Cultural humility in designing supports (e.g., role expectations in collectivist vs. individualist contexts); 3. Strengths-based approaches that leverage grandparents' resilience and racial/ethnic identity while mitigating structural inequities. The symposium calls for policies and programs that bridge gaps between evidence-based practices and the lived realities of custodial grandfamilies, prioritizing accessibility, identity affirmation, and multi-level support systems. Grandparents as Caregivers Interest Group Sponsored Symposium

Abstract citation ID: igaf122.886

### TRAUMA INFORMED PARENTING: TAILORING INTERVENTIONS FOR CUSTODIAL GRANDPARENTS

Laura Shillingsburg, Danielle Nadorff, Maia McLin, and Amara Mason, *Mississippi State University, Starkville, Mississippi, United States*

The rising number of custodial grandparents reflects a growing population navigating unique challenges. Grandparents often assume caregiving roles following traumatic events (e.g., parental substance use, incarceration), which may contribute to grandchildren's behavioral or emotional difficulties. This abrupt transition, compounded by navigating legal, educational, and developmental demands, heightens stress for grandparents adapting to late-life parenting. Existing parenting interventions rarely address custodial grandparents' distinct needs, particularly trauma-informed strategies and relational support. This mixed-methods study explored custodial grandparents' experiences, stressors, and intervention preferences. First, grandparents shared insights into the circumstances prompting their caregiving role, its emotional impact, and ongoing challenges. Second, they will reviewed PC-Care—a trauma-informed parenting intervention emphasizing caregiver-child relationships—and evaluated its relevance, preferred delivery methods (e.g., virtual, in-person), and identified unmet support needs (e.g., legal guidance, peer networks). Data was collected via surveys and semi-structured interviews, transcribed, coded, and analyzed thematically to identify priorities such as accessibility, cultural sensitivity, and relational skill-building. Findings will inform adaptations of PC-Care and the development of tailored interventions addressing systemic barriers (e.g., school advocacy) and trauma-related parenting strategies. By centering grandparents' voices, this study bridges gaps between evidence-based practices and intergenerational caregiving realities, aiming to reduce caregiver burden while fostering resilient grandparent-grandchild relationships. Outcomes will guide practitioners in designing flexible, trauma-responsive supports aligned with custodial grandparents' lived experiences.

Abstract citation ID: igaf122.887

### RURAL-URBAN DIFFERENCES IN THE SOCIAL DETERMINANTS OF HEALTH AMONG CUSTODIAL GRANDPARENTS, 2018-2023

Tenesha Littleton[1], Orion Mowbray[2], and Joana Okine[1], *1. The University of Alabama, Tuscaloosa, Alabama, United States, 2. University of Georgia, Athens, Georgia, United States*

Custodial grandparents report worse mental and physical health than their non-caregiving counterparts. Prior research indicates that custodial grandparents' health and well-being are adversely impacted by the social determinants of health (SDOH), including economic hardship and social isolation. However, prior research on the SDOH among custodial grandparents is limited by small sample sizes and a lack of focus on geographic variations in these experiences. Compared to custodial grandparents in urban settings, rural dwelling custodial grandparents may face additional challenges that impede health due to remote location and lack of social services within rural settings. The purpose of this study was to examine rural-urban differences across multiple SDOH indicators (social support, economic hardship, neighborhood built and social environment) and determine if these indicators were stable across time. Data on custodial grandparents were obtained from the 2018-2023 National Survey of Children's Health (n = 11,355). About 16% of the sample lived in rural areas. The average age was 60 and most of the sample was female (79%). Controlling for demographics, a multivariate logistic regression model examined the association between rural/urban residence and SDOH indicators. Custodial grandparents in rural areas showed lower odds of better neighborhood-built environment (OR = 0.75) and higher odds of better neighborhood social environments (OR = 1.11). From 2018 to 2023, changes in built and social environment scores among custodial grandparents in both rural and urban areas were not significant. Improving the built environment for rural custodial grandparents is crucial to enhancing well-being and guiding caregiver-focused interventions, policies, and programs.

Abstract citation ID: igaf122.888

**UNDERSTANDING KOREAN AND THE U.S. CUSTODIAL GRANDPARENTS THROUGH THE LENS OF ROLE THEORY**

Youjung Lee[1], and Nancy Mendoza[2], *1. Binghamton University, Binghamton, New York, United States, 2. The Ohio State University, Columbus, Ohio, United States*

This study explored grandparent caregiving experiences during and post-pandemic in Korea and the United States. Using a phenomenological approach, semi-structured interviews were conducted with 20 custodial grandparents—10 from each country. Among the Korean grandparents, the mean age was 73 years, two were grandfathers, and on average, they had been raising 1.4 grandchildren for 6 years. Reasons for raising their grandchildren included divorce (7 instances), death (1), substance abuse (1), or financial hardship (1) of the grandchildren's parent(s). U.S. interviewees identified as female (10), White (9) or African American (1) and had a mean age of 59 years. On average, they had been raising 1.8 grandchildren for 8.1 years. Reasons for grandparents raising grandchildren included parents' substance abuse (7), parent's leaving grandchildren after a divorce/relocation (2), or supporting a single parent (the grandparent's child (1). Under the guidance of role theory, the caregivers' experiences were explained by themes of role consensus, role conformity, and role conflict. Despite Korean and U.S. grandparent groups performing a similar role, relative differences were observed, including Korean grandparents with increased role conformity and U.S. grandparents with increased role conflict. The distinction highlights the importance of understanding grandparent caregiving in cultural contexts and ensuring that interventions are culturally responsive. Regardless of cultural context, the grandparent caregivers disclosed relatively positive experiences of the pandemic, testifying to their resilience. Lastly, critical role supporters were identified at various levels, demonstrating that developing support systems can be a key in empowering grandparent caregivers.

Abstract citation ID: igaf122.889

**RESILIENCE, RACIAL IDENTITY, AND MEMORY IN MARGINALIZED CAREGIVING GRANDPARENTS: THE ROLE OF PHYSICAL ACTIVITY**

Maia McLin, Danielle Nadorff, Laura Shillingsburg, and Amara Mason, *Mississippi State University, Starkville, Mississippi, United States*

Prospective memory failures in aging populations may reflect reduced engagement in protective behaviors like physical activity. Ethno-racially marginalized caregiving grandparents face intersecting stressors exacerbating memory challenges. While resilience and ethnic identity may buffer effects, their links to prospective memory remain underexplored. This study examines: 1) resilience-prospective memory links, 2) ethnic identity (resolution/exploration) moderation of resilience-physical activity pathways, and 3) physical activity's mediation. A nationwide sample of 84 Black/Hispanic/multiracial grandparents (ages 45-54) completed surveys assessing resilience (MIIRM), prospective memory (PMQ; higher scores = more failures), ethnic identity (EIS_R resolution, EIS_E exploration), and physical activity. Moderated mediation analyses revealed resilience predicted more frequent memory failures (PMQ: $b=0.52$, $p<.001$), potentially explained by cognitive load theory (heightened responsibility increasing self-reported lapses). Physical activity mediated this link inversely ($b = 2.86$, $p<.001$): resilience increased activity, reducing failures among those with stronger ethnic identity resolution ($B = 0.04$, $p<.01$), aligning with social identity theory. Exploration (EIS_E) showed weaker moderation ($B = 0.017$, $p=.023$), with non-significant mediation, suggesting identity exploration offers fewer stabilizing benefits. Moderated mediation was significant for resolution (index=0.12, 95%CI[0.047,0.23]). Interventions fostering resilience and ethnic identity commitment may mitigate structural inequities' cognitive toll through protective behaviors.

### SESSION 2345 (SYMPOSIUM)

Abstract citation ID: igaf122.890

**NEIGHBORHOOD AND ENVIRONMENTAL CONTEXT OF HEALTH AND AGING: MECHANISMS AND HETEROGENEITY**

Chair: Jeffrey Stokes
Co-Chair: Heather Farmer
Discussant: Martina Brandt

There is a rich literature establishing the importance of neighborhood and environmental context for successful aging, including physical, mental, and cognitive health in later life. Yet, to date, the mechanisms underlying these associations are not fully understood, nor has research thoroughly examined heterogeneity in the role(s) neighborhood and environmental context play. This research has also been limited, overlooking diversity within the U.S. as well as outside it. This symposium addresses this gap by examining implications of multiple levels of environment across diverse and international samples of older adults. Dr. Wang will assess associations of neighborhood social and built environment with ADRD diagnosis. Dr. Álvarez will discuss mechanisms linking neighborhood stressors with cognition among the aging population in Brazil. Dr. Choi will highlight aging-related immune phenotypes as potential biological mechanisms for effects of neighborhood characteristics on long-term health. Dr. Thierry focuses on the unique experience of Black adults in the U.S., examining neighborhood stress and cognitive functioning variations within and outside of the South and across urban/rural settings. Finally, Dr. Cantu examines whether environment – exposure to extreme heat – is associated with cognitive health and related behaviors among older Mexican Americans. As Discussant, Dr. Brandt will synthesize the contributions made by these papers concerning mechanisms underlying place effects on health and heterogeneity across diverse groups within the U.S. and internationally. This symposium holds the potential to shape policy efforts aimed at structural influences on successful aging and to provide scholars with a more nuanced understanding of contextual factors and health.

Abstract citation ID: igaf122.891

### WHERE YOU LIVE MATTERS: NEIGHBORHOOD ENVIRONMENT AND DEMENTIA RISKS AMONG U.S. OLDER ADULTS

Haowei Wang, *Syracuse University, Syracuse, New York, United States*

Alzheimer's Disease and Related Dementias (ADRD) are a growing public health crisis among older adults. Emerging research suggests that neighborhood environment plays a significant role in ADRD risks. This study explores the association between various aspects of neighborhood environment and the risk of ADRD in the US older adult population. We used a large scale, nationally representative data from the All of Us research program, including N = 15,683 respondents aged 60+. Respondents provided information about their neighborhood environment and their ADRD diagnosis was accessed using Electric Health Records. Using factor analysis, we identified four aspects of neighborhood environment, including physical environment disorders, accessibility of facilities, relationship with neighbors, and neighborhood safety. We estimated structural equation models (SEM) with latent variables to analyze the relationship between ADRD risk and multiple aspects neighborhood environment, controlling for respondents' sociodemographic and behavioral characteristics. SEM results showed that older adults had higher risks of ADRD diagnosis if there were more environmental disorders and fewer accessible facilities in the neighborhood. Perceived neighborhood safety was also associated with a lower risk of ADRD diagnosis among older adults. Understanding how neighborhood environments contribute to ADRD risk is crucial for developing targeted public health interventions and promoting healthy cognitive aging for older adults in the US.

Abstract citation ID: igaf122.892

### THE ROLE OF NEIGHBORHOOD STRESSORS ON COGNITIVE FUNCTIONING IN URBAN-DWELLING BRAZILIAN ADULTS

Lourdes Romañach-Álvarez[1], Jordana Breton[1], Mateo Farina[2], and Elizabeth Muñoz[2], *1. The University of Texas at Austin, Austin, Texas, United States, 2. University of Texas at Austin, Austin, Texas, United States*

Exposure to neighborhood stressors is linked to lower cognitive functioning in older adulthood, potentially through poor mental and physical health. However, most research has focused on high-income settings, overlooking unique patterns in low- and middle-income countries. In Brazil, this link may be shaped by neighborhoods with high deprivation and significant socioeconomic and racial segregation. Using data from 6,051 urban respondents in the 2016 Brazilian Longitudinal Study of Aging (ELSI-Brazil), we assessed the association between neighborhood physical disorder and neighborhood inaccessibility with global cognitive functioning, and whether depressive symptoms and mobility limitations mediated this association. Fully-adjusted structural equational models showed that greater physical disorder was linked to better cognitive functioning ($\beta = .043$, $p < .001$), while greater inaccessibility was associated with worse cognitive functioning ($\beta = -.074$, $p < .001$). Bootstrapped analysis indicated an indirect effect of neighborhood inaccessibility on worse cognitive functioning through greater depressive symptoms ($\beta = -.011$, 95% CI [-.019,-.004]) and mobility limitations ($\beta = -.059$, 95% CI [-.079,-.040]). Results for physical disorder showed a negative indirect effect on worse cognitive functioning through greater depressive symptoms ($\beta = -.005$, 95% CI [-.010,-.002]), and a positive indirect effect on better cognitive functioning through fewer mobility limitations ($\beta = .007$, 95% CI [.003,.011]). Findings highlight the unique role of neighborhood stressors on cognitive function in urban Brazil and its differential effects on cognitive functioning. Given their associations with mental health, mobility, and cognitive functioning, addressing neighborhood stressors may support independence and overall well-being in later life.

Abstract citation ID: igaf122.893

### NEIGHBORHOOD SOCIOECONOMIC ENVIRONMENT ASSOCIATED WITH AGING-RELATED IMMUNE PHENOTYPES

Eun Young Choi[1], Gokul Seshadri[2], Bharat Thyagarajan[2], and Jennifer Ailshire[1], *1. University of Southern California, Los Angeles, California, United States, 2. University of Minnesota, Minneapolis, Minnesota, United States*

Neighborhood disparities in health and mortality in later life are well-documented, yet their biological underpinnings are not fully understood. Emerging evidence suggests that immune aging, the age-related gradual decline of the immune system, may be a key mechanism driving these disparities. However, little is known about how neighborhood socioeconomic status (NSES) contributes to immune aging. This study examines how neighborhood affluence, deprivation, and specific NSES indicators are associated with immune cell composition, focusing on T-and B-cell subsets. We analyzed individual-level data from a nationally representative sample of adults aged 56+ in the 2016 Health and Retirement Study Venous Blood Study (N = 7,660), merged with neighborhood-level data from the 2014-2018 American Community Survey. We used multilevel regression models, adjusting for census tract clustering and sociodemographic and health-related covariates (e.g., education, comorbidities, CMV seropositivity). Residents of the most affluent neighborhoods had higher CD4+ naïve T cells (45.6% vs. 43.1%, $p < 0.001$) and lower CD4+ central memory T cells (35.8% vs. 36.8%, $p < 0.05$) than those in the least affluent neighborhoods. Those in the most deprived neighborhoods had higher IgD- memory B cells (11.9% vs. 11.1%, $p < 0.05$). Among NSES indicators, neighborhood-level poverty and higher education rates were associated with immune cell profiles less and more capable, respectively, of responding to foreign stressors. Affluent neighborhoods may support immune resilience through lower chronic stress and better access to healthcare, while deprivation may accelerate immune aging potentially through greater antigen exposure or chronic immune activation. Our findings offer important insights into the immunological mechanisms underlying long-standing neighborhood-driven health disparities.

Abstract citation ID: igaf122.894

### GEOGRAPHIC VARIATION IN NEIGHBORHOOD STRESS, DISCRIMINATION, AND COGNITION IN BLACK MIDLIFE AND OLDER ADULTS

Amy Thierry[1], and Kyler Sherman-Wilkins[2], *1. Xavier University of Louisiana, New Orleans, Louisiana, United States, 2. Missouri State University, Springfield, Missouri, United States*

Background: Black Americans have twice the risk of developing Alzheimer's disease (AD) than white adults. Historical racial inequities in neighborhood environments and experiences of discrimination among Black people together may exacerbate risk for AD, with variation across geographic contexts. This study examines if discrimination and neighborhood stressors interact to negatively affect cognitive function among Black older adults by region and urbanicity. Methods: Data are from Black adults >50 years of age (n = 1,690) in the 2010-2016 waves of the Health and Retirement Study. To determine geographic differences in lifetime discrimination (i.e., unfair experiences in housing, employment, and policing), perceived neighborhood stressors (disorder and social discohesion), and cognitive function, descriptive statistics stratified by US region (South versus non-South) and urbanicity (rural versus non-rural) were used. Linear regression models tested the association between cognitive function and two interactions terms: neighborhood disorder*discrimination and neighborhood discohesion*discrimination. Results: Older Black adults living outside of the South experienced more discrimination events and reported greater neighborhood disorder than those residing in the South, while those living in non-urban areas had lower cognitive function than those in urban areas. Statistically significant interaction terms indicate that among Black adults living in urban areas, greater neighborhood disorder and discohesion along with greater lifetime discrimination was associated with lower cognitive function. Discussion: Lifetime discrimination may increase Black older adults' vulnerability for poor cognitive function especially if they reside in stressful, under-resourced neighborhoods. Further exploration of life course factors such as migration and timing of discrimination events will enhance understanding of these findings.

Abstract citation ID: igaf122.895

### EXTREME HEAT AND BEHAVIORAL SYMPTOMS OF COGNITIVE DECLINE IN OLDER MEXICAN AMERICANS

Phillip Cantu[1], and Sadaf Milani[2], *1. UTMB Health, The University of Texas Medical Branch, Galveston, Texas, United States, 2. University of Texas Medical Branch, Galveston, Texas, United States*

Extreme temperatures are associated with worse cognitive functioning among older adults. It remains unknown if these patterns are consistent for behavioral symptoms of cognitive decline. Mexican American older adults have historically lived mostly in the southwestern United States in states that are particularly vulnerable to high heat. We examined the association of extreme heat, as measured by days of heat indices above 90 degrees a year, with informant reports of behavioral symptoms of cognitive decline, as measured by the Neuropsychiatric Inventory (NPI), in a large representative sample of older Mexican Americans, the Hispanic Established Population for the Epidemiologic Study of the Elderly. Using OLS regression, we found that individuals who experienced more days of extreme heat had more reports of behavioral symptoms of cognitive impairment, net of actual cognitive functioning. Additionally, we found that the relationship between behavioral symptoms and extreme heat were stronger for people with cognitive impairment, defined as a Mini-Mental Status Examination score of less than <21. We also found that extreme heat days were associated with specific NPI symptoms including engaging in repetitive activities, losing interest in usual activities, excessive happiness (mania), low spirits (depression), and hearing voices. These results suggest that the behavioral manifestations of cognitive impairment may be related to the physical environment older adults live in. Future research should examine the extent to which the built environment and physical structures may mediate the relationship between extreme heat and behavioral symptoms of cognitive decline.

### SESSION 2350 (PAPER)

### WORK, WORTH, AND WELL-BEING IN LATER LIFE

Abstract citation ID: igaf122.896

### AGING WITH MEANING: WHAT MOTIVATES THE OLDER ADULTS TO END-OF-LIFE CARE VOLUNTEERING

Asenath Lin, Vivian Lou, Gloria Chun, and Felix Mak, *The University of Hong Kong, Hong Kong, China (People's Republic)*

Objective: This study examines the motivations and impacts of end-of-life care (EoLC) volunteering among older adults (aged ≥60), addressing critical gaps in aging societies where cultural taboos around death often limit engagement. Method: Under The Jockey Club End-of-life Community Care Project, a mixed-methods approach was employed, combining quantitative data from 212 participants and qualitative interviews with 11 volunteers. Findings: Using the Volunteer Functional Inventory (VFI)—a framework identifying six motivations (value, understanding, enhancement, protective, career and social). A Latent Profile Analysis revealed three distinct motivational profiles: a "Uniformly Low" group (40.5%) with minimal motivation across all factors, a "Uniformly High" group (41.8%) demonstrating strong engagement in all domains, and a "Protective-Career Oriented" group (17.7%) primarily driven by self-protective and career-related motives. Qualitative data revealed transformative impacts: volunteers gained EoLC knowledge, reported heightened meaning, reduced death fear, and contributed to breaking cultural taboos in Chinese communities. The "Uniformly High" profile aligns with qualitative intrinsic rewards (e.g., meaning, happiness), whereas "Protective-Career Oriented" underscores the fulfilment of personal needs in sustaining participation. Notably, participants motivated by altruism demonstrated sustained engagement and resilience in navigating service-related challenges. Conversely, those driven by protective motivations (e.g., alleviating negative emotions) were more susceptible to burnout overtime. Conclusion: Despite minimal quantitative shifts, findings highlight EoLC volunteering's societal value in normalizing death discussions and its significance for older adults. Practical implications include tailoring recruitment and training to motivational profiles and leveraging volunteers as cultural change agents.

Abstract citation ID: igaf122.897

### EMOTIONAL REACTIONS TO THE AGED FUTURE SELF MATTER: A 6-MONTH LONGITUDINAL STUDY ON RETIREMENT PLANNING

Dannii Yeung[1], and Edwin Ka Hung Chung, *City University of Hong Kong, Hong Kong, China (People's Republic)*

The experimental manipulation of exposing the individuals to their aged future selves is found to be effective in increasing retirement planning. However, it is questionable whether the emotional reactions to the aged future-self image play a role in strengthening the future-self continuity. This study therefore aims to address this research gap using a mixed-research method consisting of an experimental manipulation, a 5-day diary study, and a 6-month follow-up assessment. A total of 203 Hong Kong Chinese workers (Mage = 41.01, SD = 11.34) participated in the experiment where they were randomly assigned to future-self (FS) or current-self (CS) conditions. FS participants were presented with their aged-morphed photos, while CS participants viewed their current photos. Following the experiment, a 5-day diary study assessed participants' plans to improve their future well-being and retirement activities at the 6-month follow-up. Results from the sequential mediation analysis indicated that, compared with CS participants, FS participants experienced more negative emotions towards their future selves at the experiment, resulting in lower perceived closeness to future self during the 5-day diary period and fewer retirement planning activities at the 6-month follow-up [Financial domain: B= -0.02, SE = 0.01 (-0.04, -0.00); Health domain: B= -0.02, SE = 0.01 (-0.05, -0.00); Social domain: B= -0.02, SE = 0.01 (-0.05, -0.00); Psychological domain: B= –0.02, SE = 0.01 (-0.04, -0.00)]. These findings suggest that promoting a positive image of aging can strengthen working adults' preparation for retirement. A diary approach with positive messages can maintain the connection to the future self and thus facilitate long-term retirement preparation.

Abstract citation ID: igaf122.898

### LATENT SEMANTIC ANALYSIS OF LIFE NARRATIVES OF CULTURAL AND ETHNIC EXPERIENCES IN OLDER BLACK AND WHITE AMERICANS

Hsiao-Wen Liao[1], Siddhi Pandare[1], Nikhil Shanbhogue[1], Sashank Varma[1], Wei Xu[1], Hongchen Wu[1], and Thomas Oltmanns[2], *1. Georgia Institute of Technology, Atlanta, Georgia, United States, 2. Washington University in St. Louis, St. Louis, Missouri, United States*

The life narratives people tell about themselves are conducive to well-being and a sense of self-continuity. This study utilized natural language processing methods to address between and within-group differences in the cultural and ethnic experiences that were remembered by a cohort of older Black and White Americans (N = 457; 50% Black) who grew up during the civil rights movement. The history-graded influence differentiated the lives of these two groups. We predicted that (a) when all the memories are combined and analyzed, two clusters would emerge with each cluster respectively reflecting experiences shared by the majority of individuals in each racial group, (b) idiosyncratic experiences would also be recalled, resulting in within-group differences, and (c) the affective qualities of cultural memories (i.e., event valence, use of emotion words, overall sentiment) are shaped by racial background and what was recalled. Latent semantic and cluster analyses identified two meaningful clusters: one cluster encompasses memories about racial division mostly from Black Americans (70.9%); the other cluster contains memories about family ancestry largely from White Americans (72.2%). Chi-square tests and independent t-tests revealed that Black (vs. White) Americans and the racial division (vs. family ancestry) cluster presented greater memory negativity. Notably, Black Americans who recalled divergent experiences had lower memory negativity than their same-race counterparts who recalled racial division. White Americans who recalled divergent experiences showed greater memory negativity than their same-race counterparts who recalled family ancestry. In discussing these findings, we draw on the master narrative framework and the life script theory.

Abstract citation ID: igaf122.899

### MEANINGFUL ACTIVITY FOR RESIDENTS WITH DEMENTIA IN ASSISTED LIVING: RESULTS FROM THE MAC-4-BSD PILOT STUDY

Sarah Holmes[1], Elizabeth Galik[1], Barbara Resnick[2], Sorah Levy[2], Erin O'Brien[1], Stephanie McCorvey[3], Merve Gurlu[1], and Shijun Zhu[1], *1. University of Maryland School of Nursing, Baltimore, Maryland, United States, 2. University of Maryland, Baltimore, Maryland, United States, 3. University of Maryland at Baltimore, Baltimore, Maryland, United States*

Engagement in meaningful activity is associated with increased sense of purpose, improved mood, and decreased behavioral and psychological symptoms. Despite these benefits, there are limited opportunities for assisted living (AL) residents with dementia to engage in activities that are personally valued and meaningful. This study evaluated the feasibility and preliminary efficacy of a theory-based approach, Meaningful Activity for Managing Behavioral Symptoms of Distress (MAC-4-BSD). Using a cluster randomized controlled trial with repeated measures design, 66 residents with dementia from 5 AL communities were included. AL communities were randomized to either MAC-4-BSD or education-only control group. Outcomes were evaluated at baseline and 4 months. It was hypothesized that exposure to MAC-4-BSD will demonstrate increased meaningful activity engagement, improved quality of life, and reduced behavioral symptoms compared to the control group. Descriptive statistics and linear mixed models were used. Most participants were female (n = 49, 74%), and White (n = 58, 88%) with a mean age of 85 years (SD = 8.4). MAC-4-BSD was feasible to implement based on evidence of delivery and receipt of the intervention. From baseline to 4 months, residents exposed to MAC-4-BSD had significant improvements in meaningful activity engagement (b = 7.8, p=.008, Cohen's d=.74) and decreased number of behavioral symptoms (b=-2.3, p=.010, Cohen's d=-.79) relative to the control group. These findings demonstrate that engagement in meaningful activity is a promising approach that is feasible and can help to address behavioral and psychological symptoms for AL

residents with dementia. Future research is needed to support the long-term sustainability and adoption of MAC-4-BSD in AL communities.

Abstract citation ID: igaf122.900

**SOCIAL PATTERNING OF SUBJECTIVE LIFE EXPECTANCY IN LATE MIDLIFE**

Anu Siren, *Tampere University, Tampere, Pirkanmaa, Finland*

Subjective life expectancy (SLE), an individual's personal estimate of their lifespan, impacts various aspects of life, including retirement planning and health behaviors. Due to increasing longevity and changing institutions such as pension systems and statutory insurances, individuals are increasingly compelled to adapt to the prospect of longevity and weigh the risks of aging. However, the ability to envision a long life is socially stratified and influenced by factors such as gender, socioeconomic status, and health. Notwithstanding, relatively little is known about the social patterning of SLE. Using various measures of lifestyle such as social participation, cultural consumption, and health lifestyle, this study investigates how SLE is determined in late midlife and how this determination is socially patterned. The data comes from a nationally representative survey (n = 3327) in Finland among people born in 1960, 1965, and 1970. We employ principal components analysis and logistic regression and find that, in addition to female gender, good health, and high income, certain lifestyles and cultural consumption patterns independently predict higher SLE. The results contribute to the literature on the social stratification of SLE, suggesting that while more socially varied populations live longer, the prospect of a long life seems imaginable only for rather select subgroups of people. Increasing longevity prompts societal negotiation of risks related to long life courses. Nevertheless, it is important to acknowledge that a great variation exists at the individual level in people's ability and willingness to commit to the long futures and to manage the risks of aging.

Abstract citation ID: igaf122.901

**WHO REDUCES WORKING HOURS BEFORE RETIREMENT? A LONGITUDINAL STUDY ON PLANS AND BEHAVIORS IN THE NETHERLANDS**

Camilla Marabini[1], Marleen Damman[2], and Kene Henkens[1], *1. Netherlands Interdisciplinary Demographic Institute, The Hague, Zuid-Holland, Netherlands, 2. Utrecht Universiy, Utrecht, Utrecht, Netherlands*

As retirement ages increase, reducing working hours before retirement is seen as a way to facilitate longer working lives of those workers struggling with work demands. The current study analyzes individual, household, job, and organizational factors that shape the decision to reduce work hours before retirement. Three key questions are examined: Who plans to reduce working hours before retirement, who is actually reducing working hours, and what explains the discrepancy between the two? Using two waves of the NIDI pension panel survey, we examine the plans to reduce work hours of 4243 employees aged 60-63 nested in 1086 organizations, and track their actual behavior after retirement. Results suggest that around 20% of the sample plans to reduce working hours in the years before retirement age. Older workers with poor health, and higher income were more likely to plan to reduce working hours. In the follow-up years, 30% of the older workers actually reduced working hours. Plans proved to be strong predictors of actual behavior. Within those who plan to reduce working hours before retirement, around half actually reduces them; around 30% of those who were uncertain ends up reducing work hours; and a fifth of those who planned to work full-time until retirement eventually reduces work hours. Findings from this study contribute to scientific debates about agency during later working lives and to policy debates about gradual retirement pathways.

### SESSION 2355 (SYMPOSIUM)

Abstract citation ID: igaf122.902

**POLICY SERIES: AGING AND HEALTH POLICY UPDATE: A VIEW FROM WASHINGTON**

Chair: Brian Lindberg

Discussant: Patricia D'Antonio

Leading policy advocates will present their findings and viewpoints regarding aging and health care policy during a divided Congress. Issues will include behavioral health, social isolation, health care workforce, telehealth, hospice policy, elder justice and nursing home care, home and community-based services, and Medicare and Medicaid.

### SESSION 2360 (SYMPOSIUM)

Abstract citation ID: igaf122.903

**SOCIAL CONNECTION AND BRAIN FUNCTION: FROM MEASUREMENT TO CLINICAL TRIALS**

Chair: Hiroko Dodge

Co-Chair: Brea Perry

Over five decades of gerontological studies have shown the negative impact of social isolation on health, including an increased risk of dementia. The most recent Lancet Commission on Dementia reported that social isolation contributes to 5% of dementia cases, higher than the 2% attributed to physical inactivity or diabetes. Reducing social isolation could significantly impact dementia prevalence. However, compared to other behavioral interventions like exercise and diet, fewer randomized controlled trials (RCTs) have been conducted on social isolation/connectedness. Social isolation is multifactorial, affecting cognition through various pathways, including tangible and intangible factors, multiple types of cognitive stimulation, and emotional bonding. Thus, RCTs require careful consideration of theoretical frameworks to clarify and identify the different potential mechanisms. This symposium aims to highlight key factors in developing behavioral interventions for social isolation/connectedness through four presentations: 1) Perry will discuss egocentric social network methodology to assess social connectedness, which operationalizes social bridging and bonding theories. 2) Yu will present the results of her systematic literature review on psychosocial and physiological pathways linking social isolation/connectedness to brain health. 3) Krendl will examine whether the theory of mind mediates the relationship between older adults' social connectedness and cognition, both cross-sectionally and longitudinally. 4) Dodge will present the theoretical framework and findings from the I-CONECT project, which aimed to enhance cognition by providing frequent conversational interactions as cognitive stimulation among socially isolated older adults. Together, this symposium will offer critical components for developing targeted interventions that address social isolation/connectedness and support cognitive resilience.

Abstract citation ID: igaf122.904

### LEVERAGING EGOCENTRIC SOCIAL NETWORK METHODOLOGY TO UNDERSTAND RISK AND RESILIENCY IN ALZHEIMER'S DISEASE

Brea Perry, and Siyun Peng, *Indiana University Bloomington, Bloomington, Indiana, United States*

Research suggests social connectedness reduces dementia risk and helps older adults with neuropathology maintain cognitive functionality and quality of life. However, relatively little empirical evidence identifies the specific underlying social and biological mechanisms. This presentation provides an overview of potential pathways through social bridging (i.e., cognitive enrichment through expansive social networks) and social bonding (i.e., neuroendocrine benefits of integration in cohesive social networks). We discuss egocentric social networks, which focus on the structure, function, and composition of ties between a single individual (i.e., the "ego") and the people they are directly connected to (i.e., "alters"). This methodology is ideal for collecting relational data to operationalize social bridging and bonding theories and test hypotheses regarding risk and resilience in cognitive aging. We illustrate the advantages of egocentric network methods using results from a cohort study of older adults that combines social network data with repeated assessments of general cognitive function and neuroimaging biomarkers. These findings provide insight into specific etiological mechanisms and have important implications for cognitive health disparities that can be leveraged to inform policies and programs that support brain health and cognitive function in older adults.

Abstract citation ID: igaf122.905

### UNRAVELING THE UNDERLYING MECHANISMS OF LONELINESS, SOCIAL ISOLATION, AND COGNITIVE DECLINE: A SYSTEMATIC REVIEW

Kexin Yu[1], Karina Van Bogart[2], Jee Eun Kang[3], Harry Taylor[4], Karra Harrington[5], Lisa Silbert[6], Ihab Hajjar[1], and Hiroko Dodge[7], *1. UT Southwestern Medical Center, Dallas, Texas, United States, 2. Northwestern University, Chicago, Illinois, United States, 3. The Pennsylvania State University, University Park, Pennsylvania, United States, 4. University of Toronto, Toronto, Ontario, Canada, 5. Cogstate, Moses Lake, Washington, United States, 6. Oregon Health & Science University, Portland, Oregon, United States, 7. Massachusetts General Hospital, Harvard Medical School, Boston, Massachusetts, United States*

Despite extensive evidence linking loneliness and social isolation to cognitive decline in later life, the underlying pathophysiological and psychosocial mechanisms remain unclear. This systematic review synthesizes existing research to clarify these pathways. A comprehensive search was conducted across PubMed, Web of Science, EMBASE, and PsycINFO until October 2024. Using Covidence for literature management, we included peer-reviewed studies in English involving human participants aged 50+. Studies examining psychosocial pathways required mediation analyses, whereas studies on physiological pathways were included if outcomes were related to dementia pathology. From 4,314 unique articles, 40 met the inclusion criteria. Identified pathophysiological mechanisms included amyloid-beta and tau levels, grey matter atrophies, white matter hyperintensities, dementia-related gene expression, reduced brain-derived neurotrophic factor (BDNF), hypothalamic-pituitary-adrenal (HPA) axis dysregulation, and impaired cerebral blood flow. Psychosocial mediators encompassed depression, anxiety, sleep disturbances, physical inactivity, personality traits, and diminished sense of control. The presentation will contrast the unique pathways for loneliness and social isolation. Evidence suggests loneliness and social isolation are linked to decreased resiliency to neuropathological changes and to behavioral and psychological factors that accelerate cognitive decline. However, existing research relies heavily on self-report measures and does not account for potential confounders like pre-existing health conditions, which limits causal inference. By integrating evidence across disciplines, our findings underscore the multifaceted impact of social disconnection on cognitive health. Future research should prioritize rigorous longitudinal designs, including multi-modal biomarkers, both subjective and objective psychosocial measures, and potential confounders in analyses. Such work would inform targeted interventions that promote cognitive resilience.

Abstract citation ID: igaf122.906

### SOCIAL CONNECTEDNESS AND COGNITIVE RESILIENCE: THE ROLE OF SOCIAL COGNITION

Anne Krendl[1], Lucas Hamilton[2], and Brea Perry[1], *1. Indiana University Bloomington, Bloomington, Indiana, United States, 2. Augustana University, Sioux Falls, South Dakota, United States*

Social connectedness is a modifiable lifestyle factor that may also delay the progression of Alzheimer's disease. However, the mechanism by which this occurs is not well-understood. Our work examines whether older adults' social cognitive function is the mechanism by which social connectedness confers resilience against cognitive decline. We focus specifically on theory of mind – the ability to infer what others are thinking and feeling – because it is a core social cognitive skill that is impaired in healthy aging. We examined whether theory of mind mediated the relationship between older adults' social connectedness and cognition using data collected from 305 community-dwelling older adults. 110 of those participants completed follow-up social network interviews and cognitive assessments about 1.5 years later to determine whether baseline social connectedness and theory of mind predicted cognitive change. Results revealed that theory of mind accounted for 32% of the relationship between social connectedness and overall cognition, even when covarying age, gender, education, and a control task. The effects were particularly robust for episodic memory and language. Longitudinal analyses replicated this pattern. An additional 55 other older adults completed an experimental manipulation in which participants were asked to describe a task to a close and distant network member. We found that older adults with larger, less dense social networks provided more details to distant versus very close network members. Together, these results suggest that theory of mind may provide the mechanism through which social connectedness confers cognitive resilience.

Abstract citation ID: igaf122.907

### THE INTERNET-BASED CONVERSATIONAL ENGAGEMENT TRIAL (I-CONECT): THEORETICAL FRAMEWORK AND LATEST FINDINGS.

Hiroko Dodge[1], Chao-Yi Wu[1], Liu Chen[1], and Kexin Yu[2], 1. Massachusetts General Hospital, Harvard Medical School, Boston, Massachusetts, United States, 2. UT Southwestern Medical Center, Dallas, Texas, United States

Social isolation is a risk factor for dementia. In the recently completed randomized controlled trial, I-CONECT (www.i-conect.org; ClinicalTrials.gov: NCT02871921), we investigated the impact of frequent conversational interactions—a key component of social interactions—on cognitive functions. We hypothesized that engaging in frequent conversations enhances compensatory neural activity and helps maintain cognitive function, a concept framed by Park et al. (2013) as "scaffolding," similar to Stern's (2012) cognitive reserve theory. To specifically target the benefits of social bridging (Perry, 2012) separate from social bonding, we rotated interviewers each week. Participants in the experimental group engaged in semi-structured conversations with trained interviewers, prompted by daily themes and pictures, four times a week (30 minutes/session) for 6 months using user-friendly video-chat devices. The control group received brief (~10-minute) weekly phone check-ins. A total of 186 socially isolated older adults aged 75 and older (86 with normal cognition, 100 with mild cognitive impairment [MCI]) were randomized. We previously reported that global cognitive function (the primary outcome), measured by the Montreal Cognitive Assessment (MoCA), improved by nearly 2 points in the MCI experimental group compared to the control group at 6 months (effect size: Cohen's d = 0.73) (Dodge, 2024, doi: 10.1093/geront/gnad147). Functional MRI data suggested a trend toward increased connectivity in the dorsal attention network favoring the experimental group. Social satisfaction improved in both groups. During the symposium, we will explain the theoretical framework, recruitment, intervention approach, and findings to date, which can serve as a foundation for future behavioral trials of this kind.

### SESSION 2365 (SYMPOSIUM)

Abstract citation ID: igaf122.908

### LOGISTICAL CHALLENGES ASSOCIATED WITH CAREGIVER COORDINATION OF IN-PERSON MEDICAL APPOINTMENTS

Chair: Lauren Moo
Co-Chair: Victoria Ngo
Discussant: Elizabeth Marfeo

Arranging to bring a medically complex older person to an in-person medical visit involves lots of planning and disruption of daily routines. Family caregivers coordinating and navigating such visit-associated logistics must incorporate such challenges into their already strained schedules, potentially adding to caregiver burden and burnout. Virtual visits may reduce some of these challenges, but these benefits are difficult to catalog as a formal assessment of these visit-associated logistics has not been previously undertaken. This symposium will include five talks. The first will describe qualitative data from interviews with caregivers of people living with dementia (PLWD) about their experience bringing the PLWD to medical visits, including preparing for visits and visit-related time and travel. The second will describe a novel visit-associated logistics (VAL) framework that includes 6 domains: scheduling, preparing for the visit, visit-related time and travel, caregiver independence, routine disruption, and overall feelings & impressions. The third talk will share VAL results from a web-based national survey of > 500 informal caregivers of older Veterans regarding the most recent in-person medical visit they helped their care recipient attend. The fourth talk will discuss the additional logistical challenges faced by caregivers coordinating medical visits for older adults living in rural areas. The fifth talk will describe the VAL assessment results from 20 family caregivers of PWLD in the context of their qualitative reflections on the merits of in-person versus video visits.

Abstract citation ID: igaf122.909

### VISIT-ASSOCIATED LOGISTICS FRAMEWORK

Lauren Moo[1], Victoria Ngo[2], Elizabeth Marfeo[3], Steven Shirk[2], and Elizabeth Chamberlin[2], *1. VA Bedford Healthcare System, Bedford, Massachusetts, United States, 2. VA Bedford Health Care System, Bedford, Massachusetts, United States, 3. Tufts University, Medford, Massachusetts, United States*

Coordinating in-person clinical visits requires complex logistical planning for the family caregivers of older adults. We developed a conceptual framework integrating concepts from geriatric research and visit logistics that conceptualizes how visit-related challenges can impact caregiving and attendance of medical appointments. In this session, we will introduce the Visit-Associated Logistics (VAL) Framework that includes domains of Scheduling, Preparing for the visit, Visit-related time and travel, Caregiver independence, Routine disruption, and Caregiver feelings and impressions.

Abstract citation ID: igaf122.910

### EXAMINATION OF CAREGIVER LOGISTICS IN A NATIONAL SAMPLE OF FAMILY CAREGIVERS OF OLDER VETERANS

Victoria Ngo[1], Steven Shirk[1], Elizabeth Marfeo[2], Elizabeth Chamberlin[1], Bret Hicken[3], Maria Venegas[4], Leah Christensen[5], and Lauren Moo[6], *1. VA Bedford Health Care System, Bedford, Massachusetts, United States, 2. Tufts University, Medford, Massachusetts, United States, 3. VHA Office of Rural Health, Salt Lake City, Utah, United States, 4. Department of Veterans Affairs, Bedford, Massachusetts, United States, 5. Veterans Health Administration, Washington, District of Columbia, United States, 6. VA Bedford Healthcare System, Bedford, Massachusetts, United States*

Family caregivers of older Veterans face challenges in coordinating visit-associated logistics. Based on a national survey we developed, we were able to learn more about some of these challenges. In this talk, we share results as we examined caregiver logistics of attending a medical visit. Descriptive statistics including means, standard errors, and percentages, were used to summarize the sociodemographic characteristics of caregiver respondents. Associations between caregiver and care recipient characteristics and caregiver burden were examined in bivariate analyses using chi-square tests of independence for categorical variables and t-tests for continuous variables. Relationships between variables related to ADL and IADL and visit-associated logistics were examined using t-tests.

Abstract citation ID: igaf122.911

### BRINGING PERSONS WITH DEMENTIA TO HEALTH CARE VISITS: CAREGIVER PERSPECTIVES

Megan Gately[1], Jaye McLaren[2], and Lauren Moo[2], *1. VA Bedford Health Care System, Bedford, Massachusetts, United States, 2. VA Bedford Healthcare System, Bedford, Massachusetts, United States*

Caregivers of persons living with dementia (PLWD) face many care challenges including the need to provide increasing support to PLWD day-to-day needs as the disease progresses. Caregivers' management of PLWD health care is an acknowledged aspect of their role, and includes scheduling appointments, managing medications, and communicating with clinicians. Yet little is known about the full scope of that support particularly in the realm of attending health care visits with PLWD. This study presents factors associated with attending health care visits with PLWD. Factors were identified by caregivers of PLWD residing in the community during semi-structured qualitative interviews (N = 24). Content analysis of interview data revealed three categories related to the experience of attending health care visits with PLWD: 1) Preparing for the Visit, 2) Visit-Related Time and Travel, and, 3) In-visit Experience, highlighting associated barriers and facilitators within each category. Barrier statements generally reflected the negative impact of cognitive impairment, such as PLWD anxiety, confusion, and repetitive questioning about the purpose of the visit, at times well in advance of the visit. Facilitators statements generally reflected the benefit of attending visits as opportunities for social participation and providing caregivers an opportunity to engage with care teams. Taken together, these factors elucidate the experience of taking PLWD to health care visits from the perspectives of caregivers, highlighting opportunities to mitigate more negative aspects of that experience.

Abstract citation ID: igaf122.912

### URBAN AND RURAL DIFFERENCES IN LOGISTICS WHEN ATTENDING IN-PERSON APPOINTMENTS

Elizabeth Chamberlin[1], Steven Shirk[1], Victoria Ngo[1], Elizabeth Marfeo[2], and Lauren Moo[3], *1. VA Bedford Health Care System, Bedford, Massachusetts, United States, 2. Tufts University, Medford, Massachusetts, United States, 3. VA Bedford Healthcare System, Bedford, Massachusetts, United States*

The lack of long-term formal care for older adults and the wish to age in place has increased the use of informal caregivers. These caregivers take on many responsibilities, including assisting their care recipients with in-person healthcare visits. A national survey of informal caregivers of military Veterans (N = 511), included the VAL to assess logistical differences between urban (n = 253) and rural (n = 258) caregivers when assisting in in-person health care appointments. There was no significant difference between urban and rural Veterans' level of functioning. For the six domains of VAL (1) scheduling, 2) preparing for the visit, 3) visit-related time and travel, 4) work, therapeutic, academic, and recreational impact, 5) routine disruption, and 6) overall feelings/impression), preparing (2), time (3), and impact (4) were significantly different for urban and rural caregivers. Gathering veterans' information (e.g., complete forms, medication lists) ($p = .02$) and helping the Veteran prepare for the appointment beyond their usual routine ($p = .04$) took rural caregivers significantly more time than urban caregivers. Time to get to the appointment ($p = .03$), as well as the total round-trip time for all trips (e.g., dropping off at childcare, picking up Veteran) ($p = .003$) and total distance ($p = <.0001$) was significantly greater for rural caregivers than their urban counterparts. Rural caregivers also missed more work ($p = .003$) than urban caregivers. These findings suggest that rural caregivers need additional assistance to deter burnout. Increasing programs and services supporting informal caregivers in assisting care recipients is necessary.

Abstract citation ID: igaf122.913

### VIRTUAL GROUPS ADDRESS VISIT-ASSOCIATED LOGISTICAL CHALLENGES BUT REQUIRE DEDICATED TECHNICAL SUPPORT

Maureen O'Connor[1], Jaye McLaren[2], Andrew Nguyen[3], Kendra Pugh[4], Lauren Moo[2], Steven Shirk[4], and Samantha Harrington[5], *1. Bedford Veterans Health Care System, Bedford, Massachusetts, United States, 2. VA Bedford Healthcare System, Bedford, Massachusetts, United States, 3. Department of Veterans Affairs, Bedford, Massachusetts, United States, 4. VA Bedford Health Care System, Bedford, Massachusetts, United States, 5. New England Geriatric Research, Education & Clinical Center, Bedford, Massachusetts, United States*

The demanding nature of caregiver often acts as a barrier to accessing in-person care services and programs. Many caregivers find it difficult to travel to and from in-person support and education programs. Additionally, many caregivers are uncomfortable leaving the care recipient home alone and/or are unable to find respite care. These difficulties are often intensified for rural carers. For example, compared to their urban counterparts, rural dementia caregivers typically know of fewer available services and specialty providers and must travel significantly farther to find services. Telehealth services, provided via video or phone, can reduce these barriers. The current study adapted an in-person educational intervention to teach dementia caregivers how to manage neuropsychiatric symptoms to telehealth, named TeleCARE. The intervention took place over 8 weeks, 1 hour a week, over Webex. Twenty-four mostly white (100%), female (76%), spousal (82%) caregivers with an average age of 72 ($M = 71.8$, $SD = 12.6$) completed the study. Seventy percent of caregivers had some type of difficulty using telehealth technology and we quickly realized personalized help was necessary to support intervention engagement. Over the 8 sessions, less support was needed with each passing session as caregivers became more fluent with using the technology. At week 8, none of the caregivers required technology assistance. By the end of the intervention, caregivers reported a sense of accomplishment and increased comfort with the technology. At 3-month follow-up, technology support was again required. This talk will focus on the lessons learned as they are broadly applicable to telehealth interventions for dementia caregivers.

### SESSION 2370 (SYMPOSIUM)

Abstract citation ID: igaf122.914

### UNTANGLING AGEISM: INSIGHTS FROM DIVERSE PERSPECTIVES, METHODS OF INQUIRY, AND CONTEXTS

Chair: Liat Ayalon

Ageism, defined as stereotypes, prejudices, and discrimination, has been studied for over five decades now. Yet, it is still more prevalent than the other big "isms" such as racism or sexism but is less recognized than other "isms." In this symposium, we present research conducted in various socio-cultural contexts including Canada, China, Germany, Israel, and the United States to highlight the complexity and multi-faceted nature of ageism. Levy and Martin present research from the Health and Retirement Survey showing the importance of positive views on aging in reversing and preventing the development of mild cognitive impairment. Xi and colleagues highlight the paradox of digital ageism in two different cultural contexts: the United States and China, demonstrating differential age and culture effects of digital ageism on technology use . Ayalon and colleagues examine shifts in society and their potential effects on ageism. They demonstrate a reduction in ageism following the Sward of Iron war in Israel. de Paula Couto & Rothermund examine the interaction between chronological age and disengagement norms, showing a reduction in the reports of age discrimination in those older persons who are more likely to hold disengagement norms. Finally, Gans & Chasteen discuss intersectional identities in relation to prescriptive norms, demonstrating that older White men are the ones most expected to step aside for younger generations and most negatively judged as moral violators if they fail to comply with prescriptive age norms. The studies highlight the importance of context and the distinction between prescriptive and descriptive age norms.

Abstract citation ID: igaf122.915
**"THE WAR ON AGEISM" OR HOW AGEISM IS REDUCED DURING WARTIME**
Liat Ayalon, Sarit Okun, and Assaf Suberry, *Bar-Ilan University, Ramat-Gan, HaMerkaz, Israel*
Objectives: The study evaluated the perceptions of lay people in Israel concerning the presence of ageism during the Swards of Iron war. Methods: An online survey was administered to 911 Israelis during the month of April 2024. Respondents provided responses to two open-ended questions concerning their general views on ageism during the war in general as well as their own personal experiences with ageism during the war. Results: In total, 50% of the responses discussed a reduction or non-existence of ageism during the war in general, whereas 72% of the responses discussed such a reduction or non-existence of ageism based on their own personal experience during the war. This was attributed to: a) growing collaboration between young and old, b) younger people's willingness to sacrifice their lives, and c) older persons' contribution during the war. Discussion: The findings are discussed in the context of the contact theory and the theory of successful aging. It is of note that a horrific situation such as the war and neo-liberal values that advocate for activity and contribution may have unexpected positive consequences in the form of reduced ageism.

Abstract citation ID: igaf122.916
**BLIND TO BIAS: THE IMPACT OF PRESCRIPTIVE AGE NORMS ON PERCEIVED AGE DISCRIMINATION**
M. Clara de Paula Couto[1], and Klaus Rothermund[2], *1. Friedrich-Schiller-University Jena, Jena, Thuringen, Germany, 2. Friedrich-Schiller University Jena, Institute of Psychology, Jena, Thuringen, Germany*
Age discrimination is prevalent in many societies, impacting psychological well-being, self-esteem, and health. It can be experienced personally (directly) or socially (by observing others being targeted). A key factor shaping these experiences is the presence of prescriptive age norms. For example, when older adults are expected to withdraw from social roles to make way for younger generations (the disengagement norm), violating these expectations may lead to age discrimination and feelings of guilt or shame. This study investigates whether the endorsement of prescriptive age norms influences the perception of age discrimination across age groups. Strong adherence to these norms may cause older individuals to not perceive or trivialize age discrimination and to internalize blame for their experiences instead of attributing it to external factors. The sample was drawn from the Aging-as-Future questionnaire study, which included 2,902 participants aged 40 to 90 years. Moderated regression analyses examined the relationship between endorsement of disengagement norms and perceived age discrimination, with age as a moderator. Results showed that stronger endorsement of disengagement norms was linked to fewer reports of age discrimination, particularly among older adults. In conclusion, this study highlights the complex role of prescriptive age norms, suggesting that strong endorsement of disengagement norms may reduce sensitivity for age discrimination. Understanding these norms' psychological impact is crucial for addressing age discrimination across different age groups.

Abstract citation ID: igaf122.917
**INTERSECTIONAL AGEISM: PRESCRIPTIVE STEREOTYPES AND MORAL JUDGMENTS OF OLDER ADULTS**
Hannah Gans, *University of Toronto, Toronto, Ontario, Canada*
Research on ageism has primarily focused on descriptive stereotypes, while prescriptive expectations—societal beliefs about how older adults should behave—have received less attention, particularly in relation to intersecting identities. When older adults do not conform to these expectations, they may experience negative social consequences, and drawing from intersectionality research, these consequences are likely to vary among older adults with different intersecting identities. To examine these dynamics, we surveyed 355 young adults on societal pressures placed on older adults with intersecting identities. Using a modified Succession, Identity, and Consumption scale (North & Fiske, 2013), we varied the target's race (Black, White) and gender (man, woman). In a separate analysis, participants also rated the perceived immorality of these targets in society. We found that succession-based prescriptive stereotypes—specifically the expectation that older adults should make room for younger generations—varied by intersectional identity, such that older White men were most strongly expected to adhere to this stereotype. A separate analysis of moral violation ratings revealed that perceptions of immorality also varied across intersectional targets, with older White men rated as the greatest moral violators in society. These findings demonstrate that prescriptive age stereotypes and moral judgments are shaped by intersecting identities. Older White men were most strongly expected to step aside for younger generations and were also rated as the greatest moral violators in society. This highlights the need for further research on how intersectional biases shape perceptions of aging and the distinct social consequences older adults face in different domains.

Abstract citation ID: igaf122.918
**THE PARADOX OF DIGITAL AGEISM: CULTURAL AND AGE DIFFERENCES IN TECHNOLOGY USE BETWEEN CHINA AND THE U.S.**
Wanyu Xi[1], Ittay Mannheim[2], Yuxiang (Chris) Zhao[3], Ella Cohn-Schwartz[2], and Yaacov G. Bachner[2], *1. Nanjing University of Chinese Medicine, Nanjing, Jiangsu, China (People's Republic), 2. Ben Gurion University of the Negev, Be'er Sheva, HaDarom, Israel, 3. Nanjing University, Nanjing, Jiangsu, China (People's Republic)*

Digital ageism—the stereotyping, prejudice, and discrimination against older adults in digital contexts—is an increasing challenge alongside global digitalization. As ageism differs between Eastern and Western cultures and digital progress varies worldwide, this study reveals a paradox in digital ageism between China and the US, examining its impact on technology use. In 2024, we surveyed 410 Chinese participants (ages 18-86, Mage=51.49, SD = 17.86, 65.9% women) and 398 Americans (ages 18-85, Mage=51.63, SD = 17.00, 48.5% women) using both online and paper questionnaires. Measurements included digital ageism, measured with the Attitudes Towards Older Adults Using Technology (ATOAUT) scale, daily technology use, and demographic covariates. A two-way ANOVA revealed significantly higher levels of digital ageism among Chinese participants overall. Paradoxically, older Chinese exhibited higher digital ageism than younger Chinese, while older Americans displayed less ageism than younger Americans. Moreover, a regression analysis showed that, after controlling for demographic factors, higher digital ageism may suppress daily technology engagement for middle-aged and older Chinese (rather than younger adults), yet it unexpectedly boosted younger and middle-aged Americans' technology use (rather than older adults). These findings suggest that middle-aged and older adults in China, which is undergoing rapid digitalization, are more likely to internalize digital ageism, despite filial piety valuing elder respect. In contrast, older adults in America, where individualism is emphasized, may defy self-ageism, thus avoiding following negative impact. By illuminating digital ageism's cultural and age-specific dynamics, this research highlights the need for targeted interventions to promote inclusive technology use across diverse societies.

Abstract citation ID: igaf122.919

### MILD COGNITIVE IMPAIRMENT RECOVERY:ROLE OF RESISTING AGEISM AND EMBRACING POSITIVE AGE BELIEFS

Becca Levy[1], and Martin Slade[2], *1. Yale School of Public Health, New Haven, Connecticut, United States, 2. Yale University, New Haven, Connecticut, United States*

It is widely assumed that individuals who develop mild cognitive impairment (MCI) will not recover. Yet nearly half of older persons with MCI regain normal cognition. The reason for this improvement is not well understood. This study is the first to consider whether a culture-based factor—positive age beliefs—contributes to MCI recovery. In previous experimental studies with older persons, positive age beliefs reduced stress caused by cognitive challenges, increased self-confidence about cognition, and improved cognitive performance. We therefore hypothesized that older individuals with positive age beliefs would demonstrate higher and faster rates of cognitive recovery compared to those with negative age beliefs. Data from the Health and Retirement Study provided a cohort of 1716 participants, aged 65 and older, all of whom had baseline MCI. As predicted, participants with positive age beliefs had a 30.2% higher likelihood of cognitive recovery and transitioned from MCI to normal cognition more rapidly (hazard ratio, 1.26; 95% CI, 1.08-1.46; p = .003). Additionally, those with positive age beliefs showed lower rates of developing MCI over a 12-year period. These results underscore the potential of addressing ageism by promoting positive age beliefs at both individual and societal levels, suggesting that such interventions could enhance cognitive recovery and reduce MCI prevalence.

### SESSION 2375 (SYMPOSIUM)

Abstract citation ID: igaf122.920

### FAMILY CAREGIVING ACROSS THE LIFE COURSE: PATTERNS, CONSEQUENCES AND SUPPORT STRATEGIES

Chair: Yiqing Qian
Co-Chair: Katherine Miller
Discussant: Katherine Ornstein

Caregiving for aging families represents an important life transition, intersecting with many life domains such as family relationships, education, employment, and social engagement. Despite its long-term implications on health and well-being, fewer studies have investigated caregiving as a dynamic lifespan experience and its impacts on late-life outcomes. This symposium integrates multiple data sources and approaches to build evidence on family caregiving across the life course and seek solutions to enhance family caregiver support through policy and technology. First, we will examine the life histories of family caregivers using panel data from the Health and Retirement Study. Dr. Miller describes the educational and economic status of youth caregivers as older adults compared to individuals who never become caregivers. Dr. Johnson explores how early-life relationships with parents and grandparents differ between caregivers and those who never become caregivers. Next, we focus on gendered patterns of caregiving for older adults and their implications for work and health using nationally representative data from the National Study of Caregiving. Dr. Patterson reports national trends regarding the impact of caregiving on work by gender. Dr. Gimm examines gender differences in caregiving tasks and strain among older spousal caregivers. Finally, with a focus on solutions to support caregivers in self-care, social participation, and navigating emerging challenges, Dr. Hu presents the pilot testing of a novel user-centered mobile health application to assist caregivers. Dr. Ornstein, as the discussant, will synthesize the implications for research and policies to support the growing needs of diverse family caregivers in the US.

Abstract citation ID: igaf122.921

### THE LONG-TERM SOCIOECONOMIC OUTCOMES OF YOUTH CAREGIVERS: EVIDENCE FROM THE HEALTH AND RETIREMENT STUDY

Katherine Miller[1], Elise Parrish[2], Yiqing Qian[3], and Regina Shih[4], *1. Johns Hopkins Bloomberg School of Public Health, Baltimore, Maryland, United States, 2. University of Pennsylvania, Philadelphia, Pennsylvania, United States, 3. Johns Hopkins University School of Nursing, Baltimore, Maryland, United States, 4. Emory University Rollins School of Public Health, Atlanta, Georgia, United States*

Family (unpaid) caregivers are the backbone of the long-term care delivery system in the United States. The economic consequences of providing unpaid care are overwhelming negative. However, the existing evidence excludes youth family caregivers. Youth caregivers typically have lower educational attainment and worse mental and physical health outcomes than non-caregiving peers, yet the long-term economic outcomes for youth caregiving are unknown. We describe the associations between youth caregiving (aged <25 at time of first caregiving episode) and later-life economic and caregiving outcomes compared to individuals who never report caregiving in their lifetime. We use the Health and Retirement Study Core and Leave Behind Supplement. Among youth caregivers, respondents reported two caregiving episodes (providing unpaid care for ≥6 months) on average over the life course and 15% became a caregiver again after age 50. Compared to individuals who never became caregivers, youth caregivers are significantly more likely to be female (77% vs 51%); have lower levels of life-time educational attainment (25% have bachelor's or higher versus 34%); and were less likely to initiate post-secondary education within 2 years of graduating high school (-14.2 percentage points). Youth caregivers' predicted social security value at age 50 is approximately $23,000 less than never caregivers ($75,071 vs. $98,271). Youth caregiving before age 25 is associated with later life socioeconomic status and likelihood of becoming a caregiver in later life. Further research to disentangle the causal effects of caregiving across the life course is necessary to inform targeted policy supports to mitigate long-term adverse economic consequences.

Abstract citation ID: igaf122.922

### EARLY-LIFE EXPERIENCES AND CAREGIVER STATUS IN MID- AND LATER ADULTHOOD

Florence Johnson[1], Maria Roche-Dean[2], Yiqing Qian[3], Jacobs Moyosoreoluwa[4], Richard Gonzalez[5], and Sheria Robinson-Lane[1], *1. University of Michigan School of Nursing, Ann Arbor, Michigan, United States, 2. Kirkhof College of Nursing, Grand rapids, Michigan, United States, 3. Johns Hopkins University School of Nursing, Baltimore, Maryland, United States, 4. Wayne State University Department of Psychology, Detroit, Michigan, United States, 5. University of Michigan, Ann Arbor, Michigan, United States*

The selection of caregivers within families is a complex process influenced by various early-life factors, such as parental relationships, a history of physical abuse, and care provided by grandparents. Understanding how these contextual factors lead to the role of the adult family caregiver is crucial for developing effective support systems for caregivers. Using data from the 2019 Health and Retirement Study Life History survey, this study described early family life experiences below age 18 among caregivers and non-caregivers. Among the 2,849 respondents, we found 23% (n = 650) reported ever providing unpaid care to an adult family or friend for at least 6 months. Female respondents (29%) were more likely to be caregivers than male respondents (15%). We found respondents who lived with grandparents before 18 (27%) were more likely to be caregivers. Notably, about 32% of respondents who reported physical abuse before age 18 identified as caregivers, higher than the percentage of caregivers among those who did not report abuse (24%). Furthermore, we found those with good relationships with mothers were less likely to be caregivers (23% vs 27%). No difference in relationships with fathers was found (24%). Overall, these results highlight caregivers' diverse backgrounds and experiences, underscoring the need for targeted support and resources to address the unique challenges different caregiving populations face. Further research is needed to understand the underlying factors driving these caregiving patterns and to develop interventions that can alleviate the burden on caregivers, particularly those with histories of trauma or living in multigenerational households.

Abstract citation ID: igaf122.923

### TRENDS IN CAREGIVING, WORK, AND CAREGIVING-RELATED WORK CONSEQUENCES FOR CAREGIVERS OF OLDER ADULTS

Sarah Patterson[1], Vicki Freedman[1], Jennifer Cornman[2], and Jennifer Wolff[3], *1. University of Michigan, Ann Arbor, Michigan, United States, 2. Columbus, Ohio, United States, 3. Johns Hopkins University - School of Public Health, Baltimore, Maryland, United States*

Both men and women continue to have high rates of employment at the same time that more family and unpaid caregivers are providing help to an older adult. We use the National Study of Caregiving (NSOC) 2011 - 2023 to investigate trends among working caregivers to older adults. Findings reveal that the number of working caregivers to older adults grew from 7.8 million in 2011 to 9.6 million in 2023. Women comprised more than 70% of working caregivers in both years. The percentage of working caregivers assisting older adults with dementia decreased for both men and women, from about half in 2011 to slightly more than a third in 2023. Among working caregivers, women were more likely than men to experience distractions at work related to caregiving, or "presenteeism" in 2011 (7.5. vs. 3.0; p<.01); but men experienced a significant increase in presenteeism from 2011 to 2023 (3.0 to 7.1; p<.05), which closed the gap (7.1 men, 9.1 women, n.s.). Similar patterns were found for work productivity loss (a measure combining absenteeism and presenteeism). Trends in the percentage providing specific types of care converged for working men and women for personal care, chores, ordering medications, and health-care related activities (with men's provision of these types of care no longer significantly lower than women's provision). Results have implications for policies and programs aimed at supporting working caregivers of older adults.

Abstract citation ID: igaf122.924

### ASSESSING GENDER DIFFERENCES IN CAREGIVER STRAIN AND ACTIVITIES AMONG OLDER ADULT SPOUSAL CAREGIVERS

Gilbert Gimm[1], Yiqing Qian[2], Mary Louise Pomeroy[3], and Katherine Ornstein[4], *1. George Mason University, Fairfax, Virginia, United States, 2. Johns Hopkins University School of Nursing, Baltimore, Maryland, United States, 3. Johns Hopkins Bloomberg School of Public Health, Baltimore, Maryland, United States, 4. Johns Hopkins University, Baltimore, Maryland, United States*

Male spousal caregivers have received less attention in the aging literature despite playing an important and growing role. Although prior studies have found male adult caregivers have lower average burden scores compared to female adult caregivers, less is known about whether such gender differences are associated with caregiving activities or hours. This study examined differences in caregiving activities and hours of male vs. female spousal caregivers of community-dwelling Medicare beneficiaries. Using nationally representative data on 451 spousal caregivers from Round 12 of the National Study of Caregiving (NSOC), we conducted weighted bivariate analyses to assess differences in caregiving roles by gender. We found that males represented almost one-half (45.5%) of the 5.2 million spousal caregivers. Among racial/ethnic subgroups, only non-White Hispanics had a larger share of male vs female spousal caregivers (12.8% vs. 6.3%). Although male spouses were less likely to experience physical strain (18.6% vs. 60.4%, $p < 0.01$) and emotional strain (18.7% vs 37.9%, $p < 0.01$) than female spouses, average caregiving hours did not significantly differ by gender. For caregiving activities, male spouses were more likely to provide transportation assistance than female spouses (81.9% vs. 61.4%, $p < 0.01$), but were less likely to assist with health behavior management (52.3% vs 69.8%, $p < 0.05$) or health system logistics (46.9% vs 79.6%, $p < 0.01$). Overall, males represented nearly one-half of spousal caregivers. These results suggest the need for additional studies on male caregiver activities and non-pharmacological interventions (online peer support) that can be tailored to address the unique experiences and needs of male caregivers.

Abstract citation ID: igaf122.925

### IMPROVING SUPPORT THROUGH TECHNOLOGY: PILOT TESTING OF A USER-CENTERED MOBILE HEALTH APP FOR FAMILY CAREGIVERS

Haomin Hu[1], I Made Agus Setiawan[2], Zara Ambadar[3], Andi Saptono[2], Heidi Donovan[1], Brad Dicianno[3], Bambang Parmanto[2], and Yong Kyung Choi[1], *1. University of Pittsburgh, Pittsburgh, Pennsylvania, United States, 2. University of Pittsburgh Department of Health Information Management, School of Health and Rehabilitation Sciences, Pittsburgh, Pennsylvania, United States, 3. University of Pittsburgh Department of Physical Medicine and Rehabilitation, School of Medicine, Pittsburgh, Pennsylvania, United States*

Family caregivers (caregivers) often begin their caregiving roles unexpectedly without adequate training or support. They prioritize caregiving over self-care, leading to compromised health outcomes. In response, we employed user-centered approaches to interview caregivers, co-design solutions, and develop a multi-component Family Caregiver App. While many digital solutions focus solely on caregiving skills, our Family Caregiver App uniquely integrates self-care alongside caregiving education. This study aimed to evaluate the app's usability, acceptability, and feasibility in a three-week cohort with 12 caregivers averaging 44.5 (±13.69) years of age. Usability was measured with the Mobile Health App Usability Questionnaire (MAUQ), ranging from 1 (disagree) to 7 (agree), resulting in an average score of 5.49 (±0.92). Acceptability, assessed through MAUQ subscales (ease of use: 5.62 ± 0.72, usefulness: 5.11 ± 1.35) and qualitative feedback guided by the Technology Acceptance Model (TAM), revealed positive user perceptions. Caregivers highlighted the app's simple and accessible user interface, intuitive prompts, and straightforward navigation as key facilitators of ease of use. Additionally, cross-platform compatibility and tailored caregiver-focused features contributed significantly to the perceived usefulness and participants' intentions for continued app use. Feasibility metrics indicated high engagement, with all caregivers completing the study and meeting predefined app usage criteria (average usage >1/3 days across more than four modules). Our findings consistently demonstrate that the Family Caregiver App is usable, acceptable, and feasible. Integrating user-centered and inclusive design principles offers a promising avenue to support caregivers, holistically addressing their caregiving responsibilities and well-being.

### SESSION 2380 (COMMUNITY ENGAGED SCHOLARSHIP SYMPOSIUM)

Abstract citation ID: igaf122.926

### SUSTAINABLE COMMUNITY ENGAGED EXERCISE AND PHYSICAL ACTIVITY FOR UNDERREPRESENTED OLDER ADULTS

Chair: Rebecca Lassell
Co-Chair: Laura Baehr
Discussant: Brian Andonian

This symposium aims to showcase three approaches to community-engaged research for exercise and physical activity intervention development and implementation for underrepresented older adults. Presenters will provide a how-to guide with examples of recruitment and study materials and community engagement strategies as well as lessons learned and recommendations for early career investigators. Four presenters will provide examples from their research. Presentations will include a co-design process to identify community challenges and solutions to design a nature lifestyle intervention for Chinese American older adults, a community-engaged fitness facility recreational needs assessment to enhance participation by older adults; and the implementation of a peer-supported intervention for Latinx older adults with Parkinson's. This symposium will cover 1) How to build trust and relationships communities with two examples from institutions with varying levels of infrastructure and resources 2) different methodological approaches to understanding community needs ranging from needs assessments to a six-step co-design process, 3) strategies to engage underrepresented older adults in exercise and physical activity intervention design and implementation, and 4) new learnings and a how-to guide from early career investigators to start and implement exercise and physical activity interventions in the NIH Stage Model Stages 0-1. Fitness, Exercise and Wellness Interest Group Sponsored Symposium

Abstract citation ID: igaf122.927

### CO-DESIGNING A GREEN ACTIVITY PROGRAM WITH CHINESE AMERICAN PEOPLE LIVING WITH MEMORY LOSS

Jun Luo[1], Shih-Yin Lin[2], Jennifer Wong[1], Lorna Thorpe[1], Stella Yi[1], Abraham Brody[3], Hayley Belli[1], and Rebecca Lassell[4], *1. New York University, New York, New York, United States, 2. New York University Rory Meyers College of Nursing, New York, New York, United States, 3. NYU Rory Meyers College of Nursing, New York, New York, United States, 4. Indiana University Bloomington, Bloomington, Indiana, United States*

Chinese American adults have the highest incidence of Alzheimer's disease among Asian American groups. Few programs exist to address cognitive decline that are designed with their input. We sought to co-design a nature or 'Green' Activity Program (GAP) to promote physical activity tailored to the needs of Chinese American people living with memory loss (PLML) or dementia. Participants were recruited in Brooklyn, New York. Co-design (engaging end-users in design) occurred separately with Chinese American PLML, professional care partners, and outdoor professionals across three 60-90-minute focus groups.

Sessions were recorded, transcribed, translated, and analyzed using directed content analysis. A priori Program Design codes were: 'preferred nature activities,' 'session frequency, duration, delivery mode'; Research Design codes were: 'outcomes,' 'recruitment strategies,' and a 'referral pathway.' Participants included Chinese American PLML (n = 4), Chinese American professional care partners (n = 5), and outdoor professionals (n = 6). Program Design preferences were: 1) session duration: 30 minutes, 2) frequency: 4-8 across 12-weeks, and 3) delivery: in-person and virtual. 'Existing nature activities' included dance and walking, while 'preferred nature activities' included walking, Tai Chi, and Wuxing Cao (5-element exercises). Research Design preferences included 'outcomes' of delaying memory loss, improving physical function, and socializing. 'Recruitment strategies' included in-person events, with 'preferred terms' being "memory loss" and "brain health." 'Referral pathways' involved champions at family health and older adult centers. 'Barriers' included physical limitations, inconveniences, and cost. 'Facilitators' were a personalized approach. Co-design led to a culturally tailored GAP with/for Chinese American PLML to guide piloting the program in preparation for efficacy testing.

Abstract citation ID: igaf122.928

### RECRUITMENT OF LATINX OLDER ADULTS WITH PARKINSON DISEASE FOR A REMOTE PHYSICAL ACTIVITY INTERVENTION TRIAL

Cristina Colón-Semenza, *University of Connecticut, Storrs, Connecticut, United States*

Recruitment for a completely remote, culturally and linguistically tailored, peer supported, physical activity intervention for older Hispanic/Latinx adults with Parkinson disease (PD) was evaluated. We employed both direct and indirect recruitment methods. Direct methods included direct contact by a study team member that was bilingual/bicultural (Spanish and English). Direct contact occurred via individualized telephone calls or emails from the study team (previous research participants), individualized letters from their treating neurologist (research team member), or research team member presentations in support group meetings. Indirect methods were also targeted at Hispanic/Latinx populations and available in Spanish and English but included general announcements or education that were presented to large audiences. This included recruitment flyer postings on social media, a news article within a university publication, organizational newsletters or listservs, large general community events in Latinx communities, shared with healthcare providers and within healthcare systems, and national webinars. Thirty-six individuals expressed interest in the study over a 6-month period (6 individuals/month). Fifty-six percent (20/36) were recruited through direct methods and 44% (16/36) through indirect methods. Twenty-nine individuals were screened, with 34.5 % consented. Of the 10 individuals that participated, 80% (8/10) were recruited through direct methods. The recruitment method that was most effective was direct contact by a research team member from within the target community. All 10 individuals that passed the screening and were consented were retained for the 12-week study duration. Direct methods, although labor intensive, resulted in greater numbers of underrepresented participants recruited and retained in this physical activity intervention.

Abstract citation ID: igaf122.929

### ADVANCING HEALTH EQUITY OF OLDER ADULTS THROUGH A COMMUNITY-ENGAGED RECREATIONAL NEEDS ASSESSMENT IN PHILADELPHIA

Anna Grasso, and Laura Baehr, *Drexel University, Philadelphia, Pennsylvania, United States*

Most older adults are living with at least one chronic health condition that can be positively modified through regular exercise, but less than 15% of this population achieve national physical activity guidelines for health. Several social, organizational, and environmental barriers contribute to the physical activity participation gap. Physical inactivity is highest among older adults with chronic disease and disability, reporting non-white race or Hispanic ethnicity, and lower socioeconomic and educational levels. Inaccessible recreational environments also limit community-based exercise and physical activity by older adults. While it is a federal mandate for all public buildings to comply with Americans with Disabilities Act (ADA) accessibility standards, there are additional recreational considerations for older adults that are rarely addressed or corrected. The purpose of this project is to evaluate the recreational accessibility of the Drexel Recreation Center, a public health club in the heart of an urban university campus that is proximate to some of the most socioeconomically disadvantaged older adult residents in the United States as defined by the Area Deprivation Index. Using the Person-Environment-Occupation-Performance (PEOP) model, the team completed a recreational needs assessment inclusive of the Accessibility Instruments Measuring Fitness and Recreation Environments (AIMFREE) tool, physical therapy and occupational therapy clinical knowledge, and community advisory board member input. A voluntary electronic survey was sent to older adults who are current or prospective members of the recreation center to determine use, barriers, and facilitators to participation. Data collection is ongoing, with a planned townhall to distribute results to all invested community members.

### SESSION 2385 (SYMPOSIUM)

Abstract citation ID: igaf122.930

### THE HISTORY OF SOCIAL AND BEHAVIORAL RESEARCH IN GERONTOLOGY

Chair: Soomi Lee

Discussant: Karl Pillemer

Celebrating GSA's 80th anniversary, we launched the inaugural session of "History of Gerontology" at GSA 2024, which generated significant interest and enthusiasm among members and attendees. The session highlighted the importance of continuing this initiative to provide an educational and scholarly exchange platform rooted in historical perspectives. Building on this momentum, we propose a second symposium in this series, focusing on the history and importance of social and behavioral research in gerontology. This theme aligns well with this year's focus, "Innovative Horizons in Gerontology," fostering discussions on how advancement in social and behavioral research can generate novel insights into disease prevention and well-being promotion. While social and behavioral research in gerontology encompasses many significant topics, our three panelists will focus on their expertise in long-term care, caregiving, and stress research, sharing valuable insights and experiences. Dr. Kathleen Wilber will reflect on lessons from over five decades of long-term care program and policy development. Dr. Steven H. Zarit will discuss the evolution of caregiving research and the challenges of capturing the complexity of caregiving. Dr. David Chiriboga will examine the history of stress research and its contributions to advancing gerontology. The discussant, Dr. Karl Pillemer, will engage panelists and the audience in exploring the role of social and behavioral research in Translational Geroscience. This symposium provides an opportunity for the Academy for Gerontology in Higher Education (AGHE) and the broader GSA community to reflect on the lasting impact of social and behavioral research in enhancing support for the aging population. This is a collaborative symposium between the Age Inclusivity in Higher Education and Generativity and Aging Interest Groups.

Abstract citation ID: igaf122.931

### CAREGIVING THROUGH ANOTHER LENS: THE HISTORY OF LONG-TERM CARE

Kathleen Wilber, *University of Southern California, Los Angeles, California, United States*

Long-term care has been a key focus of gerontological research for decades. Passage of the Medicaid program in 1965 led to a boom in the nursing home industry. In 1971 the White House Conference on Aging called for the development of non-institutional services. States launched demonstration programs in home and community- based services (e.g., adult day health care, case management) and seminal research (Weissart,1979; the Channeling Demonstration) assessed “nursing home alternatives.” Although studies failed to demonstrate diversion of high-risk people from placement, policymakers, undeterred, began to enact Medicaid waivers to “rebalance” services for aging in place. Efforts also focused on improving care in nursing facilities. The 1987 Nursing Home Reform Act sought to provide systematic data, expand monitoring, and require input from residents and families. Private sector investments in Assisted Living, offered more home-like environments for those with means. Oversight and litigation expanded. In the early 2000s, culture change programs sought to overhaul how care is provided. This presentation will offer lessons from over five decades of program and policy development. The long-term care landscape includes innovative models and pioneering programs. Yet scandals continue; the COVID pandemic revealed heroes, villains, and examples of horrifying care, facility ownership and hence responsibility is often hidden. On the “alternatives” side, those seeking home and community-based services may find that they are not eligible. Those that are eligible may encounter long waiting lists. What are the lessons learned, where are we, how can we do better?

Abstract citation ID: igaf122.932

### HISTORY AND DIRECTION OF GERONTOLOGY AND CAREGIVING

Steven Zarit, *The Pennsylvania State University, University Park, Pennsylvania, United States*

There has been so much research on caregiving in recent years that we do not recognize that it once was a backwater in the field. My goals are to consider where we have gotten, what we should emphasize, and next steps: (1) What is the patient’s disorder? The impact on patient and caregiver can be very different for different illnesses. (2) Most studies only look at caregivers. Karen Lyons has emphasized that we need to look at caregiver and patient as a dyad and analyze their findings together to understand benefits and potential harms. (3) Lyons’ also addresses another issue often overlooked in interventions, using measures that can potentially show change. A common example is using depression as an outcome but many of the caregivers in studies may not be depressed at the outset and so can’t show improvement. (4) Another issue is the family network and how that affects caregiver and patient. Family dynamics and support have a major effect on the primary caregiver. Bringing together family members in a therapeutic model has been one of the more promising interventions. (5) The caregiver’s social network. Support groups have become less common, but they can have considerable benefit in sharing ideas, learning skills, and learning about community resources. They may be effective but the early studies including my own may have used the wrong outcomes. (6) For group and family interventions, the leaders should have clinical training. There are excellent models of intervention that could achieve positive outcomes.

Abstract citation ID: igaf122.933

### HISTORICAL PERSPECTIVES ON THE EVOLUTION OF STRESSOR RESEARCH

David Chiriboga, *University of South Florida, Tampa, Florida, United States*

This presentation will focus on changes in how stressors, the things that cause distress, have been studied. Early work was generally of two types (1): lab studies where people, often students, were exposed to stressful film, electric shock, or stressing situations; (2) studies of how people responded to natural disasters, bereavement, etc. These studies primarily focused on post-stress reactions, and on how people coped. During the 60s the development of inventories of potentially stressful events broadened the scope of stress research to include so-called “life events”. Several inventories were developed, with the Holmes and Rahe (1967) Schedule of Recent Events the best known. However, a number of inventories were developed, with several including events in later life. Underlying this second wave of research was the idea that how people perceived the stressful nature of events was associated with outcomes, although there was an initial, erroneous assumption that most people agree about how much disruption the various events caused. In addition to this research, there were a number of researchers who looked at what might be called micro events (e.g., daily hassles), or macro events like economic downturns or the threat of war. While for many years there was general agreement about the adequacy of specific measures, their use became less consistent over the past two decades. Today, the approach to stressor measurement is much more varied, with little consensus as to which is the most effective. Overall, however, stressors continue to demonstrate their relevance to the study of behavioral outcomes.

### SESSION 2390 (PAPER)

### VULNERABILITY AND VISIBILITY: EXPLORING AGING THROUGH THE LENS OF STRESS, IDENTITY, AND FINANCIAL STRAIN

Abstract citation ID: igaf122.934

### ASSOCIATIONS BETWEEN SEXUAL ORIENTATION AND SELF-PERCEPTIONS OF AGING: THE WITHERING ROLE OF STRESS

Anyah Prasad[1], and Serena Sabatini[2], *1. Vanderbilt University, Boston, Massachusetts, United States, 2. University of Barcelona, Barcelona, Catalonia, Spain*

Self-perceptions of Aging (SPAs) are an overarching term encompassing different aspects of people's awareness of aging, including their own internal aging processes, changes in their social roles/relationships, and the broader cultural notions of aging. Generally, people with more positive SPAs tend to be healthier and live longer. This study uses nationally representative data from the German Ageing Survey (wave 2021) to investigate the differences between sexual minority and heterosexual respondents on multiple dimensions of SPAs and examine the mediating role of stress. Two hundred and fifty seven sexual minority respondents were matched one-to-one with heterosexual respondents based on their age (mean 72 years) and sex (45% women). Cross-sectional linear regression and mediation models were estimated. Sexual minority respondents had more negative attitudes towards own aging (Standardized $\beta$=-0.14, $p = 0.001$) and reported more perceived social losses ($\beta = 0.11$, $p = 0.017$) than heterosexual respondents. Sexual orientation was not significantly associated with felt age, perceived physical losses, personal growth, and self-knowledge. Stress fully mediated the cross-sectional association of sexual orientation with attitudes towards own aging, and perceived social losses. Although causality cannot be inferred, higher stress may explain why sexual minority respondents may experience more negative attitudes towards own aging and social losses. SPAs can be used as a screening tool to identify sexual minority older adults at most risk. Interventions to reduce stress and improve SPAs may help in supporting sexual minority individuals' future wellbeing.

Abstract citation ID: igaf122.935

### ASSOCIATIONS BETWEEN SOCIAL NETWORK RELATIONSHIPS, COGNITIVE FUNCTIONING, AND FINANCIAL EXPLOITATION

Gali Weissberger[1], Shira Peleg[1], and Ella Cohn-Schwartz[2], *1. Bar-Ilan University, Ramat Gan, HaMerkaz, Israel, 2. Ben Gurion University of the Negev, Be'er Sheva, HaDarom, Israel*

Financial exploitation (FE) of older adults usually involves a social interaction between the older adult and the exploiter. However, few studies have comprehensively examined social network compositions of older adults who experienced FE. In this study utilizing data from the Survey of Health, Ageing, and Retirement in Europe and Israel (SHARE) Wave 8, we examined whether social relationships, cognitive functioning, and their interactions, are associated with FE in a nationally representative sample of Israeli older adults. Participants ($N = 704$; M age=71.90, SD = 8.08, Range=53-103) self-reported whether or not they experienced FE over the past 12 months. They also completed social network and cognitive assessments. A global composite score of cognition was calculated based on scores across four cognitive tests. A logistic regression model regressed FE on the presence/absence of children, spouses, other family, and friends in networks, global cognition, and covariates. Interactions between global cognition and relationship types were also tested. The results demonstrated that the presence of children and friends in one's close network were associated with a lower likelihood of experiencing FE (Children: OR = 0.57, $p = 0.011$; Friends: OR = 0.30, $p < 0.001$). Interactions revealed that the association of having friends in the network with FE was significant for those with average or above average global cognition, but not for those with below average global cognition. Findings suggest that friends may play a critical role in protecting against FE when cognition is intact, possibly because family members have less involvement in financial decisions in such circumstances.

Abstract citation ID: igaf122.936

### ASSOCIATIONS BETWEEN SUBJECTIVE AGE AND HISTORY OF INCARCERATION IN A POPULATION-BASED SAMPLE OF OLDER ADULTS

Rodlescia Sneed, and Sabrina Blank, *Wayne State University, Detroit, Michigan, United States*

The number of older adults with history of incarceration (HOI) has increased significantly since the 1990s; however, the psychological effects of incarceration on older adults are understudied. Using pooled data from the 2012 and 2014 waves of the Health and Retirement Study, we examined associations between HOI and older subjective age and explored potential mediators. Participants were considered to have older subjective age if they reported feeling older than their chronological age. We used logistic regression to evaluate the association between HOI and older subjective age, adjusting for demographic characteristics. To examine mediation effects, we performed a Karlson-Holm-Breen (KHB) decomposition analysis using logistic regression. Potential mediators included depressive symptoms, number of chronic conditions, income, number of difficulties with activities of daily living (ADLs), and years of education. We observed a significant association between HOI and older subjective age. (OR: 1.72; 95% CI: 1.44-2.05). Fifty-seven percent of the observed effect of HOI on older subjective age was accounted for by these mediators. Depressive symptoms were the primary mediator, accounting for 72.26% of the mediation effect. Number of chronic conditions, years of education, and number of ADLs accounted for 11.40%, 11.02%, and 7.02% of the mediation effect, respectively. Income did not contribute significantly. Our findings underscore the need to address the mental health needs in this population to promote healthier aging. Further, the significant impact of multiple mediators highlights the interplay of social, physical, and psychological vulnerabilities in this population.

Abstract citation ID: igaf122.937

### INCIDENT TRAUMATIC EXPERIENCES AND POOR FINANCIAL WELLBEING: A DOUBLE HIT FOR COGNITIVE AGING?

Katrina Kezios[1], and Eleanor Hayes-Larson[2], *1. Boston University, Boston, Massachusetts, United States, 2. University of Southern California, Los Angeles, California, United States*

Traumatic experiences and financial hardship may jointly affect cognitive aging through shared stress-related pathways. Using data from the Health and Retirement Study ($N = 3432$), we quantified the effect of incident traumatic experiences (ITE, occurring 2006-2010 or 2008-2012), defined as experiencing ≥1 events from the Lifetime Traumas index (death of a child, major natural disaster, firing/being fired upon in combat, spouse/partner/child addicted to drugs/alcohol, serious physical attack/assault, life-threatening illness/accident, or life-threatening illness/accident in spouse/child). We examined effect modification by poor financial wellbeing (FWB), defined as reporting ≥3 of 7 subjective (financial dissatisfaction, low financial control, difficulty paying bills, taking less medication because of money) and objective (low income, low non-housing wealth, no housing wealth) indicators of financial hardship. Memory was assessed biennially from 2010/2012 to 2020 via a composite score computed from direct memory assessments and proxy reports. We used linear mixed models to estimate the effect of ITE on ~10-year memory decline overall and stratified by FWB. Models used follow-up time as the timescale and adjusted for baseline age, practice effects, sex/gender, race, educational attainment, parental education, Southern birth, childhood SES, marital status, self-rated health, and interactions between covariates and time. Overall, having ≥1 ITE was associated with accelerated memory decline (β_anyITE*time=-0.007, [95% CI:-0.012, -0.002]). The magnitude of this association was larger in the presence of poor FWB (β_anyITE*time|FWB≥3=-0.014, [-0.030, 0.003] vs. β_anyITE*time|FWB < 3=-0.006, [-0.012, -0.001]). Our results suggest financial wellbeing may modify the effects of traumatic experiences and may be a policy intervention point to delay memory decline in older adults.

Abstract citation ID: igaf122.938

**OBJECTIVE AND SUBJECTIVE VISION ABILITIES AND FINANCIAL EXPLOITATION AMONG OLDER ADULTS**

Nava Mironi[1], Gali Weissberger[1], and Yoav Bergman[2],
*1. Bar-Ilan University, Ramat Gan, HaMerkaz, Israel,*
*2. Ashkelon Academic College, Ashkelon, HaDarom, Israel*

Financial exploitation (FE) of older adults, including frauds and scams, has significant negative social/health implications for older adults. Thus, identifying risk factors of FE is imperative for preventing FE and enhancing older adults' well-being. Subtle visual cues in fraudulent transactions (e.g., emails, websites) may be missed in those who have reduced visual abilities. Poor vision may also increase dependence, thereby intensifying FE vulnerability. However, to our knowledge, no study has examined the relationship between objective/subjective vision abilities and FE. Accordingly, we examined the relationship between vision abilities and FE vulnerability among 97 community-dwelling older adults (M = 70.26, SD = 5.61). Participants completed self-report questionnaires assessing subjective vision difficulties, FE vulnerability, and history of FE experiences. Additionally, participants underwent objective vision assessments (near, distant, and contrast sensitivity) conducted by a certified gerontological optometrist. Multiple linear regression and logistic regression models regressed FE vulnerability and FE, respectively, on subjective and objective vision abilities, covarying for background characteristics. While self-reported vision difficulties were associated with FE vulnerability (β =.355, p<.001) and FE (OR = 7.866, p= .005), objective measures of vision were not. Findings suggest that self-perceived vision difficulties/declines are more strongly associated with FE vulnerability and FE experiences than similar objective measures. It is possible that subjective vision reflects a person's cognitive averaging of vision ability that is not fully captured in well-controlled environments characteristic of objective assessments. These findings advance our understanding of FE risk factors and suggest practical approaches for identifying and supporting at risk older adults.

Abstract citation ID: igaf122.939

**SUBJECTIVE FINANCIAL STRAIN AND OBJECTIVE FINANCIAL IMPACT AMONG CAREGIVERS IN THE UNITED STATES**

Yujun Zhu[1], Francesca Falzarano[2], Susanna Mage[3], Donna Benton[3], Kathleen Wilber[2], and Susan Enguidanos[2],
*1. Department of Family Medicine, Keck School of Medicine of USC, Los Angeles, California, United States, 2. University of Southern California, Los Angeles, California, United States, 3. University of Southern California Leonard Davis School of Gerontology, Los Angeles, California, United States*

Currently more than 41.8 million people in the U.S. provide care to an aging family member. These family caregivers provide critical unpaid care at significant personal expense to both financial and emotional well-being. However, limited research has examined the factors contributing to caregivers' financial burden. This study aimed to investigate factors associated with both subjective financial strain and objective financial impact among caregivers of older adults in the U.S. Using cross-sectional data from the nationally representative "Caregiving in the U.S. 2020" survey, we analyzed data from 1,330 caregivers of older adults 50 years and older. We conducted hierarchical logistic regression models to explore key factors associated with subjective financial strain and objective financial impact. Younger care recipients (OR = 0.97, p < 0.001; OR = 0.98, p = 0.001) and the voluntary nature of the caregiving role (OR = 1.72, p < 0.001; OR = 1.32, p = 0.050) differentially influenced subjective and objective financial strain. In adjusted models, providing assistance with more instrumental activities of daily living (IADLs) was significantly associated with higher odds of experiencing subjective financial strain (OR = 1.19, p < 0.001) and objective financial impact (OR = 1.13, p = 0.007). Additionally, caregivers who reported time-consuming financial management tasks had higher odds of experiencing subjective financial strain (OR = 2.45, p < 0.001) and objective financial impact (OR = 1.46, p = 0.002). Our findings highlight the financial vulnerability of family caregivers heavily involved in IADLs, particularly those managing finances, and targeted financial support should be provided to those caregivers. Further, future research is needed to test the effectiveness of novel support programs in reducing caregiver's financial strain.

### SESSION 2395 (SYMPOSIUM)

Abstract citation ID: igaf122.940

**PSYCHOSOCIAL AND EMOTIONAL WELLBEING OF OLDER ADULTS WITH HIV**

Chair: Anna Rubtsova
Co-Chair: Paul Nash
Discussant: David Vance

In the era of modern antiretroviral therapy (ART) when treated people with HIV enjoy improved health outcomes and live nearly normal lifespans, psychosocial and emotional wellbeing of older people with HIV (OPWH) increasingly comes into focus. First, studies are emerging that show that in OPWH with well-controlled HIV important health outcomes, such as aging-related comorbidities, may be less related to HIV and increasingly associated with social and psychosocial determinants of health. Second, there is a growing realization that quality of life, emotional wellbeing, and mental health are important outcomes for whole-person care of OPWH. This symposium explores both these themes. Our first presentation is based on a scoping review of published research examining social network structure, social support, and isolation among older women with HIV and highlights the associations between social relationships and wellbeing. The second presentation discusses results of a qualitative study and explores emotional wellbeing and trust among older adults relative to younger adults with HIV during COVID-19 pandemic in New York City. Our third presentation showcases significant associations between perceived stress, loneliness, social support and frailty in women with HIV, based on statistical analysis of data by the Women's Interagency HIV Study (WIHS). Lastly, our final presentation focuses on loneliness and social engagement as targets for public health interventions in OPWH, reporting results related to the development and pilot testing of B'ACTIV behavioral activation intervention. Together, these presentations provide a broad perspective on ongoing research and future directions for exploring psychosocial and emotional wellbeing in OPWH. HIV, AIDS and Older Adults Interest Group Sponsored Symposium

Abstract citation ID: igaf122.941

**PSYCHOSOCIAL CORRELATES OF FRAILTY IN WOMEN WITH HIV IN THE UNITED STATES**

Anna Rubtsova[1], Deborah Gustafson[2], Michael Plankey[3], Anajali Sharma[4], Maggie Wang[1], C. Christina Mehta[1], Andrew Edmonds[5], and Gina Wingood[6], *1. Emory University, Atlanta, Georgia, United States, 2. SUNY Downstate Medical School, Brooklyn, New York, United States, 3. Georgetown University, Washington, District of Columbia, United States, 4. Albert Einstein College of Medicine, Bronx, New York, United States, 5. The University of North Carolina at Chapel Hill, Chapel Hill, North Carolina, United States, 6. Columbia University Mailman School of Public Health, New York, New York, United States*

Untreated or poorly controlled HIV is associated with frailty, while the use of antiretroviral therapy is protective. Independent of HIV-related factors, whether psychosocial factors are associated with frailty among women with HIV (WWH) has been underexplored. We examined associations of frailty with social support, loneliness, and stress among WWH aged >40 years enrolled in the Women's Interagency HIV Study (WIHS). We included the latest frailty assessment collected in 2017-2019. Frailty was defined via the Fried Frailty Phenotype as meeting ≥3 of 5 criteria: 1) slow walking speed (4-meter timed gait assessment), 2) reduced grip strength (measured by a dynamometer), 3) low physical activity (self-report), 4) exhaustion (self-report), and 5) unintentional weight loss (self-report). Psychosocial assessments were measured with validated scales (R-UCLA Loneliness, MMOS-SS, PSS-10) and collected in 2015-2017. Separate multivariable logistic regression models assessed for association with each psychosocial measure, adjusting for variables earlier found to be associated with frailty in the WIHS: age, income, obesity, history of cancer, and history of tuberculosis. Our sample included 1250 WWH: mean age 53.4 (SD = 7.59) years, 72% Black, 62% with an annual household income of USD<18,000. Frailty was significantly associated with loneliness (aOR=1.24, 95% CI = 1.11-1.37), perceived stress (aOR=1.04, 95% CI = 1.01-1.06), and emotional social support (aOR=0.99, 95% CI = 0.985 -0.998), but not with instrumental social support (aOR=0.996, 95% CI = 0.989-1.002). These findings suggest that loneliness, perceived stress, and emotional social support may be important factors worth further investigation. Future longitudinal research is needed to examine the relationships between psychosocial factors and frailty among WWH.

Abstract citation ID: igaf122.942

**A SCOPING REVIEW OF SOCIAL NEEDS AND NETWORKS OF OLDER WOMEN LIVING WITH HIV**

Jasmine Manalel[1], and Alvin Gao[2], *1. Hunter College, CUNY, New York, North Carolina, United States, 2. Hunter College, New York, New York, United States*

Older women living with HIV require adequate support from their social networks to meet their informal care needs and promote psychosocial well-being as they age. Understanding the social resources available to them and their effectiveness in meeting care needs is essential. The purpose of this scoping review is to summarize findings and identify gaps in research on informal care needs and networks of older women living with HIV. We conducted a scoping review using key search terms related to aging, women, HIV, social relationships, and care needs. We included English-language empirical work that focused on women with HIV aged 45 and older and explicitly discussed social relationships or informal care needs. Thematic content analysis was used to analyze the data. Out of 3,108 screened articles, 25 were included. Eighteen studies were based in the United States. Selected articles included 17 qualitative studies (including two longitudinal), 6 cross-sectional quantitative studies, and 3 mixed-methods studies. Guided by the convoy model of social relations, we focused on network structure, support, and isolation. The most common relationships discussed were with romantic partners (13 studies) and children (9 studies). Social relations and informal care needs were shaped by caregiving roles (13 studies), HIV stigma (13 studies), and had wide-ranging impacts on psychosocial outcomes (4 studies). Findings highlighted that social relationships can be both a source of stress and resilience. This review documented the complexity of the care networks for women aging with HIV, shaped by stigma and changing social roles, and their effects on well-being.

Abstract citation ID: igaf122.943

**OLDER ADULTS WITH HIV DISPLAYED ADVANTAGES IN WEATHERING THE PANDEMIC**

Jennifer Kaufman[1], Yiyi Wu[2], Jasmine Manalel[3], Alvin Gao[2], Luis Scaccabarrozzi[4], Vera Antonios[4], Jerome Ernst[4], and Mark Brennan Ing[2], *1. Brookdale Center for Healthy Aging at Hunter College, New York, New York, United States, 2. Hunter College, New York, New York, United States, 3. Hunter College, CUNY, New York, North Carolina, United States, 4. Amida Care, New York, New York, United States*

The COVID-19 pandemic disrupted health care access, challenging care engagement among people living with HIV (PLWH). The pandemic was also a time when social connections frayed, threatening emotional well-being. As part of a qualitative study on care experiences of PLWH during the pandemic, we examined the differences between older and younger participants. We recruited 40 PLWH, aged 23-64, through a Medicaid managed care plan in New York City; 58% were 50 or older. Half the participants were non-Hispanic Black and 28% were Hispanic/Latinx; 55% were cisgender men, 25% cisgender women, and 20% transgender/nonbinary. Almost all the cisgender women were 50 or older. Average time since HIV diagnosis was 22 years. We conducted semistructured phone interviews during fall 2023 regarding the pandemic's impact on care engagement, community program participation, and health and well-being. Overall, participants aged 50 and older reported strong bonds and often long-term relationships with their doctors and health care teams, as well as active community participation. They also expressed relative emotional stability during the pandemic, as behavioral health conditions were fairly well controlled. Participants in their 30s and 40s, by contrast, frequently reported experiences with anxiety, depression, and other behavioral health concerns, often coinciding with lower trust in the health care system. Building physician-patient trust may enhance both care engagement and emotional well-being. On the medical side, older adults, who have more comorbid conditions, reported difficulty seeing specialists and expressed an appreciation for coordinated care. These findings highlight the growing importance of care coordination as PLWH age.

Abstract citation ID: igaf122.944

### B'ACTIV BEHAVIORAL ACTIVATION, NUTRITION, AND ACTIVITY INTERVENTION FOR PERSONS AGING WITH HIV

Ann Gruber-Baldini[1], Laura Nnadi[1], Renee Pepin[2], Elizabeth Dennis[1], Nancy Latham[3], and Denise Orwig[4], *1. University of Maryland School of Medicine, Baltimore, Maryland, United States, 2. Geisel School of Medicine at Dartmouth, Lebanon, New Hampshire, United States, 3. Brigham and Women's Hospital, Boston, Massachusetts, United States, 4. University of Maryland, Baltimore, Baltimore, Maryland, United States*

B'ACTIV is a pilot study of a 12-week Behavioral Activation intervention targeted to decrease loneliness and increase social engagement, that was augmented with specific content to increase home exercise and address poor nutrition among persons aging with HIV (PAWH). The BA intervention includes a coach that focuses participants on their values to align them with behaviors to increase engagement. Recruitment included those ages 50+ with HIV from the University of Maryland THRIVE program. This work has been completed in three stages: Stage 1: A community engagement team of 5 PAWH provided feedback on the intervention, including preferences for delivery mode, recruitment strategies and brochures, measures, other study materials, and inclusion of the physical activity and nutrition modules. Recommendations included use of telephone/in-person intervention, deletion of "HIV" in materials to avoid stigma, and inclusion of foods to help with medication side effects of nausea, lack of appetite, and difficulty swallowing. Stage 2: We worked with HIV clinical and research experts in exercise, nutrition, and psychological functioning to tailor the content of the material and resources for the coaches to meet the needs of PAWH. Stage 3: Following the community engagement and tailoring the intervention for PAWH, we tested the feasibility and acceptability of the intervention. There are currently 7 enrolled (5 active, 1 completed, 1 withdrew). Mean age is 60.0 (SD = 7.5, range 51-75); 5 females/2 males; 6 AA/1 white. This study tests the feasibility of a much-needed intervention to improve functioning and health outcomes among a vulnerable group of older adults.

### SESSION 2400 (SYMPOSIUM)

Abstract citation ID: igaf122.945

### ADVANCING STUDIES ON NEIGHBORHOODS AND BRAIN HEALTH: NEW METHODOLOGIES AND UNDERREPRESENTED GROUPS

Chair: Lilah Besser
Co-Chair: Jessica Finlay
Discussant: Jana Hirsch

Evidence is building for associations between neighborhood social/built environments and brain health in older adults. Studies show that individuals in more deprived neighborhoods have faster cognitive decline and increased dementia risk, while those in neighborhoods with more health-promoting resources (e.g., parks, destinations) have slower cognitive decline and lower dementia risk. However, prior studies had some methodological limitations by not considering critical periods of exposure, underrepresented groups, biological measures of brain health, neighborhood environments as structural determinants of health, or neighborhood environments as effect modifiers. Additionally, the field would benefit from incorporation of technologies such as GPS tracking, which characterize how frequently and in what manner individuals are exposed to community environments. This symposium presents five studies that address these scientific gaps. Dr. Magid will present on five domains of spatial social polarization (SSP) and the projected change in dementia incidence should SSP be eliminated. Dr. Moored will present on the differential findings for an intervention to improve memory among older adults (Experience Corps) based on their neighborhood resources. Dr. Hyun will present on associations between GPS-derived activity space measures of community environment exposure and cognitive function in the Einstein Aging Study. Dr. Sadler will present on the development of structural built environment measures across the life course, which will be linked to cognitive outcomes in the Healthy Aging in Neighborhoods of Diversity across the Life Span cohort. Lastly, Dr. Besser will present on associations between neighborhood greenness in midlife and late-life brain imaging measures in the Multi-Ethnic Study of Atherosclerosis.

Abstract citation ID: igaf122.946

### NEIGHBORHOOD GREENSPACE IN MIDLIFE AND LATE-LIFE BRAIN IMAGING OUTCOMES: MULTI-ETHNIC STUDY OF ATHEROSCLEROSIS

Lilah Besser[1], Jana Hirsch[2], Susan Heckbert[3], Peter James[4], Marcia Pescador Jimenez[5], Kari Moore[6], Joel Kaufman[3], and Timothy Hughes[7], *1. University of Miami, Boca Raton, Florida, United States, 2. Drexel University, Philadelphia, Pennsylvania, United States, 3. University of Washington, Seattle, Washington, United States, 4. University of California, Davis, Davis, California, United States, 5. Boston University, Boston, Massachusetts, United States, 6. Drexel University Dornsife School of Public Health, Philadelphia, Pennsylvania, United States, 7. Wake Forest University School of Medicine, Winston-Salem, North Carolina, United States*

We investigated associations between residential neighborhood greenspace in midlife and late-life magnetic resonance imaging (MRI) outcomes. We used data on 1,141 participants without dementia in the Multi-Ethnic Study of Atherosclerosis (MESA), restricting to those with address history covering midlife. Geocoded addresses (1980-2009) were used to derive midlife residential greenness using annual (summer:~July 1) normalized difference vegetation index (NDVI) values from LANDSAT satellite imagery (range=0-1, closer to 1=greener) and annual values of % tree canopy and open/park space linearly interpolated from 2001-2011 National Land Cover Datasets. We calculated 10-year mean neighborhood greenspace values spanning midlife (45-54 years). MRI measures included hippocampal and white matter hyperintensity volumes and total gray matter volume (GMV) in Alzheimer's disease (AD) prone regions of interest (ROI), which accounted for head size differences (e.g., (hippocampal volume/intracranial volume) x 100%). Multivariable linear regression estimated associations between midlife 10-year mean greenspace measures and later-life MRI outcomes, controlling for site, demographics (e.g., age, ethnoracial group, gender, education), neighborhood socioeconomic status, and comorbidities (e.g., hypertension, diabetes), measured at the time of MRI. Participants were 70.1±6 years old at MRI and 54% were women. Greater midlife residential greenness and % open/park space (but not % tree canopy) were associated with greater late-life GMV (%) in AD ROI (estimates per interquartile range (IQR), respectively=0.09, 95% CI = 0.04-0.14; estimate=0.09, 95% CI = 0.05-0.12). In a diverse US cohort, individuals living in greener neighborhoods in midlife exhibited greater late-life GMV (%) in AD ROI, possibly leading to a small reduction in AD risk that should be explored further.

Abstract citation ID: igaf122.947

**POPULATION INTERVENTION EFFECTS OF SPATIAL SOCIAL POLARIZATION ON DEMENTIA DISPARITIES**

Hoda Abdel Magid, Mengya Xu, Wei-Lin Wang, Evelyn Gonzalez, Jennifer Ailshire, and Julie Zissimopoulos, *University of Southern California, Los Angeles, California, United States*

Spatial social polarization (SSP), the unequal geographic distribution of privilege and deprivation, may contribute to disparities in dementia prevalence and incidence. This study used the Index of Concentration at the Extremes (ICE) to measure SSP across five domains: race, income, education, primary language, and homeownership. Using a 20% sample of 2019 Medicare claims data (N > 4 million), we applied Population Intervention Models (PIMs) with the parametric g-formula to estimate dementia outcomes under hypothetical scenarios where SSP disparities were eliminated. These counterfactual simulations compared current exposure distributions to idealized scenarios, such as universal privilege or universal deprivation. Dementia prevalence was 8%, with incidence at 3.5%, disproportionately affecting Black populations. In the hypothetical scenario where all individuals lived in high-income neighborhoods, dementia incidence was projected to decrease by 0.4%, whereas a scenario where all individuals lived in income-deprived neighborhoods was projected to increase incidence by 0.5%. In a scenario where all individuals lived in predominantly White neighborhoods, dementia incidence was projected to decrease by 0.1%, whereas residence in predominantly Black neighborhoods was projected to increase incidence by 0.3%. Similarly, a scenario where all individuals lived in neighborhoods with higher educational attainment was associated with a projected 0.4% reduction in dementia incidence, while lower educational attainment corresponded to a projected 0.8% increase in prevalence. These findings illustrate potential population-level impacts of SSP disparities under counterfactual scenarios and provide insight into how neighborhood factors may shape dementia risk.

Abstract citation ID: igaf122.948

**EFFECT OF THE BALTIMORE EXPERIENCE CORPS INTERVENTION ON MEMORY: MODERATION BY NEIGHBORHOOD CHARACTERISTICS**

Kyle Moored[1], Michael Desjardins[2], Vijay Varma[3], Qian-Li Xue[2], and Michelle Carlson[1], *1. Johns Hopkins Bloomberg School of Public Health, Baltimore, Maryland, United States, 2. Johns Hopkins University, Baltimore, Maryland, United States, 3. Clinical and Translational Neuroscience Section, National Institute on Aging, Baltimore, Maryland, United States*

Neighborhood socioeconomic status (nSES) and walkability may promote cognitive health in later life by providing opportunities for active engagement. Yet, structural disparities have led many older adults to reside in more disadvantaged neighborhoods, especially minoritized groups. Experience Corps was developed to encourage sustained activity for older adults through intergenerational volunteering in local elementary schools. We examined whether Experience Corps improved memory performance and whether this association was moderated by nSES and walkability. Participants were from the Baltimore Experience Corps Trial (n = 659, mean±SD age=67±5.9), which tested the 2-year efficacy of Experience Corps versus an active control. Neighborhood measures included tract-level walkability (e.g., transit access, service/commercial density) and nSES (e.g., poverty, unemployment). Individuals were categorized into combined walkability/nSES groups using median splits of each index: low walkability/high nSES (39%), high walkability/high nSES (12%), high walkability/low nSES (38%), low walkability/low nSES (11%). Memory was assessed using the Rey Auditory Verbal Learning Test (delayed recall) at baseline and 24 months (post-intervention). We fit mixed effects models with individual-level random intercepts and slopes and adjusted for age, sex, education, and health conditions. Experience Corps had no effect on memory performance in the overall sample (p>.05). While the intervention effect on memory did not differ significantly across all neighborhood strata (p-interaction=.12), it was significantly greater for the low walkability/low nSES group versus the low walkability/high nSES group, 1.2 (95% CI: -0.2, 2.6) vs. -0.6 (95% CI: -1.3, 0.2), p=.03. Experience Corps may provide structured opportunities that promote memory especially for aging adults in less resourced neighborhoods.

Abstract citation ID: igaf122.949

**BEYOND NEIGHBORHOOD BOUNDARIES: GPS-BASED ACTIVITY SPACE, BUILT ENVIRONMENT, AND COGNITIVE HEALTH**

Jinshil Hyun[1], Nelson Roque[2], Gina Lovasi[3], Dean Hosgood[1], Mindy Katz[1], Richard Lipton[1], and Charles Hall[1], *1. Albert Einstein College of Medicine, Bronx, New York, United States, 2. The Pennsylvania State University, University Park, Pennsylvania, United States, 3. Drexel University, Philadelphia, Pennsylvania, United States*

Neighborhood effects on late-life cognition have predominantly focused on built environment features within residential Census Tracts. However, individuals are active agents who, guided by personal goals and preferences, navigate environments beyond their residential context. GPS technology allows us to more accurately capture individuals' true spatiotemporal exposure to built environment features relevant for cognitive, social, and physical activities. Here, we compare how built environment features within residential Census tracts versus person-specific GPS-derived activity spaces are associated with late-life cognitive outcomes. Participants (N = 126) were from Bronx, NY in the Einstein Aging Study (mean age=76; 63% females; 37% non-Hispanic Blacks). Person-level activity space was assessed using minimum convex hull areas derived from GPS data collected over a 14-day period. Five cognitive domains (episodic memory, language, executive function, processing speed, visuospatial) were derived by averaging a subset of scores from 13 standard neurocognitive tests, with final scores in T-score units. Results from linear regression suggested that a higher availability of built environment features supporting physical and cognitive activities within Census tracts were associated with better speed (p=.032), visuospatial (p = 0.012), and executive function (p = 0.010). Person-specific activity spaces showed more consistent associations; individuals whose daily environments provided greater opportunities for physical, social, and cognitive activities had better cognitive performance in episodic memory (ps=.000~.018), language (ps=.001~.043), executive function (ps=.000~.005), and processing speed (ps=.035~.038). Person-specific activity space measures may more accurately reflect individuals' true exposure to neighborhood environments that influence cognitive health. Further investigation into behavioral mechanisms is warranted to identify potential targets for cognitive health interventions.

Abstract citation ID: igaf122.950

### CONSTRUCTING ARCHIVAL GEOSPATIAL DATASETS TO LINK HISTORICAL STRUCTURAL RACISM TO COGNITIVE & FUNCTIONAL DECLINE

Richard Sadler[1], Danielle Beatty Moody[2], Ayla Novruz[3], Allie Akmal[2], Rachel Davis[2], Graham Mooney[4], Kevin Henry[5], and Robert Belli[6], *1. Michigan State University, East Lansing, Michigan, United States, 2. Rutgers University, New Brunswick, New Jersey, United States, 3. University of Maryland, Baltimore County, Baltimore, Maryland, United States, 4. Johns Hopkins University, Baltimore, Maryland, United States, 5. Temple University, Philadelphia, Pennsylvania, United States, 6. University of Nebraska-Lincoln, Lincoln, Nebraska, United States*

More accurately measuring lifecourse environmental exposures requires working with data not readily available from digitized sources. Here we detail the processes for scouring city archives to uncover aspects of structural racism in the built environment. In our initial non-human subjects work in Baltimore, we have built datasets on the built environment and prepared methods to collect residential histories, which together allow us to study impacts of residential exposures across the lifecourse for this longitudinal cohort of aging adults. We discuss the iterative process of archive visits—working with team members who are experts in this field—to obtain the full complement of features in the built environment driven by structurally racist policies (i.e. redlining, blockbusting, restrictive covenants, urban renewal, freeway construction, predatory lending). We then discuss how residential histories are collected from our cohort, giving us lifecourse perspectives on where individuals have lived. These allow for the assignment of age- and period-specific exposures (e.g. one might expect developmental exposure to proximal neighborhood change to be more impactful than adult exposure to stable neighborhoods). These environmental data will eventually be spatially and temporally joined to our health data in GIS software, and multilevel models will allow us to examine what features of the environment and durations of exposure matter most to cognitive decline. Such data allow us to move 'beyond redlining' and other simplistic methods to consider more complex exposure frameworks.

### SESSION 2405 (SYMPOSIUM)

Abstract citation ID: igaf122.951

### THE ROLE OF RELIGION IN INTERGENERATIONAL RELATIONS: FINDINGS FROM THE LSOG AND WFDS MULTIGENERATIONAL STUDIES

Chair: J. Jill Suitor
Co-Chair: Megan Gilligan
Discussant: Justin Hendricks

This symposium brings together scholarship that explores how religion shapes intergenerational relations in later-life families. The literature has documented that religion plays important roles in older adults' health and well-being. However, little attention has been given to how religion shapes another major predictor of older adults' well-being—the quality of their relationships with adult children and grandchildren. Papers in this symposium use data from two multi-wave panel studies of intergenerational relations (the Longitudinal Study of Generations and the Within-Family Differences Study) to examine how religion affects relationships between members of different generations. First, Silverstein and colleagues use LSOG data from 2016 and 2022 to examine the association between intergenerational religious discordance (e.g., regarding denomination, intensity, participation, belief in God) and closeness, contact, and support. Next, Ogle and colleagues use WFDS-III data to compare the association between adult children's perceptions of religious value similarity to their mothers and relationship quality in later-life and mid-life families. Third, He and colleagues use data from the WFDS to explore stability in adult children's perceptions of religious value similarity with their mothers and the role of similarity in the quality of parent-child relationships between 2008-2021. Last, Lakomy and colleagues use seven waves of the LSOG to study whether strength of religious values shapes grandparents' provision of instrumental and emotional support to grandchildren, and the roles that lesser importance attributed to self-interest and individualistic goals play in mediating these associations. Hendrix will discuss these papers in the context of religion and family across the life course.

Abstract citation ID: igaf122.952

### INTERGENERATIONAL RELIGIOUS DISCORDANCE AND RELATIONSHIP QUALITY BETWEEN OLDER PARENTS AND ADULT CHILDREN

Merril Silverstein[1], Woosang Hwang[2], Maria Brown[1], and Wencheng Zhang[3], *1. Syracuse University, Syracuse, New York, United States, 2. Sookmyung Women's University, Seoul, Republic of Korea, 3. Purdue University, West Lafayette, Indiana, United States*

In this analysis we examined intergenerational religious discordance and its association with emotional closeness, conflict, frequency of contact, and emotional support exchanged between older parents and their adult children. We considered discordance as absolute difference scores in four dimensions of religiosity: denomination, intensity, frequency of service attendance, and belief in God. Data from the Longitudinal Study of Generations collected in 2016 and 2022 were used in four sub-samples of dyads: 157 mother-son, 180 mother-daughter, 124 father-son, and 113 father-daughter dyads. The results of this analysis will pinpoint whether adverse impacts of religious differences between older parents and their middle-aged adult children have their basis in denominational identification, congregational practice, self-concept, or deistic beliefs. A sub-analysis focuses on whether children have more strained relationships with their parents if they married outside their parents' religious denomination.

Abstract citation ID: igaf122.953

### PERCEIVED RELIGIOUS VALUE SIMILARITY AND MOTHER-ADULT CHILD RELATIONS: EXPLORING VARIATIONS BY GENERATION

Destiny Ogle[1], J. Jill Suitor[1], Ranran He[1], Robert Frase[2], and Megan Gilligan[3], *1. Purdue University, West Lafayette,*

*Indiana, United States, 2. Southern Illinois University, Carbondale, Illinois, United States, 3. University of Missouri, Columbia, Missouri, United States*

Research on interpersonal relations has emphasized the importance of value similarity in the formation and maintenance of ties across the life course. Consistent with this body of theoretical and empirical work, religious similarity has been highlighted as a predictor of the quality of relationships between parents and adult children. However, most studies have conceptualized religious similarity in terms of denominational congruence, rather than perceptions of religious similarity. Moreover, despite evidence that religion is less salient for younger generations, no consideration has been given to the role that generation plays in the relationship between perceptions of religious value similarity and parent-child relationship quality. In this paper, we explore the association between perceptions of religious value similarity and mother-child closeness and tension using data collected from adult children in two generations of the same 147 families as part of the Within-Family Differences Study-III. Specifically, we compare the associations between perceptions of religious value similarity and relationship quality in ties between older mothers (Mage=88) and their midlife adult children (Mage=59), and midlife mothers (Mage=59) and their young adult offspring (Mage=29). Regression analyses revealed that adult children's perceptions of religious value similarity were associated with mother-child closeness and tension in both generations. However, these associations were stronger for ties between midlife children and their mothers than for ties between younger adult children and their mothers. These findings highlight the importance of religious value similarity for the quality of mother-adult child ties across generations and the greater impact of such similarity in later-life families.

Abstract citation ID: igaf122.954

### HOW DOES RELIGIOUS VALUE SIMILARITY SHAPE MOTHER-ADULT CHILD RELATIONS OVER TIME IN LATER-LIFE FAMILIES

Ranran He[1], J. Jill Suitor[1], Destiny Ogle[1], Robert Frase[2], Megan Gilligan[3], and Di Wang[1], *1. Purdue University, West Lafayette, Indiana, United States, 2. Southern Illinois University, Carbondale, Illinois, United States, 3. University of Missouri, Columbia, Missouri, United States*

Fueled by increasing political polarization and broader trends toward secularization, generational value discrepancies in religious beliefs expanded in the United States over the past decade. Despite these general trends, it remains unclear whether parents and adult children within the same families changed their perceptions regarding the extent to which they shared religious values. Further, it is not known whether similarity of religious values played the same role in the quality of parent-adult child relations in later-life families across this period. To address these questions, we used data collected from 291 adult children from Time 2 (2008–2011) and Time 3 (2020–2021) of the Within-Family Differences Study to examine (1) patterns of stability and changes in midlife adult children's perceptions of religious similarity to their older mothers, and (2) changes in the effects of perceived religious similarity on mother-child closeness and tension. Our findings suggest that adult children's perceptions of religious similarity with their mothers remained relatively stable over time in later-life families. Perceived religious value similarity with mothers had a positive effect on closeness with mothers at both waves. Although similarity in religious values had no discernible effects on mother-child tension at T2, a strong negative association was found at T3. There were no differences in the associations by child's gender for either closeness or tension at T2, or for closeness at T3; however, at T3, religious similarity was negatively associated with tension for daughters, but not sons.

Abstract citation ID: igaf122.955

### ARE RELIGIOUS GRANDPARENTS MORE INVOLVED GRANDPARENTS BECAUSE THEIR VALUES ARE LESS INDIVIDUALISTIC?

Martin Lakomy[1], Merril Silverstein[2], Tianqi Zhou[2], and Jeewon Oh[2], *1. Mendel University in Brno, Brno, Czech Republic, 2. Syracuse University, Syracuse, New York, United States*

This investigation examined whether middle-aged and older adults who hold stronger religious values are more likely to provide instrumental and emotional support to their grandchildren, and whether this relationship is mediated by lesser importance attributed to self-interested or individualistic values. We used data from Waves 4-10 of the Longitudinal Study of Generations to examine the link between religious values, individualistic values, and support provided using mixed models with mediating effects. The operational sample consisted of 3,568 observations from 1,340 respondents nested within 305 families. Values were measured with eight items from the Rokeach Values Survey a scale ranking various intrinsic goals, ordered by their degree of importance. The scale's dimension of individualism-collectivism is used to assess the importance of self-aggrandizing goals associated with individualism—such as achievement and skills—as opposed to social goals associated with collectivism—such as friendship and community. Religious intensity—a measure of the religious self-concept—is considered as a lagged indicator taken prior to the respondent becoming a grandparent. While we expect individualistic values to weaken with aging, the degree of the connection between religion and those values may be manifest in greater support that directly benefits grandchildren and indirectly their adult children.

### SESSION 2410 (AWARD LECTURE)

Abstract citation ID: igaf122.956

### JOSEPH T. FREEMAN AWARD AND EXCELLENCE IN REHABILITATION OF AGING PERSONS AWARD PRESENTATIONS AND LECTURES

Chair: Debra Bakerjian

The Joseph T. Freeman Award lecture will feature an address by the 2025 Freeman Award recipient Jeff Williamson, MD, FGSA and the 2025 Excellence in Rehabilitation of Aging Persons Award recipient Sajay Arthanat, PhD. The Joseph T. Freeman Award is a lectureship in geriatrics awarded to a prominent clinician in the field of aging, both in research and practice. The award was established in 1977 through a bequest from a patient's estate as a tribute to Dr. Joseph T. Freeman. The Excellence in Rehabilitation of Aging Persons Award is designed to acknowledge outstanding contributions in the field of the rehabilitation of aging individuals.

Abstract citation ID: igaf122.957

**CROSS-DISCIPLINARY MENTORING AND COLLABORATION TO ADVANCE PREVENTIVE GERONTOLOGY**

Jeff Williamson, *Wake Forest University School of Medicine, Winston-Salem, North Carolina, United States*

This lecture will present the impact of cross disciplinary research mentoring and collaboration on improving outcomes for all of us as we age. Dr. Williamson will present the impact of the landmark concept article by Dr. Jim Fries, published in the New England Journal of Medicine ("Aging, Natural Death, and the Compression of Morbidity") as a framework for his career. Among others, Dr. Williamson credits Dr. Linda Fried, his original mentor in this area, as he pursued a career focused on improving the health and well-being of older adults. The fruit of Drs. Fries's work and Fried's advice culminates in the work that he accomplished with his colleagues as part of the SPRINT and SPRINT MIND clinical trial. The lecture will encourage listeners to further advance the exploration of preventive gerontology as the best way to ensure that the care we provide is truly aligned with the needs of an aging population.

Abstract citation ID: igaf122.958

**SYSTEMATIC CO-DESIGN OF TECH INNOVATIONS FOR AGING IN PLACE IN A SMART WORLD**

Sajay Arthanat, *University of New Hampshire, Durham, New Hampshire, United States*

Rapid innovations in information communication technologies, home automation, robotics, wearables and digital health platforms offer tremendous promise in helping sustain the independence of older adults in the community. However, the design and intended application of these technologies are often not in sync with proven foundations of aging, consumer dispositions and skillset of older consumers. Dr. Arthanat will emphasize the significance of co-designing technological innovations with older adults based on a series of exploratory, multifactorial, and empirical research studies he has conducted over a span of a decade. The presentation will feature an ongoing project to co-design an AI-framework for socially assistive robots to provide personalized care for individuals with Alzheimer's disease and related dementia at home

### SESSION 2415 (SYMPOSIUM)

Abstract citation ID: igaf122.959

**DECIPHERING THE BLACK BOX OF MIDLIFE AGING: UNRAVELLING FUNCTIONAL CHANGES IN THE AGING PROCESS**

Chair: Maayan Agmon

Midlife is a pivotal yet understudied phase of the aging process, offering a unique opportunity to identify early markers of functional decline and implement interventions that promote healthy aging. This symposium presents multidisciplinary research examining the influence of personal and environmental factors as key determinants of midlife function and aging trajectories. Professor Agmon will provide an overview of the spectrum of personal and environmental factors studied in her lab, emphasizing newly developed behavioral markers that can be used to quantify function in midlife. Roy Tzemah-Shahar and Merav Asher will each present studies exploring the effects of gait characteristics, sensory responsiveness, and emotion regulation on midlife function, comparing different aging markers (biological age vs. physical capacity). Khalil Iktilat will present an examination of the impact of environmental factors, such as exposure to violence, on health disparities, manifested as accelerated biological aging in midlife. Finally, we will present the Midlife Aging and Performance Study (MAPS) which introduces a novel functional assessment battery linked to traditional biological age evaluation. Presented studies highlight the importance of integrating physiological, psychological, and behavioral markers to advance our understanding of midlife function and its long-term health implications. The findings emphasize the need for deeper scientific focus on midlife and targeted preventive interventions ensuring tailored strategies that enhance active aging and reduce disparities in diverse populations.

Abstract citation ID: igaf122.960

**THE ROLE OF GAIT CHARACTERISTICS IN UNDERSTANDING AGING IN MIDLIFE**

Roy Tzemah-Shahar, Merav Asher, and Maayan Agmon, *University of Haifa, Haifa, HaZafon, Israel*

Gait quality is a hallmark of aging, with its decline signalling cognitive deterioration, fall risk, and mortality. Gait has been linked to Biological Age (BA), a quantitative measure of aging, estimated by physiological or behavioral markers such as Physical Capacity (PC). However, most studies of BA focus on older adults, leaving midlife largely unexplored. This study examines the relationship between gait characteristics and BA in midlife. We conducted a cross-sectional study of healthy midlife adults ($N=95$, mean age 45.6±0.7, 48% women). BA was estimated using the Klemera-Doubal method with physiological biomarkers and a PC battery assessing muscular strength, endurance, flexibility, balance, and agility. Gait speed and variability were evaluated under single- and dual-task walking conditions. PC composite scores were negatively associated with BA when controlling for age, education, and smoking (adjusted $R^2=0.19$; $b=-0.42$, $p<0.001$), with endurance, flexibility, and balance remaining significant predictors. Gait speed and stride length variability were not significantly associated with BA (Pearson's $r=0.17$-$0.01$, $p=0.10$-$0.99$). However, gait speed in both single- and dual-task conditions was significantly correlated with PC scores (Pearson's $r=0.61$, $p<0.001$ and $r=0.51$, $p<0.001$, respectively). While gait is a key aging marker in older adults, these findings suggest that it may not fully capture physiological aging in midlife. A comprehensive PC assessment may better detect subtle aging-related changes, offering a valuable tool for early interventions to promote healthy aging.

Abstract citation ID: igaf122.961

**EMOTIONAL REGULATION, SENSORY OVER-RESPONSIVENESS AND DEVELOPMENTAL COORDINATION DISORDER IN MIDLIFE**

Merav Asher, and Maayan Agmon, *University of Haifa, Haifa, HaZafon, Israel*

Developmental Coordination Disorder (DCD) is a prevalent neurodevelopmental condition that causes lifelong challenges across all aspects of life. Research has predominantly examined DCD in children, establishing connections with sensory over-responsiveness (SOR) and emotional dysregulation. However, evidence regarding these relationships in midlife adults remains unexplored. This study investigated the relationships between SOR and DCD in midlife, considering the role of emotion regulation, highly relevant for healthy aging, as a mediator. Data were collected from 89 middle-aged adults (mean age 45.59±0.68; 47% women) using the Adult Developmental Coordination Disorders/Dyspraxia Checklist (ADC), Sensory Responsiveness Questionnaire Intensity Scale (SRQ-IS), and Difficulties in Emotion Regulation Scale (DERS). A mediation analysis examined the role of emotional dysregulation in the relationship between SOR and DCD symptoms. Results showed that SOR significantly predicted DCD symptoms ($\beta = 0.303$, $p = 0.006$). However, when emotional dysregulation was included, the direct effect of SOR on DCD symptoms became non-significant ($\beta = 0.106$, $p = 0.278$), while the indirect effect via emotional dysregulation was significant ($\beta = 0.197$, $p < 0.05$, 95% CI [0.082, 0.343]). This finding suggests complete mediation, indicating that SOR increases emotional dysregulation ($\beta = 17.971$, $p = 0.001$), which in turn exacerbates DCD symptoms ($\beta = 0.011$, $p < 0.001$). Our findings demonstrate a developmental shift wherein emotional regulation becomes the primary pathway linking sensory processing to motor coordination in midlife, rather than the direct sensory-motor connections observed in childhood. This suggests that interventions targeting emotional regulation may be more effective than those focusing solely on sensory processing to address persistent coordination difficulties in midlife adults.

Abstract citation ID: igaf122.962

### BIOLOGICAL AGE AND PSYCHOLOGICAL DISTRESS: HEALTH DISPARITIES BETWEEN MIDLIFE JEWISH AND ARAB ADULTS

Khalil Iktilat[1], Merav Asher[2], Roy Tzemah-Shahar[2], and Maayan Agmon[2], *1. Tel Aviv University, Tel Aviv, Tel Aviv, Israel, 2. University of Haifa, Haifa, HaZafon, Israel*

Health disparities between Jewish and Arab populations in Israel are well-documented, particularly in contexts of chronic diseases, aging, and socioeconomic conditions. These gaps are widening and could be related to increasing violence in Arab society and the associated increases in stress. However, most research on health disparities focuses on older adults who already have multiple diagnoses, overlooking midlife individuals who can provide valuable insights into the aging process and the underlying patterns of these disparities. This study examines middle-aged individuals, using Biological Age (BA) as an objective health marker and considering the role of psychological distress in these two populations. This cross-sectional study included 221 participants (age 43.8±1.6, 57% female, 112 Jewish, 109 Arab). BA was estimated using the Klemera-Doubal algorithm. Executive function was assessed using the Trail Making Test (TMT) and Stroop Test, psychological distress via the six-item Kessler Scale (K6), and socioeconomic status based on education and income levels. No significant difference in chronological age between groups were observed; however, BA was significantly higher among Arabs ($p < 0.001$), suggesting accelerated aging. Additionally, Arabs exhibited higher psychological distress ($p < 0.001$), lower socioeconomic status ($p < 0.01$), and poorer executive function ($p < 0.001$) than Jews. These findings highlight the need for early intervention to address disparities before they escalate. Elevated BA and psychological distress among Arabs underscore the urgent need for action to reduce violence and address socioeconomic and mental health inequalities, which may help reduce premature aging and improve health equity in Israel.

Abstract citation ID: igaf122.963

### MIDLIFE AGING AND PERFORMANCE STUDY: ADOPTING PHYSICAL CAPACITY TESTS FOR BIOLOGICAL AGE ASSESSMENT

Roy Tzemah-Shahar, Merav Asher, and Maayan Agmon, *University of Haifa, Haifa, HaZafon, Israel*

Aging is a risk factor for the development of chronic diseases and functional decline; however, it is heterogenous. Measuring aging in midlife is commonly done using physiological markers to estimate Biological Age (BA). This method limits the possibilities of developing personalized behavioral interventions to promote healthier aging. To address this gap, we have developed the Midlife Aging and Performance Study (MAPS), a cross-sectional study aiming to examine the association between a comprehensive measure of physical capacity, relying on behavioral markers, and BA, measured by physiological markers. A total of 112 individuals (age 44.5±1.4, 47% women) were included in MAPS; BA was estimated using the Klemera-Doubal method. Physical capacity (PC) was measured using tests covering five behavioral domains (muscle strength, endurance, balance, agility, and flexibility). Higher performance in strength, endurance, balance and flexibility domains was correlated with younger BA (Pearson's r 0.33-0.49, $p < 0.001$); a composite score of physical capacity, including five domains, was significantly negatively associated with accelerated aging in a logistic regression (where biological age>chronological age). The composite physical capacity score had an odds ratio of 0.40 (95% CI 0.25-0.64) in predicting accelerated aging, each incremental increase in physical capacity corresponded with a 60% reduction in the risk of being in an accelerated aging state. The proposed physical capacity battery also includes a functional behavioral assessment for aging state during midlife. Measuring physical capacity may help in population wide risk-screening assessment, presenting both easily conveyed intervention goals and a means to evaluate temporal changes of health independent of laboratory tests.

### SESSION 2417 (BIOLOGICAL SCIENCES INVITED SYMPOSIUM)

Abstract citation ID: igaf122.964

### INNOVATIVE APPROACHES TO EMERGING GEROSCIENCE CHALLENGES

Chair: Mark McCormick

Abstract citation ID: igaf122.965

### MACRONUTRIENT BALANCE AND LONGEVITY: MECHANISTIC INSIGHTS OF DIETARY PROTEIN REGULATION ON AGING PHYSIOLOGY `MACRONUTRIENT BALANCE AND LONGEVITY: MECHANISTIC INSIGHTS OF DIETARY PROTEIN REGULATION ON AGING PHYSIOLOGY

Cristal Hill, *University of Southern California, Los Angeles, California, United States*

Neuroendocrine systems play a vital role in homeostatic processes that regulate growth, metabolism, and stress responsiveness, among others. In addition, dietary interventions such as CR or altering macronutrients are reported to modify neuroendocrine signaling to benefit health-span and lifespan. There is substantial evidence that reducing dietary protein intake (dietary protein restriction-DPR), without reducing caloric intake, improves glucose homeostasis, increases energy expenditure, and remodels white adipose tissue (WAT) by inducing thermoregulatory markers such as UCP1. Data from our lab shows that DPR robustly improves metabolic health via increased liver-derived FGF21 action in the brain and that DPR increases thermoregulatory genes, thus DPR represents a purely physiological model to study adipocyte biology. Specifically, the experiments in our lab are focused on identifying the potential mechanisms through which reducing protein intake alters cellular and molecular function in adipose tissue that impact metabolic health. The central focus of this work is to further connect the effects of protein intake on the impact of neuroendocrine signaling on aging physiology.

Abstract citation ID: igaf122.966

### MULTIMORBIDITY AS A PREDICTOR AS MORTALITY IN COMPANION DOGS

Jessica Hoffman, *Augusta University, Augusta, Georgia, United States*

Multimorbidity, the co-occurrence of two or more diseases, increases with age and is a predictor of mortality in humans; however, modeling multimorbidity in laboratory models is difficult if not impossible. Here, we utilize the companion dog as a translational model of multimorbidity, tracking over 50,000 dogs in the Dog Aging Project (DAP) for up to five years. Similar to humans, we found that the accumulation of multiple conditions is associated with an increased risk of mortality in dogs. In addition, dogs with a diagnosis of osteoarthritis or other degenerative joint diseases at baseline entry into the DAP exhibited significantly higher diagnosis rates and a higher likelihood of developing multimorbidities. Interestingly, we then determined that specific co-morbid disease combinations were more likely to be associated with mortality than individual disease effects. For example, dogs with several periodontal diseases and heart murmurs were at an increased risk of death compared to dogs with either condition on its own. Overall, our study is the first to investigate the effects of multimorbidity on mortality in companion dogs, and future studies will attempt to determine if circulating biomarkers can predict future multimorbidity.

Abstract citation ID: igaf122.967

### WHEN WHAT YOU SEE ISN'T WHAT YOU GET: HOW ENVIRONMENTAL CUES MODIFY LONGEVITY AND BEHAVIOR

Scott Leiser, *University of Michigan, Ann Arbor, Michigan, United States*

Research in geroscience has identified interventions that can extend lifespan across model organisms. These interventions often involve exposure to mild environmental stressors that activate stress-response pathways to promote longevity. However, exposure to environmental stress can also change an animal's behavior and mental state. Our research focuses on the environmental cues and signaling pathways that affect longevity cell non-autonomously, and how they feedback to regulate mental state or behavior. This presentation will focus on the dietary restriction (DR) induced nematode longevity gene, fmo-2, whose expression is regulated by food cues and whose activity modifies behavior. We find that multiple food cues repress fmo-2 induction in the DR state through well-conserved bioamine signaling pathways. We further find that loss of fmo-2 and overexpression of fmo-2 each lead animals to exhibit altered sensory perception and decision-making relative to wild-type (WT) controls. These behavioral changes likely represent metabolic feedback to the nervous system, and interventions that rescue behavior also rescue some, but not all of the other health and longevity phenotypes of the animals. These results establish a feedback loop for environmental response and demonstrate the importance of examining pleiotropic effects in promising longevity interventions.

Abstract citation ID: igaf122.968

### COMBINED DOWNREGULATION OF INSULIN AND MITOCHONDRIAL SIGNALING LEADS TO SYNERGISTIC LIFE- AND HEALTHSPAN EXTENSION IN CAENORHABDITIS ELEGANS

Sinclair Emans[1], Nicole Sthur[2], Sophia DiPaola[3], Zoe Nathwani[4], and Alexander Soukas[5], *1. Harvard Medical School, Boston, Massachusetts, United States, 2. University of Southern California, Los Angeles, California, United States, 3. Eckerd College, St. Petersburg, Florida, United States, 4. Harvard University, Boston, Massachusetts, United States, 5. Massachusetts General Hospital, Boston, Massachusetts, United States*

The Caenorhabditis elegans insulin/IGF-1 signaling (IIS) pathway functions as both a rheostat for nutrient levels and as an evolutionarily conserved mechanism likely to shed light on its functions and regulation in higher organisms. Early studies in C. elegans demonstrated that IIS receptor homolog daf-2 downregulation led to a drastic increase in lifespan, from a maximum of 25 to 60 days. Over the following decades many other manipulations were shown to increase lifespan in worms including interventions in diet, AMPK, mTOR signaling, and mitochondrial homeostasis, to name just a few. However, combinatorial lifespan studies still remain few and far between. Herein, we demonstrate that combining a daf-2 hypomorph with adenine nucleotide translocase 1.1 (ant-1.1) knockdown leads to a four-fold increase in lifespan and a concomitant healthspan increase. We further demonstrate that this increase is not dependent on the mitochondrial unfolded protein response. Moreover, we show that a distinctive transcriptional signature could explain this distinctive synergistic phenomenon. In sum, we describe a combinatorial treatment that can quadruple lifespan and produce a similar healthspan increase through a potentially unique transcriptional program.

### SESSION 2420 (SYMPOSIUM)

Abstract citation ID: igaf122.969

### APPLYING RESEARCH ON CULTURAL NARRATIVES ABOUT LIVING WITH DEMENTIA TO SOCIAL POLICY AND PRACTICE

Chair: Nancy Berlinger
Discussant: Nancy Berlinger

This symposium presents a 2025 report on cultural narratives about dementia, funded by the National Endowment for the Humanities. Cultural narratives are stories expressed through metaphors and other representations that circulate through social groups to make meaning out of observations. By shaping common knowledge, cultural narratives influence social policy and practice concerning responses to societal needs. Papers will show how recognition of cultural narratives, practices of narrative repair, and the generation of new narratives are integral to supporting people with dementia in a society and communities. The first paper analyzes selected cultural narratives, showing how they convey ideas about improving dementia care. It presents recommendations for improving policy development. The second paper draws on findings from community-based participatory research (CBPR) with North American Indigenous communities that reveal community-level narratives about aging and dementia. It explains strategies that respect these narratives. The third paper considers the experiences of older Black women in the context of elevated risk of dementia, precursor conditions, later-life economic precarity, and caregiving. It suggests narrative repair strategies to better represent and support these experiences. The fourth paper explores the concept of brain health as it applies to Black men, a group at elevated risk of dementia and precursor conditions. It draws on findings from CBPR and community health initiatives to consider new narratives about brain health that include Black men. The fifth paper offers insights from research and practice on an innovative dementia-friendly initiative for congregations. It explains how dementia-friendliness narratives can evolve from awareness to inclusion.

Abstract citation ID: igaf122.970

**BEYOND "LIVING WELL": TOWARD SOCIALLY JUST CULTURAL NARRATIVES ABOUT LIVING WITH DEMENTIA**

Janelle Taylor[1], and Nancy Berlinger[2], *1. University of Toronto, Toronto, Ontario, Canada, 2. The Hastings Center, Garrison, New York, United States*

This paper considers how cultural narratives function when an aging society is reflecting on itself and what it wants for fellow members of this society, specifically, people facing dementia and people who are dementia caregivers. It explores the moral imagination required to develop socially just cultural narratives for aging societies that will endure under non-ideal conditions. Moral imagination is the capacity to think about current and future challenges in ways that aim at better lives and greater justice. Socially just cultural narratives are the stories embedded in these tasks that convey ideas and values about how to support better lives for disadvantaged members of a society, alongside other important goals such as effectiveness and sustainability. When used and acted upon by those with the power to drive change, these narratives should help to produce opportunities for better lives and greater justice under real-world conditions. The author, an anthropologist, studies the social, cultural, and political dimensions of illness and medicine in North America, with special attention to dementia and care.

Abstract citation ID: igaf122.971

**INTERWEAVING CULTURAL NARRATIVES, HONORING DIVERSITY: INDIGENOUS AND WESTERN CONCEPTS OF DEMENTIA**

Jordan Lewis, *Center on Aging, Thompson School of Social Work & Public Health, University of Hawai'i at Manoa, Honolulu, Hawaii, United States*

This paper discusses Indigenous concepts of the causes and meaning of dementia, and how these concepts differ from Western concepts. It employs a two-eyed seeing approach, defined as seeing the strengths of the Western knowledge systems through one eye and the strengths of Indigenous value systems through the other eye; both are critical to seeing the whole picture and blending the two systems together. Drawing on the author's extensive community-engaged participatory research in North American Indigenous communities, the paper explains key features of cultural narratives of Indigenous peoples, which focus on the person and who they were before the onset of dementia symptoms, emphasizing their status as Elders within families and communities, and show how these narratives many also reflect Western narratives focusing on challenges associated with living with dementia. The author provides examples from community-focused collaborations to show how narrative interweaving can support community members through effective, culturally attuned education about dementia.

Abstract citation ID: igaf122.972

**MAKING THE INVISIBLE VISIBLE: NARRATIVE REPAIR IN REPRESENTING EXPERIENCES OF BLACK WOMEN IN DEMENTIA RESEARCH**

H. Shellae Versey, *Fordham University, New York, New York, United States*

This paper draws on research insights and analysis of personal narratives to consider cultural narratives about Black women's experiences of aging, dementia, and caregiving. Black women face elevated risks for dementia, associated health conditions, and financial vulnerability in later life. Attention to cultural narratives about Black women and aging, as these narratives circulate within health care and within communities, may reveal positive yet unrealistic assumptions about what Black women should be able to endure or manage, for themselves or for others. This paper presents an argument for narrative repair, relevant to dementia research, that recognizes culturally meaningful narrative features concerning strength, while providing better recognition of and support to Black women navigating significant challenges in later life.

Abstract citation ID: igaf122.973

**THE EVOLVING NARRATIVE OF BLACK MEN'S BRAIN HEALTH: IMPLICATIONS FOR DEMENTIA RESEARCH AND PUBLIC ENGAGEMENT**

Darlingtina Esiaka, *University of Kentucky - College of Medicine, Lexington, Kentucky, United States*

This paper considers the concept of "brain health"—the optimal state of brain functioning, encompassing cognitive, sensory, social-emotional, behavioral, and motor aspects -- as it has been deployed as an empowering narrative within the broader discourse of "healthy aging," emphasizing individual agency and preventative approaches to maintaining cognitive function and reduce dementia risk. Drawing on community-based participatory research and examples from community health initiative, the author considers the tension between this empowering narrative and the stark risk of dementia and precursor conditions within Black communities, focusing on Black men. The author demonstrates the need for narratives about brain health to recognize that Black men's brain health outcomes are not solely the result of individual choices but are deeply influenced by historical and ongoing inequities. The author explains current efforts to construct a new, strength-based narrative about brain health that can empower and support Black men.

Abstract citation ID: igaf122.974

**INNOVATION WITH PEOPLE LIVING WITH DEMENTIA: CREATING NEW NARRATIVES ON MEANING, BELONGING, COMMUNITY, AND PLACE**

Fayron Epps[1], Jordan Lewis[2], Alison Phinney[3], and Marigrace Becker[4], *1. UT Health San Antonio, San Antonio, Texas, United States, 2. Center on Aging, Thompson School of Social Work & Public Health, University of Hawai'i at Manoa, Honolulu, Hawaii, United States, 3. The University of British Columbia, Vancouver, British Columbia, Canada, 4. UW Medicine, Seattle, Washington, United States*

This paper shares insights about innovation resulting from collaboration between dementia researchers and community-based organizations in North America, and how these collaborations can foster new ways of thinking and doing with people with dementia. The author focuses on examples from work with Black congregations to better support community members with dementia, with attention to institutional support for this type of collaboration, building trust with community partners, the role of effective marketing in extending impact beyond pilot projects, and the challenges of leading innovation.

### SESSION 2430 (SYMPOSIUM)

Abstract citation ID: igaf122.975

### BRIDGING AGING AND DISABILITY RESEARCH: WHERE TO GO FROM HERE?

Chair: Michelle Putnam
Discussant: Susan Stark

This session discusses future directions for bridging aging and disability research, building from their work published in a special issue of The Gerontologist. The first presenter will provide a historical overview of efforts to bridge aging and intellectual disability, discussing trends in population aging, research and policy initiatives that have forwarded bridging over time, and examples of evidence-based knowledge and practice interventions that demonstrate realized outcomes of bridged aging and disability research such as future planning and health promotion and areas where more bridged research is needed including areas of family support. The second presenter will discuss the TechSage Technology Intervention Model, which designs for the intersection of aging and disability, emphasizing interdisciplinary problem solving. Looking forward to new technologies, opportunities and challenges to utilizing this model and implications and recommendations for bridging aging and disability research will be identified. The third presenter will discuss the evolution of aging and disability research over time, conceptual and methodological challenges to engaging in research that bridges aging and disability domains, and opportunities for reconciling these to support future bridging work. The session's discussant will reflect upon where bridging work goes from here by highlighting gaps that need to be addressed, examples of success, and considering how researchers can more actively engage in bridging research work. Lifelong Disabilities Interest Group Sponsored Symposium

Abstract citation ID: igaf122.976

### BRIDGING THE GAP BETWEEN AGING AND DISABILITY RESEARCH

Michelle Putnam, *University of Massachusetts Boston, Boston, Massachusetts, United States*

Efforts to bridge aging and disability research date back over forty years. Increased longevity of persons experiencing early and mid-life disability has prompted bridging work, but conceptual and methodological differences reflecting disciplinary-specific approaches to age-based disability-related research have research restricted synergy across fields. This presentation describes the evolution of efforts to bridge aging and disability research over time, historical and current variances in how aging and disability research has approached the study of disability, and what resultant challenges for aligning and bridging aging and disability research are. Issues identified include use of medical vs social models of disability, operationalization and measurement of disability, and interpretation of disability-related data. Legal, social and historical factors that have shaped approaches to investigating aging and disability are also discussed. Recommendations regarding how to address long-standing gaps in knowledge and barriers to bridging work are made.

Abstract citation ID: igaf122.977

### BRIDGING AGING AND INTELLECTUAL DISABILITIES RESEARCH: FROM PAST LESSONS TO FUTURE DIRECTIONS

Tamar Heller, *University of Illinois Chicago, Chicago, Illinois, United States*

This presentation provides a historical overview of efforts to bridge aging and intellectual disability, highlighting key moments in more than 40 years of research. It discusses trends in aging among persons with intellectual disabilities and what increased longevity has meant for families and communities. Research and policy initiatives that have forwarded bridging over time are identified. Examples of evidence-based knowledge and practice interventions that demonstrate realized outcomes of bridged aging and disability research such as future planning and health promotion are provided. Recommendations for the future focus on addressing knowledge gaps and clarifying why bridged research is needed in areas such as family support, health care, and participation.

Abstract citation ID: igaf122.978

### DESIGNING FOR THE INTERSECTION OF AGING AND DISABILITY: APPLICATION OF THE TECHSAGE TECHNOLOGY INTERVENTION MODEL

Laura Rice[1], Tracy Mitzner[2], Jon Sanford[3], Elena Remillard[4], and Wendy Rogers[5], *1. University of Illinois College of Applied Health Sciences, Urbana, Illinois, United States, 2. Person in Design, Atlanta, Georgia, United States, 3. Georgia State University, Atlanta, Georgia, United States, 4. Georgia Institute of Technology, Atlanta, Georgia, United States, 5. University of Illinois Urbana-Champaign, Champaign, Illinois, United States*

As people live longer with disabilities acquired early in life, the additive effects of aging create unique challenges at the intersection of aging and disability. Technology interventions can minimize barriers and create facilitators to support performance of activities integral to health and quality of life. The absence of a theoretical framework to guide such interventions, in either gerontology or rehabilitation, created gaps in the knowledge base required to meet the needs of these individuals. We proposed the TechSAge Technology Intervention Model (TechSAge-TIM) to support activity engagement of older adults aging with long-term disabilities through technology design that bridges the gap between intrinsic capabilities and functional abilities (Mitzner et al., 2018). We have since utilized the model to advance understanding of technology-based supports for persons aging with long-term mobility, hearing, and vision disabilities. We describe herein applications involving people with mobility disabilities. We identified unmet needs by exploring lived experiences and used the TechSAge-TIM to guide research and development of a seated tele tai chi program for exercise/social engagement, smart bathroom technologies, and an automatic fall detection system for wheelchair users. These applications advanced the field of aging and disability and provided a roadmap for future research and development efforts.

### SESSION 2435 (SYMPOSIUM)

Abstract citation ID: igaf122.979

### FALLS AMONG OLDER ADULTS – INSIGHTS ON ASSESSMENT AND PREVENTION FROM SINGAPORE AND THAILAND

Chair: Rahul Malhotra
Co-Chair: Vanessa Koh

Falls, and fall-related injuries, are a major public health challenge for ageing populations worldwide; but especially in Asia, where the proportion of older adults, aged 60 years and older, has already surpassed the rest of the world. Yet, current international fall prevention guidelines are not tailored for Asian populations where significant ethnic, cultural and environmental differences in fall risk factors have been identified. This symposium informs fall assessment and prevention practice and policy for Asian populations by presenting five novel ongoing studies from two rapidly ageing Asian nations, Singapore and Thailand. The first presentation introduces TARGET, a Singapore-based prospective cohort of community-dwelling older adults designed specifically for assessing fall and fracture risk. Analyzing data from TARGET, the second presentation highlights the need for resilience-boosting interventions among older adults to improve functional and psycho-social outcomes. The third presentation introduces SAFE-TECH, a tailored, multi-component community-based fall prevention program developed in Singapore, aimed at improving physical performance, falls efficacy, and promoting preventive behaviors. From Thailand, the fourth presentation introduces the Thai-STEADI program, adapted from the CDC's falls prevention model, utilizing community health workers and care managers to deliver scalable interventions in low-resource settings. The final presentation introduces a simulation model designed to identify policy bottlenecks and optimal strategies for implementation of a national falls prevention framework in Singapore. Together, this symposium discusses individual, community, and policy-level falls assessment and prevention strategies, and explores cross-country learnings for healthcare professionals, researchers, and policymakers. Aging Among Asians Interest Group Sponsored Symposium

Abstract citation ID: igaf122.980

### TARGET: A SINGAPORE NATIONAL COHORT FOR ASSESSING GERIATRIC FALL AND FRACTURE RISK IN THE COMMUNITY

Kok Yang Tan[1], Angelique Chan[1], Rahul Malhotra[1], Abhijit Visaria[1], Vanessa Koh[1], Lakkhina Troeung[1], David Matchar[1], and William Taylor[2], *1. Duke-NUS Medical School, Singapore, Singapore, 2. Institute for Biomechanics ETH Zürich, Zürich, Zurich, Switzerland*

The Targeted Assessment and Recruitment of Geriatrics for Effective fall prevention Treatments (TARGET) study is a national prospective cohort study in Singapore, integrating novel health technology approaches, to develop cost-effective and scalable frameworks for early detection and prevention of falls and fractures in community-dwelling older adults. TARGET combines epidemiological survey data with data from novel wearable sensor, clinical imaging and virtual reality (VR) technology to predict the risk of incident falls and hip fractures. Between October 2022 and October 2024, a total of 2,291 Singapore residents aged 60 years and older were enrolled. Participants underwent a baseline fall risk assessment (T0) comprising (i) an interview to capture sociodemographic, anthropometric, physical, functional, and psychosocial measures, (ii) gait assessment using wearable inertial measurement unit motion sensors, (iii) dual-energy X-ray absorptiometry and whole-body 3D scans, and (iv) VR-based spatial navigation assessment. While all participants underwent (i) and (ii), a subset (13%-39%) underwent (iii) to (v). Prospective follow-up of participants – every three months for two years (T1-T8) to monitor incident falls, fractures and cognitive status – is ongoing. Electronic medical records are also being linked, providing objective longitudinal data on fall-related morbidity, mortality, healthcare utilisation and costs. Statistical analyses focus on (i) developing and validating personalised risk prediction models to identify fallers, (ii) developing hip fracture risk prediction models for clinical decision-making, (iii) assessing the predictive utility of VR technology for detecting cognitive decline and fall risk, and (iv) understanding the psychological and sociodemographic correlates of fall risk in older adults.

Abstract citation ID: igaf122.981

### RISING ABOVE FALLS: RESILIENCE, FEAR OF FALLING, AND IMPACT ON FUNCTIONAL AND PSYCHOSOCIAL OUTCOMES

Rahul Malhotra, Lakkhina Troeung, Abhijit Visaria, Vanessa Koh, Kok Yang Tan, Wen Huey Lim, and Angelique Chan, *Duke-NUS Medical School, Singapore, Singapore*

Falls are associated with multiple adverse outcomes for older adults including loss of independence, social isolation, and increased mortality risk. The fear of falling (FoF) is common among older adults and has been shown to significantly predict post-fall outcomes across numerous international studies. This study examines the proposed causal pathways between resilience, falls, FoF, and functional and psychosocial outcomes using a moderated-mediation approach. Structural equation modelling (SEM) using the TARGET cohort (n=2,291) showed that self-reported fall count in the prior 12-months significantly predicted poorer functional independence (Modified Barthel Index; MBI, Instrumental Activities of Daily Living; IADL), greater depression (Patient Health Questionnaire-9; PHQ-9) and poorer quality of life (EuroQol-5D; EQ5D). FoF (Iconographical Falls Efficacy Scale) fully mediated the paths between falls and MBI, IADL and EQ5D, and partially mediated the path between falls and PHQ-9. Resilience (Brief Resilience Scale; BRS) significantly moderated the effects of falls on FoF, and FoF and all outcomes. The effect of falls on FoF was largest for the Low resilience ($\beta=4.52$, $p<0.002$) compared to Normal ($\beta=1.39$, $p<0.000$) and High ($\beta=1.20$, $p=0.034$) groups. Similarly, FoF had the largest indirect effects on outcomes in the Low resilience group, PHQ-9 ($\beta=0.46$, $p=0.044$), MBI ($\beta$=-2.34,p=0.006), IADL ($\beta$=0.79,p=0.003), and EQ5D ($\beta$=-0.05,p=0.008). In contrast, FoF was not significantly predictive of all four outcomes in the High resilience group. Thus, resilience is an important protective mechanism for older adults. This presentation will discuss clinical implications, notably the importance of developing targeted "resilience-boosting" interventions to improve post-fall outcomes.

Abstract citation ID: igaf122.982

### A TAILORED, MULTI-COMPONENT FALLS PREVENTION INTERVENTION FOR COMMUNITY-DWELLING OLDER ADULTS IN SINGAPORE

Jing Wen Goh[1], Vanessa Koh[1], Kok Yang Tan[1], Angelique Chan[1], Navrag Singh[2], and David Matchar[3], 1. Duke-NUS Medical School, Singapore, Singapore, 2. Institute for Biomechanics ETH Zürich, Zürich, Zurich, Switzerland, 3. Duke-NUS Medical School Programme in Health Services & Systems Research, Singapore, Singapore

Despite strong evidence supporting personalized, multi-component falls prevention programs, many existing programs in Singapore focus primarily on exercise and lack individualization. This study aims to address this gap by assessing the effectiveness of a tailored multi-component program, integrating both exercise and education, to reduce falls and injurious falls among community-dwelling older adults at high risk for falls. A 12-month, randomized controlled trial was conducted, where the intervention group participated in a tailored exercise and education program twice a week for 12 weeks, while the control group received falls prevention booklet. Intention-to-treat (ITT) and per protocol analyses (PPA) were conducted to assess the impact on number of fallers and injurious fallers, physical performance (assessed using the Short Physical Performance Battery, SPPB), falls efficacy (assessed using the Falls Efficacy Scale, FES), and falls prevention behaviors (assessed using the Falls Behavioral Scale, FaB). Preliminary results from both ITT and PPA analyses demonstrated that the intervention group had positive improvements in the SPPB lower limb strength subscale compared to the control group, along with improvements in falls efficacy and falls prevention behaviors. The PPA analysis also revealed a positive improvement in the SPPB total score and balance subscale for the intervention group. These preliminary findings highlight the need for a multi-component, tailored falls prevention program, as such a program not only improves physical performance, but also increases awareness and knowledge about falls and falls prevention; resulting in positive behavioral changes and more proactive approach to safety.

Abstract citation ID: igaf122.983

### THAI-STEADI PROGRAM: ROLES OF COMMUNITY HEALTH WORKERS AND CARE MANAGERS TO PREVENT FALLS IN LMICS

Ladda Thiamwong[1], Rui Xie[1], Boon Ng[1], Sirinart Tongsiri[2], and Jom Suwanno[3], *1. University of Central Florida, Orlando, Florida, United States, 2. Mahasarakham University, Maha Sarakham, Maha Sarakham, Thailand, 3. Walailak University, Tasala, Nakhon Si Thammarat, Thailand*

One-third to half of older adults in low and middle-income countries (LMICs), including Thailand, have falls, fear of falling, and physical inactivity. There is a lack of pragmatic and cost-effective multi-factorial fall prevention interventions embedded within primary care settings led by trusted and trained local health personnel to ensure interventions reach older adults who need them the most and enhance the intervention's sustainability. This leaves clinical practice bereft of knowledge to prevent falls in LMICs. Our team worked closely with community partners in Thailand in 4 steps of cultural adaptation and pilot-testing the CDC's Stopping Elderly Accidents, Deaths, and Injuries (STEADI) with input from Thai older adults, community health workers, and care managers. Our pilot study ($n = 27$) indicated that Thai-STEADI delivered by community health workers and care managers is feasible, appropriate, and acceptable for low-income older adults with limited health care access. The Thai-STEADI group had a significant reduction in fall risk from pre-intervention ($M = 11.60$, $SD = 1.52$) to post-intervention ($M = 10.80$, $SD = 1.64$) but not the control group. Thai-STEADI also group improved dynamic balance and the mean of the Timed-Up and Go test scores decreased from 20.24 ($SD = 23.58$) to 19.60 ($SD = 23.31$), but the decrease was not statistically significant. Fear of falling was reduced in the Thai-STEADI group from 100% to 80%, whereas 100% of the control group still had a fear of falling. Using a combination of community health workers and care managers with increasing stakeholder influence is one of the most vital strategies to prevent falls at a system level in LMICs.

Abstract citation ID: igaf122.984

### FALLS AND FALLS PREVENTION IN SINGAPORE: POLICY INSIGHTS FROM A DYNAMIC SIMULATION MODEL

Vanessa Koh[1], John Ansah[2], Angelique Chan[1], and David Matchar[3], *1. Duke-NUS Medical School, Singapore, Singapore, 2. Case Western Reserve University, Cleveland, Ohio, United States, 3. Duke-NUS Medical School Programme in Health Services & Systems Research, Singapore, Singapore*

Falls among older adults have complex etiologies, requiring a multi-system approach for effective prevention. In Singapore, 85% of geriatric trauma cases presented in the emergency department result from falls. While multi-component falls prevention programs demonstrate evidence in reducing falls, their implementation remains challenging. Currently, Singapore lacks a national strategy to tackle falls, hence this study examines how such a strategy should be implemented to significantly avert falls. A Systems Dynamics model was developed to simulate the current reality of falls among older adults in Singapore. The model represents the resident population, patient-care continuum of falls prevention and capacity for screening and prevention services. Through different policy experiments, we aim to provide policy insights into the bottlenecks for intervention, and a combination of strategies that should be prioritized to optimize the impact of falls prevention programs. The model was simulated from 2010 to 2040, to project the impact of policies across a 30-year period. Five key bottlenecks in the current environment for falls have been identified. Policy experiments simulated changes in patient uptake, program quality, and capacity of services to alleviate these barriers. Findings suggest that an appropriate combination of investing resources for capacity expansion of screening and primary prevention programs, coupled with increased older adult engagement, improves the utilization of falls prevention services and health outcomes within 5-10 years after policy implementation. These findings demonstrate the need for a coordinated, resource-optimized approach to falls prevention, providing critical insights to inform policymakers in designing a national falls prevention strategy.

### SESSION 2440 (SYMPOSIUM)

Abstract citation ID: igaf122.985

### NAVIGATING RESEARCH ACROSS LONG-TERM CARE SETTINGS: STRATEGIES FOR PARTNERSHIP, RECRUITMENT, AND DATA COLLECTION

Chair: Jasmine Altizer
Discussant: Abraham Brody

Long-term care settings have long been known as spaces where recruitment of participants and engagement in research including implementation of interventions, surveys, and interviews are challenging. In the same vein, research in these settings is necessary to improve the care of older adults and those who care for them. This symposium will examine innovative methods for conducting research across long-term care settings amid challenges. Key topics include building partnerships with long-term care communities and agencies, adapting research approaches for hard-to-reach participants, and optimizing participant recruitment and data collection that minimizes burden on staff and residents and maintains research rigor and integrity. The session will offer practical insights from researchers working across the long-term care continuum --- in nursing homes, adult day centers, assisted living, home health agencies, and hospice. Dan David will present an innovative strategy to support resident recruitment and facility partnership in Assisted Living through the Facility-Embedded Nurse Researcher model. Tina Sadarangani will share successful approaches to recruitment, intervention design, and implementation of technology in adult day centers. Andreina Jimenez will present strategies to improve research engagement of Black, Latino, and White nursing home residents living with dementia, family care partners, and staff. Chenjuan Ma will present key strategies for navigating challenges related to recruitment, data collection, and stakeholder engagement among providers and aides in home health care. Ab Brody will serve as the discussant for the panel integrating the insights shared across the presentations and adding in approaches to research engagement in Hospice settings. Research in Quality of Care Interest Group Sponsored Symposium

Abstract citation ID: igaf122.986

### INNOVATIVE STRATEGIES FOR RECRUITMENT IN ASSISTED LIVING: LESSONS FROM QUALITATIVE AND PILOT INTERVENTION STUDIES

Daniel David, *New York University, New York, New York, United States*

Recruiting assisted living (AL) residents for research presents unique challenges, including resident mistrust, cognitive impairment, and low research engagement. This abstract outlines innovative strategies to recruitment developed through qualitative and pilot intervention studies in three AL facilities. Key to successful recruitment was the Facility-Embedded Nurse Researcher model, where a researcher spent 1 month prior to study initiation to build rapport with staff, residents, and families, enhancing trust and study visibility. This approach established familiarity and interest in the project before the study launch and integrated the principal investigator as part of the residential community. The following aspects will be discussed: 1. Securing support from administration early in the process facilitated recruitment by ensuring frontline staff were informed and engaged with research efforts. 2. Pre-recruitment community events – The investigator led a weekly facility community event to engage residents in discussions about their health needs, goals and values – "Health Talk with Dr. X". 3. Pre-recruitment facility needs assessment – through staff focus groups, staff identified the need for training on dementia care, palliative care, and resident education on health topic which created reciprocal value and encouraged administrative and staff buy-in. 4. A Community Advisory Board (CAB) helped refine intervention development, recruitment materials, and messaging to align with resident and facility priorities. These approaches improved recruitment efficiency, increased resident participation, and strengthened facility-researcher partnerships. Future studies should integrate these strategies to enhance research feasibility and impact in AL settings.

Abstract citation ID: igaf122.987

### ENGAGING STAFF, FAMILY, AND OLDER ADULTS LIVING WITH DEMENTIA IN NURSING HOME RESEARCH

Andreina Jimenez, *New York University, New York, New York, United States*

Despite the importance of inclusive research, conducting studies in nursing home communities with Black, Latino, and White older adults living with dementia, family care partners, and nursing home staff poses unique research engagement challenges such as recruitment, consent, and implementation of study activities. The significant strain the COVID-19 pandemic placed on the nursing home workforce has further hindered research engagement efforts. This presentation will discuss the obstacles encountered and strategies implemented in research engagement activities within our ongoing research with Black, Latino, and White older adults living with dementia, family care partners, and nursing home staff. It will emphasize the importance of collating traditional recruitment tactics (i.e. email, phone calls, and snowball methods) with a more modern approach using text, referral bonuses, and social media. Leveraging findings from 12 nursing home interactions, we will highlight the importance of transparency, culturally sensitive approaches, and trust-building strategies in overcoming research engagement challenges with nursing home executive leadership and nursing home research participants. These insights are important for informing future recruitment strategies, research methodologies, and approaches.

Abstract citation ID: igaf122.988

### RECRUITING AND COLLECTING DATA FROM HOME HEALTH CARE PROVIDERS: EXPERIENCES AND LESSONS

Chenjuan Ma[1], Hillary Dutton[1], Sora Aikawa[1], Ann Lee[2], Jeanmarie Moorehead[3], and Jenna Blind[3], *1. New York University, New York, New York, United States, 2. 1199SEIU Training and Employment Funds, New York, New York, United States, 3. New York University Langone Health, New York, New York, United States*

As home health care (HHC) becomes increasingly pivotal in serving millions of homebound Americans, the need for empirical evidence to inform quality HHC delivery is imperative. However, conducting HHC research has unique challenges related to recruitment, data collection, and stakeholder engagement. Based on our experience conducting a study involving licensed HHC providers and HHC aides, this abstract presents key strategies for navigating these challenges to enhance research feasibility and quality. First, building partnerships with clinical and non-clinical HHC stakeholders facilitates access to participants. Identifying a dedicated site liaison, engaging them early in the research process, and incorporating their recommendations strengthens recruitment and fosters trust among participants. Fair and transparent compensation approaches that consider participant preferences incentivizes participation while maintaining ethical standards. While virtual approaches are feasible for data collection, they may require more structured facilitation to maintain smooth and meaningful interactions. Planning for potential technological challenges and providing anticipatory guidance ensures smoother execution of interviews. Flexibility in data collection methods—including focus groups and individual interviews—accommodates participant needs, and tailoring interview language based on participants' professional backgrounds enhances response quality. While using technology for live transcription may expedite data processing, manual quality checks remain essential for accuracy. Lastly, leveraging multilingual research team members broadens inclusivity and representation. By integrating these strategies, researchers can enhance the rigor of HHC studies, ensuring meaningful participant engagement while capturing the complexities of their experiences. Future research should continue refining these approaches to strengthen data collection and partnership efforts in HHC settings.

Abstract citation ID: igaf122.989

### BRIDGING THE DIGITAL DIVIDE: COMMUNITY-ENGAGED STRATEGIES FOR IMPLEMENTING TECHNOLOGY IN RURAL ADULT DAY CENTERS

Tina Sadarangani, *New York University, New York, New York, United States*

Successfully embedding technology into long-term care settings requires intentional, evidence-based community engagement strategies that address the unique needs of providers, caregivers, and persons living with dementia. This presentation will explore the implementation of CareMobi, a HIPAA-compliant mobile health application designed to enhance communication between adult day centers and family caregivers, within a rural, under-resourced adult day center setting and identify key components of a community-engaged approach to technology implementation that prioritized collaborative design, staff training, and iterative adaptation to fit the center's workflows. Drawing from qualitative and quantitative data, we identify successful approaches to recruitment, intervention design, and implementation of CareMobi that address digital literacy among staff and caregivers, infrastructure limitations, and the importance of trust-building in rural communities. We identified strategies that supported sustained engagement, including co-developing implementation protocols and recruitment strategies with frontline staff, an iterative user centered process for designing the app, leveraging existing caregiving networks, and aligning technology use with reimbursement structures and regulatory requirements. Findings from 2 centers and n = 17 participants, suggest that CareMobi improved information-sharing between adult day center staff and families, enabling earlier identification of health changes and care coordination needs. By providing insights into real-world application, this presentation will contribute to the broader discussion of how researchers and practitioners can effectively introduce and sustain technology in community-based long-term care settings. The lessons learned from this rural implementation will offer a framework for scalable, equitable adoption of digital health tools across diverse adult day center environments.

### SESSION 2445 (SYMPOSIUM)

Abstract citation ID: igaf122.990

### HOME SWEET HOME: TRANSFORMING AGING IN PLACE THROUGH THE 5MS

Chair: Beth Fields
Discussant: Leslie Scheunemann

Aging in place remains a high-priority goal for older adults. Achieving this goal requires innovative solutions to address common problems of aging. This symposium will explore cutting-edge approaches and technologies that support aging in place, focusing on the 5Ms framework (What Matters, Medication, Mentation, Mobility, and Multicomplexity). Presenter 1 will introduce CAPABLE (Community Aging in Place—Advancing Better Living for Elders) program, which combines home visits by an occupational therapist, a nurse, and a handyperson to prioritize "What Matters" to the individual and to enhance "Mobility" through home modifications for aging in place. Presenter 2 will discuss medication management for older adults with Alzheimer's disease and related dementias and multiple chronic conditions, highlighting strategies to reduce polypharmacy and its associated risks, emphasizing "Medication" and "Multicomplexity" in maintaining engagement in health-promoting daily activity. Presenter 3 will present 3D modeling for remote home assessments, enhancing "Mobility" and "Mentation." Finally, Presenter 4 will share her work on a mobile app for home assessment that crosscuts the 5Ms, using augmented reality to evaluate and modify living environments, making them safer and more supportive for aging in place. This symposium will conclude with a discussion on integrating these innovative approaches and technologies into existing healthcare systems to promote aging in place, providing practical insights into applying the 5Ms framework to enhance the lives of older adults, ensuring they can live independently and with dignity in their own homes. Environmental Gerontology Interest Group Sponsored Symposium

Abstract citation ID: igaf122.991

### BECOMING MORE CAPABLE: PRELIMINARY RESULTS OF IMPLEMENTING A PERSON-CENTERED INTERVENTION FOR AGING IN PLACE

Pamela Toto[1], Ava Giatras[1], Kate Perepezko[2], Morgan Blanchflower[1], and Beth Fields[3], *1. University of Pittsburgh, Pittsburgh, Pennsylvania, United States, 2. Miami University, Oxford, Ohio, United States, 3. University of Wisconsin–Madison, Madison, Wisconsin, United States*

Community Aging in Place, Advancing Better Living for Elders (CAPABLE) is an evidence-based intervention that combines occupational therapy, nursing, and low-cost home modifications to target "what matters" to community older adults. CAPABLE has been demonstrated to promote aging in place by delaying disability, preventing hospitalizations, and avoiding institutionalization, yet limited information exists on its real-world application through existing home and community-based services (HCBS). This study descibed preliminary results of a pilot providing CAPABLE through an area agency on aging (AAA). An effectiveness-implementation hybrid trial type 1 study was completed implementing CAPABLE with 90 older adults. Participants were recruited through 3 HCBS agencies affiliated with an AAA and received up to 10 visits and $1300 in home modifications over a 5-month period to address self-identified functional goals. Descriptive statistics were conducted to assess the program's feasibility, cost, and acceptability. 69% (n = 62) participants completed CAPABLE. Primary reasons for non-completion included health decline, death, or move to a skilled nursing facility. On average, participants identified and set 4.5 goals (range 1 -6). Most common goal areas included bathing, functional mobility, and pain. Home modification costs averaged $943 per participant with most common modifications including grab bars (33%), railings (16%) and lighting (15%). Program satisfaction scores indicated high satisfaction with # of visits, length of program, home modifications and goal attainment. Preliminary outcomes suggest that delivering CAPABLE through an AAA and HCBS is a feasible and acceptable approach to supporting older adults in achieving self-identified goals for aging in place through low-cost home modifications.

Abstract citation ID: igaf122.992

### CAREGIVER-REPORTED BARRIERS AND FACILITATORS TO DEPRESCRIBING IN OLDER ADULTS WITH DEMENTIA: A SYSTEMATIC REVIEW

Tara Klinedinst[1], Sarah Joseph[1], Te-Lien Ku[2], April Schweikhard[3], Beth Fields[4], and Lee Jennings[5], *1. The University of Oklahoma Health Sciences Center, Oklahoma City, Oklahoma, United States, 2. University of Wisconsin-Madison, Madison, Wisconsin, United States, 3. The University of Oklahoma, Norman, Oklahoma, United States, 4. University of Wisconsin–Madison, Madison, Wisconsin, United States, 5. University of Oklahoma Health Sciences, Oklahoma City, Oklahoma, United States*

Many older adults with ADRD are at heightened risk for inappropriate medication prescription, due to the complexity of managing dementia in addition to comorbid medical conditions. Deprescribing medications that are harmful or no longer beneficial can prevent illness and injury, and caregivers play a crucial role in this process. Unfortunately, guidance on decision-making and practical supports for caregivers interested in deprescribing is limited. Previous low-touch interventions have not significantly reduced prescriptions, indicating that older adults with ADRD and their caregivers may need more intensive supports and resources during the deprescribing process. To address this gap, we used systematic review methodology to characterize barriers and facilitators to deprescribing as reported by caregivers of older adults with ADRD. We used thematic analysis to synthesize qualitative study results (n = 8 articles). Barriers to deprescribing included challenges such as limited caregiver knowledge and skills, reliance on medications to regulate behavior, fear of “giving up” on treatment, and lack of collaborative relationship with providers. Facilitators included a trusting alliance with providers and proactive communication (framing deprescribing as “routine”); worsening of care recipient health and negative effects of medication also triggered deprescribing conversations. Findings suggest that caregiver engagement and confidence in deprescribing is influenced by multiple factors, with communication and trust playing a central role. Addressing caregiver hesitancy through improved provider communication, clearer deprescribing guidelines, and targeted education may enhance confidence and willingness to deprescribe. Future initiatives should prioritize incorporating caregiver perspectives into deprescribing strategies to enhance care recipient outcomes and support informed medication management.

Abstract citation ID: igaf122.993

### NEXT-LEVEL REMOTE HOME ASSESSMENTS WITH 3D TECHNOLOGY

Jaewon Kang[1], Mi Jung Lee[2], Consuelo Kreider[3], Kimberly Findley[4], Keith Myers[5], and Sergio Romero[5], *1. Colorado State University, Fort Collins, Colorado, United States, 2. UTMB Health, The University of Texas Medical Branch, Galveston, Texas, United States, 3. University of Florida College of Public Health and Health Professions, Gainesville, Florida, United States, 4. Malcom Randall VA Medical Center, Gainesville, Florida, United States, 5. Veterans Rural Health Resource Center-Gainesville (VRHRC-GNV), Gainesville, Florida, United States*

Most older adults want to remain in their homes as they age. However, their homes may become unsafe or inaccessible, especially for those with disabilities or chronic conditions. Occupational therapists conduct home assessments to identify barriers that impact safety and accessibility for aging in place. Unfortunately, older adults, especially those in rural or remote areas, often face delays or are unable to receive these services due to logistic challenges posed by long distances and a shortage of trained clinicians. To improve access to home assessment services, videoconferencing has been recently adopted in practice. However, clinicians often report challenges such as unstable internet connections, limited field of view, difficulty measuring home environments, and heavy reliance on older adults and their care partners, which impact the quality of assessments. Our project tested 3D modeling technology, commonly used in real estate, to overcome these limitations. Occupational therapists in the study reported that 3D models allowed them to independently observe home environments from multiple perspectives, including 360-degree views, bird’s-eye views, and floor plans, while also enabling accurate remote measurements. By reducing reliance on high-speed internet and care partners, clinicians can conduct more comprehensive assessments anytime and anywhere, providing older adults with better access to essential services for aging in place.

Abstract citation ID: igaf122.994

### REVOLUTIONIZING HOME SAFETY AND INDEPENDENCE FOR OLDER ADULTS WITH THE AUGMENTED REALITY HOME ASSESSMENT TOOL

Emily Thompson, Jung-hye Shin, Kevin Ponto, and Beth Fields, *University of Wisconsin–Madison, Madison, Wisconsin, United States*

The number of older adults is rising due to medical advancements, with one in four U.S. households including older adults in 2019. Many of these older adults have a strong desire to remain in their homes safely and independently for as long as possible. However, fewer than 1% of homes are equipped to accommodate their needs, increasing their risk of falls and healthcare service use. The Augmented Reality Home Assessment Tool (ARHAT), a mobile-based app, addresses this gap by using 3D scanning and augmented reality capabilities to assess home environments. ARHAT has been accepted and deemed appropriate by key end users, including occupational therapists, housing professionals, and older adults, for identifying and addressing functional limitations and barriers in home environments. In partnership with a community-based organization, this study aims to evaluate the feasibility of implementing ARHAT and its impact on reducing fear of falling and healthcare service use among 75 community-dwelling older adults in the Midwest. Descriptive and inferential statistics will be conducted using STATA software. Preliminary data to be presented include recruitment, retention, readiness to implement, adherence, demographics, fear of falling, and healthcare service use. “What Matters” to older adults is the ability to age in their home environment safely and independently. ARHAT aligns with this goal by providing a practical, acceptable, and appropriate tool for enhancing home safety and supporting aging in place.

### SESSION 2450 (SYMPOSIUM)

Abstract citation ID: igaf122.995

### ROADMAP TO INNOVATION: CHARTING CALIFORNIA’S PATH TO STRENGTHENING CAREGIVING SERVICES AND SUPPORTS

Chair: Rachael Fulp-Cooke
Co-Chair: Heather Young

The landscape of family caregiving is evolving, shaped by demographics and changing care needs. In California, nearly seven million family caregivers form the foundation of the state’s Long-Term Services and Supports (LTSS) system, enabling older adults and people with disabilities to remain in their homes and communities. As the population ages, and more individuals live with chronic conditions or functional and cognitive limitations, the demand for caregiving is increasing while the number of available caregivers is declining. Caregivers span all ages, and represent diverse backgrounds, experiences, and identities. While caregivers share common challenges, factors such as income, language, and geographic location shape their caregiving experience in distinct ways with significant policy implications.

The California Department of Aging (CDA) partnered with the Family Caregiving Institute (FCI) at the UC Davis Betty Irene Moore School of Nursing to develop the California Caregiver Equity Roadmap ("Roadmap"). Launched in July 2023, this initiative aims to strengthen publicly funded caregiver services and supports advancing the fourth goal of the California Master Plan for Aging: Caregiving That Works. This project also assessed how California's caregiving efforts align with the 2022 National Strategy to Support Family Caregivers and identified promising practices from other states. This symposium will explore the Roadmap's development, including the community engagement process and data analyses that shaped it. Additionally, presenters will discuss caregiving strengths and opportunities in California and examine innovative caregiving initiatives nationwide to inform efforts to strengthen caregiver services and supports. Family Caregiving Interest Group Sponsored Symposium

Abstract citation ID: igaf122.996

### CALIFORNIA'S APPROACH TO THE 2022 NATIONAL STRATEGY TO SUPPORT FAMILY CAREGIVERS

Rachael Fulp-Cooke[1], Janice Bell[1], Quynh Vo[2], and Heather Young[1], *1. Betty Irene Moore School Of Nursing at UC Davis, Sacramento, California, United States, 2. University of California, Davis, Davis, California, United States*

Family caregivers provide essential support for older adults and people with disabilities across California and the United States. The 2022 National Strategy to Support Family Caregivers ("National Strategy") outlines five goals, 27 outcomes, and hundreds of actions that federal, state, and local governments, businesses, and communities can implement to strengthen caregiver support. To identify strengths and opportunities for expanding caregiving support in California in line with national priorities, we identified publicly funded caregiving initiatives in the state and assessed their alignment with the National Strategy. Caregiving initiatives were identified using the Data Dashboard for Aging, the Master Plan for Aging Implementation Tracker, the AARP Long-Term Services and Support Scorecard 2023, and additional internet research. Each initiative was mapped to one or more National Strategy outcomes, with results summarized in visual matrices. Findings indicate that California demonstrates strong alignment with the National Strategy offering a diverse range of caregiving support initiatives. Key strengths include 1) a robust policy framework, 2) a statewide caregiver resource center network, 3) a centralized caregiver database, and 4) a focus on research and education. However, the depth, reach, and sustainability of these initiatives vary, presenting opportunities for further planning and investment. Key areas for growth include 1) increasing caregiver awareness, 2) supporting employed caregivers, 3) improving program evaluation, 4) expanding public-private partnerships, and 5) workforce development. Research implications in California include exploring ways to further align existing caregiving infrastructure with national priorities to ensure that services are accessible, inclusive, and responsive to caregiver needs.

Abstract citation ID: igaf122.997

### PROMISING PRACTICES IN CAREGIVING: LEARNING FROM INNOVATIVE INITIATIVES ACROSS U.S. STATES

Rachael Fulp-Cooke[1], Janice Bell[1], Quynh Vo[2], and Heather Young[1], *1. Betty Irene Moore School Of Nursing at UC Davis, Sacramento, California, United States, 2. University of California, Davis, Davis, California, United States*

There is growing recognition of the essential role that family caregivers play in families, communities, and society, as well as the need for comprehensive, tailored support. While California offers some of the most comprehensive publicly funded caregiver services and supports in the country, opportunities remain for the state to better align with priorities outlined in the 2022 National Strategy to Support Family Caregivers. To provide insights to inform California's approach to caregiving support, we identified innovative and promising caregiving programs and initiatives from other U.S. States with similar population sizes or demographic profiles to California based on the AARP Long-Term Services and Supports Scorecard 2023. As research progressed, additional states with promising practices were identified and included. All initiatives were compiled into an inventory organized by state. Building on a prior analysis of gaps in California's publicly funded caregiving services and supports, key initiatives addressing the needs of California caregivers were identified. Findings indicate that multiple states have developed and implemented innovative approaches to caregiving support. Notable initiatives from New York, Washington, Texas, and Georgia were recognized as particularly relevant to California's caregiving landscape. These models offer valuable insights for potential adaptation and enhancement in California and in other settings. Research implications include assessing how these initiatives can be adapted to and integrated into California's caregiving infrastructure and developing strategies to improve accessibility, sustainability, and effectiveness while strengthening alignment with national priorities.

Abstract citation ID: igaf122.998

### LEVERAGING ANALYTICS TO SHAPE CALIFORNIA'S CAREGIVER EQUITY ROADMAP

Shikha Bhurtel[1], Rachael Fulp-Cooke[2], Heather Young[2], and Janice Bell[2], *1. University of California, Davis, Davis, California, United States, 2. Betty Irene Moore School Of Nursing at UC Davis, Sacramento, California, United States*

Shifts in population and other factors are reshaping caregiving in California. To ensure access and effective support, publicly funded caregiver services and supports must be responsive to population trends and caregiver characteristics. The California Department of Aging (CDA) partnered with the Family Caregiving Institute (FCI) at UC Davis Betty Irene Moore School of Nursing to develop a roadmap to outline strategies and actions to improve publicly funded caregiver support over five years. This study identified data sources and analytical approaches to develop the California Caregiver Equity Roadmap ("Roadmap"). Data sources included administrative records from publicly funded caregiver support programs, caregiver assessments from the California Caregiver Resource Centers, and population-based surveys. Analyses integrated these data sources to examine population trends and caregiver characteristics. Key sociodemographic and health characteristics included age distribution, geographic patterns, race and ethnicity, poverty level, broadband access, care dependency ratios, care receiver needs, and attributes of the caregiving role. Analyzing the intersection of population trends and caregiver characteristics provided a foundation for the Roadmap's development. The findings provide a deeper understanding of caregiving in California, insights for improving services and supports across the state, and offer a framework for other states to follow. Research implications include using data to better understand caregiver needs, improve service delivery, and guide policy decisions and program development.

Abstract citation ID: igaf122.999

**A ROADMAP TO STRENGTHEN CAREGIVER SERVICES AND SUPPORTS IN CALIFORNIA**

Rachael Fulp-Cooke[1], Janice Bell[1], Shikha Bhurtel[2], Quynh Vo[2], Pauline Martinez[3], Lorena Garcia[1], Kathleen Kelly[4], and Heather Young[1], *1. Betty Irene Moore School Of Nursing at UC Davis, Sacramento, California, United States, 2. University of California, Davis, Davis, California, United States, 3. Betty Irene Moore School of Nursing, University of California, Davis, Davis, California, United States, 4. Family Caregiving Alliance, San Francisco, California, United States*

Family caregivers provide essential support for older adults and people with disabilities across California. However, many caregivers face barriers to accessing programs and services that support their own health and well-being. Demographic shifts and increasing care demands are reshaping California's caregiving landscape. To address these challenges, the California Department of Aging (CDA) partnered with the Family Caregiving Institute (FCI) at UC Davis to develop the California Caregiver Equity Roadmap ("Roadmap"). This initiative aims to improve publicly funded caregiver services and supports in California and advances the fourth goal of the Master Plan for Aging: Caregiving That Works. Community engagement was central to the Roadmap's development. Input was regularly collected from a wide range of constituent groups including a statewide advisory group, a steering committee, and leadership from Caregiver Resource Centers and Area Agencies on Aging. These constituent groups were comprised of family caregivers and individuals from caregiving service organizations. Strategic goals and actions were developed, tested, refined, and revised through an iterative process including regular meetings and two surveys distributed to constituents statewide. The final Roadmap outlines five strategic goals and 24 supporting actions to strengthen California's caregiving infrastructure, focusing on five key areas: 1) awareness, 2) access, 3) supporting health and social service providers, 4) using evidence to strengthen services and supports, and 5) using data to inform service improvement. Research implications include evaluating the Roadmap's impact on caregiving services and supports. Findings could serve as a model for other states aiming to strengthen family caregiver support.

Abstract citation ID: igaf122.1000

**ADVANCING EQUITY FOR FAMILY CAREGIVERS: RURAL-URBAN DIFFERENCES IN DEMANDS, RESOURCES, AND OUTCOMES**

Everlyne Ogugu[1], Orly Tonkikh[2], Jessica Famula[3], Heather Young[1], Janice Bell[1], and Bronwyn Fields[4], *1. Betty Irene Moore School Of Nursing at UC Davis, Sacramento, California, United States, 2. University of Haifa, Haifa, HaZafon, Israel, 3. University of California, Davis, Davis, California, United States, 4. Sacramento State University, Sacramento, California, United States*

In 2020, 55.8 million US adults were ≥65 years old, making up 16.8% of the US population, and this number is expected to rise to 80.8 million by 2040. With increases in the proportion of older adults, the demand for caregiving increases, particularly in rural communities with a higher proportion of older adults. An examination of rural versus urban caregiver needs, and resource availability is a crucial initial step toward providing effective and equitable support services for caregivers. This study examined caregiver experiences, caregiving intensity, caregiver resources (availability of paid or unpaid help, satisfaction with social support) and caregiver health outcomes (self-rated health, depressive symptoms) for rural-dwelling caregivers in comparison with suburban/urban-dwelling caregivers. We used assessment survey data of caregivers from the California Caregiver Resource Centers (CCRC) online platform, CareNav (n = 5,782). We used logistic regression models to assess association between place of residence and caregiving intensity, caregiver resources and health outcomes and negative binomial models to explore the association of place of residence and count outcomes (caregiving tasks, memory, and behavioral problems). The proportion of rural dwelling caregivers was 4.4% and 95% of caregivers identified as the primary caregiver. Rural dwelling caregivers reported managing 9% more memory and behavior problems whereas urban dwelling caregivers were 25% more likely to report experiencing difficulties in managing medical-surgical nursing tasks. There were no statistically significant differences between rural and suburban/urban-dwelling caregivers in caregiving intensity, caregiver resources or health outcomes. This study highlights differences in caregiving demands with implications for program delivery.

### SESSION 2455 (SYMPOSIUM)

Abstract citation ID: igaf122.1001

**GLOBAL PATHS TO AFU DESIGNATION: EXAMPLES FROM THE ASIAN AND NORTH AMERICAN REGIONS**

Chair: Katarina Friberg-Felsted
Co-Chair: Jacqueline Eaton
Discussant: M. Aaron Guest

The Age-Friendly University Global Secretariat promotes and processes applications to the Age-Friendly University Global Network (AFUGN). Membership indicates institutional commitment to endorsing the 10 Principles and implementing age-inclusive practices to promote older adult value and engagement in academic settings. New this year is the Guidebook For Endorsing the 10 Principles of an Age-Friendly University, created to assist institutions of higher education in becoming a designated Age-Friendly University. This symposium provides an overview of the endorsement guidebook, addressing the four steps of the process, and examples from a variety of institutions who will share their individual journeys in obtaining the AFU designation. Anderson, representing the University of Mississippi, will describe the challenges and opportunities in seeking AFU membership at an R1 university located in a rural, diversely populated state. Borrero will demonstrate the alignment between the AFU application and the University of Indianapolis's new mission and vision "Anytime. Anywhere. For Life." The holistic approach taken by the Chinese University of Hong Kong, including the Actionable Social Knowledge initiative, will be outlined by Chen. Perry will explore the team-based, goal-oriented, principle driven approach taken at Frontier Nursing University which led to sustainable system level efforts. Aaron Guest, Chair of the Age Friendly University Global Secretariat, will serve as discussant.

Abstract citation ID: igaf122.1002

**BECOMING AN AGE-FRIENDLY UNIVERSITY: THE ONGOING JOURNEY OF THE UNIVERSITY OF MISSISSIPPI**

Keith Anderson, *University of Mississippi, Oxford, Mississippi, United States*

The University of Mississippi (UM) joined the Age-Friendly University (AFU) Global Network in 2024, approximately two years after this GSA Fellow arrived on campus. The first step in the process was to educate key personnel on the meaning and importance of being an AFU. We quickly learned that most university and community members did not fully understand the concept, but there was genuine interest. We then formed a committee and inventoried age-related initiatives. We found that programs and resources existed (e.g., gerontology courses, older adult learning programs); however, utilization was often limited due to format (e.g., in-person, online), access (e.g., parking, cost), and lack of awareness. In the final step in the application process, we identified strengths and, equally important, deficits and unmet opportunities. While our efforts to join the AFU Global Network were ultimately successful, much work is left to be done. UM and Mississippi are unique in many ways. UM is a large, R1 university located in a rural and diversly populated state. Building a network of age-friendly organizations, including those that serve diverse populations (e.g., HBCUs, rural health departments) is key to reaching all older adults across the state. UM's main campus is located over two hours away from UM's Medical Center, a major resource for age-related programming, education, and research. Closing this distance through effective communication and collaboration is essential. Finally, embedding the AFU concepts into the fiber of UM and Mississippi, rather than in a few champions, will ensure continued progress toward our age-friendly goals.

Abstract citation ID: igaf122.1003

**TEAMWORK MAKES THE DREAM WORK: A TEAM-BASED APPROACH TO ACHIEVING AFU DESIGNATION**

Audrey Perry, *Frontier Nursing University, Versailles, Kentucky, United States*

The university created an Age-Friendly University (AFU) application team representative of the diverse services and stakeholders that assess, evaluate, support, and create Age-Friendly university and community opportunities. After creating a diverse team, reviewing AFU principles, and establishing goals and team agreements, university staff, faculty, and community partners effectively and swiftly contributed to the AFU application using principles of effective teams. The team submitted a successful application based on past and future Age-Friendly collaborative efforts. Additionally, the team-based, goal-oriented, principle-driven approach clarified future collaboration between the university and the community. Establishing a diverse application team resulted in short-term and sustained systems-level AFU efforts.

Abstract citation ID: igaf122.1004

**BRIDGING GENERATIONS: REDEFINING AGE-FRIENDLY UNIVERSITIES THROUGH INNOVATION AND INCLUSION**

Zhuolin Chen, Long Ki Fung, Wai Tong Lee, Helene Fung, and Jean Woo, *The Chinese University of Hong Kong, Hong Kong, China (People's Republic)*

The Chinese University of Hong Kong (CUHK) has been recognized as an Age-Friendly University (AFU) for its comprehensive and innovative approach to integrating older adults into academic and campus life. In preparation for submitting our AFU application, CUHK conducted a thorough assessment of existing programs, policies, and practices to ensure alignment with the AFU principles. This included engaging stakeholders across the university and community to identify areas for enhancement. Guided by AFU principles, CUHK actively involves older adults in teaching, research, and campus activities, while promoting public discourse on aging-related issues. The university offers specialized programs in gerontology and its Jockey Club Institute of Ageing, leads research and community projects on critical areas such as developing tailor-made framework and implementing activities under the principles of integrated care of older people, tackling the impact of climate change on older people, and using human-centric AI and robotics technology for geriatric care. Initiatives like the Actionable Social Knowledge (CU-ASK) program in the Motivation and Emotion Laboratory exemplify CUHK's commitment to bridging generational gaps and developing evidence-based solutions. Additionally, intergenerational projects such as the Age-Friendly City program and Elder Academy foster mutual understanding and collaboration between generations. Our survey findings on the AFU underscore the critical importance of recognizing the range of education needs of older adults. Moving forward, CUHK will prioritize the development and integration of health and wellness programs specifically designed for older adults and foster intergenerational learning opportunities, thereby enhancing the university's commitment to inclusivity and lifelong learning.

Abstract citation ID: igaf122.1005

**CHAMPIONING SECOND CAREERS AND ONLINE EDUCATION AT AN AGE-FRIENDLY UINDY**

Lisa Borrero, and Marwa Noureldin, *University of Indianapolis, Indianapolis, Indiana, United States*

The University of Indianapolis's (UIndy) path to becoming a member of the Age-Friendly University (AFU) Global Network reflects a strategic commitment to fostering a more inclusive educational environment for individuals across the lifespan. As part of the plan to prepare for AFU membership, UIndy established a campus-wide task force to assess the university's strengths and areas of opportunity in promoting age-inclusive practices. Additionally, through a series of surveys targeting UIndy's alumni and retired employees and focused listening sessions with current faculty and staff, the university collected valuable insights on engagement and opportunities for improvement. These findings helped shape the university's approach to the AFU application, which emphasized principles #2 and #5 of the AFU framework—principles that align with UIndy's new mission to champion lifelong learning and vision to provide educational opportunities "Anytime. Anywhere. For life." UIndy's commitment to these principles is further demonstrated through the development of the new UIndy Online and Sease Institute, which aims to expand access to online education, support second careers, and foster lifelong learning. This holistic approach underscores UIndy's dedication to intergenerational connection and meeting the needs of an aging population while advancing its mission of inclusive, flexible, and lifelong educational opportunities.

### SESSION 2460 (SYMPOSIUM)

Abstract citation ID: igaf122.1006

### ZOOMING IN ON CEREBRAL AMYLOID ANGIOPATHY IN THE CONTEXT OF AMYLOID-RELATED IMAGING ABNORMALITIES

Chair: Mo-Kyung Sin
Discussant: Yuan Luo

The significance of cerebral amyloid angiopathy (CAA) has been heightened since anti-amyloid therapies were approved. Amyloid-related imaging abnormalities (ARIA) are a major side effect of anti-amyloid therapies, and CAA is the major risk factor for ARIA. CAA is highly prevalent in Alzheimer's disease, and both are driven by impaired amyloid β clearance. In addition, the imaging and pathological manifestations of ARIA are similar to CAA, supporting both sharing a common pathway. High prevalence of CAA in AD places many patients at risk for ARIA-associated morbidity and high cost for health care. ARIA can be life-threatening in about 1% of patients, highlighting the need for better understanding of CAA. Thus, we assembled nationally and internationally well-known experts on CAA and Alzheimer's disease and related dementias research to cover the CAA accumulation pathway, risk factors and consequences of CAA in community-based clinical pathologic studies, vascular considerations for patients receiving anti-amyloid therapies, and general risk factor and management strategies. Our first presenter, Dr. Donna Wilcock will present on the time course of CAA progression in the APPSw mouse model, 2nd presenter, Dr. David Bennett on CAA risk factors and consequences from the Religious Orders Study and Rush Memory and Aging Project, and the Pathology, Alzheimer's and Related Dementias Study, 3rd presenter, Dr. Steve Greenberg on the key role of vasculature in generating ARIA, and our last presenter, Dr. Edward Zamrini on general CAA risk factors and management strategies. We believe this symposium has timely and real-life clinical and research implications.

Abstract citation ID: igaf122.1007

### CAA ACCUMULATION IN THE TG2576 APPSW MOUSE MODEL IS ASSOCIATED WITH INFLAMMATION AND VASCULAR REMODELING

Katelynn Krick[1], and Donna Wilcock[2], *1. Indiana University School of Medicine, Indianapolis, Indiana, United States, 2. Indiana University, Indianapolis, Indiana, United States*

Cerebral amyloid angiopathy (CAA) is an extremely common pathology of Alzheimer's disease (AD) included under vascular contributions to cognitive impairment and dementia (VCID). CAA has been reported in 78-98% of AD cases and has clinical significance when considering amyloid related imaging abnormalities (ARIA) that arise when using amyloid targeting immunotherapies. Despite its prevalence, studies addressing CAA mechanisms have been scarce and there are clear gaps in our understanding of how CAA progresses. This study uses Tg2576 mice, who develop CAA over time, to establish a time course of CAA progression at 8-, 14-, and 20-months of age. We identify changes in transcriptomic signatures of glial cells using NanoString nCounter and targeted protein changes using Nanostring Digital Spatial Profiling. Meso Scale Discovery and immunohistochemistry are used to establish disease progression. In this study, we saw many changes primarily associated with inflammatory response, with some changes being transient (Tnf, Lsr; VEGF) and others remaining chronically altered (Osmr, Ccl3; CTSD). Overarchingly, many of these changes relate to the perpetuation of inflammation or recruiting additional immune support, which we see in across our timepoints. Further, we identified differences in abundance of proteins (CD45, GFAP, CD31) based on presence of CAA positive vessels within a brain region. We also identified sex-specific differences in CAA burden, as well as how glial reactivity and vessel density change during disease progression. This data represents a comprehensive analysis of CAA progression and differential responses to parenchymal and vascular amyloid that could inform future basic and clinical studies.

Abstract citation ID: igaf122.1008

### RISK FACTORS FOR AND CONSEQUENCES OF CAA IN THREE COMMUNITY-BASED CLINICAL-PATHOLOGIC STUDIES

David Bennett, *Rush University Medical Center, Chicago, Illinois, United States*

Data come from the Religious Orders Study and Rush Memory and Aging Project (ROSMAP), and the Pathology, Alzheimer's and Related Dementias Study (PARDoS). To date, CAA was characterized in 2100+ ROSMAP autopsies (mean age 89, only 3 under age 65, 69% female, 95% non-Latino white). Over the past decade, we found that both APOE4, APOE2, APOEC2, were associated with CAA. In eQTL analyses using snRNAseq, microglial APOE expression was associated with CAA independent of the APOE4 locus. Microglial ITGAX and SPPI expression were also associated with CAA. LDL cholesterol and chronic kidney disease were both associated with CAA. Controlling for other pathologies, CAA was associated with a lower level of cognition but not to rate of cognitive decline. Overall, the attributable risk of AD from CAA exceeded 8% and was greater than athero- and arteriolo-sclerosis accounting for other neurodegenerative diseases. In addition, CAA was associated with cerebral microbleeds and enlarged perivascular spaces (EPVS) in the temporal and occipital lobes. CAA was also associated with epilepsy and interestingly with risk aversion. In PARDoS, more than 4000 cases were characterized for CAA (mean age 76, 1 third <65, 48% female, 58% white). Adjusted for age, CAA did not differ by sex or race. CAA was present in 5% <65 with the youngest person <40, and a quarter 65+. CAA was associated with dementia adjusted for demographics but was attenuated and no longer significant adjusting for other neuropathologies. We identified several risk factors for and consequences of CAA.

Abstract citation ID: igaf122.1009

### VASCULAR CONSIDERATIONS FOR PATIENTS RECEIVING AMYLOID IMMUNOTHERAPY

Steven Greenberg, *Harvard University, Cambridge, Massachusetts, United States*

Amyloid-related imaging abnormalities (ARIA) in the form of edema/effusions (ARIA-E) or hemorrhagic lesions (ARIA-H) are the most impactful adverse event associated with anti-amyloid immunotherapy for Alzheimer disease. Most ARIA events are mild or silent, but a small subset can be severely symptomatic or fatal. The pathophysiologic mechanisms for ARIA appear centered around interactions of β-amyloid peptide (Aβ) with the cerebrovasculature as it transits from plaques to the perivascular space or through direct antibody-Aβ interactions after deposition in arterioles as cerebral amyloid angiopathy (CAA). The key role of the vasculature in generating ARIA creates challenges for vascular clinicians, including safety of immunotherapy for individuals with CAA markers, whether immunotherapy-treated patients can also receive anticoagulants or thrombolytics, and treatment of severe ARIA. Among key vascular that have emerged from clinical trial data and adverse event reports are that 1) knowledge about ARIA should be disseminated throughout clinical teams providing stroke care, 2) ARIA risk can be stratified by pretreatment markers of CAA and incorporated into risk-benefit discussions, 3) the suggestive link between elevated blood pressure and ARIA risk supports close adherence to hypertension guidelines, 4) concomitant anticoagulation may increase risk of symptomatic ARIA-H and therefore requires considerable caution, 5) particular vigilance is required to avoid administering thrombolytics to patients with ARIA-E presenting as a stroke mimic, which has resulted in several fatalities, and 6) in addition stopping antibody infusion, high-dose corticosteroids can be considered for treatment of severe ARIA. These vascular considerations will likely evolve with ongoing post-marketing data collection efforts.

Abstract citation ID: igaf122.1010

### RISK FACTORS AND MANAGEMENT STRATEGIES FOR CEREBRAL AMYLOID ANGIOPATHY

Edward Zamrini[1], Mo-Kyung Sin[2], and Ali Ahmed[3], *1. Irvine Clinical Research, Irvine, California, United States, 2. Seattle University, Seattle, Washington, United States, 3. Washington DC VA Medical Center, Washington, District of Columbia, United States*

Cerebral amyloid angiopathy (CAA), amyloid β accumulation in the cerebral blood vessels, is a highly prevalent neuropathology and an independent risk factor for cognitive impairment in older adults. CAA is also a major risk factor for amyloid-related imaging abnormalities (ARIA), the major side effect of anti-amyloid therapies (AATs). As AATs have emerged as a treatment option for Alzheimer's disease, the importance of CAA is heightened. Better understanding of CAA risk factors without uniquely relying on hemorrhagic imaging markers is of utmost importance to inform potential prevention strategies. Thus, the objectives of this project are 1) to identify patients at high risk for CAA and 2) to provide potential prevention strategies. Most identified CAA risk factors include male gender, old age, smoking, diabetes, heart disease, lower Mini-Mental State Examination scores, APOE 4 carriers, and a deficit in semantic memory. Prevention strategies for risk factor management include lifestyle modification (no smoking, lipid management, exercise to promote cardiometabolic health), and avoiding anticoagulants. While there is no treatment specifically for CAA, any treatment modifying amyloid β accumulation and clearance as well as anti-inflammation could be considered. The need for sensitive and specific non-invasive biomarkers for CAA is also emphasized.

### SESSION 2465 (PAPER)

Abstract citation ID: igaf122.1011

### LONG-TERM CARE POLICY AND PRACTICE

Long-term care is a necessity for over 1 million older adults. This session will address both resident care issues and policy opportunities.

Abstract citation ID: igaf122.1012

### CARE TRANSITIONS AND MORTALITY AMONG SKILLED NURSING FACILITY RESIDENTS WITH OPIOID USE DISORDER

Patience Dow[1], Miriam George[2], Andrew Zullo[2], Ashley Ritter[3], and Momotazur Rahman[2], *1. Brown University School of Public Health, Providence, Rhode Island, United States, 2. Brown University, Providence, Rhode Island, United States, 3. Hunter-Bellevue School of Nursing, Hunter College, City University of New York, Yardley, Pennsylvania, United States*

There is growing demand for skilled nursing facility (SNF) services among individuals with opioid use disorder (OUD) for reasons including rising rates of chronic medical conditions and serious infections. Little is known about the characteristics and outcomes of people with OUD admitted to SNFs. We examined differences in health service use and mortality between SNF residents with and without OUD. Using 2016-2020 100% Medicare inpatient claims, we identified hospitalizations with discharge to SNFs. We matched each beneficiary with OUD with up to 5 without OUD based on age, sex, low-income subsidy status, and residential county. Outcomes were hospital readmissions, home health service use, and all-cause mortality within 180 days following hospital discharge. Inverse-probability weighting (IPW) adjusted for demographics, state, year of SNF admission, comorbidity burden, hospital length of stay, intensive care, and select principal diagnoses. Unadjusted and IPW-adjusted risk differences (RDs) were calculated. Among 30,922 beneficiaries with OUD matched to 137,454 without OUD (mean age: 71 years), those with OUD had higher unadjusted risks for readmissions (44.5% vs. 27.9%) and home health use (43.2% vs. 35.4%) but lower mortality risk (16.5% vs. 17.8%). After IPW adjustment, beneficiaries with OUD remained at higher risk for readmissions (RD = 15.8%; 95% CI:15.1%,16.5%) and home health use (RD = 9.6%; 95% CI:9.1%,10.2%) but had no difference in mortality risk (RD=-0.4%; 95% CI:-0.8%,0.1%). Best practices for providing care to individuals with OUD and co-occurring skilled nursing needs must be a near-term focus for post-acute and long-term care practice and policy to help reduce concerning differences in readmission rates.

Abstract citation ID: igaf122.1013

### CO-DESIGNING A WORKFORCE RETENTION FRAMEWORK FOR LONG-TERM CARE HOMES

Winnie Sun, and Jen Calver, *Ontario Tech University, Oshawa, Ontario, Canada*

Background: Despite the presence of long-standing staffing issues and evidence of turnover impacting quality of resident care, there is little guidance to assist long-term care (LTC) homes to create sustainable staffing stability and retention programs. The purpose of this study is to co-develop a workforce retention framework for LTC leaders to help guide staffing stability and retention programs in their nursing department. Methods: A modified Delphi study was used to obtain consensus of workplace factors for retention of nurses and personal support workers in LTC. Forty staff members shared their level of agreement through Delphi surveys. Drawing from the literature, 54 item statements for the first survey round were organized into six categories - work/life balance, scheduling policies and procedures, professional development and education, work conditions, quality of care, and communication. Results: Study reveals resilience level factors that acknowledge the challenges of LTC work and impact on individual health and wellbeing. It appreciates that the nature of the work environment can be unpredictable as resident care complexities increase, practice guidelines and ministry mandates changes, and the implications of the prolonged staffing issue. Continuous learning reflects the quality assurance and accountability of all practicing nurses to reflect on their current practice, identifying learning needs to actively update their knowledge and skills needed for continuing competence. Conclusion: This study has implications for comprehensive staff retention planning and program development in LTC. As demand for LTC services rises, comprehensive staffing retention programs are necessary to sustain skilled and experienced staff in the workforce.

Abstract citation ID: igaf122.1014

**LEVERAGING STATE-WIDE PARTNERSHIPS TO DEVELOP A CNA APPRENTICESHIP PROGRAM**

Anna Faul, Sam Cotton, Pamela Yankeelov, and Barbara Gordon, *University of Louisville, Louisville, Kentucky, United States*

Since 2015, our Geriatric Workforce Education Program (GWEP) has been committed to enhancing the skills of Certified Nursing Assistants (CNAs) across Kentucky by providing specialized training in dementia care best practices. Recognizing that CNAs are often the frontline caregivers for individuals living with dementia, we have focused on equipping them with the knowledge and skills needed to provide compassionate, evidence-based care. Through this initiative, we have trained hundreds of CNAs and fostered long-standing partnerships with key stakeholders, including nursing facilities, the Kentucky Office of Dementia Services, the Kentucky Cabinet for Health-Department of Behavioral Health, Development Intellectual Disabilities, and the Department of Public Health. These strong partnerships have not only expanded access to dementia care education but have also laid the foundation for sustainable workforce development initiatives. Building on this momentum, we are now developing a CNA apprenticeship program, designed to create a structured career pathway that integrates dementia training with hands-on mentorship and professional growth opportunities. This framework will help address Kentucky's ongoing need for a well-trained, dementia-capable workforce by providing CNAs with continued education, career advancement opportunities, and long-term retention support. This paper session will highlight the evolution of our training initiatives, the impact of our collaborations, and the next steps in developing the CNA apprenticeship program. Participants will gain insights into effective strategies for workforce development, partnership-building, and the integration of dementia-specific training into CNA education, ensuring a skilled and sustainable caregiving workforce for Kentucky's aging population.

Abstract citation ID: igaf122.1015

**NURSING HOMES AS INSURERS? THE EFFECT OF PROVIDER-LED INSTITUTIONAL SPECIAL NEEDS PLANS**

Amanda Chen[1], and David Grabowski[2], *1. University of Southern California, Los Angeles, California, United States, 2. Harvard University, Cambridge, Massachusetts, United States*

Institutional Special Needs Plans (I-SNPs) are a type of Medicare Advantage (MA) plan that allow insurers in MA to differentiate their benefits exclusively for long-term residents of nursing homes. When I-SNPs were first offered in 2006, the early I-SNP models were insurer-led as the plan was primarily at-risk for long-stay residents' Medicare spending. Today however, nursing homes have increasingly been entering the I-SNP market with I-SNP models differentiated based on the level of risk borne by the insurer and nursing home. We use a difference-in-differences design to estimate the effect of offering a provider-led I-SNP on enrollment and quality of care. Using Medicare claims and the Minimum Data Set resident assessments from 2004-2021, we find that offering a provider-led I-SNP led to a 17.0 percentage point increase in I-SNP enrollment among facility residents within four years relative to control nursing homes. We also estimate that offering provider-led I-SNPs significantly decreased hospitalizations and increased the use of hypnotic drugs and reporting of pressure ulcers by nursing homes. Relative to the baseline mean among treated nursing homes, we found relative declines of 18.2% for hospitalizations and increases of 30.3% for hypnotic drug use and 15.6% for pressure ulcers. These results suggest that risk bearing by nursing homes in the form of provider-led I-SNPs may successfully reduce utilization, but with unclear implications for quality and competition.

Abstract citation ID: igaf122.1016

**TRENDS AND FACTORS INFLUENCING NURSING HOME CLOSURES: A RETROSPECTIVE ANALYSIS (1990-2024)**

Joohyun Chung, Michael Lepore, and Tingzhong (Michelle) Xue, *University of Massachusetts Amherst, Amherst, Massachusetts, United States*

Nursing home (NH) closures have significant implications for access to long-term care, particularly for vulnerable populations reliant on public healthcare programs. This study aimed to compare the characteristics of NHs that closed versus those that remained open from 1990 to 2024. This study explores the geographic distribution, regional variations, and bed capacity of nursing home closures. An observational, quantitative, cross-sectional study design was used. De-identified data from the Centers for Medicare & Medicaid Services (CMS) Provider of Services File were analyzed. Descriptive statistics, Chi-square tests, generalized linear models (GLM), and linear regression were employed to identify trends in NH closures. A total of 114,882 records were retrieved, with 23,903 selected for Skilled Nursing Facility and Nursing Facility types. From 1990 to 2024, there were 23903 total, 14834 active NHs and 9,069 closures. Active NHs had an average of 107 beds, while closed NHs had 70 beds ($p < 0.001$). Twelve percent of active NHs ($N = 1{,}928$) had at least one ADRD bed, compared to 4.7% of closed NHs ($N = 433$) ($p < 0.001$). Ninety-four percent of active NHs participated in both Medicare and Medicaid, whereas only 55% of closed NHs did, with 17% participating only in Medicare and 28% in Medicaid. Smaller nursing homes with fewer specialized beds and lower Medicare/Medicaid participation are more likely to close. These findings suggest that changes in Medicare and Medicaid policies could affect the sustainability of resource-limited facilities, highlighting the need for targeted support to maintain access to long-term care services.

### SESSION 2470 (SYMPOSIUM)

Abstract citation ID: igaf122.1017

### CALORIC RESTRICTION AND HEALTH, AGING AND LATER-LIFE OUTCOMES IN OLDER ADULTS

Chair: Denise Houston
Co-Chair: Jason Fanning
Discussant: Stephen Kritchevsky

The prevalence of obesity (BMI ≥30 kg/m2) among older adults (65+ years) in the U.S. has almost doubled over the last three decades, with approximately 40% of older adults living with obesity. Lifestyle modifications of moderate caloric restriction (CR) have been shown to improve the metabolic and functional health in older adults (e.g., lowering blood pressure, fasting glucose, and interleukin-6, and improving cardiorespiratory fitness and gait speed) in the short-term. However, the long-term benefits and potential risks (e.g., loss of muscle and bone resulting in increased risk of sarcopenia and fractures) of CR have not been studied in older adults. This symposium will describe the outputs of an NIA-funded 3-year planning grant for use in the design of a 5-year CR trial in older adults with overweight or obesity. Fanning and colleagues will describe the feasibility and acceptability of the pilot intervention and associated self-monitoring technologies, as well as participant willingness to engage in a 5-year CR trial in older adults. Houston and colleagues will describe the metabolic adaptations in energy expenditure that occur in response to moderate CR in older adults. Hsu and colleagues will describe the effects of CR on a consensus derived panel of blood-based geroscience biomarkers that could be used as an intermediate endpoint in clinical trials of CR. Miller and colleagues will describe the development of an obesity- and aging-related health conditions index that captures the potential health effects of long-term studies of CR. Obesity and Aging Interest Group Sponsored Symposium

Abstract citation ID: igaf122.1018

### INTERVENING ON CALORIE INTAKE OR EATING TIMING IN OLDER ADULTS: LESSONS LEARNED IN THE HALLO-P TRIAL

Jason Fanning[1], Stephen Kritchevsky[2], Michael Miller[2], Denise Houston[2], Michael Walkup[2], Cynthia Stowe[2], W. Jack Rejeski[3], and Barbara Nicklas[2], *1. Wake Forest University, Winston Salem, North Carolina, United States, 2. Wake Forest University School of Medicine, Winston-Salem, North Carolina, United States, 3. Wake Forest University, Winston-Salem, North Carolina, United States*

The Health, Aging and Later-Life Outcomes Pilot (HALLO-P) trial was designed to pilot in-person and remotely-delivered caloric restriction (CR and RCR) and time restricted eating (TRE) in preparation for a long-term clinical trial. Community-dwelling older adults with obesity or overweight with an indication for weight loss were randomized to 9 months of group-based CR, RCR, or TRE supported by self-monitoring technologies [i.e., a tablet application, activity monitor (all), wireless scale (CR and RCR)]. Intervention staff recorded attendance at sessions and noted intervention-related lessons learned as they arose, and participants provided feedback related to their experiences in the trial. Participants (N = 90; 67.19±4.91 years) attended 84.4% of sessions on average, engaged with study technologies 96.4% of days, and provided daily weights on > 4 days per week across the study (CR and RCR only). Participants reported high satisfaction with the program, with RCR participants being significantly more likely than TRE participants to report being satisfied with their program overall, intending to continue their dietary change post-intervention, and being willing to sustain their dietary approach and engage with study technologies over a 5-year period. Open-ended feedback underscored the value of the group-based structure of the program and remote delivery to reduce travel burden, the need for reduced burden associated with food logging, and the need to identify methods for enhancing perceived value of TRE among older adults. An intervention designed to promote CR, primarily delivered remotely and supported by self-monitoring technologies, is both feasible and acceptable to many older adults.

Abstract citation ID: igaf122.1019

### ADAPTATIONS IN ENERGY EXPENDITURE FOLLOWING CALORIC RESTRICTION IN OLDER ADULTS

Denise Houston[1], Jason Fanning[2], James DeLany[3], Fang-Chi Hsu[1], Shyh-Huei Chen[1], Michael Miller[1], Stephen Kritchevsky[1], and Barbara Nicklas[1], *1. Wake Forest University School of Medicine, Winston-Salem, North Carolina, United States, 2. Wake Forest University, Winston Salem, North Carolina, United States, 3. AdventHealth, Orlando, Florida, United States*

Adaptations in components of energy expenditure (EE) occur in response to caloric restriction (CR) and contribute to the propensity for weight regain following weight loss. However, the magnitude of changes in EE following CR is not well known in older adults. We examined changes in total EE (TEE by doubly labeled water, DLW) and resting EE (REE by indirect calorimetry) following the 9-month HALLO-P CR interventions. The degree of adaptive thermogenesis (AT; defined as changes in EE beyond what is expected from changes in body size and composition) was also analyzed. Among 56 older adults with overweight/obesity (68±5 yrs; 61% female; 31.6±3.0 kg/m2) who were randomized to CR, the degree of CR over 9 months was estimated as 7.9% based on the intake-balance method with DLW-TEE used to estimate energy intake. This degree of CR decreased body weight by 6.4±5.4 kg (-7.0%) and fat free mass (FFM) by 2.1±1.9 kg (-4.0%). Baseline raw values of TEE and REE were 2327±449 and 1619±297 kcal/day, respectively. Both TEE and REE decreased slightly, but not significantly, following CR (TEE: Δ= -47±353 kcal/d, <0.5%; REE: Δ = -59±218 kcal/d, 3.6%). Adaptive thermogenesis for TEE and REE (adjusted for age, race, sex, and baseline height, weight, fat-free mass, fat mass, and step count) showed non-significant increases after CR (TEE-AT: 48±342 kcal/d; REE-AT: 29±185 kcal/d). We will also report the relationship of these adaptations to changes in body mass and FFM. These results suggest little adaptive thermogenesis to moderate CR over 9 months in older adults.

Abstract citation ID: igaf122.1020

### CALORIC RESTRICTION AND CHANGES IN GEROSCIENCE BLOOD-BASED BIOMARKERS IN OLDER ADULTS

Fang-Chi Hsu, Rebecca Neiberg, Michael Miller, Philip Kramer, John Stone, Denise Houston, Barbara Nicklas, and Stephen Kritchevsky, *Wake Forest University School of Medicine, Winston-Salem, North Carolina, United States*

Intermediate endpoints are needed to evaluate the effect of interventions targeting the biology of aging in clinical trials. We examined associations between randomization to caloric restriction (CR), as well as achieved weight loss, and a consensus derived panel of blood-based geroscience biomarkers in 768 older adults (mean age, 67.4±5.2 years; 68% female and 81% Caucasian). Participants were randomly selected from six completed CR trials to provide a representative subset from each randomized group (CR vs. no CR) within each trial. Plasma CRP, IL-6, TNFr1, GDF15, cystatin C and insulin were measured at baseline and 6-months post-randomization to CR or no CR by the immunoassay platform Ella. Mean±SD weight loss over 6 months was 8.0±5.8 kg in the CR group and 1.4±3.7 kg in the no CR group. CR was associated with significant improvements in CRP and insulin compared to no CR (mean difference [95% CI]: -1.1 [-0.2, -1.9] mg/L and -15.3 [-8.1, -22.6] pmol/mL, respectively); however, there were no significant differences in IL-6, TNFr1, GDF15, or cystatin C by CR group. Achieved weight loss was associated with significant decreases in CRP, IL-6, and insulin (standardized betas (95% CI) per SD weight change: log transformed CRP: -0.26 (-0.13, -0.36), $p < 0.0001$; log transformed IL-6: -0.13 (-0.03, -0.23), $p = 0.01$; and insulin: -0.25 (-0.15, -0.36), $p < 0.0001$). We will also report the relationship between CR and a composite biomarker index. These results suggest that blood-based geroscience biomarkers are responsive to short-term CR, particularly those involved in inflammation and nutrient signaling.

Abstract citation ID: igaf122.1021

### CREATION OF A MULTIMORBIDITY HEALTH CONDITIONS INDEX FOR CALORIC RESTRICTION STUDIES IN OLDER ADULTS

Michael Miller[1], Haiying Chen[1], Mark Espeland[1], Fang-Chi Hsu[1], Denise Houston[1], Anne Newman[2], W. Jack Rejeski[3], and Stephen Kritchevsky[1], *1. Wake Forest University School of Medicine, Winston-Salem, North Carolina, United States, 2. University of Pittsburgh, Pittsburgh, Pennsylvania, United States, 3. Wake Forest University, Winston-Salem, North Carolina, United States*

Using multiple sources, we provide the conceptual justification and statistical support for a multimorbidity outcome associated with 29 obesity-related conditions, which we term the Health Conditions Index (HCI). The HCI was designed to capture the health effects of multi-year studies of caloric restriction in older adults with overweight or obesity. We used a subset of participants from the Health, Aging and Body Composition Cohort Study for whom weight loss would be indicated to evaluate multiple aspects of the index and its components over five years of follow-up. The 937 participants in the subset had an average age of 73 years and BMI of 30.7 kg/m2, 53% were female and 43% were Black. The components of the HCI were consistently related to initial BMI and percent body fat on cohort entry, generally showed an increasing prevalence over the 5-yr follow-up, and, as a composite index, the HCI exhibited an association between faster progression and higher initial levels of age, BMI, and percent body fat. Further, the initial HCI was associated with a statistically significant increase in the rate of mortality over 5-years of follow-up (hazard ratio [95% CI]: 1.22 [1.04, 1.44]). By using outcomes like the HCI, clinical trials of caloric restriction in older adults may gain a better understanding of how intentional weight loss relates to future risk of multiple health conditions associated with aging.

### SESSION 2475 (SYMPOSIUM)

Abstract citation ID: igaf122.1022

### ORAL HEALTH DISPARITIES IN DEMENTIA AND MARGINALIZED COMMUNITIES: DETERMINANTS AND INTERVENTIONS

Chair: Xiang Qi
Co-Chair: Weiyu Mao
Discussant: Bei Wu

This symposium presents a comprehensive examination of oral health disparities in older adults, with a focus on those with dementia and from marginalized communities. The first presentation employs machine learning (ie., XGBoost) to analyze social and physical environmental determinants of edentulism across 38,379 census tracts, identifying social vulnerability and climate zones as key predictors. The second study investigates oral health-related quality of life among Chinese American dementia caregivers, revealing that financial hardship and fewer natural teeth are associated with poorer outcomes. The third presentation evaluates a care partner-assisted intervention using behavioral change techniques to improve oral hygiene in individuals with mild dementia, demonstrating the potential of structured coaching. Finally, in the same randomized controlled trial, the authors assessed the effectiveness of a smart toothbrush intervention with and without personalized coaching, showing sustained improvements in plaque and gingival indices with coaching. Together, these studies highlight the need for tailored, community-based approaches to address oral health disparities and showcase innovative methodologies for gerontological research. By integrating advanced analytics, mixed-methods, and clinical trials, this symposium offers robust evidence to inform interventions and policies aimed at improving oral health equity for vulnerable older adults. Oral Health Interest Group Sponsored Symposium

Abstract citation ID: igaf122.1023

### CENSUS TRACT–LEVEL PHYSICAL AND SOCIAL ENVIRONMENTAL DETERMINANTS OF EDENTULISM: A MACHINE LEARNING APPROACH

Xiang Qi[1], Jie Yao[2], Bei Wu[3], Shaoyong Su[4], Xiaoling Wang[4], and Kai Zhang[5], *1. New York University, New York, New York, United States, 2. University at Albany School of Public Health, Hampton Manor, New York, United States, 3. NYU Shanghai, Shanghai, China (People's Republic), 4. Augusta University, Medical College of Georgia, Augusta, Georgia, United States, 5. UNT Health, Fort Worth, Texas, United States*

Edentulism (i.e., complete tooth loss) disproportionately affects older US adults in marginalized communities, driven by complex social and environmental factors. There is a critical gap in understanding how these factors interact at the population level to shape oral health disparities. This study employed Extreme Gradient Boosting (XGBoost), a machine learning technique, to analyze these determinants across 38,379 census tracts using 2021 data. Social environmental determinants—such as poverty rates, education levels, and employment status—were sourced from the American Community Survey and aggregated at the census tract level to capture localized socioeconomic conditions. Physical environmental determinants, including climate zone and access to dental care, were similarly aggregated to reflect tangible surroundings impacting health. The model predicted edentulism rates among adults aged 65+ with high precision, explaining 87% of the variance. Social vulnerability and climate zone emerged as the top predictors, underscoring the interplay between socioeconomic disadvantage and geographic conditions. Census tract characteristics, like poverty and racial segregation, moderated these effects, revealing distinct risk profiles—e.g., in high-poverty tracts, social factors amplified edentulism risk differently than in racially segregated ones. These findings highlight the contextual nature of oral health and demonstrate the value of localized, data-driven analyses. By identifying key social and physical determinants, this research informs targeted interventions to reduce oral health disparities. The use of XGBoost showcases machine learning's potential to dissect complex health issues, offering actionable insights for policymakers and clinicians to improve outcomes in diverse settings.

Abstract citation ID: igaf122.1024

### CORRELATES OF ORAL HEALTH-RELATED QUALITY OF LIFE AMONG CHINESE AMERICAN FAMILY DEMENTIA CAREGIVERS

Weiyu Mao[1], Bei Wu[2], and Yaolin Pei[3], *1. University of Nevada, Reno, Reno, Nevada, United States, 2. NYU Shanghai, Shanghai, China (People's Republic), 3. The University of Texas at Austin, Austin, Texas, United States*

Oral health-related quality of life (OHRQoL) is a multidimensional concept that reflects how oral health influences one's overall well-being and daily functioning. There is scarce empirical evidence on the correlates of OHRQoL in dementia caregivers. Guided by the Andersen's model, this study investigated correlates of OHRQoL among family dementia caregivers in Chinese American communities. Data were from a pilot study on Chinese American family dementia caregivers in New York City between November 2021 and June 2022. Purposive sampling was used to recruit caregivers to participate in an online or telephone survey. Current caregivers (n = 76) were included. The dependent variable was caregiver oral health-related quality of life, measured by three items with a 5-point response set: 0 = never, 1 = hardly ever, 2 = occasionally, 3 = fairly often, and 4 = very often (Cronbach's alpha = 0.71). Summated scores were used, and higher scores suggest worse OHRQoL. Results from bivariate analyses suggest that caregivers having financial difficulty tended to report worse OHRQoL. Those caring for someone with moderate dementia tended to report better OHRQoL. Caregivers with longer length of stay and those with more natural teeth tended to report better OHRQoL. Results from multiple regressions suggest that having financial difficulty was significantly associated with worse OHRQoL in caregivers. Having more natural teeth was significantly associated with better OHRQoL in caregivers. This study highlights the importance of studying OHRQoL in dementia caregivers and offers empirical evidence to support intervention strategies to improve oral health in this vulnerable population.

Abstract citation ID: igaf122.1025

### ORAL HEALTH INTERVENTION USING BEHAVIORAL CHANGE TECHNIQUES FOR MILD DEMENTIA: A MIXED METHOD STUDY

Sophie Zhijing Xu[1], Eunjung Ko[1], Jing Wang[2], Jackie Finik[1], Shahrzad Siamdoust[1], Justine Seidenfeld[3], and Ya-Ning Chan[4], *1. New York University, New York, New York, United States, 2. University of New Hampshire, Durham, New Hampshire, United States, 3. Durham VA Healthcare System, Durham, North Carolina, United States, 4. Duke University, Durham, North Carolina, United States*

Maintaining oral health in individuals with mild dementia (PLwMD) is challenging due to cognitive impairments that hinder independent adherence to oral hygiene routines. To encourage PLwMDs' engagement in oral hygiene self-care behaviors, implementing behavioral change techniques (BCTs) are essential. Despite the effectiveness of BCT-based interventions in various health contexts, their application in PLwMD has been underexplored. To address this gap, this study evaluated the effect of a care partner-assisted intervention designed to improve oral health outcomes through structured coaching sessions incorporating BCTs. Seventeen care partners–PLwMD dyads participated in four monthly coaching sessions led by an interventionist and a dental hygienist. A mixed-methods approach was employed, including 1) deductive content analysis of coaching session transcripts to identify BCT applications and 2) quantification of BCT occurrences based on code frequency. Additionally, gingival index changes from baseline to the 3-month follow-up were used to categorize dyads as 'no change (n = 3)', 'worsened (n = 2)', and 'improved (n = 12)'. Preliminary results showed that the most prevalent BCT codes were goal setting, problem-solving, and action planning, etc. This suggests that greater use of BCTs drives positive behavior change, reinforcing better oral hygiene practices and improving oral health outcomes. The finding also highlights the potential of BCT-based interventions to enhance self-care behaviors for oral health in PLwMD. Further exploration is warranted to optimize care partner training and intervention strategies to maximize and sustain improvements in oral health outcomes for PLwMD.

Abstract citation ID: igaf122.1026

### A RANDOMIZED CONTROLLED TRIAL OF A CARE PARTNER-ASSISTED ORAL HYGIENE INTERVENTION FOR PERSONS WITH MILD DEMENTIA

Bei Wu[1], Jackie Finik[2], Catharine Mott[3], Patricia Poole[4], Melanie Bunn[5], Ruth Anderson[3], and Brenda Plassman[6], *1. NYU Shanghai, Shanghai, China (People's Republic), 2. New York University, New York, New York, United States, 3. Duke University, Durham, North Carolina, United States, 4. The University of North Carolina at Chapel Hill Adams School of Dentistry, Chapel Hill, North Carolina, United States, 5. Duke University School of Nursing, Durham, North Carolina, United States, 6. Duke University Medical Center, Durham, North Carolina, United States*

Oral hygiene contributes to oral health outcomes and quality of life. This study evaluates a care partner-assisted intervention to improve oral hygiene in community-dwelling older adults with mild dementia. We conducted a three-arm randomized controlled trial involving 60 participants with mild dementia and their care partners. Participants were randomized into three arms: (1) control (educational booklet only), (2) Treatment Group 1 (TG1: smart electronic toothbrush without coaching), and (3) Treatment Group 2 (TG2: smart electronic toothbrush plus personalized coaching). Plaque and gingival inflammation were assessed at baseline, 3 months, and 6 months using linear mixed models. All groups showed significant reductions in mean plaque and gingival inflammation from baseline. For plaque, significant reductions were observed at both 3 [$\beta$=-0.10 (95% CI, -0.15, -0.06)] and 6 months [$\beta$=-0.07 (95% CI, -0.12, -0.03)]. For gingival inflammation, significant reductions were observed at 3 months [β=-0.12 (95% CI, -0.19, -0.05)] but not at 6 months [β= -0.05 (95% CI, -0.12 to 0.02)]. In terms of absolute reduction at study follow up, TG2 showed the largest reduction from baseline in mean plaque [3 months: -0.19 TG2, -0.07 TG1, -0.06 control; 6 months -0.15 TG2, -0.07 TG1, -0.01 control] and gingival inflammation [3 months: -0.22 TG2, -0.07 TG1, -0.05 control; 6 months -0.16 TG2, -0.10 TG1, +0.11 control]. A care partner-assisted intervention may support oral health improvements in individuals with mild dementia, with potential sustained benefits. Larger studies with longer follow-up are needed to fully evaluate the efficacy of the intervention.

### SESSION 2480 (SYMPOSIUM)

Abstract citation ID: igaf122.1027

### HOME, BUT HOMEBOUND: IDENTIFYING AND SUPPORTING HOMEBOUND OLDER ADULTS

Chair: Jennifer Reckrey
Discussant: Christine Ritchie

The homebound, defined as those who never or rarely leave their homes, represent a large and growing subgroup of older adults with complex care needs. As compared to those who leave home routinely, the homebound experience high chronic disease burden, significant functional impairment, and limited access to primary care and needed social services. In this symposium, we present innovative research about how to effectively identify and support this unique population. First, McManus et al. present a scoping review that reveals persistent care gaps among the homebound, including limited access to dental care, neuropsychiatric care, healthy foods, and social and functional supports. Second, Davoudi et al. describe risk factors predicting homeboundedness identified via machine learning models within the National Health and Aging Trends Study (NHATS), including physical performance, driving, ability to walk 3 blocks, functional dependency, and having low income. Next, we present three studies that also leverage NHATS to identify subgroups within the homebound with unique care needs and experiences. Leff et al. examine heterogeneity among the homebound by examining those who are not frail. Dr. Katherine Ornstein et al characterize the nearly 2.5 million decedents who report being bedbound in the last year of life. Finally, Dr. Junxin Li et al describe associations between sleep complaints and being homebound among older adults with multimorbidity. Taken together, these findings highlight the unique care needs of the homebound and underscore the need for targeting clinical innovations and policies to improve access to needed care and supports at home.

Abstract citation ID: igaf122.1028

### PREVALENCE OF ROBUST OLDER ADULTS AMONG HOMEBOUND OLDER ADULTS

Bruce Leff[1], Katherine Ornstein[2], Mary Louise Pomeroy[3], Christine Ritchie[4], Jennifer Reckrey[5], and Orla Sheehan[6], *1. Johns Hopkins University School of Medicine, Baltimore, Maryland, United States, 2. Johns Hopkins University, Baltimore, Maryland, United States, 3. Johns Hopkins Bloomberg School of Public Health, Baltimore, Maryland, United States, 4. Massachusetts General Hospital, Boston, Massachusetts, United States, 5. NYU Grossman School of Medicine, New York, New York, United States, 6. Royal College of Surgeons in Ireland, Dublin, Dublin, Ireland*

Over seven million U.S. older adults are homebound. The majority of homebound older adults are unable to access office-based primary care. Most descriptions of the homebound imply that they are universally frail, but this hypothesis hasn't been empirically tested. We aimed to determine in a nationally representative sample of U.S. older adults the prevalence and degrees of frail and robust among the homebound using the physical frailty phenotype (PFP), and compare sociodemographic, clinical, and social characteristics of non-frail homebound to prefrail and frail homebound and using self-reported health care utilization data compare utilization across frailty subgroups. Using representative data from the National Health and Aging Trends study (NHATS) study (2011-2019, 2021-2023) we assessed the weighted prevalence of frailty among persons continuously homebound over 2 years and found that 46.3% were frail, 47.9% were prefrail and 8.1% were robust. Compared to those who were frail or prefrail, robust homebound older adults had fewer comorbidities, were more likely to be female, had higher education, income and less mental health disorders, functional and cognitive impairment. Prior work using claims-based frailty algorithms reported higher prevalence rates for the robust homebound raising a concern for misclassification in prior work due to an inability for this population to access care and generate claims. In our cohort the prevalence of the prefrail is similar to the prevalence of frailty creating the potential for targeted care interventions appropriate to the needs of the pre-frail homebound to prevent progression to frailty in this vulnerable population.

Abstract citation ID: igaf122.1029

### EPIDEMIOLOGY OF BEDBOUND STATUS AT THE END OF LIFE IN A NATIONAL SAMPLE OF OLDER ADULTS

Katherine Ornstein[1], Mary Lou Pomeroy[1], Hanna *Charankevich[1], Po-Jen Kung[1], Bruce Leff[2], and Jennifer Reckrey[3], 1. Johns Hopkins University, Baltimore, Maryland, United States, 2. Johns Hopkins University School of Medicine, Baltimore, Maryland, United States, 3. NYU Grossman School of Medicine, New York, New York, United States*

As the number of individuals living in the community at the end of life grows, many will become bedbound. This may be especially true for individuals with dementia, who have extensive care needs. Using the National Health and Aging Trends Study (NHATS), we examined bedbound status up to 12 months before death among 3,459 community-dwelling individuals ages 65+ who died between 2012–2023. We defined bedbound status using self/proxy reports of frequency, assistance, and difficulty leaving the bed/bedroom. Probable dementia status was defined using an NHATS algorithm that includes cognitive testing and diagnosis history. We estimated the predicted probability of being bedbound by dementia status using survey weights and adjusting for sociodemographic, clinical, and health characteristics. We also examined weekly hours of help received by the bedbound in the 12 months before death. Nearly 2.5 million decedents were bedbound in their last year of life. Individuals with dementia had an increased likelihood of being bedbound (Predicted probability = 23%) and required extensive help (Mean = 3.5 helpers averaging 123 hours/week in the month before death). This is the first national study of bedbound status at the end of life among older adults. A considerable portion of individuals with dementia are confined to bed in the last year of life, requiring extensive help with self-care and other activities. The high number of bedbound individuals with dementia at the end of life has important implications for the need for paid and unpaid caregiving support, hospice, and other home-based care services.

Abstract citation ID: igaf122.1030

**PREDICTING HOMEBOUND STATUS IN US OLDER ADULTS USING DATA FROM THE NATIONAL HEALTH AND AGING TRENDS STUDY**

Anis Davoudi[1], Yifan Liu[1], Jennifer Schrack[2], and Katherine Ornstein[1], *1. Johns Hopkins University, Baltimore, Maryland, United States, 2. Johns Hopkins Bloomberg School of Public Health, Baltimore, Maryland, United States*

At least 5% of U.S. older adults are homebound —defined as primarily confined to their residence— a condition associated with adverse health outcomes and increased costs. Timely and accurate prediction of homebound status can ensure effective implementation of interventions to support aging in place and long-term care planning. While several risk factors have been identified for homeboundness (e.g., dementia, low income), there is limited work investigating prediction. We aggregated data from Round 1 and Round 5 of the National Health and Aging Trends Study (NHATS), a nationally representative panel study of U.S. Medicare beneficiaries aged ≥65 years, to predict homebound status. We included 43 sociodemographic, clinical, functional and environmental factors from the NHATS surveys as predictors. Homebound was defined as "Never/rarely leaves home". We developed and evaluated CatBoost machine learning models for homebound prediction, using nested cross-validation for hyperparameter tuning and class weights to address outcome imbalance. Our final dataset included 8,651 not-homebound participants at baseline (age mean (SD): 76.6 (7.6) years, 56.2% female). 11.2% became homebound within the following four years). The final model achieved 0.75 accuracy, 0.75 specificity, 0.76 sensitivity, 0.28 precision, 0.82 AUC, and 0.41 F1-score. Short Physical Performance Battery (SPPB) score, driving, ability to walk 3 blocks, ADL dependency, and income quartile were the top 5 ranking features. These findings illustrate the feasibility of predicting homebound status among older adults, enabling focused interventions before homeboundness occurs. Moreover, identifying modifiable predictors enables the design of data-driven targeted interventions to enhance mobility and promote functional independence.

Abstract citation ID: igaf122.1031

**SHEDDING LIGHT ON AN INVISIBLE POPULATION IN GERIATRIC MEDICINE: UNMET CARE NEEDS AMONG HOMEBOUND OLDER ADULTS**

Orla Sheehan[1], Caoimhe McManus[2], Katherine Ornstein[3], Benjamin Oseroff[4], Robert Zimbroff[5], and Tiffany Riser[3], *1. Royal College of Surgeons in Ireland, Dublin, Ireland, 2. Connolly Hospital Blanchardstown, Dublin, Ireland, 3. Johns Hopkins University, Baltimore, Maryland, United States, 4. Icahn School of Medicine at Mount Sinai, New York, New York, United States, 5. University of California, San Francisco, San Francisco, California, United States*

Homebound older adults have high rates of multimorbidity, frailty, and functional decline, yet their care needs remain inadequately addressed. Using data from a scoping review we will identify and discuss care gaps in this vulnerable and growing population. The review followed the PRISMA-ScR framework, conducting systematic searches in PubMed, Embase, Web of Science, and CINAHL. A total of 159 studies met inclusion criteria. Two independent reviewers screened titles and abstracts, with a third resolving discrepancies. Six researchers extracted data using a standardized tool, capturing study characteristics, population details, and key findings. Themes were identified through consensus, resulting in four overarching categories. Studies highlight a need to address unmet care needs in dental care, nutrition, and fall prevention among the homebound. Over 90% of populations studied required dental treatment, yet fewer than 10% received care. Malnutrition was widespread, increasing frailty and hospitalization risk, yet nutritional interventions remain underutilized. Despite high fall rates, evidence on prevention strategies remains limited. Cognitive impairment and mental health conditions, particularly depression, were highly prevalent, yet access to neuropsychology services remained limited. Frailty and functional dependence were pervasive, contributing to high healthcare utilization, including emergency department visits and hospitalizations. Care gaps in advance care planning, social support, and rehabilitation were identified as barriers to improving quality of life. During the symposium, we will discuss how these gaps highlight an urgent need to transform care for homebound older adults. Expanding home-based medical care is essential to improving outcomes, reducing hospitalizations, and ensuring equitable, person-centered care.

Abstract citation ID: igaf122.1032

**ASSOCIATION BETWEEN SLEEP COMPLAINTS AND HOMEBOUND STATUS AMONG OLDER ADULTS: A COHORT STUDY USING NHATS DATA**

Rendong He, Katherine Ornstein, Claire Wang, and Junxin Li, *Johns Hopkins University, Baltimore, Maryland, United States*

Homebound status negatively impacts morbidity and quality of life in older adults. While sleep disturbances have been identified as risk factors for various adverse health outcomes, their longitudinal impact on the development of homebound status remains understudied. Using the 2011–2018 National Health and Aging Trends Study (NHATS) data, this longitudinal analysis examined the association between sleep complaints, measured by self-reported difficulties in falling or staying asleep, and the risk of transitioning to homebound status among older adults. To minimize confounding, we excluded individuals with dementia and Activities of Daily Living (ADL) and Instrumental Activities of Daily Living (IADL) impairments, as assessed using relevant NHATS assessment items. Participants who initially reported no sleep complaints at baseline were included. For participants who consistently reported no sleep complaints each year, baseline was defined at the first available wave. For those who developed sleep complaints during follow-up, baseline was redefined at the first occurrence of sleep complaints. Homebound status was defined as never or rarely leaving home. Time-varying Cox proportional hazards models were used to estimate the hazard ratios (HRs) for incident homebound status. Among 1,408 older adults, having two sleep complaints was significantly associated with an increased risk of becoming homebound in the adjusted model controlling for demographics, comorbidities, function, cognition and depression (HR = 2.17, 95% CI [1.15, 4.12]). One sleep complaint was not significantly associated with homebound status in any model. Our findings suggest that addressing sleep complaints may be a valuable strategy for preventing homebound status among older adults.

### SESSION 2485 (SYMPOSIUM)

Abstract citation ID: igaf122.1033

### FROM ISOLATION TO COMPASSION: RETHINKING PALLIATIVE AND DEMENTIA CARE IN CARCERAL SETTINGS

Chair: Raya Kheirbek
Co-Chair: Kenzie Latham-Mintus
Discussant: Raya Kheirbek

The rapid aging of incarcerated populations presents urgent challenges for correctional institutions, particularly in providing adequate geriatric, palliative, and dementia care. This symposium explores innovative, humane approaches to addressing these needs through collaborative, peer-led, and educational initiatives. Presentations will cover modified research methodologies for conducting usability testing in restrictive environments, the impact of peer-led hospice care on institutional dynamics, the unique challenges faced by individuals with frontotemporal dementia (FTD) in the criminal legal system, and the development of dementia education programs for both correctional staff and incarcerated individuals. By highlighting practical solutions and the transformative potential of compassionate care, this session aims to reframe the conversation around aging and dignity in carceral settings. Incarceration and Aging Interest Group Sponsored Symposium

Abstract citation ID: igaf122.1034

### SIMULATED USABILITY TESTING: A MODIFIED APPROACH TO HEALTHCARE RESEARCH IN PRISON

Erin Kitt-Lewis[1], Susan Loeb[2], Brandon Herbeck[3], and Barbara Walkosz[3], *1. Penn State, State College, Pennsylvania, United States, 2. The Pennsylvania State University, University Park, Pennsylvania, United States, 3. Klein Buendel, Golden, Colorado, United States*

Changes in research policies and competing priorities contribute to extended research review periods and in many cases denial of access to prisons for health-related research. To gain access, investigators must approach challenges and restrictions with an openness to modified approaches to their original study plans, while simultaneously honoring their study's aims. The purpose of this presentation is to describe a pragmatic approach to usability testing Just Care, an electronic learning program designed to prepare vetted people living in prison to assist staff in care for those needing geriatric or end-of-life care. Several Department of Corrections (DOCs) either discouraged our researchers from applying or denied the application with the original large scale usability testing plan. Researchers persevered and one DOC was willing to discuss the potential for a modified application. Collaborative brainstorming between researchers and the DOC resulted in a modified approach to present to the DOC review board. This was termed, simulated usability testing and included paper documents; participants viewing the e-learning program remotely as part of a focus group (FG) and followed by one FG discussion with staff (n = 4 at each of two prisons) and two FGs with people living in prison (n = 16 at each of two prisons). Seizing opportunities to collaborate with community partners to create pragmatic solutions to safety, security, and burden-related constraints in prisons can make accessing prison populations a reality. Gaining access while honoring the original study aims is critical to continuing supporting evidence-based solutions for those living in prison.

Abstract citation ID: igaf122.1035

### DEMENTIA FRIENDS IN INCARCERATED SETTINGS: DEVELOPMENT, IMPLEMENTATION, EVALUATION, AND EXPANSION

Jessica Bibbo[1], Sarah Nicolay[2], Marty Williman[3], Bonnie Burman[4], Elizabeth Kinzig[3], Salli Bollin[3], Susan Hazlett[5], and Lyndi Wyrostek[6], *1. Benjamin Rose Institute on Aging, Cleveland, Ohio, United States, 2. Benjamin Rose, Cleveland, Ohio, United States, 3. Ohio Council for Cognitive Health, New Albany, Ohio, United States, 4. Ohio Council for Cognitive Health, New Albnay, Ohio, United States, 5. Summa Health System, Akron, Ohio, United States, 6. MemoryLane Care Services, Toledo, Ohio, United States*

In 2021, the HRSA-sponsored Geriatric Workforce Enhancement Program, Ohio Council for Cognitive Health, Benjamin Rose Institute on Aging, and the Ohio Department of Rehabilitation and Correction (ODRC) worked together to develop Dementia Friends for Incarcerated Settings to impact the lives of those living with dementia within the correctional setting. This pilot targeted ODRC staff and furthered the development of this Dementia Friends programming. The evaluation measured staff knowledge/attitudes about dementia, as well as uncovering the experiences and needs of staff within the ODRC. This paper will present results from the online staff training finalized in January 2023, including the optional embedded pre- and post-session surveys. The results (N = 743, n = 269 completed both pre and post, n = 213 post only) underscore the need, usefulness and applicability of dementia education throughout the ODRC system, indicating that teaching effective communication techniques within a correctional facility are helpful for those working within such settings. Further, the quantitative and qualitative results indicated benefits on both a personal and professional level. In 2023, work began on creating a Dementia Friends session for people who reside in incarcerated settings in Ohio. In January of 2025, an elearning program was launched nationally through Edovo, their first program focused on memory loss and dementia. Statistics on the usage and completion of this program will be presented. The development and evolution of the collaborative partnerships necessary for successful program implementation will be presented in tandem with the usage and evaluation of the programming. Future directions for this work will also be discussed.

Abstract citation ID: igaf122.1036

### FRONTOTEMPORAL DEMENTIA & THE CRIMINAL LEGAL SYSTEM: CARE PARTNER STORIES

Victoria Helmly, *Georgia State University, Atlanta, Georgia, United States*

There is limited research on the connection between dementia and the criminal legal system, though we know that this system encounters people with dementia regularly. Prior research has established that individuals affected by frontotemporal dementia (FTD) may have symptoms, including behavioral symptoms, which violate social norms and may be considered "criminal." As a result, persons living with FTD may be more vulnerable and at risk for interactions with the criminal legal system, including with police. This session will present findings from a study investigating the experiences of people with FTD who have had contact with the criminal legal system through arrest, detention, arraignment, or incarceration through the perspective of their care partners. Preliminary analysis reveals that this group has diverse experiences and perspectives depending upon their individual circumstances. Despite this heterogeneity, they face common barriers, mostly due to challenges in receiving a correct diagnosis and the criminal legal system's lack of awareness and education about FTD. Care partners rely on shared strengths such as education, social capital, and advocacy skills to navigate these challenges effectively.

### SESSION 2490 (SYMPOSIUM)

Abstract citation ID: igaf122.1037

### CHALLENGES AND OPPORTUNITIES FOR CENTERS ON AGING: PART 2 - SUSTAINABILITY AND LEADERSHIP

Chair: Debra Dobbs

With the increase in population aging there is a continued demand for training and research in the field of aging. With a new political landscape at state and national levels, there have been several challenges to Centers in Aging programs which have made growth and sustainability tenuous. This symposium highlights challenges facing institutions of higher education across the country as well as strategies and opportunities for program growth and development during times of uncertainty and fiscal constraint. The first paper focuses on sustainable partnerships between a private University in Massachusetts and State Departments on Aging to develop innovative, skill-based online training to build a competent and confident aging workforce. The second paper presents collaborative approaches being implemented at the Center for Successful Aging at a California State University to provide research and service opportunities to increase health and social service students' knowledge and skills for working with an aging population. The third paper highlights strategies to sustain graduate research programs in a School of Aging Studies that is part of a large public University in Florida, heavily reliant on state and federal funds for their programs. The fourth paper highlights how aging centers can leverage their ability to conduct applied research as well as entrepreneurial initiatives to strengthen the value proposition of aging centers at a public University in Ohio. This symposium will serve as guidance for programs in the U.S. and other countries facing similar problems with sustainability of programs for educating future gerontologists. Directors of Aging Centers Interest Group Sponsored Symposium

Abstract citation ID: igaf122.1038

### FUNDING GRADUATE PROGRAMS IN GERONTOLOGY IN A TIME OF FISCAL CONSTRAINT

Debra Dobbs[1], and William Haley[2], *1. University of South Florida, Tampa, Florida, United States, 2. University of South Florida, University of South Florida, Florida, United States*

Graduate research programs in the United States are funded in a myriad of ways and differ based on if they are part of a public versus private institution. Such programs can face a variety of pressures, including cuts in state or federal funding, varying levels of research grant funding on certain topics, new priorities for research funding, state funding that is dependent on productivity goals (e.g., four-year undergraduate graduation rates or major headcounts). Proposed reductions in indirect cost rates for grants from the National Institute on Aging and the National Science Foundation are a new potential challenge in funding doctoral students in aging research programs. This paper presents strategies to sustain graduate student research programs through a time of federal and state economic uncertainty based on experiences with an Aging Studies graduate program from a large (55K+ student population) Florida public university that is part of the Association of American Universities (AAU) with several research doctoral programs. Strategies that have been used in the past, or that are being used currently include admitting some students part-time without financial support, increased funding of students through teaching assistantships, efforts to enhance funding from private sources such as foundations and donors and admitting smaller cohorts of students. Discussion with the panelists and the audience will include views on these and other approaches to sustaining graduate student research programs in aging given the uncertain nature of the political and economic climate.

Abstract citation ID: igaf122.1039

### STRENGTHENING THE AGING WORKFORCE: BUILDING SUSTAINABLE PARTNERSHIPS FOR INNOVATIVE, SKILL-BASED TRAINING

Bronwyn Keefe, *Boston University School of Social Work, Boston, Massachusetts, United States*

This presentation will describe workforce development partnerships between an aging Center within a large private University and state and community-based agencies that provide health and social services to older adults. The aging workforce is looking to build their competencies just as state and local agencies seek partnerships with academic institutions to offer standardized, high-quality, skill-based learning for their employees. Many practitioners in the field of aging have not received formal education in working with older adults, while those who seek services are more diverse than ever and have a complexity of medical and social concerns. Reciprocal academic and community-based partnerships are essential to prepare and retool the aging workforce, especially during a great exodus of social service practitioners. This presentation will highlight the impact of workforce development initiatives through offering innovative and evidence-informed programs. We will share state-specific examples that have led to state policy and funding to strengthen the aging workforce, which has sustained the Center's efforts for over 10 years. Training outcomes are measured through multiple mechanisms, e.g., pre-post competency assessments, impact on knowledge and skills, practice change, and overall satisfaction with the program. Results show statistically significant gains in key competency areas, including conducting assessments, identifying resources, providing culturally appropriate and person-centered care, and supporting clients during care transitions. This presentation will highlight the importance of statewide workforce development programs in providing essential skills needed to deliver high-quality care and services to older adults and how this has led to building a self-sustaining Center.

Abstract citation ID: igaf122.1040

### COMMUNITY-BASED APPROACHES TO TRAINING THE FUTURE WORKFORCE FOR AN AGING POPULATION IN CHALLENGING TIMES

Iveris Martinez, *California State University, Long Beach, Long Beach, California, United States*

In challenging times where funding is uncertain and scarce, innovative strategies must be developed to train the future workforce of health and service providers to serve the growing and diverse aging population. The Center for Successful Aging (CSA) at California State University Long Beach brings together faculty, students, and community partners to develop creative solutions for diverse aging populations, serving as a leader in community engagement, workforce development, and applied research to address disparities and promote quality of life in aging. The CSA Internship Program has taken a community-based approach to providing both research and service-oriented learning opportunities for students at the CSULB College of Health and Human Services. Service-oriented internships have included support for a service provider collaborative and student-run dementia respite program. Research-oriented internships have included a masking adherence study, an adult day-care feasibility study, and a study on the experience of older adults dining out with hearing loss. To date, thirty-seven students have completed a minimum of 120 hours each for a total of 4,400 hours. By leveraging our community partnerships, CSA has provided research and service-oriented opportunities for students to build their knowledge and skills on aging and improve attitudes towards working with older adults. In this presentation, we will describe in more detail the strategies for developing unique learning experiences, student learning outcomes, and their impact on students. Through these strategies, CSA aims to empower students to become the next generation of leaders in health and human services for an aging society.

Abstract citation ID: igaf122.1041

### NAVIGATING THE VOLATILE, UNCERTAIN, COMPLEX, AND AMBIGUOUS LANDSCAPE: SCRIPPS GERONTOLOGY CENTER'S PATH FORWARD

Katherine Abbott, *Miami University, Oxford, Ohio, United States*

The Scripps Gerontology Center at Miami University, founded in 1922, has long been a leader in aging research. With a staff of 22 and 20 affiliated research fellows, Scripps operates in an increasingly volatile, uncertain, complex, and ambiguous (VUCA) environment. As 82% of our funding comes from external state and federal sources, we are grappling with the challenges of delayed grant reviews, uncertain indirect funding rates, and the need to support a graduate program while maintaining our commitment to innovation. In 2024, Scripps secured a $2.2 million investment from the University over five years to support the hiring of three researchers and a Director of Professional Education. This investment allows us to deepen our expertise in core areas while expanding into emerging fields. Additionally, relationships with philanthropic sources infused a $1.5 million dollar investment over three years to support our signature Opening Minds through Art program as well as a focus on broader dissemination of research findings. Operationally, Agile methodologies have enhanced efficiency and collaboration. However, the Department that awards master's and doctoral degrees in gerontology faces challenges due to continued cuts to graduate assistant (GA) lines. While grant-funded GA positions remain an option, the higher costs associated with tuition waivers create financial constraints compared to hiring research staff directly. Despite these challenges, Scripps remains committed to our pillars of research, education and service. By navigating this VUCA landscape with strategic investments, operational innovation, and strong partnerships, we are positioning ourselves for continued excellence and impact in the years ahead.

### SESSION 2495 (SYMPOSIUM)

Abstract citation ID: igaf122.1042

### POLICY SERIES: FROM RESEARCH TO ACTION: ADVANCING SOCIAL CONNECTION AND ENGAGEMENT FOR OLDER ADULTS

Chair: Jillian Racoosin Kornmeier

Social isolation and loneliness (SIL) pose serious risks to the physical, cognitive, and mental well-being of older adults. While social connection is a critical determinant of health, older adults often encounter unique challenges—such as living alone, retirement, disability or illness, and reduced mobility—that heighten their vulnerability to SIL. Therefore, ensuring they have the necessary support to stay socially engaged is essential. In this symposium, experts in research, practice, policy, and philanthropy will explore the multifaceted causes of SIL in older adults, analyzing how individual, community, and systemic factors intersect. They will also examine evidence-based strategies and existing practices that promote social engagement, drawing from the Foundation for Social Connection's Systems Of Cross-sector Integration and Action across the Lifespan (SOCIAL) Framework reports on the Built Environment, Arts & Culture, and Nutrition sectors. Additionally, they will discuss key legislative efforts, including the Older Americans Act (OAA) and the Safeguarding Elderly Needs through Innovation and Occupational Resources (SENIOR) Act of 2024. Finally, they will highlight research gaps and future directions to advance solutions that foster social connection and promote healthy aging. This will include insights from the philanthropic sector into how funders can drive impact—highlighting opportunities for investment, cross-sector collaboration, and targeted funding approaches that support sustainable solutions. Attendees will leave this session equipped with practical skills, actionable strategies, and a clear understanding of how to advocate for and enact meaningful change in their communities. Loneliness and Social Isolation Interest Group Sponsored Symposium

Abstract citation ID: igaf122.1043

### EVIDENCE-BASED PRACTICES FOR ADVANCING SOCIAL CONNECTION IN AGING POPULATIONS

Jillian Racoosin Kornmeier[1], and Meredith Hanley[2],
*1. Foundation for Social Connection, Washington, District of Columbia, United States, 2. USAging, Washington, District of Columbia, United States*

This presentation will bring together research and practice experts to examine how factors at the individual, community, and systemic levels contribute to social isolation and loneliness among older adults, shaping both challenges and opportunities for social connection. They will then explore evidence-based and promising strategies for fostering social engagement through the Built Environment, Arts & Culture, and Nutrition sectors. Drawing from the Foundation for Social Connection's Systems Of Cross-sector Integration and Action across the Lifespan (SOCIAL) Framework reports and their collective expertise, they will highlight how community design, arts-based and cultural programming, and nutrition initiatives can successfully engage older adults. By combining research findings with a discussion of practical, real-world applications, this session will provide attendees with a comprehensive understanding of how these strategies can support older adults in building and sustaining meaningful connections. The session will also examine important considerations for adapting these strategies to diverse community settings and needs. Attendees will leave with a strong grasp of the research behind these approaches and inspiration to implement them in their own communities.

Abstract citation ID: igaf122.1044

### A FEDERAL LANDSCAPE ASSESSMENT AND THE PATH TOWARD A CONNECTED SOCIETY

Andrew MacPherson, *Healthsperien LLC, Washington, District of Columbia, United States*

Public policy plays a critical role in shaping the environments, systems, and structures that influence social connection. Drawing upon the Foundation for Social Connection Action Network's extensive advocacy experience, this discussion will provide attendees with a comprehensive understanding of the evolving political landscape, recent legislative advancements, and opportunities for bipartisan action to strengthen social engagement for older adults. Particular attention will be paid to the Older Americans Act (OAA), a cornerstone of aging policy that funds critical programs supporting social connection, and the Safeguarding Elderly Needs through Innovation and Occupational Resources (SENIOR) Act of 2024, which introduces new measures to address social isolation through workforce development and community-based initiatives. Attendees will also be introduced to the Action Network's Policy Priorities for the 119th Congress - an evidence-based, cross-sector agenda designed to embed social connection at the heart of public policy.

Abstract citation ID: igaf122.1045

### UNITING FOR CONNECTION: PHILANTHROPY'S ROLE IN COALITION-BUILDING AND ADVOCACY EFFORTS

Amy Eisenstein, *RRF Foundation for Aging, Chicago, Illinois, United States*

As social isolation and loneliness gain increasing recognition as urgent public health and societal challenges, philanthropy has a unique and powerful role in driving change. This discussion will explore how philanthropic organizations can strategically invest in coalition-building initiatives that advance social connection and scale evidence-based interventions. Specifically, it will focus on an example of current advocacy efforts to secure reauthorization of the Older Americans Act. Attendees will leave with a deeper understanding of how philanthropy can catalyze action and amplify impact in fostering social connection among the aging population.

### SESSION 2500 (SYMPOSIUM)

Abstract citation ID: igaf122.1046

### MORE THAN ATTENDANCE FREQUENCIES: A DEEPER LOOK AT RELIGION, SPIRITUALITY, AND AGING RESEARCH

Chair: Nirmala Lekhak
Discussant: Stephen Fogle

Recent decades of gerontological research in religion, spirituality, and aging have produced innovative approaches toward understanding the depth and diversity of older adults' lived experiences. The Religion, Spirituality, and Aging Interest Group annual symposia frequently provides a forum for dynamic conversations regarding the state of research including attention to "The Next Generation" (2019), "Forgotten Variables" (2020), "Spiritual Care" (2021) and, "Spiritual Needs" (2023). Continuing this perennial conversation, this symposium presents original contributions to provide a deeper look at emergent methods and variables of interest guiding contemporary conception and operationalization of religion and spirituality (R/S) in gerontological research. The first paper will present an examination of the extent of engagement in R/S among older adults hospitalized for acute coronary syndrome (ACS) and their association with physical health related quality of life (HRQoL). Second paper explores how death conversations and experiences contribute to one's understanding of death, including the influence of religious vocabulary, funerary customs, and after-life beliefs. Third paper offers a scoping review to understand religion and spirituality variables as found in population health data commonly used for older adult research. The last paper is a qualitative study focused on how current and former dementia caregivers perceive effects of Maitri Sambodh Dhyaan (MSD) meditation. After attending this symposium, participants will be able to 1) demonstrate knowledge of the current state of religion, spirituality, and aging research; 2) identify specific variables related to religious and spiritual practices and experiences; 3) connect emergent religion, spirituality, and aging research to health and well-being outcomes. Religion, Spirituality and Aging Interest Group Sponsored Symposium

Abstract citation ID: igaf122.1047

### MENTAL HEALTH BENEFITS OF MAITRI SAMBODH DHYAAN (MSD) MEDITATION FOR DEMENTIA CAREGIVERS

Nirmala Lekhak[1], Tirth Bhatta[1], Cecilia Fernandes[2], Karenna Tran[1], and Santosh Gupta[3], *1. University of Nevada, Las Vegas, Las Vegas, Nevada, United States, 2. PrismaHealth Children's Hospital, Columbia, South Carolina, United States, 3. P.D. Hinduja Hospital, Mumbai, Maharashtra, India*

Prior research has consistently established the role of meditation as a coping resource to mitigate the adverse impacts of stressful life situations on mental health. However, existing research on positive effects and mechanisms that transmit these positive effects of meditation for current and former dementia caregivers is limited. This qualitative study is focused on how current and former dementia caregivers perceive effects of Maitri Sambodh Dhyaan (MSD) meditation, a unique practice that integrates elements of previously researched meditation techniques into one. We used longitudinal qualitative descriptive content analysis of open-ended questions asked of 44 current and former dementia caregivers. The MSD meditation intervention was administered for 21 days and after 21 days the participants were asked to practice on their own with the video recording that was provided to them. We followed up in 30- and 60-days. Positive emotions that dementia caregivers experienced after their practice of meditation included calmness, relaxation, refreshing, content, happy, peaceful, focused, connectedness, appreciative for the meditative time and intervention. The participants also reported improvement in their self-care, mental health, sleep quality, and reaction to their care-recipient. The findings highlight the importance of integrating meditation practices into caregiver support programs to strengthen emotional resilience, enhance mental health, and improve coping mechanisms.

Abstract citation ID: igaf122.1048

### RELIGIOUS AND SPIRITUAL BELIEFS AND PRACTICES IN OLDER SURVIVORS OF A HEART ATTACK AND QUALITY OF LIFE

Hawa Abu[1], Jerry Gurwitz[1], Robert Goldberg[1], David Dosa[1], Daniel Matlock[2], barbara Kivowitz[3], Alok Kapoor[1], and David McManus[1], *1. University of Massachusetts Chan*

*Medical School, Worcester, Massachusetts, United States, 2. University of Colorado Anschutz Medical Campus, Aurora, Colorado, United States, 3. San Francisco, California, United States*

Religious and spiritual beliefs and practices (R/S) are often used by patients to cope with life-threatening events. However, the role of R/S remains understudied among older adults who survive a heart attack. The study objective was to examine the extent of engagement in R/S among older adults hospitalized for acute coronary syndrome (ACS) and their association with physical health related quality of life (HRQoL). Data was obtained from the multi-center prospective TRACE-CORE study (2011-2013). Patients aged ≥65 years hospitalized with ACS at six medical centers in Massachusetts and Georgia were included in the present study. Participants self-reported their extent of engagement in three items assessing R/S – deriving strength and comfort from religion, making petition prayers for their health, and receiving intercessory prayers. Physical HRQoL was measured with the SF-36®v2 physical component score. Linear regression models were used to examine the relationship between each R/S measure and physical HRQOL during hospitalization. Among 804 older adults hospitalized with ACS (mean age 73 years; 39% women; 87% non-Hispanic White), 88% found strength and comfort from religion, 67% prayed for their health, and 88% received intercessory prayers. The average physical HRQoL score was 40.2. After adjusting for potential confounders, greater engagement in R/S was associated with poorer physical HRQoL during hospitalization. A majority of older survivors of ACS utilize their R/S beliefs as coping mechanisms and their relationship to physical HRQoL is complex. Integrating R/S support into patient-centered care may support recovery and QoL after a heart attack.

Abstract citation ID: igaf122.1049

### RELIGIOUS AND SPIRITUAL INFLUENCE ON DEATH STORIES

Melanie Evangelista, *Yeshiva University, New York, New York, United States*

While fear and anxiety surrounding death is important to understand, it is only a piece of what shapes one's perspective on death (Alftberg et al., 2018; Gire, 2014). Death stories, defined as one's personal narrative encompassing the cumulation of their experiences that shape their view about death, provide individualized and personal insight into one's often complex thoughts and feelings about death. These stories are influenced by one's exposure and memory to death and are continually shaped throughout life (Hedtke, 2014). Current literature on spiritual and religious beliefs in relation to thoughts about death indicates that as individuals age, they often become more religious, decreasing fear of death, as fear of death is typically highest among moderately religious individuals and lowest among those who are either nonreligious or extremely religious (Ellis & Wahab, 2013; Jiang et al., 2023; Wink & Scott, 2025). Death and life after death are central tenets of religious and spiritual practices, discussed early in religious education and throughout one's life. As religion, spirituality and death are intricately intertwined, faith-based language is frequently utilized when discussing and defining one's thoughts about end-of-life across major world religions (Chakraborty et al., 2017; Gire, 2014). This session seeks to explore how these conversations and experiences contribute to one's understanding of death, including the influence of religious vocabulary, funerary customs, and after-life beliefs.

Abstract citation ID: igaf122.1050

### SPIRITUALITY AND RELIGION IN OLDER ADULTHOOD: A SCOPING REVIEW OF RESEARCH VARIABLES AND MEASURES

Shaowei Wan[1], and Stephen Fogle[2], *1. Palo Alto VA Hospital, Palo Alto, California, United States, 2. At-Large, Omaha, Nebraska, United States*

Publication metrics in epidemiological research reveal surging interest in religion, spirituality, and aging. It is important that operationalization of these dynamic and multi-faceted phenomena reflected their expressions among older adult populations. To this end, this paper offers a scoping review to understand religion and spirituality variables as found in population health data commonly used for older adult research. This study uses the Arksey and O'Malley scoping review framework to systematically search for articles in MEDLINE, PubMed, Cochrane Library, CINAHL, and PsycINFO (including Psych Test) during March of 2025. Articles published from 2015 to the present will be included for the review. Results will be presented according to the following thematic categories: religious and spiritual traditions, practices, experiences, and popular theologies. The insights offered by this scoping review provide an updated toolkit for epidemiological aging-related research in religion and spirituality. Specifically, the results illuminate the depth and diversity of religion and spirituality in the lived experiences of older adults. Finally, the paper delivers cogent recommendations for implementation of religion and spirituality research across pluralistic populations.

### SESSION 2505 (SYMPOSIUM)

Abstract citation ID: igaf122.1051

### HIGHLIGHTS FROM THE SUCCESSFUL AGING AFTER ELECTIVE SURGERY (SAGES) I STUDY: FIFTEEN YEARS OF FINDINGS

Chair: Sarinnapha Vasunilashorn

Delirium, an acute change in cognition, is common, morbid, and costly geriatric syndrome. Although the epidemiology of delirium has been well-characterized, the relationship of delirium with long-term cognitive and functional outcomes remains unclear. Moreover, less is known of the biological underpinnings of postoperative delirium and its associated long-term outcomes. Funded by an NIA program project, the five-year prospective Successful Aging after Elective Surgery (SAGES) I study was launched in 2010 to investigate novel risk factors (e.g., biomarkers, neuroimaging, reserve markers) and to examine the contribution of delirium to long-term cognitive and functional decline. The SAGES I study enrolled 560 adults age ≥70 undergoing major noncardiac surgery who did not have evidence of dementia based on detailed screening process (including a complete baseline neuropsychological test battery and a functional status battery). Fifteen years following enrollment of the first SAGES I participant, this symposium highlights the select key findings of the SAGES I study, including the: 1) relationship between functional recovery following postoperative delirium, 2) association between postoperative delirium and long-term cognitive decline 72 months post-surgery, 3) the association between a novel marker of neural injury, phosphorylated-tau 217, and delirium, and 4) identification of preoperative and postoperative blood biomarkers associated with delirium using a proteomics approach. Taken together, these studies summarize gaps in knowledge that the SAGES study has addressed and paves the way for future promising lines of inquiry in delirium research-expanding our understanding of this geriatric syndrome that threatens the independence and quality of life of older adults.

Abstract citation ID: igaf122.1052

**PROTEOME-WIDE ANALYSIS IDENTIFIES AND VALIDATES CHITINASE-3-LIKE PROTEIN 1 AS A RISK AND DISEASE MARKER OF DELIRIUM**

Sarinnapha Vasunilashorn[1], Simon Dillon[1], Tamara Fong[2], Long Ngo[1], Hasan Otu[3], Sharon Inouye[4], Edward Marcantonio[1], and Towia Libermann[1], *1. Beth Israel Deaconess Medical Center, Boston, Massachusetts, United States, 2. Beth Israel Deaconess Medical Center, Harvard Medical School, Boston, Massachusetts, United States, 3. University of Nebraska-Lincoln, Lincoln, Nebraska, United States, 4. Marcus Institute for Aging Research, Hebrew SeniorLife, Harvard Medical School, Boston, Massachusetts, United States*

Delirium (an acute change in cognition) is a common, morbid, and costly syndrome seen primarily in aging adults. Despite increasing knowledge of its epidemiology, delirium remains a clinical diagnosis with no established biomarkers to guide diagnosis or management. Advances in proteomics now provide opportunities to identify novel markers of risk and disease progression for postoperative delirium and its associated long-term consequences (eg, long-term cognitive decline and Alzheimer's disease [AD]). In a nested matched case-control study (18 delirium/no-delirium pairs) within the Successful Aging after Elective Surgery study (N = 556), we evaluated the association of 1305 plasma proteins preoperatively [PREOP] and on postoperative day 2 [POD2]) with delirium using SOMAscan. Generalized linear models were applied to enzyme-linked immunosorbant assay (ELISA) validation data of one protein across the full cohort. Multi-protein modeling included delirium biomarkers identified in prior work (C-reactive protein, interleukin-6 [IL6]). We identified chitinase-3-like-protein-1 (CHI3L1/YKL-40) as the sole delirium-associated protein in both a PREOP and a POD2 predictor model, a finding confirmed by ELISA. Multi-protein modeling found high PREOP CHI3L1/YKL-40 and POD2 IL6 increased the risk of delirium (relative risk [95% confidence interval] Quartile [Q]4 vs Q1: 2.4[1.2-5.0] and 2.1[1.1-4.1], respectively). Our identification of CHI3L1/YKL-40 in postoperative delirium parallels reports of CHI3L1/YKL-40 and its association with aging, mortality, and age-related conditions including AD onset and progression. This highlights the type 2 innate immune response, involving CHI3L1/YKL-40, as an underlying mechanism of postoperative delirium, a common, morbid, and costly syndrome that threatens the independence of older adults.

Abstract citation ID: igaf122.1053

**TRAJECTORY OF FUNCTIONAL RECOVERY AFTER POSTOPERATIVE DELIRIUM IN ELECTIVE SURGERY**

Tammy Hshieh[1], Jane Saczynski[2], Edward Marcantonio[3], Richard Jones[4], Eva Schmitt[5], Thomas Travison[6], and Sharon Inouye[7], *1. Brigham and Women's Hospital, Boston, Massachusetts, United States, 2. Northeastern University, Boston, Massachusetts, United States, 3. Beth Israel Deaconess Medical Center, Boston, Massachusetts, United States, 4. Brown University, Brown University, Rhode Island, United States, 5. Hebrew Rehabilitation Center, Boston, Massachusetts, United States, 6. Hebrew SeniorLife & Harvard Medical School, Hebrew SeniorLife, Massachusetts, United States, 7. Marcus Institute for Aging Research, Hebrew SeniorLife, Harvard Medical School, Boston, Massachusetts, United States*

Objective: Describe functional recovery after elective surgery and to determine whether improvements differ among individuals who develop delirium. Background: Limited studies of older adults have investigated whether delirium influences the trajectory of functional recovery after elective surgery. Methods: Patients age ≥70 undergoing major elective surgery were assessed daily while in hospital for presence and severity of delirium using the Confusion Assessment Method, and their functional recovery was followed for 18 months thereafter (N = 566). The Activities of Daily Living and Instrumental Activities of Daily Living Scales and the Physical Component Summary of the Short Form-12 were obtained before surgery and at 1, 2, 6, 12, and 18 months. A composite index (standard deviation 10, minimally clinically significant difference 2) derived from these scales was then analyzed using mixed-effects regression. Results: Mean age was 77 years; 58% of participants were women and 24% developed postoperative delirium. Participants with delirium demonstrated lesser functional recovery than their counterparts without delirium; at 1 month, the covariate-adjusted mean difference on the physical function composite was -1.5 (95% confidence interval [CI] -3.3, -0.2). From 2 to 18 months, the corresponding difference was -1.8 (95% CI -3.2, -0.3), an effect comparable with the minimally clinically significant difference. Conclusions: Delirium was associated with persistent and clinically meaningful impairment of functional recovery, to 18 months. Use of multifactorial preventive interventions for patients at high risk for delirium and tailored transitional care planning may help to maximize the functional benefits of elective surgery.

Abstract citation ID: igaf122.1054

**COGNITIVE TRAJECTORIES AFTER POSTOPERATIVE DELIRIUM: LESSONS LEARNED AFTER 6 YEARS OF FOLLOW-UP IN THE SAGES COHORT**

Zachary Kunicki[1], Tammy Hshieh[2], Long Ngo[3], Douglas Tommet[4], Tamara Fong[3], Thomas Travison, Richard Jones[4], and Sharon Inouye[6], *1. Warren Alpert Medical of Brown University, Providence, Rhode Island, United States, 2. Brigham and Women's Hospital, Boston, Massachusetts, United States, 3. Beth Israel Deaconess Medical Center, Boston, Massachusetts, United States, 4. Brown University, Providence, Rhode Island, United States, 6. Hebrew SeniorLife & Harvard Medical School, Boston, Massachusetts, United States*

The Successful Aging after Elective Surgery (SAGES) cohort is the longest-running prospective study of cognitive trajectories, examining how delirium can influence long-term cognitive outcomes. Two major papers from the SAGES research group — Inouye et al. (2016) and Kunicki et al. (2023) — demonstrate that delirium is associated with accelerated cognitive aging. Using a measure of general cognitive performance, derived using latent variable modeling of 11 cognitive tests spanning domains of attention, memory, language, and executive functioning, Inouye et al. (2016) illustrates a multifaceted post-surgical trajectory which is characterized by a decline one month after surgery, recovery to above the preoperative baseline at two months, followed by a gradual decline. Kunicki et al. (2023) further highlights the complexity of this trajectory by showing there is an additional period of little change until 36 months, but overall that the rate of cognitive decline in patients who experienced delirium is 40% faster than in those who did not. This symposium will review the key findings from these papers, emphasizing how delirium has a lasting and significant impact on cognitive trajectories. Lessons learned from these analyses, along with potential directions for future research, will also be discussed.

Abstract citation ID: igaf122.1055

**PLASMA GLIAL FIBRILLARY ACIDIC PROTEIN AND P-TAU217: POTENTIAL BIOMARKERS OF DELIRIUM IN OLDER ADULTS**

Tamara Fong[1], Julianna Liu[2], Edward Marcantonio[3], Richard Jones[4], Long Ngo[3], Sarinnapha Vasunilashorn[3], and Sharon Inouye[5], *1. Beth Israel Deaconess Medical Center, Harvard Medical School, Boston, Massachusetts, United States, 2. University of Rochester Medical Center, Rochester, New York, United States, 3. Beth Israel Deaconess Medical Center, Boston, Massachusetts, United States, 4. Brown University, Brown University, Rhode Island, United States, 5. Marcus Institute for Aging Research, Hebrew SeniorLife, Harvard Medical School, Boston, Massachusetts, United States*

Objective: Delirium is a common complication of hospitalization among older adults, and is associated with cognitive decline, but the underlying mechanisms are poorly understood. Delirium and dementia are closely interrelated; therefore, blood biomarkers for Alzheimer's disease (AD) and neural injury may yield insight to potential mechanisms in delirium. Methods: Data were obtained from the Successful Aging after Elective Surgery (SAGES) study (n = 530). Glial fibrillary acidic protein (GFAP), a marker of reactive astrocytosis elevated in neurodegeneration and neural injury, and tau phosphorylated at residue 217 (p-Tau217), a marker of AD pathology, were measured from plasma collected prior to surgery. Post-operative delirium incidence and severity were assessed using the Confusion Assessment Method (CAM) and CAM-S (0-19, 19 worst), respectively. Results: Higher GFAP (4th vs. 1st quartile) was associated with delirium incidence (relative risk (RR) 1.7, 95% confidence interval (CI): 1.1-2.8) and greater CAM-S severity (adjusted mean difference=0.5, 95% CI: 0.1-1.0). p-Tau217 did not significantly predict delirium incidence, though higher levels were associated with greater severity (adjusted mean CAM-S difference 1st to 4th quartile=0.6, 95% CI: 0.1-1.0). Interpretation: High pre-operative plasma GFAP was associated with higher delirium incidence and severity, whereas high p-Tau217 was associated only with higher severity. Overall, GFAP appears to be a stronger risk marker than p-Tau217 for delirium. Further work is needed to establish whether astrocytosis contributes to delirium pathophysiology.

### SESSION 2510 (SYMPOSIUM)

Abstract citation ID: igaf122.1056

**UNDERSTANDING AND MEASURING RESILIENCE: FROM BENCH TO BEDSIDE**

Chair: Chenkai Wu
Discussant: Heather Whitson

This symposium examines the multifaceted nature of resilience across diverse clinical contexts, bridging the gap between theoretical frameworks and practical applications. Resilience, the capacity to withstand and recover from stressors, is a critical concept in aging research and clinical practice, impacting patient outcomes across various health conditions. The first presentation explores the role of physical resilience in patient satisfaction following total knee replacement, demonstrating how monitoring post-surgical physical function trajectories can optimize rehabilitation and enhance long-term patient-centered outcomes. Moving from planned surgical interventions to traumatic injuries, the second and third presentations delve into the dynamics of resilience in hip fracture recovery. One presentation deconstructs physical resilience into its fundamental components—resistance and recovery—quantifying their impact on mortality. The other examines frailty trajectories as markers of resilience, highlighting their predictive value for post-fracture outcomes. Collectively, these three presentations illustrate how resilience can be operationalized and measured using longitudinal data and advanced statistical methods. By integrating insights from electronic health records, patient-reported outcomes, and functional assessments, this symposium aims to provide a comprehensive understanding of resilience, from its conceptualization to its clinical application. Attendees will gain insights into identifying individuals with lower resilience and developing targeted interventions to improve recovery and survival. This symposium bridges the gap between research and clinical practice, fostering a deeper understanding of resilience and its potential to enhance patient care.

Abstract citation ID: igaf122.1057

**THE FRAGILE PATH: RESILIENCE AND FRAILTY DYNAMICS IN HIP FRACTURE RECOVERY**

Chenkai Wu[1], Jianhong Xu[1], Yanxin Wang[1], Jonathan Ka-Long Mak[2], Emiel O. Hoogendijk[3], and Graciela Muniz-Terrera[4], *1. Duke Kunshan University, Kunshan, Jiangsu, China (People's Republic), 2. The University of Hong Kong, Hong Kong, China (People's Republic), 3. VU University Medical Center, Amsterdam, Noord-Holland, Netherlands, 4. University of Edinburgh, Edinburgh, Scotland, United Kingdom*

Hip fractures represent a significant stressor, revealing underlying resilience deficits, particularly in frail individuals. This study examines frailty trajectories surrounding hip fracture and their joint impact on mortality. Using data from 4,963 UK Biobank participants with incident hip fracture, we assessed frailty monthly via the Hospital Frailty Risk Score (derived from ICD-10 codes) for 12 months before and after hip fracture. Latent class trajectory models identified distinct frailty trajectories 12 months before and after hip fracture, separately. We used the multinomial logistic regression to examine transitions between pre- and post-fracture frailty trajectories. Survival analysis was used to determine the association between the trajectories and death. We identified three frailty trajectories in the 12 months before hip fracture: persistently robust (44.2%), moderately frail (37.1%), and progressively frail (18.7%). Frailty remained stable except in the progressively frail group, where frailty scores increased by 28.6% in the year before fracture. After hip fracture, three distinct trajectories emerged: moderate frailty with stability (63.0%), high frailty with stability (30.8%), and rapidly progressive frailty (6.2%). Higher pre-fracture frailty was strongly linked to greater post-fracture frailty severity. Pre- and post-fracture frailty trajectories were independently and jointly associated with death; higher pre-fracture frailty was linked to higher mortality within the same post-fracture category. These findings demonstrate distinct frailty trajectories surrounding hip fracture, highlighting the predictive value of pre-fracture frailty for post-fracture outcomes and mortality. Identifying high-risk individuals through these trajectories can inform targeted interventions to improve recovery and survival.

Abstract citation ID: igaf122.1058

### THE ROLE OF PHYSICAL RESILIENCE IN PATIENT SATISFACTION FOLLOWING TOTAL KNEE REPLACEMENT

Qian-Li Xue[1], Thomas Laskow[1], Mallak Alzahrani[1], Karen Bandeen-Roche[2], Ravi Varadhan[1], Jeremy Walston[3], and Frederick Sieber[1], *1. Johns Hopkins University, Baltimore, Maryland, United States, 2. Johns Hopkins Center on Aging and Health, Baltimore, Maryland, United States, 3. Johns Hopkins School of Medicine, Baltimore, Maryland, United States*

Predicting patient satisfaction after total knee replacement (TKR) remains a challenge in clinical care. While preoperative predictors are ideal, early post-surgical measures are critical for identifying intervention targets to improve long-term satisfaction. This study examined six-month trajectories of whole-body physical function and their relationships with satisfaction at 12 months in 101 patients aged 60+. Physical function was assessed at baseline, 1 month, and 6 months using the Short Physical Performance Battery and the SF-36 Physical Component Score; fatigue was self-reported using the Pittsburgh Fatigability Scale-Physical Subscale. Latent profile analysis identified distinct trajectory patterns, and logistic regression assessed their associations with high vs. lower satisfaction after adjusting for age and body mass index. Decision curve analysis (DCA) evaluated whether physical function trajectories vs. baseline measures can better guide post-TKR rehabilitation decisions compared to treating everyone or treating no one. At 12 months, 79% (n = 80) reported high satisfaction. Distinct trajectory patterns over six months were identified across all three measures, with the most favorable trajectory for SPPB, PFS, and PCS was associated with 12.6, 8.4-, and 9.8-times greater odds of high satisfaction, respectively, compared to the least favorable trajectory (P < 0.0.3 for all). DCA showed that trajectory-based decision-making provided greater net benefit than baseline measures when the risk threshold for intervention was within the range of 20–60% for SPPB, 14–35% for PFS, and 13–43% for PCS. These findings highlight the value of monitoring early post-surgical physical function trajectories to optimize patient-centered rehabilitation and improve long-term TKR satisfaction.

Abstract citation ID: igaf122.1059

### PHYSICAL RESILIENCE AFTER HIP FRACTURE: UNPACKING THE ROLES OF RESISTANCE AND RECOVERY

Chenkai Wu[1], Jianhong Xu[1], Yanxin Wang[1], Qian-Li Xue[2], and Graciela Muniz-Terrera[3], *1. Duke Kunshan University, Kunshan, Jiangsu, China (People's Republic), 2. Johns Hopkins University, Baltimore, Maryland, United States, 3. University of Edinburgh, Edinburgh, Scotland, United Kingdom*

Physical resilience—the ability to withstand, recover, or adapt after a stressor— is critical in older adults, particularly after events such as hip fractures. We conceptualize physical resilience to comprise two distinct but related components: resistance and recovery. These components were used to evaluate how individuals respond to the stress of a hip fracture, both in terms of their immediate impact and their ability to regain health over time. Using data from nearly 5,000 hip fracture participants, we used linear mixed-effects models to characterize a composite health status, derived from electronic health record diagnoses, one year before and after hip fracture for each individual. Resistance was assessed as the abrupt change in composite health status observed immediately after the fracture, while recovery represented the subsequent rate of change. We used Cox and Fine-Gray models to identify the associations of resistance and recovery with all-cause and cause-specific mortality, respectively. We observed a significant and sharp decline in the composite health measure at the time of hip fracture. Following the fracture, the rate of decline markedly accelerated when compared to the pre-fracture period. Lower resistance was associated with a 2.6-fold increase in mortality (95%CI=2.22-3.04), and slower recovery was related to a more modest, yet significant, increase in mortality (HR = 1.18, 95%CI=1.03-1.36). Low resistance was also associated with an increase in the risk of neurodegenerative mortality (sub-distribution HR = 5.78, 95%CI=3.73-8.95). Physical resilience is a key determinant of post-hip fracture outcomes. Identifying individuals with lower resilience may improve clinical interventions to enhance recovery and reduce mortality.

### SESSION 2515 (SYMPOSIUM)

Abstract citation ID: igaf122.1060

### CO-PARTNERING WITH OLDER ADULTS IN QUALITATIVE RESEARCH

Chair: Joyce Weil
Co-Chair: Jarmin Yeh
Discussant: Theresa Allison

Qualitative research with older adults is a dynamic, interactive process, enriched by rich, thick descriptions and inclusion of participants' words and images. Traditional research often separates the roles of "researcher" and "participant," creating power imbalances and limiting participant influence. By co-partnering with older adults at all stages—from planning and conceptualization to data collection, analysis, and dissemination—a more collaborative and reflexive process is achieved, with researchers no longer the sole "experts." This symposium challenges hierarchical distinctions between "researchers" and "participants" by examining methodologies distributing power and expertise among all stakeholders. We will present 1) a comprehensive model of researcher-participant partnership developed through a virtual photovoice study with older adults, detailing collaborative techniques throughout the research phases; 2) the history and key contributions of a Resource Center on Minority Aging Research, focusing on its Community Liaison and Recruitment Core's community-engaged health events in Detroit and Flint, Michigan, offering a conceptual model for advancing the science of inclusion; 3) a series of team-based photovoice projects that bridge the gap between academia and community, fostering meaningful collaborations, and informing policy through the lived experiences of diverse aging populations; and 4) findings from a participatory photovoice project documenting the lived experiences of older adults aging in place within San Francisco's single-room occupancy hotels. This symposium offers theoretical frameworks and practical strategies for reimagining power relationships in gerontological research. By centering older adults' expertise throughout the research process, these approaches generate more authentic knowledge and create pathways for meaningful community engagement and policy impact. Qualitative Research Interest Group Sponsored Symposium

Abstract citation ID: igaf122.1061

### A MODEL FOR CO-DESIGNING RESEARCH WITH OLDER ADULTS: FINDINGS FROM A VIRTUAL PHOTOVOICE STUDY

Joyce Weil, *Towson University, Towson, Maryland, United States*

Photovoice, designed as a collaborative process, offers ways to co-partner in research design with older adults. Findings from an Institutional Review Board-approved study where older adults who created photos about the meaning of home and neighborhood and were then interviewed via Zoom are used to illustrate the codesign process in a virtual photovoice study. Aging Patient-Centered Outcomes Learning Collaborative's Older Adult Subcommittee, Healthier Black Elders' Community Advisory Board, and older adults in the study, as three advisory groups, engaged in expanding the study's reach, including groups of older persons, and in the study's framing, conduction, and analysis. Data from 14 participants and a series of 20 selected photographs will be presented. These data will help illustrate the collaborative process used to create a model detailing the three co-design phases: codesign during the initial study design, throughout the study and data-collection process, and during dissemination. The model includes ways the researcher can reflect upon their role as subject and have older adults and other researchers navigate study protocols together as a form of empowerment. Findings include examples of partnering with older adults and conducting research with them, not just about them: e.g., the role of the researcher as a subject in their research, rethinking the photovoice study protocol and ways to capture desired photos within it, the role of humans in the data-collection process, and ways older adults took the study deeper in other written, narrative form and sent additional materials and research items to the researcher.

Abstract citation ID: igaf122.1062

### AGING IN SINGLE-ROOM OCCUPANCY HOTELS: USING PHOTOVOICE TO CO-CREATE KNOWLEDGE WITH OLDER ADULTS

Jarmin Yeh[1], Kattia Suarez Vargas[1], Howard Thornton[2], and Susi Stadler[2], *1. University of California, San Francisco, San Francisco, California, United States, 2. At Home With Growing Older, Berkeley, California, United States*

Single-room occupancy hotels (SROs) in San Francisco's Tenderloin provide crucial affordable housing for low-income, formerly unhoused, and marginalized populations. As residents age, these buildings are transforming into naturally occurring retirement communities, posing complex challenges for aging in place. Despite their important role, SROs frequently have neglected maintenance, deteriorating conditions, and safety concerns, and are often located in neighborhoods with higher rates of noise, crime, and substance use. While research has explored characteristics of older SRO residents, their experiences of aging in place remain understudied. This qualitative project used participatory methods to amplify the contributions of SRO residents. We formed an Advisory Council of SRO residents with lived expertise and established a partnership with two community stakeholders: a nonprofit specializing in home adaptations and a senior center. Through human-centered design activities and culturally responsive bilingual facilitation, the Advisory Council examined intersecting impacts of ageism and racism across socio-ecological levels, leading to a co-created action plan for future collaborative projects. Our subsequent photovoice study engaged twelve diverse participants in documenting their environments with cameras, producing rich visual and narrative data that highlighted the multifaceted social, spatial, cultural, and temporal dynamics of daily life in and around SROs. This approach revealed both resistance and vulnerabilities among residents while assessing systems of oppression from internalized to structural levels. By prioritizing the perspectives of marginalized older adults, this project fostered community empowerment and enhanced capacity for civic engagement. Findings offer insights for co-developing resident-centered approaches to support aging in place within these vital housing communities.

Abstract citation ID: igaf122.1063

### COMMUNITY ENGAGED RESEARCH FOR OLDER, MINORITIZED AMERICANS: MOVING THE SCIENCE OF INCLUSION FORWARD

Tam Perry[1], Amanda Horn[1], Vanessa Rorai[2], JoAnn Smith[2], James Bridgforth[2], Jamie Mitchell[3], Kent Key[4], and Cynthia Howell[5], *1. Wayne State University, Detroit, Michigan, United States, 2. Healthier Black Elders Center, Detroit, Michigan, United States, 3. University of Michigan, Ann Arbor, Michigan, United States, 4. Michigan State University, East Lansing, Michigan, United States, 5. Flint Healthier Black Elders Center, Flint, Michigan, United States*

In celebration of the recent 50th anniversary of the founding of the NIA, the presentation will outline the history and contributions of one of the originally funded Resource Centers on Minority Aging Research and highlights key contributions from the Community Liaison and Recruitment Core of its work in Detroit and Flint, Michigan. Since its inception, this Center has offered community engaged health events, Lunch & Learns, that provide opportunity for older community members to interact with researchers, service providers, and emerging scholars, obtain health screenings from a local university's mobile health unit and build community among peers. Through these engagement activities where health information is disseminated, trust is fostered as well. The participants are also invited to participate in research studies if interested. This steadfast approach at the program's Lunch & Learn programs has contributed to sustained attention to best practices for the science of inclusion. This presentation offers a conceptual model of ways to move the science of inclusion forward including values that must be held to be effective and components that will foster the science of inclusion's possibilities of sustainability. The presentation will end with a discussion of contextual challenges that may affect this conceptual model now and in the future.

Abstract citation ID: igaf122.1064

### FROM THE OUTSIDE, LOOKING IN: PHOTOVOICE AS A TRANSFORMATIVE METHOD IN COMMUNITY-ENGAGED RESEARCH

Rachel Weldrick[1], Sheila Boamah[2], Samantha Biglieri[3], Atiya Mahmood[4], and Sarah Canham[5], *1. Concordia University, Montreal, Quebec, Canada, 2. McMaster University, Hamilton, Ontario, Canada, 3. Toronto Metropolitan University, Toronto, Ontario, Canada, 4. Simon Fraser University, Vancouver, British Columbia, Canada, 5. University of Utah, Salt Lake City, Utah, United States*

Photovoice is a powerful tool in gerontological research and can bridge the gap between academia and community. Moreover, inviting participants and co-researchers to photograph their lived and embodied realities through photovoice offers researchers meaningful opportunities to learn from participants, while fostering transformative collaborations that challenge the status quo in social research. This paper explores the transformative aspects of photovoice methods in community-engaged research by synthesizing findings from three photovoice studies exploring diverse aging experiences. First, a photovoice study with older people living in temporary supportive housing illuminated the lived realities of housing precarity in later life and spurred arts-based community engagement and advocacy efforts. Second, a photovoice study with family caregivers to persons living in long-term care during COVID-19 lockdowns provided academic team members with glimpses into hidden experiences which would have been otherwise inaccessible. This process also served as a meaningful outlet for caregiver expression and connection during this time. Third, a case study in a suburban neighbourhood during COVID-19 included persons living with dementia as living experts of the embodied realities of dementia while problematizing exclusionary built environments. All three projects illuminate the transformative aspects of photographs in building partnerships and eliciting reflexivity. Findings exemplify the means through which academic and community partners can use photovoice to navigate the insider/outsider status in research and embed elements of advocacy into community-engaged research partnerships. As a critical and transformative method, photovoice can inform policy that is directly influenced by lived experiences to meaningfully address aging and health inequities.

### SESSION 2520 (SYMPOSIUM)

Abstract citation ID: igaf122.1065

### ENHANCING CARE FOR OLDER ADULTS THROUGH THE MEDICARE ANNUAL WELLNESS VISIT

Chair: Jennifer Wolff
Co-Chair: Aleksandra Wec
Discussant: Nicole Werner

The Medicare Annual Wellness Visit (AWV) is a preventive care service which collects patient-reported information and supports the creation of a personalized prevention plan to address identified patient needs. Recent studies indicate that having a billed AWV is associated with increases in preventive care services such as vaccinations, cancer screening, and dementia diagnosis. However, there is variation in patient subpopulations who receive the AWV and the opportunities to enhance implementation of the visit to better meet the needs of older adults. This session offers insights into the AWV from different contexts, including population-, health system-, and implementation-level. The first session uses a nationally representative sample of Medicare beneficiaries to provide an overview of the differential uptake of the AWV across Medicare insurance coverage type (Traditional Medicare vs. Medicare Advantage), highlighting heterogenous impact by age, race, and health status. The next session examines the AWV at a health system level, by drawing on electronic health record data to identify the frequency of follow-up actions taken after memory concerns are reported during the AWV. The third session presents a pilot study seeking to improve dementia care pathways by testing workflow enhancements via nurse-led AWVs. Attendees of this symposium will take away key insights for potential improvements to the AWV. The session discussant will offer thoughts on how the AWV can meet the needs of a diverse population of older adults while aligning with health system priorities.

Abstract citation ID: igaf122.1066

### EVALUATION OF THE ROLE OF TRADITIONAL MEDICARE AND MEDICARE ADVANTAGE PLAN IN ANNUAL WELLNESS VISITS

Zhang Zhang[1], Nancy Schoenborn[2], Katherine Miller[1], Jennifer Wolff[3], and Daniel Polsky[4], *1. Johns Hopkins Bloomberg School of Public Health, Baltimore, Maryland, United States, 2. Johns Hopkins University School of Medicine, Baltimore, Maryland, United States, 3. Johns Hopkins University - School of Public Health, Baltimore, Maryland, United States, 4. Johns Hopkins University, Baltimore, Maryland, United States*

Background: The Medicare Annual Wellness Visit (AWVs) was introduced in 2011 as a preventive services visit. Less is known about the differential uptake of the AWV by Medicare insurance coverage type - a consequence of the increasing beneficiary enrollment shifts from TM to Medicare Advantage (MA) plans. This study aims to quantify the differential effects of Medicare insurance coverage type (MA versus TM) on AWV uptake for key subpopulations. Method: We used 20% nationally representative Medicare insurance claims data from 2018-2019. Probit models assessed the likelihood of AWV uptake, with subgroup analyses by age, race/ethnicity, dual eligibility, chronic conditions, and ADRD status. We included 8,799,206 beneficiaries aged 65 and older. The outcome is whether to have an AWV; the independent variable is the Medicare insurance coverage type. Result: Over 1/3 (37.3%) of beneficiaries received an AWV in 2019. MA enrollees were 4.4 percentage points more likely to receive an AWV than TM enrollees ($p < 0.001$). Subgroup analysis showed higher AWV uptake in MA across all key subgroups of interest (all $p < 0.001$), with the largest differences among the oldest-old adults aged above 85 + (5.8 percentage points), dual eligibles (11.8 percentage points), Black beneficiaries (9.0 percentage points), and those with ADRD (6.6 percentage points). Conclusion: Enrollment in an MA plan is associated with a higher probability of AWV uptake, particularly among vulnerable populations from racial and ethnic minorities, dual eligibility, and those diagnosed with ADRD. These findings highlight MA's potential role in promoting preventive care and health equity.

Abstract citation ID: igaf122.1067

### FOLLOW-UP FOR SUBJECTIVE MEMORY CONCERNS EXPRESSED DURING THE MEDICARE ANNUAL WELLNESS VISIT

Aleksandra Wec[1], Mingche Wu[2], Danny Scerpella[1], Zhang Zhang[1], Danielle Peereboom[1], Jennifer Wolff[3], and Danielle Powell[4], *1. Johns Hopkins Bloomberg School of Public Health, Baltimore, Maryland, United States, 2. Johns Hopkins University, Baltimore, Maryland, United States, 3. Johns Hopkins University - School of Public Health, Baltimore, Maryland, United States, 4. University of Maryland, University of Maryland College Park, Maryland, United States*

The Medicare Annual Wellness Visit (AWV) includes a health risk assessment (HRA) with questions about subjective cognitive function and memory. Our objective was to identify the frequency of follow-up actions in response to newly reported memory concerns during an AWV. Using electronic health record (EHR) data from an academic health system spanning October 2017–October 2022, we identified AWVs where patients first reported memory concerns in the HRA, excluding cases with prior diagnoses of dementia or mild cognitive impairment. Using visit notes, Current Procedural Terminology (CPT) codes, and ICD-10 codes from relevant encounters, we assessed frequency of relevant follow-up actions, including memory screenings, referrals for specialist care (memory care, neurology, geriatric psychiatry), dementia diagnosis, and advanced care planning (ACP). Our sample included 1,801 AWVs for 1,411 unique patients. Patients were predominantly white (70%) and female (63%), with an average age of 78 (SD 7.48). Memory screeners (e.g., Mini-Mental Status Exam) were documented in the visit notes of 4.7% AWVs and 1.3% of AWVs resulted in a referral to a specialist. In total, 4.5% of patients had a new dementia diagnosis documented in their record during the AWV and 10.6% received a newly documented diagnosis in the 12 months following the visit. ACP was completed concurrently with 28% of AWVs. Our study demonstrates the feasibility of using the EHR to identify actions following emerging memory concerns expressed during the AWV. Health systems may use the findings to identify targets for improvement in dementia care as part of the AWV.

Abstract citation ID: igaf122.1068

### IMPLEMENTATION OF A PILOT STUDY TO ENHANCE THE ANNUAL WELLNESS VISIT FOR OLDER ADULTS WITH COGNITIVE IMPAIRMENT

Danny Scerpella[1], Danielle Peereboom[1], and Jennifer Wolff[2], *1. Johns Hopkins Bloomberg School of Public Health, Baltimore, Maryland, United States, 2. Johns Hopkins University - School of Public Health, Baltimore, Maryland, United States*

The Medicare Annual Wellness Visit (AWV) is a routine visit for patients designed to monitor ongoing health status. The AWV requires detection of cognitive impairment through a health risk assessment that probes subjective memory concerns and “direct observation” during the visit. While the AWV holds promise for overcoming dementia care gaps, completion rates lag for older adults with diagnosed dementia. The goal of this pilot project is to generate a proof-of-concept for enhanced primary care workflows to increase the effectiveness of the AWV as a component of a dementia care planning pathway. This project focuses on nurse-led AWV and involves co-development of workflow enhancements including systematic cognitive screening protocols, expanded AWV education and follow-up, creation of patient priority dashboards (patients with diagnosed dementia, patients with a high dementia risk algorithm score, patients in the high acuity clinic) and implementation of a dementia risk algorithm for patients. In collaboration with clinical partners in leadership, staffing, and education, enhancements are identified and studied using PDSA (plan, do, study, act) cycle quality improvement methodology. We discuss the process for implementation within two clinics at a large primary care health organization, including: engaging administrative and clinic-level stakeholders and champions to enhance existing dementia-specific care pathways; liaising with clinicians and staff to identify priority patients; and generating and disseminating regular data tracking reports to inform adaptations. Lessons learned reinforce the value of study design that is co-developed, adaptable, and responsive to health system workflows and priorities.

### SESSION 2525 (SYMPOSIUM)

Abstract citation ID: igaf122.1069

### LGBTQ+ AGING IN PRECARIOUS TIMES: REIMAGINING HORIZONS FROM RADICAL MARGINS

Chair: Austin Oswald
Discussant: Karen Fredriksen-Goldsen

LGBTQ+ older adults live within a dynamic sociopolitical context in which their access to human rights and freedoms across the life course is tenuous and fraught. Their experiences of political oppression are embedded in a long history of legal and social change, demonstrating the deleterious effects of anti-LGBTQ+ legislation on health while also highlighting community-based approaches to resistance. This symposium illuminates the intersectional lives of LGBTQ+ older adults to identify innovative strategies that foster resilience in the face of adversity. The first paper, presented by Dr. Oswald, employs participatory action research to illustrate how leisure can serve as both a site of oppression and a tool for resistance, demonstrating how culturally responsive leisure activities promote healthy aging among older Black lesbians. The second paper, by Dr. Prasad, utilizes statistical methods to critically examine how interpersonal network ties and environmental policy contexts shape transgender older adults’ social support resources. The third paper, by Dr. Jen, applies creative research methods to analyze life course interviews with older bisexual individuals, poetically narrating their life stories including themes of struggle, survival, and healing. These papers center the perspectives of overlooked subgroups within the LGBTQ+ community—older Black lesbian, transgender, and bisexual adults—to explore how their aging experiences both shape and are shaped by the sociopolitical context. Together, they envision new horizons in gerontology by drawing on the wisdom of those living at the margins of society, offering a critical lens to reimage health and aging.

Abstract citation ID: igaf122.1070

### RESISTING OPPRESSION AND PROMOTING HEALTH THROUGH CULTURALLY RESPONSIVE LEISURE AMONG OLDER BLACK LESBIANS

Austin Oswald[1], and Lujira Cooper[2], *1. Dalhousie University, Halifax, Nova Scotia, Canada, 2. Board Member, Brooklyn, New York, United States*

Numerous studies highlight the benefits of leisure participation for aging populations; however, the specific ways in which leisure contributes to the health and well-being of aging Black lesbians remain underexplored. Despite facing multiple forms of oppression at the intersections of age, race, gender, and sexuality, older Black lesbians actively engage in leisure as a means of self-preservation, community building, and resistance. This paper presents findings from a participatory action research project conducted with older Black lesbians to examine how leisure functions both as a site of oppression and as a tool for resistance. Guided by Black feminist theory, five Black lesbians between the ages of 65 and 75 participated in five focus groups, which were transcribed verbatim and analyzed using a reflexive thematic approach. Three themes were identified, highlighting the distinct leisure experiences of older Black lesbians and their need for culturally responsive leisure activities: (1) Reflecting realities: representation of diverse identities and experiences, (2) Belonging together: creating safe spaces for self-expression, and (3) Meeting needs: fostering culturally responsive leisure. These findings suggest that leisure is not only a source of enjoyment but also a critical mechanism for identity affirmation, social connection, and resilience. By exploring the intersections of identity, leisure, and health within the framework of structural oppression, this paper emphasizes the importance of adopting an intersectional perspective to reimagine leisure in ways that better serve marginalized and underserved populations. Implications for policy and practice interventions aimed at promoting healthy aging among older Black lesbians are discussed.

Abstract citation ID: igaf122.1071

### THE POETIC LIFE NARRATIVES OF MIDLIFE AND OLDER BISEXUAL ADULTS: STRUGGLE, SURVIVAL, AND HEALING IN THREE ACTS

Sarah Jen[1], Olivia Lafountain[1], Xavier Noriega[1], Austin Oswald[2], and Zhiqi Yi[1], *1. University of Kansas, Lawrence, Kansas, United States, 2. Dalhousie University, Halifax, Nova Scotia, Canada*

While empirical discourses surrounding bisexuality and aging often focus on challenges, stigmas, and disparities, analyses of bisexual coping and survival offer potential counternarratives of hope and possibility. The purpose of this analysis is to narrate stories of coping and survival among midlife and older bisexual individuals in the context of their life course experiences. Findings are presented in a series of three found poems, or poems created from the direct quotes of study participants, which are followed by interpretations of the quotes in the broader context of participants' lives. Each poem depicts a particular "act" of life, breaking up the narratives into three distinct stages of meaning-making. Act I: The Struggle captures early- to midlife experiences fraught with a sense of confusion, lack of belonging, mental health challenges, and interpersonal conflict. In Act II: Coping and Survival Looks Like…participants reflect on the personal strengths, skills, and relationships that allowed them to move through their most significant challenges. Act III: Healing Bisexual Futures, presents reflections on what bisexuality means to the participants at this stage of their lives and how they envision and dream of their own futures. We see this streamlined narration of an often complex and messy life history as an agentive story-telling process in which bisexual individuals seek meaning, direction, and cohesion in the telling of their life experiences. Creative research methodologies are particularly well-suited to capturing the narrative and lyrical nuance in such stories, as well as their emotional poignance.

Abstract citation ID: igaf122.1072

### STRUCTURAL AND INTERPERSONAL SOURCES OF PERCEIVED SOCIAL SUPPORT FOR TRANSGENDER OLDER ADULTS.

Anyah Prasad[1], Hailey Jung[2], and Karen Fredriksen-Goldsen[2], *1. Vanderbilt University, Boston, Massachusetts, United States, 2. University of Washington, Seattle, Washington, United States*

For minoritized and health disparate transgender older adults, perceived social support is a crucial psychosocial resource for successful aging. People form convoys of social networks to support their needs, but negative environmental context may threaten transgender older adults' perceived availability of social resources. This study uses data from the Aging with Pride: National Health and Sexuality Gender Study (NHAS) and the Movement Advance Project (MAP) to examine both the interpersonal and structural determinants of transgender older adults' perceived social support. In 2017, NHAS surveyed 189 transgender older adults on their perceived social support and asked to report the number of close ties in their social networks. For the same year MAP tracked trans supportive and anti-transgender state policies and categorized all the states in the US as high/medium and low/negative equality states. NHAS transgender respondents from 32 states, were on average 67 years old, about a third reported being racial/ethnic minority and about half were below the 200% federal poverty level. About two thirds resided in medium/high equality states and about a third resided in low/negative equality states. In the regression analysis, after accounting for the demographic variables, transgender older adults' perceived social support increased significantly with every additional close network tie ($\beta = 0.11$, $p < 0.001$). Net of social network size, transgender respondents in high/medium equality states reported feeling significantly more supported than those in low/negative equality states ($\beta = 0.34$, $p = 0.033$). These findings highlight that the new wave of anti-transgender legislations will have a lasting negative impact across transgender individuals' life course.

### SESSION 2530 (SYMPOSIUM)

Abstract citation ID: igaf122.1073

### ENGAGED AGING: DISPARITIES IN PAID AND UNPAID WORK IN LATER LIFE

Chair: Christina Matz
Co-Chair: Chin-Yi Su
Discussant: Julie Miller

As the U.S. population ages, engagement in both paid and unpaid work remains central to older adults' well-being and societal contributions. However, disparities in access, participation, and the extent to which engagement leads to positive outcomes persist across racial, ethnic, and socioeconomic contexts. This symposium examines how different forms of work—including employment, caregiving, and volunteerism—shape the aging experience, highlighting disparities and identifying opportunities for policy and workplace interventions. The first presentation examines ethnic subgroup differences in volunteerism among Asian American adults aged 50 and older, drawing on nationally representative data to explore how socio-economic and cultural factors shape engagement. The second presentation identifies caregiving typologies among older workers, revealing how caregiving responsibilities interact with employment to impact well-being. The third presentation presents findings from a 2025 nationally representative AARP study on age discrimination among multicultural older workers, documenting high rates of both overt and subtle forms of discrimination, as well as employment transitions driven by financial need. The final presentation examines the effects of productive aging activities—including education, employment, and volunteering—on cognitive health among older Hispanics, considering the moderating role of genetic inheritance. By examining these topics through diverse lenses, this symposium advances understanding of how disparities in work, caregiving, volunteering and education, shape later-life outcomes. Julie Miller, AARP Thought Leader, will synthesize findings and and discuss implications for workplace policies, financial planning, and social programs aimed at fostering greater equity in aging experiences.

Abstract citation ID: igaf122.1074

**AGING, WORK, AND CARE: TYPOLOGIES OF OLDER EMPLOYED CAREGIVERS AND THEIR WELL-BEING**

Christina Matz, *Boston College, Chestnut Hill, Massachusetts, United States*

As the U.S. population ages, the intersection of work and caregiving is becoming increasingly critical. By 2030, nearly 20% of Americans will be 65 or older, and older adults now represent a rapidly growing segment of the labor force. At the same time, over 53 million Americans provide unpaid care to loved ones, with 61% of caregivers balancing employment and caregiving responsibilities. However, little research has examined the diversity of caregiving experiences among older workers. This study analyzes data from the 2018 Health and Retirement Study (HRS) to identify distinct caregiving typologies among workers aged 50 and older. Using cluster analysis, we identify six caregiving typologies: (1) Non-Caregivers, (2) Grandchild Caregivers, (3) Spousal Caregivers, (4) Parental Caregivers, (5) Financial Caregivers to Parents, and (6) Financial Caregivers to Children. Findings reveal significant differences in sociodemographic characteristics, health outcomes, and well-being indicators across typologies. Notably, spousal caregivers experience the highest levels of stress and depressive symptoms, while grandchild and parental caregivers demonstrate resilience despite caregiving demands. These insights have substantial implications for employers seeking to support caregiving employees. Tailored workplace policies—including flexible work arrangements, mental health support, and financial planning resources—can help mitigate caregiving-related stress. Future research should explore the long-term impacts of caregiving on career trajectories and financial security. By recognizing the varied experiences of working caregivers, policymakers and employers can foster more inclusive workplaces that accommodate the needs of an aging workforce.

Abstract citation ID: igaf122.1075

**ASIAN ETHNIC GROUP DIFFERENCES IN FORMAL AND INFORMAL VOLUNTEERISM AMONG ADULTS AGED 50 AND OVER IN THE UNITED STATES**

Patrick Ho Lam Lai, *The University of Oklahoma, Moore, Oklahoma, United States*

As the aging population grows, volunteerism plays a vital role in promoting well-being and societal benefits. However, disparities in volunteerism persist across racial and ethnic groups, particularly among Asian Americans, a heterogeneous population often underrepresented or grouped homogenously in research. This study examines formal and informal volunteerism among Asian subgroups in the U.S. using data from the Civic Engagement and Volunteering Supplement of the Current Population Survey (2017, 2019, 2021). Focusing on adults aged 50-75, it investigates subgroup differences in volunteer rates and the interplay between socio-economic resources (income and education) and ethnic identity. Findings revealed significant differences across subgroups. Japanese, Koreans, and Filipinos were more likely to engage in formal volunteering compared to Chinese, while informal volunteering was higher among Filipinos and Asian Indians. Socio-economic factors, especially education, partly explained these disparities. Education had a less positive impact on formal volunteering for Koreans, Vietnamese, and other subgroups compared to Chinese, while income had a stronger influence for Filipinos. Informal volunteerism showed limited interaction effects with socio-economic resources, highlighting distinct dynamics between formal and informal engagement. This study emphasizes the importance of disaggregating data to better understand the unique experiences of Asian subgroups. Findings call for culturally responsive policies and programs to address barriers and promote equitable engagement opportunities for diverse midlife and older populations. Further research is needed to examine cultural and structural factors shaping volunteerism within and across Asian subgroups.

Abstract citation ID: igaf122.1076

**EFFECTS OF APOE ALLELES AND PRODUCTIVE AGING ACTIVITIES ON COGNITIVE HEALTH AMONG OLDER HISPANICS**

Ernest Gonzales[1], Jane Lee[2], and Cliff Whetung[3], *1. New York University, New York, New York, United States, 2. University of Hawai'i at Mānoa, Honolulu, Hawaii, United States, 3. University of Minnesota Duluth, Duluth, Minnesota, United States*

This study examined the longitudinal associations of productive aging activities – formal education, employment, volunteering – with cognitive functioning in the context of genetic inheritance (APOE alleles) among older Hispanics in the United States. Mixed effect growth curve models tested associations with respondents in the Health and Retirement Study (8,558 observations longitudinally from 2010 to 2020), controlling for known health, economic, and social covariates. A fifth of respondents carried one or two ε4 alleles. Nearly half of respondents had less than a high school diploma (46%), nearly half worked for pay (45%), approximately one quarter engaged in formal volunteering (24%), and approximately a third engaged in informal volunteering (36%). Respondents with ε4 alleles experienced lower levels of cognitive health at baseline, yet slower slopes of cognitive decline across models. Higher levels of education conferred greater cognitive health cross-sectionally and a slower cognitive decline rate over time. Although employed respondents reported lower levels of cognitive health at baseline, employment protected their cognitive health overtime relative to Hispanics not in the labor force. Formal volunteering resulted in higher levels of cognitive health, yet associations were unstable across models. Foreign born Hispanics experienced higher levels of cognitive health at baseline relative to US born Hispanics. Income, depression, and other social contexts influenced cognitive health at baseline and overtime in expected directions. Productive activities contributed to cognitive health, above and beyond genetic factors. Productive aging, income, and health policies and programs can bolster cognitive health. We will discuss areas for further research.

Abstract citation ID: igaf122.1077

**AGING, WORK, AND MULTICULTURAL POPULATIONS: FINDINGS FROM NATIONAL DATA**

Rebecca Perron, *AARP, Washington, District of Columbia, United States*

AARP is committed to the ongoing study of older workers, including within multicultural populations. A 2025 nationally representative study of older workers in the US authored by AARP revealed persistently high rates of age discrimination for African American/Black (74%), Hispanic/Latino (62%), and Asian American and Pacific Islander (67%) workers ages 50-plus. A large majority of these groups report that age discrimination is common. Beyond the more common or overt forms of age discrimination – like not getting a job due to age - subtle forms are also prevalent. These include assumptions that older workers are less tech-savvy, comments on their health related to age, beliefs that they are resistant to change, and inquiries about when they might retire. Furthermore this study found that around 2 in 5 older workers from multicultural backgrounds feel they are being pushed out of their job due to their age. Additional nationally representative data from AARP found that significant proportions of multicultural older workers expect to change jobs in the next year. Specifically, two in five of African American and Hispanic and 33% of AAPI employed older workers expect to make a job change. Money is the primary motivator of job change for all groups. Very large majorities recognize the need for some sort of help or information as they change jobs. Despite concerns about the impact of artificial intelligence on job security and age being a barrier to finding new employment, these groups remain confident in their ability to successfully change jobs.

### SESSION 2535 (SYMPOSIUM)

Abstract citation ID: igaf122.1078

### ASSOCIATIONS OF SENSORY AND MOTOR FUNCTIONS WITH NEUROPATHOLOGICAL CHANGES IN AGING

Chair: Jeannette Mahoney
Co-Chair: Jennifer Deal
Discussant: Natascha Merten

Alzheimer's disease (AD) is the most-common cause of dementia, affecting nearly 6.7 million older adults in America. The number of people with AD is projected to increase two-fold by 2050, potentially reaching 13.8 million. While AD is characterized by cognitive impairments as well as mobility disorders, it also alters sensory processing as it associated with accumulation of proteins (plaques) and tangles, which disrupt communication between brain cells. Such disruption in neural signaling leads to sensory integration issues that often precede cognitive decline. In fact, growing evidence suggests that Alzheimer's pathology manifests in sensory association areas well before appearing in neural regions involved in higher-order cognitive areas like the prefrontal cortex. As well, impairments in olfaction (loss of smell) are considered an early warning sign of Alzheimer's onset. While evidence suggests that functional changes in both sensory and motor systems modulate the progression of Alzheimer's disease, research is still in its infancy. In what follows, we will present findings from four distinct research studies examining links between sensory (auditory and olfaction) and motor (gait speed & endurance) impairments and cognitive impairments and AD biomarkers. Identification of novel, non-cognitive, non-invasive biomarkers of Alzheimer's disease and Alzheimer's-related dementias are a national priority identified by the NIH. Our continued research efforts are directly in line with this goal of identifying AD earlier and intervening as soon as possible in an effort to reduce the burden of Alzheimer's disease on individuals, their families, and our society at large. Sensory Health Interest Group Sponsored Symposium

Abstract citation ID: igaf122.1079

### MODERATING EFFECTS OF ENDURANCE ON GAIT SPEED ASSOCIATIONS WITH INCIDENT MILD COGNITIVE IMPAIRMENT AND DEMENTIA

Glen Myatt[1], Hunter Sylvester[1], Michael Griswold[2], Rebecca Gottesman[3], David Knopman[4], Kelley Pettee Gabriel[5], Kevin Sullivan[2], and B. Gwen Windham[2], *1. University of Mississippi Medical Center, Jackson, Mississippi, United States, 2. University of Mississippi Medical Center - The MIND Center, Jackson, Mississippi, United States, 3. National Institutes of Health, Bethesda, Maryland, United States, 4. Mayo Clinic Department of Neurology, Rochester, Minnesota, United States, 5. The University of Alabama at Birmingham, Birmingham, Alabama, United States*

Although slower gait speed is associated with cognitive impairment, endurance may be more predictive in older adults with faster gait speed. We examined moderating effects of endurance on the relationship of gait speed with incident mild cognitive impairment (MCI) or dementia in cognitively normal ARIC participants (n = 2242, mean age=78.6 + 4.3, 58% female, 19% Black). At the sixth exam, endurance was assessed as distance walked at a fast pace (Two Minute Walk, 2MW); usual 4-meter gait speed was also assessed. Cognitive status was adjudicated during the 6th-9th exams (mean follow-up 5.1 years). Multinomial logistic regression models with a 2MW-x-gait speed interaction term estimated relative risk ratios (RRR) of MCI and dementia relative to normal cognition per SD higher gait speed or 2MW distance, adjusting for demographic and cardiovascular factors. Results used representative continuous gait speed and continuous 2MW distance values. Longer 2MW distance was associated with lower risk of MCI and dementia at gait speed <1m/s [e.g. gait speed=0.8: MCI RRR=0.59, 95%CI(0.42, 0.83); dementia RRR=0.29, (0.13, 0.63)] and with dementia at gait speed>1m/s [gait speed=1.2: RRR=0.28, (0.09, 0.81)], but not with MCI (RRR=0.85, (0.57, 1.26)]. Conversely, faster gait speed was associated with lower risk of MCI at low 2MW [100 m; RRR=0.84, (0.74, 0.94)], but was attenuated at high 2MW (150 m; RRR=0.91, (0.84, 1.00)]. A 2MW-x-gait speed interaction term was supported (p = 0.04), primarily driven by differential MCI associations. Combining endurance and gait speed may be especially helpful in identifying cognitively normal older adults at risk for impending early-stage cognitive impairment.

Abstract citation ID: igaf122.1080

### AUDIOMETRIC HEARING AND PLASMA BIOMARKERS OF ALZHEIMER'S DISEASE PATHOLOGY AND NEURODEGENERATION IN THE ARIC-NCS

Jennifer Deal[1], James Pike[2], Priya Palta[3], and Nicholas Reed[4], *1. Johns Hopkins Bloomberg School of Public Health, Baltimore, Maryland, United States, 2. New York University, New York, New York, United States, 3. UNC School of Medicine, Chapel Hill, North Carolina, United States, 4. New York University Grossman School of Medicine, New York, New York, United States*

The mechanism linking hearing loss to Alzheimer's disease and related dementias (ADRD) is unknown. We investigated cross-sectional associations of hearing loss with plasma biomarkers of AD and neurodegeneration, hypothesizing that hearing loss is associated with biomarkers reflective of broader neurodegeneration but not with AD-specific markers. Data is from the Atherosclerosis Risk in Communities Neurocognitive Study. Pure tone air conduction hearing thresholds (frequencies 0.5-4 kHz) were obtained at Visit 6 (2016-17) and averaged, with better-ear hearing loss modeled continuously. The Quanterix SiMoA platform measured plasma biomarkers from stored specimens at the nearest visit – Visit 5 (2011-13), Visit 6 (2016-17) or Visit 7 (2018-19) – including amyloid-β 42 /amyloid-β 40 ratio, phosphorylated tau (p-tau) 181, neurofilament light chain (NfL), and glial fibrillary acidic protein (GFAP). Multivariable-adjusted linear regression estimated differences in biomarker levels, adjusting for time between audiology assessment and plasma collection, age, sex, race, center, education, APOE e4 genotype, BMI, estimated glomerular filtration rate, cigarette use, total cholesterol, high-density lipoprotein cholesterol, hypertension, diabetes, coronary heart disease and hearing aid use. Participant were 67-94 years, 60% female and 36% self-identified identified Black race. Hearing loss was positively associated with NfL, with each 10 dB increase (worse hearing) associated with 0.079 (95% confidence interval [CI] 0.007, 0.150) higher log NfL. Poorer audiometric hearing in older adults is associated with plasma biomarkers of neurodegeneration, specifically NfL, but is not associated with AD-specific markers (amyloid and tau), suggesting that pathways linking hearing and ADRD are independent of these pathognomonic Alzheimer's-related brain changes.

Abstract citation ID: igaf122.1081

### EFFECTS ON BLOOD NEURODEGENERATION BIOMARKERS OF A RANDOMIZED HEARING REHABILITATION INTERVENTION

Nicholas Reed[1], James Pike[2], Jennifer Deal[3], Priya Palta[4], and Josef Coresh[2], *1. New York University Grossman School of Medicine, New York, New York, United States, 2. New York University, New York, New York, United States, 3. Johns Hopkins Bloomberg School of Public Health, Baltimore, Maryland, United States, 4. UNC School of Medicine, North Carolina, United States*

The Aging and Cognitive Health Evaluation in Elders (ACHIEVE) study is a multicenter, parallel-arm, randomized trial (hearing intervention vs health education control) on 3-year cognitive decline among adults 70–84years with untreated hearing loss and without substantial cognitive impairment. Participants were recruited from: (1)a long-standing observational study (Atherosclerosis Risk in Communities [ARIC]), and (2)de novo community volunteers. In ACHIEVE, intervention slowed 3-year cognitive decline by 48% among ARIC participants only. To further investigate differences by treatment with respect to cognitive benefit, we tested the hypothesis that hearing intervention is associated with improved neurodegeneration blood biomarkers 3-years post-randomization. Plasma was collected in year 3 in a subsample (n = 540) while ARIC participants (n = 164) also had baseline plasma. Glial fibrillary acidic protein (GFAP) and neurofilament light (NfL) neurodegenerative biomarkers were derived using the Alamar CNS protein panel. Regression models estimated the association of treatment with 3-year biomarkers differences in the combined sample and by recruitment source and annualized change from baseline in the ARIC sample. Baseline characteristics were balanced by treatment. There were no significant differences on 3-year neurodegeneration biomarker levels by treatment in the combined or de novo groups. Among ARIC participants, intervention resulted in lower 3-year GFAP(mean:-0.415;95%CI:-0.712,-0.118) and NfL(mean:-0.349;95%CI:-0.652,-0.046). Annualized change from baseline analyses among ARIC participants revealed intervention was associated with a slower rate of increase in GFAP (difference-in-means: -0.060;95%CI:-0.112,-0.009). Hearing intervention was associated with positive 3-year effects on neurodegeneration blood biomarkers in ARIC participants which parallels cognitive decline results. Results provides objective evidence of brain changes following hearing intervention.

Abstract citation ID: igaf122.1082

### ASSOCIATION OF OLFACTORY DYSFUNCTION, SUBJECTIVE COGNITIVE DECLINE AND ALZHEIMER'S BIOMARKERS WITH DEMENTIA RISK

Mattie Derivaux[1], Xiaoqian Zhu[2], Srishti Shrestha[2], Kevin Sullivan[2], Michael Griswold[2], B. Gwen Windham[2], Priya Palta[3], and Vidyulata Kamath[4], *1. University of Mississippi Medical Center, Jackson, Mississippi, United States, 2. University of Mississippi Medical Center - The MIND Center, Jackson, Mississippi, United States, 3. UNC School of Medicine, Chapel Hill, North Carolina, United States, 4. Johns Hopkins University School of Medicine, Baltimore, Maryland, United States*

Integrating early dementia manifestations, including subjective cognitive decline (SCD, self-reported cognitive changes without objective evidence) and olfactory dysfunction (OD), with plasma biomarkers of Alzheimer's and neurodegeneration (Amyloid-β (Aβ) 42/40, phosphorylated-tau-181, Glial Fibrillary Acidic Protein, and Neurofilament Light Chain) may enhance early dementia risk stratification. Utilizing ARIC study data, we examined the combined associations of SCD, OD, and plasma biomarkers with incident dementia. We included 4,641 dementia-free participants (age=75±5y; 21% Black, 11% OD, 39% SCD, 21% mild cognitive impairment (MCI)) at ARIC Visit 5 (2011-2013) with complete information on OD (12-item Sniffin' Sticks score ≤6) and cognition (categorized as SCD, normal cognition without SCD, or MCI based on subjective reports and objective cognitive adjudication). 1,430 participants also had plasma biomarker data at Visit 5 (categorized using median cut-offs). Incident dementia was identified via expert-adjudicated cognitive evaluation and hospital discharge or death records through December 31, 2022 (median follow-up=8y). Hazard ratios (HRs) were estimated using Cox regression, adjusting for demographic and cardiovascular factors. Model 1 (olfaction/cognition) showed participants with OD/SCD had more than double the dementia risk (HR = 2.86 (95% CI:2.12–3.86)) than those with No OD/normal cognition, exceeding risks for those with OD/normal cognition (HR = 1.99 (CI:1.45–2.74)) and No OD/SCD (HR = 1.45 (CI:1.20–1.75)). Model 2 (olfaction/cognition/amyloid pathology) demonstrated that participants with OD/SCD/worse amyloid pathology (Aβ42/40

### SESSION 2540 (PAPER)

### FAMILY CAREGIVING: POLICY AND PRACTICE

Abstract citation ID: igaf122.1083

### SOLE CAREGIVING FROM AFAR: UNPACKING TRANSNATIONAL CAREGIVING AMONG THE CHINESE ONE-CHILDREN IN THE U.S.

Qi Chen[1], Leyi Zhou[2], and Julian Chun-Chung Chow[2], *1. Hunter College - City University of New York, Hunter College - City University of New York, New York, United States, 2. University of California, Berkeley, Berkeley, California, United States*

Despite growing research on transnational caregiving, little is known about how the Chinese only-child generation—who lack sibling support and face heightened filial expectations—conceptualizes and navigates their caregiving roles from abroad. This qualitative study examines how Chinese only-children in the United States define transnational caregiving and their responsibilities as transnational caregivers. We recruited participants through social media and conducted semi-structured online interviews with 25 Chinese only-children in the U.S. who had experience providing transnational care or were currently planning care for their aging parents in China. Most of the participants were female (68%), had a graduate degree (92%), an average age of 36.5 and an average stay of 11.2 years in the US. Using interpretive phenomenological analysis approach, we identified seven dimensions of transnational caregiving: (1) bridging healthcare information gaps between the two countries, (2) purchasing and mailing medical supplies, (3) providing emotional support through daily greetings and frequent video calls, (4) prioritizing self-care to reduce parents' worries, (5) educating and promoting healthy behaviors to prevent illnesses requiring intensive care, (6) offering financial support, and (7) coordinating long-distance care arrangement and provider communication. These findings advance theoretical understandings of caregiving in global contexts by illustrating how transnational caregiving extends beyond traditional frameworks of caregiving through the case of Chinese only-children in the U.S. The study sheds light on how geographical distance reshapes caregiving roles, emphasizing the need for a broader conceptualization of caregiving that incorporates digital communication, cross-border healthcare gaps and resource management.

Abstract citation ID: igaf122.1084

### EXPERIENCES OF OLDER ADULT PARENTS OF ADULT PERSONS WITH AUTISM SPECTRUM DISORDER

Yarin Cohen, Robert Hock, Abigail Reza, and Emmanuella Asabea Twum, *University of South Carolina, Columbia, South Carolina, United States*

For parents of adult children with ASD, aging is often accompanied by caregiving that creates time, financial, and lifestyle constraints. Previous research has found that support networks decrease after individuals become adults and leave school, while parents, facing health and aging challenges, may hesitate relying on family for support, risking future care crises. Yet little is known about how these parents navigate the intersection of aging and persistent caregiving. This qualitative study aimed to addressing the unique challenges and described needs, while identifying coping mechanisms that promote older adult caregiver well-being. Semi-structured interviews were conducted with 13 participants (age 54-72, 3 males, 10 females) caring for adult children with ASD (age 25-36) exploring caregiving in later life, service experiences, family relationships, and preparing for the future. These interviews were recorded and transcribed for analysis using a thematic approach. Analysis revealed five key themes: Feeling disconnected; experiencing service gaps after school transitions; anxiety about future care arrangements; prioritizing child's needs above their own; ongoing advocacy challenges. Parents often struggle finding mental health support, may feel isolated, and grapple with planning for their child's future care, a process that can be both emotionally distressing and logistically complex. However, they also find strength in established routines and skills, highlighting both challenges and resilience in long-term caregiving. Enhanced support systems are needed to assist adults with ASD in transitioning to adulthood, including accessible guidance on future planning and establishing peer networks where older caregivers can share valuable knowledge with younger families.

Abstract citation ID: igaf122.1085

### FIREARM STORAGE PRACTICES AMONG DEMENTIA CAREGIVERS: FINDINGS FROM THE BRFSS

Jiaming Liang[1], and Alexander Testa[2], *1. UTHealth Houston, San Antonio, Texas, United States, 2. UTHealth Houston, Houston, Texas, United States*

Dementia caregiving presents unique challenges that may influence firearm storage behaviors, yet little research has examined how caregiver characteristics shape firearm storage practices. This study investigates firearm ownership, and firearm storage behaviors (i.e., storing firearms loaded/unloaded; locked/unlocked) among dementia caregivers using pooled data from the 2017, 2021, 2022, and 2023 Behavioral Risk Factor Surveillance System (BRFSS). The sample included 3,436 dementia caregivers (weighted N = 444,744), of whom 40.57% reported firearm ownership. Among firearm-owning caregivers (n = 1,460), 35.79% stored a firearm loaded, and 50.78% of those with a loaded firearm kept the firearm unlocked. Weighted logistic regressions revealed that male caregivers had twice the odds of keeping firearms loaded compared to female caregivers. Older caregivers (ref: < 50 year-old) were significantly more likely to store firearms unlocked. In contrast, caregiving for more than five years and assisting with household tasks were associated with safer storage (i.e., storing a firearm loaded; locked). Interaction analyses showed that firearm storage behaviors varied by gender, income, and caregiving intensity, with male caregivers in higher-income households and those with shorter caregiving durations being more likely to store firearms loaded and unlocked. Findings underscore the need for targeted firearm safety interventions for dementia caregivers, particularly among older and male caregivers, to mitigate risks associated with firearm access in dementia-affected households.

Abstract citation ID: igaf122.1086

### USING CAREGIVER STORIES TO BETTER UNDERSTAND BURDEN AND ASSESS NEED

Aileen McGrory[1], Jacqueline Boudreau[1], Lynette Kelley[2], Marianne Desir[3], and Eileen Dryden[1], *1. Veterans Health Administration, Bedford, Massachusetts, United States, 2. Veterans Health Administration, Aurora, Colorado, United States, 3. Veterans Health Administration, Miami, Florida, United States*

While informal caregivers provide crucial care to older Veterans, they often experience high levels of personal stress and difficulties accessing support. Understanding caregiver burden is crucial to improving caregiver support, since recognition of burden is a first step in connecting the caregiver to resources. Many studies on caregiver burden focus on defining and measuring burden and its relationship with other factors, but the literature lacks consensus as to which factors are most correlated with burden. Given this divided literature, a closer qualitative look at caregivers' experiences is warranted. In our sample of 30 caregivers of rurally located older Veterans, we found that Zarit Burden scores were closely aligned with interview data about caregiver experience. Other commonly used measures that we collected, including ADLs and IADLs, were not as closely associated with Zarit Burden score. Instead, caregiving difficulty among those with high Zarit Burden scores aligned with experiential elements including 1) concerns about the caregiver and care recipient's relationship, 2) perceived lack of access to conversations about care, 3) isolation, and 4) focus on the future.

Although objective burden is often easier to collect and quantify using measurements of caregiving tasks and time commitment, those methods exclude elements that shape caregiver experience but are only apparent through a more thorough understanding of caregivers' stories. Including individual experience and subjective burden while assessing overall caregiver burden, for instance by incorporating brief caregiver "interviews," may allow more insight into caregiver needs and help improve support, and thus care and outcomes of older patients.

### SESSION 2545 (PAPER)

Abstract citation ID: igaf122.1087

### FRAILTY IN OLDER ADULTS

The assessment, trajectory and biomarkers of frailty in older adults will be presented.

Abstract citation ID: igaf122.1088

### BEYOND UNIDIRECTIONAL DECLINE: IDENTIFYING NONLINEAR FRAILTY AND MULTIMORBIDITY TRAJECTORIES IN OLDER ADULTS

Yanran Deng[1], Yan Luo[2], Yuming Chen[1], Zhou Yang[3], and Beibei Xu[4], *1. Peking University Health Science Center, Beijing, Beijing, China (People's Republic), 2. The University of Hong Kong, Hong Kong, China (People's Republic), 3. Peking University Cancer Hospital & Institute, Beijing, Beijing, China (People's Republic), 4. Peking University, Beijing, Beijing, China (People's Republic)*

Frailty and multimorbidity jointly drive adverse outcomes in older adults. However, their dynamic trajectories remain underexplored due to survival selection bias—where mortality systematically excludes high-risk individuals, distorting longitudinal progression patterns. This study aims to delineate joint trajectories of frailty and multimorbidity, and characterize their temporal synergies while accounting for the addressing selective survival effects. Using 12 waves (2011-2023) of the National Health and Aging Trends Study (NHATS), we annually assessed frailty (Fried phenotypic criteria) and multimorbidity (cumulative chronic conditions). To overcome survival selection bias inherent in aging cohorts, we extended group-based trajectory modeling through dual-process specification: a multivariate latent class model identifying progression typologies, coupled with a competing risks survival model. Shared parameters dynamically adjusted trajectory estimates for mortality-related attrition, ensuring unbiased characterization of temporal synergies. Among the 4,960 participants from the NHATS 2011 cohort included in this analysis, 2,931 (survey-weighted 57.2%) were female, and 2,070 died during follow-up (average annual attrition rate: 4.3%). Seven joint trajectories were identified, revealing three dominant patterns: stable phenotypes (Stable Low Risk, Persistent High Multimorbidity), progressive escalators (Late-Onset Frailty, Early Accelerated Frailty, Delayed Escalation), and nonlinear progressors (High Baseline Attenuation, Critical-State Reversal). Notably, Critical-State Reversal demonstrated frailty decline despite extreme multimorbidity progression, while Early Accelerated Frailty and Late-Onset Frailty diverged in progression timing despite comparable multimorbidity burdens, challenging assumptions of unidirectional frailty trajectories in aging. This study identifies seven distinct trajectories, and reveals unexpected heterogeneity in aging processes. These mortality-adjusted trajectories may offer a clinically actionable framework for risk stratification and personalized care planning.

Abstract citation ID: igaf122.1089

### HEALTH CARE USE AND PHYSICAL, PSYCHOLOGICAL, COGNITIVE, AND SOCIAL FRAILTY IN COMMUNITY-LIVING ADULTS 45-85

Lauren Griffith[1], Graciela Muniz-Terrera[2], Edwin van den Heuvel[3], Jayati Khattar[4], David Hogan[5], Megan E. O'Connell[6], Mélanie Levasseur[7], and Parminder Raina[1], *1. McMaster University, Hamilton, Ontario, Canada, 2. Ohio University, Athens, Ohio, United States, 3. Eindhoven University of Technology, Eindhoven, Noord-Brabant, Netherlands, 4. University of Toronto, Toronto, Ontario, Canada, 5. University of Calgary, Calgary, Alberta, Canada, 6. University of Saskatchewan, Saskatoon, Saskatchewan, Canada, 7. Université de Sherbrooke, Sherbrooke, Quebec, Canada*

This study examines associations between physical, psychological, social, and cognitive frailty domains with health care utilization (HCU) and the potential moderating effect of the last three domains on the association between physical frailty and HCU. A 127-item Frailty Index (FI) developed for the Canadian Longitudinal Study on Aging comprehensive cohort (n = 30,097) was used to create physical, psychological, cognitive, and social domain-specific FIs. Each FI was divided into quintiles with the highest 20% representing the frailest. Logistic regression was used to estimate unadjusted and adjusted (covariates: sex, age, income, smoking, physical activity, nutrition, and participation restriction) ORs (aORs) for frailty domains and HCU (formal/informal care, family physician visits, hospitalizations) and interactions between physical frailty and the other frailty domains. Physical frailty was associated with the highest HCU ORs in unadjusted (1.53 to 2.38) and adjusted (1.28 to 1.78) analyses, with the largest aOR for formal care (1.78, 95% CI 1.66, 1.91). For all HCU except formal care, the upper CI limits for social frailty were < 1, indicating those with higher levels of social frailty were less likely to use these services. Interactions between physical frailty and the other frailty domains were significant for only formal and informal care, with the aOR magnitudes for the other domains increasing with the level of physical frailty. Our data suggests that the drivers of HCU are multifactorial and the need to consider both frailty beyond physical characteristics and the complex relationships between frailty domains and HCU when assessing the outcomes of frailty interventions.

Abstract citation ID: igaf122.1090

### HYPOTHETICAL INTERVENTIONS ON FRAILTY TRAJECTORY AMONG OLDER GASTRIC CANCER PATIENTS USING PARAMETRIC G-FORMULA

Xinyi Xu, Yinning Guo, and Qin Xu, *Nanjing Medical University, Nanjing, Jiangsu, China (People's Republic)*

Background: Frailty is prevalent in older gastric cancer patients, seriously affecting prognosis. This study aimed to estimate the effect of hypothetical interventions on frailty heterogeneous trajectories (FHT) to design targeted intervention programs. Methods: Data from a longitudinal follow-up study of 381 gastric cancer patients ≥60 years were collected at admission, discharge, and 1, 3, 6, and 12 months post-surgery. The parametric g-formula estimated the effects of single and joint hypothetical interventions on FHT risk across physical, psychological, familial, and social factors. Results: The observed FHT risk was 43.57%. Single intervention on nutrition most effectively reduced FHT risk (RR = 0.790, 95% CI: 0.692-0.974), followed by family cohesion (RR = 0.810) and social objective support (RR = 0.822). The "all-factors" joint intervention reduced risk by 31.26% (RR = 0.323, 95% CI: 0.208-0.685). An evidence-based intervention program was designed with good precision and scientificity. Conclusions: Based on optimal intervention combinations determined from parametric g-formula, we designed a comprehensive FHT intervention program covering multiple time periods, scenarios and factors, providing theoretical and practical guidance for frailty prevention in older gastric cancer patients.

Abstract citation ID: igaf122.1091

### THE MEDIATING PATHWAY LINKING SOCIAL FRAILTY AND LATE-LIFE MALNUTRITION

Doris S. F. Yu, Mary Miao, and Chen Qiu, *The University of Hong Kong, Hong Kong, China (People's Republic)*

Background/Objectives: Social and mental health significantly influence the nutritional status of older adults. While a relationship exists between social frailty and malnutrition, the underlying mechanisms remain unclear. This study aims to explore the associations and potential psychological pathways among social frailty, depression, and malnutrition in Chinese older adults. Methods: A secondary analysis was conducted using data from the Jockey Club Pathway to Healthy Aging project, collected between May 2022 and June 2024. Structural equation modeling (SEM) was employed to examine the relationships among social frailty, depression, and malnutrition, as well as the mediating role of depression. Multi-group SEM was used to compare the hypothesized model across different BMI subgroups. Results: The study included 5,286 older adults, with a malnutrition prevalence of 0.6% and an at-risk malnutrition rate of 14.2%. The prevalence of social frailty and pre-frailty were 16.3% and 31.7%, respectively. Social frailty ($\beta$ = -0.044, $p = 0.004$) and depression ($\beta$ = -0.150, $p < 0.001$) were significant negative predictors of malnutrition scores, with depression mediating 55.1% of the total effect ($\beta$ = -0.098, $p < 0.001$). Multi-group SEM revealed differences in path effects across BMI subgroups. Conclusions: The risk of malnutrition in older adults is influenced by social and psychological factors. Preventive strategies should differ based on BMI, focusing on social factors for overweight and obese individuals while addressing negative emotions in those with normal BMI to encourage healthy dietary habits and lifestyles.

### SESSION 2550 (SYMPOSIUM)

Abstract citation ID: igaf122.1092

### PRESIDENTIAL SYMPOSIUM: THE FUTURE OF AGING EQUITY: SUSTAINING PROGRESS AND INNOVATING INCLUSION

Chair: Chivon Mingo

Aging is a fundamental aspect of identity and lived experience—yet it is often overlooked when considering equity across society. How age intersects with race, socioeconomic status, disability, LGBTQ+ identity, and geography shapes opportunities, access to care, and overall well-being throughout the lifespan. Ensuring that all individuals can age with dignity, health, and security requires a commitment to aging equity (i.e., the fair and just distribution of opportunities, resources, and support for older adults). As research, policy, advocacy, and practice efforts continue to push for inclusion, shifting funding priorities and institutional frameworks introduce new complexities that challenge the sustainability of progress. While new barriers continue to emerge, the need to sustain and advance aging equity has never been more urgent. This symposium brings together experts in policy, research, and applied practice to explore key strategies for securing the future of aging equity. Speakers will highlight how we can sustain hard-fought gains while forging new paths—leveraging innovation, collaboration, and research to drive inclusion, even in uncertain times. This session takes a firm stance on sustaining and expanding equity efforts across these intersecting dimensions of identity while developing forward-thinking strategies to ensure continued progress despite evolving challenges.

Abstract citation ID: igaf122.1093

### AGING EQUITY AND INSTITUTIONAL FRAMEWORKS: THE ROLE OF POLICY AND APPLIED INTERVENTIONS

Sarah Szanton, *Johns Hopkins University, Baltimore, Maryland, United States*

Aging equity is shaped by lifecourse institutional frameworks, including government policies, social structures, and healthcare systems, that influence social determinants of health, access to care, and the overall well-being of older adults. Research demonstrates that systemic inequities—particularly those rooted in historical injustices such as redlining—compound over the life course, leading to inequities in not just life span but health span. This session will examine how policy-driven approaches can address these disparities and promote sustainable, equitable aging solutions. A critical focus of this discussion is the CAPABLE (Community Aging in Place—Advancing Better Living for Elders) program, an evidence-based intervention designed to enhance functional independence, reduce healthcare costs, and improve quality of life among older adults. CAPABLE integrates a nurse, an occupational therapist, and a home repair specialist to support self-directed goals for aging in place. Longitudinal research has demonstrated a 7-10x return on investment, primarily through reductions in hospitalization and nursing home admissions. We will also touch on a new model called Neighborhood Nursing, which provides a nurse and community health worker, block-by-block, to all people. By integrating applied research into aging policy frameworks, findings illustrate how structural innovations can drive inclusion, reduce disparities, and sustain progress in aging equity.

Abstract citation ID: igaf122.1094

### ADVANCING AGING EQUITY IN DEMENTIA: STRUCTURAL AND PSYCHOSOCIAL FACTORS, COMMUNITY ENGAGEMENT AND INCLUSION

Crystal Glover, *University of California, Irvine, Irvine, California, United States*

Disparities in aging and dementia stem from deeply embedded structural and psychosocial determinants of health. Systemic inequities in healthcare access, socioeconomic status, and education create significant barriers to early diagnosis, treatment, and long-term support for minoritized older adults. At the same time, psychosocial determinants—including trust in research, cultural beliefs, and caregiving burdens—further shape the experiences of aging and dementia within historically underrepresented communities. Ensuring progress in aging equity requires sustained, community-driven strategies that foster inclusion, build trust, and challenge existing barriers to participation. This presentation examines how structural and psychosocial factors contribute to disparities in dementia research and care while advancing innovative, evidence-based approaches to expand equitable participation. Recent findings reinforce the critical need for participatory research models that actively engage diverse populations and address longstanding distrust. The ENGAGED model exemplifies a framework for transforming outreach and recruitment by integrating bidirectional knowledge exchange, culturally responsive engagement, and sustained community partnerships. By embedding equity at the core of both research and practice, a scalable and empirically grounded blueprint emerges—one that sustains progress, innovates inclusion, and ensures that all older adults, regardless of race, ethnicity, or socioeconomic background, are equitably represented in aging and dementia research.

Abstract citation ID: igaf122.1095

**STANDING UP FOR INCLUSION OF LGBTQIA+ OLDER ADULTS IN ALZHEIMER'S AND PARKINSON'S DISEASE RESEARCH**

Jace Flatt, *University of Nevada Las Vegas, Nevada, United States*

Over 3 million adults aged 60+ in the U.S. identify as lesbian, gay, bisexual, transgender, queer, intersex, or another identity (LGBTQIA+). Yet, despite the growing population of LGBTQIA+ older adults, there are numerous unmet health and research challenges that, if addressed, would greatly benefit this community and those who support them. We will examine the unique risks and challenges LGBTQIA+ older adults face related to neurodegenerative disorders, including Alzheimer's disease and related dementias and Parkinson's disease. Drawing on lessons learned from our national research studies with LGBTQIA+ older adults, we will discuss key insights and critical gaps in knowledge and propose steps to ensure future research efforts and how to support emerging scholars in this field. This includes describing the current challenges in continuing this important research given today's sociopolitical climate. We will also highlight the importance of collecting sexual orientation and gender identity (SOGI) data to ensure LGBTQIA+ aging communities are represented. In light of today's sociopolitical climate, it's more important than ever to advocate for inclusion of and to stand up for LGBTQIA+ aging communities and advocate for this critical research.

### SESSION 2555 (SYMPOSIUM)

Abstract citation ID: igaf122.1096

**ESPO & BUTLER-WILLIAMS SYMPOSIUM: CAREER DEVELOPMENT TO PROMOTE INNOVATIONS FROM BENCH TO BEDSIDE**

Chair: Mirella Diaz-Santos
Co-Chair: Brianna Morgan
Discussant: Ramesh Vemuri

The NIA's Butler-Williams Scholars Program and GSA's ESPO section are united in providing career development opportunities to promote leadership and innovation. This year's theme challenges our established and emerging scholars to embrace innovations addressing motivators, barriers, and facilitators to translational research with older adults from bench to bedside. Despite representing about 17% of the U.S. population, older adults remain under-represented in most clinical research and trials. This suggests that the current treatments are not fully generalizable to aging populations, and addressing this scientific gap is urgently warranted. GSA's early career professionals and alumni of the prestigious NIA Butler-Williams Scholars Program will address these gaps. In a qualitative study, Dr. Maira Castañeda-Avila will present patient-level and healthcare-level barriers and facilitators in recruiting older adults and individuals with multiple chronic conditions. Dr. Miriam J Rodriguez will extrapolate factors contributing to dementia caregiving experiences among informal caregivers and their impact on clinical trial participation, specifically in a caregiver-targeted intervention. Dr. Mirella Díaz-Santos will present the findings of a primary care champion intervention focused on engaging family medicine providers to recruit older adults and administer a dementia screening toolkit. The featured talks will engage us in expanding our collective innovations in recruitment to advance patient-centered treatments for aging populations. Dr. Benjamin F Miller, PhD will discuss his career journey in translating basic biology of aging into treatments to increase human healthspan, with a focus on proteostatic mechanisms in mitochondria to increase stress resistance for slowing aging.

Abstract citation ID: igaf122.1097

**IMPLEMENTATION OF AN ENGLISH AND SPANISH DEMENTIA SCREENING TOOLKIT IN PRIMARY CARE**

Mirella Diaz-Santos[1], Gabriela Islas Huertas[2], Samantha Shah[2], Satpal Singh Wadhwa[2], Blanca Campos[3], Marjan Dehghanian[3], Michelle Bholat[3], and Timothy Chang[2], *1. University of California, Los Angeles, Los Angeles, California, United States, 2. David Geffen School of Medicine at UCLA, Los Angeles, California, United States, 3. University of California, Los Angeles Department of Family Medicine, Los Angeles, California, United States*

Introduction: Dementia affects over 10% of individuals aged 65 and older, with Black and Hispanic/Latinos experiencing a 1.5-2.0 times higher prevalence than non-Hispanic Whites. Despite the importance of early detection, adoption of dementia screeners in primary care is challenging. This study presents a primary care champion intervention focused on providers' adoption of a brief dementia screening toolkit (DST). Methods: Intervention implemented strategies to assist physicians, and staff in the integration of the electronic DST into their routine clinical care. The DST included a three-item questionnaire and a cognitive assessment (Mini- Cog). Primary care providers recruited patients aged 60+ to complete the DST during their annual wellness visits. We compared providers' adoption of the two DST components, pre-intervention (September 2022-March 2023), and post-primary care champion intervention (April 2023-February 2024). Results: Seven out of 45 providers engaged with the DST pre-intervention, and 39 providers post-intervention. Providers enrolled 781 and 1,329 patients pre-and post-intervention, respectively. Mean age of patients was 70 (range: 60–103). Recruited patients mirrored the clinic's demographics: 80% Non-Hispanic Whites, 10% Asian/Asian Americans and 10% Black/African Americans, and 31% Hispanics. Pre-intervention, 60% completed the questionnaire while 5.3% completed the Mini-Cog. 57.1% and 26.3% completion were obtained post-intervention. Pre-post questionnaire completion analyses showed no significance (OR 1.14, $p = 0.42$). Completed Mini-Cogs significantly increased by 375% (OR 6.6, $p < 0.0001$). Discussion: Results underscore the impact of implementing a primary care champion intervention to improve providers' adoption of dementia screeners with a cognitive assessment into routine clinical care.

Abstract citation ID: igaf122.1098

### RECRUITING FOR QUALITATIVE STUDIES ON CANCER AND MULTIMORBIDITY IN PUERTO RICO

Maira Castañeda-Avila[1], Karen Ortiz-Ortiz[2], and Kate Lapane[1], *1. University of Massachusetts Chan Medical School, Worcester, Massachusetts, United States, 2. University of Puerto Rico Comprehensive Cancer Center, San Juan, Puerto Rico, United States*

Recruiting participants for qualitative research studies involving older adults and individuals with multiple chronic conditions (MCC) can present unique challenges. This abstract describes the recruitment experiences for two qualitative studies—one on monoclonal gammopathy of undetermined significance (MGUS) and the other on colorectal cancer (CRC) in Puerto Rico. The first study focused on exploring patient experiences with MGUS, a precursor to multiple myeloma, and healthcare providers' perspectives on managing this condition. Semi-structured interviews were conducted with 14 patients and 8 healthcare providers. The recruitment process involved addressing patients' uncertainties and concerns about the condition, particularly among older adults. Key themes identified included diagnosis, patient understanding and emotional impact. The second study explored the experiences of CRC patients with MCC in Puerto Rico. Semi-structured interviews were conducted with 9 oncologists and 14 patients. Barriers in care, such as insurance system complexities, limited access to specialized services, and poor communication between patients, caregivers, and providers, were highlighted. A major challenge in this study was recruiting patients who were undergoing cancer treatment. Their ongoing care demands often made participation difficult, as they were overwhelmed by the immediate medical priorities of treatment. Engaging oncologists in interviews and navigating the healthcare infrastructure in Puerto Rico, where access to care is limited, further complicated recruitment efforts. These recruitment experiences underscore the importance of overcoming logistical, social, and emotional barriers to ensure diverse and meaningful participation in qualitative research. The findings highlight the need for tailored approaches to recruitment, particularly for vulnerable populations with complex healthcare needs.

Abstract citation ID: igaf122.1099

### FACTORS CONTRIBUTING TO DEMENTIA CAREGIVING AND CLINICAL TRIAL PARTICIPATION IN AN ETHNICALLY DIVERSE SAMPLE

Miriam Jocelyn Rodriguez[1], Mariana Stavig[1], Bailey Gardner[1], Richard Holden[1], Malaz Boustani[2], Lilian Golzarri-Arroyo[1], and Jordan Hill[1], *1. Indiana University Bloomington, Bloomington, Indiana, United States, 2. Indiana University, Indianapolis, Indiana, United States*

Introduction: Informal caregiving of Alzheimer's disease and related dementias (ADRD) is impacted by various factors including race/ethnicity, socioeconomic status (SES), and care recipient behavioral and psychological symptoms of dementia (BPSD). Caregiver-targeted interventions should consider these factors as well as the impact they have on clinical trial participation. Method: First, we examined recruitment methods in a racially/ethnically diverse sample. The call-to-consent ratio was examined for recruitment occurring via 1) "Cold Call" to patients from a health system registry, and 2) "Trusted Messenger" where research staff called patients referred from a memory care physician. Second, baseline data from a large-scale clinical trial was examined (N = 167) to identify differences among various demographic groups. One-way ANOVAs were conducted to examine differences in caregiver burden, self-efficacy, perceived social support, and care recipient BPSD, and the effects of SES on these variables. The sample consisted of White (n = 121), Black/African American (n = 35) and Hispanic/Latino (n = 11) caregivers. Results: Enrollment rate was 23.88% for participants recruited via the "trusted messenger" method and 1.18% for those recruited from "cold calls" ($z = 15.11$, $p \approx 0.0000$). Hispanic participants reported less self-efficacy in obtaining respite ($p = .018$). No other significant differences across ethnic/racial groups were identified. Education level and employment status were significantly associated with caregiver burden ($p = .017$; $p = .016$), higher household income was associated to perceived social support ($p < .001$) and self-efficacy of controlling upsetting thoughts ($p = .024$). Conclusion: SES significantly impacts the caregiver experience, especially among Hispanics, and recruitment from a trusted source is effective for racial/ethnically diverse participation.

Abstract citation ID: igaf122.1100

### TRANSLATING BASIC BIOLOGY OF AGING INTO TREATMENTS TO INCREASE HUMAN HEALTHSPAN

Benjamin Miller, *Oklahoma Medical Research Foundation, Oklahoma City, Oklahoma, United States*

The focus of the Miller lab is to translate basic biology of aging into treatments to increase human healthspan. We focus on proteostatic mechanisms in mitochondria to increase stress resistance for slowing aging. These studies concentrate primarily on skeletal muscle to minimize age-associated sarcopenia. We use stable isotope approaches that are amenable to both pre-clinical models and human subjects as a primary means to translate findings from the basic biology of aging to clinical implementation. Particularly useful has been the isotope D2O that we use to measure synthetic processes of protein, DNA, and RNA to answer basic and translational questions in relevant aging models including cells, yeast, C. elegans, rodents, canines, and humans. In this session, I will discuss how I sought training to allow me to perform translational work and the skillsets needed to be able to bridge pre-clinical to clinical work. I will also discuss how our clinical work informs our more mechanistic experiments as "reverse-translation". Finally, I will present the challenges we have faced while performing work along the translational spectrum, and strategies we have used to successfully recruit and retain subjects in studies that require tissue sampling.

### SESSION 2557 (BIOLOGICAL SCIENCES INVITED SYMPOSIUM)

Abstract citation ID: igaf122.1101

### GEROSCIENCE INTERVENTIONS REIMAGINED

Chair: Nicolas Musi

Abstract citation ID: igaf122.1102

### CLINICAL TRIALS OF SENOLYTICS IN ALZHEIMER'S DISEASE TREATMENT AND PREVENTION

Miranda Orr, *Washington University in St. Louis, St. Louis, Missouri, United States*

Senolytics, agents that selectively eliminate senescent cells, have emerged as a promising geroscience-guided strategy for preventing and treating Alzheimer's disease (AD). In preclinical models, senescent cells accumulate in the aging brain and contribute to neuroinflammation, tau and amyloid pathology, and cognitive decline. Early-phase human studies have begun to evaluate the feasibility, safety, and biological activity of senolytics in both symptomatic and at-risk older adults. Two open-label Phase 1 trials, SToMP-AD and STAMINA, evaluated 12 weeks of intermittent oral dasatinib plus quercetin (D+Q) in older adults. SToMP enrolled 5 participants with symptomatic AD (mean age 72 ± 4 years, 60% female) and assessed central nervous system (CNS) penetrance, safety, CSF and plasma biomarkers, and brain imaging. STAMINA enrolled 12 older adults with mild cognitive impairment (MCI) and slow gait speed (mean age 77 ± 8 years, 58% female) and evaluated changes in cognition, physical function, and blood-based biomarkers. Plasma levels of D and Q increased in all SToMP participants, and D was detected in CSF in 80% (range 0.281–0.536 ng/mL), confirming CNS exposure; Q was not detected in CSF. In SToMP, plasma inflammatory markers—including factors of senescence associated secretory phenotype (SASP)—decreased following treatment, a trend that was similarly observed in STAMINA. In SToMP, CSF levels of IL-6 and GFAP increased, while the CTRA gene expression profile decreased in four of five participants, suggesting downregulation of systemic immune stress. In STAMINA, reductions in plasma TNF-α significantly correlated with improvements in MoCA scores ($r = -0.65$, $p = 0.02$). These Phase 1 studies support the safety, feasibility, and biological activity of senolytic therapy in older adults with or at risk for AD. Plasma-based reductions in inflammation and SASP factors, CNS penetrance of dasatinib, and early signals of cognitive benefit offer a foundation for ongoing clinical development. A Phase 2 randomized controlled trial of D+Q in AD is now underway to further evaluate target engagement and clinical outcomes; interim findings will be presented. Together, these studies contribute to growing momentum in the field to develop senolytics as a novel disease-modifying approach targeting fundamental aging processes in AD.

Abstract citation ID: igaf122.1103

### GH/IGF-1 AXIS AS A GEROTHERAPEUTIC TARGET

Sofiya Milman, *Albert Einstein College of Medicine, Albert Einstein College of Medicine, New York, United States*

While growth hormone/ insulin like growth factor-1 (GH/IGF-1) is a well-documented modulator of aging and longevity in model organisms, its role in human aging is less established. Growing epidemiologic and genetic evidence from general population and exceptional longevity cohorts is pointing to attenuated GH/IGF-1 signaling as a mediator of healthy aging and protection against age-related diseases, including cancer, diabetes, dementia, and cardiovascular disease. This evidence has led to preclinical trials of newly developed and repurposed therapeutics that diminish GH/IGF-1 signaling, with the potential to delay aging and age-related diseases in people.

Abstract citation ID: igaf122.1104

### METFORMIN TO TARGET FRAILTY IN OLDER ADULTS

Sara Espinoza and Nicolas Musi, *Cedars-Sinai Medical Center, Los Angeles, California, United States*

It has been postulated that metformin may extend healthspan, but no long-term trials have been done in humans. We conducted a randomized, double-blinded, placebo-controlled clinical trial of metformin to reduce frailty in older adults (≥65 years) with glucose intolerance. Participants who were frail at baseline (Fried criteria) were excluded. Participants were randomized to 24 months of metformin (initiated at 500 mg/day and titrated to maximum tolerated dose up to 2,000 mg/day) or matching placebo. All participants received one session of diet and exercise counseling prior to initiation of study drug. The primary outcome was frailty as measured by the frailty index based on deficit accumulation model and frailty phenotype based on Fried criteria, which was assessed every 6 months. We applied generalized estimating equations (GEE) models to examine the change in frailty per month by frailty index and Fried criteria. 145 participants were randomized; intention to treat analysis included 141 participants who took at least one dose of study drug. At baseline, participants were 48% female, 35% Hispanic/Latino, and mean age was 71.8 ±5.3 years. Metformin did not cause serious adverse events. Metformin led to a significant reduction in frailty progression rate per month assessed by frailty index compared to placebo (-0.0494 ±0.0216, 95%CI: -0.0918, -0.0071, $p=0.0222$). No difference was observed by Fried criteria (0.0116 ±0.0077, 95%CI: -0.0034, 0.0266, $p=0.1302$). Metformin administration to older adults with glucose intolerance may be useful for frailty prevention and extension of healthspan.

### SESSION 2560 (AWARD LECTURE)

Abstract citation ID: igaf122.1105

### AFAR SCIENTIFIC AWARDS: IRVING WRIGHT AWARD, TERRIE FOX WETLE HEALTH SERVICES AWARD, GEORGE MARTIN MENTORING AWARD

Chair: Steven Austad

The Irving S. Wright Award of Distinction Lecture will feature an address by the 2025 recipient, David B. Allison, PhD. The Terrie Fox Wetle Award lecture will feature an address by the 2025 recipient, Lauren Hunt, PhD, RN, FNP. The George M. Martin Lifetime Achievement in Mentoring Award will also be presented at this event to 2025 recipient, Arlan Richardson, PhD. These awards are given by the American Federation for Aging Research, Inc.

Abstract citation ID: igaf122.1106

### THE QUEST FOR TRUSTWORTHY, PROBATIVE, AND CREATIVE RESEARCH IN GEROSCIENCE

David Allison, *Baylor College of Medicine, Houston, Texas, United States*

In the field overall, in the enjoinders of luminaries and funders such as the Hevolution Foundation, AFAR, the Academy of Healthspan and Longevity Research, the National Academy of Medicine, and the strategic plans and executive orders from the NIH and federal government, there is a consistent theme of the need to increase rigor and trustworthiness in biomedical research in general and in geroscience. We are called upon to increase rapidity of translation and the probative nature of research being conducted in geroscience. In this presentation, I will discuss ways we can make geroscience ever more probative, ever more impactful, build on the tremendous momentum that exists now to promote creative ground-breaking research, and do it all while increasing the rigor and trustworthiness of our science. I will focus both on research training in this area, as well as research funding and execution.

Abstract citation ID: igaf122.1107

### DYING WITH DEMENTIA: IS HOSPICE THE ANSWER?

Lauren Hunt, *University of California, San Francisco, San Francisco, California, United States*

1 in 3 older adults die with or from dementia. The final years of life for older adults with dementia is characterized by high symptom burden, functional decline, and high costs. Hospice is the standard of care for terminally ill individuals and is known to improve end-of-life care quality and reduce costs. In recent years, hospice use has grown dramatically for older adults with dementia, who now comprise 50% of the 1.7 million hospice enrollees annually. Yet hospice was designed to address the needs of cancer patients. Additionally, the rapid growth of older adults with dementia in hospice is intertwined with increasing concerns around misuse of the Medicare Hospice Benefit. This lecture will review the history and current trends in hospice use for older adults with dementia and discuss the role of hospice and alternative care models in addressing end-of-life care needs for people with dementia and their families.

### SESSION 3000 (COMMUNITY ENGAGED SCHOLARSHIP SYMPOSIUM)

Abstract citation ID: igaf122.1108

### RESEARCH TO ACCELERATE STATEWIDE MOVEMENTS ON AGE-FRIENDLY COMMUNITIES

Chair: Emily Greenfield
Discussant: Megan Wolfe

This symposium will showcase the use of various research methods, mobilized through public-private partnership, to accelerate statewide movements on age-friendly communities. The first presentation will highlight the University of California, Davis, Family Caregiving Institute's role as technical assistance providers, evaluators, and thought partners for the California Department of Aging's (CDA) statewide Local Aging and Disability Action Planning (LADAP) grant program. They will describe how both evaluation processes and findings amplified the program, with implications for cross-sectoral partnerships for community aging initiatives nationwide. The second presentation will feature the work of the Center for Social and Demographic Research on Aging at the University of Massachusetts, Boston. They will present how their findings from an environmental scan of age-friendly state work informed the "refresh" of the State Plan on Aging for Massachusetts. The third presentation will provide an overview of university-community partnerships in New Jersey with researchers at the Rutgers School of Social Work Hub for Aging Collaboration. This presentation will demonstrate how developmental evaluation methods applied to a private grantmaking program helped to catalyze the development of a state-supported age-friendly communities program. The final presentation will feature how the University of Minnesota's Center for Healthy Aging and Innovation engaged communities to inform the multisector blueprint on aging as part of the Governor's Council for an Age-Friendly Minnesota. The discussant will address how community-engaged research with partners at the state and local levels can expand network capacity for improving aging societies and accelerate age-friendly social change for both scientific and social impact.

Abstract citation ID: igaf122.1109

### ELEVATING LOCAL VOICES: EVALUATION OF CALIFORNIA'S LOCAL AGING AND DISABILITY ACTION PLANNING GRANT PROGRAM

Elizabeth Bogumil[1], and Pauline Martinez[2], *1. University of California, Riverside, Riverside, California, United States, 2. Betty Irene Moore School of Nursing, University of California, Davis, Davis, California, United States*

The University of California, Davis, Family Caregiving Institute evaluated the California Department of Aging's Local Aging and Disability Action Planning (LADAP) grant program, a $4.5 million initiative funding 21 grantees—city and county governments, nonprofits, and consortia—to develop inclusive, community-driven aging and disability action plans. Aligned with California's Master Plan for Aging (MPA), LADAP prioritizes historically underserved communities. The mixed-methods evaluation, including surveys, document analysis, and interviews, centered community voices, ensuring their perspectives shaped local planning and state policy. Processes like iterative feedback, monthly learning labs, and office hours strengthened grantees' capacity to engage communities in meaningful ways, offering a replicable model for community-engaged scholarship. Grantees tailored strategies to local contexts, engaging cross-sector leaders and fostering partnerships with elected officials, government agencies, Area Agencies on Aging, and community-based organizations. These collaborations expanded outreach and inclusion of diverse demographic and geographic communities. Findings demonstrate how local planning informs state policy and underscore the need for sustained local-state coordination. Key strategies—leveraging trusted community leaders and fostering cross-sector partnerships—enhanced LADAP's reach and impact, ensuring policies are responsive to community needs. These insights have informed program adjustments, advancing health, well-being, and equity objectives within engaged communities. As MPAs gain traction nationwide, LADAP highlights the value of local action plans in driving equitable aging policies. The evaluation's findings and processes provide a scalable model for other states, emphasizing community-centered engagement and iterative learning. This approach strengthens state efforts, ensuring policies are inclusive, sustainable, and rooted in community-driven insights.

Abstract citation ID: igaf122.1110

### ASSESSING THE ROLE OF STATES IN THE AGE-FRIENDLY MOVEMENT: INSIGHTS AND RECOMMENDATIONS FOR POLICY DEVELOPMENT

Claire Wickersham[1], and Caitlin Coyle[2], *1. LeadingAge LTSS Center at UMass Boston, Boston, Massachusetts, United States, 2. University of Massachusetts Boston, Boston, Massachusetts, United States*

States across the U.S. are advancing age-friendly initiatives by joining networks that foster age- and dementia-friendly communities. This presentation examines state-level engagement in the age-friendly movement to inform the "refresh" of the Massachusetts' plan. A content analysis of existing state plans on aging (including age friendly plans, multisector plan, master plans on aging and state unit on aging plans) was conducted to identify key themes, strategies, and trends in age-friendly advancements across states. States were selected for this review based on having either 1) an AARP Age-Friendly State Designation; or 2) a Multisector Plan on Aging (MPA) in development, implementation, or supported by legislation or an executive order. Based on these criteria, 19 states were included: 12 with AARP Age-Friendly designations, 8 with MPAs in development or implementation, and 4 with MPAs supported by legislation or an executive order. Findings highlight diverse approaches to systems change, including legislative actions (e.g., states passing executive orders or statutory requirements for age-friendly planning), policy changes (e.g., integrating age-friendly principles into housing or transportation policy), programmatic endeavors, initiatives, advocacy efforts, and cross-sector partnerships. The research underscores the role of state governments in driving and sustaining age-friendly progress, offering insights into strategies that have successfully impacted older adults over the past 5-10 years. This presentation emphasizes the importance of shared accountability tools, strengthened cross-sector partnerships, and robust policy frameworks in accelerating and sustaining long-term impact.

Abstract citation ID: igaf122.1111

### DEVELOPMENTAL EVALUATION AS A TOOL FOR SPURRING AGE-FRIENDLY COMMUNITIES IN NEW JERSEY AND BEYOND

Natalie Pope[1], Renie Carniol[2], Julia Stoumbos[3], Cathy Rowe[4], and Emily Greenfield[1], *1. Rutgers University, New Brunswick, New Jersey, United States, 2. Grotta Fund for Older Adults, Whippany, New Jersey, United States, 3. The Henry and Marilyn Taub Foundation, Teaneck, New Jersey, United States, 4. New Jersey Advocates for Aging Well, Hamilton, New Jersey, United States*

The number of communities in New Jersey formally committed to age-friendly progress grew from two in 2015 to 27 in 2024. This paper will present a case description of how engaged research helped to spur this growth. We will describe how researchers at Rutgers University helped to catalyze a private philanthropic grantmaking program to seed the development of age-friendly community initiatives across multiple municipalities in a region of the State. The grantmakers also partnered with the researchers to conduct a developmental evaluation of the grantmaking initiative. Developmental evaluation involves studying a program model that is emergent, complex, and not fully defined, with the researchers both evaluating and contributing to the intervention's development over time. The prolonged engagement of the researchers in the initiative leaders' work–including both formal research methods (e.g.,periodic in-depth interviews) and informal background work (e.g., participant observations)–led to creating more regular opportunities to disseminate and translate these insights back to the funders and community leaders. These developments led to the founding of Age-Friendly North Jersey, a community of practice whereby age-friendly leaders meet regularly to work toward shared goals and learn together. Informed by this success and to expand it statewide, the New Jersey Department of Human Services, Division on Aging Services, recently launched a state-supported grantmaking program to similarly invest in age-friendly community planning. Presenters will describe how this "long arch" of engaged research across systems levels is helping to fuel and sustain state and local innovations on aging in New Jersey and beyond.

Abstract citation ID: igaf122.1112

### LEVERAGING COMMUNITY PARTNERSHIPS TO CRAFT MINNESOTA'S MULTISECTOR BLUEPRINT ON AGING

Farah Baig[1], and Rajean Moone[2], *1. Age-Friendly Minnesota Council, St. Paul, Minnesota, United States, 2. University of Minnesota, Minneapolis, Minnesota, United States*

In 2019, the Governor of Minnesota established the Age-Friendly Council to unite stakeholders from state agencies, academia, tribal organizations, and the public. The Council's goals were to enroll the state in the WHO's Network of Age-Friendly Communities and to guide the creation of a Multisector Blueprint for Aging. This paper examines the community-based participatory research methods used to develop the Blueprint, which engaged over 3,500 individuals statewide in meaningful discussions. Qualitative analysis of these conversations identified four key domains: Connected Communities, Emergency Preparedness, Individual Rights and Safety, Optimized Health and Longevity, and Economic Security and Vitality. The paper also explores the collaboration between the University of Minnesota's Center for Healthy Aging and Innovation, the Geriatric Workforce Enhancement Program, and public sector partners in advancing age-friendly initiatives. Additionally, it highlights strategies for engaging diverse geographic and cultural communities through action teams that define priorities, establish measurable outcomes, and implement effective strategies. By integrating voices from multiple sectors and communities through participatory methods, Minnesota's aging initiatives foster innovation and long-term sustainability. The Blueprint serves as a model for other states seeking to create inclusive environments where older adults can thrive through coordinated, evidence-based policies and programs.

### SESSION 3005 (SYMPOSIUM)

Abstract citation ID: igaf122.1113

### DELIVERY OF PSYCHOEDUCATION FOR SUPPORTING BRAIN HEALTH IN AGING: ACROSS PEOPLE, PLACES, AND SPACES

Chair: Emily Trittschuh
Co-Chair: Sarah Punshon
Discussant: Mary Wyman

There is an increased demand for information on how to support brain health in aging. Research has identified a number of healthy lifestyle behaviors and treatable health conditions that are supportive – and might help prevent dementia. Translating research study findings into actionable behavioral change for the layperson is important. This symposium was developed to highlight four health education programs which attempt to address this need, and which do so across different populations, in different settings and for unique combinations of these. AgeWISE is a 12-week highly manualized brain health education program that is rich in content developed for older adults with intact cognitive abilities and high drive to engage. Healthy Aging Project-Brain (HAP-B) is an ambulatory outpatient psychoeducational group that is less strictly manualized and only 6 sessions, developed for older Veterans and successfully deployed both in-person and via clinical video technology. Healthy Aging Project-Brain, Occupational Therapy (HAP-B OT) is designed for implementation with folks in subacute rehab, residential and/or other inpatient settings with a focus on independent sessions that can be tailored to a wider range of cognitive and physical limitations. In the UW Madison and Madison VA rural dementia care telehealth initiative, seminars on healthy brain aging are provided in rural communities to support community building and successful implementation of the telehealth initiative. This symposium will describe the development of these interventions, their evolution to serve different populations while maintaining fidelity, and the ongoing translation and dissemination of the interventions into different healthcare and community environments.

Abstract citation ID: igaf122.1114

### HEALTHY AGING PROJECT - BRAIN (HAP-B): A PSYCHOEDUCATION GROUP FOR DEMENTIA PREVENTION AND AGE-FRIENDLY HEALTHCARE

Emily Trittschuh, *VA Puget Sound Health Care System, Seattle, Washington, United States*

Healthy Aging Project-Brain (HAP-B) is an outpatient clinical psychoeducation offering developed within the Veterans Health

Administration (VHA) to encourage engagement in activities associated with successful aging. HAP-B targets sleep, socialization, physical, and cognitive activity through myth-busting, developing SMART goals, and tracking behavioral change. Study aims: 1) assess feasibility/acceptability in a Veteran population; 2) analyze pre- and post-intervention ratings to examine health and well-being; 3) explore associations between health factors and life satisfaction. We will present findings on the first 60 participants who have a mean age of 70. Results indicate self-reported improvements in life satisfaction, emotional well-being, and energy levels post-intervention. Higher ratings of life satisfaction are associated with lower depressive symptoms, higher emotional well-being, and higher self-efficacy. HAP-B has been offered as an in-person class and also via clinical video technology (CVT) without any appreciable difference in ratings of satisfaction. We did find that completion of pre- and post- QA/QI self-report ratings were significantly lower in the CVT groups, especially during the pandemic. This easily implementable education intervention can result in more positive self-appraisal with encouraging downstream effects. Healthcare providers are well-positioned to utilize classes such as HAP-B to promote patient-centric approaches to brain health.

Abstract citation ID: igaf122.1115

### SUCCESSFUL DISSEMINATION TO ADDITIONAL POPULATIONS THROUGH ADAPTATION OF THE HAP-B CURRICULUM

Sarah Punshon, *William S. Middleton Memorial Veterans' Hospital, Madison, Wisconsin, United States*

Healthy Aging Project-Brain, Occupational Therapy (HAP-B OT) is an adaptation of the original HAP-B group designed to promote health lifestyle behaviors for older persons in subacute rehab, residential and/or other inpatient settings. HAP-B OT includes four standalone sessions that can be tailored to participants with a wide range of physical and cognitive abilities. We will describe the process of adapting HAP-B to create HAP-B OT using the 5-stage group model originally developed by Mildred Ross, OTR. This model was chosen because activities are sequenced intentionally to elicit an organized behavioral response that facilitates learning. Activities for this specific group were selected because they can be tailored in the moment to varying levels of cognitive and physical demands based on the abilities of each unique set of group members. This style of group is more inclusive of the varied physical and cognitive abilities of older adults typically treated in subacute rehab, residential, and/or other inpatient settings. HAP-B OT includes the same 4 healthy aging brain topics as the original HAP-B which are sleep, physical activity, social connection, and cognitive stimulation, and provides opportunities to practice skills learned during group sessions through engagement in meaningful daily living activities and after group sessions through SMART goal setting. Outcomes measured are focused on improving perceived self-efficacy, and include the Perceived Health Competence Scale-2 and the Perceived Stress Scale-4.

Abstract citation ID: igaf122.1116

### HEALTHY BRAIN AGING EDUCATION AS A COMMUNITY ENGAGED RESEARCH STRATEGY WITH A TRIBAL NATION

Mary Wyman[1], Sarah Punshon[2], Jenna Woestman[3], Celina Whitmore[3], Kala Cornelius[4], Danielle Lennon[3], Wesley Martin, Jr.[4], and Steve Barczi[5], *1. UW–Madison and Veterans Affairs, Madison, Wisconsin, United States, 2. William S. Middleton Memorial Veterans' Hospital, Madison, Wisconsin, United States, 3. Veterans Affairs, Madison, Wisconsin, United States, 4. University of Wisconsin–Madison, Madison, Wisconsin, United States, 5. Veterans Health Administration, Madison, Wisconsin, United States*

Due to the effects of longstanding social inequities and historical trauma, Native American (NA) older adults may disproportionately experience barriers to accessing adequate healthcare, home and community-based services, and educational resources to support aging well. Efforts to address these barriers through effective collaborations are critical to support healthy aging and reduce disparities, especially in rural settings. This presentation reports on community education delivered as part of a pilot project to adapt an established telehealth dementia care protocol for implementation in one rural NA community. The project is a collaborative effort between the Tribal healthcare system, Veterans Affairs, and the University of Wisconsin. As a key strategy for community engagement and sustainment of the clinical program, brain health information is provided to older adults and their families, and through a partnership with an intertribal aging organization, wider dissemination within the region is attained. The educational sessions are conducted in-person at larger social events and include presenters both from the Tribal Nation and from external partners. Topics focus on awareness of age-related cognitive changes and lifestyle choices that can maintain or improve brain health, and have included the impacts of brain injury on cognition, healthy lifestyle behaviors that promote aging well, and an overview of available community resources. The authors describe the process of co-designing and co-delivering the educational events with NA community members and report on data from participant surveys demonstrating acceptability and satisfaction.

Abstract citation ID: igaf122.1117

### THE AGING WELL THROUGH INTERACTION AND SCIENTIFIC EDUCATION PROGRAM: AN ADAPTABLE MODEL FOR BRAIN HEALTH EDUCATION

Maureen O'Connor[1], Lauren Moo[2], Malissa Kraft[3], and Jaye McLaren[2], *1. Bedford Veterans Health Care System, Bedford, Massachusetts, United States, 2. VA Bedford Healthcare System, Bedford, Massachusetts, United States, 3. Bedford VA Medical Center, Bedford, Massachusetts, United States*

The role of lifestyle factors such as diet, exercise, and socialization in brain health outcomes has been widely acknowledged. The contribution of attitudes about aging as they specifically relate to brain health outcomes and engagement in lifestyle activities has been less of a focus. The Aging Well through Interaction and Scientific Education (AgeWISE) program is a 12-week, manualized group that provides older adults with psychoeducation about brain aging, lifestyle factors associated with successful brain aging, and strategies to compensate for age-related cognitive change. A randomized controlled pilot study of forty-nine older (mean age=73), mostly Caucasian (98%), cognitively normal male Veterans revealed increases in memory contentment and sense of control over memory decline for AgeWISE participants. AgeWISE-Action Plan (AgeWISE-AP) is an expansion program developed to capitalize on and extend AgeWISE gains in memory controllability. To date, sixty-three older Veterans have been enrolled in AgeWISE-AP as part of an ongoing randomized controlled trial, with excellent (95%) retention in the intervention arm. AgeWISE has been adapted across settings (geriatric inpatient, synchronous telehealth), populations (non-VA, mild cognitive impairment), and cultures (non-US). We will describe AgeWISE and AgeWISE-AP, with a focus on the highly adaptable AgeWISE program, to illustrate how brain health programs can be implemented in clinical settings.

### SESSION 3010 (PAPER)

### CAREGIVING, CULTURE, AND CONTINUITY: EXPLORING TRANSITIONS, NORMS, AND END-OF-LIFE SUPPORT

Abstract citation ID: igaf122.1118

### ADRD CAREGIVER AND CARE RECIPIENT RELATIONSHIP EFFECT ON BURDEN, SELF-EFFICACY, AND PERCEIVED SOCIAL SUPPORT

Mariana Stavig[1], Bailey Gardner[1], Miriam Jocelyn Rodriguez[1], Jordan Hill[1], Lilian Golzarri-Arroyo[1], Roger Zoh[1], Richard Holden[1], and Malaz Boustani[2], *1. Indiana University Bloomington, Bloomington, Indiana, United States, 2. Indiana University, Indianapolis, Indiana, United States*

Introduction: Relationship types between caregiver and care recipient with Alzheimer's disease or related dementia (ADRD) effects the caregiving experience. For example, adult child caregivers often juggle caregiving in addition to full-time employment and families of their own, increasing burden. Spouse caregivers report lower levels of social support and increased burden. Caregivers who live with the care recipient report higher rates of burden. The nature of these relationships is important to consider when examining these outcomes. Methods: Data collected at baseline from participants enrolled in a larger trial (n = 167) were analyzed. Linear regressions examined associations between the caregiver's relationship to the care recipient and caregiver burden, perceived social support, and self-efficacy. Relationship to the care recipient was categorized into four groups: spouse, child, other family members (e.g., grandchildren, children-in-law), and other (e.g., more complex spousal relationships). Results: There were no overall significant differences between a caregiver's relationship and outcome variables. Pairwise comparisons revealed that adult child caregivers and spouses reported marginally lower perceived social support than other family members (p = 0.064 and p = 0.037, respectively). Adult child caregivers had higher rates of self-efficacy in obtaining respite and responding to disruptive behaviors than did spouses (p = 0.032 and 0.014, respectively). Discussion: Our results identified marginal differences in perceived social support, which align with prior literature and suggest that caregivers in different relational roles experience caregiving in unique ways. Further research is needed to better understand how these dynamics influence caregiving outcomes and to identify targeted interventions that can provide more effective support.

Abstract citation ID: igaf122.1119

### CAREGIVING NETWORK CHANGES BEFORE AND AFTER A CARE RECIPIENT'S TRANSITION TO RESIDENTIAL CARE

Natasha Nemmers[1], Lu Qin[2], HwaJung Choi[2], Wenhua Lai[1], and Amanda Leggett[1], *1. Wayne State University, Detroit, Michigan, United States, 2. University of Michigan, Ann Arbor, Michigan, United States*

This study examines the changes in caregiver network characteristics before and after an older adult transitions from community living to residential care. Although this transition is often assumed to reduce family involvement, evidence suggests that family caregivers remain actively engaged, adapting their roles to address evolving care demands. Using the National Health and Aging Trends Study (2011-2019), our sample included 7,197 Medicare-eligible older adults living in the community at baseline, 3,360 of whom later transitioned to residential care. We employed survey-weighted linear and multinomial logistic regressions to assess changes in caregiver network size, relationship types, and care task allocation, adjusting for demographic and health-related factors. Caregiver network size and shared responsibilities for medical, self-care/mobility, and household tasks gradually increased in the years preceding residential care admission, peaking one year before the transition, followed by a slight decline. A modest increase reemerged a few years post-admission. In contrast, shared transportation tasks remained relatively stable pre- and post-admission. Key caregivers, particularly daughters and other immediate family members, remained highly involved even after admission. The use of paid caregivers gradually increased over time, while non-family caregivers (e.g., neighbors, friends) declined immediately following admission but became involved again years later. The increase in caregiving support likely mirrors declining health or reduced independence in managing daily tasks. Family caregivers remain central, complementing the growing role of paid professionals who often handle more complex tasks. These findings highlight the need for structured care coordination and policies that acknowledge family caregivers' enduring contributions during this pivotal transition.

Abstract citation ID: igaf122.1120

### THE MENTAL HEALTH IMPLICATIONS OF INFORMAL CARE RECEIPT STABILITY

Yezhen Li, Marco Angrisani, and Jinkook Lee, *University of Southern California, Los Angeles, California, United States*

Scholars have shown a growing interest in how informal care receipt shapes older adults' mental health outcomes. However, little is known about the longitudinal dynamics of informal care receipt - specifically, the stability of informal care - and their contributions to care recipients' psychological well-being. Using data from the 2010-2018 waves of the Health and Retirement Study (n = 4,160 respondents; 8,332 person-year observations), this study employs mixed-effect regressions to investigate how informal care receipt stability predicts depressive symptoms among older adults with persistent care needs, and how it buffers the stress from activities of daily living (ADL) and instrumental activities of daily living (IADL) limitations. Results suggest that receiving stable informal care, including persistent care (b = -.345; 95% CI = -.481, -.209) and partial loss of informal care sources (b = -.320; 95% CI = -.515, -.125), is significantly associated with lower depressive symptoms than not receiving informal care. Stable informal care receipt also weakens the association between IADL limitations and depressive symptoms through moderation effects, suggesting a stress-buffering pattern, though it does not play a similar role against ADL limitations. In contrast, unstable informal care receipt, including total care loss and caregiver transition, is not associated with any mental health benefits. The findings imply that informal care stability may serve as a measure of care quality through a longitudinal lens, and policies encouraging caregiving commitments may be crucial for the mental health of long-term care recipients.

Abstract citation ID: igaf122.1121

### HOSPICE USE AND SPIRITUAL SUPPORT FOR FAMILY AND FRIEND CAREGIVERS IN END OF LIFE AND BEREAVEMENT

Allison Kirkegaard, and Denise Quigley, *RAND Corporation, Santa Monica, California, United States*

As family and friend caregivers navigate the time approaching and following the care recipient's death, they may benefit from spiritual support. Though access to spiritual support for end of life and bereavement is affected by whether the care recipient receives hospice, which typically includes access to resources such as chaplains and grief support, this connection is underexplored. We use data from 315 recently bereaved caregivers in the 2023 wave of the Care Network Connections over Time (CNCT) study, a nationally representative longitudinal survey of family and friend caregivers helping adults 50 and older, to explore hospice use among care recipients and its relationship to spiritual support among caregivers. Among the 315 caregiving dyads, 60% of care recipients were on hospice at the time of death, with hospice use significantly predicted by rural residence ($OR = 0.35$, $p = 0.04$) and non-White and/or Hispanic race/ethnicity ($OR = 0.40$, $p = 0.04$). Controlling for sociodemographic and contextual characteristics, caregivers of care recipients on hospice were more likely than caregivers of care recipients not on hospice to both want ($OR = 2.66$, $p = 0.02$) and receive ($OR = 2.57$, $p = 0.04$) spiritual support before the care recipient's death. The type of spiritual support received varied by the caregiver's religious affiliation. During bereavement, there were no significant associations between care recipients' hospice use and caregivers' desire for or receipt of spiritual support. These findings suggest that hospice is an important determinant of caregivers' spiritual support, a key resource for promoting wellbeing, at the end of care recipients' lives.

Abstract citation ID: igaf122.1122

### THE JAPANESE VIEW OF "SUCCESSFUL AGING": THE IMPACT OF FAMILY NORMS ON THE DESIRE TO LIVE LONG

Saori Yasumoto[1], Junya Tsutsui[2], and Keiko Tanaka[3], *1. Osaka University, Osaka, Osaka, Japan, 2. Ritsumeikan University, Kyoto, Kyoto, Japan, 3. Meiji Gakuin University, MInato, Tokyo, Japan*

We aimed to understand how people's beliefs and expectations about family norms influence their desire to live long in Japan. Japan is the world's leading super-aged society, with the highest ratio of centenarians per 10,000 people; however, researchers report that fewer older people wish to live longer than those who do not. The most common reason for unwillingness to live long is the perceived burdensomeness of old age on their family. Such negative views on aging affect their physical and psychological health; therefore, the factors for such thoughts should be identified to reconsider it. The data used for the analysis came from 2475 people aged between 50 and 79 living in Japan and were derived from a nationwide random sample survey conducted in 2024. We used ordered logistic regression and tried to find a model that fits the data by applying family norms as an explanatory variable and other demographic variables (e.g., gender and marital status). People with a strong desire for longevity tended to have conservative views regarding family norms (e.g., younger family members should take care of older family members). Successful aging has often been defined based on the medical model, which focuses on health and independence (Rowe & Khan, 1997), and the psychological model, which emphasizes the psychological adaptation to losses in aging (Baltes & Baltes, 1990). Our findings introduce a new model of successful aging by demonstrating how culture as a social structure, especially beliefs in family norms, affects how we perceive aging.

### SESSION 3015 (SYMPOSIUM)

Abstract citation ID: igaf122.1123

### NAVIGATING FEEDING DECISIONS IN DEMENTIA CARE: CULTURALLY ADAPTED SUPPORT FOR CHINESE AMERICAN CAREGIVERS

Chair: Yaolin Pei
Co-Chair: Jing Wang
Discussant: Dena Schulman-Green

Feeding difficulties are a common and challenging aspect of advanced dementia care, requiring caregivers to make complex decisions regarding feeding options. Despite strong evidence that tube feeding does not provide clinical benefits for individuals with advanced dementia, Chinese American caregivers often opt for it due to cultural beliefs, family expectations, and healthcare interactions. To better understand the factors influencing these decisions and to develop culturally responsive support, this symposium presents findings from qualitative research involving 21 Chinese American dementia caregivers and 13 healthcare providers. The first paper examines how cultural values, family dynamics, and interactions with healthcare providers shape feeding decisions among Chinese American caregivers. The second paper explores factors that may contribute to the selection of careful hand feeding for their loved ones with dementia over tube feeding through a case study approach. The third paper delves into the broader sociocultural considerations that influence feeding decisions from healthcare providers' perspective. The fourth paper highlights health professionals' practice in supporting Chinese American dementia caregivers' decision making on feeding options. Finally, the symposium concludes with an overview of the development of a culturally adapted decision aid designed to support Chinese American caregivers in making informed and value-aligned feeding decisions. This symposium provides a comprehensive examination of decision-making in feeding options among Chinese American dementia caregivers, offering critical insights into culturally driven preferences, communication challenges, and the role of healthcare professionals. By addressing these complexities, this session aims to inform culturally responsive interventions to improve the quality of decision-making in dementia care.

Abstract citation ID: igaf122.1124

### DEVELOPMENT OF A CULTURALLY ADAPTED INTERVENTION ON FEEDING OPTIONS FOR CHINESE AMERICAN DEMENTIA CAREGIVERS

Yaolin Pei[1], Jing Wang[2], Xiang Qi[3], Dena Schulman-Green[3], Laura Hanson[4], and Bei Wu[5], *1. The University of Texas at Austin, Austin, Texas, United States, 2. University of New Hampshire, Durham, New Hampshire, United States, 3. New York University, New York, New York, United States, 4. The University of North Carolina at Chapel Hill School of Medicine, Chapel Hill, North Carolina, United States, 5. NYU Shanghai, Shanghai, China (People's Republic)*

Considering Chinese American dementia caregivers' strong preference for tube feeding and use of a family decision-making model regarding end-of-life care, it is crucial to provide culturally adapted decision support. This qualitative descriptive study aimed to inform the cultural adaptation of an existing proven decision aid intervention for Chinese American dementia families. We conducted semi-structured, in-depth interviews with 21 past and current Chinese American dementia caregivers and 13 health care providers (i.e., physicians, social workers, nurses, and speech-language pathologists). Individual interviews were audio-recorded, transcribed, and thematically analyzed. Guided by the Ecological Validity Model, which specifies eight domains of cultural adaptation (language, persons, metaphors, content, concepts, goals, methods, and context), we culturally adapted the decision aid intervention. For example, qualitative findings revealed that family harmony is central to decision-making on feeding options. To address this, the intervention's goal was refined to promote family consensus while improving informed decision-making. In the "content" domain, we add the strategies for family communication and encourage participants to discuss the decision-making on feeding options with other family members. Additionally, misconceptions equating careful handing feeding with starvation were identified. Thus, the decision aid clarifies that individuals with advanced dementia have reduced nutritional needs and distinguished this from starvation. The cultural adaptation of the decision aid intervention serves as a model to align with cultural values, address common misconceptions, and facilitate informed, family-centered decision-making in dementia care. In the next step, we will use cognitive interviews and field test to refine this culturally adapted decision aid intervention.

Abstract citation ID: igaf122.1125

### FAMILY COMMUNICATION AND DECISION-MAKING IN FEEDING OPTIONS FOR CHINESE AMERICAN PERSONS WITH ADVANCED DEMENTIA

Jing Wang[1], Jing Huang[2], Bei Wu[3], Laura Hanson[4], Dena Schulman-Green[5], and Yaolin Pei[6], *1. University of New Hampshire, Durham, New Hampshire, United States, 2. Johns Hopkins University, Baltimore, Maryland, United States, 3. NYU Shanghai, Shanghai, China (People's Republic), 4. The University of North Carolina at Chapel Hill School of Medicine, Chapel Hill, North Carolina, United States, 5. New York University, New York, New York, United States, 6. The University of Texas at Austin, Austin, Texas, United States*

Feeding difficulties commonly arise as dementia progresses to its advanced stages. Although research consistently shows that feeding tubes offer no clinical advantage, they continue to be widely used among Asian populations, particularly within the Chinese American community. This study employed a qualitative descriptive design to examine examines how family communication shaped feeding choices among 21 caregivers of Chinese American individuals with advanced dementia. Using semi-structured interviews, data were analyzed thematically to examine the influence of cultural values, family dynamics, and healthcare interactions in shaping feeding decisions. Findings highlight that feeding decisions are often made collectively, with strong deference to medical professionals and elder family members. Caregivers frequently struggle with navigating differing family perspectives, emotional distress, and decisional uncertainty, particularly in the absence of advance directives. Concerns about nutrition, prolonging life, and filial piety drive many families toward feeding tube insertion, even when caregivers express doubts. External pressures from healthcare providers and cultural expectations can further complicate decision-making. However, open family communication, shared values, and mutual understanding facilitated more cohesive decision-making, allowing families to align feeding choices with the perceived wishes of their loved ones. These findings underscore the critical need for culturally tailored decision-making support, early discussion of feeding preferences, and improved provider-family communication. Strengthening communication pathways within families and between families and healthcare providers can empower caregivers, reduce unnecessary feeding tube use, and enhance end-of-life care experiences for Chinese American families navigating advanced dementia care.

Abstract citation ID: igaf122.1126

### FAMILY-ORIENTED DECISION MAKING ON FEEDING OPTIONS: A CASE STUDY OF CHINESE AMERICAN DEMENTIA CAREGIVERS

Zexi Zhou[1], Jing Wang[2], Jing Huang[3], Dena Schulman-Green[4], Bei Wu[5], Laura Hanson[6], and Yaolin Pei[1], *1. The University of Texas at Austin, Austin, Texas, United States, 2. University of New Hampshire, Durham, New Hampshire, United States, 3. Johns Hopkins University, Baltimore, Maryland, United States, 4. New York University, New York, New York, United States, 5. NYU Shanghai, Shanghai, China (People's Republic), 6. The University of North Carolina at Chapel Hill School of Medicine, Chapel Hill, North Carolina, United States*

Making decisions about feeding options, i.e., hand feeding vs. tube feeding, for older adults with advanced dementia is a complex process shaped by cultural values. Decision-making can be particularly challenging for Chinese American families where caregivers must navigate tensions among medical recommendations, cultural norms, and familial obligations. Using a case study approach, this study explores factors that may contribute to informed and effective decision-making about feeding options for loved ones with dementia. Interviews were conducted with three Chinese American family caregivers who had cared for a parent or parent-in-law with dementia for 4–9 years. All three caregivers and their families selected careful hand feeding for their loved ones with dementia. Demographic information and thematic analyses were used to understand their decision-making process. Findings reveal that efficient intra-family communication contributed to timely and well-supported feeding decisions. Achieving consensus among family members (e.g., siblings, adult children) while maintaining family harmony was pivotal. Additionally, when the person with dementia's feeding preferences were clearly communicated pre-cognitive decline or during lucid moments, decision-making was smoother and more affirmed as compared to proxy decision-making. Access to feeding-related knowledge and effective communication with healthcare providers further facilitated informed and confident decision-making. Findings highlight family orientation as central to decision-making in Chinese American families. Advance care planning with the person with dementia as well as proactive communication with healthcare professionals also play important roles in navigating feeding options. This study supports future culturally sensitive guidance that supports dementia family caregivers to consider unique family dynamics.

Abstract citation ID: igaf122.1127

### SOCIOCULTURAL CONSIDERATIONS IN FEEDING DECISION-MAKING AMONG CHINESE AMERICANS WITH ADVANCED DEMENTIA

Peiyuan Zhang[1], Xiaoyi Zeng[2], Jing Wang[3], Bei Wu[4], Dena Schulman-Green[5], Laura Hanson[6], and Yaolin Pei[2], *1. University of Maryland, Baltimore County, Baltimore, Maryland, United States, 2. The University of Texas at Austin, Austin, Texas, United States, 3. University of New Hampshire, Durham, New Hampshire, United States, 4. NYU Shanghai, Shanghai, China (People's Republic), 5. New York University, New York, New York, United States, 6. The University of North Carolina at Chapel Hill School of Medicine, Chapel Hill, North Carolina, United States*

Feeding problems are part of the natural disease course among people with advanced dementia. Despite strong evidence that feeding tubes provide no clinical benefit, they remain widely used among Asian populations, including Chinese Americans, who have disproportionately high rates of feeding tube insertion. This qualitative study aimed to explore the sociocultural factors shaping the decision-making on feeding options among Chinese Americans with advanced dementia using healthcare providers' perspectives to understand key influences on decision-making. Researchers conducted 13 individual interviews with healthcare providers who had experience caring for Chinese Americans with advanced dementia. The average duration was 60 minutes. Approximately 70% of participants had over 10 years of clinical experience. Interviews were conducted in the preferred language of participants (e.g., mandarin or English), recorded and transcribed. Deductive thematic analysis was conducted to identify key codes and themes and the research team met weekly to resolve any discrepancies. Four major themes emerged: (1) feelings of guilt over forgoing feeding tubes influenced by filial piety; (2) the positive cultural connotation of "dying with a full stomach" in traditional Chinese culture; (3) low uptake of advance care planning due to cultural taboo of discussing death and dying and limited awareness; and (4) family-centered decision-making often overriding patients' preferences. Findings highlight the profound impact of cultural values on feeding decisions for Chinese Americans with advanced dementia. Efforts to promote culturally sensitive education and advance care planning may help support informed decision-making and align care with patients' and families' values.

Abstract citation ID: igaf122.1128

### PROFESSIONALS' PRACTICES TO SUPPORT CHINESE-AMERICAN DEMENTIA CAREGIVERS' DECISION-MAKING ON FEEDING

Xiaoyi Zeng[1], Peiyuan Zhang[2], Jing Wang[3], Dena Schulman-Green[4], Bei Wu[5], Laura Hanson[6], and Yaolin Pei[1], *1. The University of Texas at Austin, Austin, Texas, United States, 2. University of Maryland, Baltimore County, Baltimore, Maryland, United States, 3. University of New Hampshire, Durham, New Hampshire, United States, 4. New York University, New York, New York, United States, 5. NYU Shanghai, Shanghai, China (People's Republic), 6. The University of North Carolina at Chapel Hill School of Medicine, Chapel Hill, North Carolina, United States*

Health and social care providers play a crucial role in shared decision-making with caregivers, families, and older people with dementia regarding care options. However, less is known how these providers support the families of individuals with advanced dementia regarding decision-making on feeding options in Chinese American community. This qualitative study explored professionals' practices in supporting Chinese American dementia caregivers' decision-making on feeding options. We conducted individual, semi-structured interviews with thirteen professionals, with 69.2% having over ten years of professional experiences serving Chinese American older people with advanced dementia and their caregivers. Participants represented diverse disciplines and practice settings. Two bilingual researchers independently coded the transcripts and then jointly resolved discrepancies through iterative discussions. Thematic analysis identified four key professional practices: (1) assessing the patient's condition and family dynamics; (2) providing emotional support to family caregivers while advocating for patients' treatment preferences and goals of care; (3) offering continual and repeated education (e.g., on feeding options, eating management, disease progression, and end-of-life care); and (4) facilitating family communication and interdisciplinary collaboration to support informed decision-making. Decision-making was described as a dynamic, ongoing process shaped by sociocultural factors (e.g., the cultural importance of food) and family dynamics. Professionals also noted key challenges, such as time constraints and lack of advance directives. Findings highlighted the importance of provider-caregiver education, sociocultural considerations, and family-centered decision-making. They also underscored the need for dementia and end-of-life education, early advance care planning, and culturally tailored resources for Chinese American older people with dementia, their caregivers, and families.

### SESSION 3020 (PAPER)

### SOCIAL ISOLATION

Abstract citation ID: igaf122.1129

### EXPLORING THE LINK BETWEEN AGE SEGREGATION AND LONELINESS AMONG OLDER ADULTS

Seulki Kim[1], and Seung-won Emily Choi[2], *1. University of Nebraska-Lincoln, Lincoln, Nebraska, United States, 2. Texas Tech University, Lubbock, Texas, United States*

Residential segregation has been associated with various mental and physical health outcomes; however, the segregation of different age groups has garnered limited attention until recently, and its impact on health remains underexplored. Specifically, there is little knowledge about how living in age-segregated communities influences feelings of loneliness in later life. Drawing on both individual and geographic data from the Health and Retirement Study (2010-2018) and the American Community Survey, this study is among the first to explore the relationship between age segregation and loneliness among older adults, with a particular focus on gender differences. Our analytic sample includes 5,282 adults aged 65 and older in 2010, all of whom resided in the same neighborhoods throughout the study period. Results from hierarchical linear modeling indicate that individuals living in neighborhoods with higher age segregation report lower levels of loneliness. Notably, this protective effect is observed only among older women. The robust link between age segregation and loneliness highlights the importance of considering age-based residential segregation as a key neighborhood factor affecting the mental health in later life. We suggest that interventions aimed at addressing mental health disparities among older adults in age-segregated community settings consider gender differences.

Abstract citation ID: igaf122.1130

**FACTORS CONTRIBUTING TO MALE CHILDLESSNESS FROM A SOCIAL STRUCTURAL PERSPECTIVE**

Wenqian Xu[1], Chunyan Kong[2], and Fang Zhao[2], *1. Lund University, Sweden, 2. Fudan University, Shanghai, Shanghai, China (People's Republic)*

Male childlessness in late adulthood is increasingly prevalent in rural China, raising concerns about later-life care and welfare sustainability. While research has examined individual predictors and macro-level conditions, little is known about how these factors interact within the broader structure-agency context. This study, guided by a life course perspective, explores the structural factors shaping childlessness among older men in a rural area of northern China. Drawing on the life stories of 13 childless older men, the study explores how timing, institutional constraints, and life pathway interdependencies affect marriage and childbearing prospects. Structural disadvantages in early life, such as geographic constraints and education exclusion, restricted marriage opportunities. In early adulthood, the hukou system and weak rural welfare reinforced economic hardship and caregiving burdens. By midlife, these disadvantages were compounded by the urban-rural dual structure and entrenched sociocultural norms, further limiting marital prospects. Findings highlight the critical role of place and time in perpetuating socioeconomic disadvantages, as geographic constraints intersect with broader structural factors to shape lifelong trajectories. Addressing childlessness requires policies that support individuals during key life transitions and advocate for institutional reforms, including improved rural welfare, expanded employment opportunities, and reduced urban-rural disparities, to foster equitable conditions for family formation.

Abstract citation ID: igaf122.1131

**GENDER DIFFERENCES IN KINLESSNESS AND LONELINESS: PSYCHOLOGICAL RESILIENCE AS A MODERATED MEDIATOR**

Jianan Li[1], Sarah Sunghye Kang[1], Shekhar Chauhan[2], and Lené Levy-Storms[3], *1. University of California, Los Angeles, Los Angeles, California, United States, 2. Florida State University, Tallahassee, Florida, United States, 3. UCLA Luskin School of Public Affairs/Social Welfare, Los Angeles, California, United States*

Being kinless risks loneliness in later life due to having fewer close, familial relationships, but less is clear about how this relationship manifests across kinlessness types. Psychological resilience (PR) may buffer against loneliness, offsetting risks associated with kinlessness. Given the long-established relationship differences by gender, the effects of kinlessness, loneliness, and PR may vary among men and women, yet this process remains understudied. Using the 2016 and 2018 Health and Retirement Study, this study examined (1) how PR mediates the relationship between kinlessness types (having a partner and children, having children but no partner, having a partner but no children, having no partner and no children) and loneliness in later life and (2) how this mediated relationship is further moderated by gender (N = 9,384). Adjusted linear regression showed adults without kin had the highest loneliness, followed by those with children but no partner and those with a partner but no children. PR significantly mediated the relationship between kinlessness and loneliness: adults with no kin and those with children but no partner showed lower PR, leading to higher loneliness. This pathway was further moderated by gender: the association between having no kin and loneliness was nearly three times stronger for males than for females. Gender also moderated the relationship between kinlessness and loneliness: while females had similar loneliness trends and levels across kinlessness types, males with no kin experienced the highest levels of loneliness. This study highlights the critical role of a partner in PR and loneliness, especially for males.

Abstract citation ID: igaf122.1132

**IMPACTS OF DIVORCE AND WIDOWHOOD ON DEPRESSIVE SYMPTOMS BEFORE AND DURING THE COVID-19 PANDEMIC**

Erin Bisesti, and Haowei Wang, *Syracuse University, Syracuse, New York, United States*

The landscape of marital dissolution among older adults has been evolving over the last several decades in the U.S., with high and rising divorce rates and increased risk of losing a spouse during the COVID-19 pandemic. Older adults experienced more loneliness following the marital dissolution, leading to potentially greater risk of depression. We used data from the 2016-2020 Health and Retirement Study to examine the relationship between marital dissolution and depression before and during the COVID-19 pandemic. Marital status included remained married, remained divorced, remained widowed, became divorced, and became widowed between two consecutive waves (2016-2018 and 2018-2020). Depression was measured using a cutpoint of 3 or more out of eight CES-D symptoms. Our final sample included 12,432 participants aged 50+. In 2018, 15.35% of participants reported depression compared to 20.90% in 2020. We performed logistic regressions estimating the effect of marital dissolution on depression. We found that, compared to those who remained married, all other categories had higher odds of reporting depression. Specifically, the odds of reporting depression were the highest among those who became widowed (OR = 5.24) or divorced (OR = 4.62) between interview waves. This pattern was consistently found in the pre-pandemic (2016-2018) and during the pandemic (2018-2020). Additional analysis found that remaining divorced during 2020 was less detrimental to depression than remaining married (OR = 0.72). Findings suggest that mental health services should be more readily available to older adults after the loss of a spouse through divorce or widowhood.

Abstract citation ID: igaf122.1133

**PATTERNS OF SOCIAL EXCLUSION AND DEPRESSION LEVELS AMONG OLDER ADULTS IN SOUTH KOREA**

Saemi Moon, Yeonjung Lee, Sulki Chung, and Kyoungjae Park, *Chung-Ang University, Seoul, Republic of Korea*

This study examines the relationship between patterns of social exclusion and depression levels among older adults in South Korea, where rapid population aging coincides with high rates of poverty and suicide among people aged 65 and older. Older adults exhibit heterogeneous socioeconomic and health profiles, contributing to the distinct patterns of social exclusion. Social exclusion is significantly associated with individual well-being and societal cohesion. Thus, understanding complex interactions between the domains of exclusion is crucial. To examine these complex interactions, using data from the Korean Longitudinal Study of Aging in 2022, we analyzed a sample of 4,480 individuals who are aged 65 Latent Class Analysis identified four distinct patterns of social exclusion as followings: Income Exclusion; Social Relationship-Income Exclusion; Income-Health Exclusion; Multiple Exclusion. Results show that Income Exclusion was prevalent across all patterns, suggesting widespread and structural income-related challenges among older Korean adults. Additionally, people who aged 80 and older, with elementary education or less, and living alone were disproportionately represented in Multiple Exclusion group. Interestingly, urban residents showed higher levels of Social Relationship-Income Exclusion than rural residents, suggesting that urban environments may intensify social isolation despite greater service availability. Regarding the relationship between the social exclusion and depression, individuals who experience Multiple Exclusion show substantially higher depression than those with Income Exclusion. Findings demonstrate that exclusion across multiple domains significantly worsens mental health outcomes compared to single-domain exclusion, highlighting the importance of comprehensive approaches that address the interconnected nature of social exclusion dimensions rather than isolated interventions.

Abstract citation ID: igaf122.1134

### SOCIAL DEPRIVATION AND CONSUMER-REPORTED UNMET SERVICE NEEDS IN HOME- AND COMMUNITY-BASED SERVICES

Tetyana Shippee[1], Romil Parikh[2], Nicholas Musinguzi[1], Benjamin Langworthy[2], Jack Wolf[2], Stephanie Giordano[3], and Eric Jutkowitz[1], *1. University of Minnesota, Minneapolis, Minnesota, United States, 2. University of Minnesota School of Public Health, Minneapolis, Minnesota, United States, 3. Human Services Research Institute, Cambridge, Massachusetts, United States*

Greater social needs, such as limited transportation, are linked to poorer outcomes in Medicaid home- and community-based services (HCBS). However, their relationship with consumer-reported unmet HCBS needs–a key measure of quality– remains unclear. Understanding this relationship is critical for identifying barriers to service access and informing policy interventions. To address this gap, we examined the association between the Social Deprivation Index (SDI; range: 1-100) and unmet HCBS needs for personal care and homemaker services, considering both individual- and area-level factors. The SDI is a composite measure of seven area-level socioeconomic indicators from the U.S. Census Bureau, including poverty, rental housing, overcrowding, single-parent households, car access, education level, and unemployment. Our study included 6,558 community-dwelling adults aged 65 and older from the 2022-2023 National Core Indicators–Aging & Disability Adult Consumer Survey. Using logistic regression, we adjusted for individual-level sociodemographic and health factors. In all regression models, SDI score was not meaningfully associated with unmet needs for HCBS for personal care and homemaker chores ($p > 0.05$). However, individuals with better self-rated health had significantly lower odds of reporting unmet needs (odds ratios: 0.19-0.61; $p < 0.001$). Similarly, those living with family or friends (versus alone) had lower odds of unmet needs (odds ratios: 0.40-0.70; $p < 0.05$). These findings highlight the importance of person-level factors in HCBS access rather than area-level deprivation. Future research should investigate whether place-based factors predict the geographic distribution of unmet HCBS needs and other HCBS outcomes at the zip code level.

### SESSION 3025 (SYMPOSIUM)

Abstract citation ID: igaf122.1135

### EMPOWERING CAREGIVERS: INNOVATIVE TECHNOLOGY FOR SMARTER, MORE CONNECTED CARE

Chair: Walter Boot
Co-Chair: Edie Sanders

Caregivers play a critical role in supporting older adults, yet they often face significant emotional, physical, and logistical challenges. This symposium presents innovative research on technology-based interventions designed to empower caregivers and optimize care delivery. Dr. Sara Czaja will provide an overview of digital solutions for family caregivers, discussing mobile applications, web-based platforms, and sensing systems and share findings on the feasibility, acceptability, and efficacy of these tools in improving caregiver outcomes. Taekyung Kim MS will present research on the adoption of AI-assisted care technologies among formal care providers, highlighting key factors such as social influence and effort expectancy that shape technology acceptance and use in real-world caregiving environments. Dr. Felipe Jain will discuss findings from an 8-week randomized controlled trial examining the impact of mentalizing imagery therapy (MIT) delivered via a mobile app on daily happiness and stress in family caregivers of people living with dementia. Dr. Xin Yao Lin will share results from a scoping review of app-based interventions for caregivers, identifying common design features, benefits, and research gaps that must be addressed to improve intervention effectiveness. Finally, Dr. Katherine Carroll Britt will present qualitative findings from caregivers and dementia care experts to inform the development of an AI chatbot designed to support cognitive care planning for individuals living with dementia and their care partners. By showcasing these diverse approaches, this symposium will explore the potential of digital innovations to create smarter, more connected caregiving solutions that enhance well-being and care quality for both caregivers and older adults.

Abstract citation ID: igaf122.1136

### THE POTENTIAL OF DIGITAL SOLUTIONS FOR FAMILY CAREGIVERS OF OLDER ADULTS

Sara Czaja, *Weill Cornell Medicine, New York, New York, United States*

Informal caregivers, such as family members provide the bulk of support to older adults with a chronic condition such as Alzheimer's Disease or a Related Dementia (ADRD) and often do so at considerable cost to themselves. The roles of family caregivers are complex and range from assistance with daily activities and providing direct care to the care recipient to navigating complex health care and social services systems. As such, numerous studies have documented that ADRD caregiving has deleterious impacts on caregiver health and well-being. Unfortunately, for a variety of reasons, many caregivers do not avail themselves of existing services and programs and few evidenced-based programs are implemented in community settings. Digital technology solutions such as mobile applications, web-based platforms, sensing systems, and wearables hold promise in terms of enhancing the ability of caregivers to provide care and in reducing caregiver burden and distress. This presentation will discuss the potential of digital technology in providing support for family caregivers. An overview of available technology solutions will be provided with case exemplars. Additionally, data will be presented from on-going research projects regarding the feasibility and acceptability of digitally based solutions such as virtually delivered interventions, which overall show that caregivers are receptive to and able to use technology. The data from these studies also show that technology-based interventions are efficacious and improve caregiver outcomes. Finally, the potential benefits and challenges with these solutions, factors associated with the adoption of technology and recommendations for needed research in this area will also be discussed.

Abstract citation ID: igaf122.1137

### UNDERSTANDING AI-ASSISTED CARE TECHNOLOGY ADOPTION AMONG FORMAL CARE PROVIDERS: INSIGHTS FROM UTAUT

Taekyung Kim, Donghun Kang, Jiwon Park, and Moon Choi, *Korea Advanced Institute of Science & Technology, Daejeon, Republic of Korea*

This study examines the acceptance of four AI-assisted care technologies among formal care providers using the Unified Theory of Acceptance and Use of Technology (UTAUT). The Korean government has launched initiatives to develop and deploy care technologies to support independent living among older adults living alone. However, limited research has explored the intention of formal care providers—including care assistants, social workers, and visiting nurses—to adopt these technologies in delivering social and healthcare services to older adults. A total of 123 formal care providers, primarily care assistants (73.2% aged 50 or older; 92.7% women), participated in an online survey conducted in December 2024. The survey assessed performance expectancy, effort expectancy, social influence, facilitating conditions, and intention to use four technologies: (a) AI Care Call, (b) Emergency Alarm Service, (c) Companion Robots, and (d) Care-Net Platform. Path analysis revealed that technology acceptance varied across technologies. Social influence and effort expectancy were significant positive predictors of intention to use all four technologies, with social influence demonstrating the strongest effect. Performance expectancy was significantly associated only with the Emergency Alarm Service and Care-Net Platform. Given that formal care providers' perceptions and intentions influence the successful deployment and adoption of care technologies among older adults, these findings highlight the critical roles of social influence—the perceived encouragement from significant others—and effort expectancy—the perceived ease of use—in facilitating adoption.

Abstract citation ID: igaf122.1138

### INCREASING DAILY HAPPINESS IN FAMILY CAREGIVERS OF PEOPLE LIVING WITH DEMENTIA WITH A MOBILE APP-BASED PROGRAM

Felipe Jain[1], Paulina Gutierrez-Ramirez[2], Kimberly Dueck[2], Maria Galvez[2], and Liliana Ramirez Gomez[2], *1. Massachusetts General Hospital / Harvard Medical School, Boston, Massachusetts, United States, 2. Massachusetts General Hospital, Boston, Massachusetts, United States*

Family caregivers of people living with dementia are at high risk for depression and stress. Several caregiver interventions have been found to reduce symptoms of depression and stress on pre-to-post outcomes testing, but few have characterized impacts on day-to-day caregiver symptoms. Mentalizing imagery therapy (MIT) is a mindfulness and guided imagery approach that provides tools to relax and improve understanding of others (including the person living with dementia). Using ecological momentary assessments (EMA), we studied the effects of MIT on day-to-day experiences of happiness and stress of family caregivers of people living with dementia. We conducted an 8-week randomized, controlled trial of caregiver skills (CS) training with and without MIT delivered by the MGH CareDoc mobile application platform. Interventions consisted of mobile applications and optional weekly virtual groups with content tailored toward each group. EMA ratings of happiness and overall stress over the prior 24 hours were obtained daily through CareDoc from days 1 to 56. 110 caregivers were randomized ($n = 55$ in each arm). 101 completed the trial (with no difference in attrition between groups). Group X Time analyses found that those assigned to CS with MIT exhibited improvements in daily ratings of happiness ($p<.0001$) and stress ($p<.001$) relative to the CS only arm. In this randomized, controlled trial, MIT with CS exhibited superior effects to CS alone on improving daily happiness and stress. This study contributes needed data on MIT's impacts on day-to-day experiences and is among the first to show improvements in daily symptoms in family caregivers.

Abstract citation ID: igaf122.1139

### APP-BASED INTERVENTION STUDIES FOR FAMILY CAREGIVERS OF OLDER ADULTS: A SCOPING REVIEW

Xin Yao Lin[1], Francesca Falzarano[2], E-Shien Chang[1], Kristin Hon[1], Ruoying Zhang[3], Andy Hickner[1], and Sara Czaja[1], *1. Weill Cornell Medicine, New York, New York, United States, 2. University of Southern California, Los Angeles, California, United States, 3. Boston College, Chestnut Hill, Massachusetts, United States*

As the global population ages, the increasing number of individuals with chronic conditions places a growing burden on family caregivers. Behavioral interventions delivered via app-based interventions, including apps on mobile phones, tablet, or web, have emerged as a powerful tool for enhancing caregiver support. The current study aims to identify and describe app-based interventions for family caregivers of older adults with chronic conditions, focusing on their designs, features, and impact on caregiver outcomes. This review is reported according to the Preferred Reporting Items for Systematic Reviews and Meta-analyses (PRISMA). A search of publications from 2007 through December 20, 2022, was conducted across PsycINFO (EBSCO), MEDLINE ALL (Ovid), Embase (Ovid), Web of Science Core Collection (A&HCI, BKCI-SSH, BKCI-S, CCR-EXPANDED, ESCI, IC, CPCI-SSH, CPCI-S, SCI-EXPANDED, SSCI) (Clarivate), ACM Guide to Computing Literature (ACM Digital Library), and Engineering Village (Elsevier), using relevant keywords. The database search identified 9,482 studies, resulting in 290 full texts and a final 49 studies included. Forty-four unique apps were identified, demonstrating different designs and features. These studies primarily examined the impact of apps on caregiver well-being, burden, and mental health. While the majority of interventions were perceived as beneficial, several design and research limitations were noted. While many interventions demonstrate positive effects on caregiver outcomes, there are significant gaps in research design, evaluation, and reporting. Future studies should prioritize more integration of self-care practices, clarity in intervention components, rigorous adherence to behavioral intervention frameworks, and greater inclusivity in participant samples.

Abstract citation ID: igaf122.1140

### COGNITIVE CARE PLANNING FOR PERSONS LIVING WITH DEMENTIA AND THEIR CARE PARTNERS: CHALLENGES, NEEDS, AND QUESTIONS

Katherine Britt[1], Rebecca Brown[2], Xiao Luo[3], and Bin Huang[4], *1. University of Iowa, Iowa City, Iowa, United States, 2. University of Pennsylvania, Philadelphia, Pennsylvania, United States, 3. Oklahoma State University, Stillwater, Oklahoma, United States, 4. BrainCheck Inc, Austin, Texas, United States*

Dementia care partners face high levels of stress, burden, and health risks related to the complex care demands of Alzheimer's disease and Alzheimer's disease related dementias (AD/ADRD). They also struggle with coordinating care, locating AD/ADRD resources and support, and managing AD/ADRD behavioral symptoms. Cognitive care planning (CCP) is a dementia care resource that provides care recommendations based on AD/ADRD needs assessments. CCP improves quality of life and overall care management, yet care partners lack ongoing support in implementing these CCPs at home. As dementia progresses and care challenges may escalate, ongoing support, resources, and innovative solutions for older adults living with AD/ADRD and care partners are needed. We aim to develop a tailored AI-based chatbot to support dyads by first exploring potential challenges, needs, and questions dyads encounter when implementing their CCPs in daily life. We recruited older adults living at home with mild cognitive impairment or mild AD/ADRD and their care partners (N=20 dyads) and dementia care experts (N=10) from U.S. medical centers and clinics in the Pacific Northwest and the University of Pennsylvania. We conducted semi-structured interviews and will use content analysis to analyze data and report qualitative categories and subcategories denoting common and individualized questions gathered from participants. Findings will be used to inform development of an AI chatbot designed to provide continuous and real-time care support for dyads to improve quality of life, decrease care partner burden, and improve care management.

### SESSION 3030 (SYMPOSIUM)

Abstract citation ID: igaf122.1141

### THE DIGITAL AGE OF AGING: HEALTH THROUGH TELEHEALTH, DIGITAL LITERACY, AND LIFESTYLE INTERVENTIONS

Chair: Su-I Hou

This symposium explores digital health innovations for aging populations, addressing key challenges in accessibility, literacy, and engagement. Through real-world applications and research, we aim to identify best practices for integrating digital technologies into aging care and public health strategies. Drs. Park and Hou (UCF) will analyze telehealth adoption patterns using the 2022 Health Information National Trends Survey (HINTS). Findings reveal significant disparities in access, particularly among older adults, men, and rural populations, underscoring the need for broadband expansion and digital literacy initiatives. Dr. Savela (UEF) will examine digital skills and health literacy gaps among older caregivers using the Survey of Health, Ageing and Retirement in Europe (SHARE). Results highlights socioeconomic drivers of digital inequalities and the need for targeted interventions. Dr. Hou (UCF and UEF) will evaluate digital tools in dementia prevention within WW-FINGERS, the first global network of multidomain lifestyle intervention trials across 70 countries. The integration of digital tools in seven trials underscores their feasibility and the need for tailored strategies across diverse cognitive and social settings. Dr. Dino (UCF) will present findings from the MOVE Project, which leverages mixed reality (MR) to enhance older adults' physical activity. Insights reveal ways to optimize MR-based interventions for engagement and usability. Dr. Thiamwong (UCF) will explore how digital health technologies, including wearable sensors and mobile apps, enhance physical function, motivation, and mental health in low-income older adults. Collectively, these studies underscore the potential of digital health to reduce disparities, foster engagement, and promote healthy aging. This is a collaborative symposium between the International Comparisons of Healthy Aging and Technology and Aging Interest Groups.

Abstract citation ID: igaf122.1142

### DIGITAL TOOLS AND LIFESTYLE-BASED DEMENTIA RISK REDUCTION: LESSONS FROM WORLD-WIDE FINGERS

Su-I Hou[1], Parthenia Giannakopoulou[2], Miia Kivipelto[3], Francesca Mangialasche[4], Paola Padilla[5], and Alina Solomon[3], *1. University of Central Florida, Orlando, Florida, United States, 2. Imperial College London, London, England, United Kingdom, 3. Karolinska Institutet, Solna, Stockholms Lan, Sweden, 4. Division of Clinical Geriatrics, Stockholm, Stockholms Lan, Sweden, 5. FINGERS Brain Health Institute, Stockholm, Stockholms Lan, Sweden*

Introduction: The Finnish Geriatric Intervention Study to Prevent Cognitive Impairment and Disability (FINGER) was the first large, long-term randomized controlled trial to show that a two-year multidomain lifestyle intervention improved cognitive function in older adults at risk for dementia. Computer-based cognitive training was a key component. The FINGER model has been globally adapted through the World-Wide FINGERS (WW-FINGERS) network, officially launched in 2017, with growing use of digital tools. Methods: WW-FINGERS, the first global network of multimodal dementia prevention trials, includes research teams from 70 countries. We reviewed trial protocols and published results from the WW-FINGERS Global Scientific Coordinating Center, identifying 20 ongoing and 15 completed trials, including pilot studies. Results: Digital tools have been integrated into seven trials: Maintain Your Brain (MYB, Australia), FINGER (Finland), MIND-ADmini (multi-country), AgeWell.de (Germany), J-MINT (Japan), MIND-CHINA (China), and SUPERBRAIN (South Korea). Nineteen published protocols and results were analyzed. Study populations ranged from dementia-free older adults with risk factors or mild cognitive impairment. Digital tools were primarily used for cognitive training via computer programs, web platforms, or tablet applications. Some trials used wearable devices to monitor physical activity. However, discussion on digital tool implementation was limited. MYB was the first fully internet-based WW-FINGERS study, demonstrating online lifestyle interventions can improve cognition and health outcomes. Discussion: Digital tools are feasible in dementia prevention interventions across diverse populations. Their adoption has increased since the original FINGER study started in 2009. Selection should consider participants' cognitive abilities, and social engagement components may enhance usability.

Abstract citation ID: igaf122.1143
### BRIDGING THE DIGITAL DIVIDE: FACTORS AFFECTING ASYNCHRONOUS AND SYNCHRONOUS TELEHEALTH USE
Su-I Hou, and Jae Park, *University of Central Florida, Orlando, Florida, United States*

Introduction: Telehealth has transformed healthcare, offering synchronous (real-time video/phone) and asynchronous (patient portal) options. This study examines factors influencing telehealth adoption, particularly age-related differences. Methods: Data from the 2022 Health Information National Trends Survey (HINTS) 6 were analyzed, assessing asynchronous (patient portals) and synchronous (telehealth visits) use. Key variables included demographics, tech-savviness, and healthcare behaviors, with age groups categorized as young (18-49), middle-aged (50-64), and old (65+). Stepwise regression models identified significant factors. Results: Among 5,873 participants (35.3% young, 29.0% middle-aged, 35.7% old), 61.6% used asynchronous and 41.9% used synchronous telehealth. Males were less likely to use telehealth ($OR = 0.66$, $p < 0.001$), but older males favored synchronous use ($OR = 1.55$, $p = 0.002$). Hispanics preferred synchronous ($OR = 1.42$, $p < 0.001$) but used asynchronous less ($\beta$ = -0.35, $p < 0.001$). Rural/smaller urban areas had lower use (OR range: 0.63–0.78, $p < 0.001$). Tech-savviness was linked to telehealth use, especially asynchronous (e.g., internet use: $\beta = 0.53$, $p < 0.001$). Online survey use was associated with asynchronous telehealth, particularly for middle-aged ($\beta = 0.20$, $p = 0.024$) and older adults ($\beta = 0.24$, $p = 0.006$). Frequent healthcare users were more likely to use both ($OR = 1.28$, $p < 0.001$; $\beta = 0.28$, $p < 0.001$), but older adults were less likely to use asynchronous telehealth ($\beta$ = -1.21, $p < 0.001$). Discussion: Age, gender, ethnicity, location, and tech-savviness impact telehealth adoption. Addressing disparities requires broadband expansion, digital literacy programs, and age-friendly telehealth models.

Abstract citation ID: igaf122.1144
### SOCIAL INEQUALITIES IN INTERNET USE, DIGITAL SKILLS, AND HEALTH LITERACY AMONG OLDER CAREGIVERS
Roosa-Maria Savela[1], Tarja Välimäki[2], and Su-I Hou[3], *1. University of Turku, Turku, Varsinais-Suomi, Finland, 2. University of Eastern Finland, Kuopio, Pohjois-Savo, Finland, 3. University of Central Florida, Orlando, Florida, United States*

Introduction: Little is known about social inequalities in older adults' digital skills and Internet use, which may also interact with health literacy. Methods: The issue is assessed with the ninth wave of the quantitative cross-sectional panel data from the Survey of Health, Ageing and Retirement in Europe (SHARE). Data were analyzed using a Chi-squared test, and regression models were adjusted for demographic factors focusing on inequalities in Internet use, digital skills, and health literacy among older adults with and without informal care tasks. Results: Informal caregivers reported lower digital skills (63% vs. 56%, $p < 0.001$) and were less likely to use the Internet in the past week (40% vs. 35%, $p < 0.001$) than non-caregivers. Informal caregivers were also more likely to constantly than never need help interpreting written instructions from healthcare (Odds ratio [OR]=2.03, 95% Confidence Interval [CI]=1.12 to 3.67), indicating lower health literacy than non-caregivers. However, older adults who were easily making ends meet reported higher health literacy (93% vs. 65%, $p < 0.001$) or more likely good to excellent digital skills (56% vs .19% $p < 0.001$) than those who were less financially secure. Conversely, older adults who were easily making ends meet (OR 2.06, 95% CI 1.75 to 2.43) and those with high education levels (OR 3.04, 95% CI 2.72 to 3.40) were most likely to use the Internet in the past week. Discussion: Higher socioeconomic and non-caregiver status may predict better outcomes in Internet use, digital skills, and health literacy, emphasizing the need to address social ine qualities.

Abstract citation ID: igaf122.1145
### HUMAN-COMPUTER INTERACTION AMONG OLDER ADULTS IN A MIXED-REALITY TECHNOLOGY-BASED EXERCISE PROGRAM
Michael Dino[1], Ladda Thiamwong[2], and Rui Xie[2], *1. University of Central Florida, University of Central Florida, Florida, United States, 2. University of Central Florida, Orlando, Florida, United States*

Maintaining mobility becomes increasingly critical for community-dwelling older adults' overall health and independence. Recognizing this challenge, scholars and healthcare providers are gradually adopting technological innovations, such as mixed reality (MR), to support older adults' physical activity and wellness. However, emerging technologies are prone to disregard due to a lack of studies supporting their adoption in practice. More importantly, studies that examine human-computer interaction between mixed reality devices for health and older adult users remain scarce. To fill this gap, a multidisciplinary team created the Mixed Reality-Oriented Virtual Coach Experience (MOVE) Project. The program highlights the utilization of a developed "virtual coach" projected via head-mounted devices. Using principles of human-centered design in technology development, the research team examined how older adults interact with the virtual agent for technology refinement and pilot. A cognitive walkthrough design was employed to capture interaction data (e.g., gaze, fixation) from a pilot cohort of six consented community older adults. The participants were instructed to wear Tobii Pro Glasses 3™ while interacting with the virtual coach projected on a large screen. Heatmaps were generated and analyzed to reveal areas of interest (AOIs). Results revealed various user fixation patterns in reference to the virtual coach's movement and posture during physical exercise sessions and some critical insights on human-computer interaction in MR use for physical exercise programs among older adults. The outcomes of the study will be used to refine programming, movement, and physical appearance of the virtual coach to maximize interaction efficiency between the system and the user.

Abstract citation ID: igaf122.1146
### INTEGRATING DIGITAL HEALTH TECHNOLOGIES WITH PHYSICAL AND MENTAL HEALTH FOR MOTIVATING LOW-INCOME OLDER ADULTS
Ladda Thiamwong[1], Chitra Banarjee[1], Kworweinski Lafontant[1], Jethro Raphael Suarez[2], Abigail Tice[1], Dahee Kim[1], Michael Dino[3], and Rui Xie[1], *1. University of Central Florida, Orlando, Florida, United States, 2. University of Central Florida, Florida, United States, 3. University of Central Florida, University of Central Florida, Florida, United States*

Digital health technology can play a transformative role in improving overall well-being and the accuracy of frailty prediction. Digital health interventions enhance physical activity (PA) across various chronic conditions and improve depression. and mental health outcomes. However, limited research indicated that digital health technology may benefit populations experiencing health disparities by enhancing accessibility and engagement, which can reduce adverse outcomes. We examined the associations among digital health technologies, physical function, and depression in low-income older adults aged 60+ using data from our clinical trial study funded by NIH (R01MD018025). Digital health technologies, including sensor-based technologies (Balance Tracking System/BTrackS for static balance (SB), wearable accelerometers (ActiGraph) for physical activity (PA), and a mobile application with Ecological Momentary Assessment (EMA) to measure motivation for PA using a single slider-style question. Physical function was assessed using dynamic balance (Timed Up and Go: TUG) and lower limb strength (Sit-to-Stand: STS). Depression was assessed using Patient Health Questionnaire-9 (PHQ-9), and frailty was measured using the FRAIL questionnaire. We found both measures of physical function (TUG and STS) predicted motivation for PA ($pTUG < 0.001$, $pSTS = 0.054$). We also found that depression significantly predicted motivation for PA ($pPHQ < 0.001$), suggesting that motivation for PA is driven by both physical capabilities and mental health. On the other hand, frailty did not significantly correlate with PA motivation ($p = 0.471$). Integrating digital health technologies that offer reminders and progress tracking can motivate low-income older adults to stay active and enhance physical function and mental health.

### SESSION 3033 (SYMPOSIUM)

Abstract citation ID: igaf122.1147

### GET TO KNOW THE NATIONAL INSTITUTE ON AGING: NIA SESSION FOR EARLY-CAREER RESEARCHERS

Chair: Kenneth Santora
Discussant: Kenneth Santora

The National Institute on Aging (NIA) at the National Institutes of Health, Department of Health and Human Services, supports biomedical and behavioral research with a lifespan focus. NIA research seeks to understand the basic processes of aging, improve prevention and treatment of diseases in later life, and improve the health of older persons, in addition to an emphasis on Alzheimer's disease and related dementias. NIA supports a variety of training and career development opportunities for early-career investigators, including undergraduates, advanced degree students, post-docs, and junior faculty. In this session, we will provide an overview of NIA-funded research, followed by a presentation on funding mechanisms and strategies to consider when applying for extramural grants. Finally, attendees will be able to join any number of breakout group discussions led by extramural NIA training staff and program officials to discuss opportunities and resources specific to various career paths.

Abstract citation ID: igaf122.1148

### HOW TO GET AN NIA GRANT

Kenneth Santora, *National Institute on Aging, NIH, Bethesda, Maryland, United States*

Dr. Santora will provide an overview of the NIA application process, including funding mechanisms and strategies to consider when applying for extramural grants. Training and career development opportunities for early-career investigators at multiple career stages – including undergraduates, advanced degree students, post-docs, and junior faculty – will be featured.

Abstract citation ID: igaf122.1149

### OVERVIEW OF NIA MISSION AND RESEARCH

Richard Hodes, *National Institute on Aging, Bethesda, Maryland, United States*

Dr. Hodes will provide an overview of NIA's structure and mission, in addition to discussing research foci from across the Institute's scientific divisions.

### SESSION 3035 (SYMPOSIUM)

Abstract citation ID: igaf122.1150

### FOSTERING AUTONOMY: EXPLORING INNOVATIVE DEMENTIA CARE ENVIRONMENTS IN FOUR COUNTRIES

Chair: Hilde Verbeek
Co-Chair: Karin Wolf-Ostermann
Discussant: Margaret Calkins

Dementia is one of the major age-related diseases world-wide and challenges not only people living with dementia and their caregivers, but also societies and health care systems as a whole. To better meet the needs of people living with dementia, innovative care environments are being developed worldwide, as part of the wider community. Aiming to strengthen independence and slow down the cognitive decline, efforts concentrate on continuously engaging people living with dementia in activities of daily life, despite a progression of the disease. This international symposium will provide four presentations on innovative dementia care environments in four different countries, which stimulate and support autonomy of older people living with dementia in an active daily life. It examines diverse environmental elements of the care environment, including organizational, social and physical aspects, and what their impact is on residents and their caregivers. The first presentation explores Green Care Farms in the Netherlands, focusing on the impact of organizational environment, in particular culture and staff's task integration, on residents' autonomy. The second presenter discusses results from two Dementia Village models in Canada, exploring the environmental effect on residents' quality of life and staff's care practices. The third presentation examines the impact of the neighborhood-built environment on social health of older residents living with dementia in Germany, using Geographical Information Systen (GIS) analyses. Finally, the last presenter describes the effects of adaptation in the physical environment, i.e. smart, ambient bright light on residents with dementia living in nursing homes in the United States.

Abstract citation ID: igaf122.1151

### RESIDENT WELL-BEING AND STAFF PRACTICES IN DEMENTIA VILLAGES: INSIGHTS FROM TWO CASE STUDIES

Habib Chaudhury[1], Ziying Zhang[1], and Myia Wilhelm[2], *1. Simon Fraser University, Burnaby, British Columbia, Canada, 2. Simon Fraser University, Vancouver, British Columbia, Canada*

Over the last decade, "Dementia Villages" have emerged as an innovative model of long-term care, emphasizing small households and residents' access to amenities and destinations within a community. This study examines the village model's impact through two case studies in British Columbia, Canada: The Views Village and The Village Langley. Using a multi-method approach, we conducted standardized environmental assessments and staff interviews to explore how these environments influence residents' quality of life and staff's care practices. In each village, interviews were conducted with administrative and frontline staff members. Our findings show that the village model fosters resident autonomy and engagement, while requiring staff adaptation to new care approaches. For residents, the community environment offers a variety of activities and destinations, providing greater choice and flexibility in daily engagement. The small household model enhances staff-resident communication, allowing for personalized care. Environmental features like open kitchens, private bedrooms, and homelike décor promote autonomy and a sense of ownership. For staff, interviews revealed both opportunities and challenges associated with the village model. Staff experienced a transition from task-oriented to person-centered care and witnessed the significant benefits of the model for the residents. The new care model introduced higher skill requirements for frontline staff and emphasizes interdisciplinary collaboration, which requires a significant cultural shift and demands adaptation from care staff. This study contributes to the development of evidence-base in the dementia village model, offering practical insights into how innovative environments and care approaches may enhance resident well-being and staff experiences in long-term care settings.

Abstract citation ID: igaf122.1152

### FOSTERING FREEDOM AND AUTONOMY IN EVERYDAY LIFE IN GREEN CARE FARMS

Gijs Steinmann, Hilde Verbeek, Maud Hamers, and Bram de Boer, *Maastricht University, Maastricht, Limburg, Netherlands*

Green Care Farms (GCFs) integrate physical, social, and organizational aspects to promote autonomy and active daily living for people living with dementia, combining care with agricultural activities. While environmental designs have long been recognized as crucial for promoting autonomy and well-being, research has largely overlooked the organizational dimension of care. This study examined how specific organizational aspects of GCFs foster freedom and autonomy in the everyday lives of people with dementia. Ethnographic fieldwork at a Dutch GCF was conducted, including participant observations and informal conversations. Observational data was complemented with semi-structured interviews, focusing on staff and their reasoning behind work-related actions and decisions. The coding process involved open coding of observation reports and interviews, thematic coding to develop a codebook, axial coding informed by organizational theory, and abductive selective coding to refine our interpretation. Results found that the GCF environment triggered the expression of autonomy in three ways: by stimulating physical activity to retain physical functioning; by encouraging participation in meaningful activities; and by enabling residents to enjoy freedoms and risks. Findings indicated a critical role of organizational aspects in enabling autonomy and freedom. Staff mindsets and behavioral norms acted as environmental presses, fostering physical activity and purposeful engagement. Moreover, shared norms and values were central to promote freedom. Other care settings may transfer these principles to their context. Overall, the GCF's organizational culture was characterized by a distinct belief among staff that they did not work in a nursing home—implying that residents did not live in one either.

Abstract citation ID: igaf122.1153

### MAPPING DEMENTIA-FRIENDLY URBAN ENVIRONMENTS IN BREMEN USING GIS: INSIGHTS FROM THE DEN-HB PROJECT

Emily Mena[1], Janissa Altona[2], Christoph Teves[1], Benjamin Schüz[1], and Karin Wolf-Ostermann[1], *1. University of Bremen, Bremen, Bremen, Germany, 2. Institute of Public Health and Nursing Research, Bremen, Bremen, Germany*

Recent advances in dementia research highlight the critical role of the built neighborhood environment in supporting the cognitive and social health of people with dementia (PlwD). This study, part of the Den-HB project, uses Geographic Information Systems (GIS) to analyze urban environments in Bremen, Germany, focusing on neighborhoods with a high proportion of older residents (>65 years). The analysis covers districts, sub-districts, and statistical quarters and examines key indicators such as demography, green and blue spaces, noise pollution, accident hotspots, public transport accessibility, daily shopping, meeting places, and health services. Preliminary results show that multi-level GIS mapping provides crucial insights into the spatial distribution of dementia-friendly factors. For example, while an expanded public transport network can improve mobility for PlwD, it may also contribute to higher noise levels and increased accident risks. GIS enables the visualization of these trade-offs by mapping areas where benefits and challenges coexist. Incorporating thematic fields based on existing evidence allows for systematic evaluation of urban environments, identifying areas with specific needs or exemplary features. This approach highlights zones requiring targeted intervention or serving as models for dementia-friendly planning. By integrating findings from different domains, this research advances understanding of how geospatial technologies can inform evidence-based strategies for inclusive urban development. While further validation is ongoing, these initial findings provide a solid foundation for improving the dementia-friendliness of urban neighborhoods, ultimately supporting the establishment of communities tailored to the unique needs of people living with dementia.

Abstract citation ID: igaf122.1154

### SMART AMBIENT BRIGHT LIGHT FOR NURSING HOME RESIDENTS WITH DEMENTIA: NEUROBEHAVIORAL SYMPTOM IMPACT

Ying-Ling Jao[1], Diane Berish[1], Yo-Jen Liao[1], Julian Wang[1], Marie Boltz[1], Yun-Han Kuan[2], Light Ndurue[1], and Margaret Calkins[3], *1. The Pennsylvania State University, University Park, Pennsylvania, United States, 2. Far Eastern Memorial Hospital, New Taipei City, Taipei, Taiwan (Republic of China), 3. IDEAS Institute, Cleveland Heights, Ohio, United States*

Most nursing home (NH) residents with dementia experience neurobehavioral symptoms. Lighting interventions have shown some positive impact on these symptoms, yet most interventions have not incorporated natural daylight. This study developed the Smart Ambient Bright Light (SABL) and evaluated its effect on neurobehavioral symptoms in nursing home residents with dementia. The SABL is an automatic lighting system that incorporates natural daylight and delivers bright light (high circadian stimulation [CS] and light intensity [lux] levels) during the day and dim light (low CS and lux) during the night. This 13-week crossover, cluster randomized controlled trial enrolled 27 residents with dementia and agitation in two Pennsylvania NHs. Neurobehavioral symptoms were measured biweekly using the Neuropsychiatry Inventory (NPI). Participants' individual lighting exposures (CS and lux) were measured using an individual wearable light sensor clipped on their collar/shoulder all day, 7 days a week, every other week. Participants were 87.4 years old on average and primarily female (74%). Controlling for age, gender, and dementia stage, participants had significant reductions in appetite, depression, and nighttime behaviors during the intervention as compared to control periods. All other neurobehavioral symptoms also showed positive trends but did not reach significance. The lighting effect, based on individual lighting exposure, showed that higher daytime CS and intensity (lux) levels were significantly associated with lower apathy, appetite changes, delusions, and hallucinations. Individual CS levels were also significantly associated with fewer nighttime behaviors. Findings suggest that the SABL intervention has a positive effect on reducing neurobehavioral symptoms in NH residents with dementia.

### SESSION 3040 (PAPER)

### LONG-TERM SERVICES AND SUPPORTS WORKFORCE: NEW ROLES TO SOLVE AN OLD PROBLEM

Abstract citation ID: igaf122.1155

### A HOLISTIC WELL-BEING APPROACH FOR HEALTHCARE STAFF IN LONG-TERM VENTILATION CARE SETTINGS

Sharon Ost-Mor[1], Dikla Segel-Karpas[1], Jonathan Sarig[2], Gizelle Green[3], and Tammi Packer-Porat[3], *1. University of Haifa, Haifa, HaZafon, Israel, 2. State University of Florida, Jacksonville, Florida, United States, 3. Shoham Medical Center, Pardes-Hanna, HaMerkaz, Israel*

"Positive Solitude (PS) is a novel concept that redefines alone time as significant, meaningful, and potentially beneficial for healthcare workers facing stress in ventilation care units. This study examines healthcare professionals in long-term ventilation care settings, exploring the potential benefits of PS interventions in mitigating stress and promoting well-being in both professional and personal contexts. Using a qualitative descriptive research approach, a six-week PS intervention was implemented among 24 multidisciplinary staff members in the ventilation ward, incorporating structured alone time, mindfulness, and reflectiveness. Two groups of 12 participants were recruited from two similar ventilation wards. The intervention aimed to foster self-awareness, resilience, and coping strategies among participants by encouraging intentional and meaningful alone time, with qualitative interviews conducted pre- and post-intervention. Analysis of the interviews revealed themes including improved work-life balance, heightened awareness of PS benefits, and enhanced stress management skills. The intervention group reported significant improvements in using PS skills compared to the control group, though the control group reported increased awareness of their PS in general. This study contributes theoretically to understanding the advantages of PS and provides practical insights for supporting healthcare professionals' well-being and occupational experiences in high-stress environments. The findings highlight the potential of PS interventions in promoting resilience among healthcare staff, with implications for improving both worker well-being and quality of care.

Abstract citation ID: igaf122.1156

### "THEY'RE QUITE TALENTED": NURSING ASSISTANT SCOPE OF PRACTICE IN VHA COMMUNITY LIVING CENTERS

Katherine Kennedy[1], Nathalie Mcintosh[2], Sylvia Haigh[3], David Mohr[4], Hyeyoung Park[5], Eleanor McConnell[6], and Whitney Mills[7], *1. Providence VA Healthcare System, Providence, Rhode Island, United States, 2. THRIVE, Providence VA Medical Center, Providence, Rhode Island, United States, 3. VA Providence Healthcare System, Providence, Rhode Island, United States, 4. Veterans Health Administration, Mason, Ohio, United States, 5. University of Massachusetts Amherst, Amherst, Massachusetts, United States, 6. Durham VA Healthcare System, Durham, North Carolina, United States, 7. Brown University, Providence, Rhode Island, United States*

The U.S. Veterans Health Administration (VHA) operates its skilled nursing homes, known as Community Living Centers (CLCs), using different structures to support nursing assistants (NAs) and nurses' delivery of care to complex patients as compared to community nursing homes. Structures include adequate staffing, safe patient handling programs and equipment access, effective nurse supervision, higher wages with benefits, and the ability to hire and train NAs without certifications but with relevant healthcare experience in response to recruitment/retention challenges. Thus, a pilot study examined NA training aspects within 4 CLCs in the New England region. Seventeen NAs and 13 nurse leaders (i.e., nurse managers, educators, clinical resource nurse, chief nurse) completed individual interviews (July-October 2024), and a team employed qualitative content analysis using a coding frame based in the Paraprofessional Healthcare Institute (PHI) 5 Pillars of Job Quality. Four themes emerged related to NA scope of practice and care quality: (1) NAs learn advanced clinical skills in partnership with nurses (e.g., electrocardiogram, catheter placement and care); (2) Skills acquisition occurs over years of repetition; (3) Skills are context dependent (e.g., hospice, dementia, subacute); and (4) Insufficient standardization, tailored training, and training resources negatively impact care delivery. In VHA CLCs with nursing oversight and sufficient resources, NA scope of practice appears much greater than in community nursing homes. Research-practice partnerships to design and test learner-centered education using evidence-based models and technologies could benefit current and new NAs by anticipating skills needed with a new admission or using dementia care scenarios with microlearning.

Abstract citation ID: igaf122.1157

### CULTIVATING FUTURE ADVOCATES: TRAINING COLLEGE STUDENTS TO BECOME LONG-TERM CARE OMBUDSMEN

Corinne Kyriacou, and Craig Rustici, *Hofstra University, Hempstead, New York, United States*

Long-term care ombudsmen play a crucial role in advocating for vulnerable populations, mediating conflicts, and facilitating communication among residents, families, and facility staff. However, there is a shortage of volunteers trained to serve in this role. To address this long-term care need, a pilot program was launched as a collaborative initiative between a university and a state department of aging. The goals of this initiative were to develop a pipeline of certified long-term care ombudsmen, and to enrich undergraduate education by providing hands-on training in issues related to aging, disabilities, long-term care, advocacy, and conflict resolution. A committee of faculty and representatives from the state worked together over the course of nine months to develop a strategy and assess student interest. This spring, twenty undergraduate students from diverse majors, and two continuing education students enrolled in a special topics course that integrated the ombudsman training curriculum – inclusive of in-field shadowing – with a broader educational foundation in aging and disability studies, long-term care and medical communication. Preliminary findings from pre/post surveys and journal assignments indicate that students are motivated by the idea of empowering vulnerable populations, that they have limited knowledge of long-term care, and that they are interested in developing advocacy skills. Preliminary findings from stakeholder interviews with faculty and state officials show support for the pilot as a model to fill gaps in the long-term care in two ways – directly, by increasing supply of ombudsmen and indirectly, by increasing awareness of aging and disability-related career paths.

Abstract citation ID: igaf122.1158

### INFECTION PREVENTIONIST WORK PROFILES AND ORGANIZATIONAL CAPACITY FOR POLICY COMPLIANCE IN GEORGIA NURSING HOMES

Yu Jin Kang[1], Jacqueline Jordan[1], Monika Pogorzelska-Maziarz[2], Jennifer Morgan[1], and Dawn Aycock[1], *1. Georgia State University, Atlanta, Georgia, United States, 2. Villanova University, Villanova, Pennsylvania, United States*

Objective: Infection Preventionists (IPs) play a critical role in ensuring compliance with infection prevention and control (IPC) policies in nursing homes (NHs), yet workforce stability remains a challenge. This pilot study explored IP work profiles and organizational capacity for IPC policy compliance in Georgia NHs. Methods: A multi-method approach was used to survey IPs and conduct interviews with NH administrators (Feb-Dec, 2024). Recruitment was conducted through the Georgia Health Care Association, via phone and email, using snowball and purposive sampling. Work profiles were assessed via published survey tools, while facilitators and barriers to policy compliance were examined using Packard's organizational change framework. Descriptive statistics summarized survey data, and deductive content analysis was applied to the interview narrative. Results: Six survey responses were collected. Most IPs were registered nurses (83%) with dual roles in nursing, staff education, or employee health. Over half (67%) had more than five years of experience, though half had been at their current facility for less than a year. All received state or local IPC training, and two were board-certified. Half worked in non-profit facilities. All reported multiple IP turnovers within the past three years. Facilitators from one administrator interview included "supportive leadership," "organizational resources," and "policy change pressures," while barriers included "government-industry miscommunication" and "high IP turnover." Conclusions: High IP turnover, possibly due to workload and pressure, and communication gaps hinder policy compliance. Strengthening workforce stability, government-industry collaboration, and structured IP competency training may reduce turnover. These findings can inform future research using a larger sample.

Abstract citation ID: igaf122.1159

### THE EVOLVING AND EXPANDING ROLE OF PERSONAL SUPPORT WORKERS IN END-OF-LIFE CARE IN THE LONG-TERM CARE SETTING

Danielle Just[1], Hannah O'Rourke[2], Whitney Berta[1], and Lisa Cranley[1], *1. University of Toronto, Toronto, Ontario, Canada, 2. University of Alberta, Edmonton, Alberta, Canada*

Background: Personal Support Workers (PSWs) play a vital role in long-term care (LTC). Initially, their role was focused on assisting residents with activities of daily living. However, as resident care needs become increasingly complex due to a rapidly aging population, PSWs' roles have expanded. End-of-life care is now a core aspect of their responsibilities. Despite this, no formal or consistent definition of PSWs' role in end-of-life care exists. Purpose: To explore and describe the role of PSWs in providing end-of-life care in LTC. Methods: A qualitative single case study was conducted using virtual data collection methods. Data sources included study site characteristics, documentation data, archival records, demographic data, interviews, and observations of physical artifacts. Results: Sixteen participants, including residents, family members, and LTC staff, contributed to the study. Findings revealed that PSWs frequently engage in extra-role behaviors (i.e., tasks beyond their job descriptions) when providing end-of-life care. These behaviors were widely recognized and perceived as essential for improving care quality. Additionally, the strong familial relationships between PSWs and residents were key motivators for their engagement in extra-role behaviors. Conclusions: This study underscores PSWs' evolving and expanding role in end-of-life care and highlights their indispensable contributions to LTC. By formally recognizing and integrating PSWs' end-of-life care responsibilities into policies, training, and workforce planning, healthcare leaders can enhance the quality of care for residents while fostering a more supportive and sustainable work environment for PSWs. This change is critical for addressing workforce shortages and ensuring high-quality end-of-life care in the LTC setting.

### SESSION 3045 (PAPER)

### FRAILTY, COGNITION AND FUNCTION: TRENDS AND TRAJECTORIES AROUND THE WORLD

Abstract citation ID: igaf122.1160

### COMMUNITY-BASED IMPLEMENTATION OF FIT & STRONG!: FINDINGS TO INFORM STATEWIDE SCALE-UP

Julie Bobitt[1], Andrew DeMott[2], and Susan Hughes[2], *1. University of Illinois at Chicago, Chicago, Illinois, United States, 2. University of Illinois Chicago, Chicago, Illinois, United States*

Fit & Strong! (F&S) is an evidence-based group physical activity and health education program designed to improve mobility, strength, and self-efficacy among older adults with arthritis. With ARPA funding from the Illinois Department of Public Health (IDPH), community-based organizations (CBOs) successfully launched the program statewide, engaging over 600 participants. This funding enabled us to undertake a series of qualitative interviews with providers that gave us a an opportunity to gain a deeper understanding of the implementation barriers and facilitators experienced by these CBOs. This project evaluated the acceptability, feasibility, and sustainability of F&S delivery across urban and rural Illinois communities.

We conducted semi-structured interviews with staff at 20 organizations that delivered F&S. Interview data were analyzed thematically to identify key implementation factors, including organizational capacity, resource needs, staff training, and participant recruitment challenges. Findings revealed strong enthusiasm for F&S among providers, who cited ways in which F&S could be adapted to meet the needs of different communities and its positive impact on older adult health. Challenges identified included the availability of staffing (exercise instructors are scarce in rural areas), securing sustainable funding, and tailoring the program to meet the needs of rural communities with limited resources. This evaluation provides important insights to inform future strategies for sustaining and expanding F&S delivery in collaboration with community partners. Understanding implementation facilitators and barriers directly from CBOs enables more effective statewide dissemination and is an important building block in the effort to ensure long-term access to evidence-based health promotion programs for older adults.

Abstract citation ID: igaf122.1161

### DISCRIMINATION, ALLOSTATIC LOAD, AND COGNITION AMONG INDIGENOUS, BLACK, HISPANIC, AND WHITE OLDER ADULTS IN THE HRS

Cliff Whetung[1], and Ernest Gonzales[2], *1. University of Minnesota Duluth, Duluth, Minnesota, United States, 2. New York University, New York, New York, United States*

The number of Indigenous older adults (IOAs) will more than double in the next thirty years. IOAs manifest dementia more often, and earlier in life than non-Hispanic Whites. Despite this, they remain underrepresented in cognitive health research. This study examined whether associations between perceived discrimination experiences (everyday discrimination, major lifetime discrimination) and cognition were mediated by allostatic load (AL), a measure of physiological stress. We used eight years (2008-2016) of restricted Health and Retirement Study data to examine cognitive trajectories among Indigenous (n = 222), Black (n = 2,181), Hispanic (n = 1,527) and White (n = 15,007) older adults using mixed effect growth curves. Regression models were adjusted for theoretically informed covariates. Both Indigenous and Black older adults reported frequent discrimination experiences and high standardized AL scores relative to non-Hispanic Whites. In regression analysis, we found that AL partially mediated the relationship between discriminatory experiences and cognition in minimally adjusted models, but this relationship lost statistical significance in fully adjusted models. Indigenous older adults have a unique profile that places them at elevated risk for cognitive pathology in later life, and structural inequities are powerfully associated with many of these risk factors. While these risk factors were associated with higher allostatic loads, our algorithm of AL did mediate the association between these risk factors and cognition. This finding conflicts with existing minority stress hypotheses, but may be the product of AL measurement issues. We conclude that dementia research integrating measures of AL requires additional transparency on the sensitivity and reliability of the construct.

Abstract citation ID: igaf122.1162

### FUNCTIONAL LIMITATIONS OVER TIME: THE EFFECTS OF SOCIOECONOMIC STATUS IN MEXICO

Chengming Han[1], Joey Saavedra[1], and Brian Downer[2], *1. UTMB Health, The University of Texas Medical Branch, Galveston, Texas, United States, 2. UTMB, Galveston, Texas, United States*

Objective: To examine the longitudinal effects of socioeconomic status on functional limitations in Mexico by comparing two birth cohorts aged 50-58 years. Methods: Data were drawn from the Mexican Health and Aging Study (MHAS). Functional limitations were measured by the number of mobility difficulties. Cohort 1 (C1, N = 4,450) was interviewed in 2003 and followed up in 2012, while Cohort 2 (C2, N = 4,167) was interviewed in 2012 and followed up in 2021. Multilevel logistic models were used to assess the association between socioeconomic status and functional limitations over time. Results: Cluster analysis categorized participants into four socioeconomic groups in each cohort: (1) low education and low income (lower class), (2) low education and medium income, (3) high education and medium income, and (4) high education and high income. Regression results showed that the lower class in the 2012 cohort had fewer functional limitations than their counterparts in the 2003 cohort. The other three groups exhibited similar patterns across cohorts. Higher education (high school or college) was associated with fewer functional limitations. Women engaged in housework had greater functional limitations. Higher income was protective against functional limitations, but only for women. Conclusion: Over recent decades, lower-class individuals in the 2012 cohort experienced fewer functional limitations compared to those in the 2003 cohort. Educational attainment consistently showed protective effects, while income and occupational influences on functional limitations varied by gender.

Abstract citation ID: igaf122.1163

### FUNCTIONAL LIMITATIONS, TYPES OF SOCIAL SUPPORT, AND DEPRESSIVE SYMPTOMS IN YOUNG-OLD AND OLD-OLD PEOPLE IN KOREA

Haa-young Cho, and Jung-Hwa Ha, *Seoul National University, Seoul, Republic of Korea*

Instrumental Activities of Daily Living (IADLs) are essential for older adults to maintain independence and a good quality of life. The purpose of this study is to examine the effect of IADLs on depressive symptoms and moderating effects of different types of social support among young-old and old-old people. Data from the 8th and 9th waves of the Korean Longitudinal Study of Ageing were analyzed, categorizing individuals as the young-old, aged from 65 to 74(n = 1,808) or the old-old, aged 75 or older(n = 1,689). IADLs included 10 tasks such as grooming, household chores, and taking medications. Social support was classified into four types: emotional, informational, instrumental, and appraisal support. Depressive symptoms were assessed using 10 CES-D items. Hierarchical multiple regression analysis was conducted. First key finding is that in the young-old group, greater IADL limitations were significantly associated with higher depressive symptoms. However, informational support buffered IADL limitations' negative impact-individuals with the same level of IADL limitations experienced fewer depressive symptoms if they had greater informational support. Next, in the old-old group, although the direct effect of IADL limitations on depressive symptoms was not statistically significant, the negative effect of IADL limitations on depressive symptoms was significantly alleviated among those with greater instrumental support. These results suggest different types of social support may protect different age groups from a same stressor. Thus, to protect mental health of older people, it is essential not only to provide social support but also to consider what type of support is appropriate for each age group.

### SESSION 3050 (SYMPOSIUM)

Abstract citation ID: igaf122.1164
### SOCIAL NETWORKS IN DEMENTIA
Chair: Sara Moorman
Co-Chair: Alyssa Goldman
Discussant: Hui Liu

Dementia poses significant barriers to the maintenance of social network relationships while also introducing new forms of social support needs from network members. These papers examine social network characteristics of older adults living with and without dementia. Patterson examines family networks, finding that people with dementia are as likely as people without to receive care from partners and siblings, but are more likely to receive care from adult children and grandchildren. Lin et al. investigate family care networks during COVID-19, also finding that people with dementia are more likely to receive care from adult children, although rates of filial care differ by race/ethnicity, age, and education. McConnell and Haggar measure the social networks of primary dementia caregivers, finding that larger caregiver networks are associated with greater primary caregiver stress, while greater network coordination is a source of caregiver support. Moorman and Goldman focus on the last five years of life, finding that discussion network size declines in the years before death among decedents without dementia but remains smaller and stable among decedents with dementia. Torres et al. use the Health and Retirement Survey linked with Medicare data to find that socially isolated older adults are more likely to experience Medicare-based under-diagnosis of dementia when compared with survey-based measures of cognitive status. These papers highlight the importance of social network relationships in dementia care, and collectively underscore the need to prioritize familial and community-based support systems prior to dementia onset in the wake of projected increases in persons living with dementia.

Abstract citation ID: igaf122.1165
### SOCIAL NETWORK SIZE IN THE YEARS BEFORE DEATH: AN EXAMINATION BY DEMENTIA STATUS
Sara Moorman, and Alyssa Goldman, *Boston College, Chestnut Hill, Massachusetts, United States*

There is growing recognition that in addition to between-person differences in the size of older adults' social networks, within-person network growth or shrinkage also influence well-being. Relatively little attention has been paid to patterns of social network change at the end of life, when health declines can render social networks especially important for quality of life, while also potentially compromising individuals' ability to maintain social ties. Using longitudinal data from respondents of the National Health and Aging Trends Study who have died, we examine how core discussion network size changes in the years before death, with particular attention to differences in cognitive status at the end of life. Tests of mean differences indicated that respondents with normal cognition in the last interview before death had significantly larger core discussion networks ($M = 2.10$) compared to those with probable dementia ($M = 1.79$; $p < .001$). Among respondents with normal cognition in the last interview ($N = 1{,}642$), growth curve models revealed a linear decline of 0.03 social network members per year in the years before death ($p < .001$). Among respondents with probable dementia in their last interview ($N = 1{,}002$), network size did not change significantly in the years before death. Our results suggest that effective efforts to support older adults' social engagement may depend on both timing and cognitive health status. Social interventions may provide more benefits for those with dementia if implemented before the end-of-life period, while efforts implemented at the end of life may still benefit older adults with normal cognition.

Abstract citation ID: igaf122.1166
### RELATIONSHIP TYPES AND CARE RECEIVED: DIFFERENCES BY DEMENTIA STATUS
Sarah Patterson, *University of Michigan, Ann Arbor, Michigan, United States*

Families and unpaid caregivers continue to provide the majority of assistance to older Americans, including to older adults living with dementia. However, diversity in the family structures of older adults may alter the help they receive and from whom. Using the 2022 National Health and Aging Trends (NHATS) study, I find older adults with and without dementia are equally likely to report having any children or siblings. Although older adults with dementia are more likely to be unpartnered, rates of care by a partner are the same once accounting for partnership status. Older adults with dementia are significantly more likely than those without dementia to receive care from children and grandchildren, but rates of care received are similar for siblings in both groups. These findings have implications for understanding the broader care networks of older adults beyond the traditional focus on a spouse and adult children.

Abstract citation ID: igaf122.1167
### TIME HELP RECEIVED DURING CRISIS: VARIATIONS BY OLDER ADULTS' COGNITIVE FUNCTIONING
I-Fen Lin[1], Judith Seltzer[2], Janecca Chin[1], Emily Wiemers[3], and V. Hotz[4], *1. University of Missouri, Columbia, Missouri, United States, 2. University of California, Los Angeles, Los Angeles, California, United States, 3. Syracuse University, Syracuse, New York, United States, 4. The University of Chicago, Chicago, Illinois, United States*

Family caregiving is essential in meeting the care needs of older adults, particularly those with Alzheimer's disease and related dementias (ADRD). The COVID-19 pandemic further disrupted an already fragile care landscape by decreasing the desirability of nursing home care, limiting the availability of paid caregivers, and destabilizing family caregiving arrangements. Older adults with ADRD are especially vulnerable to these disruptions, which can exacerbate their care needs and well-being. In this study, we used data from the 2020 Health and Retirement Study (HRS) to compare caregiving networks for older adults with and without ADRD, examining variations by age, sex, race-ethnicity, education, and partnership status. We analyzed verbatim responses from the COVID-19 module regarding time help received, creating new variables to capture helpers' relationships beyond children and household members. Preliminary findings suggest that older adults with ADRD were more likely to include adult children in their caregiving networks, with notable demographic differences. Specifically, adults still in prime working ages (ages 55-64), men, Hispanics, and college-educated individuals with ADRD were more likely to rely on non-child helpers compared to their respective counterparts. This study advances prior research by expanding beyond the traditional focus on assistance with activities of daily living (ADLs) and instrumental activities of daily living (IADLs), as about one third of respondents with dementia in the HRS reported no ADL/IADL difficulties. Understanding the composition of caregiving networks for individuals with ADRD during a crisis is vital because these caregivers are likely to be called upon again when care needs increase.

Abstract citation ID: igaf122.1168

### UNSEEN AND UNDIAGNOSED: THE RELATIONSHIP OF SOCIAL ISOLATION TO UNDERDIAGNOSED DEMENTIA

Ashwin Kotwal[1], Irena Cenzer[1], Min Hee Kim[2], Ruijia Chen[3], Carla Perissinotto[1], Willa Brenowitz[4], and Jacqueline Torres[1], *1. University of California, San Francisco, San Francisco, California, United States, 2. Rutgers, New Brunswick, New Jersey, United States, 3. Boston University School of Public Health, Boston, Massachusetts, United States, 4. Kaiser Permanente, Portland, Oregon, United States*

Social isolation may delay recognition of cognitive decline, leading to underdiagnosis or misdiagnosis of dementia. We examined the probability of undiagnosed dementia among older adults based on social isolation status using data from the U.S. Health and Retirement Study (HRS) linked to Medicare claims. Social isolation was measured through household contacts (marital status, household size, children nearby) and community engagement (volunteering, religious attendance). Probable dementia classification followed the Power-Gianattasio Expert model, maximizing accuracy across race and ethnicity subgroups, while the Bynum algorithm was applied to Medicare claims-based diagnoses. The primary outcome was dementia diagnosis status (correctly diagnosed, undiagnosed, or overdiagnosed) based on probable dementia classification in a given HRS wave and a corresponding Medicare-based diagnosis in the three years prior or one year after. We estimated associations between social isolation and dementia diagnosis status. Preliminary estimates from 2018 and 2020 indicate that 26-27% of respondents were socially isolated, 14% had probable dementia, and 8-9% had a claims-based dementia diagnosis. In 2018, socially isolated individuals had greater undiagnosed dementia than non-isolated individuals (12.7% vs. 7.3%, $p < 0.01$), with similar overdiagnosis rates (~2%). In 2020, rates were similar by social isolation status (undiagnosed dementia: 9.8% vs. 7.4%; overdiagnosis: 3.7% vs. 2.7%). Social isolation may contribute to disparities in timely dementia diagnosis due to reduced access to care and fewer social contacts recognizing cognitive changes. These findings suggest claims-based research may underestimate dementia cases among isolated individuals. Addressing social isolation as a public health concern may help mitigate disparities in dementia detection and care.

Abstract citation ID: igaf122.1169

### ASSOCIATIONS BETWEEN DEMENTIA CAREGIVER NETWORK COMPLEXITY AND PRIMARY CAREGIVER BURDEN AND MENTAL HEALTH

William McConnell, and Katherine Haggar, *Florida Atlantic University, Boca Raton, Florida, United States*

Persons living with dementia (PLWD) are often supported by complex networks of interconnected informal and professional caregivers who perform a wide variety of caregiving-related tasks. Many primary caregivers are older adults themselves who have been thrown into a complicated and demanding care manager role with little training or support. We examine whether larger, more complex caregiver networks, including increased professional involvement, are a supporting resource or additional burden for primary caregivers at their center. We report results from a quantitative Social Network Analysis (SNA) study of 250 PLWD in the United States. SNA is a valuable tool for measuring complex networks without relying on reductive social interaction scales. We measured caregiver networks using SNA name generators to identify any contributors to ten caregiving-related tasks. Statistical analyses identified associations between caregiver network characteristics (size, composition, stability, supportiveness, and coordination) and primary caregiver well-being (PHQ-4 depression, PSS-4 stress, loneliness, and ZBI-4 burden). The sample included 131 primary caregivers age 60+ who were embedded in large networks of 9.1 secondary caregivers, on average (SD = 5.0, range=0-28). Larger caregiver networks were associated with higher primary caregiver stress ($r=.25$; $p<.01$) and burden ($r=.26$; $p<.01$). When primary caregivers had more frequent disagreements with others, they reported higher stress ($r=.20$; $p<.05$), loneliness ($r=.18$; $p<.05$), and burden ($r=.29$; $p<.001$). Professional composition and network stability were not associated with primary caregiver well-being, although more coordination was associated with perceived support and self-affirmation. Whereas others have highlighted the beneficial aspects of caregiver networks, we identify challenges in more complex caregiver systems.

### SESSION 3055 (SYMPOSIUM)

Abstract citation ID: igaf122.1170

### HONORING CULTURAL STRENGTH: NAVIGATING FORMAL & INFORMAL MUTUAL AID AMONG GRANDPARENT CAREGIVERS

Chair: Gaynell Simpson
Discussant: Wendy Lustbader

Symposium Overview: The formal and informal mutual aid of grandparent caregivers are often overlooked or underutilized, yet they serve as vital sources of support. This symposium highlights the cultural assets that African American and Latina communities have long accessed as sources of strength. Historically, underserved grandparents have leaned on mutual aid practices such as neighbors relying on each other, sharing produce from community gardens, exchanging skills and abilities, and standing together with extended families-especially when societal resources were inaccessible. Today, these networks are more critical than ever. With families becoming more geographically dispersed and formal support systems strained by reductions in government assistance, the role of community-based mutual aid has only grown in significance. Grandparents raising grandchildren must navigate these evolving challenges while drawing on deep-rooted cultural traditions of collective caregiving. Four researchers and practitioners with expertise within these communities will present insights and practical recommendations that can make a difference, informing both researchers and practitioners committed to assisting them in contemporary times. Carole Cox presents a virtual empowerment program focused on skill building, social connection and empowerment. Nancy Mendoza offers practical recommendations on how the cultural assets of Latino grandparent caregivers can enhance their caregiving experience. Tina Peterson examines mutual aid among African American grandmothers raising grandchildren, highlighting how they compensate for gaps in formal and informal services through support networks. Gaynell Simpson highlights the need for intergenerational programs and macro-level advocacy to address systematic inequalities impacting grandparent caregivers and their families.

Abstract citation ID: igaf122.1171

### STRENGTHENING SUPPORT SYSTEMS: FAMILY RESOURCES AND INTERGENERATIONAL CARE FOR GRANDMOTHER CAREGIVERS

Gaynell Simpson, *Georgia Gwinnett College, Lawrenceville, Georgia, United States*

This study examines the perceptions of family resources among seven urban African American grandmother caregivers. Using an ethnographic approach, data were gathered through participant observation, field notes, interviews, and genograms to identify available and unavailable sources of support. Findings indicate that grandmothers relied primarily on family members who had resources to share, with instrumental and socioemotional support received in areas such as household chores, financial assistance, health-related needs, respite care, and crisis intervention. Even grandmothers facing significant social challenges found family members to be reliable in times of crisis. The study underscores the importance of intergenerational programs that strengthen family systems in grandparent-headed households. While some extended family members provided essential support, others were unable to contribute due to barriers such as poverty, medical conditions, or substance use. Minimal attention has been given to extended kin or non-kin who may serve as potential sources of support. Given the cultural tradition of African American families relying on kin networks, it is critical to assess the presence, roles, and functions of support structures within these families. Beyond individual and family-level interventions, macro-level advocacy is essential to address the broader social and economic inequities that contribute to caregiving challenges. Poverty, discrimination based on race, gender, age, and socioeconomic class, and structural inequalities shape the lived experiences of these caregivers. Social workers must engage in direct practice, policy development, and advocacy efforts to address the systemic barriers affecting African American grandparent caregivers and their families.

Abstract citation ID: igaf122.1172

### LATINO GRANDPARENTS RAISING GRANDCHILDREN: CULTURAL ASSETS AND SUPPORT STRATEGIES

Nancy Mendoza, *The Ohio State University, Columbus, Ohio, United States*

The prevalence of Latino grandparents raising grandchildren continues to rise. In 2019, an estimated 1 out of 7 Latino children lived with a grandparent, and 1.6% were being raised solely by a grandparent, with no parent in the home. Despite the high prevalence of these grandfamilies, they remain under-researched and underserved. Limited research shows that cultural values such as familismo, machismo, and marianismo significantly impact their experiences. Effective supports should consider and incorporate Latino cultural values. In a climate where formal support services are becoming more limited and are often inappropriate for Latino grandparent caregivers, there is a greater reliance on and need for mutual aid. This is crucial as there is often reluctance to engage with formal service networks due to mistrust and fear of repercussions. By understanding how Latino culture influences the lived experiences of these grandparents, researchers and practitioners can become trusted allies. They can help Latino grandparent caregivers leverage their cultural assets to address the service and support gaps they may be experiencing. This presentation will begin with a summary of how Latino cultural practices such as multigenerational living, the grandparent role in helping to raise grandchildren, and a woman's role in caring for the family can impact the experiences of Latino grandparent caregivers' experiences both positively and negatively. It will conclude with suggestions on ways Latino cultural values can be incorporated as cultural assets for effective supportive strategies for Latino grandparents raising grandchildren.

Abstract citation ID: igaf122.1173

### REIMAGINING MUTUAL AID TO ADVANCE HEALTHY OUTCOMES AMONG OLDER GRANDPARENT CAREGIVERS

Tina Peterson, *University of Cincinnati, Cincinnati, Ohio, United States*

African American grandparents raising grandchildren vary in their use of informal and formal services. These differences in service utilization can emerge from societal influences that limit access to needed services and resources. African American grandparents raising grandchildren may compensate by developing mutual aid support networks. Yet, little is known about mutual aid observed among African American grandparents raising grandchildren. This research reports on mutual aid described by older African American grandparents raising grandchildren. The sample included eleven African American grandparent caregivers who participated in two separate qualitative research studies between 2015 and 2024. All grandmothers were primary caregiver for a grandchild and 40 years or older. In-depth interviews were audiotaped, transcribed verbatim, and analyzed for themes. A common theme among these African American grandmothers was the intersection between caregiving, hypertension, and stress. Mutual aid evolved from historical experiences with informal and formal services. African American grandmothers linked caregiving processes to past role models of caregiving within their family of origin. Mutual aid also consisted of exchanges with family, friends, coworkers, neighbors, and peers. Multiple forms of mutual aid manifested as information sharing to encourage and prevent health-related crises, sharing financial resources, coping with the demands of raising grandchildren, volunteering, identifying healthier recipes and meal exchanges, and distributing vegetable from personal gardens. More emphasis is needed to identify the distinct sources and contributions of mutual aid systems. Mutual aid can help to advance healthy outcomes when resource and service gaps exist in informal and formal social support networks.

Abstract citation ID: igaf122.1174

### EMPOWERMENT AS MUTUAL AID

Carole Cox, *Fordham University, New York, New York, United States*

Empowerment programs for grandparent caregivers have traditionally been conducted in-person, allowing participants to interact directly. However, COVID 19 pandemic made such gatherings impossible, where isolation was essential. Concomitantly, grandparents raising grandchildren faced heightened challenges, requiring assistance both for themselves and their caregiving roles. The goals of empowerment practice are to help clients achieve a sense of personal power, become more aware of connections between individual and community problems, develop skills, and be able to work collaboratively toward social change. A virtual empowerment group was developed to meet the needs of these families with the program under the auspices of the NY City Department for the Aging. The program consisted of 7 classes offered through Zoom. The only prerequisites to participating were to be a grandparent caregiver and have access to WIFI. The program graduated 95 grandparents, almost all were Black and low income living in New York public housing. Throughout the program, the participants in each cohort developed ties as they listened to and learned from each other. They exchanged phone numbers and emails and often contacted each other during the week. Given the isolation that many were experiencing, they benefited from a solid network as they interacted with others going through the same experiences. Even in normal times, raising a grandchild can be isolating as it can limit socialization. Consequently, virtual groups can be an important means to fostering ties and building social inclusion.

### SESSION 3060 (SYMPOSIUM)

Abstract citation ID: igaf122.1175

### SOCIAL DETERMINANTS OF COGNITIVE HEALTH AND COGNITIVE HEALTH CARE PLANNING

Chair: Alexa Allan
Co-Chair: Abigail Stephan
Discussant: Elizabeth Muñoz

Social determinants are critical to shaping our path toward health equity. In fact, research has highlighted healthcare, education access, social and community context, economic stability, and neighborhood and built environment as key factors in health and well-being across the lifespan. This symposium highlights contextual and healthcare-based determinants to advance our understanding of their role in shaping cognitive health, emphasizing midlife and older adulthood. This symposium will include presentations using data from the Healthy Aging in Neighborhoods of Diversity Across the Life Span Sleep Study (HANDLSleep) and a project through the South Carolina Alzheimer's Disease Research Center (SC-ADRC) that examines Black/African American families' intergenerational discussions around Alzheimer's disease and related dementias (ADRD). These studies explored the psychosocial and contextual factors of sleep and cognition in a sample of Black and White middle-aged and older adults in Baltimore, Maryland, and the psychological and social factors influencing conversations and decision-making in cognitive healthcare across generations of Black/African American adults in South Carolina. The objectives of the proposed symposium are the following: (1) discuss the associations observed between neighborhood, leisure, and cognition using ambulatory data, and (2) discuss the role of social support and healthcare planning in cognitive health. Allan and colleagues explored the association between neighborhood quality and cognitive function using ambulatory measures. Sardina and colleagues examined the coupling of leisure activities and cognition. Simon and colleagues explored whether researcher-facilitated family conversations increased ADRD knowledge. Lastly, Stephan and colleagues explored family members' perspectives on cognitive healthcare by rurality.

Abstract citation ID: igaf122.1176

### DAILY ASSOCIATIONS OF NEIGHBORHOOD QUALITY AND COGNITIVE FUNCTION

Alexa Allan[1], Roland Thorpe, Jr.[2], Lesley Ross[3], Christopher Engeland[1], Orfeu Buxton[1], and Alyssa Gamaldo[3], *1. The Pennsylvania State University, University Park, Pennsylvania, United States, 2. Johns Hopkins University, Baltimore, Maryland, United States, 3. Clemson University, Clemson, South Carolina, United States*

Research has indicated that measures of positive and negative neighborhood quality relate to cognitive function in opposing ways. However, limited studies have examined the role that daily reports of neighborhood quality may play in daily cognitive functioning. Thus, this study examined the daily associations between neighborhood quality and cognitive function in a sample of Black and White adults from the HANDLSleep study in Baltimore, MD. Measures of neighborhood quality (e.g., litter, buildings being repaired) and cognitive function (Symbol Search [processing speed] and Dot Memory [working memory]) were collected using daily mobile phone surveys over a 7-day period. Multilevel linear models tested the within- and between-person associations among reported positive and negative neighborhood characteristics and cognitive function, adjusted for age, sex, race, reading literacy, poverty status, depressive symptomology, and medical condition history. The analysis included 229 midlife to older adults (Mage = 62.65, SDage = 8.63; 72% female; 66% Black). Of the 18 items, on average, participants reported 1.73 (SD = 2.52) and 1.34 (SD = 2.93) positive and negative neighborhood characteristics, respectively. ICC results revealed that 59.7% of the variance in reports of positive neighborhood characteristics and 39.9% of variance in reports of negative neighborhood characteristics could be attributed to within-person differences. No significant associations were observed between positive and negative neighborhood characteristics and cognitive function. While significant associations were not observed, the findings underscore the importance of considering methodology when capturing between- and within-person variability in neighborhood characteristics within aging adults.

Abstract citation ID: igaf122.1177

### FAMILY SYSTEMS AND COGNITIVE HEALTH: EXPERIENCES OF BLACK MIDLIFE AND OLDER ADULTS IN SOUTH CAROLINA

Abigail Stephan[1], Lena Simon[1], Angie Sardina[1], Jeff Meadows[2], Marsha Hampton[1], Lesley Ross[1], and Alyssa Gamaldo[1], *1. Clemson University, Clemson, South Carolina, United States, 2. Clemson University Institute for Engaged Aging, Clemson, South Carolina, United States*

The family is one of the most impactful contexts for development across the lifespan. Systems theories posit that families can act as sources of support or strain that impact individual outcomes, while individual members' health and well-being simultaneously have meaningful effects on entire family units. This is exceedingly true for families navigating—or preparing for—changes in older members' cognitive function. At the same time, families are embedded within larger systems that influence beliefs and behaviors, therefore requiring consideration of the broader context of the social determinants at play when families engage in cognitive healthcare planning. Accordingly, the current investigation highlights the voices of Black midlife and older adults within family systems—consisting of both relatives and fictive kin—from rural and suburban communities in South Carolina who participated in a focus group about their families' approaches to discussing and making decisions on matters pertaining to aging, such as Alzheimer's disease and related dementias (ADRD). Our team conducted seven focus groups (three in rural and four in suburban communities) with 5-7 participants in each group (N = 40, M = 70.25 years, 45-89 years, 97.5% Black, 82.5% female). Preliminary findings from our thematic analysis highlight similarities (e.g., salience of personal experiences) and differences (e.g., beliefs about support structures and obligations) in families' perspectives of ADRD planning by rural v. suburban locale. These findings support the importance of taking a nuanced, integrated approach when considering the role played by family and place, both with their own historical, structural, and social complexities, in cognitive healthcare.

Abstract citation ID: igaf122.1178

### MEASURING ADRD KNOWLEDGE AND OPINIONS OF AFRICAN AMERICAN FAMILIES IN SOUTH CAROLINA

Lena Simon[1], Abigail Stephan[1], Angie Sardina[1], Jeff Meadows[2], Marsha Hampton[1], Lesley Ross[1], and Alyssa Gamaldo[1], *1. Clemson University, Clemson, South Carolina, United States, 2. Clemson University Institute for Engaged Aging, Clemson, South Carolina, United States*

Black Americans are at a disproportionate risk for being diagnosed with Alzheimer's disease or related dementias (ADRD), which can negatively impact care planning. Although Black families are a pivotal source for care planning, limited research has explored ADRD knowledge and influential factors to seek ADRD care among Black families. This study (1) explores the relationships between perceptions of aging/ADRD research participation and ADRD care planning, and (2) explores changes in ADRD knowledge following participation in the facilitated focus group session among family members about their ADRD care planning. The sample included 40 South Carolinians (age range: 45–89, 97.5% black, 82.5% female), who completed surveys on perceptions of aging/ADRD research participation, and ADRD care planning prior to the focus group session. The Basic Knowledge of Alzheimer's Disease (BKAD) scale was assessed before and 1-week after the focus group. Results prior to the focus group indicated that seeking care for a family member with suspected ADRD was positively correlated with recommending future research studies to others ($r = .596$, $p = <.001$). Recommending participating in aging research to others was also positively correlated with higher anticipation the focus group session would be helpful ($r = .48$, $p = .002$). Additionally, ADRD knowledge significantly increased between the pre- ($M = 24.44$, $SD = 4.47$) and 1-week post-focus group assessment ($M = 26.27$, $SD = 3.7$; $p < .05$). These preliminary analyses suggest that focus groups might be an acceptable method to increase ADRD literacy, conversation, and research participation for Black Americans.

Abstract citation ID: igaf122.1179

### THE DAILY ASSOCIATION BETWEEN LEISURE ACTIVITY ENGAGEMENT AND COGNITIVE FUNCTION

Angie Sardina[1], Alexa Allan[2], Lesley Ross[1], and Alyssa Gamaldo[1], *1. Clemson University, Clemson, South Carolina, United States, 2. The Pennsylvania State University, University Park, Pennsylvania, United States*

Regular leisure activity engagement (LAE) is a protective factor for maintaining cognition and reducing Alzheimer's disease and related dementia (ADRD) risk. However, limited research has explored the association between daily LAE and cognition despite evidence of both LAE and cognition varying daily in sociodemographic heterogenous aging populations. Thus, this study examined 1) intraindividual variability in daily LAE; 2) the association between LAE and cognitive functioning (within-person and between-person); and 3) whether the LAE and cognitive association varies by sociodemographic characteristics. Data from the Healthy Aging in Neighborhoods of Diversity Across the Life Span (HANDLS) Sleep sub-study were utilized. Using a mobile device, participants (N = 211; age range=46-83; 68% Black; 73% female) reported their daily participation in 56 leisure activities (e.g., choir, reading, jogging, watching tv) across a 7-day period. Participants also completed brief cognitive assessments of memory (DOT memory) and processing speed (Symbol Search). Participants, on average, engaged in seven leisure activities/day (SD = 3.5). Multi-level models revealed significant intraindividual variability observed in LAE with 50% of LAE observed at both within- and between-person levels. Individuals who reported more daily LAE tended to perform significantly better on the memory task [$b=-0.04$, $SE = 0.02$, $p = 0.04$]. No significant within-person associations between LAE and cognition were observed. No significant interactions between LAE and sociodemographic characteristics were observed for either cognitive measure. These findings emphasize the importance of routine, daily, LAE on everyday cognition, which may elicit longer-term protective effects against cognitive decline and ADRD. Future research should explore psychosocial and contextual factors that potentially moderate these relationships.

### SESSION 3065 (SYMPOSIUM)

Abstract citation ID: igaf122.1180

### APPLICATION OF LARGE LANGUAGE MODELS (LLMS) TO GERIATRIC PRACTICE AND ITS EVALUATION AT 4 VA GRECCS

Chair: Huai Cheng
Co-Chair: Juliessa Pavon
Discussant: Mo-Kyung Sin

LLMs application to clinical practice is growing fast. However, LLMs are less studied in geriatrics practice but are urgently needed. This symposium will address whether LLMs allocation to geriatric practice can be trusted via five approaches. 1) LLMs generated gender and race-biased outputs. We will demonstrate whether LLMs generated age-biased output by assessing their geriatric attitude evaluated by social workers. 2). LLMs passed USMLE and other examinations. We will demonstrate whether LLMs can pass geriatrics knowledge competence tests evaluated by geriatricians 3). LLMs performed well on clinical vignettes from different clinical disciplines. We will demonstrate whether LLMs can perform well on geriatrics 5M-based vignettes of older adults evaluated by clinical providers and trainees 4) LLMs reviewed and summarized clinical charts. We will demonstrate whether LLMs can review geriatrics and general medicine notes to extract Mobility (one of Geriatrics 5Ms) documentation evaluated by geriatricians 5). LLMs can generate deprescribing recommendations, tapering schedules, and patient education materials. We will demonstrate their accuracy, safety, and appropriateness compared to recommendations from a multidisciplinary team of pharmacists, geriatricians, and nurses. Specifically, this symposium will address the following topics: 1) Geriatric Attitude of ChatGPT4.o and Its Evaluation by Social Workers. 2) ChatGPT4.o Geriatrics Knowledge Competency and Its Evaluation by Geriatricians. 3) LLMs application to geriatrics 5Ms evaluated by clinical providers and trainees. 4) Using LLMs to Extract and Assess Mobility Documentation for Age-Friendly Health System evaluated by geriatricians. 5) Using LLMs to generate medication deprescribing recommendations compared to clinician-led deprescribing recommendations.

Abstract citation ID: igaf122.1181

### GERIATRIC ATTITUDE OF CHATGPT4.O AND ITS EVALUATION BY SOCIAL WORKERS

Huai Cheng[1], and Jill Vinge[2], *1. Minneapolis VA Hospital, Minneapolis, Minnesota, United States, 2. Minneapolis VA Health System, Minneapolis, Minnesota, United States*

Ageism is global and prevalent in healthcare. Previous studies have shown that ChatGPT generated gender and racial biased outputs. This study aimed to explore ChatGPT4.o geriatric attitude using validated UCLA geriatric attitude instrument and to compare it with health trainees and providers and to examine whether hospital social workers agree or disagree with ChatGPT 4.o responses to geriatric attitude statements. Six of 16 statements reflected positive geriatric attitudes, such as “Most old people are pleasant to be with”. Ten statements reflected negative geriatric attitudes, such as “Treatment of chronically ill old patients is hopeless”. ChatGPT4.o responses and social workers’ evaluation of ChatGPT4.o responses were graded on a Likert scale of 1-5 (1=strongly disagree, 5 =strongly agree). Positive and negative geriatric attitudes scores were calculated to compare them to the medical students, ED residents, and neurologists from the previously published studies. The positive geriatric attitude score by ChatGPT4.o was 4.2 and higher than humans (higher scores indicated a more positive geriatric attitude), which was agreed by 40 social workers as 4.1 on a Likert scale of 1-5. The negative geriatric attitude score by ChatGPT4.o was 1.8 and lower than humans (lower scores indicated a less biased aging attitude), which was slightly agreed by 40 social workers as 3.2 on a scale of 1-5. In conclusion, ChatGPT4.o had a better geriatric attitude than humans, which is consistent with our previous findings using ChatGPT3.5. Social workers had modest agreement with ChatGPT4.o geriatric attitude. It suggests ChatGPT4.o is less likely to generate age-biased outputs.

Abstract citation ID: igaf122.1182

### CHATGPT4.O GERIATRICS KNOWLEDGE COMPETENCY AND ITS EVALUATION BY GERIATRICIANS

Iriana Hammel[1], Natasha Resendes[2], Dominique Tosi[3], Gokhan Demir[4], and Huai Cheng[5], *1. Miami VA Medical Center, Miami, Florida, United States, 2. Miami VA GRECC, Miami, Florida, United States, 3. Miami VA Heathcare System, Miami, Florida, United States, 4. University of Miami, Coral Gables, Florida, United States, 5. Minneapolis VA Hospital, Minneapolis, Minnesota, United States*

ChatGPT has passed USMLE and other medical knowledge exams, demonstrating competence in the medical field. It is less studied in Geriatric Medicine specifically. This study aimed to evaluate the geriatric competency of ChatGPT4.o by examining its performance on the validated UCLA Geriatrics knowledge tests, comparing its performance with that of trainees and exploring whether geriatricians agree with ChatGPT 4.o’s performance. 18 UCLA Geriatrics knowledge questions were answered. “Correct answer” was graded as 1, “incorrect answer” as -1 and “don’t know” as 0. The total score was between -18 to + 18. Test scores were calculated to compare ChatGPT and trainees (medical students, internal medicine residents and Geriatric medicine fellows) from previously published studies. ChatGPT4.o responses were evaluated by participants and graded on a Likert scale of 1-5 (1=strongly disagree, 5=strongly agree). Score of Geriatric knowledge by ChatGPT 4.0 was 18, higher than all trainees (9.9, 9.5, 13.6, 13,2, 14.7, 14.9. and 17.5 for MS1, MS2, MS3, PGY 1, 2 and 3 IM residents, and Geriatrics fellows respectively). Six geriatricians (four faculty and two fellows) rated its performance at 4.2 on a 1-5 Likert scale. In conclusion, ChatGPT4.o outperformed the trainees in Geriatric knowledge tests. Geriatricians were in agreement with ChatGPT4.o performance. It suggests ChatGPT4.o has competency in Geriatric knowledge. Concurrent control trainees’ testing is underway, as their scores may differ than those of the historical controls’ scores due to the trainees’ own use of artificial intelligence.

Abstract citation ID: igaf122.1183

### APPLICATION OF CHATGPT4.O TO GERIATRICS 5MS AND ITS EVALUATION BY CLINICAL PROVIDERS AND TRAINEES

Huai Cheng, *University of Minnesota, Minneapolis, Minnesota, United States*

ChatGPT did well on clinical vignettes from different disciplines. Our previous study showed ChatGPT had good geriatric knowledge and could help reduce polypharmacy and falls in two geriatric vignettes. Geriatrics 5M is a new geriatric framework and foundation for Age-Friendly Health System and the CMS new quality metrics for hospitalized older adults. This study aims to explore the responses to 8 geriatric 5Ms-based clinical questions by ChatGPT, clinical providers, and trainees. These 8 case-based clinical questions were integrated with two seminars on ChatGPT application to geriatric practice for 307 clinical providers and trainees. ChatGPT performance on these 8 geriatric 5Ms-based questions were evaluated by clinical providers and trainees. 8 geriatric 5Ms-based clinical questions were provided consequentially such as what is the simple instrument you will recommend evaluating the memory decline for this patient? What is the simple assessment instrument you will recommend assessing physical function for this patient? What is the simple instrument you will recommend evaluating potentially inappropriate medications (PIM) such as drug-drug, drug-disease interaction, and renal function adjusted medications? What is the simple instrument you will recommend predicting life expectancy for this patient? During 2 geriatric seminars, ChatGPT responded to these 8 questions well and were like clinical providers and trainees. More than 90% clinical providers’ and trainees’ responders agreed or strongly agreed with ChatGPT performance on 8 geriatrics 5Ms-based clinical questions. These results suggest ChatGPT could be applied to geriatric 5Ms practice as an assistant tool.

Abstract citation ID: igaf122.1184

### USING LARGE LANGUAGE MODELS TO EXTRACT AND ASSESS MOBILITY DOCUMENTATION FOR AGE-FRIENDLY HEALTH SYSTEMS

Huai Cheng[1], Betsy Yang[2], Anders Westanmo[3], Amy Gravely[4], Deborah Kado[2], and Howard Fink[5], *1. Minneapolis VA*

*Hospital, Minneapolis, Minnesota, United States, 2. GRECC at Palo Alto VA Health System, Palo Alto, California, United States, 3. Minneapolis VA Health System, Minneapolis, Minnesota, United States, 4. Research Service at Minneapolis VA Health Care System, Minneapolis, Minnesota, United States, 5. Minneapolis VA Health Care System, Minneapolis, Minnesota, United States*

The Age-Friendly Health System (AFHS) uses the geriatric 4Ms framework (mind, mobility, medications, matters most) to improve health outcomes in older adults. Achieving AFHS Level 2 recognition in outpatient settings requires documentation of annual 4Ms care, but current methods to assess documentation, manual chart reviews or template from electronic health records are time-consuming and prone to inconsistency and errors. This study evaluates whether Large Language Models (LLMs) can accurately extract mobility-related documentation from patient charts, comparing their performance to manual reviews conducted by two geriatricians. Mobility documentation includes seven key components, including falls, activities of daily living (ADLs), instrumental ADLs, fall related injuries, gait and balance assessments, assistive device, and home safety. First, two geriatricians independently reviewed 26 structured geriatric consult notes (with a 4M-structured template) and 78 unstructured general medicine notes and reached consensus on mobility documentation. The LLMs prompts were fine-tuned to extract the same information from the structured notes. Next, we tested the prompts on the same 26 structured notes pooled with the 78 unstructured general medicine notes. We then assessed consensus on mobility documentation in these 104 notes between the two geriatricians (Cohen's kappa 0.82), and geriatrician vs. LLMs (Cohen's kappa 0.65), respectively, indicating moderate to substantial agreement. Our findings show that LLMs demonstrated high agreement with geriatricians across both note types, suggesting that LLMs can reliably chart review for mobility documentation in AFHS. This automation has the potential to streamline chart reviews, reduce documentation burden, enhance documentation compliance, and support the broader adoption of AFHS.

Abstract citation ID: igaf122.1185

**EVALUATING GENERATIVE AI FOR DEPRESCRIBING: ACCURACY, SAFETY, AND CLINICAL UTILITY**

Juliessa Pavon[1], Cara McDermott[2], Marc Pepin[3], William Bryan[4], Ivuoma Igwe[4], and Cathleen Colon-Emeric[5],
*1. Durham VA Medical Center, Durham, North Carolina, United States, 2. Duke University, Durham, North Carolina, United States, 3. Durham VA Health Care System, Durham, North Carolina, United States, 4. Durham VA Health Care System, Geriatric Research Education and Clinical Center (GRECC), Durham, North Carolina, United States, 5. Duke University School of Medicine, Durham, North Carolina, United States*

Limited geriatrics and pharmacy resources in VA Medical Centers (VAMCs) necessitate innovative strategies to enhance deprescribing efforts. Generative AI platforms, such as OpenAI, have the potential to generate deprescribing recommendations, tapering schedules, and patient education materials by synthesizing information from medical literature and drug interaction databases. When integrated into deprescribing programs, these platforms could enhance scalability and sustainability by providing real-time, context-aware decision support. However, before implementation, it is essential to assess their safety, accuracy, and potential risks, including errors, omissions, and confabulations. Using the VA LLM platform TryOpen AI 3.5, this project assesses AI-generated deprescribing recommendations compared to those generated by an interprofessional team of pharmacists, geriatricians, and nurses, using de-identified case scenarios (N = 100) from our VA deprescribing program. We assess AI performance through content analysis, identifying recurring themes (e.g., medication selection, tapering regimens, side effects, and patient education) using the HELM criteria (Holistic Evaluation of Language Models), which assesses accuracy, uncertainty, efficiency, fairness, and bias. Findings will inform safe AI integration in deprescribing programs, identifying appropriate applications and potential safety risks to enhance medication management for older Veterans.

### SESSION 3070 (SYMPOSIUM)

Abstract citation ID: igaf122.1186

**POLICY SERIES: CAREGIVING IN THE US 2025: EMERGING THEMES AND ACTIONABLE POLICY AND PRACTICE SOLUTIONS**

Chair: Selena Caldera
Co-Chair: Rita Choula

Every five years, the National Alliance for Caregiving and AARP's Caregiving in the US (CGUS) survey provides a snapshot of family caregivers, their care recipients, care tasks, help with caregiving, supports received in health systems, and the work and financial impacts of caregiving. This symposium presents emerging themes from the 2025 CGUS survey and highlights actionable policy and practice solutions in each area. Family caregivers are now providing help with more activities of daily living and instrumental activities of daily living than in the past, yet few receive training on how to perform these tasks. Presenters 1 and 2 discuss these findings and the Caregiver Training Services (CTS) Codes introduced for Medicare providers in 2024. They share qualitative work examining provider experiences supporting caregivers in health systems and offer a practical guide for using the new CTS codes. Caregivers report greater availability of supportive workplace policies but continue to struggle balancing work and care responsibilities. Presenter 3 overviews these new findings and caregivers' ongoing struggle to balance work and care responsibilities despite increased support at work. While many caregivers experience financial pressures due to caregiving, certain groups disproportionately face these challenges. Presenter 4 summarizes the financial impact of caregiving, focusing on younger caregivers and other groups most affected by financial impacts. Presenters 3 and 4 highlight progress in introducing policies to support working caregivers and innovative new policy solutions to support caregivers financially. Presenter 5 shares the newest innovation in CGUS: state-level data on caregivers and their experiences.

Abstract citation ID: igaf122.1187

**FINANCIAL PRESSURES OF FAMILY CAREGIVING**

Selena Caldera, *AARP, Washington, District of Columbia, United States*

As in the 2020 Caregiving in the US survey, one in five family caregivers experienced high financial strain due to their caregiving responsibilities. This strain may result from out-of-pocket care costs, reduced work hours, or a combination of factors. The 2025 Caregiving in the US report provides a detailed look at the financial impacts that family caregivers experience. Caregivers report a range of financial impacts, including debt, savings, and housing-related issues. Lower-income family caregivers (those with less than $50,000 in household income) are significantly more likely to experience all thirteen financial impacts identified in the survey. Younger caregivers also face more financial challenges due to caregiving. Both groups often start their caregiving journey with fewer savings and assets, making them more vulnerable to long-term financial insecurity. This presentation will summarize the financial strain and impact measures reported by caregivers in 2025 and highlight the groups most likely to experience financial pressures. Caregivers overwhelmingly support policy solutions such as caregiver income tax credits, paid family leave, and programs to pay family caregivers for their care. Understanding both the overall financial pressures and the caregivers hardest hit is critical to developing effective policy solutions to support all caregivers financially.

Abstract citation ID: igaf122.1188

### IMPLEMENTING CAREGIVER TRAINING IN HEALTH SYSTEMS

Juliana Cuozzo, *AARP Public Policy Institute, Washington, District of Columbia, United States*

The introduction of Caregiver Training Services (CTS) codes in Medicare's Current Procedural Terminology (CPT) marks a significant step forward in formalizing support and education for family caregivers. In 2024, the Centers for Medicare & Medicaid Services (CMS) allowed Medicare to reimburse practitioners for caregiver training under the Physician Fee Schedule (PFS). Data from Caregiving in the US shows that 50% of caregivers need training, underscoring the significance of this policy change. This body of work explores the implementation and impact of these codes in the first year of their introduction. Our research explores the utilization of five new CPT codes (97550, 97551, 97552, 96202, and 96203) in their first year, as well as the Health Risk Assessment established for caregivers in 2017 (96161), analyzing Medicare claims data to identify trends and gaps in their use. Additionally, qualitative research captures the perspectives of providers and caregivers on the benefits, challenges, and potential improvements of these codes. This work highlights the importance of translating policy into practice to ensure adequate support without creating additional barriers. By examining the practical applications and outcomes of the new CPT codes, we aim to provide insights into optimizing these policy changes for caregivers, patients, and providers.

Abstract citation ID: igaf122.1189

### CAREGIVING IN THE USA -- A FIRST LOOK AT STATE LEVEL FINDINGS

Terri Guengerich, *AARP, La Plata, Maryland, United States*

For the first time in the history of the National Alliance for Caregiving and AARP's Caregiving in the US survey, the survey sample size was increased radically to oversample caregivers at the state level. The purpose of these oversamples was to obtain the prevalence of caregivers in each state and to provide more detailed information about caregivers and their experiences at the state level. Information shared during this session will include the sample plan and weighting strategy as well as the prevalence of caregivers at the state level. In addition, summary information will be provided about caregivers and their experiences at the state level.

Abstract citation ID: igaf122.1190

### INTEGRATING CAREGIVERS IN HEALTH CARE DELIVERY MODELS

Yadira Montoya, *National Alliance for Caregiving, Washington, District of Columbia, United States*

NAC's presentation will outline strategies for integrating family caregivers into healthcare delivery models, with emphasis on implementing reimbursable Caregiver Training Services (CTS). We will highlight family caregivers' roles in cancer care, present a workflow for CTS integration, and provide a six-point checklist for program development. The presentation will showcase case studies from Northwell Health and RUSH University Medical Center, demonstrating real-world considerations for CTS implementation in geriatric and serious illness care, complemented by practical tools and evidence-based resources for effective caregiver integration.

Abstract citation ID: igaf122.1191

### WORKING WHILE CAREGIVING IN 2025

Alessandra Raimondi, *AARP, Washington, District of Columbia, United States*

Roughly three-fifths of family caregivers work at some point while caregiving. Fortunately, caregivers report that employers increasingly offer workplace flexibilities and policies to help balance their responsibilities at work and at home. Many states now have paid leave laws extending protections to family caregivers. The 2020 Caregiving in the US report noted that only eight states and DC had enacted paid family leave; currently, 13 states and DC have active paid family and medical leave laws, and 15 states and DC have enacted paid sick leave laws. The nature of employment significantly influences access to caregiver-friendly benefits. Just over half of working caregivers are paid hourly, while 40 percent are salaried workers. Caregivers in salaried employment are significantly more likely to have access to a range of supportive workplace benefits than hourly-wage caregivers. Benefits access also differs for caregivers working full-time compared to those working part-time. Despite increased access to caregiver-friendly benefits and policies, most working caregivers still struggle to balance work and care, often facing significant impacts on their professional and personal lives. The share of caregivers reporting work impacts, ranging from going in late or leaving early to giving up work entirely, remains unchanged from the 2020 survey. This points to persistent gaps in meeting the needs of working family caregivers. This panel will explore access to caregiver-friendly workplace benefits, disparate access by employment type, and the common work impacts reported by caregivers trying to strike a balance between care responsibilities and work.

### SESSION 3075 (PAPER)

### NEW TECHNOLOGIES FOR HEALTH AND CARING: BRINGING IT ALL BACK HOME

Abstract citation ID: igaf122.1192

### CONSTRUCTION AND APPLICATION OF THE INTERNET PLUS HOME-BASED CASE MANAGEMENT SERVICE MODEL IN HEALTH MANAGEMENT

Wanling Li, Xianmei Cui, Qian Zhang, Huali Miao, Wenjuan Zhu, Linying Wang, and Lihua Wu, *Shanxi Bethune Hospital, Taiyuan, Shanxi, China (People's Republic)*

Objective: China's existing "Internet Plus Nursing Services" primarily focus on single nursing procedures and are insufficient for the multifaceted health needs of elderly patients. This study incorporates health management into existing services, developing the Precision-Safety-Homogenization (PSH) "Internet Plus Health Management" model and evaluating its effectiveness. Methods: Conducted in a large general hospital in China, this study provided on-demand health management services for elderly patients. Precision: Supported by an MDT, comprehensive health assessments were conducted across pre-, in-, and post-hospital phases. The MDT formulated individualized health management plans, which were implemented and monitored by nurses. Safety: During home visits, nurses assessed patients' health risks and collaborated with the MDT team to provide coordinated health management, optimizing intervention strategies. Homogenization: Nurse training and oversight adhered to standardized in-hospital protocols, standardizing service scope, medical supply use, and pricing. Results: From June 2021 to September 2024, the PSH health management model was applied to 1,244 patients. After six months, key health indicators showed significant improvements: The HbA1c target achievement rate in diabetes patients increased from 45.2% to 72.8% (P = 0.04). The blood pressure control rate in hypertensive patients improved from 50.3% to 78.9% (P = 0.03). The Barthel Index in stroke patients increased from 48.2 to 68.7 (P = 0.01).Lung function (FEV⁴⁰%) in COPD patients improved from 58.6% to 72.4% (P = 0.01).A total of 191 adverse events were identified and managed, with 100% patient satisfaction reported. Conclusion: The PSH health management model effectively identifies diverse health issues in elderly patients, supports structured and evidence-based health management, helps slow disease progression, and enhances patient safety.

Abstract citation ID: igaf122.1193

### ETHICAL ISSUES IN EMPIRICAL STUDIES ON SMART HOME TECHNOLOGIES: A SCOPING REVIEW

Agnes Jihae Kim, Taekyung Kim, Jiwon Park, and Moon Choi, *Korea Advanced Institute of Science & Technology, Daejeon, Republic of Korea*

Smart home technology is increasingly employed to enhance independence, participation, and healthcare for older adults and individuals with disabilities. Given its close integration into daily life, ethical concerns surrounding these technologies have been widely discussed. However, limited research has examined how ethical considerations are applied in empirical studies and how existing conceptual models inform their use. This scoping review provides a comprehensive analysis of the integration of ethical frameworks in empirical studies on smart home technologies for these populations. Following PRISMA-ScR guidelines, two databases (Web of Science and SCOPUS) were searched, identifying 373 articles after duplicate removal. Using Covidence software, reviewers screened articles in pairs, resulting in 37 eligible studies. The findings indicate that 15 studies focused on smart home technologies for social or healthcare applications, with integrated systems being the most prevalent technology type, identified in 20 studies. Privacy was the most frequently addressed ethical issue, appearing in 34 of 37 studies. While privacy concerns vary based on user characteristics and context, broader ethical considerations—including autonomy, trust, bias, and stigma—remain underexplored. Moreover, ethical concerns evolve across different stages of technology development, yet relatively few studies address these issues in early phases. This review highlights the need for a systematic and comprehensive approach to ethical considerations in smart home technology research. It moves beyond theoretical discussions to examine how ethical issues are addressed in the actual processes of developing, adopting, and using smart home technologies based on empirical evidence.

Abstract citation ID: igaf122.1194

### TELEHEALTH USE AMONG OLDER ADULTS RECEIVING HOME- AND COMMUNITY-BASED SERVICES

Dana Urbanski[1], Romil Parikh[2], Jack Wolf[2], Benjamin Langworthy[2], Chanee Fabius[3], Janette Dill[2], Eric Jutkowitz[4], and Tetyana Shippee[4], *1. Indiana University Bloomington, Bloomington, Indiana, United States, 2. University of Minnesota School of Public Health, Minneapolis, Minnesota, United States, 3. Johns Hopkins University, Baltimore, Maryland, United States, 4. University of Minnesota, Minneapolis, Minnesota, United States*

Telehealth was essential for maintaining care continuity during the COVID-19 pandemic, leading to its rapid expansion and adoption in the United States. Since then, telehealth has been increasingly viewed as a strategy to reduce health disparities among vulnerable populations. However, data on telehealth use among socially and financially vulnerable older adults are limited, leaving unanswered questions about the role of telehealth in promoting health equity for older Americans. This study examined individual and structural factors associated with post-pandemic telehealth use among older recipients of publicly funded home- and community-based services (HCBS; an indicator of social and financial vulnerability). Using cross-sectional data from the 2021–2022 National Core Indicators-Aging and Disability Adult Consumer Survey, the analytic sample included 3,680 HCBS users aged ≥ 65 years without intellectual or developmental disability, 40% of whom used telehealth. Complete-case multivariable logistic regression, adjusting for sociodemographic and health-related factors with state-level random intercepts, revealed significantly lower odds of telehealth use associated with older age, male gender, Black race, non-metropolitan residence, and receipt of Older Americans Act services. Individuals with dementia had higher odds of telehealth use (OR, 1.33, $p < .05$). Home internet access was strongly associated with telehealth use (OR, 2.51, $p < .001$). Follow-up bivariate analyses showed younger age, female gender, and White race were associated with higher internet access. These findings highlight differences in telehealth use among older HCBS users associated with both individual and structural factors, offering valuable insights for efforts aimed at addressing barriers to telehealth adoption among vulnerable older adults.

### SESSION 3080 (SYMPOSIUM)

Abstract citation ID: igaf122.1195

### AGE-FRIENDLY CITIES AND COMMUNITIES: AN INTERNATIONAL PERSPECTIVE

Chair: Kathrin Boerner
Discussant: Nina Silverstein

Given population aging and urbanization, cities and communities must evolve meeting the needs and foster the potential of older adults. The Age-Friendly Cities framework, launched by the World Health Organization in 2007, provides a structured approach to assessing and enhancing urban environments for aging populations. It specifies eight interconnected domains of urban life: Outdoor Spaces and Buildings, Transportation, Housing, Social Participation, Respect and Social Inclusion, Civic Participation and Employment, Communication and Information, Community Support and Health Services. This symposium brings together international research on developing and implementing age-friendly initiatives, offering insights from different countries. Grenz and colleagues present a study using the Age-Friendly Cities and Communities Questionnaire (AFCCQ) in Germany, identifying four distinct subgroups of older adults based on socio-demographic profiles and evaluations of age-friendliness. Marston and colleagues assess the AFCCQ in the UK, demonstrating its intergenerational applicability and revealing financial disparities as a key factor in age-friendliness perceptions. Ayalon and colleagues examine the AFCCQ in Israel, highlighting variations in satisfaction across cities, with social inclusion and respect emerging as critical concerns. Siebecker and Coyle present findings from the second cycle of Age-Friendly Boston, illustrating how older adults' priorities have shifted over time from housing and economic security to social connection and civic engagement. By considering findings across different national and cultural contexts, this symposium provides a cross-cultural view and comparison of age-friendly city initiatives. The discussion will emphasize the importance of data-driven urban planning, targeted interventions, and international collaboration in creating inclusive and sustainable environments for aging populations worldwide.

Abstract citation ID: igaf122.1196

### HOW AGE-FRIENDLY IS YOUR CITY TO YOU: EXPERIENCES FROM A MID-SIZED GERMAN CITY

Adele Grenz[1], Michael Weinhardt[2], Moritz Hess[3], Joost van Hoof[4], Jeroen Dikken[5], and Kathrin Boerner[6], *1. The Carl von Ossietzky University of Oldenburg's School of Medicine and Health Sciences, Oldenburg, Niedersachsen, Germany, 2. German Centre of Gerontology, Berlin, Berlin, Germany, 3. University of Applied Sciences Niederrhein, Krefeld, Nordrhein-Westfalen, Germany, 4. The Hague University of Applied Sciences, Wrocław University of Environmental and Life Sciences, The Hague, Zuid-Holland, Netherlands, 5. The Hague University of Applied Sciences, The Hague, Zuid-Holland, Netherlands, 6. Carl von Ossietzky University of Oldenburg, Oldenburg, Niedersachsen, Germany*

Urbanization and demographic change pose social challenges, underscoring the need for a systematic approach to assessing the age-friendliness of urban environments and the experiences of older adults within them. The purpose of this study was to provide a valid German version of the Age-Friendly Cities and Communities Questionnaire (AFCCQ), which aligns with the WHO's eight domains of age-friendliness, and to examine personal and contextual correlates of perceived age-friendliness in a mid-sized city. A representative sample of older adults (N = 905) assessed their city's age-friendliness, with average scores reflecting at least moderate satisfaction in all domains, but also significant interindividual variation. A cluster analysis identified several distinct groups, including a group of rather vulnerable individuals facing health challenges and socioeconomic disadvantage — who reported the lowest age-friendliness scores — as well as a more affluent, healthier residents who rated their environment positively across all domains. Late life phase was also a factor, with very old adults overrepresented in the less satisfied groups. Narrative responses to open-ended follow-up questions specifically highlighted challenges in mobility, healthcare access, and caregiving support. Findings indicate that health conditions, living arrangements and socio-economic status may critically shape how older adults experience urban environments. These results emphasize the need for targeted policies to ensure urban environments are inclusive and accessible for all older adults. Additionally, this study underscores the value of systematic evaluations in shaping and monitoring urban policies that align with age-friendly principles.

Abstract citation ID: igaf122.1197

### THROUGH THE LOOKING GLASS: MEASURING AGE-FRIENDLINESS IN THE UK

Hannah Marston[1], Jeroen Dikken[2], Verina Waights[3], Lorna Rouse[3], Louise Wallace Wallace[3], and Joost van Hoof[4], *1. The Open University, Milton Keynes, England, United Kingdom, 2. The Hague University of Applied Sciences, The Hague, Zuid-Holland, Netherlands, 3. The School of Health, Wellbeing and Social Care, Faculty of Wellbeing, Education & Languages, The Open University, Miltin Keynes, England, United Kingdom, 4. The Hague University of Applied Sciences, Wrocław University of Environmental and Life Sciences, The Hague, Zuid-Holland, Netherlands*

Age-friendly cities is an important initiative set out by the World Health Organization and many researchers and policy makers aim to create age friendly environments to support their ageing populations. This presentation will present findings from the UK through the validation of the age-friendly cities and communities questionnaire (AFCCQ-UK) deployed across the UK. The sample (n = 426) completed a survey answering a 23-item measure deployed online in a bid to reach broader audiences throughout the years 2023 and 2024. Employing a life course perspective, the survey targeted people aged 18-60+ years through mailing lists, social media posts, and word of mouth. The AFCCQ-UK demonstrates validity and reliability across two age groups (18-59 and 60+ years) showing for the first time globally that age-friendliness can be measured intergenerationally rather than just among those aged 60+. Respondents demonstrated a moderately positive overall perception of age-friendliness. Data shows no significant differences between the age groups suggesting consistency of age-friendliness across the demographics. However, financial differences were observed to be of influence especially relating to the domains of social participation, and community support and health services. A current case study will illustrate how the AFCCQ-UK is used by stakeholders to measure the age-friendliness across the city of Milton Keynes, a new city developed in the 1960s, to demonstrate how the findings have fed into the strategic priorities of the local authority to make significant changes to improve the city for its residents.

Abstract citation ID: igaf122.1198

### THE AGE FRIENDLY CITIES AND COMMUNITIES QUESTIONNAIRE: A VALIDATION STUDY OF THE HEBREW VERSION IN ISRAEL

Liat Ayalon[1], Shvat Eilat[1], Jeroen Dikken[2], and Joost van Hoof[2], *1. Bar-Ilan University, Ramat Gan, HaMerkaz, Israel, 2. The Hague University of Applied Sciences, The Hague, Zuid-Holland, Netherlands*

In this presentation, I will report research concerning the reliability and validity of the Hebrew version of the Age Friendly Cities and Communities Questionnaire (AFCCQ). The study was conducted in four Israeli cities, all of which were recognized as age-friendly during the time of the study in June-July 2023, namely: Tel Aviv-Jaffa, Herzliya, Kfar Saba, and Jerusalem. A total of 223 Hebrew speakers over the age of 65 participated in the study. We conducted confirmatory factor analysis to establish structural validity and established the reliability of the measure. Slight dissatisfaction was noted regarding respect and social inclusion across cities. There also was variability across the four cities with Jerusalem being perceived as worse off compared with the other three cities. The findings are discussed considering qualitative interviews with city planners concerning their reflections on the meaning of age friendly cities and communities. Issues of universalism versus age blindness, and age awareness are discussed.

Abstract citation ID: igaf122.1199

### COMING BACK AROUND: LESSONS FROM THE SECOND CYCLE OF AGE FRIENDLY BOSTON PLANNING

Megan Siebecker, and Caitlin Coyle, *University of Massachusetts Boston, Boston, Massachusetts, United States*

This presentation demonstrates the evolution of community reengagement in Boston through the second iteration of the Age-Friendly Boston planning process. Boston became a member of the World Health Organization's Age-Friendly Cities network in 2014, and its first Age-Friendly Boston Action Plan was published in 2017. Through a combination of community forums, surveys, and other community engagement efforts, this presentation highlights key shifts in the needs and priorities of older residents over time with evidence from the 2025 Age-Friendly planning process. Results from a mixed methods analysis of 35 community forums, 1,308 surveys representing 11 languages across the 21 unique neighborhoods in Boston will be presented. Notably, data suggests the shifting needs of Boston's older adults over time reflects the success of the City's efforts in instituting the 2017 action plan. While earlier conversations focused primarily on issues like housing and economic security, more recent discussions have increasingly centered on social connection, a sense of belonging, and civic engagement. The evolving needs of the community reflect a growing recognition of the importance of social fulfillment and communication. Additionally, new insights suggest that neighborhood-level differences have emerged as a critical focus. This presentation will highlight the progress made in Age-Friendly Boston's efforts, while also offering insights into the changing needs and expectations of older Bostonians as they navigate a changing urban environment.

### SESSION 3085 (COMMUNITY ENGAGED SCHOLARSHIP SYMPOSIUM)

Abstract citation ID: igaf122.1200

### PROMOTING PARTICIPATION OF OLDER BLACK ADULTS IN ALZHEIMER'S DISEASE RESEARCH

Chair: Rebecca Lassell
Co-Chair: Christopher Carey

Symposium will be here

Abstract citation ID: igaf122.1201

### ADAPTING A GREEN ACTIVITY PROGRAM WITH OLDER BLACK PEOPLE LIVING WITH MEMORY CHALLENGES

Christopher Carey[1], Lola Sample[2], Laura Gitlin[3], Jaroslaw Harezlak[4], Richard Holden[4], NiCole Keith[2], Kathleen Unroe[5], and Rebecca Lassell[4], *1. Indiana University School of Medicine, Indianapolis, Indiana, United States, 2. Indiana University School of Public Health Bloomington, Bloomington, Indiana, United States, 3. Drexel University, Philadelphia, Pennsylvania, United States, 4. Indiana University Bloomington, Bloomington, Indiana, United States, 5. Indiana University School of Medicine, Indianapolis, Indiana, United States*

Few physical activity programs are designed with Black older adults living with mild cognitive impairment and dementia (memory challenges) to address their needs. Applying the Cultural Adaptation Process model and the Framework for Modification and Adaptations, we sought to adapt a nature or 'Green' Activity Program for Black people living with memory challenges (PLMC) and study partners. Participants were recruited from Eskenazi Health in Indianapolis, Indiana. A modified 5-step co-design process occurred with six 90-minute sessions with four teams separately (n = 21 total, n = 5-6 each). Participants were Black PLMC (n = 5), Black study partners (n = 6), outdoor professionals (n = 5), and healthcare providers (n = 5). Recorded design sessions were analyzed. Challenges for PLMC included: 1) noise (construction, traffic), 2) weather 3) aches and pain; Study partners: 1) health challenges/behaviors, 2) transportation/accessibility; 3) finances. Solutions identified by PLMC were 1) wearing headphones, 2) weather appropriate clothing, 3) activity modifications; Study partners: 1) accessibility/motivation, 2) transportation services and convenience, and 3) free/lower cost activities. Surface cultural adaptions (language) included preferred terms of 'memory challenges' for dementia and 'Black.' Deep cultural adaptations (values/beliefs) included adding family, awareness/exposure to nature activities. 'Content' adaptations included preferred nature activities of water Zumba, fishing, and camping. 'Contextual' modifications for program delivery were a 'green activity pill box' to schedule weekly activities as a visual reminder, a buddy system, and monetary incentives for motivation. Referral pathways included direct referrals in electronic health records, or in-person from healthcare providers. Findings can guide piloting the program and cultural adaptation of other interventions.

Abstract citation ID: igaf122.1202

### EMPOWER!: BRAIN HEALTH EDUCATION TO PROMOTE EARLY DETECTION OF ALZHEIMER'S AND COGNITIVE SCREENING

Veronica Derricks[1], Sarah VanHeiden[2], Jane Musema[3], Miriam Jocelyn Rodriguez[4], Patricia Garcia[5], Sophia Wang[3], and Christopher Carey[5], *1. University of Colorado Boulder, Boulder, Colorado, United States, 2. Indiana Alzheimer's Disease Research Center, Indianapolis, Indiana, United States, 3. Indiana Alzheimer's Disease research center, Indianapolis, Indiana, United States, 4. Indiana University Bloomington, Bloomington, Indiana, United States, 5. Indiana University School of Medicine, Indianapolis, Indiana, United States*

Introduction: Black older adults are twice as likely to develop Alzheimer's disease and related dementias (ADRD). They are also more likely to have delayed or missed diagnoses of ADRD, because of lower cognitive screening rates during the earliest stages of the disease. To address these gaps, we developed the EMPOWER! program to provide education about the importance of early detection of ADRD for those communities at higher risk of this disease. Methods: Educational materials about brain health and the importance of cognitive screening were co-developed with community stakeholders. Qualitative interviews were conducted with Black older adults who had cognitive concerns and <16 years of education, their close contacts, and community center staff working closely with communities at risk of ADRD (N = 15). Thematic analysis identified key themes related to barriers, facilitators, and recommendations for how to improve relevance and accessibility to information about brain health, AD, and cognitive screening. Results: Study results highlighted the critical factors influencing cognitive health awareness and research participation among Black older adults. Findings emphasize the importance of trust, social support, culturally relevant communication, and targeted outreach in encouraging engagement with cognitive screening and brain health initiatives. Discussion: Our preliminary data identified factors which may increase acceptability of culturally tailored materials to engage communities most at risk of ADRD to learn more about brain health and the importance of early detection. Future efforts will further assess content and attitudes towards cognitive screening and adapting EMPOWER! for use in other communities at risk of ADRD.

### SESSION 3090 (SYMPOSIUM)

Abstract citation ID: igaf122.1203

### NOVEL RESEARCH OF DEMENTIA PREDICTION AND MONITORING: WHEN AI MEETS TEXT DOCUMENTS

Chair: Hiroko Dodge
Co-Chair: Liu Chen
Discussant: W. Quin Yow

Early prediction and monitoring of dementia provide a longer window for interventions, risk factor management, and future disease-modifying therapies. Various data types, such as blood samples and neuroimaging, have been explored for predicting early dementia risk, showing promising results. More recently, text documents, such as conversation transcriptions and health records, have gained attention due to their potential for AI-driven analysis and practical advantages. The accessibility, scalability, cost-effectiveness, continuous monitoring, and information-rich nature of text documents make them particularly compelling for widespread early detection. Thus, AI-driven analysis of text documents for dementia prediction deserves further investigation. This symposium highlights four novel research directions in applying AI to text documents for dementia risk prediction. Presentations will feature: 1) Quin will present an exploration evaluating the use of machine learning and AI-driven measurements for cognitive impairment detection, revealing insights into detection performance and the value of various measurement sets. 2) Hoang will present a temporal harmonization technique for longitudinal conversation, showing enhanced mild cognitive impairment (MCI) detection accuracy on data collected by I-CONECT study. 3) Jiankun will present a dementia risk prediction pipeline that combines conventional machine learning with large language models (LLMs), showing that incorporating LLM significantly improves performance. 4) Chen will present the difference in linguistic characteristics between MCI and individuals with normal cognition and MCI individuals' changes after they participated in the I-CONECT intervention for 6 months. The symposium collectively demonstrates the promising and multifaceted potential of incorporating AI with text documents for early dementia prediction.

Abstract citation ID: igaf122.1204

### MACHINE LEARNING CLASSIFICATION MODEL PERFORMANCE IN DETECTING COGNITIVE IMPAIRMENTS FROM MULTIMODAL EMBEDDINGS

W. Quin Yow, Adharsha Sam Edwin Sam Devahi, Lihan Zuo, and Ka Lon Sou, *Singapore University of Technology and Design, Singapore, Singapore*

In the fast-evolving field of AI for healthcare, there is a growing trend of combining acoustic and linguistic features from speech data to improve ML model performance for cognitive impairment detection. This paper evaluates a hyperparameter-tuned SVM model trained on three feature sets derived from pretrained Transformer-based models: acoustic CrisperWhisper embeddings, linguistic BERT embeddings, and both. Using 150 data points (94F, ages 51-99) equally distributed across healthy, MCI, and dementia classes, sourced from DementiaBank and challenge datasets from the University of Edinburgh and Carnegie Mellon University, no statistically significant differences ($\chi^2$=3.45, p = 0.178) were found in the accuracy scores of the model trained on the three feature sets across 30 validation datasets. The mean validation accuracy scores of the model trained on acoustic, linguistic, and combined embeddings were 72% (SD = 0.233), 63% (SD = 0.221), and 57% (SD = 0.202), respectively. However, there were statistically significant differences ($\chi^2$=58.067, $p < 0.001$) in the accuracy scores of the model trained on the feature sets across 30 training datasets. Among significant pairwise comparisons, the training accuracy scores of the model trained on acoustic embeddings (M = 1, SD = 0) were significantly higher than those using linguistic embeddings (M = 0.847, SD = 0.008; W = 0, n = 30, $p < 0.001$) and those using acoustic+linguistic embeddings (M = 0.875, SD = 0.012; W = 0, n = 30, $p < 0.001$). The results suggest that while acoustic embeddings alone yielded the highest accuracy, hyperparameter tuning failed to mitigate overfitting, and acoustic+linguistic embeddings did not improve model performance. However, given the small dataset size, these findings should be interpreted with caution. Future directions for improving model performance will be discussed during the presentation.

Abstract citation ID: igaf122.1205

### IMPROVED DETECTION OF MILD COGNITIVE IMPAIRMENT FROM TEMPORAL LANGUAGE MARKERS: I-CONECT STUDY

Jiayu Zhou[1], Siqi Liang[1], Yijiang Pang[2], Hiroko Dodge[3], and Bao Hoang[2], *1. University of Michigan, Ann Arbor, Michigan, United States, 2. Michigan State University, East Lansing, Michigan, United States, 3. Massachusetts General Hospital, Harvard Medical School, Boston, Massachusetts, United States*

Background: Mild Cognitive Impairment (MCI) is an early stage of Alzheimer's disease, where timely detection can significantly improve intervention outcomes and quality of life. Language markers from routine conversations offer a promising, accessible method to identify MCI. Current research primarily aggregates multiple conversations, potentially masking valuable dynamic cognitive fluctuations over time. Additionally, individual differences in speech styles complicate cognitive assessments. We address this by proposing a novel "temporal harmonization" method, enhancing MCI detection accuracy through personalized language analysis. Method: Using 6,771 conversation samples from 74 older adults participating in the Internet-Based Conversational Engagement Clinical Trial (I-CONECT, ClinicalTrials.gov#: NCT02871921), we analyzed linguistic indicators including vocabulary diversity, grammatical complexity, and conversational response patterns collected monthly over 12 months. Our innovative harmonization technique leverages advanced machine learning methods to distinguish cognitive changes from personal speaking styles, thus increasing the accuracy and reliability of detecting early cognitive impairment. Result: Our analysis demonstrates that assessing language markers as temporal sequences significantly improves MCI detection accuracy compared to traditional, single-conversation methods. With temporal harmonization, the predictive accuracy increased notably (AUC=0.721 vs. 0.648 without harmonization), underscoring the value of considering personalized conversational patterns over time. Conclusion: Routine conversational language patterns analyzed longitudinally can effectively signal early cognitive impairment. Our temporal harmonization method further improves predictive precision by controlling individual speaking differences. Such conversational screening holds potential as a practical, non-intrusive, and ecologically valid tool for social workers and geriatric professionals supporting cognitive health in older adults.

Abstract citation ID: igaf122.1206

### CHANGES IN CONVERSATIONAL PATTERN AS A CLINICAL TRIAL OUTCOME USING THE I-CONECT DATA

Liu Chen, Chao-Yi Wu, and Hiroko Dodge, *Massachusetts General Hospital, Harvard Medical School, Boston, Massachusetts, United States*

Background: Linguistic measures derived from spontaneous conversations are behavioral indicators linked to cognitive functions. We investigated a natural language processing measure, semantic noise (SN), to quantify conversational patterns and monitor intervention-induced changes. Method: We analyzed transcriptions from semi-structured conversations in the I-CONECT project (ClinicalTrials.gov: NCT02871921). Participants engaged in 30-minute semi-structured conversations four times per week via online video chats for six months. Mean SN (MSN) from the second and 24th weeks' conversations represented participants' conversational patterns at baseline and month six (M6), respectively. Two-tailed t-tests and paired t-tests are used for statistical analysis. Results: Among participants who completed baseline and M6 video chat interventions, the MCI group's MSN was significantly higher than the NC group's (MCI: 2.09 ± 0.34, NC: 1.87 ± 0.35; p = 0.024) at baseline. After six months of intervention, no significant difference was observed between groups (MCI: 1.81 ± 0.47, NC: 1.95 ± 0.47; p = 0.280). In the MCI group, baseline MSN was significantly higher than M6 MSN (paired t-test: p = 0.009), but no significant change occurred in the NC group (paired t-test: p = 0.426). Conclusions: We demonstrated a conversational pattern difference between the MCI and NC groups at baseline. After six months of conversational interaction, the MCI group's MSN decreased, shifting from being significantly higher than that of the NC group at baseline to no significant difference at M6. This suggests that following the intervention, the conversation pattern of the MCI group resembled that of the NC group.

Abstract citation ID: igaf122.1207

### AUGMENTED RISK PREDICTION FOR ALZHEIMER'S ONSET FROM ELECTRONIC HEALTH RECORDS WITH LARGE LANGUAGE MODELS

Jiankun Wang[1], Sumyeong Ahn[2], Taykhoom Dalal[3], Xiaodan Zhang[2], Bin Chen[2], Hiroko Dodge[4], Fei Wang[3], and Jiayu Zhou[1], *1. University of Michigan, Ann Arbor, Michigan, United States, 2. Michigan State University, East Lansing, Michigan, United States, 3. Weill Cornell Medicine, New York, New York, United States, 4. Massachusetts General Hospital, Harvard Medical School, Boston, Massachusetts, United States*

Background: Alzheimer's disease and related dementias (ADRD) are among the top causes of mortality and disability in older adults. Early detection is critical for timely clinical intervention, caregiver planning, and patient enrollment in clinical trials. Electronic health records (EHRs) offer a readily accessible data source for automated dementia risk screening. However, existing predictive models often struggle to accurately identify nuanced or complex patient presentations, limiting their clinical utility. Recently, large language models (LLMs)—advanced artificial intelligence systems proficient at reasoning and interpreting medical data—have demonstrated promising capabilities for improving diagnostic accuracy. Methods: We propose a novel dementia risk prediction pipeline that integrates traditional supervised machine learning methods (SLs) with advanced LLMs. Our method uses SLs to accurately classify clear-cut dementia risk cases from structured EHR data, while leveraging LLMs' sophisticated reasoning capabilities to handle ambiguous or challenging patient records. We evaluated this collaborative approach using a large, real-world EHR dataset from Oregon Health & Science University (OHSU), consisting of over 2.5 million patient records spanning more than 20 million clinical encounters. Results: Our integrated approach significantly outperformed traditional predictive models alone, demonstrating improved accuracy and reliability in dementia risk prediction. The combined use of SLs and LLMs effectively captured subtle clinical indicators that traditional methods overlooked. Conclusion: Integrating advanced language models into existing machine learning approaches markedly improves dementia screening from routine clinical data. This method shows substantial potential for enhancing early ADRD detection, offering physicians powerful tools for proactive, personalized patient management.

### SESSION 3095 (SYMPOSIUM)

Abstract citation ID: igaf122.1208

### CONNECTING SOCIAL DETERMINANTS OF HEALTH TO CANCER CARE: ACCESS TO CANCER CARE TO PATIENT EXPERIENCES OF OLDER ADULTS

Chair: Mackenzie Fowler

Social determinants of health (SDOH) have emerged as important predictors of outcomes in cancer. SDOH are the "conditions in which people are born, grow, learn, work, play, live, and age, and the broader set of structural factors shaping the conditions of daily life". These are generally described in five domains: economic stability, education access/quality, healthcare access/quality, neighborhood/built environment, social/community context. SDOH span all levels of the social ecological framework from health policy to the individual. However, limited evidence exists examining the relationship between SDOH among older adults with cancer. Our symposium brings together researchers to evaluate SDOH across domains and levels of influence and their relationship with outcomes among older adults with cancer. First, Dahal will present the policy level of influence and healthcare access/quality domain by describing access to colon cancer care for vulnerable Medicare-Medicaid dual enrollees, specifically plan enrollment at diagnosis. Second, Fowler will present the community level and neighborhood/built environment domain describing the adjusted association between neighborhood-level social vulnerability and its differential effect on cancer survival among older versus younger adults. Finally, Swaim will discuss the individual level and social/community context domain to understand how daily experiences may fluctuate within a person day-to-day. Understanding how SDOH across domains and levels of influence affect outcomes among older adults with cancer will help facilitate opportunities for intervention specific to the unique needs of this population.

Abstract citation ID: igaf122.1209

### DIFFERENTIAL EFFECTS OF SOCIAL VULNERABILITY INDEX AMONG OLDER AND YOUNGER ADULTS WITH CANCER

Mackenzie Fowler, Geetanjali Saini, Elizabeth Baker, Mahak Bhargava, and Ritu Aneja, *The University of Alabama at Birmingham, Birmingham, Alabama, United States*

Social determinants of health, (e.g. social vulnerability index [SVI]), are associated with adverse cancer-related outcomes. Most cancer cases occur among older adults (≥65). Effects of SDOH may differ between older and younger adults. Our objective was to examine the role of SVI on all-cause mortality among adults with cancer in Alabama. We included adults (≥18) with incident breast, prostate, colorectal, or lung cancer between 1/1/2010 and 12/31/2019 from the Statewide Cancer Registry. The exposure was census tract-level SVI in quartiles. The outcome was overall survival (OS) from diagnosis to death or end of follow-up (12/31/2021). We performed Cox proportional hazards models to estimate associations adjusting for age, race, sex (if applicable), stage, census-tract rurality (Rural-Urban Commuting Area Code), and subtype (breast). We assessed interaction and stratified by age group at diagnosis (<65, ≥65). We further adjusted for insurance status. Living in higher SVI quartiles was associated with significantly higher hazard of mortality across all cancer types examined; this association was stronger for those <65 at diagnosis as interaction with age was significant for breast, prostate, and colorectal cancer (p-values: breast: 0.025; prostate: <0.001; colorectal: 0.031). Adjusting for insurance status, the association between SVI remained significant among older adults but not among younger adults. Insurance status had a stronger independent association with mortality among younger adults. These results indicate neighborhood-level SVI and individual-level insurance status may have greater negative effect on OS among younger adults whereas other unmeasured factors may affect OS in cancer among older adults.

Abstract citation ID: igaf122.1210

### PLAN ENROLLMENT OF MEDICARE-MEDICAID DUAL ENROLLEES AT COLON CANCER DIAGNOSIS: INSIGHTS FROM SEER-MEDICARE

Roshani Dahal[1], Sayeh Nikpay[1], and Helen Parsons[2], *1. University of Minnesota School of Public Health, Minneapolis, Minnesota, United States, 2. University of Minnesota, Minneapolis, Minnesota, United States*

Medicare-Medicaid dual status is associated with poorer quality of cancer care and reduced survival rates. Understanding plan enrollment patterns may provide insight into access to cancer care. Our study examined plan enrollment among Medicare beneficiaries diagnosed with cancer nationally. We created a cohort of colorectal cancer patients aged 66 and older, diagnosed between 2016 and 2019, and enrolled in Medicare Parts A and B in the diagnosis month. We linked Surveillance, Epidemiology and End Results data and Master Beneficiary Summary File. Our cohort included 108,947 Medicare beneficiaries diagnosed with colorectal cancer. Approximately 18% (n = 19,634) were Medicare-Medicaid dual enrollees, with median age at diagnosis of 76 years, 57.2% female, predominantly White (68.1%) followed by Black (18.2%), and mostly residing in metropolitan counties (61.7%). Among these, 71.2% were full-benefit duals and 28.8% were partial-benefit duals. At diagnosis, most duals were enrolled in Traditional Medicare (59% overall; 63.9% full-benefit duals), while others in Medicare Advantage plans (41% overall; 53% partial-benefit duals). Notably, half of colorectal cancers had unknown or missing staging, while the remaining cases were stage II (14.2%), stage III (13.2%), stage IV (12.2%), and stage I (10.7%). Our results indicate that most Medicare-Medicare duals with colorectal cancer are enrolled in Traditional Medicare (known for wide provider networks and limited prior authorization). Future studies should compare access to comprehensive cancer care (including palliative care, supportive therapies, social support, end-of-life care for advanced-stage cancers) between full-benefit and partial-benefit duals in Traditional Medicare versus Medicare Advantage, with attention to missing staging and treatment pathways.

Abstract citation ID: igaf122.1211

### CONTROL BELIEFS & AWARENESS OF AGE RELATED CHANGE IN MIDDLE-AGED AND OLDER ADULTS WITH CANCER

Kathryn Swaim, and Shevaun Neupert, *North Carolina State University, Raleigh, North Carolina, United States*

Control beliefs have been defined as the belief that one can achieve their goals. As people age, the likelihood of experiencing physical changes, and serious illness increases which has been found to impact control over time. While aging and illness have been seen as normative parts of aging, how people perceive these changes may impact health and well-being overtime. Awareness of age related change (AARC), highlights not only losses (e.g., physical limitations, illness) but gains (e.g., greater social connections, knowledge and experience, engagement in hobbies) that come with aging. Experiencing daily AARC gains may impact how adults with cancer may feel in control day-to-day. The objective of this study was to investigate moderating variables such as daily uplifts (i.e., any activity or connection that brings joy) in the potential relationship with daily AARC gains and control beliefs in adults with cancer. A sample of 440 U.S. adults aged 50-85 participated in a 14-day daily diary study with 48 reported having been diagnosed with cancer. It was found that on days when adults with cancer reported more AARC gains, they reported feeling more in control ($t = 3.29$, $p = 0.0017$). It was also found that on days people reported having few AARC gains and but engaged in more uplifts, they reported feeling more control ($t = -2.47$, $p = 0.0167$). Therefore, not only focusing on gains associated with aging, but engaging in uplifting activities can be substantially beneficial for adults navigating cancer and aging.

### SESSION 3100 (SYMPOSIUM)

Abstract citation ID: igaf122.1212

### NAVIGATING LIFE TRANSITIONS AS A STRATEGY FOR PREVENTING LATE-LIFE SUICIDE

Chair: Catherine Ju
Discussant: Amy Fiske

Older adults have higher rates of suicide than the general population. Life transitions and changes unique to aging may contribute to this increased risk of suicide. This symposium will feature research examining the identification of suicide risk factors and prevention of suicide in older adults, with a particular focus on the role of changes in functioning and health. First, Catherine Ju will present research investigating how strategies older adults might use to compensate for functional impairment (e.g., seeking help) might play a role in risk for suicidal ideation among people who highly value their own autonomy. Second, Dr. Kimberly Van Orden will present results of a clinical trial testing whether an intervention addressing barriers to social connection (produced by functional impairment and health problems) results in reductions in suicidal ideation as measured via ecological momentary assessment. Last, Dr. Briana Mezuk will describe research employing large language models to identify and characterize the structure of life transitions of older suicide decedents using the National Violent Death Reporting System. Dr. Amy Fiske will serve as discussant.

Abstract citation ID: igaf122.1213

### SUICIDAL IDEATION IN OLDER ADULTS WHO HIGHLY VALUE AUTONOMY: THE ROLE OF COMPENSATORY STRATEGIES

Catherine Ju, and Amy Fiske, *West Virginia University, Morgantown, West Virginia, United States*

Greater value placed on one's autonomy is related to suicidal ideation in older adults, but no research has examined the mechanisms underlying this relationship. The Motivational Theory of Lifespan Development contends that as individuals age and functional impairment increases, relying on one's own effort may not be enough to obtain important goals. Therefore, older adults might use compensatory strategies (e.g., seeking help) to achieve their goals. However, older adults who highly value their autonomy may engage in fewer compensatory strategies, resulting in failure to achieve goals. This could contribute to feelings of hopelessness and suicidal ideation in older adults. This presentation will introduce research that examined whether (a) compensatory primary control strategies and (b) depressive symptoms mediated the relationship between value placed on autonomy and suicidal ideation and whether these effects were moderated by functional status in older adults. Older adults (N = 419) completed an online survey with questions about functional ability, value placed on autonomy, control strategy usage, depressive symptoms, and suicidal ideation. Results showed that compensatory primary control strategy usage and level of depressive symptoms partially mediated the relationship between value placed on autonomy and suicidal ideation. Unexpectedly, functional impairment did not moderate these relationships. Findings suggest that non-engagement in compensatory strategies is one reason value placed on autonomy is related to suicidal ideation in older adults. Investigating what may promote usage of these compensatory strategies could increase the field's understanding of what factors lead to greater risk for suicide in older adults.

Abstract citation ID: igaf122.1214

### TARGETING HEALTH AND FUNCTIONING BARRIERS TO SOCIAL CONNECTION TO REDUCE SUICIDE RISK

Kimberly Van Orden, *University of Rochester Medical Center, Rochester, New York, United States*

Changes in health and functioning can introduce barriers to social engagement for older adults. Social disconnection—low social engagement, isolation, and loneliness—is associated with suicide ideation (SI), attempts, and deaths in later life. However, it is not known if promoting social connection is an effective strategy to prevent suicide in later life because studies have not directly tested this mechanism. This presentation will describe results from a clinical trial testing a brief behavioral intervention to increase social engagement among older adults experiencing clinically significant loneliness. Severity of SI via 10 days of ecological momentary assessment (EMA) at baseline and 3-month follow-up will be presented. The Geriatric Suicide Ideation Scale, Screening Version was used in EMA (4 items rated 1-3; possible scores 4-12). Currently, 29 out of 30 planned subjects have been assigned to the study intervention and completed the EMA protocol at baseline; 13 subjects completed follow-up EMA. The sample has an average age of 72.26 years (range 60-96); 5 subjects are male, 19 female, and 1 intersex. Just under half the sample (48%) reported suicide ideation (lifetime) at baseline. SI severity (GSIS) was 6.71 (std 1.71) via EMA at baseline and decreased to 6.40 (std 2.13) at follow-up ($p = 0.001$). Presentation results will include the full sample and effect sizes to illustrate magnitude of effects. Results indicate reliable reductions in suicide ideation severity after 10 sessions of one-on-one social engagement coaching to address functioning and health-related barriers, consistent with potential benefits of this approach for late-life suicide prevention.

Abstract citation ID: igaf122.1215

### THE STRUCTURE OF MAJOR LIFE TRANSITIONS AMONG OLDER SUICIDE DECEDENTS: AN APPLICATION OF LARGE LANGUAGE MODELS

Briana Mezuk[1], Viktoryia Kalesnikava[2], David Jurgens[1], Lily Johns[1], and Kara Zivin[3], *1. University of Michigan, Ann Arbor, Michigan, United States, 2. Institute for Social Research, Ann Arbor, Michigan, United States, 3. University of Michigan Medical School, Ann Arbor, Michigan, United States*

The role of life events in shaping suicide risk for older adults is unclear. Structure and meaning of events are interconnected: structure shapes the relationship between event elements (e.g., timing), with meaning emerging through their dynamic interactions. Large language models (LLM) provide an opportunity to analyze textual data at scale, but they have not yet been widely used to understand the contributing circumstances of suicide. In this study, we applied LLMs to narrative texts from the National Violent Death Reporting System (2003-2022), a nationwide registry of suicide deaths, to (1) identify salient events in the lives of suicide decedents aged 50+ (n = 164,240), (2) classify aspects of the structure of these events (e.g., timing, sequence, expectedness), and (3) explore variation by age. Mean age of decedents was 63 years, 24% were female, 88% were non-Hispanic White; 54% did not have a known mental health problem at the time of their death. Most narratives described sequences of life events involving interlinked health or family problems that often escalated days before suicide (e.g., mobility loss after a recent surgery); less frequently, narratives mentioned isolated events, referring to financial or relationship crises. For decedents aged 65+, events often involved legal issues or relationship loss (e.g., widowhood), while those aged <65 events typically related to employment or relationship discord. Preliminary analyses demonstrate the feasibility of identifying structural elements of life events using LLMs; next steps will include refining prompts to improve classification of complex transitions from these texts.

### SESSION 3105 (SYMPOSIUM)

Abstract citation ID: igaf122.1216

### SARCOPENIA AMONG OLDER ADULTS: EPIDEMIOLOGY, PROTEOMICS, TECHNOLOGY AND BIOMARKERS

Chair: Josef Coresh
Co-Chair: Shoshana Ballew
Discussant: Jennifer Schrack

This session delves into research in sarcopenia, a condition characterized by the loss of muscle mass and function in older adults, from epidemiology, proteomics, technology and biomarkers. The session begins with a discussion on the challenges and methodologies in defining sarcopenia within a population-based study of the oldest adults, highlighting the importance of accurate and practical diagnostic criteria in understanding its prevalence and impact. Following this, data on the incidence of sarcopenia from age 66 to 90+ years, using the ARIC cohort from 2011 to 2023, is presented to provide insights into the age-related progression of sarcopenia and its public health implications. The session then explores the use of actigraphy monitoring among older adults with sarcopenia, showcasing innovative approaches to tracking physical activity patterns that offer new avenues for early detection and management. The proteomics of sarcopenia is examined next, focusing on both shared and distinct biological pathways that contribute to its prevalence and future risk, shedding light on the molecular underpinnings and potential targets for intervention. Finally, longitudinal changes in the creatinine muscle index, a novel blood marker of muscle mass, among older adults are discussed, emphasizing the importance of continuous monitoring and early intervention in mitigating the adverse effects of muscle loss. Overall, the session provides a wide-ranging set of results in sarcopenia research and a discussion of the implications of this common and treatable condition in aging populations.

Abstract citation ID: igaf122.1217

### LONGITUDINAL CHANGES IN CREATININE FILTRATION AS A NOVEL INDEX OF MUSCLE MASS AND MORTALITY RISK

Shoshana Ballew[1], Aditya Surapaneni[1], Jordan Weiss[2], Hubert Leo[1], Morgan Grams[1], Marcus Goncalves[1], and Josef Coresh[1], *1. New York University, New York, New York, United States, 2. NYU Grossman School of Medicine, New York, New York, United States*

Sarcopenia, defined by low muscle mass, is common among older adults and is associated with adverse outcomes. Quantifying muscle mass is challenging in routine clinical practice as the gold standard measures are expensive, cumbersome, and time consuming. A single timepoint of estimated glomerular filtration of creatinine (eGFcr=eGFR using serum cystatin C x serum creatinine) as an index of muscle mass has been shown to be associated with frailty, mortality, and muscle imaging measurement. Cox regression was used to estimate associations of change in eGFcr from visit 5 (2011–2013) to visit 6 (2016-2017) of a community-based prospective cohort, the Atherosclerosis Risk in Communities (ARIC) Study, with mortality. Models were adjusted for demographics, clinical variables, and comorbid conditions. Among the 2943 participants (average age 75 years, 56% women), 2210 participants had a decline in eGFcr (76.8% of men and 73.4% of women) and 280 participants had a decrease of > 5% per year (9.7% in men and 9.3% in women). There were 407 deaths over 4.5 years of follow-up. In models adjusted for baseline covariates, women with a decrease in eGFcr had an increased risk of mortality (HR 1.01 CI: 0.96-1.06 and HR 1.08 CI: 1.03-1.37 per 1% decline in eGFcr in men and women, respectively). When accounting for the last eGFcr measure, the change was no longer significant. The eGFcr can be used in research and clinical practice to assess muscle mass in older adults, and the trajectories over time may be informative for risk when considering prevention of muscle loss.

Abstract citation ID: igaf122.1218

### DEFINING SARCOPENIA IN A POPULATION BASED STUDY OF OLDEST ADULTS

Josef Coresh[1], Shoshana Ballew[1], Jordan Weiss[2], Morgan Grams[1], B. Gwen Windham[3], Lynne Wagenknecht[4], Marcus Goncalves[1], and Hubert Leo[1], *1. New York University, New York, New York, United States, 2. NYU Grossman School of Medicine, New York, New York, United States, 3. University of Mississippi Medical Center - The MIND Center, Jackson, Mississippi, United States, 4. Wake Forest University School of Medicine, Winston-Salem, North Carolina, United States*

Sarcopenia is common in older age and is associated with increased risk of mortality and other complications. Sarcopenia definitions vary, but consensus suggests the following should be present: (1) low muscle mass; (2) impaired strength; and (3) decreased performance. We sought to assess the agreement and correlates of candidate input variables in 6,515 Atherosclerosis Risk in Communities (ARIC) Study participants at visit 5 (2011-2013, age 66+ years). The input variables were fat free mass (FFM kg/m2) using bioimpedance analysis (BIA, lower limb leads), grip strength (grip, kg), and walking speed (WS, 4-meter walk, m/s). Age correlations were: -0.03 for FFM, -0.18 for grip and -0.23 for WS. Correlations between variables were 0.03 in men/-0.05 in women (FFM and grip), -0.05/-0.32 (FFM and WS) and 0.25/0.21 (grip and WS), respectively. Grip and WS were each strongly related to mortality and hospitalization. Having both weak grip and slow WS associated with a doubling in mortality risk (hazard ratio=2.3 (95% CI 2.0-2.6)) compared to having neither, supporting the sarcopenia definition put forward by the Sarcopenia Definitions and Outcomes Consortium. FFM association with mortality was weak and inconsistent across strata of grip and WS, suggesting limitations in the use of BIA in defining sarcopenia. In summary, we detail differences across the sarcopenia input variables by demographics, as well as their implications for risk associations. More robust measures of muscle mass (e.g. DXA vs. BIA with lower limb leads) may add value to sarcopenia definitions.

Abstract citation ID: igaf122.1219

**SARCOPENIA INCIDENCE FROM AGE 65 TO 90+ YEARS FROM 2011 TO 2023 IN THE ARIC COHORT**

Jordan Weiss[1], Shoshana Ballew[2], Hubert Leo[2], Morgan Grams[2], B. Gwen Windham[3], Lynne Wagenknecht[4], Josef Coresh[2], and Marcus Goncalves[2], *1. NYU Grossman School of Medicine, New York, New York, United States, 2. New York University, New York, New York, United States, 3. University of Mississippi Medical Center - The MIND Center, Jackson, Mississippi, United States, 4. Wake Forest University School of Medicine, Winston-Salem, North Carolina, United States*

Sarcopenia, characterized by progressive loss of skeletal muscle mass and function, is a major contributor to disability and mortality in aging populations. Incidence rates at the oldest ages from recent studies are limited and needed to identify at-risk populations and inform interventions and policy. We estimated age-specific sarcopenia incidence by race and sex in the Atherosclerosis Risk in Communities (ARIC) study. Sarcopenia was defined using Sarcopenia Definitions and Outcomes Consortium (SDOC) criteria: low grip strength (<35.5 kg for males, <20 kg for females) and slow gait speed (<0.8 m/s). Among 5,985 adults (mean age, 75.4 [SD, 5.1] years; 57.9% female; 21.9% Black) at baseline (visit 5, 2011–2013), 665 (11.1%) had prevalent sarcopenia with prevalence rising from 5.0% at age 65-69 to 36.0%at age 85-89 years. Incident cases were identified through visit 10 (2022–2023). Over 30,727 person-years (PY), 900 incident cases were identified (incidence rate [IR], 2.92 per 100 PY; 95% CI, 2.74–3.13). Incidence increased with age. Among participants aged 60–69 at baseline, IRs were highest in Black males (2.57; 95% CI, 1.55–4.27) and lowest in Black females (1.13; 95% CI, 0.64–1.99). By ages 80–89, all rates rose to approximately 5% a year except for Black females (2.87; 95% CI, 1.73–4.76). Targeted prevention and early interventions are needed to mitigate disparities and reduce sarcopenia-related disability in aging populations.

Abstract citation ID: igaf122.1220

**SARCOPENIA AND DIGITALLY MEASURED FREE-LIVING PHYSICAL ACTIVITY IN OLDER ADULTS**

Amal Wanigatunga[1], Lacey Etzkorn[1], Brian Buta[2], Sunan Gao[2], Josef Coresh[3], and Jennifer Schrack[1], *1. Johns Hopkins Bloomberg School of Public Health, Baltimore, Maryland, United States, 2. Johns Hopkins University, Baltimore, Maryland, United States, 3. New York University, New York, New York, United States*

This study examined the association between sarcopenia (age-related muscle mass and strength loss) with digitally measured free-living physical activity patterns—measures that summarize biological, physiological, behavioral, and environmental effects on mobility. Among 853 participants in the Atherosclerosis Risk in Communities (ARIC) Study, sarcopenia at visit 9 was defined as slow gait speed (<0.8 m/s) and low hand grip strength (<20 kg/women, <35.5 kg/men). Accelerometry was collected using a 7-day, 24-hour wrist-worn protocol and summarized into activity amount (total activity counts/d), intensity (mean activity counts in the consecutive five most active minutes/d), endurance (activity bouts lasting ≥10 minutes/d), and accumulation (physical activity fragmentation %) patterning metrics. Linear regression was used to estimate cross-sectional associations between sarcopenia and each physical activity metric adjusted for age, sex, body mass index, gait speed, and grip strength. Mean sample age was 83 years, 58% were women, and 20% (n = 198) were classified as living with sarcopenia. After covariate adjustment, sarcopenia was associated with lower total activity (β=- 21,8158 counts/d, SE = 60,436; p < 0.001) and endurance (-24 minutes in activity bouts ≥10 minutes, SE = 10; p = 0.02). Conversely, sarcopenia was not associated with daily mean activity intensity (p = 0.13) or the way daily activity was accumulated throughout the day (p = 0.18). Results indicate that sarcopenia, above and beyond its individual components, is primarily associated with lower amounts of daily physical activity and endurance for movement related to everyday living. Our findings help define how sarcopenia connects to physical activity behavior in older adults and provides insight into potential preventative mechanisms.

Abstract citation ID: igaf122.1221

**PROTEOMICS OF SARCOPENIA PREVALENCE AND FUTURE RISK: SHARED AND DISTINCT PATHWAYS**

Hubert Leo[1], Aditya Surapaneni[1], Shoshana Ballew[1], Jordan Weiss[2], Morgan Grams[1], Marcus Goncalves[1], and Josef Coresh[1], *1. New York University, New York, New York, United States, 2. NYU Grossman School of Medicine, New York, New York, United States*

Sarcopenia is a state of low muscle mass and function that is known to be related to systemic, low-grade, chronic inflammation. We screened over 5,000 proteomic measures to identify changes preceding sarcopenia incidence to inform development of therapies and risk stratification. We defined sarcopenia using the SDOC definition of weak grip strength and slow walking speed at the 2011–2013 (Visit 5, N = 6,538, mean (SD) age 76 (5)) and 2016-2017 (visit 6, N = 4,003) visits of the Atherosclerosis Risk in Communities (ARIC) study. We quantified aptamer measures of over 4,000 unique proteins from Visit 5 plasma using the SomaLogic platform. Sarcopenia was present in 665 participants (11.3%) at baseline and new incident cases developed in 391 (9.8%) by Visit 6. Among the 5,272 aptamers tested, 114 were associated with sarcopenia prevalence and 9 were associated with sarcopenia incidence after adjustment for age, sex, eGFRcr-cys, and multiple comparisons (Bonferroni p < 9.5E-6). The proteins associated with sarcopenia incidence (PXDN, TIMP4, RNASE1, GDF15, SVEP1, THBS2, SVEP1, ANGPT2, EGFR) were all associated with sarcopenia prevalence (eight at p < 4E-6; TIMP4 p = 1E-4). Pathway analysis on proteins associated with sarcopenia prevalence showed enrichment for Antigen Processing and Presentation, Apoptosis, Carbohydrate Digestion and Absorption, and Fanconi anemia pathways. Sarcopenia incidence prediction had an AUC of 0.713 for nine proteins, 0.709 for age and clinical factors, and 0.749 combined. This is the largest proteomic screen of incident sarcopenia and provides new insights into proteomic alterations that both precede and predict risk of sarcopenia.

### SESSION 3110 (SYMPOSIUM)

Abstract citation ID: igaf122.1222

**ORAL HEALTH & COGNITIVE IMPAIRMENT: BIOLOGICAL, CLINICAL, AND SOCIAL PERSPECTIVES**

Chair: Bei Wu
Co-Chair: Zhang Zhang
Discussant: Huabin Luo

This symposium offers an in-depth exploration of the complex interplay between oral health and cognitive function in older adults, integrating biological, clinical, and social perspectives to deliver observational and behavioral insights. The first presentation investigates the biological underpinnings, examining how oral bacteria moderate the link between tooth loss and cognitive performance. It reveals that a high periodontal bacterial load significantly worsens cognitive dysfunction, even with minimal tooth loss, emphasizing oral hygiene's critical role. The second presentation provides clinical evidence, assessing how multiple oral health issues—such as tooth loss, dental plaque, and periodontal symptoms—contribute to domain-specific cognitive decline, notably impacting memory and executive function. The third presentation explores behavioral dimensions, analyzing how cognitive impairment affects dental care utilization across insurance types. It highlights reduced access among those with no insurance or Medicare-only, signaling a need for policy reform. The fourth presentation adopts a life course perspective, exploring how edentulism and social mobility jointly shape cognitive trajectories, with effects varying by age. Collectively, these studies illuminate the bidirectional relationship between oral health and cognitive function, offering actionable insights for prevention, clinical intervention, and policy enhancements to improve older adults' quality of life.

Abstract citation ID: igaf122.1223

### THE MODERATING EFFECTS OF ORAL BACTERIA ON THE ASSOCIATION BETWEEN TOOTH LOSS AND COGNITIVE PERFORMANCE

Huabin Luo[1], Angela Kamer[2], Zhijing Xu[2], and Bei Wu[3], *1. East Carolina University, Greenville, North Carolina, United States, 2. New York University, New York, New York, United States, 3. NYU Shanghai, Shanghai, China (People's Republic)*

There is growing evidence that tooth loss is a risk factor for cognitive dysfunction. Oral microbiome may also influence brain health and contribute to cognitive decline. Yet little evidence exists on the potential modifying role of oral microbiome in the relationship between tooth loss and cognitive performance. Using the 2011-2012 National Health and Nutrition Examination Survey data, we investigated the interaction effects between tooth loss and oral dysbiotic status on cognitive performance. The sample included 670 adults aged 60 years or older. Cognitive performance was assessed by the Consortium to Establish a Registry for Alzheimer's Disease: Immediate Word Recall (IWR), Delayed Recall (DWR), Animal Fluency Test (AFT), and Digit Symbol Substitution Test (DSST). Significant tooth loss was defined as a loss of 10+ permanent teeth (out of 28). A high dysbiotic index (DI) was defined by the upper tertile of the ratio of periodontal bacteria (Treponema, Porphyromonas, and Tannerella) to healthy bacteria (Rothia and Corynebacterium). Multivariate regression model results showed a significant interaction between tooth loss and the DI (b=-1.76, P = 0.04) on AFT; this interaction was approaching significance for word recall test scores (sum of IWR and DWR) (b=-1.67, P = 0.07). This interaction was due to the significant difference in cognitive performance between low and high DI at low tooth loss but not at significant tooth loss. At low tooth loss, cognitive dysfunction is driven by high load of periodontal bacteria. Oral health awareness and good oral hygiene practice should be further promoted among older adults in the community.

Abstract citation ID: igaf122.1224

### ORAL HEALTH CONDITIONS AND DOMAIN-SPECIFIC COGNITIVE DECLINE IN OLDER ADULTS: BLSA 2004-2024

Xiang Qi[1], Huabin Luo[2], and Bei Wu[3], *1. New York University, New York, New York, United States, 2. East Carolina University, Greenville, North Carolina, United States, 3. NYU Shanghai, Shanghai, China (People's Republic)*

Associations between poor oral health and cognitive impairment have been documented, yet research examining associations between various oral health conditions and domain-specific cognitive decline remains limited. Using data from the Baltimore Longitudinal Study of Aging (2004-2024), we investigated the association between cognitivelymultiple oral health conditions and cognitive decline across domains in adults aged 50+ who were cognitive intact at baseline. Cognitive function was assessed across language, memory, attention, executive function, and visuospatial ability domains, with domain scores calculated as means of standardized cognitive test scores. Oral health was evaluated through objective assessment of tooth loss and dental plaque, alongside self-reported periodontal symptoms at baseline. The study included 980 participants (49.3% women, 66.5% White) with a mean age of 68.2 years (SD = 10.1), followed for about 7.8 years (SD = 4.9). Significant tooth loss showed associations with greater declines in global cognitive function (β=-0.0024; 95% CI: -0.0037, -0.0010), and across all cognitive domains, including language (β=-0.0035; 95% CI: -0.0049, -0.0020), memory (β=-0.0041; 95% CI: -0.0060, -0.0021), attention (β=-0.0028; 95% CI: -0.0045, -0.0011), executive function (β=-0.0018; 95% CI: -0.0032, -0.0003), and visuospatial ability (β=-0.0037; 95% CI: -0.0052, -0.0021). Moderate/significant dental plaque was associated with declining in global cognitive function (β=-0.0041; 95% CI: -0.0075, -0.0008) and memory (β=-0.0051; 95% CI: -0.0097, -0.0005), compared with no/hardly visible plaque, while moderate/severe periodontal symptoms solely predicted executive function decline (β=-0.0013; 95% CI: -0.002, -0.0003). Poor oral health significantly impacts memory and executive function, with tooth loss showing broader cognitive effects compared to other oral health conditions.

Abstract citation ID: igaf122.1225

### THE EVALUATION OF COGNITIVE IMPAIRMENT ON DENTAL CARE UTILIZATION: COMPARATIVE ANALYSIS OF INSURANCE TYPES

Zhang Zhang[1], and Bei Wu[2], *1. Johns Hopkins Bloomberg School of Public Health, Baltimore, Maryland, United States, 2. NYU Shanghai, Shanghai, China (People's Republic)*

Background: Dental care is critical for overall health and quality of life, yet barriers to access persist, particularly for individuals with disabilities. This study examines the relationship between cognitive impairment and dental care utilization, focusing on differential effects across insurance types. Method: Using the Health and Retirement Study (HRS) from 1996 to 2018, we constructed a longitudinal sample cohort of 25,201 participants aged at or above 65 with 127,217 observations. We adopted an individual fixed-effect model to measure the relationship between cognitive impairment and the probability of using dental care in the past two years and examined the heterogeneous effect across insurance types. We defined cognitive impairment using the Langa–Weir classification method. Results: 4.4 % have no insurance, 38.4% are in Medicare only, 9.8% are dual-eligible in Medicare and Medicaid, and 47.4% are in Medicare and private insurance. Compared with the healthy group, cognitive impairment was significantly associated with reducing the probability of dental care utilization by 7.6 percentage points ($p < 0.01$, 95% CI: -0.084, -0.068). Moreover, cognitive impairment was significantly associated with declining dental visits by 7.7 percentage points ($p < 0.01$, 95% CI: -0.151, -0.003) among no insurance and by 1.9 percentage points ($p < 0.01$, 95% CI: -0.035, -0.003) among Medicare-only, but no significant association among dual eligibles or those with private insurance. Conclusion: This study reveals the relationship between cognitive impairment and reduction in dental care utilization among heterogeneous insurance groups, particularly among non-insurance and Medicare-only. These findings underscore the urgent need for Medicare reforms to include dental coverage.

Abstract citation ID: igaf122.1226

### EDENTULISM, SOCIAL MOBILITY, AND COGNITIVE AGING: A LIFE COURSE PERSPECTIVE

Ruotong Mona Liu[1], Huabin Luo[2], Xiang Qi[1], Zhijing Xu[1], and Bei Wu[3], *1. New York University, New York, New York, United States, 2. East Carolina University, Greenville, North Carolina, United States, 3. NYU Shanghai, Shanghai, China (People's Republic)*

While edentulism and social mobility have each been linked to cognitive decline among older adults, few studies have examined their interactive effects or how these relationships differ across age groups. This study investigated the joint effects of edentulism and social mobility on cognitive function and age-specific patterns. We analyzed data from the Health and Retirement Study (2006–2020), comprising 23,158 adults aged 51+. Cognitive function was assessed using modified Telephone Interview for Cognitive Status. Social mobility was represented by intergenerational education mobility, and edentulism was determined through self-reporting. Linear mixed-effects models with lagged time-varying covariates were used to examine how these factors affected cognitive changes over time. At baseline, 13.2% of participants were edentulous. We classified participants into four social mobility groups: stable low socioeconomic position (SEP) (29.6%), stable medium/high SEP (34.3%), upward mobility (17.1%), or downward mobility (19.1%). Edentulism was associated with accelerated cognitive decline, whereas all non–low-SEP groups had higher baseline cognition. The stable medium-high SEP group also demonstrated slower cognitive decline and greater resilience against the negative effects of edentulism over time. The interaction effects between edentulism and social mobility were most pronounced in older adults aged 65-74, while neither tooth loss or social mobility significantly affected cognitive outcomes in those 75 and older. The results highlight that edentulism and social mobility play an important role in cognitive trajectories both independently and jointly, with effects varying across age groups. Targeting oral health, especially among adults aged 51–74, may help mitigate cognitive decline within diverse SEP.

### SESSION 3115 (SYMPOSIUM)

Abstract citation ID: igaf122.1227

### CHALLENGES TO GETTING OLDER ADULTS GERIATRIC CARE: LESSONS FROM A NATIONAL RURAL GERIATRICS PROGRAM

Chair: Lauren Moo
Co-Chair: Eileen Dryden

Rural older adults have higher rates of chronic diseases and have more complex care needs than their urban counterparts. Interdisciplinary geriatrics teams have the necessary expertise to meet these complex needs but access to geriatric care in rural areas is challenging. It is critical to address the needs of aging rural older adults, so that many can successfully age in place. Information on regional needs of the older adult population and availability of existing resources is critical to informing efforts to reach older rural Veterans and the appropriate mechanisms to support these efforts. Within the Veteran healthcare system, the GRECC Connect program has improved access to geriatric care for older rural veterans using telehealth strategies while providing support to rural frontline teams as they care for complex older adults and their families. While current steps to ensure sustainment of these services are underway, we share the lessons learned on developing geriatric care services in rural areas-- highlighting the limited access to geriatric care in rural areas, developing and advocating for developing geriatric care services in resource poor areas, potential and limitations of a hub and spoke model for geriatric care and barriers and successes in sustaining and integrating geriatric care services to serve rural areas.

Abstract citation ID: igaf122.1228

### LEGITIMACY AND GERIATRICS: MAKING SPACE FOR GERIATRIC CARE IN A NATIONAL HEALTHCARE SYSTEM

Jessica Riley[1], Eileen Dryden[2], Lauren Moo[3], Meaghan Kennedy[4], Camilla Pimentel[4], and William Hung[5], *1. Veterans Health Administration, Quincy, Massachusetts, United States, 2. Veterans Health Administration, Bedford, Massachusetts, United States, 3. VA Bedford Healthcare System, Bedford, Massachusetts, United States, 4. VA Bedford Health Care System, Bedford, Massachusetts, United States, 5. James J Peters VA Medical Center, New York, New York, United States*

This presentation uses the lens of medical anthropology to better understand how geriatric services are established and maintained within the Veterans Health Administration (VHA) system. Specifically, we look to Margaret Locks theoretical concept of 'biomedical legitimacy' in biomedicine to examine the challenges and strategies involved in implementing and maintaining geriatric programs aimed at serving rural Veterans within a complex healthcare landscape. Particularly we build on Lock's biomedical legitimacy concept by considering how contextual factors, within a national healthcare system setting, influence the availability of geriatric services. We conducted semi-structured interviews with 13 staff members from four VA Clinical Resource Hubs (including a range of professional backgrounds such as psychiatry, family medicine, and geriatrics) and 11 former national, regional, and facility leaders in the VHA representing over 250 cumulative years of experience. Data were initially analyzed through team-based rapid qualitative analysis and then examined using the construct of "legitimacy." Some of the findings include facilitators and barriers staff experienced while advocating for geriatric care as part of their region's service line, some of which included leadership support and availability of clinicians to provide specialized geriatric care. This analysis provides a broader understanding of the systemic and logistical considerations that impact the delivery of geriatric care and highlights potential pathways for strengthening support for aging Veterans within the VA system and beyond.

Abstract citation ID: igaf122.1229

### UNCERTAINTY ABOUT GERIATRICIANS' ROLES LIMITS ACCESS TO ESSENTIAL CARE FOR RURAL VETERANS

Eileen Dryden[1], Jessica Riley[2], Lauren Moo[3], Meaghan Kennedy[4], Camilla Pimentel[4], and William Hung[5], *1. Veterans Health Administration, Bedford, Massachusetts, United States, 2. Veterans Health Administration, Quincy, Massachusetts, United States, 3. VA Bedford Healthcare System, Bedford, Massachusetts, United States, 4. VA Bedford Health Care System, Bedford, Massachusetts, United States, 5. James J Peters VA Medical Center, New York, New York, United States*

Tele-health hub-and-spoke models, where urban specialists provide care for rural patients via telemedicine platforms, are one way to address the dearth of specialty care clinicians in rural areas. Two examples at the VA are GRECC Connect (GC), a program that provides rural patients access to multidisciplinary geriatrics teams, and Clinical Resource Hubs (CRH), a program that provides remote access to individual primary, specialty, and mental health clinicians. Both rely on clinicians making referrals to them for their older, medically complex patients. Built into this model is the assumption that referring clinicians understand what geriatricians do and place value on interdisciplinary geriatric care. As part of an ongoing evaluation of GC, between 2020-2024 we conducted qualitative interviews with directors, clinicians, and staff from 17 GC hub sites, four CRHs, and 13 referring clinics about these geriatric telemedicine services. We explored barriers and facilitators to establishing virtual geriatric services, promoting their uptake, and sustaining their use. While those that had used geriatric services for their patients generally found them beneficial, many potential referring clinicians and staff from 'spoke' sites seemed unsure what value geriatricians or geriatric teams would provide beyond their own knowledge. Given this, clinicians and staff from the CRH and GC 'hubs' provided substantial amounts of ongoing geriatric education and marketing to 'spoke' clinicians and staff to maintain use of their geriatric services. Promoting understanding of what geriatric services are and how they can benefit patients is essential for ensuring older, rural Veterans have access to evidenced- based geriatric services.

Abstract citation ID: igaf122.1230

### LESSONS LEARNED: IMPLEMENTATION & SUSTAINMENT OF A GERIATRIC CARE MODEL WITHIN A CHANGING HEALTHCARE LANDSCAPE

Michelle Rossi[1], Steve Barczi[2], and William Hung[3], *1. VA Pittsburgh Health Care System, Pittsburgh, Pennsylvania, United States, 2. Veterans Health Administration, Madison, Wisconsin, United States, 3. James J Peters VA Medical Center, New York, New York, United States*

Americans in rural communities tend to be older and in poorer health than their urban counterparts. Telemedicine programs like GRECC Connect, within the VA healthcare system, have proven to reduce healthcare barriers such as limited availability of specialists in rural areas, cost, and travel time for frail older patients. During the initiation of this program, telehealth was widely supported within VA. Even with this support, implementation and dissemination was a challenge with rural staff turnover and limitations of technology and Wi-Fi access as examples. GRECC Connect has become a mature program which has been focusing in recent years on maintaining the positive gains GRECC Connect has made in educating rural primary care clinicians and caring for older medically complex rural Veterans despite the changing landscape of healthcare inside and outside of VA. We will discuss lessons learned in our efforts to sustain GRECC Connect clinical efforts and how these may inform implementation and sustainment of Geriatric models in context of a Value based payment system.

Abstract citation ID: igaf122.1231

### RURAL URBAN DISPARITIES IN GERIATRIC CARE RESOURCES AND USAGE

Orna Intrator[1], Sara Elgayar[2], Porooshat Dadgostar[3], Lynette Kelley[4], Emily Fanzosa[1], and William Hung[1], *1. James J Peters VA Medical Center, New York, New York, United States, 2. The University of Alabama at Birmingham, Birmingham, Alabama, United States, 3. University of Rochester, Rochester, New York, United States, 4. Veterans Health Administration, Aurora, Colorado, United States*

Disparities in availability of geriatric care may lead to limited access of geriatric care for older adults, especially those residing in rural areas. Mapping locations of geriatric resources and needs will identify incongruities between supply and demand. Through the use of data from multiple sources including Medicare data and from the Veteran Healthcare System, we used an annual repeated cohort approach of older adults in the Veteran Healthcare system using VA and Medicare data to identify usage of geriatric care services across health systems. Older adults are categorized by their geographic location using RUCA codes matched to their residential zip-code, categorized into urban, rural and highly rural areas. We compare and contrast the characteristics of older adults who utilize geriatric care services using complexity criteria previously established including risk of hospitalization or mortality, high cost utilization, and long term institutionalization, frailty, and specific geriatric conditions including dementia and falls. We also identify the distribution of care providers in geriatric specialties using the National Provider Identifier comparing rates in rural and urban areas.

### SESSION 3117 (SYMPOSIUM)

Abstract citation ID: igaf122.1232

### GSA JOURNALS PANEL WITH THE EDITORS-IN-CHIEF: RECENT TRENDS, DEVELOPMENTS, AND OPPORTUNITIES

Chair: Jessica Kelley
Co-Chair: Lewis Lipsitz

The Gerontological Society of America is committed to cultivating excellence in interdisciplinary aging research and education to advance innovations in practice and policy. This session brings together the seven Editors-in-Chief of the GSA journal portfolio, The Journals of Gerontology, Series A: Biological Sciences and Medical Sciences, The Journals of Gerontology, Series B: Psychological Sciences and Social Sciences, Innovation in Aging, The Gerontologist, and Public Policy & Aging Report, to discuss the scope and vision of each journal. Judie Lieu, Vice President for Publishing and Professional Resources, will present an overview of the GSA publishing portfolio and strategic initiatives to continue to publish the top science in aging. Then each Editor will discuss scientific priorities for their journal, upcoming opportunities for publishing, and new trends. The panel will address recent developments in grant funding and science, and how the journals are responding. Ample time for audience questions will be reserved. This session will be useful for emerging scholars seeking to publish in the GSA journals, as well as seasoned scholars who are interested in new developments in GSA publishing priorities and efforts.

Abstract citation ID: igaf122.1233
### INNOVATION IN AGING
Michelle Putnam, *University of Massachusetts Boston, Boston, Massachusetts, United States*

Innovation in Aging is an interdisciplinary, Open Access journal of the Gerontological Society of America (GSA). Innovation in Aging publishes innovative, conceptually sound, methodologically rigorous research on aging and the life course that has high potential for translating into positive individual, community, and societal outcomes in later life. Submissions are encouraged from a wide range of disciplines including technology, engineering, architecture, economics, business, law, political science and public policy, education, public health, social and psychological sciences, social work, biomedical and health sciences, and the humanities and arts. Innovation in Aging seeks to forward gerontological science by identifying emerging issues, advancing new frameworks for understanding complex problems, presenting cutting-edge methodological approaches and data analysis techniques, and supporting creative thinking about long-standing as well as novel issues and areas of study. The journal also seeks submissions that deepen and broaden understanding of aging, the life course, and later life experiences in new ways. Submissions to Innovation in Aging should articulate what is innovative about the work being presented and how it is translatable and useful for relevant stakeholders and constituencies.

Abstract citation ID: igaf122.1234
### PSYCHOLOGICAL SCIENCES SECTION OF THE JOURNALS OF GERONTOLOGY SERIES B
Duke Han, *University of Southern California, Los Angeles, California, United States*

The Psychological Sciences section of The Journals of Gerontology, Series B publishes articles on development in adulthood and old age that advance the psychological science of aging processes and outcomes. Articles in the journal have clear implications for theoretical or methodological innovation in the psychology of aging or contribute significantly to the empirical understanding of psychological processes and aging. Areas of interest include, but are not limited to, attitudes, clinical applications, cognition, education, emotion, health, human factors, interpersonal relations, neuropsychology, neuroscience, perception, personality, physiological psychology, social psychology, and sensation. Applied research with theoretical significance is welcome, as are conceptually interesting examples of cutting-edge analytic approaches. Manuscripts reporting work that relates behavioral aging to neighboring disciplines are also appropriate. The Journal publishes three types of articles: (a) Research Articles, reportings on original research, preferably with multiple studies and/or samples, (b) Research Reports, for brief presentations generally of single studies, and (c) New Directions in Aging Research—reviews of cutting-edge topics with theoretical or methodological implications. See word and page limitations below. All submissions are peer-reviewed, with final decisions made by the Editor.

Abstract citation ID: igaf122.1235
### THE GERONTOLOGIST
Joseph Gaugler, *University of Minnesota, Minneapolis, Minnesota, United States*

The Gerontologist®, first published in 1961, features applied, multidisciplinary research and analysis of social issues related to human aging. Our journal informs the broad community of disciplines and professions involved in understanding the aging process and providing care to older people. Contributions are welcome from scholars from many fields, including social and psychological sciences, social work, health sciences, the humanities and arts, education, public health, technology, political science, and public policy. The Gerontologist® prioritizes submissions that feature, refine, or create new conceptual/theoretical models that advance our understanding of aging. The Gerontologist® encourages manuscript submissions of various types: research articles; review articles; measurement articles; forums; and those commissioned by the Editors including book and media reviews, International Spotlights, and award-winning lectures. The Gerontologist®'s robust, interdisciplinary articles and issues are augmented by a series of timely special issues and collections on key topics of relevance to gerontological scholarship.

Abstract citation ID: igaf122.1236
### BIOLOGICAL SCIENCES SECTION OF THE JOURNALS OF GERONTOLOGY SERIES A
Gustavo Duque, *McGill University, Montreal, Quebec, Canada*

The Biological Sciences section of The Journals of Gerontology Series A publishes articles on the biological aspects of aging in areas such as translational gerontology, biomarkers of aging, biochemistry, biodemography, cellular and molecular biology, comparative and evolutionary biology, endocrinology, exercise science, genetics, immunology, neuroscience, nutrition, pathology, pharmacology, physiology, and the biological mechanisms of late life diseases. Our primary focus is on mechanisms and novel insights in aging biology from diverse models such as cultured cells, invertebrates, vertebrates, and mammalian species including humans. The journal is particularly interested in publishing research on translational gerontology including interventions designed to enhance longevity and healthspan.

Abstract citation ID: igaf122.1237
### PUBLIC POLICY AND AGING REPORT
Michael Lepore, *University of Massachusetts Amherst, Amherst, Massachusetts, United States*

All manuscripts submitted to Public Policy & Aging Report should address practice and/or public policy implications and aging. The journal publishes three types of articles. (1) Policy Analysis Articles. Public Policy & Aging Report is committed to publishing high quality analyses of public policy and aging issues. Original research is welcome, or articles can provide expert analysis of and commentary on key policy issues drawing on past studies. (2) Policy Spotlight Articles. Public Policy & Aging Report is committed to raising awareness of important public policy and aging issues. The goal of Policy Spotlight Articles is to provide a short overview of key policy issues. (3) Point-Counterpoint Articles. Public Policy & Aging Report is committed to heightening scholarly engagement with key public policy and aging issues. The goal of Point-Counterpoint Articles is to engage different scholarly perspectives on public policy and aging issues addressed in Public Policy & Aging Report. Point-Counterpoint Articles should respond to and reference one or more article(s) in a prior issue of Public Policy & Aging Report and should provide alternate perspectives on the topic including a clear statement of the counterargument.

### SESSION 3120 (AWARD LECTURE)

Abstract citation ID: igaf122.1238

### THE CLARK TIBBITTS AWARD AND HIRAM J. FRIEDSAM AWARD LECTURES AND PRESENTATIONS

Chair: Tina Newsham

The Clark Tibbitts Award Lecture will feature an address by the 2025 award recipient Rona Karasik, PhD, FAGHE, FGSA and the Hiram J. Friedsam Award Lecture will feature an address by the 2025 award recipient Sean Curran, PhD, FGSA. AGHE's Clark Tibbitts Award was established in 1980 and named for an architect of the field of gerontological education. The award is given each year to an individual or organization that has made an outstanding contribution to the advancement of gerontology and geriatrics education. Hiram J. Friedsam was the professor, co-founder, and director of the Center for Studies in Aging and dean of the School of Community Service at the University of Northern Texas. Dr. Friedsam was an outstanding teacher, researcher, colleague, and mentor to students, faculty, and administrators, as well as a past president of AGHE. The purpose of this award is to recognize those who emulate Dr. Friedsam's excellence in mentorship.

Abstract citation ID: igaf122.1239

### LIFE LESSONS IN LEARNING: CONTINUITY & CHANGE IN GERONTOLOGICAL EDUCATION

Rona Karasik, *Saint Cloud State University, Saint Cloud, Minnesota, United States*

The title of Robert Atchley's introductory textbook Aging: Continuity & Change (1983,1987) reflects two enduring themes in gerontological education. On the one hand, it is essential, albeit daunting, to keep abreast of the rapidly emerging advances in age-related science, technology, health care and public policy. Change is inevitable and on-going preparation a necessity for gerontological educators. Ultimately, no two offerings of a particular gerontology course are ever exactly the same, nor should they be. On the other hand, there are also inescapable constants to contend with such as ageism, inequity, a lagging workforce, and the never-ending battle to attract students to the field of aging. This presentation examines the paradox of continuity and change in gerontological education over the past thirty years and considers potential trajectories for the next thirty.

Abstract citation ID: igaf122.1240

### EVOLUTIONS IN MENTORING TO ENGINEER OPPORTUNITY

Sean Curran, *University of Southern California Leonard Davis School of Gerontology, Los Angeles, California, United States*

The Hiram J. Friedsam Award lecture will feature an address by the 2025 award recipient, Sean Curran, PhD, FGSA, Hiram J. Friedsam was the professor, co-founder, and director of the Center for Studies in Aging and dean of the School of Community Service at the University of Northern Texas. Dr. Friedsam was an outstanding teacher, researcher, colleague, and mentor to students, faculty, and administrators, as well as a past president of AGHE. The purpose of this award is to recognize those who emulate Dr. Friedsam's excellence in mentorship.

### SESSION 3125 (PAPER)

### AGING IN PLACE IN A WIDE RANGE OF PLACES

Abstract citation ID: igaf122.1241

### FOOD INSECURITY IN AGING COMMUNITIES: THE IMPACT OF NEIGHBORHOOD AGE COMPOSITION AND DISADVANTAGE ON FOOD ACCESS

Samuel Van Vleet[1], Madeline Webber[1], Bailee Brekke[2], and Erreannau Zellous[1], *1. Miami University, Oxford, Ohio, United States, 2. Miami University, Waddington, New York, United States*

Food insecurity among older adults is a growing public health concern, yet little research has examined how the proportion of older residents in a neighborhood relates to food resource availability. This study investigates whether census tracts with higher proportions of people have access to food resources and explores how neighborhood disadvantage impacts this relationship. Using nationally representative data from the National Neighborhood Data Archive (NaNDA), we assess food insecurity risk across varying levels of neighborhood age composition, neighborhood disadvantage and other confounding factors. Findings indicate that census tracts with higher concentrations of older adults have reduced access to food resources, and this disparity is exacerbated in areas with greater neighborhood disadvantage. Furthermore, the interaction between neighborhood disadvantage and age composition suggests that older adult-dense areas experience compounded food insecurity when disadvantage is high. These results highlight the critical role of place-based disparities in shaping food access for aging populations. This study has significant implications for policymakers, researchers, and practitioners focused on addressing nutritional deficits in older adults. By identifying geographic disparities in food access, this study highlights the need for targeted interventions and policy solutions that consider both neighborhood age composition and disadvantage. Future research should explore community-driven strategies to improve food security and reduce disparities in disadvantaged aging communities.

Abstract citation ID: igaf122.1242

### HOME, IDENTITY, AND AGING: THE IMMIGRANT EXPERIENCE

Rong Fu[1], and Marco Psyllos[2], *1. Siena University, Loudonville, New York, United States, 2. Bard College, Loudonville, New York, United States*

The need to facilitate aging in place for the growing aging population has gained increasing global attention in both policy and practice. However, there is a significant gap in research regarding older immigrants' perceptions of home and identity, which are essential for understanding both individual- and community-level influences on aging in place. This study systematically synthesizes qualitative evidence on older immigrants' conceptualizations of home and their positive and negative experiences of aging in a foreign country. A systematic review of literature was conducted by searching JSTOR, Medline, PsycINFO, and PubMed. Eligible studies were appraised using the JBI Critical Appraisal Checklist for Qualitative Research, resulting in the inclusion of 21 studies. Findings indicate that older immigrants have diverse experiences regarding their sense of home and belonging within their current communities. Home is conceptualized in multiple ways, including as a physical dwelling, a sense of belonging and security, the presence of family, and the preservation of cultural identity. Negative experiences were associated with factors such as insecurity, inadequate support, and social isolation, whereas positive experiences were shaped by the presence of significant others and enhanced access to social welfare. This review highlights the importance of fostering supportive and inclusive communities to better align with the needs of older immigrants aging in place.

Abstract citation ID: igaf122.1243

**REDISCOVER NEIGHBORHOOD: A SYNTHESIS OF LIVED EXPERIENCES FOLLOWING DEMENTIA DIAGNOSIS THROUGH WALKING INTERVIEW**

Wenjin Wang[1], Ziying Zhang[2], Xi Chen[1], and Chanam Lee[1], *1. Texas A&M University, College Station, Texas, United States, 2. Simon Fraser University, Burnaby, British Columbia, Canada*

Around 85% of older adults with dementia in the US reside within the community (NHATS, 2019). However, many do not venture far from their homes due to fears of getting lost or the loss of their driving ability. Therefore, the immediate neighborhood environment plays a crucial role in supporting their independence. Walking interview is an effective in-situ, participatory method to capture the lived experiences and environmental interactions of persons with dementia (PwDs), yet studies on walking interviews with PwDs is often limited by small sample sizes and a narrow focus. A systematic review and meta-synthesis can bridge these gaps and offer a critical, comprehensive view of neighborhood experiences after dementia diagnoses. We included 17 papers that involved 172 PwDs from four countries. Guided by the Contexts for Development and Aging (CODA) framework, we found that environmental features (e.g., street crossings, sidewalks, and landmarks) can either support or challenge PwDs' outdoor mobility, yet many adapt by developing unique strategies, modifying daily routines, or relying on others. From the social perspective, PwDs form a vital, reciprocal network with family, friends, neighbors, and even familiar "strangers". Technology further expands PwDs' physical and social experiences by facilitating location tracking and virtual interactions, despite potential accessibility barriers and misuse. The study revealed how PwDs address vulnerability and adapt with resilience in neighborhoods as dementia progresses. More research is needed on the neighborhood experiences of PwDs, focusing on translating their daily realities into actionable frameworks for dementia-friendly communities.

Abstract citation ID: igaf122.1244

**INTERGENERATIONAL SOLIDARITY IN APPALACHIAN OHIO: EXPLORING SOCIAL CONNECTEDNESS AND EXTREME WEATHER RESPONSE**

Fiona Doherty[1], Smitha Rao[2], and Holly Dabelko-Schoeny[3], *1. University of Tennessee, Nashville, Tennessee, United States, 2. Ohio University, Columbus, Ohio, United States, 3. The Ohio State University, Columbus, Ohio, United States*

Older adults (aged 65+) and emerging adults (aged 18-29) face vulnerability to two converging crises: social disconnectedness and extreme weather attributable to climate change. These challenges compound each other, creating a cycle of risk. Disconnected communities struggle to respond collectively to extreme weather, increasing the likelihood of harm. Additionally, extreme weather can disrupt social connectivity by altering local environments. While older and emerging adults share vulnerabilities and agency, generational divides hinder cooperation, highlighting the need to build intergenerational capacities and connections. This study employed an intergenerational solidarity framework in Athens County, a rural and underserved area in Appalachian Ohio, to 1) compare older and emerging adults' experiences of social connectedness and extreme weather, and 2) examine outcomes from intergenerational and intragenerational conversations focused on enhancing social connectedness and extreme weather adaptive capacity. Sixteen older and emerging adult participants engaged in Participatory Photo Mapping from May to September 2024. Data collection included generating photos and spatial maps of social connectedness factors and conducting photo-and-map elicitation interviews and focus groups. Data analysis included iterative thematic and comparative analyses. Older adults' connectedness assets were closer to home while emerging adults' assets were dispersed across the county. Older adults were more experienced and confident in handling extreme weather situations. In the intergenerational focus group, participants valued the other generation's perspectives. Conversely, the intragenerational group showed a mix of curiosity and skepticism about the other generation's contributions to extreme weather response. Findings highlight significant potential for knowledge exchange and trust-building through participatory intergenerational dialogues.

### SESSION 3127 (BIOLOGICAL SCIENCES INVITED SYMPOSIUM)

Abstract citation ID: igaf122.1245

**LEVERAGING DATA SCIENCE TO UNDERSTAND AGING TRAJECTORIES**

Chair: Jonathan Wren

Aging is heterogeneous: individuals follow distinct cognitive, cardiometabolic, and neurodegenerative trajectories shaped by genetics, environments, and care contexts. This symposium showcases bioinformatics/data-science strategies for modeling that heterogeneity and extracting actionable signals for prevention and treatment. Talks will span (i) longitudinal and genetically informed models linking adversity, social determinants, and polygenic variation to cognitive aging; (ii) machine-learning methods for multimodal biomedical data (e.g., digital pathology and imaging), emphasizing interpretability and clinical utility; and (iii) translational insights from large, deeply phenotyped cohorts to improve risk stratification and trial enrichment in dementia.

Abstract citation ID: igaf122.1246

### HOW EPIGENETIC CLOCKS TICK: UNPACKING THE BLACK BOX BY DECIPHERING BIOLOGICAL PATHWAYS AND DOWNSTREAM TRANSCRIPTOMIC SIGNATURES OF ACCELERATED AGING

Thalida Arpawong, and Eileen Crimmins, *University of Southern California, Los Angeles, California, United States*

Epigenetic clocks derived from DNA methylation data are often used to assess biological aging and predict health outcomes and mortality. Several epigenetic clocks have been developed to date, although the basis for their differential predictive power on health outcomes remains unclear due to the "black box" nature of the clocks and lack of information about the specific biological processes they assess. DNA methylation is biologically significant due to its role in regulating gene expression, but the degree to which these clocks reflect biological gene expression pathways as well as common or unique biological processes is not well understood. We utilized data from 3,227 individuals in the US Health and Retirement Study, measuring gene expression via RNA sequencing and epigenetics through DNA methylation. Epigenetic clocks including the Horvath, Hannum, PhenoAge, GrimAge, and DunedinPACE clocks, were used as independent variables in an 80% training sample (n=2,584) to conduct differential gene expression analyses. Differentially expressed genes were identified for each clock, then functionally annotated to identify shared and unique biological processes and pathways underlying each clock. In a 20% hold-out test sample (n=645), we evaluated transcriptomic signatures, derived from differentially expressed genes from each clock, alongside the epigenetic clocks for their association with mortality and multiple aging-related health outcomes. The results revealed more unique than common biological processes, shedding light on why each clock predicts different health and aging phenotypes with varying strength. Findings clarify the internal mechanisms of these clocks, offering biological insights and paving the way for translation into potential clinical applications aimed at predicting aging-related health trajectories.

Abstract citation ID: igaf122.1247

### MULTI-OMIC DISSECTION OF ALZHEIMER'S DISEASE: DISENTANGLING AGING TRAJECTORIES, NEURODEGENERATION AND PATHOPHYSIOLOGICAL CONFOUNDERS

Agustin Ruiz Laza, and Sudha Seshadri, *Glenn Biggs Institute for Alzheimer's and Neurodegenerative Diseases, San Antonia, Texas, United States*

Alzheimer's disease (AD) is a heterogeneous, age-related neurodegenerative condition in which genetic risk, exposome, and disease biology interact in complex and non-linear ways. Multi-omic technologies now allow us to dissect this complexity at scale. We integrate genomics, CSF proteomics (SomaScan 7k), CSF lipidomics (LC–MS/MS), ancillary CSF measurements, and clinical phenotyping across deeply characterized individuals to identify latent molecular axes that shape CSF composition. Two orthogonal components — CSF turnover and blood–brain barrier integrity — explain ~75% of CSF omic variance and drive strong artifactual inflation in biomarker signatures if not accounted for. Adjustment for these cryptic biological states improves classification performance, reveals more faithful disease-linked molecular signals, and allows a clearer separation of natural aging and disease trajectories. Our data show that multi-omic integration is not only descriptive — it is necessary to deconvolve the causal layers that structure AD phenotypes. This supports a precision therapeutics agenda in which molecular endophenotypes become the operative unit for disease modeling, biomarker development, and targeted intervention.

Abstract citation ID: igaf122.1248

### MULTIMODAL AI FOR BIOMARKER AND ETIOLOGICAL ASSESSMENT OF DEMENTIAS

Vijaya Kolachalama, *Boston University, Boston, Massachusetts, United States*

Dementia differential diagnosis and biomarker assessment pose significant challenges due to symptom overlap and limited access to advanced imaging like PET for detecting amyloid beta (Aβ) and tau (τ) in neurodegenerative conditions such as Alzheimer's disease (AD). In this talk, I will highlight our lab's recent work on multimodal AI frameworks that fuse diverse data to identify dementia etiologies, handle mixed dementias and incomplete cases, and estimate Aβ/τ PET status. This approach promises scalable screening for personalized dementia management, AD therapies, and clinical trials.

### SESSION 3130 (AWARD LECTURE)

Abstract citation ID: igaf122.1249

### DONALD P. KENT AWARD, ROBERT W. KLEEMEIER AWARD, AND JAMES JACKSON OUTSTANDING MENTORSHIP AWARD LECTURES

Chair: Marilyn Gugliucci

The Donald P. Kent Award lecture will feature an address by the 2024 Kent Award recipient Peter Lichtenberg, PhD, FSGA of the Wayne State University. The Robert W. Kleemeier Award lecture will feature an address by the 2024 Kleemeier Award recipient Jiska Cohen-Mansfield, PhD, FGSA of Tel Aviv University. The James Jackson Outstanding Mentorship Award lecture will features an address by the 2024 Jackson Award recipient Lisa Barnes, PhD, FGSA of Rush University. The Kent Award is given annually to a member of the Gerontological Society of America who best exemplifies the highest standards of professional leadership in gerontology through teaching, service, and interpretation of gerontology to the larger society. The Kleemeier Award is given annually to a member of the Gerontological Society of America in recognition for outstanding research in the field of gerontology. The Jackson Award is given annually to a member of the Gerontological Society of America in recognition outstanding commitment and dedication to mentoring minority researchers in the field of aging.

Abstract citation ID: igaf122.1250

### INTERPRETING FINANCIAL CAPACITY RESEARCH TO THE LARGER SOCIETY

Peter Lichtenberg, *Wayne State University, Detroit, Michigan, United States*

Scholarship and service regarding financial capacity assessment and best practices led to a program of research on financial capacity. This lecture will focus on key elements of that research including financial decision-making, financial exploitation and personal financial behaviors and how each of these link to cognitive functioning and early memory loss in older adults. Our expansion of this work into interventions with older adult victims of scams and identity theft lead us to interpreting our work to older adults and the professionals who work with them. The creation and continual expansion of our website https://olderadultnestegg.com gave us one platform for offering training, intervention and risk assessment to older adults, caregivers and professionals alike. Using a prevention science approach, we created and piloted a prevention of financial exploitation program. This presentation will tie together how leadership principles guide our work.

Abstract citation ID: igaf122.1251

**PARADIGM SHIFTS: THE IMPACT OF CLINICAL RESEARCH ON UNDERSTANDING PERSONS WITH DEMENTIA AND THEIR CARE**

Jiska Cohen-Mansfield, *Tel Aviv University, Tel Aviv, Tel Aviv, Israel*

The conceptualization of dementia has traditionally been bleak, assuming that little can be done for individuals with dementia, that their behaviors and life experiences are dictated by the disease and that psychoactive medications must be used to control them – at least until effective medication emerge to prevent, alleviate, or cure the disease. However, the last decades of clinical research have revealed a markedly different perspective – one in which past paradigms give way to new possibilities. This lecture will highlight key paradigm shifts in understanding dementia and potential avenues for improved care, including insights into the etiology of behavioral challenges, the preservation of self-identity, improved methods for detecting pain, and strategies for enhancing well-being. By integrating these advances into practice, we can move beyond limiting assumptions and advance toward a more holistic approach that offers opportunities to enhance the life experiences of people living with dementia, and concomitantly, their caregivers.

Abstract citation ID: igaf122.1252

**MY PATH FROM BEING MENTORED TO BECOMING A MENTOR, AND EVERYTHING IN BETWEEN**

Lisa Barnes, *Rush University Medical Center, Chicago, Illinois, United States*

Eliminating health disparities and ensuring health equity for all is an important public health imperative. In order to advance knowledge and innovation through research, we must change the healthcare and research workforce so that they are reflective of the communities that face the most health challenges. My goal has always been to be a successful research scientist, and I received excellent training from an institution that was highly ranked in my discipline. But I never received training on being a mentor. Instead, I benefitted from having strong mentoring relationships throughout my academic journey. Relationships that were built on trust, respect, and knowledge-sharing. This presentation will describe my path from being mentored myself to becoming a mentor of others, including students, trainees, post-docs, and junior faculty. I will include insights I've learned along the way and offer guidance on how best to support young investigators, especially those under-represented in academia.

### SESSION 3135 (SYMPOSIUM)

Abstract citation ID: igaf122.1253

**ESPO AND HEALTH SCIENCES SECTION SYMPOSIUM: A MULTIDIMENSIONAL VIEW OF AGING: UTILIZING INNOVATIVE APPROACHES IN GERONTOLOGY**

Chair: Kate Perepezko
Co-Chair: Tingzhong (Michelle) Xue
Discussant: Hanzhang Xu

As our aging population grows, understanding the aging process is increasingly critical for developing strategies to enhance the health and quality of life of our population. Aging is a highly individualized process shaped by biological, interpersonal, and social factors. Therefore, addressing the complexities of aging requires innovative methodological and analytical approaches to explore unanswered questions in gerontology. This symposium will showcase cutting-edge strategies for studying key issues affecting the aging population, emphasizing innovative research methods and interdisciplinary collaboration. This session highlights the work of early career scientists who utilize advanced methodological and analytical approaches to explore key topics in gerontology. Dr. Kate Singer will present her work exploring how recalling stressful caregiving experiences affects caregiver stress levels and cognitive performance using functional near-infrared spectroscopy (fNIRS). Drs. Hyojin Choi and Kate Perepezko will present their work examining the interdependence of frailty among caregiving dyads, comparing homebound dyads to non-homebound dyads. Dr. Tingzhong Xue will present a study aimed to examine the roles of behavioral symptoms and vision loss on physical resilience among nursing home residents with dementia after hip fracture. Jiameng Yuan will present her evaluation of the implementation of a telemedicine program, EAGLEcareACT (Enhancing Advance Care Planning, Geriatrics, and End-of-Life Care Acute Care Team) in Singapore. This symposium will conclude with a discussion led by Dr. Hanzhang Xu who will synthesize key insights from these presentations, exploring common methodological advancements, their implications for intervention design, and future directions for aging research.

Abstract citation ID: igaf122.1254

**LINKED LIVES: HOW HOMEBOUND STATUS SHAPES PHYSICAL HEALTH INTERDEPENDENCE IN CARE DYADS**

Hyojin Choi[1], and Kate Perepezko[2], *1. University of Vermont, Burlington, Vermont, United States, 2. Miami University, Oxford, Ohio, United States*

Homebound older adults - those who cannot leave home without someone's help – tend to have multiple health conditions, substantial functional limitations, and complex care needs. Family caregivers play an instrumental role in supporting these adults and may experience greater physical strain and an increased risk of frailty. Given the strong connection between caregiver and older adult health outcomes, their frailty may also be interdependent. The current study examined this interdependence and if it varied by older adult homebound status. Using dyadic data from the National Health and Aging Trends Study and the National Survey of Caregiving (2015 and 2017), the sample was divided into three groups: becoming homebound (n = 108; not homebound in 2015, homebound in 2017), long-term homebound (n = 235; homebound in 2015 and 2017) and never homebound (n = 166; not homebound in 2015 or 2017). Actor-Partner Interdependence models (APIM) were employed to examine the interdependence of frailty in caregiving dyads. In long-term homebound and never homebound dyads, APIM models revealed partner effects from caregiver to care recipient, indicating when caregivers were frail at baseline, frailty of care recipients declined in the following wave. Interestingly, in becoming homebound dyads, a care recipient to caregiver partner effect was observed, with baseline care recipients' frailty associated with subsequent caregiver frailty. These preliminary findings demonstrate how frailty interdependence can vary by homebound status. These findings have implications for creating interventions aiming to prevent and reduce frailty for older adults and their caregivers, suggesting dyadic interventions may have greater success.

Abstract citation ID: igaf122.1255

**REDUCING ED VISITS: OPTIMIZING AN INTERDISCIPLINARY TELEMEDICINE PROGRAM IN SINGAPORE NURSING HOMES**

Jiameng Yuan[1], Gayathri Devi Nadarajan[2], Marcus Ong[3], Jean Mui Hua Lee[4], and Hanzhang Xu[1], *1. Duke University, Durham, North Carolina, United States, 2. Singapore General Hospital, Singapore, Singapore, 3. Duke-NUS Medical School, Singapore, Singapore, 4. Sengkang General Hospital, Singapore, Singapore*

Emergency department (ED) utilization among nursing home (NH) residents is reportedly high, with one study showing 42.4% of visits leading to hospital admission. This could be particularly pronounced in rapidly aging societies like Singapore. Prior research has suggested that many ED visits from NHs residents were are often preventable. Our prior work has developed and pilot tested a telemedicine program (EAGLEcareACT [Enhancing Advance Care Planning, Geriatrics, and End-of-Life Care Acute Care Team]) that connected ED physicians with NH staff to triage patients from NHs and address care needs within NHs. Preliminary evaluation has suggested EAGLEcareACT reduces avoidable ED visits among older adults from NHs. To broadly implement EAGLEcareACT through a large-scale, pragmatic trial, we sort to conduct a qualitative pre-implementation study, guided by the Consolidated Framework for Implementation Research (CFIR). We plan to conduct semi-structured qualitative interviews with 2 NH managers, 4 NH staff, 2 ED physicians, and 4 family members of NH residents. We will comprehensively assess the current practice at each targeted NH and the complexity of care needs for NHs residents. We will explore perceived barriers and facilitators of this interdisciplinary telemedicine program and seek input on person-centered outcomes that should be measured in this pragmatic trial. All interviews will be recorded and transcribed verbatim, with fieldnotes supplementing the interview transcription. We will analyze data in Nvivo 14 using both deductive/inductive coding based on CFIR domains. This information would optimize the implementation and expansion of EAGLEcareACT as well as enhance its sustainability and effectiveness in NH settings.

Abstract citation ID: igaf122.1256

**EXPLORING THE ROLE OF STRESS RUMINATION IN CAREGIVER COGNITIVE PERFORMANCE**

Kate Singer, *Miami University, Oxford, Ohio, United States*

Caregiving is a rewarding and strenuous activity affecting physiological, psychological, and cognitive processes. While caregiving stress has been studied previously, these studies have overlooked rumination and its effect on cognition. The present study explored how recalling stressful caregiving experiences affected caregiver stress levels and executive functions using the AX-CPT. Neural correlates underlying cognitive performance were also examined using functional near-infrared spectroscopy (fNIRS). 36 caregivers were recruited for this study, including both formal and informal caregivers of persons diagnosed with a variety of chronic conditions. Our findings indicated that longer lifetime durations of caregiving activities, not recalling stressful experiences, were associated with poorer performance on the AX-CPT in the present sample of caregivers, though no changes in neural correlates were observed. Future studies should continue to examine the impact of momentary stress recall on caregiver outcomes and the role of caregiving duration their executive functions.

Abstract citation ID: igaf122.1257

**ROLES OF DEMENTIA AND VISION LOSS ON PHYSICAL RESILIENCE IN PERSONS WITH DEMENTIA AFTER HIP FRACTURE**

Tingzhong (Michelle) Xue[1], Wei Pan[2], Cathleen Colón-Emeric[3], Michael Cary Jr.[2], and Eleanor McConnell[2], *1. College of Nursing, UMass Amherst, Amherst, Massachusetts, United States, 2. Duke University, Durham, North Carolina, United States, 3. Duke University School of Medicine, Durham, North Carolina, United States*

Older adults with dementia residing in nursing homes (NHs) are more likely to experience worse health outcomes after a hip fracture than those without dementia. How chronic stressors such as dementia and vision loss interact to influence physical resilience after acute injury is not well understood. Behavioral symptoms, common among NH residents with dementia, may worsen with the acute stressor of hip fracture, affecting rehabilitation and function recovery. However, the mechanisms of how vision loss and behavioral symptoms together affect physical function recovery after hip fracture are poorly understood. This study aimed to examine the roles of behavioral symptoms and vision loss on physical function recovery over one year among those with dementia after hip fracture by conducting a secondary analysis of 2,824 long-stay residents with dementia and behavioral symptoms from the Nursing Home Minimum Data Set 3.0 from 2017-2019. Longitudinal parallel process modeling was used to examine the longitudinal relationships among the trajectories of cognition, behavioral symptoms, vision loss, and physical function. Results showed that the initial status of cognitive impairment was associated with the initial status ($\beta$=-0.31, $p<0.0001$) and rate of change ($\beta$=-0.26, $P<0.0001$) of physical function. Behavioral symptoms partially mediated the longitudinal relationship between the initial status of cognitive impairment and the initial status ($\beta$=-0.01, $p=0.002$) and rate of change ($\beta$=-0.01, $P<0.001$) of physical function. Vision loss did not moderate such longitudinal relationships. The findings add new knowledge of how dementia and vision loss might longitudinally affect physical resilience among persons with dementia after a hip fracture.

### SESSION 3140 (SYMPOSIUM)

Abstract citation ID: igaf122.1258

**CONNECTING GERONTOLOGY AND HUMANITIES THROUGH THE LEGACIES OF W. ANDREW ACHENBAUM**

Chair: Stephen Fogle
Discussant: James Dowd

Inspired by the legacies of W. Andrew Achenbaum, this symposium features innovative visions for exchange between humanities and gerontological imaginations. With presentations from medical, theological, political, and anthropological disciplines the symposium incorporates contributions that reflect the range of influence that Achenbaum provided during his over four decades of scholarship. Thematically, the symposium moves from reflection to application of Achenbaum's scholarship. The session begins with a reflective response to Achenbaum's essay, "Being a Humanistic Patient" to consider what it means to be a Humanistic Clinician. The second paper presents a practical theology of aging called Living Elderhood inspired by Achenbaum's religion, spirituality, and aging writing. The third paper forwards Achenbaum's upstanding convictions for democracy through applied theoretical analysis of political narratives and older adults' political action. The final paper manifests creative potential of incorporating humanities in gerontological research, a cause championed by Achenbaum, through a community engaged research project utilizing Journey Mapping and Graphic Novel-style illustrations of older adults' health histories. The discussant will provide commentary regarding how the legacies of W. Andrew Achenbaum, as presented by symposium papers, uncover new perspectives, foster collaborations, and address the challenges and opportunities that arise in humanities and gerontological futures. Attendees for this symposium will be able to 1) stimulate provocative conversations about the dynamic ways humanities are manifested in old age 2) garner deeper appreciation of W. Andrew Achenbaum's legacies, and 3) bring forward new visions of old age to inspire fresh directions of exchange between humanities and gerontological imaginations.

Abstract citation ID: igaf122.1259

### LIVING ELDERHOOD: A PRACTICAL THEOLOGY OF OLD AGE ACCORDING TO W. ANDREW ACHENBAUM

Stephen Fogle, *At-Large, Omaha, Nebraska, United States*

Collaboration between Humanities & Gerontology is poised for a breakthrough. After decades of research operating through Successful and Positive Aging paradigms, gerontologists dedicated to understanding the 'good stuff' of old age are charting a complementary course for, "Innovative Horizons in Gerontology." Inspired by the religion, spirituality, and aging writing of W. Andrew Achenbaum, this paper forwards collaborative exchange between Humanities & Gerontology through presentation of a practical theology of old age called Living Elderhood. Composed in three parts, the Living Elderhood framework focuses attention to 1) recognition of old age; 2) responsibility to love; 3) relationship with Divinity. The paper opens with a brief historical review of Elderhood as a concept of interest across interdisciplinary perspectives from humanities, social sciences, and medicine in gerontological literature. The paper then dives deep into the religion, spirituality, and aging writing of W. Andrew Achenbaum to explicate the dimensions of Living Elderhood expressed through his personal and professional reflections. In doing so, the paper seeks to include religion and spirituality as a part of Achenbaum's legacy of connecting gerontological and humanities imaginations. The paper concludes with describing how the three dimensions of the Living Elderhood framework are manifested in the lived experiences of older Jesuit priests. Attendees for this paper will 1) become conversant in broader currents of gerontological and humanities collaboration; 2) gain appreciation of W. Andrew Achenbaum as a religious and spiritual thinker; 3) understand application of Living Elderhood in researching older adults' lived experiences.

Abstract citation ID: igaf122.1260

### THE HUMANISTIC CLINICIAN

James Powers, *Vanderbilt University Medical Center, Nashville, Tennessee, United States*

The Humanistic Clinician will invite audience participants to achieve new insights to enrich their appreciation for how we define the human condition including our capacity for empathy, care, compassion, vulnerability, resilience, and mutual support. The presentation will address changes in medicine, healthcare expectations of older adults, tensions between autonomy and interdependence surrounding healthcare decisions and guide-posts of humanistic practice.

Abstract citation ID: igaf122.1261

### MIND THE GENDER AND GENERATION GAPS - HOW THE DRIVERS OF POPULATION CHANGE AFFECT DEMOCRACY

Gemma Carney, *Queen's University Belfast, Belfast, Northern Ireland, United Kingdom*

Political participation of older people has not been extensively researched in gerontology. Nor has age been a concern of political scientists. The resulting gap in the literature has been filled by supposition, such as the claim that all Trump voters are aged. These conspiracy theories proliferate during a period of intense political polarization, often referred to as 'the culture wars.' The culture wars focus on identity, but age has not emerged as an identity with an accompanying political movement like class or gender. As a result, democracies with older populations lack policies appropriate to an ageing population despite an active, older electorate. Yet older voters are influencing electoral outcomes. Cultural Backlash theory (Norris and Inglehart, 2019) makes an empirical link between the ageing of the electorate and the rise of socially conservative leaders. In this paper, I set out a research agenda which rises to broader, long-term challenges for democracy in ageing societies. I outline the importance of political demography, which analyses the political implications of population ageing, for gerontology. I argue that the drivers of population change – fertility, migration and extended life expectancy - all have important implications for democracy. We can see this in the pro-natalist ideology of Italy's Prime Minister Giorgia Meloni and in the success of anti-migrant populist parties around the world. Most urgently, we must pay urgent attention to who is deciding not to vote, namely women and younger cohorts. The paper concludes that a focus on political demography in gerontology is urgently needed.

Abstract citation ID: igaf122.1262

### CO-CREATING A HEALTH EQUITY JOURNEY: PARTICIPATORY ACTION RESEARCH WITH OLDER BLACK ADULTS

Laura Heinemann[1], and LaShaune Johnson[2], *1. Creighton University, Omaha, Nebraska, United States, 2. Tilman J. Fertitta Family College of Medicine, University of Houston, Houston, Texas, United States*

We present the story of an interdisciplinary participatory-action research project for addressing health-inequities among older Black adults who had experienced hospitalization. Melding social science, humanities, arts-based approaches, and local epistemologies, we presupposed project outcomes required guidance and partnership from older adults with direct knowledge and lived experiences of the consequences of health inequities. Working within a historically Black Midwestern urban community impacted by redlining and disinvestment, our multigenerational team included a public health-trained faith leader and gerontological social worker, alongside faculty and students from social science, studio art, public health, and medicine. Honoring the African tradition of griots (travelling storytellers), narrative interviews about hospitalization segued into weekly conversation hours and arts-based activities where community residents imparted insights from their broader life journeys. Seeking to ensure non-harmful, culturally responsive representation of study findings, we applied Peak and Anderson's (2018) Journey Scroll technique by offering spoken and graphic-novel-style visual retelling of participants' collective stories. During community data "share-back" events, community residents were invited to see, discuss, interpret, and potentially challenge or reframe study findings. Together with local organizations, residents, and leaders long-committed to Black community wellbeing, the project co-created emerging methodologies for inquiry and action. Attendees for this paper will be able to 1) recognize W. Andrew Achenbaum's legacy of connecting humanities and gerontology through community-participatory collaborations; 2) appreciate lessons and new possibilities for weaving together social science and humanistic approaches to support older Black adults' well-being; 3) leverage incorporation of intergenerational and multidisciplinary research teams toward addressing health-inequities.

### SESSION 3145 (SYMPOSIUM)

Abstract citation ID: igaf122.1263

### ENGAGING OLDER ADULTS IN CLIMATE ACTION: INSIGHTS INTO ATTITUDES, MOTIVATIONS, AND CONTRIBUTIONS

Chair: Paola Zaninotto
Co-Chair: Karl Pillemer

Older adults are among the most vulnerable to the effects of climate change, yet their perspectives, behaviours, and potential contributions to climate action remain underexplored. This symposium brings together new research on attitudes, motivations, and engagement with climate change among older adults, offering insights into how this growing demographic perceives climate risks and contributes to environmental sustainability. The session will open with findings from the English Longitudinal Study of Ageing (ELSA), highlighting distinct attitudes towards climate change risk among older adults in England and their demographic, social, and economic correlates. Next, we explore eco-generativity, how exposure to nature fosters both social and environmental responsibility, and its implications for promoting pro-environmental behaviours across the lifespan. We then turn to older climate activists, examining the key motivational pathways driving their engagement and the role of social connections, emotional ties to nature, and lifelong learning in sustaining their activism. A comparative study of older adults in Italy and Sweden further contextualises these themes, shedding light on the interplay between environmental attitudes, generational responsibilities, and activism. Finally, we present a new set of validated measures designed to assess climate knowledge, concerns, and actions among older adults, providing tools to better integrate climate change into ageing research and policy. By weaving together these diverse yet complementary perspectives, this symposium advances our understanding of older adults as active agents in climate resilience and sustainability, challenging assumptions of disengagement and highlighting strategies to enhance their involvement in environmental action. Climate and Aging Interest Group Sponsored Symposium

Abstract citation ID: igaf122.1264

### ATTITUDES TO CLIMATE CHANGE RISK AMONG OLDER PEOPLE: NEW EVIDENCE FROM THE ENGLISH LONGITUDINAL STUDY OF AGEING

Giorgio Di Gessa, and Paola Zaninotto, *University College London, London, England, United Kingdom*

Public perceptions of climate change's risks are essential to ensure support for the strategies necessary to achieve carbon emissions goals. Although older people are often considered most vulnerable to the effects of climate change and extreme weather, their perspectives have often been overlooked. Therefore, in this study, we aim to describe public attitudes towards climate change risk among older people in England, using data from the latest wave of the English Longitudinal Study of Ageing (2023/24) that assessed, for the first time, attitudes towards climate change risk. We applied latent class analysis to categorise respondents according to their level of agreement with six statements on climate change risks. Multinomial regression modelling was then used to investigate the extent to which demographic, socioeconomic, health, and social engagement were associated with different classes of perceptions. In our preliminary analysis, we identified four distinct attitude classes: the "Sceptical" (6%), the "Very Worried" (33%), the "Slightly Concerned" (40%), and the "Unsure" (21%). Results suggest that people aged 70 and older and men were more likely to be sceptical or unsure about climate change than those in their 50s and women. Also, wealthier and highly educated older people were more likely to be worried, with respondents actively participating in political and religious lives being the most likely to be worried about climate change. These initial findings suggest that public information campaigns regarding climate change risk are still necessary and should be tailored specifically to those older people who remain sceptical or unaware of these issues.

Abstract citation ID: igaf122.1265

### ENVIRONMENTAL LEGACY ACROSS THE LIFESPAN: ECO-GENERATIVITY, NATURE EXPOSURE, AND PRO-ENVIRONMENTAL BEHAVIOR

Selma Korlat, Mathew White, and Jana Nikitin, *University of Vienna, Vienna, Wien, Austria*

Generativity and the legacy one leaves behind have been proposed as strong developmental motivators for older adults to engage in actions that mitigate the negative effects of climate change (Diehl, 2022). However, only a handful of studies have specifically examined social generativity (care for people) and eco-generativity (care for nature) as distinct motivations for preserving humankind and its resources. Moreover, previous research suggests that nature exposure promotes prosocial and environmentally sustainable behaviors. The goal of this study was to investigate the facilitating role of nature exposure in social and eco-generativity across the lifespan. In Study 1, data from a nationally representative survey in the UK were analyzed ($N=18{,}633$). The results showed that, for older adults, nature exposure was more strongly associated with social generativity, whereas the relationship between nature exposure and eco-generativity did not vary significantly across age groups. Study 2 followed up on these findings, investigating whether momentary nature exposure increases social and eco-generativity, and whether these effects translate into pro-environmental action across the lifespan. Results from an experience sampling study ($N=103$, Mage = 47.53, SDage = 17.96, age-range = 19–79) showed that momentary nature exposure increases both momentary social and eco-generativity and provided support for a partial mediation of eco-generativity in the relationship between momentary nature exposure and momentary pro-environmental behavior across the lifespan. Using different approaches and study scope, both studies consistently highlight the benefits of nature exposure in fostering care for environmental preservation and encouraging pro-environmental action in all age groups.

Abstract citation ID: igaf122.1266

### MOTIVATIONS OF OLDER CLIMATE CHANGE ACTIVISTS: FIVE MAJOR PATHWAYS TO ENGAGEMENT

Haojing Zhu, and Karl Pillemer, *Cornell University, Ithaca, New York, United States*

Mobilizing older environmental volunteers can provide a powerful solution to addressing both the environmental crisis and the aging society—two global challenges critical to achieving the United Nations' sustainability development goals. Understanding how to motivate this demographic to engage in environmental volunteering is essential for harnessing the benefits of this solution. However, limited research directly addresses this topic. This study conducted semi-structured interviews with 54 older environmental activists to examine their motivations and patterns of engagement. Coding and analysis of the interviews identified five key motivational themes: 1) problem-solving, involving movement from issue identification to action; 2) the desire to contribute and make an impact; 3) immediate personal considerations; 4) beliefs and values; and 5) emotional motivations. Key findings include: 1) a strong desire to expand knowledge and challenge oneself to learn new skills; 2) an emphasis on the quality of social relationships rather than using volunteering to combat loneliness; and 3) both positive and negative emotions about nature motivating action. Future directions for research are discussed, including the emotional regulation role of nature and motivational differences across various volunteer programs, modes, and intensities. Also presented are practical insights for organizations to design more effective volunteer programs and infrastructures, helping recruit and retain older volunteers for environmental and climate change action.

Abstract citation ID: igaf122.1267

**MEASURING CLIMATE CHANGE KNOWLEDGE, ATTITUDES, BEHAVIOURS AND ACTIVISM AMONG OLDER ADULTS**

Paola Zaninotto[1], and Matthew Prina[2], *1. University College London, London, England, United Kingdom, 2. Newcastle University, Newcastle upon Tyne, England, United Kingdom*

Older adults are particularly vulnerable to climate-related hazards such as extreme heat, flooding, and severe storms, yet their perspectives and contributions to climate resilience remain underrepresented in research. We highlight the importance of incorporating climate-related themes into ageing studies and propose a set of short, validated questions to assess older adults' knowledge, concerns, preparedness, behaviours, and involvement in climate action. These questions have undergone cognitive testing with diverse participants to ensure clarity, accessibility, and relevance. Expert review helped refine content, simplifying wording, response scales, and framing to better capture lifetime actions rather than limited timeframes. Designed for flexibility across research contexts, they are easy to implement via face-to-face interviews, telephone, or self-completion. By integrating these measures, ageing research can provide valuable insights to inform policies and initiatives that reduce climate risks while promoting sustainable and healthy ageing. Recognising older adults' voices and capacities is crucial in fostering climate resilience.

Abstract citation ID: igaf122.1268

**ENVIRONMENTAL ATTITUDES AND (IN)ACTIONS THROUGH TIME: OLDER ADULTS' PERSPECTIVES IN ITALY AND SWEDEN**

Federica Previtali, Malin Bäckman, and Virpi Timonen, *University of Helsinki, Helsinki, Uusimaa, Finland*

The climate crisis compels reflection on the future and the environments left for future generations. While public discussions often focus on youth as drivers of change, ageing societies bring forth the crucial role of the growing older population in addressing environmental issues. Later life can serve as a space for exploring tensions between environmental concerns, generational responsibilities, and related (in)actions. Later life is a period of time-related tensions: while a shrinking future time perspective may lead to a focus on individual goals, the ecological generativity theory highlights that transcendent goals gain prominence, extending beyond family legacies to include involvement in ecological activities. To explore these interrelated topics, we analyse 30 in-depth, semi-structured interviews with older adults (aged 60 and over) conducted in Italy and Sweden as part of the Legacies project, using a Constructivist Grounded Theory (CGT) approach. The analysis reveals the complexity surrounding environmental engagement throughout individual life courses, shaped by a caring relationship with the environment, access to ecological practices, concerns for grandchildren and intergenerational contact. By looking at older adults' lived experiences and future-oriented perspectives, this study reflects dynamic positioning along a continuum between activism and cynicism in response to environmental crises in daily life.

### SESSION 3150 (SYMPOSIUM)

Abstract citation ID: igaf122.1269

**LEVERAGING THE AGE-INCLUSIVITY DOMAINS OF HIGHER EDUCATION MODEL FOR PERSONNEL AND COMMUNITY ENGAGEMENT**

Chair: Katherina Nikzad-Terhune
Co-Chair: Allyson Graf
Discussant: Joann Montepare

At a time of contraction, return to work orders, and shrinking budgets, universities must be innovative in supporting age-diverse employees and sustaining employee morale. The age-inclusivity domains of higher education (AIDHE) model provides a framework for how institutions of higher education may consider age-inclusive practices across seven institutional domains, including personnel and community engagement. This symposium highlights ways in which these two components of the AIDHE model can help institutions craft a university work environment that is responsive to the needs of an age-diverse workforce. Nikzad-Terhune and colleagues (Northern Kentucky University) evaluated caregiving needs in a campus-wide assessment and focus here on data-driven recommendations on how universities can support family caregiving beyond FMLA. A study by Swanson and June (Central Connecticut State University) furthers the work of the community engagement domain at their Age-Friendly University by exploring the utilization and experience of those aged 62+ years who have registered for a course through the tuition waiver program in the past three years. Graf and colleagues (Northern Kentucky University) will apply the AIDHE model to results from a survey of mid-career faculty to illustrate how institutions can apply an age-inclusive lens to employee retention, development, and support. Kaskie and colleagues (University of Iowa) will present data on age-inclusive management strategies (the AIMS project), which seeks to educate employers about the aging workforce and support the adoption of management strategies targeting older employees. Joann Montepare (Lasell University), Chair of the GSA Age Inclusivity in Higher Education Workgroup, will be the discussant. Age Inclusivity in Higher Education Interest Group Sponsored Symposium

Abstract citation ID: igaf122.1270

### EMPOWERING UNIVERSITY EMPLOYEES THROUGH SUPPORT FOR FAMILY CAREGIVING

Katherina Nikzad-Terhune[1], Allyson Graf[1], and Lydia Manning[2], 1. Northern Kentucky University, Highland Heights, Kentucky, United States, 2. Scripps Gerontology Center, Oxford, Ohio, United States

Informal caregiving continues to increase, and caregivers are becoming more diverse, prompting continued efforts to examine the impact of informal caregiving. Focusing on caregivers in the higher education landscape is beginning to gain more traction as the impact of population aging and caregiver diversity becomes more evident. Supporting faculty and staff caregivers in higher education is essential for fostering a more inclusive and equitable environment, allowing them to more effectively balance professional and caregiving responsibilities. The evidence-based age-inclusivity domains of higher education (AIDHE) model guides institutions of higher education in assessing age-inclusive practices that impact faculty and staff across seven domains of institutional function. Data from a campus-wide caregiving study revealed that nearly one in four employees (24.6%) identified as caregivers, most commonly to aging parents and spouses. Stress from caregiving, work conflicts, and care for others in the household were cited as limiting caregiving ability. Approximately half (47.5%) experienced low quality of life due to lacking caregiver support. The majority (58.5%) were unaware of support services for caregiving. The experiences of faculty and staff caregivers highlight the need for better recognition and support services from institutions of higher education. Based on study findings and framed through the lens of the AIDHE model, recommendations will be made for universities to capitalize on pre-existing campus programs and resources, and to develop new age-inclusive programs and practices to effectively respond to the needs of faculty and staff caregivers.

Abstract citation ID: igaf122.1271

### UTILIZATION AND EXPERIENCE OF AN OLDER ADULT TUITION WAIVER PROGRAM

Helena Swanson, Andrea June, Erica Dewey, Yein Cho, Cailee Sheehan, and Isabella Krumm, *Central Connecticut State University, New Britain, Connecticut, United States*

Outreach and Engagement is a core domain within the Age Inclusivity Domains of Higher Education model. Central Connecticut State University furthers the work of this domain at their Age-Friendly University by advertising and advocating for the use of a tuition waiver available to Connecticut residents aged 62 and older. This waiver allows residents to enroll in courses after minimal fees, assuming open seats in the section and either meeting pre-requisites or obtaining permission from the instructor. While this program has been available for many years, the utilization and experience of those participating has not been explored. Utilization data was obtained with support from the Bursar's office and the Registrar's office. Between Spring 2023 and Spring 2025, the program accounted for 1073 credits (61% graduate, 37% undergraduate, and 2% doctoral) across 272 unique individuals. Students were enrolled in courses across the various schools of the campus, though courses in the college of liberal arts and social sciences were more heavily represented. Classes ranged from 1 to 4 credits. More than half of the students (63%) were not pursuing a degree. Only 11 students were enrolled full-time, and all were seeking graduate degrees. Students who have utilized the program were also invited to participate in a semi-structured interview that was recorded and transcribed. Major themes from the interview will be discussed. Taken together, the results of the study will be used to strengthen participation in the waiver program and to provide additional support for the benefits of program to the university.

Abstract citation ID: igaf122.1272

### AGE INCLUSIVE STRATEGIES TO ADDRESS MID-CAREER FACULTY PERCEPTIONS

Allyson Graf[1], Darcey Powell[2], Deanne Buffalari[3], Meagan Patterson[4], John Edlund[5], Sadie Elder[6], and Crystal Quillen[7], *1. Northern Kentucky University, Highland Heights, Kentucky, United States, 2. Texas A&M University, Corpus Christi, Corpus Christi, Texas, United States, 3. Westminster College, New Wilmington, Pennsylvania, United States, 4. University of Kansas, Lawrence, Kansas, United States, 5. Rochester Institute of Technology, Rochester, New York, United States, 6. Working Wardrobe, High Point, North Carolina, United States, 7. Rutgers Institute for Teaching, Innovation, & Inclusive Pedagogy, New Brunswick, New Jersey, United States*

Given a dearth of research and programming targeting mid-career faculty, higher education institutions are largely not responsive to variable career stage needs or age-related changes. The experiences and perceptions of mid-career faculty interpreted through the lens of promoting age-inclusivity can guide institutions in strengthening support at mid-career and beyond. In a sample of self-identified mid-career faculty ($N = 94$, Mage=45.67, $SD = 6.48$), 73.9% considered leaving their institution; reasons, which were rated from not at all (1) to to a great extent (3), included reducing stress ($M = 2.41$, $SD = 0.67$), institutional concerns ($M = 2.37$, $SD = 0.66$), increasing salary ($M = 2.24$, $SD = 0.75$), and seeking a supportive work environment ($M = 2.04$, $SD = 0.76$). Despite a high percentage of participants considering a move, they also indicated on a 7-point Likert type scale that they were somewhat unlikely to leave in the next three years ($M = 2.75$, $SD = 1.64$). We examined psychosocial factors including feeling valued, respected, excluded, isolated, belonging, and defeated within one's department, institution, and discipline as well as career satisfaction across multiple domains. Isolation, respect, and overall satisfaction with current position were significantly associated with both consideration and likelihood of leaving one's institution. Isolation, especially within the context of the broader institution, was higher among those who considered leaving ($p=.001$, $g=-0.837$) and was also associated with a higher likelihood of leaving within the next three years ($r=.31$, $p=.006$). Additional associations will be highlighted for discussion. Strategies for enhancing age-inclusivity among personnel, such as mentorship and recognition, may combat mid-career isolation and bolster feelings of respect. These will be discussed further during the presentation.

Abstract citation ID: igaf122.1273

### AGE INCLUSIVE MANAGEMENT STRATEGIES: APPLYING A PUBLIC POLICY FRAMEWORK TO INSTITUTIONS OF HIGHER EDUCATION

Brian Kaskie, *University of Iowa, Iowa City, Iowa, United States*

The Age Inclusive Management Strategies Advisory Council, established in 2024, comprised 20 representatives from diverse employment sectors including two institutions of higher education. These employment leaders offered their perspectives about the best ways to recruit, retain and promote workplace inclusivity for aging employees. Quarterly meetings and workgroups identified actionable policy alternatives, focusing on the Workforce Innovation and Opportunity Act (WIOA), the Senior Community Service Employment Program (SCSEP), caregiving policies, and age discrimination. The Council built this agenda upon foundational research from the AIMS initiative which identified gaps in current employer practices relative to need and demand among aging employees and surveyed employer awareness of workforce policies and programs designed for employees over 50. The council identified several alternatives to address the recruitment and retention of an aging workforce, aligning each with an established policy platform operating within state agencies including aging, labor, human rights, and the governor's office. Relative to the aging workforce within institutions of higher education, we highlight how federal and state programs support employment of older workers through skills training and job placement programs. We then examine how institutions of higher education have adopted such age inclusive management strategies and focus on how caregiving challenges faced by older workers have been addressed through employee assistance programs and other supportive services. Supporting the employment of older persons who are able and want to work corresponds with several benefits. Employers address labor force needs and older adults retain income and benefits that promote financial security, health, and successful aging.

### SESSION 3155 (COMMUNITY ENGAGED SCHOLARSHIP SYMPOSIUM)

Abstract citation ID: igaf122.1274

### EVALUATION AND DISSEMINATION STRATEGIES FOR COMMUNITY-ENGAGED RESEARCH

Chair: Sarah Dys
Co-Chair: Liza Behrens
Discussant: Elinor Schoenfield

Community-engaged research and evaluation strategies have not been widely discussed or documented, especially those focused on older adults and the communities in which they live. To address the needs of the gerontological research community, this symposium describes evaluation, measurement, and dissemination strategies commonly used in community-engaged research and evaluation through real world examples. Three presentations highlight diverse strategies to perform community-engaged research, conduct evaluations with community partner input, employ meaningful measurement practices, and share findings back with community partners involved in the work. The first presentation showcases multi-level evaluation of a unique community-based and intergeneration nutrition program in New York City. This program emphasizes the intersection of multisector partnerships, multiple methodologies, and a combination of participatory engagement, process evaluation, and outcomes assessments. The second presentation showcases a Patient Centered Outcomes Research Institute (PCORI) project to develop a comprehensive tool to measure engagement activities in community-engaged research and evaluation and how these activities impact research outcomes. The third presentation focuses on dissemination activities in community-engaged work, primarily working with a community-based advisory board to develop a communication and dissemination plan at the outset of a dementia care needs and assets assessment among African immigrant community members in Minnesota. Finally, the session concludes with a moderated discussion to provide a forum for participants and presenters to co-construct practice-based strategies to apply to their own community-engaged research and evaluation projects centering older adults and the communities in which they live. Participants are encouraged to engage in discussion to generate applications for future dissemination. Community-Engaged Research Interest Group Sponsored Symposium

Abstract citation ID: igaf122.1275

### AN INNOVATIVE AND COMPREHENSIVE EVALUATION STRATEGY OF AN ALL-AGES AND WHOLE OF COMMUNITY NUTRITION PROGRAM

Dalila Lanza[1], Madison LeCroy[1], Luisa Cardenas[2], R. Gabriela Barajas-Gonzalez[1], Nelson Lin[3], Stella Yi[4], Celine Chan[1], and Lan Doan[5], *1. New York University Langone Health, New York, New York, United States, 2. New York Academy of Medicine, New York, New York, United States, 3. Joint Medical Program UC Berkeley Public Health, Berkeley, California, United States, 4. New York University, New York, New York, United States, 5. NYU Langone Health, New York, New York, United States*

Harvest Share is a partnership between 11 multi-sector partners including NYU Langone Health, four farming/gardening organizations, three community-based organizations, two elementary schools, and a food pantry. The goal is to implement a whole-of-community intervention suitable for all ages to improve diet and social/built environments for English-, Chinese-, Spanish-, and Bangla-speaking community members living in Sunset Park and surrounding neighborhoods in Brooklyn, NY. This is achieved through a community-centered, equity-driven multi-level strategy centered on five main pillars: food access, nutrition education, experiential learning, food policy, and economic security. Much of the extant nutrition research is evaluated quantitatively and with clinical outcomes, which are limited in its ability to capture nuanced and social outcomes, especially over shorter time periods and for outcomes at the interpersonal or community levels. Furthermore, for community-engaged research or community-based participatory research programs, an important goal is to build equitable partnerships to address health outcomes and reduce disparities, which requires a multi-faceted evaluation strategy beyond individual-level outcomes examining participants. We describe Harvest Share's innovative and comprehensive evaluation strategy to understand intended and unintended impacts the program had on community partners and program participants of all ages. The evaluation strategy included five mixed methods at multiple levels: 1) surveys and skin carotenoid assessment; 2) semi-structured interviews stratified by language and income level; 3) ripple effects mapping — a participatory method to collect untold stories and behind-the-scene activities that can ripple out from a specific program; 4) partnership evaluation and social networks analysis; and 5) process evaluation.

Abstract citation ID: igaf122.1276

### MEASUREMENT MATTERS: MEASURING ENGAGED RESEARCH IN THE REAL WORLD

Erin McGaffigan[3], Marc Cohen[1], Tam Nguyen[2], Airia Papadopoulos[3], Myrna Finn[4], Melissa Destrampe[3], Sophia

Webber[3], and Alexa Fleet[1], *1. University of Massachusetts Boston, Boston, Massachusetts, United States, 2. Boston College William F. Connell School of Nursing, Chestnut Hill, Massachusetts, United States, 3. Collective Insight, North Reading, Massachusetts, United States, 4. Osher Lifelong Learning Institute, Pittsfield, Massachusetts, United States*

Engaging older adults and individuals with chronic conditions to drive research design, implementation, and dissemination is gaining increased attention. Many researchers lack clarity on how to operationalize engagement frameworks, creating frustrations among those who try and within the communities they seek to engage. To address this gap, the Measurement Matters project, funded by a Patient Centered Outcomes Research Institute (PCORI) Research Award (SOE-2022C2-28570), created and tested the PCOR-EM, a comprehensive tool to measure engagement activities and their potential impact on research outcomes. Collective Insight, LeadingAge LTSS Center @UMass Boston, Boston College Connell School of Nursing, in partnership with our Technical Advisors, Steering Committee, and Pilot Partner Subcommittee, developed the PCOR-EM through expert consensus methods, focus groups, and cognitive testing in 2024. In 2025, we tested the PCOR-EM with approximately 250 engaged research projects from the National Patient-Centered Clinical Research Network (PCORnet) – a network of diverse healthcare institutions across the U.S. Through this presentation, we will share the methods used and findings formulated from this multi-phase process, including our revised conceptual definition of engagement and key domains important for measurement as described by engaged researchers, engagement facilitators, and patient partners themselves. We will share the piloted PCOR-EM core concepts and discuss preliminary findings from our pilot testing with engaged research projects. These findings, as well as lessons learned on the use of advisory structures inclusive of patients and community partners, will provide a foundation for operationalizing key domains to support and evaluate researchers' meaningful community engagement practices.

Abstract citation ID: igaf122.1277

### COMMUNITY DISSEMINATION OF A COMMUNITY-ENGAGED RESEARCH PROJECT: APPROACHES AND LESSONS LEARNED

Truphosa (Posa) Aswani[1], Wynfred Russell[2], Tetyana Shippee[3], Joseph Gaugler[3], and Manka Nkimbeng[4], *1. University of Washington, Seattle, Minnesota, United States, 2. Strengthening African Resilience Towards Excellence, Brooklyn Park, Minnesota, United States, 3. University of Minnesota, Minneapolis, Minnesota, United States, 4. University of Minnesota School of Public Health, Minneapolis, Minnesota, United States*

Beginning in September 2019, we conducted a dementia care needs and assets assessment with the African immigrant community in Minnesota. Project advisory board members informed the development of a community conversation guide. Findings from the community conversations were used to develop a survey that was subsequently implemented through various community partner organizations. As a community-based participatory research project, there was a project dissemination plan that included presentations to community and scientific audiences. Our presentation will describe a community-engaged approach to disseminating the findings of the needs and assets assessment with the African immigrant community. Community dissemination of the findings was done through diverse events, activities, and partnerships both in person and remotely. In-person presentation of the findings was done at partner churches, mosques, and community fairs, while remote presentations were done via social media and Zoom. Finally, we partnered with another project to disseminate key findings to the community in person and through booklets. Project findings have been disseminated to over 1000 members of the community. Project findings have also been disseminated to scientific audiences through three journal publications, with one more under review. Our approach to dissemination, while successful, has not been without challenges. As such, we will also present lessons learned in the process of disseminating project findings to the community.

### SESSION 3160 (SYMPOSIUM)

Abstract citation ID: igaf122.1278

### EAST MEETS WEST: ADDRESSING FRAILTY AND SARCOPENIA

Chair: Sean Leng
Co-Chair: Cuntai Zhang
Discussant: Barbara Resnick

Frailty and sarcopenia are two critically important geriatric syndromes. As China and the US each face large and fast-growing old adult populations, substantial efforts have been devoted to study frailty and sarcopenia in both countries. This East Meets West symposium will present most recent and exciting research progress by senior investigators from China and in the US. Dr. Lin Kang will present results from a randomized trial demonstrating the feasibility and beneficial impact of a 6-month intervention of WHO ICOPE care pathways on health outcomes measures including vitality and mobility in frail and pre-frail older adults. Dr. Kathryn Callahan will describe an electronic frailty index (eFI) as a passive digital marker for frailty. Dr. Sean Leng will present the work from his group on bidirectional relationship between frailty and viral infections in older adults. Dr. Yaomin Hu will present her effort using self-developed AI diagnostic system and other technological innovations to establish a database and biobank for sarcopenia in China. Dr. Zhen Liang will present data suggesting that KLF13 restrains Dll4-muscular Notch2 axis to improve sarcopenia in animal models. Dr. Roger Fielding will describe the ongoing efforts towards a global definition and the potential for therapeutic interventions for sarcopenia in older adults. Dr. Cuntai Zhang, President of Chinese Medical Association Geriatrics Branch (CMA-GB) will serve the Co-Chair of this symposium. Dr. Barbara Resnick, President Emeritus of the Gerontological Society of America (GSA) will serve as the discussant.

Abstract citation ID: igaf122.1279

### GERONTOLOGIZING THE LEARNING HEALTH SYSTEM – THE ELECTRONIC FRAILTY INDEX AS A PASSIVE DIGITAL MARKER FOR FRAILTY

Kathryn Callahan, *Wake Forest University School of Medicine, Winston-Salem, North Carolina, United States*

Whether defined as a phenotype (Fried et al.) or through a frailty index (Rockwood et al., age-associated deficit accumulation), frailty metrics capture a unique description of an individual's, or population's, health in aging. Frailty has been slow to implement in healthcare systems, likely due to burdensome assessments. Building upon the Rockwood frailty index and the Clegg et al. electronic health record (EHR) based frailty index (eFI) in England, our team developed and implemented an eFI within the Wake Forest University School of Medicine (WFUSM) EHR, derived from personalized data collected during routine clinical care. Consistent with established methods, we compiled 54 age-associated deficits. eFI scores are reported as a simple proportion, with cut-points denoted as "Fit" (eFI≤0.10), "Pre-Frail" (0.100.21). Across 22+ published articles in multiple healthcare domains, eFI scores are consistently associated with adverse health outcomes for older adults. In primary care, the c-statistics for frailty include (adjusted for age, sex, race/ethnicity, past utilization) 0.79 for mortality, 0.74 for acute utilization, 0.79 for emergency department visits, 0.74 for injurious falls. Post-operative frail (eFI>0.21) older adults experienced an elevated hazard of six-month mortality (HR 2.86, 1.84-4.44), and elevated odds of 30-day readmissions (OR 2.46, 1.72-3.52) and post-acute services (OR 1.68, 1.36-2.08). Frail older adults with solid tumors undergoing chemotherapy experienced greater hazard of hospitalization (HR 3.23, 2.23-4.69) and lower mean overall survival (19m in frail vs 54m in fit). We are testing care pathways to improve quality of life and health outcomes in frailty.

Abstract citation ID: igaf122.1280

### SARCOPENIA: TOWARDS A GLOBAL DEFINITION AND THE POTENTIAL FOR THERAPEUTIC TREATMENTS

Roger Fielding, *Tufts University, Medford, Massachusetts, United States*

The age-related loss of skeletal muscle mass and function, sarcopenia, is associated with well-characterized functional limitations, physical disability, and distal clinically relevant outcomes such as falls, fractures, and death. Underlying these age-related changes are physiological changes in the force/power generating capacity of skeletal muscle that appear to be driven by changes in skeletal contractile protein function, metabolic derangements and alterations in neuromuscular activation. Biologically-relevant age-associated changes in skeletal muscle biology include alterations in gene transcription, mitochondrial stability, anabolic capacity, cellular senescence and metabolic flexibility. Underlying molecular targets have been identified in skeletal muscle that are potential sites for the development of therapeutic interventions. In my presentation, I will provide a state-of-the-art update on key therapeutic targets for components of the sarcopenia syndrome. Two major classes of therapies, selective androgen receptor activation and inhibition of myostatin signaling, have been proposed as targets for treatment of sarcopenia. However, more recently, additional relevant novel pathways have been uncovered that show promise. These include approaches that target skeletal muscle excitation-contraction coupling such as the selective activation of skeletal muscle troponin proteins, and targets that stimulate biogenesis or stability of mitochondria and their upstream activators. I will summarize the available clinical trials data on these emerging novel pathways and discuss the barriers towards regulatory approval for these indications which include the need to develop an international consensus definition of sarcopenia and establishment of treatment guidelines.

Abstract citation ID: igaf122.1281

### IMPLEMENTATION AND IMPACT OF THE WHO INTEGRATED CARE FOR OLDER PEOPLE PROGRAM IN OLDER ADULTS WITH FRAILTY IN CHINA

Lin Kang, *Peking Union Medical College Hospital, Beijing, Beijing, China (People's Republic)*

Fragmentation of services increases health and social care burden as people live longer with higher prevalence of diseases, frailty and dependency. Evidence from China and around the world indicates that development and implementation of person-centered integrated care are urgently needed to improve senior health and achieve healthy ageing. To test the feasibility and impact of World Health Organization's (WHO) Integrated Care for Older People (ICOPE) approach in China, we designed a randomized controlled trial to evaluate its impact on health outcomes and health resource utilization. Community-dwelling older adults screened as at-risk of functional declines (pre-frailty or frailty) were randomized into intervention (n = 537) and control (n = 1611) groups between September 2020 and February 2021.A 6-month intervention program following WHO ICOPE care pathways implemented by integrated care managers compared to standard care. After 1 to 1 propensity score matching, participants in intervention and control groups (totally 938) had comparable baseline characteristics, demonstrated feasibility of implementing ICOPE with satisfaction by participants (97–99%) and providers (92–93%). All outcomes showed improvements after a 6-month intervention, with statistically significant least-squares mean differences (control-intervention) in vitality as measured by mini-Nutritional Assessment Short Form to measure vitality (−0.21, 95% CI, −0.40–0.02, worse in control group), mobility by Short Physical Performance Battery (−0.29, 95% CI, −0.44–0.14), and psychological health by 5-item Geriatric Depression Scale (0.09, 95% CI, 0.03–0.14). These results support the feasibility and impact of this intervention on health outcomes of frail older adults as well as its acceptance among key stakeholders in China.

Abstract citation ID: igaf122.1282

### ADVANCING SARCOPENIA MANAGEMENT THROUGH INTEGRATED CLINICAL-RESEARCH TRANSLATION AND INNOVATION

Yaomin Hu, *Renji Hospital, Shanghai, Shanghai, China (People's Republic)*

Sarcopenia is closely linked to aging, driving our comprehensive clinical and scientific efforts to address this condition through a multidimensional integration of clinical translation and technological innovation. We established a database and biobank for sarcopenia, which formed the foundation for leading the development of the 2024 Chinese Expert Consensus on the Construction of Standardized Biobank and Database for Sarcopenia. This initiative has fostered a collaborative medical consortium network for sarcopenia management. Our self-developed AI diagnostic system, Muscle and Fat Intelligent Analysis System (MFIAS), automates CT-based muscle quantification, significantly improving analytical efficiency compared to the traditional manual approach, while shifting from single-parameter to multidimensional metrics analysis. The newly constructed skeletal muscle rehabilitation unit employs isokinetic dynamometry testing and training systems, demonstrating short-term improvements in physical performance scores and metabolic parameters through personalized treatment protocols. In addition, in collaboration with the School of Basic Medical Sciences, we have established a basic science research team to investigate the molecular mechanisms underlying skeletal muscle aging. These innovations align with global trends in precision geriatrics, offering scalable solutions for promoting healthy aging for the ever-growing older adult population. By redefining sarcopenia as a modifiable aging trajectory rather than an inevitable decline, our work provides an actionable framework for early intervention and multidisciplinary care, addressing one of the most pressing challenges in gerontological health.

Abstract citation ID: igaf122.1283
### KLF13 RESTRAINS DLL4-MUSCULAR NOTCH2 AXIS TO IMPROVE SARCOPENIA
Zhen Liang, *Peking University Shenzhen Hospital, Shenzhen, Guangdong, China (People's Republic)*

Krüppel-like factor 13 (KLF13), a central regulator of cellular energy metabolism, is highly expressed in skeletal muscle and implicated in the pathogenesis of several diseases. This study investigated the role of KLF13 in sarcopenia. In a dexamethasone-induced muscle atrophy mouse model, compared to the control group, the KLF13 knockout group had decreased overall muscle strength (1.77 ± 0.10 vs. 1.48 ± 0.16, $P<0.01$), muscle weight (%) 76.0 ± 5.69 vs. 60.7 ± 7.23, $P<0.001$ for gastrocnemius (Gas) and 75.8 ± 6.21 vs. 67.5 ± 5.01, $P<0.05$] for tibialis anterior, and exhaustive running distance (m) (495.5 ± 64.8 vs. 315.5 ± 60.9, $P<0.05$). KLF13 overexpression preserved muscle mass (Gas: 100 ± 6.38 vs. 120 ± 14.4, $P<0.01$) and exhaustive running distance (423.8 ± 59.04 vs. 530.2 ± 77.45, $P<0.05$) in an in vivo diabetes-induced skeletal muscle atrophy model. Clofoctol protected against dexamethasone-induced muscle atrophy. Myotubes treated with dexamethasone were aggravated by KLF13 knockout, but anti-atrophic effects were achieved by inducing KLF13 overexpression. Transcriptome analyses and luciferase reporter assays demonstrate that delta-like 4 (Dll4) was a novel target gene of KLF13. KLF13 suppressed Dll4 expression, leading to the inhibition of the Dll4-Notch2 axis and prevention of muscle atrophy. Dexamethasone inhibited KLF13 expression by inhibiting myogenic differentiation 1-mediated KLF13 transcriptional activation and promoting F-Box and WD repeat domain containing 7-mediated KLF13 ubiquitination. These findings provide novel insights into mechanisms underlying skeletal muscle atrophy and sarcopenia with potential therapeutic targets.

### SESSION 3165 (SYMPOSIUM)

Abstract citation ID: igaf122.1284
### HONORING THE VOICES OF OLDER ADULTS: CBPR APPROACHES TO BUILD COMMUNITY GUIDED INITIATIVES
Chair: Jordan Lewis

Much of the past research conducted with BIPOC communities was conducted under false narratives, or as "helicopter research," where researchers would enter the community, gather data, and leave the community without ever discussing or consulting with community members. These past unethical practices, with no community involvement, and no reporting to the communities on how the data was used or published have created significant mistrust. Community Based Participatory Research is an approach conducted as an equal partnership between community members, organizational representatives, and researchers. This symposium will offer a panel of presentations highlighting the importance of community-engaged research studies with BIPOC communities. The first presentation focuses on a study with a southern African American community that created a training program for clinicians on how to communicate in a culturally congruent and respectful way with older southern, African Americans with serious illness and at end of life. The second paper discusses the community engaged practices that developed a culturally relevant, community-delivered intervention to improve social connections among Black caregivers. The third presentation focuses on experiences and lessons learned when developing Elder-based research approaches to Indigenous healthy aging research. The final presentation, discusses the importance of building local and national partnerships to address the crucial need of sharing culturally sensitive knowledge related to brain health, resources and support for dementia caregivers, and dementia knowledge in remote Alaska Native communities. The presenters will discuss the partnership building process and its impact on communities on the family, the community, and at the state level. This is a collaborative symposium between the Community-Engaged Research and Generativity and Aging Interest Groups.

Abstract citation ID: igaf122.1285
### BLACK AMERICANS TRAIN CLINICIANS WHO PROVIDE CARE TO OLDER BLACK AMERICANS: PROGRAM DEVELOPMENT AND PILOT OUTCOMES
Ronit Elk[1], Shena Gazaway[2], Alvin Reaves[3], and Michael Barnett[4], *1. The University of Alabama at Birmingham, Birmingham, Alabama, United States, 2. School of Nursing, Birmingham, Alabama, United States, 3. US Acute Care Solutions, Rockville, Maryland, United States, 4. Four Seasons Hospice, Flat Rock, North Carolina, United States*

Lack of appreciation of cultural differences during serious illness compromises care for seriously ill older African American patients. Training programs for clinicians in providing culturally appropriate care for older adults of different cultures is lacking. The goals of this study were to partner equitably with a southern African American community to create a training program for clinicians on how to communicate in a culturally congruent and respectful way with older southern, African Americans with serious illness and at end of life, and to test program delivery feasibility. Community Based Participatory Research guided the equitable partnership. The Community Advisory Board (CAB) guided the creation and implementation of two focus groups (caregivers and pastors). Based on focus groups' results on important cultural values and respect for lived experiences, the CAB created 4 training videos. Each video highlighted a key message for clinicians- cultural, religious, and demand for equal care. The videos were incorporated into a training program based on Kolb's Adult-based Experiential Learning Theory, and a training guide created for the 3-hour training that can be delivered via zoom or in person. Donabedian's Structure-Process-Outcome model was used to determine program delivery feasibility. Two pilot training sessions were held, with high rate of clinician attendance. At three months post-training clinicians reported a high rate of adhering to the community recommendations. The first training program developed by African American community members to train clinicians was feasible to create and implement, and resulted in clinicians changing their practice to adhere to the community's recommendation.

Abstract citation ID: igaf122.1286

### IMPROVING SOCIAL CONNECTIONS AMONG BLACK DEMENTIA CAREGIVERS THROUGH COMMUNITY ENGAGEMENT

Sandhya Seshadri[1], Phyllis Jackson[2], Paula Alio[3], Sarah Morgan[4], Angela Contento[5], and Benzi Kluger[4], *1. University of Rochester, Rochester, New York, United States, 2. Common Ground Health, Rochester, New York, United States, 3. University of Rochester School of Medicine & Dentistry Department of Public Health, Rochester, New York, United States, 4. University of Rochester, School of Medicine & Dentistry, Rochester, New York, United States, 5. University of Rochester Warner School of Education, Rochester, New York, United States*

Limited access to resources, economic inequality, and cultural beliefs about dementia may lead to increased social isolation and loneliness among Black caregivers of persons living with dementia (PLwD). On the other hand, religious activities and spiritual connectedness are integral to Black caregiver wellbeing. To improve social connections among Black caregivers of PLwD we collaborated with a community advisory board (CAB) comprising members of the Interdenominational Health Ministry Coalition (IHMC), a local faith-based organization. The study was guided by the Dimensions of Social Connection Model to promote social connections in dementia caregivers (Van Orden & Heffner, 2022). The study aims were to (1) conduct semi-structured interviews with Black caregivers of PLwD to understand their caregiving experiences and social needs, and (2) co-develop the Congregational Compassionate Care (CCC) intervention. Our community-engagement practices were based on trust, respect, and mutual learning. Findings from the first aim informed the co-development of the six-week intervention that will be delivered by trained IHMC member-facilitators. The interactive Reach, Effectiveness, Adoption, Implementation, Maintenance (RE-AIM) toolkit guided CCC co-development. Researchers and CAB members held 5 workshops to co-develop the CCC intervention topics and content, a member-facilitator training program, resources for participants and volunteers, measures to assess feasibility, acceptability, social isolation, loneliness, and intervention fidelity, and manuals for participants and facilitators. In the next step we will collaboratively train member-facilitators and test the intervention. Our community engaged practices facilitated the development of a culturally relevant, community-delivered intervention that fostered community ownership of efforts to improve social connections among Black caregivers.

Abstract citation ID: igaf122.1287

### DISSEMINATION OF A CBPR STUDY WITH ALASKA NATIVE ELDER ADVISORY COMMITTEE MEMBERS

Lena Thompson[1], Steffi Kim[2], Marcelo Quinto[3], and Jordan Lewis[4], *1. University of Alaska Fairbanks, Fairbanks, Alaska, United States, 2. University of Alaska Anchorage, Anchorage, Alaska, United States, 3. Alaska Native Brotherhood, Juneau, Alaska, United States, 4. Center on Aging, Thompson School of Social Work & Public Health, University of Hawai'i at Manoa, Honolulu, Hawaii, United States*

Historically, unethical research practices with American Indian and Alaska Native communities has left community members feeling distrustful of Western research institutions. While the scientific community has made improvements in fostering community participation in the designing and analyzing of research, community participants are often left out of dissemination efforts. Dr. Lewis and his research team have worked to build healthy relationships with Alaska Native communities through an 18-year Community-based participatory research study exploring successful aging among Alaska Native communities. To disseminate study findings, the research team worked with two Alaska Native Elder Advisory Committees (EAC) in Southeast and Interior Alaska to conceptualize, plan, and carry out dissemination efforts locally, nationally, and internationally. In addition to peer-reviewed manuscripts and conference presentations, the study team worked with Elders to plan community presentations and discussions, short videos, and products that could be worn and used in the community, such as sweatshirts and berry buckets with the study logo. Elders presented knowledge gained though the study at an international Arctic conference. Opportunities like this include community members beyond local dissemination efforts to receive international recognition of their community and scholarly efforts. EAC members described feeling grateful for opportunities to travel and were excited to disseminate through videos and products to engage youth. Community participation in dissemination efforts is a key aspect to conducting responsible Community-based participation research. Dissemination efforts should find balance between meeting community needs and grant/academic requirements. Creative dissemination efforts, such as products and videos can help findings reach a wider audience.

Abstract citation ID: igaf122.1288

### FOSTERING COMMUNITY PARTNERSHIPS

Steffi Kim, *University of Alaska Anchorage, Anchorage, Alaska, United States*

The importance of building local and national partnerships to address the crucial need to share culturally sensitive knowledge related to brain health, resources and support for dementia caregivers, and dementia knowledge in remote Alaska Native communities is well known. This presentation will discuss the partnership-building process and its impact on communities at the family, community, and state levels. Remote Alaska Native communities have unique needs in addressing health disparities, access to resources, and the latest health-related knowledge. With increasing numbers of Alaska Native people being diagnosed with dementia, it is crucial to continue to share information on brain health, warning signs of dementia, and what dementia is, as well as to develop resources that specifically speak to the needs of those living in these communities. Alaska Native culture has shown to be a factor that protects people caring for someone with dementia from stigma. Knowledgeable and responsive Alaska Native communities minimize the strain on caregivers and increase the ability of the Elder to age within the community. Through collaboration, the AN CARE Lab seeks to advance knowledge and engage community members in developing resources. Community partners such as the Alzheimer's Resources of Alaska, the Commission on Aging, the Aleutian Pribilof Island Association, the Alzheimer's Association Alaska Chapter, and the Alaska Department of Health are engaging with communities, supporting community projects, and participating in state-level advocacy to include the needs of Alaska Native community members.

### SESSION 3170 (SYMPOSIUM)

Abstract citation ID: igaf122.1289

### ENHANCING TRANSPORTATION IN RURAL COMMUNITIES THROUGH INNOVATIVE SOLUTIONS

Chair: Renee St. Louis
Discussant: Renee St. Louis

Transportation remains one of the biggest barriers to maintaining health and quality of life in rural areas. While the challenges are familiar and have been well-documented, solutions are scarce. The presentations in this session collectively highlight successful examples of technology deployment, collaboration, and other innovative approaches to address challenges and improve accessibility for older adults. Marcus will discuss insights from an analysis of national data on older adults' requests for rides in rural areas. Freund will describe the results of a study of ITNCountry's national network of nonprofit community transportation services and the effects of the network on rider experience and trip volume. Jansen will present the results of an evaluation of Montachusett Regional Transit Authority (MART) initiatives to improve coordination among local Council on Aging transportation providers. Ramsey will summarize preliminary results of a pilot project in rural Iowa that has enabled medical patients to call public transportation rides on demand after their doctors' visits have concluded. Brooks will introduce UZURV's model for providing accessible, safety-conscious ridesharing services in rural areas and review insights from their rider data on remaining mobility challenges and opportunities. St. Louis will conclude as discussant, relating key themes across presentations and proposing next steps for research and practice. Transportation and Aging Interest Group Sponsored Symposium

Abstract citation ID: igaf122.1290

### CREATING A GEOGRAPHY OF ACCESS FOR RURAL OLDER ADULT MOBILITY

Cara Marcus, *National Rural Transit Assistance Program, Woburn, Massachusetts, United States*

Older adults in rural areas often lack access to transportation that is available in cities, suburbs, and large towns. Challenges to providing transportation in rural areas include lack of funding and resources, long distances, and difficult-to-navigate terrain. National Rural Transit Assistance Program (National RTAP) analyzed data on the breakdown of rural older adults asking for assistance in locating rides between 2016 and 2019 (n = 31). Findings indicated that 38% needed transportation for medical appointments, 23% needed general transportation, and the remainder was evenly distributed between transportation to grocery stores, assisted living facilities, hospitals, dialysis centers, and leisure destinations. We also found that rural communities are working towards creating more access for older adults through innovative transportation solutions, such as ride-sharing, transportation brokerages, feeder routes, nutrition shuttles, and more. Through collaboration with organizations like Area Agencies on Aging, community action councils, mobility managers, elected officials, and others, rural transit agencies can work to address the full spectrum of mobility needs of this important population. National RTAP has created guidance for all stakeholders on assisting people with finding transportation that includes situational assessment, research processes, databases and tools, and recommendations for relaying information to older adults.

Abstract citation ID: igaf122.1291

### ITNCOUNTRY: TRANSPORTATION FOR RURAL AND SMALL COMMUNITIES

Katherine Freund[1], and Aaron Meuser[2], *1. ITNAmerica, Westbrook, Maine, United States, 2. ITN America, Westbrook, Maine, United States*

Providing transportation in rural and small communities presents unique challenges -- resources diminish as distances and costs increase, while efficiency declines. This research demonstrates ITNCountry on the Salesforce platform in small communities in California, Maine, Kentucky, Florida, and New York to address these challenges. The demonstration occurred in two phases. Phase One: (1) rebuild ITNRides software on Salesforce; (2) develop marketing strategies; and (3) collaborate with ITN affiliates to establish ITNCountry in small and rural communities. Phase Two: (1) transition demonstration communities to ITNRides2.0; (2) train communities in ride services and coordination; (3) utilize the Online Learning Center for training; and (4) market and implement innovative payment programs. Data were collected over 12 months, with an additional 8 months as service continued. Methods included community profiles, stakeholder interviews, and pre- and post-surveys of stakeholders, riders, and volunteer drivers. Rides increased steadily, and rider and volunteer surveys showed high satisfaction. Over 60% of rides were for healthcare, 44% of riders used a mobility aid and 29% required door-through-door assistance. No membership was required, but survey results indicated that riders valued membership for its sense of belonging, community and security. ITNCountry volunteer transportation in rural and small communities shows promise in meeting the mobility needs of older adults and people with mobility challenges.

Abstract citation ID: igaf122.1292

### REGIONAL TRANSIT AUTHORITY EFFORTS TO SUPPORT COA TRANSPORTATION IN RURAL AREAS IN MASSACHUSETTS

Taylor Jansen[1], Shayna Gleason[2], Nina Silverstein[1], Caitlin Coyle[1], and Scott Rich[3], *1. University of Massachusetts Boston, Boston, Massachusetts, United States, 2. International Transportation Learning Center / Transit Workforce Center, Washington, District of Columbia, United States, 3. Montachusett Regional Transit Authority, Fitchburg, Massachusetts, United States*

The Montachusett Regional Transit Authority (MART) serves both urban and rural areas within its 624-square-mile region, encompassing parts of Northern Worcester and Western Middlesex Counties in Massachusetts. MART, in cooperation with Councils on Aging (COA) in their region, sought to improve transportation services for older adults and people with disabilities and enable them to access a greater array of destinations, through the deployment of software for trip booking and management. Trip data were analyzed for N = 10 communities in June and September 2024 after the new software was introduced to COAs to assist with dispatching and reporting requirements. In addition, zoom interviews were conducted with N = 9 dispatchers, N = 6 drivers, N = 5 COA directors, and phone interviews with N = 21 older adult riders. An in-person focus group of riders was also held. Key findings included that dispatchers and directors appreciated being able to link rider information automatically, having voice call and text reminders to riders, and moving from "paper and pen" to more efficient reporting, though some software challenges remained. Overall, trip data revealed a slight increase in life-sustaining trips from June to September, while riders voiced an interest in also having more social and recreational destinations or "life-enriching" trips provided. MART's interest in increased coordination across the towns remains a challenge while COAs have varying operations and town policies for travel across jurisdictions. Recommendations for the future include more robust training on the software and uses of its data for drivers and dispatchers, as well as incentivizing and enabling trip coordination between COAs.

Abstract citation ID: igaf122.1293

### HEALTH CONNECTOR: BRIDGING THE GAP BETWEEN HEALTHCARE AND TRANSPORTATION

Jenna Kubiak, *Flexlynqs, Iowa, United States*

Access to healthcare remains a significant challenge for many individuals due to transportation barriers. The Health Connector program addresses this by integrating transportation services with healthcare appointment scheduling, creating a seamless mobility solution for all populations. This initiative directly links transit resources with healthcare appointments through a unified platform. To rigorously assess program effectiveness during the ongoing 18-month pilot, Iowa State University analyzed specific performance measures developed during the research phase. Data collection, including rider surveys, trip data analysis, and interviews with healthcare and transit staff provided continuous insights into the program's impact and effectiveness. Preliminary findings suggested positive trends in patient satisfaction with transportation services. This project continues to highlight the critical importance of robust middleware integration and strong stakeholder communication for successful implementation. Innovative transportation solutions play a vital role in advancing health access, and data from the Health Connector project demonstrates the impact of integrated mobility strategies on healthcare outcomes. Attendees will explore how leveraging technology, stakeholder engagement, and data-driven decision-making can create sustainable, accessible transportation options that improve health outcomes and overall community well-being.

Abstract citation ID: igaf122.1294

### HOW ADAPTIVE TNCS CAN IMPROVE TRANSPORTATION QUALITY FOR OLDER ADULTS AND STRETCH RESOURCES FOR SMALLER COMMUNITIES

Ron Brooks, Steve Cummings, and Susan Starwalt, *UZURV, Richmond, Virginia, United States*

Established in 2017, UZURV is an adaptive Transportation Network Company (TNC) aimed at enhancing mobility services for older adults and individuals with disabilities. It collaborates with public transit agencies, social service organizations, and communities to deliver millions of door-to-door trips annually across approximately 25 communities in 15 U.S. states. UZURV's innovative model involves tailoring its technology, driver onboarding processes, and service methods to address specific community needs. This has set a standard in the rideshare industry, paving the way for other providers to emulate this approach by adjusting to the unique service demands and regulatory requirements of smaller communities. This presentation explores UZURV's adaptations of the rideshare model to meet the needs of those requiring door-to-door service, including wheelchair accessibility, service animal accommodations, and options for people who do not have, or cannot use, smartphones. We will address how UZURV aligns its operations with agency contractual and compliance mandates, detailing the implementation of background checks, employee training, and substance abuse testing and reporting. Furthermore, this presentation will illustrate how compliant rideshare providers like UZURV can augment traditional community transport systems and enhance service coordination. We will present operational and financial data demonstrating that rideshare services can offer safe, accessible, and dignified transportation at lower costs compared to traditional paratransit options. This evidence will highlight the viability of the rideshare model in expanding transit opportunities for underserved populations while maintaining rigorous safety and accessibility standards.

### SESSION 3175 (SYMPOSIUM)

Abstract citation ID: igaf122.1295

### COGNITIVE AGING AND DEMENTIA RESEARCH: EXPANDING HORIZONS ON SOCIAL AND ENVIRONMENTAL CONTEXTS

Chair: Jacqui Smith
Discussant: Dawn Carr

This symposium showcases new cross-disciplinary research into proposals about early-life and midlife contexts associated with later-life cognition and dementia. The early-career speakers utilize data from the Carolina African American Twins Study of Aging (CAATSA), Health and Retirement Study (HRS), and Panel Study of Income Dynamics (PSID) and apply advanced analysis methods. Presentations consider the influence of education, early adult and midlife neighborhood poverty, and pre-retirement work contexts in cognitive aging. Early-life education is well-known as a predictor of dementia. Johnson expands this research by revealing a unique role for education beyond zygosity and socio-environmental factors. Kalousová examines the relevance to late life cognition of early-to-late life residential contexts characterized by neighborhood poverty. Using sequence analysis, she identifies distinct patterns of exposure to neighborhood disadvantage over time. Cumulative exposures to areas with limited resources increased the likelihood of late-life cognitive impairment. Retirement from the workforce often contributes to increased stress, loneliness, and reduced cognitive engagement. Kayser, Xiang, and Smith apply cross-wave dynamic change models to HRS multi-wave data to disentangle bidirectional associations between changes in cognition and loneliness after retirement. They report that over the longer-term both cognitive decline and loneliness increase. Working longer and different retirement trajectories may mitigate cognitive decline. Oh used sequence analysis to identify six work-related trajectories prior to retirement. He finds that working longer and engaging in so-called "encore" careers reduce the probability of later-life dementia. The session concludes with an integrative discussion by Carr of the implications of these studies for future research and policy.

Abstract citation ID: igaf122.1296

### DISENTANGLING NATURE AND NURTURE IN COGNITIVE AGING AMONG AFRICAN AMERICAN TWINS

Kimson Johnson[1], and Keith Whitfield[2], *1. University of Michigan Institute for Social Research, Ann Arbor, Michigan,*

*United States, 2. University of Nevada, Las Vegas, Las Vegas, Nevada, United States*

Higher educational attainment is linked to better cognition in later life, with twin studies helping to disentangle genetic and environmental contributions. Few studies have examined African American twins, particularly in relation to memory performance. This study examines the relationship between education, early-life factors, and cognitive outcomes in older African American twins, focusing on genetic and environmental contributions, stratified by zygosity. Data from the Carolina African American Twins Study of Aging (n = 138, 69 twin pairs) were used to assess cognitive outcomes in adults aged 50 and older, focusing on educational attainment and genetic influences. Correlation analyses examined the relationship between education and cognition (global functioning, working memory, processing speed), stratified by monozygotic (MZ) and dizygotic (DZ) twins. Analyses adjusted for sociodemographic factors, parental education, and childhood health. Overall, findings suggest education-cognition relationships vary slightly across twin types. For global functioning, MZ twins had a slightly higher correlation with education (r = 0.58) than DZ twins (r = 0.55). In fluid reasoning, DZ twins (r = 0.46) had a slightly higher correlation for education compared to MZ twins (r = 0.44). In logical reasoning, the correlation was stronger for MZ twins (r = 0.42) than DZ twins (r = 0.35). Genetics had little impact on the education-cognition relationship. Age showed a small negative correlation with cognition, while parental education had a modest positive impact. Education's positive impact on cognitive function highlights the importance of improving educational opportunities to support cognitive health in older adults, potentially reducing cognitive aging regardless of genetic predispositions.

Abstract citation ID: igaf122.1297

### NEIGHBORHOOD POVERTY EXPOSURE IN ADULTHOOD AND COGNITIVE OUTCOMES IN LATER LIFE

Lucie Kalousova, *Vanderbilt University, Nashville, Tennessee, United States*

Dementia risk is shaped not only by biological and individual factors but also by broader structural conditions, including neighborhood disadvantage. I examine the long-term impact of neighborhood poverty on cognitive health, focusing on when in the life course exposure matters most. Using longitudinal data from the Panel Study of Income Dynamics (PSID: N = 1,957), I reconstruct respondents' neighborhood poverty trajectories from early adulthood to late life. I apply sequence analysis to classify individuals into distinct patterns of exposure and use marginal structural models with inverse probability of treatment weights to estimate the effects of neighborhood poverty on dementia risk while accounting for time-varying confounding. My findings show that cumulative exposure to neighborhood poverty is associated with higher odds of cognitive impairment in later life (OR: 1.39; [1.28, 1.51]. Early adulthood exposure appears more consequential (OR: 1.24; [1.06, 1.45]) than later life exposure (OR:1.05; [0.98, 1.13]. The results suggest that interventions aimed at reducing dementia risk must begin earlier in the life course and contribute to the growing literature on the social determinants of cognitive health by clarifying the role of neighborhood disadvantage across different life stages. They also highlight the need for policies that address structural inequalities in housing and neighborhood conditions long before cognitive decline becomes apparent.

Abstract citation ID: igaf122.1298

### THE TEMPORAL DYNAMICS OF LONELINESS AND COGNITIVE FUNCTION IN OLDER ADULTS

Jay Kayser[1], Xiaoling Xiang[2], and Jacqui Smith[1], *1. University of Michigan, Ann Arbor, Michigan, United States, 2. University of Michigan-Ann Arbor, Ann Arbor, Michigan, United States*

The life course stress model suggests that stressors, such as loneliness, can accumulate and shape long-term cognitive trajectories, particularly in later life. While loneliness has been linked to cognitive decline, few studies have examined whether this relationship is bidirectional. This study investigated associations between loneliness and cognitive function over time. Data were drawn from four waves (2008–2022) of the Health and Retirement Study. The analytic sample included 12,144 adults aged 50 and older. A random-intercept cross-lagged panel model was used to separate within-person fluctuations from stable between-person differences, and a dual-process latent growth curve model examined long-term patterns in loneliness and cognitive function. Loneliness was measured using the 11-item UCLA Loneliness Scale, and cognition was assessed via immediate and delayed word recall tasks. Cross-lagged effects pathways between loneliness and cognitive function were not statistically significant, suggesting no within-person bidirectional relationship. However, the random intercepts for cognition and loneliness were negatively correlated (r = -0.22, p < .001), suggesting that individuals with higher loneliness tended to have lower cognitive function at baseline. Additionally, the random slopes for cognition and loneliness were also negatively correlated (r = -0.30, p < .001), indicating that individuals with greater declines in cognition also tended to experience increases in loneliness over time. These findings suggest that loneliness and cognitive function are interconnected, even in the absence of short-term directional effects. Understanding their long-term association can inform interventions aimed at preserving cognitive health and reducing loneliness in older adults.

Abstract citation ID: igaf122.1299

### INFLUENCE OF WORK-TO-RETIREMENT TRAJECTORIES ON COGNITIVE OUTCOMES IN LATER LIFE

Yun Taek Oh, *University of Nevada, Reno, Reno, Nevada, United States*

Previous studies report that working longer is associated with better cognitive health. Yet, few studies evaluated the cognitive outcomes from various ways of working longer, such as remaining in career jobs longer, pursuing new careers, and gradually reducing work hours. While working in career jobs longer provides cognitive benefits, pursuing new careers in later life (i.e. encore careers) involves learning and training of different skills and knowledge that possibly can provide significantly more cognitive benefits by stimulating cognitive functionality. I use the Health and Retirement Study (HRS; n = 1,765) to evaluate how various work-to-retirement trajectories influence the cognitive outcomes of older workers during their later-phase of work lives (ages 60-71) and post-retirement lives (ages 70-81). I first use sequence analysis to categorize work-to-retirement trajectories into six groups. Then, I use latent growth modeling and competing risk analyses to examine the changes in cognitive health, separately by gender. The results show that working longer, regardless of making career transitions, is associated with better cognitive outcomes during the later-phase of work life (Slopes = 0.023 and

0.245 for pursuing new careers and taking phased exits respectively), and pursuing new careers in later life is associated with lower probability of getting dementia for men during post-retirement lives (Sub-distributional Hazard Ratio = 0.332). These results suggest that provision of learning and training opportunities for older workers, especially the skills and knowledge outside their career jobs, can contribute to preserving better cognitive health in later life.

### SESSION 3180 (SYMPOSIUM)

Abstract citation ID: igaf122.1300

### BLACK MEN'S HEALTH AND AGING ACROSS THE LIFE COURSE

Chair: Roland Thorpe
Co-Chair: Carl Hill

Much of the research on Black men tend not to focus on concepts that have been found to be important determinants of health for Black men. Nor, do they seek to identify sources of resilience that may be protective of health. This symposium contains a collection of papers that discuss some key social determinants of health (SDOH) that can provide insights to advance our understanding of Black men's health and aging. Esiaka investigated whether active coping moderates the association between neighborhood segregation and Subjective cognitive decline (SCD) in Black American men. Findings indicate the association between neighborhood segregation and SCD depends on active coping levels. Bruce and colleagues examined how heterogeneity of income influences correlates of cognitive impairment among Black men using data from the 2016 Health and Retirement Study. These investigators found that age and poor health were significant for men reporting incomes below the national median while post-secondary education and depressive symptoms were associated with any cognitive impairment among Black men with incomes over $50,000. Jones examined equity-focused approaches to preserve brain health in aging Black men with HIV to reduce disparities in cognitive and cardiovascular outcomes. His work highlighted the importance of the need for an interdisciplinary team to advance the understanding of the link between HIV, cardiovascular outcomes and cognitive outcomes. These presentations will bolster our knowledge on SDOH among Black men across the life course.

Abstract citation ID: igaf122.1301

### ACTIVE COPING MODERATES THE LINK BETWEEN NEIGHBORHOOD SEGREGATION & COGNITIVE DECLINE IN BLACK AMERICAN MEN

Darlingtina Esiaka, *University of Kentucky - College of Medicine, Lexington, Kentucky, United States*

Black American men experience disproportionate exposure to neighborhood segregation, which may contribute to cognitive health disparities. Subjective cognitive decline (SCD) is an early indicator of potential cognitive impairment, yet protective factors such as active coping may buffer adverse effects. This study examines whether active coping moderates the association between neighborhood segregation and SCD in Black American men. Data were collected from a sample of 94 Black American men (Mean age = 43.71, SD = 8.59). A moderated regression analysis was conducted using the PROCESS Model 1 (Hayes, 2022) in SPSS V.30. The model examined the interaction between neighborhood segregation and active coping in predicting SCD while controlling for covariates. Neighborhood segregation was marginally associated with increased SCD (B = -0.94, p = .052) and active coping was significantly associated with lower SCD (B = -0.95, p = .008). A significant interaction between neighborhood segregation and active coping was observed (B = 0.03, p = .017), suggesting that the association between neighborhood segregation and SCD depends on active coping levels. At lower levels of active coping, neighborhood segregation was unrelated to SCD (p = .688). However, at moderate (p = .038) and high (p = .004) levels of active coping, greater neighborhood segregation was associated with increased SCD. Our findings highlight the complex interplay between neighborhood stressors and coping mechanisms in cognitive health. Also, the results suggest that while active coping may be protective in certain contexts, it may also exacerbate the cognitive health of people in segregated environments.

Abstract citation ID: igaf122.1302

### HETEROGENEIETY AND COGNITIVE STATUS AMONG OLDER BLACK MEN

Marino Bruce[1], Roland Thorpe, Jr.[2], Gillian Marshall[3], Charles Udo[1], Kiara Spooner[1], and Bettina Beech[1], *1. University of Houston, Houston, Texas, United States, 2. Johns Hopkins University, Baltimore, Maryland, United States, 3. Dynamic and Innovative Research Solutions, Seattle, Washington, United States*

Persistent and cumulative inequality have contributed to disproportionate levels of cognitive impairment and decline among Black men. The association between inequality and cognitive outcomes among this population is deemed complex and recent studies have suggested that heterogeneity can be an important consideration. The purpose of this study was to examine how heterogeneity influences correlates of cognitive impairment among Black men in the Health and Retirement Study (HRS). Data were drawn from participants who completed the Core and Leave Behind Questionnaire items in the 2016 HRS. Any cognitive impairment was the outcome of interest. Income status was the primary contextual variable indicating whether particpants had incomes above or below the national medican income ($50,000). Income status distinctions were observed for every variable in the study. There was a significant difference in any cognitive impairment as the proportion of Black men with incomes less than $50,000 (48.2%) more than doubled the corresponding percentage for their more affluent peers (18.4%). Results from stratified Modified Poisoon Regression models indicated that correlates associated with any cognitive impairment varied by income status. Age (PR = 1.02: CI 1.00 – 1.03) and poor health (PR = 1.43: CI 1.08 – 1.90) were significant for men reporting incomes below the national median while post-secondary education (PR = 0.22: CI: 0.09 – 0.51) and depressive symptoms (PR = 1.19 CI: 1.02 – 1.39) were associated with any cognitive impairment among Black men with incomes over $50,000. Additional studies are needed to advance our understanding how heterogeneity can impact research examining cognitive impairment and decline among Black men.

Abstract citation ID: igaf122.1303

### AGING, HIV, AND VASCULAR HEALTH: IMPLICATIONS FOR BLACK MEN'S BRAIN HEALTH

Raymond Jones, *The University of Alabama at Birmingham, Birmingham, Alabama, United States*

As Black men with HIV age, they face compounded risk of neurocognitive decline and cardiovascular disease, driven by chronic inflammation, vascular dysfunction, and structural inequities. HIV accelerates biological aging, increasing cerebrovascular injury and cognitive impairment. Hypertension, diabetes, and other risk factors compound these effects, disproportionately affecting Black men due to barriers to healthcare access. Emerging work underscores the role of endothelial dysfunction and cerebrovascular injury in HIV, aging, and cognitive impairment. Given the intersectionality of these conditions, addressing brain and health in aging Black men with HIV requires a multidisciplinary, culturally tailored approach. Lifestyle modifications – physical activity and diet – show promise in reducing neurovascular damage. Community-based interventions, culturally tailored health education, and improved access to care are critical for promoting healthy aging. This presentation will highlight the need for interdisciplinary, equity-focused approaches to preserve brain health in aging Black men with HIV and reduce disparities in cognitive and cardiovascular outcomes.

### SESSION 3185 (SYMPOSIUM)

Abstract citation ID: igaf122.1304

### PSYCHOLOGICAL SCIENCES SECTION OF THE JOURNALS OF GERONTOLOGY SERIES B: FEATURED 2024 EDITOR'S CHOICE ARTICLES

Chair: Duke Han

The psychological sciences of the aging process are vast in scope and complexity. Methodological approaches that are interdisciplinary, innovative, and inclusive can make these enigmatic processes more tangible. This symposium showcases articles in the Psychological Sciences section of The Journals of Gerontology, Series B: Psychological Sciences and Social Sciences that were selected as Editor's Choice in 2024 because they exemplify these ideals. Bey et al. presents evidence on the interaction between psychosocial risk and neighborhood disadvantage among older Black adults. Huo et al. provides evidence for associations between caregiver ambivalence and psychological and cognitive symptoms of patients with Alzheimer's. Doyle et al. sheds light on characteristics of Hispanic SuperAgers. Ly et al. presents evidence for the impact of discrimination on metamemory.

Abstract citation ID: igaf122.1305

### NEIGHBORHOOD DISADVANTAGE, BIOLOGICAL AGING, AND PSYCHOSOCIAL FACTORS IN HEART FAILURE AMONG BLACK PERSONS

Ganga Bey[1], James Pike[2], Anthony Zannas[1], Qian Xiao[3], Amil Shah[4], and Priya Palta[5], *1. The University of North Carolina at Chapel Hill, Chapel Hill, North Carolina, United States, 2. New York University, New York, New York, United States, 3. The University of Texas Health Science Center, Houston, Texas, United States, 4. UT Southwestern Medical Center, Dallas, Texas, United States, 5. UNC School of Medicine, Chapel Hill, North Carolina, United States*

Deprived living environments contribute to greater heart failure (HF) risk among non-Hispanic Black persons, who disproportionately occupy disadvantaged neighborhoods. The mechanisms for these effects are not fully explicated, partially attributable to an insufficient understanding of the individual factors that contribute additional risk or resilience to the impact of neighborhood disadvantage on health. The objective of this study was to clarify the complex pathways over which such exposures act to facilitate more targeted, effective interventions. Given the evidence for a mediating role of biological age and a moderating role of individual psychosocial characteristics in the neighborhood disadvantage-HF link, we tested a moderated mediation mechanism. Using multilevel causal moderated mediation models, we prospectively examined whether the association of neighborhood disadvantage with incident HF mediated through accelerated biological aging, captured by the GrimAge epigenetic clock, is moderated by hypothesized psychosocial risk (negative affect) and resilience (optimism) factors. Among a sample of 1,448 Black participants in the shared Jackson Heart Study-Atherosclerosis Risk in Communities cohort (mean age 64.3 years), 334 adjudicated incident hospitalized HF events occurred over a median follow-up of 18 years. In models adjusted for age and sex, the indirect (GrimAge-mediated) effect of neighborhood disadvantage was moderated by psychosocial risk such that for every standard deviation increase in negative affect the hazards of HF was 1.18 (95% confidence interval = 1.05, 1.36). No moderated mediation effect was detected for optimism. Findings support the necessity for multilevel interventions simultaneously addressing neighborhood and individual psychosocial risk in the reduction of HF among Black persons.

Abstract citation ID: igaf122.1306

### DYADIC AMBIVALENCE AND LIFE SATISFACTION IN COUPLES MANAGING EARLY-STAGE ALZHEIMER'S DISEASE

Meng Huo[1], Megan Gilligan[2], Kyungmin Kim[3], Nicole Richards[4], Karen Fingerman[4], and Steven Zarit[5], *1. University of California, Davis, Davis, California, United States, 2. University of Missouri, Columbia, Missouri, United States, 3. Seoul National University, Seoul, Republic of Korea, 4. The University of Texas at Austin, Austin, Texas, United States, 5. The Pennsylvania State University, University Park, Pennsylvania, United States*

Caring for a spouse with Alzheimer's disease (AD) can elicit considerable distress but there are also positive moments. A growing body of work has examined caregivers' ambivalence in the care relationship and linked it to negative caregiver outcomes such as depression, but dyadic assessments of both parties' perspectives are missing. We examined ambivalence in both people with AD and their spousal caregivers, seeking to identify the correlates and well-being outcomes of such ambivalence in this unique context. Participants included 72 couples managing early-stage AD. People with AD and spousal caregivers independently self-reported positive and negative relationship qualities (used to indirectly calculate their ambivalence) and life satisfaction. Caregivers reported both partners' demographic characteristics and their spouses' behavioral and psychological symptoms of dementia (BPSD), focusing on memory-related behaviors and psychological symptoms. Path analyses revealed that the number and frequency of psychological symptoms in people with AD were positively associated with their own and caregivers' ambivalence. Caregivers' distress ratings of memory-related behaviors and psychological symptoms were positively associated with their ambivalence. Greater ambivalence was associated with lower life satisfaction in both spouses. BPSD directly affected both spouses' life satisfaction but there were also indirect effects via ambivalence. This study utilizes a dyadic approach to assess ambivalence in dementia care. Findings reveal the conflicting emotions that couples experience as they cope with early-stage AD, identify sources of such ambivalence, and shed light on the development of dyadic interventions that can promote positive outcomes in both partners.

Abstract citation ID: igaf122.1307

### CORRELATES OF SUPERAGING IN TWO POPULATION-BASED SAMPLES OF HISPANIC OLDER ADULTS

Cassidy Doyle, *University of South Florida, Pittsburgh, Pennsylvania, United States*

"SuperAgers" are defined as people 80+ years old with episodic memory performance comparable to those 20 years younger. Limited knowledge exists to describe characteristics of SuperAgers, with even less known about Hispanic SuperAgers. We examined indicators of cognitive, physical, and psychological resilience in relation to the likelihood of being a SuperAger using data from 2 population-based studies of Hispanic older adults (Puerto Rican Elderly: Health Conditions [PREHCO] Study; Health and Retirement Study [HRS]). SuperAgers were defined as (1) ≥80 years old, (2) recall scores ≥ the median for Hispanic respondents aged 55–64, and (3) no cognitive impairment. Overall, 640 PREHCO participants and 180 HRS participants were eligible, of whom 45 (7%) and 31 (17%) met SuperAging criteria. Logistic regressions controlling for age and sex demonstrated that higher education (PREHCO: $p < .001$; HRS: $p = .044$) and fewer instrumental activities of daily living limitations (PREHCO: $p = .019$; HRS: $p = .077$; cognitive resilience), fewer activities of daily living limitations (PREHCO: $p = .031$; HRS: $p = .068$; physical resilience), and fewer depressive symptoms (PREHCO: $p = .015$; HRS: $p = .007$; psychological resilience) were associated with SuperAging, although not all results reached threshold for statistical significance. Known indicators of physical health (e.g., chronic conditions and self-rated health) did not relate to SuperAging. Increasing access to education and recognizing/treating depressive symptoms represent potential pathways to preserve episodic memory among older Hispanic adults.

Abstract citation ID: igaf122.1308

### INVESTIGATING THE EFFECTS OF DISCRIMINATION EXPERIENCES ON EVERYDAY METAMEMORY ACCURACY

Timothy Ly, *The University of Alabama, Tuscaloosa, Alabama, United States*

Lifetime and daily experiences of discrimination contribute to impaired performance on cognitive assessments. However, the underlying mechanism by which discrimination negatively impacts cognition is unclear. Recent research investigating stress-induced impairment of metamemory may address the relationship between discrimination experiences and cognitive impairment. The aim of this study was to determine the relationship of lifetime and daily experiences of discrimination, daily affect balance, baseline objective cognitive performance, and sociodemographic variables (age, race, ethnicity, and sex) with metamemory accuracy across the lifespan (ages 20-75). Impaired metamemory accuracy was defined by the number of subjective cognitive complaints. Diary data from the Midlife in the United States (MIDUS Refresher 1) Daily Diary Project ($N = 782$) was used for these analyses. Results from linear mixed model analyses showed significant within-person effects of daily discrimination, where people who reported more daily discrimination also reported lower metamemory accuracy, and daily affect balance, where people who reported very negative affect also reported lower metamemory accuracy. Additionally, linear mixed model analyses revealed significant between-person effects of race on metamemory accuracy, with individuals from minoritized racial groups generally reporting poorer metamemory accuracy. Daily discrimination experiences also interacted with other variables in predicting day-to-day metamemory accuracy. These findings add to our understanding of how psychosocial stress in the form of daily discrimination experiences may impair metamemory processes contributing to increased subjective cognitive complaints. Future research should consider the contribution of daily experiences of discrimination across the lifespan to poor cognitive outcomes in later life.

### SESSION 3190 (SYMPOSIUM)

Abstract citation ID: igaf122.1309

### SUPPORTING OLDER ADULTS USING LONG-TERMS SERVICES AND SUPPORTS: SUPPORTING NEEDS AND ENHANCING THE WORKFORCE

Chair: Stephanie Giordano
Co-Chair: Lindsay Dubois
Discussant: Joe Caldwell

The demand for home- and community-based services (HCBS) continues to grow, due to population aging and increased desire to receive services in the community rather than in congregate settings. Despite this demand, there are still significant workforce shortages, contributing to unmet need and reliance on unpaid natural supports. The COVID-19 pandemic exacerbated and brought greater attention to the workforce shortages, while also requiring rapid innovation to the ways in which services were delivered. One important change to ensure older adults and people with disabilities were able to continue to get the services and supports they need was effectuated through Appendix K, allowing for family to be paid as caregivers. This symposium focuses on how service needs of older adults receiving long-term services have changed since COVID-19, the important role of family caregivers in support people to achieve good outcomes, and national benchmarks highlighting challenges direct service providers face in recruiting and retaining a stable workforce. These presentations not only provide a foundation for understanding the needs of older adults, from the perspective of people receiving services, but also highlight implications for ways to use data to accelerate policy and practice innovations to improve services and supports for older adults receiving long term services.

Abstract citation ID: igaf122.1310

### THE CONNECTION BETWEEN PAID AND UNPAID SUPPORTS WITH PERSON-CENTERED OUTCOMES FOR OLDER ADULTS WITH DISABILITIES

Lindsay Dubois[1], Rosa Plasencia[2], Stephanie Giordano[1], and Nilufer Isvan[1], *1. Human Services Research Institute, Cambridge, Massachusetts, United States, 2. ADvancing States, Arlington, Virginia, United States*

As funding for long-term services and supports shifts from institutional settings to home and community-based services (HCBS), the need for consumer-reported outcomes data is paramount. This study uses data from National Core Indicators—Aging and Disabilities™ (NCI-AD™) Adult Consumer Survey (ACS) to investigate the connection between types of providers (e.g., paid staff, paid family, unpaid supports)

on self-reported service experiences of adults receiving HCBS, controlling for relevant service and recipient characteristics. The unadjusted likelihood of having sufficient help with activities of daily living (ADL) is highest among people whose primary caregiver is a paid family member compared to paid nonfamily staff (OR = 2.26, p < 0.001) and lowest for unpaid family caregivers (OR = 0.71, p < 0.001). Likewise, the unadjusted likelihood of reporting that services and supports meet needs was highest among those whose primary caregiver is a paid family member compared to paid nonfamily staff (OR = 1.42, p < 0.001) and lowest for unpaid family caregivers (OR = 0.77, p < 0.001). Adjusting for covariates, the likelihood of having sufficient help with ADL and services meeting needs remains significantly higher for those with paid family caregiver relative to paid staff (aOR=1.66, p < 0.001, and aOR=1.42, p = 0.002 respectively). These data suggest that even with the influence of demographic and service-related factors, paid family caregivers are associated with positive outcomes for older adults and people with disabilities using HCBS. These results can support policy and practice that workforce needs, improving benefits to reduce turnover, and implementing caregiver support initiatives.

Abstract citation ID: igaf122.1311

### CHANGES IN SERVICE UTILIZATION AND UNMET NEEDS IN HCBS IN THE UNITED STATES DURING THE COVID-19 PANDEMIC

Romil Parikh[1], Tetyana Shippee[2], Jack Wolf[1], Eric Jutkowitz[2], and Stephanie Giordano[3], *1. University of Minnesota School of Public Health, Minneapolis, Minnesota, United States, 2. University of Minnesota, Minneapolis, Minnesota, United States, 3. Human Services Research Institute, Cambridge, Massachusetts, United States*

More than 5 million older adults in the United States use publicly-funded home-and community-based services (HCBS) which were disrupted during the COVID-19 pandemic. We evaluated changes in HCBS use and consumer-reported unmet HCBS needs during the pandemic (2021-2022) versus pre-pandemic (2018-2019). We evaluated six commonly used HCBS: personal care, homemaker, meal delivery, adult day, transportation, and caregiver respite/support services. We used mixed effects logistic regression modeling to estimate adjusted odds ratios (OR) and 95% confidence intervals (CI), adjusting for consumers' sociodemographic and health-related characteristics, with random intercepts for state. We included community-dwelling, HCBS consumers (age, ≥65 years) from 11 states that participated in both- 2018-2019 and 2021-2022 survey waves. Compared to 2018-2019, service use increased for personal care (OR, 1.24; 95% CI, 1.09, 1.40) and caregiver respite/support (OR, 1.28; 95% CI, 1.00, 1.63) but decreased for homemaker services (OR, 0.69; 95% CI, 0.60, 0.79) and meal delivery (aOR, 0.81; 95% CI, 0.70, 0.93). In 2021-2022, unmet HCBS needs significantly increased for personal care (OR, 1.23; 95% CI, 1.03, 1.46) and meal delivery (OR, 1.26; 95% CI, 1.01, 1.56) but significantly decreased for caregiver respite/support (OR, 0.49; 95% CI, 0.35, 0.70). During the pandemic, changes in service utilization and consumer-reported unmet service needs differed between various types of HCBS. These findings can inform disaster preparedness efforts for assuring continuity of HCBS during future public health emergencies.

Abstract citation ID: igaf122.1312

### NCI STATE OF THE WORKFORCE - AGING AND DISABILITIES: RESULTS AND IMPLICATIONS FOR POLICY & PRACTICE

Dorothy Hiersteiner[1], Lindsay Dubois[2], and Rosa Plasencia[3], *1. Human Services Research Institute (HSRI), Cambridge, Massachusetts, United States, 2. Human Services Research Institute, Cambridge, Massachusetts, United States, 3. ADvancing States, Arlington, Virginia, United States*

There are approximately 5 million direct service workers (DSWs) providing supports to older adults and people with disabilities in the US. While this number seems high, the demand for DSWs often outstrips the supply; combined with high levels of turnover at direct care provider agencies, the workforce crisis is intensifying and has a substantial impact on the quality of supports. This presentation will showcase key findings from the National Core Indicators (NCI) State of the Workforce Aging and Disabilities 2023 survey of more than 1,200 provider agencies in 6 states. Altogether, these provider agencies employ more than 1.4 million DSWs. Among the states that participated, the average turnover ratio was 48%, with 1 out of ever 3 agencies reporting that they turned away or stopped accepting referrals due to staffing issues. The median hourly wage across states was $16.00 per hour, and 99% of agencies pay a median hourly wage that is more than $0.50 below living wage in their state. Finally, just 55% of agencies offered paid time off to DSWs, 41% offered health insurance, and 38% offered an employer-sponsored retirement plan. The low wages and limited benefits offered to DSWs are likely contributors to the high rates of turnover. These data highlight the unsustainable working conditions for many DSWs, and point to tangible quality improvement opportunities for state and service providers.

### SESSION 3195 (SYMPOSIUM)

Abstract citation ID: igaf122.1313

### FAMILY TIES AND LATE-LIFE WELL-BEING: EVALUATING PATTERNS ACROSS SOCIOCULTURAL AND WELFARE STATE POLICY CONTEXTS

Chair: Deborah Carr
Discussant: Nekehia Quashie

Older adults' well-being is powerfully shaped by the instrumental, socioemotional, and material supports received by spouses and adult children. However, the extent to which family ties enhance specific dimensions of well-being may vary across national contexts. Cultural factors, including normative expectations regarding family support, and policy contexts, including the level of public supports for older adults' economic and physical well-being, may moderate the effects of family support on late-life well-being. These four papers use cross-national data and quantitative methods to evaluate the role of sociopolitical contexts in the lives of older adults. Wang and Yang use data from the Health and Retirement Study (HRS) and China Health and Retirement Longitudinal Study (HRS) and find that marital histories affect loneliness and social participation differently for older adults in the U.S. vs. China. Jessee and Carr use Survey of Health, Ageing and Retirement in Europe (SHARE) data to examine linkages between parent-child disconnectedness and older adult mental health, and find pronounced effects in Southern Europe, where familism is a pervasive value. Azar, Wahrendorf and Cheng use

SHARE data to document linkages between intergenerational financial support and older adults' depressive symptoms, and find patterns moderated by welfare regime characteristics. Mahmoud and Carr use SHARE data to examine associations between marital status and place of death, and find that the protective effects of marriage, especially for women, differ across contexts, although marital history is unrelated to place of death in Nordic states with generous welfare regimes. Implications for policy and practice are highlighted.

Abstract citation ID: igaf122.1314

### THE IMPACT OF MARITAL HISTORY ON LATE-LIFE LONELINESS AND SOCIAL PARTICIPATION: A U.S.-CHINA COMPARISON

Leping Wang[1], and Yang (Claire) Yang[2], *1. Vanderbilt University, Nashville, Tennessee, United States, 2. The University of North Carolina at Chapel Hill, Chapel Hill, North Carolina, United States*

In this paper, we use HRS and CHARLS data to investigate the relationship between marital history over the life course and loneliness and social participation in late life among older adults aged 65 and over across the U.S. and China, and the gender differences therein. We show that in the U.S., marital trajectories are more heterogeneous than in China, and non-normative marital transitions are more prevalent. U.S. and Chinese older adults exhibit compositional differences in social participation. Chinese older adults predominately meet up with friends, whereas more U.S. older adults volunteer or participate in organized group meetings. Marital history is associated with loneliness and social participation differently by country and by gender. Off-time marital transition is associated with worse socio-emotional well-being than on-time transition, represented by the higher likelihood of loneliness among Chinese widowed prematurely. On-time transition, featured by mid-life divorce in the U.S., is otherwise linked to less loneliness. U.S. Women widowed prematurely and staying lifelong single have more active social participation than men, whereas never-married Chinese women are less active in social participation than men. Our findings reveal the impact of marital histories, including the states and transitions, on the socio-emotional well-being of older adults from two interconnected themes: the life course framework and the normative transition theory. We also highlight the importance of studying the holistic marital history (rather than current marital status) and its socio-psychological consequences over the life course, with particular attention to non-normative marital transitions that standardized measures of marital status tend to overlook.

Abstract citation ID: igaf122.1315

### PARENT-CHILD DISCONNECTEDNESS AND MENTAL HEALTH IN OLDER EUROPEANS: REGIONAL DIFFERENCES IN SHARE

Deborah Carr[1], and Lisa Jessee[2], *1. Boston University, Boston, Massachusetts, United States, 2. University of Cologne, Cologne, Nordrhein-Westfalen, Germany*

Parent-child disconnectedness – or the lack of contact to at least one child – can negatively affect older adults' mental health. However, studies have largely overlooked how the psychological impact varies across cultural contexts. In regions with a strong cultural emphasis on family ties, like Southern Europe, the psychological impact of disconnectedness may be more severe, while in societies with more individualistic cultural norms, the consequences could be less pronounced. To address this gap, we used pooled data from eight waves (1, 2, 4, 5, 6, 7, 8, 9) of the Survey of Health, Ageing and Retirement in Europe (SHARE) and compared the association of parent-child disconnectedness and parents' depressive symptoms among N = 216,573 older Europeans across four regions: Northern, Eastern, Southern, and Western Europe. We also test for gender and marital status differences therein. Descriptive analyses show that parent-child disconnectedness is rare in Europe, ranging from 1% among women and men in Southern Europe to 5% among women and 6% among men in Northern Europe. Multivariable pooled OLS show that parent-child disconnectedness is linked to higher depressive symptoms across all regions, with the greatest mental health burden in Southern Europe. These trends are consistent for men and women, but vary across different marital statuses. Our results align with theories suggesting that the psychological toll of disconnectedness is greater in cultures where it is less common, leading to a lack of support and stigma for parents. However, we also find evidence of a more modest burden in other European regions.

Abstract citation ID: igaf122.1316

### INTERGENERATIONAL FINANCIAL SUPPORT AND MENTAL HEALTH: A COMPARISON OF WELFARE POLICY CONTEXTS IN EUROPE

Kent Jason Cheng[1], Ariel Azar[2], and Morten Wahrendorf[3], *1. University of Massachusetts Boston, Boston, Massachusetts, United States, 2. Purdue University, West Lafayette, Indiana, United States, 3. Heinrich-Heine-University Düsseldorf, Düsseldorf, Nordrhein-Westfalen, Germany*

As Europe faces a rapidly aging population, understanding the interplay between family and welfare state support becomes critical for addressing mental health concerns among older adults. This study examines the association between financial transfers from descendants and depressive symptoms, exploring variations across European welfare state contexts with differing levels of pension generosity and gender differences in the associations explored. Using data from 20 European countries included in the Survey of Health, Ageing, and Retirement in Europe (SHARE), waves 3–9, we examined predictors of depressive symptoms measured by the EURO-D scale. Key predictors included receiving financial transfers of EUR 250 or more from children or grandchildren, the net replacement rate of pensions, and the interaction between these variables. Receiving financial support from children or grandchildren is associated with higher depressive symptoms, signalling that the transfer often arises due to the older adult's need. Gender differences were notable: men experienced a greater increase in depressive symptoms when receiving transfers, likely due to their stronger ties to labour force participation and the impact of pension adequacy. Robust welfare states amplified the association between receiving transfers and depressive symptoms, highlighting a complex dynamic between state support and family assistance. This may reflect situations where dual sources of support indicate severe underlying needs. These findings align with contingency exchange theories and crowding-in, emphasizing that family and the welfare state often work in tandem rather than cancelling each other out to address significant hardships faced by the most vulnerable segments of the population.

Abstract citation ID: igaf122.1317

### MARITAL STATUS AND PLACE OF DEATH AMONG OLDER ADULTS: A COMPARISON OF EUROPEAN COUNTRIES

Kafayat Mahmoud, and Deborah Carr, *Boston University, Boston, Massachusetts, United States*

Marital status differences in mortality are well-established, but it is unclear how marriage shapes place of death. While most older adults prefer home deaths, hospital or hospice deaths may be necessary for those desiring intensive medical care or palliation, respectively. Associations between marital status and place of death may vary across national contexts, reflecting cultural norms and healthcare policies. We use harmonized data from waves 2 through 9 of SHARE (Survey of Health, Ageing, and Retirement in Europe), to document regional differences in the associations between marital status and place of death (as reported by proxy) in 18 European countries classified into four welfare regimes: Continental (Austria, Belgium, France, Germany, Luxembourg, Netherlands, Switzerland), Eastern (Czech Republic, Estonia, Hungary, Poland, Slovenia), Southern (Greece, Italy, Poland, Spain), and Nordic (Denmark, Sweden). Marital status is not related to place of death in Nordic countries, distinguished by strong social welfare systems. In other regions, we detect strong associations, with slight differences for men and women. For instance, in Continental Europe, never married adults were more likely than married and widowed to die in nursing home whereas widowed adults were more likely to report home deaths relative to married and divorced adults. However, home deaths were especially rare for never married and divorced women. Similarly, in Southern Europe, divorced persons, especially women, were more likely to die in nursing homes or hospice, relative to their married or widowed counterparts, who were more likely to die at home. We discuss the implications for social policy.

### SESSION 3200 (PAPER)

### CARE, CONTEXT, AND COMMUNICATION IN DEMENTIA: INSIGHTS INTO EQUITY, DESIGN, AND LIVED EXPERIENCE

Abstract citation ID: igaf122.1318

### BUILT ENVIRONMENTS AND DEMENTIA CARE: ARCHITECTURAL LAYOUTS AS DETERMINANTS OF RESIDENT WELL-BEING

Yifat Rom[1], Ido Morag[2], Yuval Palgi[1], and Michal Isaacson[1], *1. University of Haifa, Haifa, HaZafon, Israel, 2. Shenkar College of Engineering and Design, Ramat Gan, HaMerkaz, Israel*

The fast-growing number of individuals with dementia worldwide has prompted the establishment of specialized dementia units, yet these facilities may not always meet their residents' well-being needs. This study, therefore, examined correlations between the architectural layout of seven dementia units and their residents' well-being. Physical well-being was measured in terms of comfort and stimulation, while social well-being was measured in terms of status, behavioral confirmation, and affection. The residents' verbal and physical agitated behaviors were also observed and documented. Diverse correlations were found between the five well-being dimensions and agitated behaviors. Negative correlations were seen between layouts that support residents' physical well-being, verbal agitation, and all agitated behaviors combined. Yet unexpectedly, inconsistencies were seen between layouts that support residents' social well-being and agitated behaviors, with increased agitated behaviors occurring in layouts that support status and affection. This exploratory study offers insights into the significance of the architectural layout of dementia units in augmenting residents' well-being. Furthermore, this novel research sheds light on the potential of employing quantitative tools for comprehensively examining the spatial configurations of such units. Incorporating a descriptive well-being dimension into quantitative investigations can enhance our understanding of such environments, offering a promising avenue for optimizing residents' overall well-being.

Abstract citation ID: igaf122.1319

### ASSOCIATIONS OF EARLY-LIFE RISK FACTORS WITH RACIAL DISPARITIES IN INCIDENT DEMENTIA AMONG OLDER AMERICANS

Yi Wang[1], Yuting Qian[2], Zhuoer Lin[3], and Xi Chen[1], *1. Yale University, New Haven, Connecticut, United States, 2. Yale School of Public Health, New Haven, Connecticut, United States, 3. University of Illinois Chicago, Chicago, Illinois, United States*

Early-life risk factors, such as exposure to trauma or receiving less and poor-quality education, have been linked to brain development and academic achievement, both of which may influence dementia risk later in life. However, there remains a limited understanding of the associations between these early-life risk factors and incident dementia, as well as their role in driving racial disparities in dementia incidence. This study aimed to examine the associations between early-life risk factors and incident dementia, and to assess how these factors mediate racial disparities in dementia incidence. Using data from the 2000-2020 Health and Retirement Study (HRS), we analyzed a sample of 5,578 participants aged 70 years and older, excluding those with dementia at baseline. We determined dementia status using previously validated algorithms (the Expert Model) designed for racial/ethnic disparities research in HRS. Our survival analyses suggest that the age- and sex-adjusted hazard ratio (HR) for incident dementia was 2.12 (95% CI, 1.76-2.55) for Black compared to White individuals. Mediation analyses showed that attending any segregated schools before college explained 42.2% of this racial disparity, while being born in the US South and having less than a high school education accounted for 36.8% and 21.9% of the disparity, respectively. These findings emphasize the crucial role of early-life factors in the screening, prevention, and intervention of dementia onset, and suggest that educational experiences and early-life environments are key mechanisms contributing to racial disparities in dementia incidence.

Abstract citation ID: igaf122.1320

### PERSONAL END-OF-LIFE HEALTHCARE PREFERENCES AMONG BEREAVED SURROGATES TO PEOPLE WITH DEMENTIA: A VIGNETTE STUDY

Rachel Bloom[1], and Karen Siedlecki[2], *1. Yale University, New York, New York, United States, 2. Fordham University, New York, New York, United States*

As the number of people living with dementia in the U.S. grows, more people are taking on the role of surrogate medical decision-making. Bereaved dementia surrogates have been uniquely exposed to end-of-life decision-making, observing what can go right and wrong and forming judgments around quality-of-life. This project utilizes a lifespan developmental perspective to gain greater insight into bereaved dementia surrogates' preferences and attitudes towards end-of-life healthcare. Using vignettes, this study compared the end-of-life preferences of bereaved dementia surrogates (n = 108), bereaved non-dementia surrogates (n = 187), and non-surrogate controls (n = 697). Significant differences between the three groups were found in responses to the second vignette on memory loss and feeding, $\chi 2(8, n = 992) = 33.88$, $p < .001$, the third vignette on aspiration pneumonia, $\chi 2(8, n = 992) = 46.15$, $p < .001$, and the fourth vignette on feeding tube placement, $\chi 2(8, n = 992) = 42.50$, $p < .001$. Both surrogate groups were less likely to select the most aggressive treatment option in the second, third, and fourth vignettes. Controls were more likely than all surrogates to opt for aggressive treatment and less likely to opt for comfort care in the third vignette. Dementia surrogates were more likely to indicate they want measures to help them die peacefully in the fourth vignette, and less likely to respond "I don't know." These findings demonstrate how serving as a dementia surrogate can impact personal end-of-life healthcare preferences, with public health implications for an aging population.

### SESSION 3205 (PAPER)

Abstract citation ID: igaf122.1321

### GERIATRIC SYNDROMES ACROSS SETTINGS

Research on geriatric syndromes across settings from the community to acute care setting. Research will be presented on obesity, delirium, dementia and falls.

Abstract citation ID: igaf122.1322

### FIVE ASPECTS MEAL MODEL OF EATING EXPERIENCES IN OLDER PEOPLE WITH DEMENTIA: THEORY ANALYSIS AND EVALUATION

Zih-Ling Wang[1], and Basia Belza[2], *1. University of Washington School of Nursing, Seattle, Washington, United States, 2. University of Washington, Seattle, Washington, United States*

Eating experiences significantly impact the quality of life and nutritional status of people living with dementia (PLWD). The concept of eating experiences encompasses both subjective perceptions and functional aspects of mealtimes, offering a broader perspective than traditional approaches focused solely on nutrition or feeding/eating difficulties. This paper presents a theory analysis of the Five Aspects Meal Model (FAMM) as applied to eating experiences in older people with dementia, using Walker and Avant's systematic approach. The FAMM provides a comprehensive framework examining five interconnected dimensions of meals: room, meeting, product, management control system, and atmosphere. The analysis examines the model's origins, meaning, logical adequacy, usefulness, generalizability, parsimony, and testability in the context of eating experiences for PLWD. Findings indicate that the FAMM effectively conceptualizes how physical environment (room), social interactions (meeting), food qualities (product), organizational factors (management control system), and multisensory elements (atmosphere) collectively shape eating experiences. The interrelationships between these five aspects offer valuable insights into enhancing mealtime satisfaction and nutritional intake. While the FAMM has been widely used in hospitality and food service research, it has yet to be fully applied within gerontological nursing, particularly dementia care. This model has significant potential for application in long-term care settings, where holistic mealtime approaches are increasingly recognized as essential to person-centered care. This analysis contributes to nursing science by providing a theoretical foundation for developing comprehensive interventions that address multiple dimensions of eating experiences for PLWD.

Abstract citation ID: igaf122.1323

### IMPACT OF HOME HEALTH CARE ON END-OF-LIFE OUTCOMES FOR MEDICARE BENEFICIARIES ACROSS RACIAL AND ETHNIC GROUPS

Olga Jarrín[1], Babatope Ogunjesa[2], Andre Rosario[3], Dione Sandiford[4], Roland Thorpe[5], Anum Zafar[1], Haiqun Lin[6], and Paul Duberstein[3], *1. Rutgers, The State University of New Jersey, New Brunswick, New Jersey, United States, 2. University of Illinois Urbana-Champaign, Champaign, Illinois, United States, 3. Rutgers University, New Brunswick, New Jersey, United States, 4. Rutgers University, Newark, New Jersey, United States, 5. Johns Hopkins Bloomberg School of Public Health, Baltimore, Maryland, United States, 6. Rutgers, The State University of New Jersey, Newark, New Jersey, United States*

End-of-life care is crucial for Medicare beneficiaries, yet racial and ethnic disparities persist in access and use of hospice care and place of death. The aim of this study was to evaluate the impact of home health care on racial/ethnic inequities in end-of-life outcomes for Medicare beneficiaries with and without dementia. Analyzing patterns in 2,065,300 beneficiaries who died in 2019, logistic models were used to explore the impact of home health care use during the last three years of life on end-of-life care outcomes. Home health care use was associated with a lower predicted probability of hospital death without hospice and a higher probability of home death with hospice across all racial/ethnic groups. Home health care use (vs. no home health care use) increased the probability of home death with hospice more for White, Asian American/Pacific Islander, and American Indian and Alaska Native beneficiaries compared to Hispanic and Black beneficiaries. Among patients with dementia, similar trends were observed, but the effect was smaller relative to patients without dementia. For example, among Black decedents with dementia, home health care was associated with a 5.1% increase in the probability of home death with hospice vs. an 8.2% increase among those without dementia. This study underscores the role of home health care in enhancing end-of-life quality, especially in facilitating the use of home hospice care. However, targeted interventions to ensure equitable access to hospice care are needed to optimize end-of-life care and meet the diverse needs of Medicare beneficiaries.

Abstract citation ID: igaf122.1324

### PREVALENCE OF THE RECENTLY PROPOSED CLINICAL OBESITY AMONG OLDER ADULTS FROM THE HEALTH AND RETIREMENT STUDY

Weiyang Ding[1], Ngai Kwan[2], and Laurie Milliken[1], *1. University of Massachusetts Boston, Boston, Massachusetts, United States, 2. Institute for Community Inclusion, Boston, Massachusetts, United States*

Obesity is traditionally defined as a body mass index (BMI) $\geq 30\,kg/m^2$ (CurDef), but this may overlook health risks, especially in older adults. The Lancet Diabetes and Endocrinology Commission on Obesity recently proposed a new definition (NewDef) that classifies individuals with BMI ≥30 kg/m² as "clinical obese" only if they also have high adiposity (measured by waist circumference, WC) and an obesity-related chronic condition. This study compared the prevalence of obesity using NewDef versus CurDef in a nationally representative sample of older adults (≥50 years) from the Health and Retirement Study (HRS) (waves 13 and 14; n = 13,852; 58% female). The prevalence of obesity was 45.9% (95% CI: 45.1–46.7) using CurDef and 40.4% (95% CI: 39.6–41.3) using NewDef, reducing the number of obese individuals by 754. Among those reclassified as non-obese, 26.6% (18.4% female) had BMI ≥30 and low WC, 19.6% (18.9% female) had BMI ≥30, low WC, and ≥1 chronic condition, and 73.3% (53% female) had high BMI and WC but no chronic condition. The NewDef lowers the prevalence of obesity and may better identify older adults at higher health risk by focusing on meaningful health consequences rather than BMI alone. This refined approach could improve clinical care and resource allocation for older adults. However, the lack of detailed information on whether chronic conditions are obesity-related limits the accuracy of the NewDef, highlighting the need for improved health condition documentation in older adult populations.

Abstract citation ID: igaf122.1325

### SOCIAL INTEGRATION, FUNCTIONAL INDEPENDENCE, AND DEPRESSIVE SYMPTOMS: LONG-TERM EFFECTS AFTER TBI IN OLDER ADULTS

Wonkyung (Kelly) Jung[1], Dabin Hwang[2], and Janiece Taylor[3], *1. Boston College, Chestnut Hill, Massachusetts, United States, 2. Boston College William F. Connell School of Nursing, Chestnut Hill, Massachusetts, United States, 3. Johns Hopkins University, Baltimore, Maryland, United States*

Traumatic Brain Injury (TBI) is a leading cause of disability worldwide, particularly among older adults. The resulting disabilities can significantly affect social integration, functional independence, and contribute to depressive symptoms. Addressing these factors is vital for promoting healthy aging in older individuals post-TBI. However, research on the interplay between social integration, functional independence, and depressive symptoms in this population is limited. This study examines the reciprocal associations between these factors over 5 years post-injury. We conducted a secondary data analysis using the Traumatic Brain Injury Model Systems National Database (TBIMS-NDB). Structural equation modeling (SEM) was used to explore the causality and directionality of the relationships between social integration, functional independence, and depressive symptoms in older adults post-TBI. A cross-lagged analysis (n = 2101) assessed longitudinal changes in these variables at 1-, 2-, and 5-years post-injury, adjusting for covariates. Social integration was associated with improved functional independence but did not predict depressive symptoms. Greater functional independence was linked to better social integration and fewer depressive symptoms. Higher levels of depressive symptoms were associated with poorer functional independence. Cross-lagged relationships between social integration and functional independence, as well as between functional independence and depressive symptoms, suggest reciprocal effects that accumulate over time. These findings emphasize the interconnectedness of these factors and the importance of addressing them in interventions aimed at improving long-term well-being in older adults post-TBI.

Abstract citation ID: igaf122.1326

### THE ALIVIADO DSD PROGRAM: A TIME-SERIES STUDY ON ENHANCING FAMILY CAREGIVER DELIRIUM KNOWLEDGE

Shih-Yin Lin[1], Yong Kyung Choi[2], Donna Fick[3], and Abraham Brody[4], *1. New York University Rory Meyers College of Nursing, New York, New York, United States, 2. University of Pittsburgh, Pittsburgh, Pennsylvania, United States, 3. The Pennsylvania State University, University Park, Pennsylvania, United States, 4. NYU Rory Meyers College of Nursing, New York, New York, United States*

Delirium superimposed on dementia (DSD) refers to delirium occurring in individuals with existing dementia. Due to the overlapping symptoms between delirium and dementia, family caregivers play an important role in detecting DSD. They can assist in DSD detection by informing clinicians whether the cognitive disturbances in their care recipients are typical day-to-day fluctuations in dementia symptoms or indicative of something beyond the normal. However, many family caregivers are unfamiliar with DSD. To assess the impact of the Aliviado DSD Caregiving Mastery Program on caregivers' delirium knowledge, we conducted a single-group, time-series study with data collected at baseline, 2 weeks, and 6 weeks. Delirium knowledge was assessed using the Caregiver Delirium Knowledge Questionnaire. Descriptive statistics summarized participant characteristics, while a mixed-effects regression model analyzed the delirium knowledge outcome, accounting for repeated measures across time points. A random intercept was included to account for individual baseline differences. Thirty primary caregivers participated, with 26 completing the study (retention rate: 86.7%). At 2 weeks, delirium knowledge significantly increased compared to baseline (mean difference = 3.80, $p < 0.001$). At 6 weeks, knowledge remained significantly higher than baseline (mean difference = 3.63, $p < 0.001$). The intervention effect sizes from baseline to 2 weeks and baseline to 6 weeks were large (Cohen's $d = 0.8$ and 0.76, respectively). No significant difference in delirium knowledge was observed between 2 and 6 weeks, suggesting knowledge maintenance. The study findings provide preliminary evidence supporting the positive impact of the Aliviado DSD program on improving caregivers' delirium knowledge.

### SESSION 3210 (SYMPOSIUM)

Abstract citation ID: igaf122.1327

### POLICY SERIES: GSA CONGRESSIONAL UPDATE

Chair: Brian Lindberg
Co-Chair: Patricia D'Antonio

This session provides Hill staff perspectives on what has happened in the aging and health care space during the first ten months of the 119th Congress. Of particular importance will be the Older Americans Act Reauthorization, including funding for the Center for Research and Demonstration in the Aging Network, as well as an update on Medicare Advantage, nursing home regulations, and hospice and palliative care legislation.

### SESSION 3215 (SYMPOSIUM)

Abstract citation ID: igaf122.1328

### HARNESSING TECHNOLOGY TO FOSTER SOCIAL CONNECTION AND ENHANCE WELL-BEING AMONG OLDER ADULTS

Chair: Walter Boot
Co-Chair: Edie Sanders

Existing and emerging technologies offer powerful tools to foster social connection and enhance well-being among older adults. From digital communication platforms to personalized digital health tools, these innovations can help reduce loneliness, support mental health, and promote meaningful engagement. As the older population grows and diversifies, leveraging technology to meet their social and emotional needs has become both an opportunity and a necessity. This symposium brings together researchers advancing our understanding of how technology can support connection and quality of life in later life. Dr. Kalon Sou will present findings from a study evaluating older adults' experiences with an age-friendly, AI-powered chatbot, identifying key factors—such as empathy, sociability, and agency—that shape willingness to use social chatbots. Nicole Memmer will explore how different types of Internet use, especially for social purposes, relate to older adults' offline participation, with digital skills and local context influencing the benefits gained. Dr. Paul P. Freddolino will highlight a multi-year initiative to increase digital health literacy among hard-to-reach older adults using community-based strategies, emphasizing the role of trust and early outcomes related to technology engagement. Dr. Ines Simbrig will examine how aging-in-place technologies affect older adults' perceptions of age-related change, revealing a complex trade-off between feelings of safety and potentially negative shifts in self-perception. Finally, Dr. Yong Kyung Choi will introduce the Adult Wellbeing CheckUp, a web-based platform that offers multi-domain assessments and personalized recommendations to help older adults and care partners improve health, engagement, and quality of life.

Abstract citation ID: igaf122.1329

### OLDER ADULTS' ATTITUDE TOWARDS AND INTENT TO USE OF AI-POWERED CONVERSATIONAL AGENTS FOR SOCIAL SUPPORT

Ka Lon Sou, Matthew Zhi Yuan Lau, Fuxi Ouyang, and W. Quin Yow, *Singapore University of Technology and Design, Singapore, Singapore*

Social isolation affects nearly one-fourth of those aged 65+ in the U.S., and imposes risks to one's health. Recent advancement of large language model (LLM) suggests that artificial intelligence (AI)-powered chatbot may foster social support. Our lab, thus, developed an age-friendly LLM-powered chatbot, Ami Chat, that aims to provide social support for (isolated) older adults. In this study, we aim to identify the attitude-related factors and AI-related characteristics that influence older adults' intent to use an AI-powered chatbot. Thirty-two participants (21 females, Mage=72.28, range=66-85) interacted with Ami Chat for around 20 minutes, and then completed questionnaires that evaluated their attitudes towards Ami Chat, their perception on Ami Chat' AI-related characteristics, as well as their intent to use of Ami Chat in their daily lives. Our results showed that the AI social interaction quality (AISI) was significantly predicting intent to use of the chatbot ($\beta = 1.30$, $p<.001$). AI anthropomorphism was negatively predicting intent to use ($\beta$=-0.65, p=.004). Health ($\beta = 0.48$, p=.043), sociability ($\beta = 0.81$, p=.007), agency ($\beta$=-0.78, p=.025) and perceived empathy ($\beta = 0.76$, p=.004) significantly predicted AISI. Attitude towards Ami Chat was only significantly predicted by perceived empathy ($\beta = 1.39$, p=.0032). Our results highlight the importance of social interaction closeness, sociability, agency, and perceived empathy in shaping older adults' attitude towards and intent to use AI-powered chatbots in their daily lives. Notably, our results also suggest that overly human-like AI may not always appeal to older users. These insights emphasize that there are age-specific needs in the design of the AI-driven solutions for social support.

Abstract citation ID: igaf122.1330

### BRIDGING THE DIGITAL-ANALOG GAP IN LATER LIFE: TESTING THE INTERRELATION OF INTERNET USE AND SOCIAL PARTICIPATION

Nicole Memmer[1], Anna Schlomann[2], and Hans-Werner Wahl[1], *1. Heidelberg University, Heidelberg, Baden-Wurttemberg, Germany, 2. Network Aging Research, Heidelberg, Baden-Wurttemberg, Germany*

We target the question whether Internet use enables or impedes social participation in older adults' analog world. Doing so, we test a rather comprehensive model that includes multiple predictors (i.e., cognitive attitude, affective attitude, and stereotype endorsement) of Internet use, different types of Internet use (i.e., social use, information seeking, entertainment), and a multi-indicator assessment of social participation, encompassing leisure activities as well as perceived social support and loneliness. Data come from the baseline assessment of the randomized controlled SMART-AGE trial. The analytical sample consists of 649 community-dwelling adults aged 67–93 (mean = 75.8; female = 52%). Structural equation modeling was applied to estimate the association of predictors with different types of Internet use and the relation of these usage patterns with social participation. Additionally, the study examines the moderating effect of digital skill level and socio-structural meso-context, i.e., two cities in southwestern Germany, on these relationships. Results show that stereotype endorsement and cognitive attitude were significantly related to different types of Internet use. Social Internet use exhibited the strongest positive association with social participation, while the path from information seeking to social participation was weaker, and entertainment revealed a null effect. Individuals with higher digital skills were found to derive greater social benefits from Internet use. Multi-group comparison revealed a weak but statistically meaningful impact of socio-structural meso-context on the inter-relations among Internet use and social participation. We conclude that a differential perspective is needed to understand the link between Internet use and social participation.

Abstract citation ID: igaf122.1331

### THREE COMMUNITY-TESTED STRATEGIES TO INCREASE HARD-TO-REACH OLDER ADULTS' DIGITAL HEALTH LITERACY

Paul Freddolino, Fei Sun, Ha-Neul Kim, Ayaka Lingard, and AnnMarie Schneider, *Michigan State University, East Lansing, Michigan, United States*

In most parts of the world, technology use among older adults is increasing, yet remains low. Lowest user rates are among the oldest old, less affluent, and less educated groups of older adults.

Many older adults suffer from social isolation and loneliness, often alongside multiple chronic diseases. Enhancing digital health literacy, which includes improving digital skills and awareness of the resources available through telehealth, presents a promising strategy to address these issues. This session will highlight the results of a four-year effort to develop and test models designed to increase hard-to-reach older adults' digital health literacy. Three successful strategies have emerged: 1) Trained volunteer coaches go directly to home delivered meals recipients' homes for coaching; 2) Coaches present at congregate meal sites and senior center special programs; and 3) Local groups develop unique strategies that fit their preferences and resources, using manuals and videos freely shared with agencies. Process evaluation points to the role of trust in getting technology resistant older adults to be willing to learn about devices and telehealth. Preliminary outcome data from trials using all three models show improved digital literacy and increased telehealth awareness. However, results on loneliness and isolation measures have not shown statistically significant improvement, likely due to relatively positive scores on these measures at baseline and the relatively low intensity of the interventions. Long-term effects may be more likely to appear. Resources developed for the models to support digital literacy among the aging population will be illustrated.

Abstract citation ID: igaf122.1332

### RELATIONSHIPS BETWEEN TECHNOLOGY USE AND VIEWS ON AGING: RESULTS FROM A RANDOMIZED CONTROLLED TRIAL

Ines Simbrig[1], Felix Piazolo[2], and Hans-Werner Wahl[3], *1. UMIT TIROL – Private University of Health Sciences and Health Technology, Hall in Tirol, Austria, 2. University of Innsbruck, Innsbruck, Tirol, Austria, 3. Heidelberg University, Heidelberg, Baden-Wurttemberg, Germany*

Previous research has shown that views on aging affect self-regulatory behavior and may thus also influence technology use. However, trials assessing the effect of technology use on older adults' quality-of-life have rarely considered the role of subjective aging. Our objective was to investigate the associations between Awareness of Age-Related Change (AARC) and the use of compensatory aging-in-place technologies as part of the i-evAALution randomized controlled field study. The analytical sample contained 72 community-dwelling older adults (mean age: 76.7). 36 participants were randomly assigned to an intervention arm, using technologies like an emergency watch and a tablet with various functions, across 6-month and 13-month follow-up assessments. The 36 controls received no intervention. Primary outcomes were different quality-of-life variables, whereas AARC-Gains and -Losses were assessed as secondary outcomes. Here, we focus on the latter. Participants in the intervention group revealed a decrease in AARC-Gains during the first six months, followed by an increase up to the 13-month assessment. In contrast, participants in the control group showed an increase first, followed by a decrease. No significant difference between both conditions at the 13-month assessment and no main effect for AARC-Losses were observed. Furthermore, participants who used the emergency watch and the tablet more frequently perceived more AARC-Losses than persons who used them less frequently. Aging-in-place technologies with a strong compensatory focus may result in a dilemma such as increasing feelings of safety on the one hand, but in parallel impacting negatively the subjective experience of one's aging.

Abstract citation ID: igaf122.1333

### LEVERAGING THE ADULT WELLBEING CHECKUP PLATFORM TO ENHANCE SOCIAL CONNECTION AND WELL-BEING AMONG OLDER ADULTS

Yong Kyung Choi[1], Diana Yin[2], Ronald Kaiser[2], and James Firman[3], *1. University of Pittsburgh, Pittsburgh, Pennsylvania, United States, 2. BetterAge, Sunnyside, New York, United States, 3. BetterAge, Arlington, Virginia, United States*

The Adult Wellbeing CheckUp (AWC) is an innovative web-based platform, designed and built by BetterAge, to assess older adults' well-being and deliver personalized recommendations to enhance health, social engagement, and quality of life. Supported by the National Institute on Aging (NIA) SBIR program, AWC integrates a multi-domain assessment covering physical, mental, and social health, generating customized action plans based on individual priorities. The platform applies behavioral change principles, curated peer support, and local service referrals to help older adults, including care partners (CPs), take actionable steps toward well-being. AWC was developed using a user-centered design approach through three focus groups (N = 77; 40 general older adults (OAs), 37 older adult CPs) across senior centers in PA, MA, and TX. Findings revealed key differences in well-being challenges and technology use. Social isolation was a major concern among OAs, with participants citing limited engagement opportunities, mobility barriers, and difficulty accessing meaningful activities. While many relied on family or close friends, they expressed a need for structured support and community engagement. CPs, who also experience social isolation, highlighted emotional exhaustion, time constraints, and lack of self-care due to caregiving demands. Phase I pilot testing (N = 48; 24 OAs, 24 CPs) is currently underway and involves four weeks of engagement with AWC. Preliminary findings suggest high acceptability, as participants value tailored recommendations and ease of navigation. These findings underscore the importance of digital tools in addressing the unique well-being needs of OAs and CPs, particularly in reducing social isolation and supporting self-care.

### SESSION 3220 (SYMPOSIUM)

Abstract citation ID: igaf122.1334

### MEASUREMENTS IN DEMENTIA CARE AND OUTCOMES: ADVANCING RESEARCH AND PRACTICE WITH INNOVATIVE METHODS

Chair: Anju Paudel
Co-Chair: Wen Liu
Discussant: Ann Kolanowski

Many individuals living with dementia in the U.S. receive care and support across settings of care. Growing research has focused on optimizing the quality of dementia care and outcomes through behavioral interventions and utilizing validated measures to assess the impact of behavioral interventions on quality of care and outcomes. This symposium describes different methodologies (Delphi Survey, Tool Modification, Structural validation, Electronic Medical Record Validation) to develop and validate measures assessing important aspects of dementia care and outcomes (care interactions, mealtime behaviors, rejection of care, and pain) in nursing home (NH), hospital, and assisted living (AL) settings. The first paper describes the development and refinement of the Quality of Interactions Inventory (QUALII) tool that assesses positive care interactions in dementia care in

ALs. The second paper discusses the development, validity, and reliability of the Pain Competency Evaluation in Dementia (PACED) scale in NH. Next, the adaptation of an existing tool - Cue Utilization and Engagement in Dementia (CUED) - into three observational scales to assess NH residents' mealtime behaviors is presented. The fourth paper describes the Modified Quality of Interactions Schedule (MQuIS) that assesses staff-resident interactions and differences in staff-resident interactions by racial concordance versus discordance in ALs. The final presentation describes validation of a practical protocol using electronic medical record nurse charting data against a gold-standard tool of rejection of care. We will compare the methodologies based on research questions, identify directions for future research in measurements in dementia care and outcomes, and explore practice implications of discussed measures.

Abstract citation ID: igaf122.1335

### OPTIMIZING DEMENTIA CARE INTERACTIONS WITH THE QUALITY OF INTERACTIONS INVENTORY (QUALII)

Anju Paudel[1], Elizabeth Galik[2], Beth Howd[1], and Marie Boltz[1], *1. The Pennsylvania State University, University Park, Pennsylvania, United States, 2. University of Maryland School of Nursing, Baltimore, Maryland, United States*

To optimize daily care interactions and enhance quality of care and quality of life of residents with dementia, a comprehensive measure is needed that can guide the desired practice of positive care interactions and evaluate the impact of practice. Such a comprehensive measure of staff-resident interactions in dementia care in assisted living (AL) settings is lacking. Thus, we developed the QUALity of Interactions Inventory (QUALII)— a five domain 21-item comprehensive tool with items representing approaches to positive care interaction in dementia care in AL. This study aims to describe the development and refinement of QUALII. QUALII was developed based on communication theory and the review of the literature. It was revised with input from a panel of N = 10 clinical and academic experts in dementia care and communication utilizing the Delphi survey approach. Following three rounds of Delphi survey, the five domain 21-item QUALII was reduced to a five domain 19-item tool. The revised QUALII demonstrated good content validity based on Content Validity Index (CVI) of individual items (Item relevance I-CVI range= 0.90 to 1.00; Item clarity I-CVI range= 0.80 to 1.00) and the overall scale (S-CVI Average= 0.93). The tool's theoretical foundation, evidence base, and demonstration of content validity suggests its significant potential to guide and evaluate positive care interactions in dementia care and warrants further psychometric testing.

Abstract citation ID: igaf122.1336

### PAIN COMPETENCY EVALUATION IN DEMENTIA (PACED) SCALE – VALIDITY AND RELIABILITY

Yo-Jen Liao[1], Ying-Ling Jao[1], Marie Boltz[1], Nai-Ching Chi[2], Diane Berish[1], and Terrence Murphy[1], *1. The Pennsylvania State University, University Park, Pennsylvania, United States, 2. University of Iowa, Iowa City, Iowa, United States*

People with dementia experience undermanaged pain and often rely on nurses for pain management. Therefore, it is essential to understand nurses' pain management competency in dementia. Existing pain competency scales are mostly subjective and lack focus on people with dementia. This study describes the development and establishment of validity and reliability of the Pain Competency Evaluation in Dementia (PACED) scale. PACED is an observational scale developed from a review of relevant literature, protocols, and guidelines. It includes 24 items in 5 categories. Each item is rated from 0=not performed to 3=accurate. Total score is calculated as History Takingx15% +Documentationx15% +Pain Assessmentx30% +Physical and Functional Assessmentx20% +Pain Treatmentx20%. Content validity was established with pain and/or dementia experts using the Delphi survey approach. We calculated Item-level Content Validity Index (I-CVI; range = 0-1, higher is better) focused on relevance and clarity. Undergraduate nursing students and Registered Nurses completed two virtual case scenarios in nursing home (NH) setting. Two raters were trained to observe and rate nurses' pain management competency using the PACED scale to establish inter-rater and intra-rater reliability. Seven pain and dementia experts completed two rounds of Delphi Survey, and PACED was reduced to 16 items with relevance I-CVI=1.0 and clarity I-CVI=0.99. The raters were trained until interclass correlation (ICC)>0.7 at least 3 times. The raters demonstrated excellent inter-rater (ICC=0.971) and intra-rater (ICC=0.958) reliability for PACED. Next, the internal consistency of PACED will be examined. PACED will be used to evaluate nursing home nurses' pain management competency in dementia.

Abstract citation ID: igaf122.1337

### MEALTIME BEHAVIORS IN NURSING HOME RESIDENTS WITH DEMENTIA: VALIDATING CLINICALLY PRACTICAL SCALES

Wen Liu[1], Yelena Perkhounkova[2], and Maria Hein[2], *1. Penn State University, University Park, Pennsylvania, United States, 2. University of Iowa, Iowa City, Iowa, United States*

Optimizing mealtime behaviors (i.e., positive behaviors, functional impairments, resistive behaviors) is critical to improve enjoyment of mealtimes, nutrition, and function in residents with dementia. Few tools are valid and clinically practical to measure resident mealtime behaviors. We adapted the Cue Utilization and Engagement in Dementia (CUED) video-coding scheme, a novel, valid, yet resource-intensive tool, into three observational scales to assess resident mealtime behaviors and examined their structural validity. N = 425 videos of dyadic mealtime interactions capturing n = 44 residents in 10 nursing homes were coded using CUED. Coded resident mealtime behaviors (i.e., items) were categorized based on frequency: 0 (not observed), 1 (observed 1-2 times), and 2 (observed ≥3 times). Some behaviors were combined based on content relevance, redundancy, and distributions, resulting in 8 items for positive behaviors, 6 items for functional impairments, and 8 items for resistive behaviors. A total of 20 random splits of 425 videos were created for exploratory (EFA) and confirmatory factor analyses (CFA) of each item set. EFA supported 1-factor structure for the three scales. Based on model fit and conceptual interpretability, three items were selected for the positive behaviors scale (Cronbach alpha=.65), four items were selected for the functional impairments scale (alpha=.38), and four items were selected for the resistive behaviors scale (alpha=.44). The 3-item positive behaviors scale, 4-item functional impairment scale, and 4-item resistive behaviors scale were developed for pragmatic use in research and practice. Future testing is needed through direct observations of mealtime care in nursing homes to establish validity and clinical utility.

Abstract citation ID: igaf122.1338

### EVALUATING CARE INTERACTIONS AND CHARACTERISTICS: THE MODIFIED QUALITY OF INTERACTION SCALE

Rachel McPherson[1], Barbara Resnick[2], Sarah Holmes[1], Anju Paudel[3], and Elizabeth Galik[1], *1. University of Maryland School of Nursing, Baltimore, Maryland, United States, 2. University of Maryland, Baltimore, Maryland, United States, 3. The Pennsylvania State University, University Park, Pennsylvania, United States*

While there is growing recognition of the importance of measuring care quality in long-term care settings, there has been limited focus on assessing care interactions. Existing measurement tools capture only a few aspects of these interactions. The Quality of Interactions Scale, an existing measure that examines characteristics (e.g., location and interpersonal distance during interactions) and quality (e.g., positive, negative, and neutral) of staff-resident interactions, was modified to assess a broader range of interaction characteristics, including staff and resident demographics, which may influence care quality. This study aimed to (1) test the reliability and validity of the Modified Quality of Interactions Scale (MQuIS), and (2) evaluate differences in the quality of care interactions (positive, neutral, or negative) based on staff-resident racial concordance (i.e., shared racial background) versus discordance (i.e., different racial background). Data was collected in four assisted living facilities, and the sample included 152 staff-resident interactions. Results showed strong reliability (item reliability = 0.98) and supported validity of MQuIS, with significant positive associations between active resident engagement and positive interactions. Care interactions were significantly more positive when staff and residents shared the same race ($M=5.0$, $SD=2.0$) compared to different racial backgrounds ($M=4.2$, $SD=1.9$), $F(1,150) = 3.51$, $p = .032$. These findings support the use of MQuIS to evaluate care interactions and highlight the importance of considering staff-resident demographics in assessments of dementia care and outcomes. Our findings also suggest that simple modifications to existing measures can help to capture and examine important aspects in dementia care and outcomes.

Abstract citation ID: igaf122.1339

### VALIDATING ELECTRONIC MEDICAL RECORD DOCUMENTATION OF REJECTION OF CARE: THE EMROC PROTOCOL

Clarissa Shaw[1], Gregory Issac[2], Lisa Weimar[3], and Caitlin Ward[4], *1. University of Iowa College of Nursing, Iowa City, Iowa, United States, 2. University of Minnesota, Minneapolis, Minnesota, United States, 3. University of Iowa, Iowa City, Iowa, United States, 4. University of Minnesota School of Public Health, Minneapolis, Minnesota, United States*

Rejection of care (RoC) in people living with dementia can lead to harmful practices including physical and chemical restraints. While the Resistiveness to Care scale (RTC-DAT) is the gold standard for measuring RoC through trained observation of 13 behaviors during care encounters, a more pragmatic measurement approach is needed. This study aimed to validate electronic medical record (EMR) nurse charting data against RTC-DAT scores to develop the EMRoC protocol. Eighty-eight care encounters from 16 hospitalized patient with dementia were observed and scored using the RTC-DAT. EMR flowsheet data was extracted for the corresponding observation days, yielding 35 unique shifts in which 24 (68.6%) included RoC. Analysis identified 16 EMR variables that demonstrated complete concordance with the RTC-DAT, successfully classifying 12 of 35 shifts. The EMRoC decision tree was developed to categorize the remaining 23 shifts. The EMRoC was then applied to a large dataset from one hospital spanning 4-years of nurse flowsheet data, encompassing 1,503 patients across 2,141 admissions and 31,598 shifts. Multiple EMRoC configurations were evaluated using area under the curve (AUC) analysis and assessed against data completeness to determine optimal performance. The final EMRoC protocol included 20 EMR variables (16-variables meeting complete concordance and a 4-variable decision tree) and achieved an in-sample accuracy of 91.4% identifying RoC in 57.9% ($N=18{,}282$) of shifts. The validation process revealed which specific nurse-charted EMR variables indicated RoC presence or absence. This study provides a methodological framework for validating nurse flowsheet data against gold-standard tools and offers a practical approach to measuring RoC.

### SESSION 3225 (SYMPOSIUM)

Abstract citation ID: igaf122.1340

### RETIREMENT, WORK AND HEALTH OUTCOMES IN OLDER AGE

Chair: Wei Yang
Discussant: Karen Glaser

As populations age and workforce participation among older adults shifts, understanding the effects of retirement on health and economic activity has become increasingly critical. This symposium brings together emerging research exploring the multidimensional impacts of retirement and work transitions on cognitive function, mental health, physical well-being, and economic inactivity. Drawing on data from the English Longitudinal Study of Ageing (ELSA) and Survey of Health, Ageing, and Retirement in Europe (SHARE), the presentations examine key questions: How do different retirement pathways influence cognitive decline and mental distress? To what extent does ill health—particularly disability and mental distress—contribute to economic inactivity among older adults? How do working conditions shape employment trajectories after the onset of disability? What are the short- and long-term cardiovascular effects of retirement? Findings from these studies indicate that full retirement is often linked to declines in cognitive and mental health, while semi-retirement—especially with a job change—may mitigate some of these negative effects. Economic inactivity among older adults in the UK has risen post-pandemic, with mental distress and disability playing a crucial role, yet the full picture remains unclear. Working conditions significantly influence employment responses to disability, underscoring the need for workplace policies that support older employees. Finally, retirement appears to increase the risk of cardiovascular disease over time, potentially due to declines in mental health.

Abstract citation ID: igaf122.1341

### THE IMPACT OF DIFFERENT RETIREMENT PATHWAYS ON COGNITIVE AND PHYSICAL HEALTH AMONG OLDER PEOPLE IN ENGLAND

Wei Yang, *King's College London, London, England, United Kingdom*

Understanding the health implications of retirement is essential for policymaking and aging research. This study explores how different retirement pathways impact cognitive function, mental health, and physical well-being among older adults, drawing on data from the English Longitudinal Study of Ageing (ELSA) wave 1 to wave 10. We apply instrumental variable models with fixed individual and time effects, using state pension age, early retirement, and state pension receipt as instruments. Our findings indicate that retirement generally has a negative impact on cognitive abilities (particularly memory), mental health, and physical functioning in daily life. However, semi-retired individuals—especially those who change employers after reaching retirement—experience better memory, mental health, and physical well-being compared to those who remain fully employed or stay with the same employer. Conversely, full retirement is associated with declines in cognitive function (orientation and memory) and mental health, while having no significant effect on physical health. These results underscore the importance of gradual retirement pathways, enabling older adults to remain engaged in work while mitigating the potential adverse health effects of full retirement.

Abstract citation ID: igaf122.1342

**THE EFFECT OF RETIREMENT ON CARDIOVASCULAR DISEASE: EVIDENCE FROM EUROPEAN COUNTRIES**

Jiaru Liu, *King's College London, London, England, United Kingdom*

Retirement marks a pivotal life stage for many individuals. As older adults transition into retirement, changes in lifestyle, socioeconomic status, income, and social relationships can significantly influence their health and well-being. This study investigates the short- and lagged-term relationship between retirement and cardiovascular disease (CVD) - one of the most commonly diagnosed conditions among older adults. Using individual-level data from the Survey of Health, Ageing, and Retirement in Europe (SHARE), we employ an Instrumental Variable Fixed Effects (IV-FE) approach to estimate both the immediate and lagged impact of retirement on CVD and further explore the underlying mechanisms and potential heterogeneity in effects. Our findings suggest that retirement does not exert a significant impact on heart attacks in the short term, but the probability of having a heart attack increases by almost 7% within two years. We further explore several underlying mechanisms through which retirement may influence CVD. While we found no significant associations between metabolic or behavioral risk factors and CVD as potential mediators, our findings highlight a notable exception: a decline in mental health, a key psychosocial risk factor, appears to significantly contribute to adverse cardiovascular outcomes following retirement. This highlights the importance of incorporating mental health support and social engagement initiatives into retirement planning and community programs for older adults.

Abstract citation ID: igaf122.1343

**THE EMPLOYMENT CONSEQUENCES OF DISABILITY AFTER AGE 50: DO WORKING CONDITIONS MATTER?**

Constance Beaufils, *King's College London, King's College London, England, United Kingdom*

This study examines how employment responses to disability vary with working conditions. We use data from the English Longitudinal Study of Aging and focus on individuals aged 50+ who experience the onset of one or more difficulties with activities of daily living (ADL) or instrumental activities of daily living (IADL) between two waves (N = 992). We combine coarsened exact matching and entropy balancing with logistic modelling to estimate the impact of the onset of disability on the probability of leaving paid work, working part-time, changing jobs or looking for a new job. We examine the moderating role of gender and working conditions (job autonomy, job demands, job physicality). Disability significantly increases the probability of leaving paid work, working part-time and looking for a new job. This effect is stronger for people who had a physically demanding job. This suggests the importance of working conditions in shaping older workers' employment behaviours.

Abstract citation ID: igaf122.1344

**CHANGES IN HEALTH AND THEIR RELATIONSHIP TO PAID WORK ACROSS OLDER COHORTS IN ENGLAND**

Karen Glaser, *King's College London, London, England, United Kingdom*

This paper investigates the role of mental distress and other forms of ill-health/disability as drivers of increases in economic inactivity among older workers. Economic inactivity among 50–64-year-olds has risen since the pandemic (2019-2022) and has yet to recover to pre-pandemic levels (ONS 2023). This rise is not seen in any other high-income country and reverses the rise of UK employment in this age group since the mid-1990s. It has been suggested that this rise in economic activity is due to increased levels of poor health; however, such findings are based on responses to questions about the 'main reasons' for economic inactivity rather than health-related questions. Thus, we know little about whether changes in mental distress and other forms of ill-health (if any) are driving economic inactivity levels among older adults and for which groups (e.g., low-income households). Employing data from the English Longitudinal Study of Ageing, we investigate changes across cohorts in: (1) mental distress and other forms ill-health/disability (drawing on work and health-related measures) and their relationship with paid work; (2) the nature of long-term sickness (and individual and family circumstances), and implications for paid work; and (3) how people respond to ill-health/disability through multiple types of work transitions (e.g., exiting employment, reducing work hours, changing jobs) and depending on working conditions.

### SESSION 3230 (SYMPOSIUM)

Abstract citation ID: igaf122.1345

**INNOVATING COMMUNICATION WITH SOCIAL TIES: DIVERSITY IN OLDER ADULT INFORMATION AND COMMUNICATION TECHNOLOGY USE**

Chair: Jess Francis-Levin
Discussant: Toni Antonucci

When it comes to understanding information and communication technology (ICT) use among older adults, context matters. This symposium will explore such varied contexts with diverse methodologies and samples all centered around the theme of ICT-facilitated social engagement among older adults. First, Suh and colleagues analyzed the Health and Retirement Study and identified four distinct ICT user profiles; they found that user profile membership served as a moderator between social support and future wellbeing. Next, Francis-Levin and Dogari, employ a mixed-methods approach to explore the motivations for social media use among LGBTQ+ older adults (55+) and younger

(18-54) adults. Their findings indicate a difference in motivation as older adults are significantly more motivated than younger adults to use social media for social bonding and in responses to family request. Qualitative findings will also be discussed. Third, Ajrouch and Francis-Levin will present findings from their online survey of older Facebook users (65+) who live alone. Findings from their analysis of the different modes of Facebook communication and the impact on perceived social support indicate a significant and positive association with the frequency of direct communication on Facebook and perceived social support. Finally, Choi and colleagues will present their findings from a scoping review on the use of technology for access to social programs and care during the Covid-19 pandemic among older adults with cognitive impairments. Their findings illuminate benefits and challenges, as well as contextual factors impacted by the increased exposure to technology use to access both social and health support.

Abstract citation ID: igaf122.1346

### INTERGENERATIONAL DIFFERENCES IN SOCIAL MEDIA USE AMONG LGBTQ+ ADULTS

Jess Francis-Levin[1], Bolu Dogari[2], and Emily Briggs[3], *1. University of Michigan, ANN ARBOR, Michigan, United States, 2. University of Michigan, Ann Arbor, Michigan, United States, 3. University of Michgian, University of Michigan, Michigan, United States*

Older adults in who identify as LGBTQ+ are at an increased risk of becoming socially isolated. Members of the LGBTQ+ community have been shown to be innovative in their ability to create community through social media use. Although the research related to younger LGBTQ+ folks is increasing, there is still little known regarding how older adults use social media and how that compares to their younger counterparts. We explored differences in motivations to use social media among older adults (aged 55+; n = 88) and young adults (aged 18-54; n = 253) who identify as members of the LGBTQ+ community. Participants completed an online survey, rating their agreement with statements assessing their motivations for social media use on a 7-point scale. Older adults were significantly more likely to use social media for social bonding (M = 3.66) compared to younger adults (M = 3.33), Welch t (142.7) = 2.8, p = 0.005, 95% CI: (0.11, 0.60). Similarly, family requests serve as a stronger motivation for older adults (M = 2.58) than for younger adults (M = 2.32), Welch t (176.2) = 2.3, p = 0.02, 95% CI: (0.04, 0.51). These findings suggest that LGBTQ+ older adults are more likely than LBGTQ+ younger adults to use social media to maintain relationships, particularly with family members. We discuss these results further along with supporting qualitative findings from brief response survey items.

Abstract citation ID: igaf122.1347

### FACEBOOK USE AND PERCEIVED SOCIAL SUPPORT AMONG OLDER ADULTS WHO LIVE ALONE

Kristine Ajrouch[1], and Jess Francis-Levin[2], *1. University of Michigan, Ann Arbor, Michigan, United States, 2. University of Michigan, ANN ARBOR, Michigan, United States*

Older adults (aged 65+) living alone are at especially high risk of experiencing social isolation. Prior research has shown that Facebook communication is associated with social connectedness, serving as a protective resource against the negative influences of social isolation. Yet, Facebook communication is complex, offering various domains of engagement. This study will assess the impact of three separate of Facebook communication domains (broadcasting, directed, consumption) and their association with perceived social support among older adults who live alone. Data were collected through an online survey of older Facebook users who were living alone, unmarried, and without children (n = 517; M age=70). Participants were asked to respond to survey questions related to social support, Facebook use, and socio-demographics. We also asked participants to provide responses to an open-ended item inquiring about the role of Facebook in their daily life. Approximately 50% were women who reported on average some level of college education. Multiple regression analysis showed a positive association between the frequency of Facebook communication and perceptions of social support (β =.88; p = .001). Specifically, our findings indicate that the frequency of directed communication has a significant and positive association with social support (β =.22; p = .01). There were no significant associations between frequency of broadcasting communication or consumption communication with social support. Findings will be discussed through the lens of social relations theories to elaborate the benefits and barriers of Facebook use for facilitating perceived social support among older adults who live alone.

Abstract citation ID: igaf122.1348

### OLDER ADULTS' COMMUNICATION CHANNEL PROFILES: CORRELATES & IMPLICATIONS FOR PROTECTIVE EFFECTS OF SOCIAL SUPPORT

JungYeon Suh[1], Ha Young Choi[2], and Shannon Mejia[1], *1. University of Illinois Urbana-Champaign, Champaign, Illinois, United States, 2. University of Illinois Urbana-Champaign, Urbana, Illinois, United States*

Social support is essential to health and well-being in later life. Age-related declines in social network size heightens the importance of social support. In this study we examine the implications of Information and Communication Technology (ICT) for the protective effects of social support. Using the 2016/2018 Health and Retirement Study (n = 11,068; aged 51-102), we employed latent profile analysis to identify four communication profiles: (1) email dominant (23.4%); (2) SNS dominant (13.4%); (3) in-person/phone contact (41.1%); and (4) high interaction across all channels (22.3%). Multinomial logistic regression and path analyses were conducted to identify sociodemographic predictors (age, gender, education, living alone status, and emotional and geographical closeness) of profile membership and how the profiles moderate relationships between social support and well-being (life satisfaction and loneliness). Compared to the email dominant profile, SNS profile tended to be women, younger, more educated, have more emotionally and geographically close family, and fewer friends. Meanwhile, in-person/phone contact profile was more likely to be older, men, living alone, and have lower education, more emotionally close children, and family and friends who were geographically, but not emotionally close. The protective effect of support from children on well-being was strongest for the SNS dominant profile. Conversely, the email dominant profile dampened the effects of support from family and friends. Our findings imply that older adults adopt different communication strategies, shaping the protective effects of social support. Additionally, SNS interactions with specific social networks should be differentiated from general use, when assessing its effect on older adults' well-being.

Abstract citation ID: igaf122.1349

**TECHNOLOGY IN CARE ACCESS AND USE AMONG ADULTS WITH COGNITIVE LIMITATIONS DURING THE PANDEMIC – SCOPING REVIEW**

HwaJung Choi, Mohsen Najar, Rahat Naseem, Hisham Irshaid, and Tina Mainieri, *University of Michigan, Ann Arbor, Michigan, United States*

Many care services and social programs were halted or reduced during the COVID-19 pandemic. Online platforms for care delivery and social connections were introduced. It is important to understand the experience with the new technology by those with cognitive limitations in accessing care and support services. We reviewed 68 published articles that examined and discussed technology in the context of care availability and utilization among adults with cognitive limitations during the COVID-19 pandemic. There was overwhelming evidence of increased exposure to technology in care services and social programs – physician consultation, community programs, and contact with family and friends. Some suggest that remote care services played a significant role in filling the gap, such as prescriptions, during the pandemic. However, about 30% of the articles mentioned limitations in the use of technology. Adapting to a remote care system was challenging for adults living with cognitive impairments, including cognitive fatigue and difficulties with navigation. Additional health problems, in particular hearing and vision impairments, exacerbated the challenge with online consultation and social engagements. Family caregivers also experienced limitations and challenges due to their lack of familiarity with technology. The use of some services (e.g., telemedicine) declined as the state of emergency was lifted. A prompt shift to remote care delivery system, assisted with new technology, was necessary during the pandemic. While it played a substantial role in filling the gap in care services and social programs, careful assessments of the effectiveness of remote systems are needed specifically for those with cognitive limitations.

### SESSION 3235 (SYMPOSIUM)

Abstract citation ID: igaf122.1350

**MEDICAL SCIENCES SECTION OF THE JOURNALS OF GERONTOLOGY SERIES A EDITOR'S CHOICE ARTICLES OF 2024: THE EFFECT OF LIFE EXPERIENCES ON AGING AND DISEASE.**

Chair: Lewis Lipsitz

Discussant: Raya Kheirbek

This symposium will present four 2024 "Editor's Choice" articles from the Medical Sciences section of The Journals of Gerontology, Series A: Biological Sciences and Medical Sciences that focus on the effect of life experience and life-style influences on aging and disease. Maddison Mellow and her colleagues, in their article "Intensity and Domain-Based 24-Hr Activity Patterns Differ in Their Links to Cognitive and Cardiometabolic Health" examined how differences in the intensity and timing of physical activities can influence cognitive function and cardiometabolic health in older adults. So Yeon Jeon and her coauthors, in "Caregiving-Related Depression Increases Neuroinflammation in older adult spousal caregivers to Individuals With Cognitive Impairment: A Longitudinal Study" look at the neurobiological impact of caregiving to individuals with cognitive impairments and how it contributes to neuroinflammation, a well-known risk factor for various diseases. In "Sociodemographic Determinants of Extreme Heat and Ozone Risk Among Older Adults in 3 Sun Belt Cities", Peter Crank and colleagues surveyed how older adults are affected by extreme heat and ozone pollution inside and outside of their homes across select cities in the United States. Christopher Kaufmann and his co-authors investigated the association between previous incarceration and various chronic health conditions in older adults in "Links of Previous Incarceration With Geriatric Syndromes and Chronic Health Conditions Among Older Adults in the United States". Raya Kheirbek, our discussant, will draw upon her own research studying the unique needs of marginalized and medically vulnerable individuals to highlight commonalities and lessons learned from these studies.

Abstract citation ID: igaf122.1351

**INTENSITY AND DOMAIN-BASED 24-HR ACTIVITY PATTERNS DIFFER IN THEIR LINKS TO COGNITIVE AND CARDIOMETABOLIC HEALTH**

Maddison Mellow[1], Dorothea Dumuid[2], Timothy Olds[3], Ty Stanford[2], Hannah Keage[1], Frini Karayanidis[4], and Ashleigh Smith[2], *1. University of South Australia, Adelaide, South Australia, Australia, 2. Alliance for Research in Exercise, Nutrition and Activity (ARENA) Research Centre, University of South Australia, Adelaide, South Australia, Australia, 3. Allied Health and Human Performance, University of South Australia, Adelaide, South Australia, Australia, 4. Functional Neuroimaging Laboratory, University of Newcastle, Newcastle, New South Wales, Australia*

Each day is made up of a composition of "time-use behaviours." These can be classified by their intensity (eg, light or moderate–vigorous physical activity [PA]) or domain (eg, chores, socialising). Intensity-based time-use behaviours are linked with cognitive function and cardiometabolic health in older adults, but it is unknown whether these relationships differ depending on the domain (or type/context) of behaviour. This study included 397 older adults (65 ± 3 years, 69% female, 16 ± 3 years education) from Australia. Time-use behaviours were recorded using the Multimedia Activity Recall for Children and Adults (MARCA), cognitive function was measured using the Addenbrooke's Cognitive Examination III (ACE-III) and Cambridge Neuropsychological Test Automated Battery, and systolic and diastolic blood pressure, total cholesterol, and waist–hip ratio were also recorded. Two 24-hour time-use compositions were derived from each participant's MARCA, including a 4-part intensity composition (sleep, sedentary behaviour, light, and moderate–vigorous PA) and an 8-part domain composition (Sleep, Self-Care, Chores, Screen Time, Quiet Time, Household Administration, Sport/Exercise, and Social). Linear regressions found significant associations between the domain composition and both ACE-III ($p = .010$) and waist–hip ratio ($p = .009$), and between the intensity composition and waist–hip ratio ($p = .025$). Isotemporal substitution modelling demonstrated that the domains of sedentary behaviours and PA impacted their associations with ACE-III, while any PA appeared beneficial for waist–hip ratio. Findings suggest the domain of behaviour should be considered when aiming to support cognitive function, whereas, for cardiometabolic health, it appears sufficient to promote any type of PA.

Abstract citation ID: igaf122.1352

### CAREGIVING-RELATED DEPRESSION AND NEUROINFLAMMATION: A LONGITUDINAL STUDY

So Yeon Jeon[1], Bo Ran Son[2], Hee Won Yang[2], Jimin Baek[2], and Jeong Lan Kim[2], *1. Seoul Metropolitan Government-Seoul National University (SMG-SNU) Boramae Medical Center, Seoul, Republic of Korea, 2. Chungnam National University Hospital, Daejeon, Republic of Korea*

Background: The caregiving burden of the spousal caregivers (SCGs) to individuals with cognitive impairment poses public health challenges with adverse psychosocial and physiological effects. However, few studies have investigated the neurobiological impact of caregiving, particularly through the investigation of neuroinflammation and neurodegeneration. Methods: Using data from a longitudinal cohort at Chungnam National University Hospital, the relationship between caregiving burden, neuroinflammation and neurodegeneration was examined in 38 older adult couples over a 16-month period. Caregiving burden was assessed through a multifaceted approach. For factors related to the care recipient, we assessed cognitive function and neuropsychiatric symptoms. Factors regarding the SCGs included the measurement of perceived depression. Glial fibrillary acidic protein (GFAP) was used as a plasma biomarker for neuroinflammation and neurofilament light chain (NfL) for neurodegeneration. Regression analyses were adjusted for age, sex, apolipoprotein E status, follow-up interval, vascular risk factors, and physical activity. Results: Changes in depression among SCGs were significantly correlated with increased GFAP levels ($p = 0.003$), indicating that greater depressive symptoms during caregiving are associated with increased neuroinflammation. In contrast, no significant correlations were found between changes in cognitive function or neuropsychiatric symptoms in care recipients and the plasma biomarker levels of SCGs. Additionally, there was no significant association between changes in depression and NfL levels in SCGs. Conclusions: The psychological stress experienced by SCGs while caring for partners with cognitive impairment actively contributes to neuroinflammation, a well-known risk factor for various diseases. This study emphasizes the need to address psychological stress experienced by older adult caregivers.

Abstract citation ID: igaf122.1353

### SOCIODEMOGRAPHIC DETERMINANTS OF EXTREME HEAT AND OZONE RISK AMONG OLDER ADULTS IN 3 SUN BELT CITIES

Peter Crank[1], Cassandra O'Lenick[2], Amir Baniassadi[3], David Sailor[4], Olga Wilhelmi[5], and Mary Hayden[6], *1. University of Waterloo, Waterloo, Ontario, Canada, 2. The University of North Carolina at Chapel Hill, Chapel Hill, North Carolina, United States, 3. Hebrew SeniorLife, Boston, Massachusetts, United States, 4. Arizona State University, Tempe, Arizona, United States, 5. National Center for Atmospheric Research, Boulder, Colorado, United States, 6. University of Colorado Colorado Springs, Colorado Springs, Colorado, United States*

Background: Vulnerable populations across the United States are frequently exposed to extreme heat, which is becoming more intense due to a combination of climate change and urban-induced warming. Extreme heat can be particularly detrimental to the health and well-being of older citizens when it is combined with ozone. Although population-based studies have demonstrated associations between ozone, extreme heat, and human health, few studies focused on the role of social and behavioral factors that increase indoor risk and exposure among older adults. Methods: We conducted a household survey that aimed to understand how older adults are affected by extreme heat and ozone pollution inside and outside of their homes across Houston, Phoenix, and Los Angeles. We examine contributing factors to the risk of self-reported health effects using a generalized linear mixed-effects regression model of telephone survey data of 909 older adults in 2017. Results: We found an increased occurrence of self-reported symptoms for extreme heat with preexisting respiratory health conditions and a lack of air conditioning access; self-reported ozone symptoms were more likely with preexisting respiratory health conditions. The risk of heat-related symptoms was slightly higher in Los Angeles than Houston and Phoenix. We found several demographic, housing, and behavioral characteristics that influenced the risk of heat- and ozone-related symptoms. Conclusions: The increased risk among older adults based on specific social and behavioral factors identified in this study can inform public health policy and help cities tailor their heat and ozone response plans to the specific needs of this vulnerable population.

Abstract citation ID: igaf122.1354

### LINKS OF PREVIOUS INCARCERATION WITH GERIATRIC SYNDROMES AND CHRONIC HEALTH CONDITIONS AMONG OLDER ADULTS IN THE US

Christopher Kaufmann[1], Alexander Testa[2], Dylan Jackson[3], Meghan Novisky[4], Carmen Gutierrez[5], Jack Tsai[2], Adam Spira[6], and Roland Thorpe[6], *1. University of Florida, Gainesville, Florida, United States, 2. UTHealth Houston, Houston, Texas, United States, 3. Johns Hopkins University, Baltimore, Maryland, United States, 4. Cleveland State University, Cleveland, Ohio, United States, 5. The University of North Carolina at Chapel Hill, Chapel Hill, North Carolina, United States, 6. Johns Hopkins Bloomberg School of Public Health, Baltimore, Maryland, United States*

This study investigated the association between previous incarceration and various geriatric and chronic health conditions among adults 50 and older in the United States. Data came from the National Longitudinal Study of Adolescent to Adult Health—Parent Study (AHPS) collected in 2015–2017, including 2,007 individuals who participated in the parent study (Parent Sample) and 976 individuals who participated in the spouse/partner study (Spouse/Partner Sample). Multiple logistic regression was used to investigate the relationship between previous incarceration and geriatric syndromes (dementia, difficulty walking, difficulty seeing, difficulty with activities of daily living) and chronic health conditions (self-reported poor/fair health, diagnosis of cancer, hypertension, diabetes, heart disease, stroke, chronic lung disease, depression, and alcohol use [4 or more drinks per week]). In adjusted analyses, respondents with previous incarceration in the AHPS had significantly higher odds of reporting difficulty walking, activities of daily living difficulty, cancer diagnosis, depression diagnosis, and chronic lung disease (adjusted odds ratios [aORs] = 2.21–2.95). Respondents in the AHPS spouse/partner study reported higher odds of difficulty seeing, cancer, depression, chronic lung disease, and heavy alcohol use (aORs = 1.02–2.15). Previous incarceration may have an adverse impact on healthy aging. Findings highlight the importance of addressing the enduring health impacts of incarceration, particularly as individuals transition into older adulthood.

### SESSION 3240 (SYMPOSIUM)

Abstract citation ID: igaf122.1355

### INNOVATION IN EDUCATION ACROSS THE CARE CONTINUUM

Chair: Kristen Neises

The Geriatric Academic Career Award (GACA) has been a critical catalyst for advancing geriatrics education, developing future healthcare professionals, and addressing the growing needs of an aging population. GACA-supported scholars have led initiatives that integrate geriatrics into medical, nursing, and allied health curricula, expand workforce training, and improve care for underserved older adults. These efforts have enhanced clinical training, developed novel educational methodologies, and strengthened interprofessional collaboration, ensuring that learners are prepared to deliver high-quality care to older adults across various healthcare settings. Despite its success, sustaining these innovations remains a challenge due to a lack of structured, long-term funding pathways. In this symposium, six GACA recipients will showcase the innovative educational programs they have developed, highlighting how their work has transformed workforce training and improved health outcomes for older adults. Presenters will discuss re-envisioning geriatrics education in nursing using oral history methodology, addressing care gaps for aging homeless populations, developing interdisciplinary training models, and creating geriatrics curricula for nursing home-based education for nursing and medicine. By reflecting on the successes and challenges of implementing and sustaining their programs, panelists will illustrate the need for continued investment in geriatrics education and stronger collaboration between clinicians, educators, and researchers. This session will explore emerging strategies to embed geriatrics education across disciplines and settings, ensuring that innovations in training continue to shape the future of aging-related care.

Abstract citation ID: igaf122.1356

### ADVANCING AGE-FRIENDLY CARE THROUGH LIFESTYLE MEDICINE EDUCATION

Kristen Neises, *University of Louisville, Louisville, Kentucky, United States*

The integration of age- and dementia-friendly principles into healthcare education is essential for preparing a workforce capable of delivering high-quality care to older adults. Traditional training often lacks a preventive, interdisciplinary approach that addresses the role of lifestyle factors in aging and cognitive health. Through my Geriatric Academic Career Award (GACA)-funded initiative, I have developed a comprehensive geriatrics curriculum that embeds lifestyle medicine into interprofessional education, ensuring that learners across disciplines gain the skills needed to support healthy aging and dementia prevention. This project incorporates case-based virtual learning, a micro-credentialing course in age- and dementia-friendly lifestyle medicine, and the infusion of lifestyle medicine principles into interprofessional education involving medicine, nursing, social work, paraprofessionals, pharmacy, and other disciplines. By fostering collaboration and strengthening workforce competency, this curriculum enhances learners' knowledge, confidence, and ability to apply lifestyle-based interventions in older adult care. This presentation will highlight the impact of this educational approach, discuss challenges and opportunities in sustaining and scaling geriatrics training, and explore how embedding lifestyle medicine into workforce education can improve patient outcomes and better prepare healthcare professionals to address the needs of an aging population.

Abstract citation ID: igaf122.1357

### ENHANCING GERIATRIC KNOWLEDGE FOR INTERPROFESSIONAL HEALTH SCIENCES STUDENTS AND SKILLED NURSING FACILITY STAFF

Vania Leung, *University of Illinois Chicago, Chicago, Illinois, United States*

My GACA was designed to provide clinical geriatrics and skilled nursing facility training for an interprofessional group of learners to address the need to improve geriatrics care for the skilled nursing facility population. The primary goal of this curriculum is to enhance bidirectional geriatric training and curriculum for health sciences training programs (medicine and nursing) and nursing home staff. The aim is to educate on holistic geriatrics concepts including social determinants of health and disparities in aging, the basic tenets of geriatric clinical medicine and the importance of age-friendly health systems frameworks. This novel, interprofessional approach achieves multiple aims: 1) bridge the gap between what is taught in the classroom and the clinical setting for students and trainees; 2) offer opportunities for existing nursing home staff to better understand and apply core geriatrics knowledge; and 3) provide direct exposure to possible career opportunities for trainees. This presentation will focus on the impact thus far of this program for trainees as well as skilled nursing facility staff and discuss the challenges of implementation. It further discusses the translational aspect of this program on a systemic level across a healthcare system.

Abstract citation ID: igaf122.1358

### DEVELOPING A NOVEL GERIATRICS PERIOPERATIVE CASE CONFERENCE SERIES

Evan Henricks, *Medical College of Wisconsin, Milwaukee, Wisconsin, United States*

This session aims to demonstrate how support from career development awards enables the creation and implementation of educational interventions that improve knowledge base and change practice in medical professionals. With support from the Geriatric Academic Career Award (GACA) this project developed a novel, recurring, geriatrics perioperative case conference series. Using the Kern method of curriculum development, targeted providers (physician, advanced practice provider, and nursing) were identified and asked to complete a voluntary, anonymous self-assessment of needs, ranking interest in six different topics of age-friendly elements (4Ms) in the perioperative care of older adults. A case conference series was then created and implemented with preliminary learner competence data collected using an anonymous self-assessment survey. 24 participants (36% of sample) completed the needs assessment survey. After creation and implementation of the two of three planned case-series, 9 learners' post-session evaluation (response rate 22%), rated level of comfort on the topic higher post-session (mean 4.11) compared to pre-session (mean 3.0) with nearly 90% of participants replying "would recommend this session" to others. All attendees were either "somewhat likely" or "extremely likely" to utilize geriatrics templates, screening tools, and recommend interventions after attending sessions. Perioperative providers are interested in learning about age-friendly health elements, specifically regarding mentation, medications, and mobility. The targeted curriculum increased the level of comfort in clinical care of older adults, with participants likely to recommend the session. Development and implementation of this curricular intervention was possible through the support of the GACA funding.

Abstract citation ID: igaf122.1359

### SEEING AGING ANEW: USING ARTS-BASED TEACHING TO ENRICH INTERPROFESSIONAL GERIATRICS EDUCATION

Mariah Robertson[1], and Kelseanne Breder[2], *1. Johns Hopkins School of Medicine, COLUMBIA, Maryland, United States, 2. New York University, New York, New York, United States*

Arts and humanities-based pedagogy encourages a human-centered approach to learning that is valuable to all health professions education (HPE). This type of teaching promotes empathy, tolerance of ambiguity, capacity to wonder, observation and communication skills, and assists with dialogue across differences. These are all priority skills for people who serve older adults across the educational continuum. Arts-based education can also help to reframe aging, building interest and investment in geriatrics among non-geriatrics trained clinicians. In this presentation, we review the uses of arts-based teaching implemented across a variety of educational settings including the home and nursing home for interprofessional learners from paramedicine, medicine, nursing and public health. We will specifically highlight the core aspects of two arts-based curricular innovations focused on geriatrics education and will assist participants in identifying opportunities to use components of these curricula in their own HPE.

Abstract citation ID: igaf122.1360

### CREATING THE GERIAPOLIS – HOW A SMALL AWARD CAN BUILD A CITY

Benjamin Rosenstein, *University of Minnesota, Minneapolis, Minnesota, United States*

The University of Minnesota Medical School has no geriatrics focused training such as rotations, clinics, or a fellowship program. Additionally, we have limited geriatrics teaching faculty. The time and money necessary to develop geriatrics education in medicine, and collaborations with interdisciplinary health professions schools, does not exist. Unless it is mandated by a grant. My GACA is aimed at overcoming the long-standing barriers to furthering geriatrics education and care at our institution. The GACA provides me with the time and finances necessary to develop and design new curriculum and establish collaborations. I aim to develop geriatrics education vertically (from medical school to practice) and horizontally (across health professions), creating a foundation for ongoing geriatrics education where it had not previously existed. This presentation will highlight the challenges I faced in promoting geriatrics education and how the GACA has provided me with support to achieve success. I will provide examples by reviewing current projects including establishing dementia and delirium education, collaboration with our longitudinal rural care program, and the wide-spread networking needed to develop a long-term care medical student experience. I will then focus on questions that may arise with future plans such as establishing an interprofessional geriatrics clinic, creating inpatient geriatrics experiences, and using the ECHO platform to maximize educational reach.

### SESSION 3245 (SYMPOSIUM)

Abstract citation ID: igaf122.1361

### FROM RISK TO RESILIENCE: MULTIDIMENSIONAL INFLUENCES ON HEALTH AND WELL-BEING IN LATER LIFE

Chair: Ann Nguyen
Co-Chair: Katrina Ellis

This symposium examines risk and resilience factors that shape the health and well-being of middle-aged and older adults using a health equity lens. The first paper reports the findings for the association between stressful social roles and poor sleep quality among women experiencing menopause. The findings demonstrate that stress from social roles negatively impacts sleep health. The second paper investigates how the interplay between neighborhood characteristics and education impacts cognitive decline. The results indicate that the combination of low educational attainment and living in a disadvantaged environment is a risk factor for cognitive decline. The third paper examines the financial exploitation among people living with dementia using a scoping review methodology. The results identify risk and protective factors and intervention strategies for financial exploitation. The fourth paper interrogates the relationship between resilience and health in older African Americans living with advanced chronic kidney disease. The findings indicate that resilience may be a response to managing multiple health conditions. The fifth paper examines the interactive effect of social support and depressive symptoms on mortality risk among older adults living with type 2 diabetes. The results demonstrate the importance of leveraging social support and managing depressive symptoms in diabetes management. Overall, these papers will provide important insights into modifiable psychosocial factors that influence health and well-being in later life. Modifiable psychosocial factors have broad translational importance in promoting health equity. The findings will be discussed with attention to important socio-cultural and contextual factors and highlight implications for gerontological practice and research.

Abstract citation ID: igaf122.1362

### THE ASSOCIATION BETWEEN STRESSFUL SOCIAL ROLES AND SLEEP QUALITY AMONG PERI- AND POST-MENOPAUSAL WOMEN

Luciana Giorgio, Erick Scherf, and Michelle Favero, *The University of Alabama, Tuscaloosa, Alabama, United States*

Poor sleep, particularly during menopause, is associated with an increased risk of cognitive and cardiovascular disease in women's later life. Although hormonal changes partially explain poor sleep quality during menopause, stress from performing multiple social roles may exacerbate poor sleep quality. This study examines the association between the number of social roles perceived as stressful and sleep quality among midlife women. Using survey data from peri/post-menopausal women participating in the Study of Women Across the Nation Wave 1 (n = 1,690), we conducted linear regressions to test the effects of social role stress on sleep quality. The number of social roles identified as stressful were summed across social role categories (e.g., employment, family caregiving, being a spouse/partner, and childrearing). Sleep quality was measured using the Women's Health Initiative Insomnia Scale (WHIIRS). Models were adjusted for sociodemographic characteristics, health conditions, menopausal status, number of social roles. A final adjustment for depressive symptoms was made. Participants had Mage=47.14 (SD = 2.73) and most identified as non-Latina White (48.46%), spoke English (87.04%) and were perimenopausal (97.22%). On average, participants reported WHIIRS of 5.18 (SD = 3.89) and 31% had clinically significant depressive symptoms. Each additional role perceived as stressful was associated with a 0.09 unit increase in WHIIRS (SE = 0.01, p < 0.001). This association was attenuated when controlling for depressive symptoms (β = 0.05, SE = 0.01, p < 0.001). Our findings suggest that reducing stress from social roles may improve sleep quality among mid-life women. Future studies should leverage longitudinal designs to test the directionality of these associations.

Abstract citation ID: igaf122.1363

### A SCOPING REVIEW: FINANCIAL EXPLOITATION AMONG OLDER PEOPLE LIVING WITH DEMENTIA

Wenxing Wei, and Sarah Balser, *Case Western Reserve University, Cleveland, Ohio, United States*

Financial exploitation is among the most commonly reported forms of elder abuse, with older adults with dementia facing a heightened risk due to various vulnerabilities. However, limited research has specifically addressed this critical issue. Guided by the methodological framework of Arksey and O'Malley, this scoping review aims to summarize the existing literature to enhance current knowledge and raise awareness about financial exploitation in this vulnerable population. Following rigorous methods, we systematically searched four scientific databases (Ageline, Medline, CINAHL, and PsycINFO) for peer-reviewed articles published in English up to July 2024, identifying 21 articles for inclusion. A comprehensive data extraction process identified six key themes, including the prevalence of financial exploitation, the relationship between financial exploitation and cognitive function, risk factors and warning signs, case descriptions, preventive and intervention strategies, and barriers and facilitators to safeguarding. The findings emphasize the necessity for a multi-level safeguarding framework to prevent and address financial exploitation at the individual, professional, institutional, and systemic levels. This review also identifies critical gaps in existing literature, such as the need for research on protective factors, adverse effects, and barriers to implementing safeguarding measures. Research in this field is diverse yet fragmented, with limited studies directly addressing the intersection of financial exploitation and dementia. These insights underscore the need for future research, policy-making, and intervention development to better support and protect this at-risk population.

Abstract citation ID: igaf122.1364

### NEIGHBORHOOD PHYSICAL DISADVANTAGE, EDUCATION, AND COGNITIVE DECLINE TRAJECTORIES IN U.S. OLDER ADULTS

Kedong Ding, *Case Western Reserve University, Cleveland, Ohio, United States*

One in four U.S. older adults experiences cognitive decline before death, which can significantly affect their quality of life and independence. Given the important role of neighborhood environments in cognitive health, it remains unclear whether living in physically disadvantaged neighborhoods has a long-term impact on cognitive trajectories. This study aims to identify the trajectories of cognitive decline and examine the associations between the trajectories and neighborhood physical disorder, and whether these associations differ by educational background. We used data from 7,460 participants aged 50+ in the Health and Retirement Study (HRS) from 2006 to 2020. We used group-based trajectory models to identify latent groups for trajectories of cognitive decline over time. Multinomial logistic analyses were adjusted for sociodemographic and health covariates. We identified five trajectory groups: Group One ("high start, stable"), Group Two ("medium-high start, stable"), Group Three ("medium-high start, slight decrease"), Group Four ("medium start, sharp decrease"), and Group Five ("low start, slight decrease"). We found that neighborhood physical disadvantage was associated with higher odds of belonging to Group One (RRR=1.17, P < 0.01), Two (RRR=1.28, p < 0.001), Three (RRR=1.33, p<.001) and Four (RRR=1.23, p < 0.01), all with lower baseline cognition and faster decline than Group One, which had the best cognitive trajectory. Additionally, participants with more than a high school education who lived in disadvantaged neighborhoods were more likely to be in groups with lower baseline cognition and faster decline than Group One. Our findings highlighted the important role of neighborhood physical disadvantage and education in older adults' cognitive function trajectories.

Abstract citation ID: igaf122.1365

### EXPLORING RESILIENCE IN OLDER AFRICAN AMERICANS WITH ADVANCED CHRONIC KIDNEY DISEASE

Tyrone Hamler[1], Seungjong Cho[2], Tabitha Pederson[1], and Pilar Ingle[1], *1. University of Denver, Denver, Colorado, United States, 2. Texas Tech University, Lubbock, Texas, United States*

Chronic kidney disease (CKD) affects about 1 in 7 individuals in the U.S., with prevalence increasing with age. Nearly 50% of those over 75 have CKD. Beyond age, racial disparities impact disease progression, with Black individuals nearly four times more likely to develop kidney failure than Whites. This study examined resilience among older African Americans with advanced CKD. While resilience is recognized as a positive adaptation to adversity, researchers highlight the need to explore contextual factors influencing resilience, as chronic social stressors can negatively affect health. No known studies have examined resilience in older Black adults with CKD. Data were drawn from a larger study on decisional conflict in CKD among older Black adults recruited from an outpatient nephrology clinic in the Midwest. Among participants (N = 124), resilience levels were typical (M = 3.10, SD = .56). At the bivariate level, resilience correlated with more comorbidities (r = .20, p < .001). An OLS regression confirmed this association, while resilience was unrelated to other clinical or demographic variables. These findings suggest that resilience may be a response to managing multiple health conditions rather than a protective factor against comorbidities. Future research should explore mechanisms underlying resilience and their implications for health outcomes. Understanding these relationships can inform targeted interventions that foster resilience while addressing the unique challenges faced by older Black adults with CKD. Addressing both social and clinical determinants of health is critical in addressing disparities and improving well-being in this population.

Abstract citation ID: igaf122.1366

### DIRECT SOCIAL SUPPORT AND 19-YEAR MORTALITY AMONG OLDER ADULTS WITH TYPE 2 DIABETES

Weidi Qin[1], Emily Nicklett[2], and Deven Murray[2], *1. University of Wisconsin–Madison, Madison, Wisconsin, United States, 2. The University of Texas at San Antonio, San Antonio, Texas, United States*

While existing research suggests that social support improves diabetes management and outcomes, the long-term impact on longevity remains unaddressed. To bridge this gap, the present study examines two questions: 1) Is direct social support associated with mortality among older adults with diabetes? 2) Is the interaction between social support and depressive symptoms associated with mortality? Older adults with type 2 diabetes were selected from the Health and Retirement Study's 2003 Diabetes Study ($n = 1{,}139$). The year of death was ascertained by exit interview or spouse respondents through 2022. Direct social support was assessed with an 8-item scale indicating whether participants could count on their family or friends to help with diabetes management. Depressive symptoms were measured using CESD-8. Cox proportional hazards models were performed to test the study aims. From 2003 to 2020, 75.43% of participants died. Compared to low social support, high social support was associated with 1.58 times higher hazards of death ($p = 0.016$). The interaction between social support and depressive symptoms was significantly associated with mortality (hazard ratio = 2.39, $p = 0.033$). Specifically, among older adults with diabetes who had low social support, those with no depressive symptoms had lower hazards of mortality compared to those with mild depressive symptoms. The findings suggest that high social support is related to higher hazards of mortality among older adults with type 2 diabetes. It is important for diabetes management plans to incorporate strategies for leveraging social support to prolong the life expectancy among older adults.

### SESSION 3250 (SYMPOSIUM)

Abstract citation ID: igaf122.1367

### ARTIFICIAL INTELLIGENCE IN THE LONGEVITY ECONOMY: WHAT DO WE KNOW?

Chair: Taylor Brennan
Discussant: Taylor Brennan

Artificial intelligence (AI) is transforming our society. As AI in the U.S. increasingly intersects with the growing longevity economy, it will continue influence public opinion and disrupt how consumers across the lifespan access information, work, care, and age (Coughlin, 2017). Aligned with GSA's 2025 theme "Innovative Horizons in Gerontology," this symposium presents findings from four MIT AgeLab studies exploring Americans' attitudes towards AI in key gerontological domains. Using mixed methods and unique samples, the studies examine AI's impact on older workers, family caregivers, aging in the latest phase of life, and information-seeking behavior at different ages. The first presentation highlights AI's potential to support older workers, drawing on national interviews with HR professionals and company executives. The second presentation reports on a survey conducted with family caregivers exploring their use of and trust in AI for family caregiving. The third discusses how adults aged 85+ perceive and use AI for managing aging in the latest phase of life. The final presentation shares insights from a national public opinion survey on attitudes toward AI for information and advice, emphasizing age-related differences. Together, these presentations will deepen our understanding of how Americans engage with AI across diverse contexts of aging. A facilitated discussion will follow, encouraging attendees to reflect on key themes and emerging issues at the intersection of AI and the longevity economy.

Abstract citation ID: igaf122.1368

### EXPLORING AI IN THE MULTIGENERATIONAL WORKPLACE: OPPORTUNITIES, CHALLENGES AND BEST PRACTICES

Samantha Brady, and Lauren Cerino, *Massachusetts Institute of Technology, Cambridge, Massachusetts, United States*

Artificial Intelligence (AI) is rapidly changing the nature of work across nearly all industries. With the potential to enhance efficiency, decision-making, and innovation, many workplaces are adopting a range of AI tools and solutions. In light of the rapid expansion of AI in the workplace as well as the aging of the U.S. workforce, it is imperative to understand the ways in which AI can support older workers while also presenting new challenges for this growing segment of workers. To understand the opportunities and challenges AI presents for workers across generations, we conducted in-depth interviews with human resource managers and executives across a variety of industries in the U.S. Interviews highlighted potential benefits of AI for older workers including greater productivity, work quality improvements, and the ability to shift from administrative and logistical tasks to more human-centered work. Additionally, AI can assist with the documentation process necessary for knowledge transfer, especially in organizations with a large population of older workers. However, HR professionals also identified key challenges, particularly in multigenerational workplaces. These include training and education gaps, usability concerns, data security risks, and differing mental models of work that may complicate AI adoption. This session will examine both the opportunities and obstacles AI presents across generations and offer best practices for organizations seeking to integrate AI effectively. By considering the unique needs of older workers, organizations can create a more inclusive and adaptive workplace for the future.

Abstract citation ID: igaf122.1369

### FACTORS IMPACTING FAMILY CAREGIVERS' ADOPTION OF DIGITAL AND AI-ENABLED TECHNOLOGIES FOR CARE-RELATED TASKS

Niels Wu[1], Adam Felts[1], Alexa Balmuth[1], Chaiwoo Lee[1], Lisa D'Ambrosio[1], Samantha Brady[1], Sophia Ashebir[1], and Joseph Coughlin[2], *1. Massachusetts Institute of Technology, Cambridge, Massachusetts, United States, 2. Massachusetts Institute of Technology AgeLab, Cambridge, Massachusetts, United States*

Family caregivers are critical to the delivery of care for older adults, but may experience physical, financial, and emotional strains from the caregiving role. Although existing research has established the capacity for digital technologies to ease these strains, adoption remains limited for some technologies that could support care-related tasks, especially for newer, AI-enabled technologies, indicating a need to explore where caregivers encounter barriers related to usability, cost, availability, or value. In this study, an online questionnaire was fielded to members of the MIT AgeLab's CareHive Panel, an open membership panel of informal caregivers who either currently provide or formerly provided care to an adult family member. Current caregivers (n = 192) were asked about their reasons for avoidance or cessation of use for four AI-enabled technologies: home monitoring and security systems, digital assistants or smart speakers, medication management platforms or smart dispensers, and health/fitness metric trackers. In general, caregivers who reported high levels of physical and financial strain reported more barriers to technology adoption. Those with lower levels of income and education also reported having more difficulties understanding or using technologies for caregiving. Male caregivers were more likely to report finding technologies to be not useful for caregiving. Caring for an individual with certain conditions such as memory or emotional/mental health problems, or developmental/intellectual disorders, was associated with adoption of fewer technologies. Lastly, living apart from one's care recipient had a mixed effect on caregivers' behaviors, being associated with higher levels of adoption for some technologies but lower levels for others.

Abstract citation ID: igaf122.1370

### HUMAN FIRST, MACHINE SECOND: PERSPECTIVES FROM THE AGE 85+ ON ARTIFICIAL INTELLIGENCE

Taylor Brennan[1], Niels Wu[2], Sophia Ashebir[2], and Lisa D'Ambrosio[2], *1. MIT, Boston College, Cambridge, Massachusetts, United States, 2. Massachusetts Institute of Technology, Cambridge, Massachusetts, United States*

Rapid advancements in the capabilities of artificial intelligence (AI) have sparked interest in its potential to support an aging population. Minimal work has examined how the oldest of older adults engage with and think about AI in their lives, despite this demographic being one that might especially benefit from AI technologies. Leveraging data from focus groups (N = 14) and a questionnaire (N=25), this study examines a sample of the age 85+ and their attitudes toward, experiences with, and trust in AI. Participants were recruited from the MIT AgeLab's 85+ Lifestyle Leaders Panel, a longitudinal study of octogenarians and nonagenarians. Initial findings indicate participants had low familiarity and experience with AI; greatest levels of familiarity were around AI-powered voice assistants. While participants expressed limited experience, many showed openness to learning about AI's potential to support health and care management, cognition and memory, technology troubleshooting, and personal security. Participants preferred AI that enhanced rather than replaced human capabilities. Their reported concerns about AI centered on its surveillance capabilities, data security, and potential to reinforce social biases, particularly ageism. These findings underscore opportunities to better educate the age 85+ about AI, the advantages of involving them in AI design, and the need to consider how AI may contribute to or bridge digital divides within this population.

Abstract citation ID: igaf122.1371

### OLDER ADULTS' PERSPECTIVES ON ARTIFICIAL INTELLIGENCE AS A SOURCE OF ADVICE

Lauren Cerino[1], Adam Felts[1], Alexa Balmuth[1], Taylor Brennan[2], Chaiwoo Lee[1], and Joseph Coughlin[3], *1. Massachusetts Institute of Technology, Cambridge, Massachusetts, United States, 2. MIT, Boston College, Cambridge, Massachusetts, United States, 3. Massachusetts Institute of Technology AgeLab, Cambridge, Massachusetts, United States*

A large proportion of Americans use generative AI tools to seek information and advice. Prior research suggests that older people are less likely than their younger counterparts to use generative AI (NORC, 2023), but older adults' attitudes toward AI technologies remain relatively obscure. Drawing on data from a national online survey study of American adults between ages 18 to 96 (N = 1008), this study examines older adults' experiences with AI and openness to receiving advice from AI-enabled tools. The survey included questions about experiences with AI and using AI-enabled chatbots for general use and advice. Preliminary findings indicate that most older adults had some level of familiarity with AI-enabled technologies, with nearly three-quarters (73.7%) of respondents aged 60 and older at least "a little" familiar. However, fewer (28.3%) in this age group reported having used AI-enabled technology. Older adults exhibited greater skepticism toward AI-generated advice than younger respondents; compared to younger age groups, fewer older respondents (36.0%) considered AI to be a valid source of advice, and 34.7% of older respondents indicated that they did not at all trust AI-enabled tools at all to give good advice. Mean trust scores also declined with age. These findings contribute to understanding about attitudes toward and adoption of AI across age groups. As generative AI becomes an increasingly common presence in everyday life, knowing how different generations engage with these tools is essential for understanding how they may be adopted, the extent of their disruptive potential, and the emergences of disparities of access.

### SESSION 3255 (SYMPOSIUM)

Abstract citation ID: igaf122.1372

### THE INTERSECTION OF EVALUATION AND GERIATRIC EDUCATION

Chair: Rhiannon Williams
Co-Chair: Christina Cauble
Discussant: Miranda Moore

The intersection of geriatric education and evaluation plays a critical role in shaping high-quality geriatric learning environments, ensuring that educational programs effectively prepare learners to advance the quality of care for older adults while strategically communicating their value to key stakeholders who can drive investment and prioritization. The number of older adults in the United States continues to grow, while many healthcare professionals are inadequately trained to provide care to this population. Through the use of cases, this session explores how evaluative thinking drives design, implementation, and outcomes of geriatric education programs. Unfortunately, evaluation is often just used to determine outcomes. Evaluative thinking and the use of a logic model provides a critical lens and framework for program development. First, we provide a brief overview of what a logic model is and how it can be used from inception of a learning program to the end. Next, we focus on a rubric tool development and use for geriatric education products/ outputs such as written work, presentations, or other projects. Then, we examine the assessment of short-term student learning outcomes, using a reflective tool as a means of both facilitating student learning as well as assessing their learning. Finally, we address how to create and share evaluation output or outcome data in a visual format that diverse stakeholders can utilize to ensure transparency, accountability, and collaboration. Attendees will leave with actionable insights on how to systematically evaluate educational efforts and drive meaningful improvements in geriatric education.

Abstract citation ID: igaf122.1373

### LOGIC MODEL DEVELOPMENT AND USE IN GERIATRIC EDUCATION PROGRAMS

Rhiannon Williams, *University of Minnesota, Minneapolis, Minnesota, United States*

This presentation will provide a brief overview of what a logic model is and how it can be used to support the development, implementation, and assessment of one's geriatric education program/ projects. A logic model is a picture or visual representation of how a program does its work – the theory and assumptions underlying the program, the activities it undertakes, and how those actions are thought to lead to desired results/outcomes. For those that design and use a logic model, it provides a shared understanding around an initiative/program, is flexible, and can be updated, as well as serve as a framework for evaluation: one can evaluate any of the logic model steps or the linkages between steps. The presentation will provide attendees with the components of a logic model, a description of each component; inputs/ resources, activities, products/outputs, short-term outcomes, and long-term outcomes, using the case of the interprofessional geriatric case competition. We will give tips on how to create and use one in the development and checking of logic of a program. Finally, the presentation will explain how subsequent presentations provide examples of evaluation at different points along the logic model. Attendees will have a clear understanding of the value of a logic model and how they could use one with their geriatric education program.

Abstract citation ID: igaf122.1374

### UNIVERSITY OF MINNESOTA: RUBRIC DEVELOPMENT FOR GERIATRIC EDUCATION PROGRAMMING

Christina Cauble, *University of Minnesota, Minneapolis, Minnesota, United States*

This presentation focuses on the development and use of a rubric to assess geriatric education products/ outputs such as written work, presentations, or other projects. This presentation focuses on a case example that was used to evaluate team performance in an interprofessional geriatric case competition. The rubric was created to assess key competencies such as teamwork, clinical reasoning, patient-centered care, interprofessional engagement, and application of geriatric principles. Many existing rubrics are generic, pre-designed tools with vague criteria that fail to provide meaningful guidance for students or accurately measure learning outcomes. This highlights the need for geriatric educators to apply evaluative thinking when designing programs and assessments to ensure clarity, relevance, and alignment with learning objectives. The session will cover best practices for rubric development, including ensuring reliability and validity, incorporating stakeholder feedback, and adapting assessment tools for different educational settings. Strategies for piloting, refining, and implementing rubrics effectively will also be discussed, along with common challenges and solutions. By the end of the session, participants will have a deeper understanding of how to create and apply a rubric into one's geriatric program. They will leave with actionable strategies for developing rubrics that foster transparency, drive continuous improvement, and support meaningful learning in geriatric education.

Abstract citation ID: igaf122.1375

### IMMERSION SHADOWING: NURSING STUDENT REFLECTIONS OF POST-ACUTE AND LONG-TERM CARE (PALTC)

Jinmyoung Cho[1], Susan Elliott[2], and Marla Berg-Wedger[3], *1. Saint Louis University, Saint Louis University, Missouri, United States, 2. Saint Louis University School of Medicine, St. Louis, Missouri, United States, 3. Saint Louis University, Saint louis, Missouri, United States*

This presentation focuses on the assessment of short-term student learning outcomes, using a reflective tool as a means of both facilitating student learning as well as assessing their learning in a geriatric nursing program. Great geriatric care requires a deep understanding of the complex physical, cognitive, and psychosocial needs of older adults. As the demand for well-trained professionals in post-acute and long-term care (PALTC) increases, effective educational strategies are essential. This presentation examines an experiential learning case where the use of reflective assessment was used to evaluate short-term student learning outcomes in geriatric nursing education. As part of an experiential learning program, 163 upper-level undergraduate nursing students shadowed staff in PALTC settings to gain first hand exposure to geriatric and dementia care. The program integrated two key learning components: (1) a structured session on Age-Friendly Health System (AFHS) concepts and the 4Ms framework, and (2) direct observation and clinical experience in a PALTC facility. To assess learning, students completed pre and post knowledge assessments and engaged in structured reflection to explore their evolving perceptions of geriatric care, interprofessional collaboration, and the realities of working in PALTC settings. This session will highlight how reflective tools can both facilitate student learning and serve as a critical assessment strategy within geriatric education. Attendees will gain practical insights into designing reflective assessments that capture meaningful learning outcomes and inform program development to better prepare future healthcare professionals for geriatric practice.

Abstract citation ID: igaf122.1376

### EFFECTIVELY SHARING DATA, DATA VISUALIZATION AND SHARING THE STORY

Frances Yang[1], Katie Krueger[2], Dan English[2], Bradley Newell[3], and Teri Kennedy[2], *1. University of Kansas, University of Kansas Medical Center, Kansas, United States, 2. University of Kansas Medical Center, Kansas City, Kansas, United States, 3. University of Kansas, Wichita, Kansas, United States*

This presentation focuses on the sharing of evaluation data in a visual format that diverse stakeholders can utilize to ensure transparency, accountability, and collaboration. Effective data sharing of outcomes is essential for advancing the quality and impact of geriatric education, ensuring transparency, accountability, and collaboration among diverse stakeholders. Through an illustrative case, this presentation highlights the Kansas 4M Geriatric Workforce Enhancement Program (GWEP) Dashboard as an example of optimizing data visualization to drive meaningful insights and improve program outcomes. The dashboard applies FAIR (Findable, Accessible, Interoperable and Reusable) principles to transform complex data into a meaningful story. Using a cloud-based portal, called mySidewalk.com, access to data is streamlined through a keyword-driven search engine, and integrates an AI-powered assistant to support dynamic dashboard creation. Interactive visualizations, including color-coded workforce maps and correlation matrices linking the "4Ms" of Age-Friendly Health Systems – What Matters, Medications, Mentation, and Mobility – enhance interoperability. Additionally, automated saving of searches, templates and reports fosters efficient knowledge sharing on an ongoing basis. By aligning evaluation efforts with strategic data visualization, this dashboard demonstrates how geriatric education initiatives can leverage data to communicate to diverse stakeholders in order to drive sustainability and prioritization of geriatric education, attendees will gain insights into how systematic data-sharing practices can enhance geriatric workforce development and drive investment.

### SESSION 3260 (SYMPOSIUM)

Abstract citation ID: igaf122.1377

### PRESIDENTIAL SYMPOSIUM: TRANSFORMING AGING THROUGH TECHNOLOGY: A PRACTICAL GUIDE FOR EARLY SCHOLARS

Chair: Sohyun Kim
Co-Chair: Katherine Britt

The intersection of technology and aging provides opportunities to enhance research, practice, education, and policy. As the global aging population grows, leveraging technology to improve health outcomes, quality of life, and system efficiencies has become increasingly vital. This symposium will explore cutting-edge, technology-driven approaches in aging fields, offering early scholars and professionals practical insights into integrating these innovations into their careers. Speakers will highlight emerging technologies such as artificial intelligence, sensing technology, AI-powered robots, intergenerational technology interventions, and person-centered interventions transforming aging research and practice. Additionally, the session will provide practical guidance on effectively implementing and evaluating technology-based solutions with careful consideration of the needs of older adults across diverse professional settings, including academia, clinical practice, policy development, and community engagement. This symposium aims to empower attendees with actionable strategies to incorporate technology into their work by bridging the gap between technological innovation and its real-world application. Participants will leave with a deeper understanding of how to harness technological advancements to drive progress in aging-related disciplines.

Abstract citation ID: igaf122.1378

### INTERGENERATIONAL TECHNOLOGY PROGRAMS IN HIGHER EDUCATION: FROM RESEARCH TO PRACTICE

Jill Juris, *Appalachian State University, Boone, North Carolina, United States*

This presentation will guide attendees through the presenter's early career journey of securing funding and developing a practical tool- the InterGenerational Technology Engagement Checklist for Higher Education (IGTECH). Designed for early career scholars and professionals, this session introduces an innovative approach to creating an intergenerational technology program in higher education. Emerging research highlights the benefits of intergenerational technology programs, which bring together multiple generations for collaborative technology learning. Intergenerational technology programs have been shown to reduce social isolation among older adults while also addressing their technology needs. At the same time, younger participants gain professional skills and develop more positive attitudes toward aging. Developed through an RRF-funded multi-stage qualitative study, the IGTECH serves as a guide for university representatives to establish programs that foster meaningful intergenerational relationships through shared technology learning experiences.

Abstract citation ID: igaf122.1379

### NAVIGATING INNOVATION RESEARCH: PRACTICAL STRATEGIES FOR EARLY-CAREER SCHOLARS

Lillian Hung, *University of British Columbia, Vancouver, British Columbia, Canada*

Integrating new technologies into long-term care (LTC) presents exciting opportunities and significant challenges. As the aging population grows, there is increasing interest in how artificial intelligence (AI)-enabled robots can support residents' well-being and improve care delivery. Drawing on my research with AI-powered social robots such as Aether, Paro, and Lovot, this presentation examines the practical considerations of adopting these innovations in LTC. Robotic technologies offer many benefits, including enhanced engagement, cognitive stimulation, companionship, and reduced loneliness. These tools may also help alleviate caregiver burden by assisting with repetitive tasks or providing entertainment and therapeutic interactions. However, implementation presents challenges. Ethical concerns—such as ensuring equitable access, avoiding substitution of essential human care, and preserving dignity and autonomy—must be carefully navigated. Practical barriers, including staff training, infrastructure adjustments, cost, and acceptance by residents and care providers, also affect successful adoption. This presentation highlights key lessons learned and offers strategies for conducting successful innovation research. Early-career scholars should seek diverse funding sources, secure pilot study grants, and align projects with practice needs. Networking within aging, technology, and healthcare communities fosters mentorship, partnerships, and knowledge exchange. Publishing in both academic and practitioner-oriented outlets bridges research and real-world application. By ensuring responsible implementation, AI-enabled robots can be used ethically and effectively to support resident well-being, promote person-centered care, and complement rather than replace human interactions in LTC settings.

Abstract citation ID: igaf122.1380

### INTERPLAY OF TECHNOLOGY AND APPLICATION OF GERONTOLOGIC BIOSTATISTICS AND DATA SCIENCE FOR CAREER IN AGING

Michelle Shardell[1], Joanna Lynn Borgogna[2], Fatemeh Adelnia[3], Chixiang Chen[1], Shuo Chen[1], and Jennifer Schrack[4], *1. University of Maryland School of Medicine, Baltimore, Maryland, United States, 2. Montana State University, Bozeman, Montana, United States, 3. Vanderbilt University Medical Center, Nashville, Tennessee, United States, 4. Johns Hopkins Bloomberg School of Public Health, Baltimore, Maryland, United States*

The term 'gerontologic biostatistics' was introduced in 2010 and recently updated to include data science to describe a collection of analytical methods that addresses the challenging features of research data in studies of older adults. Many of the advances in gerontologic biostatistics and data science (GBDS) that have occurred in the past ~15 years were motivated by new data modalities generated from recently developed technologies. As a result, the ability of early career scientists to leverage these data for innovative research depends on their understanding of the underlying technologies that produced these data. Herein, we will describe three such technologies, the data they generate, and examples of studies conducted by analyzing these data using GBDS methods. Specifically, we describe 16S rRNA amplicon sequencing technology that characterizes relative abundance of microbial taxa, accelerometers that provide objective measurements of physical activity, and magnetic resonance imaging-based multimodal neuroimaging that measures both brain structure and function. Specific examples include longitudinal cervicovaginal microbiota and reproductive aging among 750 women enrolled in the Human Papillomavirus in Perimenopause study, the role of physical activity in maintaining muscle oxidative capacity among 384 adults enrolled in the Baltimore Longitudinal Study of Aging, and age-related differences in whole-brain connectome networks among 39,675 adults enrolled in UK Biobank and 499 adults enrolled in the Lifespan Human Connectome Project in Aging study. These research studies exemplify advantages for scientists—at any career stage—to understand technological developments, the data they generate, and the application of GBDS for aging research.

Abstract citation ID: igaf122.1381

### SUPPORTING HEALTHY AGING IN THE ERA OF AI: PRACTICAL INSIGHTS FOR AGING AND TECHNOLOGY RESEARCH

Jane Chung, *Emory University, Atlanta, Georgia, United States*

As artificial intelligence (AI) and digital technologies rapidly evolve, there is a growing opportunity to leverage these innovations to support healthy aging in older adults. However, translating technological advancements into real-world solutions for aging populations requires careful consideration of accessibility, usability, digital literacy, and ethical implications. This panel presentation will share insights from the research on AI-driven and digital technology-based interventions designed to enhance functional assessment, social connectedness, and self-management. Also, the presentation will focus on developing equitable technology solutions and implementing them for sustained use to promote healthy, independent living among community-dwelling older adults including those experiencing health disparities. Drawing on extensive experience in engaging older adults in technology co-design, navigating interdisciplinary collaborations, and securing research funding, this presentation will provide practical guidance for early-career scholars and professionals interested in applying technology to community-based aging research. Key topics will include strategies for designing inclusive and effective digital interventions, overcoming barriers to technology adoption and use among older populations, and fostering meaningful engagement with communities of interest in AI technology development and implementation. By centering the conversation on both innovation and inclusivity, this discussion will equip early-career scholars with actionable strategies to leverage AI for aging research while addressing persistent health disparities.

Abstract citation ID: igaf122.1382

### TECHNOLOGY: A FRIEND OR FOE IN THE CONTEXT OF PERSON-CENTEREDNESS?

Sheryl Zimmerman, *University of North Carolina at Chapel Hill, Chapel Hill, North Carolina, United States*

As is true for all cohorts, technology holds promise to improve quality of care and quality of life for older adults, their families, and their care providers. At the same time, technology can negatively affect quality of care and life, such as by increasing burden on record-keeping with no demonstrable benefit to care and outcomes. More so, while literature is replete with studies about innovative technologies, it rarely examines them using a lens of person-centeredness, which emphasizes how technology serves an individual's interests, values, goals of care, and needs. This presentation will provide examples from clinical trials in long-term care illustrating how technology can be either agnostic or responsive to person-centeredness, and key issues identified through related discussion groups with experts in long-term care services and supports, technology design and evaluation for older persons, and aging-related health and function. Challenges identified from the discussion groups include that (1) technology can both facilitate and impede person-centeredness; (2) designing technologies in a person-centered manner requires participation of potential users in every stage of development; (3) ease of use and adoption of a new technology should be at the forefront in advanced design and implementation planning; and (4) implementation of new technologies should progress in a manner that reduces racial, geographic, and income-related disparities. Potential mitigating factors include involving users in design; considering return on investment and value-based measures; understanding the interaction between care providers and technology; and making technology more equitable. These topics will be discussed and audience perspectives invited.

### SESSION 3265 (SYMPOSIUM)

Abstract citation ID: igaf122.1383

### PERSPECTIVES ON FACILITATORS AND BARRIERS TO SUCCESSFUL AGING AMONG LATINOS IN THE UNITED STATES

Chair: Phillip Cantu
Co-Chair: Angela Gutierrez
Discussant: Elizabeth Vásquez

Latinos are the fastest-growing segment of the older adult population in the U.S. Latinos' reliance on informal care, well-documented barriers to health care access, and the continued immigration of older Latinos underscore the need for multifactorial, interdisciplinary approaches to address their unique aging trajectories. This symposium brings together perspectives from public health, sociology, occupational therapy, educational psychology, and health policy to examine psychosocial, caregiver, migration, and intervention factors contributing to healthy aging among Latinos. Researchers will highlight how health systems are innovating interventions to better serve Latinos, how caregiver-care recipient dyads' stress are shaped by health conditions, the role of neighborhoods and loneliness in shaping cognitive trajectories, and how migration factors influence health outcomes. Taken together, these presentations will highlight strategies to improve health in late life for older Latinos while also accounting for unique barriers that are associated with adverse health outcomes. Since 2001, the International Conference on Aging in the Americas (ICAA) has been a driving force in advancing research on the experiences of aging Latinos in the U.S. and Mexico. What began as a primarily Southwestern U.S.-based network has since expanded into a national collective of senior researchers and emerging scholars. With research led by ICAA emerging scholars and RCMAR scientists, this session showcases diverse methodological approaches—qualitative research, quantitative analysis, and interventions—across various regional contexts in the U.S. to provide a comprehensive perspective on the facilitators, barriers, and policy recommendations for healthy aging at multiple ecological levels.

Abstract citation ID: igaf122.1384

### NEIGHBORHOOD AND PSYCHOSOCIAL PREDICTORS OF COGNITIVE FUNCTION AMONG LATINOS IN THE UNITED STATES

Angela Gutierrez[1], Alejandra Marroig[2], Courtney Thomas Tobin[3], María Aranda[4], David Camacho[5], Roland Thorpe[6], Barış Sevi[7], and Graciela Muniz-Terrera[8], *1. Western University of Health Sciences, Pomona, California, United States, 2. Universidad de la Republica Uruguay, Montevideo, Montevideo, Uruguay, 3. University of California, Los Angeles Fielding School of Public Health, Los Angeles, California, United States, 4. University of Southern California, Los Angeles, California, United States, 5. University of Illinois Chicago, Chicago, Illinois, United States, 6. Johns Hopkins University, Baltimore, Maryland, United States, 7. MEF University, Maslak, Istanbul, Turkey, 8. Ohio University, Athens, Ohio, United States*

Prior research highlights the independent roles of neighborhood and psychosocial risk and protective factors for accelerated cognitive decline. However, the combined role of risk and protective neighborhood and psychosocial factors for cognitive aging among middle-aged and older Latinos in the U.S. remains unclear. Utilizing data from Latinos aged 50 and older ($n = 2,321$) in the Health and Retirement Study (2006 - 2016), this study aimed to examine the role of neighborhood (perceived physical disorder, neighborhood social cohesion) and psychosocial (loneliness) factors in shaping baseline cognitive function and change in cognitive function. Linear mixed models were used to estimate baseline and rate of change in cognitive function, adjusting for sociodemographic factors. Neighborhood social cohesion was positively associated with baseline cognitive function ($p < 0.001$), while perceived neighborhood social disorder and loneliness were both negatively associated with baseline cognitive function ($p < 0.001$). However, none of these factors were significantly associated with cognitive trajectories over time. These findings highlight the role of psychosocial and neighborhood risk and protective factors in baseline cognitive function among Latino adults. To promote healthy aging, health promotion efforts should consider implementing culturally relevant strategies to strengthen neighborhood social cohesion and reduce loneliness.

Abstract citation ID: igaf122.1385

### CULTURALLY ADAPTING THE IPROACTIF INTERVENTION FOR SPANISH-SPEAKING LATINX ADULTS WITH HYPERTENSION AND DIABETES

Dalmina Arias, and Mansha Mirza, *University of Illinois Chicago, Chicago, Illinois, United States*

Diabetes is highly prevalent in the U.S. Latino population, with adults over age 18 having nearly twice the prevalence of diabetes compared to non-Hispanic whites. Likewise, hypertension is an important comorbid condition with significantly higher prevalence in the Latino community compared to non-Hispanic whites. While several effective self-management interventions have been developed to improve health outcomes of older adults with diabetes, few have been culturally adapted to address the specific needs of older Latinos. This study adapts the Integrated PRimary care and Occupational therapy for Aging and Chronic disease Treatment for Independence and Functioning (IPROACTIF) intervention for Spanish-speaking Latino older adults with hypertension and diabetes using the Ecological Validity Framework. We interviewed 16 older Latinos residing in the Chicago Area who were living with diabetes and/or hypertension about their experiences managing their chronic conditions and the role of healthcare services. Stage two of this project will interview service providers about their experiences with older Latino populations. Through focus groups and thematic analysis, we identify barriers to health management and adapt the intervention's language, content, and context. Preliminary results have identified understanding of medication, insurance and continuity of care, and language as barriers to self-management of chronic conditions for older Latinos. We these barriers in mind, we work to adapt the IPROACTIF to patient experiences and a culturally tailored intervention. This work addresses health disparities by improving chronic disease management in Latino older adults.

Abstract citation ID: igaf122.1386

### CAREGIVING INTENSITY, STRESS, AND SOCIAL RELATIONSHIPS IN MEXICAN AMERICAN DEMENTIA AND NON-DEMENTIA CAREGIVERS

Suyoung Kim[1], Anna Bokun[2], Sunshine Rote[3], Flavia Andrade[4], Phillip Cantu[5], and Jacqueline Angel[6], *1. The University of Chicago, Chicago, Illinois, United States, 2. The University of Texas at Austin, Boston, Massachusetts, United States, 3. University of Louisville, Louisville, Kentucky, United States, 4. University of Illinois--Urbana-Champaign, Urbana, Illinois, United States, 5. UTMB Health, The University of Texas Medical Branch, Galveston, Texas, United States, 6. The University of Texas at Austin, Austin, Texas, United States*

Mexican American caregivers often face intensive caregiving demands, which may be further complicated or protected by social and family dynamics. This study examines the mediating roles of social relationships, including support, negative interactions, and familism, in the relationship between caregiving intensity and perceived stress. Using the stress process paradigm, we assess whether these factors increase or reduce caregiver perceived stress and explore differences between dementia and non-dementia caregivers. Data from the Hispanic Established Populations for the Epidemiologic Study of the Elderly (Wave 9, $N = 457$) were analyzed using Structural Equation Modeling. Social support, negative interaction, familism, and perceived stress were modeled as latent constructs. A multiple-group SEM approach assessed differences in caregiving paths between dementia and non-dementia caregivers. Caregiving intensity was negatively associated with social support and familism but positively linked to negative interactions. Social support significantly buffered the relationship between caregiving intensity and perceived stress, whereas negative interactions exacerbated it. Dementia caregivers reported a stronger association between caregiving intensity and perceived stress than non-dementia caregivers. Social support helped reduce stress more for non-dementia caregivers, while negative interactions had a greater impact on dementia caregivers. High-intensity caregiving is associated with increased stress, particularly among dementia caregivers in Mexican American communities. Social relationships play a complex role, both alleviating and exacerbating caregiver burden. These findings underscore the need for interventions that strengthen support networks, reduce negative family dynamics, and address the unique challenges facing dementia caregivers, ultimately improving caregiver well-being and care sustainability.

Abstract citation ID: igaf122.1387

**MECHANISMS OF LATINO MIGRANT RETENTION: LIVED EXPERIENCES FROM THE THIRD AGE AND IMPLICATIONS FOR HEALTH**

Sarah Salas[1], Michal Engelman[2], and Johanna Nunez[1], *1. University of Wisconsin–Madison, Madison, Wisconsin, United States, 2. University of Wisconsin-Madison, Madison, Wisconsin, United States*

Recent decades have seen significant growth in immigrant populations in non-traditional U.S destinations. Latinos are Wisconsin's largest minority group at 8% of the population; immigrants comprise 27% of this group. As most research on immigrant acculturation focuses on those in early and midlife, the experiences of older Latino immigrants remain unclear. We conducted semi-structured life history interviews with 33 Latino immigrants aged 60 years or older living in Wisconsin. Our analysis examines how immigrants' varied backgrounds shape their migration experiences, and how they relate to their health outcomes in later life. We draw on theories of immigrant identity formation and cultural ties across countries to examine Latinos' lived experiences and migration histories. We find that reasons for migration (including loose social ties, family reunification, and health concerns) interplay with immigrants' attitudes towards living in the U.S. to influence one's retention pathway. Transnational older adults have mixed dispositions about being in the U.S. and can be on either constrained or classic retention pathways. While constrained retention involves external motivators for staying in the U.S. and largely includes those who wish to return to their home country, classic retention involves more aspects of traditional assimilation and acculturation. The heterogeneity in retention factors differentially influences immigrants' sense of belonging as they continue to live and age in Wisconsin, either accepting or rejecting the assimilation process. Our findings underscore the fluidity of transnational identities and how sense of social cohesion and perceived well-being shape older Latino immigrant health behaviors, resulting in disparate health outcomes.

### SESSION 3267 (BIOLOGICAL SCIENCES INVITED SYMPOSIUM)

Abstract citation ID: igaf122.1388

**AGING REPROGRAMMED: NUTRITION, METABOLISM, AND HEALTH**

Chair: Rozalyn Anderson

Abstract citation ID: igaf122.1389

**FASTING MIMICKING DIET CYCLES, REGENERATION, BIOLOGICAL AGE, AND DISEASE**

Valter Longo, *USC Leonard Davis School of Gerontology, Los Angeles, California, United States*

Fasting mimicking diets (FMDs) are low calorie and protein and high fat compositions lasting 4-7 days emerging as periodic dietary interventions with the potential to improve healthspan and decrease the incidence of age-related diseases. In animal studies, FMD cycles promote stem cell activation, cellular reprogramming and autophagy leading to longevity extension but also to the regression of multiple diseases and conditions. Recent randomized clinical studies consistently indicate that FMD cycles reverse insulin resistance and promote pre-diabetes and diabetes regression and result in lower diabetes and hypertension drug use by a mechanisms not correlated with weight loss. The latest studies indicate that FMD cycles can improve clinical response to autoimmune disease drugs and reverse chemosensory dysfunction raising the possibility that the multi-system regenerative effects demonstrated in mice and rats are conserved in humans, as also supported by a 2.5 years of biological age reduction associated with 3 FMD cycles in 2 different clinical trials.

Abstract citation ID: igaf122.1390

**METABOLIC AND MICROBIAL MASTERY: UNVEILING THE SECRETS OF NAKED MOLE-RAT LONGEVITY**

Shelley Buffenstein, *University of Illinois, Chicago, Chicago, Illinois, United States*

Naked mole-rats (Heterocephalus glaber) stand out among mammals for their extraordinary longevity, showing no increase in mortality risk with age and maintaining robust physiological and molecular stability throughout their 40-year lifespan. These subterranean herbivorous rodents have evolved remarkable adaptations for thriving in low-oxygen environments, including the ability to switch from glucose to fructose metabolism, supporting sustained anaerobic activity. Elevated glycogen stores provide a vital energy reserve for anaerobic ATP production, while increased glutathione levels offer a strong defense against oxidative stress, contributing to their exceptional health span and cancer resistance. Metabolomic analyses reveal significant distinctions between naked mole-rats and laboratory mice, notably in their lower plasma levels of methionine pathway amino acids, akin to longevity-associated states such as calorie restriction. Additionally, they exhibit enriched lipid sub-pathways related to membrane composition and sterols, enhancing biological resilience. Elevated concentrations of anti-aging metabolites like α-tocopherol and N8-acetylspermidine further underscore their unique metabolic profile. Unlike mice, naked mole-rats' gut microbiome remains stable with age, showing minimal changes in microbial populations, suggesting a robust microbiome beneficial for sustained good health. The one exception is the Archaea, Methanomassiliicoccus intestinalis, which is absent in the mouse microbiome, and increases in abundance in aging mole-rats. This comprehensive metabolomic and microbiome signature reflects evolutionary adaptations surpassing typical age-related changes, offering valuable insights into their longevity and disease resistance, with potential implications for human health interventions.

Abstract citation ID: igaf122.1391

### PILOTING NOVEL NUTRITIONAL INTERVENTIONS FOR MECHANISTIC STUDIES OF HUMAN AGING

Corby Martin[1], and Leanne Redman[2], *1. Pennington Biomedical Research Center, 2. Pennington Biomedical Research Center, Baton Rouge, Louisiana, United States*

Calorie restriction (CR) and time-restricted eating (TRE) have demonstrated efficacy for improving healthspan and biological age, yet long-term adherence remains problematic. Adaptive interventions may promote adherence in longer trials. This 24-week, parallel-arm, randomized controlled pilot trial compared adaptive and traditional versions of TRE and CR against a no-intervention control. Seventy (n = 70) healthy adults with body mass index 22.0-29.9 kg/m2 were randomized to one of five groups: control, traditional TRE with an 8-hour window, adaptive TRE, traditional CR targeting 25% energy restriction, or adaptive CR. The study aimed to examine intervention feasibility and effect sizes for primary aging and healthspan. Participants were 38±3 y (Mean+SD), 77% female, with mean BMI 26.0±2.6 kg/m2. The CR arms achieved 48% (traditional) and 77.6% (adaptive) of the CR target. TRE participants adhered to the 8-hour eating window on 74.5% (traditional) and 40.1% (adaptive) of days, respectively (86% adherence reflected the goal of following TRE 6 days per week). Weight change compared to control was greatest in adaptive CR (-8.8 kg, P < 0.001) followed by traditional CR (-5.8 kg, P < 0.001), traditional TRE (-2.3 kg, P = 0.05) and adaptive TRE (-1.4 kg, P = 0.15). Compared to control, biological age (Klemera-Doubal) effect sizes were modest over 24-weeks, with adaptive CR having the largest change (-1.8 y, P = 0.10; Cohen's d = 0.24). Compared to control, healthspan (Metabolic Syndrome Score) improved in the CR arms (traditional -2.2, P < 0.01; adaptive -1.8, P = 0.02) but not in the TRE arms (traditional -0.6, P = 0.26; adaptive -0.7, P = 0.21). Intervention feasibility was highest in the adaptive groups.

Abstract citation ID: igaf122.1392

### THE METABOLOME AS A BIOMARKER OF HEALTH AND AGING IN COMPANION DOGS

Daniel Promislow, *Jean Mayer USDA Human Nutrition Research Center on Aging, Boston, Massachusetts, United States*

The companion dog is an exceptional translational model for understanding the causes and consequences of aging. Among breeds, we observe high levels of variation not only in morphology and behavior, but also in lifespan and the risk of specific diseases, many of which are common in humans. Dogs share our environment and, like us, have a sophisticated healthcare system. Their shorter lifespan relative to humans means that lessons that might take decades to learn from human longitudinal studies can be gleaned in just a few years by observing aging in dogs. Here, we share recent findings from the Dog Aging Project, a long-term longitudinal study designed to identify the genetic and environmental factors that influence healthy aging in dogs and the mechanisms by which they do so. As part of the Dog Aging Project, we collect annual measures of the plasma metabolome for nearly 1,000 dogs from across the United States. Age-related changes in the metabolome point to underlying mechanisms that might account for breed variation in lifespan. We have also identified individual metabolites that predict mortality risk and found that the sign and magnitude of their effects are significantly correlated with patterns observed in comparable human studies. Finally, we discuss how metabolome profiles can serve as a bridge linking upstream genetic and environmental factors with downstream traits related to aging. As such, metabolome profiles provide a more complete picture of the causes and consequences of aging and related disease.

### SESSION 3270 (SYMPOSIUM)

Abstract citation ID: igaf122.1393

### DYADIC APPROACHES TO THE STUDY OF ADULT DEVELOPMENT AND AGING: EMOTION REGULATION, ATTITUDES, AND WELL-BEING

Chair: Gloria Luong

Dyadic approaches to the study of aging provide unique opportunities to examine interpersonal processes that may shape psychological development. These four presentations include complementary methodological approaches to some of the latest advances in dyadic processes and adult psychological development. First, English and colleagues investigated interpersonal emotion regulation (IER), whereby regulators attempt to influence the emotions of a target person across two studies including daily diary and lab methods. Greater IER was associated with better emotional and relational well-being, and such effects were more pronounced for older people. Next, Luong examined how married heterosexual couples navigate the transition into older adult housing facilities together. Husbands tended to show greater positive affect and lower depressive symptoms than wives, but they also exhibited lower empathic perspective taking across the 3-5 year longitudinal study. Using three large international panel study datasets, Yang and colleagues demonstrate that romantic couples with different political orientations tend to show a convergence effect over time, such that their political views become closer aligned with their partner's views. Partners who changed their views and/or showed more moderate political views to begin with, were more likely to have partners who changed their views in line with theirs. Finally, Chopik and colleagues used novel methodological techniques to understand how couples "match" on their love languages, and the implications that matching may have on relationship success. These talks highlight advantages to dyadic approaches to studying aging for both individuals and couples. Dyadic Health Research Interest Group Sponsored Symposium

Abstract citation ID: igaf122.1394

### DYADIC ADAPTATION TO MOVING INTO A SENIOR HOUSING FACILITY: CHANGES IN PSYCHOLOGICAL WELL-BEING

Gloria Luong, *Colorado State University, Fort Collins, Colorado, United States*

For older adults, moving to a new housing facility (e.g., independent living, assisted living) has been linked to poorer well-being, including greater negative affect, loneliness, and depressive symptoms. Much of this work has focused on individuals, with less research examining how romantic partners navigate this major life event together. In the current study, we investigated how married heterosexual couples (N = 41 couples, 82 individuals) adapted to the transition into housing facilities for older adults. Participants ranged in age from 40-95 years old. Using a prospective longitudinal measurement burst design spanning approximately 3-5 years, participants in the Relocation and Transitional Experiences (RELATE) study completed ecological momentary assessment

(EMA) surveys over 8 consecutive days, questionnaires, and health and cognitive assessments at each of 5 bursts. We found that over the 5 bursts, husbands tended to show greater trait positive affect and fewer depressive symptoms than wives. Moreover, husbands showed decreased empathy (perspective taking) over time, compared to wives. Implications for dyadic approaches to developmental transitions in later life are discussed.

Abstract citation ID: igaf122.1395

### LOST IN TRANSLATION: MATCHING ON LOVE LANGUAGES EXPLAINS LITTLE VARIATION IN RELATIONAL OUTCOMES IN MID AND LATE LIFE

William Chopik[1], Louis Hickman[2], Rebekka Weidmann[3], Mariah Purol[4], and Hyewon Yang[1], *1. Michigan State University, East Lansing, Michigan, United States, 2. Virginia Tech, Blacksburg, Virginia, United States, 3. Brigham Young University, Provo, Utah, United States, 4. Union College, Schenectady, New York, United States*

Love languages—and the degree to which people “match” on them—have captivated lay audiences searching for explanations for why some relationships do or do not work out. Previous work formally testing whether matching on love languages translates to better relationship success has been relatively rare, provided mixed results, and has tested matching in non-optimal ways. In the current study, we examined whether methodological variation in matching procedures (e.g., difference scores, response surface analysis, and machine learning) can help characterize whether love languages have implications for relationship success in 954 male/female couples (Mage = 55.42, SD = 16.07; MRelationshipLength = 27.39 years). Across all these approaches and all possible love language pairings (via machine learning methods), we found virtually no evidence that matching on love languages explained additional variation in outcomes above and beyond individual and spousal personality traits and attachment orientations. Further, expression (highest r = .795) and preferences (highest r = .774) between love languages were occasionally and problematically, highly correlated. Based on the results of the current study, it is worth revisiting whether love languages tap into distinct relationship preferences and behaviors or if they reflect broader responsiveness or positive relationship behaviors regardless of matching. The presentation will conclude with a critical discussion of the personal and relational characteristics most likely to lead to resilience in the context of close relationships.

Abstract citation ID: igaf122.1396

### INTERPERSONAL EMOTION REGULATION EFFORT IN ROMANTIC COUPLES ACROSS ADULTHOOD

Tammy English[1], Claire Growney[2], Jocelyn Lai[1], and Junyuan Luo[1], *1. Washington University in St. Louis, St. Louis, Missouri, United States, 2. Stanford University, Stanford, California, United States*

Extrinsic interpersonal emotion regulation (IER) refers to when an individual (the regulator) attempts to regulate the emotions of another person (the target). Outcomes of IER are theorized to depend on both the strategy one deploys and the regulator’s motivational strength or effort put into IER. Greater effort is expected to lead to greater success in helping the target resolve the stressor and may strengthen the regulator-target relationship. However, there may be personal costs to the regulator due to the demands of IER. Given age-related shifts in motivational priorities and accumulated life experience, we expected IER effort to be more effective and less costly for older adults. To test these ideas, we recruited romantic couples (Study 1 = 134, Study 2 = 121) across the adult lifespan (age 23-92) to complete 9 days of daily diaries and engage in an emotional conversation in the lab. Using actor-partner interdependence modeling, we found that greater IER effort was generally associated with more positive emotional and relational outcomes in both partners. However, IER effort was also linked to greater negative emotion in the regulator, especially when this support was under-acknowledged. IER was more strongly linked to wellbeing outcomes for relatively older targets. Results contribute to a deeper understanding of the motivational aspect of emotion regulation across adulthood. Putting in greater effort to manage the emotions of a close partner may have mixed consequences for both younger and older adults.

Abstract citation ID: igaf122.1397

### A HOUSE DIVIDED: INTERPOLITICAL COUPLES ACROSS ADULTHOOD AND THEIR CO-CHANGE IN POLITICAL ORIENTATIONS

Hyewon Yang[1], Gabriel Olaru[2], Rebekka Weidmann[3], Mark Brandt[1], Shree Vallabha[1], and Abigail Cassario[1], *1. Michigan State University, East Lansing, Michigan, United States, 2. Tilburg University, Tilburg, Noord-Brabant, Netherlands, 3. Brigham Young University, Provo, Utah, United States*

Many studies demonstrate assortative mating in political orientation in romantic relationships, but the long-term relationship dynamics of politically heterogeneous couples remain underexplored. To better understand how partners with different political standings change over time, we examined interpolitical couple members’ political orientation trajectories and their co-change over 14 to 24 years using three large panel data from the Netherlands, Switzerland, and Germany (Ninterpolitical couples = 3,980; age range 14-93). Growth curve models showed that the dissimilarity between partners decreased over time. Specifically, the relatively left-wing individuals within each couple generally moved right, whereas the relatively right-wing partners moved left (i.e., a convergence effect). Moreover, the less politically extreme their partners started off, the quicker the individuals converged. Lastly, partners’ rates of change correlated with each other—if one partner changed over time, so did the other. These findings highlight the dynamic nature of political development in adulthood and later life, shedding light on the role of the partner’s political dissimilarity in shaping individuals’ political orientations over time. By placing political attitude change within a lifespan and dyadic framework, this study adds to a larger knowledge of socio-political development in aging populations.

### SESSION 3275 (SYMPOSIUM)

Abstract citation ID: igaf122.1398

### AGING AND DECISION MAKING: NEW INSIGHTS REGARDING INFORMATION EVALUATION AND MANAGEMENT

Chair: Julia Nolte
Co-Chair: Corinna Löckenhoff
Discussant: JoNell Strough

Age-related cognitive shifts in information processing and memory capacities are associated with more frugal information management: Older adults engage with some rather than all available information before making a decision. At the same time, motivational shifts prompt older adults to selectively engage with information that is personally meaningful or emotionally salient. The individual talks in this symposium document how these phenomena manifest themselves across a range of decision-related contexts. With respect to personal meaningfulness, Nolte, Shavit, and Ng demonstrate that older adults express a stronger preference for information or options that reaffirm personally held beliefs than is the case for younger age groups. Wild, Lachs, and Löckenhoff show that by comparison, older adults are more likely to prepare for healthcare decisions by developing written agendas of concerns and questions that are personally meaningful to them. With respect to emotional salience, Liao, Lu, Ma, Guo, Zhou, Sun, Li, and Zhang document that older adults process age-related stereotypes differently depending on valence: When exposed to positive rather than negative age-related stereotypes, older adults expect to encounter lower levels of risk within financial decisions. By contrast, Chen, Toh, Shavit, and Carstensen observe that older adults' financial decisions are susceptible to negatively valenced information: Compared to middle-aged adults, older adults make larger donations when faced with the negative consequences of their failure to donate (versus the positive consequences of donating). To integrate insights across these studies and identify directions for future research, Strough will contextualize the presentations against the extant aging and decision-making literature. Judgement and Decision Making Interest Group Sponsored Symposium

Abstract citation ID: igaf122.1399

### OLDER AGE IS ASSOCIATED WITH CONFIRMATION BIAS IN CLIMATE-RELATED DECISION MAKING

Julia Nolte[1], Yochai Shavit[2], and Wee Qin Ng[3], *1. Tilburg University, Tilburg, Noord-Brabant, Netherlands, 2. Stanford University, Stanford, California, United States, 3. Nanyang Technological University, Singapore, Singapore*

Prior findings suggest that older adults are more likely to underuse and avoid negative or upsetting information. Yet, the extent to which they demonstrate higher levels of "confirmation bias"- the preference to engage with or assign more meaning to belief-consistent information and options - is still unclear. An online experiment examined the above research gap in the context of climate change, a divisive topic among older adults. A US adult lifespan sample (N = 206, 19–88 years, M = 51.20, SD = 17.77, 52% women, 28% Non-Hispanic White) engaged with information about three pro- and three anti-environmental fictional candidates running for a US office. Individual differences in confirmation bias were indexed as: (1) searching for more belief-consistent than -inconsistent information, (2) ranking candidates with belief-consistent campaigns more highly, and (3) perceiving belief-consistent information as more persuasive. In addition, participants responded to assessments of their sociodemographic background, goals, physical and mental health, affect, personality, cognition, and knowledge about climate change. In pre-registered analyses, we found evidence for confirmation bias in all three bias indices, which was positively associated with older age in the contexts of information search ($p = .018$) and candidate rankings ($p = .016$). Of all covariates considered, only older adults' relatively better mental health accounted for older adults' more biased information search behavior (but not their ranking decisions). Together, findings suggest that older adults may be more susceptible to disregarding belief-inconsistent information or options when making climate change-related decisions, and that this bias may serve emotional well-being goals.

Abstract citation ID: igaf122.1400

### AGE DIFFERENCES IN THE USE OF WRITTEN MATERIALS DURING HEALTHCARE VISITS

Tess Wild[1], Mark Lachs[2], and Corinna Löckenhoff[1],
*1. Cornell University, Ithaca, New York, United States,*
*2. Weill Cornell Medicine, New York, New York, United States*

Prior to healthcare choices, people often search for information online, compile self-collected health data, or write down questions and priorities. Such patient-generated lists and agendas have potential benefits for streamlining healthcare decisions but may also derail patient-physician communication by introducing extraneous detail. It remains unclear whether older adults are more likely than younger adults to prepare written materials, as they are generally less likely to engage in pre-decisional information-seeking, but more likely to use external memory aids, and more likely to manage complex health conditions that would benefit from written documentation. To address this question, an adult lifespan sample ($n = 457$; Mage = 51.50; SDage = 19.00, aged 18-99 years) completed a pre-registered online survey assessing demographics, personality, health status, health-related attitudes, the timing of their most recent healthcare visit, and whether they brought written material to the visit. Logistic regressions revealed that those who brought written materials were significantly older ($p < .01$) and more likely to be male ($p < .05$). They also scored higher on neuroticism and reported more recent healthcare visits, a higher importance of being informed about their health, but a lower understanding of their health condition (all $ps<.05$). None of the other covariates showed significant associations with using written materials. Further research is needed to examine potential implications of older adults' use of written materials for healthcare outcomes and patient satisfaction.

Abstract citation ID: igaf122.1401

### THE IMPACT OF AGE-RELATED STEREOTYPES ON RISKY DECISION-MAKING IN THE BALLOON ANALOGUE RISK TASK

Shuyao Liao[1], Yi Lu[2], Gu Ma[1], Zheng Guo[1], Yatian Zhou[3], Yue Yang Sun[3], Tianyuan Li[3], and Xin Zhang[1], *1. Peking University, Beijing, Beijing, China (People's Republic),*
*2. Cornell University, Ithaca, New York, United States,*
*3. The Chinese University of Hong Kong, Shenzhen, Shenzhen, Guangdong, China (People's Republic)*

Previous research has suggested that older adults are more loss-averse in risky decisions, and their performance can be susceptible to age-related stereotype threats. However, prior findings have predominantly focused on negative stereotypes, and the underlying cognitive mechanisms remain unclear. This study expands the literature by examining the impact of both positive and negative age-related stereotypes and its cognitive underpinnings in the Balloon Analogue Risk Task (BART), a well-established measure of experience-based risky decision-making. We randomly assigned 159 older adults (M = 67.09, SD = 5.22) to receive information containing either positive or negative age-related stereotypes, or the same neutral information as 53 younger adults (M = 20.55, SD = 2.04) received. Participants completed the BART by pumping virtual balloons.

Computational models were applied to decompose the underlying cognitive dimensions influencing decision behaviors. Consistent with prior findings, modeling results revealed that compared to younger adults, older adults exhibited lower learning rates and greater loss aversion. Contrary to our expectations, further analyses suggested that age-related stereotypes impacted primarily on the prior belief of risk. Older adults exposed to positive stereotypes held lower prior beliefs of risk compared to their counterparts receiving negative stereotypes, while there was no significant priming effect of age-related stereotypes on loss aversion. Our findings elucidate age differences in the cognitive underpinnings of risky decisions in the BART and underscore that age-related stereotypes impact older adults' decision performance by distinctively influencing their prior beliefs about risk instead of their risk or loss preferences.

Abstract citation ID: igaf122.1402

### AGE DIFFERENCES IN THE EFFECT OF FRAMING ON PROSOCIAL GIVING DECISIONS

Enna Chen[1], Wei Xing Toh[2], Yochai Shavit[1], and Laura Carstensen[1], *1. Stanford University, Stanford, California, United States, 2. Nanyang Technological University, Singapore, Singapore*

According to the Federal Reserve, Americans ages 55 and over hold an increasing percentage of US wealth, reaching 73.2% in 2024 compared with 55.8% in 2000. Promoting charitable giving across the adult life span, therefore, presents significant opportunities to improve communities. Although older adults are more prosocial than younger adults, consistent with Socioemotional Selectivity Theory, they also give more selectively to familiar causes and recipients, challenging efforts to increase charitable giving. Based on findings of age differences in the framing effect on health decision making, the present study examines the effect of positive and negative framing on older and younger adults' prosocial giving decisions. Participants (N=293) aged 40 to 86 were randomly assigned to one of three conditions: positive framing (vignette emphasizing beneficial outcomes of donations), negative framing (vignette emphasizing adverse consequences of donor inaction), and a neutral condition (no vignette). Participants then allocated a hypothetical windfall between education funds for younger relatives and under-resourced non-kin. Age was positively associated with giving to non-kin. Although there was no main effect of framing, a significant interaction effect between age and framing indicates that negative framing is more persuasive for older adults than middle-aged adults. Furthermore, older adults rated negative framing more positively and less negatively than middle-aged adults. Findings suggest that older adults may perceive the negative information less as a source of distress and more as an opportunity to assist. Understanding these differences may inform strategies to encourage prosocial behavior across different age groups.

### SESSION 3280 (SYMPOSIUM)

Abstract citation ID: igaf122.1403

### PRIVATE EQUITY ACQUISITIONS OF ASSISTED LIVING

Chair: Kali Thomas
Co-Chair: Momotazur Rahman
Discussant: Sheryl Zimmerman

Private equity (PE) is a growing modality of investment in healthcare and affecting various populations, including older adults. However, within the long-term care sector, previous studies only focus on PE's impact on nursing homes. To meet the unmet gap in the literature, this panel assembles five presenters who will discuss their research to identify and understand PE's activities in assisted living (ALs) - the largest residential provider of long-term care in the US. The first presenter will share findings from a systematic review describing the measurement of PE ownership of healthcare and associated potential measurement issues. In order to identify PE ownership in ALs, the second presenter will share a novel approach to tease out complex corporate structures of ALs -- an essential step to measure PE's ownership. Because PE acquisitions mostly focus on chain-affiliated providers and yet AL chain information is not available, the third presenter identifies and compares the corporate chain ownership of AL communities. Using the innovations described by the second and third presenters, the fourth panelist shares findings from identifying PE ownership in ALs and outlines the growing trends over the past decade. The fifth presenter provides valuable, qualitative information from extensive in-depth semi-structured interviews with PE and AL stakeholders to provide a comprehensive understanding of PE involvement in AL. The discussant is the director of the Center for Excellence in Assisted Living at UNC and will contextualize these panelists' findings in the current research and regulatory landscape and discuss implications for resident care. This is a collaborative symposium between the Assisted Living and Research in Quality of Care Interest Groups. Assisted Living Interest Group Sponsored Symposium

Abstract citation ID: igaf122.1404

### MEASUREMENT OF PRIVATE EQUITY OWNERSHIP IN HEALTHCARE RESEARCH: REVIEW AND OPPORTUNITIES

Sean Huang[1], Jennifer Bunker[2], Cassandra Hua[3], Yashaswini Singh[4], Momotazur Rahman[5], and Kali Thomas[2], *1. Georgetown University, Washington, District of Columbia, United States, 2. Johns Hopkins University, Baltimore, Maryland, United States, 3. University of Massachusetts Lowell, Lowell, Massachusetts, United States, 4. Brown University School of Public Health, Providence, Rhode Island, United States, 5. Brown University, Providence, Rhode Island, United States*

Because ownership reporting requirements vary by healthcare entity types, PE ownership may not be publicly reported and researchers must rely on varying sources to measure PE ownership. We aim to understand measurement considerations relevant for examining PE in health care, including how researchers operationalize PE ownership of healthcare organizations, and to what extent the heterogeneity of private equity transaction (e.g., by boutique vs. general PE firms, different deal, or amount of debt financing) is studied in the literature. We systematically reviewed quantitative studies focused on PE and healthcare providers in the US published in years 2008-2023. We find that proprietary commercial databases and news/web searches are the two major approaches researchers used to identify private equity transitions. About 48% of the papers rely on commercial databases, and 14% of the papers use news releases and web searches to identify PE transitions. Additional 39% use a combination of commercial databases and news searches to construct their samples. Most papers often rely on a binary indicator of PE ownership, but do not study the heterogeneity effects of PE transitions. Overall, we find researchers increasingly rely on multiple commercial datasets to identify private equity transitions. However, most of the literature doesn't discuss the potential discrepancy of PE definitions among data vendors. A more transparent and uniform database of PE and non-PE transactions, can facilitate studies on understanding nuance and heterogeneity that can facilitate a better design and regulation of PE transitions for all sectors of healthcare, including assisted living.

Abstract citation ID: igaf122.1405

### IDENTIFYING CORPORATE OWNERSHIP IN ASSISTED LIVING: LINKING COMMUNITIES TO PARENT COMPANIES

Lindsey Smith[1], John Bowblis[2], Momotazur Rahman[3], Kali Thomas[4], Gauri Gadkari[3], Cassandra Hua[5], Yashaswini Singh[6], and Leya Kurzhiparambil[5], *1. Oregon Health & Science University, Portland, Oregon, United States, 2. Miami University, Oxford, Ohio, United States, 3. Brown University, Providence, Rhode Island, United States, 4. Johns Hopkins University, Baltimore, Maryland, United States, 5. University of Massachusetts Lowell, Lowell, Massachusetts, United States, 6. Brown University School of Public Health, Providence, Rhode Island, United States*

Corporate ownership in assisted living (AL), including private equity investment, has drawn increasing attention due to potential implications for care quality, financial practices, and resident outcomes. However, ownership data for AL communities is complex and fragmented, as different companies often own the property, manage the AL, and operate the AL. This makes it difficult to study how corporate structures shape operations. We developed and validated a method for systematically linking AL communities to corporate owners, operators, and managers–resulting in the first database linking AL communities to corporate ownership structures. We compiled a dataset of 14,866 AL communities (25+ units) from 2023 state licensing data and merged it with business records from Data Axle (n = 181,797), the National Investment Center for Senior Housing (n = 20,805), and corporate hierarchies from Duns & Bradstreet (n = 19,955 businesses linked to 307 parent companies). Using natural language processing (TF-IDF, Levenshtein distance, Jaccard index) and geospatial methods (geocoding, spatial joins) we matched 97% of AL communities to business characteristics and 86% to corporate ownership. A manual review of a random 10% sample confirmed 98% match accuracy. This approach provides a systematic way to study private equity and other ownership trends in AL, improving data reliability and reproducibility. Our findings highlight the need for transparency in ownership data and offer groundwork for evaluating how financial structures affect AL communities on a national scale. These approaches may be useful for studying ownership in other healthcare settings and for policymakers considering approaches to oversight and regulation.

Abstract citation ID: igaf122.1406

### CHARACTERISTICS AND GEOGRAPHIC VARIABILITY OF UNITED STATES ASSISTED LIVING COMMUNITIES BY CHAIN STATUS

Cassandra Hua[1], Leya Kurzhiparambil[1], Lindsey Smith[2], Gauri Gadkari[3], John Bowblis[4], Momotazur Rahman[3], and Kali Thomas[5], *1. University of Massachusetts Lowell, Lowell, Massachusetts, United States, 2. Oregon Health & Science University, Portland, Oregon, United States, 3. Brown University, Providence, Rhode Island, United States, 4. Miami University, Oxford, Ohio, United States, 5. Johns Hopkins University, Baltimore, Maryland, United States*

Previous studies indicate chains operate nursing homes with wealthier, higher-acuity residents, though state policies like Medicaid generosity create variation. We analyzed the distribution and characteristics of chain-affiliated versus non-chain ALs. We connected AL chain status (2021 data from National Investment Center, Argentum, and Dun & Bradstreet) to community characteristics (American Community Survey) and AL resident characteristics (Medicare enrollment and claims). In state-level analysis, we compared the number of chain vs. nonchain ALs per 100,000 adults aged 65+ using a scatterplot and Pearson's r. In AL-level analysis, we compared characteristics by chain status using t-tests and chi-squared tests. Our sample included ALs with a capacity of 25+ beds with at least 10 fee-for-service Medicare beneficiaries, including 6,409 ALs across the 48 contiguous U.S. states. We found a modest correlation between the number of chain and nonchain AL beds per 100,000 older adults in each state ($r = 0.29$, $p = 0.047$). Some states, including Utah, Indiana, and Idaho, were "chain dominant." Utah, for example, had 1,480 chain operated AL beds per 100,000 older adults compared to just 16 nonchain beds. Residents in chain-owned ALs were more likely to have dementia than those in nonchain ALs (36% vs. 30%, $p < 0.001$). Significantly more residents in nonchain ALs were dually eligible for Medicare and Medicaid (40% vs. 21%, $p < 0.001$). County characteristics (e.g., median income) did not differ significantly by chain status. Findings suggest that, similar to nursing homes, chains may serve residents with greater care needs and less financial disadvantage.

Abstract citation ID: igaf122.1407

### TRENDS IN REPORTED ASSISTED LIVING FACILITY ACQUISITIONS BY PRIVATE EQUITY FIRMS FROM 2006-2023

Jennifer Bunker[1], Gauri Gadkari[2], Yashaswini Singh[3], John Bowblis[4], Sean Huang[5], Lindsey Smith[6], Momotazur Rahman[2], and Kali Thomas[1], *1. Johns Hopkins University, Baltimore, Maryland, United States, 2. Brown University, Providence, Rhode Island, United States, 3. Brown University School of Public Health, Providence, Rhode Island, United States, 4. Miami University, Oxford, Ohio, United States, 5. Georgetown University, Washington, District of Columbia, United States, 6. Oregon Health & Science University, Portland, Oregon, United States*

Assisted living (AL) facilities provide a supportive alternative to nursing homes for approximately 1 million older adults across 34,000 U.S. facilities. While private equity (PE) investment in nursing homes has grown, its impact on AL remains unclear. This research examines PE acquisitions of ALs from 2006-2023, analyzing transaction volume, value, and geographic trends. Using LevinAssociates databases amended with SEC reports, Pitchbook, S&P NetAdvantage, press releases, and company websites, we identified 254 PE transactions involving 711 AL facilities. Transactions ranged from 1 to 134 ALs, with 70.9% (n = 180) involving a single facility. ALs were located in 46 states, with Florida (n = 69), California (n = 62), and Texas (n = 46) comprising 25% of acquisitions. Transaction value was reported for 66.9% (n = 170) of deals (n = 558 ALs), ranging from $750,000 to $1.8 billion (median $29.1M, mean $79.6M). Average value per facility ranged from $750,000 to $95.5M (median $22.5M, mean $25.6M). Reported transactions increased over time, peaking at 35 in 2021, with 65.7% occurring between 2015-2021. AL acquisitions grew annually, from two in 2008 to a peak 184 in 2015. Reported PE investment in AL has risen significantly in the past decade. This study provides a novel dataset to understand PE trends in AL, offering insights into its growing role in this important sector of long-term care.

Abstract citation ID: igaf122.1408

### PERSPECTIVES ON PRIVATE EQUITY INVESTMENT IN ASSISTED LIVING

Paula Carder[1], Jacklyn Kohon[1], Sarah Dys[1], John Bowblis[2], Yashaswini Singh[3], Sean Huang[4], Momotazur Rahman[5], and Kali Thomas[6], *1. Portland State University, Portland, Oregon, United States, 2. Miami University, Oxford, Ohio, United States, 3. Brown University School of Public Health, Providence, Rhode Island, United States, 4. Georgetown University, Washington, District of Columbia, United States, 5. Brown University, Providence, Rhode Island, United States, 6. Johns Hopkins University, Baltimore, Maryland, United States*

This qualitative study on the role of private equity (PE) in assisted living mergers, acquisitions, and development, included open-ended interviews with 26 senior housing professionals, 10 state agents, and 7 ombuds offices. Participants, identified through a combination of standard qualitative approaches, including referrals from study team networks, snowball sampling, grey literature review, and state agency, senior housing and investment firm websites. The senior housing professionals included 8 owner/operators, 5 PE fund managers, 5 lenders, 2 real estate attorneys, 2 senior housing consultants, 2 senior housing professional organizations, a real estate developer, and an insurance broker. Interviews averaged 60 minutes, were conducted online, audio-recorded and transcribed. We used "practical" thematic analysis (Saunders, 2022): first, independently reading transcripts, developing and applying descriptive codes, and writing summary memos; and second, as a team, discussing data saturation, reviewing coding, and co-constructing themes. All participants were asked to define PE, and describe challenges and benefits of PE involvement. We present overarching themes that describe areas of alignment and divergence across participant types. Themes that describe areas of alignment: Local operators provide higher quality care compared to national firms, AL combines real estate and healthcare, AL faces significant workforce challenges and capital investments result in improvements. Themes that describe divergence: Changes following a PE-funded acquisition are positive or negative, PE investment impacts affordability, the demand for PE-backed senior housing, and what attracts investors. This paper provides a unique perspective on how AL communities are developed and operated, and on mergers and acquisitions.

### SESSION 3285 (SYMPOSIUM)

Abstract citation ID: igaf122.1409

### INNOVATIONS IN ECONOMICS OF AGING: HOUSEHOLD FINANCIAL FACTORS AND WELL-BEING IN LATER LIFE

Chair: Jessica Kelley
Discussant: Dawn Carr

Income and wealth are broadly understood to shape what it means to age well. Other household financial factors, however, receive less attention and may have more daily impact on well-being. For instance, the daily strain of having sufficient food or managing debt in older adulthood can diminish cognitive, emotional, or physical health. This symposium brings together scholars who utilize innovative measures about financial constraints or challenges and their relation to later-life outcomes. Topics include: midlife food insecurity and SNAP receipt in midlife, neighborhood proximity to "bricks and mortar" banks, debt-to-asset ratios, and later life mortgage borrowing. Dawn Carr will serve as discussant as we explore ways to expand inquiry on the economic-health linkage over the life course.

Abstract citation ID: igaf122.1410

### BANKING ON THE NEIGHBORHOOD? EXAMINING THE ROLE OF PROXIMITY TO LOCAL BANKING IN OLDER ADULTS' WELL-BEING

Alyssa Goldman[1], and Megan Bea[2], *1. Boston College, Chestnut Hill, Massachusetts, United States, 2. University of Wisconsin–Madison, Madison, Wisconsin, United States*

Access to local banking represents an understudied dimension of neighborhood-based inequalities that could significantly influence older adults' perceptions of their neighborhood spaces in ways that matter for disparities in well-being. Building on a 2024 publication by the authors, we evaluate disparities in banking access and then examine how local banking access informs older adults' perceptions of their neighborhood, which hold important implications for well-being. We use nationally representative data from older adults in the United States who were interviewed at Round 3 of the National Social Life, Health, and Aging Project, linked with data on banks establishments in respondents' residential and surrounding census tracts from the National Establishment Time Series database, in a series of regression analyses. White older adults have significantly higher rates of banks in their local areas compared with Black and Hispanic older adults, and older adults with higher levels of education have significantly greater local banking access than those with less education. Higher rates of local banking institutions are associated with significantly lower perceptions of neighborhood danger, but not with perceived collective efficacy. This finding emerges when accounting for neighborhood concentrated disadvantage and physical disorder. Local banks may represent neighborhood investment and the broader economic vitality of a community, as well as the ability of communities to meet older adults' everyday needs in ways that enhance older residents' feelings of safety. Increasing access to local financial institutions may help attenuate neighborhood-based contributors to inequalities in health and well-being among the aging population.

Abstract citation ID: igaf122.1411

### HOUSEHOLD FOOD INSECURITY AND SNAP DYNAMICS: IMPLICATIONS FOR COGNITIVE HEALTH AND WELL-BEING IN LATER LIFE.

Zuo Dongmei[1], and Colleen Heflin[2], *1. Emory University, Atlanta, Georgia, United States, 2. Syracuse University, Maxwell School of Citizenship and Public Affairs, Syracuse, New York, United States*

This research examines how food insecurity (FI) and public assistance exposure are associated with later-life cognitive health and economic well-being from a life course perspective. Using Health and Retirement Study (1995–2020) data, we find that midlife FI can be persistent, with longer exposure during one's fifties significantly linked to lower cognitive functioning after age 60. These results underscore how early economic disadvantage can have enduring health consequences and highlight the potential protective role of SNAP participation against cognitive decline. In a complementary analysis, we find that as individuals transition into cognitive impairment, they face substantial barriers to enrolling in SNAP, which we attribute to potential difficulties with administrative burdens. Our results suggest that the administrative burden inherent in SNAP enrollment may contribute to lower take-up rates among cognitively impaired seniors. The efforts to simplify eligibility and recertification processes may help improve access to food assistance for this vulnerable group. Extending the inquiry into the familial domain, we assess how intergenerational and intragenerational dynamics influence SNAP usage. Evidence from the Panel Study of Income Dynamics (1975–2019) shows that early exposure to SNAP during adolescence, along with parental and sibling participation, is significantly associated with an individual's own increased likelihood of enrollment. This indicates that family networks play a pivotal role in mitigating stigma and administrative burdens associated with public assistance, while also buffering against food insecurity—particularly among disadvantaged populations. Encouraging whole-family outreach and educational programs may strengthen benefit communication and access, particularly in communities where familial influence is especially pronounced.

Abstract citation ID: igaf122.1412

### RACE AND COGNITIVE HEALTH AMONG OLDER AMERICANS: THE ROLE OF DEBT AND ASSET PROFILES DURING PRERETIREMENT AGE

Chioun Lee[1], Dana Glei[2], and Soojin Park[1], *1. University of California, Riverside, Riverside, California, United States, 2. Georgetown University, Washington, District of Columbia, United States*

Objectives: Low-cost debt can potentially enhance wealth and indirectly benefit health, yet Black Americans disproportionately lack this type of debt, which may constrain their ability to accumulate wealth throughout their lives and across generations. Our objectives are to develop a novel debt–asset measure, use it to quantify the Black–White differential in debt–asset profiles, and estimate its contribution to the racial gap in cognition. Methods: Using the Health and Retirement Study (1998–2020), we grouped individuals based on debt and asset information during the preretirement period of ages 55 to 61, including the absence of debt and the relative amount of debt compared to assets. Linear mixed models were used to examine the extent to which cognition in later life (ages 62–80) differs across these debt–asset profiles and its role in explaining the racial disparity in cognition. Results: Compared with Whites, Blacks were more likely to fall into categories characterized by high debt-to-asset ratio (DAR) or limited asset ownership. Low-asset non-borrowers displayed the poorest cognition, followed closely by high-DAR borrowers. The Black–White differential in debt–asset profiles contributed to the racial gap in cognition. Discussion: There were two unfavorable debt–asset profiles: high debt relative to assets and little or no debt due to a lack of assets, which was more prevalent among Blacks than Whites. We discuss how institutional and structural racism shapes Black–White disparities in debt–asset profiles, such as limited access to borrowing opportunities, thereby contributing to health inequalities, including cognition.

Abstract citation ID: igaf122.1413

### MORTGAGE BORROWING AND CHRONIC DISEASE OUTCOMES IN OLDER AGE: EVIDENCE FROM BIOMARKER DATA IN THE HRS

Cäzilia Loibl[1], Alec Rhodes[2], Stephanie Moulton[1], Donald Haurin[1], Matthew Pesavento[1], Madison Hyer[1], and Joshua Joseph[1], *1. The Ohio State University, Columbus, Ohio, United States, 2. University of Wisconsin–Madison, Madison, Wisconsin, United States*

The medical diagnosis of a disease is common in older age and can carry significant financial costs. For many older adults, equity in a home is their primary component of wealth; however, housing wealth is illiquid. We analyze the relationship between the liquidation of housing wealth through mortgage borrowing on older homeowners' ability to successfully control a disease. We use data on homeowners aged 65 and older from the 1998–2016 waves of the Health and Retirement Study (N = 3,457). We use biomarkers and physical health indicators to measure disease control following a medical diagnosis of diabetes, heart condition, high blood pressure, lung disease, or cancer. Descriptively, 28% of older homeowners who borrow against home equity are not controlled on their disease, compared to 33% of non-borrowers. Panel data instrumental variable regressions show that each $10,000 borrowed from home equity after diagnosis is associated with a 17-percentage-point reduction in the probability of the disease not being controlled. Many older adults are not able or willing to liquidate housing wealth, and the ability to borrow also depends on changes in home values. Thus, housing wealth is not a uniform social determinant of health but is shaped by older adults' participation in financial markets. In follow-up research, we have focused on older adults with diabetes and document the role of access to financial resources for the ability of older adults to manage diabetes.

### SESSION 3290 (SYMPOSIUM)

Abstract citation ID: igaf122.1414

### RESOURCE CENTERS FOR MINORITY AGING RESEARCH (RCMAR) FOSTERING COMMUNITY ENGAGEMENT

Chair: David Lee
Co-Chair: Yaolin Pei

The Resource Centers for Minority Aging Research (RCMAR) Program has been continuously funded by the National Institute on Aging (NIA) since 1997. The RCMAR Program has two primary objectives: to support the career development of early-career scientists conducting social, behavioral, psychological, and economic research related to aging, health disparities in older adults, and/or Alzheimer's Disease and Alzheimer's Disease-related dementias (AD/ADRD), and to advance scientific discoveries related to aging. This includes efforts to eliminate health disparities and inequities and to improve the health and well-being of older adults. There are 18 RCMAR Centers in the United States, 10 are focused on AD/ADRD and 8 are focused on aging.

Abstract citation ID: igaf122.1415

### BEYOND THE CLINIC WALLS: HOW COMPREHENSIVE DEMENTIA CARE FOSTERS COMMUNITY ENGAGEMENT

David Lee[1], Stacy Castellanos[2], Xi Zhu[2], Gery Ryan[3], Ron Hays[4], Luisa Blanco[5], and David Reuben[6], *1. University of California,*

*Los Angeles David Geffen School of Medicine, Los Angeles, California, United States, 2. University of California, Los Angeles Fielding School of Public Health, Los Angeles, California, United States, 3. Kaiser Permanente Southern California, Pasadena, California, United States, 4. University of California, Los Angeles Department of Medicine, Division of General Internal Medicine & Health Services Research, Los Angeles, California, United States, 5. Pepperdine School of Public Policy, Malibu, California, United States, 6. University of California, Los Angeles, Los Angeles, California, United States*

Comprehensive dementia care is a holistic approach to the complex medical and social needs of persons living with dementia and their caregivers. Six major evidence-based care models are being disseminated, each demonstrating psychosocial and healthcare-related benefits for patient and caregiver. This qualitative study explores how these models are delivered and create an environment that fosters community engagement. Semi-structured virtual interviews with at least two stakeholders (n = 44 interviewees) per organization (n = 22 organizations) across the six dementia care models were conducted, ranging from 29-58 minutes. Participants included managers (25%), care providers (43.2%), and individuals who held both roles (31.8%). Most organizations were healthcare systems (54.5%), while 45.5% were community-based. Qualitative codes were developed inductively and deductively, and thematic analysis was conducted. The following themes emerged indicating that some programs 1) Enhanced care coordination through structured community partnerships and having staff deliver services directly in the community (e.g., improving access to resources like meal delivery services and adult day services, using community-health workers who did home visits) (community embeddedness); 2) Used virtual communication methods to expand service coverage, enabling more frequent and broader community engagement (community expansion); 3) Created social support and educational opportunities for patients and caregivers to provide emotional support, foster caregiving self-efficacy, and promote community among other patients and caregivers (e.g., support programs, dementia education programs/virtual calls, memory cafes) (community creation). These findings highlight how comprehensive dementia care programs foster care coordination, enhance accessibility, and provide essential social and educational support for patients and caregivers.

Abstract citation ID: igaf122.1416

### ADDRESSING THE NEEDS OF CHINESE AMERICAN DEMENTIA CAREGIVERS IN FEEDING DECISIONS THROUGH COMMUNITY ENGAGEMENT

Yaolin Pei[1], Bei Wu[2], Terri Fried[3], Dena Schulman-Green[4], and Laura Hanson[5], *1. The University of Texas at Austin, Austin, Texas, United States, 2. NYU Shanghai, Shanghai, China (People's Republic), 3. Yale University School of Medicine, New Haven, Connecticut, United States, 4. New York University, New York, New York, United States, 5. The University of North Carolina at Chapel Hill School of Medicine, Chapel Hill, North Carolina, United States*

Feeding difficulties are common among individuals with advanced dementia. Dementia family caregivers have to make difficult decisions about feeding options. Despite limited clinical benefit, over 50% of Chinese individuals with advanced dementia receiving tube-feeding. Little is known about Chinese American dementia caregivers' attitudes towards tube feeding and their decision-making needs regarding feeding options. In collaboration with CaringKind, a community agency serving dementia caregivers in NYC, we conducted a multi-method study among Chinese American dementia caregivers consisting of an original survey to capture knowledge/attitudes about feeding decisions and a qualitative descriptive study to inform a culturally adapted decision aid. We calculated descriptive statistics for quantitative data and used thematic analysis for qualitative data. The survey (n = 63) showed that participants had limited knowledge about tube feeding; 35% opted for tube feeding in an end-of-life scenario, while 41% were uncertain. Qualitative interviews with 21 Chinese American dementia caregivers and 13 healthcare providers indicated that: 1) caregivers value family-based and harmony-oriented decision-making and often misunderstand that careful hand feeding equates to starvation; and 2) Chinese American persons with dementia prefer indirect communication regarding their end-of-life care wishes. Based on study results, we culturally adapted an appropriate decision aid titled "Making choices: feeding options for patients with dementia" successfully used with dementia caregivers. We are collecting feedback from caregivers and health care providers to make refinements. Next, we will pilot test the feasibility, acceptability, and preliminary efficacy of the culturally adapted decision aid intervention to better address the needs of our caregiver group.

Abstract citation ID: igaf122.1416a

### BRIDGING BRAIN HEALTH AND COMMUNITY TRUST: A COMMUNITY-ENGAGED PROGRAM FOR DEMENTIA AWARENESS AND EARLY DETECTION IN LOS ANGELES COUNTY

Mirella Diaz-Santos, *University of California, Los Angeles, Los Angeles, California, United States*

Feeding difficulties are common among individuals with advanced dementia. Dementia family caregivers have to make difficult decisions about feeding options. Despite limited clinical benefit, over 50% of Chinese individuals with advanced dementia receiving tube-feeding. Little is known about Chinese American dementia caregivers' attitudes towards tube feeding and their decision-making needs regarding feeding options. In collaboration with CaringKind, a community agency serving dementia caregivers in NYC, we conducted a multi-method study among Chinese American dementia caregivers consisting of an original survey to capture knowledge/attitudes about feeding decisions and a qualitative descriptive study to inform a culturally adapted decision aid. We calculated descriptive statistics for quantitative data and used thematic analysis for qualitative data. The survey (n = 63) showed that participants had limited knowledge about tube feeding; 35% opted for tube feeding in an end-of-life scenario, while 41% were uncertain. Qualitative interviews with 21 Chinese American dementia caregivers and 13 healthcare providers indicated that: 1) caregivers value family-based and harmony-oriented decision-making and often misunderstand that careful hand feeding equates to starvation; and 2) Chinese American persons with dementia prefer indirect communication regarding their end-of-life care wishes. Based on study results, we culturally adapted an appropriate decision aid titled "Making choices: feeding options for patients with dementia" successfully used with dementia caregivers. We are collecting feedback from caregivers and health care providers to make refinements. Next, we will pilot test the feasibility, acceptability, and preliminary efficacy of the culturally adapted decision aid intervention to better address the needs of our caregiver group.

Abstract citation ID: igaf122.1416b

### FEASIBILITY OF CARE PARTNER-ASSISTED CONTINUOUS GLUCOSE MONITORING IN OLDER CHINESE AMERICANS WITH MCI

Yaguang Zheng[1], Richard Lipton[2], Katharine Lawrence[3], Susan Zweig[3], Linda Siminerio[4], Jessica Zwerling[2], Eric Chen[1], and Bei Wu[5], *1. New York University, New York, New York, United States, 2. Albert Einstein College of Medicine, Bronx, New York, United States, 3. NYU Grossman School of Medicine, New York, New York, United States, 4. University of Pittsburgh, Pittsburgh, Pennsylvania, United States, 5. NYU Shanghai, Shanghai, China (People's Republic)*

Mild cognitive impairment (MCI) can increase care burden for type 2 diabetes (T2D), and has been evident in the older Chinese Americans. Continuous glucose monitoring (CGM) offers a promising solution but is underutilized in this population, we thus aimed to examine the feasibility of using CGM among them. Older Chinese Americans with MCI and T2D (N=11) wore a CGM for 10 days and real-time CGM data were shared with their care partners for immediate support of their diabetes management. Both participants and their care partners received education on CGM data use. Additionally, care partners assisted patients in completing a diary for daily food intake, physical activity, and antihyperglycemic medication. Older Chinese Americans with MCI and T2D had a mean age of 74.5 ± 5.2 years, with 63.6% female, 100% retired, 36.4% college education, and 90.9% family income<$30,000. The care partners were 59.4 ± 20.6 years old, 63.6% female, 54.5% retired, and 54.5% college education. The relationship of care partners to participants was couples (45.5%) and adult children (36.4%). The primary language was Chinese. Most of them (90.9%) had no prior experience using CGM. All participants adhered to the 10-day CGM use with the retention rate of 100%. Most participants (90.1%) indicated they would like to continue receiving CGM-guided intervention. Participant enrollment and data collection were completed in two months. CGM use in care partner-assisted diabetes management among older Chinese Americans with MCI is feasible. Findings are informative for applying CGM interventions in this population to address their care burden.

### SESSION 3295 (SYMPOSIUM)

Abstract citation ID: igaf122.1417

### USING STAKEHOLDER PERSPECTIVES TO IMPROVE END-OF-LIFE TRAINING OPPORTUNITIES

Chair: Britteny Howell
Co-Chair: Raven Weaver
Discussant: Tina Newsham

Health and human services professionals are often underprepared to support patients with end-of-life (EOL) decision-making and supporting the bereaved, largely due to gaps in their professional training. Colleges and universities can fill this gap by providing courses to increase the knowledge of the future workforce around issues related to death and dying, but faculty teaching these courses may themselves also be underprepared for the realities of teaching this challenging material. In this symposium, presenters from several different fields and universities will address two goals: (1) fill some remaining knowledge gaps regarding how to teach about advance care planning (ACP) and EOL discussions and (2) suggest resources and self-care strategies for those teaching courses on death and dying. The session will begin with three presentations presenting process data and stakeholder perspectives on death education. The first paper will present preliminary process data on designing and implementing a pilot program where medical and health professional students facilitate ACP discussion events in rural communities. The next presentation will discuss data collected from a study on faculty perspectives of the benefits and challenges of teaching and talking about death and dying. Lastly, the third paper will present medical students' and older adult patient's perspectives regarding the need for EOL training and teaching strategies. The session will wrap up with a final presentation from a multidisciplinary team who will provide some personal benefits and self-care strategies from those who teach death and dying courses.

Abstract citation ID: igaf122.1418

### FACULTY PERSPECTIVES ON TEACHING DEATH AND DYING: EXPLORING PERSONAL BENEFITS, CHALLENGES, & LESSONS LEARNED

Autumn Decker[1], Raven Weaver[2], and Cory Bolkan[3], *1. Rede Group, Pullman, Washington, United States, 2. Washington State University, Pullman, Washington, United States, 3. Washington State University, Vancouver, Washington, United States*

Faculty are expected to carefully navigate sensitive and complex topics related to death and dying, often prioritizing students' comfort over their own. This creates space for students to reflect on their own beliefs, values, and attitudes toward mortality, fostering resilience and promoting compassionate care in both personal and professional contexts. However, less is known about faculty comfort with the topic. Through content analysis of secondary data collected using a phenomenological research approach, we explored faculty perspectives on teaching death and dying. This paper highlights their personal lessons learned to inform others teaching similar courses in U.S.-based higher education settings. Key informant semi-structured interviews (n=27) yielded insights into common benefits, challenges, and effective teaching strategies. More specifically, three key themes emerged regarding faculty health and well-being: (1) the importance of engaging in formal therapy and counseling; (2) having a strong support system (e.g., family, friends, colleagues); and (3) investing in meaningful self-care for both them and their students. Participants also stressed that faculty teaching these courses must be deeply invested in ensuring students receive the best and safest experience when engaging with sensitive material. They also emphasized the importance of boundary setting and referring students to school counseling services when needed. Lastly, participants recommended sharing university, local, and national resources, including opportunities for mental health first aid training, with instructors. These lessons learned offer valuable insights into best practices and are crucial for advancing death education on a broader scale.

Abstract citation ID: igaf122.1419

### MEDICAL STUDENTS' INSIGHTS ON END-OF-LIFE TRAINING AND EFFECTIVE TEACHING STRATEGIES

Cory Bolkan[1], Katelyn Costanza[2], Raven Weaver[3], Anna Roman[2], Angelique King[2], Kaela Kyaw[2], Logan Patterson[4], and Autumn Decker[5], *1. Washington State University, Vancouver, Washington, United States, 2. Washington State University, Spokane, Washington, United States, 3. Washington State University, Pullman, Washington, United States, 4. Washington State University Elson S. Floyd College of Medicine, Spokane, Washington, United States, 5. Rede Group, Pullman, Washington, United States*

Medical students typically encounter death during their training, an experience that profoundly shapes their professional aspirations, coping strategies, and personal reflections on mortality. This study explored the emotional impact of these encounters, along with students' attitudes about death and their experiences with end-of-life (EOL) training. Undergraduate medical students completed an online survey (n = 45) and then a sub-sample (n = 10) participated in in-depth interviews focusing on how EOL encounters or training influenced their professional and personal growth. Using a reflective research methodology, four additional medical student researchers contributed to the narrative analysis of transcribed interviews via an inductive, open coding approach. Over 70% of survey respondents (n = 32) reported both personal and professional experiences with death and 90% wanted more education (i.e., elective course offering). Follow-up interviews highlighted gaps in essential skills like goals-of-care conversations and trauma stewardship. Key themes from the narrative analysis included: (1) developing personal strategies for coping with death, (2) exploring how to comfort others, and (3) conceptualizing what it means to "die well." Students also emphasized the importance of peer and institutional support to build resilience, manage emotions, navigate EOL care, and prevent burnout. However, students also perceived hesitancy among mentors to discuss EOL options or palliative care, attributing their discomfort to knowledge gaps. These findings highlight the need for comprehensive EOL training in medical curricula to help improve future patient care and to foster resilience, compassion, and well-being in future physicians.

Abstract citation ID: igaf122.1420

### EMPOWERING FUTURE HEALTHCARE PROVIDERS: A PILOT EDUCATIONAL PROGRAM FOR ADVANCE CARE PLANNING IN RURAL COMMUNITIES

Raven Weaver[1], Cory Bolkan[2], Amanda Lamp[3], Kaela Kyaw[3], and Katelyn Costanza[3], *1. Washington State University, Pullman, Washington, United States, 2. Washington State University, Vancouver, Washington, United States, 3. Washington State University, Spokane, Washington, United States*

Aging and end-of-life knowledge are critical for healthcare providers, as the majority of high healthcare utilizers are middle-aged or older adult patients. Given population aging, it is essential that healthcare professionals are skilled in facilitating advance care planning (ACP) discussions. Further, Medicare covers ACP services, yet many providers still do not bill for them, citing insufficient preparation for facilitating ACP discussions with patients. To address this gap, our pilot educational project engages and trains interprofessional health students as facilitators of informal community-based ACP discussions in rural settings, where barriers such as geographic isolation, provider shortages, and limited awareness of end-of-life options are significant. Using experiential and competency-based learning frameworks, we trained student volunteers (n = 8) to conduct three community-based ACP events in underserved rural communities. We report on the process evaluation of developing the training, implementing community events, and how we employed a community-based participatory research framework to promote equitable and culturally sensitive ACP. As the project continues, we will also evaluate the overall effectiveness in improving ACP engagement and preparedness via surveys of community participants, student competencies (e.g., communication skills, empathy), and students' focus-group interviews. Ultimately, this initiative seeks to enhance end-of-life curricula/training for interprofessional health students, empower future healthcare providers with essential ACP communication skills, improve access to ACP in rural areas by scaling up this program for wider dissemination, and address healthcare disparities through patient-centered, community-aligned care.

Abstract citation ID: igaf122.1421

### MAKING SURE WE DON'T THINK ABOUT DEATH ALL DAY: SOME TIPS FROM HEALTH SCIENCE & PSYCHOLOGY INSTRUCTORS

Britteny Howell, Corrie Whitmore, and Calista Kern-Lyons, *University of Alaska Anchorage, Anchorage, Alaska, United States*

Courses about death and dying can take an emotional toll on students and instructors alike. While instructors may develop resources and prepare strategies to care for their students, they often neglect their own psychological needs. Cumulative stress and collective grief can lead to faculty burnout and other adverse outcomes, suggesting a need to share resources, strategies, and information with other instructors. In this presentation, we share some self-care strategies we have found helpful to continue this important work. Personal benefits to teaching courses on death and dying include addressing a pressing workforce need for this type of education, as well as the opportunity to confront our own mortality, better understand grief and cultural views of death, and prepare ourselves and our loved ones for end-of-life situations. However, the emotional burden of this work means we also need to intentionally incorporate mindfulness, activities that bring joy, and exercise into our daily lives, nurturing our support networks, and protecting boundaries for working on the course and/or end-of-life planning so it does not overwhelm us. We also welcome ideas and suggestions from participants in this session.

### SESSION 3300 (SYMPOSIUM)

Abstract citation ID: igaf122.1422

### ADVANCING ELDER MISTREATMENT RESEARCH THROUGH THEORY: EXPLORING MECHANISMS AND DEVELOPING MODELS

Chair: Kelly Marnfeldt
Co-Chair: Susanna Mage
Discussant: Kathleen Wilber

Elder mistreatment (EM) is a critical yet under-addressed public health issue, with family caregivers among the most likely to perpetrate mistreatment. This symposium underscores the importance of strong theoretical frameworks in advancing EM research and developing effective prevention strategies. Historically described as "data rich and theory poor," the field must strengthen its theoretical foundations to drive more robust, evidence-based approaches. Dr. Wilber will serve as the discussant, guiding a conversation on integrating theory to enhance research and practice. Dr. Meyer will begin by describing the iterative integration of Resourcefulness and Quality of Life Theory into the KINDER intervention to establish a theory-based mechanism of change. Next, Dr. Marnfeldt will present a case analysis of the COACH program, emphasizing the need to move beyond identifying risk factors to understanding why mistreatment occurs and how interventions work. Dr. Avent will then discuss findings from the Better Together Couples' Study, applying the web of violence framework and life course theory to explore how past abuse shapes caregivers' recognition of mistreatment and their caregiving experiences, strengthening support for trauma-informed, lifespan-focused interventions. Following, Dr. Rosen will introduce a theoretical model outlining multiple pathways through which EM may increase Medicaid expenditures, identifying eight intervention targets to guide research on Medicaid costs, eligibility, and transitions. Lastly, Dr. Bloemen will examine the complex ways EM affects families, illustrating how family systems and resiliency theory can enhance acute care responses for victims. Collectively, these presentations advance EM research by strengthening theoretical foundations and informing more effective prevention strategies. Abuse, Neglect and Exploitation of Older Persons Interest Group Sponsored Symposium

Abstract citation ID: igaf122.1423

### INTEGRATION OF A THEORETICAL MECHANISM INTO AN INTERVENTION TO PREVENT ELDER MISTREATMENT IN FAMILY CAREGIVING

Kylie Meyer[1], Jaclene Zauszniewski[1], Wenxing Wei[1], Zachary Gassoumis[2], Alexander Gonzales[3], Elliane Irani[1], Jeanine Yonashiro-Cho[3], and Donna Benton[4], *1. Case Western Reserve University, Cleveland, Ohio, United States, 2. UTHealth Houston, Houston, Texas, United States, 3. University of Southern California, Los Angeles, California, United States, 4. University of Southern California Leonard Davis School of Gerontology, Los Angeles, California, United States*

Approximately half of family caregivers of persons living with Alzheimer's Disease and Related Dementias (AD/ADRD) engage in elder mistreatment (EM), with psychological EM being among the most common types. The KINDER (Knowledge and Interpersonal Skills to Develop Enhanced Relationships) intervention aims to prevent psychological EM and support high quality care by enhancing caregiving relationships. Initially, KINDER lacked a clearly defined theoretical framework, a known barrier to intervention translation. This study describes the iterative integration of Resourcefulness and Quality of Life Theory© (RQoLT) into KINDER to establish a plausible theory-based mechanism of change. Two studies were conducted. Study 1 involved qualitative interviews with seven caregivers who participated in the first KINDER intervention. Thematic analysis identified the use of personal resourcefulness skills, and, to a lesser extent, social resourcefulness skills. Based on these findings, KINDER was refined to include small group discussions to promote the use of social resourcefulness, and to emphasize the development of each type of resourcefulness. Study 2 tested the revised intervention with 71 caregivers, using qualitative interviews and surveys to assess the applicability of RQoLT. Caregivers reported using personal and social resourcefulness skills to improve care relationships and prevent EM. The revised intervention demonstrated continued applicability of RQoLT, with caregivers showing increased reliance on personal resourcefulness skills. Findings suggest RQoLT is a plausible theory of intervention for KINDER, supporting the development of such skills to prevent psychological EM and promote high-quality caregiving. Further research is needed to rigorously test resourcefulness as a mechanism and identify effective intervention components.

Abstract citation ID: igaf122.1424

### BEYOND RISK FACTORS: LESSONS FROM AN ELDER MISTREATMENT PREVENTION INTERVENTION

Kelly Marnfeldt[1], Susanna Mage[1], Susan Enguidanos[2], Zachary Gassoumis[3], and Kathleen Wilber[2], *1. University of Southern California Leonard Davis School of Gerontology, Los Angeles, California, United States, 2. University of Southern California, Los Angeles, California, United States, 3. UTHealth Houston, Houston, Texas, United States*

Elder mistreatment (EM) remains a critical yet under-addressed public health concern, particularly among people living with dementia (PLWD). Since caregiver stress is strongly linked to EM risk, community-based prevention efforts have primarily focused on reducing caregiver burden, depression, and anxiety. However, empirical findings suggest that the pathways leading to EM are more complex. The Comprehensive Older Adult and Caregiver Help (COACH) intervention is one of the few rigorously tested programs that challenge these assumptions. Despite successfully reducing EM, COACH did not significantly impact the hypothesized risk factors, raising questions about the mechanisms underlying its effectiveness. To explore this, we conducted a retrospective case study using the intervention itself as the unit of analysis. Findings suggest that COACH's impact may have stemmed from its ability to enhance caregivers' capacity to navigate challenges in real-time. Three key components were identified as potential contributors to its success: (1) tailored emotional and practical support that helped caregivers sustain their role; (2) care navigation and resource provision that facilitated access to essential services; and (3) caregiver education that provided problem-solving strategies for managing difficult situations. While COACH was effective, the lack of an explicit theoretical framework makes it difficult to pinpoint which elements drove its success. Strengthening future interventions with a theory-driven approach could improve their adaptability and predictability across diverse settings. These insights can help guide the development of caregiver support programs that mitigate EM risk while ensuring interventions remain effective and scalable.

Abstract citation ID: igaf122.1425

### LIFE COURSE THEORY, THE WEB OF VIOLENCE, AND ELDER MISTREATMENT: INSIGHTS FROM THE BETTER TOGETHER COUPLES' STUDY

Elizabeth Avent[1], Renee Garbe[2], Laura Mosqueda[3], and Zachary Gassoumis[4], *1. National Opinion Research Center at The University of Chicago, Chicago, Illinois, United States, 2. The Pennsylvania State University, University Park, Illinois, United States, 3. University of Southern California, Pasadena, California, United States, 4. UTHealth Houston, Houston, Texas, United States*

Elder mistreatment (EM) interventions often focus on mitigating immediate risk factors for perpetration and victimization, yet they frequently overlook the broader patterns of violence and abuse across the lifespan. Drawing on the web of violence framework and life course theory, this study underscores how EM is rarely an isolated occurrence but rather part of an interconnected trajectory of polyvictimization, intergenerational transmission of violence, and the co-occurrence of multiple forms of abuse over time. The Better Together Couples' Study applies these theoretical frameworks to examine how early-life family violence and intimate partner violence (IPV) shape experiences of EM within dementia caregiving dyads. Qualitative interviews with caregivers revealed three key findings: (1) at least one partner in the dyad had a history of abuse, (2) past experiences of violence influenced their ability to recognize and define mistreatment, and (3) these histories shaped how they navigated caregiving for an abusive partner. These findings highlight the urgent need for EM prevention and intervention strategies that account for the long-term consequences of violence and abuse. Recognizing these patterns is critical for developing interventions that address the cumulative and relational nature of mistreatment across the life course. In addition to addressing immediate risk factors, interventions must incorporate a lifespan perspective, acknowledging how past trauma informs current caregiving dynamics and shapes vulnerability to mistreatment. Addressing these interconnections is essential for providing effective, trauma-informed support for older adults and their families with complex histories of abuse.

Abstract citation ID: igaf122.1426

**THEORETICAL MODEL TO GROUND RESEARCH ON MEDICAID COST SAVINGS FROM ELDER MISTREATMENT INTERVENTION**

Tony Rosen[1], Elaine Gottesman[2], Alyssa Elman[3], Yongkang Zhang[3], Yiyuan Wu[3], Jiani Yu[3], Zachary Gassoumis[4], and Kenneth Steinman[5], *1. Weill Cornell Medicine / NewYork-Presbyterian Hospital, New York, New York, United States, 2. New York-Presbyterian Hospital, New York, New York, United States, 3. Weill Cornell Medicine, New York, New York, United States, 4. UTHealth Houston, Houston, Texas, United States, 5. The Ohio State University, Columbus, Ohio, United States*

Measuring the costs associated with elder mistreatment (EM) and identifying potential downstream cost savings from intervention and prevention efforts are essential research goals to influence policy. Given that Medicaid has a central role in funding healthcare for poor and institutionalized older adults, we hypothesized that EM and response may have a substantial impact on associated costs. We developed a theoretical model describing in detail the multiple pathways by which EM may lead to additional Medicaid expenditures, including through: (1) unplanned high-intensity, high-cost acute health care, (2) inability set up/maintain adequate home and community-based services, (3) financial exploitation diverting and older adult's income and draining assets, (4) long-term care (LTC) placement, and (5) spend down of private assets after LTC admission. We used the model to identify 8 discrete targets for intervention. This model may guide future research examining the impact of EM on total per capita Medicaid cost and whether EM increases the likelihood of: qualifying for Medicaid, conversion from community to institutional Medicaid, and/or spending down from private payment in an LTC to Medicaid. Also, the impact of intervention and prevention efforts at specific targets may be measured. This theoretical model also informs research strategies, which may include close examination of natural experiments, longitudinal examination of a cohort of older adults experiencing EM before and after identification/intervention, and comparative studies using control groups.

Abstract citation ID: igaf122.1427

**FAMILY SYSTEM AND TRAUMA-INFORMED CARE FOR ELDER ABUSE CONSULTATION TEAM RESEARCH**

Elizabeth Bloemen[1], Sarah Tietz, MD[2], Sarah Cox[3], and Daniel Lindberg[2], *1. University of Colorado, Aurora, Colorado, United States, 2. University of Colorado School of Medicine, Aurora, Colorado, United States, 3. University of Colorado Hospital, Aurora, Colorado, United States*

Elder abuse consultation teams are a promising model of care with initial evidence showing both an improvement in the recognition of abuse and the care that is provided to patients. By using a family system and trauma-informed approach, our team has grounded both our direct care and our measurement in theory. Our team approaches elder abuse cases through the lens of a family system, with abuse impacting an entire family rather than a single victim-harmer dyad. To this end, we strive to identify other possible victims in the family system and put into place interventions that improve the resiliency of both the patient and the family. This impacts both our clinical care and measurement as some of our critical outcomes include the identification of other potential victims and resources implemented at discharge. We also prioritize trauma-informed care for all members of the family system and reflect this in our research priorities through tracking of patient-centered discharge planning and harm reduction utilization. Through this session, we will describe the patient, family, and system-level variables we have measured throughout our 5 years of elder abuse consultations and the theoretical framework these are based upon. Initial data will be reported within this framework.

### SESSION 3305 (SYMPOSIUM)

Abstract citation ID: igaf122.1428

**HOUSING, COMMUNITY, AND EQUITY: STRENGTHENING DISASTER RESILIENCE AMONG OLDER ADULTS**

Chair: Zhirui Chen
Discussant: Tam Perry

Climate change is intensifying disasters and disproportionately affects older adults, particularly those who are low-income, racial minorities, or living with disabilities. This symposium employs quantitative, qualitative, and mixed methods to explore disaster preparedness and response of older adults, focusing on the role of housing and neighborhood resources in shaping vulnerability and resilience. The first presentation examines the associations between housing characteristics (housing type, ownership, crowding, and cost, assessed at both the individual and community level) and disaster preparedness, highlighting the needs of older adults with low income facing housing vulnerability. The second presentation explores older adults' and community partners' experience with extreme heat and identifies best practices and barriers to effective response in affordable rental housing. The third presentation investigates preparedness in low-income housing, emphasizing the heightened risks for isolated or disabled older adults and presenting a toolkit to enhance disaster preparedness in affordable housing communities. The fourth presentation examines housing insecurity following climate disasters, underscoring systemic disparities affecting low-income Black and Hispanic communities. Finally, the fifth presentation examines the relationship between disasters and loneliness in older adults, as well as the mediating roles of ageism and social participation (e.g., social ties and activities of social nature). Together, these studies highlight the critical need for targeted, neighborhood-level interventions to enhance disaster preparedness and resilience among older adults. The symposium will conclude with implications for research, practice, and policy, advocating for equitable resource distribution, improved housing policies, and community-based strategies to support aging populations in disaster contexts. Disasters and Older Adults Interest Group Sponsored Symposium

Abstract citation ID: igaf122.1429

**HOUSING CHARACTERISTICS AND DISASTER PREPAREDNESS IN LOW-INCOME POPULATIONS: AGE AS A MODERATOR**

Zhirui Chen[1], Samantha Teixeira[2], and Rebekah Coley[3], *1. Boston College, Framingham, Massachusetts, United States, 2. Boston College School of Social Work, Chestnut Hill, Massachusetts, United States*, 3. *Boston College, Chestnut Hill, Massachusetts, United States*

This study examined associations between individual- and community-level housing characteristics and disaster preparedness among low-income adults. Given the increased vulnerability of older adults to climate disasters, we also tested the moderating role of age. Data from three panels (2021-2023) of FEMA's National Household Survey were pooled and merged with CDC's community-level Social Vulnerability Index. The analytic sample included 6,442 low-income adults nested within 4,376 zip codes. Weighted multilevel models examined associations between housing characteristics (housing type, ownership, crowding, and cost, all assessed at both the individual and community level) and three types of disaster preparedness (perceived, actual, and financial preparedness), adjusted for individual demographics. Results showed that both individual- and community-level housing characteristics were associated with individual disaster preparedness. Specifically, renters reported lower perceived and financial preparedness relative to homeowners. Compared to individuals in single-unit homes, those in mobile home reported lower financial preparedness. Those residing in communities with higher proportions of multi-unit housing or crowding also reported lower disaster preparedness. Older adults generally exhibited better perceived and financial preparedness than younger people, but their advantages diminished when experiencing housing cost burden, residing in multi-unit apartment, or living in crowded communities. Our findings highlight the important role of housing characteristics in shaping disaster preparedness and the special needs of low-income older adults facing housing vulnerability. Social workers and community practitioners can draw on these findings to develop locally-tailored and age-responsive strategies to reduce disaster risk among low-income individuals and communities while enhancing climate and disaster resilience in an aging society.

Abstract citation ID: igaf122.1430

### A COMPREHENSIVE APPROACH TO ENHANCE OLDER ADULTS' PREPAREDNESS FOR EXTREME HEAT: COPE -ENGAGE

Atiya Mahmood, Leticia Zhu, Cindy Wei, and Theresa Pauly, *Simon Fraser University, Vancouver, British Columbia, Canada*

Climate change has resulted in increase in the frequency of extreme heat days and older adults are disproportionately affected due to physiological vulnerabilities and systemic barriers to adaptation. In this study, we conduct focus group interviews with older adults and semi-structured interviews with community partners (e.g., healthcare and housing providers, city planners and community service organizations) to identify best practices and challenges in existing heat response strategies. Focus groups findings provide rich, narrative-driven insights into lived experiences, enabling us to understand how older adults interpret and respond to heat-related risks as they navigate extreme heat and access community services. Questions are asked about awareness and preparedness; perception of vulnerability; socioeconomic influence; thermal comfort and heat relief. Findings from the semi-structured interviews provide in-depth information on how public organizations and service sectors deliver service and programs during these events. The community partners are asked about access and quality of community facilities, services and programs to mitigate and manage extreme heat events. Preliminary findings demonstrate that older adults who are supported through specific community-based organizations during heat events have better awareness about risk factors and are able to access and use services more effectively. Transportations play a key role in utilization of services like cooling centres. Community organizations emphasize the need for more multi-sectorial collaboration to better serve older adults. Both groups highlight the need for better communication and awareness campaigns around risks factors of extreme heat event. A multi-year approach integrates co-creation workshops to refine intervention strategies based on study findings.

Abstract citation ID: igaf122.1431

### A MIXED METHODS CASE STUDY ON EXTREME WEATHER PREPAREDNESS AMONG OLDER ADULTS IN LOW-INCOME HOUSING

Holly Dabelko-Schoeny[1], Smitha Rao[2], Anthony Traver[1], Josie Sarabia[1], Emma Rademacher[3], Max Stokey[1], and Marisa Sheldon[1], *1. The Ohio State University, Columbus, Ohio, United States, 2. Ohio University, Columbus, Ohio, United States, 3. The Ohio State University College of Public Health, Columbus, Ohio, United States*

Disasters and extreme weather events are no longer considered rare; they routinely disrupt daily routines and challenge people's ability to maintain productivity and health. Older adults and individuals with disabilities living in low-income housing often bear the brunt of these occurrences, including extreme heat, cold, winter storms, hurricanes, and excessive precipitation. Using the Aging in the Right Place (AIRP) and cumulative disadvantage frameworks, this convergent mixed methods case study examined older adults' experiences with extreme weather, focusing on preparedness and response in low-income housing. Data from survey responses (N=40) and focus group discussions (N=26) were analyzed using constant-comparison analysis. Residents' understanding of extreme weather preparedness and response was mapped onto elements of the disaster risk management cycle, informed by the theory of planned behavior. Despite differing views on extreme weather risks, residents considered preparedness vital for safety, especially for isolated or disabled individuals. The built environment, information sources, and social connections influenced attitudes and actions related to extreme weather. Based on study findings, a toolkit was developed for use by human services professionals working in low-income affordable housing properties, including identification of resident risk, resident-informed informational flyers, and space assessment worksheets. This study supports the co-development of community-informed regional disaster preparedness plans, particularly for individuals in affordable housing communities.

Abstract citation ID: igaf122.1432

### HOUSING INSECURITY AND CLIMATE-RELATED DISASTERS IN UNDERSERVED COMMUNITIES: A CALL FOR ACTION

Carlos Fuentes[1], Quenette Walton[2], Brenda Tijerina[3], Lauren Gilbert[4], and Omolola Adepoju[4], *1. Tilman J. Fertitta Family College of Medicine, University of Houston, Houston, Texas, United States, 2. University of Houston, Houston, Texas, United States, 3. West Street Recovery, Houston, Texas, United States, 4. University of Houston College of Medicine, Houston, Texas, United States*

Although natural disasters are equal opportunity events, research suggests that they place a disproportionate burden on minoritized communities. Examining the effects of these disasters is critical to increasing preparedness among our most vulnerable. This study aims to explore community experiences related to housing following natural disasters. Participants included members from three historically underserved Houston communities with Social Vulnerability Index rankings in the 80th percentile. Three town hall-style conversations were held within each community. Participants engaged in facilitated round-table discussions with open-ended questions. Notes were captured in real-time by both an assigned note-taker and a professional graphic recorder. Reflexive thematic analysis was used to identify themes, supported by research triangulation, reflexivity, and member checking for trustworthiness. Analysis identified seven key themes:1) Successive Disasters Exacerbate Problems Driven by Gentrification, 2) Inequitable Distribution of Resources Post-Disaster Results in Reliance on Community-Led Support, 3) Systemic Delays in Relief Services for Underserved Communities must be Addressed by Government Officials, 4) Growing Distrust in Local Government to Address Evolving Post-Disaster Needs, 5) Navigating Complex Insurance Policies While Being Drained by a Disaster, 6) Trickle-Down Unpreparedness Starts at a City Level, and 7) Steps to Prepare for Future Disasters. Systemic inequities in disaster preparedness and response affecting low-income Black and Hispanic communities are evident. Addressing these disparities requires prioritizing equitable resource distribution, infrastructure investments, and community-driven planning and building resilience. Implementing transparent, inclusive strategies to reduce vulnerability and ensure these communities are better supported before, during, and after disasters is crucial.

Abstract citation ID: igaf122.1433

### AGEISM MEDIATES THE RELATIONSHIP BETWEEN CLIMATE AND MAN-MADE DISASTERS AND LONELINESS

Liat Ayalon, and Sayani Das, *Bar-Ilan University, Ramat Gan, HaMerkaz, Israel*

This paper addresses two global phenomena, which pose a great challenge and threat to society at large as well as to individuals, namely natural and man-made disasters (e.g., disasters) and loneliness (defined as the subjective experience of having inadequate ties). Both the incidence and the impact of disasters and of loneliness are growing in our lives with substantial ominous prospects for the younger and older generations. Relying on data from the Longitudinal Ageing Study in India (LASI; Wave 1 (2017–18), we examine the relationship between disasters and loneliness in older persons over the age of 60. Our findings show that after controlling for demographic and environmental variables, the relationship between the two is fully mediated by ageism (e.g., stereotypes, prejudices, and discrimination towards people because of their age) and social participation (e.g., social ties and activities of social nature). Results are discussed considering theories of intergenerational relationships, stressing the need for solidarity and cooperation especially in times of threat and scarce resources.

### SESSION 3310 (SYMPOSIUM)

Abstract citation ID: igaf122.1434

### RECENT TRENDS IN THE LABOR MARKET FOR OLDER WORKERS AND THEIR IMPACT ON RETIREE WELL-BEING

Chair: Joseph Quinn
Co-Chair: Kevin Cahill
Discussant: Christine Bishop

The labor market for older workers continues to change, and these changes can have far-reaching impacts on the well-being of retirees. This symposium covers some of the key trends in the labor force participation of older workers, with an emphasis on gender and racial/ethnic differences and wealth outcomes later in life. The first paper explores how trends in labor force participation have differed historically for men and women, and how these gender differences appear to have stabilized since 2010. The second paper explores the impact of sandwich caregiving on labor force participation, and how these impacts differ by key subgroups. The third paper explores labor force participation in the unique context of Turkey, where the pension system has developed all in living memory. The fourth paper connects labor market experiences with retirement outcomes, by examining structural barriers to racial/ethnic equity in retirement. The fifth paper rounds out the symposium by exploring the pivotal relationships between cognitive decline, financial decision making, and the wealth outcomes of older households. The flexibility of the labor market has real benefits for workers generally and for older workers in particular. Indeed, the ubiquitousness of gradual retirement reflects how older workers customize their transitions out of the labor force. The flexibility of the labor market comes with clear trade-offs, however, as structural challenges have left some individuals behind. This symposium provides much-needed context on the labor market outcomes of older workers and how these outcomes impact the financial well-being of older individuals into their retirement years. Economics of Aging Interest Group Sponsored Symposium

Abstract citation ID: igaf122.1435

### HAVE WE REACHED A STEADY STATE BETWEEN OLDER MALE AND FEMALE LABOR FORCE PARTICIPATION RATES?

Kevin Cahill[1], Michael Giandrea[2], and Joseph Quinn[3], *1. FTI Consulting, Inc., Boise, Idaho, United States, 2. U.S. Bureau of Labor Statistics, Washington, District of Columbia, United States, 3. Boston College, Chestnut Hill, Massachusetts, United States*

Older workers' labor force participation rates have stabilized in recent years, after increasing for more than two decades. This apparent change in trend contrasts with one among younger workers, who have experienced increases in labor force participation in the wake of the Covid-19 pandemic. A narrative that older workers are the exception to reductions in labor force participation generally may be coming to an end. This paper explores changes in older workers' labor force participation rates, with a focus on gender differences, to assess whether gender differences have also stabilized in recent years. We find that a change in trend occurs among older women around 2010, with a less prominent one occurring among older men around the same time. Year-on-year increases in older women's labor force participation rates are now more or less aligned with those of older men, the product of post-World War II trends, with large numbers of women entering the labor force, having run their course. Policymakers interested in promoting continued work later in life among older workers may need to reflect on gender differences in new ways, at least with respect to overall trends.

Abstract citation ID: igaf122.1436

### IMPACTS OF SANDWICH CAREGIVING ON LABOR MARKET OUTCOMES

Siavash Radpour[1], Aida Farmand[2], and Jessica Forden[3], *1. Stockton University, Galloway, New Jersey, United States, 2. Office of the New York City Comptroller, New York, New York, United States, 3. The New School, New York, New York, United States*

This study examines the dynamics of caregiving and its impact on labor market outcomes, with a focus on the often-overlooked segment of sandwich caregivers defined as individuals engaged in both childcare and adult care. Utilizing data from the American Time Use Survey (ATUS) spanning the years 2012 to 2022, our findings reveal significant trends: women of all ages are more likely than men to be sandwich caregivers, individuals with higher educational attainment tend to assume sandwich caregiving roles later in life, and black workers are more inclined to provide sandwich care at an earlier age. Inverse Probability Weighted Regression Adjustment (IPWRA) is used to estimate unbiased treatment effects while propensity score matching is utilized to model interactions between sandwich caregiving and gender. Results indicate that sandwich caregivers are 5.7 percent less likely to participate in the labor force and, for those employed, work 5 hours less per week compared to non-caregiving workers, with more pronounced effects on women. This research contributes to a deeper understanding of the impacts of sandwich caregiving responsibilities on extensive and intensive margins, highlighting the need for policies that address the unique challenges faced by sandwich caregivers.

Abstract citation ID: igaf122.1437

### TURKISH OLDER WORKERS IN AN EMERGING PENSIONS AND RETIREMENT LANDSCAPE

Loretta Platts, and Sima Karakullukçu Erdem, *Stockholm University, Stockholm, Stockholms Lan, Sweden*

Pension claiming and retirement behaviors in middle-income countries offer rich insights for studies on the economics of ageing. In recent decades, Türkiye has experienced rapid urbanization, industrialization, and formalization of its economy. Its contributory pension funds have developed within living memory. Pension eligibility is based on days of registered employment, gender and cohort; chronological age is largely irrelevant. Interviews with 23 working older adults living in north-west and north-east Türkiye explored pension saving, economic strategies in later life and the meaning of retirement. Translated transcripts were analyzed using constant comparative analysis, in which categories were described and refined by writing analytical memos. Pensions generally played a minor role in safeguarding economic security in later life; lifestyle changes and rental and labor income were more central. Generally, pensions were low and viewed as providing extra income from mid-life, rather than heralding a retirement phase. An image of a leisure-filled retirement was generally absent, instead participants expressed a strong work ethic, in which working is what humans do and how one lives in a society with others. Retirement involved risks of idleness and unproductivity. Pensions are gradually becoming part of the taken-for-granted institutional set-up in Türkiye: in discussing their children's pensions, participants expressed a growing acceptance of paying premiums and trusting pensions as a reliable income source in a capricious economy. Ceasing paid work—with no link to chronological age and little link to pension claiming—was highly individualized, a direction in which high-income countries may also be headed.

Abstract citation ID: igaf122.1438

### STRUCTURAL OBSTACLES IN SAVING FOR RETIREMENT BY RACE/ETHNICITY

Christian Weller[1], Dania Francis[1], and Michele Tolson[1],
*1. University of Massachusetts Boston, Boston, Massachusetts, United States*

Many people of color have a lot less saved for retirement than White workers, a gap which has remained large for decades. We use the most recent available data to document three structural obstacles to racial retirement equality. Many people of color have less access to an employer-based retirement benefit. They also have lower earnings, which translate into lower retirement benefit contributions. And, they face more widespread and costlier chances of economic setbacks throughout their careers. The data show that the racial gaps in these obstacles are smaller among union members than non-union members. This suggests that greater union membership is one possible policy venue to lower racial retirement wealth inequality. We will discuss other potential employer approaches and policy solutions to increase retirement savings among all groups of households, but especially among Black and Latino householders as well as householders of other/multiple races/ethnicities.

Abstract citation ID: igaf122.1439

### COGNITIVE DECLINE, FINANCIAL DECISION-MAKING, AND WEALTH OF OLDER HOUSEHOLDS

Rashmita Basu, *East Carolina University, Greenville, North Carolina, United States*

Cognitive ability can impact older adults' economic well-being. This study investigated the relationship between cognitive decline and switching financial responsibility within the household and its impacts on household wealth using the Health and Retirement Study (HRS) from 2006-2020. Cognitive decline and financial responsibility were measured for each HRS respondent aged 50 years or older. A decrease in objective test scores measured cognitive decline. Two primary outcomes included switching financial responsibility within the household and financial wealth. The analysis focused on the extent to which individuals' cognitive decline was related to switching the financial responsibility within the household and if there was any impact on changes in financial wealth due to cognitive decline. Between two consecutive waves from 2006 to 2020, approximately 35% of respondents experienced cognitive decline, 35% improved, and 15% received the same cognitive score. Thirty-five percent did not switch to the role of financial responsibility within the household. Compared to those with no change in cognitive scores, those who experienced improvement or decreased scores were 26% and 27% more likely not to switch the financial responsibility role within the household. Further analysis examined if self-reported memory changes versus the objective value of cognitive scores influenced the switching decision within the household and its impacts on household wealth over time. This study provides an expanded profile of the variation in individual characteristics of a financial respondent within the household that leads to the switching decision of the financial responsibility role and its impact on household wealth.

### SESSION 3315 (SYMPOSIUM)

Abstract citation ID: igaf122.1440

### MAKING HOME CARE WORK FOR ALL: REAL-WORLD CONSIDERATIONS FOR EFFECTIVE, AFFORDABLE CARE POLICIES

Chair: Jennifer Reckrey
Co-Chair: Emily Franzosa
Discussant: Joanne Spetz

Older adults increasingly rely on paid care from home health aides and other home care workers to age in place. There is broad agreement that home care is too costly for most families and pay is too low for home care workers, jeopardizing access to these vital services. However, there is less consensus on the most impactful policy solutions to make home care more effective and affordable. This symposium explores how cost and finances shape paid care delivery and highlights successes and challenges of models that aim to address financial barriers to care. First, Miller et al share a study examining support for paid caregiving policy across political affiliations, finding bipartisan support for expanding financial access to affordable home care and increasing the capacity of the paid caregiving workforce. Reckrey and Shen then use data from the Health and Retirement Study to illustrate the limited ability of most people living with dementia to pay for needed home care after diagnosis. Franzosa and colleagues examine home care within the integrated Veterans Health Administration, finding that even with minimal or no co-payments, means testing and administrative burden can create access barriers. Finally, Angell et al discuss how well-intentioned wage increases for direct care workers may reduce their eligibility for public benefits, erasing financial gains. Taken together, these presentations highlight the complexity of policies seeking to improve access to paid care and highlight the need for nuanced approaches to ensure affordable access to these critical services for those who need them most. Paid Caregiving Interest Group Sponsored Symposium

Abstract citation ID: igaf122.1441

### DOES SUPPORT OF POLICIES TO SUSTAIN THE CARE ECONOMY DIFFER BY POLITICAL AFFILIATION OR CAREGIVING RESPONSIBILITY?

Katherine Miller[1], Jennifer Wolff[2], Karen Shen[3], Sandro Galea[4], and Catherine Ettman[1], *1. Johns Hopkins Bloomberg School of Public Health, Baltimore, Maryland, United States, 2. Johns Hopkins University - School of Public Health, Baltimore, Maryland, United States, 3. Johns Hopkins University, Baltimore, Maryland, United States, 4. Washington University in St. Louis, St. Louis, Missouri, United States*

Identifying effective and financially viable strategies to meet the care needs of individuals with impaired function is a key policy challenge for the United States. The 2022 National Strategy to Support Family Caregivers identifies a range of actions to support caregivers while family-oriented policies to promote the affordability of care gain increasing attention. Using a nationally representative survey of adults in 2024, we examine public perceptions of federal policies to support older adults, adults living with disabilities, and their family caregivers by political affiliation and caregiving status. We find one-fifth of U.S. adults (20%) report caregiving responsibilities for adult(s) with disability, with no significant difference across political affiliation. We find widespread support for policies to sustain the care economy across demographic characteristics, caregiver status, and political affiliation. Endorsement was highest for policies to make care in facilities (79.0%) and homes (75.4%) more affordable, expand eligibility for financial access to care (77.3%), and increase capacity of the paid caregiving workforce (78.3%) – and was lower for expansion of paid family leave (65.4%) and payment of family caregivers (61.2%). Endorsement by political affiliation was most similar for policies of making care more affordable and least similar for paid family leave. Political affiliation is a major driver of endorsement of policies to support the care economy that is stronger in magnitude than sociodemographic characteristics, e.g., gender or caregiving. Public support of the care economy is strong—across caregivers and non-caregivers and political affiliations, suggesting policy action is a promising priority with bipartisan support.

Abstract citation ID: igaf122.1442

### BENEFITS CLIFFS AND FINANCIAL STABILITY FOR THE DIRECT CARE WORKFORCE

Sarah Angell[1], Susan Chapman[2], Jiyeon Kim[1], and Stephen McCall[1], *1. PHI, New York, New York, United States, 2. University of California, San Francisco, San Francisco, California, United States*

Our research examined the risk of benefits cliffs among direct care workers (DCWs) in Florida, Kansas, Massachusetts, New Mexico, and Virginia by analyzing effective marginal tax rates (EMTRs), which measure how much of a worker's additional earnings are lost due to higher taxes and reduced public benefits. A benefits cliff occurs when the EMTR exceeds 100%, meaning workers end up financially worse off despite earning more. Using the American Community Survey (ACS) to identify DCWs, we estimated earnings thresholds for benefits cliffs with the Federal Reserve Bank of Atlanta's Policy Rules Database (PRD), which calculates benefits and taxes based on family characteristics. We modeled three consecutive $5,000 wage increases and calculated overall resources as take-home family income plus benefits. We find that DCWs face the risk of benefits cliffs. In Virginia, 8.8% of workers would encounter a cliff after a first $5,000 raise, 6.4% after a second, and 5.3% after a third, with 16.3% experiencing at least one cliff across raises. Median EMTRs range from 35.4% to 36.2% across raises. These cliffs and high EMTRs can leave DCWs with few financial resources even after wage increases. Given low wages, unstable work hours, and high poverty rates, policies to improve economic stability for DCWs are critical. To mitigate the effects of benefits cliffs and improve financial stability for DCWs, policymakers can make changes to public benefits programs including adjusting income limits and asset limits, while employers can engage DCWs in strategic discussions about wage increases and scheduling changes.

Abstract citation ID: igaf122.1443

### FINANCIAL RESOURCES AND MEDICAID ENROLLMENT AFTER DEMENTIA ONSET: FINDINGS FROM THE HEALTH AND RETIREMENT STUDY

Jennifer Reckrey[1], and Karen Shen[2], *1. NYU Grossman School of Medicine, New York, New York, United States, 2. Johns Hopkins University, Baltimore, Maryland, United States*

People living with dementia (PLWD) frequently require high levels of paid home care for extended periods of time so that they can remain living at home. While Medicaid may cover some care costs for those who qualify, PLWD throughout the income spectrum experience significant financial burden as they pay out-of-pocket for home care. In this study, we use longitudinal data from the Health and Retirement Study between 2006-2018 to: 1) characterize financial resources available to fund home care among people with new-onset dementia, and 2) identify the proportion who go on to receive Medicaid following dementia onset. We operationalize financial resources as the ability to pay for full-time home care (40 hours per week, $16.00/ hour) over time considering self-reported income and annuitized wealth. We identified 6,228 unique PLWD with new-onset dementia between 2006-2018, 24% of whom had Medicaid at dementia onset. Among those without Medicaid, 51% had financial resources such that they could afford full-time home care for 6 months. 43%, 35%, 24% could afford full-time home care for 1, 2, and 5 years respectively. Two years after dementia onset, 7% now received Medicaid. Four years later, that proportion had grown to 14%. Few PLWD have the financial resources to support long-term paid care in the community. While Medicaid enrollment is one pathway to receive care, further work is needed to identify and characterize innovative policy and programmatic solutions to ensure that all PLWD—regardless of financial resources—can have their care needs in the community.

Abstract citation ID: igaf122.1444

**PAID CAREGIVING IN AN INTEGRATED SYSTEM: ACCESS TO THE VETERANS HEALTH ADMINISTRATION'S HOME HEALTH AIDE BENEFIT**

Emily Franzosa[1], Lauren Hall[2], and Margaret Ding[2], *1. Bronx VA, Bronx, New York, United States, 2. James J Peters VA Medical Center, New York, New York, United States*

The Veterans Health Administration (VHA) is unique in offering home health aide services (HHA) at no or minimal cost to veterans. Serving 180,000 veterans, HHA is VHA's fastest growing and most widely used home and community-based service. To understand factors affecting veterans' HHA use, we conducted semi-structured, multi-perspective interviews with 60 key stakeholders at 5 geographically diverse VHA medical centers (10-15 per site, including VA HHA managers, primary care teams, and contracted home health agency administrators and aides). Interviews were analyzed through directed content analysis and translated to visual process maps to identify best practices, roadblocks and modifiable intervention points. We identified 4 key concepts: 1) HHA benefits were considered "part of a larger plan" including other VHA HCBS (respite, caregiver support, adult day, Veteran-directed care), Medicaid, or state programs, with VHA staff helping determine the most effective service mix; 2) some veterans paid privately for services VHA does not cover (e.g., transportation, housekeeping); 3) sites requiring veterans to verify income cited this as a barrier due to paperwork delays and veterans' reluctance or inability to provide financial information; 4) some participants felt even minimal co-pays or fear of co-pays caused veterans to decline services, while others felt they increased veterans' perceived value of the benefit. Our results suggest that navigating paid caregiving is complex even within an integrated health system, highlighting the need for close case management, patient education, reducing cost barriers, and closing gaps for those unable to pay for supplemental care.

### SESSION 3320 (SYMPOSIUM)

Abstract citation ID: igaf122.1445

**FROM EARLY-LIFE TO LATE-LIFE: PATTERNS AND DETERMINANTS OF MENTAL HEALTH IN OLDER CHINESE ADULTS**

Chair: Qian Song

Discussant: Michelle Putnam

Depressive symptoms are highly prevalent among Chinese older adults, with studies indicating that nearly a quarter experience such symptoms. By the 2010s, this figure had risen to nearly 40%, alongside a 13% prevalence of cognitive impairment. This symposium brings together five papers that explore the early- and late-life determinants of psychological and cognitive health in older Chinese adults. The research topics span childhood food insecurity, job loss, late-life living arrangements, social engagement, and end-of-life hospice care. Wuyi examines the long-term impact of childhood food insecurity on depression trajectories, considering the roles of hukou status and the timing of food insecurity. Dr. Song et al. analyze the effects of involuntary job loss on late-life depressive symptoms, focusing on the rural-urban hukou divide and work ownership differences. Dr. Zhang et al. assess how late-life living arrangement transitions impact depressive symptoms, particularly among rural and urban older adults. Zhang et al. explore the effects of reduced social engagement on cognitive function during the COVID-19 pandemic, emphasizing gender and urban-rural disparities. Lastly, Yi et al. investigate depression and anxiety symptom networks in Chinese hospice patients, comparing different symptom profiles. Together, these studies deepen our understanding of the social determinants of mental and cognitive health in older Chinese adults, offering insights for policy and intervention development. Chinese Gerontology Studies Interest Group Sponsored Symposium

Abstract citation ID: igaf122.1446

**THE (NON)IMPACT OF EARLY-LIFE JOB LOSS ON LATER-LIFE DEPRESSIVE SYMPTOMS: CITIZENSHIP STATUS AND WORK OWNERSHIP**

Qian Song, *University of Massachusetts Boston, Boston, Massachusetts, United States*

This study examines the impact of non-fault, involuntary job loss experience on depressive symptoms in later life among older Chinese adults. We investigate the heterogeneity of impacts along two important social axes in China – the rural/urban citizenship divide and work ownership in urban China. Utilizing the 2011-2018 China Health and Retirement Longitudinal Study and the 2014 Life History survey, we employed growth curve models with inverse-probability of weighting. Results show that urban citizens experienced heightened depressive symptoms at age 50 ($\beta = 0.75$, $p < .05$), with effects persisting into older age, whereas rural migrants (i.e., rural citizens) showed no significant impacts. Further, only job loss from public sectors adversely affected urban citizens' mental health in later life ($\beta = 0.91$, $p < .05$), whereas private-sector losses had no impact. Notably, job loss during the 1990s to mid-2000s, the period of critical market transition and massive public-sector layoffs, had the most profound impact on the mental health of older Chinese.

Abstract citation ID: igaf122.1447

**THE LASTING IMPACT OF CHILDHOOD FOOD INSECURITY ON DEPRESSION TRAJECTORIES: THE ROLE OF CITIZENSHIP AND TIMING**

Wuyi Dong, *University of Massachusetts Boston, Boston, Massachusetts, United States*

Using 2011-2018 China Health and Retirement Longitudinal Study and the 2014 Life History survey, this study examines the association between childhood food insecurity (before age 17) and later-life depression trajectories among Chinese older adults. It further explores the role of the rural-urban hukou divide and the timing of food insecurity across three developmental stages—early childhood (ages 0–5), middle childhood (ages 6–12), and adolescence (ages 13–17) shaping this relationship. The final sample includes 27,235 observations from 6,818 respondents aged 50 and older with the experience. Growth curve models with age centered at 55 were used. 81.3% of respondents experienced childhood food insecurity, with higher prevalence among rural hukou holders (82.9%) than urban counterparts (75.1%). Regression analyses show that childhood food insecurity is significantly associated with higher depressive symptoms in later life ($\beta=2.14$, $p < .001$) but not with changes in symptom growth over time among the overall sample. Stratified analyses reveal that only among rural hukou holders, food insecurity is significantly associated with higher depressive symptoms at age 55 ($\beta=3.06$, $p < .001$), though symptom growth slowed with age ($\beta$

= -0.21, p < .01). Further, among rural hukou holders, those exposed to food insecurity in adolescence (ages 13–17), a critical transition period marked by significant physical, emotional, and social development, exhibited higher depressive symptoms at age 55 ($\beta$=2.95, p < .01) than those exposed in early childhood (0–5).

Abstract citation ID: igaf122.1448

### THE IMPACT OF LIVING ARRANGEMENT TRANSITIONS ON DEPRESSION AMONG OLDER ADULTS: HOW TRANSITIONS AND WHO LEAVES MATTER

Yalu Zhang[1], Xintong Zhao[2], and Meijun Wan[2], *1. Stony Brook University, Stony Brook, New York, United States, 2. Southwest Medical University, Luzhou, Sichuan, China (People's Republic)*

Many older adults experience transitions in living arrangements, particularly shifts from cohabitation to living alone due to widowhood or children moving out. These changes can impact mental health by increasing depression risks due to reduced social support. While prior research examines living arrangements and mental health, fewer studies focus on how different transitions to living alone affect depression, particularly between losing a spouse versus children. Using data from the China Health and Retirement Longitudinal Study (CHARLS) 2011–2020 and fixed-effects models, this study first examined the effect of transitioning from cohabitation to living alone on depression among older adults. Second, it compared the mental health effects between those who transition from living with a spouse to living alone and those who transition from living with children to living alone. Third, it further explored disparities in these effects between rural and urban older adults. The results indicate that transitioning to living alone is significantly associated with increased depressive symptoms. In particular, the older adults who transition from living with a spouse to living alone experience a notable rise in depression ($\beta = 0.528$, $p < 0.001$), while those transitioning from living with children to living alone exhibit an even greater increase in depression ($\beta = 1.036$, $p < 0.001$) in the national sample (N = 5,176). These patterns persist across rural and urban subsamples, with rural older adults (n = 4,126) experiencing more pronounced effects. These findings highlight the need for targeted social and mental health interventions that account for the nature of living arrangement transitions.

Abstract citation ID: igaf122.1449

### KEY DEPRESSION AND ANXIETY SYMPTOMS IN HOSPICE PATIENTS: A NETWORK ANALYSIS COMBINED WITH LATENT PROFILE ANALYSIS

Zhiqi Yi[1], Shuo Xu[2], Jing Zhao[3], and Hongmei Wang[3], *1. University of Kansas, Lawrence, Kansas, United States, 2. Renmin University of China, Beijing, Beijing, China (People's Republic), 3. Beijing Sereniturn Palliative Care Development Center, Beijing, Beijing, China (People's Republic)*

Depression and anxiety are prevalent among hospice patients. A detailed understanding of the symptom comorbidity and key symptoms of depression and anxiety among Chinese hospice patients facilitate targeted interventions. This study investigates the depression and anxiety symptom network and compares networks in different symptom profiles in 388 Chinese hospice patients (Mean age=73.62). Patient Health Questionnaire-9 and the Seven-Item Generalized Anxiety Disorder Scale were used to measure depression and anxiety. Psychometric network analysis and latent profile analysis were conducted using R. Hopelessness and anhedonia in depression, and excessive worry and nervousness in anxiety symptoms were identified as the most central symptoms. Hopelessness, nervousness, and irritability were identified as the bridging symptoms. Latent profile analysis identified two profiles based on sixteen symptoms: "mild-symptom" and "severe-symptom" profiles. Significant global strength differences were found between the networks of mild-symptom profile and severe-symptom profile. In the mild-symptom network, hopelessness, excessive worry, reduced concentration, and anhedonia were the central symptoms, while excessive worry, insomnia, and hopelessness were the bridging symptoms. In the severe-symptom network, hopelessness, difficulty relaxing, reduced concentration, and nervousness were the central symptoms, while hopelessness, irritability, and nervousness were the bridging symptoms. These findings highlight that hopelessness should be the key intervention target to reduce the overall depression and anxiety symptoms in Chinese hospice patients, combining intervening in other key symptoms, including anhedonia, excessive worry, and nervousness. Intervening in hopelessness, nervousness, and irritability helps reduce the concurrence between depression and anxiety. Nuanced intervention strategies for hospice patients with different symptom severity should be adopted.

Abstract citation ID: igaf122.1450

### POST-COVID DECLINE IN SOCIAL ENGAGEMENT AND COGNITIVE FUNCTION AMONG CHINESE OLDER ADULTS

Wei Zhang, *University of Hawai'i at Mānoa, Honolulu, Hawaii, United States*

Purpose: This study examines the impact of reduced social engagement on cognitive function among older adults in China during the COVID-19 pandemic, with a focus on gender and urban-rural differences. Methods: Using data from the China Health and Retirement Longitudinal Study, we employed OLS regression models to assess changes in cognitive function before and after the pandemic, focusing on how these changes are linked to changes in social engagement, which was measured across a variety of activities, including both physical and cognitively stimulating tasks. Findings: Significant cognitive declines were observed across all subgroups, with urban females experiencing the greatest reduction. Reduced participation in moderate physical activities appeared to have a protective effect, while decreased engagement in cognitively stimulating activities, such as playing games, was associated with greater cognitive decline. Conclusions: The study highlights the importance of maintaining social engagement, particularly in cognitively stimulating activities, to mitigate cognitive decline among older adults. The observed differences in cognitive health outcomes by gender and urban-rural status suggest a need for targeted interventions to support older populations in the post-pandemic context.

### SESSION 3325 (SYMPOSIUM)

Abstract citation ID: igaf122.1451

### FROM TRADITION TO INNOVATION: THE FUTURE OF AGING IN KOREAN AND KOREAN AMERICAN COMMUNITIES

Chair: Kyeongmo Kim
Co-Chair: Giyeon Kim
Discussant: Nan Sook Park

As aging populations grow in both Korea and Korean American communities, the intersection of tradition and innovation plays an important role in shaping the health and well-being of older adults. This interdisciplinary symposium highlights research in Korean and Korean American communities, bridging traditional practices and modern innovations to address health behaviors, mental health, digital inclusion, and novel interventions. The main objectives of this symposium are to (1) examine how traditional values and emerging innovations influence aging experiences among Korean and Korean American older adults, (2) identify key social and technological determinants of well-being, and (3) discuss policy and intervention strategies that can support older adults in these communities. This session features experts in gerontology, social work, and public policy to offer a nuanced understanding of aging in Korean and Korean American communities. Dr. Yi examines Korean American older adults' reliance on traditional complementary and alternative medicine (TCAM) due to healthcare access barriers, highlighting individual and geographic influences. Dr. Lim-Soh investigates the impact of post-retirement workforce re-entry on depressive symptoms in Korea, revealing mental health benefits of bridge employment. Dr. Kang analyzes digital skills trajectories among middle-aged and older Koreans, identifying key social and demographic determinants influencing adaptation. Finally, Dr. Kwon assesses the feasibility of a virtual reality-based psychological intervention for older Korean immigrants in the U.S., addressing mental health challenges linked to racial discrimination and the COVID-19 pandemic. By bridging tradition and innovation, this discussion provides insights into culturally responsive policies and interventions that support aging populations across borders. Korean and Korean American and Aging Interest Group Sponsored Symposium

Abstract citation ID: igaf122.1452

### TRADITIONAL COMPLEMENTARY AND ALTERNATIVE MEDICINE USE AMONG KOREAN AMERICAN OLDER ADULTS: A MULTILEVEL ANALYSIS

Grace Yi[1], and Yuri Jang[2], *1. California State University, Fullerton, Fullerton, California, United States, 2. University of Southern California, Los Angeles, California, United States*

Korean American older adults (KAOAs) often face challenges in accessing the conventional healthcare system due to limited knowledge of the healthcare system, financial constraints, and cultural or linguistic differences that may affect their health beliefs and behaviors. As a result, many KAOAs turn to traditional complementary and alternative medicine (TCAM). However, research on TCAM use among this population remains limited. To address this knowledge gap, this study examined individual and geographic level factors associated with TCAM utilization. Data were drawn from the Study of Older Korean Americans (SOKA), a cross-sectional survey conducted between 2017 and 2018 across five states (California, New York, Texas, Hawaii, and Florida) (N = 2,150). Binomial logistic and multilevel logistic regression analyses were conducted. Findings from binomial logistic regression showed that several individual-level factors were positively associated with TCAM use, including health/well-being factors [arthritis (OR = 1.55, $p<.001$), health needs (OR = 1.12, $p<.01$), and lack of interest in work/leisure (OR = 1.74, $p<.01$)]; acculturation factor (use English frequently, OR = 1.45); demographic factors [gender (OR = 1.31), spirituality (OR = 1.31), perception Korean community density (OR = 1.38)]. Multilevel analysis further revealed significant state-level differences in TCAM use after controlling for the individual factors. These findings suggest that age-related physical/emotional changes may drive TCAM utilization, highlighting its potential role as a preventive health measure among ethnic minority older adults. Variations in TCAM use across states reveal the influence of community and geographic factors that shape the health-promoting behaviors of ethnic minorities, calling for culturally tailored healthcare services.

Abstract citation ID: igaf122.1453

### TRADITIONAL RETIREMENT OR BRIDGE EMPLOYMENT? TRAJECTORIES OF DEPRESSIVE SYMPTOMS AFTER RETURNING TO WORK IN KOREA

Jeremy Lim-Soh[1], Hui Xiang Chia[2], Shannon Ang[3], Rahul Malhotra[1], and Pildoo Sung[4], *1. Duke-NUS Medical School, Singapore, Singapore, 2. Lee Kuan Yew School of Public Policy, National University of Singapore, Singapore, Singapore, 3. Nanyang Technological University, Singapore, Singapore, 4. Hanyang University, Seoul, Republic of Korea*

Along with an increase in life expectancy and healthy life expectancy among aging populations globally, there is a re-evaluation of the traditional concept of retirement and the value of work in later life. This study explores Korean workers' exit and re-entry into employment, and their associated trajectories of change in depressive symptoms. Nine waves of the Korean Longitudinal Study of Ageing provided data on the depressive symptoms (Center for Epidemiologic Studies – Depression scale; CES-D) of individuals 45 years and older who left work (mean age after leaving work was 66 years). Group-based trajectory modeling captured heterogeneous changes over time in depressive symptoms, describing the diverse experiences of population sub-groups. We estimated associations between the trajectories and time-invariant individual characteristics and also considered the time-variant role of returning to work. Three trajectory groups were identified: Consistently Low Depressive Symptoms (18%), Consistently Moderate Depressive Symptoms (64%), and Clinically Relevant Depressive Symptoms (19%). The Clinically Relevant Depressive Symptoms group had a CES-D score above the clinical cutoff and were more likely to be self-employed (before retirement), have lower education, and live in non-metropolitan areas. For this group, returning to work had a strong ameliorative effect on depressive symptoms. These findings underscore the dignity of work and the importance of productive aging to mental health. Aging societies should reconsider the overly fixed notion of a universal retirement age and explore policy innovations such as bridge employment and flexible re-employment.

Abstract citation ID: igaf122.1454

### THE TRAJECTORIES OF DIGITAL SKILLS AND THEIR DETERMINANTS AMONG MIDDLE-AGED AND OLDER KOREANS

Miseon Kang[1], and Hyo Jung Lee[2], *1. Symbiotic Life-TECH Institute, Seoul, Republic of Korea, 2. Yonsei University, Seoul, Republic of Korea*

The digital divide manifests within and between the people in diverse aspects. This phenomenon became more evident during COVID-19 pandemic, which profoundly accelerated digitalization. In this context, individuals exhibit diverse trajectories in their digital skills, shaped by differing life choices, social positions, and environments. Recognizing this, this study explored distinct patterns of digital skills trajectories among middle-aged and older Koreans and examined the key determinants influencing these trajectories. 'The Korea Media Panel Survey' (2018-2021) was used, and the sample included 2,291 middle-aged Koreans (aged 45-64) and 1,528 older Koreans (aged 65 and above). Latent class growth analysis and multinominal logistic regression were employed. The analysis identified five digital skills trajectory types among middle-aged adults: 'Digitally advanced (50.1%)', 'Digitally shrinking (11.8%)', 'Digitally transforming (18.0%)', 'Digitally adapting (16.0%)', and 'Digitally stalled (4.1%)'. Among older adults, three digital skills trajectories were identified: 'Digitally skilled (5.6%)', 'Digitally adapting (26.8%)', and 'Digitally dragging (67.6%)'. For middle-aged adults, significant determinants of digital skills trajectory types were age, gender, educational level, employment status, monthly income, residential area, need for cognition, and smartphone ownership. For older adults, age, gender, educational level, monthly income, residential area, marital status, need for cognition, and smartphone ownership predicted the types of digital skills trajectories. These findings highlight the heterogeneity of digital skills trajectories in middle-aged and older adults and underscore the importance of these specific determinants in designing tailored interventions to promote digital skills and competencies for these populations.

Abstract citation ID: igaf122.1455

### FEASIBILITY AND USER EXPERIENCE OF VIRTUAL REALITY POSITIVE PSYCHOLOGICAL INTERVENTION IN OLDER KOREAN IMMIGRANTS

Soonhyung Kwon, *University of South Florida, Tampa, Florida, United States*

Older Korean immigrants in the U.S. face significant mental health challenges, particularly discrimination-related stress, anxiety, and depression, exacerbated by anti-Asian racism during the COVID-19 pandemic. Limited access to culturally and linguistically appropriate mental health care necessitates innovative approaches, such as virtual reality (VR)-based positive psychological interventions. This study examined the feasibility, acceptability, and user experience of a culturally tailored VR-based positive psychological intervention for older Korean immigrants during the pandemic. A mixed-methods approach was employed. The three-week (four-module) intervention assessed feasibility through recruitment, retention, adherence, and dropout rates. Acceptability and user experience were evaluated using surveys and semi-structured interviews. Feasibility was supported by high retention (96.9%) and engagement. Participants reported positive emotional engagement, cultural relevance, and ease of use. Participants found the VR intervention engaging, particularly enjoying the immersive nature-based environments and interactive exercises, which fostered a sense of achievement, gratitude, and a positive mindset. However, some faced technical difficulties and physical discomfort, such as pressure from the headset and eye strain due to blurred screens. A VR-based culturally tailored positive psychological intervention is a feasible and acceptable tool for addressing mental health disparities in older Korean immigrants. Future research should refine usability and assess long-term effectiveness for broader implementation.

### SESSION 3330 (SYMPOSIUM)

Abstract citation ID: igaf122.1456

### THE VA NORMATIVE AGING STUDY: REFLECTING SIX DECADES OF AGING RESEARCH AND INNOVATING FORWARD

Chair: Lewina Lee
Co-Chair: Carolyn Aldwin
Discussant: David Almeida

Deeply-phenotyped longitudinal cohort studies have been the cornerstone of aging research. They collect rich data on biopsychosocial processes, including biomarker and neurocognitive assessments, and embed time-intensive experience sampling protocols that enable rigorous designs to inform etiological knowledge and developmental processes underlying health and well-being outcomes. The Veterans Affairs Normative Aging Study (NAS), initiated to distinguish between illness and normal aging, is among first longitudinal aging studies initiated in the US in the mid-20th century and has evolved into a deeply-phenotyped study over 60+ years. This symposium will consider the scientific contributions of NAS to the fields of stress, personality, and aging. It will also examine recent innovations aimed to augment the scientific value of aging cohorts nearing the end of participants' lifespan. Dr. Spiro will describe the evolution of NAS from its inception in 1963 to become an exemplar deeply-phenotyped study through substantial efforts to cultivate a robust psychosocial research program. Dr. Aldwin will synthesize findings and lessons learned from four decades of stress and coping research using NAS. Turning to methodological developments, Dr. Mroczek will illustrate how a multi-study coordinated analysis framework can leverage the strengths of aging cohort studies to enhance scientific replicability. Dr. Marino will demonstrate the use of administrative data linkages to enrich NAS with historical data on childhood environmental (lead) exposure and adding siblings into the cohort. Dr. Almeida, an expert on studying stress and aging using intensive longitudinal designs, will discuss the collective wisdom gleaned and help envision the next generation of research. Aging Veterans: Effects of Military Service across the Life Course Interest Group Sponsored Symposium

Abstract citation ID: igaf122.1457

### THE VA NORMATIVE AGING STUDY: THE AGING OF A LONGITUDINAL STUDY OF AGING

Avron Spiro, *Boston University - Chobanian & Avedisian School of Medicine, Waban, United States*

In 1963, the Normative Aging Study (NAS) was initiated at the Boston VA Outpatient Clinic. Over the next several years, 2280 men, mostly veterans, were enrolled into a life-long study of aging. To better understand the distinction between aging per se vs. the progression of disease, these men were selected for good health, regardless of age. The intention was to examine, over their lifespan, the interplay between aging and the development and progression of disease. Men returned to the clinic every few years for examinations and completed periodic surveys that assessed their health, disease, anthropometry, and health behaviors. In this talk, we will discuss the origins of the NAS and its place in the first wave of longitudinal studies of aging (e.g., Framingham, Baltimore, Duke). We will then focus on the growth of the psychosocial research program at NAS, which was nascent in the initial decades but expanded rapidly during the 1980's. This program examined social support, stress and coping, mental health, personality and cognition. We will also mention recent work in this deeply phenotyped study that focuses on epigenetics and its role in psychosocial aging and health. In concluding, we offer some observations on the complexities of establishing and maintaining longitudinal studies and their contributions to the study of aging and development.

Abstract citation ID: igaf122.1458

### FORTY YEARS OF STRESS & COPING RESEARCH AT THE VA NORMATIVE AGING STUDY

Carolyn Aldwin, *Oregon State University, Corvallis, Oregon, United States*

The VA Normative Aging Study (NAS) began collecting data in the 1980s on trauma, stressful life events (SLEs), hassles, and coping.

At that time, the idea that psychosocial stress affected health was controversial, with critics arguing that stress was simply confounded with personality. Our first study showed that neuroticism did predict stress 10 years later, but both SLEs and hassles had independent effects on mental health. Further, combat exposure (CE) had long-term effects on mental health. WWII Veterans with moderate CE were 13 times more likely to experience PTSD symptoms 45 years later, especially among those who reported negative appraisals of their military service, poor social support and negative homecoming experiences. While CE did not predict mortality, those who also experienced civilian trauma were 16% more likely to die prematurely. However, CE also had positive effects on well-being in late life, especially for those with positive appraisals of their military service. While SLEs and hassles generally declined with age, there were marked individual differences in patterns of stress across the lifespan; those with consistently elevated SLEs had a 42% greater risk for premature mortality, while those with consistently strong reactions to hassles had a 63% greater risk. Coping strategies also decreased with age, but coping efficacy was usually maintained, suggesting more efficient coping in late life. Those with elevated coping effort had a 14% greater risk of premature mortality. Thus, NAS research showed conclusively that stress and coping processes had marked effects on health in later life, independent of personality.

Abstract citation ID: igaf122.1459

### MULTI-STUDY COORDINATED ANALYSIS TO PROMOTE REPLICABILITY: EXAMPLE USING SELF-RATED HEALTH

Daniel Mroczek, *Northwestern University, Evanston, Illinois, United States*

Self-reported health is a non-invasive and low-cost way of identifying who is at risk of poor health in later life. We examined the extent to which self-rated health was associated with cognitive health outcomes, both cross-sectionally and longitudinally. Using the coordinated data analysis methodological framework to promote robustness an replicability in our findings, we identified 14 longitudinal datasets worldwide that met analytic inclusion criteria. Our pre-registered analyses tested whether self-rated health was associated with cognitive function both concurrently and at longitudinal follow up, as well as with odds of developing cognitive impairment or dementia over the course of the study, net of demographic and health covariates. Results indicated that, across nearly all datasets, higher self-rated health was associated with better cognitive function (both concurrently and longitudinally) and lower odds of developing dementia, over and above the effects of objective health (chronic conditions). Age moderated some of these effects, suggested that these associations may be strongest among older adults.

Abstract citation ID: igaf122.1460

### LEVERAGING ADMINISTRATIVE DATA LINKAGES TO STUDY WATERBORNE LEAD EXPOSURE IN LONGITUDINAL AGING COHORT STUDIES

Victoria Marino[1], Joseph Ferrie[2], Mina Antic[3], Ashley Dorame[4], Avron Spiro[5], Robert Waldinger[6], Daniel Mroczek[2], and Lewina Lee[7], *1. VA Boston Health Care System, Boston, Massachusetts, United States, 2. Northwestern University, Evanston, Illinois, United States, 3. Massachusetts General Hospital, Boston, Massachusetts, United States, 4. Boston University - Chobanian & Avedisian School of Medicine, Boston, Massachusetts, United States, 5. Boston University - Chobanian & Avedisian School of Medicine, Waban, United States, 6. Harvard University, Cambridge, Massachusetts, United States, 7. VA National Center for PTSD & Boston University Chobanian & Avedisian School of Medicine, Boston, Massachusetts, United States*

Lead (Pb) is a neurotoxin with lasting cognitive, behavioral, and physiological effects, particularly when exposure occurs in early childhood. Nearly half of the U.S. population today experienced Pb exposure during this critical developmental period, yet few studies have examined its long-term health consequences using a life course perspective. We aim to demonstrate the feasibility of aging cohort studies to construct a phenotype of early childhood Pb exposure via administrative data linkages. The Boston Early Adversity and Mortality Study integrated municipal, state, and federal records with data from three Boston-based, socioeconomically diverse longitudinal cohorts (the Normative Aging Study [NAS] and the Grant and Glueck [GG] cohorts of the Harvard Study of Adult Development) and added siblings (NBEAMS=13,151; nNAS=9,972, nGG=3,179). Pb exposure was operationalized using linkage-derived information on water service line materials (non-Pb, Pb, mixed Pb and non-Pb) and municipal water pH (continuous) anchored to participants' residential home address at birth. We examined Pb exposure patterns by childhood SES status. We identified birth locations for 99% of the 12,559U.S.-born BEAMS participants, spanning 688 unique city-state combinations. Pb exposure could be estimated for 52% of 688 birth locations, covering 93% ($n = 11,654$) of U.S.-born BEAMS participants. Pb exposure followed a socioeconomic gradient, $\chi 2(4)=1,035.9$, $p<.0001$, such that 90%, 66%, and 37% of the lowest- (Glueck), middle- (NAS), and highest- (Grant) SES cohorts, respectively, had the highest Pb exposure levels. We discuss the value of administrative data linkages in reconstructing environmental exposures and advancing life course research.

### SESSION 3335 (SYMPOSIUM)

Abstract citation ID: igaf122.1461

### GOT A QUESTION ABOUT PUBLISHING GLOBALLY IN GERONTOLOGY? ASK THE EDITORS!

Chair: Edward Miller
Co-Chair: Elizabeth Simpson

Global aging has proceeded at an unprecedented and accelerating rate. The aging of the population creates both opportunities and challenges for older adults, their families, and society. Importantly, there is substantial variation in the effects of and response to global aging both within and across nations depending, in part, on prevailing cultural expectations and values, political and economic imperatives, and social and demographic characteristics. Thus, while some regions and countries have responded with innovative policies and programs to better enable the growing cohort of older adults to remain active and engaged in the community, other regions and countries have struggled with their response or barely begun to plan for the rising population of older adults. This symposium assembles editors at five leading gerontological journals to demonstrate the role that peer-reviewed scholarship can play in disseminating knowledge that informs gerontological research, policy, and practice internationally. The editors include: Edward Alan Miller, PhD, Journal of Aging & Social Policy; Jeffrey Stokes, PhD, Research on Aging; Debra Dobbs, PhD, Journal of Applied Gerontology; Julie Hicks Patrick, PhD, International Journal of Aging and Human Development; and Sandra Torres, PhD, Ageing & Society. Each presenter will review the scope, content, and focus of their journals and the role and opportunities for international scholarship.

Abstract citation ID: igaf122.1462

**GLOBAL PUBLISHING OPPORTUNITIES AT THE JOURNAL OF AGING & SOCIAL POLICY**

Edward Miller[1], Elizabeth Simpson[1], Michael Gusmano[2], and Pamela Nadash[1], *1. University of Massachusetts Boston, Boston, Massachusetts, United States, 2. Lehigh University, Bethlehem, Pennsylvania, United States*

Policymakers, practitioners, and researchers need a balanced, thoughtful, and analytical resource to meet the challenge of global aging at a rate that's historically unprecedented. The Journal of Aging & Social Policy (JASP), which was founded in 1989, serves this role by drawing contributions from an international panel of policy analysts and scholars who assume an interdisciplinary perspective in examining and analyzing critical phenomena that affect aging and the development and implementation of programs for older adults from a global perspective. Study settings extend beyond the United States to include Europe, the Middle East, Australia, Latin America, Asia, and the Asia-Pacific rim. This presentation will document the scope, content, and focus of JASP, including the rise of international submissions, which now account for approximately half of articles published. Opportunities for publishing in JASP will be discussed; so too will strategies for navigating the peer-review process successfully.

Abstract citation ID: igaf122.1463

**RESEARCH ON AGING: THE INTERNATIONAL VIEW FROM THE EDITORS' DESKS**

Jeffrey Stokes[1], Kyungmin Kim[2], Changmin Peng[1], and Jeffrey Burr[1], *1. University of Massachusetts Boston, Boston, Massachusetts, United States, 2. Seoul National University, Seoul, Republic of Korea*

We review the scope, content, and focus of the peer-reviewed journal, Research on Aging (SAGE), publishing its 47th volume this year. We will discuss how scholarship produced from researchers around the globe has changed over the years. Data on submissions, acceptance rates, and the important role of an international editorial board will be presented. The review process will be described, along with suggestions on how to increase chances of success when submitting original research. Although Research on Aging is sometimes considered to focus primarily on social gerontology, the scope in recent years has widened considerably, with manuscripts in aging studies published from such fields as economics, psychology, demography, public health, and public policy, as well as from sociology, and social work, among others. One of several special issues forthcoming in the journal will be described to demonstrate the possibilities for international impact. Finally, new directions and changes undertaken by the incoming editorial team will be highlighted.

Abstract citation ID: igaf122.1464

**THE JOURNAL OF APPLIED GERONTOLOGY: AN INTERNATIONAL PERSPECTIVE**

Debra Dobbs, *University of South Florida, Tampa, Florida, United States*

The mission of applied gerontology is to bridge science and practice to benefit the health and well-being of older people, their families, their communities, and other contexts. Journal of Applied Gerontology features original investigations or meta-analyses/systematic reviews with significant clinical, policy, and/or practice implications. This presentation will provide insights from Journal of Applied Gerontology and its commitment to publish and disseminate scholarship with international application. Following an overview of the growing internationalization of peer-reviewed submissions to Journal of Applied Gerontology on a variety of topics and from a range of perspectives, the presentation will highlight opportunities to apply gerontological scholarship to aging contexts worldwide. Concluding comments will examine how outlets for dissemination and authors themselves can better position their work to enhance their influence on aging in an international context.

Abstract citation ID: igaf122.1465

**PUBLISHING WITH THE INTERNATIONAL JOURNAL OF AGING AND HUMAN DEVELOPMENT**

Julie Hicks-Patrick[1], and Danielle Nadorff[2], *1. West Virginia University, Morgantown, West Virginia, United States, 2. Mississippi State University, Starkville, Mississippi, United States*

For more than 50 years, under the leadership of four editors and two publishers, The International Journal of Aging and Human Development (IJAHD) has featured multidisciplinary scholarship related to aging processes and older adults. With the publication of eight issues a year and over 1000 pages of scientific content, the IJAHD places emphasis upon psychological and social studies of aging and the aged. However, the Journal also publishes research that integrates observations from other disciplines that illuminate the "human" side of gerontology. A more recent focus includes midlife development, as well. About half (47%) of the publications in the IJAHD are from international colleagues. IJAHD is delighted to support new investigators as they navigate dissemination. This presentation will discuss tips for both international and US-based scholars for ensuring timely reviews and positive decisions for manuscript submissions, including such areas as key words, suggesting unbiased reviewers, formatting, writing mechanics, clearly-articulated methods, and a sound theoretical basis.

Abstract citation ID: igaf122.1466

**PUBLISHING IN AGEING & SOCIETY: TOP TEN TIPS FROM THE EDITOR-IN-CHIEF**

Philip Taylor[1], and Sandra Torres[2], *1. University of Warwick, Coventry, England, United Kingdom, 2. Uppsala University, Uppsala, Uppsala Lan, Sweden*

Understanding the differences between academic writing genres is crucial to nailing down the craft that is writing for peer-review journal publication. Thus, this presentation will bring attention to the different publication outlets there are, the different readers these cater to, and how these differences impact how we craft manuscripts about the research we conduct. Using this as a starting point, as well as allusions to how data collected in specific national settings can be presented so that it can be used to contribute to ongoing international debates in social gerontology, this presentation will offer insight into an editor-in-chief's top 10 tips. By bringing specific attention to the routines that established international peer-review journals, such as Ageing & Society utilize, and the expectations that editors place on the different sections of a manuscript, this presentation aims to give attendees valuable insights into how to write for publication in such journals.

### SESSION 3340 (SYMPOSIUM)

Abstract citation ID: igaf122.1467

### PIONEERING AI INNOVATIONS TO TRANSFORM AGING AND GERONTOLOGICAL CARE

Chair: Walter Boot

The rapid advancement of artificial intelligence (AI) is reshaping society, offering unprecedented opportunities for innovation. In aging and gerontological care, AI holds transformative potential to enhance research, improve clinical decision-making, and support the health and well-being of older adults. This symposium highlights cutting-edge work applying AI to challenges in aging populations. Dr. Ian B. Kwok will introduce Clinical SmartReporter™, an AI-powered tool designed to enhance serious illness communication through real-time, patient-facing transcripts of family meetings. Yijung Kim will present an analysis of social determinants of health among homebound older adults, demonstrating how large language models like GPT-3.5 can support—but not fully automate—social needs identification. Dr. Amy M. Schuster will share survey findings showing that older adults' attitudes toward AI significantly influence their likelihood of using it in healthcare, with implications for improving acceptance through targeted interventions. Taekyung Kim will discuss age-related stereotypes in GPT-4o-generated content, revealing lower perceived competence in descriptions of older adults and calling for strategies to reduce ageism in AI systems. Dr. Bo Xie will present a multi-agent large language model framework for streamlining systematic reviews of AI interventions for older adults, showing how hybrid human–AI collaboration can accelerate evidence synthesis. Collectively, these talks explore how AI can be thoughtfully integrated into aging research and care to promote equity, effectiveness, and inclusion. Technology and Aging Interest Group Sponsored Symposium

Abstract citation ID: igaf122.1468

### MULTIMODAL AI FOR SERIOUS ILLNESS COMMUNICATION: OPPORTUNITIES AND BEST PRACTICES

Ian Kwok[1], and Kimberly Murdaugh[2], *1. New York-Presbyterian Hospital/Weill Cornell Medicine, New York, New York, United States, 2. New York-Presbyterian Hospital, New York, New York, United States*

Patients and families face complex communication challenges in the setting of serious illness. These difficulties are compounded for individuals who are non-English-speaking, have low health literacy, have visual/auditory impairments, or possess unique learning styles. We hypothesize that augmenting standard verbal communication modalities with a multimodal communication approach (integrating written, visual, and other modalities) can improve medical information-sharing. In the first use of artificial intelligence (AI) for patient-facing serious illness communication, we developed Clinical SmartReporter™, a program capable of generating real-time transcribed reports of family meetings for patients, their caregivers, and other clinicians. Clinical SmartReporter™ uses machine learning, natural language processing, and speaker diarization modules to accurately integrate medical terminology, translate between multiple languages, and distinguish between a large number of different speakers. In this presentation, we will describe our model of multimodal communication, present results of our user-centered design process based on patient, caregiver, and clinician interviews, and demonstrate the use of Clinical SmartReporter™ in the setting of a simulated family meeting (professionally filmed video). We will outline technology-based opportunities for the advancement of communication science in palliative care research, quality improvement, and medical education. We will also facilitate a discussion of practical and ethical challenges, as well as the need to establish field-wide best practices for AI implementation in geriatrics and palliative care settings. These include data security considerations, partnership with institutional offices for technology licensing and privacy/security, and protection of the integrity of intimate patient-clinician relationships, emphasizing the need for clinician advocacy and cross-institutional collaboration.

Abstract citation ID: igaf122.1469

### USING HOME VISIT NOTES TO PREDICT SOCIAL NEEDS AMONG HOMEBOUND OLDER ADULTS: LESSONS FROM MEDSTAR HOUSE CALL PROGRAM

Yijung Kim[1], Valeria Londono[2], Christine Chow[2], Lauren Bangerter[3], and Karl De Jonge[4], *1. Medstar Health Research Institute, Columbia, Maryland, United States, 2. Georgetown University School of Medicine, Washington, District of Columbia, United States, 3. Medstar Health, Columbia, Maryland, United States, 4. MedStar Washington Hospital Center, Washington, District of Columbia, United States*

Over four million U.S. adults are homebound, facing challenges related to social determinants of health (SDoH). Despite their impact on health outcomes, SDoH data for this population are often underrepresented in electronic health records. To assess prevalence and variability in social needs among homebound older adults, we analyzed unstructured text data from the MedStar Washington Hospital House Call Program, identifying SDoH patterns and evaluating automation using large language models. Our dataset included 1,823 home visit notes from 671 homebound older adults (mean age=88.03; 77% female; 83% Black/African American) written between 2021-2024. Using keyword-based identification, we extracted 702 SDoH-related sentences, manually coding them into nine categories. We examined correlations between identified SDoH patterns and patient characteristics, including demographics, baseline diagnoses, and emergency department (ED) use in the 12 months post-enrollment. To assess scalability, we evaluated SDoH identification using an instruction-tuned large language model (GPT-3.5). Among SDoH categories, excellent care/support was most frequently documented (44%), followed by caregiver burden (21%), social isolation (20%), unsafe environment (11%), lacking transportation (9%), substance abuse (9%), economic instability (7%), poor nutrition (5%), and grief (5%). Correlation analysis revealed excellent care/support was negatively associated with other adverse SDoH factors. In a regression model adjusting for demographics and baseline diagnoses, poor nutrition significantly predicted increased ED utilization. GPT-3.5 demonstrated moderate performance in SDoH identification (precision=0.85, recall=0.74, F1=0.76). Our findings highlight diverse SDoH patterns among homebound older adults. While GPT-3.5 showed promise for automation, we discuss policy and regulatory challenges in implementing the model for clinical use.

Abstract citation ID: igaf122.1470
**THE RELATIONSHIP BETWEEN OLDER ADULTS' ATTITUDE TOWARDS AI AND LIKELIHOOD OF USING AI IN HEALTHCARE**
Amy Schuster[1], Gwendolyn Watson[2], Shubham Agrawal[1], and Shelia Cotten[1], *1. Clemson University, Clemson, South Carolina, United States, 2. Auburn University, Auburn, Alabama, United States*
Artificial Intelligence (AI) is changing the way many fields, including healthcare, operate. The adoption of AI depends on both technological innovation and users' acceptance and use. Understanding factors that influence acceptance, like attitudes about AI, is critical for incorporating AI into healthcare. Older adults, compared to those younger, are more likely to have chronic medical conditions and use more healthcare services. We need to understand older adults' attitudes about AI as they may be more likely to encounter AI used in healthcare. This study explored the relationship between older adults' (60 years and older) attitudes towards AI (AI in general, human vs. AI interaction, and impact on well-being) and their likelihood of using AI in healthcare controlling for age, gender, race, level of education, relationship status, and self-reported health status. Data collected through an online survey (N = 405) were analyzed using multiple linear regression. Results indicate that older adults with more positive attitudes about AI in general and who were in more agreement that AI can have positive impacts on well-being were more likely to use AI in healthcare. Whereas older adults who had a higher preference of interacting with a human compared to an AI system were less likely to use AI in healthcare. Future research should explore factors contributing to older adults' negative attitudes towards AI and design interventions to improve these attitudes and, thereby, increase their likelihood of using AI in healthcare.

Abstract citation ID: igaf122.1471
**AN EXPLORATORY SEMANTIC ANALYSIS OF AGE-RELATED STEREOTYPES IN OPENAI'S GPT 4O MODEL**
Wan Hong, and Moon Choi, *Korea Advanced Institute of Science & Technology, Daejeon, Republic of Korea*
Generative artificial intelligence, particularly large language models (LLMs), has become a powerful tool for navigating information, potentially shaping users' ways of thinking and influencing their perceptions of different social groups. While a growing body of research has examined the presence of racism and sexism in LLM-generated content, the issue of ageism remains relatively underexplored. This study investigates age-related stereotypes in texts generated by LLMs utilizing natural language processing (NLP) techniques. To ensure methodological rigor, extensive pilot testing was conducted to develop a neutral prompt that did not fish for bias but still generated coherent responses. The final prompt followed the structure: "Describe the personality of a [AGE]-year-old person." Text responses were collected using OpenAI's GPT-4o API in February 2025, with the AGE variable ranging from 10 to 90 in increments of 10. The analysis was guided by the Stereotype Content Model, which evaluates social cognition along two key dimensions: warmth (sociability, morality) and competence (ability, agency). Findings indicate that descriptions of individuals aged 60 and older exhibit relatively lower competence than those of younger individuals. These findings suggest that OpenAI's GPT 4o model might embed age-related stereotypes, even when using mostly positive terms, potentially causing their users to be repeatedly exposed to characterizations containing these stereotypes. Future research should explore the underlying mechanisms driving age-related stereotypes in LLMs and develop strategies to mitigate them, ensuring more age-inclusive AI-generated content.

Abstract citation ID: igaf122.1472
**DOING SYSTEMATIC LITERATURE REVIEWS WITH ARTIFICIAL INTELLIGENCE TOOLS: WHAT, WHY, AND HOW**
Bo Xie[1], Kimia Tuz Zaman[2], Wordh Ul Hasan[2], and Juan Li[2], *1. The University of Texas at Austin, Austin, Texas, United States, 2. North Dakota State University, Fargo, North Dakota, United States*
Systematic reviews are indispensable to evidence-based practice but require considerable time and effort, particularly in screening large numbers of studies. Recent advances in large language models (LLMs) offer a promising avenue to reduce this burden through partial automation of the process. We present a two-stage multi-agent framework that leverages LLMs—to supplement human expertise—in conducting systematic literature reviews of AI interventions for older adults. In Stage 1, specialized LLM agents independently assess titles and abstracts against seven predefined criteria—including population age (≥65 years), AI focus, care setting, study design, outcome measures, older adult participation, and empirical evidence. An aggregator LLM agent then synthesizes these evaluations into a composite inclusion score. In Stage 2, a set of specialized LLM agents extracts structured data from full-text articles based on targeted research questions addressing technology types, outcome effectiveness, user acceptance, and ethical considerations. The extracted data are then merged into a comprehensive dataset that supports both qualitative and quantitative synthesis. Although our multi-agent system significantly reduces the time and labor traditionally required for manual screening and data extraction—while enhancing consistency through task specialization—human oversight remains essential for resolving ambiguous cases and making necessary adjustments. While the complete results of applying this framework will be reported elsewhere, this paper focuses on the method, its rationale, and its potential to accelerate evidence synthesis in the face of an ever-growing biomedical literature base. This innovative, scalable approach exemplifies how hybrid human–AI methods can advance the rigor and timeliness of systematic reviews.

### SESSION 3345 (SYMPOSIUM)

Abstract citation ID: igaf122.1473
**MEASURING STRUCTURAL RACISM TO ADVANCE HEALTH EQUITY IN AGING**
Chair: Karen Bandeen-Roche
Co-Chair: Boeun Kim
Discussant: Roland Thorpe
Structural racism is a fundamental determinant of health inequities among older adults, yet its complexity poses significant challenges for research. This symposium brings together a panel of interdisciplinary researchers with expertise in biostatistics, nursing, epidemiology, sociology, and public health to explore innovative approaches to measuring structural racism and understanding its impact on aging. Dr. Paris Adkins-Jackson of

Columbia University will discuss an autoethnographic study reflecting on a multidisciplinary team's approach to measuring structural racism, highlighting challenges and opportunities in this research. Dr. Karen Bandeen-Roche of Johns Hopkins University will present a novel modeling framework for measuring structural racism that analyzes racial disparities in healthcare, education, and policing to provide a better understanding of systemic inequities. Dr. Boeun Kim of University of Iowa will examine the impact of residential segregation and economic deprivation on mental health among older adults, highlighting how racialized economic segregation differentially affects Black and White individuals across the life course. Dr. Erik Westlund of Johns Hopkins University will highlight challenges in processing and sharing publicly available data to study structural racism, addressing issues such as recency bias and data integration. Dr. Roland J. Thorpe Jr. of Johns Hopkins University will serve as the discussant, leading a discussion on bridging research, policy, and practice to advance structural racism research in aging. This session will provide critical insights into methodological advancements, interdisciplinary collaboration, and the translation of research into action to drive meaningful change in aging and health equity.

Abstract citation ID: igaf122.1474

### MEASURING STRUCTURAL RACISM TO IMPROVE OLDER ADULT HEALTH USING PUBLICLY DERIVED DATA

Karen Bandeen-Roche[1], Erik Westlund[2], Sierra Grey-Coker[3], Boeun Kim[4], Roland Thorpe[2], and Sarah Szanton[3], *1. Johns Hopkins Center on Aging and Health, Baltimore, Maryland, United States, 2. Johns Hopkins Bloomberg School of Public Health, Baltimore, Maryland, United States, 3. Johns Hopkins University, Baltimore, Maryland, United States, 4. University of Iowa, Iowa City, Iowa, United States*

Racial disparities in older Americans' health have been extensively documented, hence must urgently be investigated and addressed. Among potential causes, this presentation concerns structural racism— "the totality of ways in which societies foster [racial] discrimination, via mutually reinforcing [inequitable] systems (e.g., in housing, education, ..., etc.)," (Bailey ZD et al., 2017). It aims to measure structural racism using publicly available data that were freely downloaded from websites of government agencies or social justice organizations, and that characterized geographical units nationwide. We report a partial hierarchy of models by which discrimination in mutually reinforcing systems might erode health. Five county-level indicators measuring Black-White disparity in healthcare, education, and policing were analyzed to illustrate the modeling framework and attendant data challenges. Factor analysis and cluster (latent profile) analysis agreed in identifying two major dimensions underlying the indicators: One in which disparities in preventive health care, educational progression, and remaining un-incarcerated tended to be lower or higher all together; and a second where benefits and harms coexisted, most strongly contrasting education and incarceration. Correlation of a z-score index of the indicators with the first factor exceeded 0.97 but was negligible with the second factor; clusters distinguished individuals whose indices were similar and vice versa. Correlations among measures differed substantially by geographic regions, indicating different systemic reinforcement networks. These findings suggest that meaningful signals can be derived from publicly derived data and a modeling hierarchy adds value over simple-sum indices. It aims to equip researchers with improved measures to elucidate, and address, health inequities.

Abstract citation ID: igaf122.1475

### JOINT IMPACT OF RESIDENTIAL SEGREGATION AND POVERTY ACROSS THE LIFE COURSE ON MENTAL HEALTH

Boeun Kim[1], Sarah Szanton[2], Laura Samuel[2], Karen Bandeen-Roche[3], Paris Adkins-Jackson[4], Erik Westlund[5], Sierra Grey-Coker[2], and Roland Thorpe, Jr.[2], *1. University of Iowa, Iowa City, Iowa, United States, 2. Johns Hopkins University, Baltimore, Maryland, United States, 3. Johns Hopkins Center on Aging and Health, Baltimore, Maryland, United States, 4. Mailman School of Public Health, Columbia University, New York, New York, United States, 5. Johns Hopkins Bloomberg School of Public Health, Baltimore, Maryland, United States*

Racially segregated communities often experience concentrated poverty, yet the impact of racialized economic segregation on mental health across the life course remains understudied. This study examined associations between life stage-specific, county-level racialized economic segregation and the number of mentally unhealthy days over the past 30 days among U.S. adults aged 50 and older. Racialized economic segregation during young and middle adulthood was measured using the Index of Concentration at the Extreme (ICE), which quantifies spatial social polarization between deprived (Black individuals in poverty) and privileged (White individuals not in poverty) groups. ICE scores (range: -1 to1) were derived from U.S. Census (1970–2000) and the American Community Survey (2010–2020) data and categorized into quintiles. Life-course addresses were geocoded and linked to county-level data corresponding to their ages. Negative binomial regression models were fitted separately for each life stage and racialized group. This analysis included 365 Black and 885 White participants (56.1% male; 24.3% aged 80+). After adjusting for age, gender, and population density, living in a county in the 4th or 5th ICE quintile during young adulthood —but not middle adulthood—was associated with a 61% or 56% lower likelihood of currently experiencing poor mental health days among White participants (IRR = 0.39, 95% CI: 0.17, 0.91; IRR = 0.44, 95% CI: 0.19, 1.00), compared to those in the 1st quintile. This pattern was not observed among Black participants. This finding contributes to research delineating sensitive periods of exposure to racialized economic segregation and mental health in older adults.

Abstract citation ID: igaf122.1476

### BETTER SCIENCE ENOUGH? AUTOETHNOGRAPHY OF A COLLABORATION TO MEASURE STRUCTURAL RACISM

Paris Adkins-Jackson[1], Emily Haozous[2], Boeun Kim[3], Alicia Cooke[4], Melissa Hladek[4], Laura Samuel[4], Deidra Crews[4], and Sarah Szanton[4], *1. Mailman School of Public Health, Columbia University, New York, New York, United States, 2. Pacific Institute for Research and Evaluation, Beltsville, Maryland, United States, 3. University of Iowa, Iowa City, Iowa, United States, 4. Johns Hopkins University, Baltimore, Maryland, United States*

Addressing structural racism remains a critical challenge in health and social sciences, despite significant methodological advances over recent decades. This autoethnographic study explores a multidisciplinary collaboration's approach to measuring structural racism through an intentional anti-racist praxis. Comprising biostatisticians, nurse scientists, epidemiologists, gerontologists, social scientists, and a nephrologist from diverse racial backgrounds, multi-career stages, and institutions, the research team employed innovative methodological approaches to examine structural racism's complex dimensions. Our collaborative efforts included conducting life course history interviews with older adults racialized as Black, developing an infrastructure for structural racism measurement, organizing an interdisciplinary symposium, and producing a multi-season podcast exploring varied perspectives on racism measurement. Key themes emerging from our work centered on understanding structural racism through lived experiences, institutional infrastructures, and comprehensive measurement strategies. By critically reflecting on our collaborative process, we illuminate both the methodological challenges and potential transformative approaches to understanding and addressing systemic racialized inequities in health research.

Abstract citation ID: igaf122.1477

### LESSONS FROM THE PROCESSING AND SHARING OF PUBLIC DATA SETS FOR THE STUDY OF STRUCTURAL RACISM

Erik Westlund[1], Boeun Kim[2], Sierra Grey-Coker[3], Karen Bandeen-Roche[4], and Sarah Szanton[3], *1. Johns Hopkins Bloomberg School of Public Health, Baltimore, Maryland, United States, 2. University of Iowa, Iowa City, Iowa, United States, 3. Johns Hopkins University, Baltimore, Maryland, United States, 4. Johns Hopkins Center on Aging and Health, Baltimore, Maryland, United States*

As part of a larger study of structural racism, we collected 50 publicly available data sets containing geographic measures. These data sets covered over 100 years of history, six geographic units, and nine domains of inquiry (civics, credit/income/wealth, education, employment, environment, healthcare, media/marketing, neighborhoods, and policing). Structured metadata about each data set were compiled and used to standardize data files with shared conventions, allowing analysts to combine data files to study structural racism. To allow researchers to assess the potential value of these data in relation to their areas of inquiry, we created dashboards that summarize key measures in each data set, including the geographic level of measurement, the years covered by the data, and the extent of missingness. This process made clear several problems researchers seeking to use public data files to study structural racism will face, such as recency bias, temporal and geographic mismatch of data sources, and data missingness. We also developed data transformation pipelines to process each data file to follow shared conventions that allow reliable linking of data files across spatial units and time periods. To help outside researchers to use these data in their own projects, we created a public repository of structured metadata about the form and content of data files, accompanied by tools to allow users to procure data files, process them, and analyze them. This effort provided insight into the challenges faced by researchers trying to follow best practices with respect to open science, particularly around data licenses and data custody.

### SESSION 3350 (SYMPOSIUM)

Abstract citation ID: igaf122.1478

### POLICY SERIES: FIFTY PLUS: ELDER ABUSE - PAST, PRESENT AND FUTURE

Chair: Bridget Penhale
Discussant: Pamela Teaster

This symposium welcomes inquiry and insights between gerontology, geriatrics, law, policy and elder abuse/adult protective services as areas for exploring knowledge and understanding of violence and abuse in later life. In bridging these contributions across violence intervention and prevention, we aim to celebrate 50 years of inquiry in the US on elder abuse – encompassing research, practice, and policy. For this interactive symposium, we have invited contributions to explore previous traditions and inquiry in the field, examine approaches currently being used, and uncover new and future perspectives. Through exploration, discussion, and interaction, we aim to foster collaborations and address challenges and opportunities arising within the field, how these occurred in the past, how they are managed currently, and in the next 10 years - including those that intersect with other areas in gerontology. Laura Mosqueda, M.D. will focus on clinical issues considering several key areas of medical/healthcare practice, including what is known (past work), what is currently happening (present) and what we need to know. From the social work perspective, Georgia Anetzberger, PhD, will explore some of the major developments in this field relating to research and practice, identifying strengths and shortcomings together with potential future initiatives. Robert Blancato, MPA will consider policy issues and the policy framework in elder abuse and elder justice – past, present and future. Finally, Erica Costello, JD will explore legal matters relating to elder abuse, and adoption of criminal and civil responses including examination of perspectives on fatality review teams and restorative justice.

Abstract citation ID: igaf122.1479

### CLINICAL ISSUES IN ELDER ABUSE RESEARCH

Laura Mosqueda, *University of Southern California, Pasadena, California, United States*

There are several areas in the clinical arena that have come a long way in recent decades yet still have a long way to go. Medical forensic markers of abuse/neglect, i.e. those symptoms, signs, and data that may show evidence of a crime are notoriously difficult to distinguish from normal and common age-related changes of aging. While bruises and some types of fractures have been studied, other markers such as pressure sores and behavioral indicators deserve to be researched as well. Another important clinical aspect relates to going upstream, to the prevention of abuse/neglect. There are well-documented risk and associated factors for elder mistreatment as an umbrella term, and it is time to distinguish among the sub-types of abuse as we study causes and consequences. For example, it may be that the risk factors for neglect are quite different than those for physical abuse which would then have significant implications for prevention. As the field matures, we ought and now need to develop a more nuanced understanding and approach to our work and future research can help to achieve this. This brief presentation will consider what is known about this field (from past work), what is happening now (current work and initiatives) and what do we need to know (possible work in future).

Abstract citation ID: igaf122.1480

### SOCIAL WORK'S ROLE IN ELDER ABUSE RESEARCH

Georgia Anetzberger, *Case Western Reserve University, Cleveland, Ohio, United States*

Social work's interest in understanding and addressing elder abuse predates the 1975 recognition of the mistreatment of older adults as a problem. Indeed, the discipline was the earliest lead in the development and evaluation of adult protective services, today still the dominant intervention for elder abuse. This presentation will explore social work research, both past and present. It will briefly examine collective inquiries to date on adult protective services and other social services directed at elder abuse, with the aim to identify relevant strengths and shortcomings and to offer suggestions for improvement and future research in this domain.

Abstract citation ID: igaf122.1481

### POLICY ISSUES IN ELDER ABUSE

Bob Blancato, *Elder Justice Coalition, Washington, District of Columbia, United States*

My work in elder abuse and elder justice has been at the national policy level for almost the entire 50-year history of elder abuse research. This includes more than 10 years as a Subcommittee Staff Director on the House Select Committee on Aging, when the first hearings on elder abuse in the early 1980's were initiated and held. Since 2003, I have been the National Coordinator of the bipartisan Elder Justice Coalition, which led the advocacy efforts on behalf of the landmark Elder Justice Act of 2010. Research in elder abuse and its proper dissemination has and continues to make important contributions to both legislative and administration policy in elder justice. This brief presentation will consider what has been achieved in the policy domain relating to elder abuse, both past and present. It will explore initiatives to date on elder justice and other programs concerned with elder abuse, neglect and exploitation. The aim is to identify the relevant strengths and weaknesses/challenges of approaches and initiatives already taken, both past and present and to offer suggestions for future improvements and potential research to support this important area of public policy.

Abstract citation ID: igaf122.1482

### REFLECTING ON THE DIVERSE LEGAL RESPONSES TO ELDER ABUSE IN THE UNITED STATES

Erica Costello, *American Bar Association, Washington, District of Columbia, United States*

Elder abuse is a complex and pervasive problem affecting countless older adults throughout the United States. It is estimated that at least one in ten community dwelling older adults have experienced some form of abuse in the past year, including physical abuse, emotional abuse, financial abuse, and neglect by family members or caregivers. This presentation will highlight the diverse legal responses that have been adopted over the past fifty years to reduce and prevent elder abuse, including the creation of elder abuse fatality review teams and multi-disciplinary teams, and the adoption of criminal and civil responses to abuse. Restorative justice practices will also be discussed as a unique viable alternative to criminal or civil responses when older adults are abused by family members or caregivers. Rooted in Native American culture, the concept of restorative justice provides older adults with the opportunity to address elder abuse and resolve challenging disputes within families.

### SESSION 3355 (PAPER)

### INNOVATIVE AND INCLUSIVE APPROACHES TO DEMENTIA AND CHRONIC CAREGIVING SUPPORT

Abstract citation ID: igaf122.1483

### DEVELOPMENT OF A CONCEPTUAL FRAMEWORK FOR ART MAKING INTERVENTION IN CHRONIC PAIN MANAGEMENT: A NARRATIVE REVIEW

Kyung Soo Kim, Clara Baldus, and Harleah Buck, *University of Iowa, Iowa City, Iowa, United States*

Chronic pain is a significant public health concern requiring holistic, multidimensional interventions. Art making has emerged as a promising approach to pain management, yet its theoretical foundations remain underdeveloped. Establishing a clear framework is essential for advancing research and ensuring scientific rigor. This narrative review synthesizes existing literature to explore how art making influences chronic pain and proposes a framework for its potential mechanisms. We conducted a narrative review with thematic analysis using a predetermined codebook. A systematic search of Medline, CINAHL Plus with Full Text, and Art Full Text identified articles published from January 2010 to March 2024. Studies were included if they examined art making interventions aimed at improving health outcomes such as pain or mental health and discussed their theoretical foundations. Exclusion criteria included a focus on art appreciation, non-English language, or lack of full-text availability. From 21 identified articles, 19 addressed theoretical foundations of art making interventions. Most studies ($n = 12$) relied on a single predetermined concept, such as creation of art, creativity, self-expression, or distraction, to explain their intervention's foundation. This review suggests that art making can improve the multidimensional pain experience: 'distraction' may alleviate the physical aspect of pain, while 'self-expression' supports psychological well-being. However, the role of 'creativity,' a key factor in subjective well-being and neuroplasticity, remains underexplored. Further research is needed to integrate creativity into a more comprehensive theoretical framework for chronic pain management.

Abstract citation ID: igaf122.1484

### DEVELOPMENT OF NEW FAMILY CAREGIVING MOTIVATION SCALE

Dingyue Wang[1], Qing Yang[2], Michael Cary[2], Tamryn Gray[3], and Cristina Hendrix[2], *1. Duke University, Seattle, Washington, United States, 2. Duke University, Durham, North Carolina, United States, 3. The University of North Carolina at Chapel Hill, Chapel Hill, North Carolina, United States*

Caregiver motivation refers to the underlying reasons individuals choose to take on or continue in the role of a family caregiver for those with chronic illnesses or disabilities. Motivation for caregiving varies among individuals. Some naturally embody the role of a family caregiver or feel a strong calling to take on this responsibility, while others may not find caregiving to be aligned with their personality or strengths. Existing measures do not fully capture the multifaceted nature of caregiving motivation. This study aimed to develop and validate the Caregiver Motivation Scale (CMS). A total of 227 family caregivers were recruited through Amazon CloudResearch's supervised panel. The 13-item CMS was developed based on concept analysis, an in-depth qualitative study (caregiver interviews, n=20), and an expert panel (n=4). Validity was assessed using the Old Caregiver Motive Scale (for convergent validity) and the Caregiver Preparedness Scale (for divergent validity). Items with a factor loading above 0.45 were retained. Principal Axis Factoring identified two distinct factors: (1) Intrinsic motivation (e.g., love, emotional connection, appreciation, sense of purpose) and (2) Obligatory motivation (e.g., external expectations, duty, responsibility, lack of choice). Nine items loaded onto Factor 1, and four onto Factor 2. The CMS demonstrated good psychometric properties (S-CVI = 1, Cronbach's alpha = 0.764, Kaiser's MSA = 0.875, RMSR = 0.046) and serves as a valid and reliable measure of caregiver motivation, distinguishing between intrinsic and obligatory motivations. Future research should explore its use in longitudinal studies and diverse caregiving populations.

Abstract citation ID: igaf122.1485

### ENGAGING A COMMUNITY COUNCIL FOR LATINO DEMENTIA RESEARCH: FINDINGS FROM A MIXED-METHODS STUDY

Sara Masoud[1], Janna Lesser[1], Juana Escareno[1], Belinda Flores[1], Byeong Yeob Choi[1], Carole White[1], and Karla Daniela López-Lorenzo[2], *1. UT Health San Antonio, San Antonio, Texas, United States, 2. Texas A&M University, College Station, Texas, United States*

Latino communities are disproportionately affected by Alzheimer's disease, yet they remain underrepresented in dementia research. To address this issue in alignment with community engaged frameworks, we formed a Steering Council (SC) (N=16) of caregivers, individuals living with dementia, community health workers, health and social service providers, and researchers. The SC worked with the study team to design and implement six "community pláticas" among different South Texas communities. These gatherings were carefully designed to integrate culturally-specific components (e.g., music, food, language translation), with a key component being reciprocal group discussions aimed at identifying research priorities and patient-centered outcomes. To assess whether strategies to engage the SC were effective, an explanatory sequential mixed-methods design study was employed. The Research Engagement Survey Tool (REST) was administered at three time points, revealing statistically significant increases in quality of engagement over time ($p<0.05$ across multiple engagement principles) and strengthened levels of engagement (collaboration and partnership). Additionally, 10 in-depth interviews with SC members provided insight into the dynamics of engagement, emphasizing the evolution of trust, co-learning, and collective leadership over time. Integrated analysis confirmed that iterative, community-driven engagement strategies contribute to stronger, more sustainable research partnerships. This study offers a replicable framework for inclusive research engagement. Findings reinforce the need for culturally-aligned, patient- and family-centered research models that reflect the needs, perspectives, and experiences of populations typically excluded from research.

Abstract citation ID: igaf122.1486

### LIVED EXPERIENCES VERSUS ARTIFICIAL INTELLIGENCE TO REDUCE HOSPITAL READMISSION: A QUALITATIVE STUDY

Elham Mahmoudi, *University of Michigan, Ann Arbor, Michigan, United States*

Frequent hospitalization including hospital readmissions are expensive and harmful to older adults, particularly those with dementia. Despite efforts to predict and reduce hospital readmissions through novel applications of machine learning (ML), readmission remains elusive. This study stemmed from our prior unsuccessful work and work of others attempting to use ML to predict and, by acting upon that, reduce hospital readmission among people with dementia. To understand what was missing in our ML models, we used a qualitative descriptive study and interviewed 21 participants (n=11 caregivers of people with dementia; n=10 hospital staff including nurses, discharge planners, and social workers). Participants took part in a semi-structured interview. To analyze the data, we used thematic text analysis to identify overarching themes. We identified five themes related to high risk of readmission among patients with dementia: (1) caregivers' burdens of providing dementia care, (2) lack of or inadequate support for caregivers, (3) lack of evidence-based educational materials for caregivers, (4) inadequate dementia-care protocols and guidelines across all healthcare settings, and (5) shortage of trained work force for dementia care. Mainly, in the absence of accessible, affordable, and dementia-specific outpatient medical care and respite centers, emergency departments and hospitals are caregivers only choices when care at home becomes too challenging. Recommendations provided by the caregivers and hospital staff to reduce readmission for patients with dementia include: (1) specialized wards in hospitals for dementia patients; (2) dementia-specific inpatient/discharge protocol with educational materials for caregivers, (3) at-home visits/follow-up, and (4) accessible and affordable home care services.

Abstract citation ID: igaf122.1487

### PARTICIPANT PERCEPTIONS OF A TRAUMA-INFORMED CARE PLANNING INTERVENTION: RESULTS FROM QUALITATIVE INTERVIEWS

Christine Kimpel[1], Victoria Finamore[2], Lorely Chavez[3], Christian Ketel[4], and Kate Clouse[3], *1. The Ohio State University, Columbus, Ohio, United States, 2. Vanderbilt University Medical Center, Nashville, Tennessee, United States, 3. Vanderbilt University, Nashville, Tennessee, United States, 4. The University of Alabama at Birmingham, Birmingham, Alabama, United States*

Low advance care planning (ACP) participation among subsidized housing residents may result from a higher prevalence of adverse life experiences and traumatic responses to threatening topics, necessitating a trauma-informed approach to foster safety and trust. Using a qualitative descriptive design, we aimed to understand participant perceptions of a trauma-informed advance care planning (TI-ACP) intervention - a one-time, one-to-two-hour conversation guided by a discussion checklist and brief trauma education (symptom recognition and re-traumatization avoidance). In a southeast Nashville subsidized housing community, we conducted one-time, individual, face-to-face, five-to-twenty-minute, semi-structured interviews with adult residents after the intervention. Two coders individually and iteratively coded transcripts, resulting in a hierarchical codebook. Final themes emerged from an iterative thematic analysis process by inductively grouping codes and quotes and deductive application of the SAMHSA trauma-informed care principles. Participants had a median age of 60 (IQR=56-64), and the majority identified as men (52%) and African American/Black (62%)

(n = 29). Three themes (ten sub-themes) emerged, including people (participant psychological readiness and capacity for understanding; interventionist's down-to-earth demeanor and professionalism); process (nonjudgmental, therapeutic, and direct communication; personalized style; and private and comfortable setting), and products (trust: social connection; safety: feelings of ease and relaxation; and raised awareness and empowerment). Our novel approach provided a participant-informed model of how TI-ACP is operationalized. Clinicians and community health workers should promote safety and trust for low SES patients by reinforcing privacy, assessing psychological readiness, and responding with compassion and friendliness. Recommendations for research, policy, and practice are provided.

Abstract citation ID: igaf122.1488

### RECRUITING PERSONS WITH DEMENTIA IN A MEMORY CLINIC WORKFLOW: LESSONS FROM A PHYSICAL ACTIVITY TRIAL

Lijuan Yin, Woojin Song, Maria Caceres, Kyle Jennette, Elise Hu, Neil Pliskin, Jason Soble, and Naoko Muramatsu, *University of Illinois Chicago, Chicago, Illinois, United States*

Recruiting diverse populations remains a significant challenge in Alzheimer's Disease and related dementias (ADRD) research. Memory clinics (MeC) are uniquely positioned to recruit patients directly into research and promote behavioral change; however, integrating recruitment into clinical workflows is rare, with limited knowledge about feasibility. To address this gap, this study examined facilitators and challenges in recruiting patients diagnosed with mild cognitive impairment or mild dementia for a physical activity trial at a MeC serving racially and economically diverse populations. A total of 161 potential participants were identified through neuropsychological evaluations during their MeC visit or from patient records (March 2023–December 2024). Of these, 28 were enrolled (17.4% enrollment rate; ages 50-90; 61% women; 54% Black, 18% Latino). Unexpected challenges emerged, including 1) declining referrals within the same health system, linked to the recent retirement of referring physicians and the Geriatrics Department relocation; 2) moderately high no-show rates for scheduled patients; 3) difficulty reaching patients by phone. Nevertheless, several key factors facilitated successful recruitment. Neuropsychologists were able to initiate conversations with patients about study participation at the time of evaluation, leveraging their established, trusted relationships and cognitive health expertise. Motivated by the mission of improving care and health for patients with ADRD, the multidisciplinary clinic leadership provided full support; all members committed to successful study implementation. Recruitment strategies were co-developed by the clinic and research teams, adapting to emerging challenges. This study demonstrates the potential of integrating recruitment into MeC workflows and offers strategies for engaging hard-to-reach populations in ADRD research.

### SESSION 3360 (SYMPOSIUM)

Abstract citation ID: igaf122.1489

### DAILY VIEWS ON AGING, POSITIVE AND NEGATIVE EXPERIENCES – FINDINGS FROM DIVERSE CONTEXTS AND COUNTRIES

Chair: Anna Kornadt
Co-Chair: Dwight Tse

Views on aging have recently been investigated regarding short term fluctuations and in relation to daily experiences. In the current symposium, we draw on international samples of the Subjective AGES consortium. We focus on how adverse and positive experiences are related to daily views on aging, such as subjective age and awareness of age-related changes. Senyk and colleagues investigate two samples of older adults from Ukraine and Israel find that war exposure increases subjective age. In a daily diary study with older adults from four different countries, Shrira et al. find that feeling closer to death on a given day decreases well-being, and that the strength of this relation was moderated by religious affiliation. Watson and Tse more broadly investigate the role of daily hassles and uplifts for subjective age among UK older adults. They find that especially uplifts contribute to a younger subjective age, potentially mediated by daily health problems. Finally, Wu and colleagues address the association between daily hassles and uplifts and views on aging among Hong Kong Chinese. Mattering and non-essential beliefs about aging were found to increase the positive impact of uplifts and attenuating the negative role of stressors on awareness of age-related changes. Taken together, our symposium shows that the study of daily life experiences and the mechanisms that relate them to views on aging enhance our understanding of psychological processes and dynamics among views on aging, contextual factors, and developmental outcomes.

Abstract citation ID: igaf122.1490

### STRESSING THE IMPORTANCE OF UPLIFTS: THE ASSOCIATIONS AMONG STRESSORS, UPLIFTS, HEALTH PROBLEMS AND SUBJECTIVE AGE

Dwight Tse, *University of Strathclyde, Glasgow, Scotland, United Kingdom*

Subjective age refers to the age a person feels and is linked to healthy aging. Studies suggest stressors and uplifts influence subjective age, although the mechanisms remain relatively unclear. Given that health problems are linked to feeling older and vary daily, this study examines the indirect effects of daily stressors and uplifts on subjective age through daily health problems (DHPs). Using a 14-day diary design, 85 UK participants aged 50-85 completed a baseline survey and daily questionnaires on stressors, uplifts, health symptoms, and felt age, yielding 933 entries (78% completion). Multilevel mediation analysis results showed that within individuals, more stressors and fewer uplifts predicted more DHPs and older subjective age, with significant indirect effects of stressors and uplifts on subjective age through DHPs. At the between-person level, uplifts—but not stressors—predicted lower DHPs and, in turn, younger subjective age. Compared to daily stressors, uplifts consistently supported healthier aging at both levels, highlighting the short-term and long-term benefits of everyday positive events. In parallel with the subjective weathering hypothesis, uplifts may initiate a "fortification" process that helps older adults maintain and enhance well-being and functioning.

Abstract citation ID: igaf122.1491

### THE IMPACT OF EXPOSURE TO WAR AND WAR-RELATED STRESSORS ON SUBJECTIVE AGE AND ACCELERATED AGING:

Anna Kornadt[1], Oksana Senyk[2], Ehud Bodner[3], Yuval Palgi[4], Amit Shrira[3], Lee Kimron Greenblatt[5], and Kvitoslava Khorob[6], *1. University of Luxembourg, Esch-sur-Alzette,*

*Grevenmacher, Luxembourg, 2. WSB Merito University in Gdansk, Gdansk, Pomorskie, Poland, 3. Bar-Ilan University, Ramat Gan, HaMerkaz, Israel, 4. University of Haifa, Haifa, HaZafon, Israel, Israel, 5. Ariel University, Ariel, HaMerkaz, Israel, 6. Ukrainian Catholic University, Lviv, L'vivs'ka Oblast', Ukraine*

Previous research shows that the experience of adversity and extreme life events can have an impact on the experience of aging (e.g., Avidor et al., 2016; Hoffman et al., 2022). Still, empirical evidence is sparse regarding the impact of war experiences, especially in short-term and daily contexts. We thus investigated the role of exposure to war-related events, stressors, and their experience on subjective age and subjective accelerated aging in two samples of older adults exposed to war. The first sample includes N = 71 adults aged 50-78 years from the Ukraine, who reported on their exposure to war-related events as well as their daily experience of aging, and daily war-related stressors over 14 days during the third year of the Russian invasion of Ukraine. Multilevel regression analyses found that there was no effect of daily war-related stressors on daily subjective age (b = .45, p = .14) or accelerated aging (b = .11, p = .08), but on the between-person level, more exposure to war-related events was associated with feeling older (b = 1.10, p <.001). We will expand these findings with a second sample of Israeli adults aged 45 to 70, who reported on the same variables weekly over six weeks during the Israel-Hamas war in early 2025. Our findings inform theoretical models aimed to understand how extreme adversity and disasters affects older adults' experience of aging.

Abstract citation ID: igaf122.1492

### DAILY SUBJECTIVE NEARNESS TO DEATH AND WELL-BEING AMONG OLDER ADULTS OF DIFFERENT RELIGIOUS AFFILIATIONS

Amit Shrira[1], Shevaun Neupert[2], Dwight Tse[3], Reyyan Can[2], and Yuval Palgi[4], *1. Bar-Ilan University, Ramat Gan, HaMerkaz, Israel, 2. North Carolina State University, Raleigh, North Carolina, United States, 3. University of Strathclyde, Glasgow, Scotland, United Kingdom, 4. University of Haifa, HaZafon, Israel*

Subjective nearness to death (SNtD) refers to how close one perceives oneself to death. It is related to well-being indices and is even associated with actual life expectancy. While daily variations were demonstrated in different subjective views of aging, whether SNtD fluctuates and covaries with well-being in short-term time frames remains unclear. Moreover, although SNtD variations and concomitants may be affected by religious belief systems, such effects are still underexplored. We capitalized on data from the Subjective AGES (Aging within Global Everyday Ecological Studies) project examining daily changes in SNtD and their covariation with well-being indices: somatic symptoms, memory failures, and negative affect. We further assessed daily changes and covariations among older adults with various religious affiliations: Christians (from the UK and USA), Muslims (from Türkiye), and Jews (from Israel). SNtD showed significant daily fluctuations (within-person variance ranged from 20% to 35% across the different religious affiliations). It further correlated with daily well-being so that on days when participants felt closer to death than usual, they reported more somatic symptoms, more memory failures, and higher negative affect. In addition, significant moderation effects for religious affiliation showed that the daily covariation between SNtD and some well-being indices was weaker among Muslims but stronger among Jews. The findings indicate that SNtD exhibits significant daily variations, which coincide with older adults' daily physical, cognitive, and emotional experiences. While SNtD was relevant to the well-being of older individuals with varying religious affiliations, religious belief systems, including beliefs about the afterlife, modify the SNtD-well-being relationship.

Abstract citation ID: igaf122.1493

### IMPACT OF DAILY UPLIFTS AND HASSLES ON DAILY AWARENESS OF AGE-RELATED CHANGES IN LATE LIFE

Yiwen Wu, Hongmei Lin, and Helene Fung, *The Chinese University of Hong Kong, Hong Kong, China (People's Republic)*

Background: Awareness of Age-Related Changes (AARC), which refers to individuals' awareness of age-related gains and losses, is a crucial contributor to subjective aging experiences. While previous research has focused on exploring factors related to AARC at the trait level, little is known about the contributors to AARC at the daily level. Purpose: This study aims to investigate the relationship between daily uplifts and hassles and daily AARC and to explore potential moderators. Methods: A total of N = 286 older participants, aged 50 to 80 years (M = 60.8, SD = 6.1), were recruited in Hong Kong. Participants reported their daily AARC, as well as daily hassles and uplifts, every night over 14 consecutive days, along with baseline and demographic measures. Results: The results showed that participants were not only more aware of age-related losses on days with more daily hassles but also, surprisingly, on days with more daily uplifts. This suggests that even positive life experiences might become sources of awareness related to loss in old age. Additionally, trait-level non-essentialist beliefs about aging and sense of mattering may serve as protective factors, buffering the impact of daily experiences on age-related loss awareness. Finally, neither daily uplifts nor daily hassles were significantly related to AARC gains at the daily level. Conclusion: This study reveals the complex dynamics between daily events and subjective aging. Moreover, non-essentialist beliefs about aging and a sense of mattering may serve as protective factors, buffering the impact of daily experiences on age-related loss awareness.

### SESSION 3365 (SYMPOSIUM)

Abstract citation ID: igaf122.1494

### THE ANTECEDENTS AND CONSEQUENCES OF EXTENDED NURSING STAFF WORK HOURS IN NURSING HOMES

Chair: Rohit Pradhan
Discussant: Robert Weech-Maldonado

A key determinant of nursing home (NH) performance, particularly quality, is the adequacy and the expertise of nursing staff [registered (RNs), licensed practical nurses (LPNs), and certified nursing assistants (CNAs)]. Nursing staff serve as primary caregivers in NHs, directly influencing resident care and outcomes. Yet, burnout remains a significant concern, and NHs experience extraordinary levels of turnover. Extended work hours among nursing staff may be an important factor contributing to this challenge. While existing research shows that prolonged work hours can negatively affect healthcare workers' well-being—leading to poorer quality outcomes and higher turnover rates—little is known about how extended nursing staff work hours specifically affect NH outcomes. NHs differ substantially from acute care settings for various reasons, such residents' need for sustained and continuous care. Therefore, it is important to understand the implications of extended work hours on resident and staff outcomes in NHs. The overarching goal of this symposium is to explore the antecedents and consequences of extended work hours nursing staff in NHs. As part of the symposium, we have included studies examining how extended work hours relate to quality, turnover, the organizational and environmental factors associated with extended work hours, and overall NH costs. The findings and discussions may offer valuable insights for managerial decision-making and policy and regulatory formulation. They can help address the systemic challenges of nursing staffing in NHs and improve NH performance, particularly regarding quality of care and turnover.

Abstract citation ID: igaf122.1495

**EXTENDED WORK HOURS FOR NURSING STAFF AND THE IMPACT ON NURSING HOME QUALITY**

Rohit Pradhan[1], Akbar Ghiasi[2], Ganisher Davlyatov[3], and Robert Weech-Maldonado[4], *1. Texas State University, San Marcos, Texas, United States, 2. University of the Incarnate Word, San Antonio, Texas, United States, 3. The University of Oklahoma, Oklahoma City, Oklahoma, United States, 4. The University of Alabama at Birmingham, Birmingham, Alabama, United States*

Nursing staff—including registered nurses (RNs), licensed practical nurses (LPNs), and certified nursing assistants (CNAs)—are the primary caregivers in nursing homes (NHs), and the quality of care largely depends on their adequacy and expertise. Previous studies, primarily conducted in acute care settings, suggest that extended work hours among nursing staff can lead to more medical errors and lower care quality. This study examined the association between extended work hours among nursing staff and NH quality. It utilized multiple secondary datasets, including the Payroll-Based Journal and Care Compare: Five-Star Quality Rating System (Five-Star QRS) (2020-2022). The study focused on the quality star rating (1-5 scale) from the Five-Star QRS as the dependent variable. The independent variable was extended work hours, measured as the percentage of nursing staff exceeding 50 work hours per week, averaged across the year to calculate the annual facility-level rate. A Multivariable ordinal logistic regression with two-way (facility and year-level) fixed effects was employed with appropriate control variables (42,743). Results indicated that extended work hours were associated with lower odds of achieving a higher star rating. Specifically, for RNs, each additional hour was linked to a 1 percentage point decrease in the odds of being in a higher star rating category. For LPNs and CNAs, the decrease was 2 percentage points per additional hour. All results were statistically significant at $p < .0001$. Policy and managerial efforts should focus on nursing staff retention and recruitment though workload regulations may be required to ensure sustainable, high-quality NH care.

Abstract citation ID: igaf122.1496

**ORGANIZATIONAL AND ENVIRONMENTAL DRIVERS OF EXTENDED WORK HOURS AMONG NURSING STAFF IN NURSING HOMES**

Gregory Orewa[1], Akbar Ghiasi[2], Justin Lord[3], and Rohit Pradhan[4], 1. The University of Texas at San Antonio, San Antonio, Texas, United States, 2. University of the Incarnate Word, San Antonio, Texas, United States, 3. Louisiana State University Shreveport, Shreveport, Louisiana, United States, 4. Texas State University, San Marcos, Texas, United States

Prior research has shown that extended nursing staff [registered nurses (RNs, licensed practical nurses (LPNs), and certified nursing assistants (CNAs) work hours can lead to more medical errors, declines in care quality, and higher turnover rates. This study examined the organizational and environmental factors associated with extended work hours among nursing staff in nursing homes (NHs). The study utilized multiple datasets such as Care Compare: Five-Star Quality Rating System and Payroll Based Journal, and Data were modeled using multivariable linear regression with year and state-levels fixed effects (2023, $n = 42{,}743$). Three separate models were run for for RNs, LPNs, and CNAs. The dependent variable was extended work hours, measured as the percentage of nursing staff exceeding 50 work hours per week, averaged across the year to calculate the annual facility-level rate. The key variable of interest was a categorical variable that captured the intersection of ownership status and chain affiliation: for-profit-chain (FPC), for-profit independent (FPI), not-for-profit chain (NFPC), and not-for-profit independent (NFPI). Results suggested that FPIs and FPCs experienced the highest level of extended work hours, with the largest increases observed for RNs and LPNs. Higher wages correlated positively with extended work hours for RNs and LPNs, but negatively associated for CNAs. NH size was positively associated with extended work hours across all nursing staff. All results were significant at $p < 0.001$. These findings highlight the need for targeted policy and regulatory interventions to mitigate extended work hours, particularly in for-profit NHs, to improve workforce sustainability and care quality.

Abstract citation ID: igaf122.1497

**THE RELATIONSHIP BETWEEN EXTENDED WORK HOURS AND TURNOVER AMONG NURSING HOME STAFF IN NURSING HOMES**

Ganisher Davlyatov[1], Gregory Orewa[2], and Rohit Pradhan[3], *1. The University of Oklahoma, Oklahoma City, Oklahoma, United States, 2. The University of Texas at San Antonio, San Antonio, Texas, United States, 3. Texas State University, San Marcos, Texas, United States*

High turnover rates among nursing staff including registered nurses (RNs), licensed practical nurses (LPNs), and certified nursing assistants (CNAs) have been a long-standing concern in nursing homes (NHs). Nursing staff turnover has been associated with poorer NH quality outcomes such as a higher number of quality of care deficiencies and increased mortality among residents. This study examined the association between extended work hours among nursing staff and NH turnover. It utilized multiple secondary datasets, including the Payroll-Based Journal and Care Compare: Five-Star Quality Rating System (Five-Star QRS) (2023). The dependent variable was nursing staff (RNs, LPNs, CNAs) turnover calculated by dividing the number of staff who left during the year by the average number of staff employed. The independent variable was extended work hours, measured as the percentage of nursing staff exceeding 50 work hours per week, averaged across the year to calculate the annual facility-level rate. A mixed-effect maximum likelihood regression was conducted with appropriate control variables (14,279).

Results indicated that extended work hours significantly increased turnover for RNs (β = 0.075, p < 0.001) and LPNs (β = 0.091, p < 0.001), but not for CNAs. Policy and managerial efforts should focus on nursing staff retention and recruitment though workload regulations may be required to ensure sustainable, high-quality NH care. These findings highlight the need to regulate excessive work hours to reduce nursing staff turnover. Administrators should implement workload management strategies, such as capping overtime and ensuring adequate staffing, to improve retention and maintain care quality in NHs.

Abstract citation ID: igaf122.1498

**EXAMINING THE IMPACT OF EXTENDED NURSING STAFF WORK HOURS ON NURSING HOME COSTS**

Akbar Ghiasi[1], Rohit Pradhan[2], Justin Lord[3], and Robert Weech-Maldonado[4], *1. University of the Incarnate Word, San Antonio, Texas, United States, 2. Texas State University, San Marcos, Texas, United States, 3. Louisiana State University Shreveport, Shreveport, Louisiana, United States, 4. The University of Alabama at Birmingham, Birmingham, Alabama, United States*

Nursing home (NH) care is labor intensive with total labor costs constituting nearly 70% of an average facility's operating costs. This study aimed to assess the relationship between nursing staff [registered nurses (RNs), licensed practical nurses (LPNs), certified nursing assistants (CNAs)] extended work hours and NH financial performance, specifically their operating costs. The study utilized four datasets: Care Compare: Five-Star Quality Reporting System, Medicare Cost Reports, LTFocus.org, and the Area Health Resource Files (2020-2022). A multivariable linear regression model with two-way fixed effects (year and state) was employed to analyze the data (N = 38,966). Separate regression models were estimated for RNs, LPNs, and CNAs. The dependent variable was operating costs, which include all costs incurred in direct resident care. The independent variable was extended work hours, measured as the percentage of nursing staff exceeding 50 work hours per week, averaged across the year to calculate the annual facility-level rate. After controlling for appropriate organizational and environmental level factors, the analysis did not find significant association between nursing staff extended work hours and NH operating costs. The findings suggest that extended work hours may be a convenient strategy for NH administrators to meet resident care demands, as it does not appear to significantly impact operating costs in our study. However, relying on extended hours could have unintended consequences, such as increased nursing staff burnout, reduced job satisfaction, and potential declines in care quality. While this approach may seem financially neutral, its long-term effects on resident outcomes and workforce stability warrant closer attention.

### SESSION 3370 (SYMPOSIUM)

Abstract citation ID: igaf122.1499

**CARE DYNAMICS OF PUERTO RICO'S OLDER ADULTS: DEMENTIA, CAREGIVING, MENTAL HEALTH, AND OUTMIGRATION**

Chair: Michael Crowe
Co-Chair: Maricruz Rivera-Hernandez
Discussant: Michael Crowe

Puerto Rico is experiencing a rapidly aging population due to outmigration and other demographic changes. Older adults in Puerto Rico have a high prevalence of chronic conditions including higher rates of diabetes, heart disease, dementia and depressive symptoms compared to older adults in the US mainland (CDC, 2020). However, compared to the US mainland, Puerto Rico receives lower funding from federal programs including Medicare and Medicaid, which may limit access to quality care, services and support for older adults. This may also impact the health and well-being of caregivers. Understanding the complex relationships between mental health, dementia, caregiving, and social support among older Puerto Rican adults is needed to guide interventions to improve health and well-being of older adults and caregivers. This symposium will feature 5 presentations using various comprehensive datasets including rich demographic and clinical information that provide novel insights and deeper understanding about health and care dynamics faced by older adults in Puerto Rico. Individual presentations will examine: 1) The Prevalence of Alzheimer's And Related Dementia Among Medicare Advantage Enrollees in Puerto Rico; 2) The Role of Subjective Memory in the Link Between Chronic Pain, Cognitive Decline, and Depressive Symptoms In Older Puerto Rican Adults; 3) Depressive Symptoms in Dementia Caregivers in Puerto Rico: The Role of Caregiver Burden and Symptoms of Dementia; 4) The Association between Social Support and Mental Health-Related Quality of Life among Older Adults in Puerto Rico; and 5) Dementia Diagnosis and Outmigration of Older Puerto Rican Fee-for-Service Beneficiaries.

Abstract citation ID: igaf122.1500

**PREVALENCE OF ADRD AMONG MEDICARE ADVANTAGE ENROLLEES IN PUERTO RICO COMPARED TO US COUNTERPARTS**

Maricruz Rivera-Hernandez[1], Yanru Liao[2], Yoojin Lee[3], and Michael Crowe[4], *1. Brown University School of Public Health, Providence, Rhode Island, United States, 2. Brown University Center for Gerontology and Healthcare Research, Providence, Rhode Island, United States, 3. Brown University, Providence, Rhode Island, United States, 4. University of Alabama at Birmingham, Birmingham, Alabama, United States*

There is limited information regarding Alzheimer's Disease and Related Dementia Diagnosis (ADRD) in Puerto Rico (PR), making it difficult to draw comparisons of the quality of care for people with ADRD between PR and the US mainland. In fact, data from the Mapping Medicare Disparities by Population Tool from Medicare only shows prevalence rates for fee-for-service Medicare enrollees. Given that about 90% of PR beneficiaries are enrolled in Medicare Advantage (MA), these rates may not reflect the full extent of older adults with ADRD on the island. Thus, the purpose of the study was to compare ADRD prevalence rates among Hispanic MA enrollees in PR to those of their US mainland counterparts. We used 20% Medicare beneficiaries (carrier file claims) and applied up to one-year look-back period. We focused on adults who were 65 and older. Our study sample included 123,609 and 547,978 Hispanic MA enrollees in PR and the US mainland in 2019. Approximately, 54% and 56% of enrollees in PR and the US mainland were female. The ADRD rates were 15% compared to 9% for PR and enrollees in the mainland. Our results show that MA enrollees in PR have six percentage points higher ADRD rates than their US counterparts. This highlights the importance of reporting ADRD rates, including MA beneficiaries. Further research on the characteristics and healthcare utilization for the people with

ADRD in PR is required. This information is needed to improve the care and supports for people with ADRD and their caregivers (ASPE, 2022).

Abstract citation ID: igaf122.1501

### SUBJECTIVE MEMORY: A CLINICAL MARKER FOR COGNITIVE DECLINE AND DEPRESSION IN OLDER PUERTO RICANS WITH CHRONIC PAIN

Tyler Bell[1], Caitlin Northcutt[2], Sadaf Milani[3], Nathalie Gider[1], and Michael Crowe[4], *1. University of California, San Diego, San Diego, California, United States, 2. University of Kentucky, Lexington, Kentucky, United States, 3. University of Texas Medical Branch, Galveston, Texas, United States, 4. University of Alabama at Birmingham, Birmingham, Alabama, United States*

Chronic pain has been linked to subjective memory problems in older Puerto Rican adults, yet its relationship to objective cognitive decline and depressive symptoms remains unclear. This study examined these associations using data from Waves 1 (2002/2003), 2 (2006/2007), 3 (2020/2021), and 4 (2022/2024) of the Puerto Rican Elderly: Health Conditions Study (PREHCO), a longitudinal, population-based study of Puerto Rican adults aged 60 and older. The sample included individuals who were cognitively unimpaired at baseline with at least one follow-up (n = 2,630, Mage=71.33, SD = 8.25; 59.5% female). Chronic pain was defined as bodily pain reported at both Waves 1 and 2. Subjective memory was assessed using the Health and Retirement Study item, "How would you rate your memory at the present time?" (1 = excellent to 5 = poor). Cognitive function and depressive symptoms were measured using the Mini-Mental Caban and the 15-item Geriatric Depression Scale, respectively. Structural equation modeling examined how chronic pain at Wave 2 influenced subjective memory concerns at Wave 3 and cognitive decline at Wave 4, accounting for prior cognitive function. Chronic pain was associated with worse subjective memory problems (β=.31, p<.001) and greater depressive symptoms (β=.31, p<.001) at Wave 3. By Wave 4, chronic pain predicted increases in depressive symptoms (β=-.08, p=.038). Bootstrap methods confirmed that subjective memory problems mediated the relationship between chronic pain and cognitive decline (β=-.27, p=.035) and depressive symptoms (β=.23, p=.008). These findings highlight subjective memory as a potential intermediatory step between declines in cognitive and emotional health in aging Puerto Rican adults.

Abstract citation ID: igaf122.1502

### DEPRESSIVE SYMPTOMS IN DEMENTIA CAREGIVERS IN PUERTO RICO: THE ROLE OF CAREGIVER BURDEN AND SYMPTOMS OF DEMENTIA

Junyub Lim[1], Ross Andel[2], Frank Puga[3], María Aranda[4], Maricruz Rivera-Hernandez[5], Ana Luisa Davila-Roman[6], and Michael Crowe[3], *1. Arizona State University, Phoenix, Arizona, United States, 2. Arizona State University, Arizona State University, Arizona, United States, 3. University of Alabama at Birmingham, Birmingham, Alabama, United States, 4. University of Southern California, Los Angeles, California, United States, 5. Brown University School of Public Health, Providence, Rhode Island, United States, 6. University of Puerto Rico, San Juan, Puerto Rico, United States*

Background/Objectives: Dementia is more prevalent in Puerto Rico than on the U.S. mainland, increasing demands for caregiving. However, research on the mental health impact of dementia caregiving among island Puerto Ricans is sparse. We examined caregiver burden and depressive symptoms among Puerto Rican dementia caregivers and a potential moderating role of behavioral and psychological symptoms of dementia (BPSD). Methods: Using data from the Puerto Rican Elder: Health Conditions (PREHCO) study (Wave 3), 132 caregivers of PREHCO participants completed an ancillary data collection, of whom 101 were dementia caregivers with complete data. Measures included the 15-item Geriatric Depression Scale (depressive symptoms), Neuropsychiatric Inventory Questionnaire (NPI-Q; BPSD), and Zarit Burden Interview (ZBI-6; caregiver burden). Results: Average caregiver age was 63±10 years and 77% were women. Exploratory factor analysis yielded two BPSD factors—Emotional Dysfunction/Behavioral Instability and Perceptual Disturbances/Apathy. In separate multivariable linear regression models adjusted for age, sex, and education, caregiver burden (standardized coefficient [β]=0.53, p<.001) was positively associated with depressive symptoms, as was Emotional Dysfunction/Behavioral Instability (β = 0.54, p<.001) and Perceptual Disturbances/Apathy (β = 0.55, p<.001). Emotional Dysfunction/Behavioral Instability (β = 0.20, p=.037) and Perceptual Disturbances/Apathy (β = 0.26, p=.009) moderated this association such that the caregiver burden-depressive symptoms link grew significantly stronger as BPSD increased. Discussion: These findings indicate that BPSD may influence caregiver mental health in Puerto Rican dementia caregivers, and that the effect of BPSD on this association follows a non-linear pattern. This highlights the need for tailored interventions to alleviate caregiver burden and depression based on managing BPSD in the care recipient with dementia.

Abstract citation ID: igaf122.1503

### THE ASSOCIATION BETWEEN SOCIAL SUPPORT AND MENTAL HEALTH-RELATED QUALITY OF LIFE AMONG OLDER ADULTS IN PUERTO RICO

Ebenezer Martey[1], Maricruz Rivera-Hernandez[2], Ross Andel[3], Olivio Clay[1], Ana Davila[4], and Michael Crowe[1], *1. The University of Alabama at Birmingham, Birmingham, Alabama, United States, 2. Brown University School of Public Health, Providence, Rhode Island, United States, 3. Arizona State University, Arizona State University, Arizona, United States, 4. University of Puerto Rico, San Juan, Puerto Rico, United States*

Emigration is a major concern for Puerto Rico residents as they lose younger people to better economic opportunities on the US mainland. This has probably led to reduced caregiving availability and social support for older adults, but limited research exists on how social support and sociodemographic factors are related to quality of life (QoL) among older Puerto Ricans. We examined the relationship between social support and mental health-related QoL using data from a long-term follow-up of the Puerto Rican Elder Health Conditions (PREHCO) Study (Wave 3; n = 617). QoL was measured with the Veterans RAND 12-Item Health Survey (VR-12) mental component summary. Social support network (SSN) was assessed using the 6-item Lubben Social Network Scale. Multiple regression was used to examine associations between QoL and social support while adjusting for age, sex, education and living alone. The average age of participants was 84 years and 63% were women. Participants had an average of 9.2 years of education and 37% were living alone. SSN was positively associated with education but not with other demographic factors. QoL was higher among women but showed no significant associations with other demographic factors. In the multivariable regression model, stronger SSN was associated with higher QoL score ($\beta = 0.11$, $p = 0.006$). The association between QoL and SSN was similar in people who lived alone versus not alone. These findings suggest the importance of maintaining a strong SSN as it appears to play a significant role in the mental health-related QoL of older adults in Puerto Rico.

Abstract citation ID: igaf122.1504

### DEMENTIA DIAGNOSIS & OUTMIGRATION OF OLDER PUERTO RICAN MEDICARE FEE-FOR-SERVICE BENEFICIARIES, 2012-2019 TRENDS

Jeung Hyun Kim[1], Yoojin Lee[2], Yanru Liao[3], Monica Colon-Vargas[2], and Maricruz Rivera-Hernandez[4], *1. Brown University, Brown University, Rhode Island, United States, 2. Brown University, Providence, Rhode Island, United States, 3. Brown University Center for Gerontology and Healthcare Research, Providence, Rhode Island, United States, 4. Brown University School of Public Health, Providence, Rhode Island, United States*

Prior research shows that older Puerto Ricans may be migrating to the US mainland, which may include those with an Alzheimer's Disease and Related Dementia (ADRD) diagnosis (Rivera-Hernandez, 2022; Kim et al., 2024). The purpose of this research was to examine yearly out-migration trends among older Puerto Ricans by examining their movement patterns from 2012-2019, which included the period of Hurricane Maria. The analysis used data from the Master Beneficiary Summary File and the American Community Survey, and focused on Medicare fee-for-service enrollees in Puerto Rico (65+). The Chronic Conditions Warehouse algorithm was used to ascertain ADRD diagnosis, and migration was defined as the change of (end-of-year) residence from one year to the next. A linear probability model was used to calculate differences in the rate of outmigration between beneficiaries with and without ADRD. There were about 44,000~66,000 beneficiaries each year and around 15~16% with ADRD. Outcomes showed that, overall, older Puerto Ricans previously diagnosed with ADRD were slightly more likely to move to the US mainland compared to those without an ADRD diagnosis across the years (Adjusted differences ranged, $b = 0.0014 \sim 0.0041$), with the highest proportion migrating during the year of Hurricane Maria (over 2.5% in 2017). However, the difference was not statistically significant for some years. In sensitivity analyses across groups with sociodemographic and clinical characteristics, the likelihood of outmigration was higher among those with ADRD compared to those without ADRD. Our results suggest that older adults with complex health needs appear to be out-migrating following their ADRD diagnosis.

### SESSION 3375 (SYMPOSIUM)

Abstract citation ID: igaf122.1505

### EARLY CAREER TRAINING CAREER EXPERIENCES AND INSIGHTS IN DEMENTIA CARE SCIENCE

Chair: Joseph Gaugler
Co-Chair: Allycia Wolff
Discussant: Abraham Brody

Recent summits and national reports have emphasized the need for more rigorous methodological approaches when designing, evaluating, and disseminating/implementing dementia care interventions. The Advanced Behavioral Intervention Design in Dementia Care (ABIDDC) training and professional development program is designed to: 1) Deliver a graduate curriculum in advanced dementia care intervention science; 2) Establish a robust mentoring program to support early career investigators (pre-doctoral and post-doctoral fellows) in dementia care science; and 3) Leverage existing support at the University of Minnesota to position trainees to launch successful, independent careers in dementia care science. In addition to summarizing the ABIDDC program and its progress as a scientific training and professional development program, this symposium will summarize the training and mentoring experiences of current ABIDDC Fellows in similar initiatives to provide insights and recommendations to inform the refinement and creation of future scientific mentoring efforts in dementia care. Ultimately, this symposium will inform the infrastructure, training, and support necessary to cultivate a cadre of early career investigators and other investigators to conduct state-of-the-art dementia care science.

Abstract citation ID: igaf122.1506

### THE ADVANCED BEHAVIORAL INTERVENTION DESIGN IN DEMENTIA CARE TRAINING PROGRAM: INSIGHTS AND OUTCOMES

Dana Urbanski[1], Allycia Wolff[2], Manka Nkimbeng[3], Emily Mroz[4], Maria Quiñones-Cordero[5], Quinton Cotton[6], Mayra Sainz[4], and Joseph Gaugler[2], *1. Indiana University Bloomington, Bloomington, Indiana, United States, 2. University of Minnesota, Minneapolis, Minnesota, United States, 3. University of Minnesota School of Public Health, Minneapolis, Minnesota, United States, 4. Emory University, Atlanta, Georgia, United States, 5. University of Rochester School of Nursing, Rochester, New York, United States, 6. University of Pittsburgh, Pittsburgh, Pennsylvania, United States*

Non-pharmacologic behavioral interventions are increasingly recognized as essential to address the needs of people living with dementia and their family caregivers. However, university programs are not organized around providing the training needed to prepare early-career investigators to develop, test, and refine such interventions. The Advanced Behavioral Intervention Design in Dementia Care (ABIDDC) program addresses this gap by 1) Delivering an advanced curriculum in dementia care intervention science; 2) Establishing a robust, structured peer mentoring program to support a cohort of early-career investigators in dementia care science; and 3) Leveraging resources at the University of Minnesota (e.g., Center for Healthy Aging and Innovation, aging-related special interest groups, idea-hatching and grant review workshops) to enrich training in dementia care intervention science. This presentation will summarize the key components and outcomes of the ABIDDC during its first two years. We will discuss the ABIDDC curriculum, peer mentoring program, and plans for collaborative projects. Additionally, we will highlight how the ABIDDC facilitates engagement with other programs that offer pilot funding and professional development in dementia care intervention science, such as the Resource Centers for Minority Aging Research Center and the National Institute on Aging IMbedded Pragmatic Alzheimer's disease (AD) and AD-Related Dementias Clinical Trials Collaboratory. Preliminary outcomes related to trainee engagement, research productivity, and career trajectories will also be shared, offering insights into the ABIDDC's impact. The session will conclude with recommendations for integrating similar training models into other institutions to prepare the next generation of dementia care intervention scientists.

Abstract citation ID: igaf122.1507

### ALZHEIMER'S ASSOCIATION INTERDISCIPLINARY SUMMER RESEARCH INSTITUTE (AA-ISRI): INSIGHTS FROM PROGRAM ALUMNI

Andrew Pickett[1], Quinton Cotton[2], Emily Mroz[3], Kalisha Bonds Johnson[3], and Manka Nkimbeng[4], *1. Indiana University School of Public Health- Bloomington, Bloomington, Indiana, United States, 2. University of Pittsburgh, Pittsburgh, Pennsylvania, United States, 3. Emory University, Atlanta, Georgia, United States, 4. University of Minnesota School of Public Health, Minneapolis, Minnesota, United States*

Held annually since 2021, the Alzheimer's Association Interdisciplinary Summer Research Institute (AA-ISRI) provides an immersive, no-cost professional development opportunity for dementia researchers. The week-long institute provides training on innovations in dementia science, offers peer group support and networking, and structured mentorship centered around grant writing and career coaching to ensure success in a research career. Organized across two overarching research tracks (i.e., psychosocial, public health) and by participant interests, daily peer group meetings (4-6 people) are led by established mentors. Presenters in this session have all completed the AA-ISRI program and will share experiences and insights related to participation. Presenters' experiences broadly aligned with three overarching themes: (1) an interdisciplinary approach to dementia research training, (2) emphasis on peer and mentorship networking, and (3) opportunities to interact with field-leaders and funding agency representatives. AA-ISRI, by definition, is interdisciplinary—accepting participants from a variety of educational backgrounds (e.g., MD, PhD) and experiences (e.g., public health, nursing, social work, medicine). As such, the program curriculum introduces the state-of-the-science across multiple subdisciplines within the wider field of dementia research (e.g., pathology/ treatment, trial design, participant recruitment, implementation science). In small group meetings, participants receive feedback on an in-progress grant proposal and learn to develop an effective 'elevator pitch' for their research. Finally, each participant has one-on-one mentorship meetings with leading experts in their area and opportunities to interact with research funders. Thus, AA-ISRI provides an impactful opportunity for early career and new-to-the-field investigators, as well as more established researchers transitioning into dementia research.

Abstract citation ID: igaf122.1508

### DEMENTIA IN AFRICAN AMERICAN DYADS AND BLACK IMMIGRANT FAMILIES: LESSONS FROM IMPACT-AD

Kalisha Bonds Johnson[1], Manka Nkimbeng[2], Joshua Grill[3], Rema Raman[4], Kenneth Hepburn[1], Wizdom Powell[5], Carol Whitlatch[6], and Karen Lyons[7], *1. Emory University, Atlanta, Georgia, United States, 2. University of Minnesota School of Public Health, Minneapolis, Minnesota, United States, 3. University of California, Irvine, Irvine, California, United States, 4. University of Southern California, Los Angeles, California, United States, 5. University of Connecticut School of Medicine, Farmington, Connecticut, United States, 6. Benjamin Rose Institute on Aging, Cleveland, Ohio, United States, 7. Boston College, Chestnut Hill, Massachusetts, United States*

The goal of IMPACT-AD is to "train the next generation of ADRD clinical trialists, to ensure that investments in discovery yield outcomes for patients and families." This program offers a weeklong summer workshop with curated sessions. In this presentation, scholars (Summer 2021 and 2022) will discuss the program's impact on their work and career. Lessons learned include: 1) study design for the development of a clinical trial for dementia dyads (described below) and 2) identifying analytic approaches for behavioral interventions with dementia care partners. Specifically, we will offer outcome data to highlight how IMPACT-AD facilitated the successful implementation of phase one of a mixed-methods intervention to understand the decision self-efficacy (DSE) of dementia care dyads. Data from 39 African American parent-adult daughter dementia dyads was analyzed using multilevel modeling. On average, parents' DSE was significantly lower than their adult daughters' DSE. Parents experienced significantly better DSE when they perceived a more positive family decision-making process, reported a positive interaction within the dyadic relationship with their daughter, and had daughters who reported an "intense motivation to succeed." Adult daughters experienced significantly better DSE when they reported better general family functioning and had greater confidence in their parent's patient-provider relationship. However, daughters experienced significantly worse DSE when their parents perceived a more positive family decision-making process. Not all scholars' projects are in the data analysis phase. Cumulatively, scholars gained skills from participation in IMPACT-AD and its annual scientific retreats that they continue to implement in current and planned clinical trial projects.

Abstract citation ID: igaf122.1509

### EMORY CAREGIVER RESEARCH INTEREST GROUP: ADVANCING DEMENTIA CAREGIVER RESEARCH FOR SCHOLARS

Maria Quiñones-Cordero[1], Zachary Baker[2], Emily Mroz[3], Kalisha Bonds Johnson[3], Kenneth Hepburn[3], and Fayron Epps[4], *1. University of Rochester School of Nursing, Rochester, New York, United States, 2. Arizona State University, Phoenix, Arizona, United States, 3. Emory University, Atlanta, Georgia, United States, 4. UT Health San Antonio, San Antonio, Texas, United States*

Dementia caregiving occurs within diverse contexts, so there are ongoing opportunities to develop and test behavioral interventions to promote context-specific caregiver mastery. The NIA-supported Emory Roybal Center for Dementia Caregiving Mastery established the Caregiver Research Interest Group (CRIG) to advance behavioral intervention research in untargeted caregiving contexts and to support the career development of early-stage investigators nationwide. CRIG convenes monthly virtual discussions to identify research gaps, generate innovative intervention strategies, and foster interdisciplinary collaboration. These meetings serve as an incubator for novel intervention concepts, a forum for refining research methodologies, and a networking platform that facilitates cross-disciplinary engagement. By exploring the intersections between diverse caregiving environments and behavioral intervention research, scholars refine research questions that address the unique challenges faced by dementia caregivers. CRIG also provides vital career advancement opportunities, including discussions with National Institute on Aging Program Officers on funding priorities, exposure to training programs for early-career investigators, and individualized consultations on research trajectories. Compared to other programs in this symposium CRIG is particularly flexible, possessing less formal targeted outcomes. CRIG includes 21 scholars (post-doctoral through professor). Feedback from scholars will be discussed in this session including highlighted accomplishments (i.e., collaborations in publications and abstracts; leadership and group feedback on grant proposals leading to successful funding) and areas of improvement such as increasing diversity in expertise and fostering more peer collaborations. Through leadership-driven engagement, CRIG fosters a dynamic research community dedicated to enhancing dementia caregiver mastery science and expanding the impact of intervention research in diverse caregiving contexts.

Abstract citation ID: igaf122.1510

### THE DEMENTIA PALLIATIVE CARE CLINICAL TRIALS TRAINING PROGRAM

Zachary Baker[1], Emily Mroz[2], Jean Kutner[3], Kathryn Pollack[4], Christine Ritchie[5], and Ana-Maria Vranceanu[5], *1. Arizona State University, Phoenix, Arizona, United States, 2. Emory University, Atlanta, Georgia, United States, 3. University of Colorado School of Medicine, Aurora, Colorado, United States, 4. Duke University School of Medicine, Durham, North Carolina, United States, 5. Massachusetts General Hospital, Boston, Massachusetts, United States*

The Dementia Palliative Care Clinical Trials Training Program (DEM-PCCT) is a 10-month intensive program designed to equip early- and mid-career researchers with the skills necessary to design and conduct clinical trials in the dementia palliative care space. The program integrates large cohort-wide meetings, monthly small-group mentoring, and a five-day in-person institute. Scholars receive hands-on training in trial methodology, grant writing, and research ethics while building a strong professional network. A core component is the development of a competitive funding proposal for a dementia-related palliative care clinical trial. Since its inception, 53 scholars across three cohorts have completed DEM-PCCT. Of these, a growing number have secured funding, initiated collaborations, contributed to dementia palliative care research, and increased confidence and comfort with conducting clinical trials ($p<.05$). This presentation highlights two concrete examples of submissions to the National Institute on Aging: (1) the first author's R01 application, currently under review, and (2) the second author's K22 application, which has been successfully funded. Both applications marked first-time submissions for the Principal Investigators, underscoring the program's role in fostering emerging researchers. DEM-PCCT's success is driven by several key elements: a) Structured mentorship providing individualized guidance, b) Access to previously funded trial materials and methods, allowing scholars to build on successful frameworks, c) Regular deadlines and peer feedback to ensure steady progress toward grant submission, and d) Statistical mentorship, a unique feature often missing from other dementia training programs. As part of a broader symposium, this talk will examine how DEM-PCCT differs from other programs.

### SESSION 3380 (SYMPOSIUM)

Abstract citation ID: igaf122.1511

### NOVEL APPROACHES TO UNDERSTANDING EDUCATION AND LATER-LIFE COGNITIVE HEALTH

Chair: Lindsay Kobayashi
Discussant: Jennifer Manly

Education is the strongest known modifiable factor that reduces dementia risk. However, there remain several unanswered questions about this relationship, which limit the design of policies to optimize the role of educational systems in promoting the cognitive health of populations. This symposium presents a suite of epidemiological studies using complementary designs and analytical approaches to uncover new insights into the complex role of education in later-life cognitive health across diverse older populations. First, Emma Nichols will present a series of analyses designed to probe the counterintuitive finding of faster cognitive decline amongst older adults with higher educational attainment in a population-based cohort study of aging in India. Results demonstrate how regression to the mean may bias longitudinal studies of education and cognitive decline. Second, Mateo Farina will present on the interactive roles of relative and absolute educational attainment in shaping later-life cognitive function among men and women in five high- and middle-income countries, using an intersectionality framework. Third, Vicky Chen will examine the role of cerebrovascular and neurodegenerative mediators linking educational attainment with cognitive decline among men and women in a comparative study of older adults in Korea and the United States. Fourth, Sirena Gutierrez will investigate how literacy, numeracy, and educational attainment contribute to cognitive aging disparities between Indigenous and non-Indigenous older adults in Mexico. Jennifer Manly will serve as the discussant, integrating findings to discuss the complex role of education and later-life cognitive health across these globally diverse aging populations.

Abstract citation ID: igaf122.1512

### ASSOCIATIONS BETWEEN EDUCATION AND COGNITIVE DECLINE REVISITED: EVIDENCE FROM INDIA

Emma Nichols[1], Alden Gross[2], Richard Jones[3], Erik Meijer[1], Lindsay Kobayashi[4], and Jinkook Lee[1], *1. University of Southern California, Los Angeles, California, United States, 2. Johns Hopkins Bloomberg School of Public Health, Baltimore, Maryland, United States, 3. Brown University, Brown University, Rhode Island, United States, 4. University of Michigan, Ann Arbor, Michigan, United States*

Educational attainment is consistently associated with cognitive level, but the evidence for cognitive decline is mixed, with most research suggesting no association. However, most evidence comes from high-income contexts. We used nationally representative data from the first two waves of the Longitudinal Aging Study in India – Diagnostic Assessment of Dementia (LASI-DAD) ($N=3{,}673$) to estimate the association between educational attainment and cognitive decline using linear mixed effects models. We evaluated the consistency of findings across a range of generalized estimating equations (GEE) models with inverse probability weights to adjust for sample selection, attrition, and mortality. We further explored the role of practice effects, selective survival, and regression to the mean using a range of supplementary analyses, including simulation analysis. Compared to those with no education, those with less than primary school (difference of -0.03 SD units/year; 95% CI -0.04 to -0.01), primary school (-0.04; -0.06 to -0.03), middle-secondary school (-0.06; -0.07 to -0.04), and higher secondary school education and up (-0.05; -0.07 to -0.03) had steeper cognitive decline after adjustment for demographic and early-life socioeconomic factors. Neither selective survival nor practice effects could explain findings. However, evidence suggested that due to the strong association between education and baseline cognition, educational attainment may act as a proxy for cognitive level and lead to regression to the mean in models adjusting for educational attainment. Regression to the mean may impact findings on the association between education and cognitive decline more broadly, particularly in contexts with larger associations between education and cognitive level.

Abstract citation ID: igaf122.1513

### CROSS-NATIONAL INTERACTIONS BETWEEN ABSOLUTE AND RELATIVE EDUCATION ACROSS SEX/GENDER

Mateo Farina[1], Chloe Eng[2], Arthur Wang[3], Laura Zahodne[4], Usha Dhakal[5], Justina Avila-Rieger[6], Lindsay Kobayashi[4], and Jennifer Manly[6], *1. University of Texas at Austin, Austin, Texas, United States, 2. Stanford University, Stanford, California, United States, 3. University of Southern California, Los Angeles, California, United States, 4. University of Michigan, Ann Arbor, Michigan, United States, 5. Georgetown University, Washington, District of Columbia, United States, 6. Columbia University Irving Medical Center, New York, New York, United States*

The global burden of dementia is expected to grow in the coming decades, impacting the lives of millions. However, the determinants of dementia and the strengths of their associations are not universal but likely vary across country contexts. Understanding this variability in key determinants of cognitive health like education is paramount for understanding the population dynamics of this growing public health crisis. Specifically, the association between education and cognitive health can vary due to distribution, quality, and opportunities within countries. Using data from the Harmonized Cognitive Assessment Protocol, we examine variability in the association between absolute and relative levels of education, and their interaction, for older men and women in the United States, United Kingdom, India, Mexico, and South Africa. We use Multilevel Analysis of Individual Heterogeneity and Discriminatory Accuracy (MAIHDA), which allows for a complex modeling approach that accounts for additive and multiplicative interactions across country context. We examine these models across global cognitive functioning as well as specific domains (memory, language, executive functioning). Preliminary findings show strong main effects of relative and absolute levels of education on global cognitive functioning and across domains. We also find that strong evidence of interactive effects across country context. For example, women with high relative education and high absolute levels overperformed in Mexico, while women with high relative education regardless of absolute levels underperformed in South Africa. Overall, these findings point to the importance of considering country context (i.e. educational quality, socioeconomic opportunities, and larger policy environment) when examining determinants of dementia.

Abstract citation ID: igaf122.1514

### SEX/GENDER DIFFERENCES IN BRAIN HEALTH PATHWAYS LINKING EDUCATION TO COGNITIVE TRAJECTORIES

Diefei Chen[1], C. Shaaban[2], Ileana De Anda-Duran[3], Dahyun Yi[4], Shannon Risacher[5], Paul Crane[6], and A Kraal[7], *1. Johns Hopkins Bloomberg School of Public Health, Baltimore, Maryland, United States, 2. University of Pittsburgh, Pittsburgh, Pennsylvania, United States, 3. Tulane University, New Orleans, Louisiana, United States, 4. Seoul National University, Seoul, Republic of Korea, 5. Indiana University School of Medicine, Indianapolis, Indiana, United States, 6. University of Washington, Seattle, Washington, United States, 7. Columbia University, New York, New York, United States*

Educational attainment may protect against poor cognitive health outcomes in later-life potentially via cerebrovascular and neurodegeneration pathways implicated in Alzheimer's disease (AD). Underlying brain health mechanisms may vary by sex/gender and ethnicity, given group differences in educational opportunities. This study aims to characterize sex/gender differences in brain health pathways linking education to later-life cognition in Korean and US research study cohorts. Participants comprised older adults without dementia from Korea (KBASE: N=434, age=70±8, education=11±5, 57% women) and the US (ADNI: N=375, age=71±7, education=17±2, 53% women; all non-Latinx white). Latent growth models tested sex/gender differences in the indirect effects of education on four-year cognitive trajectories via markers of cerebrovascular disease (white matter hyperintensities (WMH)) and neurodegeneration (hippocampal volume and cortical thickness of AD-signature regions), stratified by sex/gender and cohort, adjusting for age, APOE genotype, intracranial volume, and cardiometabolic conditions. Among women in KBASE, lower WMH burden mediated the associations of higher education with better executive function and memory levels and slower executive function decline. Larger hippocampal volume mediated the associations of higher education with better memory and executive function levels and slower memory decline. Among women in ADNI, hippocampal volume mediated the association between education and memory level, but not memory change. Mediation was not observed in men in KBASE and ADNI. Cortical thickness did not mediate associations. Findings suggest potential sex/gender differences in neurobiological pathways linking education to cognition in older adulthood. Future research is needed to characterize sociocultural factors that may shape educational opportunities and brain health across diverse populations.

Abstract citation ID: igaf122.1515

### THE ROLE OF NUMERACY, LITERACY, AND EDUCATION ON COGNITION AMONG INDIGENOUS AND NON-INDIGENOUS MEXICAN OLDER ADULTS

Sirena Gutierrez[1], Iris Strangmann[2], Zachary Kunicki[3], Alexa Gonzalez[4], Gelan Ying[5], Jet Vonk[6], Emily Briceño[7], and Miguel Rentería[8], *1. University of California, San Francisco, San Francisco, California, United States, 2. Columbia University Medical Center, New York, New York, United States, 3. Warren Alpert Medical of Brown University, Providence, Rhode Island, United States, 4. University of Houston, Houston, Texas, United States, 5. University of Florida, Gainesville, Florida, United States, 6. UCSF Memory and Aging Center, San Francisco, California, United States,*

*7. University of Michigan Medical School, Ann Arbor, Michigan, United States, 8. Taub Institute for Research on Alzheimer's Disease and the Aging Brain, Columbia University College of Physicians and Surgeons, New York, New York, United States*

Bilingualism is generally associated with better cognitive performance; however, Indigenous-Spanish bilingual older adults tend to perform worse than their non-Indigenous monolingual counterparts. Older Indigenous populations in Mexico, particularly in rural areas, face inequities in education, healthcare access, and pervasive discrimination. It is unclear if factors beyond education, like literacy and numeracy, influence brain aging in Indigenous adults. This analysis included 1,958 adults ages 55+(mean 68.1 [8.9±SD] years) from the Mexican Health and Aging Study Ancillary Study on Cognitive Aging (2016). Indigeneity was assessed via self-reported indigenous language spoken. Linear regression models estimated the relationship between speaking an indigenous language and cognitive function factor scores, adjusting for early-life sociodemographic covariates. Mediation analysis decomposed the effects of Indigeneity into direct and indirect effects through numeracy, literacy, and education. Among participants, 11% spoke an indigenous language. These individuals had lower educational attainment (3.2 vs. 5.7 years) and were more likely to reside in rural areas (71.4% vs. 39.6%). Speaking an indigenous language was associated with lower memory (β[95% CI]= -0.31 [-0.40, -0.21]), language (-0.65 [-0.75, -0.55]), executive function (-0.60 [-0.73, -0.47]), and orientation (-0.31 [-0.44, -0.18]) scores. Numeracy mediated 2%-9%, literacy 11%-26%, and education 31%-61% of the total effect of speaking an indigenous language on domain-specific cognition. Numeracy and literacy may be important mechanisms underlying cognitive disparities, alongside education, and efforts to improve them could help reduce these gaps. Future research should explore other factors, such as health conditions and racism, contributing to poorer cognitive outcomes among Indigenous populations in Mexico.

### SESSION 3385 (SYMPOSIUM)

Abstract citation ID: igaf122.1516

### THE MANY FACES OF PRECARITY: RESEARCH ON SOCIAL SECURITY AND DIVERSE POPULATIONS

Chair: Jennifer Kaufman
Discussant: Siavash Radpour

The New York Retirement and Disability Research Center (RDRC) was one of two new RDRCs created by a cooperative agreement with the Social Security Administration in 2023. The center aimed to focus on structural health, wealth, and life course processes that yield both opportunities and social and economic stratification. Although the center was defunded in early 2025, its first year of work brought together public health professionals, demographers, sociologists, urban planners, economists, and gerontologists to study a breadth of populations using both quantitative and qualitative methods. This symposium presents a range of work that examines how people get by in the face of disability, disaster, and aging on a low income. Our first presentation employs a new poverty measure to illuminate the effects of homeownership, health and social insurance benefits, and other transfers (especially rental assistance) on poverty among older adults. Our second presentation investigates the work and income situations of older adults displaced by disasters. Our third presentation examines how Social Security disability decisions affect health insurance status, poverty, and out-of-pocket medical costs. Our final presentation is a qualitative study exploring the experience of formerly incarcerated older adults in applying for disability benefits. Findings from this symposium demonstrate the complexity of economic insecurity and highlight the role of public benefits in alleviating poverty among older adults.

Abstract citation ID: igaf122.1517

### POVERTY AT OLDER AGES: INSIGHTS FROM A PILOT PRINCIPAL POVERTY MEASURE

Rosemary Hyson, and Sanders Korenman, *Baruch College, CUNY, New York City, New York, United States*

This paper estimates poverty rates and impacts of government benefits on poverty according to a pilot Principal Poverty Measure (PPM) for 2020-2023 using March CPS data. The PPM is a revision of the Census Bureau's Supplemental Poverty Measure (SPM) and was recommended by a 2023 NAS report. Our pilot PPM poverty line uses policy-based needs standards following an approach developed for the Health Inclusive Poverty Measure (Korenman and Remler 2016): a benchmark silver plan from ACA Health Insurance Marketplaces or Medicare coverage, Fair Market Rents from HUD, and dietary needs in USDA's Thrifty Food Plans. PPM resources include cash income, in-kind benefits including health insurance, and, for homeowners, an implicit net resource flow from their home: avoided rental payments minus homeowner costs. Compared to the SPM, PPM poverty rates are lower among homeowners and those with health insurance benefits. Accordingly, the PPM estimates greater differences in poverty between children and older persons. PPM poverty gap analyses reveal that, together, social insurance, means-tested transfers and tax credits close about 96% of the pre-transfer PPM poverty gap for persons 65+, while substantial gaps remain for children. The PPM also finds large anti-poverty impacts of rental assistance: among older people who receive it, rental assistance reduces poverty by more than 50 percentage points according to the PPM, but only 34 percentage points according to the SPM. We will also project impacts on poverty of changes in federal healthcare, housing and food programs currently under discussion.

Abstract citation ID: igaf122.1518

### DISASTER DISPLACEMENT IN THE U.S.: UNDERSTANDING PATTERNS AND IMPLICATIONS FOR OLDER ADULTS

Jenna Tipaldo[1], Deborah Balk[2], and Frank Heiland[2], *1. City University of New York, New York, New York, United States, 2. Baruch College, CUNY, New York City, New York, United States*

This research project utilizes the new nationally representative, rapid-turnaround Household Pulse Survey (HPS) data to investigate the prevalence and vulnerability of natural disaster displacement in the United States. We analyze data from all HPS cycles from December 2022 through September 2024, focusing on individuals ages 60 and older, making comparisons within groups of older adults as well as to the general population. Key preliminary findings are that 1.2% of the general population reports displacement due to a natural disaster in the past year (asked from late 2022 through Q3 of 2024). Importantly, displacement is not distributed evenly and varies by state – e.g., Florida has a much higher share of disaster displacement and a relatively larger share of older adults – income classes, race/

ethnicity, and type of disaster. The oldest adults seem more likely to be displaced due to multiple disasters, and many older adults who are displaced face health hazards and disability: such as food and water shortages, loss of electricity, and unsanitary conditions, isolation and mental health impacts, and both cognitive and physical disabilities. Preliminary results also suggest that working older adults resemble the general population in terms of work disruptions: those who report displacement in the past year are also more likely to report recent loss of income or work. These compositional issues, as well as plausible mechanisms, are also analyzed and discussed.

Abstract citation ID: igaf122.1519

### HEALTH INSURANCE, POVERTY, AND MEDICAL COSTS BEFORE AND AFTER DISABILITY APPLICATION

Jennifer Kaufman[1], Jasmine Manalel[2], Jenna Tipaldo[3], Na Yin[4], and Ruth Finkelstein[5], *1. Brookdale Center for Healthy Aging at Hunter College, New York, New York, United States, 2. Hunter College, CUNY, New York, North Carolina, United States, 3. City University of New York, New York, New York, United States, 4. Baruch College, CUNY, New York City, New York, United States, 5. Hunter College, New York, New York, United States*

Social Security Disability Insurance (SSDI) and Supplemental Security Insurance (SSI) programs are designed to provide a safety net after disability onset. SSDI qualifies an individual for Medicare after a two-year waiting period, and SSI qualifies an individual for Medicaid almost immediately. Yet applying for benefits is a lengthy process. Disabled workers lacking health insurance while they wait may experience deteriorating health and financial insecurity, compounding economic and health disparities. To learn how health insurance, financial security, and out-of-pocket costs changed from before disability application to the years after, we analyzed disabled workers aged 51-64 using data from the HRS linked to Social Security administrative records. Our findings show that disability benefits are significantly associated with higher rates of health insurance coverage. By the second wave (two to four years) after the disability decision, those with SSI and Medicaid eligibility had lower out-of-pocket costs than those rejected for benefits. For the group awarded SSDI only, out-of-pocket costs were higher at baseline, spiked at the wave (within two years) following the decision, and remained substantially higher than for the other groups. Poverty rates were markedly different between groups at baseline and showed no significant change across waves. Our findings confirm that disability benefits generally work as intended to provide a safety net; Medicaid eligibility is particularly important. Yet many adults with disabilities experience poverty as they approach old age. Future research should examine longer-term outcomes and whether transitioning into Medicare evens things out for those who do not receive disability benefits.

Abstract citation ID: igaf122.1520

### THE REENTRY AND DISABILITY APPLICATION EXPERIENCES OF FORMERLY INCARCERATED OLDER ADULTS

Christian González-Rivera[1], and Ruth Finkelstein[2], *1. Brookdale Center for Healthy Aging, Hunter College, CUNY, New York, New York, United States, 2. Hunter College, New York, New York, United States*

This study investigates barriers that formerly incarcerated older adults (FIOA) face when applying for Social Security Disability Insurance (SSDI) and Supplemental Security Income (SSI), which are among the most important benefits that they rely on when integrating into society after prison. Justice-involved older adults often struggle to access these benefits due to systemic inefficiencies, inadequate pre-release assistance, and misinformation. Using a community-based participatory research approach, we conducted semi-structured interviews with 28 FIOA aged 55-75 and two key informants from reentry service organizations in New York State. Interviews explored participants' experiences with Social Security Administration (SSA) processes, employment barriers, and interactions with agency personnel. Our findings revealed a range of structural, personal, and interpersonal hurdles: respondents frequently lacked essential documentation upon release, received inconsistent guidance from SSA staff, and experienced prolonged application delays averaging six months to a year. Many felt that SSA employees distrusted their disability claims, perceived discrimination based on their race or incarceration history, and faced digital barriers due to lack of knowledge about technology. Participants who received advocacy support from reentry organizations experienced smoother application processes. The study's findings suggest a need for implementing pre-release benefit application assistance, enhanced SSA staff training, expanded support from advocacy organizations, trauma-informed reentry services, and clearer guidance on eligibility and documentation requirements. These implications may lead to policy and practice changes that would smooth the transition of older adults—especially those who served long sentences—from incarceration to community life.

### SESSION 3390 (SYMPOSIUM)

Abstract citation ID: igaf122.1521

### PRESIDENTIAL SYMPOSIUM: NATIONAL INSTITUTE OF AGING P30 RESULTS IN AI AND TECHNOLOGY FOR HEALTHY AGING AND PERSONS WITH ADRD

Chair: Pamela Cacchione
Co-Chair: Elizabeth Vásquez
Discussant: Pamela Cacchione

The National Institute on Aging funded three P30's focused on Artificial Intelligence (AI) and Technology in ADRD and healthy Aging. University of Pennsylvania, Penn AITech, collaborating universities in Massachusetts, Mass AITC, and Johns Hopkins University, Hopkins AITC are in their last year of funding and have supported transdisciplinary 1 year pilot awards for 4 years for over 40 pilot awardees. This year completed these P30's funding. The P30s are supported by the a2 collective a coordinating center dedicated to hosting the annual pilot award competition and enhancing communications across the three P30s. Each of the P30s have a different focus for their pilot funds and will share their successes and opportunities in running a P30. Each of the P30s funded pilot awards from industry, technology start-up companies, and academic institutions. Penn AI Tech focuses on technology and AI development to advance the science of caring for older adult or those with ADRD living in the community. Mass AITC spans 5 institutions and focuses on technologies and AI for successful aging and care of PLwD at home. Hopkins AITC focuses on the implementation of new AI and technology solutions to improve the health of older adults and those with ADRD with an emphasis on physical function.

Each PI, George Demiris, PhD, (Penn AI Tech), Niteesh Choudhry, MD, PhD (Mass AITC), and Peter Abadir, MD, (Hopkins AITC) will describe their successful awardees' accomplishments as well as challenges for the P30's and the some of their pilot awardees over the first 3 years.

Abstract citation ID: igaf122.1522

**THE PENNAITECH EXPERIENCE: PROMOTING USE OF ARTIFICIAL INTELLIGENCE TO SUPPORT AGING**

George Demiris, *University of Pennsylvania, Philadelphia, Pennsylvania, United States*

The overarching goal of the Penn Artificial Intelligence and Technology (PennAITech) Collaboratory for Healthy Aging is to identify, develop, evaluate, commercialize, and disseminate innovative technology and artificial intelligence (AI) methods and software to support older adults and those with Alzheimer's Disease (AD) and Alzheimer's Disease and Related Dementias (ADRD) in their home environment. The Collaboratory is motivated by the need for a comprehensive pipeline from technology-based monitoring of older adults in the home, collection and processing monitoring data, integration of those data with clinical data from electronic health records, analysis with cutting-edge AI methods and software, and deployment of validated AI models at point of care for decision support. A central focus of the PennAITech Collaboratory is to advance this vision through the solicitation, review, and funding of pilot grants focused on technology and AI development to advance the science of care management and aging in place for vulnerable older adults or those with AD/ADRD receiving skilled home and community-based services. Funded pilot projects are supported through cores focused on administration, stakeholder engagement, technology identification and training, clinical translation and validation, networking, and ethical and policy issues. We will present our funded portfolio of funded projects by academic sites, industry and health systems, ranging from robotic tools for home assistance to predictive algorithms to support diagnostic excellence, smart home solutions and generative AI tools to facilitate coping and mental health services for older adults and their caregivers.

Abstract citation ID: igaf122.1523

**ARTIFICIAL INTELLIGENCE AND TECHNOLOGY FOR AGING: INSIGHTS FROM THE JOHNS HOPKINS AITC**

Peter Abadir, *Johns Hopkins University School of Medicine & Wisting School of Engineering, Baltimore, Maryland, United States*

The National Institute on Aging (NIA) funds the Johns Hopkins Artificial Intelligence and Technology Collaboratory for Aging Research (Hopkins AITC) to develop engineering innovations, technologies, and devices aimed at increasing independence in older adults. The Hopkins AITC is dedicated to bridging AI and biomedical advancements to support and enhance physical and cognitive health, ensuring that emerging technologies translate into practical, scalable solutions for aging populations. An important focus of the Hopkins AITC is the biological basis of physical and cognitive decline in older adults. Many of its pilot projects explore the integration of AI and novel technologies to assess, monitor, and intervene in age-related deterioration. These projects leverage wearable technology, smart devices, remote patient monitoring, and AI-driven analytics to provide insights into mobility, sleep, frailty, and cognitive function. Additionally, the Hopkins AITC has supported pilot projects focusing on caregivers and training the geriatrics workforce. These efforts include developing home monitoring devices to enhance caregiving, AI-driven tools to assist with care management, and technologies targeting social isolation in older adults. By addressing both direct patient needs and caregiver support, the Hopkins AITC aims to create a comprehensive framework for aging care. This session in the symposium will highlight key Hopkins AITC pilot projects and their contributions to aging research. Discussions will address the challenges of translating AI and engineering innovations into clinical and home settings, overcoming barriers to adoption, and ensuring ethical AI implementation.

Abstract citation ID: igaf122.1524

**MASS AITC SUPPORTS TECHNOLOGIES INCORPORATING MACHINE LEARNING AND AI TO SUPPORT SUCCESSFUL AGING AND CARE OF PLWD**

Niteesh Choudhry, *Harvard Medical School, Boston, Massachusetts, United States*

MassAITC supports pilot studies on using mobile technologies, wearables, and contactless sensors that incorporate emerging machine learning and AI approaches to support successful aging and the care of individuals with ADRD in their home environments. We will provide an overview of studies supported by our Center, which have addressed a broad range of topics including the early diagnosis of cognitive impairment, cognitive rehabilitation, longitudinal monitoring of chronic conditions of aging, falls and safety detection, balance and mobility training, cardiovascular risk assessment and management, and caregiver support. We will describe some of the early successes resulting from these awards including the formation of new start-up companies (e.g. Billion Labs Inc.), company acquisitions (e.g. Kinto by Rippl and TellUs You Care by AIP Healthcare), venture capital funding (e.g. Butlr and Moneta Health), capital and business alliances (e.g. Butlr and Ricoh), and design innovation awards (e.g. MindMics). We will also discuss some of the unique challenges arising from the funding of predominantly industry investigators within the context of a NIH granting mechanism. These have included the timelines and requirements for regulatory oversight, including IRB approvals, the management of conflicts of interest and intellectual property interests, the need to provide mentorship about study design and the conduct of human subjects research, difficulties recruiting individuals with ADRD for investigators not working in environments without an established pool of potential participants, and limitations of the amount of data available to build ML/AI models within the constraints of the size of the individual awards.

### SESSION 3395 (SYMPOSIUM)

Abstract citation ID: igaf122.1525

**TRANSFORMING GERONTOLOGY: THE POWER OF MINORITY AGING RESEARCH**

Chair: Elizabeth Muñoz
Co-Chair: Tyrone Hamler

As the field of gerontology continues to evolve, minority aging research remains essential in shaping its future by promoting health equity and advancing inclusive aging scholarship. This session convenes esteemed scholars—past recipients of the James Jackson Outstanding Mentorship Award—who have made significant contributions to minority aging research. Dr. Jacqueline

Angel, Dr. Maria Aranda, Dr. Tamara Baker, Dr. Lisa Barnes, and Dr. Roland Thorpe will share insights from their careers, reflecting on the challenges, breakthroughs, and impact of their work. Through a moderated discussion, panelists will provide key advice for emerging scholars seeking to advance research in this area, offering perspectives on navigating the field, fostering mentorship, and driving meaningful change. Attendees will gain a deeper understanding of the complexities and opportunities within minority aging research, learning from the experiences and lessons of leading experts. By drawing from the collective wisdom of these distinguished scholars, this session aims to inspire, support, and equip the next generation of gerontologists to advance equity in aging research and practice.

Abstract citation ID: igaf122.1526

### THE POWER OF INCLUSION: ADVANCING MINORITY AGING RESEARCH IN COGNITIVE HEALTH AND ALZHEIMER'S DISEASE

Lisa Barnes, *Rush University Medical Center, Chicago, Illinois, United States*

Lisa L. Barnes, PhD is the Alla V. and Solomon Jesmer Professor of Gerontology and Geriatric Medicine and a cognitive neuropsychologist within the Rush Alzheimer's Disease Center at Rush University Medical Center. She is also the Associate Director of the Rush Alzheimer's Disease Research Center. She received her PhD from the University of Michigan in biopsychology and completed a post-doctoral fellowship in cognitive neuroscience at the University of California, Davis. She joined the faculty of Rush as an assistant professor in 1999. Dr. Barnes has received many NIH grants and has published over 300 manuscripts. She is internationally recognized for her contributions to minority aging and minority health. Her research interests include disparities in chronic diseases of aging, cognitive decline, and risk factors for Alzheimer's disease. She is the Principal Investigator of two longitudinal community-based studies of older African Americans, including the Minority Aging Research Study (MARS), which has been funded by NIA since 2004 and the African American Clinical Core which she has led since 2008. She advocates for recruitment of under-represented groups into clinical studies and has received many awards and fellowships from universities and organizations throughout the U.S.

Abstract citation ID: igaf122.1527

### THE POWER OF EQUITY: ADDRESSING DISPARITIES IN PAIN MANAGEMENT FOR OLDER ADULTS

Tamara A. Baker, *The University of North Carolina at Chapel Hill, Chapel Hill, North Carolina, United States*

Dr. Tamara A. Baker is a Professor in the Department of Psychiatry at the University of North Carolina at Chapel Hill. She is an appointed member of the US Department of Veterans Affairs' Geriatric and Gerontology Advisory Committee, a member of the National Ins tutes of Health's Interagency Pain Coordina ng Commi ee, and a member of the Helping to End Addiction Long-Term (HEAL) Pain Strategic Plan's Execu ve Commi ee. Dr. Baker is a member of Taylor & Francis' editorial Advisory Board and Editor-in-Chief of Ethnicity & Health. She is formerly the Gerontological Society of America's (GSA) Secretary and has served as Chair of the Commi ee on Minority Issues in Gerontology and Chair of the Behavioral and Social Sciences (BSS) section. She is a GSA Fellow, founder and co-convener of GSA's Historically Black Colleges and Universi es (HBCU) Collaborative Interest Group, and appointed GSA Board of Governor's Vice President (2025) and GSA President (2026). Her background in Gerontology, Psychology, and Biobehavioral Health has evolved into an active research agenda that focuses on understanding the behavioral and psychosocial predictors and outcomes of chronic pain and pain and symptom management among older adults from historically marginalized populations. She also examines health disparities and inequities in pain management, and access and availability to pain management resources among older adults. Broadly, her research includes health disparities and health equity, cultural diversity/ sensitivity, and social determinants of health. Dr. Baker is recently funded in developing pipeline programs to address diversifying the aging research workforce.

Abstract citation ID: igaf122.1528

### THE POWER OF INTERVENTION: TACKLING ALZHEIMER'S AND MENTAL HEALTH DISPARITIES IN AGING POPULATIONS

María Aranda, *University of Southern California, Los Angeles, California, United States*

María P. Aranda, PhD, MSW, MPA, LCSW is an internationally-recognized social worker and sociobehavioral scholar in the fields of social work, gerontology, and health equity. She is Professor of Social Work with a joint appointment in Gerontology at the University of Southern California, and holds the Margaret W. Driscoll/Louise M. Clevenger Professorship in Social Policy and Administration. Dr. Aranda specializes in the area of medical and psychiatric comorbidity in adult and older adult populations specifically in the areas of Alzheimer's disease, depression, psychosocial interventions for individuals and family care partners, health disparities and ADRD. Dr. Aranda is the Executive Director of the USC Edward R. Roybal Institute on Aging. She has extensive experience in randomized trials, ethnographic methods, large-scale epidemiological research, stakeholder engagement, and innovative recruitment and retention protocols with underrepresented racial and ethnic groups in the US and international populations.

Abstract citation ID: igaf122.1529

### THE POWER OF POLICY: SUPPORTING LATINO AGING AND FAMILY CARE ACROSS GENERATIONS

Jacqueline Angel, *The University of Texas at Austin, Austin, Texas, United States*

For over three decades at The University of Texas at Austin, I have mentored emerging scholars committed to addressing the health and well-being of Latino aging populations. As Wilbur J. Cohen Professor of Health and Social Policy and Mentoring and Principal Investigator of the NIH R13-funded International Conference on Aging in the Americas (ICAA), my mentoring philosophy integrates rigorous training with contextual understanding of policy, culture, and family systems. Through the ICAA's structured, research-driven mentoring model now embedded in the Texas RCMAR I have guided emerging scholars and early-career underrepresented scholars in successfully publishing in top-tier journals, securing NIH funding (R01, R03, R21, K01), and advancing into academic and policy leadership positions. The ICAA mentorship framework emphasizes cross-national, policy-relevant research using large-scale studies such as the Hispanic EPESE, Mexican Health and Aging Study, and Health and Retirement Study. Recent initiatives include mentoring on pilot projects examining formal and informal caregiving among Medicare-Medicaid dually eligible Latino and non-Latino older adults. At ICAA meetings, I lead program development for juried poster competitions, speed-mentoring sessions, and manuscript development workshops all designed to translate research into policy impact. In this presentation I will reflect on how sustained mentorship rooted in evidence, collaboration, and cultural competency can shape research agendas and policy solutions that promote healthy aging and intergenerational caregiving within Latino communities. Angel has received several recognitions for mentoring research on aging, among which are the 2023 GSA James Jackson Outstanding Mentoring Award, UT Austin Faculty Mentor, President's Award for Global Learning, 2020. She was elected GSA Fellow in 2000.

Abstract citation ID: igaf122.1530

**THE POWER OF REPRESENTATION: ADVANCING HEALTH EQUITY FOR AFRICAN AMERICAN MEN ACROSS THE LIFE COURSE**

Roland Thorpe, *Johns Hopkins Bloomberg School of Public Health, Baltimore, Maryland, United States*

Roland Thorpe, Ph.D. is a Professor in the Department of Health, Behavior, and Society in the Bloomberg School of Public Health where he also serves as Deputy Director of the Hopkins Center for Health Disparities Solutions, and a Director of the Johns Hopkins Alzheimer's Disease Resource Center for Minority Aging Research. He holds joint appointments in the Division of Geriatric Medicine and Gerontology and the Department of Neurology in the School of Medicine, and the Department of Sociology in the Krieger School of Arts and Sciences. Dr. Thorpe is a social epidemiologist and gerontologist, whose research focuses on how race, socioeconomic status, and segregation influence health and well-being for African Americans, particularly African American men. His work has been funded by the NIH and he has published extensively. He is the recipient of numerous awards that recognize his commitment and valuable contributions to student and faculty mentoring, including the Johns Hopkins Bloomberg School of Public Health Advising, Mentoring, and Teaching Recognition Award, the inaugural 2018 NHLBI OHD PRIDE Roland J. Thorpe, Jr. Mentoring Award, the 2020 Johns Hopkins Bloomberg School of Public Health Dean's Award of Distinction in Faculty Mentoring, and the 2020 Minority Issues in Gerontology Outstanding Mentorship Award. Dr. Thorpe earned a bachelor's in theoretical mathematics from Florida A&M University, a master's in statistics, and a Ph.D. in clinical epidemiology with a graduate minor in gerontology from Purdue University.

### SESSION 3397 (BIOLOGICAL SCIENCES INVITED SYMPOSIUM)

Abstract citation ID: igaf122.1531

**SEX DIFFERENCES AND AGING**

Chair: Brittany Lasseigne

Sex differences are critical for deciphering normal biological function, including the aging process. However, the biological influence of sex, including metabolic, genomic, epigenetic, immunological, hormonal, and other differences between males and females, has been under-addressed in biomedical research. This gap has prompted key policies, notably the National Institutes of Health's Sex as a Biological Variable (SABV) and the Sex and Gender Equity in Research (SAGER) guidelines, designed to motivate researchers to consider sex differences in their studies. This session focuses on the impact of sex across the lifespan. Our presentations and discussion will center on identifying and characterizing sex-specific molecular signatures of aging, their role in developmental programming, and their influence on tissue-specific aging. We will also examine how biological sex modifies the effects of lifestyle interventions like exercise on the aging trajectory.

Abstract citation ID: igaf122.1532

**TISSUE- AND SEX-SPECIFIC MOLECULAR SIGNATURES OF AGING AND THE IMPACT OF DEVELOPMENTAL PROGRAMMING**

Michael Olivier, *Wake Forest University School of Medicine, Winston-Salem, North Carolina, United States*

Aging, even in healthy individuals, induces a wide range of molecular changes in cells, tissues and organs, resulting in (and mediating) the age-related decline in health. Many of these changes are influenced by both genetic and lifestyle factors, including early exposures during fetal development. We have used baboons, a non-human primate species highly similar in genetics, physiology and nutrition to humans, to characterize age-correlated molecular changes in organs and tissues using in integrated multi-omics approach. We have demonstrated that different organs show early age-related changes at different timepoints, and in a sex-specific manner, highlighting differences in aging and age-related changes to molecular and cellular functions. Also, early molecular changes are tissue-specific, with different genes, proteins, metabolites and pathways affected. Examining the adult offspring born to mothers that are either under-nourished or over-nourished during pregnancy, we also have evidence that these early exposures substantially alter the aging trajectory, lead to different molecular and pathway changes on the cellular level, and result in unique characteristic age-related changes in organ function. This suggests that developmental programming (the exposure in utero) not simply accelerates aging, but fundamentally alters the molecular mechanisms mediating age-related effects in these animals.

Abstract citation ID: igaf122.1533

**COMPARATIVE ANALYSIS OF SEX-SPECIFIC AGING**

Nicole Riddle, *University of Alabama at Birmingham, Alabama, United States*

Women and men show many differences in how they age, evident for example in the longer average lifespan seen in women. Sex-specific aging also occurs in many animal species. However, females do not always live longer. In some species males live longer than females, and in some species, there are no differences in lifespan between females and males despite other sex differences in aging. The Integration Initiative: Sex, Aging, Genomics, and Evolution (IISAGE) brings together researchers working in diverse species to exploit this naturally occurring variation in sex-specific patterns of aging to gain insights into the mechanistic basis of sex-specific aging and their evolution. The IISAGE team utilizes organismal, physiological, and genomics measures from various Drosophila species, moths and butterflies, fishes, geckos, turtles, snakes, bats, and mice. Using common methodologies allows us to carry out comparative studies across these diverse species. We present the results from several studies, including comparative transcriptomic analyses.

Abstract citation ID: igaf122.1534

**SINGLE-NUCLEUS RNA-SEQUENCING REVEALS SEX-DIMORPHIC MOLECULAR REMODELING BY RUNNING AND TFEB-OVEREXPRESSION IN SKELETAL MUSCLE**

Constanza Cortes, *University of Southern California, Los Angeles, California, United States*

Endurance exercise induces complex structural and functional remodeling in skeletal muscle, promoting metabolic efficiency and geroprotective effects. However, to date, our understanding of how these pathways may differ by sex remains poorly understood. We have previously shown that TFEB-overexpression in skeletal muscle (cTFEB;HSACre transgenic mice) recapitulates multiple aspects of running-activated metabolic remodeling across the lifespan. To determine the degree to which running and TFEB-overexpression transcriptionally remodels skeletal muscle, we performed single-nuclei RNA sequencing on tibialis anterior muscle from male and female mice across young sedentary, voluntary wheel running, and muscle-specific TFEB-overexpressing groups. Our results reveal significant sex- and condition-specific changes in myonuclei subtypes and non-myonuclear populations, including fiber-type switching, extracellular matrix remodeling, mitochondrial function, and immune signaling pathways. Importantly, TFEB overexpression recapitulates many transcriptional features of endurance exercise, with convergent yet distinct transcriptional responses across sexes, highlighting shared molecular pathways involved in muscle adaptation. This study advances our understanding of the molecular mechanisms driving sex-specific skeletal muscle plasticity in response to endurance running and demonstrates that TFEB overexpression effectively mimics key aspects of exercise-induced transcriptional remodeling in a sex-dependent manner. Moreover, it positions cTFEB;HSACre transgenic mice as a valuable experimental system for the discovery and prioritization of novel exercise-mimetic therapeutics aimed at promoting geroprotective muscle adaptations.

### SESSION 4000 (SYMPOSIUM)

Abstract citation ID: igaf122.1535

**MIDDLE AGE AS A WINDOW ON HEALTHY AGING: THE MIDLIFE IN THE UNITED STATES STUDY OVER 30 YEARS**

Chair: Margie Lachman
Co-Chair: David Almeida

The John D. and Catherine T. MacArthur Foundation provided 10 years of funding for the Research Network on Successful Midlife Development in 1995. The network included 12 scholars and 10 junior affiliates who designed the first wave of the Midlife in the United States (MIDUS) study. The initial study included a random digit dial sample of over 7000 adults ages 25 to 75 from the 48 contiguous United States. With funds from the National Institute on Aging, the study continues today with new samples added. MIDUS has garnered significant attention as the most frequently downloaded study at the National Archive of Computerized Data on Aging. Its extensive measures of biopsychosocial resources have highlighted resilience in the face of adversity as a central theme. In this symposium that marks the 30-year anniversary of MIDUS, the speakers, who have all been with the study since its inception, will present the history and rationale of the study along with recent innovations and some of the most intriguing findings that have shed light on the aging process. A longitudinal focus on early risk and protective factors during midlife is invaluable for optimizing health and well-being in later life. Almeida presents the history, design, and collaborative nature of the study. Mroczek discusses MIDUS as an exemplar of intergenerational science. Lachman highlights long-term effects of physical and psychosocial factors in midlife for later life health. Carr covers processes that contribute to social disparities in health. Ryff shows the importance of psychological well-being for health and longevity.

Abstract citation ID: igaf122.1536

**DEVELOPMENTAL TRAJECTORIES OF MIDUS: HISTORY AND EVOLVING DESIGN INNOVATIONS OF TEAM SCIENCE**

David Almeida, *The Pennsylvania State University, University Park, Pennsylvania, United States*

The Midlife in the United States (MIDUS) study has continuously evolved since its launch in 1995 with initial funding from The MacArthur Foundation. Originally designed to examine how psychosocial factors shape health and aging, MIDUS was groundbreaking in its inclusion of population-based daily diary studies, which provided novel insights into how everyday stressors accumulate to influence long-term well-being. With the first P01-funded expansion from the National Institute on Aging (NIA), MIDUS shifted beyond surveys to integrate biomarkers, cognitive testing, and affective neuroscience measures, marking a pivotal transition toward biopsychosocial integration. The addition of a diverse Milwaukee sample strengthened its generalizability, while new methodological innovations linked socioeconomic conditions to neurobiological health outcomes. In 2011, the MIDUS Refresher was introduced to address emerging societal challenges, such as the Great Recession, and to enhance statistical power for studying intersectional factors like race, gender, and socioeconomic status. More recently, the study has focused on maintaining sample representativeness by re-engaging past participants, a strategy that allows researchers to assess the long-term effects of early vulnerability markers on aging trajectories. As MIDUS moves into its next phase- the second and fourth waves of collection for the refresher and core samples, it continues to exemplify team science, fostering interdisciplinary collaboration to explore the complex interplay of daily experiences, social inequalities, and biological aging. By continuously adapting to new scientific priorities, MIDUS remains at the forefront of lifespan health research, providing a powerful model for studying adult development in a changing world.

Abstract citation ID: igaf122.1537

**YOUNG SHRUBS: PROMOTION OF EARLY CAREER SCHOLARS IN MIDUS**

Daniel Mroczek, *Northwestern University, Evanston, Illinois, United States*

From its earliest days, MIDUS leadership involved and included early career scholars. Access to MIDUS data and networking with senior members promoted the careers of those first generation MIDUS associates in the 1990s. This role modeling has passed down to later generations whereby that first cohort of beneficiaries has mentored later generations in the early 2000s, 2010, and now the 2020s. Second and Third generation MIDUS mentees are now active. This international system of mentoring is exemplified by recent articles that include scholars who were involved at the MIDUS inception as well as those who are much more recent PhDs (2021 or later). Examples of work that not only makes use of three waves of MIDUS data but includes MIDUS first generation scholars as well as much later generations of scholars will be highlighted. The success of long-term longitudinal studies like MIDUS are dependent on such intergenerational connections.

Abstract citation ID: igaf122.1538

### WHAT HAPPENS IN MIDLIFE DOESN'T STAY IN MIDLIFE: EXPLORING LONGITUDINAL PATHWAYS TO HEALTH IN MIDUS

Margie Lachman, *Brandeis University, Waltham, Massachusetts, United States*

There is growing evidence that physical and psychosocial factors in midlife play an important role in later life functioning. Blood pressure, lung function, glucose levels, cholesterol, sleep, and weight in midlife are strong predictors of health and cognition in later life. Psychosocial factors such as the sense of control, purpose in life, and social support in midlife also are important as they can motivate health-promoting behaviors that contribute to maintaining functional and cognitive health and reducing chronic conditions in later life. The relationships between midlife functioning and later life health and cognition are moderated by educational attainment and mediated by biomarkers including inflammation and allostatic load. With the rich Midlife in the United States (MIDUS) data we can examine the long-term cumulative effects and pathways to health over 30 years, showing differential trajectories in the transition from midlife into older ages. Health in midlife provides a window on later life and can inform preventative interventions to optimize functioning and health span. There is much interest in identifying modifiable lifestyle factors that can promote healthy aging. Results from multiple studies using the MIDUS dataset provide convincing evidence that addressing risk factors in midlife and adopting adaptive behaviors such as engaging in physical and cognitive activity can have long-term benefits for health outcomes and longevity. The discussion will focus on middle age as an ideal time to make lifestyle changes to increase health span throughout later life.

Abstract citation ID: igaf122.1539

### BEYOND SOCIAL STRUCTURES: USING MIDUS TO UNDERSTAND PSYCHOLOGICAL PROCESSES AND SOCIAL INEQUALITIES

Deborah Carr, *Boston University, Boston, Massachusetts, United States*

MIDUS is a large multi-wave multicohort longitudinal study of midlife and aging in the United States. The large sample enables critical explorations of intersectionality, or the ways that gender, race, age, disability status, socioeconomic status, obesity, and other social identities intersect to shape key outcomes spanning physical, emotional, and cognitive health. MIDUS is distinct from other U.S.-based and international studies of aging because it collects extensive and novel data on psychosocial processes that link social and structural factors to individual-level outcomes. This presentation will focus on one subset of these questions: perceived discrimination. Perceptions of both structural/institutional discrimination, like blocked opportunities in hiring and workplace advancement, and interpersonal discrimination, which encompasses microaggressions like being treated condescendingly, are key mechanisms linking structural positions to health and well-being. This presentation will feature key publications from the MIDUS and new empirical analyses that demonstrate the value of understanding institutional and interpersonal discrimination, and how these experiences are shaped by social contexts. For example, results from new analyses reveal: (a) how the impacts of disability status on workplace discrimination vary based on workplace requirements as defined by O*NET data, and (b) the complex ways that disability status and obesity intersect to shape experiences of interpersonal and institutional discrimination. These studies build on prior work demonstrating how workplace discrimination mediates the association between disability status and psychological well-being over the life course. Study results can inform policies and practices to enhance population health.

Abstract citation ID: igaf122.1540

### MIDUS AS MULTIDISCIPLINARY SCIENCE: CONNECTING WELL-BEING TO EVERYTHING

Carol Ryff, *University of Wisconsin–Madison, Madison, Wisconsin, United States*

A central objective of MIDUS is to study human development as an integrative process that links sociodemographic factors with experiential and psychosocial factors as well as biological factors and health. Such multidisciplinary science is illustrated with decades of research on a eudaimonic model of psychological well-being. First noted are studies showing how well-being is contoured by demographics, such as age, gender, race, and educational status. Other inquiries used aspects of well-being to predict stress hormones, inflammatory markers, and cardiovascular risk factors as well as chronic conditions (morbidity) and length of life (mortality). Still other studies have investigated well-being as mediators or moderators (intervening pathways) between indicators of inequality and health, broadly defined. A final group of studies have probed underlying mechanisms, such as how well-being is linked with brain processes (reward circuitry, amygdala activation, volumetric measures) or genomics (gene expression, epigenetic age acceleration). The historical stage on which MIDUS has unfolded has brought further emphasis to widening inequality, which is compromising the well-being of disadvantaged segments of the population. These problems have been exacerbated by hardships of the Great Recession, and more recently, hardships of the COVID-19 pandemic. Critical questions going forward are whether and how such cumulative hardships combined with persistently low well-being will compromise the health and longevity of less educated adults.

### SESSION 4005 (SYMPOSIUM)

Abstract citation ID: igaf122.1541

### ROBOT ASSISTED PERSON-CENTERED DEMENTIA CARE

Chair: Sajay Arthanat
Co-Chair: Jing Wang

This symposium explores the development, implementation, and ethical considerations of socially assistive robots (SARs) in dementia care, highlighting their potential to enhance caregiving and support aging in place for individuals with Alzheimer's disease and related dementias (ADRD). Through an interdisciplinary collaboration between researchers from health science, computer science, and nursing, this work presents a novel SAR equipped with smart sensing capabilities to deliver individualized care protocols tailored to the needs of persons living with dementia (PLWD) and their care partners. First, overview of the project will be shared, highlighting the feasibility, preliminary effectiveness, and user acceptance of SARs in home settings. Second, we will examine the SAR's technical framework, focusing on the integration of artificial intelligence and perception-action models that enable real-time decision-making in caregiving environments. Third, the symposium will explore the potential of SARs to deliver home-based exercise programs, addressing barriers to physical activity access among older adults, highlighting the use of AI to teach and track exercises and implications for SAR-led rehabilitation interventions. Fourth, we will discuss person-centered SAR care protocols, focusing on care partner feedback and real-world interactions. Findings will highlight design modifications that improve usability, personalization, and engagement for PLWD. Finally, we will address ethical challenges in SAR implementation, emphasizing strategies to balance autonomy with safety, protect privacy, and foster trust in SAR-assisted caregiving. These presentations will provide a comprehensive perspective on how SARs can be effectively designed, implemented, and evaluated to enhance dementia caregiving while maintaining ethical and person-centered care principles.

Abstract citation ID: igaf122.1542

### FEASIBILITY OF A NOVEL SOCIALLY ASSISTIVE ROBOT WITH SMART SENSING TO CARE FOR INDIVIDUALS WITH ADRD

Sajay Arthanat, *University of New Hampshire, Durham, New Hampshire, United States*

Several studies have highlighted the scope of socially assistive robots (SARs) to care of individuals with ADRD in supervised or institutionalized settings. However, the feasibility and effectiveness of SARs to independently support aging-in-place at homes and retirement facilities are yet to be explored. This overview section will highlight the significance of our project to support caregiving and daily living routines of individuals with ADRD. We will elucidate the technological framework used to develop a novel SAR, Mobile Assistive Robot with Smart Sensing (MARSS), with multi-modal capabilities to deliver care protocols. We will present findings from an ongoing mixed-method pilot study with five caregiver-care recipient dyads who utilized MARSS in the community for 3-6 months. Findings pertaining to feasibility, preliminary effectiveness and acceptance of the SAR will be presented through analysis of in-depth qualitative data (pre-and post-intervention) as well as monthly data gathered using Goal Attainment Scaling. Qualitative content analysis of data gathered through semi-structured interviews (based on the Unified Theory of Acceptance and Use of Technology) prior to the introduction, during usage, and end of trial is currently underway. The Goal Attainment Scaling involves individualized care goals set with the caregiver that considers the degree of accomplishment of each goal, importance of each goal (weight), and the difficulty with the goal at baseline. The project employs the NIH Stage Model for Intervention Development to identify and validate the target behaviors among care partners leading to potential adoption of MARSS, and to iteratively refine the technology towards a randomized controlled trial.

Abstract citation ID: igaf122.1543

### SOCIALLY ASSISTIVE ROBOTS FOR DEMENTIA CARE: A HUMAN-IN-THE-LOOP APPROACH TO PERSON-CENTERED CARE PROTOCOLS

Jing Wang, Sajay Arthanat, Momotaz Begum, Ola Ghattas, Marzan Alam, Moniruzzaman Akash, and Dain LaRoche, *University of New Hampshire, Durham, New Hampshire, United States*

The integration of Socially Assistive Robots (SARs) in dementia care offers innovative solutions to support aging in place and enhance autonomy for persons living with dementia (PLWD). This study explores the collaborative development of SAR care protocols using human-in-the-loop (HITL) and person-centered design approaches to support PLWD and their care partners in home settings. Grounded in interdisciplinary discussions between robotics scientists and health scientists, notes from the care protocol tailoring meetings with participants, and ongoing pilot study deploying SARs in real-world home environments with five caregiver-care recipient dyads over six months, this study highlights how direct user involvement and iterative refinement enhance SAR functionality and usability. Key design modifications included enhanced voice interaction, simplified task reminders, and personalized care routines, ensuring that SARs remained intuitive and responsive to cognitive and sensory limitations. To improve usability, the docking system was redesigned to function in various lighting conditions, while a tablet interface was incorporated for interactive guidance. User feedback was crucial in optimizing medication adherence prompts, mobility assistance, and social engagement features. Findings emphasize the importance of collaborative, person-centered refinement to create adaptive robotic systems that align with PLWD preferences and caregiving workflows. This study offers insights into real-world SAR deployment and provides a framework for integrating user-centered iterative design into assistive technology development. Future research should focus on scalability, ethical considerations, and long-term engagement to maximize SAR effectiveness in dementia care.

Abstract citation ID: igaf122.1544

### AI-POWERED SOCIALLY ASSISTIVE ROBOTS: REVOLUTIONIZING IN-HOME THERAPEUTIC EXERCISE FOR OLDER ADULTS

Dain LaRoche[1], Momotaz Begum[1], Sajay Arthanat[1], Jing Wang[1], and Paul Gesel[2], *1. University of New Hampshire, Durham, New Hampshire, United States, 2. PickNik Robotics, Boulder, Colorado, United States*

Physical activity for older adults is an important countermeasure to reduce the risk for chronic disease, falls, osteoporosis, depression and dementia while serving to preserve mobility and independence in activities of daily living. Access to structured physical activity and rehabilitation services is hampered by a deficit of licensed physical and occupational therapists, transportation challenges, and proximity to care services, particularly for older adults with disability and those who live in rural settings. Artificially intelligent SAR have the potential to bridge the care access gap by providing home exercise programs that can be tailored and autonomously delivered to older adults. A learning from demonstration algorithm allows therapists to teach robots exercises without computer programming, and computer vision can be used to recognize, quantify, and assess the quality of the exercises performed by older adults in their home, allowing for the identification of movement deficiencies and tracking of progress over time. We will illustrate the process by which therapists can teach robots exercises using AI, how the exercises are encoded and delivered by the robot, a framework for tracking performance, and results of a survey of 110 licensed therapists who were asked to evaluate the potential for robot-led therapy in home settings. We will discuss the emerging potential of SAR as an in-home adjunct therapy to supplement clinical exercise programs as well as the barriers to implementation including the anatomical structure of current robots, ability to provide real-time, physical, corrective feedback, potential loss of human connection, user acceptance and cost.

Abstract citation ID: igaf122.1545

### TECHNICAL FRAMEWORK FOR A CARE ROBOT

Momotaz Begum, Ola Ghattas, Marzan Alam, Moniruzzaman Akash, Noushad Sojib, and Mostafa Hussein, *University of New Hampshire, Durham, New Hampshire, United States*

A robot that cares for older adults in their home is tasked with making reliable sequential decisions subjected to uncertainties of human behaviors and environments. This is highly non-trivial due to a plethora of challenges involved with real-time processing of perception-action pipeline. The state of the art in AI robotics offers efficient tools to solve individual problems that may arise along this pipeline. An integrated solution, however, is still non-existent. We will present our ongoing work on developing an open-source control architecture that implements the entire perception-action pipeline for home care robots in a platform agnostic manner. The core of this architecture is a lightweight ROS2-based AI planner that can generate task plans executable by any robot – equipped with appropriate actuators – while sensing the current state of the environment and the care recipient (ROS: Robot Operating System; AI: Artificial Intelligence). Tasks typically included care services – e.g., reminding an older adult to take the medication or encouraging him/her to perform physical exercise – defined based on the needs of a care recipient. We integrate state of the art algorithms in robotics and computer vision – such as navigation, tracking, face recognition, natural language processing, etc. – in an innovative way with this lightweight AI planner to realize real-time planning and execution of a wide range of services that are critical for older adults to age gracefully in their own homes. We will report some data from our ongoing field studies.

Abstract citation ID: igaf122.1546

### ETHICAL CHALLENGES AND STRATEGIES IN IMPLEMENTING SOCIAL ASSISTIVE ROBOTS IN DEMENTIA CARE

Jing Wang, Sajay Arthanat, Momotaz Begum, and Dain LaRoche, *University of New Hampshire, Durham, New Hampshire, United States*

The integration of social assistive robots (SARs) in dementia care presents both opportunities and challenges in enhancing support for persons living with dementia (PLWD) and their care partners. From the initial stages of development through deployment, this study highlights how ethical considerations were embedded in the interdisciplinary collaboration between researchers from engineering, health sciences and nursing to ensure SARs align with the needs, values, and autonomy of PLWD. Drawing on data from baseline and follow-up interviews, participant-robot interaction observations, and interdisciplinary discussions, we examine how ethical awareness shaped SAR design, functionality, and implementation strategies. Key ethical considerations included balancing autonomy with safety in SAR interventions, addressing privacy concerns related to smart sensing technologies, and ensuring transparency in decision-making. Findings reveal that co-designing SAR care protocols with PLWD and their care partners through person-centered design fostered trust and engagement. Adaptations based on user feedback allowed for greater responsiveness, ensuring that SAR interactions remained meaningful and respectful of individual preferences. Additionally, strategies such as user-driven customization, ongoing ethical oversight, and structured feedback loops reinforced participant agency while mitigating potential biases in technology deployment. This study underscores the critical role of interdisciplinary collaboration in navigating the ethical complexities of SAR integration in dementia care. By prioritizing ethical vigilance throughout development and deployment, SARs can be implemented in a manner that upholds dignity, inclusivity, and person-centered care, ultimately enhancing their effectiveness in supporting aging in place.

### SESSION 4007 (SYMPOSIUM)

Abstract citation ID: igaf122.1547

### GENERATIVE AI IN GERONTOLOGICAL EDUCATION: INNOVATIVE APPROACHES AND EMERGING TOOLS

Chair: Carrie Andreoletti

Technology is increasingly shaping the landscape of gerontological education, offering novel ways to enhance learning experiences, professional training, and care provision. From virtual patient simulations and Telehealth applications to dementia sensory experiences and continuing education platforms, artificial intelligence (AI) is transforming the field. This symposium, sponsored by Gerontology & Geriatrics Education, explores the role of generative AI in educating current and future gerontology and geriatrics practitioners, with a focus on innovative applications, challenges, and best practices. Schumacher will open the session by providing an overview of current applications of generative AI in higher education, setting the stage for its integration into gerontological instruction. Brown will examine the use of Microsoft Co-Pilot and ChatGPT in the classroom, discussing both opportunities for enhancing learning and challenges related to student misuse of AI tools. Hawes will introduce AI-driven strategies for teaching nursing home regulations in healthcare management courses. Ogletree will highlight ways to optimize the use of online data dashboards, such as the HDPulse dashboard, to enhance data-driven learning in gerontology education. Finally, Katz will present research on attitudes toward AI, addressing common concerns and resistance while offering strategies to promote AI adoption in the classroom and beyond. This session will provide valuable insights into the evolving role of generative AI in gerontological education, fostering discussions on evidence-based best practices and ethical considerations for integrating AI tools into curricula. Attendees will gain practical strategies for leveraging AI to enhance educational experiences and professional training in the field.

Abstract citation ID: igaf122.1548

**GERONTOLOGY INSTRUCTION MEETS ARTIFICIAL INTELLIGENCE: WHAT DO FACULTY NEED TO KNOW?**

John Schumacher, *University of Maryland, Baltimore County (UMBC), Baltimore, Maryland, United States*

Generative AI represents a major paradigm shift for higher education and society. Ever-evolving tools like ChatGPT, Gemini, Perplexity, Claude, and others confront faculty with emergent pedagogical challenges as well as exciting opportunities. Faculty now face new questions about effective instructional design and meaningful integration of these technologies into gerontology curricula. How can educators balance traditional approaches with innovative, AI-driven strategies to enhance learning outcomes? This presentation provides a comprehensive overview of the generative AI landscape, including clear explanations and tips for using key technologies, offering practical information and insights designed to strengthen educators' generative AI skills and AI literacy.

Abstract citation ID: igaf122.1549

**CREATING GERONTOLOGICAL SPACE FOR GENERATIVE AI IN THE CLASSROOM**

Candace Brown[1], and Meredith Troutman-Jordan[2], *1. University of North Carolina, University of North Carolina, Charlotte, North Carolina, United States, 2. University of North Carolina Charlotte, Charlotte, North Carolina, United States*

The discussion of Generative AI (GAI) and its uses, purposes, and misuse have exponentially increased in the last several years, highlighting the need for instructors to learn more about GAI tools and how they can effectively navigate the GAI space in their classroom. The University of North Carolina, Charlotte supports ethical and responsible use of artificial intelligence to enhance academic teaching and learning of faculty and students who engage in this technology while responsibly sustaining high standards of integrity. This presentation will: 1) discuss challenges and opportunities from faculty and student perspectives of GAI; 2) exemplify GAI development and use in gerontology courses; 3) provide resources for attendees interested in developing GAI into their course curriculum; and, 4) challenge participants to consider how they may build faculty capacity for GAI integration in their own institutions.

Abstract citation ID: igaf122.1550

**INTEGRATING AI IN HEALTHCARE MANAGEMENT EDUCATION**

Frances Hawes, *University of Wisconsin-Eau Claire, University of Wisconsin-Eau Claire, Wisconsin, United States*

This study explores AI's role in healthcare education, focusing on nursing home regulatory citations. The study aimed to assess students' ability to interpret F-tag citations, apply them in quality improvement strategies, and distinguish between AI-generated data and manual findings. Students first researched assigned F-tags using the CMS State Operations Manual. In the second phase, they used AI to retrieve and analyze citation information, adjusting queries to evaluate AI output variations. Learning outcomes were assessed through two completed worksheets (n = 32), class discussions, and student reflections on AI's role in understanding citations and regulatory management. Results indicate that AI simplifies complex regulations, making them more accessible and helping students identify gaps in their initial understanding. Students found AI useful for citation management, recognizing its potential to automate analysis, identify trends, and assist in corrective action planning. AI was also seen as a valuable tool for compliance monitoring and training, providing real-time updates and tracking regulatory changes. However, concerns emerged regarding AI's limitations, including inaccurate responses, the risk of over-reliance, and the need for human oversight to ensure accuracy and ethical use. Ethical discussions particularly focused on AI-generated corrective action plans and the importance of maintaining human responsibility in regulatory decision-making. This study highlights AI's potential in healthcare education and its broader applications in policy analysis and patient safety.

Abstract citation ID: igaf122.1551

**COMPLEMENTING ONLINE DATA DASHBOARDS WITH GENERATIVE AI TO ENHANCE GERONTOLOGY EDUCATION**

Aaron Ogletree, *Virginia Tech, Blacksburg, Virginia, United States*

The universe of online data dashboards focused on presenting and visualizing population health data is rapidly growing. At the same time, educators are grappling with how best to use generative AI to support gerontology education. Despite the promise of these two innovative technologies for advancing the study of older adults, educators may be unsure about using them in practice. The goal of this presentation is to (a) introduce a range of online data dashboards that can be leveraged for the study of older adults, and (b) demonstrate how generative AI can support educators in using these dashboards to address gerontology competencies. We conducted a landscape analysis of data dashboards to identify those with information on older adults aged 65 and older. Focusing on a subset of dashboards, we then manually created a crosswalk between AGHE competencies for gerontology education and features available in those dashboards to develop curated (i.e., gerontologist drafted) classroom prompts. Separately, we used generative AI tools, like ChatGPT, to develop similar prompts. Comparison of manually curated classroom prompts with those originating from generative AI highlighted overlap between the two. That is, several of the manually curated prompts were also provided by generative AI tools. In some cases, the prompts originating from generative AI tools captured applications that we had not considered but that were nonetheless topical and relevant. Despite these strengths, limitations are noted. Our findings illustrate how educators in gerontology can leverage generative AI to teach about population health using online data dashboards.

Abstract citation ID: igaf122.1552

**FACTORS SHAPING PERCEPTIONS OF AI TOOLS AMONG A NATIONALLY REPRESENTATIVE SAMPLE OF US ADULTS**

Benjamin Katz[1], Norhan Abdelgawad[2], Daniel Friedberg[2], Patrick Roberts[2], and Shalini Misra[2], *1. Virginia Tech, Blacksburg, Virginia, United States, 2. Virginia Tech, Arlington, Virginia, United States*

Individuals throughout the life span are increasingly faced with challenging decisions regarding the adoption of generative AI tools in a variety of workplace contexts. In this nationally representative study of N = 500 US adults collected via the Prolific platform, we examined how a variety of demographic factors, thinking dispositions, and industry-types, including both K-12 and higher education (N = 37), influenced how individuals considered risk and utility of generative AI tools in their work. AI-relevant scales included the General Attitudes towards AI scale, an AI risk and benefits scale, AI frequency and expertise, and a scale for the assessment of non-experts' AI literacy. While higher education and K-12 industry status was not linked to differences in differential AI perceptions, age was closely linked to a variety of outcomes, including perceived benefits of AI, perceived risk of AI, and the optimal role of AI in workplace applications. For example, older individuals were in some cases more likely to agree strongly with statements emphasizing the potential benefits of AI and were somewhat less likely to agree with statements emphasizing the risks of AI in workplace contexts. Further analyses identified nuanced links with thinking dispositions including one's likelihood to engage in cognitively challenging activities and how susceptible one was to everyday cognitive failures. These findings may have implications for future curricula and programming designed to help individuals throughout the life span manage the proliferation of generative AI tools in workplace contexts, including within gerontology education.

### SESSION 4008 (SYMPOSIUM)

Abstract citation ID: igaf122.1553

### HORIZONS IN GERONTOLOGY: A CHAT WITH NIA SENIOR LEADERSHIP

Chair: Kenneth Santora
Discussant: Kenneth Santora

The National Institute on Aging (NIA) at the National Institutes of Health, Department of Health and Human Services, supports research to understand the nature of aging and to extend the healthy, active years of life. In the decades since its establishment, NIA has propelled advances and innovations across multiple domains of aging research, including basic biology, neuroscience, geriatrics and clinical gerontology, and behavioral and social science. NIA is also the federal leader in Alzheimer's disease research. This multidisciplinary research portfolio affords a wide array of opportunities in aging science, for early-career and established investigators alike. This symposium will provide a forum for discussion between the research community and NIA leadership, explore research foci of NIA's scientific divisions, and review opportunities and resources available to the research community.

Abstract citation ID: igaf122.1554

### OVERVIEW OF THE NATIONAL INSTITUTE ON AGING (NIA)

Richard Hodes, *National Institute on Aging, Bethesda, Maryland, United States*

Dr. Hodes will provide an overview of NIA, its research mission, and key NIA programs and initiatives.

Abstract citation ID: igaf122.1555

### NIA DIVISION OF EXTRAMURAL ACTIVITIES

Kenneth Santora, *National Institute on Aging, NIH, Bethesda, Maryland, United States*

Dr. Santora will discuss the work of the NIA Division of Extramural Activities. Dr. Santora will also be available for small group discussion.

Abstract citation ID: igaf122.1556

### NIA DIVISION OF BEHAVIORAL AND SOCIAL RESEARCH

Elena Fazio, *National Institute on Aging, Bethesda, Maryland, United States*

Dr. Fazio will discuss research foci of the NIA Division of Behavioral and Social Research. Dr. Fazio will also be available for small group discussion.

Abstract citation ID: igaf122.1557

### NIA DIVISION OF GERIATRICS AND CLINICAL GERONTOLOGY

Chhanda Dutta, *National Institute on Aging, Rockville, Maryland, United States*

Dr. Dutta will discuss research foci of the NIA Division of Geriatrics and Clinical Gerontology. Dr. Dutta will also be available for small group discussion.

Abstract citation ID: igaf122.1558

### NIA DIVISION OF NEUROSCIENCE

Jennie Larkin, *National Institute on Aging, Bethesda, Maryland, United States*

Dr. Larkin will discuss research foci of the NIA Division of Neuroscience. Dr. Larkin will also be available for small group discussion.

Abstract citation ID: igaf122.1559

### NIA DIVISION OF AGING BIOLOGY

Viviana Perez Montes, *National Institute on Aging, Bethesda, Maryland, United States*

Dr. Perez Montes will discuss research foci of the NIA Division of Aging Biology. Dr. Perez Montes will also be available for small group discussion.

Abstract citation ID: igaf122.1560

### NIA INTRAMURAL RESEARCH PROGRAM

Eleanor Simonsick, *National Institute on Aging, Baltimore, Maryland, United States*

Dr. Simonsick will discuss the work of the NIA Intramural Research Program. Dr. Simonsick will also be available for small group discussion.

### SESSION 4010 (PAPER)

Abstract citation ID: igaf122.1561

### GEROPSYCHIATRIC CARE OF OLDER ADULTS: SUBSTANCE USE, DELIRIUM AND DEPRESSION

In this session the participants will gain insights into research addressing substance use, delirium, depression and suicide in older adults

Abstract citation ID: igaf122.1562

### EPIDEMIOLOGY OF NEUROPSYCHIATRIC SYMPTOMS AND AD/ADRD RISK AND SURVIVAL IN OLDER U.S. ADULTS

Igor Akushevich[1], Arseniy Yashkin[1], Larry Tupler[1], Meishuo Ouyang[2], and Julia Kravchenko[2], *1. Duke University, Durham, North Carolina, United States, 2. Duke University School of Medicine, Durham, North Carolina, United States*

Alzheimer's disease and related dementias (AD/ADRD) pose a critical public health challenge, further complicated by the high prevalence of neuropsychiatric symptoms (NPS). Using a 5% Medicare sample, we quantified the epidemiology of NPS in relation to AD/ADRD onset and survival. We identified the onset dates for nine NPS—aggression, delirium, wandering, restlessness/agitation, hallucinations, anxiety, demoralization/apathy, sleep disturbances, and psychosis—and estimated their prevalence, the hazard ratios for developing AD and non-AD ADRD, and the hazard ratios for mortality following AD/ADRD diagnoses. We found that prevalence increases with age; maximum values at AD diagnosis were detected for anxiety (38.3%), psychosis (33.1%), and delirium (17.6%). Hazard ratios for AD/ADRD risk were evaluated using the Cox model with predictors measured with a one-year lag to avoid accounting ties between NPS and AD/ADRD onsets. The highest effects were detected for restlessness and agitation (HR = 4.30, CL = 4.21-4.39), psychosis (4.16; 4.12-4.20), and demoralization and apathy (4.15; 3.28-5.26). The effect of any NPS was 2.47 (2.45-2.48). Finally, we calculated the death hazard ratios for cohorts of individuals with diagnoses of AD and ADRD using a left-truncation design and age as the time-scale variable. The highest effect was detected for restlessness and agitation (1.61; 1.59-1.62) and delirium (1.52; 1.51-1.53). The death hazard ratio for any NPS was (1.46; 1.45-1.47). Our robust and biologically interpretable estimates provide new knowledge on underlying pathological processes—including an improved understanding of NPS and their treatment, and additional clues to potential underlying neuropathology—and to facilitate applications at individual-patient and population levels.

Abstract citation ID: igaf122.1563

### EXPERIENCES OF HEALTH CARE INTERACTIONS AMONG OLDER ADULTS IN GEROPSYCHIATRIC TREATMENT FOLLOWING SELF-HARM

Maja Sjöberg[1], Sara Hed[2], Stefan Wiktorsson[2], Anne Ingeborg Berg[2], Jennifer Strand[2], Sabrina Doering[2], and Margda Waern[2], *1. University of Gothenburg, Sahlgrenska University Hospital, Gothenburg, Vastra Gotaland, Sweden, 2. University of Gothenburg, Gothenburg, Vastra Gotaland, Sweden*

Physical illness and functional disability are common in older adult populations and strongly linked to suicidal behavior. The aim was to explore how older adults who engaged in self-harm experienced their interactions with healthcare providers. Two separate semi-structured interviews were conducted with each participant. Transcripts were analyzed using Interpretative Phenomenological Analysis. The participants were recruited among consecutive Swedish-speaking patients in outpatient treatment following self-harm within the last 3-36 months. Exclusion criteria were personality disorder, ongoing psychosis, aphasia, delirium, clinical dementia, or Montreal Cognitive Assessment score indicating moderate/severe cognitive impairment. Out of 22 eligible, nine accepted participation (4 women and 5 men, age range 71-92 years). Prior to their engagement in self-harm, all had their main care contact in primary care and all but one were on antidepressants. Participants described interactions with health care services that amplified their feelings of alienation, loneliness, worthlessness and self-stigma. Difficulties accessing care increased their sense of powerlessness. Some participants were cognizant of their mental health needs but experienced obstacles that hindered them from managing their illness. These situations increased frustration and hopelessness and contributed to the development of suicidal behavior. On the other hand, feeling listened to in trustful and validating relationships helped restore self-respect and agency, and fostered engagement in their individual suicide preventative strategies. Finding can inform clinical approaches to the care of older adults with symptoms of common mental disorders. Exploring experiences of care interactions before and after suicidal acts across different clinical settings and cultures could be areas for future research.

Abstract citation ID: igaf122.1564

### IMPROVING CARE FOR SKILLED NURSING FACILITY RESIDENTS WITH OPIOID USE DISORDER: A QUALITATIVE STUDY

Ashley Ritter[1], Corinne Roma[2], Charlie Merrick[2], Shivani Nishar[3], Katherine Kennedy[4], Jon Soske[5], Andrew Zullo[2], and Patience Dow[3], *1. Hunter-Bellevue School of Nursing, Hunter College, City University of New York, Yardley, Pennsylvania, United States, 2. Brown University, Providence, Rhode Island, United States, 3. Brown University School of Public Health, Providence, Rhode Island, United States, 4. Providence VA Healthcare System, Providence, Rhode Island, United States, 5. Brown University Health, Providence, Rhode Island, United States*

Skilled nursing facilities (SNFs) face financial, safety, and legal risks when transitions from hospitals are poorly managed and care practices fail to meet the needs of people with opioid use disorder (OUD). We examined strategies to optimize hospital-to-SNF transitions and access to medications for OUD (MOUD). From March to October 2023, we conducted semi-structured interviews with 29 administrative and clinical leaders involved in admissions from 27 SNFs across 19 states. Using descriptive thematic analysis, we identified five themes: (1) Variation in facility experience and stigma, and readiness: Resources and willingness to care for people with OUD varied substantially. (2) Conflation of OUD with pain management: Participants struggled to distinguish between opioids for pain, OUD, and physiologic dependence, highlighting knowledge differences about OUD. (3) Navigating information transfer: SNF staff often sought potential challenges that could negatively impact patient care before admission and noted that hospitals sometimes omitted important details to secure placement. (4) Siloed regulations and care delivery: Participants described how fragmented regulatory structures hindered admissions of people with OUD and limited their access to MOUD in SNFs. (5) Building trust and managing expectations during transition: The hospital-to-SNF transition and onboarding process was a crucial period for developing trust between people with OUD and SNF staff. Improving transitions and care for people with OUD requires education about OUD and stigma, enhanced information transfer and care coordination, and regulatory reforms to expand MOUD access in SNFs. Persistent challenges in the care for people with OUD who require SNF care require urgent action.

Abstract citation ID: igaf122.1565

### PRIMARY CARE PROVIDER PERSPECTIVES ON DEPRESSION MANAGEMENT IN OLDER ADULTS WITH MULTIMORBIDITY

Irina Mindlis[1], Dimitris Kiosses[2], and M. Carrington Reid[2], *1. Rutgers University, New York, New York, United States, 2. Weill Cornell Medicine, New York, New York, United States*

Primary care remains the main setting for depression treatment among older adults with multimorbidity (OAMM), who are also more likely to experience persistent depression. We sought to characterize primary care providers' (PCPs) perceived barriers to effective depression management for OAMM receiving primary care. We conducted a cross-sectional survey of 100 PCPs across New York City metro area health systems on satisfaction with resources, barriers, use of evidence-based approaches, and confidence in treating depression in OAMM. Descriptive statistics were summarized using SPSS. Most respondents (58%) were somewhat to very dissatisfied with available resources to manage depression in OAMM. Confidence levels in managing depression in this target population were high, yet rates of measurement-based care for depression were low, with only 19% using validated questionnaires to track symptom trajectories. Competing priorities and limited time within visits were noted as important barriers to depression management in OAMM. PCPs overwhelmingly endorsed a need for better access to mental health providers (85%), especially providers accepting Medicare/Medicaid, and those who offered long-term treatment. Even in the high-resource context of New York City, an area with one of the highest rates of mental health providers in the country, PCPs were dissatisfied with their ability to manage depression in OAMM, reported poor rates of measurement-based care for depression, and advocated for improved access. Future studies with national samples are needed to understand the landscape of depression management for OAMM in primary care. Our findings highlight the need for interventions to improve depression management for OAMM in primary care.

Abstract citation ID: igaf122.1566

### STNFR1 IS ASSOCIATED WITH DEPRESSIVE SYMPTOMS IN COMMUNITY-DWELLING OLDER ADULTS: A LOGISTIC REGRESSION ANALYSIS

Russell Calderon[1], Nada Lukkahatai[2], Jing Huang[2], Melissa Hladek[2], and Junxin Li[2], *1. Johns Hopkins School of Medicine, Baltimore, Maryland, United States, 2. Johns Hopkins University, Baltimore, Maryland, United States*

Inflammation has been implicated in the pathophysiology of depression, but specific inflammatory pathways may differentially contribute to depressive symptoms in older adults. This study examines the relationship between inflammatory biomarkers and depression in 91 community-dwelling older adults without dementia [Montreal Cognitive Assessment ≥17] using baseline data from a randomized clinical trial (NCT03959202). Depression was assessed using the Geriatric Depression Scale, with scores >5 indicating depression. Plasma-derived inflammatory biomarkers, including C-reactive protein (CRP), interleukin-6 (IL-6), tumor necrosis factor-alpha (TNF-α), and soluble TNF receptor-1 (sTNFR1), were Z-score normalized. A logistic regression model examined associations between biomarkers and depression, adjusting for age, sex, body mass index, and mild cognitive impairment (MCI) status. Participants were 70.2±6.2 years old, 80.2% females, 19.8% with MCI. Overall, 20.8% of participants (n = 19) were classified as having depression. In adjusted analyses, sTNFR1—a key mediator for TNF signaling—was significantly associated with depression (OR = 2.89, 95% CI [1.20, 5.60], p = 0.009), indicating that individuals with higher sTNFR1 levels had nearly three times the odds of depression. However, CRP, IL-6, and TNF-α were not significantly related to depression. These findings support sTNFR1 as a potential inflammatory biomarker linked to late-life depression, independent of demographics and cognitive status. Clinically, measuring sTNFR1 levels in routine visits may help in identifying older adults with risk for depression and informing early intervention strategies. Given its role in TNF-mediated inflammation, sTNFR1 warrants investigation as a potential target for interventions addressing late-life depression. Larger longitudinal studies should further elucidate the causal role of TNF signaling pathways in geriatric depression.

### SESSION 4015 (SYMPOSIUM)

Abstract citation ID: igaf122.1567

### LAYING THE GROUNDWORK FOR DEMENTIA SERVICE INNOVATIONS: DEVELOPING AND VALIDATING MEASURES OF DEMENTIA CAREGIVING

Chair: Yuanjin Zhou
Discussant: Lauren Bangerter

Caregivers of persons living with dementia face significant challenges in managing their complex health and care needs, often due to insufficient training and support. A major barrier to addressing these challenges is the lack of targeted and validated measures that capture the complex process of dementia caregiving. This symposium presents five papers focused on developing and validating novel measures to enhance caregiver support and service innovation. The first paper introduces the development of the Caregiver Outcomes of Psychotherapy Evaluation, Individualized Needs and Goals (COPE-ING) assessment tool, which offers promise for rapidly identifying caregivers' needs in clinical settings. The second paper aims to develop and establish the content validity of the Delirium Caregiving Self-Efficacy Scale using a Delphi Expert panel. This scale can be used to enhance dementia caregivers' adherence to delirium prevention. The third paper describes the development and content validation of the Fall Risk Management Scale (FRMS), which can be used to engage dementia caregivers in fall risk management effectively. The fourth paper presents the data supporting the reliability and validity of a new Caregiving Style (CG-STYLE) measure. Findings support its utility in tailoring support to family caregivers' unique caregiving styles. The fifth paper examines the structural validity of two clinical practical tools to evaluate person- and task-centered dementia mealtime care in nursing homes, with findings supporting the progression to establish these tools' clinical utility. This discussant, Dr. Lauren Bangerter, will highlight the research and clinical implications of these novel measures, especially how they contribute to dementia service innovations.

Abstract citation ID: igaf122.1568

### ASSESSING THE INDIVIDUAL NEEDS AND GOALS OF DEMENTIA CAREGIVERS: DEVELOPMENT OF THE COPE-ING MEASURE

Alyssa Aguirre[1], Jared Benge[2], and Rovianne Rovianne[2], *1. The University of Texas at Austin Dell Medical School, Austin, Texas, United States, 2. The University of Texas at Austin, Austin, Texas, United States*

The experiences of caregivers supporting persons living with dementia (PLWD) vary widely, yet existing measures often fail to accurately identify and address their unique needs and goals. This study aimed to develop the COPE-ING (Caregiver Outcomes of Psychotherapy Evaluation, Individualized Needs and Goals); an assessment tool designed to capture the diverse priorities of dementia caregivers in clinical settings. In the initial stage, we refined 16 items and sought input from 28 caregivers and 24 professional experts to evaluate item importance and provide qualitative feedback. All items were deemed important and revised accordingly. A final version was administered to 100 caregivers representing eight types of dementia, including 40% non-spousal caregivers, 18% non-white participants, and 25% male caregivers. Analysis revealed significant heterogeneity in caregivers' top priorities, with no single item being prioritized by fewer than 5% of participants. Top priorities included strategies to improve caregiver mood, finding community activities for PLWD, and increasing dementia knowledge. Importance ratings varied, with dementia knowledge, caregiver mood support, and communication strategies being rated highest. These findings outline the initial development of the COPE-ING, a novel measure designed to capture the individualized needs and goals of dementia caregivers. Our findings challenge the assumption that caregivers with similar clinical profiles have similar needs. The COPE-ING tool offers promise for identifying caregiver needs rapidly in clinical settings, with implications for theory and practice, particularly in tailoring interventions and informing caregiving support strategies. Further research is needed to validate the tool across diverse populations and settings.

Abstract citation ID: igaf122.1569

### AN E-DELPHI STUDY TO DEVELOP AND VALIDATE A DELIRIUM CAREGIVING SELF-EFFICACY SCALE

Shih-Yin Lin[1], Zachary Kunicki[2], Abraham Brody[1], and Donna Fick[3], *1. New York University Rory Meyers College of Nursing, New York, New York, United States, 2. Warren Alpert Medical of Brown University, Providence, Rhode Island, United States, 3. The Pennsylvania State University, University Park, Pennsylvania, United States*

Delirium is an acute neurocognitive disorder that commonly occurs in older adults, especially those with dementia. Multicomponent non-pharmacological interventions are the most effective for delirium prevention. While caregiver-administered multicomponent interventions show promise, adherence to different components varies. Self-efficacy may play a role in determining adherence. To explore this relationship, a validated delirium caregiving self-efficacy scale is needed. This study aimed to develop such a scale and establish its content validity using an e-Delphi expert panel. First, a rapid review using the NIDUS Delirium Bibliography identified delirium experts (corresponding authors of relevant publications) to invite and key domains of multicomponent interventions to guide the development of candidate scale items. Two rounds of e-Delphi surveys followed, during which experts rated the relevance, clarity, and importance of candidate items using Likert-type scales. Content validity was assessed using the content validity index (CVI, cutoff: 0.8) and content validity ratio (CVR, cutoff: 0.49). The rapid review identified 16 domains—leading to the development of 167 candidate items, and 70 experts. Twenty identified experts participated, with 18 completing both rounds. In Round 1, 61 items (36.5%) met both CVI and CVR cutoffs and were retained. Experts also suggested 25 new items. In Round 2, four of the 25 new items met the CVI and CVR criteria, resulting in a final 65-item scale. These findings support the content validity of the 65-item scale. Future research should refine it through cognitive interviews with caregivers to ensure comprehension and conduct item reduction analysis to create a more concise measure.

Abstract citation ID: igaf122.1570

### MEASURING DEMENTIA CARE PARTNERS' FALL RISK MANAGEMENT BEHAVIORS: DEVELOPMENT AND CONTENT VALIDATION

Yuanjin Zhou[1], Xiaoyi Zeng[1], Amanda Kilcrease[1], Noah Meah[1], Seung Choi[1], and Elizabeth Phelan[2], *1. The University of Texas at Austin, Austin, Texas, United States, 2. University of Washington, School of Medicine, Seattle, Washington, United States*

Family/friend care partners play a critical role in fall risk management (FRM) for older people with dementia via adopting FRM behaviors. No measure exists to assess these behaviors, hindering the development of effective strategies for engaging dementia care partners in FRM. The study aimed to develop a Fall Risk Management Scale (FRMS) for dementia care partners and improve its content validity. Based on our prior formative research, we drafted the 46-item FRMS that consists of eight sub-scales that assess multiple domains of FRM behaviors. To enhance the content validity, we performed two main activities: (1) we conducted a self-administered, written survey with 22 healthcare professionals to rate each item's clarity, relevance, and importance using a four-point ordinal scale, followed by interviews to gather additional feedback; and (2) we carried out two rounds of cognitive interviews with 21 dementia care partners to explore their understanding of each item and overall scale. The Item-Content Validity Indices (I-CVI) from the survey, along with the issues identified in the interviews using the Question Appraisal System, guided the iterative revision of the scale. Although only 19 items had I-CVIs lower than 0.78, all items showed at least one problem that required revision. Commonly identified problems included unclear/vague wording and inappropriate assumptions. Care partners generally understood items as intended but requested adding examples to ensure their understanding. Over the two-round process, we added eight items, removed one, and revised all items. Our study improved the FRMS's content validity for the next steps in its psychometric evaluation.

Abstract citation ID: igaf122.1571

### VALIDATING THE CG-STYLE MEASURE IN FAMILY CAREGIVERS OF INDIVIDUALS LIVING WITH DEMENTIA

Amanda Leggett[1], Jonathan Troost[2], Jennifer Miner[2], Sophia Tsuker[1], Jin-Shei Lai[3], and Noelle Carlozzi[2], *1. Wayne State University, Detroit, Michigan, United States, 2. University of Michigan, Ann Arbor, Michigan, United States, 3. Northwestern University, Evanston, Illinois, United States*

Family caregivers for individuals with dementia provide care based on unique caregiving styles. Using mixed-methods approaches, we developed a measure representing five caregiving style domains: understanding, adaptability, emotional expression (positive, negative), orientation to self-or-other, and behavioral management strategies (adaptive, maladaptive). We present data supporting the reliability and validity of this new Caregiving Style (CG-STYLE) measure. The sample included 209 family/friend caregivers for a community-dwelling individual living with dementia. Participants completed the CG-STYLE measure and comparator measures. We explored floor and ceiling effects (criterion ≤20%), convergent validity (correlations between similar domains should be moderate: 0.36 - 0.67 or high: 0.68 - 0.89), discriminant validity (correlations between dissimilar domains should be small: ≤ 0.3), and known groups validity (there should be significant differences between groups known to be different; determined by high versus low scores [median split] on caregiver readiness, competence, criticism, and active management). The CG-STYLE domains were devoid of floor or ceiling effects apart from a slight ceiling effect (30.3%) for maladaptive behavioral management strategies. There were moderate to high correlations between the CG-STYLE domains and known comparators supporting convergent validity, and negligible to small correlations between the CG-STYLE domains and an unrelated construct (i.e., pain intensity) supporting discriminant validity. There were significant group differences for all CG-STYLE domains (Cohen's d's range from 0.38 to 1.68). Findings support the preliminary reliability and validity of the CG-STYLE measure suggesting its utility in research, community, and clinical practice to target and tailor supports to caregiver's unique caregiving styles.

Abstract citation ID: igaf122.1572

### MEASURING PERSON- AND TASK-CENTERED DEMENTIA MEALTIME CARE: VALIDATING CLINICALLY PRACTICAL TOOLS

Wen Liu[1], Yelena Perkhounkova[2], and Maria Hein[2], *1. Penn State University, University Park, Pennsylvania, United States, 2. University of Iowa, Iowa City, Iowa, United States*

Person-centered mealtime care (PCMC) is assistance prioritizing accommodations of individual abilities, needs, and preferences and engaging individuals in eating through dyadic interactions and environmental stimulations. Task-centered mealtime care (TCMC) is assistance prioritizing completion of eating activities without adequate considerations of individual needs and preferences. Maximizing PCMC and minimizing TCMC is critical to optimize mealtime care quality and outcomes for people with dementia. Few tools are valid and clinically practical to measure PCMC and TCMC. We adapted the Cue Utilization and Engagement in Dementia (CUED) video-coding scheme, a novel and valid but resource-intensive tool, into two observational scales and examined their structural validity to assess PCMC and TCMC. 424 videos of dyadic mealtime interactions (mean duration=25.8 minutes, range=1-142 minutes) were coded using CUED, capturing 66 staff (age mean=39.6 years; 84.9% female; 22.7% non-white; 16.9% Hispanic) in 10 nursing homes. Staff behaviors were categorized based on frequency: 0 (not observed), 1 (observed 1-2 times), and 2 (observed ≥3 times). Several behaviors were combined based on content relevance and redundancy, resulting in 16 PCMC items and 5 TCMC items. For PCMC, 20 random splits of videos were created for exploratory (EFA) and confirmatory factor analyses (CFA): 1- and 2-factor structures with 8-9 items were suggested by EFA; an 8-item 1-factor scale (Cronbach alpha=.75) was chosen based on conceptual interpretability, CFA model fit, and internal consistency. A 3-item TCMC scale (Cronbach alpha=.32) was identified. Future testing is needed using the scales for direct observations of mealtime care in nursing homes to establish clinical utility.

### SESSION 4020 (COMMUNITY ENGAGED SCHOLARSHIP SYMPOSIUM)

Abstract citation ID: igaf122.1573

### INNOVATIONS IN COMMUNITY: LEVERAGING COMMUNITY NETWORKS TO REDUCE SOCIAL ISOLATION AND LONELINESS

Chair: Amy Eisenstein
Discussant: Jillian Racoosin Kornmeier

Meaningful connection is a fundamental part of what constitutes a good life at any age. Just as the causes of social isolation and loneliness are multi-faceted, and in many cases interrelated, addressing these issues is equally complex. Existing services and supports within the community can lay the groundwork to build innovative solutions. This symposium will bring together three community-centered research projects examining implementation of innovative approaches to address social connection in community settings. Presenters will share lessons learned from working within the community to address this issue. The first presenter will describe the initial steps of a community-engaged initiative to adapt and implement a Social Prescribing Decision Support Tool for use across diverse settings, and will discuss lessons learned from community partnerships necessary to make this work successful. The second presenter will discuss the role of Parks and Recreation professionals and the creation of a Healthy Aging in Parks Framework (HAIPF), along with plans and preliminary work to test the implementation of the HAIPF in four communities across the country. The third presenter will discuss implementation outcomes of Connect ME: a Brief Behavioral Activation intervention to increase social connectedness among homebound Meals on Wheels clients living in rural Maine. The session will be pulled together with a discussion on how we can take these lessons learned to continue to implement and disseminate innovative programs to reduce loneliness and create meaningful connections within existing community networks.

Abstract citation ID: igaf122.1574

### ADAPTING A SOCIAL PRESCRIBING DECISION SUPPORT TOOL FOR COMMUNITY AND HEALTH ORGANIZATIONS

Ashwin Kotwal[1], Carla Perissinotto[1], Katrina Hough[1], Nandini Singh[1], and Shannon Fuller[2], *1. University of California, San Francisco, San Francisco, California, United States, 2. Johns Hopkins University, Baltimore, Maryland, United States*

Social connection is a critical determinant of health, yet loneliness and social isolation remain pervasive challenges among older adults. Social prescribing—an evidence-based approach to matching individuals with social interventions tailored to their needs—holds promise in bridging the gap between healthcare and community-based services. However, there is a lack of structured, cross-sector tools to support this process. This presentation describes the initial steps of a community-engaged initiative to adapt and implement a Social Prescribing Decision Support Tool for use across diverse settings, including senior centers, health systems, and community-based organizations. Guided by a social-ecological conceptual framework, we plan to use qualitative methods and implementation science to adapt an existing Social Prescribing Decision Support Tool through an iterative process with feedback from older adults, community program staff, healthcare providers, and national experts. The resulting tool will account for intake and referral workflows, as well as the capacity to implement social prescribing in each setting. It will facilitate structured assessment and individualized matching of older adults to social interventions that address key dimensions of social connection (structure, function, and quality). We will present preliminary findings from the adaptation process, discuss lessons learned from community partnerships, and outline plans for a pilot implementation in two community-based organizations. This work aims to advance community-engaged scholarship by co-designing scalable, pragmatic solutions that enhance social prescribing efforts, promote interdisciplinary collaboration, and improve social well-being outcomes for older adults.

Abstract citation ID: igaf122.1575

### IMPLEMENTING CONNECT ME: PARTNERING WITH MEALS ON WHEELS AGENCIES TO IMPROVE SOCIAL CONNECTEDNESS

Renee Pepin, *Geisel School of Medicine at Dartmouth, Lebanon, New Hampshire, United States*

Aim: To test the reach and fit of Brief Behavioral Activation for Improving Social Connectedness, an intervention to increase social connectedness among homebound older adults living in rural Maine. Methods: We partnered with two Maine Area Agencies on Aging (AAA) that deliver Meals on Wheels (MoW) to conduct a study examining the implementation of Connect ME, a program to improve social connectedness which has two components 1) systematic loneliness screening for all clients during routine MoW assessments and 2) Brief Behavioral Activation for Improving Social Connectedness, a 6-session intervention, which was embedded in one partner AAA and delivered by their AAA staff. We assembled a blended research team, which included academic researchers and AAA staff. To test reach, we collected data on screening and recruitment. To test fit, we conducted qualitative interviews with AAA staff. Results: Over 2,000 MoW clients were screened, with 18.8% screened as lonely. Of those, 21% opted to participate in additional eligibility assessment for the Behavioral Activation component. The blended research team identified opportunities to improve procedures in real time. AAA staff reported that screening for loneliness and embedding Brief Behavioral Activation for Improving Social Connectedness were a good fit for AAA organizations. Staff identified key organizational factors that contributed to screening and intervention delivery success. Staff identified significant barriers regarding engaging MoW clients in the Behavioral Activation intervention along with potential solutions for future implementation. Implications: Key findings of organizational factors related to success and challenges can be applied to future Aging Service implementation.

Abstract citation ID: igaf122.1576

### PARKS AND RECREATION: A SOCIAL DRIVER OF HEALTHY AGING

Dianne Palladino, Austin Barrett, Natalia Ospina, Colleen Pittard, Allison Colman, Melissa May, and Eydie Kramer-Kostecka, *National Recreation and Park Association, Ashburn, Virginia, United States*

According to NRPA's Supporting Older Adults Through Parks and Recreation report, 92% of park and recreation (P&R) agencies offer resources and programs for older adults, and 95% consider improving social connection and reducing social isolation the top benefits of older adult programming. However, 30% of agencies currently offer intergenerational programming, identifying an opportunity for P&R agencies to fill a gap in this need for supporting older adults' physical and mental health. With support from RRF Foundation for Aging, NRPA responded to the need for more research on community-based solutions through P&R that increase intergenerational connectedness. Along with the survey of park and recreation agencies, focus groups of interdisciplinary experts in the healthy aging space revealed seven key pathways to advance health equity and social and intergenerational connectedness through P&R (centering equity, stakeholder engagement, equitable partnerships, co-location of services/spaces, empowerment/reciprocity, key messaging/mindsets, diversity of programs). We also conducted structured interviews with five diverse P&R agencies currently offering programs focused on encouraging connection between older adults and youth, resulting in case studies and summaries that highlight implementation, successes, challenges, best practices, and lessons learned. Through these findings, we created the Healthy Aging Framework, A Guide to Supporting Healthy Aging Through P&R. With continuing support from RRF, NRPA is conducting a follow-up intervention study to test the framework with four diverse P&R agencies that wish to establish sustainable programming to promote intergenerational connectedness and physical and mental health in older adults in the communities in which they live, learn, work, play, and age.

### SESSION 4025 (SYMPOSIUM)

Abstract citation ID: igaf122.1577

### USE OF CAUSAL INFERENCE METHODS IN EXPLORING GEROTHERAPEUTIC BENEFITS OF FDA-APPROVED MEDICATIONS

Chair: Jatupol Kositsawat
Co-Chair: George Kuchel
Discussant: Karen Bandeen-Roche

Randomized controlled trials (RCTs) represent the "gold" standard for validating gerotherapeutics for their potential delaying the onset and progression of varied chronic diseases. However, observational studies designed to guide the design of expensive and lengthy RCTs are subject to nearly unavoidable biases. Causal inference methods permit inference of causal relationships between exposures and outcomes, serving as a methodological link spanning the gap between traditional associational studies and RCTs. Causal inference benefits include ability to move from association to causality, decreased bias, and opportunities for sensitivity analyses. Mendelian randomization (MR), in the development of gerotherapeutics, uses genetic variants strongly associated with a proxy of drug exposure to mimic the drug's actions. Comparing genotype groups is analogous to comparing different treatment groups in RCTs for the long-term effects on outcomes. This symposium proposal includes a talk by Dr. Kuchel, who will present updated results of our 2022 Aging Cell article "Geroscience-guided repurposing of FDA-approved drugs to target aging: A proposed process and prioritization," including the first proposal for Cochrane-like assessments of the quality of traditional observational studies, while Dr. Chia-Ling Kuo will focus on Mendelian randomization studies, prioritizing evidence for gerotherapeutic potential based on study design and statistical considerations. Dr. Kositsawat will present MR data evaluating the benefits of metformin in Alzheimer's disease (AD) prevention. Dr. Karen Bandeen-Roche would be a Discussant to seek common threads between traditional observational epidemiology and causal inference methods. This meeting will highlight this critical topic with a particular emphasis on assessing medications with potential gerotherapeutic benefits. Geroscience Interest Group Sponsored Symposium

Abstract citation ID: igaf122.1578

### BENEFITS OF METFORMIN REPURPOSED FOR ALZHEIMER'S DISEASE PREVENTION, EVIDENCE FROM MENDELIAN RANDOMIZATION

Jatupol Kositsawat, *UConn Health, Farmington, Connecticut, United States*

Previous observational studies reported a decreased incidence of dementia in type 2 diabetic patients who take metformin compared to other hypoglycemic drugs. The possible neuroprotective effects of metformin also showed dose-dependent effects, either the daily dosage or the accumulative dosages taken. However, the Mendelian randomization (MR) study, one of the causal inference methods robust to confounders and reverse causation, poses some challenges because no specific target genes are attributable to metformin. Instead, researchers have based their MR study on a downstream biomarker such as A1c. This talk will review current evidence of MR studies on metformin exposure that look through the mechanistic effects of metformin other than glycemic control reflected by A1c in more detail. A recent study showed evidence of metformin gene expression in reducing the risk of Alzheimer's disease (AD) in the general population. Plausible mechanisms in AD protection include mitochondrial function and decreased mitochondrial complex 1-related gene expression. Another mechanistic link might be through a reduction in biological aging, an important process underlying AD pathogenesis. The MR studies of metformin effects showed benefits on phenotypic age and leukocyte telomere length. Our future goal is to thoroughly investigate the causal inference of metformin and other repurposed drugs for AD prevention through MR studies. A large number of observational study participants enable various sensitivity analyses that can show how these protective effects differ in different population characteristics and thus help identify patients who would benefit most from metformin following the Precision Gerontology concept.

Abstract citation ID: igaf122.1579

### MENDELIAN RANDOMIZATION EVIDENCE FOR GEROTHERAPEUTICS

Chia-Ling Kuo, *UConn Health, Farmington, Connecticut, United States*

Aging-related diseases and functional decline present major public health challenges. Mendelian randomization (MR) offers a robust approach for evaluating gerotherapeutic candidates by leveraging genetic variants as instrumental variables for drug exposures to infer their causal effects on aging outcomes. Additionally, the increasing availability of genetic instruments from genome-wide association studies (GWAS) has enhanced the ability to conduct rigorous MR analyses in this field. Successful MR studies require well-defined drug exposure proxies and careful selection of genetic instruments, such as biomarker levels or target gene expression. However, challenges remain, including limited knowledge of target genes, the restriction of protein GWAS to blood samples, and the lack of genetic instruments for RNA expression in highly expressed tissues. To ensure valid MR analyses, several statistical considerations must be addressed to uphold key MR assumptions. Strong genetic instruments are crucial for robust causal inference. Horizontal pleiotropy should be minimized to avoid bias, and sample overlap bias must be assessed to prevent inflated estimates. Finally, consistency across multiple MR methods strengthens the reliability of causal inferences. This presentation aims to provide a framework to assess MR studies on candidate gerotherapeutics by using genetic instruments linked to drug exposures to evaluate their effects on lifespan, healthspan, and aging-related traits. Systematically assessing the evidence for gerotherapeutic candidates is critical to identifying promising interventions, refining drug development strategies, and advancing the field of geroscience toward effective and targeted therapies for aging-related diseases.

Abstract citation ID: igaf122.1580

### GEROSCIENCE-GUIDED REPURPOSING OF FDA-APPROVED DRUGS TO TARGET AGING: A 2025 UPDATE

George Kuchel, *UConn Health, Farmington, Connecticut, United States*

Common chronic diseases represent the greatest driver of rising healthcare costs, as well as declining function, independence, and quality of life. Geroscience-guided approaches seek to delay the onset and progression of multiple chronic conditions by targeting fundamental biological pathways of aging. This approach is more likely to improve overall health and function in old age than treating individual diseases, by addressing aging the largest and mostly ignored risk factor for the leading causes of morbidity in older adults. Nevertheless, challenges in repurposing existing and moving newly discovered interventions from the bench to clinical care have impeded the progress of this potentially transformational paradigm shift. In a 2002 Aging Cell article, we proposed the creation of a standardized process for evaluating FDA-approved medications for their geroscience potential. Criteria for systematically evaluating the existing literature that spans from animal models to human studies will permit the prioritization of efforts and financial investments for translating geroscience and allow immediate progress on the design of the next Targeting Aging with MEtformin (TAME)-like study involving such candidate gerotherapeutics. This talk will provide an update on scientific developments which have taken place over the last 3 years with a particular emphasis on potential "off-target" gerotherapeutic benefits of more recent diabetes drugs such as SGLT2 inhibitors and GLP1R agonists on conditions as varied as congestive heart failure, kidney disease, frailty, and vulnerability to severe COVID-19. Moreover, a novel Cochrane-like approach to evaluating the quality of varied observational and causal inference studies will also be discussed.

### SESSION 4030 (PAPER)

### COGNITIVE AGING ACROSS THE LIFE COURSE: MECHANISMS, MEASUREMENT, AND CONTEXTUAL INFLUENCES

Abstract citation ID: igaf122.1581

### ASSOCIATION OF HEARING LOSS AND COGNITIVE DECLINE IN THE ACTIVE LONGITUDINAL STUDY

Nathalie Gider[1], Caitlin Northcutt[2], Cynthia Felix[3], George Rebok[4], Karlene Ball[5], Michael Crowe[5], and Tyler Bell[1], *1. University of California, San Diego, San Diego, California, United States, 2. University of Kentucky, Lexington, Kentucky, United States, 3. University of Pittsburgh, Pittsburgh, Pennsylvania, United States, 4. Johns Hopkins University, Baltimore, Maryland, United States, 5. The University of Alabama at Birmingham, Birmingham, Alabama, United States*

Hearing loss has been identified as a potential risk factor for dementia, but findings remain inconsistent due to methodological limitations, such as failure to account for hearing aid use and comorbidities like depressive symptoms. This study examined the relationship between hearing loss and cognitive decline using data from the Advanced Cognitive Training for Independent and Vital Elderly (ACTIVE) Study (n = 2,802; Mean age=73.63, SD = 5.91; 52.9% women; Mean education=13.35, SD = 2.66). Linear mixed models assessed whether baseline hearing loss predicted changes in Mini-Mental State Examination (MMSE) scores, processing speed (Useful Field of View), reasoning (Letter Series, Number Series, Letter Sets), and memory (Hopkins Verbal Learning Test, Rey Auditory Verbal Learning Test, Rivermead Behavioral Memory Test - Paragraph Recall) over 12 years, adjusting for age, sex, education, race, depressive symptoms, and intervention effects. At baseline, 48.8% of participants (n = 1,205) reported hearing loss, with only 23.2% (n = 280) using hearing aids. Over time, individuals declined in memory (b=-.043, p<.001). Those with hearing loss had worse baseline memory (b=-.256, p<.001) and experienced faster memory decline (b=-.013, p<.001). However, hearing loss was not associated with differences in reasoning or processing speed at baseline or over time (ps>.05), and hearing aid use did not moderate these associations (ps>.05). These findings suggest that hearing loss, regardless of hearing aid use, is linked to worsening memory but not other cognitive domains. Preventing hearing loss may be critical for preserving memory and reducing dementia risk. Addressing hearing loss in aging populations could enhance daily functioning and quality of life.

Abstract citation ID: igaf122.1582

### COGNITIVE MECHANISMS UNDERLYING MNEMONIC DISCRIMINATION ACROSS AGE AND CULTURAL GROUPS

James Qian[1], Yu-Ling Chang[2], Xiaodong Liu[1], Joshua Goh[2], and Angela Gutchess[1], *1. Brandeis University, Waltham, Massachusetts, United States, 2. National Taiwan University, Taipei, Taipei, Taiwan (Republic of China)*

Mnemonic discrimination, differentiating between similar items, declines with age. Declines may result not only from reduced hippocampal function but also from changes in executive function (EF). This study examines how memory and EF contribute to mnemonic discrimination across age and cultural groups. A total of 201 participants (98 Americans: 43 older, 55 younger; 103 Taiwanese: 50 older, 53 younger) completed the Mnemonic Similarity Task (MST) and a culturally adapted neuropsychological battery. The MST consisted of an encoding phase and a recognition phase, in which participants discriminated old items from similar and new items. Exploratory factor analysis derived memory and EF factors from the neuropsychological tests. General linear models examining the effects of memory, EF, age, culture, and two-way interactions on MST performance were significant, $F(5, 179) = 21.23$, $p < .001$, adjusted R2 = .35. Main effects of memory, age (younger), and culture (American) were observed (Ps < .01). A significant interaction between EF and age emerged, $t(179) = 2.93$, $p < .01$, such that EF correlated with MST performance in younger ($p = .02$) but not older adults ($p = .11$). These findings suggest that while memory processes support mnemonic discrimination, EF serves a compensatory role that diminishes with age, as declines in EF limit its ability to offset deficits. The results are consistent with an age-related shift toward pattern completion mechanisms. When EF can no longer compensate for declines in pattern separation, older adults rely more on pattern completion – a process for which EF is less effective.

Abstract citation ID: igaf122.1583

### CO-TWIN CONTROL MODELS INDICATE GENETIC CONFOUNDING BETWEEN EDUCATION AND LONGITUDINAL CHANGES IN COGNITIVE AGING

Deborah Finkel[1], Malin Ericsson[2], Ida Karlsson[2], Miriam Mosing[2], Marianne Nygaard[3], Chandra Reynolds[4], Margaret Gatz[1], and Brian Finch[1], *1. University of Southern California, Los Angeles, California, United States, 2. Karolinska Institutet, Solna, Stockholms Lan, Sweden, 3. University of Southern Denmark, Odense M, Syddanmark, Denmark, 4. University of Colorado Boulder, Boulder, Colorado, United States*

Education is perhaps the most widely examined early life protective factor for cognitive decline. Potential mechanisms for the relationship include direct causation, indirect causation (education may reflect a more advantaged early life environment), or genetic confounding (genes associated with educational attainment may also impact cognitive aging). Twin studies offer a method for testing causal hypotheses by incorporating within and between twin pair differences in longitudinal latent growth curve models (LGCM) of cognitive aging on both level of functioning and rate of change with age. Nine longitudinal twin studies of aging from the IGEMS consortium (N = 23,269) included up to 27 years of follow-up on measures of cognitive function in the domains of memory, perceptual speed, verbal ability, and semantic fluency. Pair means (between family effect) and within pair differences (within family effect) were included as covariates of both intercept and slopes in age-based quadratic LGCM (centered at age 65). LGCM results were compared across full sample, monozygotic twins, and dizygotic twins. Results differed across cognitive tasks, but generally MZ within pair effects of education were strongly attenuated compared with DZ within pair effects, indicating some genetic confounding. Some sex differences in the results were identified, likely a result of the differential access to education among the birth cohorts represented in the sample (birthyears 1900-1948). Thus, results suggest shared genetic factors explain some of the association between education and longitudinal change in cognitive function. In other words, genes that impact educational achievement also influence cognitive aging.

Abstract citation ID: igaf122.1584

### RETIREMENT, COGNITIVE HEALTH, AND NEIGHBORHOOD ENVIRONMENT: EVIDENCE FROM THE HEALTH AND RETIREMENT STUDY

Debasree Das Gupta[1], Uma Kelekar[2], David Wong[3], Kallol Kumar Bhattacharyya[4], and Jeein Law (Jang[5], *1. Utah State University, Logan, Utah, United States, 2. Marymount University, Arlington, Virginia, United States, 3. George Mason University, Fairfax, Virginia, United States, 4. The University of Memphis, Memphis, Tennessee, United States, 5. University of Massachusetts Boston, Boston, Massachusetts, United States*

The mental retirement hypothesis suggests that retirement may lead to cognitive decline due to reduced intellectual engagement and decreased cognitive reserve. Engaging retirees in mentally stimulating environments is encouraged, particularly within their neighborhoods. However, current empirical research has not adequately examined how neighborhood factors influence the relationship between retirement and cognitive health. To address this gap, we utilized data from two waves (2016-2018) of the Health and Retirement Study and analyzed whether perceived neighborhood environment moderates the association between retirement and cognitive health over a two-year follow-up period, after adjusting for covariates (retirement duration, baseline sociodemographic, socioeconomic, and health factors). The study sample (n = 2,040) consisted of non-institutionalized adults, age 51-70 years with no prior history of stroke or baseline cognitive impairment. Perceived neighborhood environment was measured using self-reported data on four separate neighborhood indicators that included social cohesion (e.g. friendliness/trustworthiness of neighbors), physical disorder (e.g. graffiti/vacant houses in neighborhood), social ties (friends/relatives in neighborhood), and safety. In the adjusted regression models, mean cognitive function at follow-up was significantly lower (β=-6.79; 95%CI=-13.06,-0.52) among those retired, compared to those fully or partly engaged in the workforce. However, this gap decreased by 1.02 units (β=-1.02; 95%CI=-0.06,-1.99) with each unit increase in perceived social cohesion. The remaining three neighborhood indicators did not significantly moderate the retirement—cognitive health relationship. Our findings indicate that greater social cohesion positively moderated the adverse relationship between retirement and cognitive health. Neighborhood resources that promote social and mental engagement may help protect the cognitive health of retirees.

Abstract citation ID: igaf122.1585

### THE LONG-TERM IMPACT OF CHILDHOOD EXPERIENCES ON COGNITIVE FUNCTION IN LATER LIFE: EVIDENCE FROM THE HCAP DATASET

Yujun Liu[1], Thomas Smith[2], and Jamie Mayer[2], *1. Northern Illinois University, Naperville, Illinois, United States, 2. Northern Illinois University, DeKalb, Illinois, United States*

Objectives: This study aims to explore the long-term impact of childhood experiences on cognitive function in later life and examines the moderating role of physical activity and educational attainment in adulthood. Methods: Using the first wave of the Harmonized Cognitive Assessment Protocol (HCAP) data, our sample included adults aged 65 or older in the U.S. (N = 3,496). We used multiple regression to model the relationships involving childhood experiences (parental education, childhood financial situation, and childhood health), physical activity and educational attainment in adulthood, and cognitive function in later life, specifically, memory (MEM), executive function (EF), visual-spatial cognition (VS), language and fluency (LF), and global cognition (GC). Results: The findings indicated that higher levels of financial situation in childhood and parental education positively predicted EF, LF, and GC. Participants with higher level of childhood health had higher levels of EF, GC, and VS. Participants with higher levels of educational attainment and physical activity in adulthood showed higher levels of all five cognitive domains. There was a significant positive buffering effect of physical activity on the relationship between parental education and LF. Educational attainment also significantly and positively moderated the relationship between parental education and both EF and LF. We also found a gender difference in the relationship between childhood health and LF and VS. Discussion: This study has important implications for developing recommendations and interventions that target behavioral and social factors associated with cognitive change among older adults with the greater goal of delaying or preventing Alzheimer's diseases and related dementia.

### SESSION 4035 (SYMPOSIUM)

Abstract citation ID: igaf122.1586

### TEACHING TRICKS OF THE TRADE: SHAKING UP GERONTOLOGY CURRICULUM THROUGH PEDAGOGICAL INNOVATION

Chair: L.B. Bouchard
Discussant: Rona Karasik

The Academy for Higher Education in Gerontology (AGHE) provides comprehensive competencies for gerontology education that can guide pedagogical innovations in gerontology curriculum. With these guidelines in mind, this symposium will showcase fresh activities, strategies, and partnerships that promote meaningful student engagement and learning outcomes. Speaker one will discuss a community based learning program that trains students to gain beneficial volunteer experience as resident advocates for older adults in long term care. Speaker two will discuss a course project where students created podcast episodes about familial relationships based on interviews they conducted with older adults. Speaker three will discuss an ungrading approach that promotes detailed, qualitative feedback and reflection instead of traditional numerical grading. Speaker four will discuss a rich departmental partnership to prepare students for practicum utilizing poetic portraiture and application of virtual reality tools. Speaker five will discuss a role playing game to promote deeper understanding of cumulative inequality among undergraduate gerontology students.

Abstract citation ID: igaf122.1587

### ROLLING INTO OLDER ADULTHOOD: ILLUSTRATING CUMULATIVE (DIS)ADVANTAGE WITH A DICE-BASED ROLE PLAYING GAME

L.B. Bouchard, *Western Oregon University, Monmouth, Oregon, United States*

Undergraduate curriculum can benefit from active, creative classroom participation aligned with the Academy for Gerontology in Higher Education (AGHE) competencies. In order to achieve student comprehension of AGHE standard I. 4. 2 (i.e., assess the impact of inequality on individual and group life opportunities throughout the lifespan/course impacting late-life outcomes), a role playing game (RPG) was developed. This activity was designed for an upper level undergraduate course called Intersectionality: Inequalities and Vulnerabilities in Older Adulthood. RPGs are being incorporated into higher education curriculum to encourage engaged student learning, foster empathy, and promote an understanding of deeper level concepts. RPGs are also useful to solidify challenging concepts while encouraging deeper social interaction, and this pedagogical modality lends itself to complex life narratives. Students begin by rolling for a case study, and each group is randomly assigned a personal history of a five year old child. Utilizing dice from table top role playing games, such as Dungeons and Dragons, students progress through the journey of life in adulthood with a combination of chance, choice, and circumstances outside their control. The instructor takes an active role to guide the scenarios, elicit reactions, and determine the outcome of student decision making. This presentation will focus on the basics of RPGs in higher education, explain instructions and parameters for this competency based activity, and explain next steps for instructors implementing this activity or other RPGs into their gerontology courses.

Abstract citation ID: igaf122.1588

**A FAMILY RELATIONSHIPS CLASS PROJECT: CREATING PODCAST EPISODES FROM OLDER ADULT INTERVIEWS**

Melinda Heinz, *University of Northern Iowa, Cedar Falls, Iowa, United States*

Students enrolled in a Family Relationships course created a podcast episode based on three interviews with older adults. After asking students to complete self-reflections on interviewing and technology strengths, student pairs were created, matching strong interview skills with savvy technology abilities. Together, students generated questions about what they might ask their older adult, focusing on family relationships. Students met their older adult interviewees on Zoom and recorded the meetings. After the first interview, each group discussed what the theme of their podcast episode might be and proceeded to ask follow-up questions at their next two meetings with the older adult. Student pairs were also required to act as podcast hosts by recording their own dialogue with each other, introducing content, navigating to interview segments with the older adult, and expanding on topics mentioned by the older adult and relating it to course content. At the end of the semester, each student pair created a singular podcast episode that was shared and discussed in class. Overall, students reported that they enjoyed the creativity and agency with creating their own podcast episode, but at times faced barriers with technology. Students also reported that they enjoyed learning how family relationships evolved and changed over time (e.g., role reversal with caring for a parent) directly from the older adult. Instructors may want to consider partnering with another class who has more advanced video editing skills or bringing a guest in to teach basic audio editing techniques.

Abstract citation ID: igaf122.1589

**IMPLEMENTATION OF INTERGENERATIONAL POETIC PORTRAITURE AND VIRTUAL REALITY PROGRAMMING AS COMPANION ACTIVITIES**

Kelly Munly[1], Erin Murphy[2], and Nick Smerker[2], *1. Penn State Altoona, Altoona, Pennsylvania, United States, 2. The Pennsylvania State University, University Park, Pennsylvania, United States*

Human Development and Family Studies (HDFS) and English faculty at a small campus collaborated to support advanced students of Adulthood Across the Lifespan and Social Perspectives of Aging (N = 28) to be trained in both poetic portraiture and application of virtual reality tools to support exploration of the life course context of older adults they prepared to work with in a long-term care practicum experience. This effort to prepare students for intergenerational programming is part of a larger effort toward Age Friendliness at this campus and in the surrounding community. Life course theory is the foundation of project aims and design. After HDFS faculty established a plan for collaboration with the English program, English faculty provided students with training in poetic portraiture as both a potential qualitative research method and as a foundation for conversations with older adults served in the practicum. Subsequently, Media Commons trainers provided classes with training in using virtual reality headsets to support older adults' opportunity to reengage with prior experiences (e.g., farm life or travel) and to take on exploration of new travel destinations, adding to life course experience. The poetic portraiture activity provided foundational conversations and insights into lived experiences, informing the subsequent explorations through virtual reality. Students followed their practicum training and experiences with reflections and forum discussions on their Canvas course online platform, with opportunities to share and analyze what they learned in the context of life course understanding.

Abstract citation ID: igaf122.1590

**INTEGRATING COMMUNITY-BASED LEARNING INTO THE CLASSROOM: STUDENTS BECOME LONG-TERM CARE RESIDENT ADVOCATES**

Katherine Kwong[1], and Claire Benedict[2], *1. Connecticut College, New London, Connecticut, United States, 2. The Holleran Center for Community Action and Public Policy, Connecticut College, New London, Connecticut, United States*

Community-based learning (CBL) is a teaching method that develops critical and reflective thinking on social and civic issues by combining academic instruction with community work by partnering with local community groups. The most successful CBL courses are built around a partnership between all participants (community partners, faculty, and students). CBL provides students with practical experiences of their academic studies and an understanding of ethical community engagement. In 2024 KFF notes that there were approximately 1.2 million individuals residing in certified nursing home facilities. In the state of Connecticut, KFF estimates that there are approximately 20,000 residents, with the majority being older adults. The Connecticut Long-Term Care Ombudsman Program (CT-LTCOP) provides an essential service as advocates for individuals who reside in long-term care facilities (LTC) as the eight Regional Ombudsman work to resolve complaints and concerns of residents and offer assistance and support. Their critical work is aided by Volunteer Resident Advocates (VRAs) assigned to select buildings. Elder Abuse was a course offered in partnership with CT-LTCOP with the goal of integrating CBL into the classroom, resulting in students becoming certified Volunteer Resident Advocates at the completion of the course. The main goals for integrating CBL into the classroom were: (1) provide an opportunity for undergraduate students to connect concepts learned in the classroom to their work within the LTC community; and, (2) to develop a model for faculty on building an effective CBL curriculum that benefits the students and the community.

### SESSION 4040 (SYMPOSIUM)

Abstract citation ID: igaf122.1591

**BRIDGING GENERATIONS: SOCIAL PERCEPTIONS AND SUPPORT IN INTERGENERATIONAL NETWORKS**

Chair: Claire Growney
Co-Chair: Laura Carstensen
Discussant: Merril Silverstein

As life expectancy increases and family structures evolve, opportunities for intergenerational relationships are expanding, creating new possibilities for social connections, support exchanges, and mutual influence across generations. These relationships can foster social integration, promote well-being, and shape perceptions of aging. Understanding the support behaviors that characterize intergenerational relationships and the perceptions that shape these connections is essential for identifying when and how they provide benefits. This symposium brings together studies examining intergenerational dynamics using diverse methodologies, including family and social network analysis, surveys, and experimental design, to examine implications for older and younger generations. Growney will present findings from the St. Louis Personality and Intergenerational Network study examining associations between socioemotional engagement with grandchildren and well-being outcomes for grandparents. De Fries will explore patterns of social support in older adults using latent class analysis, highlighting how different configurations of intergenerational and non-intergenerational relationships relate to social support and loneliness. Pot will discuss an experimental study about age-based stereotypes, suggesting that representations of older adults as individuals are associated with more positive and less negative stereotyping, which in turn relate to intergenerational affinity. Suitor will examine the intergenerational transmission of parental favoritism and disfavoritism, showing that midlife adults who perceive favoritism from their own parents are more likely to differentiate among their own children in similar ways. The session will conclude with a discussion led by Merril Silverstein, integrating insights from these studies and exploring broader implications for intergenerational connections, well-being, and social cohesion.

Abstract citation ID: igaf122.1592

### SOCIOEMOTIONAL ENGAGEMENT WITH GRANDCHILDREN IS ASSOCIATED WITH BENEFITS FOR GRANDPARENTS

Claire Growney[1], Thomas Oltmanns[2], Patrick Hill[3], Ryan Bogdan[2], and Laura Carstensen[1], *1. Stanford University, Stanford, California, United States, 2. Washington University in St. Louis, St. Louis, Missouri, United States, 3. Washington University in St. Louis, Saint Louis, Missouri, United States*

As life expectancy has increased, grandparenting has become a normative social role, yet its implications for older adults' well-being remain understudied. The present study examined well-being as a function of the amount and type of engagement grandparents have with their grandchildren. Participants (N = 1,002) aged 66–79 in the St. Louis Personality and Intergenerational Network (SPIN) study completed questionnaires about physical health, health practices, loneliness, and subjective memory and indicated whether they were grandparents. The 533 who identified as grandparents also answered questions about their involvement with grandchildren and related emotional experiences. There were minimal differences between grandparents and non-grandparents. However, among grandparents, those who were relatively more engaged socially and emotionally with their grandchildren reported less loneliness, more engagement in healthful behaviors, and better subjective memory. Socioemotional engagement with grandchildren was also associated with more positive and less negative emotional experience. Grandparents who reported providing relatively high levels of both instrumental support and socioemotional engagement reported better overall physical health-related quality of life. In contrast, providing relatively more instrumental support for grandchildren was associated with higher levels of loneliness and unrelated to healthful behavior engagement, subjective memory, or emotional experiences. Social and emotional ties to grandchildren are associated with cognitive, physical, social functioning among older adults, highlighting the importance of considering potential benefits and costs of grandparental involvement. We suggest that socioemotional engagement with grandchildren represents a particularly meaningful social connection, fostering a sense of purpose.

Abstract citation ID: igaf122.1593

### A LATENT CLASS ANALYSIS OF OLDER ADULT RELATIONSHIP PATTERNS AND THE ASSOCIATION WITH SOCIAL SUPPORT

Carson De Fries[1], and Kaipeng Wang[2], *1. University of Utah, Salt Lake City, Utah, United States, 2. University of Denver, Denver, Colorado, United States*

Social isolation leads to worse health and social outcomes for older adults. Social capital theory purports that different types of relationships offer different types of social support to an individual. Intergenerational relationships provide benefits to older adults, however the associations with social support have not yet been explored. The aim of this study was to explore how different types of relationships (e.g., intergenerational vs non-intergenerational) are associated with different types of perceived social support for older adults. A latent class analysis (LCA) was used to identify social support patterns within this population to create classes of older adults and then compare the differences between each class in social support and loneliness outcomes using ANOVA. 307 older adults (50+) completed an online survey describing their relationships, perceived social support, and feelings of loneliness. The LCA resulted in three classes of older adults: 1) Low amounts of intergenerational, familial, close, and reciprocal relationships (LOW), 2) Low amounts of intergenerational and familial relationships and high amounts of close and reciprocal relationships (LOWHIGH), and 3) High amounts of intergenerational and familial relationships and low amounts of close and reciprocal relationships (HIGHLOW). Results of one-way ANOVA showed the LOW class had significantly lower appraisal, tangible, belonging, and overall social support than both the LOWHIGH and HIGHLOW classes. Additionally, the LOW class reported significantly higher levels of loneliness than the LOWHIGH class ($p < .01$), potentially indicating that for loneliness, closeness and reciprocity of the relationship may be more important than age or relation of the other person.

Abstract citation ID: igaf122.1594

### TALKING 'BOUT YOUR GENERATION: YOUNG ADULTS' BELIEFS ABOUT OLDER ADULTS

Anna Pot, *Stanford University, Stanford, California, United States*

Most research on age stereotyping is based on surveys that ask participants to imagine "an older person" and respond to questions about this hypothetical person. In the present study, we examine whether younger adults' responses differ when they are asked to imagine hypothetical older people versus presented with an image of a single individual. We also examine how ratings differ when participants evaluate individuals versus generalized representations of older people. Additionally, we examine how these age-based stereotypes are tied to feelings of intergenerational affinity. 288 young participants, aged 18-35 years, were randomly assigned to one of four conditions and asked to rate six positive and six negative traits in response to experimental cues. Experimental cues varied by condition. In two conditions, participants viewed 36 faces presented as either individuals or representatives of their generation. In two other conditions, participants provided ratings about text cues, i.e., an older person or the older person's generation. Additionally, participants completed a measure of intergenerational affinity. Presentation condition significantly influenced beliefs. Individual presentations elicited less negative beliefs than generalized representations, with individual faces yielding the least negative ratings across all conditions. Conversely, responses to hypothetical representations were less positive than to individual faces. Across conditions, positive perceptions of older people were associated with greater intergenerational affinity. These findings suggest that beliefs about individuals tend to be more positive than beliefs about the category of "older people." Thus, age integration may more effectively reduce age stereotyping than general discussions about older people.

Abstract citation ID: igaf122.1595

**INTERGENERATIONAL TRANSMISSION OF PARENTAL FAVORITISM AND DISFAVORITISM IN MIDDLE AND LATER ADULTHOOD**

J. Jill Suitor[1], Megan Gilligan[2], Robert Frase[3], Destiny Ogle[1], Ranran He[1], and Yifei Hou[4], *1. Purdue University, West Lafayette, Indiana, United States, 2. University of Missouri, Columbia, Missouri, United States, 3. Southern Illinois University, Carbondale, Illinois, United States, 4. School of International and Public Affairs, Shanghai, Shanghai, China (People's Republic)*

Drawing from theories of socialization and agency, we use mixed-method data on 187 multigenerational families from the Within-Family Differences Study to examine whether midlife sons' and daughters' (G2) self-perceptions of their mothers' (G1) favoritism and disfavoritism shape their own patterns of differentiation among their offspring (G3). Prior research has suggested that parents' differential provision of support may be transmitted intergenerationally. The present study extends this work by focusing on the role of adult children's self-perceptions of their mothers' relational favoritism and disfavoritism in shaping their likelihood of differentiating among their own offspring. We study these processes late in the life course when G1 mothers are in their late 80s and 90s, and their children are in later midlife—a point by which it might be expected that differentiation among offspring would be less common. We concentrate on dimensions of relational differentiation that we have found to have the greatest impact on adult children's well-being—emotional closeness, conflict, and disappointment. Our findings revealed that adult children (G2) who perceived that their mothers (G1) currently differentiated on these relational dimensions were more likely to differentiate among their own offspring (G3) on the same dimensions. Further, patterns of differentiation in both generations appeared to be fueled by the same structural and socioemotional factors, with children's gender, perceived value similarity, and children's violations of expectations shaping which offspring were favored and disfavored. Thus, the findings indicate that both patterns and predictors of relational favoritism and disfavoritism are similar across generations.

### SESSION 4045 (SYMPOSIUM)

Abstract citation ID: igaf122.1596

**FROM ASSESSMENT TO IMPLEMENTATION: INNOVATIONS TO DECREASING DEMENTIA RISK IN RURAL SETTINGS**

Chair: Lisa Wiese
Co-Chair: Heather Fuller
Discussant: Cassandra Ford

Though rural U.S. counties comprise 85% of the "older-age counties," where more than 20% of residents are >65, innovative interventions to minimize the age-related risk of dementia are not well established. This symposium offers advances in assessment, recruitment, data collection, and interventions among rural U.S. regions. Fergen and colleagues share findings from their approach of using merged ethnographic and socioecological frameworks to identify dementia-focused themes that can be applied when developing interventions across the home, clinic, and community in rural northern Minnesota. Second, Cook et al. reveals new findings regarding the intersection of social isolation and environment in the rural community, through novel smartwatch-based measures of socialization and physical activity and how they relate to cognitive performance in older racially/ethnically diverse adults living in a rural southern Florida farmworker region. Third, Ghazi and colleagues compare the level of dementia risk as measured by the Montreal Cognitive Assessment (MoCA) across rural, micropolitan, and urban samples as a means for informing future innovations. Wiese's team concludes the symposium by demonstrating the effectiveness of adapting a faith-based framework for cancer detection and diagnosis, to a new focus area; cognitive decline. This model, tested in a southern, rural, racially/ethnically diverse cohort, can serve as an upstream approach to increase primary (education), secondary (screening), and tertiary (treatment) of dementia in other rural settings. Finally, as discussant, Ford will synthesize implications of these findings for policy and practice. Combined, these abstracts provide a pathway for diminishing the threat of cognitive decline in rural communities.

Abstract citation ID: igaf122.1597

**UNRAVELING THE RURAL LIVED EXPERIENCE WITH DEMENTIA: PATHWAYS TO IMPROVED CARE.**

Joshua Fergen[1], Dana Ketcher[1], Melinda Dertinger[1], Kelsie Larson[2], Kirsten Cruikshank[2], Amy Otto[2], Wayne Warry[1], and Kristen Jacklin[1], *1. University of Minnesota Medical School, Duluth Campus, Duluth, Minnesota, United States, 2. University of Minnesota Medical School Memory Keepers Medical Discovery Team, Duluth, Minnesota, United States*

This study describes findings from a qualitative, community-based participatory research project with rural communities. Using ethnographic techniques and a phenomenological approach, our aim was to collect and analyze data focused on the lived experience of dementia in rural communities to inform appropriate program development and interventions. Community-based researchers conducted 63 interviews with formal dementia care providers and community-dwelling older adults in northern rural Minnesota. Data were organized and analyzed using QSR NVivo. Findings identify the socio-cultural and environmental aspects of rural spaces, along with rural Alzheimer's disease and related dementias (ADRD) care accessibility, as cross-cutting themes to guide a socio-ecological model of care. Results provide insight into needs, barriers and opportunities at the level of the home, clinic and community, that require policy intervention across those three levels. For example, technological solutions in the home, dementia education in the clinic, and addressing spatial isolation in the community. Centering community voices and perspectives revealed gaps in our understanding how rural contexts shape community experiences with ADRD, which can inform actionable strategies and interventions. Results from this study identify the need for educational interventions and clinical approaches to improve care, unique challenges and adaptations that could facilitate aging in place, and assets in rural communities that can support persons living with dementia and their caregivers. These results can be used to create highly tailored programming, interventions, and policies for rural populations that are impacted by ADRD.

Abstract citation ID: igaf122.1598

### WATCH-SENSED SOCIALIZATION AND COGNITIVE TASK PERFORMANCE FOR OLDER ADULTS IN A RURAL COMMUNITY

Diane Cook[1], Bryan Minor[1], Christine Williams[2], Janet Holt[2], Juyoung Park[3], Lilah Besser[4], Diana Mitsova[2], and Lisa Wiese[2], *1. Washington State University, Pullman, Washington, United States, 2. Florida Atlantic University, Boca Raton, Florida, United States, 3. University of Arizona, Tucson, Arizona, United States, 4. University of Miami, Boca Raton, Florida, United States*

An extensive body of research links social isolation, physical inactivity, and rural residence with cognitive decline. The long-term goal of this work is to analyze the contribution of PM2.5 exposure during agricultural burn seasons to social isolation and cognitive function. As a step toward this goal, we investigated how smartwatch-based measures of socialization and physical activity relate to cognitive performance in older multicultural adults living in a rural southern Florida farmworker region. A total of $n = 36$ older adult participants (29 female, 7 male, mean age = 64.28) wore Apple Watches for two weeks to track movement and location, while cognitive function was assessed daily using a 45-second n-back shape test delivered via the watch. We quantified socialization as the percentage of time spent outside the home and defined activity level as the mean magnitude of the acceleration vector. Daily markers for socialization and activity level were extracted. Given individual differences in cognitive performance, we calculated daily deviations from each participant's mean n-back score. We then assessed correlations between these cognitive deviations and behavior markers. Across 798 data points, we found a small but statistically significant correlation between n-back score deviations and time spent outside the home ($r = 0.190$, $p < .001$). In contrast, the correlation between n-back score deviations and physical activity level was not significant ($r = .033$, $p = .354$). This ongoing work highlights the potential of continuous sensing to evaluate the influence of environmental and social factors on ADRDs and design community-specific interventions to promote brain health.

Abstract citation ID: igaf122.1599

### TRENDS IN DEMENTIA RISK VS COGNITIVE FUNCTION: RURAL, MICROPOLITAN & URBAN AREAS

Ladan Ghazi Saidi, *University of Nebraska Kearney, Kearney, Nebraska, United States*

Cognitive aging is influenced by environmental, demographic, and healthcare factors. Rural populations may face a higher risk of cognitive decline due to healthcare access and socioeconomic conditions. We assessed cognitive function across rural, micropolitan, and urban populations in the United States using the Montreal Cognitive Assessment (MoCA) in a sample of 150 adults aged 55 and older. Participants were clustered into rural (<5,000 residents, $n = 50$), micropolitan (5,000–50,000 residents, $n = 50$), and urban (>50,000 residents, $n = 50$). Statistical analyses included descriptive statistics and independent samples t-tests, with significance set at $p < 0.05$. The overall MoCA score averaged 25.46 ($SD = 2.88$, range = 14–30), aligning with prior research on aging populations and mild cognitive impairment thresholds. While no significant differences were observed in MoCA scores across rural ($M = 25.52$, $SD = 3.124$), micropolitan ($M = 25.33$, $SD = 2.987$), and urban ($M = 25.58$, $SD = 2.663$) groups, the proportion of participants scoring below the MoCA cutoff (<26) followed a trend of rural (43.3%) > micropolitan (38.5%) > urban (33.3%). Age distributions varied, with micropolitan participants being the oldest ($M = 69.82$ years) compared to rural ($M = 67.2$ years) and urban ($M = 66.41$ years) participants. Although MoCA scores did not significantly differ by residential classification, the higher proportion of rural participants scoring below the cutoff suggests an increased risk for cognitive impairment. Findings highlight the need for further research into modifiable dementia risk factors to inform policy development regarding healthcare access, education, and lifestyle differences.

Abstract citation ID: igaf122.1600

### ADAPTING THE "FAITH MOVES MOUNTAINS" APPROACH TO INCREASE DEMENTIA TREATMENT IN A RURAL COHORT

Lisa Wiese[1], James Galvin[2], Jennifer Lingler[3], Ishan Williams[4], Janet Holt[1], and Nancy Schoenberg[5], *1. Florida Atlantic University, Boca Raton, Florida, United States, 2. University of Miami, Coral Gables, Florida, United States, 3. University of Pittsburgh, Pittsburgh, Pennsylvania, United States, 4. University of Virginia, Charlottesville, Virginia, United States, 5. University of Kentucky, Lexington, Kentucky, United States*

Rurality confers a heightened risk for Alzheimer's disease and related dementias (ADRD). Recent demographic shifts have fueled rural, small-town growth, reshaping the composition of rural communities with increased representation of older and racially and ethnically diverse individuals, whose ADRD risk is compounded by other factors. Using a community-based participatory approach, we tested the hypotheses that (1) a faith-based, resident-led approach would increase ADRD knowledge and screening, and (2) older age, female gender, lower educational levels, and more years lived rural would predict number of referrals, new dementia diagnoses, and treatment. An adaptation of Schoenberg's (2009) Faith Moves Mountains model, previously successful in detection and management of other chronic illnesses in rural settings, guided this community-based participatory research. Local faith community members were trained as research assistants to recruit, administer surveys, conduct brief memory assessments, teach brain health strategies, and follow-up with residents. Outreaches were offered virtually during the pandemic, then in-person monthly at rotating church sites, and repeated ~1 year later. This rural sample was 74.5% non-White, with 28% reporting

### SESSION 4050 (SYMPOSIUM)

Abstract citation ID: igaf122.1601

### EXPERIENTIAL DIVERSITY: NEW MEASUREMENT APPROACHES AND NEW INSIGHTS INTO DAILY SLEEP AND COGNITION

Chair: Soomi Lee
Co-Chair: Rachel Koffer
Discussant: Martin Sliwinski

Research on experiential diversity (i.e., variety) has burgeoned in recent years, yet most studies remain at the between-person analyses (e.g., comparing individuals with greater activity diversity to those with lower activity diversity). With the rise of intensive longitudinal data, researchers now have the opportunity to quantify daily experiential diversity and examine its associations with daily health outcomes. These methodological advancements coincide with theoretical advancements highlighting the importance of sociohistorical context, and invite new approaches to assessing daily experiential diversity beyond the between-person entropy and other diversity indices. This symposium brings together five presentations to understand the dynamics among daily activity diversity, nightly sleep, and ambulatory cognition, while also exploring emerging domains and innovative methods for assessing daily experiential diversity. Lee presents the associations of nightly sleep quantity and quality with day-level activity diversity, including age-related differences in these associations. Drewelies examines the associations between daily subjective working memory and within- and between-person activity diversity in older adults. Bielak uses 14-day momentary objective cognitive tests (4 times/day) and their associations with activity variety (a composite sum) at the between-person and within-person levels. Clancy focuses on a new domain of experiential diversity, diversity in emotional support, and examines its sociohistorical, and age-related trends. Koffer proposes a sequence analysis approach to daily activity diversity, showing how daily sequences are associated with entropy, age, and sleep as a daily health indicator. The Discussant, Sliwinski, will integrate these findings and discuss the implications of experiential diversity for health and well-being across the adult life span.

Abstract citation ID: igaf122.1602

### ASSOCIATIONS BETWEEN NIGHTLY SLEEP AND DAILY ACTIVITY DIVERSITY AMONG OLDER ADULTS

Soomi Lee[1], Sandra Duezel[2], Denis Gerstorf[3], Ilja Demuth[2], Ulman Lindenberger[4], Rachel Koffer[5], and Johanna Drewelies[3], *1. The Pennsylvania State University, University Park, Pennsylvania, United States, 2. Charité - Universitätsmedizin Berlin, Berlin, Berlin, Germany, 3. Humboldt University Berlin, Berlin, Berlin, Germany, 4. Max Planck Institute for Human Development, Berlin, Berlin, Germany, 5. Arizona State University, Phoenix, Arizona, United States*

Greater activity diversity—broad and even participation across daily activities—is found to promote psychological well-being, cognitive functioning, and brain health. Sleep is a key lifestyle factor important for health, but it often competes for time with daily activities. Little is known about its relationship with daily activity diversity in older adults. This study examined the bidirectional associations of nightly sleep quantity and quality with daily activity diversity, as well as age-related differences in these associations. Data came from 127 retired older adults (Mage=76.88, Range=67–88) who participated in a 7-day experience sampling study (6 times/day) embedded into the Berlin Aging Study II. Participants reported momentary engagement in 16 activities across 7 domains (i.e., social, physical, mental, productive, household chores, self-care, and leisure), which were used to calculate a daily activity diversity score using Shannon's entropy. They also reported nightly sleep duration and quality. Multilevel models, adjusted for age, gender, education, morbidity, perceptual speed, negative affect, and daily pain, tested bidirectional associations between nightly sleep and daily activity diversity. Results showed no significant main association between previous night's sleep duration/quality and the next-day activity diversity, or vice versa. However, age moderated the association between prior night's sleep quality and the next-day activity diversity: for the oldest-olds (≥80), higher sleep quality predicted greater activity diversity, whereas for the younger-olds (≤73), the association was negative. These findings highlight the nuanced role of nightly sleep quality in shaping daily activity participation across different age groups.

Abstract citation ID: igaf122.1603

### DIVERSE DAILY ACTIVITY PATTERNS: EXAMINING DAILY ACTIVITY SEQUENCES AND ACTIVITY DIVERSITY IN OLDER ADULTS

Rachel Koffer[1], Sandra Duezel[2], Denis Gerstorf[3], Ilja Demuth[2], Ulman Lindenberger[4], Soomi Lee[5], and Johanna Drewelies[3], *1. Arizona State University, Phoenix, Arizona, United States, 2. Charité - Universitätsmedizin Berlin, Berlin, Berlin, Germany, 3. Humboldt University Berlin, Humboldt University Berlin, Berlin, Germany, 4. Max Planck Institute for Human Development, Berlin, Berlin, Germany, 5. The Pennsylvania State University, University Park, Pennsylvania, United States*

While daily activity diversity has been widely associated with healthy aging outcomes, further examination of daily activity patterns can uncover daily time use that is most beneficial for older adults. This study identified distinct daily activity sequences and examined whether they were associated with better quality sleep that night. Using a sample of older adults who participated in an experience sampling study embedded into the Berlin Aging Study II (N = 140, Mage = 77.09, range: 67–88 years), we applied sequence analysis to self-reported activity data (social, mental, leisure, physical, self-care, domestic, and productive activities) collected six times per day over seven consecutive days (N = 879 daily sequences). Distance metrics identified how dissimilar each day's sequence was from each other day. Using cluster analysis of the distance metrics, we identified 8 common sequence patterns. Days with activity patterns dominated by physical and domestic work predicted better quality sleep than days with patterns dominated by leisure (B = -2.10, p = .004), self-care (B = -5.33, p < .001), social (B = -3.65, p < .001), and mental activities (B = -2.91, p = .01). Findings controlled for comorbidities, age, gender, education, and weekday. Further, the likelihood of each sequence pattern differed across age and traditional measures of activity diversity. Daily activities in later life vary not just in diversity of types but also in the sequential patterns in which they occur throughout the day. Findings highlight the benefit of examining daily activity sequences in addition to activity diversity.

Abstract citation ID: igaf122.1604

### WORKING MEMORY AND ACTIVITY DIVERSITY IN OLDER ADULTS: EXAMINING WITHIN- AND BETWEEN-PERSON ASSOCIATIONS

Johanna Drewelies[1], Denis Gerstorf[2], Sandra Duezel[3], Ilja Demuth[3], Ulman Lindenberger[4], Rachel Koffer[5], and Soomi Lee[6], *1. Humboldt Universität zu Berlin, Berlin, Berlin, Germany, 2. Humboldt University Berlin, Humboldt University Berlin, Berlin, Germany, 3. Charité - Universitätsmedizin Berlin, Berlin, Berlin, Germany, 4. Max Planck Institute for Human Development, Berlin, Berlin, Germany, 5. Arizona State University, Phoenix, Arizona, United States, 6. The Pennsylvania State University, University Park, Pennsylvania, United States*

Cognitive function and activity diversity have been linked, yet it remains unclear whether this association holds at the daily level. This study examined associations between daily working memory and within- and between-person activity diversity in older adults. Data were collected six times per day for seven days from 127 participants in Berlin (Mage = 76.59, range: 67–88). Participants reported their activity engagement across social, physical, mental, productive, household chores, self-care, and leisure domains; a daily activity diversity score was calculated using Shannon's entropy. Multi-level models adjusted for age, gender, education, morbidity, perceptual speed, and negative affect and loneliness. Results showed that, at the between-person level, individuals with greater overall activity diversity exhibited better working memory. However, at the within-person level, on days when individuals engaged in more varied activities than usual, working memory was lower. Negative affect and loneliness moderated these associations: higher negative affect buffered the within-person effect of activity diversity on working memory, while loneliness amplified it. These findings suggest that although engaging in diverse activities may support working memory over the long term, daily fluctuations in activity diversity could be cognitively taxing. The interplay between emotional states and activity diversity highlights the complexity of daily cognitive functioning in older adults.

Abstract citation ID: igaf122.1605

### ASSOCIATIONS BETWEEN AVERAGE ACTIVITY VARIETY AND AVERAGE COGNITIVE PERFORMANCE ACROSS 14 DAYS

Allison Bielak[1], Jacqueline Mogle[2], and Martin Sliwinski[3], *1. Colorado State University, Fort Collins, Colorado, United States, 2. RTI Health Solutions, Durham, North Carolina, United States, 3. The Pennsylvania State University, University Park, Pennsylvania, United States*

There is some evidence that greater diversity of activity across the day is positively associated with that day's working memory performance. However, past work only permitted choosing a single, currently engaged in activity at each assessment, providing a limited index of activity variety. Using tablet-based assessments, 81 community-dwelling participants 41-94 years of age (M = 61.26, SD = 12.12) reported up to 20 activities they participated in the past 3-4 hours, and concurrently completed 3 cognitive tasks (2-back working memory, spatial working memory, processing speed) 4 times per day for 14 days. Multilevel models covarying age, gender, education, and retirement status showed that within-person fluctuations in average activity variety per session did not covary with average cognitive performance per session. Greater between-person average activity variety was associated with faster 2-back working memory on day 1, but those with higher average activity variety also showed less improvement over days for both working memory tasks. A detrimental association was also found for those in their 40s where greater average activity variety was associated with lower day 1 processing speed and spatial working memory. The limited associations with average activity variety and average cognitive performance were unexpected, along with variation in findings by cognitive outcome and age group. Associations with average activity variety appear to be more relevant than daily fluctuations in activity variety. Other work has showed short-term covariation between activity and cognition over the day when a frequency-based activity measure was used. Further inquiry into the type of activities engaged in may yield clearer results.

Abstract citation ID: igaf122.1606

### DIVERSITY IN EMOTIONAL SUPPORT, AND ITS SOCIAL, HISTORICAL, AND AGE-RELATED TRENDS

Erin Clancy, and Rachel Koffer, *Arizona State University, Phoenix, Arizona, United States*

In this study, we introduce the concept of emotional support diversity: the breadth and balance of emotional support given and received from different individuals in one's social network. Further, we test how developmental (i.e., age), historical (i.e., birth cohorts), and social (i.e., sexual minority status) contexts relate to differences in emotional support diversity. We utilized data from two independent cohorts of the Midlife in the United States (MIDUS) study (1995/1996 cohort: n = 5,492 adults aged 24-75 years; 2013/2014 cohort: n = 2,495 adults aged 23-76, stratified by age and gender to match the 1995-96 cohort). An overall emotional support diversity score was calculated based on 6 social roles (e.g., spouse or partner, in-laws) across 2 pathways (i.e., giving and receiving emotional support) using the Gini index. General linear models controlling for gender, race, education, self-rated health, and network size (i.e., partnership and parental status), revealed that older adults reported less emotional support diversity ($b = -0.005$, $p < .001$). The later-born cohort experienced less overall emotional support diversity ($b = -0.022$, $p = 0.035$). An interaction between age and cohort effects revealed that those in early midlife experienced less overall emotional support diversity in the later-born cohort than in the earlier-born cohort ($b = 0.002$, $p = 0.025$). There were no differences in emotional support diversity by sexuality (ps >.05). Implications of this new construct and differences in the hours of support received and given by specific social roles will be discussed in the context of age, cohort, and sexual minority status.

### SESSION 4055 (PAPER)

Abstract citation ID: igaf122.1607

### WHAT WE CAN LEARN FROM STUDIES OUT OF CHINA

This session includes five diverse studies out of China addressing life expectancy, exercise prescriptions, smoking and depression, cardiovascular risk in HIV and psychological resilience and cognitive frailty.

Abstract citation ID: igaf122.1608

### DYNAMIC LINKS BETWEEN CAREGIVING TRAJECTORIES AND SUBJECTIVE LIFE EXPECTANCY IN CHINESE OLDER CARE RECIPIENTS

Xin Sun[1], and Zi Yan[2], *1. Fudan University, Shanghai, Shanghai, China (People's Republic), 2. Waseda University, Tokyo, Tokyo, Japan*

Subjective life expectancy (SLE), an individual's self-assessed lifespan prediction shaped by health, psychological, and sociodemographic factors, serves as a critical metric for evaluating caregiving efficacy and long-term health trajectories; however, how longitudinal caregiving trajectories dynamically shape SLE among care recipients remains underexplored. Based on data from 3,350 adults aged 60 and above across four waves (2011, 2015, 2018, and 2020) of the China Health and Retirement Longitudinal Study (CHARLS), group-based multi-trajectory modeling (GBTM)was employed to identify distinct caregiving trajectory groups. Latent growth curve modeling (LGCM) was then applied to examine how these trajectory types were associated with longitudinal changes in SLE. Four types of care trajectories were identified through GBTM: Sustained-Independence, Spouse-Anchored Gradual Transfer, Multi-to-child Shift, and Multi-Source Intensification. Latent growth curve modeling revealed that all care-receiving groups had lower baseline SLE than the 'Sustained-Independence Group'. While the 'Sustained-Independence Group' maintained the highest baseline SLE, the 'Multi-to-child Shift' and 'Spouse-Anchored Gradual Transfer Group' exhibited converging trajectories over time. Notably, despite its initially lowest SLE levels, the 'Multi-Source Intensification Group' progressively narrowed the disparity with the 'Sustained-Independence Group' across waves. Our findings suggest that care dependency is not inherently linked to reduced perceived future time but is instead mediated by care provision dynamics. The study highlights the need for integrated, embedded, and modular support systems to alleviate mortality anxiety associated with perceived life constraints, offering actionable insights for developing interventions to enhance care recipients' sense of agency and future orientation.

Abstract citation ID: igaf122.1609

### IMPACT OF FAMILY DOCTOR CONTRACT SERVICES ON HEALTHCARE UTILIZATION AMONG OLDER ADULTS IN CHINA

Sijiu Wang, and Tingyu Lei, *Tsinghua University, Beijing, Beijing, China (People's Republic)*

As China's population ages, ensuring access to primary care for older adults is a growing priority. Family doctor contract services have been introduced to strengthen primary care and promote stable patient-provider relationships, yet their effectiveness in improving healthcare access for older adults remains unclear. This study examines the impact of family doctor contract services on healthcare utilization and expenditures among middle-aged and older adults, with a focus on rural-urban disparities. Using data from the sixth (2018) and seventh (2023) Shandong Province Health Service Survey, which employed multi-stage stratified cluster sampling across 20 cities, we analyzed a sample of 36,027 adults aged 45 and older. A panel data approach was used to compare healthcare utilization before and after contract enrollment. Probit and OLS regression models assessed service utilization and expenditures, while instrumental variable analysis, using regional contract signing rates, addressed potential endogeneity. Results indicate that family doctor contracts significantly increased outpatient utilization (6.73%, $p<0.01$), with stronger effects in rural areas (13.95%, $p<0.01$) than urban areas (2.56%, $p<0.05$). Inpatient service use also increased modestly (2.48%, $p<0.05$). While outpatient spending initially decreased (10.72%, $p<0.1$), this effect became insignificant under IV estimation. Findings underscore the role of family doctors in expanding healthcare access for older adults, particularly in rural regions where primary care is traditionally weaker. Policymakers should prioritize strengthening rural healthcare infrastructure, increasing provider incentives, and addressing disparities to ensure equitable access to primary care as China's population continues to age.

Abstract citation ID: igaf122.1610

### IMPLEMENTATION GAP AND INFLUENCING FACTORS OF EXERCISE PRESCRIPTION FOR OLDER ADULTS: A NATIONWIDE SURVEY IN CHINA

Qin Zhang, Chengfan Qin, Nan Hua, and Jing Chen, *The First Affiliated Hospital, Zhejiang University School of Medicine, Hangzhou, Zhejiang, China (People's Republic)*

Objective: This study aimed to assess the knowledge, attitudes, and practices of exercise prescription among healthcare professionals in China, and to examine factors influencing exercise prescription for older adults. Methods: A nationwide survey was conducted to recruit healthcare professionals in geriatric care in China. Descriptive statistics summarized their knowledge, attitudes, practices, and barriers to implementation. Thematic analysis was used to analyze participants' insights and suggestions. Ordinal logistic regression examined factors associated with prescription practices, and mediation analysis tested whether knowledge and attitudes mediated the effect of training on practice. Results: A total of 1,890 participants were included. Most doctors and nurses (43%–59%) reported a slight awareness of exercise prescriptions. Over half had never received training, yet nearly 95% rated prescribing exercise for older adults as quite to very important. Written prescriptions were uncommon across tertiary hospitals (9%), non-tertiary hospitals (4%), and primary healthcare institutions (10%). The perceived main barriers were time constraints, lack of knowledge, safety concerns, and insufficient family support. Trained doctors ($OR=4.26$, 95% CI: 3.36–5.64, $p<0.001$) and nurses ($OR=2.52$, 95% CI: 1.91–3.32, $p<0.001$) were more likely to provide written prescriptions. Knowledge and attitudes mediated the relationship between training and prescription practices. Conclusions: Chinese healthcare professionals had limited knowledge of exercise prescriptions, with low usage of written prescriptions. They recognized the importance of exercise prescriptions and maintained a positive attitude. Knowledge and attitudes mediated the impact of training on prescription practices.

Abstract citation ID: igaf122.1611

### PSYCHOLOGICAL RESILIENCE AND COGNITIVE FRAILTY PROGRESSION AMONG OLDER ADULTS: EVIDENCE FROM CHINA IN 2002-2018

Ruoxi Ding[1], and Yanan Luo[2], *1. Peking University, Beijing, Beijing, China (People's Republic), 2. Peking University School of Public Health, Beijing, Beijing, China (People's Republic)*

Objectives: Psychological resilience (PR) may protect against cognitive frailty, yet its relationship remains unexplored. This study examines the association between PR and cognitive frailty progression among older adults in China using 16 years of nationally representative cohort data. Methods: Data from the Chinese Longitudinal Healthy Longevity Survey (CLHLS, 2002–2018) were analyzed. Cognitive frailty was defined as coexisting physical frailty and cognitive impairment without dementia. Cognitive function was assessed using the Chinese Mini-Mental State Examination (CMMSE), and physical frailty was measured through daily living, instrumental activities, and functional limitations. Fixed-effect models, Latent Class Growth Models, and multinomial logistic and Cox regression analyses were used to explore PR trajectories and their links to cognitive frailty progression. Results: A 1-point increase in PR score reduced cognitive frailty risk by 9% (RRR=0.91, 95% CI: 0.90, 0.93), with stronger effects in younger, non-illiterate, and married individuals. Compared to the high-level stable PR group, those with moderate-level declining or low-level rising PR faced higher risks of mild cognitive impairment (MCI) with accelerated frailty (RRR=1.34, 95% CI: 1.15, 1.56; RRR=2.10, 95% CI: 1.39, 3.16, respectively) and high-probability MCI and frailty trajectories (RRR=1.59, 95% CI: 1.26, 2.02; RRR=3.49, 95% CI: 2.13, 5.72). Conclusions: Higher PR levels are linked to reduced cognitive frailty risk, particularly among younger, married, and non-illiterate older adults. Lower PR trajectories predict greater cognitive and physical decline, underscoring the importance of PR interventions in secondary prevention strategies for cognitive impairment and disability, and in promoting healthy aging.

Abstract citation ID: igaf122.1612

### SEX DIFFERENCES IN CARDIOVASCULAR DISEASE RISK AMONG CHINESE OLDER ADULTS LIVING WITH HIV

Junwen Yu[1], Bei Wu[2], Xiang Qi[3], Lu Shao[4], Ting Xu[5], Hongzhou Lu[6], and Zheng Zhu[1], *1. Fudan University, Shanghai, Shanghai, China (People's Republic), 2. NYU Shanghai, Shanghai, China (People's Republic), 3. New York University, New York, New York, United States, 4. University of Hong Kong, Guangzhou, Guangdong, China (People's Republic), 5. Nanjing Medical University, Nanjing, Jiangsu, China (People's Republic), 6. The Third People's Hospital of Shenzhen, Shenzhen, Guangdong, China (People's Republic)*

People living with HIV have twice the risk of developing cardiovascular disease (CVD) compared to HIV-negative individuals, yet sex differences remains unclear. This study aimed to address this gap by examining sex differences in CVD risk among Chinese older adults living with HIV. We analyzed data from hospitalized HIV-infected individuals aged 50 years or older at a major hospital in Shenzhen, China, from 2017 to 2022. The 10-year CVD risk was estimated using the Pooled Cohort Equations. Logistic regression and marginal analyses were performed, and a segmented regression model was used to analyze the relationship between high CVD risk prevalence and age, grouped into 5-year intervals. Among the 576 participants, 214 (37.2%) had high CVD risk (9.6% in females and 43.9% in males). The average CVD risk was significantly higher in males than females (8.79% vs. 3.58%, P < 0.001). High CVD risk was significantly associated with CD4 counts < 200 cells/μL (OR = 1.832, 95% CI = 1.101-3.050, P = 0.020). The predicted probability of low CD4 counts was higher in males (46.8%) than females (35.5%, P = 0.016). The prevalence of high CVD risk increased significantly in males after age 50, whereas in females, the breakpoint occurred at age 65. Males exhibited a significantly higher CVD risk, emphasizing the need for early monitoring from a younger age. Although females demonstrated a later onset of high CVD risk, menopause-related lipid metabolism changes highlight the need of early prevention and integrating female-specific factors into risk assessment tools. Future research should further investigate the underlying mechanisms to improve precise prevention strategies.

### SESSION 4060 (PAPER)

Abstract citation ID: igaf122.1613

### ARTIFICIAL INTELLIGENCE AND TECHNOLOGY IN AGING

This session provides insight into the use of artificial intelligence in disease and functional assessments.

Abstract citation ID: igaf122.1614

### ACCURACY OF DEMENTIA CLASSIFICATION ALGORITHMS IN LMICS AND HICS: A SYSTEMATIC REVIEW AND META-ANALYSIS

Wenjie Cai[1], Tsai-Chin Cho[2], Chihua Li[2], Kelvin Zhang, Sneha Mani[1], Lindsay Kobayashi[2], and Alden Gross[1], *1. Johns Hopkins Bloomberg University School of Public Health, Baltimore, Maryland, United States, 2. University of Michigan School of Public Health, Ann Arbor, Michigan, United States*

Compared with using clinical diagnosis information, dementia algorithms are a cost-effective alternative to classify dementia status. Numerous studies have developed dementia classification algorithms; however, many rely on specialized diagnostic information, such as MRI, PET, and blood/CSF biomarkers, that are costly and more available in high-income countries (HICs) compared with low- and middle-income countries (LMICs). In this systematic review and meta-analysis, we screened and synthesized the diagnostic accuracy of dementia classification algorithms in HIC and LMIC contexts based on demographics, health history, cognitive assessment, and/or informant reported cognitive and functional ability. We searched PubMed and PsycINFO for papers published between January 01, 2013 and February 28, 2025. After screening abstracts and full texts, 39 studies were included, of which 7 were conducted in LMICs. Fifteen studies reported sensitivity, specificity, and number of dementia cases, and thus were included in a meta-analysis: 53% (8/15) were community-based, and 80% (12/15) used machine learning algorithms. A hierarchical summary receiver operating characteristic (HSROC) model was used to construct summary ROC curves in LMICs (AUC=0.83) and HICs (AUC=0.95) separately. Dementia classification algorithms showed excellent performance in distinguishing dementia from normal cognition or mild cognitive impairment in both HICs and LMICs. The sparsity of published algorithms from LMICs highlights the need for further research on culturally and linguistically appropriate dementia classification algorithms in LMICs.

Abstract citation ID: igaf122.1615

### AI-DRIVEN FRAILTY DETECTION IN LONG-TERM CARE USING ACCELEROMETER-MEASURED GAIT AND DAILY PHYSICAL ACTIVITY

Xiaoping Zheng[1], Ziwei Zeng[1], Kimberley van Schooten[2], and Yijian Yang[1], *1. The Chinese University of Hong Kong, Hong Kong, China (People's Republic), 2. University of New South Wales Sydney, Kensington, New South Wales, Australia*

Frailty affects over 50% of older adults in long-term care (LTC), and early detection is critical due to its potential reversibility. Wearable sensors enable continuous monitoring of gait and physical activity, and machine learning (ML) has shown promise in detecting frailty among community-living older adults. However, its applicability in LTC remains underexplored. Furthermore, dynamic gait outcomes (e.g., gait stability, symmetry) may offer more sensitive frailty indicators than traditional measures like gait velocity, yet their potential remains largely untapped. This study investigated whether gait and daily physical activity data collected from a single accelerometer can effectively identify frailty in LTC residents. Fifty-one LTC residents (85.1±9.2 years; 24 women, 27 men; 26 non-frail, 25 frail per FRAIL-NH scores) wore a 3D accelerometer near the L3 vertebrae for about one week during daily life. Thirty-seven dynamic and spatiotemporal gait outcomes, three physical activity outcomes, and six demographic outcomes were extracted and trained XGBoost ML models, employing a leave-one-out cross-validation approach. Model performance was evaluated using accuracy and area under the receiver operating characteristic curve (AUC). Explainable AI (XAI) techniques were applied to identify key predictive outcomes. The XGBoost model achieved an accuracy of 86.3% and an AUC of 0.92. XAI analysis revealed that frail older adults exhibited more irregular gait patterns characterized by higher variability, increased asymmetry, and reduced predictability. These findings suggest that dynamic gait outcomes may serve as more sensitive indicators of frailty than gait velocity in LTC settings, which offers new insights to support frailty detection and management in LTC.

Abstract citation ID: igaf122.1616

### SYMPTOM TRAJECTORY AND READMISSION RISK PREDICTION IN CHRONIC RESPIRATORY FAILURE VIA LATENT CLASS GROWTH MODEL

Rong Zhang, *Shanxi Bethune Hospital, Taiyuan, Shanxi, China (People's Republic)*

Objective: This study employs the Latent Class Growth Model(LCGM) to identify symptom trajectory categories in patients with chronic respiratory failure(CRF)and examines the association between distinct trajectory categories and readmission risk. Methods: A retrospective cohort study was conducted, encompassing CRF patients hospitalized at a tertiary comprehensive hospital between January 2022 and December 2024. Data on demographic characteristics, clinical indicators, symptom scores, and readmission rates were collected.LCGM was applied to model symptom trajectories and identify latent trajectory categories.Additionally, multivariate Cox proportional hazards models were utilized to examine the relationship between different trajectory categories and readmission risk. Results: A total of 512 CRF patients were included.LCGM analysis identified three distinct symptom trajectory categories: stable, fluctuating, and deteriorating. Patients in the stable category exhibited minimal variations in symptom scores over time, while those in the fluctuating category demonstrated periodic fluctuations. Conversely, patients in the deteriorating category experienced a continuous escalation in symptom scores. Multivariate Cox regression analysis revealed that, compared to the stable trajectory, patients classified under the fluctuating and deteriorating categories exhibited significantly increased readmission risks, with hazard ratios of 1.56 and 2.34, respectively. Moreover, patients in the deteriorating category were found to have substantially longer hospital stays and significantly higher medical expenditures than those in the other two categories. Conclusion: This study successfully identified three distinct symptom trajectory categories in CRF patients through the application of LCGM and established that fluctuating and deteriorating symptom patterns were significantly correlated with an increased risk of readmission. The findings highlight the necessity of prioritizing clinical attention toward patients exhibiting fluctuating and deteriorating symptom trajectories, implementing early interventions, and adopting personalized management strategies to mitigate readmission rates and enhance patient outcomes.

Abstract citation ID: igaf122.1617

### UTILIZING MACHINE LEARNING TO IDENTIFY GUT METABOLITES ASSOCIATED WITH ALZHEIMER'S DISEASE

Joshua Chuah, *Union College, Schenectady, New York, United States*

The prevalence of Alzheimer's Disease (AD), which encompasses the vast majority of dementia cases, increases drastically in adults age 65 or older. In recent years, researchers have investigated the physiological relationships between the gut microbiome and the progression of AD. Furthermore, the early diagnosis of AD is important to improve patient outcomes. As such, the goal of this study is to develop a machine-learning based diagnostic test using gut metabolite data in order to facilitate earlier diagnosis of AD. Publicly available gut metabolite data was utilized, which included 104 gut-microbiome associated metabolites. These metabolites were measured from serum samples collected from 108 early mildly cognitively impaired (EMCI) and 145 AD patients. From these 104 metabolites, recursive feature elimination (RFE) feature selection identified 14 metabolites as being highly predictive between EMCI and AD status. A Logistic Regression classification model was trained using the data from these selected features and resulted in a 10-fold cross-validation accuracy of 91.34%. Moreover, robustness analysis of this classifier revealed that this classifier could still perform well with some missing values, maintaining above an 80% diagnostic threshold accuracy with approximately 13% of the data randomly re-sampled from a normal distribution (e.g., if missing values were randomly imputed). Overall, the predictive performance of this classifier indicates that it is both an effective and, more importantly, generalizable diagnostic test to distinguish between AD and EMCI individuals using gut metabolite measurements.

Abstract citation ID: igaf122.1618

### VALIDATION OF A LOW-COST VIDEO-BASED COMPETENCY SCORE FOR ASSESSING SAFE FALLING MOVEMENT PATTERNS IN OLDER ADULTS

Lingjun Chen[1], Tobia Zanotto[1], James Fang[1], Joohyun Lee[2], Neil Alexander[3], and Jacob Sosnoff[1], *1. University of Kansas Medical Center, Kansas City, Kansas, United States, 2. University of Kansas Medical center, Kansas City, Kansas, United States, 3. University of Michigan, Ann Arbor, Michigan, United States*

Here is growing interest in enabling older adults with skills to reduce fall-related injury. One approach involves teaching older adults safely falling movements, such as tuck and roll, to minimize fall-related injury risk. However, the current methods for evaluating fall movement patterns require biomechanical assessments and are not clinical feasible. To address this gap, Safe Falling Competency Score was developed, a standardized and low-cost approach to assess key evidence-based safe falling movement patterns: 1) active attempt of squatting (to lower the center of mass); 2) trunk rotation and rolling (to reduce vertical impact); 3) appropriate upper limb use (to avoid outstretched hand landing); 4) active and successful chin tuck (to prevent head impact). This study validated the competency score using

264 video-recorded experimental falls from 24 older adults at risk for falls (72.53 ± 5.39 years), who completed the pre- and post-training assessments in a Safely Falling Training Project (R21AG07892). Two independent reviewers scored the falls, with discrepancies resolved via discussion (excellent inter-rater reliability, ICC = 0.713). Head and hip acceleration of each fall were extracted from motion capture data using custom MatLab script. Spearman's correlation revealed that higher competency scores were associated with the reduced head ($\rho$ = -0.3705, $p < 0.0001$) and hip accelerations ($\rho$ = -0.293, $p < 0.0001$), supporting the score's validity. These findings suggest the Safe Fall Competency Score provides a reliable and valid tool for clinicians to assess fall movement patterns and fall-related injury risk.

### SESSION 4065 (PAPER)

Abstract citation ID: igaf122.1619

### EXPLORING THE IMPACT OF SENSORY IMPAIRMENT

In this session the association of sensory impairment, vision, hearing and olfaction are discussed including impact on caregiving, falls, cognition and cost.

Abstract citation ID: igaf122.1620

### EFFECT OF CATARACT SURGERY ON RISK OF FALLS OR FRACTURES IN MEDICARE BENEFICIARIES WITH CATARACTS

Atalie Thompson[1], Lindsey Abdelfattah[1], Emilie Duchesneau[1], Amol Joshi[2], and Amresh Hanchate[1], *1. Wake Forest University School of Medicine, Winston-Salem, North Carolina, United States, 2. Wake Forest University School of Business, Winston-Salem, North Carolina, United States*

Falls and fractures are a significant public health concern linked to visual impairment. Cataracts are the most common cause of visual impairment in older adults, and can be corrected with cataract surgery. We evaluated whether cataract surgery reduces the one-year risk of falls/fractures in Medicare beneficiaries. Using 2019-2021 claims data from a nationally representative cohort of 1 million Medicare fee-for-service enrollees (65+ years), we identified those with an ICD-10 diagnosis of cataract in 2019. Cataract surgery in 2020 was identified using procedure codes and participants were followed for one year from the surgery date; individuals with cataracts who did not receive surgery were included as controls and assigned a random pseudo-index date. We used propensity score weighting to standardize covariates (demographics, frailty, comorbidities, eye diseases, socioeconomic indicators) among controls (N=240,164) to resemble those with cataract surgery (N=16,957). We applied a propensity score weighted linear regression model to estimate the impact of surgery on 1-year risk of falls/fractures. Without surgery, the estimated likelihood of falls/fractures was 8.92% ((95% CI:8.64-9.21%), $p < 0.001$). The estimated effect of surgery was a reduction by -0.32 percentage points ((95% CI: 1.22-0.59), $p = 0.494$). Sensitivity analyses treating death as a competing risk, adjusting for prior falls/fractures, and evaluating number of falls/fractures yielded similar results. Thus, cataract surgery did not significantly impact the one-year risk of having falls/fractures in Medicare beneficiaries. Future studies should evaluate fall risk over a longer follow-up period or the impact of surgery on self-reported falls not captured by the healthcare system.

Abstract citation ID: igaf122.1621

### EXPLORING THE LONGITUDINAL ASSOCIATION BETWEEN VISUAL FUNCTION AND MEMORY PERFORMANCE AMONG OLDER ADULTS

Jonathan Thomas[1], Shu Xu[2], and Joshua Ehrlich[2], *1. Texas A&M University Health Science Center, Bryan, Texas, United States, 2. University of Michigan, Ann Arbor, Michigan, United States*

Older adults with a visual impairment (VI) are more likely to experience cognitive decline; however, limited longitudinal studies exist on how changes of specific visual function influence the trajectories of cognitive performance, including memory. Therefore, the purpose of this study is to examine the longitudinal association between visual function measures and memory performance among U.S. older adults. We analyzed three-year longitudinal data (2021-2023) from Medicare beneficiaries ≥ 71 years in the National Health and Aging Trends Study. Visual function was assessed based on presenting binocular near visual acuity [NVA], distance visual acuity [DVA], and contrast sensitivity [CS]. Any VI was defined as either near or distance VI [>0.30 logMAR], or CS impairment [< 1.55 logCS]. Memory performance was measured using immediate and delayed word recall, scored from 0-20, with higher scores indicating better memory. Longitudinal associations between visual function and memory were analyzed with growth curve model. Among 3,162 participants, 34% had any VI. Older adults with any VI (7.34) had lower baseline memory scores than those without any VI (9.19). Growth curve models showed significant between-person effects for all visual function measures (DVA: B=-2.73, p<.001; NVA: B=-2.89, p<.001; CS: B=-2.60, p<.001); however, only NVA (B=-1.16 p<.001) and CS (B=-0.77 p<.01) showed significant within-person effects. In conclusion, older US adults with worse overall visual function have lower memory performance, and a decline in NVA and CS over time is associated with a faster decline in memory performance. Findings highlight the importance of vision health in cognitive aging.

Abstract citation ID: igaf122.1622

### HEARING IMPAIRMENT AND FRAILTY: THE HIDDEN PSYCHO-COGNITIVE-SOCIAL MEDIATING PATHWAY

Doris S. F. Yu, and Chen Qiu, *The University of Hong Kong, Hong Kong, China (People's Republic)*

Hearing impairment is highly prevalent problem affecting more than two-third of older adults worldwide. With more evidence to indicate its relationship with frailty, it is imperative to understanding the underlying mediating pathway to inform comprehensive care planning. Based on the biopsychosocial model, the objectives of this study were to: i) identify the relationship between hearing impairment and frailty, and ii) examine the mediating roles of depression, cognitive impairment and social loneliness in the relationship, if any From Dec 2023 to July 2024, a total of 2332 older adults were recruited from 7 geographic districts in Hong Kong. The Hearing Handicap Inventory for the Elderly-Screening version (HHIE-S) was used to assess the hearing impairment. The Edmonton Frail Scale (EFS) measured frailty from a multi-dimensional perspective. The 5-minute Montreal Cognitive Assessment Test (MoCA-5), Geriatric Depression Scale (GDS-15) and UCLA Loneliness Scale (UCLA3) were administered to measure the potential mediators. Structural equation model was conducted to test the hypothesized mediating model. The mean age of the participants was72.2 (SD = 7.1). The prevalence of hearing impairment and frailty were 13% and 16.4% respectively. The SEM result indicated that hearing impairment was directly associated with severe frailty [β = 0.147,95%CI(0.106-0.186)], and its relationship is also mediated through poor cognitive function [β = 0.011,95%CI(0.003-0.021)], social loneliness [β = 0.045, 95%CI(0.031-0.061)] and depression [β = 0.034,95% CI(0.023-0.048)]. The indirect effect explained 38% of the total effect. This study recommended a holistic approach to manage late-life hearing impairment, with emphasis on compensatory communication strategies, brain health and social engagement optimization, and mental health support.

Abstract citation ID: igaf122.1623

### INFORMAL CAREGIVING FOR INDIVIDUALS WITH DEMENTIA AND CONCURRENT SENSORY IMPAIRMENT: A SYSTEMATIC REVIEW

Shaoqing Ge[1], Katie Trainum[2], Yaolin Pei[3], Wonshik Chee[4], Yuting Song[5], and Bo Xie[3], *1. University of Texas at Austin School of Nursing, Austin, Texas, United States, 2. University of Pennsylvania, Philadelphia, Pennsylvania, United States, 3. The University of Texas at Austin, Austin, Texas, United States, 4. University of Texas at Austin, Austin, Texas, United States, 5. Qingdao University, Qingdao, Shandong, China (People's Republic)*

Caregivers of individuals with dementia often face the dual challenge of managing dementia alongside sensory impairments (hearing and/or vision loss). Despite the significant burden, this issue remains underexplored, with existing research largely overlooking the impact of sensory impairments on caregivers' experiences. This systematic review examines the challenges and needs of caregivers managing both conditions. A comprehensive literature search across three databases identified 12 studies published before May 2024 in five countries. The review revealed that caregivers face increased challenges when managing both dementia and sensory impairments. Unmet needs, particularly in accessing tailored support and assistive technologies, were prevalent. Caregivers reported difficulties in communication, increased stress, and a higher risk of burnout due to the compounded effects of managing both cognitive and sensory impairments. The review highlights the importance of multidisciplinary care and interventions to address both cognitive and sensory needs. Effective strategies include integrating sensory assessments into dementia care plans, providing training for caregivers on managing sensory impairments, and ensuring access to affordable assistive technologies. Additionally, caregivers expressed a need for more comprehensive support systems, including respite care and mental health services, to alleviate the emotional and physical strain of caregiving. Enhanced caregiver education on the use of assistive devices and techniques to improve communication with sensory-impaired dementia patients is essential. Addressing these disparities is critical for providing effective care and improving the quality of life for both caregivers and sensory-impaired dementia patients. This review underscores the urgent need for targeted interventions and policies to support this vulnerable population.

Abstract citation ID: igaf122.1624

### PRELIMINARY RESULTS ON A COST-EFFECTIVENESS ANALYSIS OF THE ACHIEVE HEARING INTERVENTION

Emmanuel Garcia Morales[1], Victoria Sanchez[2], Haley Calloway[2], Laura Sherry[3], Michelle Arnold[2], James Pike[4], and Nicholas Reed[5], *1. New York University Langone Health, New York, New York, United States, 2. University of South Florida, Tampa, Florida, United States, 3. Johns Hopkins University School of Medicine, Baltimore, Maryland, United States, 4. New York University, New York, New York, United States, 5. New York University Grossman School of Medicine, New York, New York, United States*

This study evaluates the cost-effectiveness of a best-practices hearing intervention in mitigating cognitive decline, measured by Mini-Mental State Examination (MMSE) scores, over three years in the ACHIEVE trial (NCT03243422). ACHIEVE, a randomized controlled trial, compared a hearing intervention to a successful aging health education control group among older adults with hearing loss. We enrolled 977 adults (70-84 years) with untreated mild-to-moderate hearing loss from four U.S. sites. Participants were randomized to the intervention (N = 490) or control group (N = 487). The intervention included audiologist-fitted hearing aids, assistive devices, counseling, and ongoing support, while the control group received health education on aging. Costs were assessed from the provider's perspective, covering professional services and technology. Three years post-randomization, MMSE scores declined less in the intervention group by 0.28 points (95% CI: 0.01-0.55). The intervention cost an average of $3,900.53 per participant, including $692.22 for professional services and $3,208.31 for technology. The incremental cost-effectiveness ratio (ICER) was estimated at $13,917.76 per MMSE point preserved. The ACHIEVE intervention, a non-pharmacologic, low-risk approach, showed a modest cognitive benefit at a cost of ~$14K per MMSE point over three years. Comparatively, anti-amyloid drug treatments have reported an ICER of $60,516.82/MMSE over 76 weeks. These findings support the potential role of hearing interventions in cognitive health strategies and inform evidence-based decision-making.

### SESSION 4070 (PAPER)

### FROM FRAILTY TO FRIENDSHIP: EXPLORING SOCIAL ISOLATION AND WELL-BEING IN OLDER ADULTS

Abstract citation ID: igaf122.1625

### "I FEEL LIKE A STRANGER IN THE COMMUNITY:" SOCIAL ISOLATION OF OLDER AFRICAN IMMIGRANTS

Dolapo Adeniji[1], Onome Osokpo[2], Gifty Ashirifi[3], and Margaret Adamek[4], *1. Indiana State University, United States, 2. University of Illinois Chicago, Chicago, Illinois, United States, 3. Central State University, Wilberforce, Ohio, United States, 4. Indiana University Indianapolis, Indianapolis, Indiana, United States*

Social isolation—limited interactions, relationships, and engagements with others—negatively impacts health outcomes, particularly among older adults, leading to an increased risk of anxiety and depression. Yet little is known about social isolation amongst older African immigrants, a population at increased risk for social isolation. Using a qualitative narrative design, we explored the social isolation experiences of a purposive sample of African immigrants aged ≥60 years recruited across the US (N = 11). In-depth video/phone, audiotaped interviews were completed using a semi-structured guide. Data were analyzed using an inductive thematic approach. The sample was 55% female, 63–79 years old, mostly Nigerian (n = 7), with no primary/secondary education (n = 4), widowed (n = 4), and spouse not in the US (n = 4). All lived with family, and most (n = 10) have lived in the US for < 10 years. We identified two major themes: 1) minimal social engagement outside the family, with participants feeling like strangers in their communities; and 2) barriers to social engagement outside the home, including security concerns, a lack of individuals to socialize with, language barriers and cultural differences, and transportation challenges. Findings indicate that feeling like a stranger, lacking social connections, cultural differences, and lack of transportation contribute to feelings of isolation amongst older African immigrants. African immigrant communities should be educated about the challenges faced by their older adults. Likewise, practitioners and policymakers should prioritize interventions that encourage meaningful engagement of older African immigrants to improve their health and quality of life.

Abstract citation ID: igaf122.1626

### MULTILEVEL FACTORS ASSOCIATED WITH SOCIAL ISOLATION TRAJECTORY AMONG OLDER CHINESE IMMIGRANTS

Ke Li[1], Fengyan Tang[2] BoRin Kim[3], Wendi Da[4], and Yanping Jiang[5], *1. Ohio University, Athens, Ohio, United States, 2. University of Pittsburgh, Pittsburgh, Pennsylvania, United States, 3. University of New Hampshire, University of New Hampshire, New Hampshire, United States, 4. Rutgers University, New Brunswick, New Jersey, United States, 5. Rutgers, The State University of New Jersey, New Brunswick, New Jersey, United States*

Social isolation is particularly prevalent among older immigrants due to language and cultural barriers. However, the factors contributing to social isolation among this population remain understudied. This study aims to identify the patterns of social isolation change trajectory over eight years and to examine the factors at multiple ecological systems that are relevant to social isolation among older Chinese immigrants. Data came from five waves of the Population Study of Chinese Elderly in Chicago, with a study sample of 2,835 Chinese immigrants aged 60 years and older. Social isolation was measured by the social disconnectedness index composed of social network size and range, household size, living arrangement, and marital status. The examined multilevel factors included socio-demographic and health conditions, interpersonal relationships, immigration, and neighborhood characteristics. Latent class growth analysis (LCGA) identified four distinct change trajectories of social isolation, including "Persistent Low", "Persistent Medium", "Persistent High", and "Increasing" social isolation. The results of multinominal logistic regression models indicated the unequal contributions of different factors to social isolation. Among older Chinese immigrants, factors including age, sex, social support, social strain, years in the U.S., sense of community, social cohesion, and neighborhood physical disorder were significantly associated with different social isolation change trajectories. These findings inform the need for culturally sensitive services that consider the effects of multifaced factors when addressing the issue of social isolation among older immigrants.

Abstract citation ID: igaf122.1627

### SOCIAL FRAILTY AND INCIDENT DEMENTIA RISK AMONG COMMUNITY-DWELLING OLDER ADULTS

Claire Wang, Sofia Liu, Anna Beeber, Katherine Ornstein, Rendong He, and Junxin Li, *Johns Hopkins University, Baltimore, Maryland, United States*

While social engagement is increasingly recognized as a key determinant of cognitive aging, the broader construct of social frailty—which includes dimensions such as social support, engagement, and resource access—remains understudied as a predictor of dementia risk among community-dwelling older adults. This study draws on data from the National Health and Aging Trends Study (NHATS) collected at Round 12 (baseline) and a one-year follow-up to examine whether baseline social frailty predicts incident dementia. We adapted Makizako's (2018) social frailty framework by selecting items from its five original domains: (1) being alone; (2) reduced outings; (3) infrequent visits with friends/family; (4) a diminished sense of usefulness; and (5) a lack of daily conversation. Participants were classified as robust (score=0), socially pre-frail (score=1), or socially frail (score=2-5). Among 4,804 participants without dementia at baseline, 5.71% progressed to incident dementia within one year. In the unadjusted logistic analysis, compared to the robust group, social pre-frailty was significantly associated with 38% higher odds of dementia, whereas social frailty had a near 151% higher odds of dementia. In the fully adjusted model, which accounted for demographic and baseline health conditions, this association was attenuated but remained significant for the frail group (OR = 1.41, 95% CI: 1.02–1.93). These findings underscore the importance of social frailty as a predictor of dementia risk. Future research should extend the follow-up period to better understand the mechanisms linking social frailty to cognitive decline and explore innovative interventions that may mitigate dementia risk among community-dwelling older adults.

Abstract citation ID: igaf122.1628

### SOCIAL ISOLATION AND DEPRESSION IN COMMUNITY DWELLING OLDER ADULTS

Loretta Anderson[1], Lenis Chen-Edinboro[2], Denise Orwig[3], and Fernando Wagner[4], *1. University of Maryland School of Medicine, Baltimore, Maryland, United States, 2. The University of North Carolina at Wilmington, Wilmington, North Carolina, United States, 3. University of Maryland, Baltimore, Baltimore, Maryland, United States, 4. University of Maryland, Baltimore County, Baltimore, Maryland, United States*

Studies show that 25% of older adults experience social isolation and approximately 28% experience depression, and both are linked to increased risk of chronic health conditions and poor health outcomes. This study examines the association between social isolation and depression in a nationally representative sample of community-dwelling older adults. Using the 2011 National Health and Aging Trends Study (NHATS), logistic regression models assessed the cross-sectional association between social isolation and depression, accounting for common sociodemographic factors and health conditions. Social isolation was measured using a previously validated measure in NHATS, and depression was measured using the PHQ-2. The analytic sample for this study included 6,802 participants aged 65 and older. Fully adjusted models showed mild social isolation having 24% greater odds of depression [OR = 1.24 (95%CI: 1.07,1.43), p < 0.01] compared to those without social isolation. For severe social isolation, bivariate analysis showed borderline significance of 37% greater odds of depression compared to those without social isolation [OR = 1.37 (95%CI: 0.99,1.89), p = 0.054], and became insignificant when controlling for sociodemographic and health factors. Mild social isolation was associated with depression in community-dwelling older adults, but severe social isolation was not. Longitudinal studies are warranted to confirm this difference between mild and severe social isolation and their association with depression in older adults. These results remain important as an increasing number of older adults in the United States experience social isolation. These findings may help inform clinical and policy efforts to improve depressive symptoms by helping to reduce social isolation in older adults.

Abstract citation ID: igaf122.1629

### SOCIAL ISOLATION PREVALENCE ESTIMATES AND TRENDS OVER TIME: IT DEPENDS ON WHAT YOU MEASURE

Yulin Yang[1], Karla Renata Flores Romero[1], Irena Cenzer[1], Carla Perissinotto[1], Mary Thoma[1], Ruijia Chen[2], Jacqueline Torres[1], and Ashwin Kotwal[1], *1. University of California, San Francisco, San Francisco, California, United States, 2. Boston University School of Public Health, Boston, Massachusetts, United States*

Social isolation is common among older adults and worsened during COVID-19, but its population-level trends surrounding the pandemic remain unclear. Variations in the measures of social isolation across surveys may contribute to discrepancies in prevalence estimates over time. This study examines social isolation prevalence using two population-based studies, comparing pre-pandemic and pandemic periods and evaluating variation across population subgroups. We analyzed data from the Health and Retirement Study (HRS, 2016–2022) and the National Health and Aging Trends Study (NHATS, 2019–2022). Social isolation was measured across three domains: household and core contacts, social network interaction, and community engagement. We estimated survey-weighted prevalence, adjusting for socio-demographic covariates, and evaluated subgroup differences by socio-demographics (e.g., race and ethnicity). In the HRS, social isolation prevalence increased from 12% in pre-pandemic years (2016, 2018) to 12.9% in 2020 and 13.5% in 2022. In the NHATS, prevalence rose from 4.1% in 2019 to 9.2% in 2020, then declined to 5.6% in 2021–2022. Subgroup differences in social isolation varied by study and time period. In the HRS, non-Hispanic white participants had the highest prevalence compared to non-Hispanic Black and Hispanic participants both before and during the pandemic. In contrast, Black and Hispanic participants had higher prevalence in the NHATS, with disparities shifting over time. Social isolation is a pressing public health challenge for older adults, yet its prevalence varies widely across studies due to measurement differences. Measurement differences should be reconciled to accurately track trends, identify at-risk populations, and inform targeted interventions.

Abstract citation ID: igaf122.1630

### WHEN LAUGHTER HURTS AND HELPS: ASSOCIATIONS BETWEEN GELOTOPHOBIA, GELOTOPHILIA, KATAGELASTICISM AND WELL-BEING

Robert Reinecke[1], Diana Mazzarella[2], and Jürgen Maurer[1], *1. University of Lausanne, Neuchâtel, Vaud, Switzerland, 2. University of Neuchâtel, Neuchâtel, Neuchatel, Switzerland*

Laughter is commonly seen as a source of positive emotion and a facilitator of social bonding, yet its perception varies based on individual characteristics. Three constructs capture individual differences in how laughter is perceived: gelotophobia (fear of being laughed at), gelotophilia (joy of being laughed at), and katagelasticism (joy in laughing at others). Although these constructs have been predominantly studied in younger adults, their roles in older populations remain underexplored, despite unique social, emotional, and health challenges – including increased social avoidance behavior. This study examined how these constructs relate to well-being – specifically, life satisfaction, social engagement, and mental health – in 1,163 Swiss respondents aged 60 and older from Wave 9 (2021/2022) of the Survey of Health, Ageing and Retirement in Europe (SHARE). The PhoPhiKat-9 questionnaire measured gelotophobia, gelotophilia, and katagelasticism. Life satisfaction, social connectedness, friendship approach and avoidance behaviors, activity frequency, and social support were assessed alongside loneliness (UCLA-3) and depression (EURO-D). Regression models controlling for social, health, and regional factors revealed that higher gelotophobia is linked to lower life satisfaction, greater friendship avoidance, elevated loneliness, and increased depression. Conversely, higher gelotophilia correlates with stronger social connectedness, more frequent approach behaviors, increased activity participation, and a greater likelihood of receiving support. Katagelasticism is associated with reduced social connectedness, lower friendship approach, lower social support, and less frequent social engagement. These findings underscore the significance of laughter-related dispositions for older adults' well-being and suggest that targeted interventions may enhance social behaviours and mental health in later life.

### SESSION 4075 (PAPER)

### INCREASING WORKFORCE READINESS FOR HEALTH SUPPORT WITH CREATIVE AGING EDUCATION AND TRAINING STRATEGIES

Abstract citation ID: igaf122.1631

### AN INNOVATIVE APPROACH IN RURAL AGING EDUCATION: EXTENSION'S IMPACT THROUGH HEALTHY AGING WEBINARS

Christine Fruhauf, Natalie Bachmeier, Ginger Williams, and Sue Schneider, *Colorado State University, Fort Collins, Colorado, United States*

While rural communities possess unique strengths, limited access to aging services can detrimentally impact individuals' health-related outcomes. Colorado State University Extension focused its engaged education on innovative approaches to Health Extension and learned that county-level Extension specialists need additional training to effectively support older adults and improve services in rural communities. Thus, in this presentation we will describe how we increased the confidence of interdisciplinary Extension specialists and community partners to respond to aging-related needs in rural communities through our Advancing Healthy Aging (AHA) webinar series. Webinar topics were identified during a needs assessment and delivered in fall 2024 and spring 2025. Of the eight webinars, to date, a total of five AHA webinars were delivered online with 193 attendees. Highest attended webinars addressed reducing ageism ($n = 60$), reducing social isolation and increasing belonging ($n = 43$), and avoiding caregiver burnout ($n = 36$). A total of 75 attendees completed an evaluation administered after each webinar. Data indicate that 93% ($n = 70$) of respondents reported an increase in knowledge and ability to apply knowledge gained. Additionally, 95% ($n = 71$) of respondents reported increased confidence in educating people about healthy aging and in directing people to additional resources; comments gathered from open-ended questions support the findings. Limitations include more than half (61%) of attendees did not complete the evaluation and may have different views on the webinars' impact. Despite limitations, the AHA webinars have potential to further impact rural communities as they were recorded and posted publicly.

Abstract citation ID: igaf122.1632

### BUILDING WORKFORCE CAPACITY TO DELIVER EFFECTIVE BEHAVIORAL HEALTH SUPPORT FOR OLDER ADULTS WITH HOARDING & CLUTTER

Bronwyn Keefe[1], Jordana Muroff[2], Annalee Wilson[2], Crystal Carrington[2], and Megan Nizza[2], *1. Boston University School of Social Work, Boston, Massachusetts, United States, 2. Boston University, Boston, Massachusetts, United States*

The goal of this project was to develop and implement capacity-wide training and programming focused on hoarding and clutter to build behavioral health supports and services to address this growing concern. This collaboration between a private University and an Area Agency on Aging (AAA) in a large urban area focused on addressing the urgent and growing priority area of hoarding and clutter among culturally and linguistically diverse older adults. The training aimed to enhance the understanding of hoarding as a mental health challenge, teach evidence-based assessments and intervention strategies, and apply effective communication and engagement techniques and community approaches. We delivered and evaluated a blended hoarding training (online and in-person) to a broad group of AAA providers ($n = 130$) working with individuals with hoarding, e.g., case managers, nurses, management staff, caregiver advisers, and direct care staff. The blended training includes an innovative online interactive self-paced course and "live" practice-based training sessions delivered by hoarding experts. Before and after the training program, learners self-reported their skill level on nine competencies related to working with individuals with a hoarding disorder. Learners showed statistically significant ($p < 0.05$) improvements in all competencies. Learners also rated their confidence (How confident do you feel working with people with hoarding?) and comfort (Do you feel comfortable with clients with hoarding?) before and after the training program. Upon completing the in-person training session, learners were significantly ($p < 0.05$) more confident and comfortable working with clients with hoarding disorder. These findings highlight the impact of providing high-quality training and effective hoarding-related interventions.

Abstract citation ID: igaf122.1633

### ENHANCING WORKFORCE READINESS FOR AGING AND DISABILITY NETWORKS: A NO WRONG DOOR STATE TRAINING INITIATIVE

Bronwyn Keefe[1], Annalee Wilson[2], and Erin Verdile[3], *1. Boston University School of Social Work, Boston, Massachusetts, United States, 2. Boston University, Boston, Massachusetts, United States, 3. New York State Office for the Aging, Albany, New York, United States*

This project focused on enhancing the competencies of information and assistance specialists and options counselors working in New York's No Wrong Door (NWD) system by providing innovative, skill-based online training. Responding to a need for specialized person-centered training, the New York State Office for the Aging (NYSOFA) and the Center for Aging and Disability Education and Research (CADER) at Boston University partnered with the goals of developing and implementing a standard training program for NWD staff, and ensuring that staff have the knowledge and skills to support older adults. 465 learners completed the training, which included coursework in aging and disability issues and networks, performing assessments, person-centered planning, mental health, and ethics. Two customized courses were developed: a course describing resources for older adults offered by three NY state offices, and a video scenario course where actors depicted situations that NWD staff may encounter. Learners self-reported their skill level on course competencies before and after each course and showed statistically significant ($p < 0.05$) improvements on all competencies. On a pre-post workforce readiness assessment, which included statements such as "I know how to review eligibility criteria for various resources," there was a statistically significant ($p < 0.05$) change in learners' agreement with 18 out of the 20 statements, indicating that the training had a positive impact on their ability to effectively support older adults navigating the NWD system. Future directions include the development of a state policy requiring that NWD staff complete the training program upon hire and recertify every three years.

Abstract citation ID: igaf122.1634

### SIMULATION PROMOTES NURSE PRACTICE READINESS IN AGE-FRIENDLY HEALTH SYSTEM CARE

Eleanor McConnell, Dana Convoy, Valerie Sabol, and Nicole Blodgett, *Duke University, Durham, North Carolina, United States*

As the Age-Friendly Health System (AFHS) framework becomes the accepted standard in caring for older adults across the United States, health professions schools seek effective approaches to learning so their graduates are competent to provide care consistent with AFHS principles. The Duke University Nurses Advancing Geriatric Care Excellence (NU-AGE) Simulation program offers an innovative approach to preparing pre-licensure and advanced practice nursing students to care for older adults using simulations focused on assessment and management of challenges associated with mentation, mobility, medications, and what matters most to older adults. We developed, deployed, and evaluated a combination of commercially-available simulations using the Embodied LabsTM virtual reality platform, combined with simulations developed by our team. The new simulations were enacted using videotaped simulated patients, using professional actors. These cases were deployed using the hologram technology. Student experience and clinical performance were evaluated with 20 students, (15 pre-licensure RN, 5 nurse practitioner students). Students rated the experience very positively, with mean +/- SD of 99 +/- 1.2 and 98.1 +/- 4.1 for the virtual reality and hologram simulations respectively. Themes from open-ended evaluation responses included the value of an empathy experience for cognitive and sensory impairments, and opportunity to experience diagnostic dilemmas and how best to respond prior to going into a clinical rotation. Simulation education using advanced technology platforms represent a promising, innovative approach to preparing pre-licensure and advanced practice nursing students to mastering the competencies needed to practice within the AFHS framework upon graduation.

Abstract citation ID: igaf122.1635

### TELEHEALTH WITH OLDER ADULTS CURRICULUM DEVELOPMENT AND IMPLEMENTATION

Joan Ilardo, and Angela Zell, *Michigan State University, East Lansing, Michigan, United States*

The SEMCOAV Telehealth curriculum was developed based on insights gathered from focus groups with older adults in both urban and rural areas. These groups provided valuable input on what doctors should know to conduct telehealth visits that effectively meet the needs of older patients. Their feedback directly shaped the curriculum to ensure it addressed real-life needs of older adults. Content aligns with the six telehealth competencies outlined by the Association of American Medical Colleges. We designed six digitally recorded modules: 1) Setting Expectations, 2) Patient Needs and Preferences, 3) Communication via Telehealth, 4) Techniques for Evaluating Older Patients During a Telehealth Visit, 5) Technology for Telehealth, and 6) Ethics and Legal Considerations of Telehealth. Each module is paired with a facilitator guide, ensuring ease of use and effectiveness in teaching. Residents from four family and internal medicine programs completed pre- and post-surveys to assess their knowledge before and after viewing the modules. Feedback was overwhelmingly positive. Residents reported the modules were helpful, and residency program directors expressed satisfaction with the added value these modules brought to their curriculum. The modules provided both new information and reinforced residents' clinical experiences conducting telehealth visits with older adult patients. Our project has the potential to improve access to quality, convenient care, reduce no-show rates, lower readmissions, minimize unnecessary emergency visits, and enhance care plan adherence, ultimately leading to improved clinical outcomes, higher patient and provider satisfaction, and reduced patient costs, such as transportation and time off work.

### SESSION 4080 (SYMPOSIUM)

Abstract citation ID: igaf122.1636

### HARNESSING THE POTENTIAL OF ARTIFICIAL INTELLIGENCE IN SUPPORTING OLDER ADULTS

Chair: Wendy Rogers

The landscape of aging and technology is changing dramatically. Uptake of technology is increasing among aging adults; technology is increasingly considered a solution to support needs of aging adults; and more technology products are marketed to older adults. Deployment of technology in healthcare and day-to-day activities is increasing and advances in technology such as artificial intelligence (AI) are increasingly aimed at supporting older adults. Yet, aging adults are often ignored in design, and robust research evaluating the usability, safety, and efficacy of these systems with older adults is limited. Even among older technology adopters, rapid changes in technology pose challenges in terms of the constant need for adaptation and continual learning. AI has the potential to provide support for older adults and enhance quality of life. We will present four projects aimed at harnessing this potential in myriad ways to provide support for older adults with and without cognitive impairment. Sara Czaja will focus on virtual reality as a method for supporting cognition, activities, social engagement, and technology proficiency. Walter Boot will share findings from evaluations of generative AI systems to provide decision support for older adults across a range of domains. Neil Charness will focus on the AI potential for the specific – and complex – task of Medicare decision making. Wendy Rogers will provide insights about the needs of older adults that can be supported by AI through an adaptive system. Together, this collection will provide valuable insights for AI to enhance everyday activities for older adults.

Abstract citation ID: igaf122.1637

### UNDERSTANDING USER NEEDS FOR AN ADAPTIVE SYSTEM TO SUPPORT PERSONAL ACTIVITIES AND REINFORCE COGNITION (SPARC)

Wendy Rogers[1], Raksha Mudar[2], Tracy Mitzner[3], Sara Czaja[4], Walter Boot[4], and Neil Charness[4], *1. University of Illinois Urbana-Champaign, Champaign, Illinois, United States, 2. University of Illinois Urbana-Champaign, University of Illinois Urbana-Champaign, Illinois, United States, 3. Person in Design, Atlanta, Georgia, United States, 4. Weill Cornell Medicine, New York, New York, United States*

The likelihood of developing a cognitive impairment (CI) increases with age. Older adults with CI experience difficulties performing a range of everyday activities and are at risk for social isolation. Technology-based interventions have the potential to improve everyday functioning. Artificial intelligence capabilities can be harnessed to tailor solutions to the unique and changing needs of older adults with CI. Most prior efforts focused on cognitive training or rehabilitation but have not included other aspects of functioning such as everyday activities or social engagement. Our goal is to develop and evaluate an innovative intelligent adaptive software system to support personal activities and reinforce cognition (SPARC). The system will be designed to adapt to the needs and abilities of the user with CI, following the CREATE model of user-centered design. We started with interviews with subject matter experts to assess older adults' needs for activity support in their homes and identify potential technology solutions. We interviewed both clinicians and technical experts to obtain guidance for the initial system design. In this presentation, we will present results of focus group interviews with older adults who have CI wherein we present prototype images of SPARC and explore their perceptions of usefulness, ease of use, preferences for instructional support, and ideas for specific content that would be of interest to them. This multi-faceted needs assessment approach will guide design of SPARC to support everyday activities, primarily in the home environment. Our findings inform design of other technologies and interventions to support older adults with CI.

Abstract citation ID: igaf122.1638

**THE POTENTIAL OF GENERATIVE AI TO SUPPORT MEDICARE DECISION MAKING**

Neil Charness[1], Walter Boot[1], Sara Czaja[1], Wendy Rogers[2], Joseph Sharit[3], Emily Langston[4], and Xin Yao Lin[1], *1. Weill Cornell Medicine, New York, New York, United States, 2. University of Illinois Urbana-Champaign, Champaign, Illinois, United States, 3. University of Miami, Coral Gables, Florida, United States, 4. Florida State University, Tallahassee, Florida, United States*

In 2022, ~ 99% of American adults age 65+ yr were enrolled in Medicare, a complex, difficult to use insurance system. We describe a multi-pronged approach to assessing the potential value of AI to support Medicare decision-making processes that includes querying subject matter experts who provide Medicare advice, assessing Medicare users' knowledge, preferences, and abilities through interviews and while interacting with knowledge sources, and evaluating existing AI tools for accuracy, reliability, and conciseness. In this presentation we present findings about the accuracy and reliability of digital assistants in answering Medicare questions, a new tool for assessing Medicare knowledge, and a study to assess preferences and performance with Medicare information sources. Generative AI (ChatGPT, Bard) were highly accurate (>90%) and reliable as well as superior to the average Medicare beneficiary, and much superior to digital home assistants (Alexa and Google Home Assistant). The Medicare Proficiency Questionnaire proved to be a short, reliable, and valid scale of older adults' Medicare knowledge, differentiating between Medicare enrollees and non-enrollees. Prior access to the Medicare website and the Medicare and You Handbook mediated the relationship between knowledge and education level as well as knowledge and Medicare enrollment status, suggesting that understanding Medicare information sources may be critical for designing AI decision support tools and training. We also describe early results from an observational study with Medicare-enrolled and unenrolled older adults examining preferences for, and performance with, the Medicare.gov website, the Medicare and You Handbook, and Gemini (a Large Language Model).

Abstract citation ID: igaf122.1639

**WHAT DO OLDER ADULTS THINK ABOUT VIRTUAL REALITY: FINDINGS FROM A PREFERENCE STUDY**

Sara Czaja[1], Walter Boot[1], Neil Charness[1], Shenghao Zang[1], George Mois[2], Pallabi Bhowmick[3], and Wendy Rogers[3], *1. Weill Cornell Medicine, New York, New York, United States, 2. University of Georgia, Athens, Georgia, United States, 3. University of Illinois Urbana-Champaign, Champaign, Illinois, United States*

Social and cognitive engagement are critical elements of successful aging. Yet many older adults, especially those who live alone or have a chronic condition or disability, lack social connectivity, experience loneliness, and lack opportunities for cognitive engagement. Virtual Reality (VR) applications can play a key role in enhancing social and cognitive engagement and decreasing loneliness among older adults. VR provides an immersive and interactive experience, which enables users to feel "present" in an environment, and have the ability to interact solely or with others in a virtual space or activity (e.g., museum, gardening). The presentation will focus on data from a cross-site study that examined perceptions of value, enjoyments and usability (comfort, safety) of VR applications as well as preferences for VR apps among a sample of 48 of community dwelling older adults aged 65 years and older. Sixteen pre-selected apps that focused on social, cognitive and productive engagement were examined. Each participants received a brief training on the use of VR and were exposed to eight apps either interactively or in a video format (apps were counter-balanced across participants). They rated each app after exposure, completed the Attitudes Toward VR Scale, A Virtual Reality Activity Inventory, and an Opinion Interview. The findings indicated that the older adults rated the experience of VR as valuable and highly enjoyable. There were also low ratings of discomfort and high ratings of usability. Most participants indicated that they would like to engage with VR in the future.

Abstract citation ID: igaf122.1640

**A FRAMEWORK FOR EVALUATING AI-POWERED VIRTUAL ASSISTANTS TO SUPPORT OLDER ADULTS' INFORMATION-SEEKING NEEDS**

Walter Boot[1], Emily Langston[2], Varitnan Hattakitjamroen[3], Mario Hernandez[1], Hye Soo Lee[4], Hannah Mason[1], and Willencia Louis-Charles[3], *1. Weill Cornell Medicine, New York, New York, United States, 2. Florida State University, Tallahassee, Florida, United States, 3. University of Illinois Urbana-Champaign, Champaign, Illinois, United States, 4. University of Illinois--Urbana-Champaign, Champaign, Illinois, United States*

Older adults often face the challenge of searching for critical health, financial, and resource-related information to make complex decisions, a process further complicated by age-related cognitive changes that impact information processing and decision-making. Artificial intelligence (AI)-powered virtual assistants may help by providing concise, easy-to-understand information, yet their accuracy and effectiveness remain unclear. This presentation will introduce a general framework for evaluating AI's potential to support important decisions of older adults and provide a case example illustrating this approach. To examine the accuracy and utility of AI-powered virtual assistants, we assessed the responses of Alexa, Google Assistant, Bard, and ChatGPT-4 to queries related to Medicare, long-term care insurance, and resource access. Findings showed that Large Language Model (LLM)-based assistants (Bard, ChatGPT-4) were more accurate than non-LLM systems, with Bard producing 6% inaccurate responses compared to Alexa's 60%. They also provided more supplemental details, with Bard offering high levels of additional information in 79% of responses, compared to 37% for ChatGPT-4 and under 20% for others. However, response variability was observed over time. While LLM-powered virtual assistants may be useful tools for older adults seeking health and financial information, potential inaccuracies, response complexity, and variability must be considered. We will outline key challenges in conducting this research and implementing AI solutions, emphasizing the need for further refinement and user training to enhance reliability and usability for older users.

### SESSION 4085 (PAPER)

### MULTIDIMENSIONAL PERSPECTIVES ON DEMENTIA CAREGIVING: COGNITION, CONTEXT, AND CAREGIVER WELL-BEING

Abstract citation ID: igaf122.1641

**ACCEPTANCE AS A FOUNDATIONAL SKILL FOR MANAGING AMBIGUOUS LOSS IN DEMENTIA CAREGIVING**

Gavin Green, Josey Batura, Heather Kelley, and Elizabeth Fauth, *Utah State University, Logan, Utah, United States*

Background: Dementia caregiving presents unique challenges, particularly ambiguous loss, in which caregivers witness the gradual psychological absence of their loved ones despite their continued physical presence. Objectives: This paper has three primary aims: (1) to conceptualize acceptance as a cognitive foundation that shapes how caregivers perceive, interpret, and respond to stress; (2) to examine the role of acceptance in fostering emotional resilience, highlighting its influence on psychological flexibility, stress mitigation, and caregiving effectiveness; and (3) to introduce acceptance as a clinical heuristic, providing a structured framework for therapeutic interventions and psychoeducational programs. Theoretical Framework & Approach: This theoretical analysis integrates Family Stress Theory (FST), specifically the ABCX and Family Adjustment and Adaptation Response (FAAR) models, with a synthesis of empirical literature on acceptance-based interventions such as Acceptance and Commitment Therapy (ACT). Key Contributions: We propose that fostering acceptance enhances psychological flexibility, reduces caregiver stress and burnout, and improves caregiving effectiveness. By positioning acceptance as a cognitive and clinical foundation, this paper provides a structured framework for guiding evidence-based caregiver interventions. Clinical Implications: We underscore the importance of acceptance-based psychoeducational programs, structured interventions, and therapeutic approaches in caregiver support. Conclusion: As dementia prevalence rises, equipping caregivers with evidence-based tools for resilience is essential for sustaining well-being and long-term caregiving effectiveness.

Abstract citation ID: igaf122.1642

### AN ECOLOGICAL-DEVELOPMENTAL MODEL OF GRANDPARENT CAREGIVING

Bert Hayslip[1], and Julian Montoro-Rodriguez[2], *1. University of North Texas, Denton, Texas, United States, 2. The University of North Carolina at Charlotte, Charlotte, North Carolina, United States*

While a stress and coping as well as a contextual perspective have informed grandparent caregiving, we lack an integrative model that reflects both contextual influences (e.g. service use, neighborhood quality, housing) and developmental change. We present a new and more comprehensive model here stressing the interaction between an evolving social-ecological context (e.g. health vulnerability and service access) and intraindividual developmental change in the grandparent (e.g. late young adulthood, midlife, later life) and the grandchild (e.g. infancy, childhood, adolescence, young adulthood). Key elements of this interactive model, presented with exemplar supportive literature, are a) sociocultural historical shifts defining different cohorts of grandparent caregivers, on par with work identifying grandparent cohort effects, b) emergent uniquenesses of different generations of grandparents, dependent upon when, developmentally, they first become caregivers, be it on-time/age normative or off-time/nonnormative in nature, and c) the impact of caregiving on developmentally significant life domains (e.g., health, work/retirement, grandchild maturation and psychosocial development). How such interrelated influences are navigated will yield different life trajectories for each grandparent cohort, subject to the negative or positive interaction with differential levels of context, e.g. social policy, service access, relationships with age peers, grandchildren, and the biological parent, all of which are shaped by historical change. As grandparent caregiving is countertransitional and whose antecedents are often nonnormative, our developmental-ecological model is grounded in lifespan theory stressing the ongoing interaction of micro-and macro-ecological influences over time, individual differences in grandparent caregiving, and multidimensional developmental outcomes, i.e. health, well-being, mediated by numerous idiosyncratic and historical influences.

Abstract citation ID: igaf122.1643

### DEVELOPMENT OF CAREGIVING TYPOLOGIES: UNDERSTANDING DECISION MAKING AND PRIORITIZATION IN DEMENTIA CAREGIVING

Henrietta Bennett[1], Vicki Winstead[2], and Carolyn Pickering[3], *1. University of Alabama at Birmingham, Birmingham, Alabama, United States, 2. Cizik School of Nursing at UTHealth Houston Texas, Houston, Texas, United States, 3. UTHealth Houston, Houston, Texas, United States*

Family caregivers of individuals with dementia navigate complex and often competing care demands, requiring them to make difficult decisions about task prioritization. As the disease progresses and care needs intensify, balancing immediate concerns with long-term goals becomes increasingly challenging, both emotionally and practically. The study aimed to assess how caregivers prioritize care tasks through typologies of caregiving to understand their decision-making processes and approach to caregiving. 20 family caregivers who lived with and provided unpaid care to a family member living with dementia were purposefully selected from a national study on caregiver decision-making. Semi-structured interviews were conducted to explore how caregivers prioritized and navigated decisions regarding daily care tasks. Using a grounded theory approach, two qualitative coders coded and compared codes at each stage of coding and collapsed codes into thematic categories of caregiving. We identified two primary caregiving typologies: Proactive and Reactive, based on whether caregivers plan or initiate decisions in arising care situations. The proactive-reactive typology captures the heterogeneity of caregiving experiences and provides better understanding of the mechanisms behind caregiver decision-making process. The study revealed that both typologies are influenced by multiple individual, interpersonal and structural factors including caregiver-care recipient relationship type and quality, caregiving values, and support. Findings from the study provides a useful framework for understanding family caregivers decision-making processes and care prioritization. Given the dynamic and context-dependent nature of daily caregiving, targeted interventions should consider the variability between these typologies, ensuring that caregivers are equipped with resources to mitigate stressors associated with crisis-driven decision-making.

Abstract citation ID: igaf122.1644

### REDUCED PREPARATORY RESPONSES IN PEOPLE WITH DEMENTIA PREDICT CAREGIVER MENTAL HEALTH IMPROVEMENTS AFTER CARE ENDS

Julian Scheffer[1], Kuan-Hua Chen[2], Claire Yee[3], Jenna Wells[4], Sandy Lwi[5], Enna Chen[6], Jennifer Merrilees[7], and Robert Levenson[8], *1. University of Western Ontario, London, Ontario, Canada, 2. University of Nebraska Medical Center, Omaha, Nebraska, United States, 3. Mayo Clinic, Scottsdale, Arizona, United States, 4. Cornell University, Ithaca, New York, United States, 5. VA Northern California Health Care System, Fairfield, California, United States, 6. Stanford University, Stanford, California, United States, 7. University of California, San Francisco, San Francisco, California, United States, 8. University of California, Berkeley, Berkeley, California, United States*

Caregivers for people with neurodegenerative disease (PWNDs) often experience mental and physical health problems, especially when caring for PWNDs who have deficits in emotional functioning. We studied 65 PWNDs and their caregivers both during active caregiving and after caregiving had ended to determine: (a) how caregiver health changes after caregiving ends; and (b) whether PWNDs' emotional functioning predicts these changes. PWND emotional functioning was assessed in the laboratory by measuring their generation of preparatory physiological activity (e.g., increased heart rate) to an upcoming emotional event (i.e., emotion eliciting films). Caregiver mental and physical health were measured using the Short-Form Health Survey (SF36) during active caregiving and again after caregiving had ended. Results indicated that, overall, caregivers reported increases in mental health, ($t(36) = 2.58$, $p = .014$, 95% CI [1.31, 10.93]) but decreases in physical health ($t(36) = -2.35$, $p = .025$, 95% CI [-7.26, -0.53]) across the two measurement periods. Using latent change score models, PWNDs' preparatory physiological response impairments were associated with better caregiver mental health trajectories ($\beta = -0.37$, $SE = 0.04$, $p = .003$) such that smaller PWND preparatory responses predicted greater improvements in caregiver mental health ($\beta = 9.92$, $SE = 2.84$, $p < .001$). There was no association with changes in caregiver physical health. Smaller preparatory physiological responses in PWNDs may indicate diminished emotional responding, which is linked with lower caregiver mental health during active caregiving. Once caregiving ends, these caregivers may be most likely to "rebound" and show improvements in mental health.

Abstract citation ID: igaf122.1645

### THE "LINKED HEALTH" OF DIVERSE CAREGIVERS: INSIGHTS FROM THE NATIONAL SOCIAL LIFE, HEALTH, AND AGING PROJECT

Lissette Piedra[1], Selena Zhong[2], Melissa Howe[3], and Nell Compernolle[3], *1. University of Illinois at Urbana-Champaign, Urbana, Illinois, United States, 2. NORC, Chicago, Illinois, United States, 3. NORC at the University of Chicago, Chicago, Illinois, United States*

This study explores the health of caregivers across five dimensions—physical, cognitive, mental, sexual, and sexual health—using the "linked health" concept, which suggests that changes in one health aspect affect others. We examine the correlation between caregivers' ethnoracial and linguistic backgrounds and their overall health, with a focus on social networks. Data from 1,309 caregivers in Rounds 1-3 of the National Social Life, Health, and Aging Project were analyzed. Health was assessed through self-reports and clinical measures. The findings show that Black and Hispanic caregivers had poorer cognitive health and lower self-rated health compared to White caregivers, although functional health levels were similar. Larger social networks were linked to better cognitive outcomes. Black caregivers reported greater social strain and less support, while Spanish-speaking Hispanic caregivers experienced better social health and fewer mental health symptoms than English-speaking caregivers. These results underscore the need for targeted interventions to address health disparities among caregivers.

Abstract citation ID: igaf122.1646

### THE WILL ADAPTS, THE MIND STAYS SHARP, BUT THE BODY KEEPS SCORE: LIFESPAN UNPAID CAREGIVING TRAJECTORIES AND HEALTH

Janecca Chin, and I-Fen Lin, *University of Missouri, Columbia, Missouri, United States*

Unpaid caregiving can have lasting health consequences. While extensive research has examined the link between caregiving and health, much of it treats caregiving as an isolated event rather than a cumulative life course process. This study adopts a life course perspective to examine how lifespan caregiving trajectories—defined by age at caregiving onset, duration, recurrence, and overlapping responsibilities—shape later-life health across physical, psychological, and cognitive domains. Using data from the Health and Retirement Study and its Life History Mail Survey, latent class analysis identified four distinct caregiving trajectories, and their health outcomes were compared with those of non-caregivers. Multivariable logistic and multinomial regression models assessed the link between caregiving and later-life health. Among women, Early Enduring and Immersed Caregivers (early and prolonged caregivers) exhibited worse physical health than non-caregivers (AME = +0.27 and +0.43 chronic conditions, $p < .001$). However, depressive symptoms were comparable, except among Immersed Caregivers who reported more distress than non-caregivers (AME = +0.24, $p < .05$). In contrast, caregiving appeared protective against cognitive impairment, as Common and Recurrent Caregivers (short-term or intermittent caregivers) had lower impairment risks than non-caregivers (AME = -0.06 and -0.05, $p < .01$). Among men, caregiving had fewer health consequences. Findings challenge one-dimensional portrayals of caregiving, instead highlighting its trajectory-based and multidimensional health effects. While moderate lifespan caregiving may not compromise well-being, the most intensive caregivers—particularly women—face enduring physical health risks. These findings underscore the need for interventions that support high-risk caregivers while recognizing the cognitive benefits of caregiving.

### SESSION 4090 (PAPER)

Abstract citation ID: igaf122.1647

### PHARMACOLOGICAL INTERVENTIONS IN OLDER ADULTS

This session describes opportunities for pharmacological management in older adults as well as deprescribing in older adults.

Abstract citation ID: igaf122.1648

### A 2-YEAR TRIAL OF METFORMIN TO REDUCE FRAILTY IN OLDER ADULTS WITH GLUCOSE INTOLERANCE

Sara Espinoza[1] Chen-pin Wang[2], and Nicolas Musi[1], *1. Cedars-Sinai Medical Center, Los Angeles, California, United States, 2. UT Health San Antonio, San Antonio, Texas, United States*

It has been postulated that metformin may extend healthspan but no long-term trials have been done in humans. We conducted a randomized, double-blinded, placebo-controlled clinical trial of metformin to reduce frailty in older adults (≥65 years) with glucose intolerance. Participants who were frail at baseline (Fried criteria) were excluded. Participants were randomized to 24 months of immediate release metformin (initiated at 500 mg/day and titrated to maximum tolerated dose up to 2,000 mg/day) or matching placebo. All participants received one session of diet and exercise counseling prior to initiation of study drug. The primary outcome was frailty as measured by the frailty index based on deficit accumulation model and frailty phenotype based on Fried criteria, which was assessed every 6 months. We conducted generalized estimating equations (GEE) models to examine the change in frailty per month by frailty index and Fried criteria. 145 participants were randomized; intention to treat analysis included 141 participants who took at least one dose of study drug. At baseline, participants were 48% female, 35% Hispanic/Latino, and mean age was 71.8 ±5.3 years. Metformin did not cause serious adverse events. There were no differences in adverse events by treatment group. Metformin led to a significant reduction in frailty progression rate per month assessed by frailty index compared to placebo (-0.0494 ±0.0216, 95%CI: -0.0918, -0.0071, p = 0.0222). No difference was observed by Fried criteria (0.0116 ±0.0077, 95%CI: -0.0034, 0.0266, p = 0.1302). Metformin administration to older adults with glucose intolerance may be useful for frailty prevention and extension of healthspan.

Abstract citation ID: igaf122.1649

### ENGAGING PATIENTS AND CAREGIVERS IN CO-DESIGN OF A PATIENT PORTAL-BASED DEPRESCRIBING INTERVENTION

Tobie Taylor-McPhail[1], Kathy Gleason[1], Andrea Daddato[1], Rosalphie Quiles[2], Jennifer Portz[3], Elizabeth Bayliss[1], and Ariel Green[2], *1. Kaiser Permanente Colorado, Aurora, Colorado, United States, 2. Johns Hopkins University School of Medicine, Baltimore, Maryland, United States, 3. University of Colorado School of Medicine, Aurora, Colorado, United States*

Successful deprescribing requires older adult and caregiver engagement, yet few deprescribing interventions have been co-designed with people with cognitive impairment and caregivers. We convened an advisory panel with 19 older adults with cognitive impairment and their caregivers from across the United States in four listening sessions (June 2024–March 2025) to refine the language and appearance of a patient portal-based deprescribing intervention involving an outreach message and educational video. We co-designed messaging, video content, and optimal patient/caregiver preparation for a deprescribing conversation. Each session followed a discussion guide, and participants received session summaries and incentive gift cards. The collaborative design process provided key insights, emphasizing: 1) reassure viewers the older adult's prescribing provider will be involved in the deprescribing process, 2) highlight the caregiver's role in the deprescribing conversation, and 3) describe the role of clinical pharmacists in clinical care. Based on feedback, we simplified video messaging, focused on accurate character portrayals, and added action steps. While most found the video motivating, they preferred discussing deprescribing during visits with prescribing clinicians rather than scheduling separate appointments. As patient portals become more universal, designing interventions that resonate with older adults and caregivers is increasingly important. The intervention will be piloted as a next step to inform a pragmatic deprescribing intervention. Engaging an advisory board with lived experience in the co-design of a portal-based deprescribing intervention for people with cognitive impairment led to important modifications of the message and video.

Abstract citation ID: igaf122.1650

### LONG-TERM EFFECTIVENESS OF PATIENT-DIRECTED EDUCATION TO SUSTAIN DEPRESCRIBING: A HYBRID IMPLEMENTATION TRIAL

Amy Linsky[1], Katie Jones[1], Kelly Stolzmann[1], Jacquelyn Pendergast[1], Christopher Miller[1], Michael Still[1], Amy Rosen[1], and Jolie Wormwood[2], *1. VA Boston Health Care System, Boston, Massachusetts, United States, 2. University of New Hampshire, Durham, New Hampshire, United States*

Deprescribing, stopping or dose-reducing medication, can avoid harms from potentially inappropriate medications (PIMs). We examined the effectiveness, sustainability, and safety of a patient-engagement strategy in a hybrid effectiveness-implementation pragmatic trial at three Veterans Affairs facilities. Subjects were mailed brochures for one of three PIMs (proton pump inhibitors, high-dose gabapentin, diabetes agents with hypoglycemia risk) if they had chronic active prescriptions before a primary care provider (PCP) visit. Control subjects received usual care. We determined PIM deprescribing (cessation or any dose reduction) 6- and 12-months after the visit, categorizing participants into four patterns. A multinomial mixed effect logistic regression with patients nested in PCPs, adjusting for intervention cohort, PIM, and site, tested the intervention effect on patterns. We assessed for possible adverse drug withdrawal events (ADWEs). Compared to controls (n = 2,448), intervention patients (n = 2,480) were significantly older (M = 70.8 vs. M = 70.0, p = 0.012) and less likely to be White (82% vs. 83%, p = 0.017). Intervention patients had more sustained deprescribing (6 and 12 months: 19.3% vs 15.1%), but comparable short-term (6 months only: 9.8% vs 10.5%) and delayed (12 months only: 13.9% vs 14.5%) deprescribing. In the adjusted regression, intervention patients were significantly more likely to exhibit sustained deprescribing (OR = 1.32 [95%CI=1.13, 1.55]) but not short-term (OR = 0.96 [95%CI=0.79, 1.16]) or delayed (OR = 0.99 [95%CI=0.84, 1.17]) deprescribing. There were five potential ADWEs (0.2% incidence rate); three assessed as not related and two as possibly related to the intervention. Despite the low intensity, patient-directed education materials are an effective implementation strategy to promote and sustain deprescribing with minimal ADWEs.

Abstract citation ID: igaf122.1651

### SSRI-ASSOCIATED CLINICAL OUTCOMES IN OLDER ADULTS WITH BEHAVIORAL AND PSYCHOLOGICAL SYMPTOMS OF DEMENTIA

Chun-Ting Yang[1], James Wilkins[2], Dae Hyun Kim[3], and Joshua Lin[1], *1. Brigham and Women's Hospital, Boston, Massachusetts, United States, 2. McLean Hospital, Belmont, Massachusetts, United States, 3. Hebrew SeniorLife, Boston, Massachusetts, United States*

Citalopram, escitalopram, and sertraline are three selective serotonin reuptake inhibitors (SSRIs) that are commonly prescribed in older adults with behavioral and psychological symptoms of dementia (BPSD), but evidence about their comparative safety remains limited. This retrospective cohort study used Medicare 2013-2018 and Optum Clinformatics® 2013-2024 to examine the risks of clinical outcomes between different SSRIs in older adults with BPSD. BPSD individuals aged≥65 years and newly prescribed citalopram, escitalopram, or sertraline were included. Propensity score matching was employed to balance baseline covariates. Outcomes included all-cause mortality, all-cause hospitalization, and hospitalization reasons. The on-treatment effect was measured using Cox proportional hazards model. Analyses were conducted separately in the two databases, and the effect estimates were pooled by fixed-effect meta-analysis. A total of 15,562 citalopram-sertraline and 17,180 escitalopram-sertraline matched pairs were analyzed.

Compared with sertraline, both citalopram and escitalopram showed non-significantly different risks of all-cause death (hazard ratio [95% CI]: 0.96 [0.91-1.01] for citalopram; 0.95 [0.91-1.01] for escitalopram), inpatient delirium (1.05 [0.97-1.15]; 1.03 [0.95-1.11]), falls (1.04 [0.94-1.16]; 1.04 [0.94-1.14]), stroke (1.05 [0.88-1.26]; 1.13 [0.95-1.33]), ventricular arrhythmia (0.63 [0.21-1.94]; 1.00 [0.39-2.53]), and hyponatremia (1.00 [0.87-1.16]; 1.02 [0.90-1.17]). A marginally increased risk of all-cause hospitalization was observed in citalopram users versus sertraline users (1.05 [1.01-1.09]), but this increase was not statistically significant for escitalopram users (1.05 [1.00-1.09]). The modest magnitude of the HR estimate, along with the marginal significance, may suggest limited clinical relevance. Our findings suggest the comparable safety profiles between the use of citalopram, escitalopram, and sertraline in older adults with BPSD.

Abstract citation ID: igaf122.1652

### USE OF ATYPICAL ANTIPSYCHOTICS FOR POST-HOSPITALIZATION DELIRIUM AND ASSOCIATED CLINICAL OUTCOMES IN OLDER ADULTS

Chun-Ting Yang[1], James Wilkins[2], Kevin Pritchard[1], Xiaojuan Liu[1], Dae Hyun Kim[3], and Joshua Lin[1], *1. Brigham and Women's Hospital, Boston, Massachusetts, United States, 2. McLean Hospital, Belmont, Massachusetts, United States, 3. Hebrew SeniorLife, Boston, Massachusetts, United States*

Atypical antipsychotic medications (APMs) are commonly prescribed to manage delirium symptoms among hospitalized older adults, but their comparative safety beyond hospital discharge remains unknown. This retrospective cohort study utilized Medicare 2013-2018 and Optum Clinformatics® 2013-2024 to examine the risks of clinical outcomes between quetiapine, risperidone, and olanzapine use among older adults following hospitalization. We included patients aged≥65 years without psychiatric disorders who newly filled quetiapine, risperidone, or olanzapine within 30 days of hospital discharge. The index date was the first dispensing date of the APM. Propensity score overlap weighting was employed to balance baseline covariates. Outcomes included all-cause mortality, all-cause rehospitalization, and specific rehospitalization causes. The on-treatment effects were measured using Poisson regression model. Analyses were conducted in the two databases separately and the effect estimates were pooled by fixed-effect meta-analysis. A total of 32,209, 9,732, and 6,363 quetiapine, risperidone, and olanzapine users were identified. The mortality rate (per 1000 person-years) was 478.1 for quetiapine (reference), 544.3 for risperidone (rate ratio [95% CI]: 1.11 [0.96-1.29]), and 726.2 for olanzapine (1.49 [1.30-1.71]). The rehospitalization rate was 1,408.6 for quetiapine (reference), 1,540.6 for risperidone (1.07 [0.97-1.17]), and 1,703.2 for olanzapine (1.16 [1.06-1.29]). Olanzapine also showed a significantly higher risk of death (1.34 [1.17-1.53]) compared to risperidone. Among rehospitalization reasons, olanzapine was associated with an increased risk of delirium compared to quetiapine (1.34 [1.12-1.60]) and risperidone (1.20 [1.01-1.42]). Our findings suggest that olanzapine increased the risks of adverse outcomes compared to risperidone and quetiapine among older adults prescribed with APMs after hospital discharge.

### SESSION 4095 (PAPER)

Abstract citation ID: igaf122.1653

### SOCIAL DETERMINANTS OF HEALTH IN OLDER ADULTS

Social determinants of health impact a variety of health outcomes in older adults. and often start early in life. This session provides insight into the impact of the environment, nutrition and support systems can affect health in older adults.

Abstract citation ID: igaf122.1654

### DEMOGRAPHIC AND SOCIOECONOMIC RISK FACTORS FOR PAIN PROGRESSION AND RECURRENCE IN MIDDLE-AGED AND OLDER ADULTS

Mikaela Bloomberg, and Andrew Steptoe, *University College London, London, England, United Kingdom*

Chronic pain is a major contributor to disability and reduced quality of life, particularly for older adults. Many pain conditions are also intermittent or progressive, with repeated episodes of pain worsening, subsiding, and recurrence. Identifying the demographic and socioeconomic factors that put individuals at risk of pain worsening or recurrence is therefore crucial for designing targeted early interventions and addressing disparities in pain management. Even so, the current evidence base is mixed and mostly uses methods that do not allow for nonlinear trajectories of pain. In this study, we used multistate models to examine demographic and socioeconomic risk factors for transitioning between pain states (moderate-severe, mild, or no pain) in 9,369 adults aged 50-98 years from the English Longitudinal Study of Ageing (study years: 2002/03-2021/23). This approach allowed us to capture the dynamic and recurrent nature of long-term pain. Findings highlighted the importance of gender and socioeconomic position: for women (compared with men) pain was less likely to improve in severity (hazard ratio [HR]=0.84, 95% confidence interval=0.74-0.96) and more likely to recur (HR = 1.45, 1.25-1.68). Higher education level was associated with pain improvement (HR = 1.43, 1.16-1.76), and lower risk of pain worsening (HR = 0.52, 0.42-0.64) and recurrence (HR = 0.67, 0.52-0.85); similar patterns were observed for wealth. These results emphasise the variability of long-term pain over time and support policies aimed at improving access to pain management for women and individuals with fewer socioeconomic resources. The findings also point to the importance of systematic long-term follow-up of vulnerable groups to enable timely intervention.

Abstract citation ID: igaf122.1655

### DISPARITIES IN PAIN AMONG NURSING HOME RESIDENTS: RACE, COGNITIVE IMPAIRMENT, AND NURSING HOME RACIAL COMPOSITION

Cassandra Dictus[1], Matthias Hoben[2], Kali Thomas[3], Tamara A. Baker[4], Baiming Zou[4], Ruth Anderson[1], Ashley Leak Bryant[4], and Anna Beeber[3], *1. Duke University, Durham, North Carolina, United States, 2. York University, Toronto, Ontario, Canada, 3. Johns Hopkins University, Baltimore, Maryland, United States, 4. The University of North Carolina at Chapel Hill, Chapel Hill, North Carolina, United States*

Pain is common among nursing home (NH) residents and can significantly impact quality of end-of-life experiences, yet inequities in pain assessment and treatment persist and are less studied among racial groups with smaller population sizes. This study examined associations between resident race, cognitive impairment, and NH racial composition with any pain documented in the last five days. We conducted a longitudinal multi-level logistic regression using MDS 3.0, LTCFocus, and MBSF data. We focused on residents who died in 2018 or 2019, regardless of place of death (n = 617,922). Analyses were stratified by staff- and self-reported pain, controlling for resident age, function, comorbidity, gender, and NH percent Medicaid, bed number, staffing, ownership, and rurality. In the initial adjusted models, both staff- and self-reported pain increased over the year before death. Staff- and self-reported pain decreased as cognitive impairment increased, and this was more dramatic in the self-reported model. In both models, pain varied by race, with American Indian or Alaskan Native residents having the highest rates, followed by Hispanic and White residents at similar rates; Black residents had slightly lower rates, followed by Asian residents, and Native Hawaiian or other Pacific Islander residents at the lowest. Pain was more frequently reported in predominately White NHs than in more racially diverse NHs, especially in the staff-reported model. Disparities may reflect underassessment of pain and inappropriate pain assessment methods, particularly at the NH-level in facilities that included more racially minoritized residents. Further research is needed to clarify these disparities and ensure equitable pain management.

Abstract citation ID: igaf122.1656

### IMPACT OF DISCRIMINATION IN HEALTHCARE ON PATTERNS OF DOCTOR VISITS OVER TIME

Michael Green[1], Qing Yang[2], Hanzhang Xu[2], Radha Dhingra[1], Heather Farmer[3], Roland Thorpe[4], and Matthew Dupre[1], *1. Duke University School of Medicine, Durham, North Carolina, United States, 2. Duke University, Durham, North Carolina, United States, 3. University of Delaware, Newark, Delaware, United States, 4. Johns Hopkins Bloomberg School of Public Health, Baltimore, Maryland, United States*

Discrimination in healthcare negatively affects patient care. We examined how perceived discrimination in healthcare settings influenced long-term patterns of doctor visit among 17,642 Black and White middle-aged and older adults (ages 50-80 at baseline), using data from the 2008-2020 Health and Retirement Study. Discrimination in healthcare was assessed by asking participants how often they experienced poorer treatment from doctors or hospitals. Group-based trajectory models identified longitudinal patterns of doctor visits over 14 years of follow-up. Multinomial logistic regression models assessed whether perceived discrimination at baseline was associated with different trajectories of subsequent doctor visits, and whether sociodemographic factors, health behaviors, and health status attenuated the associations. We identified 5 trajectories of doctor visits: Low (~2 visits/year; 33.5%), Frequent (~4 visits/year; 33.1%), Frequent-to-High (~4.5 visits/year increasing to ~9; 13.7%), High-to-Frequent (~13.5 visits/year decreasing to ~6; 10.3%), and High (~14 visits/year decreasing to ~11; 6.3%). Groups with more doctor visits had poorer health, greater social vulnerability, and higher reported discrimination. Discrimination was associated with significantly greater risks of exhibiting trajectories with High (relative risk ratio [RRR]=1.62; P<.0001) and High-to-Frequent (RRR=1.38; P<.001) utilization relative to Low utilization after accounting for participants' background factors. Overall, we found discrimination in healthcare was associated with trajectory groups exhibiting high doctor visits at baseline that subsequently declined. This suggests that among middle-aged and older adults with intensive healthcare needs, patient disengagement is more likely after experiencing discrimination. Health systems should consider the quality of care for patients with greater healthcare needs.

Abstract citation ID: igaf122.1657

### UNSEEN BURDENS: HOW ACES AND CITIZENSHIP STATUS SHAPE HEALTH IN AGING LATINX POPULATIONS

Alein Haro-Ramos, *University of California, Irvine, Irvine, California, United States*

Background and Objectives: Citizenship (or the lack thereof) and adverse childhood experiences (ACEs) are both independent predictors of poor self-rated health (SRH), but we know little about their cumulative effects among older Latinx adults. Using a life course perspective, we examine 1) the relationship between ACEs, citizenship status, and poor SRH and 2) whether race-ethnicity-citizenship status combinations modify the association between ACEs and SRH. Research Design and Methods: Latinx and White adult respondents age 50+ were selected from the 2021 and 2022 California Health Interview Survey (n = 20,491). Generalized linear models (logit link, binomial family) estimated the relationship between ethnicity-citizenship status, ACEs, and SRH. We use an interaction between ethnicity-citizenship and ACEs to test for a moderating effect on SRH. Results: High ACE exposure and noncitizen status were each significant predictors of poor SRH, adjusting for covariates. In the mutually adjusted model, including ACEs and citizenship status, the magnitude of the coefficient for naturalized and noncitizen Latinx adults and for each ACE score increased. The association between ACEs and poor SRH was strongest among noncitizen Latinx adults. Discussion and Implications: ACEs and citizenship status are significant predictors of poor SRH, and the relationship between ACEs and SRH is pronounced among noncitizen Latinx older adults. Our findings underscore the importance of addressing early and later life experiences to understand and improve the health and well-being of older Latinx individuals, particularly those lacking citizenship.

Abstract citation ID: igaf122.1658

### VALIDATING OF A NUTRITION AND FOOD SECURITY SCREENING TOOL FOR OLDER ADULTS

Lauri Wright[1], Jenna Pennella[2], Jenifer Ross[2], and Paul Fuglestad[2], *1. University of South Florida, Tampa, Florida, United States, 2. University of North Florida, Jacksonville, Florida, United States*

Food insecurity affects many older Americans due to factors such as financial constraints, transportation access, physical mobility, and bodily functioning. However, the most widely used measure for food insecurity, the USDA Household Food Security Survey Module (FSSM), primarily assesses financial security, overlooking other critical domains. This study aimed to develop and validate a screening tool that captures additional dimensions of food insecurity among older adults. We surveyed 72 older adults (ages 66–87). Each participant met with a registered dietitian nutritionist (RDN), who conducted a comprehensive nutritional assessment and administered both the USDA-FSSM and a newly developed scale based on the World Health Organization's (WHO) International Classification of Functioning, Disability, and Health. Based on the nutritional assessment, the RDN categorized individuals as food secure, food insecure, or at risk for food insecurity. The FSSM explained only 34% of the variance in dietitian assessments, suggesting it does not fully capture food insecurity in this population. The newly developed scale demonstrated strong internal reliability (Cronbach's alpha = .77). Factor analysis using Principal Axis Factoring with Oblimin rotation identified four key factors: mobility/physical limitations, access limitations, financial limitations, and bodily function issues. The overall scale score correlated strongly with the dietitian assessment (r = .69) and moderately with the FSSM (r = .51). These findings highlight that food insecurity in older adults extends beyond financial constraints. The newly developed screening tool effectively captures physical and functional challenges that impact food access and consumption, offering a more comprehensive assessment than the FSSM.

### SESSION 4100 (SYMPOSIUM)

Abstract citation ID: igaf122.1659

### PRESIDENTIAL SYMPOSIUM: LOOKING OVER THE HORIZON: STATES AS THE LABORATORY FOR POLICY INNOVATIONS

Chair: Howard Degenholtz

Supreme Court Justice Louis Brandeis suggested that states are the laboratories of democracy. In a year in which so much attention is at the federal level, this year we will focus on the ways in which we turn our gaze to the states as sources to learn about innovations in policies that support older adults. States have long been the incubators for new ideas that have had dramatic impact. For example, the notion of using Medicaid dollars to provide long-term services and supports in people's own homes rather than in institutional settings had its start through state demonstrations. This symposium will feature innovative state programs that support caregivers for people with dementia, the creation of a long-term care insurance program, and models for paying family caregivers. Speakers will describe how states have created these programs and learned from each other, notable accomplishments and challenges in implementing them, and what they expect to see moving forward. They will also touch on where and how researchers can play an important role in studying and advancing state-level innovations.

Abstract citation ID: igaf122.1660

### SUPPORTING STATE INNOVATION IN ALZHEIMER'S RESEARCH

Joseph Gaugler, *University of Minnesota, Minneapolis, Minnesota, United States*

The State Alzheimer's Research Support Centers (StARS) is a research support center for state's dementia care policies. The center is developing and implementing an approach to partnering with a wide range of programs to monitor activities, provide logistical, funding, and coordination support, and establish a robust outreach and dissemination strategy. This presentation will provide an overview of the StARS Centers and examples of state policies and programs that are being evaluated under this initiative.

Abstract citation ID: igaf122.1661

### STATE POLICIES TO SUPPORT FAMILY CAREGIVING

Rita Choula, *AARP, Washington, District of Columbia, United States*

Family and friends provide 64% of caregiving hours in the US. While uncompensated, the value of this care is estimated to be $600 billion per year. This presentation will share experience of AARP in developing policies to support family caregivers, with example form selected promising state and local programs.

Abstract citation ID: igaf122.1662

### THE PROMISE AND CHALLENGE OF STATE-BASED LONG-TERM CARE INSURANCE

Alicia Munell, *Boston College, Chestnut Hill, Massachusetts, United States*

The cost of Long-Term Care is unaffordable for most people. Paid home care for an individual with significant needs can easily exceed $35,000 per year which is more than the total annual income of about half of older Americans and more than the total net worth for about one-fifth. The state of Washington launched We Care, a publicly funded long-term care insurance policy in 2019. The first benefits will be paid in 2026. This presentation will address some of the challenges that the designers of the program faced, for example managing adverse selection, improving fairness and allowing some people to opt-out. The Washington program has some important lessons for other states that are exploring similar policies, but state variation in tax rates and benefit levels raise new questions about what level of benefit is adequate to support people who need long-term care.

### SESSION 4105 (PAPER)

### TRAINING STRATEGIES TO EXPLORE EMPATHY, ATTITUDES, PERCEPTIONS, AND VALUE-CONGRUENCE TOWARD OLDER ADULT CARE

Abstract citation ID: igaf122.1663

### ATTITUDES TO CONDUCT RESEARCH AMONG PHYSICIANS SPECIALIZING IN OLDER ADULT CARE: LONGITUDINAL STUDY

Jenny van der Steen[1], Anna van Ede[1], Sytske van Bruggen[2], Wing H. Tong[3], Annemarie Moll-Jongerius[1], Victor Chel[1], Maaike N. Scheffers-Barnhoorn[1], and Belinda W.C. Ommering[4], *1. Leiden University Medical Center, Leiden, Zuid-Holland, Netherlands, 2. Leiden University Medical Center, The Hague, Zuid-Holland, Netherlands, 3. LUMC, Rotterdam, Zuid-Holland, Netherlands, 4. HU University of Applied Sciences Utrecht, Utrecht, Utrecht, Netherlands*

Attitudes regarding conducting research may be shaped by experiences during medical training and residency. Supervised performing of scientific research might motivate physicians to contribute to the evidence base on medical treatment of older people. We conducted a longitudinal study (2019-2024) to assess perception and attitudes towards conducting research of physicians specializing in older adult care ("elderly care medicine") in the Netherlands who conducted a mandatory training study on pain and discomfort in dementia. Residents completed validated scales for intrinsic and extrinsic motivation, self efficacy and perception of research at the beginning, midway and end of the 3-year program. Random effect models assessed changes across study phases. Separately, residents' personal reflection reports were analyzed inductively and, subsequently, against a Self Determination Theory framework with qualitative thematic analysis. Of 78 invited residents, 41 (53%) participated. Changes–in particular a decrease of intrinsic motivation scores–were greatest when developing a research question or data analyses, and in the later phases, mostly returned to initial levels. Self efficacy was the most stable. Starting during the pandemic, being native Dutch and little experience was associated with lower scores. The qualitative results indicated that many appreciated collaborative working in itself as educational. Further, intrinsic motivation for research appeared positively associated with perceived autonomy, competence and relatedness. Both types of data suggest that attitudes regarding doing research are shaped mostly before residency, factors such as a feeling of being in control and relatedness to the research may be used to encourage development of positive attitudes towards conducting scientific research.

Abstract citation ID: igaf122.1664

### ATTITUDES TOWARDS AGING AND RELATED FACTORS: A CROSS-SECTIONAL COMPARISON OF GERONTOLOGY AND OTHER UNDERGRADUATES

Shiyu Chen, and Yongfang Tang, *Hunan Women's University, Changsha, Hunan, China (People's Republic)*

Evaluating the young generation's attitudes towards the older people is important to investigate on ageism, especially in higher education. This study aims to compare the attitudes towards older adults and aging between Chinese undergraduates from either an established gerontology program or other undergraduate programs, as well as to explore the influencing factors. 285 participants currently enrolled in Hunan Women's University were recruited either from the gerontology program ($n = 164$) using purposive sampling, or other undergraduate programs($n = 121$) using snowball sampling. The Kogan's attitudes toward old people scale (KAOP), Anxiety about Aging Scale (AAS), and Aging Semantic Differential (ASD) were used to measure the students' attitudes towards the older people and aging. ANCOVA analysis adjusted for sociodemographic covariates was used to examine the difference between majors. The KAOP score was significantly higher among gerontology students, compared to other students (152± 18.3 vs 146± 18.0, $p < 0.01$). The KAOP score was significantly associated with students' current close relationship with older relative(s) ($p < 0.05$) and experience of growing up with older relative(s) ($p < 0.05$). Although AAS score were not different between programs, it was also significantly associated with students' current close relationship with older relative(s) ($p < 0.01$) and experience of growing up with older relative(s) ($p < 0.05$). Our results suggest that gerontological higher education as well as previous intergenerational experience are beneficial in improving young adults' attitudes towards aging and the older people, and further actions should be taken to enhance aging related education in a wider spectrum.

Abstract citation ID: igaf122.1665

### EXPLORING THE PERCEPTIONS OF PARTNERS OF PERSONS WITH DEMENTIA ON VALUE-CONGRUENT CARE

Teuta Kadiu, *University of California, Davis, Davis, California, United States*

The purpose of this study was to explore caregivers of PwD perceptions of receiving value congruent care in encounters with healthcare professionals throughout the progression of dementia. The method was an interpretative descriptive study that included 25-partners of persons with dementia. Participants were partners caring for persons with dementia during different stages of the disease. Interviews were conducted in-person and online. Interview questions were designed based on the psychosocial caregiver integrity model, the development of which has been reported elsewhere. Several screening tools were also used to explore the patient with dementia and the partners' health concerns as well as any relationship concerns. Interview questions explored caregivers' perceptions of the healthcare provider's ability to address the various concerns. Data was transcribed using AliceAI. Transcripts were interpreted using the role theory framework. From this, the results were descriptions of the elements of value congruent care, as they emerged from interviews with partners of persons with dementia. Three component themes were identified: 1) acknowledging relational concerns, 2) assessing what mattered most to the dyad, and 3) creating meaning for both. Lack of value congruent care had implications for caregivers' socialization into the caregiving role, as well as utilization of healthcare resources. Awareness of these factors can help providers be more effective when helping socialize partners of patients with dementia into the role of a long-term caregiver.

Abstract citation ID: igaf122.1666

### TRANSFORMING EMPATHY THROUGH SIMULATION: A MIXED-METHODS STUDY ON DEMENTIA EDUCATION

Suzanne Parkman, Julie Larouche, Ashely Condon, and Abou El-Makarim Aboueissa, *University of Southern Maine, Portland, Maine, United States*

Dementia, including Alzheimer's disease, poses substantial challenges due to its progressive and multifaceted nature. Addressing these complexities requires healthcare students to undergo specialized training emphasizing empathy and equipping them with the skills necessary to provide compassionate, patient-centered care. This study used an embedded mixed-methods, one-group quasi-experimental design to evaluate empathy levels in pre-licensure nursing and graduate occupational therapy students ($N = 125$) using the Kiersma-Chen Empathy Scale-Revised (KCES-R). Empathy data was gathered at three intervals: before, immediately after, and six weeks following a virtual dementia simulation. Additionally, 36 focus groups (comprising 3-4 students each) were conducted during post-simulation debriefings to collect qualitative insights. The findings demonstrated significant increases in empathy scores across all time points, $F(2,124) = 17.02$, $p = 0.000$. Thematic analysis of the qualitative data revealed five core themes: 1) The Illusion of Empathy, 2) Building Empathetic Competence, 3) Facing Difficult Realities, 4) The Eureka Moment: Transformative Insights, and 5) Empathy within Power Dynamics. Collectively, the results indicate that simulation-based dementia education effectively fosters empathy among healthcare students while inspiring them to deliver higher-quality care. Experiential learning approaches like these are vital for preparing the next generation of healthcare professionals to meet the growing needs of individuals living with dementia.

### SESSION 4107 (BIOLOGICAL SCIENCES INVITED SYMPOSIUM)

Abstract citation ID: igaf122.1667
**LEVERAGING BIOMARKERS OF AGING**
Chair: Nathan LeBrasseur

There is considerable interest in using diverse forms of data—e.g., routine and advanced laboratory tests, diagnostic images, physiological measures, and wearable outputs—to assess states of biological vs. chronological age and rates of biological vs. chronological aging. Without question, biomarkers offer incredible potential in research and clinical practice as predictors of future health and as informers of what, how, when, and in whom exposures, behaviors, and interventions affect aging. This symposium will provide an update on the latest advances in, and strengths and weaknesses of, biomarkers of aging and their application.

Abstract citation ID: igaf122.1667a
**EMERGING BIOMARKERS OF MITOCHONDRIAL HEALTH**
Anthony Molina, *University of California, San Diego, La Jolla, California, United States*

Advances in human mitochondrial bioenergetic profiling have improved our understanding of how cellular metabolism can drive a wide array of age-related conditions, including changes in physical and cognitive performance with advancing age. This presentation will review new techniques and approaches that we are applying in clinical studies to better understand age-related bioenergetic decline and its consequences. As mitochondrial therapeutics are increasingly recognized to have potential benefits for promoting healthy longevity, biomarkers of mitochondrial health have an important role in clinical trials evaluating promising gerotherapeutic interventions.

Abstract citation ID: igaf122.1668
**BIOMARKERS OF AGING - A CONSORTIUM STORY**
Mahdi Moqri, *Harvard University, Cambridge, Massachusetts, United States*

The search for biomarkers that quantify biological aging has intensified in recent years. Such biomarkers could predict aging-related outcomes and could serve as surrogate endpoints for the evaluation of interventions promoting healthy aging and longevity. However, no consensus exists on how biomarkers of aging should be validated before their translation to the clinic. I will present the work on the Biomarkers of Aging Consortium over the past three years to address this unmet need.

Abstract citation ID: igaf122.1669
**BRINGING GEROSCIENCE TO THE BEDSIDE: LEVERAGING BIOMARKERS OF AGING IN CLINICAL RESEARCH**
Sara LaHue, *University of California, San Francisco, San Francisco, California, United States*

Biomarkers of aging, including epigenetic and proteomic “clocks” and senescence-associated secretory phenotype (SASP) factors, offer powerful tools for potentially understanding and predicting outcomes in older adults, yet their application in patient-facing clinical research remains limited. In this talk, I will share practical lessons from integrating geroscience markers into patient cohorts, focusing on delirium and other complications that follow traumatic injuries in older adults, to show how aging biomarkers may both illuminate mechanisms of vulnerability and serve as predictors of adverse clinical outcomes. I will highlight strategies for measuring these markers in clinical settings and overcoming implementation challenges to demonstrate that geroscience biomarkers are not only feasible to integrate but also essential for advancing precision approaches to aging-related conditions.

Abstract citation ID: igaf122.1670
**DECODING BIOLOGICAL AGE: EMERGING INSIGHTS, PERSISTENT CHALLENGES, AND OUR JOURNEY TOWARD DE-DEVELOPMENT OR RELIABLE AGING BIOMARKERS**
Andrea Cipriano, *Stanford University, Palo Alto, California, United States*

Biological age provides a more accurate measure of healthspan and disease risk than chronological age, yet the development of robust and clinically actionable biomarkers remains an ongoing challenge. In this presentation, I will outline emerging insights that are reshaping our understanding of the aging process, while highlighting the persistent technical and conceptual barriers that hinder biomarker validation and standardization. Drawing on work from the Biomarkers of Aging Consortium, I will share our collaborative efforts to define, benchmark, and harmonize key readouts of biological age, with a particular focus on clinical immune aging biomarkers as a case study for translating discovery into real-world applications.

### SESSION 4110 (SYMPOSIUM)

Abstract citation ID: igaf122.1671
**FROM BEHAVIOR TO THE BUILT ENVIRONMENT: THE ROLE OF THE EXPOSOME ON COGNITIVE HEALTH**
Chair: Olivia Atherton

The exposome – the psychological, social, lifestyle, and physical environments where people live, connect, and work – plays an important role in supporting cognitive health across the lifespan. However, we know little about the mechanisms linking psychosocial, lifestyle, and built environment factors to cognitive health, as well as roles of the exposome across socioculturally-diverse populations. This symposium showcases innovative research that fills these gaps by leveraging longitudinal study designs (up to 14 years), multi-method data (e.g., self-report, geographical linkages, cognitive batteries), lifespan perspectives (from adolescence through older adulthood), and socioculturally-diverse populations (including samples of Mexican-origin and Latino individuals, and an adoption/twin study). First, Dr. Emily Willroth uses data from a 14-year longitudinal study of Mexican-origin adults to examine how well-being (i.e., life satisfaction, optimism) is related to modifiable dementia risk factors and cognitive health. Second, Dr. Graciela Muniz Terrera uses longitudinal data from a Latino sample to demonstrate how social cohesion and loneliness are associated with cognitive function and trajectories. Third, Dr. Chandra Reynolds uses longitudinal data from an adoption-twin study to demonstrate the role of neighborhood Cognability on cognitive performance in midlife, while also considering demographic, developmental, and neighborhood covariates. Finally, Dr. Olivia Atherton uses geographical linkages to the Health and Retirement Study to characterize discrepancies between health behavior engagement and built environment opportunities, as well as the impact of behavior-opportunity gaps on cognitive health. We conclude with a general discussion of future directions in understanding the role of the exposome on cognitive health and healthy aging in place.

Abstract citation ID: igaf122.1672
**WELLBEING TRAJECTORIES AND DEMENTIA RISK AMONG MEXICAN-ORIGIN ADULTS LIVING IN THE U.S.**
Emily Willroth[1], Olivia Atherton[2], Angelina R. Sutin[3], and Richard Robins[4], *1. Washington University in St. Louis, St. Louis, Missouri, United States, 2. University of California*

*Riverside, Riverside, California, United States, 3. Florida State University College of Medicine, Tallahassee, Florida, United States, 4. University of California, Davis, Davis, California, United States*

Higher wellbeing is consistently associated with better cognitive health. However, little is known about how long-term trajectories of wellbeing are associated with cognitive health or modifiable dementia risk factors, particularly among populations at high risk for dementia. To address these open questions, we used data from The California Families Project, a 14-year longitudinal study of Mexican-origin adults living in the United States (analytic N = 1,082), to estimate long-term trajectories of wellbeing (i.e., life satisfaction and optimism) and whether the level and/or slope of wellbeing was associated with later cognitive health (i.e., cognitive function and impairment status, self- and informant-rated memory) and modifiable dementia risk factors (i.e., hearing loss, hypertension, higher body weight, smoking, depression, social isolation, physical inactivity, diabetes, alcohol use, poor sleep quality, inadequate or excessive sleep, inadequate health insurance, poor overall health). Higher life satisfaction level was associated with better self- and informant-reported memory, and more positive life satisfaction slope was associated with better self-rated memory. Higher optimism level was associated with better cognitive function and better self-rated memory, and more positive optimism slope was associated with better cognitive function. Life satisfaction and optimism levels and change were also associated with several modifiable dementia risk factors. These findings provide evidence that long-term trajectories of life satisfaction and optimism are associated with later levels of several cognitive health outcomes and modifiable dementia risk factors in Mexican-origin adults living in the United States, an ethnic group that experiences disproportionate rates of dementia.

Abstract citation ID: igaf122.1673

### ARE THERE SEX DIFFERENCES IN HOW SOCIAL COHESION AND LONELINESS RELATE TO COGNITIVE DECLINE IN LATINOS?

Graciela Muniz-Terrera[1], Alejandra Marroig[2], Angela Gutierrez[3], Courtney Thomas Tobin[4], and Barış Sevi[5], *1. Ohio University, Athens, Ohio, United States, 2. Universidad de la Republica Uruguay, Montevideo, Montevideo, Uruguay, 3. Western University of Health Sciences, Pomona, California, United States, 4. University of California, Los Angeles Fielding School of Public Health, Los Angeles, California, United States, 5. MEF University, Maslak, Istanbul, Turkey*

Latinos are the largest minoritized population in the US, and therefore, understanding cognitive decline in this population is paramount. Previous literature has identified exogenous factors, such as social cohesion and endogenous factors, such as loneliness, as associated with cognitive decline in older men and women. To improve our understanding of the role of social cohesion and loneliness on cognitive decline in Latino older adults, we fitted independent linear mixed effects models to cognitive scores from men and women aged 50 and older (n = 2,321) who participated in the Health and Retirement Study (2006 - 2016), accounting for both, social cohesion and loneliness. Models were also adjusted for sociodemographic factors. In men and women, social cohesion was positively associated with baseline cognitive function ($p < 0.001$), while loneliness was negatively associated with baseline cognitive function ($p < 0.001$). The effect size of loneliness on baseline cognition in men was 3-fold the effect of social cohesion in men, while in women, it was 2-fold. However, none of these factors were significantly associated with cognitive trajectories over time. These findings highlight the importance of the role of exogenous and endogenous domains of the exposome in cognitive function among Latino adults. Health promotion initiatives should focus on implementing culturally appropriate strategies that enhance social cohesion within neighborhoods and help reduce feelings of loneliness.

Abstract citation ID: igaf122.1674

### NEIGHBORHOOD COGNABILITY AND COGNITIVE PERFORMANCE APPROACHING MIDLIFE IN THE CATSLIFE STUDY

Chandra Reynolds[1], Jessica Finlay[1], Elizabeth Muñoz[2], Robin Corley[1], Soo Rhee[1], and Sally Wadsworth[1], *1. University of Colorado Boulder, Boulder, Colorado, United States, 2. University of Texas at Austin, Austin, Texas, United States*

Neighborhood features may support cognitive health in later life through social and behavioral pathways such as physical activity, social connection, and cognitive engagement, as well as reduced exposure to pollution and fast-food. Whether such neighborhood features—conceptualized as Cognability—are associated with cognitive functioning before midlife is unknown. In the present study, we evaluated Cognability and cognitive performance associations at midlife and tested for possible selection effects by controlling for adolescent cognitive performance. We analyzed data from 1061 participants from the Colorado Adoption/Twin Study of Lifespan behavioral development and cognitive aging (CATSLIfe; Mage= 33 years, range 28-49; 53% female, 10% non-white/Hispanic; tested between 2015-2021) with available year 16 IQ assessments. Cognability scores (2017) and Social Vulnerability Index SES scores (SVI-SES, 2018) were aligned. WAIS-III IQ scores indexed cognitive performance. In generalized estimating equations, we regressed current IQ on Cognability, adjusting for SVI-SES, age, sex, race/ethnicity, adoption status, and sibling similarity, then tested whether entering adolescent IQ changed the observed associations. Results revealed that higher neighborhood Cognability was associated with higher full-scale IQ at midlife ($B = 0.9894$, $p = 0.0100$), even when adjusting for neighborhood SES and individual sociodemographics. However, the Cognability–IQ association was attenuated by 74% when adolescent IQ was entered ($B = 0.2537$, $p = 0.2249$). Similar patterns were observed for verbal and performance IQ. We observed positive associations between neighborhood Cognability and IQ at midlife even when adjusting for current neighborhood SES and individual sociodemographics. However, neighborhood selection may partly underlie these associations, as accounting for adolescent IQ diminished the associations.

Abstract citation ID: igaf122.1675

### BEHAVIOR-OPPORTUNITY GAPS AND COGNITIVE HEALTH: FINDINGS FROM A GEOGRAPHICALLY-LINKED LONGITUDINAL STUDY

Olivia Atherton[1], Priscilla Whang[2], and Emily Willroth[3], *1. University of California Riverside, Riverside, California, United States, 2. University of California, Riverside, Riverside, California, United States, 3. Washington University in St. Louis, St. Louis, Missouri, United States*

Approximately 40% of Alzheimer's Disease cases in the U.S. are due to modifiable lifestyle factors such as physical inactivity, heavy alcohol use, and smoking. As such, researchers have begun to focus on how to change individuals' lifestyle behaviors to improve cognitive health and reduce dementia risk. However, sustained engagement in healthier behaviors can be hindered by environmental constraints (or promoted by environmental opportunities) such as living in an area with few sidewalks, parks, and gyms, or more liquor stores and bars. We refer to this potential discrepancy between health behavior engagement and environmental opportunities as a behavior-opportunity gap. Using data from a large longitudinal study of U.S. older adults (Health and Retirement Study) with geographical linkages, we aimed to characterize behavior-opportunity gaps and their associations with cognitive health in two domains: physical activity and substance use (N = 20,289 participants nested within 5,874 census tracts; 58% female). Using multilevel models, we found that approximately 82% of the variance in cognitive health is due to within-tract variation (rather than between-tract variation). Further, self-reported physical activity behavior was related to cognitive health at both the individual- and neighborhood levels, but there were no effects of the built environment (physical activity structures) or behavior-opportunity gaps on cognitive health. Likewise, there were no significant associations for substance use behavior, built environment structures, or behavior-opportunity gaps. We discuss the implications of these results for identifying prevention and intervention targets for promoting healthy cognitive aging in place.

### SESSION 4115 (SYMPOSIUM)

Abstract citation ID: igaf122.1676

### EXPANDING HORIZONS: RE-VIEWING AGING AND THE ARTS

Chair: Justine McGovern
Co-Chair: James Powers
Discussant: Desmond O'Neill

Across four presentations, this symposium explores new horizons in gerontology in terms of content and format. Not only does it examine representations of old age in the arts and the role of engaging in the arts into later life from a research perspective, but also it provides a platform for older artists to discuss their visual practice and introduce their work to new audiences. By blurring the lines between gerontology research and arts practice, the symposium challenges ingrained hierarchies and categories of knowledge production and dissemination, and belonging. As a result, the symposium is in itself an example of innovative gerontology. The presentations encourage the audience to seek creative ways of generating knowledge that combine traditional research, gerontology practice, and the arts. Moreover, including the voices of older artists puts their practice on equal footing with research in knowledge-building processes. Takeaways from the session include a deepened understanding of the impact of arts engagement across the life span, increased awareness of how to generate gerontology knowledge in new ways, and an improved outlook on the possibilities of later life.

Abstract citation ID: igaf122.1677

### VISUALIZING AGING

Justine McGovern, *CUNY--Lehman College, Bronx, New York, United States*

This presentation aims to heighten awareness of ageism and provide alternatives to negative beliefs about older adults by offering a new look at lived experiences of aging. Justine has spent most of her career as a social work practitioner, educator and scholar exploring and expanding visual methods in gerontology practice, education and research. Now in her 60s, her many decades of experience documenting the lives of others in words and images and teaching students and colleagues how to engage with photography in social work practice, have taken a new turn. Borrowing from the humanities, she refers to herself as an imagistic phenomenologist. Her visual practice seeks to capture and communicate the life course of people, places and things, combining art, research, and advocacy to poke holes in deficit narratives about the passage of time and advanced age. Using her own images, Justine will discuss her process in becoming an artist later in life, as well as how to integrate a visual practice in gerontology education, research and practice.

Abstract citation ID: igaf122.1678

### CREATIVE ENGAGEMENT AND COGNITIVE FUNCTION: IMPLICATIONS FOR GERONTOLOGY PRACTICE

Janelle Fassi, *University of Massachusetts Boston, Boston, Massachusetts, United States*

This presentation will discuss scholarly work on 1) the relationship between engagement in creative leisure activities and cognitive functioning among older adults and 2) whether education plays a moderating role in this relationship. Significantly, individuals who demonstrated the highest level of creative engagement (four activities) had the best cognitive functioning. In sum, this research highlights the importance of remaining engaged in creative pursuits in later life and is just one example of the innovative ways to bridge gerontology research and practice. The sample (n = 2,462) consisted of adults aged 65 years and older who completed either the 2016 or 2018 waves of the Leave Behind Questionnaire (LBQ) and the 2015 wave of the Consumption and Activities Mail Survey (CAMS) as part of the Health and Retirement Study. Cognitive function was measured using the Telephone Interview for Cognitive Status (TICS-M) and a new conceptualization of creative engagement was created using four leisure activities from the LBQ and CAMS. Results from linear regression models revealed that engagement in creative activities was associated with better cognitive functioning scores. Education (measured in years) was also positively associated with cognitive function; however, education did not have a moderating effect in the relationship between creative engagement and cognitive function.

Abstract citation ID: igaf122.1679

### VISUAL ART INFLUENCES ON ATTITUDES OF AGING

LaVona Traywick[1], and Erica Fields[2], *1. University of Oklahama, Oklahoma City, Oklahoma, United States, 2. University of Arkansas Division of Agriculture, Little Rock, Arkansas, United States*

The interplay between visual art and perceptions of aging is a powerful yet often overlooked avenue for fostering age-inclusive attitudes. Visual art influences both its creators and viewers, shaping societal narratives about aging. Historically, famous depictions of older adults in visual art have perpetuated stereotypes—some even subconscious—that reinforce negative perspectives on aging. These representations affect how society views older adults, often contributing to ageism. However, intentional shifts on how aging is portrayed can transform these perceptions. This presentation explores how visual art creation can be a catalyst to reshape the narrative of aging, by both professional artists and educational interventions for the public.

The power of visual art to influence societal views of aging will be highlighted through showing historical and contemporary art examples. Additionally, a 4-H youth educational program will be shared that encompasses hands-on participation in visual art for the purpose of gerontological literacy. The youth reimagined their grandparents' talents and skills as superpowers, cultivating a deeper appreciation for the unique contributions of older adults in their lives, as reflected through their artwork. Finally, the session will emphasize the critical role of gerontology professionals and educators in our everyday choice of imagery used in the classroom. Attendees will leave with a new awareness and actionable insights on how visual arts can promote, or hinder, positive perceptions of aging.

Abstract citation ID: igaf122.1680

**I LIKE IT HERE: A LIFE IN FILMMAKING**

Ralph Arlyck, *Columbia Journalism, Germantown, New York, United States*

Ralph Arlyck, age 85, has been making acclaimed and award-winning movies since the mid-60s, when he was a graduate student at San Francisco State. His most recent film, "I Like It Here" explores death and the fear of being left alone. Ralph wanders through his neighborhood and his own past to understand what's happening within his body and brain — and those of the friends he encounters along the way. Topics swing from the mundane to the existential, seeking answers to complex questions, ie what it's like to feel your joints and thoughts stiffen but to be more relaxed about getting ahead or keeping up. The film's title has a double meaning: "here" refers to both the filmmaker's green, rural surroundings and the preciousness of life itself. Ralph will share footage from the film and discuss his perspective on what it means to be an older artist actively engaged in a arts practice that has lasted over 60 years.

### SESSION 4120 (SYMPOSIUM)

Abstract citation ID: igaf122.1681

**NEW DIRECTIONS IN THE INVESTIGATION OF LONELINESS AND SOCIAL CONNECTION ACROSS TIME SCALES AND CONTEXTS**

Chair: Karina Van Bogart
Co-Chair: Eileen Graham
Discussant: Susan Charles

This symposium will focus on novel methodology and approaches to investigating loneliness and social connection across the lifespan and influences on health and aging. Although loneliness has been shown to be a risk factor for poor health outcomes and greater social connection has been linked to better health and well-being, more research is needed on nuances underlying these associations. In keeping with the GSA program theme this year (Innovative Horizons in Gerontology), such work is needed to push the field forward and help elucidate temporal and contextual factors that may be important targets for future interventions that aim to promote social connection and offset related negative health outcomes. This symposium will begin with a brief introduction to the value of the overall topic (Graham), followed by four empirical talks that will highlight different approaches to better understanding social connection in later life. The first talk will present a coordinated analysis examining the link between loneliness and social isolation with cognitive health span across 11 datasets (Yoneda). A second talk will present a coordinated analysis examining bivariate change in social asymmetry (discordance between loneliness and social isolation) and cognitive function across midlife and older adulthood (Van Bogart). The third talk will focus on how trait-like loneliness relates to fluctuations in loneliness and social threat detection in daily life (Shao and Ong). The final talk will focus on a novel conceptualization of social connection dynamics in daily life (Rush). The discussant (Susan Charles) will highlight the importance and innovation of this work.

Abstract citation ID: igaf122.1682

**DOES CHANGE IN SOCIAL ASYMMETRY PREDICT COGNITIVE FUNCTIONING AND DEMENTIA RISK ACROSS ADULTHOOD?**

Karina Van Bogart[1], Emorie Beck[2], Tomiko Yoneda[3], Kathryn Jackson[4], Sijing Shao[5], Anthony Ong[6], and Eileen Graham[4], *1. Northwestern University, Chicago, Illinois, United States, 2. University of California, Davis, Davis, California, United States, 3. University of California Davis, Davis, California, United States, 4. Northwestern University, Evanston, Illinois, United States, 5. FIU, Miami, Florida, United States, 6. Cornell University, Ithaca, New York, United States*

Abundant evidence exists on the separate links between loneliness, social isolation, and cognitive decline. However, little work has investigated the relative contribution of loneliness in relation to social isolation on cognitive decline across the adult lifespan. Understanding how change in this relative contribution (i.e., social asymmetry) relates to cognitive decline and dementia risk may shed light on differences in susceptibility to loneliness and key timepoints across the lifespan for intervention. In the current research, we use coordinated data analysis (CDA) incorporating several existing longitudinal samples across 21 different countries (ages ~18-95) to examine how trajectories of social asymmetry relate to cognitive functioning and dementia risk across the lifespan using growth curve modeling. We examine how change in social asymmetry relates to change in cognitive functioning, and how change in social asymmetry relates to odds of dementia diagnosis at long-term follow-up. Preliminary results from one sample show a significant association between social asymmetry and cognitive functioning at longitudinal follow-up. Additional models will be expanded to assess bivariate change in social asymmetry and cognitive functioning in multiple samples. We will highlight the extent of replicability and generalizability of our findings, and discuss challenges and considerations of using CDA to longitudinally explore the novel index of social asymmetry.

Abstract citation ID: igaf122.1683

**LONELINESS, ISOLATION, COGNITION, AND MORTALITY RISK: HARMONIZATION WITHIN A COORDINATED ANALYSIS FRAMEWORK**

Tomiko Yoneda[1], Kathryn Jackson[2], Katy Bedjeti[2], Graciela Muniz-Terrera[3], Daniel Mroczek[2], Andrew Steptoe[4], Anthony Ong[5], and Eileen Graham[2], *1. University of California Davis, Davis, California, United States, 2. Northwestern University, Evanston, Illinois, United States, 3. Ohio University, Athens, Ohio, United States, 4. University College London, London, England, United Kingdom, 5. Cornell University, Ithaca, New York, United States*

Loneliness and social isolation are associated with several negative health outcomes in older adulthood, including adverse cognitive outcomes and increased mortality risk. Between-study variability in how these constructs are defined and modeled poses a major challenge, potentially contributing to inconsistencies in previous findings. In response, this registered report leveraged a coordinated data analysis framework, applying independent but harmonized operational definitions and statistical models across 11 longitudinal studies of aging ($N > 110{,}000$) representing 20 countries. Using Cox regression, logistic regression, and multistate survival models (MSMs), we systematically examined how loneliness and social isolation—both separately and in combination—are associated with cognitive aging outcomes (no cognitive impairment, mild cognitive impairment, severe cognitive impairment) and mortality risk. Preliminary synthesized results based on random-effects meta-analysis show that loneliness and isolation both predict increased risk of dementia and death in Cox and logistic models (all $ps < 0.001$), with isolation showing a particularly strong link to mortality. In MSMs, however, loneliness (but not isolation) was associated with an increased risk of transitioning from no impairment to mild cognitive impairment. Loneliness and isolation were also associated with an increased risk of transitioning from mild cognitive impairment to death, but this was only true for isolation when loneliness was included in the model. Conversely, isolation appears to be most important for the transition from mild to severe cognitive impairment. Discussion will focus on the evidence for and against the generalizability and variability in associations among social disparities, cognitive outcomes, and mortality risk across individuals, time, and countries.

Abstract citation ID: igaf122.1684

### DOES LONELINESS MODULATE SOCIAL THREAT DETECTION IN DAILY LIFE?

Sijing Shao[1], Emorie Beck[2], Zoe Hawks[3], Karina Van Bogart[4], Eileen Graham[5], and Anthony Ong[6], *1. FIU, Miami, Florida, United States, 2. University of California, Davis, Davis, California, United States, 3. Brooklyn Health, New York, New York, United States, 4. Northwestern University, Chicago, Illinois, United States, 5. Northwestern University, Evanston, Illinois, United States, 6. Cornell University, Ithaca, New York, United States*

Loneliness is theorized to heighten sensitivity to social threats, reinforcing maladaptive behaviors that perpetuate social isolation. This study used experience sampling methods (ESM) to examine how loneliness modulates social threat detection and social behaviors. A sample of 123 participants (46–74 years, $M = 56.02$, $SD = 8.3$, 69.1% female) completed five daily assessments over 20 days. A two-part Dynamic Structural Equation Model revealed small to medium autoregressive effects, suggesting both stability and within-person fluctuations in loneliness and perceived social threat. Concurrent effects showed a reciprocal relationship, where feeling lonely significantly increased the level of perceiving social threat at the same time point (est. effect = 0.335, 95% CrI [0.285, 0.0.386]), and vice versa. Greater fluctuations in loneliness were associated with greater fluctuations in perceived social threat (est. effect = 0.22, 95% CrI [0.14, 0.30]). However, trait loneliness did not significantly moderate these effects. Generalized linear mixed models indicated that within-person loneliness predicted lower self-disclosure (est. effect = -0.382, 95% CrI [-0.495, -0.274]), whereas trait loneliness showed no significant main effect. However, an interaction suggested that highly lonely individuals were less affected by momentary loneliness in their self-disclosure (est. effect = 0.144, 95% CrI [0.053, 0.237]). Interaction partner diversity had no effect on subsequent loneliness. Our findings reveal that loneliness heightens sensitivity to social threat, reducing social engagement and deepening social isolation. Understanding these dynamics can inform interventions targeting maladaptive cognitive and behavioral patterns associated with chronic loneliness.

Abstract citation ID: igaf122.1685

### CONCEPTUALIZING DAILY DYNAMICS OF SOCIAL CONNECTION IN AN ADULT LIFESPAN SAMPLE

Jonathan Rush[1], Eric Cerino[2], Jennifer Piazza[3], David Almeida[4], and Susan Charles[5], *1. University of Victoria, Victoria, British Columbia, Canada, 2. Northern Arizona University, Flagstaff, Arizona, United States, 3. California State University, Fullerton, Fullerton, California, United States, 4. The Pennsylvania State University, University Park, Pennsylvania, United States, 5. University of California, Irvine, Irvine, California, United States*

Social connection is central to many aspects of healthy aging, including increased emotional well-being and a reduced risk of mortality. Existing studies examining the link between social connection and health typically conceptualize social connection as a static, trait-like variable. Yet similar to other daily experiences, feeling socially connected fluctuates from day-to-day, and may provide additional insight into how social connection and health are linked. The present research examines three aspects of daily social connection: one structural (positive interactions) and two related to quality: feeling a sense of belonging; and responsivity (i.e., how sense of belonging varies in response to a positive interaction). Using eight days of daily diary data ($N = 2{,}022$), we examine the psychometric properties and utility of these three aspects of daily social connection. Each day, participants reported whether they had a positive interaction; their sense of belonging; and their closeness to others over the past 24 hours. Multilevel factor analyses revealed that daily feelings of belonging and closeness to others reflect a reliable unified social connection construct at both the within- and between-person levels (between-person factor loadings=.98, .87; within-person loadings=.67, .65, respectively; reliability=.92). Nearly 40% of the variability in daily social connection is within-person. Furthermore, these aspects of daily social connection demonstrated convergent validity to global indicators of social well-being. We discuss the value of utilizing daily social connection measures to effectively capture experiences of social connection within and between individuals, and how they can be used for future research examining the links between social connection and health.

### SESSION 4125 (SYMPOSIUM)

Abstract citation ID: igaf122.1686

### DISCRIMINATION, STRESS, AND HEALTH ACROSS THE LIFE COURSE

Chair: Roland Thorpe
Discussant: Carl Hill

There is a paucity of research that seeks to understand why race disparities in health across the life course remain elusive. Two such explanations that have been garnering attention are stress and discrimination. The symposium includes several papers addressing the effects of discrimination and stress on health disparities across the life course. Cobb used latent class analysis to identify discrimination subgroups among Black men aged 50 and older, finding that those in the Race/Ancestry Discrimination group had significantly lower odds of hospital visits. Thomas Tobin examined the association between everyday discrimination and multimorbidity among Black Americans, revealing that the discrimination-multimorbidity association was strongest among young adults and decreased among middle-aged and older adults. Kim conducted a multi-methods study on food insecurity and food environments, finding that residing in neighborhoods with low-income, low-vehicle access, and higher low-fat milk prices was associated with a greater risk of incident dementia. Qualitatively, food environmental factors relevant to healthy food purchasing and eating—both protective against dementia. Whilby characterized racial discrimination trajectories and identified correlates of the trajectories among Black adults, identifying individuals reporting higher levels of major lifetime experiences of discrimination and greater neighborhood social cohesion were associated with membership in the "moderate" and the "persistently high and increasing" racial discrimination trajectory groups. These findings highlight the variability in the cumulative patterning of racial discrimination and stress among midlife and older Black adults, providing insights into how these factors impact health outcomes in middle to late life.

Abstract citation ID: igaf122.1687

### SILENT BARRIERS: THE HIDDEN COST OF DISCRIMINATION IN BLACK MEN'S HEALTH CARE UTILIZATION

Ryon Cobb, *Rutgers University, New Brunswick, New Jersey, United States*

Background: Everyday discrimination is a well-documented risk factor for poor health outcomes, yet its impact on healthcare utilization among older Black men remains understudied. While discriminatory experiences shape healthcare behaviors, little is known about how distinct patterns of discrimination influence hospital visits. This study employs latent class analysis to identify discrimination subgroups and examines their association with healthcare utilization, addressing a critical gap in minority health research. Methods: Using data from the 2010/2012 Health and Retirement Study, I conducted a latent class analysis to identify discrimination patterns among Black men aged 50 and older ($N = 843$). Negative binomial regression models assessed the association between discrimination classes and hospital visits, adjusting for sociodemographic and health factors. Results: The mean number of hospital visits was slightly under 13. Three distinct discrimination classes emerged: Low Discrimination (35.85%), Race/Ancestry Discrimination (32.4%), and Broad Discrimination (31.75%). Compared to men in the Low Discrimination class, those in the Race/Ancestry Discrimination group had significantly lower odds of hospital visits. No significant differences were observed between the Broad Discrimination and Low Discrimination groups. Implications: Findings highlight the need for healthcare interventions that address multiple forms of discrimination. Providers should implement culturally responsive practices and integrate discrimination screening into clinical care. Future research should investigate the mechanisms through which discrimination shapes healthcare-seeking behaviors, informing targeted interventions to reduce disparities and improve health outcomes for older Black men.

Abstract citation ID: igaf122.1688

### MULTIMORBIDITY AND EVERYDAY DISCRIMINATION AMONG YOUNG, MIDDLE-AGED, AND OLDER BLACK ADULTS

Courtney Thomas Tobin[1], Heather Farmer[2], Angela Gutierrez[3], Xumeng Yan[1], Hans Oh[4], and Roland Thorpe[5], *1. University of California, Los Angeles Fielding School of Public Health, Los Angeles, California, United States, 2. University of Delaware, Newark, Delaware, United States, 3. Western University of Health Sciences, Pomona, California, United States, 4. University of Southern California, Los Angeles, California, United States, 5. Johns Hopkins Bloomberg School of Public Health, Baltimore, Maryland, United States*

Multimorbidity, the presence of two or more chronic conditions, disproportionately affects Black Americans and contributes to racial disparities in disability and mortality. Everyday discrimination is a chronic psychosocial stressor associated with multimorbidity. Although prior research suggests chronic stress accumulates and manifests differently over time, it remains unclear whether the association between discrimination and multimorbidity varies across adult life course age groups. Using data from the Nashville Stress and Health Study ($n = 627$), this study examined the association between everyday discrimination and multimorbidity and whether it varied significantly among young (ages 18-35), middle-aged (ages 36-49), and older (ages 50+) Black Americans. Multimorbidity was based on the sum of 11 chronic physical health conditions, and we used the Everyday Discrimination Scale to assess discrimination exposure. We estimated negative binomial regression models with an interaction term between discrimination and age. Results showed a greater likelihood of multimorbidity among older adults (IRR=3.21, 95% CI=2.64-3.86, $p > 0.001$) and a positive association with everyday discrimination (IRR = 1.03, 95% CI=1.01-1.05, $p < .05$). A significant interaction term ($F(2, 256) = 13.75$, $p < 0.001$) indicated the discrimination-multimorbidity association was strongest among young adults and decreased incrementally among middle-aged and older adults. This suggests discrimination may accelerate multimorbidity among younger adults, whereas its impact diminishes in later life as other factors impact the aging process. These findings underscore the need for early-life interventions to mitigate the health consequences of discrimination and prevent the early onset of multimorbidity among Black Americans.

Abstract citation ID: igaf122.1689

### FOOD INSECURITY, LOCAL FOOD ENVIRONMENTS, AND INCIDENT DEMENTIA AMONG OLDER ADULTS

Boeun Kim[1], Sarah Szanton[2], Suyoung Kwon[3], Wen Liu[4], Laura Samuel[2], Roland Thorpe[2], and Paris Adkins-Jackson[5], *1. University of Iowa, Iowa City, Iowa, United States, 2. Johns Hopkins University, Baltimore, Maryland, United States, 3. George Mason University, Fairfax, Virginia, United States, 4. Penn State University, University Park, Pennsylvania, United States, 5. Mailman School of Public Health, Columbia University, New York, New York, United States*

It is well known that food insecurity contributes to disparities in dementia risk; however, no studies have examined the role of food environments in exacerbating the relationship between food insecurity and dementia risk in older adults. We conducted a multi-methods study to examine the associations of food insecurity and food environments with dementia risk and explore how food environments affect food purchasing and healthy eating. We analyzed data from 6,551 participants aged 50+ without dementia in 2014 from the Health Care and Nutrition Study. We conducted individual interviews with 16 community-dwelling adults aged 50+. Food insecurity was assessed in 2013 using the six-item Short Form US Household Food Security Survey Module. Food environmental factors included the county-level price of low-fat milk, as well as low-income, low-food access, and low-vehicle access at Census-tract-level. Incident dementia from 2016 to 2020 was identified using a validated algorithm. Discrete-time proportional hazard models were fitted, adjusting for age, gender, education, racialized group, survey mode, living arrangement, and urbanicity. Residing in neighborhoods characterized by low-income, low-vehicle access, and higher low-fat milk prices was associated with a greater risk of incident dementia. Our qualitative findings further demonstrated food environmental factors relevant to healthy food purchasing and eating—both protective against dementia—including convenience, portion variety, prices, quality, safety, supply, mobility aids, proximity, and transportation. However, food environment factors did not modify the association between food insecurity and dementia risk. These findings underscore the significant role of both food environments apart from food insecurity in shaping dementia risk.

Abstract citation ID: igaf122.1690

### LONGITUDINAL PATTERNS OF RACIAL DISCRIMINATION IN MIDLIFE AND OLDER BLACK ADULTS AND IMPLICATIONS FOR HEALTHY AGING

Kellee White Whilby[1], Shuo Huang[2], and Bethany Bell[3], *1. University of Maryland College Park, College Park, Maryland, United States, 2. University of Maryland Institute for Health Computing, Baltimore, Maryland, United States, 3. University of Virginia, Charlottesville, Virginia, United States*

Single cross-sectional assessments of racial discrimination may overlook its dynamic, cumulative patterns. Capturing these trajectories could provide deeper insight into age-related health risks and resilience. However, few studies have examined racial discrimination trajectories, especially among midlife and older Black adults. This study characterizes racial discrimination trajectories and identified correlates of the trajectories among a sample of middle-aged and older black adults over a 12-years. Using data from the Health and Retirement Study (2006–2020, N = 1,710, 50+), repeated measures latent profile analysis was employed to identify racial discrimination trajectories which were constructed based on the Everyday Discrimination Scale. Multinomial logistic regression models were used to identify predictors of membership for each racial discrimination trajectory class adjusting for sociodemographic, health behaviors, social and psychological resilience characteristics at baseline. Three racial discrimination trajectories were identified: low-stable (70%), moderate (23%), and persistently high and increasing (7%). Individuals reporting higher levels of major lifetime experiences of discrimination and greater neighborhood social cohesion were associated with membership in the “moderate” and the “persistently high and increasing” racial discrimination trajectory groups. Those reporting positive social support and psychological resilience were less likely to be in the “moderate” or the “persistently high and increasing trajectory” groups. These findings suggest variability in the cumulative patterning of racial discrimination among midlife and older Black adults. Discrimination trajectories may offer more precise estimates of the health consequences of cumulative exposure. Future studies should explore whether membership in specific discrimination trajectory groups confers differential risk for age-related conditions.

### SESSION 4130 (SYMPOSIUM)

Abstract citation ID: igaf122.1691

### RESEARCH TO OPTIMIZE THE ACTION AND IMPACT OF LOCAL AGE-FRIENDLY COMMUNITY EFFORTS

Chair: Mildred Warner
Co-Chair: Natalie Pope
Discussant: Bill Armbruster

This symposium features four empirical studies that explore how local-level age-friendly community (AFC) action and impact are created using a range of methodological approaches to understand the experiences and perspectives of AFC leaders across diverse settings. The first two papers use survey methods, with one focused on examining the role of mobility managers for advancing transportation alternatives as part of AFC progress in Ohio. Results highlight the importance of training and education, regional planning, and inter-organizational collaboration to amplify impact. The second paper presents findings from a survey of municipal leaders across the country regarding planning, the built environment, and services. Findings indicate age-friendly action is stalled at the municipal level, suggesting that more attention to challenges faced in suburban and rural environments is needed. The third paper presents a study based on qualitative interviews with older adults across four states to identify the developmental strengths they leverage to advance local age-friendly implementation. Findings highlight assets such as their long-standing community relationships, personal experiences of aging, and relevant professional skills. The fourth paper showcases a project that engaged AFC initiative leaders across multiple countries to create a novel assessment tool to measure the impact of AFC initiatives in terms of “spillover” or unexpected positive outcomes from local AFC efforts. Discussant remarks will expand on the importance of promoting age-friendly praxis wherein knowledge (production) and practice dynamically advance insights into the contexts, facilitators, processes, and outcomes of local-level age-friendly work to strengthen the movement in the U.S. and worldwide.

Abstract citation ID: igaf122.1692

### MATCHMAKER OF RESOURCES, INFLUENCERS OF CHANGE: OHIO MOBILITY MANAGERS IN ACTION

Holly Dabelko-Schoeny, and Phoebe Allebach, *The Ohio State University, Columbus, Ohio, United States*

Few alternative transportation (AT) options exist in many communities, and older adults have low rates of alternative transportation utilization, which is associated with higher levels of social isolation and depression. Developing and sustaining responsive AT options for older adults is a crucial step in the creation of age-friendly communities. Mobility managers work to improve transportation access for residents in their service areas and may play an important role in supporting AT development. Through a statewide survey of Ohio’s mobility managers (N = 39), we explored the role mobility managers play in supporting AT use and the infrastructure challenges and opportunities they perceive in AT utilization. Mobility managers reported that nearly half (41%) had not worked in transportation before becoming a mobility manager, and 48% did not receive any training on mobility management before starting their positions. Respondents believe their most important job responsibilities were leading advisory groups (63%), attending community meetings to advocate for transportation options (59%), and providing training to individual residents about transportation options (37%). Despite advocating for improved services, respondents perceived significant service barriers to AT use. These included infrequent service and limited hours (52%), insufficient service coverage area (48%), rides needed outside of the county (41%), and transportation services at capacity (41%). The findings highlight policy and practice implications for how AT options and utilization could be improved in Ohio, such as standardized education and training for mobility managers, regional-level transportation planning, and collaboration with local organizations (e.g., area agencies on aging).

Abstract citation ID: igaf122.1693

### PLANNING FOR AGING: ARE WE MAKING PROGRESS?

Mildred Warner[1], and Xue Zhang[2], *1. Cornell University, Ithaca, New York, United States, 2. The Pennsylvania State University, University Park, Pennsylvania, United States*

Did increased recognition of the need to address aging, prompted by the COVID-19 pandemic, and increased funding provided by the American Rescue Plan lead to more age-friendly action at the community level in the U.S.? We present data from two nationally representative cross-sectional surveys we conducted of U.S. municipalities with the International City/County Management Association in 2019 (N = 1290) and 2023 (N = 896). These surveys of city managers measure if U.S. communities include attention to the needs of older adults in their plans, built environment and services. We find only slight improvement in attention to aging in economic development and emergency plans, and no improvement in comprehensive or transportation plans. The built environment is harder to change in the short term, and we find little movement. We also find little improvement in the number of older adult-focused services at the community level. We discuss possible explanations for why municipal attention to aging overall was stagnant, such as the long-time course requisite for implementing municipal changes, societal ageism, and the overall centering of urban communities in the global age-friendly communities framework. We further discuss these findings in light of predominant frameworks for age-friendly communities, championed by high-level organizations such as AARP and the American Planning Association, suggesting that these frameworks have not yet materialized into widespread social change. Future research needs to give more attention to age-friendly initiatives in suburban and rural communities.

Abstract citation ID: igaf122.1694

### OLDER ADULTS' STRENGTHS AS LEADERS OF AGE-FRIENDLY COMMUNITY INITIATIVES

Emily Greenfield, Uri Amir-Koren, and Natalie Pope, *Rutgers University, New Brunswick, New Jersey, United States*

The global age-friendly movement has long positioned older residents as central to age-friendly community (AFC) initiatives at the local level, yet there has been limited empirical exploration and theorizing into the ways older adults are positioned to advance this work in actual practice. To address this gap, our study aimed to understand the strengths older adults bring, from their own perspectives, to their age-friendly involvement. We explored this area using data from qualitative interviews with 23 older adults across four states in the United States. All study participants were in a leadership capacity for their community's age-friendly initiative. Based on iterative rounds of reflexive thematic analysis, findings indicated how older adults leverage a variety of developmental strengths to advance age-friendly efforts at the local level, such as long-standing relationships with key community leaders and volunteers, personal experiences of challenges and opportunities for aging in the context of their communities, and past professional skills of relevance to advancing age-friendly goals and activities. We interpret these findings through transdisciplinary theoretical perspectives from social gerontology and beyond, such as socioemotional selectivity theory and continuity theory. We also discuss these findings in terms of their value in helping to identify and promote more optimal opportunities for older adults' civic contributions through AFC initiatives, as well as how this research helps to address simultaneous attention to concerns about devolution and opportunities for empowerment as part of the AFC movement.

Abstract citation ID: igaf122.1695

### DEVELOPMENT OF A SPILLOVER EFFECT ASSESSMENT TOOL FOR AGE-FRIENDLY COMMUNITIES

Sophia Casale[1], Caitlin Coyle[2], Shayna Gleason[2], and Kathy Black[3], *1. University of Massachusetts Boston, Red Bank, New Jersey, United States, 2. University of Massachusetts Boston, Boston, Massachusetts, United States, 3. University of South Florida, Tampa, Florida, United States*

Efforts to measure the impact of age-friendly initiatives are just beginning to take shape. Knowledge to date suggests that there are unexpected positive outcomes of the age-friendly process that go unreported. Addressing this gap, we present the development of a conceptual framework and corresponding practice assessment tool for identifying, documenting, and disseminating age-friendly 'spillover'. Our framework was developed through the triangulation of multiple data collection methods. A comprehensive inductive review of the age-friendly literature (n = 213); a representative cross-national sample of progress reports (n = 44); and insights from conversations with local, state, regional, and global stakeholders (n = 9) informed the building of the conceptual model and tool creation. Additionally, global pre-testing of the spillover assessment tool was conducted through interviews and feedback questionnaires from an internationally representative sample of age-friendly community country affiliates (n = 23) and additional community-level leaders. Results from this research suggest that although the necessary elements of spillover are alluded to, the full realization of the spillover process as a function of systems change is under-recognized. Pre-testing the tool for assessing spillover in age-friendly community work revealed diverse cultural perspectives on spillover outcomes, but highlighted the ubiquitous nature of spillover as a measurable dimension of impact. This framework and assessment tool development offers cross-national implications for age-friendly practice, systems change policy, and future research and evaluation. The tool demonstrates promise for improving the ability of age-friendly initiatives across international settings with differing resource levels and cultural contexts to assess previously underreported effects that demonstrate systems change.

### SESSION 4135 (PAPER)

### END OF LIFE CARE: DYING WITH DEMENTIA

Abstract citation ID: igaf122.1696

### END-OF-LIFE CARE OF ASSISTED LIVING RESIDENTS WITH ADVANCED DEMENTIA: VIEWS OF INFORMAL CAREGIVERS

Molly Perkins[1], Michael Lepore[2], Mi-Kyung Song[3], Louise Savoye[1], Shermetria Massingale[1], Jeffrey Lentz[1], Nidhi Ramprasad[1], and Alexis Bender[3], *1. Emory University School of Medicine, Atlanta, Georgia, United States, 2. University of Massachusetts Amherst, Amherst, Massachusetts, United States, 3. Emory University, Atlanta, Georgia, United States*

Most persons with dementia in the U.S. die in long-term care communities. Assisted living (AL), the fastest growing long-term care option in the U.S., is a primary provider of this end-of-life (EOL) care. To date, little is known regarding the caregiving experiences of informal caregivers (relatives and friends) who are providing care for the growing population of AL residents with advanced dementia at end of life. Using data from interviews with 25 informal caregivers of recently deceased AL residents with advanced dementia enrolled in a 5-year NIA-funded (RF1AG069114/R01AG069114) longitudinal project, this study begins to address this important knowledge gap. Semi-structured interviews were digitally recorded, transcribed verbatim, and analyzed using thematic analysis. Most participants (mean age = 64) were female (88%), Non-Hispanic White (88%), and had some college education or higher (84%). Most residents (96%) were on hospice at the time of their death. Some participants described the "teamwork" they observed among AL staff and hospice workers as important to the quality of EOL care residents received. Others described a lack of coordination and collaboration, including some conflict, among these care providers as their biggest challenge. A need for more communication between informal caregivers, AL staff, and hospice regarding everyone's role during EOL was noted by some, including concerns about inadequate pain management and insufficient monitoring of vital signs, and other care needs. Findings have important implications for interventions to improve the EOL experience of AL residents with advanced dementia and their care partners.

Abstract citation ID: igaf122.1697

### NURSING HOMES IMPACT HOSPICE CARE FOR CONNECTICUT MEDICAID DECEDENTS WITH SERIOUS ILLNESSES

Ellis Dillon, Chae Man Lee, Doreek Charles, Wenqi Gan, Germine Soliman, and Julie Robison, *University of Connecticut, Farmington, Connecticut, United States*

Hospice care is underutilized in nursing home (NH) populations for unclear reasons. This mixed methods study analyzed Connecticut Medicaid, Medicare, Minimum Data Set, and NH characteristics data; and thematically analyzed 14 in-depth interviews with hospice and NH professionals about hospice care in NHs. The cohort included 39,633 Medicaid-insured decedents with serious illnesses in Connecticut from 2017-2023. Overall, 24,512 (61.9%) individuals had NH stays and 18,428 (46.5%) received hospice care ≤ 6 months of death, 7,265 (39.4%) with short hospice length of stay (≤ 7 days). Individuals with NH stays (versus without) had higher rates of hospice use (49.0% versus 42.4%). However, in multivariable analysis long-term NH stays were associated with reduced odds of hospice use. Among those with NH stays, the odds of receiving hospice care were higher for individuals with stays at NHs that were part of a chain, had an Alzheimer's unit, and had lower CMS quality ratings. Interviews revealed barriers to hospice use including the focus on rehabilitation, policy disincentives, NH workflows/priorities, and staffing: "They miss the fact that someone's declining if it's a slow decline or if there's a lot of different nurses caring for residents." Facilitators include acute care use triggering hospice discussions, hospice benefitting NH patients and staff, and leveraging care planning meetings for hospice education/discussions. Overall, Connecticut Medicaid individuals with NH stays had lower odds of receiving hospice care, but certain NH characteristics, workflows, education, and policies influenced the likelihood of receiving hospice care and could be mechanisms for policy and practice change.

Abstract citation ID: igaf122.1698

### PALLIATIVE CARE FOR COMMUNITY-DWELLING PERSONS LIVING WITH DEMENTIA: A CONCEPT ANALYSIS

Xuefan Ji, *Columbia University, New York, New York, United States*

Background: Palliative care is underutilized by patients with neurological conditions, especially persons living with dementia (PLWD) residing in community settings in the U.S. Palliative care has not been defined for community-dwelling PLWD, and misconceptions that palliative care is only applicable when the patient is dying may have led to underutilization in this population. Aim: To clarify and provide a refined definition of the concept of palliative care for community-dwelling PLWD. Method: A concept analysis using Rodger's method was completed. Three databases were searched for literature that described the antecedents, defining attributes, and consequences of palliative care in the context of community-dwelling PLWD. A model case of the concept was identified from the literature. Results: A total of 16 studies were included. Palliative care differs from hospice and does not require PLWD to be near end of life. Antecedents included acute care use, poor prognosis, and primary care provider referral. Defining attributes encompassed comprehensive care, care coordination, and serious illness communication. Consequences included more home death, less hospitalization and other burdensome acute care use, and patients and caregivers electing less aggressive care option. Discussion: Antecedents of acute care use and poor prognosis may suggest late utilization of palliative care among PLWD and lack of palliative care needs screening. Defining attribute of care coordination suggest primary care providers (PCPs) play a crucial role in coordinating palliative care for PLWD. Future study should investigate the concept of palliative care needs in PLWD and effectiveness of primary care based palliative care models for PLWD

Abstract citation ID: igaf122.1699

### PALLIATIVE CARE NEEDS ASSESSMENT IN NY NURSING HOMES: PSYCHIATRIC SYMPTOMS

Molly Nowels[1], Rose Carlson[1], Mark Unruh[2], M. Carrington Reid[1], Catherine Riffin[1], Manali Saraiya[3], Evan Plys[4], and Daniel Shalev[1], *1. Weill Cornell Medicine, New York, New York, United States, 2. Joan and Sanford I. Weill College of Medicine, Cornell University, New York, New York, United States,*

*3. New York-Presbyterian Hospital, New York, New York, United States, 4. Massachusetts General Hospital, Boston, Massachusetts, United States*

Palliative care is specialized medical care aimed at improving quality of life for individuals with serious illnesses. Serious illness is prevalent among nursing home (NH) residents but integration of palliative care in NHs remains limited. Psychiatric comorbidities predict NH placement among older adults with serious illnesses, so the psychiatric domain of palliative care may be particularly critical in this setting. We conducted a palliative care needs assessment survey of 597 staff across seven NHs in New York. Our survey assessed staff experiences with specific domains of palliative care. Here, we present findings pertaining to the psychiatric components of palliative care: what proportion of respondents' patients would benefit from psychiatric symptom management, the frequency with which respondents addressed psychiatric symptoms, and respondents' comfort managing psychiatric symptoms. Over half of respondents (60.5%) identified psychiatric symptom management needs in at least half of their patients. Nearly half of respondents treated psychiatric symptoms at least weekly (49.2%) and felt comfortable treating psychiatric symptoms (48.7%). Staff with professional palliative care experience were likelier than those without to identify psychiatric needs in their patients (aOR 1.62 [1.07, 2.43]), treat psychiatric symptoms at least weekly (aOR 1.97 [1.29, 3.01]), and report comfort with treating psychiatric symptoms (aOR=1.54 [1.01, 2.35]). Professional experience in palliative care was was associated with increased recognition of psychiatric symptoms, and increased frequency and comfort in managing these symptoms. This suggests that workforce development in palliative care for NH staff may improve capacity in managing challenging psychiatric comorbidities.

Abstract citation ID: igaf122.1700

### SOCIAL DETERMINANTS OF QUALITY OF LIFE IN THE LAST YEAR OF LIFE AMONG OLDER ADULTS: A COMPARATIVE MIXED METHODS STUDY

Raeann LeBlanc, Sangduan Ginggeaw, and Joohyun Chung, *University of Massachusetts Amherst, Amherst, Massachusetts, United States*

Quality of life (QOL) in the last year of life declines among community-dwelling older adults with multimorbidity, with social determinants playing a critical role. However, social capital remains underexamined in nursing research, particularly in end-of-life care. This study explores how social determinants, especially social capital, influence QOL in this population. A convergent mixed-methods design was used. The quantitative component analyzed data from 230 participants in the National Health and Aging Trends Study (NHATS) using linear regression and path analysis to examine social capital's mediating role between social determinants and QOL. The qualitative component involved thematic analysis of semi-structured interviews with seven family caregivers. Findings were integrated through joint displays and meta-inferences. Results showed that QOL in the last year of life was generally low, as was social capital. Mental conditions and social capital were key predictors of QOL, with social capital partially mediating the relationship between mental conditions and QOL. Thematic analysis identified six key themes: financial well-being, access to resources through social networks, supportive relationships, acceptance of death, positive emotions, and manageable physical symptoms. Meta-inferences revealed that mental health and social capital significantly influence QOL. Social capital not only mediates the relationship between mental conditions and QOL but also extends to socioeconomic factors, physical symptoms and disability. Family caregivers, acting as bridging social capital, connect patients to broader support networks, positively impacting QOL and highlighting the need for care strategies that enhance social capital to improve end-of-life QOL for both patients and caregivers.

### SESSION 4140 (SYMPOSIUM)

Abstract citation ID: igaf122.1701

### THE DAILY EXPERIENCES OF HISPANIC AND LATINO DEMENTIA CAREGIVERS: FINDINGS FROM THE NUESTROS DÍAS STUDY

Chair: Frank Puga
Co-Chair: Natashia Bibriescas
Discussant: Krystal Kittle

Hispanic and Latino family caregivers of people living with Alzheimer's disease and related dementias (ADRD) experience high levels of stress, which can negatively impact their mental health and caregiving behaviors. However, little is known about the day-to-day experiences that contribute to these outcomes. The Nuestros Días (Our Days) study, a fully remote daily diary observational cohort study, addresses this gap by examining daily stress, mood, and well-being among Hispanic and Latino ADRD caregivers. This symposium presents an overview of the Nuestros Días Study, key findings, and implications for developing culturally responsive interventions for H&L caregivers. Drs. Bibriescas and Puga will provide an overview of the study's methodology, sample characteristics, and initial findings on daily variations in caregiving-related stress and mental health. Ms. Rodriguez Colmenares will discuss how coping strategies influence daily caregiver mental health, highlighting the link between dysfunctional coping and increased depression, anxiety, and stress. Ms. Mildrum Chana will discuss the relationship between social media use and caregiver well-being, emphasizing the risks of passive social media engagement for mental health. Ms. Poe will present findings on how caregivers' involvement in care-related decision-making influences daily anxiety and depressive symptoms. Finally, Dr. DiFiglia will present qualitative findings on caregivers' daily challenges and the strategies they use to manage stress, offering insights into culturally relevant approaches to stress reduction. Findings from Nuestros Días provide critical insights into the daily experiences of Hispanic and Latino dementia caregivers, offering implications for interventions that enhance well-being and resilience.

Abstract citation ID: igaf122.1702

### EXAMINING DAILY MENTAL HEALTH IN HISPANIC AND LATINO DEMENTIA CAREGIVERS USING AN INTENSIVE LONGITUDINAL DESIGN

Natashia Bibriescas[1], Loreli Alvarez[2], and Frank Puga[1], *1. University of Alabama at Birmingham, Birmingham, Alabama, United States, 2. The University of Alabama at Birmingham, Birmingham, Alabama, United States*

Caregiving for individuals with dementia is a dynamic experience characterized by daily variations in stress and mental health. However, most research relies on cross-sectional data, which may overlook these variations. This is especially true for Hispanic and Latino (H&L) caregivers, who experience disproportionate caregiving burden and stress. This study examined the effectiveness of recruitment strategies, the feasibility of repeated daily assessments, and mental health trajectories using data from the Nuestros Días (Our Days) Study. Participants (n = 180) completed daily diary surveys for 21 consecutive days at baseline, 6 months, and 12 months. The effectiveness of recruitment strategies was assessed using enrollment data, while feasibility was assessed using retention and completion data. Multilevel mixture modeling was used to identify distinct trajectories of depression and anxiety symptoms over time. Digital marketing was the most successful recruitment strategy, accounting for 76.5% of total enrollments. Retention rates were high, with 98.9% of expected participants completing wave one, 84.3% completing wave two, and 84.8% completing wave three. Preliminary mixture model analyses from wave one (n = 178) revealed distinct depression and anxiety symptom trajectories among H&L dementia caregivers. Three patterns emerged for both depression and anxiety symptoms: 1) caregivers with high symptoms that decreased over time, 2) caregivers with high symptoms that increased over time, and 3) caregivers with consistently low symptoms. These findings underscore the heterogeneity in H&L caregiver mental health over time and highlight both the feasibility of intensive daily dairy data collection and its value in capturing the dynamic nature of caregiving experiences.

Abstract citation ID: igaf122.1703

### SOCIAL MEDIA USE AND MENTAL HEALTH AMONG HISPANIC/LATINX DEMENTIA CAREGIVERS

Sofia Mildrum Chana[1], Natashia Bibriescas[2], Andres Azuero[2], Michael Crowe[2], and Frank Puga[2], *1. The University of Alabama at Birmingham, Birmingham, Alabama, United States, 2. University of Alabama at Birmingham, Birmingham, Alabama, United States*

Loneliness and poor mental health are commonly observed among Hispanic & Latinx (H&L) family dementia caregivers given caregiving-related stress, frequent reliance on informal caregiving, and resource underutilization. Given the ubiquitous presence of social networking sites (SNS, i.e., social media), caregivers may turn to these platforms for coping and social support. However, no research has yet evaluated the impact of SNS use on caregivers' mental health. Therefore, this study examines variations in daily SNS use and mental health experiences of H&L caregivers employing daily diary surveys from an ongoing national level study entitled "Nuestros Días (Our Days)." Participants (n = 80) primarily identified as female (85.0%) with an average age of 56.86 years (SD = 9.94) and preferred Spanish language (82.7%). About 68.3% of caregivers had been providing care for three or more years to their biological parent (60.1%) and reported using SNS an average of 152.53 minutes (SD = 69.66) daily. Data were analyzed using multilevel modeling where days were nested in people. Two separate models were conducted where within-person variations in hours of daily passive (i.e., consumption of SNS content without active engagement) and active SNS use (i.e., engagement with content and deliberate interaction with other users) predicted daily depressive and anxiety symptoms. A significant positive association was observed between caregivers' daily passive SNS use and depressive symptoms (B = 0.75, p<.001) and anxiety symptoms (B = 0.66, p<.01). However, there were no significant associations between daily active SNS use and depressive or anxiety symptoms. Findings highlight the detrimental contributions of passive SNS use to caregivers' mental health.

Abstract citation ID: igaf122.1704

### HISPANIC AND LATINO DEMENTIA CAREGIVER COPING AND ASSOCIATIONS WITH DAILY STRESS, DEPRESSION, AND ANXIETY

Nury Rodriguez Colmenares[1], Natashia Bibriescas[2], and Frank Puga[2], *1. The University of Alabama at Birmingham, Birmingham, Alabama, United States, 2. University of Alabama at Birmingham, Birmingham, Alabama, United States*

Hispanic and Latino (H&L) caregivers of people living with dementia experience significant emotional and psychological strain, yet little is known about how their coping strategies influence daily mental health. This study examined coping strategies used by H&L caregivers and associations with daily stress, depression, and anxiety symptoms. Data from 166 participants were analyzed using multiple linear regression. Over 21 days, caregivers completed daily surveys assessing coping strategies (Brief-COPE inventory), depressive and anxiety symptoms (PROMIS Emotional Distress Short Form Depression and Anxiety), and stress related to behavioral symptoms (adapted Neuropsychiatric Inventory-Questionnaire). Most caregivers identified as female (87.3%), with a mean age of 57 years (SD = 9.7), and were adult children caring for a biological parent (62.7%). The depression model was statistically significant, with dysfunctional coping predicting higher depression symptoms ($\beta = 1.06$, SE = 0.09, t = 10.80, $p < 0.001$). Similarly, the anxiety model was significant, with dysfunctional coping predicting higher anxiety symptoms ($\beta = 1.00$, SE = 0.10, t = 9.46, $p < 0.001$). The stress model was also significant with both dysfunctional coping ($\beta = 1.02$, SE = 0.51, t = 1.99, $p < 0.05$) and providing care for someone other than a parent or a spouse ($\beta = 7.27$, SE = 3.27, t = 2.22, $p < 0.05$) predicting higher stress levels. These findings highlight how dysfunctional coping influences caregiver well-being and underscore the need for interventions that promote adaptive coping strategies among H&L dementia caregivers.

Abstract citation ID: igaf122.1705

### QUALITATIVE ANALYSIS OF DAILY DIARY RESPONSE DATA FROM HISPANIC AND LATINO ADRD FAMILY CAREGIVERS

Stephanie DiFiglia[1], Elisha Underwood[2], Sarah Khalidi[2], Loreli Alvarez[2], Michael Crowe[3], and Frank Puga[3], *1. Metropolitan Jewish Health System, Inc. Institute for Innovation in Palliative Care, New York, New York, United States, 2. The University of Alabama at Birmingham, Birmingham, Alabama, United States, 3. University of Alabama at Birmingham, Birmingham, Alabama, United States*

Hispanic and Latino (H&L) family caregivers of individuals living with dementia experience worse mental health outcomes than non-caregiver peers. The Nuestros Dias (Our Days) study examines multi-level factors influencing their daily and distal mental health outcomes using a multi-wave assessment. Eligible H&L caregivers were >18 years who provide unpaid care to a family member living with dementia (assisting with >1 Activity of Daily Living [ADL] or > 2 Instrumental ADLs). The purpose of this study was to examine preliminary themes emerging from participants' qualitative responses to the open-ended question, "Did anything else happen today?". This question was provided in each Wave 1 daily diary survey for 21 days. Participants included 11 caregivers who completed >1 open-ended response via REDCap. From the 231 daily diary surveys sent to the 11 participants, 88 qualitative responses were recorded. Two coders independently coded 88 responses, reaching consensus with a third coder. Participants had an average age of 55.2 years; most were female (n = 10), and half (n = 5) were of Mexican background. Most provided care to a parent (n = 7). Preliminary themes included emotional challenges of providing care (n = 10 participants), engagement/support from family/friends/formal caregivers (n = 5), engagement in wellness/self-care activities (n = 4), and struggles managing dementia-related behaviors (n = 4), as well as positive daily experiences (n = 6). Initial data show that H&L caregivers report both positive and negative caregiving experiences, with unmet needs in managing challenging behaviors and emotional distress. These findings provide early insights into the daily experiences of H&L caregivers that can inform culturally responsive interventions to support mental health.

Abstract citation ID: igaf122.1706

### FINANCIAL DECISION-MAKING INVOLVEMENT AND DAILY ANXIETY AMONG HISPANIC DEMENTIA CAREGIVERS

Abigail Poe[1], Loreli Alvarez[1], Liang Shan[1], Karen Heaton[1], Verna Keith[1], Michael Crowe[2], and Frank Puga[2], *1. The University of Alabama at Birmingham, Birmingham, Alabama, United States, 2. University of Alabama at Birmingham, Birmingham, Alabama, United States*

This study examined the association between financial decision-making involvement (DMI) and daily anxiety symptoms among Hispanic and Latinx (H&L) dementia caregivers of people living with dementia (PLWD). Data were drawn from the ongoing Nuestros Días (Our Days) Study, a multi-wave daily diary study investigating the daily mental health experiences of H&L dementia caregivers. A linear mixed model (LMM) was used to examine the relationship between DMI (Decision-Making Involvement Scale) at baseline and daily anxiety symptoms (PROMIS Short Form Depression and Anxiety measures adapted for daily diaries), adjusting for gender, age, and days. A sample of 95 H&L caregivers was included in the analysis. Most caregivers identified as female (85.9%), with a mean age of 55.69 years (SD = 15.34). Approximately 30.6% were employed full-time. Results indicated that greater involvement in everyday decision-making related to finances (i.e., decisions related to what the PLWD spends their money on) was significantly associated with increased daily anxiety symptoms (B = 1.367, 95% CI [0.267, 2.466], p =.015). Model fit statistics supported the inclusion of a random intercept to account for between-person variability in baseline anxiety (variance = 34.984, SE = 6.785, p < .001). However, overall decision-making involvement across all domains was not significantly associated with daily anxiety (p = .420). These findings suggest that involvement in financial decision-making may increase the risk of experiencing daily anxiety symptoms among H&L dementia caregivers. Future research should examine targeted interventions to alleviate financial-related caregiver stress and assess how other DMI domains may influence mental health.

### SESSION 4145 (SYMPOSIUM)

Abstract citation ID: igaf122.1707

### TRANSFORMING ADULT DAY SERVICES: INNOVATIVE SOLUTIONS FOR CAPTURING AND IMPROVING HEALTH OUTCOMES

Chair: Tina Sadarangani
Co-Chair: Clara Scher
Discussant: Keith Anderson

Adult day services (ADS) are an essential, but overlooked, part of the long-term care continuum, offering structured activities, social interaction, and health services in a community-based setting. However, the absence of uniform regulations has led to a shortage of standardized data, making it challenging to assess the overall health impact of ADS. This symposium summarizes recent research advancing both how care is delivered in ADS and the ways outcomes are measured–both of which are grounded in community partnered and person-centered approaches that elevate the voices of ADS providers and persons with dementia. The first presentation presents the quality-of-life domains most impacted by ADS according to the perspectives of people with dementia and their caregivers across 4 ADS sites in the U.S. The second presentation describes findings from a feasibility and usability study of CareMobi™, a centralized mhealth platform for family caregivers and ADS to exchange information about a person with dementia's day-to-day health. The third presentation showcases preliminary findings on health outcomes and healthcare utilization among 1,000 unique ADS participants from 40 ADS providers across 11 U.S. states from the National Adult Day Services Association's dataset. The fourth presentation summarizes the structures, processes and accomplishments of a learning community developed to implement best practices for dementia-capable ADS as part of a collaboration between 7 ADS sites in California. Discussant remarks will contextualize results with prior research on ADS and highlight implications for future research, policy and practice seeking to amplify the health impact of ADS.

Abstract citation ID: igaf122.1708

### PERSON-CENTERED IMPACT OF ADULT DAY SERVICES FOR PEOPLE LIVING WITH DEMENTIA AND THEIR CAREGIVERS

Clara Scher[1], Tina Sadarangani[2], Shahrzad Siamdoust[2], Keith Anderson[3], and William Zagorski[4], *1. Weill Cornell Medicine, New York, New York, United States, 2. New York University, New York, New York, United States, 3. University of Mississippi, Oxford, Mississippi, United States, 4. American Senior Care Centers, Inc., Nashville, Tennessee, United States*

Researchers and advocates have recognized the untapped potential of adult day services (ADS) for providing person-centered care for people living with dementia (PLWD) and their caregivers. Yet, valid and reliable outcome measures that reflect PLWD's values, preferences, and strengths are needed to advance our understanding of how ADS can enhance quality-of-life for this population. This study sought to address this gap by exploring the quality-of-life domains most impacted by ADS from the perspectives of PLWD and their caregivers. Six focus groups were conducted at five ADS sites with 57 total participants (51 PLWD and 6 caregivers). Located across the United States (Tennessee, New York, California), the ADS sites served a diverse population of PLWD and their caregivers, such as by racialized identity (e.g., Black, Hispanic, Chinese) and socioeconomic status (e.g., low income). Reflexive thematic analysis of focus group transcripts was guided by Kitwood's (1997) conceptual approach to cultivating personhood in dementia care. Results revealed that PLWD and their caregivers perceive ADS as improving quality-of-life by fostering inclusion (e.g., relationships with ADS members and staff), attachment (e.g., feeling safe at the ADS), comfort (e.g., trusting ADS members and staff), occupation (e.g., a sense of purpose by attending the ADS), and identity (e.g., promotion of personhood and culture). Findings have implications for developing person-centered outcome measures to systematically evaluate the impact of ADS on quality-of-life outcomes for PLWD and their caregivers living across the United States and beyond.

Abstract citation ID: igaf122.1709

### FACTORS THAT ENHANCE THE FEASIBILITY OF IMPLEMENTING INNOVATIVE TECHNOLOGY IN RURAL ADULT DAY CENTERS

Tina Sadarangani, *New York University, New York, New York, United States*

Adult Day Services (ADS) play a critical yet underutilized role in chronic disease management and care coordination. However, communication barriers—such as reliance on paper-based systems, voicemail, and non-interoperable electronic health records—impede effective information exchange between ADS, family caregivers, and healthcare providers. This presentation introduces CareMobi™, a user-centered smartphone application designed to bridge these gaps by providing a centralized platform for family caregivers and ADS to track and share health updates, medication adherence, vital signs, and care-related documentation.A mixed methods feasibility and usability study (n = 24) was conducted in two sites (one rural, one urban) to assess CareMobi™'s impact on communication and workflow efficiency within ADS. Preliminary findings indicate that caregivers find the app intuitive and beneficial for monitoring loved ones' well-being, facilitating real-time data sharing with ADS staff, and improving engagement with healthcare providers. Moreover, ADS staff report that CareMobi™ reduces unnecessary phone calls and enhances transparency in care coordination. This presentation will discuss study outcomes, implementation challenges and successes, and the broader implications of integrating digital tools into ADS settings. By leveraging innovative interventions like CareMobi™, ADS can enhance care continuity, caregiver confidence, and overall participant well-being.

Abstract citation ID: igaf122.1710

### PRAGMATIC USE OF STANDARD OUTCOMES MEASURES AND DATA COLLECTION IN ADULT DAY SERVICES

William Zagorski, *American Senior Care Centers, Inc., Nashville, Tennessee, United States*

The National Adult Day Services Association (NADSA) represents the growing Adult Day Services (ADS) industry, with over 5,000 providers nationwide. To advance the quality of care in ADS, NADSA has established standardized outcome measures, published in the Journal of Applied Gerontology in 2018. Through an innovative public-private collaboration, NADSA has since implemented a secure, national data entry portal to systematically track these outcomes. This dataset represents 40 ADS providers across 11 states, with the capacity to scale to 25,000 participants nationwide. Preliminary results from n = 995 participants across 27 ADS sites indicate high acuity among attendees. Of those assessed, 42% were at high fall risk (Hendrich II), 36% required ambulatory assistive devices, and 77% had high nutritional risk (DETERMINE Checklist). Dementia diagnoses were prevalent (78% per Saint Louis University Mental Status-SLUMS), with significant functional dependence—96% being dependent on assistance with instrumental activities of daily living (iADLs) (Lawton), and 65% with activities of daily living (ADLs) (Katz). Additionally, 24% exhibited signs of geriatric depression (Geriatric Depression Screening-GDS-15). This session will provide insight into NADSA's evidence-based measures and the largest ADS-specific outcomes dataset in the nation, including change over time in functional status, and health outcomes (results pending). The data are available to academic and institutional partners to advance research on ADS benefits for participants, caregivers, payers, and communities. Discussion will focus on how to leverage this novel dataset to improve standards of care in ADS.

Abstract citation ID: igaf122.1711

### INNOVATIONS OF A LEARNING COMMUNITY TO OPTIMIZE DEMENTIA-CAPABLE ADULT DAY SERVICES

Orly Tonkikh[1], Jessica Famula[2], Janice Bell[3], Shikha Bhurtel[2], Quynh Vo[2], and Heather Young[3], *1. University of Haifa, Haifa, HaZafon, Israel, 2. University of California, Davis, Davis, California, United States, 3. Betty Irene Moore School Of Nursing at UC Davis, Sacramento, California, United States*

Adult day services (ADS) offer social and healthcare services to enhance quality of life of persons with Alzheimer's disease and related dementias (ADRD) and their family caregivers. Sustainable approaches to standardized dementia-capable ADS are vital. This study describes the structures, processes and accomplishments of a learning community developed to advance and integrate best practices for dementia-capable ADS as part of Cal-COMPASS (California Community Program for Alzheimer's Services and Supports) program, involving seven sites. We collected learning community documentation and conducted 14 focus groups with 16-24 staff and directors from each organization participating in Cal-COMPASS at baseline and upon completion. The data were analyzed using qualitative descriptive methods. To develop an optimal dementia-capable ADS model, five workgroups focused on structural and process-related elements required for effective dementia-capable ADS including staffing and professional training, financing and sustainability, screening and measurement, and caregiver support practices. The learning community developed a comprehensive set of guidelines and resources to promote the standardization and sustainment of dementia-capable services and to make them accessible to the diverse populations of persons with ADRD and their care partners in California. Each workgroup had accomplishments, including recommendations for assessment and care. The participants identified substantial long-term expertise, a wide range of skill, strength, and capacity mix as facilitators of collaborative knowledge sharing and action. Future research could explore how learning communities promote quality of care, as well as long-term adoption and impact of the established practices for persons with ADRD and their caregivers.

### SESSION 4150 (SYMPOSIUM)

Abstract citation ID: igaf122.1712

### CONSIDERING CULTURAL AND CONTEXTUAL RESOURCES IN LONELINESS AND HEALTH STUDIES

Chair: David Camacho
Co-Chair: Naomi Adjei
Discussant: Mélanie Levasseur

Loneliness is a significant public health problem for older adults around the world. Cultural and contextual resources influence the experience of loneliness, and its impact on health outcomes. Yet, there is a dearth of literature that examines the experience of loneliness in non-westernized cultures, rural spaces, or under resourced settings. This symposium brings together gerontological scholars from counseling, occupational therapy, healthcare informatics, and social work to examine loneliness in older adults navigating French-Canadian, Latin American, and US contexts. Researchers will highlight unique cultural (e.g., linguistic, familism, gender) and contextual factors (e.g., rural, transitory housing, nascent geriatric healthcare infrastructure) that may contribute to the presence of loneliness and challenge intervention implementation. Researchers will discuss findings from projects that: 1) examine the relationship of loneliness and smoking behaviors in older adults living in transitory housing in the US; 2) explore physical health, social, and technology factors that individually and jointly enhance or assuage loneliness in older adults living in rural Indiana; 3) examine health factors that facilitate social participation, and barriers and facilitators to implementing socialization interventions to reduce isolation and loneliness in low-income French-Canadian older adults; and 4) synthesize findings on quantitative studies investigating the relationship of loneliness and cognitive health in more than 26,000 Latin American older adults. Together, these presentations acknowledge critical cultural and contextual challenges that influence the impact of loneliness on health. Further, presentations will inform loneliness prevention and treatment efforts and highlight unique strategies to reach and include underrepresented older adults.

Abstract citation ID: igaf122.1713

### LONELINESS AND COGNITIVE FUNCTION IN OLDER ADULTS LIVING IN LATIN AMERICA: A SYSTEMATIC REVIEW

David Camacho[1], Pamela Vega[2], Fernando Wagner[3], Carolina Santamaria-Ulloa[4], Amanda Lehning[5], and Joe Gallo[6], *1. University of Illinois Chicago, Chicago, Illinois, United States, 2. Instituto Nacional de Geriatría, Mexico City, Distrito Federal, Mexico, 3. University of Maryland, Baltimore County, Baltimore, Maryland, United States, 4. University of Costa Rica, San Pedro de Montes de Oca, San Jose, Costa Rica, 5. University of Maryland Baltimore, Baltimore, Maryland, United States, 6. Johns Hopkins University, Baltimore, Maryland, United States*

Extant systematic reviews with samples from high-income countries have found an inverse relationship between loneliness and cognitive function. Considering that cultural and contextual resources influence the experience of loneliness and cognitive health, we conducted a systematic review analyzing quantitative studies exploring the relationship between loneliness and cognitive function in older adults in Latin America. Following PRISMA guidelines, we used five databases (PubMed, PsycInfo, Scopus, LILACS, and SciELO). Inclusion criteria were: a) quantitative research examining the relationship between loneliness and cognitive health, b) descriptions of loneliness and measures of cognitive function, c) English or Spanish language peer-reviewed articles, and d) a sample of older adults in Latin America (≥60 years). We assessed bias using the Risk of Bias Instrument for Cross-Sectional Surveys of Attitudes and Practices. Seven of the 1,887 studies (all cross-sectional) met the inclusion criteria, comprising 26,440 participants from Brazil or Mexico. Most, but not all, found a significant inverse association between loneliness and cognitive function after controlling for salient health and psychosocial factors. Measures and conceptualizations of loneliness and cognitive function, as well as theoretical explanations linking these concepts, varied. Two studies had a high risk of bias. Current evidence suggests a possible cross-sectional association between loneliness and cognitive function in older adults in these countries. Further research is needed to examine the possible bidirectional relationship using representative samples and longitudinal designs; test pathways linking dimensions of loneliness (e.g., chronicity) to cognitive function (e.g., Alzheimer's disease continuum), and explore Latin American diversity (e.g., countries, indigenous peoples, sexual minorities).

Abstract citation ID: igaf122.1714

### EXPLORING THE ROLE OF PLACE IN RURAL OLDER ADULTS' SOCIAL ISOLATION: A LIFE-COURSE PERSPECTIVE

Pallabi Bhowmick[1], and Erik Stolterman Bergqvist[2], *1. University of Illinois Urbana-Champaign, Champaign, Illinois, United States, 2. Indiana University Bloomington, Bloomington, Indiana, United States*

Loneliness among older adults is a public health concern, significantly affecting their mental health and well-being. While technology interventions to foster social connections have been widely studied, the role of place – a specific location or community – remains underexplored. This interview study conducted with 12 older adults, aged 66-85 (M: 75.2; SD: 7.1) applies a life-course perspective to explore how older adults' perceptions of place, shaped by personal histories, social networks, and environments, contribute to feelings of loneliness. Focusing on a rural community in Indiana, USA, the study examines how both physical and social aspects of place interact with aging to either foster or alleviate loneliness. Preliminary findings suggest that rural settings often contribute to loneliness due to geographic distance from neighbors/family and limited access to services. However, the sense of belonging and community found in these rural settings, rooted in long-standing social networks and practices, can reduce loneliness. The study further reveals that older adults in rural areas, particularly those at different life-course stages, may experience loneliness in varying ways. For instance, older individuals who have lived in the community for decades may have stronger social ties, while newcomers or those with fewer local connections may experience more loneliness. By applying a life-course perspective, this research underscores the importance of understanding how aging and place intersect over time. The findings offer insights into how technology can bridge social gaps created by geographic isolation, fostering social engagement and enhancing the quality of life for older adults in rural communities.

Abstract citation ID: igaf122.1715

**LONELINESS, AND SMOKING STATUS: A PILOT STUDY OF EXTENDED-STAY HOTEL RESIDENTS IN THE UNITED STATES**

Naomi Adjei[1], Terri Lewinson[2], Abhirupa Dasgupta[3], and Wambui Onsando[3], *1. Dartmouth College, The Dartmouth Institute for Health Policy & Clinical Practice, New Hampshire, United States, 2. Geisel School of Medicine at Dartmouth, Lebanon, New Hampshire, United States, 3. The Dartmouth Institute for Health Policy and Clinical Practice, Lebanon, New Hampshire, United States*

As the housing industry continues to struggle to meet the growth demands in the United States, more people have resorted to extended-stay hotels (ESH) as a housing alternative. ESH residents may experience complex psychological issues such as loneliness due to the transient nature of their living environment. Research shows a bidirectional relationship between loneliness and smoking status regardless of place. However, previous research found that both ESH residents viewed smoking as a form of community socialization. Furthermore, research asserts that aging and the female gender have a greater association with loneliness. Thus, in this pilot study, we examined the relationship between loneliness, measured by the DeJong Loneliness Score, and smoking status, measured by a yes or no response to smoking in ESH residences. The analysis, based on a sample of 77 adults (age range 23-72), indicated that the overall model did not significantly explain variation in loneliness ($F=1.44$, $p=0.23$). However, a significant interaction effect between smoking status and gender emerged (coefficient = 1.10, $p=0.037$), suggesting that smokers experience greater loneliness regardless of age. Additionally, women who smoked reported feeling less lonely than men, regardless of age differences, suggesting that age does not meaningfully affect the outcome (coefficient = -1.38, $p=0.032$). These findings highlight smoking status, especially with gender, as a key factor in loneliness among ESH residents. Future research with larger, longitudinal samples is needed to further explore the link between loneliness and smoking across the lifespan in this population.

Abstract citation ID: igaf122.1716

**LINKING PEOPLE AND PLACE: FACILITATORS & BARRIERS TO IMPLEMENTING LIFESTYLE REDESIGN IN FRENCH-CANADIAN CONTEXT**

Marie-Hélène Lévesque, and Mélanie Levasseur, *Université de Sherbrooke, Sherbrooke, Quebec, Canada*

Lifestyle Redesign is a well-recognized group-based occupational therapy approach to foster healthy lifestyles and reduce social isolation and loneliness among older adults. Mainly studied for its effects and cost-effectiveness, little is known about what influences its implementation. Drawing on insights from occupational therapists (OTs), OT students and expert-partners, this pre-implementation study explored facilitators and barriers to implementing the French-Canadian version (Remodeler sa vie®). Guided by the Consolidated Framework for Implementation Research, an action research involved 58 purposefully recruited participants, including experienced OTs from public and private sectors, OT students in entrepreneurial stream, and expert-partners with diverse backgrounds (e.g. gerontology, decision-making, and community services). Eleven focus groups were conducted using two semi-structured interview guides led by two OTs trained in qualitative methods. Participants (86.2% women, aged 20 to 72; mean = 43.2, SD=12.5) identified 36 influential factors, with public-sector OTs encountering the most barriers to implementation (17) compared to those in private practice (12) and expert-partners (7). Key enablers were the approach's strong evidence, relevance to aging populations, engagement of key actors, and growing interest in health self-management. Barriers stemmed from approach complexity (e.g., duration), resource constraints (e.g. human), socio-political challenges, implementation demands, and uncertainty around older adults' engagement. Equitable access was a common concern, notably regarding socioeconomic status, geographical location and disability profiles. Findings supported a detailed implementation plan with 7 key axes, 20 objectives, and 34 actions to help Remodeler sa vie reach its full potential in promoting healthy lifestyles and reducing social isolation and loneliness.

Abstract citation ID: igaf122.1717

**SOCIAL PARTICIPATION OF MARGINALIZED OLDER ADULTS: INFLUENCE OF URBAN CONTEXT AND SOCIAL ENVIRONMENT**

Mélanie Levasseur[1], Stéphanie Meynet[1], Mélissa Généreux[1], Shareck Martine[1], Sébastien Lord[2], and Ruth Ndjaboue[1], *1. Université de Sherbrooke, Sherbrooke, Quebec, Canada, 2. Université de Montréal, Montréal, Quebec, Canada*

To optimally promote health, it is essential to better consider the heterogeneity of older adults, especially in a context of a revitalization that can lead to various forms of gentrification and exacerbate the social exclusion of those at risk of marginalization. Using a participatory action research design, this study aimed to describe and explore the influence of three actions to foster the social participation of 20 older adults at risk of marginalization in a revitalizing urban city center. Guided tours of the local library were organized to share information about services and activities aimed at seniors and to help the library develop its tour offerings. Co-designed by, for, and with residents, 5 coffee meetings were held to provide older women living in a downtown low-income housing building with an opportunity to gather, access information, rebuild connections, and engage socially. Finally, in collaboration with the local fine arts museum, 4 artistic mediation workshops were implemented in the same building to increase participants' social participation through various artistic techniques and to enable the museum to establish initial contact with a population that rarely attends its activities. The effects of these actions were explored through questionnaires and focus groups with older adults and stakeholders. The results highlight the importance of considering social dimensions during revitalization. Beyond physical improvements, it is crucial to integrate accessible and sustainable actions tailored to the abilities and needs of older adults at risk of marginalization, fostering a sense of community belonging and social participation.

### SESSION 4155 (SYMPOSIUM)

Abstract citation ID: igaf122.1718

**"I NEED SOMEONE TO TALK TO" - PERSPECTIVES FROM LOW INCOME OLDER ADULTS IN ASSISTED LIVING AND FEASIBILITY PILOT**

Chair: Daniel David

Medicaid-funded assisted living programs (ALPs) provide essential housing and care for low-income older adults in New York City. However, many residents experience significant barriers to serious illness discussions and advance care planning (ACP), including social isolation, mistrust of healthcare providers, and limited access to resources. Findings from 72 qualitative interviews reveal that ALP residents often enter care through medical or social crises, shaping their perspectives on serious illness and end-of-life planning. Many express ambivalence toward ACP due to past healthcare experiences, uncertainty about options, and a lack of trusted discussion partners. However, structured conversations with trusted personnel provide an opportunity to reframe these discussions, empowering residents to make informed decisions aligned with their values. In this symposium we will present qualitative findings from ALP resident perspectives on: 1. Arrival Stories - How did they get to ALP? 2. Addressing Serious Illness Needs 3. Surviving Aging – Finding late-life meaning in ALP 4. Telehealth Ambivalence and Serious Illness Conversations To address these challenges, we developed "Someone to Talk To", a community-driven pilot initiative integrating trained community health workers (CHWs) under nurse supervision to facilitate structured conversations about serious illness. This program builds on five years of collaboration with ALP residents, incorporating insights from a resident-led community advisory board (CAB), staff and assisted living administration. The CAB emphasized the need for a strengths-based approach that prioritizes autonomy, meaning, and proactive decision-making. We will present preliminary findings of a 3 facility serious illness conversation pilot trial.

Abstract citation ID: igaf122.1719

### "SOMEONE TO TALK TO" - AN INTERVENTION SUPPORTING SERIOUS ILLNESS CONVERSATIONS IN MEDICAID-FUNDED ASSISTED LIVING

Daniel David[1], Kimberly Hadson[1], Brianna Morgan[2], Kelseanne Breder[1], and Neha Tungaturthy[1], *1. New York University, New York, New York, United States, 2. New York University Langone Health, New York, New York, United States*

Medicaid-funded assisted living programs (ALPs) provide essential support for under-resourced older adults who wish to age in place. However, these facilities lack structured support for serious illness management and advance care planning (ACP). As a result, residents experience significant health-related risks, including social isolation, unaddressed medical needs, avoidable hospitalizations, and premature nursing home placement. In this presentation we will present the findings from "Someone to Talk To," a community health worker (CHW)-led intervention designed to facilitate serious illness conversations and introduce ACP among ALP residents. Through a five-year partnership with three NYC-based Medicaid-supported ALPs, qualitative research involving 75 low-income older adults and focus groups with staff and administrators major care gaps were identified. A resident-led Community Advisory Board (CAB) informed program development, emphasizing a strengths-based approach that prioritizes autonomy, trust-building, and meaningful engagement. "Someone to Talk To" pairs residents with CHWs under nurse supervision for three structured one-hour sessions addressing medical and social needs. The intervention incorporates the PREPARE for Your Care program, an evidence-based ACP tool designed for low-health-literacy populations. Key outcomes include: 1. Resident engagement in ACP discussions 2. Communication of ACP preferences with care teams 3. Development of a scalable, sustainable model for ACP integration in ALPs This pilot study assesses feasibility and "lessons learned", laying the foundation for a broader implementation strategy to improve ACP engagement among low-income, high-risk older adults in ALPs.

Abstract citation ID: igaf122.1720

### "I HAD NO CHOICE. I HAD TO COME SOMEWHERE" - THE JOURNEY TO ASSISTED LIVING FOR LOW-INCOME OLDER ADULTS

Kimberly Hadson, Neha Tungaturthy, and Daniel David, *New York University, New York, New York, United States*

Assisted living provides support for older adults to age in a home-like environment. Traditionally associated with clients paying for services "out of pocket", New York's Assisted Living Program (ALP) extends access to those who are Medicaid/Medicare dually-eligible. The purpose of this study is to describe the transition experience and "arrival story" of dually-eligible older adults into New York's ALP and identify the influence of social determinants of health (SDOH). Low-income residents (n = 72), largely from underserved racial and ethnic backgrounds residing in three New York City ALPs were interviewed. Narrative thematic analysis is uncovering themes related to social relationships, and access to housing and healthcare. Preliminary results suggest individuals arrive via three trajectories: 1. Well-resourced individuals after a financial set-back 2. Life-long low-income older adults who cannot afford other care 3. Indigent and marginally housed individuals who develop stability through ALP support Many arrive after a medical or social emergency that led to a hospitalization or skilled nursing stay. Interactions between individual health and housing needs, interpersonal relationships and networks, community support, and policy factors lead people to choose ALP placement with some ambivalence. Residents reluctantly view their ALP placement as necessary; however, desire alternative options when few are available. Understanding how and why people enter assisted living and the influence of SDOH at multiple levels will support the implementation of policies and nursing care interventions in these lower resourced environments for healthy aging in a historically marginalized population.

Abstract citation ID: igaf122.1721

### "WHAT GOOD IS THAT?" – PERSPECTIVES OF LOW-INCOME OLDER ADULTS USING TELEHEALTH FOR SERIOUS ILLNESS CONVERSATION

Kelseanne Breder[1], Christine Jacob[2], and Daniel David[1], *1. New York University, New York, New York, United States, 2. Albany Medical College, Albany, New York, United States*

Telehealth offers an avenue for older adults to access providers for serious illness conversations. However, telehealth may be embraced by some and not by others. Urban-dwelling, low-income older adults have unique challenges in accessing continuous care, engaging with the healthcare system and receiving support to address serious illness care needs. To investigate telehealth acceptance, we conducted interviews with 46 residents of 3 Medicaid-funded assisted living facilities which provide social, functional, and clinical support to historically underserved populations. Interview transcripts were analyzed using conventional content analysis. We identified four qualitative themes. 1. Benefit: Telehealth Offers Convenience – Participants highlighted how telehealth facilitates access to healthcare appointments, making it easier to receive care. 2. Barrier: Technology Fluency and Access is Not Universal – Many described physical and technological barriers that limit their ability to use telehealth effectively. 3. Preference: General Distaste for Telehealth – This theme captures participants' overall attitudes and gut reactions to telehealth, ranging from enthusiasm to skepticism. 4. Concern: Mistrust in Clinical Connection – Patients expressed concerns about trust, security, and the ability to build meaningful connections with providers through telehealth. A key finding is that telehealth, which is intended to expand access to care, may diminish trust in the therapeutic relationship between urban-dwelling low-income older adults and providers. We offer guidance to revise screening tools that assess urban-dwelling older adults' comforts and readiness to engage with telehealth technology. We suggest amendments to the Department of Health and Human Services' telehealth guidelines to improve equitable provision of telehealth services.

Abstract citation ID: igaf122.1722

### "BY HOOK OR BY CROOK" - MEANING IN LIFE FOR OLDER ADULTS RESIDING IN MEDICAID-FUNDED ASSISTED LIVING FACILITIES

Brianna Morgan[1], Kelseanne Breder[2], and Daniel David[2], *1. New York University Langone Health, New York, New York, United States, 2. New York University, New York, New York, United States*

Meaning in life (MiL) is the comprehension of one's life and its purpose and is associated with well-being. Wong (2021) characterizes four categories of MiL: Enjoyment, responsible action, purpose, and understanding. Older adults residing in Medicaid-funded assisted living programs (ALP) have unique experiences that may influence how they describe MiL. This qualitative descriptive study aims to characterize MiL for older adults residing in ALP, illuminating their psychosocial needs. We applied a five-phase qualitative data analysis process from Bingham et al., (2023) to semi-structured interviews with older adults. We used trustworthiness methods of co-coding, peer debriefing, and community advisor board review. Older adults described experiences across all four MiL categories with cross-cutting themes: 1. Remaining connected 2. Feeling (in)dependent 3. Engaging with (in)justice 4. Keeping the faith Second, structural and contextual factors, such as systemic racism and ageism, financial and housing instability, and lack of trust and privacy influenced how participants experienced MiL. These findings highlight that older adults residing in ALP may experience structural and contextual challenges, such as inconsistent access to resources. Yet, they have robust MiL experiences characterized by meaningful connections, fierce independence, engagement with justice, and strong faith. These results inform policy and practice in ALF settings. Adapting individual-level meaning-making interventions using participatory co-design methods can incorporate the unique needs of ALP residents. System-level programs should facilitate connection, independence, engagement in justice, and faith while addressing contextual barriers. Overall, engaging older adults with how they experience MiL has the potential to enhance well-being for all.

### SESSION 4160 (SYMPOSIUM)

Abstract citation ID: igaf122.1723

### POLICY SERIES: DEMENTIA CARE POLICY AND PRACTICE: EARLY DETECTION, LIVING ALONE, AND NONPHARMACOLOGICAL INTERVENTIONS

Chair: Michael Lepore
Co-Chair: Tara McMullen
Discussant: Ian Kremer

Dementia is a public health priority with a wide range of implications for policy and practice. Nations, states, and local communities address dementia in many different fields of policy and practice. This symposium builds on a recent thematic issue of Public Policy & Aging Report to address important opportunities and challenges for policy and practice regarding dementia, including arguments for and against early detection, gaps in care for people with dementia who live alone, and measuring outcomes of nonpharmacological interventions. The symposium comprehensively reviews the policy landscape for dementia and by focusing on unique challenges and opportunities related to dementia care, this symposium brings to light more and less successful policy and practice initiatives, highlights emerging issues and concerns, and provides a forum for discussion and policy debate, including discussion of alternative policy options. This symposium will serve as a launchpad for disseminating new knowledge on dementia care policy and practice, facilitating dementia care policy and practice comparisons and collaboration, and shaping the future of policy and practice regarding care for people living with dementia.

Abstract citation ID: igaf122.1724

### A COMPREHENSIVE POLICY LANDSCAPE FOR ALZHEIMER'S AND RELATED DEMENTIAS: PAST, PRESENT, AND FUTURE DIRECTIONS

Lauren Stratton[1], Robert Egge[1], Rachel Conant[1], Jennifer Rosen[1], Matthew Baumgart[1], Carl Hill[2], Lycia Neumann[1], and Katie Evans[3], *1. Alzheimer's Association, Chicago, Illinois, United States, 2. Alzheimer's Association, Chicago, Illinois, United States, 3. Alzheimer's Association, Chicago, Indiana, United States*

The Alzheimer's Association, as the leading voluntary health organization in Alzheimer's care, support, and research, presents a detailed analysis of the evolving policy landscape surrounding Alzheimer's and related dementias (ADRD) in the United States. Federal and state level policies have been pivotal in advancing ADRD research. The passage of the National Alzheimer's Project Act (NAPA) marked a central moment, establishing the first National Plan to Address Alzheimer's Disease and creating the Advisory Council on Alzheimer's Research, Care, and Services, and has since led to subsequent policy achievements. Additionally, this framework has been furthered by three key reauthorization acts: the NAPA Reauthorization Act, the Alzheimer's Accountability and Investment Act, and the BOLD Infrastructure for Alzheimer's Reauthorization Act. These legislative successes have resulted in unprecedented increases in federal research funding and substantial improvements in public health infrastructure. This presentation will explore the current policy implications and future directions through multiple lenses, including research and science, public health initiatives, and care and support advancements. Emphasizing the importance of comprehensive care models and equitable access to resources, strategic initiatives to sustain momentum in ADRD research, risk reduction, diagnosis, and access to treatment will be discussed. These initiatives include increased funding, nationwide awareness campaigns, and standardized care protocols. The session will conclude with a discussion on potential federal and state legislative actions and funding priorities to support ongoing and future efforts in combating ADRD.

Abstract citation ID: igaf122.1725

### BRIDGING THE CARE GAP FOR PEOPLE LIVING ALONE WITH DEMENTIA

Heather Menne, Kate Singer, Emerson McSparran, and Matt Nelson, *Miami University, Oxford, Ohio, United States*

Researchers, practitioners, and policymakers are drawing attention to the experiences of people living alone with dementia.

Research in the US and UK describes the common characteristics and service needs of people who are living alone with dementia. Community-based providers are identifying and serving this often overlooked and complex population, and policymakers in federal and state governments are acknowledging the role of policies and programs to address any gaps in care for people living alone with dementia. Using data from the National Health and Aging Trends Study (NHATS), the Health and Retirement Study (HRS) and the National Survey of Older Americans Act Participants (NSOAAP), this analysis considers the role of home- and community-based services as one mechanism to support the ongoing independent, community-living of people living alone with dementia. Preliminary results indicate that less than 1% of the NHATS and HRS samples of adults aged 65 and older were living alone with dementia; in comparison, 1-6.4% of Older Americans Act (OAA) homemaker, case management, and home-delivered meal clients were living alone with dementia. Our findings demonstrate that community-based services play a role in supporting people living alone with dementia, and this is reinforced with the recent CMS Guiding an Improved Dementia Experience (GUIDE) Model which includes connections to community-based services. Encouragement of CMS GUIDE participating providers to connect older adults to qualified community-based providers and further investment in OAA and other community-based services are crucial steps to address the care and support needs of people living alone with dementia.

Abstract citation ID: igaf122.1726

### POLICY AND PRACTICE IMPLICATIONS OF OUTCOME MEASURES OF NONPHARMACOLOGICAL INTERVENTIONS

Kalisha Bonds Johnson[1], Daniel Siconolfi[2], Heather Menne[3], Nicole Batsch[4], William Zagorski[5], Kate Perepezko[3], Julie Robison[6], and Regina Shih[7], *1. Emory University, Atlanta, Georgia, United States, 2. RAND Corporation, Santa Monica, California, United States, 3. Miami University, Oxford, Ohio, United States, 4. Consultant, Florida, United States, 5. American Senior Care Centers, Inc., Nashville, Tennessee, United States, 6. University of Connecticut, Farmington, Connecticut, United States, 7. Emory University Rollins School of Public Health, Atlanta, Georgia, United States*

Nonpharmacological interventions often measure the wellbeing of persons living with dementia (PLWD) and their family care partners by assessing their quality of life (QOL). These interventions use various outcome measures to evaluate and assess improvement in the QOL of PLWD and their care partners. Quality of life has been operationalized as a multidimensional concept, which makes comparing outcome measures across interventions challenging. In this presentation, we provide an in-depth examination of the outcome measures used in 37 nonpharmacological interventions in a resource identified by the National Alzheimer's and Dementia Resource Center at the request of the Administration for Community Living (ACL), which includes both evidence-based and evidence-informed interventions based on the ACL requirements. We organized the outcome measures from these 37 interventions into four main sectors identified by Lawton's multidimensional concept of QOL —behavioral competence (e.g. activities of daily living and cognitive functioning), perceived QOL (e.g. relationships and health), objective environment (e.g. neighborhood characteristics and social networks), and psychosocial wellbeing (e.g. emotions and mental health). We also highlight who the outcome measures were about as well as who completed the measures in each intervention (e.g., the person living with dementia, the care partner, or both). Findings provide opportunities to showcase how focusing on a more multidimensional approach to measuring the impact of interventions on QOL may reveal challenges and policy ramifications that influence one or both members of the dementia dyad.

Abstract citation ID: igaf122.1727

### BENEFIT OR HARM? ARGUMENTS FOR AND AGAINST EARLY DETECTION OF DEMENTIA IN PRIMARY CARE.

Heather Schickedanz[1], Anna Chodos[2], Jarmin Yeh[2], and Freddi Segal-Gidan, *1. Harbor-UCLA Family Medicine, Harbor City, California, United States, 2. University of California, San Francisco, San Francisco, California, United States, 3. University of Southern California, Los Angeles, California, United States*

There is an ongoing debate over the value of early dementia detection in primary care settings. Routine screening of adults aged 65+ aligns with the Affordable Care Act's Annual Wellness Visit requirements but is in opposition to the 2020 U.S. Preventive Services Task Force's "insufficient evidence", and therefore negative, recommendation for universal screening. Proponents emphasize that over half of dementia cases go undiagnosed with people from racial and ethnic minority backgrounds disproportionately affected. They argue that primary care providers' long-standing relationship with patients positions them well to identify subtle cognitive changes and implement early interventions, such as hearing correction, which may slow cognitive decline regardless of etiology. Early detection enables patients and families to better manage symptoms, access treatments or therapies, and engage in care planning for future needs. Opponents supports the U.S. Preventive Services Task Force's position against routine screening, citing insufficient evidence of benefit. They argue that primary care providers face significant time constraints, and without clear post-diagnosis interventions, screening may be premature. They emphasize fundamental gaps in provider preparedness to identify cognitive impairment and provide support and resources. Opponents contend that widespread screening without robust dementia-capable infrastructure risks causing potential harm to patients and caregivers. This debate illuminates critical tensions between the potential benefits of early detection and healthcare system limitations. A full discussion, however, should extend beyond clinical considerations to encompass broader questions of health equity, workforce development, and system readiness. These complexities necessitate careful consideration of how to ensure the earliest actionable detection of dementia.

### SESSION 4165 (SYMPOSIUM)

Abstract citation ID: igaf122.1728

### LGBTQ+ WELL-BEING IN RURAL US: BARRIERS AND PROMOTION OPPORTUNITIES

Chair: Bryce Van Vleet

LGBTQ+ aging adults across geographies have disproportionately poor well-being outcomes compared to aging non-LGBTQ+ adults. However, rural communities pose additional structural challenges for individuals across the LGBTQ+ spectrum. This mixed-methods symposium highlights burgeoning research on understudied, and often under resourced, LGBTQ+ rural adults. Van Vleet and Fuller introduce LGBTQ+ disparities across queer identities in rural areas and find that social support does not buffer discrimination's effects on mental health. Johnson investigates disproportionate material-need insecurity among diverse, rural Texan LGBTQ+ adults and poses suggestions for investigating LGBTQ+ populations in public datasets. The last two papers investigate barriers to social support and adequate services among LGBTQ+ older adults in rural areas. Perone and Zhou highlight the need for culturally-informed social services and increased need for LGBTQ+ gathering spaces. Bietsch further affirms these findings in rural New England, suggesting LGBTQ+ older adults have disproportionate access to social support and culturally-responsive healthcare. This symposium investigates LGBTQ+ well-being across rural contexts within the United States and offers strategies to promote well-being among adult and aging LGBTQ+ populations. Strategies for researching LGBTQ+ topics and developing interventions in rural areas are also discussed.

Abstract citation ID: igaf122.1729
### DISCRIMINATION AND SOCIAL SUPPORT EXPERIENCES FOR QUEER, RURAL ADULTS

Bryce Van Vleet[1], and Heather Fuller[2], *1. Eastern Mennonite University, Harrisonburg, Virginia, United States, 2. North Dakota State University, Fargo, North Dakota, United States*

Minority stress theory (MST) suggests that queer people exhibit worse health outcomes due to increased discrimination. The following study investigates MST and social support in queer, rural adults (age range=18-60) utilizing the TransPop and Generations combined dataset. Rural adults who self-identified as a sexual or gender minority ($n = 224$) were recruited from across the US. Survey measures included: the Everyday Discrimination Index; mental health (Kessler 6 – Mental Distress); self-rated physical health; and the Multidimensional Scale of Perceived Social Support. ANOVA was used to compare differences in discrimination, health, and social support across three gender categories: cismen, ciswomen, and gender diverse. Only mental health significantly differed by gender ($F(2)=3.44$, $p=.034$, $\eta 2=0.02$) with gender diverse people ($M = 10.63$) indicating significantly worse mental health than sexual minority cisgender men ($M = 7.82$; $p=.029$). As social support is a touted benefit of rural communities, a mediation was conducted using SPSS PROCESS to test MST. Discrimination was found to partially mediate the relationship between four types of social support (general, significant others, family, friends) and mental health, but not physical health, lending support to MST. Finally, moderation analyses investigating buffering effects of social support on the association between discrimination and mental distress were not significant. These findings suggest that queer identities are mostly equally marginalized in rural areas, but that mental health is particularly poor for gender diverse adults. Results affirm MST, but suggest that queer people in rural areas may not benefit from supportive social networks. Thus, queer, rural adults face unique obstacles across the life span.

Abstract citation ID: igaf122.1730
### EXAMINING MATERIAL NEED INSECURITY AMONG LGBTQ+ OLDER ADULTS ACROSS FRONTIER, RURAL & URBAN SOUTH TEXAS

Ian Johnson, *The University of Texas at San Antonio, San Antonio, Texas, United States*

Material-need insecurity—unthreatened access to tangible, basic human needs—is a key social determinant of health. LGBTQ+ individuals experience disproportionately higher rates of such insecurity (e.g., food, housing, medication) across their lifespans compared to cisgender, heterosexual age peers. With 30% of older Texans residing in rural areas, understanding the material needs and protective factors of rural LGBTQ+ older adults is crucial. This presentation examines material-need insecurity among LGBTQ+ adults aged 55 and over in South Texas's 38 counties. Data includes English and Spanish survey responses collected via area agencies on aging and LGBTQ+ community centers, assessing material security across five domains: housing, food, utilities, technology, and prescription medication. Surveys also gathered information on perceived health, diagnosed health conditions, social isolation, and loneliness. Key informant interviews with survey participants and organizational professionals who assisted in survey distribution supplement the survey data. Findings reveal (1) variations in material security across frontier, rural, and urban communities; (2) health conditions as a moderating factor for both insecurity and isolation; (3) the role of social networks in supporting rural aging queer and trans populations; and (4) community-identified solutions to material insecurity, including promotora-model peer-led interventions. The presentation also offers future research ideas using this dataset in conjunction with publicly-available datasets lacking sexual orientation and gender identity measures.

Abstract citation ID: igaf122.1731
### CHALLENGES AND OPPORTUNITIES FOR LGBTQ+ OLDER ADULTS IN RURAL CALIFORNIA: A COMMUNITY-ENGAGED FOCUS GROUP STUDY

Angela Perone, *University of California, Berkeley, Berkeley, California, United States*

Research on rural older adults underscores social, health, and economic disparities (Cohen & Greaney, 2022), which mirror many disparities emerging from research on LGBTQ+ older adults (e.g., Fredriksen-Goldsen & Hoy-Ellis, 2017; Lampe et al., 2024; Rowan et al., 2022). Research on LGBTQ+ older adults in rural communities, however, is sparse, and scholars have called for more research on this population (e.g., Guest, Hunger & Schoenberg, 2023; Dakin, Williams, & MacNamara, 2020; Daken, Levy, & Williams, 2022). This community-engaged study incorporates 23 focus groups with 208 LGBTQ+ older adults in California to examine challenges, thriving and surviving strategies, and recommendations regarding health, housing, social services, and caregiving. Community partners collaborated in refining the focus group protocol, recruitment, and dissemination. Of the 23 focus groups, 17 were inter-categorical (included both rural and non-rural participants) ($n = 164$) and 6 were intra-categorical (included only rural participants) ($n = 44$). Thematic analysis revealed six interrelated challenges regarding health, housing, technology, transportation, caregiving, and community for LGBTQ+ older adults in rural communities. Informal support networks and community resources also played a vital role in wellbeing and social connectedness. Three themes emerged around policy and practice suggestions to enhance the lives of LGBTQ+ older adults in rural communities: improve access to culturally responsive services, increase opportunities for community gathering spaces, and address transportation barriers and service gaps that may disproportionately impact LGBTQ+ older adults in rural communities. These findings underscore important challenges and opportunities for researchers, policymakers, and practitioners on how to best support LGBTQ+ older adults in rural areas.

Abstract citation ID: igaf122.1732

**RURAL LGBTQIA+ AGING IN NEW ENGLAND: DEMAND FOR SERVICE CONNECTION AND SOCIAL SUPPORTS**

Breana Bietsch, *Bridgewater State University, Glastonbury, Connecticut, United States*

In the New England region of the United States, approximately 5% of people aged 18 and over identify as Lesbian, Gay, Bisexual, Transgender, Queer, Intersex, and Asexual (LGBTQIA+) (Conron & Goldberg, 2020), with no statistics specific to LGBTQIA+ people aged 50 and over or who live in a rural area of this region. This demographic information is much needed to better understand the extent of the health needs for rural LGBTQIA+ adults, as rural compared to urban LGBTQIA+ adults often face worse health outcomes, lack access to health care, have limited community supports, experience social isolation, and discrimination (McKay et al., 2024). In the current study, which asked participants in focus groups (n = 33) and individual interviews (n = 15) about their lived experiences regarding what impacts their health, participants echoed these concerns of rural, older LGBTQIA+ adults. Themes of Lack of Services and Attachments and Social Connections showed that rural areas of New England were fundamentally worse off compared to participants in urban settings. For example, rural participants spoke about limited access to affirming LGBTQIA+ services, lack of health care service coordination, and sparse social connection opportunities. Participants suggested solutions for services to be more interconnected, receive attention for innovation, and LGBTQIA+ aging competent. Participants also called for more positive, face-to-face programming that allowed for mentorship within LGBTQIA+ communities, such as intergenerational programs or congregate meals. Implications of the current study point to building programs in rural areas that are more interconnected, affirming, and increase mentorship opportunities to improve health.

### SESSION 4170 (SYMPOSIUM)

Abstract citation ID: igaf122.1733

**INNOVATIVE METHODS IN CHRONIC DISEASE: ADVANCING QUALITATIVE, INTERSECTIONAL, AND COMMUNITY-BASED STRATEGIES**

Chair: Weiyu Mao
Co-Chair: Hanzhang Xu
Discussant: Bei Wu

Despite the growing diversity in older Americans, engaging older adults from underrepresented communities in research remains challenging. This presents opportunities for enhancing diversity within aging research through innovative methodological approaches. To address significant disparities in representation, this symposium collectively investigated innovative methodologies that promote inclusivity, diversity, and engagement involving qualitative, intersectional, and community-based strategies. The first study utilized reflexive thematic analysis of three qualitative studies among Chinese Americans on different health topics. It recognized patterns of participant responses that lacked depth from qualitative interviews and discussed practical strategies and important considerations to amplify diverse participant voices, thereby preventing or reducing potential biases in data collection and analysis. The second study compared methodology and recruitment strategies in a qualitative study and a randomized controlled trial on Chinese and Korean American dementia caregivers. It highlighted the importance of understanding the influences of methodology on recruitment strategies and participant characteristics. The third study employed an intersectionality framework to analyze cardiovascular-kidney-metabolic syndrome stages and associated risks in the All of Us program. Findings from this study demonstrated varied risks by ethnic and racial designation, gender, and disadvantaged area of residence. The fourth study contrasted recruitment strategies of Chinese Americans with chronic conditions through electronic health records versus community engagement. The findings suggested differing participant characteristics by recruitment methods and highlighted the need for complementary approaches to ensure representativeness in clinical research. Together, these studies offered unique insights into advancing innovative research methods and highlighting practical implications on recruitment, engagement, and representation for vulnerable populations.

Abstract citation ID: igaf122.1734

**IMPROVING THE QUALITY OF QUALITATIVE INTERVIEWS THROUGH THE LENS OF HEALTH EQUITY**

Weiyu Mao[1], Jing Wang[2], Yaguang Zheng[3], Yaolin Pei[4], and Bei Wu[5], *1. University of Nevada, Reno, Reno, Nevada, United States, 2. University of New Hampshire, Durham, New Hampshire, United States, 3. New York University, New York, New York, United States, 4. The University of Texas at Austin, Austin, Texas, United States, 5. NYU Shanghai, Shanghai, China (People's Republic)*

The contributions of qualitative research to health equity research are increasingly recognized. There remain challenges in incorporating diverse voices of underserved populations into aging research. Although qualitative interviews are valuable for understanding differences in health behaviors and perspectives, they may not adequately capture the complexities of inequities within marginalized communities. To illustrate, we conducted reflexive thematic analysis of three qualitative studies involving Chinese Americans on topics, such as oral health, diabetes technology use, and advanced care planning. Across the studies, we frequently encountered challenges in eliciting detailed responses, and some participants provided only brief or non-informative responses. This issue persisted despite time, efforts, and methodological considerations, such as training and cultural competence of interviewers, pilot testing of instruments, and use of intentional interview techniques. The lack of depth in participant responses presents a risk of overlooking key insights during data collection and analysis, potentially limiting the identification of underlying factors contributing to health inequities. We recognized the need to amplify the voices of individuals from vulnerable backgrounds, such as low socioeconomic status, rural residence, limited language proficiency, acculturation, and health literacy. This highlights the importance of examining how cultural norms, language preferences, personality traits, and communication styles may shape the nature and depth of participants' responses. This also suggests that inclusive sampling should be enhanced by fostering meaningful engagement with participants. Ensuring data saturation and nuanced understandings of participant diversity requires not only capturing various perspectives but also mitigating interpretative biases that may arise from the underrepresentation of certain subgroups.

Abstract citation ID: igaf122.1735

### COMPARING METHODOLOGY AND RECRUITMENT STRATEGIES IN TWO STUDIES ON CHINESE AND KOREAN AMERICAN DEMENTIA CAREGIVERS

Eunjung Ko[1], Karen Rose[2], Mary Mittelman[3], and Bei Wu[4], *1. New York University, New York, New York, United States, 2. The Ohio State University, The Ohio State University, Ohio, United States, 3. New York University Grossman School of Medicine, New York, New York, United States, 4. NYU Shanghai, Shanghai, China (People's Republic)*

Recruiting individuals from East Asian cultures for research presents unique challenges, which vary based on study methodologies, recruitment strategies, and cultural sensitivities. We compared methodologies and recruitment strategies in two studies targeting Chinese and Korean American dementia caregivers—a completed qualitative study and an ongoing psychosocial randomized controlled trial (RCT). The qualitative study explored participants' help-seeking behaviors for their mental health, while the ongoing year-long RCT is implementing a culturally adapted, validated intervention. Both used purposive sampling and offered participant compensation. However, the former study included first or second-generation foreign-born individuals fluent in English, regardless of age or health risks, whereas the latter is targeting individuals aged 50 or older with one or more cardiometabolic disease risk factors, regardless of language proficiency. In the qualitative study, social media, a research registry, and word-of-mouth were used to recruit 20 participants across the US over eight months. In contrast, community engagement, social service agencies, social media, and word-of-mouth are being used in the RCT; over 170 people living within the New York metropolitan area have been recruited since 2023. These different recruitment strategies appeared to influence participants' characteristics. All participants in the qualitative study had tertiary degrees, with 40% earning over $100,000 annually. However, over a third of RCT participants had a high school education or lower, most reported low levels of English proficiency, and fewer than 8% earned $100,000 or more. This comparison indicates that recruitment strategies and study methodology can influence participant demographics, suggesting the need for tailored recruitment approaches.

Abstract citation ID: igaf122.1736

### CARDIOVASCULAR-KIDNEY-METABOLIC SYNDROME STAGES VIA INTERSECTIONALITY IN THE ALL OF US PROGRAM

Xiang Qi[1], Junyu Sui[2], and Bei Wu[3], *1. New York University, New York, New York, United States, 2. Arizona State University, Nursing and Health Innovation, Pheonix, Arizona, United States, 3. NYU Shanghai, Shanghai, China (People's Republic)*

Cardiovascular-kidney-metabolic (CKM) syndrome, a newly defined, multistage disorder by the AHA, integrates cardiovascular, kidney, and metabolic dysfunction. While disparities in CKM stages across race/ethnicity, and sex are known, an intersectional lens is essential to uncover how these factors, combined with contextual deprivation, influence disease risk. This cross-sectional study analyzed differences in CKM stages among 126,892 adults aged 18-79 from the All of Us Research Program. Exposures included race/ethnicity (White, Black, Hispanic, Asian, Middle Eastern and North African, Other), sex, and contextual deprivation, measured via the Neighborhood Deprivation Index. CKM stages (0-4) were assessed using electronic health records, laboratory tests, and physical measurements, with stages 3-4 indicating advanced disease. Overall, 23.5% of participants had advanced CKM syndrome, specifically: stage 0 (10.0%), stage 1 (20.8%), stage 2 (45.7%), stage 3 (9.5%), and stage 4 (14.0%). Adjusted analyses revealed higher odds of advanced stages among Black (OR 1.97, 95% CI 1.90-2.03), Hispanic (OR 1.72, 95% CI 1.66-1.79), Native Hawaiian or Pacific Islander (OR 2.07, 95% CI 1.55-2.75), and Other race/ethnicity participants (OR 1.40, 95% CI 1.31-1.49) compared to White participants, while Asian and Middle Eastern or North African groups showed similar odds. Males (OR 1.58, 95% CI 1.55-1.62) and individuals in more deprived areas (OR 1.14, 95% CI 1.11-1.18) also faced elevated risk. Intersectional findings highlighted Black or Pacific Islander males in deprived contexts with the highest risk, contrasting with lower risk among Asian or Middle Eastern females in less deprived settings. These disparities emphasize the need for targeted, equity-focused interventions.

Abstract citation ID: igaf122.1737

### RECRUITING CHINESE AMERICANS WITH CHRONIC CONDITIONS THROUGH ELECTRONIC HEALTH RECORDS AND COMMUNITY ENGAGEMENT

Yaguang Zheng[1], Susan Zweig[2], Katharine Lawrence[2], Keer Chen[3], and Bei Wu[4], *1. New York University, New York, New York, United States, 2. NYU Grossman School of Medicine, New York, New York, United States, 3. University of California Health, San Diego, California, United States, 4. NYU Shanghai, Shanghai, China (People's Republic)*

Clinical trials are essential for evaluating the effects of new technologies and treatments on health outcomes; however, varied recruitment approaches may introduce biases that affect the representativeness of study samples, particularly recruiting disproportionately low participation of Asian Americans, including Chinese Americans. This study thus aimed to compare the characteristics of Chinese Americans with chronic conditions recruited through different approaches. In Study I, we recruited 11 Chinese Americans with type 2 diabetes (T2D) via electronic health records, while in Study II, we recruited 11 older Chinese Americans with comorbid T2D and mild cognitive impairment (MCI) through community engagement (e.g., Adult Day Care Center). In Study I, participants' mean age was 55.6 ± 9.5 years (ranging from 42-77), with 72.7% being male, 81.8% employed, 81.8% at least a college education, 100.0% of family income>$50,000, and 81.8% of primary language being English. In contrast, in Study II, participants had a mean age of 74.5 ± 5.2 years (ranging from 67-86), with 63.6% being female, 100% retired, 36.4% at least a college education, 90.9% family income<$30,000, and 100.0% of primary language being Chinese. Our findings indicate that participants recruited via electronic health records tend to have higher socioeconomic status and fewer language barriers than those recruited through community engagement. Complementary approaches are crucial to recruiting a minoritized population in clinical studies to achieve representativeness. Additionally, linguistically sensitive programs, providers, and healthcare systems are essential for community engagement.

### SESSION 4175 (SYMPOSIUM)

Abstract citation ID: igaf122.1738

### AGING AND VISION LOSS: BIOPSYCHOSOCIAL PERSPECTIVES

Chair: Silvia Sörensen
Discussant: Su-I Hou

The risk of severe vision problems increases significantly with age. Older adults with vision impairment are at increased risk for falls and hospitalization; they find it more difficult to take their medications properly, and they are more likely to be socially isolated, placing them at greater risk for physical health problems. In addition, low vision reduces individuals' ability to engage in valued and enjoyable activities, thus increasing their vulnerability to depression and anxiety. Vision rehabilitation that assesses the patients' current abilities and goals and provides rehabilitative training is available to those of working age with legal blindness, but access is more limited to adults 65+ and to those with low vision, but who are not legally blind. Furthermore, emotional adjustment to vision loss is less consistently addressed in rehabilitation contexts, placing the vision-impaired older adult at risk for unrecognized mental health problems. In this interdisciplinary symposium, Donaher will describe the changes in the eye that occur with aging. Sörensen will describe the psychological effects of vision loss in late life, interventions that can ameliorate these effects, and cultural adaptation of such interventions to a highly underserved population, Latino older adults. Chang investigates the potential factors contributing to loneliness among vision impaired older adults. Cai and Powers will report changes among treatment and control groups in dimensions of psychological well-being in a clinical trial of a psychosocial intervention. The discussant will reflect upon the implications of these findings for clinical care and future research needs.

Abstract citation ID: igaf122.1739

### VISION LOSS IN LATE LIFE: AN OVERVIEW

Brooke Donaher, *University of Rochester, Rochester, New York, United States*

Vision loss is not synonymous with aging, but the risk for vision impairment increases with age. By 2050 there will be 1.28 billion older people with avoidable vision impairment (De la Fuente-Núñez et al ., 2023). The purpose of this presentation is to provide an overview of the medical and ophthalmological issues that contribute to vision loss among older adults. The most common causes of vision loss are refractive error and diseases associated with aging (but not necessarily caused by aging), namely cataracts, glaucoma, age-related macular degeneration (the leading cause of blindness in those over 60), and diabetic retinopathy (leading cause of blindness in those 20-74). Refractive error and cataracts can be treated and fully corrected in most cases. Vision loss from glaucoma, macular degeneration and diabetic eye disease can be prevented when identified and treated early. However, these age-related eye diseases cannot be cured and a late diagnosis can result in permanent vision loss. Macular degeneration and diabetic retinopathy are associated with central vision loss or blind spots in the visual field, whereas glaucoma causes loss of peripheral vision. Vision and eye health screenings and comprehensive eye exams for older individuals are recommended every one to two years to reduce the risk for permanent vision loss. Such preventive measures contribute to healthy aging. De la Fuente-Núñez V, Bouzanis K, Joseph S. Connecting healthy ageing and vision: Report summary; 2023.

Abstract citation ID: igaf122.1740

### PSYCHOLOGICAL EFFECTS OF VISION LOSS AND CULTURAL ADAPTATIONS OF RESILIENCE BUILDING INTERVENTIONS

Silvia Sörensen, *University of Rochester Warner School of Education and Human Development, Rochester, New York, United States*

Affecting an estimated 19.8 million Americans aged 40 and older (about 12.6% of the population), late life vision impairment (VI) presents significant challenges to everyday functioning, social interactions, engaging in valued activities, and self-care. Depression occurs in about 1/3 of adults who develop VI. Although Latinos will constitute almost 21% of the US adult population by 2050, they are less likely than other racial/ethnic groups to access new or available treatments and interventions and are thus underserved regarding vision care and vision research. Supportive Interventions for people with VI exist in English but are not available in Spanish; many Latinos are not comfortable discussing health matters in English, so they remain undertreated. In the past 18 months we have translated our 10-week evidence-based Preventive Problem Solving Program for older adults with vision loss into Spanish and have worked with an Advisory Board of older adults to incorporate Latino cultural norms. We are currently testing the adapted intervention called "Mejorar Su Bienestar" with 8 community dwelling older Latinos who will complete all stages of the program and provide feedback. Next steps will be to build community capacity for designing and implementing a clinical trial with this community. In this presentation I will describe the psychological effects of late life VI, how interventions can address the emotional effects of vision loss, and the steps we are taking in adapting the intervention to Latino older adults in our community, with the goal to develop a national model of engagement.

Abstract citation ID: igaf122.1741

### PATHWAYS TO LONELINESS AMONG OLDER ADULTS WITH VISION LOSS: A MACHINE LEARNING STUDY

Ya-Han Chang[1], and Silvia Sörensen[2], *1. University of Rochester, Rochester, New York, United States, 2. University of Rochester Warner School of Education and Human Development, Rochester, New York, United States*

Loneliness is more prevalent among visually impaired than sighted older adults. However, the complex interplay of the factors in shaping loneliness remains unclear. This study applies a machine learning (ML) approach to examine the role multiple variables in predicting loneliness among older adults with age-related macular degeneration (AMD). We used baseline data from adults with AMD ($N = 210$, Mean Age = 80.1) participating in a depression prevention intervention (Sörensen et al., 2015). We implemented a machine learning decision tree approach to explore the if-then conditions leading to loneliness using emotional responses to vision loss (ERVL), legal blindness, psychological conditions, satisfaction with social life, future planning, optimism, and sociodemographic variables as potential predictors. All variables' variance inflation factor scores were below 5, indicating a low risk of multicollinearity. Decision tree models with the strongest predictive performance (Train AUC =.88, Test AUC = .78) suggested that legal blindness, ERVL, social life satisfaction, anxiety, depression, and optimism strongly predicted loneliness. Decision trees capture combinations of variables that determine pathways that increase or decrease the risk for loneliness. For example, if social life satisfaction and optimism are low, then the loneliness classification is high (above mean, 24% of cases). If social satisfaction is high and the person is legally blind, then loneliness is low (below mean, 9% of cases). These models effectively capture the relationships between variable combinations that influence loneliness. Decision tree models extend beyond regression to show combinations of variables that describe different if-then pathways leading to loneliness.

Abstract citation ID: igaf122.1742

### GROWTH TRAJECTORIES IN PSYCHOLOGICAL WELL-BEING: A CLINICAL TRIAL FOR OLDER ADULTS WITH MACULAR DEGENERATION

Piaopiao Cai[1], Timothy Powers[1], and Silvia Sörensen[2], *1. University of Rochester, Rochester, New York, United States, 2. University of Rochester Warner School of Education and Human Development, Rochester, New York, United States*

Age-related macular degeneration (AMD) affects over 20% of aging populations (Lim et al., 2012). AMD is a significant cause of visual impairment and severe vision loss (Mitchell et al., 2018), affecting around 37 million Americans aged 50 and above (Pelletier et al., 2016). Our study aims to understand growth trajectories of psychological well-being (autonomy, environmental mastery, personal growth, positive relationships, and self-acceptance), utilizing a five-wave clinical trial study (Sörensen et al., 2015), that randomized participants into two groups: Treatment (Preventive Problem-Solving Intervention (PREPSI, n = 90)); Social Contact Control ("Life and Health Review Intervention" (LIHRI, n = 90)). Mixed-model ANOVAs showed a significant decline in perceptions of autonomy, environmental mastery, purpose in life, and self-acceptance after five waves (in both Intention to Treat (ITT) and Completed Case (CC) analysis), but stability in positive relationships with others and a marginally significant quadratic effect for personal growth in the CC analysis. Treatment and control groups differed significantly in trajectories of environmental mastery and self-acceptance; PREPSI respondents had significantly higher scores on these two outcomes after wave 4, while those in the control group exhibited a marked decrease. The minimum and maximum turning points of these trajectories were identical in CC analysis. Additionally, the strong variation and significant cubic change in the trajectories of purpose in life for both groups indicate that the PREPSI did not significantly enhance the purpose in life trajectory. The steady decline in wellbeing among people with vision loss and underlying individual and contextual influences require further investigation to guide future intervention development.

### SESSION 4180 (PAPER)

### NEW TECHOLOGIES FOR HEALTH AND CARING: NOTHING ARTIFICIAL ABOUT CARE

Abstract citation ID: igaf122.1743

### CONVERSATIONAL ANALYSES OF OLDER ADULTS' SOCIAL ENGAGEMENT WITH AI-DRIVEN SOCIALLY ASSISTIVE ROBOTS

Othelia Lee[1], Narae Choi[2], and Do-Hyung Park[2], *1. The University of North Carolina at Charlotte, Charlotte, North Carolina, United States, 2. Kookmin University, Seoul, Republic of Korea*

Objectives: The digital divide and limited digital literacy hinder technology adoption among older adults in low-resource communities. This study examines how socially assistive robots (SARs) can facilitate social engagement and emotional well-being among older adults by analyzing conversational interactions with the AI-driven Hyodol SAR. Methods: Multimodal data—including log-based usage patterns and conversational voice data—were collected through SAR-embedded sensors. Pre- and post-surveys gathered information on demographics and health status. Human-robot conversations were categorized into nine emotional and topical categories and six types of activity participation. To explore user engagement patterns, K-means clustering was applied to identify distinct user personas. Results: Among participants, 44.6% engaged in conversations with the SARs, with 30.2% discussing their activity participation. Three personas emerged: Social Butterflies (n = 19, 28.35%) maintained balanced engagement in social and personal activities, with positive emotional exchanges but limited long-term impact on well-being. Lone Wolves (n = 28, 41.79%) had low social engagement, yet showed notable improvements in emotional well-being through conversational interactions with the SAR. Emotional Peacocks (n = 20, 29.85%) displayed high emotional and sensory engagement with the SAR, demonstrating the greatest reduction in loneliness among the three groups. Discussion: Findings suggest that SARs helped mitigate social isolation, especially among older adults with limited social engagement. Furthermore, the integration of large language models with SAR technology enables autonomous, dialogue-based AI companions delivering personalized, emotion-sensitive interactions. Future research should explore adaptive AI learning models, ethical considerations in caregiving, and the long-term effects of SAR-facilitated engagement on well-being.

Abstract citation ID: igaf122.1744

### EVALUATING THE CULTURAL RELEVANCE OF AI THERAPIST RESPONSES FOR CHINESE AMERICAN CAREGIVERS OF OLDER ADULTS

Yanjing Liang[1], Jingyi Li[2], Serena Jinchen Xie[1], Shumenghui Zhai[3], Xuehong Fan[1], and Weichao Yuwen[2], *1. University of Washington, Seattle, Washington, United States, 2. University of Washington Tacoma, Tacoma, Washington, United States, 3. Pacific Lutheran University, Seattle, Washington, United States*

Chinese American caregivers underuse professional mental health services despite the high demand for caregiving support. A key barrier is the lack of culturally and linguistically appropriate programs. Large Language Model (LLM)-based AI chatbots offer potential mental health support, but many lack cultural adaptation, limiting effectiveness and acceptance. Advances in Natural Language Processing (NLP) provide opportunities for cost-effective cultural adaptations. This study evaluates the cultural relevance of LLM-generated responses with (WCC) and without (NCC) cultural context. We previously developed a chatbot to support Chinese American caregivers for their self-care. Community partners created culturally rich client inputs, which we used to generate chatbot's responses. The research team finalized 40 prompts, which were used to generate responses via GPT-4o. Chinese American caregivers evaluated these responses using the Cultural Competency Measure (CCM), Empathy Scale, and Cultural Relevance Questionnaire (CRQ) via Qualtrics. Data from 36 participants (collected until March 11, 2025) were analyzed using descriptive statistics and the Wilcoxon signed-rank test. Results showed that WCC responses had significantly higher CCM, empathy, and CRQ median scores than NCC ($p < 0.05$). These findings suggest that GPT-4o, when combined with cultural context prompts, generates more culturally appropriate responses. This study highlights the importance of culturally adapted AI-driven interventions in addressing mental health disparities among Chinese American caregivers, offering an accessible solution to enhance caregiver support. By demonstrating the effectiveness of culturally contextualized LLM responses, these findings underscore the potential for integrating AI tools into gerontological nursing to improve caregiver well-being and, ultimately, the quality-caring for older adults.

Abstract citation ID: igaf122.1745

**HOW DO DIGITAL INCLUSIVE PROGRAMS PROMOTE HEALTHY AGING: THE CASE OF HONG KONG**

Bobo Hi Po Lau[1], Alex Kin-shing Chan[1], Gigi Lam[1], Alex Chi-keung Chan[2], Rick Yiu-cho Kwan[2], Daniel Dick-man Leung[2], Alex Pak-ki Kwok[3], and Fan Lui[1], *1. Hong Kong Shue Yan University, Hong Kong, China (People's Republic), 2. Tung Wah College, Tsim Sha Tsui, Hong Kong, China (People's Republic), 3. The Chinese University of Hong Kong, Hong Kong, China (People's Republic)*

Metropolises worldwide have adopted community-based support for older adults to foster digital inclusiveness, thereby facilitating their well-being and societal engagement. Hong Kong ranks seventh on the IMD Digital Competitiveness Index in 2024. The government has been funding community-based digital support targeting older adults since early 2010. This study content-analysed 39 digital inclusiveness programs for older adults between 2013 and 2025 under five key government schemes. We coded their content by the digital divide levels, social and intermediary determinants of health, and aspects of healthy aging they affected. Our findings show that 20.5% of the programs enhanced material access through renting or lending smart devices to older adults; 59.0% facilitated device operation skills, 84.6% taught strategic skills (e.g., telehealth, cognitive training), and 35.9% exposed older adults to devices beyond smartphones including wearable health devices and aerial cameras. These programs fostered health management (20.5%) and social service utilization (15.4%), thereby enhanced healthy aging domains including health and cognition (33.3%), psychological and spiritual well-being (25.6%), and community engagement (17.9%). Another survey of our team with 491 participants aged 60 or older found older adults use smart technologies for only 3.1 life domains ('communication' (82.7%), 'economics' (13.2%), and 'daily practical' (12.2%)); usage variety is constrained by their age and education. As device ownership among older adults had peaked in most metropolises post-pandemic, the digital divide shifted to be one related to the extensiveness and flexibility of usage. Our findings will illuminate areas of technology usage that require additional input for enhancing digital inclusiveness.

Abstract citation ID: igaf122.1746

**LAUNCHING THE ACL INNOVATION LAB: DEMONSTRATING A CORE COMPONENTS APPROACH FOR REDUCING OLDER ADULT FALLS**

Emily Nabors[1], Liz Noble[2], and Matthew Smith[3], *1. National Council on Aging, Arlington, Virginia, United States, 2. Mission Measurement, Chicago, Illinois, United States, 3. Texas A&M University, College Station, Texas, United States*

Falls among older adults are common, and while evidence-based falls prevention programs are available, they can be challenging for organizations with limited capacity to offer and sustain, and they may not resonate with all populations. To address these challenges, the Administration for Community Living (ACL) funded the National Council on Aging to become the ACL Innovation Lab. In partnership with Impact Genome and several academic institutions, the Lab developed the first research-based falls prevention taxonomy to identify core components of interventions that address falls risk factors among older adults. A core components approach allows practitioners to adapt interventions to fit different populations or settings by integrating components that have shown relationships to positive outcomes in research. The Lab conducted a literature review on falls prevention interventions and coded the literature to draft the falls prevention taxonomy and its four frameworks of core intervention components, intended recipients, contextual elements, and outcomes. After applying the frameworks to the evidence base to build a coded and structured dataset, the Lab conducted a taxonomic meta-synthesis to identify core components based on their relationship to falls prevention outcomes. This session will define a core components approach and how this methodology can be applied to other aspects of gerontology. Presenters will introduce the core components approach and falls prevention taxonomy; describe how selected core components are being piloted in community-based settings to test the feasibility, flexibility, acceptability, and sustainability of this approach to reducing falls among older adults; and outline the research questions guiding this work.

### SESSION 4185 (PAPER)

### FAMILY AND INTERGENERATIONAL RELATIONSHIPS IN AGING: IMPLICATIONS FOR MENTAL HEALTH AND PSYCHOSOCIAL WELL-BEING

Abstract citation ID: igaf122.1747

**"IT TOOK ME 61 YEARS TO FIND WHAT I REALLY LOVE": OLDER ADULT VOLUNTEERS' ENGAGEMENT IN SCHOOLS**

Youjung Lee[1], Laura Bronstein[1], and Francie Keefe[2], *1. Binghamton University, Binghamton, New York, United States, 2. Broome County Office for Aging, Binghamton, New York, United States*

Older adult volunteers in schools benefit multiple generations, including older adults themselves. Given multiple positive impacts of classroom volunteering with youth in one's later years, it is important to explore older adult volunteers' experiences in schools. Using a phenomenological qualitative approach, 30 in-depth interviews with older adult classroom volunteers in New York State were conducted (20 in 2019 and 10 in 2024). The interviewees were recruited from the Foster Grandparent Program in the AmeriCorps Seniors, where older adults provide support and mentorship to children. The 2024 interviews included additional questions about volunteers' experiences related to the pandemic. Interviewees included three African Americans, one Asian, and 16 White older adults (mean age: 63). Two volunteers were male. The qualitative data analyzed using a thematic analysis revealed: (1) Intergenerational engagement benefits youth and their teachers; (2) Older adult volunteers' benefit from their social engagement in schools; and (3) The volunteers' proposal for enhancing the Foster Grandparent Program. Findings from this study disclose that the older adult classroom volunteering offers multi-layered engagement: intergenerational engagement for children, older adults, and school professionals, as well as social engagement for older adults from having a new role and identity in later years. The older adult volunteers' desire for social engagement were exemplified by their eagerness to return to schools during the pandemic. In an aging society, schools can play an innovative role in supporting multiple generations, including older adults, which can be understood from a social capital building perspective.

Abstract citation ID: igaf122.1748

### AN ATTACHMENT-BASED SUPPORT IN ADULT CHILD-PARENT DYADS AND PARENTAL MENTAL HEALTH: A RESPONSE SURFACE ANALYSIS

Ella Carasso[1], Dikla Segel-Karpas[2], Shira Barzilay[2], and Roi Estlein[2], *1. University of Haifa, Cambridge, Massachusetts, United States, 2. University of Haifa, Haifa, HaZafon, Israel*

Intergenerational relationship between older parents and adult children offers a crucial framework for support and social belonging in older age. However, support dynamics that do not align with individual expectations and needs might have harmful effects on their mental health, causing feelings of burdensomeness and loneliness, considered significant risk factors for suicide among older adults. This study employed a dyadic attachment perspective to examine the dynamics of support exchanged between adult children and their parents, focusing on two attachment-based support forms: secure base and safe haven. We aimed to explore how the (dis)similarity in the provision of these support types between parents and adult children were associated with self-perceived burdensomeness and loneliness in older parents. A total of 129 adult child-parent dyads (parents' age: M = 69.63; adult children's age: M = 42.02) were recruited. We employed Structural Equation Modeling (SEM) and Response Surface Analysis (RSA) as our analytical methods. The findings show that parents experienced lower perceived-burdensomeness and loneliness when they and their children were more similar in providing both a secure base and a safe haven for each other. These results indicate that parents' ability to rely on their children for a secure base and a safe haven promotes their mental health, when they were also able to reciprocate similar support. This emphasizes the meaningful role that parents ascribe to continuous involvement in their child's life and the provision of support into adulthood, as well as the potential contribution of this involvement to older adults' health.

Abstract citation ID: igaf122.1749

### ATTACHMENT-BASED SUPPORT AND DEPRESSION IN ADULT CHILD-PARENT DYADS: THE ROLE OF SECURE BASE AND SAFE HAVEN

Ella Carasso[1], Dikla Segel-Karpas[2], Shira Barzilay[2], and Roi Estlein[2], *1. University of Haifa, Cambridge, Massachusetts, United States, 2. University of Haifa, Haifa, HaZafon, Israel*

The adult child-parent relationship is a key source of mutual support, promoting psychological well-being in both generations. This study examined the dynamics of support between adult children and their parents through an attachment framework, focusing on how two forms of attachment-based social support, secure base and safe haven, are associated with depressive symptoms in both generations. A total of 129 adult child-parent dyads (parents' age: M = 69.63; adult children's age: M = 42.02) participated. Multilevel modeling (MLM) was used to assess associations between depressive symptoms and attachment-based support dynamics. Results showed that parents' provision of a safe haven and secure base was negatively associated with their own depressive symptoms, but not with their children's. Adult children's provision of a secure base, but not a safe haven, was negatively associated with their own depressive symptoms. These findings suggest that providing a secure base and safe haven may help parents preserve their parental role, enhancing resilience in older age and mitigating depression. Moreover, adult children's provision of a secure base to aging parents may support their mental health by balancing the support of their parents' autonomy with their own life goals.

Abstract citation ID: igaf122.1750

### CROSS-NATIONAL COMPARISONS OF INTERGENERATIONAL RELATIONS BETWEEN OLDER PARENTS AND ADULT CHILDREN

BoRin Kim[1], and Ke Li[2], *1. University of New Hampshire, University of New Hampshire, New Hampshire, United States, 2. Ohio University, Athens, Ohio, United States*

This study aims to identify and compare various patterns of intergenerational relations as well as to examine their associations with depressive symptoms among older adults across three countries, which have different family cultures and policies. Guided by the intergenerational solidarity framework, the present study explored the multidimensional portrayal of intergenerational relations including structural, associational, functional, and normative solidarity. Data came from the 2018 harmonized datasets of the Gateway to Global Aging from China, South Korea, and the United States. The sample was restricted to respondents over 65 years with at least one child, and one respondent per household was selected (China N = 4,368; South Korea N = 3,093; the US N = 2,153). Latent Profile Analysis was used to identify patterns of intergenerational relations with nine variables including the number of sons and daughters, distance to the geographically closest child, contact frequency (face-to-face and remote), and physical and financial help from/to children. OLS regressions were used to analyze the associations between intergenerational relations and depressive symptoms among older parents. Five clusters were identified as the most optimal classification: Disconnected, Distant-Interacted, Interdependent, Close-Helping, and Coresiding-Dependent Parents. The proportions and the characteristics of each intergenerational profile significantly differed across countries. The associations between clusters and depressive symptoms also varied. This study confirmed that intergenerational relations are influenced by the interplay between micro-level individual and family attributes as well as macro-structural policies and cultures. Our findings offer valuable insights into cultural norms, societal structures, and policy implications related to family dynamics and aging.

Abstract citation ID: igaf122.1751

### INTERGENERATIONAL RELATION TRAJECTORIES IN MULTI-CHILD FAMILIES AND DEPRESSIVE SYMPTOMS OF OLDER PARENTS IN CHINA

Chang Yu[1], Jiaowei Gong[2], and Emma Zang[3], *1. University at Buffalo, SUNY, Getzville, New York, United States, 2. Harvard University, Cambridge, Massachusetts, United States, 3. Yale University, New Haven, Connecticut, United States*

Objectives: While close intergenerational relationships are linked to fewer depressive symptoms in older parents, less is known about how these relationships evolve over time, how different aspects of these relationships (e.g., emotional closeness, economic and housework support, frequency of contact) function together, and how relationships with multiple children in a family shape mental health. This study examines trajectories of intergenerational relationships in multi-child families and their associations with depressive symptoms among older Chinese parents across socioeconomic (SES) groups. Methods: Using nationally representative panel data from the China Longitudinal Aging Social Survey (2014–2020, N = 1,637), we applied group-based trajectory modeling to track changes in intergenerational relationships over six years, considering overall, best, and worst child-parent relationships within families. Linear regressions assessed how these relationship trajectories relate to depressive symptoms among adults aged 60+, with attention to differences by SES (education, income, hukou). Results: Across average, best, and worst relationships, we identified four trajectories: persistently strained, slight decline with sustained positivity, steadily strengthening, and rapidly strengthening. Compared to persistently strained relationships, steadily and rapidly strengthening relationships were associated with fewer depressive symptoms, particularly for low-SES parents. The worst relationship trajectory best predicted depressive symptoms, while the average trajectory lost significance when controlling for best and worst relationships. Discussion: Findings underscore the mental health impact of relationship dynamics, especially for low-SES parents, highlighting the need for government-supported caregiving programs in China.

Abstract citation ID: igaf122.1752

### RACE DIFFERENCES IN PATTERNS AND CONSEQUENCES OF MOTHERS' ADVICE TO MIDLIFE CHILDREN

Robert Frase[1], J. Jill Suitor[2], Megan Gilligan[3], Destiny Ogle[2], Ranran He[2], and Di Wang[2], *1. Southern Illinois University, Carbondale, Illinois, United States, 2. Purdue University, West Lafayette, Indiana, United States, 3. University of Missouri, Columbia, Missouri, United States*

Although advice is often conceptualized as a positive dimension of intergenerational support, a growing body of research has examined the potentially adverse effects of advice from older parents on adult children's well-being. However, no attention has been given to exploring race differences in this association. Drawing from theories of intergenerational solidarity and using data from 670 midlife children nested in 276 later-life families who participated in wave 2 of the Within-Family Differences Study, we extend this scholarship by examining whether the association between advice from mothers and adult children's depressive symptoms differs for Black and White adult children. On one hand, advice is a potential source of interpersonal tension, and some research indicates that negative facets of family relationships (e.g., arguments, disappointment) have stronger adverse effects on the well-being of Black than White adults, suggesting that the association between receiving advice from mothers and adult children's depressive symptoms would be amplified among Black adults. On the other hand, research indicating higher levels of intergenerational solidarity and more frequent exchanges of support in Black than White families would suggest that Black adult children would be more accepting of advice from mothers, attenuating the effects of advice on Black children's well-being. Findings from multilevel models revealed that the association between receiving advice from older mothers and depressive symptoms was stronger among Black than White midlife children. Our results both augment existing scholarship on the potentially harmful effects of advice on well-being and underscore the importance of considering social-structural characteristics in this research.

### SESSION 4190 (SYMPOSIUM)

Abstract citation ID: igaf122.1753

### INNOVATIVE STRATEGIES FOR OPTIMAL AGING: FUNCTIONAL AND BRAIN HEALTH RESEARCH THROUGH COMMUNITY-BASED APPROACHES

Chair: Patricia Heyn
Co-Chair: Catherine Diaz-Asper
Discussant: J Taylor Harden

The Marymount Center for Optimal Aging (MCOA) is committed to advancing inclusive, interdisciplinary aging research that informs policy and practice. This symposium will highlight recent research initiatives in falls prevention, mental health, artificial intelligence (AI) for cognitive health, and community-based health promotion participatory research. Each presentation will discuss findings, methodological advancements, and strategies to enhance equity in aging-related interventions. Falls prevention remains a public health priority, with disparities influenced by socioeconomic status, geographic accessibility, and racial and ethnic backgrounds. Social isolation, mental health conditions, and cognitive impairment are strongly associated with falls, yet their mediating and direct relationships with fall risk remain insufficiently understood. Natural language processing (NLP) is gaining recognition as a digital biomarker for analyzing text and speech data, potentially enabling early detection and monitoring of Alzheimer's disease. A key highlight is the Arlington Longitudinal Optimal Healthy Aging Study (ALOHA), a community-driven research initiative designed to track and improve health trajectories among diverse aging populations. ALOHA integrates fall prevention, cognitive health monitoring, and personalized wellness strategies into a scalable, interdisciplinary framework for low-funded yet high-impact aging research. Additionally, MCOA research examines challenges and solutions for supporting older adults to age in place and within their communities by exploring inequities in fall prevention programs, mental health's role in fall risk, and AI-driven speech analysis tools as emerging biomarkers for cognitive impairment. The presentations will inform evidence-based interventions, enhance interdisciplinary approaches, and address critical gaps in aging research and community-based health promotion services.

Abstract citation ID: igaf122.1754

### DEMOGRAPHIC DIFFERENCES IN FALLS PREVENTION OUTCOMES: INSIGHTS FROM EVIDENCE-BASED FALLS PREVENTION PROGRAMS

Uma Kelekar[1], Mahederemariam Dagne[1], Sara Pappa[1], Catherine Diaz-Asper[1], J Taylor Harden[2], Shelly Aboagye[1], Erin Staker[3], and Patricia Heyn[1], *1. Marymount University, Arlington, Virginia, United States, 2. Marymount University, Fairfax Station, Virginia, United States, 3. Center for Optimal Aging, Arlington, Virginia, United States*

Falls remain a leading cause of injury among older adults in the United States, with prevalent disparities influenced by demographic factors. Race/ethnicity was reported to be one factor influencing overall falls risk. The Administration for

Community Living (ACL) launched nationwide Evidence-Based Falls Prevention Programs (EBFPPs) to counter falls among older adults. This provided a unique opportunity to analyze program effectiveness across the U.S. among racial and ethnic minorities. For this study, we utilize the results from the large-scale EBFPP datasets to assess variations in falls prevention outcomes related to race/ethnicity. The participant data (N = 203,838) is representative of various racial and ethnic groups, with a majority of participants identifying as NH White (79.9%), followed by NH Black (9.03%), Hispanic (5.12%), and other races or ethnicities. Across all participants, total falls declined by 20%, fear of falls declined by 30%, and injurious falls declined by 10%. There were statistically significant differences amongst the racial-ethnic groups in all metrics analyzed, with NH white and Hispanic participants consistently reporting the highest reductions across all falls metrics while NH Black participants reported lower reduction rates. This work provides valuable insights for researchers, policymakers, public health officials, and aging service providers to optimize falls prevention interventions to better serve diverse and high-risk populations indicating the need for incorporating targeted outreach efforts, culturally competent program modifications, and state-level policy considerations to enhance equity in falls prevention.

Abstract citation ID: igaf122.1755

### SAVING CLAIRE: EFFECTIVENESS OF AN INNOVATIVE APPROACH TO PREVENTING FALLS IN THE COMMUNITY

Sara Pappa[1], Cathy Elrod[1], Mahederemariam Dagne[1], Elizabeth Terhune[1], Erin Staker[2], and Patricia Heyn[1], *1. Marymount University, Arlington, Virginia, United States, 2. Center for Optimal Aging, Arlington, Virginia, United States*

Falls are a major concern for older adults. According to the Centers for Disease Control and Prevention (CDC), about 3 million emergency room visits among older adults are due to fall injuries and over 300,000 hospitalizations are due to hip fractures. Several community-based interventions are being implemented yearly including evidence-based falls prevention programs. Additionally, several behavioral interventions are also implemented to increase awareness of falls and fall injuries. One novel approach to community-based falls prevention efforts is the Saving Claire (SC) project, an event that combines storytelling, a documentary film viewing, and an expert-led panel discussion. We implemented the SC project in the Northern Virginia region starting in September 2023. The expert panel comprised members of the community, including a physical therapist, an occupational therapist, a home modification expert, and other health professionals to provide advice and answer questions on falls prevention strategies. We also provided an optional survey to capture program effectiveness starting in April 2024 to evaluate the effectiveness and use of the project as a falls prevention education tool. We collected 306 surveys between April - October. Our preliminary data analysis showed that the majority of participants reported that the film and panel increased their understanding and awareness of falls prevention strategies. To date, the SC project has reached over 1,000 individuals. This study takes a deeper dive into the data, including participants' intent to have a home safety check; get their hearing, vision, medications, and balance evaluated; and participate in an exercise program.

Abstract citation ID: igaf122.1756

### ASSOCIATIONS BETWEEN MENTAL HEALTH CONDITIONS AND FALLS IN OLDER ADULTS: AN UMBRELLA REVIEW OF SYSTEMATIC REVIEWS

Mahederemariam Dagne[1], Shelly Aboagye[1], Elizabeth Terhune[1], Erin Staker[2], Malaz Hassan[2], Samia Jones[2], Aderonke Aderonmu[2], and Anita Rizvi[3], *1. Marymount University, Arlington, Virginia, United States, 2. Center for Optimal Aging, Arlington, Virginia, United States, 3. University of Ottawa, Ottawa, Ontario, Canada*

Falls are a leading cause of injury and reduced quality of life in older adults, with evidence suggesting a bidirectional relationship between falls and mental health conditions. Depression, anxiety, and psychosocial factors may influence fall risk and outcomes, yet findings across studies remain inconsistent. This umbrella review synthesizes evidence from systematic reviews to clarify these associations and identify potential moderating and mediating factors. We conducted a comprehensive search across MEDLINE, EMBASE, Web of Science, CINAHL, PsycINFO, LILACS, Cochrane, and KSR Evidence. Eligible reviews examined relationships between mental health conditions (e.g., depression, anxiety) and falls in adults aged ≥60 years. Dual independent screening and data extraction will be performed, with quality assessment using AMSTAR 2. Approximately 1780 articles were obtained from MeSH keyword searches. Preliminary findings suggest a strong association between depression and increased fall risk, with psychosocial factors, including loneliness, as potential moderators. Variability in mental health assessment tools and fall reporting methods contributed to heterogeneity in results. This review will synthesize the current body of evidence on the association between mental health and falls, assess methodological quality, and highlight gaps for future research. The findings will inform clinical guidelines and intervention strategies to improve fall prevention and mental health management in aging populations.

Abstract citation ID: igaf122.1757

### ARTIFICIAL INTELLIGENCE (AI) AS A COGNITIVE FUNCTION DIGITAL BIOMARKER: ANALYZING SPEECH IN OLDER INDIVIDUALS

Catherine Diaz-Asper[1], Mahederemariam Dagne[1], Elizabeth Terhune[1], Erin Staker[2], and Patricia Heyn[1], *1. Marymount University, Arlington, Virginia, United States, 2. Center for Optimal Aging, Arlington, Virginia, United States*

Artificial intelligence (AI) technologies designed to analyze speech have demonstrated considerable potential as screening tools for Alzheimer's disease (AD), enabling the detection of subtle alterations in the content and structure of natural speech. The most common AD markers in speech include word finding difficulties, reduced coherence, and longer pauses between words and sentences. Natural Language Processing (NLP) and acoustic analysis can identify subtle linguistic and structural changes associated with cognitive decline. However, most AI models are trained on English-speaking populations, limiting their global applicability. In fact, most NLP research focuses on about 20 of the 7,000 languages spoken worldwide, ignoring the roughly billion speakers who speak other, low resource languages —languages that are underrepresented, under-researched, and under-computerized. In the United States alone, over nine million older adults speak a language other than English at home, potentially limiting their access to speech-based cognitive screening tools. Additionally, due to the limitation in current datasets most NLP platforms do not account for different dialects and accents, further limiting their uses even to English speakers with intonations that differ from the reference speaking style. To reduce disparities and ensure AI models can be applicable to more individuals, it is imperative to prioritize research into low-resource languages in the development of AI-driven screening technologies. Thus, this research focuses on developing inclusive AI-driven cognitive screening tools by integrating diverse linguistic datasets and analyzing speech patterns among non-English-speaking older adults. Findings will contribute to equitable AI applications in dementia screening and cognitive health research.

Abstract citation ID: igaf122.1758

### ARLINGTON LONGITUDINAL OPTIMAL HEALTHY AGING STUDY (ALOHA): A FRAMEWORK FOR AGING RESEARCH WITH LIMITED RESOURCES

Patricia Heyn[1], Erin Staker[2], Mahederemariam Dagne[1], Catherine Diaz-Asper[1], Uma Kelekar[1], Shelly Aboagye[1], Sara Pappa[1], and J Taylor Harden[3], *1. Marymount University, Arlington, Virginia, United States, 2. Center for Optimal Aging, Arlington, Virginia, United States, 3. Marymount University, Fairfax Station, Virginia, United States*

As the population of older adults increases, so does the need for sustainable, inclusive, and interdisciplinary aging research, particularly in resource-limited settings. The Arlington Longitudinal Optimal Healthy Aging Study (ALOHA) offers a scalable, community-based framework for tracking health trajectories, cognitive function, and mobility among diverse older adults in Northern Virginia. Led by an interdisciplinary team, ALOHA identifies modifiable risk factors related to aging, such as frailty, cardiovascular health, and cognitive decline. Participants receive personalized results through a “Health Passport”, a toolkit that provides individualized health goals, wellness resources, and behavior change recommendations to support optimal aging. The study focuses on engaging underrepresented populations in a region that mirrors the growing diversity of the United States. ALOHA addresses disparities in access to physical activity, health promotion resources, and preventative care, especially among underserved older adults. Longitudinal data will be used to evaluate participant health trajectories and refine the Health Passport, to expand this model to other communities and research centers. Northern Virginia, recognized nationally for its high community health rankings and demographic diversity, serves as a unique “living laboratory” for developing equitable, accessible, and translational aging interventions. Despite operating with limited funding, ALOHA demonstrates how community partnerships, participant-centered approaches, and interdisciplinary collaboration can drive impactful aging research. This model can inform future policy, enhance healthy aging outcomes, and strengthen research infrastructure in both academic and community settings.

### SESSION 4195 (PAPER)

### IDENTITY AND ISOLATION IN AGING: EXPLORING PURPOSE, PERSONALITY, AND PSYCHOSOCIAL WELL-BEING

Abstract citation ID: igaf122.1759

### FRIENDSHIP AND LONELINESS IN DEMENTIA CAREGIVING: DAILY EXPERIENCES OF HIGH AND LOW BURDEN CAREGIVERS

Yee To (Crystal) Ng[1], Angela Turkelson[2], Anna Kratz[1], and Kira Birditt[1], *1. University of Michigan, Ann Arbor, Michigan, United States, 2. University of Michigan Institute for Social Research, Ann Arbor, Michigan, United States*

Dementia caregivers are particularly vulnerable to loneliness compared to non-dementia caregivers and non-caregivers. Grounded in the Social Convoy and Stress Process Models, this study examined whether (a) dementia caregivers with fewer close friends experience greater loneliness; (b) daily interactions with friends are linked to reduced momentary loneliness, and if the quality and closeness of these interactions matter; and (c) whether such links vary by caregiver burden. The sample included 223 dementia caregivers from the Stress and Well-Being in the Everyday Lives of Caregivers Study. They completed a baseline interview assessing their demographics, caregiving, and social network characteristics, followed by a five-day ecological momentary assessment (EMA), reporting their social interactions and loneliness every three hours. Findings revealed that greater number of close friends was associated with reduced momentary loneliness compared to those with fewer close friends in social networks. Caregivers reported interactions with friends in 22% of EMA surveys. Multilevel linear models showed that caregivers experienced lower loneliness during interactions with friends—especially positive interactions and with non-close friends—compared to when they did not interact with friends. Furthermore, friend interactions were associated with lower momentary loneliness for caregivers with higher burden but not lower burden. Findings suggest that caregivers with fewer friends experience more loneliness, and daily interaction with friends helps reduce momentary loneliness, particularly for high-burden caregivers. These results highlight the need for interventions that promote friendship connections, to alleviate loneliness and enhance dementia caregiver well-being.

Abstract citation ID: igaf122.1760

### LATE LIFE DIVORCE AND EMOTIONAL EXPERIENCES THAT FOLLOW: AN INTERGENERATIONAL FAMILY PERSPECTIVE

Chaya Koren[1], Yafit Cohen[1], and Naor Demeter[1], *1. University of Haifa, Haifa, HaZafon, Israel*

Late life divorce is rising in Western societies, and accordingly, more scientific knowledge is required regarding emotional experiences of being alone following late life divorce. Our aim was to examine emotional experiences following late life divorce from an intergenerational family perspective, considering late-life divorce consequences for the wellbeing of ex-spouses and their adult children. Data was collected through 51 semi-structured qualitative, in-depth interviews, comprised of 7 family units (n = 33) and 9 parent-child dyads (n = 18), using principles of thematic-analysis and dyadic interview-analysis. Findings reveal emotions related to loneliness and freedom. Different ex-family members’ perceptions regarding these experiences were identified. Most ex-spouses were willing to pay the price of loneliness to gain freedom. However, their adult-children mainly described the disadvantages of late-life divorce, emphasizing loneliness, and perceiving both loneliness and freedom as negative. These gaps could be explained in two ways: (1) The adult children see their parents in their parental role and less as individuals enjoying freedom outside of the family context following late life divorce. Therefore, their adult children have difficulty seeing the benefits of freedom. (2) The adult children feel responsibility to relief their parents’ loneliness, which causes them an emotional burden. Unique aspects of freedom and loneliness in old age along with coping strategies of both generations are discussed. Implications for families and family professionals are addressed.

Abstract citation ID: igaf122.1761

### LONELINESS TRAJECTORIES AND DEMENTIA RISK AMONG MEXICAN AMERICAN OLDER ADULTS: A LATENT GROWTH MODEL APPROACH

Suyoung Kim, *The University of Chicago, Chicago, Illinois, United States*

Objective: Loneliness is an important yet understudied psychosocial determinant of health among Mexican American older adults. This study examines whether loneliness trajectories predict dementia risk in this population, considering the role of functional ability, socioeconomic factors, and psychological well-being. Method: Data were drawn from Waves 7, 8, and 9 of the Hispanic Established Populations for the Epidemiologic Study of the Elderly (HEPESE). A latent growth model was used to estimate baseline loneliness and its change over time. Predictors included marital status, living arrangement, Instrumental Activities of Daily Living (IADL) limitations, financial strain, and depressive symptoms. Dementia status was assessed at Wave 9 using the Mini-Mental State Examination (MMSE). Results: On average, loneliness was low at baseline but increased over time. Living alone and depressive symptoms were associated with higher initial loneliness, while IADL limitations and financial strain predicted steeper increases. Both higher baseline loneliness and greater increases over time were linked to a higher risk of dementia at Wave 9, even after adjusting for covariates. Discussion: These findings suggest that loneliness trajectories contribute to dementia risk among Mexican American older adults. Addressing loneliness, particularly among individuals living alone or experiencing financial and functional difficulties, may be key to mitigating cognitive decline in this population.

Abstract citation ID: igaf122.1762

### OLDER ADULTS' IDENTITY DEVELOPMENT AND MENTAL HEALTH AFTER THE COVID-19 PANDEMIC: A 4-YEAR LONGITUDINAL STUDY

Lauren Mitchell[1], and Jessica Finlay[2], *1. Emmanuel College, Boston, Massachusetts, United States, 2. University of Colorado Boulder, Boulder, Colorado, United States*

The COVID-19 pandemic introduced substantial challenges to older adults' identities and self-concepts as they navigated social isolation, health risks, bereavement, and other disruptions. This mixed-methods study examined how older adults perceived their identities being affected by the pandemic, and how such identity shifts related to trajectories of well-being. Older adults (N=2248; mean age=68) completed 12 monthly surveys in Year 1 (April, 2020-April, 2021) and annual surveys in Years 2-4 (2022-2024) of the COVID Coping Study (Kobayashi et al., 2021). Identity dynamics were assessed with open-ended questions addressing how the pandemic affected participants' self-concepts, sense of purpose, and expectations for the future. Life satisfaction, loneliness, depressive symptoms, anxiety, and self-rated health were assessed with quantitative measures. Thematic analysis was used to identify common patterns among the open-ended identity responses, and latent growth curve modeling was used to test the association between identity themes and well-being outcomes. Initial findings suggest most participants emphasized positive identity adjustments, such as changing their priorities in life, or realizing their strength in the face of adversity. A small subset (6%) indicated identity disruption, or a loss of identity coherence and self-continuity, due to the pandemic. Identity disruption was associated with lower trajectories of mental health on average, and increasing levels of loneliness across the pandemic period. Findings suggest that, although most older adults have coped effectively with the identity-related challenges of the pandemic, some may continue to experience negative consequences that can hinder their social engagement and harm their mental health.

Abstract citation ID: igaf122.1763

### PERSONALITY PROFILES AND LIFE SATISFACTION IN AGING: A GROWTH MIXTURE MODELING APPROACH

Yeon Ji Ryou[1], and Momoho Kakuta[2], *1. Iowa State University, Ames, Iowa, United States, 2. The University of Osaka, Suita, Osaka, Japan*

This study examined life satisfaction trajectories among individuals 65 years and older using growth mixture modeling (GMM) with pre-identified personality profiles from latent profile analysis (LPA) and covariates. The targeted sample (N = 2,424) was from the merged data of the 2020 RAND Health and Retirement Study (HRS) and 2022 Core data, covering three time points (T1=2012/2014, T2=2016/2018, T3=2020/2022). Given the alternating design of data collection, each point pooled two consecutive waves for the complete sample for psychosocial questions. Three personality profiles: Resilient Extraverts (53.26%), Balanced (40.18%), and Sensitive Introverts (6.56%), were identified and used to predict life satisfaction trajectories. Results found significant baseline differences across the sub-groups. Resilient Extraverts showed the highest life satisfaction at T1 ($B = 1.984$, $SE = .44$, $p < .001$), followed by the Balanced group ($B = 1.695$, $SE = .44$, $p < .001$) and Sensitive Introverts ($B = 1.405$, $SE = .44$, $p < .01$). Despite these initial differences, all groups demonstrated an increasing trend in life satisfaction over time ($p < .01$). Better subjective health, older age, and being married were positively associated with life satisfaction at baseline. However, older age was linked to a slower rate of change. These findings suggest that psychosocial resources could involve sustaining well-being. Although aging was associated with a slower rate of growth, the overall upward trend suggests the possibility of continued psychological well-being, emphasizing the need for targeted interventions that utilize individual personality strengths and social resources to enhance well-being in later life.

Abstract citation ID: igaf122.1764

### PURPOSE IN LIFE AND LONELINESS AMONG AFRICAN AMERICAN WOMEN: AN APPLICATION OF LATENT GROWTH MIXTURE MODELING

Eunbea Kim[1], Carolyn Cutrona[2], and Daniel Russell[2], *1. Auburn University, Auburn, Alabama, United States, 2. Iowa State University, Ames, Iowa, United States*

Purpose in life has been widely proposed as a significant predictor of positive health outcomes. However, few studies have employed longitudinal methods to address the effects of purpose in life on loneliness. Furthermore, studies have rarely addressed the role of purpose in life in levels of loneliness among people of color. This study investigated the extent to which purpose in life predicted African American women's loneliness over time. Using data from 661 African American women (Mage = 44.92, SD = 8.10) across four waves of the Family and Community Health Study (FACHS), latent growth mixture modeling was employed to explore the trajectories of loneliness across approximately 10 years and whether level of purpose in life was a significant predictor of the trajectories. This study also accounted for potent influential factors such as positive and negative social support, religiosity, racial discrimination, and financial strain as covariates. Findings revealed significant between-individual differences in loneliness trajectories, with individuals reporting a higher initial level of purpose in life tending to have lower levels of loneliness over time. Thus, greater purpose in life predicted lower loneliness among African American women, highlighting the importance of these factors in their psychosocial well-being.

Abstract citation ID: igaf122.1764a

### PERSONALITY PROFILES AND LIFE SATISFACTION IN AGING: A LATENT GROWTH CURVE MODELING APPROACH

Yeon Ji Ryou[1], and Momoho Kakuta[2],
*1. Iowa State University, Ames, Iowa, United States,*
*2. The University of Osaka, Suita, Osaka, Japan*

This study examined life satisfaction trajectories among individuals 65 years and older using growth mixture modeling (GMM) with pre-identified personality profiles from latent profile analysis (LPA) and covariates. The targeted sample (N = 2,424) was from the merged data of the 2020 RAND Health and Retirement Study (HRS) and 2022 Core data, covering three time points (T1=2012/2014, T2=2016/2018, T3=2020/2022). Given the alternating design of data collection, each point pooled two consecutive waves for the complete sample for psychosocial questions. Three personality profiles: Resilient Extraverts (53.26%), Balanced (40.18%), and Sensitive Introverts (6.56%), were identified and used to predict life satisfaction trajectories. Results found significant baseline differences across the sub-groups. Resilient Extraverts showed the highest life satisfaction at T1 (B = 1.984, SE = .44, $p < .001$), followed by the Balanced group (B = 1.695, SE = .44, $p < .001$) and Sensitive Introverts (B = 1.405, SE = .44, $p < .01$). Despite these initial differences, all groups demonstrated an increasing trend in life satisfaction over time ($p < .01$). Better subjective health, older age, and being married were positively associated with life satisfaction at baseline. However, older age was linked to a slower rate of change. These findings suggest that psychosocial resources could involve sustaining well-being. Although aging was associated with a slower rate of growth, the overall upward trend suggests the possibility of continued psychological well-being, emphasizing the need for targeted interventions that utilize individual personality strengths and social resources to enhance well-being in later life.

### SESSION 4200 (SYMPOSIUM)

Abstract citation ID: igaf122.1765

### INNOVATIVE MONITORING TECHNOLOGIES TO ENHANCE THE HEALTH, INDEPENDENCE, AND WELL-BEING OF OLDER ADULTS

Chair: Walter Boot

Advancements in monitoring technologies offer new opportunities to enhance the health, independence, and well-being of older adults. This symposium presents innovative, non-invasive solutions for the early detection of functional decline, fall prevention, and support for aging in place. Dr. Megan Huisingh-Scheetz will discuss how hip accelerometry and machine learning models can detect and forecast frailty decline in older adults, enabling proactive interventions. Dr. Jeannette Mahoney will introduce CatchU... Before You Fall, a multisensory digital health tool designed to predict fall risk—particularly in individuals with preclinical Alzheimer's disease—while promoting independence and facilitating provider-initiated falls counseling. Dr. Clara Berridge will explore the Let's Talk Tech (LTT) decision aid, a web-based tool that helps individuals with memory loss and their caregivers make informed decisions about digital health technologies, highlighting its potential value to both family members and providers by supporting the sharing of technology preferences beyond the care dyad. Dr. Elinor Schoenfeld will present on the development and deployment of contactless sensors for Monitoring Vital Signs and Movement, advancing real-time health monitoring to support aging in place. Finally, Dr. Jane Chung will introduce a Wi-Fi Sensing-Based Deep Learning Solution that recognizes daily activities in older adults, offering insights into functional decline and cognitive health. By showcasing these cutting-edge approaches, this symposium highlights the transformative potential of monitoring technologies in aging research, emphasizing user-centered and scalable solutions to support older adults' health and independence.

Abstract citation ID: igaf122.1766

### FREE-LIVING HIP ACCELEROMETRY DETECTS AND FORECASTS FRAILTY DECLINE IN A SAMPLE OF COMMUNITY-DWELLING OLDER ADULTS

Megan Huisingh-Scheetz[1], Benjamin Kramer[2], Yanan Long[1], Michelangelo Pagan[1], Sylvia Brown[3], and Andrey Rzhetsky[1],
*1. The University of Chicago, Chicago, Illinois, United States, 2. Case Western Reserve University, Cleveland, Ohio, United States, 3. National Opinion Research Center at The University of Chicago, Chicago, Illinois, United States*

Frailty is a geriatric syndrome combining reduced strength, exhaustion, slow gait, weight loss, and low activity, foreboding adverse health outcomes. Early detection of frailty is critical for mitigating risk, but the standard frailty detection is time-consuming. Non-invasive, wearable accelerometers capture physical activity and sleep patterns and may detect frailty changes. In this retrospective study, 7-days of free-living data from hip accelerometers were used to both detect concurrent frailty phenotype (range 0-5, nonfrail=0 vs pre-frail or frail=1-5 points) and forecast frailty increase (1+ point worse frailty score vs no change or improved score) over 1 year in community-dwelling older adults. We extracted 115 accelerometry features from the hip accelerometry readings (activity, sleep, harmonic variables) using open-source code. Five machine learning models included all accelerometry features plus demographic and health characteristics. The models were trained on 85% of the sample and were tested on 15% of the sample. In this presentation, we will share the best-performing cross-sectional model (F1=0.84, AUROC=0.84) and the best performing longitudinal model (F1=0.87, AUROC=0.84). These findings suggest that wearable sensors' data may be useful for real-time frailty screening and monitoring. Integrating accelerometry into clinical care could facilitate earlier detection of at-risk individuals and improve outcomes.

Abstract citation ID: igaf122.1767

### CATCHU. . . BEFORE YOU FALL: A QUANTITATIVE DIGITAL HEALTH APP FOR PREDICTING AND MONITORING FALLS IN OLDER ADULTS

Jeannette Mahoney[1], Claudene George[2], and Joe Verghese[1],
*1. Stony Brook University, Stony Brook, New York, United States, 2. Albert Einstein College of Medicine, Bronx, New York, United States*

Thirty-three percent of Americans aged 65 and over will fall each year at an annual cost of over $50 billion to US healthcare. Falls are the leading cause of injury & injury-related death among older adults. In fact, over 3 million older adults will require an ED visit because of fall-related injuries. People living with Alzheimer's disease (AD) are at even greater risk for mobility declines and falls. Our research suggests that the magnitude of multisensory integration, which is crucial for independent functioning & completion of daily activities, is linked to falls, balance, gait, cognition and amyloid pathology. The CDC recommends routine fall-risk screening annually, but this is not well-integrated into clinical practice. There is a large unmet need for quantitative, research-based digital health tools that raise falls awareness, while monitoring and improving wellness - especially for high-risk older adults living with preclinical Alzheimer's disease. CatchU®…Before You Fall, a 10-minute multisensory digital health assessment grounded in over 15 years of research, uniquely fills this void by assessing visual-somatosensory integration performance in conjunction with existing medical comorbidities, in an effort to detect the propensity of falling in the near future. The vision and mission of CatchU® is to alleviate disability, promote independence, and increase quality of life for older adults and all people by promoting provider-initiated falls counseling and easy access to personalized recommendations scientifically proven to reduce falls.

Abstract citation ID: igaf122.1768

### SHARING PATIENT TECHNOLOGY PREFERENCES WITH DEMENTIA CARE NETWORKS: THE LET'S TALK TECH DECISION AID

Clara Berridge[1], Natalie Turner[1], William Lober[1], George Demiris[2], and Jeffrey Kaye[3], *1. University of Washington, Seattle, Washington, United States, 2. University of Pennsylvania, Philadelphia, Pennsylvania, United States, 3. Oregon Health & Science University, Portland, Oregon, United States*

Let's Talk Tech (LTT) is a self-administered web intervention for people with memory loss and their care partners that supports decision-making about digital health technologies. In past work, study participants wanted to share LTT technology preference reports with their larger care networks. This study aims to understand with whom care dyads want to share their technology preference reports and why, and if and how clinicians want to receive them. Together, fifteen dyads of people living with MCI or early-stage dementia (n = 15) and a care partner (n = 15) completed LTT and two survey questions. Care partners completed independent follow-up interviews, and 32 clinicians at four Alzheimer's Disease Research Center-affiliated clinics viewed an LTT report and completed a 10-question survey. We used descriptive statistics for survey responses and thematic analysis for interviews. Two-thirds of care partners (n = 10) wanted to share the report with family members. Half (n = 8) wanted to share it with clinicians to keep them informed about the dyad's planning and facilitate conversations about technology options. Clinicians were highly receptive to accessing technology preference reports in EHRs and to having discussions about technology be a part of advance care planning. Thirty of 32 clinicians reported they would want their patients' technology preferences reports, with 25 wanting to access it via the EHR. Findings demonstrate potential value to both family members and providers of sharing technology preferences beyond the care dyad. Future research should test integration in the EHR and the potential of sharing technology preferences to support person-centered technology choices.

Abstract citation ID: igaf122.1769

### MONITORING VITAL SIGNS AND MOVEMENT USING CONTACTLESS SENSORS: BUILDING A HOME OF THE FUTURE

Elinor Schoenfield[1], Elinor Schoenfeld[2], Mengjing Liu[3], Isac Park[3], Suvab Baral[3], Tristan Vozzolo[3], Jason Mathew[3], and Fan Ye[3], *1. Stony Brook University Renaissance School of Medicine, Stony Brook, New York, United States, 2. Renaissance School of Medicine at Stony Brook University, Stony Brook, New York, United States, 3. Stony Brook University, Stony Brook, New York, United States*

As Americans live longer, there is a growing need to develop and implement technologies to monitor changes in health, supporting aging in place. We have installed privacy-preserving ultra-wideband wireless sensors in a model home to facilitate sensor and algorithm development for future home deployment. These sensors monitor vital signs and provide localization/tracking and activity recognition. We have developed a protocol to continuously collect and measure activities of daily living, location, and gait. We developed a multi-modal app using videos, audio, and texts to guide individuals and track activity completion time. To protect data privacy, raw sensor data is sent to a research cloud, and identifiable image data is sent to a university-supported HIPAA-compliant Box app. We spent >10 months developing, evaluating, stress testing, and improving our system and cloud infrastructure stability to ensure <1% data loss. We completed 19 sessions with volunteers to test our app and protocol, provide feedback, and continue to update our procedures and app before beginning data collection with individuals post-stroke. Recovery progress in post-stroke study participants will complete 3 study visits over 14 weeks to capture clinical and sensor measurements. We continue to refine our home deployment protocols for older adults with diverse technological knowledge. This presentation highlights our progress in developing and deploying our home monitoring system. We will discuss the challenges and procedures to collect, analyze, and interpret continuous physiologic and movement data in real-time, providing information, knowledge, and insights that will become vital to supporting the health of our expanding aging population.

Abstract citation ID: igaf122.1770

### DEVELOPING A WI-FI SENSING-BASED DEEP LEARNING SOLUTION FOR RECOGNIZING DAILY ACTIVITIES IN OLDER ADULTS

Jane Chung[1], MD Touhiduzzaman[2], Ingrid Pretzer-Aboff[2], John Karlsen[2], Megan Vain[2], and Eyuphan Bulut[2], *1. Emory University, Atlanta, Georgia, United States, 2. Virginia Commonwealth University, Richmond, Virginia, United States*

Assessing older adults' daily activities and detecting early changes is important for identifying those at risk of functional decline and enabling timely interventions. Given the limitations of existing sensing technologies, there is a need for discreet, affordable system that quantifies daily activity levels and patterns with minimal user engagement. We aimed to develop a Channel State Information (CSI)-based device-free Wi-Fi sensing system using deep neural network (DNN) classification models to localize and recognize different in-home activities in older adults. We deployed three transmitter-receiver pairs in participants' residences and collected Wi-Fi CSI data over 5-7 days. Ground truth annotations for activities such as entering or exiting the residence, entering or exiting a bedroom, working in the kitchen, and opening or closing the refrigerator were derived from video recordings. We trained DNN models with a one-dimensional convolutional layer for each transmitter-receiver pair. To improve accuracy, we combined predictions from different pairs using class-weighted linear regression. Finally, we applied majority voting across consecutive predictions to enhance reliability. The prediction accuracy provided by our models using the six activities ranges between 75-90% for different participants (average accuracy = 83%). Participants generally had no to minimal concerns about privacy and obtrusiveness but differed in their views on the system's usefulness, due to the lack of clear information on how the sensing data could benefit their health and wellbeing. This sensing system's ability to distinguish human activities has the potential to enable early detection of functional decline, a potential indicator of cognitive impairment.

### SESSION 4205 (PAPER)

Abstract citation ID: igaf122.1771

### INNOVATIVE PROGRAMS IN AGING

To address the increasing complexity in care of older adults, frameworks and innovative programs will be presented during this session.

Abstract citation ID: igaf122.1772

### ENGAGING COMMUNITY-BASED ORGANIZATIONS IN THE SCALE AND SPREAD OF WALKON!

Jaime Hughes[1], Laura Jakiela[1], Barbara Nicklas[1], Elizabeth Kemp[2], Courtney Hayes[1], Justin Moore[1], Case Peters[1], and Michelle Simpson[3], *1. Wake Forest University School of Medicine, Winston-Salem, North Carolina, United States, 2. Atrium Health Wake Forest Baptist Medical Center, Winston Salem, North Carolina, United States, 3. Advocate Aurora Health, Milwaukee, Wisconsin, United States*

Few community-based walking programs exist within rural areas. Scaling WalkOn!, an evidence-informed walking program for older adults with mobility limitations, may require adaptations to intervention components and implementation strategies to best align with the needs, preferences, and capacity of rural communities. Informed by principles of implementation science and guided by the Consolidated Framework for Implementation Research, we engaged diverse community-based organizations (CBOs) in exploring implementation determinants, organizational readiness, and program adaptations. Project invitations were sent to 93 CBOs across four diverse service sectors: faith-based, fitness/recreation, community services (e.g., libraries), and aging organizations. Twenty-four (26%) CBOs indicated interest, 13 (14%) attended an informational session, and 8 (9%) enrolled. While 7 (88%) have implemented new programs, one-quarter reported no exercise or older adult (OA) programs. Information sessions revealed CBOs' heavy interest in adopting WalkOn! despite varied staffing models ranging from a part-time director to 250+ staff/leaders/volunteers (S/L). CBOs participated in quantitative surveys (S/L, N=21; OA, N=102) to assess site readiness, barriers and facilitators to adoption and sustainment, and desired program adaptations. CBOs' motivation for adopting WalkOn! included enhancing access to evidence-based programs (N=19, 90%) and responding to community needs (N=15, 71%). S/L respondents reported high organizational readiness and only endorsed an average of 1.2 barriers (competing demands, funding) when asked to rank 8 potential implementation barriers. OAs' most desired intervention adaptations included home exercise instruction (N=53, 52%) and accountability buddies (N=38, 37%). This session will conclude with best practices for engaging diverse CBOs in the planning, adaptation, and scale-up of effective programs.

Abstract citation ID: igaf122.1773

### HAVING HARD CONVERSATIONS: LEVERAGING PRIMARY CARE SOCIAL WORKERS TO IMPROVE TAKE-UP OF VA HOME HEALTH AIDE SERVICES

Lauren Hall[1], Margaret Ding[1], and Emily Franzosa[2], *1. James J Peters VA Medical Center, New York, New York, United States, 2. Bronx VA, Bronx, New York, United States*

The Veterans Health Administration's (VHA) home health aide benefit (HHA) serves 180,000 Veterans annually and is VHA's fastest growing and most widely used home and community-based service. Like other payers, VHA purchases HHA care from community providers, which can make navigating the benefit a complex process for VHA staff and veterans. We conducted multi-perspective, semi-structured interviews at five geographically diverse VHA medical centers with key informants (n=61) who connect veterans to HHA services, including primary care providers (geriatricians, social workers, nursing staff), HHA program managers, and home health agency administrators and aides to identify best practices and modifiable intervention points to improve take-up of VA HHA services. Interviews were recorded, transcribed, and analyzed using inductive thematic analysis. Findings across all participants emphasized the importance of primary care social workers in assisting veterans in making the most informed decisions about whether to use HHA. We identified 3 main themes that facilitated Veterans' use of HHA: 1) social workers often are the primary care team members who know the veteran and their needs best; 2) social workers use rapport-building, goal alignment, and priority setting to persuade veterans to take up services, and 3) valuing veterans' autonomy helps establish trust and support, encouraging veterans' use of HHA. Leveraging the central role that social workers play in geriatric primary care teams, their relationships with older patients, and the recognition of veterans' autonomy and preferences can support age-friendly care and improve older adults' connection to the supports they need to live independently.

Abstract citation ID: igaf122.1774

### HCAP FACTOR STRUCTURE: RESULTS FROM THE NORTHERN IRELAND COHORT FOR THE LONGITUDINAL STUDY OF AGEING

Nicola Ann Ward[1], Calum Marr[1], Leeanne O'Hara[1], Charlotte Neville[1], Michael McAlinden[1], Claire Potter[1], David R Weir[2], and Bernadette McGuinness[1], *1. Queen's University Belfast, Belfast, Northern Ireland, United Kingdom, 2. University of Michigan, Ann Arbor, Michigan, United States*

Dementia affects nearly one million people in the United Kingdom, with Northern Ireland (NI) projected to experience the highest increase due to rapid population aging. Accurate prevalence estimates are essential for public health planning and predicting future societal and economic needs. The Harmonized Cognitive Assessment Protocol (HCAP) enables standardized cross-country comparisons of dementia prevalence. HCAP was implemented as a substudy of the NI Cohort for the Longitudinal Study of Aging (NICOLA). The current study aimed to validate the cognitive factor structure of HCAP, previously implemented in HRS-HCAP, in a representative Northern Irish population using confirmatory factor analysis (CFA). The NICOLA-HCAP subsample included 1,037 randomly selected community-dwelling adults aged ≥65 who completed neurocognitive tests. CFA was conducted in STATA(v15.1) to evaluate single and multi-domain factor structures. All factor scores were presented as mean ± SD. A preliminary five-factor model encompassing memory (MEM: -0.16 ± 0.82), executive functioning (EXF: -0.15 ± 0.64), visuospatial (VIS: -0.02 ± 0.11), orientation (ORI: -0.01 ± 0.05), and language (LAN: -0.10 ± 0.43), with a higher-order general cognition factor (GCOG: -0.15 ± 0.64), was identified (SRMR = 0.043, RMSEA = 0.055, CFI = 0.932). GCOG strongly influenced MEM, EXF, and ORI (loadings >0.80) and had moderate associations with VIS (0.70) and LAN (0.67). The model fits well, aligning with other HCAP models. Next steps will involve selecting the normative HCAP sample, standardizing scores by age and gender, and establishing diagnostic cutoffs for impairment (>1.5SD below the mean in two domains derived from the model with informant-rated deficits).

Abstract citation ID: igaf122.1775

### IMPLEMENTING EVIDENCE-BASED CREATIVE ARTS THERAPIES IN FACILITIES CATERING FOR OLDER ADULTS

Daphna Kalir[1], Shoshi Keisari[2], Hod Orkibi[2], Reem Nashef-Hamuda[3], and Neta Singer[2], *1. University of Haifa, Tel Aviv, Tel Aviv, Israel, 2. University of Haifa, Haifa, HaZafon, Israel, 3. University of Haifa, Tel Aviv University, Haifa, HaZafon, Israel*

Implementing Evidence-Based Creative Arts Therapies in Facilities catering for Older Adults Daphna M. Kalir, Shoshi Keisari, Hod Orkibi, Rim Abu-Nashef, Neta Singer Abstract Creative Arts Therapies (CATs) utilize various art forms to foster psychological growth, particularly among older adults. The current study explored the implementation patterns of CATs in facilities that cater to older adults' needs in Israel, and especially the barriers and facilitating factors of the implementation processes. Our research consisted of two studies: a qualitative study aimed to identify barriers and facilitators to CATs implementation, and a quantitative study that included a survey to characterize CATs implementation in Israel. The qualitative sample (n = 18) included managers, social workers, CATs therapists, and older adult clients in community settings. The quantitative study distributed a structured survey to 62 CAT therapists treating older adults, analyzing therapist demographics, practice settings, and key variables. Subgroup comparisons highlighted differences in training, supervision, and work environments, offering a comprehensive view of CATs implementation. Barriers to CATs implementation included a lack of legally established standards, insufficient funding, preconceptions about CATs and mental health among older adults, and decision makers, and limited awareness of the field. Facilitating factors included prior familiarity of managers and policymakers with CATs, recognition of their effectiveness, therapists' strong motivation, flexible intervention strategies, and implementation frameworks. Findings underscore the need to raise awareness of CATs' benefits and ensure accessible mental health care for community-dwelling older adults. Addressing misconceptions about CATs and mental health treatment is essential for improving attitudes among older adults and policymakers.

### SESSION 4210 (PAPER)

### NEW HORIZONS IN HOUSING RESEARCH

Abstract citation ID: igaf122.1776

### AGING WELL IN THE HEART OF AMERICA: OPTIMIZING HOUSING OPTIONS THROUGH POLICY REFORM

Lana McKinney, *University of Nebraska Omaha, Chicago, Illinois, United States*

This research study investigates the growing housing availability and affordability challenges facing older adults in Kansas City, Missouri. Using the capability approach and the rational comprehensive model as theoretical frameworks, the study analyzes the current state of housing and proposes improvements to address availability and affordability concerns for older adults. A qualitative research design was employed, involving semi-structured interviews with 15 participants, including public officials, experts, and community members. Thematic analysis was used to analyze the interview transcripts, identifying recurring patterns and themes related to the participants' perspectives on housing concepts, policies, and recommendations for improvement. The analysis revealed four key themes: affordability and availability deprivation, policy gaps, the need for targeted capital resources and rational decision-making, and potential policy solutions. The findings highlight a critical gap between existing housing policies and the needs of older adults, underscoring the necessity for increased affordability and availability of housing options. The study proposes several policy recommendations, including streamlining the regulatory process, enhancing public and private partnerships, directed capital for housing from local, state, and federal government, using implementation science to enact policy. These recommendations aim to address the identified challenges and ignite policy reform on behalf of older adults providing suitable and supportive housing.

Abstract citation ID: igaf122.1777

### ASSOCIATIONS OF HOUSING DISREPAIR WITH FUNCTIONAL DISABILITY AMONG OLDER ADULT HOUSEHOLDS IN THE UNITED STATES

Safiyyah Okoye[1], Karlin Moore[1], Amruta Patil[1], Daniel Vader[1], Craig Pollack[2], and Laura Gitlin[1], *1. Drexel University, Philadelphia, Pennsylvania, United States, 2. Johns Hopkins University, Baltimore, Maryland, United States*

Housing disrepair (leaking roofs/walls, broken plumbing/heating) is increasingly common among older adults who often struggle to repair and maintain their homes. Unknown is whether housing disrepair is associated with rates of functional disability and if this association varies for higher and lower income households. Drawing on cross-sectional data (N = 17,610 households with older adults ≥65 years) from the U.S. Census Bureau's American Housing Survey national survey (2023), we characterized housing disrepair and its associations with functional disability. Housing disrepair was measured as occupant report of at least one of 40 problems across 8 categories (electricity, heating, inside structural, bathroom, kitchen, outside structural, water, sewer). Overall, 36.2% of older adult households reported disrepair; 38.5% among low-income and 34.9% among moderate/high-income households. Functional disability (measured as older adult occupant with difficulty bathing or dressing, doing errands, concentrating or remembering, or climbing or walking stairs) was more prevalent among households with disrepair than without (36.3% vs 24.8%). In a fully adjusted logistic regression model, the odds of an older adult occupant having a functional disability was 1.81 (95% CI 1.65, 1.98) times as high in households with vs without disrepair. In additional adjusted models stratified by income, the odds ratios for the association between disrepair and functional disability were similar in low-income (OR 1.87; 95% CI 1.65, 2.12) and moderate/high-income households (OR 1.77; 95% CI 1.55, 2.02). Further research is needed to establish a causal relationship between housing disrepair, income, and disability and identify causes and interventions to decrease disrepair among older adult households.

Abstract citation ID: igaf122.1778

### ENVIRONMENT AND SOCIAL SERVICE FOR AGING IN PLACE DECISIONS: A DIFFERENT SCENARIO BY HEALTH STATUS

Junmin Park, and Yeonjung Lee, *Chung-Ang University, Seoul, Republic of Korea*

In the era of longer life spans, Aging in Place (AIP) has become a global topic. AIP goes beyond staying in the community and requires meeting various needs. A well-developed social service environment is key to effective AIP strategies, yet Korea still lacks sufficient support. This study examines the role of social services in Korea, considering the influence of the environment. Specifically, it examines how awareness of social services moderates the relationship between both physical and social environments and the planning for AIP, considering health status. Logistic regression was conducted using data from the 2023 National Survey of Older Koreans (n = 9,770). Those with higher satisfaction with both physical and social environment are more likely to AIP, when they are in good health. These relationships were stronger for people who have higher awareness of social services. However, when they are in deteriorating health, having higher satisfaction with physical environment was related to less intention to AIP, and these associations were only significant for older adults with more knowledge of social services. The results show that improving the suitability of physical and social environments for older people is essential. In addition to the need for diverse social services to support AIP regardless of health status, older adults must also be aware of the availability and accessibility of these services. Lastly, Korean older adults, who are generally reluctant to burden their children, require a tailored AIP strategy that emphasizes the need for a Respite Care system.

Abstract citation ID: igaf122.1779

### HOUSING ADEQUACY AND HOME MODIFICATION IMPLEMENTATION AMONG HOUSEHOLDS WITH OLDER ADULTS LIVING IN THEM

Bernard Steinman[1], Sung-Jin Lee[2], Randall Cantrell[3], Denise McAllister[4], Gina Peek[5], and Andrew Carswell[6], *1. University of Wyoming, Laramie, Wyoming, United States, 2. North Carolina Agricultural and Technical State University, Greensboro, North Carolina, United States, 3. University of Florida, Gainesville, Florida, United States, 4. Texas State University, San Marcos, Texas, United States, 5. Oklahoma State University, Stillwater, Oklahoma, United States, 6. University of Georgia, Athens, Georgia, United States*

Given the significance of aging in place (AIP) for fulfilling preferences of older adults and guiding policy options to improve their safety and wellbeing, it is important to understand relationships between amenable factors that may facilitate aging at home. The quality of homes in which older adults live can be a facilitator or a barrier to AIP. Housing adequacy is a measure of physical housing quality, which includes characteristics such as whether units have hot/cold running water, flush toilets, or electrical problems. Home modification (HM) is one potential strategy to improve the physical quality of the home environment and to accommodate functional changes experienced by many older people. This study employed data from the American Housing Survey to examine the relationship between housing adequacy and implementation of HMs during a two-year period (2017 through 2019). We controlled covariates for household-level demographics (e.g., age of householder, disability) and housing characteristics (e.g., housing type, year built) within a logistic regression model. Results show that housing adequacy was not associated with HM implementation. Among household demographic variables, education level and presence of at least one person with a disability predicted implementation of HMs in 2019. Among housing variables, age of home structure, metropolitan status, and whether HMs had been implemented previously were statistical predictors of HMs in 2019. Findings provide insights into household-level associations with HM implementation and may be used to inform better understanding of precursors as to why older people may choose to adapt their homes for AIP.

Abstract citation ID: igaf122.1780

### PARTNERING TO SUPPORT NEEDS-APPROPRIATE HOUSING FOR PHYSICALLY DISABLED AND OLDER VETERANS

Anushka Sista[1], Jennifer Palmer[1], and Nathan Boucher[2], *1. Veterans Administration, Bedford, Massachusetts, United States, 2. Duke University, Durham, North Carolina, United States*

The number of homeless Veterans aged 55 and older increased by 150% from 2010 to 2023, creating a significant housing stability crisis. While the Veterans Health Administration (VHA) provides programs like Housing and Urban Development-Veterans Affairs Supportive Housing (HUD-VASH) to support Veterans transitioning out of homelessness; older Veterans and those living with physical disabilities require specialized assistance to maintain independence. This VHA quality improvement project aimed to identify barriers and facilitators to HUD-VASH teams and community collaboration as they support Veterans living in least restrictive settings and to develop an implementation blueprint to guide optimal collaboration. We conducted qualitative interviews with 18 regional HUD-VASH geriatric specialists across VHA nationally and facilitated user-centered design (UCD) sessions with VHA staff and community agency representatives at two VA medical centers. Our integrated analysis from the interview and UCD datasets used Shepherd et al.'s framework for interagency collaboration, including elements such as Memoranda of Understanding, Shared Goals, Policies and Procedures, Role Descriptions, Information Sharing, and Regular Meetings. Key challenges included communication barriers, administrative complexity, resource limitations, partnership difficulties, and Veteran-specific obstacles. Geographic diversity, unclear role definitions, and lack of centralized information sharing were major barriers to effective collaboration. Many findings applied to multiple framework elements. Our integrated data informed an implementation blueprint for potential implementation by VHA HUD-VASH teams nationwide. This project offers a structured approach to addressing the complex housing needs of aging and physically disabled Veterans through improved community collaboration. Future research should evaluate the effectiveness of the implementation blueprint in practice.

Abstract citation ID: igaf122.1781

### SUPPORTING AGING-IN-PLACE: AN INNOVATIVE PROGRAM FOR OLDER ADULTS LIVING IN SUBSIDIZED HOUSING

Uri Amir-Koren, Jasmine Akman, Karen Zurlo, and Ayse Akincigil, *Rutgers University, New Brunswick, New Jersey, United States*

Traditional Assisted Living Facilities (ALFs) remain financially out of reach for many low-income older adults. This study examines an innovative assisted living model that was developed in NJ, which decouples supportive services from residential facilities, allowing seniors to receive personal care, health monitoring, and social services while remaining in subsidized public housing. By leveraging existing public housing infrastructure, this approach provides a cost-effective alternative to institutional care, promoting aging in place while maintaining access to essential services. This study utilizes a SWOT (Strengths, Weaknesses, Opportunities, and Threats) analysis to evaluate the model's effectiveness, feasibility, and scalability. Findings indicate the program's strengths include affordability, flexibility, and resident autonomy, while the weaknesses highlight challenges related to care coordination, funding sustainability, and workforce capacity. The opportunities for expansion are significant given the policy environment unique to the state. However, threats such as lack of price control, reimbursement challenges, labor costs and workforce dynamics, and regulatory challenges must be addressed to ensure long-term success. By systematically evaluating this model, this paper contributes to the growing body of research on alternative long-term care strategies and provides policy and practice recommendations to enhance its implementation. Expanding this model has the potential to reshape aging policy, reduce institutionalization rates, and improve quality of life for low-income older adults, making assisted living more accessible and sustainable.

### SESSION 4215 (PAPER)

### SLEEP DISRUPTION AND SOCIAL VULNERABILITY: IMPLICATIONS FOR COGNITIVE AND PHYSICAL HEALTH IN AGING

Abstract citation ID: igaf122.1782

### CAREGIVER STRESSORS PREDICT SUBJECTIVE SLEEP QUALITY IN AFRICAN AMERICAN FAMILY CAREGIVERS

Miranda McPhillips[1], Glenna Brewster[2], and Elliane Irani[3], *1. Villanova University, Villanova, Pennsylvania, United States, 2. Emory University, Atlanta, Georgia, United States, 3. Case Western Reserve University, Cleveland, Ohio, United States*

There is a known association between caregiving stress and sleep quality in the general caregiving population. This study aims to identify specific caregiving stressors that predict subjective sleep quality among African American family caregivers of adults with chronic or disabling conditions. By focusing on this understudied population, we seek to uncover potential intervention targets to improve their sleep quality and overall health. This is a secondary data analysis of a community-engaged study about the social determinants of sleep health and cardiovascular risk in African American family caregivers. The Pittsburgh Sleep Quality Index was the subjective measure of sleep quality. Four measures of caregiver stress were used: three Caregiver Reaction Assessment subscales 1) lack of family support, 2) financial impact, 3) schedule disruption, and 4) relationship quality with the care recipient, measured by two items from the National Study of Caregiving. Data were analyzed using bivariate analyses and linear regression. Participants (n = 100) were primarily female (85%), caring for a parent (52%) and on average 59 years old. Three quarters of the sample (76%) had poor sleep quality. Lack of family support (r = 0.260), financial impact (r = 0.290), schedule disruption (r = 0.482) and negative relationship (r = 0.339) were positively correlated with sleep quality ($p < 0.01$). In the multivariate model, after controlling for age, gender and co-residence with the care recipient, schedule disruption (b = 1.651; p = 0.005) and relationship quality (b = 0.552; p = 0.039) remained statistically significant predictors of sleep quality. Tailored interventions aimed at managing caregiving schedules and promoting positive caregiving relationships can improve sleep quality in African American family caregivers.

Abstract citation ID: igaf122.1783

### PERSON-PLACE FIT AND QUALITY OF LIFE AMONG OLDER ADULTS IN DISADVANTAGED AREAS IN SWEDEN

Marianne Granbom[1], Afsaneh Taei[1], and Anders Kottorp[2], *1. Lund University, Lund, Skane Lan, Sweden, 2. Malmo University, Malmo, Skane Lan, Sweden*

Disadvantaged residential areas (DAs), often affected by territorial stigmatization, influence the health and quality of life in older adults. Nevertheless, older adults may prefer to stay in these areas due to emotional bonds. With the Person-Place Fit Measure for Older Adults (PPFM-OA), older adults self-assess how their home and community environments suit their needs. The aim of the study was to explore associations between person-place fit and quality of life among older adults living in different types of DAs. Survey data were collected from 459 older adults living in urban areas with low socioeconomic status and depopulated rural areas in Sweden. Person-place fit was measured using the Swedish 19-item version of the PPFM-OA and quality of life was assessed with WHOQOL-BREF. Mean age was 76 years (SD 7), with 51% being women and 13% were not born in Sweden. Linear regression models demonstrated significant associations between person-place fit and all aspects of quality of life (overall QoL, overall health, physical health, psychological health, social relationships, and environment) even after adjusting for sex, gender and ADL performance. No differences were found between residents of urban and rural DAs. The study shows that older adults' perceptions of how well their home and community environments meet their needs are associated with their quality of life. Furthermore, the results suggest that residential areas that, from an outsider perspective are considered non-age-friendly, may not be perceived as such by older the older residents.

Abstract citation ID: igaf122.1784

### MULTIDIMENSIONAL SLEEP PROBLEMS AS MEDIATORS BETWEEN RECESSION HARDSHIPS AND CHANGES IN PHYSICAL HEALTH

Julie Kirsch, *University of Wisconsin–Madison, Madison, Wisconsin, United States*

The 2008 economic Recession was associated with physical health declines and poor sleep outcomes. This study tests a novel composite measure of poor sleep as a mediator of Recession-related changes in physical health in a longitudinal sample of adults from the Midlife in the United States study (n = 2,674). Participants reported sleep problems across five dimensions (Regularity, Satisfaction, Alertness, Efficiency, and Duration) and functional health and total chronic conditions at pre-recession (2004-2006) and post-recession (2013-2014). Hardships included financial, housing, and job events attributed to the 2008 Recession. Adjusting for relevant covariates and pre-Recession sleep problems, regression models showed that recession hardships were positively associated with total post-Recession sleep problems (a = 0.05, 95% CI [0.03, 0.07], $p < .001$) and were positively associated with functional limitations (c = 0.03, 95% CI [0.02, 0.04], $p < .001$) and chronic conditions (c = 0.10, 95% CI [0.05, 0.15], $p < .001$). Further, post-recession sleep problems were associated with increases in functional limitations (b = 0.11, 95% CI [0.08, 0.14], $p < .001$) and chronic conditions (b = 0.48, 95% CI [0.38, 0.58], $p < .001$). The bootstrap estimate of the indirect effects of Recession hardships on health outcomes via post-Recession sleep problems were statistically significant (Functional limitations a*b = .004, 95%CI [.002, .006]; Chronic conditions a*b= 0.01, 95% CI [.006, .02]). More sleep health problems may be a key pathway through which Recession hardships lead to poorer health in middle and older adulthood and may be important targets for intervention.

Abstract citation ID: igaf122.1785

### OBJECTIVE COGNITIVE PERFORMANCE MODIFIES THE ASSOCIATION BETWEEN POOR SLEEP HEALTH AND SUBJECTIVE MEMORY DECLINE

Muhammad Thalil, Martin Sliwinski, and Soomi Lee, *The Pennsylvania State University, University Park, Pennsylvania, United States*

Poor sleep health has been linked to subjective memory decline in older adults, yet the underlying mechanisms remain unclear. This study examined how objective cognitive performance may modify the relationship between sleep health and subjective memory decline in two cohorts of adults across adulthood. Two-wave longitudinal data were drawn from the National Health and Aging Trends Study (NHATS 2013 & 2014, n = 911; age 65+) and the Midlife in the United States Study (MIDUS 2004-2005 & 2013-2014, n = 2,421; age 33–84). A composite measure of sleep health, based on the self-reported Ru-SATED dimensions (regularity, satisfaction, alertness, timing, efficiency, duration), was used (higher score=poorer sleep health). Objective cognitive performance was assessed using episodic memory and executive function in MIDUS, with the addition of orientation in NHATS. After adjusting for baseline subjective memory and socio-demographic and health covariates, adults with poorer sleep health at baseline had higher odds of subjective memory decline in both the NHATS (OR: 1.21, 95% CI: 1.01-1.46) and MIDUS (OR: 1.24, 95% CI: 1.10-1.41) samples. In the NHATS only, the odds of subjective memory decline associated with poorer sleep health were more pronounced for older adults with worse cognitive performance at baseline (interaction p = 0.041). Middle-aged and older adults with poorer sleep health were at a higher risk of declining subjective memory. Those with both poorer sleep health and poorer objective cognitive performance were at particularly higher risk of subjective memory decline. These findings emphasize the importance of sleep-focused interventions for aging populations to help mitigate the risks of cognitive decline.

### SESSION 4220 (SYMPOSIUM)

Abstract citation ID: igaf122.1786

### PRESIDENTIAL SYMPOSIUM: COMPELLING EVIDENCE TO ADVANCE GERONTOLOGY EDUCATION ON LGBTQIA+ AGING

Chair: Laura Donorfio
Co-Chair: Brian Chapman

The goal of the 2025 AGHE Presidential Symposium is to enhance awareness, foster interdisciplinary dialogue, and promote evidence-based strategies to support aging sexual and gender minority (SGM) populations within gerontology and geriatric education, practice, and research. This symposium will provide innovative approaches, pedagogical strategies, applied research, and evidence-based tools to advance inclusive curricula, policies, and services. These efforts aim to address the unique challenges faced by LGBTQIA+ older adults and to prepare future professionals to meet their needs. The first presentation shares work focused on using the film Gen Silent, as an education tool to increase aging providers knowledge and empathy for LGBTQIA+ older adults. The second presentation showcases semi-structured interview data from transgender, non-binary, and intersex (TNBI) older adults to enrich gerontology and geriatrics education by incorporating real-world narratives. The third presentation examines the unique challenges faced by older adults living with HIV in medical settings and how these experiences can be effectively incorporated into gerontology curricula through medical rhetoric frameworks. The fourth presentation explores the development of the LGBTQ Aging Toolkit and the HIV & Aging Toolkit and how they can provide ready-to-use interprofessional modules to support faculty in integrating LGBTQIA+ aging content into their curricula. We conclude with a lively discussion exploring how to support aging SGM populations by taking action through education across GSA member groups.

Abstract citation ID: igaf122.1787

### FINDINGS FROM THE GEN SILENT SURVEY PROJECT: A MIXED METHODS PRE-POST DESIGN

Alison Rataj[1], Kristen Porter[2], and Beth Dugan[2], *1. College of Health and Human Services, Center on Aging and Community Living, Durham, New Hampshire, United States, 2. University of Massachusetts Boston, Boston, Massachusetts, United States*

Providers lack access to accessible, virtual, cost-effective training resources to safely serve LGBTQ+ older adults. The purpose of this study was to evaluate change in knowledge, attitudes, and anticipated behaviors of providers after watching the film Gen Silent through a quasi-experimental pretest-posttest survey design. Primary data was collected from aging service providers (e.g., long-term care ombudsmen, nurses, social workers, site supervisors) across one in-person (pre-pandemic) and two-virtual (during pandemic) screenings. Data were analyzed using the Wilcoxon signed-rank test as well as, linear and logistic regression models. Most participants (79%) improved their survey score after watching Gen Silent. Scores on eight out of nine measures of knowledge, attitudes, and anticipated behavior improved after watching the film. Between group differences were found. Baby Boomers were significantly more likely to show improvement on posttest scores ($B = 0.19$, $p < .05$) compared to participants from other generations. Additionally, LGBTQ+ individuals were significantly more likely to score higher on the pretest ($B = 0.26$ $p < .01$), similarly, participants caring for a LGBTQ+ individual were more likely to have higher pretest scores ($B = 0.20$, $p > .05$). Eighty-three (83%) of participants indicated that their thoughts and views changed after watching the film due to deepened understanding of LGBTQ+ aging issues. Findings imply that the use of Gen Silent is a relevant educational tool that can be utilized virtually to increase providers' awareness of the lived experiences of, and empathy toward, LGBTQ+ older adults.

Abstract citation ID: igaf122.1788

### USING THE VOICES OF TRANSGENDER, NON-BINARY AND INTERSEX OLDER ADULTS TO ENHANCE GERONTOLOGY/GERIATRICS EDUCATION

Nik Lampe[1], and Alexandra "Xan" Nowakowski[2], *1. University of South Florida, Tampa, Florida, United States, 2. Florida State University College of Medicine, Tallahassee, Florida, United States*

In this presentation, we utilize semi-structured interview data from transgender, non-binary, and intersex (TNBI) older adults (N = 50) to enrich gerontology and geriatrics education. By incorporating real-world narratives, educators can provide students and professionals with a deeper understanding of the healthcare needs, systemic and interpersonal barriers, and resilience strategies of TNBI older adults. This presentation aligns with the 2025 AGHE Presidential Symposium's goal of advancing LGBTQIA+ aging education by showcasing applied research and pedagogical strategies that promote critical discussions on researching and delivering evidence-based care. Attendees will gain practical tools for integrating case studies into curricula, ensuring a more inclusive, research-informed approach to addressing the needs and experiences of TNBI older adults.

Abstract citation ID: igaf122.1789
**"WE WERE NOT SUPPOSED TO LIVE": TEACHING MEDICAL RHETORIC THROUGH THE LENS OF OLDER ADULTS LIVING WITH HIV**
Nels Highberg, *University of Hartford, Avon, Connecticut, United States*
The HIV/AIDS epidemic has evolved from a terminal diagnosis to a chronic condition, resulting in a generation of survivors who were "not supposed to live" now navigating healthcare systems as older adults. This presentation examines the unique challenges faced by older adults living with HIV in medical settings and how these experiences can be effectively incorporated into gerontology curricula through medical rhetoric frameworks. Drawing on classical rhetorical analysis (ethos-pathos-logos), this session demonstrates how faculty can facilitate the critical examination of healthcare policies, institutional communications, and medical discourse affecting older HIV-positive adults. As documented in advocacy resources such as those provided by Lambda Legal, particular attention will be given to discrimination in long-term care facilities and healthcare services. The presentation offers strategies for developing and adapting case studies that remain pedagogically effective even in educational environments where DEI content faces restrictions and explores how AI tools can help create or translate case studies to address regional policy variations while maintaining essential learning objectives. Participants will leave with concrete teaching strategies that promote student understanding of healthcare discrimination against older adults with HIV, rhetorical frameworks for analyzing medical texts that transcend political constraints, and adaptable approaches to case-based teaching in changing educational landscapes.

Abstract citation ID: igaf122.1790
**INTEGRATING LGBTQ AGING RELATED CONTENT INTO POST-SECONDARY CURRICULA**
Rajean Moone, *University of Minnesota, Minneapolis, Minnesota, United States*
Everyone deserves access to high-quality, compassionate care as they age. However, many LGBTQ older adults have faced discrimination when seeking services and often experience greater health, economic, and social disparities compared to their peers. With smaller informal support networks, they are more likely to rely on formal services—yet fear and past discrimination can discourage them from doing so. It is essential for students, especially those in healthcare-related fields, to understand the unique needs and challenges of LGBTQ aging. Despite this, few courses address the topic, and many faculty members have not been exposed to LGBTQ aging in their academic training. To bridge this gap, the Minnesota Northstar Geriatric Workforce Enhancement Program (GWEP) developed two resources: the LGBTQ Aging Care Toolkit (2023) and the HIV & Aging Toolkit (2024). These community-developed, peer-reviewed toolkits provide ready-to-use interprofessional modules to support faculty in integrating LGBTQ aging content into their curricula. This session will explore the development of these toolkits and strategies for incorporating them into existing aging-related courses.

### SESSION 4225 (PAPER)

Abstract citation ID: igaf122.1791
**TECHNOLOGY IN AGING**
Digital tools in Aging including telehealth, smart phones and video capture research are described.

Abstract citation ID: igaf122.1792
**VALIDITY RESEARCH ON ECOLOGICAL MOMENTARY ASSESSMENT IN OLDER ADULTS: A SCOPING REVIEW**
Thanakrit Jeamjitvibool[1], Wirampa Tanglai[2], and Krisada Suamchaiyaphum[3], *1. University of Illinois at Chicago, College of Nursing, Chicago, Illinois, United States, 2. University of Illinois Chicago College of Nursing, Chicago, Illinois, United States, 3. The University of Alabama at Birmingham, Birmingham, Alabama, United States*
Ecological momentary assessment (EMA) is a longitudinal self-report method used to assess fluctuating symptoms and health behaviors in real time within individuals' natural environments during daily activities. Increasing use of technologies such as mobile applications has enhanced EMA research. However, ensuring EMA's validity and reliability for older populations remains challenging. This study summarized the literature on validity testing of EMA in older adults. We employed the JBI Scoping Review Framework and reporting guidelines. We systematically searched PubMed, Web of Science, CINAHL, PsycInfo, and Embase for relevant articles published through March 10, 2025. Included studies were empirical research published in English and measured behavior or compared EMA to other methods. We excluded review articles, study protocols, and research exclusively utilizing ambulatory assessments without self-reported symptom data. We identified 16 studies, most involving cognitive testing, physical activity, daily activity, depression, pain, alcohol consumption, and fatigue. Most studies (15 of 16) were published after 2016. Sample sizes ranged from 20 to 311 participants. Of the studies, 75% employed mobile applications, while the others utilized Actiwatchs and email. Adherence rates varied between 61% and 95%, with study durations ranging from 5 to 316 days. Leveraging the advantages of EMA design, most studies assessed construct validity, particularly convergent validity, along with criterion and ecological validity. EMA demonstrated satisfactory validity in research involving older adults. The sampling methods and assessment tools used in the studies enhanced EMA's effectiveness in capturing real-time data on multiple symptoms and health-related behaviors within this population.

Abstract citation ID: igaf122.1793
**ENHANCING COGNITIVE CARE PLANNING: HEALTHCARE PROVIDERS' INSIGHTS ON PAPER-BASED VS. DIGITAL TOOLS**
Shaoqing Ge[1], Xaviera Xiao[2], Katherine Britt[3], and Bin Huang[4], *1. University of Texas at Austin School of Nursing, Austin, Texas, United States, 2. Brain Check, Austin, Texas, United States, 3. University of Iowa, Iowa City, Iowa, United States, 4. BrainCheck Inc, Austin, Texas, United States*
Cognitive care planning (CCP) is an effective intervention to manage and improve health among patients with cognitive impairments like Alzheimer's Disease and Related Dementias (ADRD). Despite its benefits, CCP is underutilized in clinical settings. The Alzheimer's Association (AA) has developed paper-based guidelines to promote CCP. Online tools are also emerging to support healthcare providers and patients in implementing CCP, automatically generating assessment results and care plans. This study examines the perceptions and impact of a comprehensive online platform compared to AA's paper/pdf-based guideline through 17 interviews with healthcare providers, focusing on providers' perceptions of using these tools in clinical settings. Our findings demonstrate several perceived advantages of the all-inclusive digital cognitive assessment and care planning tool. Such advantages include the necessity and value of a computerized assessment that can be easily adapted to existing electronic medical record systems. Additionally, the time-saving potential of an automated assessment and automatically generated care plan was also mentioned. Healthcare providers also mentioned challenges that may arise from an all-inclusive platform wherein assessments and surveys are carried out uniformly amongst patient populations, causing concerns surrounding the lack of nuance and "humanness". Finally, concerns about technology acceptance amongst older adult patients and older adult caregivers and reimbursement-related issues were also highlighted. In conclusion, the digital cognitive assessment and care planning tool is acknowledged by providers as beneficial for promoting CCP. Our study provides insights into the impact of digital platforms in supporting older adults with cognitive decline and AD/ADRD and their caregivers in clinical care settings.

Abstract citation ID: igaf122.1794

### REMOTE SYMPTOM ASSESSMENT IN PALLIATIVE CARE: PATIENT AND CLINICIAN PERSPECTIVES

Irina Mindlis[1], Emily Bivona-Maldonado[2], Maureen Ekwebelem[2], Catherine Samaniego[2], Samprit Banerjee[2], Zilong Yu[2], and Daniel Shalev[2], *1. Rutgers University, New York, New York, United States, 2. Weill Cornell Medicine, New York, New York, United States*

Palliative care (PC) aims to improve quality of life by managing physical and psychological symptoms. High symptom burden is a primary driver of PC referral, yet gaps in symptom assessment limit the impact of PC interventions. Given that ambulatory PC visits often occur weeks apart, day-to-day symptom patterns may go unrecognized and recall bias during appointments may further hinder the quality of data on which treatment decisions are based. Remote symptom assessment (RSA), the collection of symptom data outside of clinical visits via digital or telephonic methods, offers a potential solution but remains underutilized. We aimed to understand the perspectives of PC patients and clinicians on RSA to improve symptom monitoring in ambulatory PC. We conducted a descriptive-exploratory qualitative study using semi-structured interviews with 10 PC patients and 9 PC clinicians at a single ambulatory PC program. Thematic analysis, using hybrid inductive-deductive coding, identified key themes regarding RSA's benefits, desired features, and challenges. Participants viewed RSA as a tool to empower both patients and clinicians in managing symptoms. Desired features included facile user interfaces, structured reminder systems, and alerts for clinical changes. Participant concerns included technical complexity, time burden, and information burden. Ultimately, both patients and clinicians felt that RSA could enhance symptom management in the ambulatory PC setting, but only if developed with high usability and minimized burden. These findings highlight the need for user-centered RSA tools tailored to the ambulatory PC setting. Future research should focus on co-designing and piloting RSA platforms to optimize feasibility and clinical integration.

### SESSION 4227 (BIOLOGICAL SCIENCES INVITED SYMPOSIUM)

Abstract citation ID: igaf122.1795

### MICROBIOME, HOST-PATHOGEN INTERACTIONS, AND AGING

Chair: Cristal Hill

Abstract citation ID: igaf122.1796

### PARITY AFFECTS THE GUT MICROBIOME, GUT HOMEOSTASIS, AND GLUCOSE METABOLISM IN AGED FEMALE MICE

Megan Wong[1], Audrey Pierce[1], Kerry Wellenstein[2], and Jennifer Lee[3], *1. Tufts University School of Medicine, Medford, Massachusetts, United States, 2. JM-USDA HNRCA at Tufts University, Boston, Massachusetts, United States, 3. The JM-USDA Human Nutrition Research Center on Aging at Tufts University, Boston, Massachusetts, United States*

Metabolic health declines with age. Parity, a history of prior reproduction, is implicated in regulating long-term metabolic health. Despite nearly 75% of women having a history of parity, virtually all preclinical studies use nulliparous mice, thus limiting the translation potential to improve women's health. The gut microbiome and gut epithelium contribute to regulating host metabolism, but their roles in the context of parity remain unclear. Thus, this study aimed to determine parity effects on the gut microbiome, gut epithelium, and glucose metabolism in aged female mice. We metabolically phenotyped 16-21-month-old nulliparous and parous chow-fed C57BL/6 female mice. In the same mice, we sequenced the cecal metagenome, measured the cecal metabolome by mass spectrometry, and measured ileal expression of genes related to inflammation and cellular senescence. Compared with nulliparous mice, age-matched parous mice were more glucose tolerant despite having greater body weight and lean mass. Analyses of the cecal metagenome and metabolome identified several microbial species, including Lactobacillus reuteri, and specific metabolic pathways pointing to a unique signature of lipid metabolites, that were differentially regulated by parity. The expression of pro-inflammatory T-cell markers and hallmark cellular senescence genes were reduced by up to 10-fold in the ileum of parous mice versus nulliparous controls. Altogether, these data indicate an effect of parity on the gut microbiome and gut epithelium that likely regulates glucose metabolism in aged female mice. Identifying parity-dependent changes in the gastrointestinal tract may reveal novel gut-based targets with translational potential to better support women's health outcomes across the lifespan.

Abstract citation ID: igaf122.1797

### SEROTONIN SIGNALING IN HOST-PATHOGEN RESPONSES

Sean Curran[1], and Tripti Nair[2], *1. University of Southern California Leonard Davis School of Gerontology, Los Angeles, California, United States, 2. University of Southern California, Los Angeles, California, United States*

When an organism encounters a pathogen, the host innate immune system activates to defend against pathogen colonization and toxic xenobiotics produced. C. elegans employ multiple defense systems to ensure survival when exposed to Pseudomonas aeruginosa including activation of the cytoprotective transcription factor SKN-1/NRF2. Although wildtype C. elegans quickly learn to avoid pathogens, here we describe a peculiar behavior in animals with constitutive activation of SKN-1 whereby they choose not to leave the pathogenic environment even when a non-pathogenic and healthspan-promoting food options are available. SKN-1 activation, specifically in neurons and intestinal tissues, orchestrates a unique transcriptional program which leads to defects in serotonin signaling that is required from both neurons and non-neuronal tissues. Serotonin depletion from SKN-1 activation limits pathogen defenses capacity, drives the pathogen-associated apathy behaviors and induces a synthetic sensitivity to selective serotonin reuptake inhibitors. We further define the serotonin receptors required for this behavioral response to Pseudomonas. Taken together, our work reveals interesting insights into how animals perceive environmental pathogens and subsequently alter behavior and cellular programs to promote survival.

Abstract citation ID: igaf122.1798

### LONGITUDINAL DYNAMICS OF THE GUT MICROBIOME AND SERUM PROTEOME OVER THE MOUSE LIFESPAN

*Yanjiao Zhou[1], Yair Dorsett[2], Ruixuan Li[2], and Kun Chen[2], 1. UConn Health, Farmington, Connecticut, United States, 2. University of Connecticut Health Center, Farmington, Connecticut, United States*

In mice, the gut microbiota undergoes dynamic changes from early development through aging. Parallel to these microbial dynamics, the host serum proteome reflects systemic physiological states and responses to microbial and environmental cues. However, longitudinal studies integrating both the gut microbiome and serum proteomics in the same animals remain limited. We collected stool samples from 15 mice every two weeks and blood every three months, from young adulthood until death at around 2–3 years of age. Principal component analysis and DE-SWAN analysis revealed five waves of microbiome changes across the lifespan, with a major compositional shift occurring in middle age. Longitudinal clustering identified four distinct patterns of microbial dynamics. Proteomic analysis of serum markers revealed that different functional groups of host proteins exhibited distinct age-related trajectories. Inflammatory cytokines and chemokines rose sharply in late middle age, whereas central nervous system (CNS)-associated markers increased predominantly in old age. Increases in CNS serum markers correlated most strongly with expansions in Proteobacteria and, even more so, with Cyanobacteria. Such integrated analyses enable the identification of coordinated host–microbe changes, offering a systems-level view of aging biology and potential biomarkers for age-associated dysfunction.

Abstract citation ID: igaf122.1799

### HARNESSING PROTEIN INTAKE AND MICROBIOME INTERACTIONS TO IMPROVE HEALTHSPAN

Cristal Hill[1], and Prerana Vaddi[2], *1. University of Southern California, Los Angeles, California, United States, 2. University of Southern California, Leonard Davis School of Gerontology, Los Angeles, California, United States*

Substantial evidence demonstrate that gut dysbiosis affects metabolic health, particularly while advancing in age. Notably, females tend to experience greater age-related comorbidities such as diabetes and neurodegeneration, many of which are driven by metabolic dysfunction and cellular senescence. Previous work from our lab has demonstrated that dietary protein restriction (DPR) induces fibroblast growth factor 21 (FGF21) to improve metabolic health and extend lifespan in male mice. However, the effects of DPR on gut health in aged female mice remains unclear. In this study, we investigate how DPR influences metabolic health and the gut microbiome in aged female mice. Female mice subjected to DPR at 16 months until 22 months of age exhibited notable improvements in metabolic health. Relative abundances of Clostridium were reduced, while Faecalibaculum rodentium and Kineothrix were increased. Gene set enrichment analysis (GSEA) revealed that bacterial groups associated with membrane integrity and metal ion binding were most enriched during DPR.

### SESSION 4230 (SYMPOSIUM)

Abstract citation ID: igaf122.1800

### RISK AND PROTECTIVE FACTORS RELATED TO MENOPAUSE, WOMEN'S HEALTHY AGING, AND COGNITION

Chair: Amber Watts
Co-Chair: Amy Costa
Discussant: Amber Watts

About 1.3 million women per year in the U. S. experience the menopause transition. Given modern life expectancies, women spend more than one-third of their lifetime post-menopause. The menopause transition is a critical window for positive and negative influences on women's healthy aging and cognition. This symposium proposes to bring together research from five laboratories investigating risk and protective factors for menopause health and women's healthy aging including lifestyle (sleep, exercise), environment (pollutants, greenspace), and psychological risks (childhood adversity, depression) that occur throughout women's lifelong development. The projects present a range of methodologies including clinical intervention, cognitive and brain health assessment, survey design, and narrative review. Results of these projects demonstrate that aging trajectories differ by sex and that characteristics of the menopause transition are important for physical, psychological, and cognitive health outcomes related to aging. Results inform the development of effective interventions for healthy menopause and aging in women. They add to theoretical knowledge regarding the key contributors to healthy menopause and aging in women. Additionally, this symposium provides an opportunity for junior scholars (pre-doctoral, early career) and senior scholars (mid- to late-career) to connect to advance scholarship and to build representation of menopause research at GSA, a topic that has recently been under the public spotlight in news media, as well as privately and federally funded research. Women's Issues Interest Group Sponsored Symposium

Abstract citation ID: igaf122.1801

### THE MENOPAUSAL MIND: HOW SLEEP AND IMMUNE FUNCTION SHAPE COGNITIVE HEALTH

Ashley Curtis, Amy Costa, Natasa Billeci, Melanie Stearns, Fareeha Hussaini, Amy Brown, and Christina McCrae, *University of South Florida, Tampa, Florida, United States*

Sleep health and poor immune functioning are linked to worse cognition. There are known sex differences in both functions (2x higher insomnia prevalence and increased low grade inflammation in aging women). However, whether interactive associations impact cognition across the menopausal transition remains understudied. This study examined interactive associations between insomnia symptoms and immune status on cognition in mid-life women. Middle-aged women with insomnia and cognitive complaints (N = 51, aged 42-64 years) completed the Insomnia Severity Index, Immune Status Questionnaire, Cognitive Failures Questionnaire (CFQ) and Everyday Memory Questionnaire at baseline and 3-month follow-up. Multiple regression analyses examined interactive associations between proportional changes in insomnia severity, immune status and cognition, and evaluated whether these were further moderated by menopause status (STRAW +10 pre/peri/post staging), controlling for age. Menopause status significantly moderated interactive insomnia severity/immune functioning associations with CFQ-distractibility (R-squared=.08, p=.04), with a trend for CFQ-Total (R-squared=.07, p=.07). Specifically, worsening of insomnia severity was associated with increased distractibility only in women who experienced greatest decline in immune functioning and were either peri-menopausal (B=.43±.18, p=.02) or post-menopausal (B=.53 ± =.22, p=.02). Similarly, worsening insomnia severity was associated with increased global cognitive complaints in peri-menopausal women with moderate (B=.33±.16, p=.047) or greatest (B=.50±.20, p=.02) decline in immune functioning, and in peri-menopausal women with greatest immune functioning decline (B=.57±.25, p=.02). Sleep and immune responses may synergistically interact with sex-hormone disruption during menopausal transition and impact neurocognitive health. Findings point to sex-specific sleep and associated mechanistic targets for early monitoring and intervention to mitigate risk of cognitive decline and/or neurodegeneration.

Abstract citation ID: igaf122.1802

### SMART BRAIN: STUDY OF MENOPAUSE AND RESISTANCE TRAINING ON BRAIN HEALTH

Cindy Barha, *University of Calgary, Calgary, Alberta, Canada*

Two-thirds of all persons with dementia are females and females have a 2-fold greater lifetime risk of developing dementia than males. Exercise is a promising lifestyle intervention for promotion of cognitive function. We have shown that this is particularly true for older, postmenopausal females compared with older males. Perimenopause – one of the most significant endocrine transitions in a female's life – negatively impacts the female brain and is believed to be a key contributor to a female's greater lifetime risk for dementia. Thus, perimenopause may be a critical window to implement exercise interventions for the promotion of female brain health. There is a dearth of randomized controlled trials that examine the impact of exercise of any type on brain health in midlife (i.e., 40 to 60 years of age). Resistance training, specifically, may provide multi-system benefits for females as they transition through menopause. In a small pilot study, 35 perimenopausal and early postmenopausal participants underwent a 2x/week, 9-month resistance training intervention. Cognition was measured with the NIH Toolbox, a valid neuropsychological test battery, and task-induced hemodynamic response in the brain was measured using functional near infrared spectroscopy at baseline and trial-completion. Compared with the waitlist control group, a significant improvement was seen in cognitive flexibility in the intervention group. Non-significant improvements were also seen in the intervention group in processing speed, inhibitory control and verbal episodic memory delayed recall. These findings indicate that resistance training is a promising intervention for promoting cognition in midlife.

Abstract citation ID: igaf122.1803

### IMPACT OF GREENSPACE AND AIR POLLUTANTS ON WOMEN DURING MENOPAUSE

Sneha Dhanavanthri Muralidhara[1], and Candace Brown[2], *1. The University of North Carolina at Charlotte, Charlotte, North Carolina, United States, 2. University of North Carolina, University of North Carolina, Charlotte, North Carolina, United States*

Previous research has highlighted the diverse experiences of women during menopause, revealing significant variations in health outcomes both during midlife and later years. While much attention has been given to the role of individual lifestyle and social factors in contributing to these disparities, the impact of environmental factors on menopause and related health outcomes among middle-aged women remains limited. This narrative review aimed to summarize and interpret the literature on the environmental impact of greenspace, air pollution, and particulate matter on menopause and related health outcomes among middle-aged women. A systematic search of PsychInfo, Pubmed, Web of Science, CINAHL, and ProQuest was conducted for relevant scientific literature published between January 2014 and November 2024. Following inclusion and exclusion criteria, 18 articles were selected out of 9805 articles obtained. Emergent themes related to health and women's aging included: 1) impact of greenspace; 2) air pollution and cardiovascular health; and 3) air pollution and the evolution of menopause. Greenspace was more beneficial for women with lower socioeconomic status (SES) and worse health status than those with higher SES. Higher exposure to resident ambient particulate matter was associated with accelerated atherosclerosis in women during early midlife, whereas long-term exposure contributed to elevated risk of atherosclerosis among post-menopausal women. Particulate matter exposure may also elevate the risk of cardiovascular disease in menopausal women, thereby accelerating aging. Inconsistent findings, due to diverse methods employed, require inquiry to further understand and identify environmental factors that substantially contribute to menopause and related outcomes.

Abstract citation ID: igaf122.1804

### FEASIBILITY AND ACCEPTABILITY OF BRIGHT LIGHT THERAPY GLASSES FOR OLDER WOMEN

Linlin Ding[1], Meina Zhang[2], Chelsea Howland[1], and Chooza Moon[1], *1. University of Iowa, Iowa City, Iowa, United States, 2. UT Health, United States*

Women's later years bring notable shifts in mood, sleep, and cognitive abilities influenced by menopause. Bright light therapy (LT) may help regulating circadian rhythms, improving sleep patterns, as well as enhancing emotional and cognitive functions. This study aimed to evaluate the feasibility of using bright light therapy glasses, contributing to preventive strategies that promote women's health. The intervention group participants were instructed to wear Re-Timer glasses (500 lux light at the eye) for at least 30 minutes each morning upon waking for two weeks. The control group participants received a sleep health education booklet. Participants completed an acceptability questionnaire and open-ended structured questions to receive feedback. A total of 9 participants (mean age (SD) = 61.78 (8.47)) were randomly assigned to either the intervention group (n=4) or control group (n=5) and successfully completed the protocol. The majority (77.78%, n=7/9) expressed strong satisfaction with involvement in the study. Among those in the intervention group, 100% (n=4/4) participants strongly agreed that the Re-Timer glasses were acceptable. Additionally, 75% (n=3/4) reported using Re-Timer glasses daily with an average of 32.23 minutes per day (range: 30-60 minutes). Participants reported improvements in focus and feeling refreshed throughout the day. However, some noted eye fatigue, difficulty using the glasses in low-light conditions, and issues with fit. Overall, participants reported the study was feasible and acceptable. Additional well-structured randomize controlled trial with larger sample sizes are needed to further validate their effectiveness.

Abstract citation ID: igaf122.1805

### ADVERSE CHILDHOOD EVENTS ARE ASSOCIATED WITH PHYSICAL, PSYCHOLOGICAL, AND COGNITIVE HEALTH IN MENOPAUSE

Emma Muller[1], Pilar Thangwaritorn[1], Jaime Perales-Puchalt[2], Melanie Meister[3], and Amber Watts[1], *1. University of Kansas, Lawrence, Kansas, United States, 2. University of Kansas Medical Center, Kansas City, Kansas, United States, 3. University of Kansas School of Medicine, Kansas City, Kansas, United States*

Adverse childhood experiences (ACEs) can have long term impacts on women's physical and psychological health and cognition. Despite growing evidence linking ACEs to negative health outcomes, the specific effects on women in midlife are underexplored. We present findings from an online survey of women (N=98) hypothesizing that higher ACES would be associated with more symptoms reported during the menopause transition. Measures include the ACE-Q, PROMIS depression and anxiety scales, self-reported cognitive complaints, and Pittsburgh Sleep Quality Index (PSQI). Our approach uniquely compares methods of characterizing childhood adversity (number vs. type of exposures). Women from the purposive sample (53.1% non-Hispanic white, 24.5% Hispanic/Latina, 17.3% African American, 8.2% rural-dwelling, 63.3% college-educated and above) were between the ages of 40-62 years of age (M age = 49.47). Our cross-sectional analysis showed higher ACE scores were associated with increased odds of reporting poor memory ($p=0.012$, OR=1.347) and difficulty sleeping ($p=0.009$, OR=1.44) adjusting for covariates. ACE scores and Anxiety scores showed a weak, but statistically significant positive correlation ($r=0.226$, $p=0.036$) that was attenuated below the level of significance in multivariate models. Specific adverse experiences such as emotional neglect and abuse were significantly associated with menopause symptoms, including difficulty concentrating and crying spells, and other symptoms including bladder issues, pain, bloating, anxiety and depression in univariate models ($r=0.316$–$0.626$, $p<0.05$). Our findings align with lifespan theoretical perspectives suggesting early life events have lifelong cumulative effects. They also have implications for clinical practice in the menopause transition, including screening for psychological and cognitive symptoms.

### SESSION 4235 (SYMPOSIUM)

Abstract citation ID: igaf122.1806

### BEYOND WORDS: CREATIVE ARTS THERAPIES AS PATHWAYS TO COGNITIVE AND MENTAL HEALTH IN LATER LIFE

Chair: Shoshi Keisari

Creative arts therapies are rooted in spontaneity, imagination, and creativity. They allow individuals to move beyond predictable patterns, engaging in novel and often surprising interactions. By fostering active participation, creative arts therapies allow people to engage as productive contributors to their communities. The arts can also bring to life the embodied and sometimes muted aspects of human experience that cannot be fully captured through verbal expression alone. This symposium brings together interdisciplinary research exploring how playful interactions, drama therapy, participatory theater, and phototherapy contribute to psychological resilience and cognitive health in later life. The first presentation investigates the impact of playfulness and improvisational theater on cognitive functioning and social engagement of older adults. It demonstrates how brief playful interactions can enhance executive functioning and positive emotions, exploring the physiological mechanisms underlying these effects. The second presentation reports on an RCT study examining tele-drama therapy for older adults with constricted mobility, highlighting its role in fostering psychological well-being and reducing depressive symptoms. The third presentation explores Theater-Based Participatory-Action-Research with caregivers, illustrating how theatrical methods help women caring for spouses with dementia navigate evolving relational roles and strengthen their sense of agency. The fourth study delves into phototherapy interventions for older women, revealing how visual self-representation can serve as a means of redefining societal perceptions of the aging body. Together, these studies underscore the significance of arts in aging research and practice. The discussion highlights practical applications for integrating these approaches into interventions that promote cognitive functioning and mental health in aging. Mental Health Practice and Aging Interest Group Sponsored Symposium

Abstract citation ID: igaf122.1807

### PLAYFUL MINDS: USING IMPROVISATIONAL THEATRE TO ENHANCE COGNITIVE FUNCTIONING IN OLDER ADULTHOOD

Shoshi Keisari[1], Yulia Golland[2], and Boaz Ben-David[3], *1. University of Haifa, Haifa, HaZafon, Israel, 2. Reichman University, Herzliya, HaMerkaz, Israel, 3. Reichman University, Herzliya, Tel Aviv, Israel*

Social playfulness, characterized by spontaneity and mutual enjoyment, allows individuals to step away from routine roles and engage in novel and surprising exchanges. Emerging evidence suggests that social playfulness is a promising approach for supporting cognitive functions in aging in a joyful and engaging way. This study examined how brief playful interactions that are based on improvisational theatre-based interactions enhance cognitive functions in older adults. Across three experimental studies, 150 participants aged 75 to 100 engaged in two types of interactions: playful interactions and a control activity (e.g., structured conversations or routine exercises). Sessions were conducted either face-to-face or online (Zoom). Cognitive tests and self-report measures were administered before and after each interaction. Findings indicate that brief playful interactions improved cognitive functions, including digit span performance, word fluency, and response times on the Flanker test, compared to control activities. Playful interactions also enhanced social closeness and increased positive emotions. Rooted in drama therapy principles, improvisational theatre offers a natural, engaging alternative to traditional cognitive training. Unlike structured cognitive interventions, playful interactions tap into spontaneity, co-creation, and embodied engagement, making them both enjoyable and accessible. These findings suggest that integrating playful interactions into routine care could offer a simple, scalable approach to supporting cognitive health in older adults.

Abstract citation ID: igaf122.1808

### TELE-DRAMA THERAPY FOR OLDER ADULTS WITH CONSTRICTED LIFE-SPACE MOBILITY: A RANDOMIZED CONTROLLED TRIAL

Talia Elkarif, Shoshi Keisari, and Hod Orkibi, *University of Haifa, Haifa, HaZafon, Israel*

Drama therapy is a mental health profession that aims to promote well-being by systematically using dramatic tools and techniques, such as storytelling, dramatic improvisation, and role-play. Research on drama therapy demonstrates its effectiveness in enhancing the mental health and well-being of older adults. Ensuring access to such interventions for older adults with constricted life-space mobility is particularly important, as they are at greater risk for social isolation and poor psychological well-being. The current mixed-methods, randomized controlled trial (RCT), aimed to explore the transition to group tele-drama therapy for older adults with constricted life-space mobility. The quantitative arm of the study examined the impact of a 12-week tele-drama therapy intervention on mental health indices, and the relationship between in-session change factors and changes in outcome measures. The qualitative arm of the study explored the experiences of participants, drama therapists and community coordinators. A total of 111 older adults (aged 63-102) with constricted life-space mobility were randomly assigned to treatment or control groups. The intervention, integrating life review therapy and drama therapy techniques, was delivered via Uniper Care, a social platform with accessible technology designed for the aging population. Findings revealed a significant Time X Group interaction, demonstrating the intervention's efficacy in reducing depressive symptoms and enhancing social connectedness, well-being, and personal growth. Thematic analysis of semi-structured interviews helped account for these quantitative findings, offering insights into treatment acceptability. These findings point to tele-drama therapy as an effective intervention for older adults with constricted life-space mobility, with implications for broader implementation.

Abstract citation ID: igaf122.1809

### THEATER-BASED PARTICIPATORY ACTION RESEARCH WITH WOMEN CARING FOR SPOUSES WITH DEMENTIA

Meira Medina-Junge[1], Shoshi Keisari[1], and Dovrat Harel[2], *1. University of Haifa, Haifa, HaZafon, Israel, 2. Tel-Hai College, Qiryat Shemona, HaZafon, Israel*

Spouses caring for partners with dementia navigate complex challenges shaped by their marital life course. This study explored the experiences of older women caring for spouses with dementia and examined how Theater-Participatory Action Research (T-PAR) can serve as a tool for self-exploration and transformation. Over 10 sessions, seven female caregivers engaged in theatrical methods to examine the personal, familial, and social transitions they were undergoing. Guided recall interviews were conducted while reviewing video-recorded sessions, and the data were analyzed using an inductive reflexive thematic approach, integrating the Six-Keys model for analyzing dramatic scenes. Three main themes emerged: (a) redefining roles within the couple's relationship, (b) redefining roles within their immediate environment, and (c) redefining roles in relation to healthcare institutions and authority figures. These themes were further captured through three experiential continua: (a) integration vs. splitting, (b) loneliness vs. shared experiences, and (c) suppression vs. activism. Findings highlight how theatrical methods provided a space for caregiver-women to explore shifting identities, rehearse coping strategies, and reimagine ways of relating to their partners and broader support systems. The discussion will focus on how drama-based participatory methods can empower spousal caregivers to navigate their evolving roles with agency and resilience.

Abstract citation ID: igaf122.1810

### DEVELOPING PHOTOTHERAPY-BASED INTERVENTIONS FOR OLDER WOMEN: INSIGHTS FROM THERAPISTS' EXPERIENCE

Silvia Piol, Shoshi Keisari, and Hod Orkibi, *University of Haifa, Haifa, HaZafon, Israel*

Phototherapy is a therapeutic approach in which trained therapists use photography and photographic materials to facilitate psychological growth and therapeutic change. This qualitative study explores how professionals employ phototherapy with older women, focusing on their perspectives on creative processes related to the aging female body. Aging women often navigate the impact of gendered ageism - the intersection of ageism and gender bias - which contributes to shape their self-perceptions and body image. In Western societies, where youthfulness is highly valued, older women may experience internalized ageist stereotypes that influence their sense of self. This study seeks to understand how phototherapy can serve as a means of reflection, validation, and self-expression in the face of these challenges. To investigate this, 16 international therapists working with middle-aged and older women through phototherapy were interviewed. Using reflexive thematic analysis, four key themes were generated: (1) encountering the aging body in photographs: navigating ageism and self-ageism; (2) exploring the aging body through visual metaphors and symbols; (3) photographs as a space for visibility and validation; and (4) claiming agency through active engagement in photography. The findings provide insights into how phototherapy techniques can be used to support aging women in exploring and expressing their embodied self-representations. These insights will inform the development of an arts-based intervention designed to address the psychosocial needs of older Italian women, fostering self-acceptance and agency through narrative and phototherapy practices.

### SESSION 4240 (SYMPOSIUM)

Abstract citation ID: igaf122.1811

### INNOVATIVE APPROACHES TO HEALTHCARE ACCESS, COMMUNICATION, AND CULTURALLY RESPECTFUL CARE FOR LGBTQ+ OLDER ADULTS

Chair: Korijna Valenti
Discussant: Jennifer Carnahan

LGBTQ+ aging research increasingly underscores the necessity of inclusivity, targeted provider education, and systemic responsiveness in healthcare settings. This symposium integrates findings from four complementary studies addressing these crucial areas among older LGBTQ+ populations. Dr. Valenti and colleagues apply Minority Stress Theory and Queer Gerontology frameworks to highlight affirming healthcare experiences of older gay men living with serious illnesses in the Deep South. Their analysis identifies clinician-patient connection, respectful communication, and sexuality disclosure as key facilitators of genuinely patient-centered care. Dr. Bybee presents an innovative educational method employing Advance Directive Mini-Plays, demonstrating that interactive theater significantly enhances healthcare providers' understanding and confidence in facilitating advance care planning discussions with LGBTQ+ patients and their support networks. Dr. Lee and colleagues offer results from a nationwide landscape assessment of the VA's Home-Based Primary Care program, identifying substantial gaps in staff training and preparedness to support older sexual and gender minority veterans effectively. Although clinicians expressed general awareness of unique LGBTQ+ healthcare needs, findings underscore a critical demand for enhanced, tailored training resources to promote affirming care practices. Dr. Hilgeman's formative evaluation utilizing the Long-Term Care Equality Index in a Veterans Community Living Center provides measurable evidence of improvements in inclusivity across resident services, employee benefits, and community engagement through structured assessments, comprehensive staff training, and targeted policy enhancements. Collectively, these presentations highlight advancements achieved, barriers remaining, and strategies required to systematically ensure equitable and affirming healthcare experiences for LGBTQ+ older adults, offering practical implications for future research, policy, and clinical practice improvements. Rainbow Research Group Interest Group Sponsored Symposium

Abstract citation ID: igaf122.1812

### RETHINKING BIAS: POSITIVE HEALTHCARE EXPERIENCES OF OLDER GAY MEN LIVING WITH SERIOUS ILLNESS IN THE DEEP SOUTH

Korijna Valenti[1], Stacy Smallwood[2], Ronit Elk[1], and Michael Barnett[3], *1. The University of Alabama at Birmingham, Birmingham, Alabama, United States, 2. Wake Forest University School of Divinity, Winston-Salem, North Carolina, United States, 3. Four Seasons Hospice, Flat Rock, North Carolina, United States*

Background: While research on health disparities for sexual and gender minority (SGM) older adults is progressing, less is known about positive experiences for those living with serious illness. This community-based participatory research study examines the perspectives of older cisgender gay men and caregivers from the Deep South, highlighting affirming encounters that challenge dominant narratives of discrimination. Applying Minority Stress Theory and a Queer Gerontology approach, we explore how inclusion, communication, and identity disclosure shape healthcare experiences for older gay men and discuss implications for systemic changes in care. Methods: Semi-structured interviews were conducted with cisgender gay men (n = 12) aged 50+ living with serious illness and caregivers (n = 4). Results: Thematic analysis identified three main themes: (1) experiences of inclusion and visibility—meaningful connections with clinicians and compassionate care; (2) positive communication—clear, respectful dialogue enhancing trust and engagement; and (3) sharing sexuality and its effect on care—while disclosure varied, recognition of partnerships improved healthcare interactions. Notably, participants overwhelmingly described positive experiences, thus raising questions about whether discrimination has shifted from overt to more covert forms, or if cultural humility efforts and clinician training may be aiding in successfully improving care. Discussion: Findings suggest healthcare professionals may be more adept at demonstrating inclusion, yet gaps remain in how systemic biases manifest in subtler ways. Future research should explore whether positive experiences reflect genuine progress, self-selection bias in research participation, or shifting forms of discrimination. Understanding what drives affirming care can inform best practices to ensure all older SGM adults receive equitable, patient-centered care.

Abstract citation ID: igaf122.1813

### MINI-PLAYS: AN INNOVATIVE METHOD TO IMPROVE CLINICIAN CONFIDENCE IN ADVANCE DIRECTIVE DISCUSSIONS

Sara Bybee, Jacqueline Telonidis, Jacqueline Eaton, Nicole Fleming, and Katherine Supiano, *University of Utah, Salt Lake City, Utah, United States*

For health care clinicians, there are few training opportunities on end-of-life or advance directive conversations with patients and families, especially with lesbian, gay, bisexual, transgender, queer, and other sexual and gender minority (LGBTQ+) persons. To address these gaps, the Hospice Foundation of America's Advance Directive Mini-Plays were produced during a hybrid continuing education event. Mini-plays ("Lily" and "Room 402") written by playwright Bryan Harnetiaux, were performed by two actors during a continuing education event for clinicians working in hospice-related contexts. Afterwards, a brief discussion was held, and attendees completed a REDCap questionnaire regarding their satisfaction and confidence level using a 5-point scale (1=not at all true and 5=completely true). Fifty attendees completed post-surveys. The majority were social workers (62.0%), followed by other mental health clinicians (14.0%), other health care clinicians (10.0%), chaplains (6.0%) and nurses (2.0%). Nearly all (96%) reported they would recommend this event to colleagues. Clinicians (84%) reported "very true" or "completely true" in response to the statement, "The mini-plays were an engaging way to learn about advance directives," and 80% reported "very true" or "completely true" in response to the statement, "The mini-plays improved my understanding of advance care planning." Implications include that advance directive mini-plays may be effective in conveying the importance of advance care planning and in training clinicians to foster these discussions with patients and their chosen support persons. Open-ended responses illuminated the difficulties in providing this content electronically, which may suggest the need for future productions of the mini-plays to be performed in-person.

Abstract citation ID: igaf122.1814

### PARTICIPATION IN THE LONG-TERM CARE EQUALITY INDEX TO SUPPORT OLDER LGBTQ+ MILITARY VETERANS

Michelle Hilgeman[1], Sylvia Haigh[2], Jessica Wilkins[1], Elizabeth Western[1], Teddy Bishop[1], Jamie Key[1], David Hollingsworth[3], and Whitney Mills[4], *1. Tuscaloosa VA Medical Center, Tuscaloosa, Alabama, United States, 2. VA Providence Healthcare System, Providence, Rhode Island, United States, 3. Fielding Graduate University, Washington, District of Columbia, United States, 4. Brown University, Providence, Rhode Island, United States*

There are more than 500,000 older LGBTQ+ Veterans currently living in the United States. The US Veterans Health Administration (VHA) aspires to align care preferences to individualized goals and to offer high quality care to all Veterans. Consistent with these priorities, a growing number of VHA Community Living Centers (CLCs) have participated in the Long-Term Care Equality Index (LEI) developed by the Human Rights Foundation and the national Advocacy & Services for LGBTQ Elders group (SAGE). We used mixed methods to describe the formative evaluation process used to complete the LEI tool in 2023 and again in 2025 in a 150-bed CLC in the Deep South that specializes in long-term care, dementia care, mental health recovery, and hospice/palliative care. LGBTQ+ Veterans, CLC residents, clinicians, and administrators contributed to the LEI completion in 2023 resulting in a High Performer designation. Scores across all 4 domains of the LEI were maintained at 100% (foundational policies) or increased in demonstrated best practices from 2023 to 2025 (i.e., 60% to 73% in resident services/supports, 58% to 67% in employee benefits/policies, and 14% to 29% in resident/community engagement). Online trainings were completed by more than 50% of CLC staff (N = 212) with 59.1% (n = 127) completing an introductory course and 56.3% (n = 120) completing modules on sexual orientation or care for older gender diverse Veterans. In conclusion, the LEI offers an opportunity for residential communities to identify, measure, and share best practices within healthcare systems to improve care quality for all residents.

Abstract citation ID: igaf122.1815

### NATIONAL LANDSCAPE ASSESSMENT OF SEXUAL AND GENDER MINORITY INCLUSIVITY OF THE VA'S HOME BASED PRIMARY CARE SERVICES

Joy Lee[1], Michelle Hilgeman[2], Tetla Menen[3], Nicholas Sweeney[4], Lorraine Stigar[5], and Jennifer Carnahan[6], *1. Mass General Brigham, Boston, Massachusetts, United States, 2. Tuscaloosa VA Medical Center, Tuscaloosa, Alabama, United States, 3. Roudebush VA Medical Center, Indianapolis, Indiana, United States, 4. University of Massachusetts Chan Medical School, Worcester, Massachusetts, United States, 5. Purdue University, West Lafayette, Indiana, United States, 6. Indiana University Center for Aging Research, Indianapolis, Indiana, United States*

Background: Sexual and gender minority (SGM) Veterans are more likely to require home care services than non-SGM Veterans. However, fear of discrimination deters many from seeking these supports. This landscape analysis seeks to assess team readiness and experiences with older SGM veterans within the U.S. Department of Veterans Affairs' (VA) Home Based Primary Care (HBPC) program. Methods: Using a multi-method approach, we conducted a survey of VA HBPC staff nationwide and five focus groups with staff at select sites. Results: Of approximately 10,000 HBPC staff invited to participate, 321 responded. Respondents included clinicians (44%; e.g., nurses and physicians), social workers (20%), and other staff (31%; e.g., nutritionists). Most reported minimal training on SGM care, with over half (54%) receiving less than three hours in the past five years; 12% received no training at all. Despite this, 43% strongly agreed they were aware of the unique challenges faced by SGM Veterans and 46% somewhat agreed. Regarding community resources for SGM Veterans, 71% endorsed confidence in finding facilities safe from discrimination, but only 61% endorsed confidence finding an affirming one. In focus groups, staff reported limited experience with SGM veterans and raised concerns about the inclusivity of community resources. They also noted a strong desire for training in SGM care. Conclusion: Our landscape assessment found that HBPC staff had concerns about the inclusivity and affirming nature of both VA and external services. Further training and resources are needed to enable the VA to deliver exemplary, affirming care for older SGM Veterans.

### SESSION 4245 (SYMPOSIUM)

Abstract citation ID: igaf122.1816

### WORK IN LATER LIFE: STRUCTURES AND SPACES/BARRIERS AND BENEFITS

Chair: Anne Barrett
Co-Chair: Dawn Carr

Despite the prevailing image of later life as centered on leisure, work – whether paid or unpaid – remains a core institution and activity in the lives of older adults. This symposium brings together multidisciplinary research conducted at both macro- and micro-levels to explore a range of contemporary issues surrounding work in later life, including the legal system's role in protecting older workers, the impact of technology on paid and unpaid work, the influence of the built environment on work patterns, and the health effects of paid work in later life. Focusing on the macro-level, Nathan examines how the legal framework meant to protect older workers can sometimes reinforce inequities, a concern that is growing with the rise of artificial intelligence in the workplace. Also focusing on the macro-level, Barrett and colleagues analyze how U.S policy documents depict age tech as a tool for reducing work and delivering better care, despite some evidence suggesting the opposite. DeJohn and colleagues examine spatial patterns in work, finding that public transit investments benefit employed older adults, while inadvertently displacing retirees. Addressing work at the micro-level, Cao and Carr investigate the effect of occupational autonomy on cognitive health and find that its benefits are greatest among more educated older adults. Together, these studies illuminate how work, as both an institution and activity, structures older adults' lives and impacts their quality of life.

Abstract citation ID: igaf122.1817

### AGING IN THE WORKPLACE: THE ROLE OF THE ADEA, ADA, AND AI IN SHAPING THE FUTURE OF WORK

Rima Nathan, *Florida State University, Tallahassee, Florida, United States*

Despite the protections of the Age Discrimination in Employment Act (ADEA) and the Americans with Disabilities Act (ADA), age discrimination remains deeply embedded in workplace structures. While these laws were designed to prevent bias, they often fall short due to weak enforcement and the intersectional nature of workplace discrimination. Older workers—especially women—face unique challenges, including compounded bias based on gender and caregiving responsibilities, disparities in promotions and pay, and systemic barriers to reemployment after workforce exits. This presentation will examine how the legal framework meant to protect older workers sometimes reinforces inequities instead. It will explore the disproportionate impact on women in mid-to-late career stages and the challenges for all of proving age bias under current law. Additionally, with the rise of artificial intelligence in hiring and employment decisions, new forms of age-based exclusion are emerging, further complicating legal and policy responses. Through a critical analysis of these issues, this presentation will highlight areas for policy reform, stronger enforcement mechanisms, and employer best practices to create truly inclusive workplaces. Attendees will gain a deeper understanding of how legal structures, workplace culture, and evolving technologies intersect to shape the experiences of older workers—and what can be done to dismantle these barriers.

Abstract citation ID: igaf122.1818

### BETTER CARE AND LESS WORK? FRAMINGS OF CAREWORK IN U.S. AGE TECH POLICY DOCUMENTS

Anne Barrett, Brianna Soulie, and Hope Mimbs, *Florida State University, Tallahassee, Florida, United States*

As technologies aimed at enhancing older adults' quality of life continue to advance, so does the optimism surrounding their potential to improve care. This framing appears in state policy documents that address technology and aging, but relatively few studies have examined them. Our study addresses this gap by analyzing age tech policy documents published over the past decade by major institutional actors in U.S. aging policy (e.g., Office of Science and Technology Policy; Department of Health and Human Services). Our analyses revealed that age tech is framed not only as providing older adults with better care, defined as more effective, more efficient, and safer care, but also as reducing care providers' work by making it less time-intensive and demanding. Critical analysis of these framings, however, revealed that age tech, especially digital forms centering on monitoring and surveillance, can have the opposite effect. It expands the work of care providers, paid and unpaid, by creating new tasks, like "tech support," and reduces time spent in more emotionally rewarding aspects of carework, like providing support in face-to-face interactions. Age tech also creates new forms of "self-care work" for older adults, like collecting and tracking biometric health data. These shifts can diminish rather than improve quality of care. We interpret these intertwined illusions of better care and less work through an analysis of the political economic underpinnings of technology's application to carework, which reveals how it contributes to the exploitation, alienation, and commodification of care workers, as well as care recipients.

Abstract citation ID: igaf122.1819

### IMPROVING ACCESS AND ABILITY TO AGE IN PLACE? EFFECTS OF LIGHT RAIL TRANSIT ON NEIGHBORHOOD WORKERS AND DEMOGRAPHICS

Amber DeJohn[1], Matthew Suandi[2], and Matthew Palm[3], *1. Florida State University, Tallahassee, Florida, United States, 2. Charles River Associates, Washington, District of Columbia, United States, 3. The University of North Carolina at Chapel Hill, Chapel Hill, North Carolina, United States*

Public transit may be a key intervention to address declining mobility in later life. Access to transit is related to health and well-being among older adults; however, older adults have relatively lower transit ridership compared to younger cohorts. Transit projects inherently reshape the built environment, influencing access to community amenities that support aging in place. Disentangling the true consequences of transit construction from pre-existing trends is challenging, since planners design transit systems based on political and economic considerations. While older adults may be a factor in transit planning, it remains unclear whether new transit infrastructure attracts or displaces them. To overcome reverse causality challenges, we identify counterfactual station locations, or alternative alignments, specified by planners in Environmental Impact Statements. These alignments then constitute control areas as part of a difference-in-differences estimation strategy. We include extensions associated with light rails systems opening since 2009. Ultimately, we find that transit investments may primarily serve working older adults, but retirees may be deterred from these neighborhoods. Specifically, the construction of light rail causes the proportion of older adults to decline in the immediate, walkable area of the new station. This effect is particularly significant in larger metropolitan areas included in our analysis, like Los Angeles. When considering the number of workers over 55 who live near newly built stations, we find a growing population as well as increasing numbers of workers with high salaries. In sum, not all older adults reap benefits from new transit investments.

Abstract citation ID: igaf122.1820

### OCCUPATIONAL AUTONOMY AND LATER LIFE COGNITIVE CHANGE: THE MODERATING ROLE OF EDUCATION

Katy Cao, and Dawn Carr, *Florida State University, Tallahassee, Florida, United States*

As a component of enriching occupational environments, work autonomy has been associated with workers' better physical and mental health. However, few longitudinal studies have connected autonomous work with later-life cognition. Furthermore, although education selects people into different career paths, few studies examined whether the cognitive health impact of autonomous work depends on education. To address these gaps, we ask the following research question: Is the association between autonomous work and later-life cognitive maintenance robust across different levels of education? Using the longitudinal Health and Retirement Study and Occupational Information Network (O*NET) linked dataset, we examine participants' 8-year cognitive change associated with occupational autonomy in mid-life (age 51-60), N=4179. Interaction effects were tested using Ordinary Least Squares (OLS) regression with robust standard error. Five occupational autonomy measures were selected to comprehensively evaluate different dimensions of autonomy: (1) freedom to make decisions, (2) frequency of decision-making, (3) unstructured work, (4) schedule regularity, and (5) work value of independence. Findings from OLS and post-estimation Wald tests show that education significantly moderates the association between cognitive maintenance and three occupational autonomy measures: Freedom of decision-making ($\beta=0.05$, $p<0.01$), unstructured work ($\beta=0.05$, $p<0.01$), and work value of independence ($\beta=0.02$, $p<0.10$). A margins plot shows that freedom to make decisions was positively associated with cognitive maintenance among people with 14 or more years of education but not among those without. Results imply that enhancing the employment quality among individuals with less education calls for a multiple-dimensional approach, including but not limited to improving their occupational autonomy.

### SESSION 4250 (SYMPOSIUM)

Abstract citation ID: igaf122.1821

### THE MOSAIC INITIATIVE: ADDRESSING MULTIMORBIDITY AMONG BLACK AMERICANS THROUGH COMMUNITY-ENGAGED RESEARCH

Chair: Courtney Thomas Tobin
Co-Chair: Angela Gutierrez
Discussant: Roland Thorpe

Multimorbidity, the co-occurrence of two or more chronic conditions, disproportionately affects Black Americans and remains an urgent public health challenge. The M.O.S.A.I.C. (Multimorbidity Outcomes & Solutions for African/Black Americans in California) Initiative is a community-engaged research collaborative dedicated to understanding and addressing multimorbidity among Black populations. This session brings together studies that examine key determinants of multimorbidity and strategies for intervention, providing a comprehensive foundation for the MOSAIC Initiative's efforts. Yan's study leverages data from the California Health Interview Survey to explore life course patterns of multimorbidity, highlighting age- and gender-specific disparities that call for targeted prevention efforts. Robinson's study examines the role of socioeconomic status in shaping multimorbidity, demonstrating that higher SES does not consistently translate to better health for Black Americans, particularly Black men. Mills' study investigates how perceived neighborhood racial composition influences multimorbidity, revealing significant age-specific associations that underscore the importance of place-based interventions. Finally, the MOSAIC Initiative's own research identifies best practices in health communication strategies for addressing multimorbidity among Black Los Angeles residents, emphasizing the need for tailored dissemination approaches across different age groups. Together, these studies provide critical insights into the structural, social, and environmental factors contributing to multimorbidity in Black communities. By integrating these findings, the MOSAIC Initiative aims to develop evidence-based, community-driven solutions to reduce multimorbidity disparities and improve health outcomes for Black Americans.

Abstract citation ID: igaf122.1822

### LIFE COURSE PATTERNS OF MULTIMORBIDITY AMONG BLACK RESIDENTS IN THE CALIFORNIA HEALTH INTERVIEW SURVEY

Xumeng Yan[1], Angela Gutierrez[2], Bo-Kyung Elizabeth Kim[3], and Courtney Thomas Tobin[1], *1. University of California, Los Angeles Fielding School of Public Health, Los Angeles, California, United States, 2. Western University of Health Sciences, Pomona, California, United States, 3. University of California, Los Angeles, Los Angeles, California, United States*

Multimorbidity, defined as having two or more chronic conditions, may exacerbate aging-related health disparities. However, the patterns of multimorbidity among Black Americans (BAs) over the life course remain less understood. Utilizing data from the 2023 California Health Interview Survey (CHIS), this study examined age- and gender-specific patterns of multimorbidity among BAs (n = 971) across six chronic conditions: diabetes, hypertension, asthma, stroke, heart disease, and high cholesterol. Logistic and Poisson regression models were used, adjusting for education level, poverty level, marital status, employment status, and health insurance status. Overall, 32.6% of BAs reported multimorbidity, and they consistently exhibited higher prevalence and mean counts of multimorbidity across all age groups compared to non-Hispanic White adults. In young adulthood (18-34 years), 11.1% of BAs reported multimorbidity, with hypertension and asthma being the most common co-occurring conditions. In middle adulthood (35-49 years), multimorbidity prevalence increased to 18.2%, with hypertension and diabetes emerging as the most frequent combination. By ages 50-64 years, 49.1% of BAs experienced multimorbidity, with hypertension and high cholesterol being the most prevalent co-occurring conditions. In older adulthood (65+ years), the multimorbidity burden remained high (45.7%), with hypertension commonly co-occurring with diabetes, high cholesterol, and asthma. Gender differences were also observed: men had higher multimorbidity rates in early and middle adulthood, while women faced a greater burden of multimorbidity later in life. These findings highlight the disproportionate burden of multimorbidity among Black women aged 50-64 years, underscoring the need for targeted preventive interventions tailored to age and gender.

Abstract citation ID: igaf122.1823

### SES AND MULTIMORBIDITY AMONG BLACK WOMEN AND MEN: A CASE OF DIMINISHING RETURNS?

Millicent Robinson[1], Courtney Thomas Tobin[2], Xumeng Yan[2], Christy Erving[3], Hans Oh[4], and Angela Gutierrez[5], *1. The University of North Carolina at Chapel Hill, Chapel Hill, North Carolina, United States, 2. University of California, Los Angeles Fielding School of Public Health, Los Angeles, California, United States, 3. The University of Texas at Austin, Austin, Texas, United States, 4. University of Southern California, Los Angeles, California, United States, 5. Western University of Health Sciences, Pomona, California, United States*

Higher socioeconomic status (SES) is typically associated with better health, but research suggests that Black Americans do not experience the same health benefits from SES as other groups. While multimorbidity (MM) is a growing public health concern, few studies have examined how SES influences MM among Black Americans. Using data from the Nashville Stress and Health Study, we investigated the relationship between several SES indicators—education, household income, occupational prestige, and a combined SES index—and MM among Black women (n = 325) and men (n = 296). Full sample and gender-stratified negative binomial regression models indicate limited associations between SES and MM, with effects primarily observed among men. Among women, no significant associations between SES and MM were found. Among men, having some college education was linked to higher MM (IRR=1.50, 95% CI: 1.06-2.12, p = 0.024), while a bachelor's degree or higher was protective (IRR=0.58, 95% CI: 0.38-0.89, p = 0.013). Middle-income men had lower MM (IRR=0.66, 95% CI: 0.46-0.96, p = 0.031), and occupational prestige was inversely associated with MM (IRR=0.99, 95% CI: 0.99-1.00, p = 0.022). These findings support the diminishing returns hypothesis, demonstrating that SES does not uniformly translate to better health for Black Americans, particularly Black women. The absence of significant associations for women highlights the need for further research on gendered SES-health relationships to address MM inequities in this population.

Abstract citation ID: igaf122.1824

### PERCEIVED NEIGHBORHOOD RACIAL COMPOSITION AND MULTIMORBIDITY AMONG YOUNG, MIDDLE-AGED, AND OLDER BLACK AMERICANS

Corina Mills[1], Courtney Thomas Tobin[2], Angela Gutierrez[3], Aisha Fletcher[4], Reeya Patel[4], James Huynh[5], and Roland Thorpe[1], *1. Johns Hopkins Bloomberg School of Public Health, Baltimore, Maryland, United States, 2. University of California, Los Angeles Fielding School of Public Health, Los Angeles, California, United States, 3. Western University of Health Sciences, Pomona, California, United States, 4. University of California, Los Angeles, Los Angeles, California, United States, 5. University of Michigan, Ann Arbor, Michigan, United States*

Multimorbidity, the co-occurrence of two or more chronic health conditions, is a major public health challenge in the U.S., disproportionately affecting Black Americans. Neighborhood conditions are crucial determinants of health disparities, affecting healthcare access, exposure to stressors, and availability of resources for disease management. However, the impact of individuals' perceptions about the residents of their community on multimorbidity remains underexplored. This study examined the association between perceived racial neighborhood composition (PNRC) and multimorbidity among young (18-35 years), middle-aged (36-49 years), and older (50 – 69 years) Black Americans. Data were drawn from 627 Black adults in the Nashville Stress and Health Study. Multimorbidity was based on the sum of 11 chronic physical health conditions. PNRC was derived from participants' assessment of the relative proportions of Black and White people in their neighborhoods [(0) Mostly Black, (1) About Half Black, (1) Mostly White]. Age-group-stratified negative binomial regression models were estimated, adjusting for gender, SES, and marital status. Results indicate that young adults who perceived living in half Black neighborhoods had more chronic conditions [incident rate ratio (IRR) = 1.81, 95% confidence interval (CI): 1.01-3.25] than young adults who perceived living in mostly Black neighborhoods. Older adults who perceived living in mostly White neighborhoods reported fewer chronic conditions [IRR = 0.77, 95% CI: 0.61-0.97] than older adults living in mostly Black neighborhoods. Findings demonstrate the distinct impact of PNRC across age groups, underscoring the need for age-specific, place-based interventions to address multimorbidity among Black Americans.

Abstract citation ID: igaf122.1825

### BEST PRACTICES IN ADDRESSING MULTIMORBIDITY AMONG BLACK LOS ANGELES RESIDENTS: INSIGHTS FROM THE MOSAIC INITIATIVE

Angela Gutierrez[1], Kyeongwon Kim[2], Anna Lucas-Wright[3], Bo-Kyung Kim[2], Sonya Brooks[4], and Courtney Thomas Tobin[2], *1. Western University of Health Sciences, Pomona, California, United States, 2. University of California, Los Angeles Fielding School of Public Health, Los Angeles, California, United States, 3. Charles R. Drew University of Medicine and Science, Los Angeles, California, United States, 4. University of California, Los Angeles, Los Angeles, California, United States*

The M.O.S.A.I.C. (Multimorbidity Outcomes & Solutions for African/Black Americans in California) Initiative is a community-engaged research collaborative focused on understanding and addressing multimorbidity among Black communities. A key component of this initiative is developing effective health communication strategies to bridge the gap between research and real-world impact. Despite growing efforts to address persistent health disparities among Black Americans, there remains a critical need to identify effective health communication strategies for disseminating health information about multimorbidity to Black communities. This study aimed to identify best practices in health communication for developing and sharing multimorbidity health information for Black Los Angeles residents and to determine key components for inclusion in a virtual toolkit. Data were collected through a consensus-building session with a Community Advisory Board (CAB) comprising healthcare providers, organizational leaders, and community stakeholders with 20+ years of community health experience. Structured group discussions were followed by a consensus-building survey. The top-ranked health communication strategy was printed pamphlets, followed by in-person education sessions. A three-way tie emerged for third place: virtual presentations, social media posts, and videos. Social media and podcasts were preferred for young adults (18–29) and early midlife adults (30–44). Printed materials and in-person education were preferred for adults 45 and older. These best practices inform The MOSAIC Initiative's efforts to develop tailored, community-driven health communication strategies for Black communities in Los Angeles. By aligning dissemination methods with age-specific preferences, these findings offer a foundation for more effective, culturally responsive multimorbidity interventions.

### SESSION 4255 (SYMPOSIUM)

Abstract citation ID: igaf122.1826

### EAST MEETS WEST: TAI CHI AND HEALTH PROMOTION

Chair: Timothy Kauffman
Co-Chair: Gong Chen
Discussant: Timothy Kauffman

East Meets West will focus on Tai Chi as an exercise activity that promotes health and wellness. The 1st paper explores the role of Tai Chi in promoting healthy cardiovascular and neuromuscular systems, metabolic processes and delaying aging declines in a university community. This intervention yielded significant improvements in sleep duration and knees osteoarthritis symptoms. The next presentation focuses on Yin-Yang theory in Tai Chi and emphasizes the balance between stability and mobility, energy flow and mental coordination. The 3rd presentation employs qualitative research to study the benefits of Tai Chi in community health. The findings indicate positive effects in lifestyle changes on physical activity, diet, sleep and psychological resilience. The 4th speaker will provide evidence of the benefits of Tai Chi on persons with ankylosing spondylitis. A 12-week intervention showed statistically significant benefits on pain/stiffness (BASDAI), function (BASFI) and spinal mobility. The final speaker investigates the impact of Tai Chi on balance/falls, cognition and motor health in older adults. Emphasis will be given to outcomes from dual task conditions that inform the cognitive-motor interdependence. There will be an opportunity to learn and perform Tai Chi.

Abstract citation ID: igaf122.1827

### A CASE STUDY OF TAI CHI EXERCISE PRESCRIPTION AND ACTIVE HEALTH COMMUNITY DEVELOPMENT AT PEKING UNIVERSITY

Dongmin Wang, Ning Kang, and Gong Chen, *Peking University, Beijing, Beijing, China (People's Republic)*

This study explores the role of scientific Tai Chi exercise in promoting active health within university communities, advancing the implementation of the "Integration of Sports and Public Health"

model to support the Healthy China strategy. Tai Chi has demonstrated significant benefits in cardiovascular health, musculoskeletal function, metabolic processes, and delaying aging.

Abstract citation ID: igaf122.1828

### INTEGRATING YIN-YANG CONCEPTS IN TAI CHI FOR GERIATRIC HEALTH: A HOLISTIC APPROACH TO MIND-BODY HARMONY

Hao "Howe" Liu, *Louisiana State University, Baton Rouge, Louisiana, United States*

This presentation will focus on applying yin-yang theory in Tai Chi to improve the health and well-being of older adults. It will emphasize the balance between stability and mobility, energy flow, and mental coordination—key elements in supporting aging populations. Tai Chi's dynamic balance offers a low-impact exercise that can enhance functional independence, circulation, and cognitive health for older individuals.

Abstract citation ID: igaf122.1829

### THE ROLE OF TAI CHI IN COMMUNITY HEALTH MANAGEMENT: AN EMPIRICAL STUDY ON RESIDENTS' HEALTH BEHAVIORS

Ning Kang, Gong Chen, and Dongmin Wang, *Peking University, Beijing, Beijing, China (People's Republic)*

Findings indicate that Tai Chi plays a multifaceted role in community health management. First, it enhances residents' health awareness, encouraging them to be more attentive to their well-being and adopt proactive health management strategies. Second, long-term Tai Chi practice fosters the development of healthy behaviors, including the establishment of regular exercise routines and positive lifestyle changes in diet and sleep habits. Additionally, Tai Chi demonstrates significant psychological benefits, effectively alleviating anxiety and stress while enhancing psychological resilience. As a group-based activity, Tai Chi also fosters social interactions within the community, strengthening neighborhood ties and contributing to the formation of a supportive health environment. This, in turn, facilitates a mutual-assistance model of community health management. Furthermore, some communities have integrated Tai Chi into their health management frameworks, combining it with health consultations and chronic disease management services, thereby expanding its application in health promotion. Consequently, Tai Chi serves not only as a form of physical exercise but also as a valuable tool for community health management, improving residents' overall health at multiple levels. Conclusion: Tai Chi is more than just an exercise; it is a strategic tool for health management that fosters healthy behaviors, enhances residents' physical and mental well-being, and strengthens social capital within communities. It is recommended that community health administrators leverage the advantages of Tai Chi by incorporating it into health promotion and chronic disease management programs. Optimizing health management strategies through Tai Chi can further improve the overall health of community residents.

Abstract citation ID: igaf122.1830

### EMPIRICAL RESEARCH ON THE INTERVENTION OF ANKYLOSING SPONDYLITIS WITH TAI CHI

Sinuo Chen, *The School of the Art Institute Of Chicago, China (People's Republic)*

This study aims to explore the intervention effect of Tai Chi on patients with ankylosing spondylitis. The experimental group received a 12-week Tai Chi intervention. The Tai Chi exercise prescription was designed based on the pathological characteristics of ankylosing spondylitis. The study indicates that the Tai Chi exercise prescription intervention can effectively improve the symptoms and functions of patients with ankylosing spondylitis and enhance their quality of life.

Abstract citation ID: igaf122.1831

### THE IMPACT OF TAI CHI ON BALANCE, FALLS AND COGNITIVE AND MOTOR HEALTH IN OLDER ADULTS.

Peter Wayne, *Brigham and Women's Hospital, Boston, Massachusetts, United States*

As a mind-body exercise, Tai Chi has the potential to impact both cognitive and motor function. This presentation will highlight recent research regarding the effectiveness of Tai Chi in improving outcomes related to cognition, gait, balance and fall prevention. Drawing on both randomized trials cross-sectional studies with seasoned tai Chi experts, emphasis will be given to outcomes assessed under experimental dual task conditions that inform cognitive-motor interdependence.

Abstract citation ID: igaf122.1832

### META-ANALYSIS OF THE INFLUENCE OF TAIJIQUAN EXERCISE ON THE DISEASE SEVERITY AND BALANCE FUNCTION OF PATIENTS WITH MILD TO MODERATE PARKINSON'S DISEASE UNDER DIFFERENT CONTROL MEASURES

Yan Zhang, and Yunhua Cao, *North China Electric Power University, Beijing, Beijing, China (People's Republic)*

Objective: To explore the influence of Taijiquan exercise on the disease severity and balance function of patients with mild to moderate Parkinson's disease under different control interventions. Method: As of February 15, 2025, a literature search was conducted in four databases, including PubMed. The inclusion and exclusion criteria for the literature were formulated, and a literature information form was created. Two experienced evaluators independently completed the literature screening and data extraction. Stata18.0 software was used for data processing such as heterogeneity testing and subgroup analysis. Results: A total of 15 randomized controlled trials were included, with a total of 17 groups of research data and 784 participants. The results showed that the combined intervention of different control measures and Taijiquan had a significant difference in the improvement of disease severity as a whole ([WMD = -3.804, 95%CI(-5.345, -2.263), $P < 0.001$]). At the same time, there was a significant difference in the improvement of the balance function of patients with mild to moderate Parkinson's disease compared with various control measures ([WMD = -3.498, 95%CI(-5.096, -1.901), $P < 0.001$]). Conclusion: Taijiquan exercise intervention can significantly improve the disease severity and balance function of patients with mild to moderate Parkinson's disease. It is recommended that patients with mild to moderate Parkinson's disease actively engage in Taijiquan exercise. Since the degrees of influence of various interventions (such as drugs, routine nursing in the department of neurology, etc.) combined with Taijiquan exercise vary, more in-depth research is still needed on the impact of Taijiquan intervention on Parkinson's disease.

### SESSION 4260 (PAPER)

### LONG-TERM SERVICES AND SUPPORTS WORKFORCE: POLICY AND PRACTICE

Abstract citation ID: igaf122.1833

### AN ASSESSMENT OF THE ROLE OF WAGES AND FRINGE BENEFITS ON NURSE AIDE TURNOVER IN NURSING HOMES

Christopher Brunt[1], John Bowblis[2], and Robert Applebaum[2], *1. Georgia Southern University, Statesboro, Georgia, United States, 2. Miami University, Oxford, Ohio, United States*

Nurse aide (NA) turnover remains a persistent challenge with significant implications for care quality in nursing homes. While wages have been cited as a primary factor influencing turnover, limited research has examined the role of total compensation, including fringe benefits. This study evaluates the relationship between NA turnover and compensation as measured through wages, fringe benefits, and the specific fringe benefits offered across different nursing home ownership types (for-profit, not-for-profit, and government-run). Utilizing national data from 2022 and 2023, we construct a comprehensive dataset from the Centers for Medicare & Medicaid Services' (CMS) Medicare Cost Reports, Payroll-Based Journal files, and Care Compare archives. We employ regression models controlling for facility and market characteristics, including ownership, payer mix, staffing levels, and state-fixed effects. The results indicate that while higher wages modestly reduce NA turnover, investment in fringe benefits is significantly more effective. While a 10% increase in NA wages reduces turnover by 0.34-0.36 percentage points, a 10% increase in fringe benefit generosity is associated with a 3.5-3.6 percentage point reduction in turnover, with for-profit facilities experiencing even greater effects. Cost-effectiveness analyses indicate that investing the same dollar amount in fringe benefits yields three to sixteen times greater reductions in turnover than an equivalent investment in wages. Specific fringe benefits, such as daycare assistance and tuition reimbursement, further mitigate turnover, while other fringe benefits such as life insurance) are not associated with turnover. These results suggest that policymakers and nursing home administrators seeking to improve workforce stability should prioritize comprehensive compensation strategies.

Abstract citation ID: igaf122.1834

### EVALUATION OF THE CALGROWS INNOVATION FUND DIRECT CARE TRAINING AND STIPENDS PROGRAM

Jacqueline Miller, Timothy Bates, Xenia Mendez, and Joanne Spetz, *University of California, San Francisco, San Francisco, California, United States*

Research Objective: The California Growing a Resilient and Outstanding Workforce (CalGrows) program distributed $150 million in grant funding to provide free training to direct care workers (DCWs). DCWs could earn up to $7,000 in training stipends. This study evaluated the design and delivery of CalGrows training courses and DCWs' and caregivers' attitudes about their training experiences. Study Design: A survey measuring DCWs' and caregivers' perceptions of their CalGrows training experience was sent to training participants. Interviews were conducted with training providers, DCWs, and program administrators to understand program design and delivery, perceptions of training's impact on confidence in skills, and quality of care. Principal Findings: CalGrows funded 76 organizations with about 850 different training courses offered in varying formats. More than 18,500 DCWs completed at least one training course. Approximately 90% of participants indicated that the training made them feel better prepared across a range of knowledge and skill areas. Over 90% agreed that the training made them want to remain in their current job or the caregiving field. More than 95% felt that the CalGrows training improved the quality of care they provide and their confidence. Grantees who provided training to their own employees described dramatic improvements in employee retention. Training providers emphasized the importance of promoting their training through a trusted network in the home and community-based services sector and the need for sustainable funding. Conclusions: The CalGrows program demonstrated the need for and value of providing free and accessible training to DCWs.

Abstract citation ID: igaf122.1835

### POLICY INITIATIVES REGARDING THE LONG-TERM CARE IMMIGRANT WORKFORCE AND ASSOCIATED OUTCOMES: A SCOPING REVIEW

Moroni Fernandez Cajavilca[1], and Lei Chen[2], *1. University of Utah, Salt Lake City, Utah, United States, 2. University of California, San Francisco, San Francisco, California, United States*

It is widely acknowledged that a significant shortage of nurses and direct care workers willing to work with older adults in long-term care (LTC) settings exists. The LTC industry has addressed this issue by advocating for expanding the immigrant labor force to strengthen the nursing workforce. However, there has been insufficient attention to immigration reform that could optimize the contributions of immigrant workers in the LTC sector while protecting their health and well-being, which may impact the care they provide. This scoping review examined how policy initiatives, or lack thereof, influence retention, protection, and/or health outcomes of the LTC immigrant workforce. We conducted a literature search with a librarian to tailor searches for five databases: Medline, CINAHL, ABI/INFORM, PAIS Index, and Web of Science. Inclusion criteria included articles globally and between 2010-2024 that focused on national, state, or organizational policy initiatives related to the LTC immigrant workforce and/or their health outcomes. We used Covidence, which automatically excluded duplicates (n = 387). Two reviewers screened by text and abstract (n = 1149), followed by full-text screening (n = 113). We identified 46 articles that met our inclusion criteria. The findings suggest there is a dearth of policy initiatives designed to retain or protect the LTC immigrant workforce. Moreover, the health outcomes of the LTC immigrant workforce are generally negative, owing to the absence of comprehensive policy initiatives. Studies largely reported the need for specific policy initiatives (e.g., language access, payment restructures, antiracism policies), which may be innovative approaches to encouraging, supporting, and fostering the LTC immigrant workforce.

Abstract citation ID: igaf122.1836

### REGISTERED NURSE EMPLOYMENT AND EARNINGS IN LONG-TERM CARE

Joanne Spetz, and Timothy Bates, *University of California, San Francisco, San Francisco, California, United States*

Objectives This study identifies RN characteristics associated with employment in LTC and factors associated with wage differences between RNs working in LTC versus other settings.

Methods We analyzed data from the 2022 US National Sample Survey of Registered Nurses. We calculated descriptive statistics and conducted statistical tests of differences in the characteristics of RNs employed in LTC versus other settings and of their hourly wages. We then estimated a logistic regression to identify demographic, human capital, and employment characteristics that predict LTC employment. We estimated a linear regression with the logarithm of wage as the dependent variable to measure wage differences between LTC and other RNs, holding other characteristics constant. Results Our study sample had 14,216 RNs, of whom 1,334 were employed in LTC settings. RNs employed in LTC were older than RNs in other settings, and the racial-ethnic composition was similar between the groups. LTC-employed RNs were more likely to have completed an associate degree versus a bachelor's degree, and to have been educated outside the US. RNs in LTC earned an average of 9.5% less than other RNs. Among RNs in LTC settings, there were significantly lower hourly wages for those with management and education job titles, compared with staff nurse titles, and no differences by age group, race/ethnicity, education, or gender. Conclusions Because RNs in LTC earn lower wages than RNs in other settings, policies to minimize pay inequities are needed to support the RN workforce providing LTC services.

Abstract citation ID: igaf122.1837

### THE COST OF DIRECT CARE WORKFORCE INSTABILITY: ECONOMIC AND HEALTH IMPACTS OF HIGH TURNOVER IN PENNSYLVANIA

Sarah Aviña, Amanda Kreider, and Howard Degenholtz, *University of Pittsburgh, Pittsburgh, Pennsylvania, United States*

Pennsylvania's direct care workforce crisis is intensifying, with annual turnover rates between 44–65% and an estimated shortfall of over 37,000 workers by 2026. While workforce shortages in long-term services and supports (LTSS) are well-documented, the financial burden of turnover remains underexplored. This study quantifies the economic and health costs of workforce instability, focusing on avoidable emergency department (ED) visits, hospitalizations, and long-term care admissions linked to staffing shortages. Using longitudinal workforce and wage data from the Bureau of Labor Statistics (BLS), PHI, and the Pennsylvania Department of Human Services (DHS) 2025-2026 Blue Book, along with Pennsylvania Medicaid data from 2024-2025, we assess turnover trends and cost implications. We find that direct care workforce shortages are associated with a 15-20% increase in preventable ED visits and a 12-18% increase in hospitalizations, adding as much as $75 million in excess Medicaid expenditures annually. Comparing Pennsylvania's LTSS workforce trends against national data suggests that that the state's crisis is disproportionately severe, surpassing the national average by 10%, with rural and low-income populations disproportionately affected. Findings indicate that each direct care worker turnover costs providers an estimated $4,200, leading to millions in annual financial losses and service instability. Policy recommendations include retention-based Medicaid reimbursements, expansion of direct care worker roles in interdisciplinary care teams, and pipeline initiatives. This study underscores the need for evidence-based policy reforms to address workforce instability and prevent excess healthcare costs in the Medicaid LTSS system.

### SESSION 4265 (PAPER)

### ALZHEIMER'S DISEASE AND OTHER RELATED DEMENTIAS: INTERVENTIONS AND HEALTH OUTCOMES

Abstract citation ID: igaf122.1838

### ANTIHYPERTENSIVE DRUG USE: A RACIAL DECOMPOSITION ANALYSIS BASED ON THE LARGEST ALZHEIMER'S DISEASE REGISTRY DATA

Kevin Lu[1], Xiangxiang Jiang[1], and Gang Lv[2], *1. University of South Carolina, Columbia, South Carolina, United States, 2. Chinese PLA General Hospital, Beijing, Beijing, China (People's Republic)*

Background: Mortality from Alzheimer's Disease and Related Dementias (ADRD) has more than doubled in the past two decades, with South Carolina leading the nation in crude ADRD death rates. African Americans face a disproportionately higher risk of ADRD, with widening racial disparities, partly driven by comorbid conditions like hypertension. However, limited research has quantified the contribution of racial disparities to healthcare costs and utilization. This study addresses this gap by analyzing racial differences in healthcare expenditures, utilization, and antihypertensive medication (AHM) use among South Carolina Medicaid beneficiaries with ADRD, leveraging data from the South Carolina Alzheimer's Disease (AD) Registry. Methods: A longitudinal analysis was conducted using SC Medicaid claims data linked to the SC AD Registry, the nation's oldest and most comprehensive statewide ADRD database. The study included ADRD patients aged 65 and older (2005–2021). Healthcare costs were adjusted to 2024 dollars. The Blinder-Oaxaca decomposition method was used to quantify racial disparities in inpatient, outpatient, emergency room, and medication expenditures. Results: Among 7,865 ADRD Medicaid beneficiaries, racial differences accounted for 23.52%–34.37% of disparities in inpatient, outpatient, and medication expenditures ($P < 0.001$). Racial disparities explained 28.95% ($P < 0.001$) of AHM use differences. Conclusion: Racial disparities significantly contribute to variations in healthcare costs, utilization, and medication use among ADRD patients. Addressing these disparities is critical for ensuring equitable access to care and improving outcomes.

Abstract citation ID: igaf122.1839

### LIFE REVIEW INTERVENTION DELIVERED BY CAREGIVERS OF PERSONS LIVING WITH DEMENTIA IMPROVES DEPRESSION

Christina Miyawaki[1], Angela McClellan[1], Erin Bouldin[2], Cheryl Brohard[1], and Mark Kunik[3], *1. University of Houston, Houston, Texas, United States, 2. University of Utah, Salt Lake City, Utah, United States, 3. Baylor College of Medicine, Houston, Texas, United States*

Approximately 40% of older Americans (≥65 years) living with dementia experience depression, yet behavioral interventions remain scarce. We developed a 6-week intervention, Caregiver-Provided Life Review (C-PLR) for people with dementia and depression and their family caregivers. Life review is an evidence-based depression intervention, in which patients retell their life stories to therapists chronologically. We trained caregivers in life review interview skills virtually, and caregivers conducted life reviews with their loved ones at home. This paper reports on the intervention outcomes and C-PLR's acceptability and feasibility for participants. We used a mixed-methods design with 45 caregiver-care recipient dyads (N = 90). We measured care recipients' depression (primary outcome), life satisfaction, caregiving rewards, caregiver burden, and dyads' relationship quality (secondary outcomes) quantitatively. We asked about caregivers' experiences conducting life reviews qualitatively. Caregivers were 58 years old on average, married, college-educated, working, female, and in good/excellent health while care recipients were 81 years old, widowed, retired, female, and in poor/fair health. Care recipients' depression significantly improved ($p < 0.001$), as did caregiving rewards ($p = 0.029$), and relationship quality ($p = 0.041$) without affecting caregiver burden ($p = 0.519$). Caregivers supported the quantitative results in their interviews and confirmed the feasibility of virtual training and confidence in conducting life reviews. Training family caregivers to conduct life reviews may be an acceptable and cost-effective way to improve depression among people with dementia while C-PLR appeared to be a comprehensible and convenient depression intervention for their loved ones. Thus, C-PLR has the potential to reach a wide range of caregivers and people living with dementia.

Abstract citation ID: igaf122.1840

### LONG-RANGE PLANNING FOR GROUP HOME DEMENTIA CARE FOR ADULTS WITH INTELLECTUAL DISABILITY

Matthew Janicki, *University of Illinois Chicago, Rockport, Maine, United States*

With the increasing prevalence of dementia among adults with intellectual disability (ID), there is a growing need for specialized, sustainable community-care models. Community-based group homes offer an alternative to institutional settings, providing trained staff, dementia-friendly environments, and individualized support. However, long-term planning is crucial for resource allocation, service intensity, and continuity of care. This longitudinal study (2011–present) examined the operation of three 5-person dementia-capable group homes, tracking 15 adults with ID/dementia and 15 matched non-dementia controls. At entry, five participants had Down syndrome, nine were male, and their mean age was 59.1 years. The dementia cohort exhibited higher mortality, with one survivor compared to five in the control group. Over 14 years, 35 adults cycled through the homes, with an average length of stay (LOS) of 4.75 years prior to death. LOS was shorter for older adults with complex health conditions. Admission age patterns revealed a tri-modal distribution (50.5, 57.0, and 66.5 years), influencing care needs and longevity. Comorbidities increased over time (8.6 at entry to 12.1 at death), with fluctuations per home based on resident turnover. A key management strategy involved grouping residents by dementia stage, with one home dedicated to advanced dementia and the other two generally similar as to care needs. These findings highlight the importance of age at admission, dementia stage, morbidity trajectories, and mortality-driven transitions in long-term planning. By integrating these factors, providers can optimize staffing ratios, enhance care quality, and ensure the sustainability of dementia-capable group homes for adults with ID.

Abstract citation ID: igaf122.1841

### SLEEP AND THE SPEED OF PROGRESSION FROM MILD COGNITIVE IMPAIRMENT TO DEMENTIA

Ning Zhang, Lichuan Ye, and Jack Johnson, *Northeastern University, Boston, Massachusetts, United States*

Background: The factors contributing to the progression from mild cognitive impairment (MCI) to Alzheimer's Disease Related Dementia (ADRD) remain unclear. Increasing evidence suggests sleep disturbances are a significant risk factor for the rate of developing ADRD. This study aims to explore how sleep issues affect the speed of cognitive decline from MCI to ADRD. Methods: This secondary analysis used data from the Health Retirement Survey (HRS, 2006-2020), a biennial survey of Americans aged 50 or older. Sleep disturbances included self-reported sleep complaints and short sleep duration (< 6 hours). The speed of cognitive decline was measured by the number of years an individual with MCI remained in the MCI stage before transitioning to ADRD, with assessments conducted every two years. MCI was evaluated using standardized cognitive test scores. Joint regression analyses, including logistic and survival analyses, were used to determine the association between sleep disturbances and cognitive progression from MCI to ADRD. Results: Among 5,228 MCI participants, 45% reported sleep disturbances. Those with sleep complaints (OR = 1.14) or short sleep duration (OR = 1.37) were more likely to develop ADRD. Among those who developed ADRD, sleep disturbances predicted a faster transition from MCI to dementia—2 years for sleep complaints and 4 years for short sleep duration. Conclusions: Sleep disturbances in the early stages of Alzheimer's may indicate a faster cognitive decline. Addressing these disturbances through targeted interventions could help delay or prevent cognitive impairment in older adults.

Abstract citation ID: igaf122.1842

### THE ASSOCIATION OF PAIN AND THE USE OF PAID CARE IN OLDER ADULTS WITH DEMENTIA

Katherine Miller[1], Emerald Jenkins[2], and Tamar Rodney[2], *1. Johns Hopkins Bloomberg School of Public Health, Baltimore, Maryland, United States, 2. Johns Hopkins, University, Baltimore, Maryland, United States*

Chronic pain and dementia are major public health issues and risk factors for multiple comorbidities and all-cause mortality. Over half of persons living with dementia (PLWD) experience daily pain, yet pain among PLWD is often inadequately recognized and treated, which can lead to adverse health outcomes. Paid carers have been shown to prevent or mitigate behavioral and psychological symptoms, identify health status changes, and implement non-pharmacological interventions. Yet, few studies examine the intersection of pain and dementia. We use the National Health and Aging Trends study (2017-2019) to identify PLWD to examine the association of chronic pain (having bothersome or activity-limiting pain) with receipt of any paid care and intensity of paid care receipt using logistic and linear regressions, respectively, adjusting for sociodemographic and clinical characteristics (e.g., age, gender, race, depression, chronic conditions). Fifty-six percent of PLWD reported any pain. Compared to PLWD without pain, PLWD with pain are more likely to be female, Hispanic, screen positive for depression, and have more chronic conditions. Nearly 60% of PLWD with pain reported activity-limiting pain. Among PLWD, after adjusting for covariates, having pain is associated with a 5 percentage-point increase in the probability of having any paid helper ($p < 0.05$) but not with hours of care received. However, the association of activity-limiting pain specifically is significantly associated with an increase in the number of helpers and hours of both paid and unpaid care. These findings can help inform future interventions targeting paid and unpaid caregivers to help PLWD successfully managed pain.

### SESSION 4270 (SYMPOSIUM)

Abstract citation ID: igaf122.1843

### DYNAMICS OF DAILY AGEISM AND ATTITUDES FOR HEALTHY AGING: CROSS-CULTURAL ANALYSIS OF THE SUBJECTIVE AGES PROJECT

Chair: Shevaun Neupert
Co-Chair: Reyyan Can

The proposed symposium provides a global perspective regarding antecedents, correlates, and consequences of views on aging based on the Subjective AGES (Aging within Global everyday Ecological Studies) consortium. This culture-informed approach highlights the contextual and dynamic influences of daily ageism, attitudes, and behaviors across different temporal perspectives. We enrich the existing body of knowledge by including a broad variety of cultures and investigating how daily ageism and attitudes connect to daily indicators of health and well-being. First, Neupert et al. characterize the amount of day-to-day fluctuation in daily ageist attitudes across daily diary studies from 10 countries and show cross-cultural differences in the impact of those fluctuations on daily memory functioning. Wirth et al. use daily diary data from Germany, Israel, Türkiye, and USA and find that people with more age-related losses endorse more ageist attitudes than those with fewer losses. Lee et al. examine three exposure contexts as potential sources of daily ageist attitudes in Canada, finding that greater overall social media use, but not TV viewing or neighborhood age composition, was significantly associated with greater experiences of ageism in daily life. Röcke et al. connect fluctuations in subjective age with mobility in Switzerland, showing that participants who report higher flexibility concerning environmental and personal challenges travel further away from home on days when feeling younger than their actual age. Our findings shed light on country-specific and global relationships between views on aging and development. We showcase how experiences of aging are shaped by contextual and dynamic influences.

Abstract citation ID: igaf122.1844

### DAILY AGEIST ATTITUDES, SUBJECTIVE AGE, AND MEMORY FAILURES: A MULTI-COUNTRY INTEGRATED DATA ANALYSIS

Shevaun Neupert[1], Reyyan Can[1], Shalini Mukherjee[1], Jana Nikitin[2], Oksana Senyk[3], Christiane Hoppmann[4], Kvitoslava Khorob[5], and Minxia Luo[6], *1. North Carolina State University, Raleigh, North Carolina, United States, 2. University of Vienna, Vienna, Wien, Austria, 3. WSB Merito University in Gdansk, Gdansk, Pomorskie, Poland, 4. University of British Columbia, Vancouver, British Columbia, Canada, 5. Ukrainian Catholic University, Lviv, L'vivs'ka Oblast', Ukraine, 6. University of Zurich, Zürich, Switzerland*

Ageist attitudes reflect negative views about others' aging and are negatively associated with health and well-being. However, the ways in which ageist attitudes may fluctuate on a daily basis, and how those fluctuations are coupled with everyday memory functioning, are underexplored. Applying a cross-cultural perspective and leveraging 14-day daily diary data from the USA, Switzerland, Türkiye, Germany, India, Ukraine, Israel, Austria, Czech Republic, and Canada, the goal of the current study was to identify within-person fluctuations in daily ageist attitudes and connect those fluctuations to everyday memory functioning. Multilevel models were conducted across a mixture of integrated data analysis (for data that could be shared) and coordinated data analysis (standardized models applied to locally controlled data) to maximize the inclusion of countries. Estimates of within-person variability in daily ageist attitudes ranged from 13% (Germany) to 44% (India). Conditional models indicated varying patterns across countries. For example, in the US and Germany, daily increases in ageist attitudes were associated with increases in memory failures (reflecting lower levels of everyday memory functioning). However, in Canada and Türkiye, daily ageist attitudes were not associated with memory failures. The current results suggest that ageist attitudes move to varying degrees on a daily basis, and that their importance for everyday memory functioning may depend on cultural context.

Abstract citation ID: igaf122.1845

### OLDER ADULTS' EXPOSURE TO AGEISM IN DAILY LIFE – ON THE ROLE OF TV, SOCIAL MEDIA, AND NEIGHBORHOOD COMPOSITION

Juhyeong Lee[1], Yoonseok Choi[2], and Christiane Hoppmann[3], *1. The University of British Columbia, Vancouver, British Columbia, Canada, 2. Simon Fraser University, Vancouver, British Columbia, Canada, 3. UBC, Vancouver, British Columbia, Canada*

Experiences of ageism undermine well-being in old age but the underlying everyday mechanisms are not well understood. Our study focused on three potential exposure contexts: a) TV, b) social media, and c) neighborhood composition that may contribute to the experience of everyday ageism. We aimed to investigate the association between TV viewing, social media use, and neighborhood age composition with ageism reports. We analyzed up to 14 days of end of day diaries from 76 older adults living in Canada (Age: M=72.01, SD=9.47; 80% female), with data collection still ongoing. Participants reported their daily TV viewing, social media use, and exposure to ageism each evening over fourteen consecutive days. To determine the age composition of the neighborhoods where participants resided, we used the first three digits of their postal codes. We pre-registered the study on the Open Science Framework. We employed hierarchical linear models to account for the nested data structure. As hypothesized, result indicate that greater overall social media use was significantly associated with greater experiences of ageism in daily life ($b=0.141$, $p < .01$). However, TV viewing and neighborhood age composition showed no significant association with ageism over and above social media use. The results suggest that social media serves as a primary context where older adults experience ageism in their daily lives. Future analyses will replicate the findings using the complete dataset and a larger sample, and will explore potential moderators of the social media–everyday ageism nexus.

Abstract citation ID: igaf122.1846

### PERSONAL AGING EXPERIENCES AND AGING-RELATED BELIEFS – PROJECTION PROCESSES IN DAILY LIFE AND MULTIPLE COUNTRIES.

Maria Wirth[1], Anna Kornadt[2], Shevaun Neupert[3], Reyyan Can[3], Amit Shrira[4], Yuval Palgi[5], and Klaus Rothermund[1], *1. Friedrich Schiller University Jena, Jena, Thuringen, Germany, 2. University of Luxembourg, Esch-sur-Alzette, Grevenmacher, Luxembourg, 3. North Carolina State University, Raleigh, North Carolina, United States, 4. Bar-Ilan University, Ramat Gan, HaMerkaz, Israel, 5. University of Haifa, Haifa, HaZafon, Israel*

Views of aging, including personal aging perceptions and beliefs about characteristics and behaviors of older adults, impact behavior and developmental outcomes in later life. While most research focuses on how aging-related beliefs become internalized into personal aging experiences, aging perceptions can also affect aging-related beliefs, indicating a projection or “externalization” of personal aging experiences. Put differently, aging-related beliefs are not immune against one’s own age-related experiences. This study aimed to understand how personal aging experiences affect aging-related beliefs in a daily context and across multiple countries. Participants in Germany (N=199, 50-91 years), Türkiye (N=50, 50-90 years), USA (N=71, 54-85 years), and Israel (N=78, 50-88 years), reported on their daily experiences of age-related gains and losses, and on their endorsement of ageist attitudes for 14 consecutive days. Projecting own aging experiences should influence endorsement of aging-related beliefs. Specifically, experiencing more losses and fewer gains should result in endorsing more ageist attitudes, both daily and overall. Our results indicated partial support for the projection hypothesis. Across investigated countries, reporting above-average losses was related to higher endorsement of ageist attitudes. In contrast to the idea of projection, on days with more losses, endorsement of ageist attitudes was lower. Gains were unrelated to endorsement of ageist attitudes in all countries. Our results highlight the impact of negative own aging experiences for shaping beliefs about (other) older adults. Interventions targeting aging-related beliefs, thus, need to consider personal aging experiences.

Abstract citation ID: igaf122.1847

### MOBILITY AND ITS PSYCHOLOGICAL CORRELATES: SUBJECTIVE AGE AND MOBILITY-RELATED BEHAVIORAL FLEXIBILITY

Christina Roecke, Minxia Luo, Zhiyong Zhou, and Robert Weibel, *University of Zurich, Zürich, Switzerland*

Mobility is a key determinant of healthy aging and has been shown to be associated with older adults’ health and well-being. However, the psychological antecedents of mobility have been understudied. This study examined daily mobility in relation to two psychological constructs that have been found to be associated with older adults’ everyday engagement, including daily subjective age and trait-like attitudes of mobility-related behavioral flexibility. We examined data from 101 older participants (aged 65-88 years) who carried a GPS sensor and completed smartphone experience sampling surveys over two weeks. Participants reported their momentary subjective age seven times per day and reported the farthest place they travelled during the day each evening. Moreover, daily distance travelled from home was extracted from the GPS data. At baseline, participants completed a questionnaire on their mobility-related behavioral flexibility. Results from multilevel modelling showed that neither self-reported nor GPS-derived daily mobility was associated with daily subjective age within persons on average. However, moderation analysis indicated that for participants reporting higher flexibility concerning environmental and personal challenges, on days when feeling younger than their actual age, they reported to have travelled further away from home. The results remained the same after controlling for age, sex, subjective health, and health conditions. Higher mobility-related flexibility in combination with feeling younger on a day are associated with participants’ greater reported out-of-home mobility on the same day.

### SESSION 4275 (PAPER)

Abstract citation ID: igaf122.1848

### BIOLOGY OF AGING: ANIMAL AND HUMAN MODELS

Research addressing biology of aging and disease through model and human models will be presented.

Abstract citation ID: igaf122.1849

### AGE-RELATED DIFFERENCES IN SENESCENCE MARKER EXPRESSION DURING CUTANEOUS WOUND HEALING

Maria Shvedova, Chenyu Chu, and Daniel Roh, *Boston University, Boston, Massachusetts, United States*

Impaired wound healing in older patients represents a significant and escalating clinical and economic challenge. Transient upregulation of cellular senescence signaling during wound healing is an important physiological mechanism that facilitates tissue repair. It is essential to differentiate senescent cells that accumulate with aging from the temporary upregulation of senescence signaling during acute wound healing. In this study, we compared cutaneous wound healing rates in 2-month-old (roughly corresponds to the age of 18 years in humans) and 24-month-old (roughly corresponds to the age of 70 years in humans) C57Bl/6 wild type male mice and examined differences in senescence marker expression. 24-months-old mice exhibited delayed wound healing and significantly reduced senescence marker expression, with a lack of p16, p21, and SA-β-gal upregulation, as well as altered inflammatory marker expression. Single-cell sequencing of wound tissue on day 6 after wounding - the peak of p16 expression - revealed that 91% (in 2-months-old mice) and 93% (in 24-months-old mice) of p16-positive cells were fibroblasts with an anti-inflammatory, extracellular matrix-producing phenotype. Notably, 24-month-old mice had 50% fewer p16-positive cells at this stage of wound healing. Given the essential role of fibroblasts in extracellular matrix deposition and tissue remodeling, these findings suggest that senescent fibroblasts may influence wound healing in a complex manner, with transient senescence playing a crucial role and aging impairing this physiological response. In conclusion, delayed wound healing in old mice is associated with decreased senescence signaling, highlighting a potential therapeutic target for improving wound repair in aging.

Abstract citation ID: igaf122.1850

### MORE THAN A LOADING CONTROL:STUDYING THE ACTIN CYTOSKELETON FOR NOVEL ANTI-AGING MECHANISMS.

Maxim Averbukh, Naibedya Dutta, Daniel Hicks, Gilberto Garcia, and Ryo Higuchi-Sanabria, *University of Southern California, Los Angeles, California, United States*

The actin cytoskeleton is a three-dimensional scaffold of proteins that is a regulatory, energy-consuming network with dynamic properties to shape the structure and function of the cell. Proper actin function is required for many cellular pathways, including cell division, autophagy, chaperone function, endocytosis, and exocytosis. Deterioration of these processes manifests during aging and exposure to stress, which is in part due to the breakdown of the actin cytoskeleton. However, the regulatory mechanisms involved in preservation of cytoskeletal form and function are not well understood. Thus, we performed a multi-pronged, cross-organismal screen combining a whole-genome CRISPR-Cas9 screen in human fibroblasts with in vivo C. elegans synthetic lethality screening to identify novel regulators of actin health. We identified the bromodomain protein, BET-1, as a key regulator of actin function and longevity. Overexpression of bet-1 preserves actin function at late age and promotes lifespan and healthspan in C. elegans. These beneficial effects are mediated through actin preservation by the transcriptional regulator function of BET-1. Interestingly, we also find that BRD4 (homolog of bet-1) is required for senescent cell survival. Interestingly, upon neuron-specific overexpression of bet-1, we identified a novel neuron-to-body communication driven by dopamine signaling that extends lifespan by over 70%. This non-autonomous signaling drives peripheral protein homeostasis to drive lifespan in an HSF-1-dependent, but actin-independent mechanism. Together, our discovery assigns a key role for BET-1 in promoting longevity through actin homeostasis when acting autonomously in peripheral tissue, but a unique actin-independent method to promote protein homeostasis when actin non-autonomously through dopaminergic neurons.

Abstract citation ID: igaf122.1851

**SEX-DIMORPHIC GUT MICROBIOTA MODULATION BY ICARIIN IN OLDER MICE**

Xiaoang Li, and Liping Duan, *Peking University Third Hospital, Beijing, Beijing, China (People's Republic)*

This study investigates the sexually dimorphic effects of icariin—a bioactive flavonoid from Epimedium—on gut microbiota remodeling in older C57BL/6J mice, evaluate its impact on microbial diversity, structure, and metabolic functions, and analyzes the role of sex differences in its intervention effects. Through 16S rRNA sequencing (V3-V4, Illumina NovaSeq) and PICRUSt2-based metabolic prediction, we demonstrate that 15 days icariin treatment (100 mg/kg/day) preferentially restores youthful microbial profiles in older mice (24-month-old), with females exhibiting 25% greater α-diversity improvement (Shannon index, $p<0.05$) and 40% closer β-diversity proximity to young controls (Bray-Curtis, $p=0.008$) compared to males. Sex-stratified analysis revealed distinct taxonomic shifts: aged females showed Lactobacillus enrichment (4.1-fold) linked to estrogen metabolism, while males displayed Akkermansia amplification (3.9-fold, $p=0.012$) associated with mucin degradation. Functionally, icariin upregulated steroid biosynthesis pathways 3.2-fold in females versus 1.8-fold in males ($q<0.05$), correlating with phytoestrogen-mediated microbial regulation. These findings not only establish icariin as a sex-specific modulator of gut microbiota aging but also provide mechanistic insights into sexual dimorphism in host-microbiome interactions, advocating for gender-precise nutraceutical strategies to counteract age-related dysbiosis.

Abstract citation ID: igaf122.1852

**TARGETING KYNURENINE METABOLISM IN AGING AND AGE-ASSOCIATED DISEASE**

George Sutphin, *University of Arizona, Tucson, Arizona, United States*

Tryptophan metabolism through the kynurenine pathway becomes dysregulated during normal aging and is implicated in many age-associated diseases, including chronic inflammation, atherosclerosis, neurodegeneration, and cancer. Kynurenine pathway enzymes and metabolites influence a range of molecular processes critical to healthy aging, including regulation of inflammatory and immune responses. Kynurenine metabolism is active in immune cells and activated in response to proinflammatory cytokine signaling. We discovered that elevating physiological levels of the kynurenine pathway metabolite 3-hydroxyanthranilic acid (3HAA) via either direct supplementation or inhibition of the enzyme that degrades 3HAA, 3HAA dioxygenase (HAAO), extends lifespan and delays age-associated health decline in both roundworms and mice. In recent work, we find that elevating physiological 3HAA can beneficially enhance both stress resilience and the immune response of C. elegans to bacterial pathogens during aging. In the absence of HAAO, 3HAA accumulates in lysosome related organelles (LROs) in the intestinal cells of C. elegans, the same subcellular compartment that contains engulfed bacteria. LROs are a cellular repository for both iron and zinc, and we further find that iron chelation or zinc supplementation dramatically enhances the bactericidal properties of 3HAA. Here we present a mechanistic model in which age-dependent accumulation of 3HAA in intestinal LROs combines with changes to iron and zinc homeostasis to enhance stress and bacterial pathogen resistance with age in C. elegans.

Abstract citation ID: igaf122.1853

**TRANSCRIPTS INVOLVED IN MITOCHONDRIAL ENERGETICS ARE ASSOCIATED WITH VO2PEAK IN OLDER ADULTS**

Bumsoo Ahn[1], Gregory Tranah[2], Tyler Mansfield[2], Paul Coen[3], Russell Hepple[4], Steven Cummings[2], and Stephen Kritchevsky[5], *1. Atrium Health Wake Forest University School of Medicine, Winston-Salem, North Carolina, United States, 2. California Pacific Medical Center Research Institute, San Francisco, California, United States, 3. AdventHealth, Orlando, Florida, United States, 4. University of Florida, Gainesville, Florida, United States, 5. Wake Forest University School of Medicine, Winston-Salem, North Carolina, United States*

Abstract Mitochondrial dysfunction plays an important role in age-related mobility disability. The study of Muscle, Mobility and Aging (SOMMA) is a cohort study designed to identify muscle characteristics associated with worsening mobility. Using vastus lateralis muscle biopsies ($n=724$; ages 70+ years; 56% women), we performed bulk RNAseq and used DESeq2 to test whether the expression of preselected muscle RNA transcripts related to mitochondrial energetics were associated with physical traits including cardiorespiratory fitness (VO2peak), leg power, and 400 m walk speed. In our analysis of 288 transcripts from both nuclear and mitochondria-encoded genes, several transcripts were strongly and positively associated with VO2peak. Among the top 20 differentially regulated genes (DEGs, adjusted $p<0.05$) were genes involved in oxidative phosphorylation: IDH2, FH, SUCLG1 (Krebs cycle), NDUFA8 and NDUFA9 (complex I), SDHB and SDHC (complex II), CYC1 (complex III), COX5A (complex IV), and ATP5F1B, ATP5F1C, ATP5MC1, ATP5MC3, ATP5PB, and ATP5F1A (complex V). All mitochondrial DNA encoded OXPHOS complex transcripts were significantly associated with VO2peak and mitochondrial energetics. Among the genes we tested, a smaller proportion of the selected transcripts reached statistical significance (adjusted $p<0.05$) for leg power and walking speed, with the highest proportion observed for $VO_2$peak: $VO_2$peak (66%), leg power (29%), and walking speed (11%). In summary, transcripts involved in mitochondrial energetics show a strong association with VO2peak, a trait highly dependent on mitochondrial function.

### SESSION 4280 (SYMPOSIUM)

Abstract citation ID: igaf122.1854

**METHODOLOGICAL CHALLENGES AND OPPORTUNITIES IN POPULATION AGING RESEARCH**

Chair: Eleanor Hayes-Larson
Co-Chair: Ruijia Chen
Discussant: Delany Salazar

Population health research on aging is complicated by methodological issues such as measurement challenges and error, competing risks, and selective attrition, each of which may manifest in multiple ways as sources of bias and affect reproducibility of results. Novel methods have been developed to address these issues. The session features cutting-edge research that incorporates novel epidemiological methods to address key methodological challenges in population aging research. Speakers will address (1) measures to crosswalk between cognitive instruments to better compare results across studies, (2) use of simulation to understand sources of bias such as measurement error in biomarker analyses, (3) the impact of mortality as a competing risk in aging-related causal questions, (4) approaches to account for inter-wave mortality in cohort studies with high mortality, and (5) use of multiverse analysis to understand importance of operational definition of constructs like social isolation for replication of study results.

Abstract citation ID: igaf122.1855

### METHODS TO CROSSWALK BETWEEN COGNITIVE TEST SCORES USING DATA FROM THE ALZHEIMER'S DISEASE NEUROIMAGING COHORT

Sarah Ackley[1], Jingxuan Wang[2], Ruijia Chen[3], Tanisha Hill-Jarrett[4], L Paloma Rojas-Saunero[5], Andrew Stokes[6], Sachin Shah[7], and M. Maria Glymour[6], *1. Brown University, Providence, Rhode Island, United States, 2. University of California, San Francisco, San Francisco, California, United States, 3. Boston University School of Public Health, Boston, Massachusetts, United States, 4. University of California Memory and Aging Center, San Francisco, California, United States, 5. University of California, Los Angeles, Los Angeles, California, United States, 6. Boston University, Boston, Massachusetts, United States, 7. Massachusetts General Hospital, Boston, Massachusetts, United States*

Studies use multiple instruments to measure dementia-related outcomes, making comparisons of intervention or exposure effects challenging. For example, trials of anti-amyloid drugs like TRAILBLAZER-AD 2 (donanemab), CLARITY-AD (lecanemab), and GRADUATE I & II (gantenerumab) report changes in the clinical dementia rating sum of boxes (CDR-SB) as the primary outcome, while earlier trials used measures such as the Mini-Mental State Exam (MMSE) or the Alzheimer's Disease Assessment Scale-Cognitive Subscale. Comparing or meta-analyzing findings requires integrating effect estimates from different outcome measures. Psychometric methods can harmonize these measures but rely on unverifiable assumptions and do not work with summary statistics. To address this gap, we developed two methods to crosswalk estimated treatment effects on cognitive outcomes that are flexible, broadly applicable, and do not rely on strong distributional assumptions. We present two methods to crosswalk effect estimates using one measure to estimates using another measure, illustrated with global cognitive measures from the Alzheimer's Disease Neuroimaging Initiative (ADNI). Specifically, we develop crosswalks for the following measures and associated change scores over time: the clinical dementia rating scale sum of box (CDR-SB), Montreal cognitive assessment (MoCA), and mini-mental state exam (MMSE) scores. Finally, a setting in which crosswalking is not appropriate is illustrated with plasma phosphorylated tau (p-tau) concentration and global cognitive measures. Given the inconsistent collection and reporting of dementia and cognitive outcomes across studies, these crosswalking methods offer a valuable approach to harmonizing and comparing results reported on different scales.

Abstract citation ID: igaf122.1856

### SIMULATION METHODS TO INFORM STUDY DESIGN AND UNDERSTAND POTENTIAL BIAS: THE EXAMPLE OF RANDOM ERROR IN AD BIOMARKERS

Eleanor Hayes-Larson[1], and Sarah Ackley[2], *1. University of Southern California, Los Angeles, California, United States, 2. Brown University, Providence, Rhode Island, United States*

Novel blood-based biomarkers for Alzheimer's disease pathology are increasingly available and are appealing options for inclusion in population-based studies due to numerous advantages related to cost and low invasiveness. However, they are subject to more random measurement error (i.e., noise) relative to more traditional biological measures such as neuroimaging or CSF. We leverage data from the Alzheimer's Disease Neuroimaging Initiative and use a simulation study to highlight potential implications of random measurement error for power and sample size requirements when using blood-based biomarkers (e.g., p-tau 181) vs. neuroimaging (amyloid PET) to understand the effects of risk factors like age on biomarker levels, and additional implications for bias when using the biomarker as an independent variable to evaluate the relationship between biomarkers and cognitive outcomes (e.g. Clinical Dementia Rating Sum of Boxes). More broadly, we highlight the benefits of simulation studies to inform study design, understand sources of bias, and investigate other methodological issues in population aging research.

Abstract citation ID: igaf122.1857

### CAUSAL ESTIMANDS FOR AGING-RELEVANT OUTCOMES IN THE PRESENCE OF DEATH AS A COMPETING OR TRUNCATING EVENT

L Paloma Rojas-Saunero, Yixuan Zhou, and Elizabeth Rose Mayeda, *University of California, Los Angeles, Los Angeles, California, United States*

Research aiming to identify causal mechanisms and intervention targets to prevent dementia and other aging-related outcomes often faces challenges when death acts as a competing or truncating event. For example, some studies have reported inverse associations between cigarette smoking and dementia risk, which could be attributable to "survival bias." Substantial progress has been made in developing statistical estimators to address this issue, but less attention has been given to aligning research questions and appropriate estimators, an essential step for meaningful interpretation of results. This work evaluates potential causal contrasts (i.e., estimands) relevant to incident outcomes, such as dementia diagnosis, as well as longitudinal outcomes, such as cognitive change, in the presence of death as a competing or truncating event. We compare these estimands, discuss potential sources of selection bias, and clarify the interpretation of estimates through simulations grounded in population-based cohort studies. Our findings aim to guide researchers in selecting estimators that align with their research questions, ultimately improving the validity of inferences in aging research.

Abstract citation ID: igaf122.1858

### APPROACHES TO ACCOUNT FOR INTER-WAVE MORTALITY IN COGNITIVE AGING STUDIES IN HIGH MORTALITY SETTINGS

Emma Nichols[1], Alden Gross[2], Erik Meijer[1], and Jinkook Lee[1], *1. University of Southern California, Los Angeles, California, United States, 2. Johns Hopkins Bloomberg School of Public Health, Baltimore, Maryland, United States*

A myriad of approaches exist to account for selective attrition from a study sample due to inter-wave mortality. When studies have longer inter-wave intervals or higher mortality, the importance of accounting for this potential bias increases. We compared mortality adjustment methods using data from Waves 1 (2017-2019) and 2 (2022-2024) of the Longitudinal Aging Study in India – Diagnostic Assessment of Dementia (LASI-DAD) (N = 3,546), given the high mortality in this setting, with 23.9% mortality during the 5-year inter-wave period. We estimated cognitive decline by age-group

(60-69/70-79/80+) using a generalized estimating equations model and compared results to subsequent analyses applying inverse probability weights to account for death, using a joint model for decline and survival, and imputing cognitive status before death using informant data from end-of-life interviews. Given concerns of reporting biases in the exit interview data, we also considered adjustments to this data prior to imputation. Compared to the base model with no adjustment, estimated cognitive decline in the 80+ age group was 42.2%, 17.1%, 14.2%, and 42.1% greater for the model with IPW, joint model, model with imputations, and model with adjusted imputations, respectively. IPW-based estimates were more variable (i.e., had larger standard errors) than other methods. The percentage difference was smaller for age groups with lower mortality (e.g., 13.9% for the model with adjusted imputations in the 60-69 age group). Results highlight the potential impact of accounting for mortality. Comparisons of methods suggest some heterogeneity in estimates and highlights potential of using imputations with adjusted end-of-life interview data.

Abstract citation ID: igaf122.1859

### SOCIAL ISOLATION AND COGNITIVE AGING: ARE OPERATIONAL DEFINITIONS CREATING A REPRODUCIBILITY CRISIS?

Ruijia Chen[1], Dylan Tran[2], Jingxuan Wang[3], Erin Ferguson[3], Mary Thoma[3], Ashwin Kotal[3], Jacqueline Torres[3], and M. Maria Glymour[2], *1. Boston University School of Public Health, Boston, Massachusetts, United States, 2. Boston University, Boston, Massachusetts, United States, 3. University of California, San Francisco, San Francisco, California, United States*

Research on social isolation and health shows conflicting findings, potentially due to methodological differences in defining social isolation. We conducted multiverse analyses to examine how varying definitions affect associations with cognitive function and decline. Using the 2010-2020 Health and Retirement Study (n=12,975), we defined social isolation using 16 items related to marital status, living arrangements, social interaction frequency, and participation in social activities. We created continuous and binary composite scores based on all possible combinations of these items, using top tertile and quartile cutoffs for binary definitions. Cognitive function was measured using the Telephone Interview for Cognitive Status, with scores standardized to baseline. Linear mixed-effects models evaluated associations between each definition and cognitive level/decline, adjusting for age, sex, race, and education. Across 196,587 model choices, most models indicated a negative association between social isolation and cognitive function level, with point estimates ranging from very large to nearly null (-0.31 to 0.01 standard deviations average difference across binary social isolation specifications). Most models also suggested social isolation was associated with faster cognitive decline (-0.001 to 0.03 standard deviations average annual difference across binary specifications). While social isolation appears associated with lower cognitive function levels and faster cognitive decline, the magnitude of these associations varies widely depending on how social isolation is defined.

### SESSION 4285 (SYMPOSIUM)

Abstract citation ID: igaf122.1860

### SOCIAL CONVOYS ACROSS THE LIFESPAN AND LINKS WITH LATER-LIFE WELL-BEING AND SOCIETAL TRUST

Chair: Rita Hu
Co-Chair: Noah Webster
Discussant: Elaine Wethington

Social relationships evolve across the lifespan and have great potential to shape individual and societal outcomes. This symposium examines how early-life bonds, caregiving experiences, and social network characteristics influence aging trajectories and their broader implications for macro-level societal trust. Drawing on multiple population-level and longitudinal studies, this set of papers together highlight recent innovations in social convoy research. Garcia and Antonucci examine how childhood closeness to grandparents predicts midlife depressive symptoms, stress and self-efficacy. Their findings demonstrate the lasting emotional benefits of intergenerational relationships. Hu and Lee investigate earlier-life caregiving histories and their long-lasting influence on later-life social disconnectedness and loneliness. Results suggest caregiving is linked to greater social participation and less loneliness, particularly among men, underscoring positive effects on caregivers' later-life social well-being. Iveniuk and Ricketts examine how social network turnover affects mental health. Their findings reveal race and gender differences in patterns of network turnover. They also found that the loss of weaker ties is linked with more depressive symptoms, while gaining new close confidants is associated with fewer depressive symptoms. Their study provides new insights into how network stability and change influence mental health disparities. Webster and colleagues explore how social network structure and composition shape macro-level personal trust. They found that larger inner circles and more non-family ties foster societal trust, linking interpersonal relationships to broader social cohesion. Discussant Elaine Wethington, a leading expert in social relationships and translational research, will synthesize these findings and discuss implications for interventions and policies that foster lifelong social convoys.

Abstract citation ID: igaf122.1861

### CAREGIVING HISTORY'S ASSOCIATIONS TO LATER-LIFE SOCIAL DISCONNECTEDNESS AND LONELINESS

Rita Hu[1], and Ji Hyun Lee[2], *1. The University of Chicago, Chicago, Illinois, United States, 2. Montana State University, Bozeman, Montana, United States*

Throughout adulthood, many people provide care for loved ones. While research has documented both the stressors and rewards of caregiving, less is known about caregiving experiences over the life course and their long-term implications for later-life social well-being. This study examines whether earlier-life caregiving experiences predict later-life social disconnectedness and loneliness. Using the Health and Retirement Study (HRS)'s retrospective Life History Mail Survey (2015/2017/2019; N=6,373), we identified individuals who had ever provided unpaid care for six or more months to people with chronic conditions. Social disconnectedness (indexed by close network size, network diversity, contact frequency, and social participation) and loneliness (11-item UCLA scale) were measured in the 2016/2018 HRS Psychosocial Leave-Behind Questionnaire. Covariates included later-life material status, memory, and physical and mental health. Results showed that individuals with any caregiving history (vs. non-caregivers) were less likely to experience social disconnectedness ($\beta$ = -0.08, p < .001) and general loneliness ($\beta$ = -0.02, p = .04) in later life. Further analysis revealed that caregiving history was associated with higher social participation ($\beta$ = -0.07, p < .001) but not with close network size, network diversity, or contact frequency. For loneliness, caregiving predicted lower collective loneliness ($\beta$ = -0.06, p < .001), with stronger effects for men. Stratified analyses showed that both genders benefited from lower collective loneliness; caregiving was also associated with lower social loneliness among men only (β = -0.044, p = .002). Findings highlight the enduring positive social health outcomes of earlier-life caregiving, particularly for men.

Abstract citation ID: igaf122.1862

### THE ROLE OF SOCIAL NETWORKS IN FACILITATING PERSONAL TRUST

Noah Webster, Kristine Ajrouch, and Toni Antonucci, *University of Michigan, Ann Arbor, Michigan, United States*

Trust is a core component of social cohesion and a strong predictor of older adults' well-being. Most research on trust focuses on others who are geographically proximate, with less focus on those in one's country more generally, i.e., macro-level personal trust. Larger-scale trust is critical for maintaining social norms, ensuring effective institutions, and civic engagement. In this study, we integrate two perspectives on social capital by examining the association between social network structure (e.g., size, composition) and trust of 'people in the U.S.' and the extent that trust clusters within close social networks. Data are from adults age 60 and older from Wave 3 of the Detroit-based Social Relations Study collected in 2015 (n = 303). Respondents identified social network members based on varying levels of closeness and provided characteristics on up to the first 10 people mentioned. Additionally, up to three network members age 18 plus were interviewed and also asked about personal trust. Networks with at least one person age 60 and older were selected for analysis (n = 162 networks; 425 respondents). Regression analyses controlling for age, gender, marital status, race, and education indicate having a larger inner circle size (i.e., more people one cannot imagine life without) and a greater proportion of friends in one's network were significantly associated with greater trust. Further, examination of a random intercept model predicting trust indicated significant clustering of trust within close social networks (intra class correlation = .21). Findings suggest fostering close non-family ties can be an effective approach to building societal-wide trust.

Abstract citation ID: igaf122.1863

### CHILDHOOD GRANDPARENT CLOSENESS AND MIDLIFE MENTAL HEALTH

Cheyenne Garcia, *University of Michigan, Ann Arbor, Michigan, United States*

We propose to examine the association between childhood closeness to a grandparent and mid-life depressive symptoms and stress. We used data from the Social Relations study, a longitudinal representative study of individuals in Detroit. We collected Wave 1 data in 1992 and follow-up data in 2005 (Wave 2) and 2015 (Wave 3). We examined closeness to a grandparent in childhood and later depressive symptoms, self-esteem, self-efficacy, and stress in 205 children (51.7% girls, 61.6% white). We defined closeness as being within the grandchild's closest 10 social relationships. The median age (SD) was 10.4 years old (1.4) in Wave 1, 23.3 (1.5) in Wave 2, and 33.4 (1.5) in Wave 3. In Wave 1, 77 children (37%) were close to at least one grandparent. After controlling for age and sex, childhood closeness to a grandparent was almost significantly associated with lower depressive symptoms in Wave 3 ($F(1,109)=3.90$, $p = 0.05$), but not at all in Wave 2($F(1, 140) = 0.88$, $p = 0.35$). We found that self-efficacy mediated much of this relationship, such that grandparent closeness in childhood was associated with higher self-efficacy in Wave 2 and higher self-efficacy was associated with greater depressive symptoms in Wave 3. Children who were close to their grandparent were bothered by fewer hassles in Wave 2 (13% vs. 18%). Closeness to a grandparent influences self-efficacy, which mediates the relationship with depressive symptoms and, possibly, stress. Promoting grandparent involvement in childcare could benefit children's mental health in midlife.

Abstract citation ID: igaf122.1864

### CONVOYS, NETWORK ADVANTAAGE, AND MENTAL HEALTH IN OLDER ADULTHOOD

James Iveniuk[1], and I'deyah Ricketts[2], *1. National Opinion Research Center at The University of Chicago, Scarborough, Ontario, Canada, 2. NORC at the University of Chicago, Chicago, Illinois, United States*

The convoy model is one of the most influential theories in gerontology of how social network change shapes psychological well-being over the life course. However, it is rare that researchers have the data to observe changes in social networks by emotional closeness to network members – especially in nationally-representative data. In this paper, we draw on three waves of the National Social Life Health and Aging Project, describe social network change at different levels of emotional closeness to the respondent, and then explore associations between network change and levels of depression. We find that a) Black respondents are far more likely to experience turnover (gains and losses) among their closest confidants, b) women are less likely to experience turnover in not-so-close confidants, and c) losing not-so-close confidants was associated with greater depression, and d) gaining new extremely close confidants was associated with lower levels of depression. We close with implications for dialogue between convoy theories, and theories of network advantage.

### SESSION 4290 (SYMPOSIUM)

Abstract citation ID: igaf122.1865

### MULTIDISCIPLINARY APPROACHES TO BETTER UNDERSTANDING THE HEALTH IMPLICATIONS OF MIGRATION ACROSS THE LIFE-COURSE

Chair: Yesenia Cruz-Carrillo
Co-Chair: Frank Infurna
Discussant: Kyriakos Markides

Migration influences health and aging trajectories across the life course. The goal of this symposium is to bring together a collection of papers that utilize multidisciplinary perspectives on trajectories and social determinants of health among middle-aged and older adult immigrants in the United States and Mexico. Cruz-Carrillo and Infurna use data from the Health and Retirement Study (HRS) to examine historical changes in and explanatory factors of self-rated health, episodic memory, and depressive symptoms; successive cohorts of immigrants demonstrated historical improvements compared to earlier-born cohorts. García and Garcia use data from the American Community Survey to investigate geographic variation in cognitive functioning among Latino immigrants across 24 states. The probability of reporting cognitive difficulties was higher among those in Northeastern states compared to other regions; differences by Latino heritage groups were also observed. Sheftel and colleagues use data from the HRS to estimate the projection of poverty and uninsurance rates among unauthorized Latino immigrants. By age 76, 56% of unauthorized foreign-born are expected to be in poverty and 59% uninsured. Saenz and Burns use data from the Mexican Health and Aging Study to examine differences in cognition levels among individuals from rural areas who stayed and those who migrated. The cognitive advantages of urban-dwelling inform our understanding of healthy migration. The discussion by Markides will integrate the four papers by highlighting novel findings that contribute to our understanding of migration across the life course and insights gained on the implications of population-level health trends in the U.S. and Mexico.

Abstract citation ID: igaf122.1866

### HISTORICAL CHANGES IN SELF-RATED HEALTH, MEMORY, AND DEPRESSIVE SYMPTOMS AMONG IMMIGRANTS IN THE UNITED STATES

Yesenia Cruz-Carrillo, and Frank Infurna, *Arizona State University, Tempe, Arizona, United States*

Empirical evidence has documented historical declines in midlife health, well-being, and cognition, whereas historical improvements have been observed among older adults in the U.S. Less is known about whether recent cohorts of immigrants are doing better or worse than their earlier-born peers and how this relates to broader trends that have been transpiring in the U.S. We examined whether self-rated health, depressive symptoms, and memory have changed across cohorts of middle-aged and older adult immigrants living in the U.S. We also explored the moderating role of gender, race, education, and age at immigration. Multilevel models were applied to longitudinal data from the Health and Retirement Study to examine historical changes in trajectories of self-rated health, episodic memory, and depressive symptoms among immigrants (N=5,131, 57% women, birth year: M=1945, range: 1890 to 1973) in midlife and old age. Historical improvements in self-rated health, historical stability in episodic memory, and historical decreases in depressive symptoms were observed across birth cohorts of immigrants in midlife and old age. Race and education moderated historical change. For example, attaining more years of education was associated with less strong historical decreases in depressive symptoms among successive cohorts. Our findings contrast the broader trends observed in the U.S. population; successive cohorts of immigrants demonstrated improvements in each of the included outcomes. Our discussion focuses on potential explanations for these trends, such as migration selectivity, the consideration of the historical context in our understanding of immigrant health, and the protective effects of cultural assets.

Abstract citation ID: igaf122.1867

### COGNITIVE DIFFICULTIES AMONG IMMIGRANT LATINOS: WITHIN GROUP VARIATION ACROSS U.S. STATES

Catherine Garcia, and Marc Garcia, *Syracuse University, Syracuse, New York, United States*

This study examined how U.S. state contexts shape disparities in cognitive disability risk among immigrant Latino adults, highlighting geographic variation in cognitive functioning. Using data from the 2008–2019 American Community Survey (ACS), we estimated a series of binary logistic regression models with clustered standard errors to assess differences in self-reported cognitive difficulties across (1) all Latinos by state, (2) immigrant Latinos by state, and (3) immigrant Latinos by country of origin and state. The analysis adjusted for gender, age, $age^2$, educational attainment, poverty status, and survey year. The sample included 901,016 self-identified immigrant Latinos aged 45 and older residing in one of 24 U.S. states that collectively account for 95% of the U.S. Latino population. Findings indicate substantial state-level variation in cognitive difficulties among foreign-born Latinos. Specifically, the probability of reporting cognitive difficulties was significantly higher among foreign-born Latinos residing in Northeastern states compared to other regions. Additionally, island-born Puerto Ricans exhibited the highest prevalence of cognitive difficulties relative to other Latino heritage groups across states. These results demonstrate how geographic contexts contribute to disparities in cognitive health among aging immigrant Latino populations. The observed variation underscores the role of structural and contextual factors in shaping cognitive disability risks, emphasizing the need to account for state-level influences when addressing Latino health inequities.

Abstract citation ID: igaf122.1868

### AGING INTO PRECARITY: POVERTY AND HEALTH INSURANCE COVERAGE AMONG OLDER UNAUTHORIZED LATINO IMMIGRANTS

Mara Sheftel[1], Ashton Verdery[2], Jennifer Van Hook[2], James Bachmeier[3], and Alexis Santos[2], *1. Rutgers University, New Brunswick, New Jersey, United States, 2. The Pennsylvania State University, University Park, Pennsylvania, United States, 3. Temple University, Philadelphia, Pennsylvania, United States*

The US immigrant population, including those who are unauthorized, is quickly aging in place. Ineligible for Social Security and Medicare, unauthorized immigrants may age at increased risk of poverty and uninsurance. Due to data limitations, the scale of their potential disadvantage remains unknown. We pioneer and validate an innovative approach to infer legal status for foreign-born respondents in the Health and Retirement Study (HRS), based on linkage to Social Security administrative records. Using this method, we find that by age 76, 56% of unauthorized foreign-born Latinos are expected to be in poverty and 59% uninsured, rates that are considerably higher than their authorized foreign-born peers, for whom 30% are estimated to be in poverty and 4% uninsured. We combine these estimates with high quality projections to examine their population-level implications. Projection results show that 20% of the growth in older adult poverty (1.1 million) and 64% of the growth in older adult uninsurance rates (1.3 million) are attributable to increases in the unauthorized population. This is notable considering the unauthorized population contributes only 6% of the growth in the total population age 65 and older in the next 20 years. Our findings provide striking evidence that legal status is a critical source of stratification for older immigrants with implications for the US population as a whole. The consequences of continued exclusion from public programs will have cascading effects, potentially impacting second- and third-generation immigrants and pressuring health systems, thereby extending far beyond the health and wellbeing of older immigrants themselves.

Abstract citation ID: igaf122.1869

### URBANIZATION, LIFE-COURSE MIGRATION, AND LATE-LIFE COGNITIVE HEALTH WITHIN THE MEXICAN CONTEXT

Joseph Saenz[1], and Shane Burns[2], *1. Arizona State University, Phoenix, Arizona, United States, 2. University of Michigan, Ann Arbor, Michigan, United States*

Urban-dwelling is linked with better cognition across diverse settings. Whether urban advantages can be attributed to urban-dwelling (e.g., health promoting resource access) or health-related migrant selection from rural-to-urban areas is unclear. Using data from the 2003–2012 Mexican Health and Aging Study, we analyzed differences in cognition levels, and cognitive decline, among individuals who self-reported growing up in rural areas, distinguishing between those who stayed in their childhood communities ("stayers") versus "movers" and objective late-life community size (rural/urban). We accounted for demographics and early-life health/socioeconomic position. Results indicated community urbanization may benefit cognition levels, as stayers who lived in communities that urbanized throughout their lives had better cognition than stayers whose communities remained rural. However, rural-to-urban movers had better cognition levels than rural-to-urban stayers, but this was only observed for men. Our findings suggest that cognitive advantages of urban-dwelling are due to a combination of urban-dwelling itself and healthy migration.

### SESSION 4295 (SYMPOSIUM)

Abstract citation ID: igaf122.1870

### IMPROVING DIETARY GUIDANCE FOR OLDER AMERICANS: BRIDGING THE RESEARCH GAPS

Chair: Kyla Shea
Co-Chair: Sarah Booth
Discussant: Sameera Talegawkar

The Scientific Report of the 2025 Dietary Guidelines Advisory Committee was recently released (in December 2025). This report is a vital resource in shaping the Dietary Guidelines for Americans, which are updated every five years. It highlighted older adults as a key population requiring updated dietary guidance to support their health and longevity. However, several critical knowledge gaps were identified regarding how to best address older adults' unique nutritional needs. For example, frailty and cognitive impairment are increasingly common in older age, yet there remain significant gaps in our understanding of the specific role diet plays in either mitigating or exacerbating these conditions. Furthermore, studies that focus on racially and ethnically diverse populations are limited. These gaps must be addressed before dietary recommendations for older adults can be appropriately tailored. Meal timing has emerged as an important dietary factor to consider for healthy aging. Yet, the impact of meal timing on lifespan is uncertain. This is an important question to clarify to begin to develop reliable guidance about meal timing. Little is known about how diet quality varies among racial and ethnic groups across older adulthood, highlighting a gap in developing culturally appropriate dietary recommendations to improve diet quality in older age. This symposium aims to present and discuss recent research that addresses these gaps in our understanding of the dietary needs of older adults to help inform the development of evidence-based dietary guidelines that can support the health and well-being of older adults from diverse backgrounds.

Abstract citation ID: igaf122.1871

### DIETARY PATTERNS, FRAILTY AND PLASMA PROTEOMIC MEDIATORS IN THE JACKSON HEART STUDY

Toshiko Tanaka[1], Luigi Ferrucci[1], Yichen Jin[2], Xiyuan Zhang[3], Katherine Tucker[3], and Sameera Talegawkar[2], *1. National Institute on Aging, Baltimore, Maryland, United States, 2. Milken Institute School of Public Health, Washington, District of Columbia, United States, 3. University of Massachusetts Lowell, Lowell, Massachusetts, United States*

Following a balanced and healthful diet is an important modifiable risk factor to slow the rates of aging, however, the mechanisms underlying this association is unclear. We examined this question in the 3645 participants from the Jackson Heart Study. A 34-item frailty index (FI) was constructed that showed association with risk of mortality (HR: 1.48; 95%CI:1.31-1.66). Adherence to four dietary patterns was measured: Mediterranean diet score (MDS), Dietary Approaches to Stop Hypertension (DASH) diet, Healthy Eating Index 2010 (HEI), and Alternate HEI 2010 (AHEI). Greater adherence to DASH ($\beta$=-0.063±0.014, $p < 0.0001$), HEI ($\beta$=-0.046±0.015, $p = 0.003$), or AHEI ($\beta$=-0.075±0.014, $p < 0.0001$) was associated with lower FI while no significant association was observed for MDS ($\beta$=-0.022±0.014, $p = 0.122$). Using plasma proteomic data from a subset of 1,406 participants, we identified associations between dietary patterns and protein levels: 25 proteins linked to the DASH diet, 20 to the AHEI diet, and 1 to the HEI diet. Notably, four proteins (CNTN4, EGFR, KYNU, SEMA6A) were associated with the DASH diet, nine proteins (CHL1, CNTN4, PI3, EGFR, IL1RAP, KYNU, MMP2, MAP2K2, NCAM1) with the AHEI diet, and one protein (EGFR) with the HEI diet. These proteins significantly mediated the relationship between dietary patterns and FI. Among the mediating proteins, six (CNTN4, SEMA6A, CHL1, MMP2, MAP2K2, NCAM1) are known to be involved in axon development and guidance. These findings suggest that adherence to specific dietary patterns influences plasma protein abundances and that the regulation of the nervous system may be a key mechanism linking diet to frailty.

Abstract citation ID: igaf122.1872

### DIETARY PATTERNS AND RISK OF AGE-RELATED COGNITIVE DECLINE AND DEMENTIA: ENTHUSIASM BEFORE EVIDENCE?

Sarah Booth, *Tufts University, Medford, Massachusetts, United States*

With respect to brain aging, there has been particular emphasis in the literature on three named diet patterns, DASH, the "Mediterranean" diet, and the MIND diet. Between 1980 and 2024, 139 human observational or intervention studies were published that examined the role of these individual dietary patterns in brain aging. When systematically reviewed using the USDA Nutrition Evidence Systematic Review method and an a priori established protocol, a common theme emerged: diets characterized by higher consumption of vegetables, fruits, legumes, nuts, fish, seafood, and unsaturated vegetable oils, alongside lower intake of red and processed meats and sugar-sweetened beverages, are consistently associated with a reduced risk of cognitive decline and dementia. However, a significant knowledge gap remains regarding the critical timing and duration of dietary pattern consumption in early adulthood for the prevention of cognitive decline. Furthermore, the biological mechanisms responsible for the cognitive protective effects of these diets remain unclear. Ultimately, it is important to understand the underlying mechanisms of select nutritional factors found within healthy dietary patterns that are not sufficiently consumed in the U.S. population yet show promise in delaying various aspects of brain aging.

Abstract citation ID: igaf122.1873

### ASSOCIATION BETWEEN EATING WINDOW AND LONGEVITY: A POPULATION-BASED STUDY

Samaneh Farsijani[1], Ziling Mao[1], Haley Grant[1], Stephen Kritchevsky[2], and Anne Newman, *1. University of*

*Pittsburgh, Pittsburgh, Pennsylvania, United States, 2. Wake Forest University School of Medicine, Winston-Salem, North Carolina, United States*

Time-based diets are popular for their health benefits, but their long-term effects on longevity remain unclear, with evidence largely from short-term trials and animal studies. Therefore, we determined the association between eating window and mortality risk in US adults. We conducted a retrospective cohort study using NHANES 2003–2018 data, linked to mortality records through December 2019. The analysis included 33,052 adults (>19 years) with two complete dietary and mortality records. Dietary data were collected via two 24-hour recalls, defining the eating window as the time between first and last intake of any food/beverage containing >0 calorie within 24 hours. We used survey-weighted Cox regression with Restricted Cubic Spline (RCS) modeling to assess nonlinear associations. Models adjusted for demographics, socioeconomic factors, lifestyle behaviors, health status, BMI, and diet quality. Subgroup analyses were conducted by age, sex, and race/ethnicity. Over eight years, 4,158 (8.9%) all-cause, 1,277 (2.6%) cardiovascular, and 989 (2.2%) cancer deaths occurred. The fully adjusted RCS model showed a U-shaped relationship ($p = 0.004$), with the lowest mortality risk at ~11-12 hours/day. Eating windows ≤8 hours/day were linked to ≥ 30% higher all-cause mortality, especially in older adults, and >50% higher cardiovascular mortality in older adults, men, and Whites. Extended eating windows (≥15 hours/day) were associated with 25% higher all-cause mortality, particularly among Whites, driven by cardiovascular mortality. Our study suggests that a moderate eating window (~11-12 hours/day) is linked to the lowest mortality risk, highlighting the need for personalized dietary recommendations. Long-term studies are essential to confirm causality and underlying mechanisms.

Abstract citation ID: igaf122.1874

### RACE AND ETHNIC DIFFERENCES IN DIET QUALITY ACROSS OLDER ADULTHOOD

Kyla Shea[1], and Sarah Booth[2], *1. Tufts University, Boston, Massachusetts, United States, 2. Tufts University, Medford, Massachusetts, United States*

Research on diet quality among different racial and ethnic groups across older adulthood is limited. This study aimed to examine diet quality in non-institutionalized U.S. adults aged 60-69 (n = 11,520), 70-79 (n = 7,202), and 80 + (n = 4,114) using NHANES data from 2005 to March 2020. Participants were categorized by race/ethnicity: non-Hispanic white (n = 11,792), non-Hispanic black (n = 5,038), Hispanic (n = 4,590), and 'other' (n = 1,416). The Healthy Eating Index 2015 (HEI) and component scores were calculated using the population ratio method, with results presented as weighted means (95% confidence intervals). Total HEI scores (possible range 0-100) were similar across race/ethnic groups in all age categories [range 61.9(60.5, 63.2) to 68.0(62.1, 73.8)]. However, significant differences in HEI components were observed. In all age groups, Non-Hispanic blacks had significantly lower scores for dairy [60-69: 3.6(3.4, 3.8); 70-79: 3.7(3.4, 4.0); 80+: 4.3(3.8, 4.8)], Hispanics had significantly lower scores for refined grains [60-69: 5.1(4.6, 5.6), 70-79: 5.0(4.4, 5.6), 80+: 4.8(3.89, 5.7)], and sodium scores were significantly lowest in those categorized as 'other' [60-69: 1.9(0.8, 3.0), 70-79: 2.5(1.4, 3.6), 80+: 1.2(0.2, 3.1)]. Non-Hispanic whites had significantly lower scores for greens and beans in the 70-79 and 80+ age groups [2.8(2.6, 3.1) and 2.1(1.9, 2.4), respectively]. Mean scores for total protein, seafood and plant protein, and whole fruit met the recommendations for all race and ethnic groups in all age groups. This study revealed racial and ethnic differences in HEI component scores despite similar overall diet quality, highlighting the need for culturally tailored interventions to improve diet quality in older adults.

### SESSION 4300 (SYMPOSIUM)

Abstract citation ID: igaf122.1875

### AGING AND PROSOCIALITY

Chair: Molly Lin
Co-Chair: Helene Fung

Aging has been found to positively correlate with prosocial behaviors, such as charitable donations and volunteering. Older adults not only engage in more prosocial behavior but also derive greater benefits from it compared to activities focused on self-development. Socioemotional selectivity theory explains this by categorizing prosocial goal as one of the 'emotionally meaningful goals' that increase with age. Yet, questions remain unknown from this perspective about what makes prosociality so meaningful and significant to older adults, and whether positive emotions drive or result from older adults' prosocial behaviors. This symposium addresses three questions: 1. WHETHER older adults are more prosocial than younger adults under any circumstances (i.e., what the boundaries of this age-related effect are), 2. WHY this is the case (i.e., what the underlying mechanisms are), and 3. HOW prosocial behavior can contribute to positive aging. Five presentations tap into these questions using theoretical, experimental, and ecological momentary approaches. They examined the relative importance of prosocial versus pro-closeness values, compared the factors associated with prosocial behaviors from adolescence to late adulthood, and explored the anti-loneliness effects of volunteering in older age. This symposium will shed light on future studies to further explore the mechanisms and outcomes of older adults' prosocial behavior.

Abstract citation ID: igaf122.1876

### AGE DIFFERENCES IN SOCIAL DISCOUNTING AND CHARITABLE GIVING IN A U.S. SAMPLE

Yi Lu, and Corinna Löckenhoff, *Cornell University, Ithaca, New York, United States*

Social discounting describes the tendency to show less generosity as social distance to the recipient increases. Prior studies examining age differences in social discounting suggest that older age is associated with greater generosity toward distant others (Pornpattananangkul et al., 2019; Gong et al., 2019). However, the generalizability of these findings is limited due to small sample sizes, comparisons of extreme age groups, and an exclusive focus on Asian samples. To address these gaps, a pre-registered study examined age differences in social discounting and charitable giving in a U.S. adult life-span sample (N = 432, aged 18-93, Mage = 51.23, SDage = 18.92, 50% female, 55% non-Hispanic White). We predicted that older age would be associated with lower social discounting and increased generosity across social distances and towards charities. The social discounting measure was adapted from Jones and Rachlin (2006) and involved five social distances (1/5/10/50/100, ranging from 0 = closest, 100 = most distant). For each social distance, participants chose between giving $100 to the other person and receiving $0/$20/$40/$60/$80/$100/$120 for themselves alone. They made the same decision for their favorite charity. Contrary to predictions, there were no significant age differences in social discounting rates, generosity across social distances, and charitable giving (ps >.05). Regardless of age, charitable giving was positively associated with generosity across social distances ($\rho$ = .39, p < .001). This pattern of effects remained robust after controlling for demographics. Findings suggest that age differences in social discounting may be sensitive to cultural setting and methodological factors.

Abstract citation ID: igaf122.1877

### THE INFLUENCE OF MOTIVE AND OUTCOME ON AGE-RELATED DIFFERENCES IN PROSOCIAL EVALUATION

Hongmei Lin, and Helene Fung, *The Chinese University of Hong Kong, Hong Kong, China (People's Republic)*

Prosocial behavior increases with age, but how older (vs. younger) adults' prosocial behaviors are evaluated remains unclear. Across two pre-registered studies, younger and older participants evaluated prosocial behaviors stemming from various motives (material, social, emotional, other's benefit) and resulting in different outcomes (positive vs. negative). Study 1, which did not disclose actor's age, included 155 older (54% female, Mage = 59.90, SDage = 3.88) and 175 younger adults (51% female, Mage = 28.20, SDage = 3.94). Study 2, which identified the actor as either younger or older, included 279 older (55% female, Mage = 59.50, SDage = 4.18) and 340 younger adults (58% female, Mage = 30.40, SDage = 3.43). Both studies revealed that, compared to actions driven by material and social motives with positive outcomes, younger and older participants rated prosocial acts as more prosocial when they are motivated by emotional or other-oriented benefits, even if the outcome was negative. Furthermore, when the actor's age was disclosed, both older and younger participants applied similar standards in their prosocial evaluations when outcome was positive. However, with negative outcome, older participants showed a bias: they were more lenient towards older actors performing prosocial acts for social benefits compared to younger actors, but less lenient when those acts were performed for material benefits. These findings suggest that emotional value is perceived as a genuine motive for prosocial behavior across ages. Moreover, older adults demonstrate greater flexibility in prosocial evaluations, adjusting their standards based on actor's age and motives when negative outcomes occur.

Abstract citation ID: igaf122.1878

### FROM POSITIVE YOUTH DEVELOPMENT TO POSITIVE AGING: A CASE ON PROSOCIALITY

Dwight Tse, *University of Strathclyde, Glasgow, Scotland, United Kingdom*

Developmental psychologists often focus research on specific stages across the lifespan, leading to theories that address unique challenges, opportunities, and developmental contexts at each stage. However, this raises the question of whether models designed for one stage can be applied to others. Positive Youth Development (PYD), which emphasizes the potential for growth and positive developmental trajectories in youth, challenges the view of adolescence as a period of "storm and stress." Similarly, aging has historically been framed as functional decline and frailty, but increasing research highlights the role of prosocial behaviors (e.g., volunteerism) in fostering positive aging outcomes. One prominent PYD theory is Lerner's 5Cs model (later expanded to 6Cs and 7Cs), identifying caring, confidence, competence, character, and connection as key PYD facilitators. These factors are also linked to prosociality, and growing evidence suggests they play a crucial role in positive aging. In this presentation, I will map how the 5Cs model can be applied in prosocial programs and explore how these same factors are acknowledged in existing aging theories. Using prosociality as an example, I will demonstrate the potential for adopting the 5–7Cs model to the study of positive aging. Finally, I will conclude by discussing the challenges and future directions for studying prosociality in positive aging, within the lifespan perspective, alongside research on positive development at specific life stages.

Abstract citation ID: igaf122.1879

### SELF- AND OTHER-ORIENTED MOTIVES, PROSOCIALITY, AND WELL-BEING IN OLDER ADULTS: AN ESM STUDY

Jeanne Nakamura[1], and Ajit Mann[2], *1. Claremont Graduate University, Claremont, California, United States, 2. Loyola Marymount University, Los Angeles, California, United States*

In past research on other-related and self-related motives for volunteering, other-related motives have been associated with higher wellbeing. However, some theories of motivation would suggest that self-oriented motives, notably immediate experiential rewards, will co-occur with other-oriented motivation for older adults engaging in prosocial activity. We report research that contributes to the understanding of motivation, prosociality, and well-being in three ways. We examined the conjunction of self- and other-oriented motives in addition to their separate occurrence, and compared how these motives were linked to well-being. Because prosocial behavior, motivation, and well-being fluctuate intra-individually, we examined their relationship using the experience sampling method. Finally, given challenges with researcher classification of volunteering motives as self- or other-oriented, we studied participant-reported motives. Data came from a sample of older, high-commitment volunteers and leaders in social-purpose programs in the US (n of responses=5,501; N=165, 58% female, age: M=71.1, SD=5.7). Consistent with past research, in moments when only other-oriented motivation was reported, positive correlates including eudaimonic well-being and positive affect were higher than when only self-oriented motivation was reported. Notably, a similar pattern held on occasions when other-oriented motivation co-occurred with self-oriented motivation. Theoretical and applied implications of the findings are discussed.

Abstract citation ID: igaf122.1880

### PROSOCIAL ENGAGEMENT REDUCES LONELINESS AMONG OLDER ADULTS THROUGH: THE HEAL-HOA DUAL RANDOMIZED CONTROLLED TRIAL

Da Jiang[1], Dannii Yeung[2], Lisa Warner[3], Namkee Choi[4], Rainbow TinHung Ho[5], Jojo YanYan Kwok[5], and Kee-Lee Chou[1], *1. The Education University of Hong Kong, Hong Kong, China (People's Republic), 2. City University of Hong Kong, Hong Kong, China (People's Republic), 3. MSB Medical School Berlin, Berlin, Germany, 4. The University of Texas at Austin, Austin, Texas, United States, 5. The University of Hong Kong, Hong Kong, China (People's Republic)*

The COVID-19 pandemic has exacerbated loneliness among older adults, highlighting the critical need for targeted interventions to reduce loneliness among older adults. Adopting an

"peer-support framework", we conducted a dual randomized controlled trial (HEAL-HOA RCT) to evaluate the effects of structured volunteering on reducing loneliness in older adults. Participants aged 50–70 (N = 375) were randomized into either volunteering or a psychoeducation group. Volunteers received training and then delivered one of three telephone-based psychosocial interventions (mindfulness, behavioral activation, or befriending) to lonely seniors aged 65+ who were living alone, below the poverty line and without internet. Volunteers committed to intervention delivery 2 hours/week for six months. Primary and secondary outcomes were assessed at baseline, 6 months (T2), and 12 months (T3). Results revealed significant loneliness reductions among volunteers at T2 (medium/large effect sizes) measured by the UCLA Loneliness Scale and De Jong Gierveld Loneliness Scale. Sustained benefits at T3 were only observed in the participants who maintained >2 hours/week of volunteering. Volunteering engagement also improved secondary outcomes, including (Lubben Social Network Scale & Multidimensional Scale of Perceived Social Support), life satisfaction (Satisfaction with Life Scale), and psychological well-being (Psychological Wellbeing Scale) at T2. These findings underscore the dual value of prosocial acts: addressing community needs while improving older adults' own mental health. Structured, sustained volunteering programs are essential, as benefits depend on continued engagement beyond initial commitments. This study bridges gaps in understanding causal effects of volunteering and offers actionable strategies to promote healthy aging through purposeful engagement.

### SESSION 4305 (SYMPOSIUM)

Abstract citation ID: igaf122.1881

### THE HEALTHY HOME LABORATORY: IMPLEMENTING SMART TECHNOLOGY TO PROMOTE AGING IN PLACE

Chair: Pamela Toto
Discussant: Steven Handler

Older adults prefer to remain in their homes as they age. However, according to U.S. Census Bureau, only about 10% of homes are "age-ready." Many older homes were not designed to support age-related changes and pose unique barriers for aging-in-place. To tackle these challenges, the University of Pittsburgh launched the Healthy Home Laboratory (HHL). HHL is a university-community laboratory established in a 107-year-old home that typifies older housing. An interprofessional team and community partners collaborate in this space to design, develop, evaluate and deploy smart technology solutions for aging-in-place. Representative of real-world challenges in homes, HHL reduces the knowledge-to-practice gap between science and everyday living and accelerates the implementation of solutions that are acceptable, sustainable and effective. This symposium provides an overview of the HHL and showcases smart technologies being implemented through the HHL to complement traditional home modifications for community impact. An introduction to Home Safe and Smart will share facilitators and barriers to integrating smart technology into a person-centered intervention offered through a health plan. Presentations of work funded by the Department of Housing and Urban Development will share results of efforts to implement a smart technology air quality assessment program within existing home and community-based services and work-in-progress to implement a smart technology fall prevention package and medication adherence solutions into low-income senior housing apartments. The final presentation will share results of a pilot supported by the Veterans Affairs to develop a technology preparedness assessment to help Veterans access care through telehealth.

Abstract citation ID: igaf122.1882

### ADDING ASSESSMENT OF HOME ENVIRONMENTAL HEALTH HAZARDS TO HOME- AND COMMUNITY-BASED SERVICE MODELS

Steven Albert, Jenn McCartney, Joey Engelmeier, Jemima Ohwobete, James Fabisiak, Sarah Haig, and Pamela Toto, *University of Pittsburgh, Pittsburgh, Pennsylvania, United States*

Aging in place refers to an older adult's ability to safely and independently carry out their desired, necessary, and expected daily activities within their home and community. Home- and community-based services (HCBS) support aging in place by developing care plans based on assessment of disability and the safety and accessibility of homes, but these assessments mostly ignore environmental health hazards, such as poor air quality (PM2.5, $CO_2$, radon) and mold, which are commonly reported by residents. To address this problem, the University of Pittsburgh's Healthy Home Lab (HHL) developed an air quality assessment and mitigation program, the Healthy Home Assessment Tool (H-HAT), in partnership with four regional HCBS agencies. H-HAT includes assessor observations, resident appraisals, consumer-grade indoor air quality monitoring, pathogen and mold analysis (DNA species extracted from dust). In a pilot with 43 low-income older adult (median age 75) households, 23% reported mold odor, and water damage was observed in 48.7%. While EPA standards for indoor air quality are evolving, almost all homes had PM2.5 values over 9 ug/m3. Fungal species with potential clinical significance were also identified. Care managers at the agencies received a packet reporting H-HAT results, which they discussed with residents. The care managers found the reports useful, which in some cases led them to alter care plans. Residents received resources for mitigation as well as an air purifier as part of the program. Follow-up assessments will determine success for mitigation and help in the design of wider dissemination.

Abstract citation ID: igaf122.1883

### HOME SAFE AND SMART: A PILOT PROGRAM INTEGRATING SMART TECHNOLOGY AND HOME MODIFICATIONS FOR AGING IN PLACE

Jack Fried, Meredith Hughes, Portia Singh, and Pamela Toto, *University of Pittsburgh, Pittsburgh, Pennsylvania, United States*

Smart technology is an effective solution for improving person-environment fit and eliminating barriers to aging in place. Adding smart technology to traditional home modifications can be cost-effective and provide powerful solutions for creating a healthy home. Home Safe and Smart (HSS) is an evidence-informed program delivered by an occupational therapist and a handy person integrating low-cost smart technologies and home modifications to reduce disability, increase safety and promote aging in place for community-dwelling older adults. Objective: Describe procedures and preliminary results of a pilot program delivering Home Safe and Smart (HSS) in partnership with a regional health plan. Methods: HSS was developed and piloted (N = 100) with nursing facility (NF) clinically eligible and NF ineligible persons 60+ invited by their health plan. The in-home assessment included standardized measures of self-report and observed performance of activities of daily living (ADL), fear of falling, readiness for change and environmental assessment to set personalized goals for function and health. HSS participants were eligible to receive select smart technology, home modifications and up to 5 in-home visits for associated training. Results: Participants demonstrated 89% ADL improvement, 91% increase in readiness for change and clinically meaningful reduction in fear of falling. Average cost for smart technology and home modifications was $567 per participant. Most common smart technology provided include Amazon Echo Show, smart lighting, smart health devices and smart doorbells. Conclusion: Preliminary outcomes indicate HSS and smart technology can be an acceptable and feasible prevention program for aging in place offered through a regional health plan.

Abstract citation ID: igaf122.1884

### ENHANCING FALL PREVENTION IN PUBLIC SENIOR HOUSING THROUGH SMART HOME SAFETY TECHNOLOGY

Yong Kyung Choi, Patricia Karg, Portia Singh, Haomin Hu, Julie Faieta, Meredith Hughes, Jon Pearlman, and Steven Handler, *University of Pittsburgh, Pittsburgh, Pennsylvania, United States*

Falls are the leading cause of injury among older adults, accounting for over 32,000 deaths and three million emergency department visits annually in the U.S. Older adults in public housing face increased fall risks due to environmental hazards, limited accessibility modifications, and restricted access to preventive interventions. Despite the potential of smart home safety technologies to mitigate these risks, their adoption in public housing remains limited due to usability concerns, cost barriers, and lack of implementation strategies. This study aims to identify and evaluate smart home safety technologies that can reduce fall risks and enhance safety for older adults in public senior housing. Using a community-based participatory research approach, we engage residents and housing authorities to assess fall-related hazards, technology usability, and implementation feasibility. The study consists of three phases: (1) a needs assessment to identify key fall risk factors, existing mitigation strategies, and policy barriers; (2) expert evaluation and usability testing of smart home safety technologies with older adults in a controlled simulation lab; and (3) pilot implementation of a tailored smart home intervention in public housing units. Technologies under evaluation include motion-activated lighting, environmental sensors, wearable fall detection devices, and smart medication management tools. At this conference, we will present preliminary findings, providing insights into older adults' perceptions of smart home safety technologies and the specific technologies identified and evaluated. This research aligns with national aging-in-place initiatives and has significant implications for housing policy, health equity, and digital inclusion.

Abstract citation ID: igaf122.1885

### HUMAN CENTERED DEVELOPMENT OF THE VETERAN'S VIDEO CONNECT MATCH (VVCMATCH) ASSESSMENT

Julie Faieta[1], Timothy Bober[1], Steven Handler[2], and Dan Ding[1], *1. University of Pittsburgh, Pittsburgh, Pennsylvania, United States, 2. Veterans Health Administration, Pittsburgh, Pennsylvania, United States*

The Veteran's Healthcare Administration (VHF) was an early adopter of broad telehealth use, however, telehealth implementation within the VHF continues to be limited by digital literacy barriers. Many of the Veterans who would benefit most from the option of telehealth due to heath needs and contextual barriers are also those who carry very limited technology experience. Individualized, and Telehealth-specific digital literacy, evaluation is needed to guide successful telehealth implementation. Therefore, the Veteran's Video Connect Match (VVCMatch) was developed through task analysis of the telehealth process. The VVCMatch utilizes both objective cognitive evaluation and a novel self-report assessment of the physical, cognitive, and digital literacy-related competencies needed for successful engagement with telehealth. The objective of our study was to gather preliminary Veteran feedback on the content and structure of the VVCMatch (Aim 1) and to gather initial exploratory data on the ability of the VVCMatch to identify Veterans who require education or support prior to adopting telehealth. Our initial findings point to the VVCMatch's ability to identify cases in which Veterans are not prepared to use telehealth. However, it also flagged cases in which the Veterans were able to complete a call with fair ability. These findings are being used to improve the specificity of the measure in preparation for larger scale psychometric and efficacy testing.

### SESSION 4310 (SYMPOSIUM)

Abstract citation ID: igaf122.1886

### UTILITY OF DIGITAL TECHNOLOGY FOR AMBULATORY ASSESSMENT OF EXPOSURES AND HEALTH IN OLDER ADULTS

Chair: Carol Derby
Co-Chair: Mindy Katz
Discussant: Lesley Ross

Digital technologies including wearable censors and smartphones facilitate the real-time assessment of psychological and physiologic states, behavioral patterns, and cognitive functioning, as well as person-level environmental exposures in the course of daily life. Further, these methods allow for continuing follow-up of older adults when in-person assessments are not possible, such as during infectious disease outbreaks, or when older adults are not able to travel to a study clinic. This session will illustrate the application of digital technology to the study of cognitive aging within the Einstein Aging Study (EAS) cohort of community residing adults. The EAS utilizes an integrative approach that combines ecologically valid ambulatory measures of both exposures and cognition with traditional clinic-based assessments in a diverse community-based sample of adults aged 60 and above. The session will illustrate the feasibility and utility of this approach for: 1) maintaining ongoing follow-up in older adults and obtaining real-time longitudinal data during environmental events (Dr. Roque), 2) using GPS data to derive person-specific indices of neighborhood environment exposures as individuals navigate everyday life (Dr. Hyun), 3) understanding how glucose regulation impacts cognition in older adults with diabetes (Dr. Hoogendoorn), 4) examining real time associations between subjective cognitive concerns and cognitive performance (Dr. Garcia De La Garza), and 5) prospectively tracking psychological health during the COVID-19 pandemic (Dr. Wang).

Abstract citation ID: igaf122.1887

### WEARABLE TECH FOR HEALTH MONITORING OF ENVIRONMENTAL EVENTS: CASE STUDIES IN EXPERIMENTAL MEDICINE

Nelson Roque[1], Mindy Katz[2], and Carol Derby[2], *1. The Pennsylvania State University, University Park, Pennsylvania, United States, 2. Albert Einstein College of Medicine, Bronx, New York, United States*

The COVID-19 pandemic underscored the need for adaptability in both daily life and scientific research. Using data from the Einstein Aging Study, we present case studies demonstrating the value of mobile and wearable technologies for behavioral monitoring in rapidly changing environmental conditions. First, we examine data availability during the COVID-19 pandemic, highlighting how smartphone-based assessments enabled real-time tracking of cognitive and psychosocial health measures—such as stress, affect, and social relationships—alongside the impact of different viral strains on cognition and behavior. Second, we explore the feasibility of low-cost wearable air quality monitors in capturing environmental exposures during two distinct events—Canada wildfires of March 2023 and holiday fireworks. Third, we examine how seasonal variations impact cognitive function measurement, highlighting the importance of considering temporal context in behavioral assessments. Our findings suggest that mobile monitoring methods offer a reliable approach to data collection in unpredictable and rapidly changing environmental conditions. Future research should identify the minimum data required for accurate outcome prediction, determining the threshold at which data remains meaningful.

Abstract citation ID: igaf122.1888

### GPS-BASED EXPOSURE TO NEIGHBORHOOD DEPRIVATION AND LATE-LIFE COGNITION

Jinshil Hyun[1], Nelson Roque[2], Mindy Katz[1], Richard Lipton[1], Carol Derby[1], and Charles Hall[1], *1. Albert Einstein College of Medicine, Bronx, New York, United States, 2. The Pennsylvania State University, University Park, Pennsylvania, United States*

Prior literature shows deleterious effects of neighborhood deprivation on cognitive and brain health. Most studies focus on neighborhood deprivation within individuals' residential areas, but people often travel across multiple neighborhoods. The use of GPS technology allows us to track person-specific exposure to deprived areas as individuals navigate their everyday environments. The aim of this study was to examine associations between GPS-derived exposure to deprived areas and different domains of cognition in late life. Participants include 126 older adults from Bronx, NY enrolled in the Einstein Aging Study (mean age=76; 63% females; 37% non-Hispanic Blacks). GPS signal was measured every minute for up to two weeks. GPS coordinates were coded at the census block group level and linked to publicly available Area Deprivation Index (ADI) scores. The final variable of interest was time-weighted exposure to deprived areas across entire study days for each participant (range = 0 to 9). Five cognitive domains (episodic memory, language, executive function, processing speed, visuospatial) were derived by averaging a subset of scores from 13 standard neurocognitive tests, with final scores in T-score units. Results from linear regression indicated that greater exposure to deprived areas in everyday life was associated with lower levels of executive function (estimate= -1.52, p=.007) and language ability (estimate= -1.41, p=.012) after controlling for age, sex, race/ethnicity, education, and total number of GPS collection days. We did not find significant associations between residential-level ADI and cognition. Everyday exposure to neighborhood deprivation, beyond residential deprivation, may influence cognitive health in older adults.

Abstract citation ID: igaf122.1889

### WEARABLE CONTINUOUS GLUCOSE MONITORING AND COGNITIVE PERFORMANCE IN OLDER ADULTS WITH TYPE 2 DIABETES

Claire Hoogendoorn[1], Angel Garcia De La Garza[2], Nelson Roque[3], Cuiling Wang[2], Richard Lipton[2], and Jeffrey Gonzalez[1], *1. Yeshiva University, New York, New York, United States, 2. Albert Einstein College of Medicine, Bronx, New York, United States, 3. The Pennsylvania State University, University Park, Pennsylvania, United States*

Limited work has evaluated continuous glucose monitoring (CGM) in relation to cognitive performance, and this has been conducted largely in type 1 diabetes. We explore associations between CGM and cognition among older adults with type 2 diabetes (T2D). Data are from community-residing adults over age 60 enrolled in the Einstein Aging Study, who self-reported T2D. Participants wore blinded Abbott FreeStyle Libre 3 sensors for 14 days and completed the WAIS III Digit Span, Trails A and B, Multilingual Naming(MINT), and WAIS III Block Design. CGM metrics include mean glucose, variability(coefficient of variation), %time in range(70-180 mg/dL), %time low(<70 mg/dL), %time high(181-250 mg/dL), and %time in very high(>250 mg/dL) glucose. Linear regression models were adjusted for age, sex, education and race/ethnicity. Interim analysis of the first 30 participants(73.9±7.4yrs, 56% female) indicate that they spent 73.8% of their time in range, 16.4% in high, 8.5% in very high, and 1.3% in low glucose. Less time in low glucose was associated with better Block Design scores($\beta = 0.35$, p=0.041). More variability showed a trend with worse MINT scores($\beta = 0.36$, p=0.058) and more time in high showed a trend with slower Trail B performance($\beta = 0.37$, p=0.097). Metrics were not associated with Digit Span or Trail A. A wearable CGM protocol is feasible in older adults and identified clinically relevant metrics that were associated with cognitive performance in T2D. Time in hypo- and hyperglycemia, and variability, were most predictive, with each linked to different cognitive functions including visuospatial processing, language, and executive function. Analyses will be extended to the larger sample.

Abstract citation ID: igaf122.1890

### PERCEPTION TO PERFORMANCE: LINKING COGNITIVE LAPSES & SMARTPHONE COGNITIVE ASSESSMENTS IN THE EINSTEIN AGING STUDY

Angel Garcia De La Garza[1], Carol Derby[1], Cuiling Wang[1], Jacqueline Mogle[2], Richard Lipton[1], Nelson Roque[3], Mindy Katz[1], and Laura Rabin[4], *1. Albert Einstein College of Medicine, Bronx, New York, United States, 2. Clemson University, Clemson, South Carolina, United States, 3. The Pennsylvania State University, University Park, Pennsylvania, United States, 4. Brooklyn College and the Graduate Center, The City University of New York, Brooklyn, New York, United States*

There is limited literature examining the relationship between subjective cognitive concerns (SCC) and objectively measured cognitive performance. In the Einstein Aging Study, we collected digital diary data on 18 types of cognitive lapses reported daily over 14 days from 297 participants (mean age = 77.54, SD = 4.82; 68% female; 48% Non-Hispanic White, 41% Non-Hispanic Black, 11% Hispanic). Participants completed four mobile cognitive tests assessing processing speed, visual short-term memory binding, spatial working memory, and working memory precision. SCC was analyzed by calculating the average daily number of reported lapses related to memory (12 items; mean = 1.58, SD = 1.48), executive functioning (6 items; mean = 0.84, SD = 1.07), and total cognitive lapses (18 items; mean = 2.42, SD = 2.17), with higher scores indicating greater self-reported cognitive difficulties. We calculated both average daily performance and within-day variability for all cognitive tests. Within-day synchronous associations between SCC and cognition were assessed using linear mixed-effects models, adjusting for age, sex, ethnicity, depression, and education. Findings showed that for each 1 standard deviation decrease in processing speed performance, participants reported 0.09 additional memory lapses ($p = 0.013$), 0.07 executive functioning lapses ($p = 0.005$), and 0.14 total lapses ($p = 0.003$). For each 1 standard deviation decrease in working memory precision, participants reported 0.15 additional memory lapses ($p < 0.001$), 0.11 executive functioning lapses ($p < 0.001$), and 0.24 total lapses ($p < 0.001$). Similar associations were found with within-day cognitive variability, where greater variability in both working memory precision and processing speed corresponded to increased reported daily cognitive lapses.

Abstract citation ID: igaf122.1891

### AMBULATORY ASSESSMENT FACILITATES LONGITUDINAL ASSESSMENT OF THE MENTAL HEALTH IMPACT OF COVID-19 IN OLDER ADULTS

Cuiling Wang, Chenxin Zhang, Qi Gao, Angel Garcia De La Garza, Richard Lipton, Carol Derby, and Mindy Katz, *Albert Einstein College of Medicine, Bronx, New York, United States*

The impact of the COVID-19 pandemic on the mental health of community-dwelling older adults from diverse racial and ethnic backgrounds is not well known, partly due to a lack of longitudinal studies with systematic and continuous assessments before, during and after the pandemic. In the Einstein Aging Study (EAS), both conventional and ambulatory digital assessments of various clinical and behavioral measures, including mental health, were administered annually since 2017. The ambulatory assessment consisted of a 2-week burst of digital survey and cognitive measures obtained 6 times per day. Using ambulatory assessment of mood, stress, anxiety, loneliness, depression, we evaluated changes in mental health through stages of the COVID-19 pandemic while taking account of age-related changes. The impact of age, sex and race/ethnicity, as well as various factors including social economic status (SES), social network, personality, were also examined. Multi-level mixed effects models for the intensive longitudinal mental health outcomes were applied to examine simultaneously the trajectories during follow-up time and changes over the calendar time based on phases of the COVID-19 pandemic. Among $n = 320$ EAS participants, we observed resilience in mental health as shown by quick recovery after an initial worsening at the beginning of the COVID-19 pandemic as indicted by the calendar time factor, and there was an overall decline trend during follow-up time. The role of demographics, SES, social network, personality, and behaviors during COVID-19 pandemics are discussed.

### SESSION 4315 (PAPER)

### FAMILY CAREGIVING ACROSS GENERATIONS: ROLES, RESPONSIBILITIES, AND CULTURAL CONTEXTS IN LATER LIFE

Abstract citation ID: igaf122.1892

### BRIDGING CULTURES AND GENERATIONS IN THE CAREGIVING OF MIDLIFE CHINESE IMMIGRANT WOMEN

Bo Jian, Merril Silverstein, and Catherine Garcia, *Syracuse University, Syracuse, New York, United States*

This study explores the well-being of midlife Chinese immigrant women as they navigate the complexities of intergenerational caregiving within the intersecting contexts of immigration, family obligations, and aging. Specifically, it examines how these women adapt to dual caregiving roles—supporting their children's education while caring for aging parents—amid the constraints of their immigrant experience. Using an ethnographic approach, this study draws on in-depth interviews and field notes from 20 immigrant Chinese women aged 35–50, analyzed through reflexive thematic analysis. This qualitative methodology captures the nuanced experiences of caregiving across transnational contexts, highlighting how cultural expectations, economic precarity, and shifting caregiving norms shape midlife immigrant women's daily lives. Findings reveal that caregiving responsibilities contribute to significant role strain, stress, and health consequences, including anxiety, emotional distress, premature hair graying, breast conditions, and migraines. Many participants prioritize their children's academic success over their own aspirations, further intensifying their caregiving burden. To cope, they turn to ethnic community networks, adopt flexible caregiving strategies, and advocate for culturally responsive resources. However, structural barriers such as language access, employment precarity, and policy inaccessibility persist, limiting their ability to seek formal support. This study underscores the urgent need for culturally inclusive policies and community-based interventions that recognize the intersection of gender, caregiving, and immigration. Addressing these challenges can help midlife immigrant women balance caregiving responsibilities while safeguarding their well-being.

Abstract citation ID: igaf122.1893

### GRANDPARENTHOOD, CARING FOR GRANDCHILDREN, AND MORTALITY IN CHINA

Linghan Ge[1], Ming Wen[1], and Yuying Tong[2], *1. The University of Hong Kong, Hong Kong, China (People's Republic), 2. Chinese University of Hong Kong, Hong Kong, China (People's Republic)*

This study addresses the research gap concerning the relationship between grandparenthood, intergenerational caregiving, and mortality in non-Western contexts. Existing theories propose both benefits and drawbacks: role enhancement theory highlights the protective effects of increased emotional support, physical activity, and cognitive stimulation, whereas role strain theory points to the risks posed by excessive responsibilities, intergenerational conflicts, and energy depletion. Utilizing data from the 2011-2018 China Health and Retirement Longitudinal Study, encompassing 9,336 respondents aged 50 and above in 2011 (34,976 observations), we applied logistic regression with random effects to examine the impact of grandparenthood and caregiving patterns, including no grandchildren, having grandchildren without caregiving, and caring for grandchildren, on mortality, exploring mediation by living proximity and meeting frequency with children. Grandparenthood significantly reduces mortality risk versus having no grandchildren. Grandparents not caregiving had 26.3% lower odds of death (OR = 0.737, p = 0.041), while caregiving grandparents had 31.7% lower odds (OR = 0.683, p = 0.012). KHB mediation analysis indicated that reduced living distance and increased meeting frequency with children partly explain this effect. Living proximity mediated 24.2% (β=-0.074, p < 0.001) and 10.6% (β=-0.040, p = 0.001) of the effect for non-caregiving and caregiving grandparents, respectively; meeting frequency mediated 15.9% (β=-0.049, p = 0.001) and 5.1% (β=-0.019, p = 0.026). This research underscores the protective effects of grandparenthood and caregiving on mortality among older Chinese adults, with living proximity and meeting frequency as critical mediators, offering insights into family mechanisms promoting healthy aging in non-Western contexts.

Abstract citation ID: igaf122.1894

### GRANDPARENTING STYLES IN THE U.S.: PATTERNS OF INTERGENERATIONAL SUPPORT AND THEIR PREDICTORS

Hyeonji Cho, and Catherine Garcia, *Syracuse University, Syracuse, New York, United States*

Diversity in aging and recent demographic changes could contribute to heterogeneous grandparenting patterns. We classified grandparenting styles in the U.S. based on intergenerational support dynamics and examined predictors of grandparenting style membership. Using data from the Health and Retirement Study (2012–2018), we analyzed 12,435 households where the respondent or their spouse had at least one child and grandchild. Latent class analysis was conducted on five intergenerational support indicators: providing financial support, providing grandchild care, living with children, receiving financial support, receiving ADL/IADL assistance. A three-class model provided the best fit. "Active Providers (28%)" provided both financial and childcare support while receiving minimal assistance from their children. "Financially Supportive but Detached (55%)" grandparents primarily offered financial support but had lower overall engagement. "Co-residing Recipients (17%)" were grandparents who live with their children and were more likely to receive both financial and instrumental support. Multinomial logistic regression examined predictors of grandparenting style membership, with "Financially Supportive but Detached" as a reference group. Higher household income (RRR=1.09, p<.001) and better health (RRR=1.17, p<.001) were associated with a greater likelihood of being in the "Active Providers" group, while lower household income (RRR=0.94, p<.001) and poorer health (RRR=0.55, p<.001) were associated with membership in the "Co-residing Recipients" group. Additionally, households with non-White respondents were more likely to be classified as "Active Providers" and "Co-residing Recipients" compared to White grandparents. Findings illustrate how financial resources, health, and race shape grandparenting roles. Recognizing these patterns can help inform policies and programs for aging families and multigenerational households.

Abstract citation ID: igaf122.1895

### INTERRUPTED SOCIAL ACTIVITIES AND BURNOUT IN CAREGIVERS: THE ROLE OF ACTIVITY VALUE

Jeong Eun Lee[1], Eunbea Kim[2], Yulri Kim[1], and Joseph Svec[3], *1. Iowa State University, Ames, Iowa, United States, 2. Auburn University, Auburn, Alabama, United States, 3. St. Joseph's University, Patchogue, New York, United States*

Caregivers increasingly experience disruptions to their social activities due to their caregiving responsibilities. While previous studies have highlighted the negative impact of such disruptions on caregivers' quality of life, the longitudinal relationship between disrupted activities and the values caregivers place on those activities remains underexplored. Using data from the National Study of Caregiving (NSOC) across three waves (N = 2328), this study examines how disrupted activities and their value change over time and their interaction with caregiver outcomes. Findings from multilevel modeling reveal that caregivers who experienced significant disruptions to their activities while attaching a high value to them reported a rapid increase in negative relationships and burnout over time. Conversely, caregivers who experienced high disruption but placed lower value on these activities showed a more moderate increase in both negative relationships and burnout. Caregivers with lower disruption and lower attachment to activities experienced only a slight increase in these outcomes. These findings underscore the critical role that the interruption of activities and the value placed on those activities play in shaping caregivers' mental health outcomes. From a practical perspective, this study highlights the importance of considering the meaning caregivers attach to their activities when developing interventions to support their mental well-being. Practitioners should be aware that caregivers who find meaning in their social activities may be at greater risk for burnout and adverse relational outcomes when disrupted. Thus, strategies should focus on providing support for both activity continuity and emotional attachment to activities.

Abstract citation ID: igaf122.1896

### PERCEPTIONS OF GRANDMOTHERS' FAVORITISM: CONSEQUENCES FOR ADULT GRANDCHILDREN'S WELL-BEING

Destiny Ogle[1], J. Jill Suitor[1], Megan Gilligan[2], and Shawn Bauldry[1], *1. Purdue University, West Lafayette, Indiana, United States, 2. University of Missouri, Columbia, Missouri, United States*

The consequences of maternal differential treatment on adult children's psychological well-being are well-documented. However, no consideration has been given to grandmothers' differentiation among their adult grandchildren and its consequences for the well-being of members of the youngest generation. Drawing from theories of socioemotional selectivity and gender-role socialization, this study uses mixed-methods data collected from 221 adult grandchildren nested within 81 families from the Within-Family Differences Study-III to investigate the association between adult grandchildren's perceptions of grandmothers' favoritism and psychological well-being. Forty-nine (28.5%) of the respondents perceived themselves as the grandchildren to whom their grandmothers were most emotionally close. Multilevel regression analyses suggest that perceiving oneself as the grandchild to whom grandmothers were the most emotionally close was associated with higher depressive symptoms among granddaughters, but not grandsons. Qualitative analyses revealed that these gender differences emerged from variations in the meaning granddaughters and grandsons ascribed to being the favored grandchild. In particular, granddaughters were much more likely than grandsons to explain their role as their grandmothers' emotional caregiver—a role that has been found to contribute to adult daughters' distress when their mothers face age-related challenges in later life. These findings contribute to the literature on differential treatment in intergenerational families by demonstrating that perceptions of grandmothers' emotional closeness are both common and consequential for the psychological well-being of adult granddaughters.

Abstract citation ID: igaf122.1897

**THE "SECOND EMPTY NEST": THE LIVED EXPERIENCES OF OLDER WOMEN WHOSE INTENSIVE 'GRANDMOTHERHOOD' ENDED**

Yarin Cohen[1], Gabriela Spector-Mersel[2], and Sharon Shiovitz-Ezra[3], *1. University of South Carolina, Columbia, South Carolina, United States, 2. Sapir College, Sderot, HaDarom, Israel, 3. The Hebrew University of Jerusalem, Jerusalem, Yerushalayim, Israel*

In Israel, grandmothers constitute the primary source of informal childcare. This intensive caregiving refills "the empty nest" and aligns with core Israeli cultural values: it reinforces the active Sabra ethos (contrasting with the stereotype of the Diaspora Jew as passive), and maintains the grandmother`s feminine identity through childcare—central to womanhood in Israel's pro-natalist society. This study examined the lived experiences of Israeli older women whose intensive grandmotherhood has diminished or ceased. Through in-depth interviews with 11 grandmothers and Moustakas' three-phase phenomenological analysis, we identified three key themes: a view of the active grandmother role as a second motherhood, the difficulties experienced following cessation of intensive caregiving, and the strategies employed to cope with the resulting void. Participants' experiences showed both positive and negative aspects of the intensive grandmotherhood. After cessation of their caregiving role, some showed remarkable similarities to mid-life mothers experiencing the "empty nest" syndrome. However, the "second empty nest" seems to pose more profound identity challenges, as it represents a final passage into what is culturally constructed as passive, genderless old age. Accordingly, grandmothers are required to employ various strategies to address these challenges. The "second empty nest" pertains to the experience of older women in many countries, but seems particularly intense within Israeli society, given the cultural emphasis on activity as characteristic of "worthy" (non-old) individuals and childcare as a defining element of (non-old) femininity. These findings demonstrate how deeply personal experiences of older adults are rooted in the socio-cultural construction of old age.

### SESSION 4320 (SYMPOSIUM)

Abstract citation ID: igaf122.1898

**REIMAGINING REMOTE CARE: ADVANCING TELEHEALTH TO SUPPORT AN AGING POPULATION**

Chair: Walter Boot

As telehealth continues to evolve, it holds immense potential to support the health and well-being of older adults. However, barriers related to digital literacy, accessibility, and user preferences must be addressed to ensure equitable access and effectiveness. This symposium brings together experts to examine the challenges and opportunities of telehealth in diverse aging populations, with a focus on digital inclusion, usability, and the future of remote healthcare solutions. Dr. Sohyun Kim will explore the role of video chat interventions in Long-Term Services and Supports (LTSS) settings, highlighting their potential to enhance social engagement and family relationships for persons living with dementia while addressing technological and logistical barriers. Sungjae Hong, MS, MPH, will present an exploratory study on telehealth adoption among Korean older adult immigrants, revealing key factors influencing digital healthcare use and the role of healthcare providers in shaping adoption. Dr. Paul P. Freddolino will introduce a comprehensive cost-benefit model assessing the impact of telehealth awareness and digital literacy training for direct care workers, examining the economic, social, and functional value of these initiatives for multiple stakeholders. Dr. Renato Ferreira Leitao Azevedo will discuss findings from an online feasibility study investigating older adults' preferences for different telehealth communication mediums, evaluating their effectiveness in enhancing comprehension, emotional engagement, and risk perception. By addressing critical issues in telehealth adoption and design, this symposium will provide valuable insights into optimizing remote care for aging populations.

Abstract citation ID: igaf122.1899

**VIDEO CHATS AS TOOLS FOR FAMILY CONNECTIONS IN LONG TERM SERVICE AND SUPPORT SETTINGS: A MULTIPLE CASE STUDY**

Sohyun Kim, *The University of Texas at Arlington, Arlington, Texas, United States*

Having regular video family visits in Long Term Service and Support (LTSS) can support persons living with dementia and their family caregivers in promoting social engagement, family relationship quality, and quality of life. This study explored family perspectives on video family visit intervention for persons living with dementia in three LTSS and their family caregivers, focusing on barriers and benefits. A thematic analysis was conducted through post-intervention interviews with three family caregivers of persons living with dementia in long-term service and support settings. Data was collected between June 5th and December 16th, 2024. Four main themes emerged: (1) Facilitating family connections and emotional well-being, (2) Technology as a barrier and bridge, (3) The need for structured support, and (4) Adaptability of scheduling. Families shared their experiences, noting that video family visits provided an opportunity to bridge physical distance and maintain family relationships. Video family chats can serve as a valuable tool to facilitate family connections in long-term service and support settings, particularly when personalized approaches are employed. By addressing technological barriers, incorporating structured support, and adapting to the specific needs of families and persons living with dementia, video family chats can enhance the quality of life for both patients and their families. Further research is needed to explore more personalized approaches and technological support to improve family relationships, enhance quality of life, and reduce feelings of loneliness and isolation.

Abstract citation ID: igaf122.1900

**NARRATIVES IN TELEHEALTH (NON-)ADOPTION AMONG KOREAN OLDER ADULT IMMIGRANTS: AN EXPLORATORY STUDY**

Sungjae Hong[1], Ha Young Choi[2], and Shannon Mejia[1], *1. University of Illinois Urbana-Champaign, Champaign, Illinois, United States, 2. University of Illinois Urbana-Champaign, Urbana, Illinois, United States*

Stay-at-home orders during the COVID-19 crisis accelerated the transition from in-person to digital healthcare. However, older adult immigrant populations remain among the least likely to adopt digital healthcare services. This study explored narratives from older adult immigrants about their experiences with digital healthcare services during the COVID-19 crisis and adoption practices in the years that followed. Our purpose is to reveal hidden components of digital healthcare transition among older adult immigrants in the United States to inform future research on this topic. A total of 22 older Korean adults were recruited from the Chicago metropolitan area during Spring 2024. Semi-structured interviews collected narratives of participants' digital healthcare service use during and after the COVID-19 crisis. We employed inductive and latent thematic analysis to extract contexts of (not) using digital healthcare. Emergent themes included: 1) selective transition to digital healthcare, 2) phone-based remote healthcare as a potential competitor of digital healthcare, 3) healthcare providers as a key actor determining digital healthcare adoption and use, and 4) healthcare visits as a potential life routine for older adults. This study asks gerontechnologists to consider different modalities of digital health and recognize the roles of diverse stakeholders, including healthcare providers, in shaping digital healthcare adoption and use. Also, this study contributes to digital healthcare inclusivity by shedding light on the nuanced healthcare experiences in later life and health crisis contexts, aspects which might not be fully replaced by digitalized healthcare services.

Abstract citation ID: igaf122.1901

### A COMPREHENSIVE MODEL FOR COST-BENEFIT ANALYSIS OF TELEHEALTH AWARENESS AND DIGITAL LITERACY TRAINING

Paul Freddolino[1], and Ying (Jessica) Cao[2], *1. Michigan State University, East Lansing, Michigan, United States, 2. University of Wisconsin–Madison, Madison, Wisconsin, United States*

Even before the COVID-19 pandemic telehealth use was increasing globally. The pandemic supercharged this trend due to prohibitions against in-person services. Practitioners and patients found that telehealth provided increased access to primary and specialty care, overcame transportation barriers, permitted better-coordinated chronic condition management, and potentially reduced healthcare costs. In the post-pandemic world telehealth use is stabilized at around 20% of all outpatient encounters. However, many older adults still face barriers, particularly limited IT literacy. Direct care workers (DCWs) who provide personal care and support can help address these barriers if trained. While some DCWs receive digital training, direct evidence of cost and benefits of such digital health literacy training is still limited. This presentation aims to fill this knowledge gap based on insights from two community projects. The first utilizes several coaching models to enhance digital literacy including telehealth awareness for older adults. The second provides similar skill-building to DCWs of older adults with the intent that DCWs will share this new knowledge with their clients and family caregivers. We will present a comprehensive model for assessing the costs and benefits of telehealth awareness and digital literacy training for DCWs through the standpoint of four stakeholder groups: direct care workers, patients, unpaid family caregivers, and providers/employers of DCWs (e.g., hospitals). For each group the benefit model explores functional value/benefit, economic value, social value, and emotional value. The cost model includes hiring, training, workload, cognitive burden, and other factors. Potential measurement strategies for model components will also be reviewed.

Abstract citation ID: igaf122.1902

### REIMAGINING REMOTE CARE: AN ONLINE FEASIBILITY STUDY OF TELEHEALTH COMMUNICATION MEDIUMS FOR OLDER ADULTS

Renato FerreiraLeitao Azevedo, and George Mois, *University of Georgia, Athens, Georgia, United States*

With the growing integration of artificial intelligence (AI) in healthcare, it remains unclear which communication system best enhances comprehension, emotional engagement, and health outcomes for older adults. While audio-based systems like Voice User Interfaces (VUIs) offer accessible and cost-effective solutions, more advanced modalities — such as interactive data visualizations, conversational agents (CAs), and social robots — may provide additional benefits to remote care by incorporating gestures, emotional cues, and interactive feedback. In this presentation, we will share findings from an initial online feasibility study examining older adults' preferences and responses to various communication mediums when receiving clinical test results and medication instructions. Our study aims to (1) identify which communication medium yields the most favorable outcomes by assessing whether these systems should function solely as informational tools or also as relational agents mimicking healthcare provider interactions, and (2) evaluate how these systems impact comprehension, emotional responses, and risk perception compared to traditional text-based formats. Findings will inform the development of user-centered telehealth solutions that improve health communication for aging populations, optimizing accessibility and effectiveness in remote care settings.

### SESSION 4325 (PAPER)

### LANGUAGE, IMMIGRATION AND GLOBAL HEALTH: EUROPE, ASIA AND AFRICA

Abstract citation ID: igaf122.1903

### A SCOPING REVIEW ON TOPICS RELATED TO WELL-BEING IN LATER LIFE IN EUROPE

Dilare Ecenur Irbık, and Bram Vanhoutte, *Universite Libre de Bruxelles, Brussels, Brussels Hoofdstedelijk Gewest, Belgium*

Ageing policy has evolved over the last decades through consecutive policy frameworks, such as healthy ageing, active ageing, or age-friendly environments. Each framework emphasises different aspects of what "ageing better" means and shifts the policy spotlight on new and often previously neglected aspects, sequentially improving the landscape of policies for later life. Going beyond these emphasised aspects of each framework, this paper aims to detect, from the current research literature, topics of ageing supporting well-being in later life which are missing from these policy frameworks. We used Arksey and O'Malley's five-stage methodological framework to map the available evidence, reported according to the PRISMA Extension for Scoping Reviews (PRISMA-ScR) Checklist. Three databases were used to identify the peer-reviewed quantitative, qualitative, and mixed-methods empirical articles published between January 2002 and January 2024. Articles were included if they were about older adults (Population), reported practices and/or recommendations that support well-being in later life (Concept) and were conducted in EU Member States (Context). Our search string resulted in 12,206 articles, of which 110 papers were retained for analysis. Results show that topics regarding well-being in later life that are missing in the main ageing policy frameworks are older prisoners, transnational families with older members, COVID-19 and emergency response regarding social isolation of older people, and LGBT+ older people. We believe this scoping review will trace the limits of the current policy frameworks and help us to understand which challenges could become central in future ageing frameworks.

Abstract citation ID: igaf122.1904

### AGING IN DEMOCRACY: ACCOMPLISHMENTS AND CHALLENGES OF LONG-TERM CARE POLICY IN POST-AUTHORITARIAN TAIWAN

Mo'e Yaisikana, *University of California, Berkeley, Berkeley, California, United States*

Taiwan has one of the fastest-growing aging populations in the world. As a rapidly aging society, the Taiwan government implemented a national universal Long-Term Care (LTC) service program in the early 2000s. The enactment of the Long-Term Care Services Act in 2017 was a milestone in further institutionalizing LTC into a centralized delivery network. The democratic political system played a crucial role in establishing LTC through legislation and parliamentary scrutiny. This paper investigates how democratic politics contributes to the accomplishments of elder care policy in the recently democratized Taiwan with archival data and ethnography conducted in rural villages in an extended case study approach. Findings suggest that older adults and their needs comprised a nascent social identity with unique interests that merge elders, caregivers, and business owners in the aging society. Amplified through their population size in multi-level electorate participation, they further propelled the advocacy for national-level policies and public service. Nevertheless, without a robust social insurance system, the tax-based governmental budget implied the financial insecurity and solid regulations of LTC. These regulations created incompatibilities between the local knowledge of caregiving and public services administration that may inhibit service development. The democratization established channels for private interests to be institutionalized, bridging a bottom-up path for welfare development, but it also depoliticizes elders' care in administration and turns older adults into the governable population. Localization and diversification have become LTC's following goals in Taiwan. This case study offers critical insights for developing sustainable care policies in aging democratic states worldwide.

Abstract citation ID: igaf122.1905

### ASSOCIATION BETWEEN FOOD INSECURITY AND PSYCHOLOGICAL DISTRESS AMONG OLDER KENYAN WOMEN

Beatrice Oppong[1], and Gabrielle Maloney[2], *1. University of Texas Rio Grande Valley, Oxford, Ohio, United States, 2. Drexel University, Philadelphia, Pennsylvania, United States*

Objective: Food insecurity is a major influencing factor of chronic psychological distress among older Kenyan women. However, these women experience food insecurity differently, with various factors influencing how it affects them. The aims of this study were as follows: (1) identify distinct subgroups of food insecurity among older Kenyan women based on indicators of food-related hardships, (2) examine the demographic and socioeconomic factors associated with food insecurity cluster membership, and (3) investigate the relationship between food insecurity cluster membership and psychological distress. Method: 274 older Kenyan women were recruited from four rural and urban areas in the summer of 2022. We collected sociodemographic, socioeconomic (including wealth indicators), global health, psychological health, and food availability data. We conducted a two-step cluster analysis using SPSS to identify potential distinctions among groups. A binary logistic regression was also conducted to identify the demographic characteristics of the clusters. Results: No significant differences were found in demographic and socioeconomic factors between the "often food insecure" and "sometimes food insecure" clusters. Binary logistic regression results also indicated that a unit increase in material wealth is associated with a decrease in food insecurity among older Kenyan women. Conclusion: Our study examined psychological distress and food insecurity, two areas that are tremendously impacting older Kenyan women. It shines a light on factors that are affecting the mental and physical well-being of this population.

Abstract citation ID: igaf122.1906

### GLOBAL PAIN TRENDS AND DISPARITIES: A LONGITUDINAL STUDY OF ADULTS 50+ ACROSS EUROPE, ASIA, AND THE AMERICAS

Esteban Calvo, Jose Medina, and Antonia Diaz-Valdes, *Universidad Mayor, Santiago, Region Metropolitana, Chile*

Pain is a prevalent condition among older adults, significantly impacting quality of life and healthcare systems. Understanding cross-national pain trends and disparities can inform targeted interventions and policies. This study examines long-term pain trends and social disparities in 22 countries across three continents. Using harmonized longitudinal data from six surveys, we analyzed 212,904 adults aged 50+ observed repeatedly between 1998 and 2018. Pain prevalence trends from 2006 to 2016 were estimated, adjusting for survey differences. Disparities by sex, education, and age were assessed using logistic regression models. The unadjusted pain prevalence across all countries and years was 43.2%. Instrument-adjusted prevalence varied from 26.7% (Netherlands, 2006) to 59.2% (France, 2016). Pain prevalence increased in 15 countries (mean annual increase: 1.21 percentage points; $p < 0.05$) and decreased only in China (-0.66 percentage points per year; $p < 0.05$). Pain was higher among women, less educated individuals, and older adults, with disparities varying significantly across countries and subtly over time. South Korea exhibited the largest disparities, while Denmark had the smallest. Some countries (e.g., Spain, Italy) showed widening disparities, whereas others (e.g., Denmark, Sweden) exhibited stable or decreasing trends. The substantial cross-national variation in pain prevalence and disparities suggests that pain can be mitigated through policy and intervention efforts. Identifying high-disparity regions enables targeted strategies, while lessons from low-disparity regions may inform best practices.

Abstract citation ID: igaf122.1907

### PREVALENCE OF NON-COMMUNICABLE DISEASES AND HEALTHCARE UTILIZATION AMONG OLDER ADULTS IN MONGOLIA

Zoljargalan Gantumur, and Yifan Lou, *Virginia Commonwealth University, Richmond, Virginia, United States*

Non-communicable diseases (NCDs) pose significant health burdens in Mongolia, a lower-middle-income landlocked country in Central Asia, where 16% of the population is projected to be of retirement age by 2045. NCDs are the leading causes of morbidity, mortality, and disability in later life, yet NCD-related research in Mongolia remains limited. Using nationally representative data, we investigated the prevalence, predictors, and health service utilization outcomes among older Mongolians. Data were from 1,989 adults aged 50+ in Mongolia's National NCD Risk Factor Surveillance Survey (W4; 2019). NCDs were measured by the diagnosis of hypertension, raised cholesterol, cardiovascular diseases, and diabetes. We also consider the number of NCDs (0, 1, 2+). Logistic and multinomial logistic regression were used to understand the demographic and socioeconomic status (SES) predictors of NCDs and their relationships with healthcare utilization (doctor visits, outpatient hospital visits, and hospitalization). 64% of sampled older Mongolians had an NCD, with hypertension being the most prevalent. Nearly 49% received their diagnosis within the past year, and 24.1% had 2+ NCDs. Females are more likely to have 2+ NCDs. Employment status is also a strong predictor of having comorbidity. Socio-demographic predictors for each NCD vary, with low SES being the strongest predictor for cardiovascular diseases, but not for hypertension. Having 2+ NCDs increased all types of healthcare utilization compared to having none or one NCD, with each type of NCD driving greater use of different healthcare services. The findings inform targeted NCD management programs in Mongolia, particularly for women with multiple diagnoses.

### SESSION 4330 (PAPER)

Abstract citation ID: igaf122.1908

### MILD COGNITIVE IMPAIRMENT AND ALZHEIMER'S DISEASE AND RELATED DISORDERS

This session will address both biological and clinical biomarkers of MCI and Alzheimer's Dementia and Related Dementias

Abstract citation ID: igaf122.1909

### LONELINESS AMONG STROKE SURVIVORS WITH MILD COGNITIVE IMPAIRMENT AND THEIR FAMILY CAREGIVERS: A QUALITATIVE STUDY

Jiahui Xu[1], Ruotong Chu[1], Jing Wang[2], Juan Li[1], and Bei Wu[3], *1. Huashan Hospital Fudan University, Shanghai, Shanghai, China (People's Republic), 2. University of New Hampshire, Durham, New Hampshire, United States, 3. NYU Shanghai, Shanghai, China (People's Republic)*

Loneliness is a significant challenge for stroke survivors with mild cognitive impairment (MCI) and their family caregivers, yet little is known about their unique experiences within social networks. This study explores how stroke survivors with MCI navigate loneliness, their psychological needs, and the strategies they use to cope. A descriptive qualitative study was conducted using semi-structured, in-depth interviews with 20 purposively selected stroke survivors with MCI and their family caregivers. All interviews were recorded, transcribed, and analyzed using thematic analysis. Five key themes emerged: (1) Enduring loneliness despite family presence. Survivors felt isolated even when surrounded by family due to a lack of meaningful interaction; (2) Concealing illness due to stigma. Fear of being judged or burdening others led many to hide their condition, further deepening isolation; (3) Limited awareness and neglect of cognitive impairment. Both survivors and caregivers often overlooked or misunderstood MCI symptoms, delaying support seeking; (4) Emotional vulnerability and defensive behaviors. Survivors struggled with feelings of shame, leading to withdrawal or defensive coping mechanisms; (5) Barriers to rehabilitation and social reintegration. A lack of personalized rehabilitation guidance and accessible social opportunities hindered survivors from rebuilding their confidence and social connections. The emotional barriers and social withdrawal experienced by both survivors and caregivers contribute to a cycle of isolation. Understanding these self-perceptions and the changing dynamics of social engagement is crucial for caregivers and healthcare providers to design interventions that foster meaningful connections and mitigate loneliness in this vulnerable group.

Abstract citation ID: igaf122.1910

### LONGITUDINAL RELATIONSHIP BETWEEN A NOVEL ALZHEIMER'S DISEASE METHYLATION INDEX AND COGNITIVE FUNCTION

Nwanyieze Jiakponnah[1], S. Michal Jazwinski[2], and Sangkyu Kim[3], *1. Tulane University School of Medicine / Wake Forest University School of Medicine, New Orleans, Louisiana, United States, 2. Tulane University School of Medicine, New Orleans, Louisiana, United States, 3. Tulane University, New Orleans, Louisiana, United States*

Alzheimer's disease (AD) remains the leading cause of dementia. While existing biomarkers provide insights into AD pathology, they often fall short in early detection and tracking cognitive decline. This study examines the longitudinal relationship between the Alzheimer's Disease Methylation Index (AD-DMI), a novel, disease-specific epigenetic biomarker, and cognitive trajectories preceding AD diagnosis. Data from 541 ROSMAP participants (mean age = 88 years; 308 AD cases, 233 controls) with up to 16 years of cognitive assessments were analyzed. AD-DMI was developed using dorsolateral prefrontal cortex DNA methylation via elastic-net logistic regression, which selected 100 CpGs that best distinguished AD cases from controls (AUC = 0.79). AD-DMI scores were computed as the average methylation across these CpGs. Multivariable linear mixed-effects models assessed AD-DMI's association with episodic memory, semantic memory, perceptual speed, visuospatial ability, working memory, and global cognition, adjusting for covariates. Higher AD-DMI scores were significantly associated with steeper decline across all domains ($\beta$ range: -0.15 to -0.37, $q < 0.001$). Functional enrichment analysis of AD-DMI CpG-mapped genes identified biological processes related to AD, synaptic function, and autophagy. Eight of these genes overlapped with AD-associated genes previously identified in GWAS. Additionally, AD-DMI scores were significantly associated with increasing methylation at CpGs mapped to APOE, ANK1, and HOXA3 ($\beta$ range: 0.56 to 2.13, $q < 0.01$), previously reported as hypermethylated in AD in EWAS. AD-DMI tracks epigenetic signatures linked to AD, supporting its potential for disease monitoring. Future studies should validate AD-DMI in diverse cohorts and assess its non-invasive potential.

Abstract citation ID: igaf122.1911

### WHO RECEIVES AN MCI DIAGNOSIS?

Elyse Couch, Munachimso Ugoh, Lauren Thomas, and Emmanuelle Belanger, *Brown University, Providence, Rhode Island, United States*

Mild cognitive impairment (MCI) is a key risk factor for future dementia and represents an opportunity for early engagement in treatment and decision-making. Although MCI affects more people than dementia, little is known about MCI diagnosis rates. This study aimed to determine the proportion of people with MCI who received a formal diagnosis and identify patient characteristics associated with an MCI diagnosis in a nationally representative sample of Medicare beneficiaries in the United States. To achieve this aim, we used data from the National Health and Aging Trends Study (NHATS) linked with Medicare claims data. We included beneficiaries enrolled in the 2015 round of NHATS, who developed symptoms of cognitive impairment consistent with MCI according to a validated algorithm of NHATS measures of cognition, and were continuously enrolled in Medicare fee-for-service. Beneficiaries were followed up until 2022. Beneficiaries with diagnosed MCI were identified in Medicare claims data using ICD-9 and ICD-10 codes for MCI. Univariate and multivariate logistic regressions were used to identify patient characteristics associated with diagnosed MCI. Of 2.7 million continuously enrolled Medicare beneficiaries with symptoms of cognitive impairment consistent with MCI, only 10.6% had a diagnosis of MCI recorded in claims. Multivariate analyses found the odds of diagnosed MCI were higher among women, beneficiaries with a bachelor's degree or higher, and lower among beneficiaries who attended doctor visits alone. The findings of this study suggest that MCI is significantly under-diagnosed. Future research and policy initiatives are needed to increase MCI diagnosis rates.

Abstract citation ID: igaf122.1912

### PLASMA PROTEOMIC PROFILE OF DUAL DECLINES IN COGNITIVE AND PHYSICAL PERFORMANCE

Alan Rathbun, Chixiang Chen, and Michelle Shardell, *University of Maryland School of Medicine, Baltimore, Maryland, United States*

Dual declines in cognitive (or memory) and physical performance multiplicatively increase dementia risk, yet shared biological mechanisms remain unclear. This study investigated plasma proteins and biological mechanisms underlying dual cognitive-physical decline in older adults. Men and women ($n = 774$, age $\geq 60$ years) enrolled in the Invecchiare in Chianti study were classified into three phenotypes—no decline, physical decline only, and dual decline—using group-based trajectory modeling of up to 15 years of cognitive or memory function (Mini-Mental State Examination) and 4-meter gait speed. Proteins were measured using aptamer-based proteomics (SomaLogic). Multinomial regressions, adjusted for potential confounders (age, sex, marital status, education, self-rated health, and body mass index), identified proteins that differed across phenotypes. Additionally, Reactome-based functional enrichment analysis highlighted pathways associated with dual decline. Eight proteins (q-value < 0.05) predicted dual cognitive and physical declines, five of which also predicted memory and physical declines. Elevated PI3, GDF15, TFF3, CCL15, TNNT2, and AGRP were associated with dual decline, while higher CKM and GHR were linked to no decline. Proteins differentiating dual decline from no decline were enriched in pathways regulating insulin-like growth factor (IGF) transport, IGF binding, and post-translational phosphorylation. Findings support a multi-system model in which inflammatory, metabolic, and muscle-related disruptions in IGF regulation and protein phosphorylation contribute to key hallmarks of aging. These findings can guide geroscience-driven interventions to prevent dual cognitive-physical decline and reduce dementia risk.

Abstract citation ID: igaf122.1913

### SLEEP IN OLDER ADULTS

Glenna Brewster, *Emory University, Atlanta, Georgia, United States*

This session shares research on the association and impact of sleep in older adults.

Abstract citation ID: igaf122.1914

### EPIDEMIOLOGY OF HIGH-RISK SLEEP PHENOTYPES IN A DIVERSE COHORT OF OLDER ADULTS

Brienne Miner[1], Jarett Talarczyk[1], Clemence Cavailles[2], Carrie Peltz[3], and Kristine Yaffe[2], *1. Yale University, New Haven, Connecticut, United States, 2. University of California, San Francisco, San Francisco, California, United States, 3. Northern California Institute for Research and Education, San Francisco, California, United States*

Background: Insomnia with objective short sleep duration (ISSD) and objective long sleep duration (LS) are high-risk sleep phenotypes that have not been examined in a diverse group of older adults. Methods: We studied 1001 adults in the DORMIR study (average age 64.4[8.6] years, 65.7% female; 28.4% Black, 31.6% Hispanic, 40.1% non-Hispanic White). Insomnia was defined as self-reporting any of the following >=3 times/week: trouble getting to sleep within 30min, waking in the middle of the night/early morning or taking sleep medication. Objective overnight sleep duration was assessed by actigraphy (averaged over 5.9[0.9] days). We examined ISSD (insomnia and sleep duration< 6h), LS (sleep duration>8h), and normal sleep (NS; no insomnia and sleep duration=6-8h). Using ANOVA F-tests or chi-square, we assessed phenotype prevalence overall and according to race/ethnicity, and also examined, within each race/ethnicity group, whether comorbidities (e.g., depression, anxiety, dementia status, physical impairment [Short Physical Performance Battery< 10]) differed among phenotypes. Results: ISSD prevalence was 8.1% overall and was higher among Blacks (13.4%; $p < 0.001$). LS prevalence was 22.1% overall and was higher among Whites (27.2%; $p < 0.001$). Compared to NS, depression was higher in ISSD and LS across all groups. Anxiety was higher in Hispanics and Whites with ISSD and LS compared with NS. Across all groups, physical impairment was significantly higher in LS compared with NS. Dementia status was not different across phenotypes. Conclusion: The prevalence of high-risk sleep phenotypes differs across race/ethnicity groups, while comorbidities associated with phenotypes were similar. Future work should examine the association of phenotypes with adverse outcomes.

Abstract citation ID: igaf122.1915

### EVALUATING BASELINE SLEEP AND FEEDBACK ON THE ABCS OF ZZZ, A SLEEP PROGRAM IN BLACK CHURCHES

Glenna Brewster[1], Daum Chung[1], Miranda McPhillips[2], Elliane Irani[3], and Fayron Epps[4], *1. Emory University, Atlanta, Georgia, United States, 2. Villanova University, Villanova, Pennsylvania, United States, 3. Case Western Reserve University, Cleveland, Ohio, United States, 4. UT Health San Antonio, San Antonio, Texas, United States*

Sleep disparities disproportionately affect Black/African American communities, with high rates of insomnia and poor sleep quality often going unaddressed. The ABCs of Zzz is a four-hour, in-person sleep education program co-developed with church ambassadors and a community advisory board. The program was delivered at Alter-affiliated, predominantly

Black/African American churches in Georgia. This study presents baseline sleep characteristics of participants and their initial feedback on the program. Participants completed demographic information and validated sleep measures: Insomnia Severity Index (ISI), Pittsburgh Sleep Quality Index (PSQI), and Dysfunctional Beliefs and Attitudes about Sleep (DBAS). Following the session, participants provided feedback on program satisfaction. Descriptive statistics were used for analysis. The sample (N = 134) was predominantly female (77.4%) and Black/African American (97.0%). Participants ranged in age from 18 to 91 years (M = 62.77, SD = 13.05), with half of the participants being older than 64 years. Participants had mean baseline ISI score of 9.52 (SD = 6.80), indicating mild to moderate insomnia symptoms. The mean PSQI score was 9.44 (SD = 4.21), suggesting poor sleep quality. DBAS scores averaged 4.12 (SD = 2.31), suggesting flawed sleep beliefs. Session feedback was positive;100% of participants would refer a friend or family member; 84.1% were "very satisfied" with the content; and 98.9% agreed the session met their expectations. Baseline data highlight significant sleep challenges among participants from predominantly Black/African American churches, emphasizing the need for targeted sleep interventions in this community. The overwhelmingly positive feedback underscores the value of community-driven sleep education programs.

Abstract citation ID: igaf122.1916

### INTERACTION OF PHYSICAL ACTIVITY, SLEEP, AND COGNITIVE FUNCTION IN STROKE: SEM AND ML APPROACHES

Mo Yi[1], Lan Gao[2], Li Chen[3], Junxin Li[4], and Tangsheng Zhong[2], *1. Peking University, Beijing, Beijing, China (People's Republic), 2. First Hospital of Jilin University, Changchun, Jilin, China (People's Republic), 3. Jilin University, Changchun, Jilin, China (People's Republic), 4. Johns Hopkins University, Baltimore, Maryland, United States*

Cognitive impairment is prevalent among stroke survivors, highlighting the importance of exploring modifiable lifestyle factors such as physical activity and sleep, along with their biological underpinnings. This study aimed to investigate how physical activity and sleep influence cognitive function in stroke survivors, examining potential biological mediators. Cross-sectional data collected at hospital admission from 262 stroke survivors (First Hospital of Jilin University, 2024) were analyzed. Measures included self-reported weekly physical activity (exercise frequency/duration), nighttime sleep (duration, latency), cognitive function (Montreal Cognitive Assessment [MOCA]), emotional status (Hamilton Depression Rating Scale [HAMD]), and Serum neurological (homocysteine [Hcy]) and inflammatory (hs-CRP) biomarkers. Participants were aged 65.4±8.2 years, 43% female, and 58% showed cognitive impairment. Correlation and regression analyses examined associations, and structural equation modeling (SEM) explored causal pathways. Machine learning (ML) methods identified key predictors of cognitive impairment. Frequent physical activity ($r = 0.35$, $p < 0.001$) and adequate sleep ($r = 0.25$, $p < 0.001$) significantly correlated with higher cognitive scores. Depression severity ($r = -0.40$, $p < 0.001$) and elevated Hcy ($r = -0.36$, $p < 0.001$) negatively correlated with cognition. Regression analyses confirmed exercise frequency ($\beta = 0.34$, $p < 0.001$) and sleep duration ($\beta = 0.29$, $p < 0.001$) as independent positive predictors of cognitive function. SEM demonstrated direct beneficial effects of physical activity ($\beta = 0.41$, $p < 0.001$) on cognition and indirect effects mediated by improved sleep and reduced inflammation (CFI>0.9). ML predicted cognitive impairment (accuracy>80%), identifying age, education, Hcy level, physical activity, and sleep as crucial predictors. Physical activity and sleep synergistically enhance cognition in stroke survivors via inflammatory and neurological pathways. Promoting regular exercise, optimizing sleep, and managing biomarkers may preserve cognitive function.

Abstract citation ID: igaf122.1917

### SLEEP OF MEDICALLY ILL HOSPITALIZED OLDER ADULTS: DO SLEEP MEDICATIONS MAKE A DIFFERENCE?

Juliana Smichenko, Tamar Shochat, and Anna Zisberg, University of Haifa, Haifa, HaZafon, Israel

Sleep disturbances are common among medically ill older adults during acute hospitalization, often leading to adverse outcomes. Despite frequent use of sleep medications, their effectiveness remains uncertain. This study examined changes in sleep parameters from home to hospital and evaluated the impact of physical symptom burden and sleep medications burden daily trajectories as key factors. A prospective multicenter study was conducted across four Israeli hospitals. The study included 683 cognitively intact older adults who completed an admission interview and at least one follow-up. Sleep parameters—including total sleep time (TST), sleep efficiency (SE), sleep quality (SQ), and number of awakenings (NOA)—were assessed daily, along with sleep medication use and burden (dosage and quantity). Physical symptom burden and additional covariates were analyzed using a repeated measures mixed models approach. Participants (54% male, mean age 77.31±6.60) experienced reduced TST (329.73±111.94 vs. 377.03±101.06 minutes), lower SE (71.49±19.28% vs. 76.14±15.53%), and poorer SQ in the hospital compared to home. Sleep medication use was not associated with any sleep parameter, while sleep medication burden correlated with increased NOA. Physical symptom burden had significant effects on SE, SQ, and NOA. A time-dependent interaction showed that higher symptom burden was most strongly linked to reduced TST at the first in-hospital follow-up, stabilizing thereafter. Sleep deteriorates during hospitalization, with minimal benefit from sleep medications, however stronger effect of physical symptoms severity. Prioritizing symptom management may be key to improving sleep in hospitalized older adults.

Abstract citation ID: igaf122.1918

### SLEEP QUALITY MODERATES THE ASSOCIATION BETWEEN ROUTINE DISRUPTION STRESS AND AFFECT IN MIDLIFE AND LATE ADULTHOOD

Eunjin Tracy[1], Jichan Kim[2], Pei-Shu Chao[1], Shadi Ansari[1], and Eunjung Kim[1], *1. University of Missouri, Columbia, Missouri, United States, 2. Liberty University, Lynchburg, Virginia, United States*

Disruptions in daily routines can misalign circadian rhythms, reduce structure and predictability, and contribute to emotional dysregulation. Individuals with poor sleep quality may be particularly vulnerable to these stressors due to preexisting circadian misalignment, yet few studies have examined these associations at the daily level or the potential role of sleep in these relationships. This study analyzed data from 1,011 participants (M age = 55.34, range = 34–84) in the Midlife in the United States (MIDUS) Daily Stress Project, assessing daily stress related to routine disruptions and affect over eight days, along with global sleep quality. Multilevel modeling revealed significant within- and between-person effects: higher stress related to daily routine disruption was associated with greater negative affect and lower positive affect at both within- and between-person levels. Sleep quality significantly moderated these associations for negative affect at both levels. At the within-person level, routine disruption stress increased negative affect only among those with poor sleep quality ($z=4.49$, $p < .001$ vs. $z=1.12$, $p = .26$ for better sleep quality). At the between-person level, the association between routine disruption stress and negative affect was stronger for those with poorer sleep quality ($z=7.37$, $p < .001$) compared to those with better sleep quality ($z=3.58$, $p < .001$). These findings underscore the role of daily routines in emotional regulation in midlife and suggest that better sleep quality may buffer against stress-related affective disturbances. Interventions targeting routine stability and sleep quality may enhance emotional well-being.

### SESSION 4340 (AWARD LECTURE)

Abstract citation ID: igaf122.1919

### THE M. POWELL LAWTON AWARD LECTURE AND PRESENTATION

Chair: Christine Mueller

The M. Powell Lawton Award Lecture will feature an address by the 2024 recipient Stephen M Golant, PhD, FGSA, of the University of Florida. This session will also include the presentation of the 2025 M. Powell Lawton Award to recipient Joseph E. Gaugler, PhD, FGSA. The M. Powell Lawton Award is presented annually to an individual who has made outstanding contributions from applied research that has benefited older people and their care. The Lawton Award is generously funded by the Polisher Research Institute of Abramson Senior Care.

Abstract citation ID: igaf122.1920

### RETHINKING LAWTON'S ENVIRONMENTAL DOCILITY HYPOTHESIS AS OLDER PEOPLE ADOPT GERONTECHNOLOGY SOLUTION

Stephen Golant, *University of Florida, Gainesville, Florida, United States*

Lawton's "environmental docility hypothesis" proposed that older people with physical or cognitive limitations have a greater share of their behaviors explained by the qualities of their residential environments. Occupying dwellings, neighborhoods, or communities with restrictive or unsafe physical or social aspects will more likely constrain or endanger their behaviors. The validity of this proposed relationship is troublesome given the large projected worldwide growth of older people with functional limitations threatening their ability to realize both their obligatory and discretionary needs. However, this lecture argues that these controlling environmental relationships will be less apparent as older people—even at advanced ages—actively cope with their limitations by introducing digital and sensor solutions informed by artificial intelligence into their homes. Having more ways to act proactively—their limitations are less predictive of adverse individual outcomes. Lawton would be pleased—his mission was to improve the "good life" of the less able.

### SESSION 4345 (PAPER)

### STUDENTS' PERSPECTIVES AND ATTITUDES TOWARD CREATIVE TRAINING PROGRAMS TO PREPARE THEM FOR WORKING WITH OLDER ADULTS

Abstract citation ID: igaf122.1921

### AGEISM IN TRAINING: MEASURING ATTITUDES AMONG MEDICAL, SOCIAL WORK, AND PA STUDENTS

Euna Cho[1], and Diane Martin[2], *1. University of Maryland School of Medicine, Baltimore, Maryland, United States, 2. University of Maryland, Baltimore County, Baltimore, Maryland, United States*

Ageism in healthcare negatively affects older adults' health outcomes and increases healthcare costs. Research shows that healthcare professionals such as physicians and social workers hold ageist attitudes. However, there is limited research on the attitudes toward older adults of healthcare students. This study aimed to assess ageist attitudes among first- and second-year graduate and professional students. Fifty-four students (14 medical, 14 social work, and 26 physician assistant) completed the Fraboni Scale of Ageism (FSA) — a validated tool measuring three components of ageist attitudes: Antilocution (negative language; 10 items); Discrimination (unequal treatment; 9 items); Avoidance (social distancing; 10 items). Ageist attitudes were present across healthcare student disciplines, with no significant differences between groups. While the three groups scored in the low-range for antilocution, they all notably scored in the mid-range for discrimination (medical students $M=15.33$, $SD=2.09$; physician assistant students $M=15.52$, $SD=2.94$, social work students $M=16.1$, $SD=3.21$), suggesting a moderate belief in limiting older adults' roles and abilities. Additionally, avoidance scores for medical students ($M=17.20$; $SD=4.41$) and physician assistant students ($M=17.45$; $SD=3.85$) indicate a moderate tendency to distance themselves from older adults. These findings illustrate the need to design and incorporate healthcare education and training that challenges age-related stereotypes, reduces social distancing behaviors, and promotes a more accurate and compassionate understanding of aging and older adults across professional healthcare disciplines.

Abstract citation ID: igaf122.1922

### IMPACT OF A VIRTUAL, ARTS-BASED PROGRAM ON STUDENTS' ATTITUDES TOWARD OLDER ADULTS

Jenny Kwon[1], Meghan Young[2], Yue Li[2], and Amy Elliot[2], *1. University of California, San Francisco, San Francisco, California, United States, 2. Miami University, Oxford, Ohio, United States*

As a response to COVID-19, a gerontology service-learning course transitioned from an in-person format to a virtual one, ensuring intergenerational connections could persist despite physical distancing. The course adapted Opening Minds through Art (OMA), an intergenerational art-based program designed to foster relationships between college students and individuals living with dementia while reducing ageism. Through the virtual OMA course, college students and older adults with and without dementia engaged in artistic activities via Zoom. The purpose of this study was to assess changes in students' perceptions of individuals living with dementia before and after participating in a virtual OMA course. The Allophilia scale measured affection, social comfort and kinship, engagement and enthusiasm toward individuals living with dementia. Self-reported survey data were collected from 130 students enrolled in the course between Fall 2020 and Spring 2022. Pre- and post-assessments were conducted at the start and end of each semester. Results from the paired-sample t-tests show significant increases in overall Allophilia scores ($p < .001$) and three subscale scores ($p < .001$) after students' interaction with individuals living with dementia. Findings from multiple linear regression indicate that pre-scores and prior experience with individuals living with dementia were significant predictors of higher post-Allophilia scores across all domains ($p < .001$). The effect of pre-score on Allophilia scores was stronger among students without prior experience with individuals living with dementia ($p < .05$). These findings highlight the effectiveness of virtual intergenerational courses in reducing ageism and shaping positive perceptions of older adults living with dementia.

Abstract citation ID: igaf122.1923

### PREPARING HEALTH PROFESSIONAL STUDENTS FOR HOME VISITS: LEVERAGING TECHNOLOGY AND INTERPROFESSIONAL LEARNING

Bronwyn Keefe[1], Jordana Muroff[2], Craig Slater[2], Karen Jacobs[2], and Megan Nizza[2], *1. Boston University School of Social Work, Boston, Massachusetts, United States, 2. Boston University, Boston, Massachusetts, United States*

Home visits are a key part of being a health and social service professional. However, students often do not receive hands-on, interprofessional learning in class to prepare them for home assessments at field placements or after graduation. This leaves home assessment training to agencies, which can lead to a variety of training depth, standardization, and quality. In this interprofessional project between social work and rehabilitation health sciences, we created six innovative online modules and a live session that guided students through AI-simulated scenarios of home assessments for people with disabilities and older adults. 33 students in social work, occupational therapy, physical therapy, nutrition, and speech-language pathology programs participated in the interprofessional home-based assessment training. Students completed a retrospective pre-/post-assessment of their knowledge related to each of the module's learning objectives. At the end of the live session, students completed the 10-item Student Perceptions of Interprofessional Clinical Education Revised (SPICE-R). Students answered two Likert-type questions: 1) how helpful the training was in learning about the home setting, and 2) how likely they are to pursue future employment in home-based care. Results show that students had statistically significant pre-post gains related to the learning objectives ($p < 0.05$), developed positive perceptions of interprofessional practice, and felt more prepared to conduct home-based assessments following the training. Students gained valuable, hands-on experience performing home visits in a low-stakes environment, better preparing them for fieldwork and their careers. These findings support the training's effectiveness in enhancing knowledge about home-based assessment and comfort with home visits.

### SESSION 4347 (BIOLOGICAL SCIENCES INVITED SYMPOSIUM)

Abstract citation ID: igaf122.1924

### ESPO AND BIOLOGICAL SCIENCES SECTION SYMPOSIUM: EXPLORING NEW HORIZONS IN GEROSCIENCE

Chair: Nisi Jiang
Co-Chair: Sarah Ashiqueali

Over the past year, the field of gerontology has made significant strides, deepening our understanding of the biological mechanisms underlying aging and paving the way for potential interventions. Breakthroughs in cellular and molecular research have provided new insights into the fundamental drivers of aging, while emerging therapeutic approaches show promise in extending healthspan. Advances in genetics, epigenetics, and metabolic regulation are shaping novel strategies to slow down or even reverse aspects of aging. Concurrently, clinical studies are actively evaluating novel interventions that could translate these scientific discoveries into real-world applications. Join us to explore the latest frontiers in aging research, gain insights from leading experts, and connect with the next generation of scientists driving innovation in this rapidly evolving field.

Abstract citation ID: igaf122.1925

### THE EFFECT OF ISOCALORIC MACRONUTRIENT COMPOSITIONS ON HEPATIC CELLULAR SENESCENCE IN AGING MICE

Samantha Jezak[1], Tamara Pulpitel[2], Amanda Brandon[2], Stephen Simpson[2], and Christopher Wiley[3], *1. Tufts University, Medford, Massachusetts, United States, 2. The University of Sydney, Sydney, New South Wales, Australia, 3. Jean Mayer HNRCA at Tufts University, Boston, Massachusetts, United States*

Macronutrients (proteins, carbohydrates, and fat), independent of caloric intake, impact health span (HS) and lifespan (LS) in mice. A low-protein: high-carbohydrate diet yields the longest lifespans in both male and female mice. The cellular mechanisms responsible for mediating this relationship have not been thoroughly investigated, but cellular senescence may play an important role. In response to stress and upon natural aging, cellular senescence manifests as an essentially irreversible cell cycle arrest, accompanied by an often pro-inflammatory senescence-associated secretory phenotype (SASP), which damages the surrounding environment and contributes to accelerated aging and degenerative pathology. To better understand how isocaloric macronutrient compositions affect hepatic cellular senescence in aging mice, we measured markers of cellular senescence in the liver at four different timepoints in males and females fed 20 different diets. To quantify the synergistic impact of all three macronutrients, we used the Geometric Framework for Nutrition (GFN) – a multi-dimensional modeling system that allows n-nutrients to be considered for a particular outcome. Females had increased expression of all cell cycle arrest markers compared to males. We found that three senescence cell cycle arrest markers (p16INK4a, p15INK4b, and p21WAF1) responded differently to diet. p16INK4a expression was lowest on a high-protein: high-carb: low-fat diet. However, p21WAF1 expression was lowest on a low-protein: low-carb: high-fat diet.

p15INK4b was more similar to p21WAF1 but displayed less dramatic variation as macronutrient distributions changed. This data identifies macronutrients' influence on the cell cycle arrest arm of senescence and may provide new insights on mechanisms mediating diet and aging.

Abstract citation ID: igaf122.1926

### MITOCHONDRIAL DNA AFFECTS METABOLOME, LIPIDOME, AND PROTEOME IN A SEX SPECIFIC MANNER IN OLD HETEROGENOUS RATS

Hoang Van Nguyen[1], Chloe Fender[2], Manuel Garcia-Jaramillo[2], Ahsan Nagib[3], Norman Hord[4], Steven Austad[5], Arlan Richardson[6], and Michael Stout[7], *1. The University of Oklahoma Health Sciences, Oklahoma City, Oklahoma, United States, 2. Oregon State University, Corvallis, Oregon, United States, 3. The University of Oklahoma, Norman, Oklahoma, United States, 4. Oklahoma State University, Stillwater, Oklahoma, United States, 5. The University of Alabama at Birmingham, Birmingham, Alabama, United States, 6. The University of Oklahoma Health Sciences Center, Oklahoma City, Oklahoma, United States, 7. Oklahoma Medical Research Foundation, Oklahoma City, Oklahoma, United States*

The effect of mitochondria-haplotype (mt-haplotype) on aging was studied using a unique rat model (OKC-HETB/W), which has a heterogenous nuclear background and mitochondria from either Brown Norway (B-haplotype) or Wistar Kyoto (W-haplotype) rats that differ in 94 nucleotides. The impact of mt-haplotype on aging was studied using an unbiased multi-omics approach to analyze the plasma from 9- and 26-month-old male and female OKC-HETB/W rats. Of the 280 metabolites, 961 lipids, and 230 proteins that changed significantly with age, 50% to 80% were mt-haplotype specific, occurring in either OKC-HETB or OKC-HETW rats. Interesting, the majority (78-93%) of the changes that were mt-haplotype specific were also sex specific, occurring in either male or female rats but not both. Our data are the first to show the potential importance of mt-haplotype in aging and that this mt-haplotype difference is largely sex dependent.

### SESSION 4350 (COMMUNITY ENGAGED SCHOLARSHIP SYMPOSIUM)

Abstract citation ID: igaf122.1927

### BRAIN HEALTH AND DEMENTIA CARE SERVICES: COMMUNITY-LED INNOVATION WITH AND FOR INDIGENOUS PEOPLES

Chair: Mary Wyman
Co-Chair: Sarah Punshon
Discussant: Jordan Lewis

Compared to other groups of older adults, Indigenous peoples are at higher risk for developing cognitive disorder as they age, due to health and healthcare inequities. However, indigenous communities also have a rich history of resilience and creativity, as well as many cultural traditions that support brain health and best practice care for persons with dementia. This symposium brings together several indigenous-focused projects across the US working within collaborative community-engaged frameworks, all of which center meaningful interactions with community members throughout the scholarly process to attain goals of improved community understanding of brain health and dementia, cultural tailoring of best practice models, and innovative approaches to improve services to help Indigenous elders age in place. Thompson et al. report on a community-led effort to enhance the GSA-developed Kickstart, Assess, Evaluate, and Refer (KAER) model for brain health education, tailoring it for elders in the Meskwaki Settlement in Tama County, Iowa. Kim and colleagues joined with community members to develop accessible, community-driven online educational resources focused on dementia prevention and supporting dementia caregivers in remote Alaska Native communities. Punshon and co-authors describe a novel partnership between multiple healthcare systems to support implementation of specialty dementia diagnosis and management services via telehealth within a rural Native American community. All presentations will outline innovative, successful strategies to strengthen community-held knowledge and practices that can be emphasized and built upon to expand resources for dementia prevention and care.

Indigenous Peoples Interest Group Sponsored Symposium

Abstract citation ID: igaf122.1928

### WEAVING TOGETHER: ENHANCING KAER TO OPTIMIZE CONVERSATIONS BETWEEN PROVIDERS & AMERICAN INDIAN FAMILY CAREGIVERS

Maria Donohoe[1], Anne Helene Skinstad[2], Sato Ashida[1], and Lena Thompson[3], *1. University of Iowa, Iowa City, Iowa, United States, 2. University of Iowa College of Public Health, Iowa City, Iowa, United States, 3. University of Alaska Fairbanks, Fairbanks, Alaska, United States*

American Indian and Alaska Native (AI/AN) populations experience higher rates of dementia than other racial/ethnic groups in the U.S. Toolkits such as Kickstart Assess Evaluate, and Refer (KAER) can help primary care providers start conversations with people experiencing cognitive decline and their families, facilitating dementia diagnosis and care. Yet, preliminary conversations with members from a midwestern tribe highlighted cultural incongruence in KAER contents. To conduct this SAMHSA-funded project to enhance the KAER model, the research team engaged two members from a Midwest Tribe who had cared for someone with dementia, one provider from the midwestern tribe, and a Native American Elder justice specialist. Participants completed cognitive interviews, in-depth interviews, and document reviews to enhance KAER. Along with enhancing KAER, the research team responded to participant feedback that a toolkit was needed for AI/AN caregivers to begin conversations with providers about dementia. The Communication Assess Review Essential referrals and Self-care (CARES) toolkit was developed for AI/AN caregivers. The CARES and enhanced KAER included mental and physical health topics, language modifications, Tribe-specific community resources, and a section on self-care. CARES and enhanced KAER also included case studies sharing experiences of caregivers from the midwestern tribe to provide support and examples to AI/AN caregivers and their providers. Enhancements to existing toolkits such as KAER can support AI/AN caregivers and reduce ADRD disparities. Including community members in early conversations and assessments can support community-engaged intervention enhancement by informing culturally relevant programs and tools. Future work to test the adapted toolkit is needed.

Abstract citation ID: igaf122.1929

### BUILDING ACCESSIBLE AND CULTURALLY SENSITIVE RESOURCES: ALASKA NATIVE COMMUNITY-ENGAGED AGING RESEARCH LAB

Hannah Rebadulla, Murat Demir, Alexandra Timmins, Edmond Arroyo, Zoey Hilderbrand, Prince Alejo, and Steffi Kim, *University of Alaska Anchorage, Anchorage, Alaska, United States*

Kim and colleagues joined with community members to develop accessible, community-driven online educational resources focused on dementia prevention, dementia knowledge, and dementia caregiver resources and support in remote Alaska Native communities. The presentation will outline community engagement strategies and efforts to strengthen community-held knowledge and practices that can be emphasized and built upon in Alaska Native dementia care practices and provide increased awareness of brain health and dementia knowledge. Presenters will outline the strengths and benefits of a collaborative that consists of various community partners engaging rural and remote communities related to aging, dementia, and caregiving. Furthermore, presenters will describe the unique engagement practices vital to sharing available resources and materials to rural villages in Alaska, including radio shows, attendance of cultural festivals, and community presentations.

Abstract citation ID: igaf122.1930

### IMPROVING ACCESS TO DEMENTIA CARE FOR NATIVE OLDER ADULTS THROUGH TELEHEALTH: A COMMUNITY-LED IMPLEMENTATION

Kala Cornelius[1], Jenna Woestman[2], Danielle Lennon[2], Celina Whitmore[2], Wesley Martin[1], Steve Barczi[3], Mary Wyman[4], and Sarah Punshon[5], *1. University of Wisconsin–Madison, Madison, Wisconsin, United States, 2. Veterans Affairs, Madison, Wisconsin, United States, 3. Veterans Health Administration, Madison, Wisconsin, United States, 4. UW–Madison and Veterans Affairs, Madison, Wisconsin, United States, 5. William S. Middleton Memorial Veterans' Hospital, Madison, Wisconsin, United States*

The Native American population is rapidly aging and is disproportionately impacted by dementia. Native older adults and their family caregivers, especially those in rural areas, often face significant barriers to accessing dementia diagnosis, care, management and support services. Efforts to address these barriers through effective collaborations are critical to support aging in place. This presentation reports on a partnership between one Midwest Tribal Nation, Veterans Affairs, and the University of Wisconsin to tailor and implement comprehensive dementia care telehealth services. We report on our approach to developing collaborations with Tribal partners, delivery of geriatric education activities and telehealth implementation planning to date. We describe the implementation of dementia diagnosis and management services, delivered in the Tribal Health Clinic over telehealth by specialty geriatrics providers from the external healthcare systems and guided by a community engaged research approach. Infrastructure development and sustainment are facilitated through geriatrics-specific trainings for local Tribal Clinic staff and healthy aging education for community members. The authors report on development of the Tribal Telehealth Collaboration Roadmap (TTCR), a toolkit to guide other healthcare systems in dementia care telehealth delivery in the rural Tribal Clinic setting, based on our learned experience. The TTCR can support future dissemination efforts, minimize barriers to implementation, and increase access to comprehensive dementia care for Native older adults. Facilitators and barriers to implementation of a clinical program in the context of community engagement will be discussed.

### SESSION 4355 (SYMPOSIUM)

Abstract citation ID: igaf122.1931

### INNOVATIONS IN PROMOTING AND ADVANCING AGE-FRIENDLY PRINCIPLES AFTER ACHIEVING AFU DESIGNATION

Chair: Katarina Friberg-Felsted
Co-Chair: Jacqueline Eaton
Discussant: M. Aaron Guest

Receipt of the Age-Friendly University designation from the AFU Global Network (AFUGN) allows institutions to further progress in the development of age-friendly practices in a variety of academic and community settings. Institutional representatives from various parts of the world describe the unique ways they have advanced age-friendly and age-inclusive contributions and share concrete ways forward. Themes include multifaceted strategies and activities to increase visibility on campus and in the community, as well as ways to foster an inclusive and supportive environment for all ages. Peters-Beumer will describe the age-friendly/age-inclusive processes undertaken by Concordia University's Center for Gerontology, including facilitating programs with community partners (such as Reframing Aging); Doron will outline how the University of Haifa's eight impactful initiatives align with the domains of the Age Inclusivity Domains of Higher Education (AIDHE) model; Elfenbein will explore how AFU status led to the creation of the University of North Georgia Institute for Healthy Aging; Shovali will share Eckerd College's multifaceted approach to raise awareness and highlight the value of working in an age-diverse environment; and Waterhouse will showcase age-friendly practical strategies and specific projects successfully executed by the University of Colorado Anschutz Medical Campus. A discussion focusing on advancing and AFU initiatives will be led by AFUGN Secretariat Chair, Aaron Guest.

Abstract citation ID: igaf122.1932

### BUILDING AWARENESS AND ENGAGEMENT: LAUNCHING ECKERD COLLEGE'S AGE-FRIENDLY UNIVERSITY STATUS

Tamar Shovali, Alexis Ramsey-Tobienne, and Kat Robinson, *Eckerd College, St. Petersburg, Florida, United States*

Eckerd College, a small, private liberal arts college in St. Petersburg, FL, earned the Age-Friendly University (AFU) designation in 2019. Guided by the evidence-based Age Inclusivity Domains of Higher Education (AIDHE) model, we adopted a multifaceted approach to launch our AFU status and foster campus-wide awareness and engagement across the seven domains. The AIDHE model outlines that campus-wide strategies for this launch should focus on raising awareness, providing information and education, and assessing campus constituents. Our first goal was to raise awareness of age diversity, friendliness, and inclusivity on campus. A multi-tiered approach began by forming a coalition with The Inspired Network center for teaching, learning, and faculty development and engaging in conversations with administration and key staff from various domains, including advancement, OLLI, the Academy of Senior Professionals, admissions, and human resources. This was followed by an AFU Lunch and Learn for faculty, staff, and community members to raise broader awareness about our designation, highlight the value of working in an age-diverse environment, and assess attendees' specific resource needs to inform future practices. To broaden student awareness, a Careers in Aging Month event was planned to provide information about AFUs and opportunities in the field of aging. Subsequently, an assessment of non-traditional student experiences at the college led to the development of a student club designed for this population. Our overarching strategy, other Age-Friendly initiatives aiding in this launch, and specific resource needs will be discussed.

Abstract citation ID: igaf122.1933

### ADVANCING AN AGE-FRIENDLY ECOSYSTEM: OUTSIDE IN AND INSIDE OUT

Lisa Peters-Beumer, *Concordia University Chicago, River Forest, Illinois, United States*

Concordia University Chicago (CUC), an Age-Friendly University (AFU), participated in a 2020-2021 AFU study (Whitbourne et al., 2024) and subsequently was redesignated part of the AFU Global Network in 2023. Recommendations resulting from the study led the Center for Gerontology to spearhead the university's AFU efforts and encompass a variety of initiatives in an effort to address ageism, increase awareness, and foster community engagement. Notably, the university held an Ageism Awareness Day (AAD) with a virtual event and campus-based booth, accompanied by a declaration from the CUC president. Additionally, CUC facilitated programs such as "Reframing Aging" with community partners, Careers in Aging Lunch and Learns, and Age Inclusive Higher Education in-person focus groups. Collaboration with leaders in this movement has enriched this work and driven internal age-inclusive prioritization discussions with campus constituents. These initiatives have garnered significant participation both in-person and virtually. Outcomes to date include increased awareness and growing engagement from faculty, staff, and students, with an increasing commitment to addressing ageism in academia. The focus of next steps includes enhancing the AFU ecosystem and its implications for future educational models that prioritize inclusivity and age diversity.

Abstract citation ID: igaf122.1934

### ADVANCING AGE-FRIENDLY INITIATIVES IN HIGHER EDUCATION: INSIGHTS FROM CU ANSCHUTZ MEDICAL CAMPUS

Jodi Waterhouse, *CU Anschutz Medical Campus, Aurora, Colorado, United States*

The successful implementation of Age-Friendly Work across higher education institutions hinges on the collaboration of diverse campus stakeholders, including supporters, decision-makers, doers, and creative thinkers. These key players are instrumental in helping the campus Age-Friendly Champion establish a robust foundation for age-friendly initiatives, identify impactful projects, and deliver results that foster an age-friendly campus culture. The CU Anschutz Medical Campus, one of only four medical campuses worldwide to receive the prestigious age-friendly designation, will present its journey in identifying key stakeholders and forming an effective Age-Friendly Task Force. This presentation will detail the composition of the Task Force and how it strategically aligned the three age-friendly university domains—Research and Innovation, Encore Work, and Intergenerational Learning—to integrate age-friendly principles throughout the campus. Attendees will gain insight into the framework developed by CU Anschutz Medical Campus to create, implement, deliver, and evaluate initiatives within each of these domains. The presentation will showcase specific projects that have been successfully executed, demonstrating how they support age-friendly principles and benefit visitors, faculty, staff, and students across all Schools and Colleges. Join us to learn how CU Anschutz Medical Campus has become a model for age-friendly work in higher education and discover practical strategies for fostering an inclusive and supportive environment for all ages.

Abstract citation ID: igaf122.1935

### FOSTERING AGE INCLUSIVITY IN HIGHER EDUCATION: BRIDGING THEORY AND PRACTICE AT THE UNIVERSITY OF HAIFA

Malka Doron[1], Sigal Naim[2] Michal Elboim-Gabyzon[3], Tal Kohli-Hailovski[3], Stav Barouch[2], Orit Hirsch-Matsioulas[1], [2], and Anna Zisberg[3], *1. The Center for Research and Study of Aging, University of Haifa, Israel, 2. The Center for Research and Study of Aging, University of Haifa, Haifa, HaZafon, Israel, 3. University of Haifa, Haifa, HaZafon, Israel*

The Age Inclusivity Domains of Higher Education (AIDHE) model guides universities in embedding age-friendly practices across seven key domains: Teaching and Learning, Services and Resources, Student Affairs, Physical Environment, Research, Personnel, and Outreach and Engagement. This presentation demonstrates how the University of Haifa has operationalized the AIDHE model since joining the global network of age-friendly universities last year through eight targeted, impactful initiatives, that align with the AIDHE's seven domains: (1) a student volunteer project with older adults, fostering intergenerational connections and reducing old age related stereotypes; (2) faculty-led lectures for older adults, promoting lifelong learning and university-community collaboration; (3) designating parking spaces for older persons, enhancing campus accessibility; (4) promoting the transformation of the university into an age-friendly workplace; (5) partnering with the university's sports center to develop a course certifying fitness trainers in older adult wellness; (6) collaborating with the city municipality to promote an age-friendly city; (7) developing an age-friendly university app that centralizes studies and activities for older adults, advancing digital inclusion; and (8) collaborating with the Ministry for Social Equality and Advancement of the Status of Women of Israel to integrate Government Resolution 127: 'National Indicators Framework for Optimal Aging', preparing for longer lifespans and supporting optimal aging. Some of these initiatives are accompanied by research involving students and the older adult community. The presentation provides a roadmap for adapting the model to the university's unique academic, social, cultural, and environmental characteristics, highlighting the reciprocal benefits for students, faculty, staff, and the broader older community.

Abstract citation ID: igaf122.1936

### AGE-FRIENDLY UNIVERSITY DESIGNATION: RECOGNITION, SYNERGY, AND UNLIMITED POSSIBILITIES

Pamela Elfenbein, *University of North Georgia, Dahlonega, Georgia, United States*

Immediately upon becoming recognized as an Age-Friendly University (2019) by the Age-Friendly University Global Network, the University of North Georgia Institute for Healthy Aging (IHA) was created. The IHA was tasked with a three-part mission: enhance the university's academic gerontology programming, develop research partnerships and initiatives, and engage with community stakeholders to improve the quality of life for older adults in our region. With the recognition and synergy provided by our AFU designation the Institute for Healthy Aging has developed multiple programs and initiatives to meet each of our mission mandates, including increasing gerontology course offerings and in collaboration with the Georgia Division of Aging Services creating an enhanced associate's degree to directly meet workforce needs, the development and facilitation of multiple gerontology focused grants and contracts, and, with community partners developed intergenerational programs and programs for older adults throughout our service region. This presentation will offer a timeline of the Institute for Healthy Aging's academic, research, and community-based activities and programs in direct alignment with the Age-Friendly University Global Network Principles of an Age-Friendly University and the seven Domains of Age-Inclusive Practices.

### SESSION 4360 (SYMPOSIUM)

Abstract citation ID: igaf122.1937

### LONELINESS, GENDER, AND RELATIONSHIPS IN LATER LIFE

Chair: Jeffrey Stokes
Co-Chair: Heather Farmer
Discussant: Katherine Fiori

Loneliness is a major public health concern among the older population. However, not all lonely persons are socially isolated, and increasingly, research has examined loneliness as a relational experience with implications for social partners. For instance, loneliness can be "contagious" within couples and social networks, and can generate stress, leading to adverse outcomes for intimate partners. Moreover, the experience, determinants, and implications of loneliness are gendered in later life. This symposium explores the relational causes and consequences of loneliness for older women and men. First, Stokes and colleagues discuss how verbal fluency and memory problems in an intimate partner predict spouses' loneliness longitudinally, finding stronger effects among women and older partners. Second, Ermer and colleagues explore dyadic partners' loneliness and friendship characteristics over time, finding gendered patterns, where women's loneliness was associated with reduced friendship support among men four years later. Third, Rippon and colleagues focus on minoritized older couples, finding that men's loneliness affects their partners' life satisfaction as well as their own. Lastly, Patterson and colleagues explore the role of friendship characteristics for older adults' sense of companionship and isolation with age. As Discussant, Dr. Fiori will contextualize these studies within the broader literature on loneliness, friendship, and relationships among the aging population, and will suggest fruitful avenues for future research. This symposium will provide new insights into the interpersonal consequences of loneliness and highlights how gender may play an important role in determining risk, which has implications for future studies and interventions seeking to reduce loneliness.

Abstract citation ID: igaf122.1938

### DON'T YOU FORGET ABOUT ME: GENDERED ASSOCIATIONS OF DYADIC COGNITION AND LONELINESS IN SHARE

Jeffrey Stokes[1], Heather Farmer[2], Lisa Jessee[3], Martina Luchetti[4], and Angelina R. Sutin[4], *1. University of Massachusetts Boston, Boston, Massachusetts, United States, 2. University of Delaware, Newark, Delaware, United States, 3. University of Cologne, Cologne, Nordrhein-Westfalen, Germany, 4. Florida State University College of Medicine, Tallahassee, Florida, United States*

Recent research has sought to contextualize the experience of loneliness within social networks as well as within intimate couples. Loneliness is influenced not only by the number but also by the quality of individuals' close relationships, and it can have health consequences for intimate partners, as well, including their cognitive functioning. However, little attention has been paid to potential reciprocal effects of cognition on loneliness. That is, does having a partner experiencing cognitive decline lead to greater loneliness over time as well? We use longitudinal dyadic data from Waves 6 (2015) and 9 (2022) of the Survey of Health, Ageing, and Retirement in Europe (SHARE; N = 16,392) to examine whether individuals' own and/or their partners' cognitive functioning at baseline – measured using verbal fluency and episodic memory – are associated with changes in loneliness over the 7-year period. Moreover, we examined potential variation in effects by gender and age. Multilevel longitudinal dyadic lagged dependent variable regression models revealed that (a) poorer verbal fluency and episodic memory at baseline were associated with increased loneliness over time; (b) a partner's poorer verbal fluency at baseline was associated with increased loneliness over time for women, but not for men; and (c) a partner's poorer memory at baseline was associated with increased loneliness among those 70 and older, but not among younger respondents; this effect was strengthened at older ages. We discuss the implications of these findings for theory and future research concerning gender and relationships, cognition, and loneliness within aging couples.

Abstract citation ID: igaf1ss22.1939

### LONELINESS AND LIFE SATISFACTION IN OLDER ETHNIC MINORITY COUPLES IN THE UK: A DYADIC ANALYSIS OF UKHLS DATA

Isla Rippon[1], Kimberley Smith[2], and Christina Victor[3], *1. Brunel University of London, Uxbridge, England, United Kingdom, 2. University of Surrey, Guildford, England, United Kingdom, 3. Brunel University London, Guildford, England, United Kingdom*

Loneliness is independently associated with a range of health outcomes including reduced life satisfaction. Although there is a substantial body of research about loneliness in older adults in the UK, there is a significant evidence gap reporting experiences of loneliness of older people from ethnic minorities and how this influences measures of wellbeing such as life satisfaction. Spouse/partner interpersonal relationships may result in the loneliness experienced by one member of the couple influencing their partner's life satisfaction as well as their own. We investigate self and partner experiences of loneliness on life satisfaction in older couples from ethnic minority communities in the UK. We focus upon the experiences of loneliness for 611 heterosexual couples from ethnic minority communities from wave 13 (2021-23) of the UK Household Longitudinal Study (UKHLS). Loneliness was measured using the 3-item UCLA scale and life satisfaction using a study specific item where higher scores indicate greater satisfaction with life. 58% of participants identified as Asian and around a third of couples were of mixed ethnicity. 24% of males and 29% of females were lonely (UCLA score of 6+) and 46% had high life satisfaction (mostly or completely satisfied with their life). Data were analysed using the Actor-Partner Interdependence Model framework. Loneliness was associated with poorer life satisfaction for both male and female partners. Male partners loneliness affected the life satisfaction of their partner. Interventions to combat loneliness in men may benefit both themselves and their partners.

Abstract citation ID: igaf122.1940

### LONGITUDINAL ASSOCIATIONS BETWEEN FRIENDSHIP SUPPORT, STRAIN, AND LONELINESS AMONG OLDER ADULT COUPLES

Ashley Ermer[1], and Jeremy Kanter[2], *1. Montclair State University, Montclair, New Jersey, United States, 2. University of Illinois Urbana-Champaign, Urbana, Illinois, United States*

Friendship plays an important role in later life. How friendship operates in the context of marriage, along with its links to loneliness, is underexplored. The life course perspective, which highlights time and linked lives, serves as a theoretical framework for this study. Specifically, the role of historical time (i.e., Great Recession), chronological time, and linked lives (spouses' friendship support, friendship strain, and loneliness) will be considered. The purpose of this study is to examine the longitudinal associations between spouses' friendship support, friendship strain, and loneliness over 12 years. Waves 8 (2006), 10 (2010), 12 (2014), and 14 (2018) of the Leave-Behind Questionnaire in the Health and Retirement Study were utilized (N = 849). Using two dyadic, random-intercept cross-lagged panel model (RI-CLPM; separate models for friendship strain and friendship support), out of a total of 96 associations, 21 actor and partner effects were statistically significant across both models. For example, women's greater loneliness in 2010 was associated with men's lower friendship support in 2014. Men's greater friendship strain in 2010 was associated with women's greater loneliness in 2014. Further analyses will probe whether significant effects are the same over time and/or differ between spouses. Implications regarding the role that individuals' social networks play in their spouse's loneliness will be discussed, along with future directions for research.

Abstract citation ID: igaf122.1941

### OLDER ADULTS' CLOSE FRIENDSHIPS AND FEELINGS OF LACKING COMPANIONSHIP AND ISOLATION

Sarah Patterson[1], Yee To (Crystal) Ng[1], Emily Lerner[1], Erica Solway[1], Matthias Kirch[1], Sydney Strunk[1], Jeffrey Kullgren[1], and J. Scott Roberts[2], *1. University of Michigan, Ann Arbor, Michigan, United States, 2. University of Michigan School of Public Health, Ann Arbor, Michigan, United States*

Having close friendships (henceforth, "friends") in later life and their characteristics (e.g., contact, support) may impact an older adult's feelings of companionship and isolation. We used data from the 2024 National Poll on Healthy Aging with adults ages 50 and older (N = 3,181). Logistic regression analyses indicated that the perception of not having enough friends mediates the relationship between having any friends and experiencing feelings of companionship or isolation, even after adjusting for covariates. In fully adjusted models among older adults with friends (N = 2,877), having fewer friends, less than weekly contact, and feeling that you do not have enough friends or the right amount of contact with them were each associated with being more likely to feel a lack of companionship or to feel isolated. These findings highlight the importance of satisfaction with friendships and their quality, in addition to the number of friends one has in later life.

### SESSION 4365 (SYMPOSIUM)

Abstract citation ID: igaf122.1942

### IMPACT OF RESTRICTING VISITORS TO NURSING HOMES DURING THE PANDEMIC: CAUSAL INFERENCES AND QUALITATIVE ANALYSIS

Chair: Huiwen Xu
Discussant: Edward Miller

Early in the COVID-19 pandemic, federal and state governments implemented visitation restrictions to nursing homes to reduce the risk of infections. However, those restrictions also removed a significant amount of personal care and social support provided by family and friends of nursing home residents. Later in 2020, these restrictions were lifted. In this symposium, we leveraged unique visitation data and family satisfaction surveys, and novel causal inference methods to understand the impact of visitation restrictions on resident infections and quality of life. The first study analyzed facility-level visitation data to examine whether allowing visitors was associated with increased resident COVID-19 infection rates. After weighting facilities by the inverse of the probability of allowing visitors, we found no relationship between nursing homes' allowing visitors and COVID-19 infections among their residents. The second study utilized a difference-in-differences event study framework to compare changes in nursing home COVID-19 infection rates in the 4 weeks before vs. 8 weeks after the lifting of the federal visitation restriction on September 17, 2020. Study results suggest that lifting the federal visitation restriction had a negligible impact on nursing home infection rates. The third study qualitatively analyzed open-ended comments in a statewide family satisfaction survey in Ohio to identify the impacts of visitation bans. The majority of survey commenters reported negative social and emotional impacts on both residents and family members. In summary, allowing visitors back did not increase the risk of COVID-19 infections, but may improve quality outcomes for residents and family members.

Abstract citation ID: igaf122.1943

### NURSING HOME VISITOR POLICY AND COVID-19 INFECTION RATES: A MARGINAL STRUCTURAL MODEL ANALYSIS

Huiwen Xu[1], John Bowblis[2], Shuang Li[3], Jennifer Heston-Mullins[2], Yong-Fang Kuo[4], and James Goodwin[3], *1. Emory University, Atlanta, Georgia, United States, 2. Miami University, Oxford, Ohio, United States, 3. UTMB Health, The University of Texas Medical Branch, Galveston, Texas, United States, 4. University of Texas Medical Branch, Galveston, Galveston, Texas, United States*

During the COVID-19 pandemic, Ohio was the only state that collected facility-level visitation data after rescinding its ban on visitors. This study examines the association of allowing outside visitors with COVID-19 infection rates among nursing home residents. We assembled a cohort of Ohio nursing homes over 9 weeks (11/1/2020 to 1/3/2020). For each week, we obtained whether a facility allowed visitors, any COVID-19 infections among residents, community infection rates, and other facility characteristics. Marginal structural models examined the association of allowing visitors with resident infections, weighted by the inverse of the probability of allowing visitors. Of the 677 nursing homes with visitation data, the number of nursing homes that allowed visitors during any week from 10/29/2020 to 1/3/2021 ranged from 226 to 327, and the number with bans in place was between 316 and 448. Marginal models substantially improved the balance in both time-varying and time-invariant covariates between facilities that allowed vs. banned visitors. In the marginal models, allowing visitors was not associated with the unadjusted rates or adjusted odds of new infection among residents (odds ratio=0.92, 95% confidence interval: 0.78, 1.08). The result was similar in sensitivity analyses on the lagged effect of allowing visitors (odds ratio=1.02, 95% confidence interval: 0.86, 1.22). Allowing visitors in the context of adequate PPE and social distancing was safe, even in a period with large increases in community infection rates.

Abstract citation ID: igaf122.1944

### LIFTING OF FEDERAL VISITATION RESTRICTIONS AND COVID-19 INFECTIONS IN NURSING HOMES

John Bowblis[1], Shuang Li[2], Yong-Fang Kuo[3], Jennifer Heston-Mullins[1], James Goodwin[2], and Huiwen Xu[4], *1. Miami University, Oxford, Ohio, United States, 2. UTMB Health, The University of Texas Medical Branch, Galveston, Texas, United States, 3. University of Texas Medical Branch, Galveston, Galveston, Texas, United States, 4. Emory University, Atlanta, Georgia, United States*

Early in the COVID-19 pandemic, the federal government recommended restricting visitors from entering nursing homes (NH) to reduce the spread of the virus. To date, there is limited evidence to suggest that visitation restrictions reduced the spread of the virus. Using the lifting of federal restrictions on visitation in September 2020, this study compared NH COVID-19 infection rates for states that were only subject to the federal restriction on visitation bans to those that lifted their bans earlier. This statistical framework determines whether lifting visitation restrictions lead to increased infection rates, and thus visitation restrictions were effective at reducing the spread of the virus. Our regression analysis found no statistically significant increase in NH infection rates or the community-adjusted infection rates associated with the lifting of the federal visitation restrictions. NH infections rates were closely tied to community infection rate throughout the study period, suggesting that community spread was the driving factor in infections among NH residents. Visitation restrictions took a physical and emotional toll on residents and family members, and limited the ability of visitors to monitor care. Our results suggest that allowing NHs the freedom to choose whether to allow does not necessarily mean that COVID-19 infections will increase. Thus, future policies should focus on providing general guidance that allows NHs to make the decision on when visitation is or is no appropriate for their facility based on their available resources instead of implementing a universal visitation restriction.

Abstract citation ID: igaf122.1945

### "THE GOVERNMENT IS KEEPING ME FROM MY MOTHER": IMPACTS OF VISITATION RESTRICTIONS AS REPORTED BY FAMILY MEMBERS

Jennifer Heston-Mullins, and Lirisha Tuladhar, *Miami University, Oxford, Ohio, United States*

The Ohio Nursing Home Family Satisfaction Survey (FSS) is a self-administered mailed questionnaire with an online option conducted every two years per state mandate. Data collection for the tenth implementation of the FSS occurred between August 2021 and May 2022, while some nursing facilities in the state were still restricting visitation based on occurrences of COVID-19 infections. While the 2021-22 FSS questionnaire did not ask about family members' experiences with visitation restrictions during COVID-19, respondents were provided the opportunity to leave general comments at the end of the survey and to participate in a separate, online COVID-19 survey where they could provide input about their experiences during the pandemic. A total of 13,010 family members of nursing facility residents responded to the 2021-22 FSS and 764 nursing home family members responded to the COVID-19 survey. Using content analysis, researchers from Scripps Gerontology Center at Miami University analyzed 2,890 open-ended comments from the FSS and COVID-19 surveys and identified 269 comments pertaining to visitation restrictions, which were then coded and analyzed for themes. While some family members reported appreciation or neutrality regarding visitation restrictions, the majority of commenters reported negative social and emotional impacts on both residents and family members and negative physical and cognitive impacts on residents, particularly those living with dementia. This session will share findings from the qualitative analysis and considerations for policy makers in the event of future infectious disease outbreaks.

### SESSION 4370 (PAPER)

### FRAILTY, COGNITION AND FUNCTION: MEDIATING AND INTERVENING TO IMPROVE OUTCOMES

Abstract citation ID: igaf122.1946

### GARDENING AND HEALTHY AGEING: A STUDY ON HEALTH BENEFITS

Amir Chwa, Eliza Khoo, Sarah Goh, Donovan Lim, Chen Yunru, Jing Xuan Tan, Clement Chia, and Cynthia Chen, *National University of Singapore, Singapore, Singapore*

This study explores the health benefits of gardening, particularly its relevance in an ageing population. As societies age, promoting sustainable and accessible activities that enhance physical and mental well-being is crucial. Gardening, a widely enjoyed activity, may offer significant health benefits. A cross-sectional study was conducted among 386 participants aged 30 to 99 years, assessing sociodemographic, gardening habits, and physical and mental health through a structured questionnaire. Participants were categorized into two groups based on gardening frequency: those who do not garden or garden occasionally and those who garden daily (26.4%). The primary motivation for gardening was "happiness or satisfaction", while "insufficient time" was the most cited barrier. Analysis revealed that daily gardening was associated with 43% lower odds of poor health (OR = 0.57, 95% CI: 0.32-0.99, p = 0.0499), defined as having either anxiety, health limitations, or both. Although not statistically significant, results suggest daily gardening may also reduce the odds of anxiety and health limitations. These findings highlight gardening's potential as a simple, cost-effective intervention for promoting healthy ageing. By fostering physical activity, relaxation, and social engagement, gardening could play a key role in enhancing overall well-being and improving quality of life. Given its accessibility, policymakers and healthcare professionals should consider incorporating gardening into public health initiatives. Future research could conduct a randomised controlled trial to evaluate the effectiveness and cost-effectiveness of gardening interventions, providing stronger evidence for its role in preventive health strategies for ageing populations.

Abstract citation ID: igaf122.1947

### MEDIATING EFFECTS OF SARCOPENIA ON FRAILTY, COGNITION, AND IADL DISABILITY IN COMMUNITY-DWELLING ELDERS

Bei Cheng, Ping He, Jing Ge, Wenhan Li, Aihong Liu, Ling Li, Tangmeng Guo, and Kemeng Zhang, *Union Hospital, Tongji Medical College, Huazhong University of Science and Technology, Wuhan, Hubei, China (People's Republic)*

Background: Frailty, cognitive decline, and disability are prevalent among older adults and significantly impact the health of community-dwelling elderly individuals. This study investigates the associations between frailty, cognition and IADL disability and explores mediating factors affecting frailty in this population. Methods: This cross-sectional study included 571 older adults (aged >65 years) from the Junshan community. Frailty (Fried phenotype), IADL disability (IADL Scale), and cognition (Mini-Cog) were assessed alongside sarcopenia (skeletal muscle mass index, SMI). Spearman correlation analysis was conducted to examine the associations between frailty severity and various factors, multiple single-mediation models were constructed to explore the mediating roles of sarcopenia in the relationships. Results: Age, BMI, SMI, IADL Score and cognitive function were significantly related with frailty. Mediation analysis revealed that sarcopenia played significant mediating roles in the effect of frailty on cognitive function ($\beta$=-0.4247, $p<0.0001$) and the reciprocal effect of cognitive function on frailty ($\beta$=-0.5818, $p<0.0001$). Similar mediation effects were observed in the relationship between frailty and IADL disability ($\beta$=-0.6256, $p<0.0001$) and the reciprocal effect of IADL disability on frailty ($\beta$=-0.6170, $p<0.0001$). Conclusions: The findings indicate that sarcopenia plays a crucial mediating role in the interactions between frailty, cognitive decline, and IADL disability. Implementing comprehensive interventions that focus on enhancing muscle function may help disrupt this vicious cycle, thereby slowing the progression of frailty, preserving cognitive function, and mitigating disability among community-dwelling older adults.

Abstract citation ID: igaf122.1948

### OPTIMIZING VOLUNTEER ENGAGEMENT AND VOLUNTARY WORK DESIGN FOR HEALTHY AGING

Shiyu Lu, *The University of Hong Kong, Hong Kong, China (People's Republic)*

Volunteering is crucial for healthy aging, yet research has often prioritized volunteering quantity over role quality (e.g., psychological states where individuals are dedicated to their activities). This study investigates how volunteering engagement, one-year volunteering hours, and voluntary work design (VWD) influence subjective well-being (SWB) and cognition in older adults. The cross-sectional study involved 457 participants aged 55 and above in Hong Kong from 2023 to 2024. Volunteer engagement was evaluated using the adapted Chinese Utrecht Work Engagement Scale. The VWD Questionnaire assessed aspects like skill variety, task identity, feedback, and social support and recognition within the volunteer roles. Results from structural equation models indicated that volunteer engagement ($\beta=0.06$, $p=0.007$), recognition ($\beta=0.24$, $p<0.001$), task identity ($\beta=0.80$, $p=0.029$), and social support within volunteer roles ($\beta=1.35$, $p=0.016$) positively associate with SWB. Both volunteering engagement and hours positively affect cognition, with cognitive benefits of feedback ($\beta=0.12$, $p=0.044$) and skill variety ($\beta=0.10$, $p=0.046$) mediated by volunteering hours, and cognitive benefits of social support within volunteer roles ($\beta=0.17$, $p=0.049$) by volunteer engagement. Cognitive benefits of recognition are jointly mediated by the quantity and quality of volunteering ($\beta=0.032$, $p=0.002$). These findings underscore the importance of role engagement in late-life volunteering and pinpoint the key voluntary work characteristics (e.g., skill variety, task identity, feedback, and social support and recognition) to create supportive volunteer environment as part of social prescribing for healthy aging.

Abstract citation ID: igaf122.1949

### TRAJECTORY OF CARDIOMETABOLIC DISEASE-RELATED HOSPITALIZATIONS BEFORE AND AFTER DEMENTIA DIAGNOSIS

Sakura Sakakibara, Abigail Dove, Michelle Dunk, Johan Fastbom, Giulia Grande, Ulrika Akenine, and Weili Xu, *Karolinska Institutet, Solna, Stockholms Lan, Sweden*

Cardiometabolic diseases (CMDs) including type 2 diabetes, heart disease, and stroke often co-occur with dementia, but the impact of dementia on hospitalizations related to CMDs remains unknown. We aimed to map the trajectory of CMD-related hospitalization before and after dementia diagnosis and to further identify factors of CMD-related hospitalization. Within the Swedish Twin Registry, 1657 participants aged ≥65 with incident dementia were matched with 1657 dementia-free participants using propensity scores. Participants were followed for up to 20 years to detect CMD-related hospitalizations. Dementia and CMD-related hospitalizations (planned and unplanned) were identified from medical records. Potential related factors included age, sex, education, marital status, smoking, drinking, physical activity, body mass index, and hypertension. Data were analyzed using Poisson regression and generalized estimating equation models. Compared to the controls, the incidence of CMD-related hospitalization among people with dementia started to increase from 4 years before (incidence rate ratio [IRR] 1.25, 95% confidence interval [CI] 1.00-1.57) and started to decrease 3 years after, the diagnosis (IRR 0.74, CI 0.58-0.94). People with dementia had a greater number of CMD-related hospitalizations (IRR 1.19, CI 1.04-1.36) and longer cumulative length of stay (IRR 1.27, CI 1.04-1.56) in the five years before dementia diagnosis, but these rates decreased after the diagnosis. Male sex, smoking, obesity, and hypertension were associated with further increases in CMD-related hospitalization among people with dementia. Our findings suggest that the healthcare burden increases even before the diagnosis of dementia, and CMD-related hospitalization may be an early sign of the future onset of dementia.

### SESSION 4375 (SYMPOSIUM)

Abstract citation ID: igaf122.1950

### LET'S GET DIGITAL: DIGITAL BIOMARKERS FOR UNDERSTANDING ADRD

Chair: Jennifer Schrack
Co-Chair: Adam Spira
Discussant: Amal Wanigatunga

Digital biomarkers from wearable sensors are emerging as sensitive, novel predictors of cognitive health. A growing body of evidence links elements of sleep and physical activity with both current cognitive status and longer-term dementia risk. To this end, "digital biomarkers" from wearable sensor data may enhance our ability to better detect and understand changes in brain health through remote assessments. Yet, many aspects of digital data remain under-addressed, including how to best leverage the multi-dimensional time series data produced by most sensors into clinically meaningful metrics. This symposium will focus on quantifying and defining digital measures of physical activity and sleep, and their associations with various features of brain and cognitive health in the Atherosclerosis Risk in Communities (ARIC) Neurocognitive Study. Dr. Schrack will present evidence on the association of long-term cognitive trajectories with subsequent physical activity. Mr. Gao will the discuss how time of day activity varies across these cognitive trajectories. Dr. Dougherty will describe the use of compositional data analysis to investigate the interplay between daily active and sedentary behaviors and their association with measures of cortical thickness. Dr. Chamberlin will present evidence on inter-daily variability of physical activity intensity and the rest-activity cycle with dementia risk. Finally, Dr. Spira will present the association of rest-activity rhythms with measures of brain volumes, white-matter hyperintensity volume, and the presence of infarcts and microbleeds. Collectively, these presentations will highlight novel pathways by which digital biomarkers can be used to shed novel insights into cognitive health and ADRD.

Abstract citation ID: igaf122.1951

### TRAJECTORIES OF COGNITIVE DECLINE AND SUBSEQUENT PHYSICAL ACTIVITY

Jennifer Schrack[1], Sunan Gao[2], Ciprian Crainiceanu[2], Anis Davoudi[2], Ryan Dougherty[3], Lacey Etzkorn[1], Amal Wanigatunga, and Adam Spira[1], *1. Johns Hopkins Bloomberg School of Public Health, Baltimore, Maryland, United States, 2. Johns Hopkins University, Baltimore, Maryland, United States, 3. Rutgers University, New Brunswick, New Jersey, United States*

Physical activity (PA) is a modifiable risk factor for dementia, but whether a faster rate of cognitive decline adversely affects subsequent PA is unknown. We investigated the association of cognitive trajectories over 8.2((SD = 1.6), visits 5-9) years with late-life PA (visit 9) in 810 (mean age73.1(SD = 4.0)years, 60.4%, female) Atherosclerosis Risk in Communities study participants. PA was assessed using 24-hour/7-day wrist-worn accelerometry and defined using measures of daily activity volume, fragmentation, and variability. Overall and domain-specific cognitive function were derived using mixed-effects models and divided into tertiles of cognitive decline (fast, slow, stable(ref)). Associations between cognitive trajectories and PA were analyzed using multivariable linear regression adjusted for relavant covariates. At baseline, fast cognitive decliners were more likely to have low education, smoking, and ≥1 APOE ε4 allele compared to those in the slow-decline and stable groups. For overall cognition, compared to those in the stable group, those in the fast- and slow-decline groups had lower daily activity (β=-35.4(SE = 9.8) min/day, β=-24.3(SE = 8.6)min/day, respectively), higher fragmentation (β = 2.9%(SE = 0.7), β = 1.3%(SE = 0.6), respectively), and lower variability of activity (β=-127.6(SE = 18.4), β=-62.9(SE = 16.2), respectively) ($p < 0.01$ for all). In domain-specific analyses, results for executive function were similar to overall cognition, but significance for language and memory results varied. Across all domains, variability was consistently lower among those in the fast-decline group compared to the stable group ($p < 0.05$ for all). Collectively, these results suggest those with faster cognitive decline have lower and more variable daily PA, independent of baseline cognition. Notably, variability of PA may be an indicator of adverse cognitive change across domains.

Abstract citation ID: igaf122.1952

### COGNITIVE DECLINE TRAJECTORIES AND TIME-OF-DAY PHYSICAL ACTIVITY PATTERNS: THE ARIC NEUROCOGNITIVE STUDY

Sunan Gao[1], Ciprian Crainiceanu[1], Anis Davoudi[1], Ryan Dougherty[2], Lacey Etzkorn[3], Amal Wanigatunga[3], Adam Spira[3], and Jennifer Schrack[3], *1. Johns Hopkins University, Baltimore, Maryland, United States, 2. Rutgers University, New Brunswick, New Jersey, United States, 3. Johns Hopkins Bloomberg School of Public Health, Baltimore, Maryland, United States*

Cognitive decline may influence physical activity (PA) patterns in late life, but how cognitive trajectories differentially impact PA throughout the day remains unexplored. We established cognitive trajectories in 2,140 initially cognitively unimpaired Atherosclerosis Risk in Communities participants (mean age 74.1±4.5 years, 59.9% female) over 7.9±1.7 years of follow-up (visits 5-9). Trajectories were derived using linear mixed-effects models assessing overall cognition, language, memory, and executive function across visits. In 810 participants with subsequent accelerometry data, we examined the association between cognitive trajectories and time-of-day PA patterns measured using wrist-worn devices (visit 9). Function-on-scalar regression revealed time-dependent associations between cognitive trajectories and minute-by-minute PA. After adjustment for demographics, health factors, and self-reported PA, each 10% slower annual cognitive decline was associated with 1.5-2.0% higher midday physical activity (noon: 29 counts/minute higher for overall cognition, 40 for executive function, $p < 0.001$), with minimal associations during evening/overnight hours ($p > 0.05$). Executive function decline showed the most temporally extensive association (significant across 11.8 hours/day versus 4.3 hours for language). Multivariable linear regression demonstrated that one percent less decline relative to the median decline rate in overall cognitive function was associated with more favorable PA metrics during daytime hours, particularly 12-6pm ($p < 0.001$): higher total activity (0.035 SD, SE = 0.007), reduced sedentary time (-0.028 SD, SE = 0.007), and lower activity fragmentation (-0.033 SD, SE = 0.007). All cognitive domains showed similar relationships with afternoon PA patterns. These findings suggest that monitoring afternoon physical activity patterns may provide a non-invasive approach for detection of individuals at risk for cognitive decline in aging populations.

Abstract citation ID: igaf122.1953

### ACCELEROMETRY-DERIVED SEDENTARY BEHAVIOR & PHYSICAL ACTIVITY WITH BRAIN STRUCTURE: A COMPOSITIONAL DATA ANALYSIS

Ryan Dougherty[1], Xinkai Zhou[2], Jill Rabinowitz[1], Lin Yee Chen[3], Vadim Zipunnikov[2], Adam Spira[4], Amal Wanigatunga[4], and Jennifer Schrack[4], *1. Rutgers University, New Brunswick, New Jersey, United States, 2. Johns Hopkins University, Baltimore, Maryland, United States, 3. University of Minnesota School of Medicine, Minneapolis, Minnesota, United States, 4. Johns Hopkins Bloomberg School of Public Health, Baltimore, Maryland, United States*

Higher levels of physical activity and lower sedentary behavior in mid-to-late adulthood are each linked to better brain health outcomes, but their combined influence on brain structure remains unclear. We used compositional data analysis to investigate how the distribution of device-measured physical activity and sedentary behavior is associated with MRI measures of brain cortical thickness in 290 adults free of dementia (mean age 76.0±3.7 years, 60% women) from the ARIC study who wore a wrist accelerometer for one week and completed an MRI scan. Averaged-log-ratios between activity intensity categories (sedentary, low-light [lower-intensity light], high-light [higher-intensity light], moderate-to-vigorous) were constructed for compositional data analysis to examine covariate-adjusted associations with cortical thickness in the frontal and temporal lobes. There were significant associations with all log ratios of moderate-to-vigorous physical activity and temporal lobe cortical thickness, such that allocating more time to moderate-to-vigorous activity instead of sedentary, low-light, and high-light activity was associated with greater cortical thickness ($^4$range=.019-.026, all FDR $p<0.05$). Further, the low-light to high-light log ratio was significantly associated with greater temporal cortical thickness ($^4$=.083, FDR $p=0.002$). In contrast, there were no observed associations with frontal lobe cortical thickness (all FDR $p>0.05$). These findings underscore the potential importance of physical activity intensity in maintaining brain cortical thickness, particularly in the temporal regions. Moderate-to-vigorous physical activity was linked to the best outcomes, but even slightly higher levels of activity intensity (i.e., low-light to high-light) showed potential neuroprotective associations. Longitudinal studies are needed to elucidate the temporality of the associations and biological mechanisms.

Abstract citation ID: igaf122.1954

### INTERDAILY REST-ACTIVITY VARIABILITY AND RISK OF DEMENTIA: THE ARIC NEUROCOGNITIVE STUDY

Keran Chamberlin[1], Lacey Etzkorn[1], Anis Davoudi[2], Ryan Dougherty[3], Priya Palta[4], Amal Wanigatunga[1], Adam Spira[1], and Jennifer Schrack[1], *1. Johns Hopkins Bloomberg School of Public Health, Baltimore, Maryland, United States, 2. Johns Hopkins University, Baltimore, Maryland, United States, 3. Rutgers University, New Brunswick, New Jersey, United States, 4. UNC School of Medicine, Chapel Hill, North Carolina, United States*

Given mixed findings regarding the relationship between interdaily rest-activity variability and dementia risk, we hypothesize that interdaily variability of physical activity (PA) intensity and of rest-activity cycle each have distinct associations with dementia risk. We included 2332 dementia-free participants who had ≥3 days of ZioXT accelerometer data from the ARIC Neurocognitive Study Visit 6 (79±4.6 years, 58% female, 24% Black). Interdaily variability of PA intensity was operationalized as two measures: median absolute deviation (MAD) of total 24-hour activity and MAD of total activity during the most active 10 hours (M10). Interdaily variability of rest-activity cycle was defined in two ways: MAD of the start time of M10 and MAD of the start time of the least active 5 hours. Participants were followed until incident dementia, death, last contact, or December 31, 2022. After a median 5.2-year follow-up, 319 (13.7%) individuals developed dementia. Comparing the lowest (Q1) to the highest quartile (Q4) of MAD of total 24-hour activity, the cause-specific hazard ratio (HR) of dementia was 2.3 (95% confidence interval [CI]: 1.56, 3.46), with similar findings for MAD of total activity during M10. In contrast, the lower MAD of the start time of M10 was associated with lower risk of dementia (cause-specific HR for Q1 vs. Q4: 0.7 [95% CI: 0.51, 0.97]). The associations persisted after further adjusting for total 24-hour activity. Our results suggest that higher intensity PA (leading to higher interdaily variability) appears protective for dementia risk, while greater inconsistency of rest-activity cycle may induce greater risk.

Abstract citation ID: igaf122.1955

### CIRCADIAN REST/ACTIVITY RHYTHMS AND MRI-DERIVED MEASURES OF BRAIN HEALTH IN OLDER ADULTS

Adam Spira[1], Lacey Etzkorn[1], Amal Wanigatunga[1], Wendy Wang[2], Bruce Wasserman[3], Daniel Callow[4], Jennifer Schrack[1], and Lin Yee Chen[5], *1. Johns Hopkins Bloomberg School of Public Health, Baltimore, Maryland, United States, 2. UT Southwestern Medical Center, Dallas, Texas, United States, 3. University of Maryland School of Medicine, Baltimore, Maryland, United States, 4. Johns Hopkins School of Medicine, Baltimore, Maryland, United States, 5. University of Minnesota School of Medicine, Minneapolis, Minnesota, United States*

Altered circadian rest/activity rhythms (RARs), measured by accelerometers, have been linked to dementia risk, but associations with neuroimaging measures of brain health remain unclear. We studied cross-sectional associations of RARs with magnetic resonance imaging (MRI) measures of brain volumes, white-matter hyperintensity (WMH) volume, and the presence of infarcts and microbleeds in 396 cognitively unimpaired Atherosclerosis Risk in Communities Study participants (mean±SD age = 78.9±4.6; 57.1% women; 28.0% Black) who wore the Zio XT ECG monitor (containing an accelerometer) for 13.0±2.1 days at Visit 6 (2016-17) and had 3-Tesla MRI scans at Visit 6 or 7 (2016-19). RAR indices of rhythm strength (relative amplitude; RA), fragmentation (intradaily variability; IV), and day-to-day stability (interdaily stability; IS) were derived from accelerometer data. After adjustment for demographic and clinical characteristics, ApoE e4 allele carriage, and intracranial volume, each SD increase in RA was associated with 1.0% greater frontal, temporal, and parietal lobe volume, 0.9% greater total brain volume, a 13.4% smaller WMH volume, and a 28.2% lower odds of microbleeds (all $p<0.05$); RA was not, however, associated with odds of an infarct ($p=0.4$). In addition, higher IV was associated with lower frontal lobe volume across models ($p<0.05$), but it was not associated with other outcomes in fully adjusted models. There were no significant associations of IS with MRI variables. Findings support links of weaker and more fragmented RARs with poorer brain health in cognitively unimpaired older adults. Prospective studies are needed to further evaluate the potential for a causal link.

### SESSION 4380 (SYMPOSIUM)

Abstract citation ID: igaf122.1956

### UNDERSTANDING THE CHALLENGES OF INCREASED CARE BURDEN IN DEMENTIA CAREGIVING AFTER HOSPITALIZATION

Chair: Ashley Kuzmik
Discussant: Lycia Tramujas Vasconcellos Neumann

For persons living with dementia and their care partners, the post-acute transition period presents unique challenges. Increased care needs, medical complexities, and limited formal support can heighten care partner burden, ultimately affecting care quality and patient outcomes. This symposium explores key factors contributing to care partner burden during post-hospital transitions, emphasizing the role of education, health literacy, dyadic characteristics, and burden's impact on post-acute outcomes. The first presentation will examine how education and health literacy function as pathways to burden, specifically assessing the mediating role of health literacy on the relationship between education and burden. The second presentation will introduce a typology of dementia care dyads, identifying distinct care partner and care recipient groups and highlighting differences in burden across these groups. The third presentation will explore the association between care partner burden and negative post-acute outcomes, including emergency visits, hospital readmissions, falls, and injuries, with an emphasis on burden levels at discharge and changes over the post-discharge period. The symposium will conclude with a discussion on the implications of these findings for future research, interventions, and policy, with a focus on strategies to mitigate care partner burden and improve post-acute transitions in dementia care.

Abstract citation ID: igaf122.1957

### EDUCATION, HEALTH LITERACY, AND BURDEN AMONG DEMENTIA CARE PARTNERS AT HOSPITAL DISCHARGE

Ashley Kuzmik, *Alzheimer's Association, Chicago, Illinois, United States*

Care partners of hospitalized persons with dementia often experience significant burden at the time of discharge. This study examined whether dementia care partner health literacy mediates the relationship between education and burden at hospital discharge. Data from 277 care partners in the Family-centered Function-focused Care (Fam-FFC) trial were analyzed using mediation analysis to assess indirect effects of education on burden (Short-Form Zarit Burden Interview [ZBI-12]) through health literacy (Rapid Estimate of Adult Literacy in Medicine-Short Form [REALM-SF]). Care partners had an average age of 60.4 years (SD = 14.3), with 71.8% identifying as female and the majority being White (53.1%) or Black (43.7%). Education levels were categorized as follows: 8.3% had low education (less than high school), 53.1% had medium education (high school, some college, or trade school), and 38.6% had high education (college degree or above). For medium education (B = -0.012; 95% CI = -0.019, -0.003), the indirect effect accounted for 18.5% of the total effect, while for high education (B = -0.018; 95% CI = -0.026, -0.006), it accounted for 21.4%, indicating partial mediation. Findings underscore the role of health literacy in reducing care partner burden and highlight the need to address education and literacy disparities to provide effective support at hospital discharge.

Abstract citation ID: igaf122.1958

### WHO EXPERIENCES BURDEN? A TYPOLOGY OF DEMENTIA CARE DYADS DURING POST-HOSPITAL TRANSITION

Marie Boltz, *Pennsylvania State University, University Park, Pennsylvania, United States*

Dyads of persons living with dementia and their care partners represent diverse populations with unique characteristics and needs. The everyday stress faced by care partners is compounded by new burdens after hospitalization of the person with dementia. This study aimed to develop a typology of dyads based on characteristics of the care partner, as well as their care receivers, to gain a better understanding of the association of dyad typology with care partner burden during transition from the hospital. A latent class analysis was used to detect different dyad types based on intrinsic characteristics of 451 dyads of care partners and their care receivers, persons living with dementia. The care partners' average age was 61.8 (SD = 14.1); 291 (63%) were white; 154 (33%) were Black and the majority were daughters (n = 172, 37.3%) or spouses (n = 132, 28.6%), who lived with the care receiver (60%) and perceived their health as good or better. Care receivers' average age was 81.6 (8.4); they demonstrated moderate cognitive impairment (MoCA mean=11.6, SD = 7.0) and 4 (SD = 2.8) co-morbidities on average. A 5-class model was identified. The classes could be differentiated based on care partner- care receiver key characteristics (gender, age, kinship, living situation, health). There were significant differences with regard to burden. The verification of different types of dyads strengthens the need to develop tailored dyad-centered interventions to support the care partner of the person with dementia post-hospitalization.

Abstract citation ID: igaf122.1959

### IS CARE PARTNER BURDEN ASSOCIATED WITH NEGATIVE POST-ACUTE OUTCOMES IN OLDER ADULTS WITH DEMENTIA?

Diane Berish, *Pennsylvania State University, University Park, Pennsylvania, United States*

Informal care partners are asked to care for individuals living with dementia with little formal training or supports. During the period after hospitalization, care partners face multiple demands, including increased care dependency of the person with dementia and increased likelihood for adverse events in the care receiver. This secondary analysis of Fam-FFC trial data examined the association between care partner burden, both level and change, and negative outcomes, including falls, emergency department transfers, injuries, and hospital admissions, following acute care hospitalization in a sample of 339 care partner/care recipient dyads. The average age for care partners was 61 years and 81 years for care recipients. Most care partners were female (72%) and married (61%). Burden, assessed using the Zarit Short Form, increased from discharge (M = 9.2, SD = 9.0) to two-months (M = 12.6, SD = 9.9) with average change being 3.5 (SD = 8.7, $p < 0.0001$). Emergency room visits were the most frequent negative outcome at 27% (93/339), followed by falls (24%, 83/339), hospitalizations (21%, 72/339) and injuries (12%, 40/339).

Logistic regression models were created for level at discharge and change in care partner burden (discharge to two-months) predicting negative outcomes 6-months post discharge. Intervention effects, care partner, and care recipient factors were included as control variables. No measures of care partner burden were predictive of negative outcomes at 6-months. These findings suggest that the first two months after discharge may be a critical period when caregiver burden intensifies, highlighting the importance of early support to help caregivers navigate this transition and maintain their ability to provide care.

### SESSION 4385 (SYMPOSIUM)

Abstract citation ID: igaf122.1960

### SLEEP, COGNITIVE AGING, AND DEMENTIA RISK: INSIGHTS FROM LONGITUDINAL COHORT STUDIES

Chair: Junxin Li
Co-Chair: Jing Huang
Discussant: Christopher Kaufmann

Healthy sleep is recognized as a key determinant of cognitive aging; however, its precise role remains unclear due to the heterogeneity of health conditions in older adults. This symposium brings together five studies leveraging large-scale, longitudinal cohort data to examine how sleep, independently or in combination with other conditions, influences cognitive aging and dementia risk. Additionally, it explores how cognitive performance profiles predict sleep and key health outcomes for independent living in older adults without dementia. The first presentation introduces a novel approach to measuring long-term sleep variability in adults aged 80 and older, providing insights into sleep patterns in the oldest-old population. The second study investigates how sleep disturbances and physical frailty jointly impact dementia risk in individuals with early cognitive concerns. The third study examines the combined effects of poor sleep and depression on dementia onset, emphasizing the need for multidimensional interventions. The fourth presentation explores sleep as a predictor of motoric cognitive risk in older adults, an early indicator of dementia. Finally, the last study identifies distinct cognitive profiles across six cognitive domains and evaluates their predictive value for key health outcomes essential for independent living, including sleep difficulties, depressive symptoms, falls, and daily functional impairment. Together, these studies highlight the critical role of sleep in cognitive aging and dementia prevention, identifying key risk factors and intervention targets across preclinical and early symptomatic stages of dementia.

Abstract citation ID: igaf122.1961

### LATENT COGNITIVE PROFILES PREDICT ONE-YEAR HEALTH OUTCOMES IN OLDER ADULTS WITHOUT DEMENTIA

Junxin Li, and Jiaying Li, *Johns Hopkins University, Baltimore, Maryland, United States*

Traditional classifications of cognitive status often overlook domain-specific cognitive deficits in older adults. This study aims to identify distinct cognitive profiles in older adults without dementia and evaluates their one-year predictive value for key health outcomes, including falls, sleep difficulties, depressive symptoms, and daily functioning. We analyzed data from 2,219 adults aged ≥65 years in the National Health and Aging Trends Study. Baseline cognitive performance, assessed across six domains at Round (R) 12 (with supplemental data from R11), included episodic memory, executive function, orientation, psychomotor function, visual attention, and working memory. Latent profile analysis identified five cognitive subgroups, and logistic regression models examined associations with one-year outcomes at R13, excluding pre-existing events.The five profiles were: (1) Overall intact (50.5%); (2) Isolated moderate orientation impairment (15.6%); (3) Mild global impairment with preserved orientation (22.0%); (4) Mild global impairment with significant orientation impairment (5.5%); (5) Moderate global impairment (6.2%).At one-year follow-up, compared to Profile 1, Profile 5 had higher odds of falls (OR 1.98), Profile 4 was linked to prolonged sleep onset (OR 2.04), and Profiles 4 and 5 had higher odds of depressive symptoms (OR 2.55–2.69). Functional difficulties were observed in Profile 2 (shopping: OR 1.79; banking: OR 2.84) and Profile 5 (medication management: OR 2.98; laundry: OR 2.11; banking: OR 3.42). Nearly half of dementia-free older adults exhibit distinct cognitive profiles with varying risks for key outcomes. These findings highlight the need for domain-specific cognitive assessment to guide targeted interventions that support independent living and aging in place.

Abstract citation ID: igaf122.1962

### COGNITIVE IMPAIRMENT RISK IN SUBJECTIVE COGNITIVE DECLINE: ROLES OF SLEEP, PHYSICAL FRAILTY, AND THEIR INTERACTION

Jing Huang[1], Katie Stone[2], Chakra Budhathoki[1], and Junxin Li[1], *1. Johns Hopkins University, Baltimore, Maryland, United States, 2. California Pacific Medical Center Research Institute, Castro Valley, California, United States*

Subjective cognitive decline (SCD)—a self-perceived decline in cognition without objective impairment—may signal early Alzheimer's disease or dementia. Sleep disturbances and physical frailty are linked to cognitive decline, but their roles in the progression from SCD to objective cognitive impairment and dementia, and their potential interaction, remain unclear. This study examined the associations of sleep disturbance, physical frailty, and their interaction with incident cognitive impairment and dementia over a 10-year follow-up period using six waves of Health and Retirement Study (HRS) data (2010–2020). While analysis on sleep used all 3,014 individuals with SCD at baseline, analyses involving frailty data included a subsample of 1,141. Sleep disturbance was assessed using the modified Jenkins Sleep Scale, and physical prefrailty/frailty was defined by the Fried frailty phenotype. Cognitive impairment and dementia incidence were determined using validated HRS cognitive cutoffs. Discrete-time survival analyses, adjusted for sociodemographic and health factors, found no independent association between baseline sleep disturbance and subsequent cognitive impairment or dementia. However, physical prefrailty/frailty was significantly associated with increased risk of cognitive impairment (adjusted odds ratio [aOR] = 1.44, 95% CI: 1.15–1.80) and dementia (aOR = 1.89, 95% CI: 1.23–2.93). A significant interaction (aOR = 0.90, 95% CI: 0.81–1.00, $p < 0.05$) suggested that sleep disturbance was more strongly associated with cognitive impairment in non-frail older adults than in those who were prefrail or frail. In conclusion, physical prefrailty/frailty is a significant predictor of cognitive impairment in older adults with SCD, while the effect of sleep disturbance may vary by frailty status.

Abstract citation ID: igaf122.1963

### LONG-TERM SLEEP DATA AMONG OLDER ADULTS: ATHEROSCLEROSIS RISK IN COMMUNITIES NEUROCOGNITIVE STUDY

Erin Spaulding[1], Henry Zhao[2], Jie Ding[2], Pamela Lutsey[3], Brendan Lucey[4], Katherine Ornstein[1], Josef Coresh[5], and Seth Martin[2], *1. Johns Hopkins University, Baltimore, Maryland, United States, 2. Johns Hopkins University School of Medicine, Baltimore, Maryland, United States, 3. University of Minnesota, Minneapolis, Minnesota, United States, 4. WashU Medicine Department of Neurology, St. Louis, Missouri, United States, 5. New York University, New York, New York, United States*

The American Heart Association recommends 7-9 hours of sleep per night, based primarily on self-reported data, to promote cardiovascular and cognitive health. Due to limited objective, long-term sleep data in adults aged 80 years and older, we sought to objectively characterize sleep in this group. Fitbit Charge 6 devices were used to collect long-term data on sleep among Atherosclerosis Risk in Communities Neurocognitive Study (ARIC NCS) participants with a smartphone in 2024-2025. Nighttime sleep (8 pm-8 am) with ≥8 hours of heart rate data, ensuring consistent Fitbit wear, was analyzed to assess sleep duration and efficiency. Multiple linear regression was used to assess independent predictors of sleep efficiency, including age, sex, and race. A total of 301 ARIC NCS participants (84.0±3.2 years [36% ≥85 years], 60% women, 82% White) contributed a median of 112 (IQR: 144) nights of sleep data (~3.7 months). This sample of older adults aged 79-95 years spent a median of 301 (IQR: 115) minutes asleep (~5 hours) and 450 (IQR: 104) minutes in bed (~7.5 hours) per night. Median sleep efficiency was 68% (IQR: 15), with 85% having fair or poor sleep (efficiency <80%). Men (-4.28%; 95% CI: -7.11%, -1.45%) and Black adults (-5.40%; 95% CI: -9.01%, -1.80%) had significantly lower sleep efficiency compared to women and White adults. In conclusion, adults 80 years and older, particularly Black adults and men, experience poor sleep. Future research could examine long-term sleep variability in this population and reasons for sleep deficits among Black older adults and men.

Abstract citation ID: igaf122.1964

### IMPACT OF DEPRESSIVE AND INSOMNIA SYMPTOMS ON MEMORY DECLINE AND DEMENTIA RISK IN OLDER ADULTS

Sofia Liu, Claire Wang, and Junxin Li, *Johns Hopkins University, Baltimore, Maryland, United States*

Depressive symptoms (DS) and insomnia symptoms (IS) often co-exist in older adults, yet their combined impact on cognition is understudied. This study investigates the association between baseline coexistence of depressive and insomnia symptoms on memory and dementia incidence over a one-year follow-up among community-dwelling older adults. We analyzed data from 4474 participants in the National Health and Aging Trends Study (NHATS) using Round (R) 12 (baseline) and R13 (follow-up). The sample included 70.48% aged 75 and older, 57.38% female, 64.30% White without dementia at baseline. Participants were classified as (1) neither condition (reference), (2) IS only, (3) DS only, and (4) both DS and IS at baseline. At follow-up, 41.72% experienced memory decline, and 2.12% converted to dementia. Generalized estimating equations models suggest those with both conditions showed a poorer memory at follow-up compared to the reference (B = -0.08, CI: -0.12, -0.04), adjusting for baseline sociodemographic and health factors. Logistic regression shows that individuals with only DS (OR = 3.80, CI: 1.71,8.45) and those with both conditions (OR = 2.39, CI: 1.31, 4.39) were associated with higher odds of developing dementia at follow-up compared to those with neither condition. The coexistence of depressive and insomnia symptoms is associated with worse cognitive performance over time and increases the risk of dementia. These findings highlight the potential compounding effect of coexisting depressive and insomnia symptoms on cognitive health. Addressing both conditions concurrently may mitigate dementia risks. Studies over longer periods of time are needed to confirm these findings and inform targeted interventions.

Abstract citation ID: igaf122.1965

### LONGITUDINAL ASSOCIATION OF SLEEP QUALITY AND DURATION WITH MOTORIC COGNITIVE RISK SYNDROME IN CHINESE OLDER ADULTS

Yiming Qiu, Youngmin Cho, and Junxin Li, *Johns Hopkins University, Baltimore, Maryland, United States*

Motoric Cognitive Risk Syndrome (MCR), defined by the co-occurrence of subjective cognitive complaints and slow gait, is an established risk factor for dementia and other adverse health outcomes in older adults. While sleep characteristics have been cross-sectionally linked to MCR, their longitudinal impact remains unclear. This study examines the association between sleep characteristics and MCR risk using data from 4,359 participants (median age: 66; 50% female) without MCR at baseline from the China Health and Retirement Longitudinal Study (CHARLS), spanning baseline (2011), Wave 2 (2013), and Wave 3 (2015). Self-reported poor sleep was categorized as rarely (≤2 days/week), occasionally (3–4 days/week), and frequently (5–7 days/week); nighttime sleep duration was classified as < 6 hours/night, 6–8 hours/night, and >8 hours/night. Using Cox proportional hazard models, we found that frequent poor sleepers had higher odds of developing MCR compared to those with rare poor sleep (HR = 1.30, 95% CI: 1.00–1.69). Short sleep duration (<6 h/night) was also associated with increased MCR risk compared to 6–8 h/night (HR = 1.33, 95% CI: 1.06–1.66). Participants experiencing both frequent poor sleep and short sleep duration had the highest risk of developing MCR (HR = 1.49, 95% CI: 1.10–2.03) compared to those who rarely experience poor sleep and with 6–8 h/night sleep. These findings highlight the importance of both self-perceived sleep quality and sleep duration in MCR risk and suggest that addressing both aspects through targeted sleep interventions may help prevent mobility and cognitive decline in aging populations.

### SESSION 4390 (SYMPOSIUM)

Abstract citation ID: igaf122.1966

### CANNABIS AND OLDER PERSONS: CHARTING THE COURSE FROM THE CLINIC TO THE DISPENSARY

Chair: Brian Kaskie

Since 2020, the Cannabis and Older Persons symposium has featured panelists representing distinct academic disciplines offering unique perspectives about cannabis and aging. This year our panel features the latest work from individuals trained in clinical psychology, health services and policy, psychiatry, and public health. Erin Bonar and Erica Solway rely on the National Poll on Healthy Aging to discuss their latest findings about older adults'

attitudes and use of cannabis. Madeline Meier presents the DNA-methylation profiles of long-term cannabis users participating in the longitudinal Dunedin Study, a five-decade long study based in New Zealand. Ofir Livne presents differences in detailed cannabis use patterns and Delta-9 THC intake between older adults and younger age groups, and associations with cannabis use disorder. Cyril D'Souza considers how cannabis use corresponds with presentation of psychiatric symptoms among older persons. Divya Bhagianadh explores linkages between location of Medical Cannabis Dispensaries (MCDs) across 12 states with comprehensive medical cannabis programs and socioeconomic, and health characteristics of the neighborhood in which they are located. Together, these studies reflect how scientific understanding of cannabis use among older persons has been informed by multiple disciplinary perspectives, and how researchers ask and answer unique questions about a common topic with varied methodological approaches. This symposium offers researchers a state-of-the-science cannabis use among older adults.

Abstract citation ID: igaf122.1967

### LICENSED MEDICAL CANNABIS DISPENSARIES: EXPLORING LINKAGES WITH NEIGHBORHOOD CHARACTERISTICS

Divya Bhagianadh[1], Rebecca Ellis[1], and Julie Bobitt[2],
*1. University of Arkansas, Fayetteville, Arkansas, United States, 2. University of Illinois at Chicago, Chicago, Illinois, United States*

This study explores the linkages between location of Medical Cannabis Dispensaries (MCDs) across 12 states with comprehensive medical cannabis programs and socioeconomic, and health characteristics of the neighborhood in which they are located. We mapped the location of 1,912 MCDs that were licensed to operate in January 2025 to 885 (12%) zip codes. The remaining 6,319 (88%) zip codes constitute the control group. We merged this dataset with zip code-level data on community characteristics from the social vulnerability index and county-level characteristics from the 2024 county health rankings. Multivariate logistic regression models showed that uninsured rates (OR 1.02, p-value<0.05); single parent household rates (OR 1.01, p-value<0.01); housing burden (OR 1.03, p-value<0.001); Hispanic population (OR 1.02, p-value<0.01), and being a medium metro ( vs. large central metro) (OR 1.78, p-value<0.01) were associated with higher odds of having an MCD. Households without internet (OR 0.97, p-value<0.001); being non-core or rural area (OR 0.40, p-value<0.01); fair or poor population health status (OR 0.88, p-value<0.05), and higher food environment index values (OR 0.74, p-value<0.05) were associated with lower odds of MCD. We found no association between number of physicians, drug overdose death rates, and smoking and excessive drinking rates in the area and presence of MCDs. These results contradict previous studies that found lower proportion of licensed dispensaries in locations with higher levels of socio-economic vulnerabilities. Our finding that licensed dispensaries and subsequently quality assured licensed product availability might be lower in areas with poorer health status and rural areas warrants further exploration.

Abstract citation ID: igaf122.1968

### DNA METHYLATION PROFILES OF LONG-TERM CANNABIS USERS IN MIDLIFE: AN EVALUATION OF PUBLISHED MARKERS

Madeline Meier, *Arizona State University, Tempe, Arizona, United States*

Long-term cannabis use has been linked to a number of health problems, including respiratory problems, cardiovascular diseases, cognitive decline, and mental illness. Epigenetic responses to cannabis use could underlie cannabis-related health problems. We examined the DNA-methylation profiles of long-term cannabis users in midlife, re-evaluating a set of 246 previously published cannabis-associated methylation markers. Data were from the Dunedin Study, a five-decade longitudinal study of a birth cohort (analytic n = 787). Peripheral whole blood was drawn when the cohort was age 45, and DNA methylation was assayed using the EPIC 850K BeadChip. Analyses compared long-term cannabis users with non-users and, for a benchmark comparison, with long-term tobacco users. Results showed that long-term cannabis use was associated with sixteen of the previously published 246 cannabis-related methylation markers. All cannabis-related methylation markers were also associated with long-term tobacco use. Nonetheless, after adjusting for long-term tobacco use and other covariates, including childhood SES, family history of substance dependence, and long-term alcohol use, long-term cannabis use was robustly associated with hypomethylation of nine markers: cg05575921, cg21566642, cg03636183, cg21161138, cg01940273, cg17739917, cg05086879, cg02978227, cg23079012. Six of the nine cannabis-related markers were associated with gene expression levels, suggesting meaningful biological associations. Cannabis quitters showed less extreme DNA hypomethylation than long-term cannabis users. Long-term cannabis use could affect the epigenome similarly to tobacco use, possibly though smoke inhalation. Cannabis cessation, like tobacco cessation, may reverse altered DNA methylation.

Abstract citation ID: igaf122.1969

### OLDER ADULTS' PERSPECTIVES ON AND EXPERIENCES WITH CANNABIS USE: RESULTS FROM THE 2024 NATIONAL POLL ON HEALTHY AGING

Erin Bonar[1], Erica Solway[1], Lianlian Lei[1], Matthias Kirch[1], Dianne Singer[1], Sydney Strunk[1], J. Scott Roberts[2], and Preeti Malani[1], *1. University of Michigan, Ann Arbor, Michigan, United States, 2. University of Michigan School of Public Health, Ann Arbor, Michigan, United States*

Cannabis use is increasing among older adults, concurrent with regulations allowing medical and/or recreational consumption, increases in the THC potency of cannabis products, and declining public perceptions of cannabis-related risks. Using a nationally representative poll of 3,379 adults age 50 and older, we described the prevalence of cannabis use and associated characteristics (e.g., motives for consumption, cannabis use disorder [CUD] symptoms, discussions with healthcare providers). One-fifth (21%) of older adults reported past-year cannabis use (once/twice: 9%, at least monthly: 12%). Frequently endorsed motives for consumption included: relaxation (81%), sleep (68%), enhancement (i.e., enjoyment, celebration, feel good; 64%), pain (63%), and mental health/mood (53%). Among respondents who consumed cannabis at least monthly, 44% had not discussed their use with healthcare providers and many reported CUD symptoms including tolerance (17-22% across items) and craving (13%). Greater understanding of older adults' motives for and experiences with cannabis use can guide recommendations and interventions.

Abstract citation ID: igaf122.1970

**AGE-RELATED ACUTE SUBJECTIVE, BEHAVIORAL, COGNITIVE AND CARDIOVASCULAR EFFECTS OF TETRAHYDROCANNABINOL**

Deepak Cyril D'Souza, *Yale University, New Haven, Connecticut, United States*

There has been a relatively recent increase in recreational and medical cannabis use in older adults compared to younger adults which is projected to further increase4 and is associated with legalization of cannabis. Further, there are higher odds of cannabis-related problems in those ≥55 years including a significant increase in emergency department visits for acute consequences of cannabis. However, although there is a wealth of studies of cannabis effects in younger populations, the acute effects of cannabinoids in older adults have not been well-studied. Little is known about their effects in older individuals despite growing use of cannabis in this demographic. Age-related changes in 1) the endocannabinoid system, the target of THC, 2) cognitive function, 3) psychomotor function, 4) postural stability, 5) cardiovascular reserve, and 6) drug metabolism may render older adults more vulnerable to THC and cannabis. Data from a controlled double-blind, placebo-controlled crossover study on the subjective, behavioral, cognitive, psychomotor and cardiovascular effects of THC in older vs. younger adults will be presented. Age-related differences in the pharmacokinetics of THC will also be explored. On immediate recall, older individuals recalled fewer words vs. younger controls under the influence of THC relative to placebo both on immediate and delayed recall. Furthermore, relative to younger individuals, older individuals reported greater THC-induced anxiogenic effects. These preliminary data support further research on the benefits and risks of cannabis an its principal active constituent THC, in the aging population.

Abstract citation ID: igaf122.1971

**AGE DIFFERENCES IN PATTERNS OF CANNABIS USE AMONG U.S. ADULTS WHO CONSUME CANNABIS FREQUENTLY**

Ofir Livne[1], Alan Budney[2], Jacob Borodovsky[2], and Deborah Hasin[3], *1. Columbia University Irving Medical Center, New York, New York, United States, 2. Dartmouth College, Hanover, New Hampshire, United States, 3. Columbia University, New York, New York, United States*

Cannabis use has risen disproportionately among middle-aged and older U.S. adults, groups vulnerable to adverse effects, including cannabis use disorder (CUD). While consumption patterns have diversified, age-related differences in such patterns remain understudied. This study examined variations in cannabis behaviors and the relationship between quantity—measured in milligrams of THC (mgTHC)—and CUD in regular users. A total of 4,134 U.S. adults who reported daily cannabis use completed an online survey assessing consumption patterns and DSM-5 CUD criteria. Comparisons across sex, reasons for use, methods of consumption, CUD severity, criteria count, and mgTHC were conducted across three age groups (18–49, 50–64, 65+). Regression models, adjusted for sex and reasons for use, analyzed age-specific associations between mgTHC and CUD. Over 70% of participants used cannabis for both medical and recreational purposes. Middle-aged adults reported medical use more frequently than younger (18.1% vs. 13.7%; $p < 0.001$) and older adults (14.1%; $p = 0.027$), while older adults more often reported recreational-only use than middle-aged adults (15.8% vs. 10.5%; $p = 0.002$). Smoking buds was the most common method, with high-potency concentrate use declining with age. Daily mgTHC was positively associated with CUD severity, though middle-aged and older adults endorsed fewer CUD criteria than younger users at all mgTHC levels, with no significant age effects. Findings highlight age-related differences in consumption and reasons for use. Despite consuming less than younger users, older adults remain at risk, underscoring the need for targeted prevention strategies.

### SESSION 4395 (SYMPOSIUM)

Abstract citation ID: igaf122.1972

**REFRAMING AGING IN THE ERA OF DISASTERS: STRENGTHENING DISASTER PREPAREDNESS, RESPONSE, AND RECOVERY**

Chair: Omolola Adepoju
Co-Chair: Katie Cherry
Discussant: Nancy Kusmaul

With the increasing global occurrence of severe weather events and other conflicts, researchers, policymakers, and organizations are focusing on risk, and also strengths and resilience factors of the aging population to mitigate adversity and ease the recovery of older adults. First, Dr. Assaf Suberry will present data on how informal and formal social gatherings enhance coping among older populations with the Israel-Gaza conflict. Second, Piper Bordes will address how positive psychological factors can contribute to post-disaster mental health in younger and older adults using data collected after the 2021 Hurricane Ida. Third, Dr. Katie Cherry will discuss health-related quality of life predictors after exposure to severe weather events. Fourth, Yanjun Don will present FEMA data related to disaster roles, resilience, and recovery. Finally, Dr. Hunter will examine the health impacts of extreme environmental events on older adults from the consideration of the rural-urban continuum. Taken together, these papers provide new behavioral evidence concerning post-disaster health along with new programmatic approaches to disaster mitigation. While age-related changes can make an older person more at risk in a disaster event older adults bring resilience, lived experience, and additional contributions to disaster planning, response, and recovery.

Abstract citation ID: igaf122.1973

**MUSIC IN DISPLACEMENT: COMMUNAL GATHERINGS AS ANCHORS OF RESILIENCE FOR OLDER PERSONS**

Assaf Suberry, and Liat Ayalon, *Bar-Ilan University, Ramat-Gan, HaMerkaz, Israel*

This study aims to explore how musical gatherings serve as a source of psychological support for displaced older Israelis following the October 7, 2023, Hamas attacks. After 60,000 Israeli citizens were relocated to safer and more remote locations, musicians and artists generously held weekly musical performances. Additionally, community initiatives organized a variety of traditional and innovative events and activities. Through thematic analysis of 17 semi-structured interviews, participants describe these gatherings as temporary "bubbles in time" that provide emotional relief and social reconnection. The findings highlight that these gatherings not only offered a brief escape from daily hardships but also enabled the continuation of traditional ceremonies in an intergenerational setting, preserving cultural continuity despite displacement. Within these shared experiences, a sense of communitas emerged, reinforcing social bonds and collective resilience. Given their profound impact, communal gatherings should be integrated into crisis response protocols to support the well-being of displaced older persons.

Abstract citation ID: igaf122.1974

### PSYCHOLOGICAL WELL-BEING IN YOUNGER AND OLDER ADULTS AFTER HURRICANE IDA

Piper Bordes, and Katie Cherry, *Louisiana State University, Baton Rouge, Louisiana, United States*

Hurricane Ida slammed into the coast of south Louisiana on August 29, 2021 sixteen years to the day after Hurricane Katrina crippled the US Gulf Coast. Ida is considered the second most destructive hurricane known to the state of Louisiana following Katrina. The present study is part of a larger research program on risk and resilience factors in younger and older adults after the 2021 Hurricane Ida. In this study, we assessed symptoms of depression using the PHQ-9 and post-traumatic stress using the PCL-C in a sample of 128 community-dwelling adults. Half of the sample was recruited from three parishes (counties) where Ida damage was more severe and the other half was sampled from two parishes farther inland with less damage. All were tested individually, either in person or virtually via Zoom platform in a single session where they responded to individual difference measures, including state hope (agency and pathways subscales) and a structured storm questionnaire. Correlation analyses revealed that agency and pathways were negatively related to symptoms of depression and post-traumatic stress, while hurricane stressors and prior lifetime trauma were positively associated both mental health indicators. Regression analyses confirmed that only agency, pathways, and prior lifetime trauma significantly predicted depression symptoms, while agency and prior lifetime trauma significantly predicted symptoms of post-traumatic stress. Implications of these data for identifying positive factors that may promote long-term recovery and foster post-disaster psychological health will be discussed.

Abstract citation ID: igaf122.1975

### PREDICTORS OF HEALTH-RELATED QUALITY OF LIFE IN OLDER ADULTS AFTER A FLOOD

Katie Cherry, Piper Bordes, Luke Miller, Matthew Calamia, and Emily Elliott, *Louisiana State University, Baton Rouge, Louisiana, United States*

Multiple severe weather events may threaten health and well-being for those who live in disaster-impacted communities. In 2005, coastal residents of south Louisiana experienced catastrophic damage in Hurricane Katrina which forced many to permanently relocate inland to higher and presumably safer ground. In August of 2016, historic flooding brought widespread destruction across a 22-parish (county) area, creating additional loss for those who had moved to Baton Rouge, Louisiana after Katrina. This study is part of a larger longitudinal research project on health indicators after multiple disasters. Here we examined health-related quality of life assessed by the SF-36 Health Survey (Ware & Sherbourne, 1992). Three flood exposure groups were compared across two waves of testing: non-flooded (controls), single disaster (flooded in 2016) and double disaster (flooded in 2005 and again in 2016). The flood exposure groups did not differ in physical health. Mental health was poorer for those whose homes flooded compared to the non-flooded controls, although their scores improved somewhat by the second wave of testing. Regression analyses confirmed that age, prior lifetime trauma, and intolerance of uncertainty (IUS) at Wave 1 predicted physical health at Wave 2, although IUS lost its significance when agency was added as a predictor. In contrast, age and IUS predicted mental health and these variables held their significance when agency was accounted for. These data show that persons who experience difficulty tolerating ambiguous or uncertain situations may be at greater risk for post-disaster distress when navigating the challenges of long-term flood recovery.

Abstract citation ID: igaf122.1976

### DISASTER PREPAREDNESS DISPARITIES: THE IMPACT OF SELF-EFFICACY, INFORMATION, RISK PERCEPTION AMONG OLDER ADULTS

Yanjun Dong, *SUNY at Albany, Albany, New York, United States*

Background: Disaster preparedness is essential for mitigating risks among older adults, yet disparities persist in how individuals prepare based on prior disaster experience and psychosocial factors. This study examines how experiencing a natural disaster influences preparedness actions through self-efficacy, information access, and risk perception, with a focus on racial differences between BIPOC and non-BIPOC older adults. Method: Using FEMA's 2023 National Household Survey data (N = 2,225), we employ mediation analysis to assess both direct and indirect pathways linking disaster experience to preparedness behaviors among individuals aged 60 and older. Results: Findings indicate that disaster experience enhances preparedness both directly and indirectly. Self-efficacy (BIPOC: $\beta = 0.11$, $p < 0.01$; non-BIPOC: $\beta = 0.15$, $p < 0.001$) and information access (BIPOC: $\beta = 0.07$, $p < 0.05$; non-BIPOC: $\beta = 0.10$, $p < 0.001$) mediate this relationship, underscoring the role of confidence and information-seeking behavior in disaster readiness. While influenced by disaster experience, risk perception does not predict preparedness, suggesting that knowledge and self-efficacy are more critical drivers. Total effects show that BIPOC older adults ($\beta = 0.69$, $p < 0.001$) exhibit a stronger overall relationship between disaster experience and preparedness than their non-BIPOC counterparts ($\beta = 0.62$, $p < 0.001$), primarily due to a stronger direct effect. Discussion: These findings highlight the importance of enhancing self-efficacy and improving access to preparedness information, particularly for BIPOC older adults. Disaster preparedness programs should incorporate culturally tailored strategies to reduce disparities and ensure equitable resilience across diverse aging populations.

### SESSION 4400 (SYMPOSIUM)

Abstract citation ID: igaf122.1977

### ADDRESSING THE GERO-BEHAVIORAL HEALTH WORKFORCE SHORTAGE: INSIGHTS FROM OREGON

Chair: Lindsey Smith
Discussant: Brenda Sulick

Older adults experience significant behavioral health (BH) challenges, including substance use disorders (SUD), serious mental illness (SMI), and other conditions such as depression and anxiety. While BH prevalence rates are lower among older adults than younger cohorts, aging-related complexities, comorbidities, and diagnostic overshadowing contribute to severe impacts on health and quality of life. The growing demand for BH services is compounded by systemic barriers, including workforce shortages, stigma, and disparities in access to care—particularly for racially and ethnically diverse populations, rural residents, and those in long-term care settings. This panel will provide a summary of the national-level needs and current efforts before describing the work of the Oregon Center of Excellence for Behavioral Health and Aging (OCEBHA), the first state-level center of excellence in the United States specifically focused on addressing the intersectional needs of older adults with behavioral health needs. Specifically, we will share the structure and funding of OCEBHA, as well as feedback and successes from our inaugural behavioral health and aging conference. A key component of OCEBHA's work is its partnerships with state agencies, healthcare providers, community-based organizations, and advocacy groups. These collaborations have strengthened our ability to address systemic challenges, promote evidence-based practices, and elevate the voices of older adults and their caregivers. Finally, our discussant will facilitate a conversation about lessons for other states, remaining needs, future directions, and policy implications.

Abstract citation ID: igaf122.1978

**THE COMPLEX DETERMINANTS OF OLDER ADULT BEHAVIORAL HEALTH: A STATE LEVEL OVERVIEW AND RESPONSE**

Walter Dawson[1], Paula Carder[2], Allyson Stodola[2], Lindsey Smith[1], Karen Cellarius[2], and Leah Brandis[1], *1. Oregon Health & Science University, Portland, Oregon, United States, 2. Portland State University, Portland, Oregon, United States*

To address the multiple, complex determinants of older adult behavioral health, Oregon established the first state-level center of excellence in the U.S. with a specific focus on this population through a state-university-community partnership. Previous research has shown that comprehensive care for older adults with behavioral health needs is impeded by the workforce shortage of generalist BH clinicians and a severe workforce shortage of clinicians trained to work with older adults. In the years ahead it is projected there simply will not be enough behavioral health providers who specialize in gero focused-fields to meet the needs of an increasingly aging population. This lack of trained professionals, in turn, leads to a lack of awareness, screening, and proper assessment of the unique behavioral health needs of older adults. Building on Oregon's long history of innovation with the services and care of older adults, the Oregon Center of Excellence for Behavioral Health and Aging (OCEBHA) was conceptualized by the state health authority and initiated using block grant funds from the Substance Abuse and Mental Health Services Administration (SAMHSA). OCEBHA seeks to expand the capacity of health and social programs and providers to deliver behavioral health services for older adults with serious mental illness and substance use disorders through translational research, workforce development, and policy innovation. The focused approach taken by Oregon can serve as a catalyst for further innovation in behavioral health and aging service delivery.

Abstract citation ID: igaf122.1979

**THE FORMATION OF OREGON'S CENTER OF EXCELLENCE FOR BEHAVIORAL HEALTH & AGING**

Allyson Stodola[1], and Walter Dawson[2], *1. Portland State University, Portland, Oregon, United States, 2. Oregon Health & Science University, Portland, Oregon, United States*

As Oregon's population rapidly increases, there is an increased need for improved access to behavioral health resources. In 2024, the Oregon Health Authority funded a collaboration of Portland State and Oregon Health & Science University to establish Oregon's Center of Excellence for Behavioral Health and Aging (OCEBHA) to address growing mental health and substance use disorders among older adults by improving access to behavioral health care services and supports. OCEBHA serves as a backbone organization to support partners in aging, behavioral health, and disability services by advancing workforce development, policy innovation, and translational research. The Center draws on models such as the State-University Partnership Learning Network using a multidisciplinary approach to serious mental illness, substance use, health equity, and best practices in care. Guided by a local steering committee—including individuals with lived experience—and a national advisory board, OCEBHA works to bridge policy and practice through training, resource development, and system-wide improvements.

Abstract citation ID: igaf122.1980

**MEASURING SUCCESS: KEY FINDINGS FROM THE OCEBHA ANNUAL CONFERENCE SURVEY**

Julia Unsworth[1], and Lindsey Smith[2], *1. OHSU-PSU School of Public Health, Portland, Oregon, United States, 2. Oregon Health & Science University, Portland, Oregon, United States*

In assessing training needs, a strong demand was identified for specialized behavioral health training for professionals working with older adults, as well as targeted aging-related training for those working in the behavioral health field. To address this need, OCEBHA held its first statewide conference in 2024, selling out and drawing over 150 professionals. The two-day conference covered topics related to integrative behavioral health care for older adults. The OCEBHA evaluation team collected surveys (60% response rate) and conducted onsite interviews. Most attendees worked in direct care or social work, primarily within behavioral health or aging and disability services. Nearly all survey respondents (92%) were likely to apply conference knowledge in their work and 96% planned to share insights with colleagues. The majority of respondents gained valuable knowledge on substance abuse in older adults (88%), challenges for older adults with SMI (89%), and geriatric care (87%). All respondents (100%) reported learning at least a little about these core conference learning objectives. Attendees appreciated the conference's focus on behavioral health and aging, an often neglected issue highly relevant to the challenges of many attendees' work. Respondents valued the event's focus on diverse topics, expert speakers, networking opportunities, and the overall organization and engagement of the event. Overall, the conference team exceeded their goal of 100 attendees but aims to expand engagement across Oregon and beyond.

Abstract citation ID: igaf122.1981

**CONVENING PARTNERS: LESSONS FOR SUSTAINING STATE-UNIVERSITY & COMMUNITY PARTNERSHIPS IN AGING & BEHAVIORAL HEALTH**

Paula Carder[1], Karen Cellarius[1], Lindsey Smith[2], Allyson Stodola[1], Walter Dawson[2], and Leah Brandis[2], *1. Portland State University, Portland, Oregon, United States, 2. Oregon Health & Science University, Portland, Oregon, United States*

OCEBHA is a state-university-community agency partnership in Oregon, a state with a long history of policy successes driven by advocates, policy entrepreneurs, public agency employees and community organizations. These efforts have resulted in innovative home and community-based services for older adults, and an expansion of non-institutional Medicaid-funded and private pay services. We describe how this tradition of partnerships advanced older adult behavioral health on the state policy agenda over time. In 2015, a workgroup composed of aging services and mental health agencies, health care providers, quality improvement, and advocacy organizations identified gaps in and recommended changes to the behavioral health and aging arena, resulting in the establishment of the Older Adult Behavioral Health Initiative (OABHI). The success of this initiative, which included timely access to care from qualified providers who collaborate to provide coordinated, quality, and culturally responsive BH and wellness services, established OCEBHA's foundation. The organizational structure includes multiple state, university, and community organizations, a national advisory council with experts in workforce, aging, mental health, substance use, and public policy, and a local steering committee including individuals with lived experience. We describe how these partnerships were created and supported, including setting expectations, sharing values and defining goals. In addition, we provide examples of tools, agreements and planning processes that others may use to create and sustain state-university-community partnerships that advance the health and well-being of older adults, especially those with behavioral health needs.

### SESSION 4405 (SYMPOSIUM)

Abstract citation ID: igaf122.1982

### POLICY SERIES: 3 PILOT COLLABORATIONS TO EMPOWER NURSING HOME RESIDENTS AND STAFF: TAKING ACTION WITH THE MOVING FORWARD COALITION

Chair: Mairead Painter
Dr. Discussant: Anna Fisher

The Moving Forward Nursing Home Quality Coalition brings together a diverse group of nursing home stakeholders to advance recommendations from "The National Imperative to Improve Nursing Home Quality," a landmark 2022 report by the National Academies of Sciences, Engineering, and Medicine (NASEM). A core part of the Coalition's mission is designing, testing, and spreading innovative and accessible solutions to support and empower nursing home residents and direct-care staff. This symposium presents three such initiatives that translate national recommendations into actionable strategies with implications for quality improvement and long-term care policy. A partnership with the Kansas State University Center on Aging supports up to 30 nursing homes in implementing tools to assess and act on residents' goals of care, promoting alignment between care delivery and individual priorities. A second initiative, led in collaboration with the Connecticut Long-Term Care Ombudsman Program, strengthens the role of resident councils by promoting statewide adoption, disseminating best practices, and integrating resident feedback into nursing home quality improvement processes. The third initiative convenes a national learning network of Geriatrics Workforce Enhancement Program (GWEP) grantees to develop shared standards for certified nursing assistant (CNA) apprenticeship programs, with the aim of strengthening the direct care workforce and improving job quality. Presenters will share insights from their first year of implementation, including consensus-building strategies, early outcomes, and lessons learned. Together, these efforts reflect the Coalition's broader commitment to making change and system-level improvement through a collaborative process.

Abstract citation ID: igaf122.1983

### LEARNING FROM RESIDENT COUNCILS: HOW RESIDENTS COUNCIL CAN PROVIDE VITAL DATA FOR INTERNAL AND EXTERNAL RESEARCH

Mairead Painter[1], Daniel Beem[1], and Alice Bonner[2], *1. State of Connecticut, Hartford, Connecticut, United States, 2. Institute for Healthcare Improvement, Boston, Massachusetts, United States*

Resident Councils serve as vital sources of process and outcomes data in nursing homes, offering researchers and organizational leaders valuable insights into care quality, resident well-being, and systems-level improvement opportunities. This paper explores structured approaches that enhance meaningful resident participation, generating actionable data for quality improvement. We will first examine Resident Councils as data sources, discussing methods for inclusively collecting and analyzing resident-driven feedback—including process measures such as engagement levels, issue tracking, and advocacy effectiveness. Next, we will explore how nursing homes can integrate Resident Council insights and direct participation into their federally required Quality Assurance and Performance Improvement (QAPI) programs to drive measurable improvements in care delivery, resident satisfaction, and operational efficiency. Finally, we will consider broader research and policy implications, identifying how resident council engagement can inform quality benchmarks, innovative long-term care research efforts and approaches, and policy initiatives at local, state, and national levels. The paper will highlight lessons learned from ongoing initiatives—specifically the Moving Forward Nursing Home Quality Coalition and its ongoing Strengthening Resident Councils pilot—and showcase real-world examples of how resident voices contribute to evidence-based quality improvement. By leveraging Resident Councils as an inclusive, action-oriented, and valuable source of key information, nursing homes can foster a more responsive, resident-directed approach to care.

Abstract citation ID: igaf122.1984

### DEVELOPING NATIONAL STANDARDS AND BEST PRACTICES FOR NURSING HOME REGISTERED APPRENTICESHIP PROGRAMS

Robyn Stone[1], and Molly Carpenter[2], *1. Leading Age, Washington, District of Columbia, United States, 2. The LeadingAge LTSS Center at University of Massachusetts, Washington, District of Columbia, United States*

Under the Biden Administration, registered apprenticeships emerged as a promising strategy for healthcare providers to recruit, train, and retain a qualified workforce, supported by expanded access to federal and state funding. Yet, nursing homes and other aging services providers have been slow to adopt this model, often facing barriers related to regulatory complexity, limited infrastructure and resources, and lack of tailored guidance. This paper describes how the federally funded Geriatrics Workforce Enhancement Program (GWEP) is working to close this gap by supporting the development of registered apprenticeship programs for direct care professionals in long-term care settings. First, we outline the structure and distinguishing features of registered apprenticeships and describe how GWEP grantees are helping introduce and adapt this model within nursing homes. Second, we present the formation and early activities of a national GWEP learning network convened by the Moving Forward Nursing Home Quality Coalition and focused on developing common standards and best practices for apprenticeship implementation for nursing home certified nursing assistants (CNAs). Drawing on stakeholder input and early field experiences, we highlight considerations related to program design, partnership building, and alignment with quality improvement and other organizational goals. Finally, we discuss the broader policy and regulatory implications of expanding apprenticeships in the long-term care workforce and offer recommendations to support their sustainable growth.

Abstract citation ID: igaf122.1985

**ADDRESSING NURSING HOME RESIDENTS' GOALS, PREFERENCES, AND PRIORITY: A LOCAL AND NATIONAL IMPERATIVE**

Laci Cornelison[1], and Addison Van Zutphen[2], *1. Kansas State University, Manhattan, Kansas, United States, 2. Kansas State University Center on Aging, Manhattan, Kansas, United States*

The regulatory F-tag F656—Comprehensive Care Plans—is consistently among the top ten most cited deficiencies in nursing home surveys, highlighting a widespread challenge: nursing homes often lack the resources or processes to meaningfully address residents' goals, preferences, and priorities (GPPs). To address this gap, we developed a practical, user-friendly guide to help nursing home staff engage residents in conversations about what matters most to them and integrate those insights into care plans and ultimately into daily care provided. In partnership with the Kansas State University Center on Aging, we are piloting this guide and process in up to 30 nursing homes. The pilot includes education on goal-concordant care, structured action planning for individualized quality improvement (QI) projects, and evaluation of implementation progress. Findings from this pilot will inform revisions to the guide and underlying process to prepare for broader dissemination. In addition to improving person-centered care at the nursing home level, we share preliminary lessons that could inform the design of health information technology (HIT) systems capable of capturing and exchanging resident GPPs across care settings. We also consider how structured approaches to GPPs could contribute to the development of goal-concordance measures with relevance for federal regulation and quality monitoring. This paper shares the design, implementation strategy, and early insights from this ongoing initiative, along with its broader implications for systems change in long-term care.

### SESSION 4410 (SYMPOSIUM)

Abstract citation ID: igaf122.1986

**PREPARING FOR LONGEVITY: CONSUMER PERSPECTIVES ON FINANCIAL PREPAREDNESS, RISKS, AND DECISION-MAKING**

Chair: Lauren Cerino
Co-Chair: Lisa D'Ambrosio

As Americans live longer, financial preparedness is increasingly critical for ensuring stability and independence in later life. However, individuals' perceptions of financial planning timelines, risk management products and strategies, and overall preparedness vary widely based on factors such as age, life stage, and income. Understanding these differences is essential for designing effective tools, policies, and interventions that support long-term financial security. This symposium presents findings from three MIT AgeLab studies exploring key aspects of financial decision-making across the lifespan. The first presentation explores how individuals at different life stages perceive the ideal timeframe for retirement preparation, comparing prospective (i.e., pre-retirement) and retrospective (i.e., post-retirement) insights on planning windows. The second presentation examines consumer attitudes, knowledge, and usage of financial products such as annuities and life and disability insurance designed to hedge against different financial risks. The third presentation explores how financially prepared middle-income Americans feel for later life, including the planning they engage in, and what kinds of opportunities or supports would support their future security. Together, these studies offer a comprehensive view of how people envision financial security, navigate risks, and engage with financial tools and products to support their later-life well-being. The session will provide valuable insights into the evolving landscape of longevity planning, followed by a discussion addressing implications for financial professionals, policymakers, and researchers in gerontology.

Abstract citation ID: igaf122.1987

**MIDDLE-INCOME AMERICANS AND PREPARING FOR LONGEVITY**

Lisa D'Ambrosio, Alexa Balmuth, Lauren Cerino, Adam Felts, and Niels Wu, *Massachusetts Institute of Technology, Cambridge, Massachusetts, United States*

With longer lives, people face greater complexity in financial planning and management. While a range of services and products exist to address the needs of wealthy Americans, there remain significant needs and opportunities to support middle-income Americans in planning for their financial futures. To explore this, an online survey was fielded to a nationwide US sample of adults ages 18+ with household incomes between $50,000 and $200,000. Survey items included people's financial wellbeing, financial information sources, financial knowledge, and demographic characteristics. Results indicated that people were generally positive about being on the path to meeting their future financial goals, but were less confident about being financially prepared for older age. Most thought that they could be doing a better job of planning for their financial futures. Data indicated that everyday Americans valued financial security (88.5%) over moving up the income ladder (11.5%). People reported consulting a range of different sources for financial information and advice, with internet/web searches being the most common source, followed by family and friends and financial professionals. Online sources were least trusted. Participants also reported a relatively high degree of confidence in their financial knowledge and acumen, although within this income band women and people earning less reported were more likely to display lower levels of financial literacy and have less confidence in their financial knowledge. The results point toward opportunities to support everyday Americans around building financial knowledge and connecting them to planning resources.

Abstract citation ID: igaf122.1988

**MANAGING LONGEVITY RISKS: CONSUMER ATTITUDES TOWARD AND EXPERIENCES WITH FINANCIAL PRODUCTS**

Chaiwoo Lee[1], Kathryn Warren[2] Lisa D'Ambrosio[1], Sophia Ashebir[1], and Joseph Coughlin[3], *1. Massachusetts Institute of Technology, Cambridge, Massachusetts, United States, 2. MIT AgeLab, Cambridge, Massachusetts, United States, 3. Massachusetts Institute of Technology AgeLab, Cambridge, Massachusetts, United States*

Financial products such as annuities, life insurance, and disability insurance are positioned to play a key role in future planning, including retirement, by providing financial security; hedging against different kinds of financial risks, including illness, injury, or death; and maintaining a steady income stream throughout older age. A national online survey of employed American adults ages 25 to 67, with medium to high income or investable assets, explored consumers' sense of preparedness for their financial futures and lives in older age. Items included people's overall financial well-being; attitudes toward and usage of various financial products; attitudes toward different longevity risks; and expectations for retirement. Data on demographic characteristics, recent and anticipated life events, and behavioral characteristics such as risk attitudes and optimism were also gathered to allow for multidimensional comparisons. Preliminary results indicate that while concerns around costs related to health conditions and disability, income stability, and spending flexibility are prevalent among consumers, adoption rates for annuities and disability insurance remain low. Across the three products covered in the survey, usage was significantly correlated with knowledge of and familiarity with the product. Many people were learning about these products from informal sources such as family and friends, and through internet searches, rather than from financial institutions or employers. This presentation will provide a comprehensive look into how different financial products and concerns to manage different longevity risks fit into people's future planning, and how people's preferences and experiences differ by their family situation or by individual characteristics.

Abstract citation ID: igaf122.1989

### WHEN SHOULD I START PLANNING FOR RETIREMENT?: COMPARING PERCEPTIONS OF RETIREMENT PLANNING TIMELINES

Kathryn Warren[1], Adam Felts[2], Lisa D'Ambrosio[2], and Joseph Coughlin[3], *1. MIT AgeLab, Cambridge, Massachusetts, United States, 2. Massachusetts Institute of Technology, Cambridge, Massachusetts, United States, 3. Massachusetts Institute of Technology AgeLab, Cambridge, Massachusetts, United States*

The demographic shift toward longer life expectancy changes how people are planning for retirement, necessitating both financial and lifestyle preparation for an increasing period of time beyond one's career. The anticipated duration of life without employment income affects retirement savings goals and the length of working life, making early and strategic planning essential. To explore people's attitudes toward the time necessary to prepare for retirement, the MIT AgeLab conducted a national online survey (N = 1152, ages 18-89), stratified by age and gender. Respondents were asked when they believed a person should begin to plan for life in retirement and when they expected they would (or, if already retired, when they did) retire. Among respondents who had not yet retired (N = 884), younger respondents anticipated a shorter preparation period. The 18-29 age group, on average, believed planning should begin 16.1 years before retirement, whereas those aged 60-69, still pre-retirement, reported an average preparation window of 30.0 years. These findings suggest that as individuals near their expected retirement age, their ideal timeframe for preparation lengthens. Similarly, retired respondents (N = 268) indicated that planning should begin at a younger age compared to their non-retired counterparts, reinforcing the importance of early preparation. This session will explore these shifting perceptions of retirement readiness, discuss potential psychological and behavioral factors influencing planning timelines, and highlight strategies for encouraging proactive financial and lifestyle planning across the lifespan.

### SESSION 4415 (SYMPOSIUM)

Abstract citation ID: igaf122.1990

### INNOVATIONS TO ADDRESS DEPRESSION IN HIGH RESOURCE NEEDS OLDER ADULTS

Chair: Jason Burnett
Co-Chair: Jennifer Beauchamp
Discussant: Melba Hernandez-Tejada

This presentation highlights the implementation and preliminary outcomes of multiple studies aimed at improving mental health care access and treatment for older adults. A four-arm randomized controlled trial (RCT) examines behavioral activation (BA) and falls prevention (FP) interventions for homebound older adults with depression, delivered via video conferencing. Recruitment challenges include self-stigma and physical health crises, but qualitative feedback has been positive. Additionally, a feasibility study evaluated the use of Amazon Alexa Show 8 (AES8) devices among cognitively impaired homebound older adults, finding improvements in technology acceptance, depression, and social engagement. These findings informed a subsequent pilot study on remote AES8 installation to combat loneliness and isolation. Another RCT explores tele-delivered BA for post-stroke depression (PSD) prevention in low-income stroke survivors with subthreshold depression. Participants receive five weekly sessions and two booster calls, while the control group receives usual care. The study assesses PSD progression, anxiety, quality of life, and healthcare utilization. Lastly, a study on self-neglect (SN) in older adults—commonly linked to untreated depression—investigates whether intergenerational social connections improve mental health referral acceptance. Preliminary data suggest that pairing APS SN clients with health professional students for weekly calls fosters meaningful connections, leading to increased treatment engagement. Collectively, these studies underscore the role of technology, social engagement, and behavioral interventions in addressing geriatric mental health challenges. Findings support innovative, scalable approaches to enhance access to mental health care, reduce isolation, and improve outcomes for high-needs older adult populations.

Abstract citation ID: igaf122.1991

### INTERGENERATIONAL SOCIAL CONNECTIONS ENHANCE DEPRESSION TREATMENT ACCESS FOR OLDER ADULTS WITH SELF-NEGLECT

Jason Burnett[1], Jordan Brousard[1], Melba Hernandez-Tejada[1], and Ron Acierno[2], *1. UTHealth Houston, Houston, Texas, United States, 2. The University of Texas Health Science Center, Houston, Texas, United States*

Self-neglect (SN) is the most reported issue to Adult Protective Services (APS) nationwide, linked to high morbidity and mortality. An estimated 50-60% of individuals with SN also suffer from untreated depression, which impairs self-management of chronic conditions. While treating depression could improve self-care, many older adults with SN decline APS-offered services, including mental health referrals. Increasing referral acceptance is crucial for improving outcomes. Social disconnection contributes to both depression and healthcare avoidance. Our study explored whether fostering meaningful social connections could increase acceptance of depression treatment referrals. We developed an eight-week intergenerational phone-calling program pairing health professional students with APS SN clients. Students screened participants weekly using the PHQ-2, offering referrals if depression was detected in two consecutive weeks. Preliminary data show that 77% of APS SN clients screened positive for depression at baseline. Among 74 pairings, 54% qualified for a referral, 98% were offered a referral, and 80% agreed to be referred and receive a contact from a treatment center and were referred. These findings strongly support intergenerational connections as a supportive strategy demonstrating that newly developed empathy-based social connections can enhance mental health referral acceptance and improve treatment access for older adults living with SN.

Abstract citation ID: igaf122.1992

**BEHAVIORAL ACTIVATION FOR PREVENTION OF POST-STROKE DEPRESSION IN LOW-INCOME OLDER STROKE SURVIVORS (LIVE-WEL)**

Jennifer Beauchamp, *UTHealth Houston, Houston, Texas, United States*

Post-stroke depression (PSD) affects approximately one-third of survivors and is associated with recurrent stroke, poor recovery and quality of life, and mortality. The efficacy of antidepressants to treat PSD is based on mixed results and can be problematic for older survivors who can be sensitive to adverse effects and drug-drug interactions. Behavioral activation (BA) has been shown to be efficacious in treating depression in a range of populations and can be adapted for stroke survivors. This randomized effectiveness study was designed to examine the effectiveness of tele-delivered BA delivered by community health workers to prevent PSD in low-income (single person income ≤ 45,000), older (≥ 55 years) adults with subthreshold depression (SD; 24-item Hamilton Rating Scale for Depression [HAMD] between 5 to 8) within 3 months of a first-time ischemic or hemorrhagic stroke. Eligible survivors will be randomized (1:1) to the tele-delivered BA or treatment-as-usual. Tele-delivered BA will consist of 5-weekly videoconferences, homework, and 2-monthly booster calls. Treatment-as-usual includes an educational packet and a stroke practitioner evaluation within 6 weeks of hospital discharge that includes PSD screening. Antidepressant prescribing and/or referral to mental health care may be done according to practitioner practices as well as risk factor and complication assessments, stroke education, and care management. The primary outcome is the difference in the proportion of survivors that transition from SD to PSD at 4-months, assessed using generalized linear modeling. Secondary outcomes include anxiety, emotional distress, healthcare visits, quality of life, and disability. Mediating variables include self-efficacy, motivation, and activity engagement.

Abstract citation ID: igaf122.1993

**BEHAVIORAL ACTIVATION AND FALLS PREVENTION FOR HOMEBOUND OLDER ADULTS WITH DEPRESSION**

Namkee Choi[1], Kelly Vences[2], Angelina Gutierrez[1], and Brian Fons[1], *1. The University of Texas at Austin, Austin, Texas, United States, 2. The University of Texas School of Social Work, Austin, Texas, United States*

This presentation focuses on the implementation and preliminary outcomes of an ongoing four-arm randomized controlled trial of short-term behavioral activation (BA) and multi-component falls prevention (FP) for homebound older adults, age 50 and older, with depression (24-item Hamilton Rating Scale for Depression [HAMD] >15). Both BA and FP are delivered by trained lay counselors via video-conferencing. iPads with a video-conferencing platform and an exercise app, "Keep on Keep up" are loaned to the participants. The study arms are BA only, FP only, combined BA and FP (TBF), and telephone support call which serves as attention control. BA is designed to increase engagement in value-based activities, and FP uses the CDC's STEADI tool kits and the exercise app. The expected enrollment goal is 320 by March 1, 2026. We have used a variety of recruitment Methods: referrals from Meals on Wheels case workers, distribution of the study fliers with Meals on Wheels, and presentations at local senior apartment complexes. Barriers to recruitment and retention included mental illness self-stigma and related denial of depression, lack of motivation, chronic pain, and physical health crises. Each study participant goes through baseline assessment, weekly sessions over nine weeks, and three follow-up assessments over nine months. The average age of the participants is 68 years; 70% female and 30% male; 55% Black or Hispanic and 45% non-Hispanic White; and the baseline HAMD scores of 23. We will present 12- and 24-week outcomes. Qualitative feedback from participants in BA, FP, and TBF have been highly positive.

Abstract citation ID: igaf122.1994

**USE OF VIRTUAL ASSISTANT DEVICES TO IMPROVE DEPRESSION IN COGNITIVELY-IMPAIRED HOMEBOUND OLDER ADULTS**

Jessica Lee[1], Jason Burnett[2], Prisha Patel[2], and Matthew Nunez[2], *1. The University of Texas Health Science Center at Houston, Houston, Texas, United States, 2. UTHealth Houston, Houston, Texas, United States*

Technology is progressively playing a larger role in individual health and wellness, with increasing use seen in older adults. However, older adults can face uncertainty and inadequate understanding of the features of such devices. Homebound older adults, in particular, often have cognitive or physical impairments that may heighten barriers to technology use. Therefore, we wanted to assess the feasibility and acceptance of virtual assistant device use in cognitively impaired homebound older adults. We partnered with our local Meals on Wheels to evaluate whether the Amazon Alexa Show 8 (AES8) could be appropriately utilized and potentially improve depression in clients aged 60 and older. Participants went through three phases of the study: 6 weeks of meal deliveries alone (control), followed by 6 weeks of meals+AES8 basic usage, and lastly 6 weeks of meals+AES8 advanced usage. Participants had improvements in technology acceptance and associations with improvements in depression and memory by the end of the study, despite no specific health programming. Participants anecdotally enjoyed being able to play music, set reminders, and access spiritual content on the devices. These results have led to a subsequent pilot study evaluating the feasibility of remote installation of the AES8 with evaluations of loneliness and social isolation. Preliminary data indicates that cognitively intact homebound older adults are successfully able to install the devices with telephone assistance. Being able to reach larger numbers of homebound older adults with these virtual assistant devices, may mean increased potential to improve the mental health of high-needs homebound older adults.

### SESSION 4420 (SYMPOSIUM)

Abstract citation ID: igaf122.1995

### OLDER ADULTS' ADAPTATION TO SMART MOBILITY TECHNOLOGY: BRIDGING THE DIGITAL DIVIDE FOR INDEPENDENT MOBILITY

Chair: Moon Choi

Transportation services are increasingly digitalized and moving toward automation. As a result, digital skills are essential for accessing and utilizing navigation and rideshare applications, as well as for understanding and making informed choices regarding automated vehicles. However, older adults, often referred to as digital immigrants, face challenges in adopting these technologies, as they emerged later in their lives. This symposium session examines older adults' adaptation to smart mobility technology, with a particular focus on the digital divide. The session will explore the following key questions: (a) How do older adults perceive safety-related messages of automated vehicle (AV) technologies compared to younger adults? (b) To what extent does training enhance older adults' ability to use mobile applications for demand-responsive transport (DRT) services? (c) Is a tutorial designed to support older adults with cognitive impairments effective in improving their ability to use navigation and rideshare applications in usability tests? (d) How is the use of navigation technologies associated with perceived constraints and travel behavior among older adults? Discussions on the findings from these four studies will contribute to a deeper understanding of age-related differences in perceptions, digital literacy, and the use of smart mobility technologies. Moreover, the session will provide evidence on the effectiveness of interventions aimed at improving digital literacy related to smart mobility services and their potential impact on the well-being of older adults. This is a collaborative symposium between the Technology and Aging and Transportation and Aging Interest Groups.

Abstract citation ID: igaf122.1996

### PERCEPTIONS OF SAFETY WITHOUT EXPERIENCE: AGE DIFFERENCES IN THE INFLUENCE OF SAFETY-RELATED MESSAGES FOR AVS

Ryan Best, *West Virginia University, Morgantown, West Virginia, United States*

The introduction of automated vehicle (AV) technologies to the roadways has the potential to improve road safety and reduce adverse outcomes. Benefits of AVs can play an outsized role for older adults considering age is commonly associated with a reduction in driving. As a cutting-edge technology where few have personal experience, individuals' initial perceptions of the safety of AVs will likely stem from communications originating from several industry stakeholders, including AV companies themselves, outside regulators, and governing bodies. A survey instrument was developed to both implicitly and explicitly estimate how personal perceptions of AV safety are influenced by safety messages from different sources. Data was collected (n = 2,203) from the RAND American Life Panel, a nationally representative online probability sample. The implicit measure revealed that increasing age was generally associated with lower perceptions of AV safety, b=-.14, t=-6.48, p<.001. Compared to adults of higher ages, younger adults were particularly influenced by positive safety-related information provided by average AV crash rates, b=-.03, t=-4.03, p<.001, and official statements from AV companies, b=-.02, t=-3.90, p=.003. When measured explicitly, increased age was associated with a larger preference for safety-related messages from safety advocacy groups, ρ=.18, p<.001, and a decreased preference for information from average crash rates, rho=-.06, p=.005, and AV companies, rho=.14, p<.001. Results are discussed in terms of how safety related messages from various stakeholders might be utilized to affect perceptions of AVs across adulthood.

Abstract citation ID: igaf122.1997

### EMPOWERING OLDER ADULTS THROUGH DIGITAL TRAINING: FACILITATING DEMAND-RESPONSIVE TRANSPORT ADOPTION

Ern Chern Khor, Donghun Kang, Jiwon Park, Wan Hong, Jungwoo Song, and Moon Choi, *Korea Advanced Institute of Science & Technology, Daejeon, Republic of Korea*

Demand-responsive transport (DRT) services effectively address mobility needs in rural and low-density areas where public transportation options are limited. In South Korea, Shucle, a DRT service, offers shared rides within designated zones, dynamically adjusting routes based on demand. However, as these services rely on smartphone apps, older adults—who often constitute the majority of residents in such areas—face challenges in accessing them due to limited digital literacy. This intervention study aimed to develop and evaluate a training program to enhance older adults' ability to use the Shucle app. Participants (n = 57, aged 61–81) were categorized into three groups based on their digital literacy. The training followed a structured sequence: (a) pre-assessment, (b) step-by-step guidance, (c) hands-on practice with Naver Map (the most popular digital map) and the Shucle app, (d) real-ride experience, and (e) post-assessment. Given that digital map literacy is essential for DRT adoption, the training prioritized navigation skills. Initially, nearly half of the participants struggled with Naver Map; however, post-training, over 90% successfully used it. Once proficient in digital mapping, most participants adapted quickly to the Shucle app, confirming the foundational role of navigation skills in DRT usage. Notably, those with moderate digital literacy demonstrated the greatest improvement in app proficiency and attitudes toward DRT services. These findings highlight the importance of tailored interventions to support older adults with varying digital competencies, ensuring equitable access to emerging mobility solutions.

Abstract citation ID: igaf122.1998

### USABILITY OF TUTORIALS TO AUGMENT MOBILITY FOR OLDER ADULTS WITH COGNITIVE IMPAIRMENT

Edie Sanders[1], Dorota Kossowska-Kuhn[2], Michael Prevratil[2], Hunhui Na[2], Walter Boot[3], and Neil Charness[3], *1. Exponent, Atlanta, Georgia, United States, 2. Florida State University, Tallahassee, Florida, United States, 3. Weill Cornell Medicine, New York, New York, United States*

The ability to navigate and travel within one's community is critical to maintaining independence and quality of life but can be difficult for older adults experiencing cognitive impairments (CIs). Navigation and rideshare applications can support these needs but are complex and present usability challenges for this population. Under the Augmenting User Geocoordinates & Mobility with ENhanced Tutorials (AUGMENT) project, we developed and tested the usability of technology-based tutorials to support the use of Google Maps and Uber by older adults with

CIs. Eight older adults with CIs due to stroke, mild cognitive impairment, or traumatic brain injury completed the tutorials while thinking aloud, then completed the System Usability Scale. One week following usability testing, participants were quizzed on material from the tutorials. Usability scores suggested room for improvement (Google Maps M = 67.2%, SD = 30.1%; Uber M = 60.6%, SD = 26.1%). After modification to the tutorials, four new participants completed a second round of usability testing. Scores now indicate very good usability (Google Maps M = 83.8%, SD = 15.6%; Uber M = 88.8%, SD = 13.1%). In both rounds, participants remembered over half of the tested material. Results validate following an iterative user-centered design process for designing effective tutorials for older adults with CIs. The next phase of the project will test the efficacy of the tutorials in helping this population learn how to use complex navigation and rideshare apps.

Abstract citation ID: igaf122.1999

### BEYOND WAYFINDING: THE ROLE OF NAVIGATION TECHNOLOGY IN SUPPORTING EMOTIONAL WELL-BEING

Bisola Adeleke, and Shannon Mejia, *University of Illinois Urbana-Champaign, Champaign, Illinois, United States*

The ability to navigate successfully is essential for maintaining independence, autonomy, and social engagement, which are fundamental to healthy aging. However, age-related change in cognitive and physical function can reduce confidence and limit older adults' willingness to commute, thereby affecting their quality of life. While modern navigation technologies offer potential solutions to support independence, the role in enhancing emotional well-being in older adults remains underexplored. This study, grounded in the Technology Acceptance Model (TAM), explored the implications of navigation use for perceived constraints, travel, and affect among older adults. Using data from the 2022 Health and Retirement Study (N = 4,074), we applied structural equation modeling (SEM) to test reduction in perceived constraints on personal control and travel/commuting yesterday as serial mediators of the association between frequency of navigation use and yesterday's positive affect, with age, financial strain, and self-reported ongoing health problems as covariates. Findings revealed that more frequent navigation technology use was directly associated with greater positive affect. A significant indirect effect ($\beta = 0.064$, $p = 0.001$) indicated that navigation technology use also supported positive affect through lower perceived constraints, which increased the likelihood of travel/commuting yesterday, and in turn by increasing the likelihood of travel through lower perceived constraints on personal control. Taken together, our findings suggest that digital navigation tools play a role beyond wayfinding, and also support well-being through psychological pathways. Attention to preservation of autonomy may play a critical role in enhancing perceived usefulness and support sustained adoption and use of navigation technologies.

### SESSION 4425 (PAPER)

Abstract citation ID: igaf122.2000

### ACUTE CARE OF OLDER ADULTS

In this session the researchers will share their insights into the challenges of research in the acute care setting and their research outcomes.

Abstract citation ID: igaf122.2001

### ADAPTING A SYSTEMS-BASED INTERVENTION TO IMPROVE OLDER ADULT PATIENT MOBILITY IN HOSPITAL SETTINGS

Barbara King[1], David Tucker[1], and Linsey Steege[2], *1. University of Wisconsin–Madison, Madison, Wisconsin, United States, 2. University of Wisconsin–Madison School of Nursing, Madison, Wisconsin, United States*

Hospitalized older adults often experience functional decline due to low mobility. Acute care nurses are responsible for maintaining patient functional status but often do not walk patients due to multiple system barriers. To address system barriers, we developed Mobilizing Older adults Via a systems-based INtervention (MOVIN) a multicomponent mobility model of care. However, implementing multicomponent mobility interventions in hospitals can be complex often requiring adaptations to meet unique needs of inpatient units. A cluster randomized controlled trial design was used paired with a participatory approach to implementing MOVIN. Four inpatient units across 2 hospitals served as study sites. Adaptations of MOVIN to meet the unique needs of each intervention unit were made. A Fidelity rating tool was used to evaluate adherence to the core components of MOVIN. An interrupted time series was used to evaluated changes in percent ambulation and ambulation distance across pre, intervention, and post intervention periods. Adaptations for MOVIN occurred in psychomotor training, mobility resources, and communication tools due to staff turnover, physical layout of the unit, and unique unit workflows. Adaptations occurred during the planning (proactive adjustment) as well as the active (reactive to ongoing barriers) intervention period. Fidelity of all MOVIN components was maintained with very good to excellent ratings. Unit ambulation outcomes (percent ambulation and ambulation distance) significantly increased across pre, intervention, and post-intervention periods and sustained for all four units. Further research is needed on navigating complexities in implementing multicomponent interventions in hospital settings to improve older adult patient mobility.

Abstract citation ID: igaf122.2002

### ALIGNING-CARE IN GERIATRIC SURGERY CO-MANAGEMENT: PRE-OPERATIVE PHASE

Stephanie Chow[1], Yihan Wang[2], William Hung[3], Abigail Baim-Lance[4], Ko Harada[1], Danialle Coyne[2], and Fred Ko[5], *1. Icahn School of Medicine at Mount Sinai, New York, New York, United States, 2. Mount Sinai Hospital, New York, New York, United States, 3. James J Peters VA Medical Center, New York, New York, United States, 4. Veterans Health Administration, New York, New York, United States, 5. James J. Peters Department of Veterans Affairs Medical Center, New York, New York, United States*

Older patients comprise more than half of surgeries, with greater risk for death, loss of function and quality of life. Geriatric Surgery Co-Management programs aim to assess peri-operative risks and improve surgical outcomes, via comprehensive assessments and interprofessional interventions. Our ALIGN-CARE pilot explored the outcomes and potential for pre-operative intervention in older frail adults. Surgical candidates 65+ years screened pre-frail or frail by surgeons were included. Enrolled patients underwent a comprehensive geriatric and frailty assessment, and an ALIGN-CARE framework of measurements ("7Ms") to assess and intervene on important geriatric domains.

Patients were offered pre-operative interventions and followed to surgery. Data was collected from January 2022 to May 2024. ALIGN-CARE enrolled 51 patients, mean age 78.5 years, 45.1% female, 49% reported primary spoken language other than English. Surgeons from 7 specialties were included with Urology comprising majority (58.5%) of referrals. Of those enrolled, 42% were clinically frail. Of the 7Ms framework, recommendations and interventions included 19% of PCPs outreached by ALIGN-CARE, 50.8% recommended for medication adjustment, 54% advised for physical therapy or pre-rehabilitation, 15.9% newly diagnosed with MCI or dementia, 22.2% recommended for further cognitive testing, 46.0% completed healthcare proxy form, 20.6% discussed advance care planning, and 44.4% scheduled for a social work visit. ALIGN-CARE assessment and interventions spanned important domains of geriatric care, signaling need for interprofessional incorporation of medical and psychosocial care in older frail persons. Further data on patient and provider experience in this co-management model, combined with extension into post-operative phases of co-management, is needed.

Abstract citation ID: igaf122.2003

### CARE MANAGEMENT FOR SEVERELY FRAIL OLDER ADULTS IN ACUTE & COMMUNITY SETTINGS: A QUALITATIVE PERSPECTIVE FROM NURSES

Betsy Seah[1], Xuan Min Ng[1], Nandhini Nadarajah[1], and Myint Myint Than[2], *1. National University of Singapore, Singapore, Singapore, 2. Alexander Hospital, Singapore, Singapore*

Background: Despite nurses playing a crucial role in the continuity of care for severely frail older adults from hospital to home, there is limited understanding towards their care delivery across these settings. Aim: To explore the factors influencing nurses' care management for severely frail older adults in acute and community care settings. Methods: In this descriptive qualitative study, 18 inpatient and community care nurses who cared for older adults with a clinical frailty score ≥7 were purposively selected from a hospital in Singapore. Individual in-depth interviews were conducted, audio-taped and transcribed verbatim. Data was analysed using reflexive thematic analysis. Results: The three identified themes are 'addressing intricacies of care', 'navigating care in the community', and 'engaging caregivers in care'. Other than providing holistic, dignified care and working in multidisciplinary teams, having specialised training and cultural competency were vital in managing intricate care needs. Despite existing workstreams to integrate health and social care, participants faced financial and structural barriers and experienced limited resources when navigating care. Participants highlighted the importance and challenges of engaging caregivers in caring for the severely frail at home and provided insights on rapport-building and communication. Conclusion: This study provided insights and strategies to improve care delivery for severely frail older adults. The findings revealed the need to normalise serious illness conversations, increase interdisciplinary training, develop care-specific guidelines for severe frailty, develop cultural competence among internationally educated nurses, enhance community nurses' competencies, improve efficiency and flexibility of community care services, and develop rapport-building strategies for caregivers.

Abstract citation ID: igaf122.2004

### EVALUATING THE POPPY FLOWER PROGRAM: A PERSON-CENTERED COMMUNICATION TOOL FOR DEMENTIA CARE

Cassandra Dictus[1], Christy Knight[2], and Eleanor McConnell[2], *1. Duke University, Durham, North Carolina, United States, 2. Durham VA Healthcare System, Durham, North Carolina, United States*

Hospitalization can be challenging for individuals with dementia, as interacting with unfamiliar environments and new caregivers often exacerbates cognitive and behavioral symptoms. These symptoms can contribute to challenges such as falls, elopement, and resistance to care. While staff may learn patient preferences over time, this knowledge is not always effectively elicited or shared. The Poppy Flower Program was introduced at the Durham VHA Medical Center to address this gap by improving access to patient-specific information, with support from the VHA's Innovation Network's Spark-Seed-Spread initiative. The Poppy Flower, a dry-erase board featuring a symbolic poppy of remembrance, was placed in patient rooms on four acute care medical-surgical units. Each petal included prompts (e.g., "I am upset by..." or "Important people and pets to me are...") to facilitate communication and enhance person-centered care. This evaluation gathered staff and volunteer input to inform the spread phase of the program by describing the frequency of Poppy Flower use, how staff use the tool, and the perspectives of frontline staff and leadership on the program's value and effectiveness. Preliminary findings suggest that while some staff view the Poppy Flower as a useful tool for communication, its use has been inconsistent. Barriers include the placement of the Poppy in the room, workflow integration challenges, and a task-driven care culture where staff were less accustomed to engaging with patient background information. Identifying barriers and facilitators will help determine whether to continue, modify, or expand the program to better support staff in providing high-quality dementia care.

Abstract citation ID: igaf122.2005

### TUBE FEEDING IN OLDER ADULTS WITH DELIRIUM: A MULTICENTER STUDY ON HOSPITAL OUTCOMES

Marlon Juliano Romero Aliberti[1], Ezemir Dantas Fernandes Junior[2], and Thiago Junqueira Avelino-Silva[3], *1. University of Sao Paulo Medical School, Baltimore, Maryland, United States, 2. University of Sao Paulo Medical School, Sao Paulo, Sao Paulo, Brazil, 3. University of California, San Francisco, University of California, San Francisco, California, United States*

Delirium is common in hospitalized older adults and is associated with malnutrition and dehydration. Enteral nutrition, including tube feeding, is often considered to ensure adequate intake, yet its impact on delirium and clinical outcomes remains debated. This study assessed the association between tube feeding and adverse hospital outcomes in hospitalized older adults with delirium. We conducted a prospective cohort study using data from the multicenter CHANGE (Creating a Hospital Assessment Network in Geriatrics) Study, which included patients aged ≥65 years diagnosed with delirium at hospital admission via the Confusion Assessment Method across 43 centers in five countries. Those receiving enteral or parenteral nutrition before hospitalization were excluded. The primary exposure was the initiation of tube feeding during hospitalization. Propensity score modeling and inverse probability of treatment weighting (IPTW) were applied to balance covariates. Among 566 participants (mean age=83±8 years; women=58%), 149 (26%) received tube feeding. Median hospital length of stay was 8 days (IQR=5-13) for those without tube feeding and 14 days (IQR=8-24) for those receiving tube feeding. Hospital mortality was 20% and 50%, respectively. Nosocomial pneumonia occurred in 9% of patients without and 35% with tube feeding. After IPTW adjustment, tube feeding was associated with higher in-hospital mortality (OR = 2.01; 95%CI=1.23-3.28) and nosocomial pneumonia (OR = 2.58; 95%CI=1.46-4.54), but not with length of hospital stay (OR = 0.99; 95%CI=0.94-1.04). In older adults with delirium, tube feeding was associated with higher in-hospital mortality and nosocomial pneumonia, without reducing length of stay. These findings warrant careful risk-benefit assessment when considering tube feeding in this population.

### SESSION 4430 (PAPER)

### END OF LIFE CARE: NEW HORIZONS FOR RESEARCH

Abstract citation ID: igaf122.2006

### COMPARING MATCHING STRATEGIES TO ADDRESS SELECTION INTO PALLIATIVE CARE AMONG VETERANS

Brystana Kaufman[1], Sandra Woolson[2], Paul Dennis[3], Joshua Thorpe[4], Susan Hastings[5], David Bekelman[6], and Courtney Van Houtven[1], *1. Duke University, Durham, North Carolina, United States, 2. Department of Veterans Affairs, Durham, North Carolina, United States, 3. VA Durham Health Care System, Durham, North Carolina, United States, 4. The University of North Carolina at Chapel Hill, Chapel Hill, North Carolina, United States, 5. Durham VA Healthcare System, Durham, North Carolina, United States, 6. Department of Veterans Affairs, Auroroa, Colorado, United States*

Selection bias influences observational studies of palliative care because many of the reasons for palliative care referral are not documented in administrative data sources, making it difficult to control for unobserved confounders. In observational studies, matching and weighting methods aim to control for unobserved confounding that is correlated with observed characteristics. This study aimed to quantify residual selection bias due to unobserved factors by comparing 3 strategies (combinations of exact match, Mahalonobis distance, and propensity weighting) applied to multi-payer data (2014-2017) to support causal inference approaches. The national cohort included veterans with life limiting conditions who were new users of VA specialty outpatient PC (treat) and matched veterans with non-PC specialty outpatient encounter (active comparator). Residual unobserved confounding was measured by comparing survival curves between PC and non-PC groups at 90 days. Of 5820 unique new PC users, 1527, 2811, and 4381 pairs were matched in strategy 1, 2, and 3, respectively. Survival at 90 days were 14.1, 15.8, and 17.8 percentage points lower compared to matched veterans in strategies 1,2, and 3 respectively. In subgroup analyses, the smallest difference in survival between groups was among veterans with prior inpatient PC (4.4 percentage points). Matching on observed characteristics was not sufficient to control for selection into PC, as illustrated by differences in survival following an initial PC compared to non-PC specialty encounter among veterans with life limiting conditions. Further work is needed to understand the impact of residual confounding on estimates for the effect of PC on veteran outcomes.

Abstract citation ID: igaf122.2007

### MEDICARE ADVANTAGE PRIOR-AUTHORIZATIONS ARE LINKED TO RACIAL DISPARITIES IN END-OF-LIFE HOME HEALTH USE

Tessa Jones[1], Mohamed Benyamine[1], Evan Bollens-lund[1], David Meyers[2], Sean Morrison[1], and Claire Ankuda[1], *1. Icahn School of Medicine at Mount Sinai, New York, New York, United States, 2. Brown University, Providence, Rhode Island, United States*

In Medicare Advantage (MA), Hispanic and Asian or Pacific Islander decedents are less likely to receive Medicare home health (HH) compared to their counterparts in traditional Medicare. However, the impact of MA plan features, such as requirements for referrals or prior authorizations, on these disparities is unclear. We aim to investigate what MA plan features contribute to racial and ethnic disparities in HH use at the end of life. We used a 100% Medicare cohort of adults age ≥66 who died in 2019 and were enrolled in MA in their last year of life. We first fit a linear probability model with and without a MA plan-level fixed effects to determine if racial disparities were driven by within- vs. between- plan differences. We then tested interactions between decedent race/ethnicity and two MA plan characteristics: prior authorization and referral requirements for HH. All models adjusted for individual and regional characteristics. In our sample of N = 410,351, 259,175 were in MA plans that required prior authorization and 153,439 that required referral for HH. Racial and ethnic differences were primarily driven by between-plan differences, with prior authorization requirements increasing disparities. For example, prior authorization was associated with a 1.49% (95% CI -.02 – 0.05) decrease in HH use for non-Hispanic Whites, 7.17% (95% CI .10 - .05) decrease for Hispanics; and a 3.64% (95% CI .07-.05) decrease for Asian/Pacific Islanders. Our findings suggest that prior authorizations and referrals for HH, contribute to racial and ethnic disparities in the MA program.

Abstract citation ID: igaf122.2008

### MULTI-LEVEL FACTORS IMPACTING IMPLEMENTATION OF OUTPATIENT PALLIATIVE CARE: PERCEPTIONS OF VA PROVIDERS

Brystana Kaufman[1], Tiera Lanford[2], Nina Sperber[2], Jessica Ma[2], Joshua Thorpe[3], Susan Hastings[4], David Bekelman[5], and Courtney Van Houtven[1], *1. Duke University, Durham, North Carolina, United States, 2. Department of Veterans Affairs, Durham, North Carolina, United States, 3. The University of North Carolina at Chapel Hill, Chapel Hill, North Carolina, United States, 4. Durham VA Healthcare System, Durham, North Carolina, United States, 5. Department of Veterans Affairs, Auroroa, Colorado, United States*

Strategies to support implementation and sustainability of palliative care (PC) are needed to better meet palliative needs for older adults living in the community. Applying an implementation science framework, this qualitative study explores barriers and facilitators impacting the implementation of outpatient PC, as perceived by PC team members in the Veterans Health Administration (VHA). Provider perceptions were explored using semi-structured group interviews with 9 outpatient specialty PC

teams. Participants (n = 26) included outpatient PC team members and team leaders. Barriers and facilitators were identified for key implementation and sustainability constructs within the provider, organizational context, and external levels. At the provider level, providers reported barriers including limited staffing and fragmented teams; however, they also felt that interdisciplinary and cohesive teams were facilitators to high quality PC. Teams reported that referrals they receive are not always appropriate for specialty PC, and education of referring providers was critical. At the organization level, teams embedded within a specialty or primary care clinics viewed co-location as a facilitator for referrals and care coordination. At the external environment level, most felt that traditional methods of health system performance tracking (relative value units) may undervalue the role of PC in supporting Veterans. Improved performance measures are needed to document and incentivize appropriate referral and use of PC throughout the continuum of care. Key considerations for health systems seeking to implement or expand outpatient PC include addressing misperceptions, co-location with referring clinical teams, improved performance measures, and building the PC workforce to support interdisciplinary PC teams.

Abstract citation ID: igaf122.2009

### PATIENT AND CAREGIVER EXPERIENCES OF NAVIGATING THE "SEESAW" OF HOSPICE LIVE DISCHARGE

Cara Wallace[1], Stephanie P. Wladkowski[2], Todd Becker[3], Joseph Trinitas[4], Ruaa Al Juboori[5], Leslie Hinyard[6], and Verna Hendricks-Ferguson[7], *1. Saint Louis University, Saint Louis, Missouri, United States, 2. Bowling Green State University, Bowling Green, Ohio, United States, 3. WashU Medicine, St. Louis, Missouri, United States, 4. Saint Louis University, St. Louis, Missouri, United States, 5. University of Mississippi, Oxford, Mississippi, United States, 6. Saint Louis University School of Medicine, St. Louis, Missouri, United States, 7. Trudy Busch Valentine School of Nursing, St. Louis, Missouri, United States*

The receipt of quality hospice care may prompt changes in patient eligibility under Medicare's prognostic requirement of 6 months or less. In 2022, 224,900 hospice beneficiaries across the United States were discharged alive from hospice care, with 6.1% discharged due to "no longer being terminally ill." This critical care transition is associated with losing important hospice-based supports and questions if to re-enroll back onto hospice care. Previous research is limited on how patients and caregivers navigate countervailing positive and negative reactions that shape this phenomenon. The current study aimed to address this gap by exploring the experience of service coordination and potential impacts to quality-of-life post-discharge. With a purposive sample of 32 patient-caregiver dyads, in-depth, semi-structured interviews were analyzed via thematic analysis. Results were characterized by navigating a process of ups and downs with varying questions about whether the patient was maintaining eligibility, akin to a seesaw. Specifically, caregivers described an uncertainty regarding how hospice patients managed to "get better" to warrant discharge, when their needs for assistance remained the same. Other caregivers cast doubt on if discharge was necessary or even appropriate. Whereas nearly half of participants referred to live discharge as incongruent with their goals for care, the experiences associated with navigating live discharge (both positive and negative) led others to question if they would want the patient to re-enroll in hospice in the future. These results suggest a need to develop and examine robust services to support patient and caregiver transitions from hospice care.

### SESSION 4435 (PAPER)

### GOIN' DOWN THE ROAD: NEW HORIZONS IN TRANSPORTATION RESEARCH

Abstract citation ID: igaf122.2010

### A NEW LOOK AT OLDER ADULTS' USE OF RIDE SHARE SERVICES: INSIGHTS FROM A NATIONAL SURVEY

Alycia Bayne, Alexa Siegfried, and Lindsey Witt-Swanson, *National Opinion Research Center at The University of Chicago, Chicago, Illinois, United States*

Ride share services are a promising transportation solution for older adults. While research has documented that ride share use among older adults has generally increased since 2015, no studies have examined national trends in their use among older adults across age groups and by geography. To address this research gap, NORC conducted a nationally representative survey of 1,010 U.S. adults aged 50+ using the NORC Foresight 50+ Panel, which is drawn from the NORC national sample frame that has over 97% coverage of U.S. addresses. The survey was fielded in May 2024 and included questions about ride share use, trip purpose, frequency of use, and factors affecting use. This session describes findings from our descriptive analysis to identify the characteristics of older adults who had ever used a ride share service, including age, education, and income, trip purpose, frequency of use, and factors affecting use. We also examined variation in the availability of ride share services. This session describes our findings, including that nearly half of older adults (45.6%) reported using a ride share service, with most using it monthly or less (92.8%). Geographic differences in use also emerged. Factors affecting use include convenience, safety, and affordability. This study provides a clear picture of the characteristics of older adults who use ride share services. Further, this study has implications for public health practitioners and transportation planners developing transportation programs that meet the health and mobility needs of older adults.

Abstract citation ID: igaf122.2011

### ARE OLDER ADULTS PLANNING FOR THEIR TRANSPORTATION FUTURES? A NATIONAL SURVEY ON DRIVING RETIREMENT

Alycia Bayne, Alexa Siegfried, and Lindsey Witt-Swanson, *National Opinion Research Center at The University of Chicago, Chicago, Illinois, United States*

Driving a personal vehicle is the preferred mode of transportation among older adults. However, many older adults will outlive their ability to drive due to age-related changes in vision, cognition, and physical function. Despite the impact of transportation on health, wellbeing, independence, and mobility, few older adults plan for their driving retirement (i.e., the time when they permanently stop driving). Research is needed to understand how older adults plan for their driving retirement, including who they trust to talk with them about this transition. To explore this topic, NORC conducted a nationally representative survey of 1,010 U.S. adults aged 50+ using the NORC Foresight 50+ Panel (May 2024), which is drawn from the NORC national sample frame that has over 97% coverage of U.S. addresses. We found that older adults trust a spouse or partner (51%), adult child

(48%), or primary care provider (41%) to have conversations with them about driving retirement. Few older adults (31%) have a plan in place for how they will travel when they can no longer drive. Adults aged 75+ are more worried about not being able to drive in the future, compared to adults aged 50 to 64. Demographic factors affect driving retirement planning. This study provides new data that can be used by public health, transportation practitioners, caregivers, and others to support older adults in proactively planning for their transportation futures.

Abstract citation ID: igaf122.2012

### EVALUATION OF A SELF-DRIVING SHUTTLE SERVICE TO IMPROVE MOBILITY OF OLDER ADULTS AND PEOPLE WITH DISABILITIES

Renee St. Louis[1], Jennifer Zakrajsek[2], Nicole Zanier[2], Dillon Funkhouser[2], and James Sayer[2], *1. University of Michigan Transportation Research Institute, Ann Arbor, Michigan, United States, 2. University of Michigan, Ann Arbor, Michigan, United States*

This study centers on a one-year pilot program launched in June 2024 in Detroit, Michigan called Accessibili-D. This initiative provides a self-driving shuttle service for individuals aged 62 and older and people with disabilities, addressing traditional transit service gaps and providing an innovative solution to enable continued mobility. Accessibili-D operates six days a week across 128 pre-programmed stops, encompassing medical centers, grocery stores, libraries, and entertainment venues. Participants schedule free rides via a mobile app or by phone. The vehicles operate autonomously on specified routes but are supervised by onboard safety operators. The pilot program enrolled 298 participants in the first three months. Participant evaluations include a baseline survey and three follow-up surveys to assess travel behavior, shuttle utilization, trust in automated vehicle (AV) technologies, and opinions on how to improve the service. Results to date revealed that those who have ridden the shuttle agreed more strongly than non-riders that AVs are dependable ($\chi2=12.61$, p=.01), reliable ($\chi2=13.73$, p=.01), that they can trust AVs ($\chi2=12.18$, p=.02), and that AVs will be useful in meeting their transportation needs ($\chi2=10.27$, p=.04). Key findings from the qualitative analysis of open-text responses revealed the desire for an expanded service area and the importance of the safety operator to the participants' riding comfort. Details of the shuttle service and outcomes of the baseline and follow up surveys to address participant experience and changes over time will be discussed. Insights from this pilot will inform scalable approaches to AV deployment, promoting safer and more equitable transportation systems.

Abstract citation ID: igaf122.2013

### NAVIGATING THE ROAD AHEAD: A SCOPING REVIEW OF CARE PARTNER INVOLVEMENT IN DRIVING DECISIONS FOR PEOPLE WITH DEMENTIA

KJ Hansmann[1], Mark D'Alesio[2], Anthony Mcdonald[1], and Beth Fields[1], *1. University of Wisconsin–Madison, Madison, Wisconsin, United States, 2. University of Pittsburgh Medical Center, Pittsburgh, Pennsylvania, United States*

The prevalence of dementia is steadily increasing among older adults, raising concerns about safe driving as cognitive and functional decline impact the ability to drive safely. People with dementia often rely on care partners (e.g., family, friends) for support with daily activities and health care management due to the progressive nature of the disease. However, their role in driving-related decisions and transitions remains less understood. This scoping review aims to identify and characterize how care partners are involved in research on driving for people with dementia. We included articles if they (a) were full-text articles describing qualitative, quantitative, or mixed methods research; (b) investigated driving for people with dementia; and (c) directly involved care partners in the study. Multiple reviewers completed abstract and full-text screening, and completed a qualitative descriptive analysis to summarize themes drawn from the included articles. We identified 15 research articles that included care partners in studies investigating people with dementia's driving. Of these, 12 specifically focused on driving cessation. Our analysis revealed three key themes describing care partners' involvement: (1) sharing their emotional experiences related to driving restriction and cessation, (2) serving as decision-makers about driving restriction and cessation, and (3) providing insights into the driving behaviors of people with dementia. Care partner's inclusion in driving research and interventions facilitates addressing safety concerns. In addition, care partner involvement in the research process can support the development of innovative approaches that extend beyond driving cessation to support broader community mobility needs for people with dementia.

Abstract citation ID: igaf122.2014

### OLDER ADULTS' CURRENT AND MAXIMUM ACCEPTABLE TRAVEL TIMES FOR PRIMARY CARE: RESULTS FROM A NATIONAL SURVEY

Tabasa Ozawa, Ying Liu, and Soeren Mattke, *University of Southern California, Los Angeles, California, United States*

Long travel times may create obstacles to care, particularly for older adults who may need frequent care and assistance with transportation. However, little data exists on how much travel time might prevent care-seeking. We surveyed 5,453 individuals aged 50+ in the nationally representative Understanding America Study about their willingness to travel for primary care and examined the factors associated with preference. Most respondents (80%) currently traveled 0-30 minutes for primary care, 14% 31-60 minutes, 2% more than 60 minutes, with 3% having no primary care provider. The vast majority (94%) traveled by car. Asked about the maximum travel time before delaying or forgoing care, 28% responded 0-30 minutes, 42% 31-60 minutes, and 30% more than 60 minutes. Most (77%) were willing to travel longer than their current time. Logistic regression showed older age, higher income, belonging to non-Hispanic white group, higher education, poorer self-rated health, and shorter current travel time were significantly associated with greater willingness to travel longer than current. Income and race/ethnicity had strong effects: 83% of individuals with income ≥$100,000 were willing to travel longer, versus 69% of those with income ≤$39,000 (odds ratio [OR]=2.41, $p < 0.0005$); 81% for non-Hispanic White versus 67-68% for racial/ethnic minority groups (OR = 2.14-2.22, $p < 0.0005$). The findings suggest that most older adults would be accepting of longer travel times rather than forgoing care, but there are notable differences by demographic groups. Further research should investigate whether these stated preferences correspond to actual behavior, and whether and how much travel times limit access.

### SESSION 4440 (PAPER)

### HEALTH EQUITY AND AGING

Abstract citation ID: igaf122.2015

### ADHERENCE TO VARIOUS DIETARY QUALITY INDICES IN THE NEW ENGLAND CENTENARIAN STUDY

Erfei Zhao[1], Emma Schluter[2], Naglaa El-Abbadi[3], Kyla Shea[4], Stacy Andersen[5], Thomas Perls[5], Paola Sebastiani[6], and Andres Ardisson Korat[4], *1. Human Nutrition Research Center on Aging, Tufts University, Boston, Massachusetts, United States, 2. Harvard University, Boston, Massachusetts, United States, 3. Jean Mayer USDA Human Nutrition Research Center on Aging, Boston, Massachusetts, United States, 4. Tufts University, Boston, Massachusetts, United States, 5. Boston University, Boston, Massachusetts, United States, 6. Tufts Medical Center, Boston, Massachusetts, United States*

Centenarian offspring (CO) in the New England Centenarian Study (NECS) typically show survival and health advantages, yet little is known about their dietary intake patterns. In this study, we characterized participants' diets according to four established patterns: Alternative Healthy Eating Index (AHEI), Healthy Eating Index (HEI), Mediterranean-DASH (Dietary Approaches to Stop Hypertension) Intervention for Neurodegenerative Delay (MIND), and Planetary Health Diet Index (PHDI) in CO and a referent group and compared them to national dietary surveys. NECS participants (335 CO; 128 controls; mean age: 73.6 years; 55.1% women) completed the Harvard 131-item food frequency questionnaire in 2005. We assessed each index according to published scoring systems and performed linear regression to examine differences by CO status, sex, age, and education, and compared mean scores to nationally representative data (e.g., National Health and Nutrition Examination Survey [NHANES ]) for older individuals. We found no significant differences between CO and referents across all four indices. However, women had slightly higher AHEI and HEI scores than men ($P < 0.05$). Younger participants scored higher on AHEI, while education was positively associated with all indices ($P < 0.01$). Compared to NHANES participants of similar ages, NECS participants scored modestly higher on all indices; however, intakes of whole grains and dairy were below national averages, while seafood protein and added sugar were higher. In summary, CO showed no advantage in dietary quality compared to controls, whereas sociodemographic factors were significant predictors of eating patterns. Future research should explore the diet-physiology-lifestyle interaction and its combined effect on CO's survival advantage.

Abstract citation ID: igaf122.2016

### EARLY LIFE TRAUMA AND IMMUNE AGING: EVIDENCE OF A BIOLOGICAL THRESHOLD IN OLDER WOMEN

Grace Noppert, Philippa Clarke, and Kate Duchowny, *University of Michigan, Ann Arbor, Michigan, United States*

Immunosenescence is implicated in aging-related decline and disease and can be shaped by physical and social environments. However, whether early life trauma is directly associated with immune aging in later life remains unclear. Using 2016 venous blood data from the U.S. Health and Retirement Study (HRS), we examined associations between early life trauma and five markers of immunosenescence among adults aged 60 + (n = 8,373). We estimated gender-stratified linear regression models to assess differences in standardized immune measures across trauma exposure categories (0, 1–2, 3, 3+ traumas before age 18). Strikingly, significant associations were observed only among women, and only for those experiencing 3+ traumas compared to none. For instance, women reporting 3+ traumas had a 0.19 higher standardized value of CD4+ EMRA:Naïve (95% CI: 0.08, 0.14) and a 0.16 higher CD8+ EMRA:CD4+ Naïve ratio (95% CI: 0.05, 0.27), adjusting for age. Results were robust to adjustment for trauma in other life stages and parental education, supporting a direct association. Importantly, evidence also suggests a threshold effect: we observed no significant associations when comparing individuals with 1–2 or 3 traumas to those with none, but differences were evident when comparing individuals with 3+ traumas to all other groups, including those with fewer traumas. These findings highlight a threshold beyond which early trauma exerts lasting biological consequences for immune aging. Given the critical role of immune function in healthy aging, our results underscore the importance of early life social environments as key determinants of later-life immune health.

Abstract citation ID: igaf122.2017

### FOOD INSECURITY AND ITS ASSOCIATION WITH DEPRESSIVE SYMPTOMS: EVIDENCE FROM OLDER ADULTS IN INDIA

Sindhu Vasireddy, *Mahindra University, Hyderabad, Telangana, India*

This study is novel in examining the association between both the prevalence and incidence of food insecurity and depressive symptoms among older adults in India. For this, we use two waves of data from the Study of Global AGEing and Adult Health (SAGE) conducted in 2007 and 2015. Logistic regression analysis is employed to examine the prevalence of depression, while a discrete-time event history model is used to study the incidence of depression and determine the influence of food insecurity on the onset and offset of depression. We find that severe food insecurity is strongly associated with increased odds of depression, and that this association strengthened across the waves. The incidence models find that relative to those who do not experience any transitions, when people transition from being insecure to secure about food over the waves, they are twice (OR: 2.035, CI: 1.53-2.69) as likely to move out of depression. Similarly, those who experience the onset of food insecurity are more likely to transition into depression over the waves. In addition to health counselors and psychological support, this study fortifies the necessity for policies and interventions targeted to lessen hunger and improve the affordability of food to alleviate depressive symptoms in Indian older adults.

Abstract citation ID: igaf122.2018

### HOW DOES CHRONIC STRESS IMPACT BIOAGE AND INFLAMMATION AMONG OLDER BLACK AND WHITE ADULTS?

Lauren Brown[1], Patricia Louie[2], Calley Fisk[1], Cameron Brown[1], and Kenya Luna[1], *1. University of Southern California, Los Angeles, California, United States, 2. University of Washington, Seattle, Washington, United States*

Prior research suggests that exposure to stressful events contributes to disparities in biological health in older adulthood. Yet, to understand the extent to which Black older adults bear a disproportionate stress and health burden, we consider Black-white differences in not only stress exposure, but also stress appraisal—how upsetting the exposures are perceived to be across five domains (health, financial, residential, relationship and caregiving). Data come from 4,912 adults ages 56+ from the 2016 Health and Retirement Venus Blood Study. Black older adults are more exposed to chronic stressors than white older adults and are more likely to rate health, financial, and housing stressors as upsetting. White older adults are more likely to rate relationship and caregiving stressors as upsetting. Stratified models show greater exposure to stressors and appraising stressors as upsetting are associated with increased bioage and inflammation for White older adults. For Black older adults, exposure and appraisal was not predictive of bioage or inflammation. Domain specific analyses show exposure to health, financial and housing strain drives the association between stress, bioage and inflammation among White older adults. For Black older adults only health problems are associated with increased bioage and inflammation. Findings suggest exposure and appraisal have varying consequences for biological health. Black older adults continue to demonstrate remarkable fortitude in the face of chronic stressors. Alternatively, our data may not capture the stress process for Black older adults, suggesting we need to recalibrate our measures to include culturally relevant approaches to measuring stress and bioage.

Abstract citation ID: igaf122.2019

**ROLE OF DISTANCE IN THE USE OF THE EMERGENCY DEPARTMENTS FOR DENTAL CONDITIONS AMONG OLDER ADULTS IN MARYLAND**

Uma Kelekar[1], Shillpa Naavaal[2], Debasree Das Gupta[3], and Sidney Turner[4], *1. Marymount University, Arlington, Virginia, United States, 2. Virginia Commonwealth University, Richmond, Virginia, United States, 3. Utah State University, Logan, Utah, United States, 4. Fors Marsh Group, Arlington, Virginia, United States*

Disparities in access to oral health in the US are evident in the utilization of Emergency Departments (ED) for non-traumatic dental conditions (NTDCs). However, evidence regarding the role of distance in ED utilization for NTDCs, particularly among older adults (≥65) remains limited. To fill this gap, our objective was to compare how ED utilization among different age-groups varied based on the location of nearest EDs and community dental providers. We used State Emergency Department Database and American Dental Association provider data files for 2017-2021 for Maryland. Using negative binomial regressions, we estimated ED utilization rates for different age-groups as a function of two types of distances—1) patient zip codes and nearest EDs and 2) patient zip codes and dental providers, while controlling for confounders. Compared to other age-groups, older adults reported the lowest ED visits (296 per 100,000) and lived the farthest from the nearest ED. The regression results revealed no statistically significant differences in ED utilization rates for NTDCs across the different age-groups. Nevertheless, among older adults, the average NTDC utilization rate decreased as the distance to the nearest ED increased (β=-0.22; 95%CI: -0.31,-0.13), while it increased with greater distances to the nearest dental provider (β = 0.19; 95%CI: 0.08,0.30). Together, these results indicate that the 65+ are less likely to use emergency services for NTDCs when they are farther away from an ED, particularly if a dental provider is nearby. Thus, our findings highlight the complex interplay of access-based factors with perceived needs of dental care among the 65+.

Abstract citation ID: igaf122.2020

**THE ROLE OF COMMUNITY AND SOCIAL SUPPORT IN THE LINK BETWEEN SOCIAL DETERMINANTS OF HEALTH AND SELF-RATED HEALTH**

Samuel Van Vleet[1], Jeein Law (Jang[2], and John Bowblis[1], *1. Miami University, Oxford, Ohio, United States, 2. University of Massachusetts Boston, Boston, Massachusetts, United States*

As the older adult population in the United States grows, understanding how social determinants of health, neighborhood environment, and social networks influence health outcomes in later life is crucial. Marginalized populations often face cumulative disadvantages, making community support essential for improving health. This study explores the impact of social determinants of health, neighborhood environment, and social networks on self-reported health among older adults with multiple chronic conditions, using data from the National Health and Aging Trends Study (N = 5,356). Survey-weighted logistic regression models assessed relationships between income, rurality, food insecurity, transportation, meal assistance, neighborhood disorder, and built environment issues, with self-rated health as the outcome. The moderating effects of community support and social network size were also examined. Results indicate that food insecurity, transportation difficulties, limited meal assistance, and high physical disorder were associated with lower self-rated health, while higher community support was associated with better self-rated health. Notably, community support buffered the negative impact of food insecurity and high physical disorder on self-rated health. Additionally, social network size positively moderated the negative impact of high physical disorder on self-rated health. These findings emphasize the significant role of social determinants of health and social support in shaping health outcomes in later life. Policies should prioritize strengthening community support systems and social networks, particularly for food-insecure and physically disadvantaged older adults, to improve self-rated health and mitigate health disparities. The study contributes to understanding how social and environmental factors intersect to affect health disparities among aging populations.

### SESSION 4445 (SYMPOSIUM)

Abstract citation ID: igaf122.2021

**ADAPTING THE PATIENT PRIORITIES CARE FRAMEWORK: INNOVATIONS IN TECHNOLOGY, SURGERY, CULTURE, AND CLINICAL CARE**

Chair: Melissa Hladek
Discussant: Mary Tinetti

The Patient Priorities Care (PPC) framework aligns healthcare decisions with the values, goals, and care preferences of older adults. In geriatric primary care, multiple studies have shown that it is feasible, reduces treatment burden, improves collaborative communication and improves health outcomes. As a result, there is increased interest in applying this framework to other communities and situations. This symposium presents four interrelated adaptations of PPC—technological, surgical, cultural, and clinical—that extend its applicability beyond traditional chronic disease management in primary care to address decision-making, digital inclusion, and underrepresented populations. The VISTA (Values Informed Solutions for Technology Adoption) study applies PPC principles in the digital space, helping older adults learn technology to support their goals, thus broadening PPC's reach beyond clinical settings. Similarly, in surgical decision-making, the Patient Priorities-Aligned Intervention Decisions (PPAID) tool provides structured guidance to ensure that procedures align with patient-defined outcome goals, offering a model for value-driven intervention choices. Recognizing the role of cultural context, a web-based adaptation of the "My Health Priorities" program is being tailored to older African Americans with multiple chronic conditions, addressing usability and accessibility concerns to ensure equitable patient engagement. Lastly, a PPC-focused geriatric consultative model is being implemented to improve care alignment for older, rural-living veterans with multiple chronic conditions. These adaptations demonstrate the flexibility and scalability of the PPC framework, reinforcing its role in fostering patient-centered decision-making across diverse healthcare settings.

Abstract citation ID: igaf122.2022

### ADAPTING PATIENT PRIORITIES CARE TO BRIDGE THE DIGITAL DIVIDE: A PATIENT-CENTERED, VALUES-BASED APPROACH

Melissa Hladek[1], Olivia Rubio[2], Samantha Curriero[1], Elisa Letts[3], and Sarah Szanton[1], *1. Johns Hopkins University, Baltimore, Maryland, United States, 2. The Pennsylvania State University, University Park, Pennsylvania, United States, 3. Johns Hopkins University School of Nursing, Baltimore, Maryland, United States*

Digital literacy is essential for accessing health information, maintaining social connections, and enhancing quality of life. However, many older adults face barriers to digital engagement particularly those with frailty and from historically marginalized communities, exacerbating healthcare and social disparities. Traditionally, the Patient Priorities Care framework is used in primary care to systematically elicit patient's values, goals, and healthcare preferences and guide healthcare decision-making and medical treatment options. However, the framework can be applied not only to other healthcare settings, but to broader research and wellness-related fields in order to center the patient's voice in all aspects of research design and implementation science. To that end, we conducted a randomized control pilot trial enrolling diverse older adults with varying frailty status and digital literacy (N = 20). Over 4 to 6 in-home sessions, interventionists collaborated with participants to identify priorities and co-develop personalized digital strategies and tools based on their goals. Changes in digital literacy and self-efficacy will be reported. Key motivators for technology use included enhancing quality of life, improving health, and connecting with family and friends. Unexpected benefits included engagement with new online communities based on personal interests (e.g., playing chess with global peers). We will also discuss research design-related insights gained from applying the PPC framework in non-traditional ways. Co-designing solutions with participants offers a scalable model to reduce the digital divide and promote equity in aging. Future plans include testing this intervention in a larger trial.

Abstract citation ID: igaf122.2023

### ACCEPTABILITY AND FEASIBILITY OF A SELF-DIRECTED HEALTH PRIORITIES PROGRAM FOR SOUTHERN OLDER AFRICAN AMERICANS

Deborah Ejem[1], Marie Bakitas[1], Raegan Durant[2], Tamara Nix Parker[3], Kwaku Duah Oppong[4], J. Odom[5], Kenneth Boockvar[1], and Mary Tinetti[6], *1. The University of Alabama at Birmingham, Birmingham, Alabama, United States, 2. The University of Alabama at Birmingham Heersink School of Medicine, Birmingham, Alabama, United States, 3. The University of Alabama at Birmingham School of Nursing, Biriminggham, Alabama, United States, 4. The University of Texas at Austin, Austin, Texas, United States, 5. The University of Alabama at Birmingham School of Nursing, Birmingham, Alaska, United States, 6. Yale University, New Haven, Connecticut, United States*

Acceptability and feasibility of a self-directed health priorities program for Southern older African Americans were assessed in a mixed-methods formative evaluation (NIH Stage 1a). The study explored patient and family caregiver (FCG) experiences with the "My Health Priorities" (MHP) web-based program, a component of the Patient Priorities Care Approach. Fifteen older African Americans (≥65 years, ≥2 chronic conditions) and their adult African American FCGs (≥18 years) from a southeastern U.S. primary care clinic participated. Thematic analysis of interviews and usability assessments via the System Usability Scale (SUS) were conducted. Patients generally found the program acceptable but faced navigation challenges on non-computer devices. Recommendations included culturally adapting avatars, optimizing mobile functionality, and providing strategies for patient-clinician interactions. Patients rated usability at 75.3 ± 14.6 (good), whereas FCGs rated it at 30.1 ± 4.3 (poor). Study measures took 30 minutes to complete, while the intervention took 60 minutes, with 81% of study measures completed. Findings suggest that web-based programs may be acceptable for African American patients and caregivers, but usability and cultural relevance require improvement. Differences in patient and caregiver usability ratings highlight the need for tailored design considerations.

Abstract citation ID: igaf122.2024

### PATIENT PRIORITIES-ALIGNED INTERVENTION DECISIONS (PPAID) APPROACH

Aanand Naik, *UTHealth Houston, Houston, Texas, United States*

Decision-making for surgery and procedures is challenging for older adults with multiple chronic conditions (MCCs) due to uncertain benefits and heightened risks. These individuals vary in the health outcomes they desire most, necessitating discussions that go beyond 'fixing' a clinical problem. However, intervention decisions usually do not consider the likelihood of achieving patient-identified outcome goals. The Patient Priorities Care (PPC) approach reduces treatment burden and improves alignment with patient goals and priorities in managing chronic conditions. We adapted PPC principles for discrete intervention decisions into a tool that can be completed with clinician guidance or self-directed with probes. The Patient Priorities-Aligned Intervention Decisions (PPAID) approach was developed through an iterative process incorporating clinician expert and patient input. Unlike traditional decision aids, PPAID centers discussions on specific, realistic and actionable goals rather than procedural choices, ensuring decisions align with patient outcome goals. The PPAID approach builds from four recurring goal categories that broadly capture the outcomes that patients desire from surgery. These categorical goals anchor subsequent deliberations, resulting in the identification of a specific and realistic outcome goal for the given discrete intervention. Future research will evaluate tool feasibility, acceptability, and effectiveness in clinical settings using an artificial intelligence enabled interface. Metrics will include pre-procedure goal agreement, decision regret, and patient satisfaction. Integrating the tool into clinical workflows is crucial for its adoption. By emphasizing goal identification and deliberation, PPAID approach supports age-friendly, patient goals-aligned care as required by the new CMS age-friendly measure.

Abstract citation ID: igaf122.2025

### ALIGNING CARE WITH THE PRIORITIES OF RURAL VETERANS: EVALUATION OF A PPC-FOCUSED GERIATRIC CONSULTATIVE MODEL

Katherine Ritchey[1], Gwen Bernacki[1], Amy Thomas[1], Linnaea Schuttner[1], Karl Brown[1], and Erica Martinez[2], *1. VA Puget Sound Health Care System, Seattle, Washington, United States, 2. Department of Veterans Affairs, Seattle, Washington, United States*

Patient Priorities Care (PPC) is an effective approach to identifying and aligning 'what matters' with healthcare for patients with multiple chronic conditions (MCC). The enrollment of older, rurally-based veterans with MCC will increase throughout Puget Sound, placing additional strain on VA clinics lacking geriatric specialty care. Therefore, we developed a geriatric consultation that utilizes the PPC approach to improve the identification of rehabilitative or social services and support rural VA clinics in their ability to care for persons with MCC. Patients were referred to a Geriatric PPC Consultation clinic received recommendations to health care. Multiple linear regression was used to correlate total number of recommendations with Jen frailty (JFI) scores. Surveys and semi-structured interviews with providers, patients and/or caregivers explored experience with the consultation service. On average, patients received four recommendations per consult. Frailty scores had no effect on number of recommendations for both rural ($p = 0.85$) and non-rural ($p = 0.35$) groups. Veteran and care partner experience with the consultation was positive and enhanced the clinical-patient encounter. Results from surveys and interviews suggest that this approach may improve Veteran perception of person-centered care on aspects that 'matter most' and the quality of VA healthcare encounters. Most providers indicated that consultation increased veteran access to VA and community-based services and was work-load neutral. The PPC approach identifies unmet health care needs for rural, patients with MCC and is supportive to patients, care partners and providers. Future work is needed to clarify how to scale this model across VA.

### SESSION 4450 (PAPER)

Abstract citation ID: igaf122.2026

### PHYSICAL FUNCTION IN OLDER ADULTS

In this session, research will be presented from bench to bedside to provide insights into functional decline in aging.

Abstract citation ID: igaf122.2027

### HOW PROBABLE COGNITIVE IMPAIRMENT SHAPES FALL RISK AND PHYSICAL FUNCTION AFTER 12 MONTHS

Laura Himmelmann, Tania Zieschang, and Tim Stuckenschneider, *The Carl von Ossietzky University of Oldenburg's School of Medicine and Health Sciences, Oldenburg, Niedersachsen, Germany*

Older adults with cognitive impairment (OACI) experience more severe fall-related sequelae and have a higher risk of recurrent falls than cognitively healthy older adults (OACH). Despite the high risk of decline in physical function following fall-related emergency department (ED) visits, targeted falls prevention interventions remain limited. Therefore, this study examines the trajectories of physical function and recurrent falls in OACH and OACI. Older adults (≥60 years) were recruited shortly after a severe fall that required an ED visit and were followed up for 12 months. Follow-up included monthly telephone calls and three home visits to assess recurrent falls, functional performance measured by self-preferred, three-meter overground gait speed (m/s), and cognition (MoCA). After twelve months, 116 OACI (mean age 77.1± 8.4; MoCA: 21.8±1.9) experienced more recurrent falls ($n = 61/116$, $p = 0.01$) compared to 126 OACH (mean age 70.7±7.8; MoCA: 26.8±1.4; recurrent falls 46/126). ANCOVA indicated a significant difference between the groups with worse performance in OACI ($p < 0.001$), although both groups showed cognitive improvement over time ($p < 0.001$). Physical function including gait speed remained stable, with both groups demonstrating persistently slow gait speed at 12 months (OACI: 1.0±0.3; OACH: 1.1±0.3 m/s; $p = 0.51$). Since physical function remained unchanged, the high fall rate may be more closely linked to deficits in attention or executive function. Nevertheless, a longer follow-up is needed to identify the clinical relevance of these findings. Future studies should explore cognitive training and dual-task exercise interventions to mitigate fall risk in this vulnerable population.

Abstract citation ID: igaf122.2028

### MEASURING FUNCTIONAL DEPENDENCY AMONG OLDER ADULTS: VALIDATION OF THE PHYSICAL ABILITY QUESTIONNAIRE IN ARIC STUDY

Wuyang Zhang[1], James Pike[2], Pablo Martinez-Amezcua[1], Shoshana Ballew[2], Katherine Ornstein[3], Martha Abshire Saylor[4], Erin Kent[5], and Anna Kucharska-Newton[6], *1. Johns Hopkins Bloomberg School of Public Health, Baltimore, Maryland, United States, 2. New York University, New York, New York, United States, 3. Johns Hopkins University, Baltimore, Maryland, United States, 4. Johns Hopkins School of Nursing, Baltimore, Maryland, United States, 5. University of North Carolina at Chapel Hill, Chapel Hill, North Carolina, United States, 6. The University of North Carolina at Chapel Hill, Chapel Hill, North Carolina, United States*

Functional independence is critical to the wellbeing of older adults. We examined the psychometric properties of the Physical Ability Questionnaire (PAQ) in the Atherosclerosis Risk in Communities (ARIC) Study, an ongoing population-based cohort. We derived and validated a functional dependency score based on the Disablement Process Framework. The study population consisted of ARIC participants ($N = 3{,}492$; median age: 78 [range: 71-94]; 58% female; 22% Black) with complete PAQ assessment at Visit 6 (2016-2017). We conducted exploratory factor analyses of the 17 PAQ items and identified a one-factor model to derive a score of functional dependency. The construct validity of this score was examined against physical function (Short Physical Performance Battery), physical activity (moderate-to-vigorous, Baecke questionnaire), Fried frailty phenotype, all-cause hospitalization risk, and all-cause mortality using multivariable generalized linear regression and Cox proportional hazard models. The derived factor score exhibited moderately high internal consistency (Cronbach's α = 0.85) and test-retest reliability (Intraclass Correlation Coefficient=0.69), along with moderate-to-strong cross-sectional associations with constructs of physical function (β=-1.57; 95% CI: -1.68, -1.47), physical activity (β=-56.08; 95% CI: -65.11, -47.05), and frailty (robust-to-prefrail: RRR=0.50; 95% CI: 0.45, 0.57; frail-to-prefrail: RRR=4.09; 95% CI: 3.27, 5.11). During a 5-year follow-up period, a one-unit increase in the functional dependency score was associated with elevated risks of hospitalization (HR = 1.43; 95% CI: 1.34, 1.52) and all-cause mortality (HR = 1.79; 95% CI: 1.59, 2.01). The derived functional dependency score can facilitate future research on aging-related outcomes in the ARIC cohort, and may serve as a basis for harmonization of functional assessment across studies.

Abstract citation ID: igaf122.2029

### MULTIMORBIDITY AND FUNCTIONAL IMPAIRMENTS IN CHRONIC LIFE STRESSORS: EVIDENCE FROM THE HEALTH AND RETIREMENT STUDY

Ayse Malatyali[1], Tom Cidav[1], Abigail Tice[1], Rui Xie[1], Abbie Ashton[1], Michael Dino[2], and Lisa Wiese[3], *1. University of Central Florida, Orlando, Florida, United States, 2. University of Central Florida, University of Central Florida, Florida, United States, 3. Florida Atlantic University, Boca Raton, Florida, United States*

Chronic Life Stressors (CLS) significantly impact the well-being of older adults, yet their health-related determinants remain underexplored. This study examines associations between multimorbidity, functional impairments, and CLS exposure. Using logistic regression models, we analyzed cross-sectional data from 2,822 older adults in the 2020 Health and Retirement Study. CLS was assessed across seven domains: physical-emotional stress, substance use in the family, work-related stress, financial strain, housing instability, strained relationships, and caregiving burden and was categorized as no exposure, moderate exposure (1–2 stressors), and severe exposure (3+ stressors). Multimorbidity was defined as having up to seven chronic conditions. Functional impairments included difficulties with Instrumental Activities of Daily Living (IADL) and mobility limitations. Older adults ≥75 years had lower odds of severe CLS than those aged 65–74. Women had a significantly higher risk of severe CLS exposure (OR = 1.20) than men. African American individuals had lower odds of moderate CLS (OR = 0.61) but no significant difference in severe CLS. Hispanic ethnicity was not significantly associated with CLS. Multimorbidity significantly predicted CLS; Individuals having three or more chronic conditions were 2.68 times more likely to report severe CLS exposure, and those with two conditions were 2.55 times more likely to report severe CLS exposure. Individuals with IADL difficulties had a 2.34 times increased risk of severe CLS, while those with mobility limitations had 2.07 times higher odds of severe CLS exposure. These findings underscore the need for targeted interventions to address multimorbidity and functional limitations, reducing the burden of CLS in aging populations.

Abstract citation ID: igaf122.2030

### NOCIPLASTIC FEATURES ASSOCIATE WITH POORER FUNCTION IN AGING VETERANS WITH CHRONIC LOW BACK PAIN

Victoria Powell[1], Jinkyung Kang[1], Andrzej Galecki[1], Daniel Clauw[1], Pooja Lagisetty[1], Maria Silveira[1], Caroline Logue[2], and Sarah Krein[1], *1. University of Michigan, Ann Arbor, Michigan, United States, 2. LTC Charles S. Kettles VA Medical Center, Ann Arbor, Michigan, United States*

Chronic low back pain (CLBP) is a common cause of disability in aging veterans. CLBP is increasingly recognized as one of several overlapping pain conditions where nociplastic pain is likely the predominate mechanism underlying pain and associated disability. Nociplastic features include pain that is chronic, widespread, and accompanied by additional symptoms such as fatigue, sleep disorders, and mood disturbance. However, to what extent clinically significant nociplastic features interfere with function in aging veterans is unknown. We conducted a nationally representative cross-sectional survey of veterans 50-89 years old with ≥2 outpatient encounters for low back pain. The Veterans RAND 12-Item Health Survey was used to measure general functioning. The Fibromyalgia Survey Questionnaire was used to quantify nociplastic features. Of n = 800 invited veterans, 45.1% returned surveys. Nociplastic features were common, with over one-third (35.8%) reporting nociplastic features severe enough to screen positive for fibromyalgia. More severe nociplastic features were significantly associated with poorer physical (β =-0.30 (-0.49, -0.12), p < 0.0001) and psychological functioning (β=-0.639 (-0.93, -0.35), p < 0.0001) after adjusting for demographics, comorbidities, pain intensity, and opioid use. Our findings support that in aging Veterans with CLBP, nociplastic features are commonly present, and in one-third of cases severe enough to meet criteria for fibromyalgia, the prototypical nociplastic pain disorder. Reporting more nociplastic features was independently associated with worse physical and psychological function in a dose-dependent manner. Future studies, including trials of new pain interventions aimed at improving or maintaining functional status, should examine whether the severity of nociplastic features predicts treatment response.

Abstract citation ID: igaf122.2031

### PREHABILITATION UTILIZATION FOR ELECTIVE LOWER EXTREMITY JOINT REPLACEMENT AMONG OLDER MEDICARE BENEFICIARIES

Jenni Reiff[1], Jennifer Wolff[2], John McGready[3], Jennifer Schrack[3], Tracy Mroz[4], and Lisa Reider[3], *1. Veteran's Administration (Atlanta), Decatur, Georgia, United States, 2. Johns Hopkins University - School of Public Health, Baltimore, Maryland, United States, 3. Johns Hopkins Bloomberg School of Public Health, Baltimore, Maryland, United States, 4. University of Washington School of Medicine, Seattle, Washington, United States*

This retrospective cohort study presents the first national characterization of preoperative rehabilitation ("prehabilitation") for lower extremity joint replacement among older Medicare Fee-for-Service beneficiaries. Using claims data from a 20% sample of Medicare beneficiaries, we included beneficiaries ages 65 and older undergoing incident elective inpatient hip or knee replacement between January 1, 2016, and December 31, 2019, and identified those receiving physical or occupational therapy services initiated within 30 days preceding surgery. Using statistical testing and multivariate linear regression, we estimated utilization trends and characteristics associated with 1) individual receipt of prehabilitation, 2) inpatient facility delivery of prehabilitation, and 3) prehabilitation services (timing, volume, and setting). Of 196,690 included older adult Medicare beneficiaries (mean age 73, 45.4% non-frail, 37.5% hip surgery), 18,739 (9.53%) received prehabilitation. Prehabilitation prevalence increased from 8.03% in 2016 to 11.14% in 2019. Stratifying by operative joint and frailty status, greatest increase was noted among non-frail knee replacement patients (+3.64% between 2016 and 2019). Most prehabilitation patients (77%) received a single visit, primarily in the outpatient physical therapy setting. Likelihood of receiving prehabilitation varied by patient race, gender, financial and health characteristics. More than half (55.7%) of inpatient facilities delivered prehabilitation to at least one patient during the study period; likelihood of delivering prehabilitation varied by facility location, size, and financial characteristics. Findings demonstrate growth in prehabilitation utilization over time, supporting further investigation of why and how providers are implementing prehabilitation, what factors impact patient access, and what strategies may maximize the utility of this service delivery innovation.

### SESSION 4455 (PAPER)

### PREDICTING AND PROMOTING COGNITIVE HEALTH: EVIDENCE FROM DIVERSE AGING POPULATIONS

Abstract citation ID: igaf122.2032

### BIDIRECTIONAL RELATIONSHIP BETWEEN DEPRESSION AND COGNITIVE FUNCTION: EVIDENCE FROM A PSYCHOSOCIAL INTERVENTION

Lucy Xi Lu[1], Dara Kiu Yi Leung[1], Stephanie Ming Yin Wong[1], Wai Chi Chan[1], Gloria Hoi Yan Wong[2], and Terry Lum[1], *1. The University of Hong Kong, Kowloon, Hong Kong, China (People's Republic), 2. University of Reading, Reading, United Kingdom*

Depression is a modifiable predictor of dementia, but its underlying relationships with cognition is unclear. Most research studied their relationships in spontaneous contexts, lacking insight into their interactions during interventions. This research examined the longitudinal relationships between depressive symptoms (DS) and cognitive function (CF) across psychosocial intervention in older adults with intact cognition and mild cognitive impairment (MCI). Data were from JC JoyAge, a community stepped-care program for late-life mental health. Participants (≥ 60 years) with mild to moderate DS (n = 4448) completed assessments of DS (PHQ-9) and CF (MoCA 5-min) before and after the program. Bivariate latent change score models were computed to examine the interactions between DS and CF by modeling changes as latent variables and testing individual-level relationships, with multigroup modeling for comparisons. Participants experienced improvements in DS and CF. For both participants with intact cognition and MCI, higher baseline CF predicted greater reduction in DS (est = -0.04, se = 0.01, z = -3.83, p < .001), lower baseline DS predicted greater improvement in CF (est = -0.04, se = 0.01, z = -3.76, p < .001). Greater reduction in DS was associated with greater improvement in CF (est = -0.51, se = 0.11, z = -4.65, p < .001). The findings evidenced the bidirectional relationships between depressive symptoms and cognitive function, and showed their hand-in-hand improvements during psychosocial intervention. Results suggested the benefits of treating depressive symptoms for both mental health and cognitive function, no matter at the stages of intact cognition or MCI.

Abstract citation ID: igaf122.2033

### EFFECTS OF PHYSICAL ACTIVITY TRAJECTORIES ON TRANSITIONS ACROSS COGNITIVE STATES: A MULTINATIONAL COHORTS STUDY

Chenlu Hong[1], and Yanan Luo[2], *1. Peking University, Beijing, Beijing, China (People's Republic), 2. Peking University School of Public Health, Beijing, Beijing, China (People's Republic)*

Abstract Background Identifying modifiable factors that prevent dementia progression is crucial. However, the impact of long-term physical activity (PA) trajectories on cognitive transitions remains understudied. This study examines the association between PA trajectories and cognitive state transitions in late life. Methods We analyzed data from HRS, ELSA, SHARE, MHAS (2004–2018), and CHARLS (2011–2018). PA trajectories were classified into two groups using GBTM based on PA intensity and frequency. A three-state model was applied (State 1 = Not Cognitively Impaired; State 2 = Mild Cognitive Impairment (MCI); State 3 = Dementia). The MSM model assessed cognitive transitions, with PA trajectory as a covariate to measure the HRs. A meta-analysis provided an overall effect size across cohorts. Results A higher PA trajectory was associated with a 25% lower risk of transitioning from no impairment to MCI (pooled HR = 0.75; 95% CI: 0.65–0.87) and a reduced risk of MCI progressing to dementia (pooled HR = 0.87; 95% CI: 0.77–0.98), but had no significant effect on cognitive recovery from MCI. Individual study results were largely consistent, though some heterogeneity existed. HRS, SHARE, and MHAS showed significantly lower risk of State 1 to State 2 transition. Only HRS showed a reduced risk of State 2 to State 3, while ELSA reported a significantly higher likelihood of cognitive recovery. Conclusion Our multinational study confirms the protective effect of higher PA trajectories in delaying MCI and dementia onset. Findings suggest even physically inactive individuals may achieve neurocognitive benefits by increasing PA after age 60.

Abstract citation ID: igaf122.2034

### HIGHER ANTIPSYCHOTIC USAGE AMONG SEXUAL MINORITIES WITH COGNITIVE IMPAIRMENT: EVIDENCE FROM THE "ALL OF US" STUDY

Addam Reynolds, *University of Southern California Leonard Davis School of Gerontology, Los Angeles, California, United States*

Antipsychotics are often used to treat the behavioral and psychotic symptoms of Alzheimer's disease and related dementia (ADRD); however, such use comes with increased risk for adverse events, such as death and cognitive impairment. Absent from this literature are considerations that sexual minorities are more likely to have a treatment history with antipsychotics due to comorbid mental health diagnoses. The current study addresses this gap in the literature by testing if sexual minorities are more likely than their heterosexual counterparts to have a treatment history of antipsychotic usage. This study leverages data from the All of Us Research Program. We restricted our sample to those 65 and over, and those with a clinical finding of impaired cognition, or a diagnosis of mild cognitive impairment or ADRD listed at least twice in participant's electronic health records (N = 9,358). We use linear regression to test the study's hypothesis. Sexual minorities with cognitive impairment, in comparison to heterosexuals, have 31.2% (95% CI: 1.063 – 1.6120; p < .05) higher odds of having a treatment history of antipsychotics, even after controlling for demographic information (e.g. race/ethnicity, age, sex assigned at birth, and education) and co-morbid mental health diagnoses. Sexual minorities with cognitive impairment are more likely to have a treatment history of antipsychotics, in comparison to their heterosexual counterparts. Future research is needed to determine why sexual minorities with cognitive impairment are more likely to be prescribed drug class that places them at higher risk for adverse events.

Abstract citation ID: igaf122.2035

**HOUSEHOLD ASSETS, EXPENDITURES, AND LONGITUDINAL MEMORY CHANGES AMONG OLDER ADULTS IN RURAL SOUTH AFRICA**

Chuwen Zhong[1], Xuexin Yu[2], and Lindsay Kobayashi[1], *1. University of Michigan, Ann Arbor, Michigan, United States, 2. Columbia University Mailman School of Public Health, New York, New York, United States*

The relationships between cognitively-stimulating types of household assets and expenditures with cognitive function and decline among older adults in low-income settings are unclear. We investigated how household assets and expenditures relate to cognitive function and decline over time among 3,942 participants aged ≥40 years in the population-representative HAALSI cohort study in rural Agincourt, South Africa (three study time points over 8 years from 2014/15 to 2022). Household assets were categorized as the ownership of each of a radio, cellphone, smartphone, refrigerator, vehicle(s), and stove (yes/no for each) and monthly expenditures were on each of food, communication, transportation, and entertainment (measured continuously in units of 1000 RAND). Latent memory z-scores were derived from immediate and delayed recall trials at each time point. Multivariable-adjusted linear mixed-effects regression models were applied to examine associations. Mean baseline age was 63 years (SD: 12.5 years); 53% were female (2,098/3,942). Ownership of a refrigerator, a smartphone, or a vehicle, and higher expenditures on food, communication, and transportation were each associated with higher baseline memory scores. Owning a refrigerator, a vehicle, a radio, and having greater expenditures on food, communication, and transportation were each associated with a slower rate of cognitive decline over time. Consistent with cognitive reserve theory, findings indicate that improvements in household assets and expenditures, especially related to food storage, communication, and transportation, may help to delay cognitive decline in this low-income setting.

Abstract citation ID: igaf122.2036

**SPECIFICITY EFFECTS OF THE COUPLING AUDITORY RHYTHM ON NEURAL OSCILLATIONS OF OLDER ADULTS**

Yilin Chen[1], Tommy Lam[2], Davynn G. H. Tan[2], and Karen P. Y. Liu[2], *1. The Hong Kong Polytechnic University, Hong Kong, China (People's Republic), 2. The Hong Kong Polytechnic University, Hong Kong, Hong Kong, China (People's Republic)*

Sensory entrainment may enhance cognitive performance by modulating abnormal neural oscillations associated with cognitive impairment, potentially alleviating healthcare burdens. The study investigated the effectiveness of different auditory rhythms in altering neural oscillations in the aging brain. We compared changes (in mean power spectral density [PSD]) within the frontal and parietal lobes in response to the single rhythm (3 or 40 Hz.) and coupling rhythm (3 and 40 Hz. coupling). Twelve participants (69.17 ± 0.79 years) underwent electroencephalography recordings during three rhythm conditions in one session. Each condition included a 120-second auditory exposure state, with 30-second pre- and post-exposure resting states. Conditions were randomly ordered and blinded to participants. PSD during exposure was normalized by subtracting the pre-exposure PSD. The 3 Hz. PSD change was significantly higher for 3 Hz. condition (mean: 3.58 dB, range: -2.24 to 10.60) compared to the coupling condition (mean: 1.91 dB, range: -5.01 to 11.42) at the parietal lobe ($p < 0.001$). The 40 Hz. PSD change was significantly lower for the 40 Hz. condition (mean: -0.45 dB, range: -11.06 to 4.32) compared to the coupling condition (mean: 1.40 dB, range: -17.90 to 9.58) at the frontal lobe ($p = 0.014$). The coupling rhythm showed stronger effectiveness on 40 Hz. PSD but weaker effects on 3 Hz. PSD, demonstrating the specific effects on 40 Hz. PSD. This provides a novel approach to enhance targeted neural oscillations in aging. Further studies should investigate the long-term effects of oscillation modulation on various cognitive functions in the elderly.

Abstract citation ID: igaf122.2037

**THE HEALTHY BRAIN PROJECT: EMBEDDING THE EARLY IDENTIFICATION OF COGNITIVE IMPAIRMENT IN AREA AGENCIES ON AGING**

Maria Brown[1], and Sharon Brangman[2], *1. Syracuse University, Syracuse, New York, United States, 2. SUNY Upstate Medical University, Syracuse, New York, United States*

Almost seven million older Americans live with Alzheimer's disease and related dementias (ADRD). The Healthy Brain Project evaluates the practicality and impact of embedding evidence-based brief cognitive screens in case manager workflows in seven Area Agencies on Aging (AAAs) in Central New York State. The project team trained 50 case managers to administer an evidenced based brief cognitive screening tool (Mini-Cog™) and refer clients with scores (0-2 of 5) suggesting cognitive impairment for comprehensive assessment at the Central New York Center of Excellence in Alzheimer's Disease (CEAD). Data collection began November 1, 2024, and concludes October 31, 2025. As of February 28, 2025 (presentation will include final data), AAAs have offered Mini-Cog™ screenings to 569 clients during routine client assessments; 301 (53%) have accepted screening. Of those screened, 117 (39%) had scores indicating potential cognitive impairment, 102 were offered referrals, 33 accepted a referral, 17 have been seen in the CEAD, and 14 have been diagnosed - more than half (8) were diagnosed with mild cognitive impairment. Early identification of cognitive impairment allows clinicians to identify treatable causes of dementia-like symptoms, provide access to treatments to help manage ADRD progression and symptoms, empower patients to make their own care decisions, and plan for supports to maximize independence and quality of life. Incorporating brief cognitive screens in routine AAA client assessments can identify older adults with previously unidentified and untreated cognitive issues who would benefit from these opportunities.

### SESSION 4460 (PAPER)

Abstract citation ID: igaf122.2038

**RESEARCH METHODS IN AGING RESEARCH**

In this session research designs and methods are described to help inform clinicians and researchers in distinguishing impact.

Abstract citation ID: igaf122.2039

**ALIGNING DIETARY INTAKE AND FOOD PURCHASES IN U.S. OLDER ADULTS: INSIGHTS FROM NHANES AND CIRCANA CONSUMER NETWORK**

Naglaa El-Abbadi[1], Marcus Lehr[1], Frederick Nusetor[1], Cristina Moraga Franco[1], Andrea Carlson[2], Kyla Shea[3], Chao-Qiang Lai[4], and Joshua Rutt[1], *1. Jean Mayer USDA Human Nutrition Research Center on Aging, Boston, Massachusetts, United States, 2. U.S. Department of Agriculture, Washington, District of Columbia, United States, 3. Tufts University, Boston, Massachusetts, United States, 4. USDA ARS, Human Nutrition Research Center on Aging at Tufts University, Boston, Massachusetts, United States*

Diet quality is critical for healthy aging, yet assessing dietary intake remains complex. Nationally representative datasets provide valuable but distinct perspectives. The National Health and Nutrition Examination Survey (NHANES) captures self-reported consumption, while the Circana Consumer Network (Circana) records grocery purchases of approximately 60,000 households. The objective of this analysis was to explore whether dietary patterns observed in NHANES 2017-2018 align with food purchase trends in Circana 2018 in U.S. adults 55 years and older. Dietary intake data were derived from single 24-hour recalls (NHANES n = 2132), or grocery food purchase from 1- and 2-person households (Circana n = 26432). We also conducted stratified analyses by sex and age group (55-64, 65-74, 75+). Diet quality was scored using the 2020 Healthy Eating Index (HEI), and food group composition was evaluated by % weight (g) and energy (kcal). The mean±SE total HEI in NHANES was 52.1±0.9. Though overall HEI scores improved with age in both datasets, neither dataset suggests that older adults consistently meet dietary recommendations. Older adults were below HEI component adequacy/moderation recommendations for Fruit, Greens/Beans, Whole Grains, and Saturated Fats; these also showed greatest variation by age. While food group proportion by mass aligned between consumption and purchase for various food groups, there were key disparities in Dairy, Mixed Dishes, Condiments, and Snacks/Sweets; and Vegetables by caloric energy. These observations underscore the potential for integrating food purchase data with dietary intake assessments to enhance understanding of older adults' dietary behaviors. Further research should explore demographic and socioeconomic factors influencing these patterns.

Abstract citation ID: igaf122.2040

**DIFFERENCES IN QUALITY OF CARE INTERACTIONS ACROSS CARE TASKS IN ASSISTED LIVING**

Rachel McPherson[1], Barbara Resnick[2], Sarah Holmes[1], Anju Paudel[3], Sorah Levy[2], and Elizabeth Galik[1], *1. University of Maryland School of Nursing, Baltimore, Maryland, United States, 2. University of Maryland, Baltimore, Maryland, United States, 3. The Pennsylvania State University, University Park, Pennsylvania, United States*

The quality of care interactions is crucial for optimizing the engagement of residents in personal care activities such as bathing and dressing. Limited research has explored how the quality of care interactions varies across different care tasks such as personal care (e.g., bathing, dressing), supportive/ambulatory care, or other care. As assisted living (AL) residents often require staff assistance in care tasks, it is critical to assess differences in the quality of care interactions that residents receive during the care tasks. This study aimed to determine whether the quality of care interactions differs by care task type in AL communities. This was a descriptive study done in four AL communities in Maryland. A total of 152 staff-resident care interactions were observed across various care tasks (e.g., intimate personal care, dietary care) using the Modified Quality of Interaction Scale. Descriptive statistics and a two-tailed analysis of variance with a post hoc Tukey HSD test were conducted. The majority of staff-resident interactions were positive (58%), and the remaining interactions were neutral (23%) or negative (19%). Most interactions occurred during supportive/ambulatory care (34%), followed by dietary care (24%), clinical care (21%), and intimate personal care (21%). There was a significant difference such that care interactions during supportive/ambulatory care were significantly more negative than interactions during personal care (p=.022). There were no other differences in quality of care interactions between care tasks. Findings from this study suggest that training AL staff on how to improve interactions during ambulation assistance or supportive care would be beneficial.

Abstract citation ID: igaf122.2041

**INFLUENCE OF PRAGMATIC DESIGNS ON ESTIMATION OF CLINICAL EFFECTS AMONG RANDOMIZED TRIALS IN AGING**

Dana Weisenfeld[1], and Thomas Travison[2], *1. Hebrew SeniorLife, Boston, Massachusetts, United States, 2. Hebrew SeniorLife & Harvard Medical School, Hebrew SeniorLife, Massachusetts, United States*

There is broad acceptance that an intervention's 'real-world' effectiveness is attenuated relative to its efficacy, but few articulations of what degree of reduction may be expected. We sought to determine the proportionate decrease in effects measuring effectiveness relative to efficacy, as well as the degree to which pragmatic trials may be underpowered in estimating clinically relevant effects in at-risk populations. To do so, we conducted a systematic search identifying 200 two-arm pragmatic trials, as well as 171 explanatory trials, published in high-impact biomedical journals from 2010-2024. Of these, 57 (29%) concerned aging populations and/or aging-related illnesses; twenty-eight (14%) described pharmacologic agents, while the remainder dealt with non-pharmacologic interventions. For each trial, the hypothesized 'planning' effect informing its power computation was isolated and the standardized treatment effect it estimated obtained. Of the pragmatic trials, relatively few were preceded by a companion efficacy trial. A total of 83 (42%) pragmatic trials demonstrated statistically significant evidence in favor of an intervention's effect relative to control, while the corresponding proportion was 94 (55%) among the efficacy trials. Among the efficacy trials, the median ratio of the estimated effect to the planning effect was 0.60 (interquartile interval 0.07 to 1.07), while in the pragmatic trials this ratio was diminished, at 0.44 (interquartile interval 0.06 to 0.89). This review provides a first estimation of the impact of pragmatism on measures of effectiveness relative to efficacy in contemporary pragmatic trials in aging. Implications for the design of pragmatic trials in aging populations will be discussed.

Abstract citation ID: igaf122.2042

**MECHANISMS OF GENERATING HEALTH DISPARITIES IN THE RISK OF ALZHEIMER'S DISEASE**

Igor Akushevich[1], Arseniy Yashkin[1], and Julia Kravchenko[2], *1. Duke University, Durham, North Carolina, United States,*

*2. Duke University School of Medicine, Durham, North Carolina, United States*

Disparities in Alzheimer's disease (AD) and related dementias (ADRD) persist across race/ethnicity, sex, and U.S. regions, yet few quantitative studies clarify how specific predictors drive these differences. Traditional methods often fall short by not addressing both the higher prevalence (exposure) and the increased risk (vulnerability) associated with a predictor. We applied two advanced approaches—the Powers-Yun decomposition technique and our recent PAF decomposition method—to quantify the exposure and vulnerability effects of each predictor using Medicare claims data from a nationally representative sample of U.S. adults aged 70, 75, 80, and 85. The analysis focused on six types of disparities: Black-White, Hispanic-White, Native American-White, Asian-White, Female-Male, and Stroke-Belt vs. non-Stroke-Belt states. Predictors included low-income status (ascertained through Medicare/Medicaid dual eligibility) and ten AD/ADRD-related diseases. We found that low income and vulnerability to arterial hypertension were the primary contributors to AD/ADRD disparities, with cerebrovascular diseases and depression as notable secondary predictors. The exposure effect dominated for income-related disparities, while hypertension's effect was largely driven by increased vulnerability. Racial disparities (Black-White, Hispanic-White) were most affected by low income and hypertension, while Female-Male and Stroke-Belt disparities were less influenced by these predictors. Our findings indicate that different intervention strategies are needed to address AD/ADRD disparities. Low income-related disparities require targeting exposure (e.g., socioeconomic improvements), while hypertension-related disparities suggest a focus on managing vulnerability (e.g., better control of hypertension). The developed methodology offers a robust framework for explaining disparities and designing targeted interventions.

Abstract citation ID: igaf122.2043

### SELECTION BIAS IN ANALYSES OF PHYSICAL AND COGNITIVE FUNCTION: THE STUDY OF WOMEN'S HEALTH ACROSS THE NATION

Lauren MacConnachie[1], Jillian Baker[2], Alexis Reeves[3], Michelle Hood[2], Kelly Ylitalo[4], Aleda Leis[1], Sherri-Ann Burnett-Bowie[5], and Carrie Karvonen-Gutierrez[1], *1. University of Michigan, Ann Arbor, Michigan, United States, 2. University of Michigan School of Public Health, Ann Arbor, Michigan, United States, 3. Stanford Medicine - Stanford University, Stanford, California, United States, 4. Baylor University, Waco, Texas, United States, 5. Mass General Research Institute, Boston, Massachusetts, United States*

The bi-directional relationship between physical and cognitive functioning is well-known, but longitudinal investigations may produce biased estimates due to differential loss-to-follow-up caused by poor health or mortality. We hypothesize that selection bias significantly affects estimates of the association between gait speed and cognitive function in the Study of Women's Health Across the Nation (SWAN). SWAN is a community-based, longitudinal study of 3,302 women. At visit 15, 59.4% (n = 1,345) of participants completed a timed four-meter gait test, East-Boston Memory Test (immediate and delayed), Symbol Digit Modalities Test (SDMT), and Digit Span Backwards test. Linear regressions analyzed the relationship between gait speed and each cognitive test, incorporating inverse probability weighting to correct for selection bias. Models were analyzed with and without weights and stratified by financial strain and education to examine moderation. Weighted models yielded larger effect estimates than unweighted models, especially among disadvantaged women. Among women with a high school education or less, the unweighted model estimated increased gait speed is associated with 17.8 higher SDMT score (β:17.8, 95%CI:11.7, 23.9), while the weighted model estimated the effect as 16.4% larger (β:21.26, 95%CI:15.11, 27.42). Among women with a college degree or more, the weighted effect estimates were only 9.1% larger than unweighted estimates. A similar pattern was observed in models stratified by financial difficulties and for other cognitive outcomes. Selection bias in the SWAN study resulted in underestimation of the association between gait speed and cognitive function, especially among disadvantaged groups. Addressing this bias is essential for accurate estimation of these relationships.

### SESSION 4465 (PAPER)

### UNDERSTANDING ELDER ABUSE: PREVALENCE, DISPARITIES, AND PATHWAYS TO PREVENTION

Abstract citation ID: igaf122.2044

### EVALUATING STRATEGIES USED BY FAMILY CAREGIVERS IN RESPONSE TO DAILY BEHAVIORAL SYMPTOMS OF DEMENTIA.

Wesley Browning, *UTHealth Houston Cizik School of Nursing, Houston, Texas, United States*

Alzheimer's disease and related dementias (ADRD) are marked by behavioral symptoms (BSD) that often complicate their care by family members. These symptoms include care refusals, delusions, mood difficulties, along with inappropriate, compulsive, and safety-related behaviors (i.e., property damage, aggression, and suicidality). These behaviors are frequently associated with caregiving behaviors, including elder mistreatment, and ultimately, worse caregiving outcomes. Potentially inappropriate medication (PIM) use is one form of mistreatment that has been studied in long-term care settings, but has received little attention in family caregiving research. This study (N = 85, observations=2012) evaluated descriptive data from an ongoing study investigating daily caregiving strategies, including pharmacological and nonpharmalogical strategies, to determine what strategies were most commonly used in response to BSDs. Caregivers frequently reported using nonpharmacological strategies (70-91%) to manage BSDs; however, medications were frequently used to stop safety-related (19.9%), aggressive (27.2%), and inappropriate (29.6%) behaviors or make them easier to manage. Caregivers frequently engaged in neglectful behaviors in response to physical aggression by their relative with dementia (32.1%). These findings indicate that while caregivers are aware of nonpharmalogical strategies to manage BSDs, and frequently use them as their first treatment, aggression and inappropriate behaviors may result in a breakdown of these strategies, where caregivers are more likely to revert to using medications as an alternative. These findings suggest emphasis should be placed on educating family caregivers on nonpharmalogical strategies that better manage aggressive behaviors. Work is needed to determine how frequently caregivers provide medication to manage behaviors and what influences their use of PIM.

Abstract citation ID: igaf122.2045

### PREVALENCE OF ELDER ABUSE AND SUB-TYPES IN SOUTH ASIA: A SYSTEMATIC REVIEW AND META-ANALYSIS

Aman Shrestha[1], Saruna Ghimire[2], Krishna Prasad Sapkota[2], Isha Karmacharya[2], Indrajit Ghosh[3], Ahmed Danquah[2], Nicole Shelawala[1], and Emily Gorman[1], *1. University of Maryland, Baltimore County, Baltimore, Maryland, United States, 2. Miami University, Oxford, Ohio, United States, 3. Eli Lilly and Company, Indianapolis, Indiana, United States*

Elder abuse is a critical public health and human rights issue, particularly in South Asia, where patriarchal norms, family-centered caregiving and inadequate institutional support exacerbate the issue. Despite global focus, regional data on elder abuse in South Asian countries are limited. This systematic review and meta-analysis estimated the prevalence of community-based elder abuse and its subtypes among older adults (≥60 years) in South Asia and explored the gender differences in abuse experiences. A comprehensive search of six databases and gray literature identified 33 relevant studies from Bangladesh, India, Nepal, Pakistan, and Sri Lanka. Data were extracted using Covidence, and study quality was assessed with the modified Newcastle-Ottawa Scale. Meta-analyses were conducted using random-effects models, with heterogeneity evaluated via the $I^2$ statistic. Most studies were from India (n = 21) and Nepal (n = 7), with an overall elder abuse prevalence of 31.8%, ranging from 25.8% in India to 49.9% in Nepal. Women experienced higher abuse rates (33.0%) than men (24.3%). Caregiver neglect and psychological abuse (around 20% each) were the most common, disproportionately affecting women. Physical abuse (3.9%), financial exploitation (8.0%), and sexual abuse (0.7%) were less frequent, with women experiencing higher abuse in all categories except sexual abuse. This study underscores the significant prevalence of elder abuse in South Asia, with women being disproportionately affected, emphasizing the urgency for culturally appropriate interventions. Targeted policies and community-driven initiatives are essential to combat elder abuse, enhance eldercare, and safeguard vulnerable populations in the region.

Abstract citation ID: igaf122.2046

### THE MENTAL HEALTH IMPACT OF FINANCIAL MISTREATMENT: THE MEDIATING ROLE OF PERCEIVED STRESS

Juyoung Park[1], and Yuri Jang[2], *1. Emory University, Georgia, United States, 2. University of Southern California, Los Angeles, California, United States*

Financial mistreatment is a public health concern with severe consequences for older adults' mental well-being. While prior research has established a direct link between financial mistreatment and depressive symptoms, the mechanisms underlying this relationship remain underexplored. Guided by the Stress Appraisal Model, this study examined the mediating role of perceived stress in the association between financial mistreatment and depressive symptoms. Using data from the National Social Life, Health, and Aging Project Round 3 (N = 2,409, Mean age = 75.8, SD = 6.94), we conducted multivariate regression and mediation analyses to examine the dynamics among financial mistreatment, perceived stress, and depressive symptoms. Results demonstrated that financial mistreatment (B [SE] = 1.09 [.22], p < .001) and perceived stress (B [SE] = .54 [.04], p < .001) had significant direct effects on depressive symptoms. We also found a significant indirect effect (B [SE] = .20 [.07], bias-corrected 95% bootstrap confidence interval = 0.06, 0.35), where the mental health impact of financial mistreatment was mediated through perceived stress. These findings suggest that financial mistreatment contributes to depressive symptoms both directly and indirectly by intensifying perceived stress levels. As a critical life stressor, the experience of financial mistreatment may elevate perceived stressfulness, which in turn may lead to depressive symptoms. While prevention efforts are essential, our findings highlight the critical need to expand access to stress management resources and services for those financially mistreated.

### SESSION 4470 (PAPER)

### CONTEXTUALIZING COGNITIVE HEALTH: SOCIAL, ENVIRONMENTAL, AND CULTURAL PERSPECTIVES WITHIN AGING POPULATIONS

Abstract citation ID: igaf122.2047

### BETTER PERCEIVED SENIOR CENTER QUALITY WAS ASSOCIATED WITH LESS SUBJECTIVE COGNITIVE DECLINE

Hui "Jimmy" Xie[1], Xiao Zhang[1], Cinthia Camacho[1], Maryna Bankovska[1], Bing Han[2], and Deborah Cohen[2], *1. California State University, Northridge, Northridge, California, United States, 2. Kaiser Permanente Southern California, Pasadena, California, United States*

Senior centers offer various programs and activities that are beneficial to brain health such as exercise classes, arts and craft, and social activities. Despite that, there is a dearth of research on the role of senior center in older adults' cognitive health. Using data collected at two time points, this study examined the association between perceived senior center quality (PSCQ) and older adults' subjective cognitive decline (SCD). PSCQ was measured using an 8-item, 5-point scale adapted from Park Quality Scale, addressing senior center's built/social environment and programming. SCD was measured using the 14-item Cognitive Function Instrument. Control variables included gender, race/ethnicity, age, marital status, education, senior center attendance frequency, and physical and mental health (measured using SF-12 questionnaire). Data were collected in 2023 (PSCQ, demographics, physical/mental health) and 2024 (SCD, attendance frequency) from 152 older adults at 6 senior centers in Los Angeles and neighboring counties. Linear mixed effects model was used to examine the relationship between PSCQ and SCD. Of all the participants (mean age = 74), 78% were female, 39% were married, and 44% had a college or higher degree. As for race/ethnicity, 33% were White, 31% were Asian and 26% were Hispanic. After controlling for demographics, attendance frequency, and physical/mental health, PSCQ was negatively associated with SCD (b=-.89, p<.05). The results highlight the importance of senior center quality to older adults' cognitive health. Future studies should examine potential mechanisms underlying the relationship between PSCQ and cognitive health.

Abstract citation ID: igaf122.2048

### URBAN-RURAL AND GENDER DIFFERENCES IN COGNITIVE IMPAIRMENT AMONG OLDER ADULTS IN INDIA

Ratna Patel[1], Shekhar Chauhan[2], Waad Ali[3], and Boadi Agyekum[4], *1. International Institute for Population Sciences, Mumbai, Maharashtra, India, 2. Florida State University, Tallahassee, Florida, United States, 3. Sultan Qaboos*

*University, Muscat, Masqat, Oman, 4. University of Ghana, Accra, Central, Ghana*

The urban-rural divide in health outcomes has long been a subject of policy discussion, yet cognitive impairment among older adults across urban and rural settings remains underexplored. This study examines urban-rural and gender differences in cognitive impairment among older adults in India, using data from 14,109 individuals aged 60 years and above from the Longitudinal Ageing Study in India (LASI) Wave-1. Logistic regression models with interaction terms were employed to assess how gender (male vs. female) and residence (urban vs. rural) influence cognitive impairment. Additionally, Fairlie decomposition analysis was used to quantify the contribution of key factors to these disparities. Findings reveal that females have a significantly higher likelihood of cognitive impairment than males (OR = 2.63, $p < 0.01$). Urban residence was associated with a lower likelihood of cognitive impairment compared to rural areas (OR = 0.36, $p < 0.01$). Interaction analysis showed that females were 1.3 times more likely to report cognitive impairment than males (OR: 1.33; CI: 1.17–1.51), while urban older adults were significantly less likely to report cognitive impairment than their rural counterparts (OR: 0.77; CI: 0.67–0.88). These findings underscore the need for targeted interventions to address cognitive health disparities by gender and residence. Improving access to mental health care and cognitive health resources in rural areas and among older women could help mitigate these inequalities. This study highlights the importance of tailoring cognitive health policies to the socio-cultural and geographic contexts of aging populations in India.

Abstract citation ID: igaf122.2049

### COGNITIVE IMPAIRMENT, MULTIMORBIDITY, AND MOBILITY LIMITATIONS AMONG MEXICAN-ORIGIN OLDER ADULTS

Nicholas Bishop[1], Rebeca Wong[2], Fernando Riosmena[3], Ana Quiñones[4], and Steven Haas[5], *1. University of Arizona, Tucson, Arizona, United States, 2. The University of Texas Health Science Center at San Antonio, San Antonio, Texas, United States, 3. The University of Texas at San Antonio, San Antonio, Texas, United States, 4. Oregon Health & Science University, Portland, Oregon, United States, 5. The Pennsylvania State University, University Park, Pennsylvania, United States*

Age-related cognitive impairment (CI), multimorbidity (MM; 2+ chronic conditions), and mobility limitations (MLs) are burdensome, develop concurrently, and lead to disablement. Examining disablement processes among Mexican-origin older adults in the US and Mexico is critical given the rapid aging and persistent health challenges faced by these groups, and migration status may modify disablement processes. Using harmonized data from the Health and Retirement Study and Mexican Health and Aging Study (2012 & 2018), we estimated cross-lagged panel models to investigate concurrent change in cognitive function (CF; average proportion of maximum score on study-specific cognitive items, 0–10), MM (count of diagnosed diabetes, hypertension, heart attack, stroke, cancer, arthritis), and MLs (count of 11 functional limitations) among Mexican-origin adults aged ≥ 65. Study/country of residence and migration status were identified (HRS: US-born [n = 215], US-immigrant [n = 173]; MHAS: non-migrant [n = 3,770], and return migrant [n = 542]). Covariates (age, sex, education, marital/partnership status, obesity, rural residence, doctor visits) were harmonized at baseline (2012). Greater baseline MM preceded increasing MLs and faster decline in CF, greater baseline MLs were associated with faster MM accumulation, and greater baseline CF was associated with slower progression of MLs. Compared to US-born respondents, MHAS respondents experienced faster accumulation of MLs and faster decline in CF, but slower MM accumulation. Greater baseline MLs were associated with faster decline in CF among the US-born, but slower decline for the other groups. These findings indicate bidirectional associations between CF, MM, and MLs, and suggest disablement processes are patterned by migration status among Mexican-origin older adults.

Abstract citation ID: igaf122.2050

### IDENTIFYING SELF-REPORTED HUMOR STYLE AS A POTENTIAL NEW DIRECTION IN COGNITIVE HEALTH: A PILOT STUDY

Nikki-Anne Wilson[1], Moyra E. Mortby[2], Justine M. Gatt[3], Fiona Kumfor[4], Jill Bennett[2], Henry Brodaty[2], and Kaarin J. Anstey[2], *1. University of New South Wales Sydney/ Neuroscience Research Australia, Kensington, New South Wales, Australia, 2. University of New South Wales Sydney, Kensington, New South Wales, Australia, 3. Neuroscience Research Australia, Randwick, New South Wales, Australia, 4. The University of Sydney, Camperdown, New South Wales, Australia*

Humor is a central aspect of social communication yet little research has explored how its use in everyday life may relate to cognitive performance in older adults. Building on extant literature investigating humor styles and psychosocial health, we examine the association between positive (affiliative, self-enhancing), and negative (self-defeating, aggressive) humor styles and cognitive function in a cross-sectional pilot study. Ninety-five older adults (90% White; 84% culturally Australian, mean age 76 years) completed the Humor Styles Questionnaire and an online cognitive battery including Paired Associates Learning (PAL), Pattern Recognition Memory (PRM), and Spatial Working Memory (SWM) tasks. Multiple linear regression models controlled for age, gender, culture, education, and depressive symptoms. Higher positive humor was significantly associated with better episodic memory ($\beta = 0.30$, $p = .020$). For negative humor style, a significant gender by negative humor interaction ($p = .013$) showed more negative humor was associated with poorer episodic memory in males ($\beta = -0.43$, $p = .030$) but a trend towards better performance in females ($\beta = 0.23$, $p = .079$). Both males and females showed an association between higher negative humor and better executive function (SWM, $\beta = -2.97$, $p = .014$), however, the significance of this relationship weakened in upper ages (69 years, $\beta = -0.44$, $p \leq .001$; 80 years, $\beta = 0.04$, $p = .795$). We show that self-reported humor style is significantly associated with domain specific cognitive processes. This has important implications for understanding the ways humor use may reflect broader cognitive performance and establishing new directions in cognitive health research.

Abstract citation ID: igaf122.2051

### LIVING WELL WITH COGNITIVE IMPAIRMENT: SOCIAL CONNECTION, WELL-BEING, AND COGNITION IN VERY OLD AGE

Selina Vogel, and Susanne Zank, *University of Cologne, Cologne, Nordrhein-Westfalen, Germany*

Research emphasizes that social relationships protect against cognitive impairment. However, their role for individuals already experiencing cognitive impairment is less understood. For them, social connections may be crucial to maintain well-being. Therefore, the present study examined the associations between cognitive impairment, social relationships, and psychological well-being in very old adults (80+ years), a high-risk group for cognitive decline. We analyzed data from proxy- and target-person interviews of 1.561 participants recruited for the Study on Quality of Life and Well-Being in North-Rhine Westphalia (NRW80+). Participants were classified as having no cognitive impairment (66.80%), mild cognitive impairment (16.00%), or major cognitive impairment (17.20%). We compared the groups on three social factors (close network, social activities, and loneliness) and two well-being measures (depressive symptoms and positive affect). Moreover, we investigated whether cognitive impairment moderated the associations between social factors and well-being. As anticipated, cognitive impairment was associated with lower social outcomes and reduced well-being. Additionally, social factors were associated with higher well-being, and these associations were partly moderated by cognitive status. Notably, the association between social engagement and depressive symptoms was more pronounced for individuals with major cognitive impairment compared to the other groups. The findings underscore the significance of social relationships for psychological well-being, especially in the presence of major cognitive impairment. They also demonstrate the general risk of reduced social and mental health faced by individuals with cognitive impairment. Further research is needed, as the topic is increasingly relevant due to the growing rates of cognitive impairment in our aging society.

### SESSION 4473 (BIOLOGICAL SCIENCES INVITED SYMPOSIUM)

Abstract citation ID: igaf122.2052

### THIS IS THE YEAR THAT WAS: THE BEST OF THE BIOLOGICAL SCIENCES SECTION OF THE JOURNALS OF GERONTOLOGY SERIES A

Chair: Gustavo Duque
Co-Chair: Fabrisia Ambrosio

Following the tradition started in 2023, this symposium will present the best papers published in 2024 in the Biological Sciences section of The Journals of Gerontology, Series A: Biological Sciences and Medical Sciences. This will be a great opportunity for the authors of the top ranked papers to interact with the audience, receive feedback and answer to their questions in an interactive manner.

Abstract citation ID: igaf122.2053

### MURINE MYOBLASTS EXPOSED TO SYUIQ-5 ACQUIRE SENESCENCE PHENOTYPE AND DIFFERENTIATE INTO SARCOPENIC-LIKE MYOTUBES, AN IN VITRO STUDY

Laura Gerosa[1], Giovanni Lombardi[2], Guiseppe Banfi[2], Amir Malvandi[2], Marta Gomarasca[2], Chiara Verdelli[2], Veronica Sansoni[2], and Martina Faraldi[2], *1. IRCCS Ospedale Galeazzi, Milan, Italy, 2. IRCCS Ospedale Galeazzi - Sant'Ambrogio, Milan, Italy*

Aging profoundly affects the musculoskeletal system, where bone and skeletal muscle function as an integrated unit. The accumulation of senescent cells contributes to functional decline in both tissues, but the underlying molecular mechanisms remain poorly understood, partly due to the lack of reliable in vitro models. To address this, we developed a novel in vitro model of muscle cell senescence using mouse C2C12 myoblasts that, when subjected to differentiation, the resulting myotubes showed sarcopenic features. Senescence was induced by SYUIQ-5, a quindoline derivative that inhibits telomerase activity and triggers a senescent phenotype characterized by cell cycle arrest, increased p21 expression, enhanced senescence-associated β-galactosidase (SA-β-gal) activity, and phosphorylation of p53 and histone H2AX. These senescent cultures displayed impaired differentiation capacity, forming myotubes with sarcopenic features including elevated ubiquitin ligases, ATROGIN-1 and MURF1, reduced mitochondrial content, and increased secretion of myostatin, a negative regulator of muscle growth, suggesting that a greater burden of senescent muscle cells could contribute to sarcopenia. This developed model enables the investigation of the dynamic crosstalk between muscle and bone during aging, shedding light on the pathological mechanisms that jointly contribute to the deterioration of both tissues specifically exploring the role of muscle cell senescence. In this framework, we employed untargeted metabolomic and lipidomic profiling to examine metabolic alterations in senescent muscle cells and examined the contribution of the senescence-associated secretory phenotype (SASP) and extracellular vesicles (EVs), and their miRNA content, as mediators of altered inter-tissue communication. Overall, these findings present a robust and well-characterized in vitro model of muscle cell senescence, offering a powerful platform for uncovering the molecular drivers of musculoskeletal aging. Indeed, the first studies conducted using this system are already uncovering potential molecular pathways linking muscle senescence to bone dysfunction, paving the way for the identification of novel biomarkers and therapeutic targets associated with sarcopenia, frailty, and age-related musculoskeletal disorders.

Abstract citation ID: igaf122.2054

### USING DRUGS TO SLOW AGING IN THE NEMATODE CAENORHABDITIS ELEGANS

David Gems, and Aihan Zhang, *University College London, London, United Kingdom*

With its 2-3 week lifespan, C. elegans is a convenient animal model for testing drug effects on aging. We have used liposome-mediated drug delivery to reduce confounding effects of interactions between drugs and the nematodes' bacterial food source, improve compound uptake, and greatly reduce drug quantities needed for trials. We tested six compounds previously reported to extend lifespan: vitamin C, N-acetylcysteine, glutathione (GSH), trimethadione, thioflavin T (ThT), and rapamycin. Life extension was reproduced for the latter four. However, for GSH and ThT, antibiotics abrogated life extension, implying a bacteria-mediated effect; in the latter case, life extension was due to an antibiotic effect of ThT, which protected elderly nematodes against life-limiting infection. For rapamycin, treatment throughout adulthood had a dose-dependent effect, causing a maximal 21.9% increase in mean lifespan, but shortening of lifespan at the highest dose, suggesting drug toxicity. Notably, treatment initiated late in life, from day 16 (or ~70 years in human terms), robustly increased lifespan. The rapalog temsirolimus extended lifespan similarly to rapamycin. As in mouse, rapamycin had mixed effects on age-related pathologies, inhibiting one (uterine tumor growth) but not several others, suggesting a segmental antigeroid effect.

Abstract citation ID: igaf122.2055

### EPIGENETIC AGE ACCELERATION AND CHRONOLOGICAL AGE: ASSOCIATIONS WITH COGNITIVE PERFORMANCE IN DAILY LIFE

Stacey Scott[1], and Daisy Zavala[2], *1. Stony Brook University, Stony Brook, New York, United States, 2. Florida State University College of Medicine, Tallahassee, Florida, United States*

DNA methylation-derived epigenetic clocks offer the opportunity to examine aspects of age acceleration, which differ among individuals and may better account for age-related changes in cognition. Leveraging existing ambulatory cognitive assessments in daily life from a sample of 142 middle-aged adults, we examined associations between measures of epigenetic age acceleration and performance on tasks of processing speed and working memory. Covarying for chronological age, we used multilevel models to examine associations of epigenetic age acceleration with both average level and variability of cognitive performance. Positive age acceleration was associated with poorer mean processing speed and working memory. Greater chronological age was associated with poorer mean processing speed and working memory performance. Further, positive age acceleration was associated with greater intraindividual variability, and greater chronological age was associated with less. Our results suggest that epigenetic age acceleration may account for additional risk and inter-individual variation in cognitive performance above chronological age.

Abstract citation ID: igaf122.2056

### DIFFERENTIAL RESPONSES TO AGING AMONG THE TRANSCRIPTOME AND PROTEOME OF MESENCHYMAL PROGENITOR POPULATIONS

Jack Feehan[1], Nicholas Tripodi[2], Dmitry Kondrikov[3], Tissa Wijeratne[4], Jeffrey Gimble[5], William Hill[3], Vasso Apostolopoulos[6], and Gustavo Duque[7], *1. Royal Melbourne Institute of Technology, Melbourne, Australia, 2. Victoria University, Melbourne, Victoria, Australia, 3. Medical University of South Carolina, Charleston, South Carolina, United States, 4. Western Health, University of Melbourne, Melbourne, Victoria, Australia, 5. Tulane University School of Medicine, New Orleans, Louisiana, United States, 6. RMIT University, Melbourne, Victoria, Australia, 7. McGill University, Montreal, Quebec, Canada*

Cellular senescence, a hallmark of aging, results in a senescence-associated secretory phenotype (SASP) with an increased production of proinflammatory cytokines, growth factors, and proteases. Evidence from nonhuman models demonstrates that SASP contributes to tissue dysfunction and pathological effects of aging. However, there are relatively few human studies on the relationship between SASP and aging-related health outcomes. Proteins from the SASP Atlas were measured in plasma using aptamer-based proteomics (SomaLogic). Regression models were used to identify SASP protein associations with aging-related traits representing multiple aspects of physiology in 1 201 participants from 2 human cohort studies (BLSA/GESTALT and InCHIANTI). Traits examined were fasting glucose, C-reactive protein, interleukin-6, alkaline phosphatase, blood urea nitrogen, albumin, red blood cell distribution width, waist circumference, systolic and diastolic blood pressure, gait speed, and grip strength. Study results were combined with a fixed-effect inverse-variance weighted meta-analysis. In the meta-analysis, 28 of 77 SASP proteins were significantly associated with age. Of the 28 age-associated SASP proteins, 18 were significantly associated with 1 or more clinical traits, and 7 SASP proteins were significantly associated with 3 or more traits. Growth/differentiation factor 15, Insulin-like growth factor-binding protein 2, and Cystatin-C showed significant associations with inflammatory markers and measures of physical function (grip strength or gait speed). These results support the relevance of SASP proteins to human aging, identify specific traits that are potentially affected by SASP, and prioritize specific SASP proteins for their utility as biomarkers of human aging. Several of the identified proteins, specifically of the IGFBP protein family, were also identified as biomarkers of aging in human tissues from healthy aging individuals (as Senescence Network tissue mapping efforts): IBFBP proteins were aging biomarkers in aged human tissues originating from human ovary, spinal cord, and lung.

Abstract citation ID: igaf122.2058

### PROTEOMIC ANALYSIS OF THE SENESCENCE-ASSOCIATED SECRETORY PHENOTYPE: GDF-15, IGFBP-2, AND CYSTATIN-C ARE ASSOCIATED WITH MULTIPLE AGING TRAITS

Birgit Schilling[1], Daniel Evans[2], Danielle Young[3], Mark Watson[1], Toshiko Tanaka[4], Nathan Basisty[5], Stefania Bandinelli[6], and Francesca Duncan[7], *1. Buck Institute for Research on Aging, Novato, California, United States, 2. California Pacific Medical Center Research Institute, San Francisco, California, United States, 3. California Pacific Medical Center, San Francisco, California, United States, 4. National Institute on Aging, Baltimore, Maryland, United States, 5. National Institute on Aging, Bethesda, Maryland, United States, 6. Azienda Sanitaria Firenze, Milan, Italy, 7. Feinberg School of Medicine, Northwestern University, Chicago, Illinois, United States*

Abstract citation ID: igaf122.2059

### THE MEDIATING ROLE OF KYNURENINE PATHWAY METABOLITES ON THE RELATIONSHIP BETWEEN INFLAMMATION AND MUSCLE MASS IN OLDEST–OLD MEN

Megan Hetherington-Rauth[1], Eileen Johnson[2], Eugenia Migliavacca[3], Lisa Langsetmo[4], Russell Hepple[5], Terence Ryan[5], Luigi Ferrucci[6], and Denis Breuillé[7], *1. Champalimaud Foundation, Lisbon, Portugal, 2. California Pacific Medical Center Research Institute, San Francisco, California, United States, 3. Nestlé Research, Lausanne, Vaud, Switzerland, 4. University of Minnesota, Minneapolis, Minnesota, United States, 5. University of Florida, Gainesville, Florida, United States, 6. National Institutes of Health, Gaithersburg, Maryland, United States, 7. Nestlé Research, Lausanne, Switzerland*

Tryptophan (TRP) metabolites along the kynurenine (KYN) pathway (KP) have been found to influence muscle. Proinflammatory cytokines are known to stimulate the degradation of TRP down the KP. Given that both inflammation and KP metabolites have been connected with loss of muscle, we assessed the potential mediating role of KP metabolites on inflammation and muscle mass in older men. Five hundred and five men (85.0 ± 4.2 years) from the Osteoporotic Fractures in Men cohort study with measured D3-creatine dilution (D3Cr) muscle mass, KP metabolites, and inflammation markers (C-reactive protein [CRP], alpha-1-acid glycoprotein [AGP] and a subsample [n=305] with interleukin [IL-6, IL-1β, IL-17A] and tumor necrosis factor-α [TNF-α]) were included in the analysis. KP metabolites and inflammatory markers were measured using liquid chromatography-tandem mass spectrometry and immunoassays, respectively. 23%-92% of the inverse relationship between inflammatory markers and D3Cr muscle mass was mediated by KP metabolites (indirect effect $p < .05$). 3-hydroxyanthranilic acid (3-HAA), quinolinic acid (QA), TRP, xanthurenic acid (XA), KYN/TRP, 3-hydroxykynurenine (3-HK)/3-HAA, QA/3-HAA, and nicotinamide (NAM)/QA mediated the AGP relationship. 3-HAA, QA, KYN/TRP, 3-HK/XA, HKr ratio, 3-HK/3-HAA, QA/3-HAA, and NAM/QA mediated the CRP. KYN/TRP, 3-HK/XA, and NAM/QA explained the relationship for IL-6 and 3-HK/XA and QA/3-HAA for TNF-α. No mediation effect was observed for the other cytokines (indirect effect $p > .05$). KP metabolites, particularly higher ratios of KYN/TRP, 3-HK/XA, 3-HK/3-HAA, QA/3-HAA, and a lower ratio of NAM/QA, mediated the relationship between inflammation and low muscle mass. Our preliminary cross-sectional data suggest that interventions to alter D3Cr muscle mass may focus on KP metabolites rather than inflammation per se.

### SESSION 4475 (SYMPOSIUM)

Abstract citation ID: igaf122.2060

### HARNESSING TECHNOLOGY AND DATA TO UNLOCK ACTIONABLE INSIGHTS IN IMPLEMENTATION RESEARCH - SLEEP RESEARCH EXAMPLES

Chair: A. Lynn Snow
Discussant: Rosa Baier

Effective implementation of cutting-edge technology and data is dependent upon the development of solutions that are responsive to the unique needs of older adults and their environments. This symposium includes five presentations from the world of sleep research reflecting on efforts to develop and refine technology and data solutions for real-world implementation. The first and second presentations describe how interdisciplinary collaborations can result in new ways of bundling and using packages of multiple technologies to solve implementation complexities in the challenging arena of sleep environments. By using iterative pilot testing and multidisciplinary collaboration to inform technological development, the final technologies are much more acceptable and feasible than would be possible without collaborative iterative design. The third presentation reviews commercially available sleep tracking technologies, describes how technologies have evolved over time, and considers their implementation advantages and disadvantages. The final two presentations consider innovative ways of bringing data out of the analyst's office and into the field so it can be dynamically used to inform and shape quality improvement and research in real time. The discussant, Dr. Rosa Baier, will reflect on the implications of these presentations for implementation research and quality improvement practice. Using the complexities of sleep research as a backdrop, this group of presentations offer an opportunity to consider how new insights can be stimulated through iterative field-informed packaging of technologies and data.

Abstract citation ID: igaf122.2061

### DEVELOPING TECHNOLOGY FOR NURSING HOME IMPLEMENTATION: AN ENGINEERING AND SOCIAL SCIENCE COLLABORATION

A. Lynn Snow[1], Todd Freeborn[1], Kimberly Lim[1], Brian Cox[2], Vanessa Aguilar[1], and Hope Carwile[3], *1. The University of Alabama, Tuscaloosa, Alabama, United States, 2. Alabama Research Institute on Aging, Tuscaloosa, Alabama, United States, 3. Vivage, Lakewood, Colorado, United States*

Although temperature, light, and noise are known to be key factors for a good night's sleep, they are difficult to measure in the nursing home. We report on a multidisciplinary collaboration (engineering, psychology, nursing, social work) to develop a flexible, unobtrusive, and affordable a measurement solution feasible for nursing home use. We piloted commercially available instruments that measured volume and light in two nursing homes. We trialed different solutions for powering these devices, downloading the data, and placing the devices in resident rooms and common areas. We concluded that commercially available instruments were too large, noticeable, and expensive. We found that: 1) large noticeable microphones led to privacy concerns even though the devices were only measuring sound levels and were not recording; 2) strategic and customized device placement was key to success – in some resident rooms devices did best when placed above doors, and in other resident rooms the device could be successfully placed on a piece of furniture like a chest of drawers; 3) connecting to nursing home wifi was not typically possible, so data download solutions that relied on removable memory cards worked best. Based on pilot work, the second and third authors developed a small bespoke device on a raspberry pi platform that is wall mountable with heavy-duty Velcro and measures sound, temperature, and light using removable SD memory cards and has both battery and AC power options. The device is being used to supplement wrist actigraph/light meters. We report on nursing home feasibility and acceptability.

Abstract citation ID: igaf122.2062

### EXTRACTING HEART RATE AND TEMPERATURE DATA FROM COMMERCIAL AVAILABLE SLEEP TRACKING DEVICES: PROOF OF CONCEPT PILOT

Ellen McCreedy[1], Ji Won Chung[2], Corinne Roma[2], Ann Reddy[2], and Melissa Simonian[3], *1. Brown University, School of Public Health, Providence, Rhode Island, United States, 2. Brown University, Providence, Rhode Island, United States, 3. Program of All-Inclusive Care for the Elderly, Riverside, Rhode Island, United States*

One of the barriers to using commercially available sleep tracking devices is that each company maintains proprietary sleep algorithms and individual users are unable to download the data that feed those algorithms. Minute-by-minute heart rate and temperature data are important raw data to sleep researchers, as they may be used to help identify people with potential circadian rhythm disruptions and evaluate the effect of promising interventions. In an ongoing pilot, we are using a man-in-the-middle (MiTM) software program to extract user data in real-time from two commercially available sleep tracking devices – a biometric wearable (smartwatch) and a sleep tracking mat. To date, the software has been tested in four community dwelling adults with dementia, each of whom used both devices for three consecutive nights. Caregivers also completed a nightly sleep diary. For the smartwatch, we were able to extract minute-by-minute heart rate and temperature data for three of the four participants. However, despite promising lab tests, the sleep tracking mat failed to connect to our MiTM software in the field. Median differences between caregiver- and device-reported sleep and wake times were similar for both devices. Both devices were feasible and acceptable to all four participants and their caregivers, with a slight caregiver preference for the mat. While data collection is ongoing, we hope to share our MiTM methods and some early lessons learned. Our goal is to increase the transportability and generalizability of research findings across sleep tracking devices, especially given frequent changes in device availability in the marketplace.

Abstract citation ID: igaf122.2063

### FROM DATA TO BETTER CARE: CONSUMER WEARABLES FOR SLEEP AND CIRCADIAN RHYTHM MONITORING IN NURSING HOMES

Kathy Richards[1], Brian Cox[2], Vanessa Aguilar[3], Robert Morgan[4], A. Lynn Snow[3], and Christine Hartmann[5], *1. The University of Texas at Austin, Austin, Texas, United States, 2. Alabama Research Institute on Aging, Tuscaloosa, Alabama, United States, 3. The University of Alabama, Tuscaloosa, Alabama, United States, 4. The University of Texas Health Science Center, Houston, Texas, United States, 5. University of Massachusetts Lowell, Lowell, Massachusetts, United States*

The expanding availability of consumer-grade sleep tracking devices, like smartwatches, fitness bands, and smart rings, has been driven by advancements in wearable technology, miniaturized sensors, and artificial intelligence-driven analytics. This presentation reviews the state of the science for application of wearable technology to measure and monitor sleep and circadian rhythms in nursing home residents and describes how technologies have evolved over time. Key characteristics, including sensors, feedback to consumers, cloud service, raw data access, accuracy, cost, and capability will be discussed in light of the inherent opportunities and challenges for implementation of this technology in both nursing home research and practice. As these technologies become more sophisticated and widely available, they present new possibilities for integrating sleep and circadian rhythm tracking into both research and practice and may significantly improve residents' health and well-being.

Abstract citation ID: igaf122.2064

### DATA WITH DIALOGUE: EMPOWERING NURSING HOME TEAMS THROUGH SMALL DATA REPORTS

Vanessa Aguilar[1], Brian Cox[2], Hope Carwile[3], David Farrell[1], Jennie Keleher[1], Catherine McGuire[4], Angela Westergard[5], and A. Lynn Snow[1], *1. The University of Alabama, Tuscaloosa, Alabama, United States, 2. Alabama Research Institute on Aging, Tuscaloosa, Alabama, United States, 3. Vivage, Lakewood, Colorado, United States, 4. Caraday Health, San Marcos, Texas, United States, 5. National HealthCare Corporation, Murfreesboro, Tennessee, United States*

40Winks, a pragmatic trial aimed at improving nighttime sleep quality and daytime activity among nursing home residents, leverages wearable technology to translate small data into actionable insights. Individual resident data from wrist actigraphy devices is compiled into a care team report carefully designed for easy interpretation by non-researchers. The report visually represents residents' 24-hour sleep, activity, and light exposure across seven days. This helps nursing home staff identify both trouble areas for intervention and bright spots where care is succeeding. The report is specifically designed to be used in whole team discussions that include front-line staff, fostering dialogue and collaboration across role and department silos. The reports aid the whole team in intervention planning to minimize nighttime sleep disruptions (e.g., light or noise) and increase daytime light exposure and activity. By providing an accessible visual and easily understandable and digestible amounts and types of data, the whole team including front-line staff can pinpoint areas needing attention while also recognizing effective care practices. For residents undergoing multiple assessments, these reports provide a concrete way for teams to track progress, test and refine interventions, and sustain successful strategies over time. Training nursing home teams on small data and how to use it for powerful team dialogue enhances the quality of resident care, provides concrete evidence for the effectiveness of trialed interventions, and supports implementation of resident-centered care improvements over time. By integrating small data into daily practice, nursing home teams can drive continuous improvement, leading to sustained enhancements in resident-centered care.

Abstract citation ID: igaf122.2065

### APPLYING ITERATIVE RE-AIM TO TRANSLATE EVALUATION DATA INTO REAL-TIME ADAPTATIONS

Amy Recker[1], Ashley Smith[2], Sophia Geisser[3], Vanessa Aguilar[2], Amanda Piechota[3], Rosa Baier[1], Ellen McCreedy[4], and A. Lynn Snow[2], *1. Brown University School of Public Health, Providence, Rhode Island, United States, 2. The University of Alabama, Tuscaloosa, Alabama, United States, 3. University of Alabama, Tuscaloosa, Alabama, United States, 4. Brown University, School of Public Health, Providence, Rhode Island, United States*

Iterative RE-AIM provides a novel approach to evaluating interventions in real-time and translating those insights into actionable adaptations. Using the principles of iterative RE-AIM, we conducted a midpoint survey with the members of the coaching intervention team and the implementation evaluation team for 40Winks, a nursing home-based sleep trial. We convened a facilitated meeting with these two teams to discuss results and priorities, evaluate existing adaptations, review the effectiveness of the current intervention plan, propose future adaptations, and formulate a plan for implementation. The aggregated survey data were displayed and discussed by the two teams together as part of this process. While iterative RE-AIM outlines a process for evaluating an intervention's adherence to the RE-AIM model, we applied this framework to evaluate the intervention's components rather than the RE-AIM dimensions. This modification allowed us to bring all levels of the intervention and evaluation teams together to have a combined voice in the direction and evaluation of the intervention, and to make critical, real-time adaptations to enhance the effectiveness and reach of the intervention. This presentation will provide a roadmap of the process our team developed, guided by the principles of iterative RE-AIM, to display the evaluation survey and data and discuss strategies for implementing the results of this survey to lead real-time adaptations. This novel methodology allows the teams to learn from and act upon their own data in real time, allowing for iterative and systematic improvements in an intervention as a trial progresses.

### SESSION 4480 (SYMPOSIUM)

Abstract citation ID: igaf122.2066

### BALANCING AUTONOMY AND SAFETY: DEVELOPING AND PILOT TESTING DIGNITY PROGRAM IN RURAL NURSING HOMES

Chair: Liza Behrens
Co-Chair: Marie Boltz

Person-centered care that honors individual preferences enhance the health and well-being of nursing home (NH) residents living with Alzheimer's disease and related dementias (ADRD). However, preferences such as going outside independently are often seen as "risky" by NH staff and subsequently restricted. There is an urgent need for shared decision-making strategies to help NH staff balance perceived health and safety risks with the residents' right to make autonomous choices, particularly in under-resourced rural NH communities. This symposium will present the preliminary results of a multi-site cluster randomized controlled pilot study to co-design with key stakeholders

(Stage 1a), implement, and assess (Stage 1b) the acceptability and feasibility of an innovative risk management program: DIGNITY (Decision making in aging and dementia for autonomy). The first presentation will describe the early development of DIGNITY intervention, establishing the rationale and theoretical underpinnings of the staff-focused behavioral intervention. The second presentation will highlight the program manual and tool development within formal consultation with a community advisory board from the perspective of an attending physician. The third presentation will present results from community engagement studios with rural NH stakeholders from the perspective of a NH leader. The fourth presentation will share a case study implementing the DIGNITY process in a risky preference situation commonly encountered in all NH communities. Finally, the session will conclude with a fifth presentation reporting acceptability, feasibility, and preliminary efficacy of implementing DIGNITY within four rural NH communities.

Abstract citation ID: igaf122.2067

### INITIAL DEVELOPMENT OF THE DIGNITY PROGRAM: BALANCING AUTONOMY AND SAFETY IN DEMENTIA CARE

Liza Behrens[1], Kimberly Van Haitsma[1], Jennifer Kraschnewski[2], and Marie Boltz[1], *1. The Pennsylvania State University, University Park, Pennsylvania, United States, 2. Penn State College of Medicine, Hershey, Pennsylvania, United States*

In the name of safety, nursing Home (NH) residents living with dementia often have their preferences for care and daily activities disregarded by NH staff. This impinges on residents' rights to exercise autonomy in clinical decision-making and leads to dissatisfaction with care comprising quality of life and well-being. No comprehensive evidence-based risk management strategies exist to assist NH staff to identify and support care choices of residents while attempting to balance autonomy with safety. Thus, the purpose of this project was to use community engagement intervention mapping techniques to develop a multilevel intervention designed to support shared decision-making with NH residents living with dementia thus promoting resident health and autonomy. This presentation will describe the early planning phase of intervention mapping including the construction of a logic model of the problem, a review of literature identifying behavioral, normative, and control beliefs associated with NH staff intentions to honor residents' preferences, and a set of training goals and objectives. This project resulted in a multi-component intervention that guides shared decision-making in NHs to support person-centered dementia care called DIGNITY (Decision-making in aging and dementia for autonomy). It includes an adapted care planning protocol and staff training to help direct-care NH staff negotiate intrinsic and cultural factors in preference situations that carry a risk to residents' health and safety. Initial development of this intervention sets the stage for initial program manual and tool development necessary for pilot testing the intervention in the future.

Abstract citation ID: igaf122.2068

### DIGNITY: INITIAL MANUAL AND TOOL DEVELOPMENT WITH COMMUNITY ADVISORY BOARD

Nicole Osevala[1], Liza Behrens[2], Susan Ryan[3], Jennifer Kraschnewski[4], Kimberly Van Haitsma[2], and Marie Boltz[2], *1. Penn State Health, Hummelstown, Pennsylvania, United States, 2. The Pennsylvania State University, University Park, Pennsylvania, United States, 3. Center for Innovation, Linthicum, Maryland, United States, 4. Penn State College of Medicine, Hershey, Pennsylvania, United States*

Successful translation of scientific discoveries into health improvements requires community involvement in all research stages. Recruiting nursing home (NH) stakeholders has been challenging due to systemic issues highlighted during the COVID-19 pandemic. This project leverages existing NH community stakeholders to co-develop the DIGNITY program for future testing. A community advisory board (CAB) of NH stakeholders was established using purposive sampling. Semi-structured sequential focus groups collected stakeholder opinions on the development, refinement, implementation, and evaluation of the DIGNITY program for rural NH environments. Stakeholders completed a demographic survey and reviewed the drafted DIGNITY program intervention manual. Fourteen NH stakeholders, including academic and clinical experts (n = 5), NH administrators/leaders (n = 3), direct care workers (n = 2), regulators (n = 1), and ombudsman (n = 3), participated in four focus group sessions between October and December 2022. Qualitative content analysis of transcript data indicated that stakeholders found the DIGNITY program relevant and feasible for assisting rural NH staff in honoring residents' risky preferences for care and activities. Stakeholders identified programmatic areas for modification within six themes: (1) DIGNITY manual formatting, (2) communication, (3) expanding staff roles, (4) addressing residents' decision-making capacity, (5) potential barriers to implementation, and (6) potential facilitators to implementation. Discussions with stakeholders indicated a need to involve other key stakeholders in rural areas, including residents, family members, direct-care staff, and regulators, in the initial development of DIGNITY. Presented by a participating physician clinician, the discussion will provide a pragmatic participatory lens for improvements made to the DIGNITY program.

Abstract citation ID: igaf122.2069

### DIGNITY: FEEDBACK FROM RURAL NURSING HOME STAKEHOLDERS VIA COMMUNITY ENGAGEMENT STUDIOS

Susan Ryan[1], Liza Behrens[2], Kimberly Van Haitsma[2], Jennifer Kraschnewski[3], and Marie Boltz[2], *1. Center for Innovation, Linthicum, Maryland, United States, 2. The Pennsylvania State University, University Park, Pennsylvania, United States, 3. Penn State College of Medicine, Hershey, Pennsylvania, United States*

Prior intervention development work identified the need to involve rural nursing home (NH) stakeholders in the initial development of DIGNITY. To meet this need, Community Engagement (CE) Studios were held to obtain feedback on study design and procedures (intervention protocol, program materials, implementation and evaluation plan). Four rounds of CE Studios were conducted with self-identified NH leaders, staff, regulators, and resident advocates (n = 2), and rural NH community staff, residents with dementia, and family members (n = 2). All CE studios were held in person and followed institutional guidelines. Each CE studio followed a similar format including: a welcome and ice breaker exercise, an introduction to the DIGNITY program, a risky preference card sort activity, and a detailed review of relevant DIGNITY program manual procedures and tools. Audio recordings and written field notes recorded participant responses. Rapid content analysis provided themes for reporting results in a standardized feedback loop. In total, 39 Community Experts participated in CE studios between July 2022 and August 2024. The Experts represented NH leadership (n = 3), nursing staff (n = 10), social work (n = 1), activities staff (n = 1), residents with dementia (n = 4), family members (n = 4), surveyors

(n=15), and a federal regulator (n=1). Just under half of the participants were working/living in a rural NH community (n=16). Experts primarily suggested changes to program materials and procedures, including the DIGNITY Practice Model, Personal Risk Portfolio, Risk and Impact Worksheet, and Decision-Making Capacity Assessment Tool. Experts were also concerned about program costs. Future work needs to implement all expert suggestions into the intervention manual.

Abstract citation ID: igaf122.2070

**DIGNITY: CASE STUDY OF PERSON-CENTERED RISK MANAGEMENT IN DEMENTIA CARE**

Caroline Madrigal[1], Joanne Roman Jones[2], Mary Lou Ciolfi[3], Karolus Wangi[4], Susan Ryan[5], Nicole Osevala[6], and Liza Behrens[7], *1. VA Boston Healthcare System, Boston, Massachusetts, United States, 2. University of Massachusetts Boston, Boston, Massachusetts, United States, 3. UMaine Center on Aging, Orono, Maine, United States, 4. The Pennsylvania State University, University Park, Pennsylvania, United States, 5. Center for Innovation, Linthicum, Maryland, United States, 6. Penn State Health, Hummelstown, Pennsylvania, United States, 7. Pennsylvania State University, UNIVERSITY PARK, Pennsylvania, United States*

While developing the DIGNITY Pilot program, nursing home regulators, leaders, and staff all expressed difficulty with balancing resident autonomy with safety for intimacy preferences of residents living with dementia. Intimacy preferences are challenging to honor in nursing homes due to staff comfort levels with residents' ability to make autonomous choices, societal stigma surrounding sexuality in older adults, and restrictive policies. The DIGNITY program empowers nursing home staff to collaborate with residents, their families, and other interdisciplinary team members to identify and mitigate risks to the health and safety of residents. This presentation will provide a case exemplar to demonstrate the use of the DIGNITY program for a resident living with dementia who is perceived by staff to be making a risky choice to co-sleep with another resident living with dementia. The scenario will employ the six components of the DIGNITY program, highlighting areas of concern for all involved, namely the resident's capacity to make informed choices. We will explore the resident's preferences, values, and capacity for decision-making, utilizing DIGNITY assessment tools and care planning process; and discuss the importance of assessing a resident's capacity to make informed choices, focusing on their ability to understand, appreciate, and reason about their preferences, distinct from the legal determination of competency. We will discuss potential practice and legal implications for nursing home staff to adopt and implement the DIGNITY process.

Abstract citation ID: igaf122.2071

**DEMONSTRATING FEASIBILITY OF THE DIGNITY INTERVENTION FOR DELIVERY IN RURAL NURSING HOME COMMUNITIES**

Erin Kitt-Lewis[1], Liza Behrens[1], Kalei Kowalchick, Caroline Madrigal[2], Terrence Murphy[1], Jennifer Kraschnewski, Kimberly Van Haitsma[1], and Marie Boltz[1], *1. The Pennsylvania State University, University Park, Pennsylvania, United States, 2. VA Boston Healthcare System, Boston, Massachusetts, United States*

The DIGNITY intervention is a newly co-developed risk management strategy aimed at reducing barriers and enhancing staff engagement in risk mitigation to support residents' care and activity preferences. This comprehensive staff-focused behavioral intervention remains untested. Therefore, a pilot test was conducted to evaluate its acceptability, appropriateness, feasibility, and preliminary efficacy among direct-care nursing home (NH) staff members. A 12-week, two-arm cluster randomized controlled trial was conducted in four rural Pennsylvania NHs. Staff (N=54) and residents (N=57) were recruited. All staff participated in a 60-minute introductory session and received a training manual. They were asked to refer to the manual during routine care planning for enrolled residents and while participating in six bi-weekly web-based coaching sessions. Staff rated their intention to honor residents' risky preferences, as well as the acceptability, feasibility, and appropriateness of the intervention through surveys with closed and open-ended questions. Structured interviews collected residents' satisfaction with preference fulfillment. Staff had an 83% retention rate and an 80% survey response rate. In the DIGNITY group, average scores for acceptability (4.19), appropriateness (4.24), and feasibility (3.99) exceeded benchmarks with supporting qualitative remarks, indicating high feasibility, acceptability, and appropriateness. Positive trends were seen in staff intent to honor residents' preferences. A t-test revealed a statistically significant difference in resident satisfaction between study arms ($p = .04$). The DIGNITY intervention is feasible and shows promise in supporting NH staff engagement in risk mitigation for person-centered dementia care. Future research should assess DIGNITY's efficacy across a larger sample of NHs.

### SESSION 4485 (SYMPOSIUM)

Abstract citation ID: igaf122.2072

**DEMENTIA CARE AND WELL-BEING: DYADIC AND RELATIONAL CONSIDERATIONS**

Chair: Hanamori Skoblow
Co-Chair: Megan Gilligan
Discussant: Christine Proulx

Caregiving in the context of dementia exerts profound impacts on personal well-being, for both the person living with dementia and their care partners. Dementia care is inherently relational as care is often provided by a variety of care partners, most of whom are unpaid family members and nonrelatives. The well-being of people with dementia and their care partners are also interconnected, emphasizing the need to understand the broader social context in which caregiving takes place. This symposium brings together four studies that explore how dementia care shapes well-being among family caregivers and people living with dementia. This research highlights a diverse range of family relationships and relational processes, including siblings navigating parent care together, couples managing early-stage Alzheimer's disease, adult daughters caring for a parent with dementia, and caregivers raising children while caring for an older adult. Dr. Skoblow will examine the implications of discrepancies in siblings' views of their parents' dementia symptoms on caregiver burden. Dr. Bonds Johnson will investigate life course factors that are associated with quality of life in both members of African American parent-adult daughter dementia dyads. Ms. Lee will consider how dementia knowledge, empathy, and marital quality relate to incongruence in romantic partners' perceptions of care needs. Dr. Nemmers will look at psychological well-being in sandwiched caregivers and the older adults receiving care, highlighting differences by dementia status. The discussant, Dr. Proulx, will contextualize these findings within the broader literature on family caregiving, cognitive impairment, and dyadic research.

Abstract citation ID: igaf122.2073

### (DIS)AGREEMENT IN SIBLINGS' PERCEPTIONS OF PARENTS' DEMENTIA SYMPTOMS: IMPACT ON CAREGIVER BURDEN

Hanamori Skoblow[1], Megan Gilligan[1], Destiny Ogle[2], and Audrey Deyoung[1], *1. University of Missouri, Columbia, Missouri, United States, 2. Purdue University, West Lafayette, Indiana, United States*

More severe behavioral and psychological symptoms of dementia (BPSD) are associated with higher caregiver burden. Typically, BPSD is measured using reports from one caregiver although disagreements between multiple caregivers may exacerbate the challenges of care. However, the consequences of caregivers' disagreements regarding BPSD symptoms remain unknown. Therefore, we examined whether adult child caregivers' (dis)agreements on total BPSD symptoms and subscales for depression, disturbance, and memory were associated with sibling reports of caregiver burden. We used data from 91 sibling dyads (mean age = 57.30, SD = 9.32) who were caring for a parent with dementia (mean age = 82.30, SD = 7.74). Actor-partner interdependence models revealed that sibling dyads who reported minor disagreements (within 1 SD) regarding their parent's total BPSD symptoms reported higher caregiver burden compared to siblings who were in agreement about their parent's symptoms (b = 2.67). This effect was primarily driven by disagreements on the parent's depressive symptoms, where major disagreements (>1 SD) were associated with heightened caregiver burden (b = 1.81), relative to siblings who agreed on their parent's symptoms. In contrast, disagreements on the parents' disturbance and memory symptoms were not linked to sibling caregiver burden. These findings suggest that when siblings' perceptions of their parent's depressive symptoms are aligned, they report lower caregiver burden. This research has implications for health and long-term care providers, whose contact regarding the parent's symptoms may be limited to only one caregiver. Ensuring consistency and alignment across the care network may help promote better caregiver well-being.

Abstract citation ID: igaf122.2074

### LIFECOURSE FACTORS INFLUENCING THE QUALITY OF LIFE OF AFRICAN AMERICAN PARENT-ADULT DAUGHTER DEMENTIA DYADS

Kalisha Bonds Johnson[1], Kenneth Hepburn[1], Wizdom Powell[2], and Karen Lyons[3], *1. Emory University, Atlanta, Georgia, United States, 2. University of Connecticut School of Medicine, Farmington, Connecticut, United States, 3. Boston College, Chestnut Hill, Massachusetts, United States*

African American adult daughters are the most common care partners of African American persons living with dementia. Yet little is known about how these African American parent-adult daughter dementia dyads (i.e., parent living with dementia and their adult daughter care partner) navigate this disease process or how their quality of life (QOL) is influenced. Guided by the NIA Health Disparities Research Framework, Black Family Social-Ecological Context Model, and Superwoman Schema, this presentation identifies lifecourse factors (e.g., behavioral, sociocultural, and environmental) significantly associated with the quality of life of these dyads. In this sample of 39 African American parent-adult daughter dementia dyads, data were analyzed using multilevel modeling to control for the interdependence of the data. On average, the parents' QOL did not significantly differ from the adult daughters' QOL. Parents experienced significantly better QOL when the adult daughters reported a more positive family decision-making process but experienced significantly worse QOL when the adult daughters reported greater cultural justification for caregiving. Adult daughters experienced significantly better QOL when they reported greater family social support and had a more positive family decision-making process. However, adult daughters reported significantly worse QOL when their parents had appointment with healthcare professionals within 30 days of the study visit compared to those parents who did not. Findings suggest taking a lifecourse perspective may hold promise in improving the QOL of African American parent-adult daughter dementia dyads. Findings will be discussed considering this first phase of an explanatory sequential mixed methods intervention design.

Abstract citation ID: igaf122.2075

### SPOUSAL DISCREPANCY IN PERCEIVED IMPORTANCE OF CARE NEEDS AMONG COUPLES MANAGING EARLY-STAGE ALZHEIMER'S DISEASE

Eunju Lee[1], Kyungmin Kim[1], and Meng Huo[2], *1. Seoul National University, Seoul, Republic of Korea, 2. University of California, Davis, Davis, California, United States*

Understanding the care needs of people with Alzheimer's disease (AD) is important for caregivers to make informed care decisions and prevent unmet needs of people living with AD (PLWD). Existing interventions have targeted promoting dementia knowledge in caregivers to help them better acknowledge PLWD's common needs. Yet, caregivers' empathy, their ability to understand and share PLWD's emotions may be central as they assess their PLWD's unique needs. This study examined discrepancies between PLWD and their spousal caregivers in perceived importance of PLWD's care needs and how these discrepancies were associated with caregivers' dementia knowledge and empathy (i.e., empathic concern, perspective taking, and personal distress). We also examined whether couple relationship quality moderated these associations. Data was from 65 couples coping with early-stage AD. PLWD self-reported the importance of physical, psychological, and social needs, and caregivers provided proxy reports on the perceived importance for PLWD. PLWD perceived addressing physical needs as more important than their caregivers, while the two parties showed agreements about perceived importance of PLWD's psychological and social needs. Couples where caregivers reported greater empathic concern showed smaller discrepancies in importance of physical needs. Caregivers' personal distress was negatively associated with the discrepancy in social needs. Caregivers' dementia knowledge was not significantly associated with the discrepancy in importance across three types of needs, but dementia knowledge was positively associated with the discrepancy in perceived importance of psychological needs when relationship quality was low. The findings highlight the importance of caregivers' emotional responsiveness and spousal dynamics in meeting PLWD's needs.

Abstract citation ID: igaf122.2076

### DEMENTIA AND SANDWICHED CAREGIVING: EFFECTS ON CAREGIVER AND OLDER ADULT PSYCHOLOGICAL WELL-BEING

Natasha Nemmers[1], Sarah Patterson[2], Yuchen Zhang[3], and Virginia Gallagher[4], *1. Wayne State University, Detroit, Michigan, United States, 2. University of Michigan, Ann Arbor, Michigan, United States, 3. University of Pittsburgh School of Nursing, Pittsburgh, Pennsylvania, United States, 4. University of Virginia, Charlottesville, Virginia, United States*

The population of sandwiched caregivers—those supporting both an older adult and a minor child—is increasing yet understudied particularly regarding dementia care. Existing research on this group often focuses on adult children, neglecting other caregivers (e.g., neighbors or grandchildren). This study examines psychological well-being in sandwiched versus non-sandwiched caregivers and their older adult care recipients by dementia status [person living with dementia (PLWD) versus non-PLWD]. Data from 1,514 caregivers and 1,109 older adults were analyzed using the 2022 National Health and Aging Trends Study and National Study of Caregiving. Among caregivers, 253 had a child under 18: 99 cared for PLWD and 154 for non-PLWD. Non-sandwiched caregivers (n=1,261) included 524 caring for PLWD and 737 non-PLWD. Multivariate linear regression models assessed both caregiver and older adult well-being, adjusting for demographic and care-related covariates. Models were weighted and adjusted for NHATS/NSOC's complex survey design. For caregiver well-being, sandwiched caregivers had significantly higher well-being ($B=1.85$, $p<.01$) relative to non-sandwiched. However, care-recipient dementia status was not significantly associated with caregiver well-being. The interaction between dementia status and being sandwiched was significant ($B=-2.18$, $p<.01$), indicating a reduced well-being advantage for sandwiched caregivers of PLWD. Among older adults, PLWD had significantly lower well-being than non-PLWD ($B=-7.36$, $p<.001$). These findings suggest that caregivers with children may experience protective benefits if their older adult care-recipient does not have dementia, whereas dementia negatively impacts well-being among both members of the caregiving dyad. Future research should focus on tailored strategies to improve dyadic well-being in these complex caregiving situations.

### SESSION 4490 (SYMPOSIUM)

Abstract citation ID: igaf122.2077

### HEALTH AND WELL-BEING TRAJECTORIES IN DIVERSE SOCIAL CONTEXTS

Chair: Laura Buchinger
Co-Chair: Denis Gerstorf
Discussant: Toni Antonucci

This symposium aims to bring together a collection of papers that consider both social presence and absence, as well as different types of social ties, to provide a nuanced perspective on the role of interpersonal connections for late-life health and well-being. Rauer and colleagues challenge singlehood stereotypes by examining how desire for a romantic partner, gender, and age shape satisfaction with being single. Lewis and colleagues examined the role of co-exercise in enhancing the mood benefits of physical activity. They found that exercising with a close social partner regardless of relationship type amplified the positive effects of physical activity on mood. Birditt and colleagues examined caregiving experiences across relationship types, age groups, and dementia caregiving status, finding that younger spousal caregivers for individuals with dementia experience the highest strain. Buchinger and colleagues analyzed the longitudinal links between physical health constraints and perceived stress, finding that maintaining strong social ties can mitigate stress associated with frailty and functional decline. The studies employ rigorous methodologies, leveraging large-scale nationally representative data, advanced statistical modeling, daily life ambulatory assessments, and the integration of self-reported and objective measures. They examine multiple dimensions of health and well-being, including subjective well-being, physician-assessed physical health, and health behavior. Together, these studies underscore the critical role of social contexts in shaping psychological and physical health across adulthood and late life. Discussant Toni Antonucci will provide an integrative perspective on the broader implications for social and aging research.

Abstract citation ID: igaf122.2078

### SOCIAL INTEGRATION MODERATES LONGITUDINAL LINKS BETWEEN HEALTH CONSTRAINTS AND PERCEIVED STRESS IN OLDER ADULTS

Laura Buchinger[1], Johanna Drewelies[2], Valentin Vetter[3], Ilja Demuth[3], and Denis Gerstorf[4], *1. Humboldt-Universität zu Berlin, Berlin, Berlin, Germany, 2. Max Planck Institute for Human Development, Berlin, Berlin, Germany, 3. Charité - Universitätsmedizin Berlin, Berlin, Berlin, Germany, 4. Humboldt University Berlin, Humboldt University Berlin, Berlin, Germany*

Stress is a normative experience that likely imposes considerable individual and societal costs due to its strong links with accelerated aging, early retirement, and numerous health conditions. As life expectancy increases, more individuals reach advanced ages that are often characterized by declining physical abilities and social resources. These age-related changes can in turn heighten stress vulnerability, making effective coping more and more difficult. In this preregistered study, we applied longitudinal multilevel and bivariate growth curve models to investigate associations of trajectories of four (performance-based) measures of physical health (morbidity, frailty, grip strength, and the timed-up-and-go (TUG) test) with trajectories of perceived stress. We examined social integration as a potential moderator. Longitudinal data from the Berlin Aging Study II ($N=1,598$, age range: 60–94) span 11 years, with perceived stress assessed ten times and physical health assessed three times during this period. Findings indicate statistically significant between-person and within-person associations. For example, steeper increases in frailty and more pronounced declines in the TUG test are each associated with steeper increases in perceived stress. Results also revealed that social integration moderated within-person effects of frailty and TUG performance on perceived stress, thereby alleviating the negative impact of health constraints on stress. Our findings underscore the interplay between physical health and stress in old age, highlight the protective role of social integration, and emphasize the need for interventions that enhance social resources to support well-being in later life.

Abstract citation ID: igaf122.2079

### THE COMPLEXITY OF SINGLEHOOD: DESIRE FOR A PARTNER, GENDER, AND AGE DIFFERENCES

Amy Rauer[1], Nadja Kelle[2], and Oliver Huxhold[2], *1. University of Tennessee, Knoxville, Tennessee, United States, 2. German Centre of Gerontology, Berlin, Berlin, Germany*

Singlehood is often framed through societal stereotypes that portray singles as less happy or living a less fulfilled life than individuals in romantic relationships. However, this paper challenges such narratives by examining the diverse experiences of singlehood and the factors influencing satisfaction with being single. Using autoregressive models on longitudinal data from the German Aging Survey—a representative study of the German population aged 40 to 85—this study explores the role of the desire for a romantic partner, alongside gender and age, in shaping satisfaction with singlehood among the currently unpartnered. The findings reveal a significant negative association between the desire for a partner and satisfaction with singlehood across midlife and late adulthood. Furthermore, women and older individuals were less likely to express a desire for a partner than men and younger individuals. Interaction effects for gender and age did not moderate the association between desire for a partner and satisfaction with singlehood, suggesting a stable influence across demographics. Further analyses will incorporate relevant social factors, such as levels of social support and experiences of social exclusion, to investigate their role in satisfaction with singlehood. This study highlights the complexity of singlehood, showing that it is perceived negatively only when individuals desire a romantic partner—a desire less common among women and older individuals. This variation in partner-seeking helps explain differences in satisfaction with singlehood. The findings emphasize the need to challenge societal perceptions, advocating for a more nuanced and positive understanding of single life.

Abstract citation ID: igaf122.2080

### CO-EXERCISE AND MOOD: THE ROLE OF RELATIONSHIP TYPE IN THE LINK BETWEEN PHYSICAL ACTIVITY AND POSITIVE AFFECT

Nathan Lewis[1], Yoonseok Choi[2], Maureen Ashe[1], Ken Madden[1], Rachel Murphy[1], Wolfgang Linden, [1]Denis Gerstorf[3], and Christiane Hoppmann, [4], *1. The University of British Columbia, Vancouver, British Columbia, Canada, 2. Simon Fraser University, Vancouver, British Columbia, Canada, 3. Humboldt University Berlin, Humboldt University Berlin, Berlin, Germany, 4. UBC, Vancouver, British Columbia, Canada*

Physical activity is known for its positive health benefits, including improvements in mood. While research supports the association between physical activity and elevated positive affect, the possibly unique role of exercising with close social connections (i.e., co-exercise), such as spouses, friends, and family, in enhancing these mood benefits remains less understood. This study used ambulatory assessment data from 140 dyads, each consisting of an older adult (60+) and a close social connection (56.6% spouses, 30.2% friends or family members; aged 21-95 years, M = 68.59 years, SD = 10.56). Participants completed a 10-day assessment period with morning and evening surveys on affect (0-100), along with self-reported and accelerometer-measured physical activity. Multilevel models examined the relationship between physical activity and end-of-day positive affect and whether these associations varied by relationship type. A moderation analysis assessed whether the effect of co-exercise on mood differed by relationship type (spouse/partner vs. friend/family). Participants reported engaging in physical activity with their study partner on 35% of days. Higher self-reported physical activity was associated with higher positive affect ($b = 0.13$, $SE = 0.01$, $p < .001$), and this effect was stronger on days when participants exercised with their study partner ($b = 1.44$, $SE = 0.69$, $p = .039$). However, the effect of co-exercise on positive affect did not differ by relationship type. Future analyses will explore whether a similar pattern is seen with accelerometer-measured physical activity. These findings suggest that co-exercise may enhance the mood benefits of physical activity, regardless of relationship type, and may be valuable in interventions supporting the physical and mental health of older adults.

Abstract citation ID: igaf122.2081

### DIVERSITY OF CAREGIVING EXPERIENCES IN THE UNITED STATES: FINDINGS FROM THE NATIONAL UAS-CLEAR STUDY

Kira Birditt[1], Marco Angrisani[2], Angela Turkelson[3], Angelica Eagle[1], Crystal Ng[1], and Joan Monin[4], *1. University of Michigan, Ann Arbor, Michigan, United States, 2. University of Southern California, Los Angeles, California, United States, 3. University of Michigan Institute for Social Research, Ann Arbor, Michigan, United States, 4. Yale, Yale, Connecticut, United States*

Given the rising number of older adults with disability and dementia, unpaid caregivers are increasingly vital in the long-term care of older adults. Caregiving is often provided by multiple types of social ties (e.g., family, and non-family); yet, most research focuses on spouse and adult child caregivers and lacks information regarding caregivers across the adult lifespan who may have unique caregiving experiences. The purpose of this study is twofold: 1) describe the frequency of caregivers by relationship type (care for spouse, parent, other family, non family), dementia status, and age of caregiver, and 2) examine whether there are differences in their reports of caregiving intensity (i.e., number of caregiving tasks), caregiver burden, and rewards. We used data from UAS-CLEAR; a national study of caregivers in the U.S. (N = 2202; 67% women; 56% white; aged 18 to 102; M = 50). Caregivers were most likely to care for other family, followed by parent, spouse, and non-family. A total of 22% were caring for an individual with dementia. Non-family caregivers reported the least burden and lower caregiving intensity, whereas spouse caregivers reported the greatest burden and caregiving intensity. Older caregivers reported less burden and less caregiving intensity than younger caregivers. Individuals who cared for individuals with dementia reported greater burden and caregiving intensity, and fewer rewards than those caregiving for individuals without dementia. In sum, caregiving is not universally burdensome and younger spousal caregivers for those living with dementia may be at greatest risk.

### SESSION 4495 (SYMPOSIUM)

Abstract citation ID: igaf122.2082

### BIOMARKER INSIGHTS INTO BRAIN HEALTH, AGING, AND HEALTHSPAN

Chair: Breno Diniz
Co-Chair: Qu (Teresa) Tian

There has been a surge in the novel biomarker approach to identify early predictors of diseases, understand multiple disease etiology, and, more importantly, the effect of biological aging on health and disease dynamics. In this symposium, we will present novel biomarker data to reveal novel pathways related to brain health, dementia, and biomarker signatures of longevity and healthspan. This symposium features 5 presentations of recently conducted studies using biomarker data from US and European cohorts. First, Dr. Diniz will present recent results suggesting that a history of major depression is causally associated with biological aging acceleration using Mendelian randomization approaches. Second, Dr. Tian will present data about developing a novel biomarker composite score based on eye-movement measures and its association with age-related cognitive and mobility decline. Third, Dr. Evans uses data from the Longevity Consortium to examine metabolomic signatures of human aging and longevity. Firth, Dr. Liu shows a novel protein-based signature predictive of cumulative frailty index, the proteomic frailty index (pFI), and examines the association of pFI with multiple health indicators in three independent cohorts, including physical, clinical and cognitive measures, dementia, and mortality. Finally, Dr. Kuo will show the development of a healthspan proteomic score (HPS) to identify proteomic signatures of healthspan and its association with mortality and multiple age-related adverse health outcomes. Based on the symposium presentations, we seek to generate discussions of how biomarkers can aid the understanding of novel risk factors for accelerated biological aging, dementia risk, and healthspan.

Abstract citation ID: igaf122.2083

### CAUSAL ASSOCIATIONS BETWEEN MAJOR DEPRESSION AND ACCELERATED BIOLOGICAL AGING.

Breno Diniz[1], Shangshu Zhao[1], Richard Fortinsky[2], George Kuchel[1], and Chia-Ling Kuo[1], *1. UConn Health, Farmington, Connecticut, United States, 2. University of Connecticut, Farmington, Connecticut, United States*

Over the past decades, there is growing evidence from clinical and epidemiological studies that history of major depression is associated with adverse health outcomes, including higher incidence of Alzheimer's disease, cardiovascular diseases, and mortality. The mechanisms for such associations are unclear, but points to the fact that major depression is associated with abnormalities in multiple hallmarks of biological aging. Therefore, this analysis aimed to investigate if major depression was causally linked with accelerated biological aging, based on proteomic measures. We carried out a bidirectional Mendelian randomization analysis to evaluate the causal relationships between these two phenomena. We focused on 3 proteomic measures of biological aging acceleration: The Proteomic Aging Clock (PAC), the Healthspan Proteomic Score (HPS), and the Brain-specific Proteomic Aging (BsPA). We found that MDD was causally associated with biological aging acceleration (Major Depression Biological Aging) for all 3 measures (PAC: MR-IVW $\beta = 0.07$, $p = 0.02$; HPS: MR-IVW $\beta$=-0.02, $p < 0.001$; BsPA: MR-IVW $\beta = 0.09$, $p = 0.002$). However, we found no statistical evidence that biological aging was causally linked with major depression (Biological Aging Major Depression). Our results provides robust evidence that genetically determined risk of major depression is causally associated with accelerated biological aging and provides a mechanistic perspective for the worse adverse health outcomes observed in these individuals. Also, it highlights the importance of prevention and treatment of major depression across the lifespan to mitigate the speed of biological aging acc eleration.

Abstract citation ID: igaf122.2084

### UNIQUE FEATURES OF EYE MOVEMENTS LINK TO COGNITIVE AND MOBILITY DECLINE

Qu (Teresa) Tian[1], Yang An[1], Susan Resnick[1], and Luigi Ferrucci[2], *1. National Institute on Aging, Baltimore, Maryland, United States, 2. NIA, Bethesda, Maryland, United States*

Eye movement or ocular function, quantified by eye tracking devices, is a vital indicator of neurodegenerative diseases and behavior. However, existing knowledge is limited to patient samples or cross-sectional analysis. Little is known about eye movement associations with age-related cognitive and mobility decline. We investigated a diverse array of eye movement features in association with longitudinal changes in cognition and mobility in community-dwelling adults from the Baltimore Longitudinal Study of Aging (n = 543, mean age=71 years, 58% women, 25% Black, 5% cognitive impairment). Of 130 eye movement features derived from a portable eye-tracking device (Neurolign Dx100), 81 with <20% missing data were used. Of these 81, 40 features were significant predictors of age using LASSO regression and were used to create composite scores by category (saccade, smooth pursuit, vergence, optokinetic nystagmus). We examined the associations between eye movement composite scores and longitudinal changes in cognition, mobility, balance, manual dexterity, and gesture imitation up to 20 years using linear mixed-effects models, adjusted for age, sex, and race. Higher saccade and vergence scores were associated with less decline in executive function (Trail Making Test-part B), processing speed (Digit Symbol Substitution Test), mobility, balance, manual dexterity (Pegboard), and gesture imitation performance (all $p < 0.05$). A higher smooth-pursuit score was associated with less decline in mobility and balance. A higher OKN score was associated with less decline in balance. Selected eye movement features link to age-related cognitive and mobility decline in an aging population. Future studies are warranted to investigate underlying neuroimaging markers and brain pathology.

Abstract citation ID: igaf122.2085

### METABOLOMICS OF HUMAN AGING AND LONGEVITY

Daniel Evans[1], Oliver Fiehn[2], Nicholas Schork[3], and Steven Cummings[4], *1. University of California, San Francisco, San Francisco, California, United States, 2. University of California, Davis, Davis, California, United States, 3. The Translational Genomics Research Institute (TGen), Phoenix, Arizona, United States, 4. California Pacific Medical Center Research Institute, San Francisco, California, United States*

Metabolites associated with longevity in a prospective study could lead to predictive longevity biomarkers. The Longevity Consortium (LC) performed a prospective case-cohort metabolomics study of longevity from four cohort studies of elderly individuals: Study of Osteoporotic Fractures (SOF), Osteoporotic Fractures in Men (MrOS), Health, Aging, and Body Composition (Health ABC) Study, and the Cardiovascular Health Study (CHS). Longevity cases were defined by reaching the age corresponding to the 98th survival percentile from United States Social Security Administration life tables. This longevity definition for the 1910 US birth cohort for males is 95 years and for females is 99 years. There were 827 longevity cases and 2,555 members of the subcohort that did not achieve longevity over follow-up. GC-TOF MS was used to measure 322 serum metabolites. Metabolite associations with age were identified with sex-specific regression models adjusted for race, clinic site, study, body mass index (BMI), and estimated glomerular filtration rate (eGFR). The same covariates were included in sex-specific regression models for longevity associations, but age was additionally adjusted for. In a meta-analysis of age-association results from both sexes, older age was associated with lower levels of tryptophan ($P = 4x10-5$) and higher levels of cystine ($P = 4x10-4$) and hypotaurine ($P = 9x10-3$). In a meta-analysis of prospective longevity results from both sexes, higher levels of tryptophan were associated with higher odds of longevity ($OR = 2.7$, $P = 6x10-5$), and higher levels of cystine were associated with lower odds of longevity ($OR = 0.81$, $P = 0.02$). These metabolites could serve as biomarkers for human aging and longevity.

Abstract citation ID: igaf122.2086

### A FRAILTY-BASED PLASMA PROTEOMIC SIGNATURE CAPTURING OVERALL HEALTH AND WELL-BEING IN OLDER ADULTS

Fangyu Liu[1], Sanish Sathyan[2], Toshiko Tanaka[3], Nir Barzilai[4], Sofiya Milman[5], Keenan Walker[3], and Joe Verghese[2], *1. National Institute on Aging, United States, 2. Stony Brook University, Stony Brook, New York, United States, 3. National Institute on Aging, Baltimore, Maryland, United States,*

*4. Institute for Aging Research, Bronx, New York, United States, 5. Albert Einstein College of Medicine, Albert Einstein College of Medicine, New York, United States*

Frailty is an age-related syndrome characterized by an increased vulnerability to adverse health outcomes in the face of stressors. By deriving a blood-based proteomic signature for frailty, the current study aimed to enhance the understanding of frailty biology and derive a person-specific predictor for risk of frailty and other adverse age-related health outcomes. Penalized regression method was used to derive a 25-proteins signature (proteomic frailty index [pFI]) predictive of the cumulative frailty index (FI) in a training set of participants from the LonGenity cohort (N = 440). The pFI was validated with measured FI at baseline (r = 0.58) in the validation set (N = 440) from the same cohort and in two other independent cohorts: the Atherosclerosis Risk in Communities (ARIC) study (N = 5195, r = 0.61, p < 0.001) and the Baltimore Longitudinal Study of Aging (BLSA, N = 654, r = 0.45, p < 0.001). In all three cohorts, the pFI showed significant associations with age-related conditions including hypertension and diabetes, clinical measures of cholesterols, glucose and lipids, and functional measures of gait speed and grip strength (p < 0.001), after adjusting for demographic factors. In ARIC, the pFI were also significantly associated with cognitive scores (p < 0.001) and incident dementia (HR [95% CI] = 1.07 [1.05-1.09], follow-up: 7.22 [1.82] years). The pFI were also associated with mortality (LonGenity: HR [95% CI] = 1.12 [1.07-1.18]; ARIC: HR [95% CI] = 1.13 [1.12–1.14]; BLSA: HR [95% CI] = 1.11 [1.05–1.17]). In conclusion, we derived and validated a 25-protein signature of frailty that also captures overall well-being, health, and risk for key age-related diseases.

Abstract citation ID: igaf122.2087

### A PROTEOMIC SIGNATURE OF HEALTHSPAN

Chia-Ling Kuo[1], Peiran Liu[2], Richard Fortinsky[3], George Kuchel[1], and Breno Diniz[4], *1. UCONN HEALTH, Farmington, Connecticut, United States, 2. FDA, Silver Spring, Maryland, United States, 3. University of Connecticut, Farmington, Connecticut, United States, 4. UConn Health, Simsbury, Connecticut, United States*

Despite significant efforts to develop biomarkers of aging, few studies have focused on biomarkers of healthspan. We developed a proteomics-based signature of healthspan (healthspan proteomic score (HPS)) using proteomic data from the Olink Explorer 3072 assay in the UK Biobank Pharma Proteomics Project (53,018 individuals and 2920 proteins). Unlike previous biological age predictors trained to predict chronological age, mortality, or organ/disease-specific predictors, HPS was developed to evaluate the risk of a major chronic disease or mortality based on a healthspan definition. HPS outperformed chronological age and other biological age measures in predicting mortality and various chronic diseases. Subgroup analyses revealed that individuals at higher risk for adverse health outcomes—such as males, older adults, current or former smokers, obese individuals, and those with hypertension or hypercholesterolemia—had lower HPS values, indicating a less healthy systemic biological status in these groups. Additionally, the biological processes associated with HPS align with the hallmarks of biological aging, particularly immune response, inflammation, cellular signaling, and metabolic regulation, providing a strong biological validation of HPS as an integrative measure of biological aging. HPS serves as a novel proteomic aging measure, complementing existing proteomic and epigenetic measures. Combining HPS and a proteomic aging clock developed to predict mortality strengthens the associations with mortality and multiple adverse health outcomes. Overall, HPS is a valid biological aging measure, having strong clinical, predictive, and biological validity, making it a valuable tool for assessing healthspan in humans and potentially guiding the development of geroscience-based interventions in the future.

### SESSION 4500 (SYMPOSIUM)

Abstract citation ID: igaf122.2088

### DIGNITY OF RISK ATTUNED CARE: A KNOWLEDGE TRANSLATION INITIATIVE VIA EDUCATION WITH HEALTHCARE STAFF

Chair: Catherine-Anne Murray
Co-Chair: Kailey Durette

The Dignity of Risk approach is an important human rights-based approach to older adult care. It recognizes that daily life is inherently uncertain and taking risks can lead to positive outcomes in quality of life, health and well-being. When living with frailty, dementia or changing abilities, older adults can be supported to take reasonable risks that uphold their preferences and values. Unfortunately, current healthcare practice with older adults poses challenges to the implementation of Dignity of Risk principles. These can include ageism, overprotective attitudes, misconceptions regarding liability, and a hyperfocus on "keeping safe". These underlying biases and attitudes contribute to an interdisciplinary care culture that can unintentionally restrict and remove autonomy and independence of older adults. This practice-based research and education initiative aims to: 1. Explore healthcare staff's attitudes, beliefs, barriers, and facilitators surrounding the application of Dignity of Risk to care 2. Develop and deliver education workshops that aim to enhance staff's knowledge, awareness, and skills in integrating Dignity of Risk principles into care of older adults. 3. Use key findings and lessons learned to inform evolution of ongoing education methods and implications to healthcare policy and quality improvement. Understanding how to shift culture towards supporting older adults and their caregivers using a risk management approach to care planning can have a positive effect not only on maintaining older adults' quality of life, wishes, and values, but also on the healthcare system as it faces increasing demands to meet the needs of older adults living with frailty.

Abstract citation ID: igaf122.2089

### DEVELOPING DIGNITY OF RISK EDUCATION: EXPLORATORY NEEDS ASSESSMENT WITH HEALTHCARE STAFF

Catherine-Anne Murray[1], Elizabeth Gillis[1], Karen Nicholls[1], and Connor Dawe[2], *1. Nova Scotia Health, Halifax, Nova Scotia, Canada, 2. Nova Scotia Health Implementation Science, Halifax, Nova Scotia, Canada*

The Dignity of Risk (DoR) concept is being applied more to the care of older adults living with frailty, as healthcare systems are being challenged to broaden perspectives and policy to include a human rights lens to care provision. This initiative aims to develop and provide education for health care staff, based on a needs assessment and DoR evidence from the literature. To explore staff perspectives on DoR in the care of older adults, more than 600 surveys were completed at acute care and community sites across Nova Scotia. Main findings include: Most staff have little to no previous knowledge of the DoR, however most felt it moderately-very important to implement into care. Most staff identified the following barriers to implement DoR into practice: a hyperfocus on safety culture, paternalistic attitudes, fear of liability, and ageism. Other challenges included cognitive issues of the older adult and lack of support from manager and team members. Staff identified DoR facilitators as: policies and guidelines reinforcing same, skills in risk management conversations with caregivers, and understanding how decision-making abilities of the older adult can affect DoR application. Education via participatory workshops was developed and provided. Topics included defining DoR and its value in older adult care, ageism, overprotective attitudes, liability, using an informed strength-based risk management approach in care planning, and building confidence in difficult conversations with caregivers. Post workshop surveys were completed and main findings shared in following abstract.

Abstract citation ID: igaf122.2090

### DIGNITY OF RISK EDUCATION WITH HEALTHCARE STAFF: PRELIMINARY RESULTS, LESSONS LEARNED, AND FUTURE DIRECTIONS

Catherine-Anne Murray[1], Elizabeth Gillis[1], Connor Dawes[2], Caroline King[2], Karen Nicholls[1], and Kailey Durette[3], *1. Nova Scotia Health, Halifax, Nova Scotia, Canada, 2. Nova Scotia Health Implementation Science, Halifax, Nova Scotia, Canada, 3. Nova Scotia Health, Nova Scotia, Canada*

Following the Dignity of Risk (DoR) adult participatory education workshops with healthcare staff, post workshop surveys were completed by over 200 participants (response rate ~30%). The aims were: 1. To measure effectiveness of in person education with healthcare staff regarding the application of a DoR approach in the care of older adults living with frailty. 2. Use key findings and lessons learned to further inform education methods and implications to healthcare policy and quality improvement. Results showed an increase in staff confidence in applying DoR into practice and having DoR and risk management conversations with caregivers. Several main themes emerged as "takeaways": staff perceived older adults often value autonomy over safety, risk can be included as a part of a supportive care plan, and the importance of a balanced, broadened and strength-based approach to risk management in care planning. Additionally, staff identified further learning needs to enhance skills in risk management care planning and conversations. There were notable lessons learned that continue to guide DoR initiatives, including an inpatientpilot with DoR/Frailty experts embedded onsite and supporting staff. Knowledge translation and staff behaviour change were measured via the Behaviour Change Wheel methods, and the Consolidated Framework for Implementation Research. In terms of behaviour change in practice that leads to measurable outcomes for older adults, engaged leadership (managers and team leads) is required to support and reinforce this culture shift. Additionally, enhanced interprofessional collaboration that focuses on a united approach to prioritize autonomy of the older adult first, followed closely by risk management. Policies (i.e. Falls Prevention) and guidelines need to incorporate human rights language and strength-based risk management considerations. Only when healthcare staff feel competent and empowered to support positive risk with older adults, will those older adults likely be able to return home from hospital or remain home longer despite living with frailty and changing abilities.

### SESSION 4505 (SYMPOSIUM)

Abstract citation ID: igaf122.2091

### EXPLORING STRUCTURAL AND BIOLOGICAL DETERMINANTS OF COGNITIVE HEALTH AND AGING: INSIGHTS FROM DIVERSE POPULATIONS

Chair: Ruotong Mona Liu
Co-Chair: Xiang Qi

Cognitive health and aging are influenced by a complex interplay of social, environmental, and biological factors, yet disparities in these determinants remain underexplored across diverse populations. This symposium brings together four studies that examine how secondhand smoke exposure, central adiposity, racial disparities in autism spectrum disorder (ASD) diagnosis and care, and neighborhood environments shape cognitive and physical health outcomes in aging populations. The first study investigates the impact of secondhand smoke exposure on cognitive function among never-smoking older adults in the U.S., using biomarker-based assessments to highlight the cognitive risks associated with even moderate exposure. The second study explores how body fat distribution modifies the association between obstructive sleep apnea and frailty, revealing significant racial disparities in vulnerability and underscoring the role of central adiposity in aging-related health risks. The third study conducts a scoping review on racial and ethnic disparities in ASD diagnosis and healthcare access among older adults, demonstrating inequities in mental health comorbidities, treatment access, and healthcare utilization. The final study examines the role of cognitively supportive neighborhood environments in slowing cognitive decline among older Chinese Americans over an 8-year period, emphasizing the protective effects of social and environmental resources. Together, these studies contribute to a broader understanding of the structural and biological determinants of cognitive health and aging, offering critical insights for public health policies, community-based interventions, and personalized healthcare strategies aimed at promoting health equity and well-being across diverse aging populations.

Abstract citation ID: igaf122.2092

### SECONDHAND SMOKE EXPOSURE AND COGNITIVE FUNCTION IN U.S. NEVER-SMOKING OLDER ADULTS

Sophie Zhijing Xu[1], Jinyu Hu[1], Jackie Finik[1], Ruotong Liu[1], Yaguang Zheng, and [1]Bei Wu, [2], *1. New York University, New York, New York, United States, 2. NYU Shanghai, Shanghai, China (People's Republic)*

While active smoking is a known risk factor for cognitive decline, the impact of secondhand smoke (SHS) on cognitive function in never-smoking adults remains understudied. This study examines the association between SHS exposure and cognitive function in U.S. never-smoking older adults using data from 615 individuals aged 60+ from the NHANES 2011–2014. SHS exposure was measured via serum cotinine levels and categorized as low (0.01–0.02 ng/mL), moderate (0.02–0.04 ng/mL), and high (≥0.04 ng/mL), with never-smokers defined as those reporting fewer than 100 lifetime cigarettes. Cognitive function was assessed using the CERAD-WL immediate and delayed recall, Animal Fluency Test, Digit Symbol Substitution Test (DSST), and Global Cognitive Score. Multivariable regression models adjusted for demographic, lifestyle, and socioeconomic factors. The mean age was highest in the unexposed group (69.50, SD = 6.69 years), followed by the low, moderate, and high exposure groups. Moderate SHS exposure was significantly associated with lower scores in CERAD-WL immediate recall ($\beta$ = -1.94, $p < 0.05$), delayed recall ($\beta$ = -0.87, $p < 0.05$), DSST ($\beta$ = -3.81, $p < 0.05$), and Global Cognitive Score ($\beta$ = -0.31, $p < 0.01$), while no significant associations were observed in the low and high exposure groups. These findings suggest that even moderate SHS exposure may contribute to worse cognitive performance in never-smoking older adults, emphasizing the need for public health efforts to minimize SHS exposure and preserve cognitive health in aging populations.

Abstract citation ID: igaf122.2093

### THE ROLE OF CENTRAL ADIPOSITY IN THE LINK BETWEEN OBSTRUCTIVE SLEEP APNEA AND FRAILTY: INSIGHTS FROM NHANES 2015-2018

Ting Xu[1], Xichenhui Qiu[2], Qiqi Hang[3], Xiang Qi[4], Haokun Mei[5], Minghui Ji[1], Qin Xu[1], and Bei Wu[6], *1. Nanjing Medical University, Nanjing, Jiangsu, China (People's Republic), 2. Shenzhen University, Nanshan, Jiangsu, China (People's Republic), 3. The Second Clinical School, Nanjing Medical University, Nanjing, Jiangsu, China (People's Republic), 4. New York University, New York, New York, United States, 5. The Fourth Clinical School, Nanjing Medical University, Nanjing, Jiangsu, China (People's Republic), 6. NYU Shanghai, Shanghai, China (People's Republic)*

We investigated how body fat distribution, specifically central adiposity measured by the A Body Shape Index (ABSI), modifies the association between obstructive sleep apnea (OSA) and frailty and explored racial /ethnic differences, particularly focus on non-Hispanic White individuals. We used data from 4,154 adults in the NHANES 2015–2018. Frailty was measured using the 36-item Frailty Index. We applied logistic regression models to investigate the association between OSA and frailty, grouping participants into three levels (tertiles) based on their ABSI scores, with particular focus on the role of non-Hispanic White individuals. Among the 4,154 participants, 2,376 (57.2%) had OSA, and 469 (19.7%) were classified as frail. Individuals with OSA had a 1.5 times higher likelihood of frailty ($P < 0.001$). However, this association varied based on central adiposity levels. In participants with the lowest ABSI, no significant relationship was found between OSA and frailty. In contrast, the association was significant among those with moderate (OR = 1.564, 95% CI: 1.062–2.303, P = 0.049) and high (OR = 1.681, 95% CI: 1.105–2.556, P = 0.038) ABSI levels, indicating that greater central adiposity amplifies the OSA-frailty link. Racial differences were also evident. Non-Hispanic White participants exhibited a stronger association between OSA and frailty (OR = 1.829, 95% CI: 1.148–2.914, P = 0.009) compared to other racial/ethnic groups in the intermediate ABSI group. These findings emphasize the role of central adiposity in frailty risk among individuals with obstructive sleep apnea and underscore the need for race/ethnicity-specific risk assessments.

Abstract citation ID: igaf122.2094

### AGING WITH AUTISM: RACIAL DISPARITIES IN DIAGNOSIS, AND CARE AMONG OLDER ADULTS WITH ASD

Jiameng Yuan[1], Tianming Guo[2], and Hanzhang Xu[1], *1. Duke University, Durham, North Carolina, United States, 2. University of Rochester, Rochester, New York, United States*

Autism Spectrum Disorder (ASD) is a neurodevelopmental condition affecting cognition, psychological well-being, and daily life. As individuals with ASD age, their unique healthcare needs also increased. Racial and ethnic disparities in health and healthcare access have been well-documented, yet little is known about how racial and ethnic disparities affect older adults with ASD. We conducted a scoping review to examine literature on older adults with ASD, focusing on racial and ethnic disparities in diagnosis and healthcare access. We searched PubMed, PsycINFO, and Scopus for peer-reviewed, data-based articles published before December 3, 2024. Two independent raters screened articles based on prespecified inclusion and exclusion criteria and extracted relevant data from each article. Ten eligible articles were included in the final analysis. Compare to White older adults with ASD, Black older adults have higher prevalence of chronic conditions and are less likely to receive diagnoses of their mental health comorbidities. Hispanic older adults with ASD have fewer outpatient mental health visits and are less likely to receive psychotropic medications than White older adults. Older adults with ASD who are Asian/Pacific Islanders were less likely to be dual-eligible for Medicaid and Medicare than their White counterparts across different age groups. Furthermore, ASD is more frequently diagnosed in younger individuals, leading to underdiagnosis in older adults, further exacerbating their healthcare challenges. Recognizing the intersection of aging, autism, and racial disparities is essential for advancing healthcare equity and promoting health aging for older adults with ASD.

Abstract citation ID: igaf122.2095

### COGNITIVELY SUPPORTIVE NEIGHBORHOODS SLOW COGNITIVE DECLINE: AN 8-YEAR FOLLOW-UP AMONG OLDER CHINESE AMERICANS

Yanping Jiang[1], Wendi Da[2], Jessica Finlay[3], and Fengyan Tang[4], *1. Rutgers, The State University of New Jersey, New Brunswick, New Jersey, United States, 2. Rutgers University, New Brunswick, New Jersey, United States, 3. University of Colorado Boulder, Boulder, Colorado, United States, 4. University of Pittsburgh, Pittsburgh, Pennsylvania, United States*

Neighborhoods play a critical role in influencing cognitive health, particularly as most older adults prefer to age in place. Research on this topic has largely focused on composite features relevant to socioeconomic and racial composition. Specific neighborhood features, such as libraries and senior centers that can support cognitive stimulation, exercise, and social engagement, have received less attention, particularly among racially and ethnically diverse older adults. This study tests the effect of Cognability, a neighborhood index, on cognitive health in a large sample of Chinese immigrants aged 60 years and older (N = 2,787) living in metro Chicago. We computed Cognability using 13 indicators to capture cognitively supportive resources at the Census tract level using item response theory. Specifically, these13 indicators reflected availability of neighborhood resources, walkability (e.g., transit), availability of broadband internet, and availability of healthcare service. Cognitive function was assessed using various cognitive tests at baseline in 2011-2013 and every other year during 2013-2020. We included key covariates (e.g., sex, age, education) in the mixed-effects models. Results indicate that neighborhood Cognability was not associated with overall cognitive function at baseline. However, participants who lived in neighborhoods with higher Cognability scores exhibited slower cognitive decline over 8 years. This result was robust to the exclusion of participants who relocated. To conclude, our findings indicate the cognitive health benefits of living in stimulating and supportive neighborhoods in a sample of older immigrant adults. These results can inform the development of community-based interventions and policies to reduce dementia risk.

### SESSION 4510 (SYMPOSIUM)

Abstract citation ID: igaf122.2096

### IMPROVING DEATH LITERACY THROUGH INTERDISCIPLINARY RESEARCH-PRACTICE PARTNERSHIPS AND EXPERIENTIAL LEARNING

Chair: Kelly Melekis
Discussant: Kelly Melekis

Death literacy is considered both an outcome of one's experiences of and learning about death and dying, and a resource for strengthening individual and community capacity for end-of-life care. In this symposium, a group of interdisciplinary faculty will share ways they have utilized research-practice partnerships and experiential learning to improve death literacy. The symposium will begin with an overview of death literacy and the development of a multi-site collaboration to enhance death literacy through 1) research practice partnerships between community-run social model hospice homes and thanatology researchers, and 2) a Community Action, Research, and Education (CARE) experiential learning program whereby participants care for hospice patients in their last months of life. The second presentation will highlight opportunities available to students at a rural liberal arts college, discussing the potential for building death literacy and social capital for enhancing community capacity for end-of-life care. In the third presentation, a prior CARE fellow will share her experience of developing death literacy through a community-based participatory research (CBPR) project and present the results of a mixed methods study of hospice patients' experiences with hyperactive delirium in the last week of life. A fourth presentation will describe CBPR and the use of an expansive qualitative caregiver narrative database to develop stage plays aimed at improving death literacy. An excerpt will be presented along with strategies for dissemination and community engagement. The symposium will conclude with a discussion of ways to improve death literacy through interdisciplinary research-practice partnerships and experiential learning.

Abstract citation ID: igaf122.2097

### INTERDISCIPLINARY PATHWAYS TO DEATH LITERACY: SOCIAL MODEL HOSPICE HOMES AS A SITE FOR EXPERIENTIAL LEARNING

Kelly Melekis[1], and Carol Weisse[2], *1. University of Vermont, Burlington, Vermont, United States, 2. Union College, Schenectady, New York, United States*

Over the last century, major shifts have occurred in the way people die, including where they die and who provides most of the care. Most people wish to die at home, and while home deaths are more likely to result in patient-centered, goal-concordant care (Khandelwal et al., 2017), only 30% of people in the U.S. die at home (Cross & Warraich, 2019). Several barriers prevent individuals from dying in their preferred place, including caregiver and/or housing instability as well as a lack of knowledge and skills needed to make informed decisions. In this presentation, social model hospice homes will be discussed as a resource for addressing gaps in end-of-life care and improving death literacy. Death literacy, or knowledge and understanding about death and end-of-life care, impacts access to quality care for dying people (Collins et al., 2020) and allows a community approach to supporting those experiencing death, dying, and bereavement. As a community-driven response to end-of-life care needs, social model hospice provides access to hospice services in a home setting with support of volunteer community caregivers (Kliewer et al., 2023). This presentation will share lessons learned from a 10-year, multi-site collaboration to enhance death literacy through 1) research practice partnerships between community-run social model hospice homes, undergraduate student caregivers, and thanatology researchers, and 2) an experiential learning Community Action, Research, and Education (CARE) program whereby participants care for hospice patients in their last months of life. Strategies for developing interdisciplinary research-practice partnerships and experiential learning opportunities will be discussed.

Abstract citation ID: igaf122.2098

### BUILDING DEATH LITERACY AND SOCIAL CAPITAL THROUGH EXPERIENTIAL LEARNING IN RURAL COMMUNITIES

Leah Rohlfsen, *St. Lawrence University, Canton, New York, United States*

According to Noonan et al. (2016), the four facets of 'death literacy' are knowledge, skills, experiential learning, and social action. This presentation will highlight a variety of opportunities available to undergraduate students at a liberal arts college in a rural community. Experiences like the CARE fellowships, letter exchanges, community-based placements at skilled nursing facilities, and a variety of relevant internships not only provide students the opportunity to achieve 'death literacy,' but each experience builds social capital within and beyond the local community. Within the local communities, this social capital contributes to a community's capacity to develop and share knowledge, sustain caring support systems, and problem solve (Horsfall, Noonan, & Leonard, 2012). When students move to other communities and into a variety of occupations, social capital becomes a more widely distributed 'public good' that demonstrates 'caring' and can be used to serve other needs. This is particularly valuable given the desire for alternatives to medicalized dying and demographic changes in many rural communities. This presentation will highlight valuable commonalities in experiential learning opportunities such as regular contact with older adults and caregivers, strong mentors in the community, reflection and introspection on the experience, and peer support. Data from student experiences indicates that improved death literacy among young people contributes to building compassionate, caring communities that support those in old age and at the end-of-life. Indeed, when young people have more contact with older adults, they have lower ageism and less death anxiety (Barnett & Adams, 2018).

Abstract citation ID: igaf122.2099

### HYPERACTIVE DELIRIUM AMONG HOME HOSPICE PATIENTS: IMPROVING DEATH LITERACY THROUGH COMMUNITY-BASED RESEARCH

Jordan Whittier[1], and Carol Weisse[2], *1. Union College, Strafford, New Hampshire, United States, 2. Union College, Schenectady, New York, United States*

Hyperactive delirium (HD), characterized by restlessness, agitation, hallucinations, and delusions, can be distressing to patients and their caregivers. Symptoms have been reported to increase as death approaches in palliative care and inpatient settings, but less is known about HD occurring in home hospice settings where care is often provided by informal caregivers. This study was conducted through an experiential learning program, that included serving as a caregiver to hospice patients experiencing HD. Using a mixed methods approach, a data registry containing caregiver narratives of 101 hospice patients was queried to gain a deeper understanding of the frequency and severity of HD occurring during patients' last week of life. Severity was assessed using a modified version of 2 delirium scales (CRS; NU-DESC), coding symptoms as mild (1) or severe (2) based on references to aggression, distress, and safety threats and accompanying descriptors (e.g. extremely) about intensity/duration. Results indicated 23.7% of patients (N = 24) experienced HD, with behavioral symptoms documented more than cognitive ones. Severe symptoms were reported in 95.8% of patients, and incidents peaked four days before death with the most severe symptoms occurring five days before death. Content analysis highlights themes of caregiver challenges, barriers to quality care, and situations placing caregivers at risk for burnout. Results indicate that HD can be frequent and severe during home hospice care and underscore the need for additional support at the end-of-life. The author will share firsthand experience managing HD and discuss the interplay between research and practice.

Abstract citation ID: igaf122.2100

### FINAL SCENES: DEATH LITERACY THROUGH DEVISED THEATER

Carol Weisse, *Union College, Schenectady, New York, United States*

Most people wish to die in the comfort of home, but few possess a realistic understanding of the challenges patients and families face when death occurs outside of a medical setting. Through well-established research practice partnerships with several hospice residential care homes, we have curated an extensive collection of caregiver narratives describing the day-to-day end-of-life journeys of over 350 deceased hospice patients. These care narratives provide a special lens to the dying experience in a raw, holistic, detailed manner, offering a rare opportunity to examine and interrogate the death and dying process when it occurs in a home setting. This presentation will describe a community-based participatory artistic endeavor through which an interdisciplinary team of students, faculty, local theater experts, and community caregivers examined hospice caregiver narratives with the goal of developing a series of ten, 10-minute plays capturing the diverse final journeys of terminally ill patients and those caring for them. This presentation will describe the collective creation process of devised theater and how a team-based approach for devising plays about death and dying provides an opportunity for joint examination of the challenges associated with accessing and receiving home-based hospice care. There will be a discussion of the use of caregiver narratives in developing public performances aimed at improving death literacy, and the presentation will close with a brief reading of a devised play and the play's accompanying discussion questions developed for use in facilitating community conversations about death and dying.

### SESSION 4515 (SYMPOSIUM)

Abstract citation ID: igaf122.2101

### EMOTION IN DYADS ACROSS ADULTHOOD

Chair: Claudia Haase
Co-Chair: Tabea Meier
Discussant: Tammy English

Emotions in close relationships play an important role in well-being, health, and longevity across adulthood. While research has uncovered important insights into how individuals experience and express emotions in close relationships, emotional processes within dyads have received less attention. This symposium brings together four contributions that provide novel insights into antecedents and consequences of emotional functioning in dyads across adulthood. Contributions examine multiple emotion response systems (i.e., subjective emotional experiences, behaviors, physiological functioning, language), multiple emotion contexts (i.e., positive and negative), diverse dyads (i.e., healthy married couples, dementia care dyads), and different study designs (i.e., laboratory-based observations of dyadic interactions, ambulatory assessments, longitudinal studies). Dr. Meier and colleagues will present findings from a laboratory-based study on marital interaction, showing how socioeconomic status as an important macro-level factor shapes physiological linkage in middle-aged married couples. Fu and colleagues will present findings showing that language that conveys validation or invalidation during dyadic interactions can predict spouses' emotional experiences, drawing from a laboratory-based study of married couples. Dr. Yoneda and colleagues will present findings from a large dyadic ambulatory assessment study on positivity resonance (i.e., co-experienced positive emotions) and links with momentary cortisol in spouses' daily lives, highlighting important benefits of positivity resonance at a biomarker level in older couples. Dr. Chen and colleagues will combine data from two studies on dementia care dyads, revealing important links between physiological linkage and caregiver mental health and well-being across both laboratory and real-life contexts. Dr. English will be the discussant.

Abstract citation ID: igaf122.2102

### CONNECTED AT HEART? SOCIOECONOMIC STATUS AND PHYSIOLOGICAL LINKAGE DURING MARITAL INTERACTIONS

Tabea Meier[1], Aaron Geller[2], Kuan-Hua Chen[3], and Claudia Haase[2], *1. University of Zurich, Zürich, Switzerland, 2. Northwestern University, Evanston, Illinois, United States, 3. University of Nebraska Medical Center, Omaha, Nebraska, United States*

Connections with our loved ones play a critical role in our health, and emotions during marital interactions in mid-life can predict the development of health problems in later life. Research has documented greater interdependence among people from lower socioeconomic status (SES) backgrounds, but little is known about whether greater interdependence also emerges at a physiological level in low-SES contexts. This laboratory-based study examined physiological linkage (i.e., coordinated changes in partners' interbeat intervals, the time between two successive heart beats) in 48 married couples (96 spouses; M = 43.38, SD = 9.25, range: 21-68 years) from highly diverse SES and racialized backgrounds across two marital interaction contexts

(conflict and pleasant conversations). We analyzed both in-phase (i.e., coordinated changes in the same direction) and anti-phase (i.e., coordinated changes in opposing directions) physiological linkage. The overall number of observations was N = 192 (48 couples with four repeated measures; i.e., in-phase and anti-phase linkage in conflict and pleasant conversations). Repeated measures analyses showed that, across both conversations, spouses from lower (vs. higher) SES backgrounds showed greater in-phase and lower anti-phase physiological linkage. That is, their heart rates were more likely to change in similar ways and less likely to change in opposite ways. These findings provide first evidence linking socioeconomic status—a key macro-level factor shaping resources, rank, and societal hierarchy—with physiological linkage in married couples. Viewed through the lens of physiological linkage as an amplifier of emotions, this study prompts further research on the risks and resources for healthy aging in couples from lower-SES backgrounds.

Abstract citation ID: igaf122.2103

### ASSENT AND NEGATION LANGUAGE AND EMOTIONAL EXPERIENCES IN MARRIED COUPLES

Lillian Fu[1], Tabea Meier[2], and Claudia Haase[3], *1. University of California, Irvine, Evanston, Illinois, United States, 2. Northwestern University, School of Education and Social Policy, Evanston, Illinois, United States, 3. Northwestern University, Evanston, Illinois, United States*

Emotional processes in marital interactions predict late-life well-being and health outcomes, but few studies have examined how words that convey validation or invalidation may shape the emotional climate of marital interactions. In a laboratory-based dyadic interaction study with 49 married middle-aged couples (N = 98 individuals; ages 21-65) from diverse socioeconomic and racial/ethnic backgrounds, we examined links between assent (e.g., "mhm", "yes") and negation (e.g., "no", "never") language and emotional experiences. Couples engaged in 10-minute videotaped conflict and pleasant conversations. Then, they individually reported on the intensity of their subjective negative and positive emotional experiences. The relative frequency of assent and negation words during each conversation was calculated using the Linguistic Inquiry and Word Count software. Linear mixed models revealed that individuals' assent language and partners' assent language were both associated with lower levels of negative emotional experiences. Moreover, partners' negation language was associated with higher levels of own negative emotional experiences, and a conversation interaction effect revealed that this association was driven by conflict conversations. Results generalized across gender and remained stable when controlling for age, socioeconomic status, negation/assent word frequency, and total word count. These findings highlight the dyadic interplay between spouses' use of assent and negation words and emotional experiences. Implications for dyadic interaction research with couples across the lifespan and large-scale text analysis will be discussed.

Abstract citation ID: igaf122.2104

### BETTER TOGETHER: MOMENTARY PHYSIOLOGICAL BENEFITS OF CO-EXPERIENCED POSITIVE EMOTIONS IN LATE LIFE

Tomiko Yoneda[1], Nathan Lewis[2], Theresa Pauly[3]NIlam Ram[4], Denis Gerstorf[5], Claudia Haase[6], and Christiane Hoppmann[7], *1. University of California Davis, Davis, California, United States, 2. The University of British Columbia, Vancouver, British Columbia, Canada, 3. Simon Fraser University, Vancouver, British Columbia, Canada, 4. Stanford University, Stanford University, California, United States, 5. Humboldt University Berlin, Humboldt University Berlin, Berlin, Germany, 6. Northwestern University, Evanston, Illinois, United States, 7. UBC, Vancouver, British Columbia, Canada*

Although positive emotions tend to be experienced most frequently and intensely within social interactions, most research has focused on positive emotions at the individual level. Yet, emerging research shows that shared positive emotions predict health and longevity over and above individually experienced positive emotions. However, the extent to which relationship partners co-experience positive emotions in daily life remains poorly understood. Moreover, whether positive emotions "get under the skin," shaping proximal biomarkers in everyday life, is unclear. Drawing on Positivity Resonance Theory, we examined the links between co-experienced positive emotions and momentary cortisol levels in 321 older couples (ages 56–89). Findings showed that both relationship partners reported higher-than-usual positive emotions during 38% of the occasions they were together. Additionally, co-experienced positive emotions were linked to lower cortisol levels at the same occasion, adjusting for individually experienced positive emotions and several additional individual-level and time-varying covariates. Findings did not differ across age, sex, or relationship satisfaction, suggesting that the physiological benefits of co-experienced positive emotions are not dependent on individual or relationship characteristics. Notably, co-experienced positive emotions were also associated with lower cortisol at the subsequent assessment (but not vice versa), indicating that the benefits of co-experienced positive emotions extend beyond the immediate moment. This study highlights the important role that shared positive emotions play in promoting physiological health in older adults, providing evidence that co-experienced emotions offer benefits beyond individual experiences.

Abstract citation ID: igaf122.2105

### PHYSIOLOGICAL LINKAGE AND CAREGIVER MENTAL HEALTH AND WELL-BEING IN DEMENTIA CARE DYADS

Kuan-Hua Chen[1], Casey Brown[2], Alice Verstaen[3], James Casey[4], Jennifer Merrilees[5], and Robert Levenson[4], *1. University of Nebraska Medical Center, Omaha, Nebraska, United States, 2. Georgetown University, Washington, District of Columbia, United States, 3. Lyra Health, Burlingame, California, United States, 4. University of California, Berkeley, Berkeley, California, United States, 5. University of California, San Francisco, San Francisco, California, United States*

When people share emotions (e.g., laughing together), their physiological responses can become momentarily "linked" (i.e., changing in coordinated ways)—a phenomenon referred to as "physiological linkage". Reduced physiological linkage has been observed in social interactions between people with dementia (PWD) and their family caregivers. To expand this work, in two independent samples of caregiver-PWD dyads, we examined the relationship between physiological linkage—measured in laboratory and real-world settings—and caregiver mental health and well-being. In study 1, 64 dyads had a 10-minute conversation about an area of disagreement in the laboratory with six measures of physiological responses continuously monitored. In study 2, 22 dyads wore wristwatch devices in their homes that provided a continuous measure of one physiological measure (activity) over seven days. Physiological linkage was computed as the covariation of dyads' physiological responses during the conversation (Study 1) or waking hours (Study 2). Caregivers reported their emotional well-being (Study 1) and anxiety symptoms (Study 2) using well-established questionnaires. Findings revealed that lower physiological linkage in the laboratory was associated with lower caregiver emotional well-being ($r = 0.27$, $p = .03$). Similarly, lower physiological linkage in the home was associated with higher caregiver anxiety ($r = -.48$, $p = .02$). Findings remained statistically significant after accounting for individual-level physiological responses. We believe that caregiver-PWD dyads with lower physiological linkage may have weaker emotional connections, which can increase vulnerability in caregivers to declines in mental health and well-being.

### SESSION 4520 (SYMPOSIUM)

Abstract citation ID: igaf122.2106

### LIFE COURSE APPROACHES TO FAMILY RELATIONSHIPS AND HEALTH IN LATER LIFE: THE LONG-LASTING SHADOW OF CHILDHOOD TRAUMA

Chair: Jooyoung Kong
Co-Chair: Stephanie Robert
Discussant: Karen Roberto

Childhood trauma can have lasting effects on individuals' lives, influencing relationships, health, and well-being. This symposium presents four studies that explore the intersection of childhood trauma, as assessed by Adverse Childhood Experiences (ACEs), family dynamics, and later-life functioning and health through a life course framework. Drawing on longitudinal data from the Midlife in the United States (MIDUS), National Longitudinal Study of Adolescent to Adult Health (Add Health), and Wisconsin Longitudinal Study (WLS), these studies examine how ACEs shape sibling relationships, intergenerational caregiving and support, and social isolation in adulthood. Study 1 investigates the impact of childhood sibling relationships and ACEs on cognitive functioning in late adulthood, emphasizing the role of adult sibling closeness and contact. Study 2 examines the daily experiences of adult-child caregivers, exploring how ACEs influence daily caregiving outcomes and the role of family support and strain. Study 3, grounded in the support bank model, explores how ACEs may affect adult children's provision of financial or instrumental support when caregiving needs arise in aging parents. Study 4 investigates the association between cumulative ACEs and social isolation in adulthood, examining how family support and strain mitigate or exacerbate these effects. Together, these studies underscore the significance of family relationships across the life course, offering insights into how childhood trauma may influence later-life social functioning and health. This symposium contributes to a deeper understanding of the complex interplay between ACEs, family dynamics, and health, emphasizing the importance of trauma-informed approaches to promoting resilience and well-being across the life course.

Abstract citation ID: igaf122.2107

### ADVERSE CHILDHOOD EXPERIENCES AND INTERGENERATIONAL SUPPORT TO AGING PARENTS

Jooyoung Kong[1], Amy Liu[1], Stephanie Robert[1], and Xing Zing[2], *1. University of Wisconsin–Madison, Madison, Wisconsin, United States, 2. Arizona State University, Phoenix, Arizona, United States*

Background and Objective: Using the support bank model, the current study examined aging mothers' receipt of support from their adult children, focusing on the roles of adult children's emotional closeness and aging mothers' needs. We also explored the long-term impacts of adult children's reports of adverse childhood experiences (ACEs) on intergenerational support patterns. Methods: Data were drawn from 2,162 aging mothers (average age 63 years) who participated in the Add Health Parent Study Phase I and their adult-child respondents from Waves I, III and IV of the National Longitudinal Study of Adolescent to Adult Health. We conducted a series of logistic regressions to predict a binary outcome of adult children's assistance (financial and instrumental) to aging mothers. Results: Mothers' poor health and not being married or partnered were significantly associated with receiving financial and instrumental assistance from their adult children. Among adult daughters, the number of ACEs interacted with mothers' depressive symptoms, such that having a greater number of ACEs exacerbated the negative impact of mothers' depressive symptoms on the receipt of assistance. Specifically, with each additional adult child's ACE, the effect of mothers' depressive symptoms on the odds of receiving assistance weakened – decreasing from 60% to 44%. Conclusions: Intergenerational support for aging mothers may be hampered by adult daughters' ACEs histories. Our focus on the life course factor of ACEs can contribute to a holistic view of later-life intergenerational support patterns. Future research could further explore the changes or stability in family dynamics for individuals with ACEs histories.

Abstract citation ID: igaf122.2108

### LIFE COURSE ASSOCIATIONS OF SIBLING RELATIONSHIPS AND COGNITIVE FUNCTIONING IN LATE ADULTHOOD

Sara Moorman[1], Jooyoung Kong[2], Gina Lee[2], and Michal Engelman[3], *1. Boston College, Chestnut Hill, Massachusetts, United States, 2. University of Wisconsin–Madison, Madison, Wisconsin, United States, 3. University of Wisconsin-Madison, Madison, Wisconsin, United States*

Background/objective: Relying on the linked lives concept within the life course perspective, the current study examined the lifelong associations of sibling relationships and their effects on cognitive functioning in late adulthood. Methods: Using data from the sibling cohort of the Wisconsin Longitudinal Study, we estimated a mediational model, examining the effects of childhood sibling interactions and adverse childhood experiences (ACEs), as proxy measures for the parent-child relationship, on cognitive functioning in late adulthood through adult sibling closeness and contact. Respondents' cognitive functioning was assessed using the Telephone Interview for Cognitive Status-modified (TICS-m) when they were in their early 80s, on average. We conducted multilevel structural equation modeling. Results: Positive childhood sibling interactions (e.g., hugging, helping) were associated with increased adult sibling closeness and contact, whereas higher cumulative ACEs were associated with decreased adult sibling closeness and contact. We found significant indirect associations: (a) positive sibling interactions during childhood were associated with more frequent sibling contact across respondents' mid-50s through early 70s, which was in turn associated with higher TICS-m scores in their early

80s; (b) higher cumulative ACEs were associated with less frequent sibling contact, which was in turn associated with lower TICS-m scores. Conclusions: The results suggest that childhood experiences within the family of origin may have lasting effects on adult sibling relationships and the cognitive functioning of older adults. This study highlights the potential cognitive benefits of frequent contact with adult siblings, underscoring the importance of nurturing sibling relationships throughout one's life.

Abstract citation ID: igaf122.2109

### DAILY HEALTH AND WELL-BEING AMONG CAREGIVERS WITH MULTIPLE ADVERSE CHILDHOOD EXPERIENCES

Yin Liu[1], Jooyoung Kong[2], David Almeida[3], and Stephanie Robert[2], *1. Utah State University, Logan, Utah, United States, 2. University of Wisconsin–Madison, Madison, Wisconsin, United States, 3. The Pennsylvania State University, University Park, Pennsylvania, United States*

Background/objective: Caregiving for aging parents is a prevalent experience for middle- and older adults in the US. Utilizing a life course perspective to family caregiving, this study examined the associations between providing care to parents and daily well-being outcomes, while also testing the moderating roles of adverse childhood experiences (ACEs) and current family support or strain. Methods: Data were drawn from the National Study of Daily Experiences (NSDE) 3, a series of daily diary surveys over eight consecutive days. A study sample included 434 caregivers and their 1,123 daily diary records. The daily outcomes included daily negative and positive affect, physical symptoms, and sleep quality. A multilevel modeling approach was employed to account for days nested within individuals. Results: Having three or more ACEs significantly moderated the within-person association between caregiving for parents and higher daily negative affect. Among respondents with three or more ACEs, positive family support buffered the associations between daily caregiving to parents and higher negative affect, lower positive affect, and poorer sleep quality. Family strain exacerbated the effects of daily caregiving to parents on higher negative affect, lower positive affect, more physical symptoms, and poorer sleep quality. Conclusions: ACEs play a crucial role in contextualizing caregivers' daily health outcomes. This study highlights the importance of trauma-informed support tailored to these caregivers who have faced multiple ACEs. Future research should focus on exploring the specific coping mechanisms and cumulative disadvantages of these caregivers to inform more effective, targeted interventions.

Abstract citation ID: igaf122.2110

### CUMULATIVE ADVERSE CHILDHOOD EXPERIENCES AND ADULT SOCIAL ISOLATION: THE ROLES OF FAMILY SUPPORT AND STRAIN

Stephanie Robert, Jooyoung Kong, and Weidi Qin, *University of Wisconsin–Madison, Madison, Wisconsin, United States*

Background/objectives: Childhood trauma can have lasting effects on various aspects of adult life, one of which is the potential impairment in the ability to form healthy, stable relationships. Despite this, little research has explored the long-term effects of such trauma on social isolation in adulthood. This study investigates the association between adverse childhood experiences (ACEs) and adult social isolation, with a focus on how family support and strain may impact this relationship. Methods: Data were drawn from three-wave longitudinal surveys of the Midlife in the United States (MIDUS) study. Social isolation was measured using a composite scale that included partnership status, contact with family and friends, and participation in social activities. Growth curve modeling was used to assess the effects of ACEs, family support, and family strain on social isolation while controlling for covariates such as age, sex, race, education, and self-rated health. Results: ACEs were positively associated with social isolation, though the time effects of ACEs were not statistically significant. Both family support and strain were associated with lower levels of social isolation. For those with higher ACEs, the protective effect of family support was weakened. When excluding partnership status from the social isolation measure and including it as a covariate, most results remained consistent. Conclusions: ACEs contribute to increased social isolation in adulthood and may attenuate the protective role of family support in reducing social withdrawal. Further research is needed to identify underlying mechanisms, which could inform targeted interventions to promote social connectedness for adults with childhood trauma.

### SESSION 4525 (SYMPOSIUM)

Abstract citation ID: igaf122.2111

### INTEGRATING LIFESTYLE MEDICINE AND THE GERIATRIC 5MS FOR SUSTAINED BEHAVIOR CHANGE IN OLDER ADULTS

Chair: Rebecca Lassell
Co-Chair: Brian Andonian

This symposium introduces a new framework of Lifestyle Medicine (LM) that acknowledge the complex contexts and care needs of older adults to promote and sustain behavior change. The framework of Lifestyle Medicine for Older Adults integrates their lived experiences (e.g., social determinants of health, built environment), the 5M framework of geriatric medicine (i.e., Mind, Mobility, Medications, Multicomplexity, Matters Most) and the six pillars of LM (i.e., nutrition/diet, physical activity/exercise, restorative sleep, stress management, avoidance of risky substances, positive social connections) through a novel transdisciplinary approach. Guided by the framework, we will describe: 1) lifestyle recommendations for older adults: challenges and opportunities for practice, research, and policy; 2) how applying the framework using a transdisciplinary approach might address these challenges, and 3) recommendations for prescribing lifestyle for older adults tailored to their daily contexts and experiences. Five presenters will provide clinical and research examples that embody the framework. Presentations will range from the current evidence for sustained behavior change in older adults, different methodological approaches for LM analyses in publicly available data, observational studies, and community-based interventions.

Abstract citation ID: igaf122.2112

### EMPOWERING PHYSICAL THERAPISTS TO ADDRESS THEIR PATIENT'S INADEQUATE PHYSICAL ACTIVITY

Mariana Wingood[1], Jaime Hughes[1], Kritchevsky Stephen[1], Jennifer Brach[2], and Moore Justin[1], *1. Wake Forest University School of Medicine, Winston-Salem, North Carolina, United States, 2. University of Pittsburgh, Pittsburgh, Pennsylvania, United States*

One in ten older adults are inadequately active, doubling their falls and frailty risk. Despite this, 65% of physical therapists

(PTs) do not assess or address patients' physical activity (PA). We are addressing this gap by implementing the Physical Activity Vital Sign (PAVS) and Brief Action Planning (BAP), an evidence-based behavior change technique. Our work is guided by the Exploration, Preparation, Implementation, and Sustainment (EPIS) Framework. In the exploration phase, 51 PTs completed a needs assessment that examined potential implementation determinants, including: 1) individual capability and opportunity; 2) PAVS and BAP's characteristics; 3) clinic culture and infrastructure; and 4) health system's attitudes and partnerships. During the preparation phase, an implementation team consisting of PTs, clinic supervisor, older adults, and principal investigator co-developed an implementation plan that aims to address three barriers identified in the needs analysis and prioritized based on feasibility and importance. These barriers are lack of knowledge, confidence, and documentation infrastructure. The plan includes training, audit and feedback, site champions, facilitation, and electronic health record-based implementation strategies. We are evaluating the plan in an outpatient physical therapy clinic, where 20 PTs are participating in a year-long implementation pilot. Using a pre-post knowledge assessment and a confidence rating, we identified that our training increased PTs' knowledge and confidence in assessing and addressing inadequate PA ($p<0.001$). Three months into the project, 50% of PTs used the PAVS and 25% completed BAP with >25% of older adult patients. Currently, the implementation team is refining strategies to further increase the adoption rates.

Abstract citation ID: igaf122.2113

### THE EFFECTIVENESS OF LIFESTYLE INTERVENTIONS FOR OLDER ADULTS ACCORDING TO PUBLICLY AVAILABLE DATA

Kevin Pritchard[1], Pureum Jeon[2], Eun-Young Yoo[3], and Ickpyo Hong[2], *1. Brigham and Women's Hospital, Boston, Massachusetts, United States, 2. Yonsei University Mirae Campus, Wonju, Kangwon-do, Republic of Korea, 3. Yonsei University College of Software and Digital Healthcare Convergence, Seoul, Republic of Korea*

We used publicly available data from the "Well Elderly 2 Study" to compare the effectiveness of a lifestyle intervention in improving mental and physical health. The original study's intention-to-treat (ITT) analysis was criticized for its small treatment benefit, likely because low adherence diluted the treatment's effect. We re-analyzed the data to estimate the benefit of treatment adherence. This retrospective analysis included 424 older adults aged 60-95 in the U.S., who were randomly assigned from 2004 to 2008 to an occupational therapist-led lifestyle intervention or a no-treatment control, with follow-up starting at treatment assignment. To evaluate the effect on SF-36v2, we pooled data from participants who crossed over treatment every six months during the four-year period and analyzed them using generalized estimating equations (GEE) with robust standard errors. An ITT analysis estimated the effect of treatment assignment regardless of adherence. An AST (As-Treated) analysis estimated the effect of treatment adherence, accounting for selection bias from treatment deviation by using inverse probability of censoring weights. The ITT analysis showed a significant improvement in the mental composite ($B=0.54$, 95% CI [0.15, 0.93]) but no significant effect on the physical composite ($B=0.71$, 95% CI [-0.02, 0.03]). In contrast, the AST analysis showed improvements in both the mental ($B=0.77$, 95% CI [0.49, 1.04]) and physical ($B=2.28$, 95% CI [2.48, 2.75]) composites. While ITT analysis risks exposure misclassification, AST analysis is susceptible to selection bias if treatment deviation is intentional. Presenting both estimates improves clarity in findings.

Abstract citation ID: igaf122.2114

### MOBILITY IN COMPLEX ENVIRONMENTS: INVESTIGATING THE DIFFERENCES IN AGING TRAJECTORIES

Sudeshna Chatterjee[1], David Clark[2], Patricia Shewokis[1], Lynnette Montgomery[1], Jennifer Plumb[1], Diana Robins[1], and Hasan Ayaz[1], *1. Drexel University, Philadelphia, Pennsylvania, United States, 2. University of Florida, Gainesville, Florida, United States*

Age-related decline in executive function, balance, and movement control are associated with fall risk, activity avoidance, and lower engagement in physical activity which may restrict functional independence and participation in life roles. The deficits in control mechanisms are particularly amplified in complex environments at home and in the community, such as uneven surfaces, poor ambient conditions, and/or distractions. Nearly 35% of older adults report falling in an outdoor environment, and 59% of the falls occur due to trips and slips. Using mobile brain imaging with functional near-infrared spectroscopy, we have reported that age, executive function, and their interaction are associated with the magnitude of prefrontal brain recruitment during obstacle negotiation ($N=50$, $R^2=0.34$, $p=0.01$). Specifically, lower executive function in adults aged <75 years was associated with greater prefrontal recruitment during obstacle negotiation when compared to peers with higher executive function ($p=0.03$). Interestingly, older adults aged ≥75 years exhibited a ceiling of prefrontal recruitment during obstacle negotiation, regardless of their executive function ($p=0.87$). It is currently not clear how aging with neurodevelopmental conditions such as autism impacts mobility in complex environments. Since adults with autism are twice as susceptible to injurious falls as their peers and have a higher risk of developing conditions such as Parkinson's disease and dementia, we are investigating the differences in executive function, balance, and movement control in adults with autism aged 18 to 64 years. Our findings will provide novel insights into mobility function in this understudied population and guide future lifestyle interventions.

Abstract citation ID: igaf122.2115

### BUILDING CAPACITY FOR MEANINGFUL AND SUSTAINABLE EXERCISE RESEARCH PARTNERSHIPS WITH COMMUNITY ORGANIZATIONS

Laura Baehr[1], Megan George[1], Janet Bettger[2], and Michael Wolf[3], *1. Drexel University, Philadelphia, Pennsylvania, United States, 2. The University of North Carolina at Chapel Hill, Chapel Hill, North Carolina, United States, 3. Northwestern University, Evanston, Illinois, United States*

Older adults should engage in lifestyle physical activity (aerobic, strength, and balance tasks) to maintain health, manage chronic conditions, and reduce frailty, yet only 11% achieve minimal weekly recommendations. Older adults with social considerations such as mobility limitations and/or socioeconomic disadvantage are at highest risk for physical inactivity and subsequent poor health outcomes compared to age matched peers. There is strong evidence that community-based exercise programs promote physical activity in older adults, yet the needs and perspectives of those most in need of these interventions aren't prioritized during development, assessment, and implementation. The goal of this capacity building project is to employ the Explore, Prepare, Implement, Sustain (EPIS) Framework to the systematic assessment of community-based organizations (CBOs) providing free or low cost health promotion programs in Philadelphia, home to some of the most socioeconomically disadvantaged Americans with poverty rates nearly four times higher than suburban counties. To achieve this goal, we first explored CBOs to determine appropriateness for research collaborations by creating an exhaustive list through multiple inputs. The team leveraged known partnerships through local universities as well as web-based searches. A list of key search words was generated to capture CBOs, health promotion, and older adults. Repeated web-based searches inclusive of public databases were performed to collect CBO information, until a final list of potential CBOs (n = 217) was identified. The next step (ongoing) applies the Preparation phase of the EPIS Framework to interview all identified CBOs to determine goals, and interest in partnership to prepare for future collaborations.

Abstract citation ID: igaf122.2116

### PHYSICAL ACTIVITY PROMOTION IN AGING ADULTS WITH RHEUMATIC DISEASES: PERSPECTIVES FROM AGING ADULTS WITH LUPUS

Sarah Lieber[1], Sarah Young[2], Dongmei Sun[1], Carol Sames[3], Lisa Mandl[1], Sara Czaja[2], M. Carrington Reid[2], and Iris Navarro-Millan[2], *1. Hospital for Special Surgery, New York, New York, United States, 2. Weill Cornell Medicine, New York, New York, United States, 3. Upstate Medical University, Syracuse, New York, United States*

Aging adults with systemic rheumatic diseases, including systemic lupus erythematosus (SLE), face unique challenges related to physical activity (PA) engagement, including mobility limitations and multicomplexity, and may benefit from tailored approaches to PA promotion. PA may be especially important in aging adults with SLE, who have high rates of cardiovascular disease and multimorbidity, but low rates of PA. In this qualitative study, we elicited perspectives of adults with SLE on PA promotion, including delivered via digital health technologies. Adults ≥50 years of age with SLE took part in semi-structured interviews, drawing in part from a socioecological framework and the technology acceptance model. We obtained self-reported data on sociodemographic and disease characteristics and asked participants to offer insights on PA promotion strategies, particularly related to digital health technology. We performed a thematic data analysis. Among 30 participants with SLE, who were 93.3% female with a mean age of 62.3 years, themes included PA intervention suggestions, digital literacy and access, and usefulness and ease of use of technology in the context of PA promotion. Participants recommended incorporating friends and family into PA promotion efforts. We identified interest among participants in tailored PA strategies for SLE, potentially incorporating digital health technology and social connection. Designing, testing, and disseminating tailored interventions to enhance lifestyle changes, including regular PA, among aging adults with SLE, as well as other systemic rheumatic diseases, could lead to improved health and wellbeing in conjunction with disease-directed therapies in this growing patient population.

### SESSION 4530 (SYMPOSIUM)

Abstract citation ID: igaf122.2117

### NEIGHBORHOODS AND AGING: HOW PLACE-BASED ASSETS AND CHALLENGES SHAPE MENTAL HEALTH AND WELL-BEING

Chair: Megan Nizza
Co-Chair: Clara Scher

Neighborhood environments play a critical role in shaping the mental health and well-being of older adults, particularly those with intersecting minoritized identities such as race, cognitive status, and socioeconomic position. This symposium examines how neighborhood-based assets and barriers contribute to disparities in later-life health, emphasizing both protective (e.g., neighborhood cohesion and accessible infrastructure) and adverse factors (e.g., poor housing conditions and experiences of discrimination). The first presentation explores how neighborhood contexts support and challenge the health and well-being of older adults racialized as Black and living with mild cognitive impairment or dementia. The second presentation examines geographic and age-related variations in experiences of discrimination among self-identified Black women, discussing the extent to which neighborhood racial composition affects the reporting frequency of discrimination. The third presentation examines how perceived neighborhood disorder and cohesion are associated with later-life cognitive function, underscoring the mediating role of psychological resilience. The fourth presentation investigates how public housing quality and broader neighborhood contexts interact with age to shape mental health outcomes, offering implications for housing interventions to mitigate disparities. Finally, the fifth presentation explores neighborhood-level facilitators and barriers to social connectedness for older people living with cognitive impairment or mild dementia, proposing community-driven solutions to neighborhood designs. Through this interdisciplinary lens, presenters will conclude with a discussion on research, practice, and policy implications, emphasizing the need for neighborhood-level innovations to improve the mental and psychosocial well-being of older adults with intersectional social positions.

Abstract citation ID: igaf122.2118

### NEIGHBORHOOD ASSETS AND CHALLENGES FOR BLACK OLDER ADULTS WITH DEMENTIA OR MILD COGNITIVE IMPAIRMENT

Clara Scher, *Weill Cornell Medicine, New York, New York, United States*

Due to the detrimental impacts of structural racism, many Black older adults living with dementia or mild cognitive impairment in the United States are aging in communities with unequal access to essential resources. While previous research has examined how neighborhood environments impact those aging in community with dementia or mild cognitive impairment, few studies have specifically focused on experiences of older adults racialized as Black. Guided by the Black Family Socioecological Context Model, this study explored how neighborhood factors support or challenge the well-being of this population in the context of an urban city. Researchers recruited eight Black adults 65 and older with self-reported memory loss from social adult day centers across two New York City boroughs. Participants engaged in both a sit-down interview and a walking interview, which involved the participant guiding the researcher on a tour throughout their neighborhood. Reflexive thematic analysis and memoing were used to analyze interview transcripts and field notes. Findings emphasized how place-based community assets—such as social adult day centers, churches, and transportation services—enhanced participants' well-being by providing access to essential resources, fostering emotional support, and strengthening social connections. Yet, community-level hazards—including crime and safety concerns, structural racism and ageism, and insufficient government investment—created barriers to accessing services and contributed to distress. Future participatory research should include larger, more geographically diverse samples to explore how community assets can be leveraged to enhance well-being and reduce the negative effects of neighborhood-based barriers among Black older adults with dementia or mild cognitive impairment.

Abstract citation ID: igaf122.2119

### POOR HOUSING QUALITY AND MENTAL HEALTH IN PUBLIC HOUSING: AGE DIFFERENCES AND NEIGHBORHOOD CONTEXTS

Zhirui Chen[1], Yilin Wang[2], Samantha Teixeira[3], and Rebekah Coley[2], *1. Boston College, Framingham, Massachusetts, United States, 2. Boston College, Chestnut Hill, Massachusetts, United States, 3. Boston College School of Social Work, Chestnut Hill, Massachusetts, United States*

Poor housing quality presents a significant challenge for public housing residents in the U.S., which impedes their health and wellbeing. This study examined the association between housing quality and mental health among public housing residents and how age and neighborhood contexts moderated this relationship. Data come from the first wave of a mixed-methods evaluation of public housing redevelopment, the Housing Opportunity and Mobility Experiment (HOME), located in a large public housing community. The analytic sample included 448 adults (ages 18-97), with data collected through in-person and online surveys between October 2022 and January 2024. Results of multiple linear regression showed that poor housing quality (e.g., mold, pests) was associated with increased symptoms of anxiety, depression, and perceived stress among public housing residents. Older adults who experienced lower housing quality reported worse mental health relative to their younger counterparts. Additionally, neighborhood problems, as a risk factor, was significantly associated with worse mental health outcomes and further exacerbated the links between housing quality and mental health more for older adults than for younger populations. These findings have implications for the development of age-responsive mental health interventions tailored to the unique contexts of public housing communities. As many public housing developments are undergoing redevelopment, often with the goals of enhancing supportive services, there is an opportunity to incorporate targeted mental health services, particularly for older residents who are more vulnerable to environmental stressors. To better support residents' mental health, initiatives are needed to address community stressors such as drug presence and violence.

Abstract citation ID: igaf122.2120

### NEIGHBORHOOD CHARACTERISTICS TO ENHANCE SOCIAL CONNECTEDNESS FOR OLDER PEOPLE WITH DEMENTIA

Xiaoyi Zeng, Yuanjin Zhou, Lailea Noel, Catherine Cubbin, and Namkee Choi, *The University of Texas at Austin, Austin, Texas, United States*

This qualitative study explored the perspectives of older people with mild cognitive impairment (MCI) or dementia and their care partners on: (1) neighborhood-level facilitators and barriers influencing social connectedness, and (2) essential characteristics of dementia-friendly neighborhoods addressing the social connectedness needs of older people with dementia. Seventeen semi-structured interviews, each lasting an average of 77 minutes, were conducted with nine older adults with MCI or mild dementia (mean age = 76.4 years, 55.6% female) and eight care partners (mean age = 68.5 years, 75% female) in a large metropolitan area in a Southwestern state in June to August, 2024. Thematic analysis steps were employed for data analysis. Five key facilitators that emerged were: (1) safe and walkable environments, (2) access to essential services and social activities, (3) strong neighborhood ties, (4) stable and familiar surroundings, and (5) dementia-friendly community programs. Barriers were: (1) safety concerns, (2) transportation, (3) limited access to suitable activities, (4) lack of meaningful interactions with others in the neighborhood, and (5) public stigma surrounding dementia and aging. Participants emphasized neighborhood environments that protect older people with dementia's autonomy, interests, safety, and accessibility. Findings underscore the importance of expanding inclusive and affordable community programs for older people with dementia and empowering care partners and neighbors to facilitate social connectivity through senior-friendly transportation, assistance with adaptations to changes in the neighborhood environment, and public stigma reduction education. These community-driven insights provide actionable guidance for developing and advancing dementia-friendly neighborhoods that foster social connectedness and support aging in place.

Abstract citation ID: igaf122.2121

### DOES DISCRIMINATION DISCRIMINATE BY AGE AND ZIP CODE? AREA-LEVEL EFFECTS AMONG BLACK WOMEN

Brionna Colson-Fearon, and H. Shellae Versey, *Fordham University, New York, New York, United States*

Black women experience everyday discrimination (ED) across multiple levels and social positionalities (e.g., race, gender, and gendered racism). Yet research exploring how and whether age intensifies the effects of ED is limited. Moreover, prior literature suggests that reports of discrimination may vary according to spatial indices (i.e., neighborhood racial composition, segregation). Among a national sample of Black women (N = 944) aged 18-80, we examine whether reports of ED frequency vary by age and place. We also explore whether area-level ethnic-racial similarity (e.g., neighborhood-level segregation) plays a role in reporting frequency. That is, does the density of Black residents within an area (e.g., higher percentage of Black racial composition) moderate this association? Using hierarchical linear models and data from the Our Story, Our Voices Survey of Black Women collected between 2019-2020, we find significant associations between age and ED where older Black women generally report lower frequencies of discriminatory experiences, compared to younger Black women. Findings regarding area-level effects are mixed; it is unclear whether area-level density of majority Black residents functions as a buffer against frequency of ED. Results reveal future directions for research in intersectional and gerontological research. Implications for the growing diverse older adult population are discussed.

Abstract citation ID: igaf122.2122

### HOW NEIGHBORHOOD COHESION AND DISORDER SHAPE LATE-LIFE COGNITION: THE MEDIATING ROLE OF PSYCHOLOGICAL RESILIENCE

Guoping Jin, *University of Pittsburgh, Pittsburgh, Pennsylvania, United States*

Although prior research has linked neighborhood environments to cognitive health in later life, the mechanisms under these associations are less well understood. Drawing on the theoretical concept of resilience, this study examined whether psychological resilience mediated the effects of perceived neighborhood cohesion and disorder on cognitive functioning among older adults. Using the Health and Retirement Study 2010-2018 Leave Behind Questionnaire and RAND Data File, this study included 4,708 respondents aged 65 and older at baseline. Cognitive functioning was assessed using a modified 35-point Telephone Interview Cognitive Screen, while psychological resilience was measured with a simplified resilience scale based on prior research. Using path analyses, this study examined the potential direct and indirect effects of Time 1 neighborhood environment (predictor) and Time 2 psychological resilience (mediator) on Time 3 cognitive functioning (outcome), adjusting for sociodemographic and health-related covariates. The results indicated that greater neighborhood cohesion was significantly associated with higher levels of cognitive functioning ($\beta = 0.03$, $p < .001$), with psychological resilience partially mediating this effect (indirect effect = 0.01, $p < .001$). In contrast, neighborhood disorder was not significantly associated with cognitive functioning ($\beta = 0.1$, $p > .05$). These findings advance our understanding of the mechanisms through which neighborhood environments influence cognitive health in later life. Enhancing resilience may help leverage the cognitive benefits of neighborhood cohesion. Other implications for practice and future research are discussed.

### SESSION 4535 (SYMPOSIUM)

Abstract citation ID: igaf122.2123

### REMOTELY DELIVERED NONPHARMACOLOGIC TREATMENTS OF CHRONIC PAIN: INSIGHTS FROM EMBEDDED PRAGMATIC CLINICAL TRIALS

Chair: Kushang Patel
Discussant: M. Carrington Reid

Chronic pain increases with advancing age and contributes to decreased functioning, reduced quality of life, and high healthcare costs. Given the limited efficacy of analgesic medications and greater risk of adverse effects with advancing age, clinical practice guidelines recommend nonpharmacologic treatments. While evidence for nonpharmacologic pain treatments in older adults is accumulating, access to and uptake of nonpharmacologic treatments remain limited, particularly among rural-dwelling and minoritized populations. In the proposed symposium, we will report findings from 4 NIH-funded pragmatic clinical trials that are evaluating the implementation and effectiveness of remotely delivered nonpharmacologic treatments for chronic pain among middle-aged and older adults in health systems across the United States. First, Eric Roseen will present key challenges and solutions to implementing remotely delivered tai chi for knee osteoarthritis in 4 health systems in 4 geographic regions. Second, Elise Hoffman will present the adaptation and implementation of a telehealth delivered nurse care management program, involving care coordination, cognitive-behavioral therapy, and tele-exercise for rural primary care patients who have chronic pain. Third, Natalia Morone will present findings from a telehealth delivered, group-based mindfulness intervention in primary care patients with chronic low back pain. Fourth, Kushang Patel will report on age differences in the effects of remotely delivered self-paced and group-based mindfulness interventions (versus usual care) among Veterans who have chronic pain. Lastly, Cary Reid will deliver a critical discussion of the research findings and provide recommendations for future embedded pragmatic clinical trials, highlighting special considerations for improving pain management in older primary care patients.

Abstract citation ID: igaf122.2124

### PILOT IMPLEMENTATION OF A TELEHEALTH NURSE CARE MANAGEMENT MODEL FOR RURAL PRIMARY CARE PATIENTS WITH CHRONIC PAIN

Elise Hoffman, Sebastian Tong, Brennan Keiser, Basia Belza, and Kushang Patel, *University of Washington, Seattle, Washington, United States*

Prevalence of chronic pain is higher in rural areas than in urban areas. Rural residents have limited access to evidence-based chronic pain care, including non-pharmacologic treatments. Healthcare shortages, a lack of specialty care services, and aging populations in rural areas place a high demand for pain management on primary care providers. To help address this challenge, we implemented a telehealth nurse care management model that integrates care coordination, cognitive behavioral therapy, and referral to a tele-exercise program for rural primary care patients with chronic pain. We partnered with two rural-serving health systems in Northeast Washington and Central North Carolina to pilot the intervention. We trained two care managers to deliver the intervention to patients for 6 months. Among 31 participants who were enrolled, the mean age was 57.2 years (SD = 11.9), 20 (65%) were female, 23 (74%) had an annual household income <$50,000, and 26 (84%) were not employed. The mean baseline pain intensity and interference (0-10 rating) score was 6.8 (SD = 1.8). Exit interviews with participants, care managers, and local champions will be completed in March-April 2025. Data analysis will be guided by the COM-B Model for Behavior Change and Framework for Reporting Adaptations and Modifications (FRAME). We will present the patient-reported outcome results from the pilot study, as well as qualitative data on the experience of rural patients and nurse care managers with the intervention. Lastly, we will discuss adaptations made to the intervention in preparation for a larger pragmatic trial in 416 patients from 4-5 rural-serving health systems.

Abstract citation ID: igaf122.2125

### BARRIERS AND FACILITATORS TO REMOTE TAI CHI FOR KNEE OSTEOARTHRITIS IN LARGE HEALTHCARE SYSTEMS: A QUALITATIVE STUDY

Eric Roseen[1], Lance Laird[1], Rocky Reichman[1], Ludovic Trinquart[2], Brian Mittman[3], Helen Lavretsky[4], Robert Saper[5], and Chenchen Wang[2], *1. Boston University, Boston, Massachusetts, United States, 2. Tufts University, Medford, Massachusetts, United States, 3. Kaiser Permanente Southern California, Pasedena, California, United States, 4. University of California, Los Angeles Healthcare, Los Angeles, California, United States, 5. Cleveland Clinic, Cleveland, Ohio, United States*

While clinical practice guidelines from the American College of Rheumatology strongly recommend Tai Chi for treating knee osteoarthritis (OA), adoption of Tai Chi for Knee OA in healthcare systems is low. Our aim is to identify multi-level barriers and facilitators to embed a remote Tai Chi intervention for knee OA in four large healthcare systems. We conducted 30- to 60-minute interviews among individuals from four healthcare systems in California, Ohio, Florida, and Massachusetts from September 2023 to June 2024. Recorded semi-structured interviews were transcribed verbatim. Participants were asked about factors influencing use of in-person or remotely delivered Tai Chi. Major themes were identified using thematic content analysis and an a priori codebook based on the Consolidated Framework for Implementation Research. We interviewed 59 participants (Median age=58 [range=36-85] years; 54% women; 42% White) across four large healthcare systems (13 patients, 16 primary care providers, 12 Tai Chi instructors, 18 health system leaders). Logistical challenges (e.g., transportation, scheduling, technology access) and lack of insurance coverage were considered main barriers. PCPs were also unsure how to describe Tai Chi and did not have a mechanism for making referrals or communicating with Tai Chi instructors via the electronic health record. These results have informed recruitment efforts to address known barriers in an ongoing multi-site embedded pragmatic trial of remote Tai Chi for 480 patients with knee osteoarthritis. However, long-term adoption may also rely on addressing policy barriers such as insurance coverage for tai chi or credentialling of tai chi instructors in large healthcare systems.

Abstract citation ID: igaf122.2126

### MINDFULNESS GROUP VISITS FOR CHRONIC LOW BACK PAIN IN PRIMARY CARE: THE OPTIMUM PRAGMATIC CLINICAL TRIAL

Natalia Morone[1], Tra Nguyen[2], Keturah Faurot[3], Susan Gaylord[3], Kathleen McTigue[4], Kristina Astone[5], Janice Weinberg[5], and Carol Greco[4], *1. Boston University - Boston Medical Center, Boston, Massachusetts, United States, 2. Boston Medical Center, Boston, Massachusetts, United States, 3. The University of North Carolina at Chapel Hill, Chapel Hill, North Carolina, United States, 4. University of Pittsburgh, Pittsburgh, Pennsylvania, United States, 5. Boston University, Boston, Massachusetts, United States*

Mindfulness is recommended as a first-line non-pharmacologic treatment for chronic low back pain (cLBP). However, accessibility is challenging. The OPTIMUM trial (Optimizing Pain Treatment In Medical settings Using Mindfulness) compared a telehealth-delivered group mindfulness program for primary care patients with cLBP to usual care. Here we describe the methods of this investigation, and report on baseline sample characteristics, intervention acceptability, and retention. The intervention is an 8-session program based upon Mindfulness-Based Stress Reduction (MBSR) led by an experienced MBSR instructor and co-led by a primary care provider who participated in sessions and met briefly with each participant. 451 adults with cLBP were randomized to the intervention or to usual medical care, across three sites. The primary outcome was the PEG (Pain intensity, Enjoyment, General Activity). Participants also completed measures of physical and psychological function, medications, and healthcare utilization, at baseline, week-8, and 6- and 12-months. Participants' mean age=52.1 years (SD 14.7), 315 (70%) female, 176 (39%) are Black, 202 (45%) White, 253 (56%) not employed, 140 (31%) income < $25,000, 332 (74%) have pain ≥ 5 years duration. Participants' pain intensity/pain interference was moderate at baseline, with PEG score=6 (SD 2.4). Retention was 89% at 12-month follow-up. OPTIMUM successfully enrolled and retained primary care patients with cLBP into a telehealth group mindfulness program. The program holds promise for integration into routine primary care medicine.

Abstract citation ID: igaf122.2127

### AGE DIFFERENCES IN THE EFFECTIVENESS OF TELEHEALTH MINDFULNESS-BASED INTERVENTIONS FOR CHRONIC PAIN

Kushang Patel, *University of Washington, Seattle, Washington, United States*

Mindfulness-based interventions (MBIs) are evidence-based treatments for managing chronic pain in adults. However, few studies have assessed heterogeneity in the effectiveness of remotely delivered MBIs by age. Given the high burden of pain among older adults and the potential scalability of telehealth MBIs, we explored potential age differences in the effectiveness of two 8-week telehealth MBIs. The primary outcome was pain interference as measured by the Brief Pain Inventory scale (higher scores are worse) collected at baseline, 10 weeks, 6 months, and 12 months. A total of 811 Veterans with moderate-to-severe chronic pain were randomized to either synchronous group-based MBI (n = 270), asynchronous self-paced MBI (n = 271), or usual care (n = 270). The numbers of older participants (age ≥65 years) by treatment assignment were 59 (21.9%) in group-based MBI, 71 (26.2%) in self-paced MBI, and 58 (21.5%) in usual care. Adjusting for gender, study site, and baseline pain interference and self-efficacy, older participants in self-paced MBI reported significantly less pain interference compared with those in usual care at 10 weeks (mean difference=-0.7 [95% CI: -1.3, -0.1]), but there were no significant between-group differences at 6 and 12 months. In contrast, there were no significant differences in pain interference between older participants in group-based MBI versus usual care at any post-treatment timepoint. Among younger participants (<65 years old), both MBIs significantly decreased pain interference at each post-treatment timepoint compared with usual care. Findings suggest that self-paced MBI can improve pain interference among older Veterans. Larger trials of telehealth MBIs in older adults are needed.

### SESSION 4540 (SYMPOSIUM)

Abstract citation ID: igaf122.2128

### MEASUREMENT CHALLENGES AND SOLUTIONS IN CROSS-NATIONAL COGNITIVE AGING RESEARCH

Chair: Alden Gross
Co-Chair: Lindsay Kobayashi
Discussant: Emma Nichols

Measurement precedes science and hypothesis testing. Particularly for cross-national aging research, there are profoundly important measurement issues around whether instruments measure what they are supposed to measure (validity), whether instruments provide comparable measurement across countries and contexts (invariance), and shifting reliability of measures. Presentations in this symposium highlight common challenges and propose innovative solutions to many such intractable problems with cross-national data, related to construct validation, measurement invariance, and differential measurement precision, which will be highlighted in this symposium. The first presentation by Cho and colleagues reports on a systematic review of dementia algorithms, several developed in lower and middle income countries, with a goal to provide guidance on the rubrics to evaluate dementia classification algorithms and how well they represent the construct of dementia around the world. Cantu and colleagues present an analysis of differential item functioning to uncover measurement differences between the US and Mexico in informant reports of cognitive functioning. Also speaking to the importance of evaluating measurement invariance, Gonzalez and colleagues examine measurement differences by literacy in India and Mexico, where they found differences in the measurement of language ability by literacy status in both countries. Finally, a presentation by Andrews will illustrate the value of simulation to evaluate whether differences in measurement precision of neuropsychological test batteries across country, as well as changes in precision within a country, provides biased inferences in cross-national cognitive aging research. Dr. Emma Nichols, Methods Lead for the Gateway to Global Aging Exposome Coordinating Center, will be the discussant. This is a collaborative symposium between the Brain, Epidemiology of Aging, International Comparisons of Healthy Aging, and Measurement, Statistics, and Research Design Interest Groups.

Abstract citation ID: igaf122.2129

### DIFFERENTIAL ITEM FUNCTIONING OF INFORMANT MEASURES OF COGNITIVE FUNCTIONING IN THE U.S. AND MEXICO

Phillip Cantu[1], Joce Jaen[2], Douglas Tommet[3], Richard Jones[3], and Alden Gross[4], *1. UTMB Health, The University of Texas Medical Branch, Galveston, Texas, United States, 2. Rush University, Chicago, Illinois, United States, 3. Brown University, Providence, Rhode Island, United States, 4. Johns Hopkins Bloomberg School of Public Health, Baltimore, Maryland, United States*

Population studies of cognitive functioning rely on survey questionnaires to estimate the prevalence of dementia in nationally representative samples. In addition to direct tests of cognitive functioning, the international studies of the Harmonized Cognitive Assessment Protocol also include informant questionnaires, in which family members or other persons close to the research participant are asked about the participant's cognitive functioning. While international harmonized measures of directly assessed cognitive have been developed, there are no harmonizations of informant assessments. This study investigates differences in how informants respond to questions about cognitive functioning in the US and Mexico. Using data from the Harmonized Cognitive Assessment Protocol (HCAP) of the Health and Retirement Study (HRS) (n = 3,183) in the United States and the Mexican Cognitive Aging Study (MexCOG) (n = 1,259) in Mexico, we examined informant reports of cognitive functioning of the Community Screening Instrument for Dementia (CSID). We test for differential item functioning (DIF) using structural equation modeling (SEM) to develop a confirmatory factor analysis (CFA) model of the CSID informant questionnaire in Mexico and the U.S. We find that a harmonized measure of the CSID has fixed factor loadings between countries but different item thresholds. Our harmonized measure of informant reports of cognitive functioning suggests that informants report less cognitive impairment for older adults in Mexico when controlling for directly assessed cognitive functioning. Our results suggest that either the harmonized direct assessment of cognition may underestimate cognitive functioning in Mexico or that informants in Mexico are systematically underreporting symptoms of cognitive decline.

Abstract citation ID: igaf122.2130

### EXAMINING MEASUREMENT INVARIANCE IN THE ASSESSMENT OF LANGUAGE ACROSS LITERACY STATUS IN INDIA AND MEXICO

Alexa Gonzalez[1], Gelan Ying[2], Pranali Khobragade[3], Miguel Arce Rentería[4], Zachary Kunicki[5], and Emily Briceño[6], *1. University of Houston, Houston, Texas, United States, 2. University of Florida, Gainesville, Florida, United States, 3. University of Southern California, Los Angeles, California, United States, 4. Columbia University Irving Medical Center, New York, New York, United States, 5. Warren Alpert Medical of Brown University, Providence, Rhode Island, United States, 6. University of Michigan Medical School, Ann Arbor, Michigan, United States*

Accurate neuropsychological assessment is essential for evaluating cognitive decline, particularly across culturally and linguistically diverse populations with varying levels of formal education. Ensuring cognitive tests measure the same constructs across those with varying levels of literacy is crucial, especially when assessing language abilities. Therefore, we evaluated the measurement invariance properties of the language domain across literate and illiterate older adults in India and Mexico. Data were drawn from the Longitudinal Aging Study in India–Diagnostic Assessment of Dementia's (LASI-DAD) and the Mexican Health and Aging Study Cognitive Aging Ancillary Study's (Mex-Cog) Harmonized Cognitive Assessment Protocol (HCAP). In each cohort, a multiple-group confirmatory factor analysis was used to determine the level of invariance achieved in the latent language factor, composed of items measuring verbal fluency, item naming, and comprehension. As demonstrated by acceptable fit, results supported configural invariance in LASI-DAD (CFI=.94; RMSEA=.03; SRMR=.03) and Mex-Cog (CFI=.93; RMSEA=.04; SRMR=.03). However, relative fit statistics significantly differed between configural and metric invariance in LASI-DAD ($\Delta\chi^2$=49.88; $\Delta$df = 13; $p < .001$) and Mex-Cog ($\Delta\chi^2$=39.62; $\Delta$df = 9; $p < .001$); therefore, full metric invariance is not supported, suggesting language measurement differs by literacy status. This study highlights the need for careful evaluation of measurement properties when assessing language function across culturally and linguistically diverse adults with varied levels of literacy. Future studies leveraging these HCAP cohorts should adjust the language domain due to these measurement differences driven by literacy and evaluate whether other cognitive domains are similarly affected.

Abstract citation ID: igaf122.2131

### LITERATURE REVIEW OF ALGORITHMS FOR CLASSIFYING DEMENTIA AND MILD COGNITIVE IMPAIRMENT

Tsai-Chin Cho[1], Chihua Li[2], Kelvin Zhang[1], Wenjie Cai, Kenneth Langa[1], Lindsay Kobayashi[1], and Alden Gross[3], *1. University of Michigan School of Public Health, Ann Arbor, Michigan, United States, 2. University of Macau, Macao, Macao, Macau, 3. Johns Hopkins University Bloomberg School of Public Health, Baltimore, Maryland, United States*

Demand is increasing for research algorithms that classify mild cognitive impairment and Alzheimer's disease and related dementias (AD/ADRD) based on a set of rules and data features. Rules may include well-established criteria, such as the NINCDS-ADRDA Alzheimer's Criteria, Diagnostic and Statistical Manual of Mental Disorders (DSM), Clinical Dementia Rating (CDR), or National Institute on Aging and Alzheimer's Association (NIA-AA) framework, as well as ad hoc machine learning methods. Data used in algorithms may contain one or multiple features, such as cognitive performance scores, functional performance scores, informant reports, or brain imaging data. Despite the availability of numerous algorithms, there remains a lack of guidance on the rubrics to evaluate dementia classification algorithms built upon clinical or observational data and validated by different gold standards. We thus conducted a systematic review and meta-analysis of existing dementia algorithms to summarize and compare their construction. We identified 4,565 published articles from January 01, 2013, to February 28, 2025 from PubMed and PsycINFO. After screening abstracts and full text, 451 articles were included. Existing algorithms relied on similar data sources: 48.8% leveraged the clinical and imaging data from the Alzheimer's Disease Neuroimaging Initiative (ADNI). Almost all articles evaluated algorithmic criterion validity against a gold standard, most commonly clinical or consensus diagnosis (67.9% of algorithms). This critical appraisal of algorithms for distinguishing dementia and mild cognitive impairment from neurocognitively healthy individuals in published studies will help guide future investigation into developing and implementing algorithms of dementia classification.

Abstract citation ID: igaf122.2132

### EXAMINING THE CONSEQUENCES OF NEUROPSYCHOLOGICAL TEST BATTERY CHANGES ON MEASURING COGNITIVE DECLINE

Ryan Andrews[1], Brandon Gavett[2], Natalia Gomes Goncalves[3], Emma Nichols[4], Kendra Sims[5], Douglas Tommet[6], Yingyan Wu[7], and Dan Mungas[2], *1. Columbia University, New York, New York, United States, 2. University of California, Davis, Davis, California, United States, 3. Universidade de São Paulo Medical School, São Paulo, Sao Paulo, Brazil, 4. University of Southern California, Los Angeles, California, United States, 5. Boston University, Boston, Massachusetts, United States, 6. Brown University, Providence, Rhode Island, United States, 7. University of California, Los Angeles, Los Angeles, California, United States*

Large, representative studies of older adults have collected longitudinal cognitive data, but cognitive batteries often change over time. When cognitive batteries change, changes in measurement properties may result in misleading findings about cognitive trajectories. Using the Health and Retirement Study (HRS) as an example, we used simulation methods to assess the magnitude of bias in estimating cognitive decline induced by changing test batteries over time. We first simulated true cognition values using non-linear models of cognitive change derived from four harmonized longitudinal cognitive aging studies. We then used item response theory (IRT) methods to simulate measured test results for different cognitive testing batteries, including the introduction of the Harmonized Cognitive Assessment Protocol (HCAP) for a subset of HRS participants. We then estimated blended cognitive trajectories, artificially introducing changes of the simulated test to measure cognition. To illustrate the impact of these changes, we used linear mixed-effects models to estimate 11-year cognitive trajectories, overall and by quartile of true decline. Using instruments with the highest measurement precision led to estimated cognitive trajectories that best matched the truth; however, estimated trajectories using less informative test versions did not deviate from the truth by an appreciable amount. As such, we conclude that in practice, changes in test batteries over time may not meaningfully affect estimates of cognitive decline when IRT scoring is used.

### SESSION 4545 (SYMPOSIUM)

Abstract citation ID: igaf122.2133

### REDUCING SOCIAL ISOLATION AND LONELINESS THROUGH CREATIVE AGING INITIATIVES

Chair: Candace Cramer
Discussant: Robyn Stone

This symposium aligns with the Social Research, Policy, and Practice Section's focus on social, economic, and environmental contexts of aging by examining the role of arts-based interventions in fostering social connectedness among historically marginalized individuals and communities. Convened by Goddard House, a non-profit provider of assisted living and memory care which also offers creative aging programs in underserved neighborhoods throughout Greater Boston, this session will contribute to ongoing discourse on the implementation and evaluation of community-engaged interventions that enhance quality of life for older adults. Candace Cramer, MA, MBA, CEO of Goddard House, will moderate the symposium and provide an overview of Goddard House's philosophy of creative aging programs. Katherine Richman, PhD, and Caitlin Coyle, PhD, will summarize evaluation findings of MusicWorks, a program offered at affordable senior housing sites in Boston that fosters community through music that resonates with a culturally and economically diverse group of older adults. Eileen Tell, MPH, and Jacob Watson, EdM, will provide a summary of their comprehensive literature review and environmental scan—the first since 2015—highlighting the evidence base on the effectiveness of creative engagement strategies in reducing social isolation and loneliness. This summary, in conjunction with the findings of the MusicWorks evaluation, demonstrates new and promising approaches to reduce social isolation and loneliness among older adults. Robyn Stone, DrPH, will serve as a discussant, providing a critical analysis of how community-based participatory arts programs can inform broader policy and practice to enhance the well-being of older adults who face systemic barriers to social inclusion.

Abstract citation ID: igaf122.2134

**CULTIVATING CREATIVITY AND CONNECTION: GODDARD HOUSE'S INNOVATIVE CREATIVE AGING PROGRAMS**

Candace Cramer, *Goddard House, Brookline, Massachusetts, United States*

Creative Aging Connections (CAC), a Goddard House initiative, embodies the principles of creative aging, a concept substantiated by Dr. Gene Cohen's 2006 landmark study which highlights the significant benefits of arts-based learning for the health and wellness of older adults. CAC's programs leverage these insights to reduce social isolation among under-resourced older adults in Greater Boston, fostering individuality, expression, social connection and belonging through diverse artistic mediums. CAC operates and sponsors creative aging programs that are offered in affordable senior housing buildings and community-based sites in Greater Boston. These programs are informed by our experience engaging with older adults in our assisted living community, and by a literature review and environmental scan of the role and efficacy of art-based interventions in older adult populations, both of which confirm the importance of participant voice and choice, cultural relevance, and high-quality instruction. These elements are crucial in enhancing physical and emotional well-being among older adults. This presentation will show how CAC integrates these research-backed strategies into our creative aging programs, focusing on the positive outcomes of engaging diverse older adults in art forms that resonate with their experiences and needs. One of our sponsored programs, MusicWorks, will present results from a recent qualitative analysis suggesting that music programming offers an outlet for connection as well as positive physical and cognitive outcomes. We will also discuss the challenges and considerations in replicating and scaling such programs, emphasizing the impact of duration, dosage, and participant engagement on the success of these initiatives.

Abstract citation ID: igaf122.2135

**CREATIVE AGING LITERATURE REVIEW: THE ROLE AND EFFICACY OF ARTS-BASED INTERVENTIONS IN OLDER ADULT POPULATIONS**

Eileen Tell[1], and Jacob Watson[2], *1. ET Consulting, LLC, Bemont, Massachusetts, United States, 2. The Practice, Chicago, Illinois, United States*

The term "creative aging" comes from a 2006 landmark study by Dr. Gene Cohen (The Creativity and Aging Study) which evidenced the promise for arts-based learning to support overall health and wellness of older adults. As lifespans increase, there is a need for interventions that sustain the well-being of older adults living longer lives. This presentation reviews the relevant literature and offers an environmental scan of best practices in creative aging to provide design considerations and practical recommendations for those interested in bring creative aging initiatives into their practice. Specifically, we explore prior research across various art forms and the outcomes identified regarding: cognitive and physical health; mental and emotional well-being; and health care spending and/or utilization. The literature review summarizes measurement practices, study designs, research limitations, and key findings. The environmental scan describes the variety of sites and program types where creative aging takes place including: senior/assisted living communities; arts-based organizations (museums, theatres, galleries) and public and community spaces. We present evidence that participant voice and choice, cultural relevance and quality of instruction have a critical impact on the success of the intervention and the social, emotional, and cognitive benefits to participants than any single medium or art form. We conclude with considerations for program design and challenges regarding replicability and bringing programs to scale, such as duration and dosage, choice of art form, active vs. passive participation, instruction quality, and research design.

Abstract citation ID: igaf122.2136

**DEPTH OR BREADTH? ASSESSING THE IMPACT OF MUSICWORKS, A CREATIVE AGING MUSIC PROGRAM**

Katherine Richman[1], Julie Burkley[2], and Caitlin Coyle[1], *1. University of Massachusetts Boston, Boston, Massachusetts, United States, 2. FriendshipWorks, Boston, Massachusetts, United States*

Creative aging programs are essential for promoting cognitive health, social engagement, and emotional well-being among older adults. They provide opportunities for self-expression, help to combat isolation, and enhance memory. By fostering creativity through music, and storytelling, these programs empower older adults to stay active, connected and fulfilled. This presentation shares results from a mixed methods evaluation of MusicWorks, a program that uses music and music-related activities to create opportunities for residents of low-income buildings to socialize and interact with neighbors, thereby reducing isolation and loneliness. Through the integration of music-related activities (e.g. singing, dancing, music bingo), the program also seeks to promote cognitive stimulation and physical health. Data was collected via 9 focus groups (4 in Spanish) at 7 program sites around Boston ($n=92$). Staff from 6 sites were also interviewed. Attendance records from 6 of the sites revealed that for most sites, there were first-time participants in attendance as well as those who attend regularly. There was some variation in terms of which sites had more frequent participation compared to others and this trend seemed to align with the frequency of programming (e.g., sites with monthly programming had less regular attendance compared to those with weekly programming). Results from the qualitative analysis suggest that music programming offers an outlet for connection as well as positive physical and cognitive outcomes. Deep social connections and endearment to the program comes with repeat participation, suggesting that depth is achieved by creating group dynamics. Facilitators and barriers to these outcomes will be discussed.

### SESSION 4550 (SYMPOSIUM)

Abstract citation ID: igaf122.2137

**DIRECT CARE WORKERS AND JOB QUALITY: AMPLIFYING VOICES AND IDENTIFYING EMPOWERMENT PRACTICES**

Chair: Jennifer Morgan
Co-Chair: Philip Taylor
Discussant: Deke Cateau

Direct care workers across the long-term care sector experience low compensation, few benefits (e.g. paid leave, affordable health insurance, affordable mental health coverage), and heavy workloads that leave these workers prone to both burnout and injury. These job-related challenges layer on other vulnerabilities that are keyed to social position and resource constraints. The implementation of person-centered care is dependent on the capacity and stability of direct care workers across the long-term care sector. Trends in where older adults receive care, including those living with dementia, are shifting and rely on collaboration with unpaid care partners. Direct care workers are pivotal to the ability of long-term care services and supports to deliver high quality care to older adults and people with disabilities. This symposium seeks to amplify the voices of direct care workers across the long-term care sector and identify empowerment practices that can be supported through policy and practice. The first paper, the authors provides an overview of the employment trends of direct care workers by employment and occupation. In the second paper, the authors engage a life course perspective to identify social position and relational factors that nursing assistants find relevant to their perceptions of job satisfaction and intent to stay. The third paper focuses on giving voice to direct care workers in assisted living as they characterize their work. The fourth paper identifies coping strategies and elaborates organizational policies and practices that could support direct care workers. The final paper explores these issues in the VA context.

Abstract citation ID: igaf122.2138

### TRENDS IN THE U.S. DIRECT CARE WORKFORCE, 2013 TO 2023

Christopher Kelly, and Jerome Deichert, *University of Nebraska Omaha, Omaha, Nebraska, United States*

We used data from the 2013-2023 American Community Survey to examine trends among direct care workers (DCWs) in both occupation and industry. We found the number of medical aides (i.e., nursing assistants, psychiatric aides, and home health aides) decreased 20.9%, from 2.31 million in 2013, to 1.83 million in 2023, while the number of nonmedical aides (i.e., personal and home care aides) increased 19.2%, from 1.19 million to 1.42 million over the same 10-year period. Medical aides were more likely than nonmedical aides to work in an institutional setting (i.e., hospitals, nursing homes, assisted living), to be under age 25, to be Latina(o), to be African American, and to be never married or recently married. From 2013 to 2023, there were increases in the number of DCWs employed by home health care services (8.9%) and individual and family services (34.4%). There were decreases in the number of DCWs employed by skilled nursing facilities (35.1%), outpatient care centers (29.0%), hospitals (20.0%), assisted living facilities (10.1%), and private households (15.6%). DCWs employed in institutional settings were more likely to be medical aides, to be male, to be under age 25, to be African American, to be never married, and to work year-round and full-time. These findings suggest two major shifts in the direct care workforce between 2013 and 2023. Direct care workforce jobs are trending towards non-medical aides and to home and community-based services. Further, the DCWs employed as medical or non-medical aides, or in institutional or non-institutional settings, are increasingly distinct.

Abstract citation ID: igaf122.2139

### USING A LIFE COURSE APPROACH TO UNDERSTAND NURSING ASSISTANT PERSPECTIVES ON JOB SATISFACTION AND INTENT TO STAY

Waqar Ahmad[1], Jennifer Morgan[2], Elisabeth Burgess[3], Antonius Skipper[2], and Eric Wright[2], *1. A.G. Rhodes, Atlanta, Georgia, United States, 2. Georgia State University, Atlanta, Georgia, United States, 3. University of North Georgia, Dahlonega, Georgia, United States*

Nursing home staff, particularly direct care workers, experience low job quality which includes low levels of compensation, few benefits, heavy workloads and very little career mobility. This study uses a life course framing to incorporate and amplify CNAs voices on how their working lives unfold in nursing homes (NHs). Using a modified grounded theory approach, we analyze interview data from 25 certified nursing assistants (CNAs) in two NHs as structured by previous in-depth quantitative analysis of the predictors of job satisfaction and intent to stay for nursing home staff. Our analysis provides valuable insights into a range of factors affecting CNAs' working lives as well as the quality of care in long term care settings. We highlight how workers' social locations are linked to job satisfaction and retention. We find evidence for the role of underexplored contingency factors, e.g., single breadwinner, health insurance, and coworker support in staying in the job and meaningful care partnership. We further find that broader life course factors and processes in the workplace, e.g., supportive leadership, equity and equality, shared culture and values, continuous education & training, and relationships in NHs significantly influence CNAs retention and interest in how care is delivered in NHs. Our findings suggest they need for key changes to organizational practice including increased access to mental health services, professional support, increased leave for caregiving and respite, improved access to high-quality health insurance and benefits that assist this workforce to deal with contingency factors and promote career mobility.

Abstract citation ID: igaf122.2140

### "A LOT OF PEOPLE CAN'T DO THIS": VOICES OF DIRECT CARE WORKERS IN ASSISTED LIVING

Candace Kemp, and Jennifer Morgan, *Georgia State University, Atlanta, Georgia, United States*

In this paper, our focus is on giving voice to and understanding the lived on-the-job experiences of assisted living (AL) staff, including direct care workers and engagement staff. We present analysis of qualitative interviews with staff (n = 77) collected as part of a study focused on meaningful engagement and quality of life for AL residents with dementia. We collected data in 8 diverse care communities located in and around Atlanta, Georgia, each studied for one year between 2019 and 2024. Staff voices unified around having "heart" for care work being drawn to and remining in the job for the "love of residents." Most characterized their work as "hard," requiring "patience," and "not something just any can do." Consequently, many viewed the material rewards and status of their work as being incommensurate with its importance and level of difficulty. One care worker explained, "I wish it pays more" because "wiping behinds and dealing with different personalities . . . I should be eating off of a silver platter, because a lot of people can't do this." Most reported ongoing job quality and workload challenges. Additional challenges arose from residents' family members being uninvolved or well-intentioned yet lacking sufficient dementia knowledge. Empowerment practices that increase worker capacity and improve job quality and career mobility are needed across assisted living. The role of training, competency development, specifically in the area of dementia care, and employer of choice practices to support career development across this sector are discussed.

Abstract citation ID: igaf122.2141

**NAVIGATING THE CHALLENGES: IDENTIFYING COPING STRATEGIES TO EMPOWER DIRECT CARE WORKERS IN NURSING HOMES**

Alfred Boakye[1], and Jennifer Morgan[2], *1. University of Maryland, Baltimore, Baltimore, Maryland, United States, 2. Georgia State University, Atlanta, Georgia, United States*

A growing body of literature examines the challenges direct care workers face in the nursing home setting. Workforce shortages, low job quality, high workloads, and rising acuity make this context particularly fraught. Less attention has been devoted to exploring how nursing home staff can manage the everyday trauma they experience to improve their quality of life and work in the light of the multilayered social-based vulnerabilities inside and out of nursing homes. This study examines the various individual, organizational, and community coping strategies to help mitigate the challenges impacting direct care workers in nursing homes. Using a narrative exploratory design, we conducted 25 semi-structured interviews with certified nurse assistants recruited from two nursing home sites in the southeastern part of the United States. Member-checking or respondent validation was used to ensure accuracy and credibility. We find that individual strategies (e.g., faith/religion, personality traits/learned behaviors, recreational/self-care activities, and family history/experiences), organizational strategies (e.g., better compensation, developmental training & extracurricular/self-care activities), and community/family strategies (e.g., support groups, i.e., family & non-kin family) were instrumental in quality of life outcomes for certified nurse assistants as well as person-centered care for residents. Adopting and implementing coping strategies is foundational to building support for staff and ensuring they can understand and manage trauma and strengthen relationships. The study findings have implications for how to address multiple layers of factors (e.g., policy, organizational practices, and culture change initiatives) that impact carework and resident care in ways that support resilience-building and, ultimately, empowerment practices.

Abstract citation ID: igaf122.2142

**FACILITATORS OF NURSING ASSISTANT JOB COMMITMENT AND CAREER RETENTION IN VHA COMMUNITY LIVING CENTERS**

Katherine Kennedy[1], Nathalie Mcintosh[2], Sylvia Haigh[3], David Mohr[4], Ciaran Phibbs[5], and Whitney Mills[6], *1. Providence VA Healthcare System, Providence, Rhode Island, United States, 2. THRIVE, Providence VA Medical Center, Providence, Rhode Island, United States, 3. VA Providence Healthcare System, Providence, Rhode Island, United States, 4. Veterans Health Administration, Mason, Ohio, United States, 5. Health Economics Resource Center, VA Palo Alto Health Care System, Menlo Park, California, United States, 6. Brown University, Providence, Rhode Island, United States*

Job satisfaction matters for the retention of long-term care aides; however, we know little about this in the Veterans Health Administration (VHA) setting. This study examined the facilitators and barriers to NA job commitment in VHA Community Living Centers (CLCs; VHA nursing homes). We recruited 4 CLCs and interviewed 6-9 staff at each (N = 30), of which 17 were NAs and 13 were nurse leaders. We employed qualitative content analysis based on codes relating to Paraprofesional Healthcare Institute's (PHI) Five Pillars of Direct Care Job Quality. 100% (N = 17/17) of NAs reported intention to remain employed at the CLC in the next 12 months and/or reported high job satisfaction. Interviewees reported many factors related to job commitment and satisfaction. Facilitators included VA benefits (e.g., healthcare, retirement), passion for NA work, relationships with the Veterans, favorable work conditions (e.g., workload, safe patient handling equipment, training, steady work), and a supportive work environment (e.g., supportive managers/coworkers, teamwork). Suggestions for improvement in retention included addressing dissatisfaction with pay (e.g., non-competitive, pay caps), scheduling, career advancement, staffing (e.g., shortage, human resource (HR) challenges), management practices (e.g., mandatory overtime, inability to take requested leave, floating), staff- and resident-to-staff interactions (e.g., disrespect), and structural supports (e.g., no onsite HR). Job commitment and satisfaction are multilayered. Despite desiring certain changes, NAs remain committed to their roles. VHA nursing home care has been affected by general nursing workforce shortages. Promoting policy change related to NA wages and pay caps concurrent with supportive training, scheduling, and employee wellness may improve retention.

### SESSION 4555 (SYMPOSIUM)

Abstract citation ID: igaf122.2143

**AGE INCLUSIVITY IN HIGHER EDUCATION: ENSURING EXCELLENCE FOR STUDENTS, FACULTY, AND STAFF**

Chair: Jacqueline Eaton
Co-Chair: Rebekah Perkins
Discussant: Nina Silverstein

Age inclusivity has positive outcomes for learning, including improving health, reducing social isolation, and decreasing ageism. Yet in higher education, age-stratification is often reinforced in how we view traditional vs nontraditional students and role expectations based on individual definitions of age. This symposium explores an initiative to assess age-friendly practices and beliefs and iteratively develop opportunities to improve educational excellence by addressing age-friendly practices. Eaton et al., discuss the use of the Age-Friendly Inventory and Campus Climate Survey (ICCS) to explore the congruence between practice and perceptions in a College of Nursing. Perkins et al., describe the process of planning and implementing focus groups, within a College of Nursing, to understand faculty, staff, and student needs specific to age inclusivity. Hart et al., detail the impact stemming from the "Excellence Across the Lifespan Champions" program, developed in partnership with the National Center to Reframe Aging to cultivate institutional culture change. Friberg-Felsted et al., explore the process of seeking accreditation through the Accreditation for Gerontology Education Council. Dr. Nina M. Silverstein will facilitate discussion on the process of assessing and developing initiatives to foster age inclusivity in higher education.

Abstract citation ID: igaf122.2144

**EXPLORING THE CONGRUENCE BETWEEN AGE FRIENDLY PRACTICE AND PERCEPTIONS IN A COLLEGE OF NURSING**

Jacqueline Eaton, Katarina Friberg-Felsted, Rebekah Perkins, Joshua Seabury, Kara Dassel, and Sara Hart, *University of Utah, Salt Lake City, Utah, United States*

Fostering an age-friendly environment is imperative to gerontology and nursing education. In an effort to advance University of Utah College of Nursing (CON) educational excellence, we implemented the Age-Friendly Inventory (AFI) and Campus Climate Survey (ICCS) to identify strengths and weaknesses in our College. The purpose of this presentation is to describe age friendly practices across the University of Utah and how these align with perceptions from CON students, faculty, and staff. Fourteen units across campus completed individual sections of the AFI. CCS surveys were distributed electronically to gather CON perceptions of age-friendly practices (N = 240). The AFI was analyzed to identify practices across seven domains. Congruence between AFI and CCS responses was analyzed to identify agreement between awareness and practice. Age friendly practices were most present in the domain of Physical Environment (94%), and Personnel (78%) and least present in Teaching and Learning (50%). Overall congruence demonstrated low match between practice and perception (M = 0.37, SD = 0.13). The highest congruence was faculty response to the Research domain (M = 0.70, SD = 0.40) while the lowest congruence was staff response to the domain of Outreach and Engagement (M = 0.06, SD = 0.09). Practice and perception alignment was low overall, demonstrating a need to improve awareness of age-friendly practices across faculty, staff, and students in the CON. We are using these results to create professional development opportunities targeting CON needs.

Abstract citation ID: igaf122.2145

### INSIGHTS FROM STAFF AND FACULTY FOCUS GROUPS TO ADVANCE AGE-INCLUSIVE PROFESSIONAL DEVELOPMENT

Rebekah Perkins, Joshua Seabury, Katarina Friberg-Felsted, Kara Dassel, Sara Hart, and Jacqueline Eaton, *University of Utah, Salt Lake City, Utah, United States*

Ensuring age-inclusivity in higher education is essential for fostering professional development among staff and faculty, supporting student success, and strengthening workforce sustainability. However, higher education remains largely age-stratified, often overlooking the professional development needs of a multigenerational workforce and student body. Applying a lifespan lens, the purpose of this presentation is to describe the process of planning and implementing focus groups, within a College of Nursing, to understand faculty, staff, and student needs specific to age inclusivity. Baseline results from the Age-Friendly Inventory and Campus Climate Survey (ICCS) (n = 240) and two student focus groups (n = 13) exploring non-traditional (25+) student experiences informed the focus group interview guide. Three focus groups with staff and faculty (n = 15) and one validation focus group with staff leadership (n = 8) were conducted to gather feedback on ICCS and student focus group results. Analyzed using Braun and Clarke's thematic analysis, six themes were identified: Mentorship Needs emphasized structured onboarding support. Support Resources highlighted gaps in student success strategies. Succession Planning addressed knowledge transfer. Intergroup Collaboration revealed a need to strengthen engagement across faculty, staff, and students. Healthy Aging Representation emphasized work-life balance considerations. Policy Considerations emphasized institutional policies with a multigenerational workforce Findings highlight the need for lifespan awareness in professional development. Addressing these training needs supports student retention, staff and faculty engagement, and institutional sustainability. Informing the Champions Across the Lifespan training initiative, staff and faculty will apply an age-inclusive lens, enhancing educational excellence and professional growth.

Abstract citation ID: igaf122.2146

### EXCELLENCE ACROSS THE LIFESPAN CHAMPIONS: A PARTNERSHIP WITH THE NATIONAL CENTER TO REFRAME AGING

Sara Hart[1], Rebekah Perkins[1], Patricia D'Antonio[2], Katarina Friberg-Felsted[1], Kara Dassel[1], and Jacqueline Eaton[1], 1. University of Utah, Salt Lake City, Utah, United States, 2. Gerontological Society of America, Washington, District of Columbia, United States

To promote age-friendly policies within a College of Nursing, the "Excellence Across the Lifespan Champions" program was developed in partnership with the National Center to Reframe Aging. This initiative aimed to cultivate a cohort of faculty and staff champions to lead institutional culture change. The program utilized a professional development framework to incentivize participation. Champions received education and mentorship while conducting age-inclusivity mini-projects aligned with their roles. Peer-mentored group publications were encouraged to foster collaboration and knowledge dissemination. Program design involved collaborative meetings with the Center and institutional partners. A synchronous online format was chosen, limited to six faculty and six staff to ensure project diversity. Recruitment targeted staff leadership teams and faculty through a custom flyer and interest survey. The program included four online training sessions followed by a 3-month project implementation phase, with individualized feedback and mentorship throughout. This innovative partnership demonstrates a commitment to professional development that challenges ageist attitudes and hidden biases while promoting age-inclusive practices. The "Excellence Across the Lifespan Champions" program serves as a model for organizations seeking to reframe aging and foster an age-friendly ecosystem. This session will detail champion projects and their early impact.

Abstract citation ID: igaf122.2147

### EXPANDING OUR HORIZONS: THE PROCESS OF SEEKING ACCREDITATION FROM AGEC

Katarina Friberg-Felsted, Jacqueline Eaton, Kara Dassel, Sara Hart, Rebekah Perkins, Gail Towsley, and Marla De Jong, *University of Utah, Salt Lake City, Utah, United States*

As the final part of the age-inclusivity initiative to enhance excellence in education at the University of Utah College of Nursing (CON), the Gerontology Interdisciplinary Program (GIP) sought to elevate the Master of Science in Gerontology (MSG) curriculum to a standard level of national accreditation. The GIP is unique in that it has a 43-year history as the only interdisciplinary degree housed in the College of Nursing (CON). Although the CON has a long tradition of offering accredited programs of nursing, we facilitated purposeful collaboration across programs to align standards and apply for inaugural accreditation of the MSG. The abstract presenter will transparently describe the process of working with the Accreditation for Gerontology Education Council (AGEC) to demonstrate how the MSG meets accreditation standards for gerontology education and how graduate students achieve gerontology competencies. Across two years, we conducted a program self-study, delineating our programmatic structure, student learning goals and outcomes, and ensuring alignment with Academy for Gerontology in Higher Education (AGHE) competencies. We established support documents such as evidence of competency alignment, program policies, and faculty credentials. Following submission of the self-study, we responded to review team feedback and prepared for a 2-day site visit. Multiple faculty dedicated substantial time to prepare materials; coordinated meetings with university leaders, community partners, and faculty; and hosted site visitors. Participating in the process of accreditation increased our collaboration across the College of Nursing, improved our visibility with university leadership, and augmented investment from community partners both internal and external to the university.

### SESSION 4560 (PAPER)

### FALLS, FUNCTION, AND ENVIRONMENT: CONTEXTUALIZING RISK AND RESILIENCE IN AGING POPULATIONS

Abstract citation ID: igaf122.2148

### BREAKING THE CYCLE OF INACTIVITY: THE IMPACT OF EXERCISE ON PAIN, DISTRESS, AND FUNCTIONALITY IN KNEE OSTEOARTHRITIS

Donya Nemati[1], Jacob Jahn[2], Levi Travis[2], Ryan Rizk[2], Eric Kholodovsky[3], Navin Kaushal[4], and Thomas Best[3], *1. Ohio State University, Columbus, Ohio, United States, 2. University of Miami Miller School of Medicine, Miami, Florida, United States, 3. University of Miami, Coral Gables, Florida, United States, 4. Indiana University, Indianapolis, Indiana, United States*

Introduction: Knee osteoarthritis (KOA) is a chronic condition marked by joint degeneration that leads to pain, functional limitations, and diminished quality of life. The cyclical nature of KOA symptoms creates a self-perpetuating loop: reduced physical activity raises body mass index (BMI), increasing joint stress and worsening symptoms, further discouraging exercise. The purpose was to investigate the relationship between clinical measures of KOA and physical activity-related variables. Methods: Patients ($n = 704$; mean age 56.42) with symptomatic KOA (K-L grades 0–4) were evaluated at the University of Miami's sports medicine clinic from 2020-2024. Clinical measures of pain, psychological distress, and functional limitations were assessed using the VAS, PCS, and WOMAC. Validated exercise variables included Physical Activity (PA), Social Support (SS), Exercise Intention (EI), and Exercise Self-Efficacy (ESE). Multivariable linear regression assessed the relationship between clinical and exercise measures, while ANOVA was used for gender comparisons in exercise-related measures. Results: PA was associated with VAS ($\beta$=-0.037, p<.001), PCS scores ($\beta$=-0.124, p<.001), and WOMAC scores ($\beta$=-0.364, p<.001). SS for exercise was associated with lower VAS ($\beta$=-0.017, $p = 0.042$) and WOMAC scores ($\beta$=-0.203, p<.001). ESE was associated with reduced PCS ($\beta$=-0.061, $p = 0.017$) and WOMAC scores ($\beta$=-0.267, p<.001). Gender comparisons revealed that males scored higher than females in EI: $F(1, 734)$=4.69, ($p = 0.031$), ESE: $F(1, 733)$=7.36, ($p = 0.007$), PA: $F(1, 289)$=6.03, ($p = 0.015$), and SS $F(1, 734)$=4.32, $p = 0.038$. Conclusion: Higher PA significantly reduced pain and functional limitations in KOA patients. Gender discrepancies in self-efficacy and intention to exercise highlight the need for exercise interventions tailored for males and females.

Abstract citation ID: igaf122.2149

### COUNTRY DIFFERENCES IN FALL RISK AMONG OLDER ADULTS: A COMPARATIVE STUDY OF INDIA AND THE USA

Shekhar Chauhan, and Dawn Carr, *Florida State University, Tallahassee, Florida, United States*

Falls represent a critical health concern for older adults, with risk factors varying across socio-demographic and lifestyle contexts. Despite significant implications for public health, few studies have compared fall risk between older adults in different countries. This study examines how socio-economic status and socio-demographic factors influence fall risk among older adults (age 65+) in the U.S and India. Using a harmonized cross-sectional dataset we created using data drawn from the Longitudinal Ageing Study in India (LASI 2017-18) and the Health and Retirement Study (HRS, 2016 & 2018), we used logistic regression models to identify factors that predict fall risk, and interaction tests to evaluate if fall-related risk factors are moderated by country (i.e., USA vs. India). Results show that older adults in the USA have almost three times the odds of experiencing a fall compared to their counterparts in India ($p < 0.001$). Regarding moderators, we find that more years of education was associated with higher fall risk in the US ($p < 0.001$) (but not in India), and females have elevated risk of Falls in India ($p < 0.001$), but not in the US relative to their male counterparts. Although rural-living older adults face elevated fall risk in both countries, rural-living older adults in the US adults face particularly high risk of falling. We conclude with a discussion about implications for country-specific fall prevention strategies.

Abstract citation ID: igaf122.2150

### IADL LIMITATIONS LINKED TO MORTALITY AND COGNITIVE IMPAIRMENT IN OLDER PUERTO RICAN ADULTS

Caitlin Northcutt[1], Tyler Bell[2], Sadaf Milani[3], and Michael Crowe[4], *1. University of Kentucky, Lexington, Kentucky, United States, 2. University of California San Diego, San Diego, California, United States, 3. University of Texas Medical Branch, Galveston, Texas, United States, 4. University of Alabama at Birmingham, Birmingham, Alabama, United States*

Limitations in instrumental activities of daily living (IADL) are an important clinical indicator of dementia severity and risk factors for mortality. Research suggests that IADL limitations are a predictor of future cognitive decline. A greater understanding of this longitudinal association is needed, especially in underrepresented samples with a high risk of Alzheimer's disease, such as older adults in Puerto Rico. This study examined these associations using data from Waves 1 (2002/2003), 2 (2006/2007), 3 (2020/2021), and 4 (2022/2024) of the Puerto Rican Elder: Health Conditions Study (PREHCO), a longitudinal, population-based study of Puerto Rican adults aged 60 and older. The sample included adults who were cognitively unimpaired at baseline (Mini Mental Cabán score of 11 or higher) with at least one wave of follow-up data ($n = 2{,}630$, mean age=71.33, $SD = 8.25$; 59.5% female). Participants self-reported limitations on seven IADLs and were screened for cognitive impairment at each wave. National Death Index data was integrated. Overall, increased baseline IADL limitations were associated with an increased risk of death [hazards ratio (HR)=1.05, p<.001], as were increases in IADL limitations over time ($HR = 1.04$, p<.001). When accounting for this mortality risk using inverse-probability weighting, increases in IADL limitations remained significantly related to a heightened risk of cognitive impairment (HR = 1.08, p<.001). In contrast, baseline IADL limitations did not (p=.102). Exploratory analyses revealed that these associations were moderated by sex and education. Findings highlight the need for early screening and interventions tailored to older Puerto Rican adults' specific needs and lived experiences to sustain functional independence in mid-life.

Abstract citation ID: igaf122.2151

### NEIGHBORHOOD CHARACTERISTICS, FUNCTIONAL LIMITATIONS, AND FALLS AMONG OLDER ADULTS

Jeein Law (Jang[1], Debasree Das Gupta[2], and Uma Kelekar[3], *1. University of Massachusetts Boston, Boston, Massachusetts, United States, 2. Utah State University, Logan, Utah, United States, 3. Marymount University, Arlington, Virginia, United States*

Falls among older adults (≥65 years) pose a significant public health concern, leading to increased morbidity, loss of independence, and substantial healthcare costs. While prior research has largely focused on individual health factors, less is known about how perceived neighborhood environment influences fall risk, particularly among older adults with functional limitations. We examined whether the relationship between neighborhood environment and falls was moderated by functional limitations. Using data from the Health and Retirement Study, we analyzed two waves (2014-2016 baseline and 2016-2018 follow-up) (n = 2,486). Perceived neighborhood social cohesion and physical disorder were assessed at baseline. Functional limitations were classified into three categories (no/mild/severe) based on difficulties in daily and instrumental activities of daily living and Nagi strength and mobility tasks. Falls were self-reported at follow-up. Survey-weighted logistic regression models estimated the associations between neighborhood characteristics and falls, controlling for sociodemographic and health-related factors. Interaction terms examined whether the effects of neighborhood characteristics varied by functional status. Results showed perceived physical disorder but not perceived social cohesion was significantly associated with fall risk. However, higher levels of physical disorder were associated with decreased fall risk among older adults with mild and severe functional limitations, which may be indicative of reduced mobility or outdoor engagement of these groups when exposed to higher levels of disorder. These findings underscore the importance of neighborhood context in shaping fall risk, particularly among older adults with functional limitations. Policymakers and urban planners should prioritize neighborhood enhancements, such as maintaining clean, hazard-free public spaces for healthy aging.

Abstract citation ID: igaf122.2152

### NEIGHBORHOOD PERCEPTION AND PHYSICAL ACTIVITY IN LATER LIFE: DOES AUTONOMY MATTER?

SaeMi Park, and W. Quin Yow, *Singapore University of Technology and Design, Singapore, Singapore*

This research examines the role of neighborhood perception in shaping physical activity engagement among Singaporean older adults, applying the Transactional Model of Stress and Coping. This study proposes a serial mediation model where neighborhood perception influences physical activity through perceived health and autonomy, with autonomy serving as a key coping resource. Using data from the 7th wave of the World Values Survey, this study analyzed a Singaporean society (N = 2007, Mage = 47.78, Range=21-91). PROCESS macro is used to test serial mediation as well as moderated mediation. Serial mediation analysis reveals that neighborhood perception indirectly influences physical activity through perceived health and autonomy (OR = 1.015, p = 0.012), though no direct effect of neighborhood perception on physical activity is observed. Furthermore, moderation analysis indicates that the moderating role of autonomy on perceived health is stronger among older adults (β=.018, p=.001), compared to younger adults, suggesting that autonomy is particularly important in later life to take advantage of the health benefits of favorable neighborhood environments. Findings highlight the cyclical linkage between perceived health and autonomy, which perpetuates and reproduces well-being in neighborhood among older adults. These findings confirm the erosion model within a stress and coping model, underscoring the deleting impacts of chronic exposure to stressor on psychological resources as well as contribute to the aging-in-place theory by highlighting the significance of autonomy in mitigating environmental stressors. Interventions fostering a sense of autonomy may enhance physical activity engagement among older adults, ultimately supporting healthier aging.

Abstract citation ID: igaf122.2153

### THE ASSOCIATION OF SYMPTOM BURDEN WITH THE RISK FOR FALLS IN COMMUNITY-DWELLING OLDER ADULTS.

Michelle McKay[1], Paul Bernhardt[1], Melissa O'Connor[2], Jessica Yarnall[1], and Suzanne Leveille[3], *1. Villanova University, Villanova, Pennsylvania, United States, 2. Hunter College, New York, New York, United States, 3. University of Massachusetts Boston, Boston, Massachusetts, United States*

One in four older adults (≥65 years) suffer a fall each year. However, falls are not a part of normal aging and are often preventable. An underexplored area of research is the role of symptom burden as a risk for falls in older adults. The purpose of this study was to determine whether symptom burden, measured as symptom severity classes based on subjective severity ratings of pain, balance, endurance, weakness, sleep, hearing, anxiety, and vision, was associated with time to first fall and experiencing a fall within one year. A secondary analysis of the MOBILIZE Boston dataset, which examined novel risk factors for falls in a population-based cohort of older adults (n = 765), was conducted. Initially, latent class analysis identified 4 symptom severity classes across all symptoms (mild, moderate, moderate-severe, severe). Descriptive statistics, cox proportional hazards modeling (hazard ratios, HR), and logistic regression (odds ratios, OR) were used for further analysis of symptom severity classes and falls. In the unadjusted Cox PH models for time to first fall, the hazard of falling was found to increase with each symptom severity level [HR = 1.44 (1.30, 1.61)]. Similar results were noted with logistic regression modeling; each level increase in symptom severity class was associated with an increased risk for falls within the first year [OR = 1.91 (1.55, 2.38)]. Symptom burden, as a subjective measure, may enable identification of older adults at risk for falls. Using a symptom-based treatment approach may lead to fewer falls and the associated negative consequences.

### SESSION 4565 (PAPER)

### FAMILY CAREGIVING: CAREGIVER OUTCOMES

Abstract citation ID: igaf122.2154

### ANALYSIS OF CORRELATES AND TRENDS IN CAREGIVERS' MENTAL HEALTH PRE- AND COVID-ONWARD FROM THE HINTS, 2018-2022

Jingxin Yao[1], Xiayu Chen[2], Sasha Zhou[3], and Minakshi Raj[1], *1. University of Illinois Urbana-Champaign, Urbana, Illinois, United States, 2. University of Central Florida, Urbana, Illinois, United States, 3. Wayne State University, Detroit, Michigan, United States*

Objectives: To compare correlates of mental distress among caregivers versus non-caregivers, and evaluate trends in caregivers' mental distress pre- versus COVID-onward. Methods: This cross-sectional study used nationally representative data from multiple cycles of the Health Information National Trends Survey (2018 to 2022; n = 15,318). We characterized sociodemographic, health, and caregiving characteristics and estimated logistic regression models evaluating correlates of mental distress among the full sample and pre- and COVID-onward. Results: Caregivers reported a higher mean PHQ-4 score than non-caregivers overall (|t|=2.88, p<.01). Caregiving status was significantly associated with mental distress pre-COVID (OR = 1.47; 95%CI=1.13-1.92; p<.01) and COVID-onward (OR = 1.51; 95%CI=1.01-2.26; p<.05). Being in fair or poor health status was associated with a higher likelihood of mental distress pre-COVID (OR = 2.58, 95%CI=1.31-5.09, p < 0.01) while having a college education or higher was associated with a lower likelihood of mental distress COVID-onward (OR = 0.46, 95%CI=0.21-0.99, p< .05). Conclusions: Caregiving status continues to be a significant risk factor for mental distress. Factors significantly associated with mental distress among caregivers change over time. Findings suggest the COVID-19 pandemic may continue to present risks to caregivers' mental wellbeing, requiring development and implementation of policies and programs to address caregiver mental health.

Abstract citation ID: igaf122.2155

### CAREGIVER QUALITY OF LIFE WHEN PERSONS LIVING WITH DEMENTIA SPEND TIME IN HEALTHCARE SETTINGS

Cassandra Dictus[1], Justine Seidenfeld[2], Wenhan Zhang[1], Kevin McKenna[1], Nathan Boucher[1], Megan Shepherd-Banigan[1], Susan Hastings[2], and Courtney Van Houtven[1], *1. Duke University, Durham, North Carolina, United States, 2. Durham VA Healthcare System, Durham, North Carolina, United States*

Dementia significantly impacts the quality of life (QoL) of both persons living with dementia (PLWD) and their family caregivers. Efforts are underway to develop person-centered measures that reflect both PLWD and caregiver well-being. Caregivers play a crucial role in helping PLWD remain at home, which is associated with higher PLWD QoL. However, recent research suggests that measuring PLWD home time (days spent at home versus facility-based care) does not fully reflect caregiver QoL. Understanding how PLWD time in different health care settings affects caregiver QoL is essential for developing meaningful, caregiver-centered measures. We interviewed 46 family caregivers (mean age 68 years, 70% female, 96% spouses) of PLWD with experiences in emergency, inpatient, outpatient, post-acute, and assisted living settings. Using a qualitative descriptive approach with directed content analysis, we identified key factors influencing caregiver QoL. These include caregiver financial/social resources, transportation, view of PLWD's well-being during the stay, time spent staying with PLWD in the setting, as well as setting-specific elements such as the quality of care, communication with staff, physical environment, and access to specialists. Caregivers' experiences were complex with positive and negative QoL impacts such as relief from worry, new stressors, improved sleep, exhaustion from driving back and forth, respite from caregiving, and frustration with new care tasks, such as managing insurance or care coordination. Findings highlight the need for refined caregiver QoL measures beyond home time. Understanding caregiver perspectives is vital to developing person-centered metrics that inform interventions and policies aimed at supporting both PLWD and their caregivers.

Abstract citation ID: igaf122.2156

### CAREGIVER-PROVIDER COMMUNICATION AND UNMET CARE NEEDS AMONG OLDER ADULTS: EVIDENCE FROM NSOC AND NHATS

Jiaming Liang[1], and Rafael Samper-Ternent[2], *1. UTHealth Houston, San Antonio, Texas, United States, 2. University of Texas Health Science Center at Houston, Houston, Texas, United States*

Effective caregiver-provider communication is crucial for high-quality care in older adults, yet little is known about factors influencing communication quality (CQ) and its relationship with unmet care needs. Using data from the 2021–2023 National Study of Caregiving (NSOC) and the National Health and Aging Trends Study (NHATS), this study examined caregiver (N = 3,673) and care recipient (N = 2,439) characteristics associated with CQ and its impact on unmet care needs. CQ was assessed using three items: (1) whether the doctor listened to the caregiver, (2) whether the doctor checked if the caregiver understood, and (3) whether the doctor inquired about the caregiver's need for help. Unmet care needs were categorized as none, one, or two or more. A mixed-effects model identified factors associated with CQ, and ordered logistic regression examined its association with unmet care needs among older adults. Higher CQ was reported among caregivers with frequent provider communication, Black and Hispanic caregivers (vs. non-Hispanic White), adult children caregivers (vs. spouses), those caring for older adults with dementia, and those with family support and social service use. Lower CQ was observed among caregivers aged 75 + (vs. < 55), those with higher education, and those reporting depression and anxiety. Higher CQ was associated with lower odds of unmet care needs (OR = 0.92, p < 0.001). Findings highlight the need to improve caregiver-provider communication, particularly for caregivers lacking family support and social resources or experiencing mental health difficulties, to reduce unmet care needs and improve care outcomes for older adults.

Abstract citation ID: igaf122.2157

### HEALTH OUTCOMES OF CAREGIVER-FRIENDLY WORKPLACE POLICY INTERVENTIONS: A SYSTEMATIC REVIEW

Maryssa Pallis, *University of Massachusetts Boston, Boston, Massachusetts, United States*

Support for caregiver-friendly workplace policies (CFWPs) is on the rise. Recent attention has grown around workplace interventions of CFWPs and related health outcomes. However, little research has been done synthesizing findings across varying intervention types. This systematic review aimed to identify CFWP intervention studies, comparing the methods, types of interventions, and outcomes related to caregiver health. The search, conducted in December 2024, originally yielded 25 articles overviewing a CFWP intervention. Following eligibility screening, 10 articles were included in the final review. In terms of methods, six were randomized control trials (some cluster-randomized control trials and some random assignments to a waitlist control condition), three were pre-and post-tests, and one was a pilot study. From low to high-quality intervention studies, results showed improvements in physical health (cardiometabolic risk factors, self-rated health, and sleep) and mental health (depression, anxiety, and self-efficacy) post-implementation of CFWPs. Across intervention types and study design variations, CFWP interventions were associated with improvements in both physical and mental health. Evidence points to CFWPs being health-promoting, with worksites offering new capabilities for advancing the health of caregivers managing dual roles of work and informal caregiving responsibilities. Findings also point to the need for additional intervention studies examining CFWPs and increased coordination around methods and intervention types.

Abstract citation ID: igaf122.2158

### SERVING UP SUPPORT: UNDERSTANDING THE NEEDS AND EXPERIENCES OF SANDWICH CAREGIVERS

Autumn Decker[1], Raven Weaver[2], Nancy Swanger[2], and Darcie Bagott[2], *1. Rede Group, Pullman, Washington, United States, 2. Washington State University, Pullman, Washington, United States*

In the United States, there are an estimated 2.5 million adults living as "sandwich caregivers," or people who are simultaneously caring for aging parents and children. These caregivers face similar struggles and difficulties to other family caregivers (e.g., high levels of stress, burnout), but report even higher levels of financial and emotional difficulty, yet less is known about the unique hopes, fears, and needs of these carers. Using Atlas.ti software, we conducted inductive content analysis of the 30 top posts each month, posts that had the highest engagement including views, comments, and reactions, in a Facebook support group for sandwich caregivers from the past 6 months (August 2024-February 2025; we anticipate updating results through August 2025). This evolving group (n = 32,633) includes members from 91 different countries who are majority Women (96%) and those between the ages of 45-54 (45%) and 55-64 (32%). Analysis of 180 monthly posts revealed several themes including needing instrumental advice, seeking emotional support, or wanting help navigating family relationships and priorities. Common topics of posts included end-of-life care needs, grief, financial, physical, and mental health concerns, and healthcare and insurance navigation. Posts were often emotionally charged and sometimes included humor, highlighting the ups and downs of family caregiving. It is critical to identify and address caregivers' needs throughout their caregiving journey; our findings offer insights into the types of services and supports that should be more accessible, available, and affordable.

Abstract citation ID: igaf122.2159

### WILLINGNESS TO CONTINUE CAREGIVING AMONG INFORMAL CAREGIVERS OF OLDER ADULTS IN SOUTHWESTERN NIGERIA

Eniola Cadmus[1], Oluwakayode Ilugbo[2], Onyinye Mbanefo[3], Aderonke Alabi[4], Oluwaseun Ajayi[2], Eme Owoaje[1], Lawrence Adebusoye[1], and Pamela Teaster[3], *1. University of Ibadan, Ibadan, Oyo, Nigeria, 2. University College Hospital, Ibadan, Oyo, Nigeria, 3. Virginia Tech, Blacksburg, Virginia, United States, 4. University College Hospital Ibadan, Ibadan, Oyo, Nigeria*

Informal caregiving of older persons is a time-consuming and demanding role often entered into with little preparation. Understanding the willingness of caregivers to continue in this role is essential for long-term care provision for older persons, especially in low-resource settings. This study assessed the willingness of informal caregivers to continue providing care for community-dwelling older persons in Oyo State, Southwestern Nigeria. A cross-sectional study was carried out using quantitative methods,, with data collected from 554 informal caregivers through a semi-structured, interviewer-administered questionnaire. The Caregiver Continuation Willingness Scale, Zarit Burden Interview, and a 15-item physical status assessment tool for older persons were used. Data were analyzed using SPSS version 25 at a significance level of $p < 0.05$. The caregivers had a mean age of 37.24 (±12.54) years, with most being female (83.6%), married (76.9%), and employed (93.5%). The older adults had a mean age of 79.81 (±12.28) years and were predominantly female (76.5%). Overall, 80.3% of caregivers expressed willingness to continue caregiving. Factors positively associated with willingness included being older, married, a close relative, experiencing mild caregiver burden, prolonged caregiving duration, emotional attachment, obligation to care, and caregiver satisfaction. Perceived ability to care (OR = 4.36; 95% CI [2.19, 8.68]; $p < 0.001$) and caregiver satisfaction (OR = 5.75; 95% CI [2.98, 11.08]; $p < 0.001$) were the strongest predictors of willingness to continue. These findings highlight the need for caregiver support programs and training to enhance caregiving skills and satisfaction, ultimately improving long-term care for older adults.

### SESSION 4570 (PAPER)

### INNOVATIONS IN COGNITIVE FUNCTION AND AWARENESS: EXPLORING TOOLS, CONTEXTS, AND INDIVIDUAL DIFFERENCES

Abstract citation ID: igaf122.2160

### EFFECT OF THE MIND DIET INTERVENTION ON COGNITION IN OLDER ADULTS WITH CLASS 2 AND 3 OBESITY

Shannon Halloway[1], Lisa Barnes[2], Neelum Aggarwal[2], Konstantinos Arfanakis[2], Frank Sacks[3], and Klodian Dhana[2], *1. University of Illinois Chicago, Chicago, Illinois, United States, 2. Rush University Medical Center, Chicago, Illinois, United States, 3. Harvard School of Public Health, Boston, Massachusetts, United States*

With increasing prevalence of obesity in the United States, dietary interventions that promote weight loss have high public health significance. In the recently completed clinical trial (i.e., MIND diet trial), both the MIND diet intervention and control diet groups received mild caloric restriction and showed clinically significant weight loss and improved cognition over 3 years. In the current study, we examined whether the effect of the MIND diet intervention on cognition differs across people with different body mass index (BMI) at enrollment. The MIND trial enrolled older adults without cognitive impairment but with a family history of dementia, BMI>25, and a suboptimal diet. A linear mixed-effect model was used to examine if BMI modified the effect of MIND on cognition, specifically annual change in a global cognition score derived from a battery of 12 neurocognitive tests. Participants (N = 604) were, on average, 70.4 years old, 65.5% female, and with a BMI of 33.9 kg/m2 at baseline. In a multivariable-adjusted model, there was a significant interaction between intervention x BMI x time (p= .011); as such, among individuals with BMI>35 kg/m2 (n = 213), those within the MIND diet group showed a slower rate of decline in global cognition by 0.040 (SE = 0.017, p=.018) standardized units per year when compared to people in the control diet group. In people with BMI< 35, there were no differences between the MIND diet and the control group in global cognition. Future work should examine underlying mechanisms that explain the cognitive benefits of the MIND diet among people with obesity.

Abstract citation ID: igaf122.2161

### EVERYDAY FUNCTIONAL SELF-AWARENESS PATTERNS IN OLDER PEOPLE LIVING WITH HIV: A MULTINOMIAL LOGISTIC ANALYSIS

David Vance[1], Michael Crowe[1], Victor Del Bene[2], Pariya Fazeli[1], Despina Stavrinos[3], and Alexandra Jacob[4], *1. University of Alabama at Birmingham, Birmingham, Alabama, United States, 2. The University of Alabama at Birmingham, Birmingham, Alabama, United States, 3. The University of Alabama, Tuscaloosa, Alabama, United States, 4. University of Kentucky, Lexington, Kentucky, United States*

Nearly 30% of people living with HIV (PLWH) have some mild-to-moderate cognitive impairment which can impact performance on instrumental activities of daily living (IADLs). Some PLWH also experience impaired self-awareness in their ability to perform IADLs. In fact, differences between self-reported and objectively measured IADL performance reveals some PLWH lack accurate awareness of functional abilities. This cross-sectional study examined self-awareness of everyday functioning in 260 middle-aged and older PLWH (40+ years) of whom most were African Americans (82.5%). Participants completed a comprehensive assessment, including cognitive performance testing and subjective and objective measures of everyday functioning. IADL domains, such as financial management, grocery shopping, medication management, and telephone use, were assessed by comparing self-reported performance on the Lawton and Brody IADL Questionnaire with objective performance from the Timed Instrumental Activities of Daily Living (TIADL) test. Three groups emerged based on differences between self-reported and objective performance for each IADL domain: accurate reporters, over-reporters (underestimating their abilities), and under-reporters (overestimating their abilities). Multinomial logistic regression models identified correlates of self-awareness for each IADL domain. Results indicated that under-reporting was more common than over-reporting, especially for grocery shopping (48.6%), medication management (23.2%), and telephone use (22.6%). Higher depressive symptoms predicted over-reporting in financial management and grocery shopping while lower executive function and word-reading scores predicted under-reporting. Men were more likely to under-report grocery shopping difficulties. Individuals who under-reported problems with IADLs may also under-utilize compensatory strategies. These results emphasize the importance of examining self-awareness to support functional independence, especially as PLWH age.

Abstract citation ID: igaf122.2162

### LOCUS OF CONTROL MODERATES THE EFFECT OF SEGREGATION ON EVERYDAY FUNCTIONING OF BLACK OLDER ADULTS IN THE ACTIVE STUDY

Abbey Hamlin[1], Alexandra Weigand[2], Ashley Chikkala[1], Michael Marsiske[3], Shannon Sisco[4], Wassim Tarraf[5], Kelsey Thomas[6], and Alexandra Clark[1], *1. University of Texas at Austin, Austin, Texas, United States, 2. University of California, San Francisco, San Francisco, California, United States, 3. University of Florida, Gainesville, Florida, United States, 4. Michael E. DeBakey VA Medical Center, Houston, Texas, United States, 5. Wayne State University, Detroit, Michigan, United States, 6. University of California, San Diego, La Jolla, California, United States*

Residential segregation has been linked to accelerated declines in everyday functioning for Black older adults, and there is a need to better understand how individual-level resilience factors may help mitigate the impact of structural inequities. Locus of control (LOC) is the extent to which individuals believe life outcomes are determined by internal or external forces. Higher internal LOC has been associated with better cognition, whereas higher external LOC has been linked to poorer outcomes. Given LOC may be an important psychological mechanism to consider in the face of adversity, the present study examined whether LOC moderates the effects of segregation on everyday functioning. Data came from 682 Black older adults from the ACTIVE study who completed a self-report measure of LOC. Baseline residential addresses were used to characterize segregation as measured by tract level dissimilarity (uneven spatial distribution of racial groups) and interaction (likelihood of contact between racial groups). Everyday functioning was measured at baseline, post-intervention, 1-, 2-, 3-, 5-, and 10-year follow-ups. Latent growth curve models, adjusted for demographic, health, and study characteristics, revealed a significant LOC x dissimilarity effect (B=-.45, p=.002); lower external LOC was associated with higher levels of everyday functioning for those living in more segregated versus less segregated tracts. No significant LOC x interaction effect on everyday functioning was observed (Bs ≤ .25, ps ≥ .43). Results suggest that LOC is an important psychological factor to capitalize on while we continue to address unjust and unfair policies that have led to inequalities.

Abstract citation ID: igaf122.2163

### THE DIGITAL ASSESSMENT OF COGNITION: INFORMANT RATINGS OF FUNCTIONAL DISABILITIES AND PSYCHIATRIC SYMPTOMS

David Libon[1], Rod Swenson[2], Sheina Emrani[3], Terri Ginsberg[1], Mitchel Kling[4], Kevin Overbeck[4], Lenny Powell[4], and Adaora Okoli-Umeweni[1], *1. Rowan University, Glassboro, New Jersey, United States, 2. North Dakota Medical School, Grand Forks, North Dakota, United States, 3. University of Pennsylvannia, Philadelphia, Pennsylvania, United States, 4. Rowan-Virtua School of Osteopathic Medicine, Stratford, New Jersey, United States*

Background: The Digital Cognitive Assessment (DAC) is a 7-minute, iPad administered/scored protocol that assesses verbal episodic memory, verbal working memory, and language-related skills. The current research examined relations between DAC-memory and executive indices and family ratings for neurocognitive decline, instrumental activities of daily living (IADL) impairment, and psychiatric symptoms. Methods: 179 memory clinic patients were assessed with the DAC. Spouses or knowledgeable family members rated the severity of neurocognitive impairment, IADL decline, and psychiatric symptoms using the Everyday Cognition Scales (Ecog); the Functional Assessment Questionnaire (FAQ) and the Instrumental Activities of Daily Living – Compensation Scale (IADL-C); and the Neuropsychiatric Inventory (NPI). Results: Partial correlations controlled for age, education, and sex found that greater informant total Ecog, FAQ and IADL-C scores were associated with lower DAC Memory and Executive summary scores. Greater IADL-C Money/Self-Management difficulty was associated with a lower DAC Memory summary score; while greater IADL-C Social Skills difficulty was associated with a lower DAC Executive summary score. A lower DAC Executive summary score was associated with greater informant rated NPI-apathy. Conclusions: The Digital Assessment of Cognition is powerful tool to assess neurocognitive abilities. The relations between DAC summary scores, total informant FAQ and IADL- scores; and IADL-C subscales and selected NPI-defined psychiatric problems suggest that DAC assessment of memory and executive abilities are both sensitive to gross IADL decline, and specific to differing IADL-C difficulty and selected psychiatric problems. Combining the DAC with informant IADL ratings is an effective strategy to assess for emergent MCI and dementia syndromes.

### SESSION 4575 (PAPER)

### MEDICAL DECISION-MAKING AND PROGNOSTIC INSIGHT IN LATER LIFE: ETHICS, ECONOMICS, AND ACCESS

Abstract citation ID: igaf122.2164

### COST-UTILITY ANALYSIS OF THUMB REPLANTATION IN WORKING PATIENTS AGED BETWEEN 65-70 IN CHINA

Jing Bai, *Yale University, New Haven, Connecticut, United States*

Thumb replantation plays a critical role in restoring hand function after traumatic amputation, significantly impacting grip strength, dexterity, and quality of life. While typically recommended for younger patients, its utility in actively working individuals aged 65–70 remains underexplored. This study evaluates the cost-utility of thumb replantation compared to revision amputation in this demographic in China, focusing on economic and functional outcomes. A decision-tree-based cost-effectiveness analysis was conducted from a societal perspective using data from Shanghai Jiao Tong University Affiliated Sixth People's Hospital. Patients aged 65–70 who underwent thumb replantation or revision amputation were included. The primary outcome was the incremental cost-effectiveness ratio (ICER), with functional recovery assessed via Disabilities of the Arm, Shoulder, and Hand (DASH) score and total active motion (TAM) degree. Cost data included surgical, rehabilitation, and indirect costs (e.g., lost wages). Sensitivity analyses (one-way and probabilistic) were performed to assess the robustness of the results. Replantation demonstrated superior functional outcomes with lower DASH scores (mean 25.31 vs. 29.75) and higher TAM degrees (mean 106.78 vs. 44.62) compared to revision amputation (both $p < 0.001$). Replantation also incurred lower total costs (183,195.36 CNY vs. 198,055.67 CNY). The ICER for replantation was -3346.28 CNY per unit improvement in DASH score and -239.08 CNY per unit improvement in TAM degree, indicating cost savings alongside better outcomes. Thumb replantation is a cost-effective intervention for actively working elderly patients in China, offering superior functional outcomes and lower overall costs compared to revision amputation.

Abstract citation ID: igaf122.2165

### CULTIVATING PROGNOSTIC AWARENESS IN ACUTE CARE: THE ROLE OF SOCIAL WORK IN INTERPROFESSIONAL PALLIATIVE CARE TEAMS

Arden O'Donnell[1], Juliet Jacobsen[2], Rennie Bimman[2], and Judith Gonyea[1], *1. Boston University, Boston, Massachusetts, United States, 2. Massachusetts General Hospital, Boston, Massachusetts, United States*

For patients with life-limiting illnesses, informed medical decision-making depends on an understanding of disease prognosis and trajectory. Prognostic awareness—the ability to recognize and integrate knowledge about one's illness—has been associated with reduced psychological distress, improved quality of life and enhanced bereavement outcomes. There is growing recognition that cultivating prognostic awareness is an iterative, interprofessional process involving multiple disciplines. Using interpretive description methodology and thematic analysis, this study explores the clinical skills PSWs use to support prognosis-related discussions. The Prognostic Awareness Integration Scale (PAIS) serves as the conceptual framework for understanding both the cognitive and emotional processing of prognosis. Semi-structured interviews conducted with 17 matched pairs of palliative social workers and physicians across the United States. Findings affirm the PSW's role in cultivating prognostic awareness, identifying specific interventions that support both cognitive understanding and emotional processing of serious illness discussions. PSWs enhance cognitive understanding by assessing knowledge gaps, providing education on disease trajectory, translating medical terminology, repeating key medical details, and offering anticipatory guidance. They also address emotional aspects by assessing coping capacities, evaluating the psychosocial impact of symptoms, modulating the pace and intensity of discussions, facilitating emotionally challenging conversations, centering emotions over medical details, and promoting a trauma-informed approach. The PAIS tool offers critical insights into the specific skills and strategies PSWs use to navigate complex, emotionally charged conversations about prognosis. The results of this study can be used in further defining the advanced skills of specialty palliative social work as well as in education and training.

Abstract citation ID: igaf122.2166

### DISTINCT PREDICTORS OF SOCIAL VISITATION VS CARE COORDINATION VISITATION OF LTC FACILITY RESIDENTS

Caroline Collins-Pisano[1], and Rachel Weiskittle[2], *1. University of Colorado Colorado Springs, Colorado Springs, Colorado, United States, 2. University of Colorado, Colorado Springs, Colorado Springs, Colorado, United States*

Loneliness is pervasive among older adults residing in long-term care (LTC) facilities despite frequent familial visitation. Extant research on LTC visitation assess social visitation (e.g., talking with the resident about their day) and care coordination (e.g., provision of personalized care) as one construct. It is possible these two forms of interaction impact resident loneliness differently. Past studies have identified barriers to overall visitation. The purpose of the current study was to investigate if predictors of overall LTC visitation were associated with social visitation distinct from care coordination. 175 community-dwelling adults aged 18 and older with a close friend or relative residing in a LTC facility were recruited via ResearchMatch to complete an online survey regarding demographics, LTC visiting behaviors, and barriers of engagement with LTC residents. Multiple regressions were conducted to assess the relationships between known predictors of overall LTC visitation with social visitation and care coordination. Our collective findings support our hypothesis that social visitation and care coordination are distinct types of LTC visitation. LTC facility characteristics and residents' communication and functional abilities predicted social visitation but not care coordination. Characteristics of the participant and resident relationships predicted both social visitation and care coordination. Demographic variables and participants' perceptions of aging, LTC facilities, and the resident's quality of life predicted neither social visitation nor care coordination. This paper presentation will also discuss the clinical implications of these preliminary findings, such that programs targeting resident loneliness may warrant tailoring towards specific predictors of social visitation over care coordination.

Abstract citation ID: igaf122.2167

### THE HIDDEN COSTS OF CARING: MAPPING PATHWAYS TO DEPRESSION AMONG INFORMAL CAREGIVERS IN EUROPE

Marco Albertini, and Eva Bei, *University of Bologna, Bologna, Emilia-Romagna, Italy*

Europe is experiencing a demographic shift characterized by increasing longevity and declining birth rates, leading to rising long-term care (LTC) demands. As informal caregivers become the backbone of LTC systems, understanding the societal pathways linking caregiving intensity to mental health outcomes such as depression becomes crucial. Using data from the European Social Survey (ESS) rounds 7 and 11, this study employed Structural Equation Modeling (SEM) with maximum likelihood estimation to examine pathways between socioeconomic status (modeled as a latent construct with education, income, and employment indicators), caregiving intensity, and depression. We analyzed 23,799 individuals reported providing informal care. The caregiving stress process model framed our study, with care intensity hypothesized as a key mediator between SES and depression. SEM revealed significant associations between key SES indicators and caregiving patterns. Lower household income emerged as the strongest SES indicator of care intensity ($\beta$=-0.690, p<.001), followed by unpaid work status ($\beta$=-0.502, p<.001) and lower levels of educational attainment ($\beta$=-0.476, p<.001). Gender differences were pronounced, with women reporting higher levels of both caregiving intensity ($\beta$=0.069, p<.001) and depression ($\beta$=0.085, p<.001). SEM analysis confirmed caregiving intensity as a significant predictor of depression ($\beta$=0.070, p<.001), supporting its mediating role between all SES predictors and depression. Additionally, satisfaction with healthcare services negatively predicted both caregiving intensity ($\beta$=-0.086, p<.001) and depression ($\beta$=-0.106, p<.001), highlighting healthcare system importance for caregivers. These findings contribute to understanding how socioeconomic determinants stratify care intensity as a mediator pathway to depression, offering valuable insights for policymakers to support informal care across Europe.

### SESSION 4580 (PAPER)

### PSYCHOSOCIAL RESILIENCE AND BEHAVIORAL HEALTH PATHWAYS IN AGING

Abstract citation ID: igaf122.2168

### COGNITIVE, PERSONALITY AND PSYCHOLOGICAL ASSOCIATIONS OF HABIT FORMATION AND CHANGE IN AGEING

Anangsha Pathak[1], Daniel Hermens[1], Daniel Fassnacht[2], Thomas Pace[1], Sanne de Wit[3], and Sophie Andrews[1], *1. University of the Sunshine Coast Thompson Institute, Birtinya, Queensland, Australia, 2. University of the Sunshine Coast, Sunshine Coast, Queensland, Australia, 3. University of Amsterdam, Amsterdam, Noord-Holland, Netherlands*

All behaviour can be influenced by automatic, context-dependent habits. The ability to form and modify these habits is crucial for healthy ageing. Whilst cognition and personality can impact habit-related behaviours, their specific roles remain unclear. This study aimed to explore the relationships between cognitive, psychological, and personality factors in habit formation and change among older adults. A total of 125 healthy adults (Mage = 72.8, range = 60–87 years, SD = 7.25, 50.4% female) participated. Participants completed a computerised task comprising a habit learning phase and a habit switching phase (Symmetrical Outcome Revaluation Task), along with assessments of personality (IPIP-NEO), cognitive function (HVLT, Trails B), mood (DASS-21), and single-item scales for motivation, enjoyment, and satisfaction. Backward stepwise structural equation modelling was conducted and yielded an overall good model fit (indicated by, for example, the Comparative Fit Index =.956, reflecting the proportion of variance explained compared to a baseline model, with values above 0.90 indicating a good fit). The analysis revealed that cognitive and psychological factors significantly influenced habit learning and behavioural flexibility. Specifically, lower age, faster processing speed, better execute function, more enjoyment, motivation and lower depression was associated with improved habit learning accuracy ($R^2$ = .3). Additionally, better verbal recognition memory, higher conscientiousness, apathy and faster discrimination accuracy were linked to better performance in switching from habitual to goal-directed responding ($R^2$ = .11). These findings suggest that a combination of cognitive and psychological factors influence habit learning and behaviour change, with important implications for promoting healthy habits in ageing.

Abstract citation ID: igaf122.2169

### FEELING YOUNGER EVERY DAY? DAILY ACTIVITIES AND SUBJECTIVE AGE IN THE HEALTH AND RETIREMENT STUDY

Wassim Tarraf[1], Sangwoo Ahn[2], Kimson Johnson[3], Yeonwoo Kim[4], Kafayat Mahmoud[5], Samuel Van Vleet[6], Jiao Yu[7], and Briana Mezuk[8], *1. Wayne State University, Detroit, Michigan, United States, 2. The Pennsylvania State University, University Park, Pennsylvania, United States,*

*3. University of Michigan Institute for Social Research, Ann Arbor, Michigan, United States, 4. The University of Texas at Arlington, Arlington, Texas, United States, 5. Boston University, Boston, Massachusetts, United States, 6. Miami University, Oxford, Ohio, United States, 7. Yale University, New Haven, Connecticut, United States, 8. University of Michigan, Ann Arbor, Michigan, United States*

Subjective age (i.e., how old one "feels") reflects beliefs about aging, both positive and negative, and is consequential to health and well-being. The mechanisms through which subjective age is linked to health are likely through age-salient social interactions and engagement in health promoting daily activities. This study examined the relationship between subjective age and activities in the prior 24 hours. We used data from n = 4,251 adults aged >50-years who completed the 2020 Health and Retirement Leave-Behind Questionnaire. Weighted generalized linear models (logistic and Poisson) tested whether, and to what extent, feeling younger than one's age (i.e., discrepancy between chronological and subjective age) is associated with (a) engagement in 19 typical daily relevant activities (No/Yes) and (b) time spent doing these activities (No time to 7+hours). All models adjusted for demographics, number of chronic conditions (r = 0-8), and limitations in activities of daily living (r = 0-5). On average, people felt 8.7 years older (SD: +11.8) than their actual age. Feeling younger was positively associated with volunteering, walking, exercising, socializing, reading, and religious activities, as well as more time spent doing these activities. Feeling younger was also linked to increased time spent traveling, running errands, and seeking quiet time (e.g. meditating). Controlling for health burden and functional impairments, feeling younger is associated with greater engagement in, and time spent doing, cognitive, physical, and social activities, offering opportunities for scalable, community-based programming focused on these activities to promote health and well-being.

Abstract citation ID: igaf122.2170

### LONGITUDINAL ASSOCIATIONS BETWEEN NEIGHBORHOOD DISORDER, DIABETES, AND DEPRESSIVE SYMPTOMS IN LATER LIFE

Seungjong Cho[1], and Jiao Yu[2], *1. Texas Tech University, Lubbock, Texas, United States, 2. Yale University, New Haven, Connecticut, United States*

Neighborhood environments are increasingly recognized as determinants of mental health, yet the biological pathways linking neighborhood stressors to depressive symptomatology remain underexplored. Chronic exposure to neighborhood physical disorder, characterized by visible signs of neglect such as vandalism and poorly maintained infrastructure, may activate physiological stress responses that disrupt metabolic regulation, elevating the risk of diabetes and related complications. Moreover, diabetes-related metabolic dysregulation has been connected to the development of depressive symptoms, yet the underlying mechanisms warrant further investigation. To address this gap, we examined whether metabolic dysregulation, measured by hemoglobin A1c (HbA1c), mediates the association between perceived neighborhood physical disorder and depressive symptoms. Using longitudinal data from the Health and Retirement Study (HRS) Left-Behind Questionnaire, we analyzed three waves spanning 2006 to 2016 (N = 5,576). Perceived neighborhood physical disorder (2006/2008) was hypothesized to influence depressive symptoms (2014/2016) through HbA1c levels (2010/2012). Covariates included baseline depressive symptoms, age, gender, education, income, and marital status. A structural equation modeling path model (SRMR=.020) revealed a significant indirect path: greater neighborhood physical disorder was associated with higher HbA1c (Coef=.051, SE=.013, p<.001), which in turn predicted elevated depressive symptoms (Coef=.057, SE=.028, p=.046). These findings suggest that diabetes and related complications may serve as a physiological pathway through which neighborhood stressors impact mental health. Given the role of HbA1c in both metabolic and mental health, interventions targeting neighborhood disorders could have broader health benefits beyond social and psychological well-being. Future research should investigate additional biological pathways and explore interventions that address neighborhood stressors to promote healthier aging.

Abstract citation ID: igaf122.2171

### PHYSICAL ACTIVITY'S ROLE IN REDUCING NEUROPSYCHIATRIC SYMPTOMS AMONG OLDER ADULTS WITH DEMENTIA

Lin-Na Chou[1], Niying Li[2], Yongseop Kim[3], and Anne Thackeray[4], *1. University of Utah, Saratoga Springs, Utah, United States, 2. University of Georgia, Athens, Georgia, United States, 3. University of Utah, University of Utah, Utah, United States, 4. University of Utah, Salt Lake City, Utah, United States*

Neuropsychiatric symptoms (NPS) pose significant challenges in dementia care. With limited pharmacological treatments, identifying behavioral targets is crucial for developing effective interventions. This study examines the associations between Alzheimer's Disease and Related Dementia (ADRD) and physical activity (PA) with NPS among individuals aged 70 years and older, using data from the 2021 National Health and Aging Trends Study (NHATS). NPS assessed included depression (PHQ-2), anxiety (GAD-2) and sleeping problems (single-item question on difficulty falling asleep). ADRD was determined using the NHATS algorithm, and PA was measured through self-reported questions on walking for exercise and vigorous activities in the last month. Data from 3388 subjects (21% with ADRD) were analyzed. In multivariable logistic regression models adjusted for sociodemographic factors and medical conditions, ADRD was associated with increased risks of depression (OR, 95% CI: 2.05, 1.41-2.97) and anxiety (2.69, 1.76-4.11). Walking was associated with decreased risks of depression (0.70, 0.51-0.97) and anxiety (0.67, 0.46-0.97), while vigorous activities were associated with decreased risks of depression (0.59, 0.40-0.87) and sleep problems (0.70, 0.52-0.94). Subgroup analysis among the oldest old adults (age ≥85 years) revealed that walking reduced the risk of anxiety (0.52, 0.27-0.99). This study underscores the benefits of walking and vigorous activities in reducing NPS risk. Incorporating PA into regular care practices is highly encouraged for healthcare providers and caregivers to enhance the well-being of individuals with dementia.

Abstract citation ID: igaf122.2172

### TEMPORARY SETBACK OR LASTING CHALLENGE? IMPACT OF TRANSIENT AND PERSISTENT FUNCTIONAL DISABILITY ON WELLBEING

Thibault Kohler, and Bram Vanhoutte, *Universite Libre de Bruxelles, Brussels, Brussels Hoofdstedelijk Gewest, Belgium*

According to the hedonic treadmill theory, people's wellbeing returns to a set-point after experiencing either positive or negative shocks. In the context of functional disability, studies differ on the degree of adaptation following this event. This study adds nuance to this debate by adopting a multidimensional approach to wellbeing in later life, differentiating between affective, cognitive, and eudemonic wellbeing, and examining how wellbeing trajectories are influenced by temporary functional disability, distinguishing between persistent and transient cases. We reordered longitudinal panel data from waves 4 to 8 of the Survey of Health, Ageing and Retirement in Europe (SHARE), from more than 3500 Europeans to align on the transition from no limitation to at least one limitation in ADL. We used linear spline growth models separately for each wellbeing measure (using Euro-D, Life Satisfaction and CASP) to examine non-linear trajectories. We observed a substantial decline in all three wellbeing measures at the onset of functional disability, with life satisfaction less affected (standardised mean differences = -0.11) than quality of life (-0.23) and depression (-0.27). On average, short-term disability led to a return to the initial level of wellbeing, while long-term disability led to a pronounced decline during the transition, with much less adaptation. Our findings highlight the significant impact that functional disability can have on wellbeing, revealing distinct patterns across various dimensions. Persistent disability often marks a crucial stage in the wellbeing of older people, whereas transient cases are characterised by a subsequent return to previous levels of wellbeing.

### SESSION 4585 (PAPER)

Abstract citation ID: igaf122.2173

### RISK FACTORS AND PREVENTIVE STRATEGIES FOR ALZHEIMER'S DISEASE AND RELATED DEMENTIAS

This session provides insights into trajectories of risk factors for dementia as well as potential targets for Alzheimer's and Related Dementia prevention.

Abstract citation ID: igaf122.2174

### LIFE COURSE MODIFIABLE RISK FACTORS FOR DEMENTIA STRATIFIED BY APOE-4 STATUS

Victoria Williams[1], Ralph Trane[1], Kamil Sicinski[2], Carol Roan[2], Pamela Herd[3], Michal Engelman[4], and Sanjay Asthana[1], *1. University of Wisconsin–Madison School of Medicine and Public Health, Madison, Wisconsin, United States, 2. University of Wisconsin–Madison, Madison, Wisconsin, United States, 3. University of Michigan, Ann Arbor, Michigan, United States, 4. University of Wisconsin-Madison, Madison, Wisconsin, United States*

The 2024 Lancet Commission identified 14 modifiable risk factors reliably associated with increased risk for dementia. However, dementia is a complex condition influenced by both genetic predisposition and environmental factors, with gene by environment interactions often overlooked in risk models. The apolipoprotein (ApoE-4) allele confers the strongest genetic risk for Alzheimer's disease, while also shown to modify the effects of environmental exposures on the dementia phenotype. We applied the Lancet risk factor model to a single cohort of 5,526 participants in the Wisconsin Longitudinal Study (WLS). Risk factors were defined by Lancet cut-points and life-course timing criteria, drawing from 70 years of prospectively collected WLS data. We used logistic regression with multiple imputation to model dementia outcomes by the Lancet model risk factors (also controlling for age and sex), stratified by ApoE-4 carrier status. Replication of the 2024 Lancet model in a single cohort revealed only a restricted subset of modifiable risk factors that associated with late-life dementia status. Among APoE-4 carriers only, we found that mid-life diabetes increased dementia odds, whereas mid-life hypertension reduced risk for dementia. Among ApoE-4 non-carriers, only mid-life hearing loss and diabetes associated with increased dementia odds. Overall, we observed slightly divergent dementia risk profiles based on ApoE-4 status, such that mid-life diabetes emerged as the primary risk factor for dementia among ApoE-4 carriers, whereas mid-life hearing loss was found to be most strongly associated with dementia risk among ApoE-4 non-carriers. Resultant findings highlight the importance of considering gene-by-environment interactions when formulating dementia risk models.

Abstract citation ID: igaf122.2175

### LIFETIME EXPOSURE TO ESTROGEN AND NEUROIMAGING OUTCOMES IN OLDER WOMEN IN THE IGNITE TRIAL

Amber Watts[1], Robyn Honea[2], Shannon Donofry[3], Hayley Ripperger[4], Sarah Aghjayan[4], Chaeryon Kang[4], and Kirk Erickson[5], *1. University of Kansas, Lawrence, Kansas, United States, 2. University of Kansas School of Medicine, Kansas City, Kansas, United States, 3. RAND Corporation, Santa Monica, California, United States, 4. University of Pittsburgh, Pittsburgh, Pennsylvania, United States, 5. AdventHealth Research Institute, Orlando, Florida, United States*

Lower lifetime estrogen exposure is associated with age- and Alzheimer's-related reductions in brain volume, while estrogen-based therapies are associated with larger volume. We investigated associations between hormone-based medication use and structural neuroimaging markers, hypothesizing that hormone-based therapies would be associated with larger brain volumes. We investigated baseline associations in Investigating Gains in Neurocognition in an Intervention Trial of Exercise (IGNITE), a multi-site, randomized exercise trial. We report structural data from magnetic resonance imaging. We processed the T1-MPRAGE using SPM12 and CAT12 software, adjusting for total intracranial volume. We focused on regions related to Alzheimer's disease and higher concentrations of estrogen-beta receptors, dominant in the hippocampus. In 459 women (ages 65-80), we evaluated associations between retrospective recall of hormone-based medication use (birth control, menopausal hormone therapy) and brain volumes accounting for age, education, SES, and APOE4 carrier status. Birth control use (average start age 22) was associated with larger right parahippocampal volume ($\beta = 0.100$, $p = 0.034$). APOE4 carriers had smaller volumes in estrogen-beta-receptor regions ($\beta$=-0.096, $p = 0.045$). APOE4 carriers using birth control had smaller volumes than non-carriers using birth control ($\beta$=-.242, p=.028). Younger age when starting menopausal hormone-therapy was associated with greater white matter volume ($\beta$=-0.150, $p = 0.037$). These cross-sectional findings support the critical window hypothesis, i.e., timing of estrogen exposure is important for healthy brain aging. Our study uniquely links estrogen exposure in early adulthood to later-life brain structure. Future research should investigate mechanisms by which APOE4 may moderate estrogen's influence on brain aging. Findings have clinical implications for estrogen therapies throughout the lifespan.

Abstract citation ID: igaf122.2176

**PREVALENCE OF REVERSIBLE AND POTENTIALLY REVERSIBLE COGNITIVE FRAILTY IN OLDER ADULTS: A META-ANALYSIS**

Jinwei Bian, Zi Chen, Ying Gao, Fung Yee Lau, Daniel Yee Tak Fong, Edmond Pui Hang Choi, and Pui Hing Chau, *The University of Hong Kong, Hong Kong, China (People's Republic)*

Background: Potentially reversible cognitive frailty (PRCF) and reversible cognitive frailty (RCF), characterized by concurrent declines in physical and cognitive function, may be modifiable or responsive to interventions. Understanding their epidemiology can inform research and shape public health strategies. However, reviews on their prevalence remain scarce. This study aimed to estimate the pooled prevalence of PRCF and RCF among older adults without dementia. Methods: A search of 6 literature databases was conducted from inception to August 15, 2024. Cohort and cross-sectional studies reporting PRCF or RCF prevalence in older adults aged 60+ without dementia were included. Random-effects meta-analyses with logit-transformed prevalence were used, alongside subgroup analyses and meta-regression to examine heterogeneity. Risk of bias was assessed using the Joanna Briggs Institute Critical Appraisal Checklist. Results: Of the 13,100 articles identified, 51 studies from 14 countries were eligible, with 50 studies on PRCF and 10 studies on RCF. The pooled prevalence of PRCF in communities, hospitals and nursing homes was 17% (95% CI: 13%-21%), 32% (95% CI: 23%-42%), and 32% (95% CI: 1%-99%), respectively, while the pooled RCF prevalence was 21% (95% CI: 15%-29%), 48% (95% CI: 45%-51%), and 15% (95% CI: 12%-19%), respectively. Studies with suboptimal sample sizes reported higher PRCF prevalence compared to those with optimal sample sizes. Conclusion: The prevalence of PRCF and RCF among older adults is substantial and varies across settings, highlighting the need for early detection and intervention. Further research in underrepresented regions, along with prior sample size calculations and appropriate assessment tool selection, is essential.

### SESSION 4590 (PAPER)

### TECHNOLOGY, AGING, AND CARE: INNOVATIONS, ADOPTION, AND IMPLEMENTATION CHALLENGES

Abstract citation ID: igaf122.2177

**ANTICIPATED IMPACT OF EXOSKELETON USE ON OLDER WORKERS' OCCUPATIONAL SELF-EFFICACY: A SURVEY STUDY**

HeeSun Choi, Sahar Khanpour, Yves Valentin, Jessica Alquist, and Jonathan Singer, *Texas Tech University, Lubbock, Texas, United States*

Exoskeletons are wearable assistive technology that can reduce users' physical strain and enhance performance, having significant health and safety implications for older manual labor workers with age-related declines. However, their psychosocial impact remains understudied; exoskeleton use may enhance occupational self-efficacy (OSE) – beliefs in one's ability to perform work tasks, by helping to regain functional capabilities and successfully meeting day-to-day work demands. This survey study explored whether older manual labor workers anticipated changes in OSE with exoskeleton use, using a hypothetical scenario. Additionally, we examined whether perceived embodiment (i.e., perceiving exoskeletons as an extension of their body vs. an external tool) influenced the impact of exoskeletons on OSE. Forty-four participants aged 55+ (M = 62.59, SD = 6.24) rated their baseline OSE, followed by watching an exoskeleton introduction video. They were then asked to indicate their anticipated OSE, assuming their workplace implemented exoskeletons and they had been using them for 12 months. A repeated measures ANCOVA revealed a significant main effect, with post-video OSE ratings (M = 25.59, SD = 6.55) lower than baseline (M = 30.32, SD = 3.39), $F(1,42) = 17.84$, $p < .001$, $\eta p^2 = .30$. Additionally, perceived embodiment moderated this effect, $F(1,42) = 10.18$, $p = .003$, $\eta p^2 = .20$. Workers with low embodiment anticipated reduced OSE, while those with high embodiment did not. Findings suggest exoskeletons may negatively impact older workers' OSE, particularly if perceived as external tools. Future research should explore this effect in larger samples and assess real-world changes following hands-on experience.

Abstract citation ID: igaf122.2178

**DIGITAL MINDFULNESS APP UTILIZATION AND PERCEIVED STRESS AMONG CAREGIVERS OF PERSONS LIVING WITH DEMENTIA**

Michael P. Williams[1], Morgan Seward[1], Elizabeth M. Allen[1], Raquel G. Tatar[2], Jennifer Huberty[3], Ana-Maria Vranceanu[1], and Evan Plys[1], *1. Massachusetts General Hospital, Boston, Massachusetts, United States, 2. Healthy Minds Innovation, Madison, Wisconsin, United States, 3. Fit Minded Inc., Phoenix, Arizona, United States*

Mindfulness digital health interventions (DHIs) can reduce stress among caregivers of persons living with dementia (PLWD). Little is known about how mindfulness DHI utilization relates to stress reduction. This study is a secondary analysis of a feasibility pilot RCT of Healthy Minds Program for Caregivers (HMP-C), a mindfulness stress-reduction DHI for caregivers of PLWD. Forty-seven participants randomized to the intervention condition (HMP-C) in the primary trial were included the secondary analysis. Participants were instructed to use HMP-C for a minimum of 52.5 minutes per week. We describe HMP-C utilization and the association between HMP-C usage and reductions in perceived stress (PSS-10) between baseline and post-test using linear and logistic regression. Median usage of HMP-C was 30 minutes per week (IQR = 30; Range= 1-110). Every 10 additional minutes of HMP-C usage per week was associated with a 1.2 (CI = 0.4,1.9) point decrease in perceived stress. Using HMP-C for 52.5 minutes or more was associated with an 8-point reduction in stress (CI = 3.2,13.2). These findings suggest that utilization of HMP-C is related to reductions in perceived stress. However, there was significant variability in utilization, with few caregivers sufficiently using HMP-C to see clinical reductions in perceived stress. Future research is needed to explore strategies for improving utilization among low-usage caregivers and to identify the optimal balance of feasibility and clinical benefit for mindfulness DHIs among caregivers.

Abstract citation ID: igaf122.2179

**EXPLORING AI-ASSISTED QUERY REFORMULATION FOR OLDER ADULTS SEEKING TECHNOLOGY SUPPORT**

Debaleena Chattopadhyay[1], Hasti Sharifi[2], Homaira Huda Shomee[2], and Sourav Medya[1], *1. University of Illinois Chicago, Chicago, Illinois, United States, 2. University of Illinois at Chicago, Chicago, Illinois, United States*

Digital applications and services have become essential for full participation in society, yet older adults often struggle to use them effectively. While quality technology support can help, many face difficulties articulating their problems due to unfamiliarity with technical terminology and age-related changes in verbal abilities. Effective communication is crucial when seeking technology support, whether from people (e.g., customer service representatives, friends, family) or systems (e.g., search engines, chatbots). This study examines these communication challenges and explores AI-driven solutions to improve older adults' access to technology support. We conducted an online diary study with English-speaking, community-dwelling older adults (n = 27, median age = 66), to analyze asynchronous technology-related queries. Reflexive thematic analysis identified four key barriers to resolution: verbosity, incompleteness, over-specification, and under-specification. To address these issues, we designed and evaluated a few-shot prompt-chaining approach using GPT-4o. The model first elicited necessary contextual details, then paraphrased the query, and finally generated a solution. Reformulated queries significantly improved solution accuracy (69%) compared to verbatim queries (46%). Even when used solely for paraphrasing, reformulated queries yielded more accurate Google search results (69%) than verbatim ones (35%). A separate survey of digitally skilled adults (n = 48, median age = 28) found that rephrased queries were significantly more comprehensible (93%) than verbatim ones (67%). Participants also reported significantly greater confidence and ease in understanding the reformulated queries. These findings highlight the potential of AI-driven query reformulation to empower older adults in navigating digital environments more independently.

Abstract citation ID: igaf122.2180

### FAMILY CAREGIVERS' NEEDS FOR INTEGRATING HOME CARE TECHNOLOGY AND THEIR EVERYDAY CAREGIVING

Tomoko Wakui[1], Sakiko Itoh[2], Hiroyasu Miwa[3], and Kentaro Watanabe[3], *1. Tokyo Metropolitan Institute for Geriatrics and Gerontology, Tokyo, Tokyo, Japan, 2. Institute of Science Tokyo, Tokyo, Tokyo, Japan, 3. National Institute of Advanced Industrial Science and Technology, Kashiwa, Chiba, Japan*

Beyond addressing care recipients' needs, family caregiving factors also play a crucial role in the adoption of home care technologies. This study explores the factors influencing family caregivers' preferences for integrating such technologies into daily caregiving. We surveyed 2,002 family caregivers of community-dwelling older adults, selected based on the national percentage of older adults requiring long-term care under Japan's public long-term care insurance program. The primary outcome was caregivers' preferences for 17 types of home care technologies, such as bathing aids, toileting prediction, mobility support, medication management, and GPS tracking. We controlled for care recipient characteristics (age, dementia status, ADLs), and examined family caregiver characteristics (age, marital/employment status, income, health), and caregiving situations (frequency, duration, co-residence, burden). Logistic regression analyses revealed that higher caregiving burden was significantly associated with preferences for bathing aids (OR: 1.04, $p < .01$), toileting prediction (OR: 1.03, $p < .01$), GPS trackers (OR: 1.03, $p = .004$), and shopping assistance tools (OR: 1.03, $p = .01$). Live-out caregivers were more likely to prefer communication robots (OR: 1.47, $p = .03$). Higher household income was linked to preferences for bathing aids, toileting support, pill dispensers, fall detection monitors, and social robots ($p < .05$). Our findings highlight that family caregivers' circumstances—not just care recipients' needs—significantly shape technology adoption preferences, emphasizing the need for caregiver-centered solutions.

Abstract citation ID: igaf122.2181

### HUMAN VS. AI IN CONDUCTING SCOPING REVIEWS: EVALUATING LARGE LANGUAGE MODEL ACCURACY ACROSS ARTICLE AND TASK TYPE

Chang Yu[1], Mo Han[2], Rui Huang[2], Hanna Grol-Prokopczyk[2], and Gongda Yu[2], *1. University at Buffalo, SUNY, Getzville, New York, United States, 2. University at Buffalo, Buffalo, New York, United States*

Objectives: Large language models (LLMs), such as ChatGPT's, are increasingly used to assist with health- and aging-related scoping reviews, which are often very time-consuming when done by humans alone. However, empirical evidence on the reliability of LLMs in extracting data from peer-reviewed literature (content coding) remains limited. This study evaluates the accuracy of ChatGPT-4o's content coding across different article types (systematic reviews, narrative reviews, and meta-analyses) and task types (e.g., single-select vs. multiple-select questions) by comparing its results to human coding from an existing scoping review. Methods: We selected 26 articles from a previously human-coded scoping review of 398 articles on social disparities (including age-related disparities) in pain. We then used ChatGPT-4o's Application Programming Interface (API) to extract and code eight characteristics of each article, including article type, independent variable(s), dependent variable(s), mechanism(s), and research findings, based on predefined options. For single-select questions, agreement between human and AI codings was categorized as same or different. For multiple-select questions, additional categories captured partial agreement. Results: Human-AI agreement was higher for single-select questions (71%) than for multiple-select questions (29%). By article type, agreement was highest for meta-analyses (85% and 45% for single- and multiple-select questions, respectively), and much lower for systematic reviews in single-select questions (38% agreement) or narrative reviews in multiple-select questions (17% agreement). Discussion: ChatGPT is helpful for straightforward coding tasks in scoping reviews, but its reliability declines with more complex tasks and diverse article types. These findings highlight the need for careful consideration when incorporating LLMs into scoping reviews.

### SESSION 4595 (SYMPOSIUM)

Abstract citation ID: igaf122.2182

### THE BLUES AND OLDER MINORITY MUSICIANS -- MORE THAN JUST MUSIC XXXII

Chair: John Migliaccio
Co-Chair: Michael Marcus

The Blues may have emerged in the Mississippi Delta but its influence and music is mightily represented today in the Puget Sound! Seattle's Blues Bash, Blues Awards, and the Rhapsody Project make intergenerational cultural connections between the Blues and the community by tuning into cultural heritage exploration and social justice. With a lively array of bars, clubs, and iconic Blues musicians with insights into their lives, influences, resilience, and productive aging, this popular session draws an enthusiastic crowd of GSA members for a lecture/interview and "mini-performance" with a leading older musician. But Wait! There's more- later that evening, a complete performance and party at a local Blues joint guarantees good music, dancing, libations, and Blues swag. This is a "don't miss" combo with 32 years of GSA history.

Abstract citation ID: igaf122.2183

### THE BLUES AND OLDER MINORITY MUSICIANS, NUMBER XXXII

Michael Marcus, *University of Maryland Baltimore County, Raleigh, North Carolina, United States*

The Blues may have emerged in the Mississippi Delta but its influence and music is mightily represented today in the Puget Sound! Seattle's Blues Bash, Blues Awards, and the Rhapsody Project make intergenerational cultural connections between the Blues and the community by tuning into cultural heritage exploration and social justice. With a lively array of bars, clubs, and iconic Blues musicians with insights into their lives, influences, resilience, and productive aging, this popular session draws an enthusiastic crowd of GSA members for a lecture/interview and "mini-performance" with a leading older musician. But Wait! There's more- later that evening, a full performance and party at a local Blues joint guarantees good music, dancing, libations, and Blues swag. This is a "don't miss" combo with 31 years of GSA history.

Abstract citation ID: igaf122.2184

### THE BLUES AND OLDER MINORITY MUSICIANS -- MORE THAN JUST MUSIC XXXII

Michael Marcus, *University of Maryland Baltimore County, Raleigh, North Carolina, United States*

The Blues may have emerged in the Mississippi Delta but its influence and music is mightily represented today in the Puget Sound! Seattle's Blues Bash, Blues Awards, and the Rhapsody Project make intergenerational cultural connections between the Blues and the community by tuning into cultural heritage exploration and social justice. With a lively array of bars, clubs, and iconic Blues musicians with insights into their lives, influences, resilience, and productive aging, this popular session draws an enthusiastic crowd of GSA members for a lecture/interview and "mini-performance" with a leading older musician. But Wait! There's more- later that evening, a complete performance and party at a local Blues joint guarantees good music, dancing, libations, and Blues swag. This is a "don't miss" combo with 32 years of GSA history.

### SESSION 4600 (PAPER)

### UTILIZING VARIOUS CURRICULUM STRATEGIES TO HELP EMPOWER OLDER ADULTS, CAREGIVERS, AND AGE FRIENDLY HEALTH SYSTEMS

Abstract citation ID: igaf122.2185

### ATTRACTING NURSES TO PRACTICE IN SKILLED NURSING HOME SETTINGS WITH AGE-FRIENDLY CURRICULUM

Diane Brown[1], Sue Hazelett[2], Susan Fosnight[2], Jennifer Drost[2], Cynthia Hovland[3], Margaret Sanders[4], Lori Kidd[1], and Donna Barrett[5], *1. The University of Akron, Akron, Ohio, United States, 2. Summa Health System, Akron, Ohio, United States, 3. Cleveland State University, Cleveland, Ohio, United States, 4. Northeast Ohio Medical University, Rootstown, Ohio, United States, 5. Summit County Public Health, Akron, Ohio, United States*

Skilled nursing home quality of care and staffing are positively correlated. Staffing shortages have been a long-standing problem with more than one in four US nursing homes reporting nurse shortages. We developed a 1-credit course for undergraduate nurse seniors with 12 online modules based on Age-Friendly Health Systems care, interprofessional teamwork, and other skilled nursing setting relevant topics. Modules included a short video lecture, pre-post quizzes, discussion boards, and supplemental materials. Trainees also spent 10 hours shadowing the interprofessional team in a skilled nursing facility. Forty students completed the course and pre/post course surveys to measure outcomes. Statistical path analysis was done using survey items regarding practice environment factors and interprofessional teamwork attitudes to assess for predictors of increasing a student's willingness to work with older adults, or to work in the skilled nursing setting. Results identified if giving psychological support was rated with high importance, the student had an 85% chance to increase their interest in the care of older adults post-course. If a student rated making clinical decisions with high importance, they were 88% more likely to increase their interest in practicing in a nursing home setting post-course. Qualitatively, students cited the negatives that deter interest in nursing home work most commonly as nurse-patient ratios, low pay, emotional toll of frequent deaths, and working with dementia clients. These results can help identify ways to increase recruitment of nurses to work with older adults and in skilled nursing facilities.

Abstract citation ID: igaf122.2186

### BRIDGING THE GAP: INTEGRATING PAIN INTO THE AGE-FRIENDLY HEALTH SYSTEMS 4MS FRAMEWOR

Beth Hogans[1], and Raya Kheirbek[2], *1. Johns Hopkins School of Medicine, Baltimore, Maryland, United States, 2. University of Maryland School of Medicine, Baltimore, Maryland, United States*

Background: Pain is a prevalent yet often underrecognized factor in geriatric healthcare, significantly influencing quality of life, functional independence, medication use, and cognitive health. The 4Ms framework of Age-Friendly Health Systems (AFHS)—What Matters, Medication, Mentation, and Mobility—has redefined geriatric care by emphasizing patient-centered approaches. However, pain is not explicitly addressed within this model, despite its profound impact across all four domains. We hypothesize that pain has bidirectional relevance to each M and plays a crucial role in geriatric multicomplexity, the proposed '5th M'. Objective: This review explores the relationship between pain, the 4Ms, and geriatric multicomplexity, evaluating how pain influences each domain individually and collectively while assessing the potential consequences of its exclusion from AFHS frameworks. Methods: A comprehensive literature synthesis was conducted, analyzing peer-reviewed studies, clinical guidelines, and policy documents on pain, aging, and the 4Ms/5Ms models. Key focus areas included pain-associated conditions, barriers to effective management, pharmacological and non-pharmacological treatments, and cognitive and mobility impairments. Results: Pain profoundly affects patient priorities, increases polypharmacy risks, contributes to cognitive decline, and exacerbates functional limitations. It is both a consequence and a driver of multimorbidity, influencing healthcare utilization and social determinants of health. Conclusions: Integrating pain into AFHS models as a core component is essential for optimizing geriatric care. Recognizing its role across the 4Ms will lead to more effective healthcare policies, improved patient-centered treatment strategies, and enhanced quality of life for older adults. A policy shift toward routine pain assessment and multimodal management strategies is urgently needed.

Abstract citation ID: igaf122.2187

### EMPOWERING CAREGIVERS THROUGH TECHNOLOGY: AN INTERGENERATIONAL APPROACH TO DIGITAL LITERACY & CONNECTION

Cathy Tompkins[1], Emily Ihara[1], Skye Leedahl[2], and Jill Juris[3], *1. George Mason University, Fairfax, Virginia, United States, 2. University of Rhode Island, Kingston, Rhode Island, United States, 3. Appalachian State University, Boone, North Carolina, United States*

Many older adult caregivers struggle to find reliable resources due to limited digital literacy, increasing their stress. Intergenerational digital literacy programs, like the University of Rhode Island's (URI) Cyber-Seniors, help bridge this gap by enhancing older adults' tech skills and fostering student connections. This mixed-methods research presentation explores pretest survey findings from a pilot program modeled after URI Cyber-Seniors. Six college students work with nine family caregivers to improve technology skills and access caregiving resources. A pretest survey, using a modified COCOA measurement tool, Geriatrics Attitudes Scale, and Relational Aging Anxiety Scale, assesses perceptions of aging and intergenerational relationships. Open-ended questions explore students' views on aging, career interests, and older adults' impressions of younger generations. The program includes five hours of training and runs for five weeks, with weekly student-caregiver meetings. Three students are in an MSW program, two are undergraduate social work majors, and one is a doctoral student. All identify as female, aged 18-29; 33% are white, and 33% multiracial. Caregivers range from 50-89 years old, 78% female, and 55% white. Using grounded theory analysis, emerging student themes range from discomfort working with older adults to strong enthusiasm for intergenerational engagement. Caregivers frequently describe younger generations by referencing technology proficiency but often include negative descriptors such as "challenged," "unaware," or "entitled." This discussion will explore generational perceptions, shared strengths, and motivations for engaging in intergenerational programs.

Abstract citation ID: igaf122.2188

### GROWTH MIXTURE MODELING OF LONELINESS TRAJECTORIES IN OLDER ADULTS: THE ROLE OF COGNITIVE FUNCTION

Elnaz Abaei, and Peter Martin, *Iowa State University, Ames, Iowa, United States*

Loneliness is a growing public health concern among older adults in the United States, with significant implications for cognitive function. This study utilized conditional growth mixture modeling (GMM-CV) to investigate the longitudinal trajectories of loneliness and how cognitive function (measured by immediate word recall, delayed word recall, and serial 7s) predicted these trajectories. Data were drawn from a sample of 3,944 older adults (Mage = 68.74) using eight waves of the Health and Retirement Study (HRS) data from 2006–2020. Data from every two years were pooled to increase the sample size and create four time points. GMM-CV in Mplus identified three distinct loneliness trajectories best fit the Data: LL= -25,495.269, BIC=51,214.097, SSABIC=51,128.303, Entropy=0.814, Adj. LMR-LRT (.52)=245.630, BLRT(.51)=255.518. The three identified classes were: Class 1: Low-Stable Loneliness (75.6%), Class 2: High-Decreasing Loneliness (13.1%), and Class 3: Moderate-Increasing Loneliness (11.3%). The mean group differences revealed significant group differences in immediate word recall at time 1 (F = 3.19, p=.04) and time 4 (F = 3.9, p=.02), delayed word recall at time 1 (F = 3.31, p=.037) and time 4 (F = 3.11, p=.045), while no significant effect was found for serial 7s at time 1 (F = 2.33, p=.098) and time 4 (F=.068, p=.934). Immediate word recall at time 4 exhibited a significant negative effect on the loneliness slope, indicating that higher cognition scores were associated with a less steep increase in loneliness over time. These findings suggest enhancing cognitive function as a potential intervention to reduce loneliness in later life. Future research should explore underlying mechanisms and moderating factors.

### SESSION 7000 (POSTER)

### ACUTE CARE

Abstract citation ID: igaf122.2189

### EVALUATING THE UNMET NEEDS OF OLDER ADULTS TO PROMOTE FUNCTIONAL RECOVERY AFTER THE ICU: THE LANTERN STUDY

Allison Cho[1], Nicole Bouranis[1], Janet Truebig[1], Nalani Walker[1], Amy Shelton[1], Andrea Benjamin[1], Jason Falvey[2], and Lauren Ferrante[1], *1. Yale School of Medicine, New Haven, Connecticut, United States, 2. University of Maryland School of Medicine, Baltimore, Maryland, United States*

Each year, nearly two million older adults survive a critical illness in the U.S., with half experiencing increased functional disability thereafter. No studies have focused on improving post-ICU functional recovery among older adults. Preliminary data suggests unmet needs within four domains (home environment, skilled rehabilitation, hearing/vision, caregiving) hinder functional recovery. LANTERN is a longitudinal study enrolling older ICU survivors. The objectives are to (1) identify unmet needs in these four domains 2-4 weeks after the patient returns home and evaluate associations with disability, rehospitalizations, and mortality over the subsequent six months, and (2) determine facilitators and barriers to addressing unmet needs. At enrollment, the home is thoroughly examined for barriers to functional recovery, such as needing grab bars in the shower. Participants are asked about needs in skilled rehabilitation and caregiving services. They undergo subjective and objective hearing/vision assessments, and a comprehensive geriatric assessment to inform covariates. Six monthly follow-up phone interviews ascertain disability in 15 functional activities, rehospitalizations, vital status, and changes to the domains of unmet needs. Data extraction from the electronic medical record (EMR) supplement the interviews. After the six-month visit, participants and caregivers are interviewed to determine facilitators and barriers to addressing unmet needs. To date, we have screened 1462 patients:

770 were eligible, 238 consented in-hospital, and 92 enrolled. Of enrolled participants, the mean age (SD) was 74.8 (7.1). This study elucidates whether unmet needs among older ICU survivors are associated with subsequent disability burden, rehospitalizations, and mortality, informing future interventions to support functional recovery.

Abstract citation ID: igaf122.2190

**GERIATRICIAN-LED INTERDISCIPLINARY ROUNDS TO DECREASE HOSPITAL READMISSIONS**

Julio Defillo Draiby[1], Michael Capilupi[2], Ka Ly[3], Barbara Mckenzie[4], and Wen-Chih Wu[3], *1. VA Providence Healthcare System, Palm City, Rhode Island, United States, 2. Brown University, Providence, Rhode Island, United States, 3. VA Providence Healthcare System, Providence, Rhode Island, United States, 4. VA Providence Health Care System, Providence, Rhode Island, United States*

Background: Age-Friendly Health Care Systems (AFHS) framework is used as a way to care for Older Adults[1]. Older patients occupy nearly half of the inpatient beds in community hospitals[2]. We wanted to determine if a Geriatrician-led interdisciplinary team was associated with changes in key performance indicators. Methods: Veterans 75 years or older assigned to a ten-bed virtual capacity, inpatient medical ward evaluated by a Geriatric led interdisciplinary (GEC) team utilizing the mentation, mobility, what matters, medications framework (4Ms) from the AFHS, generating a Geriatric Assessment note. Patients were excluded if admitted with a decompensated psychiatric disease or needed intensive or step-down level of care. After the initial evaluation, the GEC discussed daily until discharge. A total of 233 Veterans admitted to the AFHS was compared against 418 age-matched Veterans not admitted to AFHS (non-AFHS) during the same period (10/1/2023 – 6/30/2024; n = 651 total) on their length-of-stay (LOS), and 30-day readmission and mortality rates using multivariate linear and logistic regression where applicable adjusted for Charlson comorbidity and care assessment needs (CAN) scores. Results: Veterans admitted to the AFHS had a similar age but significantly higher Charlson and CAN scores. LOS, 30-day readmission, and mortality rates were similar between the two groups. Qualitative interviews of both patients and caregivers were also positive. Conclusion: In this Geriatric led AFHS improvement teamwork, we found that it had similar rates of mortality and readmissions in older veterans admitted to the medical ward despite the AFHS having a sicker population.

Abstract citation ID: igaf122.2191

**INVOLUNTARY HOSPITALIZATION OF PEOPLE LIVING WITH DEMENTIA: A COMPARATIVE POLICY ANALYSIS**

Clark Benson[1], Lea Gardiner[2], Laura Block[3], and Andrea Gilmore-Bykovskyi[4], *1. University of Wisconsin–Madison School of Medicine and Public Health, Madison, Wisconsin, United States, 2. University of Wisconsin–Madison, Madison, Wisconsin, United States, 3. University of Wisconsin-Madison, Madison, Wisconsin, United States, 4. University of Wisconsin-Madison School of Medicine and Public Health, Madison, Wisconsin, United States*

Neuropsychiatric symptoms, such as agitation and aggression, affect virtually all individuals with dementia and can sometimes be challenging to manage. These symptoms can result in crises characterized by psychological or physical threat to self and others. In some cases, these crises lead to involuntary hospitalization, a controversial policy typically used for people living with mental illness that involves the person being hospitalized and treated without their consent. Involuntary hospitalization policies vary by state, requiring local approaches to understanding their impact on people living with dementia. Rates of involuntary hospitalization among people with dementia and evaluation of existing policies for this practice are largely unknown. As a first step, this study conducts a comparative analysis of Wisconsin and Idaho state policies to lay the groundwork for subsequent research on their application. Wisconsin has required use of an "Emergency Protective Placement" policy since 2012 as involuntary hospitalization was declared unconstitutional for people living with dementia. In contrast, Idaho created and implemented a "Crisis Hold Policy" in 2024 to provide acute crisis stabilization services for people living with neurological conditions, including dementia. Comparative analysis reveals both state policies allow people living with neurological conditions to be hospitalized for a limited amount of time. While Wisconsin's policy has strict facility and treatment restrictions, Idaho's policy is more flexible and systematically collects data. This analysis generates three dementia crisis policy evaluation domains for consideration in future research and evaluation, including access to acute stabilization facilities, barriers to acute treatment, and data provision and government accountability.

Abstract citation ID: igaf122.2192

**POTENTIALLY PREVENTABLE HOSPITALIZATIONS AMONG DIFFERENT SUBPOPULATIONS OF OLDER ADULTS WITH DEMENTIA**

Elham Mahmoudi, *University of Michigan, Ann Arbor, Michigan, United States*

Potentially preventable hospitalizations (PPHs) are expensive and harmful to people. We examined the risk of PPH among (1) everyone with dementia, (2) those in disadvantaged neighborhoods, and (4) those in rural/micropolitan areas. We used a 20% random sample of 2017-2018 Medicare claims data to select dementia patients, with at least one hospitalization (n = 67,110). Using multilevel regressions, we adjusted for age, sex, race/ethnicity, frailty index, disadvantaged neighborhood index, rurality, and number of primary care physicians and hospitals at the county. One-third of all hospitalizations were PPH, with the urinary tract infection (33.84%) being the most common. In our complete sample, PPH risk in disadvantaged neighborhoods was 18% higher than in non-disadvantaged (OR = 1.18; 95% CI: 1.08-1.30). Females were at 24% higher risk of PPH than males (OR = 1.24; 95% CI: 1.19-1.29). Black (OR = 1.15; 95% CI:1.08-1.22) and Hispanic (OR = 1.07; 95% CI: 1.00-1.16) patients had 15% and 7% higher risk of PPH than Whites, respectively. Limiting our sample to disadvantaged areas, risk of PPH for females than males increased to 35% (OR = 1.35; 95% CI: 1.12-1.62). In disadvantaged areas, there were no racial/ethnic or geographic differences in PPH risk; however, adjusted predicted PPH risk increased to 25% (95% CI:0.17-0.36) for everyone. Similarly, limiting the sample to rural/micropolitan areas, females PPH risk increased (OR = 1.30; 95% CI: 1.17-1.43) and adjusted PPH risk for disadvantaged neighborhoods increased to 27% (95% CI: 0.22-0.32). Disadvantaged and rural/micropolitan areas have substantially fewer primary care physicians. For dementia patients, practical and affordable access to primary care is one critical key to reduce PPH.

Abstract citation ID: igaf122.2193

### PRE-INJURY MULTI-DIMENSIONAL HEALTH AND 6-MONTH OUTCOME AFTER GERIATRIC TRAUMATIC BRAIN INJURY

Roy Tzemah-Shahar[1], Yael Rosen-Lang[2], Ava M. Puccio[3], Esther L. Yuh[4], Michele Diaz Nelson[4], Kristine Yaffe[4], and Raquel C. Gardner[2], *1. University of Haifa, Haifa, HaZafon, Israel, 2. Sheba Medical Center, Ramat Gan, Tel Aviv, Israel, 3. University of Pittsburgh, Pittsburgh, Pennsylvania, United States, 4. University of California, San Francisco, San Francisco, California, United States*

Older adults with traumatic brain injury (oaTBI) are, on average, at greater risk for morbidity and mortality compared to younger adults. Most existing models predicts recovery measured by Glasgow Outcome Status Extended (GOSE, scored 1-8 while 1 means death and 8 returning to pre-injury state) based primarily on age and injury severity, and may not be optimized for oaTBI. In this study we tested the performance of four existing models developed to predict GOSE< 5 (unfavorable outcome, severe disability or worse) 6-month following the injury), in oaTBI cases. We additionally quantified the added prognostic value of pre-injury multi-dimensional health metrics: activities of daily living, comorbidity burden, cognitive status, and frailty. The analytic cohort included N = 112 participants from the TRACK-GERI study, a 2-center prospective cohort study of adults aged 65 years and older presenting to Level 1 Trauma Centers with acute TBI who undergo head CT (95% with Glasgow Coma Scale 13-15, 33% with ICU admission). The CRASH Basic model had the highest accuracy for discriminating GOSE< 5 (AUC 0.74, 95% CI 0.64,0.83). Incorporating pre-injury characteristics improved the model's ability to discriminate favorable vs. unfavorable outcome, however the change was statistically insignificant. Frailty, measured using the Groningen Frailty Indicator, was an independent predictor of poor outcome (OR 1.30, 95% CI 1.06,1.61). These findings demonstrate the role pre-injury characteristics play in oaTBI recovery process; integrating such factors in new outcome prediction models may improve clinical decision-making and individualized outcome prediction.

Abstract citation ID: igaf122.2194

### THE HUMAN FACTOR: HOW STAFF ATTITUDES SHAPE MOBILITY IN HOSPITALIZED OLDER ADULTS

Anna Zisberg, Ksenya Shulyaev, Nurit Gur-Yaish, Juliana Smichenko, and Efrat Shadmi, *University of Haifa, Haifa, HaZafon, Israel*

In-hospital mobility is crucial for older adults' recovery, post-hospital function, and quality of life. While patient characteristics influence mobility, the impact of organizational factors, specifically unit workload and staff attitudes, remains underexplored. This study examined their effects on mobility among hospitalized older adults. We analyzed mobility data from 610 cognitively intact, ambulatory older adults (mean age 77.6±6.8, 54.1% male) across 20 units in four Israeli hospitals. Mobility was self-reported daily (up to 7 days) using the Mobility Rating (MR). Unit workload was assessed via nurse-to-patient ratio, patient turnover, and ventilated patient count. Staff attitudes (nurses, nursing assistants, physical therapists, and physicians) were measured using the Barriers to Early Mobility questionnaire. Covariates included age, pre-hospital ADL, cognitive status (MMSE), and acute illness severity (NEWS). Mixed-model analysis (5306 observations, 1079 data points) revealed that positive staff attitudes ($\beta = 2.16$, $p = 0.046$), higher ADL ($\beta = 0.078$, $p < 0.0001$), and better cognition ($\beta = 0.099$, $p = 0.027$) were associated with greater mobility. Conversely, older age ($\beta = -0.06$, $p = 0.002$) and higher acute illness severity ($\beta = -0.211$, $p = 0.0012$) predicted lower mobility. Notably, workload factors were not significantly associated with daily mobility. Findings highlight the critical role of staff attitudes in preserving mobility during hospitalization. Promoting a mobility-supportive hospital culture may be key to improving outcomes for older adults.

Abstract citation ID: igaf122.2195

### THE IMPACT OF MITT AND TRADITIONAL RESTRAINT USE ON LENGTH OF STAY IN HOSPITAL DEMENTIA CARE

Clarissa Shaw[1], Gregory Isaac[2], Lisa Weimar[3], and Caitlin Ward[2], *1. University of Iowa College of Nursing, Iowa City, Iowa, United States, 2. University of Minnesota School of Public Health, Minneapolis, Minnesota, United States, 3. University of Iowa, Iowa City, Iowa, United States*

The regulation of physical restraints in dementia care varies between hospitals and nursing homes, creating a gap in how devices like mitts are classified. Mitts are padded gloves that prevent finger and thumb movement. Traditional physical restraints are wrist, ankle, or vest devices that secure a patient to their bed or chair. While nursing homes classify mitts as restraints, hospital practices vary widely, with some designating them as "restraint alternatives" that do not require a provider order or frequent monitoring. This regulatory discrepancy creates discharge barriers for patients in mitts, as many nursing homes cannot accept patients that are actively restrained, potentially prolonging hospitalizations. This study examined whether mitt use impacts length of stay similarly to traditional restraints. A retrospective analysis of electronic medical record data from a single university hospital that treats mitts as restraint alternatives was conducted. The data included 1,503 patients with dementia admitted to medical-surgical units across 2,141 hospital stays with an average length of stay of 8.3 days. Mitts were utilized in 15.9% of hospital stays and traditional physical restraints were used in 12.5% of stays. Linear mixed modeling revealed that mitt use was associated with a 4.4-day increase in length of stay ($SE = 0.29$, $p < .001$) compared to patients without mitts. Similarly, traditional restraint use was associated with a 3.9-day increase ($SE = 0.33$, $p < .001$) compared to patients without traditional restraints. Despite having comparable impacts on length of stay as traditional restraints, mitts remain under-recognized in hospital settings while being regulated as restraints in nursing homes.

### SESSION 7005 (POSTER)

### AGEISM

Abstract citation ID: igaf122.2196

### AGE AND OTHER INDIVIDUAL DIFFERENCE VARIABLES PREDICT AGEISM AWARENESS

Carly Pullen, and Julie Hicks-Patrick, *West Virginia University, Morgantown, West Virginia, United States*

One in every two people endorses ageist attitudes (WHO, 2021). Identifying the 50% who do and do not endorse such attitudes may reduce bias in Society. We examined beliefs about ageism among an age-diverse sample, including 231 US-based adults (51.9% female; Mage= 47.51, SD = 16; 67.5% WNH).

A hierarchical regression examined how prominent an issue people believe ageism to be (M = 2.38 out of 4, SD = 0.88). In Step 1, age, sex, and race were entered into the model. Results were significant, F (3,227) = 9.13, p < .001, but only 10.8% of the variance in how prominent an issue people believe ageism to be was explained. Age and sex uniquely contributed to the model, (βage = .26, p< .001) and (βsex = .16, p < .05). In Step 2, hostile and benevolent ageism were added to the model to determine if a person's attitude regarding older adults impacted their perception of ageism as a problem. Results were significant, F (5, 225) = 9.6.40, p < .001, with only 12.4% of the variance in how prominent an issue people believe ageism to be was explained. Lower hostile ageism uniquely contributed to the model (β = -0.12, p < .05), while benevolent ageism was non-significant (β = .07, p = .26). Results of the current study call attention to ageism as a problem and draw attention to the impact it has on older adults is a step forward in combatting stereotypes, prejudice, and discrimination.

Abstract citation ID: igaf122.2197

**AGE DISCRIMINATION AND SLEEP QUALITY IN OLDER ADULTS: DEPRESSION AS MEDIATOR**

YoungBin Koh, and Yeonjung Lee, *Chung-Ang University, Seoul, Republic of Korea*

Though Korean society has traditionally emphasized respect for older adults, these values are fading in contemporary times, resulting in widespread age discrimination and hostility toward older adults. Stress process model suggests that life events such as discriminatory experience is a substantial stressor and contributes to chronic strain, which, in turn, those have been linked to a number of adverse outcomes in older adults. Previous studies suggest that perceived age discrimination adversely affects older adults' mental, physical, and social health. Additionally, there is a growing interest that discrimination may be a contextual contributor to sleep problems in later life. However, little attention has been paid to the stress process of age discrimination, particularly whether depression mediates the relationship between perceived age discrimination and sleep problems. The purpose of the study is to examine the pathway of how age discrimination contributes to depression, ultimately affecting sleep quality. Using data from the 2023 Senior Citizen Survey (N = 9,818), we applied PROCESS Macro Model 4 to assess the mediating role of depression. Results show that age discrimination is significantly associated with increased depression, which, in turn, negatively impacts sleep quality. The results suggest that the effects of age discrimination on sleep operate indirectly through heightened depression. These findings have implications for policy and practice, emphasizing the need for strengthening the laws to prevent age discrimination and expanding depression management programs to raise awareness of the consequences of age discrimination.

Abstract citation ID: igaf122.2198

**AGEISM IN STORYTELLING: HOW AGE BIAS INFLUENCES PERCEPTIONS OF STORY QUALITY**

Annalise Evans, and Nicole Alea, *University of California, Santa Barbara, Santa Barbara, California, United States*

Ageism permeates many facets of an older adult's life, even, perhaps, the way that the autobiographical stories they tell are perceived. Older adults are often criticized for going "off-target" or including too many story asides (i.e., tangentially-related information) when telling stories. The paradox is that their stories are also sometimes rated as higher in quality, compared to younger adults. This study aimed to determine if ageism contributes to story quality, depending on the age of the storyteller and how many story asides they used. College-aged participants (N = 123, M = 19.01, 21% male) rated the quality (i.e., entertainment, emotion, memorability, originality, imagery, and engagement) of eight real autobiographical stories that experimentally varied by age of the storyteller (young, old) and amount of story asides (few, many). They were also given an ageism questionnaire. Higher levels of ageism was associated with lower quality ratings for the older adults' autobiographical stories, regardless of whether those stories were low or high on story asides. Ageism, however, was not associated with the quality of either of the young adults' stories. Results suggest that the driver of younger adults' perceptions about the quality of stories may not be the amount of story asides, but bias against older adult storytellers.

Abstract citation ID: igaf122.2199

**DEHUMANIZATION OF OLDER UNHOUSED ADULTS: EXAMINING AGE BIASES AND THE LIMITS OF EMPATHY**

Brooke Dolenc Nott, Dan Dowhower, and Melissa Cannon, *Western Oregon University, Monmouth, Oregon, United States*

The population of older individuals experiencing homelessness is growing in the U.S. and understanding the psychological factors influencing their treatment is crucial. Previous research highlights consistent dehumanization of unhoused individuals and the impact of ageism on the care older adults receive. However, limited work examines the compounded dehumanization faced by older unhoused adults. This study explored age-based dehumanization of unhoused individuals and whether empathy interventions could mitigate these biases. Participants (N = 399; M age = 22.5) were randomly assigned to read scenarios about unhoused adolescents or adults, with varying empathy prompts (no empathy, perspective-taking, or empathy conformity). Measures of dehumanization and helping intentions were then assessed. Results revealed older unhoused individuals faced significantly higher dehumanization than younger ones, with males and younger participants demonstrating stronger biases. Empathy interventions reduced dehumanization toward adolescents but had limited effects for older adults. However, older participants showed greater willingness to help overall. Findings suggest that intersectional biases heighten dehumanization against older unhoused individuals, potentially influencing policies and services for this population. Addressing these biases is strategic to fostering more inclusive and supportive services for all unhoused populations.

Abstract citation ID: igaf122.2200

**DIFFERENCES IN TRAINEES' AGEIST ATTITUDES AND AGING ANXIETY BY QUANTITY AND QUALITY OF CONTACT WITH OLDER ADULTS**

Grace Caskie, and Maria Tice, *Lehigh University, Bethlehem, Pennsylvania, United States*

Ageist attitudes and aging anxiety have been identified as explanations for psychology trainees' low interest in geropsychology. Frequent, high-quality, intergenerational contact between trainees and older adults appears key to reducing ageism (Allport, 1954; Levy, 2018). However, little is known about the types or quality of contact trainees have with older adults or how quantity and quality may interact. In this study, trainees (N = 213; aged 21-35) from clinical/counseling psychology doctoral programs completed ageism, aging anxiety, and contact measures. Frequent contact occurred most often as brief (78.9%) or long (62.9%) conversations with older adults; frequent contact in other domains was less common (22.1%-54.5%). High quality contact significantly associated with more frequent contact for 10 of 11 domains. Only significant main effects of quantity and quality of contact were found for ageist attitudes and aging anxiety. Ageist attitudes were lower for those reporting frequent long talks with an older adult; aging anxiety was lower for trainees report frequent contact in five domains (long talk, older adult discussed life with them, helped older adult, older adult helped them). Ageist attitudes were lower for those with positive contact in four domains (discussed problem with older adult, helped older adult, older adult helped them, older adult coworker); aging anxiety was lower for those with positive contact in 10 of 11 domains. Encouraging intergenerational contact that occurs in one-on-one settings, involves collaboration, and includes sharing of personal information may be most likely to be associated with less ageist attitudes and aging anxiety among psychology doctoral trainees.

Abstract citation ID: igaf122.2201

### EVERYDAY AGEISM: PERSPECTIVES FROM PEOPLE AGED 85+ USING PARTICIPATORY PHOTOVOICE METHODOLOGY

Sophia Ashebir[1], Taylor Brennan[2], Niels Wu[1], and Lisa D'Ambrosio[1], *1. Massachusetts Institute of Technology, Cambridge, Massachusetts, United States, 2. MIT, Boston College, Cambridge, Massachusetts, United States*

While research on ageism is increasingly well-documented, little attention has been paid to how people may personally experience ageism during specific age periods in their older adulthood. Methods leveraging approaches from participatory action research to explore how the oldest of older adults (those aged 85+) experience ageism are even more scarce. This study addresses this gap by employing mixed methods, including PhotoVoice, a qualitative method leveraging participant-taken photographs, and virtual semi-structured interviews, to investigate ageism through the perspectives of a sample of adults aged 85+. Participants (N = 30) were recruited from the MIT AgeLab 85+ Lifestyle Leaders panel, a longitudinal study of U.S. octogenarians and nonagenarians, the MIT AgeLab Volunteer Database, and snowball sampling. Using an inductive, team-based thematic analysis approach, we identified a spectrum of experiences related to ageism, including covert and overt manifestations, positive and negative encounters, and external versus internalized perceptions. Participants highlighted challenges related to ageism in specific domains such as product design, healthcare, and the built environment. Additionally, instances of positive ageism—where aging was met with preferential treatment or social advantage—were particularly salient in this sample. The use of PhotoVoice as a visual storytelling tool offered a richer, multidimensional understanding of experienced ageism, further supplementing findings from interviews. Findings underscored the importance of co-creating knowledge with older adults and exploring alternative methodologies to understand lived experiences. The study contributes to both theoretical and applied discussions on ageism, emphasizing the value of inclusive, participatory research.

Abstract citation ID: igaf122.2202

### HOW NON-ESSENTIALISM BELIEFS OF AGING SHAPES AWARENESS OF AGE-RELATED CHANGES: A CROSS-CULTURAL PERSPECTIVE

Yiwen Wu, and Helene Fung, *The Chinese University of Hong Kong, Hong Kong, China (People's Republic)*

Non-Essentialism Belief of Aging (NEBA), the beliefs that the aging process is malleable, is closely related to subjective aging and motivation in old age. While previous studies have shown that NEBA predicts more positive subjective aging experiences, few studies have explored the underlying mechanisms. We propose that one crucial pathway might be attributional styles, the dispositional tendency to attribute negative health events to specific factors (e.g. lifestyle) or to general causes as old age. This study aims to examine the potential mediating effect of attribution styles on the relationship between NEBA and subjective aging, measured by awareness of age-related changes (AARC). N = 766 older adults aged 55-75 years (Mage = 61.8 ± 4.9 years) in China (N = 524), the United Kingdom (N = 122), and the United States (N = 120) responded to online survey instruments measuring NEBA, attribution styles, and AARC. Results showed that higher NEBA was associated with more specific attributional styles, was positively related to more AARC gains and fewer AARC losses across all three cultures. Moreover, age attribution styles fully mediated the relationship between NEBA and AARC gains and partially mediated the relationship between NEBA and AARC losses. Taken together, our findings reveal that attribution styles might be an important pathway through which NEBA influences subjective aging and well-being in old age.

Abstract citation ID: igaf122.2203

### IMPROVING PERCEPTIONS OF AGING AND DEMENTIA USING CREATIVE STORYTELLING: IMPLICATIONS FOR PEDAGOGY

Melannie Pate, and Kent Guion, *The University of North Carolina at Wilmington, Wilmington, North Carolina, United States*

Background: Negative stereotypes and ageism toward older adults with dementia remain a problem, proving to reduce quality of life and can contribute to negative health outcomes. Depictions of dementia are often associated with negative stereotypes. Training an adequate workforce for this population is essential as there is concern with the attitudes and knowledge that students and future public health professionals hold about individuals living with dementia. Description: Using TimeSlips, a creative storytelling method, undergraduate and graduate students participated in community sites serving individuals living with dementia. Students facilitated the creative storytelling sessions weekly at each site. Pre and post-evaluations measuring perceptions, outcomes, satisfaction, and knowledge were administered. Lessons Learned: • Results of the evaluation provide opportunities to view the importance of field work for students with individuals living with dementia • Creative engagement lessens negative stereotypes for students • Students enjoy undergraduate and graduate aging courses more and feel more positively about older adults when they participate in community-based field work • Pedagogical approaches that include field work to reduce negative stereotypes around aging and dementia are needed Implications • Lead to a greater understanding of students' attitudes toward aging and dementia • Inform educational interventions to improve quality of life • Extend creative storytelling as part of the pedagogy for courses in aging and public health • Improve perceptions of dementia, resulting in less resistance and fear of working with the population

Abstract citation ID: igaf122.2204

### IN THEIR OWN WORDS: AN INNOVATIVE APPROACH TO INTERGENERATIONAL LEARNING IN AGING COURSES

Carly Chak, Eleni Vodantis, and Nicole Alea, *University of California, Santa Barbara, Santa Barbara, California, United States*

Educational initiatives that encourage younger adults to interact with older adults is one way to reduce negative age-related stereotypes in university students. Incorporating intergenerational interactions into aging-related courses can be challenging when the class size is large. To address this challenge, this project included the development of pre-recorded "Aging Insight Videos". The videos feature five to six different older adults of varying backgrounds and lived experiences. The older adults answer instructor-developed questions that link with course concepts (e.g., social relationships, cognitive aging). These videos provide students the opportunity to learn about course material directly from the life experiences of older adults, in their own words. The current presentation focuses on: how older adult interviewees were chosen, the development of the questions, how the videos were edited, and ideas for implementing the videos into aging courses. Initial evidence, from week one of our adult development and aging course (N = 125), in which the videos were not included, suggests this instructional pedagogy is needed. Students held implicit ageist attitudes: older adult faces were rated significantly lower across five positive traits (warmth, competence, likeability, physical health, memory ability) compared to middle-aged and younger adult faces. We anticipate perceptions to positively change by the end of the course. However, the discussion will focus on how inclusion of the Aging Insight Videos as part of the course curriculum, and having students learn from and interact with older adults through these videos, in a next iteration of the course, should further reduce age-related negative stereotypes.

Abstract citation ID: igaf122.2205

### INTERSECTIONAL DISCRIMINATION AND MENTAL HEALTH IN LATER LIFE: AGEISM AS A CORE DIMENSION

Yi Wang[1], Yifan Lou[2]Huei-wern Shen[3], and Ernest Gonzales[4], *1. University of Iowa School of Social Work, Iowa City, Iowa, United States, 2. Virginia Commonwealth University, Richmond, Virginia, United States, 3. University of North Texas, Denton, Texas, United States, 4. New York University, New York, New York, United States*

Despite extensive literature that examines the relationship between discrimination and health, less is known about specific discrimination attributions and how they may differentially affect health outcomes. To address this gap, the current study investigated the latent typology of the discrimination attributions, and the effects of intersectional attributions on mental health in later life. Data came from 6,282 respondents in the 2016 Psychosocial Leave-Behind Questionnaire of the Health and Retirement Study. Participants ascribed their everyday discrimination experiences to a list of ten potential reasons (e.g., ethnicity, ancestry, gender, race, age, religion, financial). Latent class analysis (LCA) was performed to identify discrimination attribution typologies. Regression models with marginal effects were conducted to explore differential mental health effects of different attribution typologies. Five distinct typologies were identified: few discrimination experiences (33%), discrimination with no specified attributes (5%), discrimination due to age (48%), discrimination due to age, race, ethnicity (8%), and discrimination due to age and explicit characteristics (5%) such as physical disability and appearance. Regression analysis revealed significant associations between discrimination and depressive symptoms, cognitive function, and loneliness. Discrimination involving more than just age, especially explicit characteristics, had stronger negative effects on mental health. Ageism emerged as a core dimension and prevalent theme across multiple discrimination experiences, and often co-occurs with other characteristics, highlighting the intersectionality of discrimination. The negative mental health effects were most pronounced for those who experienced discrimination related to intersectional attributions. Implications for practice and research were discussed.

Abstract citation ID: igaf122.2206

### KNOWLEDGE IS POWER: CHALLENGING AGEIST ATTITUDES AS A PATHWAY TO CAREERS IN AGING

Hannah Friedland, Jillian Crocker, Amanda Morgan, Jessica Ruiz, Soledad Arguelles-Borge, and Ashley Stripling, *Nova Southeastern University, Fort Lauderdale, Florida, United States*

Ageist bias is underrecognized and contributes to misdiagnosis, delayed treatment, and a range of adverse mental health outcomes for older adults. Aging-related coursework and awareness of bias may lead to reductions in ageist attitudes. Therefore, the current study aims to examine the impact of implicit bias assignments and subsequent knowledge from an eight-week adult and older adult development course taught by a geropsychologist. Participants consisted of 26 first-year psychology doctoral students asked to complete pre/post measures of the Age Harvard Implicit Association Test (IAT) and a written reflection. Results of the IAT were coded on a 7-point Likert scale to assess the prevalence and change of preferences for young people or older people. The reflections were analyzed using constructivist grounded theory methodology. Descriptive statistics revealed that 77.8% of participants had a baseline preference for young people. Notably, a paired-samples t-test indicated that pre-test scores ($M = 2.58$, $SD = 1.137$) were significantly different than post-test scores ($M = 3.38$, $SD = 1.134$), $t(25)=-3.035$, $p=.003$, suggesting a shift in scores towards age neutrality. On the baseline reflection, participants reported a range of emotional responses (e.g., shock, disbelief), rationalizations (e.g., fear of aging, lack of exposure to older adults, sociocultural influences), and acknowledged the impact of bias on professional practice. At post-test, participants reported a shifted perspective on aging due to course content, with some indicating novel career interest in geropsychology. Implications of the current presentation emphasize the importance of implementing aging-related coursework and awareness of bias early in graduate training to develop geriatric interest and competency of care.

Abstract citation ID: igaf122.2207

### LEARNING ACROSS GENERATIONS: THE DUAL BENEFITS OF INTERGENERATIONAL MENTORING FOR STUDENTS AND SOCIETY

Emily Ihara[1], Cathy Tompkins[1], Madeline McIntyre[2], and Shannon Arnette[2], *1. George Mason University, Fairfax, Virginia, United States, 2. Virginia Commonwealth University, Richmond, Virginia, United States*

Despite efforts to encourage careers in aging, fewer than 1% of first-year medical fellows choose geriatrics, and only about 3% of master's-level social work students enroll in an aging concentration. Intergenerational mentoring programs present a unique opportunity to foster meaningful student-older adult connections, enhance learning outcomes, and challenge societal ageism. Such programs are particularly valuable for medical and social work students, helping them develop empathy, cultural humility, and a deeper understanding of aging to provide effective, person-centered care. This study examines attitudes toward aging among medical (n = 192) and social work (n = 7) students before program implementation. Using a modified COCOA measurement tool (Hollar et al., 2011), the Geriatrics Attitudes Scale (Reuben et al., 1998), the Relational Aging Anxiety Scale (Gendron et al., 2020), and open-ended responses, students reflected on their perceptions of aging and working with older adults post-degree. Preliminary analysis of social work students' open-ended questions reveals a range of perspectives, from optimism and aging as a source of wisdom to anxiety about physical decline and societal ageism. Among medical students, over 87% agreed they could learn from older adults, but only 14% expressed strong interest in geriatrics. While nearly 60% felt confident working with older adults, only 23% found it deeply fulfilling. Structured mentoring may shift these perspectives by offering experiential learning that challenges biases and enhances professional interest. Future analysis will assess post-program attitude shifts and their impact on aging-related career pathways.

Abstract citation ID: igaf122.2208

### METHODOLOGICAL DIFFERENCES IN MEASURING AGE BIAS AMONG DOCTORAL PSYCHOLOGY TRAINEES

Benjamin Johnson, and Grace Caskie, *Lehigh University, Bethlehem, Pennsylvania, United States*

Nearly 90% of ageism measures utilize explicitly negative statements about older adults. Accurately measuring ageism with explicitly ageist statements may be hampered by social desirability bias, particularly among doctoral psychology trainees. To compare different methodological approaches to measuring ageism, 80 doctoral psychology trainees (23-33 years) completed explicit (FSA, ASD, AmbAS Hostile), implicit (Age IAT), and indirect (FAQ Negative Bias) ageism measures online, as well as a social desirability scale. Normalized to a 0-10 range for direct comparability, age bias was highest on the Age IAT (M = 2.70), followed by explicit measures (ASD, M = 2.67; FSA, M = 2.14; AmbAS Hostile, M = 2.10), and was least on the indirect measure (FAQ Negative Bias, M = 1.90). Follow-up analyses indicated the FSA Stereotypes subscale showed higher (M = 3.30) age bias than any full-scale score. Correlations of the Age IAT with explicit and indirect measures were non-significant. However, age bias measured indirectly (FAQ Negative Bias) correlated significantly with age bias measured explicitly (FSA total, FSA Stereotypes), even after partialling out social desirability; these correlations strengthened after controlling for social desirability. The indirect ageism measure overlapped more with explicit measures than the implicit measure and generally indicated less age bias, while explicit measures and the implicit measure, which focused on ageist stereotypes, produced higher age bias. Ageist stereotypes can operate unconsciously, which may pose harmful consequences in doctoral psychology trainees' future work with older adult clients. Thus, different methodological approaches for measuring ageism influence the amount of self-reported ageism, suggesting that researchers and educators carefully consider their methodological approach when assessing ageism.

Abstract citation ID: igaf122.2209

### OPINIONS TOWARDS AGING IN THE MARSHALLESE COMMUNITY RESIDING IN NORTHWEST ARKANSAS

Shakshi Sharma[1], Gohar Azhar[2], Karen Coker[3], Sheldon Riklon[4], Philmar Kabua[4], Pearl Mcelfish[4], and Jeanne Wei[2], *1. University of Arkansas for Medical Sciences, Little Rock, Arkansas, United States, 2. University of Arkansas for Medical Sciences, Little Rock, AR, LITTLE ROCK, Arkansas, United States, 3. University of Arkansas for Medical Sciences, Department of Geriatrics, Donald W. Reynolds institute of Aging, 4301 West Markham Street, Slot 748, little Rock, AR, 72205-7199, USA, Little Rock, Arkansas, United States, 4. University of Arkansas for Medical Sciences Northwest, Fayetteville, Arkansas, United States*

The Marshallese is a Pacific Islander community located around the US with much of the population primarily residing in Northwest Arkansas. The perception of aging in this community might be influenced by different cultural traditions, health conditions as well as health information and education. We sought to develop an understanding of how Marshallese view aging. An anonymous survey exploring perspectives and beliefs regarding aging was administered to fifty subjects (mean age 46.7±11.1 SD). This was a convenience sample with 68% of the participants between 18-50 yrs. and 32% between 51-89, with 72% females and 28% males. When we asked participants if aging was a natural process, 86% agreed, 6% disagreed and 8% were uncertain. Furthermore 52% agreed when asked if getting older was associated with fewer activities, 22% disagreed and 26% were uncertain. When asked if aging was a possible punishment or sin, 18% agreed, 59% disagreed and 22% were uncertain. Additionally, 12 % believed that Alzheimer's disease was a punishment for sin. Although the majority of our participants were younger adults, only 38% believed they were young, whereas 32% classified themselves as being "old" and 30% were uncertain. The results from our survey highlighted the somewhat unfavorable beliefs of Marshallese towards aging and dementia. Understanding these perspectives in the context of their traditions and culture will assist with developing educational materials to promote a healthier and more positive image of aging while improving the quality of life of the older adult Marshallese community members.

Abstract citation ID: igaf122.2210

### PREPARING THE HOME AND COMMUNITY BASED WORKFORCE FOR REMOTE MONITORING TECHNOLOGY: LESSONS FROM A QUALITATIVE STUDY

Erica Perryman[1], Kathleen Buckwalter[2], Emily Jones[3], Jennifer Heck[3], Diana Sturdevant[3], and Frances Wen[4], *1. The University*

*of Oklahoma Health Sciences Center, Tulsa, Oklahoma, United States, 2. University of Iowa, Iowa City, Iowa, United States, 3. The University of Oklahoma Health Sciences Center, Oklahoma City, Oklahoma, United States, 4. The University of Oklahoma, Norman, Oklahoma, United States*

The use of remote monitoring technology for care coordination among community-dwelling older adults (age ≥ 65) increased during the COVID-19 pandemic and continues today. Remote patient monitoring is valuable for older patients because it eliminates barriers to healthcare access and care coordination, better allowing them to age in place. Yet, many professionals in healthcare believe that their older patients are unwilling or unable to use remote patient monitoring technology. Healthcare professionals are challenged to overcome ageist beliefs that contribute to the digital divide among older adults. Likewise, health systems are challenged to develop new approaches to care delivery. Remote patient monitoring offers a new approach to home and community-based services care coordination, but its implementation is less understood. A qualitative descriptive study using multi-perspective interviewing (N = 34, resulting in 7 triads) was conducted to better understand the perspectives of remote monitoring technology among older adults (n = 15), their informal caregivers (n = 8), and their home and community-based service providers (n = 11). Synchrony was found among the three groups about the benefit of remote patient monitoring for the older adult's care coordination, yet ageist beliefs most often prevented the HCBS provider from introducing remote monitoring technologies to their older adult clients. Lessons from this study inform the workforce development efforts intended to prepare home and community-based service providers for remote patient monitoring as a new approach to care coordination and delivery.

Abstract citation ID: igaf122.2211

### ROLE OF SOCIAL CAPITAL IN THE ASSOCIATION BETWEEN AGEISM AND DEPRESSIVE SYMPTOMS AMONG OLDER ADULTS IN SOUTH KOREA

Bokyoung Choi[1], Jihwan Lee[1], Jihee Choi[2], Eunhee Choi[2], Chang-O Kim[3], and Soong-nang Jang[1], *1. Chung-Ang University, Seoul, Republic of Korea, 2. Institute for Community Care and Health Equity, Seoul, Republic of Korea, 3. Dolbom Clinic, Seoul, Republic of Korea*

Background: Ageism is a known risk factor for mental health in older adults, but the role of social capital in this relationship remains unclear. This study examines how bridging and bonding social capital moderate the association between perceived ageism and depressive symptoms. Methods: Using data from the 2023 National Survey of Older Koreans, we analyzed the association between perceived ageism (interpersonal and institutional) and depressive symptoms (SGDS-15) among adults aged 65 and older. Perceived ageism was categorized into interpersonal ageism and institutional ageism based on the context in which discrimination occurred, including public institution, hospital, workplace, and home. We also examined the moderating effects of bridging social capital (e.g., participation in senior welfare centers) and bonding social capital (e.g., membership in social or hobby clubs). Prevalence ratios (PR) and 95% confidence intervals (CI) were calculated. Results: Among participants, 47.5% reported interpersonal ageism, while 27.5% reported institutional ageism. Interpersonal ageism was associated with 2.92 times higher odds of depressive symptoms, and institutional ageism with 1.98 times higher odds. However, the relationship between ageism and depressive symptoms varies depending on the type of social capital. Bridging social capital attenuated this association to non-significance, whereas bonding social capital exacerbated it, amplifying the adverse impact of ageism on depressive symptoms. Conclusion: Bridging social capital may act as a protective factor against the negative effects of ageism, whereas bonding social capital may reinforce them. These findings underscore the need for expanding older adults' social networks beyond close-knit groups to mitigate the psychological impact of ageism.

Abstract citation ID: igaf122.2212

### THE ASSOCIATION OF AGEISM, COPD-RELATED STIGMA, AND PSYCHOSOCIAL HEALTH IN CHRONIC OBSTRUCTIVE PULMONARY DISEASE

Seoyoon Woo[1], Jeonghwa Lee[2], Susan Glose[2], and Yeoun Soo Kim-Godwin[2], *1. University of North Carolina Wilmington, Wilmington, North Carolina, United States, 2. The University of North Carolina at Wilmington, Wilmington, North Carolina, United States*

Rationale/Objectives: People with chronic obstructive pulmonary disease (COPD) may experience an accelerated aging process that affects both their health and social roles. This study aimed to examine the relationship between stigma related to aging and that associated with COPD, and the psychosocial health of individuals living with COPD. Methods: A cross-sectional study using an online survey method was conducted. Participants who self-reported having COPD (N = 101; mean=61.4 years) completed a series of questionnaires, including the Everyday Ageism Scale, the COPD-related Stigma Scale, the Attitudes to Aging Scale (AAQ), the PROMIS Anxiety (SF-8a), the Social Isolation Scale, and questions on unplanned doctor visits due to acute exacerbations, and age. ANOVA and multivariate regression models were used to analyze the data. Results: There were no significant differences in ageism (mean=14.65±5.13) and COPD-related stigma (mean=2.37±0.57) among different age groups. Using Wilks' criterion, the combined ageism and COPD-related stigma were significantly associated with the following factors: the AAQ-psychosocial loss scale ($F(2, 94)=9.75$, $p < 0.001$; partial $\eta^2=0.17$), anxiety ($F(2, 94)=4.26$, $p = 0.017$; partial $\eta^2=0.08$), social isolation ($F(2, 94)=4.21$, $p = 0.018$; partial $\eta^2=0.08$), and acute exacerbation episodes ($F(2, 94)=4.89$, $p = 0.010$; partial $\eta^2=0.09$). However, there was no significant relationship with age ($F(2, 94)=2.42$, $p = 0.094$; partial $\eta^2=0.05$). Univariate analyses indicated that anxiety and age were significantly associated with ageism, but not with COPD-related stigma. Conversely, social isolation was significantly associated with COPD-related stigma, but not with ageism. Conclusion: Findings enhance the understanding of the relationships between aging-related and COPD-related stigma experiences and psychosocial factors in individuals with COPD.

Abstract citation ID: igaf122.2213

### THE POTENTIAL EFFECTS OF THERAPIST AGEISM ON OLDER ADULTS' THERAPY OUTCOMES: A SCOPING REVIEW

Lea Beresford, and Richard Zweig, *Ferkauf Graduate School of Psychology, Bronx, New York, United States*

Ageism has profound effects on the mental health of older adults. Further, psychotherapists are not immune to holding ageist beliefs and ageism in therapists may affect older adults' therapy outcomes. Data selection included searches (using key words e.g. "ageism," "aging," "age stereotype," "older adult," "access," "therapeutic alliance," "prognosis," and "therapy outcome") across Google Scholar and PsycInfo from 2000 on and yielded 15 studies about older adults' experience of ageism, therapists' attitudes toward treating older adults, and therapists' expectations about therapy with older adults. Analogue studies of psychotherapists indicated that they were less willing to treat older adults. Analogue and survey studies of therapist biases and rapport suggested that psychotherapists who held stereotypical attitudes were less willing to work with the subjects of their beliefs and had worse expectations for the therapeutic alliance, while the patients reported poorer working relationships with these therapists. Analogue studies of therapists revealed that they treated older adults differently by offering dissimilar, potentially less effective therapies and expected poorer prognoses and outcomes. A major limitation is that analogue studies may not accurately predict attitudes and behaviors in the real world. In conclusion, ageism in therapists may negatively affect older adults' access to therapy, therapists' alliances with older adult patients, the type and quality of therapy therapists offer older adults, and therapists' expectations of older adults' prognoses and outcomes.

### SESSION 7010 (POSTER)

### ALZHEIMER'S DISEASE AND RELATED DEMENTIAS I

Abstract citation ID: igaf122.2214

### A SUMMARY OF ORGANIZATIONS PROVIDING EVIDENCE-BASED DEMENTIA CAREGIVING PROGRAMS IN BEST PROGRAMS FOR CAREGIVING

Zoe Fete[1], Megan Huth[2], David Bass[2], Rachel Cannon[2], Sara Powers[1], and Morgan Minyo[2], *1. Benjamin Rose, Cleveland, Ohio, United States, 2. Benjamin Rose Institute on Aging, Cleveland, Ohio, United States*

Best Programs for Caregiving (BPC) is a free online resource of 45 evidence-based dementia caregiving programs. The Public Version of BPC, launched in 2024, enables family/friend caregivers to use a zip code search to find BPC programs available in their communities. Data were analyzed from a structured survey of 206 organizations identified by program developers as delivering their BPC program and used to populate the Public Version. The most common types of delivery organizations were healthcare or single-service community organizations (37; 18.0%), AAAs (37; 18.0%), multi-service community organizations (31; 15.0%), and senior centers/meal programs (28; 13.6%). There were 28 (13.6%) organizations offering a BPC program nationwide to caregivers living anywhere in the US, and 178 offering a program locally or statewide (86.4%). Most organizations were delivering a BPC program partially or fully remote, with only 34 (16.5%) exclusively in-person. An overwhelming majority were delivered exclusively by professionals (160; 77.7%) and were free (175; 85%). Nearly a quarter of programs (49; 23.8%) were adapted for caregivers identifying with diverse communities, most commonly Hispanic and Latino/Latina, Black/African American and LGBTQ. There were 73 (35.8%) programs delivered in languages other than English, with the most frequent offering being Spanish (69). Findings demonstrate that caregivers throughout the US, including those who identify with underserved populations or live in underserved areas, have evidence-based support programs available in their communities, with many being free. Findings also illustrate variation in the characteristics of programs, enabling caregivers to choose options that most closely match their preferences.

Abstract citation ID: igaf122.2215

### ASSOCIATIONS OF METALS WITH COGNITIVE FUNCTION AND BLOOD-BASED AD BIOMARKERS IN MIDLIFE

Sithara Vivek[1], Shannon Sullivan[1], Jesse Seegmiller[1], Eric Grodsky[2], Chandra Muller[3], John Warren[1], and Bharat Thyagarajan[1], *1. University of Minnesota, Minneapolis, Minnesota, United States, 2. University of Wisconsin–Madison, Madison, Wisconsin, United States, 3. The University of Texas at Austin, Austin, Texas, United States*

Dysregulation of essential metal homeostasis and metalloproteinase activity contribute to AD pathology. While previous studies have examined metal concentrations and AD biomarkers in CSF, their associations in blood remain largely unexplored. In this study, we investigated the association between circulating metals and blood-based biomarkers of AD and cognitive function in the High School and Beyond (HS&B:80/21) study ($n = 4,150$, mean age= 58 ± 1 years). We analyzed whole-blood levels of copper, manganese, selenium, zinc, arsenic, and lead (measured using inductively coupled plasma mass spectrometry) with composite cognitive function score and blood-based AD biomarkers (p-Tau 181, NfL, GFAP) measured using Quanterix Simoa system. Metals and AD biomarkers were log-transformed and standardized. Linear regression models were adjusted for age, sex, race, smoking status, education, BMI, eGFR and APOE e4 allele status. Benjamini-Hochberg correction was used for multiple testing. Several essential metals were positively associated with cognitive function, including selenium ($\beta = .062$, $p < 0.0001$, manganese ($\beta = .051$, $p < 0.0001$), and zinc ($\beta = .025$, $p = .03$), while copper $\beta = -.107$, $p < 0.0001$) was inversely associated. Additionally, several essential metals were inversely associated with AD biomarkers. Higher selenium and copper levels were associated with lower p-Tau 181 (Se: ($\beta = -.043$, $p < 0.0001$; Cu: ($\beta = -.116$, $p < 0.0001$). Selenium was also inversely associated with NfL ($\beta = -.036$, $p = 0.03$), while zinc showed a marginal inverse association with GFAP ($\beta = -.035$, $p = 0.05$). Our findings highlight the complex role of metals in AD and suggest that essential metals, particularly Selenium, may protect against cognitive decline and neurodegeneration.

Abstract citation ID: igaf122.2216

### CARE PARTNER VIEWS ON A PROGNOSIS-INFORMED DEMENTIA ROADMAP

Nancy Schoenborn, and Halima Amjad, *Johns Hopkins University School of Medicine, Baltimore, Maryland, United States*

Almost all older adults with Alzheimer's disease and related dementias (ADRD) receive help from care partners. An important unmet need in ADRD care partner support is anticipatory guidance and individualized prognostic information. Given a growing compendium of prognostic tools for older adults, including specifically for people living with dementia (PLWD), the science is ripe for developing a prognosis-informed roadmap in PLWD. However, prognostic tools will have little or negative impact if they are not relevant or acceptable to end-users. We aimed to explore care partners' ADRD prognosis information needs and preferences using qualitative interviews. The study is actively recruiting, with 9 completed interviews thus far. Interviews were analyzed using qualitative thematic analysis. We found that care partners often reported not receiving adequate guidance at the time of ADRD diagnosis. Care partners consider multiple prognostic outcomes important, including life expectancy, likelihood of unsafe behaviors, need for supervision and personal care assistance, and likelihood of mobility challenges. Outcomes that impact living arrangements and safety were the highest priority. Care partners were uniformly accepting of the potential uncertainty inherent in predictions and would nonetheless find the prognostic information useful. Some preferred more personalized predictions while others preferred more general guidance. Care partners reported that the prognosis information would impact healthcare decisions as well as finances, living arrangements, and family members' life plans. Our preliminary findings demonstrate that ADRD care partners strongly desire prognosis information for anticipatory guidance despite potential uncertainties and prioritize predictions about safety challenges and care needs as the most important.

Abstract citation ID: igaf122.2217

### COGNITIVE IMPAIRMENT AND PAIN DISCREPANCIES IN ADRD: EVALUATING PAIN RESPONSES TO TDCS INTERVENTION

Juyoung Park, Chiyoung Lee, Jason Hoang, and Hyochol Ahn, *University of Arizona, Tucson, Arizona, United States*

Assessing pain in individuals with Alzheimer's disease and related dementias (ADRD) is challenging due to cognitive impairments that reduce the reliability of self-reported pain ratings, such as the Numerical Rating Scale (NRS). While caregiver-observed assessments, such as the Mobilization-Observation-Behavior-Intensity-Dementia-2 (MOBID-2), provide options, discrepancies between self-reported and observer-rated pain across cognitive impairment levels remain understudied. This study examines how cognitive function, stratified by the Montreal Cognitive Assessment (MoCA-30), influences pain reporting and response to transcranial direct current stimulation (tDCS), a non-pharmacological pain intervention. In this double-blind, randomized, sham-controlled trial, 40 older adults with ADRD and chronic pain (tDCS: $n = 20$, Sham: $n = 20$) received home-based tDCS (2mA, 20 minutes/session) or sham stimulation for five consecutive days. Pain intensity was assessed at baseline, post-intervention, and 1-, 2-, and 3-month follow-ups using NRS and MOBID-2. Participants were categorized into mild (18–25) and moderate (10–17) cognitive impairment groups. The sample included 29 females, 36 White participants, and 23 married individuals. Results showed that cognitive impairment significantly moderated discrepancies between NRS and MOBID-2 ($F(4,35) = 3.364$, $p = .020$, $\eta^2 = .278$). A significant Time × MoCA Severity interaction ($p = .012$) indicated that participants with lower MoCA scores demonstrated greater discrepancies in pain ratings. MOBID-2 consistently detected pain reduction following active tDCS, whereas NRS was less sensitive to these changes, particularly in those with greater cognitive impairment. These findings highlight the importance of cognitive-adaptive pain assessments and reinforce tDCS as a promising nonpharmacological intervention for pain management in ADRD.

Abstract citation ID: igaf122.2218

### COLLABORATIVE EXCELLENCE FOR PUBLIC HEALTH DEMENTIA EDUCATION

John Shean[1], Elma Johnson[2], Mickal Lewis[3], Alexandra Nordyke[4], Madiha Shakir[5], Kayleigh Jones[6], and Eva Jackson[1], *1. Alzheimer's Association, Chicago, Illinois, United States, 2. University of Minnesota, Minneapolis, Minnesota, United States, 3. Alzheimers Association, Chicago, Illinois, United States, 4. New York University Langone Health, New York, New York, United States, 5. Ullu Learning Design, LLC, Atlanta, Georgia, United States, 6. Emory University, Atlanta, Georgia, United States*

As the prevalence of Alzheimer's disease and related dementias continues to increase, public health action is necessary across the primary, secondary and tertiary levels of prevention. In 2020, the Centers for Disease Control and Prevention (CDC) funded three Building Our Largest Dementia Infrastructure (BOLD) Public Health Centers of Excellence to focus on these three levels. Working with the three Centers of Excellence, the Alzheimer's Association developed and released modules as part of a free, online, interactive public health curriculum for current and future public health professionals. In this session, attendees will learn more about A Public Health Approach to Dementia, including the 3-part curriculum suite focused on the prevention levels and how this resource can be used by faculty and students. This suite explores and addresses the following topics: Public Health and Dementia Risk Reduction; Public Health and Early Detection of Dementia and Diagnosis; and Public Health and Dementia Caregiving. Attendees will learn about the innovative applications of a virtual, self-paced curriculum that incorporates learner-centered user experience design principles. The curriculum can be used as stand-alone modules or as a suite. Attendees will also experience use of the instructor guide that enhances virtual and classroom learning experiences. Dementia is a public health issue because the prevalence and costs are substantial, the impacts are serious and there are public health solutions. Now is the time to use innovative, collaborative methods such as this curriculum suite to equip the current and future public health workforce.

Abstract citation ID: igaf122.2219

### CONTRASTING APPROACHES IN ADVANCE CARE PLANNING IN DEMENTIA AS PERCEIVED BY GENERAL PRACTITIONERS

Jenny van der Steen, Xu Jingyuan, Willemijn Tros, and Jeanet W. Blom, *Leiden University Medical Center, Leiden, Zuid-Holland, Netherlands*

Various approaches to advance care planning are in use and generally acceptable also for persons with dementia and their care partners. Two contrasting approaches involve a highly scripted, predominantly medical approach to decide on specific treatments in advance versus a more flexible psychosocial, coping-based approach that comprises global care goal setting. We aim to assess for whom and when either approach is preferred from the perspective of general practitioners. General practitioners in the Netherlands participated in the CONT-END randomized controlled trial and we interviewed six practitioners trained in the medical approach and four in the psychosocial approach. We explained the other approach during the interview. Twelve other general practitioners were interviewed after viewing video vignettes of the two approaches shown in random order. With inductive qualitative content analyses, we identified four attributes distinguishing situations in which either approach is preferred: understanding, trust, readiness and momentum. For the medical approach, understanding, trust, and readiness on the part of person and care partner were prerequisites for the right momentum. This may be achieved in a follow-up conversation, but such conversations may also be triggered by urgent medical reasons. In contrast, the psychosocial approach would help understand the person, foster trust and create readiness also in a first conversation early in the course of the disease. Without a clear trigger, momentum would need to be created. We will visualize the contrasting attributes and discuss implications for practice of advance care planning with persons with dementia and their care partners in primary care settings.

Abstract citation ID: igaf122.2220

### DAYS AT HOME FOLLOWING HIP FRACTURE AMONG OLDER ADULTS WITH DEMENTIA

Rebecca Rodin[1], Alexander Smith[2], Siqi Gan[2], John Boscardin[2], Edie Espejo[2], Lauren Hunt[2], and Sean Morrison[3], *1. Mount Sinai Medical Center, New York, New York, United States, 2. University of California, San Francisco, San Francisco, California, United States, 3. Icahn School of Medicine at Mount Sinai, New York, New York, United States*

Hip fracture is common among older adults and nearly twice as common among those with dementia, who may be particularly vulnerable to their adverse effects. Time spent away from home may be an important indicator of such effects, but this outcome has not been previously examined in older adults with dementia (PWD) and hip fracture. We aimed to assess the impact of hip fracture hospitalization on days alive at home among community-dwelling PWD age 65+ compared to those without dementia utilizing 100% Medicare claims data (2012-2021). We did not report p values or confidence intervals due to their limited utility in very large sample sizes. We identified 1,853,295 older adults (mean age [SD] 82.5 [8.1]); 1,307,640 [70.6%] female) with a hip fracture hospitalization, of whom 556,765 (30.0%) had dementia. PWD had fewer days at home within 1-year post-hip fracture compared to those without dementia (adjusted mean 181 days vs. 267 days; difference 86 days), including more nursing home days (adjusted mean 37 days vs. 11 days; difference 26 days), skilled nursing facility days (adjusted mean 33 days vs. 24 days, difference 9 days), and days after death (adjusted mean 103 days vs. 51 days; difference 52 days). These findings indicate that older adults with dementia had 52 fewer days alive and spent >1 month more in long-term care or post-acute rehabilitation/skilled-nursing facilities in the year following hip fracture than those without dementia.

Abstract citation ID: igaf122.2221

### DESIGNING A PALLIATIVE CARE PATHWAY FOR ENHANCED DEMENTIA CARE

Christine Jensen[1], and Jenny Inker[2], *1. Riverside Martha W. Goodson Center, Williamsburg, Virginia, United States, 2. Virginia Commonwealth University, Richmond, Virginia, United States*

Riverside Health has developed a new palliative care pathway to serve as an innovative standard of person-centered care for persons living with dementia (PLWD) and their care partners. Building on an existing interdisciplinary palliative care team (PCT) of a physician, nurse practitioner, and intake/nurse navigator, the positions of social worker, geriatric pharmacist, and spiritual care advisor were added to the team. The PCT have completed extensive training in dementia-specific palliative care, including risks for those who live alone, and those with developmental disabilities. Further, educational programs, in the form of microlearning lessons, have been designed and delivered to PLWD, their care partners, the PCT, and community-based partners. These microlearning lessons (brief bursts of targeted educational content) highlight the key elements of palliative care, which are often inaccurately equated with hospice care, and ways to address stigma associated with both a dementia diagnosis and enrolling in palliative care. The enhanced PCT has served more than 175 PLWD and their family caregivers, with resulting increases in quality of life and well-being for both PLWD and care partners. PLWD and care partners have expressed gratitude for social work support in connecting to resources, as well as the meaning that is provided through engaging with a spiritual care advisor. Additionally, comprehensive medication reviews provided by the geriatric pharmacist have resulted in a decrease in medication burden and an increase in adherence. Application of palliative care principles and lessons learned will be referenced for other health systems to consider.

Abstract citation ID: igaf122.2222

### EFFECTS OF FUNCTIONAL IMPAIRMENT ON DEMENTIA CAREGIVER BURDEN ACROSS ETHNIC GROUPS.

Miriam Jocelyn Rodriguez[1], Mariana Stavig[1], Bailey Gardner[1], Jordan Hill[1], Lilian Golzarri-Arroyo[1], Roger Zoh[1], Richard Holden[1], and Malaz Boustani[2], *1. Indiana University Bloomington, Bloomington, Indiana, United States, 2. Indiana University, Indianapolis, Indiana, United States*

Introduction: Functional impairment among individuals with Alzheimer's disease and related dementias has been associated with increased behavioral and psychological symptoms of dementia (BPSD) and greater caregiver burden. Previous studies have identified greater functional impairment among African American and Hispanic individuals when compared to White non-Hispanic groups. The aim of this study was to examine associations between functional impairment to caregiver burden and depressive symptoms across racial/ethnic groups. Method: One-way ANOVAs were conducted to compare scores on the Functional Activities Questionnaire (FAQ) between three racial/ethnic groups: White ($n=121$), Black/African American ($n=35$) and Hispanic/Latino ($n=11$). Linear regression analyses were conducted to examine associations between FAQ scores and care recipient BPSD, caregiver burden and depressive symptoms. Results: The mean of FAQ scores was $M=23$ with an $SD=6.51$. No significant differences in FAQ scores were found between groups and no associations between FAQ and caregiver burden or depressive symptoms. Significant associations were identified between FAQ scores and care recipient BPSD for the overall sample ($F(1,167)=7.93$, $p<.01$; $R2= 0.05$). Findings were not significant between these two variables and the race/ethnicity of the caregiver. Discussion: Our findings are consistent with previous research and identified significant associations between functional impairment and BPSD. However, these associations did not impact caregiver burden or depression in our sample. Our sample had limited variation in FAQ scores. Future studies should examine these associations among a sample with a wider range of functional impairment. Our findings have implications for the development of dementia caregiver interventions to best address caregivers' needs.

Abstract citation ID: igaf122.2223

### FEASIBILITY OF RECRUITING PEOPLE WITH ALZHEIMER'S DISEASE AND RELATED DEMENTIAS IN HEALTHCARE RESEARCH

Rashmita Basu, Donna Roberson, Rodriguez Gonzalez, and Shivrajo Patil, *East Carolina University, Greenville, North Carolina, United States*

With Alzheimer's Disease and Related Dementia (ADRD) increasing in the U.S., more research is needed to deliver patient-centered care. However, recruiting people with ADRD is challenging—this pilot study aimed to test the feasibility of collecting self-reported health data from people with ADRD. The primary data collection site was a geriatric clinic in eastern North Carolina. Potential patients were identified based on dementia diagnoses from the electronic medical record system (EPIC). After the clinic visits with the healthcare providers, we consented to those with "mild" or "moderate" dementia using a short portable mental status questionnaire. Participants self-reported (in person or over the phone) survey questions focused on their health and well-being. Thirty-five patients with ADRD consented to the study, and 25 participants completed the survey (>70% completion rate). The sample average age was 71; 62% were female, 42% were White, 56% were African American, and 54% had limitations in at least one daily activity. Participants were satisfied with their life circumstances, with an average score of 4.5 (range 0-5). Qualitative interviews indicated the need for improvements in dementia care, including better person-centered care and resources. None expressed difficulty with the survey completion. Although recruiting people with ADRD for healthcare research is challenging, it can be done. Partnering with healthcare providers and community support groups was an effective recruitment strategy. Our results indicate that community-dwelling individuals with ADRD can complete the surveys to report their health and well-being. Self-reporting was feasible and should be used in research to design person-centered care.

Abstract citation ID: igaf122.2224

### P. GINGIVALIS-LPS ACTIVATES THE ALZHEIMER'S DISEASE-ASSOCIATED AMYLOID SECRETASE PATHWAY VIA CASPASE-4

Ambika Verma[1], Gohar Azhar[2], Pankaj Patyal[1], Xiaomin Zhang[1], and Jeanne Wei[2], *1. University of Arkansas for Medical Sciences, Little Rock, Arkansas, United States, 2. University of Arkansas for Medical Sciences, Little Rock, AR, LITTLE ROCK, Arkansas, United States*

Porphyromonas gingivalis, a periodontal pathogen, has been implicated in the pathogenesis of Alzheimer's disease (AD) and related dementias (ADRD) through its virulent lipopolysaccharide (LPS) component. Amyloid pathologies, characterized by the accumulation of amyloid-beta (Aβ), are central features of AD/ADRD. Our previous work demonstrated that P. gingivalis-LPS treatment activated the amyloid precursor protein (APP) and leading to the production of Aβ1-42 and Aβ1-40 peptides in SH-SY5Y cells. Caspase-4, an inflammatory mediator that directly senses and binds to cytosolic LPS, plays a critical role in inflammatory signaling. However, the involvement of caspase-4 in P. gingivalis-LPS induced amyloid pathologies remains underexplored. We hypothesized that P. gingivalis-LPS contributes to AD/ADRD pathogenesis by activating the Alzheimer's disease-associated amyloid secretase pathway mediated by caspase-4. To investigate this, we performed caspase-4 knockdown in SH-SY5Y cells using caspase-4 specific siRNA and the caspase-4 inhibitor Ac-LEVD-CHO. Presenilin-1 (PS1), a key γ-secretase component, is essential for amyloidogenic APP processing and Aβ1-42 production, was also examined. Our findings demonstrated that P. gingivalis-LPS treatment activated both APP and PS1 in SH-SY5Y cells, with caspase-4 silencing significantly reducing the expression of these proteins compared to negative control siRNA ($p < 0.05$). Furthermore, P. gingivalis-LPS induced elevated Aβ1-42 and Aβ1-40 peptide release was reversed upon treatment with caspase-4 siRNA or Ac-LEVD-CHO ($p < 0.05$). These results provide compelling evidence that caspase-4 mediates P. gingivalis-LPS induced neuroinflammation and highlight the complex interactions between caspase-4, APP, PS1 and Aβ in the pathogenesis of AD/ADRD.

Abstract citation ID: igaf122.2225

### PERSON-CENTERED CARE REDUCES PSYCHOTROPIC MEDICATION USAGE IN RANDOMIZED, CONTROLLED, ALZHEIMER'S TRIAL

Sunnie Kenowsky[1], Yongzhao Shao[2], Qiao Zhang[2], Sloane Heller[3], Martin Sadowski[2], Thomas Wisniewski[2], Arjun Masurkar[2], and Barry Reisberg[2], *1. New York University Langone Health, NYU Grossman School of Medicine, Center for Cognitive Neurology, New York, New York, United States, 2. New York University Langone Health, New York, New York, United States, 3. Columbia University Irving Medical Center, New York, New York, United States*

Behavioral and psychological symptoms of dementia (BPSD) are prevalent and associated with poor health outcomes. Psychotropic medications, prescribed to treat BPSD, have limited efficacy and deleterious effects including stroke and death (ncbi.nlm.nih.gov/books/NBK551552). To evaluate whether comprehensive, individualized, person-centered management (CI-PCM) could improve healthcare for persons diagnosed with moderate-to-severe Alzheimer's disease (PAD) we conducted a 28-week and a 24-week extension trial in community-residing, 65-year and older PAD (doi.org/10.1159/000455397). Twenty PAD-carepartner dyads were randomized to receive usual community care (UCC) or CI-PCM. BPSD were assessed using the Behavioral Pathology in Alzheimer's Disease-Frequency-Weighted Severity Scale which showed significant benefit in PAD receiving CI-PCM at 52-weeks ($p = 0.01$, doi:10.1002/alz.090018). BPSD and medication usage were assessed at baseline, and weeks 4, 12, 28 and 52. Differences in psychotropic medications taken were compared by calculating the percent maximum daily dose of each medication taken cumulatively throughout each observation period and examining the ratio of daily medication usage in each group using the Wilcoxon Rank Sum Test. At baseline, PAD receiving CI-PCM consumed significantly less psychotropic medication than those receiving UCC ($p = 0.017$, doi.org/10.1002/alz.056048). Therefore, we analyzed the magnitude of daily medication used in each group on a per day average from weeks 4 to 52, compared to baseline. PAD receiving CI-PCM consumed significantly less medication on a per day average ($p = 0.002$) and PAD receiving UCC consumed significantly higher total amounts of psychotropic medication ($p < 0.0001$) from weeks 4 to 52, relative to baseline. CI-PCM is readily implemented, reduces BPSD, reduces psychotropic medication usage, and improves PAD health outcomes.

Abstract citation ID: igaf122.2226

### PHYSICAL ACTIVITY AND COGNITIVE FUNCTION AMONG OLDER ADULTS: EVIDENCE AND INTERVENTION

Yanan Luo, *Peking University School of Public Health, Beijing, Beijing, China (People's Republic)*

Physical activity is an important modifiable risk factor for cognitive decline and dementia in older adults. This study aims to analyze the relationship between physical activity and the levels of cognitive function and its decline rates as well as the association between physical activity intensity, type, and cognitive function in older adults. Additionally, guided by the integrated model of health behavior, this study explores the construction and application of digital medical exercise interventions for patients with mild cognitive impairment. The main findings are as follows: firstly, older adults who engage in moderate or vigorous physical activity daily have higher cognitive function scores and the slower cognitive decline with age; secondly, moderate or vigorous physical activity also helps reduce the decline in cognitive function levels in patients with multimorbidity, significantly slowing cognitive decline in patients with cardiometabolic multimorbidity; thirdly, aerobic training and reaching 1000-1499 MET-min of physical activity per week are significantly associated with the higher cognitive function levels in older adults; fourthly, under the guidance of the integrated model of HAPA-TPB and integrating guidelines such as the ACSM exercise testing and prescription guide along with Delphi expert consultation, an exercise intervention program for elderly patients with mild cognitive impairment was developed. The intervention program showed high satisfaction and feasibility in a preliminary pilot study, and future research will further validate its effectiveness based on randomized controlled trials. The study suggests that a certain level of physical activity is crucial for preventing cognitive decline in older age and controlling dementia.

Abstract citation ID: igaf122.2227

### POPULATION-LEVEL IMPACT OF INSOMNIA ON DEMENTIA AMONG OLDER ADULTS IN THE U.S.

Yuqian Lin[1], Fei Wu[2], Yuhan Wu[2], Wanru Liu[3], Goodarz Danaei[2], and Ruijia Chen[4], *1. Massachusetts General Hospital, Boston, Massachusetts, United States, 2. Harvard T.H. Chan School of Public Health, Boston, Massachusetts, United States, 3. Brown University, Providence, Rhode Island, United States, 4. Boston University School of Public Health, Boston, Massachusetts, United States*

Insomnia has been identified as a plausible modifiable risk factor for dementia, yet no studies have quantified its population-level impact on dementia risk in the United States. Using data from 2022 National Health and Aging Trends Study and relative risks from a published meta-analysis, this study estimated the population attributable fraction (PAF) of dementia cases due to insomnia among U.S. adults aged 65 and older, overall and stratified by age and sex. Among 5,899 participants (44.7% aged ≥80 years; 57.9% female), 28.7% reported having experienced sleep-onset insomnia, sleep-maintenance insomnia, or both. The prevalence of probable dementia was 6.6%, increasing with age for both sexes. The estimated PAF of probable dementia due to any type of insomnia was 12.8% (95% CI: 1.1%, 27.1%), with higher estimates in females (13.4%) than males (12.0%). Among females, insomnia had the largest contribution to dementia in the youngest age group (ages 65–69, 14.4%). For males, the greatest contribution was observed in the 70–74 age group (12.8%). An estimated 471,229 (95% CI: 42,313, 1,014,816) dementia cases in the U.S. in 2022 could have been prevented if insomnia had been eliminated among adults aged 65 and older. Approximately 13% of probable dementia cases, almost half a million cases per year, among older adults in U.S. could be attributable to having any types of insomnia, an estimate comparable to the well-established risk factors such as hearing loss. These findings underscore the potential for interventions aimed at addressing insomnia to reduce dementia risk in aging populations.

Abstract citation ID: igaf122.2228

### PREDICTING LONG-TERM CARE DEMANDS AND OPTIMAL STRATEGIES FOR OLDER PERSONS WITH DEMENTIA IN CHINA TO 2040

Peijia Zhu, Qifan Yang, Jie Chen, Liangwen Zhang, and Ya Fang, *Xiamen University, Xiamen, Fujian, China (People's Republic)*

Objective: Limited evidence exists on the future trajectory of long-term care (LTC) demands for older persons with dementia in China. This study aims to quantify projected changes in the dementia population from 2020 to 2040 and provide evidence for national dementia LTC planning and informal care support. Methods: Using multi-source data (CLHLS 2011/2014/2018, UN projections, official statistics, surveys, interviews), we developed and validated a Markov model with age, gender, and urban-rural differences, with a 1-year cycle. Using dementia transition probabilities, the model projected trends in the dementia population from 2020 to 2040. Scenario analyses (low and high standards) were conducted to explore demand characteristics in care workforce and costs from both formal and informal care perspectives. Results: The dementia population is projected to rise by 51.2%, from 19.73 million in 2020 to 29.83 million in 2040, with mild dementia at 60.16%. Informal care workforce remains dominant, gaps range from 23.34 million (low standard) to 38.33 million (high standard) by 2040. Dementia LTC costs as a share of GDP are notable (0.26%-0.64%), with formal care costs exceeding informal care, the main driver is increasing new dementia cases due to population aging. Conclusion: Informal care plays a critical role in dementia LTC and needs to be supplemented with diverse formal care services. Additionally, expanding community-based screening and intervention for high-risk populations with mild dementia, along with providing tiered support and corresponding subsidies, can ensure that resources are precisely allocated to meet the care needs across different stages of dementia.

Abstract citation ID: igaf122.2229

### PRELIMINARY RESULTS OF A COGNITIVE TRAINING PROGRAM IN A MEMORY CLINIC

Dev Ashish[1], and Molly Maxfield[2], *1. Banner Alzheimer's Institute, Tucson, Arizona, United States, 2. Arizona State University, Phoenix, Arizona, United States*

Background: Patients with mild cognitive impairment (MCI) or early stages of dementia often search for treatments to improve cognition and help manage deficits. Care partners also seek information to provide better care. Multi-component non-pharmacological interventions provided to dementia patients with caregivers have similar benefits as pharmacological treatments but without side effects. Here, we present information about the Multicomponent Comprehensive Intervention with Care-partners (MCI-Care) at Banner Alzheimer's Institute in Tucson. Method: Twenty-one sessions for dyads (patients with MCI or early stages of dementia and their care-partners) include psychoeducation, training, and problem-solving for 1) healthy brain behaviors and 2) compensatory strategies, combined with

3) cognitive training for attention, processing speed, executive functioning, and memory domains. Sixteen dyads started the treatment. Brief pre- and post-assessments included a cognitive screen and self-report questionnaires. Dyads completed a brief exit survey about their experiences. Results: Eleven of 16 patients completed training. Patients demonstrated improvement in objective cognition and self-reported measures. Care-partners endorsed improvement in patients' cognition, function, and their caregiver burden. All patients who completed the treatment found it informative and helpful; all indicated they were "very likely" to recommend this training. Three patients with MCI did not complete the training after their mood improved. Two patients with dementia discontinued, needing more support with caregiving resources. Conclusion: MCI-Care is best tolerated by patients without significant mood issues or need for caregiving resources. There is evidence for the acceptability and effectiveness of the intervention. Future research will establish statistical and clinical significance of dyadic and group interventions.

Abstract citation ID: igaf122.2230

**RECRUITMENT OF INFORMAL CAREGIVERS OF PERSONS WITH DEMENTIA INTO CLINICAL TRIAL: THE POWER OF THE MESSENGER.**

Zayn Boustani[1], Mariana Stavig[2], Hanani Ndebele[2], Jordan Hill[2], Miriam Jocelyn Rodriguez[2], Lilian Golzarri-Arroyo[2], Richard Holden[2], and Malaz Boustani[3], *1. Indiana University Bloomington, Chicago, Illinois, United States, 2. Indiana University Bloomington, Bloomington, Indiana, United States, 3. Indiana University, Indianapolis, Indiana, United States*

Intro: The success of a randomized controlled trial depends on the timely and effective recruitment of participants. We aimed to investigate the effect of a trusted messenger on the recruitment process into a randomized controlled trial testing the efficacy of a mobile caregiver intervention in reducing caregiver burden and patient behavioral and psychological symptoms of dementia (BPSD). Methods: A total of n = 4630 potential participants were contacted. Recruitment occurred via (1) "Cold Call," where patients from a health system registry were called by research staff, and (2) "trusted messenger," where research staff called patients who were referred directly from a memory care physician. The outcome measure was the call-to-consent ratio. A two-population proportion Z-test compared the ratios between the two recruitment methods. Results: The enrollment rate was 23.88% for participants recruited via the "trusted messenger" method and 1.18% for those recruited from the "cold calls" method ($z = 15.11$, $p \approx 0.0000$). Conclusions: "Trusted Messenger" was 20 times more effective than "Cold Calls" in recruiting human subjects into a clinical trial. Trusted Messenger leverage preexisting relationship. Although "Cold calls" is perceived as easy for the researcher to complete, it yields fewer enrollments per points of contact. Future studies should examine and compare other recruitment methods among ethnically diverse samples such as community outreach, flyer posting, and door-to-door solicitation.

Abstract citation ID: igaf122.2231

**RECRUITMENT OF PEOPLE WITH COGNITIVE IMPAIRMENT AND CAREGIVERS FOR A RANDOMIZED CLINICAL TRIAL**

Pei-Shiun Chang[1], Amy Katz[2], Richard Passey[3], Ziyi Yang[3], Sujuan Gao[3], Liana Apostolova[4], and Yvonne Lu[2], *1. Indiana University Bloomington, Bloomington, Indiana, United States, 2. Indiana University School of Nursing, Indianapolis, Indiana, United States, 3. Indiana University School of Medicine, Indianapolis, Indiana, United States, 4. Indiana University, Indianapolis, Indiana, United States*

Recruiting older people with cognitive impairment (PwCI) and their caregivers (CG) for research studies is an ongoing challenge, which was magnified by the onset of COVID-19. This study describes the unique recruitment of PwCI-CG dyads for a Randomized Control Trial using a tailored Daily Engagement in Meaningful Activity (DEMA) intervention to improve the quality of life for both PwCI and their CGs. Instead of traditional in-person recruitment, telephone-based recruitment was a driving factor for successfully contacting participants. Using a state-wide healthcare system database, we identified 4133 potential participants who met study criteria and could identify a primary caregiver. By mailing study information to potential PwCI-CG dyads, we observed a significant increase in initial contact, returned calls, and general interest in study participating. In-person recruitment, direct referrals from physicians in the clinic setting, and partnered local agencies were among the least successful strategies for establishing initial contact and recruiting dyads. After implementing new recruitment strategies, we recruited 152 dyads in 16 months (mean = 10 dyads/month). We found that establishing a monthly recruitment goal was a beneficial strategy. The healthcare system database, followed by an organizational registry, demonstrated higher rates of making initial contact and reaching eligible dyads (46% and 37%, respectively). Specific dyadic recruitment challenges included finding a suitable CG to participate and the extended follow-up timeframe from initial contact to screening and enrollment. There is a growing need to better understand successful strategies for didactic recruitment, particularly among older PwCI and their CGs.

Abstract citation ID: igaf122.2232

**RELATIONAL AND COMMUNICATION STRATEGIES UTILIZED BY UNDERSERVED CARE PARTNERS AND FAMILY MEMBERS WITH DEMENTIA**

Carissa Coleman[1], and Amanda Runyan[2], *1. University of Kansas Medical Center, Kansas City, Kansas, United States, 2. Robert J. Dole VA Medical Center, Wichita, Kansas, United States*

Dementia burden is disproportionally carried by underserved communities resulting in higher intensity in-home caregiving and greater unmet needs. Communication is a challenge for family dementia care dyads and underserved ADRD populations face greater challenges due to lack of access. Identifying evidence for strategies facilitating or impeding communication and gaps in education and technology needs is needed for an inclusive communication intervention. This study expands on our existing communication research, extending to a diverse sample to understand their unique experiences. Ten, one-hour, mixed-method interviews were conducted with dementia care dyads. Eight dyads were African American and two were Hispanic/Latino. All care partners were female (daughter or spouse). The people with dementia were mostly female (80%) with Alzheimer's (50%) categorized as severe (40%). Operational definitions for relational strategies and verbal/nonverbal communication behaviors were developed in Dedoose and inter-rater reliability was excellent (Kappa = .86, .81). During analysis of the transcripts, more complex strategies were identified. The most common relational strategies included personalized care (29.6%), managing emotions (27.2%), and tending to the environment (20.2%). Orienting (34.6%) and clarifying (23.4%) were the most common verbal strategies. Utilizing time (25.4%) and the environment (22.4%) were the most common nonverbal strategies. Preferred educational modalities were those that facilitated face-to-face connections with others versus technology-based modalities. In-interview survey responses were notably different than spontaneous responses indicating dementia dyads identified their own strategies without prompting. Based on these findings, family dementia care communication education will be developed tailored to the needs of diverse dementia dyads and their communities.

Abstract citation ID: igaf122.2233

### RESPONDING TO THE CHALLENGES OF AGING: A REVIEW AND OUTLOOK ON CHINA'S DEMENTIA CARE POLICY SINCE 2011

Jiakang Zhu[1], Qingwei Wang[2], and Jiawei Cao[3], *1. The University of North Carolina at Chapel Hill, Chapel Hill, North Carolina, United States, 2. Xi'an Jiaotong-Liverpool University, Suzhou, Jiangsu, China (People's Republic), 3. Nanjing University of Chinese Medicine, Nanjing, Jiangsu, China (People's Republic)*

China is facing a pressing public health challenge as its population ages rapidly, leading to a significant rise in dementia prevalence. This article systematically reviews China's dementia care policies to identify gaps and provide recommendations for future policy design. We examine three key years—2011, 2016, and 2024—as benchmarks for shifts in policy focus and implementation. In 2011, dementia was officially recognized in policy documents, marking the beginning of governmental attention towards this growing issue. Early policies aimed to improve detection rates and provide financial subsidies to families affected by dementia. However, these measures fell short of addressing comprehensive care. By 2016, the introduction of the Healthy China 2030 plan emphasized the need for effective dementia interventions, aligning with the World Health Organization's Dementia Global Action Plan. Despite these advancements, the implementation of policies encountered practical challenges. By 2024, the Chinese government incorporated proactive dementia prevention and treatment interventions into its five-year plans. This effort culminated in the release of the most comprehensive action plan to date, representing an important milestone in China's approach to manage dementia. Our analysis identifies two major issues: an unclear delineation of departmental responsibilities and inconsistency terminology used in policy documents. The evolution of China's dementia care policies provides valuable lessons for other aging societies confronting similar challenges. Clearer roles and a more consistent language are crucial for effective policy implementation in the future.

Abstract citation ID: igaf122.2234

### THE EFFECT OF PATIENT AGE AND SEX ON DECISION-MAKING AROUND AN ALZHEIMER'S DISEASE BLOOD TEST: QUIP II STUDY RESULTS

Mark Monane[1], Kim Johnson[2], Daniel Parker[2], Robert Carlile[3], Darren Gitelman[4], Leslie Jacobs[1], Tim West[1], and Joel Braunstein[1], *1. C2N Diagnostics, St. Louis, Missouri, United States, 2. Duke University, Durham, North Carolina, United States, 3. Palmetto Primary Care Physicians, Summerville, South Carolina, United States, 4. Advocate Health, Park Ridge, Illinois, United States*

Blood biomarker (BBM) tests for Alzheimer's disease represent accurate and accessible tools to aid healthcare providers (HCPs) in the evaluation of patients presenting with signs or symptoms of mild cognitive impairment or dementia. The objective of this analysis was to examine the effects of patient age and sex on clinical decisions using a commercially available mass spectrometry BBM test (PrecivityAD2™ test) yielding the Amyloid Probability Score 2 (APS2), which informs on the likelihood of a positive amyloid PET scan result. This secondary analysis of the QUIP II Study (NCT06025877) included 203 patients (average age 74 years, 53% female) evaluated by 12 HCPs representing 8 outpatient sites. Clinical decision-making was recorded by survey pre- and post-BBM testing. The composite primary endpoint, defined as a change in AD diagnostic certainty, AD medications, or additional brain amyloid evaluation pre- and post-BBM testing, was 75% ($p < 0.0001$ versus pre-specified threshold of 20% clinically meaningful change). The relationship between APS2 and composite endpoint was highly significant ($p < 0.0001$), yet was not significantly different when stratified by age ($p = 0.94$) or sex ($p = 0.337$). In a subgroup analysis of the three individual components of the composite endpoint, the only significant finding was that the magnitude of change in pre- and post-BBM AD diagnostic certainty was lower in the Negative APS2 patients aged 60-69 versus Negative APS2 patients aged 80 and older ($p = 0.01$), likely related to a lower pre-test AD probability among younger patients. These study results support the usefulness and generalizability of this BBM test in clinical care.

Abstract citation ID: igaf122.2235

### WALKING THE TALK FOR DEMENTIA: A CATALYST FOR CHANGE IN THE GLOBAL DEMENTIA LANDSCAPE

Walter Dawson[1], Sherril Gelmon[2], Fernando Aguzzoli Peres[3], Jonathan Adrian Zegarra Valdivia[4], Kate Irving[5], and Lingani Mbakile-Mahlanza[6], *1. Oregon Health & Science University, Portland, Oregon, United States, 2. Portland State University, Portland, Oregon, United States, 3. Blobal Brain Health Institute, Dublin, Dublin, Ireland, 4. Achucarro Basque Center for Neuroscience, Bilbao, Pais Vasco, Spain, 5. Dublin City University, Dublin, Dublin, Ireland, 6. University of Botswana, Goborone, South-East, Botswana*

Walking the Talk for Dementia (WTD) is an innovative global initiative that challenges the stigma surrounding dementia, fosters intergenerational and multidisciplinary dialogue, and inspires a renewed sense of purpose among participants. The annual event combines a four-day, 40 km pilgrimage to Santiago de Compostela, Spain, with a two-day symposium, creating an immersive space to reflect on the physical, emotional, spiritual, and societal dimensions of dementia. WTD prioritizes multicultural and multistakeholder engagement, elevating the voices and improving the quality of life of people living with dementia, care partners, and advocates while promoting inclusive and sustainable solutions for global brain health. WTD integrates professional and lived experiences, fosters collaboration to address stigma, increase awareness, and build an empathetic community. Data collection occurred before, during, and after the 2024 WTD through surveys and personal reflections from participants ($n = 79$) representing 27 countries. Post-event surveys demonstrated high levels of satisfaction. Participants rated the effectiveness of the unique WTD format highly, and reported the experience as superior to the traditional academic conference model regarding networking and engaging with people with lived experience. Thematic analysis of qualitative responses highlighted the transformative nature of WTD. Walking the Talk for Dementia exemplifies how dementia changemakers across disciplines and regions can be inspired. By integrating physical activity, interpersonal dialogue, and potentially emotional experiences, WTD empowers participants to transform stigma into understanding, and challenges them to create opportunities for global advocacy. Participants' voices underscore its profound impact, identifying WTD as a powerful catalyst for change in the global dementia landscape.

Abstract citation ID: igaf122.2236

### WHAT MATTERS TO YOU? A QUALITATIVE STUDY ON THE LIVED EXPERIENCES OF COMMUNITY-LIVING INDIVIDUALS WITH DEMENTIA

Rashmita Basu, and Donna Roberson, *East Carolina University, Greenville, North Carolina, United States*

With Alzheimer's Disease and Related Dementia (ADRD) increasing in the U.S., more research is needed to deliver patient-centered care. However, recruiting people with ADRD is challenging. This study examines what matters to individuals living with dementia in their community through the conceptual lens of person-centered care. We conducted seven qualitative interviews with individuals who have early or mid-stage dementia and live in the community. Two researchers used an inductive line-by-line approach to perform thematic data analysis independently and then as a team. The analysis revealed one overarching theme with four sub-themes. The overarching theme was holistic person-centeredness in dementia care. The four sub-themes were: (1) understanding the interplay between disease and daily-life activities; (2) participating in meaningful activities based on one's preferences; (3) incongruence between the person with dementia's preferences, needs, and the care received from healthcare providers; and (4) concerns of lost income due to job loss, burdening the family, and lack of comprehensive dementia care supports. There is a clear incongruence between individual preferences and needs and a demand to operationalize person-centeredness in dementia care, including the healthcare provider's role. This group of participants wants the "incongruence" resolved to improve person-centered care. Healthcare providers should focus on encouraging individuals with dementia to participate in meaningful activities in daily routines. As a start to person-centeredness in care delivery, healthcare providers need to know and share resources based on their patients' needs and preferences for care.

### SESSION 7015 (POSTER)

### ARTIFICIAL INTELLIGENCE (AI)

Abstract citation ID: igaf122.2237

### A NEURAL FRAMEWORK FOR AGE-RELATED MULTI-OMICS DISCOVERY

Bartek Nogal, John Earls, and Nathan Price, *The Buck Institute, Navato, California, United States*

Aging is a dynamic, multifactorial, and nonlinear process, shaped by genetics, environment, and stochastic forces. Polygenic risk scores (PRS) provide a powerful framework, with advancing genomic technologies capturing greater trait variance and enhancing their utility in aging research. Nonetheless, many studies rely on linear analytical approaches that can overlook the complex and variable nature of aging biology. GWAS-based phenotypes often mask sub-phenotypes, limiting the granularity of PRS-driven insights. Also, they don't account for when in the life course genetic risk manifests into disease, and what aging-related mechanism makes the vulnerability arise. A single age-related PRS, for instance, may align with multiple omics features in deeply phenotyped cohorts, which can propel biomarker discovery. Yet, relying on linear methods, PRS often explain relatively modest variance and can be confounded by aging—losing predictive power once age is incorporated. To address these challenges, we leverage a perturbable variational autoencoder (VAE) trained on nearly 2,000 individuals with over 100 PRS and 1,200 multi-omics features, explicitly modeling age-related effects. This unsupervised neural architecture captures complex relationships, enabling exploration of nonlinear aging trajectories. We uncovered both established and potentially novel associations, including metabolic and mitochondrial pathways in Alzheimer's disease, lysosomal processes in Parkinson's disease, and steroid sulfation in frailty and longevity phenotypes. To contextualize these findings, we integrated a Retrieval-Augmented Generation (RAG) system, coupling large language models with structured database queries. By combining PRS, omics-based modeling, and LLM-driven interpretation within a perturbable VAE framework, we enable robust inference and advance mechanistic discovery of aging-related traits.

Abstract citation ID: igaf122.2238

### ADVANCING GERIATRIC CARE: HARNESSING AI AND HUMAN EXPERTISE TO ENHANCE CNA TRAINING

Suzanne White[1], Stephanie Trotter[2], Robin McAtee[2], and Valerie Bearden[3], *1. College of Nursing - University of Arkansas for Medical Sciences, Little Rock, Arkansas, United States, 2. University of Arkansas for Medical Sciences, Little Rock, Arkansas, United States, 3. Oaklawn Center on Aging, Hot Springs, Arkansas, United States*

Staff of the Arkansas Geriatric Education Collaborative (AGEC), a HRSA-funded Geriatric Workforce Enhancement Program, employed a strategic collaboration between human expertise and artificial intelligence (AI), specifically ChatGPT, a Large Language Model (LLM) to develop a robust 40-hour Advanced Geriatric Specialty curriculum for Certified Nursing Assistants (CNAs). Subject matter experts (SMEs) first conducted a thorough review of the Arkansas State CNA curriculum, identifying opportunities to supplement geriatric knowledge and practice. Based on this analysis, they developed a list of learning module topics to include in the specialty curriculum, beginning with an introduction to the 4Ms of Age-Friendly Care – What Matters, Medication, Mentation, and Mobility. ChatGPT was then employed to synthesize information from reputable sources for each topic and accelerate the generation of content for each module, linking content across modules to the 4Ms where relevant. Each module included a scenario-based application activity or a reflection exercise to reinforce learning and encourage critical thinking. Pre/post tests were also generated to assess the learning of key concepts. Subject matter experts reviewed and refined all AI-generated content to ensure accuracy, clinical relevance, and alignment with best practices in geriatric care. This innovative integration of AI-driven content development with expert oversight allowed for the accelerated creation of a high-quality, evidence-informed curriculum to enhance geriatric competency for CNAs.

Abstract citation ID: igaf122.2239

### AI-DRIVEN ANALYSIS OF NEGLECT IN DEMENTIA CAREGIVING: INSIGHTS FROM UNSTRUCTURED CLINICAL NOTES

Jessica Hernandez-Chilatra, Carolyn Pickering, and Xiaoqian Jiang, *UTHealth Houston, Houston, Texas, United States*

Understanding neglect in the dementia caregiving context requires more than detection; it demands insight into caregiving scenarios, risk factors, and potential preventive strategies. Clinical notes contain rich, unstructured data, yet traditional methods fail to efficiently extract patterns that can inform caregiving prevention interventions. This study leverages AI-driven natural language processing (NLP) techniques to analyze dementia patient clinical notes, uncovering key caregiving challenges, neglect risks, and early intervention opportunities. We analyzed 379 clinical notes from 18 dementia patients, applying MPNet embeddings to assess similarity and detect highly redundant notes. Cosine similarity scores identified 1,146 highly similar notes, which were then grouped at the patient level. GPT-4 was subsequently used to extract structured neglect insights, categorizing risks into medical, emotional, and financial domains while providing caregiving recommendations. A general summary was generated to identify common neglect patterns, risk factors, and potential interventions. Recurrent neglect patterns included unmanaged pain, dehydration, and medication nonadherence; social isolation and unaddressed distress behaviors; and missed healthcare due to financial strain. Caregiver burnout, gaps in follow-up care, and lack of support were common challenges. GPT analysis synthesized high-risk neglect patterns, anonymized case examples, and targeted interventions to inform prevention strategies. A final structured report highlighted high-risk neglect patterns, real-life examples (anonymized quotes), and suggested care interventions. This study demonstrates how AI-driven text analysis can provide actionable insights for identifying and mitigating dementia caregiving neglect. Future work may explore risk-scoring models using embeddings and structured insights to support early detection and intervention strategies in clinical practice.

Abstract citation ID: igaf122.2240

### ARTIFICIAL INTELLIGENCE AIDS IN THE DEVELOPMENT OF MULTIDISCIPLINARY AGE-FRIENDLY EDUCATION

Suzanne White[1], Robin McAtee[2], and Laura Spradley[2], *1. College of Nursing - University of Arkansas for Medical Sciences, Little Rock, Arkansas, United States, 2. University of Arkansas for Medical Sciences, Little Rock, Arkansas, United States*

This work describes a structured approach to leveraging Artificial Intelligence (AI), specifically ChatGPT, a Large Language Model (LLM), to streamline and accelerate the development of educational content while maintaining accuracy and adherence to clinical best practices. Goals were developed to meet the learning objectives of the targeted learners and then AI was introduced. The process began by prompting ChatGPT to generate an e-learning module with specific instructional design elements based on goal-oriented outcomes while integrating age-friendly content into specialized geriatric modules for healthcare professionals. For example, the prompt for "Introducing the 4Ms of Age-Friendly Care" included: "Generate a structured 15-minute e-learning module introducing the 4Ms Framework to licensed and unlicensed healthcare professionals, 8th grade reading level, include an introductory paragraph, key sections with structured content, a conclusion paragraph, key takeaways, and a scenario-based application activity. Use content from the IHI website and the John A. Hartford Foundation only". In seconds, ChatGPT generated the requested e-learning content. The output was reviewed and refined to ensure the desired competencies were appropriately addressed. Subject matter and educational experts conducted a final review to ensure accuracy, adherence to the desired instructional design parameters, and applicability to real-world healthcare settings. After the final review, ChatGPT was prompted to generate knowledge assessments to test key concepts. Key takeaways included: ChatGPT, when coupled with human expertise and oversight for educational content development, was a useful tool to streamline module creation, ensure alignment with instructional design principles, integrate age-friendly care elements, and generate knowledge assessments to evaluate learning.

Abstract citation ID: igaf122.2241

### ARTIFICIAL INTELLIGENCE-ENABLED COMPREHENSIVE ELECTRONIC HEALTH RECORD PHENOTYPING AT A LARGE SCALE

Niels Turley[1], Marta Fernandes[2], Aditya Gupta[1], Manohar Ghanta[1], Haoqi Sun[1], Robert Thomas[1], Sahar Zafar[2], and M. Brandon Westover[1], *1. Beth Israel Deaconess Medical Center, Boston, Massachusetts, United States, 2. Massachusetts General Hospital, Boston, Massachusetts, United States*

The Electronic health record (EHR) contains rich and ever-growing information, especially for the gerontologic population with multiple comorbidities. With the advent of powerful artificial intelligence (AI) tools, we can perform accurate EHR phenotyping, which is the foundation for downstream analyses. Here, we performed EHR phenotyping of ten diseases in a large multi-site clinical dataset of 145,787 unique patients, including epilepsy (and subtypes), ischemic stroke, subarachnoid hemorrhage, subdural hematoma, Alzheimer's diseases and related dementias, Parkinson's disease, cardiac arrest, traumatic brain injury, brain tumor, and congestive heart failure. We used AI-enabled natural language processing that extracts the presence of keywords from unstructured clinical notes while considering negations, as well as structured diagnosis codes (ICD) and medications. We used logistic regression, random forest, and XGBoost. The dataset was constructed from two sites by sampling an equal number of participants in people with or without ICD or medication in the EHR, which forms four groups: ICD+Med+, ICD+Med-, ICD-Med+, and ICD-Med-. We manually annotated each case as positive or negative by reading the clinical notes. In this way, the dataset has a roughly balanced positive/negative ratio to ease training. We then did leave-one-site-out cross-validation. The areas under the receiver operator curve are higher than 0.95 across the diseases. The areas under the precision-recall curve are higher than 0.83 across the diseases. For each disease, we conducted detailed error analyses for both false positives and negatives. The results lead to accurate phenotyping and insights from patient phenotypes at both population and individual levels.

Abstract citation ID: igaf122.2242

**BRIDGING CONNECTION & CARE: AI-MODIFIED ACTIVITIES FOR RESIDENTS**

ZaKiyah Timmons-Crear[1], and Melinda Heinz[2], *1. University of Northern Iowa, waterloo, Iowa, United States, 2. University of Northern Iowa, Cedar Falls, Iowa, United States*

This study explored the effectiveness of using artificial intelligence (AI) to modify activities in care facilities based on vision, hearing, mobility impairments, and for those living with dementia. AI has become a widespread tool, although its applications in care facilities remains unexplored. Two programs (e.g., Co-Pilot and ChatGPT) were used to explore their effectiveness in modifying activities from four different care facilities (e.g., independent living, assisted living, nursing home, and memory care). Actual activity calendars from each facility were used. In the first iteration, our prompt was "In a [type of care facility] how would you modify [type of activity] for someone who has [dementia, mobility, hearing, or vision impairment]. We noted that ChatGPT provided significantly more detail about how the activity could be modified and why that would be important (e.g., explaining why using bright colors and textures might help to provide increased sensory stimulation for those living with dementia). In a second iteration, we asked Co-Pilot and ChatGPT to modify the activities if the facility was short-staffed. Both AI platforms suggested allowing residents more independence with the activities. However, for some areas, such as memory care units, it may not be feasible for residents to complete activities independently due to their cognitive functioning. These findings suggest that AI may be most useful for providing employees guidance with tailoring activities to individual needs. However, AI lacked critical thinking when suggesting that residents could complete activities with more independence if the facility were short staffed.

Abstract citation ID: igaf122.2243

**BRIDGING EVIDENCE GAPS IN GERIATRIC CARE: LARGE LANGUAGE MODELS AND KNOWLEDGE GRAPHS FOR DECISION SUPPORT**

Shuqi Yang[1], Zheng Zhu[1], Shuai Wang[2], Jiaqing Wang[1], Zongan Huang[1], and Tiantian Hu[1], *1. Fudan University, Shanghai, Shanghai, China (People's Republic), 2. Dali University, Dali, Shanghai, China (People's Republic)*

The increasing prevalence of complex chronic conditions among older adults has highlighted critical gaps in the application of evidence-based practices in geriatric care. While large language models (LLMs) have demonstrated remarkable potential in medical decision support, they are prone to generating hallucinations. To address these challenges, this study developed an intelligent decision support system that integrated LLMs and knowledge graphs (KGs) to enhance evidence-based practice. The system was constructed through a multi-phase construction process: First, a high-quality geriatric symptom knowledge graph was built using structured evidence sources, including clinical guidelines and symptom networks derived from longitudinal patient data. Next, an LLM was fine-tuned with domain-specific knowledge and optimized using retrieval-augmented generation (RAG) to ensure its responses aligned with validated evidence. Finally, an interactive validation mechanism cross-referenced LLM-generated outputs with the KG, reinforcing decision accuracy and minimizing misinformation. Evaluation results demonstrated that the proposed system achieved an accuracy of 87.6%, exceeding that of a domain-specific fine-tuned LLM without KG integration (82.5%) and GPT-4 (78.3%). In terms of classification performance, the system obtained an F1 score of 85.2%, outperforming the fine-tuned LLM (80.4%) and GPT-4 (74.9%). Furthermore, the system significantly reduced the hallucination rate to 3.4% and provided rationales with citations in 92.1% of cases, ensuring enhanced transparency and reliability. These findings demonstrated that anchoring AI-generated insights in validated knowledge structures increases trust, optimizes care strategies, and supports healthcare professionals in delivering high-quality, individualized geriatric care.

Abstract citation ID: igaf122.2244

**COMPARATIVE ASSESSMENT OF PREDICTIVE MODELS FOR OLDER ADULTS' HEALTH OUTCOMES: LOGISTIC REGRESSION, LASSO, ML, LLM**

Feng Wei, Yuanhao Liu, and Jiayu Zheng, *Johns Hopkins University, Baltimore, Maryland, United States*

Accurate prediction of health outcomes in older adults is crucial for tailoring interventions and informing public health strategies. Despite advancements in predictive methodologies, significant gaps remain in understanding how these models perform across various health indicators and demographic segments. This study evaluates four predictive methodologies—conventional logistic regression, regularization represented by LASSO, machine learning ensembles (decision trees, random forests, support vector machines), and AI LLM (DeepSeek and GPT)—focusing on two critical health outcomes: depression and mortality. Using nationally representative data from the Health and Retirement Study (HRS) and the China Health and Retirement Longitudinal Study (CHARLS), we evaluate these models' performance and fairness comparatively: (1) evaluate model performance through traditional metrics such as accuracy, precision, recall, F1-score, and ROC-AUC; (2) explore whether performance discrepancies are consistent across these health outcomes; (3) investigate fairness by examining predictive performance disparities among elderly subpopulations defined by geographic location, gender, and socioeconomic status, using fairness metrics like statistical parity, equalized odds, and predictive parity. Through this multidimensional comparison, our study provides a comprehensive evaluation of the progression from traditional to AI-based predictive models, contributes to understanding the current capabilities and limitations in predicting elderly health outcomes, and identifies the trade-offs between model complexity, predictive accuracy, and fairness. These potential outcomes are expected to inform further research, and provide practical guidance for policymakers, educators, and healthcare practitioners in deploying equitable and effective predictive strategies for elderly health outcomes.

Abstract citation ID: igaf122.2245

**COMPARING ROBOTIC AND REAL CAT COMPANIONSHIP IN MITIGATING LONELINESS AMONG ELDERLY POPULATIONS**

Bingwen Wang[1], and Kaiting Ma[2], *1. Duke University, Durham, North Carolina, United States, 2. The Education University of Hong Kong, Hong Kong, China (People's Republic)*

Aims: This study evaluates the effectiveness of a newly developed robotic cat compared to live cat therapy in reducing loneliness among elderly individuals. It aims to determine whether robotic feline companionship can provide similar emotional and mental health benefits to real cat interactions, offering a scalable solution for socially isolated older adults. Method: A randomized controlled trial was conducted with 180 elderly participants experiencing chronic loneliness. Participants were assigned to one of three groups: interaction with a robotic cat, interaction with a real therapy cat, or a control group receiving standard care. The study lasted six months, with outcomes measured using the UCLA Loneliness Scale, Geriatric Depression Scale, and physiological stress indicators (cortisol levels and heart rate variability). Semi-structured interviews explored participants' subjective experiences and attachment to the cats. Results: Both intervention groups showed significant improvements in loneliness and mental health compared to the control group. The robotic cat group experienced a 38% reduction in loneliness scores and a 28% improvement in mental health metrics, while the real cat group reported a 45% reduction in loneliness and a 35% improvement in mental health. Qualitative feedback indicated that robotic cat users valued its constant availability and ease of care, while real cat users highlighted emotional warmth and authenticity. Conclusion: The robotic cat presents a promising alternative to live cat therapy, significantly reducing loneliness and enhancing well-being. Its practicality and accessibility make it a viable, scalable intervention, though further research is needed to optimize design and assess long-term effects.

Abstract citation ID: igaf122.2246

### ENHANCING EMOTIONAL EXPERIENCE IN AI-ENABLED ROBOTICS FOR OLDER ADULTS: A KANSEI ENGINEERING APPROACH

Chorong Park, and Rua Williams, *Purdue University, West Lafayette, Indiana, United States*

As the global population ages, there is a growing need for technologies that not only assist older adults in their daily lives but also enhance their emotional well-being and social engagement. While assistive robots have been widely developed for functional support, their ability to foster meaningful human-robot interactions remains limited. This study explores how Kansei Engineering for Older Adults, a design methodology focused on emotional experience of older adults, can improve the way older adults engage with AI-enabled robotic companions. Through ethnographic research and interaction studies, we examine how four AI-powered robots, designed with Kansei Engineering principles—including facial expressions, voice modulation, and haptic feedback—affect social engagement and emotional connection in older adults. We measure engagement through interaction frequency, duration, and qualitative user feedback, providing insight into how emotional responsiveness in robots influences trust, companionship, and long-term acceptance. Preliminary findings suggest that older adults respond more positively to robots that express emotions naturally and adapt their interactions based on user feedback. Participants reported feeling more connected and engaged, highlighting the potential for emotionally intelligent robotics to reduce loneliness and improve quality of life. This study contributes to the growing field of human-centered AI and gerontechnology, offering a framework for designing emotionally aware robotic systems that foster social and psychological well-being in aging populations. As technology continues to evolve, integrating Kansei-driven AI interactions may shape the future of companion robotics, caregiving technology, and digital social engagement for older adults.

Abstract citation ID: igaf122.2247

### EVALUATING LARGE LANGUAGE MODELS FOR MILD COGNITIVE IMPAIRMENT: A BILINGUAL COMPARISON OF CHATGPT, GEMINI, AND KIMI

Yexuan Xiao, Qianhui Pan, Nan Jiang, Haoyuan Liu, Yilin He, Yuhe Zhang, and Tingmei Wang, *Tsinghua University, Beijing, Beijing, China (People's Republic)*

Background: Mild Cognitive Impairment (MCI) is a key stage between normal aging and Alzheimer's Disease (AD), with early intervention crucial for slowing progression. Large Language Models (LLMs) offer promising support by providing accessible, evidence-based information for non-specialist healthcare professionals and care partners. However, concerns about accuracy and limited multilingual evaluations remain. Objective: This study explores the potential of LLMs in managing MCI, examines their support for non-specialist healthcare professionals and care partners, and compares English and Chinese responses to MCI-related queries, considering language-specific nuances and effectiveness. Methods: We submitted 72 open-ended questions related to MCI management to ChatGPT-4o, Gemini, and Kimi, assessing their responses based on accuracy, comprehensibility, specificity, and actionability using a five-point Likert scale. Statistical analyses, including Intraclass Correlation Coefficients and Mann-Whitney U tests, were conducted to examine response across models. Result: LLMs' performance in MCI management was evaluated, with the Symptoms and Diagnosis domain scoring highest. Healthcare professionals' needs were better met than care partners', particularly in accuracy, comprehensibility, and actionability. English responses outperformed Chinese in comprehensibility and specificity. Conclusion: Based on the results, LLMs demonstrate potential in assisting non-specialist healthcare professionals and care partners, particularly in the domains of symptoms and diagnosis. However, there is a need for further optimization in Chinese medical corpora, as English responses outperformed Chinese ones due to corpus disparities. Tailored models for care partners, focusing on reliability and clarity, are essential to address their unmet needs and enhance their experience in managing MCI.

Abstract citation ID: igaf122.2248

### EXPLORING LLM-BASED GENERATIVE RECOMMENDER SYSTEMS: CORPORA, CUSTOMIZATION, AND EVALUATION INSIGHTS

Shuqi Yang[1], Mingrui Jing[1], Shuai Wang[2], Jiaqing Wang[1], Weijie Xing[1], Yan Hu[1], and Zheng Zhu[1], *1. Fudan University, Shanghai, Shanghai, China (People's Republic), 2. Dali University, Dali, Shanghai, China (People's Republic)*

Large Language Model-Driven Generative Recommender Systems (LLM-GRSs) are increasingly transforming healthcare, particularly in question-answering systems. This study systematically reviewed their corpora sources, customization techniques, and evaluation metrics. A search of PubMed/MEDLINE, Embase, Scopus, and Web of Science identified 61 studies (2021–2024) using LLM-GRSs for medical information delivery. Corpus sources were categorized into real-world clinical resources (n = 24), literature materials (n = 34), open-source datasets (n = 33), and web-crawled data (n = 11), with 44 studies integrating multiple sources. Key model customization strategies included pre-training, prompt engineering, retrieval-augmented generation (RAG), fine-tuning, in-context learning, and offline learning. Fourteen studies used a single customization technique, while 41 studies combined these methods during model development. The evaluation metrics were classified into three main domains: 1) process metrics, 2) usability metrics, and 3) outcome metrics. The outcome metrics could also be divided into two categories: model-based outcomes and expert-assessed outcomes. The study identified critical gaps in corpus fairness, contributing to biases from geographic, cultural, and socio-economic factors. The reliance on unverified or unstructured data highlights the need for better integration of evidence-based clinical guidelines. Future research should focus on developing a tiered corpus architecture with vetted sources and dynamic weighting, while ensuring model transparency. Additionally, the lack of standardized evaluation frameworks for domain-specific models calls for comprehensive validation of LLM-GRSs in real-world healthcare settings.

Abstract citation ID: igaf122.2249

### FOSTERING NEXT-GEN GERONTOLOGISTS: AI-POWERED RESEARCH TRAINING FOR UNDERGRADUATES

Catheryn Koss, *California State University, Sacramento, Sacramento, California, United States*

Artificial intelligence (AI) is increasingly being integrated into academic and professional training as educators explore its potential to enhance student learning. By lowering barriers to entry, AI tools can help students who are intimidated by research gain hands-on experience in the research process, enabling them to produce high-quality, professional-level outputs such as research posters. This presentation will demonstrate how AI can be effectively incorporated into undergraduate curricula, drawing from experience using these tools in a research course focused on aging and the life course. Participants will learn practical strategies for leveraging different AI models to support student engagement in research, from understanding and evaluating academic literature to synthesizing key findings. Additionally, the session will showcase how AI tools, particularly ChatGPT, can assist students in conducting statistical analyses, interpreting results, and visualizing data through graph generation. By integrating AI into coursework, educators can help students develop critical thinking skills, improve their ability to navigate complex research, and gain confidence in quantitative methods. The presentation will include hands-on examples of AI applications in research assignments, discussions on ethical considerations, and best practices for balancing AI assistance with traditional learning methods. Attendees will leave with concrete strategies for integrating AI into their teaching to better prepare students for an evolving technological landscape in gerontology and related fields.

Abstract citation ID: igaf122.2250

### GENAI OUTPERFORMS HUMAN REVIEWERS IN CLASSIFYING ALZHEIMER'S DISEASE AND RELATED DEMENTIA RESEARCH

Duo Wei, Riya Goyal, Tasnim Raisa, Jeannine Elmasri, and Jessica Fleck, *Stockton University, Galloway, New Jersey, United States*

Categorizing research literature is critical in aging-related studies [1], particularly for Alzheimer's disease and related dementias (ADRD), where articles are typically classified into screening, diagnosis, and intervention [2]. This study compares the performance of human reviewers and GenAI in classifying ADRD literature. The human group included three trained individuals with computer science backgrounds and expertise in systematic reviews on mental health for older adults, while the AI group comprised DeepSeek (AI1), ChatGPT (AI2), and Google Gemini (AI3). Sixty-six PubMed papers were analyzed to evaluate congruence and accuracy. Congruence was measured using mutual information, a metric from information theory that quantifies shared information between variables. Results revealed strong agreement among AI models, with scores of 0.910 for AI1 and AI2, 0.686 for AI2 and AI3, and 0.776 for AI1 and AI3. In contrast, human congruency scores averaged 0.45 or below ($p < 0.05$). Accuracy, assessed using a majority vote and domain expert review as the gold standard, showed that human reviewers achieved an average accuracy of 0.495, while AI models performed with average of 0.757, with AI1 achieving the highest accuracy at 0.818. These findings highlight AI's superior classification performance over human reviewers ($p = 0.0019$). However, challenges such as AI interpretability must be addressed. Future research should explore integrating human expertise with AI to improve the classification of ADRD literature. This study underscores the potential of AI in advancing aging-related research while emphasizing the need for careful consideration of its limitations.

Abstract citation ID: igaf122.2251

### PIXELS OF POSSIBILITY: AI ART AND THE VISUAL REIMAGINATION OF SUCCESSFUL AGING

Jiayu Chen[1], and Qingwei Wang[2], *1. National University of Singapore, Singapore, Singapore, 2. Xi'an Jiaotong-Liverpool University, Suzhou, Jiangsu, China (People's Republic)*

This article explores AI art's cultural implications for reimagining successful aging. Despite AI's increasing presence in artistic and everyday visual culture, its role in challenging dominant aging narratives and broadening representations of later life remains largely underexplored. This research takes an interdisciplinary approach, bridging gerontology, visual cultural studies, and AI research to investigate whether and how AI art can serve as a medium for shaping the narratives around successful aging. Through a qualitative case study of Auntieverse, an AI art project featuring elderly female figures, this research employs an innovative three-study design to examine the intersections of AI aesthetics, artistic intention, and audience reception. The findings reveal that while Auntieverse aims to disrupt ageist stereotypes by portraying older women with extreme autonomy, it also uncovers algorithmic biases in AI aesthetics and the interpretive gaps between artist and audience. There main themes emerged through iterative analysis of multimodal Data: "Re-seeing Age Identity," which explores how AI-generated imagery reshapes representations of aging; "Re-thinking the Aging Body," which examines AI aesthetics' role in mediating perceptions of physical aging; and "'Re-pAInting' Successful Aging," which considers AI art's potential to either reinforce or redefine prevailing narratives of autonomy in later life. By positioning AI art as an active cultural force, this study demonstrates AI art's potential role in contributing to gerontology through an in-depth case study. It advocates for a more critical and intentional engagement with AI's influence on aging representation, through which the visual discourse surrounding successful aging could be challenged and reimagined.

Abstract citation ID: igaf122.2252

### PREDICTING NIH TOOLBOX COGNITION SCORES IN OLDER ADULTS WITH MCI USING WEARABLE SENSORS: A MACHINE LEARNING APPROACH

Assma Habadi, Miloš Žefran, Naoko Muramatsu, Lijuan Yin, Woojin Song, Maria Caceres, and Elise Hu, *University of Illinois Chicago, Chicago, Illinois, United States*

Early detection of cognitive changes enables timely treatment, intervention, and care planning. However, comprehensive neuropsychological evaluations may not be always feasible. The current study examined the utility of physiological biomarkers for assessing cognitive function among older adults diagnosed with mild cognitive impairment or mild dementia. Participants (N = 23) wore Empatica EmbracePlus, a health-monitoring smartwatch, while completing the NIH Toolbox Cognition Battery (NIHTB-CB). EmbracePlus collected physiological biomarkers continuously throughout the assessment, including blood volume pulse, electrodermal activity, skin temperature, and tri-axial accelerometry. Machine learning techniques, including supervised learning and ablation analysis, were used to examine correlations between physiological data and NIHTB-CB scores. The analysis showed that tri-axial accelerometry and electrodermal activity were the leading markers in predicting processing speed tasks scores. For working memory scores, blood volume pulse and temperature were particularly informative. In inhibitory control tasks, both blood volume pulse and electrodermal activity emerged as key markers. Overall, the predicted scores were highly correlated with participants' actual test scores, achieving correlations as high as 0.88 (Spearman's rho). Studies with larger samples and additional sensor modalities may further improve predictive accuracy. These findings suggest that health-monitoring smartwatches may serve as a real-time, non-invasive marker of cognition. In turn, such methods can help with timely clinical decision making and offer the possibility of novel clinical interventions.

Abstract citation ID: igaf122.2253

### PREDICTIVE VALUE OF THE PROTEOMIC AGING CLOCK AND APOE GENOTYPE FOR INCIDENT ALZHEIMER'S DISEASE RISK

Longjian Liu, *Drexel University, Philadelphia, Pennsylvania, United States*

Aims: This study aimed to develop a novel proteomic aging clock (PAC) and evaluate its predictive value, along with APOE-ε4, for Alzheimer's disease (AD) and AD-related dementias (AD/ADRD) risk in a cohort of adults ages 45–64 at baseline (1987–1989) with a median follow-up of 30 years. Methods: We analyzed data from 469 participants in the Atherosclerosis Risk in Communities (ARIC) Study, including plasma proteomic measurements (n = 460) and APOE genotyping. Aging-related proteomic features were identified using machine learning-based elastic net and Lasso regression. Cox proportional hazards models were used to retrospectively estimate hazard ratios (HRs) for AD/ADRD associated with PAC. Results: With a total follow-up of 9,366 person-years, the incidence rates (95% CI) of AD/ADRD per 1,000 person-years were 11.1 (8.8–13.9) in individuals without APOE-ε4, 15.9 (11.7–21.7) in those with one copy, and 23.7 (8.9–63.0) in those with two copies of APOE-ε4. Among the 460 candidate plasma proteomics, 43 were identified as key predictors. After adjusting for chronological age and other key covariates, the adjusted HRs (95% CI) for incident AD/ADRD by PAC quantiles (Q1 as the reference group) were: Q2: 1.63 (0.78–3.38), Q3: 1.13 (0.50–2.55), Q4: 2.48 (1.10–5.59), and Q5: 3.24 (1.43–7.34). African Americans had a significantly higher AD/ADRD risk than Whites (HR = 2.14, 95% CI: 1.39–3.31). Conclusions: Plasma proteomics are strong predictors of incident AD/ADRD risk. The identified proteomic markers provide new insights for developing novel risk prediction models, innovative therapeutics, and targeted treatment strategies. The observed racial disparities in AD/ADRD risk warrant further investigation.

Abstract citation ID: igaf122.2254

### PROMPT ENGINEERING AND CHATGPT: DELIVERING QUALITY INFORMATION TO DEMENTIA CAREGIVERS

Stephanie Bennett[1], Alex Fedorov[1], Rasheeta Chandler[1], Kenneth Hepburn[1], and Fayron Epps[2], *1. Emory University, Atlanta, Georgia, United States, 2. UT Health San Antonio, San Antonio, Texas, United States*

Dementia caregivers rely on the internet for access to quick information. Increasingly, they are turning to prompt-based large language models (PBLLMs) like ChatGPT to improve outcomes for persons living with dementia (PLWD). However, researchers highlight significant limitations in ChatGPT's responses to health-related questions, including contextual understanding, emotional awareness, readability, and transparency. To overcome these shortcomings, prompt engineering (PE) —the practice of crafting specific, detailed prompts to guide a PBLLM—has emerged as promising strategy. In this novel study, we used web content analysis to evaluate 15 responses from ChatGPT. Specifically, we assessed the effectiveness of using PE with ChatGPT based on aforementioned limitations by comparing responses to five general caregiving questions and two iterations (10) of PE statements. Questions focused on five topics: websites, specialists, behavioral issues, tests, and medications. We maintained the topics but revised each question using Cumming's PE method, which required details about reference websites, ChatGPT's role, caregivers' goals, context, and tasks. The first set of PE-statements (5) included context about the PLWD. The second set (5) incorporated the additional context of race. Study findings showed that responses to general questions promoted engagement by including follow-up questions. Although the PE responses lacked these follow-ups, they provided contextually relevant information to help caregivers achieve their goals and fostered emotional awareness. These responses demonstrated improved readability and included links to credible sources. Our findings suggest that PE methods can enhance ChatGPT's limitations, improving responses for caregivers of PLWD and paving the way for future methodological research in AI-assisted communication.

Abstract citation ID: igaf122.2255

### PUBLIC ATTITUDE TOWARD ELDER FAMILY FINANCIAL EXPLOITATION: ANALYSIS OF SOCIAL MEDIA DATA

Tina Kilaberia, Weicheng Zeng, and Ruopeng An, *New York University, New York, New York, United States*

Underreporting of privacy-laden family financial exploitation of older adults and Federal underinvestment in response to elder mistreatment can adversely influence older people's help-seeking, health, and mental health outcomes. This study compared public attitudes toward family financial exploitation by focusing on one victim (21 YouTube videos), one perpetrator (8), and a case of family financial abuse generally (19), totaling 3,796 comments included in the analysis. The victims and perpetrators in the videos were not related. Three levels of analysis were conducted: sentiment and emotion analyses using advanced natural language processing (NLP) methods and a human-coded subsample. Sentiment analysis showed that among the three cases, a greater share of negative (about 40%) and positive (nearly 60%) comments was attributed to the perpetrator and victim, respectively. Emotion analysis found no shared emotions between the victim and perpetrator cases. Disapproval, surprise, disgust, and confusion were expressed solely concerning the perpetrator, whereas anger and fear were expressed solely concerning the victim. Caring, love, and gratitude were shared emotions across the victim and the general cases. Sadness, admiration, curiosity, annoyance, and approval were five emotions shared across all three cases. Human-coded sub-sample analysis showed expressions of sarcasm, and microaggressions directed at the perpetrator's race and gender. Although NLP methods did not capture victim-perpetrator background-related attitudes in relation to family financial abuse, they delineated relatively clear-cut attitudes toward victim and perpetrator status, reflecting a public concern toward elder family financial exploitation. Public attitudes may be harnessed for stakeholder engagement to increase reporting and investment in elder mistreatment.

Abstract citation ID: igaf122.2256

### RESULTS OF A SYSTEMATIC LITERATURE REVIEW ON AI IN AGING: EVIDENCE AND INSIGHTS

Kimia Tuz Zaman[1], Wordh Ul Hasan[1], Juan Li[1], and Bo Xie[2], *1. North Dakota State University, Fargo, North Dakota, United States, 2. The University of Texas at Austin, Austin, Texas, United States*

The rapidly aging population necessitates innovative healthcare solutions. This review synthesizes current evidence on artificial intelligence (AI) applications in older adult care to inform stakeholders about clinical, functional, and psychosocial outcomes. (Details of our AI-assisted systematic literature review method are reported elsewhere; here we focus solely on the findings.) Following PRISMA 2020, an initial PubMed search yielded 857 records, with 57 meeting inclusion criteria after automated filtering and manual validation. Data were extracted using AI agents addressing 12 predetermined research questions on AI technologies, outcome measures, stakeholder perspectives, and implementation challenges. Most studies employed machine learning and natural language processing in home-based and telehealth settings. Thematic analysis identified five key domains: (1) health monitoring and prediction, where fall risk models and chronic disease predictors achieved accuracy rates above 70%; (2) social and emotional support, with AI-driven voice assistants and reminiscence therapy significantly reducing loneliness; (3) cognitive assessment, as sensor-based gait analysis demonstrated high sensitivity (0.961) and AI-driven music therapy improved cognitive scores ($p < .05$); (4) variable implementation effectiveness across studies; and (5) challenges including technical infrastructure, data privacy, and regulatory issues. Notably, several studies reported improvements in balance outcomes, while others documented moderate accuracy in detecting atrial fibrillation and diabetes, underscoring the diversity of AI applications. Cost-effectiveness, privacy concerns, and integration with care systems were recurrent themes. These findings underscore the potential of AI to enhance older adult care, although short follow-up periods limit long-term conclusions. Future research should address these barriers to facilitate broader AI integration in healthcare.

### SESSION 7020 (POSTER)

### BIOBEHAVIORAL HEALTH

Abstract citation ID: igaf122.2257

### ADVANCING BEHAVIORAL HEALTH INTEGRATION IN THE PROGRAM OF ALL-INCLUSIVE CARE FOR THE ELDERLY (PACE)

Alexa Fleet[1], Liane Wardlow[2], Daniel Shalev[3], Michelle Moses-Eisenstein[4], William McGuire[5], Kristen Hartlieb[2], and Harold Pincus[6], *1. University of Massachusetts, Boston, Massachusetts, United States, 2. West Health Policy Institute, San Diego, California, United States, 3. Weill Cornell Medicine, New York, New York, United States, 4. New York State Psychiatric Institute, New York, New York, United States, 5. New York State Office of Mental Health, New York, New York, United States, 6. Columbia University, New York, New York, United States*

Older adults enrolled in the Program of All-Inclusive Care for the Elderly (PACE) face significant barriers to accessing behavioral health (BH) services despite high rates of BH comorbidities (60%). Integrating BH into PACE programs is critical to achieving truly comprehensive, person-centered care. This poster introduces a conceptual framework for Behavioral Health Integration in PACE (BHI-PACE) and presents current BH practices and challenges within PACE Organizations (POs) from a nationwide assessment. A mixed-methods sequential explanatory approach was used to assess BHI across POs. This study employed (1) a national survey distributed to 148 POs (38 responses, representing 119 PACE sites and 25,806 participants) and (2) semi-structured interviews with 15 POs (representing 58 sites and 10,029 participants). The BHI-PACE framework comprises four domains: (1) Person/Family-Oriented Care, (2) Clinical Functions, (3) Workforce to Support Clinical Functions, and (4) Structures to Support Clinical Functions. Key findings highlight that 60% of PACE participants experience BH comorbidities, with depression (92.1%), behavioral and psychological symptoms of dementia (57.9%), and anxiety disorders (55.3%) as the most common referral reasons. Workforce shortages, insufficient funding structures, and limited integration of BH specialists into POs were identified as primary barriers. However, successful integration strategies include the use of telemedicine, interdisciplinary team collaboration, and formalized external partnerships. This work contributes to both theory and practice by presenting a replicable framework for BH integration in complex care settings for older adults. It informs future policy initiatives, workforce development strategies, and implementation research aimed at improving BH outcomes in aging populations.

Abstract citation ID: igaf122.2258

### CANNABIS-INVOLVED SUBSTANCE USE TREATMENT ADMISSIONS AGE 50+, 2000-2021: CHANGES AND CORRELATES

Namkee Choi, and C. Nathan Marti, *The University of Texas at Austin, Austin, Texas, United States*

Given the rapidly increasing medicinal and recreational cannabis use among U.S. older adults, we examined older adults' cannabis-involved substance use treatment admissions. We used the 2000-2021 concatenated Treatment Episode Data Set-Admissions (TEDS-A) age 50 + (N = 5,593,004) and joinpoint regression models to examine changes in cannabis-involved admissions and multinomial and binary logistic regression models to examine the demographic and treatment-related correlates of cannabis-primary and -secondary/tertiary admissions. The results show that cannabis-involved admissions ages 50-64 increased significantly between 2000 and 2012 (annual percentage changes [APC]=4.36 and 6.62 for the 50-54 and 55-64 age groups, respectively) and then decreased between 2012-2021 (APC=1.84 and 1.27 for the 50-54 and 55-64 age groups, respectively). In the 65+ age group, there was a steady increase between 2000 and 2016 (APC=5.2), followed by no significant changes between 2016 and 2021. Compared to no-cannabis admissions, the likelihood of both cannabis-primary and -secondary/tertiary admissions was higher among males, Blacks, American Indians/Alaska Natives, residents of states where medical or recreational cannabis use was legal, and referrals from healthcare providers and court/criminal legal systems. The likelihood of cannabis-primary admissions was higher among those age 65+, Blacks, Hispanics, residents of states with medical cannabis laws, and those who were referred by healthcare providers and court/criminal legal systems. More effective regulations and enforcement of delta-9-tetrahydrocannabinol potency and research on cannabis harms and poly-substance use are needed to protect the health of older adults who turn to cannabis for its purported health benefits. Increased availability and accessibility of treatment infrastructure are also needed.

Abstract citation ID: igaf122.2259

### CONCEPT ANALYSIS OF CAREGIVER ATTACHMENT: IN THE CONTEXT OF HEART DISEASE

Jinseon Hwang, *University of Washington, Seattle, Washington, United States*

Caregiver attachment significantly impacts patient well-being, particularly in chronic illnesses like heart disease, yet its role in caregiving remains underexplored despite extensive study in psychology. Unlike general adult attachment, caregiver attachment is shaped by illness-related stress and evolving caregiver-patient interactions, and a clearer understanding of it can enhance caregiving effectiveness, patient adherence, and emotional resilience. This study clarifies caregiver attachment in heart disease by identifying its defining attributes, antecedents, and consequences through a structured framework. A concept analysis was conducted using Walker and Avant's (2014) eight-step method to refine its definition in heart disease caregiving, and a literature review was performed to explore theoretical foundations and clarify conceptual boundaries. Antecedents include caregiver's perceived responsibility, attachment history, patient dependency, relationship quality, psychological resilience, social support, and disease severity, while the four defining attributes are providing a Secure Base, Attunement, Responsiveness, and a Safe Haven. Secure caregiver attachment results in lower stress, improved adherence, and better emotional well-being, whereas insecure attachment leads to burnout, emotional detachment, and poor treatment compliance. Caregiver attachment is defined as a dynamic, emotionally attuned response fostering security, emotional stability, and mutual motivation in caregiving relationships, and recognizing it informs nursing interventions such as psychosocial support programs, caregiver education, and emotional regulation training, with future research needed to refine measurement tools and integrate attachment-based approaches into clinical practice to improve both caregiver and patient outcomes.

Abstract citation ID: igaf122.2260

### CONNECTIONS BETWEEN DIABETES, DEPRESSION, AND PURPOSE IN LIFE IN OLDER ADULTS

Jonathan Sundby[1], Tiana Broen[1], and Leilani Feliciano[2], *1. University of Colorado Colorado Springs, Colorado Springs, Colorado, United States, 2. University of Colorado Colorado Springs Department of Psychology, Colorado Springs, Colorado, United States*

Purpose in life is defined as the belief that one's life has goals, direction, and meaning. In older adulthood, purpose in life is an important component of well-being and previous literature has found it to be associated with more "successful aging," including inner strength and social integration (AshaRani et al., 2022). Our project aimed to examine whether the experience of living with diabetes impacts purpose in life in older adulthood. Diabetes is a chronic disease that requires regular monitoring of blood sugar levels, as well as lifestyle changes (dietary, exercise, medication), to prevent serious medical complications or fatal consequences. We hypothesized that the experience of living with diabetes erodes a person's purpose in life, partially explained through the mechanism of increased risk of depression (Cho et al., 2024). Data were analyzed from older adults (65+ years; n = 1358) in the third wave of the Midlife in the United States (MIDUS) dataset. A series of linear regressions indicated that the presence of diabetes predicted lower purpose in life [$\beta = -.098$, $p < .001$], as well as a higher likelihood of experiencing depression [B=.372, $p = .025$]. Running both variables together in a hierarchical linear regression led to a smaller, but still significant, role for diabetes within the model [$\beta = -.086$, $p = .001$]. These results emphasize the connections between the presence of chronic disease and purpose in life and highlight that effective treatment of physical conditions alongside their mental health comorbidities could help increase the well-being of older adults.

Abstract citation ID: igaf122.2261

### DEATH CIRCUMSTANCES OF OLDER ADULTS WITH CANNABINOID EXPOSURES

Armiel Suriaga, *Florida Atlantic University, Boca Raton, Florida, United States*

Results from the 2021 and 2022 National Drug Use and Health Survey reported that 7.7 million older adults (60+) used cannabis. Reasons for cannabis use were to feel good, relax, improve sleep, and pain relief. However, studies on the long-term effects of cannabis in the older population remain limited. Our cross-sectional study aimed to report the characteristics and the death circumstances of people (65+) with cannabinoid exposures as determined by the medical examiners through autopsy and toxicology results. We used descriptive statistics to examine the de-identified data from the Florida Department of Law Enforcement (2020-2022). There were 10,851 (23.37%) out of 46,437 cases with cannabinoid exposures; 576 (5.31%) were older adults. 482 (83.68%) were males, whites (496 or 86.11%), and 576 (94%) happened in urban counties. 213 used a single drug, while 363 (63.02%) had polydrug use. 180 older decedents had cardiac-related conditions, 57 with respiratory-related conditions, and 14 with diabetes. The manner of death were accidents (n = 247 or 42.88%), homicide (n = 12 or 2.08%), natural cause (n = 177 or 30.73%), suicide (n = 133 or 23.09%), and undetermined cause (n = 7 or 1.22%). 115 older adults with cannabinoid exposures died from intoxication, while 28 (4.64%) from motor vehicular accidents. Using univariate logistic regression, cannabis users had 60% odds of dying from intoxication compared to non-users, OR=.604, 95% CI, .57-.63, p < 0.001. After adjusting for confounders, the odds of experiencing toxicity decreased, OR=.35, 95% CI, .33-.37, p < 0.001. Our study provided evidence of the harm from cannabinoid exposures, underscoring the need for tailored interventions for public safety.

Abstract citation ID: igaf122.2262

### EPIGENETIC AGING AND LINKS BETWEEN CHILDHOOD ADVERSITY AND COGNITION IN LATER LIFE

Marrium Mansoor, and Benjamin Katz, *Virginia Tech, Blacksburg, Virginia, United States*

Adverse childhood events (ACEs) have been linked to worse outcomes throughout life, from poorer academic and workplace performance to compromised physical health, depression and cognitive impairment. Epigenetic age acceleration (EAA) refers to a combined set of factors causing DNA methylation that may help explain these links While these links have been explored in younger and middle aged adults they remain largely unexamined in large, nationally representative samples of older adults. This study utilized data from 3490 older adults in the United States (Mage = 69.5 years, 58.5% female, 75% white) from the Health and Retirement Study. Childhood adversity was measured through an aggregate score including whether participants had trouble with police, repeated a year of school, experienced substance abuse or physical abuse from parents. EAA was quantified using GrimAge, an epigenetic clock that measures the level of DNA methylation to provide an estimate of mortality and morbidity risk. Mediation models were run via PROCESS to test if GrimAge acted as a mediator between ACEs and cognitive performance measured by Serial 7s and Number Series (executive function measures) along with Immediate and Delayed Recall (memory measures). GrimAge fully mediated the association between ACEs and performance on Serial 7s, Number Series, and Delayed Recall, while controlling for age, gender, race, years of education, and depressive symptoms. This supports the hypothesis that childhood adversity may impact cognitive performance in later life through EAA. These results underscore the importance of interventions and policies to support individuals with ACEs as they age.

Abstract citation ID: igaf122.2263

### EXERCISE IMPROVES CLOCK DRAWING TEST IN OLDER ADULTS WITH KIDNEY DISEASE

Diego Redondo-Sáenz[1], and Ulf Bronas[2], *1. Columbia University School of Nursing, New York, New York, United States, 2. Columbia University, New York, New York, United States*

Older patients with chronic kidney disease (CKD) are at high risk of cognitive impairment. We have previously demonstrated a coexistence between higher levels of fitness and psychomotor speed and executive control in older adult patients with CKD. This study investigated the impact of a 6-month home-based walking intervention Vs usual care on the clock drawing test (CDT). The CDT evaluates select cognitive domains, including executive function. Participants (n = 39, mean age of 67.6 (5.0) years; 66.7% women, 71.8% Black) were randomized to intervention or usual care. The intervention included a wearable activity monitor, weekly didactic phone meetings, interactive tools (e.g., reminders), and monthly coach-delivered feedback. The compliance rate was 78%, the mean minutes of exercise per week was 159.9 (149.2) minutes/week. The CDT was scored using Libon's criteria and ANCOVA was used for analysis adjusting for age and education. CDT total score and executive function error score trended higher in the intervention group, compared to the control group at 6-months with a mean difference of 0.48 (95% CI of -0.02-0.97, p=.06), and a mean difference of -.012 (95% CI -.122 to .098. p=.08) respectively. The effect sizes were large at $\eta^2$=.11 and $\eta^2$=.079 respectively. Total CDT score at 6-months was correlated with 6-minute walking distance (r=.36, p=.04) and executive function error score at 6-months was correlated with change in step count (r=-39, p=.03). These findings suggest that in older adults with CKD, a 6-months of home-based exercise walking may be associated with an improvement in executive function and constructional praxis.

Abstract citation ID: igaf122.2264

### IMPACT OF FAMILY DOCTOR SYSTEM ON CHRONIC PATIENTS WITH DISTINCT SERVICE UTILIZATION PATTERNS

Guanggang Feng[1], and Lingxue Sun[2], *1. Anhui University of Finance and Economics, Bengbu, Anhui, China (People's Republic), 2. Shanghai Administration Institute, Shanghai, Shanghai, China (People's Republic)*

This study evaluates the impact of China's Family Doctor System (FDS) on healthcare utilization and costs among chronic patients using group-based trajectory modeling (GBTM) and difference-in-differences (DID) analysis. Data from 306,464 chronic patients (mean age 60.37, 45.6% female) in City S (2018-2023) was analyzed, with 75.9% enrolled in FDS (intervention) and 24.1% non-enrolled (control). Four service utilization trajectory groups were identified through GBTM: low utilization (18.7%), high utilization (23.5%), high community visits (32.2%), and high hospital visits (25.6%). After propensity score matching (PSM), DID analysis revealed FDS significantly increased primary care visits by 28.3% (p < 0.01) and costs by 19.7% (p < 0.05), while reducing secondary/tertiary hospital visits by 15.2% (p < 0.01) and costs by 12.5% (p < 0.05). Subgroup analysis showed the largest effects among historically high hospital users, with a 21.3% reduction in hospital costs (p < 0.001). The SUEST test confirmed model consistency across trajectory groups (p = 0.68). Ongoing regression results further validate FDS effectiveness in optimizing resource allocation by shifting care to primary settings. Policymakers should strengthen FDS through financial incentives and capacity building to enhance chronic disease management. This research provides empirical evidence for deepening hierarchical medical system reforms by demonstrating FDS's role in promoting primary care utilization and reducing costly hospital dependency.

Abstract citation ID: igaf122.2265

### PHOTO-CONVERSION METHODOLOGY IN ZEBRAFISH NEURAL MAPPING

Alejandro Quezada[1], and Adam Roberts[2], *1. California State University, Fullerton, Santa Ana, California, United States,*

*2. California State University, Fullerton, Fullerton, California, United States*

Zebrafish possess unique attributes that make them ideal for brain imaging techniques, particularly those that enable in vivo tracking of neural activity. Their genetic modifiability, translucency, and ease of maintenance compared to other models make them well-suited for such research. Using a fluorescent protein called Kaede, real-time neural activity can be recorded by monitoring its fluorescence. Kaede fluorescence can be observed in two different states—green and red—through a process known as photo-conversion. Exposure to UV light induces an irreversible shift from green to red fluorescence, allowing researchers to track neural activity dynamically. This project aims to optimize the parameters involved in photo-conversion to improve efficiency. Enhancing these parameters will lead to better imaging quality and reduced phototoxicity, ultimately refining our ability to study neural circuitry and the molecular mechanisms underlying disordered behavior.

Abstract citation ID: igaf122.2266

### PHYSICAL ACTIVITY AMOUNT IS ASSOCIATED WITH BETTER ATTENTION AND LESS DAYTIME SLEEPINESS IN OLDER ADULTS

Pin-Shiuan Lee, Yi-Ling Chen, and Wan-Ju Cheng, *National Health Research Institutes, Zhunan Town, Miaoli, Taiwan (Republic of China)*

The natural aging process and inadequate physical activity represent modifiable and non-modifiable risk factors for cardiometabolic conditions, dementia progression, and sleep pathologies. Increased physical activities may reduce the risk of cognitive deterioration and dementia progression. While the association between sleep disorders and cognitive impairment has been established, limited research exists on the relationship between exercise, cognition, and sleep in older adults. This study recruited 147 community-dwelling older adults (62 males: 70.4 ± 6.2 years; 85 females: 69.0 ± 5.6 years) and evaluated their physical activity and sleep patterns using actigraphy. Sleep patterns were measured using Pittsburgh Sleep Quality Index and Epworth Sleepiness Scale and cognitive function was measured by psychomotor vigilance tests. Melatonin level was determined by saliva samples. Circadian rhythm measurements were analyzed by cosinor analysis, generalized additive model, interdaily stability (IS), and intradaily variability (IV). Objective sleep and physical activity pattern (power law exponent alpha, beta coefficient of a linear regression line in log-log space (GGIR), the average activity during the least active 5-h period (L5) and the most active 10-h period (M10), relative amplitude (RA), weekly vigorous, moderate, and sedentary activity, and lag 1440 mins) were retrieved from 14-days actigraphy recordings of activity counts. Results showed that participants with high-amount physical activities manifested higher IS, IV, RA, and 24-h autoregression correlation. Higher relative amplitude is associated with fewer lapses. This study reveals the positive impact of physical exercise on cognitive function and sleep, demonstrating that physical activity amount is associated with individual cognitive function and sleep qualities.

Abstract citation ID: igaf122.2267

### SEDENTARY BEHAVIOR AND SLEEP DURATION: DEPRESSION RISK FACTORS IN OLDER ADULTS PRE- VS POST-PANDEMIC

Anna Egbert[1], and Isa-Marie Kreuzinger[2], *1. St. Joseph's University New York, Brooklyn, New York, United States, 2. St. Joseph's University, Brooklyn, New York, United States*

As the older adult population grows, identifying modifiable lifestyle factors that support mental health is increasingly crucial. The COVID-19 pandemic further disrupted their well-being, exacerbating vulnerabilities. Given their high levels of sedentary behavior and poor sleep—both changeable factors—it is essential to reassess their effects on mental health pre- and post-pandemic. This study examined the relationship between self-reported depressive symptoms (Patient Health Questionnaire–PHQ9), sedentary behavior, and sleep duration using National Health and Nutrition Examination Survey (NHANES) data from U.S. adults aged 60–80 years in 2017-2018 (n = 1,442) and August 2021-August 2023 (n = 1,893). The number of older adults showing minimal to severe levels of depressive symptoms remained rather stable from pre- to post-pandemic (Chi2=2.887, p=.577), with moderate to severe cases increasing from 8% to 9%. Hierarchical regression models showed that race/ethnicity–a significant factor before the pandemic–lost significance post-pandemic, while younger age, female gender, lower education, living alone, and lower economic status remained significant across both periods. Importantly, more time spent on sedentary activities (B=.003, p<.001, CI95%=.002, .004) and inadequate sleep (>7h or < 9h; B = 1.439, p<.001, CI95%=1.038, 1.840) were slightly more strongly linked to depressive symptoms post-pandemic. These findings highlight the need to target sedentary behavior and sleep patterns as modifiable risk factors. Promoting healthier habits could help mitigate mental health risks and improve well-being in this growing population.

### SESSION 7025 (POSTER)

### BRAIN

Abstract citation ID: igaf122.2268

### AN ESTIMATE OF THE PACE OF AGING FROM A SINGLE BRAIN SCAN PREDICTS CHRONIC DISEASE, DEMENTIA, AND MORTALITY

Ethan Whitman[1], Maxwell Elliott[2], Annchen Knodt[1], Terrie Moffitt[1], Avshalom Caspi[1], and Ahmad Hariri[1], *1. Duke University, Durham, North Carolina, United States, 2. University of Minnesota, Minneapolis, Minnesota, United States*

Neuroimaging is a common, non-invasive measure, making it a desirable target for developing aging biomarkers. Current MRI-based aging biomarkers (i.e., brain-age gap) are estimated from cross-sectional associations with chronological age - akin to "first-generation" epigenetic clocks. DunedinPACE, a "third-generation" epigenetic measure, has advantages over first-generation epigenetic clocks because it directly estimates longitudinal aging. We extend this approach to neuroimaging with a "next-generation" neuroimaging-based biomarker of the rate of biological aging. The pace of whole-body aging was determined by tracking physiological decline of 6 organ systems over 20 years in Dunedin Study members. An elastic-net regression model was trained to estimate the pace of whole-body aging using neuroimaging data collected at age 45 from 860 Study members. We call this measure the Dunedin Pace of Aging Calculated from NeuroImaging or "DunedinPACNI." We exported DunedinPACNI to the UK Biobank and ADNI to test associations with disease and decline. DunedinPACNI estimated whole-body aging with a cross-validated accuracy of $r = 0.42$ in Dunedin Study members. In 1,737 ADNI participants, people with faster DunedinPACNI had greater cognitive impairment (MCI: $\beta = 0.27$; $p < 0.001$; AD: $\beta = 0.81$; $p < 0.001$) dementia risk

(HR = 1.76, p < 0.001), and hippocampal atrophy (β=-0.15; p < 0.001). In 42,583 UK Biobank participants, people with faster DunedinPACNI also had greater hippocampal atrophy (β=-0.09; p < 0.001), frailty (β = 0.17; p < 0.001), disease risk (HR = 1.14, p = 0.01), and mortality risk (HR = 1.32, p < 0.001). DunedinPACNI was superior or similar to brain-age gap at predicting clinical outcomes. DunedinPACNI, a novel, exportable MRI biomarker for longitudinal whole-body aging, offers improved opportunity to measure aging from brain structure.

Abstract citation ID: igaf122.2269

### ASSOCIATIONS OF CUMULATIVE VASCULAR RISK FACTORS WITH CEREBRAL PERFUSION

Nazm Rahat[1], Chad Blackshear[2], A.J. Spychalla[3], Farwa Ali[3], Kevin Sullivan[2], Michael Griswold[2], Prashanthi Vemuri[3], and B. Gwen Windham[2], *1. University of Mississippi Medical Center, Jackson, Mississippi, United States, 2. University of Mississippi Medical Center - The MIND Center, Jackson, Mississippi, United States, 3. Mayo Clinic, Rochester, Minnesota, United States*

Lower cerebral perfusion is associated with adverse outcomes, but risk factors are poorly understood. This study examined associations of cumulative systolic blood pressure [SBP], diabetes, and BMI from mid- to late-life with late-life cerebral perfusion, using whole-brain MRI Arterial Spin Labeling (ASL). Cumulative exposures were defined as either average continuous SBP or BMI across visits or number of years with diabetes, hypertension, or obesity. Multiple regression, adjusted for age and gender, estimated ASL (β: 95% CI) among participants without prevalent conditions at baseline in participants with ASL data from the Atherosclerosis Risk in Communities Study (ARIC; Jackson, MS; n = 100; age=81±3 years; 65% Female; 100% Black participants; 34 years retrospective risk factor assessments (1987-2021); pulsed ASL, 8/2021-1/2024) and the Mayo Clinic Study of Aging (MCSA; Rochester, MN; n = 537; age=71±13 years; 36.3% Female, 100% White participants; 18 years retrospective risk factor assessments (2005-2023); pseudo-continuous ASL 2/2023-3/2024). A 10 mmHg higher cumulative SBP was associated with a 0.04 lower ASL (MCSA β=-0.04; -0.01, -0.07; ARIC β=-0.04; -0.09, 0.01). A 10 kg/m2 higher cumulative BMI was associated with a 0.30 lower ASL (-0.51, -0.08) in ARIC and 0.03 lower ASL (-0.16, 0.10) in MCSA participants. Every five additional years of diabetes exposure was associated with a 0.03 (-0.06, -0.01) lower ASL in ARIC and 0.11 (-0.27, 0.05) lower ASL in MCSA participants. High BP, obesity, and diabetes may contribute to poor cerebral perfusion over extended exposures from midlife. Longer risk factor assessment periods and different ASL techniques may explain differences in the cohorts.

Abstract citation ID: igaf122.2270

### CHARACTERIZATION OF MULTISCALE REGULATION OF THE BRAIN IN AGING AND AGE-RELATED CONDITIONS: A COMPREHENSIVE REVIEW

Haoru Li[1], Brad Manor[2], Lewis Lipsitz[3], and Junhong Zhou[4], *1. Vanderbilt University, Nashville, Tennessee, United States, 2. Hebrew SeniorLife/Harvard Medical School, Boston, Massachusetts, United States, 3. Hebrew Senior Life, Boston, Massachusetts, United States, 4. Harvard Medical School Hebrew SeniorLife, Roslindale, Massachusetts, United States*

As the core for many important functions, the brain activities are regulated by numerous underlying neuro-biophysiological elements communicating and interacting over multiple temporospatial scales. Studies have emerged to characterize such complex multiscale regulation of the brain (i.e., brain complexity), especially under the influences of aging and age-related conditions/diseases, via advanced signal-processing techniques, and obtained exciting insights into the pathology of brain-related neurodegenerative diseases that cannot be characterized using traditional single-scale measures. We thus performed a literature review to comprehensively summarize these achievements/progress in this area. Seven electronic databases were searched. The key information (e.g., participant cohort) of 43 eligible studies was extracted. These studies were all cross-sectionally designed with relatively small sample size. The brain signals in older adults with (n = 40) (i.e., Parkinson's disease (n = 5), Alzheimer's disease (n = 31) and other types of conditions (n = 4)) and without (n = 3) age-related conditions were recorded using electroencephalogram (n = 23), functional MRI (n = 10) and near-infrared-spectroscopy (n = 2), and magnetoencephalography (n = 8). The brain complexity was measured by multiscale entropy, transfer entropy (TE) or partial TE (i.e., quantifying the efficiency of the information exchange between brain regions). Compared to younger-age control or those with normal aging, individuals in older age and/or with age-related conditions had altered multiscale regulation of the brain (e.g., lower brain complexity), and lower complexity was associated with advanced disease stage and poorer functional performance. The evidence level was relatively low. It is thus highly demanded to confirm these findings in studies with larger sample size, and explore the causal relationship between brain complexity and functions.

Abstract citation ID: igaf122.2271

### CLINICAL CORRELATION OF VISUOSPATIAL EVALUATION WITH DEMENTIA DIAGNOSIS IN MEMORY CLINIC

Rui Huang, Malini Nair, Viviana Obando, Surya Sunil, and Anil Nair, *ADC, Braintree, Massachusetts, United States*

Background: We attempted to find if different level of abilities to draw three-dimensional cube (stereopsis) as measured by cube-copying test can be used as an early quick screening tool for preclinical dementia diagnosis. Methods: The study was conducted at the Alzheimer Disease Center, a suburban memory clinic serving the south shore of Boston MA. We collected retrospective data by chart review on patients during the period July 2024 to March2025. A retrospective database search included subjects with memory impairment with a diagnosis code and stereopsis score. All data analyses were performed using SPSS. Pearson Correlation was performed for correlations of age and education. Results: We identified 42 subjects with stereopsis and diagnosis code. Mean Stereopsis score was 8.08 (SD 3,80) for blood Tau negative patients, 8.19 (SD 3.58) for blood Tau positive patients. Stereopsis score was statistically positively significantly related to sex (t = 2.34, p = 0.025) and education (t = 2.66, P = 0.029). Mean age was 67.5 (SD 7.32), mean education 13,64 years (SD 1.92), 45.2% Caucasians and 64.30% females. We found a insignificant association of stereopsis to the initial diagnosis code in the clinic (p = 0.896). Conclusion: Performance on the cube-copying test can be influenced by factors such as sex, education, The cube-copying test remains to potential screening tool and is not a definitive diagnostic tool for AD or other dementias and should be used in conjunction with other cognitive assessments.

Abstract citation ID: igaf122.2272

### MULTI-FOCAL SYMPTOM-BASED NEUROMODULATION FOR MEMORY AND DEPRESSION IN DEMENTIA

Jasmine Morgan[1], Alisha Syed[2], Wanting Yu[2], Judy Zheng[3], Alexander Opitz[4], Brad Manor[5], Alvaro Pascual-Leone[6], and Davide Cappon[6], *1. Hebrew SeniorLife - Marcus Institute for Aging Research, Boston, Massachusetts, United States, 2. Hebrew SeniorLife, Boston, Massachusetts, United States, 3. Marcus Institute for Aging Research, Roslindale, Massachusetts, United States, 4. University of Minnesota, Minneapolis, Minnesota, United States, 5. Hebrew SeniorLife/Harvard Medical School, Boston, Massachusetts, United States, 6. Hebrew SeniorLife & Harvard Medical School, Boston, Massachusetts, United States*

Major depressive disorder in the context of dementia (MDD + D) is highly prevalent, on the rise, and a leading cause of impairment in older adults. It accelerates functional decline, worsens health outcomes, hastens long-term care admission, increases caregiver burden, and raises mortality risk. MDD+D involves brain network dysfunction affecting mood and memory. Transcranial direct current stimulation (tDCS) improves mood in MDD, while transcranial alternating current stimulation (tACS) enhances memory in dementia. Combining these neuromodulation techniques offers a promising dual-targeted intervention. This study evaluates the safety and feasibility of a home-based, caregiver-administered tACS + tDCS intervention. Ten participants (8 female, average age 73 ± 7.13) underwent a 4-week intervention consisting of 20-minute sessions, five days per week, targeting the left prefrontal cortex (tDCS) and left angular gyrus (tACS). Caregivers, trained by study staff, administered the stimulation with initial tele-supervision before continuing independently with remote monitoring. Participants completed questionnaires to assess safety and feasibility. Following the intervention, participants assessed their experience. All ten participants completed the study, with eight adhering to 100% of sessions, one completing 95%, and one 90%. Across 197 total sessions, the most reported side effect was scalp sensations (tingling, itching), mild in 55%, moderate in 21%, and severe in 1%. 90% of participants expressed willingness to repeat the program and continue for a longer duration. A home-based tACS + tDCS program is feasible, safe, and well-tolerated, with high adherence and satisfaction, supporting further investigation into at-home tACS + tDCS treatment for MDD+D.

Abstract citation ID: igaf122.2273

### SPATIAL NAVIGATION INTERVENTION INFORMED BY REGION-SPECIFIC BRAIN ACTIVATION FOR INDIVIDUALS AT RISK FOR DEMENTIA

Pierfilippo De Sanctis[1], Sunil Agrawal[2], Jiawei Chen[2], and Joe Verghese[3], *1. Albert Einstein College of Medicine, Bronx, New York, United States, 2. Columbia University, New York, New York, United States, 3. Stony Brook University, Stony Brook, New York, United States*

Up to a third of dementia cases may be preventable by engaging in protective behaviors, such as staying cognitively active, according to observational data. Yet, current cognitive training protocols to delay dementia onset often fall short. This project focuses on spatial navigation (SN), the ability to travel familiar/unfamiliar environments. Tau and amyloid-beta accumulation starts in regions subserving SN. Even though SN difficulties present an important target, there are few clinical trials aimed at SN. We developed a full-immersive virtual-reality (VR) maze which participants learn to navigate and use Mobile Brain Body Imaging (MoBI) to record body movement and EEG to record and analyze brain activity during active movement through space. We designed VR mazes to induce different navigational strategies (allocentric and egocentric) at different periods (Stand/Encode and Walk/Navigate) of maze learning. Allocentric and egocentric spatial strategies rely on mediotemporal and posterior parietal cortex regions, respectively. So far, we collected data in 10 individuals showing that Stand/Encode time increases as maze complexity increases. Furthermore, we show a significant increase in theta power as participants navigate towards an intersection that requires a memory-based directional decision. Region-specific modulations in theta (3-7Hz) and alpha (8-12Hz) power we enable us to identify, dissociate, and track participants' brain dynamics applying mediotemporal-based allocentric and posterior parietal-based egocentric navigational strategies and test relationships with improvements in SN. This pilot study will position us to design a future randomized clinical trial to test efficacy to improve navigational abilities, and thereby delay cognitive decline in older adults at-risk for dementia.

Abstract citation ID: igaf122.2274

### THE MENTAL HEALTH CONSEQUENCES OF TRAUMATIC BRAIN INJURY AMONG ADULTS 50 AND OLDER

Laura Mehegan, G. Rainville, and Rachel Lazarus, *AARP, Washington, District of Columbia, United States*

In September 2024, AARP fielded a nationally representative survey to explore the prevalence and mental health outcomes among adults 50 and older who have experienced a traumatic brain injury (TBI) (N = 3,657). The identification of possible/probable TBI from nine different causes of head injury (e.g., falls, car wrecks, sports injuries) is based on a new scoring system developed by scientists at the Centers for Disease Control and Prevention (Daugherty et al., 2024). Previously, the prevalence of TBI was based on hospital records but the new scoring system identifies TBIs treated in other settings or not treated at all and are based on self-report. Overall, the results show that three in 10 (30%) adults 50-plus have likely experienced a TBI in their lifetime. Adults 50-plus who have had one or more possible/probable TBI at any point in their life fare worse on mental health outcomes -- higher levels of anxiety and depression and lower mental well-being scores -- compared to adults who have not experienced a head injury or have experienced a head injury unlikely to be TBI. Adults who have experienced multiple TBIs or a TBI after age 50 fare even worse in these mental health domains. Because adults in this age group are under-studied, this research represents a step forward in understanding the prevalence of TBI in this age group as well as the consequences for mental health outcomes. It also highlights the need for strategies to prevent TBI at any age with emphasis on fall prevention among older adults.

### SESSION 7030 (POSTER)

### CARE VALUES AND PREFERENCES

Abstract citation ID: igaf122.2275

### CARE NETWORKS, UNMET NEEDS, AND WELL-BEING IN OLDER ADULTS: EVIDENCE FROM THE NATIONAL AGING TRENDS STUDY (NHATS)

Sophie Arnold, *Boston University, Boston, Massachusetts, United States*

A growing body of research suggests the potential benefits of receiving help from multiple caregivers, yet little is known about whether more extensive support networks reduce care gaps and improve well-being among people aged 65 and older. Using cross-sectional data from Round 13 National Health and Aging Trends Study (NHATS), this study investigates whether having more than one primary caregiver, including both informal and formal supports, is associated with fewer unmet needs and better psychosocial outcomes. The analytic sample comprises community- and facility-dwelling participants aged 65 and older who reported needing assistance with at least one activity of daily living (ADL) or instrumental activity of daily living (IADL). Estimations include the prevalence of having one versus multiple caregivers and compare rates of self-reported ADL/IADL unmet needs and indicators of psychosocial well-being (self-reported health, feelings of pleasure, loneliness, depression, anxiety, and worry). Multivariable logistic regression models will be used to adjust for sociodemographic and health covariates to examine whether care network size independently predicts these outcomes. I hypothesize that older adults with multiple caregivers report lower levels of unmet needs, with variations in psychosocial well-being among care recipients and care provider relationship types—such as family, paid aides, friends, or unpaid care providers. These findings contribute to understanding how caregiver arrangements shape care sufficiency and psychosocial outcomes, with implications for optimizing caregiver coordination and resource allocation. Furthermore, addressing gaps in knowledge regarding multiple caregivers and older adult well-being to inform policies that improve care quality and accessibility in aging populations.

Abstract citation ID: igaf122.2276

### CAREGIVING BARRIERS IN PARKINSON'S DISEASE WITH COGNITIVE IMPAIRMENT: A QUALITATIVE STUDY

Yuncheng Huang[1], Yijing Hua[1], Jing Wang[2], Bei Wu[3], and Juan Li[1], *1. Huashan Hospital Fudan University, Shanghai, China (People's Republic), 2. University of New Hampshire, Durham, New Hampshire, United States, 3. NYU Shanghai, Shanghai, China (People's Republic)*

Background: Parkinson's disease (PD) is often accompanied by cognitive impairments (CI), which lead to a decline in the quality of life for patients and increasing pressure on caregivers. However, there are currently few qualitative studies exploring the care barriers for patients with Parkinson's disease and cognitive impairment (PD-CI). Objective: This study aimed to identify unmet care needs, analyze caregiving challenges, and propose solutions by triangulating perspectives from people with Parkinson's, caregivers, and healthcare professionals. Methods: Semi-structured interviews were conducted with ten people with Parkinson's and cognitive impairment, ten family caregivers, and eleven healthcare professionals in the Huashan Hospital, Fudan University in Shanghai, (2024.10-2025.1). Participants were recruited through purposive sampling, with cognitive impairment identified by healthcare professionals. Interviews were audio-recorded, transcribed, and analyzed using reflexive thematic analysis following Bronfenbrenner's ecological systems theory. Results: The study extracted four themes: ①Microsystem - diagnostic uncertainty, stigma and self-identity, self-management challenges, and motivation difficulties; ② Mesosystem - caregivers' struggles (communication barriers, household dynamics, caregiving stress, and difficulties in care coordination); ③ Exosystem - Social challenges; ④ Macrosystem - the influence of cultural and structural factors (care access and resource distribution). Conclusion: The care pressure of patients with PD-CI is great, and they face many challenges. It is possible to try to build a "patient-medical staff-family member" co-participatory care model to improve the quality of care and quality of life of patients with PD-CI.

Abstract citation ID: igaf122.2277

### PILOT OF A PRIORITIES ALIGNED DECISION-MAKING MICROSKILLS CURRICULUM

Jennifer Oullet[1], Eliza Kiwak[1], Loni Belyea[2], Brenda Nettles[3], Mallory Brown[4], Mary Tinetti[5], Timothy Farrell[6], and Natalie Sanders[6], *1. Yale School of Medicine, New Haven, Connecticut, United States, 2. Hello Alpha, Southern Pines, North Carolina, United States, 3. Johns Hopkins School of Nursing, Baltimore, Maryland, United States, 4. The University of North Carolina at Chapel Hill, Chapel Hill, North Carolina, United States, 5. Yale University, New Haven, Connecticut, United States, 6. University of Utah, Spencer Fox Eccles School of Medicine, Salt Lake City, Utah, United States*

Patient Priorities Care (PPC) provides an approach for identifying patient health care priorities and aligning care with those priorities. Training in PPC has demonstrated high overall satisfaction and its implementation is associated with positive patient centered outcomes. Yet, the current training approach is time and resource intensive. This work describes the adaptation and piloting of a PPC curriculum to address these barriers. An interdisciplinary group of clinician educators collaborated to divide PPC content into 8 core skills and organize it into graphic power point and PDF documents. Educators are piloting materials with learners. A baseline survey was administered to pilot participants (educators and learners). Participants will complete a post-pilot survey to assess feasibility and ease of use of materials. Eighteen learners have completed the baseline survey. Most learners were from either Internal Medicine (22%) or Geriatric Medicine (61.1%) disciplines. More than half (55.6%) had some prior training in priorities-aligned care. On a scale of 1-5 (1 not confident and 5 extremely confident), learners reported being moderately confident (range 22.2%-44.4%), quite confident (range 27.8%-44.4%) or extremely confident (range 11.1%-22.2%) in the 8 core skills. A high level of baseline confidence in the core skills will allow assessment of content feasibility. Next steps include evaluating ease of use of materials and refining the materials and assessment methods based on feedback. We will then disseminate curricular materials and assessment methods to educators nationally. We hope to demonstrate that the adapted curriculum will ensure durable implementation of PPC across multiple health care settings.

Abstract citation ID: igaf122.2278

### SOCIAL SUPPORTIVE DAY SERVICES: A PROMISING, YET UNDERUTILIZED RESPITE OPTION FOR DEMENTIA CAREGIVERS

Adriana Hernandez, Setarreh Massihzadegan, and Caitlin Coyle, *University of Massachusetts Boston, Boston, Massachusetts, United States*

Social supportive day programs (SSDP) offer socialization and activities in a group setting for individuals who require a more structured setting, but need minimal assistance with activities of daily living. SSDPs primarily serve older adults with Alzheimer's disease and related dementias (ADRD), as well as those with physical and functional limitations. Along with positive physical and psychological outcomes for participants, SSDPs provide a form of respite for caregivers. Although promising, utilization rates of SSDPs are varied as are the models of delivery. SSDP may be an essential respite option for dementia caregivers if adequate attention, funding, and resources is given to these programs. In this paper we detail recent efforts by researchers to assess demand for SSDP services and identify barriers and facilitators to SSDP participation. Taking a mixed-methods approach, data from both a survey of residents age 50 and older (N = 1,076) and in-depth interviews with current and past participant families, nonparticipant families, and program staff. Results of this analysis indicate that nearly half of caregivers are providing fewer than 10 hours of care per week and still working full-time. As well, lack of awareness about the target population for the program and financial barriers were identified. Implications of these findings for the models of delivery of supportive day programs will be presented. These findings highlight SSDP as an underdeveloped form of respite and a growing demand for more respite options for dementia caregivers, particularly in smaller towns that provide access to SSDP.

Abstract citation ID: igaf122.2279

### UNDERSTANDING DIFFERENCES IN TREATMENT BURDEN AMONG OLDER ADULTS WITH MULTIPLE CHRONIC CONDITIONS

Anne Wang[1], Jie Liang[2], Lea Kiefer[1], Marcia Mecca[3], Lilian Dindo[1], Terri Fried[4], and Aanand Naik[2], *1. Michael E. DeBakey VA Medical Center, Houston, Texas, United States, 2. UTHealth Houston, Houston, Texas, United States, 3. Yale School of Medicine, New Haven, Connecticut, United States, 4. West Haven VA Medical Center, West Haven, Connecticut, United States*

Older adults managing multiple chronic conditions (MCCs) often need to balance various medications, treatments, and interventions, so this group of older adults is expected to experience healthcare burden. In this study, we examined the treatment burden of a group of 397 veterans who were older than 65 with three or more chronic conditions and/or ten or more medications. Of this group, 115/397 surprisingly reported no burden (NB) on the Treatment Burden Questionnaire (overall score = zero) while 282/397 reported an average score of 50 during baseline data collection. A chi-square test was run on different demographic variables to determine differences between participants reporting NB versus some burden (SB). NB and SB groups had older adults of similar race, gender, ethnicity, housing status, and educational level. However, older adults reporting NB more frequently reported perfect scores on the PROMIS questionnaire (NB: 48.7%; SB: 17.4%) and a 27/30 (90%) or better on the WBS questionnaire (NB: 52.2%; SB: 19.9%). Older adults reporting SB more frequently reported using assistive devices (NB: 20%; SB: 34.8%) and feeling as if every task was effortful (NB: 23.5%; SB: 40.8). These findings suggest that despite similar background and management of MCCs, older adults with minimal functional and mobility limitations often report NB. Furthermore, perceptions of treatment burden are related to perceptions of wellbeing and quality of life.

### SESSION 7035 (POSTER)

### CLIMATE AND AGING

Abstract citation ID: igaf122.2280

### CHILDHOOD ADVERSITY AND NATURAL DISASTER EXPOSURE INTERACT TO IMPACT OLDER ADULT COGNITIVE FUNCTIONING

Karen Lawrence, and Yeon Jin Choi, *University of Kentucky, Lexington, Kentucky, United States*

The frequency and severity of natural disasters are increasing due to climate change; these events may be perceived as stressful or traumatic. Studies have shown that childhood trauma is associated with poorer cognitive function in older adults yet research on the impact of natural disasters on this relationship is rare. Leveraging data from the Health and Retirement Study, we investigated whether having experienced a natural disaster moderates the relationship between childhood adversity (range: 0–4 adversities) and cognitive function in 12,995 adults 50+ years of age [Mage=64.6, SD = 10.1]. Test scores from total word recall, serial 7's test, and backwards counting were summed to calculate an overall cognitive function score (range: 0-27). At least one childhood adversity was experienced by 35.6% and a natural disaster by 17.9% of the sample. In a weighted regression model, adjusted for sociodemographic characteristics and health conditions, the interaction between childhood adversity and natural disaster exposure was associated with .25 points lower cognitive function score [$\beta$ = -.25, 95%CI: -.45, -.05]. Respondents with childhood adversity had significantly lower cognitive function than those without, when exposed to a natural disaster. Among those who experienced a natural disaster, the estimated cognitive function score was 15.56 for those without childhood adversity. Those who experienced one, two, three, or four adversities had lower cognitive function at 15.17, 14.78, 14.39, and 14.00, respectively. These findings suggest that natural disaster exposure is a potential environmental stressor that can exacerbate the effects of childhood trauma on cognitive function outcomes later in life.

Abstract citation ID: igaf122.2281

### OLDER AGE PREDICTS BETTER PSYCHOLOGICAL WELL-BEING EVEN AFTER EXPERIENCING A DISASTER

Esha Azhar, and JoNell Strough, *West Virginia University, Morgantown, West Virginia, United States*

Climate change is associated with the increased prevalence of extreme weather and disasters, which can pose negative consequences for well-being. Yet, post-disaster benefits of aging for well-being have been found, such that the benefits were more pronounced among those who had experienced a disaster versus not (Strough et al., 2023). Aiming to replicate this finding, adult age differences in psychological well-being were compared among people who had experienced a disaster versus not in 2023. An exploratory, secondary analysis of data from the Understanding America Study (UAS), a national US panel, was conducted. Data from two surveys, UAS 588 (N = 10,193) and UAS 592 (N = 9,591), containing panelists' self-reported experience with disasters (e.g. wildfires, hurricanes, earthquakes, etc.), and self-reported psychological well-being in the last 14 days (e.g. "Feeling nervous, anxious, or on edge", "Feeling down, depressed, or hopeless", etc.) respectively, were merged and analyzed. A multiple linear regression analysis predicting psychological well-being was significant, $F(2,9202) = 328.37$, $R^2 = .10$, p

< .001. Significant predictors of worse psychological well-being were younger age, disaster exposure, annual household income below $75k, education less than a bachelor's degree, and being female. A significant age by disaster interaction indicated that among those who had experienced a disaster, the association between older age and better psychological well-being was even stronger compared to those who had not experienced a disaster. We find that older adults possess better psychological well-being overall and post-disaster, indicating this group's resilience and ability to cope with adverse circumstances.

Abstract citation ID: igaf122.2282

### SOCIETIES IN MOTION: UNDERSTANDING AGE DIFFERENCES IN THE PERCEIVED IMPACT OF SOCIETAL TRANSITIONS

Patrick Klaiber, John Bechara, Loes Abrahams, Bell Piyasinchai, Tom Junker, Meeke Hoedjes, and Stefan Bogaerts, *Tilburg University, Tilburg, Noord-Brabant, Netherlands*

Societal transitions such as climate change and digitalization are reshaping daily life, yet individuals differ in how they experience their impact. Despite growing research on these changes, little is known about age differences in their perceived impact. Using data from the Tilburg University Monitor of Social and Behavioral Issues, a representative sample of the Dutch population, we analyzed responses from 4,891 participants aged 16-96 (M = 54.75, 52% female) who completed an online questionnaire in December 2024. Participants rated the perceived impact of six societal transitions: (1) climate change and sustainability, (2) digitalization, (3) diversity and inclusion, (4) demographic changes, (5) international conflicts, and (6) lack of living space. Structural equation modeling was used to examine age as a predictor of both a generalized sense of impact (common factor) and the specific impact of each transition. Our findings showed clear age-related differences in the perception of societal changes. Older adults generally perceived societal transitions as less impactful than younger adults. However, when adjusting for this general trend, older adults felt more affected by climate change, demographic shifts, and international conflicts, while they felt less impacted by digitalization, diversity and inclusion, and housing shortages. These findings suggest that while older adults may feel generally more detached from societal change, they do recognize the influence of transitions that pose broad, existential challenges. This study provides first insights into generational differences in the experience of societal change, with important implications for designing inclusive policies that address the concerns of all age groups.

Abstract citation ID: igaf122.2283

### THE EFFECT OF TROPICAL CYCLONE ON COGNITIVE FUNCTION IN OLDER ADULTS: A LONGITUDINAL STUDY FROM THAILAND

Fei Sun[1], Jin Ke[2], and Phatchanun Vivarakanon[3], *1. Michigan State University, East Lansing, Michigan, United States, 2. Huazhong University of Science and Technology, Wuhan, Hubei, China (People's Republic), 3. Boromarajonani College of Nursing, Nakhon, Lampang, Thailand*

In the context of climate change, tropical cyclones (TCs) represent an increasingly severe threat to human health, particularly among vulnerable populations such as older adults. This study investigated the impact of TC exposure on cognitive function among older adults in Thailand, focusing on specific cognitive domains and exploring potential underlying mechanisms. Data were obtained from two key sources: the Health, Aging, and Retirement in Thailand (HART) survey and the Emergency Events Database (EM-DAT). These datasets were integrated to assess both the short- and long-term effects of TC exposure using a fixed-effects model. Cognitive function was measured across three key dimensions: memory, calculation, and time orientation. Additionally, the study examined depression, hypertension, and social isolation as potential pathways through which TCs might affect cognitive health. The findings revealed that exposure to TCs had a significant and lasting negative impact on the calculation dimension of cognition, whereas the effects on memory and time orientation were either absent or short-lived. Specifically, individuals exposed to TCs demonstrated significantly lower calculation scores on the day of exposure, and this decline persisted for up to four years post-exposure. Further analyses suggested that depression, exacerbation of hypertension, and increased social isolation were contributing factors to this cognitive decline. These findings underscore the long-term cognitive risks associated with extreme weather events and highlight the need for targeted public health interventions. Policies and practices to mitigate the cognitive impact of TCs among older adults, particularly in climate-vulnerable regions are needed.

Abstract citation ID: igaf122.2284

### THE RELATIONSHIP BETWEEN ENERGY POVERTY AND DEPRESSION: FOCUSING ON AGE AS MODERATOR

Haeun Kim, Nayeong Gwon, and Yeonjung Lee, *Chung-Ang University, Seoul, Republic of Korea*

Climate change, including more frequent temperature changes and extreme weather conditions affects this society in many different ways. Given that older adults are disproportionally vulnerable to climate change, it has brought attention to another aspect of poverty, which is energy poverty. Energy poverty (EP) refers to the inability to access and use essential levels of energy for daily living such as heating and air conditioning for cooling. Studies showed that older adults experience higher levels of depression, and they can be more vulnerable to EP than younger counterparts. This study examines the association between EP and depression, and how this relationship will vary by age. Though the suicide rate and poverty among older adults in Korea are extremely high compared to other countries, no studies have paid attention to the potential harmful association between the difficulty in affording energy necessary for daily living and depression. Hierarchical regression analysis and linear prediction were performed using the 2023 Korea Welfare Panel Study (N = 12,986). Results show that those who experienced EP are likely to show higher levels of depression. However, the relationship between EP and depression was different by age. While EP was positively associated with depression for the age group who are 65 and older, the relationship was negative for the younger age group who are under 65. Findings have implications for supporting older adults who lack the necessary energy for daily living, helping to prevent the exacerbation of their depressive symptoms.

Abstract citation ID: igaf122.2285

### WHO WORRIES MORE?: GENERATIONAL DIFFERENCES IN CLIMATE ANXIETY IN SOUTH KOREA

Giyeon Kim[1], Takashi Yamashita[2], and Daniel Jimenez[3], *1. Chung-Ang University, Seoul, Republic of Korea, 2. University of Maryland, Baltimore County, Baltimore, Maryland, United States, 3. University of Miami Miller School of Medicine, Miami, Florida, United States*

Climate change poses a growing threat to public health, disproportionately impacting older adults who are more vulnerable to extreme weather events such as heatwaves and floods. The present study aims to examine generational differences in climate anxiety among South Korean adults using nationally representative data from the 2024 Social Survey. We analyzed climate change prevalence and associated factors across three age groups: young adults (aged 20-39), middle-aged adults (aged 40-59), and older adults (aged 60 and older). Results indicate a significant increase in climate anxiety from 2022 to 2024, with 53.2% of respondents expressing anxiety. Among them, 41.1% reported moderate anxiety, while 12.1% experienced severe anxiety. Climate anxiety levels showed generational differences: Climate anxiety levels were higher among middle-aged adults (57.5%) and younger adults (52.15%) than older adults (50.3%). Additionally, across all three age groups, those who are younger, women, and living in urban areas tended to have higher levels of climate anxiety. This suggests possible generational differences in risk perception, adaptation, or psychological resilience. As climate-related disasters become more frequent, these findings underscore the need for targeted public health interventions. Policies should prioritize psychological resilience-building and developing age-sensitive risk communication strategies. Future research should explore coping mechanisms, long-term mental health impacts, and policy implications for different generations in the context of climate challenges.

### SESSION 7040 (POSTER)

### COGNITION I

Abstract citation ID: igaf122.2286

### AIR POLLUTION, BLOOD LIPIDS, AND COGNITIVE PERFORMANCE FROM THE MULTI-ETHNIC STUDY OF ATHEROSCLEROSIS (MESA)

Xi Pan[1], Robyn McClelland[2], Lilah Besser[3], Jana Hirsch[4], Joel Kaufman[2], Jing Cao[5], and Timothy Hughes[6], *1. Texas State University, San Marcos, Texas, United States, 2. University of Washington, Seattle, Washington, United States, 3. University of Miami, Boca Raton, Florida, United States, 4. Drexel University, Philadelphia, Pennsylvania, United States, 5. UT Southwestern Medical Center, Dallas, Texas, United States, 6. Wake Forest University School of Medicine, Winston-Salem, North Carolina, United States*

The current study aimed to identify the role of blood lipids in the air pollutant-cognition relationship. The study sample consisted of 1,518 adults aged 45+ in the Multi-Ethnic Study of Atherosclerosis (MESA). Outdoor residential concentrations of PM2.5, NO2, and NOx between Exams 1 (2000-2002) and 5 (2010-2012) were assessed using validated spatiotemporal models. Outcomes were three cognitive test scores of Cognitive Abilities Screening Instrument (CASI), the Digit Symbol Coding (DSC), and the Digit Span (DS) tests at Exam 6 (2016-2018). Total cholesterol (TC) and low-density lipoprotein cholesterol (LDL-C) concentrations in blood at Exam 5 were evaluated as mediators or modifiers using linear regression models. The inverse odds ratio weighting approach was used for mediation analyses. Models assessed gender and race/ethnicity related heterogeneity in air pollutant-cognition and blood lipid-cognition associations. The direct effect of NO2 and NOx on DSC scores was significant (ß=-2.96 for NO2; ß=-3.20 for NOx, per interquartile range increase), but there was no evidence of mediation by cholesterol levels. Interaction terms of air pollutants and LDL-C were significantly associated with CASI scores (ß = 0.02, $p = 0.01$ for PM2.5; ß = 0.03, $p < 0.001$ for NO2; ß = 0.02, $p < 0.01$ for NOx). Associations of DSC scores with NO2 and NOx were only significant among Black participants (ß = -7.11 for NO2; ß = -6.18 for NOx). Findings of the study suggest that air pollution monitoring and control may be effective for protecting brain health and delaying the onset of cognitive impairment and dementia in late adulthood.

Abstract citation ID: igaf122.2287

### CAN SOCIAL ACTIVITY PARTICIPATION ATTENUATE AGED-RELATED COGNITIVE DECLINE? A 12-YEAR LONGITUDINAL ANALYSIS

Layla Katharine Santana[1], Hongdao Meng[1], Debra Dobbs[1], Yujun Liu[2], Angela Grippo[3], and Jamie Mayer[3], *1. University of South Florida, Tampa, Florida, United States, 2. Northern Illinois University, Naperville, Illinois, United States, 3. Northern Illinois University, DeKalb, Illinois, United States*

As populations age, the risk of cognitive decline presents significant public health challenges. While prior research highlights the role of lifestyle factors in cognitive health, less is known about the long-term protective effects of social engagement. Our previous cross-sectional study demonstrated that social participation moderates the relationship between aging and cognitive function in older adults. Building upon these findings, this study investigates how social participation influences cognitive trajectories in aging populations using data from the Health and Retirement Study (HRS) from 2008 to 2020. The sample included 6,109 adults aged 50 and older. Cognitive function scores were assessed across four waves (2008, 2012, 2016, 2020), and social activity participation was measured using a validated 10-point scale. A linear mixed-effects regression model, implemented in Stata, was used to estimate changes in cognition while accounting for key sociodemographic and health-related covariates. The mean participant age was 65.4 years ($SD = 8.87$); 57.8% were female, and 83.8% were White. Findings revealed that higher social activity participation predicted better cognitive function, with particularly beneficial effects observed in older age. Notably, the interaction effect indicated that increasing social engagement over time may slow cognitive decline. By examining these dynamics longitudinally, we provide deeper insight into the long-term protective effects of social participation. These findings can inform targeted interventions and policies aimed at supporting cognitive resilience in aging populations. Addressing barriers to social engagement remains critical for enhancing not only social well-being but also cognitive health among older adults.

Abstract citation ID: igaf122.2288

**COMMUNITY-BASED E-HEALTH PROGRAM ON SELF-CARE AND COGNITION OF OLDER ADULTS: MULTI-SITE CLUSTER-CONTROLLED TRIAL**

Vivien Xi Wu[1], Xin Yi Yap[1], Poh Choo Tan[2], and Wai SanWilson Tam[1], *1. National University of Singapore, Singapore, Singapore, 2. Changi General Hospital, Singapore, Singapore*

Objective: The growing prevalence of chronic diseases among the older population has become a global public health concern. This study aims to examine the effects of a 12-week Community-based eHealth Program (CeHP) in enhancing the self-care, cognition and health outcomes for community-dwelling older adults with chronic diseases. Design, setting and participants: A non-randomized cluster-controlled trial was conducted. Participants were recruited from 11 Active Ageing Centers (AACs) affiliated with Community Health Posts located in the Eastern region of Singapore. Six AACs served as the intervention group where five AACs served as control. The CeHP consisted of seven in-person sessions at the AACs and five virtual sessions through Care4Senior App. The outcome measures included self-care abilities of chronic illness, cognition, health literacy, healthy ageing practice, empowerment, social support and physical function at baseline, immediate post-intervention and 6-month follow-up. Repeated measure analysis was conducted using linear mixed effect models. Results: A total of 108 participants (n = 61 in intervention; n = 47 in control) completed the study. The CeHP intervention resulted in significant effect on the cognition among community-dwelling older adults with chronic diseases. However, no significant difference was observed on other health outcomes. Conclusion/Implications: eHealth solutions are gaining prominence in improving quality of care and changing how individuals manage their health. However, disparities in digital access, technological proficiency and eHealth literacy continue to impact on the vulnerable older population. Further research could continue to explore eHealth interventions and support system to minimize the barriers to eHealth uptake among the older adults.

Abstract citation ID: igaf122.2289

**DID YOU CARE FOR THE ORCHIDS THIS WEEK? OUTCOMES OF PERSON-CENTERED COGNITIVE THERAPY FOR PARKINSON'S DISEASE**

Magdalen Balz, *University of Massachusetts Boston, Boston, Massachusetts, United States*

Motor deficits in people with Parkinson's disease (PwPD) are recognized by healthcare professionals as a hallmark feature of the diagnosis. However, PwPD also experience cognitive decline that negatively impacts quality of life and communication. Cognitive treatment in PwPD has received less attention in clinical care and research. This intervention study aimed to increase functioning in PwPD by applying cognitive compensatory strategies in a telehealth, group therapy program, BrainFit. Based on schedule availability, 3 participants elected to begin BrainFit treatment immediately, and 4 participants elected to join the waitlist, non-treatment group. All participants (N = 7, 56-74 years old) completed a comprehensive cognitive assessment and executive functioning questionnaire. BrainFit participants generated measurable, personally relevant goals using Goal Attainment Scaling (GAS). Goals targeted diverse objectives, from increasing consistency with medication management to following a schedule to care for household orchids. All participants, including the non-treatment group, repeated assessments at post-treatment and again at 3-month post-treatment. At each assessment, BrainFit participants also measured their status with personal goals. Descriptive analyses were applied to examine pre, post, and 3-month post-treatment change. BrainFit participants improved in at least one personal goal from pre to post-treatment. Two out of 3 BrainFit participants maintained gains with a personal goal at 3-month post-treatment. Cognitive assessment results varied across all participants at pre, post, and 3-month post-treatment. This study was built on work from Spencer and colleagues (2020) and further supports cognitive compensatory strategy intervention for PwPD. Findings provide novel contributions by investigating a telehealth, group treatment modality for PwPD.

Abstract citation ID: igaf122.2290

**DOES AN INNOVATIVE PT PROGRAM TARGETING MOBILITY WORK FOR VETERANS WITH EXECUTIVE FUNCTION DEFICIT?**

Elisa Ogawa[1], Rebekah Harris[1], Ildiko Halasz[2], William Milberg[2], and Jonathan Bean[3], *1. VA Boston Healthcare System, Boston, Massachusetts, United States, 2. VA Boston Health Care System, Boston, Massachusetts, United States, 3. Harvard Medical School, Boston, Massachusetts, United States*

People with executive function problems may respond differently to Physical Therapy (PT) treatment compared to those without executive function problems. The study aimed to examine the treatment response after a novel PT treatment among Veterans with and without executive function deficits (EFD+/EFD-). This study was a preplanned secondary analysis of a Randomized Controlled Trial among middle- and older-aged Veterans (≥50yrs) with slow gait speeds. Changes in mobility (Short Physical Performance Battery (SPPB), gait speed) and executive function (Delis-Kaplan Executive Function System Verbal Fluency (letter fluency, category fluency, category switching, category switching accuracy) after 8-week PT treatment were evaluated based on baseline EFD status. One-hundred-and-one Veterans who completed the 8-week PT treatment were included in this study (mean age 71) and 15% (n = 15) were EFD+. There was no evidence of differences in mobility outcomes based on EFD status, both exceeding clinically meaningful improvements (Δ SPPB: EFD+ 1.5±1.4 vs. EFD- 1.1±1.6, $p = 0.43$; Δ gait speed: EFD+ 0.9±9.14 vs. EFD- 0.10±0.15, $p = 0.86$). We observed statistically significant differences in the change of category switching (EFD+ 2.7±3.1 vs. EFD- -0.5±3.0, $p < 0.001$) and category switching accuracy (EFD+ 2.9±3.3 vs. EFD- -0.2±2.9, $p < 0.001$), favoring EFD+. LLWS treatment may be appropriate for improving mobility in individuals with and without EFD. LLWS treatment may also have benefits in aspects of executive function such as cognitive flexibility, particularly for Veterans with EFD+. Future research is needed to evaluate rehabilitation treatment responses based on cognition on a larger sample, given the high prevalence of cognitive impairment among patients with mobility problems.

Abstract citation ID: igaf122.2291

**EDUCATION AND MIDLIFE COGNITIVE FUNCTIONING: DIFFERING ASSOCIATIONS BY CHILDHOOD SOCIOECONOMIC STATUS?**

Fabio Bolz[1], John Warren[2], Eric Grodsky[3], and Chandra Muller[4], *1. University of Minnesota, United States,*

*2. University of Minnesota, Minneapolis, Minnesota, United States, 3. University of Wisconsin–Madison, Madison, Wisconsin, United States, 4. The University of Texas at Austin, Austin, Texas, United States*

Educational attainment has consistently been identified as an important determinant of later-life cognitive functioning. Recent studies, moreover, suggest that additional aspects of the educational process, particularly school quality, may be related to later-life cognition. Another strand of research has examined the association between childhood conditions and later-life cognitive functioning and found strong associations between childhood socioeconomic status and later-life cognition. A substantial part of this association is often found to be mediated by educational attainment. What is not known, however, is whether the associations between the different aspects of the educational process and later-life cognitive functioning vary by childhood socioeconomic status: Is education more beneficial for the cognitive functioning of individuals from lower or higher socioeconomic backgrounds? Using long-spanning nationally representative longitudinal data from the High School & Beyond (HS&B) cohort study we examine whether the associations between school quality, educational attainment, and midlife cognitive functioning vary by childhood socioeconomic status. HS&B includes prospectively collected data on the high schools that the respondents attended and allows us to construct measures of school quality at the school level. The findings will have implications for our understanding of the life course processes underlying disparities in later-life cognitive functioning. Preliminary results suggest that the associations may not vary by childhood socioeconomic status.

Abstract citation ID: igaf122.2292

### ENERGY INSECURITY AND COGNITIVE FUNCTION: A PRELIMINARY ANALYSIS OF THE OFFSPRING STUDY

Gabriella Meltzer[1], Qian Li[2], Justina Avila-Rieger[3], Diana Hernández[2], and Jennifer Manly[3], *1. American University, United States, 2. Columbia University Mailman School of Public Health, New York, New York, United States, 3. Columbia University Irving Medical Center, New York, New York, United States*

Older adults are highly susceptible to energy insecurity (EI), the inability to adequately meet basic household energy needs. While burgeoning research has focused on the health implications of EI for vulnerable older adults, its relationship with cognition remains unknown. This analysis examines the association between EI and cognitive health in the Offspring Study of Racial and Ethnic Disparities in Alzheimer's Disease, which studies the risk factors of cognitive function disparities among middle-aged adults with and without parents with Alzheimer's in the Washington Heights Inwood Columbia Aging Project parent cohort. Based on 14 indicators capturing physical, economic, and coping EI, $N = 318$ Offspring participants (median age 54; IQR 46, 62) were characterized as energy secure ($n = 89$, 28.0%) or moderately ($n = 144$, 45.3%), severely ($n = 66$, 20.8%), or extremely ($n = 19$, 6.0%) energy insecure. The Kruskall-Wallis rank sum test was used to compare median scores on the Selective Reminding Test (total + delayed), Animal/Vegetable Fluency test, Letter Fluency test, and Digit Span test (forward + backward) across EI categories, stratified by gender. Among men ($n = 101$), vegetable fluency was the lowest among those experiencing severe EI compared to those who were energy secure (10.0 vs 14.0, $p = 0.045$). Among women ($n = 217$), extreme EI was marginally associated with lower average SRT scores than the other EI groups (51 vs. 57, $p = 0.059$). Energy insecurity is a salient socio-environmental risk factor to consider in future studies on the burden of cognitive health disparities.

Abstract citation ID: igaf122.2293

### EVIDENCE FOR THE AGE-RELATED POSITIVITY BIAS IN AUTOBIOGRAPHICAL MEMORIES OF THE 2020 U.S. PRESIDENTIAL ELECTION

Karen Siedlecki[1], Francesca Falzarano[2], Neshat Yazdani[3], and Jillian Minahan Zucchetto[1], *1. Fordham University, New York, New York, United States, 2. University of Southern California, Los Angeles, California, United States, 3. College Access: Research & Action, New York, New York, United States*

The age-related positivity bias refers to the finding that older adults recount events more positively (or less negatively) as compared to younger adults (i.e., a main effect of age on memory valence). This bias is closely related to the positivity effect, which reflects an interaction between age and valence of information to be remembered. We examined the age-related positivity bias and positivity effect using a one-year longitudinal design with a sample that spanned adulthood ($N = 374$; age range 19-90; $M = 47.41$; $SD = 16.75$). Participants answered questions regarding their memories of learning about the outcome of the 2020 U.S. presidential election. Analyses examined the association between age and valence ratings (positive, negative) and ratings of feelings (happy, elated, upset, and shaken) at Time 1, as well as the association with age between change scores for each of those variables, while controlling for who the participant voted for in the election. Results indicate that increased age was associated with reporting feeling less negative at the time of the event, and also remembering feeling more positive (elated and happy) when reconstructing the event one year later, thereby providing evidence of the positivity bias. There was no evidence of an age by valence interaction in a 2 (Valence) x 3 (Age) mixed ANCOVA on the positive and negative change scores, indicating there was not a positivity effect. Depressive symptoms partially mediated the relationship between age and valence variables, indicating that depressive symptoms may be one mechanism for explaining the age-related positivity bias.

Abstract citation ID: igaf122.2294

### HARMONIZATION OF COGNITIVE FUNCTION IN LONGITUDINAL INTEGRATIVE DATA ANALYSIS: RESULTS FROM TWO CHINESE DATASETS

Chang Yu, *University at Buffalo, SUNY, Getzville, New York, United States*

Objective: Integrating data from multiple longitudinal studies is crucial for understanding cognitive aging across different population groups. However, common harmonization methods, such as z-scores or percentiles, often fail to account for differences in measurement properties and sample demographics, potentially introducing bias. This study applies Item Response Theory (IRT) to harmonizing general and domain-specific cognitive function across two major Chinese national longitudinal aging studies. By developing a harmonized framework, this study aims to enhance comparability while ensuring reliability and validity in pooled analyses. Method: The sample consists of individuals who completed at least one wave of cognitive testing from two major datasets. The Chinese Longitudinal Healthy Longevity Survey

(CLHLS) (N = 38,760; 1998–2018; 8 waves) primarily focuses on older adults (65+), while the China Health and Retirement Longitudinal Study (CHARLS) (N = 19,150; 2011–2018; 4 waves) includes adults aged 45 and older. This study applies factor analysis to identify common latent cognitive constructs and employs IRT-based scale linking to align cognitive scores. Differential Item Functioning (DIF) analysis is conducted to examine variations in different datasets. Results: Preliminary findings suggest that factor score harmonization improves measurement alignment compared to traditional standardization techniques. However, differences in item properties and demographic distributions pose challenges, requiring further refinement of linking procedures. Discussion: This study advances longitudinal integrative data analysis in cognitive aging research, demonstrating the feasibility of factor score-based harmonization while highlighting potential limitations. Future work will refine linking approaches and assess how harmonization impacts cognitive trajectory analyses.

Abstract citation ID: igaf122.2295

### LINKS BETWEEN BURDEN OF COMMON INFECTIONS AND COGNITIVE DECLINE OVER MORE THAN A DECADE

Chunyu Liu[1], David Sosnowski[1], Yiwei Yue[1], Alden Gross[1], Keenan Walker[2], Robert Yolken[3], Brion Maher[1], and Adam Spira[1], *1. Johns Hopkins Bloomberg School of Public Health, Baltimore, Maryland, United States, 2. National Institute on Aging, Baltimore, Maryland, United States, 3. Johns Hopkins School of Medicine, Baltimore, Maryland, United States*

A growing literature links common infections to dementia. We previously found, among participants in Wave 4 of the Baltimore Epidemiologic Catchment Area (ECA) Study that increased levels of antibodies to a greater number of common pathogens (Herpes Simplex Virus-1, Cytomegalovirus, Epstein-Barr virus, Varicella-Zoster virus, and Toxoplasma gondii) were cross sectionally associated with a greater number of errors on the Mini-Mental State Examination (MMSE). Here, we examined the association between infection burden and performance on the MMSE and verbal memory >10 years later in 291 ECA participants (mean age: 54.3±8.8 years; 63.4% women; 37.2% Black) who gave blood and completed cognitive testing at Wave 4 (2004-06) and repeated cognitive testing at Wave 5 (2016-22). We performed antibody tests for the aforementioned pathogens and summed the number of pathogens for which participants tested positive at Wave 4. After adjustment for age, sex, race, education, APOE genotype, and Wave 4 MMSE score, individuals with more infections at Wave 4 had a greater number of errors on the MMSE at Wave 5 ($B = 0.134$, $p = 0.008$). In similar analyses adjusting for Wave 4 word-list memory performance, more infections at Wave 4 were associated with poorer Wave 5 word-list delayed recall ($B = –0.057$, $p = 0.041$); associations with immediate recall ($B = –0.042$, $p = 0.088$) and recognition ($B = –0.031$, $p = 0.080$) did not reach significance. Results link infection burden to long-term global cognitive performance and memory. Studies investigating potential mechanisms linking infection burden to cognitive outcomes are needed.

Abstract citation ID: igaf122.2296

### LONGITUDINAL ASSOCIATIONS BETWEEN PAIN TRAJECTORIES AND COGNITIVE FUNCTION: THE ROLE OF NEIGHBORHOOD CONTEXT

Eunbea Kim[1], Jeong Eun Lee[2], and Peter Martin[2], *1. Auburn University, Auburn, Alabama, United States, 2. Iowa State University, Ames, Iowa, United States*

Physical pain is a prevalent issue among older adults and a potential factor exacerbating age-related cognitive impairment. However, the longitudinal relationship between pain and cognitive function has yet to be fully accomplished. While previous research has focused on the direct impact of pain on cognitive outcomes, less attention has been given to the role of social and environmental contexts that may moderate cognitive decline associated with pain. This study examines the association pain trajectories and cognitive function and explores whether neighborhood characteristics (neighborhood social cohesion and physical environment) moderate this relationship. Using data from the Health and Retirement Study (HRS), we analyzed a sample of adults aged 50 and older ($n = 6,983$, $Mage = 44.92$) across 16 years. Growth mixture modeling identified groups of pain trajectories and examined their relationships with cognitive function. This study also tested the interaction effect between pain trajectories and neighborhood characteristics on cognitive function. The results indicated that individuals with persistent moderate- and severe- levels of pain exhibited lower cognitive function compared to those with mild level of pain over time. While neighborhood characteristics did not significantly moderate this relationship, group-specific regression revealed that higher neighborhood social cohesion and lower neighborhood physical disorder were related to higher level of cognitive function among individuals with mild pain trajectory. These findings suggest that social context may play a protective role in cognitive function when pain levels are low but may have limited impact in cases of severe pain, where cognitive decline may already be progressing.

Abstract citation ID: igaf122.2297

### RELATIONSHIP BETWEEN PHYSICAL FRAILTY, DEPRESSIVE SYMPTOMS AND COGNITIVE ABILITY AMONG OLDER INDIANS

Muhammad Thalil[1], Manish Kumar[2], Shobhit Srivastava[2], and Waad Ali[3], *1. The Pennsylvania State University, University Park, Pennsylvania, United States, 2. International Institute for Population Sciences, Mumbai, California, United States, 3. Sultan Qaboos University, Muscat, Masqat, Oman*

With a rapidly aging population, physical frailty has become a significant public health concern globally. We examined the associations of physical frailty with depressive symptoms and cognitive functioning among older Indian men and women, while also exploring how the frailty-cognition link differs between those with and without depression. We used data from the Longitudinal Aging Study in India (2017-19), with a sample of 14,652 males and 15,899 females aged ≥ 60 years. Frailty was assessed using a modified version of Fried's frailty phenotype. Linear regression models were used to examine the associations. The prevalence of frailty was higher in older women than that in older men (32.2% vs. 27.4%). Physical frailty was significantly associated with higher levels of depressive symptoms ($\beta = 0.51$; 95% CI: 0.39, 0.64), and poor cognitive performance ($\beta = -1.06$; 95% CI: -1.37, -0.75). Frail older women performed worse on cognitive tests than did frail older men ($\beta = -2.14$; 95% CI: -2.40, -1.87). In addition, non-frail older women had poorer cognitive performance than frail older men ($\beta = -0.77$; 95% CI: -1.22, -0.32). Conversely, stratification by depressive status showed that frailty was associated with cognitive ability, with no difference between individuals with and without depression. We found that frail older individuals, particularly women, have significant mental and cognitive deficits compared with their non-frail counterparts. Appropriate policies and programs should be implemented to reinforce the strength of pre-frail and frail older adults, and women in particular, and maintain improved mental health and cognition in older adults.

Abstract citation ID: igaf122.2298

### SOCIOECONOMIC STATUS ACROSS THE LIFE COURSE AND OLD AGE COGNITION

Michael Topping, *University of Wisconsin–Madison, Madison, Wisconsin, United States*

While linkages between socioeconomic conditions and cognition are well-documented, how socioeconomic conditions operate across the whole life course as a fundamental cause of disparities in cognition is less clear. Furthermore, many prior studies disproportionately rely on retrospective reports of socioeconomic status from earlier in life, and also utilize unitary, rather than multidimensional constructs of socioeconomic status. Drawing on rich prospective data from the Wisconsin Longitudinal Study over six decades, I constructed latent measures of socioeconomic status for individuals for the entire cohort across key life course periods: early life, adolescence, early adulthood, mid-life and later life, to examine both the independent and joint impact of socioeconomic status on cognition in old age. Results from linear regression analyses show that socioeconomic status at each life course period has a positive impact on cognition at age 80. However, upon modeling all life course periods, all are completely mediated, save for those corresponding to early and later life, which are substantially reduced. This implies that the effects of early life socioeconomic status operate directly and indirectly across the life course to shape global cognitive functioning. Additional results using sequence analysis revealed those who had trajectories of downward mobility or those who faced persistent, prolonged exposure to low socioeconomic status across the life course had the lowest levels of cognition, relative to those who experienced continuous socioeconomic advantage throughout their lifetimes. These findings have profound implications for estimating the impact of how socioeconomic conditions operate independently and in-tandem to shape later life cognition.

Abstract citation ID: igaf122.2299

### SUBJECTIVE COGNITIVE IMPAIRMENT AND MILD COGNITIVE IMPAIRMENT: RISK FACTORS IN COMMUNITY-DWELLING OLDER ADULTS

Neyda Mendoza Ruvalcaba[1], Melina Rodríguez-Díaz[2], and Karla Patricia Vazquez-Nuñez[2], *1. Universidad de Guadalajara, Guadalajara, Jalisco, Mexico, 2. Universidad de Guadalajara, Tonala, Jalisco, Mexico*

Introduction: Subjective cognitive impairment (SCD) and mild cognitive impairment (MCI) are preclinical states in the natural history of Major Neurocognitive Disorder or dementia. SCD has been studied as an indicator of MCI and future dementia, it is defined as persistent self-experienced decline in cognitive ability despite having normal performance on standardized tests. Objective: To identify the prevalence and risk factors for SCD and MCI in community-dwelling older adults from the Metropolitan Area in Guadalajara, Mexico. Method: Cross-sectional study, participated n = 1190 older adults (from 2018-2024). Mean age 71.44 years(SD = 7.5), education 9.24 years(SD = 4.8), 78% women(n = 927), 53% without a partner. After informed consent, trained gerontologists administered face-to-face interviews, measures of global cognitive functioning with the MMSE, meta-memory, and measurements of risk variables: sociodemographic data, physical and mental health. Data were analyzed with SPSSv28 and Odds Ratios (95% CI) were calculated. Results: Of the total participants 72.1% presented SCD, 11.6% MCI and 18.1% had normal cognitive functioning. The significant risk factors for SCD were age(OR = 1.31,CI=1.09-1.72,p=.025), sex(OR = 2.05, CI = 1.54 – 2.73, p < .001), education(OR = 1.21, CI = 1.03 – 1.53, p = .049), recent life crises(OR = 1.55, CI = 1.16 – 2.08, p = .002), loss of a loved one(OR = 1.32, IC = 1.02 – 1.74, p = .023), cancer(OR = 2.46 CI = 1.40 – 4.34, p < .001) and anxiety diagnosis(OR = 1.27, CI = 1.02 – 1.71, p = .049). The risk factors for MCI were presenting SCD(OR = 1.60, CI = 1.48 – 1.74, p < .001), having a previous diagnosis of depression(OR = 1.54, CI = 1.10 – 2.30, p = .016), while having no memory complaints was a protective factor(OR = .622, CI = .432-.895, p = .007). Conclusions: Detection of subjective cognitive impairment as an early manifestation of cognitive pathology is relevant to prevent or delay progression to MCI and dementia, allowing planning interventions to reverse/reduce the rate of conversion and its consequences on people's lives and public health.

Abstract citation ID: igaf122.2300

### THE THINKING IN MOTION THEORETICAL MODEL: EVALUATION OF COGNITIVE LOAD IN MOTOR-COGNITIVE TRAINING

Shiri Embon Magal[1], and Maayan Agmon[1], *1. University of Haifa, Haifa, HaZafon, Israel*

Motor-cognitive training is recognized for its role in preserving and enhancing cognitive function in aging populations. However, while sequential motor-cognitive training and dual-task paradigms are well-defined, Motor Training with Incorporated Cognitive Tasks (MTICT), where cognitive demands are integrated within movement execution, remains conceptually and practically underdeveloped, regardless of its high potential as an ecologically valid training. This gap limits research consistency, hinders comparative analysis, and reduces the ability to identify mechanisms underlying cognitive benefits of MTICT training. Furthermore, the absence of structured criteria in MTICT limits the ability to distinguish cognitively demanding interventions from general motor activities, posing challenges for both research standardization and practical application. To address these limitations, the Thinking in Motion theoretical model (TIM™), was developed. This model stems from a comprehensive review of existing theories and empirical studies examining key factors influencing cognitive load in motor learning. The TIM™ model defines five core cognitive load components: novelty, speed, variability, control complexity, and learning mode. By integrating these dimensions into standardized rating scales, TIM™ offers a structured framework for assessing both cognitive load intensity and its expected impact on cognitive domains. TIM™ refines the classification of MTICT interventions and equips clinicians and researchers with a structured tool for optimizing and evaluating cognitive stimulation in a given MTICT interventions. Standardizing cognitive load assessment in MTICT is critical for advancing both scientific methodologies and real-world applications. This presentation will outline the model's rational, theoretical foundation, and implications for refining motor-cognitive training strategies in both clinical and community-based settings.

### SESSION 7045 (POSTER)

### COMPARATIVE AGING RESEARCH

Abstract citation ID: igaf122.2301

### EVOLUTIONARY CONSERVATION OF THE EFFECTS OF SEX AND AGE IN THE DROSOPHILA METABOLOME

Avani Mital[1], Jessica Hoffman[2], Ben Harrison[3], Daniel Raftery[3], and Daniel Promislow[1], *1. Jean Mayer USDA Human Nutrition Research Center on Aging, Boston, Massachusetts, United States, 2. Augusta University, Augusta, Georgia, United States, 3. University of Washington, Seattle, Washington, United States*

Sex plays a crucial role in aging, influencing health trajectories, longevity, and disease susceptibility. Understanding the underlying mechanisms of these sex differences, and the extent to which they are evolutionarily conserved, remains a key challenge in aging research. Metabolites, the small molecules that mediate biochemical processes and link genetic variation with phenotypic traits, offer a powerful lens through which to explore sex-specific biology. Using a comparative framework across 11 Drosophila species, representing 50 million years of evolution, we investigate sex differences in the metabolome of young and old flies to identify patterns of age- and sex-related metabolomic changes. We find that male and female metabolomic profiles are highly correlated with each other across species, with this correlation weakening slightly with age. Despite this, numerous metabolites remain consistently sexually dimorphic across species and age groups, suggesting that sex-specific metabolic regulation is deeply evolutionarily conserved. We propose that endophenotypes like the metabolome can provide valuable insights into the mechanisms underlying sex differences in diverse traits such as age-related morbidity and mortality. Moreover, conserved and species-specific patterns can reveal why these differences have arisen and been maintained over evolutionary time.

Abstract citation ID: igaf122.2302

### PHYLOGENETIC RECONSTRUCTION OF AGING RATES IN THE PRIMATE LINEAGE

Eugene Melamud[1], Wendy Newton[2], and Joseph Kemnitz[3], *1. Calico Labs, Calico Labs, California, United States, 2. CleMetric, Madison, Wisconsin, United States, 3. University of Wisconsin–Madison, Madison, Wisconsin, United States*

Median lifespans of primates show nearly 10-fold variation, ranging from ~8 years in marmosets to ~80 years in humans. The molecular mechanisms that govern this variation and how they evolved remain poorly understood. In this study, we implemented a novel phylogenetic Gompertzian survival framework to leverage the evolutionary history of primates in order to estimate parameters of aging for 38 captive primate species. We find that baseline hazards (at the time of sexual maturity) and adult aging rates display significant variation, and aging rates are more evolutionarily conserved than baseline hazards (Pagel lambda=0.96 vs lambda=0.34, respectively). Furthermore, we find that aging rates and baseline hazards do not show a pattern of phylogenetic covariation, suggesting differential evolutionary pressures may act on these traits. Aging rates were strongly correlated with body weights, with a notable exception within the Ape family, where aging rates remained approximately unchanged despite large differences in body weight. Based on the reconstruction of ancestral aging rates, we estimate that the ancestor of Apes was likely aging at a similar rate to modern humans.

Abstract citation ID: igaf122.2303

### PREDICTION OF POPULATION SIZE AND AGEING IN CHINA BASED ON THE LESLIE MATRIX MODEL

Juan Luo[1], Keting Xia[2], and Chiyue Huang[2], *1. Shanghai University of Engineering Science, Shanghai, Shanghai, China (People's Republic), 2. Shanghai University of Engineering Science, Shanghai, Shanghai, China (People's Republic)*

Objective To better understand the changes in population size and ageing trends in China and to provide theoretical and policy support for China's future response to the various impacts triggered by heavy population ageing, we predict China's future population and the degree of ageing. Methods The age-specific fertility and mortality data of the China Population and Employment Statistical Yearbook from 2006 to 2023 were collated, three fertility scenarios—low, medium, and high—were established, and their future values were predicted by using EXCEL; the age-specific population data published by the National Bureau of Statistics (NBS) for the year 2022 were used to predict the future total population of China by using the Leslie Matrix Model in conjunction with the migration rate. Total population to make predictions. Conclusion Under the three scenarios, the total population of China showed a downward trend. Under the middle plan, the total population will fall below 1.4 billion in 2036 and 1.3 billion in 2049. The decline trend changes from a gentle decline in 2023-2039 to a steep decline around 2040, showing a rapid downward trend. The total size of China's population will continue to decline in the next 30 years; The degree of ageing continues to deepen, and will exceed 21 per cent in 2032, entering a severely ageing society.

Abstract citation ID: igaf122.2304

### THE ELITE PORTAL: A FAIR DATA RESOURCE FOR HEALTHY AGING OVER THE LIFE SPAN

Milan Vu, *Sage Bionetworks, Seattle, Washington, United States*

Exceptional longevity (EL) is a rare phenotype characterized by an extended health span and sustained physiological function. Various domain-specific factors contribute to EL, influencing the maintenance of key physiological systems (e.g., respiratory, cardiovascular, immune) and functional domains (e.g., mobility, cognition). Studying the impacts of protective genetic variants and cellular mechanisms associated with EL may facilitate the identification of novel therapeutic targets that may replicate their beneficial effects. The Exceptional Longevity Translational Resources (ELITE) Portal (eliteportal.synapse.org) is a new, open access repository for disseminating data and other research resources from translational longevity research projects. The portal supports diverse data types including genetic, transcriptomic, epigenetic, proteomic, metabolomic, and phenotypic data from longitudinal human cohort studies and cross-species comparative biology studies of tens- to hundreds- of nonhuman species; data from longevity-enhancing intervention studies in mouse and cell models; access to web applications and software tools to support exploration of EL-related research outcomes; and a catalog of publications associated with the National Institute on Aging (NIA)-funded translational longevity research projects. The portal also features integration with the external Trusted Research Environment (TRE) CAVATICA and is poised to support future integrations with additional data resources like Terra. All resources hosted in the ELITE Portal are distributed under FAIR (Findable, Accessible, Interoperable, and Reusable) principles. The ELITE Portal is funded by the NIA 5U24AG078753 and 2U24AG061340.

### SESSION 7050 (POSTER)

### COVID-19 PANDEMIC

Abstract citation ID: igaf122.2305

### AGE AND GENDER DISPARITIES IN PROCESSED MEAT CONSUMPTION DURING THE COVID-19 PANDEMIC

Lillie Monroe-Lord, Azam Ardekani, Phronie Jackson, and Weitman Cassidy, *University of the District of Columbia, Washington, District of Columbia, United States*

The COVID-19 pandemic significantly altered dietary habits, including processed meat consumption, which holds critical health implications. This cross-sectional study utilized the Dietary Screening Tool (DST) to analyze age- and gender-related differences in processed meat consumption among 10,050 U.S. adults aged 40–100 years. Three age groups (40–60, 61–80, and 81–100 years) were compared using binary logistic regression to assess the odds of decreased processed meat consumption by age and gender. Results revealed significant disparities: women were 23.6% less likely than men to report reduced processed meat consumption. Participants aged 40–60 years were 47% more likely to reduce processed meat consumption compared to those aged 61–80 years and over twice as likely compared to those aged 81–100 years. The difference between the 61–80 and 81–100 age groups was not statistically significant. These findings suggest that younger adults, particularly those aged 40–60 years, were more likely to decrease processed meat consumption, possibly due to heightened health awareness during the pandemic. In contrast, women exhibited a lower likelihood of reduced processed meat consumption, underscoring potential gender-specific challenges in dietary habits. This study highlights the need for tailored dietary interventions addressing processed meat consumption. Public health strategies should focus on promoting healthier dietary patterns through targeted messaging for diverse demographic groups, particularly during public health crises.

Abstract citation ID: igaf122.2306

### COGNITIVE FUNCTION AND DELAYED MEDICAL CARE DURING COVID-19: DOES FAMILY SOCIAL SUPPORT MODERATE THIS ASSOCIATION?

Brittany McFeeley, *University of Massachusetts Boston, Boston, Massachusetts, United States*

During the pandemic, healthcare facilities postponed or cancelled elective procedures and patients delayed their care to avoid the infection. Little is known about the role of cognitive function for older adults who delayed care. Further, social support from spouses and children may have protected against delays in medical care during the pandemic. This study used the 2020 wave of the Health and Retirement Study (N = 4,087) to examine the relationship between cognitive function and delaying medical care and whether positive or negative social support from family had a moderating effect. Cognitive function was measured with the Telephone Interview for Cognitive Status and delaying medical care was a binary variable (1=delayed care). Positive and negative support scales from spouses and children were converted to z-scores. Results showed that 30% of older adults reported delaying their medical care. Those with better cognitive function (OR = 1.03, 95%CI=1.01-1.05, p=.001) and those who reported more negative support from children (OR = 1.14, 95%CI=1.05-1.24, p=.002) were more likely to delay care. The moderation results were not statistically significant. Older adults with better cognitive function may have better understood the social distancing guidelines and threats of Covid-19. Older adults who felt unsupported by their children may be less likely to share health concerns or seek care. Future research should examine whether specific domains of cognitive function are related to seeking medical care during public health crises.

Abstract citation ID: igaf122.2307

### FAMILY DYNAMICS AND MENTAL VULNERABILITY OF OLDER ADULTS DURING THE COVID-19: EVIDENCE FROM A NATIONAL STUDY IN CHINA

Zhe Chen, and Senhu Wang, *National University of Singapore, Singapore, Singapore*

Mental vulnerability poses a serious threat to older adults, especially during the COVID-19 pandemic, underscoring the need to understand protective factors that mitigate such risks. Intergenerational family relationships have been shown to play a pivotal role in safeguarding older adults' well-being, yet limited research has specifically examined their impact on mental vulnerability during the pandemic. This study investigates how family dynamics influenced the mental vulnerability of older adults in China during the COVID-19 outbreak, utilizing data from the 2020 wave of the China Health and Retirement Longitudinal Study (CHARLS). Employing ordered logit regression models, we analyze the effects of adult children's gender, education level, and the quality of intergenerational relationships on older parents' mental vulnerability. Results indicate that older adults with daughters, children holding college degrees, or strong intergenerational relationships experienced significantly lower levels of mental vulnerability compared to those with sons, children with lower educational attainment, or weaker intergenerational ties. The subgroup analyses further reveal that these effects vary by the gender of the older parent and are more pronounced among those living with a partner, suggesting that adult children complement the protective role of spousal support. These findings underscore the importance of family in mitigating mental vulnerability among older adults and call for targeted social policies that strengthen the protective functions of families for older adults.

Abstract citation ID: igaf122.2308

### LONG-TERM PSYCHOSOCIAL IMPACTS OF THE COVID-19 PANDEMIC ON OLDER ADULTS

Riva Rainier[1], Jaclyn Bergstrom[1], Rebecca Daly[1], Colin Depp[1], Danielle Glorioso[1], and Anthony Molina[2], *1. University of California San Diego, San Diego, California, United States, 2. University of California San Diego, La Jolla, California, United States*

The psychosocial impact of the COVID-19 pandemic on older adults is not fully understood. This study aims to explore the differences in emotional measures pre- and post-pandemic to assess the emotional burden of the COVID-19 pandemic on this population. We hypothesize that older adults will have a significant decrease in emotional wellbeing status post pandemic, with the highest impact on the 75+ older adult population. UCSD Successful Aging Evaluation (SAGE) longitudinal study is a prospective cohort study with a focus on the cognitive and emotional aspects of aging. This analysis utilized validated emotional measures: anxiety (BSIA), depression (CESD), self-compassion (NSCS), resilience (CDRS), and loneliness (UCLA Loneliness Scale). 248 community-dwelling participants were found eligible to be included in this study. Across all participants we observed a significant increase in anxiety (p = 0.0403), depression

($p = 0.0707$), and loneliness ($p = 0.0111$) and a significant decrease in self-compassion ($p = 0.0441$) and resilience ($p = < 0.0001$). The 50-74 population had higher levels of anxiety ($p = 0.0055$), depression ($p = 0.0189$), and loneliness ($p = 0.0317$) pre-pandemic than the 75+ population. Post-pandemic, the 75+ population experienced a greater change in emotional characteristics with significant changes in anxiety ($p = < 0.0001$), depression ($p = < 0.0001$), loneliness ($p = 0.0099$), self-compassion ($p = 0.0006$), and resilience ($p = < 0.0001$) with no significant changes in the 50-74 population. The 75+ population was significantly more affected by the psychosocial impact of the pandemic, indicating greater vulnerability of this population. Uncovering the emotional vulnerability of this population is critical to promote greater awareness and provide insight into potential intervention strategies to aid in full emotional recovery.

Abstract citation ID: igaf122.2309

### NURSING HOME ADMISSION DECISIONS DURING THE COVID-19 PANDEMIC: A RESOURCE DEPENDENCE PERSPECTIVE

Zihan Chen[1], Talia Benheim[1], Courtney Hawes[1], Joan Brazier[1], Amy Meehan[1], and Emily Gadbois[2], *1. Brown University, Providence, Rhode Island, United States, 2. Brown University School of Public Health, Providence, Rhode Island, United States*

During the COVID-19 pandemic, hospitals needed to efficiently discharge stabilized patients who still required care. Although nursing homes were potential care settings, most facilities did not admit COVID-19 patients during the pandemic. This study used qualitative methods to examine environmental factors that shaped nursing home admission strategies for COVID-19 patients. We conducted 156 in-depth, semi-structured qualitative interviews with administrators from 40 nursing homes in eight U.S. healthcare markets. Each participant participated in four interviews between July 2020 and December 2021. The research team created and iteratively refined a coding scheme and applied that scheme to the transcripts of audio recordings. Using Resource Dependence Theory, we identified key themes related to nursing home environments, relationships with external stakeholders, and responses to market uncertainties. First, the pandemic led to decreased concentration in regulatory authority, and to shortages in critical resources, such as staffing and physical capacity. Second, nursing homes navigated competing pressures from key actors, including hospitals, regulators, patients' families, and the broader community. Some urged facilities to admit COVID-19 patients, while others strongly opposed it. Third, to manage market uncertainties and conflicting demands, many nursing homes implemented new admission strategies, such as halting admissions early in the pandemic, or forming collaborations with other nursing facilities to coordinate patient referrals and discharges. Our findings highlight policies to improve care for vulnerable populations in future pandemics, such as disseminating consistent regulations and supporting communication channels among providers.

Abstract citation ID: igaf122.2310

### THE IMPACT OF COVID-19 ON COGNITIVE FUNCTION

Adriana Hernandez, and Jeffrey Stokes, *University of Massachusetts Boston, Boston, Massachusetts, United States*

The present study examined the association between COVID-19 infection and cognitive function among adults aged 50 and older. Specifically, we investigated whether individuals who contracted COVID-19 in 2020 experienced greater declines to cognitive functioning by 2022 compared to individuals who did not contract COVID-19. This study used data from the 2020 and 2022 wave of The Health and Retirement Study. The sample consisted of 7,349 participants aged 50 and older, with 6,989 participants reporting "no" to testing positive for COVID-19 and 360 participants reporting "yes." Cognitive function was determined using episodic memory scores from the HRS-adapted version of the Telephone Interview for Cognitive Status (TICS). A series of logistic and linear regression models were employed to estimate the association between cognitive function and COVID-19 infection. Results indicated that individuals who tested positive for COVID-19 showed greater declines in memory over two years than people who tested negative for COVID-19. However, COVID-19 was not randomly distributed and was associated with factors that also lead to declines in cognition such as: age, depressive symptoms, and employment status. Therefore, cognitive decline reflected other causal factors rather than a direct association with COVID-19 infection.

Abstract citation ID: igaf122.2311

### THE MEDIATING ROLE OF HISTORY OF HEART DISEASE IN THE RELATIONSHIP BETWEEN COVID-19 AND DEMENTIA

Reza Amini, and Jahanvi Patel, *University of Michigan-Flint, Flint, Michigan, United States*

This study used data from the National Health and Aging Trends Study (NHATS) spanning 2011-2012 to explore the long-term health impacts of COVID-19, with a focus on cardiovascular and cognitive outcomes. We hypothesized that COVID-19 exacerbates the risk of cardiovascular disease (CVD), which then leads to increased hospitalizations and cognitive decline. Employing Structural Equation Modeling (SEM), we analyzed the interrelationships among COVID-19 severity, history of CVD, hospital stays, and dementia across two waves. Our results confirm that severe COVID-19 significantly escalates the risk of a history of CVD. These conditions serve as crucial mediators that not only increase the probability of hospitalization but also contribute to the risk of dementia. The SEM model demonstrated that COVID-19 indirectly impacts cognitive function through its effect on cardiovascular health, with significant pathways linking heart disease to higher hospitalization rates and increased risk of dementia (Standardized $\beta = 0.161$, $p < 0.001$ for hospitalization; $\beta = 0.037$, $p = 0.030$ for cognitive decline). The optimal fit of the model (RMSEA ~0.00, CFI ~1.00) indicates an excellent replication of the data structure, affirming the model's accuracy in capturing the relationships among the study variables. These findings highlight the imperative of cardiovascular monitoring in COVID-19 recovery plans and suggest that addressing cardiovascular and cognitive health in tandem may enhance outcomes for COVID-19 survivors. This research contributes to a deeper understanding of the pandemic's multifaceted impact on public health and underscores the necessity for integrated healthcare strategies that consider the interplay between infectious diseases and chronic conditions.

Abstract citation ID: igaf122.2312

### WHEN TIME STANDS STILL: AGE DIFFERENCES IN TEMPORAL DISINTEGRATION DURING THE COVID-19 PANDEMIC

Teagan McCune[1], Meghan Elliot[2], Kayley Estes[2], Alison Holman[2], and Roxane Silver[2], *1. University of*

California, Davis, Los Gatos, California, United States, 2. University of California, Irvine, Irvine, California, United States

The COVID-19 pandemic was globally disruptive and life-altering. During collective traumas like the pandemic, some individuals experience temporal disintegration (TD), defined as a distorted sense of the passage of time (e.g., speeding up, slowing down, stopping). Numerous studies have shown that TD is associated with psychological distress following trauma exposure. While research on age differences in TD is limited, some studies suggest older people may experience less TD. The current study explored self-reported TD over the first two years of the pandemic among a nationally representative U.S. sample of adults (ages 18-97) who completed two online surveys (Sept-Oct 2020, N=5,654; Nov 2021, N=4,856). Weighted linear regressions assessed associations between TD, negative life events, emotional distress, and age groups. Both middle-aged and older adults experienced less TD compared to younger counterparts. Higher TD was also associated with having previously experienced more negative life events. While higher TD was associated with greater emotional distress, the relationship was significantly weaker in older adults compared to young adults; middle-aged adults did not show this weakening relationship with age. Results suggest that older adults may experience less distress in response to distorted time perceptions, perhaps because they have age-related positivity biases, suggesting greater adaptation to disruptions in time perception. These age-group differences may also be a result of increased self-continuity and time stability with age, a topic that future research should further explore. Future studies should also explore potential mechanisms underlying the age differences in TD.

### SESSION 7055 (POSTER)

### DEATH, DYING, AND BEREAVEMENT

Abstract citation ID: igaf122.2313

### DOCUMENTING GRIEF AND BEREAVEMENT EXPERIENCES AMONG OLDER CHINESE IMMIGRANTS IN LA: A PHOTOVOICE PILOT STUDY

Qianyun Wang, and Cindy Sangalang, *University of California, Los Angeles, Los Angeles, California, United States*

This pilot study explored grief and bereavement experiences among older Chinese immigrants in Los Angeles using a Photovoice approach. The COVID-19 pandemic not only triggered global waves of grief but also intensified anti-Asian racism, disproportionately affecting older Asian immigrants with severe symptoms of depression and anxiety. Older Chinese immigrants faced unique challenges in grief adjustment due to cultural and linguistic barriers, limited social support, and discrimination in healthcare settings. This study employed Photovoice, a participatory action research strategy, to authentically capture and discuss these experiences through visual data. The research, conducted in collaboration with the Chinatown Service Center, addressed the following research questions: 1) What were the lived experiences of grief and bereavement among older Chinese immigrants? 2) How did intersecting factors like age, immigration status, and ethnicity impact their experiences? 3) What challenges and support did they encounter in adapting to grief? A purposive sample of 20-30 participants in total aged 65 or older, born outside the United States, and having lost a loved one over six months ago will be recruited. The methodology included two individual interviews and a focus group discussion, where participants took and discussed photographs representing their grief experiences. Preliminary results were documented and will be presented in a community photographic exhibition. This study aimed to provide insights into the nuanced grief experiences of older Chinese immigrants, contributing to culturally sensitive support frameworks.

Abstract citation ID: igaf122.2314

### END-OF-LIFE PERSPECTIVES AND NEEDS FOR HOSPICE CARE AMONG RURAL CHINESE OLDER ADULTS IN ISOLATED MOUNTAIN AREAS

Heshuo Yu, *Fudan University, Shanghai, Shanghai, China (People's Republic)*

Hospice care has become more prevalent in China since 2017; however, it remains largely inaccessible in isolated rural mountain areas. Discussions about death are often considered cultural taboos, and older adults in these remote regions face significant disparities in end-of-life care due to lower socioeconomic status, limited transportation, and inadequate healthcare infrastructure. Few studies have examined how this population perceives "good death" or their willingness to receive hospice care. This study explores the expectations and needs of rural Chinese older adults regarding "good death" and their perceptions of hospice care. Using a qualitative descriptive approach, we conducted semi-structured, open-ended interviews with 21 older adults and analyzed the data inductively through thematic analysis. Five key themes emerged: being accompanied by family, attaining inner peace, experiencing no physical pain, departing with dignity, and preferring to pass away at home. Cultural values deeply rooted in the Chinese context shape these perceptions. Many participants expressed a strong attachment to their home villages and a preference for in-home death, reflecting the belief that "fallen leaves return to their roots." Fear of pain, particularly from terminal diseases like cancer, was common, and participants showed a strong willingness to use hospice care if affordable. The presence of adult children at the end of life was deemed crucial despite geographic challenges. Future research should explore cross-national perspectives on hospice care utilization and definitions of "good death" across cultures. Further study is also needed to incorporate the perceptions of other key stakeholders, including family caregivers and local healthcare providers.

Abstract citation ID: igaf122.2315

### EXPLORING COMBINATIONS OF DUAL FUNCTIONALITY, EDUCATION, AND MORTALITY: NEW FINDINGS FROM MIDUS

Jonghwa Do, Jieun Song, and Carol Ryff, *University of Wisconsin–Madison, Madison, Wisconsin, United States*

There has been growing attention on the role of functionality in older adults, as diseases become less lethal and more chronic over time. While lower cognition and poorer physical health are well-established predictors of mortality risk, less is known about their combined effects. This study examines mortality risks across dual functionality, assessed by cognition and physical functioning, among adults aged 60 and older using data from Midlife in the U.S. (MIDUS) (N=1,578). Cognition and physical functioning were assessed at MIDUS III (2013-2014) via Brief Test of Adult Cognition by Telephone (BTACT) and SF-36. Mortality data were obtained via National Death Index 2023 files. Dual functionality groups were identified based on quartiles of cognitive and physical functioning: Both High (n = 245), Both Mid (n = 445), Both Low (n = 494), Cognition High–Physical Low (n = 170), and Cognition Low–Physical High (n = 224). Results from Cox proportional hazard models showed that imbalance between cognitive and physical functionality predicted greater mortality risks, compared to Both High or Both Mid functioning. Specifically, mortality risks were significantly greater in Cognition High–Physical Low (HR = 3.06; 95% CI = 1.52–6.16) and Cognition Low–Physical High (HR = 3.91; 95% CI = 1.95–7.85) groups, compared with Both High group, and somewhat comparable with Both Mid group (HR = 2.59; 95% CI = 1.35-4.97). Further, the detrimental effects of imbalanced functionality on mortality risks were observed regardless of educational attainment. The findings suggest substantial impacts of imbalanced functionality on mortality risks. It warrants future studies to examine the pathways through which imbalanced functionality increases mortality, which can shed light on possible interventions.

Abstract citation ID: igaf122.2316

### FACTORS INFLUENCING LOCATION OF DEATH

Lauren Montemuro Rode, *Bryn Mawr College, Bryn Mawr, Pennsylvania, United States*

This research aims to explore contributing factors to location of death, with a focus on how the social network can impact where end-of-life (EOL) occurs. This analysis includes four primary locations of death: home, hospital, nursing home/assisted living facility (NH/ALF), and hospice in-patient center (IPC). The research theoretically draws from Life Course Perspective, which names the various influences of biological, psychological, and sociological spheres, as well as The Convoy Model, which specifically names consideration of closeness, quality, function, and structure when analyzing a network. The analysis includes demographic variables, health related variables, and social network variables through progressive modeling. A multinomial logistic regression analysis is applied, using longitudinal data from the Health Retirement Study ranging from 2000 through 2018 (maximum sample size of 10,414). Social Network Factors demonstrate the largest impact related to likelihood of EOL in SC/ALF vs home. Larger household size, increased number of children, discussing EOL wishes, increased time spent with children, and higher frequency of religious service attendance all decreased likelihood of EOL in SC/ALF relative to home. Length of time of terminal illness and heart, circulatory, and blood condition as COD (reference group, Cancer) appears to be a common barrier to death at home (vs hospital, SC/ALF, and hospice IPC). Household size is a consistent support to death at home. Programs focusing on supporting family caregivers for long-term conditions and education for family caregivers surrounding care for heart, circulatory, and blood conditions are suggested.

Abstract citation ID: igaf122.2317

### PERCEPTION OF GOOD DEATH AND DEATH PREPARATION AMONG COMMUNITY-DWELLING OLDER ADULTS IN SOUTH KOREA

Bomgyeol Kim[1], Hun Kang[2], Seongmi Choi[3]JaeWon Hyun[2], and JiYeon Choi[2], *1. Yonsei University College of Nursing, Seoul, Republic of Korea, 2. Yonsei University, Seoul, Republic of Korea, 3. Daegu Catholic University, Daegu, Republic of Korea*

As societies age, preparing for a good death is becoming a critical public health concern. This study investigated how perceptions of a good death shape engagement in death preparation among community-dwelling older adults in South Korea. We conducted a secondary analysis of the 2023 National Survey of Older Koreans. The final sample included 9,140 older adults (≥65 years), excluding proxy responses and severe cognitive impairment (K-MMSE~2 ≤17). Death preparation was assessed based on engagement in eight specific activities, categorized into practical and conceptual death preparation. Perceptions of a good death were measured using a five-item scale evaluating key end-of-life aspects. Participants were predominantly female (60.0%) and in the 65-74 -year-old group (60.7%). Among participants, 51.3% engaged in practical death preparation, while 5.5% in conceptual death preparation. Results of multiple logistic regression revealed that those who valued organizing personal affairs (aOR = 1.12, 95% CI = 1.05–1.21), minimizing the burden on family (aOR = 1.18, 95% CI = 1.11–1.26), and preferring to die at home (aOR = 1.06, 95% CI = 1.01–1.12) were more likely to engage in practical death preparation. Conceptual death preparation was most strongly associated with preferring to die at home (aOR = 1.52, 95% CI = 1.35–1.70), whereas being surrounded by loved ones was negatively associated (aOR = 0.77, 95% CI = 0.67–0.88). These findings highlight the need for tailored policies and community-based interventions to help older adults align end-of-life preferences with preparation, promoting meaningful aging and a dignified death.

Abstract citation ID: igaf122.2318

### THE IMPACT OF COVID-19 ON RACIAL DISPARITIES IN DEATH QUALITY: THE MODERATING ROLE OF HOSPICE AND PALLIATIVE CARE

Clifford Ross[1], Brina Ratangee[1], Emily Schuler[1], Zheng (Noah) Lian[1], Benmun Damul[1], Deborah Carr[2], and Lucie Kalousova[1], *1. Vanderbilt University, Nashville, Tennessee, United States, 2. Boston University, Boston, Massachusetts, United States*

Objectives: This study investigates how the COVID-19 pandemic affected death quality and whether these impacts differed for White and Black decedents. We also evaluate the extent to which hospice and palliative care moderated these associations. Methods: Data are from the Health and Retirement Study (HRS) Core and Exit Interviews conducted between January 2018 and May 2021. Our analytic sample included 1,595 decedents (281 Black, 1,314 White). Outcomes include proxy-reported perceived death quality and whether decedents' healthcare wishes were consistently met. Multivariable regression models with three-way interactions were used to evaluate the effects of race and hospice/palliative care usage on death quality during and before the COVID-19 pandemic. Results: Deaths during the pandemic were associated with lower subjective death quality scores and reduced odds of healthcare wishes being met. Proxies for Black decedents rated overall death quality higher than White proxies, although they also reported Black decedents were less likely to have their healthcare wishes fulfilled. Hospice care during the pandemic was linked to decreased death quality for Black decedents, likely reflecting disparities in the effectiveness of hospice/palliative care. Discussion: Death during (versus prior to) the COVID-19 pandemic was associated with worse death quality. The use of hospice/palliative care during COVID-19 was associated with poorer quality deaths for Blacks. Our results suggest that purportedly protective healthcare practices may be implemented less effectively for Black patients, underscoring the need for cultural competence training and sufficient structural resources for potentially underserved healthcare settings.

### SESSION 7060 (POSTER)

### DELIRIUM

Abstract citation ID: igaf122.2319

### EDUCATION AND IMPLEMENTATION CO-DESIGN APPROACHES FOR READI-SET-GO USING CFIR FRAMEWORK

Zahra Karimi[1], Priyanka Shrestha[2], Jessica Fleming[1], Erica Husser[1], Donna Fick[1], Edward Marcantonio[4], and Chia Jou Lin[5], *1. The Pennsylvania State University, University Park, Pennsylvania, United States, 2. The George Washington University, Washington, District of Columbia, United States, 4. Beth Israel Deaconess Medical Center, Boston, Massachusetts, United States, 5. Taipei Medical University, Taipei City, Taipei, Taiwan (Republic of China)*

Background: Delirium in hospitalized older adults can lead to severe outcomes, including falls, antipsychotic use, and misdiagnosis as Alzheimer's disease, contributing to cognitive decline and mortality. Early detection is critical, yet implementation barriers include lack of clinical prioritization, competing demands, time constraints, discomfort with screening tools, and limited leadership support. This study explores education and implementation strategies for integrating the Ultra-Brief Confusion Assessment Method (UB-CAM) into the electronic health record (EHR) to enhance delirium screening and management. Method: Using a stepped-wedge design across three hospitals over 42 months, nurses conduct daily UB-CAM screenings, with new units launching every six months. Implementation strategies are guided by RE-AIM and CFIR frameworks and include: (1) EHR-integrated app-based screening, (2) stakeholder engagement, (3) co-designed education (in-person and online), (4) hands-on case-based practice with feedback, and (5) addressing organizational factors such as leadership, culture, and workflow alignment. Results: In the first intervention block, 100% of eligible nurses completed the education program (65% in-person, 35% online), utilizing video-based case studies for hands-on learning and feedback. We describe champion selection, use of screening feedback reports, leadership engagement, and co-design of clinical strategies to enhance adherence. Discussion: UB-CAM integration into EHR, combined with co-designed education and multifaceted implementation strategies, has improved engagement and adherence. Hybrid training, technology-driven formats, and organizational leadership involvement have optimized adoption. Future research should assess the sustainability of these approaches for delirium screening.

Abstract citation ID: igaf122.2320

### POSTOPERATIVE INFLAMMATORY MARKERS IN OLDER ADULTS: A LONGITUDINAL STUDY OF DELIRIUM AFTER SPINE SURGERY

Jeongeun Choi, and Hyangkyu Lee, *Yonsei University, Seoul, Republic of Korea*

Systemic inflammation is linked to postoperative delirium (POD) and adverse postoperative outcomes in older adults undergoing spine surgery. Previous studies have primarily focused on cytokine changes, limiting clinical applicability. This study aimed to examine changes in blood-based clinical inflammatory biomarker levels over time in older adults with and without POD. This study included 536 participants aged 70 and older who underwent elective spine surgery between November 2019 and May 2023. Serum inflammatory markers, including the neutrophil-to-lymphocyte ratio (NLR), platelet-to-lymphocyte ratio (PLR), systemic immune-inflammation index (SII), and C-reactive protein (CRP), were measured at three time points: pre-operation (Time 1), postoperative day 1 (Time 2), and discharge (Time 3). The trajectories of these markers were analyzed using linear mixed models, adjusting for age, sex, body mass index, Charlson Comorbidity Index (CCI), American Society of Anesthesiologists (ASA) score, operative level, and baseline inflammatory marker levels. 95 participants (17.7%) out of 536 developed POD. Compared to the non-delirium group, participants with delirium exhibited significantly higher inflammatory marker levels with different patterns. In particular, NLR and SII levels were significantly elevated at Time 2 (both $p < 0.001$), while PLR levels were elevated at both Time 2 ($p < 0.001$) and Time 3 ($p = 0.021$), and CRP levels gradually increased over time and remained high at Time 3 ($p = 0.006$). These findings suggest that elevated inflammatory marker levels in older adults experiencing POD persist until discharge, highlighting the need for timely monitoring to reduce delirium risk and improve postoperative recovery.

Abstract citation ID: igaf122.2321

### PREDICTION OF DELIRIUM IN ICU PATIENTS AND DEVELOPMENT OF A CDSS MODEL USING CLINICAL DATA WAREHOUSE

Sun Ju Kim, *Chungnam National University Se-Jong Hospital, Se-Jong, Republic of Korea*

Delirium in intensive care unit (ICU) patients is a critical condition associated with increased hospital stay, mortality, and healthcare costs. Using a Clinical Data Warehouse (CDW), key risk factors influencing the occurrence of delirium in ICU patients are identified and a Clinical Decision Support System (CDSS) for early prediction and prevention developed. From May 2015 to May 2024, a retrospective cohort study involving adult patients (≥18 years) admitted to the ICU of a tertiary hospital in Daejeon, South Korea was conducted. Patients who developed or did not develop delirium within five days of ICU admission were classified as the experimental and control group, respectively. Predictive models were developed and compared using machine learning techniques, including logistic regression, decision tree, and XGBoost. Demographics, biometric indicators, vital signs, and past medical history were key predictive variables. The decision tree model demonstrated high sensitivity, whereas the XGBoost model exhibited high accuracy (0.66) and specificity, thereby making it an effective tool for delirium prediction. Logistic regression provided easy interpretability but limitations were observed due to lower sensitivity (0.22). GCS-Verbal, GCS-Eye, age, blood pressure, and oxygen saturation were the most significant predictive variables. Accordingly, a CDSS was designed to proactively assess the risk of delirium in ICU patients. The performance of machine learning-based models for delirium prediction was compared and the feasibility of integrating these models into a CDSS explored. The proposed CDSS can facilitate early detection and intervention strategies for ICU delirium. Future research should focus on model optimization and real-time clinical implementation.

Abstract citation ID: igaf122.2322

**SEDATIVE AND ANTICHOLINERGIC BURDEN AS PREDICTORS OF DELIRIUM IN HOSPITALIZED OLDER ADULTS**

Hadas Shachaf[1], Juliana Smichenko[2], Rami Klayn[1], Rabab Awad[3], Ron Oliven[4], and Anna Zisberg[2], *1. Bnai Zion Medical Center, Haifa, Hefa, Israel, 2. University of Haifa, Haifa, HaZafon, Israel, 3. University of Haifa, Haifa, Hefa, Israel, 4. Bnai Zion Hospital, HaifA, HaZafon, Israel*

Delirium is a prevalent yet underdiagnosed acute cognitive disorder among hospitalized older adults, contributing to prolonged hospital stays, increased morbidity, and higher mortality rates. While medications with sedative and anticholinergic properties are known risk factors, their cumulative impact and potential as early indicators for delirium remain unclear. This study aims to assess whether sedative burden, anticholinergic burden, and polypharmacy can predict delirium in hospitalized older patients and serve as a trigger for early intervention. A retrospective cohort analysis was conducted on 260 patients (aged 65+) from three internal medicine wards. Patients were categorized based on delirium status (positive: n = 150, negative: n = 110) using RADAR and MOTYB screening tools. Sedative and anticholinergic medication burden was quantified using the Drug Burden Index (DBI), based on medication data from the first hospitalization day. Covariates included age, gender, illness severity (NEWS), comorbidities (Charlson Index), polypharmacy (≥5 medications), and physical status (Norton Scale). Logistic regression examined associations between medications burdens at admission and delirium risk. Results showed that patients screening positive for delirium had significantly higher sedative burden (OR = 2.93 CI [1.42-6.06], p=.004), while no significant differences were observed in anticholinergic burden. Physical status protected from (OR = 0.59 CI [0.51-0.69], p=.000) while polypharmacy (OR 3.63 CI [1.34-9.83], p = 0.011) and older age (OR = 1.12 CI [1.063-1.18], p=.000) significantly predicted delirium. These findings emphasize the need for systematic medication review upon hospital admission, with a focus on minimizing sedative load to mitigate delirium risk. Integrating delirium screening with pharmacological assessments may enhance early identification and intervention strategies, ultimately improving patient outcomes.

### SESSION 7065 (POSTER)

### DISABILITIES, LIFELONG

Abstract citation ID: igaf122.2323

**AGING & ADVOCACY ONLINE: DISABLED FASHION BLOGGERS AND THE PURSUIT OF WELLNESS.**

Allison Kabel[1], and Kerri McBee-Black[2], *1. Towson University, Towson, Maryland, United States, 2. University of Missouri, Columbia, Missouri, United States*

The goal of this study is to explore techniques used to advocate for social inclusion by fashion bloggers who are aging with disabilities and chronic health conditions. Online platforms have allowed people living with disabilities to work around establishment media barriers, share their perspectives, and educate the public, however, blog-based advocacy has been known to struggle with the required task of reaching its target audience, especially an audience that is not already committed to disability-related issues, such as accessibility in clothing, housing and transportation. The online forums discussed here include video logs, or 'vlogs', and weblogs, or 'blogs', associated with topics related to female disability-focused fashion. These data were publicly available social media platforms in accordance with ethical guidelines. Key findings/results included acknowledgement of the influential power of social media to reframe issues of aging and disability; Discovery of techniques for using fashion to raise awareness of social and policy issues, particularly those associated with older adult women and; Content creation as an advocacy tool for combating ageism and ableism. Implications for further research and education include the importance of working across disciplines, such as the social sciences, rehab health professions, design fields, education and communication professions, especially those specializing in social media.

Abstract citation ID: igaf122.2324

**ASSOCIATION OF FUNCTIONAL LIMITATIONS (ADLS) AND SEXUAL IMPORTANCE IN MIDDLE-AGE AND OLDER U.S. ADULTS.**

Lucas Prieto, Claire Leonard, and Gilbert Gimm, *George Mason University, Fairfax, Virginia, United States*

Although prior studies have examined functional limitations (ADLs) in older adults, relatively few U.S. studies examine the association of disability and importance of sex in middle-age and older adults. Sexual health is a vital part of overall well-being in adults, therefore, understanding how older adults prioritize sex is essential. Given this gap, the goal of this study was to analyze a national sample of Wave 3 NSHAP participants and assess the relationship between disability as measured by the count of functional limitations (ADLs) and having any sexual importance among U.S. adults (50 years or older). Using respondent data from 4,371 NSHAP participants, we conducted bivariate and multivariate analyses of the likelihood of reporting sex as important among middle-age (50-64 years) and older U.S. adults (65 years or more). Nearly one-quarter (24.5%) of participants had any ADL limitations and a majority (55.5%) reported sex as important. Compared with middle aged adults (50-64 years), older adults (65+ years) had lower odds (OR = 0.30, p<.001) of reporting sex as important. Also, participants with 3 or more ADLs had lower odds of expressing sexual importance compared to zero ADLs (OR=.64, p=.005). Other factors that were significant with sexual importance included gender, education, race/ethnicity, relationship type, and health. These findings suggest that health care providers should include sexual health questions in their interactions with older adult patients regardless of sociodemographics. Results suggest the need to assess the link between functional limitations and sexual importance among middle-age and older U.S. adults to maintain well-being and healthy aging.

Abstract citation ID: igaf122.2325

**AUTONOMY AS CONVEYED BY FOOD ENVIRONMENTS: NOURISHMENT VS RESTRICTION AMONG NEURODIVERSE OLDER ADULTS**

Sophie Koestner[1], Shari Brotman[2], Laura Pacheco[3], Aglaé Mastrostefano[2], Marie-Hélène Deshaies[4], Camille Demers[5], Daniel Dickson[6], and Samuel Ragot[2], *1. University of California, Los Angeles, Los Angeles, California, United States, 2. McGill University, Montreal, Quebec, Canada, 3. Memorial University of Newfoundland, St. John's, Newfoundland and Labrador, Canada, 4. Université Laval, Quebec, Quebec, Canada, 5. Université du Québec à Montréal, Montreal, Quebec, Canada, 6. University of Saskatchewan, Saskatoon, Saskatchewan, Canada*

Food serves as a lens to understand identity, memory, and belonging. However, in the context of care, food often reflects a mechanism of social control (Davies, 2007; Gerber, 2020). Feminist scholars have critiqued conceptualizations of autonomy as "self-sufficient independence" for overlooking how individuals and their contexts are intertwined (Salami & Lashewicz, 2015, p. 93). Narrow conceptualizations of autonomy, which emphasize functional independence, hold sway in many caregiving environments, shaping food experiences (Dickson et al., 2022; Boyle, 2008). This study explores food-related experiences among neurodiverse older adults (NDOA) in Quebec. We collected intersectional life story narratives (Brotman et al., 2021) of 21 neurodiverse older adults, 15 family caregivers, and 27 service providers about challenges encountered and agency enacted across the life course. Findings highlight the interplay between NDOAs' food experiences, identities, memories, and relationships. Our observations reveal how formal and informal carers across sectors enact controls over food consumption, schedules, and daily choices, restricting NDOAs' autonomy. We illuminate structures of dominance and expressions of resistance, highlighting how NDOAs internalize messages conveyed through their food experiences, leading to feelings of empowerment or constraint. Findings highlight how experiences of nourishment versus restriction affect neurodiverse people into older age. We encourage carers across settings to support autonomous eating to promote agency and well-being. Such inclusive recommendations would serve to safeguard NDOAs' autonomy and rights.

Abstract citation ID: igaf122.2326

### LONG-TERM SERVICES & SUPPORTS AVAILABLE TO OLDER INDIVIDUALS WITH INTELLECTUAL AND DEVELOPMENTAL DISABILITIES

Akwasi Adjei Gyimah[1], Eric Frimpong[2], Samuel Asante[3], and Mathias Adjei[4], *1. Miami University Scripps Gerontology Center, Oxford, Ohio, United States, 2. University of Massachusetts Boston, Boston, Massachusetts, United States, 3. Northeastern State University, Tahlequah, Oklahoma, United States, 4. Miami University, Oxford, Ohio, United States*

This study investigates the Long-term Services and Supports (LTSS) provided to individuals with intellectual and developmental disabilities (IDD) at two selected Special Schools. The primary objective was to enhance our understanding of services available to older adults with IDD from the perspective of the headteacher, housemothers, family members of IDD, and older adults. Using a robust qualitative case study approach, an in-depth interview was conducted to gather the insights of participants on services available to older individuals with IDD. A purposive sampling technique was employed, and interviews were conducted with all participants. The interview guide for older adults with IDD had graphical images to enhance comprehension and easy answering of questions. The study used the cumulative advantage and disadvantage model as a conceptual framework. The findings showed that family members of older adults with IDD have little influence in helping their loved ones achieve independence through some LTSS programs. The burnout for the housemothers was severe and a faith-based mechanism was the only coping strategy employed. Older adults with IDD felt more comfortable and included in daily activities in school than at home. The home environment was tagged as a place of stigma and stereotypes for them and felt like an unwanted environment. The government officials felt they had no academic knowledge on how to help older adults in the Special schools. This study makes a significant contribution to the academic discourse on demonstrating how to make LTSS more responsive to the needs of older individuals with IDD.

Abstract citation ID: igaf122.2327

### MEANING OF PARK-GOLF EXPERIENCE AMONG OLDER ADULTS WITH PHYSICAL DISABILITIES: A SALUTOGENIC APPROACH

Rokbit Lee, Sua Im, Yaeeun Yoo, and Jinmoo Heo, *Yonsei University, Seoul, Republic of Korea*

Park-golf is a simplified and casual version of golf that has been popular among older adults in Asian countries. Park-golf has been appealing to older adults due to its shallow learning curve. This phenomenological study explored the meaning of park-golf experience using Salutogenesis Theory. Salutogenesis is a concept that focuses on origins of health, and it focuses on the resources that contributes to promoting health. The participants in this study were older park-golf players with physical disabilities in South Korea. A total of eight older adults were selected through snowball sampling who were members of local park-golf clubs and involved in playing park golf at least 5 years (mean age = 66.1). Semi-structured in-depth interviews were conducted with the participants. Analysis of the transcripts revealed the following three main themes: comprehensibility, manageability, and meaningful activity. The result demonstrated that Park-golf plays an important role in enhancing the comprehensibility of older adults' lives, leading to increased predictability in life. Participants perceived that they had the ability and resources to cope with difficult moments in park golf, which contributed to effectively managing challenges in their daily lives. Participants have established their identity as accomplished park-golf players, and actively engage in social activities, contributing to meaningful and active aging within their local communities. This study emphasizes park-golf as a valuable leisure activity for older adults with physical disabilities, helping them manage daily stress, maintain a positive mindset, and build resilience for a meaningful life.

### SESSION 7070 (POSTER)

### ECONOMICS OF AGING

Abstract citation ID: igaf122.2328

### BRIDGING THE GAP: EXPLORING THE SYNERGY BETWEEN PET ECONOMY AND SUPER-AGED SOCIETIES FOR SUSTAINABLE WELL-BEING

Chen-Chang Su, *National Chung Cheng University., Minxiong, Chiayi, Taiwan (Republic of China)*

As global populations rapidly age, the social and economic challenges of super-aged societies—such as loneliness, rising healthcare costs, and fragmented community ties—demand innovative solutions. Simultaneously, the pet economy, driven by companion animals and pet-related technologies, has emerged as a potential catalyst for addressing these challenges. This study investigates the dual role of pets as emotional companions and economic drivers in aging populations, employing a mixed-methods approach combining quantitative analysis of national health surveys (N = 5,000 elderly participants) and qualitative interviews with stakeholders (pet owners, industry leaders, and policymakers). Preliminary findings indicate that pet ownership is significantly associated with reduced loneliness scores

($\beta = -0.32$, $p < 0.01$) and lower annual healthcare expenditures (15% decrease compared to non-owners). Furthermore, case studies from Japan and Germany highlight successful models of pet-inclusive senior housing and robotic pet adoption programs. The research proposes a framework for "pet-integrated aging ecosystems," emphasizing cross-sector collaboration among healthcare, technology, and urban planning. This study contributes to gerontology and consumer behavior literature by bridging the gap between silver economy strategies and pet industry innovations, while offering actionable insights for policymakers aiming to leverage pet-related interventions in aging societies.

Abstract citation ID: igaf122.2329

### CAREGIVING AND FINANCIAL HARDSHIP AMONG MIDDLE-AGED AND OLDER ADULTS

Seokmin Kim[1], and Kingsley Mbam[2], *1. University of Massachusetts Boston, Quincy, Massachusetts, United States, 2. University of Massachusetts Boston, Boston, Massachusetts, United States*

Studies have shown a negative association between caregiving and financial hardship. However, relatively few studies examined this association among middle-aged and older individuals, despite the increasing age of caregivers. Using data from the 2018 Health and Retirement Study, this study investigated the financial impact of caregiving in middle adulthood and later life. The respondents include individuals aged 51 years and older ($n = 4569$). Caregiving is assessed as a binary measure: 1= provided care at least once a month, 0 = did not provide care in the last month. Financial hardship is assessed utilizing four hardship indicators - difficulty paying bills, ongoing financial stress, medication reduction due to cost, and food insecurity. Respondents are considered to be in financial hardship if they experienced two or more of the four hardship indicators. Results from logistic regression models showed that providing care in middle and old age is associated with a substantially higher likelihood of experiencing financial hardship (OR: 1.36; 95% CI: 1.14-1.62). These findings call for policy interventions to help middle-aged and older caregivers maintain financial stability and security. Out-of-pocket cost reimbursement programs can be developed to reimburse caregivers for expenses incurred in their provision of care.

Abstract citation ID: igaf122.2330

### DYNAMIC MULTIDIMENSIONAL ANALYSIS OF ACTIVE AGING IN ITALY: EFFECTS OF REGIONAL ECONOMIC GROWTH

Davide Lucantoni[1], Valerio Intraligi[1], Bernard Steinman[2], Mirko Di Rosa[1], Giovanni Lamura[1], Francesco Barbabella[1], Marco Socci[1], and Andrea Principi[1], *1. INRCA - National Institute on Health and Science of Aging, Ancona, Marche, Italy, 2. University of Wyoming, Laramie, Wyoming, United States*

The United Nations and European policy bodies encourage national governments to promote and implement active aging (AA), to address social and economic challenges linked to population aging. A crucial feature of AA as a construct is its multidimensionality. Indeed, a measuring tool of AA is the Active Ageing Index (AAI), a composite index which encompasses four domains (i.e., 1. Employment; 2. participation in society; 3. independent healthy and secure living; and 4. capacity and enabling environment for AA) for a total of 22 indicators within those domains. The aim of this study was to assess whether and to what extent AA was implemented in a multidimensional way within all the (20) Italian regional governments, over the period 2007-2018. An Italian ad-hoc version of the AAI was used. First, it was measured the percentage contribution of each AA domain to the overall growth of AA during the study's time-frame. Then, ordinary least squares regression models were employed, to assess whether regional economic growth was correlated with the observed changes in the different AAI domains. Results show that changes in the AAI scores in Italy's administrative regions are predominantly driven by changes in the employment domain. Thus, the implementation of multidimensionality was mostly neglected. Moreover, regional economic growth resulted to be positively associated with changes in the employment domain and negatively associated with changes in the participation in society domain. These results entail important implications for policy-making, as they highlight the need to promote AA multidimensionality more extensively.

Abstract citation ID: igaf122.2331

### ECONOMIC BENEFITS OF CARE COORDINATION FOR OLDER ADULTS

Erblin Shehu[1], and Brian Kaskie[2], *1. State University of New York at Cortland, United States, 2. University of Iowa, Iowa City, Iowa, United States*

In response to rising costs associated with providing healthcare services to Americans over 65 years old, policymakers have called for the expansion of care coordination programs to reduce total spending while improving patient outcomes. This study estimates the financial impact of the Iowa Return to Community (IRTC) initiative, a care coordination program designed to identify at-risk older adults and support their transitions from nursing homes and hospital settings to community living. We apply a Markov chain model with Monte Carlo simulations to capture all possible transitions among hospitals, nursing homes, and community settings. Using de-identified data from the State of Iowa ($N = 523$; 2020–2022) and a statistically matched control group from the National Health and Aging Trends Study (NHATS, 2020–2021), we compare IRTC outcomes with projections of what would occur without the program. Matching criteria include race, ethnicity, gender, income, living status, and 12 measures of instrumental (IADLs) and basic (ADLs) activities of daily living. Findings suggest IRTC participation is associated with reduced healthcare service utilization, resulting in per capita cost savings of $7,920.24 over five years. Subgroup analysis reveals that including informal care partners (ICPs) further increases these savings, as their involvement was linked to lower inpatient hospitalizations and deferred nursing home admissions. Findings call for IRTC expansion as a viable strategy to reduce aggregate healthcare spending while supporting aging in place.

Abstract citation ID: igaf122.2332

### FINANCIAL BURDEN AND ITS IMPACT ON THE LIVES OF FAMILY CAREGIVERS WITH AN OLDER LONG-TERM CARE RECIPIENT

Ayumi Honda, *St. Mary's College, Kurume, Fukuoka, Japan*

This study used a cross-sectional design to examine caregiver characteristics and financial burden associated with the cost of utilizing long-term care services for an older relative among 427 family caregivers. The proportion of family caregivers experiencing financial burden was significantly higher among those living with an older care recipient compared to those whose older care recipient was in a care facility (68.2% vs. 57.3%; $p = 0.049$).

Among 198 family caregivers living with an older care recipient, greater severity of functional disability in the recipient was significantly associated with financial burden and its impact on their lives ($p = 0.004$). On the other hand, among 229 family caregivers of an older care recipient utilizing institutional care, those bearing the costs of long-term care were more likely to experience financial burden and its impact on their lives ($p < 0.001$). Among the family caregivers, experiencing financial burden and its impact on their lives related to poorer health and greater caregiver burden, along with a lower annual household income and bearing the caregiving costs. We found that financial shortages in older care recipients had a negative impact on their family caregivers' work, private lives, and care choices. Community-based family care plays a crucial role in supporting stress coping for family caregivers facing financial hardship.

Abstract citation ID: igaf122.2333

### FINANCIAL CAPABILITY AND ECONOMIC WELL-BEING IN LATER LIFE: A POPULATION-BASED STUDY

Yu-Chih Chen, *National Taiwan University, Taipei, Taipei, Taiwan (Republic of China)*

Financial capability, the interaction of financial literacy, access, and behavior, can influence individuals' ability to manage finances and build economic stability over the life course. However, empirical evidence linking financial capability and economic well-being in old age is limited. We examine the components and mechanisms of the financial capability framework and investigate the differences between middle-aged (aged 50–64) and older adults (aged 65+) using nationally representative data in the United States. 12,840 individuals aged 50 and over were selected from the population-based 2018 National Financial Capability Study. Structural equation modeling and multigroup comparison were used to examine the mechanisms of financial capability on economic well-being and the moderating effects of age. Results showed that financial literacy and access were positively associated with financial behavior and subsequent economic well-being. The mediation results showed that financial literacy and access were equally important in contributing to economic well-being in later life. However, age had no moderating effect, suggesting these associations operated similarly in middle-aged and older adults. Findings suggest that strategies to promote economic well-being in later life through effective finance should be multifaceted, targeting each aspect of financial capability. Practices such as financial coaching and guidance, credit counseling, and financial profile assessments should be integrated into human and social services. Policies and programs that expand financial inclusion, particularly investments in accessible and appropriate financial products, should be developed to reach and cover the financially underserved, especially those with limited financial literacy and restricted access to mainstream financial services.

Abstract citation ID: igaf122.2334

### OCCUPATIONAL LICENSING IN LATER LIFE: WORK AND RETIREMENT DECISIONS BEFORE AND AFTER THE COVID-19 PANDEMIC

Yun Taek Oh, *University of Nevada, Reno, Reno, Nevada, United States*

Attaining occupational licenses is considered a career development option for younger workers because the attainment cost outweighs the benefits for older workers. Contrary to this expectation, a significant proportion of workers attain licenses in their later lives. However, the reasons and outcomes of later-life attainment of occupational licenses are not well-documented. This study investigates and compares the sociodemographic and economic predictors of later-life attainment of occupational licenses and the post-attainment work and retirement decisions before and after the COVID-19 pandemic. Using the sample of respondents aged between 55 and 63 from the Current Population Studies 2018-2019 (men = 1,927 and women = 1,576) and 2023-2024 (men = 1,501 and women = 1,182), I first conduct logistic regressions to examine the predictors of later-life attainment. Then, I use clustered competing risk analysis with propensity score matching to compare the choice of retirement pathways of newly licensed and unlicensed workers in pre- and post-pandemic cohorts. The results show that men's license attainment decreased ($OR = 0.439$) after the pandemic while women's attainment increased ($OR = 8.399$). Furthermore, newly licensed women are more likely to move to different employers than unlicensed women before and after the pandemic (SHR = 1.50 and 1.23 respectively), while newly licensed men are more likely to switch to part-time than unlicensed men after the pandemic (SHR = 3.75). These results imply that the purpose of later-life attainment of occupational licenses varies by gender and period yet contribute to longer work lives by taking on alternative retirement pathways.

Abstract citation ID: igaf122.2335

### PROJECTED HEALTH AND ECONOMIC IMPACT OF LONG-TERM LIFESTYLE INTERVENTIONS AMONG OLDER ADULTS

Xueying Guo, and Cynthia Chen, *National University of Singapore, Singapore, Singapore*

Singapore's rapidly aging population presents an urgent challenge for healthcare sustainability. As chronic disease prevalence rises, proactive policy interventions are essential to mitigate future healthcare costs and disease burdens. Using the Future Elderly Model, a dynamic Markov microsimulation, we projected chronic disease trends and hospitalization costs from 2020 to 2050 while evaluating four HealthierSG-inspired interventions: (i) improved blood pressure management, (ii) increased physical activity, (iii) sodium reduction, and (iv) all combined. Without intervention, diabetes, heart disease, hypertension, stroke, obesity, and disability will increase, placing greater strain on Singapore's healthcare system. Lifetime hospitalization costs were projected to be highest for Indians (US$93,900), followed by Chinese (US$75,700) and Malays (US$70,000). Although Malays had a higher chronic disease burden, their lower lifetime costs were due to shorter life expectancy. Implementing interventions could save up to US$505 million, with the Chinese benefiting the most, followed by Malays and Indians. These savings highlight the economic value of long-term preventive measures. This study underscores the critical role of preventive care in reshaping Singapore's healthcare landscape. Investing in sustainable lifestyle interventions can curb rising disease rates, reduce financial strain, and improve long-term health outcomes. Policymakers must prioritize prevention strategies tailored to diverse population needs, ensuring a healthier and more sustainable future for Singapore.

Abstract citation ID: igaf122.2336

### THE ECONOMIC IMPLICATIONS OF THE COVID-19 PANDEMIC ON OLDER ADULTS IN THE U.S

Michael Vetter, *MGH - Center for Optimal Aging and Serious Illness, Danvers, Massachusetts, United States*

Older adults were among those most disproportionately affected by the Covid-19 pandemic. Research over previous years has outlined the changes to health, loneliness, and long-term well-being outcomes, in addition to economic impacts. This project recognizes and addresses a gap in the literature pertaining to the role of government economic impact payments (EIP) and labor force participation during the pandemic for older adults. Leveraging the Health and Retirement Study's 2020 Core Survey and 2021 "Perspectives on the Pandemic" supplement, results highlight the demographic characteristics of the population of respondents (n = 2167) and the distribution of changes to economic and labor outcomes resulting from the pandemic. Most respondents noted having received an EIP and used this payment to primarily pay off existing debt, followed closely by contributing to savings. The majority were not working at the time of receipt and saw few changes to their economic situation throughout the pandemic. However, a stratified analysis of the payment amount showed that women received, overall, 31% less than men. Findings indicate that while labor force participation among this group was low, use of the economic payment indicates a need to address personal debt. Moreover, the gendered difference in total amount received may be the result of several factors, including the division of labor and labor force participation in older generations and the responsibilities of caregiving duties falling to women rather than men over the life course.

### SESSION 7075 (POSTER)

### EDUCATION AND TRAINING: WORKFORCE DEVELOPMENT

Abstract citation ID: igaf122.2337

### EVALUATING A PROGRAM ADDRESSING THE CERTIFIED NURSING ASSISTANT (CNA) AND HOME HEALTH AIDE (HHA) WORKFORCE SHORTAGE

Brittany McFeeley[1], Natasha Bryant[2], Verena Cimarolli[2], and Robyn Stone[2], *1. University of Massachusetts Boston, Boston, Massachusetts, United States, 2. LeadingAge, Washington, District of Columbia, United States*

The Gateway-In Project© (TGIP©) was established to expand the CNA and HHA workforce by recruiting students, providing free training, supports, and job placement assistance for new CNAs and HHAs in California. To assess TGIP©'s impact, we surveyed Year 1 graduates for employment status and career plans 12-18 months post-graduation. We also surveyed Year 2 students before (baseline) and after training completion to determine changes in students' views of working in aging services, their financial and employment status. Students of both cohorts were mostly women of color, 35 years or younger, and had low household incomes. Over 80% of Year 1 students (N = 271), were employed as CNAs, HHAs, or both with close to 70% working in skilled nursing facilities. Working as a CNA/HHA was predominantly (74%) perceived as "a steppingstone to furthering their career" over "a career" or "job to make money." Most students (73%) reported planning to advance to another occupation, particularly to a higher nursing degree/occupation. Year 2 students (N = 146) showed significantly lower interest in working as a CNA/HHA (t=-4.923, p=.004) and in working with older adults (t=-2.78, p=.006) after training completion compared to baseline. There was a significant increase in household income (t = 2.140, p=.034). Most were employed as CNAs. TGIP© evaluation demonstrates employment gains, important career advancement aspirations, and increased household incomes. Decreases in interest in working as CNAs/HHAs and with older adults may be due to the job's demands becoming clearer during training. The ongoing evaluation efforts will provide further insights into TGIP©'s long-term impact.

Abstract citation ID: igaf122.2338

### FUTURE-READY CARE: INTEGRATING GEROSCIENCE INTO HEALTH PROFESSIONS TRAINING

Gregory Hartley[1], Patricia Cuff[2], and Cathy Maxwell[3], *1. University of Miami, Coral Gables, Florida, United States, 2. National Academies of Sciences, Engineering, and Medicine, Washington, District of Columbia, United States, 3. University of Utah, Salt Lake City, Utah, United States*

With increasing life expectancy and a growing aging population, healthcare systems must shift from a reactive, disease-centered approach to one that promotes healthy aging. It is the role of health professionals and their educators to move society in this direction. Such a transformation requires a unified approach to integrating principles of geroscience, interprofessional collaboration, and proactive aging strategies. A national call to action (unpublished National Academy of Medicine Perspective), collectively written by health professional educators from six health professions, directs colleagues on steps they can take to advance the principles and promote provider competence in the care of older adults. Calls to action were developed within three domains: Shifting Societal Norms; Focusing on Healthspan; and Educating a Competent Workforce. Each domain promotes four calls to action, with the top priorities listed here. • To shift societal norms, health professionals and educators must advocate for policy reforms that incentive a holistic approach to healthy aging. Demonstrating the financial return on investment will be key to successfully promoting policy reforms. • To influence individual patients' lives, health professional providers should become educated on georscience and healthspan principles, and effectively communicate evidence to patients. Those who have limited understanding of the aging process should pursue professional development in geroscience and caring for older patients. • To educate the future generation of care providers, educators must incorporate into curricula, evidence-based data and information on geroscience and best practices in caring for older adults. Educating interprofessionally will strengthen learners' understanding of the wider health system.

Abstract citation ID: igaf122.2339

### PROFESSIONALIZING GERONTOLOGY: WHY IT IS ESSENTIAL TO CREDENTIAL THE EDUCATION OF GERONTOLOGISTS

Judith Sugar[1], and Donna Schafer[2], *1. University of Nevada, Reno, Reno, Nevada, United States, 2. National Association for Professional Gerontologists, Healdsburg, California, United States*

The National Association for Professional Gerontologists (NAPG) has credentialed the education and professional expertise of practicing gerontologists since 2007, in the absence of licensing in any U.S. state or Canadian province. To date, nearly 400 professionals have received a credential. Today it is more important than ever to credential gerontologists and professionalize gerontology. While many disciplines produce practitioners who support older adults and their families, it is graduates of gerontology degree programs who have the interdisciplinary breadth of knowledge, informed by a set of key gerontology competencies, to respond holistically to the multifaceted challenges experienced by older adults. First developed more than 30 years ago, the key gerontology competencies are periodically updated by GSA's Academy of Gerontology in Higher Education (AGHE) and widely used by gerontology programs. Given the projected increase in the older population, the need for more trained gerontologists is apparent. However, the field has not kept pace with the need for qualified, trained gerontology practitioners by producing more graduates. As data presented in this poster indicate, there are no more gerontology degree programs today than there were 20 years ago. The poster also presents data derived from a survey of almost 100 credentialed NAPG members. Survey responses identify barriers to employability and career mobility, including an inadequate understanding on the part of employers about a trained gerontologist's skills and competencies. The poster concludes with suggestions for enhancing the professional stature of credentialed gerontologists.

Abstract citation ID: igaf122.2340

### RETHINKING DEMENTIA AWARENESS TRAINING: HOW ELAPSED TIME SHAPES KNOWLEDGE RETENTION AMONG BUSINESSES

Elena Ionescu[1], and Monit Cheung[2], *1. California State University, Long Beach, Houston, Texas, United States, 2. University of Houston, Houston, Texas, United States*

Dementia-Friendly Businesses (DFBs) play a crucial role in fostering inclusive environments for individuals living with dementia. While workforce training aims to enhance dementia-friendly awareness, little research has examined how training mode or time elapsed since training may influence long-term knowledge retention and behavioral adaptation. This study assessed dementia knowledge retention among 510 employees in DFB settings, evaluating whether training mode or time elapsed had a greater impact on knowledge retention for longer-term dementia-friendly awareness. Guided by the Theory of Planned Behavior (TBP) and Dementia-Centered Person Care (DCPC), findings show that the training mode does not significantly vary workforce dementia awareness. A two-way ANOVA examined the effects of training mode and time elapsed on dementia awareness scores. The training mode was insignificant ($F(2, 501) = .709$, $p = .492$), suggesting that in-person, hybrid, and online training were equally effective. However, time elapsed significantly impacted awareness ($F(2, 501) = 17.547$, $p < .001$), with employees trained over a year ago exhibiting higher scores ($M = 132.93$, $SE = 1.43$) than those trained 6–12 months ago ($M = 128.46$, $SE = 1.27$, $p < .001$) or < 6 months ago ($M = 119.89$, $SE = 1.68$, $p < .001$). Post-hoc analyses confirmed that awareness increases over time, regardless of training mode. Findings reinforce the importance of sustained engagement and exposure in strengthening dementia awareness, aligning with the TBP's emphasis on sustained normative influences and DCPC's focus on continued, person-centered learning. Future research should explore optimal reinforcement strategies to sustain dementia awareness in business settings.

Abstract citation ID: igaf122.2341

### SUPPORTING THE SEXUAL HEALTH OF PEOPLE WITH DEMENTIA: DEVELOPING CONFIDENT CARE TEAMS THROUGH ONLINE EDUCATION

Birgit Pianosi[1], Katelynn Aelick[2], Lori Schindel Martin[3], Kristy McKibbon[4], Rosemarie Mangiardi[5], and Em Thielking[6], *1. Laurentian University, Sudbury, Ontario, Canada, 2. North Bay Regional Health Centre, Sudbury, Ontario, Canada, 3. Toronto Metropolitan University, Toronto, Ontario, Canada, 4. Hamilton Health Sciences, Hamilton, Ontario, Canada, 5. Ontario Health, Toronto, Ontario, Canada, 6. North Bay Regional Health Centre, North Bay, Ontario, Canada*

Healthcare providers (HCPs) often feel unprepared to respond to the sexual health needs of people with dementia. Behavioural Supports Ontario's (BSO) Sexual Expression and Dementia working group developed a 3-part e-learning series to address the need for HCP education in this domain. The program, Supporting the Sexual Health of People with Dementia, informed by expert consultation and over 300 academic sources, is designed to equip HCPs with the tools and resources they need to support people with dementia, specifically those residing in long-term care (LTC) homes. The first module of the program challenges common myths about sexuality and dementia. Module 2 provides practical tools and strategies for communicating with people living with dementia in LTC homes about their sexual health needs. The third module guides learners through assessing the risk of sexual expressions and outlines strategies to reduce risk in a manner that upholds LTC residents' dignity. Throughout the series, learners engage in case studies of diverse people living with dementia. Learners follow LTC residents' journeys as HCPs and care partners collaborate to understand and respond to residents' unmet needs in person-centered ways. Following the launch of the online program in February 2025, it was evaluated using optional pre- and post-learning surveys. The purpose of the evaluation was to measure change in learners' confidence and competence in supporting the sexual health of people with dementia. The results of this evaluation will be used to inform the development of future resources and education for healthcare providers on this topic.

Abstract citation ID: igaf122.2342

### USING COMMUNITIES OF PRACTICE IN THE HOME AND COMMUNITY-BASED SERVICE WORKFORCE

Sandi Lane[1], Caroline Yoon[2], Zavera Basrai[2], Yisel Pomier Maren[3], Trish Farnham[4], Kezia Scales[5], Gwen Tanner[6], and Nathan Boucher[2], *1. The University of North Carolina at Charlotte, Charlotte, North Carolina, United States, 2. Duke University, Durham, North Carolina, United States, 3. National Domestic Workers Alliance, Charlotte, North Carolina, United States, 4. North Carolina Court of Appeals, Wilmington, North Carolina, United States, 5. PHI, New York, New York, United States, 6. Paraprofessional Healthcare Institute, New York, New York, United States*

Older adults and people living with disabilities receive home- and community-based services (HCBS) from approximately 115,000 often inadequately supported direct service workers (DSW) in North Carolina (NC). The demand for these workers is increasing rapidly yet fragmented long-term services and support system (LTSS) deter new entrants into these careers. The previous stage of this Centers for Medicare/Medicaid Services (CMS)-funded project identified communication and relationship building as key competencies that DSW need to build career pathways, elevate skills and recognition for their work, and improve job quality and retention. Our project team then conducted a pilot DSW training program: asynchronous virtual training modules, virtual facilitated learning labs, and communities of practice (CoPs) led by direct service workers. The training program was designed to build knowledge and skills to support better communication with family caregivers through active listening, self-awareness, and self-management. A total of 50 DSWs from the categories of Home Health Aides, In-Home Aides providing limited assistance, personal care aides, direct support professionals, and self-directed direct care workers participated. Upon completion of asynchronous training, participants engaged in a two-hour learning lab facilitated by direct service workers followed by a series of DSW-facilitated peer learning groups called CoPs. These combined learning experiences encourage workers with lived experiences to engage in shared learning, peer support, and collective problem-based learning, which is crucial for reinforcing and contextualizing their knowledge leading to sustainable skills application in the workplace. We will discuss learnings from our process and the opportunity to scale the CoPs model for DSWs.

### SESSION 7080 (POSTER)

### EMPLOYMENT AND OLDER WORKERS

Abstract citation ID: igaf122.2343

### A DECADAL SHIFT IN TIME USE AMONG CHINESE OLDER ADULTS: A COMPARATIVE ANALYSIS OF THE 2008 AND 2018 TIME USE SURVEYS

Jing Ye, and Hongyan Yang, *Wuhan University, Wuhan, Hubei, China (People's Republic)*

China is undergoing rapid population aging, with 22% of its population aged 60 and above in 2024 and an increasing dependency ratio. In response to mounting labor shortages and pension pressures, the government initiated a progressive extension of the retirement age in September 2024. Against this backdrop, this study investigates the evolving time-use patterns of older adults (aged 60 and above) by analyzing data from the 2008 and 2018 China Time Use Surveys. Findings reveal that, for paid labor, the employment duration of older adults increased significantly (with workdays rising by 0.28 hours and rest days by 0.30 hours), while weekday household production participation rate declined from 36% to 24%. This reduction in total labor time underscores tensions with delayed retirement policies. Regarding unpaid labor, although overall housework participation fell from 79% to 70% on workdays, time devoted to childcare increased by 0.12 hours, with persistent gender disparities—women engaged in 1.53 more hours of housework than men. Leisure and socialization activities shifted notably, with internet use growing by 16% on mobile devices and leisure time expanding by 1.37 hours on workdays, though traditional activities such as television viewing and fitness remained dominant. Additionally, self-care practices improved, as indicated by increased time spent on sleep and meals. These results suggest a transition toward lighter labor burdens and enriched leisure activities among older adults, while also reinforcing intergenerational support despite enduring traditional gender roles. The study offers empirical insights to inform policies on retirement reform and caregiving infrastructure in aging societies.

Abstract citation ID: igaf122.2344

### HOW DOES AGE DISCRIMINATION LIMIT THE MENTAL HEALTH BENEFITS OF ACTIVE AGING?

YoungBin Koh, and Yeonjung Lee, *Chung-Ang University, Seoul, Republic of Korea*

Activity theory suggests that participating in social activity or having a job improves overall physical and mental health in older people. Meanwhile, perceived age discrimination is a substantial stressor because they can adversely affect mental health. It is known that specific major events of discrimination are often tied to life-course experiences that are less likely to occur in later life. However, as the benefits of active aging have been emphasized, more older adults are engaged in employment and social activity, which they might be exposed to the potential stressor of experiencing discrimination due to their age. Since the Senior Employment and Social Activity Program (SESAP) was introduced in Korea, many studies have evaluated the effects of programs in various ways. However, not much attention has been paid to whether age discrimination maybe get in the way of benefitting from participation in SESAP. The purpose of this study is to examine the moderating effect of perceived age discrimination on the relationship between participation in SESAP and depression. Data from the Senior Citizen Survey in 2020 was (N = 9,201). Results show that the association between participation in SESAP and depression is significant and negative, but only at a low level of age discrimination. Essentially, age discrimination negates the mental health benefits of participating in work and social activity in later life. Findings have implications for policy and practice such as strengthening the laws to prevent age discrimination and expanding education programs to raise awareness of the consequences of age discrimination.

Abstract citation ID: igaf122.2345

### OVERCOMING CAREER PLATEAUS: ENHANCING SOCIAL PARTICIPATION AND WELL-BEING AMONG MIDDLE-AGED AND OLDER ADULTS

Pei-Ying Yu[1], Ai-Tzu Li[2], and Wen-Bing Gau[2], *1. National Chung Cheng University, Taiwan (Republic of China), 2. National Chung Cheng University, Minxiong, Chiayi, Taiwan (Republic of China)*

Amid the global movement toward active and productive aging, there is a growing emphasis on promoting sustained employment among middle-aged and older adults to enhance social participation. In Taiwan, the combined effects of population aging and declining birth rates have led to continuous negative population growth and structural demographic shifts. These trends underscore the importance of developing and utilizing the middle-aged and older workforce, not only to enhance individual well-being but also to address labor demands in a super-aged society and foster positive social development. This study examines the career trajectories of middle-aged and older individuals, explores the challenges they encounter, and investigates career plateaus along with corresponding coping strategies. Using a qualitative research approach with purposive sampling(N = 12), semi-structured, in-depth interviews were conducted, examining three key perspectives. The thematic analysis revealed three key findings: (1) Middle-aged and older employees exhibit strengths such as workplace stability and professional expertise, with adaptability and continuous learning being crucial for career development; (2) Employment challenges are multifaceted, encompassing psychological well-being, job skills, and workplace interactions; (3) Effective strategies for overcoming career plateaus should integrate personal career planning with external support systems. The findings provide insights for adult education institutions, emphasizing the importance of continuous, personalized career development and goal setting, enhancing learning motivation, fostering an age-inclusive environment, and establishing support networks that facilitate intergenerational learning. For businesses, fostering a fair workplace culture, offering diverse and flexible employment options, and promoting lifelong learning mechanisms are crucial for building an inclusive and supportive work environment.

Abstract citation ID: igaf122.2346
### SELF-EMPLOYMENT AND COGNITIVE FUNCTION AMONG OLDER INDIVIDUALS: EVIDENCE FROM THE HEALTH AND RETIREMENT STUDY

Kingsley Mbam[1], and Yan-Jhu Su[2], *1. University of Massachusetts Boston, Boston, Massachusetts, United States, 2. The Pennsylvania State University, University Park, Massachusetts, United States*

A large body of research has shown that employment is related to cognitive functioning. However, how self-employment impacts cognition among older individuals is largely unaddressed. This study looks to address this gap by examining whether cognitive function changes over time among self-employed older individuals. This longitudinal study used data from 1998 to 2016 waves of the Health and Retirement Study (HRS) for participants who completed at least two waves and provided valid responses to the cognitive function scale (N = 19,242). Participants had 1998, 2004, and 2010 as baseline waves and were followed from these baseline waves until the 2016 wave. Cognitive function was a time-variant variable measured at the baseline and over time and was accessed with the modified version of the Telephone Interview for Cognitive Status (TICS). Self-employment was a time-invariant variable measured at the baseline only, which differed for participants depending on the wave they entered the study. Linear mixed-effects models were utilized to examine the impact of self-employment on cognitive function at the baseline and over time. Adjusted models results show that self-employed participants had higher cognitive function scores ($b = 0.63$, $p = 0.001$) than not self-employed participants at the baseline wave(s). The results also revealed that the interaction term between self-employment and time was statistically significant ($b = -0.07$, $p < 0.001$), suggesting variation in cognitive decline rate between self-employed individuals and not self-employed individuals. Strengthening existing socioeconomic policies to protect self-employed older individuals will ensure they remain cognitively active with self-employment for improved cognitive health.

Abstract citation ID: igaf122.2347
### WORK CONDITIONS AND LIFE SATISFACTION AMONG OLDER AMERICAN ADULTS: THE MODERATING ROLE OF MARITAL STATUS

Kingsley Mbam, and Jeffrey Burr, *University of Massachusetts Boston, Boston, Massachusetts, United States*

Although past studies have explored the connection between work conditions and life satisfaction among older adults, whether this association varies by marital status is largely unaddressed. This study aimed to examine the relationship between work conditions and life satisfaction among older American adults and to investigate whether marital status moderates this association. This study analyzed models of life satisfaction utilizing data from the 2020 wave of the Health and Retirement Study for respondents aged 55-75 (N = 937). Linear regression models were utilized to analyze the association between a variety of subjective work conditions and life satisfaction and whether marital status conditions this association. Life satisfaction was assessed utilizing Diener's measure of life satisfaction. Marital status was measured as follows: 1=married, 0=not married. The results of the linear regression models showed that some work conditions - better mood at work due to family or personal life ($b = 3.81$, $p = 0.000$), better mood at home due to job ($b = 2.94$, $p = 0.000$), work gives energy to do things with family and other people ($b = 2.86$, $p = 0.000$), and job worries distraction when not at work ($b = -4.03$, $p = 0.000$) - are significantly related to life satisfaction, controlling for relevant covariates. The results also revealed that the interaction of job worries distraction when not at work and married for life satisfaction was statistically significant ($b = 3.35$, $p = 0.04$). These results suggest that fostering supportive and favorable working conditions for older workers can enhance their life satisfaction, which is important to improving their overall health and well-being.

Abstract citation ID: igaf122.2348
### WORK, FATIGUE, AND WELL-BEING: THE MEDIATING EFFECT OF TIREDNESS ON LIFE SATISFACTION IN OLDER ADULTS

Hyejoong Kim, and Jaeon Yoo, *Gachon University, Seongnam, Kyonggi-do, Republic of Korea*

This research examines the relationship between older adults' labor participation and life satisfaction, with a focus on whether tiredness mediates this relationship. South Korea has the highest labor force participation rate among older adults across OECD countries, surpassing Japan (25.6%), the United States (18.9%), and the United Kingdom (10.5%). The average retirement age in South Korea is 72.3 years, which is 12.3 years higher than the statutory retirement age of 60, making it the highest among OECD nations. Even after reaching the national pension eligibility age of 63, many older adults remain in the labor market and continue to be economically active. As the elderly population grows, the number of older individuals participating in economic activities is expected to increase, making it critical to understand how work participation affects their life satisfaction. This study analyzes data from the 2019 Korea Time Use Survey, using a sample of 6,570 individuals (3,855 women (58.7%) and 2,726 men (41.5%)). The findings reveal two key insights. First, tiredness mediates the relationship between work hours and life satisfaction, meaning that longer work hours reduce life satisfaction primarily by increasing fatigue. Second, total work hours alone do not significantly impact life satisfaction, reinforcing tiredness as a key mediator. These results suggest that reducing work-related fatigue is essential for improving life satisfaction among older adults. Workplace interventions, such as flexible working hours, regular rest breaks, and fatigue management strategies, could help mitigate the negative effects of prolonged work hours and support the overall well-being of older workers.

Abstract citation ID: igaf122.2349
### WORKPLACE DISCRIMINATION AND MENTAL HEALTH IN KOREAN BABY BOOMERS: THE ROLE OF WORK ENGAGEMENT

Gaeun Han, and Giyeon Kim, *Chung-Ang University, Seoul, Republic of Korea*

As Korean Baby Boomers, born between 1955 and 1974, continue to participate in the workforce actively, they face workplace discrimination, which negatively affects their mental health. Work engagement can mitigate the negative effects of workplace discrimination but is also influenced by those experiences. Using data from the 7th Korean Working Conditions Survey (KWCS) conducted in 2023, this study investigates the mediating and moderating effects of work engagement on the relationship between workplace discrimination and mental health among wage-employed Korean Baby Boomers (N = 12,113). A moderation and mediation analysis were conducted by PROCESS macro using SPSS 28.0. Results show that: first, workplace discrimination was negatively associated with both mental health (B=-.534, p<.001) and work engagement (B=-.441, p<.001), indicating that an increase in workplace discrimination experiences have a negative impact on mental health and work engagement. Second, a significant indirect effect of work engagement was found (B=-.501, CI=-.621, -.382), while no direct effect was observed, indicating an indirect-only mediation effect on the relationship between workplace discrimination and mental health. Third, work engagement moderates the relationship between workplace discrimination and mental health, with the effect being significant only for the high work engagement group (B=-.101, p<.05). However, work engagement exacerbated the negative impact of workplace discrimination on mental health. These findings contribute to the Job Demands-Resources (JD-R) model by uncovering the complex characteristics of work engagement among aging workers. The study underscores the need for targeted interventions to reduce workplace discrimination and enhance mental health among Baby Boomers, particularly through work engagement strategies.

### SESSION 7085 (POSTER)

### FAMILY CAREGIVING I

Abstract citation ID: igaf122.2350

### ASSOCIATED FACTORS OF CAREGIVER BURDEN IN PARKINSON'S DISEASE: A SYSTEMATIC REVIEW AND META-ANALYSIS

Wilfred Wing Fung Sin, Lily Man Lee Chan, Christine Tsz Ying Ng, Edmond Pui Hang Choi, Kris Yuet Wan Lok, Daniel Yee Tak Fong, and Jojo Yan Yan Kwok, *The University of Hong Kong, Hong Kong, China (People's Republic)*

Informal caregiving for Parkinson's disease (PD) patients could be challenging and often results in severe caregiver burden. This systematic review and meta-analysis, guided by the Stress-Appraisal Model, aimed to identify factors associated with caregiver burden. A search of five databases from inception to February 2025 identified 66 studies involving 30,533 patients and 30,419 caregivers. Of 106 factors identified (78 patient-level, 28 caregiver-level), 41 were included in random-effects meta-analyses. Patients' neuropsychiatric symptom severity (r = 0.57, 95% CI = 0.35–0.73, p < 0.001) and caregivers' psychological distress (r = 0.46, 95% CI = 0.38–0.53, p < 0.001) showed the strongest, albeit moderate, associations with greater caregiver burden. Other factors—including patients' advanced disease stage, disability, disease duration, motor symptoms, and caregivers' caregiving hours, social support, and care dyad demographic characteristics—demonstrated weak associations with caregiver burden. Meta-regression indicated stronger correlation coefficients among studies from Southeast Asia and Western Pacific regions These findings highlight the multidimensional nature of caregiver burden of caregiver burden in Parkinson's disease informal caregivers, primarily associated with patients' neuropsychiatric symptom severity and caregivers' psychological distress, with cultural contexts amplifying burden in certain regions. Further research on psychological resilience, A paradigm shift is need to prioritize modifiable traits and protective factors among PD caregiver-patient dyads, and to explore system-level determinants to inform the development culturally adapted interventions for Parkinson's disease caregiving dyads.

Abstract citation ID: igaf122.2351

### CAREGIVER SPOUSES OF PERSONS WITH DEMENTIA WHO ENGAGE WITH AN EMERGENCY PREPAREDNESS TOOLKIT INTERVENTION

Anna Satake[1], Rebecca Boxer[1], Andrea Daddato[2], Kris Wain[2], Blythe Dollar[2], and James Lagrotteria[2], *1. University of California, Davis, Davis, California, United States, 2. Kaiser Permanente Colorado, Aurora, Colorado, United States*

Hospitalizations of a spousal caregiver (SC) of a person with dementia (PWD) can significantly disrupt caregiving and negatively impact the SC. This study conducted a low-touch intervention by offering a SC an emergency preparedness toolkit (EPT) to prepare for their own unexpected hospitalization. In January 2023, 1,062 caregivers of PWD who live together and are not institutionalized were invited to request the EPT and use it to prepare for their own healthcare emergency. Of caregivers notified, 489(46%) requested the EPT. Caregivers who didn't request the EPT n = 573(54%) were more likely to be male [261(46%) vs 188(38%);p=.02], have higher Elixhauser comorbidity scores (3.0±2.6 vs 2.5±1.9, p<.01), have more ED visits[132(23%) vs 88(18%)p=.04], and hospitalizations [77(13%) vs. 34(7%);p = <.01] in the prior 12 months compared to caregivers who requested the EPT. Caregiver outcomes by EPT request were measured 6-month after the EPT was sent. Caregivers who didn't request the EPT were no different in number of hospitalizations [52(9% vs 34(7%), p=.2] than those who did; however, they had a higher number of deaths [13(2%) vs 2(.4%), p=.01] at 6 months. For the caregiver, there were no significant differences in outcomes such as ED visits/hospitalizations between the two groups. Findings indicate that the health severity of caregivers of PWD was associated with the caregiver's response to a supportive intervention. Outreach and engagement to caregivers may need to be tailored to a range of caregiver health conditions and severity to be successful. Health of caregivers and their uptake of support and resources warrants further study.

Abstract citation ID: igaf122.2352

### DEPRESSIVE SYMPTOMS AMONG CHINESE AND KOREAN IMMIGRANT FAMILY DEMENTIA CAREGIVERS: A PILOT STUDY

Mina Lee[1], Kun Wang[2], Daniel Park[3], Jueon Kim[4], Ian Choi[5], Abigail Choi[6], and Yeoeun Kim[4], *1. Binghamton University, Binghamton, New York, United States, 2. University of Alabama at Birmingham, Birmingham, Alabama, United States, 3. Hackensack Meridian School of Medicine, Netley, New Jersey, United States, 4. East Coweta Highschool, Sharpsburg, Georgia, United States, 5. Riverside High School, Greenville, South Carolina, United States, 6. Northview High School, Johns Creek, Georgia, United States*

Taking care of family members with Alzheimer's Disease/Alzheimer's disease and related dementias (AD/ADRD) places a huge burden on caregivers and such a burden puts them at risk of developing depressive symptoms. Older Chinese and Korean immigrant caregivers who face cultural and language barriers may face even greater risks. Despite these challenges, little research has explored the depressive symptoms and determinants among this high-risk and underserved population. Preliminary data were collected from 42 participants (average age: 71 years; 52% female) and the study is ongoing. Online and paper-pencil surveys in English, Mandarin, and Korean were administered. Eligibility to participate are (1) self-identified as Chinese or Korean, (2) aged 50 or older, and (3) caregiving for a family member with AD/ADRD. Most reported being U.S. citizens or permanent residents, half reported being the spouse of care recipient, and 70% lived with the care recipient. More than 70% reported that they had experienced depressive symptoms at least a day the week before the survey was administered. In the preliminary analysis, depressive symptoms were significantly related to the level of caregiving burden, general health status, self-reported and cognitive decline. This pilot study highlights that many Asian American family caregivers for people with AD/ADRD suffer from depressive symptoms. As much as caring for people with dementia matters, it is also equally as important to understand and acknowledge that caregiving work takes a huge physical and emotional toll. Culturally tailored support for older dementia caregivers is necessary to prevent these family caregivers from experiencing depression.

Abstract citation ID: igaf122.2353

### DEVELOPMENT OF THE BAJJAJJA INTERVENTION DESIGNED TO MEET GRANDMOTHER-CAREGIVERS' NEEDS IN UGANDA

Schola Matovu[1], Jacqueline Eaton[2], Lee Ellington[2], and Heather Young[3], *1. University of Utah College of Nursing, Salt Lake City, Utah, United States, 2. University of Utah, Salt Lake City, Utah, United States, 3. Betty Irene Moore School of Nursing at UC Davis, Sacramento, California, United States*

The financial, social, health and overall well-being of Ugandan grandmothers who care for their grandchildren has been significantly affected by years of ongoing socioeconomic, political, and historical HIV/AIDS-related challenges. We engaged a Community Advisory Board (CAB) of key community partners to co-develop a culturally responsive Building A Joint Action for JaJJAs (BAJJAJJA) intervention to address the most salient financial, social, and health challenges. Using a community-engaged and qualitative research approach, we worked with a CAB (N = 10) that consisted of grandmother-caregivers, local council leaders, income generating activity experts, and health providers from local health centers in a peri-urban setting of central Uganda. This participatory approach ensured that intervention development aligned with the Uganda National Council for Science and Technology's priorities for community engagement while also promoting acceptability and sustainability. The developed BAJJAJJA manual details three core intervention components: (1) Income-Generating Activities (IGA)—training in small business skills and cooperative saving practices to enhance financial security; (2) Social Support Groups—peer-led gatherings to strengthen emotional resilience and shared problem-solving; and (3) Health Coaching—education on chronic disease prevention, mental well-being, and self-care strategies with culturally relevant materials tailored for low-literacy populations. This study highlights the effectiveness of a community-engaged approach in designing interventions that address the multidimensional needs of aging grandmother-caregivers. Findings from this study will inform future research that will refine, adapt and test the efficacy of similar interventions in both low-resource and other global settings such as rural areas in the US.

Abstract citation ID: igaf122.2354

### DOES FAMILY CAREGIVER SELF-IDENTIFICATION MATTER? AN ANALYSIS OF SURVEY RESPONSES

Pamela Nadash[1], Eileen Tell[2]Chang Pu Liang[1], and Marc Cohen[1], *1. University of Massachusetts Boston, Boston, Massachusetts, United States, 2. ET Consulting, LLC, Bemont, Massachusetts, United States*

One of the five goals identified as a priority by the National Strategy to Support Family Caregivers is Goal 1, which centers on raising awareness of family caregiving. While this goal concerns awareness-raising on all fronts, raising awareness among caregivers themselves may be the most important, given its presumed role in accessing supports. As part of an evaluation supporting the Administration for Community Living, 4,573 representative family caregivers across 10 states were surveyed on a range of issues in the Fall/early Winter of 2024. This study focuses on the extent to which they self-identified as caregivers, using both quantitative and qualitative data. It found that while only 66% of respondents initially identified themselves as caregivers, after interviewers defined the term, 74% did. An open-ended question asking respondents to define caregivers found that participants regularly mentioned paid caregivers, rarely mentioned end of life or dementia caregiving, and frequently referred to the daily nature of caregiving and types of tasks performed. Multivariate analyses found that self-identification was less likely amongst older and retired caregivers, men, lower income caregivers, and caregivers with less than a college education, although not among Hispanic caregivers. Controlling for factors including age and education, we found high-awareness caregivers were more likely to be confident about providing care and accessing services and supports than less aware caregivers were, and were more likely to agree that government and communities should play a role supporting caregivers. These findings underline the importance of caregiver self-identification in accessing services and in making change.

Abstract citation ID: igaf122.2355

### DUAL CAREGIVERS: STRESS, PHYSICAL STRAIN, AND SELF-RATED HEALTH

Sarah Clem[1], Stephanie McCorvey[2], and Joan Davitt[3], *1. University of Maryland School of Social Work, La Junta, Colorado, United States, 2. University of Maryland at Baltimore, Baltimore, Maryland, United States, 3. University of Maryland, Baltimore County, Baltimore, Maryland, United States*

While research on the "sandwich generation" emphasizes the impact of simultaneously caregiving for both an older parent and a child, other contexts of dual caregiving require further exploration. Relying on a convenience sample of caregivers to vulnerable adults in one east coast state, this study examined caregiving stress, physical strain, and self-rated health between caregivers of one person (n = 1,161) and dual caregivers. In addition to caregiving for a vulnerable adult, dual caregivers included in the study were: a) those who also care for a child under the age of 18 (n = 364), b) those caring for another adult with a developmental disability (n = 151), and c) those caring for another older adult (n = 427). Associations were examined using ordered logistic regression, controlling for race, gender, caregiver age, caregiving difficulty, and service utilization. Dual caregivers with a child under the age of 18 had 55% ($p<.01$) greater odds of caregiving stress, 106% greater odds of increased physical strain ($p<.001$), and 94% greater odds of poorer self-rated health (p<.001) compared to caregivers for an individual adult. Similarly, the odds of poorer self-rated health were also greater for those caring for another older adult (OR = 1.68, p<.05) or another adult with a developmental disability (OR = 1.61, p<.01). Though, no associations were identified between these two types of dual caregivers compared to caregivers of a single vulnerable adult when exploring stress and physical strain. Results highlight how different contexts of dual caregiving impact negative indicators of caregiver wellbeing. Implications for systems supporting caregivers will be discussed.

Abstract citation ID: igaf122.2356

### STRATEGIES TO SUPPORT UNIVERSITY EMPLOYEE AND STUDENT CAREGIVERS OF OLDER ADULTS

Jodi Southerland[1], Erin Mauck[1], Shimin Zheng[1], Esther Osime[1], Kwangman Ko[1], and Matthew Smith[2], *1. East Tennessee State University, Johnson City, Tennessee, United States, 2. Texas A&M University, College Station, Texas, United States*

A growing number of university employees and students provide care to older adults. These individuals often face unique challenges navigating their professional, academic, and caregiving responsibilities. While universities can adapt their culture to meet caregivers' diverse needs, limited research identifies caregivers' perspectives about university-based support strategies necessary to promote caregiver-friendly environments. The study objective was to explore strategies for supporting caregivers in a university setting using a mixed methods approach. A Qualtrics survey was administered to 143 employees and 123 students who provide care to an individual >55 years at an Appalachian university. Questions examined demographic characteristics, caregiving context, and support strategies. Survey responses were analyzed for descriptive statistics. Individuals who completed the survey were invited to participate in semi-structured interviews to gain further insight into their responses. Sixteen individuals (9 employees and 7 students) participated in interviews. Thematic analysis was used to identify key support strategies. Flexibility (63.9%), general resources for caregivers (39.1%), information about university resources (37.6%), self-care (27.8%), and counseling (19.9%) were the most cited assistance that universities could provide to help caregivers. Employees were more likely to report needing information about general and university-specific resources. Qualitative analysis supported these findings and revealed new concepts such as a need to promote awareness about caregivers as a "hidden" group. Findings highlight the needs of employee and student caregivers and identify actionable support strategies to enhance caregiver well-being on campus. Strategic planning and administrative support are needed to implement such strategies and create caregiver-friendly environments in university settings.

Abstract citation ID: igaf122.2357

### THE IMPACT OF DIVORCE ON INTERGENERATIONAL SUPPORT FOR OLDER ADULTS IN CHINA

Tianqi Zhou[1], Merril Silverstein[1], Ying Xu[1], and Xiaoyu Fu[2], *1. Syracuse University, Syracuse, New York, United States, 2. Syracuse University, Los Angeles, California, United States*

Studies of the long-term effects of parental divorce on intergenerational relationships in later life suggest that divorce may weaken parent-child ties, reducing the likelihood of support from children. However, alternative theories propose that the disadvantaged status of divorced parents may prompt greater support, particularly in a familistic culture that prioritizes intergenerational responsibility. This study examines the relationship between parental marital status and financial and instrumental support provided by adult children in China. Using data from the 2022 Chinese Family Panel Studies, we analyze responses from 2,088 adult children with living fathers and 2,198 with living mothers, both parents aged 60+. Logistic regression models reveal that adult children are more likely to provide financial support to divorced mothers than to married mothers, while no such effect is observed for divorced fathers. Divorced parents, irrespective of sex, are more likely to receive instrumental support, such as help with household chores. Notably, divorced mothers and fathers are no less likely to receive either type of support than their widowed counterparts despite the former's greater emotional distance from children. Our study extends research on the relationship between marital status and support in societies with strong filial norms; family support is more likely to be directed toward vulnerable unmarried older parents whose financial and caregiving needs cannot be met by spouses, regardless of how their unmarried status was achieved. These findings highlight the enduring strength of filial responsibility in China, demonstrating its prominence over emotional closeness in shaping intergenerational support.

Abstract citation ID: igaf122.2358

### THE IMPACT OF SPOUSAL CAREGIVING ON FINANCIAL WELL-BEING: AN ANALYSIS OF SUBJECTIVE FINANCIAL STRAIN

Susanna Mage[1], Michael Hurlburt[2], Yujun Zhu[3], Kathleen Wilber[4], Donna Benton[1], and Kylie Meyer[5], *1. University of Southern California Leonard Davis School of Gerontology, Los Angeles, California, United States, 2. University of Southern California Suzanne Dworak-Peck School of Social Work, Los Angeles, California, United States, 3. Department of Family Medicine, Keck School of Medicine of USC, Los Angeles, California, United States, 4. University of Southern California, Los Angeles, California, United States, 5. Case Western Reserve University, Cleveland, Ohio, United States*

Caregiving for a spouse or partner often imposes a significant financial burden, straining household resources and affecting overall financial well-being. This study leverages data from the Health and Retirement Study (2012-2018) to examine the relationship between spousal caregiving and subjective financial strain, focusing on the ability to pay monthly expenses and financial satisfaction. Descriptive findings from regression analyses indicate that a significantly higher proportion of caregivers reported difficulty paying bills (39% vs. 29%, p < 0.001) and dissatisfaction with their financial situation (24% vs. 19%, p = 0.008). Results from population-averaged logistic regression models showed that caregivers had 26% lower odds of reporting the ability to meet their monthly expenses than non-caregivers (OR = 0.74, 95% CI [0.61, 0.89], p < 0.001). Although caregivers had 12% lower odds of reporting financial satisfaction than non-caregivers, this association was not statistically significant (OR = 0.88, 95% CI [0.69, 1.12], p = 0.151), suggesting possible adaptation or coping mechanisms. These findings highlight the financial vulnerabilities of spousal caregivers and underscore the need for targeted policies and interventions to alleviate their economic burdens.

Abstract citation ID: igaf122.2359

### THE ROLE OF MOTHERS' COGNITIVE HEALTH IN SIBLING SUPPORT, STRAIN AND PSYCHOLOGICAL WELL-BEING DURING CAREGIVING

J. Jill Suitor[1], Megan Gilligan[2], Robert Frase[3], Ranran He[1], Destiny Ogle[1], Di Wang[1], Hanamori Skoblow[2], and Viridian Verbanac[1], *1. Purdue University, West Lafayette, Indiana, United States, 2. University of Missouri, Columbia, Missouri, United States, 3. Southern Illinois University, Carbondale, Illinois, United States*

A growing number of studies have shown that caregiving is usually a "family affair" navigated among siblings. Most of this research has focused on the detrimental consequences of negative interactions with siblings. As a result, less is known about the potential benefits of sibling ties for caregivers. We extend previous research by considering the differential impact of sibling support and strain on psychological well-being in the context of maternal care provision. We use mixed-methods data collected from 307 adult children nested within 150 families collected as part of the Within-Family Differences Study-III. On average, these adult children were 59 years old, and their mothers were 89 years old. We considered whether the impact of sibling support (i.e. siblings create feelings of love and care) and strain (i.e. siblings create arguments and disagreements) on psychological well-being varied by whether older mothers had symptoms of cognitive impairment (CI). Findings indicated that when adult children provided care to mothers who did not have symptoms of CI, sibling support was associated with increased psychological well-being, whereas sibling strain had no effect. In contrast, when adult children provided care to mothers with symptoms of CI, sibling strain was associated with decreased psychological well-being, whereas sibling support had no impact. These findings suggest that when mothers do not have CI, sibling support is beneficial to caregivers' psychological well-being; however, when CI is present, caregivers do not experience these benefits from sibling's positive interactions, yet experience the detrimental effects of negative interactions.

Abstract citation ID: igaf122.2360

### USABILITY AND ACCEPTABILITY OF A DIGITAL HEALTH ACTIVITY PROGRAM AMONG OLDER HEART FAILURE FAMILY CARE PARTNERS

Dawon Baik[1], Catherine Jankowski[1], Larry Allen[1], Colleen McIlvennan[1], Heather Coats[1], and Blaine Reeder[2], *1. University of Colorado, Aurora, Colorado, United States, 2. University of Missouri, Columbia, Missouri, United States*

Family care partners of persons with heart failure (HF-FCPs) face significant stress and exhaustion from prolonged caregiving, impacting their health. As they age, their increased burden and limited support raise the risk of poor health outcomes for both themselves and the care recipients. To support older HF-FCPs' health management, we developed a tailored digital health physical activity (PA) program, called TPA4You, that includes videoconferencing with a health coach, wearable sensors, and motivational messaging. We conducted a 1-week, two-phase, iterative field test with 11 participants to evaluate the usability and acceptability of TPA4You. Modifications were made based on Phase 1 participants' feedback (i.e., reducing weekly PA sessions from three to two, replacing the automated messaging system with manually sent motivational messages). All participants in both phases (mean age 68.2±7.1 years, 91% female, 82% spouses) attended 100% of Zoom PA sessions. Key themes from Phases 1 and 2 were similar. Overall, participants found TPA4You acceptable and useful. One key theme was TP4AYou is a digital tool that builds confidence in PA capabilities by increasing awareness and motivation. Another key theme was TPA4You's ability to provide emotional and psychosocial benefits by reducing caregiver stress and facilitating human contact with a health coach. A third theme was TPA4You served as a valuable reminder to take care of themselves. In Phase 2, manual motivational messages led to higher satisfaction, less confusion, and greater PA engagement as compared to Phase 1 automated messaging. These findings will inform further revisions and a pilot RCT of TPA4You.

Abstract citation ID: igaf122.2361

### UTILIZATION AND PERCEIVED NEED FOR CAREGIVER SUPPORT SERVICES: IMPLICATIONS FOR EMOTIONAL WELLBEING

Tochukwu Okolie[1], and Heather Menne[2], *1. University of Connecticut, Mansfield Center, Connecticut, United States, 2. Miami University, Oxford, Ohio, United States*

The demonstrable efforts of family members providing care to an older person in the United States continue to make research headlines. Nonetheless, family caregivers providing hours of unpaid care are disproportionately at risk of declining emotional wellbeing. One of the protective mechanisms against this decline may be caregiver programs and services including: caregiver education/training, counseling, and support services. Hence, the aim of the study was to explore the differences in emotional wellbeing associated with: (i) attending caregiver support services, and (ii) a perceived need for caregiver support services. Data from the 2023 wave of the National Survey of Older Americans Act Participants (NSOAAP) was used ($n = 1004$). Univariate, bivariate, and regression techniques assessed the relationship between emotional wellbeing and the predictors of interest. The regression results show that compared to those who have attended counseling services, emotional wellbeing was about 0.17 points higher among those who have not attended, controlling for other variables in the model ($\beta = 0.166$, $p = 0.004$). Also, compared to those who perceived the need for counseling services, emotional wellbeing was about 0.3 points higher among those who do not, controlling for other variables in the model ($\beta = 0.312$, $p < .0001$). Also, being a male or a Black/African American were each significantly associated with an increase in emotional wellbeing, while accounting for other factors. The results suggest a need to understand the motivations and barriers to counseling services. There is also a need to sensitize caregivers on the need for these services.

### SESSION 7090 (POSTER)

### FRAILTY

Abstract citation ID: igaf122.2362

### A METABOLITE-WIDE ASSOCIATION STUDY OF FRAILTY IN OVER 200,000 ADULTS

Julian Mutz, *King's College London, London, England, United Kingdom*

Frailty is an age-related medical syndrome associated with increased mortality risk. Identifying metabolomic profiles linked to frailty may provide insights into its biological causes and consequences. The UK Biobank is a multi-centre observational health study that recruited over half a million middle-aged and older adults. The Nightingale Health platform was used to quantify circulating plasma metabolites. We derived the two most common measures of frailty: the frailty phenotype and the frailty index. The analytical sample included over 200,000 participants (mean age = 56.44 years, SD = 8.12). Most metabolite levels varied with chronological age, showing considerable evidence of non-linear relationships. Of the metabolites statistically significantly associated with age ($P < 0.05/168$), 95% were also associated with the frailty index and 89% with the frailty phenotype. A metabolite-predicted age (MileAge) older than one's chronological age was associated with higher frailty index scores ($\beta = 0.023$, 95% CI 0.021–0.024, $P < 0.001$, comparing individuals with a MileAge delta at least one standard deviation above and below the mean). This group difference was comparable to an 18.3-year chronological age difference in frailty index scores. Individuals with a metabolite-predicted age older than their chronological age were also more likely to be physically frail (OR = 1.29, 95% CI 1.23–1.35, $P < 0.001$). These findings highlight a considerable overlap in the metabolites associated with age and frailty. Metabolite-based age predictions are correlated with both the frailty index and the frailty phenotype, suggesting they have potential for identifying individuals at risk of developing frailty.

Abstract citation ID: igaf122.2363

### ASSESSING CLINICAL FRAILTY IN AN EMERGENCY DEPARTMENT CONTEXT USING ELECTRONIC HEALTH RECORDS

Jasmine Su[1], Ula Hwang[1], Natalia Sifnugel[2], Inessa Cohen[3], Debra Tomasino[5], and Ling Hang[3], *1. NYU Grossman School of Medicine, New York, New York, United States, 2. NYU Langone Medical Center, New York, New York, United States, 3. Yale University, New Haven, Connecticut, United States, 5. New York University, New York, New York, United States*

Frailty is a key predictor of health outcomes and mortality in older adults, yet physical assessments are time- and labor-intensive. The Claims Frailty Index (CFI) is a validated tool that estimates frailty with claims data, removing the need for physical assessments (Kim, 2017). However, its feasibility in emergency departments (EDs)—where older adults are frequent visitors—remains unclear. This study examines how frailty can be measured using electronic health records (EHR) from ED visits. We calculated CFI for 7,173 older adults (65+) who visited the ED at NYU Langone Health in December 2023, applying a one-year lookback of ICD-10 and CPT codes from prior encounters (PE) and historical diagnoses (problem list [PL], previous medical history [PMH]). The average CFI was low (0.121, SD ± 0.016), with the majority (95%) classified as non-frail (CFI< 0.15). Most historical diagnosis inputs came from PE (80%), while PMH (12%) and PL (16%) contributed less. Source-specific CFIs showed minimal variation: with a mean of 0.115 (SD ± 0.013) using only PE, 0.112 (SD ± 0.010) with PMH, and 0.109 (SD ± 0.012) with the PL alone. These findings align with prior research using inpatient and outpatient claims, which also reported low CFIs among older adults (CFI< 2.0). While CFI provides a scalable, data-driven approach to frailty assessment without requiring physical exams, its limitations include potential underestimation due to incomplete EHR documentation. Future research should refine CFI for ED settings and explore its use in predicting geriatric health outcomes, improving patient care, and enhancing ED decision-making.

Abstract citation ID: igaf122.2364

### ASSOCIATION BETWEEN BLOOD PRESSURE VARIABILITY AND FRAILTY IN CHRONIC KIDNEY DISEASE

Danya Pradeep Kumar, Tobia Zanotto, Kate Young, and Aditi Gupta, *University of Kansas Medical Center, Kansas City, Kansas, United States*

Blood pressure (BP) dysregulation is a common early sign of cardiovascular dysfunction and contributes to the progression of chronic kidney disease (CKD). BP variability (BPV), reflecting physiological BP fluctuations, is a marker of cardiovascular, physical, and cognitive aging. This single center cross-sectional study examines the association between frailty, and BPV and nocturnal BP dipping in CKD. We recorded 24-hour ambulatory blood pressure (ABP) in individuals with CKD. We assessed frailty using the FRAIL scale. BPV was examined for the 24-hour duration, daytime and nighttime hours (based on individual participant bedtimes). Nocturnal dipping for systolic and diastolic BP, and their association with frailty were examined. Twenty-eight individuals (46% female; age: 69.8±8.67 years; eGFR: 34.4±8.6 mL/min/1.73m2) with CKD completed the assessments. Based on the FRAIL scale, 10 participants were non-frail and 18 were pre-frail/frail. Nocturnal dipping was lower in the pre-frail/frail (systolic: 6.65±7.8%; diastolic: 11.2±8.7%) compared to the non-frail group (systolic: 13.1±9.0%; diastolic: 18.5±8.8%). Systolic BPV did not differ in the pre-frail/frail group (24-hour: 13.0±2.4%; day: 12.8±2.7%; night: 10.4±3.1%) compared to the non-frail group (24-hour: 14.4±2.8%; day: 12.9±2.4%; night: 11.2±3.5%). Lack of nocturnal BP dipping is associated with worse cardiovascular outcomes and higher mortality. Here we show that lack of nocturnal dipping is also associated with frailty. While cardiovascular causes and lack of nocturnal dipping could impact frailty, we cannot prove causality with this study. Addressing underlying factors for frailty and attenuation of nocturnal dipping, such as autonomic dysfunction, volume overload, and sleep disorders, may be important for improving outcomes in CKD.

Abstract citation ID: igaf122.2365

### COMPARING COGNITIVE FUNCTION TOOLS AND THEIR RELATIONSHIPS WITH PHYSICAL FRAILTY MEASURES

Abigail Tice, Janet Lopez, Dahee Kim, Rui Xie, and Ladda Thiamwong, *University of Central Florida, Orlando, Florida, United States*

Declining cognitive function and physical frailty can independently decrease quality of life and increase dependence and mortality in older adults. While previous works demonstrate relationships between physical and cognitive frailty, studies investigating differences between cognitive function tools and their associations with physical frailty in older adults are lacking. This study aimed to investigate the relationship between various cognitive tools and physical frailty markers in community-dwelling older adults residing in low-income settings. Older adults (≥60 years old) living in the community were recruited (n = 118). Participants completed cognitive functioning assessments, including, Digital Symbol Substitution Test (DSST), Trail Making Test (TMT)-A, TMT-B, Rowland University Dementia Assessment Scale (RUDAS), and Memory Impairment Screen (MIS). Physical frailty was assessed using the Fatigue, Resistance, Ambulatory, Illness, and Loss of weight (FRAIL) questionnaire, short physical performance battery (SPPB), BTracks Balance system, timed-up-and-go (TUG), 30-second sit-to-stand (STS), and handgrip strength (HGS). Spearman correlations were performed ($p < 0.05$) using GraphPad Prism. FRAIL score was significantly correlated with TMT-A, TMT-B, and DSST. SPPB total was significantly correlated with all cognitive tests. SPPB balance was significantly correlated with RUDAS and MIS, gait speed with TMT-A, TMT-B, and DSST, and chair stand with RUDAS, MIS, TMT-A, and TMT-B. STS was significantly correlated with RUDAS, TMT-A, TMT-B, and DSST. TUG was significantly correlated with TMT-A, TMT-B, and DSST. HGS was significantly correlated with TMT-A and DSST. Findings suggest that tools measuring different aspects of cognitive function have varying relationships with physical frailty. Future work should be done to dissect these relationships better.

Abstract citation ID: igaf122.2366

### COMPREHENSIVE FRAILTY ASSESSMENT IN CHRONIC KIDNEY DISEASE

Danya Pradeep Kumar[1], Nicole Oblon[1], Jacob Hiss[1], Mary Piedad[2], Kate Young[1], and Aditi Gupta[1], *1. University of Kansas Medical Center, Kansas City, Kansas, United States, 2. Kansas City University, Kansas City, Kansas, United States*

Individuals with chronic kidney disease (CKD) are at a high risk of frailty, a multidimensional geriatric syndrome of physiological decline. The ideal method to assess frailty in individuals with CKD is unknown. In this single center cross-sectional study of individuals with CKD stage 3-5, we compared frailty as assessed by Fried's Frailty Phenotype (FFP) and the Comprehensive Frailty Index (CFI) in individuals with CKD. FFP is comprised of 5 criteria – slowness, weakness, low-activity, exhaustion and weight-loss. CFI ranges from 0-1 and was calculated using 50 health deficits from various health domains based on standardized procedures. Forty-nine individuals (59% female; age: 68.8 ± 8.86 years; eGFR: 34.8 ± 8.53 mL/min/1.73m2) with CKD completed the assessments. With FFP, 20, 25, and 4 participants were non-frail, pre-frail, and frail respectively. With the CFI, 16, 23, and 10 participants were non-frail, pre-frail, and frail respectively. There was a discrepancy in frailty status by FFP vs. CFI in 26 participants (53%). Domain-wise comparison also showed a discrepancy between FFP and CFI. Of the 49 participants, 39 (80%) had discrepancy in exhaustion, 22 (45%) in low-activity, and 6 (12%) in slowness. There were no direct comparison for weakness or weight loss. These data show that frailty status for an individual can differ depending on the frailty assessment used. Compared to FFP, CFI may provide more details on the cause of frailty. Clinical frailty assessments need to be refined to identify suitability of different interventions in managing frailty.

Abstract citation ID: igaf122.2367

### DIGESTIVE DISEASES AND FRAILTY IN MIDDLE-AGED AND OLDER CHINESE ADULTS: A LONGITUDINAL STUDY

Hongmei Jiao, and Jiaxin Li, *Peking University First Hospital, Beijing, Beijing, China (People's Republic)*

Frailty, characterized by reduced physiological reserves and diminished stress response, is linked to chronic diseases. However, its association with nonneoplastic digestive diseases remains underexplored. This study investigates this relationship among middle-aged and older Chinese adults using data from the 2011–2018 China Health and Retirement Longitudinal Study (CHARLS). Frailty was assessed using a 32-item frailty index ($FI > 0.25$), and nonneoplastic digestive diseases were identified through self-reported diagnoses. Cox proportional hazards models and Restricted Cubic Splines (RCS) were employed to examine associations, while mediation analysis evaluated the roles of inflammation-related markers (leukocytes, platelets, platelet-to-leukocyte ratio [PWR], and C-reactive protein [CRP]) and nutrition-related markers (triglycerides [TG] and glycosylated hemoglobin [HbA1c]). Among 8,507 non-frail participants (mean age 59.9 ± 9.7 years, 56.4% female), 24.0% reported nonneoplastic digestive diseases. Over a 7-year follow-up, 2,397 frailty cases were documented. Nonneoplastic digestive diseases independently increased frailty risk (HR = 1.250, 95% CI: 1.100–1.430, $P < 0.001$). RCS revealed a non-linear relationship. Mediation analysis indicated that TG and HbA1c mediated -0.998% and -2.26% of the association, respectively, while inflammation-related markers showed no significant mediation. These findings underscore the link between nonneoplastic digestive diseases and frailty, highlighting the importance of early intervention and nutritional strategies to mitigate frailty risk in older persons.

Abstract citation ID: igaf122.2368

### DISTINCT MULTIOMIC SIGNATURES REVEAL METABOLIC MECHANISMS OF FRAILTY INDEPENDENT OF CHRONOLOGICAL AGE

Noa Rappaport[1], Dantong Zhu[2], Lance Pflieger[3], Heeju Noh[2], Alex Carr[2], Kengo Watanabe[4], Max Robinson[2], and Alice Kane[2], *1. Institute for Systems Biology, Buck Institute, Phenome Health, Seattle, Washington, United States, 2. Institute for Systems Biology, Seattle, Washington, United States, 3. Phenome Health, Seattle, Washington, United States, 4. Tokushima University Graduate School of Biomedical Sciences, Tokushima, Tokushima, Japan*

Frailty is a heterogeneous clinical syndrome characterized by decline in the functioning of multiple physiological systems. Despite its prevalence, the molecular mechanisms mediating frailty remain poorly understood. This study aimed to identify biological mechanisms underlying frailty that are distinct from chronological aging by leveraging multiomic data integration in a deeply phenotyped wellness cohort. We analyzed data from >3000 participants in the Arivale program with comprehensive multiomic profiling (plasma metabolomics, proteomics, chemistries and gut microbiome) and constructed three complementary deficit accumulation frailty indices: self-reported (35 items), laboratory-based (34 items), and combined. Using a novel multiomic adapted version of the Weighted Gene Co-expression Network Analysis (WGCNA) we identified distinct multiomic modules significantly correlated with frailty independent of chronological age ($p < 0.001$), with notable sex-specific differences. Metabolic signatures characterized by elevated HOMA-IR, HbA1c, and triglycerides were strongly associated with frailty status, supporting the existence of an "obese frail" phenotype that challenges traditional frailty paradigms. Nutritional biomarkers, particularly low vitamin D, calcium, and potassium levels, were also significantly associated with higher frailty indices. Microbiome features explained approximately 9% of frailty variance, with butyrate production capacity inversely correlated with frailty scores. We identified specific gut microbiome enterotypes that differentially affected frailty trajectories following lifestyle intervention. Additionally, we demonstrated that lifestyle interventions significantly improved frailty trajectories, with effects moderated by polygenic risk scores for frailty. Finally, we validated our findings in external cohorts, including UKBB. Our findings reveal that frailty has distinct biological underpinnings from chronological aging, opening new avenues for personalized interventions.

Abstract citation ID: igaf122.2369

### FRAILTY DEVELOPMENT IN OLDER ADULTS: INSIGHTS FROM AN EIGHT-YEAR LONGITUDINAL STUDY

Khalil Iktilat[1], Aviad Tur-Sinai[2], Netta BenturNetta Bentur[1], and Debbie Rand[1], *1. Tel Aviv University, Tel Aviv, Tel Aviv, Israel, 2. University of Haifa, Haifa, HaZafon, Israel*

Frailty is a key marker of biological aging, associated with adverse health outcomes and loss of independence. Most studies focus on risk factors using cross-sectional data, limiting understanding of individual transitions. This study utilizes an eight-year longitudinal follow-up across European countries to assess how demographic, socioeconomic, and health factors influence frailty progression. Data of adults aged 65–84 were drawn from Waves 4-8 (2011-2020) of the SHARE survey, across 27 countries. Frailty was assessed using Fried's criteria (grip strength, walking speed, weight loss, exhaustion, and physical activity). Participants were classified as non-frail, pre-frail, or frail based on cumulative deficits in these domains. Key predictors included age, sex, chronic conditions, socioeconomic status, self-rated health, and physical activity levels. Frailty progression followed a cumulative pattern. Over two years, 65.96% of 18,331 participants remained non-frail, 32.10% became pre-frail, and 1.94% transitioned to frailty. By eight years, only 57.44% remained non-frail, while 37.55% became pre-frail and 5.02% developed frailty. Older age (OR = 1.098, p < 0.001) and poor self-rated health (OR = 0.751, p < 0.001) were the strongest predictors. Physical activity was protective, particularly indoor (OR = 1.294, p < 0.001) and outdoor activity (OR = 1.157, p = 0.019), while financial distress (OR = 1.143, p = 0.005) increased frailty risk. These findings emphasize the high risk of frailty progression in older adults without intervention, leading to increased health deterioration. Identifying key risk factors can guide targeted strategies to slow frailty onset and reduce its impact. Promoting physical activity, social engagement, and financial stability may help delay frailty progression and improve health outcomes in aging populations.

Abstract citation ID: igaf122.2370

### FRAILTY IS LINKED TO A LOWER SUBJECTIVE LIFE EXPECTANCY AMONG KOREAN MIDDLE-AGED AND OLDER ADULTS

Juhyeong Lee[1], Yeonsoo Shin[2], and Giyeon Kim[3], *1. The University of British Columbia, Vancouver, British Columbia, Canada, 2. The University of Texas at Austin, Austin, Texas, United States, 3. Chung-Ang University, Seoul, Republic of Korea*

Frailty in later life can lead to an identity crisis, potentially limiting individuals' perception of their future and opportunities. This study examines differences in subjective life expectancy (SLE) between frail and non-frail Korean middle-aged and older adults. The sample was drawn from the Wave 1 of the Korean Longitudinal Study of Aging (N = 10,254). SLE was assessed based on respondents' perceived likelihood of surviving for another 10 to 15 years, given their current age. Frailty was measured using 33 items, including self-rated health and chronic disease diagnoses. Participants with a score of 2 or higher were classified as frail (n = 682), and those with a score below 2 as non-frail (n = 8,735). To ensure homogeneous characteristics between the two groups, we performed propensity score matching with 1:1 nearest neighbor matching. A final matched sample of 1,364 participants (M age = 73.5 years, SD = 10.0, range = 45-98; 62% female), with 682 in each group was included in the final analysis, showing no significant demographic differences. Results from a multiple linear regression analysis showed that frailty was significantly associated with lower SLE scores (B = -17.66, p < .001). Additionally, an interaction analysis indicated that the effect of age on SLE varied by frailty status (B = 0.56, p < .001). Findings suggest that frail Korean middle-aged and older adults perceive their life expectancy as lower than their non-frail counterparts. This highlights the importance of addressing frailty in older adults to support positive future outlooks.

Abstract citation ID: igaf122.2371

### FRAILTY SHOWS ENHANCED PREDICTIVE CAPACITY FOR 1-YEAR UNPLANNED READMISSION COMPARING TO REASON FOR ADMISSION

Junpeng Kan, Ying Sun, Wen Tang, Yunli Xing, and Qing Ma, *Beijing Friendship Hospital, Capital Medical University, Beijing, Beijing, China (People's Republic)*

Background: Frailty, a prevalent geriatric syndrome, predisposes older adults to adverse outcomes including disability and premature mortality. Unplanned hospital readmissions impose substantial economic burdens on families and challenge healthcare systems. Objective: To investigate the impact of frailty on 1-year unplanned readmissions among elderly inpatients and further analyze its predictive utility across subgroups stratified by admission causes (acute vs. chronic conditions). Methods: This prospective cohort study enrolled patients aged ≥65 years from the Geriatrics Department of Beijing Friendship Hospital, Capital Medical University. Frailty status was assessed using the FRAIL scale, categorizing participants into non-frail and frail groups. Participants were stratified by admission diagnosis (acute/chronic conditions). Baseline demographics, medical histories, and laboratory parameters were collected. Electronic medical records were reviewed to identify unplanned readmissions within 1 year. Results: Among 414 enrolled patients. Median age was 82 years (IQR 72~88), 58.9% (n = 244) were frail. During the 1-year follow-up period, 173 patients (42.8%) experienced unplanned readmissions. Frailty independently predicted 1-year unplanned readmissions (OR = 1.694, 95% CI:1.013–2.830, P = 0.044), with a significant interaction between frailty and admission cause (P for interction 0.006). Subgroup analysis revealed enhanced predictive accuracy of frailty comparing to chronic disease-related admissions. Conclusions: Frailty serves as an independent risk factor for 1-year unplanned readmissions in elderly inpatients, particularly among those hospitalized for chronic conditions. Systematic frailty screening at admission coupled with tailored physical rehabilitation programs may mitigate readmission risks.

Abstract citation ID: igaf122.2372

### INITIAL DAMAGE AND FRAILTY: GOING TO SLEEP HUNGRY IN CHILDHOOD

Heng Wu[1], and Kenneth Rockwood[2], *1. Dalhousie University Department of Medicine, Halifax, Nova Scotia, Canada, 2. Dalhousie University, Halifax, Nova Scotia, Canada*

Frailty deficits accumulate as individuals age, whereas resilience (the ability to repair damage) and robustness (the ability to resist damage) underlie the speed of aging from one birthday to the next. Nutritional deprivation reduces the redundancies that allow robustness and resilience. In childhood, nutritional deprivation increases the burden of initial damage. Even though the functional capacity of organs in young adults is high, changes in health are often influenced by social circumstance. Here, we studied the overall impact of childhood hunger on a population's frailty. We applied a deficit accumulation approach to construct a 47-item frailty index with domains of morbidities, cognition, lifestyle, and disabilities. We analyzed data from 10260 people aged 65+ years, participants in the Chinese Longitudinal Healthy Longevity Survey (CLHLS) 2005 cohort. They were followed in 2008, 2011, 2014, and 2018 (men=4570, age=84.6±10.6, years of education=3.5±5.6; women=6150, age=89.6±11.6, years of education=0.8±4.5). Most participants (73.0%) responded that they had slept hungry as children. A regression model using Generalized Estimating Equations showed that all predictors (sex, age, education, hungry in childhood) significantly affected the frailty index (p-value < 0.001). We found that children who had not experienced hunger had fewer health deficits (beta coefficient for "not hungry" = -0.010 (95% CI=-0.02~-0.01) versus "hungry", adjusted for age, sex, and education. Reflecting better ability to repair, as older adults, they experienced less smooth transitions in aging. In short, children who did not experience hunger on average have a slower speed of aging, with less frailty and greater resilience in old age.

Abstract citation ID: igaf122.2373

### LONGITUDINAL RELATIONSHIP BETWEEN PHYSICAL FRAILTY AND COGNITIVE DECLINE AMONG OLDER CHINESE IMMIGRANTS

Qingqing Yin[1], Guoping Jin[1], Yanping Jiang[2], Lin Chen[3], and Fengyan Tang[1], *1. University of Pittsburgh, Pittsburgh, Pennsylvania, United States, 2. Rutgers, The State University of New Jersey, New Brunswick, New Jersey, United States, 3. Fudan University, Shanghai, Shanghai, China (People's Republic)*

This study examined patterns of physical frailty changes, their sociocultural correlates, and associations with baseline cognitive functioning and cognitive decline over an eight-year observation period among older Chinese immigrants. Using five waves of data from the Population Study of Chinese Elderly in Chicago (PINE), this study included 2,835 respondents with a mean age of 72.5 years. Physical frailty was assessed using five indicators across the five waves, and repeated measures latent class analysis (RMLCA) identified four distinct frailty patterns: least frail (53%), decreased frailty (21%), increased frailty (15%), and constantly frail (11%). The associations between frailty patterns and cognitive change trajectories were evaluated using latent growth curve modeling (LGCM), adjusted for sociodemographic, health, and immigration covariates. The results indicated that, compared to the least frail class, respondents in the increased frailty class (intercept: B = -0.108, p < .05; slope: B = -0.073, p < .001) and the constantly frail class (intercept: B = -0.150, p < .01; slope: B = -0.043, p < .001) showed poorer initial cognitive functioning and faster rates of cognitive decline after controlling for covariates. No significant differences in cognitive outcomes were observed between the least frail and the decreased frailty classes. Compared to Cantonese speakers, Mandarin speakers experienced a slower rate of cognitive decline (B = 0.033, p < .001). These findings demonstrate that physical frailty is associated with cognitive decline, highlighting the need for clinical interventions with special attention to older Chinese immigrants who remain constantly frail or experience increasing frailty over time.

Abstract citation ID: igaf122.2374

### MACHINE-LEARNING-BASED PREDICTION AND RISK FOR POSSIBLE SARCOPENIA AMONG COMMUNITY-DWELLING OLDER ADULTS

Sooyoung Kwon[1], Gwang Suk Kim[2], Layoung Kim[3], and Namhee Kim[4], *1. Graduate School of Yonsei University, Seoul, Republic of Korea, 2. College of Nursing, Yonsei University, Seoul, Republic of Korea, 3. Department of Nursing, The University of Suwon, Hwaseong, Republic of Korea, 4. Yonsei University, Wonju, Republic of Korea*

To improve the health and quality of life of older adults, it is important to provide simple, accurate healthcare services that prevent sarcopenia early in the community, rather than in a hospital setting. This study developed a model to predict possible sarcopenia among older adults and to explore factors associated with possible sarcopenia. The study analyzed data from the Korean Frailty and Aging Cohort Study (KFACS) to create possible and non-possible sarcopenia classifications using the 2019 Asian Working Group for Sarcopenia criteria. Machine learning (ML) models, including Logistic Regression, Support Vector Machine, Decision Tree, and Random Forest, were developed to assess predictive performance, using the area under the curve (AUC) primarily. Analyses were conducted using Google Colab and JASP. A total of 1,761 participants (81.6±3.62 years, 46.7% male) were categorized into 500 possible sarcopenia (83.0±3.76 years, 34.4% men) and 1,261 non-possible sarcopenia (81.0±3.40 years, 51.6% men). The possible sarcopenia-prediction model built with a Random Forest algorithm achieved the highest AUC of 0.869. According to the feature-selection approach, walking speed, body mass index, age, instrumental activities of daily living, exhaustion, cognitive dysfunction, and quality of life were the most important predictors of possible sarcopenia in older adults across the four ML models. The Random Forest model was shown to be especially useful for community healthcare providers in detecting possible sarcopenia early in community-dwelling older adults. To reduce the impact of sarcopenia on the aging population, improved community screening that considers physical dysfunction as well as psychological aspects are needed.

Abstract citation ID: igaf122.2375

### PHYSICAL ACTIVITY AND WEIGHT DECLINE BEFORE FRAILTY: IMPLICATIONS FOR FRAILTY PREVENTION AND REVERSE CAUSATION

Hee-kyoung Nam[1], Jie Shen[2], Andrea Wershof Schwartz[2] Sung-il Cho[3], Changzheng Yuan[4], and Yuan Ma[2], *1. Seoul National University College of Medicine, Boston, Massachusetts, United States, 2. Harvard T.H. Chan School of Public Health, Boston, Massachusetts, United States, 3. Seoul National University, Seoul, Republic of Korea, 4. Zhejiang University, Hangzhou, Zhejiang, China (People's Republic)*

Background Physical inactivity and mid-life obesity are modifiable risk factors for frailty. However, declines in physical activity and weight loss may co-occur in the years preceding the onset of frailty. It is undetermined whether monitoring concurrent changes in physical activity and body weight can improve the early detection of frailty and to what extent their relationship is influenced by reverse causation. Methods We examined the longitudinal changes in physical activity and body weight over 8 years and the incidence of frailty over a median follow-up of 8 years among 5,759 community-dwelling older adults from the nationally representative Health and Retirement Study (US). We further assessed physical activity and BMI measured at different years (0, 2, 4, 6, and 8 years, respectively) preceding frailty assessment. Findings A total of 1293 incident frailty cases were documented. Decreased physical activity (adjusted hazard ratio [HR]:1.22; 95% CI, 1.06-1.39) and weight loss (HR = 1.20; 95% CI, 1.05-1.37) were independently associated with increased frailty risk after adjusting for initial physical activity and BMI and other covariates, with the highest risk observed in individuals experiencing both. The association between lower physical activity and increased risk of frailty appeared more pronounced when physical activity was assessed closer to frailty diagnosis, whereas the association between obesity and frailty was attenuated. Discussion Tracking changes in physical activity and body weight may be an easy and effective approach to help identify high-risk individuals for timely frailty intervention. Reverse causation may impact association estimates and causal interpretations even in non-frail older adults.

Abstract citation ID: igaf122.2376

### PREDICTORS OF PHYSICAL FRAILTY TRANSITIONS IN CHINESE OLDER ADULTS: THE ROLE OF PHYSICAL ACTIVITY AND FUNCTION

Ziwei Zeng, and Yijian Yang, *The Chinese University of Hong Kong, Hong Kong, China (People's Republic)*

Frailty is a dynamic geriatric syndrome associated with adverse health outcomes, yet its progression can be mitigated through targeted interventions. This study investigated predictors of frailty transitions in Chinese older adults, focusing on physical activity (PA) and physical performance. Using data from the China Health and Retirement Longitudinal Study (CHARLS), we examined frailty (robust, pre-frail, frail) transitions between baseline data (collected in 2011) and follow-up data (collected in 2013) among 1,014 participants aged 65 and older. Frailty was assessed using the Physical Frailty Phenotype, and PA was measured using a modified International Physical Activity Questionnaire. Physical performance was evaluated using the Short Physical Performance Battery (SPPB) and handgrip strength. Ordinal logistic regression models were used to examine the relationship between PA, physical performance, and frailty transitions. Results showed that higher PA levels and better physical performance were associated with a reduced likelihood of worsening frailty or a greater chance of transitioning to a more robust state. Among robust individuals, increased handgrip strength predicted remaining robust [Average marginal effects (AME)=1.1%, p = 0.015]. Pre-frail individuals with higher vigorous PA [AME=21.8%, p = 0.037] and handgrip strength [AME=0.6%, p = 0.003] were more likely to transition to robust state. Frail individuals with increased low-intensity PA [AME=22.5%, p = 0.038] and higher SPPB walking sub-scores [AME=27.73%, p = 0.017] had a greater probability of improving to non-frailty. These findings highlight the importance of tailored interventions based on baseline frailty status. Promoting PA and improving physical performance, particularly aspects of muscle strength and mobility, may help delay or reverse frailty progression.

Abstract citation ID: igaf122.2377

### PREVALENCE OF FRAILTY IN BRAZILIAN OLDER ADULTS: A SYSTEMATIC REVIEW AND META-ANALYSIS UPDATE

Giselle Santos[1], Gabriela Cipolli[1], and Ruth Melo[2], *1. State University of Campinas, Campinas, Sao Paulo, Brazil, 2. Universidade de São Paulo, São Paulo, Sao Paulo, Brazil*

This systematic review investigates the prevalence of frailty syndrome in Brazil, considering different factors (context, diagnosis tool, and sex). Studies that included non-institutionalized older adults (60 years old) who were recruited from different contexts were considered. Studies must include a frailty assessment and a sample size of more than 281 participants. Indexed publications, dated from 2001 to October 2024, and retrieved from different databases were considered. COVID-19 related studies were excluded. Studies were independently selected by two reviewers and, in case of disagreements, a third reviewer was consulted. Prevalence data of included studies were meta-analyzed using the PERSyst-MA tool. Thirty-seven studies were included, totaling 82,955 participants. Studies were concentrated in the Southeast region (54%) and used the frailty phenotype (56.7%) as a diagnostic measure. The estimated prevalence of frailty was 21.6%, which was influenced by the assessment method and recruitment context. Frailty prevalence was lower when assessed with frailty phenotype criteria (16.7%) than Edmonton Frail Scale (27.7%). Higher frailty prevalence was observed in health services (32.3%) compared to primary care (23.2%) and community (16.1%). Frailty prevalence did not differ between men and women. These findings reveal significant variability in frailty prevalence among Brazilian older adults, influenced by the assessment tool and recruitment context. The present estimated prevalence underscores the substantial burden of this frailty in the Brazilian population. The results highlight the need for standardized frailty assessment methods and targeted public health strategies to address frailty across different settings.

Abstract citation ID: igaf122.2378

### PROGNOSTIC ACCURACY OF FRAILTY & VULNERABILITY SCREENING IN OLDER ED PATIENTS: A SYSTEMATIC REVIEW & META-ANALYSIS

Nai-Wen Ku[1], Yu-Chi Hsu[2], Jasmine Mudhur[1], Lusine Abrahamyan[1], Chu-Lin Tsai[2], Alibhai Shabbir[3], and Martine Puts[1], *1. University of Toronto, Toronto, Ontario, Canada, 2. National Taiwan University Hospital, Taipei, Taipei, Taiwan (Republic of China), 3. University Health Network, Toronto, Ontario, Canada*

Frailty is associated with increased mortality and morbidity in older adults, highlighting the need for effective screening in emergency departments (EDs). However, the optimal tool for predicting adverse outcomes remains uncertain. Therefore, our review's question is: which frailty and vulnerability screening instrument has the highest prognostic accuracy for adverse outcomes in older adults visiting EDs? Studies were eligible for inclusion if they included patients aged ≥60 admitted to the ED, used frailty or vulnerability instruments, and reported sensitivity, specificity, and area under the curve (AUC). Databases searched until January 25, 2025, included MEDLINE, EMBASE, Cochrane Library, CINAHL, and CNKI. Study quality was assessed using the updated Quality in Prognosis Studies (QUIPS) tool. Two investigators independently reviewed abstracts and full-texts. A meta-analysis was conducted for tools with consistent cutoffs in $\geq 4$ studies. Sixty-seven papers described 62 studies using 40 screening instruments. The Identification of Seniors at Risk (ISAR) was the most frequently used ($n = 25$), followed by the Clinical Frailty Scale (CFS) ($n = 15$) and the Triage Risk Screening Tool (TRST)($n = 11$). Most instruments showed high sensitivity but low specificity. Meta-analysis was feasible for ISAR and TRST. ISAR's pooled sensitivity, specificity, and AUC for in-hospital mortality were 92% (95% CI, 88-95), 26% (95% CI, 21-33), and 0.82. For ED revisits, ISAR's AUC was 0.64, while TRST's was 0.60. Frailty screening instruments in EDs exhibit high sensitivity but low specificity, limiting clinical utility. Future research should enhance specificity by incorporating domains beyond geriatric conditions and known prognostic indicators into frailty instruments in large-scale studies.

Abstract citation ID: igaf122.2379

### SARCOPENIA TO FRAILTY AND MALNUTRITION AND THEIR PREDICTIVE ROLE FOR POSTOPERATIVE OUTCOMES IN OLDER GASTRIC CANCER

Yinning Guo, Qin Xu, Ting Xu, and Xinyi Xu, *Nanjing Medical University, Nanjing, Jiangsu, China (People's Republic)*

Objectives: This study aimed to investigate the prevalence of preoperative sarcopenia, frailty, and malnutrition in older gastric cancer patients, the correlation between sarcopenia and frailty as well as malnutrition, and the predictive role of them on postoperative outcomes. Methods: Older gastric cancer patients who were proposed to undergo radical surgery were selected and data were prospectively collected at admission and 3 months postoperatively. Regression models were used to explore the correlation between sarcopenia and frailty and malnutrition, as well as the predictive role of all three on functional quality, quality of life, and death at 3 months postoperatively. All models were adjusted. Results: 624 patients were enrolled at admission and 595 completed follow-up 3 months after surgery. 46.3% patients with preoperative frailty, 44.7% with nutritional risk, and 9.5% with sarcopenia. There was a very strong correlation between sarcopenia and physical frailty, as well as nutritional risk and malnutrition($p<0.001$). In terms of prediction, sarcopenia was a good predictor of quality of life and mortality at 3 months postoperatively, the three frailty subtypes of physical-social frailty, psychosocial frailty, and multidimensional frailty were good predictors of functional status, quality of life, and mortality, and malnutrition was effective in predicting quality of life. Conclusion: Sarcopenia was no less predictive of postoperative outcomes than malnutrition, but slightly less so than frailty. In the future, the interaction and joint predictive value of the three can be further analyzed to determine an objective, time-consuming, and predictive preoperative health risk assessment system for older cancer patients.

Abstract citation ID: igaf122.2380

### THE CAUSES AND CONSEQUENCES OF FRAILTY: A COMPARATIVE COHORT STUDY IN COSTA RICA AND THE UNITED STATES

Amanda Lehning[1], Carolina Santamaria-Ulloa[2], Ericka Mendez-Chacon[2], and Jihyeong Jeong[1], *1. University of Maryland Baltimore, Baltimore, Maryland, United States, 2. University of Costa Rica, San Pedro de Montes de Oca, San Jose, Costa Rica*

Frailty is a common condition among older adults that results from aging-related declines in multiple systems and increases vulnerability to negative health outcomes. The aim of this study is to compare the causes and consequences of frailty among older adults from Costa Rica and the United States. Using data from community-dwelling older adults from the Costa Rican Longevity and Healthy Aging Study (CRELES, $n = 1,790$) and the National Health & Aging Trends Study (NHATS, $n = 6,680$), first we estimated Cox proportional hazard models to examine the association between frailty and all-cause mortality 8 years later. The death hazard for frail compared to non-frail older adults was three-fold in Costa Rica ($HR = 3.14$, $p < 0.001$) and four-fold in the US ($HR = 4.02$, $p < 0.001$). At baseline 21.9% of NHATS respondents met the criteria for frail compared to 16.2% of CRELES respondents. Second, we estimated multinomial regression models, stratified by sex, to examine frailty transitions (i.e., worse, same, or better) between two waves. Among men in the Costa Rican sample, respondents who were older had higher odds of becoming more frail; those who reported lower income, had fewer years of education, or had been diagnosed with arthritis had higher odds of becoming less frail. In the United States, however, there were no significant associations between sociodemographic characteristics, health and functioning, and frailty transitions except for diabetes/hypertension. Findings indicate differential pathways to frailty between these two countries, and indicate that social, environmental, and policy influences should be examined in future research.

### SESSION 7095 (POSTER)

### HEALTH BEHAVIOR CHANGE

Abstract citation ID: igaf122.2381

### A HOME AND COMMUNITY-BASED MULTICOMPONENT PHYSICAL ACTIVITY INTERVENTION FOR OLDER ADULTS

Jing Chen, Nan Hua, and Qin Zhang, *The First Affiliated Hospital, Zhejiang University School of Medicine, Hangzhou, Zhejiang, China (People's Republic)*

Feasible physical activity (PA) interventions to promote multicomponent PA among community-dwelling older adults in China are limited. We aimed to develop and test the implementation feasibility of a multicomponent PA intervention for community-dwelling older adults. The intervention was developed by a multidisciplinary working group based on behavior change theories and techniques. A 12-week feasibility study was conducted in a community in Hangzhou, China. Feasibility was tested by Bowen's framework for assessing acceptability, demand, implementation, practicality, adaptation, integration, expansion, and limited efficacy of the PA intervention program. The PA intervention combined face-to-face group exercise (twice a week) in a community center and home-based exercise assisted by a WeChat applet or exercise manuals (at least once a week) with a weekly goal of engaging in multicomponent exercise at least three days per week. Overall, 19 participants (95%) completed the intervention (mean age of 77 years) with 12 participants using the applet and seven using the exercise manuals for home-based exercises. The intervention satisfied all Bowen's feasibility criteria. In the third month, 93% of the applet users and 79% of the non-applet users adhered to weekly exercise goals. Significant improvements were observed in grip strength

(+1.4 kg, 95% CI 0.1 to 2.7) and chair stand test (-3.5 seconds, 95% CI -4.9 to -2.2). The findings suggested that the PA intervention program was feasible in real-world settings, demonstrating its potential for promoting multicomponent PA among community-dwelling older adults. A large-scale randomized controlled trial is needed to test the long-term effectiveness.

Abstract citation ID: igaf122.2382

### COGNITIVE SYMPTOMS ACROSS THE MENOPAUSE TRANSITION ARE NOT EXPLAINED BY ACUTE OR CHRONIC STRESS

Kavya Jhaveri[1], Teresa Facchetti[1], Sandra Rizer[1], Jadira Cuevas[1], Sarah Tom[1], Jillian Joyce[2], Mary Rosser[1], and Stephanie Cosentino[1], *1. Columbia University Irving Medical Center, New York, New York, United States, 2. University of Southern California, Los Angeles, California, United States*

Subjective cognitive difficulties are increasingly recognized as an early marker of Alzheimer's disease. Cognitive complaints in women are often attributed to elevated stress, particularly during the menopausal transition. However, physiological changes during menopause may impact cognition. Here, we hypothesized that subjective cognitive difficulties would predict objective cognition independently of acute or chronic stress — acknowledging that while stress significantly impacts cognition, it does not fully explain the relationship between subjective and objective cognition. 112 women aged 45 + (mean age = 61.9, SD = 10.1, range=45-85) at different stages of menopause participated in the study. Subjective cognition was measured with a 6-item scale (SCoPE). The Linus Health Core Cognitive Evaluation, a digital cognitive health assessment, measured objective cognition (Digital Clock and Recall) and acute stress ("In the last month, there has been a significant increase in stress in my life"). Chronic stress was measured by the Ongoing Chronic Stressors Questionnaire (OCSQ). Linear regression showed SCoPE negatively predicted DCR (B=-.596, p=.008). Women with subjective complaints (N = 62) reported higher acute (Mann-Whitney U test: p = 0.043, U = 1251.0, Z=-2.024) and chronic stress (Mann-Whitney U test: p=.045, U = 1210.5, Z=-.2003) than those without (N = 50). In multiple regression adjusting for stress, SCoPE emerged as an independent predictor of DCR (B=-.564, p=.016) while stress was non-significant (OCSQ: B=-.031, p=.317; LHQ Q4: B=.043, p=.852). These findings suggest that while stress is elevated with subjective cognitive complaints, it does not fully account for their objective cognitive deficits, highlighting the need for multifaceted approaches to assess and intervene during menopausal transition to reduce dementia risk.

Abstract citation ID: igaf122.2383

### DEVELOPMENT AND VALIDATION OF PROACTIVE HEALTH CAPACITY MEASUREMENT SCALE FOR OLDER ADULTS IN CHINA

Mengya Zhu, Qianhui Pan, Yexuan Xiao, and Nan Jiang, *Tsinghua University, Beijing, Beijing, China (People's Republic)*

Objective Proactive health capacity refers to individuals' ability to actively engage in and manage their own health. However, existing proactive health capacity measurement tools have not been specifically developed for older adults. This study aims to fill the gap in China, which can guide the development of targeted self-health management interventions. Methods The scale was developed following COSMIN guidelines. Initially, an item pool was created through literature review, and the scale was developed using Delphi expert consultation. The items were categorized based on Self-Management Theory. A cross-sectional survey of 390 older adults (aged≥60) in China was conducted to refine the scale, employing exploratory factor analysis (EFA), followed by reliability and validity testing. Results After two rounds of Delphi consultation, a scale with 5 dimensions and 26 items was developed. Familiarity coefficients were 0.80 and 0.77, authority coefficients were 0.86 and 0.84, and Kendall's W values were significant (P < 0.05). EFA (n = 390) showed a KMO of 0.844, with two items removed, yielding a final scale of 5 dimensions and 24 items (Cronbach's α = 0.911). The dimensions included health problem-solving, self-determination, access and utilize resources, communication and action planning capacity. Discussion The PHCMS enables clinicians to assess older adults' key self-management capacity, thus guide personalized interventions, evaluate intervention effectiveness, and inform public health strategies. Ultimately, this tool may improve older adults' well-being by promoting proactive engagement in health and preventive care.

Abstract citation ID: igaf122.2384

### ESTIMATION TO INTUITION: HOW DIGITAL TOOLS SUPPORT DIET & WEIGHT CONTROL IN MIDDLE & OLDER ADULTS

Sarah Hubner[1], and Julie Blaskewicz Boron[2], *1. Oregon Health Authority, Portland, Oregon, United States, 2. University of Nebraska Omaha, Omaha, Nebraska, United States*

Weight dysregulation is associated with increased morbidity and mortality across the lifespan. Age-related changes make weight control more challenging for middle-aged/older adults, highlighting the importance of self-management. Improving dietary balance may help some adults regulate weight; however, making healthy food choices remains difficult. Various methods/tools aim to facilitate healthier eating, including calorie information on menus (CI) and real-time calorie summation (CS) tools. Although understudied in aging adults, CS tools may be especially useful in digital environments for reducing healthy eating barriers (e.g., cognitive load, health literacy). This study explored CI/CS use, expanding on Gustafson & Zeballos (2019). Participants (N = 529; MAge = 56.1 ± 8.4, Range = 45-80; Female = 66.9%) completed an online survey, building and ordering a hypothetical sandwich, then blindly estimating its total calories. Participant condition was randomized (CI, n = 264; CS, n = 265). All participants were provided with calories-per-sandwich-ingredient; however, CS-participants could also view an auto-updating calorie total. Results revealed actual and estimated calories were positively correlated (r = 0.54, p < 0.001) and significantly different (t = 9.9, p < 0.001). Comparing conditions, CI-participants ordered 15.4 more calories (CI = 606.2, CS = 590.8; t = 1.2, p = 0.24), but estimated 35.1 fewer (CI = 510.9, CS = 546.0; t=-2.2, p < 0.001). This led to nearly double the absolute calorie discrepancy (CI = 164.9, CS = 86.3; t = 7.6, p < 0.001). CI-participants also underestimated calories more frequently (CI = 71.6%, CS = 47.9%). Despite not significantly reducing calories ordered, the CS tool improved estimation accuracy, suggesting it may increase awareness in food choice scenarios. While CS tools may not prompt immediate changes to habit/diet, improving calorie estimation could foster intuitive eating. Future research should explore ways to engender this critical skill in middle-aged/older adults, as it may help individuals regulate weight, mitigate disease, and improve aging outcomes.

Abstract citation ID: igaf122.2385

**NUTRITION AND FOOD INSECURITY SCREENING IN A PRESCRIPTION PRODUCE PROGRAM**

Katherine Falls[1], Ana Diallo[2], Kimberly Battle[1], Rachel Regal[1], Natalie Mansion[1], Genevieve Beaird[1], Johnathan Williams[3], and Leland Waters[1], *1. Virginia Commonwealth University, Richmond, Virginia, United States, 2. Virginia Commonwealth University School of Nursing, Richmond, Virginia, United States, 3. Virginia Commonwealth University Center for Translational Research, Richmond, Virginia, United States*

Understanding the complex social drivers that impact access to nutritious foods and dietary behaviors is essential to inform interventions that ultimately improve access to healthier options for older adults experiencing food and nutrition insecurity. The Prescription Produce Plan (PPP) is a Food is Medicine intervention embedded in the Mobile Health and Wellness Program, a wellness program serving low-income older adults. The PPP offers 6 goal-setting sessions and bags of fresh fruits and vegetables weekly or bi-weekly. The goal-setting sessions focus on behavior change to support chronic disease self-management. We conducted a pre-post analysis to measure the effects of the PPP on nutrition security and baseline descriptives of food security and access. Eighty-one participants enrolled, and 71 (88%) completed the program. On average, participants were 67 years old and reported five chronic diseases, with the most prevalent being hypertension (75%), type 2 diabetes (38%), and hypercholesterolemia (36%). At baseline, 43 participants (53%) screened positive for food insecurity, while 51% reported nutrition insecurity and 55% reported using food pantries to access groceries. The program was significantly associated with an 18% decrease in nutrition insecurity at the post-test (Z= -2.4, p = 0.02). These findings suggest that the PPP effectively addresses nutrition insecurity among older adults, emphasizing the importance of community-based interventions in tackling food access challenges. Additionally, using nutrition and food insecurity screening tools provides a deeper understanding of the complex nature of food access for older adults with significant health inequities.

Abstract citation ID: igaf122.2386

**THE IMPACT OF PHYSICIAN ADVICE ON LIFESTYLE CHANGES AMONG HYPERTENSIVE OLDER ADULTS IN THE U.S.**

Joseph Phillips, and Jun Chu, *University of Maryland, Baltimore County, Baltimore, Maryland, United States*

Hypertension affects half of the U.S. population and increases the risk of heart attacks, strokes, and kidney failure. Managing the progression of hypertension requires medication adherence and lifestyle changes, yet little is known about the prevalence of such changes after receiving medical advice from a physician. Additionally, variations in adherence across racial/ethnic groups remain unclear. Our objective was to measure the changes in medication intake and four other lifestyle measures (reducing alcohol consumption, reducing salt intake, eating healthier, and increasing exercise) among older hypertensive patients in the U.S. using the 2017 Behavioral Risk Factor Surveillance System's Hypertension Awareness module. We estimated unadjusted and adjusted percentage point changes in groups with and without a physician's advice using linear probability models, controlling for predisposing, enabling, and need factors. Our findings indicate that physician advice significantly increased rates of all five lifestyle changes across all tested racial/ethnic groups. Non-Latino Black patients exhibited the highest overall adherence, with or without advice. Among non-Latino White patients, the largest increases were in changing eating habits (27.02 percentage points), reducing salt intake (25.06 percentage points), reducing alcohol consumption (27.63 percentage points), and increasing exercise (22.21 percentage points). Non-Latino Black patients experienced notable increases in changing eating habits (20.58 percentage points) and increasing exercise (16.79 percentage points). This study demonstrates the importance of physician advice in managing hypertension changes among older adults, finding they are more likely to make lifestyle changes after receiving direct advice from medical providers.

Abstract citation ID: igaf122.2387

**VARIABILITY IN HEALTH BEHAVIORS OVER 14 YEARS PREDICTS LATER COGNITIVE OUTCOMES**

Elayna Seago, Benjamin Katz, and Laura Sands, *Virginia Tech, Blacksburg, Virginia, United States*

Engaging in healthy behaviors is important for maintaining cognitive functioning throughout the lifespan. We assessed whether variability in physical exercise and hours of sleep over a fourteen year period are associated with subsequent cognitive performance among Health and Retirement Study (HRS) participants aged 50-69 in 2004. We calculated variability in exercise and sleep across 7 biennial assessments by dividing the standard deviation of each participant's biennial scores by the mean of the participant's exercise or sleep score over the 14 year interval. Linear Regression analyses of participants aged 50-59 (n = 2370) at baseline revealed that higher initial cognition and educational levels, and female gender were associated with higher cognitive scores 14 years later. Although baseline levels of exercise and mobility were not associated with cognitive scores 14 years later, older age, more chronic conditions at baseline, and higher variability in exercise scores over the 14 years were negatively associated with cognitive scores in 2018. Similar results were found for participants aged 60-69 (n = 2738). We replicated the analyses described above to assess the association between variability in hours of sleep and subsequent cognitive functioning and found very similar findings. Importantly, we also found that the greater variability in biennial assessments of sleep was negatively associated with cognitive functioning 14 years later for those aged 50-59 (n = 743) at baseline, and 60-69 (n = 889) at baseline. The findings reveal the importance of considering variability in health behaviors when assessing their impact on future cognitive functioning and the importance of maintaining health behaviors throughout adulthood.

### SESSION 7100 (POSTER)

### HEALTH PROMOTION

Abstract citation ID: igaf122.2388

**AGING RESEARCH IN FOCUS: SCHOLARLY OUTPUT OF THE DE TORNYAY CENTER FOR HEALTHY AGING NETWORK**

Wenting Peng, Paige Bartlett, Yanjing Liang, Priscilla Carmiol-Rodriguez, and Basia Belza, *University of Washington, Seattle, Washington, United States*

Since 1998, the de Tornyay Center for Healthy Aging at the School of Nursing, University of Washington has served as a catalyst for promoting healthy aging research. The purpose of our project was to analyze publications authored by scholars with aging interests and affiliated or associated with the de

Tornyay Center over the past five years. We searched Web of Science for publications authored by 41 faculty and scholars, published between January 1, 2020, and December 31, 2024. Conference abstracts were excluded. The R-package BibliometrixRuan was used to perform analyses. Among 480 identified publications, 394 were research articles, 48 were review articles, 25 were editorial material, 7 were letters to the editor, and 6 were corrections. The most frequently published journal was Biological Research for Nursing (N = 14). Overall, the publications had 2,221 co-authors and an average of 8.7 co-authors. International collaborations accounted for 28.0% of the publications. These works were cited in 4,849 articles, contributing to a total of 5,113 citations, with an average of 10.67 citations per publication. The H-index for these articles was 28, suggesting that 28 papers had each been cited at least 28 times. The most frequent keywords in the publications, with dementia being the most common, followed by HIV, irritable bowel syndrome, depression, and older adults. These findings highlight the de Tornyay Center's impact in mentoring scholars and supporting innovative research addressing key aging challenges. The center's interdisciplinary contributions reinforce its role in advancing knowledge and promoting healthy aging across the lifespan.

Abstract citation ID: igaf122.2389

### BARRIERS AND FACILITATORS OF INDOOR NO-SMOKING POLICY IMPLEMENTATION AMONG OLDER CHINESE AMERICAN WOMEN

Chien-Ching Li[1], Tingfei Hu[1], Samantha Wu[2], Sharon Mei[2], and Alicia K. Matthews[3], *1. Rush University, Chicago, Illinois, United States, 2. University of Illinois Chicago, Chicago, Illinois, United States, 3. Columbia University, New York, New York, United States*

Objective: Asian American female non-smokers face nearly twice the risk of lung cancer compared to their White counterparts. Among Chinese Americans, smoking is predominantly a male behavior, influenced by cultural norms, with approximately 25% of Chinese American men being current smokers. Given that over 88% of them are married, Chinese American women face heightened exposure to secondhand smoke (SHS), a known risk factor for lung cancer. Family and cultural dynamics may shape SHS exposure patterns, influencing the adoption of indoor no-smoking policies. This study examines the barriers and facilitators to implementing such policies among Chinese American women using the Health Belief Model. Methods: Our study recruited 86 Chinese American women aged 50 and older who had ever lived with a smoker. Participants completed a survey assessing indoor smoking policies at home, with selected questions developed based on the Health Belief Model. The survey measured perceived susceptibility to secondhand smoke (SHS), perceived severity of SHS, perceived benefits of a smoke-free environment, self-efficacy in implementing no-smoking policies, and cues to action. Results: Binary logistic regression results showed that Chinese women who expressed higher levels of self-efficacy were 1.8 times more likely to implement no-smoking policies at home after controlled for covariates. Conclusion: Higher self-efficacy significantly increased the likelihood of implementing indoor no-smoking policies among Chinese American women. Culturally tailored interventions that enhance self-efficacy may be key to reducing secondhand smoke exposure and lung cancer risk in this population.

Abstract citation ID: igaf122.2390

### IMPACT OF HEALTH BEHAVIOR PRACTICES ON EMERGENCY DEPARTMENT UTILIZATION AMONG OLDER ADULTS WITH DIABETES

Suhyun Kim[1], and Ye-Ji Lim[2], *1. Kyungpook National University, Daegu, Republic of Korea, 2. Kyungpook National University Hospital, Daegu, Republic of Korea*

Background: Older adults with diabetes are at heightened risk for emergency department (ED) visits due to disease-related complications. Identifying modifiable factors, such as health behaviors, that contribute to ED utilization is crucial for developing targeted interventions to enhance self-management and reduce healthcare burdens. Objective: This study examines the association between demographic and disease characteristics, health behavior practices, and ED visits among older adults aged 65 years and older diagnosed with diabetes. Methods: A descriptive correlational study was conducted using data from 956 older adults diagnosed with diabetes, drawn from the Korean Healthcare Panel Annual Data (2014–2017). Data analysis was performed using descriptive statistics, Mann-Whitney U tests, chi-square analysis, and univariate regression analysis in SPSS 26.0. Results: The findings revealed that older age, activity limitations due to disability, depression, and a greater number of chronic comorbidities significantly increased the likelihood of ED visits among older adults with diabetes. Additionally, fewer days of moderate physical activity and walking were associated with a higher risk of ED utilization. Conclusion: Promoting sustained physical activity is essential in mitigating ED visits among older adults with diabetes. Healthcare providers should implement tailored interventions that enhance mobility and address psychological and physical health barriers to improve self-care management and reduce acute healthcare utilization. Implications for Clinical Practice: Integrating physical activity counseling into routine care, alongside comprehensive chronic disease management strategies, may help prevent ED visits and improve overall well-being in this population.

Abstract citation ID: igaf122.2391

### MIND-BODY PRACTICE, CARDIOVASCULAR HEALTH, AND WELL-BEING: A 20-YEARS STUDY IN US ADULTS

Kallol Kumar Bhattacharyya, and Sharmila Acharya, *The University of Memphis, Memphis, Tennessee, United States*

Mind-body practice is associated with various trajectories of health. However, the exact role of cardiovascular health in the association between mind-body practice and holistic well-being in mid and late life is still a growing field of research. We examined participants enrolled in waves 1-3 (1995–2015) of the Midlife in the United States (MIDUS) study (N = 2,536) and merged data from the main self-administered questionnaire (SAQ) and biomarker projects. Holistic well-being was assessed by flourishing score, a composite construct of emotional, psychological, and social well-being. We used structural equation models to examine whether persistent mind-body practice across two waves (1–2) or intermittent practice at one wave is associated with better flourishing over 20 years, compared to no practice, while controlling for prior wave flourishing and covariates (baseline socio-demographics, health, and functional status). Additionally, we assessed if ideal cardiovascular health, which was measured using the parameters of Life's Essential 8 defined by the American Heart Association, mediates the above associations. Findings suggested that persistent mind-body practice has a significant positive effect (b = 1.052; SE = 0.531; $p < .05$) on flourishing. Ideal cardiovascular health status also has a significant positive effect (b = 0.303; SE = 0.141; $p < .05$) on flourishing; however, it does not mediate the association between persistent mind-body practice and flourishing. More research is necessary to support future policy and practice recommendations validating the current findings to enhance the well-being of more vulnerable populations, such as long-term care residents.

Abstract citation ID: igaf122.2392

### THE IMPACT OF FORMAL AND INFORMAL VOLUNTEERING PATTERNS ON HEALTH IN OLDER ADULTS

Huei-wern Shen[1], Peter Sun[2], and Yi Wang[3], *1. University of North Texas, Denton, Texas, United States, 2. Bar-Ilan University, Ramat Gan, Tel Aviv, Israel, 3. University of Iowa School of Social Work, Iowa City, Iowa, United States*

In later life, many individuals participate in both formal and informal volunteering. While the health benefits of volunteering are well-documented, little is known about the impact of patterns of formal and informal volunteering on health outcomes among older adults. Using five waves of data (2010–2018) from the Health and Retirement Study, multichannel sequence analysis and cluster analysis were applied to identify volunteering patterns among 3,579 individuals aged 65 and older in 2010 who remained alive throughout the study period (17,895 observations). Four distinct clusters (C1-C4) were identified: (C1) Decreasing Informal Volunteering (37%), (C2) Steady Non-Involvement (31%), (C3) Decreasing High-Intensity Dual Involvement (17%), and (C4) Decreasing Low-Intensity Dual Involvement (15%). Then, controlling for sociodemographic characteristics, three regression models were used to explore the relationships between these clusters and three health indicators: cognitive function, self-rated health (SRH), and depressive symptoms (CESD). Compared to Cluster 2 (consistently non-volunteering older adults), those who engaged in volunteering (either formal or informal) during the study period (Clusters 1, 3, and 4) exhibited better cognitive function and fewer depressive symptoms, even as their involvement declined over time. However, only those in Cluster 3 (decreasing high-intensity dual involvement) reported significantly better self-rated health than the non-volunteering group. These findings highlight the lasting health benefits of volunteering in later life. Future research should explore the mechanisms underlying these relationships and investigate their relevance across diverse populations to provide further insights into the long-term effects of volunteering patterns on health.

### SESSION 7105 (POSTER)

### IMPLEMENTATION SCIENCE

Abstract citation ID: igaf122.2393

### A SCOPING REVIEW OF STUDIES UTILIZING THE DELPHI METHOD IN AGING RESEARCH FROM 2018 TO 2024

Aaron Ogletree[1], Shannon Jarrott[2], Shelbie Turner[3], and Michelle Demetres[3], *1. Virginia Tech, Blacksburg, Virginia, United States, 2. The Ohio State University, Columbus, Ohio, United States, 3. Weill Cornell Medicine, New York, New York, United States*

The Delphi method is a structured process used to obtain expert input on the development of evidence-based tools and resources like quality indicators and clinical guidelines. Despite an estimated 250% increase in the number of studies utilizing the Delphi method since 2014, no published literature has explored its use in aging research. The goals of this scoping review were to characterize applications of the Delphi method in aging research and identify areas for improvement in its application. We searched multiple databases for published literature from January 2018 through November 2024, identified eligible studies using predefined criteria, and extracted key information (e.g., scientific topics and alignment with best practices). We identified 89 Delphi studies in aging research that met inclusion criteria. The most frequent topics included models of care (n = 9), technology (n = 8), and bone health (n = 7). Delphi studies were seldom used to develop materials for subpopulations of older adults (e.g., racial/ethnic minority groups). Experts were mostly clinicians/care providers (n = 78) and researchers (n = 52); older adults (n = 8), unpaid caregivers (n = 3), and advocacy and education groups (n = 5) were least represented. Few studies (n = 9) adhered to guidance on Delphi best practices (e.g., CREDES), highlighting that most studies omitted checklists used to ensure transparent reporting of Delphi conduct. Our findings document trends in use of the Delphi method in aging research and document gaps in representation of experts and focal populations. Opportunities to improve Delphi application in aging research include identification and promotion of best practices for researchers and journals focused on aging.

Abstract citation ID: igaf122.2394

### ADVANCING DIGITAL EQUITY: A QUALITATIVE CASE STUDY USING THE IMPLEMENTATION RESEARCH LOGIC MODEL

Josie Santilli[1], Skye Leedahl[1], Jill Juris[2], Erica Liebermann[1], and Casey McGregor[1], *1. University of Rhode Island, Kingston, Rhode Island, United States, 2. Appalachian State University, Boone, North Carolina, United States*

Older adults without digital access, who are attempting to manage their lives within a digital landscape often struggle to maintain their independence. Technology is transforming the world around them, but they are excluded from participating. The purpose of the research was to explore how an evidence-based, university-led program for older adults, the iPad Project, was implemented to empower its participants with digital literacy. This qualitative case study used semi-structured interviews with 30 study participants and collaborators, a document review, and an Implementation Research Logic Model (IRLM) to investigate the interaction of elements, determinants, strategies, and mechanisms that impacted the outcomes. Interview questions were derived from relevant constructs in the Consolidated Framework for Implementation Research and the Expert Recommendation for Implementing Change strategies to explore the perspectives of project participants and collaborators. A thematic analysis of the responses was completed and informed the development of the IRLM which displayed how the project was implemented. The IRLM presented the key core and adaptable elements of the iPad Project that contributed to the project's outcomes. Environmental determinants were found to impact these elements by challenging or facilitating project progress. Strategic approaches used to address these changing effects were identified and reflected how the project was managed. The interplay between hypothesized mechanisms and contextual determinants was examined and revealed the underlying influences that shaped the outcomes. The implementation outcomes were highlighted and illustrated the effect of this evidence-based intervention. This study provides guidance for future digital literacy program implementations.

Abstract citation ID: igaf122.2395

### IMPLEMENTATION AND QUALITY IMPROVEMENT STRATEGIES IN ASSISTED LIVING: A SCOPING REVIEW

Cameron Ulmer[1], Jennifer Leeman[1], Sheryl Zimmerman[2], and Mark Toles[1], *1. The University of North Carolina at Chapel Hill, Chapel Hill, North Carolina, United States, 2. University of North Carolina at Chapel Hill, Chapel Hill, North Carolina, United States*

Gaps persist in the quality of care in assisted living (AL), yet little is known about the best approaches for closing these gaps. Guided by Proctor's Framework for Implementation Research, this scoping review examined studies of quality improvement strategies and outcomes in AL. In collaboration with a health sciences librarian, we systematically searched PubMed, CINAHL, and Scopus to identify English-language, empirical, peer-reviewed, quality improvement and implementation studies in AL published before May 2023. Of 204 studies identified, 9 met inclusion criteria. The clinical focus of papers included behavioral expressions of persons with dementia (n = 2), activities of daily living (n = 2), and antibiotic stewardship (n = 1), among others. Strategies used to implement new clinical practices included staff training (n = 9), the addition of new staff and/or new tools (n = 8), and rapid cycles of improvement (n = 5). Studies reported outcomes related to both feasibility (n = 7), (e.g., intervention adherence, acceptability), and intervention effectiveness (n = 7), (e.g., pre- and post-intervention testing, objective observation). While sample sizes tended to be small, most interventions demonstrated preliminary evidence of effectiveness (n = 6), with only one (n = 1) reporting statistically significant outcomes. Common gaps in study reports included estimates of statistical power, details regarding missing data and unintended consequences, and the cost of the interventions. In summary, few implementation or quality improvement studies have been published on the AL setting and more empirical research is necessary to understand strategies for closing gaps in AL care quality.

Abstract citation ID: igaf122.2396

### IMPLEMENTATION AND SUSTAINABILITY OF AUTONOMY INITIATIVES IN LONG-TERM CARE SETTINGS

Ashley Smith[1], A. Lynn Snow[1], and Michelle Hilgeman[2], *1. The University of Alabama, Tuscaloosa, Alabama, United States, 2. Tuscaloosa VA Medical Center, Tuscaloosa, Alabama, United States*

Resident autonomy is a core component of person-centered care in nursing homes yet autonomy-focused interventions face implementation and sustainability challenges (Boumans et al., 2021). The Montessori approach promotes autonomy through individualized choice, engagement, and environmental modifications and is increasingly applied in long-term care settings (Yan & Traynor, 2023). Collective autonomy (e.g., shared decision-making) represents an important but often underexplored aspect of autonomy. This secondary data analysis examines changes in resident autonomy during implementation of Montessori staff training in a cluster randomized study in 8 US Veterans Health Administration Community Living Centers (VA nursing homes). The Montessori Organizational Self-Assessment (MOST) was used to assess four autonomy domains across 5 timepoints: 1) Ask the Resident, 2) Connecting with Others, 3) Prepare the Environment, and 4) Create Community, rated on a scale from 0 (Not There) to 2 (In Action). At baseline, sites rated themselves below the midpoint in all domains, with the highest scores in Domain 1 (M = .88, SD = .07) and Domain 3 (M = .82, SD = .10). Linear mixed-effects models showed significant increases from baseline to 12 months across all domains. However, only Domain 1 demonstrated sustained improvement at each follow-up. Other domains improved by 12 months but showed variability across intermediate timepoints. To our knowledge, this is one of the first studies to examine MOST scores as an indicator of collective autonomy over time after implementation of a complex intervention. Findings suggest collective autonomy progress may be challenging to sustain. Future research should investigate strategies to reinforce autonomy across all domains and the relationship between environmental and process-related autonomy initiatives.

### SESSION 7110 (POSTER)

### LGBTQIA+

Abstract citation ID: igaf122.2397

### ALZHEIMER'S DISEASE AND RELATED DEMENTIAS AMONG TRANSFEMININE ADULTS: A COHORT STUDY

Ethan Cicero[1], Jace Flatt[2], Vin Tangpricha[1], Darios Getahun[3], Courtney McCracken[4], Michael Silverberg[5], Suma Vupputuri[6], and Michael Goodman[1], *1. Emory University, Atlanta, Georgia, United States, 2. University of Nevada Las Vegas, Nevada, United States, 3. Kaiser Permanente Southern California, Pasadena, California, United States, 4. Kaiser Permanente Georgia, Atlanta, Georgia, United States, 5. Kaiser Permanente Northern California, Oakland, California, United States, 6. Kaiser Permanente Mid-Atlantic States, Washington, District of Columbia, United States*

Understanding of Alzheimer's disease and related dementias (ADRD) among transfeminine (TF) adults is limited. Kaiser electronic health records (01/2006–03/2023) were used to identify a cohort of 2,362 TF individuals aged 45+ enrolled in three integrated health systems. Each transgender individual was matched to 10 cisgender men (CM) and 10 cisgender women (CW) on birth year, race/ethnicity, study site, and enrollment at index date (first evidence of transgender status in TF cohort members). Enrollment time-adjusted odds ratios (aOR) and 95% confidence intervals (CI) were calculated to examine the odds of ADRD among TF adults compared to CM and CW referents. TF adults had a higher ADRD prevalence (1.8%) relative to CM (0.8%) and CW (0.9%). The aOR (95% CI) estimates of ADRD among TF were 1.61 (1.14–2.25) and 1.37 (0.98–1.91) relative to CM and CW, respectively. When restricting analyses to TF with evidence of gender-affirming hormone therapy (GAHT) and their referents, the associations were only slightly stronger than in the overall analysis with aOR (95% CI) estimates of 1.82 (1.20–2.75) and 1.29 (0.54–3.11) related to CM and CW, respectively. Results restricted to cohort members of color were similar, but imprecise due to small numbers of ADRD cases. TF adults have significantly higher odds of ADRD compared to CM and CW referents; this association does not appear to be attributable to GAHT, although hormone levels should be explored. Modifiable risk factors for ADRD should be examined to prevent or delay the onset of ADRD in transgender persons.

Abstract citation ID: igaf122.2398

**ANXIETY, DEPRESSION, AND PTSD AMONG A COHORT OF MIDDLE AGED AND OLDER TRANSGENDER ADULTS**

Ethan Cicero[1], Jace Flatt[2], Vin Tangpricha[1], Darios Getahun[3], Courtney McCracken[4], Michael Silverberg[5], Suma Vupputuri[6], and Michael Goodman[1], *1. Emory University, Atlanta, Georgia, United States, 2. University of Nevada Las Vegas, Nevada, United States, 3. Kaiser Permanente Southern California, Pasadena, California, United States, 4. Kaiser Permanente Georgia, Atlanta, Georgia, United States, 5. Kaiser Permanente Northern California, Oakland, California, United States, 6. Kaiser Permanente Mid-Atlantic States, Washington, District of Columbia, United States*

Systematic studies of mental health status among older transgender adults are largely lacking. Kaiser electronic health records (01/2006–03/2023) were used to identify a cohort of 2,362 transfeminine (TF) and 1,185 transmasculine (TM) individuals aged 45+ years enrolled in three integrated health systems. Each transgender individual was matched to 10 cisgender men (CM) and 10 cisgender women (CW) on birth year, race/ethnicity, study site, and enrollment at index date (first evidence of transgender status in TF/TM cohort members). Enrollment time-adjusted odds ratios (aOR) along with 95% confidence intervals (CI) were calculated to examine prevalence of anxiety, depression, and post-traumatic stress disorder (PTSD) among TF and TM adults compared to their respective CM and CW referents. In the analyses comparing TF cohort members to CW referents the aOR (95% CI) estimates were 2.01 (1.84–2.20) for anxiety, 2.66 (2.43–2.90) for depression, and 3.27 (2.80–3.81) for PTSD. When TF individuals were compared to CM referents the aOR was 4.13 (3.77–4.52) for anxiety, 5.46 (4.99–5.97) for depression, and 7.53 (6.33–8.96) for PTSD. The aORs were more pronounced for TM relative to CM and attenuated for TM relative to CW. All associations were stronger in the sub-analyses restricted to persons of color whereas the sub-analyses focusing on gender-affirming hormone therapy recipients showed no discernable difference from the overall results. Older TF and TM adults experience a higher burden of mental health conditions compared to their cisgender counterparts. This burden appears to disproportionally affect transgender persons of color.

Abstract citation ID: igaf122.2399

**BISEXUAL INDIVIDUALS ENVISIONING AGING FUTURES: EMERGENT OUTLOOKS ON LATER LIFE**

Zhiqi Yi[1], Olivia Lafountain[1], Xavier Noriega[1], Austin Oswald[2], and Sarah Jen[1], *1. University of Kansas, Lawrence, Kansas, United States, 2. Dalhousie University, Halifax, Nova Scotia, Canada*

The outlooks individuals envision their future shape their expectations and approach to later life. While bisexual older adults report distinct life experiences from lesbian, gay, and heterosexual counterparts, their outlook on later life remains unclear. Guided by the queer aging futures perspective (Sandberg & Marshall, 2017), this study examines future outlooks among 21 bisexual-identified adults in the U.S. (aged 51–71, Mean=58.6) through semi-structured life review interviews analyzed via inductive thematic coding. Four future outlooks emerged, forming two contrasting but not mutually exclusive pairs: Exploratory vs. Settling-Down and Affirmation vs. Fraught. Individuals with exploratory outlooks anticipated continued fluidity in their own sexuality, gender, and social connections, embracing the potential of rich and flexible social networks and communities in the future. Individuals with settling-down outlooks desire to settle into a comfortable and consistent day-to-day experience with their current families and loved ones. Affirmation-oriented individuals expressed optimism, envisioning futures of acceptance, belonging, and LGBTQ+ activism and aging-supportive communities. Conversely, fraught outlooks entailed concerns about aging, end-of-life challenges, and potential setbacks in LGBTQ+ rights. Consistent with the life course perspective, these outlooks reflect earlier life experiences that may shape individuals' anticipated futures. We see the exploratory outlook as reflecting particular strengths of bisexual communities, such as a capacity for openness, fluidity, and flexibility that could inspire adaptability in anticipated later lives, not just for bisexual aging but for aging populations broadly. These findings illustrate possible multiplicities and complexities of queering aging futures with distinctly bisexual interpretations of what later life might entail.

Abstract citation ID: igaf122.2400

**COMBINING HEALTH INFORMATION WITH SOCIAL NETWORKING FOR LGBTQIA+ OLDER PERSONS**

Jennifer Smith[1], Sharon Bowland[2], Joseph Winberry[3], and Namrata Mukherjee[1], *1. University of Tennessee, Knoxville, Knoxville, Tennessee, United States, 2. University of Tennessee, Knoxville, Tennessee, United States, 3. The University of North Carolina at Chapel Hill, Chapel Hill, North Carolina, United States*

The aim of this research project was to address health-related concerns and missing information in the older LGBTQIA+ population in a community where historic discrimination and stigma made culturally responsive healthcare uncertain. We used surveys to collect data about healthcare needs and concerns during five social events held in a gay-friendly coffee shop over a six-month period. During the events, we invited providers to share essential health information on topics such as mental health, caregiving, and legal aid, to promote health literacy and to provide social connection. Fifty-three people over the age of 50 filled out the survey of likert scale and open-ended questions about their healthcare experiences. Forty-six percent of participants stated that they did not know where to find culturally supportive healthcare. One key theme was the difficulty participants identified in assessing whether providers were willing to provide services to LGBTIA+ people. On the other hand, participants identified a strong sense of connection with others in the LGBTIA+ community. Ninety percent said that they were linked with other LGBTIA+ people, and 100% indicated that they were actively involved in assisting others with their needs. Eighty-four percent said they also turned to their community for help. Participants seemed to value the opportunity to meet with others and the focus on their health and emotional well-being. Social groups that focus on health literacy and provide social outlets are using sustainable strategies for enhancing social connection and health information to bolster the LGBTQIA+ community and a sense of belonging.

Abstract citation ID: igaf122.2401

**FACE TO FACE: THE KEY TO CONNECTION AND VOLUNTEER SATISFACTION FOR LGBTQ+ OLDER ADULTS**

Jenna Binaco, Jillian Crocker, Hannah Friedland, Soledad Arguelles-Borge, and Ashley Stripling, *Nova Southeastern University, Fort Lauderdale, Florida, United States*

Social isolation is a prominent issue for LGBTQ+ older adults, necessitating expanded efforts for support. LGBTQ+ volunteer programs are one such avenue of support, however, the growth of these programs relies on volunteer interest and satisfaction. Research on volunteer satisfaction offers valuable insight into the increased implementation of LGBTQ+ older adult volunteer programs. As such, the current study examines the satisfaction of volunteers visiting LGBTQ+ older adults. We hypothesized that volunteer satisfaction would be higher in individuals who had spent more time with their assigned participants. The sample consisted of 21 volunteers who completed a monthly report form in 2024. The form included questions regarding the duration and number of in-person and virtual interactions. Volunteer stress and satisfaction were ranked on a 4-point Likert scale. A series of Pearson correlations were conducted and results revealed a large, negative correlation ($r(17)=-.744$, $p<.001$) between volunteer stress ($M=1.74$, $SD=1.05$) and volunteer satisfaction ($M=3.37$, $SD=1.01$). The number of in-person contacts had a large, negative correlation with stress, $r(17)=-.481$, $p=.037$, and a large, positive correlation with satisfaction, $r(17)=.502$, $p=.029$. These relationships did not hold for virtual contacts, total number of contacts, or time spent with the client. The findings emphasize the significance of face-to-face interaction despite the availability and established benefits of virtual communication. The stress associated with volunteering may also reveal gaps for improving volunteer recruitment and retention. Implications include examining how volunteers engage with older LGBTQ+ adults to increase volunteer and participant satisfaction.

Abstract citation ID: igaf122.2402

### FROM STIGMA TO STRENGTH: TRACKING POST-TRAUMATIC GROWTH IN OLDER SEXUAL MINORITIES

Michael Vale, *Sacred Heart University, Fairfield, Connecticut, United States*

Post-traumatic growth (PTG) refers to the personal development that arises from overcoming adversity and has historically been overlooked in older sexual minorities (e.g., lesbian, gay, bisexual individuals). Throughout their lives, older sexual minorities have faced heightened vulnerability to marginalization (e.g., discrimination), which has been linked to worse health outcomes. However, over time, they may develop a sense of adaptation to these stigmatizing experiences, establishing a potential source of resilience (i.e., PTG). Yet, no existing scales specifically capture PTG in queer populations. This study aimed to (1) establish a PTG measure for sexual minorities and (2) examine PTG's correlates in younger ($N=107$, ages 18–39) and middle-aged/older ($N=216$, ages 40–90) sexual minorities. Exploratory factor analysis supported the hypothesized five-factor structure with strong internal consistency ($\alpha = .88$). Although PTG was not correlated with age ($p = .21$), it was associated with microaggressions ($r = .16$, $p < .05$), discrimination ($r = .24$, $p < .001$), and victimization ($r = .24$, $p < .001$) across age groups. PTG was also associated with other adaptive psychosocial resources, such as self-compassion ($r= .20$, $p<.05$), active coping ($r= .21$, $p<.05$), religious coping ($r= .36$, $p<.001$), and positive reframing ($r= .33$, $p<.001$), but only among the older sexual minorities. These results highlight that PTG may play a unique role in promoting well-being for this health disparity group and serve as a catalyst for future research on PTG in this population. Specifically, researchers should explore interventions that harness PTG to enhance well-being.

Abstract citation ID: igaf122.2403

### IMPROVING SOCIALLY SUPPORTED SERIOUS ILLNESS CARE FOR LGBTQIA+ PEOPLE: A FEASIBLITY STUDY

Raeann LeBlanc, and Susan Shaw, *University of Massachusetts Amherst, Amherst, Massachusetts, United States*

End-of-life doula care is non-medical care centered on positive regard and pragmatic emotional support to address social isolation, a complex issue in living with serious illness in the LGBTQIA+ context. The "Doula Pride" program centers social support for LGBTQIA+ older people with serious illness using a community engaged supportive network approach. Through training and social connection, Doula Pride aimed to decrease social isolation and bolster community based social capital. This pilot intervention used a multi-methods design to test feasibility among a cohort of LGBTQIA+ doula informed carers who completed a 4-week training matched with LGBTQIA+ persons with serious illness from a rural community. Surveys and interviews evaluated self-efficacy, social support, social isolation, social networks, and social capital at the individual and community levels. Thirteen female LGBTQIA+ doula-pride informed carers completed training. Just over half matched with an LGBTQ+ older adult in the community living with serious illness. A total of twenty-six visits were completed during the 4-month practice period. The processes of community engaged planning, curriculum design, recruiting and matching carers, and lessons learned in the process, will be discussed. The "Doula Pride" community engaged program fostered social support among LGBTQIA+ people with serious illness and their carers using an innovative local network approach to address holistic social support and decreased social isolation. Our results suggest it is feasible to sustain this community program through close LGBTQIA+ community engagement.

Abstract citation ID: igaf122.2404

### MENTAL HEALTH GENDER GAP OF GAY AND LESBIAN AGING ADULTS: THE ROLE OF AGE DISCRIMINATION AND INTERNALIZED HOMOPHOBIA

Ella Cohn-Schwartz[1], Sigal Gooldin[2], and Yaacov G. Bachner[1], *1. Ben Gurion University of the Negev, Be'er Sheva, HaDarom, Israel, 2. The Israeli Institute for Gender and LGBT Research, Tel Aviv, Tel Aviv, Israel*

Gender disparities in mental health among older adults from sexual minorities remain underexplored. This study investigated how stigma related to age and sexual orientation - both enacted by others (discrimination) and internalized – is associated with mental health outcomes among lesbian and gay adults aged 50+. The study examined 411 adults aged 50+(range: 50-85) from Israel who self-identify as lesbian women or gay men, via online questionnaires. Participants answered questions about experiencing discrimination based on their age and sexual orientation, about their internalized ageism and internalized homophobia, and about their mental health (depressive symptoms, anxiety, loneliness, and life satisfaction). Mediation analyses examined the role of stigma based on age and on sexual orientation in the association between gender and mental health. The results showed that lesbian women reported better mental health compared to gay men. The gender difference in mental health was partially explained by gendered experiences of age-based discrimination and internalized homophobia: Women experienced less age-based discrimination and reported lower internalized homophobia, and these were related to better mental health in terms of lower depressive symptoms, lower anxiety, less loneliness, and higher life satisfaction. Thus, the greater mental health challenges faced by gay men in later life may stem, in part, from higher exposure to age-based discrimination and greater internalization of negative societal attitudes toward their sexual orientation—potentially reflecting the cumulative impact of lifelong stigma. These findings underscore the need for better interventions aimed at distinct needs and experiences of aging men and women from sexual minorities.

Abstract citation ID: igaf122.2405

### REFRAMING AGING AND DIABETES: CENTERING TRANSGENDER HEALTH EQUITY

Barbara Mendez Campos[1], and Gabi Celia Ortiz[2], *1. University of Tennessee Knoxville, Knoxville, Tennessee, United States, 2. Boston College, Chestnut Hill, Massachusetts, United States*

As more transgender (trans) individuals reach older adulthood, it is essential to understand their health risks, including chronic conditions such as diabetes. Research on aging and diabetes has largely excluded trans populations, leaving gaps in knowledge about their unique experiences in healthcare access, diagnosis, and management. This study utilizes pooled data from the 2021–2023 California Health Interview Survey (CHIS) to examine predictors of prediabetes and diabetes among trans individuals 18 years and older (n = 322, weighted N = 249,822). Logistic regression, accounting for survey weights and jackknife estimation, found that adults aged 80+, those with high blood pressure, those with higher education, and those with health insurance had statistically significantly higher odds of diabetes. Findings suggest that both structural and individual factors contribute to prediabetes and diabetes risk among trans adults, with critical implications for aging. The association between health insurance and diagnosis highlights a link to healthcare access as a key aspect in diabetes detection, raising concerns about undiagnosed cases among uninsured trans individuals. Similarly, the protective effect of education may reflect greater health literacy, yet disparities in education and healthcare access persist, limiting prevention opportunities. Larger samples are needed to refine estimates, assess racial and ethnic differences, and explore key interactions shaping diabetes risk. Through centering trans adults, findings underscore the need for public health initiatives that bridge healthcare gaps and ensure culturally competent and gender-relevant diabetes prevention and management. Broadly, they emphasize the importance of including trans experiences in aging research, healthcare, and services to advance equitable health outcomes.

### SESSION 7115 (POSTER)

### MIDLIFE (AGING TO/FROM)

Abstract citation ID: igaf122.2406

### ADAPTIVE DIGITAL PROBLEM-SOLVING SKILLS FOR LIFE MANAGEMENT IN THE MIDDLE AGE

Takashi Yamashita[1], Donnette Narine[1], and Giyeon Kim[2], *1. University of Maryland, Baltimore County, Baltimore, Maryland, United States, 2. Chung-Ang University, Seoul, Republic of Korea*

Life management digital tasks, such as paying bills, checking health information, and navigating appointments online, are essential for one's life. In today's fast-changing society, adults must adapt and solve problems quickly using constantly evolving digital technologies. However, middle-aged and older adults tend to be more digitally divided than their younger counterparts. Practice engagement theory depicts that continuous skill use is crucial for maintaining and enhancing one's skills over the life course. Establishing the skill use habit in earlier adult life stages may prevent being digitally left behind in later life. Therefore, the current study examined the associations between the proficiency and utilization of adaptive digital problem-solving (ADPS) skills in middle-aged adults. We used a nationally representative sample (50 years and older; n = 1,203) drawn from the 2023 U.S. Program for the International Assessment of Adult Competencies (PIAAC) data. PIAAC provides ADPS digital skill assessment data based on the simulation of everyday life situations (e.g., planning a grocery shopping and business meeting using the digital map). The ADPS skill proficiency score (0-500 points) was regressed on the ADPS skill use and individual characteristics (e.g., age, sex, education, employment). Results showed that middle-aged adults who used ADPS skills for life management at least once a week had significantly greater ADPS skill proficiency ($b = 15.98$, $p < 0.01$) than their counterparts. Engagement with ADPS skills in middle age has implications for individual autonomy in later life in digital-technology-rich societies. Theoretical explanations and policy implications were discussed.

Abstract citation ID: igaf122.2407

### DEVELOPMENT AND VALIDATION OF RESPONSE TIME INCONSISTENCY IN THE MIDLIFE IN THE UNITED STATES (MIDUS) STUDY

Robert Stawski[1], Eric Cerino[2], Dakota Witzel[3], Margie Lachman[4], and Stuart MacDonald[5], *1. Utah State University, Logan, Utah, United States, 2. Northern Arizona University, Flagstaff, Arizona, United States, 3. South Dakota State University, Brookings, South Dakota, United States, 4. Brandeis University, Waltham, Massachusetts, United States, 5. University of Victoria, Victoria, British Columbia, Canada*

Response time inconsistency (RTI), the trial-to-trial variability on a RT-based cognitive task, is an important behavioral indicator of cognitive health, cognitive aging, and an early indicator of cognitive pathology, and central nervous system (CNS) dysfunction. Although RTI is well-studied through computer-based speeded tasks, little research has examined the validity of RTI obtained from: (1) telephone-based cognitive assessments employing voice-triggered response time protocols; or (2) midlife cohorts who are at risk for dementia. Using data from the second wave of the MIDUS study (N = 4,285; Mage=55.6, SD = 12.2, Range=28-84; 55%=women; 75% having at least some college education), participants completed the Brief Test of Adult Cognition via Telephone (BTACT), which includes tests of executive function and episodic memory abilities, as well as a RT-based stop-go switching (SGS) task. RTI was derived from 20 trials of the SGS non-switch condition and quantified as an intraindividual standard deviation (ISD) residualized for trial number. RTI was greater with age (r=.26, p<.0001), being less educated (r=-.16, p<.0001), and among women (Cohen's d=.15, p<.0001). Importantly, RTI was associated with slower task switching performance, lower scores on the BTACT, episodic memory, and executive function cognitive composites (rs=-.19 to -.41, ps<.0001), as well as poorer self-rated mental (r=-.13) and physical (-.18, ps<.0001) health. RTI was not significantly related to self-rated positive affect (r=-.02, p=.24) or negative affect (r=.02, p=.17). Discussion will focus on the validity and promise of RTI obtained from telephone-based cognitive assessments for characterizing CNS integrity and dementia risk in large-scale surveys.

Abstract citation ID: igaf122.2408

**PREVALENCE AND CORRELATES OF COGNITIVE IMPAIRMENT NO DEMENTIA (CIND) STATUS: THE MIDLIFE IN THE UNITED STATES STUDY.**

Robert Stawski[1], Dakota Witzel[2], Eric Cerino[3], and Stuart MacDonald[4], *1. Utah State University, Logan, Utah, United States, 2. South Dakota State University, Brookings, South Dakota, United States, 3. Northern Arizona University, Flagstaff, Arizona, United States, 4. University of Victoria, Victoria, British Columbia, Canada*

Cognitive impairment no dementia (CIND) status – a multi-test profile and impairment classification – is a promising indicator of cognitive health, leveraging intraindividual variability in performance across multiple domains. CIND profiles have exhibited considerable utility for differentiating adults exhibiting neuropsychological impairment (>-1SD below age- and education-adjusted norms) on one (CIND-single) or more (CIND-multiple) tasks, from those who are comparatively unimpaired or exhibit transient low scores due to temporary factors (e.g., minor illness). Research employing CIND profiles has focused on older populations, leaving questions about the prevalence, distribution, and correlates of CIND status among midlife adults, who are at risk for dementia. Using data from the MIDUS II study (N = 4,241; Mage=55.6, SD = 12.2, Range=28-84; 55%=women), participants completed the Brief Test of Adult Cognition via Telephone (BTACT), which includes tests of executive function and episodic memory abilities. CIND status prevalences were 50.2% non-CIND, 26.8% CIND-single, and 23.0% CIND-multiple. Significantly more men (p=.03), non-White (p<.0001), and lower-educated (p<.0001) adults met CIND-single and CIND-multiple criteria. Response time inconsistency (RTI), an index of central nervous system integrity and impairment/dementia risk, was greater among CIND-single (Cohen's d=.20, p<.01) and CIND-multiple (Cohen's d=.52, p<.001) participants, relative to non-CIND participants. Covariate-adjusted multinomial logistic regression models revealed RTI was associated with increased odds of CIND-single (p<.006) and CIND-multiple (p<.0001) statuses. Further, gender, education, and self-rated health also exhibited independent associations indicative of increased CIND classification risk (ps<.0001). Discussion will focus on the utility of CIND status for understanding patterns and profiles of cognitive health, and early detection of dementia risk.

Abstract citation ID: igaf122.2409

**REACTIVITY TO SPECIFIC TYPES OF DAILY STRESSORS CONFER CHRONIC DISEASE RISK IN MIDLIFE AND LATER ADULTHOOD**

Kelly Cichy[1], Dakota Witzel[2], and Robert Stawski[3], *1. Kent State University, Stow, Ohio, United States, 2. South Dakota State University, Brookings, South Dakota, United States, 3. Utah State University, Logan, Utah, United States*

Affective responses to stressors (e.g., reactivity) are malleable health risk factors in midlife and later adulthood. Less is known about the specific stressors conferring risk, whether affective responses to stressors confer general health risk, or are specific to certain types of stressors. This study uses data from the Midlife in the United States (MIDUS) study and the National Study of Daily Experiences (NSDE) to examine the prospective effect of differential affective responses to specific types of stressors on chronic disease 7-10 years later and age differences in the stress-chronic disease link. Participants included 1,211 (Mage = 55; SD = 11; range = 34-83; 56% female) who participated in the 8-day NSDE daily diary protocol and waves 2 and 3 of the MIDUS. Respondents reported on the types of daily stressors they experienced (e.g., arguments, work overloads, home overloads, network stressors), their daily affect, and self-reported on diagnosed chronic conditions. Covariate-adjusted possion regressions revealed stressor reactivity to arguments (p = .002), home overloads (p = .06), network stressors (i.e., stressors that happen to a close friend or relative; p = .002), and other stressors (p = .001) are associated with increased chronic conditions 7-10 years later. There was no evidence of linear or nonlinear age differences in any of the reactivity effects, suggesting age invariance. Findings provide insights into how reactivity to specific stressor types increases prospective risk for chronic conditions approximately 7-10 years later, suggesting stressor-specific reactivity is a potential intervention target with relevance in midlife to later adulthood.

Abstract citation ID: igaf122.2410

**SENSE OF CONTROL AND EPIGENETIC AGING: THE MEDIATING ROLE OF LIFESTYLE BEHAVIORS**

Kylie Schiloski[1], Sofia Christofi[1], Eric Kim[2], Steven Cole[3], and Margie Lachman[1], *1. Brandeis University, Waltham, Massachusetts, United States, 2. The University of British Columbia, Vancouver, British Columbia, Canada, 3. University of California, Los Angeles School of Medicine, Los Angeles, California, United States*

Psychosocial factors such as purpose in life have been found to contribute to epigenetic aging and lifestyle behaviors may mediate these associations. Sense of control is another promising protective psychosocial factor that has been associated with greater engagement in protective lifestyle behaviors and better health. The present study tested whether sense of control was related to epigenetic age and whether lifestyle behaviors (diet, exercise, sleep, alcohol consumption, and smoking history) were a mediator. We predicted that individuals with a higher sense of control would engage in more protective lifestyle behaviors which would be related to a lower epigenetic age. Data were collected from two sub-study samples, the second (M2; n = 1,255) and refresher (R1; n = 863) waves of the Midlife in the United States Study (MIDUS). We tested our hypothesis with a mediation model using the PROCESS Macro in SPSS. Results showed that the relationships between sense of control and three second-generation epigenetic clocks (DunedinPACE, GrimAge, and PhenoAge) were fully mediated by lifestyle behaviors, adjusting for chronological age, sex, education, race, and study sample. In this model, sense of control was not significantly related to two first-generation epigenetic clocks (Hannum or Horvath clocks), possibly due to their higher correlations with chronological age. Taken together, these findings suggest that sense of control is a promising modifiable psychosocial variable that could be used to promote engagement in protective lifestyle behaviors potentially slowing age-related declines.

Abstract citation ID: igaf122.2411

### THE GAP BETWEEN ACTUAL AND PREFERRED ALLOCATION OF FINANCIAL RESOURCES AMONG KOREAN MIDDLE-AGED ADULTS

Kyungun Lee, Haeun Yoo, Youngwon Nam, and Kyungmin Kim, *Seoul National University, Seoul, Republic of Korea*

As a period of "life in the middle" between generations and in the life course, middle-aged adults are required to prepare for their own future as well as respond to family needs across generations. Due to these competing demands, midlife adults often allocate financial resources to various domains in ways that do not align with their preferred priorities—which may influence their current and future well-being. Using a sample of 2,465 married middle-aged individuals who have at least one child and one living parent (aged 51–59) from the 2014 Korean Baby Boomer Panel Study, we examined actual and preferred ratios of financial resources allocated to (a) current life, (b) retirement preparation, (c) offspring support, and (d) caring for parents. On average, the actual ratios of resources allocated to current life (48% vs. 42%) and offspring support (24% vs. 20%) were higher than preferred ratios (i.e., over-allocation), whereas the actual ratios allocated to retirement preparation (22% vs. 30%) and caring for parents (6% vs. 8%) were lower than preferred ones (i.e., under-allocation). The gaps between actual and preferred allocation to each domain revealed significant associations with life satisfaction of respondents. Over-allocation to current life and under-allocation to retirement preparation were related to lower life satisfaction. Over- and under-allocation to offspring were both related to lower life satisfaction, whereas under-allocation to parents was related to lower life satisfaction. Our findings suggest the importance of balanced allocation of financial resources across domains in understanding well-being of midlife adults.

Abstract citation ID: igaf122.2412

### VALIDATING A MEASURE OF INTRINSIC CAPACITY IN THE DUNEDIN STUDY, A SAMPLE OF MIDLIFE ADULTS

J. Kathy Xie, Avshalom Caspi, Renate Houts, and Terrie Moffitt, *Duke University, Durham, North Carolina, United States*

Intrinsic capacity is a World Health Organization-supported multidimensional indicator of healthy aging that focuses on functional capacity rather than morbidity and mortality. Intrinsic capacity captures the sum of all mental and physical capabilities of the individual and includes 5 domains: locomotor, cognitive, psychological, sensory, and vitality. Consistent with the geroscience view that we must target aging processes early to extend healthspan, there is a need to validate intrinsic capacity in younger adults. Currently, the youngest participants in validation studies are 45, and most are over 65. We modelled intrinsic capacity using data from the Dunedin Study, a population-representative longitudinal birth cohort (N = 1037, Age=45). Measures comprising the 5 intrinsic capacity domains were attained using direct clinical testing and self- and other-report. Preliminary results from confirmatory factor analysis show that a bifactor model with 1 general factor (intrinsic capacity) and 5 specific subfactors (locomotor, cognitive, psychological, sensory, vitality) fit the data well, $\chi$ 2 value = 410.06 (df = 123); Comparative Fit Index = 0.93; Tucker-Lewis index=0.91; Root-Mean-Square Error of Approximation = 0.05. Future results will relate these factors to functional outcomes, such as biological aging, Alzheimer's disease risk, and life satisfaction.

### SESSION 7120 (POSTER)

### MUSCULOSKELETAL HEALTH

Abstract citation ID: igaf122.2413

### AGE OF DISEASE ONSET AND RISK OF SERIOUS INFECTION WITH ANTI-TNF USE IN OLDER ADULTS WITH RHEUMATOID ARTHRITIS

Jiha Lee[1], Sofia Pedro[2], and Kaleb Michaud[3], *1. University of Michigan, Ann Arbor, Michigan, United States, 2. National Data Bank for Rheumatic Diseases, Wichita, Kansas, United States, 3. University of Nebraska Medical Center, Omaha, Nebraska, United States*

Older age increases the risk of serious infections (SI) in rheumatoid arthritis (RA) patients using biologics like anti-TNFs. About one-third of older adults are diagnosed with RA between ages 60-65, classified as late-onset RA (LORA). LORA exhibits more acute symptoms and faster disease progression compared to young-onset RA (YORA), possibly due to age-related immune changes. This study utilized data from the FORWARD-The National Databank for Rheumatic Diseases (2001-2019) to assess SI risk differences in older adults with LORA versus YORA starting anti-TNF therapy. Patients aged ≥60 years, categorized by age at RA diagnosis (LORA ≥60; YORA < 60), were propensity-matched for demographics and health factors. Exclusions applied to those with other rheumatic diseases, cancer, or HIV preceding SI. SI was defined as infections requiring hospitalization, IV antibiotics, or causing death, linked to anti-TNF within three months after treatment cessation. The study included 1,219 LORA and 6,030 weighted YORA patients. LORA patients had fewer previous infections, lower glucocorticoid (GC) use, but higher methotrexate use. Although LORA had a higher crude SI incidence (3.6/1000 patient-years) than YORA (2.7/1000 patient-years), SI risk did not significantly differ (aHR 0.85, 95% CI 0.58-1.25). Adjusting for prior SI, smoking, and methotrexate use did not alter outcomes. Long-term GC use and older age were independent risk factors for increased SI risk. These findings indicate that SI risk from anti-TNFs does not vary by RA onset age but highlight the risky and often suboptimal reliance on long-term GC use in LORA patients, suggesting a need for improved treatment strategies.

Abstract citation ID: igaf122.2414

### DEXTERITY LIMITATIONS IN OLDER ADULTS ARE ON THE RISE: TRENDS IN HAND FUNCTION OVER 20 YEARS AND THE EFFECTS OF COVID-19

Rachel Logue Cook, Sandra Hunter, and Susan Brown, *University of Michigan, Ann Arbor, Michigan, United States*

Hand dexterity is important for completing activities of daily living and is a strong predictor of functional capacity and independence. However, the prevalence of dexterity limitations among the aging population, particularly after the onset of Covid-19, is largely unknown. We used data from the 2001-2023 cycles of the National Health and Nutrition Examination Survey (NHANES) to identify 15,222 adults aged ≥65. Participants were asked to report whether they had difficulty grasping and handling small objects and were categorized as having a dexterity limitation or no limitation. We determined the population proportion of males and females that reported limitations for each cycle and calculated percentage changes in limitations over the past 20 years and pre- and post-Covid-19. Dexterity limitations remained relatively stable from 2001-2014 for both men (~16%) and women (~19%) but significantly increased beginning in 2015 (percentage increase from 2014: 32% males, 26% females) and continued to increase through the 2021-2023 cycle. Before Covid-19 (2017-2018 cycle), 23.6% of males and 26.2% of females reported dexterity limitations and after the onset of Covid-19 (2021-2023 cycle), limitations increased to 25.1% in males(+6.6%) and 34.9% in females(+33.2%), with the greatest increases in adults aged 70-74 (+16%males, +50%females). Hand limitations are increasing among the aging population, particularly women. Covid-19 had detrimental effects on the physical health of many older adults, and our results demonstrated this extended to the hands as well. Understanding the physical limitations experienced by older adults, including the hands, is vital for developing and targeting interventions to maintain health and independence.

Abstract citation ID: igaf122.2415

### OLDER ADULTS AT RISK FOR ALZHEIMER'S DISEASE WITH LOWER COGNITIVE SCORES EXHIBIT POORER SKELETAL MUSCLE HEALTH

Ray Urbina[1], Michelle Gray[1], Anthony Campitelli[2], Megan Jones[1], Sally Paulson[3], Joshua Gills[4], and Jordan Glenn[5], *1. University of Arkansas, Fayetteville, Arkansas, United States, 2. College of Idaho, Caldwell, Idaho, United States, 3. University of Houston, Houston, Texas, United States, 4. New York University, New York, New York, United States, 5. SuppCo, Natick, Massachusetts, United States*

Alzheimer's disease (AD) is a major health concern, with early pathology often undetected due to costly assessments. Skeletal muscle health (SMH) deficiencies are strongly linked to AD progression and concurrent declines in physical and cognitive function, suggesting skeletal muscle health as an early biomarker for cognitive decline. This study examines SMH differences between low cognition (LC) and high cognition (HC) in older adults who are at risk for AD. SMH was assessed through physical function tests (PF) – handgrip strength (HG), 5-time sit-to-stand, gait assessments, 6-minute walk, and lower body power (TENDO) – and body composition (DXA). Cognitive health was evaluated using the Repeatable Battery for the Assessment of Neuropsychological Status. Individuals were grouped into low cognition (LC) or high cognition (HC) groups. Independent t-tests were used to analyze differences in means between LC and HC ($\alpha = .05$). LC consisted of 34 adults (M = 14, F = 20) and HC had 29 adults (M = 6, F = 23). LC had lower performances and poorer outcomes in 16 out of 20 measurements, with significant differences in 10m habitual walk (p=.02) and Handgrip (p=.02). Among females, seven measurements had significant differences between groups – weight (p=.04), lean mass (p=.03), chair stands (p=.02), 10m habitual (p=.02), 10m fast (p=.001), 10m DT habitual (p=.004), and peak power (p=.02). Results show that the LC group demonstrated overall poorer performance and outcomes. These findings align with current literature reinforcing that LC individuals exhibit poorer skeletal muscle health when looking into function which may suggest an increased risk for cognitive decline.

Abstract citation ID: igaf122.2416

### PREVALENCE AND BURDEN OF CHRONIC PAIN IN OLDER ADULTS

Gillian Fennell[1], Sarah Tilley[1], Sayali Dhamne[1], Emelia Benjamin[2], Margaret Clancy[2], Mary Gheller[3], Robert Edwards[4], and Tuhina Neogi[2], *1. Boston University - Chobanian & Avedisian School of Medicine, Boston, Massachusetts, United States, 2. Boston University Chobanian & Avedisian School of Medicine, Boston, Massachusetts, United States, 3. Boston University, Boston, Massachusetts, United States, 4. Brigham and Women's Hospital, Boston, Massachusetts, United States*

Over 100 million Americans experience chronic pain (i.e., pain lasting three or more months), with older adults being disproportionately affected. However, the pattern of pain locations and impact of chronic pain in much older adults have not been well-studied. Further, chronic pain itself has been associated with higher risk of mortality; thus, surviving older adults may report less pain. Greater extent of pain locations, characterized by chronic multisite (≥ 3 pain sites) and chronic widespread pain (WSP, bilateral pain above and below the waist and in the spine), is associated with worse quality of life (QOL) and more severe and activity-interfering pain. Whether these patterns hold true for much older adults is unclear. Using a large, cross-sectional, community-based sample of older adults from the Framingham Heart Study (N = 1,584; mean age, 76.1 ± 7.5; range 52-102 years), we estimated the prevalence and burden of several chronic pain phenotypes by age and sex. Overall, 36.0% had chronic multisite pain, 15.6% chronic WSP, and 10.4% bothersome/high-impact chronic pain (B-HICP) as defined by a combination of pain frequency, severity, and interference. The prevalence of extensive (i.e., multisite or WSP) pain and number of pain sites did not increase with age in men or women. However, pain interference was higher for women and the physical component of HRQOL higher for both men and women with advancing age. In summary, while extensive pain classifications were not more prevalent at older ages, pain burden was positively associated with age.

Abstract citation ID: igaf122.2417

### RE-ESCALATION OF TREATMENT IN OLDER ADULTS WITH RHEUMATOID ARTHRITIS AFTER ANTI-TNF THERAPY DE-ESCALATION

Jiha Lee[1], Jonathan Martindale[1], Una Makris[2], and Julie Bynum[1], *1. University of Michigan, Ann Arbor, Michigan, United States, 2. UT Southwestern Medical Center, Dallas, Texas, United States*

Older adults with rheumatoid arthritis (RA) treated with biologic disease-modifying antirheumatic drugs (bDMARDs), including anti-TNFs, are at an increased risk of serious infections and other adverse effects. Guidelines recommend de-escalating DMARDs for patients with low disease activity or remission to optimize the benefit-harm ratio of treatment. In a prior study, we observed that 20% of older adults with RA de-escalated anti-TNFs. This follow-up study examines the prevalence and factors associated with the re-escalation of treatment after anti-TNF de-escalation. We used 20% Medicare data from 2009-2017 to identify RA patients ≥66 years of age on anti-TNF therapy (Adalimumab, Etanercept, Certolizumab, or Golimumab), with de-escalation by cessation (> 90-day gap) or by taper (>50% effective dose reduction), and at least one rheumatologist visit pre- post-de-escalation (i.e. index-date). Re-escalation was defined as restarting the same anti-TNF or another bDMARD or increasing the effective dose by > 50% after the index date. Information on baseline patient characteristics, concomitant use of conventional synthetic DMARDs (csDMARDs), and changes in average glucocorticoids (GC) dose were collected. We identified 814 eligible Medicare beneficiaries, with an average age of 75.2 (SD 5.8), 84.5% female, 75.8% non-Hispanic white, and 43.4% with low-income subsidies (LIS). Of them, 61.1% re-escalated treatment with a median time of 160 (IQR 112-290) days since the index-date. In Cox analyses, re-escalation was more likely among blacks, Hispanics/others, and those with lower comorbidity burdens. Overall, more than half of older RA patients re-escalate treatment within a year of anti-TNF de-escalation highlights the importance of bDMARDs for disease control.

Abstract citation ID: igaf122.2418

### VALIDATING THE MINI SARCOPENIA RISK ASSESSMENT (MSRA) IN SARCOPENIA CASE-FINDING FOR HEALTHY OLDER ADULTS

Shiyun Chua, Wee Shiong Lim, Jia Qian Chia, and Justin Chew, *Tan Tock Seng Hospital, Singapore, Singapore*

AWGS 2019 consensus emphasizes screening for early identification of people at risk for sarcopenia. The short (MSRA-5) and long (MSRA-7) questionnaires were validated in Italy, but performed poorly in diagnostic accuracy among heathy older persons in Singapore. Our study aims to examine the predictive validity of MSRA in Singapore. We performed a post-hoc secondary analysis of GeriLABS-2, a prospective cohort study of 230 community-dwelling adults ≥50 years in Singapore, at baseline and Year 3. We evaluated predictive validity of MSRA for activity participation (Frenchay Activities Index, IPAQ, Life Space Assessment), patient-reported quality of life (EQ5D), and healthcare utilization (falls, ED, hospital admissions) at Year 3 using linear and negative binomial regression. Adjusted, there was no significant association between MSRA-5 and FAI (β = -0.02 (-0.08 – 0.04)), IPAQ (β = -2.85 (-47.91 – 42.20)), LSA (β = 0.02 (-0.27 – 0.30)), or EQ5D (β = 0.08 (-0.12– 0.28)). Adjusted odds ratio for composite outcome of ≥ 1 falls, ED or hospital admission was 1.03 (0.98 – 1.09). Compared to those negative in MSRA-5, adjusted relative risk is similar for falls (0.99 (0.95-1.02)), ED (1.04 (0.99-1.11)) and hospital admission (1.06 (0.99-1.15)). Results were similar for MSRA-7, indicating poor predictive validity for activity participation, quality of life and healthcare utilization at 2 years. Overall, MSRA demonstrated poor predictive validity for a comprehensive range of clinically relevant outcomes. Taken together with the low diagnostic accuracy in an earlier study, these results highlight the limited clinical utility of MSRA in healthy Asian population.

### SESSION 7125 (POSTER)

### NURSING SCIENCE

Abstract citation ID: igaf122.2419

### ASSOCIATION BETWEEN WORK ENVIRONMENT AND OUTCOMES OF PRIMARY CARE NURSE PRACTITIONERS CARING FOR DEMENTIA PATIENTS

Lusine Poghosyan[1], Maura Dougherty[1], Kyle Featherston[2], Jianfang Liu[3], and Josh Porat-Dahlerbruch[4], *1. Columbia University, New York, New York, United States, 2. Columbia University School of Nursing, New York, New York, United States, 3. Columbia University Irving Medical Center, New York City, New York, United States, 4. University of Pittsburgh School of Nursing, Pittsburgh, Pennsylvania, United States*

Nurse practitioners (NPs) play a key role in caring for patients with dementia in primary care practices and can expand the dementia workforce capacity. Yet, NPs often practice in poor work environments, lacking support and resources. We examined NP work environments and job outcomes (i.e., burnout, job dissatisfaction, and intent to leave the job in the coming year) among primary care NPs providing dementia care. We surveyed a national U.S. sample of NPs from primary care practices delivering dementia care in 2021. In total, 968 NPs from 847 practices responded. The work environment was measured using the Nurse Practitioner Primary Care Organizational Climate Questionnaire and its four subscales. While 92% of NPs reported being satisfied with their job, 35.8% reported experiencing burnout, and 21.3% reported intent to leave their current job. A one-unit increase in the NP-Administration Relations subscale score was associated with 64% decreased odds of burnout (cumulative OR = 0.36; p = < 0.0001), a three-fold increase in the odds of job satisfaction (cumulative OR = 3.42; p = < 0.0001), and 75% lower odds of intent to leave (OR = 0.25; p = < 0.0001). A one-unit increase in the Independent Practice and Support score was associated with 42% lower odds of burnout (cumulative OR = 0.58; p = 0.04) and 71% increase in the odds of having higher job satisfaction (cumulative OR = 1.71; p = 0.04). Our findings suggest that working relationships with administrators and practice support are key for promoting NP outcomes, potentially improving dementia care quality.

Abstract citation ID: igaf122.2420

### ASSOCIATION OF SHIFT PATTERNS WITH OVERWORK, TURNOVER, AND CARE QUALITY IN U.S. SKILLED NURSING FACILITIES

Fangli Geng[1], Yuqian Lin[2], Pedro Gozalo[1], and Elizabeth White[3], *1. Brown University, Providence, Rhode Island, United States, 2. Massachusetts General Hospital, Boston, Massachusetts, United States, 3. Brown University School of Public Health, Providence, Rhode Island, United States*

Workforce challenges, including high turnover and staffing shortages, persist in skilled nursing facilities (SNFs). Understanding how shift structures influence workforce stability and care quality is critical for policy and operational decisions. This study identified the dominant shift for SNFs, defined as the most frequent daily work hours for the majority of nurses (Registered Nurses, Licensed Practical Nurses, and Certified Nurse Assistants), and classified SNFs into three shift categories: predominantly 8-hour shifts, 12-hour shifts, or a mix of both. Using facility-level data from 2021, integrating Payroll-Based Journal data, Nursing Home Care Compare quality measures, and Long-Term Care Focus facility characteristics, we fit linear regression models with state fixed effects to assess the association between dominant shift type and key workforce and care quality outcomes, adjusting for facility characteristics such as ownership, size, and case mix. Of the total 13,071 facilities studied, 10,821 (83%) had a dominant 8-hour shift, 1,918 (15%) had a dominant 12-hour shift, and 332 (2%) used mixed shifts. Facilities with 12-hour or mixed-shifts had significantly higher overwork prevalence (2.1 and 1.3 percentage points higher than 8-hour facilities, respectively) and turnover rates (7.6 percentage points higher for 12-hour shifts). Quality of care was also worse in 12-hour shift facilities across 16 of 22 measures, including higher rates of pressure ulcers, falls, and depressive symptoms. Policies should address the implications of different types of shifts, and future research should explore interventions to balance staffing efficiency with staff well-being and patient care.

Abstract citation ID: igaf122.2421

### DEVELOPMENT AND VALIDATION OF AN INDEX SYSTEM FOR FAMILY CAREGIVERS' CAPACITY IN HOME-BASED DISABLED ELDERLY CARE

Shujing Suo, and Yun Li, *Zhengzhou University, Zhengzhou, Henan, China (People's Republic)*

Background: Family caregivers' capacity affects both disabled elders' health and their own experience. However, there is currently a lack of tools to assess the capacity of family caregivers of older adults with disability at home. This study aimed to establish and validate an index system of the caregiving capacity of family caregivers of the elderly with disabilities at home. Methods: A Literature review, cross-sectional survey, and qualitative interviews were used to construct an index pool. The Delphi method was used to establish the index system, and the Analytic Hierarchy Process was used to determine the weight of the indicators. Finally, the index system was transformed into a questionnaire, and a survey was conducted to validate the reliability and validity of the indicator system. Results: The effective response rate for the two rounds of expert consultation was 100%. After two rounds, 3 first-level indicators, 6 second-level indicators, and 22 third-level indicators were determined. The coefficient of expert authority for both rounds is 0.842. The first, second, and third-level indicators of Kendall's W consistency coefficient were 0.650, 0.579, and 0.2574P40.0014. The overall Cronbach's α coefficient of the index system was 0.941, and the content validity index of the index item level ranged from 0.833 to 1.000. Conclusions: The index system of family caregivers of the elderly with disabilities at home is comprehensive, scientific, and reasonable, which can provide a reference for the development of education or training of family caregivers in the future and the evaluation of its effect.

Abstract citation ID: igaf122.2422

### EMERGENCY DEPARTMENT VISIT AND REHOSPITALIZATION IN OLDER ADULTS RECEIVING HOME HEALTH NURSING: A NATIONAL STUDY

Kayoung Lee[1], and Jeongmin Lee[2], *1. Gachon University, Incheon, Republic of Korea, 2. Seoul National University Hospital, Seoul, Republic of Korea*

Older adults with chronic conditions face a high risk of emergency department (ED) visits and rehospitalization after acute hospital discharge. Home health nursing services aim to support safe transitions to home, yet their effectiveness in reducing acute care utilization remains unclear in South Korea. This study analyzes national health insurance claims data from the Health Insurance Review and Assessment Service (HIRA) 2018-2020 to examine home health nursing utilization and factors associated with 30-day rehospitalization and ED visits. Descriptive statistics and logistic regression were used to analyze client characteristics, healthcare utilization, and risk factors. The total number of home health nursing claims increased from 780,911 in 2018 to 1,034,590 in 2019 but declined to 831,947 in 2020, possibly due to COVID-19. The number of clients increased from 90,384 in 2018 to 113,589 in 2019 but decreased to 102,364 in 2020. The average client age was 79.7 years, with over 70% being female. The most common diagnoses varied by facility type, with malignant neoplasms prevalent in tertiary hospitals and mental and behavioral disorders dominant in long-term care hospitals and clinics. ED visits occurred in 0.27% of transitions, while 7.78% of clients were rehospitalized within 30 days. Male sex (OR = 1.28, $p < 0.001$), older age (OR = 1.02, $p < 0.001$), and regional disparities significantly influenced readmission risk. The region with the lowest accessibility to healthcare facilities had the highest risk of rehospitalization (OR = 3.23, $p < 0.001$). Targeted interventions are needed to reduce rehospitalization among high-risk groups and address regional healthcare disparities to improve continuity of care and patient outcomes.

Abstract citation ID: igaf122.2423

### EVALUATING NURSING WORKLOADS AND OPTIMIZING STAFFING FOR SEVERE PNEUMONIA IN ELDERLY UNDER DRGS

Rong Zhang, Yanhong Wang, and Fei Xie, *Shanxi Bethune Hospital, Taiyuan, Shanxi, China (People's Republic)*

Objective: This study evaluates nursing workload and human resource challenges for elderly severe pneumonia patients under DRG payment, proposing strategies to balance care quality and cost efficiency. Methods: A mixed-method design was applied in a tertiary hospital (2023-2024).Nursing Activities Score(NAS) and DRG data quantified workload for 150 elderly patients.Regression analysis identified workload drivers(age, comorbidities, hospital stay).Interviews assessed allocation challenges. Results: The findings revealed that the mean NAS score for elderly patients with severe pneumonia was 78.5, substantially exceeding that of non-severe cases, thereby indicating a considerable nursing workload. The DRG grouping data demonstrated that nursing costs accounted for 35% of the total medical expenses associated with severe pneumonia, with direct nursing expenditures representing the highest proportion.Multiple regression analysis identified patient age, number of comorbidities, and length of hospital stay as the principal factors influencing nursing workload ($P < 0.05$). Several critical challenges in current nursing human resource allocation were identified:(1)A lack of flexibility in workforce allocation, rendering it difficult to accommodate workload fluctuations caused by reduced hospital stays and increased patient turnover under the DRG model;(2)A growing demand for specialized nursing services, yet inadequate training resources;(3) An imperfect performance evaluation system, complicating the quantitative assessment of workload, service quality, and efficiency;(4)A misalignment between nursing workload and DRG payment adjustments, leading to delayed modifications in human resource allocation;(5)Insufficient interdisciplinary collaboration between nursing staff and the medical team, negatively affecting overall service quality. Conclusion: Against the backdrop of the DRG payment model, a significant increase in nursing workload has been observed among elderly patients with severe pneumonia, while existing nursing human resource allocation remains suboptimal.

Abstract citation ID: igaf122.2424

### EVALUATION OF THE "COGNITIVE HEALTH ACROSS SETTINGS" INTERVENTION BY CLINICIANS AND CAREGIVERS

Mariya Kovaleva, *University of Nebraska Medical center, University of Nebraska Medical Center College of Nursing, Nebraska, United States*

We created a 21-day synchronous/asynchronous transitional care intervention for caregivers of persons living with dementia (PLWD), titled "Cognitive Health Across Settings (CHASE)." CHASE's purpose is to educate and support caregivers during post-hospitalization period to reduce post-discharge complications and preventable emergency department visits and re-hospitalizations. CHASE's synchronous elements are: two 30-minute videoconferences with caregivers 1-7 and 14-21 days post-discharge, led by a registered nurse (RN), a social worker, and a pharmacist. Asynchronously, caregivers watch video lessons (selecting from a library of 50 lessons) and read caregiver manual. RN meets with caregivers before CHASE starts to assess their and PLWD's needs and create a checklist with tasks advisable to complete. We enlisted 10 stakeholders to evaluate CHASE. These included: one former and two current caregivers (for their parents); one geriatrician; one pharmacist; two social workers; and three RNs. These stakeholders provided quantitative (surveys) and qualitative (comments, focus groups) feedback on CHASE. Stakeholders evaluated the intervention mostly favorably. Criticisms included: information that is too complex and too long, not practical for busy caregivers to work with, and more suitable for health professions students. Clinicians considered the content too complex and overwhelming for caregivers – but caregivers did not perceive it as such, appreciating redundancy. Clinicians perceived presentation of skilled nursing facility experience as excessively negative. Stakeholders advised providing a glossary in the manual with medical jargon and worksheets that caregivers can pull from the manual. We are currently using this feedback to edit video lessons and manual prior to offering CHASE to caregivers.

Abstract citation ID: igaf122.2425

### IMPACT OF INCENTIVES AND CONTACT ATTEMPTS ON RECRUITMENT OF LONG-TERM CARE WORKFORCE

Soojeong Han[1], Gregory Alexander[1], Lusine Poghosyan[1], M. Schrimpf[1], Sabrina Tasnova[1], Racheal Rodriguez[1], and Hana Amer[2], *1. Columbia University, New York, New York, United States, 2. Columbia University School of Nursing, New York, New York, United States*

Depending on research topic, population, and setting, the ideal and reasonable amount of incentives and number of contact attempts are different, and even more than ten times of attempts is needed. The purpose of the parent study is to examine the impact of health information technology from the perspectives of the long-term care workforce. We encountered recruitment challenges and decided to contact participants multiple times and increase incentives to improve survey responses. This report aims to assess the trend of the survey response process among nursing home (NH) administrators. Out of 392 health information technology (HIT) maturity surveys of NH administrators who responded 'yes' to complete the survey, 206 HIT maturity surveys were returned, and 186 were not returned. Of the 206 administrators who returned the survey, 41 returned without claiming any incentive, 143 received \$25, 5 received \$50, and 17 received \$75. Our study demonstrates that approximately 64% (n = 132) returned the survey before attempts. After up to 3 attempts, 88.83% (n = 183) returned the survey. Beyond four attempts, survey return rate did not increase regardless of increased incentive. A Chi-square test revealed a significant association between the category of attempt number (Low: 0-3 vs. Medium/High:4-10+) and survey return, $\chi^2(1, N = 392) = 20.09$, $p < .0001$. Future research can build upon the findings of this study by considering contact attempts and incentives to balance recruitment efforts with the pressure or burden of multiple contact attempts during recruitment and not to compromise the voluntary nature of participation.

Abstract citation ID: igaf122.2426

### IMPACT OF RESILIENCE-BASED EXERCISE ON QUALITY OF LIFE AND CARDIAC FUNCTION IN ELDERLY CHF PATIENTS

Yanping Zhai, *Shanxi Bethune Hospital, Taiyuan, Shanxi, China (People's Republic)*

Objective: This study aims to evaluate the effects of a personalized exercise intervention, grounded in psychological resilience theory, on the quality of life and cardiac function of elderly patients with chronic heart failure (CHF). By enhancing psychological resilience, the intervention encourages active participation in exercise, thus improving overall health outcomes. Methods: In this randomized controlled trial, 80 elderly patients (aged 65 years and older) with chronic heart failure were randomly assigned to either the experimental group or the control group. The experimental group underwent a 12-week personalized exercise program, which included aerobic exercise, strength training, and psychological resilience training, while the control group received standard care. Pre- and post-intervention assessments included the SF-36 quality of life scale, cardiac function evaluations (6-minute walk test, NYHA classification, and left ventricular ejection fraction), and psychological resilience (measured using the CD-RISC scale). Results: The experimental group demonstrated significant improvements in the 6-minute walk test, left ventricular ejection fraction, and quality of life compared to the control group ($P < 0.05$). Psychological resilience scores significantly increased post-intervention ($P < 0.05$), with a positive correlation between resilience improvements and cardiac function. Conclusion: The personalized exercise intervention based on psychological resilience theory significantly improves cardiac function, quality of life, and psychological resilience in elderly patients with chronic heart failure. These findings suggest that incorporating psychological resilience strategies into treatment plans could enhance overall health and quality of life for heart failure patients.

Abstract citation ID: igaf122.2427

### PREVALENCES OF CARE PHENOMENA IN HOSPITAL AND LONG-TERM CARE – A CROSS-SECTIONAL STUDY

Nico Haller, Simone Freitag, Tobias Melms, and Steve Strupeit, *University Medicine Greifswald, Greifswald, Mecklenburg-Vorpommern, Germany*

Care dependency is a complex issue arising from physical, cognitive, or psychological conditions, collectively known as care phenomena. These phenomena can be significantly influenced by nursing interventions. While understanding the prevalence and identifying risk groups for care phenomena is crucial, there is a lack of up-to-date data in Germany. This study aims 1) to collect prevalence data on care phenomena in hospitals and long-term care settings, and 2) to investigate correlations between these care phenomena and the demographic variables age and gender. A cross-sectional study was conducted to investigate the care phenomena pain, falls, urinary and fecal incontinence, and pressure ulcers. Data collection was carried out in the setting hospital and long-term care facilities. The prevalence rates were calculated for each care phenomenon in the overall sample and for the two settings. Additionally, correlations between the care phenomena and with age- and gender were examined. In fall 2023, data was collected from 754 individuals. The results showed differences and similarities in prevalence data between long-term care and hospital settings. Similarities were observed for the prevalence of fall with 4.8%. Significant differences were found in the prevalence of urinary incontinence, with 26.6% in hospitals and 56.84% in long-term care. Moreover, significant correlations were identified between the care phenomena themselves, as well as with gender and age. This study provides current prevalence data on care phenomena in Germany. Future research should involve larger samples, in-depth analyses, and systematic investigations to enhance understanding and inform evidence-based practice, ultimately improving nursing care strategies.

Abstract citation ID: igaf122.2428

### SWIMLANE: CLINICAL WORKFLOW REDESIGN IN THE NURSING HOME

Alyce Ashcraft, Donna Owen, and Vickie Ragsdale, *Texas Tech University Health Sciences Center, Lubbock, Texas, United States*

Workflow redesign is an under utilized skill for understanding how care of older adults (OA) in the nursing home (NH) is conducted. Novel strategies are needed to improve care delivery to the 70% of OAs who would benefit from care in a NH within their lifetime. The aim of this paper is to describe the current workflow and subsequently develop the ideal workflow to improve care delivery in a NH. Data from an IRB approved observational study of NH staff and off-site primary care providers (PCPs) served as the basis for creating the redesign. Event analysis documented baseline existing point of care data collection and communication about a change in resident condition between nurses and off-site PCPs in a single NH. Swimlane and root cause analysis (RCA) methods provided an alternative view of workflow where "at a glance" snapshots were captured then evaluated using a cause-and-effect diagram. Areas for improvement were identified within an "As Is" swimlane diagram that included aspects of training, communication, technology, policies, and staffing. A best case swimlane diagram addressed gaps and areas for improvement showcasing the ideal workflow. The "To Be" swimlane offered solutions to improve workflow such as nurses' use of critical thinking skills, timely communication with the PCP, and the need to integrate technology into communication. A workflow diagram provides a map and RCA drives potential solutions. Findings suggested a roadmap for research and policy improvement of resident assessment and stronger communication skills between the clinical staff and PCPs.

### SESSION 7130 (POSTER)

### NUTRITION, EATING DISORDERS

Abstract citation ID: igaf122.2429

### ASSOCIATION BETWEEN DIETARY LIVE MICROBES, FOLATE, AND FATIGUE: A CROSS-SECTIONAL STUDY IN U.S. OLDER ADULTS

Galya Bigman[1], Amber Kleckner[2], Elizabeth Dennis[3], John Sorkin[2], and Alice Ryan[3], *1. University of Maryland, Baltimore County, Baltimore, Maryland, United States, 2. University of Maryland School of Nursing, Baltimore, Maryland, United States, 3. University of Maryland School of Medicine, Baltimore, Maryland, United States*

Fatigue is a common and debilitating condition in older adults, linked to chronic inflammation, gut-brain axis dysfunction, and impaired metabolic health. Live microbes in fermented foods and dietary fibers support short-chain fatty acid (SCFA) production, potentially alleviating fatigue by modulating these mechanisms and enhancing nutrient bioavailability. Folate, essential for energy metabolism, is associated with fatigue, and its bioavailability is influenced by the gut microbiome through live microbe intake. This study examines the relationship between live microbe intake and fatigue, with folate as a mediator. We analyzed data from 2,353 older U.S. adults (≥60 years) in NHANES (2021–2023). Fatigue, assessed via the PHQ-9, was categorized as present or absent. RBC folate, a biomarker of long-term status, was classified into tertiles. Dietary microbe intake, including probiotics, was derived from 24-hour recalls and categorized as Low (< $10^4$ CFU/g), Medium ($10^4$–$10^7$ CFU/g), or High (>$10^7$ CFU/g). Covariates included demographics, BMI, comorbidities, lifestyle, and energy intake. Multivariable regression and structural equation modeling accounted for survey design. The mean age was 69.4 years (SD = 6.3), with 46.1% male. Live microbe intake was low (28.3%), medium (45.5%), or high (26.5%), and 15.4% reported fatigue. Higher microbe intake was linked to lower fatigue odds (OR = 0.58, p = 0.021) and increased folate levels (OR = 1.38, p = 0.015), with folate mediating 21.8% of the effect. While findings suggest dietary microbes may reduce fatigue via folate, causal inference requires further study.

Abstract citation ID: igaf122.2430

### ASSOCIATION BETWEEN TOOTH LOSS AND GERIATRIC SYNDROMES IN OLDER ADULTS FROM A RURAL AREA IN EASTERN CHINA

Jing Chen[1], Caihong He[2], and Qin Zhang[1], *1. The First Affiliated Hospital, Zhejiang University School of Medicine, Hangzhou, Zhejiang, China (People's Republic), 2. The First Affiliated Hospital, School of Medicine, Zhejiang University, Hangzhou, Zhejiang, China (People's Republic)*

We aimed to explore the associations between tooth loss, denture status, and geriatric syndromes.In 2019, 1094 older adults from a rural area in eastern China were enrolled and a sub-sample of the participants (N = 690) were followed up in 2023. Logistic regression analysis was utilized to explore the association between tooth loss severity at baseline and geriatric syndromes at fourth year follow-up. The multivariable models showed that compared to those without tooth loss or with tooth loss not affecting daily life, those having tooth loss affecting daily life at baseline was associated with a 1.80-fold higher odds of sarcopenia (95% CI: 1.02 to 3.21) and a 2.31-fold higher odds of malnutrition risk (95% CI: 1.44 to 3.69) at the fourth year. Compared to those with 21 or more teeth, participants with fewer than 10 teeth had 1.87-fold odds for sarcopenia (95% CI: 1.07 to 3.26), 2.99-fold for malnutrition risk (95% CI: 1.93 to 4.62), and 1.68-fold for frailty ( 95% CI: 1.10 to 2.56). Older adults with tooth loss who did not have dentures exhibited a significantly higher risk of sarcopenia, malnutrition risk, frailty, and falls, more number of geriatric syndromes.Our findings suggest that higher severity level of tooth loss at baseline were associated with higher risk of geriatric syndromes at fourth year in older adults. Dentures might mitigate this risk but they do not fully bridge the gap compared to retaining natural teeth. Screening and intervening oral health is important for the prevention of geriatric syndromes in older adults.

Abstract citation ID: igaf122.2431

**EXPERIENCES AND PERCEPTIONS AROUND EATING AMONG OLDER ADULTS WITH SERIOUS MENTAL ILLNESS**

Erin Hubbard, and Heather Leutwyler, *University of California, San Francisco, San Francisco, California, United States*

Background: People with serious mental illness (SMI) have, on average, a shortened life expectancy of about 25 years compared to those without SMI. They experience an increased level of comorbidities such as metabolic syndrome (comprised of abdominal obesity, hypertension, impaired glucose tolerance, and hypertriglyceridemia), thus contributing to type 2 diabetes and cardiovascular disease. External circumstances such as precarious living environments and poor socioeconomic status can influence access to healthy food, and psychiatric symptoms may further impede healthy eating habits. To better understand facilitators and barriers to healthy eating among community-dwelling older adults with SMI, we report the results of a qualitative analysis. Methods: We interviewed 7 community-dwelling older adults with SMI (mean age 64.57, SD 6.705, range 59-76). A qualitative analysis using constructed grounded theory methodology was conducted to assess participants' experiences and perceptions around eating and access to food. Results: Two main themes emerged in our analyses: 1. Early life influence on eating habits and 2. Convenience. Participants indicated that the early home environment can positively or negatively influence eating habits throughout life. Cooking skills and eating habits were often acquired early in life. Participants also described how convenience guided the types of food they consume. This convenience approach to nutrition often determined whether participants were eating non-nutrient-dense food. Participants expressed interest in learning about cooking or nutrition. Conclusion: Mental health programs that incorporate cooking experiences and nutrition or health groups provide important opportunities for people to learn and practice changes to diet and eating habits.

Abstract citation ID: igaf122.2432

**EXPLORING COMMUNITY RATES OF FOOD ASSISTANCE AND DIABETES AMONG OLDER ADULTS IN MA, RI, AND CT**

Calvin Tran, Qinglin Gao, Mengshi Liu, Nachalie Rodriguez-Cruz, and Elizabeth Dugan, *University of Massachusetts Boston, Boston, Massachusetts, United States*

Food insecurity and poor nutritional intake has serious health consequences. Poor nutrition has been associated with diabetes and eating healthy is often recommended to manage blood sugar. This study aims to examine the association at the population level to explore if community rates of obtaining food benefits (SNAP) are associated with diabetes rates in adults 65+. Understanding community rates of food stamp participation can identify barriers to food access and can inform programs and interventions to address diabetes among older adults across MA, RI, and CT. The current study uses results from the 2025 Healthy Aging Data Report (healthyagingdatareports.org). Indicators were calculated data from the American Community Survey (2018-2022 MA, RI, CT) and the Centers for Medicare and Medicaid Services Beneficiary Summary File (2020-2021 MA, RI, CT). Small area estimation techniques were used to calculate age- and sex-adjusted rates, and ArcGIS software was used for mapping and geographic analyses. Results showed that state average rates for food benefits were MA 13.34% (0-48.87%), RI 14.73% (2.18-33.98%), and CT 11.04% (0-42.62%). Rhode Island had the highest average state rate but the smallest range in rates. Rural communities had the lowest rates of food stamp participation. Diabetes: MA 28.62% (15.00-48.98%), RI 32.43% (15.80-33.98%), and CT 31.78% (18.13-46.89%). Other environmental risk factors such as lack of infrastructure for physical activity or food apartheids may contribute to some of the differences observed. Understanding the difference in community rates across New England can better inform policymakers in addressing diabetes maintenance and prevention among older adults.

Abstract citation ID: igaf122.2433

**IMPACT OF HEALTHY EATING INDEX, COMPONENT-SPECIFIC EFFECTS, & SOCIOECONOMIC STATUS ON COGNITION IN OLDER ADULTS**

Samitinjaya Dhakal, and Oscar Sarasty, *South Dakota State University, Brookings, South Dakota, United States*

The Healthy Eating Index (HEI) quantifies diet quality by evaluating adherence to the Dietary guidelines for Americans. The index is calculated by scoring intake adequacy of key food groups (e.g. fruits, vegetables, whole grains) and moderation of harmful components (e.g. sodium, added sugars). This study evaluates i) the predictive capacity of the HEI scores and its components with cognitive decline, ii) the moderating role of socioeconomic status in the diet-cognition relationship. We used dietary, socioeconomic, and cognitive assessment data from NHANES 2011-2012. Logistic and probit regression models were used to identify associations by adjusting for covariates. Probit model showed higher whole fruit scores associated with improved word recall, delayed recall, and increased likelihood of above-average cognitive performance. Higher scores for whole fruits, vegetables, grains, and seafood components were associated with improved animal-fluency performance, whereas higher whole grain scores increased the probability of above-average performance, and higher fatty acid component scores reduced it. Higher overall HEI scores, along with higher scores for whole fruits, whole grains, and seafood components, also positively associated with improved performance on digit-symbo l-substitution-tests. Additionally, higher whole fruit and protein component scores increased the likelihood of individuals scoring above average in digit symbol scores. Furthermore, when stratified by socioeconomic status, higher-HEI scores increased the likelihood of above-average performance in animal-fluency and digit-symbol-tests for individuals below 185% of the poverty line. In conclusion, higher-HEI scores—particularly for whole fruits, grains, seafood, and protein components—are associated with enhanced cognitive performance, with individuals below 185% of the poverty threshold showing stronger association.

Abstract citation ID: igaf122.2434

### NUTRITION AND AGING ACROSS THE PLAINS: A QUALITATIVE STUDY

Emily Frankel[1], Lindsay Wilkinson[2], and Christopher Kelly[2], *1. University of Nebraska Medical Center, Omaha, Nebraska, United States, 2. University of Nebraska Omaha, Omaha, Nebraska, United States*

Nutrition is a vital driver of health, playing a key role in health promotion and overall well-being across the lifespan. Older adults, who face morbidity and compromised health outcomes, can leverage dietary habits that support cognitive functioning, decrease inflammation, increase longevity, and improve quality of life. Unfortunately, communities experience heterogeneity in food availability, access, and education, such that rural communities tend to be older, sicker, and economically disadvantaged, compared to urban communities. To understand the nutritional perceptions and experiences of older adults residing in rural and urban communities in Nebraska, 24 older adults (n = 8), medical providers (n = 8), and key partners (n = 8) participated in semi-structured interviews between October 2024 and November 2024. A thematic analysis revealed four main themes: 1. Relationships and Social Connectedness, 2. Education and Empowerment, 3. Balance and Wellness, and 4. Access and Independence. Ascertaining perspectives from rural- and urban-dwelling individuals demonstrated the role of the built environment on health behaviors. Rural residents were impacted disproportionately and more likely to report a lack of or diminished choice of grocery stores or healthy food establishments. Transportation also varied greatly between rural and urban communities, where rural communities often lacked public transportation. The affordability of produce was demonstrably different in rural vs urban communities and created a barrier for older adults in rural communities to access produce as readily as their urban counterparts. These findings offer innovative and valuable insight into enhancing community programming and resource development for rural and urban communities, ultimately supporting health and well-being through nutrition-based initiatives.

Abstract citation ID: igaf122.2435

### NUTRITIONAL STATUS AND ITS ASSOCIATION WITH POSSIBLE SARCOPENIA IN OLDER HOMELESS ADULTS

Phatcharaphon Whaikid[1], and Noppawan Piaseu[2], *1. Mahidol University, Bangkok, Krung Thep, Thailand, 2. Ramathibodi Hospital, Bangkok, Krung Thep, Thailand*

This study assesses nutritional status and possible sarcopenia among formerly homeless adults aged 50 years and older residing in Thai supportive housing. A total of 284 older homeless adults were screened, of whom 116 met the inclusion criteria and were selected using purposive sampling. Data were collected on sociodemographic characteristics, nutritional status (assessed using the Mini Nutritional Assessment-Short Form [MNA-SF]), and possible sarcopenia (determined using the AWGS 2019 criteria), including calf circumference (< 34 cm in men, < 33 cm in women), low handgrip strength (< 28 kg in men, < 18 kg in women), or low gait speed (≤1.0 m/s). Data were analyzed using descriptive statistics and logistic regression analysis. Among the participants, 66.4% were male, with a mean age of 59.14 years (SD = 7.79). Overall, 78.4% of participants were classified as having possible sarcopenia. Univariate analysis showed that malnutrition (OR = 6.111, 95% CI = 2.104–17.750), body mass index (BMI) (OR = 0.031, 95% CI = 0.009–0.100), and waist circumference (WC) (OR = 0.129, 95% CI = 0.049–0.342) were significant predictors of possible sarcopenia. Multiple logistic regression analysis indicated that malnutrition (OR = 3.860, 95% CI = 1.068–13.946) and BMI (OR = 0.038, 95% CI = 0.011–0.128) together accounted for 53.3% of the variance in predicting possible sarcopenia. Findings highlight a high prevalence of possible sarcopenia, with malnutrition and BMI as key risk factors. Targeted nutritional and muscle health interventions are essential to mitigate sarcopenia risk in this vulnerable population.

Abstract citation ID: igaf122.2436

### PILOT STUDY RESULTS TESTING THE IMPACT OF BLUEBERRIES IN SEDENTARY OLDER ADULTS WITH DEPRESSIVE SYMPTOMS

Courtney Millar[1], Alyssa Dufour[1], Alex Wolfe[2], Jasmin Kuo[2], Kathryn Baldyga[2], Douglas Kiel[3], and Lewis Lipsitz[4], *1. Hinda and Arthur Marcus Institute for Aging Research, Boston, Massachusetts, United States, 2. Hebrew SeniorLife, Boston, Massachusetts, United States, 3. Harvard Medical School Hebrew SeniorLife Marcus Institute, Boston, Massachusetts, United States, 4. Hebrew Senior Life, Boston, Massachusetts, United States*

Blueberries provide 2 bioactive nutrients, fiber and anthocyanins, that could modify inflammation, the gut microbiota, and clinical outcomes in older adults. We hypothesize consuming blueberries would decrease inflammation, change the gut microbiota, and improve mood, motivation, and daily step count. We conducted a randomized, double-blind, parallel pilot study in older adults with sedentary behavior and mild depressive symptoms. Participants consumed 48g/day of freeze-dried blueberry powder (~2 cups of fresh berries) or a placebo powder for 12 weeks. Outcomes were measured before and after the intervention. Shotgun sequencing of the stool evaluated gut microbial diversity, abundance, and EC (enzyme commissions). Student's t-test assessed within group differences between baseline and follow-up. Twenty-two participants completed the intervention (mean age 73 years ± 5, 77% female, 90% white, compliance >89% in both groups). Compared to baseline there was no changes in serum inflammation markers, motivation levels, or step count in either group. In a subset of individuals who provided stool samples there was no change in gut microbial diversity in either group (n = 10 blueberry; n = 8 placebo), but there was a statistically significant change in indicators of gut microbial enzymes (e.g., biotin metabolism) between baseline and follow-up in the blueberry group after adjusting for multiple comparisons. Although both groups demonstrated an improvement in mood over time (e.g., via validated questionnaire), the magnitude appeared larger in the blueberry group. Data are preliminary and more robust analyses (e.g., mixed regression) are underway. Such data are needed to determine a potential link between blueberries, the gut-microbiota, and mood.

Abstract citation ID: igaf122.2437

### PROTEIN INTAKE MEDIATES THE RELATIONSHIP OF SLEEP QUALITY WITH HANDGRIP STRENGTH IN OLDER PERSONS 60+ YEARS

Marie Fanelli Kuczmarski[1], Elizabeth Orsega-Smith[1], May Beydoun[2], Michele Evans[2], and Alan Zonderman[2], *1. University of Delaware, Newark, Delaware, United States, 2. National Institute on Aging, Baltimore, Maryland, United States*

Handgrip strength, a measure of muscle strength and biomarker for health and vitality, may be influenced by diet, physical activity, and sleep. The study objective was to determine if protein intake/body weight (gm/kg) and engagement in physical activity mediated the relationship between sleep with handgrip strength in persons ≥ 60 years examined in the HANDLS study, 2013-2017. Sleep quality was measured using the Pittsburgh Sleep Quality Index questionnaire. Handgrip strength was measured with Jamar Hydraulic Hand Dynamometer and expressed per Body Mass Index (based on measured height and weight). Protein intake was calculated from 2-24-hour recalls collected with the USDA Automated Multiple-Pass Method. Physical activity was self-reported and expressed as meeting the Life Simple 7 criterion (≥150 mins/week, 0-149 mins/week, 0 mins/wk). Mediation analysis used Hayes PROCESS macro, model #4, for SPSS Version 4.2. Before performing the analysis,138 individuals with sleep apnea were excluded, resulting in an analytical sample of 1308. Covariates that were adjusted for in models included: age (years), sex at birth (male, female), self-reported race (African American, White), poverty status (< 125% or > 125% U.S. HHS Poverty Guidelines), current cigarette smoker (yes, no), marijuana, opiate and/or cocaine user (yes, no), medical conditions of diabetes, hypertension, and/or metabolic syndrome (yes, no), and mean energy(kcal). Protein intake, but not physical activity, significantly influenced handgrip strength ($p = 0.002$) and mediated the relationship of sleep quality with handgrip ($R2 = 0.47$). Results suggest that protein is critical to preserving muscle strength and plays a role in sleep quality.

Abstract citation ID: igaf122.2438

### RANDOMIZED TRIAL OF PROBIOTICS AND NICOTINIC ACID ON GUT MICROBIOME, QUALITY OF LIFE IN PARKINSON'S

Zaw Wai Htoo[1], Lydia Zeglin[2], Linda Yarrow[2], and Mark Haub[2], *1. University of California Cooperative Extension, Novato, California, United States, 2. Kansas State University, Manhattan, Kansas, United States*

Evidence suggests that probiotics and nicotinic acid (vitamin B3) may improve symptoms and outcomes of Parkinson's disease through gut microbiome modulation. This study aimed to determine whether a 12-week placebo-controlled randomized clinical trial would result in changes in constipation, drug efficacy, neuroendocrine levels, and quality of life in people diagnosed with Parkinson's disease. Forty-eight participants were randomly assigned to one of three groups: (1) probiotics + nicotinic acid, (2) probiotics + placebo, or (3) dual placebo for 12 weeks. Constipation, depression, anxiety, quality of life, mood, symptoms, and nutrition were assessed at baseline, midpoint, and the end of the trial. Blood and stool samples were collected for blood chemistry and gut microbiome analysis using next-generation sequencing (16S rRNA genes, Illumina MiSeq). Statistical significance was set at $p < 0.05$. The results showed improvements in constipation problems, Movement Disorder Society-Unified Parkinson's Disease Rating Scale (MDS-UPDRS), Parkinson's Disease Questionnaire-39 (PDQ-39), quality-of-life scores, and communication issues in probiotics and nicotinic acid groups compared with the placebo group. Blood chemistry remained within normal reference ranges. Supplementation did not change assessments of anxiety, depression, or mood. Gut microbiome analyses showed significant differences in alpha and beta diversity, as well as distinct microbiome compositions related to different interventions, disease status, anxiety, and depression. Several differences became more pronounced after 12 weeks compared with 6 weeks of intervention. Nicotinic acid appeared to have a stabilizing effect on the gut microbiome. The findings suggest that supplementation for twelve weeks may benefit constipation symptoms, gut microbiome, and quality of life measures.

### SESSION 7135 (POSTER)

### PRIMARY CARE

Abstract citation ID: igaf122.2439

### ASSESSING FAMILY PHYSICIANS' KNOWLEDGE AND ATTITUDES TOWARD MALNUTRITION IN THE ELDERLY

Galia SheferHilel[1], Josefa Kachal[2], Danit Shahar[3], Aya Biderman[3], and Shimon Amar[3], *1. Tel-Hai College, Qiryat Shemona, HaZafon, Israel, 2. Ministry of Haelth, Ashdod, HaDarom, Israel, 3. Ben Gurion University of the Negev, Be'er Sheva, HaDarom, Israel*

Malnutrition among older populations significantly impacts healthcare, social, and aged-care systems, and often remains unnoticed and untreated in older populations. This study evaluates family physicians' knowledge and attitudes toward the diagnosis and treatment of malnutrition in this group. An online questionnaire, developed from a literature review, included seven items on knowledge and eight on attitudes concerning malnutrition in older individuals. We also explored the feasibility of including two malnutrition screening questions in regular clinic visits for people aged 70 years and above. Surveys were completed by 126 physicians (35% response rate), with an average age of 47.2±12.6 years; 15.6±12.5 years of practice; 67% females; and 92% board-certified family physicians. Notably, 77.6% agreed that diagnosing malnutrition is important in patients with decreased appetite. A majority demonstrated knowledge of nutritional screening principles (63.5%) and acknowledged that malnutrition could affect obese older individuals (83.2%). There was partial agreement (60%) that normal BMI values in the older adults differ from those in younger populations. Almost complete agreement (91%) was seen for incorporating two nutritional status questions during medical visits with physicians expressing willingness to undergo training in malnutrition identification and screening tools. Despite challenges such as time constraints and limited knowledge, there was support for biannual screening of older patients for malnutrition risk. Based on our findings. We recommend routine malnutrition screening in primary care, followed by diagnosis and referral for appropriate interventions for malnourished patients aged 70 and above.

Abstract citation ID: igaf122.2440

### HANDGRIP STRENGTH AN EASY SCREENING FOR COGNITIVE DECLINE IN PRIMARY CARE OLD PATIENTS

Constanca Paul[1], Susana Sousa[1], Laetitia Teixeira[2], and Carla Tortora[3], *1. University of Porto, Porto, Porto, Portugal, 2. ICBAS-UP, Porto, Porto, Portugal, 3. University degli Studi "G. d'Annunzio" Chieti-Pescara, Chieti, Abruzzo, Italy*

Hand grip strength (HGS) is considered a measure of overall muscular strength connected with mobility, functionality and frailty. In adition, HGS also appears associated with cognition, namely mild cognitive impairment (MCI) and Alzheimer's disease. The early diagnosis of cognitive decline should be done in

Primary Care(PC) allowing interventions to avoid or retard the progression to dementia. HGS is a quick and objective measure that seems to be be useful as a predictor of cognitive decline in Primary Care. The objective of this study is to better understand the association between HGS and usual screenings of Mild Cognitive Impairment MCI (Qmci) and dementia (MMSE). The participants of the study are 427 older adults, clients of PC. Together with cognitive tests and demographic variables, HGS was measured. The mean age of participants was 75.65 years (sd 6,76); 57.1% females with a mean of education' years of 3.3 (sd 2.4). The HGS mean of men was 27.1(sd 8.8) and of women 14.5(sd 4.8). HGS predicts Qmci results (B = 24.69 p<.001) explaining 12.5% of variance and MMSE results (B = 18.37 p<.001), explaining 13.7% of variance. Data shows that lower HGS grip is associated with a higher risk of MCI and dementia. HGS is a simple measure allowing risk-stratification and should be used in regular secreening of older adults in PC and those with lower scores being refered to further cognitive assessment and a costumised physical activity intervention that will benefit the overall muscular strength and contribute to avoid or retard the onset of dementia.

Abstract citation ID: igaf122.2441

### HOME-BASED PRIMARY CARE IN THE DEPARTMENT OF VETERANS AFFAIRS: HISTORY, LESSONS LEARNED, AND NEXT STEPS

Brooke Jespersen[1], Anaïs Tuepker[1], Elizabeth Hulen[1], Senta Wiederholt[1], Dorothea Murray[2], and Samuel Edwards[1], *1. VA Portland Health Care System, Portland, Oregon, United States, 2. Oregon Health & Science University, Portland, Oregon, United States*

Launched in 1970, the Department of Veterans Affairs (VA) Home-Based Primary Care (HBPC) Program is a distinctive, well-resourced home care model that has broadly influenced the development of HBPC practice. VA HBPC delivers team-based, comprehensive, longitudinal primary care in the homes of Veterans who are unable to access or have great difficulty accessing clinic-based care. The program's goal is to promote optimum health, while reducing the need for hospital, emergency room, and nursing home care. Overall, VA HBPC has produced favorable outcomes (e.g. reduced hospital, emergency, and nursing home utilization; high patient and caregiver satisfaction) and has lowered health care costs. Given population aging, there will be more medically complex older people who would benefit from home-based care; yet most do not have access to an HBPC program. It is critical that VA and non-VA health systems expand and improve HBPC to meet the high needs of this patient sector in a patient-centered, yet cost-effective manner. VA HBPC's fifty-five-year history contains important lessons about the provision of home-based medical care for high risk, high needs patients. In this presentation, we draw on the literature and our institutional knowledge as VA HBPC researchers to provide a narrative history of VA HBPC. We highlight key innovations, identify challenges to program expansion and care delivery, and propose next steps for research and practice, with the goal of informing home-based medical care in VA and beyond.

Abstract citation ID: igaf122.2442

### IMPROVING ADRD DIAGNOSIS IN PRIMARY CARE: TAILORING BLOOD-BASED BIOMARKER EDUCATION FOR LOW-LITERACY POPULATIONS

Ignacia Arteaga[1], Alissa Sideman[1], Cecilia Alagappan[1], Joshua Grill[2], Allyson Rosen[3], Freddi Segal-Gidan[4], Loren Alving[5], and Howie Rosen[1], *1. University of California, San Francisco, San Francisco, California, United States, 2. University of California, Irvine, Irvine, California, United States, 3. Stanford University, Stanford, California, United States, 4. University of Southern California, Los Angeles, California, United States, 5. California State University, Fresno, Fresno, California, United States*

An estimated 50–70% of symptomatic patients with Alzheimer's Disease and Related Dementias (ADRD) go unrecognized or are misdiagnosed in primary care, delaying access to supportive services, financial and residential planning, clinical trials, and potentially disease-modifying therapies. One key barrier to timely diagnosis is low educational attainment (e.g., not finishing high school), which is associated with both increased ADRD risk and higher rates of comorbidities. Blood-based biomarkers (BBMs) offer a promising, less invasive, and cost-effective tool to improve diagnosis in primary care. However, individuals with low educational attainment are underrepresented in BBM validation studies, raising concerns about their applicability to diverse populations. ADDPCP, an NIA-funded study, evaluates the accuracy and impact of ADRD diagnosis in primary care across California using clinical tools and BBMs. This presentation reviews the development and validation of educational materials to support BBM decision-making for low-literacy populations. Informed by prior PET amyloid disclosure protocols, we co-developed multi-modal pre-and post-test counseling materials using evidence-based health communication strategies and the Lexile Framework for Reading. These materials—narrated presentations and take-home brochures with pictorial aids—explain the purpose of BBM testing, the risks and benefits of learning results, and the limitations of the information provided. We are in the process of assessing their clarity and relevance through cognitive interviews with 12 matched participants naïve to the study.

Abstract citation ID: igaf122.2443

### ROLE OF HOME-BASED PRIMARY CARE IN PERCEIVED CARE SUFFICIENCY AND DEPRESSIVE SYMPTOMS IN OLDER ADULTS

Jihee Choi[1], Bokyoung Choi[2], Jihwan Lee[2], Chang-O Kim[1], and Soong-nang Jang[2], *1. Institute for Community Care and Health Equity, Seoul, Republic of Korea, 2. Chung-Ang University, Seoul, Republic of Korea*

Background With the global increase in the aging population, the importance of community-based care and Aging in Place (AIP) has been emphasized. The study aimed to investigate the role of home-based primary care in the association between perceived care sufficiency and depressive symptoms among older adults in South Korea. Methods We conducted a survey of the 'Home-Based Primary Care Cohort (HBPC). One survey has been completed by March 2024, the second survey by September 2024 and the third survey by March 2025. Study population was community-dwelling older adults aged 65 years and older who were receiving long-term care services. Perceived care sufficiency was assessed based on participant's self-reported. Depressive symptoms were measured using GDS. Additionally, this study examined how the frequency of HBPC services moderated the relationship between subjective care sufficiency and depressive symptoms. Multiple linear regression models were applied to calculate. Results Among older adults receiving HBPC services, 61.4% (N = 202) reported that their caregiving hours were insufficient, and 82% (N = 270) exhibited depressive symptoms. A significant association was found between perceived care sufficiency and depressive symptoms ($\beta = 0.89$, $p = 0.011$). However, as the frequency of HBPC service utilization increased, the relationship between perceived care sufficiency and depressive symptoms became nonsignificant. Conclusions These findings emphasize the importance of ensuring perceived care sufficiency within the community to reduce depression among older adults. HBPC can play a role in designing future community-based care systems. Furthermore, HBPC may serve as a key resource in establishing an integrated community care system.

### SESSION 7140 (POSTER)

### REHABILITATIVE CARE/PHYSICAL AND OCCUPATIONAL THERAPY

Abstract citation ID: igaf122.2444

### A HOLISTIC APPROACH TO GERIATRIC REHABILITATION: LEVERAGING THE MFOA FOR SUPERIOR PATIENT CARE

Gregory Hartley[1], Susan Leach[2], Kenneth Miller[3], Myla Quiben[4], Laura Gras[5], Marni Larkin[6], and Michelle Lusardi[7], *1. University of Miami, Coral Gables, Florida, United States, 2. University of New Mexico, Albuquerque, New Mexico, United States, 3. Medical University of South Carolina, Charleston, South Carolina, United States, 4. The University of North Texas Health Science Center at Fort Worth, Fort Worth, Texas, United States, 5. Ithaca College, Ithaca, New York, United States, 6. Gyrotonic Manhasset, Manhasset, New York, United States, 7. Sacred Heart University, Fairfield, Connecticut, United States*

The Movement Framework for Older Adults (MFOA) integrates the Geriatric 5Ms (Mind, Mobility, Medications, Multicomplexity, Matters Most) and the International Classification of Functioning, Disability, and Health (ICF) to provide a comprehensive approach to geriatric rehabilitation. This study provides a comparative analysis of two patients with Parkinson's disease, demonstrating MFOA's application in addressing their unique needs within a physical therapy context. The Geriatric 5Ms serve as the lens through which patient assessments and interventions are tailored, focusing on what is most important to the individual. The ICF model further supports this by categorizing health and health-related domains, ensuring a holistic perspective that considers all aspects of a patient's well-being. MFOA emphasizes individualized care plans, incorporating interventions and tasks specific to each patient's goals. This approach ensures that both physical and mental health are addressed to achieve optimal outcomes. The framework's application involves a thorough assessment of the patient's physical capabilities, psychological state, and social context, allowing for a comprehensive and patient-centered care plan. The integration of the Geriatric 5Ms and ICF models within the MFOA framework ensures that the care provided is aligned with what matters most to the patient, enhancing quality of life and engagement in therapy. This work underscores the novelty and utility of the MFOA in physical therapy management of older adults. By combining the Geriatric 5Ms and the ICF, the MFOA offers a unique and comprehensive approach that addresses the multifaceted needs of older adults, potentially improving geriatric rehabilitation outcomes through personalized, holistic care.

Abstract citation ID: igaf122.2445

### A MULTI-OUTCOME PROGNOSTIC MODEL FOR OLDER ADULTS ADMITTED TO SKILLED NURSING FACILITIES FOR HIP FRACTURE AND STROKE

W. James Deardorff, Siqi Gan, Bocheng Jing, John Boscardin, Alex Smith, and Sei Lee, *University of California, San Francisco, San Francisco, California, United States*

Many hospitalized older adults are discharged to a skilled nursing facility (SNF) for short-term rehabilitation following a hip/femur fracture or stroke. Mortality rates are high, and many never return home (i.e., high risk of "rehabbed to death"). To inform shared decision-making, we developed prognostic models using a 20% Medicare sample from 2017-2019 of community-dwelling adults aged ≥66 admitted to a SNF for hip/femur fracture or stroke. Within each cohort, we developed a prognostic model to predict 2 outcomes: 6-month mortality and "successful community discharge" (discharge to the community without subsequent rehospitalization or death within 30 days). Model predictors were pre-specified based on literature review: age, sex, hospital length of stay, Medicaid status, comorbidities, and hospitalizations in the past year. Model performance was assessed by discrimination (c-statistic) and calibration (calibration plots). Internal validation was performed via bootstrapping. The hip/femur fracture cohort included 52,843 individuals (mean age 83.4 years, 73% female, 3.3% Black), and the stroke cohort included 20,599 individuals (mean age 81.7 years, 60.1% female, 11.4% Black). Overall, in the hip/femur fracture and stroke cohorts, 15.5% and 24.6% died within 6 months and 60.1% and 45.4% experienced a successful community discharge, respectively. For the hip/femur fracture and stroke cohorts, the optimism-corrected c-statistics were 0.74 and 0.70 for 6-month mortality and 0.68 and 0.67 for successful community discharge, respectively. Calibration plots showed that the models were well-calibrated for both outcomes in both cohorts. Risk predictions from these models can help guide shared decision-making and future planning between SNF clinicians, patients, and caregivers.

Abstract citation ID: igaf122.2446

### EFFECTS ON COGNITIVE FUNCTION AND MOOD STATE DUE TO INTERVENTIONS INVOLVING FINGER MOVEMENTS IN HEALTHY OLDER ADULTS

Atsuko Hayashi, Momoka Ijima, Keitaro Ito, Sinan Chen, and Masahide Nakamura, *Kobe University, Kobe, Hyogo, Japan*

Previous studies have reported relationships between hand dexterity and cognitive functions. However, there are few studies examining effects of finger exercise interventions on cognitive functions. Here we administered working memory(WM) tasks involving finger movements as interventions in healthy older subjects and investigated changes in cognitive functions and mood state due to the interventions. The participants were 71 healthy older people in total (a cross-over study). They were randomly assigned to the following three intervention groups; Group A performed a WM task using numbers and Japanese kana characters accompanied by finger movements; Group B performed finger movements in accordance with the presented numbers or kana; Group C were allocated to the WM task without finger movements. Cognitive functions [Trail Making Test(TMT)-A/B, Digit/Tapping Span] and mood states (the Two-Dimension Mood Scale) were evaluated. In the comparison of before and after the intervention, group A significantly improved the time required for TMT-A/B. Group B showed a significant decrease in the time required for TMT-B. In group C, the score of Digit Span was increased and all groups showed increased arousal level and decreased mood stability significantly. In between-group comparisons, no significant changes were observed in all evaluations. These results suggest that the interventions involving finger movements may improve cognitive functions such as attention and executive functions and working memory in healthy older people. The intervention tasks might affect in mood states regardless of finger exercises. In conclusion, it is suggested that finger exercises may be used as intervention tools to improve cognitive functions.

Abstract citation ID: igaf122.2447

### EVALUATING A COMMUNITY-BASED REABLEMENT PROGRAM IN TAIWAN: A CLUSTER RCT AND USING THE RE-AIM FRAMEWORK

Hsiao-Wei Yu[1], Yen-Po Yeh[2], and Ya-Mei Chen[3], *1. National Taiwan University, Taipei, Taipei, Taiwan (Republic of China), 2. Changhua County Public Health Bureau, Changhua City, Changhua County, Taiwan (Republic of China), 3. National Taiwan University, National Taiwan University, Taipei, Taiwan (Republic of China)*

This study introduces a community-based reablement program implemented at the county level in central Taiwan using a cluster randomized controlled trial. The study has two objectives: (1) to evaluate the efficacy of the pilot program and (2) to explore its scalability using the RE-AIM framework. The pilot trial recruited 231 dyads of older adults and their family caregivers from nine adult day centers. The experimental group received a three-month intensive individualized and group-based reablement training delivered by a collaborative team of therapists and care assistants, while the control group received only group-based training. Preliminary findings indicate significant improvements in the number of hours spent performing daily activities and slight benefits in balance among participants in the experimental group compared to the control group. However, no significant changes were observed in ADLs, IADLs, cognitive function, caregiver burden, or mastery. The RE-AIM framework provides a structured approach to assess the program's generalizability and long-term feasibility. Most staff involved in the program expressed positive attitudes toward reablement training, emphasizing its potential to enhance independence. Implementation fidelity was supported by a standardized training protocol and well-trained therapists and care assistants. Additionally, outer setting consultants played a crucial role in maintaining fidelity and providing guidance to adult day centers. However, challenges remain, including the need for sustained funding and continuous incentives to support program expansion. The increased workload among care assistants delivering reablement training also highlights the importance of resource allocation and workforce support for successful integration into routine care.

Abstract citation ID: igaf122.2448

### HIGH INTENSITY-REHABILITATION IN SKILLED NURSING FACILITIES: FROM THEORY TO PRACTICE

Lauren Hinrichs-Kinney[1], Mattie Pontiff[1], Elizabeth Staton[2], Katie Butera[3], Emma Beisheim-Ryan[4], Dawn Magnusson[2], and Jennifer Stevens-Lapsley[2], *1. VA Eastern Colorado Health Care System, Aurora, Colorado, United States, 2. University of Colorado Anschutz Medical Campus, Aurora, Colorado, United States, 3. University of Delaware, Newark, Delaware, United States, 4. Naval Medical Center, San Diego, California, United States*

Implementing evidence-based, physiologic dosing of rehabilitation in skilled nursing facilities (SNFs) is essential to improve functional outcomes in a vulnerable, medically-complex population. We evaluated an implementation program to promote high-intensity resistance rehabilitation (HIR) in SNFs.

Methods: Using a prospective convergent mixed-methods design, this study 1) measured proximal (clinician knowledge, self-efficacy, and HIR perspective) and distal (HIR adoption and implementation) outcomes; 2) explored how the program influenced distal outcomes (program processes); and 3) investigated clinician factors influencing HIR implementation. Thirty-eight rehabilitation clinicians and 16 leaders in eight rural Department of Veterans Affairs SNFs participated in interviews and focus groups. Outcomes included validated questionnaires on HIR perspective (Perceived Characteristics of Intervention Scale) and adoption (Commitment to Change Scale), and study-specific questionnaires on clinician HIR knowledge, self-efficacy, and implementation. Results: The program improved clinician HIR knowledge, self-efficacy, and perspective and led to acceptable adoption rates; however, implementation was marginally affected. Only clinician HIR perspective correlated with adoption (rho = 0.47, $p = 0.004$). The program supported distal outcomes by keeping HIR at the forefront of clinicians' mind, fostering positive outcome expectations, and enhancing team cohesion and accountability. Clinician creativity, adaptability, resilience, professional discipline, and previous experience influenced implementation. Conclusion: The program influenced HIR adoption primarily by enhancing clinicians' positive perspectives of HIR. Future efforts could strengthen implementation by fostering team cohesion, accountability, and clinician creativity while assessing environmental factors influencing implementation. Strategies that enhance clinician perspective and creativity, maintain HIR top of mind, and foster team cohesion and accountability may improve adoption.

Abstract citation ID: igaf122.2449

### PREDICTING FUNCTIONAL DECLINE AND FALLS IN OLDER ADULTS PARTICIPATING IN GEROFIT

Ben Friedman[1], Jamie Giffuni[2], Megan Kelly[2], Julie Rekant[2], and Odessa Addison[3], *1. University of Maryland, Baltimore County, Baltimore, Maryland, United States, 2. Veterans Affairs, Baltimore, Maryland, United States, 3. University of Maryland School of Medicine, Baltimore, Maryland, United States*

Purpose: Functional limitations contribute to disability and fall risk in older adults. This analysis examined patterns of limitations over 12 months and if baseline function predicts falls in older Veterans participating in GeroFit, a supervised but unstructured VA exercise program. Methods: 199 Veterans (71.9±6.7 years) completed functional assessments at baseline and 12 months (12M), including Gait Speed (GS), Short Physical Performance Battery (SPPB), Four Square Step Test (FSST), and 30-Second Chair Stand (30sCS). Functional limitations were defined by published cut-off scores. Paired t-tests and Wilcoxon signed-rank test assessed changes over time, chi-square tests examined co-occurrence, and logistic regression evaluated predictors of falls at 12M. Results: At baseline, 40% of participants had limitations in at least two measures, and at 12M, 30%

remained below cut-off in multiple tests. Overall, Veterans improved significantly ($p < 0.01$) on all functional measures from baseline to 12M (SPPB: 10±2 to 11±2; GS: 1.10±0.26 m/s to 1.14±0.24 m/s; FSST: 13.12±6.90s to 11.63±6.54s; 30sCS 12±5 to 15±6 reps). Persisting below cut-offs was strongly predicted by baseline scores (OR = 2.58, 95% CI: 1.95–3.44, $p < 0.001$). Baseline 30sCS was the only significant predictor of falls at 12M (OR = 0.76, 95% CI: 0.56–0.98, $p = 0.049$), with each additional repetition in the 30sCS at baseline associated with a 24% lower odds of reporting a fall at 12 months. Conclusion: Lower extremity strength (30sCS) may be key for fall risk screening. Functional limitations frequently co-exist, and identification may guide interventions for older Veterans in exercise programs.

Abstract citation ID: igaf122.2450

### TELEREHABILITATION APPLIED BY PHYSIOTHERAPISTS TO OLDER ADULTS IN BRAZIL - A PILOT SURVEY

Roberta Oliveira Bueno de Souza[1], Fabiana Cassales Tosi[1], Judith Deutsch[2], Ruth Melo[3], and Jose Eduardo Pompeu[1],
*1. University of Sao Paulo, Sao Paulo, Sao Paulo, Brazil,*
*2. Rutgers University, New Brunswick, New Jersey, United States,*
*3. Universidade de São Paulo, São Paulo, Sao Paulo, Brazil*

This study aimed to characterize the profile and adherence rate of Brazilian physiotherapists who utilize telerehabilitation (TF) in the care of community-dwelling older adults and evaluate their satisfaction with the intervention. A cross-sectional observational study was conducted through an online survey between August and December 2024, using convenience sampling. The questionnaire investigated the physiotherapists' profile, characteristics of TF, and their satisfaction with the intervention. A total of 59 responses were obtained, with 55.9% from physiotherapists working with older adults. Most respondents were female (90.9%), with a mean age of 41.0±10.6 years, residing in the Southeast region (78.8%), and 78.8% had more than 5 years of experience. Additionally, 84.8% held a specialist title, and 78.8% adopted TF. Most TF sessions were conducted individually (55.2%), lasted between 46 and 60 minutes (65.5%), and utilized computers and/or smartphones (48.3%) with commercial softwares (82.8%). The synchronous modality was the most commonly used (96.6%), which was applied during treatment (82.8%). Regarding satisfaction perception, 69.0% rated it as satisfactory or very satisfactory. In conclusion, the use of TF in community-dwelling older adults in Brazil has a good adherence rate and is considered satisfactory or very satisfactory by Brazilian physiotherapists. Telerehabilitation for the Brazilian older adults is delivered individually and synchronously during treatment, using simple and accessible technologies. However, a larger sample is needed to enhance the robustness and generalizability of these results.

### SESSION 7145 (POSTER)

### SOCIAL DETERMINANTS OF HEALTH AND AGING

Abstract citation ID: igaf122.2451

### ASSESSING SOCIAL CAPITAL AMONG CHINESE OLDER ADULTS: DIMENSIONS AND ASSOCIATIVE FACTORS

Yuekang Li, *University of Chinese Academy of Social Sciences, Beijing, Beijing, China (People's Republic)*

Recent research highlights social capital as a crucial factor in healthy aging, with its impact varying across contexts and cultures. This study delves into the nature of social capital among older adults in China, examining its dimensions and the influence of individual and environmental characteristics. Utilizing the 2016 wave of the China Family Panel Study, the research employed factor analysis and Multiple indicators, multiple cause (MIMIC) structural equation models to dissect the components of social capital within this group. The analysis identified three key levels of social environment—family, community, and macro society—that shape the social capital of Chinese older adults. The study's findings emphasize the complex interplay between individuals and their social environments. It was observed that those with disabilities tend to have enhanced family-level social capital but diminished community-level social capital. This suggests that while families remain a crucial support system, reliance on them may limit access to broader social resources. Moreover, the community's physical environment emerged as a significant factor in determining social capital, underscoring the importance of neighborhood conditions in fostering social connections among older adults. These findings reveal the multifaceted nature of social capital in later life and highlight the need for targeted policy and practice interventions. By focusing on individuals with high family-level social capital, efforts can be made to reduce vulnerabilities and improve the capacity of older adults to engage with wider social networks, thereby enhancing their overall well-being and ability to adapt to diverse environments.

Abstract citation ID: igaf122.2452

### DISABILITY-BASED MICROAGGRESSIONS, SOCIAL SUPPORT, AND PSYCHOLOGICAL DISTRESS IN AGING ADULTS WITH DISABILITIES

Dylan Serpas[1], Daniel Ignacio[2], and Barbara Cherry[3],
*1. University of South Florida, Tampa, Florida, United States,*
*2. University of California, Los Angeles, Los Angeles, California, United States, 3. California State University, Fullerton, Fullerton, California, United States*

Introduction: Disability-based microaggressions — subtle, often unintentional discriminatory remarks or behaviors — are related to increased psychological distress among aging adults. These experiences may reinforce negative stereotypes, undermine autonomy, and contribute to feelings of exclusion. Social support is an empirically-supported moderator of the effects of discrimination, yet research examining social support and lifetime frequency of disability-based microaggressions among aging adults with disabilities (AWD) is limited. This study explored whether social support moderates the relationship between lifetime experiences of disability-based microaggressions and psychological distress. Method: A national sample of 243 aging adults with disabilities aged 40 to 70 (M = 49.13, SD = 6.28; 52.7% female; 52.3% non-Hispanic white; 39.1% heterosexual; 95.5% married; 57.6% psychiatric disability) completed the Ableist Microaggression Scale, the Multidimensional Scale of Perceived Social Support, and the Depression Anxiety and Stress Scale-21 to assess depressive and anxiety symptoms. Moderated linear regression models tested whether social support moderated the association between microaggressions and distress. Results: After controlling for relevant sociodemographic and disability characteristics, disability-based microaggressions were positively associated with anxiety symptoms ($b = 0.71$, $p=.02$), while social support was not ($b=-0.02, p=.366$). Microaggressions were not significantly correlated with depressive symptoms (b = 0.71,p=.02) when accounting for social support (b=-0.05,p=.040). No moderation effects of social support were observed. Discussion: Findings suggest disability-based microaggressions are related to anxiety symptoms, but social support did not attenuate this relationship. Results highlight the need for targeted interventions to reduce the burden of microaggressions and promote inclusive environments for aging adults with disabilities.

Abstract citation ID: igaf122.2453

### DISCUSSION WITH PROVIDERS OF MEMORY DIFFICULTIES AMONG ADULTS 45+: DEMOGRAPHIC DIFFERENCES

Drew Blasco, and Jace Flatt, *University of Nevada, Las Vegas, Las Vegas, Nevada, United States*

Subjective cognitive decline (SCD), may indicate increased risk for Alzheimer's disease—a critical area for intervention given the aging population. 2023 Behavioral Risk Factor Surveillance System data (optional SCD and Social Determinants of Health (SDoH) modules administered in 20 states and 1 territory) was utilized to understand the association between participant demographics (age, sex, race and ethnicity, education, income, health insurance, and social determinants of health) and whether participants with SCD/anyone else discussed difficulties with thinking/memory with a healthcare provider. The sample (n = 14,254) was on average 66 years old (range:45-80), majority female (~58%) and insured (~97%), with almost 25% identifying as a person of color and about 42% reporting poor/fair health. Bivariate associations demonstrated differences with those of slightly younger age, female (45% vs 38%), insured (44% vs 25%), and reporting lower incomes more likely to have discussed SCD with a provider while those who identified as Asian Americans (30%) and Native Hawaiian and other Pacific Islander (34%) compared to White (44%) and Hispanic/Latino (49%) and those with less education were less likely to have discussed with a provider. Similar patterns were found for results of the multivariable logistic regression analysis including significant demographic factors of age, sex, education, health insurance, and race and ethnicity when examining the likelihood of talking to a provider. Discussion of subjective memory concerns with a healthcare professional may aid in improving the health of middle-to-older age adults; however, important to consider are potential barriers to provider discussion which may increase health disparities.

Abstract citation ID: igaf122.2454

### DOES INTERGENERATIONAL SOCIAL MOBILITY AFFECT HEALTH AND MORTALITY RISKS AMONG OLDER ADULTS?

Yuning Xie[1], and Chen Bai[2], *1. Renmin University of China, Beijing, United States, 2. Renmin University of China, Beijing, Beijing, China (People's Republic)*

The health effects of intergenerational social mobility—particularly their existence, direction, and magnitude—remain subjects of ongoing debate, especially among older adults. This study leverages data from the 2017–2018 Chinese Longitudinal Healthy Longevity Survey – Healthy Family (CLHLS-HF) to investigate the impact of upward intergenerational occupational and educational mobility on health reproduction and to examine whether such mobility is significantly associated with mortality risk over a three-year period. The findings reveal that intergenerational social mobility exerts significant health effects, with upward occupational or educational mobility enhancing multidimensional health outcomes. Using a Cox proportional hazards model, the study finds that elderly individuals whose parents were farmers (or unemployed) and those who experienced greater absolute mobility distances exhibited significantly lower mortality risk. Additionally, a reversal in the number of chronic diseases was observed among elderly individuals whose final occupational status was "cadre," along with a protective health effect associated with short-distance downward intergenerational occupational mobility. Robustness checks and instrumental variable (IV) tests confirm the reliability of these results. Furthermore, heterogeneity analysis indicates that the health benefits of intergenerational social mobility are more pronounced among younger elderly individuals. Overall, while the long-term health benefits of upward intergenerational social mobility are evident, certain "mixed" effects persist, warranting further investigation.

Abstract citation ID: igaf122.2455

### DOES REPEATING A GRADE AFFECT LATER LIFE EXECUTIVE FUNCTIONING? FINDINGS FROM THE HEALTH AND RETIREMENT STUDY

Rachel Missell-Gray[1], Ya-Han Chang[1], and Silvia Sörensen[2], *1. University of Rochester, Rochester, New York, United States, 2. University of Rochester Warner School of Education and Human Development, Rochester, New York, United States*

Two-thirds of American adults report experiencing at least one Adverse Childhood Experience (ACE).1 ACEs demonstrate negative effects on executive functioning (EF), and EF difficulties in young children are associated with continued academic difficulties.2 In past decades, this meant repeating a grade. Studies suggest ACEs may affect adult cognition3, yet the mechanisms of this association remain underexplained. One mechanism may be that repeating a grade as a result or cause of early life stress, potentially affects later life EF. We investigated whether older adults who repeated a year of school performed poorly on Trails B, an EF task of rule switching and working memory. We examined 2,657 community-dwelling older adults from the Health and Retirement Study (HRS) with data from the 2016 Life History Survey and the Harmonized Cognitive Assessment Protocol. All respondents had education and Trails B data. Study outcomes were Trails B scores, and the primary independent variable was repeating a year of school, controlling for age, degree, and race/ethnicity. Repeating a year of school was associated with lower Trails B scores ($\beta$ = -.064, p <.001). Linear regression models found that final degree and repeating a year of school accounted for 13% of the variance in Trails B scores ($R^2$ =.13, F(1, 2649)=11.94, p <.001). Together, age, race/ethnicity, final degree, and repeating a year of school explained 25% of the variance in Trails B scores ($R^2$ =.25, F(2, 2644)=75.94, p <.001). Future research could examine possible mechanisms (e.g., decreased opportunity) by which education may impact later life EF.

Abstract citation ID: igaf122.2456

### EXAMINING EQUITY FACTORS IN HEALTHY AGING INTERVENTIONS: A SCOPING REVIEW OF HEALTHCARE STRATEGIES

Odelia Ben Harush[1], Chava Kurtz[1], Anna Zisberg[1], Stuart McLennan[2], Paola Buedo[3], and Efrat Shadmi[1], *1. University of*

*Haifa, Haifa, HaZafon, Israel, 2. Technical University of Munich, School of Medicine and Health, Munich, Bayern, Germany, 3. Technical University of Munich, Munich, Bayern, Germany*

Studies on interventions promoting healthy aging often do not address the effect of inequity on their implementation and outcomes. This scoping review aims to identify the equity-related factors explored in healthy aging strategies and interventions. A structured search of multiple electronic databases was conducted using keywords and indexed terms related to healthy aging interventions, with equity aspects including place of residence, race/ethnicity, occupation, religion, education, socioeconomic status, and social capital. A total of 5890 studies were identified and screened, of which 74 met the inclusion criteria. Approximately one-third of the studies (25, 34%) only described equity aspects in demographic characteristics but did analyze results according to these factors; Eleven studies (15%) adjusted for equity aspects; and 38 studies (51%) assessed outcomes by equity factors. Of the 38, 9 studies (24%) examined intervention effects while controlling for equity aspects, and 28 studies (74%) focused on interventions in minority or socioeconomically deprived groups. Of these 28 studies, 20 (71%) reported positive outcomes, primarily in quality of life and functioning. Only 9 studies (12%), explored multiple equity aspects (intersectionality). Equity-related aspects are rarely studied in research in health aging research. Studies examining interventions in minority or socioeconomically deprived groups generally show positive patient-reported outcomes, but none reported effects on healthcare resource use. Future research should prioritize analyzing outcomes by equity dimensions, especially intersectionality, to inform inclusive interventions that may reduce health inequities and promote healthy aging.

Abstract citation ID: igaf122.2457

### FACTORS ASSOCIATED WITH HEALTH LITERACY IN OLDER KOREAN ADULTS: KOREAN NATIONAL HEALTH AND NUTRITION SURVEY 2023

Eun Ju Park[1], Jae Jun Lee[1], Layoung Kim[2], and Gwang Suk Kim[1], *1. Yonsei University College of Nursing, Seoul, Republic of Korea, 2. The University of Suwon, Hwaseong, Republic of Korea*

Health literacy (HL) is essential for managing health and accessing healthcare, yet low HL among older adults is linked to poor health outcomes and disparities. As Korea's population ages rapidly, understanding the factors influencing HL is crucial for developing effective public health strategies. This study aimed to assess the HL status of older Korean adults and identify the influencing factors using data from the Korean National Health and Nutrition Survey 2023. A total of 1,441 individuals aged 65 and older were included after excluding cases with missing data. HL was measured using self-reported questions developed in Korea, comprising ten items rated on a 4-point Likert scale and one knowledge assessment item. Independent variables included demographic, socioeconomic, and physical and mental health-related factors. Multiple linear regression analysis identified significant predictors of HL. The mean participant age was 72.22±5.00 years, and 55% were female. The average HL score was 27.61±5.63, with only 25.9% correctly answering the knowledge item. Higher education, urban residence, higher income, spousal status, private health insurance, and employment were positively associated with HL, while older age and longer working hours were negatively associated. This study highlights significant disparities in HL among older adults, with sociodemographic and occupational factors playing crucial roles. As the workforce ages, more older adults may engage in long working hours, limiting their access to health information and potentially reducing HL. Given HL's impact on health behaviors, ensuring appropriate rest for older workers, along with targeted educational interventions, is essential to improving HL.

Abstract citation ID: igaf122.2458

### FINANCIAL STRAIN OVER THE LIFE COURSE AND HEALTH AMONG OLDER ADULTS: A RURAL-URBAN COMPARISON

Rong Fu, *Siena University, Loudonville, New York, United States*

As economic hardship and insecurity increase globally, understanding how subjective socioeconomic status influences health beyond objective measures is essential. This study examines the impact of childhood and adulthood financial strain on health among older adults in mainland China, with attention to potential rural-urban differences. Data were derived from two waves of the Chinese Longitudinal Healthy Longevity Survey (CLHLS). The final sample included 8,175 adults aged 62 and older. Statistical analyses were conducted separately for rural and urban populations. Multiple imputation techniques were used to handle missing data. The findings indicate that both childhood and current financial strain were significantly associated with poor health among urban older adults, even after adjusting for other risk factors such as household income and poverty. In contrast, among rural older adults, only current perceived financial strain was significantly linked to poor health. This study highlights the need for policies that address the specific challenges of both urban and rural residents. Expanding healthcare access for low-income populations and introducing early interventions to reduce the long-term effects of childhood poverty could make a difference.

Abstract citation ID: igaf122.2459

### GENDER DIFFERENCES IN PREDICTORS OF DEPRESSIVE SYMPTOMS AMONG OLDER HISPANIC WOMEN IN THE UNITED STATES

Jaminette Nazario-Acevedo[1], and Takashi Yamashita[2], *1. University of Maryland, Baltimore & Baltimore County, Martinsburg, West Virginia, United States, 2. University of Maryland, Baltimore County, Baltimore, Maryland, United States*

Older Hispanic women are more likely to experience depressive symptoms than older Hispanic men in the United States. Mental health challenges faced by older Hispanic adults, in general, and their gender differences, in particular, are often overlooked. Previous research highlights that a form of social relationship --- marriage, can serve as a protective factor for mental health. However, gender differences in specific factors associated with depressive symptoms among older Hispanic women and men have not been extensively examined. Given the intersectionality theory, the present study compared the known predictors of depressive symptoms among older Hispanic women and men. Nationally representative samples of Hispanic adults aged 51 years and older ($n = 2564$) are obtained from the 2018 Health and Retirement Study. Binary logistic regression shows that married older Hispanic adults (Odds ratio = 0.69, $p < 0.05$)

are less likely to be depressed than their counterparts. No gender difference in the marital status on depressive symptoms is observed. However, there is a significant gender difference in the effect of educational attainment. Older Hispanic women with a college degree or higher (Odds ratio = 0.57, $p<0.05$) are less likely to be depressed than their counterparts. Yet, educational attainment is not associated with lower depressive symptoms among men (Odd-ratio = 1.02, $p>0.05$). Findings suggest differential impacts of socioeconomic status on mental health by gender. To improve the mental health of older Hispanic adults in the United States, a more targeted and gendered public health approach may be considered.

Abstract citation ID: igaf122.2460

### GENDER, RACE/ETHNICITY, INTERPERSONAL DISCRIMINATION, AND COGNITIVE PERFORMANCE: AN INTERSECTIONAL ANALYSIS

Natalie Rivadeneira[1], Julia Libby[2], Timothy Hohman[2], and Ganga Bey[3], *1. The University of North Carolina at Chapel Hill, Chapel Hill, North Carolina, United States, 2. Vanderbilt University Medical Center, Nashville, Tennessee, United States, 3. UNC Chapel Hill, Chapel Hill, North Carolina, United States*

Previous research has shown that interpersonal discrimination affects cognitive health disparities across gender and race/ethnicity. However, the impact of belonging to multiple social groups on these experiences is rarely considered. We applied an intersectional perspective to examine how gender, race/ethnicity, and interpersonal discrimination intersect to socially pattern cognitive performance in a cohort of US adults aged 50 and older. Using pooled data from Kaiser Healthy Aging and Diverse Life Experiences (KHANDLE) and Study of Healthy Aging in African Americans (STAR) cohorts (n = 2,622), we applied multilevel analysis of individual heterogeneity and discriminatory accuracy (MAIHDA) to investigate variation in executive function, memory, and language across 24 intersectional strata defined by gender, race/ethnicity, and self-reported experiences of everyday and major lifetime discrimination. Model main effects indicate that the additive effects of gender, race/ethnicity, and discrimination experience explained most between-strata variance for all cognitive domains. Variables that define intersectional strata accounted for 17.9% of overall variance in executive function for strata that included everyday discrimination, and 17.1% of variance for strata that included lifetime discrimination. The effects of stratum-defining variables were modest for memory and language (7.7%-11.6%). However, examining by level of perceived discrimination shows cognitive performance variation within and between ethnoracially and gender-defined groups in all cognitive domains. While the effect of discrimination exposure on cognitive performance varied, the additivity of effects suggests that intervention strategies to promote equity in cognitive health should be universal while tailored to the specific needs of ethnoracially and gender-defined groups experiencing discrimination.

Abstract citation ID: igaf122.2461

### LIVING SITUATION AND HEALTHCARE UTILIZATION IN OLDER ADULTS WITH SELF-REPORTED DIABETES

Amy Van Dusen[1], Rachel Bishop[2], Zane Deliu[2], Megan O'Mara[2], Carla Obeid[2], Asef Raiyan Hoque[2], and Samantha Hahn[3], *1. Central Michigan University College of Medicine, Byron Center, Michigan, United States, 2. Central Michigan University, Mount Pleasant, Michigan, United States, 3. Central Michigan University College of Medicine, Mount Pleasant, Michigan, United States*

Diabetes is among the leading causes of morbidity and mortality in the United States, particularly in older adults. Prior research has demonstrated that social isolation is associated with worse diabetes outcomes, but the specific relationship between distinct living situations and healthcare utilization among older diabetic adults remains underexamined. This study aims to determine whether living situations, including living alone, living with a partner, or living with other adult(s), are associated with varying rates of hospitalizations, emergency department visits, and urgent care visits among older adults with diabetes. Data from the 2021 and 2022 National Health Interview Survey (NHIS) was analyzed via descriptive statistics, logistic regression, and zero-inflated negative binomial regression to investigate the relationship between the living situation and health outcomes as measured by overnight hospitalizations, number of urgent care visits, and emergency department visits of adults with diabetes over the age of 65. After adjusting for age, sex, race and ethnicity, education level, and comorbidities, participants living with a partner had significantly higher odds of visiting the emergency room when compared to individuals living alone. No significant differences were observed in urgent care visit frequency or hospitalization rates across living situations. This contrasts with the expectation that social isolation may drive increased healthcare utilization, suggesting that partners may encourage care-seeking behavior or results could reflect underlying differences in health status and access to outpatient care. Understanding factors contributing to increased ED visits is crucial for developing preventative measures to reduce the disease burden and financial burden associated with diabetes.

Abstract citation ID: igaf122.2462

### NEIGHBORHOOD COHESION'S ROLE IN SUPPORTING PERCEIVED MASTERY AND QUALITY OF LIFE IN OLDER ADULTS

Inhye Jung, and Hyo Jung Lee, *Yonsei University, Seoul, Republic of Korea*

Neighborhood cohesion has been linked to the health of older adults by influencing psychological and behavioral well-being. This study examines the role of neighborhood cohesion in older adults' health, with a particular focus on its support for perceived mastery from a relational autonomy perspective. Specifically, it investigates whether perceived mastery and self-rated health mediate the relationship between neighborhood cohesion and life satisfaction in older adults. Using data from the 2018 Health and Retirement Study (HRS) (N = 2,279), we conducted multiple mediation analyses with PROCESS Macro Model 6 to assess the mediating roles of psychological and health-related factors in this relationship. The findings reveal that higher neighborhood social cohesion is significantly associated with greater perceived mastery ($b = 0.104$, $p < .001$), which, in turn, is linked to better self-rated health ($b = 0.1172$, $p < .001$) and higher life satisfaction ($b = 0.3142$, $p < .001$). The primary mediation pathway (Neighborhood Cohesion → Perceived Mastery → Life Satisfaction) shows a significant indirect effect ($b = 0.0327$, 95% CI [0.0187, 0.0478]). Additionally, a secondary but significant mediation pathway (Neighborhood Cohesion → Perceived

Mastery → Self-Rated Health → Life Satisfaction) further contributes to the overall effect (b = 0.0022, 95% CI [0.0011, 0.0037]). The total indirect effect of neighborhood cohesion on life satisfaction is also significant (b = 0.0403, 95% CI [0.0248, 0.0569]). These results highlight the importance of fostering socially cohesive neighborhoods to enhance psychological resilience, health, and overall life satisfaction among older adults.

Abstract citation ID: igaf122.2463

### RESIDENTIAL CARE STATUS AND GENDER DIFFERENCES IN EXECUTIVE MEMORY AND DEPRESSIVE SYMPTOMS AMONG OLDER ADULTS

Idorenyin Udoh[1], and Rie Suzuki[2], *1. University of Michigan Flint, Flint, Michigan, United States, 2. University of Michigan-Flint, Flint, Michigan, United States*

Background: Older adults often experience declining memory and feelings of depression. This can further vary based on differences in their residential care status and gender. The purpose of this study is to examine if gender and residential care status differentiate in memory and depression among older adults so that caregivers and healthcare providers can tailor interventions to the specific needs of older adults. Methods: Data was analyzed from the National Health and Aging Trends Data set round 12, consisting of 6327 participants. Independent variables were residential location (community and residential facilities) and gender. Dependent variables were executive memory and depressive symptoms. Weighted descriptive statistics and two-way ANOVAs with post hoc tests were utilized. Results: Most participants were female (58.1%) and community residents (87.1%) aged between 65 and above 90 years. About 47.6% of participants reported executive memory issues, and 53.1% reported depressive symptoms. The results showed that males in residential facilities independently showed better executive memory function [F (1, 3006) = 4.446, p = .035]. The interaction between gender and residential location on executive memory function indicated that, compared to females, males living in residential care facilities showed better executive memory (p < .001). However, the direct and interaction effects did not show any mean differences in the presence of depressive symptoms. Conclusion: Findings highlight the importance of considering residential status and gender when designing interventions and support systems for older adults, enabling healthcare providers to offer more personalized and effective care.

Abstract citation ID: igaf122.2464

### SELF-RATED HEALTH AND LIVING STATUS ACROSS DIFFERENT AGES AT LATER LIFE: A POPULATION STUDY IN HONG KONG

Jasen Kin-Fung Leung, and Eliza Lai-Yi Wong, *The Chinese University of Hong Kong, Hong Kong, China (People's Republic)*

There is growing attention to the relationship of self-rated health (SRH) with living arrangements, especially when issues like social isolation are brought into the limelight, necessitating urgent social policy interventions. This study is a part of a population-level biannual patient experience survey conducted in Hong Kong to study the relationship between SRH and living arrangements across older age groups. Hong Kong residents, aged 18 or above, who stayed in a public hospital for at least a night, were contactable within two weeks after discharge, and were able to communicate in either Cantonese, English, or Mandarin, were eligible. A total of 32,562 participants, aged 51 to 109, were included from five waves of surveys between 2013 and 2023. Despite the mean of SRH scores peaking at 3.27 in 2019, it experienced a significant decline in 2023, reaching 3.03, a potential pandemic aftermath. Delving into SRH and living status, ANOVA results showed significant differences in SRH among older adults of different living arrangements. Tukey's HSD tests showed older adults aged 51-60 (means differences = -4.42, p-value = 0.00), 61-70 (means differences = -1.98, p-value = 0.00), and 71-80 (means differences = -1.39, p-value = 0.07), reported a significantly better SRH when they live with others; whilst among those who aged 81 or above, living alone is significantly associated with a better SRH (means differences = +2.62, p-value = 0.00). Future research could investigate the mechanisms underlying such discrepancies, for instance, how living and caregiving arrangements constitute older adults' subjective well-being.

Abstract citation ID: igaf122.2465

### SOCIAL DETERMINANTS OF HEALTH AND HEALTH CARE SEEKING: ANALYSIS OF A MEXICAN POPULATION-BASED SURVEY

Andrea Higareda-Garza-Ramos, Virgilio Hernández-Ruiz, José Ávila-Funes, and Juan García-Lara, *Instituto Nacional de Ciencias Médicas y Nutrición Salvador Zubirán, Mexico City, Distrito Federal, Mexico*

Disparities in social determinants of health (SDH) can hinder older people's health, regardless of their physical and functional status, for example, by limiting their access to health care. This study examines the association between SDH and the decision of older adults not to seek emergency medical care when needed, independent of their comorbidity status or intrinsic capacity. We conducted a secondary cross-sectional analysis derived from the 2021 National Survey on Health and Ageing in Mexico (ENASEM), which assesses ageing, disease impact, disability, and mortality among individuals aged ≥50 years. Weighted univariate and multivariate logistic regression models were performed to evaluate SDH as predictors of emergent healthcare-seeking behavior, adjusting for age, sex, intrinsic capacity, comorbidities, and functional status. Of 10,482 participants (median age: 71 years, IQR 65-77; 55.95% women), 560 (5.9%) had a serious health problem but did not seek medical care. For the main outcome, "being currently employed" was independently associated with higher odds of not seeking care (OR 1.64, 95% CI 1.02–2.63, p = 0.042), while "having social security" (OR 0.59, 95% CI 0.41–0.85, p = 0.004) and "benefiting from a social network" (OR 0.15, 95% CI 0.06–0.36, p < 0.0001) were found to be protective. Social programs, private transport, socioeconomic status, marital status, schooling, and rural vs urban location were not significantly associated. Our findings highlight the influence social factors may have on the access to emergency care, underscoring opportunities for advocacy for equity in the SDH.

Abstract citation ID: igaf122.2466

### SOCIAL SUPPORT, METABOLIC SYNDROME, AND WHITE MATTER HYPERINTENSITIES IN MEXICAN AMERICANS

Zoraida Garcia[1], Jordana Breton[2], Lourdes Romañach-Álvarez[2], and Elizabeth Muñoz[1], *1. University of Texas at Austin, Austin, Texas, United States, 2. The University of Texas at Austin, Austin, Texas, United States*

Mexican Americans (MA) are disproportionally affected by poor cardiovascular health, making them susceptible to brain impairment through White Matter Hyperintensities (WMH), increasing dementia risk. Metabolic Syndrome (MetS) is an early marker of cardiovascular disease, and one potential sociocultural factor that may be protective against conditions of MetS among MA is social support. We examined the link between MetS and WMH and whether social support moderated this association. Our sample consisted of 1,050 MA adults (ages 50-91) from the Health and Aging Brain Study: Health Disparities (HABS-HD). MetS was diagnosed using the Adult Treatment Panel III criteria (MetS+/MetS-), WMH volumes were measured through a 3T MRI System with higher values indicating more damage, and social support was calculated with the 12-item Interpersonal Support Evaluation List. We used linear regression to examine the independent effects of MetS and WMH volume, and an ANCOVA model to assess the interactive effects of social support on MetS and WMH volume. Models adjusted for age, sex, and education. Independent effects revealed that MetS+ status was significantly associated with higher WMH volumes ($B = 0.32$, $p < 0.05$, $R2=0.25$). This association did not vary by levels of social support WMH ($F[5,1041]=2.91$, $p=0.08$, $\eta p2<.001$). Results suggest that MetS+ exacerbates WMH, and that social support does not impact the link between MetS and WMH. Future studies should investigate other potential sociocultural resiliency factors that may buffer the impact of MetS on WMH in middle-aged and older MA adults.

Abstract citation ID: igaf122.2467

### STRESS PROCESSES, ACCULTURATION, GENDER AND MEMORY PERFORMANCE: A HABS-HD STUDY

Jordana Breton[1], and Elizabeth Muñoz[2], *1. The University of Texas at Austin, Austin, Texas, United States, 2. University of Texas at Austin, Austin, Texas, United States*

Latinos are at greater risk for dementia compared to non-Latino White adults, with women being at greater risk compared to men. Stress is a modifiable risk factor linked to cognition across gender; however, men and women experience stress differently. Higher acculturation is linked to better cognition, but it is unclear if it mitigates the negative effects of stress on cognition or whether these associations differ by gender, as acculturation levels differ by gender. We examined the interactive effects between stress exposure/appraisal (SE/SA) and acculturation on learning and memory in 260 men and 525 women who participated in the Health and Aging Brain Study: Health Disparities (Mean age= 63.16; range = 50-91). Participants self-reported gender, SE and SA, and acculturation. Outcomes included total learning and delayed recall trials from the Spanish English Verbal Learning Test. Gender stratified linear regression models examined SE/SA x acculturation on learning/memory outcomes. Gender stratified linear regression models revealed that higher SA was linked with better learning only for women with high acculturation ($B = -0.265$, $SE = 0.11$, $p = 0.016$). Higher SA was associated with better memory for men with high acculturation, but lower memory for men with low acculturation ($B = -0.432$, $SE = 0.171$, $p = 0.012$). There were no significant SE x acculturation interactions on learning/memory across gender. Findings suggest that high acculturation may be protective against the negative effects of SA for learning in women and memory in men, elucidating the heterogeneity in the protective role of sociocultural factors among Latinos.

Abstract citation ID: igaf122.2468

### THE CARE DEPENDENCY OF COMMUNITY-DWELLING OLDER ADULTS: A LATENT VARIABLE APPROACH USING ITEM RESPONSE THEORY

Alejandra Marroig[1], Fernando Massa[1], Janet Trujillo[1], Graciela Muniz-Terrera[2], and Sofia Domingorena[1], *1. Universidad de la Republica Uruguay, Montevideo, Montevideo, Uruguay, 2. Ohio University, Athens, Ohio, United States*

Background: Older adults living in the community may have functional limitations and require varying levels of assistance to satisfy their care needs. This study aimed to assess the individual degree of care needs in a population-based study of community-dwelling older adults. Using a latent variable approach, we assessed the degree of care dependency and studied its association with socioeconomic and health characteristics. Methods: We used data on adults aged 60 years and older from the Uruguayan population-based study Encuesta Longitudinal de Protección Social (ELPS) ($N = 5102$). To assess care needs, we considered 11 Activities of Daily Living and the self/proxy-reported information about the help needed to perform these activities. Using an Item Response Theory model, we estimated the dependency score (DS) used to measure the degree of care dependency. Then, we applied linear regression models to analyze the association between DS and age, sex, education, income, and health characteristics. Results: The results showed differences in the ADLs in which older adults with a low level of dependency and those with a high level of dependency needed assistance. Additionally, older women ($p = 0.002$) with multimorbidity ($p < 0.001$) and individuals with lower levels of education ($p = 0.006$) had the highest degree of care dependency. Conclusions: Social and healthcare public policies will benefit from considering the socioeconomic and health differences of older adults. The degree of care dependency should be considered for the design of services providing support to older adults with care dependency living in the community.

Abstract citation ID: igaf122.2469

### THE IMPACT OF INTRINSIC CAPACITY ON QUALITY OF LIFE IN OLDER ADULTS: A MULTIDIMENSIONAL SEM ANALYSIS

Zhen Wu[1], Lulu Xiao[1], Junyan Zeng[1], Ershuo Zhao[2], Haijun Zhao[3], Shixing Song[4], Yan Dong[5], and Linlin Hu[1], *1. Chinese Academy of Medical Sciences & Peking Union Medical College, Beijing, China (People's Republic), 2. Donghai County People's Hospital, Lianyungang, Jiangsu, China (People's Republic), 3. Ganyun County Traditional Chinese Medicine Hospital, Lianyungang, Jiangsu, China (People's Republic), 4. Guanyun County People's Hospital, Lianyungang, Jiangsu, China (People's Republic), 5. Nanjing Medical University, Nanjing, Jiangsu, China (People's Republic)*

Amidst global population aging, while the WHO's Intrinsic Capacity (IC) framework offers critical insights into multidimensional quality of life (QoL) determinants in older adults, systematic quantification of its clinical-social pathways remains limited. This cross-sectional study of 509 older adults (mean age 78.4±8.5 years) across community and institutional settings from China employed structural equation modeling with 5,000 bootstrap samples to examine pathways linking WHO-measured IC to EQ-5D-assessed QoL through frailty phenotype, Barthel Index (ADL), Visual Analog Scale (pain), Self-Rating Anxiety Scale, and Morse Fall Risk. The model demonstrated excellent fit ($\chi^2$/df=1.556, CFI=0.995, RMSEA=0.035). IC exhibited nonsignificant direct effects on QoL ($\beta$=0.019, p=0.416) versus robust indirect mediation through: Fall Risk ($\beta$=0.090, 95%CI[0.065,0.120], 92.3% mediation), Frailty($\beta$=0.045[0.029, 0.060]), ADL($\beta$=0.030[0.012, 0.050]), Pain ($\beta$=0.034[0.016, 0.050]), and Anxiety ($\beta$=0.004[0.000,0.010]). Moderation analyses revealed social support enhanced IC's effects via reduced fall risk ($\beta$=0.015[0.005, 0.025]) and Anxiety, while caregiver availability strengthened Frailty ($\beta$=0.027[0.012,0.042]) and ADL ($\beta$=0.024[0.008, 0.040]) pathways. Comorbidity burden universally amplified pathway impacts ($p<0.001$), most notably intensifying anxiety mediation by 90.0% ($\beta$=0.900[0.843, 0.957]). These findings elucidate Intrinsic Capacity's (IC) mechanisms influencing Quality of Life (QoL) through clinical mediators and social moderators. Social support and caregiver availability mitigate IC decline impacts, while comorbidity burden exacerbates effects, particularly amplifying anxiety mediation. Clinical priorities should emphasize fall risk screening, caregiver resource optimization, and comorbidity management. This study empirically validates the WHO IC framework, supporting precision health strategies that integrate biological resilience and social determinant optimization in aging populations.

Abstract citation ID: igaf122.2470

### THE SOCIAL DETERMINANTS OF SUBJECTIVE COGNITIVE DECLINE AMONG ADULTS AGE 45 AND OVER: IMPLICATIONS FOR ACTION

Iftekhar Amin, and Syeda Jesmin, *University of North Texas at Dallas, Dallas, Texas, United States*

Objective: Cognitive decline- often the first sign of dementia or Alzheimer's disease- is the gradual deterioration of one's ability to remember, learn new things, or make everyday decisions. Among older adults, subjective cognitive decline (SCD) is associated with age-related changes in the brain, injuries, substance use, and mental illnesses. Early detection and diagnosis of SCD have been prioritized in the Healthy People 2030 objectives. There is evidence that socioeconomic status is associated with cognitive decline, however, research is limited on the social determinants of SCD. Methods: We used data from the 2023 Behavioral Risk Factor Surveillance System (BRFSS). Logistic regression models have been used to examine the association between SCD and a range of social determinants among older adults aged 45 and over. Results: Several factors from the five domains of social determinants were significant predictors of SCD. Men (OR 2.9, 95% CI=2.1, 2.6), individuals without a college degree (OR 3.1, 95% CI=2.2, 3.2), and those with low income (OR 2.7, 95% CI=1.8, 2.5) were more likely to report SCD. With each additional year of age, the likelihood of SCD increased (OR 1.2, 95% CI=1.1, 1.6). People reporting isolation (OR 3.6, 95% CI=2.9, 4.1) and lower levels of social support (OR 4.2, 95% CI=3.3, 4.9) were more likely to experience SCD. Conclusion: Findings highlight the importance of increasing SCD interventions for men, individuals with low income and less education, and those who lack social support and experience isolation.

### SESSION 7150 (POSTER)

### SUCCESSFUL AGING

Abstract citation ID: igaf122.2471

### AGING POLICY ISSUES OF CONCERN TO OLDER ADULTS: A CASE STUDY OF OLDER ADULTS VOLUNTEER TEAM IN TAIWANESE HOSPITAL

Liying Chen[1], and Tsuann Kuo[2], *1. Taipei City Hospital Renai Branch, Taipei, Taipei, Taiwan (Republic of China), 2. Chung Shan Medical University, Taichung, Taichung, Taiwan (Republic of China)*

In 2021, Taiwan implemented the Aged-Society White Paper, which consisted of eleven domains including health and medical care, long-term care, social participation and learning, age discrimination, age-friendly environment, intergenerational learning, artificial intelligence technology, family and community, social security system, and research on aging society issues. However, there is limited research on the level of awareness and concerns about these issues among the older adults. . The purpose of this research was to explore the issues of concerns among hospital volunteers on the eleven domains from the Aged-Society White Paper actions. A Likert scale-based questionnaire was designed and data were analyzed to find out whether there are differences in the level of interest among different domain topics across different demographic variables. A total of 138 volunteers participated and the average age was 75. The results showed that the top concerns for older adults were health and medical care, followed by long-term care, and artificial intelligence technology. Significant differences were found in the artificial intelligence technology issue related to age. Significant differences were found in all issues related to education except long-term care, artificial intelligence technology, and research on aging society issues. Education and age were significant determinants in contributing to volunteers' awareness and interests. Furthermore, the implications for why the top issues can be expanded to older adults should be investigated. It is crucial to focus on the diversity of older adults and identify other Aged-Society policies that require further promotion across the life course.

Abstract citation ID: igaf122.2472

### CAN INCREASED EDUCATION TO THE PUBLIC OPTIMIZE AGING? RESULTS OF STRUCTURED INTERVIEWS WITH OLDER EXPERTS IN AGING

Sue Hazelett[1], and F. Michael Martin[2], *1. Summa Health System, Akron, Ohio, United States, 2. Retired, Williamsburg, Virginia, United States*

The first major cohort of experts in aging who developed the theories that guide today's best practices in geriatrics and gerontology are now experiencing aging themselves. We examined strategies used by retired aging-related experts throughout their own aging experience to determine whether the best practices that guided their professional practice impacted and are validated by their own lived aging experience. A convenience sample of experts age > 65 in the fields of gerontology, geriatrics, and/or aging were interviewed using a structured survey. Questions focused on their aging experience and how their career-associated expertise affected it. Nineteen interviews were completed with an average age of 75.4, 63% male, and 21% living alone. 16 of 19 (84%) indicated they have experienced physical or mental health issues and yet 95% consider themselves aging well. Factors rated by respondents as important contributors to this were maintaining purpose (89%), keeping/ forming relationships (100%), maintaining health (84%), coping with losses (100%), and preparing for functional decline (95%). They reported their professional knowledge allowed them to better prepare for inevitable losses through intentional physical activity, maintaining personal relationships, financial planning, and better lifestyle choices. 84% rated their previous career as important in helping them age successfully. These results suggest that greater efforts towards widespread education of community-based adults regarding common aging-related issues prior to them reaching retirement age could significantly improve the aging experience for large portions of the population by allowing them to prepare for likely age-related outcomes, just as these aging experts found for themselves.

Abstract citation ID: igaf122.2473

### CHORAL SINGING AND ITS EFFECT ON WELLNESS AMONG OLDER ADULTS: ENGAGEMENT, SELF-EXPRESSION, AND CONNECTION

Lenis Chen-Edinboro, Daniel Johnson, Hannah Catalano, and Tina Newsham, *The University of North Carolina at Wilmington, Wilmington, North Carolina, United States*

Music is an avenue for meaningful and life-long self-expression and well-being, especially for older adults. In this multiple case ethnographic study, we investigated connections between choral singing and wellness among people aged 67 to 80. We considered the effects of making music in six dimensions of holistic wellness: developmental, cognitive, physical, relational/social, emotional, and spiritual domains (Fullen, 2019). We interviewed six participants who engaged in shape note singing, a traditional American form of choral singing. We transcribed and analyzed data inductively and deductively, searching for themes within and across participants beginning with open coding to gain an overall understanding of participants' perceptions and experiences. We then conducted cross-participant analysis using focused coding to determine three emergent themes that described physiological and psychological health benefits of shape note singing: musical engagement, emotional response, and interpersonal connections. We found that older people who participated in shape note singing experienced several physiological and psychological health benefits across the considered dimensions of holistic wellness. Implications are for gerontologists to use this or other forms of making music to promote engagement, self-expression, and interpersonal connections. Other insights are to include music-based social interactions to reduce loneliness and isolation and to promote holistic wellness. Fullen, M. C. (2019). Defining wellness in older adulthood: Toward a comprehensive framework. Journal of Counseling & Development, 97(1), 62-74.

Abstract citation ID: igaf122.2474

### EXPLORING FACTORS INFLUENCING HEALTHY AGING IN FRAIL OLDER ADULTS: A MULTIDIMENSIONAL MODEL APPROACH

Rhayun Song[1], Eunna Oh[1], Jisu Seo[1], and Yuelin Li[2], *1. Chungnam National University, Daejeon, Republic of Korea, 2. University of Pittsburgh, Pittsburgh, Pennsylvania, United States*

The concept of healthy aging emphasizes maintaining independence, functional abilities, and overall quality of life, even in the presence of frailty and other age-related challenges. This study aimed to test a hypothesized model based on the multidimensional model of healthy aging and aging-related resilience theory. The model included cognitive and physical function, along with perceived health, as exogenous variables, while resilience, social support, and daily activity acted as mediators in explaining healthy aging among frail older adults. A cross-sectional study was conducted using data from a national survey of 505 frail older adults living in the community in South Korea. Data were collected between October and December 2023 and analyzed using IBM SPSS 26.0 and AMOS 26.0 for structural equation modeling. The model demonstrated a good fit, accounting for 43% of the variance in healthy aging. Resilience, cognitive function, and perceived health had both direct and indirect effects on healthy aging, while daily activity had a direct effect. Social support influenced healthy aging indirectly through daily activity, and physical function had an indirect effect via daily activity. Economic status was also identified as a significant determinant ($\beta = 0.14$, $p = .016$). These findings suggest that cognitive and physical function, mediated by resilience and social support, contribute to healthy aging by promoting daily activity. Health strategies should focus on strengthening resilience and social connectedness to enhance independence. Future research should further explore the impact of social and cultural activities in fostering active aging.

Abstract citation ID: igaf122.2475

### FACTORS ASSOCIATED WITH QUALITY OF LIFE OF INDIVIDUALS LIVING WITH PARKINSON'S DISEASE

Odunayo Arogundade, and Chelsea Spencer, *Kansas State University, Manhattan, Kansas, United States*

In the United States, Parkinson's disease (PD) is the second most common neurodegenerative disorder, and PD is related to a decreased quality of life (QoL), increased health care utilization, and economic costs. This study aimed to establish the key psychosocial factors influencing QoL in individuals with PD to help develop evidence-based interventions. A cross-sectional study involving eighty-five (85) individuals living with PD was conducted. Data were collected using validated scales, including the Parkinson's Disease Questionnaire (PDQ), Spiritual Quality of Life (SQL) scale, Personal Control (PC) scale, Illness Intrusion Rating (IIR) scale, and Quality of Life (QoL) scale. Descriptive and inferential statistics were used to analyze the data. The regression analysis showed that all the variables (personal control, illness intrusion, spiritual well-being, demographic characteristics, and social support) jointly accounted for 58.8% variance in QoL outcomes of people with PD. Higher PDQ and IIR scores were negatively correlated with QoL, while higher SQL and PC scores were positively associated with QoL. Gender and age variations also played a role in QoL outcomes. Interventions to help improve the QoL of individuals diagnosed with PD should take psychosocial factors, such as personal control, spiritual quality of life, as well as the progression of PD.: : Parkinson's Disease, quality of life, personal control, illness intrusion, spiritual well-being, social support

Abstract citation ID: igaf122.2476

### OLDER WOMEN AND SUCCESSFUL AGING: PHOTOVOICE REFLECTIONS ON ADAPTATION, COPING, AND CONNECTION

Melinda Heinz, *University of Northern Iowa, Cedar Falls, Iowa, United States*

Seven participants in a Lifelong Learning Institute engaged in a photovoice project on successful aging. Participants aged 68-80, identified as White, and had professional careers that they were retired from. They met over three class sessions to 1) receive information and an overview of the study and 2) share photographs and narratives with each other and 3) learn more about current research in the field of successful aging after data collection had concluded. During the second session, participant discussions were recorded and all photographs and narratives were retained for this study. Braun and Clarke's six-step thematic analysis approach was used for analysis. Steps include data familiarization, coding, early theme generation, refining themes, paring down themes, and results write up. Theme 1: Adapting and Coping revealed the importance of adapting to life challenges, with adjusting to widowhood as the most discussed life event. A subtheme on Challenging Yourself was noted and represented how participants enjoyed participating in lifelong learning and volunteering. Theme 2: Connections, included the diverse social resources that women had access to, such as friendships and connections with their faith/spirituality, as well as pets. Surprisingly, there was little discussion about the impact children or grandchildren had on successful aging. This finding differs from previous research and should be further explored in other groups of Baby Boomer women. It suggests that women may be more likely to consider resources outside of the home, not connected to traditional gender roles to age successfully and provides an interesting new direction for research.

Abstract citation ID: igaf122.2477

### PROMOTING LATER LIFE MEANINGFUL ENGAGEMENT: A CASE STUDY OF TECH-ASSISTED SOCIAL ENTERPRISE IN HONG KONG

Shiyu Lu, *The University of Hong Kong, Hong Kong, China (People's Republic)*

Background and Objectives: Meaningful engagement, defined as participation in enjoyable activities tailored to individual interests and abilities, is a critical factor in promoting a healthy and fulfilling later life for older adults. However, there is limited understanding of how to effectively foster such engagement in this population. Research Design and Methods: This study explores the innovative features of Happy Friend, a social enterprise project that engages older adults in diverse social and productive activities. Data were collected through one in-depth interview with the founder, two focus group interviews with staff, and six interviews with older adult participants. Thematic analysis was used to analyze the interview content. Results: The study identified a three-tiered stepped-up model for promoting meaningful engagement: 1) Joyful Community-Based Group Activities to strengthen social connections; 2) Role Transition and Empowerment, encouraging older adults to take on roles such as mentors to foster a sense of purpose; and 3) Self-Actualization and Societal Contribution, leveraging the skills of older adults who excel in leisure activities to combat ageism through collaborative partnerships. Additionally, key enablers of this model were identified, including co-production mechanisms, a low-cost technology-assisted platform to enhance autonomy for both providers and recipients, and a time-credit-based system to recognize talents and encourage sustained engagement. Discussion and Implications: These findings highlight the potential of a structured, strengths-based approach to fostering meaningful engagement in later life, offering practical insights for policymakers, practitioners, and communities aiming to enhance the well-being of older adults.

Abstract citation ID: igaf122.2478

### REWARDING VOLUNTEERING & ENHANCING PSYCHOLOGICAL MEANINGFULNESS IN VOLUNTEER ROLES BOOST LATE-LIFE WELL-BEING

Shiyu Lu, *The University of Hong Kong, Hong Kong, China (People's Republic)*

Although late-life volunteering is considered a health promotion strategy, few studies explored the characteristics of volunteering activities, including the psychological meaningfulness of volunteer roles, the perceived balance between rewards and efforts, and the types of activities that impact the healthy aging process. This study investigates how these dimensions of the volunteering experience influence subjective well-being (SWB) through the lens of belonging and empowerment processes. This cross-sectional study involved 457 volunteers aged 55 and above in Hong Kong from 2023 to 2024. The sense of belonging and general self-efficacy were utilized to capture the belonging and empowerment processes. Results from structural equation models indicated that the perceived balance between efforts and rewards in volunteer roles ($\beta = 0.83$, $p = 0.002$), sense of belonging ($\beta = 0.25$, $p < 0.001$), self-efficacy ($\beta = 0.31$, $p < 0.001$), and engagement in companionship volunteering ($\beta = 1.36$, $p = 0.005$) positively correlated with subjective well-being (SWB) among older adults. The SWB benefits of one-year volunteer hours ($\beta = 0.001$, $p = 0.032$) are mediated by the sense of belonging. Furthermore, the perceived balance between efforts and rewards ($\beta = 0.70$, $p = 0.001$) and the psychological meaningfulness in volunteer roles ($\beta = 1.09$, $p < 0.001$) are jointly mediated by both belonging and empowerment processes. These findings emphasize the significance of achieving a balanced sense of rewards and efforts and psychological meaningfulness in late-life volunteering. This study also highlights specific volunteering activities (e.g., companionship) that can create meaningful volunteering experiences for healthy aging.

Abstract citation ID: igaf122.2479

### UNDERSTANDING LATER-LIFE PLANNING IN SINGAPORE: PROFILES, SOCIAL DETERMINANTS, AND POLICY INSIGHTS

Bussarawan Teerawichitchainan, and Biying Yang, *National University of Singapore, Singapore, Singapore*

Planning for estate, long-term care (LTC), and advance care planning (ACP) is a crucial step in successful aging. However, most studies examine financial, LTC, and ACP preparedness separately, despite their interdependence. Additionally, prior research has largely focused on Western contexts, where individual autonomy and well-developed welfare states shape aging experiences. Less is known about later-life estate and healthcare planning in non-Western societies, where norms of family-based support and intergenerational dependence are deeply embedded. Singapore presents a globally relevant case due to its rapid population aging, strong filial piety norms, and proactive government initiatives in later-life planning. The country's centralized, mandatory, and multi-pillar approach integrates financial security, healthcare, housing, and social support, making it a global leader in structured aging policies. Using latent class analysis on a 2022 nationwide survey of 1,000 parents aged 50+, we identify four distinct profiles of later-life planning: comprehensive planners (9%), estate and LTC planners (31%), basic planners (32%), and the underprepared (28%). Furthermore, results from multinomial logistic regressions reveal socioeconomic inequalities, with higher socioeconomic status, Chinese ethnicity, and native-born individuals being significantly less likely to be underprepared. While marital satisfaction fosters preparedness, number of children is inversely related to planning, and social connectedness with friends and community enhances later-life preparedness. By highlighting Singapore's distinctive aging policies, our findings expand dimensions of successful aging and underscores the importance of integrated policy approaches that promote later-life preparedness across financial, LTC, and ACP domains.

Abstract citation ID: igaf122.2480

**UNDERSTANDING PHYSICAL RESILIENCE IN AGING: INSIGHTS FROM INTRINSIC CAPACITY DOMAINS**

Hyeon Jung Heselton[1], and Julie Blaskewicz Boron[2], *1. University of Nebraska Lincoln, Lincolne, Nebraska, United States, 2. University of Nebraska Omaha, Omaha, Nebraska, United States*

Maintaining a high quality of life is a central goal for aging adults, as it directly influences their well-being, independence, and overall health in later life. Older adults experience reduced physical resilience (PR), affecting their ability to recover from physical stressors. The World Health Organization's concept of intrinsic capacity (IC) captures functional potential and resilience across multiple domains. This study explored physical resilience (PR; loss of balance) through three physical IC domains: vitality (handgrip strength), sensory (hearing and vision), and locomotion (walking speed). It also investigated whether age moderated the IC and PR relationship. Forty-six healthy middle-aged (n = 25; 52% female; 55.8±7.02yrs) and older (n = 21; 47.6% female;70.14±3.55yrs) adults completed five standing trials on a force platform with perturbations to measure PR. Results indicated that higher handgrip strength and faster walking speed were significantly associated with lower loss of balance ($F(1,44)=6.03$, $p = 0.018$, $R^2=0.12$; $F(1,44)=4.88$, $p = 0.032$, $R^2=0.10$, respectively). Hearing and vision did not predict PR. Age did not significantly influence the IC and PR relationship. Results revealed consistent results from previous research that handgrip strength and walking speed are strong predictors of PR, whereas sensory IC may be more associated with compensatory mechanisms such as proprioception and vestibular function in maintaining balance. It is well-known that biological changes with age affect physical functions, but a more comprehensive understanding of IC, along with other factors (physical activity, diet, sleep) and its interaction with aging, is essential for better insight into functional resilience in later life.

Abstract citation ID: igaf122.2481

**UNDERSTANDING SUCCESSFUL AGING: THE POWER OF GROWTH MINDSETS AND OPENNESS TO EXPERIENCE**

Jessica Fleck, Katherine Wilkinson, Tanya Sharma, Mackenzie Unsworth, Ava Marie Cacia, Claire Jacobus, and Janina Costello, *Stockton University, Galloway, New Jersey, United States*

Successful aging has evolved beyond merely maintaining physical health and independence to encompass life satisfaction and overall well-being. Consequently, the elements that may contribute to successful aging have expanded to include personality traits, such as a growth mindset and openness to experiences. A growth mindset refers to the belief that an individual's actions and efforts are more influential than predetermined factors, such as age or genetics, in determining outcomes. Openness, on the other hand, reflects a person's willingness to consider new ideas and experiences and may promote successful aging, as individuals high in openness are more likely to engage in a diverse range of activities, which helps build cognitive reserve. We examined the roles of growth mindset and openness in successful aging among adults aged 60 to 80 years. We hypothesized that greater openness would be linked to increased engagement, and that both openness and growth mindset would predict cognitive and emotional aspects of successful aging. Our findings supported this hypothesis, showing that higher levels of openness and a growth mindset were associated with better aging outcomes, including improved emotion regulation and attention regulation. However, when we included both openness and growth mindset as predictors in a regression model of successful aging, only growth mindset emerged as a significant predictor. Mediation analyses revealed that openness mediated the relationship between years of education and growth mindset, and that growth mindset mediated the relationship between openness and successful aging. Our research supports the role of personality and mindsets in aging outcomes.

### SESSION 7155 (POSTER)

### TRANSPORTATION

Abstract citation ID: igaf122.2482

**BRIDGING THE GAP: TRANSPORTATION, AGING, AND ACCESS TO HEALTHCARE**

Zeve Marcus, and Katherine Freund, *ITNAmerica, Westbrook, Maine, United States*

ITNAmerica, the first national nonprofit transportation network for older adults and people with mobility challenges, is dedicated to promoting lifelong mobility. By integrating a 178-field research database into ITNRides' transportation technology, ITNAmerica has built a 1.6 million ride database to provide critical insights into how mobility influences the social determinants of health. With support from the CareQuest Institute for Oral Health, ITNAmerica is conducting a multi-phase research initiative to examine how transportation impacts healthcare access, particularly oral healthcare, for older adults. This project is in three phases: 1) migrating 1.6 million rides from 26 legacy databases into a unified, multi-tenant database on the Salesforce platform to improve data integrity and accessibility; 2) conducting a detailed statistical analysis of these rides to identify transportation patterns and disparities; and 3) planning a longitudinal study to assess the long-term impact of transportation on health outcomes. Transportation is vital for older adults to access healthcare, social engagement, and daily activities, yet barriers such as limited mobility, geographic isolation, and affordability contribute to disparities, particularly among low-income, rural, and minority populations. By leveraging decades of transportation data and engaging both a Scientific Advisory Council and a Diversity Panel, this initiative ensures that research findings reflect the needs of underserved communities while generating actionable insights to inform policies and programs that promote health equity. This project provides a unique opportunity to understand and address transportation as a critical factor in healthy aging.

Abstract citation ID: igaf122.2483

**HOMEBOUND OLDER ADULTS AND TRANSPORTATION BARRIERS TO SOCIAL AND COMMUNITY ACTIVITIES**

Kelly Vences[1], Angelina Gutierrez[1], Brian Fons[2], and Namkee Choi[2], *1. The University of Texas School of Social Work, Austin, Texas, United States, 2. The University of Texas at Austin, Austin, Texas, United States*

Although a homebound state in late life is often a result of cognitive and/or physical/functional health problems, transportation barriers may also be an important contributor. We used the 2023 National Health and Aging Trends Study (N = 7,543) to examine the associations of homebound states (defined as the past- month going-out frequency <1 a week) with driving status and self-reported transportation barriers to social/community activities. We used generalized linear models (GLM) to examine the associations. The results showed that homebound older adults comprised 5.2% of Medicare beneficiaries age 65 and older. Only 27% reported driving in the past month, and 54.2% and 32.1% reported health-related and transportation barriers to participating in social/community activities, respectively. GLM results were that homebound older adults were more likely to have stopped driving in the past year than their peers who went out 2-4 days a week (incident risk ratios [IRR]=2.28, 95% CI = 1.52-3.43) and those who went out 5+ days a week (IRR=7.20, 95% CI = 4.34-11.97). Homebound older adults were also more likely to report transportation barriers than those who went out 2-4 days a week (IRR=1.30, 95% CI = 1.06-1.58) and those who went out 5+ days a week (IRR=1.99, 95% CI = 1.50-2.65). Less than 10% of homebound older adults used taxi/ride-hailing services and public transportation. The study suggests that older adults' homebound state significantly correlates with transportation barriers. Reliable transportation services appropriate to older adults' capabilities are needed to facilitate their participation in social/community activities.

Abstract citation ID: igaf122.2484

### HOW ARE THEY DIFFERENT? COMPARISON BETWEEN OLDER ADULTS WHO LIMIT THEIR DRIVING AND THOSE WHO CEASE DRIVING

Yunji Han[1], and Yeji Hwang[1], *1. Seoul National University, Seoul, Republic of Korea*

While some older adults gradually avoid driving long distances or refrain from driving in adverse weather conditions, others completely stop driving. Lack of information is available on the differences between those who restrict their driving and those who completely stop driving. Therefore, this study aimed to identify the differences between older adults who self-restrict their driving and those who completely stop driving. This study employed a secondary data analysis using the Health and Retirement Study data from 2014 to 2018 on individuals who were 60 years and older and who self-restricted their driving at baseline (N = 629). For data analysis, participants were divided into two groups: (1) Those who continued to self-restrict their driving from 2014 to 2018 (n = 502, 79.8%), and (2) those who self-restricted their driving in 2014 but completely ceased driving in 2018 (n = 127, 20.2%). Chi-square tests and independent t-tests were performed to evaluate the differences between the two groups. The ceased-driving group were more likely to have a higher prevalence of dementia ($\chi^2$=5.880, p = 0.015) or depression ($\chi^2$=4.012, p = 0.045). Additionally, there was a significant difference in subjective health (t = 2.194, df = 626, p = 0.029), with the ceased-driving group reporting poor subjective health scores. The findings showed that the transition from limited driving to complete driving cessation in older adults is related to one's cognitive function, psychological health and subjective health. Attentions are needed for older adults who have such conditions to delay complete driving cessation and to support them with substitute measures for transportation.

Abstract citation ID: igaf122.2485

### PREPARING FOR TAKEOFF: ADDRESSING IN-FLIGHT CHALLENGES FACED BY PERSONS LIVING WITH DEMENTIA AND TRAVEL COMPANIONS

Colleen Peterson[1], Renee St. Louis[1], Tamara Statz[2], and Maria O'Reilly[3], *1. University of Michigan Transportation Research Institute, Ann Arbor, Michigan, United States, 2. Vibrant Living, St. Paul, Minnesota, United States, 3. CQUniversity Australia, Bundaberg, Queensland, Australia*

Addressing the challenges faced by travelers with dementia and their companions requires developing accessible services based on inclusive principles, dementia awareness, and a willingness to listen to the voices of those living with dementia. Although increasing awareness of the needs of travelers with dementia has seen positive changes within airports globally (e.g., the sunflower lanyard), the needs and experiences of travelers with dementia in-flight are less well understood. This poster combines findings from two similar studies conducted in the USA and Australia exploring in-flight issues from the perspective of travelers with dementia (n = 55), their companions (n = 217). We share the consistent themes on in-flight challenges that were found in both studies, with additional insight from flight crew in the Australian study (n = 21). In-flight challenges included anxiety, confusion, toilet access and use, awareness of available assistance, negative flight crew interactions, and seating arrangements. We share participants' own strategies for preventing or mitigating the issues they have faced and make additional recommendations for air travel guidelines to support safe travel.

Abstract citation ID: igaf122.2486

### THE EXPERIENCES AND JOB PERFORMANCE OF OLDER PARATRANSIT DRIVERS

Shayna Gleason, *International Transportation Learning Center / Transit Workforce Center, Washington, District of Columbia, United States*

Public transportation's frontline workforce is already considerably older than the U.S. workforce overall, and getting older. Few if any prior studies have examined the performance and experiences of older paratransit drivers, despite their growing prevalence and paratransit's growing need for qualified drivers of any age. The present study filled this knowledge gap by examining the relationships between driver age and various aspects of job performance, as well as the subjective experiences of older paratransit drivers of their jobs. The research questions for this study were: 1) What is the relationship between driver age and driver job performance? 2) What are older paratransit drivers' experiences of aging in their positions? Quantitative data from ACCESS Transportation Systems—the Pittsburgh, PA regional paratransit system—were used to answer the first research question. The associations between driver age and frequency of accidents, passenger complaints, and passenger compliments were analyzed using zero-inflated negative binomial regressions. The second research question was answered using qualitative data collected in a semi-structured focus group of eight ACCESS drivers. The quantitative results suggested that age is negatively associated with accidents, compliments, and complaints. The qualitative results indicated a high level of job satisfaction among participants, often driven by altruistic motives, though they acknowledged challenges of the job, some of which they expected to be exacerbated by age-related physical or cognitive changes. The results reveal an important and continuing role for older drivers in paratransit services, and an urgent need to focus on effective recruitment and retention initiatives for this population.

### SESSION 7160 (POSTER)

### UNDERREPRESENTED POPULATIONS

Abstract citation ID: igaf122.2487

### CHARACTERISTICS OF HIKIKOMORI CASES AGED 50-64 RECEIVING FINANCIAL AND DAILY SUPPORT FROM OLDER PARENTS IN JAPAN

Kiyoko Fukushima[1], Noriko Tsukada[2], and Asako Katsumata[1], *1. Japan Lutheran College, Mitaka, Tokyo, Japan, 2. Nihon University, Tokyo, Tokyo, Japan*

This study examines the characteristics of hikikomori cases aged 50-64 in Japan by comparing them with cases aged 15-49. Japan has approximately 613,000 socially withdrawn individuals aged 40-64, with 91,000 never leaving home. Using data from a February 2024 survey of 1,735 municipal social welfare councils (response rate: 28.4%), we analyzed 312 cases where support led to observable changes. Among them, 81 cases (26.0%) involved individuals aged 50-64. Findings show that 78.8% were male, 61.5% had work experience, and 82.3% had an illness or disability. Before support, 85.6% lived with their family, and after support, 72.7% lived with their family. Even after support, 35.8% lived with their father and 55.1% lived with their mother. Compared to the 15-49 age group, those aged 50-64 were significantly more likely to have work experience ($p<0.001$), less likely to have experienced truancy ($p<0.001$), and less likely to live with family ($p<0.001$). The duration of social withdrawal was significantly longer in the 50-64 group (17.3 years vs. 10.6 years, $p<0.001$). However, no significant differences were observed in gender, education, illness, living arrangements, or family acceptance levels (e.g., their perception of hikikomori status). Despite longer withdrawal periods, post-support recovery time showed no significant differences between age groups. These results highlight the importance of early intervention through community outreach to foster change. Strengthening trust-based relationships and multi-organizational collaboration is crucial for supporting aging parents and their withdrawn adult children in reconnecting with society.

Abstract citation ID: igaf122.2488

### DEMENTIA SCREENING INTENTION AMONG AMERICAN INDIANS: THE ROLE OF CULTURAL ORIENTATION AND SELF-EFFICACY

Heehyul Moon[1], Yeon-Shim Lee[2], and Soonhee Roh[3], *1. University of Louisville, Louisville, Kentucky, United States, 2. San Francisco State University, San Francisco, California, United States, 3. University of South Dakota, Sioux Falls, South Dakota, United States*

Up to one-third of Native American older adults may develop Alzheimer's Disease and Related Dementias (ADRD). With increasing life expectancy, the number is projected to increase nearly four-fold by 2060. Despite the benefits of early diagnosis, screening rates remain low due to barriers such as cultural beliefs and limited health literacy. This study examined the associations between cultural orientation and dementia screening intention among AI adults in the Yankton Sioux Tribe (YST), with self-efficacy as a potential mediator. A cross-sectional survey was conducted among YST adults ($N=248$) recruited through community events (e.g., powwows) using convenience sampling in September 2024. Given low screening rates, this study applied the Theory of Reasoned Action, using screening intention as a proxy for actual behavior. Participants had a mean age of 45 years (range: 18–81); 55% were female, and 65% had a bachelor's degree or less. Regression analyses revealed that stronger identification with tribal traditions significantly predicted both higher self-efficacy and greater screening intention. Self-efficacy also significantly predicted screening intention. After accounting for self-efficacy, the direct effect of cultural identity on screening intention was reduced but remained significant, indicating partial mediation. These findings suggest that cultural identity enhances screening intention in part by increasing confidence in seeking screening. Findings underscore the need for culturally responsive, self-efficacy-enhancing interventions to improve dementia screening uptake in AI communities. Future research should explore additional psychosocial factors influencing screening behaviors to develop tailored interventions that promote early diagnosis and better health outcomes.

Abstract citation ID: igaf122.2489

### LONGITUDINAL ASSOCIATIONS BETWEEN ANXIETY, FRAILTY, AND AGING PERCEPTIONS AMONG HISPANIC OLDER ADULTS

Janet Lopez[1], Dahee Kim[1], Yi Liu[1], Rui Xie[1], Abigail Tice[1], Michael Dino[2], Ayse Malatyali[1], and Ladda Thiamwong[1], *1. University of Central Florida, Orlando, Florida, United States, 2. University of Central Florida, University of Central Florida, Florida, United States*

Aging perceptions significantly impact health outcomes for older adults, especially among Hispanic populations facing unique challenges. While cross-sectional studies have examined relationships between psychological factors, physical health indicators, and aging perceptions, longitudinal relationships remain understudied, particularly among Hispanic older adults. This study aimed to examine how anxiety and frailty are associated with aging perceptions over time. We analyzed data from 87 Hispanic older adults who participated in a Technology-Based Body and Mind Intervention to Prevent Falls. They were recruited from independent living facilities and community centers in Central Florida. Generalized Linear Mixed Models (GLMMs) examined relationships between aging perceptions (B-APQ total score and five subscales: timeline-chronic, emotion representation, control-positive, consequence-positive, control consequence-negative) and key predictors, including anxiety (GAI-SF) and frailty (short FRAIL scale) across four time points. Models incorporated these predictors as fixed effects with subject-level random effects to account for repeated measures. Outcomes showed that anxiety was significantly associated with higher scores on the B-APQ Chronic subscale ($\beta=0.358$, $SE=0.138$, $p=0.010$). Both anxiety ($\beta=0.362$, $SE=0.134$, $p=0.007$) and frailty ($\beta=0.546$, $SE=0.266$, $p=0.041$) were significantly associated with increased emotional representation scores. No significant associations were found for the remaining B-APQ subscales. Our study highlights the association between psychological factors and frailty, emphasizing the need for culturally sensitive interventions to promote healthy aging. Its findings underscore the importance of addressing anxiety and frailty issues to improve aging perceptions and enhance well-being among Hispanic older adults. This approach has the potential to positively impact aging perceptions in this population, leading to improved health outcomes.

Abstract citation ID: igaf122.2490

### MIDNIGHT MUSINGS: EXPLORING SLEEP, HOME ENVIRONMENT, AND MENTAL HEALTH IN HISPANIC/LATINO COMMUNITIES

Aziza Siddiqui[1], Srujana Chekuri[2], Julie Blaskewicz Boron[1], Ibrahim Bilau[3], and Eunhwa Yang[3], *1. University of Nebraska Omaha, Omaha, Nebraska, United States, 2. University of Nebraska Omaha, Elkhorn, Nebraska, United States, 3. Georgia Institute of Technology, Atlanta, Georgia, United States*

Sleep quality is closely tied to home environment factors including safety and satisfaction with indoor environmental conditions, which significantly influence mental health outcomes. In Hispanic/Latino communities, where resource access may be limited, the home environment may disproportionately impact well-being, particularly for older adults. This study examined the interplay between sleep patterns, home satisfaction, safety, and mental health in aging Hispanic/Latino adults (N = 30, mean age=67.1, SD = 5.24, range= 60-82). A series of regression analyses were used to examine the relationship between home environmental factors and self-reported mental health outcomes. The results indicated that home safety ($p < 0.05$) and concerns about sleep problems ($p < 0.05$) were significantly associated with self-reported depression scores. Similarly, impaired quality of life ($p < 0.05$) and concerns about sleep problems ($p < 0.05$) were significantly associated with self-reported anxiety scores. In line with these findings, significant correlations emerged between poor sleep quality and higher levels of depression (r=.49, p=.003) and anxiety (r=.34, p=.03). Lower home satisfaction was associated with higher depression (r=-.39, p=.01) and anxiety (r=-.63, p<.001). These findings highlight the urgent need for innovative approaches to aging in underrepresented communities, emphasizing the critical role of the home environment in improving sleep, mental health, and overall well-being. This research underscores the need for culturally tailored innovations in housing and mental health support to improve outcomes for underserved aging populations. Future initiatives should integrate physical (e.g., adaptive home design) and emotional (e.g., social support) elements to enhance the overall aging experience.

Abstract citation ID: igaf122.2491

### THE INFLUENCE OF THE AMAZON ECHO DOT ON LONELINESS AMONG OLDER ADULTS LIVING ALONE IN UNDERSERVED COMMUNITIES

Rie Suzuki, Charlotte Tang, Jennifer Blackwood, Brent Gasser, Ashton Herrick, and Annaliese Elliott, *University of Michigan-Flint, Flint, Michigan, United States*

Loneliness is associated with a higher mortality rate in older adults. Virtual home assistants (VHAs) offer potential benefits for social engagement, but their specific impact on loneliness among older adults in underserved communities is not well documented. The purpose of this pilot study was to examine the effects of VHA use on loneliness in older adults living alone. A pre-post mixed methods study was conducted from 2024 to 2025 with participants from four senior centers in the Greater Flint Area. Inclusion criteria were being 60 years or older, living alone, self-reported isolation, smartphone use, Wi-Fi access, no prior VHA use, and a modified cognitive status telephone interview with a score of 21 or higher. Participants received an Echo Dot, a smart light, and $100 for participating in the study, with eight-week weekly follow-ups. Of the 13 who completed the study, most were white, single (divorced or widowed), female, and had some college education. At baseline, 54% of participants were dissatisfied with AI technology. In the semi-structured follow-up interview, most stated that the device had not significantly reduced loneliness in their daily lives. However, some appreciated features such as music and interaction and commented on their value to society. Others acknowledged the benefits of the device in alleviating loneliness and commented that they found it enjoyable to be 'present' and receive advice. The positive feedback from participants and belief in the future use of feasible VHAs suggest that they have the potential to tackle loneliness among older adults in underserved communities.

### SESSION 7165 (POSTER)

### WOMEN

Abstract citation ID: igaf122.2492

### ATTITUDES TOWARD AGING AMONG WIDOWED WOMEN: IDENTIFYING PROFILES AND ASSOCIATED FACTORS

Yeonsoo Shin[1], Juhyeong Lee[2], and Giyeon Kim[3], *1. The University of Texas at Austin, Austin, Texas, United States, 2. The University of British Columbia, Vancouver, British Columbia, Canada, 3. Chung-Ang University, Seoul, Republic of Korea*

Although previous research reports that widowed middle-aged and older adults' attitudes toward aging are closely linked to their psychological well-being and health status, their distinct patterns of attitudes toward aging remain understudied. This study aims to identify patterns of attitudes toward aging among widowed middle-aged and older women and examine factors associated with these profiles. Using a nationally representative sample drawn from the 2020 and 2022 Health and Retirement Study (HRS), this study analyzes 1,223 widowed women, aged 48 to 99. The dataset includes responses to the Leave-Behind Questionnaire, which was distributed to half of the participants in 2020 and the other half in 2022. Attitudes toward aging were assessed using eight items on a six-point scale. Results from Latent Profile Analysis identified three attitudinal profiles based on attitudes toward aging, with higher scores indicating a more positive perception of aging: Low (36.7%), Middle (30.7%), and High (32.5%). Results from multinomial logistic regression showed that, compared to the Low group, older age (OR = 0.95, p<.001) and higher depressive symptoms (OR = 0.75, p<.001) were associated with lower odds of belonging to the High group. In contrast, those with better self-rated health (OR = 1.57, p<.001) were more likely to be in the High group. These findings highlight the role of self-rated health in fostering positive attitudes toward aging, while older age and depressive symptoms may be linked to more negative perceptions. These results suggest opportunities for interventions to support widowed women develop a more positive perspective on aging.

Abstract citation ID: igaf122.2493

### BEYOND MARITAL DISSOLUTION: ROLE TRANSITIONS AND IDENTITY RECONSTRUCTION OF DIVORCED OLDER WOMEN

Shiau-Fang Chao[1], Yueh-Tzu Wang[1], Chien-Chou Hou[2], and Ju-Ping Lin[3], *1. National Taiwan University, Taipei, Taipei, Taiwan (Republic of China), 2. Chung Shan Medical University, Taichung, Taichung, Taiwan (Republic of China),*

*3. National Taiwan Normal University, Taipei, Taipei, Taiwan (Republic of China)*

Background: Divorced women in Taiwanese and Chinese cultures face significant social stigma, yet research on their experiences remains limited. Applying the Gendered Life Course Perspective, this study examines how divorced older women's marital transitions shape their identity reconstruction and how they establish new social roles beyond wifehood. Methods: This qualitative study used semi-structured interviews with 12 divorced older women from diverse backgrounds. Thematic analysis identified patterns in identity reconstruction and role transitions post-divorce. Findings: Divorced older women's role transitions and identity reconstruction reveal distinctive patterns:1.Traditional roles and marital adversity: Participants endured unequal labor division, extensive caregiving, and significant conflicts, including infidelity and domestic violence. Many prioritized family stability over personal wellbeing, reflecting internalized gender expectations that defined their identities through wifehood and motherhood. 2. Identity disruption and reconstruction: Post-divorce, women faced economic vulnerability, emotional strain, health complications, and social stigma, forcing them to reconstruct their social identities beyond former roles as wives. 3. Community integration as identity reinforcement: Participants actively sought community involvement, social services, and employment opportunities to validate their reconstructed identities, creating spaces where their post-divorce identities could be affirmed outside traditional family structures. Conclusions and Implications: Divorced older women undergo significant role transitions that reshape their identities and aging experiences. Their post-divorce identity reconstruction highlights the need for targeted policies addressing economic vulnerability while creating community spaces that validate their new social roles. Interventions should include emotional support systems and reminiscence programs that help process past compromises, reducing stigma while promoting community integration.

Abstract citation ID: igaf122.2494

### REDEFINING AGING WITHOUT FAMILY SUPPORT: WELFARE NEEDS & SERVICE USE AMONG NEVER-MARRIED OLDER WOMEN

Shiau-Fang Chao[1], Yueh-Tzu Wang[1], Chien-Chou Hou[2], and Ju-Ping Lin[3], *1. National Taiwan University, Taipei, Taipei, Taiwan (Republic of China), 2. Chung Shan Medical University, Taichung, Taichung, Taiwan (Republic of China), 3. National Taiwan Normal University, Taipei, Taipei, Taiwan (Republic of China)*

Background: Never-married women constitute a significant yet understudied segment of Taiwan's older adult population. Unlike their married counterparts, these women deliberately chose independence and careers over traditional marriage paths. Using the Gendered Life Course Perspective, this study examines how these early-life choices shape their aging trajectories and welfare needs. Methods: This qualitative study employed semi-structured in-depth interviews with 12 purposively sampled participants from diverse sources, ensuring variation in age and educational background. Data were analyzed using thematic analysis to identify key patterns and themes. Findings: Never-married older women's experiences reveal distinct patterns in redefining aging without traditional family support. 1. Deliberate autonomy reshaping independence: Participants intentionally remained unmarried, prioritizing careers and personal independence over societal expectations, fundamentally restructuring their aging experience. 2. Self-sufficiency as adaptive strategy: Without spousal or child support, these women developed enhanced resilience that enables them to navigate aging with greater self-determination. 3. Specialized welfare and community needs: Unlike married older adults, these women prioritize government-supported housing, formal advance care planning mechanisms, and senior communities. They express strong preferences for living arrangements that provide social opportunities and life support systems while maintaining independent living with the convenience and security of community environments—services that strategically substitute for family-based support. Conclusions and Implications: Never-married older women's deliberate life choices shape unique aging experiences and welfare needs. Policies should respond with targeted housing programs, community-based supports, and advance care planning mechanisms that substitute for family assistance—acknowledging how these women redefine aging through independence while addressing structural service gaps.

Abstract citation ID: igaf122.2495

### SEX-STRATIFIED SKELETAL TREATMENT EFFECTS FROM THE INVEST IN BONE HEALTH RANDOMIZED CLINICAL TRIAL

Kristen Beavers[1], Delanie Lynch[1], Marjorie Howard[1], Leon Lenchik[1], Ashley Weaver[1], Sarah Wherry[2], Barbara Nicklas[1], and Daniel Beavers[3], *1. Wake Forest University School of Medicine, Winston-Salem, North Carolina, United States, 2. University of Colorado Anschutz Medical Campus, Aurora, Colorado, United States, 3. Wake Forest University, Winston Salem, North Carolina, United States*

The main goal of the INVEST in Bone Health Trial (NCT04076618) was to compare effects of weight loss (WL) alone (caloric restriction targeting 10% WL), WL plus weighted vest use (WL+VEST; 8 hours/day, weight replacement titrated up to 10% total WL), or WL plus progressive resistance training (WL+RT; 3 supervised sessions/week) on bone health indicators in older adults. As women are more likely to develop osteoporosis and experience fractures, the purpose of this secondary analysis was to present 12-month skeletal treatment effects [including: volumetric bone mineral density (vBMD), areal (a) BMD, and biomarkers of bone turnover (procollagen type 1 N-terminal propeptide (P1NP) and C-terminal cross-linked telopeptide (CTX))] stratified by sex. 150 (74.7% women) older adults (66.4±4.6 years; 68.6% White) living with overweight or obesity (BMI: 33.6±3.3kg/m2) were randomized (n = 50/group) with 133 (89%) completing the trial. Baseline age, weight, height, cardiovascular disease prevalence, and physical activity energy expenditure were higher in men than women. No sex differences were noted in intervention compliance (e.g., weight loss, vest wear-time, RT session attendance). In women, WL+VEST attenuated total hip vBMD loss [estimated treatment difference (ETD): +0.0019 mg/cm3 (0.0005, 0.0034); p = 0.008] and increased P1NP [ETD: +7.6 µg/L (0.8, 14.3); p = 0.014] versus WL alone. WL+RT also increased P1NP versus WL alone [ETD: +5.3 µg/L (-0.3, 10.9); p = 0.028] in women. Conversely, in men WL+VEST exaggerated femoral neck aBMD loss versus WL alone [ETD: -0.038 g/cm2 (-0.066, -0.009); p = 0.012]. In older women, but not men, undergoing caloric restriction, weighted vest use may reduce bone loss via increased bone formation.

Abstract citation ID: igaf122.2496
**THE ASSOCIATION OF PERCEIVED DISCRIMINATION TO FUNCTIONAL LIMITATION AMONG IMMIGRANT WOMEN LIVING IN THE U.S.**
Heather Hunter, and Corey Nagel, *University of Arkansas for Medical Sciences, Little Rock, Arkansas, United States*
The purpose of this study was to examine if perceived discrimination of middle aged and older (50+) immigrant women is associated with a decline in functional status. Immigrants moving to the United States face unique challenges in meeting their healthcare needs. They have increased risk for experiencing discrimination, which impacts health negatively. One area, discrimination, may effect functional ability, which is important to maintaining independence and involvement in community. Thus, we aimed to understand if discrimination has a relationship with functional status among a sample of middle aged and older (50+) immigrant women who participated in the Health and Retirement Study (HRS) during the period 2008-2018 (n = 2,773). We used Poisson regression with robust standard errors to examine the association between perceived discrimination (using responses from the Perceived Everyday Discrimination Scale) and functional status (counts of assistance required for ADLs and IADLs). In our sample, 36% reported discrimination and 17% reported frequent discrimination. Our models indicated that immigrant women who report experiencing any discrimination have 1.25 times greater number of limitations than women who are not experiencing discrimination (RR = 1.25; 95% CI = 1.04, 1.51; p = 0.020), and those immigrant women experiencing frequent discrimination have 1.39 times greater number of limitations than women who are not experiencing frequent discrimination (RR = 1.39; 95% CI = 1.12, 1.73; p; = 0.003). Understanding the relationship between discrimination and functional health is important for healthcare professionals to anticipate the needs of immigrant women and respond with appropriate interventions aimed at improving and sustaining functional health.

Abstract citation ID: igaf122.2497
**USING THEORY TO CONDUCT QUALITATIVE RESEARCH WITH PEOPLE AGING WITH DISABILITY**
Tracie Harrison, *University of Iowa College of Nursing, Iowa City, Iowa, United States*
The use of theory to shape analysis of qualitative data while planning the study, collecting data in the field, and analyzing the data through select methods is necessary to arrive at meaningful findings in response to a research question. The lack of theoretical guidance in gerontological research within a biased interpretation of meaning and/or historical information can result in skewed findings. Although books and articles have been written about the process of using theory in qualitative research few have clarified how the study of aging populations can challenge disciplinary paradigms resulting in ethical concerns. This can leave the interpretation of complex data to a re-imagination or a best-alignment type of conclusion. In this examination of process and outcome between theory and data, the author suggests that an awareness of paradigmatic conflict must be identified or the voice of people from disparate backgrounds who have lived through multiple transitions with age will be lost, reinforcing disciplinary or analyst beliefs while disregarding complex dissention. Using data from ethnographic data collected from 133 people (age 55 and older) with 3-4 community interviews each, I demonstrate how conflict threaded along historical, individual, and disciplinary paradigms may exist. Further, the use of theory to anticipate the layering of ethical conflict within the interpretation of findings is provided.

### SESSION 7170 (POSTER)

### WORKFORCE

Abstract citation ID: igaf122.2498
**ALIGNING OPPORTUNITY AND MISSION: THE IMPACT OF HEALTH CARE CONTRACTING ON AAAS**
Elizabeth Blair[1], Suzanne Kunkel[2], and Abbe Lackmeyer[2], *1. USAging, Washington, District of Columbia, United States, 2. Miami University, Oxford, Ohio, United States*
Area Agencies on Aging (AAAs) and other community-based aging and disability organizations (CBOs) are increasingly contracting with health care entities to provide integrated care; these contracts can also increase revenue for CBOs and allow them to better serve their communities. As policymakers and other stakeholders increasingly support the formation of these contracting partnerships, it is important to understand their impact on AAAs. This session will describe AAA's experiences with contracting and its impact on their organizational culture. National data will be shared from the 2023 CBO-Health Care Contracting Survey and a qualitative study on the impact of contracting on AAAs. Thirteen individuals representing 10 AAAs participated in the 2024 qualitative study, which consisted of one in-person focus group and five individual interviews. Participants shared their contracting experiences, how contracting affected their organizational operations and culture, and their change-management strategies. Findings include: the importance of mission alignment in deciding to contract; communicating this decision to staff and leadership; and the impact of health care contracting on staffing, data infrastructure, organizational structure and culture. Findings from the CBO-Health Care Contracting Survey also show how organizational challenges related to contracting—such as staff resistance to change and integration of AAA services into health care system workflow—have changed over time. This session will discuss additional ways that policymakers and others can support AAAs in their contracting efforts, such as providing funding for AAA data infrastructure and capacity development and conducting additional research to identify the elements of a successful contracting partnership.

Abstract citation ID: igaf122.2499
**COMMUNITY RATES OF OLDER ADULT EMPLOYMENT IN NEW ENGLAND: FINDINGS FROM THE 2025 HEALTHY AGING DATA REPORTS**
Shan Qu, Taylor Jansen, Qian Song, Nina Silverstein, and Elizabeth Dugan, *University of Massachusetts Boston, Boston, Massachusetts, United States*
Human longevity makes it possible for us to keep working longer, enjoying the relationships and satisfaction of productive activity. However, for some older adults, the high cost of living may require working past age 65 to make ends meet. This study examined and compared community employment rates in four New England states: Rhode Island (RI), Connecticut (CT), New Hampshire (NH), and Massachusetts (MA). Better understanding older adult employment rates at the community level can inform local policies and services. Rates of being employed in the past year among adults 65+ were calculated from data from the 5-year files in the American Community Survey

(2012-2016 MA&NH, and 2014-2018 CT&RI). Small area estimation techniques were used to calculate age-sex adjusted community rates. Statewide rates showed that nearly one in four (24.8%) older adults in CT and NH were employed in the past year, which was higher than MA (24.3%) and RI (21.9%). However, MA reported the largest range in 65+ employment (4.5%-63.8%) compared to NH (0.0%-52.2%), CT (14.0%-44.2%), and RI (10.0%-46.3%). Aquinnah MA had the highest community rate in the four states (66.73%). Communities in these four states with the highest rates of older workers reported smaller older populations, lower rates of Hispanic/Latino adults 65+, lower rates living below the poverty line, with higher depression, ADRD, diabetes, and higher median household income (in RI and CT only). The future of work will increasingly include older workers. We think employers should consider flexible work arrangements as inducements to attract and retain older workers.

Abstract citation ID: igaf122.2500

### NAVIGATING WORK AND AGING IN OLDER ADULTS WITH SICKLE CELL DISEASE: PURSUITS, CHALLENGES, AND EARLY RETIREMENT

Rania Mohamed[1], Tamara Poole[2], Francyess Denis Oliva[1], Teagan Callaway[1], John Strouse[1], and Charity Oyedeji[1], *1. Duke University School of Medicine, Durham, North Carolina, United States, 2. Duke University School of Nursing, Durham, North Carolina, United States*

Older adults with sickle cell disease (SCD) face age-related and SCD comorbidities leading to early functional decline, making it hard to work. This forces individuals to make tough decisions about their career ambitions. There is little research on work-related experiences of older adults with SCD. This study aims to depict work-related challenges faced by older adults with SCD.19 older adults with SCD were enrolled (age ≥ 50) and conducted semi-structured interviews about their experiences living with SCD and work-related obstacles. We used conventional content analysis to analyze transcripts. The mean age was 58 years (range 50-71) and 53% were male. Nearly half were externally employed (47%), 5% unemployed, 11% retired, 32% disabled, and 5% self-employed. There were three major themes. Theme 1 was " Obstacles faced pursuing work ". Participants discussed barriers to advanced education, lack of resources to find jobs, rejection from jobs due to SCD, and narrowing job pursuits to those with limited physical demands. Theme 2 was "Challenges while working". Participants discussed the complications, SCD pain, and physical limitations that interfered with work schedules and tasks. Participants described a lack of understanding from their employers and unreasonable expectations to immediately return to work following discharge from the hospital. Theme 3 was "Decisions to retire early". Participants described needing to give up their business, retire early, or take periods of disability. This data from older adults with SCD revealed the need for greater awareness, support, and accommodation in the job market and workplace for individuals with SCD.

Abstract citation ID: igaf122.2501

### OLDER ADULT EXPERIENCES PURSING ENCORE CAREERS IN RESEARCH AT AN AGE-FRIENDLY UNIVERSITY: LONGITUDINAL EVALUATION

Kathryn Nearing[1], and Joanna Fitzgibbons[2], *1. University of Colorado Anschutz Medical Campus, Denver, Colorado, United States, 2. University of Colorado Anschutz Medical Campus, Aurora, Colorado, United States*

In 2022, the University of Colorado Anschutz Medical Campus launched a new training program to prepare older adults (average age 68; range: 54- 82) to pursue encore careers in research. The 14-week, 200-hour competency-based training program prepared Older Adult Research Specialists (OARS) for community-facing and participant-centered roles that research study coordinators typically fulfill on study teams: community outreach and engagement, initial screening for eligibility, facilitating informed consent, and addressing social needs that can impede study participation. The overall goal of the training-to-employment pathway program was to increase the age-inclusiveness of the research workforce at CU Anschutz and enhance the capacity of research teams to reach, recruit and retain older adults and other populations in clinical trials. Within 3 years, we trained six cohorts and graduated 78 OARS. This session will feature the longitudinal evaluation of this innovative initiative, focusing on interviews with OARS hired at CU Anschutz and the principal investigators of associated studies. OARS' key informant interviews were conducted by an independent program evaluator 3-, 6- and 12-months after they were hired. Key themes pertain to OARS' roles on study teams, longevity in roles, sense of preparedness for role, ongoing needs for professional development, job satisfaction, and OARS' impact on potential and enrolled study participants, research teams and research studies. This session will also explore the challenges faced by program leaders in advocating for the hiring of OARS, key barriers OARS experienced in the hiring process, and OARS' key sources of stress and coping in their demanding encore careers.

Abstract citation ID: igaf122.2502

### THE ASSOCIATION BETWEEN CAREGIVING INTENSITY AND CAREGIVER ENGAGEMENT IN PAID WORK

Shan Qu, Qian Song, and Jeffrey Burr, *University of Massachusetts Boston, Boston, Massachusetts, United States*

Framed within the Role Strain Theory, this study examined the relationship between caregiving intensity and engagement in paid work among caregivers aged 25-61 who provided care for older adults. Using data from the National Health and Aging Trends Study and the National Study of Caregiving, we estimated multilevel models to identify within- and between-person effects. Caregiving intensity was measured with number of caregiving hours provided in the previous month (logged for skewness). Paid work was dichotomized as '1=not engaged in paid work, 0=engaged in paid work.' Our results showed that as caregiving hours increased, the likelihood of not engaging in paid work increased for both within-person effects (OR = 1.01, 95%CI=1.00-1.02) and between-person effects (OR = 1.02; 95%CI=1.01-1.04). We also evaluated whether these relationships were non-linear by including a quadric term for hours of caregiving. The results showed a marginally significant non-linear relationship for both within-person effects and between-person effects, demonstrating the possibility that higher levels of caregiving intensity were more impactful in terms of not engaging in paid work than was the case for lower levels of caregiving intensity. Additional results showed that female caregivers and those with higher education were more likely to engage in paid work. Hispanic caregivers were less likely to be engaged in paid than non-Hispanic white caregivers. Sustained high-intensity caregiving increases the likelihood of caregivers not engaging in paid work over time. These findings underscore the need for programs that promote respite care and other services designed to help caregivers remain active in the paid labor market.

Abstract citation ID: igaf122.2503

### THE NOVEL INTEGRATION OF A LEARNING COMMUNITY MODEL IN OLDER ADULT WORKFORCE DEVELOPMENT RESEARCH

Jennifer Crittenden[1], Mary Lou Ciolfi[2], Lenard Kaye[3], Rachel Coleman[1], Richa Arya[4], and Catherine Taylor[4], *1. University of Maine Center on Aging, Orono, Maine, United States, 2. UMaine Center on Aging, Orono, Maine, United States, 3. University of Maine, orono, Maine, United States, 4. University of Maine, Bangor, Maine, United States*

Learning communities foster sharing of knowledge and skills in educational and workplace settings but are infrequently used in research. An AmeriCorps Seniors-funded research project at the University of Maine Center on Aging focused on older adult workforce development programs (WDPs) established a learning community (LC) to identify lessons learned and provide peer support and education across seven WDPs. Early findings shed light on the opportunities and challenges of implementing an LC model within a research initiative. Located across five U.S. states, LC members meet monthly via Zoom for discussions, small group activities, and guest speakers. Integration of the LC in the research process has yielded substantial insight into the challenges facing older adult WDPs. Challenges noted by participants include: 1) building relationships and trust between members, primarily attributed to the online modality making it difficult to form meaningful connections; 2) organizational stressors (e.g., high turnover of WDP staff, lack of time for meaningful participation); and 3) concern about program performance monitoring which may hamper candid communication. LC model adaptation and creativity have been key for overcoming these challenges. Incorporating short interactive activities at the start of meetings and regular program updates by participants with structured time for sharing and mutual assistance has facilitated relationship building, as has the presence of LC staff "champions". Learning communities can provide opportunities for growth and development for program participants and valuable insight for researchers. Responsive and adaptive strategies can increase engagement and effectiveness when using an LC in research contexts using online modalities.

Abstract citation ID: igaf122.2504

### UNDERSTANDING THE VALUE OF AGING LIFE CARE MANAGERS THROUGH THE EYES OF FAMILIES AND CARE RECIPIENTS

Carlisle Shealy[1], Pamela Teaster[1], Jullie Gray[2], and Laura Sands[1], *1. Virginia Tech, Blacksburg, Virginia, United States, 2. University of Washington, Seattle, Washington, United States*

Aging Life Care Professionals (ALCPs), also known as care managers, play a vital role in supporting families, caregivers, and older adults. With expertise in aging and disability, they help families navigate evolving care needs, including health changes, living transitions, and resource coordination. To assess the value of ALCPs, we conducted 17 virtual interviews with family members and care recipients, exploring reasons for hiring a care manager, services received, satisfaction, and perceived impact. The Aging Life Care Association (ALCA), a nonprofit promoting ALCPs, facilitated participant connections. Interviews were transcribed and thematically coded by the investigators. Families hired care managers for various reasons, including overseeing care, coordinating transitions, acting as intermediaries with medical providers, connecting clients to resources, honoring patient wishes, and supporting caregiver decision-making. Overall, care managers exceeded expectations, demonstrating attentiveness, proactive monitoring, and a strong commitment to care. Participants valued their trustworthiness, transparency, crisis responsiveness, and expertise in managing complex diseases. This study underscores the essential role of ALCPs in enhancing care quality, easing caregiver burden, and ensuring that older adults receive the support they need.

### SESSION 7175 (POSTER)

### AGE INCLUSIVITY IN HIGHER EDUCATION

Abstract citation ID: igaf122.2505

### IT'S NEVER TOO EARLY: HOW COLLEGE STUDENTS VIEW AGING AND PREPARE FOR IT

Kelly O'Sullivan[1], Raven Weaver[1], Jill Juris[2], and Cory Bolkan[3], *1. Washington State University, Pullman, Washington, United States, 2. Appalachian State University, Boone, North Carolina, United States, 3. Washington State University, Vancouver, Washington, United States*

As society ages rapidly, people must prepare for their own healthy aging trajectories. Perceptions of aging and expressions of ageism negatively influence aging experiences, contributing to poorer outcomes (e.g., chronic disease, decreased independence, earlier mortality). In contrast, aging preparedness (e.g., advanced care planning, financial security, retirement planning) leads to better outcomes, including increased autonomy and independence. This study examined how college students ($N = 277$, Mage=19.83; 66.1% White) perceived their own aging in relation to family, friends, religion, leisure, lifestyle, money, work, and health, and how these perceptions related to their preparedness for aging. We sought to answer two research questions: (1) how do college students perceive aging in these domains? and (2) how do perceptions of aging influence views of aging preparedness? Results indicated that students generally held positive aging perceptions, expressing optimism about anticipated later life experiences. Linear regression revealed significant associations between perceptions of aging preparedness in later life and perceptions of friends ($p = 0.016$), religion ($p < 0.001$), leisure ($p = 0.008$), lifestyle ($p = 0.044$), money ($p = 0.010$), and health ($p < 0.001$). These findings suggest that it is never too early for college students to prepare for aging. Students with positive perceptions of aging felt more prepared for older adulthood; thus, it is important to disrupt negative perceptions of aging early in life, which has the potential to improve health outcomes and foster greater autonomy in later years.

Abstract citation ID: igaf122.2506

### MOTIVATION, ACADEMIC ANXIETY, DEPRESSION AND WELL-BEING AMONG DOCTORAL STUDENTS OVER 50

Michelle Ackerman[1], and Bettina Shapira[2], *1. National University, Sioux Falls, South Dakota, United States, 2. National University, San Diego, California, United States*

Increased accessibility to doctoral programs has resulted in a growing trend of doctoral recipients over the age of 45 (6.9% in 2020; NCSES, 2020). Recent studies suggest doctoral students are at significantly higher risk of negative mental health outcomes such as anxiety and depression compared to the general population and current employees with high levels of education (Leveque et al., 2017; Evens et al., 2018). Older doctoral students may experience greater loneliness, more doubt in their academic abilities, have more difficulty sustaining motivation (Usher & McCormack, 2021) and may have different motivations than traditional-age students (Stehlik, 2011; Templeton, 2021). However, while there is a body of research investigating the association of age with doctoral student outcomes, few previous studies have investigated doctoral students who enrolled age 50+. Social media recruitment yielded 303 participants for this quantitative correlational study. Qualtrics online surveys included demographic questions, questions about motivation to enroll, Academic Anxiety Scale, Patient Health Questionnaire-2, Generalized Anxiety Disorder 2-item, and WHO-5 Well-Being Index. The highest reported motivation for enrolling was interest in the subject matter and love of learning (23.2%), followed by pursuing an academic career (17.9%), and to upgrade, improve or expand employment-related knowledge/ qualifications/skills (16.2%). Age, gender, number of children in the home, household income, course type and university type were associated with mental health outcomes. Discussion focuses on supporting older doctoral students to promote success and well-being. Conclusions are relevant for current and potential older adult doctoral students as well as administrators and faculty of doctoral programs.

Abstract citation ID: igaf122.2507

### TAKING US OUT OF OURSELVES: USING CLASSROOM INTERGENERATIONAL READING GROUPS TO CONNECT YOUNGER/OLDER ADULTS

Marjorie Getz, *Methodist College, Peoria, Illinois, United States*

Communities become better connected when people from different ages and backgrounds become equals working together for a common goal. We report on a service-learning project focused on intergenerational reading of books related to successful aging, which brought people with different life perspectives together for shared educational experiences. Experiential learning projects are key for the gerontology certificate program at a midwestern nursing/health science college. All courses are focused on increased understanding of course content related to aging through project reflective practices, direct contact with older adults, exposure to themes related to community health and well-being, and an improved sense of community service/civic responsibility. The service-learning opportunities for each course (and the certificate curriculum itself) fit a hierarchy leading to increased competence and skills development when working with older adults. This project incorporated data from the reading group service-learning project between undergraduate students and members of a local OLLI program (ongoing since 2018). Forty-seven students (across time, interacting with 80 OLLI participants) provided reflective data about the project experience. Qualitative analyses were used; and content analyses of students' reflections identified five themes: (a) communication skills development/enhancement (b) appreciation of older adults as vital community members; (c) processes involved in a successful reading group; (d) recognition of a variety of settings for learning opportunities in support of development as lifelong learners and for professional growth; and (e) appreciation of collaborative teamwork and expanded definition of peer. Other experiential learning activities in this joint nursing/gerontology curriculum are highlighted

Abstract citation ID: igaf122.2508

### THE IMPACT OF EMPATHY AND DEMOGRAPHIC FACTORS ON FIRST-YEAR HEALTH STUDENTS' PERCEPTIONS OF AN AGE-FRIENDLY CAMPUS

Michal Elboim-Gabyzon[1], Sigal Pearl Naim[1], Malka Doron[2], Tal Kohli-Hailovski[1], and Anna Zisberg[1], *1. University of Haifa, Haifa, HaZafon, Israel, 2. The Center for Research and Study of Aging, University of Haifa, Israel*

The concept of an "Age-Friendly-University" embodies a paradigm that fosters inclusivity and intergenerational engagement in higher education. As populations age, universities should support both traditional students and older adults, cultivating professional attitudes that value contributions from all age groups. To facilitate this transformation, it's essential to assess students' perceptions regarding age-inclusivity. We hypothesized that demographic factors and empathy influence students' these perceptions, grounded in emotional intelligence and intergroup contact theories, which suggest that higher empathy decreases ageist biases and promotes intergenerational connections. This study examined factors shaping first-year health students' views on age inclusivity and campus age-friendliness using subscales of the Age-Friendly Inventory and Campus Climate Survey (ICCS) and whether empathy place a role. A cross-sectional survey was administered to 248 health students (mean age = 23.87±4). Regression analyses of demographic characteristics including age, volunteering, working with older adults and ageistic-attitudes, revealed that age was the only significant predictor for Personal Beliefs about Age Inclusivity ($\beta = 0.193$, $p = 0.003$). Including the Interpersonal Reactivity index as a measure of empathy identified it as a central predictor ($\beta = 0.315$, $p < 0.001$), almost dabbling the explained variance. Perceptions of campus age-friendliness were significantly predicted by better economic status ($\beta = 0.109$, $p = 0.026$) and lower ageist attitudes ($\beta = 0.152$, $p < 0.01$). These findings underscore empathy's vital role in shaping age-inclusive attitudes. Developing structured, experiential learning opportunities that enhance students' empathy may serve as a transformative strategy for embedding age-friendly values within healthcare education, preparing future professionals for intergenerational settings.

Abstract citation ID: igaf122.2509

### USF AGE DISSEMINATION PROJECT: BRIDGING THE GAP FOR HEALTHY AGING THROUGH ACADEMIC RESEARCH

Jessica VanderWerf, Natalia Babenko, Lindsay Peterson, and Kathy Black, *University of South Florida, Tampa, Florida, United States*

Academic research can be difficult for older adults to access, creating barriers to raising awareness about healthy aging and improving quality of life in later years. Limited access means that older individuals may miss out on insights into disease prevention, mental well-being, and lifestyle interventions that could enhance their longevity and independence. Disseminating research in an accessible and engaging manner is essential, as it empowers older adults with knowledge, informs caregivers and policymakers, and promotes the adoption of evidence-based practices that support aging with dignity and well-being. The USF Age Dissemination Project seeks to bridge this gap by enhancing academic outreach while providing a replicable model for other universities. This initiative employs sustainable methods for distributing research to communities of older adults in a format that is both understandable and practical. This poster presents the early stages of this project, outlining its framework and offering guidelines for other institutions looking to implement similar initiatives. Specifically, the project involves the development of a product called "News We Can Use to Age Well", a structured template designed to disseminate research to older adults outside academia. The poster describes the initial steps of the process, which involved evaluating articles, analyzing them for accessibility, and translating them into an age-friendly format that is easy to understand and apply. The poster also discusses future steps aimed at expanding the project's reach, impact, and community engagement with university research. By improving access to knowledge, this initiative aims to empower older adults and support healthy, informed aging.

### SESSION 7180 (POSTER)

### ALCOHOL AND ADDICTIONS

Abstract citation ID: igaf122.2510

### A LATENT CLASS ANALYSIS OF HEALTH CHARACTERISTICS AMONG NON-DRINKING MIDDLE-AGED AND OLDER ADULTS

Sara Miller, Jennifer Maggs, and Stephanie Lanza, *The Pennsylvania State University, University Park, Pennsylvania, United States*

Despite documented health consequences of heavy alcohol consumption, there is ongoing debate around potential health benefits of low-to-moderate alcohol use. Better understanding variability in health characteristics among non-drinkers can inform discourse around the alcohol j-curve and underscore the diverse health profiles of adults who abstain in later life. The current study aimed to identify latent classes of non-drinkers based on subjective and objective health characteristics and examine person-level predictors of class membership. Participants were adults aged 50+ years in the Midlife in the United States study who did not report past-month alcohol use (N = 1,205; range=50-84 years). Dichotomous health indicators included any functional impairment, obesity, cardiometabolic risk factors (diabetes, high blood pressure, or stroke), chronic pain, chronic sleep problems, self-reported physical health (1=Fair/Poor; 2=Excellent/Very Good/Good), perceived health in comparison to others (1=Somewhat Worse/Much Worse, 2=Much Better/Somewhat Better/About the Same), chronic depression and/or anxiety, and self-reported mental health (1=Fair/Poor; 2=Excellent/Very Good/Good). Person-level predictors included age (1=Older Adult, 0=Middle-Aged), sex (1=Male, 0=Female), alcohol use history (1=Lifetime Abstainer, 0=Former Drinker), and education (1=College Degree or Higher, 0=Less Than College Degree). Five latent classes were identified: poor overall health (5.9%), poor physical and self-reported health but good mental health (17.9%), poor physical health but good self-reported and mental health (28.5%), good self-reported health but poor physical and mental health (9.8%), and good overall health (38.0%). Age, education, and biological sex, but not alcohol use history, predicted probability of class membership. Future research can explore how the underlying latent classes predict distal health outcomes.

Abstract citation ID: igaf122.2511

### EARLY LIFE ADVERSITY PREDICTS MORTALITY RISK THROUGH SUBSTANCE USE IN ADULTHOOD

Sarah Miller, Meredith Willard, and Nicholas Turiano, *West Virginia University, Morgantown, West Virginia, United States*

The lifespan effects of adverse childhood experiences (ACEs) on elevated mortality risk during adulthood are well established (Felitti et al., 1998). However, the mechanisms driving this association are less well understood. The self-medication hypothesis suggests that those experiencing ACEs could cope by using alcohol, tobacco, and drugs during adolescence and emerging adulthood. However, less is known how these behavioral patterns persist into adulthood and old age. Thus, we used data from 6,253 adults (aged 25-75) participating in the Midlife in the United States (MIDUS) study to determine if adults with higher ACEs were more likely to use substances, and subsequently be more likely to die. Mortality data was obtained from the National Death Index between 1995-2022 (deceased=2,229; Msurvival time=24.08 years). Number of alcoholic drinks consumed in an average drinking occasion during heaviest drinking year (M = 3.51), any illicit drug use (12.24% yes), and number of cigarettes smoked per day during heaviest smoking year (M = 16.92) were used as mediators of the ACE-mortality association using parallel mediation in a Cox modeling analysis. Higher ACEs and both alcohol and tobacco use were associated with an increased hazard of dying (p's < .05), with both indirect effects also significant. Those exposed to ACEs drank more alcohol and smoked more cigarettes in adulthood, which contributed to their increased risk of death. Results reveal that ACEs have a lasting impact on health well into adulthood and that these behavioral pathways should be closely monitored among populations exposed to high levels of ACEs.

Abstract citation ID: igaf122.2512

### GENDER, SOCIAL TIES, AND OLDER ADULT ALCOHOL USE

Eric Vogelsang, and Zackary Zanotelli, *California State University, San Bernardino, San Bernardino, California, United States*

There is little empirical research focusing on how community, family connections, and gender may influence the risk of older adult alcohol use. This is important, given growing concerns over (a) the long-term effects of even moderate alcohol use worldwide, (b) growing alcohol consumption among women, and (c) health impacts of social isolation. Using The Irish Longitudinal Study on Ageing (18,805 observations from 7,705 respondents), we examine how "social ties" (family characteristics and social participation) predict three measures of alcohol use and abuse among adults aged 50-plus. We also test to what extent alcohol use and its correlates may differ by gender. Our results indicate that having three or more close friends (when compared to having no close friends) is associated with regular alcohol consumption, and (for men) greater odds of possible alcohol abuse. We also find that, for women, being a member of a social organization is associated with greater odds of drinking, when compared to those who are not. Unlike similar prior studies, religious participation (which was more common among women) was negatively associated with all three types of alcohol consumption. Last, and contrary to expectations, greater education was positively associated with all three measures of alcohol use and abuse. In total, these findings suggest one potential drawback to close social ties in older ages. They also suggest ways in which a country's history and gendered social norms may influence health behavior in later life.

Abstract citation ID: igaf122.2513

**PRO AND ANTI-TOBACCO MESSAGING EXPOSURE IN A NATIONALLY REPRESENTATIVE SAMPLE OF U.S. OLDER ADULTS**

Margaret Fahey[1], and Siyuan Huang[2], *1. Middle Tennessee State University, Murfreesboro, Tennessee, United States, 2. University of Arizona, Tucson, Arizona, United States*

Adults are more impacted by the negative health consequences of tobacco use as they age, yet cigarette prevalence rates have stagnated among U.S. adults 65+ years for the past 15 years. Despite the harm reduction potential of switching from cigarettes to e-cigarettes, older adults are more likely to misperceive the relative harms of these products. With changing patterns of media consumption, updated information is needed regarding the platforms in which this age group receives tobacco messaging. Using U.S. nationally representative data from the 2022 Health Information and National Trends Survey, we measured pro- and anti-tobacco messaging exposure among adults 65+ years via internet (television/streaming, social media, websites) and non-internet (stores, billboards, pharmacies, bars/restaurants, events, radio, print) platforms. Secondarily, we assessed main effects and interactions of age (65+ vs. < 65 years) and cigarette smoking in relation to tobacco messaging platforms and harm beliefs. Pro-tobacco messaging mostly occurred via stores (46%), television/streaming (11%), and billboards (9%) among older adults who smoke cigarettes. Anti-tobacco messaging mostly occurred via television/streaming (53%), stores (28%), and print media (25%). Older (vs. younger) adults were less likely to receive pro-tobacco messaging across internet and non-internet platforms (p's<.03) and less likely to receive anti-tobacco messaging via non-internet platforms (p<.001). No age differences were found for anti-tobacco messaging via internet platforms (p>.72). Finally, older adults were more likely to report not knowing about relative e-cigarette harms (p<.001). Among this high priority population, television and streaming platforms might be a modality in which to disseminate tobacco harm reduction education.

Abstract citation ID: igaf122.2514

**RISK FACTORS FOR PRESCRIPTION OPIOID MISUSE IN OLDER ADULTS: FINDINGS FROM THE NATIONAL SURVEY ON DRUG USE AND HEALTH**

Andie MacNeil, and David Burnes, *University of Toronto, Toronto, Ontario, Canada*

Prescription opioid misuse is recognized as a significant public health concern and social problem because it significantly increases the risk of both fatal and non-fatal overdose. Although a large body of research has examined risk factors for prescription opioid misuse, this research has disproportionately focused on younger adults, even though older adults are more likely to have an opioid prescription than any other age cohort. To address this gap in the literature, this study analyzed nationally representative data from the 2023 National Survey on Drug Use and Health to identify risk factors for past-year prescription opioid misuse among older adults (n = 9,762). Stepwise binary logistic regression analyses were conducted to examine risk factors for prescription opioid misuse. In the first model, which only accounted for sociodemographics, risk factors for opioid misuse were being divorced (OR = 1.60; 95% CI: 1.15-2.22) and having an annual income less than $20,000 (OR = 1.55; 95% CI: 1.01-2.39). When physical health factors were added to the model, income was no longer associated with misuse and poor self-rated health was found to be associated with opioid misuse (OR = 1.94; 95% CI: 1.44-2.63). When mental health and other substance use factors were added in the final model, self-rated health remained significantly associated with misuse (OR = 1.70; 95% CI: 1.24-2.34) and a lifetime history of depression was also found to be associated with misuse (OR = 2.06; 95% CI: 1.47-2.88). Findings from this study help inform our basic knowledge on risk factors for prescription opioid misuse, which can help inform targeted screening and intervention.

Abstract citation ID: igaf122.2515

**VARIATION IN SPATIAL ACCESS TO SUBSTANCE USE TREATMENT FOR OLDER ADULTS: A NATIONWIDE AND KANSAS-SPECIFIC ANALYSIS**

Elizabeth Bambury[1], J. Tom Mueller[2], Elizabeth Ablah[2], and Sharon Fitzgerald Wolff[2], *1. University of Kansas Medical Center, Andover, Massachusetts, United States, 2. University of Kansas Medical Center, Kansas City, Kansas, United States*

Substance use disorder is often overlooked and undertreated among older adults – a growing population especially in rural America. Treatment programs specifically tailored to meet their age-related needs can improve recovery outcomes. This study examines spatial access to these specialized substance use treatment (SUT) programs both nation-wide and with a detailed focus on the state of Kansas. We obtain data on SUT facilities from the 2022 National Directory of Drug and Alcohol Abuse Treatment Facilities. Distances between census tract centroids and facility locations were measured, and spatial accessibility was assessed using a two-step floating catchment area method to generate a spatial access index (SPAI). For older Kansans, a second SPAI using driving distances was computed. Results are displayed nationally and for the state of Kansas, and also stratified by rurality using Rural-Urban Commuting Area Codes. Of 12,102 identified SUT facilities, 3,614 (29.9%) offered programs for older adults. Among 84,120 census tracts, 4,323 had no such facility within 50 miles, affecting 2,589,240 adults aged 65 and older. On average, the nearest older adult program was 13 miles away (SD = 19), with small rural tracts averaging 34 miles farther than urban tracts (p < 0.001). SPAI values further highlighted disparities in rural areas, even after accounting for service demand. Our findings reveal that for millions of older adults, particularly in rural areas, distance poses a significant barrier to substance use treatment. Expanding access to specialized treatment programs in underserved areas is essential to promote healthier aging among older adults.

### SESSION 7185 (POSTER)

### ALZHEIMER'S DISEASE AND RELATED DEMENTIAS II

Abstract citation ID: igaf122.2516

**ASSESSING STRESS LEVELS IN OLDER ADULTS DURING COGNITIVE AND MOTOR TESTING TO ADVANCE EARLIER DEMENTIA SCREENING**

Bernardo Villa-Sánchez, Alexandra Reed, Brianna Branson, Elyana Chacon, and Sydney Schaefer, *Arizona State University, Tempe, Arizona, United States*

Dementia is the second most feared diagnosis among adults, yet only a small percentage undergo cognitive testing for early signs during routine visits with primary care providers. It is possible that one reason for the low rate of routine screening may be patients' resistance to testing, suggesting that anticipating or completing a cognitive assessment may induce stress in older adults. The primary purpose of this study was to determine the stress responses of older adults immediately prior to and during cognitive screening. This study also compared responses between a traditional (Montreal Cognitive Assessment, MoCA) and a novel (Brief Evaluation of Cognition and Daily Function, BEAN) assessment. Twenty-one unimpaired older adults were randomly assigned to either the MoCA or BEAN group and were initially blinded to their group assignment. Test-related stress was measured using subjective (STAI-6) and objective (salivary α-amylase and cortisol) methods at four time points: baseline, in anticipation of assessment type (immediately following unblinding), and then five and 30 minutes after test initiation. Contrary to our hypothesis, neither STAI-6 scores, α-amylase, nor cortisol values indicated test-related stress for either assessment relative to baseline, based on Bonferroni-adjusted repeated-measures ANOVAs. These results are important, as cognitive screening in both clinical care and clinical trials should have minimal stress responses. Future studies should explore test-related stress using continuous measures of stress response, such as electrodermal activity, particularly in patients with cognitive decline.

Abstract citation ID: igaf122.2517

### CHILDHOOD AND RECENT STRESSORS: IMPLICATIONS FOR PHYSICAL AND COGNITIVE HEALTH IN OLDER ADULTS

Athena Chan[1], and Sun-Kyung Lee[2], *1. Texas Tech University, Lubbock, Texas, United States, 2. Arizona State University, Phoenix, Arizona, United States*

Childhood adversities and recent life stressors are less understood on their joint impact on physical and cognitive health in older adults. This study explores stressor typologies and their association with health. We analyzed data from 14,236 older adults (ages 50-101, M = 67.2) from the Health and Retirement Study (2010-2020). Using a 3-step BCH approach in Mplus, latent class analysis identified profiles based on 10 indicators: childhood adversities (e.g., repeating a grade, trouble with police, parental physical abuse) and recent life events (e.g., natural disaster, life-threatening illness/accident, homelessness). Multinomial logistic regression identified demographic predictors of profiles. Health was predicted after 10 years, adjusting for these predictors. Four latent classes emerged: Low stressors (75%), Childhood physical abuse/substance household (10%), Lifetime law enforcement/adulthood homelessness (5%), and High recent life events (9%). Compared to Low stressors, Childhood physical abuse was more likely to be White and female, while Lifetime law enforcement and High recent life events classes were more likely to be male and ethnic/racial minorities. After adjusting for demographics, no significant class differences were found in dementia risk. However, Childhood physical abuse and Lifetime law enforcement classes had lower cognitive impairment, while Low stressors had the highest. High life events class had the most chronic conditions, and Lifetime law enforcement had the highest lifestyle risk. These findings highlight how cumulative stressors impact health in older adulthood, with certain stressor types linked to cognitive decline and physical health problems. Understanding stressor history may inform tailored health interventions and care strategies for older populations.

Abstract citation ID: igaf122.2518

### CO-DESIGNING ALIVIADO CAREGIVING: AN AI-POWERED MOBILE APP FOR SUPPORTING CARE PARTNERS IN MANAGING BPSD

Moroni Fernandez Cajavilca[1], Shih-Yin Lin[2], Benedict Guzman[3], Kimberly Cheng[4], Denise Lawson[5], Narges Razavian[4], and Abraham Brody[2], *1. University of Utah, Salt Lake City, Utah, United States, 2. New York University Rory Meyers College of Nursing, New York, New York, United States, 3. New York University Langone Health, New York, New York, United States, 4. New York University, New York, New York, United States, 5. Pop Services, White Plains, New York, United States*

Care partners (CP) of persons living with dementia (PLWD) often face complex, unpaid caregiving responsibilities, particularly in managing behavioral and psychological symptoms of dementia (BPSD) such as agitation. CPs lack day-to-day access to evidence-based non-pharmacological interventions (e.g., music therapy) and decision support for daily BPSD management. Mobile health interventions offer a promising solution to aid CPs in managing BPSD. This presentation details the development of a precision heuristic machine learning algorithm and human-centered design of Aliviado Caregiving, a mobile health application designed to support CPs' self-management and prioritization of BPSD. We used the National Alzheimer's Coordinating Center Uniform Data Set to create an XGBoost model, training it on 2360 patients (80% of the final cohort) and testing it on 590 patients (20%). The model achieved an area under the curve of .829 and an average precision-recall curve of .905. We then embedded the algorithm into the Aliviado Caregiving app and established a community advisory board of diverse CPs of PLWD (N = 8). The board members participated in two focus groups and eight individual interviews to co-design and iteratively refine the Aliviado Caregiving application. We conducted a reflexive thematic analysis on open-ended responses collected from all focus groups and interviews. We identified four qualitative themes: 1) User-Interface Design Preferences and Suggestions, 2) User-Friendly Language, 3) User-Concern Prioritization, and 4) Future Application Features. Applying co-design principles and partnering with diverse CPs improved our ability to enhance the acceptability and usability of Aliviado Caregiving for future testing in a pilot project.

Abstract citation ID: igaf122.2519

### COMMUNITY RATES OF ALZHEIMER'S DISEASE AND RELATED DEMENTIAS AND SUPPORTIVE SERVICES IN CT, MA, & RI

Michelle Ward, Dongfang Hong, Nachalie Rodriguez-Cruz, Mengshi Liu, and Elizabeth Dugan, *University of Massachusetts Boston, Boston, Massachusetts, United States*

The consequences of Alzheimer's Disease and Related Dementias (ADRD) are well documented, but beyond the individual, family, and health system costs are implications for communities that are not well-studied. The aim of this study is to describe community rates of ADRD and the per capita rate of supportive services (caregiver support groups and adult day health services). Data were obtained from the 2025 Healthy Aging Data Reports (healthyagingdatareports.org) for CT, MA, and RI. Small area estimation techniques were used to calculate age- and sex-adjusted rates of ADRD for Medicare fee-for-service beneficiaries ever diagnosed (2020-2021). Caregiver support groups were obtained from the Alzheimer's Association (2023). Adult day service data sources were the CT Association of Adult Day Services, MA EOHHS, and RI Dept. of Health. Average state rates in adults 65+ were: CT 13.9% (Sherman, 6.9%; Hartford, 22.4%); MA 12.9% (Washington, 6.7%; Chelsea, 20.0%); and RI 12.0% (Jamestown, 7.3%; Central Falls, 16.3%). Per capita, RI (n = 15.9) had the most adult day centers per 100,000 adults age 65+, followed by MA (n = 12.0) and CT (n = 6.0). For ADRD caregiver support groups, CT and RI (n = 4.6) had the most groups per 100,000 older adults, followed by MA (n = 2.1). The rates of ADRD vary slightly by state, with larger variations within each state. Many communities with higher rates of ADRD do not have any caregiver support groups or adult day programs, which provide crucial respite and emotional care. Understanding disparities in access to ADRD services will enable communities to identify needs in increasing supportive services.

Abstract citation ID: igaf122.2520

**DECISION SUPPORT APP FOR MANAGING BEHAVIORAL AND PSYCHOLOGICAL SYMPTOMS OF DEMENTIA IN NURSING HOMES**

EunKyo Kim, Eunhee Cho, Minhee Yang, Sinwoo Hwang, Jungwon Cho, and SangA Lee, *Yonsei University, Seoul, Republic of Korea*

Managing behavioral and psychological symptoms of dementia (BPSD) is an increasing global challenge. This study aimed to test an app developed for managing BPSD in a nursing home setting. A cluster randomized controlled trial included 35 older adults with dementia and 28 nurses across 11 facilities. Over eight weeks, the intervention group used an app that recorded and predicted BPSD. The app analyzed BPSD patterns using machine learning and provided individualized non-pharmacological interventions such as music, reminiscence, and exercise therapy. However, the control group used the BPSD recording system without access to individualized interventions. Overall BPSD, depression, agitation, and nurses' BPSD management competencies were measured. Nurses in the intervention group recorded their narrative experiences using the app, as well as residents' reactions to it. Generalized mixed models revealed reductions in depression and agitation scores over time for both groups without a group-by-time interaction. Overall BPSD decreased in the control group, showing a group-by-time interaction. Nurses in both groups showed improvement in BPSD management competencies. Based on their experience using the app, nurses considered playing residents' preferred songs during music therapy to be helpful in maintaining focus and emotional stability. Additionally, reminiscence therapy supported cognitive engagement by connecting past experiences to the present through photographs. While the app had a user-friendly interface, technical constraints—such as slow processing and limited content—reduced its effectiveness. Future research should focus on improving technical performance, diversifying content, and adapting the app for varied care settings such as adult daycare centers.

Abstract citation ID: igaf122.2521

**DEPRESSION AND RISK OF DEMENTIA: A BURDEN OF PROOF STUDY**

Channa Buxbaum[1], Natalie Chen[2], Anh Thy Nguyen[2], Jamileh Shadid[3], Kate Gillespie[4], Damian Santomauro[3], Liane Ong[2], and Jaimie Steinmetz[2], *1. Institute for Health Metrics and Evaluation, New York, New York, United States, 2. Institute for Health Metrics and Evaluation, Seattle, Washington, United States, 3. Queensland Centre for Mental Health Research, Wacol, Queensland, Australia, 4. IHME Client Services, Seattle, Washington, United States*

Depression is widely considered a risk factor for dementia, yet its precise relationship remains difficult to quantify due to substantial variability in measurement approaches across studies. In the Global Burden of Disease (GBD) study, we estimate that the number of people living with a depressive disorder globally has more than doubled from 1990 to 2024, now affecting approximately 307 million people. Given its growing burden, clarifying its role in dementia risk is imperative. To address this, we applied the Burden of Proof framework, developed for the GBD study, to adjust for systematic biases and account for heterogeneity. We conducted a systematic review and meta-analysis of longitudinal studies in PubMed, Web of Science, and PsycInfo published between 2012–2024, building on a prior meta-analysis covering 1980–2012. Of 9,625 references, 41 met eligibility criteria, with 18 included after deduplication and removal of registry-based studies. Our initial pooled analysis found a moderate-to-strong association between depression and dementia (RR = 1.54, 95% UI: 1.32–1.79). However, when restricting to nine studies with gold-standard depression measures—excluding studies relying on symptom scales, self-report, or potentially non-representative populations—the estimated association became more uncertain (RR = 1.26, 95% UI: 0.86–1.87). These results underscore how inconsistencies in depression measurement can alter the perceived strength of the depression-dementia relationship. While depression remains a plausible contributor to dementia risk, these findings emphasize the importance of rigorous, standardized assessment in epidemiological research. As the burden of depression rises globally, ensuring methodological rigor in future studies will be essential for accurately informing dementia prevention strategies.

Abstract citation ID: igaf122.2522

**FRONTOTEMPORAL DEGENERATION (FTD) DIAGNOSIS AND IMPACT**

Esther Kane[1], Shana Dodge[1], Carrie Milliard[2], Sharon Denny[1], and Penny Dacks[1], *1. Association for Frontotemporal Degeneration, King of Prussia, Pennsylvania, United States, 2. FTD Disorders Registry, King of Prussia, Pennsylvania, United States*

Frontotemporal degeneration (FTD), a form of dementia, is the umbrella term for a range of progressive, fatal disorders that include changes in cognition, language, behavior, motor functioning, and personality. FTD is known to be particularly challenging for care partners, as it typically begins earlier than other dementias and has greater financial consequences. There are currently no approved symptomatic or disease-modifying treatments for FTD. The FTD Disorders Registry is a secure online direct-to-participant platform that allows people impacted by FTD to participate in research remotely and provides critical lived experience data to researchers. Registry enrollees, including 956 spouses/partners, family members, caregivers and friends completed the Disease Impact Survey. Nearly a third described it taking more than two years for their loved one to receive their FTD diagnosis and 31% reported that the diagnosing doctor did not explain the diagnosis. Respondents noted symptom impacts on the diagnosed person's ability to manage finances, run errands, drive or navigate transportation, prepare meals, keep track of appointments, and take their medication. They also endorsed how the FTD diagnosis had impacted their own lives, including altered relationships (81%), increased stress (73%), disrupted plans for the future (72%), and social isolation (48%). While there is a significant unmet medical need for people diagnosed with FTD, it is important for healthcare providers to recognize that FTD care partners also require significant support in navigating this difficult and stressful diagnosis.

Abstract citation ID: igaf122.2523

### HEALTH BELIEFS AND MEDITERRANEAN DIET ADHERENCE: KEY PREDICTORS OF DIETARY BEHAVIOR FOR DEMENTIA PREVENTION

Offer Edelstein, *Ben Gurion University of the Negev, Be'er Sheva, HaDarom, Israel*

Background: Dementia prevention strategies emphasize modifiable lifestyle factors, with adherence to the Mediterranean diet (MD) being a key recommendation. This study sought to (i) assess MD adherence among Israeli-born adults aged 50 and older and (ii) explore how Health Belief Model (HBM) constructs (Rosenstock, 1966, 1974) relate to dietary adherence. Method: A cross-sectional online survey was conducted in 2022–2024 with a convenience sample of 895 Israeli-born individuals aged 50 and above. MD adherence was measured using the Israeli Mediterranean Diet Adherence Screener (I-MEDAS) (Abu-Saad et al., 2018), and cognitive perceptions were evaluated using the Motivation to Change Lifestyle and Health Behaviors for Dementia Risk Reduction questionnaire (Kim et al., 2014). Results: The mean MD adherence score was 10.11 (SD = 1.36) out of 17. Multivariate regression analysis identified perceived barriers ($\beta$ = -0.301, p < .001), cues to action ($\beta$ = 0.233, p < .001), and perceived severity ($\beta$ = 0.191, p < .001) as significant predictors of dietary adherence. Additionally, being female ($\beta$ = 0.212, p < .001) and having a lower income ($\beta$ = -0.151, p < .05) were also associated with adherence. The model accounted for 22.4% of the variance in MD adherence [F(5,892) = 15.56, p < .0001]. Conclusions: These findings suggest that addressing perceptions of disease severity and perceived barriers, as well as increasing cues to action, may enhance adherence to the MD. Public health interventions should incorporate these insights to promote dietary behavior change in midlife and older adults.

Abstract citation ID: igaf122.2524

### IMPLEMENTING ANTI-AMYLOID THERAPIES IN MEMORY CLINICS: A QUALITATIVE STUDY OF CLINICIAN EXPERIENCES

Joanna Paladino[1], Anna Parks[2], Daniel Dohan[3], Seth Gale[4], Liliana Ramirez Gomez[1], Christine Ritchie[1], Sachin Shah[1], and Ayush Thacker[1], *1. Massachusetts General Hospital, Boston, Massachusetts, United States, 2. University of Utah, Salt Lake City, Utah, United States, 3. University of California, San Francisco, San Francisco, California, United States, 4. Brigham and Women's Hospital, Boston, Massachusetts, United States*

Anti-amyloid therapies (AATs) have changed the diagnosis and treatment paradigm of Alzheimer's disease (AD). Our interdisciplinary team completed a qualitative study of clinicians' experience with AAT implementation. We conducted semi-structured interviews with 27 prescribing clinicians at seven U.S. academic medical centers. We identified three themes using thematic content analysis. First, AATs affect practice norms for AD diagnosis. Clinicians feel added pressure for time-efficiency of the diagnostic process, sense expectations for more accurate diagnoses, and integrate AAT considerations into diagnostic disclosure and choice of tests. Even with increased time pressure, conversations about AATs are unfolding over multiple visits because of the time it takes for eligibility tests to return and to allow for shared decision-making. Second, the availability of AATs creates a paradigm shift with opportunities and challenges for clinicians. Clinicians describe feeling a sense of hope that is an 'antidote' to nihilism in dementia care. They also experience burdens from the increased workload and urgency of managing patients on infusion, inadequate institutional support, and reduced clinical time for patients not on AAT. Third, clinicians and institutions vary in lecanemab treatment protocols, including comfort with offering lecanemab to patients on concurrent anticoagulants or homozygous for ApoE4. While institutional protocols initially adhered closely to published appropriate use criteria regarding patient eligibility, clinicians reported modifications of eligibility criteria over time. Clinicians and clinical leaders can use findings from this study to consider structural changes to accommodate new workflows related to AAT and to inform the development of AAT decision-support interventions.

Abstract citation ID: igaf122.2525

### INTERACTION OF AGE, DISCRIMINATION, AND VIGILANCE ON MEMORY-CONCENTRATION DIFFICULTY IN A NATIONAL U.S. SAMPLE

Roger Wong, and Emily Norris, *SUNY Upstate Medical University, Syracuse, New York, United States*

Discrimination and vigilance have been linked to adverse physical and mental health conditions. There is no existing research that has examined how the intersections between age, discrimination, and vigilance may influence cognition. This study examines whether the frequency of discrimination and vigilance are associated with memory-concentration difficulty, and whether these relationships vary by age group. The 2023 National Health Interview Survey administered the five-item Everyday Discrimination Scale (e.g. receive poor customer service) and four-item Heightened Vigilance Scale (e.g. avoid social situations) for a nationally representative sample of 28,583 U.S. adults, who were categorized into four age groups: young adult (18-24), early middle-age (25-39), late middle-age (40-64), and older adult (65+). Average scores (range 0-3) were computed separately from the discrimination and vigilance scales. After applying survey sampling weights and adjusting for sociodemographic, geographic, and health covariates, the logistic regressions indicated that multiple monthly instances of discrimination and vigilance were associated with an increased odds of memory-concentration difficulty by 2.7 times (OR = 2.72, 95% CI = 2.06-3.62, p<.001) and 3.0 times (OR = 2.98, 95% CI = 2.58-3.45, p<.001) times, respectively, compared to those with no exposure. There was also a significant age-discrimination (p=.02) and age-vigilance (p <.001) interaction on memory-concentration difficulty. The highest probabilities of memory-concentration difficulty were among older adults with the most frequent exposure to discrimination and vigilance behaviors. These results suggest that discrimination and vigilance may be a factor contributing to the heightened risk for cognitive impairment among older adults. Future research is needed to examine the underlying mechanisms behind these age-related differences in memory-concentration.

Abstract citation ID: igaf122.2526

### MEDICARE PLAN SWITCHING AMONG ASSISTED LIVING RESIDENTS: THE ROLE OF MEMORY CARE

Wanru Liu[1], Gauri Gadkari[1], Jennifer Bunker[2], Kali Thomas[2], and Eric Jutkowitz[3], *1. Brown University, Providence, Rhode Island, United States, 2. Johns Hopkins University, Baltimore, Maryland, United States, 3. University of Minnesota, Minneapolis, Minnesota, United States*

Switching between Medicare Advantage (MA) and Fee-for-Service (FFS) commonly occurs after a health shock or residential relocation. However, little is known about how moving to an assisted living (AL) community influences enrollment decisions. This study examines Medicare plan switching among beneficiaries relocating to assisted living (AL) licensed for memory care versus general AL. Memory care ALs provide specialized dementia services but are approximately 36% more expensive than general ALs, potentially influencing Medicare choices. Using 2021 Medicare Beneficiary Summary Files and a national dataset of ALs, we tracked Medicare enrollment before and after moving to AL (N = 63,395) across 34 states licensing memory care ALs. A secondary analysis investigated switching among AL residents with dementia. Medicare plan switching varied based on AL licensing: FFS beneficiaries entering memory care-licensed ALs switched to MA less often than those entering general ALs (6.47% vs. 9.16%, $p < 0.05$). MA beneficiaries relocating to memory care compared to general AL were less likely to switch to another MA plan (10.95% vs. 13.08%, $p < 0.05$) or to FFS (4.31% vs. 5.55%, $p < 0.05$). Among individuals with dementia, residents with dementia in memory care ALs exhibited less FFS-to-MA switching compared to those in general ALs (3.40% vs. 4.76%, $p < 0.05$). These findings suggest that moving to an AL influences Medicare enrollment decisions; and, variation in switching exists between the type of AL (general vs memory care) and whether or not an individual has dementia.

Abstract citation ID: igaf122.2527

### MULTILEVEL ANALYSIS OF PSYCHOLOGICAL FACTORS IN CAREGIVER BURDEN TRAJECTORIES FOR LEWY BODY DEMENTIA

Zih-Ling Wang[1], Yan Su[2], and Oleg Zaslavsky[3], *1. University of Washington School of Nursing, Seattle, Washington, United States, 2. University of Massachusetts Dartmouth, Dartmouth, Massachusetts, United States, 3. University of Washington, Seattle, Washington, United States*

This study used secondary data from the parent single-arm pre-post VOCALE LBD (Virtual Online Communities for Aging Life Experiences Lewy Body Dementia) project to examine psychological factors influencing caregiver burden trajectories. The parent study implemented an 8-week asynchronous online intervention specifically designed for Lewy Body Dementia (LBD) caregivers. We analyzed a sample of 39 family/informal caregivers in the intervention group primarily recruited from the Memory and Brain Wellness Center in Seattle, Washington. Participants were predominantly female (69.2%), with a mean age of 67.6 years (SD = 9.6, range 46-91 years). Most caregivers had at least some college education, with 79.5% having completed a bachelor's degree or higher. Using multilevel regression analysis, we evaluated how caregiver burden changed across three-time points (baseline, post-intervention (8 weeks), and one-month follow-up after intervention end (12 weeks)) and identified key psychological factors influencing these trajectories. Results revealed a significant decrease in caregiver burden during the intervention period ($p = .002$), with higher self-efficacy associated with steeper decreases ($p < .01$). However, a significant increase in burden occurred during follow-up ($p = .003$), particularly associated with increased perceived stress ($p < .001$). Baseline loneliness ($p < .001$) and perceived stress ($p = .008$) significantly predicted higher initial burden levels. Age had differential effects, with older caregivers showing less improvement during the intervention but better maintenance during follow-up. These findings highlight the importance of tailoring interventions based on caregiver characteristics and psychological factors, suggesting that ongoing support after initial intervention may be necessary to maintain improvements in this understudied population.

Abstract citation ID: igaf122.2528

### PLACING PUBLIC HEALTH ON THE ADRD POLICY PLATFORM: BUILDING OUR LARGEST DEMENTIA INFRASTRUCTURE FOR ALZHEIMER'S ACT

Brian Kaskie[1], Julie Bobitt[2], Yogi Shah[3], and Sarah Khasawinah[4], *1. University of Iowa, Iowa City, Iowa, United States, 2. University of Illinois at Chicago, Chicago, Illinois, United States, 3. Broadlawns Medical Center, Des Moines, Iowa, United States, 4. Health Policy, District of Columbia, District of Columbia, United States*

The Building Our Largest Dementia Infrastructure for Alzheimer's Act (BOLD Act) of 2018 placed public health onto the Alzheimer's disease and related dementias (ADRD) public policy platform, establishing a role for state, local, territorial, and tribal public health departments to address the many challenges faced by persons living with dementia (PLwD) and those who care for them. We consider this remarkable event as another example of policy making iron triangles at both federal and state levels of government, when ADRD advocates, legislative and executive branch staff, and elected public officials who champion PLwD (i.e., a policy making iron triangle) work together to increase public investment in basic research and expand financing for care and support provided to PLwD. Upon recounting passage of the BOLD Act, we move on to highlight how 44 public health departments across the country have since been following the Centers for Disease Control and Prevention (CDC) Healthy Brain Initiative Roadmap, implementing programmatic efforts for PLwD. Referring to the BOLD Reauthorization Act of 2024, we present viable alternatives for public health leadership to sustain and expand such promising advancements bringing essential public health services to PLwD and those who care for them.

Abstract citation ID: igaf122.2529

### POPULATION ATTRIBUTABLE FRACTIONS ASSOCIATED WITH ALZHEIMER'S DISEASE RISK FACTORS

Igor Akushevich[1], Arseniy Yashkin[1], Svetlana Ukraintseva[1], Anatoliy Yashin[1], and Julia Kravchenko[2], *1. Duke University, Durham, North Carolina, United States, 2. Duke University School of Medicine, Durham, North Carolina, United States*

Although numerous risk factors for Alzheimer's disease (AD) and related dementias (ADRD) have been identified, their combined influence on risk remains uncertain. In this study, we leverage a high-power 5%-sample of the Medicare population to assess the joint impact of AD/ADRD risk-related diseases and low income—as indicated by dual Medicare eligibility—on the risk of clinical diagnosis of AD/ADRD. We leveraged the univariable and multivariable Cox models for estimating AD/ADRD risks, identified most powerful predictors, and constructed predictive multivariable models for AD/ADRD risks, and evaluated their population attributable fractions (PAFs) for the general populations of older adults and race- and sex-specific subpopulations. The identified model included nine diseases—heart failure, hypertension, arrhythmia, stroke, hypotension, renal disease, depression, traumatic brain injury, and diabetes mellitus—as primary determinants of variation in AD/ADRD risk. The fraction of the total PAF explained by the predictors increased with age. The decomposition of the total PAF in terms of disease-specific PAFs showed that hypertension, stroke, and depression provided the strongest contribution for all subpopulations: Hypertension reached 45-50% of total PAF for Black, Asian, and Hispanic subpopulations. Stroke was the primary contributor for males (>30%); depression was highest among females and White individuals (approximately 30%). Hypertension and depression were the leading contributors for Native Americans (both approximately 30%). Heart Failure was the fourth strongest contributor for all subpopulations, with the contribution exceeding 5% for Native American individuals. Additional noteworthy contributors with PAFs exceeding 3% included hypotension (males), diabetes (Asian, Hispanic), arrhythmia (males), and TBI (Black, Asian).

Abstract citation ID: igaf122.2530

### PROFILES OF BELIEFS ABOUT ALZHEIMER'S DISEASE RISK FACTORS AND PREVENTION STRATEGIES AMONG AMERICANS AGED 50+

Elyse Couch[1], Fangli Geng[1], Eric Jutkowitz[2], and Emmanuelle Belanger[1], *1. Brown University, Providence, Rhode Island, United States, 2. University of Minnesota, Minneapolis, Minnesota, United States*

Increasing knowledge about the causes of dementia is essential for motivating engagement in healthy behaviors that can lower one's risk of dementia. This study aimed to identify distinct patterns of beliefs regarding the importance of Alzheimer's disease (AD) risk factors and the effectiveness of prevention strategies among Americans aged 50+. We analyzed data from 1,819 adults who participated in a 2010 Health and Retirement Study experimental module on AD knowledge and beliefs. On a 3-point Likert scale, participants rated the importance of AD risk factors (stress and genetics) for causing AD (1 = not important; 3 = very important) and the effectiveness of prevention strategies (physical activity, mental activity, diet, vitamins)(1 = not effective; 3 = very effective). We performed an unweighted latent profile analysis to identify three distinct profiles of beliefs about AD risk factors and prevention strategies and multinomial regression to identify demographic predictors of profile membership. Profile 1 (63% of participants) were uncertain about the importance of risk factors and the effectiveness of prevention strategies; Profile 2 (15% of participants) strongly believed in the effectiveness of all prevention strategies, and Profile 3 (21% of participants) rated genetics as the most important risk factor but did not believe in the effectiveness of prevention strategies. Demographic characteristics, such as education and race, were associated with profile membership. Public health messaging about AD prevention should be tailored to the beliefs of different demographic groups.

Abstract citation ID: igaf122.2531

### PROMOTING PERSONHOOD IN FAMILY CONVERSATIONS WITH INDIVIDUALS LIVING WITH DEMENTIA

Chizobam Nweke[1], R. Amanda Cooper[1], and Hannah Brocksmith[2], *1. University of Connecticut, Storrs, Connecticut, United States, 2. University of Connecticut, Farmington, Connecticut, United States*

As the population of adults aged 65 and above keeps rising in the US and other countries around the world, so does the burden of dementia, with significant impacts on the quality of life of this population. This study explored the various ways through which family members promote the personhood of their loved ones with dementia. Thirty-one audio-recorded conversations between people with dementia, Mage 76.65 (SD 13.35), and their family members were obtained from the StoryCorps online archive and analyzed. The following were identified as the strategies through which family members affirmed personhood: reminding the people with dementia of their identities, encouraging them to share their thoughts, assessing and adjusting to their conversational needs, and cultivating relational connections with them. This study also found that the task of promoting personhood through family relationships is complex and there is a need to ensure that adequate accommodation is provided, as too much or too little can be counterproductive. Also, the findings from this study provide further evidence that people with dementia can have positive and meaningful experiences while navigating their illness at home among loved ones.

Abstract citation ID: igaf122.2532

### SUPPORT FROM DEMENTIA PATIENTS AND CAREGIVER BURDEN: EXPLORING VARIATION BY CAREGIVER PHYSICAL HEALTH

Xinyue Yang[1], Meng Huo[2], Kyungmin Kim[3], and Dahua Wang[1], *1. Beijing Normal University, Beijing, Beijing, China (People's Republic), 2. University of California, Davis, Davis, California, United States, 3. Seoul National University, Seoul, Republic of Korea*

A small but growing body of work suggests that caregivers still receive support from people living with Alzheimer's disease and related dementias (PLWD). Yet, we know little about what characteristics of the support received from PLWD benefit caregivers and whether the benefits apply to all caregivers. The current study extended prior research by examining (a) how the frequency and quality of the support that caregivers received from PLWD were associated with caregiver burden and (b) whether these associations varied by caregivers' own physical health. We analyzed data from 72 spousal caregivers of older adults with mild-to-moderate Alzheimer's disease. Caregivers reported their own and partners' demographic characteristics, the frequency and quality of support they received from PLWD, caregiver burden, and their physical health. Multiple regressions showed that receiving emotional support more often and receiving better-quality support were both associated with lower caregiver burden. These associations, however, were stronger among caregivers who self-rated better physical health. This study showcases the benefits of receiving support from PLWD and emphasizes the importance of facilitating mutually beneficial relationships in dementia caregiving dyads. Further, our findings can inform tailored interventions targeting caregivers with different physical health conditions.

Abstract citation ID: igaf122.2533

**THE EVOLUTION OF SHARE: AN EVIDENCE-BASED PROGRAM FOR PERSONS WITH EARLY-STAGE DEMENTIA AND THEIR CARE PARTNERS**

Zoe Fete[1], Silvia Orsulic-Jeras[1], Donna Salaam[1], Megan Huth[2], and Sara Powers[1], *1. Benjamin Rose, Cleveland, Ohio, United States, 2. Benjamin Rose Institute on Aging, Cleveland, Ohio, United States*

Best Programs for Caregiving (BPC) is an online database of nearly 50 evidence-based dementia caregiving programs. One such program is SHARE for Dementia, which targets care values/preferences and planning for the future, including both the person living with dementia and their care partner. Beginning in 1997 with a series of pre-intervention studies, SHARE has since gone through multiple adaptations and technological developments. In 2023, the BPC team collected online surveys from organizations (n = 8) delivering SHARE across the US about their implementation. In reviewing data, all eight organizations (100%) indicated SHARE was being delivered in-person, with two of those organizations also indicating an online component. Only one organization indicated they were delivering the program at a national level (e.g., any care dyad is eligible no matter location), with the other seven organizations offering the program locally or statewide. Results also indicated that at the time of data collection SHARE implementation sites did not identify specific diverse communities they were tailoring the delivery of the program for, including things such as adapting marketing materials and specific staff trainings. Discussion will focus on improvements to the program's ability to be offered virtually (e.g., creating a web-based application), improving accessibility to the program for dementia care dyads, as well as increasing the feasibility to implement among community-based organizations. Additionally, to better meet the needs of diverse communities, details surrounding the current development of a cultural adaptation of SHARE for Black and African American families impacted by dementia will be discussed.

Abstract citation ID: igaf122.2534

**THE PREVALENCE AND IMPACT OF ALZHEIMER'S AND RELATED DEMENTIA ON CIRRHOSIS: A RETROSPECTIVE STUDY**

Sijia Wei[1], Suyanpeng Zhang[2], Saad Hussain[2], Salva Balbale[2], Lihui Zhao[2], Nikhilesh Mazumder[3], Sanjay Mehrotra[2], and Daniela Ladner[4], *1. Northwestern University, Chicago, Illinois, United States, 2. Northwestern University, Evanston, Illinois, United States, 3. University of Michigan, Ann Arbor, Michigan, United States, 4. Northwestern University Feinberg School of Medicine, Chicago, Illinois, United States*

Alzheimer's and related dementia (ADRD) complicates cirrhosis management; particularly, its symptoms overlap with hepatic encephalopathy. However, ADRD prevalence and impact on cirrhosis remain unclear. This study examined ADRD prevalence and its association with 1-year hospitalization and 5-year mortality in cirrhosis. Using electronic health records data, this retrospective study included 36,971 adults diagnosed with cirrhosis between 2011 and 2021 across seven health systems in a large US metropolitan area. ADRD diagnosis and certainty were determined using ICD-9/10 codes, medications, specialist involvement, and diagnostic timing. Multivariable logistic regressions and the Cox proportional hazards models assessed ADRD risk factors and influence on 1-year hospitalization and 5-year mortality. Among patients with cirrhosis, 5.7% (n = 2091) had an ADRD diagnosis: 2.0% (n = 727) before cirrhosis diagnosis, 2.3% (n = 844) after, and 1.5% potentially misdiagnosed hepatic encephalopathy. Compared to those without ADRD and after adjusting for covariates, ADRD before cirrhosis was associated with increased mortality in compensated (HR = 1.72, $p < 0.001$) but not in decompensated cirrhosis (HR = 0.89, $p = 0.41$). ADRD before cirrhosis increased the likelihood of 1-year hospitalization in both compensated (OR = 3.32, $p < 0.001$) and decompensated (OR = 1.58, $p = 0.04$). ADRD after cirrhosis was associated with lower mortality in both compensated (HR = 0.33, $p < 0.001$) and decompensated (HR = 0.41, $p < 0.001$) but was not significantly associated with hospitalization. Key factors associated with ADRD diagnosis and outcomes included age, metabolic-associated alcohol-associated liver disease, alcohol-associated liver disease, and being black American. Better detection and management of ADRD may improve cirrhosis outcomes, particularly in compensated cirrhosis. Further research is needed to understand the role of ADRD diagnosis timing in cirrhosis management.

Abstract citation ID: igaf122.2535

**WHO PROVIDES HOME-BASED PRIMARY CARE TO PEOPLE WITH DEMENTIA?**

Monica O'Reilly-Jacob[1], Lusine Poghosyan[2], Madison Horton[2], Carolyn Clevenger[3], Soo Borson[4], and Jianfang Liu[5], *1. Columbia University School of Nursing, Andover, Massachusetts, United States, 2. Columbia University, New York, New York, United States, 3. Emory University, Atlanta, Georgia, United States, 4. University of Southern California, Los Angeles, California, United States, 5. Columbia University Irving Medical Center, New York City, New York, United States*

Home-based primary care (HBPC) delivery for people with dementia (PWD) is growing. Nurse practitioners (NPs) and physicians (MDs) deliver HBPC, yet how to optimize workforce resources in HBPC models is unknown. We used national 2019 Medicare claims to examine the characteristics of PWD receiving HBPC from NPs, MDs, or both from NPs and MDs. We identified PWD over age 65 who received >=4 HBPC visits extracted age, dual-eligibility for Medicare and Medicaid, race/ethnicity and 14 clinically meaningful chronic conditions. We assigned each beneficiary to a HBPC model (i.e., NP-only, MD-only, NP-MD) depending on the type of clinician providing the totality of HBPC visits. We used analysis of variance or Chi-square analyses to assess group differences. In 2019, 62,094 Medicare beneficiaries with dementia received at least four HBPC visits. Overall, almost 40% received HBPC exclusively from NPs, 33% from MDs, and 26% from NPs and MDs. Those seen by both NPs and MDs had significantly higher rates of almost all chronic conditions ($p < .001$) and were significantly more racially and ethnically diverse (Non-Hispanic White: 58.3%) than those that saw NPs or MDs exclusively (Non-Hispanic White: NP-only: 71.1%; MD-only: 65.2%, $p < .001$). The majority of HBPC for PWD is provided by NPs. HBPC provided by both NPs and MDs is more likely delivered to those with more chronic conditions and of racial and ethnic minorities. Leveraging the expertise of both NPs and MDs in HBPC models may be necessary for homebound PWD of greater clinical complexity.

### SESSION 7190 (POSTER)

Abstract citation ID: igaf122.2536

### BIOLOGY OF AGING I ANTISENSE OLIGONUCLEOTIDE-MEDIATED SRF REDUCTION AMELIORATES MITOCHONDRIAL DYSFUNCTION IN CARDIAC AGING

Pankaj Patyal[1], Gohar Azhar[2], Xiaomin Zhang[1], Ambika Verma[1], and Jeanne Wei[2], *1. University of Arkansas for Medical Sciences, Little Rock, Arkansas, United States, 2. University of Arkansas for Medical Sciences, Little Rock, AR, LITTLE ROCK, Arkansas, United States*

Cardiac aging is characterized by progressive mitochondrial dysfunction, contributing to heart failure and other age-related cardiovascular diseases. Serum response factor (SRF) is a key transcription factor regulating cardiac integrity, but its elevated expression in aging myocardium worsens mitochondrial dysfunction and heart pathology. This study examines whether antisense oligonucleotide-mediated SRF reduction can alleviate mitochondrial dysfunction in cardiac aging. A cardiac-specific antisense SRF transgenic mouse model with modestly reduced SRF levels was generated. Mice aged 12–15 months were analyzed for cardiac changes, including histology, mitochondrial function, and oxidative stress. Histological analysis revealed reduced cardiac hypertrophy and fibrosis in Anti-SRF Tg mice. Ultrastructural analysis showed well-preserved mitochondrial structure, increased mitochondrial density ($p < 0.01$), and more cristae ($p < 0.01$). Mitochondrial DNA copy number was also higher ($p < 0.05$). Western blot analysis showed increased expression of mitochondrial regulators PGC-1α ($p < 0.01$), NT-PGC-1α ($p < 0.05$), PGC-1β ($p < 0.05$), and OPA1 ($p < 0.05$), with reduced Drp1 ($p < 0.05$). High-resolution respiratory functional analysis by Oroboros O2K revealed increased activity of mitochondrial complexes I-IV ($p < 0.01$ to $p < 0.001$). This is further corroborated by western blot analyses of OXPHOS (I–V) which shows that SRF reduction resulted in upregulation of mitochondrial complex proteins of electron transport chain. SRF reduction also reduced oxidative stress, indicated by lower 4-HNE levels ($p < 0.01$) and increased MnSOD expression. In conclusion, cardiac-specific SRF reduction mitigates hypertrophy, fibrosis, mitochondrial dysfunction, and oxidative stress, suggesting its potential as a therapeutic target for cardiac aging.

Abstract citation ID: igaf122.2537

### DEVELOPING INTERDISCIPLINARY TRAINING FOR SCIENTISTS IN CANCER, AGING, AND DISPARITIES RESEARCH

Jamie Mitchell[1], Elena Flores[1], Chiranjeev Dash[2], Lucile Adams-Campbell[3], Peter Lichtenberg[4], Jason Umans[3], and Jeanne Mandelblatt[3], *1. University of Michigan, Ann Arbor, Michigan, United States, 2. Georgetown University Lombardi Comprehensive Cancer Center, Washington, District of Columbia, United States, 3. Georgetown University, Washington, District of Columbia, United States, 4. Wayne State University, Detroit, Michigan, United States*

The "Infrastructure for REsearch in Aging, Cancer and Health" (I-REACH) brings together a national coalition to increase the proportion of scientists conducting research at the crossroads of aging, disparities and cancer who are better prepared to improve the health of older cancer survivors. I-REACH developed an innovative and interdisciplinary eight module curriculum that integrates expertise across four academic hubs (Georgetown University, Wayne State/University of Michigan, UCLA, and University of Maryland). This expansive curriculum is designed to be hybrid in format, with pre-recorded lectures, live mentoring sessions, a resource library, and opportunities for early career scientists to present and receive feedback on their interdisciplinary work and pilot funding. In addition to a robust focus on cancer and aging biology, cognitive health, interventions, and social determinants of health, I-REACH also notably incorporates the lived experiences of older cancer survivors and caregivers throughout the curriculum. Scientists are mentored to conduct person-centered research that addresses stakeholder needs more effectively. Preliminary evaluation data demonstrates improvements in scholar research self-efficacy, collaborative partnerships, and grant development success. I-REACH offers a replicable model for training programs addressing complex health challenges requiring multidisciplinary approaches and meaningful stakeholder participation, with valuable insights for institutions developing similar initiatives.

Abstract citation ID: igaf122.2538

### DYSREGULATED NUCLEAR ARCHITECTURE DRIVES DEFECTIVE TRANSCRIPTION DURING MEMORY FORMATION IN OLD MICE

Jiyeon Baek, Chad Brunswick, Chad Smies, and Janine Kwapis, *The Pennsylvania State University, University Park, Pennsylvania, United States*

As life expectancy increases, there is a growing population of older adults experiencing cognitive decline, including defects in long-term memory formation. One key mechanism that may contribute to age-related memory impairments is epigenetic dysregulation of gene expression. In particular, the nuclear lamins are an attractive epigenetic target that may connect transcriptional changes to age-related memory impairments. Nuclear lamins are a cytoskeletal component located inside the nucleus that regulate gene expression by binding directly to chromatin and to other epigenetic regulators via lamin-associated proteins. Lamin expression decreases with age and decreased lamin expression has been found to alter gene expression and chromatin conformation. How lamin regulates learning-induced gene expression and chromatin reorganization in the dorsal hippocampus (a crucial brain region for spatial memory) is unknown. To investigate this, we used RNA sequencing and discovered for the first time that lamin B1 (Lmnb1) significantly decreases in the CA1-3 of the dorsal hippocampus from 3 to 18 months of age, an age at which mice show severe impairments in spatial memory. Additionally, our data also show for the first time an increased proportion of CA1 neurons with abnormally shaped nuclear lamina in 18-m.o. mice compared to 3-m.o. mice, suggesting that the reduction in lamin may dysregulate nuclear structure and, ultimately, transcriptional regulation at this age. We plan to manipulate lamin expression to causally test their role. Overall, this study identifies a novel epigenetic mechanism that regulates the transcriptional program necessary for memory formation, broadening our understanding of the biology of the aging brain.

Abstract citation ID: igaf122.2539

### DYSREGULATION OF SRF-REGULATED GENES ACCELERATES THE DEVELOPMENT OF MYOCARDIAL AGING

Xiaomin Zhang[1], Gohar Azhar[2], Sayed Hasan[1], and Jeanne Wei[2], *1. University of Arkansas for Medical Sciences, Little Rock, Arkansas, United States, 2. University of Arkansas for Medical Sciences, Little Rock, AR, LITTLE ROCK, Arkansas, United States*

Background Myocardial aging is associated with structural and functional changes that increase susceptibility to heart failure. Serum response factor (SRF), a key transcription factor regulating cytoskeletal, metabolic, and stress-response genes, plays a critical role in cardiac homeostasis. An increased expression of SRF has been observed in cardiac hypertrophy and aging. This study aims to investigate the impact of SRF dysregulation on myocardial aging. Methods We generated a transgenic mouse model (Tg) with the mild overexpression of the SRF gene in the heart. The echocardiographic and histological analyses demonstrated that the 6-month-old Tg mice manifested the phenotype of cardiac hypertrophy and aging. We analyzed the cardiac gene expression profile in Tg vs Non-Tg. Quantitative PCR and Western blotting confirmed gene expression. Bioinformatics tools were used to analyze SRF binding sites (CArG and CArG-like motifs) and categorize affected pathways. Results and conclusions Over 200 SRF-regulated genes exhibited significant changes in the Tg versus non-Tg mouse hearts ($P < 0.05$). These genes are involved in energy metabolism, lipid turnover, cardiac muscle contraction, transcriptional and translational regulation, and stress responses. Notably, the genes critical for fatty acid metabolism, lipid transport and mitochondrial function were downregulated, whereas genes involved in glycolysis were upregulated. Taken together, dysregulation of SRF-regulated genes triggers "metabolic reprogramming" leading to reduced fatty acid metabolism and mitochondrial function, as well as increased glycolysis. These changes ultimately decrease the cardiac metabolism efficiency and cardiac function. Therefore, SRF is a potential therapeutic target for improving cardiac metabolism and function in aging and pathological conditions.

Abstract citation ID: igaf122.2540

### EARLY DETECTION OF PHYSICAL FUNCTION DECLINE IN OLDER ADULTS BY IDENTIFYING GUT-MICROBIOME BASED BIOMARKERS

Sridevi Nair[1], Yi Lin[2], Shalini Jain[1], Rohit Shukla[1], Vivek Kumar[1], Robert Mankowski[2], Hongdao Meng[1], and Hariom Yadav[1], *1. University of South Florida, Tampa, Florida, United States, 2. The University of Alabama at Birmingham, Birmingham, Alabama, United States*

Decline in physical function in older adults leads to frailty, falls, fractures, reduced quality of life, and increased healthcare utilization, resulting in a significant financial burden on individuals, families, and healthcare systems. Currently, there are no effective tools to predict the risk of physical function decline in older adults. Emerging evidence suggests the gut microbiome plays a key role in maintaining muscle, joint, and overall physical function; however, its prognostic potential and underlying mechanisms remain poorly understood. In this study, we utilized data from the Florida state-wide Microbiome in Aging Gut and Brain (MiaGB) consortium to explore the relationship between the gut microbiome and physical function. We identified gut bacterial taxa and metabolic pathways associated with physical performance. Using Receiver Operating Characteristic (ROC) curve analysis, we demonstrated that specific microbial signatures accurately distinguish between individuals with low and high physical function. Furthermore, we analyzed correlations between physical function and microbial metabolic pathways, identifying operons linked to these pathways. Notably, we found that chondroitin metabolism pathways were significantly impaired in older adults with lower physical function compared to those with normal function. These findings highlight gut microbiome signatures as potential biomarkers for predicting physical function decline and suggest that impaired chondroitin metabolism may be a key microbial mechanism contributing to musculoskeletal deterioration in aging. This study provides novel insights into the microbiome's role in age-related physical decline and offers opportunities for early prognosis and development of microbiome-based interventions to prevent or mitigate these debilitating conditions in older adults.

Abstract citation ID: igaf122.2541

### EFFECTS OF CURCUMIN AND SULFORAPHANE ON C. ELEGANS HEALTHSPAN: INDEPENDENT AND COMBINED IMPACTS

R.P. Vivek-Ananth[1], Stephen Phipps[2], David Weinkove[3], and Nathan Price[4], *1. Buck Institute for Research on Aging, Novato, California, United States, 2. Thorne HealthTech Inc., New York, New York, United States, 3. Durham University, Durham, England, United Kingdom, 4. The Buck Institute, Navato, California, United States*

Aging involves a progressive decline in bodily functions, underscoring the need for interventions that enhance healthspan—the period of life spent in good health. Natural products like curcumin and sulforaphane show promise in modulating aging-related pathways; however, their combined effects remain largely unexplored. This study evaluated the healthspan-enhancing potential of nine natural products in Caenorhabditis elegans, focusing on the individual and combined effects of curcumin and sulforaphane. Using high-throughput automated phenotyping with WormGazer™, we measured movement-based healthspan metrics—the fraction of moving worms, mean speed, and distance moved—from days 2 to 7 of adulthood. Toxicological evaluations confirmed that all compounds were non-toxic at the selected concentrations. Curcumin increased the fraction of moving worms in early and late adulthood, although its positive effect on speed was limited. In contrast, sulforaphane enhanced speed and distance moved in late adulthood but had variable effects on early mobility. Notably, the combination of curcumin and sulforaphane produced effects similar to those of sulforaphane alone, with no additive benefits observed across mobility metrics. Transcriptomic analyses revealed distinct gene expression profiles for each treatment. Further AI-based analysis identified divergent molecular targets and pathways, explaining the differing effects of the compounds. These findings highlight the complementary roles of curcumin and sulforaphane in improving healthspan metrics in C. elegans. While both compounds independently enhance aspects of mobility, their lack of synergy underscores the complexity of multi-target interventions in aging biology. This study offers new insights into the molecular mechanisms modulated by natural products and supports their potential in promoting healthy aging.

Abstract citation ID: igaf122.2542

### EXPLORING ACETYL-COA-MEDIATED BETA-HYDROXYBUTYRATE ENRICHMENT IN YEAST: A MECHANISTIC LINK TO LONGEVITY

Swarup Mishra[1], Connor Oakes[1], Elisa Enriquez Hesles[1], and Jeffrey Smith[2], *1. University of Virginia, Charlottesville, Virginia, United States, 2. University of Virginia School of Medicine, Charlottesville, Virginia, United States*

Aging predisposes organisms to diseases ranging from diabetes to Alzheimer's, driving interest in interventions like Caloric Restriction (CR), which extends lifespan across species. While CR's benefits are well-documented, its metabolic mechanisms remain incompletely understood. This study investigates β-hydroxybutyrate (βHB), a ketone body, in CR-mediated longevity using Saccharomyces cerevisiae. Despite the absence of canonical ketogenesis enzymes (HMG-CoA lyase, OXCT1) in yeast, we detect βHB for the first time in stationary-phase and CR conditions via metabolomics, GC-MS, and luminescence assays. Alternative carbon sources and supplementing yeast media with acetoacetate drive βHB levels. In addition, we identify mitochondrial acetyl-CoA metabolism (ACS1) and mtFAS pathways to be critical for βHB enrichment during CR. βHB supplementation extends chronological lifespan dose-dependently, mirroring CR's effects, and combined CR + βHB yields additive lifespan extension. Transcriptomic profiling reveals 156 overlapping genes and 268 βHB-exclusive targets, suggesting shared (TCA cycle, amino acid metabolism) and βHB-specific mechanisms (mitochondrial translation, stress response). These findings resolve yeast's unexpected capacity for ketogenesis and establish it as a model for dissecting βHB's role in aging. This work also elucidates conserved nodes linking ketogenesis to longevity and positions yeast as a platform for exploring CR-mimicking therapeutics.

Abstract citation ID: igaf122.2543

### FIBROBLASTS EXPOSED TO SERUM COLLECTED AFTER LONG-TERM CALORIE RESTRICTION HAVE LOWER MITOCHONDRIAL RESPIRATION

Lek Wei Seow[1], Stephen Dozier[2], Nina Sun[2], Jaclyn Bergstrom[2], Nathan LeBrasseur[3], and Anthony Molina[4], *1. University of California San Diego School of Medicine, Division of Geriatrics, Gerontology, and Palliative Care, La Jolla, California, United States, 2. University of California San Diego, San Diego, California, United States, 3. Mayo Clinic, Rochester, Minnesota, United States, 4. University of California San Diego, La Jolla, California, United States*

Multiple lines of evidence suggest that calorie restriction may slow biological processes related to aging and extend lifespan. To determine whether calorie restriction affects mitochondrial function, we treated fibroblasts from adult donors with serum samples obtained from participants of the CALERIE (Comprehensive Assessment of Long-term Effects of Reducing Intake of Energy) study. The CALERIE study enrolled healthy, non-obese middle-aged (average 38y.o) participants to examine the effects of long-term calorie restriction. We studied the serum samples of 177 participants (calorie restriction CR = 121, ad libitum AL = 56) who had specimens available at two timepoints (12 and 24 months). We assessed the basal and maximal mitochondrial respiration of fibroblasts treated with dilute serum for 24 hours. Our results show that the baseline basal and maximal mitochondrial respiration of fibroblasts is positively correlated with the adiposity of the serum donor ($R = 0.112$, $P = 0.137$; $R = 0.183$, $P = 0.015$, respectively). Interestingly, treatment of fibroblasts with serum collected after 12 months of calorie restriction causes a decrease in both basal and maximal mitochondrial respiration ($P = 0.1854$; $P = 0.0623$, respectively). Fibroblasts treated with serum collected after 24 months of calorie restriction exhibited a further reduction in both basal and maximal mitochondrial respiration ($P = 0.0174$; $P = 0.0092$, respectively). These findings suggest that the serum of individuals on long term calorie restriction diet comprises factors that can reduce oxidative phosphorylation. This reduction in bioenergetic capacity may align with the theory that calorie restriction leads to a cellular energy-conserving state, which could have implications for cellular aging and metabolism.

Abstract citation ID: igaf122.2544

### HIV AND BACTERIAL LIPOPOLYSACCHARIDE ACTIVATE INFLAMMASOME IN HUMAN ORAL KERATINOCYTES: IMPLICATIONS FOR AGING

Md Shafayat Jamil[1], Sofia Lerma[1], Deepa Roy[1], Chun Xu[1], Hansapani Rodrigo[1], Nirakar Sahoo[2], and Upal Roy[1], *1. The University of Texas Rio Grande Valley, Brownsville, Texas, United States, 2. The University of Texas Rio Grande Valley, Edinburg, Texas, United States*

HIV infection alters the oral microbiome, elevating the presence of Gram-negative bacteria, particularly Porphyromonas gingivalis. P. gingivalis releases lipopolysaccharide (LPS), a powerful immune activator that provokes inflammation in primary human oral keratinocytes, even in antiretroviral therapy-treated people with HIV (PWH). Chronic inflammation is a characteristic of aging, and the activation of inflammasomes is pivotal in the process of inflammation. The Absence of melanoma 2 (AIM2) inflammasome, a DNA-sensing complex, is associated with age-related immunological dysregulation. We propose that HIV sensitizes human oral keratinocytes (HOK) cells to augment AIM2 activation in response to LPS, hence contributing to persistent inflammation and expedited cellular aging. To investigate this hypothesis, HOK was exposed to P. gingivalis LPS at concentrations ranging from 1 ng to 1 µg for 96 hours, and the expression of inflammasome-related genes and proteins was characterized. Concurrently, HOK cells were subjected to HIV exposure for 24 hours and then received LPS treatment for an additional 24 hours. LPS alone increased the expression of AIM2, NLRP3, CASPASE1, CASPASE4, and CASPASE5 in a dose-dependent exposure study. HIV pre-exposure markedly augmented AIM2 activation, resulting in a statistically significant increase in CASPASE 5 and AIM2 gene and protein expression in the presence of LPS. This synergistic effect indicated that HIV prepares HOK for increased inflammasome activation, potentially expediting cellular senescence and immunological aging. Our findings highlight a set of pivotal biomarkers connecting HIV-induced inflammation to cellular senescence, indicating it as a prospective therapeutic target for alleviating inflammation in PWH.

Abstract citation ID: igaf122.2545

### ISOFORM-SPECIFIC ROLES OF SIRT2 IN REGULATING MITOCHONDRIAL GENE EXPRESSION

Xiaomin Zhang[1], Jyotsna Shrivastava[1], Gohar Azhar[2], and Jeanne Wei[2], *1. University of Arkansas for Medical Sciences, Little Rock, Arkansas, United States, 2. University of Arkansas for Medical Sciences, Little Rock, AR, LITTLE ROCK, Arkansas, United States*

Sirtuin-2 (SIRT2), a member of the NAD+-dependent deacetylase family, is a key regulator of cellular processes such as metabolism, stress response and aging. We hypothesize that SIRT2 isoforms (v1, v2, v3) exert isoform-specific regulatory effects on mitochondrial-related genes, influencing mitochondrial dynamics, energy metabolism, and cellular homeostasis. To investigate this, SH-SY5Y cells, from a neuroblastoma cell line were transfected with empty vector (EV) or SIRT2 isoforms (v1, v2, v3), and the expression of mitochondrial genes, including Complex I subunits (NDUFAB1, NDUFV1, NDUFV2, NDUFS1), mitochondrial biogenesis and energy metabolism regulators (PGC-1α, PGC-1β), and mitochondrial dynamics and morphology markers (MFN-1, MFN-2, FiS1), were analyzed using quantitative RT-PCR (QuantStudio 3 Real-Time PCR System). Results revealed that SIRT2-v1 significantly upregulated mRNA levels of NDUFAB1 ($p < 0.001$), NDUFV1 ($p < 0.01$), and NDUFS1 ($p < 0.05$) compared to v2 and v3. SIRT2-v1 also increased PGC-1α expression ($p < 0.05$), numerically increased PGC-1β expression too and MFN-2 ($p < 0.01$), which are vital for mitochondrial biogenesis and mitochondrial fusion. These findings highlight the isoform-specific roles of SIRT-2 in modulating mitochondrial gene expression, with SIRT2-v1 predominantly influencing Complex I and PGC-1α. This study provides insights into the distinct regulatory effects of SIRT-2 v1 isoforms on mitochondrial function and metabolic pathways, underscoring their potential therapeutic relevance in mitochondrial-associated disorders. Additionally, the differential age-related increase in SIRT2 isoforms warrants further investigation.

Abstract citation ID: igaf122.2546

### METABOLOMICS REVEALS EXOSOME PROTECTION IN AGING MOUSE KIDNEYS VIA FERROPTOSIS ALLEVIATION

Yanqiu Xing[1], and Liu Di[2], *1. Qilu hospital of Shandong University, Jinan, Shandong, China (People's Republic), 2. Shandong University, Jinan, Shandong, China (People's Republic)*

This study evaluated the effects of human umbilical cord mesenchymal stem cell-derived exosomes (hUCMSC-exos) on renal function in naturally aging mice and explored underlying mechanisms. Twenty-four-month-old C57BL/6J mice were divided into a natural aging group (WT-AC, $n = 10$) and an hUCMSC-exos intervention group (WT-AEX, $n = 10$). Blood and kidney tissues were analyzed for biochemical markers, histological changes, and metabolomic profiles. hUCMSC-exos significantly reduced blood urea nitrogen, creatinine, and uric acid levels compared to the natural aging group ($P < 0.01$). Histological examination revealed reduced inflammatory cell infiltration, tubular degeneration, and interstitial fibrosis in the hUCMSC-exos group. Transmission electron microscopy demonstrated mitochondrial shrinkage, cristae rupture, and podocyte foot process effacement in the natural aging group, while the hUCMSC-exos group exhibited restored mitochondrial morphology, including intact cristae and reduced swelling. Aging markers (SA-β-Gal, p16INK4a, and γH2AX) were downregulated in the hUCMSC-exos group. Metabolomics identified 62 differential metabolites, with indoxyl sulfate as a potential biomarker for age-related renal injury. Enriched pathways included glutathione metabolism, glycerolipid metabolism, and aminoacyl-tRNA biosynthesis. hUCMSC-exos inhibited ferroptosis by reducing divalent iron ions and malondialdehyde levels, increasing the glutathione/oxidized glutathione ratio, and upregulating glutathione peroxidase 4 expression. These findings suggest that hUCMSC-exos improve renal function, reduce inflammation and fibrosis, and mitigate aging-related structural changes in naturally aging mice. The study highlights the protective role of hUCMSC-exos against renal aging through modulation of key metabolic pathways and ferroptosis inhibition, providing potential therapeutic targets for aging-related kidney injury.

Abstract citation ID: igaf122.2547

### SYSTEMS-LEVEL PLASMA BIOMARKERS FOR TISSUE HEALTH: INTEGRATING PROTEIN NETWORKS WITH RAG BIOLOGICAL INSIGHTS

Qiongrong Huang[1], John Earls[1], Noa Rappaport[2], Lance Pflieger[3], Kengo Watanabe[4], and Nathan Price[1], *1. The Buck Institute, Navato, California, United States, 2. Institute for Systems Biology, Buck Institute, Phenome Health, Seattle, Washington, United States, 3. Phenome Health, Seattle, Washington, United States, 4. Tokushima University Graduate School of Biomedical Sciences, Tokushima, Tokushima, Japan*

Plasma proteomics offers a promising avenue to infer tissue-specific biological processes; however, the regulatory relationships between plasma and tissues remain unclear. Using paired plasma-tissue proteomic data from 172 samples spanning muscle, ovary, and breast tissues, we we analyzed 5,415 proteins and employed correlation analyses and Lasso regression to identify plasma protein signatures predictive of tissue protein levels. To validate and contextualize these findings, we integrated a retrieval-augmented generation (RAG) model powered by large language models (LLMs) to enrich our understanding of systemic protein interactions. We uncovered robust cross-tissue regulatory protein modules independent of age and sex, primarily mediating immune system regulation and steroid hormone. Sex- and age-dependent regulatory modules were identified using weighted gene co-expression network analysis (WGCNA), revealing distinct aging effects: in males, aging impacted modules related to muscular and reproductive system development, angiogenesis regulation, and non-canonical Wnt signaling (Spearman's $\rho = 0.66$, $p < 2.2 \times 10^{4l6}$, whereas in females, aging primarily affected cell-cell interactions, angiogenesis, extracellular matrix organization, and nervous system development (Spearman's $\rho = 0.34$, $p < 2.2 \times 10^{4l6}$). Plasma-tissue modules exhibited functional parallels with minimal protein overlap, suggesting regulatory mediation via distinct proteins. Using Differential Rank Conservation (DIRAC), we further identified key plasma-tissue interactions associated with longevity. These findings provide a systems-level perspective on the plasma proteome as a noninvasive window into tissue biology, with implications for aging research.

Abstract citation ID: igaf122.2548

### VPS-34 INHIBITION AMELIORATES POSTURAL AGING BY REGULATING GABAERGIC MOTOR NEURON ACTIVITY IN C. ELEGANS

Xuhui Chen, Le Zhang, Shangbang Gao, and Cuntai Zhang, *Huazhong University of Science and Technology, Wuhan, Hubei, China (People's Republic)*

Motor aging encompasses various functional and postural phenotypes, including reduced movement speed and increased body stiffness and curvature. These phenotypes reflect physiological aging in multiple organs, such as bones (calcium loss, reduced collagen content and osteoporosis) and muscles (atrophy, decreased elasticity, reduced endurance and flexibility). However, the neurobiological mechanisms regulating motor aging, particularly the specific molecular pathways and potential interventions, are not well understood. In this study, we used Caenorhabditis elegans to model motor aging. Aging worms exhibited reduced movement speed and increased body curvature, similar to human aging phenotypes. Investigations into synaptic morphology and neurotransmission function at C. elegans neuromuscular junction revealed that decreased excitatory motor neuron activity contributed to the reduction in movement speed. Notably, an imbalance between excitatory and inhibitory signaling resulted in the increased body curvature. Mutation of the phosphoinositide 3-kinase (PI3K) VPS-34, a key regulator of endosomal trafficking, alleviated the curvature defects associated with aging. This alleviation was linked to enhanced inhibitory GABAergic motor neuron activity, suggesting a protective role of VPS-34 inhibition in postural aging. Our findings demonstrate that different motor aging phenotypes are regulated by distinct neural mechanisms and VPS-34 regulates postural aging by modulating inhibitory motor neuron activity. These results thus offer new insights into VPS-34 inhibition as a potential neuro-intervention strategy for mitigating motor aging.

### SESSION 7195 (POSTER)

### CLINICAL PRACTICE

Abstract citation ID: igaf122.2549

### CHARACTERISTICS OF NURSE-REPORTED ADVERSE EVENTS: AN EXPLORATION BASED ON FAERS DATA

Jieyu Mao, Miyae Yamakawa, and Yasushi Takeya, *Osaka University, Osaka, Osaka, Japan*

Adverse events (AEs) are undesirable experiences linked to medical product use, particularly in aging populations with polypharmacy. Nurses play a critical role in AE reporting due to their close involvement in patient care and medication administration. This study used the FDA Adverse Event Reporting System (FAERS) database to analyze the characteristics of nurse-reported AEs and highlight nurses' role in pharmacovigilance. Data from the Side Effect Resource (SIDER) database were integrated to assess the proportion of unknown adverse drug reactions in reported AEs, while report quality and completeness were evaluated using the VigiGrade scoring system, a method for assessing the quality of individual case safety reports. After extraction and deduplication, 8,653 nurse-submitted reports were analyzed, with the majority concentrated between 2012 and 2014, accounting for only 5% of all reports during this period. Nurses primarily reported AEs related to general disorders and administration site conditions, with a strong focus on tumor-related indications and specific therapies. The average time from event occurrence to reporting was 247.8 days, the shortest among all occupations. The proportion of reported AE symptoms not found in the SIDER database was 11.82%. Nurses achieved the highest VigiGrade scores across all dimensions, demonstrating the ability to provide high-quality AE reports. However, the role of nurses remains undervalued, restricting their ability to identify unknown ADRs and apply unique clinical perspectives. To strengthen the role of nurses, targeted training programs and policy improvements are necessary to reduce barriers and enhance their participation in AE reporting.

Abstract citation ID: igaf122.2550

### ENHANCING MINDFULNESS IN OLDER ADULTHOOD: MIXED-METHOD FINDINGS FROM A COMMUNITY-BASED GROUP INTERVENTION

Tiana Broen[1], Jonathan Sundby[1], Christopher Griffith[1], and Leilani Feliciano[2], *1. University of Colorado Colorado Springs, Colorado Springs, Colorado, United States, 2. University of Colorado Colorado Springs Department of Psychology, Colorado Springs, Colorado, United States*

Research indicates that mindfulness skills, such as breathing exercises and meditation, effectively reduce psychological symptoms and increase overall well-being. In older adulthood, mindfulness-based interventions are an accessible, cost-effective, and adaptable way to support emotional well-being in everyday life. The current study aimed to examine the efficacy of an 8-week mindfulness skill-building groups on older adults in a community outpatient clinic in the Rocky Mountain Region. Quantitative and qualitative data were gathered across four groups between 2023 and 2024 ($N = 22$, Mage = 72.13, $SD = 5.42$, 77.27% female). Participants completed pre- and post-measures of depression, anxiety, stress, well-being, pain, sleep, and trait-mindfulness. To provide a subjective account of specific changes participants had noticed in themselves because of their mindfulness practice, participants were asked to provide narrative feedback, which was recorded and transcribed for thematic analysis. Paired-sample t-tests indicated a significant decrease in perceived stress [$t(21) = 3.01$, $p<.01$], depressive symptoms [$t(16) = 2.09$, $p<.05$], and anxiety symptoms [$t(21) = 3.18$, $p<.01$], as well as an increase in trait mindfulness [$t(20) = -4.65$, $p< .01$] and overall well-being [$t(21) = -3.24$, $p<.01$]. Thematic analyses were conducted with moderate-good inter-rater reliability ($\kappa$=.59-.63), and revealed themes related to the primary outcome variables, in addition to themes of overall effectiveness, interpersonal functioning, group cohesion, mindfulness skills, insight, and positive self-concept. These results indicate that mindfulness-based group interventions can be effective for reducing psychological symptoms, increasing overall well-being, and providing an opportunity for meaningful connection with older adult peers in a group setting.

Abstract citation ID: igaf122.2551

### IMPACTFUL RECOMMENDATIONS FOR OLDER VETERANS: EXPERIENCES OF NEUROPSYCHOLOGISTS USING TELEHEALTH

DJ King[1], Maureen O'Connor[2], and Malissa Kraft[1], *1. Bedford VA Medical Center, Bedford, Massachusetts, United States, 2. Bedford Veterans Health Care System, Bedford, Massachusetts, United States*

Access to in-person healthcare is a barrier for older adult Veterans who live in rural areas or cannot access reliable transportation. This is especially concerning for Veterans with cognitive or neurodegenerative concerns, for which a neuropsychological evaluation is essential for diagnosis and treatment. In response, Veteran Affairs (VA) offers synchronous telehealth services through VA Video Connect (VVC) to Veterans in their homes or through Clinical Video Telehealth (CVT) at nearby Community-Based Outpatient Clinics (CBOCs). Five neuropsychologists were interviewed about their experiences conducting neuropsychological evaluations via telehealth and their challenges offering subsequent recommendations. Of the five providers, three conducted tele-neuropsychological evaluations full-time and two split their time between in-person and telehealth care. Two providers were working with a national Veteran sample, two providers with a regional sample, and one provider with a local sample, each seeing two to four cases per week on average. Reported challenges when conducting tele-neuropsychological evaluations included technological illiteracy, Veteran discomfort, distractors and background noise, differences across VA facilities, scheduling complications, and Veteran transportation issues. Common trends emerged regarding challenges to making virtual recommendations for Veterans with barriers to in-person care, including uncertainty about available VA services for remotely seen Veterans, limitations in accessing community resources, limitations in transportation services for in-person care recommendations, and variable assistance from referring providers. We will also present our manualized recommendations guide for tele-neuropsychologists, as well as preliminary feedback about the guide. Though developed for tele-neuropsychologists, the guide has potential wide scale usability for all providers working with older Veterans.

Abstract citation ID: igaf122.2552

### SGLT2 INHIBITORS IN OLDER ADULTS WITH CARDIOVASCULAR DISEASE: A SYSTEMATIC REVIEW AND META-ANALYSIS

Kota Minami[1], Rika Terashima[2], Yuji Yamada[3], and Satoshi Miyashita[4], *1. Saga University, Saga, Saga, Japan, 2. Stanford University, Stanford, California, United States, 3. Icahn School of Medicine at Mount Sinai, New York, New York, United States, 4. Cleveland Clinic Foundation, Cleveland, Ohio, United States*

SGLT2 inhibitors have shown significant cardiorenal benefits, leading to their use in various cardiovascular conditions. However, there is limited data on their effectiveness in older adults. This systematic review and meta-analysis of retrospective cohort studies (RCTs) published between January 2015 and January 2025 used MEDLINE. Eligible studies reported hazard ratios (HR) for cardiovascular outcomes in adults aged ≥65 years with cardiovascular disease. The primary outcome was a composite of hospitalization for heart failure (HHF), urgent HF visits, and cardiovascular death (CVD), while secondary outcomes included all-cause mortality, CVD, and HHF individually. Subgroup analyses were conducted for the primary endpoint in studies enrolling patients with HF, T2DM, adults aged 65-74 years, and those ≥75 years, and by SGLT2 inhibitor agent type. Nine RCTs were included. SGLT2 inhibitors significantly reduced the composite outcome (HR 0.75, 95% CI 0.67–0.83, $I^2 = 51\%$), all-cause mortality (HR 0.80, 95% CI 0.66–0.97, $I^2 = 68\%$), CVD (HR 0.78, 95% CI 0.65–0.94, $I^2 = 61\%$), and HHF (HR 0.73, 95%CI 0.65–0.83, $I^2 = 0.0\%$). Subgroup analyses showed consistent benefits in patients with HF (HR 0.76, 95% CI 0.70-0.82, $I^2 = 10\%$), T2DM (HR 0.65, 95% CI 0.44-0.95, $I^2 = 81\%$), and those ≥75 years (HR 0.71, 95% CI 0.57-0.88, $I^2 = 51\%$). No significant difference was found between SGLT2 inhibitor subgroups ($p = 0.09$). These results support the use of SGLT2 inhibitors in older adults with cardiovascular disease, with careful monitoring for age-related risks.

Abstract citation ID: igaf122.2553

### TAU POSITIVITY DID NOT CORRELATE WITH PHQ9 DEPRESSION SCORES IN A MEMORY CLINIC.

Viviana Obando, Malini Nair, Rui Huang, Surya Sunil, Kavya Chemalapatti, and Anil Nair, *ADC, Braintree, Massachusetts, United States*

Background: It is unknown if depression levels are correlated to positivity on tau blood or PET scan results in memory clinic patients. Method: We performed a retrospective analysis of memory clinic patients in the south shore of Boston from 2010 to 2025. We correlated depression screen data (PHQ9) to tau PET or Blood test positivity scores. Univariate analyses used Pearson correlation. A multivariate regression model analyzed PHQ9 to covariates of tau positivity, age, sex, education and race. Hypothesis: We hypothesized a positive correlation between depression levels scored by the PHQ9 and abnormal tau test results. Results: 1043 patients attended the memory clinic between 2010-2025; 20 had PHQ9 and tau blood test results with analyzable data. Patients were 58.9% female, 85.1 % White. Mean age was 70±14.2 years, Tau score did not correlate significantly to PHQ9 Multivariate model confirmed the lack of association of depression scores to tau abnormality (100000= 0.08, $p = 0.44$). Conclusions: Depression scores using PHQ9 during screening were not affected by tau positivity as measured as a binary outcome during tau disclosure in a memory clinic.

### SESSION 7200 (POSTER)

### COGNITION II

Abstract citation ID: igaf122.2554

### AGE-RELATED DIFFERENCES IN MORAL DECISION-MAKING: UTILITARIAN VS. DEONTOLOGICAL JUDGMENTS

Isu Cho[1], and Angela Gutchess[2], *1. Sungkyunkwan University, Seoul, Republic of Korea, 2. Brandeis University, Waltham, Massachusetts, United States*

Research has suggested that older people are more likely to make deontological (i.e., focusing on rules and duties), rather than utilitarian (i.e., focusing on the consequences to maximize welfare the most people), moral judgments compared to younger adults. Although deontological and utilitarian judgments are in independent relationships, previous studies on age differences in moral decision-making have treated them as endpoints (i.e., higher on one automatically means lower on the other). Therefore, it is uncertain whether the age-related differences come from deontological judgments, utilitarian judgments, or both. The current study compared 65 younger (aged 18 to 22) and 63 older (aged 60 and above) adults' judgments in a moral dilemma. Using a traditional moral dilemma analysis in which deontological and utilitarian judgments are considered as reciprocal, results corresponded with previous findings in that older adults made more deontological (less utilitarian) moral judgments. Using a process dissociation analysis that produces separate parameters for utilitarian and deontological judgments, results revealed that older adults made utilitarian judgments less compared to younger adults and that there was no age difference in deontological judgments. Such age differences in utilitarian judgments were mediated by working memory capacities. Results imply that the age-related declines of working memory may reduce one's ability to mentally calculate costs and benefits of actions in a moral dilemma, leading older adults to make utilitarian decisions less during moral judgments.

Abstract citation ID: igaf122.2555

### ASSOCIATION BETWEEN LEISURE ACTIVITY PATTERNS AND COGNITIVE FUNCTION FOR U.S. OLDER ADULTS

Yan-Jhu Su[1], Elizabeth Dugan[2], Jaqueline Avila[2], and Laura Hayman[3], *1. The Pennsylvania State University, University Park, Pennsylvania, United States, 2. University of*

*Massachusetts Boston, Boston, Massachusetts, United States, 3. University of Massachusetts Boston Manninng College of Nursing & Health Sciences, Boston, Massachusetts, United States*

This study explores the longitudinal impact of leisure activity patterns on cognitive function among U.S. older adults, utilizing data from the Health and Retirement Study (HRS) and the Consumption and Activities Mail Survey (CAMS). Using sequence analysis and clustering, five distinct leisure activity patterns were identified, ranging from diverse engagement to restricted or declining participation. Participants in the "All Activities" cluster demonstrated the highest cognitive scores, while those in "Gradual Cessation of Activities" or "Household-Only Activities" exhibited significant cognitive decline. Stratified analyses revealed that older adults, women, and individuals with lower educational attainment were more adversely affected by limited or inconsistent activity patterns. The findings highlight the protective role of diverse and sustained leisure engagement in cognitive health and underscore the moderating effects of demographic factors, including age, gender, and education. These results emphasize the need for tailored interventions to promote diverse leisure activities, address demographic disparities, and support healthy cognitive aging in diverse populations.

Abstract citation ID: igaf122.2556

### COGNITIVE AGING AMONG FIRST-GENERATION COLLEGE GRADUATES AND THOSE WITH DIFFERENT EDUCATIONAL BACKGROUNDS

Masahiro Toyama, and Lexi Giroski, *Marshall University, Huntington, West Virginia, United States*

Research is lacking on first-generation college students' postgraduate outcomes, particularly aging-related cognitive outcomes. While previous research suggested the positive implications of one's own and/or parents' education for cognitive aging, the present study aims to uniquely contribute to the literature by contrasting aging first-generation college graduates (FGCGs) and others with different educational backgrounds. We specifically examined whether college graduation predicted better cognitive outcomes and whether the results of FGCGs were comparable to continuing-generation college graduates (CGCGs; having parent(s) with college education). We analyzed two-wave data from the Cognitive Projects of the Midlife in the United States (MIDUS) (N = 4,206 at Time 1: mean age = 56.0, SD = 12.3; 54% female) while including education variables from the MIDUS main surveys. Our multiple regression analyses indicated that participants' college graduation, but not parents' college education, predicted verbal memory at Time 1 and its residualized change over a decade (i.e., Time 2 verbal memory controlling for its Time 1/baseline levels). In contrast, both participants' college graduation and parents' college education predicted Time 1 executive function, though neither predicted its change. There was an interaction effect, indicating that Time 1 executive function did not differ between FGCGs and CGCGs, while it differed by their parents' education among non-college graduates. In other words, the advantage of having college-educated parents appeared limited to those who did not graduate from college. By showing comparable results between FGCGs and CGCGs, this study highlights the importance of completing college education, particularly for those whose parents lacked college education.

Abstract citation ID: igaf122.2557

### COGNITIVE ASPECTS OF THE MOTOR PLANNING STAGE OF GAIT: EFFECTS OF AGING AND PARKINSON'S DISEASE

Jeffrey Hausdorff[1], Galia Shaham[2], Irina Galperin[1], Amit Salomon[1], Eran Gazit[3], Aron Buchman[4], and James Richardson[5], *1. Tel Aviv Sourasky Medical Center, Tel Aviv, Tel Aviv, Israel, 2. Tel Aviv University, Tel Aviv, Tel Aviv, Israel, 3. Tel Aviv Sourasky Medical Cetner, Tel Aviv, Tel Aviv, Israel, 4. Rush University, Chicago, Illinois, United States, 5. University of Michigan, Ann Arbor, Michigan, United States*

The response to a request to walk involves a motor planning phase followed by an execution phase. The initial phase of gait initiation, specifically the time to anticipatory postural adjustment (APA), can be viewed as a form of reaction time. However, it is not clear how to characterize the cognitive processes involved in this stage. To address this question, time-to-APA, simple and complex upper limb visuomotor reaction time (SRT, CRT), cognitive, and motor performance were evaluated in 27 people with Parkinson's disease (PD), 31 older adults (OA), and 34 young adults (YA). Our results showed that time-to-APA was significantly longer than SRT in all three groups ($p < 0.001$), indicating a more complex cognitive process. In YA, time-to-APA was significantly shorter than CRT ($p < 0.001$). In the OA and PD, time-to-APA was not significantly different from CRT. Mixed-effects analysis showed significant time ($p < 0.001$), group ($p = 0.037$), and group × time interaction effects ($p = 0.002$). Among all subjects, time-to-APA, but not APA duration, was associated with the Color-Trails Test (part B: $rs = 0.406$, $p < 0.001$). In PD, APA duration was correlated with MDS-UPDRS-part 3 (motor) scores ($rs = 0.535$, $p = 0.004$), but time-to-APA was not ($p = 0.892$). These findings suggest that time-to-APA is a cognitive process that is more complex than a SRT task and shares properties of a CRT task, especially among older adults and people with PD. In PD, this initial movement planning stage is not related to motor impairment, in contrast to APA duration. Further research is necessary to identify the factors underlying this initial stage of gait initiation.

Abstract citation ID: igaf122.2558

### COMMUNITY RATES OF INTERNET USE AND COGNITIVE CHALLENGES AMONG OLDER ADULTS IN MA & RI

Dongfang Hong, Mengshi Liu, Sophia Casale, Calvin Tran, and Elizabeth Dugan, *University of Massachusetts Boston, Boston, Massachusetts, United States*

Cognitive health is vital for aging well. Internet use may support cognitive health through social engagement and mental stimulation. This study examines the relationship between internet use and cognitive difficulties among older adults (65+) in Massachusetts and Rhode Island, highlighting potential digital inequalities. This study utilizes data from the 2025 Healthy Aging Reports (see healthyagingdatareports.org). Specific data sources: internet use 60 + (BRFSS 2018-2022), Alzheimer's disease prevalence age 65 + (CMS 2020-2021), and self-reported cognition difficulty age 65 + (ACS 2018-2022). Small area estimation techniques were used to calculate age and sex-adjusted rates at the community and state level. Results show that in MA, 70.57% of individuals aged 60+ reported internet use in the past month, with significant geographic disparities (ranging from 40.83% to

88.95%). The prevalence of Alzheimer's disease among Medicare beneficiaries aged 65+ was 12.92%, with community rates varying from 6.69% to 25.54%. Self-reported cognition difficulty in MA averaged 7.47%, with rates as high as 80.77% in certain communities. In RI, 71.43% of individuals aged 60+ reported recent internet use, with community variations from 54.75% to 88.70%. Alzheimer's disease prevalence among older adults was 12.04%, with community rates ranging from 7.25% to 16.27%. Cognition difficulty in RI was self-reported by 7.01% of the population aged 65+, with community-specific rates as high as 12.71%. Disparities exist in internet use and cognitive health among older adults. Addressing digital inequalities through targeted interventions can promote cognitive resilience, ensuring equitable access to technology's benefits for aging populations in Massachusetts and Rhode Island.

Abstract citation ID: igaf122.2559

### EFFECT OF NON-COGNITIVE TRAITS ON SUBJECTIVE COGNITIVE DECLINE AMONG BLACK AMERICAN ADULTS

Yolanda Jackson[1], Maria Clark[2], Sunkanmi Folorunsho[3], Obinna Odo[4], Candidus Nwakasi[5], and Darlingtina Esiaka[6], *1. University of Kentcuky, Lexington, Kentucky, United States, 2. University of Kentucky, Lexington, Kentucky, United States, 3. University of Nebraska-Lincoln, Lincoln, Nebraska, United States, 4. Miami University, Oxford, Ohio, United States, 5. University of Connecticut, Storrs, Connecticut, United States, 6. University of Kentucky - College of Medicine, Lexington, Kentucky, United States*

Research shows that subjective cognitive decline (SCD) is an early indicator of cognitive impairment, and it is associated with non-cognitive traits. Among Black Americans, non-cognitive traits such as grit and resilience have been found to influence their cognitive and emotional status. However, the role of grit and resilience in SCD among Black Americans remains understudied. This study examined the effects of grit and resilience on SCD among Black American men and women while controlling for age, socioeconomic status (SES), and marital status. Participants responded to standardized measures assessing SCD, grit, resilience, and demographic information. We performed multiple linear regression analyses using data from 242 participants and stratified by sex (N = 146 women; Mean age = 44.3). Among the Black American women, both grit and resilience were associated with SCD, with higher grit predicting higher SCD scores ($\beta = 0.339$, $p < .001$) and higher resilience predicting lower SCD scores ($\beta = -0.234$, $p = .007$). However, in men, only grit was associated SCD ($\beta = 0.483$, $p < .001$), with higher grit predicting higher SCD scores. Sex differences in the relationship between psychological resilience factors and SCD suggest that grit is a stronger predictor of SCD in both sexes, whereas resilience plays a protective role only in females. Understanding how non-cognitive traits impact SCD can help inform early interventions to help reduce cognitive decline risks in aging Black populations.

Abstract citation ID: igaf122.2560

### EPIGENETIC AGING MEDIATES THE ASSOCIATION OF DIET QUALITY AND COGNITION IN LATE ADULTHOOD

Marrium Mansoor, Elayna Seago, and Benjamin Katz, *Virginia Tech, Blacksburg, Virginia, United States*

Diet quality is a major factor that affects physical and cognitive health in later life. One possible mechanism for this may be through epigenetic modifications, including DNA methylation (DNAm). DNAm is associated with epigenetic age acceleration (EAA). EAA can be measured by epigenetic clocks that quantify the level of DNAm to provide an estimate of an individual's biological age. GrimAge is an epigenetic clock that is associated with cardiovascular risk factors and predicts mortality and morbidity. Links between diet quality and EAA with cognition in later life have not yet been fully examined in a representative sample of older adults in the United States. We drew from 1792 participants (Mage = 70.3, 59.3% female, 78% white) from the Health and Retirement Study to examine whether EAA mediated the relationship between diet quality and cognitive performance in later life. Diet was assessed using adherence to the Dietary Approaches to Stop Hypertension (DASH) dietary pattern that optimize cardiovascular health by prioritizing consumption of vegetables, fruits, whole grains, fish, poultry, beans and nuts. Mediation models were used to examine whether EAA mediated the association between diet quality and performance on Serial 7s, Number Series, Immediate, and Delayed Recall, while controlling for age, gender, race, and years of education. We found that EAA fully mediated the relationship between diet and cognition for all four measures, with higher DASH scores leading to lower EAA and higher cognitive performance. These results reinforce the importance of diet quality on multiple aspects of later health, including cognition.

Abstract citation ID: igaf122.2561

### EXPLORING MULTIGENERATIONAL RELATIONSHIPS AND EPISODIC MEMORY IN LATER LIFE: A STUDY OF CHILDHOOD FAMILY DYNAMICS

Kimson Johnson[1], and Maddison Linker[2], *1. University of Michigan Institute for Social Research, Ann Arbor, Michigan, United States, 2. University of Michigan, Ann Arbor, Michigan, United States*

Introduction: Understanding how childhood family dynamics shape cognitive health in later life is important for addressing cognitive decline, yet this topic remains understudied. This study investigates the impact of parental relationship quality and grandparental co-residence on episodic memory in older adults, exploring whether living with grandparents in childhood buffers the negative cognitive effects of poor parental relationships. Methods: Data from the Health and Retirement Study (HRS) 2015-2017 Life History Mail Survey, the Childhood Family and Health, and the 2020 Cross-Wave Imputation of Cognitive Functioning Measures were used (n = 3,991; Mage = 71.9, SE = 0.27). Parental relationship quality and grandparental co-residence were categorized into four groups: (1) good parental relationship, lived with grandparents; (2) good parental relationship, did not live with grandparents; (3) poor parental relationship, lived with grandparents; (4) poor parental relationship, did not live with grandparents. Episodic memory was measured using total recall (range: 0–20). Linear regression models adjusted for sociodemographic factors, childhood health, childhood SES, household size, and survey weights. Results: Compared to those with good parental relationships and grandparent co-residence, participants with good parental relationships and without grandparental co-residence had lower episodic memory scores ($\beta = -0.52$, $SE = 0.18$, $p < .01$). Participants with poor parental relationships and grandparent co-residence had even lower scores (β

= -1.02, SE = 0.47, p < .05). Conclusion: Findings suggest that poor parental relationships negatively impacts episodic memory, even with grandparental co-residence, emphasizing the importance of considering both parental dynamics and multigenerational factors in cognitive aging research.

Abstract citation ID: igaf122.2562

### HIGH-ER ORDER COGNITIVE FUNCTIONING: METACOGNITION AND CANNABIS USE IN OLDER ADULTS

Elizabeth Anquillare, Caleb Moyer, Juliamaria Coromac-Medrano, and Rachel Thayer, *University of Colorado Colorado Springs, Colorado Springs, Colorado, United States*

Previous research shows metacognition remains stable in older adulthood, despite age-related declines in other cognitive domains. Although cannabis has been demonstrated to have deleterious effects on executive function, which overlaps with metacognition in both construct and underlying neural mechanisms, metacognition has not yet been studied in the context of older adult cannabis use. Additionally, metacognition refers to several skills including self-monitoring and strategy implementation which may have unique relationships with cannabis use. This study aimed to explore associations among subjective cognitive functioning, strategy use, and objective cognitive functioning in a pilot sample of older adults using cannabis (OAUC). Participants were OAUC (N = 17, Mage=68.29, SD = 5.61, range 60-79 years), of whom 59% identified as women and 94% identified as White, with an average 15.94 years of education (SD = 2.38). Participants completed online subjective cognition surveys and a virtual neuropsychological assessment battery. Subjective cognitive functioning and strategy use were not significantly associated, r=.11, ns. Greater strategy use was associated with lower objective processing speed and executive function scores (-.60≤r≤-.40). Better subjective cognitive functioning was associated with worse verbal learning and self-monitoring scores (-.41≤r≤-.31). OAUC may require greater strategy use in the context of worse objective functioning possibly related to cannabis use. Additionally, OAUC may have inflated confidence in their cognitive ability due to successful strategy use, resulting in discrepancies between subjective and objective cognition. Considering the importance of self-monitoring and strategy use in daily functioning (e.g., medication management), more research is needed to understand the role of cannabis in metacognitive ability in older adults.

Abstract citation ID: igaf122.2563

### IMPACT OF VISUAL IMPAIRMENT AND DUAL PROCESSING ON GAIT IN OLDER PEOPLE

Maura Mlecko, Desirae Sandridge, and Evie Burnet, *College of William and Mary, Williamsburg, Virginia, United States*

Research linked visual impairment to cognitive decline in older people; it has not assessed how visual impairment affects cognitive measures, specifically dual processing tasks involving cognition and gait. To investigate whether visual impairment impacts dual processing performance, shown by velocity, stride width, and double support percent. 70 independent living residents aged 71 to 98 were assessed for visual acuity using the Snellen Test. Participants were split into normal vision (48) and people with visual impairment (22). People with visual impairment were defined as presenting or best-corrected visual acuity less than 20/40 in the better eye. They walked at their typical pace on a sensor-embedded mat and repeated this while naming animals beginning with a specific letter for dual processing. A t-test was run with SPSS with significance p < 0.05. Differences between normal vision and individuals with visual impairment were insignificant in gait velocity (p = 0.083) and stride width (p = 0.266) with normal gait, but significant in these measures during a dual processing task. Velocity was significantly faster (p = 0.016) in normal vision compared to people with visual impairment. Stride width was significantly greater (p = 0.022) in people with visual impairment compared to normal vision. There was no significant difference in double support percent in either condition. Visual impairment impacted dual processing performance shown by a slower velocity and greater stride width while performing a dual processing task. Further research is needed to determine if corrective lenses mitigate the effects of visual impairment on dual processing tasks.

Abstract citation ID: igaf122.2564

### INHIBITORY CONTROL IN AGING: A SYSTEMATIC REVIEW AND META-ANALYSIS OF THE FLANKER TASK

Sandryne Guay, and Benjamin Boller, *Université du Québec à Trois-Rivières, Trois-Rivières, Quebec, Canada*

The flanker task is a validated tool for measuring inhibitory control. The flanker effect is characterised by longer reaction times (RT) and lower accuracy on incongruent trials compared with congruent trials. While this effect is well established in young adults, the results for older adults remain inconsistent. The aim of this study is to carry out a systematic review and meta-analysis of the flanker effect in the elderly population. A search of PubMed, PsychInfo and PsycNet identified 22 studies comparing RTs of younger and older people using the flanker task. A meta-analysis of 3 versions of the task (arrows, cues and letters) was carried out. The results indicate that older adults systematically show slower RTs, particularly on incongruent trials, while the accuracy results remain heterogeneous. The various calculations of the cost of inhibition (subtraction, proportions and Inverse Efficiency Score) suggest that the slowing in processing speed in older adults may contribute to some extent to the differences observed. The results of the meta-analysis show a moderate effect of ageing in the version with arrows (g = 0.36), a large effect in the version with cues (g = 0.99) and a small effect in the version with letters (g = 0.22). These results highlight the impact of ageing on inhibition and suggest that the variability between studies could be explained by methodological differences. Standardizing measurement approaches will be crucial to better understand inhibitory control changes with age.

Abstract citation ID: igaf122.2565

### LIFE COURSE STRESSORS AND COGNITIVE FUNCTION: A LATENT PROFILE ANALYSIS APPROACH

Jean Choi[1], and Elizabeth Muñoz[2], *1. The University of Texas at Austin, Texas, United States, 2. University of Texas at Austin, Austin, Texas, United States*

Psychosocial stressors are risk factors for poor cognitive health, but existing research has largely focused on testing associations between single stressor domains or on specific life stages, overlooking the combined role of stressors across the life course. Moreover, marginalized racial/ethnic groups may be more susceptible to the effects of some exposures compared to their counterparts. We aimed to identify life course stressor profiles and examine their associations with cognitive function using the Midlife in the United States study (n = 3,684; age: 32–84). Latent profile analysis identified three profiles of life course stressors based on 11 domains: High Everyday Discrimination, High Work Stress, and High Multi-Domain Stress. Generalized estimating equations tested associations between profile membership and cognitive function. Compared to the High Everyday Discrimination profile, the High Work Stress profile had better executive function (b = 0.07, SE = 0.04, p = .016). Among non-Hispanic Blacks, the positive association between High Work Stress and executive function was weaker than for non-Hispanic Whites (b = -0.41, SE = 0.17, p = .01). The High Multi-Domain Stress profile had higher executive function among Hispanics (b = 0.64, SE = 0.26, p = .013) and greater episodic memory for both non-Hispanic Black (b = 0.44, SE = 0.21, p = .01) and Hispanics (b = 0.61, SE = 0.30, p = .04) compared to non-Hispanic Whites. These results suggest that cognitive health outcomes may be differentially predicted by distinct patterns of life course stressors, and its associations may vary by race/ethnicity.

Abstract citation ID: igaf122.2566

### NUMBER OF CHILDREN AND COGNITIVE AGING IN OLDER AMERICANS: THE ROLE OF SOCIOECONOMIC STATUS AND GENDER DIFFERENCES

Dongfang Hong, and Wuyi Dong, *University of Massachusetts Boston, Boston, Massachusetts, United States*

Evidence on the influence of the number of children on late-life cognitive functions remains inconsistent, particularly regarding its specific effects on men and women. This study analyzed data from the Health and Retirement Study (HRS) (1998–2016), a nationally representative longitudinal survey of older Americans. The sample included 28,391 individuals aged 55 and older. Cognitive function was assessed using a 27-point TICS score incorporating word recall, serial 7s, and backward counting. The number of children was categorized as none, one or two, and three or more. Linear mixed-effects models examined the association between the number of children and cognitive function, adjusting for sociodemographic, health, and lifestyle factors. In the unadjusted model, having three or more children was associated with a significant decline in cognitive functioning ($\beta$ = -0.462, p < 0.001) compared to those with no children. However, after adjusting for covariates, this association reversed, with three or more children linked to higher cognitive function ($\beta$ = 0.335, p < 0.01). Stepwise analyses indicated that the initial negative association was primarily explained away by socioeconomic status (SES), particularly education. Gender-stratified models revealed that the positive effect of having three or more children was stronger in men ($\beta$ = 0.432, p < 0.05) than in women ($\beta$ = 0.234, not significant). These findings underscore the role of SES in shaping the relationship between family structure and gender-specific cognitive trajectories. Future research should explore mechanisms such as economic stability and caregiving burden to better understand how parenthood influences late-life cognitive trajectories.

Abstract citation ID: igaf122.2567

### PROSOCIALITY, COGNITION, & HEALTH: DIVERSITY AND CONSISTENCY OF PROSOCIAL BEHAVIORS MATTERS

Morgan Taylor, and Margie Lachman, *Brandeis University, Waltham, Massachusetts, United States*

Research demonstrates that being prosocial—engaging in behaviors that benefit others—relates to better cognition and health outcomes across the lifespan. However, the effect of engaging in multiple prosocial behaviors consistently over time has yet to be investigated. Using the Midlife in the United States (MIDUS) study, we examined the relationship between three prosocial behaviors (volunteering, informally helping others, donating money), cognition (episodic memory, executive function), and functional health at Wave 3 (2013/2014). Participants (N = 2,096, ages 42-92) reported how many hours/month they volunteered and informally helped others and dollars/month they donated to organizations. We created a categorical variable for each behavior wherein participants received 0 if they didn't engage in those behaviors and 1 if they did. Replicating past work, we found those who engaged in each prosocial behavior had better cognition and functional health than those that did not, controlling for age, sex, education, race, and income. Critically, in a separate model, we found age moderated the effect of informal helping on executive function; informally helping others was more beneficial for older adults than younger adults. Furthermore, we found participants who engaged in more prosocial behaviors (2 or 3) had better cognition and health than those that engaged in fewer (0 or 1). Finally, we found those who consistently volunteered and donated over 10 years had better cognition than those who did so inconsistently. These results suggest that consistently participating in a variety of prosocial behaviors could be protective for middle and older adults' cognition and health.

Abstract citation ID: igaf122.2568

### TECHNOLOGY ACTIVITIES AND COGNITIVE TRAJECTORIES AMONG COMMUNITY-DWELLING OLDER ADULTS FROM THE NHATS 2015-2023

Erh-Chi Hsu[1], and Eric Jutkowitz[2], *1. Johns Hopkins University, Baltimore, Maryland, United States, 2. University of Minnesota, Minneapolis, Minnesota, United States*

This study examines the associations between the onsets and cessations of technology use and cognitive trajectories among community-dwelling older adults without dementia. While using digital technology is positively associated with cognitive function, the impacts of specific activities on distinct cognitive domains are underexplored. Data were collected from 5,566 older adults involved in the National Health and Aging Trends Study (NHATS) from 2015 to 2023. Assessed technology activities included online shopping, banking, medication refills, social media use, and checking health conditions online. Cognitive domains measured were episodic memory, executive function, and orientation. Linear mixed-effects models analyzed associations between technology activity transitions and cognitive outcomes, adjusting for demographic, socioeconomic, and health-related factors. Onsets of online shopping ($\beta$ = 0.044**), medication refills ($\beta$ = 0.070***), and social media ($\beta$ = 0.065**) were associated with improved episodic memory. Conversely, cessations of online banking ($\beta$=-0.083**) and social media ($\beta$=-0.064**) related to decreased episodic memory. Initiating instrumental, social, and health-related technology activities mitigated cognitive decline in orientation. Online shopping onset mitigated episodic memory decline ($\beta$ = 0.013*) while cessation worsened it ($\beta$=-0.022*); a similar trend occurred with social media cessation ($\beta$=-0.018*). More device ownership consistently linked to better cognitive functioning across activities. Higher educational attainment also correlated with superior cognitive domain performance, indicating potential disparities. Results suggest engagement in technology activities supports cognitive health in older adults. Future interventions should promote initiation and sustainment of these activities to mitigate cognitive decline in aging populations.

Abstract citation ID: igaf122.2569

### THE EFFECT OF COGNITIVE IMPAIRMENT ON MORTALITY RISK IN OLDER ADULTS

Delmarielinnette Castillo-Collazo[1], Héctor Gómez-Martínez[1], and Amílcar Matos-Moreno[2], *1. Albizu University, San Juan, Puerto Rico, United States, 2. The Pennsylvania State University, University Park, Pennsylvania, United States*

This study examined the influence of cognition on mortality among older adults in Puerto Rico, using data from the Puerto Rican Elderly: Health Conditions (PREHCO) study, collected between 2006 and 2022. Cognition has been consistently identified as a predictor of physical well-being in older adults, frequently influencing mortality. Although several studies have reported a relationship between cognitive decline and increased mortality risk in Caribbean populations, Puerto Rico has been excluded from these investigations. Given the rapid aging of this population and their high prevalence of chronic diseases, investigating how cognition influences survival outcomes among Puerto Rican older adults is essential. We analyzed longitudinal data from 2,289 participants (mean age = 74.12 years; 60.5% female) using a Cox regression model adjusted for sociodemographic and health-related covariates. Results indicated that higher cognitive scores were significantly associated with reduced mortality risk (HR = 0.945; CI: 0.924–0.967), reflecting a 5.5% decrease in risk with each incremental improvement in cognition. Additionally, advanced age, dependence in performing activities of daily living, and multiple chronic diseases significantly increased mortality risk. Men also exhibited greater mortality risk than women, while a higher level of education was associated with a reduction in mortality. Cognition emerged as a significant predictor of life expectancy among older Puerto Ricans, independently of other demographic and health factors. These findings highlight the importance of early detection of cognitive impairment and suggest the need for targeted interventions to enhance health outcomes in this population.

Abstract citation ID: igaf122.2570

### WHEN I MOVE, YOU MOVE: PHYSICAL ACTIVITY AND COGNITIVE FUNCTIONING AMONG OLDER DYADS IN EUROPE

Laura Himmelmann[1], Jeffrey Stokes[2], Tim Stuckenschneider[1], Tania Zieschang[1], and Kathrin Boerner[3], *1. The Carl von Ossietzky University of Oldenburg's School of Medicine and Health Sciences, Oldenburg, Niedersachsen, Germany, 2. University of Massachusetts Boston, Boston, Massachusetts, United States, 3. Carl von Ossietzky University of Oldenburg, Oldenburg, Niedersachsen, Germany*

Alzheimer's disease and related dementias (ADRD) impair cognitive function and well-being in older adults. While physical activity helps maintain cognitive health, less is known about the role of a partner's activity regarding cognitive outcomes. Theories of social contagion and social control suggest that having an active partner may promote greater engagement in physical activity, benefiting cognitive function. However, research using sensor-based measures to investigate physical activity in dyads remains limited. This study examines how physical activity within dyadic relationships predicts cognitive function. Specifically, we assessed how individuals' and their spouses' physical activity influenced cognitive outcomes over two years using data from the Survey of Health, Ageing, and Retirement in Europe (SHARE). A two-wave dyadic analysis was conducted with $n = 60$ older adults from 33 opposite-sex couples (mean age 67.7 ± 8.7 years) in European countries. Physical activity was measured by a three-axial accelerometer, and cognitive function was assessed through verbal fluency at baseline and follow-up. Multilevel LDV modeling with mixed effects models was performed in Stata Version 18/SE. Verbal fluency remained stable over time ($\beta = 0.62$, $p < .001$). Higher step counts predicted better cognitive function at follow-up ($\beta = 0.30$, $p < .001$). Additionally, spouses' physical activity positively correlated with individuals' cognitive function at follow-up ($\beta = 0.25$, $p < .01$). Our findings underscore the role of dyadic interactions in healthy aging. Future research should explore underlying mechanisms and assess dyadic interventions to support cognitive well-being in older adults.

### SESSION 7205 (POSTER)

### DEMENTIA I

Abstract citation ID: igaf122.2571

### ADAPTING A PAIN COPING SKILLS TRAINING INTERVENTION FOR PATIENTS WITH DEMENTIA AND THEIR CAREGIVERS

Laura Porter[1], Katherine Ramos[2], Francis Keefe[3], Deborah Weiner[4], Heather Whitson[5], Courtney Van Houtven[5], and Natalie Leary[3], *1. Duke University Medical Center, Durham, North Carolina, United States, 2. Duke University School of Medicine, Durham, North Carolina, United States, 3. Duke University School of Medicine, Durham, North Carolina, United States, 4. University of Pittsburgh, Pittsburgh, Pennsylvania, United States, 5. Duke University, Durham, North Carolina, United States*

Community dwelling persons with dementia (PWD) and their family caregivers represent a burgeoning and vulnerable population with significant unmet needs, one of which is pain management. Pain in PWD is common, causes increased distress and disability for patients, and increased stress for caregivers. Pain coping skills training (PCST) is a non-pharmacological pain management approach based on cognitive-behavioral principles that is efficacious among older adults without dementia. However, little is known about how to adapt PCST to meet the unique needs of PWD and their caregivers. In this study, we recruited six PWD (50% female, 33% Black, 77% White) and their caregivers (83% female, 67% spouses, 33% Black, 77% White) to form a Community Advisory Board (CAB). We met with them three times to obtain their input on intervention delivery and content. CAB members responded favorably to the proposed intervention length (five 45-minute sessions) and delivery to patient-caregiver dyads. They primarily preferred participation via videoconference. We presented four pain coping skills (PAINAD tool for pain assessment, relaxation, coping thoughts, and pleasant activity scheduling). Most CAB members reported perceiving the skills as highly useful, although there was variation based on the PWD's level of cognitive impairment (mild-moderate). They provided valuable input on (a) ways to tailor content and delivery for varying levels of cognitive impairment, and (b) materials (e.g., audio recordings, handouts) to promote learning and practice of skills. This feedback will be used to refine the intervention before implementing a single-arm pilot trial with 30 dyads.

Abstract citation ID: igaf122.2572

### CO-CREATING A VIRTUAL REALITY PROGRAM FOR OLDER ADULTS WITH DEMENTIA IN HOSPITAL

Lillian Hung[1], Lily Haopu Ren[2], W.Ben Mortenson[2], Angelica Lim[3], Jim Mann[2], Lily Wong[2], Christine Wallsworth[2], and Jennifer Boger[4], *1. University of British Columbia, Vancouver, British Columbia, Canada, 2. The University of British Columbia, Vancouver, British Columbia, Canada, 3. Simon Fraser University, Vancouver, British Columbia, Canada, 4. University of Waterloo, Waterloo, Ontario, Canada*

Virtual Reality (VR) is promising in improving the well-being of older adults with dementia in hospitals; however, traditional VR design and VR experience delivery to meet their diverse needs. Involving older adult patient partners, family caregivers, and staff in the co-design of gerontechnology is considered best practice, yet few researchers have adopted this inclusive approach. Appreciative Inquiry offers a collaborative framework to engage relevant users, leveraging their expertise and lived experiences for innovation. Our study aims to understand the contribution of Appreciative Inquiry in engaging multiple partners in co-creating a VR program for Older Adults with Dementia in hospitals. We co-created a VR program guided by Appreciative Inquiry principles, engaging patient partners, families, hospital staff and leaders. We adapted methods to facilitate meaningful participation, including six focus groups and interviews with 56 participants across two hospital units. Participants' insights informed the iterative development and refinement of the VR program. Patient partners, families, staff, and leaders meaningfully contributed to the design process. The collaborative process fostered a sense of ownership among participants, challenged assumptions about dementia care, and promoted positive interactions and experiences within the project team. Our approach demonstrated that using tailored methods is essential for authentic engagement, ensuring the developed technology meets real-world needs and will more likely be adopted in care practice. The study demonstrates the value of Appreciative Inquiry-informed co-design partnerships in co-creating technology. Future studies should further explore co-design methods across diverse cultural and organizational contexts with diverse older adults and other partners.

Abstract citation ID: igaf122.2573

### COMMUNITY-BASED CARE ECOSYSTEM: IMPROVING OUTCOMES FOR PEOPLE WITH DEMENTIA AND THEIR CAREGIVERS

Jill Cigliana[1], Kelli Barton[2], and Jeriel Bohall[2], *1. Memory Care Home Solutions, St. Louis, Missouri, United States, 2. University of Missouri-Kansas City, Kansas City, Missouri, United States*

The impacts of dementia diagnoses can be far-reaching, from state and federal financial burdens to strain on healthcare systems. People living with dementia (PWDs) are often cared for at home by family and friends; however, they may experience increased rates of adverse health events, higher utilization of healthcare services, and have limited access to individualized behavioral interventions. Despite overwhelming evidence that non-drug approaches are the gold standard of care, dementia programs rarely exist outside of academic trials. Care Ecosystem (CE) is one model of dementia care designed to provide personalized, cost-efficient care for people with dementia and their caregivers. A community-based dementia services organization implemented a state-wide adaptation of the CE model in Spring 2023 and used longitudinal data to measure its impact on PWDs and their caregivers. Over a 6-month enrollment phase, outcomes showed a statistically significant increase in PWD quality of life ($p = .002$, $n = 112$), measured by proxy ratings of the modified Dementia Quality of Life (DEMQOL) scale. Additionally, PWD risk for hospital admission decreased from baseline to 6 months ($p = .007$, $n = 94$), as measured by the modified LACE index. Caregiver self-efficacy was also measured across 4 items, and by 6 months, caregivers reported greater confidence getting needed services, finding help, managing caregiving challenges, and managing PWD behavioral changes ($p < .001$, $n = 130$). Feedback from semi-structured partner interviews also revealed broader benefits of CE implementation, including strengthened community partnerships, staff readiness, and organizational growth.

Abstract citation ID: igaf122.2574

### DEMENTIA ADVOCACY IN ACTION: EXAMINING SOCIAL MEDIA DURING WORLD ALZHEIMER'S MONTH

Juanita-Dawne Bacsu[1], Raymond J. Spiteri[2], Sarah Fraser[3], Allison Cammer[2], Alison L. Chasteen[4], Zahra Rahemi[5], Kate Nanson[1], and Ali Akbar Jamali[2], *1. Thompson Rivers University, Kamloops, British Columbia, Canada, 2. University of Saskatchewan, Saskatoon, Saskatchewan, Canada, 3. University of Ottawa, Ottawa, Ontario, Canada, 4. University of Toronto, Toronto, Ontario, Canada, 5. Clemson University, Clemson, South Carolina, United States*

Dementia awareness campaigns play a vital role in reducing stigma and improving the quality of life of people with dementia. However, there is a paucity of research on advocacy strategies to support dementia awareness, especially on social media. This presentation aims to: i) identify dementia advocacy strategies used to increase awareness on social media during World Alzheimer's Month; and ii) understand the role of social media in dementia awareness campaigns. Using data from the social media platform X, posts were scraped from September 1 to September 30, 2022. Filters were used to screen for duplicate posts, non-English content, and reply posts with missing content. The remaining 1,981 relevant posts were examined using thematic analysis to identify prominent dementia advocacy strategies. During our data analysis, steps were taken to support rigor such as using a standardized codebook, practice exercises, intercoder reliability checks, and team discussions to oversee coding disagreements. Based on our analysis, we identified four main advocacy strategies: i) myth-busting to address false information; ii) political advocacy to support dementia care reform; iii) tailoring messages to ethnic and cultural groups; and iv) amplifying personal narratives and lived experience of dementia. Although a range of strategies were identified, further research is needed to evaluate the effectiveness of awareness campaigns in addressing dementia-related stigma. The findings from our study have implications for community leaders, health professionals, and policymakers who are working to increase dementia awareness. Our findings provide vital insight to enhance national-level dementia advocacy and awareness campaigns on social media.

Abstract citation ID: igaf122.2575

### DESIGN CONSIDERATIONS FOR UTI DIGITAL SOLUTIONS FOR PEOPLE LIVING WITH DEMENTIA: INSIGHTS FROM CAREGIVERS

Kuan-Ching Wu[1], and Oleg Zaslavsky[2], *1. Emory University, Atlanta, Georgia, United States, 2. University of Washington, Seattle, Washington, United States*

Urinary tract infections (UTIs) are a leading cause of hospitalization and emergency room visits among people living with dementia (PLWD). Dementia caregivers often struggle to recognize early UTI symptoms and access timely guidance, increasing the risk of complications. This study aims to prototype Goodbye UTI (GUTI), a digital solution designed to support caregivers in UTI prevention and management. Following the framework of the four-steps human-centered design (HCD) approach, we identified key design considerations for GUTI. Semi-structured interviews were conducted with 11 dementia caregivers (aged 42–82, M = 68.74, SD = 11.41) from December 2023 to June 2024 to explore their needs and preferences for UTI digital solutions. Data were analyzed using inductive thematic coding with ATLAS.ti to extract insights on user needs, requirements, and context of use. Caregivers reported challenges such as emotional strain, communication difficulties, limited knowledge, and the need for accessible health information. They emphasized the importance of a simple interface with symptom tracking, reminders, and educational resources. Based on these insights, we developed a functional matrix with eight core design considerations and created ten prototype screenshots in Figma demonstrating key features. Findings highlight the need for intuitive, personalized, and actionable digital tools for UTI management in dementia care. GUTI addresses these needs by prioritizing reminder, real-time guidance, and caregiver support. Future research should focus on iterative design in prototype testing and development, usability and the effectiveness of GUTI in real-world caregiving context.

Abstract citation ID: igaf122.2576

### DIETARY SUPPLEMENT AND PRESCRIPTION MEDICATION USE AMONG OLDER ADULTS WITH COGNITIVE IMPAIRMENT.

Hao-Ping Chiang, Brady West, and Antoinette Coe, *University of Michigan, Ann Arbor, Michigan, United States*

Supplement use among older adults is associated with higher prescription medication use, leading to medication harm and hospitalizations. As patients with cognitive impairment are vulnerable to polypharmacy and these adverse outcomes, it's essential to understand how supplement use contributes to medication burden. This cross-sectional study examined the association between dietary supplement and prescription medication use among U.S. adults aged 60 and older with cognitive impairment, using the National Health and Nutrition Examination Survey (NHANES). NHANES 2011–2014 was selected as the only publicly available period with objective cognitive measurements (Cognitive Functioning Assessment) for defining impairment. The outcome was the number of prescription medications. The primary predictor was dietary supplement use (yes/no). Covariates included age, sex, race, education, insurance, and comorbidities. Mean and proportion differences between supplement users and non-users were analyzed using design-adjusted t-tests and Rao–Scott tests. Negative binomial regression models evaluated associations between the predictor variables and the number of prescription medications. Among an estimated 20,939,624 older adults with cognitive impairment (based on 1,567 NHANES respondents), an estimated 65.6% used dietary supplements. Supplement users took more prescription medications on average than non-users (4.8 vs. 4.2, $p = 0.036$). Supplement use was significantly associated with increased prescription medication use (estimated rate ratio = 1.14, 95% CI 1.01–1.29), but not after adjustment for covariates. Given the adverse outcomes of increased medication use, recognizing supplement use in older adults with cognitive impairment is suggested for medication safety. Further research is needed to clarify supplements' role in medication burden.

Abstract citation ID: igaf122.2577

### DIFFERENT TYPES OF FAMILY SUPPORT AS A MODERATOR IN THE MENTAL HEALTH OF OLDER ADULTS WITH DEMENTIA

Wan-Ling Hsu, and SangNam Ahn, *Saint Louis University, St. Louis, Missouri, United States*

Dementia impacts millions in the U.S., with family caregivers, particularly adult children and siblings, providing essential emotional support that can protect mental well-being. However, the impact of different types of family support on dementia-related mental health remains unclear. This study analyzed data from the 2016, 2018, and 2020 waves of the Health and Retirement Study, involving 14,140 U.S. participants aged 50 and older. Dementia was identified through self-reported or proxy reports. Depression was assessed using the Center for Epidemiologic Studies Depression Scale (CESD-8), and loneliness with a three-item UCLA Loneliness Scale. Family support was assessed by the number of living children and siblings. Weighted linear regression examined the association between dementia, family support, and mental health. The sample included 3,579 participants, with 562 (15.7%) having dementia. CESD-8 scores were higher among participants with dementia (+1.98), females (+0.23), non-white participants (+0.38), and those separated/divorced (+0.50) or widowed (+0.28), while those with higher education scored 0.35 points lower. A significant interaction was found between dementia and family support from children, with participants having more children reporting lower CESD-8 scores compared to those without children. On the loneliness scale, younger participants (-0.004) and those with higher education (-0.05) scored lower, while separated/divorced (+0.16) and widowed participants (+0.18) scored higher. No significant interactions were found between dementia and family support on loneliness. Enhancing family support from adult children could reduce depression symptomatology in dementia, offering a valuable approach for policymakers and healthcare providers.

Abstract citation ID: igaf122.2578

### ENROLLING PERSONS WITH DEMENTIA IN HOSPICE: A QUALITATIVE SYNTHESIS OF CAREGIVERS' EXPERIENCES

Oonjee Oh[1], Connie Ulrich[2], Lauren Massimo[1], and George Demiris[1], *1. University of Pennsylvania, Philadelphia, Pennsylvania, United States, 2. University of Pennsylvania School of Nursing - Philadelphia, PA, philadelphia, Pennsylvania, United States*

Hospice is a specialized palliative care geared towards the end of life, aiming to relieve the suffering of terminally ill individuals and their families. While persons with dementia (PwD) have high palliative care needs near death, they often receive suboptimal end-of-life care and experience challenges enrolling in hospice. Due to their progressive loss of cognitive abilities, caregivers are heavily involved in end-of-life decision-making and care transitions on behalf of PwD. Such intense involvement suggests that caregivers are in a position to offer valuable insights regarding the provision of quality end-of-life care for PwD. Yet, their experiences throughout hospice entry have not been comprehensively explored. Therefore, we conducted a systematic review of qualitative evidence that describes caregivers' experience enrolling a loved one with dementia in hospice. Based on a literature search from 5 major bibliographic databases, 22 studies were included. We conducted a thematic synthesis and identified 5 themes: 1) caregivers' perception towards late-stage dementia, 2) whether hospice is the right choice for PwD, 3) caregiving burden, 4) perception towards their loved one's death and hospice, and 5) information and hospice access. From these themes, we identified 15 barriers and 11 facilitators. There was a significant gap between the needs of caregivers caring for a seriously ill loved one with dementia and the circumstances under which resources like hospice could be offered. Current hospice referral processes and policies may not adequately reflect the complexity of severe dementia and the unique needs of caregivers at the intersection of dementia and end-of-life care.

Abstract citation ID: igaf122.2579

### EXPERIENCES OF DEMENTIA DIAGNOSTIC DISCLOSURE COMMUNICATION: A QUALITATIVE STUDY OF PATIENTS AND CAREGIVERS

Joanna Paladino, Heily Chavez Granados, Jade Connor Eruchalu, Ana-Maria Vranceanu, Deborah Blacker, and Christine Ritchie, *Massachusetts General Hospital, Boston, Massachusetts, United States*

Clinician communication at the time of a dementia diagnosis inadequately addresses patient and caregiver needs. We aimed to characterize the disclosure experiences of patients and caregivers affected by dementia to identify communication domains that will be incorporated into the co-design of a diagnostic disclosure communication intervention. We conducted thematic analysis of individual interviews of patients and caregivers using a conceptual framework for person-centered communication. Participants included 6 patients with dementia and 15 caregivers of persons with dementia recruited from the community (n = 21; n = 17 female (81%); n = 13 Caucasian (61%); n = 4 Black or African American (19%); n = 4 Latino/a (19%); n = 2 Asian). We identified five themes. First, perceptions of respectful or disrespectful communication affects the relationship with clinicians and contributes to positive or negative experiences before, during, and after disclosure of a dementia diagnosis. Second, participants described the emotional impact of sudden or unsupported disclosures, in which they felt unprepared to receive the news or emotionally abandoned after diagnosis. Third, the absence of, or ambiguity around, a definitive dementia diagnosis contributes to distress and feeling dismissed by clinicians. Fourth, mixed responses to recommendations after disclosure reveals the need for a personalized care planning process. Fifth, considerations around the timing of prognostic communication and advance care planning are necessary to meet the needs of individuals with different emotional readiness, information preferences, and cultural beliefs. Dementia diagnostic disclosure would benefit from a tailored, structured, and incremental approach that prioritizes respectful communication, emotional support, and personalized care planning to meet the needs of patients and caregivers.

Abstract citation ID: igaf122.2580

### INNOVATIONS IN DEMENTIA CARE IN NORWAY

Brooke Hollister[1], Bjørnulf Arntsen[2], and Markus Frydenlund[2], *1. Center for Care Research, south at the University of Agder, Arendal, Aust-Agder, Norway, 2. University of Agder, Grimstad, Aust-Agder, Norway*

With a growing population of people with dementia (PWD), a national health care system, and a healthy economy, Norway has the privilege of implementing innovative interventions for PWD. While this reality allows for innovation, policymakers and researchers alike struggle with funding and designing evaluations of complex interventions for PWD. Using guidelines from the Medical Research Council's Guidelines for developing and evaluating complex interventions and resources from the National Institute on Aging's IMPACT Collaboratory, researchers in Norway have taken a pragmatic approach to evaluating innovations in dementia care. Two project evaluations were designed with the aim of, 1) adequately describing complex interventions in practice; 2) conducting a process evaluation of implementation processes; 3) assessing potential outcomes on PWD, their caregivers, and health care personnel; and 4) mobilizing knowledge into policy and practice change. Evaluation of the Grimstad Dementia School, which takes a life-long learning approach to a day program for PWD in early to moderate stages, showed promising results, leading to the development and dissemination of Implementation Guidelines for replication in other Norwegian municipalities. The second innovation focused on several Dementia Villages in Norway participating in national and European networks of dementia villages and collaborating researchers. In Norway, this collaboration has resulted in the development of a Program Theory of Dementia Villages as well as several pragmatic evaluations of implementation processes and outcomes. Methods and findings from these studies will presented, in addition to lessons learned regarding replicability and dissemination in both the Norwegian and international context.

Abstract citation ID: igaf122.2581

### LISTEN TO PEOPLE WITH COGNITIVE IMPAIRMENTS: COMMUNICATION DIFFICULTIES AND STRATEGIES

Hui-chuan Hsu[1], and Shu-Nu Chang-Lee[2], *1. Taipei Medical University, Taipei City, New Taipei, Taiwan (Republic of China), 2. National Quemoy University, Jinning, Kinmen, Taiwan (Republic of China)*

Purpose: This study aimed to explore the communication skills and difficulties between people with different degrees of cognitive impairments and formal/informal caregivers to provide suggestions for effective communication for caregivers. Methods: We observed the interaction of older adults with cognitive impairment from a long-term care facility, a day-care center, and a community-based care center. Videos with 1 to 3 sessions recorded each older adult's interactions with their formal or informal caregivers; each session was 10 to 20 minutes. In total, 25 older adults participated in this study. All the videos of dialogues, facial expressions, motion, and gestures in the interactions were coded as transcripts, and then the communication strategies, difficulties, and diagnoses were analyzed. Results: Three themes were categorized: (1) Communication strategies of caregivers: The most often used strategies of caregivers included repetition, rewording, extending questions, forming assumptions, providing possible answers, following the topic, self-sharing, inappropriate questions, asking but not listening, and using assistive tools. (2) Communication characteristics of older adults:

Vocabulary difficulty, longer time to respond, incomplete sentences and hidden meaning, sharing life experience, health problems and illiteracy, changing subjects or pretending, silence or refusing to respond, inability to remember, and non-verbal language. (3) Skills to improve communication: Understanding their background and stories, conquering cohort differences, using the same language/dialects, questioning but not testing, listening and conversation extension, and being patient and sympathetic. Discussion: Caregivers' communication strategies, strengths, and weaknesses were diagnosed. General communication principles and personalized suggestions are provided.

Abstract citation ID: igaf122.2582

### SOCIAL AND MULTI-MORBIDITY PROFILES OF OLDER PERSONS WITH DEMENTIA

Amy Byers[1], Yixia Li[2], Ruth Morin[3], Daniel Jimenez[4], and Lisa Barry[5], *1. University of California, San Francisco / SFVAHCS, San Francisco, California, United States, 2. Northern California Institute for Research and Education, San Francisco, California, United States, 3. Hoag Memorial Hospital Presbyterian, Newport Beach, California, United States, 4. University of Miami Miller School of Medicine, Miami, Florida, United States, 5. University of Connecticut, Farmington, Connecticut, United States*

Despite tremendous growth in the number of persons with dementia (PWD), little is known about the distinct social and multi-morbidity profiles that characterize PWD and whether they differ by race/ethnicity. We conducted a latent class analysis examining 653,832 PWD age ≥50 years using Medicare and U.S. Department of Veterans Affairs Veterans Health Administration data from January 1, 2012, to December 31, 2020. We triangulated multiple sources of data (e.g., geocode, health factors, VA's Homeless Operations Management System) to identify distinct typologies of social risk and multi-morbidity. We found three distinct latent clusters defining multi-morbidity: (1) "Minimal Comorbidity" (49.6%); (2) "High Medical-Cardiac" (28.3%); (3) "Medical-High Depression/Sleep Disorder" (22.1%). We also found three clusters for social risk: (1) "Low-Moderate Social Risk" (92.1%); (2) "High Social Risk-Low Social Support/Housing Instability" (2.6%); and (3) "High Social Risk-Alcohol Abuse/Tobacco Dependence" (5.3%). These three-cluster multi-morbidity and social risk profiles differed by racial/ethnic groups. Notably, Non-Hispanic Black PWD had prominent diagnosis of posttraumatic stress disorder (over 50%) in multi-morbidity class 3, and additionally high drug abuse (over 60%) in social risk class 3. While Multiracial PWD had higher overall social risk, including high financial strain (over 60%) in social risk class 2, and Hispanic PWD had lower medical concerns in multi-morbidity class 3 but higher cardiac concerns in class 2. Establishing social and multi-morbidity profiles of PWD quantifies heterogeneity in this highly vulnerable population. These findings may help inform a more personalized, effective, and equitable approach to dementia care and provide opportunities to tailor intervention/prevention.

Abstract citation ID: igaf122.2583

### SOCIAL PRESCRIBING IN NURSING HOME VS COMMUNITY: EXPERIENCE WITH DOLL THERAPY AND COGNITIVE GAMES

Bobo Hi Po Lau[1], Rebecca Yung-Choy[2], and Cindy Wong[2], *1. Hong Kong Shue Yan University, North Point, China (People's Republic), 2. Golden Age Foundation, Wan Chai, Hong Kong*

Doll therapy and tablet games are often used among older adults with dementia in nursing homes and in-home rehabilitation. While both therapies may enhance mood and social interactions, doll therapy often targets a cognitively frailer clientele than cognitive games on tablets. In Smart Care Lab 2.0 and 3.0, these therapies were used in Hong Kong nursing homes and in-home rehabilitation respectively. Volunteers were trained to facilitate implementation on-site, while (in)formal caregivers were tasked with dispensing these therapies in their daily care to maximize the desired outcomes. Individual interviews, focus groups, and pre-post intervention assessments were conducted to reveal their efficacy and factors affecting their implementation. Results show that efficacies differ by contexts. For doll therapy, their use in nursing homes primarily enhanced residents' attachment to daily routines and reduced distress, yet their use in the community fostered social interactions. For cognitive games, their use in nursing homes were restricted by poor dexterity and mobility of the residents, yet their use in the community potentially bridged older adults with cognitive impairment to other technologies for their independent living. Echoing social prescribing, a professional 'link worker' is essential for identifying suitable cases, training volunteers and (in)formal caregivers for dispensing the therapy and moderating the intervention clients' changing capabilities. Through implementing the same therapies to clients in two contexts with similar volunteer training involved, this study provides a unique opportunity to compare the differences in implementation factors to inform the generalization of technologies and tools across care settings.

### SESSION 7210 (POSTER)

### DISPARITIES

Abstract citation ID: igaf122.2584

### DIGITAL CITIZENS OR DIGITAL OUTCASTS? RECENT TREND IN E-ADMINISTRATION UTILIZATION INEQUALITY AMONG OLD EUROPEANS

Jose David Lopez Blanco, and Marco Albertini, *University of Bologna, Bologna, Emilia-Romagna, Italy*

As digital technologies become central to public administration—particularly in the post-COVID-19 push for digital transformation—understanding disparities in e-administration engagement among older adults is increasingly urgent. While digitalization promises efficiency and accessibility, it may also deepen existing inequalities, particularly for older persons facing barriers to technology access and digital literacy. This study examines inequality in e-administration usage among adults aged 65+ across 27 European countries, assessing how the European Union's digital transformation agenda has shaped the digital divide. Using cross-national survey data from 2016 to 2024, we construct an index of the probability of engagement in e-administration, conditioned on key demographic and socioeconomic factors. This index, derived from a probit model, generates predicted probabilities of e-administration usage while accounting for individual characteristics over time. Our analysis explores how digital inequality has evolved, identifying the administrative levels where disparities persist and the role of demographic and social stratification. Results show that while overall disparities in digital engagement have declined, age remains a key factor, with older individuals facing persistent barriers even after adjusting for socioeconomic differences. Additionally, regional and national policies shape e-administration adoption, with digitalization efforts either mitigating or exacerbating exclusion. These findings highlight the need for inclusive digital strategies beyond infrastructure investment, actively supporting older persons through targeted training and user-friendly e-governance systems. As European governments accelerate digital transformation, ensuring equitable access to e-administration is essential to prevent the digital marginalization of aging populations.

Abstract citation ID: igaf122.2585

### DIGITAL DISPARITIES AMONG OLDER ADULTS WITH AND WITHOUT DISABILITIES IN THE U.S.: AN EXAMINATION OF THE CENSUS DATA

Xiayu Chen[1], and Kang Sun[2], *1. University of Central Florida, Urbana, Illinois, United States, 2. Southern Illinois University, Carbondale, Illinois, United States*

The rapid expansion of digital technologies has significantly reshaped the ways in which older adults communicate, access resources, and maintain independence. Despite federal efforts such as the Digital Equity Act and the Affordable Connectivity Program, substantial disparities persist, especially among older adults with disabilities. Using nationally representative data from the 2023 Current Population Survey (N = 21,152), this study investigates digital access, usage patterns, and barriers. Weighted descriptive and logistic regression analyses identified significant disparities. Among older adults, 28.77% have disabilities, and only 73.5% reported home-based digital technology use compared to those without disabilities. Smartphones are the primary device, primarily used for communication, social networking, e-commerce, financial management, and health applications. However, digital exclusion remains a pressing issue, with 26.5% of older adults with disabilities lacking home technology access versus 16.71% of those without disabilities. The most common barriers include lack of interest, financial constraints, and accessibility issues. Logistic regression demonstrated significantly lower likelihood of home digital access for older adults with disabilities (OR = 0.55, p < .001), particularly among female, economically and educationally disadvantaged individuals, and rural residents. These findings highlight the urgent need for targeted policy and community interventions, emphasizing expanded affordability programs, improved technological accessibility, and tailored digital literacy initiatives. Addressing these barriers at multiple levels will be essential for fostering digital equity and inclusion among older adult populations in the U.S.

Abstract citation ID: igaf122.2586

### EMERGENCY DEPARTMENT PROVIDER UTILIZATION IN RURAL AND URBAN SETTINGS: ROLES OF NPS, PAS, AND PHYSICIANS

Nasim Ferdows, *Northeastern University, Boston, Massachusetts, United States*

Emergency departments (EDs) are essential access points in the U.S. healthcare system, with increasing reliance on nurse practitioners (NPs) and physician assistants (PAs) due to rising patient volumes and physician shortages. However, little is known about how provider utilization varies by patient demographics, visit acuity, and geographic location. Using data from the 2017–2021 National Hospital Ambulatory Medical Care Survey, we examined provider types across 83,282 ED visits, categorized as ED physician only (74.0%), NP/PA with a physician (14.4%), and NP/PA without a physician (11.6%). Younger patients (< 15 years) were more likely to see NP/PAs without a physician (13.6%), while older adults (≥65 years) were more often treated by ED physicians only (78.9%). Non-Hispanic Black patients had the highest likelihood of NP/PA involvement (16.4%). Semiurgent/nonurgent visits had significantly higher odds of NP/PA-only care (OR: 5.39; 95% CI: 4.80–6.04), while immediate/emergent cases were primarily managed by ED physicians (78.8%). Rural EDs had higher NP/PA-only involvement (13.5%) compared to urban EDs (9.8%; OR: 1.08; 95% CI: 1.01–1.16). Public insurance holders were more likely to see NP/PAs with a physician, while self-pay patients had higher odds of NP/PA-only care (OR: 1.35; 95% CI: 1.25–1.45). These findings highlight workforce distribution patterns in EDs, with NP/PAs playing a greater role in rural and lower-acuity settings. Understanding these trends is critical for workforce planning and optimizing ED resource allocation.

Abstract citation ID: igaf122.2587

### EXAMINING THE DISPARITIES IN SENIOR CENTER BUILT ENVIRONMENT

Hui "Jimmy" Xie[1], Cinthia Camacho[1], Maryna Bankovska[1], Xiao Zhang[1], Bing Han[2], and Deborah Cohen[2], *1. California State University, Northridge, Northridge, California, United States, 2. Kaiser Permanente Southern California, Pasadena, California, United States*

Built environment has substantial influence on older adults' health and wellbeing. Despite that, there is a scarcity of research on the built environment of senior centers, an important place for many older adults to socialize and engage in leisure activities. This study examined the disparities in the built environment of senior centers in 24 communities in the Greater Los Angeles Area. Each center was jointly assessed by 2–3 trained research assistants in 2023 using the Senior Center Assessment Tool (v1.0), covering the access, exterior, and interior of the center. Additionally, statistics about communities' socioeconomic condition were retrieved from US Census Bureau database. On average, the study centers had 8.5 rooms (range: 1–19) with multi-purpose room being the most common type of room. Over half the centers had designated rooms/spaces for congregated meals, tabletop games and billiards, arts/craft, or computer use. The percentage of centers with an excellent overall condition of landscaping, exterior, and interior were 29%, 25%, and 35%, respectively. Results of bivariate correlations showed that community's median household income had a positive relationship with overall condition of the interior (r=.42, p<.05), building exterior (r=.42, p<.05), and landscaping (r=.48, p<.05), percentage of rooms in excellent condition (r=.45, p<.05), and ADA compliance (r=.45, p<.05), and had a negative relationship with presence of safety issue(s) (r=-.57, p<.01). Substantive differences were found in the quality of built environment at senior centers across communities.

Abstract citation ID: igaf122.2588

### GEOGRAPHIC DISPARITIES OF COMMUNITY-LEVEL 65+ DEPRESSION PREVALENCE IN FIVE NEW ENGLAND STATES

Elizabeth Dugan[1], Taylor Jansen[1], Qian Song[1], Yan-Jhu Su[2], Shan Qu[1], and Mengshi Liu[1], *1. University of Massachusetts Boston, Boston, Massachusetts, United States, 2. The Pennsylvania State University, University Park, Pennsylvania, United States*

Rural residents report higher depression rates than their urban counterparts. The present descriptive study will compare community and neighborhood-level 65+ "ever-diagnosed" depression rates in five New England states: Connecticut (CT), Massachusetts (MA), Maine (ME), New Hampshire (NH), and Rhode Island (RI). The Healthy Aging Data Reports (HADRs) (www.healthyagingdatareports.org) are a comprehensive tool to spur community and policy action as they calculate community-level prevalence of 38 chronic diseases for each community in a state. Using Medicare Beneficiary Summary File (2020-2021), representing 100% of traditional beneficiaries. Small area estimation techniques were used to calculate age-sex adjusted community depression rates and over 150 other health indicators. Results varied across states with NH reporting the lowest community rate of 65+ depression in the sample (20.01%) and MA reporting the highest (54.46%). CT and MA reported the largest range in 65+ depression (CT:23.68-42.65%; MA:21.90-54.46%) with the highest rate clusters located near the biggest cities, in urban areas (CT:25.62-42.65%; MA:26.39-54.46%). Conversely, in ME and NH, the highest rate clusters were found in rural areas (NH:29.60-38.08%; ME:36.99-43.40%), states with a more traditional rural/urban threshold compared to the population dense states of CT, MA, and RI. In sum, the 2025 HADRs identified geographic disparities and overall high rate of 65+ community-level depression, with some communities reporting 65+ depression rates higher than 50% (neighborhoods in Springfield & Worcester, MA). The 2025 HADRs provide visualizations to make disparities evident; in this way, they can guide policy efforts to treat and address the growing mental health needs of the aging population.

Abstract citation ID: igaf122.2589

### INTEGRATED FINDINGS ON GERIATRIC HEALTHCARE UTILIZATION AMONG OLDER JEWISH AND ARAB ADULTS IN ISRAEL

Efrat Gil[1], Rachel Kizony[2], Roy Tzemah-Shahar[2], and Maayan Agmon[2], *1. Israel Ministry of Health, Jerusalem, Yerushalayim, Israel, 2. University of Haifa, Haifa, HaZafon, Israel*

Israel's aging population presents healthcare challenges, with disparities between Jewish and Arab older adults. While 88% of those aged 65+ in Israel are Jewish and 8% Arab, the latter group has lower life expectancy due to higher morbidity, reduced preventive care, and limited healthcare use. Arabs rely more on primary care and hospitalizations, often seeking medical attention only when health worsens. This study explored geriatric healthcare utilization patterns among community dwelling Jewish and Arab older adults, identifying barriers and disparities. The research included three phases: (1) a focus group with six primary care professionals to identify barriers to geriatric service use, (2) quantitative and qualitative data collection from 401 older adults (mean age 74.9±6.4 years) assessing service utilization, and (3) a validation focus group with eight healthcare professionals. Findings reveal low awareness of geriatric services across both populations, with many respondents unsure why people seek or avoid care. Reasons for seeking services included eligibility for financial support, severe health deterioration, and referrals from healthcare providers. Barriers included lack of awareness, stigma, and misconceptions about geriatric services. Notably, Jewish participants were more proactive in seeking geriatric consultations, while Arab participants relied primarily on referrals from family doctors. Additionally, healthcare professionals and participants highlighted long waiting times, a shortage of geriatric specialists, and confusion about when to seek geriatric versus primary care, further complicating service utilization. Policy recommendations emphasize expanding multidisciplinary geriatric care, increasing accessibility and awareness, addressing healthcare disparities, and implementing proactive geriatric assessments ensuring early intervention and improve health outcomes.

Abstract citation ID: igaf122.2590

### RACIAL DIFFERENCES IN COPING STRATEGIES FOR PEOPLE LIVING WITH ALS AND THEIR CAREGIVERS

Omar Guerrero[1], Maame-Owusua Boateng[2], Ashley Nurse[2], Christina Pantzer[1], Asia Beason[2], Avery DeWitt[2], Joan Monin[3], and Chelsey Carter[2], *1. Yale University, New Haven, Connecticut, United States, 2. Yale School of Public Health, New Haven, Connecticut, United States, 3. Yale, Yale, Connecticut, United States*

People living with amyotrophic lateral sclerosis (ALS) and their caregivers face negative psychological distress. No research to our knowledge has examined the unique coping experiences of Black/Other ALS dyads. We conducted an on-line, self-report, cross-sectional study of the experiences of people living with ALS (N = 74; 60 White; 14 Black/Other) and caregivers of people living with ALS (N = 77; 57 White; 20 Black/Other). Both samples completed the Brief Cope (Carver, 1997), and we explored differences between the White and Black/Other participants for each sub-scale. We found that Black/Other caregivers (M = 2.75, SD = 1.05) used humor as a coping style more than White caregivers (M = 2.24, SD=.83; F(76)=4.93, p=.029). We also found that Black/Other people living with ALS (M = 2.18, SD=.87) reported using more religious coping than White people living with ALS (M = 1.64, SD=.55; F(73)=8.46, p=.005). Also, Black/Other people living with ALS (M = 1.68, SD=.75) used more positive coping than White people living with ALS (M = 1.24, SD=.45; F(73)=8.19). Results of this exploratory analysis suggest that future studies with larger samples replicate these findings. If replicated, these findings would have implications for the design of supportive psychoeducational interventions tailored to the needs of Black/Other caregiving dyads living with ALS. We highlight the need for advancing health equity approaches for other rare, and neurodegenerative diseases. .

Abstract citation ID: igaf122.2591

### THE LASTING IMPACT OF REDLINING ON BOSTON NEIGHBORHOODS: FINDINGS FROM THE 2025 HEALTHY AGING DATA REPORTS

Taylor Jansen, and Elizabeth Dugan, *University of Massachusetts Boston, Boston, Massachusetts, United States*

In 1933, the federally funded Homes Owner's Loan Corporation (HOLC) tasked local officials to create neighborhood maps which captured credit default risk for residents applying for mortgages. Maps were color coded and assigned grades to city neighborhoods (A "Best", B "Still Desirable", C "Definitely Declining", D "Hazardous"); "A" neighborhoods were often wealthy, majority White neighborhoods, while "D" neighborhoods were home to Black, immigrant residents in lower socioeconomic classes. Though outlawed in 1968, this practice has had a lasting impact on the racially segregated neighborhoods, access to fair housing, care, and health. This study used the HOLC map shapefiles provided by the University of Richmond Digital Scholarship Lab and overlayed them with current Boston neighborhood boundaries to examine if health disparities persisted in historically redlined neighborhoods. Descriptive statistics were estimated using data from the American Community Survey (ACS) (2018-2022) and the Medicare Beneficiary

Summary File (MBSF) (2020-2021) by HOLC grade for 23 Boston neighborhoods. Only one neighborhood in Boston (Beacon Hill) was rated "B", while 11 neighborhoods were rated C and D each. Comparing the "C" to "D" neighborhoods, "D" neighborhoods reported lower 65+ educational attainment, home and vehicle ownership, and median income. Historically "Hazardous" neighborhoods reported higher 65+ prevalence rates of current asthma, atrial fibrillation, chronic obstructive pulmonary disease (COPD), heart attack, liver disease, lung cancer, substance and tobacco use disorders, anxiety, and post traumatic stress disorder (PTSD). These findings underscore the detrimental and lasting effects of redlining on the health of older Boston residents, almost 100 years later.

Abstract citation ID: igaf122.2592

### THE ROLE OF PERCEIVED SLEEP QUALITY IN MENTAL AND COGNITIVE HEALTH AMONG OLDER RESIDENTS IN SUBSIDIZED HOUSING

Yuri Jang[1], Juyoung Park[2], Seo-Yun Choi[1], Nan Sook Park[3], David A. Chiriboga[3], Soondool Chung[4], Jung In Park[5], and Sunmin Lee[5], *1. University of Southern California, Los Angeles, California, United States, 2. Emory University, Georgia, United States, 3. University of South Florida, Tampa, Florida, United States, 4. Ewha Womans University, Seoul, Republic of Korea, 5. University of California, Irvine, Irvine, California, United States*

Given the importance of sleep health in later years and the greater likelihood of older immigrants with socioeconomic disadvantages experiencing sleep disturbances, we examined the impact of perceived sleep quality on mental and cognitive health among older Korean-American residents in subsidized housing. Data were collected from surveys with 318 older Korean Americans (Mean age = 79.5, SD = 6.66) living in subsidized housing in the Los Angeles area. Sleep quality was measured using a single-item self-rating (excellent/very good/good vs. fair/poor). Both screening tools and self-ratings were used for outcomes: mental health (measured by the Patient Health Questionnaire–9 [PHQ–9]) and cognitive health (measured by the Mini-Mental State Examination [MMSE] and self-rated cognitive health). Multivariate models assessed the mental and cognitive health risks associated with poor sleep quality. Poor sleep quality was a significant predictor in both models of probable depression, indicated by the PHQ–9, and self-rated mental health. However, the impact of sleep quality on cognitive health varied by measure: significance was found for self-rated cognitive health, but not for MMSE-based cognitive status. The discussion addresses the mental and cognitive health risks associated with poor sleep quality and potential reasons for the varied impact by measure. Findings suggest that efforts to promote health and well-being in senior housing should specifically address sleep. Improving sleep quality among senior housing residents may reduce health risks and enhance overall quality of life, empowering them to fulfill their desire for independent living and aging in place.

### SESSION 7215 (POSTER)

### EDUCATION AND TRAINING: PROGRAM EVALUATION

Abstract citation ID: igaf122.2593

### "WE CAN'T POUR WATER FROM AN EMPTY CUP": CAREGIVERS' EXPERIENCES WITH A DEMENTIA SPECIFIC TRAINING PROGRAM

Brittney Pond[1], Kattia Suarez Vargas[2], Melinda Neri[2], and Jarmin Yeh[2], *1. Sandhills Community College, North Carolina, United States, 2. University of California, San Francisco, San Francisco, California, United States*

The demand for home and community-based services continues to rise in the United States, as does the prevalence of people living with Alzheimer's Disease and Related Dementias (ADRD). However, little is known about the qualitative impacts of dementia specific training programs for California's Medicaid-Funded In-Home Supportive Services (IHSS) caregivers who provide care and services for people living with ADRD. The IHSS + ADRD Training Project implemented and evaluated a 10-week, online, dementia-specific training program offered in English, Spanish, and Chinese (Cantonese and Mandarin) for IHSS caregivers and their care recipients living with ADRD. This poster is based on open-ended qualitative responses (n = 470) drawn from a larger quantitative survey of IHSS caregivers who participated in the training program. Caregivers responded to questions about what they learned in the program, its application to everyday work and life, personal impacts, and how the training could be improved. Based on our qualitative analysis using thematic coding, there are three main findings: 1) increased dementia knowledge and skills, 2) the importance of social support from classmates and instructors, and 3) personal development related to self-care and increased confidence. This research has several implications for policy and practice, as it provides evidence to support the ongoing development and implementation of dementia-specific caregiver training programs. This work suggests that establishing policies to support training opportunities are vital for this workforce.

Abstract citation ID: igaf122.2594

### AGING "WELL" IN PLACE: A QUALITATIVE CASE STUDY THAT EXPLORES THE INFLUENCE OF A DIGITAL EQUITY PROJECT

Josie Santilli[1], Skye Leedahl[1], Jill Juris[2], Erica Liebermann[1], and Casey McGregor[1], *1. University of Rhode Island, Kingston, Rhode Island, United States, 2. Appalachian State University, Boone, North Carolina, United States*

Older adults prefer to age in the place they call home. However, home is changing as technology impacts daily living. The ability to age well while aging in place (AIP) may no longer be sustainable without digital equity. The purpose of this qualitative case study, guided by the Ecological Theory of Aging, was to explore how advancing digital equity through the iPad Project influenced older adults' ability to AIP. The participatory design of this case study sought to learn from older adults who had completed the iPad Project and crossed the digital divide and from collaborators who designed, implemented, and evaluated the intervention. A comprehensive document review was completed and semi-structured interviews were conducted with 30 older adult project participants and collaborators to explore participant experiences since completing the project. Their responses were analyzed using thematic analysis. Themes identified were expanded technology acceptance and adoption, enhanced quality of life, and increased access to healthcare. The findings suggest that AIP extends beyond the location where one lives. A contemporary perspective reflective of our digital world considers the lifestyle for how older adults live to age "well" in place (AWIP).

The iPad Project positively influenced participants' lives by supporting their goal of AWIP.

Abstract citation ID: igaf122.2595

### AN ONLINE ACCEPTANCE AND COMMITMENT THERAPY SESSION TO HELP PEOPLE MANAGE STRESS FROM THEIR DEMENTIA DIAGNOSIS

Gavin Green[1], Heather Kelley[1], Ty Aller[1], Carter Davis[2], Michael Levin[1], and Elizabeth Fauth[1], *1. Utah State University, Logan, Utah, United States, 2. VA Palo Alto Health Care System, Palo Alto, California, United States*

Background: A dementia diagnosis means that a person faces forthcoming deterioration in function and eventually, death, thus these diagnoses are accompanied by significant psychological distress. While some CBT-based interventions have been found preliminarily effective in improving well-being for this population, there are significant barriers (e.g. time and cost). This study examines the Compassion Compass, a single-session, online Acceptance and Commitment Therapy (ACT) program tailored to support individuals recently diagnosed with dementia. Methods: Eighteen participants (mean age = 66.1, SD = 11.9), diagnosed with dementia within the past year, completed Compassion Compass. Preliminary efficacy was assessed using quantitative and qualitative measures. Quantitative measures assessed changes in psychological distress and life satisfaction, while in-depth interviews provided qualitative insights into the intervention's perceived impact on participants' daily lives and well-being. Results: Quantitative findings, limited by the sample size, showed no statistically significant improvement in life satisfaction, but yielded a marginal reduction in psychological distress. Qualitative data highlighted meaningful benefits. Thematic analysis revealed increased emotional awareness, self-compassion, and values-based engagement. Participants reported feeling better able to process their diagnosis, reframe their perspective, and reconnect with meaningful activities. Conclusion: Though the study was under powered to examine efficacy quantitatively, its qualitative findings suggest that the Compassion Compass program was well-received by participants and helped support their well-being, making it a promising, low time and cost option that warrants further research. Future research will refine the intervention and explore its potential within caregiver care-recipient dyads to further improve well-being for individuals with dementia.

Abstract citation ID: igaf122.2596

### CONTENT VALIDITY OF AN AGING-RELATED PREPARATION EDUCATION PROGRAM FOR OLDER CHINESE MIGRANTS IN JAPAN

Li Yao, and Harue Masaki, *Chiba University, Chiba, Chiba, Japan*

Introduction and Objectives: This study aimed to verify the content validity of the developed aging-related preparation education program through experts in gerontological nursing. This program was provided to older Chinese migrants in Japan, focusing on long-term care, and included six modules with lectures and discussions conducted via real-time videoconferences. Methods: Nursing researchers specializing in gerontology and care providers experienced in caring for older Chinese migrants were recruited. Participants were asked to review the developed education program materials, including a booklet and six lecture videos for each module. They then partook in semi-structured interviews based on the questionnaire responses. The questionnaire focused on assessing content clarity, structure appropriateness, and consistency of the instructional content and methods using a 4-point Likert scale. Descriptive statistics were used to analyze questionnaire responses, while content analysis was used to analyze the interview data. Results: Three Chinese gerontological nursing researchers and two Chinese practicing care providers in Japan were enrolled in this study. Regarding the appropriateness of instructional content and methods, all participants evaluated modules No. 1-4 as 'appropriate' or 'somewhat appropriate', while 20% evaluated modules No. 5-6 as 'somewhat inappropriate'. Seven categories related to the evaluation reasons and revision suggestions were identified from the interview data. Conclusion: This education program was valid for older Chinese migrants in Japan, with easy-to-understand booklets and module slides, and was appropriate in terms of educational goals, structured instructional content, and volume. Moreover, case introductions through videos or photographs were requested to offer comprehensive content.

Abstract citation ID: igaf122.2597

### EVALUATING THE EFFICACY OF ONLINE AND BLENDED LEARNING TRAINING COURSES IN THE VALIDATION APPROACH

Vicki de Klerk-Rubin[1], and Harvey Sterns[2], *1. Validation Training Institute, The Hague, Zuid-Holland, Netherlands, 2. The University of Akron, Akron, Ohio, United States*

This study examines the effectiveness of online and blended learning training courses using the Validation approach as a psychosocial intervention. It focuses on pre- and post-training assessments conducted through surveys. The research aimed to measure participants' knowledge, self-efficacy, and perceived stress in relation to the Validation approach, exploring how different training formats influence these factors. Additionally, the study provides insights into the challenges of using digital survey tools and presents significant findings on the impact of this psychosocial intervention on self-efficacy and stress levels among participants.

Abstract citation ID: igaf122.2598

### GOALS OF CARE CONVERSATION EDUCATION PROGRAM: IMPACT ON RESIDENT DOCUMENTATION

Kelly Hsu[1], M. Isabel Friedman[1], Jessica Cohen[1], Ann Eichorn[1], Mark Tursi[1], Christian Nouryan[2], and Edith Burns[3], *1. Northwell Health, New Hyde Park, New York, United States, 2. Northwell Health, Great Neck, New York, United States, 3. Zucker School of Medicine Hofstra-Northwell, Manhasset, New York, United States*

Eliciting patient-centered goals of care (GOC) leads to tailored treatment plans, better outcomes, greater patient/family/provider satisfaction. Most educational approaches elicit immediate feedback on self-perceived change in knowledge and skills (Kirkpatrick level 1 and 2). Our health system implemented Goals of Care Conversation Education Program (GoCCEP) and wished to assess if GoCCEP impacted provider behavior (Kirkpatrick level 3). GoCCEP-trained resident physicians from 7 different residency programs (2 family med {FM}, 3 internal {IM}, 1 PMR, 1 surgery) compared to matched controls from same program/year of training who didn't take the course. Total number of unique patients seen by each resident with at least 1

GOC note identified (numerator) over total number unique patients seen by that resident (denominator) 6 months pre-GoCCEP and 6 months post-GoCCEP. Inpatients included were aged ≥65 or LACE ≥12. Z scores for differences calculated with 2 proportions test. Total of 2,067 patients with GOC notes located for 76 GoCCEP and 301 Control residents (total 312,895 unique patients). GoCCEP-trained residents increased documentation pre-post 0.6% to 1.1% ($p < 0.001$). GoCCEP vs. control PGY-1: 1.3% vs. 0.6% ($z=-10.17$, $p < 0.001$); PGY-2: 1.1 vs. 0.7% ($z=-2.96$, $p < 0.004$); PGY 3: 0.9% vs. 0.6% ($z=-3.78$, $p < 0.001$); PGY-3 Chief/higher: 1.1 vs.0.23% ($z=-8.1$, $p < 0.001$). Trained FM residents had significantly higher documentation than IM residents, 3.23% vs. 0.45% ($z = 13.00$, $p < 0.001$). GoCCEP associated with increased GOC documentation though overall level of documentation low regardless of year of experience. Multiple factors likely underlie these observations suggesting need for multipronged education/interventions to increase patient-centered GOCC.

### SESSION 7220 (POSTER)

### END-OF-LIFE

Abstract citation ID: igaf122.2599

### ADHERENCE TO END-OF-LIFE INSTRUCTIONS ACROSS COGNITION LEVELS: INSIGHTS FROM THE HEALTH AND RETIREMENT STUDY

Zahra Rahemi[1], Juanita-Dawne Bacsu[2], and Swann Adams[3], *1. Clemson University, Clemson, South Carolina, United States, 2. Thompson Rivers University, Kamloops, British Columbia, Canada, 3. University of South Carolina, Columbia, South Carolina, United States*

Adherence to end-of-life instructions is understudied and may vary across cognition levels in older adults, as cognitive decline can impact decision-making and the implementation of advance care planning. This study aims to examine adherence to end-of-life instructions across different cognition levels in older adults. Data from deceased participants in the Health and Retirement Study (HRS, 1994–2018) were extracted using exit files to assess adherence to end-of-life instructions. Langa Weir classification was used for cognition categorization. Adherence was measured using three indicators: proxy-reported difficulties in following written EOL instructions, proxy-reported healthcare provider difficulties in following written instructions, and instances where the patient received unwanted treatment despite family or decision-maker refusal. Individuals with dementia and impaired cognition were older at death, less likely to be married, more likely to have a high school education or less, and predominantly female. They were less likely to discuss end-of-life care prior to death or have a living will at death ($p < 0.05$) and marginally less likely to want all possible care ($p = 0.06$). Regarding adherence indicators, dementia group participants were 2.37 times more likely to receive unwanted treatment despite family/decision-maker refusal compared to normal cognition participants (OR = 2.37 [95% CI: 1.04–5.40], $p < 0.05$). Our findings highlight disparities in adherence to end-of-life instructions, with individuals with dementia being more likely to receive unwanted treatment. These results underscore the need for improved communication between individuals involved in end-of-life care and strategies to ensure that end-of-life preferences are honored across cognition levels.

Abstract citation ID: igaf122.2600

### ADVANCE DIRECTIVE PREFERENCES: COMPARING MEDICAL-SOCIAL-EMOTIONAL VS. MEDICAL-BASED APPROACHES

Ashley Shayya[1], Yuchi Young[2], Yufang Tu[3], Wan-Yu Chiu[4], and Taylor Perre[5], *1. Center for Human Services Research, University at Albany College of Integrated Health Sciences, Albany, New York, United States, 2. University at Albany College of Integrated Health Sciences, Albany, New York, United States, 3. North Dakota State University, Fargo, North Dakota, United States, 4. National Taiwan University, Zhongzheng District, Taipei, Taiwan (Republic of China), 5. Home Care Association of New York State, Albany, New York, United States*

This study explores adults' preferences for and recommendations to improve medical-based (POLST/MOLST) and medical-social-emotional-based (Five Wishes) advance directives, with a focus on age-related differences. US community-dwelling adults completed a survey on advance directives. Univariate analyses were used to assess advance directive type preferences, bivariate analyses evaluated age differences, and a thematic analysis identified recommendations for improvement. Participants, particularly young adults, preferred the Five Wishes form over the POLST/MOLST. Three key themes emerged from participants' recommendations for improving the advance directives: content, formatting, and no recommendations. A greater proportion of participants shared content recommendations for the POLST/MOLST and formatting recommendations or no recommendations for the Five Wishes form. For the POLST/MOLST, adults emphasized the need for more detailed descriptions, less medical jargon, less dense formatting, and an overall more holistic approach to end-of-life care. For the Five Wishes form, adults recommended shortening the form, adding more medical treatment questions, and altering the format of the wishes to be more user friendly. These recommendations to improve the POLST/MOLST and Five Wishes form may be useful for advance directive creators and policymakers to consider when making future adaptations to these advance directives. Preferences for medical-social-emotional-based and medical-based advance directives should be considered by medical practitioners, policymakers, and public health professionals when promoting advance directives, especially among young adults.

Abstract citation ID: igaf122.2601

### AMBIGUITIES ABOUT DISCUSSING END OF LIFE IN THE CONTEXT OF ADVANCE CARE PLANNING IN DEMENTIA

Jenny van der Steen[1], Lieve Van den block[2], Miharu Nakanishi[3], Jürgen in der Schmitten[4], Rebecca L. Sudore[5], and Ida J. Korfage[6], *1. Leiden University Medical Center, Leiden, Zuid-Holland, Netherlands, 2. Vrije Universiteit Brussel, Brussels, Brussels Hoofdstedelijk Gewest, Belgium, 3. Tohoku University Graduate School of Medicine, Sendai, Miyagi, Japan, 4. Universitätsklinikum Essen, Essen, Nordrhein-Westfalen, Germany, 5. University of California, San Francisco, San Francisco, California, United States, 6. Erasmus University Medical Center, Rotterdam, Zuid-Holland, Netherlands*

Advance care planning (ACP) in dementia has been defined as a communication process adapted to the person's capacity, which includes, and is continued with family, in our European Association for Palliative Care Delphi study Delphi study with an expert panel from 33 countries. A consensus was achieved that ACP discussions can refer to the end of life, but it is not a requirement. The study comprised four survey rounds and offered ample room for qualitative feedback. The feedback indicated ambiguity around discussing the end of life in the context of ACP. Regardless of a consensus-according to conservative criteria-to offer ACP around dementia diagnosis and to raise end-of-life issues later, there were concerns that bringing up the end of life could cause emotional harm to the person with dementia and the family. The panelists suggested that adopting a tailored, person-centered approach to ACP could help prevent such harms. On the other hand, there were concerns that being too careful could result in missed opportunities to talk about the end of life and elicit the persons' preferences when still able. Sketching a realistic scenario of the future and its uncertainties, probing attitudes of the person and their family, and offering choices may prevent possible detrimental effects of initiating ACP and discussing the end of life even early on. Taking such an individualized approach is consistent with the understanding of ACP as part of a process rather than a one-time event and may lower barriers to initiate ACP.

Abstract citation ID: igaf122.2602

### BALANCING CARE AND CHRONIC ILLNESS: A CONCEPTUAL FRAMEWORK FOR OLDER CAREGIVERS OF ADVANCED ILLNESS PATIENTS.

Hyeyoung Park, and Joohyun Chung, *University of Massachusetts Amherst, Amherst, Massachusetts, United States*

Aging caregivers are a growing yet under-recognized group. Many older caregivers provide care for individuals with advanced illness while also managing their own chronic conditions. Self-management plays a crucial role in chronic illness, and illness perception influences an individual's self-management. However, the caregiving context—particularly when caring for someone with advanced illness—may alter how caregivers perceive their own illness, subsequently affecting their self-management. Previous research has primarily categorized individuals as either patients with chronic conditions or caregivers, rather than considering these roles simultaneously. To address this gap, this paper develop a conceptual framework that integrates the caregiving context as a moderating or mediating factor between illness perception and self-management, supported by preliminary findings. We conducted an exploratory study (N=25) assessing illness perception, caregiving strain, anticipatory grief, and self-management among older caregivers of individuals with advanced illness. Descriptive statistics and non-parametric analyses were used to identify patterns. Given the small sample size, results were interpreted as preliminary insights informing theoretical development rather than definitive conclusions. Preliminary findings indicate that while illness perception among caregivers is heightened, this heightened awareness does not necessarily lead to improved or diminished self-management, pointing to the potential influence of other mediating factors. While prior research has suggested that caregiving stress may reduce illness perception due to self-neglect, our data reveal a different pattern, that caregiving stress seems to actually increase illness awareness.

Abstract citation ID: igaf122.2603

### BEREAVED NEXT-OF-KIN NARRATIVES ABOUT HOSPICE CARE IN ASSISTED LIVING

Nicole Dagen[1], Jacy Weems[2], Emily Gadbois[2], Melissa Clark[1], Grace Reed[2], Kali Thomas[3], Joan Teno[1], and Emmanuelle Belanger[1], *1. Brown University, Providence, Rhode Island, United States, 2. Brown University School of Public Health, Providence, Rhode Island, United States, 3. Johns Hopkins University, Baltimore, Maryland, United States*

There is limited evidence about the quality of end-of-life care provided in assisted living (AL), especially as related to various community-level care processes supporting dying in place. This study aimed to explore narratives of bereaved next-of-kin of deceased AL residents, focusing on end-of-life care quality and hospice utilization as a factor of AL retention policies for dying residents and staffing. We conducted 52 semi-structured telephone interviews with bereaved next-of-kin of persons who resided in AL during the last month of life across 38 states, purposefully oversampling racially and ethnically diverse ALs. We stratified qualitative data along care processes obtained through a nationally-representative survey of administrators at AL communities with 25 or more beds. Two coders applied exploratory thematic analysis to analyze narratives stratified by AL policy to retain residents in need of end-of-life care (vs. case-by-case vs. discharge) and any registered nurse (RN) on staff (yes/no). Next-of-kin cited the desire for more staff attention and avoiding late-life transitions as key motivating factors for enrollment in hospice. Even in AL communities with a policy to retain dying residents, participants reported receiving an ultimatum to either enroll their next-of-kin in hospice or move them to another setting like a nursing home. Regardless of RN availability, electing hospice enhanced staff access, particularly overnight presence and support for residents at risk of falling. Our study highlights the importance of hospice care in allowing dying residents to stay in place across AL communities with varying policies regarding end-of-life care and RN availability.

Abstract citation ID: igaf122.2604

### BLACK CAREGIVERS' PERCEPTIONS OF QUALITY OF CARE AND THE ROLE OF ADVANCE CARE PLANNING DISCUSSIONS

Jessica Yauk, Debra Dobbs, and Lindsay Peterson, *University of South Florida, Tampa, Florida, United States*

There are distinct variations in patterns of healthcare quality between minority and non-minority older adults. Black family caregivers are at the crux of the care convoy for Black older adults needing ongoing care. This study sought to understand informal Black caregivers' perceptions of care quality across the care continuum, with a specific focus on advance care planning (ACP). The National Health and Aging Trends Study last-month-of-life interview and the National Consensus Project Practice Guidelines for Quality Palliative Care framework guided the interview questions used in this study. Seven Black caregivers of seriously ill older adults were recruited in Florida. Caregivers completed one interview describing interactions with healthcare professionals and their perceptions of care quality and ACP engagement. The results identified themes including communication, care transitions, culturally relevant care, and the gaps in health literacy. Caregivers expressed a need for better communication from providers regarding diagnosis and disease trajectory and a need for ongoing relationships with care recipients to understand care needs. Care transitions were perceived as high-quality when there was clear communication between providers and family. In contrast, caregivers felt less confident in decision-making when they thought they did not have all the information or resources required to care for the care recipient. Regarding ACP, caregivers indicated that ACP was an important aspect of goal-concordant care. Practice implications to improve health care quality for Black older adults include better communication, culturally competent care and more effective care coordination. Challenges in recruiting older Black caregivers will also be addressed.

Abstract citation ID: igaf122.2605

**COST BURDEN OF END OF LIFE CARE ACROSS GRADIENTS OF COGNITIVE IMPAIRMENT AMONG NURSING HOME RESIDENTS WITH CANCER**

Long Vu[1], Siran Koroukian[1], Johnie Rose[1], David Warner[2], Sara Douglas[3], and Nicholas Schiltz[3], *1. Case Western Reserve University School of Medicine, Cleveland, Ohio, United States, 2. The University of Alabama at Birmingham, Birmingham, Alabama, United States, 3. Case Western Reserve University, Cleveland, Ohio, United States*

Compared to aggressive end of life (EOL) care, palliative and hospice care are associated with cost savings in older adults with cancer. It is not known, however, whether these savings generalize to nursing home (NH) residents, who may present with varying levels of cognitive impairment (COG-I) and complex multimorbidity. Using the 2013-2017 Surveillance, Epidemiology, and End Results Registry linked with the Minimum Data Set and Medicare claims, we identified 40,833 older NH residents who died with cancer. We examined the total costs of all Medicare payments within the last 30 days of life across gradients of COG-I, and between markers of aggressive EOL care received. 49.2% of identified NH residents were cognitively intact; 24.4% had mild COG-I; 19.7% had moderate COG-I; and 6.7% had severe COG-I. Adjusting for inflation and other clinical variables, compared to those cognitively intact, average total costs were $682 (95% confidence interval [CI]: $11-$1,353) and $1,279 (95% CI: $561-$1,997) lower for those with mild and moderate COG-I, respectively. Costs for those with severe COG-I did not differ. Any aggressive EOL care received increased average costs across all COG-I levels by $25,142 (95 % CI: $24,338-$25,946). Conversely, each additional day enrolled in hospice yielded an average savings of $546 (95% CI: $517-$577). Our results reinforce the high cost of aggressive EOL care and the savings benefit of hospice care in older NH residents with cancer. Although costs were slightly lower at increasing levels of COG-I, the most severely impaired had costs similar to their non-impaired counterparts.

Abstract citation ID: igaf122.2606

**COVID-19 PANDEMIC CARE SERVICE CHANGES AND ADVANCE CARE PLANNING ON DEATH ANXIETY AMONG FAMILY CAREGIVERS**

Jung-Nim Kim, *Tokyo University of Social Welfare, Isesaski City, Gumma, Japan*

Background: The number of family caregivers encountered significant difficulties accessing healthcare services and support from relatives during the COVID-19 pandemic due to changes in care service availability at home. Although appropriate support may reduce death anxiety and improve caregiving capacity at home, few studies have examined this relationship. This study investigated the direct associations between COVID-19 related care service changes and Advance care planning (ACP) on family caregivers' death anxiety, as well as indirect associations through decreased in care services during COVID-19 and ACP implementation. Methods: Interview surveys were conducted in 2021 and 2023 in areas near the Tokyo Metropolitan region with family caregivers of community-dwelling older adults who were certified as requiring care under Japan's long-term care insurance system. This study analyzed the combined data from each survey (n = 1,057). Results: Approximately 18.9% of family caregivers reported decreased care services during COVID-19. SEM analyses revealed that changes in care services due to COVID-19 and ACP directly influenced death anxiety. Care recipients' ADL and economic situation revealed indirect effects through ACP, but did not show any indirect effects through changes in care services during COVID-19. Multigroup SEM analyses indicated that family caregivers who engaged in ACP communication experienced more decreased in care services during COVID-19. Conclusions: Family caregivers who engaged in ACP communication and experienced reduced care services during COVID-19 may be at higher risk of death anxiety without adequate support at home. Developing strategies and policies to make home-based healthcare services and support is essential to mitigate this risk.

Abstract citation ID: igaf122.2607

**DEATH CAFES: BREWING UP CONVERSATIONS ABOUT DEATH**

Abby Schellhorn[1], and Melinda Heinz[2], *1. University of Northern Iowa, Elkader, Iowa, United States, 2. University of Northern Iowa, Cedar Falls, Iowa, United States*

Death can be a difficult topic to talk about and conversations about the idea seem taboo. Death Cafes began in the U.K. in 2011 and in the U.S. in 2016. Since 2023, 13 Death Cafes in a university town in the Midwest were offered (N = 101). Survey results revealed participants were between the ages 19 to 85, including community members, college students, and professors. Approximately 69 percent were women, 22 percent were men, 2 percent were transgender, and 7 percent were gender non-conforming. Participants reported a range of religious and spiritual beliefs. Approximately 48 percent indicated that they were not religious, but 82 percent were spiritual; 51 percent were religious, and 16 percent were not spiritual. When participants were asked about why they attended, a range of responses were given including: "Invited by a friend, sounded interesting, curiosity" and "to learn more about preparing for death." As attendees reflected, participants mentioned they enjoyed the open conversation without fear of being shut down for talking about death. One participant said, "It was great! People seemed free to discuss anything death related and everyone seemed open to discussion without judgment." Many participants also commented that they "hoped to attend more in the future." Death Cafes allow attendees the freedom to talk about difficult topics in a respectful environment where diverse viewpoints and perspectives are welcomed. These findings have implications for gerontologists and show the importance of creating safe and welcoming spaces for adults of different ages to discuss death.

Abstract citation ID: igaf122.2608

**DEMENTIA CAREGIVERS' PERCEPTION OF CAREGIVER-CENTERED HOSPICE COMMUNICATION: DOES EXPERIENCE MATTER?**

Oonjee Oh[1], Karla Washington[2], Debra Oliver[2], and George Demiris[1], *1. University of Pennsylvania, Philadelphia, Pennsylvania, United States, 2. Washington University in St. Louis, St. Louis, Missouri, United States*

While transitioning to hospice can be challenging for dementia caregivers, those new to caregiving may struggle more as they adjust to new responsibilities. Since hospice includes caregivers within the unit of care, we compared the perceptions of caregiver-centered communication with the hospice team including caregivers with different levels of experiences in caregiving and hospice. We analyzed the pre-intervention data from an ongoing cognitive-behavioral intervention targeting caregivers of hospice patients with dementia, labelled ENCODE (Empowering Caregivers of Patients with Dementia). Caregiving experience was categorized as less than 1 year, between 1 to 3 years, and over 3 years. Hospice experience was dichotomized into 4 weeks or less, and more than 4 weeks. Based on these categories, caregivers' experiences in caregiving and hospice were grouped into 6 classes. Among 281 caregivers (mean age=59.7 (standard deviation (SD)=12.7), female 82.2%), 21.4% were new to caregiving and enrolled in hospice for 4 weeks or less. The quality of the hospice team communication was measured using the caregiver-centered communication questionnaire (CCCQ). The mean CCCQ score in our sample was 117.4 (SD = 23.1). The analysis of covariance model controlled for age and sex did not demonstrate significant differences in the CCCQ scores across the 6 classes (p value=0.07). The 5 subscales of the CCCQ (e.g., exchange of information, responding to emotions) also showed no significant differences. Such results provide an evidence base supporting the hospice model in addressing the diverse needs and concerns of dementia caregivers, regardless of the level of their prior experience with caregiving and hospice.

Abstract citation ID: igaf122.2609

### DEVELOPMENT OF PAL MIND: A PALLIATIVE CARE CLINICAL TOOL TO INFORM A PILOT STUDY

Danielle Llaneza[1], and Kristopher Kern[2], *1. Rutgers Robert Wood Johnson Medical School, New Brunswick, New Jersey, United States, 2. University of Nevada, Las Vegas, Las Vegas, Nevada, United States*

As the adult population ages, chronic multimorbidity is estimated to increase by over 90%, leading to decreased quality of life. Palliative care supports chronic disease management and increases quality of life. Newer subspecialties of palliative care (e.g., neuro-palliative care and geri-palliative care) holistically address disease management and provide interventions to older adults with chronic multimorbidity and cognitive concerns to address their unique health needs in an inpatient setting. This study describes the development of PAL MIND, a clinical tool that uses a combination of intake and assessment screeners to comprehensively identify patients' psychological needs during their inpatient stay as well as inform outpatient treatment next steps; additionally, the tool grows interdisciplinary understanding of psychological services available to patients. PAL MIND was developed based on a literature review of measures validated in palliative and geriatric patient samples. Intake sections include: patient demographics, biopsychosocial spiritual evaluation, risk evaluation, mental status exam, functional evaluation, and treatment communication evaluation. Assessment sections include: orientation screener, cognitive screener, mental health screener, palliative care screener, and advanced care planning screener. Next, we will pilot-test the tool in a regional inpatient hospital setting to determine the feasibility of tool delivery in an ICU setting. The tool has the potential to identify patient needs regarding psychological services and treatment preferences, which can facilitate interdisciplinary care and targeted treatment interventions for older adults with chronic and terminal diseases in inpatient settings.

Abstract citation ID: igaf122.2610

### EFFECTIVE ADOPTION OF THE SUPPORTIVE COORDINATED TRANSITIONAL CARE PROGRAM IMPROVES END-OF-LIFE CARE FOR VETERANS

Lana Brown[1], Hallie Keller[1], Tanya Clayton[1]TaFarra Haney[2], Ramona Rhodes[3], Wanda Morrison[1], and Jane Driver[4], *1. Central Arkansas Veterans Healthcare System, North Little Rock, Arkansas, United States, 2. Eugene J. Towbin Healthcare Center, North Little Rock, Arkansas, United States, 3. UT Southwestern Medical Center, Dallas, Texas, United States, 4. VA Boston Healthcare System, Brockton, Massachusetts, United States*

The Coordinated Transitional Care (CTrac) program is a telephone-based, nurse-driven program shown to decrease hospital readmissions in acute care hospitals. This project implemented and evaluated an adapted version of CTraC, Supportive CTraC, developed at the Veteran Affairs (VA) Boston, to enhance the quality of transitional and end-of-life care for veterans with advanced disease and a life expectancy of ≤ 24 months. The Replicating Effective Programs framework, led by two registered nurse case managers, was used to adapt and implement the Supportive CTraC program at one VA facility. Program success was defined as > 30% of participating Veterans referred to palliative care/hospice, >30% enrolled in hospice, >30% with new Advance Directives, and fewer acute care deaths compared to the hospital average. Two-hundred three Veterans aged 43 to 96 enrolled in the program. Veterans were primarily male (96%), White (61%) or Black (34%), and non-Hispanic (97%). About half (52%) were urban residents compared to rural (42%) or highly rural (5%). During the 8-week program, greater than 30% of Veterans completed an Advance Directive (57%), received a hospice/palliative care consult (48%), and enrolled in hospice (39%). By the end of the program, 135 (67%) Veterans had died. Of these, fewer died in acute care compared to the hospital average (20% vs. 36% ). The remaining Veterans died at home (41%), in hospice/palliative care (36%), a nursing home or rehabilitation unit (2%), or an unknown setting (1%). The Supportive C-TraC program was successfully adapted and implemented, meeting all goals and facilitating positive end-of-life outcomes for Veterans.

Abstract citation ID: igaf122.2611

### ELEMENTS FOR SUCCESSFUL ADVANCE CARE PLANNING IMPLEMENTATION IN NURSING HOMES: A SYSTEMATIC REVIEW

Peiyuan Zhang[1], Joan Davitt[1], Nancy Kusmaul[1], Paul Sacco[2], Kathleen Unroe[3], and John Cagle[4], *1. University of Maryland, Baltimore County, Baltimore, Maryland, United States, 2. University of Maryland, Baltimore, Maryland, United States, 3. Indiana University School of Medicine, Indianapolis, Indiana, United States, 4. University of Maryland, Batimore, Maryland, United States*

Approximately 1.2 million people resided in more than 15,000 nursing homes (NHs) in the U.S., with 30% spending their last days there. Advance care planning (ACP) has well-documented benefits in improving the quality of end-of-life care and thus has significant relevance to NHs. However, the quality of ACP practices varies partly due to a lack of ACP guidelines. This systematic review aimed to identify the key elements for successful ACP in NHs, following the Preferred Reporting Items for Systematic Reviews and Meta-Analysis (PRISMA) guidelines. Three groups of terms representing ACP, implementation processes, and assessment were combined with “AND” and searched in four databases: CINAHL, PsycInfo, SocINDEX, and Medline (Ovid) in June 2024. The search yielded 2,013 studies, of which 83 were reviewed in full, and 19 met the inclusion criteria, validated through independent review and consensus by two researchers. Deductive thematic analysis of the selected studies following Donabedian’s model of quality of care identified two major themes with 11 subthemes: (1) NH structural support for ACP, including policies incorporating ACP into admission, role clarity, and resource availability (e.g., dedicated time and training for ACP, particularly among people with impaired decision-making capacity), and (2) standardized implementation processes, emphasizing resident and family engagement, trust building, discussions of care options and residents’ preferences, documentation of ACP in a standardized place, sharing ACP-related information among an interdisciplinary team, and reviewing ACP regularly. The findings highlight the need for structured support and standardized implementation processes, providing a framework for future NH ACP quality improvement initiatives.

Abstract citation ID: igaf122.2612

### EMPATHY, AUTONOMY, AND SOCIAL EXPECTATIONS IN SURROGATE DECISION-MAKING AMONG TAIWANESE ADULTS

Duan-Rung Chen, and Yu-Chen Juang, *National Taiwan University, Taipei, Taipei, Taiwan (Republic of China)*

Purpose: Surrogate decision-making involves making choices for individuals unable to express their preferences. In many Asian societies, this process is family-centered, with elders—typically sons or senior members—making decisions based on hierarchy, harmony, and collective responsibility. Tunney and Ziegler’s (2015) typology categorizes surrogate decisions into four types: (1) Substituted Judgment, reflecting the patient’s prior wishes; (2) Benevolent Decision-Making, prioritizing the patient’s best interests; (3) Egocentric Decision-Making, influenced by external pressures; and (4) Projective Decision-Making, where surrogates assume patients would choose as they would. This study examines the most prevalent decision-making type in Taiwan and the influence of gender. Methods: An online questionnaire was distributed via social media, targeting a broad demographic, including older adults. Conducted from October 2024 to January 2025, it gathered 2,000 responses, with 25% aged 65 or older. Of the respondents, 10.9% worked in medical fields, 70% held a college degree, and 51% were male. Results: Gender differences emerged in surrogate decision-making. Women prioritized Substituted Judgment, Projective, and Benevolent Decision-Making, incorporating patient preferences, empathy, and minimizing suffering. Men leaned toward Egocentric Decision-Making, influenced by societal expectations and familial opinions, prioritizing external perceptions over patient well-being. Conclusion: Cultural norms and gender roles shape surrogate decisions. Women tend to be patient-centered, while men emphasize social responsibility. Understanding these differences can enhance ethical frameworks, ensuring surrogate decisions align more closely with patient autonomy and well-being.

Abstract citation ID: igaf122.2613

### ENHANCING COMMUNICATION AND TRUST IN ADVANCE CARE PLANNING FOR OLDER ADULTS WITH SICKLE CELL DISEASE

Tamara Poole[1], Rania Mohamed[2], Francyess Denis Oliva[2], Teagan Callaway[2], John Strouse[2], and Charity Oyedeji[2], *1. Duke University School of Nursing, Durham, North Carolina, United States, 2. Duke University School of Medicine, Durham, North Carolina, United States*

Despite advancements in treatments, life expectancy for sickle cell disease (SCD) remains 20 years shorter than people without SCD, making advance care planning (ACP) important. There is a gap in literature on ACP preferences of older adults with SCD. This study aims to describe ACP preferences for older adults with SCD. We interviewed 19 older adults with SCD (age ≥ 50). Interview questions addressed comfort discussing end-of-life care, nature of prior ACP discussions, and preferences for ACP. The data was analyzed using conventional content analysis. There were three themes. Theme one was importance of family involvement. Participants described preferences to have family present during ACP. Many preferred not leaving the burden of end-of-life decisions to family, while others preferred their family making end-of-life decisions. Participants stated preferences for their healthcare proxy (HCP), but the majority reported no HCP documentation. Theme two was early ACP and appropriate timing. Participants described discussing ACP while sick in the hospital as inappropriate. Due to the unpredictably of SCD, many stated that ACP should start in early adulthood or when they’re capable of understanding their options. Theme three was limited ACP communication. Participants reported limited communication or lack thereof about ACP preferences. Most participants were open to communication with a trusted provider they perceived to be knowledgeable of their SCD to provide information on or complete ACP documents. They preferred discussing ACP in clinic without rushing or interruptions. This highlights the importance of early family-centered ACP to honor the wishes of older adults with SCD.

Abstract citation ID: igaf122.2614

### HOSPICE ADMINISTRATOR PERSPECTIVES ON SUPPORTING PATIENTS AFTER LIVE DISCHARGE

Kira Sheldon[1], Elizabeth Luth[2], Miriam Ryvicker[3], Caitlin Brennan[4], and Yongkang Zhang[5], *1. Rutgers University--New Brunswick, New Brunswick, New Jersey, United States, 2. Rutgers University, New Brunswick, New Jersey, United States, 3. VNS Health, New York, New York, United States, 4. Care Dimensions, Danvers, Massachusetts, United States, 5. Weill Cornell Medicine, New York, New York, United States*

Live hospice discharge occurs when patients are disenrolled prior to death, impacting approximately 15% of enrollees. Addressing individuals’ needs after live discharge, particularly those at increased risk for poor outcomes, is a challenge for patients and their families and a concern for hospice agencies and the Centers for Medicare and Medicaid Services. This study examined how hospices support patients discharged alive and the potential for predictive tools identifying individuals at risk for poor post-discharge outcomes (e.g., hospitalization) to help prevent these outcomes. We analyzed interviews with 10 senior level hospice administrators in roles that impact live discharge (clinical care, data science, quality assurance, and clinical operations) at three hospices in the Northeastern and Southern United

States. Participants shared that hospices support discharged patients through informal connections with healthcare providers and community organizations and formally through referral to other intra-organizational services (e.g., palliative care, GUIDE). They perceived geographic disparities in availability of post-hospice services and lack of formal infrastructure and incentives for hospices to follow-up with enrollees as contributing to poor post-discharge outcomes. They recognized the potential benefit of predictive tools to identify individuals at risk for poor post-discharge outcomes, provided concerns regarding measurement validity and end-user buy-in were adequately addressed. Careful, comprehensive discharge planning, referrals to other organization services (for larger hospices) and building connections with community services and primary care (for smaller hospices) may improve continuity of care and prevent poor outcomes following live discharge from hospice. Predictive tools may be useful if carefully specified and trusted by end-users.

Abstract citation ID: igaf122.2615

### MEANING IN LIFE OF AGING FAMILY CAREGIVERS IN HOME HOSPICE CARE

Haerim Lee, Hyun-E Yeom, Eunyoung Park, and Misook Jung, *Chungnam National University, Daejeon, Republic of Korea*

Families caring for home hospice patients directly witness end-of-life experiences, which, alongside their aging process, may lead to a reevaluation of life's meaning. As they navigate the complexities of caregiving and observe the end-of-life journey firsthand, their perceptions of life's meaning may become increasingly intricate and uncertain. This study explores how family caregivers of home hospice patients perceive and interpret life's meaning throughout the caregiving process. This qualitative phenomenological study employed face-to-face, semi-structured interviews with nine family caregivers (M age = 67 years) who cared for hospice patients at home. Data were analyzed using Colaizzi's method, leading to the identification of three key themes: (1) diminished sense of life's meaning amid physical and emotional exhaustion, (2) uncertainty and disorientation in meaning-making following bereavement, and (3) the challenging pursuit of life's meaning amid suffering. Family caregivers experienced vulnerability due to the demands of 24-hour caregiving and the physical limitations associated with aging, making it difficult to find meaning in life. They also struggled with uncertainty in redefining their lives without the patient while simultaneously grappling with an ambivalent sense of responsibility in seeking a renewed sense of purpose. These findings highlight the complex interplay between caregiving dedication, personal sacrifice, and the ongoing reconstruction of life's meaning throughout the caregiving journey. They underscore the critical need for tailored psychosocial support to assist family caregivers in navigating the challenges of post-caregiving life and age-related difficulties.: : meaning in Life; Aging; Home Care Services; Hospices; End-of-Life; Caregivers; Qualitative Research

Abstract citation ID: igaf122.2616

### NAVIGATING INTERDEPENDENCE: THE END-OF-LIFE CAREGIVING PROCESS OF ADULT ONLY CHILDREN CARING FOR THEIR PARENT

Zhiqi Yi[1], Nili Wang[2], Sarah Jen[1], and Shuo Xu[3], *1. University of Kansas, Lawrence, Kansas, United States, 2. Beijing Normal University, Beijing, Beijing, China (People's Republic), 3. Renmin University of China, Beijing, Beijing, China (People's Republic)*

The one-child policy has shaped the life experiences of millions of only children in China and providing end-of-life care to their aging parents is becoming a critical social issue. Their unique lived experiences likely lead to distinctive end-of-life caregiving experiences, however, to date no existing literature has investigated this phenomenon. This study uses a convenience sample of 15 adult only children who provided at least one month of end-of-life care leading up to their parents' death to explore their end-of-life caregiving process. Adopting a social constructionist paradigm, this study uses the "In-Vivo" approach to theory-building and thematic analysis to visualize and present findings. The emergent caregiving process encompasses four phases of interdependence depicted through the metaphor of charting a sailing voyage: 1) Sailing Out of the Harbor: Continued Interdependence; 2) Navigating Back to the Harbor: Inverted Interdependence and Shifting Life Emphasis; 3) The Collapsing Harbor: Decoupling Interdependence and Preparation for Bereaved Life; 4) The Navigator's and Harbor's Reconstruction: Divergent Interdependence and Social Life. Three turning points sequentially connect the four phases: 1) realizing the severity of the illness and caregiving should be the main task; 2) sensing the possible coming death; 3) death of or farewell to the parents. The parent-child relationship anchors the process, while sociocultural context and illness progression drive the process forward. Practitioners can provide competent psychosocial care in response to the unique dynamics of interdependence and caregiving stress in individual cases. Advocacy for death education and policy to promote hospice and palliative care are recommended.

Abstract citation ID: igaf122.2617

### PALLIATIVE CARE INTERVENTIONS AMONG HISPANIC INNER-CITY PATIENTS WITH ADVANCED LIVER DISEASE

Zachary Song[1], Mekiayla (Meki) Singleton[2], Carin van Zyl[3], Aaron Storms[4], and Susan Enguidanos[5], *1. University of Southern California Leonard Davis School of Gerontology, Los Angeles, California, United States, 2. Rutgers University, Newark, New Jersey, United States, 3. LA General Medical Center, Los Angeles, California, United States, 4. Los Angeles General Medical Center, Los Angeles, California, United States, 5. University of Southern California, Los Angeles, California, United States*

Advanced liver disease is the 9th leading cause of death in the United States and the 7th leading cause of death among Hispanic individuals. Patients suffering from end-stage liver disease frequently encounter various physical and psychosocial stressors, yet routine access to palliative care is limited. This study aimed to identify the primary needs of a predominantly Hispanic patient population with advanced liver disease and the interventions provided by a palliative care team within a hepatology clinic at a large urban public hospital. We conducted a retrospective qualitative analysis of palliative care team medical record entries from a pilot palliative care program. Participants with advanced liver disease (MELD ≥20) were recruited between 2016 and 2017. Thematic analysis was performed to identify key patient stressors, sources of patient support, and interventions conducted by the palliative care team. Of the 65 participants, 94% identified as Hispanic, with over half undocumented. Common patient stressors included physical symptoms, financial hardship, and challenges related to immigration. Family involvement and religious faith were primary sources of support. Palliative care interventions included psychosocial counseling, end-of-life care planning, social service and public benefit referrals (e.g., food, in-home support, and disability insurance), substance use management, and help navigating the healthcare system. Findings illustrate the complex interplay between social determinants of health and disease management for Hispanic patients with advanced liver disease. Integrating palliative care into hepatology clinics addresses these multifaceted needs by providing tailored psychosocial and medical support. Further research is needed to optimize and expand these interventions.

Abstract citation ID: igaf122.2618

### PROMOTING ADVANCE CARE PLANNING AMONG COMMUNITY-BASED OLDER ADULTS: A PILOT PROJECT IN CHINESE SOCIETY

Shiyu Lu[1], Ning Fan[2], and Yan Zhang[1], *1. The University of Hong Kong, Hong Kong, China (People's Republic), 2. Forget Thee Not, Hong Kong, China (People's Republic)*

While promoting advanced care planning is a priority, knowledge remains unclear about how to prepare the aging community for advanced care planning (ACP) at an early stage (EoL), particularly in Chinese societies where discussions about end-of-life care arrangements are often considered taboo. This study evaluates the effectiveness of the Community Care Plan (CCP) pilot project in Hong Kong, which is a compassionate community service model for EoL care focusing on enhancing quality of life ("good life") and establishing ACP for older individuals facing health deterioration ("good death" arrangements). The CCP pilot project is built on multidisciplinary collaborative services, including a professional team specializing in ACP, home-visit services by nursing teams, social workers from traditional community centers for older adults, and active community volunteers. The evaluation study employs a single-group pre- and post-test design to assess effectiveness. Following an 8-month pilot project, the mixed linear regression found that 63 eligible older adults showed improvements: an increased sense of community ($\beta = 0.53$, $p = 0.008$), enhanced ACP knowledge ($\beta = 0.42$, $p < 0.001$), more conversations with others ($\beta = 0.16$, $p = 0.001$), and increased readiness and self-efficacy in ACP engagement (e.g., discussing medical treatment at end-of-life with doctors and signing advanced directives). The 70 involved active community volunteers also reported improvements in quality of life, sense of community, ACP knowledge, and increased comfort initiating EoL conversations with others. The study discusses potential policy and practice implications for ACP promotion based on the preliminary findings.

Abstract citation ID: igaf122.2619

### THE MEDIATING ROLE OF PERCEIVED BENEFIT BETWEEN ATTITUDES AND DECISIONAL CONFLICT IN ADVANCE CARE PLANNING

Kyung Ah Cho[1], Jung-Ah Lee[2], and JinShil Kim[1], *1. Gachon University, Incheon, Republic of Korea, 2. University of California, Irvine, Irvine, California, United States*

Advance directives are essential for ensuring patient-centered decision-making but are often hindered by decisional conflict and varying perceptions of attitudes and benefits. Family caregivers of people with dementia play a critical role in decisions about end-of-life preferences, with their decisional conflict significantly influenced by attitudes and perceived benefits regarding advance care planning. This study examined whether perceived benefits mediate the relationship between attitudes and decisional conflict among family caregivers of people with dementia. A cross-sectional study involving 92 family caregivers was conducted, and mediation analysis was performed using the PROCESS macro for SPSS (Model 4), controlling for age, educational level, and depressive symptoms as covariates. Attitudes toward advance care planning demonstrated a significant relationship with decisional conflict, partially mediated by perceived benefits. The total effect of attitudes on decisional conflict was significant (beta = -1.198, p-value = 0.001), and the indirect effect through perceived benefits was also significant (effect = -0.371, 95% CI = -0.867, -0.033). Additionally, the direct effect of attitudes on decisional conflict remained significant (beta = -0.827, p-value = 0.034). This partial mediation model accounted for 28.2% of the variance in decisional conflict ($F = 6.755$, $R^2 = 0.282$, p-value < 0.001). These findings highlight the importance of perceived benefits in shaping the relationship between attitudes and decisional conflict. Interventions aimed at enhancing positive attitudes and increasing perceived benefits may be essential for reducing decisional conflict, ultimately improving the caregiving experience and decision-making process.

Abstract citation ID: igaf122.2620

### UNDERSTANDING SERVICE UTILIZATION & QUALITY OF LIFE POST-HOSPICE LIVE DISCHARGE

Stephanie P. Wladkowski[1], Leslie Hinyard[2], Ruaa Al Juboori[3], Verna Hendricks-Ferguson[4], and Cara Wallace[5], *1. Bowling Green State University, Bowling Green, Ohio, United States, 2. Saint Louis University School of Medicine, St. Louis, Missouri, United States, 3. University of Mississippi, Oxford, Mississippi, United States, 4. Trudy Busch Valentine School of Nursing, St. Louis, Missouri, United States, 5. Saint Louis University, Saint Louis, Missouri, United States*

Following a live discharge from hospice, patients and their caregivers often need to re-establish or transition to community resources as hospice services are no longer available. Although patients may no longer qualify for hospice, they often continue to experience complex medical and psychosocial needs. Therefore, coordinated service utilization following live discharge is critical to ensuring continuity of care, minimizing emergency room visits or caregiver burnout, and supporting quality of life (QOL) for both patients and caregivers. This study surveyed patient-caregiver dyads ($n = 24$) approximately 3 months following a live discharge due to extended prognosis, to better understand the services utilized and their QOL post-hospice care. Participants were an average age of 86 (SD 12.1), 83% female, and 79% White with 50% of participating living at home, while 42% were in long-term care. Within the first 3 months post discharge, 12.5% reported visiting an urgent care at least once, 8% reported at least one ER visit, and 8% reported at least one hospitalization. Further, 62% reported an established primary care provider while only 33% reported using home health, and less than half (46%) reported use of paid caregivers. Lastly, only 25% of patients reported "Good" QOL in the immediate post-discharge period. Results demonstrate that in the initial post-discharge period, medical service utilization is relatively low as is reported QOL. Research, clinical, and policy implications will be discussed.

### SESSION 7225 (POSTER)

### FALLS

Abstract citation ID: igaf122.2621

### ASSESSING FALL RISK FACTORS IN ADULTS 65+ LIVING IN RHODE ISLAND, MASSACHUSETTS AND NEW HAMPSHIRE

Nachalie Rodriguez-Cruz[1], Sophia Casale[2], Qinglin Gao[1], Calvin Tran[1], and Elizabeth Dugan[1], *1. University of Massachusetts Boston, Boston, Massachusetts, United States, 2. University of Massachusetts Boston, Red Bank, New Jersey, United States*

Falls are one of the leading causes of injury and death for older adults 65+. A number of risk factors for or consequences of falls include hip fractures, osteoporosis, and difficulty with ambulation. This study aims to describe/contrast disparities in community rates of hip fractures, osteoporosis, and ambulatory difficulty in adults 65+ in Massachusetts, Rhode Island, and New Hampshire. This study utilizes data from the 2025 Healthy Aging Data Reports (healthyagingdatareports.org). The hip fracture and osteoporosis rates were calculated from Medicare fee-for-service data of adults 65+(2020-2021), and ambulation rate difficulties were calculated from American Community Survey (2018-2022). Results showed that state rates for osteoporosis were MA (20.1%) [13.58; 28.6%], RI (18.9%) [13.0; 24.3%], and NH (16.3%) [9.9; 25.7%]. Difficulty with ambulation state rates were MA (18.6%) [6.12; 41.3%], RI (18.7%) [5.77; 27.9%], and NH (16.7%) [0; 42.9%]. Hip fracture state rates were MA (3.20%) [1.53; 4.59%], RI (3.09%) [2.00; 4.07%], and NH (2.79%) [2.04; 3.99%]. A trend was observed across small towns in MA experiencing the greatest rates of osteoporosis (Lenox, 28.6%; Brookline, 27.9%). New Hampshire community and state rates of hip fractures and osteoporosis were lower than MA and RI. The rates of ambulatory difficulty were lower in the southern, wealthier areas of RI. Understanding community rates of the indicators can identify areas underserved and in need of programs or services. Awareness of the disparities across states and within states can help fall prevention, physical activity programming, and hospitals assess the risks individuals within their community experience.

Abstract citation ID: igaf122.2622

### COMPARISON OF THE BURDEN OF FALLS AMONG OLDER ADULTS AGED 70 YEARS AND OVER BETWEEN GLOBAL AND CHINA

Xiaodong Chen, and Liping Li, *Shantou University, Shantou, Guangdong, China (People's Republic)*

Objective: To estimate the burden of falls in older adults globally and in China, and project the burden through 2030. Methods: Using data from the Global Burden of Disease Study 2021, we calculated age-standardized incidence rate (ASIR), mortality rate (ASMR), and disability-adjusted life years (ASDR) for falls in people aged ≥70 years from 1990 to 2021. Joinpoint regression was used to analyze the trend of falls disease burden during 1990-2021, and Bayesian age-period-cohort (BAPC) modeling was used to predict the trend of falls disease burden in global and China during 2022-2030. Results: From 1990 to 2021, the global burden of falls increased, with AAPCs of 0.60 for ASIR, 0.69 for ASMR, and 0.30 for ASDR. In China, the increase was more pronounced, with AAPCs of 2.44, 1.47, and 0.89, respectively. Projections for 2022-2030 show global ASIR will rise from 7,672.94 to 8,036.87 per 100,000, while ASMR will decrease from 106.72 to 96.96. In China, ASIR will increase from 7,143.75 to 8,757.76, and ASMR will decrease from 92.52 to 84.95. The global ASDR will decrease from 2,795.73 to 2,711.72, while China's ASDR will rise from 2,293.30 to 2,503.68. Conclusions: The burden of falls disease continues to increase globally and in China in the older adult population aged 70 years and older, with a significant rise in ASIR in China in particular. Compared to global, more attention should be paid to falls ASIR and ASDR in China in the future.

Abstract citation ID: igaf122.2623

### COST-ANALYSIS OF A GERIATRICIAN-LED FALLS PREVENTION CLINIC AMONG OLDER ADULTS AT HIGH RISK OF FUTURE FALLS

Jennifer C. Davis, Elise Wiley, Larry Dian, Kenneth Madden, Karim Khan, Naaz Parmar, Katrina Loutet, and Teresa Liu-Ambrose, *The University of British Columbia, Vancouver, British Columbia, Canada*

A geriatrician-led Falls Prevention Clinic is a best practice and evidence-based approach for falls prevention that has demonstrated feasibility and acceptability. The economic impacts of this approach, which directly impact its sustainability within the healthcare system, remain unknown. Our primary objective was to determine the costs and potential savings associated with a multi-disciplinary geriatrician-led Falls Prevention Clinic among older adults at high risk of falls. Operating costs of the Falls Prevention Clinic were determined based on operating costs (i.e., personnel, equipment, overhead), medical services plan costs, and personnel costs. Falls Prevention Clinic usage over 12 months was detailed based on monthly frequencies of new and repeat visits. Longitudinal health resource utilization was ascertained over 12 months. Cost savings from falls averted over 12 months were estimated. Main outcome measures included: operating costs of the clinic, estimated annual health resource utilization savings, number of falls over 12 months and health care costs (i.e., health care practitioner, hospital admissions, laboratory tests/investigations). A total of 543 patients were seen over a year, with 240 new and 303 follow-up patients. The total direct health resource utilization costs were 4,892 (7,767) (2024 CAD$) per person over 12 months. The annual estimated cost-saving of the clinic from fall prevention is 956,288 (2024 CAD$). The Fall Prevention Clinic provides a multi-disciplinary approach that is best practice, evidence-based for fall prevention. This approach has demonstrated feasibility and effectiveness and saves health care dollars; thus, it is an effective and economically attractive approach to consider for implementation.

Abstract citation ID: igaf122.2624

### DEVELOPMENT OF A FALL PREDICTION RISK USING MULTIDIMENSIONAL DATA FROM THE CANADIAN LONGITUDINAL STUDY ON AGING

Paulo Roberto Carvalho do Nascimento, Marla Beauchamp, Jinhui Ma, Luciana Macedo, and Lauren Griffith, *McMaster University, Hamilton, Ontario, Canada*

Falls rank first in injury prevention priorities in Canada. Approximately 40% of falls among community-dwelling older adults could be prevented with proper strategies. This study aimed to develop a fall risk index using multidimensional data from the Canadian Longitudinal Study on Aging. We selected

36 potential fall risk factors from systematic reviews and fall guidelines. Predictor variables were extracted from baseline data of older adults (≥ 65 years, n = 12,646), while incident injurious falls were retrieved from follow-up 1 (FUP 1). At FUP1, 8.07% of the participants had an injurious fall. A stepwise multivariable logistic regression model identified 14 predictors associated with injurious falls. Significant ($p < 0.05$) predictors including age, previous falls, previous injurious falls, vision impairment, pain, home dissatisfaction, comorbidities, grip strength, and use of antidepressants were used to create the final model. The dataset was split into training (70%) and test (30%) datasets for model fitting and predict the probabilities, respectively. The model's performance was modest (AUC = 0.63), with a sensitivity of 75.50%, specificity (46.77%), positive predictive value (10.07%), and negative predictive value (96.03%). Although these results limit the model's reliability in confirming high-risk cases, it suggests that the model is more effective at ruling out individuals at low risk of injurious falls. Therefore, while the model may have limited utility for identifying those at high risk, it could be valuable as a screening tool to exclude low-risk individuals and focus preventive strategies on those more likely to experience falls.

Abstract citation ID: igaf122.2625

### EFFECT OF SMALL RETROFITTING CHANGES TO DAILY LIVING ENVIRONMENTS FOR SUPPORTING THE BODIES OF FRAIL OLDER ADULTS

Satoru Handa[1], Ayano Nomura[1], Shihomi Kuribayashi[1], Koji Kitamura[2], Hisashi Kawai[3], and Yoshifumi Nishida[1], *1. Institute of Science Tokyo School of Engineering, Tokyo, Tokyo, Japan, 2. National Institute of Advanced Industrial Science and Technology, Kashiwa, Chiba, Japan, 3. Tokyo Metropolitan Institute for Geriatrics and Gerontology, Itabashi City, Tokyo, Japan*

As the aging population continues to grow, it is essential to support frail older adults in leading safer and more independent daily lives. One promising approach is to change environmental design to improve physical stability. In the previous study, we examined the effects of attaching handrails and finger-grip protrusions to desks. Our findings suggested that these design modifications could contribute to an improved standing posture. In this study, we focused on the effect of additive small changes, such as modifying the desk's surface material and adding edge protrusions of 3 mm, 5 mm, and 7 mm, to present the possibility of practical solutions in real-life settings. We conducted experiments with 59 older adults (aged 65–98), including senior car users, measuring their standing motion using the modified desks. The results were analyzed through cluster analyses, comparing desks with conventional wooden surfaces to those with alternative materials. Our findings revealed that desks with rubberized resin or polyvinyl surfaces allowed for greater forward force application compared to standard wooden desks. Additionally, when edge protrusions of 3 mm, 5 mm, or 7 mm were introduced, the cluster analyses demonstrated improved physical stability compared to desks without protrusions. Notably, some older adults were able to exert a forward force equivalent to 0.1 times their body weight when using a desk with a 7 mm edge protrusion. These results suggest that even small design modifications that can be retrofitted can effectively enhance physical stability during movement for some older adults.

Abstract citation ID: igaf122.2626

### ENHANCING USER COMPLIANCE OF WEARABLE HIP PROTECTORS IN LONG-TERM CARE THROUGH EDUCATION AND TECHNOLOGY

Yijian Yang, and Xu Song, *The Chinese University of Hong Kong, Hong Kong, China (People's Republic)*

More than 95% of hip fractures in older adults are caused by falls. In long-term care, the rates of falls and hip fractures are twice as high as those in the community. Although hip protectors (HPs) have been promoted to reduce hip fractures, the user compliance (acceptance and adherence) is generally low. This study aimed to enhance compliance of wearable HPs in long-term care through structured education and innovative design of HPs. 101 care workers from six long-term care facilities attended a 60-minute educational session. 290 residents (age: 79±10) were recruited and 152 accepted to wear HPs. Adherence with wearing HPs was recorded on weekly-basis over 12 months. Generalized linear models were used to examine factors associated with adherence. Meanwhile, we developed a novel HP through 3D-printing. Specifically, selective laser sintering technology was applied to fabricate thermoplastic polyurethane materials, and a triply periodic minimal surface structure-infill scheme was used to generate HPs. Biomechanical performance of the 3D-printed HP was tested using a drop tower. Our results showed a satisfactory adherence rate [48.0% (73/152)] of wearing HPs through education. Participants with dementia or using mobility aids were more likely to wear HPs (Odds Ratio (95% CI): 1.42 (1.10–1.83) and 1.36 (1.05–1.78), respectively). Age, sex, hypertension, osteoporosis, and use of antipsychotic were not associated with adherence ($p > 0.05$). Moreover, our 3D-printed HP is light (48g) and breathable, with 42% force attenuation. Our findings suggest that user compliance can be enhanced through education and better design of HPs with an increased comfort.

Abstract citation ID: igaf122.2627

### FALL DETECTION AND PREVENTION TECHNOLOGIES FOR OLDER ADULTS: A SCOPING REVIEW

Yong Kyung Choi, Ryan Osal, Armari Long, Haomin Hu, and Steven Handler, *University of Pittsburgh, Pittsburgh, Pennsylvania, United States*

One in three older adults experience one or more falls each year. Such falls can have severe consequences, including injuries or death, significantly impacting older adults' quality of life and independence. The economic burden is also substantial, with fall-related healthcare costs exceeding $31 billion. In response, digital and smart home safety technologies offer promising avenues for enabling timely interventions and enhancing fall detection and prevention. While prior research has developed and examined various fall-related technologies, rapid technological advancements, especially with the emergence of Artificial Intelligence (AI), necessitate exploring new and improved solutions. This scoping review examines recently published studies on digital tools designed for fall prevention and detection among older adults. A scoping review was conducted following PRISMA-ScR guidelines, identifying studies from PubMed, CINAHL, Medline (EBSCO), and Web of Science published between 2019 and 2024. This timeframe was selected to capture the most recent technological advancements and their applications in real-world settings. Studies were included if they evaluated digital tools specifically designed for fall prevention, detection, or risk assessment among older adults. From this review, 18 studies met the inclusion criteria, categorizing technologies into wearable sensors, motion capture and gait analysis tools, gamified prevention programs, robotics, smart home monitoring systems, and mobile applications. These digital tools support real-time fall risk assessment, provide personalized interventions, and integrate with health monitoring systems. However, challenges persist, including sensor synchronization issues, reliance on controlled settings, privacy concerns, and limited validation in diverse populations. Despite promising advancements, current research remains limited in scope and generalizability.

Abstract citation ID: igaf122.2628

### FALL PREVENTION FOR OLDER ADULTS WHO USE WHEELCHAIRS AND SCOOTERS

Laura Rice[1], Elizabeth Peterson[2], Toni Van Denend[2], Jacob Sosnoff[3], and Deborah Backus[4], *1. University of Illinois College of Applied Health Sciences, Urbana, Illinois, United States, 2. University of Illinois Chicago, Chicago, Illinois, United States, 3. University of Kansas Medical Center, Kansas City, Kansas, United States, 4. Shepherd Center, Atlanta, Georgia, United States*

Falls are common among the ~1 million older adults who use wheelchairs and scooters (WC/S) full-time, especially people with Multiple Sclerosis (PwMS). Falls can result in injuries and concerns about falling (CaF) that limit active engagement in the community. To address these concerns, the individualized reduction of falls (iROLL) program was developed by an interdisciplinary team to meet the unique needs of people who use WC/S. iROLL applies a self-management approach to address topics including wheelchair skills, transfer activities, seated balance exercise, management of environmental factors, post-fall management, and maintenance of assistive technology. The program is delivered online. Participants asynchronously review pre-recorded lessons and synchronously meet with a physical or occupational therapist in groups of 2-5 participants. A pre/post, mixed methods study was used to examine the preliminary efficacy of iROLL. PwMS (n = 12) who use WC/S full time, age 62+/-12 years old, 92% female, participated in the study. After exposure to the six-week iROLL program, CaF significantly decreased ($p < 0.01$) and fall prevention and management knowledge significantly increased ($p = 0.03$). Participants reported a need for the program, valued peer learning, and attention to diverse influences on fall risk. This study is the first to examine the preliminary efficacy of an online fall prevention intervention for PwMS who use WC/S. While further evaluation is needed to examine iROLL's impact on a larger and more diverse population, iROLL has good potential to meet a critical need for an underserved population of older adults aging with a disability.

Abstract citation ID: igaf122.2629

### FALL TYPES AND HOME HAZARDS: INSIGHTS INTO RISK FACTORS AMONG KOREAN OLDER ADULTS

Gwang Suk Kim[1], Min Kyung Park[1], Jae Jun Lee[1], Layoung Kim[2], Namhee Kim[3], and Seungbum Yang[4], *1. Yonsei University College of Nursing, Seoul, Republic of Korea, 2. The University of Suwon, Hwaseong, Republic of Korea, 3. Yonsei University, Wonju, Republic of Korea, 4. Graduate School of Yonsei University, Seoul, Republic of Korea*

Most falls occur at home, and individuals with a history of falls are at a higher recurrence risk. This study classified older adults' fall types and examined the association of each type with fall risk in the home environment. The data of 211 Korean older adults who had experienced falls in the past two years were analyzed. Fall risk in the home environment was assessed using the Revised Korean version of the Home Falls and Accidents Screening Tool, and fall types were identified through latent class analysis (LCA) based on fall location and cause. The highest-risk areas in the home were the bedroom (0.43±0.34), bathroom (0.35±0.14), and stairs (0.27±0.24). The most frequently reported hazards were the absence of grab rails in the bathroom (90%), absence of non-slip mats in shower areas (77.3%), presence of grab rails on only one side of the outdoor stairs (64.9%), and absence of a light switch near the bed (64.0%). The LCA identified four fall clusters: home falls due to loss of balance, home falls due to tripping, outdoor falls, and bathroom falls due to slipping. Fall risk in the home environment varied significantly by fall cluster, being the highest in home falls due to loss of balance and the lowest in bathroom falls due to slipping. Environmental hazards contributing to balance loss and subsequent falls need to be mitigated. Given that bathroom-related hazards were the most frequently reported, slip risk in bathrooms remains high among Korean community-dwelling older adults, regardless of overall home safety.

Abstract citation ID: igaf122.2630

### FALLS AMONG OLDER ADULTS WITH AND WITHOUT ALZHEIMER'S DISEASE AND RELATED DEMENTIAS IN LOS ANGELES COUNTY: 2016-2022

D'Artagnan Robinson[1], Noel Barragan[1], Emiley Chang[1], Dalia Regos-Stewart[1], Mariana Reyes[1], and Tony Kuo[2], *1. Los Angeles County Department of Public Health, Los Angeles, California, United States, 2. County of Los Angeles, Los Angeles, California, United States*

Falls are a leading cause of injury, hospitalization, and mortality for older adults in the United States. Those living with Alzheimer's disease and related dementias (ADRD) are at heightened risk for falls due to cognitive impairment and functional limitations. As part of the Los Angeles County BOLD Initiative, the Los Angeles County (LAC) Department of Public Health conducts surveillance and research on ADRD and their associated risks. Using patient discharge data from the California Department of Health Care Access and Information, this study examines fall-related hospitalizations among LAC adults aged ≥50 years during the period 2016-2022. Falls were identified using external cause of morbidity codes (ICD-10-CM: W00-W19) and ADRD status was determined via principal and secondary diagnosis codes. All falls were analyzed by admission year, age, sex, race/ethnicity and fall type (e.g., falls from same level, falls from bed), with low-frequency categories collapsed into "Other Falls." Chi-square tests were employed to compare fall type by ADRD status. A total of n = 243,136 fall-related hospitalizations occurred during this timeframe, with 20.5% involving ADRD patients. A higher proportion of ADRD patients experienced falls from beds (8.0% vs. 4.3%, $p < 0.0001$) and non-motorized mobility devices (2.4% vs. 1.2%, $p < 0.0001$), whereas a lower proportion fell from stairs and steps (1.9% vs. 4.5%, $p < 0.0001$) compared to non-ADRD patients. Study findings highlight the burden of falls among this population and underscore the need to effectively prevent and manage falls, especially through strategies that address environmental and mobility risks.

Abstract citation ID: igaf122.2631

**FEAR OF FALLING: 30 YEARS OF RESEARCH**

Helen Lach, and Joseph Trinitas, *Saint Louis University, St. Louis, Missouri, United States*

Fear of falling has the potential to reduce activity and quality of life for community-dwelling older people, and an increased risk of falling. This topic has been explored for over 30 years. As a result, the aim of this umbrella review is to explore the state of the science on fear of falling to determine implications for future research. We searched databases including CINAHL, Medline (OVID), Scopus, and Cochrane for reviews (systematic, integrative, metanalyses, metasyntheses) since 1990. Studies with a focus on fear of falling in community-dwelling older adults as the primary topic or outcome in English were included, resulting in 879 articles. After removing duplicates, 606 articles remained for title and abstract screening. Studies of specific conditions such as stroke or specific settings (e.g. nursing home) were excluded. Data was extracted to examine designs, samples, measures, and outcomes of reviews of descriptive and experimental studies for the final 47 articles. Overall, 30 years of evidence confirms that that fear of falling is a significant public health problem for older people, common even among those who have not had a fall. Fear of falling leads to negative outcomes including reduced activity, depression and increased risk of nursing home placement, and is a risk factor for future falls. Further evidence supports exercise and multifactorial interventions to reduce fear of falling and improve quality of life for older people. Future research could help target interventions to improve precision of outcomes.

Abstract citation ID: igaf122.2632

**FEASIBILITY OF A CONVERSATIONAL AGENT TO BRING HOME THE STEADI FALL PREVENTION ALGORITHM**

Chantal Kerssens[1], Brenna Phelps[2], Brian Jones[2], Madeleine Hackney[3], Ted Johnson[3], Joseph (Joe) Nocera[4], Cathleen Carroll-Sauer[4], and Ritom Gupta[1], *1. Friendi.fi Corporation, Millbrae, California, United States, 2. Georgia Institute of Technology, Atlanta, Georgia, United States, 3. Emory School of Medicine, Atlanta, Georgia, United States, 4. Emory University, Atlanta, Georgia, United States*

Accidental falls threaten the health and well-being of older adults, especially those with mild cognitive impairment (MCI) due to cognition's important role in preserving functional gait and postural stability. The STEADI (Stopping Elderly Accidents, Deaths & Injuries) Initiative by the CDC reduces fall risk and rates but traditionally requires extensive, ongoing implementation resources for impact. This study tested the feasibility of using an already-scaled conversational technology platform with tablet-based agent (pet companion avatar) in 21 cognitively-normal (CN) older adults at low fall risk (CN–, n = 6), CN older adults at fall risk (CN+, n = 8), and older adults with MCI (MCI+, n = 7). Fall risk and cognitive status were confirmed with standardized measures. Participants were majority female (76%), 75 ± 10 years old (range 54-95), and racially diverse (White, 62%; Black/African American, 24%; Asian 9%; multi-racial, 5%). All evaluated avatar-led assessments of gait, balance, and leg strength plus an exercise safety check. Users appreciated the avatar's friendliness, natural conversational tone, nonthreatening design, patience, and engaging nature. Instructions were seen as clear, concise, and easy to follow, corroborating a 9.4 ± 0.8 clarity rating out of 10. System Usability (SU) was good overall (76 ± 13) with areas of improvement noted across groups. Design recommendations included faster response times, clarifying task transitions and starts, and customizing avatar settings such as voice and volume to the individual. The agent and platform are a promising tool to implement STEADI and prevent falls. Future studies will incorporate the Otago Exercise Program.

Abstract citation ID: igaf122.2633

**GRIP STRENGTH, COGNITION, AND FALL RISK IN MEXICAN AMERICAN OLDER ADULTS: SEX DIFFERENCES**

Winrose Windsor[1], and Soham AlSnih[2], *1. UTMB Health, The University of Texas Medical Branch, Galveston, Texas, United States, 2. The University of Texas Medical Branch at Galveston, Texas, Galveston, Texas, United States*

Falls are a leading cause of injury and disability among older adults. This study investigates sex differences in the relationship between grip strength (GS) and cognitive function (CF) with fall risk among Mexican Americans aged ≥75 without history of falls at baseline. Participants (n = 913) were from the Hispanic Established Population for the Epidemiological Study of the Elderly (2004/05-2016). Measures included socio-demographics, body mass index, multi-morbidity, depressive symptoms, and pain. Cognitive function (CF) was assessed using the Mini Mental State Examination (MMSE), with scores < 21 classified as low CF and ≥21 as high CF. Low GS was defined as < 27 kg in males and < 16 kg in females. Participants were categorized into four groups by sex according to GS and CF (High GS-High CF, Low GS-High CF, High GS-Low CF, Low GS-Low CF). Generalized estimating equation models were utilized to estimate the odds ratio (OR) and 95% Confidence Interval (CI) of falls as a function of GS and CF groups. Male participants in the group of high GS-high CF had lower odds (OR = 0.52, 95% CI = 0.30-0.92) of reporting falls over time than those with low GS-Low CF group after controlling for all covariates. No significant association was found between low GS-high CF and high GS-low CF groups with falls. No significant association was found between GS and CF groups among female participants over time after controlling for all covariates. Mexican American older adults who performed high in GS and CF were at decreased odds of reporting falls over time.

Abstract citation ID: igaf122.2634

**IMPACT OF SOCIOECONOMIC STATUS ON POST-FALL RECOVERY IN OLDER ADULTS: A 12-MONTH FOLLOW-UP**

Elisa-Marie Speckmann, Tania Zieschang, and Tim Stuckenschneider, *The Carl von Ossietzky University of Oldenburg's School of Medicine and Health Sciences, Oldenburg, Niedersachsen, Germany*

Falls in older adults can be a significantly disruptive event with long-term consequences. Given that socioeconomic status may influence health outcomes in this population, assessing its potential impact on post-fall recovery is essential. Therefore, we conducted a longitudinal study with data collection at three time-points: four weeks, six months, and twelve months after a fall to assess the association of income and education with the progression of health outcomes. Data was obtained from the SeFallED study, which follows up on older adults, who were 60 years or older, attended the emergency department due to a fall, and were not admitted to the hospital. We calculated generalized least square models with first-order autoregressions to analyze the associations of education and income with physical performance, cognitive function, activities of daily living, life-space, and health-related quality of life. Age and sex were added as covariates. Over 12 months, we followed up with 267 individuals (mean age of 74.4±9 years, education: 10.2±1.9 years in school; income €2277.6±1806.3). Education was linked with physical performance ($B = 0.25$, $p < 0.05$), cognitive function ($B = 0.70$, $p < 0.05$), activities of daily living ($B=-0.42$, $p < 0.05$), and life-space ($B = 2.59$, $p < 0.05$). No further significant effects were found for either education or income. These findings show the need to account for disparities in education when designing secondary prevention programs for older adults. While education and income reflect socioeconomic differences, factors such as the subjective social status may offer additional insights and should be explored in future studies.

Abstract citation ID: igaf122.2635

### INNOVATIVE HORIZONS IN FALL RISK SELF-MANAGEMENT FOR OLDER ADULTS: AN INTEGRATIVE REVIEW AND MODEL

Angela Shanahan[1], Patricia Groves[2], and Harleah Buck[1], *1. University of Iowa, Iowa City, Iowa, United States, 2. University of Iowa College of Nursing, Iowa City, Iowa, United States*

Falls affect 1 in 4 community-dwelling older adults. Despite focusing on fall prevention factors such as knowledge about falls, mobility, and fear of falling, researchers and clinicians continue to report high fall rates and low adherence to prevention programs. The Health Belief Model (HBM) suggests unexplored factors (i.e. perceptions and beliefs), which may influence older adults' engagement in fall risk self-management. The purpose of this review was to conduct a comprehensive review of the literature examining the relationships among HBM factors: perceptions/beliefs, cues-to-action, self-efficacy, and older adults' fall risk self-management and create a conceptual model. Pubmed, PsychINFO and CINAHL were searched using the terms: perception, belief, motivation, fall risk, and community-dwelling. Inclusion criteria: English language and included perceptions or beliefs related to fall risk. Thirty studies comprised of quantitative, qualitative, and systematic reviews met the criteria. The majority of the articles ($n = 23$, 60.52%) were published within the last 10 years and conducted within the United States ($n = 18$, 60%). The average age of participants was 75.4 years. Few studies ($n = 3$) examine individual perceptions/beliefs directly as they relate to self-management. Concepts were abstracted to inform the conceptual model demonstrating how the HBM concepts can innovatively inform future studies. Understanding how an individual's beliefs: a) perception of fall risk, b) perception of injury, c) belief in ability to self-manage risk, and d) perception of benefit vs. barriers influence d) cues-to-action, and e) self-efficacy is crucial for advancing the science and developing effective strategies to support older adults in engaging in fall risk self-management.

Abstract citation ID: igaf122.2636

### MAINEHEALTH'S COMPREHENSIVE & INNOVATIVE FALL PREVENTION PRIMARY CARE INITIATIVES FOR OLDER ADULTS

Jessica Bolduc, Maureen Higgins, and Tamara Herrick, *MaineHealth, Portland, Maine, United States*

Falls are a major concern for adults over 65, with one in four experiencing a fall each year—approximately 14 million falls and 39,000 fall-related deaths nationwide. In Maine alone, over 100,000 falls were recorded in 2023 and 320 fall-related deaths in 2024. The U.S. incurs over $80 billion annually in costs associated with fall-related care for older adults. Maine ranks among the highest in the United States for fall rates and related fatalities and spends over $255 million annually. Recognizing the urgency of this issue, MaineHealth, the largest integrated health system in Northern New England, is implementing innovative and comprehensive fall prevention strategies. Using a multifaceted approach that integrates education, technology, and community engagement, these initiatives equip both care teams and patients with essential tools and resources. Embedding the Age-Friendly 4M framework alongside the CDC's STEADI initiative, MaineHealth enhances fall risk screening, assessment, and intervention through EMR integration to facilitate streamlined care delivery. MaineHealth is building infrastructure to provide A Matter of Balance: Managing Concerns about Falls-Virtual, an evidence-based fall prevention program proven to interrupt the cycle of fear of falling and increase activity levels, in order to increase access to older adults that screen at risk for falls throughout the healthcare system. Early outcomes from these efforts in ambulatory care demonstrate feasibility and ongoing opportunities for expansion. Positioned at the intersection of public health and clinical care, MaineHealth is committed to collaborative solutions to achieve our goal of working together so our communities are the healthiest in America.

Abstract citation ID: igaf122.2637

### PERCEIVED WORKLOAD VS. OBJECTIVE PERFORMANCE DURING DUAL-TASKING IN ADULTS WITH MILD COGNITIVE IMPAIRMENT

Jessica Pitts, Tanvi Bhatt, Shuaijie Wang, and Lakshmi Kannan, *University of Illinois Chicago, Chicago, Illinois, United States*

Older adults with mild cognitive impairment (OAwMCI) demonstrate reactive balance deficits (impaired responses to external perturbations) compared to cognitively intact older adults (CIOA), which are exacerbated while performing a cognitive task (i.e., dual-tasking). However, it is unknown if these cognitive-motor deficits (which could significantly increase fall risk) can even be perceived by OAwMCI. Thus, this study examined if objective performance during dual-task reactive balance control (cognitive errors, reactive center of mass (COM) stability) was associated with subjective workload in OAwMCI and CIOA. 34 OAwMCI (Montreal Cognitive Assessment (MoCA): 18-25) and 35 CIOA (MoCA ≥26) were exposed to a support surface perturbation while performing a visuomotor "Target" task, which involved rotating their head to catch virtual objects. This task was also completed while seated (single-task). Participants rated their perceived workload (0-100) on the NASA-Task Load Index (NASA-TLX) in six domains (mental, physical, temporal, performance, effort, frustration). OAwMCI reported lower mental demand, effort, and frustration than CIOA in single and dual-task ($p < 0.05$), although had higher cognitive errors and lower reactive COM stability ($p < 0.05$). Both groups reported higher workload in dual vs. single-task ($p < 0.05$). NASA-TLX scores were not correlated with objective performance in OAwMCI ($p > 0.05$), although were significantly correlated with cognitive errors and reactive COM stability in CIOA (Pearson's r: 0.3-0.5). These results suggest that OAwMCI may not be able to accurately perceive the cognitive-motor demands of challenging conditions. This impaired perception could affect the execution or scaling of reactive balance responses and contribute to increased fall risk, especially in dual task conditions.

Abstract citation ID: igaf122.2638

**PREVENTING FALLS IN THE COMMUNITY USING AN INTEGRATED HEALTHCARE AND COMMUNITY PROVIDER INTERPROFESSIONAL TEAM**

Jennifer Drost[1], Susan Fosnight[1], Brandi Chrzanowski[2], Joseph Machiano[1], Michele Gareri[1], Kristin Baughman[3], Michelle Hughes[2], and Margaret Sanders[3], *1. Summa Health System, Akron, Ohio, United States, 2. Direction Home Akron Canton Area Agency on Aging & Disabilities, Green, Ohio, United States, 3. Northeast Ohio Medical University, Rootstown, Ohio, United States*

One in four older adults fall each year resulting in significant avoidable morbidity and mortality. To address the complex interactions between fall risk factors, effective fall prevention efforts require collaboration between medical and community-based providers. Our health system and local Area Agency on Aging developed an interprofessional model called Care Management Interdisciplinary Team (CMIT). CMIT care managers (Social Workers and nurses) complete pre-team evaluations including a falls risk assessment. Cases are presented by CMs to CMIT which includes a geriatric physician and pharmacy specialists. Recommendations are implemented by the CM and consumer's primary care physician. These include home modifications, therapies, medication changes, and other prevention interventions. We evaluated pre-post team fall outcomes reported by CMs January-December 2023 (n = 64) using Chi Square. CMs reported that: 33% who had falling issues at baseline had no falls at 3 months ($p < 0.0001$), 52% who had medication concerns at baseline had those concerns resolved at 3 months ($p = 0.03$), 45% who had safety issues at baseline had those issues resolved at 3 months ($p < 0.0001$), 36% who had ED/hospital utilization issues at baseline had no utilization at 3 months ($p < 0.001$). Interprofessional, interagency collaboration can decrease fall risk by addressing both medical/pharmacy and social determinants of health. Given that healthcare costs associated with falls is currently $50 billion nationally, this integrated interprofessional and interagency model of care is highly cost-effective.

Abstract citation ID: igaf122.2639

**R/AGING PARENTS: SEEKING ADVICE ON FALL PREVENTION TECHNOLOGIES ON REDDIT**

Jaahnvi Patel, and Shannon Mejia, *University of Illinois Urbana-Champaign, Champaign, Illinois, United States*

The first generation of Reddit users are now middle-aged and their parents are aging. Accidental falls are a common and potentially life-changing event that can signal to children that their parents are aging. An open question is the role that the Reddit community plays in supporting adult children as they navigate fall prevention for their aging parents. This research conducts content analysis of the AgingParents subreddit to examine how caregivers seek and receive advice on fall prevention technology. The sample included 113 threads within the AgingParents subreddit that were posted since February 2023. A codebook was developed to categorize user posts based on the relationship of the original poster (OP) to the older adult (OA), their living situation, level of care needs, and use of fall prevention technology. We coded for key concerns, technology adoption, and types of support sought and received. Responses were analyzed to determine the types of support given, including emotional, informational, tangible, belonging, and technology-related support, as well as misinformation or conflicting advice. The findings suggest that adult children frequently seek advice on fall prevention technologies, discussing their usability and their parents' resistance. Caregivers also expressed emotional burden and family decision-making challenges, which could contribute to motivations for seeking support on Reddit. The study highlights Reddit's role as a support network and provides key insights into how Reddit functions as a support network for caregivers and highlights areas for improving fall prevention strategy discussion.

Abstract citation ID: igaf122.2640

**SCREENING DEPRESSION, FALLS, AND FEAR OF FALLING IN OLDER ADULTS: CREATE COMMUNITY PARTNERSHIPS**

Veronica Decker[1], Jethro Raphael Suarez[2], Yi Liu[1], Janet Lopez[1], Michael Dino[3], Dahee Kim[1], Chitra Banarjee[1], and Ladda Thiamwong[1], *1. University of Central Florida, Orlando, Florida, United States, 2. University of Central Florida, Florida, United States, 3. University of Central Florida, University of Central Florida, Florida, United States*

The biopsychosocial impacts of falls have been considered a public health crisis. Fear of falling (FoF) is rarely screened in practice despite its impact on healthy aging. This may cause social isolation and depression, increasing FoF and fall incidents and leading to restrictions on engaging in physical and social activities among older adults. There has been a need to investigate a multitude of factors contributing to falls, the unique stressors influencing FoF, and the progression and interplay of depression in low-income older adults. This study aimed to (1) screen for depression, falls, and FoF among older adults recruited through community center partnerships and (2) examine the associations between such variables. We screened 301 older adults ($\bar{x}$ = 74, SD = 7.29 years) for depression with the Patient Health Questionnaire-9, FoF with the Short Falls Efficacy Scale-International, and self-reported fall incident. Binary logistic regression was used to examine whether depression is significantly associated with FoF and the number of falls over the past year. Participants who experienced 2 or more falls were twice as likely to indicate depression relative to those with 0 falls (OR = 2.06). Participants with moderate FoF were over 3 times more likely to report depression (OR = 3.21) and those with a high FoF were over 7 times (OR = 7.60) more likely. Results show that a biopsychosocial approach is needed to address the complex relationship between depression, falls, and FoF. Future research should encourage partnerships with local communities to provide screenings and education to underrepresented older adults, focusing on reducing falls and FoF.

Abstract citation ID: igaf122.2641

**STRATEGIES TO MINIMIZE FALL-RELATED INJURIES IN OLDER ADULTS AT RISK OF FALLS: THE FALLING SAFELY TRAINING STUDY**

Jacob Sosnoff[1], Lingjun Chen[1], Shelley Bhattacharya[1], James Fang[1], Jianghua He[1], Abbas Tabatabaei[1], Neil Alexander[2], and Tobia Zanotto[1], *1. University of Kansas Medical Center,*

*Kansas City, Kansas, United States, 2. University of Michigan, Ann Arbor, Michigan, United States*

The Falling Safely Training (FAST) program is designed to reduce fall-related injury in older adults at risk of injury. This study evaluated its feasibility and preliminary efficacy in mitigating fall-related injury. Twenty-four older adults (72.53 ± 5.39 years) were randomized to four weeks of FAST training, which focused on progressive safe-falling strategies, or to an active control group that completed balance training via the modified Otago program. Participants underwent experimentally induced falls in a laboratory setting at baseline, post-intervention, and three-month follow-up. Head and hip accelerations were captured via motion tracking, and head impact occurrences were assessed through video screening. Eleven of 12 FAST participants completed training without adverse events. Compared to the control group, the FAST group had a greater reduction in head impact risk (odds ratio = 0.10, 95% CI: 0.02, 0.61, $p = 0.012$) and lower head acceleration post-intervention ($\Delta$ = -9.54 m/s$^2$, $p = 0.028$). Hip acceleration decreased in both groups ($p < 0.001$). Group differences in demographic variables were assessed using Mann–Whitney U and Chi-square tests, while linear mixed models examined acceleration changes and mixed logistic regression evaluated head impact occurrence. These findings suggest that FAST is a feasible and effective intervention for reducing fall-related head impact risk in older adults. By targeting safe-falling strategies, this approach addresses a critical gap in fall prevention programs and offers a promising avenue for injury mitigation. Future research should explore its long-term cost-effectiveness and community implementation.

Abstract citation ID: igaf122.2642

### STUDY PROTOCOL FOR EXPRESS FALL PREVENTION TRIAL AMONG OLDER ADULTS IN RURAL CHINA

Shaojie Li, Yao Yao, Wenjian Zhou, Yuling Jiang, Mingzhi Yu, Yifei Wu, Jing Wu, and Zhenhan Yu, *Peking University, Beijing, Beijing, China (People's Republic)*

Falls constitute a leading cause of injury-related mortality and morbidity among older adults, especially in rural China where healthcare resources are limited. To address this, we designed the EXPRESS trial (EXpress Prevent Rural oldEr adults' fallS, fractureS and dependency), a multicenter, community-based, cluster-randomized controlled trial integrating artificial intelligence (AI), village doctors, and Express Mail Service (EMS) providers. The study involves 16 villages randomized into intervention and control groups across four geographically diverse provinces in China. The intervention employs the innovative "XVV" strategy: a comprehensive ten-item fall risk screening by village doctors, AI-assisted smartphone-based gait analysis covering five biomechanical dimensions (velocity, stride variability, symmetry, stability, mobility), and five personalized, culturally adapted interventions delivered via village doctors and EMS couriers at key cultural moments (Chongyang, Spring Festival, Qingming Festival). The primary outcomes are the incidence of falls and related injuries over 12 months. Secondary outcomes include changes in quality of life (EQ-5D-5L), activities of daily living (ADL), and fall prevention literacy. Baseline data collection was completed in August 2024, with final evaluations scheduled for August 2025. A health economics evaluation will assess cost-effectiveness. By integrating AI, task-sharing, and community-based services, EXPRESS aims to demonstrate a scalable and sustainable model for fall prevention. Results will inform public health strategies globally, especially in low-resource, rural contexts, bridging the significant gap between fall prevention evidence and practice implementation. We hope to present a detailed plan for this study at the GSA conference.

Abstract citation ID: igaf122.2643

### SYNERGIC TRIAL SECONDARY OUTCOMES RESULTS ON GAIT AND FALLS

Frederico Faria[1], Surim Son[2], Teresa Liu-Ambrose[3], Quincy Almeida[4], Laura Middleton[5], Louis Bherer[6], and Manuel Montero Odasso[7], *1. Western University Lawson Health Research Institute, London, Ontario, Canada, 2. Western University, London, Ontario, Canada, 3. The University of British Columbia, Vancouver, British Columbia, Canada, 4. Wilfrid Laurier University, Waterloo, Ontario, Canada, 5. University of Waterloo, Waterloo, Ontario, Canada, 6. Université de Montréal, Montreal, Quebec, Canada, 7. University of Western Ontario, London, Ontario, Canada*

Background: Older adults with Mild Cognitive Impairment (MCI) have higher risk of gait impairments and falls, yet the effects of multimodal interventions, including combinations of exercises with cognitive training, on improving their mobility remain unclear. Objectives: To investigate the synergistic effects of aerobic-resistance exercise combined with cognitive training, with or without vitamin D supplementation, on gait performance and fall risk in older adults with Mild Cognitive Impairment (MCI). Methods: The effect of 20 weeks of aerobic-resistance exercise, cognitive training, and vitamin D supplementation (10,000 IU 3x/week) on gait and falls in older adults with MCI was evaluated in the SYNERGIC Trial, using a fractional factorial design. Results: Among 161 participants, exercise-based arms ($n = 4$) improved gait speed (+7.5 cm/s, $p < 0.001$) and reduced injurious falls by 62% (IRR=0.38, $p = 0.04$). Exercise plus cognitive training showed the greatest gains in gait speed and reduced injurious falls (IRR=0.13, $p = 0.05$). Vitamin D did not enhance outcomes and increased gait instability. Conclusion: Aerobic-resistance exercise combined with cognitive training improves gait performance and decreases the risk of injurious falls in older adults with MCI. The addition of vitamin D did not produce benefits.

Abstract citation ID: igaf122.2644

### THE VALIDITY OF VIDEO FALL DETECTION FOR ASSISTED LIVING RESIDENTS WITH DEMENTIA

London Jones[1], Scott Davis[2], Philip Sloane[2], Elizabeth Peiffer[3], Shirley Nickels[3], and Sheryl Zimmerman[2], *1. The University of North Carolina at Chapel Hill, Chapel Hill, North Carolina, United States, 2. University of North Carolina at Chapel Hill, Chapel Hill, North Carolina, United States, 3. SafelyYou, San Francisco, California, United States*

Roughly 25% of older adults fall each year, with rates almost twice as high among persons with dementia. Reducing falls in assisted living (AL) is especially important, given that AL is the residential setting housing most people with dementia. Unfortunately, many falls are unwitnessed and under reported, making it difficult to respond promptly and effectively. Video monitoring has emerged as a promising method of detecting falls, but the extent to which it can be used for research has not been evaluated. This study examined the validity of identifying a fall using recorded video events compared to an observational gold standard. A team of four researchers were trained to categorize falls using prescribed criteria, and achieved 100% accuracy.

Then, they independently reviewed 100 video clips (50 'fall' and 50 'non-fall') from a video database of "residents on the ground" and compared the ratings to the gold standard. Residents from six different AL communities and no more than four videos of the same resident were included. Overall sensitivity was 82%; raters correctly rated 41 of 50 videos that contained falls. Specificity was 93.2%; 41 of 44 videos in which the raters identified a fall met the gold standard definition of a fall. When the initiation of descent was considered, perfect agreement was achieved for descent from standing, squatting or kneeling. Challenges arose when individuals slowly descended from a bed to the floor. Based on these results, video capture of falls is a valid data source to examine, and potentially prevent, falls.

### SESSION 7230 (POSTER)

### FAMILY CAREGIVING II

Abstract citation ID: igaf122.2645

### BUFFERING CAREGIVING STRESS THROUGH ACTIVITIES: A DAILY DIARY STUDY OF DEMENTIA CAREGIVERS

Suyoung Nah[1], Jyoti Savla[2], and Karen Roberto[3], *1. Kyungpook National University, Blacksburg, Virginia, United States, 2. Dept. of Human Development and Family Science, Blacksburg, Virginia, United States, 3. Virginia Tech, Blacksburg, Virginia, United States*

Dementia caregivers often struggle to maintain daily activities such as exercising or socializing due to caregiving demands. According to the Activation Restriction model, limitations on typical or pleasurable activities can negatively impact emotional well-being. However, it remains unclear which types of daily activities are linked to caregivers' emotional health. We examined whether participation in religious, physical, and social activities is associated with better daily mood, and if these activities buffer the negative effects of caregiving stress on mood. We analyzed 7-day diary data from 158 dementia family caregivers (Mage = 65 ± 11.8 years; 89% White). Each day, caregivers reported their engagement in religious activities (e.g., attending church), physical activities (e.g., exercise), and social activities (e.g., talking with friends/family), as well as perceived stress from the care recipient's symptoms. Daily mood was measured in terms of positive and negative emotions. Using multilevel models that simultaneously accounted for the three activity types, we found that only physical activity was associated with higher positive mood ($\beta = 0.11$, $SE = 0.03$, $p = .001$) and lower negative mood ($\beta = -0.03$, $SE = 0.01$, $p = .008$). Moreover, on high-stress days, physical activity mitigated the negative impact of caregiving stress on mood. That is, on days when stress levels were elevated, caregivers who engaged in physical activity experienced lower negative mood than those who did not. Findings highlight the importance of physical activity for caregivers' emotional well-being. Interventions should incorporate strategies to include physical activity into caregivers' daily routines.

Abstract citation ID: igaf122.2646

### CAREGIVING PROFILES OF FAMILY MEMBERS ASSISTING OLDER MEXICAN AMERICAN ADULTS WITH VARYING CARE NEEDS

Karen Schlag[1], Soham Al Snih[2], and Monique Pappadis[1], *1. University of Texas Medical Branch at Galveston, Galveston, Texas, United States, 2. The University of Texas Medical Branch at Galveston, Texas, Galveston, Texas, United States*

This study analyzed population-based data to identify caregiving profiles observed among Mexican American adults who assist older relatives with varying levels of physical and cognitive limitations. Using data from the from the 2016 Hispanic Established Populations for the Epidemiological Study of the Elderly (H-EPESE) Wave 9 Caregiver supplement ($n = 460$), we conducted latent class analysis in Stata to estimate latent profiles of family caregivers. Indicators for analysis included 9 variables relaying degree of caregiving responsibility types (i.e., personal care, household tasks, medical care, financial support), patient conditions (i.e., needs assistance with activities of daily living, cognitive impairment), family dynamics (i.e., lives with care recipient, degree of conflict with them) and use of formal support services. Results supported a typology of three discernable family caregiving profiles: 1.) intense caregiving (43% of sample, e.g., high caregiving responsibility and care recipient need, likely to live with care recipient, moderate formal support); 2.) low demand caregiving (37% of the sample, minimal caregiving responsibility, moderate care recipient need, moderately likely to live with care recipient, low formal support); 3.) moderate caregiving (20% of sample, e.g., moderate caregiving responsibility, high care recipient need, less likely to live with care recipient, moderate formal support. Findings will inform a future study to investigate the associations between distinct caregiving profiles and caregiver health outcomes among family members of older Mexican American adults.

Abstract citation ID: igaf122.2647

### COMPASSION FATIGUE, SATISFACTION AND AUTOMATED MEDICATION DISPENSER: A PILOT MIXED-METHODS STUDY

Karyman Ghanem[1], Bincy Baby[1], Colleen McMillan[1], Kristen Antunes[2], Kelly Grindrod[1], Annette McKinnon[3], Linda Lee[4], and Tejal Patel[1], *1. University of Waterloo, Waterloo, Ontario, Canada, 2. Custom Health, Kelowna, British Columbia, Canada, 3. Patient Advisors Network, Toronto, Ontario, Canada, 4. McMaster University, Hamilton, Ontario, Canada*

Informal caregivers frequently support older adults with complex medication management tasks. However, most informal caregivers are inadequately trained to manage these challenging tasks and may experience caregiver burden. The study aims to evaluate the impact of an automated medication dispenser (AMD) on compassion fatigue, satisfaction, and medication administration hassles. This study recruited 7 pairs of family caregivers and their older care recipients. Caregivers completed the Family Caregiver Medication Administration Hassles Scale (FCMAHS) and the Professional Quality of Life Scale (ProQoL) at baseline, 2 weeks, and 3 months after implementing AMD in care recipients' homes. Friedman tests showed no significant change in FCMAHS subscale scores over time after Bonferroni correction ($\alpha = 0.0125$; all $p > 0.0125$). The total score (primary outcome) was assessed without correction ($\alpha = 0.05$) and was not significant. Wilcoxon Signed-Rank Tests showed a similar pattern, except for a significant reduction in total score from baseline to 3 months ($p = 0.02$). Both tests showed no significant change in scores for the subscales of ProQoL after Bonferroni correction ($\alpha = 0.0167$); all $p > 0.0167$). Caregivers were interviewed before and after using AMD. The post-intervention interviews were recorded, transcribed, and thematically analyzed. Four themes emerged from the analysis: usability and functionality, experience with remotely delivered pharmacy services, caregiver experience and wellbeing, and impact on caregivers/recipient relationships. The long-term use of AMD has the potential to be beneficial for caregiving burden related to medication management but is influenced by the caregiver's adjustment period. Future research should verify these pilot findings.

Abstract citation ID: igaf122.2648

### CONTRASTING SOCIAL/FAMILIAL NETWORKS WITH CAREGIVING NETWORKS OF OLDER ADULTS WITH COGNITIVE IMPAIRMENT

Amanda Leggett[1], Wenjing Li[1], Richard Gonzalez[2], Wenhua Lai[1], and Natasha Nemmers[1], *1. Wayne State University, Detroit, Michigan, United States, 2. University of Michigan, Ann Arbor, Michigan, United States*

A growing body of research is examining family caregivers' social networks, yet how such networks translate into care provision is underexplored. This study extends the Task Specific Model of Caregiver Selection to 1) compare social/familial network membership with caregiver network membership and 2) explore whether demographics or care needs better predict the proportion of the network assisting with care. This study includes 742 community-dwelling individuals with cognitive impairment receiving care from the 2022 wave of the National Health and Aging Trends Study. Unpaired Wilcox tests compare social/familial network members with caregivers (e.g., number of children vs. number of children caregivers). Next, Poisson regressions predict the proportion of caregivers among one's household, children, and social network members, with demographics (age, gender, race) and care needs (dementia status, functional disability, chronic medical conditions) as covariates. Significantly more household members, children, and social network members are in one's network than are providing care. In regression models, being Black or Hispanic relative to White was associated with a smaller proportion of helpers in one's household. Being older, female, Black (relative to White), and needing more transportation assistance was associated with a greater proportion of one's children serving as helpers. Finally, needing more transportation and medical assistance was associated with a greater proportion of one's social network assisting with care. Demographic characteristics of the individual with cognitive impairment related to greater proportions of family assistance, while greater care needs related to a greater proportion of one's social network assisting with care.

Abstract citation ID: igaf122.2649

### EFFICACY OF A PSYCHOEDUCATIONAL PROGRAM (CUIDANDO JUNTOS) TO SUPPORT PSYCHOLOGICAL HEALTH OF LATINO CAREGIVERS

Julian Montoro-Rodriguez[1], Charlie Reeve[1], Dolores Gallagher-Thompson[2], Roberto Velasquez[3], Ann Bilbrey[4], Jennifer Ramsey[5], Bruno Kajiyama[6], and Maria Quiñones-Cordero[7], *1. The University of North Carolina at Charlotte, Charlotte, North Carolina, United States, 2. Stanford University, Stanford, California, United States, 3. Southern Caregiver Resource Center, Chula Vista, California, United States, 4. Optimal Aging Center, Sunnyvale, California, United States, 5. York Technical College, Rock Hill, South Carolina, United States, 6. Photozig, Inc., Moffett Field, California, United States, 7. University of Rochester School of Nursing, Rochester, New York, United States*

Latinos have the highest prevalence of caring for a family member with Alzheimer's disease and have significantly worse physical and psychological outcomes than other groups. Yet, there are few culturally appropriate evidence-based psychoeducational skill-building programs for Latinos. Our team culturally adapted the online Caregiver TLC skill-building program to support Latino caregivers (Montoro-Rodriguez et al., 2024; 2025). Our goal is to examine the acceptability and efficacy of this adapted program to improve social connectedness and overall health of Latino caregivers: Cuidando Juntos. Using pre-post data from the pilot clinical trial (N = 31), we examined user-reactions to the program to assess some aspects of acceptability, as well as changes on measures of general health, care-giving self-efficacy, depression, perceived stress, and burden to evaluate program efficacy. Data was collected from the community organizations in San Diego, CA and Charlotte, NC. Findings from the analyses indicated significant improvement in scores for all five outcomes from pre- to post-program assessments. Scores on general health and self-efficacy increased, whereas scores on depression, perceived stress, and burden decreased. Effect sizes ranged from small to very large. These results indicate that the pilot program is viewed as an acceptable and useful program by the sample of Latino caregivers attending the program and it has potential for a positive impact on caregivers' self-reported general and mental health. This pilot findings suggest that Cuidando Juntos may yield mental health benefits to Latino caregivers of older adults with significant memory problems.

Abstract citation ID: igaf122.2650

### EMPOWER: A FEASIBILITY STUDY OF A REMOTE AND IN-PERSON INTEGRATIVE INTERVENTION FOR CAREGIVER SUPPORT

Katie Trainum[1], Rosa Schnyer[2], and Bo Xie[2], *1. University of Pennsylvania, Philadelphia, Pennsylvania, United States, 2. The University of Texas at Austin, Austin, Texas, United States*

Family caregivers face substantial caregiving-related burdens, needing supportive interventions, particularly those incorporating self-care strategies that can be delivered remotely with broad accessibility. We developed EMPOWER (Engage your Mind and Body to Promote your Own Wellness, Energy and Relaxation), an intervention incorporating complementary and integrative strategies like breathing exercises, Tai Chi, and self-acupressure. In Fall 2024, we conducted four sessions (3 via Zoom; 1 in-person) to evaluate EMPOWER's feasibility. Following recruitment challenges, including scammers in the first two Zoom sessions, the final sample included 8 caregivers (7 female; age range 55-90; mean = 68.38; 6 remote, 2 in-person). Each 90-120-minute session involved instruction and practice led by an experienced clinician. Participants received program handouts and audio recordings for at-home practice. Two weeks later, 75% (n = 6) completed a post-intervention questionnaire online or over the phone, including 2 from the in-person session and 4 from the third Zoom session. Data were analyzed using content analysis and paired t-tests. T-tests revealed no significant changes in caregiver burden, stress, anxiety, or quality of life from pre- to post-intervention across all groups. Content analysis of qualitative data suggested improved relaxation, stress management, empowerment, and regular practice. Participants also reported challenges such as technological barriers, physical limitations, and time constraints in both session formats. These findings support the feasibility of delivering EMPOWER remotely and in-person for caregivers. Future work should refine the intervention to overcome the identified challenges, incorporate a mobile application for shorter, more accessible sessions, and test with a larger sample.

Abstract citation ID: igaf122.2651

### EXPECTATIONS ABOUT CARE FOR AGING PARENTS AND ONE'S OWN FUTURE CARE AMONG KOREAN MIDDLE-AGED ADULTS

Haeun Yoo, Eunju Lee, and Kyungmin Kim, *Seoul National University, Seoul, Republic of Korea*

Korean adults have traditionally anticipated providing care to their aging parents, while also hoping their own children will reciprocate similar care when needed. However, as family care norms have weakened, people's expectations about care are in flux—some anticipate family care, some expect formal care (e.g., nursing homes or professional care workers), while others have no clear expectation about care. Further, people may have different expectations between care for aging parents and their own future care. This study examined middle-aged adults' expectations about (a) care for parents and (b) their own future care (i.e., family care, formal care, or uncertain) and how combinations of these care expectations were related to aging anxiety. Using data from 2014 Korean Baby Boomer Panel Study, we analyzed a sample of middle-aged adults (N = 1,748; aged 51–59), who have at least one living parent and one living child. We found that 36% expected family care for parents but formal care for themselves, followed by expecting formal care for both (32%). Expecting formal care for parents but family care for oneself was least common (1%), while expecting family care for both was also rare (6%). Additionally, 24% indicated uncertain expectations about either or both. Regression analyses revealed that individuals who expected formal care for both or who had no clear expectations experienced greater aging anxiety than those expecting family care for both. These findings highlight the importance of planning for aging parents' and own future care needs in the context of changing care norms.

Abstract citation ID: igaf122.2652

### EXPLORING CHARACTERISTICS OF CAREGIVERS WHO ARE FINANCIALLY DEPENDENT ON THEIR CARE RECEIVERS

Eleanor Batista-Malat[1], Kathleen Wilber[1], Laura Mosqueda[2], and Bonnie Olsen[1], *1. University of Southern California, Los Angeles, California, United States, 2. University of Southern California, Pasadena, California, United States*

Financial and emotional dependence of a caregiver or other trusted person on an older adult is a risk factor for elder abuse. However, there is little investigation of the personal characteristics associated with caregivers who are financially dependent on care receivers. In this secondary analysis of a convenience sample of caregivers of people living with dementia (N = 76), we examined characteristics that may be associated with financial dependence, drawing on findings on dependent caregivers and high-risk abusers. A majority of the caregivers were women (67%), Latino (50%), and lived with the care receiver (58%); a plurality was caring for a parent (47%). Bivariate test show that financially dependent caregivers (24%) had higher rates of conflict with the care receiver before dementia (28% vs 9%, p=.036), use of alcohol to cope (33% vs 5%, p=.001), and payment for caregiving (33% vs 9%, p=.009). There were no differences in the amount of caregiving, depression and anxiety symptoms, positive/neutral coping methods or smoking to cope, burden, or demographic characteristics. Financial dependence was not associated with physical or psychological mistreatment but was associated with neglect (39% vs 14%, p=.019). Our findings suggest that financially dependent caregivers provide support to people living with dementia while facing their own challenges and engaging in neglect. Policies designed to support these caregivers in building skills without removing them from their role could bolster older adults' networks of family caregivers and reduce caregiver neglect.

Abstract citation ID: igaf122.2653

### HELPING THE HELPERS: WHO ASSISTS OLD CHILDREN OF VERY OLD PARENTS?

Sein Kim, Eunju Lee, Kyungmin Kim, Joohyun Kim, and Gyounghae Han, *Seoul National University, Seoul, Republic of Korea*

With increasing life expectancy, the relationship between very old parents and their "old" children extends. Old children are the main source of support for their very old parents, but they are also likely to experience their own health problems and reduced financial and social resources. Yet, less is known about who helped old children of very old parents in the contexts of prolonged filial caregiving. This study analyzed a sample of 102 older adults (aged 65–72) who have a very old parent (aged 81–97) from the Korean Aging Together Study. This study examined old children's networks of social support (i.e., size, composition, and assistance type) and how the network characteristics were associated with depressive symptoms and caregiving burden. On average, participants listed two helpers (range = 1–5) as their social support network. The most frequent types of helpers were spouses (67%), siblings (40%), and daughters (27%). The helpers were most likely to provide financial assistance (60%), followed by listening to talk (25%) and emotional support (11%). The majority of participants (70%) included family members only in their networks of social support, while 28% included both family and non-family members. Regression analyses showed that having networks consisting of family and non-family members was related to fewer depressive symptoms and lower caregiving burden, compared to other types of networks. Our findings suggested that it is important to address unique health and social challenges that "older" child caregivers may face while taking care of their very old parents.

Abstract citation ID: igaf122.2654

### HOW ADULT CHILD CAREGIVERS DISTRIBUTE CARE TASKS FOR THEIR PARENTS WITHIN A CARE NETWORK

Eunju Lee, Haeun Yoo, Jiweon Jun, and Kyungmin Kim, *Seoul National University, Seoul, Republic of Korea*

Primary caregivers of older adults often share caregiving responsibilities with family or paid caregivers. However, less is known about how care network members engage in different roles, how tasks are distributed (e.g., jointly performed by the primary caregiver and others or solely handled by others), and which way of performing tasks is more beneficial to caregivers. This study examined how adult child caregivers perform care tasks with care network members and its association with positive and negative caregiving experiences. Adult children who are primary caregivers of their older parents/in-law (N = 767; aged 28–69) reported their parents' care needs in 16 activities and who performed each task. Among activities that need assistance, 65% were jointly managed by the primary caregiver and others, 18% were handled solely by the primary caregiver, and 17% solely by others. Besides the primary caregiver, the older parent's daughters performed various daily activities, while sons offered intermittent, mobility-focused assistance (e.g., medical visits); the parent's daughters-in-law were less likely to help with activities requiring physical contact (e.g., bathing), and paid caregivers mainly helped with parents' activities of daily living. Regression analyses showed that caregivers reported less caregiving stress and more personal time—when more tasks were handled solely by other family members. However, caregivers experienced more interference while caregiving—when they jointly managed more tasks with family members. Having more tasks solely managed by paid caregivers was associated with lower positive gain. The findings highlight the importance of considering ways of distributing care tasks for caregivers' well-being.

Abstract citation ID: igaf122.2655

### MINDFULNESS AND RUMINATION: UNCOVERING STRESS PATHWAYS IN CAREGIVERS OF PERSONS LIVING WITH DEMENTIA

Jyoti Savla[1], Marrium Mansoor[2], Lauren Hagemann[3], Katherine Lucci[3], and Mamta Sapra[3], *1. Dept. of Human Development and Family Science, Blacksburg, Virginia, United States, 2. Virginia Tech, Blacksburg, Virginia, United States, 3. Salem VA Healthcare System, Salem, Virginia, United States*

Mindfulness techniques, which encourage focusing on the present moment, have shown promise in reducing stress. However, the mechanisms through which mindfulness-based interventions alleviate stress in family caregivers, particularly those caring for persons living with dementia, remain less understood. This study aimed to determine whether a mindfulness-enhanced psychoeducational intervention could lower stress among caregivers of persons living with dementia by reducing rumination – a repetitive pattern of negative thinking. Data were drawn from a randomized controlled trial involving 142 family caregivers of veterans living with dementia (Mage = 67.2, SD = 9.8; 85% women; 82% White; 70% spouse caregivers; 90% co-residing). Participants were randomly assigned to either a standard REACH-VA multi-component skill-building intervention (n = 66) or a mindfulness-enhanced skill-building intervention, named PAACC (n = 67). Data were collected at baseline and at the end of the study. Rumination predicted higher depressive symptoms (using the Patient Health Questionnaire) and perceived stress (using the Perceived Stress Scale), but not caregiver burden (using the Zarit Burden Interview). Additionally, moderated mediation analysis showed that the relationship between anxiety (on the Generalized Anxiety Disorder Screener) and rumination (Rumination Reflection Questionnaire) was significantly stronger in the mindfulness-enhanced intervention group. These findings suggest that the mindfulness-enhanced intervention may be particularly beneficial for caregivers with higher anxiety levels by interrupting the cycle of negative, repetitive thinking, thereby reducing emotional stress even if caregiving burden remains unchanged. Incorporating mindfulness techniques may, therefore, provide meaningful benefits for caregivers by specifically targeting anxiety-driven rumination and improving emotional well-being.

Abstract citation ID: igaf122.2656

### NEEDED AND RECEIVED HOME/COMMUNITY-BASED SERVICES: EXPERIENCES OF RURAL CAREGIVERS OF OLDER VETERANS

Elizabeth Chamberlin[1], Steve Shirk[2], Victoria Ngo[1], Elizabeth Marfeo[3], and Lauren Moo[4], *1. VA Bedford Health Care System, Bedford, Massachusetts, United States, 2. Department of Veterans Affairs, Bedford, Massachusetts, United States, 3. Tufts University, Medford, Massachusetts, United States, 4. VA Bedford Healthcare System, Bedford, Massachusetts, United States*

The lack of nursing homes and assisted living facilities and the wish to age at home has led to an exponential rise in informal (family, friends) caregivers. Thus, home/community-based programs and services are vital resources to keep people in their homes. As a large population of informal caregivers and care recipients live in rural locales, it is important to determine if locality matters. A national survey of informal caregivers of older (≥65) Veterans (N = 511 [rural=258, urban=253]) found no significant difference between rural and urban caregivers' interest in types of service, with the exception that rural caregivers were more interested in respite services than urban caregivers. No difference based on location for those who actively searched for services (N = 226) (estimate = -0.11, p = 0.2058, OR = 0.80). Among the services searched for, respite was significantly different for rural and urban, with searches for this service approximately half as unlikely for those residing in urban communities, OR = 0.53. Urban caregivers reported receiving caregiver support, community, and helping services more than rural caregivers, with urban caregivers approximately three times as likely to have received support (OR = 3.01) and over twice as likely to have received community and helping services (OR = 2.35 and OR = 2.32, respectively) than their rural counterparts. These findings highlight the unevenness in services needed and those received by caregivers' locality. The lack of services in rural areas can decrease the overall health of older rural Veterans and lead to institutionalization.

Abstract citation ID: igaf122.2657

### SELF-COMPASSION AND CAREGIVING OUTCOMES: INSIGHTS FROM A NEW THEORETICAL MODEL

Claire Grant[1], and Katherine Judge[2], *1. VA, Waltham, Massachusetts, United States, 2. Cleveland State University, Cleveland, Ohio, United States*

An expansion of theoretical modeling of the stress process for caregivers of people living with dementia (PLWD) is essential for the advancement of theoretical and applied literatures. The proposed presentation will outline results from a recent study testing an adapted model of self-compassion in caregivers of PLWD (Cha et al., 2023). The model tests parallel lines of mediation by which self-compassion, a way of relating to oneself, influences outcomes including depression, anxiety, and positive aspects of caregiving through mediators including the general resource, emotion regulation, and coping strategies. Caregivers of PLWDs were recruited through CloudResearch and social media posts (N = 226). Participants were majority female (50%), White (70%) and were middle aged (M = 40.5, SD = 12). Findings demonstrated support for self-compassion impacting depression though emotion focused and dysfunctional coping (b = -.05, 95% CI [-.0877,-.0139]); b = -.15, 95% CI [-.2150, -.0874]). The general resource (b = -.09, 95% CI[-.1777, -.0018]) and dysfunctional coping

(b=-.11,95%CI[-.1964,.0583) mediated the relationships between self-compassion and anxiety. Finally, self-compassion indirectly impacted positive aspects of caregiving through problem focused coping (b=.13,95%CI[.0595,.2264]). and the emotion regulation technique of cognitive reappraisal (b=.15,95%CI[.0143,.2699]). Overall, the results highlight the role of coping as a mechanism of self-compassion to impact a range of caregiver outcomes as well as the important similarities and differences in the significant mediators and relationships. The findings may be extended in future research to further refine theoretical understanding of the caregiving process and may have implications in intervention development. Current applications of these findings as well as future implications will be discussed.

Abstract citation ID: igaf122.2658

### TESTING THE EFFICACY OF THE CAREGIVERTLC PROGRAM TO POSITIVELY IMPACT THE CAREGIVER'S PHYSICAL AND MENTAL HEALTH

Julian Montoro-Rodriguez[1], Charlie Reeve[1], Dolores Gallagher-Thompson[2], Ann Bilbrey[3], Jennifer Ramsey[4], and Bruno Kajiyama[5], *1. The University of North Carolina at Charlotte, Charlotte, North Carolina, United States, 2. Stanford University, Stanford, California, United States, 3. Optimal Aging Center, Sunnyvale, California, United States, 4. York Technical College, Rock Hill, South Carolina, United States, 5. Photozig, Inc., Moffett Field, California, United States*

Providing care for a loved-one with a chronic health condition or significant neurocognitive disorders, such as dementia, is a demanding job. The demands of this job can often have an impact on these caregivers' own physical and mental health. In this study, we examine the short-term efficacy of the Caregiver TLC psychoeducational program to attenuate this negative impact on caregivers' self-reported general, physical and mental health as assessed by the SF-12 quality of life health survey. The Caregiver TLC is a skill-building program designed to improve caregiver internal and external coping mechanisms, delivered online to a small group of caregivers over six-weekly two-hour sessions (Montoro-Rodriguez et al., 2024; 2025). Using pre-post data from a randomized controlled trial (N = 78), we conducted a two-way repeated measures ANOVA to test for differences in change in scores over time on each of the three measures of health from the SF-12. Results indicated small to medium effects, with caregivers in the intervention group reporting higher levels of improvement in scores for the 3 sub-scales of the SF-12: overall health, physical health and mental health - compared to those in the control condition. These results suggest that although the Caregiver TLC programming is targeting caregiver coping mechanisms (e.g., building resilience, and increasing emotional support), caregivers gain additional benefits on more distal self-reported health outcomes. Our findings are consistent with previous empirical studies and systematic reviews of the effectiveness of psychoeducational interventions for caregivers that report significant improvement in a variety of health outcomes.

Abstract citation ID: igaf122.2659

### THE CLOSENESS BETWEEN OLDER CARE RECIPIENTS AND FAMILY CAREGIVERS AFFECTS CAREGIVERS' DIET HABITS

Kumi Morishita-Suzuki, *Sendai Center for Dementia Care Research and Practices, Sendai, Miyagi, Japan*

In Japan, approximately 60% of family caregivers for older adults are aged ≥65 and face a higher risk of undernutrition due to caregiving burden. This study examined whether lower perceived closeness with care recipients is associated with poor dietary habits among caregivers. Data from Japan's 2024 national family caregiver panel survey were analyzed, focusing on caregivers aged ≥65 (n = 496, mean age = 70.8, SD = 5.0). Poor dietary habits were defined as a Dietary Variety Score < 4, with self-rated closeness as the independent variable. Covariates included caregivers' age, economic status, solitary eating, chronic diseases, relationship with the care recipient, level of care needed, dementia diagnosis, caregiver burden, and use of caregiving services. Binary logistic regression analysis was conducted. The study was approved by the research ethics committee (No. 24U01). Results showed that 15.4% of men and 17.7% of women reported low closeness with care recipients. Poor dietary habits were found in 42.2% of men and 24.4% of women. Multivariate analysis revealed no significant association between closeness and dietary habits in men. However, among women, lower closeness was significantly linked to poor dietary habits (odds ratio = 3.01, 95% CI [1.21, 7.54], p = .018). These findings suggest that women caregivers with lower perceived closeness to care recipients may be more vulnerable to poor dietary habits, whereas men, typically less involved in meal preparation, may be less affected. Future studies should explore how caregiver-recipient relationships influence appetite and food choices.

### SESSION 7235 (POSTER)

### FORMAL CAREGIVING

Abstract citation ID: igaf122.2660

### EFFORT–REWARD IMBALANCE AND WELL-BEING AMONG FOREIGN DOMESTIC WORKERS IN SINGAPORE

Mayo Ono[1], Yasuhiko Saito[2], Ayumi Honda[3], and Sumihisa Honda[1], *1. Nagasaki University, Nagasaki, Nagasaki, Japan, 2. Nihon University, Tokyo, Tokyo, Japan, 3. St. Mary's College, Kurume, Fukuoka, Japan*

Foreign domestic workers (FDWs) play a crucial role in providing long-term care to older adults in Singapore. This study aimed to examine the associations between (1) Effort–Reward Imbalance (ERI) and well-being among FDWs, and (2) ERI and demographic characteristics. This cross-sectional study utilized a structured, self-administered online survey. The participants were 297 female FDWs from the Philippines and Indonesia (age range, 23–59 years) who were providing live-in care for older adults in Singapore. The short version of the Effort–Reward Imbalance Questionnaire and the World Health Organization Five Well-Being Index (WHO-5) were conducted. The results showed that 55.2% of the FDWs had an effort–reward ratio > 1, indicating that their effort exceeded their reward, and 48.5% had a WHO-5 score < 13, suggesting lower well-being. Bivariate analysis revealed that a higher effort–reward ratio was associated with younger age (P = 0.005), higher education (P = 0.018), being unmarried (P < 0.001), less work experience (P = 0.03), providing care for Chinese Singaporeans (P = 0.009), giving dementia care (P = 0.037), a lack of training (P = 0.008), a lack of support (P < 0.001), and lower well-being (P < 0.001). These findings suggest that over half of FDWs experience ERI and nearly half experience poor well-being. Therefore, targeted support is needed, including caregiving training, motivational career development, mental health resources, and assistance for those lacking support.

In addition, improving working conditions among FDWs is essential.

Abstract citation ID: igaf122.2661

### FACILITATORS AND BARRIERS TO IMPLEMENTING VR IN DEMENTIA CARE TRAINING FOR FORMAL CAREGIVERS: A SCOPING REVIEW

Lillian Hung[1], Carol HokKa Ma[2], Chih Yun Huang[3], Joey OiYee Wong[1], Karen LokYi Wong[4], Keng Hao Chew[5], Lily Haopu Ren[4], and Yong Zhao[4], *1. University of British Columbia, Vancouver, British Columbia, Canada, 2. Singapore University of Social Science, Singapore, Singapore, 3. National Taiwan University Hospital, Taipei, Taipei, Taiwan (Republic of China), 4. The University of British Columbia, Vancouver, British Columbia, Canada, 5. Nanyang Technological University, Singapore, Singapore*

As the aging population grows, the need for improved dementia care training for formal caregivers is urgent. Virtual reality (VR) offers a promising approach to enhance training outcomes. This scoping review examines the facilitators, barriers, and impacts of implementing fully immersive VR in dementia care training for formal caregivers in long-term care settings. Guided by the Consolidated Framework for Implementation Research, this review followed the Joanna Briggs Institute methodology and PRISMA-ScR guidelines. A systematic search of CINAHL, MEDLINE, Embase, Scopus, Web of Science, and ProQuest identified 469 publications, with nine meeting inclusion criteria. These studies, published between 2015 and 2024, involved 362 formal caregivers aged 44.7 to 65 years. VR interventions fostered empathy through first-person perspectives and helped participants recognize behavioral triggers and apply caregiving strategies using second- and third-person perspectives. Barriers and facilitators were primarily in the innovation domain. Barriers included simulation sickness, headset discomfort, and limited immersive, interactive, and embodied experiences. Facilitators included technological advantages, highly immersive and interactive experiences, a safe training environment, individual user attributes, and structured orientation and support during training. VR training demonstrated benefits across multiple levels, from initial reactions and learning (knowledge, skills, and attitudes) to behavioral changes and systemic outcomes. This review highlights the current landscape of VR-based dementia care training. Future research should refine VR experiences and assess their impact on caregiver-resident interactions. Addressing barriers and leveraging facilitators can support the effective implementation of VR training to enhance care quality and resident well-being in long-term care settings.

Abstract citation ID: igaf122.2662

### STREET-LEVEL BUREAUCRACY AND DOCUMENTATION: ADDRESSING CONFLICTING RIGHTS IN NURSING HOMES THROUGH CARE PLANS

Angela Perone, *University of California, Berkeley, Berkeley, California, United States*

Conflicting rights are exceedingly common in nursing homes, one of the most heavily regulated industry in the United States (e.g., safety, autonomy, privacy). Staff have increasingly incorporated documentation in resident care. This study investigates how do staff use documentation to address conflicting rights in nursing homes? It employs a qualitative comparative case study with 80 in-depth interviews of staff and ethnographic observation of interactions among staff, residents, and visitors for eight months at two nursing homes. Staff referenced “care plans” as the primary tool to address conflicting rights. Front-line workers (e.g., CNAs, floor nurses) used care plans as deferential problem-solving tools and deferred to “looking in the care plan” to identify what they should do. Mid-level managers used care plans as dynamic clinical problem-solving tools for residents by identifying how to shape the care plan with various team members, including ways to change medical orders. Upper-level managers employed care plans as a liability problem-solving tool focused on the staff and organization by emphasizing the importance of documenting decisions in the care plan to insulate workers and the nursing home against liability. Care plans in this study reflect ways in which nursing home staff leveraged the documentation burdens that many often loathed to address an often vague and ambiguous policy space in nursing homes: how to navigate conflicting rights. Care plans, thus, transformed a dreaded documentation tool into a problem-solving tool that provided some staff a sense of agency and direction—albeit in different ways among different staff levels.

Abstract citation ID: igaf122.2663

### TRENDS IN INFORMAL AND FORMAL CARE USE AND UNMET NEEDS AMONG OLDER POPULATION, 2014-2023

Eun Ha Namkung, and Gyeonghee Lee, *Ewha Womans University, Seoul, Republic of Korea*

The increasing number of older adults with limitations in daily activities has significant implications for policy and social systems, particularly in rapidly aging societies. Recognizing that how care needs are met depends on both individual characteristics and societal changes, this study examines trends in informal and formal care utilization and their influencing factors among older Koreans over the past decade. Using data from four waves of the National Survey of Older Koreans (2014, 2017, 2020, and 2023), we analyze changes in care received by community-residing adults aged 65 and older with care needs (n = 1,153 to 2,902 per wave). Survey-weighted multinomial regression models show a rising proportion of older Koreans with unmet care needs, increasing from 20.8% in 2014 to 52.6% in 2023. Informal care from family and friends has declined sharply, while the modest rise in formal care use has not offset the growing care gap. Lower economic status is consistently linked to higher unmet care needs, while older age, living alone, and greater activity limitations are associated with formal care use. These findings highlight the urgent need for expanding formal care services and supporting informal caregivers. Further research is needed to understand the societal and policy shifts contributing to rising unmet care needs and to inform interventions that improve access to care for vulnerable older adults.

### SESSION 7240 (POSTER)

### GLOBAL AGING AND HEALTH

Abstract citation ID: igaf122.2664

### “I ALWAYS GET PANADOL...THERE’S NO HOPE HERE.” HEALTHCARE SERVICES ACCESSIBILITY TO INCARCERATED OLDER ADULTS.

Akwasi Adjei Gyimah[1], Samuel Asante[2], Eric Frimpong[3], and Mathias Adjei[4], *1. Miami University Scripps Gerontology*

*Center, Oxford, Ohio, United States, 2. Northeastern State University, Tahlequah, Oklahoma, United States, 3. University of Massachusetts Boston, Boston, Massachusetts, United States, 4. Miami University, Oxford, Ohio, United States*

The increasing number of Incarcerated older adults in Ghana is becoming a national issue for both policymakers and prison administrators. Currently, there are about 15,198 people incarcerated in various places in Ghana. These numbers result in having too many congestions and all these affect the quality of healthcare in prisons. The study sought to assess the quality of health care services for older adults in prisons in Ghana This study employed a qualitative approach involving an explorative descriptive design A focus group discussion was conducted among purposively selected older incarcerated adults. To be included, a participant must have been at least 50 years old and in prison for at least six months. A total of 28 older adults, comprising 18 males and 10 females were selected for participation in the study. The findings showed health care services accessibility in prison facilities remains a significant issue. Participants acknowledged that healthcare workers in prisons have limited knowledge and practice skills to effectively address their healthcare needs. They also pointed to the lack of medical equipment in prisons and stigma as additional factors that adversely affect their healthcare experiences in prisons. Participants generally noted that the quality of health services in prisons was substandard compared to that provided to the general public. The study adds to the literature on older incarcerated adults in Africa and how their healthcare accessibility can be improved. The study underscores the need for policy changes and practices that could enhance the healthcare experiences of older adults in prisons.

Abstract citation ID: igaf122.2665

### ASSOCIATION BETWEEN MULTIDIMENSIONAL ENVIRONMENTAL ENRICHMENT DOMAINS AND INTRINSIC CAPACITY AMONG OLDER ADULTS

Guanzhou Wang, and Minqing Zhang, *Duke University, Durham, North Carolina, United States*

Environmental enrichment (EE), extensively studied in animal models, integrates cognitive, physical, and social stimulation to enhance functional health, but its translation to human aging research remains limited. Intrinsic capacity (IC), defined by the World Health Organization (WHO), provides a holistic framework for assessing functional health essential for independence in older age. This study examined associations and interactions between EE domains and IC among older adults. Using cross-sectional data from 5,454 older adults in the English Longitudinal Study of Ageing (ELSA), EE domains were assessed through self-reported physical activity (high/low), cognitive engagement (frequency of stimulating activities), and social engagement (frequency of social and community interactions). IC was classified into high, medium, or low based on WHO's Integrated Care for Older People (ICOPE) guidelines. Ordinal logistic regressions, adjusted for age, sex, education, smoking, and alcohol use, were performed. Results indicated significant independent associations between each EE domain and higher IC levels: physical activity (OR = 1.35, 95% CI = 1.17–1.56, $p<.001$), cognitive engagement (OR = 1.21, 95% CI = 1.12–1.30, $p<.001$), and social engagement (OR = 1.07, 95% CI = 1.02–1.11, $p=.002$).A significant interaction between cognitive engagement and physical activity (OR = 0.77, 95% CI = 0.66–0.89, $p<.001$) indicated combined influences distinct from their individual effects. Interactions involving social engagement were not significant. Findings support translating EE concepts into human aging research, highlighting multidimensional lifestyle approaches, especially integrating physical and cognitive activities, to promote healthy aging. Future longitudinal studies and intervention trials are needed to confirm these findings and test the effectiveness of multidimensional EE interventions for optimizing intrinsic capacity and related health outcomes.

Abstract citation ID: igaf122.2666

### CHALLENGES OF REINTEGRATION AND RECIDIVISM AMONG OLDER ADULT PRISONERS IN GHANA

Mathias Adjei[1], Eric Frimpong[2], Akwasi Adjei Gyimah[3], and Spendilove Yankson[4], *1. Miami University, Oxford, Ohio, United States, 2. University of Massachusetts Boston, Boston, Massachusetts, United States, 3. Miami University Scripps Gerontology Center, Oxford, Ohio, United States, 4. University of Cape Coast, Cape Coast, Central, Ghana*

As the global aging prison population rises, understanding the challenges of reintegration for older adult prisoners is critical in developing age-responsive correctional policies. This study uses a Life Course Perspective to explore factors contributing to reintegration and recidivism among older adult prisoners in Ghana. Using a qualitative approach, data were gathered from 21 participants. This included 8 older inmates aged 60 + (with at least two prior incarcerations) and 5 prison officers from the Sunyani and Kumasi central prisons. In addition are 8 community members. Findings highlight that the lack of age-appropriate rehabilitation programs for older inmates presents a major barrier to successful reintegration. Older adult prisoners face challenges in accessing the necessary support and resources to address age-related needs such as healthcare, skill-building, and emotional well-being. Additionally, societal stigma, where former prisoners are still considered criminals, hinders their acceptance by family, communities, and potential employers. The persistence of these negative perceptions reinforces discrimination in social relationships and employment. This led some individuals to engage in criminal activities, with the intent of returning to an environment where they feel belonged. Participants further indicated that they lack the necessary resources essential to provide age-specific rehabilitation programs creating a barrier to their successful reentry into society. Policy reforms should focus on age-responsive reintegration programs, addressing healthcare, vocational training, and social support while tackling societal stigma. Adequate resources and collaboration are key to reducing recidivism and ensuring successful reentry into society.

Abstract citation ID: igaf122.2667

### DELINEATING THE TRAJECTORIES AND NORMATIVE VALUES OF MULTI-SYSTEMIC AGING METRICS IN THE UK, THE US, AND CHINA

Liming Zhang[1], Jiening Yu[1], Xueqing Jia[2], Jingyun Zhang[1], Wei Yang[3], Xi Chen[4], Emiel O. Hoogendijk[5], and Zuyun Liu[1], *1. Zhejiang University, Hangzhou, Zhejiang, China (People's Republic), 2. Zhejiang university, Hangzhou, Zhejiang, China (People's Republic), 3. King's College London, London, England, United Kingdom, 4. Yale University, New Haven, Connecticut, United States, 5. VU University Medical Center, Amsterdam, Noord-Holland, Netherlands*

Aging is a multi-dimensional process and manifests heterogeneities across different organ systems, individuals, and countries.

Here we used three large national datasets (UKB, NHANES, and CHARLS) to delineate the trajectories of 14 organ/system-representative aging metrics for persons under different sociodemographic contexts in the UK, the US, and China. The functions of all aging metrics, except for cognitive function, manifested a progressive deterioration or maintained stability after adulthood, especially after middle age. The deterioration of cognitive function was more pronounced after the age of 70 in Chinese and British populations, while not in the American populations. In the stratified analyses, males and females manifested disparities in aging trajectories involving the cardiovascular and muscle-skeletal systems. Notably, we observed substantial disparities in the aging trajectories by economic and educational levels within and between the three countries: the low-income and low-education subgroups in China manifested a more pronounced deterioration in the functioning of multiple systems (i.e., brain, muscle-skeletal, physical function, and pulmonary). However, these income or educational disparities were nonexistent in the UK and the US participants, implying health inequalities caused by regional socioeconomic disparities. This work may serve as a proof-of-concept to comprehend the multi-dimensional signature of systemic aging and call for policies to promote health equity across nations when facing dramatic global aging.

Abstract citation ID: igaf122.2668

### EXPANDED EDUCATION, BETTER HEALTH IN LATER LIFE? –EVIDENCE FROM THE MEXICAN HEALTH AND AGING STUDY (MHAS)

Chengming Han[1], Brian Downer[2], and Zhiwei Hu[3], *1. UTMB Health, The University of Texas Medical Branch, Galveston, Texas, United States, 2. UTMB, Galveston, Texas, United States, 3. the University of Texas Medical Branch, Galveston, Texas, United States*

Objective: ObjectiveThis study intends to examine the education gradients in functional limitations for older adults in Mexico by comparing effect size between two birth cohorts age 60-76 in 2001 versus 2018. Methods: Data were drawn from the Mexican Health and Aging Study (MHAS) 2001 and 2018 waves. Tobit model was used to estimate the education gradient in functional limitations. Living conditions in childhood (pluming in house, parents' education, and serious health conditions), chronic conditions, and the longest work were adjusted for. The cohort difference was tested by including interaction effects and propensity matching scores. Results: Compared to the 2001 cohort, the 2018 cohort presented lower proportion of no education, and higher proportion in each level of education, especially the secondary and tertiary education. Education gradients were significant in functional limitations for each birth cohort, but the education gradient was attenuated for the 2018 cohort compared to the 2001 cohort. Living conditions in childhood have long-term effects on functional limitations in later life. Agricultural and industrial work contribute to greater functional limitations. No different effects of primary job/work and early life conditions between the cohorts were detected. Conclusion: Disregarding the average higher levels of education within the 2018 wave birth cohort, the education gradients in functional limitations decreased compared to the early birth cohort in 2001 wave.

Abstract citation ID: igaf122.2669

### PROTECTIVE EFFECTS OF THE FOXO3 LONGEVITY GENE ON AGING-RELATED DISEASES IN THE CHAMP COHORT

Lovina Abdi[1], Richard Allsopp[1], David LeCouteur[2], and Bradley Willcox[3], *1. University of Hawai'i at Mānoa, Honolulu, Hawaii, United States, 2. ANZAC Research Institute, Sydney, New South Wales, Australia, 3. John A. Burns School of Medicine, Honolulu, Hawaii, United States*

Recent findings from the Kuakini Honolulu Heart Program cohort in Hawaii suggest that longevity-associated gene variants of FOXO3 (e.g. SNP rs2802292-G allele carriers) may confer resilience against cardiometabolic diseases (CMD) such as hypertension, diabetes, and angina, in Japanese-American men. Therefore, we studied white males from the Concord Health and Ageing in Men Project (CHAMP) cohort in Australia, focusing on the protective FOXO3 G allele and its potential impact on CMD and longevity. We found that G allele carriers with angina exhibited a significantly reduced risk of mortality, although no similar protective effect was observed in individuals with hypertension or diabetes. These data support prior findings that the FOXO3 G allele may not universally protect against CMD onset, but it may delay disease onset and lessen the severity of advanced symptomatic CMD, such as angina, highlighting its critical role in promoting cardiovascular health. The protective effects of FOXO3 are likely mediated through multiple mechanisms. This gene has been associated with enhanced cellular resilience against oxidative stress, maintenance of telomere length to preserve genomic stability, and regulation of metabolic pathways essential for cardiovascular function. Furthermore, FOXO3 influences cytokine signaling, which may mitigate systemic inflammation—a key driver of CMD risk, progression and mortality. These pathways collectively support cardiovascular integrity and overall longevity. This study reinforces FOXO3's importance as a genetic factor influencing CMD outcomes and longevity. Future studies are needed to uncover the precise molecular mechanisms and evaluate FOXO3 as a potential therapeutic target for CMD prevention and treatment.

Abstract citation ID: igaf122.2670

### THE EFFECT OF THE UNITE OF MERIDIAN FLOW CHRONOTHERAPY AND ACUPRESSURE IN IMPROVING SLEEP REVERSAL IN ELDERLY PEOPLE

Jiajia Xu, Dan Liu, Shan Li, and Yue Hu, *Shanxi Bethune Hospital, Taiyuan, Shanxi, China (People's Republic)*

Background: Global aging is accelerating, and the incidence of sleep reversal in the elderly has increased significantly, affecting the quality of life. The Chinese Meridian Flow Theory, which advocates the regulation of meridians and qi-blood at specific hours to restore physiological rhythms, has demonstrated advantages in the management of chronic diseases. Objective: To explore efficient and safe intervention programs to improve sleep inversion and to provide theoretical support and practical reference for preclinica. Methods: Intervention: For yin and yang imbalance and visceral disharmony, select the heart meridian, kidney meridian, pericardium meridian acupuncture points, and construct the "three time intervention axis"①11:00-13:00 Shenmen, Shaohai; ②17:00-19:00 Yongquan, Taixi; ③19: 00-21:00 Neiguan, Laogong. Each point is pressed using the fingertips for 3 minutes and circulated 3 times to localize a slight redness of the skin. Control: Routine sleep health promotion. RESULTS:The number of nocturnal awakenings (1.2±0.5 times ), time (28.5±10.2 minutes), and PSQI score (6.2±1.8 points) of the intervention group were significantly better than those of the control group ($P < 0.05$). The TCM evidence points were 37.5%-47.8% lower than those of the control group, and the efficacy was stable during the follow-up period without serious adverse reactions. Conclusion: This measure is effective in improving sleep regression in the elderly. It contributes Chinese medicine wisdom to the WHO Global Strategy on Aging and Health, and promotes "active and healthy" aging with the synergy of Chinese and Western medicine.

Abstract citation ID: igaf122.2671

**WHAT ABOUT OLDER PERSONS IN PALESTINE? A CALL FOR RECOGNITION, REHUMANISING AND SOLIDARITY IN GERONTOLOGY**

Yudi PamelaSuero Samboy[1], and Preetha Joseph[2], *1. Free University of Brussels (VUB), Society and Ageing Research Lab, Brussels, Brussels Hoofdstedelijk Gewest, Belgium, 2. University of Galway, Galway, Galway, Ireland*

While the impact of humanitarian crises caused by armed violence is well-documented, the specific experiences of older persons remain largely overlooked, particularly within academia and the field of gerontology. Amidst the recent genocide in Gaza, older Palestinians represent a stark example of this silence and systemic neglect. After decades of oppression under occupation, their plight is now intensified by high mortality risk, forced displacement, collapsed essential services and devastation of their communities. Our work argues that the experience of older Palestinians must serve as a wake-up call for gerontology and international policy agendas, as it exposes a failure to uphold global commitments to healthy ageing and human rights protection. Structural dispossession, exile, disrupted care and support networks, and destruction of living environments are severely undermining older Palestinians' ability to age in place or experience age-friendly conditions. Beyond reflecting on the crisis in Gaza, we critique the broader neglect of older persons hit by armed violence. We urge gerontologists to adopt an emancipatory social science approach through acts of recognition, rehumanisation, and solidarity towards older Palestinians, within teaching, research, dissemination, and scholar-activism. Against the backdrop of uncertainty following the ceasefire announced in January 2025, gerontology can play a vital role in ensuring that older persons are not left behind in efforts to rebuild a Palestine free from occupation. Breaking the silence on Palestine can reaffirm gerontology's moral purpose and legitimacy as a value-based discipline, and contribute to advancing a much-needed transformative agenda to address injustices affecting older persons around the world.

### SESSION 7245 (POSTER)

### HIV/AIDS

Abstract citation ID: igaf122.2672

**ANTIRETROVIRAL THERAPY ALTERS SKELETAL MUSCLE DNA METHYLATION IN HIV MICE AND PROMOTES CELLULAR SENESCENCE**

Alok Tripathi, Shabiha Sultana, Anthony Carrillo, Sadanand Fulzele, Huidong Shi, Eric Belin de Chantemele, Mark Hamrick, and Meghan McGee Lawrence, *Augusta University, Augusta, Georgia, United States*

HIV-associated morbidity and death have been decreased by antiretroviral therapy (ART), however age-related musculoskeletal frailty is exacerbated in people living with HIV (PLWH) and those on ART, contributing to poor healthspan. However, the combined effects of aging, HIV, and ART in musculoskeletal tissues have not been thoroughly studied. Here we investigated the effects of Emtricitabine (FTC), an FDA approved ART, or its vehicle (VEH) on skeletal muscle DNA methylation patterns in a female HIV transgenic mouse model (Tg26). Comparing the impact of HIV phenotypes in the absence of ART (i.e., WT+VEH vs. Tg26+VEH), 303 CpGs were significantly hypermethylated (methDiff≥0.1) whereas 273 were hypermethylated when comparing the same groups in the presence of ART (i.e., WT+FTC vs. Tg26+FTC). Mechanistically, the effects of FTC with TDF (Tenofovir, another ART) and TAF (Tenofovir alafenamide, a prodrug of TDF) as a combination ART (cART) were tested in primary human muscle cells. The cART treatment induced cellular senescence, (senescence-associated β-galactosidase staining) and qPCR suggested downregulated expression of genes involved in myoblast differentiation. These findings suggest that ART may have deleterious consequences on muscle function and may contribute to epigenetic changes in DNA methylation and cellular senescence, exacerbating muscle wasting and dysfunction in patients with HIV receiving ART treatments. Further research is needed to elucidate the mechanisms that drive muscle cell damage in PLWH on long-term cART and to understand contributions of altered DNA methylation to these processes. These insights could inform potential epigenetic-targeted therapeutic approaches for mitigation of musculoskeletal decline in PLWH on ART.

Abstract citation ID: igaf122.2673

**ASSOCIATIONS BETWEEN SPECIFIC IADLS AND OBJECTIVE COGNITIVE PERFORMANCE IN OLDER WOMEN WITH HIV**

David Vance[1], Lang Lang[2], Raha Dastgheyb[3], Yanxun Xu[2], and Leah Rubin[4], *1. University of Alabama at Birmingham, Birmingham, Alabama, United States, 2. Johns Hopkins University, Baltimore, Maryland, United States, 3. Johns Hopkins School of Medicine, Baltimore, Maryland, United States, 4. Johns Hopkins University School of Medicine, Baltimore, Maryland, United States*

Nearly 30% of people with HIV experience suboptimal cognitive functioning, which is expected to increase as people with HIV age. A subgroup particularly at risk for cognitive impairment is women with HIV which comprise 21% of the HIV population in the United States and 53% of the HIV population worldwide. These cognitive impairments can develop into everyday functional impairments. In the Women's Interagency HIV Study (WIHS), we examined the association between the self-rated Lawton and Brody scale of Independent Activities of Daily Living (IADL) and objective cognitive test performance in 754 older (50+) women with HIV (WWH; 84% virally suppressed). In the total sample, 73% were non-Hispanic Black (mean age = 55.03 years). To handle this longitudinal data, weighted logistic mixed effect models examined associations between cognitive domain performance (predictor) and functional outcomes (IADL item level scores). With respect to IADLs, approximately 38% of the total sample showed an overall impairment in IADLs; however, as expected this impairment (35%) was slightly less in those virally suppressed. In the total sample, poorer executive functioning was associated with impairment in planning social activities and poorer motor performance was associated with impairments in home repairs, housekeeping, and laundry. Among older virally suppressed-WWH, poorer motor performance was associated with deficits in home repair and poorer executive performance was associated with deficits in planning social activities. Since motor and executive performance were related to impairments in certain IADLs, strategies such as cognitive training targeting these domains could improve everyday functioning.

Abstract citation ID: igaf122.2674

### BASELINE CHARACTERISTICS AND OUTCOMES FOR THE 4F PILOT FOR PERSONS AGING WITH HIV

Julie Womack[1], Chloe Johnson[2], Evelyn Hsieh[2], Richard Marottoli[3], Wynnett Stewart[4], Michael Virata[2], and Lydia Aoun-Barakat[2], *1. Yale University School of Nursing, Orange, Connecticut, United States, 2. Yale School of Medicine, New Haven, Connecticut, United States, 3. Yale University School of Medicine, New Haven, Connecticut, United States, 4. Yale New Haven Healthcare System, New Haven, Connecticut, United States*

Background: More than half of people living with HIV in the US are 50+ years of age. HIV clinicians must be comfortable assessing for and managing conditions associated with aging. Methods: We trained 11 clinicians at an academic HIV ambulatory care center to assess and prevent the 4F's (polypharmacy, fragility fractures, falls) among their patients 50+ years old. We share baseline patient data and results from the HIV clinicians' knowledge, attitudes, and practices baseline assessment. Results: 125 patients were enrolled. Mean age was 63±6 years, 49% were women, 48% identified as Black. 30% were current smokers, and 30% reported ongoing marijuana use. The mean number of medications prescribed was 10±5. However, 25% experienced hyperpolypharmacy (taking 15+ medications), and 66% were prescribed potentially inappropriate medications (PIMs). Mean FRAX scores indicated low 10-year risk for fragility fractures (major osteoporotic fracture: 8.71%±6.95%; hip fracture 2.11%±3.58%). Using our serious fall risk assessment tool, we found that 27% of women and 24% of men were at high risk for a serious fall within the next 6 months. Among the clinicians participating in the study, 73% performed medication reconciliation at least once a year. 55% reported asking about falls only if the patient brought it up, and 36% had only asked their patients once about falls. 18% had never assessed their patients' fracture risk. Conclusions: Polypharmacy, high medication count, and PIMs are key risk factors for adverse outcomes among older adults. Deprescribing and fall and fracture prevention are key foci for future interventions.

Abstract citation ID: igaf122.2675

### CULTURAL VALUES AND MENTAL HEALTH SUPPORT SEEKING IN OLDER NIGERIANS LIVING WITH HIV

Candidus Nwakasi[1], and Chinelo Nduka[2], *1. University of Connecticut, Storrs, Connecticut, United States, 2. Nnamdi Azikiwe Teaching Hospital, Nnewi, Anambra, Nigeria*

Background: About 2 million people are living with HIV (PLWHIV) in Nigeria. The prevalence rate is 2.3% for suicide attempt, 2.9% for suicide ideation, 7% for alcohol abuse, and 28.2% for depression in this group. To add to our understanding of this burden and how to mitigate it, this study examined the associations between cultural values, mental health seeking attitudes and intentions among older PLWHIV in Nigeria. Methods: We recruited 150 participants (Mage = 55.2) who identified as PLWHIV. They completed a questionnaire assessing sociocultural and sociodemographic factors, mental health seeking, and health outcomes measures. Multivariate linear regression models were fitted to determine if masculinity and collectivism were associated with mental health seeking attitude (MHSA), intention to seek mental health support from family during emotional/personal problems (MHSIF-E), and when having suicidal thoughts (MHSIF-S). Age, gender, education, relationship status, and perceived stress were covariates in the models. Results: Aging is associated with decreased MHSA ($\beta$ = -0.172, $p<0.019$), those who are partnered are more likely to report higher MHSA ($\beta=3.955$, $p<0.005$) and MHSIF-E ($\beta=2.775$, $p<0.00$), and higher perceived stress is associated MHSA ($\beta=0.334$, $p<0.014$). Increased masculinity ($\beta=0.417$, $p<0.02$) and collectivism ($\beta=0.251$, $p<0.018$) are associated with increased MHSA. Increased MHSA is associated with increased MHSIF-E ($\beta=0.203$, $p<0.002$) and MHSIF-S ($\beta=0.259$, $p<0.001$). Conclusion: To strengthen access and tailor HIV-related mental health interventions in Nigeria, programs must consider cultural values, mental health seeking attitudes, and intentions to seek support.

Abstract citation ID: igaf122.2676

### EXECUTIVE FUNCTIONING TRAINING REDUCES COGNITIVE INTRA-INDIVIDUAL VARIABILITY IN OLDER ADULTS WITH HIV

David Vance[1], Hathaichanok Phaowiriya[2], Xueling Zeng[2], Maryam Rostamvand[2], Elizabeth Byrd[2], Raymond Jones[2], Pamela Bowen[2], and Pariya Fazeli[1], *1. University of Alabama at Birmingham, Birmingham, Alabama, United States, 2. The University of Alabama at Birmingham, Birmingham, Alabama, United States*

Cognitive training has been shown to enhance cognition in people with HIV (PWH). While these approaches produce some success, none have directly addressed cognitive intra-individual variability (IIV)—fluctuations in cognitive performance within the same test (inconsistency). Higher cognitive IIV is linked to cognitive decline and neurological sequelae in a way standard mean-based cognitive testing does not. The Executive Dyscontrol Hypothesis suggests that impaired executive functioning contributes to greater cognitive IIV. Strengthening executive function may reduce cognitive IIV and improve overall cognitive health. This study examined whether executive functioning training could reduce cognitive IIV in older PWH. In this 2-group baseline/posttest experimental study, we enrolled 118 PWH aged 40 to 70 who were randomized into one of two groups: 1) 20 hours of computerized executive functioning training group, or 2) no-contact control group. At baseline and posttest, participants were assessed on depression, subjective cognitive functioning, and the Connor's Continuous Performance Test (CCPT-3). The CCPT-3 has two measures of cognitive IIV: 1) HRTSD – hit reaction time standard deviation and 2) variability. In this sample, most were women (52.5%) and African American (85%) and their mean age was 53.87 years; 60 and 58 were in the training group and control group, respectively. Controlling for baseline performance, improvements in HRTSD ($F=3.448$, $p=0.067$ – trending) and variability ($F=5.163$, $p=0.026$ – significant) were observed in the treatment group compared to the no-contact control group. Results demonstrate that executive functioning training reduced cognitive IIV; this supports the Executive Dysfunction Hypothesis in a sample at-risk for cognitive problems.

Abstract citation ID: igaf122.2677

**EXPERIENCES OF NURSING CARE AMONG OLDER ADULTS LIVING WITH HIV**

Juh Shin[1], Yu-Ping Chang[2], Vanessa Cameron[3], and David Vance[4], *1. George Washington University, Ashburn, Virginia, United States, 2. University at Buffalo, Buffalo, New York, United States, 3. Vanessa Cameron Education & Consulting, Leavenworth, Washington, United States, 4. University of Alabama at Birmingham, Birmingham, Alabama, United States*

Despite advancements in HIV treatment, clinical care for people living with HIV (PLWH) remains suboptimal. To improve care delivery, it is essential to understand the lived experiences of PLWH individuals affected by HIV, particularly older PLWH adults who face unique challenges. This study aimed to explore perspectives regarding nursing care in older PLWH adults living with HIV and. Semi-structured individual interviews were conducted with 20 11 older adults living with HIVPLWH (50+). Thematic analysis was utilized to understand their experiences with nursing care. All interviews were recorded, transcribed, and systematically analyzed to identify key themes. Overall, participants described nursing care as a valuable source of support and generally high in quality. However, they also highlighted critical areas for improvement. Five key themes emerged: (1) Looking Beyond the Virus and T-Cells: the need for holistic care that addresses co-existing chronic conditions. (2) Identifying Emerging Care Needs: proactive assessments to detect evolving health concerns. (3) Unmet Psychological and Emotional Needs: addressing loneliness and social isolation. (4) Ensuring Accessibility and Culturally Appropriate Care: addressing barriers to healthcare access. (5) Supporting Patient Empowerment and Advocacy: enhancing education and resources for self-advocacy. While participants valued the nursing care they received, they emphasized the need for more comprehensive support, particularly in advocacy, social connection, care accessibility, and care coordination. These findings highlight critical gaps in current nursing practices and underscore the need for patient-centered, holistic approaches. Future research should focus on developing and implementing coordinated services to enhance the well-being of older adults with HIV.

Abstract citation ID: igaf122.2678

**FEASIBILITY OF MHEALTH TO SUPPORT SELF-MANAGEMENT IN OLDER ADULTS LIVING WITH HIV**

Gwang Suk Kim[1], Layoung Kim[2], Sooyoung Kwon[3], and Seoyoung Baek[4], *1. Yonsei University College of Nursing, Seoul, Republic of Korea, 2. The University of Suwon, Hwaseong, Republic of Korea, 3. Graduate School of Yonsei University, Seoul, Republic of Korea, 4. Korea Armed Forces Nursing Academy, Daejeon, Republic of Korea*

HIV infection is a chronic disease requiring lifelong management. Social stigma and healthcare discrimination limit access to health resources, making mobile health (mHealth) a promising intervention. However, concerns exist regarding the applicability of mHealth among older adults given potential barriers and engagement levels. This study assessed the feasibility of mHealth for older people living with HIV (PLWH). A one-arm, prospective, fully non-face-to-face mHealth trial was conducted with 24 PLWH aged ≥60 years. Participants used an mHealth app, "ESSC (Excellent Self-Supervised HIV Care)," which provided health-related information; allowed self-recording of medication adherence, mental diary, and sexual life; and enabled Q&A and comments interactions. After baseline assessments of demographics and HIV-related health, participants used the app for four weeks. Cumulative usage data were analyzed. All 24 participants were successful installing the app independently. Participant's median age was 63.88 years (range: 60–82), and all were male. Based on usage, 7 participants (29.2%) were non-users (≤2 days), while 17 (70.8%) were users (≥24 days). Among users, the 28-day average included 33.6 daily medication logs, 14.2 mental diary entries, 7.6 sexual life records, 1.5 Q&A interactions, and 9.8 comments. Notably, users exhibited significantly higher baseline depression scores (6 vs. 0), higher stigma levels (8.7 vs. 7.0), and lower health-related quality of life (70 vs. 90) than non-users. This study demonstrated the feasibility of mHealth interventions for older PLWH. Findings suggest that mHealth adoption may be particularly beneficial for older PLWH with poorer emotional health, highlighting the need for targeted interventions to enhance usability.

Abstract citation ID: igaf122.2679

**FRAGMENTED HEALTHCARE EXPERIENCES OF OLDER ASIAN AMERICANS LIVING WITH HIV**

Jen-Hao Chen[1], Cheng-Shi Shiu[2], and Wei-Ti Chen[3], *1. National Chengchi University, Taipei, Taiwan (Republic of China), 2. National Taiwan University, Taipei, Taiwan (Republic of China), 3. University of California, Los Angeles, Los Angeles, California, United States*

As the Asian American population with HIV ages, their healthcare experiences remain understudied. Understanding their challenges is critical for improving geriatric HIV care and informing culturally competent interventions for Asian American communities. We conducted qualitative interviews with 34 HIV-positive Asian American older adults in Los Angeles between 2018 and 2019. Analysis focused on identified patterns related to healthcare discontinuities, provider-patient interactions, and system navigation difficulties. Participants experienced significant healthcare fragmentation, including frequent provider changes, service disruptions, and inefficient care coordination. Many patients switched providers due to insurance changes, discrimination concerns, or communication barriers, disrupting treatment continuity. Limited English proficiency further complicated care access, as many patients relied on family members for translation, leading to misinterpretations, incomplete medical discussions, and a loss of autonomy due to the absence of professional interpreters in HIV clinics. Stigma and medical mistrust also contributed to delayed treatment, with patients avoiding care to prevent disclosure within both healthcare settings and their own communities, increasing their reliance on emergency care. Fragmentation between HIV treatment, general healthcare, and mental health services led to inconsistent medication management and poor psychosocial support. Additionally, many participants expressed concerns about discrimination in long-term care facilities, discouraging them from seeking aging-related healthcare planning. Findings highlight the need for culturally competent geriatric HIV care, including professional interpretation services, improved care coordination, and community-based aging-HIV programs to ensure equitable access and improved health outcomes.

Abstract citation ID: igaf122.2680
### PREDICTORS OF COGNITIVE INTRA-INDIVIDUAL VARIABILITY IN AGING ADULTS WITH HIV FROM THE DEEP SOUTH

Xueling Zeng[1], Hathaichanok Phaowiriya[1], Maryam Rostamvand[1], Pariya Fazeli[2], Raymond Jones[1], Frank Puga[2], Elizabeth Byrd[1], and David Vance[2], *1. The University of Alabama at Birmingham, Birmingham, Alabama, United States, 2. University of Alabama at Birmingham, Birmingham, Alabama, United States*

While conventional cognitive assessments focus on capturing mean-based performance, they often overlook naturally occurring fluctuations in cognitive performance across cognitive domains (i.e., executive functioning). Referred to as cognitive intra-individual variability (IIV), it is associated with poorer cognitive and everyday functioning, can predict cognitive impairments, and is associated with morbidity and mortality. Yet, few studies have examined cognitive IIV in aging people with HIV (PWH). Our study examined factors associated with cognitive IIV in 260 PWH aged 40+ who were administered a comprehensive cognitive and psychosocial battery. For this analysis, we used cross-sectional data to explore the correlations among demographic, HIV characteristics (e.g., CD4+ count), medication adherence, psychosocial factors (depression, personality traits, locus of control), and cognitive IIV. Cognitive IIV (iSD) was measured through the variation across cognitive domains. In this sample from Birmingham, Alabama, most were male (63.85%) and African American (82.70%), with a mean age of 51.15 years. Results showed that higher IIV was positively correlated with locus of control ($r = 0.26$), suggesting that individuals who believe outcomes are out of their control lead to less stable cognitive performance. Higher scores on openness ($r = 0.27$) and agreeableness ($r = 0.21$) were associated with lower cognitive IIV. Surprisingly, no single cognitive domain predicted cognitive IIV (r's=-0.14 to -0.22). Although exploratory, these findings suggest that cognitive IIV may have a subtle psychosocial profile, suggesting such cognitive fluctuations may have an impact of perception and behavior. Techniques to alter cognitive IIV as people age are being developed which could impact such perceptions are posited.

Abstract citation ID: igaf122.2681
### REFRAMING AGING AND HIV: INTEGRATING GERIATRIC CARE INTO PRIMARY HIV CLINICS TO CLOSE THE GAP IN ACCESS

Athina Schmidt[1], Jennifer Sergile[1], Irina Vovnoboy[2], and Archana Asundi[2], *1. Boston Medical Center, Boston, Massachusetts, United States, 2. Boston University - Chobanian & Avedisian School of Medicine, Boston, Massachusetts, United States*

The BMC HIV-Endurance (HIVE) Clinic implemented a “pop-up” geriatric care model to address the reluctance of people living with HIV (PLWH) to leave their primary care settings for geriatric services. Patients reported concerns about losing trusted providers. While systemic challenges at our clinic required an increase to the geriatric referral age to 70. The integrated HIVE model introduced specialized geriatric expertise directly into primary care settings to improve access and improve healthcare outcomes. Despite proactive referrals that originated from the patient’s primary care provider with specific concerns to address, some patients still required guidance to grasp the rationale behind their referral to a geriatric specialist. Many expressed reluctance, perceiving such care as unnecessary and associating it with an outdated notion of aging, underscoring the need for targeted education on the benefits of specialized geriatric services In this paper presentation session, we describe the implementation of the HIVE Clinic and the strategies used to address barriers experienced by PLWH in accessing geriatric care. These strategies include enhanced communication, improved scheduling, and targeted patient education on aging-related needs to keep them safe and independent at home. Additionally, partnerships with local aging services, including visiting nurse associations (VNA), adult day health programs, and meal services, provided further support to patients. Results demonstrated improved healthcare proxy documentation, addressing polypharmacy concerns, expanded referrals, and strengthened care coordination. The HIVE Clinic’s innovative model successfully improved access to geriatric care, emphasizing the value of familiar care settings and targeted patient education to address gaps in HIV-aging care.

Abstract citation ID: igaf122.2682
### TRENDS IN COMMUNITY AND STATE RATES OF HIV/AIDS AMONG ADULTS 65+ IN NEW ENGLAND

Lien Quach, Taylor Jansen, and Elizabeth Dugan, *University of Massachusetts Boston, Boston, Massachusetts, United States*

HIV/AIDS is a significant public health concern in the U.S., particularly among aging populations. While New England has lower overall HIV/AIDS incidence, disparities persist among racial and ethnic minority group members, LGBTQ+ individuals, and those affected by the opioid epidemic. This study calculates community and state HIV/AIDS prevalence trends among adults aged 65+ across Massachusetts (MA), Connecticut (CT), Rhode Island (RI), New Hampshire (NH), Vermont (VT), and Maine (ME). Data sources included: the Centers for Medicare & Medicaid Services (2012-2021), the American Community Survey (2018-2022), and the CDC’s Behavioral Risk Factor Surveillance System (2010-2022) (see healthyagingdatareports.org). Small area estimation techniques were used to calculate age and sex adjusted rates. ArcGIS was used to map community rates across the region. Results found a steady increase in HIV prevalence across all six states from 2014-2015 to 2020-2021. In 2014-2015, MA (0.17%) and CT (0.18%) had the highest prevalence, while NH (0.05%) and VT (0.08%) were lower. By 2020-2021, the increase in older adults living with HIV/AIDS was most significant in MA (+0.13%) and CT (+0.1%), while ME (+0.08%), NH (+0.05%), and VT (+0.05%) experienced slower growth. These findings highlight geographic disparities in HIV/AIDS prevalence among older adults, with MA and CT showing the fastest increases. State-level factors such as healthcare access, socioeconomic conditions, and policies may contribute. New England’s healthcare infrastructure offers a model for policy-driven prevention. Further research is needed to inform targeted public health strategies and improve outcomes for aging populations infected with HIV/AIDS.

### SESSION 7250 (POSTER)

### LONG TERM CARE I

Abstract citation ID: igaf122.2683
### “IT ONLY HURTS WHEN I MOVE”: THE MULTIDIMENSIONALITY OF PAIN IN NURSING HOME RESIDENTS

Orah Burack[1], Wingyun Mak[2], Kreshnik Hoti[3], Jeff Hughes[3], and Kimberly Bergen-Jackson[4], *1. The New Jewish Home,*

*New York, New York, United States, 2. The New Jewish Home Research Institute on Aging, New York, New York, United States, 3. PainChek, Sydney, New South Wales, Australia, 4. University of Iowa, Iowa City, Iowa, United States*

Nursing home (NH) residents often experience chronic pain and increasing cognitive deficits. In such cases effective pain assessment by staff is critical as resident self-report becomes unreliable. A consideration for staff is recognizing variability in pain due to internal and external factors. Predictable changes in pain could then be leveraged to facilitate treatment. This study examined differences in NH residents' pain in two conditions, (1) at rest and (2) after everyday type movement. Subjects were 103 NH residents with moderate to severe cognitive impairment. Ages ranged from 64 to 107 years, 29% were males; 19% Hispanic; 54% White, 29% Black, and 17% race unknown. Ninety-five percent had chronic pain, and 85% took pain medication. Participants were assessed weekly for pain under two conditions: at rest and post-movement (walking, repositioning, or range of motion) by two raters independently, one administering the Abbey Pain Scale (APS) (0-18) and one the PainChek® tool (0-42). Higher scores indicated more pain. Order of conditions was randomly assigned per assessment. Subjects participated from one to ten weeks for a total of 552 assessments. Residents displayed significantly more pain symptoms post-movement than at rest ($p < .0001$) regardless of assessment tool or condition order (APS: post-movement $M = 4.7$ ($SD = 2.91$), at rest $M = 2.9$ ($SD = 1.82$); PainChek®: post-movement $M = 8.2$ ($SD = 3.92$), at-rest $M = 5.8$ ($SD = 2.71$)). Results suggest that pain levels are dynamic depending on daily activities. Determining treatment should incorporate pain assessments across different conditions. Ways to maximize the benefits of assessing pain under variable conditions will be discussed.

Abstract citation ID: igaf122.2684

### AGENCY STAFF UTILIZATION IN NURSING HOMES AND ASSISTED LIVINGS: PATTERNS AND CONCERNS

Patrick Mese, Kennedy Berner, and John Bowblis, *Miami University, Oxford, Ohio, United States*

Since 2020, long-term care providers have been faced with a shortage of direct care workers. As a result, many nursing homes (NH) and assisted livings (AL) have turned to staffing agencies. Agency staffing is believed to be associated with worse resident outcomes, but our understanding of how and why providers use agency staff, and the implications of their use is limited, especially in ALs. Using data from the 2023 Ohio Biennial Survey (> 90% response rate), NHs and ALs that utilized agency staff for nursing aides or personal care aides during the year were identified. Nearly 68% of NHs and 38% of ALs utilized agency staff. NHs and ALs are more likely to use agency staff for hard-to-fill weekend and overnight shifts (NHs: 77%, ALs: 60%), filling in for last-minute call-offs (78%, 67%), when an employee leaves employment (76%, 64%) or is vacationing (55%, 44%), and when there is an increase in occupancy (59%, 27%). Over 55% of NHs and 39% of ALs reported using agency staff as a substitute for a directly employed aide for over 4 weeks. The most common serious concern among administrators regarding agency staff are costs, lack of dedication to building, negatively impacting the morale of directly employed staff, inadequate time to know residents' care plans, and lack of knowledge of building policies and procedures. Investment in the long-term care workforce is necessary to reduce the use of agency staff. This study provides important insights that help direct these investments.

Abstract citation ID: igaf122.2685

### BEYOND TASKS: CARE WORKERS' PERSPECTIVES ON WHEN THINGS GO WELL IN A RETIREMENT COMMUNITY SETTING

Tina Kilaberia, and Daniel David, *New York University, New York, New York, United States*

Care work in long-term care is demanding. This study asked 44 care workers about their perspectives of successful interprofessional practices to elicit an assets-based approach for the retooling and retention of workforce. Care workers delivered care on a continuum in independent living, assisted living, and a skilled nursing facility to approximately 700 residents daily. All were asked to reflect on and give examples with regard to the question: "when things work well, why do you think that happens?" The sample included professionals such as nurses, social workers, chaplains, rehabilitation specialists, dieticians, directors; and paraprofessionals such as certified nursing assistants, certified medication assistants, and environmental services workers. Two coders independently analyzed data with inductive coding first. Consensus was reached describing common themes on organizational, peer-to-peer, and resident levels. On the organizational level, communication and coordination were substantiated as positive aspects. On the collegial level, shared ethos (good heart, kindness, team spirit) was influential. On the resident level, person-centered focus stood out. Subsequently, reflecting this coding deductively through the Integrated Team Effectiveness Model (ITEM) showed greater influence of the process elements of communication and coordination, and secondary influence of the related external context (regulatory framework, accountability to families). Task-related factors were not identified as influential to working well. This suggests that in residential settings, creating effective care environments may depend more on linking organizational processes, human factors, and a resident-focused culture as components of teamwork. This has implications for the approach to workforce development and retention in long-term care.

Abstract citation ID: igaf122.2686

### CLINICIANS PRIMARILY PRACTICING IN NURSING HOMES AND CARE QUALITY FOR RESIDENTS WITH DEMENTIA

Hyunkyung Yun[1], Mark Unruh[2], Yongkang Zhang[3], Yuting Qian[4], and Hye-Young Jung[2], *1. Brown University, Providence, Rhode Island, United States, 2. Joan and Sanford I. Weill College of Medicine, Cornell University, New York, New York, United States, 3. Weill Cornell Medicine, New York, New York, United States, 4. Yale School of Public Health, New Haven, Connecticut, United States*

Physicians and advanced practitioners (APs) who primarily practice in nursing homes (NHs), often called "SNFists," provide care in more than 50% of U.S. NHs. Little is known about SNFists' impact on care quality for long-stay NH residents with dementia. We estimated the association between receipt of care from a SNFist and care quality for these residents using claims for a nationwide sample of Medicare beneficiaries, 2013–2019. The intervention of interest was resident attribution to a SNFist based on a plurality of evaluation and management visits. SNFists included generalist physicians and APs. Estimation was based on a machine learning approach incorporating a doubly-robust procedure using a generalized estimating equation with inverse probability treatment weighting. We also conducted stratified analyses for physicians and APs, and a sensitivity analysis that included physicians of any specialty, and APs. Among the 417,378 residents in our sample, 58% (242,540) were attributed to SNFists. In adjusted analyses, attribution to a SNFist vs. a non-SNFist was associated with 7% lower odds of an ambulatory care sensitive (ACS) hospitalization (OR: 0.93; 95% CI, 0.90-0.96) and 7% lower odds of an ACS ED visit (OR: 0.93; 95% CI, 0.90-0.96). In stratified analyses, attribution to a physician SNFist vs. non-SNFist physician was associated with 13% lower odds (95% CI, 0.83-0.90) and 7% lower odds (95% CI, 0.88-0.97) of an ACS hospitalization or ED visit, respectively; AP-SNFist comparisons were not statistically significant. Results of the sensitivity analysis were consistent with primary results. SNFists may enhance care quality for residents with dementia.

Abstract citation ID: igaf122.2687

### CONTINUITY OF VISITING PHARMACIST SERVICES FOR OLDER ADULTS WITH AND WITHOUT TERMINAL CANCER IN JAPAN

Reina Taguchi[1], Akira Okada[2], Rumiko Tsuchiya-Ito[3], Satomi Kitamura[1], Tomoki Ishikawa[1], and Shota Hamada[1], *1. Institute for Health Economics and Policy, Association for Health Economics Research and Social Insurance and Welfare, Tokyo, Tokyo, Japan, 2. The University of Tokyo, Tokyo, Tokyo, Japan, 3. Dia Foundation for Research on Ageing Societies, Tokyo, Tokyo, Japan*

Homebound older adults often experience polypharmacy and complex medication regimens. Visiting pharmacist services, which support in-home medication management, have been reported to help optimize medication use, but little is known about their continuity. This study aimed to examine the continuity of visiting pharmacist service and reasons for cessation in a homebound older population in Japan stratified by terminal cancer status. This retrospective cohort study used medical and long-term care claims data from older adults aged ≥65 years in Hachioji-city of Tokyo, Japan, who began to receive visiting pharmacist services between April 2014 and March 2019. Participants were followed until service cessation (absence of service claims for two consecutive months) or 12 months from initiation. Reasons for cessation were categorized into death, relocation, hospitalization/institutionalization, or discontinuation. The study included 3,952 participants, with 353 (8.9%) having terminal cancer. The median time to any-cause cessation was 2 months for participants with terminal cancer, while 51.9% of those without terminal cancer remained event-free at study end. Death was the most common reason, but the 12-month cumulative incidence for participants with terminal cancer was more than four times higher. For individuals with terminal cancer, the service typically begins months before death, requiring pharmacists to adapt to condition changes. Those without cancer often use the service for over a year, necessitating routine medication review. These findings will help pharmacists tailor care to individual patient needs and expected service duration. They can also guide countries considering similar services in response to aging populations and healthcare shortages.

Abstract citation ID: igaf122.2688

### EVALUATING CARE INNOVATIONS: A RCT OF HOME TELEASSISTANCE FOR OLDER ADULTS IN NEED OF CARE IN COSTA RICA

Alexander Chaverri Carvajal, *Universitat Autònoma de Barcelona, Bellaterra, Barcelona, Spain*

Costa Rica faces increasing pressure to develop Long-Term Care services for dependent individuals. In 2022, the government enacted a law granting care rights to dependent older adults. However, a comprehensive nationwide network of residential, home-based, or teleassistance services has yet to be implemented. In 2024, the local government of Heredia initiated a pilot of home teleassistance program for dependent older adults, assessing their situation before and after service delivery. This study aimed to evaluate the impact of the home teleassistance pilot on four key areas: (a)improving quality of life for dependent individuals, (b)reducing unnecessary hospital visits, (c)promoting correct medication use, and (d)caregiver outcomes, including burnout and workforce participation among family caregivers. A randomized controlled trial (RCT) was conducted with 634 dependent older adults and their family caregivers. Participants were randomly assigned to receive home teleassistance services or remain in a control group. A structured questionnaire assessed the four dimensions outlined in the objectives. Home teleassistance improved quality of life of care recipients, reduced caregiver burnout, improved medication use and decreased hospital visits. Additionally, the program showed limited impact in enabling labor participation among caregivers. The findings underscore the potential of teleassistance programs to enhance Costa Rica's long-term care (LTC) system. However, it is important to note that LTC systems typically integrate such programs into a broader care framework that includes home-based and residential care services. These insights offer critical guidance for the design and expansion of LTC strategies in Costa Rica and similar aging societies.

Abstract citation ID: igaf122.2689

### FUTURE LONG-TERM CARE NEEDS FOR US ADULTS: PERCEPTIONS AND PLANNING FROM A NATIONAL SURVEY

Ana Montoya[1], Erica Solway[1], Matthias Kirch[1], Dianne Singer[1], Sydney Strunk[1], J. Scott Roberts[2], Jeffrey Kullgren[1], and Julie Bynum[1], *1. University of Michigan, Ann Arbor, Michigan, United States, 2. University of Michigan School of Public Health, Ann Arbor, Michigan, United States*

Many older adults require assistance with activities of daily living as they age. Assistance can be provided as long-term care services in the home, an assisted living facility, or a nursing home. This study aimed to understand US adults' perceptions of and planning for future long-term care needs, using a nationally representative survey of U.S. community-dwelling adults aged 50 and older (N = 3,486). Respondents were asked about their perceived future need for long-term care services, knowledge about long-term care payors, and engagement in long-term care planning. Survey findings indicated that 42.6% reported they were likely to require long-term care in the future. However, over half were not confident that they would be able to afford to pay for long-term care services (50.9% home care, 57.7% assisted living, 62.3% nursing home care). Additionally, 61.6% incorrectly thought Medicare would cover the cost of their long-term care in a nursing home. Another 29.4% indicated Medicaid would pay for it, and nearly half (49.6%) indicated they would pay for it from their own financial resources. Half (50.1%) of respondents indicated they had not undertaken the usual steps for long-term care planning; only 27.2% reported having appointed a durable power of attorney for healthcare, and 23.7% had identified potential future caregivers. Most adults indicated they could not afford long-term care services if needed, and half of them have not taken steps to plan for such care. Educating and supporting aging US adults in planning is necessary to prepare for their future long-term care needs.

Abstract citation ID: igaf122.2690

**IDENTIFYING PATTERNS AND CORRELATES OF UNMET CARE NEEDS: THE ROLE OF KINSHIP NETWORKS**

Yao-Chi Shih, and Chen-Fen Chen, *National Taipei University of Nursing and Health Sciences, Taipei, Taipei, Taiwan (Republic of China)*

Given the aging population and changing family structures, there has been increasing attention on whether adequate support is provided to older individuals in need of long-term care. This study examines how unmet care needs among older persons evolve over time and in what forms. Using data from seven waves of the Taiwan Longitudinal Study on Aging (TLSA), collected between 1996 and 2019, we employed group-based trajectory models to identify developmental patterns of unmet care needs. The study sample was limited to individuals aged 60 and older, focusing on unmet needs related to six activities of daily living (ADLs) and six instrumental activities of daily living (IADLs). Distinct trajectory patterns of unmet needs were identified, including relatively low, gradually increasing, and consistently high unmet needs over time. Structural kinship network did not appear to be as strong as emotional support in explaining unmet needs, controlling for health-related covariates. Despite the apparent need for care and low prevalence of unmet needs, older individuals still reported relatively low demands for additional care. These findings highlight the importance of considering the perspectives of older individuals in understanding unmet care needs. These findings highlight the importance of understanding unmet care needs in the perspective of older people. Potential behavior adjustments and withholding demands for care may carry different policy implications and inform future care strategies.

Abstract citation ID: igaf122.2691

**IS MEDICAID PAYMENT THE KEY TO RAISING STAFFING LEVELS IN NURSING HOMES? INSIGHTS FROM A FACILITY LEVEL ANALYSIS**

Edward Miller[1], John Bowblis[2], Elizabeth Simpson[1], and Marc Cohen[1], *1. University of Massachusetts Boston, Boston, Massachusetts, United States, 2. Miami University, Oxford, Ohio, United States*

Nursing staff levels are associated with nursing home (NH) quality, but NHs face substantial challenges recruiting and retaining nursing staff. Medicaid, which does not cover the cost of care, is the primary payer for approximately two-thirds of residents, constraining NHs from increasing staff. While past work has studied state-level Medicaid payment rates, there is little work examining the relationship between staffing levels and facility-level payments. This study utilized data from the Payroll-Based Journal data along with Medicaid payment rates collected from states to examine the association between Medicaid rates and staffing levels, and to determine if this association varied by ownership (i.e., for-profit, not-for-profit, government). The study population consisted of 9,513 freestanding NHs in 44 states in 2019. Average staffing levels were calculated for total nursing staff, as well as registered nurses, licensed practical nurses, and nurse aides. Regression results accounted for facility/resident characteristics (e.g., case-mix) and state-fixed effects. Across all ownership types, higher Medicaid rates were associated with higher staffing levels for all staffing outcomes, though average nursing staff levels are lower among for-profits and government owned NHs when compared to not-for-profits. Results also indicated that for-profit and not-for-profit NHs reacted to increases in Medicaid payment rates similarly (i.e., had higher staffing levels), whereas government NHs responded more strongly to payment increases. By raising Medicaid payment rates, policymakers can address staffing shortages by providing NHs with more financial resources, which in turn allows them to invest in additional nursing staff.

Abstract citation ID: igaf122.2692

**IT'S NOT YOUR PARENTS LONG-TERM SERVICES : A 30 YEAR LONGITUDINAL STUDY OF OHIO'S SYSTEM**

Robert Applebaum, Matt Nelson, and John Bowblis, *Miami University, Oxford, Ohio, United States*

Using data from a longitudinal study of Ohio's long-term services and supports system this paper documents the monumental system changes occurring over the last three decades. The study is based on a biennial survey of nursing homes and residential care facilities in the state (92% survey response rate) and records data on participants in Ohio's home and community-based waiver program. Findings from the study document an almost un-imaginal change in how long-term services are provided. As an example, Ohio with a politically powerful nursing home industry, was widely dependent on nursing home care for older people age 60 and older needing long-term services in the early 1990's. In 1993, nine in ten older people on Medicaid needing long-term services received such support in the nursing home setting. In 2023, with the expansion of home and community-based services, that proportion had dropped to four in ten. During this time period that state's population age 85 and older increased by more than 100,000, but the number of nursing home beds actually dropped by 12%. Additionally, occupancy rate declined from 92% in 1992 to 79% in 2023. The assisted living industry (licensed as residential care facilities in Ohio) grew dramatically during this time period, increasing from less than 200 in 1992 to more than 800 in 2023. The number of short-term admissions to nursing homes also increased dramatically, going from 30,000 in 1993 to 126,500 in 2023. The paper documents these and other changes and discusses what these changes mean for the future.

Abstract citation ID: igaf122.2693

**PARTICIPATION AND EQUITY IN LONG-TERM CARE: SYSTEMIC AND SOCIETAL PERSPECTIVES**

Sandra Staudacher, Nora Peduzzi, Séverine Soiron, Katja Jungo, Norina Reiffer, Alexandra Staehli, Andrea Kaiser-Grolimund, and Franziska Zuniga, *University of Basel, Basel, Basel-Stadt, Switzerland*

As global population aging increases, so does the demand for long-term care (LTC), particularly in care homes, assisted living, and home-based care. Many older people hesitate to enter care homes, fearing a loss of autonomy and participation in society. This presentation investigates how participation of older people in LTC in Switzerland is understood, discussed, and practiced, and analyzes potential inequities within the LTC system. As part of the Swiss National Science Foundation-funded

EPICENTRE-PARTICIPATIO project, we conducted an ethnographic analysis of participation across the LTC system in Switzerland, including care homes, home-based care, assisted living, public administration, associations, and civil society. Fieldwork consisted of observations, interviews, and document analysis to examine how decisions about and with older people in LTC are made. The study reveals how different actors in the LTC system and society conceptualize participation, and where it is practiced, missed, or resisted. Findings highlight systematic inequalities in participation between social groups, shaped by factors such as cognitive or physical impairment, gender, and migration background. Socio-cultural, political, and economic contexts significantly influence participation. Addressing these disparities requires a holistic approach, including a differentiated understanding of LTC populations and consideration of external factors like policymaking and financial constraints. Applying the concept of equity provides a useful lens for LTC research on participation, emphasizing the need to address inequalities both within LTC and compared to other social groups.

Abstract citation ID: igaf122.2694

### PREDICTORS OF LIFE SATISFACTION AMONG OLDER ADULTS IN LONG-TERM CARE SETTINGS

Xiaoli Li[1], Cheng Yin[2], and Elias Mpofu[2], *1. Southern Illinois University, Carbondale, Illinois, United States, 2. University of North Texas, Denton, Texas, United States*

Resident satisfaction is a critical indicator of the quality of care in long-term care facilities. Yet, the relative importance quality of care factors in predicting resident satisfaction remains unclear for guiding resident support initiatives. This study aims to identify the predictors of satisfaction LTCF residents. This cross-sectional study enrolled a sample of 399 older adult residents from LTCFs in Shanghai, Nanjing, and Changsha, China. Hierarchical multiple regressions were utilized to examine self-report survey data on ten resident personal variables, and seven care facility service domains were examined for their relative contribution to resident care satisfaction. Moreover, the analysis included the interaction between personal factors and care service attributes.

Results: The combined personal and care service factors explained 26.1% of the variance in overall resident satisfaction. Personal factors that predicted resident satisfaction included age, level of independence, and length of stay ($\Delta R2 = 0.11$). Of the care facility factors, the significant predictors of higher resident satisfaction were spending time ($\beta = 0.60$, $p < 0.01$, $\Delta R2 = 0.09$) and environment domains ($\beta = 0.62$, $p < 0.01$, $\Delta R2 = 0.03$). Age moderated the relationship between the spending time domain and overall satisfaction, with a positive effect for residents aged 70–79 compared to those aged 60–69 ($\beta = -1.26$, $p < 0.05$). Conclusions: This study provides evidence to suggest the importance of personal and care facility characteristics to LTFC resident satisfaction. Based on the findings, improved resident satisfaction is likely with LTCF care services that provide tailored care plans using resident characteristics.

Abstract citation ID: igaf122.2695

### TEACHING FUTURE NURSES TO SUPPORT OLDER CLIENTS' AUTONOMY IN ACTIVITIES OF DAILY LIVING

Marjolein Knibbeler[1], Stan Vluggen[2], Erik van Rossum[2], Sandra Zwakhalen[1], and Petra Erkens[1], *1. Maastricht University, Maastricht, Limburg, Netherlands, 2. Zuyd University of Applied Sciences, Heerlen, Limburg, Netherlands*

Supporting older clients' autonomy in daily care is crucial for dignity and quality of life. However, research indicates that nurses often provide suboptimal autonomy-support, and little is known about how future nurses learn this skill during their education. A qualitative study examined both theoretical and practical learning. The theoretical part involved a document study of Dutch nursing curricula and teaching materials to assess how autonomy-supportive behaviour is addressed. Additionally, 20 semi-structured interviews with teachers and students explored their perspectives on autonomy in education. Practical learning was studied through 25 observations of student-supervisor interactions during activities of daily living. Furthermore, 22 follow-up interviews with students and supervisors provided deeper insights into their experiences. Findings indicate that while curricula mention autonomy, they provide little practical guidance. Teachers acknowledge its importance but rely largely on personal experience rather than structured educational methods. Students report learning autonomy-supportive behaviour primarily through hands-on training. Observations confirm that autonomy is more emphasized in practice, yet supervisors' approaches vary widely. Some prioritize autonomy, fostering client independence, while others emphasize efficiency, sometimes at the cost of client-autonomy. Given the evolving needs of an aging population, rethinking nursing education on autonomy is essential. Strengthening collaboration between teachers and practical supervisors can create a more cohesive learning environment. Additionally, integrating innovative teaching methods, such as simulation-based learning and case discussions, can bridge the gap between theoretical and practical education. Enhancing nursing education will ensure future nurses are well-equipped to support autonomy, ultimately improving person-centered elderly care.

Abstract citation ID: igaf122.2696

### THE ELEMENTS OF THE DIGNITY OF CARE STAFF WHO WORK IN LONG TERM CARE FACILITIES

Nanako Hasegawa[1], and Cynthia Jacelon[2], *1. Aichi Prefectural University, Nagoya, Aichi, Japan, 2. University of Massachusetts Amherst, Amherst, Massachusetts, United States*

There is limited research on the dignity of care staff, however, the dignity of care staff may be influenced by the work environment, the staff's personal thoughts, and care experiences with patients. In turn, the care staff's dignity is related to the delivery of dignified care. The inability to provide dignified care undermines the dignity of care staffs, and hurts their health. The purpose of this study was to clarify the elements of the individual dignity of care staff who work in long term care facilities. We integrated the findings of a systematic review focused on the individual dignity of staff with the findings of 18 hermeneutic phenomenological interviews of care staff in care at a long term care facility into a previously developed concept matrix of dignity. A total of 4242 titles were obtained from MEDLINE, Web of Science, and CINAHL using the keywords "Dignity," "Respect," and "Staff." Based on predefined criteria, eighteen ($n = 18$) articles were selected for review. The concept matrix of dignity in care was used to classify the types of staff dignity according to their characteristics. The integrated findings of the care staff interviews, and systematic review indicated the dignity of care staff could be characterized by three themes: 'The professional dignity,' 'Dignity in community,' and 'The individual dignity of the staff not affected by others.' The specific elements of dignity for care staff represent the individual and their interactions with patients, other staff, the organization and society, providing clues for bringing dignified care into practice.

Abstract citation ID: igaf122.2697

**THE IMPACT OF PERSON-CENTERED COMMUNICATION EDUCATION ON NURSING HOME REGULATORY SURVEY OUTCOMES**

Kristine Williams[1], Clarissa Shaw[2], Shelby Cooper[3], Maria Hein[4], Yelena Perkhounkova[4], Carissa Coleman[5], Frances Yang[6], and Maria Roche-Dean[7], *1. University of Kansas, Kansas City, Kansas, United States, 2. University of Iowa College of Nursing, Iowa City, Iowa, United States, 3. University of Kansas, Lawrence, Kansas, United States, 4. University of Iowa, Iowa City, Iowa, United States, 5. University of Kansas Medical Center, Kansas City, Kansas, United States, 6. University of Kansas, University of Kansas Medical Center, Kansas, United States, 7. Kirkhof College of Nursing, Grand rapids, Michigan, United States*

Elderspeak is a form of infantilizing communication commonly used with older adults in nursing homes. This study evaluated the impact of the Changing Talk Online training (CHATO) on standard nursing home annual health inspector surveys in a multi-state pragmatic clinical trial. CHATO is an asynchronous online intervention educating nursing home staff person-centered communication skills through the reduction of elderspeak. Annual health survey citations were examined for 14 F-tag deficiency categories related to resident rights, quality of care, and quality assurance one year before and one year after study participation. Sixty-six of the 142 participating nursing homes had health standard inspections both before and after the study period (intervention n = 31, control n = 35.) Prior to the study period, nine control nursing homes received 13 citations, and six intervention nursing homes received seven citations, which was not significantly different at the nursing home level (Fisher's exact test, p=.770). Following the study period, 11 control nursing homes received 14 citations, and two intervention nursing homes received three citations, which is significantly different at the nursing home level (Fisher's exact test, p = 0.015). These preliminary findings suggest that CHATO communication training may help nursing homes focus on improving person-centered care and reduce related health inspection deficiencies. While the trial's primary outcomes focus on resident-centered measures from Minimum Data Set (MDS) data that directly reflect the resident's well-being, these deficiency findings may be particularly compelling for nursing home corporations and owners given their connection to financial penalties and regulatory compliance.

Abstract citation ID: igaf122.2698

**THE PREVALENCE AND IMPACT OF VACCINATION PROGRAMS: DIFFERENCES BETWEEN NURSING HOMES AND ASSISTED LIVINGS**

Kennedy Berner, Patrick Mese, and John Bowblis, *Miami University, Oxford, Ohio, United States*

Vaccinations are a critical component in public health for preventing infectious disease outbreaks, particularly in high-risk populations living in congregate settings. Vaccinations are an important tool to protect this population; however, we have limited knowledge about vaccination programs in nursing homes (NH) and assisted livings (AL). This study utilized a unique survey of all NHs and ALs in Ohio (response rate > 90%) – the 2023 Ohio Biennial Survey of Long-Term Care Facilities – to understand the prevalence of vaccination programs and whether having a vaccination program resulted in fewer hospitalizations and outbreaks that led to the suspension of new admissions. Vaccination programs were more prevalent in NHs than ALs for flu (100% vs. 98.8%), COVID-19 (99.5% vs. 93.3%), RSV (76.7% vs. 50.5%), pneumococcal (99.6% vs. 85.0%), Tdap (58.6% vs. 37.7%), shingles (72.3% vs. 46.7%), and hepatitis B (80.9% vs. 55.8%). NHs and ALs with a vaccination program reported that residents were less likely to be hospitalized for that condition. Fewer than 5 NHs and ALs (< 0.5%) suspended admissions due to flu or RSV outbreaks, however, 9.6% of NHs and 6.9% of ALs suspended admissions due to COVID-19. Despite facing similar risks from vaccine-preventable illnesses, ALs were less likely than NHs to have vaccination programs. These programs reduce the risk of hospitalization and disruption of admissions. Furthermore, most vaccinations programs focus on respiratory diseases. Providers and policymakers may want to place greater emphasis on having programs for all vaccine preventable illnesses.

### SESSION 7255 (POSTER)

### MENTAL HEALTH I

Abstract citation ID: igaf122.2699

**ANXIETY AND DEPRESSED MOOD IN OLDER PATIENTS PERI-HOSPITALIZATION: THE ROLE OF PHYSICAL SYMPTOMS AND SLEEP QUALITY**

Juliana Smichenko, Anna Zisberg, and Tamar Shochat, *University of Haifa, Haifa, HaZafon, Israel*

Anxiety and depression are common among older adults affecting independence and well-being. However, their trajectories during the peri-hospital period remain unclear. This study examined changes in anxiety and depression from admission to discharge and one-month post-discharge, focusing on the roles of physical symptoms burden and sleep quality, and controlling for sedative-hypnotic medications burden. Data were analysed from the Hospitalization Process Effects on Mobilization Outcomes and Recovery (HoPE-MOR) study, including adults aged 65+ hospitalized in acute medical units. Assessments occurred at admission, discharge, and one-month post-discharge. A two-stage modelling approach examined mood changes from admission to discharge in 683 patients; and (out of them) from admission to post-discharge in 545 patients, controlling for demographic and baseline characteristics. Patients were aged 77.31±6.67, with 55% males. Results indicated worse anxiety at post-discharge compared to admission and discharge, and a decline in mood from admission through post-discharge. Poorer sleep quality, increased physical symptom burden (B(exp)anx=.38,p <.001;B(exp)dep=.043,p=.02), and sedative-hypnotic medication (SHM) burden (B(exp)anx=0.512,p <.001) significantly predicted anxiety and depression during and after hospitalization. Sleep quality effects varied by timepoint: poorer sleep was linked to increased anxiety post-discharge (F(2,879)=7.52,p <.001) and greater depressive symptoms during hospitalization (F(2,874)=7.99,p <.001). Additionally, higher SHM burden was associated with worsened depressive mood at discharge (F(2,874)=4.80, p<.01). Findings underscore the importance of addressing anxiety and depression during hospitalization, emphasizing the need to improve sleep quality, manage physical symptoms, and regulate SHM use. Future research should explore longer follow-up periods and modifiable factors to enhance mental well-being throughout hospitalization and recovery.

Abstract citation ID: igaf122.2700

**BEHAVIORAL HEALTH AS A KEY COMPONENT OF INTEGRATED CARE FOR DUALLY ELIGIBLE MEDICARE/MEDICAID BENEFICIARIES**

Pamela Nadash, Janelle Fassi, Elizabeth Simpson, Calvin Tran, and Marc Cohen, *University of Massachusetts Boston, Boston, Massachusetts, United States*

The integration of behavioral health into managed care programs for dually eligible individuals is understudied. Behavioral health has traditionally been siloed into separate managed care plans: only recently has policy encouraged a shift toward integration. This presentation discusses findings from a qualitative study of an innovative managed care plan, Commonwealth Care Alliance, which serves younger and older dually eligible members in separate managed care plans. Each plan enrolls high proportions of individuals with serious and persistent mental illness (SPMI): in the program serving people 18-65, 52% have an SPMI diagnosis and 35% have SUD (substance use disorder). In the older adult program, 19% have an SPMI diagnosis and 13% have SUD. Using data from 22 key informants (primarily program staff) and 41 plan members, this study analyzes program elements specific to behavioral health and member experiences. It finds behavioral health expertise woven throughout the program, along with a holistic care focus: most importantly, members with SPMI/SUD are likely to have specialized behavioral health care coordination. The plan works closely with community-based mental health providers, which operate as health homes for some members; the plan also utilizes crisis stabilization units to reduce admissions to psychiatric hospitals and integrated mobile health for crisis interventions. Members report high levels of satisfaction with their access to and quality of behavioral healthcare providers, and appreciate the plans' support for food, housing, and transportation, which enables consistent participation in treatment. Reported dissatisfaction stems from poor communication with plans, particularly due to high staff turnover.

Abstract citation ID: igaf122.2701

**BEYOND MY CONTROL: THE ROLE OF SENSE OF CONTROL ON COGNITIVE FUNCTIONING AND DEPRESSIVE SYMPTOMS IN OLDER ADULTS**

Sabine Lohmar, and Amy Fiske, *West Virginia University, Morgantown, West Virginia, United States*

Depression and depressive symptoms commonly co-occur with mild cognitive impairment (MCI) and dementia in older adults (Ismail et al., 2017; Panza et al., 2010), and may occur for various reasons, including as an emotional response to cognitive decline (Bennett & Thomas, 2014; Richard et al., 2013). Emotional responses may be explained by a decline in sense of control, or the belief in one's ability to influence their environment to achieve desired outcomes, which declines with age as cognitive and physical abilities change and limit independence (e.g., Lachman & Weaver, 1998). Using data taken from years 2006 to 2018 of the Health and Retirement Study (HRS), a mixed model regression indicated that cognitive functioning on the Modified Telephone Interview for Cognitive Status (TICS-27) was significantly associated with depressive symptoms on the Center for Epidemiologic Studies Depression (CES-D-8), $\beta = -.02$, $t(45,163) = -5.65$, $p < .001$, $R2 = .49$. Further, TICS-27 in 2016 predicted CES-D-8 in 2018, and this relation was mediated by sense of control in 2018, measured using the subscale of the Midlife Development Inventory, while controlling for demographic variables, $\beta = -.02$, SE = .004, 95% CI [-.03, -.01], $\Delta$ R2 = .11. The findings of this study confirm that cognitive function predicts depressive symptoms and highlight the importance of sense of control in explaining this emotional reaction. These findings have critical implications for preventing and treating depressive symptoms in older adults, particularly those with signs of cognitive dysfunction.

Abstract citation ID: igaf122.2702

**DECLINES IN PERCEIVED CONTROL AND SUBSEQUENT PASSIVE SUICIDAL IDEATION AMONG OLDER ADULTS**

Kallisse Dent, and Leah Richmond-Rakerd, *University of Michigan, Ann Arbor, Michigan, United States*

Perceived control tends to decline in older adulthood, while suicide rates tend to increase. More information is needed about how declines in perceived control may shape suicide risk over time. In the present study, we tested whether declines in perceived control were longitudinally associated with passive suicidal ideation (PSI). As suicide rates tend to be highest among older white men, we also tested effect modification by demographic factors. Data were from the Health and Retirement Study, a nationally-representative longitudinal cohort of adults aged 51+. Decline in perceived control was measured across a 4-year period (2010-2014 or 2012-2016) using 10 items that indexed perceived constraints on personal control and perceived mastery. Passive suicidal ideation was assessed using the Composite International Diagnostic Interview-Short Form in the subsequent wave (2016 or 2018). Logistic regression was used to test associations of decline in perceived control with PSI, with interaction terms used to test effect modification by age, sex, race, ethnicity, and education. Analyses accounted for complex survey design and adjusted for demographics, prior psychiatric diagnosis, and PSI at baseline. Preliminary data analyses found that 43.6% of older adults experienced a decline in perceived control across a 4-year period. Older adults who experienced a decline in perceived control were at higher risk for PSI (OR = 1.79, 95% CI: 1.37-2.32). There was no significant effect modification by demographic factors. Declines in perceived control precipitate passive suicidal ideation. Perceived control may be an important target for suicide prevention among older adults.

Abstract citation ID: igaf122.2703

**DEPRESSION IN OLDER ADULTS: LINKING DAILY LIVING IMPAIRMENT AND SOCIOECONOMIC STATUS**

Zeynep Abul[1], Sakeena Raza[2], and Lynn McNicoll[2], *1. Northeastern University, Boston, Massachusetts, United States, 2. The Warren Alpert Medical School of Brown University, Providence, Rhode Island, United States*

Depression is a significant public health problem among older adults. We aimed to investigate the association between depression, socioeconomic status, and activities of daily living (ADLs) in Health and Retirement Study 2018 data.A logistic regression analysis was conducted (n = 8,437). The dependent variable was an answer to a question about feeling depressed in the past week. Independent variables included education, ADL difficulties, gender, marital status, age, race/ethnicity, and household income. The model controlled for Hispanic ethnicity and racial categories, with appropriate reference groups. Study population includes 55% female, mean age of 75, 76% white individuals. ADL difficulties were associated with increased odds of depression. Each additional ADL limitation significantly increased odds of depression, with coefficients rising from $\beta = 0.87$ (1 ADL) to $\beta = 1.96$ (5+ ADLs), ($p < 0.001$). Females had higher odds of depression than males ($\beta = 0.23$, $p = 0.004$). Marital status like separation ($\beta = 0.99$ $p < 0.001$) or divorce ($\beta = 0.38$, $p = 0.002$) significantly increased depression. Hispanic ethnicity was associated with higher odds of depression compared to non-Hispanics ($\beta = 0.58$, $p < 0.001$). Lower education levels are linked to higher depression rates. Odds decrease as education increases: $\beta$=-0.32 for high school, $\beta$=-0.47 for some college, and $\beta$=-0.81 for college and above. Individuals with higher income are less likely to report depression ($\beta$ = - 0.40, $p < 0.001$). Older individuals are less likely to report depression ($\beta$ = -0.012, $p = 0.02$).Depression in older adults is associated with functional, social, and demographic factors in a complex way.

Abstract citation ID: igaf122.2704

### DEPRESSION/ANXIETY IN HOMEBOUND OLDER ADULTS: EFFECTS OF LONELINESS AND PSYCHOLOGICAL WELL-BEING

Angelina Gutierrez[1], Kelly Vences[1], Brian Fons[2], and Namkee Choi[2], *1. The University of Texas School of Social Work, Austin, Texas, United States, 2. The University of Texas at Austin, Austin, Texas, United States*

The prevalence of depression/anxiety, loneliness, and psychological distress is much higher among homebound older adults than among their non-homebound peers. In this study, we examined the direct effect of a homebound state (defined as never/rarely going outside the home in the preceding month) on depressive/anxiety symptoms and the mediation effect of loneliness and psychological well-being (assessed with purpose in life, meaning in life, confidence, desire to improve life, and satisfaction with living situation) on the associations between homebound state and depressive/anxiety symptoms. Data came from the 2023 National Health and Aging Trend Study (N = 7,447 community-dwelling Medicare beneficiaries age 65+). Results from a path model showed that depressive/anxiety symptoms were positively associated with the homebound state (B = 0.78, SE = 0.22, t = 3.54, p=.001) and loneliness (B = 0.63, SE = 0.05, t = 12.59, p<.001), but negatively associated with psychological well-being (B=-0.43, SE = 0.03, t=-12.36, p<.001). Bootstrapped results show a significant indirect effect of a homebound state on depressive/anxiety symptoms through loneliness (0.13, 95% CI = [0.03, 0.24], z = 2.47, p=.014) and psychological well-being (0.28, 95% CI = [0.18, 0.37], z = 5.62, p<.001). The ratio of the indirect effect of loneliness on a homebound state to the total effect of a homebound state on depressive/anxiety symptoms (indirect effect [0.13] + direct effect [0.78]=0.92) was 0.15. The ratio of the indirect effect of psychological well-being on a homebound state to the total effect of a homebound state on depressive/anxiety symptoms (indirect effect [0.28] + direct effect [0.78]=1.06) was 0.26. Interventions to decrease loneliness and improve psychological well-being among homebound older adults are needed to alleviate their depression/anxiety.

Abstract citation ID: igaf122.2705

### EXPLORING THE PSYCHOMETRIC PROPERTIES OF A CUMULATIVE ADVERSITY ASSESSMENT IN A DIVERSE SAMPLE OF ADULTS

Samantha Corley[1], Frank Mann[2], and Stacey Scott[1], *1. Stony Brook University, Stony Brook, New York, United States, 2. Renaissance School of Medicine at Stony Brook University, Stony Brook, New York, United States*

Cumulative adversity, defined as the accumulation of adverse life events across a lifetime, has been associated with worse mental and physical health. Black and Hispanic Americans exhibit increased risk for exposure to adverse experiences, however, these groups have been underrepresented in the development of cumulative adversity assessments. This study aimed to explore the prevalence of adverse life events and the psychometric properties of a cumulative adversity assessment, originally posited by Turner, Wheaton, and Lloyd (1995), in a racially and ethnically diverse sample (N = 260). Factor analysis models were estimated and compared, uncovering a two-factor solution, encompassing unintentional and intentional trauma. Prevalence rates of cumulative adversity were relatively low in the sample, with specific events surrounding parents and spouses being the most common. Significant associations between demographic characteristics and these factors were identified through Weighted Least Squares (WLS) regression analyses, shedding light on race/ethnicity and gender disparities for adversity, as well as relationships with age, household composition, work, and marital status. Findings contribute to a deeper understanding of cumulative lifetime adversity, providing insights by increasing the applicability of the lifetime adversity measurement in diverse populations in future studies.

Abstract citation ID: igaf122.2706

### IMPACT OF COLD WAVES ON COGNITIVE FUNCTIONS IN OLDER ADULTS: A MIXED-METHODS STUDY IN EASTERN CHINA

Qingwei Wang, Yuewen Wu, and Yue Zhou, *Xi'an Jiaotong-Liverpool University, Suzhou, Jiangsu, China (People's Republic)*

Background: As global temperatures become more unpredictable, extreme weather like heat waves and cold waves are increasingly common, creating serious health risks for older adults. Given many studies have focused on the effects of heat waves on mortality, the impact of cold waves on cognitive well-being remains unexplored. Methodology: This study employs a mixed-methods approach to examine the impact of cold waves on cognitive performance in older adults. The quantitative analysis explores correlations using data from 3,970 individuals aged 60+ in Jiangsu, Zhejiang, and Shanghai from the China Health and Retirement Longitudinal Study (CHARLS). To further identify and examine causative factors, the qualitative component includes semi-structured interviews with 11 older adults at Shanghai Yangjing Daycare Center, supplemented by observations and video recordings. By first establishing statistical correlations and then exploring causative factors through qualitative insights, this study offers a methodologically innovative and comprehensive understanding of cold waves' effects on cognitive well-being. Findings: Two studies highlighted the key roles of medical insurance, social engagement, and exercise in reducing cognitive decline among older adults during cold waves. The qualitative component further identified indoor exercise as an effective coping strategy and emphasizing the need for widely covered heating infrastructure, offering valuable insights for policy interventions despite some inconsistencies in individual responses. Conclusion: This study reveals the negative impact of cold waves on older adults' cognitive function, urging interventions to properly overcome the shortage of heating, reduced social interactions and outdoor exercises during cold waves, and finally, building their body resilience to non-optimal temper atures.

Abstract citation ID: igaf122.2707

### LONGITUDINAL ASSOCIATIONS BETWEEN FATALISM DIMENSIONS AND DIFFERENT AREAS OF HEALTH AND WELL-BEING

Masahiro Toyama, and Chloe Hooper, *Marshall University, Huntington, West Virginia, United States*

Previous research overall suggested that fatalism, closely related to external locus of control, had negative health implications. However, as older adults tend to have more uncontrollable experiences, fatalism may serve as a protective factor. For example, our previous cross-sectional research for older adults indicated that the fatalism dimension of ineluctable destiny (i.e., beliefs in fate) was associated with lower depression after controlling for helplessness as another fatalism dimension. The present study examined longitudinal associations between fatalism dimensions and different areas of health and well-being to address their directionality. Among 476 participants aged 65 + (M = 70.0, SD = 4.4; 62% female) recruited via an online crowdsourcing platform (Prolific) and included in our previous research (at Time 1 [T1]), 396 completed our six-month follow-up study (at Time 2 [T2]). We analyzed a path analysis model addressing both directions of associations: from five T1 fatalism dimensions to T2 depression, physical functioning, and purpose in life; and from T1 health/well-being measures to T2 fatalism dimensions. For each T2 outcome, its T1/baseline levels and other covariates were controlled for. The results showed good model fit indices with a robust maximum likelihood estimation; ineluctable destiny predicted lower depression over time, while depression did not predict ineluctable destiny and none of the other fatalism dimensions predicted any health/well-being outcomes. These longitudinal findings highlight the positive implications of beliefs in fate for (lower) depression. The present study contributes by adding nuance to the fatalism-aging literature and suggests future research directions (e.g., intervention studies increasing certain fatalistic beliefs).

Abstract citation ID: igaf122.2708

### MENTAL HEALTHCARE ACCESS AMONG OLDER SEXUAL MINORITY WOMEN WITH DISABILITIES

Jordan Westcott[1], Matthew Fullen[2], and Alexis Isaac[2], *1. University of Tennessee at Knoxville, Knoxville, Tennessee, United States, 2. Virginia Tech, Blacksburg, Virginia, United States*

Sexual minority older adults experience health disparities relative to cisgender, straight older adults, including related to mental health and disability (Fredriksen-Goldsen et al., 2013). This may be especially true for older sexual minority women, who experience disparities both relative to the general older adult population and to older sexual minority men (Fredriksen-Goldsen et al., 2017). Despite preliminary evidence of greater mental health needs among this population, little is known about mental healthcare (MHC) access for older sexual minority women with disabilities. Therefore, this study sought to understand how supply- and demand-side factors influenced MHC access among a sample of sexual minority women aged 55 and older who self-identified as having a disability (n = 103). Controlling for mean-centered age, we found that, when supply- and demand-side factors were collapsed into single variables, only supply-side factors predicted MHC access, R2 = .251, F(4, 98) = 8.19, p < .001. In a second analysis focused on unique demand-side factors, we found that availability (b = -.40, p = .015) and accommodation (b = .45, p = .013) of services had significant relationships with MHC access, R2 = .302, F(7, 95) = 5.88, p < .001. Finally, we found that, among demand-side factors, sexual orientation-related healthcare stereotype threat had a significant relationship (b = -.33, p = .002) with MHC access, R2 = .168, F(5, 97) = 3.94, p < .01. Implications for MHC access and service delivery will be discussed.

Abstract citation ID: igaf122.2709

### OLDER ADULTS MAKE SENSE OF THEIR SUICIDAL BEHAVIOR: A SWEDISH INTERVIEW STUDY

Sara Hed[1], Anne Ingeborg Berg[1], Stefan Wiktorsson[1], Jennifer Strand[1], Silvia Sara Canetto[2], and Margda Waern[1], *1. University of Gothenburg, Gothenburg, Vastra Gotaland, Sweden, 2. Colorado State University, Fort Collins, Colorado, United States*

This study explored how older adults (70+) understood a recent suicidal act, and what changed in the aftermath. Four women and five men (71-91 years) receiving care at a geriatric psychiatric outpatient clinic in Sweden took part in two interviews. Most of the women and none of the men had engaged in prior suicidal acts. Interpretative phenomenological analysis was employed. The suicidal act was explained as a response to losses in physical and cognitive functions, social roles and relationships that rendered previous coping strategies unviable. Participants reported being dependent on a healthcare system that they experienced as indifferent and even dismissive of their suffering. The suicidal act was described as an unplanned act of despair. Positive changes followed for participants who reported having had suicidal ideation prior to the suicidal act and had insights into its triggers. Some gained access to needed medical care; others developed greater awareness of their psychological needs and improved coping skills. Individuals who said that they had not had suicidal thoughts prior to the suicidal act and could not explain it reported no positive change in the aftermath. Participants' age-related losses were in many cases exacerbated by negative interactions with health care providers, indicating that continued attention needs to be given to implicit ageism in medical professionals. The suicidal acts were described as impulsive, which was unexpected because a dominant belief is that older adult suicidal behavior is planned. Older adult suicide planning should be addressed in larger studies across geographical and cultural settings.

Abstract citation ID: igaf122.2710

### PSYCHOTROPIC MEDICATION TRENDS BEFORE, AFTER SUICIDE ATTEMPT AMONG MID-TO LATE LIFE US ADULTS WITH BIPOLAR DISORDER

Randall Kuffel[1], Ramin Mojtabai[2], Michael Steinman[3], Matthew Growdon[3], Yixia Li[4], John Boscardin[3], G Caleb Alexander[1], and Amy Byers[5], *1. Johns Hopkins Bloomberg*

*School of Public Health, Baltimore, Maryland, United States, 2. Tulane University School of Medicine, New Orleans, Louisiana, United States, 3. University of California, San Francisco, San Francisco, California, United States, 4. Northern California Institute for Research and Education, San Francisco, California, United States, 5. University of California, San Francisco / SFVAHCS, San Francisco, California, United States*

It is unclear how medication regimens change following a non-fatal suicide attempt in mid-to late life adults with bipolar disorder. We characterized psychotropic medication use among these patients 1 year before and after a suicide attempt. Using a retrospective cohort of US veterans ages 50 and older with bipolar disorder, we matched patients attempting suicide between October 2011 and December 2019 to similar patients who did not attempt suicide. We measured quantity of fills and prevalence of use for antidepressants, antiepileptics, antipsychotics, benzodiazepines, lithium, opioids, sedative hypnotics, and polypharmacy in the 4 quarters before and after suicide attempt. Our final sample included 4,747 patients who attempted suicide and 4,747 matched controls. Mean age at index was 60 and 86% were male. At baseline, individuals attempting suicide were treated with antidepressants (60.0%), antiepileptics (48.0%), antipsychotics (41.2%), benzodiazepines (27.1%), lithium (5.4%), opioids (32.8%), and sedative hypnotics (14.9%). Many patients were on three or more psychotropic therapy classes at baseline (overall 30.8%). Psychotropic medication use was consistently greater among those attempting suicide than their matched counterparts for all medication classes except opioids. After their suicide attempt, there were large and abrupt increases in antiepileptics, antipsychotics, and sedative hypnotics, with smaller increases in antidepressants, benzodiazepines, and lithium, and reductions in opioids. These differences persisted up to six months following suicide attempt. Findings indicate patients and clinicians may make comprehensive changes to treatment plans in the short and long term. Further research is needed to understand the impact of these changes on future suicide risk.

Abstract citation ID: igaf122.2711

### RELATIONSHIPS BETWEEN DIFFICULTY WITH HEALTHCARE SERVICES AND PSYCHOSOCIAL ADJUSTMENT IN HISPANIC OLDER ADULTS

Atami De Main[1], Daphne Buitron[1], and Irina Mindlis[2], *1. Weill Cornell Medicine, New York, New York, United States, 2. Rutgers University, New York, New York, United States*

Living with multiple chronic conditions (MCC) is now the norm for older adults in the U.S. (77%), and requires adjustments across multiple life domains, including maintaining quality of life (QOL) and protecting mental health. Among older adults with MCC, depressive symptoms and poor QOL increase risk for morbidity and mortality. Hispanic/Latino older adults are more likely to sustain early onset of MCC, report poor self-rated health, and experience significant difficulties accessing health care. This study aimed to investigate the relationships between difficulties accessing healthcare services and psychological adjustment, specifically depressive symptoms and QOL, among Hispanic/Latino older adults. Data were collected from 118 participants (ages 62-92; Mean=73.6, SD = 6.33) at senior centers in New York City between July 2024 and February 2025. The sample was diverse, with 42.9% identifying as Puerto Rican, 21.4% as Dominican, and 20.4% as South American. Common chronic conditions reported included hyperlipidemia (70.7%), arthritis (65.2%), hypertension (64.1%), diabetes (51.1%), and asthma (30.4%). The results of the regression analysis indicated that poorer health status and difficulties with healthcare services were significantly associated with worsened QOL controlling for demographic factors ($R2adj=0.35$, $F(9, 83) = 5.25$, $p<.001$). In contrast, high depressive scores did not significantly correlate with difficulties accessing healthcare services ($R2adj=0.11$, $F(9, 83) = 0.99$, $p = 0.45$). These findings underscore the critical role of healthcare access in promoting the mental health and well-being of Hispanic/Latino older adults with MCC, highlighting the need for further research into the mechanisms linking MCC to poor psychosocial adjustment.

Abstract citation ID: igaf122.2712

### RELIGIOUS ATTENDANCE AND DEPRESSIVE SYMPTOMS: THE MEDIATING ROLE OF CHURCH-BASED SOCIAL TIES

Mingui Gao, and Hui Liu, *Purdue University, West Lafayette, Indiana, United States*

This study used longitudinal data from the Health and Retirement Study (HRS) to examine whether church-based social ties (e.g., friendships, relatives in the congregation) mediate the relationship between religious attendance and depressive symptoms among older adults. Three hypotheses were tested: 1) Increased religious attendance is associated with fewer depressive symptoms, 2) Greater church-based social ties are linked to fewer depressive symptoms, and 3) The relationship between religious attendance and depressive symptoms is mediated by church-based social ties. Depressive symptoms were measured using an eight-item CES-D scale, and four waves of data (2010, 2012, 2014, and 2016) were analyzed using Ordinary Least Squares, Logit regressions, and Lagged Dependent Variable models. Findings suggest that more frequent religious attendance in 2010 was associated with fewer depressive symptoms in the shorter term (2014) and the longer term (2016). Moreover, church-based friendships mediated this relationship in 2016. These results provide insights into how religious participation impacts psychological well-being, which can inform interventions aimed at reducing depressive symptoms in older adults.: : religious attendance, church-based social ties, depressive symptoms, mediation, HRS

Abstract citation ID: igaf122.2713

### REVISITING TECHNOLOGY'S IMPACT: A PRE-AND DURING-COVID STUDY ON MENTAL HEALTH IN OLDER ADULTS

Geunhye Park, and Jeonghun Kim, *SUNY Cortland, Cortland, New York, United States*

The COVID-19 pandemic has exacerbated mental health challenges among older adults, yet existing research remains methodologically limited, often relying on cross-sectional designs that fail to capture temporal changes. This study aims to fill this gap by examining individual-level mental health trajectories before and during the pandemic, contributing to deeper understanding of the long-term psychological effects of crisis events on older populations. Using a longitudinal fixed-effects probit model, we analyze data from the National Health and Aging Trends Study (NHATS) Round 9 (pre-pandemic) and Round 10 (during the pandemic), allowing us to control for unobserved heterogeneity and incorporate lagged variables that account for delayed mental health effects. The sample includes 3,257

respondents, predominantly female (58%), White American (76%), and aged 75-79 (29%). Findings reveal that increased technology use-particularly virtual communication, telehealth, and online social engagement-was associated with greater mental health deterioration among older adults. However, these effects were contingent on pre-existing mental health conditions, as those with a history of depression or anxiety experienced negative impacts. Additionally, income level emerged as a stronger predictor of mental health outcomes than technology use, particularly among individuals with severe mental health conditions, highlighting economic security as a critical determinant of resilience. These findings contribute to theoretical discussions on crisis-induced vulnerability and have significant policy and clinical implications. Policymakers must strengthen income support programs, social safety nets, and digital inclusion efforts, while clinicians should recognize that technology-driven interventions must be accompanied by financial and mental health support to effectively address disparities among older adults.

Abstract citation ID: igaf122.2714

### SELF-MANAGEMENT PRACTICES AMONG U.S. CHINESE OLDER ADULTS WITH MAJOR DEPRESSIVE DISORDER

Ying-Yu Chao[1], Jin Young Seo[2], An-Yun Yeh[3], Weiming Wang[4], and Yu-Ping Chang[5], *1. Rutgers University, Newark, New Jersey, United States, 2. Hunter College, New York, New York, United States, 3. The College of Staten Island, CUNY, Staten Island, New York, United States, 4. New York University Rory Meyers College of Nursing, New York, New York, United States, 5. University at Buffalo, Buffalo, New York, United States*

Purpose: Depression is common in older immigrants due to low levels of acculturation, language barriers, and social isolation. In Chinese communities, the cultural stigma surrounding mental health further exacerbates these challenges. This study aimed to enhance the understanding of self-management strategies and barriers among U.S. Chinese older adults living with Major Depressive Disorder (MDD). Methods: A qualitative study design was employed. Participants were recruited from a psychiatric clinic in New York City. A demographic survey was administered to collect participants' characteristics. Semi-structured individual interviews were conducted to explore their self-management strategies for depressive symptoms. Data were analyzed using descriptive statistics and thematic analysis. Results: Seventeen patients with MDD were recruited. The average age was 60.53 (SD: 6.47; range: 51-75) years, 52.9% were male, and 70.6% were married. Self-management strategies included seeking help and adhering to treatment, finding comfort in supportive relationships and social engagement, engaging in enjoyable activities, and practicing acceptance and letting go. Challenges to self-management included failing to recognize underlying emotional problems, fighting cultural stigmas and misconceptions, encountering help-seeking barriers as immigrants, and ongoing struggle with medication reliance and concerns about side effects. Conclusions/Implications: Participants developed individualized strategies to manage depressive symptoms. Health care providers should assess older adults' perceptions of depression, understand the challenges they face in self-management, and provide tailored interventions for those struggling to manage their symptoms. Moreover, efforts to deliver culturally sensitive, collaborative care should focus on enhancing mental health literacy, increasing public awareness, and implementing anti-stigma interventions for this population.

Abstract citation ID: igaf122.2715

### SOCIODEMOGRAPHICS, FUNCTIONAL ABILITY, CHRONIC CONDITIONS, AND LONELINESS IN OLDER US ADULTS IN THE 2020 WAVE OF HRS

Laurie Theeke[1], Lashawn Hutto[1], and LaTonya Smith[2], *1. The George Washington University, Washington, District of Columbia, United States, 2. The George Washington University, Westfield, New Jersey, United States*

Loneliness is a known determinant of poor health outcomes and a priority for community-dwelling older adults. This analysis describes relationships among loneliness, determinants of health, functional ability, and chronic illnesses for community-dwelling adults 50 and older in the US during 2020. SPSS was used for analysis on the Rand longitudinal data file with 4,520 cases (60.1% female) selected from Wave 15 who complete data on the UCLA-11 The mean age was 69.23 (SD 10.06), with 71.1% reported as Caucasian and 19.2% reported as Black. Majority (86.1%) were reported as not Hispanic and married (57.1%) and 82.6% had completed high school or higher. The average annual household income was less than $20,000 and 34.2% reported ongoing financial strain with 19.5% reported housing problems. Participants averaged 8.66 (SD 12.74) doctor visits since 2018 and over 2 days per month with family helpers. The median was 2 chronic conditions (range 0-8), with high prevalence for arthritis (63.3%), hypertension (62.7%), diabetes (26.9%), heart disease (24.4%), and cancer (17%). Functional impairment for basic ADLs was low except for 33.3% reporting difficulty getting up from a chair but 27.7% reported trouble walking several blocks and 14.2% reported difficulty walking one block. Though 52.2 % reported smoking in their lifetime, only 9.4% reported current smoking. Significant correlations of loneliness ($p < .001$) included depression (CES-D scores), fine and gross motor impairment, and BMI. Wellness and prevention interventions that are community-based would have potential to diminish chronic conditions and emphasize functional ability which could diminish likelihood of loneliness.

Abstract citation ID: igaf122.2716

### THE ASSOCIATION OF COMMUNITY RATES OF INTERNET USE, DEPRESSION, AND LIVING ALONE IN MASSACHUSETTS

Mengshi Liu, Dongfang Hong, Michelle Ward, Qinglin Gao, and Elizabeth Dugan, *University of Massachusetts Boston, Boston, Massachusetts, United States*

The negative health consequences of social isolation are increasingly recognized, and internet use is often cited as a potential intervention to enhance social connectivity, particularly for at-risk older adults. This study examines the relationship between internet use, depression, and the proportion of older adults living alone across 367 communities in Massachusetts (MA). Data were drawn from the 2025 Massachusetts Healthy Aging Data Reports (healthyagingdatareports.org). Internet use in the past month was derived from BRFSS data (2018–2022), the percentage of older adults living alone was obtained from the American Community Survey (2018–2022), and depression diagnoses were sourced from Centers for Medicare & Medicaid Services data (2020–2021). Across MA, the prevalence of depression ranged from 21.90% to 54.46% (state average: 34.64%), while internet use varied from 40.83% to 88.95% (state average: 70.57%). Regression analyses showed a significant negative association between community rates of internet use and depression among older adults in MA (β = -0.10, p < .000), which remained significant after adjusting for gender distribution and educational attainment. However, when stratified by the proportion of older adults living alone, this relationship was only significant in communities with a higher-than-average proportion of older adults living alone (β = -0.11, p < .001), while no significant association was observed in communities with lower proportions. These findings suggest that internet use may be particularly beneficial in reducing community rates of depression among older adults experiencing greater social isolation, highlighting its potential as a mental health resource for those living alone.

Abstract citation ID: igaf122.2717

### THE ASSOCIATION OF LANGUAGE BARRIER AND DEPRESSION AMONG NEWLY ADMITTED US NURSING HOME RESIDENTS

Natalia Nielsen, Maira Castañeda-Avila, David Dosa, Shao-Hsien Liu, William Jesdale, Yiyang Yuan, Jonggyu Baek, and Kate Lapane, *University of Massachusetts Chan Medical School, Worcester, Massachusetts, United States*

Language barriers result in negative outcomes for nursing home residents of diverse linguistic backgrounds. Yet, the relationship between language barriers and resident depression symptoms or clinical diagnosis is understudied. Coming decades will likely see a rise in older Latino adult nursing home admissions in the US. We compared the prevalence of moderate/severe depressive symptoms and clinical diagnosis of depression among English- and Spanish-speaking Latinos (L-E and L-S, respectively) and non-Latino English-speaking White residents (NLW-E). Newly admitted Latino and non-Latino White nursing home residents aged ≥50 years were included (n = 889,205). Generalized estimating equation models were used to estimate adjusted prevalence ratios (aPRs) and 95% confidence intervals (CI) for depression outcomes across racial/ethnic and language groups. Our sample was mostly White, female and ≥75 years. Of Latinos (n = 76,749), 25.8% indicated the need for a Spanish-speaking interpreter. The prevalence of moderate/severe depression was highest in Spanish-speaking Latino residents (NLW-E: 15.6%; L-E: 15.9%; L-S: 17.5%). After adjusting for covariates and accounting for facility level clustering, Latinos had lower prevalence of clinically diagnosed depression compared to non-Latino Whites (Latino vs. non-Latino White aPR: 0.93; 95% CI: 0.92-0.95) and Spanish-speaking Latinos had lower prevalence of clinically diagnosed depression compared to English-speaking Latinos (L-S vs L-E aPR: 0.95; 95% CI: 0.93-0.98). Similar results were found for moderate/severe depression (Latino vs. non-Latino White aPR: 0.93; 95% CI: 0.91-0.95; L-S vs L-E aPR: 0.95; 95% CI: 0.91-0.99). Future research to better understand the relationship between language barriers and depression in this growing population within US nursing homes is warranted.

Abstract citation ID: igaf122.2718

### THE DUAL NATURE OF SUPER WOMEN: THE NARRATIVES OF AFRICAN AMERICAN DEMENTIA CAREGIVERS

Shanae Rhodes[1], Pamela Recto[2], and Janna Lesser[2], *1. The University of Texas Health Science Center at San Antonio, San Antonio, Texas, United States, 2. UT Health San Antonio, San Antonio, Texas, United States*

African Americans are twice as likely to be affected by dementia, with most caregivers being women. However, African American women remain underrepresented in dementia research. Literature shows that while African American caregivers report worse physical health, they tend to fare better psychologically regarding caregiver burden compared to other racial groups. Despite this, inconsistencies exist regarding their mental health outcomes. Some studies suggest that African American caregivers use culturally derived coping mechanisms, such as the "superwoman" role, to suppress negative emotions. This coping strategy, driven by societal expectations and cultural norms, can provide short-term relief but contributes to long-term emotional distress and strain. This paradox underscores the need for a deeper understanding of how cultural factors influence stress and coping in caregiving contexts. The purpose of this mixed-methods study was to explore the experiences of African American female caregivers. This presentation focuses on the qualitative segment, using a descriptive approach with ethnographic strategies. Fifteen individual interviews were conducted via Zoom, generating 1,012 minutes of transcribed data. The analysis, guided by Woods-Giscombe's Superwoman Schema (SWS), identified categories aligned with each SWS domain. Additionally, a new perceived benefit, "overcoming resentment & trauma," emerged, potentially adding a new domain to the SWS. These findings provide valuable insights into the challenges and strengths of African American women caregivers, offering a foundation for culturally sensitive interventions to support this underserved group.

Abstract citation ID: igaf122.2719

### WALKING ACTIVITY AND DEPRESSIVE SYMPTOMS AMONG MEXICAN AMERICAN OLDER ADULTS AGED 80 YEARS AND OLDER

Kevin Chen[1], and Soham AlSnih[2], *1. UTMB John Sealy School of Medicine, Houston, Texas, United States, 2. The University of Texas Medical Branch at Galveston, Texas, Galveston, Texas, United States*

Physical activity is known to be protective for the development of depressive symptoms in older adults. We examined the relationship between walking activity and depressive symptoms among Mexican American older adults without a history of depressive symptoms at baseline over a 9-year period. Participants (N = 836) were Mexican American aged 80 years and older living in the southwestern United States (Arizona, California, Colorado, New Mexico, and Texas) from the Hispanic Established Population for the Epidemiologic Study of the Elderly (2007/05-2016). Measures included socio-demographics and health characteristics. Depressive symptoms were assessed with the Center for Epidemiologic Studies Depression Scale (CES-D ≥ 16). At baseline, participants were grouped into non-walkers (n = 535), walked < 150 minute/week (n = 123), and walked 150 minutes/week or more (n = 178). Generalized Estimating Equation models estimated the odds ratio (OR) and 95% Confidence Interval (CI) of depressive symptoms as a function of walking activity status controlling for all covariates. We found that those who walked ≥150 minutes/week had lower odds (OR = 0.53, 95% CI = 0.30-0.93) of developing depressive symptoms over time compared to non-walkers after controlling for all covariates. There was a non-significant relationship between walking < 150 minutes/week and depressive symptoms (OR = 0.81, 095% CI = 0.51-1.31) over time compared to non-walkers after controlling for all covariates. In conclusion,

Mexican American older adults who engaged in any walking activity were at reduced risk of developing depressive symptoms. Preventing depression in this growing population may improve their quality of life and reduce their burden of care.

Abstract citation ID: igaf122.2720

### WHAT MAKES A PEER SUPPORTER COMPETENT? A BOTTOM-UP CROSS-CULTURALLY INFORMED MULTI-METHOD STUDY

Terry Lum[1], Stephanie MingYin Wong[1], Dara KiuYi Leung[1], Eric Kwok[1], Edwin Wong[2], Wai Chi Chan[1], and Gloria HoiYan Wong[3], *1. The University of Hong Kong, Hong Kong, China (People's Republic), 2. University of Birmingham, England, United Kingdom, 3. University of Reading, England, United Kingdom*

Peer support is increasingly recognised as an important person-centred means of facilitating mental well-being among older adults. Enhancing competencies among Peer Supporters (PS) and any service provider is crucial to ensuring quality service. Yet, there is limited knowledge concerning what a PS's core competencies are from the perspectives of multiple key stakeholders and how they could be aptly assessed. We conducted a three-round bottom-up Delphi study involving 30 PS (≥50 years), 14 service users (≥60 years), and 23 social workers/project officers to develop a PS core competency framework. This was followed by constructing a self-rated Peer Supporter Competence Scale (PSCS) for the local population. The psychometric properties of the PSCS were examined in 340 trained PS from a territory-wide community-based stepped-care intervention service for older adults with depressive symptoms in Hong Kong. Thirty-five core competencies were identified with five overarching themes: 'Self and self-development', 'General work ethics', 'Work with others', 'Work with service users' (encompassing 'Interpersonal attitude and skills', 'Risk management', and 'Recovery facilitation'), and 'Peer support knowledge'. 14.3% were unique to the local context, 20% had conceptual origins in the West, and 65.7% were shared across cultures. Following standard scale development and validation procedures, the final scale for PS competence assessment comprised 16 items (PSCS-16), demonstrating good reliability ($\alpha = 0.84$) and convergent validity ($r = 0.40$ with the General Self-Efficacy Scale, $p < 0.001$). These findings have implications for enhancing peer support training and demonstrate the feasibility of developing a culturally-informed measure of PS competence using a bottom-up approach.

### SESSION 7260 (POSTER)

### NEIGHBORHOODS

Abstract citation ID: igaf122.2721

### A TYPOLOGY OF LIVABLE COMMUNITIES AND OLDER ADULTS' HEALTH IN THE U.S.

Kyeongmo Kim[1], Denise Burnette[1], Sol Baik[2], and Seon Kim[3], *1. Virginia Commonwealth University, Richmond, Virginia, United States, 2. University of Virginia, Charlottesville, Virginia, United States, 3. Sunchon National University, Suncheon, Cholla-namdo, Republic of Korea*

Neighborhoods with high-quality built environments and social environments are linked with older adults' well-being. However, research on the complex interplay of neighborhood types and health outcomes is limited, as is the role of functional limitations. This study aims to: 1) identify neighborhood types based on the World Health Organization's age-friendly community framework, 2) explore the association of neighborhood type and older adults' health, and 3) assess whether functional status affects this association. We merged data from the 2017 AARP Age-Friendly Communities Surveys and the AARP Livability Index. Our sample included 3,211 adults aged 65 and older; the majority (59%) were female. Participants identified as non-Hispanic White (81%), Hispanic (8%), Black (6%), and a member of another racial/ethnic group (2%). We conducted latent class analysis to identify neighborhood type. We then explored the relationship between neighborhood type and self-rated health, adjusting for individual sociodemographic and health-related characteristics. We identified a four-class model of neighborhood types: "Connected yet Limited Services," "Service Integrated," "Healthy Environment Zones," and "Supportive Social Engagement." Older adults in "connected yet limited services" and "service integrated" neighborhoods had worse self-rated health than those in socially supportive neighborhoods, especially among those who reported functional limitations. Our findings indicate that older adults with functional limitations particularly benefit from neighborhoods with robust health support and social engagement opportunities, highlighting the importance of designing inclusive and adaptable age-friendly environments to address diverse and changing needs.

Abstract citation ID: igaf122.2722

### HOW NEIGHBORHOOD COHESION AND DISORDER SHAPE LATE-LIFE COGNITION: THE MEDIATING ROLE OF PSYCHOLOGICAL RESILIENCE

Guoping Jin[1], Xiaoyi Zeng[2], Qingqing Yin[1], and Fengyan Tang[1], *1. University of Pittsburgh, Pittsburgh, Pennsylvania, United States, 2. The University of Texas at Austin, Austin, Texas, United States*

Although prior research has linked neighborhood environments to cognitive health in later life, the mechanisms under these associations are less well understood. Drawing on the theoretical concept of resilience, this study examined whether psychological resilience mediated the effects of perceived neighborhood cohesion and disorder on cognitive functioning among older adults. Using the Health and Retirement Study 2010-2018 Leave Behind Questionnaire and RAND Data File, this study included 4,708 respondents aged 65 and older at baseline. Cognitive functioning was assessed using a modified 35-point Telephone Interview Cognitive Screen, while psychological resilience was measured with a simplified resilience scale based on prior research. Using path analyses, this study examined the potential direct and indirect effects of Time 1 neighborhood environment (predictor) and Time 2 psychological resilience (mediator) on Time 3 cognitive functioning (outcome), adjusting for sociodemographic and health-related covariates. The results indicated that greater neighborhood cohesion was significantly associated with higher levels of cognitive functioning ($\beta = 0.03$, $p < .001$), with psychological resilience partially mediating this effect (indirect effect = 0.01, $p < .001$). In contrast, neighborhood disorder was not significantly associated with cognitive functioning ($\beta = 0.1$, $p > .05$). These findings advance our understanding of the mechanisms through which neighborhood environments influence cognitive health in later life. Enhancing resilience may help leverage the cognitive benefits of neighborhood cohesion. Other implications for practice and future research are discussed.

Abstract citation ID: igaf122.2723

### NEIGHBORHOOD CHARACTERISTICS AND INCIDENT STROKE IN US OLDER ADULTS: EVALUATION IN TWO NATIONWIDE COHORTS

Kendra Sims[1], Torsten Neilands[2], Julene Johnson[2], Loni Tabb[3], Monika Safford[4], Suzanne Judd[5], Kirsten Bibbins-Domingo[6], and M. Maria Glymour[1], *1. Boston University, Boston, Massachusetts, United States, 2. University of California, San Francisco, San Francisco, California, United States, 3. Drexel University, Philadelphia, Pennsylvania, United States, 4. Weill Cornell Medicine, New York, New York, United States, 5. The University of Alabama at Birmingham, Birmingham, Alabama, United States, 6. Journal of the American Medical Association, Chicago, Illinois, United States*

Research linking adverse neighborhood context with stroke disparities may reflect publication bias for chance associations. We compared results from the REasons for Geographic and Racial Differences in Stroke (REGARDS, n = 25,125, aged ≥ 45 years, 41% Non-Hispanic Black, 55% Stroke Belt; 2003-2020) study to the Health and Retirement Study (HRS, n = 13,197, aged > 50 years, 15% Non-Hispanic Black, 18% Stroke Belt; 2004-2020). We estimated Cox models predicting stroke for 51 American Community Survey (ACS) census tract variables, evaluating inter-cohort consistency of main, race- and region-stratified estimates. Follow-up in REGARDS (median=12.1 years; IQR: 6.5, 14.9) was similar to HRS (median=12.3; IQR: 8.0, 15.8). Cumulative stroke incidence was lower in REGARDS (5.8%) than HRS (13.9%). Twenty ACS variables had associations with incident stroke that differed by at least log(0.05) between cohorts. The percentage of vacant housing units for sale was associated with incident stroke among Stroke Belt participants in REGARDS (HR per SD: 0.92; 95% CI: 0.84, 1.02) and in HRS (HR per SD: 0.87; 95% CI: 0.73, 1.03); these associations were not observed among participants in the rest of the US in REGARDS (HR per SD: 1.02; 95% 0.96, 1.09, p for region interaction: 0.058) or HRS (HR per SD: 1.02; 95% 0.96, 1.09, p for region interaction: 0.078). None of the five other regional differences or any of the 11 Black-White differences replicated across cohorts. Inverse associations of vacancies with stroke replicated across two national studies, potentially implicating attractive housing markets as beneficial for older adult CVD health.

Abstract citation ID: igaf122.2724

### NEIGHBORHOOD ETHNIC DIVERSITY, SENSE OF COMMUNITY, AND WELLBEING AMONG NATIVE AND MIGRANT OLDER ADULTS

Max Bloem, Jane Cramm, and Anna Nieboer, *Erasmus University Rotterdam, Rotterdam, Zuid-Holland, Netherlands*

Sense of community (SoC) is a key contributor to older adults' wellbeing, fostering social support, health, and overall wellbeing. As aging-in-place policies and age-friendly community initiatives gain prominence in urban planning, understanding how SoC influences the wellbeing of older adults in diverse neighborhoods is crucial. This study examines how native and migrant older adults from Dutch, Surinamese, Turkish, and Moroccan backgrounds experience neighborhood ethnic diversity in relation to SoC and wellbeing in the Netherlands. Using data from a large-scale study on aging in urban neighborhoods, a regression model was applied to assess the relationships between neighborhood ethnic diversity, SoC, and subjective wellbeing. Ethnic diversity was measured using three indicators: outgroup size, a group-specific Herfindahl-Hirschman Index (HHI), and perceived ingroup size. Findings indicate that ethnic diversity has a limited or no effect on the wellbeing of native-Dutch older adults, while producing mixed effects for migrant groups. Of all the diversity measures, outgroup size had the most significant effect on wellbeing, though not uniformly. It had a positive effect for older adults with a Moroccan background, while a negative effect was observed for Surinamese older adults. This study highlights the importance of considering ethnic diversity in conceptualizations of age-friendly communities, recognizing differences between native and migrant older adults, and acknowledging diversity within migrant groups in research and policy.

Abstract citation ID: igaf122.2725

### REEXAMINING THE HEALTH IMPLICATIONS OF OBJECTIVE AND SUBJECTIVE NEIGHBORHOOD MEASURES: NEW INSIGHTS FROM REWARD

Meiyi Li[1], Wei Xu[2], Christina Kamis[3], Amy Schultz[1], Kristen Malecki[4], and Michal Engelman[5], *1. University of Wisconsin–Madison, Madison, Wisconsin, United States, 2. Medical College of Wisconsin, Milwaukee, Wisconsin, United States, 3. University of Illinois Urbana-Champaign, Champaign, Illinois, United States, 4. University of Illinois Chicago, Chicago, Illinois, United States, 5. University of Wisconsin-Madison, Madison, Wisconsin, United States*

Neighborhood is recognized as the key determinant of individual well-being and population health. Despite evidence on neighborhood and health, how neighborhoods should be measured remains contested. The scholarship has continuously debated over objective versus subjective measures of neighborhoods and their relative importance on health. Further, the process by which neighborhoods "get under the skin" to affect health remains to be examined. The current study draws upon longitudinal residential history and novel epigenetic biomarkers of aging from the Researching Epigenetics, Weathering, Aging, & Disadvantage Study (REWARD) to provide new insights into this ongoing debate. We aim to investigate (a) the relationship between objective neighborhood disadvantages and subjective perceptions of living in the neighborhoods and accelerated biological aging, (b) the relative importance of objective and subjective neighborhood measures, and (c) the extent to which subjective perception moderates the association between objective neighborhood disadvantages and accelerated biological aging. Preliminary results demonstrate that both objective neighborhood disadvantages and subjective perceptions are associated with accelerated biological measures, with objective neighborhood disadvantages showing stronger impacts. Further, the relationship between objective neighborhood disadvantages and accelerated biological aging cannot be moderated by subjective perceptions. Results from this study not only contribute to the ongoing debate of neighborhood measures but also shed light on the underlying pathway by which neighborhoods "get under the skin" to affect aging and health.

Abstract citation ID: igaf122.2726

### REVISITING THE HISPANIC PARADOX: NEIGHBORHOOD CHARACTERISTICS AND LIFE SATISFACTION FOR OLDER IMMIGRANTS

Morgan Stangl[1], Lauren Gil Hayes[1], Yi Wang[2], and Man Guo[1], *1. University of Iowa, Iowa City, Iowa, United States, 2. University of Iowa School of Social Work, Iowa City, Iowa, United States*

Nativity status (foreign-born, FB vs. native-born, NB) is a social determinant of well-being, yet its impact on life satisfaction may vary depending on neighborhood characteristics. Current literature discusses the Hispanic Paradox, where FB Hispanics report higher life satisfaction despite immigration challenges, attributed to strong family support. However, neighborhood characteristics remains underexamined, offering new insights. The current study seeks to explore 1) Whether the association between neighborhood characteristics (cohesion/disorder) and life satisfaction is stronger for FB middle-aged/older adults than for NB middle-aged/older adults and 2) if this association varies by race (White, Black, Hispanic). This study utilizes data from the 2018 Health and Retirement Study (HRS) Psychosocial Leave-Behind Questionnaire, a nationally representative survey of middle-aged/older adults, including both NB (N = 4,398) and FB (N = 722). Life satisfaction was regressed on neighborhood cohesion and disorder separately, controlling for demographics and socioeconomic factors. Interaction terms between neighborhood characteristics and nativity status were added to test moderation. These interaction models were then stratified by race/ethnicity. Results show that FB middle-aged/older adults are more sensitive to neighborhood characteristics, with a significant interaction between nativity and neighborhood disorder ($\beta = 0.15$, $p < 0.001$). Neighborhood cohesion shows a strong positive association with life satisfaction for FB Hispanics ($\beta = 0.38$, $p < 0.001$). Neighborhood disorder has a strong negative association for FB Hispanics ($\beta = -0.32$, $p < 0.001$). These findings suggest that neighborhood characteristics may play a role in shaping life satisfaction, particularly for FB middle-aged/older Hispanic adults. These findings provide another dimension in understanding the Hispanic Paradox.

Abstract citation ID: igaf122.2727

### SOLO AGING IN NEW ENGLAND: FINDINGS FROM THE 2025 HEALTHY AGING DATA REPORT

Sophia Casale, Mengshi Liu, Michelle Ward, Dongfang Hong, and Elizabeth Dugan, *University of Massachusetts Boston, Boston, Massachusetts, United States*

Experiencing later adulthood as a solo ager is gaining increasing research interest and adults who have never married are a major component of solo agers. This research describes community rates of adults 65+ who have never been married, the percentage of older adults who have annual incomes below the poverty level, and community medical service utilization rates (ER visits, and annual physician visits) in CT, MA, and RI. Data were from the 2025 Healthy Aging Data Reports. The indicators calculated community and state rates derived from the American Community Survey (2018-2022), and Centers for Medicare & Medicaid Services (2020-20221) data. Results showed that communities with highest percentage of never-married adults 65+ and the % of adults 65+ living below the federal poverty line are concentrated in urban areas: Hartford, CT (22.90%), Longwood (Boston), MA (57.69%) and Providence, RI (22.22%). Some of the lowest annual physician visit rates and highest annual ER visit rates are in urban/low-income communities with above state average never married 65+. Annual physician visits / year are low in Hartford, CT (6.18), Central Falls, RI (4.81), Providence, RI (6.68). Annual ER Visits / 1,000 adults 65+ are high in Hartford, CT (1,062.65), 6 Corners (Springfield), MA (1,025.32), East Boston, MA (868.89), Downtown (Worcester), MA (863.08), Chelsea, MA (822.52) and Woonsocket, RI (601.08). These findings provide important descriptive information about a group that tends to get overlooked. The findings capture evident disparities and may guide practitioners and policymakers in allocating resources to better serve the never-married 65+ population.

Abstract citation ID: igaf122.2728

### SPATIAL INCOME POLARIZATION AND ALL-CAUSE MORTALITY: LOCAL ENVIRONMENTAL ATTRIBUTES AS MEDIATORS

Ethan SiuLeung Cheung[1], and David S. Curtis[2], *1. The University of Hong Kong, Hong Kong, 2. University of Utah, Salt Lake City, Utah, United States*

Although numerous studies have reported associations between segregation and mortality, the contextual effect of income segregation, in comparison to racial segregation, is less researched. Furthermore, the extent to which local environmental features mediate this relationship is not well understood. This study examines the association between spatial income polarization with all-cause mortality, and tests local environmental features as mediators. We adopt individual data from three waves of the core sample in the Midlife in the United States study, supplemented with an oversample of African Americans from Milwaukee: Wave 1 (1995-96), Wave 2 (2004-06), and Wave 3 (2016-17). The mortality data was observed until 2022 from the National Death Index and supplemental field tracing. Our sample includes 6,460 participants with 1580 deaths throughout the study period. Spatial income polarization is measured by the Index of Concentration at the Extremes (ICE) at the tract level, further adjusted by regional price parity index. The local environmental features include area-level economic disadvantage, neighborhood trust, neighborhood safety, neighborhood disorder, PM10 concentration, heat island days, and accessibility to greenspace. Survival models with a Weibull distribution are employed to examine the associations. Results showed that one-unit increase of ICEincome is associated with 32% lower risk of mortality, highlighting the health advantage of individuals living in more income-privileged relative to income-deprived tracts. Adjustments for all environmental features, including neighborhood safety and PM10 concentration, attenuated the coefficient of ICEincome by 63%. The findings underscore the importance of the local environment in explaining the relationship between tract income concentration and mortality.

Abstract citation ID: igaf122.2729

### THE RELATIONSHIPS BETWEEN NEIGHBORHOOD ENVIRONMENT AND COGNITIVE FUNCTION IN LATER LIFE

Midori Takayama[1], Yoshiko Ishioka[2], and Ikuko Sugawara[3], *1. Keio University, Yokohama, Kanagawa, Japan, 2. O.P. Jindal Global University, Sonipat, Haryana, India, 3. Musashino University, Nishitokyo, Tokyo, Japan*

Although the role of the neighborhood environment in health is well documented, little is known about how the neighborhood affects cognitive function. This study aimed to examine how physical and social neighborhood environments affect cognitive function in later life. Furthermore, we examined the interaction effect of physical function and economic status on the relationship between the neighborhood environment and cognition. Data were obtained from a locally representative three-wave longitudinal study of older Japanese aged 74–86 years (N = 1064). We used subjective measures of two physical environmental factors (public facilities, such as community centers, and accessibility)

and two social environmental factors (availability of social participation programs and social inclusion). MMSE was used to assess cognitive functioning. The results of multilevel analyses showed that the availability of social participation programs was positively associated with cognitive function and that the degree of social inclusion was negatively associated with cognitive function. Moreover, physical functioning had an interaction effect on the relationship between social inclusion and cognitive functioning. Among older adults with lower physical function, cognitive function was lower in those living in more socially inclusive neighborhoods than in those living in less socially inclusive neighborhoods. These results suggest that creating opportunities for social participation in neighborhoods contributes to maintaining cognitive function, whereas fostering social inclusiveness in neighborhoods contributes to older adults' continued aging in place, even after their cognitive and physical function declines. These results reinforce the need for neighborhood-level interventions to maintain cognition in later life and facilitate aging in place despite cognitive decline.

Abstract citation ID: igaf122.2730

### VARYING ASSOCIATION BETWEEN CANCER CAREGIVER MENTAL HEALTH AND AREA DEPRIVATION BY HOUSEHOLD INCOME

Kyle Pitzer[1], Karla Washington[1], Darrell Hudson[2], Keisha White Makinde[1], Todd Becker[3], Debra Oliver[1], Jacquelyn Benson[1], and George Demiris[4], *1. Washington University in St. Louis, St. Louis, Missouri, United States, 2. University of Michigan, Ann Arbor, Michigan, United States, 3. WashU Medicine, St. Louis, Missouri, United States, 4. University of Pennsylvania, Philadelphia, Pennsylvania, United States*

In the United States, nearly 9 in 10 newly diagnosed cancer patients are age 50 and above; almost 6 in 10 are over 65. Consequently, older adults and their family caregivers disproportionately bear the burden of this illness, often leading to pronounced mental health concerns. While individual and interpersonal factors related to cancer caregivers' well-being have been extensively studied, little is known about how community-level factors are related to their mental health. Using data from a large randomized clinical trial of a caregiver support intervention, this secondary data analysis examined the association between area deprivation and mental health among cancer caregivers as well as differential associations based on household income. To determine area deprivation, participants were geocoded to determine their census block group and corresponding area deprivation index percentile. Linear models were then estimated to assess the association between area deprivation and anxiety and depression while adjusting for several demographic and caregiving context covariates. The interaction between area deprivation and household income was also examined. While there was no significant association between area deprivation and depression regardless of household income, models indicated a cross-over association between area deprivation and anxiety by household income. Specifically, caregivers with annual household incomes below $70,000 had greater anxiety as area deprivation increased while caregivers with household incomes above $70,000 had less anxiety as area deprivation increased, on average. These findings highlight the importance of considering both the financial situation and the living environment of cancer caregivers when developing supportive interventions.

### SESSION 7265 (POSTER)

Abstract citation ID: igaf122.2731

### PHYSICAL ACTIVITY AND EXERCISE ACCELEROMETERS REVEAL PECULIAR PATTERNS OF PHYSICAL ACTIVITY IN COGNITIVELY IMPAIRED VS. HEALTHY OLDER ADULTS

Massimiliano Pau[1], Valeria Putzu[2], Gesuina Asoni[2], Daniela Viale[2], Benedetta Brandas[1], and Maria Chiara Fastame[1], *1. University of Cagliari, Cagliari, Sardegna, Italy, 2. ASL Cagliari, Cagliari, Sardegna, Italy*

Although there is consensus regarding the association between Physical Activity (PA) and cognitive performance of older adults, quantitative data regarding amount and intensity of PA performed by those with cognitive impairments.under free-living conditions, are scarce. We investigated the effects of cognitive impairment on several PA metrics and analyzed the patterns of mobility in terms of hourly variation using accelerometers in 104 community-dwelling older adults aged 60-85 stratified into three groups: cognitively impaired living in urban area (CI, n = 27), cognitively intact living in urban area (HC, n = 33) and cognitively intact living in the Blue Zone of Sardinia (Italy), a cluster of rural communities characterized by exceptional longevity (BZ, n = 44). All participants were monitored for 7 consecutive days using a clinically validated wearable accelerometer. Raw data were processed to calculate percentage of time spent in sedentary behavior (SB), light PA (LPA) and moderate-to-vigorous PA (MVPA). The results showed that older adults of the CI groups spend 76% of the day in SB, a value significantly higher than both HC (67%) and BZ (66%), and only 3.4% in MVPA, which was significantly lower than what observed in the BZ group (12.1%). Differences were also found in the propensity to mobility during the day, as indicated by the different hourly PA patterns. Such findings suggest that CI is associated with a sedentary lifestyle. The large differences observed in MVPA with those living in the BZ, suggest that activities of sufficient intensity might contribute to mitigate the effects of cognitive deficit and contribute to longevity.

Abstract citation ID: igaf122.2732

### CHRONIC DISEASE BURDEN AND PHYSICAL ACTIVITY PROFILE IN OLDER ADULTS: A COMPOSITIONAL DATA ANALYSIS

Ethan Ouimet[1], Angeles Tepper[2], Emily Erlenbach[3], Stephanie Voss[4], Edward McAuley[5], and Neha Gothe[2], *1. Northeastern University of Bouve College of Health Sciences, Boston, Massachusetts, United States, 2. Northeastern University, Boston, Massachusetts, United States, 3. EXOS, South San Francisco, California, United States, 4. Mass General Brigham, Boston, Massachusetts, United States, 5. University of Illinois Urbana-Champaign, Urbana, Illinois, United States*

Understanding how physical activity (PA) levels differ by chronic disease (CD) status is essential for designing interventions to promote health. This study examined baseline differences in PA levels among older adults with varying chronic disease burdens. Participants (N = 142, mean age=64.2) were categorized into three groups: No CDs (N = 43), one CD (N = 42), or ≥two CDs (N = 57), with conditions such as diabetes and hypertension. PA profiles (sedentary time, light PA (LPA), moderate-to-vigorous (MVPA)) were assessed via accelerometry. Compositional data analysis was used to derive isometric log-ratio (ILR) coordinates representing PA distribution. Group differences were examined using linear mixed models. Participants with ≥two CDs had higher ILR1 values (Sedentary vs. Active) than those with no CDs (p = 0.032), indicating more sedentary behavior. Participants with ≥two CDs also had higher ILR2 values (LPA vs. MVPA, p = 0.006), suggesting a lower proportion of MVPA relative to LPA. Age was positively associated with both ILR1 and ILR2 (p = 0.021 and p = 0.012), meaning older participants engaged in more sedentary time and less MVPA. Women had significantly higher ILR2 values than men (p < 0.001), reflecting more LPA relative to MVPA. BMI was higher in those with ≥two CDs (p = 0.048). Sleep quality and age did not significantly differ across groups. Given these findings, older adults with multiple chronic diseases who are likely obese, may face additional barriers to PA, making a focus on increasing LPA along with MVPA a more feasible approach. PA interventions could target LPA to reduce sedentary time, with attention to strategies that support women and those with obesity.

Abstract citation ID: igaf122.2733

### CILIARY NEUROTROPHIC FACTOR IN SKELETAL MUSCLE OF OLDER ADULTS AND THE EFFECTS OF WEIGHT LOSS AND AEROBIC TRAINING

Alice Ryan[1], Guoyan Li[1], Colleen Lynch[2], Sausan Jaber[3], and Galya Bigman[4], *1. University of Maryland School of Medicine, Baltimore, Maryland, United States, 2. Baltimore GRECC, Baltimore, Maryland, United States, 3. Baltimore VA Medical Center, Baltimore, Maryland, United States, 4. University of Maryland, Baltimore County, Baltimore, Maryland, United States*

Ciliary neurotrophic factor (CNTF), a pluripotent cytokine which acts both centrally and peripherally, has been proposed to alter obesity, and to be modified by aging. There is limited work of CNTF in skeletal muscle in humans and its role in cardiometabolic health. The purpose of this study was to determine skeletal muscle CNTF mRNA expression and its relationship to obesity and glucose tolerance in older adults and to examine the effects of six-month weight loss (WL) and aerobic exercise training (AEX) interventions on its expression. Sixty-seven adults (age 61±3 yr, X±SD) underwent a vastus lateralis skeletal muscle biopsy, VO2max test, DXA scan, and 3-hour oral glucose tolerance test. A subset completed 6 months of WL (n = 18) or 3x/week AEX (n = 16). Skeletal muscle CNTF expression was associated with 3-hr glucose and insulin area under the curve (AUC) (r = 0.287, p = 0.03 and r = 0.289, p = 0.04) but not BMI, %body fat, and VO2max. There was a reduction in body weight (p < 0.001), %fat, glucoseAUC, and insulinAUC (all p < 0.01) after WL. VO2max increased (p < 0.001), %fat decreased (p < 0.05), and body weight, glucoseAUC and insulinAUC did not change after AEX. Muscle CNTF expression decreased after WL (6.16±2.16 vs. 4.81±1.44 AU, p = 0.01) but did not change with AEX (6.90±2.52 vs. 6.65±1.86 AU). CNTF expression in skeletal muscle is related to glucose tolerance but does not appear to be associated with body composition or fitness. Although muscle CNTF mRNA expression is reduced by weight loss, further work is needed to determine its influence on glucose metabolism and obesity in older adults.

Abstract citation ID: igaf122.2734

### COMPARING THE EFFECTS OF IMPROVISATION AND CHOREOGRAPHY ON OLDER ADULTS WITH MILD COGNITIVE IMPAIRMENT

Yunjia Yang, *University of Arizona, Tucson, Arizona, United States*

This mixed-methods study compared the impact of improvisation and choreography on the cognition and well-being of older adults with MCI.

Methods: 20 participants with MCI were randomized into either the Improvisational Dance (ID) or Choreographic Dance (CD) group. Each group had two one-hour interventions per week for 10 weeks. Quantitative assessments on attention, memory, and creativity were done before and after the interventions. Participants completed the Positive Well-Being survey before and after the 1st, 5th, 10th, 15th, and 20th classes. Qualitative methods included focus group interviews, survey answers, and observations. Results: The ID group showed a significant improvement in creativity (p = .05), with greater gains compared to the CD group (p < .05). Both groups experienced a significant increase in short-term positive well-being after the 1-hour class (p < .001); however, only the CD group demonstrated significant long-term improvement over ten weeks (p < .05). Even though quantitative data did not show a statistically significant difference, mixed data showed the CD group presented a positive indication of improving attention/reaction time, visual-spatial short-term memory, physical benefits, and self-efficacy than the ID group. The ID group showed a positive indication of promoting social interaction and personal expression compared to the BP group. Qualitative results showed that self-efficacy, emotional engagement, expression, and social interactions contribute to Positive Well-being. Semi-structured improvisation in the ID group is key to promoting creativity. Remembering and repeating the movements with timing in the CD group are the potential key factors for improving attention, reaction time, and memory.

Abstract citation ID: igaf122.2735

### EFFECTIVENESS, USABILITY, COST OF NUDGE-BASED SS-RE IN OLDER ADULTS WITH MCI: A HC-MIXED DESIGN STUDY

Xiaoyan Zhao[1], Qiaoqin Wan[2], and Yue Lan[1], *1. Peking University Health Science Center, Beijing, Beijing, China (People's Republic), 2. Peking University, Beijing, Beijing, China (People's Republic)*

Objective: This study aimed to evaluate the effect, usability, and cost-effectiveness of the nudges-based semi-supervised resistance exercise (SS-RE) program in older adults with mild cognitive impairment (MCI). Methods: A non-randomized intervention study with a historical control (HC) group [fully-supervised resistance exercise] was conducted. In the intervention group, participants engaged in the SS-RE program integrated with nudging strategies. Quantitative data on exercise adherence, cognitive function, and physical function were collected and compared between the two groups. Economic evaluation was conducted from the healthcare professional's perspective. Qualitative interviews assessed participants' experiences of the nudges. Results: The nudges-based SS-RE program demonstrated comparable exercise adherence, cognitive function, and physical function to the historical control group, with reduced professional labor costs. Almost all nudges were perceived as beneficial, expect for the precommitment. The qualitative feedback from the participants supported the quantitative findings and provided insights that may explain them. It was noteworthy that some nudges had minimal impacts, and some showed initial promise but influence diminished over time. Conclusion: The SS-RE program with nudges offered similar health benefits to fully-supervised resistance exercise at lower cost. Future large-scale randomized controlled trials are needed to validate these findings.: : Exercise, MCI, nudge, Semi-supervised intervention

Abstract citation ID: igaf122.2736

**ENHANCING ADULT WOMEN'S PELVIC HEALTH KNOWLEDGE THROUGH VIRTUAL EDUCATION: A PILOT STUDY**

LaVona Traywick[1], Alexis Gillett[2], Anna Dold[3], and Kara LaGorio[3], *1. University of Oklahama, Oklahoma City, Oklahoma, United States, 2. Alice Walton School of Medicine, Bentonville, Arkansas, United States, 3. Arkansas Colleges of Health Education, Fort Smith, Arkansas, United States*

Providing evidence-based care is essential to optimizing healthcare outcomes, particularly for senior adult women in underserved areas. Older women often face pelvic health challenges, including urinary tract infections (UTIs), pelvic floor dysfunction, pelvic organ prolapse, urinary incontinence, and chronic pelvic pain. Access to healthcare in rural areas remains a significant barrier, particularly for homebound seniors. Virtual education offers a promising avenue for addressing these disparities. This study evaluated the impact of a virtual pelvic health education session on knowledge retention among women in an underserved area. Forty-two female participants were recruited in Western Arkansas. Participants completed an online pre-session questionnaire (pre) to assess baseline pelvic health knowledge, attended a single virtual education session led by a healthcare provider specializing in pelvic health, and completed an on-line post-session (post-1) knowledge questionnaire, and one month post virtual (post-2) interview. The results demonstrate a significant increase in pelvic health knowledge immediately after the education session post-1 ($p<0.001$) and in the follow-up post-2 ($p<0.001$) assessment. Thematic interview results indicated that learning about pelvic health in a virtual environment was a positive experience. Thus, virtual education sessions appear to be a viable strategy for enhancing pelvic health knowledge, particularly among rural and homebound senior women with limited access to healthcare. This approach has the potential to empower older women to address pelvic health concerns proactively, thereby improving health outcomes and quality of life. Additionally, telehealth and virtual healthcare sessions provide significant benefits, including increased accessibility, convenience, cost-effectiveness, and the ability to reach underserved populations.

Abstract citation ID: igaf122.2737

**EXAMINING ASSOCIATIONS BETWEEN AGE, BINGE DRINKING, AND PHYSICAL ACTIVITY DURATION**

Abigail Nehrkorn-Bailey, *University of Wisconsin-Green Bay, Green Bay, Wisconsin, United States*

The existing literature suggests that engagement in one health risk behavior, such as alcohol consumption, is associated with other health risk behaviors, like physical inactivity. This association between health risk behaviors has been referred to as "clustering". However, there are gaps in the current literature: (1) a significant portion of the binge drinking research has focused on the behavior in college-aged individuals and (2) Dodge et al. (2016) found a positive association between alcohol drinking and physical activity, which contradicts the idea of clustering. A two-way ANOVA was conducted, utilizing the July 2023 Behavioral Risk Factor Surveillance System data (CDC, 2023) to examine whether age (i.e., 18-39 years, 40-59 years, 60+ years) and binge drinking categories (i.e., 0 binge drinking episodes, 1-9 episodes, 10-29 episodes, and 30 or more episodes in the past month) were related to minutes of weekly physical activity engagement. Along with significant main effects, there was a statistically significant interaction between age and binge drinking on physical activity duration, $F(6, 167478) = 3.07$, $p < .01$. The interaction results are discussed in terms of the physical activity engagement differences found for different ages at each binge drinking level, highlighting the importance of assessing binge drinking and physical activity engagement across adulthood.

Abstract citation ID: igaf122.2738

**EXERCISE MOTIVATION AND STRENGTH TRAINING AMONG OLDER ADULTS WITH AND WITHOUT A CANCER HISTORY**

Nancy Gell, and Myeongjin Bae, *University of Vermont, Burlington, Vermont, United States*

Strength training is beneficial for addressing cancer-related and age-related muscle loss. We aimed to 1) describe strength training among US older adults by cancer history and 2) examine motivating factors for exercise and their association with strength training. Nationally representative data of adults age ≥65 years from the Health Information National Trends Survey (HINTS) from 2012 and 2019 were analyzed ($n=2854$). Participants reported days of strength training/week and how much four motivating factors (appearance, enjoyment, guilt, pressure from others) reflect why they start or continue exercising regularly. Analyses included descriptive statistics and Poisson logistic regression; analyses were weighted to account for complex sampling design. Overall, 35% of older adults reported participation in any strength training. Among older adults with a cancer history, 22.8% reported strength training 2+ days/week versus 29.9% of those without a cancer history ($p=0.02$). Appearance was associated with meeting strength training recommendations among older adults without a cancer history (Incidence rate ratio [IRR] 1.6, 95% confidence interval [CI]: 1.3-1.9) but not among those with a cancer history. Enjoyment as a motivating factor was significantly associated with strength training among both groups (IRR: 2.3, 95% CI: 1.9-2.9, overall) whereas pressure from others was not associated with meeting strength training recommendations in either group. Less than half of U.S. older adults participate in strength training with significantly lower participation among older adults with a cancer history. Enjoyment is a strong motivating factor associated with meeting strength training recommendations and a potential target for exercise promotion efforts.

Abstract citation ID: igaf122.2739

**EXPLORING DYSPNEA-RELATED ANXIETY AND PHYSICAL ACTIVITY IN OLDER US VETERANS WITH COPD**

Brent Schell, Marilyn Moy, Jennifer Moye, Amy Silberbogen, Grace Rose, and Patricia Bamonti, *VA Boston Health Care System, Boston, Massachusetts, United States*

Dyspnea is a debilitating symptom in persons with chronic obstructive pulmonary disease (COPD) and has been linked to both activity restriction and psychological consequences, such as dyspnea-specific anxiety symptoms (e.g., fear of dyspnea or fear of dyspnea-inducing activities). To date, however, the association between dyspnea severity, objectively measured physical activity (PA), and dyspnea-specific anxiety symptoms has not been explored. In this study, we hypothesized that higher dyspnea-related anxiety (assessed via Breathlessness Beliefs Questionnaire-BBQ) would be associated with reduced PA (assessed via accelerometer-derived step counts), and that dyspnea severity (assessed via the University of California, San Diego Shortness of Breath Questionnaire) would mediate the relationship between dyspnea-related anxiety and PA. Forty-two Veterans were recruited (88% white, 93% male) with a mean age of 69 +/- 6 years, mean FEV1 67 +/- 21%, and mean daily step count (averaged over 14 +/- 1 days) of 4635 +/- 2115 steps. PA and BBQ scores were significantly correlated (r = -0.38, p = .014). Simple mediation analysis demonstrated a significant indirect effect of BBQ scores on PA through dyspnea severity (point estimate = -0.26, 95% CI = [-0.43, -0.11]). After accounting for indirect effects, there was no significant main effect of BBQ scores on PA (β = -0.12, p = .44), suggesting dyspnea levels completely mediated the relationship between BBQ scores and PA. Although the direction of relationship between these variables cannot be determined using cross-sectional data, this exploratory study suggests the need for longitudinal studies examining dyspnea-specific anxiety and PA in this population.

Abstract citation ID: igaf122.2740

### GAIT SPEED PREDICTS RESPONSE TO PHYSICAL ACTIVITY PROMOTION IN OLDER ADULTS WITH COPD

Patricia Bamonti[1], Stephanie Robinson[2], and Marilyn Moy[1], *1. VA Boston Health Care System, Boston, Massachusetts, United States, 2. VA Bedford Health Care System, Bedford, Massachusetts, United States*

Physical activity (PA) interventions are efficacious in individuals with chronic obstructive pulmonary disease (COPD), but not all participants improve to the same degree. Identifying characteristics that predict response to PA interventions can inform future intervention development. Gait speed is a useful indicator of physical performance, but it is unknown whether it predicts response to PA intervention. We examined whether baseline gait speed predicted response to a PA intervention. Secondary analyses included two cohorts of U.S. Veterans with COPD (N = 163 with complete data). Data from a web-based, pedometer-mediated PA intervention group (n = 89) and a control group (PA and disease self-management with or without a pedometer; n = 74) were combined. Participants completed a 243-meter walking course at their usual gait speed. Those who achieved a minimally clinically important difference (MCID) of 1000 average daily steps/day improvement from baseline to 3-month follow-up were categorized as "responders." A logistic regression model tested the association of gait speed and odds of being a responder, after controlling for age, FEV1 % predicted, baseline average daily steps (over 7-10 days), season, group assignment, and study. Participants (98% male; mean age 69±8 years) had baseline mean gait speed of 1.09±.20 m/s, and 28.8% were responders. For every 0.10 m/s increase in gait speed, equivalent to the MCID for gait speed in COPD, the odds of achieving the MCID was 34% greater (adjusted odds ratio=1.34, 95% CI [1.07, 1.70]). Baseline gait speed can inform targeted selection of individuals most likely to benefit from PA interventions.

Abstract citation ID: igaf122.2741

### IMPLEMENTING A 6-MONTH HYBRID EXERCISE INTERVENTION FOR OLDER ADULTS: FEASIBILITY OF THE SAY EXERCISE TRIAL

Stephanie Voss[1], Shayna Basu[2], Nandini Erodula[2], Emily Erlenbach[3], Edward McAuley[4], and Neha Gothe[2], *1. Mass General Brigham, Boston, Massachusetts, United States, 2. Northeastern University, Boston, Massachusetts, United States, 3. EXOS, South San Francisco, California, United States, 4. University of Illinois Urbana-Champaign, Urbana, Illinois, United States*

Hybrid programs have become ubiquitous in the post pandemic world and could help overcome challenges faced by older adults (OA)s' to engage in exercise. We present data on the feasibility, safety, and acceptability of a hybrid (in-person and virtual) yoga, aerobic and stretching exercise program for OAs. N = 145 (36 male, Mage= 64) low active OAs participated 3x/ week for 6-months in a hybrid exercise program as part of a randomized controlled trial examining exercise effects on neurocognition. Attendance, attrition, format preferences, and adverse events were documented. Participants also completed a survey to detail their experiences and preferences with the hybrid delivery model. N = 119 completed the program with attendance ranging from 74% to 79%. On average across the three exercise groups, 37% and 63% of the attended sessions were on Zoom and in-person, respectively. On post-program surveys, a majority reported a preference for hybrid (50.4%) programs in the future, with only 19% reporting a preference for a fully virtual exercise program. Nine adverse events were reported, with 3 related or potentially related to the intervention and related assessments. Collectively, these findings demonstrate that a hybrid exercise program including modalities like yoga are feasible and safe for OAs, with participants attending 1 in every 3 sessions virtually. While elements of in-person exercise programming such as socialization with peers and hands on instruction with the exercise and yoga teachers were valued, the hybrid modality allowed OAs to overcome unique barriers which are paramount for adherence to exercise programs as well as other health interventions.

Abstract citation ID: igaf122.2742

### IMPORTANCE OF CASUAL LEISURE ACTIVITIES ON COGNITIVE HEALTH IN OLDER ADULTS: THE HEALTH AND RETIREMENT STUDY

Jungsu Ryu, *Marshall University, Huntington, West Virginia, United States*

The cognitive health of older adults has become an increasing concern in our society, prompting ongoing research into factors that may positively influence it. This study aimed to examine how different types of casual leisure activities predict cognitive health in older adults. Participants (N = 5152, 59.1% female) came from a subsample of the Health and Retirement Study (HRS) and completed the psychosocial leave-behind questionnaire in 2018. Ages ranged from 50 to 102 years (M = 68.09, SD = 10.27). Descriptive statistics indicated that older adults typically engage in watching TV almost daily, reading several times a week, and walking approximately once a week. A two-step hierarchical regression analysis revealed that seven out of ten leisure activities significantly predicted cognitive health. Specifically, charity work, reading, doing word games, gardening/ maintenance, baking or cooking, and walking for 20 minutes were positively associated with cognitive health. In contrast, watching TV was negatively related to cognitive health. Among these activities, reading emerged as the strongest predictor of cognitive well-being in older adults. While previous studies have shown mixed findings regarding the impact of watching TV on cognitive health, this study emphasizes the negative relationship between watching TV and cognitive health. Although confounding factors, such as demographic variables, may influence these findings, the results highlight the importance of engaging older adults in more stimulating and active leisure activities, such as reading and walking, to maintain cognitive health. Reducing passive activities, particularly watching TV, may be beneficial if it replaces more cognitively engaging pursuits.

Abstract citation ID: igaf122.2743

### INTENSITY-SPECIFIC PHYSICAL ACTIVITY AND SUBJECTIVE WELL-BEING IN OLDER ADULTS WITH AND WITHOUT FRAILTY

Satoshi Seino[1], Yuri Yokoyama[2], Misao Kojima[2], Hiroki Mori[2], Atsuko Tanide[2], Takuya Ueda[2], Yoshinori Fujiwara[2], and Erika Kobayashi[2], *1. Yamagata University, Yamagata, Yamagata, Japan, 2. Tokyo Metropolitan Institute for Geriatrics and Gerontology, Itabashi City, Tokyo, Japan*

The health effects of physical activity (PA) may depend on both intensity and health status. This cross-sectional study examined the association between intensity-specific PA and subjective well-being (SWB) in older adults with and without frailty. This study included 32,392 non-disabled adults aged ≥65 years living in Tokyo, Japan. SWB was assessed using the Cantrill Ladder (0–10 scale). Daily durations of walking/standing (2.0 metabolic equivalents: < 1, 1–2.9, or ≥ 3 h/day) and vigorous activity (4.5 metabolic equivalents: none, < 1, or ≥ 1 h/day) were measured using the validated PA questionnaire. Frailty status was determined using the Kihon Checklist. Multivariable-adjusted partial regression coefficients for SWB were estimated for each PA intensity. Among non-frail older adults, both walking/standing and vigorous activity exhibited a positive dose-response relationship with SWB ($P < 0.001$ for trend), with coefficients of 0.07 (0.01–0.14) for 1–2.9 h/day and 0.18 (0.11–0.25) for ≥3 h/day of walking/standing (reference: < 1 h/day), and 0.16 (0.10–0.21) for < 1 h/day and 0.17 (0.11–0.23) for ≥1 h/day of vigorous activity (reference: no activity). Among frail older adults, walking/standing remained positively associated with SWB, with coefficients of 0.16 (0.07–0.25) for 1–2.9 h/day and 0.22 (0.11–0.33) for ≥3 h/day ($P < 0.001$ for trend). In contrast, vigorous activity showed a positive association only for < 1 h/day (0.13 [0.02–0.25]), with no significant association for ≥1 h/day (-0.09 [-0.22, 0.05]). While walking/standing was beneficial for SWB regardless of frailty, a frailty-dependent paradox emerged for vigorous activity. Optimizing SWB in frail older adults may require moderating vigorous activity within an appropriate range.

Abstract citation ID: igaf122.2744

### INVESTIGATING THE ASSOCIATION OF POSITIVE AFFECT AND REST-ACTIVITY RHYTHM IN MID TO LATER LIFE ADULTS

Meina Zhang[1], Jing Jiang[2], Kara Whitaker[2], Nai-Ching Chi[2], Sue Gardner[2], Boxiang Wang[2], and Chooza Moon[2], *1. UT Health, United States, 2. University of Iowa, Iowa City, Iowa, United States*

Positive affect, the experience of positive emotions, influences sleep and physical activity among midlife and older adults. However, the relationship between positive affect and rest-activity rhythm (RAR)—which specifically refers to the overall pattern of sleep and physical activity observed throughout a 24-hour period—has understudied in midlife and older adults. Further, it is unknown whether age, health, and income influence this relationship. To address this gap, we analyzed data from the Midlife in the United States Survey ($N = 308$, mean age (SD) = 54.80 (12.05)) to determine how positive affect correlates with key RAR parameters: mesor (the average activity level), amplitude (maximum activity level), acrophase (timing of maximum activity level) and R-square (a measure of rhythmicity). Participants wore the Mini Mitter Actiwatch®-64 for one week to assess RAR, and positive affect was measured using the Positive and Negative Affect Schedule questionnaire. We used interaction terms in multiple regression models to test the moderating roles of age, health, and income. After accounting for demographic factors and self-perceived health status, our results showed a significant positive association between higher positive affect and better RAR parameters, including mesor ($\beta = 16.18$, $p = 0.03$), amplitude ($\beta = 18.46$, $p = 0.02$), and R-square ($\beta = 0.03$, $p = 0.01$). Our findings also showed that higher income strengthened the positive relationship between positive affect and mesor. However, age and health did not appear to moderate these associations. These findings highlight the association between psychosocial factors like positive affect and RAR and suggest potential for longitudinal studies with diverse samples to explore causality.

Abstract citation ID: igaf122.2745

### LONGITUDINAL ASSOCIATIONS BETWEEN PHYSICAL ACTIVITY AND AFFECTIVE STATES: FINDINGS FROM A 9-YEAR NATIONAL STUDY

Angela Zhong[1], Josha Thomas[2], David Rompilla[3], Jason Levin[1], Emily Hittner[1], Jacquelyn Stephens[4], Joseph Mikels[5], and Claudia Haase[1], *1. Northwestern University, Evanston, Illinois, United States, 2. Harvard University, Cambridge, Massachusetts, United States, 3. Texas A&M University, College Station, Texas, United States, 4. Mather Institute, Illinois, United States, 5. DePaul University, Chicago, Illinois, United States*

Physical activity is widely recognized for its role in boosting positive affect, yet key questions remain regarding its nuanced longitudinal associations with affect. At the micro level, it is uncertain whether benefits extend beyond positive affect to states of varying valence and arousal. At the macro level, it is unclear whether associations generalize across diverse populations. The present study examined longitudinal associations between physical activity and positive and negative affect over nine years using a nationally representative sample of 3,100 Black and White American adults from the Midlife Development in the United States study. Affect was measured using items that varied in valence (positive or negative) and arousal (high or low) from the Positive and Negative Affect Schedule (PANAS) and Negative and Positive Affect Scale (NAPAS). Results indicated that physical activity predicted increases in positive affect over nine years, driven by high-arousal positive affect (e.g., active, enthusiastic), along with reductions in low-arousal negative affect (e.g., hopeless, everything was an effort). These findings controlled for baseline affect, along with health and sociodemographic factors. While most findings generalized across racial groups, effects for positive affect measured by PANAS were specific to White adults. Follow-up analyses probed the generalizability of these findings across different intensity levels of physical activity (light, moderate, vigorous) and across other well-being measures (e.g., life satisfaction). These findings contribute to longstanding discussions about affective benefits of physical activity and underscore the importance of including marginalized populations in research.

Abstract citation ID: igaf122.2746

### NOT ALL ACTIVITIES ARE EQUAL: DIVERSE PHYSICAL ACTIVITIES AND WELL-BEING IN INDIAN OLDER ADULTS – LASI FINDINGS

Urmimala Ghose, *Humboldt University in Berlin, Humboldt University in Berlin, Berlin, Germany*

Engagement in physical activity is widely recognized as a key factor in promoting well-being in later life. However, most research on this relationship has been conducted in Western contexts, with limited attention to diverse forms of physical activity in non-Western populations. This study examines the association between different intensities of physical activity—vigorous, moderate, and mild physical activities (e.g., Yoga, Pranayama)—and well-being outcomes, including life satisfaction and depressive symptoms, among older adults in India. Using data from the Longitudinal Aging Survey of India (LASI; N = 73,396, Mean age = 57.92, women = 57.58%, 35.96% having at least secondary education, 76.69% married), we conducted multiple regression analyses to assess both direct effects and moderation by age, gender, and health status. Results indicated that moderate physical activities were consistently associated with higher life satisfaction and lower depressive symptoms, whereas mild physical activities showed mixed effects depending on health status. Vigorous activities, while significant, had weaker and sometimes negative associations with well-being. Moderation analyses revealed that the benefits of physical activities varied by age, gender, and health, suggesting that a one-size-fits-all approach may not be optimal. These findings underscore the need to consider the diversity of physical activities in aging research, particularly in non-Western populations, to better understand how different activity intensities contribute to well-being. Tailoring physical activity recommendations to culturally relevant practices and individual health conditions may improve engagement and maximize psychological benefits for older adults.

Abstract citation ID: igaf122.2747

### PHYSICAL ACTIVITY, SLEEP, AND FUNCTION IN ASSISTED LIVING RESIDENTS WITH DIFFERING MOBILITY STATUS

Katelyn Webster-Dekker[1], Eileen Hacker[2], and Yvonne Lu[3], *1. University of Michigan School of Nursing, Ypsilanti, Michigan, United States, 2. The University of Texas MD Anderson Cancer Center, Houston, Texas, United States, 3. Indiana University School of Nursing, Indianapolis, Indiana, United States*

Physical activity (PA) is crucial for promoting health among older adults. However, those with mobility disabilities are often excluded from PA research. Our study aimed to better understand the relationship between PA and health-related characteristics in older adults in assisted living facilities with different levels of mobility. We compared those who primarily ambulated to those who primarily used a wheelchair or scooter. We recruited 35 participants aged 55 and older from five assisted living facilities. Participants wore an Actiwatch accelerometer for 7 days to monitor PA and sleep and completed physical function assessments. We used Spearman's correlations to analyze the relationships between PA, sleep, and physical function. We report correlations >0.30 rather than p-values due to the small sample size. Participants had a mean age of 75.3 (SD = 10.6) and were 86% female; 60% primarily ambulated (independently or with an assistive device), while 40% used wheelchairs or scooters. Overall, PA volume was negatively correlated with sleep time (r=-0.38). Among ambulatory residents, poorer sleep quality (increased wake after sleep onset and number of awakenings) correlated with less PA (r=-0.46 and -0.32). In participants using wheelchairs or scooters, PA showed positive correlations with handgrip strength (r = 0.47) and short physical performance battery total score (r = 0.48). In all participants, balance positively correlated with PA (r = 0.37). These findings underscore the interplay between mobility status, PA, sleep, and function in older adults in assisted living, suggesting that tailored interventions are needed to enhance health in these populations.

Abstract citation ID: igaf122.2748

### PHYSICAL EXERCISE AND BRAIN HEALTH: EXPLORING ACTIVITY TYPES TO ENHANCE COGNITION IN OLDER ADULTS

Anna Egbert[1], Isa-Marie Kreuzinger[2], and Mark Brennan Ing[3], *1. St. Joseph's University New York, Brooklyn, New York, United States, 2. St. Joseph's University, Brooklyn, New York, United States, 3. Hunter College, New York, New York, United States*

With the aging population, identifying lifestyle factors that can promote cognitive resilience and sustain independence becomes increasingly important. Physical exercise is well-established as a key contributor to brain health across all age groups, yet further research is needed to determine the most effective exercise types for maximizing cognitive benefits in older adults. This study aimed to examine the relationship between perceived cognitive difficulties (assessed via the Patient Assessment of Own Functioning Inventory–PAOFI), various forms of physical activity (walking, light, moderate, strenuous, and strength/endurance exercises), and sedentary behavior in a sample of adults aged 60–80 years (n = 42). Hierarchical regression modeling revealed that demographic, socioeconomic, and health-related factors, along with physical activity, accounted for 87% of the variance in PAOFI total scores. Higher scores on PAOFI Memory Scale were linked to male sex (B=-4.591, p=.003, CI95%=-7.518, -1.665), Caucasian White race/ethnicity (B = 12.538, p=.006, CI95%=3.961, 21.116), and engaging in more physical activities of light (B = 2.982, p=.006, CI95%=.905, 5.058) and moderate intensity (B = 14.093, p=.004, CI95%=4.736, 23.451). Higher scores on PAOFI Higher Level Cognitive and Intellectual Functioning Scale were linked to less time spent on sedentary activities (B=-18.885, p=.002, CI95%=-30.450, -7.321) and more walking (B = 3.007, p=.008, CI95%=.837, 5.177). These findings highlight potential benefits of promoting movement-based interventions and provide a basis for developing targeted programs for older adults.

Abstract citation ID: igaf122.2749

### RELOCATION, HEALTH, AND PHYSICAL ACTIVITY ENGAGEMENT IN SENIOR LIVING COMMUNITY RESIDENTS

Chiung-ju Liu, Gabriella Ulloa, and Kelly Leal, *University of Florida College of Public Health and Health Professions, Gainesville, Florida, United States*

Senior living communities offer supportive environments to promote healthy aging. However, little is known about the health status and the degree of physical activity engagement to support health in the community residents. The purpose of this study was to describe the health status and how the health status related to reasons for relocation and the use of fitness amenities. Residents who had lived in a senior living community for over 3 months in two cities of Florida were invited to complete a survey between December 2023 and November 2024. Participants answered questions about relocation reasons, fitness amenity utilization, frailty (Tilburg Frailty Indicator), sarcopenia (Strength, Assistance with walking, Rise from a chair, Climb stairs, and Falls), and cognitive changes (Ascertain Dementia 8-item Informant Questionnaire). A total of 145 residents responded to the survey (Mean age = 80 years; Female n = 104). Results indicate that 28% of respondents have frailty, 20% have sarcopenia, and 22% are at risk of cognitive impairment. Residents who relocated due to declining health (n = 25) reported greater degrees of frailty, sarcopenia, and cognitive changes relative to those due to non-health reasons (Mann-Whitney U Test, all ps < .05). Additionally, residents who used the fitness amenities more often is associated with less frailty and sarcopenia (Spearman's Rho Test, both ps < .01). Study findings suggested that residents with a relocation reason related to declining health may be at risk of poor health. Frequent use of community fitness amenities correlates with better physical health.

Abstract citation ID: igaf122.2750

### SELF-EFFICACY AND THE MAINTENANCE OF EXERCISE: A QUANTITATIVE STUDY AMONG RURAL OLDER ADULTS

Bryant O'Leary, Tasha Shaffer, Dawn Tarabochia, and Brianna Routh, *Montana State University, Bozeman, Montana, United States*

Despite the well-documented health benefits of physical activity, research shows increased sedentary behaviors among older adults (Fanning, Nicklas, & Rejeski, 2022; Leung, Sum, & Yang, 2021). A common approach to understanding exercise behaviors in high-risk populations, such as rural older adults, is to examine self-efficacy. Self-efficacy is a strong predictor of an individual's confidence in their ability to engage in exercise (Szczuka et al., 2019). This study aimed to evaluate whether older adults with more exercise facilitators also report higher self-efficacy scores. Data were collected through a survey designed to explore self-efficacy and facilitators related to physical activity. The survey questions were developed to incorporate a previously tested exercise self-efficacy measure along with a list of facilitators and barriers previously identified in research (Bethancourt et al., 2014; Patei, Sheth, & Jain, 2019). A descriptive quantitative approach was employed for data analysis to conduct standard frequency, ANOVA tests, and multiple linear regressions exploring the relationship between self-efficacy and facilitators. The results indicated that individual preferences were significantly associated with self-efficacy (p<.001). This research underscores the critical role of individual preferences in fostering exercise habits, particularly among rural older adults. Factors such as enjoyment of exercise, belief in its importance, feelings of guilt when inactive, awareness of its benefits, and the integration of exercise into daily routines all contribute to shaping exercise behaviors. These findings are pivotal in developing effective strategies to promote long-term exercise habits, emphasizing the need to consider personal preferences when designing interventions for older adults in rural communities.

Abstract citation ID: igaf122.2751

### SEX DIFFERENCES IN EXECUTIVE FUNCTIONING IN THE LEAP! RX EXERCISE STUDY

Gabrielle Lewis[1], Amanda Szabo-Reed[2], Jordan Baker[2], Jonathan Clutton[2], Mickeal Key[2], Jeff Burns[2], and Amber Watts[1], *1. University of Kansas, Lawrence, Kansas, United States, 2. University of Kansas Medical Center, Kansas City, Kansas, United States*

Executive functioning is critical in maintaining independence in older adulthood. Women's cognition tends to decline more rapidly. Previous research suggests aerobic exercise benefits executive functioning. It remains unclear whether sex moderates the cognitive benefit of exercise. We hypothesized that men and women would differ in executive function in response to the LEAP! Rx exercise intervention. We analyzed data from 219 older adults (M age=72.36) in the LEAP! Rx exercise intervention. We randomized participants to the intervention (n = 110) or control group (n = 109). We measured executive functioning using the Flanker Inhibitory Control and Attention Test (Flanker) and Dimensional Change Card Sort Test (DCCS). We conducted two 2-way repeated measures ANOVAs analyzing the effect of sex and group (intervention vs. control) on change in Flanker and DCCS scores across Baseline, 12 weeks, and 52 weeks, controlling for age and education. Results indicated no main effects of the intervention (p>.30), nor of sex (p>.40), at any time point. There was no significant interaction between sex and intervention group (p=.89). Previous results in this sample showed that although the intervention group significantly improved in cardiorespiratory fitness compared to the control group, the intervention did not appear to influence overall cognitive performance. Executive functioning scores had a ceiling effect, limiting our ability to detect improvements. Future research should include more challenging tests of executive function to investigate mechanisms by which exercise may improve executive function, such as cardiorespiratory fitness or intervention adherence. Our project contributes to examinations of individual characteristics most likely to benefit from exercise.

Abstract citation ID: igaf122.2752

### STRENGTH EXERCISE BEYOND WALKING: RISK FACTOR FOR SARCOPENIA IN EARLY OLDER ADULTS (65–75 YEARS)

Layoung Kim[1], Gwang Suk Kim[2], and Sooyoung Kwon[3], *1. The University of Suwon, Hwaseong, Republic of Korea, 2. Yonsei University College of Nursing, Seoul, Republic of Korea, 3. Graduate School of Yonsei University, Seoul, Republic of Korea*

Sarcopenia is defined as 'age-related loss of muscle mass, plus low muscle strength, and/or low physical performance.' In early older adults, it is rarely diagnosed based on physical performance, underscoring the importance of early intervention strategies. This study aimed to identify risk factors associated with sarcopenia in adults aged 65–75 years. Using the 2023 Korean National Health and Nutrition Examination Survey data, participants were categorized into three groups: sarcopenia (low muscle mass and low strength), sarcopenia risk (low muscle mass or low strength, excluding sarcopenia), and robust. The classification was based on appendicular skeletal muscle mass and handgrip strength, following the 2019 Asian Working Group for Sarcopenia criteria. Demographic characteristics and physical and psychological health-related factors were included as independent variables. Multinomial logistic regression was used to estimate odds ratios (OR) for factors associated with sarcopenia and sarcopenia risk. Among the 915 participants, the prevalence rates of sarcopenia and sarcopenia risk were 5.6% and 27.6%, respectively. Prevalence increased with age and lower BMI. Multivariate multinomial logistic regression analysis revealed that compared to the robust group, both sarcopenia and sarcopenia risk were significantly associated with not engaging in strength exercises at least once per week (OR = 2.598, OR = 1.858) and experiencing higher usual stress levels (OR = 3.034, OR = 1.710). However, not meeting the WHO's recommended 2.5 hours of weekly moderate-intensity aerobic physical activity was not significantly associated with sarcopenia or sarcopenia risk. This finding suggests that strength exercises beyond walking, should be emphasized for early older adults.

Abstract citation ID: igaf122.2753

### THE IMPACT OF PERSONALIZED EXERCISE PROGRAMS ON MUSCULAR FUNCTIONAL CAPACITY AND QUALITY OF DAILY LIFE

Semra Ercan, *Yeditepe University, Istanbul, Istanbul, Turkey*

Objective: This study evaluated the impact of a personalized exercise program on muscular functional capacity and daily life activities across various age groups and health statuses. Methods: A total of 169 participants, aged 16 to 94 with diverse health conditions, were enrolled. incorporating Entrack software and progressive resistive exercise devices by Enraf Nonius, was utilized. Specialized exercise methodology software was also developed. Each participant's maximum functional capacity across 13 muscle groups was assessed. Four questionnaires evaluated daily life activities and disease severity. Using these data, a personalized exercise regimen was formulated for each muscle group based on a mathematical model. The 12-week program consisted of thrice-weekly sessions, totaling 36 sessions. Results: Statistical analyses, including T-tests, One-Way ANOVA, Pearson correlation, and Bonferroni multiple comparisons, revealed: • Muscle Functional Capacity: A significant increase was observed (t(169) = 30.58, $p < 0.0001$), (Cohen's d = 2.35). • Questionnaire Outcomes: Paired t-tests indicated significant improvements (t(169) = 18.34, $p < 0.0001$), (Cohen's d = 1.41). • Correlation Analysis: A significant negative correlation was found between changes in muscle functional capacity and questionnaire scores (r = -0.70, $p < 0.0001$). Regression analysis yielded $R^2$ = 0.49 ($p < 0.0001$), Conclusion: Personalized exercise programs may offer potential benefits in reducing psychological and physical stress and the severity of diseases and improving quality of daily life by enhancing muscle functional capacity .This approach is particularly beneficial for older adults who may avoid physical activity due to injury fears or existing health conditions.

Abstract citation ID: igaf122.2754

### THE MEDIATING ROLE OF LEISURE-TIME PHYSICAL ACTIVITY AND MOBILITY IN THE LIFE SATISFACTION OF OLDER ADULTS

Yongseop Kim[1], Hyejin Park[2], Jaehyun Kim[3]Min Jun Hwang[4], and Junhyoung Kim[5], *1. University of Utah, University of Utah, Utah, United States, 2. University of Utah, Salt Lake City, Utah, United States, 3. East Carolina University, Greenville, North Carolina, United States, 4. Phillips Exeter Academy, Exeter, New Hampshire, United States, 5. Texas A&M University, College Station, Texas, United States*

Background: Leisure Time Physical Activity (LTPA) has been associated with numerous health benefits for older adults, including enhanced physical function and life satisfaction. However, older adults often experience physical limitations that hinder participation in LTPA. While prior research has examined the direct effects of LTPA on well-being, the mechanisms underlying this relationship, particularly the mediating roles of mobility and activities of daily living (ADL) performance, remain underexplored. Methods: This study utilized data from the 2022 National Health Interview Survey (NHIS) (N = 8,779) to examine the relationship between moderate LTPA, mobility, ADL performance, and life satisfaction in older adults. Path analysis was conducted to explore the mediating effects of mobility and ADL performance on the association between LTPA and life satisfaction. Results: Findings revealed that LTPA participation was significantly associated with improved mobility and ADL performance, both of which were positively linked to life satisfaction. Path analysis demonstrated that mobility and ADL performance mediated the relationship between LTPA and life satisfaction (Estimate = –.010, 95% CI [–.012, –.008]; Estimate = –.004, 95% CI [–.005, –.003]). Conclusion: This study highlights the importance of promoting moderate LTPA among older adults to enhance physical function and life satisfaction. Given the identified mediating effects of mobility and ADL performance, future interventions should focus on structured LTPA programs that support independence and overall well-being in aging populations. These findings provide valuable insights for policymakers and healthcare professionals in designing effective physical activity interventions for older adults.

Abstract citation ID: igaf122.2755

### THE RELATIONSHIP BETWEEN FITNESS AND WHITE MATTER USING A FIXEL BASED ANALYSIS IN OLDER ADULTS

Emma Tinney[1], Aaron Warren[2], Hannah Odom[1], Amanda O'Brien[1], Edward McAuley[3], Arthur Kramer[1], Kirk Erickson[4], and Charles Hillman[1], *1. Northeastern University, Boston, Massachusetts, United States, 2. Brigham and Women's Hospital, Boston, Massachusetts, United States, 3. University of Illinois Urbana-Champaign, Urbana, Illinois, United States, 4. AdventHealth Research Institute, Orlando, Florida, United States*

Age-related cognitive decline occurs, in part, due to diminishing white matter integrity. Higher cardiorespiratory fitness (CRF) may mitigate cognitive decline by preserving white matter structure and function, yet the mechanisms underlying these associations remain poorly understood. While prior studies have suggested that white matter microstructure mediates the relationship between CRF and cognition, they have been limited by the use of tensor-based diffusion imaging metrics, which are less sensitive to crossing fibers and complex white matter architecture. In this study, we leveraged a novel and more sensitive analytical approach, fixel-based analyses (FBA), to provide estimates of fiber density (FD), fiber cross-section (FC), and their combined measure (FDC) in multiple directions within each voxel. Using a uniquely large sample of 636 cognitively normal older adults aged 65–80 years (mean age = 69.8 years; 71% female), we hypothesized that FBA metrics would reveal robust relationships between CRF and white matter microstructure and mediate associations between CRF and cognition. In whole-brain analyses, higher CRF was associated with greater FD, FC, and FDC. Furthermore, these FBA-derived metrics statistically mediated the relationship between CRF and cognitive performance in the domains of visuospatial abilities, processing speed, working memory, and executive function/attentional control, but not episodic memory. These findings further highlight the potential for CRF to be associated with preserved cognitive function in aging through its relationship with white matter micro- and macro-structural properties, providing novel insights into the neurobiological mechanisms of fitness-related cognitive resilience.

### SESSION 7270 (POSTER)

### RESEARCH METHODS AND ISSUES: QUALITATIVE

Abstract citation ID: igaf122.2756

### DIFFERENCES IN STAFF PERCEPTIONS OF PHYSICIANS' AND ADVANCED PRACTITIONERS' CONTRIBUTIONS IN THE NURSING HOME

Brandi Peacock[1], Kira Ryskina[1], and Tetyana Shippee[2], *1. University of Pennsylvania, Philadelphia, Pennsylvania, United States, 2. University of Minnesota, Minneapolis, Minnesota, United States*

For the 1.4 million individuals who receive post-acute care in US nursing homes (NH) annually, timely access to physician or advanced practitioner (PAP) services is federally mandated. However, patient experience indicates a highly variable approach to PAP NH practice between facilities. This study aimed to qualitatively explore the perceptions of PAP contributions to NH care quality by triangulating interview data from key personnel. Purposive sampling was used to recruit 65 participants from a representative sample of 47 NHs in February - November 2023. Eligible participants included all clinical and non-clinical staff who spoke English. We used a grounded theory approach to analyze data collected from one-time semi-structured telephone interviews, divided into two groups: (1) 29 participants from 11 facilities with more than one participant; and (2) 36 solo participants from their facility. Participants included administrators (35%), PAP (17%), nurses (27%), and other staff (21%, e.g. social workers). Among participants from the same facility, 64% disagreed about the frequency of PAP visits. Two major themes emerged. First, administrators were primarily concerned with the minimum necessary presence of PAP at the facilities, whereas nurses perceived general accessibility of PAP as more important. Second, administrators, nurses, and other NH staff perceived limited roles for PAP in care coordination, whereas PAP perceived themselves to take the lead on care coordination for their patients. These findings highlight the need for transparency of expectations of PAP on-site presence and accessibility and for role clarity in care coordination to enhance the quality of PAP contributions in NHs.

Abstract citation ID: igaf122.2757

### PARTICIPATORY METHODOLOGIES, ADVANCED OLD AGE, AND POSSIBILITIES FOR THEORETICAL DEVELOPMENT

Elaine Eliopoulos, *Committee for Public Counsel Services-Private Panel Member, Weston, Massachusetts, United States*

Participatory research methodologies have received increased attention with older people but remain underutilized in research with those in advanced old age. These innovative methodologies involve a participants' collaborative co-design or co-production with the researcher. Photo-elicitation is one form of collaborative approach that enables the participant to engage with the research question through a photograph provided to them or self-initiated. This paper reports findings from a photo-elicitation study where 23 participants on the San Juan Islands of the Pacific Northwest, aged 80+, were provided digital cameras. The participants were asked to take photos that demonstrated the role their bodies played in their ability to remain social included in their communities. Their photos served as the basis for a semi structured interview which participants discussed their photographs. Participants' narratives reflected a focus on their needs and interests despite a wide range of corporeal challenges. Their focus on community and belonging, rather than bodily challenges suggests that the photo taking facilitated capture of the nuances of their daily lives beyond the physical. These findings present possibilities for re-imagining advanced old age beyond the corporeal dominant lens and looking beyond a dominant decline model of aging. Photo-elicitation with those in advanced old age may enhance our understanding of the lived experience in the ninth decade and provide the basis for theoretical development in new directions. Its' possibilities for informing and developing policy are promising.

Abstract citation ID: igaf122.2758

### PROCESS MAPPING AS AN INNOVATIVE METHOD TO IMPROVE UPTAKE OF HOME HEALTH AIDE SERVICES AMONG VETERANS

Lauren Hall[1], Margaret Ding[1], Bo Kim[2], and Emily Franzosa[3], *1. James J Peters VA Medical Center, New York, New York, United States, 2. VA Boston Health Care System, Boston, Massachusetts, United States, 3. Bronx VA, Bronx, New York, United States*

The Veterans Health Administration (VA) offers a home health aide (HHA) benefit that supports older veterans' desire to age in place. However, research from one VA medical center (VAMC) suggests only half of veterans referred to services take up the benefit. To understand reasons for this limited uptake, we conducted collaborative mapping of the process connecting veterans to HHA. Process mapping is widely used in healthcare settings to visualize steps in complex processes and identify areas for improvement. We conducted in-depth interviews with key informants of the HHA benefit at 5 VAMCs, including 15-20 primary care providers and HHA staff. We performed rapid analyses to identify process steps, barriers, and facilitators; mapped the elements using the online visual platform LucidChart; and standardized language across all VAMC-specific maps. We then held 'report back' sessions with key informants, to ensure the maps align with their firsthand understanding of the process. Salient barriers and facilitators confirmed by key informants included rurality's impact on access and the VA teams' commitment to connecting veterans to HHA services, respectively. Report back sessions also enabled expanded discussion and strategizing around identified best practices and implementation hurdles. New barriers identified in report-backs included capacity and resources of VA HHA program teams, and new facilitators included the benefit of adding a social worker perspective to the VA HHA team. Overall, this collaborative process enabled explicit delineation and shared understanding among key informants of their VAMC's veteran-to-HHA connection.

Abstract citation ID: igaf122.2759
**UNDERSTANDING PARTICIPANT-CENTERED RECRUITMENT IN AGING RESEARCH THROUGH DECISION MAKING**
Susan Passmore, Faith Ocoko, Madisin Randolph, Morgan Medina, and Carey Gleason, *University of Wisconsin–Madison, Madison, Wisconsin, United States*
Despite decades of evidence of the problem, participant samples in aging research often fail to adequately represent the population of older adults. For example, clinical research studies of Alzheimer's Disease, which disproportionately affects African American and Latine adults, frequently include over-representations of non-Hispanic White participants, limiting our full understanding of disease presentation and our ability to provide appropriate care for patients and caregivers. Authentically participant-centered research engagement offers promise to address this problem. However, few studies have specifically addressed how to create studies that participants, particularly those who are underrepresented currently, find acceptable and accessible. This presentation will include findings from a study of the real-world decision-making processes for African American adults (aged 53-77 years old) who had recently decided on their willingness to participate in the African Americans Fighting Alzheimer's in Midlife (AA-FAIM) study. Semi-structured interviews were conducted with those who eventually agreed to participate in AA-FAIM (n = 25) and those who declined participation (n = 18). Interviews were recorded and transcribed to facilitate a thematic analysis following Braun and Clarke. Findings reveal both positive and negative factors weighed in decision-making, including study team interactions, past experiences in research, personal experiences with Alzheimer's Disease, study requirements, and accessibility. Participants also offered that relationships with other study participants, a factor not reported in similar studies, figured into decision-making for many of those we interviewed.

### SESSION 7275 (POSTER)

Abstract citation ID: igaf122.2760
**RETIREMENT FACTORS DRIVING SOUTH KOREA'S HIGHEST OLDER ADULT POVERTY RATE AMONG OECD NATIONS: A DECOMPOSITION ANALYSIS**
Seoyeon Ahn[1], Eunsun Kwon[2], Ji Young Kang[3], and Sojung Park[4], *1. Duke-NUS Medical School/National University of Singapore, Singapore, Singapore, 2. Fairleigh Dickinson University, Florham Park, New Jersey, United States, 3. Chungnam National University, Daejeon, Republic of Korea, 4. Washington University in St. Louis, St. Louis, Missouri, United States*
This study investigates why South Korea has the highest older adult poverty rate (40%) among OECD countries by decomposing poverty gaps between Korea and other nations. Using data from the Luxembourg Income Study, we apply the Oaxaca-Blinder decomposition method to compare Korea with eight OECD countries (Norway, Germany, Greece, the United States, the United Kingdom, Canada, Australia, and Japan). Our findings reveal that 84–100% of the poverty rate differences are explained by structural factors. If Korea had Germany's socioeconomic structure, its poverty rate would drop from 51.9% to 5.8%. Korea's high older adult employment rate helps reduce poverty by 3.9–7.9 percentage points compared to countries like Japan and Australia. At the same time, Korea's extensive private transfer income lowers poverty by 2.9–4.9 percentage points. However, the most significant factor driving Korea's high poverty rate is its inadequate public pension system. If Korea's public pension benefits aligned with those of developed nations, poverty would decline by 24.1–50.2 percentage points. Despite mitigating effects from labor market participation and private transfers, insufficient public pension income remains the dominant cause of older adult poverty in Korea. These findings highlight the urgent challenge of ensuring income security amid rapid population aging and low fertility rates.

Abstract citation ID: igaf122.2761
**FOSTERING INTERGENERATIONAL RELATIONSHIPS AT U.S. MEN'S SHEDS**
Melinda Heinz, and Katie Cullen, *University of Northern Iowa, Cedar Falls, Iowa, United States*
Men's Sheds began in Australia during the 1990's and they now have a presence in eight other countries, including the U.S. The purpose of this study was to investigate intergenerational relationships at one of the only known intergenerational sheds in the U.S. Ten members participated in interviews about their experiences with Men's Sheds. Interviews were audio recorded and transcribed verbatim. Braun and Clark's (2020) six-step thematic approach were utilized to analyze the transcripts. Steps include data familiarization, coding, early theme generation, refining themes, paring down themes, and results write up. Results revealed, Theme 1: Intergenerational Transmission and Generativity, represented by older members commenting on a sense of purpose when sharing advice or perspectives with younger members. "There's a purpose for us being here and that is to help each other…there's several men that we are able to just share things with." Another member said, I would rather be here doing whatever needs doing encouraging other people than doing anything else." Younger members appreciated the guidance with learning how to use tools or saws in the woodshop, commenting, "So I've learned pretty well how to use everything over there." Theme 2: Community Outreach represented the community partnerships members created with youth organizations ranging from "bringing the Cub Scouts out" to helping home schooled children "build birdhouses." These findings indicate that there is potential to build meaningful intergenerational relationships at Men's Sheds that are mutually beneficial, providing a purposeful outlet for older adults and teaching younger adults practical skills such as woodworking.

Abstract citation ID: igaf122.2762
**SELF-DIRECTION IN PRODUCTIVE AGING: A QUALITATIVE STUDY**
Marilyn Cole[1], and Karen Macdonald[2], *1. Quinnipiac University, Stratford, Connecticut, United States, 2. Sacred Heart University, Fairfield, Connecticut, United States*
In this qualitative study by two occupational therapy researchers, our goal was to explore the perspectives of healthy productive agers, actively engaged in their communities. Researchers interviewed six retired older adults, ages 66-86. Using qualitative analysis, they identified three themes: hope for the future, planned performance, and purposeful social participation. Hope for the future includes a positive anticipation of future events. In order to set positive future goals, participants spoke of cognitive, emotional, and spiritual attributes. They demonstrated self-efficacy in managing their own health and some serious chronic conditions in order to maintain their meaningful personal and social activities. Planned performance required high cognitive skills in the realistic use of time and resources to accomplish needed self-care, home maintenance, and for some, caregiving responsibilities. Purposeful social participation was a dominant theme for participants who voluntarily took on many responsible social and community roles in retirement. It surprised researchers that each person valued their memberships in social groups so highly, whether families, neighborhoods, common interest groups, or charitable and religious organizations. This study highlights the importance of enabling self-direction for older adults. Given the choice and resources, each participant found ways to live productively. This guides professional service providers and policy makers to recognize the strong self-directed sense of purpose and mastery of self-management needed by older adults who seek to lead meaningful and productive lives, and to contribute to their communities and society.

Abstract citation ID: igaf122.2763

**STATUTORY RETIREMENT IN CHINA: A THEMATIC ANALYSIS OF WEIBO TRENDING POSTS IN 2024**

Jinyi Xiao, and Sonja Amadae, *University of Helsinki, Helsinki, Uusimaa, Finland*

China recently officially announced a gradual increase in the statutory retirement age for male from 60 to 63 years, and for female from 50 or 55 to 55 and 58 respectively. And the extension of contribution years based on a voluntary and flexible principle. We explored and analyzed the online public responses and discussions of increasing retirement age policy on Chinese social media (Weibo) and also examined how the themes evolved over different stages. This research retrieved 87 topics and 1,115 trending posts from Weibo across two critical policy stages (official announcement and detailed information) from July 22 to September 22, 2024. Inductive thematic analysis was employed to analyze the post content and categorize it into different themes. A frequency approach was used as supplementary to demonstrate and analyze the popularity of identified themes. Seven themes and twenty-four subthemes were identified. The most prominent theme was "subjective well-being concerns, " which was also the dominant theme in the second stage. "Public Health Concerns" and "Social Inequality" emerged as the second and third dominant themes overall, and were the most prominent themes in the first stage. Public discourse on Weibo regarding retirement age increases reveals this policy is widely perceived as unfriendly, compromising individual interests and exacerbating structural inequalities. The public views it as inherently detrimental to older workers, intensifying rather than addressing existing social disparities.

Abstract citation ID: igaf122.2764

**THE ASSOCIATION BETWEEN PHYSICAL HEALTH AND RETIREMENT PLANNING: FINDINGS FROM THE HEALTH AND RETIREMENT STUDY**

Yan-Jhu Su[1], Andrew Alberth[2], Elisabeth Stam[2], Alison Rataj[3], Shu Xu[4], and Jeffrey Stokes[2], *1. The Pennsylvania State University, University Park, Pennsylvania, United States, 2. University of Massachusetts Boston, Boston, Massachusetts, United States, 3. College of Health and Human Services, Center on Aging and Community Living, Durham, New Hampshire, United States, 4. University of Michigan, Ann Arbor, Michigan, United States*

Objectives: Previous health and anticipated retirement age studies have shown inconsistent results. One explanation for such discrepancies may result from differences in subjective and objective health across demographic groups. Therefore, this study examines whether self-rated poor health (subjective) and number of chronic illnesses (objective) were uniquely associated with anticipated retirement age, and whether this association differed by race or ethnicity for adults 50 years and older in the United States. Methods: This cross-sectional study uses data from the 2018 Health and Retirement Study (HRS). The sample included adults 50 years and older who were working for pay in 2018 (N = 5,970). The main dependent variable was respondents' predicted age of retirement and the independent variables were self-rated poor health and chronic illness. Race and ethnicity were assessed as moderators. Ordinary least squares (OLS) regression models were used. Results: The main effect of self-rated poor health on anticipated retirement age indicated less healthy people would retire sooner. However, having more chronic illnesses was not associated with predicted retirement age. Chronic illness is associated with earlier planned retirement ages among Black respondents, but racial/ethnic differences were not detected among self-rated health. Conclusions: These findings suggest older adults will remain in the workforce for a longer period of time when they are – or believe themselves to be – healthy. To support the aging workforce, enhancing chronic disease prevention efforts for Black adults should be pursued. Future research should investigate how subjective and objective health may differentially impact specific industries' workforces.

### SESSION 7280 (POSTER)

### RURAL HEALTH

Abstract citation ID: igaf122.2765

**BRIDGING DATA GAPS IN RURAL OREGON: A POPULATION-LED COMMUNITY HEALTH SURVEY PILOT**

Sarah Hubner[1], Duyen Ngo[1], Jacob Melson[1], Russ Comer[2]DaiLene Wilson[3], and Sari Hargand[1], *1. Oregon Health Authority, Portland, Oregon, United States, 2. Oregon Local Public Health Authority, John Day, Oregon, United States, 3. Oregon Local Public Health Authority, Condon, Oregon, United States*

Living in a rural area (10+ miles from a population center) is associated with increased morbidity and mortality. Rural populations are typically older, engage in fewer healthy behaviors, and have poorer access to basic resources like healthcare. Understanding the relationships between rurality, lifestyle, and health outcomes is critical to reducing disparities, particularly among older adults. Moreover, operationalizing population needs/interests supports meaningful development of actionable interventions. However, in Oregon, insufficient county-level data remains a persistent barrier to such efforts. A pilot study was therefore launched in two rural Oregon counties (Grant/Gilliam). State and local health authorities collaborated with community members to iteratively develop a comprehensive, mixed-method survey instrument (online/paper) with tailored questions on health/wellbeing, behavior, service access, and community needs. Older adults were prioritized, given Oregon's elevated proportion of residents aged 65+, especially in rural areas (US = 17.7%, Oregon=19.6%, Grant/Gilliam=30-32%). Although data collection is ongoing (2/28/25-4/20/25, 35% complete, N = 281/800), preliminary results indicate respondents were predominantly older (55% aged 65+, range=18-85+) and female (68%). Approximately 40% reported joint/muscle/mobility issues; nearly 20% reported caregiving responsibilities. Emerging themes suggest significant barriers to healthcare, recreation, and quality produce, pointing to marked regional deficiencies. There is notable demand for local health professionals/services (primary care, dentistry, telehealth, etc.), emphasizing critical gaps in access which may compound rural health disparities. Overall, results suggest aging issues are prominent in rural Oregon, potentially exacerbating individual challenges and cyclically diminishing quality of life. This pilot can inform future development and implementation of population-led assessments to improve data availability in small/rural communities.

Abstract citation ID: igaf122.2766

### BRIDGING THE DIGITAL DIVIDE: DEVELOPING TECH SUPPORT FOR RURAL VETERANS TO IMPROVE TELEHEALTH ACCESS

Elizabeth Marfeo[1], Elizabeth Chamberlin[2], Steven Shirk[2], Victoria Ngo[2], Maria Venegas[3], Cathy Cruise[4], and Lauren Moo[5], *1. Tufts University, Medford, Massachusetts, United States, 2. VA Bedford Health Care System, Bedford, Massachusetts, United States, 3. Department of Veterans Affairs, Bedford, Massachusetts, United States, 4. Veterans Health Administration, Northpoint, New York, United States, 5. VA Bedford Healthcare System, Bedford, Massachusetts, United States*

Telehealth expansion has the potential to improve healthcare accessibility, yet older rural Veterans continue to face a significant digital divide due to factors such as technology skills and comfort, as well as inadequate telehealth infrastructure. This presentation describes the challenges during the pre-implementation phases of developing the Telemedicine and Coaching for Older Adults to Connect in the Home (T-COACH) program. The T-COACH is an in-home volunteer support-based technology training program to facilitate increased telehealth access for older rural Veterans. This program aims to provide tailored education and training to empower Veterans to access their healthcare appointments via telehealth. By integrating face-to-face in-home education delivered by rurally residing trained community-based volunteers, this initiative offers a scalable approach to bridge the Digital Divide for many older Veterans living in rural areas. However, implementation in rural locales presents unique challenges, including recruiting rural volunteers with technical and interpersonal skills, transportation limitations, multi-tiered training, administrative regulations, and ensuring home-based safety and quality control. Our initial findings suggest successful deployment requires collaboration with local organizations, appropriate resource allocation, and strategic volunteer engagement. Future efforts should focus on refining training frameworks, optimizing volunteer recruitment, and securing sustainable funding models to ensure long-term program viability.

Abstract citation ID: igaf122.2767

### FACTORS PREDICTING GAPS IN ACCESS TO HEALTH-PROMOTING RESOURCES AMONG RURAL OLDER ADULTS

Molly Wylie[1], Verena Cimarolli[1], Robyn Stone[1], Regan McManus[2], and Ashley Washington[2], *1. LeadingAge, Washington, District of Columbia, United States, 2. Lutheran Services in America, Washington, District of Columbia, United States*

Many older adults living in rural communities have limited access to resources that promote health. Yet, little is known about the factors contributing to disparities in access to specific resources. Therefore, this study identified sociodemographic predictors of gaps in access to five resources (transportation, social support, food security, healthcare, and safe housing) among rural older adults (N = 795). Data was collected as part of an evaluation of the Rural Aging Action Network (RAAN) - a national collaborative that mobilizes existing and recruits new community-based services and supports. Multivariate logistic regressions examined sociodemographic predictors of gaps in resource access among RAAN users. Income-restricted older adults were twice as likely to have gaps in safe housing (95% CI [1.16, 4.78]), transportation (95% CI [1.11, 4.09]), or healthcare (95% CI [1.20, 4.25]), over three times more likely to have a food security gap (95% CI [1.44, 7.39]), and almost four times as likely to have a social support gap (95% CI [1.85, 8.37]) compared to those with adequate income. Older adults of color were over twice as likely to have a transportation gap (95% CI [1.12, 6.14]) and over three times as likely to have a food security gap (95% CI [1.30, 8.28]) compared to Whites. Veterans were over twice as likely to have a housing gap (95% CI, [1.04, 6.57]), yet 81% less likely to have a social support gap (95% CI [0.04, 0.88]) compared to non-veterans. Study findings can help providers identify rural older adults with greatest service needs.

Abstract citation ID: igaf122.2768

### FUNCTIONAL DISABILITY ON ADL AND IADL AMONG RURAL VERSUS FRONTIER OLDER ADULTS IN WYOMING

Nancy Karlin, *University of Northern Colorado, Greeley, Colorado, United States*

Given the unique challenges faced by frontier aging older adults, such as limited access to healthcare and social services, the potential for different functional disabilities as compared to rural older adults exists. A total of 142 community-dwelling older adults participated with 72 frontier and 70 rural county respondents. Participant sites were identified through the Division of Aging in Wyoming. The survey consisted of demographic questions, the Katz ADL Index, and Lawton Brody's IADL Scale. The aim of the study was to identify similarities and differences between the two cohorts on ADL and IADL functional disability along with respondent characteristics. A chi-square test of independence showed no significant association between rural and frontier respondents and the number of ADL disabilities reported, X2 (4, N = 142) = 2.18, p = .70, or in the number of IADL functional disabilities, X2 (6, N = 142) = 2.67, p = .84. A larger number of frontier residing older adults reported being widowed than rural older respondents. No significant association was evident between rural and frontier respondents and who they lived with at the time of survey, X2 (4, N = 142) = 2.77, p = .59. However, those in frontier counties were more likely to be living alone with limited ADL functional disparity, X2 (8, N = 142) = 21.9, p < .005. Rural older adults reported higher levels of education associated with few functional disabilities. These and additional findings offer insight into the aging experiences of both groups.

Abstract citation ID: igaf122.2769

### PROVIDER PERSPECTIVES ON BARRIERS AND FACILITATORS TO ENGAGING WITH CAREGIVERS OF OLDER, RURAL PATIENTS

Eileen Dryden[1], Aileen McGrory[1], Jacqueline Boudreau[1], Lynette Kelley[2], and Marianne Desir[3], *1. Veterans Health Administration, Bedford, Massachusetts, United States, 2. Veterans Health Administration, Aurora, Colorado, United States, 3. Veterans Health Administration, Miami, Florida, United States*

Over 50 million adults in the US alone are caregivers. Caregivers and the people they care for experience better health outcomes when caregivers are actively engaged with and supported by care recipients' healthcare teams. Too often, these informal caregivers (e.g. caregivers of spouses, children, friends) remain unacknowledged by the healthcare system, unengaged with care teams, and disconnected from needed support services. This can be particularly challenging for rural caregivers who more frequently report 'having no choice in taking on care' and more difficulty finding affordable services in their area and providing more hours of care per week compared with their suburban and urban counterparts. We interviewed 27 Veterans Health Administration providers about barriers and facilitators to identifying and engaging with caregivers of older, rural patients. Providers universally identified barriers to providing support to caregivers, such as lack of time and perceived scarcity of resources, and felt that more support for caregivers was needed. Many felt privacy policies limited their speaking with caregivers. Those who already discuss caregiver needs had developed their own, mostly unstandardized methods for recognizing caregivers who are struggling, initiating private time with caregivers, or asking questions to elicit needs. We highlight these provider perceptions--including how they identify caregivers, how caregiver needs and distress are determined, and systems-level barriers to information sharing--and discuss how these may be addressed in a research-informed intervention.

Abstract citation ID: igaf122.2770

### RURAL CAREGIVING AFTER STROKE: A GLOBAL QUALITATIVE EVIDENCE SYNTHESIS

Adrianne Smiley[1], Rita Jablonski[2], David Vance[2], and Elizabeth Byrd[3], *1. University of Pittsburgh, Pittsburgh, Pennsylvania, United States, 2. University of Alabama at Birmingham, Birmingham, Alabama, United States, 3. The University of Alabama at Birmingham, Birmingham, Alabama, United States*

Caregivers of stroke survivors in rural communities face geographic isolation with limited access to medical providers, which limits caregiver support and increases caregiver burden. A consolidated synthesis of the domestic and international narrative experiences of rural stroke survivor caregivers would inform interventions. We conducted a systematic search using the PRISMA 2020 framework and guidelines proposed by Bettany-Saltikov and McSherry (2016), focusing on qualitative studies published in PubMed, Embase, and PsycINFO biomedical research databases with no restrictions on location nor publication year. Two independent reviewers assessed studies for original research exploring narrative themes on caregiver burden among rural caregivers, 18 years and older, caring for stroke survivors. Eligible studies followed our indexed search strategy developed in collaboration with a health sciences librarian. Extracted data items included study identifiers, characteristics and main findings (narrative themes). A total of 13 studies representing 161 caregivers, between the ages of 21-85 years old, were eligible for inclusion in this evidence synthesis. Descriptive statistics were used to summarize data items; stratification was applied to organize data into meaningful subgroups. Thematic content analysis and open coding were used to identify patterns and themes. Rural caregivers' burden was alleviated by community and emotional support from their social networks and care providers but exacerbated by stroke survivor disability severity, unanticipated and rapid role transitions, delays in service initiation, inadequate communication from providers and mistrust in their local health system. Future interventions informed by these data may improve stroke survivors' quality of life by reducing caregiver burden.

### SESSION 7285 (POSTER)

### SEXUALITY

Abstract citation ID: igaf122.2771

### DEVELOPMENT AND APPLICATION OF A SEXUAL DYSFUNCTION ASSESSMENT SCALE FOR OLDER STROKE PATIENTS IN CHINA

Shaorui Bao, *The First Affiliated Hospital of Wenzhou Medical University, Wenzhou, Zhejiang, China (People's Republic)*

Sexual dysfunction (SD) is common among older stroke patients, affecting their physical, psychological, and social well-being. In China, SD is often overlooked due to cultural taboos around discussing sexuality in older adults. This study aimed to develop a culturally sensitive and reliable Sexual Dysfunction Assessment Scale (SDAS) for older stroke patients in China. A mixed-methods approach was used, including a literature review and expert interviews to identify factors affecting SD in this population. The SDAS was developed and tested for content validity through expert evaluation and pilot-tested on 126 older stroke patients in China. Reliability was assessed using Cronbach's alpha (0.85), and construct validity was evaluated through factor analysis. Preliminary results showed that the SDAS effectively captured critical aspects of sexual health and was practical for use in clinical settings. This scale provides healthcare professionals with a reliable tool to identify SD early, facilitate open discussions with patients, and improve patient care. The SDAS contributes to advancing sexual health research in aging populations, with potential for use in other countries with similar cultural contexts.

Abstract citation ID: igaf122.2772

### GENDER DIFFERENCES IN OLDER ADULTS' ROMANTIC RELATIONSHIP PREFERENCE AND SEXUAL SATISFACTION

William Henninger, Melinda Heinz, and Nathan Taylor, *University of Northern Iowa, Cedar Falls, Iowa, United States*

Socioemotional Selectivity Theory posits that as people age, they prioritize immediate emotional satisfaction over long-term goals due to the perception of limited time. This shift influences older adults' romantic relationships, increasing the likelihood of seeking meaningful connections and avoiding negative emotions. With healthy relationships linked to physical, psychological, and financial benefits, research is needed to further understand older adult romantic relationships. The purpose of this study is to explore gender differences in older adults' preferences for long-term and short-term relationships and their sexual satisfaction. Older adults ($n = 155$) aged 65-95 years completed surveys assessing short-term and long-term relationship preferences, current positive and negative sexual satisfaction, and predicted future positive and negative sexual satisfaction. Results indicate that males report higher preferences for long-term relationships ($t(153) = 3.86$, $p < .001$) and predicted more positive future sexual satisfaction ($t(89) = 3.01$, $p < .01$). Females were nearing significance of more negative current sexual satisfaction ($t(52) = -1.57$, $p = .06$). As the aging population increases and gray divorce is more prevalent, these findings have important implications for researchers, practitioners, and aging adults. Differences in relationship preference may result in disconnect between potential partners due to the type of relationship desired. Additionally, predicting more positive future sexual satisfaction may create expectations that could result in relationship distress and dissatisfaction. Future research is needed to explore the dyadic effects of differences in relationship and sexual preferences. Additionally, more diverse samples such as with same sex relationships and the oldest old are needed.

Abstract citation ID: igaf122.2773

### IMPROVING LGBTQ+ INCLUSIVE CARE IN LONG-TERM CARE: STAFF PERSPECTIVES, BARRIERS, AND RECOMMENDATIONS

Andrew Alberth, *University of Massachusetts Boston, Boston, Massachusetts, United States*

Lesbian, gay, bisexual, and transgender (LGBTQ+) older adults experience lifetime discrimination in healthcare settings, leading to worse health outcomes and lower trust in healthcare providers. Discrimination-related health outcomes may increase reliance on long term care (LTC) among LGBTQ+ older adults. To ensure LGBTQ+ residents get discrimination-free care, LGBTQ+ older adults often hide their sexual and gender identities. As such, little is known about how LTC staff identify and provide care to LGBTQ+ residents. This study aimed to identify LTC staff's perspectives on LGBTQ+ nursing home and assisted living facility residents' care and ways to improve that care. Semi-structured, in person, virtual, and phone-based interviews from 34 nursing home and assisted living facility direct care staff and administrators were recorded and transcribed. Thematic analysis identified common barriers and facilitators staff face when caring for LGBTQ+ residents and recommendations for improving LTC for this population. Barriers facing LTC staff include a lack of training on LGBTQ+-specific care needs, identifying LGBTQ+ residents, and navigating interactions with LGBTQ+ residents. Facilitators to staff caring for LGBTQ+ residents include a desire to provide high quality care to all residents and having LGBTQ+ staff and residents present in facilities. LTC staff recommended LGBTQ+ training for staff and residents, adding LGBTQ+ symbols in promotional materials and décor throughout the facility, and building opportunities for socialization and connection. This study explored LTC staff's perspectives on care provided to LGBTQ+ residents and recommendations to improve their care. Additional research is necessary to understand LGBTQ+ residents' perspectives in LTC.

Abstract citation ID: igaf122.2774

### SEXUALITY AND AGING: KNOWLEDGE AND ATTITUDES OF HEALTHCARE PRACTITIONERS IN COLOMBIA

Maria Reyes, and Natalie Levy, *Universidad de los Andes, Bogota, Distrito Capital de Bogota, Colombia*

In Colombia, older adults face limited access to comprehensive care that included sexual health. Despite the importance of sexuality in older adults' quality of life, healthcare professionals often overlook it in their assessments, reinforcing barriers in care. This study aims to identify the level of knowledge and attitudes of healthcare practitioners towards sexuality and aging. A cross-sectional quantitative study was conducted. The sample consisted of 150 healthcare practitioners in Colombia. The questionnaire included demographic data, education and experience, and the Aging Sexual Knowledge and Attitudes Scale, and the Staff Attitudes about Intimacy and Dementia scale.

Results: professionals reported that they occasionally or rarely address sexuality-related topics with older adults in their practice. Additionally, participants indicated that they had not received training on these topics but expressed interest in doing so. The results showed a moderately positive trend in attitudes toward sexuality in later life. The highest agreement levels were found in the importance of sexuality for quality of life, the need to address it in healthcare, and its recognition as a relevant dimension of well-being. Participants reported a lack of confidence in addressing the topic in professional practice and perceived cultural barriers to discussing it, particularly concerning older adults with dementia and those in institutional settings. Discussion The findings suggest that healthcare professionals generally have positive attitudes toward sexuality in old age but struggle to address it in their practice. This difficulty may be related to the lack of specific training on the topic, which limits their confidence and knowledge.

Abstract citation ID: igaf122.2775

### SEXUALITY AND WELL-BEING AMONG COMMUNITY-DWELLING OLDER ADULTS IN BOGOTÁ, COLOMBIA

Maria Reyes, Natalie Levy, Maria-Jose Castro, Gabriela Morales, Gabriela Garcia, Maria Ordoñez, and Angie Riveros, *Universidad de los Andes, Bogota, Distrito Capital de Bogota, Colombia*

Sexuality in late life is often overlooked, yet it plays a significant role in the well-being and mental health of older adults. We conducted a mixed methods study to explore the relationship between sexuality, well-being, and psychosocial factors among older adults in Bogotá, Colombia. The quantitative phase included 615 older adults (Mage= 66), 57.3% women, 40.9% men, and 1.4% transwomen. Surveys measured sexual well-being, depression, resilience, life satisfaction, and social support. The qualitative phase involved interviews with 19 participants (7 women, 6 men, and 6 transwomen), exploring experiences of sexuality, intimacy, and interactions with healthcare providers.

Results: multiple linear regression analysis showed that overall sexual satisfaction was predicted by age and awareness of the right to a fulfilling sexual life. Significant correlations were found between higher satisfaction with social support and perceived sexual compatibility and overall sexual satisfaction. Low social support was associated with higher distress in sexual functioning. Qualitative findings highlighted stigma surrounding aging and sexuality, particularly among transgender individuals. Participants highlighted the continued relevance of sexuality, especially for those in active relationships. While some saw no changes with aging, most reported significant shifts. Participants revealed that professionals rarely addressed sexuality. Discussion: social support, awareness of sexual rights, and resilience were positively associated with higher sexual satisfaction. Participants recognized changes in their sexuality as they aged. Cultural and religious beliefs played a crucial role in discussing sexuality, as many participants initially felt barriers to speaking openly. However, they acknowledged that sexuality is an important yet hidden topic.

### SESSION 7290 (POSTER)

Abstract citation ID: igaf122.2776

### SOCIAL ISOLATION AND LONELINESS I COGNITIVE AND PSYCHOSOCIAL BARRIERS TO SOCIAL ENGAGEMENT IN OLDER ADULTS

Molly Split[1], Sarah Prieto[1], Zachary Kunicki[2], Alyssa De Vito[1], Megan Barker[1], Louisa Thompson[2], Kathryn Devlin[3], and Maria Schultheis[3], *1. Brown University, Providence, Rhode Island, United States, 2. Warren Alpert Medical of Brown University, Providence, Rhode Island, United States, 3. Drexel University, Philadelphia, Pennsylvania, United States*

Loneliness in older adulthood can have profound effects on cognition and quality of life. While increased social engagement can alleviate loneliness, interventions to improve social connections remain limited. Developing effective interventions requires a deeper understanding of personal barriers and cognitive difficulties that may impede social engagement. We investigated these factors in a survey with cognitively healthy older adults. Participants were 167 racially and ethnically diverse older adults (M age=60.4, M education=16.1, 56% women). Participants completed a survey developed by our research team on social engagement frequency and barriers, and social life satisfaction. Online cognitive assessments of attention, working memory, and response inhibition were also administered. Spearman rank-order correlations examined associations between these measures. Common barriers to socialization included changes in interests (53%) and mood (41%). Lower social engagement was associated with more barriers (r=-.22, p=.005) and lower social life satisfaction (r=.52, p<.001), and more barriers correlated with lower social life satisfaction (r=-.30, p<.001). Worse attention (r=-.39, p<.001), working memory (r=-.18, p=.020), and response inhibition (r=-.30, p<.001) were linked to more barriers but not social engagement frequency or satisfaction. These findings highlight the need for interventions that address barriers to social engagement, such as changes in interests and mood, as well as cognitive difficulties that may limit participation in social activities. By addressing psychological and cognitive factors, tailored interventions can be developed to promote meaningful social connections and improve well-being in aging populations.

Abstract citation ID: igaf122.2777

### COGNITIVE REAPPRAISAL MODERATES THE LINK BETWEEN BURDEN AND LONELINESS IN DEMENTIA FAMILY CAREGIVERS

Ahria Dominguez[1], Darius Levan[2], Kuan-Hua Chen[1], Casey Brown[3], Jenna Wells[4], Julian Scheffer[5], Jennifer Merrilees[6], and Robert Levenson[2], *1. University of Nebraska Medical Center, Omaha, Nebraska, United States, 2. University of California, Berkeley, Berkeley, California, United States, 3. Georgetown University, Washington, District of Columbia, United States, 4. Cornell University, Ithaca, New York, United States, 5. University of Western Ontario, London, Ontario, Canada, 6. University of California, San Francisco, San Francisco, California, United States*

Caregiving for a loved one with a neurodegenerative disease is an important part of family life. However, the associated burden of experiencing declining functionality in a parent, spouse/partner, or other family member can lead to increased loneliness in caregivers. Cognitive reappraisal is the process of reevaluating one's thoughts about a situation to alter their emotional experience (e.g., finding the good in a bad situation). Using cognitive reappraisal may be a protective strategy to reduce loneliness in caregivers in the context of increased burden. We studied a total of 345 caregivers (age: M = 64.44, SD = 11.46) caring for a family member with a neurodegenerative disease (parent = 90; spouse/partner = 255) who were participating in our research on dementia caregivers. Caregivers' burden, loneliness, and likelihood of using cognitive reappraisal were assessed using well-established questionnaires. We found that greater caregiver burden was associated with greater loneliness (r = 0.436, p < 0.001). This association was moderated by caregivers' use of cognitive reappraisal (B = -0.08, p = 0.02) such that the association between burden and loneliness was less pronounced for caregivers who were more likely to use cognitive reappraisal. This moderation was maintained when controlling for potentially confounding variables (age, gender, and education). We believe that cognitive reappraisal may buffer against loneliness by helping caregivers focus on the more positive aspects of the situation (e.g., areas of preserved functioning in the person with dementia or memories of pleasurable activities in the past).

Abstract citation ID: igaf122.2778

### COMBATING SOCIAL ISOLATION: EVALUATING THE IMPACT OF AN INTERGENERATIONAL PROGRAM 'PERFECT PAIR'

Samiya Manocha[1], Elizabeth Rueppel[2], Shriya Karmarkar[3], Kamryn Casey[4], Edie Lerner[5], Madison Ebstein[6], Celene Philip[3], and Emily Lerner[3], *1. Northwestern University Feinberg School of Medicine, Chicago, Illinois, United States, 2. Icahn School of Medicine at Mount Sinai, New York, New York, United States, 3. University of Michigan, Ann Arbor, Michigan, United States, 4. University of Miami Miller School of Medicine, Miami, Florida, United States, 5. University of Michigan-Flint, Flint, Michigan, United States, 6. WashU Medicine, St. Louis, Missouri, United States*

Social isolation and loneliness are growing public health concerns that negatively impact older and younger adults. A national report found that one in four adults aged 65+ is socially isolated, while a 2023 survey revealed that 44% of college students experience depression and 37% struggle with anxiety. Perfect Pair, a national nonprofit, combats these issues by fostering intergenerational relationships between college students and older adults. Participants are paired based on shared backgrounds and interests, meeting weekly to share stories and participate in activities like language learning and painting. A retrospective survey of participants with at least 12 meeting hours demonstrated significant benefits. Among older adult respondents (N = 39), 100% felt they gained a companion/friend, 83.3% felt less lonely, 97.2% looked forward to meetings, and 81.1% felt they have closer ties to the younger generation. Additionally, 91.9% felt increased happiness after meetings and 73% reported an improvement in their mental health. Among younger adult respondents (N = 67), 96.7% learned something new from their pair, 96.7% felt an increase in happiness after meetings, 91.8% felt they better understand aging/long-term care communities, 95.1% felt they have closer ties to the older generation, and 73.4% reported an improvement in their mental health. Perfect Pair participation led to a reduction in loneliness, increased intergenerational connection, and improved mental health for both older and younger adults. Intergenerational programming like Perfect Pair demonstrates a valuable potential to combat social isolation and loneliness through building meaningful connections.

Abstract citation ID: igaf122.2779

### CONSIDERING COMMUNITY: EXAMINING CIVIC ENGAGEMENT AND WELL-BEING IN LATER LIFE

Lauren Gil Hayes[1], Morgan Stangl[1], Man Guo[1], and Yi Wang[2], *1. University of Iowa, Iowa City, Iowa, United States, 2. University of Iowa School of Social Work, Iowa City, Iowa, United States*

The socio-ecological model (SEM) highlights how well-being is shaped by individual, interpersonal, and community-level factors. While individual behaviors and interpersonal relationships are well-documented influences on well-being, civic engagement—through volunteer activities, adult learning, and special interest group participation—remains an underexamined community-level engagement factor. This study investigates whether civic engagement is associated with loneliness and health in aging adults and whether interpersonal support moderates these associations. Using data from the 2018 Health and Retirement Study (HRS) Leave-Behind Questionnaire ($n = 5{,}530$), a nationally representative survey of aging U.S. adults, we employ three survey-weighted Ordinary Least Squares (OLS) regressions with robust standard errors, adjusting for demographic and socioeconomic characteristics. Results indicate that higher civic engagement is significantly associated with lower loneliness ($\beta = -0.0436$, $p < 0.001$) and better self-rated health ($\beta = 0.1386$, $p < 0.001$). The magnitude of the association between civic engagement and self-rated health is larger than its association with loneliness. Interpersonal support from spouses, children, and friends does not significantly moderate these relationships, inviting further analysis to determine if civic engagement may be beneficial for those with limited support networks. These findings position civic engagement as a potential public health strategy, reinforcing and expanding SEM's multi-dimensional emphasis on structural and social contexts in well-being. Expanding opportunities for volunteerism, lifelong learning, and group participation may enhance both social and health outcomes, reinforcing the importance of community involvement in shaping a holistic understanding of well-being.

Abstract citation ID: igaf122.2780

### EFFECTIVENESS OF HEARING AID ADHERENCE ON SOCIAL CONNECTIVITY IN OLDER ADULTS: A SCOPING REVIEW

Fumiko Hamada[1], Charity Lewis[2], and Lindsay Peterson[2], *1. University of South Florida School of Aging, Tampa, Florida, United States, 2. University of South Florida, Tampa, Florida, United States*

Age-related hearing loss affects 65% of older adults but is widely untreated. Untreated hearing loss is associated with multiple adverse outcomes, including social isolation and loneliness. This scoping review aims to assess the effectiveness of hearing aid use on social connectivity in adults ages 50 and older living with age-related hearing loss, and the review was performed using PubMed, PsycINFO, and Web of Science databases. Search terms included older adult, hearing aid, age-related hearing loss, social connectivity, social engagement, social isolation, and loneliness. English-language studies with participants aged over 50 years diagnosed with age-related hearing loss treated with the use of hearing aids were included. A total of 375 articles were identified, of which six met the inclusion criteria. Several outcome measures were used: Three studies focused on social isolation, loneliness, or both. One study investigated the relationship between social isolation and hearing loss and cognition, while another examined the impact of hearing aid use on social participation. All studies examining social isolation and loneliness reported improved scores following the adoption of hearing aids. One study showed that hearing aid use mediated the link between hearing loss and cognitive decline, while another found that increased usage (wearing them for more hours) improved social participation. This scoping review concluded that hearing aid adherence may help to strengthen social connectivity among older adults. Given that hearing aid use is an effective treatment for age-related hearing loss, there is a substantiated need for policy and governmental involvement to improve access and aff ordability.

Abstract citation ID: igaf122.2781

### EFFECTS OF COMMUNITY ENVIRONMENTS IN LONELINESS AND DEPRESSION AMONG OLDER ADULTS IN SOUTH KOREA

Michin Hong[1], and Soondool Chung[2], *1. Indiana University, Indianapolis, Indiana, United States, 2. Ewha Womans University, Seoul, Republic of Korea*

Given its significant impact on older adults' wellbeing, community environments have gained increasing attention in gerontology. This study examined how community environments affect loneliness and depression among older adults in South Korea and explored their moderating role between the loneliness and depression relationship. We used data from a nationwide survey, focusing on adults aged 65 or older ($N = 605$). Community environments were measured through service accessibility (SA) and opportunities for community engagement (CE), along with predictors including gender, age, education, perceived health, family network, friend network, and social support. Loneliness and interaction terms for SA, CE, and loneliness were added to the depression model. All scales demonstrated high reliability. Hierarchical regressions were conducted using SPSS 29. Results showed that a greater family network, friend network, social support, SA and CE were associated with lower loneliness. Higher income, better health, a greater family network and social support, lower loneliness, and greater SA were associated with lower depression. Additionally, a moderating effect of SA on the loneliness-depression relationship was found; while living in lower-SV areas was generally associated with higher depression, the negative effect of loneliness on depression was much stronger in individuals living lower-SV areas compared to those in greater-SV areas. The results underscore the critical role of community environments in alleviating loneliness and depression among older adults. Enhancing SA and CE can mitigate loneliness and its harmful effects on depression. Community-based interventions need to be actively implemented to support older adults' wellbeing.

Abstract citation ID: igaf122.2782

**LONELINESS AMONG OLDER ADULTS IN AN INTERGENERATIONAL TELEPHONE-BASED PROGRAM**

Rebecca Mullen[1], Joanna Fitzgibbons[2], Prajakta Shanbhag[1], Mahi Mahfuza Haque[1], Sarah Tietz[1], Devin Gilhuly[1], and Jodi Waterhouse[3], *1. University of Colorado School of Medicine, Aurora, Colorado, United States, 2. University of Colorado Anschutz Medical Campus, Aurora, Colorado, United States, 3. CU Anschutz Medical Campus, Aurora, Colorado, United States*

Loneliness is a critical health concern for older adults, yet few evidence-based interventions for loneliness exist. Connecting Older Adults to Students Through Intergenerational Telecare (COAST-IT) is a telephone-based program that pairs health professional students from the University of Colorado Anschutz Medical Campus with community-dwelling older adults for unstructured conversations. This study assessed the prevalence, characteristics, and changes in loneliness in COAST-IT older adult participants. We conducted cross-sectional surveys of older adult participants at pre-intervention and post-intervention (n = 38). Loneliness was measured using the UCLA 3-item Loneliness Survey. We also conducted multivariable regression controlling for age, sex, marital status, sexual identity, duration in study, and communication frequency with student partner. The mean age of participants was 74.1 (SD = 6.98), with the majority being female (89.5%, 34/38) and White (92.1%, 35/38). The average length in the study was 7.86 months (SD = 4.52). Nearly half (47.4%, 18/38) of older adult participants communicated with their student partner less than twice per month, while 44.7% (17/38) did so more often. The prevalence of loneliness at baseline was 29.7% (11/38), compared to 18.4% (7/38) at follow-up (p = 0.41). After adjusting for covariates, loneliness was significantly associated with older age (p < 0.001), being divorced (p = 0.009), and sexual minority status (p = 0.002). In conclusion, older adults who participated in an intergenerational telephone-based program with students did not experience a significant reduction in loneliness. Future research will examine the program's impact on older adult participants' depression and anxiety symptoms in addition to student participants' loneliness and ageist beliefs.

Abstract citation ID: igaf122.2783

**LONELINESS AND DEPRESSION: THE MEDIATING EFFECT OF PERCEIVED SOCIAL STRAIN IN OLDER ADULTS WITH DIABETES**

Emma Cho, and George Demiris, *University of Pennsylvania, Philadelphia, Pennsylvania, United States*

Loneliness is a known risk factor for social strain which refers to the capability of certain social relationships to act as a source of stress. Social strain has been shown to negatively affect mental health outcomes such as depression in older adults. Older adults with diabetes are at greater risk for loneliness. However, limited research has examined there is a lack the complex relationship among loneliness, social strain, and depression in this population. The purpose of this study was to examine the relationship between loneliness and depression in older adults with diabetes and to explore the mediating role of social strain in this association. This cross-sectional study was developed as a secondary data analysis using data from the National Social Life, Health, and Aging Project in the USA. The sample consisted of 379 older adults aged 50 to 88 years. Loneliness, social strain, and depression were assessed using a multi-item survey questionnaire. Bivariate analysis and macro PROCESS in SPSS were used to analyze the data. Mediation analysis revealed a positive, direct effect of loneliness on depression (path c': $\beta = 1.61$, p <.001), and a positive, indirect effect of loneliness on depression through perceived social strain ($\beta = 0.20$, p <.001). These findings suggest that the relationship between loneliness and depression is partially mediated by social strain. Interventions that target both loneliness and the negative perceptions or interactions associated with social strain could help reduce depression in this population.

Abstract citation ID: igaf122.2784

**LONELINESS IN OLDER ADULTS WITH SERIOUS MENTAL ILLNESS**

Heather Leutwyler, and Erin Hubbard, *University of California, San Francisco, San Francisco, California, United States*

Background: Middle-aged and older adults with serious mental illness (SMI) have a lifespan that is about 25 years shorter than persons without a SMI. Tobacco use and limited social networks likely contribute to this premature mortality. Between 70-85% of people living with SMI currently smoke cigarettes. Social disconnection and limited social networks are common in people with SMI. To understand how loneliness is associated with tobacco use and symptoms among middle-aged and older adults with SMI, we report a cross-sectional analysis from the baseline assessment of a sample of middle-aged and older adults with SMI enrolled in a smoking cessation study. Methods: We analyzed baseline data from 64 participants with SMI > 40 (mean age 53.7, age range 40-73) enrolled in a smoking cessation program. We assessed loneliness with the 3-item UCLA loneliness scale, cigarette use with participants' self-report of the number of cigarettes smoked per week, and psychiatric symptoms with the Brief Psychiatric Rating Scale. Results: We identified significant positive associations between the higher ratings on the item "feeling left out" on the loneliness scale and a higher number of cigarettes smoked in the past week (p=.03). We identified a significant positive association between total psychiatric symptom score and higher ratings on the item "feeling left out" (p=.002). Conclusion: Results suggest daily cigarette use is associated with higher self-ratings of loneliness, and higher symptom burden is associated with higher self-ratings of loneliness. Future work could test integrating social connections to better support middle aged and older adults with SMI.

Abstract citation ID: igaf122.2785

**NETWORK ANALYSIS ON MULTI-DOMAIN LONELINESS FACTORS AMONG THREE AGING GROUPS WITH KINSHIP VULNERABILITY**

Jianan Li, *University of California, Los Angeles, Los Angeles, California, United States*

Close kin (i.e., a partner and children) has been identified as important supporting systems against loneliness in later life, with those with kinship vulnerability—lacking traditional family structures—facing heightened risk. While multi-dimensional factors contributing to loneliness have been studied, their associations across different kinship vulnerability groups remain unexplored. Using the 2016 and 2018 Health and Retirement Study, this study employed network analysis to construct partial correlation networks with 22 factors across six domains (demographics, socioeconomic status, physical, psychological, cognitive, and social well-being) among adults over 50 with kinship vulnerability across three groups: having a partner but no children ("Partner-only," N = 331), having children but no partner ("Children-only," N = 2635), and having no partner and no children ("kinless," N = 487). Centrality measures (betweenness, closeness, strength) showed that psychological well-being factors (depression, hopefulness) were central for Partner-only and Kinless groups, while physical health and demographic factors were prominent for the Children-only group. Ego network analyses revealed that social well-being factors had the strongest direct connections with loneliness: friend support was positively associated with loneliness in Partner-only adults (r=.296), while friend strain was the strongest predictor in Children-only (r=.270) and Kinless groups (r=.255). PageRank analysis identified self-reported health as the most influential bridge across all three networks, especially for Partner-only and Kinless groups. These findings demonstrate distinct loneliness mechanisms across kinship vulnerability groups, informing tailored interventions targeting central factors, factors with the strongest direct and indirect effects, and critical bridges between factors to effectively address loneliness in older adults based on their specific kinship vulnerability.

Abstract citation ID: igaf122.2786

### PLACE-BASED UNDERSTANDING OF SOCIAL ISOLATION AND LONELINESS IN U.S. SUBSIDIZED SENIOR HOUSING: A SCOPING REVIEW

Jihye Baek[1], Byeongju Ryu[2], and Sojung Park[1], *1. Washington University in St. Louis, St. Louis, Missouri, United States, 2. Washington University in St. Louis, Clayton, Missouri, United States*

Subsidized senior housing (SSH) provides affordable housing and social services to low-income older adults, promoting opportunities for social engagement. Despite these communal benefits, residents often experience social isolation and loneliness due to various individual and environmental challenges, including health issues, financial constraints, limited transportation options, and changes in social relationships. This scoping review examined factors associated with social isolation and loneliness through a socio-ecological framework. A systematic search of six electronic databases identified 12 studies, and a thematic analysis synthesized the evidence across multiple levels. At the individual level, residents facing physical and mental health challenges, as well as limited social skills and financial resources, faced a higher risk of social isolation and loneliness. At the interpersonal level, weakened family ties intensified social isolation, while peer relationships within SSH fostered belonging and mutual support. At the community and organizational levels, improved access to transportation, social spaces, and community cohesion served as protective factors against social isolation and loneliness. Societal factors, including the COVID-19 pandemic and changes in the digital environment, influenced social isolation and loneliness. Findings emphasize the interplay between personal and environmental factors, highlighting the need for multi-level interventions. Supporting individuals with limited personal resources while improving transportation access and community engagement may mitigate these challenges. Additionally, addressing broader societal factors, such as the impact of the pandemic and technological changes through context-specific strategies, can enhance residents' social well-being. We emphasize that a contextualized understanding of social isolation and loneliness is crucial for developing effective interventions.

Abstract citation ID: igaf122.2787

### REGIONAL FACTORS RELATED TO SOCIAL DISCONNECTION AMONG OLDER ADULTS IN SOUTH KOREA: THE ROLE OF SUBJECTIVE HEALTH

Jaeyoon Kim, and Meejung Chin, *Seoul National University, Seoul, Republic of Korea*

Despite longstanding concerns about social isolation and loneliness among older adults, research on the macro-level factors (e.g., regional characteristics) influencing these issues remain limited. Using data from 6,045 older adults (aged 59–103) drawn from the ninth wave (2022) of the Korean Longitudinal Study of Aging and the Korean Statistical Information Service, we conducted multilevel modeling to analyze the relationships between regional factors and social disconnection (i.e., social isolation and loneliness) among older adults. Additionally, we examined whether subjective health status moderates these relationships. The results revealed that social isolation and loneliness were related to different regional factors. Older adults tended to be more isolated in regions with a lower proportion of single-person households aged 65 and older. Meanwhile, they were more likely to feel lonely in regions with a higher fiscal self-reliance rate and more leisure facilities per 1,000 older adults. Subjective health status amplified the relationship between fiscal self-reliance rate and both forms of social disconnection but reversed the relationship between proportion of social welfare spending and loneliness. Although higher fiscal autonomy was associated with lower social isolation across all health groups, it was related to greater loneliness, particularly among those in poorer health. In contrast, a higher proportion of welfare spending was linked to lower loneliness among those in poor health but to higher loneliness among those in good health. These findings underscore the need to consider both regional factors and the Person-Environment Fit (P-E Fit) framework in addressing social disconnection among older adults.

Abstract citation ID: igaf122.2788

### SIBLING DISCONNECTEDNESS, SIBSHIP SIZE, AND COGNITION IN LATER LIFE

Gina Lee[1], Jooyoung Kong[1], Deborah Carr[2], and Michal Engelman[3], *1. University of Wisconsin–Madison, Madison, Wisconsin, United States, 2. Boston University, Boston, Massachusetts, United States, 3. University of Wisconsin-Madison, Madison, Wisconsin, United States*

Prior research has established that social disconnectedness can negatively impact cognition in later life. However, sibling disconnectedness and its effect on cognitive change in older adults remain unexplored. We explore how sibling disconnectedness and sibship size interact to influence cognitive change in later life. Siblings are a potentially important source of social engagement, especially as older adults experience widowhood and deaths of friends. We used four waves of data from the Wisconsin Longitudinal Study (WLS; N = 6,468) collected in 1993, 2004, 2011, and 2020. The WLS tracked graduates of Wisconsin high schools in 1957 and one randomly selected sibling, into their late adulthood. Sibling disconnectedness was measured as binary indicators (W1-W3), defined as feeling "not at all close" to their selected sibling or having zero contact over the last 12 months. Global cognition (W2-W4) was assessed by averaging four standardized cognitive performance tests. A longitudinal linear mixed model was used.

Results indicated that individuals disconnected from siblings in smaller sibships displayed an accelerated rate of cognitive decline. However, for individuals maintaining sibling connections, cognition levels did not differ across varying sibship sizes. Having more siblings appears cognitively beneficial for those experiencing sibling disconnection, potentially due to the support systems and mentally stimulating interactions with multiple network members. These findings underscore the importance of maintaining robust sibling ties, particularly for those with fewer siblings, in later life. Practitioners could leverage this knowledge to develop interventions/programs that promote family cohesion, particularly among siblings, and help mitigate cognitive decline among older adults.

Abstract citation ID: igaf122.2789

### SOCIAL ISOLATION AND METABOLIC SYNDROME AMONG COMMUNITY DWELLING, LOW-INCOME OLDER ADULTS: WHAT WE NEED TO KNOW!

Karen Bullock[1], Barbara Mendez Campos[2], Terrance Ruth[3], and Kim Stansbury[3], *1. Boston College, Chestnut Hill, Massachusetts, United States, 2. University of Tennessee Knoxville, Knoxville, Tennessee, United States, 3. North Carolina State University, Raleigh, North Carolina, United States*

During the COVID-pandemic, social determinants of health were brought to light for many gerontologists in ways that had not been given close attention in older adult communities before. Such non-medical factors that influence a person's health and wellbeing can include socio-economic status, education, social support, healthcare access, transportation and more. This study explores social isolation as a factor contributing to health and well-being among older adults. Studies show physical and emotional association between human connection and the clustering of biological factors, also known as metabolic syndrome. Such clustering of health conditions such as cardiovascular disease, arthritis, kidney disease, several types of cancers, schizophrenia and other mental health disorders constitute metabolic syndrome, which is more prevalent among older adults of racial and ethnic groups that are known to have the highest rates of morbidity. Furthermore, low educational attainment and advanced age have been associated with higher reports of social isolations and loneliness. However, less is known about the incidences of chronic illness among an older adult population living in low-income senior housing communities and social isolation, which can be associated with loneliness, which is also risk factor associated with well-being. Preliminary data is presented to highlight the importance of understanding and intervening with community-dwelling older adults to help keep them thriving and aging.

Abstract citation ID: igaf122.2790

### TESTING THE RELIABILITY AND VALIDITY OF THE SOCIAL ISOLATION VISUAL ANALOGUE SCALE AMONG OLDER ADULTS

Anne Hagan[1], Barbara Resnick[2], Nicole Brandt[3], Sarah Holmes[4], and Jennifer Klinedinst[5], *1. UMB SON, ELKRIDGE, Maryland, United States, 2. University of Maryland, Baltimore, Maryland, United States, 3. UMB School of Pharmacy, 20 N. Pine St. Baltimore, Maryland, United States, 4. University of Maryland School of Nursing, Baltimore, Maryland, United States, 5. UMB SON, 655 W. Lombard Street, Baltimore, Maryland, United States*

Background and Purpose: The purpose of this study was to test the reliability and validity of the Social Isolation Visual Analogue Scale. Methods: This was a descriptive study. Forty residents from four senior housing communities were interviewed related to social isolation, depression, and social networks at baseline and two weeks later. Results: The mean age of the participants was 78.8 (SD = 8.1), the majority were female (86%) and Black (60%). There was evidence of test-retest reliability with a significant correlation between baseline and two-week follow-up of the Social Isolation Visual Analogue Scale ($r=.74$, $p<.05$). There was a significant relationship between the Social Isolation Visual Analogue Scale and depression and social networks. Conclusions: The findings from this study provided some preliminary evidence of the reliability and validity of the Social Isolation Visual Analogue Scale in low-income senior housing residents. Keywords; Social isolation, older adults, senior housing, social networks

Abstract citation ID: igaf122.2791

### THE RELATIONSHIP BETWEEN SOCIAL ISOLATION, DEPRESSION, AND COGNITION: THE MEDIATION ANALYSIS OF DEPRESSION

Fanghong Dong[1], and Yeji Hwang[2], *1. Washington University in St. Louis, St. Louis, Missouri, United States, 2. Seoul National University, Seoul, Republic of Korea*

Social isolation is a key factor leading to cognitive decline among older adults. However, the underlying mechanisms driving this relationship remain unclear. Given the strong link between social isolation and mental health, this study aimed to examine whether depression mediates the longitudinal relationship between social isolation and cognition. This study was a 12-year longitudinal study using data from the Health and Retirement Study (2006/2008 (T1), 2010/2012 (T2), 2014/2016 (T3), 2018/2020 (T4)). Participants who were 65 years and older were selected (N = 946). Depression was assessed using the Center for Epidemiologic Studies Depression scale. Cognition was assessed via the Telephone Interview for Cognitive Status. Social isolation was measured using 9 items on contact frequency with children, family members, and friends. The longitudinal mediation model was used adjusting for age, gender, race, and education, which demonstrated excellent fit (CFI = 0.990, TLI = 0.986; RMSEA = 0.025, SRMR = 0.022). Higher baseline social isolation significantly predicted elevated baseline depression ($\beta = 0.20$, $p < .001$), which in turn negatively impacted baseline cognition ($\beta = -0.16$, $p < .001$). A significant indirect effect confirmed mediation at the intercept level ($\beta = -0.032$, $p = .002$), while social isolation retained a direct negative effect on cognition ($\beta = -0.10$, $p = .018$). This supported depression as a partial mediator at baseline levels. However, longitudinal slope paths were non-significant, suggesting no dynamic mediation over time. Future studies need to be conducted on identifying the longitudinal mechanism underlying the relationship between social isolation and cognitive decline.

### SESSION 7295 (POSTER)

### TECHNOLOGY: OLDER ADULT INTERFACE AND USE

Abstract citation ID: igaf122.2792

### ASSOCIATION BETWEEN SOCIAL DETERMINANTS OF HEALTH AND TELEHEALTH USE AMONG OLDER ADULTS

Misun Hwang, Philip Veliz, and Yun Jiang, *University of Michigan, Ann Arbor, Michigan, United States*

Telehealth holds promise to enhance health outcomes for older adults, yet significant disparities remain in access and utilization. Although examining social determinants of health (SDOH) could provide valuable insights to bridge the digital divide, little is known about how these factors affect telehealth use. This study explored the association between SDOH and telehealth use among older adults in the United States. Using logistic regression, we analyzed data from the 2023 National Health and Aging Trend Study (NHATS), a nationally representative survey including 8,597 older adults (≥65 years). According to the Healthy People 2030 SDOH framework, five dimensions of SDOH were assessed: economic stability, education, healthcare access and quality, social context, and neighborhood and built environment. Telehealth use was measured by communication with a provider, insurance handling, or health information access using the Internet or online platforms (yes/no). Findings revealed that over half of older adults (50.3%) used telehealth in the past year. Those with less than a college education (OR: 0.28, CI: 0.24-0.32), living in rural areas (OR: 0.59, CI: 0.44-0.81), lacking social networks (OR: 0.48, CI: 0.32-0.72), or with neighborhood safety concerns (OR: 0.58, CI: 0.45-0.75) were less likely to use telehealth. Economic stability (measured by food insecurity, home ownership, and ability to pay utilities) was not associated with telehealth use after controlling for other factors. While multiple dimensions of SDOH significantly affected telehealth use among older adults, addressing the impact of these factors in telehealth services and providing tailored support will improve older adults' engagement and health outcomes.

Abstract citation ID: igaf122.2793

### BARRIERS TO DIGITAL TECHNOLOGY FOR HOMEBOUND OLDER ADULTS

Sheryl Groden, MIchelle Sahli, and Donna Case, *University of Michigan-Flint, Flint, Michigan, United States*

Many homebound older adults require digital literacy to use telehealth and online programs which have been shown to be helpful in decreasing social isolation. Our research addressed homebound older adults' internet access, use of technology, and perceived barriers to digital technology. We partnered with our local Office on Aging, Meals on Wheels in two counties (Genesee and Washtenaw), and Homebound Services at the Genesee County Library. Key research questions included prevalence of internet access in homebound older adults, ways in which these older adults use technology, barriers to internet technology use, and how internet use would change if barriers were removed. We surveyed 61 homebound adults 60 years of age and older about their internet technology use and found that 24.6% did not have access to the internet. Respondents reported the following challenges to technology use: health or physical condition (44.3%), uncertainty on how to use devices (82%), unfamiliarity with options (68.9%), disinterest (42.6%), lack of device with internet access (18%), and discomfort in learning new things (50.8%). If these barriers were removed, 14.8% would start to use technology, 25.9% would use it more than they do now, 11.% still would not use technology and 27.9% technology use would not change. These findings provide evidence that internet access alone does not remove older adults' barriers to digital literacy, and may help guide professionals when planning interventions and identifying the supports that would best facilitate elderly engagement with technology.

Abstract citation ID: igaf122.2794

### BRIDGING SOCIAL NETWORKS AND WELL-BEING: THE MODERATING POWER OF TECHNOLOGY USE IN OLDER ADULTS

Manjun Kim[1], and Yesang Cho[2], *1. Florida State University, Tallahassee, Florida, United States, 2. Seoul National University, Seoul, Republic of Korea*

Social networks are widely recognized as essential to the well-being of older adults. As technology continues to advance, its use has also become important in forming and maintaining social connections. Particularly, technology use is crucial in supporting older adults and shaping their social engagement(Kim et al., 2017). While technology adoption rates among older adults have steadily increased over time (Mitzner et al., 2019), consideration must be given to how it integrates with beneficial and related factors. Within the framework of Socioemotional Selectivity Theory (SST), older adults prioritize meaningful relationships and prioritize emotionally fulfilling experiences (Carstensen et al., 1999). Technology use enhances older adults' social networking, leading to improved well-being (Baradach et al., 2021; Fang et al., 2018). This study further explores how information and communication technology (ICT)_ use impacts their well-being. Using National Health and Aging Trends Study Round 13, we employed a hierarchical multi-regression model to analyze how ICT use moderates the relationship between social network and well-being. The results indicate that ICT use moderated the relationship between social networks and well-being This effect was pronounced among individuals with lower social networking. Our findings suggest that ICT use may serve as a protective factor against declines in well-being among older adults with lower levels of social connections. Specifically, ICT users who had a smaller well-being gap associated with differences in social network size compared to non-users. These results highlight the significance of technology adoption in later life and underscore the potential of technology use to mitigate disparities in well-being.

Abstract citation ID: igaf122.2795

### BRIDGING THE DIGITAL GAP FOR OLDER ADULTS IN KOREA: DIRECT CARE WORKERS AS MEDIATORS

Jeongone Seo[1], Kyung-Zoon Hong[1], and Sol Baik[2],
*1. Sungkyukwan University, Seoul, Republic of Korea,
2. University of Virginia, Charlottesville, Virginia, United States*

In the face of rapid aging in South Korea, information and communication technologies (ICTs) hold significant potential for improving older adults' well-being and social engagement. Yet many older adults experience barriers such as low digital literacy, device anxiety, and fear of scams. This study explored direct care workers' experiences facilitating digital usage of older adults in the community. We conducted semi-structured interviews with ten direct care workers at adult daycare and home care settings in Korea. Our qualitative thematic analysis revealed that care workers played pivotal roles as demonstrators, troubleshooters, and motivators. Many older adults, especially those with mild cognitive impairments, relied on hands-on demonstrations and repeated, step-by-step guidance with emotional support from care workers. While care workers viewed tools (e.g., smartphones, tablets, and AI-based devices) as helpful in enhancing the social connections of older adults and monitoring their safety, they also expressed concerns about privacy, data security, and increased workloads. Furthermore, financial constraints and limited formal training hindered care workers' effectiveness. Findings suggest that older adults benefit most when digital solutions are tailored to their familiar interests, combined with person-centered teaching strategies. For care workers, targeted training and stronger institutional support are crucial to fostering technology adoption. By emphasizing the direct care workers' mediator role, this study contributes practical insights for better integrating digital health and social tools into long-term care settings in Korea. Future research will expand the findings with larger samples and cross-cultural comparisons to refine strategies for empowering older adults with effective digital usage.

Abstract citation ID: igaf122.2796

### BUILDING DIGITAL LITERACY IN THE OLDER ADULT NORTH NASHVILLE COMMUNITY

Jennifer Kim[1], and Cathy Maxwell[2], *1. Vanderbilt University, Nashville, Tennessee, United States, 2. University of Utah, Salt Lake City, Utah, United States*

Digital literacy (DL), the ability to understand and use digital technology, is crucial for older adults' mental and physical well-being. Using an academic and community partnership model, investigators developed an evidence-based, 8-week, in-person digital literacy program (DLP) for low-income older adults in an African American community to examine effects on participants' DL abilities. Participants were recruited from two senior centers. The DLP curriculum was based on the Mobile Device Proficiency Questionnaire (MDPQ) subscales (e.g., mobile device basics, communication, date/file creation and storage, internet access, software management). All participants received a personal electronic tablet. Reinforcement of the DLP curriculum and social support was provided through weekly engagement with volunteer student nurses. Pre/post digital literacy was measured using the MDPQ. Forty older adults participated in the program and completed an initial baseline survey on digital literacy tasks. Compared to baseline assessments, statistically significant improvements were noted for 17 tasks, which participants felt were "very easy" or "somewhat easy" to perform. Tasks with the strongest improvements included the ability to find information about local community resources (pre: 9, 25% vs. post: 20, 50%, p<.001) and about hobbies and interests (pre: 11, 27.5% vs. post: 19, 47.5%, p<.001), and the ability to enter events and appointments into a calendar (pre: 6, 15% vs. post: 16, 40%, p<.001). Digital literacy programs for older adults are wise investments for both private and public funding sponsors. The academic and community partnership model is an effective way to support nonprofit organizations that lack resources.

Abstract citation ID: igaf122.2797

### DIGITAL MENTAL HEALTH INTERVENTIONS FOR OLDER ADULTS: A META-ANALYSIS OF RANDOMIZED CONTROLLED TRIALS

Yahui Wang, and Dannii Yeung, *City University of Hong Kong, Kowloon Tong, Hong Kong, China (People's Republic)*

With growing mental health concerns among older adults and limited access to traditional services, digital interventions may offer a scalable solution. However, evidence regarding their effectiveness across different mental health outcomes remains unclear. Using multilevel modelling, a meta-analysis of randomized controlled trials examining digital interventions for older adults was conducted. Forty studies comprising 5,148 participants were analysed. Primary outcomes were depression and anxiety, with secondary outcomes including cognitive functioning, loneliness, and quality of life. Compared to control conditions, digital interventions demonstrated significant beneficial effects for depression (g = -0.28, 95% CI [-0.43, -0.13], p < .001) and anxiety (g = -0.22, 95% CI [-0.45, 0.00], p = .05). Secondary outcomes showed effects for cognitive functioning (g = 0.35, 95% CI [0.06, 0.64], p < .05) and loneliness (g = -0.29, 95% CI [-0.50, -0.08], p < .01), with effects on quality of life approaching significance (g = 0.22, 95% CI [-0.02, 0.46], p = .07). Moderator analyses for depression revealed larger effects for interventions employing guided human support and for smartphone-delivered interventions compared to unguided or internet-based approaches. Digital interventions appear effective for addressing mental health concerns in older adults, depression and anxiety, improving cognitive functioning and loneliness. The significant heterogeneity suggests that effectiveness varies based on intervention delivery method and the inclusion of human guidance. These findings not only highlight the potential of digital mental health tools for expanding psychotherapy options for older adults, but also suggest that personalized intervention addressing the individual needs can maximize the intervention outcomes.

Abstract citation ID: igaf122.2798

### ETHICAL CONSIDERATIONS OF SOCIAL ROBOTS FOR SUPPORTING MENTAL HEALTH AND WELLBEING IN OLDER ADULTS IN LONG-TERM CARE

Lillian Hung[1], Yong Zhao[2], Hadil Adfares[2], and Parsa Shafiekhani[2], *1. University of British Columbia, Vancouver, British Columbia, Canada, 2. The University of British Columbia, Vancouver, British Columbia, Canada*

Social robots are increasingly becoming utilized to address mental health challenges in older adults, such as depression, anxiety, and loneliness. However, ethical concerns surrounding their use are insufficiently explored in empirical research. This paper examines the ethical challenges and mitigation strategies for implementing social robots in long-term care settings. Drawing from insights gained over research across two Canadian studies involving Paro and Lovot, we highlight the critical role of an equity-focused approach to ensure the ethical use of social robots. We advocate for the respectful inclusion of the voices and desires of marginalized groups, such as older adults with dementia. Key ethical issues discussed include inequitable access, consent, substitution of human care, and concerns about infantilization. Our empirical work offers practical strategies to navigate these challenges, aiming to ensure that social robots promote mental health and well-being in an ethically responsible manner for older adults living in long-term care.

Abstract citation ID: igaf122.2799

### EVERYTHING, EVERYWHERE ALL AT ONCE: LIFELONG LEARNING AND THE INVISIBLE REVOLUTION

Lyn Holley[1], and Laura Donorfio[2], *1. University of Nebraska Omaha, Omaha, Nebraska, United States, 2. University of Connecticut, Farmington, Connecticut, United States*

Although it is well established that social isolation is linked to depression in older adults, many take comfort in noting that older adults can utilize a myriad of technological advances to remain connected to others and life—cell phones, various apps, direct messaging, virtual visits, holograms, etc. A visit to the supermarket provides visible evidence of older adults using this technology and commercials reinforce this by featuring older adults utilizing not only this technology, but other types that help safeguard personal safety and health, do household chores, and have conversations. While these wonderful inventions seem to have increased our quality of life and are hard won by many advocacy organizations (e.g., GSA, NCOA AARP), we may have missed what perpetual, precipitous change requires of aging persons. Perpetual technological advances have been described as "the fourth Industrial Revolution." Those who are the "oldest old" had the good fortune to age in an environment that gave them time to adapt. This fourth industrial revolution, however, is outpacing the length of time it takes to adapt, exacerbating the digital divide between generations. How can we help older adults adapt adequately to the constant daily rate of technological change? Lifelong learning may be key to help thwart the impact of perpetual, precipitous change, but other opportunities are increasingly inadequate. This paper discusses how older adults can remain and feel connected to others and life in a world of unprecedented technological change. Gerontologists are uniquely positioned to identify and deal with this threat. Shall we?

Abstract citation ID: igaf122.2800

### EXAMINING CLINICIAN OFFER OF VIDEO TELEHEALTH AT VETERANS HEALTH ADMINISTRATION: A NATIONAL SURVEY

Megan Gately[1], Steven Shirk[1], Emily Metcalf[1], Dylan Waller[2], Emma Quach[1], and Lauren Moo[3], *1. VA Bedford Health Care System, Bedford, Massachusetts, United States, 2. Center to Improve Veteran Involvement in Care (CIVIC), Portland, Oregon, United States, 3. VA Bedford Healthcare System, Bedford, Massachusetts, United States*

Though older adults have historically utilized technology less than younger adults, their use of telehealth greatly expanded in recent years. Despite these gains, a telehealth Digital Divide has led to discrepancies in use of telehealth with older adults and others with complex medical needs, creating a persistent barrier to healthcare. While evidence for how clinicians decide which patients to offer video telehealth to is generally lacking, age is frequently cited by clinicians as a barrier, suggesting an ageist attitude towards older adults. This study will present findings from a national survey of clinicians at Veterans Health Administration (VHA), the largest integrated healthcare system in the United States. The survey, which is approved for launch in March 2025, will be sent to over 297,000 inter-professional VHA clinicians, including physicians, nursing and rehabilitation professionals, psychologists, and social workers. Survey items include factors influencing clinician offer of video, such as patient factors (age, comorbidity, and caregiver availability) and organizational influences (leadership buy-in, telehealth metrics, availability of administrative/technical support), as well as clinician attitudes towards patient fitness for telehealth more broadly. Building on this first ever national survey examining factors influencing clinician utilization of video telehealth, this study aims to promote clinicians' successful integration of telehealth with older patients, thereby increasing patient access to care.

Abstract citation ID: igaf122.2801

### EXPLORING EXPERIENCES UTILIZING TECHNOLOGY OF DEMENTIA CAREGIVERS IN HOSPICE

Hannah Cho[1], Justine Sefcik[2], and George Demiris[1], *1. University of Pennsylvania, Philadelphia, Pennsylvania, United States, 2. Drexel University College of Nursing and Health Professions, Philadelphia, Pennsylvania, United States*

Technology-mediated interventions among dementia caregivers in hospice settings offer significant potential to transform end-of-life care delivery and alleviate caregiver burden. As family members navigate complex end-of-life tasks while facing unique circumstances like anticipatory grief and life reprioritization, they experience both rewards and significant stress from dementia's unpredictable trajectory. Despite technology's rapid reshaping of healthcare delivery, research examining how these technologies function specifically within dementia hospice care remains notably limited. This research aims to explore the experiences of dementia caregivers utilizing technology during hospice care. This qualitative descriptive study was guided by two frameworks Technology-enabled caregiving in the home and the Pearlin Stress Model. We conducted semi-structured interviews with 10 adult caregivers (mean age 63.6 ± 13.27, female 70%) who provided care for PWD during hospice care at home (n = 8) or in nursing homes (n = 2). Through a direct content analysis, results revealed three distinct patterns of technology use among dementia caregivers in hospice: administrative management, social support, and access to caregiving resources. Most of the caregivers emphasized that gaining online resources or training information was helpful in early dementia; however, later, during hospice care, they used technology more for practical care coordination tools such as reminders for medications and communication tools among families. These changes in technology utilization highlight the critical need for adaptive technology-mediated interventions that effectively support transitions in care, particularly assisting caregivers and their PWD as goals shift from aggressive measures to a comfort-based approach during hospice.

Abstract citation ID: igaf122.2802

### FROM ADHERENCE TO HAPPINESS: CO-DESIGNING WITH FRAIL NURSING HOME RESIDENTS TO GAMIFY TECH-BASED INTERVENTION

Mandi Tang, Ge Lin Kan, and Mingming Fan, *Hong Kong University of Science and Technology (Guangzhou), Guangzhou, Guangdong, China (People's Republic)*

Improving adherence to physio-cognitive interventions is crucial for mitigating frailty and dementia in older adults. Nursing home residents, often overlooked in both academic community and the marketplace, lack access to game-like experiences and report mental illness and poor adherence to interventions. To increase adherence, this article presents a three-phase co-design study with frail nursing home residents (medium age: 79 years) to gamify a physio-cognitive virtual reality (VR) intervention system, incorporating playful games in life-size VR streets and an indoor bike for self-directed, multisensory navigation. By addressing their needs for livingness, familiarity, and presence in VR-based gamification, the study demonstrates how playfulness effectively increases adherence (from 14.56 to 23.76 minutes), retention (from handful times to several weeks), and greater numbers of voluntary enrollment, while providing psychosocial benefits (p-value < 0.001). Design guidelines for VR-based interventions are proposed, with an unexpected emphasis on happiness, consistent with global efforts on dementia prevention.

Abstract citation ID: igaf122.2803

### HOW DOES THE USABILITY OF A DEMENTIA CARE APP DIFFER? INSIGHTS FROM COMMUNITY AND NURSING HOMES

Jungwon Cho, Eunhee Cho, Minhee Yang, Sinwoo Hwang, Eun Kyo Kim, and Sang A Lee, *Yonsei University, Seoul, Republic of Korea*

Behavioral and psychological symptoms of dementia (BPSD) is a significant factor of caregiver burden. However, symptom-specific non-pharmacological interventions have not been systematically implemented. To address this gap, the research team developed a mobile app to support caregivers in BPSD management decision-making. This study evaluated its usability in community and nursing home settings. The analysis included app usage log data from 36 dyads of people living with dementia (PLWD) and their caregivers in the intervention arm of two RCTs conducted in these settings. Participants recorded BPSD diary (type, time, duration) and performed individualized care programs. App usability was evaluated based on BPSD diary recording rates, program usage frequency, session duration, and engagement levels. While app-based interventions were feasible in both groups, differences in usability and usage patterns were observed. Usability scores were higher in the community across all measures. Music therapy was the most frequently used program in both settings. Exercise therapy was common in the community (26.7%) but rare in nursing homes (0.8%), whereas reminiscence therapy with music was more widely used in nursing homes (33.5%) than in the community (8.0%). In nursing homes, program use was limited due to the physical and cognitive conditions of PLWD and caregiver workload. Additionally, tailoring interventions to individual preferences was more challenging in nursing homes. Even with app support, person-centered care can only be effectively implemented when a minimum standard of care per person is ensured. Therefore, policies to establish this standard, along with institutional efforts from nursing homes, should be considered.

Abstract citation ID: igaf122.2804

### IMPLICATIONS OF TRANSITION IN COMMUNICATION CHANNEL PROFILES BEFORE AND AFTER THE COVID-19 CRISIS

Ha Young Choi[1]Jung Yeon Suh[2], and Shannon Mejia[2], *1. University of Illinois Urbana-Champaign, Urbana, Illinois, United States, 2. University of Illinois Urbana-Champaign, Champaign, Illinois, United States*

This study examines transitions in older adults' communication channel patterns during the COVID-19 crisis and investigates whether these transitions moderate the relationship between pre-pandemic social support and post-pandemic loneliness. Using Health and Retirement Study data (n = 2,830; aged 50-97), latent profile analysis identified four communication profiles in 2018: (1) in-person/phone contact with family, (2) social media (SNS) dominant, (3) email dominant, and (4) high interaction across all channels. By 2022, the in-person/phone profile was replaced by a "least interaction" profile. The other three profiles persisted. Latent transition analysis estimated the probability of stability and change in profile membership between the two time points. In-person/phone profile mostly transitioned to the least interaction profile. The high interaction profile largely remained stable. SNS profile was the least stable where members were most likely distributed to all other profiles. Email profile transitioned to all, except the least interaction, profiles. Path analyses tested, for each profile, the implications of change versus stability for the protective effects of 2018 social support for 2022 loneliness. Across all profiles, membership stability maintained the protective effects of social support on loneliness. However, friend support's effect disappeared when transitioning from in-person/call to email profiles. Child support's effect vanished when transitioning from SNS to least interaction profiles. Family support's effect disappeared when moving from high to least interaction profiles. These findings suggest the pandemic significantly altered older adults' communication patterns, with implications for mental health. Interventions should strengthen diverse social support networks and provide targeted assistance with digital communication adoption.

Abstract citation ID: igaf122.2805

### INTERNET USAGE AND INTRINSIC CAPACITY IN ENGLAND

Ethan Wang, and Paola Zaninotto, *University College London, London, England, United Kingdom*

The population aged 65 years and older in the United Kingdom is projected to grow from 11.8 million in 2016 to 20.4 million in 2066. However, digital disparities persist, with 46% of those aged 75 years and older in the United Kingdom not using the internet in 2020. Few studies have investigated the link between internet usage and intrinsic capacity, which is the combination of an individual's physical and mental abilities. Data was from the 2012-2013 Wave 6 of the English Longitudinal Study of Aging (ELSA). The analysis sample included 4,143 participants aged 60 years and older. Internet usage was determined by respondents' self-reported frequency in using the internet or email, categorized as high, medium, and low. Intrinsic capacity was modeled as a composite score of 14 variables: word recall, orientation in time, balance, chair rises, walking speed, upper mobility, lower mobility, eyesight, hearing, grip strength, body mass index, waist circumference, depressive symptoms, and life satisfaction. Linear regression was used to model associations of internet usage with intrinsic capacity. After full adjustment for demographics and health characteristics, participants with medium internet usage did not have an association with lower intrinsic capacity compared to the reference group (β:-0.07, 95%CI:-0.22, 0.07). Participants with low internet usage had an intrinsic capacity score 0.44 units lower compared to those with high internet usage (95%CI:-0.58, -0.29). Low internet usage is associated with lower intrinsic capacity among older adults in England. The risks of low technology usage to healthy aging should be investigated in future studies.

Abstract citation ID: igaf122.2806

### INTERNET USE AMPLIFYING OLDER ADULTS' EXISTING SOCIAL ENVIRONMENTS AND SELF-PERCEPTION OF AGING

Joonhyeog Park[1], Yanjun Dong[2], Zexi Zhou[3], and Kun Wang[4], *1. University of Pennsylvania, Philadelphia, Pennsylvania, United States, 2. SUNY at Albany, Albany, New York, United States, 3. The University of Texas at Austin, Austin, Texas, United States, 4. University of Alabama at Birmingham, Birmingham, Alabama, United States*

Negative social support, characterized by strained or harmful interactions, adversely affects older adults' self-perceptions of aging (SPA). While digital engagement, such as Internet use, has the potential to expand social networks and enhance social support, it remains unclear whether it mitigates or exacerbates the detrimental effects of negative social support on SPA. This study addresses this gap by examining how Internet use moderates the relationship between positive/negative social support and positive/negative SPA. Data were drawn from the Health and Retirement Study (HRS): 2008/2010 (T1), 2012/2014 (T2), and 2016/2018 (T3) (n = 9,304, aged 65+). Random-effect panel regressions with interactions between Internet use and positive/negative social support were conducted. The results revealed that Internet use exacerbates the detrimental effects of negative social support on negative SPA (B = .11, p < .01) but does not significantly affect positive SPA. Conversely, Internet use enhances the benefits of positive social support on positive SPA (B = .07, p < .05) but has no significant effect on negative SPA. These findings suggest that Internet use amplifies the impact of existing social environments: it strengthens the benefits of positive social support while worsening the harm caused by negative social support. This highlights the need for targeted interventions tailored to older adults' social contexts. In supportive environments, promoting digital engagement can strengthen positive age perceptions. In less supportive environments, interventions must combine digital engagement with efforts to reduce negative social interactions, ensuring the benefits of Internet use are accessible to all, regardless of social circumstances.

Abstract citation ID: igaf122.2807

### INTERNET USE PATTERNS AMONG KOREAN BABY BOOMERS: FACTORS INFLUENCING GENDER DIFFERENCES

Jeehoon Kim[1], Ji Hyun Lee[2], Hee Lee[3], Young Ji Yoon[4], and Dongwook Kim[5], *1. Idaho State University, Pocatello, Idaho, United States, 2. Montana State University, Bozeman, Montana, United States, 3. The University of Alabama, Tuscaloosa, Alabama, United States, 4. Colorado State University Pueblo, Pueblo, Colorado, United States, 5. Arizona State University, Phoenix, Arizona, United States*

Korean Baby Boomers (born between 1955 and 1963) are entering older adulthood with unprecedented educational and economic resources, enabling them to navigate the digital landscape more proficiently than previous generations. However, little is known about the patterns of Internet use and the gender-specific factors influencing their use. Using the 2012 Korean Baby Boomers Panel Study data, latent class analysis was conducted with eleven online activities. Multinomial logistic regression analysis was performed (reference: rare users) separately by gender. Predictors included health, social network size, social relationships, activity participation, loneliness, attitudes toward Internet use, and demographic variables. The participants (n = 3,227, Age M(SD)=52.8(2.6), 54% female) responded. Three use patterns were identified: rare users (46.5%), moderate users (43.9%), and maximizers (9.6%). Men were more likely to be moderate users or maximizers than women. Compared to rare users, both moderate users and maximizers engaged more in social activities, had more positive attitudes toward the Internet, higher educational attainment, and were younger. For women, moderate users and maximizers met friends/neighbors more often than rare users. Moderate users had smaller social networks, while maximizers were more likely to be single than rare users. For men, moderate users met friends/neighbors more often, while maximizers met relatives more often. Male maximizers were more likely to feel lonely than rare users. All findings had p-values less than .05. The gender differences in the relationship between social relationships, social networks, loneliness, and Internet use patterns will be discussed, along with intervention strategies for alleviating loneliness and enhancing social inclusion for maximizers.

Abstract citation ID: igaf122.2808

### LIFESTYLE HABITS AND SUBJECTIVE MEMORY COMPLAINTS IN OLDER USERS OF THE TERRAPINO MOBILE APPLICATION

Raena Nolan[1], Ross Andel[2], Jan Pavlik[3], and Jakub Hort[4], *1. Arizona State University, Phoenix, Arizona, United States, 2. Arizona State University, Arizona State University, Arizona, United States, 3. AlzheimerChain Foundation, Prague, Hlavni mesto Praha, Czech Republic, 4. Charles University, Prague, Central Bohemian, Czech Republic*

Background/objective: Subjective memory complaints (SMCs), such as difficulty recalling names or recent events despite otherwise normal cognition, can affect daily functioning and quality of life. We examined the association between lifestyle and SMCs while considering physical and mental health. Methods: Participants were 3,789 dementia-free adults aged 60+ years with complete data who used Terrapino, a free mobile application designed to promote cognitive health, launched in December 2022. SMCs were measured with 8 yes/no items on memory-related issues. Composites of physical activity (walks, exercise, sports measured), mental activity (reading, board games, mediation etc.), and health diet (nuts, fish, vegetables, berries, meat consumption [reversed]), were measured as never, sometimes, or frequently, and z scored. Average hours of sleep/day (7-8 vs. not) were also assessed. Results: Mean age was 69.6±6.4 (60-103) years, 71% were women; 85% had at least high school education, and 43% were college educated. In an ordinal logistic regression adjusted for age, sex, and education, higher physical activity (Estimate=-0.09, p=.004) and mental activity (Estimate=-0.07, p=.025), healthier diet (Estimate=-0.21, p<.001), and getting 7-8 hours of sleep/day (Estimate=-0.32, p<.001) were all associated with lower SMCs. Adding comorbidity to the model explained away the physical activity-SMCs link (p=.088); adding hearing problems had no effect, and adding ability to manage stress explained the association between physical (p=.364) and mental (p=.057) activities and SMCs, while other associations remained significant. Discussion: Lifestyle, particularly sleep, relates to memory complaints. Some associations reflected ability to manage stress.

Abstract citation ID: igaf122.2809

### LIVING ALONE, INTERNET USE, AND DEPRESSIVE SYMPTOMS IN CHINESE OLDER ADULTS: A LONGITUDINAL STUDY

Wan Wen[1], Xiayu Chen[2], and Kun Wang[3], *1. University of Illinois Urbana-Champaign, Champaign, Illinois, United States, 2. University of Central Florida, Urbana, Illinois, United States, 3. University of Alabama at Birmingham, Birmingham, Alabama, United States*

The growing trend of older adults living alone, due to rapid urbanization and family structure changes, poses unique challenges in China, where family support has traditionally been central to aging care. This shift has left many older adults with limited support from their adult children, raising concerns about increased social isolation and risk of depression. Internet use has emerged as a potential tool to mitigate these challenges by fostering social connection and engagement. However, few studies have examined the associations among internet use, living arrangement, and depressive symptoms in the Chinese context. This study examines the reciprocal relationship between Internet use and depressive symptoms and explores how this relationship varies based on living arrangements. Using two waves (2018 and 2020) of the China Health and Retirement Longitudinal Study (CHARLS), we analyzed data from 2,875 older adults (aged 60 – 95, M = 68.35 in 2018). Autoregressive cross-lagged analyses were conducted, controlling for demographic and health factors. Results revealed a unidirectional relationship among older adults living alone: more frequent Internet use predicted lower depressive symptoms two years later (B = -1.393, p = 0.003), whereas depressive symptoms did not predict Internet use. For those not living alone, no significant relationship was found. These findings suggest that older adults living alone may derive unique mental health benefits from Internet use, potentially due to its role in reducing social isolation. Policymakers and practitioners should provide more digital literacy and training programs to promote digital access among older adults, especially for those living alone.

Abstract citation ID: igaf122.2810

### LONG-TERM USE OF AN APP-BASED INTERVENTION FOR OLDER ADULTS' QUALITY OF LIFE: SMART-AGE FIRST INSIGHTS

Nicole Memmer[1], Anna Schlomann[2], and Hans-Werner Wahl[1], *1. Heidelberg University, Heidelberg, Baden-Wurttemberg, Germany, 2. Network Aging Research, Heidelberg, Baden-Wurttemberg, Germany*

With growing expectations for technology to enhance the quality of life in older adults, the ongoing SMART-AGE intervention study investigates the benefits of a multi-domain, app-based intervention designed to promote (1) social participation, (2) physical fitness, and (3) health awareness across a 6-month observation period. The design is a three-armed, randomized controlled trial, including 649 community-dwelling older adults (67+ years) with basic digital skill level, assigned to a full intervention, partial intervention, and active control condition. In addition to evaluating the effectiveness of the intervention, analyzing automatically tracked app usage data is central to the study, providing insights into adherence and engagement patterns. To this end, this poster presents initial findings on the use of one of the implemented apps, i.e., smartVERNETZT, which is a German adaptation of the proven PRISM-App in the U.S. (CREATE consortium; Czaja et al,) aimed to foster social participation. Among the 286 participants analyzed for long-term usage, 111 (39%) used smartVERNETZT weekly during the final three months, meeting the criteria for long-term users. Besides income and education, no significant baseline differences in social connectedness, technology skills, or attitudes toward technology between long-term and non-long-term users of smartVERNETZT were found. Additionally, a statistically significant decline in usage was noted, with engagement dropping from an average of 2.74 days per week in the first three months to 2.15 days per week in the last three months. Findings underline the challenge of sustaining long-term engagement in digital interventions for older adults.

Abstract citation ID: igaf122.2811

### PATTERNS OF DIGITAL DEVICE USAGE AND SERVICE NEEDS FOR DIGITAL ADAPTATION AMONG OLDER ADULTS

Sang A Lee, Eunhee Cho, Minhee Yang, Jungwon Cho, Eun Kyo Kim, and Sinwoo Hwang, *Yonsei University, Seoul, Republic of Korea*

In the digital era, digital devices play an increasingly vital role in health management beyond daily convenience. However, age-related physical changes and low digital literacy hinder older adults' technology use, widening the digital divide and impacting health. This study analyzed subgroups of older adults based on digital device usage and difficulties in digital adaptation to identify their service needs. A total 7,470 older adults were sampled from the 2023 National Survey of Older Koreans. Latent class analysis classified digital usage subgroups using 13 digital device activities (e.g., text messaging, social media, information search, photos/videos, music, games, and financial transactions) and one item on digital adaptation difficulties. Multinomial logistic regression analyses were performed to examine socio-demographic and health-related factors of each subgroup and to identify their preferred service needs. Four subgroups emerged from the analysis: digitally active (DA) (14.9%), moderately active (MA) (33.4%), text messages only (TM) (27.9%), and digitally inactive (DI) (23.9%). Compared to the DA group, the DI and TM groups were older, had more chronic diseases, lower cognitive function, and required greater assistance in daily life. Regarding service needs, compared to the DA, the other groups showed a stronger preference for age-friendly technology support (e.g., larger fonts, simplified layouts) over digital literacy education (DI: OR = 2.065, 95% CI = 1.603–2.662; TM: OR = 1.568, 95% CI = 1.259–1.953; MA: OR = 1.331, 95% CI = 1.095–1.618). By identifying subgroup-specific needs, this study provides policy and practical insights to develop targeted interventions, ensuring older adults receive the necessary support to navigate the evolving digital landscape.

Abstract citation ID: igaf122.2812

### PRIVACY, PROFICIENCY, AND THE PROMISE OF WEARABLES: INSIGHTS FROM FOCUS GROUPS WITH OLDER ADULTS

Justin Lam[1], and Walter Boot[2], *1. Donald and Barbara Zucker School of Medicine at Hofstra/Northwell, Brooklyn, New York, United States, 2. Weill Cornell Medicine, New York, New York, United States*

Older adults are increasingly using the Internet, laptops, smartphones, and other digital devices to access healthcare services and information. Additionally, wearable technologies offer the potential to continuously monitor health data, which could be used to detect and address age-related health issues. However, the adoption of these technologies remains challenging due to difficulties in using the technology and concerns about privacy. In this study, we build upon the results of Fowe and Boot's (2022) survey study, which examined older adults' attitudes toward the use of wearable and mobile technologies for predicting cognitive decline, supporting healthy behaviors, and collecting self-reported health data. The current study aimed to qualitatively explore the barriers and facilitators of wearable technology use. Data were collected through four focus group sessions with older adults. Thematic analysis revealed five main themes: (1) privacy concerns, (2) digital "nagging," (3) accuracy and reliability concerns, (4) proficiency challenges, and (5) a strong interest in monitoring personal health. These findings underscore the importance of developing resources that enhance older adults' skills, confidence, and self-efficacy in using wearable technologies safely and effectively.

Abstract citation ID: igaf122.2813

### USABILITY AND ACCEPTABILITY OF TECHNOLOGIES FOR REMOTE HOME-BASED ENVIRONMENTAL MONITORING IN OLDER ADULTS

Peyton Berning[1], Pei-An Lee[2], Wanting Yu[1], Brad Manor[3], Lewis Lipsitz[4], and Amir Baniassadi[1], *1. Hebrew SeniorLife,*

*Boston, Massachusetts, United States, 2. Hebrew SeniorLife & Harvard Medical School, Boston, Massachusetts, United States, 3. Hebrew SeniorLife/Harvard Medical School, Boston, Massachusetts, United States, 4. Hebrew Senior Life, Boston, Massachusetts, United States*

Wearable and smart-home technologies offer exciting opportunities to study environmental effects on health directly within individuals' homes. Yet, it is unclear whether older adults are capable of using these devices. This pilot study examined older adults' receptiveness and ability to use smart thermostats and watches to monitor sleep under different bedroom temperatures. Fourteen independently-living, smartphone-owning older adults (age 82.1±5.6, 7 female, MoCA score: 24-30) were equipped with cloud-controlled thermostats and given watches requiring daily syncs across two apps. Participants received 30 minutes of at-home training supplemented with graphics and could contact study staff as needed. Eleven participants were equipped with both devices in one visit, while three required additional visits. Overall, we collected 569 person-nights of data while remotely adjusting bedroom temperature. No participants needed additional thermostat training. Participants adhered to wearing the watch 96% of the time. Ten watch-related troubleshooting calls were made, six of which concerned smartphone apps. A subset of eight participants answered questions about interacting with study technology. All eight found thermostat use "very easy," as did six for watch and app use. Two found watch and app use "somewhat easy." Four attributed successful technology usage to self-motivated routine building. Critiques included discomfort sleeping with watches and non-intuitive app interfaces. All found overall participation "very easy." Older adults appear accepting and capable of using wearable and smart-home technologies with ongoing support, indicating untapped opportunities to study their environmental health. Future studies should explore wearables promoting usability and include populations with lower cognitive and technology experience levels.

Abstract citation ID: igaf122.2814

### USABILITY ASSESSMENT AND TASK PERFORMANCE OF HEALTH APPLICATION FOR OLDER ADULTS:

Suhyun Kim, Jungyeon Bae, Dahyeon Lee, Eunseo Lee, Yunseo Jang, and Daeun Jeong, *Kyungpook National University, Daegu, Republic of Korea*

Purpose: This study aimed to evaluate the usability and task performance of health applications for older adults to identify ways to enhance user experience. Methods: This study utilized an explanatory sequential mixed methods approach. The quantitative phase involved a survey of 100 older adults from senior welfare centers in D City utilizing the Mobile Application Rating Scale (MARS) to assess app usability. The qualitative phase involved in-depth individual interviews and task performance evaluations to explore functional challenges and usability barriers. Results: The overall MARS score was 3.56 out of 5, with functionality receiving the highest rating (3.60), followed by aesthetics (3.57), information (3.78), engagement (3.71), and subjective quality (3.16). The mean task performance score was 2.09 out of 3, with significant obstacles identified in app installation, information retrieval, navigation complexity, and input processes. The interviews revealed usability challenges, such as insufficient font size, lack of screen magnification, and unintuitive interface designs. Participants emphasized incorporating practical functions and in-app user guidance to enhance usability. Conclusion: Nurses can help ensure that health applications are tailored to older users' needs. Improved app usability for this population requires design modifications, including adjustable font sizes, screen magnification options, and intuitive interfaces. Additionally, user education and in-app user guides can support effective app usage and task performance.

Abstract citation ID: igaf122.2815

### WEARABLE USE, ENGAGEMENT, AND BEST PRACTICES: LESSONS LEARNED FROM THE VIRTUAL MY HEALTHY BRAIN TRIAL

Makenna Law, Mallika Saksena, and Ryan Mace, *Massachusetts General Hospital, Boston, Massachusetts, United States*

Physical activity and sleep are key lifestyle behaviors for promoting healthy aging and dementia risk reduction. Wearable activity monitors are frequently employed in research settings as an effective and valid method for objectively assessing activity and sleep. Although previous studies have investigated validity and usability for older adults, more guidance is needed on evidence-based strategies for increasing the adoption and adherence of digital health technologies embedded in behavioral dementia prevention trials. We conducted a remote randomized controlled trial with older adults (60+) with subjective cognitive decline and lifestyle risk factors comparing the 8-week My Healthy Brain program with an attention- and dose-matched educational control. We enrolled 49 older adults (M = 72, SD = 7.41; 86% female; 61% white). Participants set up a Garmin Vívosmart 5 virtually with study team assistance and were instructed to wear for baseline to post-test (9 weeks) to track step count and total sleep. Adherence surpassed our a priori feasibility benchmark, which required 70% of participants to achieve 5 of 7 days per week at a minimum of 10 hours per day (Unadjusted adherence = 78%, 91% after removing Garmin errors). Our presentation will share effective strategies to promote adherence and lessons learned such as establishing clear expectations, device use training over Zoom, addressing accessibility challenges, linking data to personal relevance, and troubleshooting Garmin errors. The feasibility data and adherence strategies will inform the design of a subsequent NIA Stage 2 trial testing the efficacy of My Healthy Brain to improve physical activity and sleep outcomes assessed via Garmin.

Abstract citation ID: igaf122.2816

### WEARABLE WELLNESS TECHNOLOGY USAGE AND CONCERNS ACROSS MIDDLE AGE AND OLDER ADULTHOOD

Laurel Mertz, and Jennifer Smith, *Mather Institute, Evanston, Illinois, United States*

Wearable technologies may enhance wellness by supporting diagnostics, self-monitoring, and behavior change. Although middle-aged to older adults may particularly benefit from wearable devices, their usage remains underexplored and may be impacted by privacy concerns, technological barriers, and affordability. This study aimed to evaluate how and why different age cohorts (adults in their 40s, 50s, 60s, and 70s) use various wearable devices and to assess their potential concerns. A total of 5,037 adults, ages 44 to 78, were recruited via an online research panel (mean age = 59.9 years, 51.7% female, 65.5% White). Participants completed a survey assessing their health monitoring using wearable technologies. Those who used wearables reported the metrics they tracked, while non-users provided reasons for their non-use. Chi-square analyses revealed that adults in their 40s used smartwatches, smart rings, and other wearables to monitor their health more than any other group. Among wearable technology users, similar proportions across age cohorts tracked blood pressure, heart rate, physical activity, sleep, and weight. However, age differences emerged in the tracking of habits, mood, screen time, and food/water intake. Common reasons for non-use across age groups included data privacy concerns, usage difficulties, cost, and lack of interest. Generally, older cohorts were more likely to report tracking their health without using wellness technologies. These results indicate that age cohorts may use wearables for different purposes, but non-users share similar concerns regardless of age. Further research is needed to explore alternative health tracking methods among older adults, and to address concerns about wearable devices.

### SESSION 7300 (POSTER)

### AGING IN PLACE

Abstract citation ID: igaf122.2817

### ACCEPTABILITY OF A BEHAVIORAL ACTIVATION INTERVENTION FOR FUNCTIONING IN OLDER VETERANS

Meaghan Kennedy[1], Sydney Ruggles[1], Christina Cardwell[2], Anastasia Canell[1], Jaye McLaren[3], Kathleen Lyons[4], Jonathan Bean[5], and Megan Kelly[6], *1. VA Bedford Health Care System, Bedford, Massachusetts, United States, 2. Lemuel Shattuck Hospital, Boston, Massachusetts, United States, 3. VA Bedford Healthcare System, Bedford, Massachusetts, United States, 4. Massachusetts General Hospital Institute of Health Professions, Boston, Massachusetts, United States, 5. Harvard Medical School, Boston, Massachusetts, United States, 6. University of Massachusetts Chan Medical School, Worcester, Massachusetts, United States*

Behavioral activation (BA) is an evidence-based psychotherapy for depression that also shows promise as a transdiagnostic intervention to support functioning. We adapted a brief BA protocol to improve physical, cognitive, and social functioning in older Veterans at risk for decline. In this study, we conducted an open pilot to assess acceptability of the adapted intervention among 10 older (age ≥65) Veterans at risk for functional decline (Vulnerable Elders Survey-13 score ≥3). Participants completed a 6-session telehealth-delivered BA intervention, baseline and post-intervention assessments, and a semi-structured interview based on the Theoretical Framework of Acceptability. Interviews were analyzed by two researchers using rapid qualitative analysis. Participants had a mean age of 79.6 years (range 69-88) and 80% were male. Nine participants completed all 6 intervention sessions and follow-up assessments; one withdrew after the second session. Mean satisfaction on the Client Satisfaction Questionnaire (CSQ-8) was 26.9±3.7 (on a scale of 8-32). Participants found the telehealth format convenient and the length and frequency appropriate. The content was highly aligned with participants' values and what mattered most to them. While most participants found value in tracking their activities, others found it tedious and recommended simplifying the process. The intervention helped participants stay organized, set goals, and break tasks into parts; increased their motivation and helped them refocus on valued activities; and provided them with skills they will continue to use to support activity engagement. Our study suggests that a brief telehealth-delivered BA intervention for functioning is acceptable, values-aligned, and perceived as effective among older Veterans.

Abstract citation ID: igaf122.2818

### AFFORDABLE SENIOR HOUSING IN RURAL MASSACHUSETTS – GAPS AND SOLUTIONS TO IMPROVE SERVICES AND SUPPORTS FOR RESIDENTS

Natasha Bryant[1], Steve Kalter[2], Robyn Stone[1], and Peter Atkins[3], *1. LeadingAge, Washington, District of Columbia, United States, 2. Acumen Marketing Research, Inc., Groton, Massachusetts, United States, 3. Fallon Health, Worcester, Massachusetts, United States*

Fallon Health, a managed care organization offering the Senior Care Options (SCO) program in Massachusetts, received a grant from the Executive Office of Health and Human Services to assess and improve supportive services in affordable senior housing in a rural part of the state. Partnering with Acumen and LeadingAge, the team conducted an evaluation of 11 properties through resident surveys (N = 327) and focus groups with property managers (N = 6) and resident service coordinators (RSCs) (N = 5). Findings revealed that most properties have operated for over 20 years and lack infrastructure suited for aging residents. Common health conditions include hypertension and arthritis, with half of residents at risk for depression and a quarter for loneliness. Social engagement is moderate. Residents most often require housekeeping and transportation assistance. While nearly all properties employ RSCs, their on-site presence is limited to adequately address resident needs. Other key challenges include RSC staffing, inconsistent assessments, and a lack of community-based service providers with whom to partner. Recommendations include increasing RSC presence, conducting periodic needs assessments to address resident needs, and strengthening community partnerships to coordinate service delivery, care integration and transportation. Massachusetts should explore using targeted Medicaid and state funds to build RSC capacity, incentivize health and social service providers to team with housing organizations and expand telehealth services. The Fallon Health Navigator program, embedded in these sites, should be strengthened and serve as a model for other SCOs. The solutions could improve the well-being of affordable housing residents and support their ability to age in place.

Abstract citation ID: igaf122.2819

### AGING IN PLACE AND HEALTHCARE EQUITY: HOW REBUILDING TOGETHER SUPPORTS LIVING LONGER IN HOMES AND COMMUNITIES

Christine McNichols[1], and Annie Rhodes[2], *1. Virginia Commonwealth University, Richmond, Virginia, United States, 2. Virginia Commonwealth University, Virginia Commonwealth University, Virginia, United States*

Aging in Place (AIP) refers to the ability to remain in one's home and community as one ages. While AIP is widely regarded as beneficial, disparities in housing stability, accessibility, and affordability create inequitable barriers. Current clinical AIP interventions focus on individual-level solutions, often overlooking broader socio-economic and structural determinants. Using a Health Disparities Framework, demographic and service data from a community-based organization, Rebuilding Together Richmond (RT-R), was analyzed. Descriptive statistics assessed the characteristics of homeowners served, the types of repairs performed, and their potential impact on AIP. Repairs were categorized as structural or occupational to evaluate their contributions to home safety and accessibility. RT-R provided repairs for 33 homes, benefiting 47 individuals, all of whom were Black or African American living in a zip code with high eviction rates. The majority (63.8%) were female, and 51% were older adults and/or had a disability. Structural repairs were more frequent than occupational modifications, reflecting both homeowner needs, service availability, and community organizational goals. Housing stability is a critical yet overlooked factor in AIP. Integrating clinical AIP interventions with community-based solutions can more effectively address health disparities, reduce institutionalization risks, and improve long-term livability. Partnerships between healthcare practitioners and organizations like Rebuilding Together are essential to advancing equity in AIP. Access to housing is not accessible housing, and to remove barriers, practitioners and community-based organizations should expand their appreciation of obstacles to include historical, contemporary, economic, and environmental factors to work toward equity in aging in place for all.

Abstract citation ID: igaf122.2820

### AGING IN PLACE IN FRONTIER WYOMING: EXPERIENCES OF OLDER ADULTS

Chelsea Davis-Hearn[1], Jeff Clark[2], and Bernard Steinman[1], *1. University of Wyoming, Laramie, Wyoming, United States, 2. State of Wyoming, Cheyenne, Wyoming, United States*

Aging in place (AIP) is a relevant concern for older adults living in remote communities, where isolation and limited resources are distinct challenges that are often faced. Many older adults prefer to remain in their homes and communities because these are more than just places to live; they are filled with memories, connections, and a strong sense of belonging. However, the ability to age in place in extremely rural settings presents significant challenges such as limited access to healthcare, transportation, and social services. Understanding these factors is essential to developing strategies that promote autonomy, safety, and well-being for older adults living in frontier regions. This study focused on the perspectives and lived experiences of 20 older adults currently aging in place in Wyoming's frontier communities, which are defined as less than 7 people per square mile. Using in-depth interviews, the study gained insights into the physical, social, and environmental factors that influence people's ability to age in place as well as the strategies they utilize to cope with the challenges faced in these remote settings. Findings suggest that strong emotional attachments to the sense of home and community may serve as powerful motivators for people remaining in place despite potential difficulties. Involvement in community activities and support from family, friends, and neighbors proved essential for older people striving to maintain their independence. These findings inform the wider conversation on aging in place and reinforce the importance of social support systems for older adults residing in frontier areas.

Abstract citation ID: igaf122.2821

### ASSESSMENT OF CORTISOL, HALITOSIS AND ORAL HEALTH IN INSTITUTIONALIZED AND NON-INSTITUTIONALIZED ELDERLY PEOPLE

Fátima CristinaCarneiro Marques, Élcio Magdalena Giovani, and Ivana Barbosa Suffredini, *UNIP, São Paulo, Sao Paulo, Brazil*

Objective: This study aims to evaluate the perception of quality of life of institutionalized elderly people (MB group) in comparison with non-institutionalized elderly people (CLIN group), in addition to comparing these results with dental indices. Study Design: Both men and women aged 60 years and older were included in the study. Anthropometric and cardiac measurements were taken, in addition to applying specific questionnaires: the GOHAI, aimed at geriatric oral health, and the SF-36, which investigates quality of life. Results: The Mann-Whitney test ($\alpha < 0.05$) was used for statistical analysis. No statistically significant differences were observed in the GOHAI domains. Despite the intensive care offered by the institution, non-institutionalized participants reported a better physical quality of life. Conclusion: The participants presented similar overall oral health conditions. The institutionalized participantsshowed a higher salivary flow. Non-institutionalized participants showed higher salivary cortisollevels. Halitosis proved to be similar. The participants in both groups predominantly used totalmaxillary prostheses, but those in the non-institutionalized group used more partial mandibularprostheses. No differences were found in self-perceived health among the participants. The non-institutionalized participants presented better physical health; however, mental health was similarbetween the two groups.

Abstract citation ID: igaf122.2822

### CAREGIVERS' IMPLEMENTATION OF HOME MODIFICATIONS FOR OLDER CARE RECIPIENTS

Mengzhao Yan[1], Kristen Glennie[2], Daniel Barbakoff[3], and Bernard Steinman[4], *1. University of Southern California, Los Angeles, California, United States, 2. Wyoming Institute for Disabilities, Laramie, Wyoming, United States, 3. Weill Cornell Medicine, New York, New York, United States, 4. University of Wyoming, Laramie, Wyoming, United States*

Home modifications can make caregiving tasks easier by enhancing safety and accessibility, yet it is not clear which factors are associated with the implementation of home modifications by caregivers on behalf of those receiving care. This study analyzed linked data from Round 12 of the National Health and Aging Trends Study and the National Study of Caregiving. Guided by the Ecology Theory of Aging and Anderson's Behavioral Model, we conducted multivariate logistic regressions to explore whether sociodemographic characteristics, the care recipients' health status (measured by the ICF framework), and caregiving activities are associated with family caregivers' actions to implement home modifications. Key findings include: (1) Compared to spousal caregivers, adult children (OR: 0.48, $p < 0.001$) and other caregivers (OR: 0.27, $p < 0.001$) were less likely to make home modifications for care recipients; (2) More restrictions in social participation (OR: 1.14, $p < 0.05$) and more physical impairments (OR: 1.30, $p < 0.001$) among care recipients were associated with higher odds of making home modifications by caregivers; (3) Caregivers' frequent assistance with self-care (OR: 2.18, $p < 0.001$), caregivers' receiving training (OR: 1.78, $p < 0.001$), and care recipients' use of rehabilitation (OR: 1.54, $p < 0.001$) were associated with higher odds of caregivers making home modifications. The findings highlight that increased caregiver responsibility and training play an important role in home modification implementation. Future research, policy, and practice to promote home modifications for aging in place should take caregivers' experiences and activities into account.

Abstract citation ID: igaf122.2823

### CHINESE HEALTHCARE PROFESSIONALS' PERSPECTIVES ON THE APPLICATION OF AI IN ELDERLY CARE

Xiuhong Zhang, and Jinxia Lu, *Shanxi Bethune Hospital, Taiyuan, Shanxi, China (People's Republic)*

Background Ageing populations, growing burden of chronic diseases, and rising costs of healthcare worldwide are posing unprecedented challenges for governments. While artificial intelligence (AI) shows considerable promise in enhancing clinical decision-making processes and optimizing healthcare efficiency, its successful implementation depends on addressing healthcare professionals' acceptance, concerns, and preferences. This study investigates health professionals' perspectives on the perceived benefits, potential risks, and implementation strategies for AI adoption in elderly care. Methodology A qualitative, multi-center study was conducted across five cities China. The study included 55 healthcare professionals—physicians, nurses, and allied health practitioners—who participated in 11 focus group discussions. Participants were recruited through purposive and snowball sampling. Semi-structured interviews explored their attitudes toward AI in elderly care, and thematic analysis was used to identify key themes. Results Participants recognized AI's potential to improve efficiency, support clinical decision-making, and enhance patient care. Many highlighted its role in reducing workload and aiding early disease detection. However, concerns about data security, ethical implications, and loss of human-centered care were common. Challenges related to trust in AI, workflow integration, and training needs were also emphasized. Preferred implementation strategies included clear regulations, human-AI collaboration, and institutional support for skill development. Conclusion Healthcare professionals view AI as a valuable tool but also state the need for ethical oversight and proper training. This study provides insights into the Chinese healthcare professionals' views and preferences to inform the delivery of an AI in elderly care in the Chinese context.

Abstract citation ID: igaf122.2824

### CLUSTERS OF HOME- AND COMMUNITY- BASED SERVICE USE AND ASSOCIATION WITH QUALITY OF LIFE IN OLDER ADULTS

Eric Jutkowitz[1], Romil Parikh[2], Jack Wolf[2], Yinfei Duan[3], Benjamin Langworthy[2], and Tetyana Shippee[1], *1. University of Minnesota, Minneapolis, Minnesota, United States, 2. University of Minnesota School of Public Health, Minneapolis, Minnesota, United States, 3. University of Alberta, Edmonton, Alberta, Canada*

Measuring the quality of home- and community-based services (HCBS) is challenging because people use a diverse array of services. To fill this gap we evaluated whether distinct clusters of older adults exist based on their patterns of HCBS use, and whether consumer-reported quality of life (QoL) varies between these clusters, with or without dementia. We included 775 Medicaid HCBS consumers in Minnesota who also participated in the 2017-2018 National Core Indicators-Aging & Disability Adult ConsumerSurvey (NCI-AD). We linked NCI-AD respondents with their Medicaid claims. A validated QoL score was constructed using factor analysis from items in the NCI-AD. The QoL score includes items reflecting care experiences, security, and autonomy. Principal component analysis identified clusters of consumers with similar patterns of HCBS use (home healthcare, non-medical transportation, personal care assistance, adult day care, durable medical equipment, and homemaker services). We evaluated differences in mean QoL scores between clusters and within each cluster among consumers with and without dementia, using linear regression. Four clusters were identified based on patterns in service type. The clusters varied in composition based on race/ethnicity, proportion with dementia, and functional dependence. In regression analyses, mean QoL scores were similar between clusters. Within each cluster, mean QoL scores were also similar between those with and without dementia. These findings indicate that despite significant heterogeneity in patterns of HCBS use, QoL scores can be used reliably as a standard quality indicator for all HCBS consumers, regardless of dementia diagnosis, to inform HCBS quality assurance efforts.

Abstract citation ID: igaf122.2825

### DEVELOPING A DIGITAL-BASED EXERCISE SNACK PROGRAM FOR COMMUNITY-DWELLING FRAIL OLDER ADULTS

Min Kyung Park[1], Gwang Suk Kim[1], Jae Jun Lee[1], Layoung Kim[2], Sooyoung Kwon[3], Eun Ju Park[1], Hayejin Yang[3], and Seungbum Yang[3], *1. Yonsei University College of Nursing, Seoul, Republic of Korea, 2. The University of Suwon, Hwaseong, Republic of Korea, 3. Graduate School of Yonsei University, Seoul, Republic of Korea*

Frail older adults' physical limitations hinder their engagement in traditional continuous exercise or group-based physical activities. A digital exercise snack program, comprising short, frequent exercises at home, may be a viable alternative. This study aimed to develop and evaluate the feasibility and acceptability of such a program. The program was developed in four stages: (1) initial design, (2) content validation by six experts, (3) refinement and a one-week pilot with the research team, and (4) a two-week pilot with three frail older adults assessing the feasibility (completion, safety, and adherence) and usability of the digital device. The item-content validity index of the program's preliminary version ranged from 0.67 to 1.00. The final program included five exercises (arm curl, marching on the spot, sideways leg lift, sit-to-stand, and calf raise), each followed by a one-minute grip ball rest, totalling ten minutes per session. Participants followed the program twice daily in the first week and thrice daily in the second week at their preferred time, with the digital device providing guidance, adherence monitoring, personalized reminders, session recordings, and pop-up notifications. All participants completed the two-week program, with no safety issues above CATAE grade 3 reported. On average, participants performed 29.3 exercise sessions, with an adherence rate of 97.4%. Digital device usability and program satisfaction were assessed on a 5-point scale, with average scores of 4.3 and 4.7, respectively. The program demonstrated adequate feasibility and adherence, highlighting its potential in facilitating physical activity among frail older adults.

Abstract citation ID: igaf122.2826

### DRAGON GRAB BARS AND BAMBOO CANES: EXPLORING HOW OLDER CHINESE PERCEIVE HOME MODIFICATIONS AND ASSISTIVE DEVICES

Mengzhao Yan[1], Maria Henke[1], Aaron Hagedorn[1], Li-Mei Chen[2], and Jon Pynoos[1], *1. University of Southern California, Los Angeles, California, United States, 2. George Mason University, Fairfax, Virginia, United States*

Aging in place continues to be the preference for most people, but the majority of homes are not equipped to handle their evolving care needs. Although home modifications and assistive devices can enable people to stay in their homes, previous research indicates that minority groups, who often have a stronger inclination to age at home, are less likely to modify their home environment. This issue is often compounded by the shortage of culturally tailored products and services. To explore how perceptions of home modifications and assistive devices are relevant to cultural backgrounds, this pilot qualitative study focuses on older Chinese Americans. Using person-environment fit and cultural adaptation as a framework, we conducted seven semi-structured, in-depth interviews in 2024 with four older Chinese Americans (aged 70 to 95) who made home modifications or used assistive devices and three of their caregivers. Using thematic analysis to examine the interview notes, we identified four themes: (1) Helpfulness. Interviewees generally appreciated supportive features that improved daily living and independence; (2) Learning Curve. Both older adults and caregivers noted the efforts required to adapt to the changes; (3) OT Support. Older adults who made home modifications acknowledged the instrumental role of occupational therapists. (4) Cultural Influence. People prefer designs that reflect their culture. Our findings suggest that, while some aspects of home modifications and assistive devices are consistently valued among older minorities, an improved cultural competency in product design and service delivery can help create person-centered solutions.

Abstract citation ID: igaf122.2827

### EXPLORING THE UNMET AND MET NEEDS OF HOMEBOUND OLDER ADULTS: A SCOPING REVIEW

Peace Kumapayi[1], Joseph Ogbuniro[2], Ravjyot Ughra[1], Benita Ugwu[2], and Michael Kalu[1], *1. York University, Toronto, Ontario, Canada, 2. University of Nigeria Nsukka, Nsukka, Enugu, Nigeria*

Homebound older adults, defined as individuals (65+) who remain indoors for extended periods without venturing outside experience significant challenges that negatively impact their physical activity, psychological health, and overall quality of life (1,2). Researchers have examined both the unmet and met needs of HB older adults, highlighting the necessity for a synthesis of this evidence. Therefore, this scoping review aims to synthesize existing evidence on the cognitive, environmental, financial, personal, physical, psychological, and social met and unmet needs of HB older adults. Method: Using Arksey and O'Malley's (2005) framework, we searched across eight databases and supplemented by hand-searching reference lists of included articles. Screening (title, abstract, full text) and data extraction were conducted in pairs using predefined criteria. We employed thematic analysis (in pairs) to identify the met and unmet needs of HB older adults. Findings: A total of 19 articles were included, of which eleven were quantitative studies, seven were qualitative, one was mixed-method studies. More than half of the included articles (n = 12) were conducted in the USA, with the remaining from China, United Kingdom, Portugal, Greece and Canada. Among the included articles, seventeen articles reported unmet needs ranging from cognitive (mild cognitive impairment), personal (lower life satisfaction), physical (functional impairment), psychological (depression), financial (financial constraints), environmental (housing needs) and social needs (loneliness). Remaining articles (n = 2) reported met needs using intervention such as Cognitive behavioural therapy (CBT) combined with Baduanjin qigong that reduced loneliness and Homebound elderly people psychotherapeutic interventions (HEPPI) that improved depression and episodic memory function.

Abstract citation ID: igaf122.2828

### FAMILY FRACTURES: A MULTILEVEL ANALYSIS OF INSTITUTIONAL DRIVERS BEHIND ELDERLY EMPTY-NESTING IN CHINA'S RUST BELT

Xiaoxuan Liang, Gong Chen, and Bo Liang, *Peking University, Beijing, Beijing, China (People's Republic)*

This study employs a multilevel institutional analysis to investigate the structural disintegration of intergenerational support systems and the rising prevalence of elderly empty-nesting in China's Rust Belt (Northeast provinces), utilizing longitudinal population census data (2000–2020) and municipal socioeconomic records. Three critical findings emerge: (1) Accelerated Family Fractures: The Rust Belt exhibits a uniquely severe trajectory of aging and empty-nesting compared to national averages. While couple-only households dominate elderly empty-nesting (reflecting weakened intergenerational cohabitation norms), the surge in solitary living arrangements—a 23% faster growth rate than couple-only cases—signals deepening familial fragmentation. (2) Spatial Inequality as Institutional Legacy: Urban empty-nesting rates persistently surpass rural levels, yet resource-depleted cities face disproportionately high rates of solitary elderly households. Geospatial clustering reveals a "peripheralization" pattern: border zones of Heilongjiang-Jilin and industrial hubs in Liaoning exhibit peak empty-nesting, mirroring the region's post-industrial decline and youth exodus. (3) Hierarchical Drivers of Institutional Failure: Mass outmigration (demographic driver) and state-led urbanization policies (economic driver) structurally erode family support capacities. The collapse of multigenerational cohabitation norms, amplified by decades of one-child policy legacies, accelerates household atomization. Solitary living correlates strongly with localized industrial collapse (e.g., mining towns), whereas couple-only households are primarily shaped by cultural inertia and demographic constraints. These findings challenge conventional narratives of filial piety decline, instead framing empty-nesting as a systemic outcome of China's Rust Belt's institutional trilemma: depopulation pressures, fragmented welfare systems, and unaddressed cultural-institutional voids.

Abstract citation ID: igaf122.2829

### HEALTH, SAFETY, AND AGING IN ELDERLY FARMERS IN THE UNITED STATES AND BEYOND: A SCOPING REVIEW

Shuang Li, *North Carolina Agricultural and Technical State University, Greensboro, North Carolina, United States*

Understanding elderly farmers' health and risk factors is fundamental to preventing agricultural injuries and illnesses as the farmer population ages. Few reviews have focused on how aging impacts farmers' health and safety. This study reviews scientific literature that characterizes aging farmers' physical and mental health, work-related risk factors, and potential interventions to support healthy work and aging. Following the framework from Arksey and O'Malley (2003), we conducted a scoping review by exclusively searching MEDLINE (via PubMed), EBSCOhost (via CINAHL), Web of Science (via Clarivate), AGRIS database, and Embase (via Elsevier). Studies that examined older farmers'

health and related risk factors as the primary outcome were included. Eligibility criteria included studies written in English and conducted in the US and globally from 1985-2024. Following PRISMA-ScR (2019) guideline, and findings were synthesized without meta-analysis. Seventy-one studies were included in the final review. Our review indicates that injuries, chronic and occupational diseases, mental health, health risk factors, aging, and retirement considerations have been reported among aging farmers. Major gaps are identified in the study of healthy aging farmers with few studies characterized the trajectory of farmers' exposure to occupational hazards and adverse health outcomes as they age. A deeper understanding of aging and farmers' health, such as through longitudinal panel studies, is needed to identify key prevention strategies that keep farmers active and productive until they reach a reasonable retirement age and guidance on societal efforts that would allow older farmers to retire when it makes sense to do so.

Abstract citation ID: igaf122.2830

### HEALTHCARE UTILIZATION IN OLDER ADULTS: THE IMPACT OF CAREGIVING ON HOSPITALIZATION OUTCOMES

Erblin Shehu[1], and Kanika Arora[2], *1. State University of New York at Cortland, United States, 2. University of Iowa, Iowa City, Iowa, United States*

While current evidence suggests that caregiver involvement may reduce hospitalizations, few studies have examined differences in hospitalization outcomes based on caregiving arrangements or the causal directionality of this relationship. Using data from the Health and Retirement Study (HRS) (2006–2018; N = 22,792 observations), this study addresses this gap by conducting a two-part model to assess the longitudinal effects of caregiving on hospitalization risk and frequency among older adults with functional limitations. First, we apply a linear probability two-way fixed-effects model to evaluate whether different types of caregiver support (no help, non-family help, family help, or combined help) influence hospitalization likelihood. Then, among those hospitalized, a Poisson two-way fixed-effects model examines the impact of caregiver support on the number of hospital stays. Interaction terms explore variation by activities of daily living (ADLs), comorbidities, depression status, urbanicity, race, and sex, with all covariates lagged by one wave. While our findings indicate that caregiver type does not significantly impact hospitalization risk, we find that combined help reduces hospital stays by 17% among hospitalized individuals. Interaction effects reveal that older adults with high depression receiving combined help are 5% less likely to be hospitalized, whereas racial minorities receiving combined help experience a higher risk of hospitalization (Black: +4%, Other minority: +11%) and increased hospital stays (+20%). These findings suggest that integrated caregiver models can reduce hospital stays but may have differential effects based on mental health status and racial background.

Abstract citation ID: igaf122.2831

### IMPACT OF POLICY REFORMS ON HOSPITAL-AT-HOME SERVICES: AN INTERRUPTED TIME SERIES ANALYSIS OF SHANGHAI

Sixian Du[1], and Yaqing Liu[2], *1. Huazhong University of Science and Technology, Wuhan, Hubei, China (People's Republic), 2. Huazhong University of Science and Technology, Hubei, Hubei, China (People's Republic)*

Background: The rapid growth of aging populations and chronic disease prevalence has increased demand for hospital-at-home (HaH) services, which offer home-based alternatives to traditional inpatient care. However, the policy impact on HaH service development in China remains understudied. Objective: To evaluate the effects of two major policy reforms on HaH services in Shanghai from 2007 to 2022. Methods: A two-stage interrupted time series (ITS) analysis with a control group was conducted. Data from Shanghai Statistical Yearbooks were used to assess changes in institutional capacity, home-based medical services, and HaH beds across hospitals and community health service centers. Eleven outcome indicators were analyzed. Results: Following the first policy in 2010, the number of HaH service providers increased significantly, particularly in community health service centers ($\beta1 + \beta3 + \beta5 + \beta7 = 8.200$, $P < 0.001$). HaH beds per 10,000 population also rose ($\beta1 + \beta3 + \beta5 + \beta7 = 0.406$, $P < 0.001$). However, home-based medical visits declined after both policies ($\beta1 + \beta3 + \beta5 + \beta7 = -90{,}214.350$, $P < 0.01$), likely due to workforce shortages and service delivery risks. After the second policy in 2020, hospital-based HaH beds decreased ($\beta1 + \beta3 + \beta9 = -76.500$, $P < 0.01$). Conclusion: While policy reforms expanded HaH service capacity, challenges remain in sustaining home-based medical visits and ensuring workforce supply. Future policies should prioritize multidisciplinary care teams and address barriers to home-based geriatric healthcare to meet the needs of China's aging population.

Abstract citation ID: igaf122.2832

### INFORMAL RURAL ELDERCARE SOLUTIONS: THE EMERGENCE AND IMPLICATIONS OF FAMILY-CARE HOMES IN SHANGHAI'S PERIPHERY

Ziqi Zhang, and Linzhi Su, *Shanghai Jiao Tong University, Shanghai, Shanghai, China (People's Republic)*

This study faces the structural tension between declining family-based care systems and inadequate formal care provisions in rural China by centering on self-organized family-care homes in Shanghai's peri-urban villages. We employ mixed methods—snowball sampling of 14 cases, 70 hours of environmental-behavioral mapping, and semi-structured interviews with 16 operators and 10 older residents—to analyze how these informal facilities navigate spatial production and regulatory constraints. Through integrated graphic analysis, thematic coding, and fsQCA, three critical dimensions were focused: (1) Spatial hybridization achieves cost-efficiency via residential retrofitting (flexible partitions, ambiguous but adaptive cohabitation units) that blend dwelling, caregiving, and livelihood functions; (2) Relational reconfiguration constructs place-based care networks through quasi-familial ties (operator-resident-local staff triads) and context-sensitive service portfolios integrating daily support, emotional bonding, and intergenerational exchange; (3) Institutional negotiation leverages policy ambiguities (exploiting ambiguities in homestead policies) and tacit trust mechanisms to bypass rigid regulatory frameworks, fostering grassroots innovation within governance gaps. The study proposes integrating such practices into an extended mutual-aid care framework for legitimacy and advocates for a community-embedded micro-institution certification system with environmental design guidelines in rural areas. It expands the theoretical understanding of "quasi-formal care spaces" in gerontological geography, as well as enriches institutional and environmental design innovation scholarship on rural aging-in-place in developing contexts.

Abstract citation ID: igaf122.2833

**LIVING ALONE, HOME HEALTH VISITS, AND FALL RISK AMONG OLDER ADULTS IN NEW ENGLAND**

Qinglin Gao, Nachalie Rodriguez Cruz, Calvin Tran, Mengshi Liu, Qian Song, and Elizabeth Dugan, *University of Massachusetts Boston, Boston, Massachusetts, United States*

Falls are a leading cause of injury and hospitalization among older adults, leading to loss of independence, increased healthcare costs, and reduced quality of life. While previous research has examined individual health and physical risk factors, studies on community-level fall rates are limited. Older adults who live alone may face delayed assistance, limited social support, and reduced access to in-home care, increasing fall risk and post-fall complications. This study compares community rates of falls, fall-related injuries, hip fractures, home health visits, and living alone in Massachusetts, Rhode Island, and Connecticut. Data were from the 2025 Healthy Aging Data Reports. Indicators were calculated using data from the Centers for Medicare & Medicaid Services Master Beneficiary Summary File (2020-2021), the American Community Survey (2016-2022), and the Behavioral Risk Factor Surveillance System (2014-2020). Fall rates were similar across states (CT: 26.3%, RI: 26.01%, MA: 26.58%), as were fall-related injuries (CT: 9.8%, RI: 10.03%, MA: 10.06%). Hip fracture rates were highest in Warren, RI (4.07%), Salisbury, CT (5.08%), and Canton, MA (4.52%). Rhode Island had the highest percentage of older adults living alone (29.27%), with Hartford, CT (41.27%) the highest statewide. Home health visits varied, with New Haven, CT (5.39 visits) the highest and Sharon, CT (1.12 visits) the lowest. Findings indicate regional differences in fall-related indicators. Areas with more older adults living alone and fewer home health visits may require targeted support. Further research is needed to explore these trends and inform potential strategies for fall prevention and aging in place support.

Abstract citation ID: igaf122.2834

**NAVIGATING INFORMAL SUPPORT IN REGIONAL DEMENTIA CARE NETWORK: A QUALITATIVE EXPLORATION IN SOUTHEASTERN GERMANY**

Zi Yan[1], Shuji Tsuda[2], Kae Ito[2], and Shuji Awata[2], *1. Waseda University, Tokyo, Tokyo, Japan, 2. Tokyo Metropolitan Institute of Geriatrics and Gerontology, Tokyo, Tokyo, Japan*

Dementia Care Networks (DCNs) play a vital role in supporting persons with dementia (PWD) and their caregivers, yet informal support mechanisms within regional DCNs remain underexplored. This study explores informal support structures in regional DCNs in southeastern Germany, identifying facilitators and barriers for effective DCNs. An exploratory-descriptive qualitative approach was used, incorporating semi-structured interviews and participant observation with 23 individuals, including social workers, physicians, nurses, volunteers, family caregivers, and PWD across two Bavarian communities. Thematic analysis and structured content analysis, framed by the Social-Ecological Model (SEM), revealed three distinct forms of informal support that enhanced resilience for caregivers, PWD, and formal service providers. Facilitators spanned multiple SEM levels: heightened dementia awareness (individual), robust volunteer traditions (interpersonal), community-level social capital(community), collaborative networking platforms (organizational), and proactive local leadership (regional). A notable unanticipated finding was a spill-over effect, wherein informal support sustained community well-being and aided former caregivers post-bereavement. Conversely, sustainability challenges stemmed from organizational fragmentation, including limited stakeholder awareness and inter-stakeholder competition. The study underscores informal support's critical role in mitigating pressures on formal care systems, particularly in resource-constrained regions. Findings offer actionable strategies for policymakers and practitioners to optimize DCN efficacy by leveraging community resources, fostering cross-sector collaboration, and addressing systemic barriers. This research contributes empirical insights to the discourse on dementia care, emphasizing the interplay of informal and formal support systems in promoting holistic, sustainable care models. Keywords Dementia, informal support; dementia care network, integrated care, care coordination, Germany

Abstract citation ID: igaf122.2835

**PREDICTIVE VALUE OF SELF-PRIORITIZED MOBILITY FACTORS ON GAIT SPEED AND LIFE SPACE IN OLDER NIGERIANS.**

Perpetua Obi[1], Isreal Adandom[2], Tyler Sun[3], Daniel Rayner[4], Francis Kolawole[5], and Michael Kalu[1], *1. York University, Toronto, Ontario, Canada, 2. The University of Alabama, Tuscaloosa, Alabama, United States, 3. McMaster University, Hamilton, Ontario, Canada, 4. Western University, London, Ontario, Canada, 5. University College Ibadan, Ibadan, Oyo, Nigeria*

Eighty-two cognitive, environmental, financial, personal, physical, psychological, and social factors significantly influence mobility decline following hospital discharge. However, assessing all these factors during the fast-paced discharge process is impractical. This study aimed to identify the factors that Nigerian older adults consider most critical and determine which factors (in combination) most realistically predict gait speed and life space among these Nigerian older adults. This is data from a cross-sectional survey that recruited 400 Nigerian older adults, 60+ years old, to rank 82 factors influencing mobility. Older adults' gait speed and life-space mobility were collected using the 10-meter Walk Test and Life Space Assessment. Multivariate binary logistic regression was used to determine the most realistic predictor of gait speed and life-space mobility. No factors were considered critical by the older adults. The life space model indicates that increased street characteristics, social cohesion, occupation, hearing, gait speed, fear of falling, and conscientiousness accounts for approximately 50% of variations in life space. The gait speed model indicates that an increase in executive function, pain, respiratory system, body composition, fatigue, social factors, racial characteristics, marital status, social network, and fear of re-injury explain about 74% of variation in gait speed. This study provides self-reported factors that could influence older adults' mobility following discharge that would allow clinicians to prioritize factors for assessment amidst multiple factors.

Abstract citation ID: igaf122.2836

**PRIORITIZING SERVICE NEEDS AND WILLINGNESS TO PAY FOR AGING IN PLACE AMONG OLDER ADULTS IN KOREA**

Bomgyeol Kim[1], Hun Kang[2], Seongmi Choi[3]JaeWon Hyun[2], and JiYeon Choi[2], *1. Yonsei University College of Nursing, Seoul, Republic of Korea, 2. Yonsei University, Seoul, Republic of Korea, 3. Daegu Catholic University, Daegu, Republic of Korea*

Aging in place (AIP) is key to healthy, independent aging, but sustaining public services is increasingly challenging. Understanding service needs and willingness to pay (WTP) is essential for effective support strategies. Using the 2023 National Survey of Older Koreans, this study classified groups based on service needs and WTP using latent class analysis and examined factors associated with group membership. Service needs and WTP were assessed across six domains: housing support, daily living support, safety support, medical services, transportation support, and counseling services. Among 9,951 community-dwelling older adults, four distinct groups were identified (p<.001): High Needs & High WTP (22.6%), Low Needs & Low WTP (21.0%), Moderate Needs & Moderate WTP (35.1%), and High Needs but Low WTP (21.4%). Individuals with higher education and income were less likely to belong to the low WTP groups. Individuals with poorer self-rated health were more likely to be in the 'High Needs & High WTP' group, as were those with three or more chronic diseases or functional limitations. In contrast, individuals with cognitive impairment were more likely to be in the 'Low Needs & Low WTP', 'Moderate Needs & Moderate WTP', or 'High Needs but Low WTP' groups. Individuals who preferred institutional care when experiencing functional dependence were more likely to be classified into the 'Moderate Needs & Moderate WTP' and 'High Needs but Low WTP' groups. These findings highlight diverse service needs and financial considerations among older adults. Public policies should prioritize affordability and accessibility for low-WTP individuals with high service needs.

Abstract citation ID: igaf122.2837

### SELF-REPORTED HEALTH, PERS, AND SOCIAL CARE USE IN OLDER ADULTS AT RISK OF FALLS: PILOT RESULTS

Nicole Zahradka[1], Jayer Chung[2], Anmol Momin[2], Alisha Williams[1], Ilse Torres Ruiz[2], and Bijan Najafi[3], *1. Best Buy Health, Boston, Massachusetts, United States, 2. Baylor College of Medicine, Houston, Texas, United States, 3. University of California, Los Angeles, Los Angeles, California, United States*

Health-related quality of life (HRQoL) is a key outcome for aging in place interventions. Decreased HRQoL is associated with increased healthcare costs. This observational pilot study included 20 older adults (72±5 years old) at high-risk of falls who had access to a personal emergency response system (PERS) with fall detection, as well as 24/7 urgent response (UR) and social care (SC) services for 6 months. Outcomes were service utilization and change in self-reported HRQoL. Utilization was measured as a percentage of days the PERS pendant was worn over the study duration and percent of patients who engaged with UR and/or SC services. Self-reported HRQoL was measured with the Healthy Days instrument (CDC HRQOL-4) at baseline and monthly during study. The number of 'unhealthy days' and general health were compared between baseline and averaged monthly responses. Median days of pendant wear was 68.0 (56.1 – 84.4)% and 70% of participants engaged with at least one of the services: UR (n = 3), SC (n = 5), or both (n = 6). Mean unhealthy days at baseline was 18.0(20.0) days [physical=13.0(13.4) days, mental=5.0(9.7) days] and decreased to 14.7(16.8) days [physical=8.4(8.9) days, mental=6.1(8.8) days]. Average general health was rated as "good" at baseline and did not change. 12 out of 20 participants reported same or better general health during study compared to baseline. While there was an HRQoL improvement overall, within-subject changes varied. Aging in place solutions that address both urgent health status changes and social determinants of health have the potential to mitigate declining HRQoL associated with aging.

Abstract citation ID: igaf122.2838

### SENSE OF BELONGING, LONELINESS, AND CIVIC ENGAGEMENT/ SOCIAL PARTICIPATION AMONG JAPANESE AND KOREAN OLDER ADULTS

Li-Mei Chen[1], Yookyong Lee[2], Sewon Kim[1], Sua Jang[1], and Duy Nguyen[3], *1. George Mason University, Fairfax, Virginia, United States, 2. The University of Alabama at Birmingham, Birmingham, Alabama, United States, 3. Sacred Heart University, Fairfield, Connecticut, United States*

Civic engagement/ social participation are not merely activities but are deeply embedded in the psychosocial well-being of older adults. While traditional models of engagement focus on institutionalized activities such as formal volunteering and political participation, they often overlook the emotional, psychological, and relational aspects that drive participation - especially among racial and ethnic minority groups. This study provides a comparative analysis of two Asian ethnic groups—Japanese and Korean older adults in the U.S. examining how cultural and psychosocial factors shape their civic engagement/ social participation and sense of belonging. Using a multi-theoretical qualitative approach and Steps for Coding and Theorization Analysis (SCAT), the in-depth interviews with Japanese (n = 6) and Korean older adults (n = 5) showed that civic engagement/ social participation are tied to their psychological sense of connection to a community. Participation in ethnic associations, informal caregiving, and mutual aid networks fosters a sense of purpose, identity, and emotional security, reinforcing the idea that civic life extends beyond structured activities. On the other hand, language barriers, cultural exclusion, intergenerational conflict, and migration-related social disruptions contribute to loneliness, which in turn diminishes opportunities for engagement and exacerbates emotional distress. Many participants described engagement as a mechanism for reducing isolation and maintaining self-worth, suggesting that civic and social participation serves both as a protective factor and as a coping strategy for aging-related vulnerabilities.

Abstract citation ID: igaf122.2839

### THE IMPACT OF HOME AND COMMUNITY-BASED SERVICES ON THE HEALTH STATUS OF OLDER ADULTS IN CHINA: A LONGITUDINAL ANALYSIS

Shibin Yan, *University of Pennsylvania, Camden, New Jersey, United States*

With China's rapidly aging population, ensuring the well-being of older adults has become a pressing concern. Home and community-based services (HCBS) play a crucial role in supporting older individuals, particularly those with chronic conditions, by fostering social engagement and promoting healthier living environments. While research has examined self-rated health among older adults, limited attention has been given to how these services influences health outcomes. Moreover, the impact of HCBS on vulnerable subgroups, such as childless and disabled older adults, remains underexplored. This study addresses this gap by analyzing Chinese Longitudinal Healthy Longevity Study (CLHLS) panel data from 2008 to 2018. Using person fixed-effect regression models, the study assesses how HCBS affects health status, measured through self-rated health, interviewer-rated health, and comparative health assessments. Unlike prior studies relying solely on self-rated health, this approach provides a more comprehensive understanding of older adults' well-being. Findings indicate a significant positive association between HCBS and improved health outcomes. Among various service types, cultural and social services exhibit the strongest effect. The results further reveal disparities based on residential location, disability status, and financial stability—with urban residents benefiting more than rural counterparts. However, no evidence suggests that childlessness moderates this relationship. This research enhances aging studies by utilizing longitudinal models and multidimensional health assessments. Findings highlight the importance of family financial support and HCBS in promoting healthy aging, offering valuable insights for policymakers.

Abstract citation ID: igaf122.2840

### THE QUALITY OF HOME SUPPORT SERVICES FOR OLDER PEOPLE IN IRELAND AND ITS IMPLICATIONS FOR CHINA

Guolei Zhang[1], and Caifang Zhang[2], *1. University College Cork, Cork, Cork, Ireland, 2. Hangzhou Normal University, Hangzhou, Zhejiang, China (People's Republic)*

Background: As global aging accelerates, home support services (HSS) are essential for enabling older adults to age in place with dignity. Ireland's well-established HSS system serves as a benchmark for high-quality elderly care. In contrast, China faces challenges such as rural-urban disparities, reliance on informal care, and insufficient caregiving standardized . This is the first study exploring how Ireland's successful HSS model can improve China's elderly care service quality. Aim: This study aims to identify practical strategies from reland's HSS model that can be adapted to improve the quality and effectiveness of HHS for older people in China. Method: The SERVQUAL model was applied to evaluate service quality across five dimensions: reliability, responsiveness, assurance, empathy, and tangibles. Data were collected researched from database (PubMed, Embasel, Cochrane ), government reports, and publicly available documents from sources such as the Irish Health Service Executive and the author's firsthand experience working in Ireland's healthcare sector. Results: Key findings highlight Ireland's success in preserving older adults' autonomy, reducing informal caregiving pressures, and alleviating financial burdens through standardized care protocols, professional staff training, effective service placement, state financial support, and rapid emergency responses. Notably, competitive compensation and clear career progression pathways for caregivers enhance job satisfaction and retention. Conclusion: These elements collectively contribute to the high-quality delivery of home-based elderly care services in Ireland.The study suggests China can improve its HSS by adopting similar strategies, including a localized standardized care protocols, caregiver certification, and government-supported financial and career incentives, to strengthen home-based elderly care.

Abstract citation ID: igaf122.2841

### THEMATIC ANALYSIS OF HOME ALONE: AN INTERVENTION FOR OLDER ADULTS LIVING ALONE WITH MEMORY IMPAIRMENT

Grace Savard[1], Robyn Birkeland[2], Amy Hobday[2], Stephanie Ingvalson[2], Laura Gitlin[3], Renee Pepin[4], Jill Cigliana[5], and Joseph Gaugler[2], *1. University of Minnesota Twin Cities, Minneapolis, Minnesota, United States, 2. University of Minnesota, Minneapolis, Minnesota, United States, 3. Drexel University, Philadelphia, Pennsylvania, United States, 4. Geisel School of Medicine at Dartmouth, Lebanon, New Hampshire, United States, 5. Memory Care Home Solutions, St. Louis, Missouri, United States*

Older adults who live alone with mild cognitive impairment (MCI) or subjective memory impairment (SMI) are at greater risk for loneliness, safety concerns, and physical and mental health concerns. These risks can lead to an increased need for support, supervision, and potentially residential care. Interventions are needed to reduce the risk of isolation and adverse health outcomes for older adults with MCI or SMI. Home Alone, a novel psychoeducational program, includes home safety education and utilizes Behavioral Activation to improve socialization and engagement among older adults with MCI or SMI who live alone. The seven-session intervention is delivered by a trained coach visiting the participant's home or via telehealth. Session foci include home safety, resource provision, motivational strategies, memory aids, and an in-depth exploration of personal values, followed by identification and scheduling of values-aligned activities. Sixty-five participants enrolled across Phase I (feasibility study, $n = 15$) and Phase II (pilot test of revised Home Alone intervention, $n = 50$) of the Home Alone study. Qualitative data were collected from participants via semi-structured interviews and open-response items on two sequential follow-up surveys. Thematic analysis revealed how the Home Alone program raised awareness and influenced engagement in socialization and meaningful activity, increased utilization of resources and daily living strategies, and prompted home safety modifications. Analyses provided nuanced insights into Home Alone's impacts on participant well-being while shedding light on 'program fit' and participant concerns for the future. These findings will inform subsequent Home Alone analyses and eventual dissemination of the intervention to community settings.

Abstract citation ID: igaf122.2842

### VILLAGES IN THE AGING SERVICES ECOSYSTEM: CASES FROM METRO BOSTON

Jennifer Molinsky, *Harvard University, Cambridge, Massachusetts, United States*

In the nearly 25 years since the founding of Beacon Hill Village, over 250 grassroots nonprofit village organizations have been established across the US, providing support and social connection for older adults living in the community, much through mutual support of members and volunteers. There are now multiple villages in the Boston metro area alone, covering geographies ranging from small urban neighborhoods (Beacon Hill Village covers an area approximately one-sixth of a square mile) to multiple municipalities (Cambridge Neighbors serves five communities), from urban to suburban locations. The concentration of villages in a single metro offers a unique opportunity to consider how geography, demographics, and service networks in each community shape villages' priorities and operations. This session will report on research conducted by the Housing an Aging Society Program at the Harvard Joint Center for Housing Studies Housing focused on village organizations in Beacon Hill, Jamaica Plain, Cambridge, Newton, Wellesley, and Brookline. The research utilizes focus groups and interviews with village staff, members, and local government aging services to gain insight into villages' place in the aging services ecosystem in the Boston area and how each is responding to the challenges and opportunities of aging in Boston's high-cost but resource-rich environment. Since villages have relatively small memberships in comparison to the older population in each community and in the region as a whole, we will explore their reach and potential for growth, particularly their capacity to serve less-resourced older adults.

### SESSION 7305 (POSTER)

Abstract citation ID: igaf122.2843

### ALZHEIMER'S DISEASE AND RELATED DEMENTIAS III A SYSTEMATIC REVIEW OF THIRD WAVE COGNITIVE BEHAVIORAL INTERVENTIONS FOR PEOPLE DIAGNOSED WITH DEMENTIA

Elizabeth Fauth, Ty Aller, Heather Kelley, and Marissa Donahue, *Utah State University, Logan, Utah, United States*

Millions of people receive a diagnosis of Alzheimer's disease or related dementia (ADRD) each year, and increased depression and anxiety often co-occur. Third wave cognitive behavioral therapies (CBT) refer to a cluster of interventions based in CBT but which focus on improving psychological and behavioral processes as a whole, rather than focusing on symptom-reduction, although symptom-reduction is a common outcome. There is extensive research on the efficacy of third wave approaches in multiple populations, but there are limited studies of its efficacy and acceptability individuals with dementia. This study is systematic review of third-wave CBT for individuals with ADRD published between 2012 and 2023. Ten studies met inclusion criteria, of which 7 were unique studies and 3 were supplemental studies using a similar dataset. There were varying designs (e.g., single-arm, randomized control trial). A total of 229 participants with ADRD were included. Mindfulness-based interventions were most used (k = 3) and interventions were primarily delivered via group sessions. Adaptations for ADRD included shortening session intensity (e.g., fewer sessions, fewer activities) and making materials more accessible (e.g., larger fonts, fewer words). The most common outcome assessed was quality of life. Results of studies were mixed; some reported improvements while others reported null or negative outcomes. We conclude that third wave behavioral interventions seem to be preliminarily feasible and acceptable for individuals with dementia, but this research is still in the early stages. Individuals with ADRD need support in their mental health. Future research should emphasize more rigorous study design and increased sample sizes.

Abstract citation ID: igaf122.2844

### A VIRTUAL SUPPORT PROGRAM FOR ASIAN FAMILY CAREGIVERS OF INDIVIDUALS WITH ALZHEIMER'S: PRACTICAL CHALLENGES

Seulgi Ryu[1], Dongmi Kim[1], Wonshik Chee[2], and Eun-Ok Im[2], *1. The University of Texas at Austin, Austin, Texas, United States, 2. University of Texas at Austin, Austin, Texas, United States*

The number of web-based virtual support programs supporting caregivers of individuals living with Alzheimer's disease has grown significantly. Several studies have examined challenges in implementing culturally tailored virtual support programs for racial/ethnic minorities in the U.S. However, there remains a critical need to understand the practical challenges in implementing such programs for Asian American women family caregivers of individuals with Alzheimer's disease. This presentation discusses the practical challenges identified in a culturally tailored virtual support program for Asian American women family caregivers. The ongoing parent study aims to develop and preliminary evaluate a virtual support program in improving the health outcomes of Asian American women family caregivers of individuals with Alzheimer's disease. The participant recruitment, program implementation, and data collection are in progress. Individual team members' research diaries documented key challenges, which were analyzed using basic content analysis. Four major challenges emerged: (a) maintaining engagement was challenging due to dropouts and non-responsiveness; (b) some participants primarily focused on obtaining information only, making it challenging to establish rapport and fully achieve the program goal of providing culturally tailored coaching and support; (c) some caregivers sought more specialized services, looking for professional institutions services; and (d) participants hesitated to share struggles, often withholding emotional or personal difficulties. These findings highlight key challenges in implementing a culturally tailored virtual support program among Asian American women caregivers. Addressing these challenges will be crucial in improving future virtual support programs for this population.

Abstract citation ID: igaf122.2845

### ADAPTATION OF AN INPATIENT CHW-LED CARE TRANSITION PROGRAM FOR PERSONS WITH DEMENTIA AND CAREGIVERS

Jung Kwak[1], Allison Salter[2], Aveen Ghodsi Jafari[2], Andrea Perry[3], Alexis Shepherd[4], Snehal Patel[4], and Sarah Stayer[4], *1. University of Texas-Austin, Austin, Texas, United States, 2. The University of Texas at Austin, Austin, Texas, United States, 3. Raymond G. Murphy Department of Veterans Affairs Medical Center, Albuquerque, New Mexico, United States, 4. Dell Medical School at The University of Texas at Austin, Austin, Texas, United States*

Persons with dementia (PWD) and their families frequently face significant challenges during hospital-to-home transitions, highlighting the need for tailored transitional care. This qualitatively driven, nested mixed-methods study applied the Framework for Reporting Adaptations-Enhanced (FRAME) to guide the adaptation of a hospital-based, community health worker (CHW)-led care transition program for hospitalized PWD and their caregivers. The goal was to improve feasibility, acceptability, and implementation fidelity. Adaptations were informed by key informant interviews (n = 5), a community advisory board of hospital providers, social services experts, and caregivers (n = 11), and a caregiver needs assessment (n = 36). Data were analyzed using thematic content analysis and descriptive statistics. Key adaptations included: (1) expanding the community health worker's (CHW) role to provide caregiver education, emotional support, and personalized coaching; (2) enhancing assessments to cover caregiver concerns, resource needs, ADL/IADL support, and home safety; (3) allowing flexible timing for initial assessments post-discharge; (3) broadening eligibility to include individuals without a formal dementia diagnosis; and (4) developing multilingual recruitment materials (English/Spanish), brief introduction videos, and hospital-based outreach through case management teams to improve participation. This presentation discusses stakeholder engagement, key intervention modifications, recruitment challenges, and practical insights for implementing and scaling CHW-led transitional care for PWD and caregivers.

Abstract citation ID: igaf122.2846

**ADRD CAREGIVER EXPERIENCES WITH AN ONLINE LEGAL AND FINANCIAL PLANNER**

Sydney Hoel[1], Richard Chunga[2], Clover Caldwell[3], Heather Schwartz[3], Matthew Zuraw[4], Christian Elliott[5], Andrew Pickett[6], and Nicole Werner[1], *1. Vanderbilt University Medical Center, Nashville, Tennessee, United States, 2. CareVirtue, New York, New York, United States, 3. Indiana University Bloomington, Bloomington, Indiana, United States, 4. Better Brains, Inc, San Diego, California, United States, 5. CareVirtue, San Diego, California, United States, 6. Indiana University School of Public Health- Bloomington, Bloomington, Indiana, United States*

Legal and financial planning pose significant challenges for family caregivers of persons living with Alzheimer's disease and related dementias (ADRD) and can lead to increased financial risk and strain. To address this, we created the CareVirtue Planner, a web-based, personalized legal and financial planning tool for ADRD caregivers. In this study, 88 ADRD caregivers completed a 3-month field test of the Planner, during which they received personalized legal and financial planning education recommendations. Participants completed semi-structured post-use interviews to share their experience of using the Planner and recommend improvements. Using an inductive team-based coding approach, we analyzed the interviews to examine participants' experiences related to navigation, perceived usefulness of the Planner, and barriers to engagement. 70 participants were women (80%), and 64 were White (73%). Participants shared that the Planner is easy to use due to its simple and intuitive interface. Some experienced minor technical issues which impacted navigation. The Planner was most useful for early-stage caregivers; for long-term caregivers, the Planner validated their prior legal and financial planning actions but would require additional and dynamic content to facilitate continued engagement. Participants found the centralized document vault feature and personalized resource recommendations particularly useful. Suggested improvements to the Planner included additional features, e.g., the ability to share access to the document vault with others, and addressing additional topics, e.g., self-care. Future iterations of the Planner can be improved by further tailoring recommendations to participants' stage of caregiving and expanding content and features to promote long-term use.

Abstract citation ID: igaf122.2847

**BINAURAL BEATS STIMULATION FOR COGNITIVE ENHANCEMENT IN ALZHEIMER'S AND RELATED DISEASES: AN UMBRELLA REVIEW**

Yawen Li, Zhaojing Chen, Jie Yu, and Kevin Zhang, *California State University, San Bernardino, San Bernardino, California, United States*

Binaural beats stimulation (BBS) is a promising neuromodulation technique that utilizes auditory stimulation to influence brainwave activity. This umbrella review synthesizes evidence from multiple systematic reviews evaluating the efficacy of BBS for cognitive enhancement in Alzheimer's disease (AD) and related neurodegenerative conditions. The methodology adhered to standard umbrella review protocols, involving systematic searches across multiple databases, including Web of Science (WOS), CINAHL, Scopus, PubMed, and PsycINFO. A total of 154 review papers were initially identified, and seven systematic reviews were included following extensive screening and critical appraisal for methodological quality using standardized tools such as AMSTAR 2 or ROBIS. Findings suggest that BBS at 40 Hz enhances cognitive function, improves memory recall, and modulates neural network connectivity. BBS also demonstrates potential in reducing symptoms of anxiety and depression, promoting relaxation, and improving attention and executive function. Physiological benefits include improved sleep quality, increased slow-wave sleep, and potential reduction in amyloid and tau pathology. Despite these promising outcomes, study heterogeneity, lack of standardized protocols, and limited large-scale clinical trials pose challenges in establishing BBS as a clinical intervention. Future research should focus on refining stimulation parameters, conducting robust randomized clinical trials, and integrating BBS with other therapeutic approaches. This review highlights the potential of BBS as a noninvasive intervention for cognitive enhancement and neuroprotection while emphasizing the need for further investigation.

Abstract citation ID: igaf122.2848

**COMMUNITY-LEVEL ALZHEIMER'S DISEASE OR RELATED DEMENTIA'S 65+ PREVALENCE IN FIVE NEW ENGLAND STATES**

Taylor Jansen[1], Nina Silverstein[1], Yan-Jhu Su[2], Shan Qu[1], Mengshi Liu[1], Yan Lin[1], Qian Song[1], and Elizabeth Dugan[1], *1. University of Massachusetts Boston, Boston, Massachusetts, United States, 2. The Pennsylvania State University, University Park, Pennsylvania, United States*

An estimated two thirds of people living with Alzheimer's disease or related dementias (ADRD) live and are cared for at home. For public health action and community programming to assist those living with ADRD, accurate disease rates must be known. The Healthy Aging Data Reports (HADR) (www.healthyagingdatareports.org) are a comprehensive tool to spur community and policy action as they calculate community-level prevalence of 38 chronic diseases for each community in a state. The present descriptive study will compare community and neighborhood-level 65+ prevalence of ADRD in five New England states: Connecticut (CT), Massachusetts (MA), Maine (ME), New Hampshire (NH), and Rhode Island (RI). Using Medicare Beneficiary Summary File (MBSF) (2020-2021), representative of 100% of traditional Medicare beneficiaries, small area estimation techniques were used to calculate age-sex adjusted community rates of ADRD and over 150 other health indicators. Results varied across states with NH reporting the lowest community rate of 65+ ADRD in the sample (5.41%) and CT reporting the highest (27.10%). CT and MA reported the largest range in 65+ ADRD (CT: 6.90-27.10%; MA: 6.69-25.54%) with the highest rate clusters located near the biggest cities, in urban areas (CT:13.11-27.10%; MA: 12.93-25.54%). Conversely, in Maine and NH, the highest rate clusters were found in rural areas (NH: 15.82-21.16%; ME: 15.33-18.98%). The 2025 HADR's found geographic disparities in 65+ community-level ADRD across New England. Communities with high rates can be targeted for additional supports like caregiver support programming, adult day health centers, and educational programming on the prevention and treatment of ADRD.

Abstract citation ID: igaf122.2849

**COMPARISON OF LOW AND MODERATE-COMPLEXITY ENROLLEES IN THE GUIDE DEMENTIA CARE MODEL**

Karina Berg[1], Richard Fortinsky[2], Julie Robison[2], Shawn Ladda[1], Michelle Wyman[1], Wendy Martinson[1], and Eleanor Miller[1], *1. UConn Health, Farmington, Connecticut, United States, 2. University of Connecticut, Farmington, Connecticut, United States*

In July 2024, the Center for Medicare and Medicaid Innovation (CMMI) launched the Guiding an Improved Dementia Experience (GUIDE) model in clinical settings. In GUIDE, people living with dementia (PLWD)-caregiver dyads are assigned to complexity tiers based on dementia severity level. Low-tier dyads receive less frequent outreach, are not eligible for respite funds, and garner a lower monthly payment from Medicare. Our clinical experience suggested that low-tier PLWD exhibit similar symptoms, and their caregivers face similar unmet needs as those in higher tiers. To empirically test our observations, we compared PLWD behaviors, caregiver burden, and unmet service needs between low-tier and higher-tier dyads. Data collected at enrollment included presence/absence of six PLWD behaviors (e.g.,irritability, paranoia); caregiver burden (Zarit Index); and nine caregiver-reported needs (e.g.,in-home services, stress management). Low vs. moderate tier dyads were compared using t-tests; the few high-tier dyads were excluded. PLWD (n = 61) were 62% female with mean(sd) age 83(9) years. Caregivers (n = 61) were 72% female with mean(sd) age 57(12) years, 47% were children, 44% spouses/partners. Thirty-two dyads (52.5%) were deemed low-tier by CMMI. Mean number of behaviors for low vs moderate-tier PLWD was 2.2 vs. 2.7, respectively (p = 0.16), mean burden scores were 35.5 vs. 34.1 (p = 0.36), and mean number of service needs was 3.4 vs. 3.8 (p = 0.26). Results indicate that low and moderate-tier dyads have similar levels of PLWD behaviors, caregiver burden, and unmet needs, suggesting that criteria for low-tier complexity should consider factors beyond dementia severity to better align GUIDE service delivery and reimbursement with care needs.

Abstract citation ID: igaf122.2850

**CULTURAL ADAPTATION OF THE CAREGIVER TLC PROGRAM TO PROMOTE LATINO DEMENTIA CAREGIVERS' WELLBEING**

Maria Quiñones-Cordero[1], Julian Montoro-Rodriguez[2], Ann Bilbrey[3], Jennifer Ramsey[4], Bruno Kajiyama[5], Claudia Kajiyama[6], Roberto Velasquez[7], and Dolores Gallagher-Thompson[8], *1. University of Rochester School of Nursing, Rochester, New York, United States, 2. The University of North Carolina at Charlotte, Charlotte, North Carolina, United States, 3. Optimal Aging Center, Sunnyvale, California, United States, 4. York Technical College, Rock Hill, South Carolina, United States, 5. Photozig, Inc., Moffett Field, California, United States, 6. Photozig, Moffett Field, California, United States, 7. Southern Caregiver Resource Center, Chula Vista, California, United States, 8. Stanford University, Stanford, California, United States*

Latino dementia caregivers provide more intensive (time and level) care, experience worse mental health outcomes, and receive less family and social support than other racial/ethnic groups. However, few evidence-based, culturally attuned behavioral interventions exist for Latino caregivers, and none focus on targeting social connectedness to improve emotional well-being. This study collaborated with a Community Advisory Board (CAB) to culturally adapt the Caregiver Thrive, Learn, and Connect intervention for Latino dementia caregivers and identify strategies to improve caregivers' social connectedness. The CAB included six Latino community leaders, serving caregivers from San Diego, Rochester, and Charlotte, who co-adapted the program following the Cultural Adaptation Behavioral Health Stage Model. We documented CAB feedback using the Framework for Reporting Adaptations and Modifications – Enhanced. CAB feedback centered on intervention content, examples, and improving social connectedness. We then conducted a Stage Ia trial (N = 5) and assessed program acceptability and satisfaction through focus groups. Data was analyzed using rapid qualitative analysis. CAB-recommended modifications included language considerations (e.g., "overwhelmed" vs. "burdened"), aligning social connectedness goals with cultural values, developing culturally relevant examples to teach skills, and condensing material to allow more time for skill-building exercises and discussions. Participant feedback from the Stage Ia trial was categorized into four themes: socially oriented activities, content augmentation, integrating self-care, and time allocation. All participants rated program satisfaction at 10/10. This collaborative adaptation process informed further refinement of the adapted intervention and a subsequent Ib early-stage trial to evaluate engagement of a social connectedness target and improvements in mental health outcomes.

Abstract citation ID: igaf122.2851

**DIGITAL LITERACY AND DEMENTIA KNOWLEDGE AMONG OLDER ASIAN AMERICANS**

Nan Sook Park[1], Yuri Jang[2], Hongdao Meng[1], Soonhyung Kwon[1], Soondool Chung[3], and David A. Chiriboga[1], *1. University of South Florida, Tampa, Florida, United States, 2. University of Southern California, Los Angeles, California, United States, 3. Ewha Womans University, Seoul, Republic of Korea*

Dementia knowledge is essential for early detection, care planning, and reducing stigma. Disparities persist among immigrant and minority populations, especially among older Asian Americans. Grounded in the digital health literacy model, this study examined how immigration and social factors, digital literacy, and dementia-related variables would be associated with dementia knowledge. Data were drawn from a survey with 507 Asian Americans aged 50 and older (M = 64.42, SD = 9.68) in central Florida. Hierarchical regression analyses estimated the impact of sequential blocks of independent variables on dementia knowledge: (1) background characteristics (age, gender, education, financial status, chronic conditions), (2) immigration-related factors (limited English proficiency [LEP], length of U.S. residence), (3) social factors (marital status, social network, activity participation), (4) digital literacy, health-information seeking behavior, and confidence in health information, and (5) dementia-related factors (having a family/friend with dementia, concerns about dementia, concerns about caregiving, dementia planning). Digital literacy emerged as a major independent predictor of dementia knowledge ($\beta = .22$, $p < .001$). Being married, having a family member/friend with dementia, and perceived importance of dementia planning as well as female gender and shorter US residence were also significant knowledge promoters. LEP was negatively associated with dementia knowledge, but its effect diminished when digital literacy was included. Findings suggest the critical role of digital literacy in reducing dementia knowledge disparities. Interventions enhancing digital and eHealth literacy, particularly among older adults with LEP, can improve access to dementia-related information. Future efforts should develop culturally tailored digital resources to bridge knowledge gaps.

Abstract citation ID: igaf122.2852

**EFFECTS OF COMBINED PHYSICAL AND COGNITIVE INTERVENTIONS IN OLDER ADULTS WITH DEMENTIA: A META-ANALYSIS**

Dandan Xue[1], Yanqiu Hu[2], and Rui Zhang[3], *1. Gongli Hospital of Shanghai Pudong New Area, Shanghai, Shanghai, China (People's Republic), 2. Eye & ENT Hospital, Shanghai, Shanghai, China (People's Republic), 3. Naval Medical University, Shanghai, Shanghai, China (People's Republic)*

The effects of combined physical and cognitive interventions in persons with dementia remains inconsistent. This review aims to compare the effects of combined interventions with those of each intervention alone on cognitive, physical, psychological, functional, and health-related quality of life outcomes for persons with dementia. Searches were conducted across 8 English and Chinese databases from their inception dates to September 10, 2024. Quality appraisal was conducted using Cochrane's risk of bias tool. Random-effects models were employed for meta-analysis, and subgroup analyses and meta-regression were performed to explore potential moderators. A total of 23 studies involving 1,716 participants were included. Combined interventions significantly improved global cognition [SMD = 0.65, 95%CI (0.35, 0.95), p<0.001], processing speed [SMD = 0.33, 95%CI (0.03, 0.63), p=0.03], functional mobility [SMD = 0.85, 95% CI (0.18–1.53), p=0.01], strength [SMD = 0.95, 95% CI (0.28, 1.61), p=0.005], depression [SMD = -1.04, 95% CI (-1.73, -0.36), p=0.003], and health-related quality of life [SMD = 0.71; 95% CI (0.24, 1.18), p=0.003] compared to active or passive controls. Combining interventions didn't provide better overall benefits than using either intervention alone. Further analysis showed that the effectiveness of combined interventions on global cognition depended on the age of the participants. In conclusion, combined interventions were beneficial for persons with dementia, and these interventions are recommended to be integrated into dementia management protocols. Future multi-arm randomized controlled trials with long-term follow-ups are warranted.

Abstract citation ID: igaf122.2853

**FOCUSING ON ALZHEIMER'S DISEASE: DATA COMPARISON AND POLICY IMPLICATIONS BETWEEN CHINA AND FOUR PROVINCES**

Wangyizhuang Lyu, and Hong Mi, *Zhejiang University, Hangzhou, Zhejiang, China (People's Republic)*

By 2050, China's population aged 65 and above is projected to exceed 400 million, with Alzheimer's disease (AD) prevalence rising sharply with age. Diagnosis and treatment remain underdeveloped, leading to underdiagnosis in early stages and significant urban-rural disparities in care resources. These gaps may widen as demographic aging accelerates, while long-term care costs pose a heavy burden on families and society, requiring early policy planning. This study, using data from the Fifth Sampling Survey on the Living Conditions of Urban and Rural Older Adults, analyzes AD prevalence across China and four provinces—Shandong, Zhejiang, Sichuan, and Heilongjiang—revealing key findings: (1) AD affects 1.58% of individuals aged 60 and above, with higher rates in rural areas (1.86%) than urban areas (1.28%). Dementia is slightly more common among urban males and rural females. (2) Prevalence rises notably after 70, peaking at 70–75 for men and after 70 for women, with men developing dementia 5–10 years earlier. (3) Regional differences exist: Zhejiang's peak occurs after 75, with women affected earlier; in Shandong, female prevalence rises faster post-75; Sichuan's peak is at 75–85; Heilongjiang shows a gender-based timing gap. (4) Rural disability rates in Zhejiang and Heilongjiang are below the national average, likely due to higher urbanization. (5) China's higher dementia rates and lower life expectancy, compared to global averages, highlight the urgent need to incorporate long-term dementia care into the social security system.

Abstract citation ID: igaf122.2854

**GENDERED DYADIC COPING AND MARITAL SATISFACTION AMONG CHINESE COUPLES NAVIGATING MILD COGNITIVE IMPAIRMENT**

Dexia Kong, *The Chinese University of Hong Kong, Hong Kong, Hong Kong, China (People's Republic)*

Background: This study investigates how dyadic coping (i.e., couples' joint efforts to manage life stressors) interacts with gender and gender ideologies to influence marital satisfaction among couples living with mild cognitive impairment (MCI) in Hong Kong. Methods: Using cross-sectional data from 202 couples living with MCI, we applied actor–partner interdependence moderation models to examine interactions between dyadic coping, ideological (dis)similarity, and the gender of the spouse with MCI. Results: Our results reveal significant gendered asymmetries. The wife's dyadic coping was related to her own marital satisfaction, while the husband's dyadic coping was not. Dyadic (dis)similarity in gender ideologies matters: Egalitarian wives with MCI reported higher marital satisfaction when their traditionalist husbands engaged in dyadic coping, whereas dyadic coping of traditionalist husbands with MCI diminished their egalitarian wives' marital satisfaction. Two-way interactions demonstrated that when persons with MCI endorse more traditional ideologies than their spouses, their dyadic coping enhanced their own satisfaction, regardless of gender. Conclusions: Dyadic (dis)similarity in gender ideologies and gender combinations of MCI-affected couples intersect to shape the dyadic coping-marital satisfaction link. The positive relational impact of dyadic coping is contingent upon deeply rooted Confucian traditions. Reconciling the gap between collaborative practices and patriarchal norms has the potential to mitigate the gendered imbalance in spousal care. The findings provide valuable insights into the development of culturally sensitive dyadic interventions to promote relational outcomes among couples navigating health challenges beyond MCI. The findings extend dyadic health theory and research in a non-Western context.

Abstract citation ID: igaf122.2855

**HIGHLY CORRELATED MULTIMORBIDITY IN ALZHEIMER'S DISEASE: AN EHR ANALYSIS**

Nai-Ching Chi[1], Kathleen Buckwalter[1], Pui Ying Yew[2], Nanle Gusen[1], Hua-Chin Chi[3], Chih-Lin Chi[2], and Scott Larson[1], *1. University of Iowa, Iowa City, Iowa, United States, 2. University of Minnesota, Minneapolis, Minnesota, United States, 3. Emory University, Atlanta, Georgia, United States*

Most multimorbidity studies focus on the prevalence of individual chronic conditions, neglecting the crucial aspect of correlated disease combinations. This study addressed this gap by investigating highly correlated multimorbidity patterns in 2,629 Alzheimer's Disease (AD) patients using de-identified electronic health records from the University of Iowa Hospitals and Clinics. Diagnoses, coded to the first three levels of ICD-10, were analyzed using the Apriori algorithm to identify significant co-occurring chronic conditions (dyads, triads, and tetrads). Leverage metrics quantified the strength of these correlations. The hypertension-lipoprotein metabolism disorders dyad (I10-E78) was most prominent, occurring 7.8% more frequently than expected (21.4% total). Other significant dyads included depression-anxiety (F33-F41; 4.5% leverage), dyslipidemia-ischemic heart disease (E78-I25; 3.9% leverage), hypertension-ischemic heart disease (I10-I25; 3.7% leverage), and type 2 diabetes-dyslipidemia (E11-E78; 3% leverage). Key triads included hypertension-dyslipidemia-depression (I10-E78-F33; 4.5% leverage), hypertension-dyslipidemia-ischemic heart disease (I10-E78-I25; 4.5% leverage), hypertension-dyslipidemia-type 2 diabetes (I10-E78-E11; 4.2% leverage), hypertension-dyslipidemia-gastroesophageal reflux (I10-E78-K21; 3.9% leverage), and hypertension-dyslipidemia-hypothyroidism (I10-E78-E03; 3.4% leverage). The hypertension-dyslipidemia-depression-anxiety tetrad (I10-E78-F33-F41; 2.6% leverage) exhibited the strongest correlation. Additional significant tetrads revealed complex interactions involving gastroesophageal reflux, sleep disorders, ischemic heart disease, and hypothyroidism. These findings demonstrate a significant clustering of cardiovascular, metabolic, and psychiatric comorbidities in AD. Identifying these correlated multimorbidity clusters will enable the development of targeted, integrated management strategies, inform evidence-based clinical guidelines, and facilitate focused clinical screening and intervention for this vulnerable population.

Abstract citation ID: igaf122.2856

### LONGITUDINAL ESTIMATION OF ADEQUATE PHYSICAL ACTIVITY LEVELS TO REDUCE THE RISK OF DEMENTIA IN OLDER ADULTS WITH MCI

Jungjoo Lee[1], Junhyoung Kim[2], Donghyuck Cho[3], and Min Jun Hwang[4], *1. Texas A&M University, Texas A&M University, Texas, United States, 2. Texas A&M University, College Station, Texas, United States, 3. Cranbrook Academy of Art, Bloomfield Hills, Michigan, United States, 4. Phillips Exeter Academy, Exeter, New Hampshire, United States*

This study estimated the level of physical activity (PA) required to reduce the risk of Alzheimer's disease and related dementias (AD/ADRD), focusing on identifying the adequate PA level to achieve significant risk reduction among older adults with Mild Cognitive Impairment (MCI). This longitudinal study investigated Health and Retirement Study data between 2012 and 2020. The sample included 9,714 individuals with MCI, screened using the Telephone Interview for Cognitive Status-27 at baseline in 2012. The dependent variable was the diagnosis of AD or ADRD between 2012 and 2020. To ensure reliability, this study included participants who responded to the diagnosis questions in a consistent chronological order. The independent variable was the average level of PA participation, including activities such as exercise and walking, from 2012 to 2020. Covariates included age, sex, education, and cognitive function. A Generalized Estimating Equation was used for analysis. Results indicated that the risk of AD/ADRD decreased by 3% at a PA level of 2.20 (OR = 0.970, 95% CI [0.958, 0.983]) and exhibited a linear declining trend with increasing PA levels: a 3.3% decrease at 2.40 (OR = 0.967, 95% CI [0.956, 0.979]), a 3.8% decrease at 2.60 (OR = 0.962, 95% CI [0.948, 0.975]), and a 4.6% decrease at 2.80 (OR = 0.954, 95% CI [0.946, 0.964]). Engaging in exercise and walking at least twice a week was identified as the threshold for achieving a significant reduction in the risk of AD/ADRD, with greater PA engagement further enhancing risk reduction in older adults with MCI.

Abstract citation ID: igaf122.2857

### NURSING HOME RESIDENTS' PREFERRED RECREATIONAL ACTIVITY ATTENDANCE AND DEPRESSIVE SYMPTOMS OVER TIME

Nahida Akter[1], Allison R. Heid[2], Michael J. Rovine[3], Karen Eshraghi[1], Katherine Abbott[4], and Kimberly VanHaitsma[5], *1. The Pennsylvania State University, University Park, Pennsylvania, United States, 2. Independent Research Consultant, Ardmore, Pennsylvania, United States, 3. University of Pennsylvania, Philadelphia, Pennsylvania, United States, 4. Miami University, Oxford, Ohio, United States, 5. pennsylvania state university, UNIVERSITY PARK, Pennsylvania, United States*

Little is known about how preference-based care impacts nursing home (NH) residents' well-being over time. The Preference Match Tracker (PMT) objectively tracks the number of recreation activities NH residents attend that match their important preferences. We explored how PMT data were linked to residents' depressive symptoms over time. The number of preferred and non-preferred activities attended and refused to attend per week were tracked with the PMT for 586 residents over 1 year. We utilized generalized linear modeling to examine the association of preferred/non-preferred attendance/refusals for three depressive symptom groups (none, minimal, depressive symptoms). After accounting for covariates, more attendance was associated with minimal depressive symptoms over time. More total activity refusals and refusals of preferred activities were associated with minimal and mild-to-severe depressive symptoms over time and, more refusals of non-preferred activities were associated with minimal symptoms. Associations were moderated by pain, count of important preferences, cognition, and length of stay. Refusing to attend preferred activities may serve as a marker of distress. Individuals who are cognitively capable and/or living in the NH for < 90 days or experiencing pain, who are refusing preferred activities should be monitored for depressive symptoms and their recreational activity participation.

Abstract citation ID: igaf122.2858

### TECHNOLOGY USE AND MONITORING PREFERENCES IN OLDER ADULTS: IMPLICATIONS FOR PASSIVE REMOTE MONITORING

Molly Lawrence[1], Sarah Prieto[2], Alexa Vasquez[2], and Alyssa De Vito[2], *1. Alpert Medical School at Brown University, Providence, Rhode Island, United States, 2. Brown University, Providence, Rhode Island, United States*

Rising Alzheimer's disease (AD) rates necessitate better detection of early behavioral changes. Passive remote monitoring (PRM), technology using sensor-based data to monitor behavior, can describe a person's day-to-day functioning. Acceptability of PRM among older adults remains uncertain. We investigated older adults' use of technology and their openness to monitoring of technology use using a 25-30-minute online survey regarding current technology use and openness to PRM of technology use. Participants were 171 older adults with normal cognition or mild cognitive impairment (mean age=56, 66% Female, 29% White, 28% Black/African American, 18% Latinx, 22% Asian, 3% Mixed/Other Race) .Most respondents reported using smartphones for: texting (95.9%), calls (95.3%), camera/photos (94.2%) and e-mail (90.6%). Fewer respondents reported using phones for games (60.2%), health monitoring (60.2%) or digital assistance (38%). Females used GPS more than males ($p < .05$). Young-old adults (age < 55) used applications for organization, digital assistance, e-mail, social media, and health tools more than Old-older adults. Most of the sample reported openness to monitoring of: mobility (86%), overall time spent on phone (80.1%) and speed/errors in typing (76.6%). Respondents were less open to PRM of app use type (69%), GPS location (63.8%) and number of incoming/outgoing calls (61.9%). No significant differences emerged regarding openness to monitoring based on age or gender ($p>.05$). These results demonstrate that older adults use technology for a range of daily tasks, indicating that PRM may be acceptable in this population. Older adults also may be willing to have some tasks monitored to detect cognitive impairment.

Abstract citation ID: igaf122.2859

### THE ROLE OF RELIGIOUS COPING AND PREPAREDNESS IN DEATH ACCEPTANCE AMONG DEMENTIA CAREGIVERS

Landon Peeples, Lauren Chrzanowski, and Benjamin Mast, *University of Louisville, Louisville, Kentucky, United States*

Religious coping is an important factor in how individuals manage aging and mortality, yet the mechanisms by which it influences acceptance of death are unclear. This study examines two competing path models assessing the relationships between positive and negative religious coping, death preparedness, and death acceptance. Data from 41 caregivers of older adults with dementia from the Resources for Enhancing Alzheimer's Caregiver Health (REACH-II) dataset were analyzed to determine the best-fitting model. The first model proposed that preparedness predicts both positive and negative religious coping, which then predict acceptance. This model showed poor fit ($\chi^2$ (1) = 11.393, $p = .001$, CFI = 0.778, TLI = -0.335), suggesting that it did not represent the data well. Preparedness significantly predicted positive ($\beta = .554$, $p < .001$) and negative religious coping ($\beta = .510$, $p < .001$), yet neither mechanism was a strong predictor of acceptance (positive: $\beta = .281$, $p = .068$; negative: $\beta = .287$, $p = .063$). Alternatively, the second model tested if religious coping predicts preparedness, which in turn predicts acceptance. This model revealed a strong fit ($\chi^2$ (2) = 0.976, $p = .614$, CFI = 1.000, TLI = 1.000). Both positive ($\beta = .403$, $p = .004$) and negative ($\beta = .322$, $p = .020$) religious coping significantly predicted preparedness, which strongly predicted acceptance ($\beta = .639$, $p < .001$). Findings suggest that religious coping facilitates preparedness and indirectly acceptance, stressing the need for interventions that enhance preparedness to foster greater acceptance of death.

Abstract citation ID: igaf122.2860

### THE ROLES OF HEALTH SERVICES ON THE ASSOCIATION BETWEEN MEDICARE ANNUAL WELLNESS VISITS AND EARLY DEMENTIA DIAGNOSES

Mukaila Raji[1], Huey-Ming Tzeng[2], Yong Shan[3], Jeong Jang[4], Peter Cram[5], and Yong-Fang Kuo[6], *1. University of Texas Medical Branch at Galveston, Galveston, Texas, United States, 2. School of Nursing, University of Texas Medical Branch, Galveston, Galveston, Texas, United States, 3. UTMB, Galvesto, Texas, United States, 4. UTMB Health, The University of Texas Medical Branch, Galveston, Texas, United States, 5. University of Maryland, Baltimore County, Baltimore, Maryland, United States, 6. University of Texas Medical Branch, Galveston, Galveston, Texas, United States*

Early recognition of cognitive impairment (CI) and timely diagnoses of mild CI (MCI) and Alzheimer's Disease and Related Dementia (ADRD) are key to optimal dementia care. Currently there are no data describing the rates of guideline-informed health services (specific blood tests – vitamin B12, thyroid stimulating hormone; specialist referrals – neurology, psychiatry; and brain images – CT, MRI) conducted after Medicare Annual Wellness Visits (AWVs) in evaluation processes leading to MCI/ADRD diagnosis. Using propensity study cohort of Texas fee-for-service Medicare enrollees, (n = 66,433 at each group), we followed health services utilization and MCI/ADRD diagnosis within one-year of the first AWV or the index date for the non-AWV group in 2018. Both groups had similar proportion of patients who received specific blood tests, specialist referrals, and brain images in the 12 months before AWV/index date. Among those with no previous specialist visits, 4.1% in the AWV group and 3.5% in the non-AWV group got specialist referrals in the follow-up period. Among those with no previous brain images, 12.4% in AWV group and 11.7% in non-AWV group got brain images in the follow-up period. AWV receipt was associated with 41% increase in MCI diagnosis—with about 20% of the increase accounted for by post-AWV specialist referrals, lab tests, and brain images. AWV receipt was also associated with an 8% increase in ADRD diagnosis—with about 75% of the increase accounted for by the three guideline-informed health services. This study suggests that receipt of Medicare AWV has larger direct effect on the identification of early CI.

Abstract citation ID: igaf122.2861

### TRUST, CULTURAL COMPETENCE, AND PROMOTORES-BASED ENGAGEMENT: EFFECTIVE OUTREACH WITH DEMENTIA FAMILY CAREGIVERS

Alma Manzo, Raheleh Bahrami, Maria Gonzalez Pyles, Lourdes Cordova, Berta Carbajal, Allison Glinka, and David Coon, *Arizona State University, Phoenix, Arizona, United States*

Hispanic/Latinos are 1.5x more likely to develop Alzheimer's disease and related dementias (ADRD) than non-Hispanic/Latino Whites. As a result, there will be a corresponding increase in Hispanic/Latino family caregivers (H/LFC) needing resources to support them through the caregiving process. As of 2022, the Hispanic/Latino population only accounts for 2% of the clinical trials on ADRD or studies funded by the National Institute of Health. For H/LFC of people with ADRD, the limited outreach is due to eligibility requirements, time constraints, and language accessibility. This research examines the barriers to H/LFC outreach and identifies effective outreach strategies to engage them in interventions. Focus groups were conducted in English and Spanish in the Phoenix metropolitan area on H/LFC (n = 26, Mage = 51) and community health workers (n = 29, Mage = 46.5). Researchers employed a community engagement research methodology, holding three separate caregiver groups and three separate community health worker groups. Transcripts were analyzed inductively through thematic content analysis identifying three main categories: (1) Cultural Stigma on Dementia & Building Trust in Community Spaces, (2) Inadequate Spanish-Speaking Services & Enhancing Cultural Competencies, and (3) Systemic Inequities & Promotores-based Engagement Strategy. Each category identifies a barrier and a strategy. These findings provide insight into how researchers, clinicians, and community health workers can collaborate to find better strategies to recruit H/LFC into programs and clinical studies. It also offers opportunities to enhance retention efforts and maintain low attrition rates in not only clinical studies, but also family education and intervention programs.

Abstract citation ID: igaf122.2862

**VARIATION IN THE MANAGEMENT OF HERPES ZOSTER AMONG US NURSING HOME RESIDENTS WITH AND WITHOUT DEMENTIA**

Peyton Free[1], Preeti Chachlani[2], Kaleen Hayes[3], Kevin McConeghy[2], H Edward Davidson[4], Lisa Han[4], Stefan Gravenstein[5], and Daniel Harris[1], *1. University of Delaware, Newark, Delaware, United States, 2. Brown University School of Public Health, Providence, Rhode Island, United States, 3. Brown University, Providence, Rhode Island, United States, 4. Insight Therapeutics, Norfolk, Virginia, United States, 5. Alpert Medical School of Brown University, Providence, Rhode Island, United States*

Herpes zoster (HZ) is a painful condition that is managed with a combination of antivirals, steroids, and pain medications. Nursing home residents with Alzheimer's disease and related dementias (ADRD) may have difficulty expressing HZ symptoms, thus affecting medication management. This study compared prescription and non-prescription medication use in nursing home residents with HZ by ADRD diagnosis. We conducted a retrospective cohort study using nursing home electronic health record data from 2017-2022. Residents with HZ and ADRD were identified using ICD-10 codes as of the HZ diagnosis date. Administration for pain medications, antivirals, and steroids were measured before and after the HZ diagnosis date in periods of time (e.g., 1-7, 8-14, and 15-30 days before/after diagnosis). To assess relative changes in medication prevalence over time, administrations 15-30 days prior to HZ represented the baseline period. We identified 4,719 residents with HZ (31.5% had ADRD). Among residents without ADRD, oxycodone increased from 1.6% to 3.4% (vs. ADRD=0.5% to 0.8%) and gabapentin from 13.4% to 33.9% (vs. ADRD=12.6% to 25.6%) between baseline and 0-7 days after HZ diagnosis. In the 0-7 days after HZ diagnosis, prednisone use was 1.74 times greater in residents without ADRD (10.3%) vs. with ADRD (5.9%), and acyclovir use was similar between groups (without ADRD=28.5%; with ADRD=27.1%). Differences in medications, especially prednisone, between residents with and without ADRD may reflect undertreatment of HZ symptoms in residents with ADRD.

Abstract citation ID: igaf122.2863

**WHAT IS KNOWN ABOUT HOSPICE ENROLLMENT FOR NURSING HOME RESIDENTS WITH COGNITIVE DISABILITIES: A SYSTEMATIC REVIEW**

Victoria Winogora[1], Patricia Stone[2], Kimberlee Grier[2], Lara Dhingra[3], and Christine DeForge[2], *1. Columbia University School of Nursing, New York, New York, United States, 2. Columbia University, New York, New York, United States, 3. Metropolitan Jewish Health System, Inc., New York, New York, United States*

Background: The population of individuals with cognitive disabilities (CD) accessing hospice care is increasing. CDs are defined as serious difficulty concentrating, remembering, and making decisions, and are often associated with neurocognitive disorders (e.g., dementias and Parkinson's disease). Individuals with CDs often live and die in nursing homes (NHs). Little is known about hospice enrollment for individuals with CDs who die in NHs. Objective: To systematically review the evidence available on hospice enrollment in NHs for individuals with CDs. Methods: Following the PRISMA guidelines, we searched for United States-based, English-language, and peer-reviewed literature on hospice enrollment in NHs for individuals with CDs. PubMed, CINHAL, Web of Science, and Google Scholar were searched for articles published before September 20, 2024. The Joanna Briggs Institute Analytical Cross-Sectional Studies Appraisal Tool to assess study quality. Results: 1,056 titles and abstracts were screened, 22 articles underwent full-text review, and 6 met inclusion criteria. The indicators of CD varied. In all studies, a significant percentage of NH decedents with CDs were not enrolled in hospice at the time of their deaths. Factors associated with increased likelihood of hospice enrollment at the time of death included female sex (n = 2), white race (n = 2), and non-CD terminal diagnosis (n = 1). Discussion: This systematic review is the first to explore hospice enrollment for NH residents with CDs. All studies focused on individuals with dementias. CD was associated with different rates of hospice enrollment for NH decedents. Future research should aim to explore interventions to improve hospice enrollment for this population.

Abstract citation ID: igaf122.2864

**WHO IS AFRAID OF COGNITIVE DECLINE AFTER AGE 50 AND WHY: REALITY OR ILLUSION?**

Jacqui Smith, and Marina Larkina, *University of Michigan, Ann Arbor, Michigan, United States*

Tips about how to remain mentally sharp abound in the media alongside reports about the personal and societal challenges of dementia. Multiple questions remain about the impact of these messages. We use 2022 panel data from the Health and Retirement Study (HRS) to examine concerns about cognitive health and proposals suggesting a mismatch between actual cognitive performance and subjective status. Eligible participants (N = 4098: Mage = 69.5; Range 50-101; 60% women) responded to five items in the Psychosocial and Wellbeing questionnaire about concerns regarding age-related challenges [staying mentally sharp, remaining in own home, health-care expenses, communication, and developing Alzheimer's Disease (AD)] together with subjective and objective assessments of cognition, health, perceptions of aging, and demographic covariates. Staying mentally sharp was the top concern (71%) followed by staying in one's own home (56%) and AD (51%). The items were intercorrelated (rs = 0.62 to 0.42). Logistic regressions predicting concerns revealed that women (ORs =1.25), people with more chronic health diagnoses (ORs =1.37), and depression (ORs =1.84) were more likely to be concerned. Objective memory performance was not associated with concerns, but people who rated their memory as poor were more likely to be concerned (ORs=1.95). Additional analyses, revealed that people who viewed their own aging more positively were less likely to be concerned (OR = 0.67). In a longitudinal HRS

subsample (N=225), 31% who were not concerned about AD in a 2010 became concerned in 2022. Future research should investigate if changes in cognition, physical health, depression, and media use contribute to concerns.

### SESSION 7310 (POSTER)

### ASSESSMENT (E.G. GERIATRIC ASSESSMENT, FUNCTIONAL ASSESSMENT, FUNCTIONAL STATUS INSTRUMENTS)

Abstract citation ID: igaf122.2865

### CAN OLDER ADULTS ACCURATELY PERCEIVE THEIR HAND FUNCTION?

Jayne Parry, Amanda Mechaber, Rachel Logue Cook, and Susan Brown, *University of Michigan, Ann Arbor, Michigan, United States*

Older adults have been shown to overestimate their ability to control balance and locomotion (Kawasaki & Tozawa, 2020; Martin et al., 2021). Despite the importance of upper limb ability for daily living, it is unknown whether older adults also overestimate manual ability. The purpose of this study was to determine whether older adults overestimate hand function compared to objective hand measures. Perception and physical measures of hand function were assessed in 28 community-dwelling older adults (mean age 75.2 ± 3.3 y/o). An adapted survey (Logue Cook et al, 2024) assessed participants' perceptions of hand function. Strength and dexterity of both hands were assessed using standard dynamometry, the Purdue Pegboard Test, and the Jebsen Taylor Hand Function Test. Two thirds of participants (66.7%) reported their current hand function to be better than the average for their age while one third (33.3%) thought their function was average. None of the participants reported their hand function as worse than average. However, 40.7% of individuals had at least one objective hand measure that fell below one standard deviation of the group mean. Of this group, 55% reported their hand function to be better than average. Objective measures showed age-related declines in hand function despite older adults perceiving their hand function as average or better than average. A tendency to overestimate hand function using self-reported surveys underscores the importance of including more objective measures when monitoring age-related changes in hand health.

Abstract citation ID: igaf122.2866

### IMPLEMENTATION OF A GERIATRICS-PRIMARY CARE CO-MANAGEMENT MODEL IN AN URBAN COMMUNITY HEALTH CENTER

Carolina Fonseca Valencia[1], and Jeremy Whyman[2], *1. Beth Israel Deaconess Medical Center, Brookline, Massachusetts, United States, 2. Beth Israel Deaconess Medical Center, Boston, Massachusetts, United States*

Vulnerable socioeconomically disadvantaged (SEcD) older adults represent a diverse and complex group with high levels of socioeconomic stressors, low health literacy, and chronic medical conditions. These complexities make diagnosis and management of geriatric syndromes difficult resulting in care delays, increased preventable costs, morbidity, and mortality. Geriatric co-management is characterized by geriatricians and non-geriatricians working collaboratively to detect, prevent, and manage geriatric syndromes. This model of care utilizes the comprehensive geriatrics assessment (CGA) as a primary tool, to frame the care of SEcD older adults, however, its impact in community health settings is not well known. To lessen healthcare inequities and understand the impact that specialized geriatrics care has in the health management of SEcD older adults, we have implemented a geriatric co-management model in an urban community health center. Our intent is to enhance early diagnosis and management of geriatric syndromes and support the primary care team by demonstrating feasibility of implementation; defining the specific characteristics of this population; and describing the prevalence of geriatric syndromes. In a 24-month period we have completed a total of 100 CGAs on patients between the ages of 53-100 years. Most patients are female (62%), non-English speaking (65%) with Cape Verdean, Creole and Spanish being the most prevalent languages. These patients are mostly moderately frail with average frailty index of 0.404, and the most common geriatric syndromes include cognitive impairment (MCI and dementia), malnutrition, polypharmacy, and gait disturbances.

Abstract citation ID: igaf122.2867

### INCREMENTAL UTILITY OF AWARENESS OF DEFICIT AS A PREDICTOR OF DEMENTIA OUTCOMES

Brenda Owe, Anthony Robinson, Destiny Weaver, and Lisa Rapport, *Wayne State University, Detroit, Michigan, United States*

Subjective report of cognitive function is often used as a proxy for actual cognitive status; however, individuals with cognitive decline frequently underestimate their impairments. Impaired self-awareness of deficits (anosognosia) is important to understand objective function and risk for multiple adverse outcomes. This study examined the incremental contribution of assessing impaired awareness of deficits (anosognosia) over self-ratings of cognitive capacity. Data were obtained from 793 participants (ages 70-110 years) in the Aging, Demographics, and Memory Study supplement to the Health and Retirement Study. Main outcome measures included clinician-report and informant-report dementia rating scales, and global cognitive status assessed using the Mini Mental State Examination (MMSE). Awareness of deficit was operationalized as the difference between self-reported memory and objective performance on a verbal memory test. Self-reported memory showed weak correlation with clinician-report and informant-report dementia rating scales and the MMSE, whereas anosognosia showed medium-to-large correlations with those outcomes. Importantly, anosognosia contributed unique variance in predicting all three outcomes, beyond that accounted for by age, education, gender, and a different objective measure of verbal memory. In contrast, parallel models testing self-reported memory indicated that it was not a significant predictor of dementia rating scales or the MMSE. Both self-perception of cognition and objective cognitive performance are necessary to understand an individual's awareness of their deficits. Information about self-perception of cognitive status is important to understand subjective well-being; however, the discrepancy between self-perception and objective capacity provided unique information about functional cognitive status, whereas self-report alone did not.

Abstract citation ID: igaf122.2868

### INTEGRATED CARE IMPROVED COGNITIVE ABILITY IN OLDER CHINESE PATIENTS WITH IMPAIRED INTRINSIC CAPACITY

Ping He, Yi Zhang, Aihong Liu, Bei Cheng, Tangmeng Guo, Jing Ge, and Yang Zhao, *Department of Geriatrics, Union*

*Hospital, Tongji Medical College, Huazhong University of Science and Technology, 1277 Jiefang Avenue, Wuhan 430022, Hubei, China, Wuhan, Hubei, China (People's Republic)*

Objectives: This study aims to investigate the effectiveness of a integrated care model for elderly patients led by geriatric care experts, in order to provide information for the management of intrinsic capacity in older Chinese patients. Methods: Between June and December 2021, 60 elderly patients with impaired intrinsic capacity were recruited from the Department of Geriatrics Union Hospital, Tongji Medical College, Huazhong University of Science and Technology. These patients were randomly assigned to either a control group or an intervention group, with 30 patients in each group. Two groups implement routine care, and the intervention group implement integrated care by a "hospital-community-family" multidisciplinary team. After 12 weeks of intervention, the differences in frailty, cognition, depression scores, comprehension social support scores, and World Quality of Life Scale scores were compared between the two groups. Results: Before the intervention, there were no significant differences between the control group and the intervention group in terms of grip strength, step speed, frailty scores, cognitive function, depression scores, social support, and quality of survival. After the intervention, both groups showed improvements in grip strength and depression scores, with no significant differences observed between the groups. However, cognitive function, comprehension social support and quality of survival scores improved only in the intervention group. Notably, cognitive ability was the only outcome that showed a significant difference between the intervention group and the control group after 12 weeks of intervention ($P < 0.05$). Conclusions: Integrated care effectively enhances patients' intrinsic capacity, with a notable improvement in cognitive abilities.

Abstract citation ID: igaf122.2869

### MEDIATING ROLE OF NUTRITION IN PSYCHOLOGICAL, SOCIAL AND PHYSICAL FRAILTY AMONG HOSPITALIZED OLDER ADULTS IN CHINA

Jing Ge, Ping He, Bei Cheng, Wenhan Li, Aihong Liu, Tangmeng Guo, Kemeng Zhang, and Yi Zhang, *Department of Geriatrics, Union Hospital, Tongji Medical College, Huazhong University of Science and Technology, 1277 Jiefang Avenue, Wuhan 430022, Hubei, China, Wuhan, Hubei, China (People's Republic)*

Background: To investigate the association between physical, psychological, and social frailty, and to explore the mediating factors that affect frailty in elderly inpatients, while providing scientific evidence for frailty intervention. Methods: A cross-sectional study enrolled 134 hospitalized older patients with geriatric diseases. Based on the Fried frailty criteria, patients were categorized into physically frail and non-physically frail groups. Psychological frailty was assessed using the MMSE and GDS-15, while social frailty was evaluated with the WHOQOL-BREF and PSSS. Spearman's correlation analyzed the relationship between the demographic variables and frailty. Mediation analyses elucidated relationships between frailty dimensions and their underlying mediators. Results: Age, self-care ability, risk of malnutrition, and comorbidity were significantly related with physical frailty. Meanwhile, psychological frailty[4]Cognitive impairment and depression[4]and social frailty[4]social support and quality of life[4]were positively correlated to physical frailty status. In addition, mediation analyses indicated that nutritional status mediated the relationship between psychological and physical frailty (MMSE: $B = -0.0671$, $p < 0.0000$; GDS-15: $B = 0.0752$, $p < 0.0001$), and between social and physical frailty (WHOQOL-BREF: $B = -0.0229$, $p < 0.0000$; PSSS: $B = -0.0165$, $p < 0.0001$). Conclusions: Physical frailty in elderly inpatients is associated with psychological and social frailty, with nutritional status mediating these relationships. Therefore, it is essential to conduct comprehensive assessments for the multidimensional aspects of frailty present in these patients, targeted interventions addressing malnutrition could disrupt the vicious cycle of multidimensional frailty.

Abstract citation ID: igaf122.2870

### OLDER ADULTS REPORT GREATER LIMITATIONS IN HAND DEXTERITY VERSUS WHOLE ARM STRENGTH TASKS

Qintong Bao, Rachel Logue Cook, and Susan Brown, *University of Michigan, Ann Arbor, Michigan, United States*

Upper extremity function, essential for most daily activities, declines with age. Such declines have been documented using validated survey instruments but to what extent these age-related changes reflect deficits in, for example, strength versus coordinated hand dexterity is unclear. Therefore, this study assessed age differences in self-reported dexterity versus strength limitations in older adults using national health survey data. Response data from 337 males and 462 females aged 60-96 years were obtained from the 2020 Health and Retirement Study wave, which included questions focused on upper extremity difficulties. We categorized these questions into two groups based on whether the activity relied predominantly on strength or hand dexterity. Group limitation scores were compared across age and gender. When adjusted for gender, older adults reported greater dexterity limitations compared to activities more dependent upon strength ($p = 0.01$). For dexterity-based activities and compared to male respondents, females reported more limitations which increased with age ($\beta = 0.038$, 95% CI: 0.012–0.066, $p = 0.005$). No significant interaction effects of age or gender on strength scores were observed ($p = 0.34$). Older adults reported greater limitations in tasks requiring dextrous hand control compared to actions involving whole-arm strength. Further, greater dexterity limitations increased with age for females only while strength limitations were relatively stable regardless of age. These findings highlight the importance of monitoring hand dexterity in addition to upper extremity muscle strength to maintain functional independence.

Abstract citation ID: igaf122.2871

### SCALES FOR HEARING IMPAIRMENT AMONG CENTENARIANS AND OFFSPRING: IMPLICATIONS FOR COGNITIVE SCREENING

Angella Lee[1], Bradley Petrowitz[1], Thomas Perls[1], Paola Sebastiani[2], and Stacy Andersen[1], *1. Boston University, Boston, Massachusetts, United States, 2. Tufts Medical Center, Boston, Massachusetts, United States*

As the need to assess cognitive function in older adults increases, it is crucial to understand how hearing may impact cognitive test performance since many tasks are administered orally. While audiometry remains the gold standard for measuring hearing loss, other methods such as self-reports are easier to collect during remote assessments and/or in large cohort studies.

Centenarian (n = 291, mean age 102.2 ± 2.2 years) and offspring (n = 481, mean age 74.0 ± 7.6 years) participants from the Integrative Longevity Omics study and Longevity Consortium Centenarian Project filled out self-rated hearing ability (Likert scale from 'excellent' to 'unable to hear') and the Hearing Handicap Inventory (HHIE, range 0-40, cutoff > =10). Examiners administered a cognitive screener (i.e, Mini-Mental State Examination [MMSE]) and indicated whether hearing impairment affected the test. In stratified analyses by generation, we used linear regressions with each measure of hearing impairment adjusting for age, sex, and education to predict MMSE score. The prevalence of hearing impairment ranged from 55% to 73% among centenarians depending on the assessment modality and from 2% to 33% for offspring. Only 10.2% of centenarians and 0% of offspring were rated as hearing impaired across all three measures. Examiner-rated validity was a significant predictor of MMSE performance ($p < 0.001$) in both generations, while self-rated hearing was a significant predictor only among centenarians ($p < 0.01$). These scales capture different aspects and levels of hearing impairment and thus both research and clinical settings should consider using multiple scales to account for the impact of hearing ability on cognitive test performance.

Abstract citation ID: igaf122.2872

### SELECTING DAY-TO-DAY FUNCTIONAL TASK ASSESSMENTS AMENABLE TO VIDEO TELEHEALTH FOR PERSONS WITH DEMENTIA

Megan Gately[1], Jaye McLaren[2], Alexandra Howard[1], Steven Shirk[1], Helen Hoenig[3], and Lauren Moo[2], *1. VA Bedford Health Care System, Bedford, Massachusetts, United States, 2. VA Bedford Healthcare System, Bedford, Massachusetts, United States, 3. Durham VA Health Care System, Durham, North Carolina, United States*

Assessment of day-to-day function is critical to prevent losses due to dementia, with observation of a day-to-day task such as toothbrushing being the gold standard. Patients with dementia (PWD) face barriers to such an assessment. In-home video telehealth may increase access, but the feasibility of its use to observe PWD completing day-to-day tasks has not been demonstrated. This project describes the process of selecting specific functional task assessments feasible for adaptation to video telehealth delivery to PWD. We sent ten subject matter experts (SMEs) from fields of occupational therapy, social work, geriatrics, neuropsychology, and nursing a list of 20 day-to-day tasks, such as toothbrushing and medication management, sourced from standardized and non-standardized tools. SMEs rated the feasibility of assessing tasks over video using 5-point Likert scales on domains of clinical importance, privacy and safety concerns, time to administer, and overall feasibility. The most feasible tasks for video administration were pouring a drink, washing hands, cleaning a counter, and toothbrushing. The least feasible tasks were meal preparation, tub/shower mobility, and toilet mobility. The tasks with the highest perceived clinical importance were medication management, tub/shower mobility, and home safety. Tasks with the most privacy and safety concerns involved more intimate activities: tub/shower mobility, toilet mobility, and dressing. Determining the feasibility of video assessment of day-to-day tasks involves weighing several complex factors. Understanding clinician perspectives of which tasks are amenable to video assessment will facilitate the adaptation of assessment processes to remote administration for PWD, thereby increasing access to care.

Abstract citation ID: igaf122.2873

### SUBJECTIVE COGNITIVE DECLINE AND EXCESSIVE SPENDING IN WALLET STUDY PARTICIPANTS

Emily Flores, LaToya Hall, and Peter Lichtenberg, *Wayne State University, Detroit, Michigan, United States*

Older adults experiencing cognitive decline are at an increased risk of financial vulnerability, yet self-awareness of this relationship remains unclear. This study examines the association between self-reported cognitive decline and excessive spending, exploring whether individuals who perceive memory loss recognize its financial impact. Data were drawn from a sample of 83 older adults in the WALLET study who shared 12 months of their checking account statements ($M = 73.54$, $SD = 8.05$), who were experiencing early memory loss. Twenty-five participants reported that memory loss was impacting their financial decision making and 58 said memory loss had no impact on their financial decision-making. Excessive spending was significantly more prevalent in those who reported that memory loss was impacting their financial decision-making, $X^2 = 9.285$, $p < .01$, $d = .34$. The group reporting that memory loss impacted financial decision-making also had significantly higher financial exploitation vulnerability scores (FEVS) than those whose memory decline was not reported to impact financial decision making, ( $t = -2.609$, $p < .05$, $d = -.55$). This pattern remained even excluding the cognition-related FEVS item, $t = -2.087$, $p < .05$, $d = -.52$. Age and education were not significantly different between groups.The results suggest that awareness of how early memory loss impacts financial decision-making might be a key factor in risk of excessive spending and financial exploitation vulnerability. Future research will examine the utility of a 3-point scale Subjective Cognitive Decline-Financial measure (i.e. no cognitive decline, cognitive decline only, cognitive decline impacting financial decision making).

Abstract citation ID: igaf122.2874

### THE RELATIONSHIP BETWEEN FEAR OF FALLING AND FUNCTIONAL MOBILITY AMONG OLDER ADULTS

Leah Houston[1], Jennifer Piazza[2], and Koren Fisher[2], *1. California State University, Fullerton, Aurora, Colorado, United States, 2. California State University, Fullerton, Fullerton, California, United States*

Fear of falling (FOF) affects 58% of older adults, irrespective of fall history. Current research indicates that FOF may have negative health effects, including an increased likelihood of falls, social isolation, and decreased physical activity and performance, including poorer balance. Similarly, functional mobility, particularly gait speed, is considered a significant predictor of disability, mortality, and fall risk in older adults. Despite the negative biopsychosocial effects, FOF is largely excluded from comprehensive geriatric assessment tools and fall prevention interventions for older adults. Thus, the purpose of this study is to investigate the relationship between FOF and functional mobility in community-dwelling older adults. This cross-sectional study conducted at the Center of Successful Aging at California State University, Fullerton examined functional mobility and FOF for 33 older adults (74.68 ± 5.380 years, 70% Female, 30% Male) using the 8 Foot Up-and-Go and the Short Falls Efficacy Scale-International (Short FES-I). Linear regression analysis indicated that a greater fear of falling was predictive of slower gait speeds, even after adjusting for age, gender, total number of comorbidities, and fall history (Beta=0.423, p=.002). Additionally, this model explained 27% of the variance for gait speed, indicating that fear of falling plays a notable role in functional mobility in older adults. These findings suggest that fear of falling may be a significant factor contributing to slower gait speeds in older adults. Thus, to improve the functional mobility of older adults, it is crucial to address FOF in holistic fall prevention and physical activity interventions in this population.

Abstract citation ID: igaf122.2875

**VALIDATION OF THE PROLONGED GRIEF DISORDER-13-REVISED IN A SAMPLE OF OLDER ADULTS**

Hannah Apostolou[1], Lindsey Jacobs[2], Rebecca Allen[2], A. Lynn Snow[2], Alexa Tullett[2], and Hyunjin Noh[2], *1. The University of Alabama, Milwaukee, Wisconsin, United States, 2. The University of Alabama, Tuscaloosa, Alabama, United States*

Older adults are at an increased risk of developing Prolonged Grief Disorder (PGD) due to the heightened chances of experiencing a loss, lower levels of cognitive functioning, and heightened rates of loneliness and depression. Currently, the Prolonged Grief Disorder-13 Revised (PG-13-R) is the only measure specifically designed to assess for PGD. The PG-13-R was developed to reduce misdiagnoses of PGD and avoid pathologizing normal grief, as research has found grief to be a distinct psychological response to bereavement. The present study compared the PG-13-R to other measures of similarly presenting mental health disorders. Our sample consisted of 120 racially and ethnically diverse adults aged 50 and older who completed measures of grief, depression, anxiety, post-traumatic stress disorder (PTSD) and loneliness using REDCap. Correlation coefficients were computed using SPSS Version 29 to investigate convergent and discriminant validity of the PG-13-R and other mental health disorder measures. The results showed the PG-13-R was strongly correlated with the PTSD measure ($r = 0.67$, $p < 0.001$), moderately correlated with the anxiety measure ($r = 0.49$, $p < 0.001$), and weakly correlated with measures of depression ($r = 0.39$, $p < 0.001$) and loneliness ($r = 0.34$, $p < 0.001$). This suggests the PG-13-R and PTSD measure are capturing aspects of a related concept. The PG-13-R could be further refined to capture unique features of grief, distinct from PTSD, to improve the quality of grief assessment. Continuing to improve existing measures further equips mental health professionals to serve the community by advancing the identification, assessment, and treatment of mental health disorders.

### SESSION 7315 (POSTER)

### BIOLOGY OF AGING II

Abstract citation ID: igaf122.2876

**ACCELERATED MOLECULAR AGING IN NEIGHBORHOOD POVERTY: A RACIAL/ETHNIC COMPARISON**

Jennifer Robinette[1], and Jennifer Smith[2], *1. Chapman University, Orange, California, United States, 2. University of Michigan, Ann Arbor, Michigan, United States*

In the US, racial/ethnic health disparities are undeniable and partially stem from residing in low SES neighborhoods. Associations between neighborhood SES and health may have some underlying molecular mechanisms reflected in the epigenome. Yet, neighborhood characteristics are not always experienced the same way for all residents, and questions remain regarding whether those most exposed to low SES neighborhoods build greater resilience to, or embody greater harmful and cumulative outcomes from, such neighborhoods. The present study tested the hypothesis that greater neighborhood poverty would relate to accelerated epigenetic aging on the Horvath, Hannum, PhenoAge, and GrimAge clocks, and that these associations would differ in strength by race/ethnicity. A national sample of 3,790 older non-Hispanic White, non-Hispanic Black, and Hispanic participants from the 2016 wave of the Health and Retirement Study and census tract poverty rates from the 2012-2016 American Community Survey were used to test these questions. Results of weighted linear regressions suggested that living in higher poverty neighborhoods was associated with accelerated PhenoAge and GrimAge (not Hannum or Horvath), adjusting for age, sex, and educational. After further considering smoking status, however, only the association with GrimAge remained. Investigating interactions with race/ethnicity suggested that poverty was more strongly associated with accelerated GrimAge among non-Hispanic White participants, but this racial/ethnic difference was completely reduced after considering smoking. These results further support the need for economic investment in low SES neighborhoods and encourage more research to determine the social and physical resources needed in such neighborhoods to attenuate accelerated epigenetic aging.

Abstract citation ID: igaf122.2877

**ASSESSMENT OF MALE REPRODUCTIVE AGING IMPLICATIONS OF SERTOLI CELL'S SENESCENCE**

Elikanah Olusayo Adegoke[1]Alexandra Montesinos[1], Reema Banarjee[2], Amit Dey[2], and Nathan Basisty[2], *1. National Institute on Aging, Baltimore, Maryland, United States, 2. National Institute on Aging, Bethesda, Maryland, United States*

Advances in cancer detection and improved treatment strategies have markedly increased survival rate for cancers (pediatric), leading to a growing population of early life cancer survivors. However, long term reproductive effects of chemotherapy (doxorubicin) and radiotherapy (irradiation) on reproductive aging has received less attention. Sertoli cells support, nourish, and protect spermatogenic cells in the testis. To evaluate the senescence inducing potential of doxorubicin and irradiation on Sertoli cells and the consequent implications in male reproductive aging, Sertoli cells in culture were exposed to doxorubicin and gamma radiation, and senescence was validated ten days after exposure by confirming the upregulation of senescence markers: CDKN2A, CDKN1A, GDF15, IL-6, IL-8. DIA proteomic analysis was conducted on the Orbitrap mass spectrometer. Data analysis was performed on Spectronaut and R. The proteomic analysis identified 6,304 proteins. Among these, 1,509 proteins were downregulated and 1,443 were upregulated in doxorubicin treatment. After irradiation, 1,090 proteins were downregulated, while 1,125 proteins were upregulated. Microtubule-based process, cell division, and sexual reproduction biological processes were enriched among downregulated proteins following both treatments, suggesting loss of key functions supporting reproduction and phagocytosis associated with Sertoli cells. Furthermore, transmembrane/cell transport pathways and immune response, were enriched among upregulated proteins in both treatments, known senescence associated pathways that are consistent with dysregulation of normal Sertoli cell paracrine functions. 45% of protein changes were overlapping between both treatments. In conclusion, we demonstrate that both doxorubicin and irradiation can induce Sertoli cells to senescence and consequently result in reproductive aging marked by decreased sexual reproduction.

Abstract citation ID: igaf122.2878

### BETA DISTRIBUTION NETWORK MODELING IMPROVES BIOLOGICAL INTEGRATION OF MULTI-OMICS DATA

Heeju Noh[1], Max Robinson[1], Lance Pflieger[2], and Noa Rappaport[3], *1. Institute for Systems Biology, Seattle, Washington, United States, 2. Phenome Health, Seattle, Washington, United States, 3. Institute for Systems Biology, Buck Institute, Phenome Health, Seattle, Washington, United States*

Correlation based clustering of multi-omics data typically results in platform-specific modules rather than biologically meaningful cross-platform associations, as technical variation and data structure differences between omics platforms dominate the correlation patterns.We introduce a novel network model that fits a Beta distribution to analyte-analyte correlations. First, correlations across different platforms are standardized by aligning Beta distribution shapes uniformly. Our approach then constructs an analyte relationship network by identifying outlier correlations against a null model background. We generated synthetic data which mimics realistic multi-omics profiles, where intra-omics correlations are generally stronger than inter-omics correlations. Next, we evaluate clustering performance using synthetic datasets, comparing our method to the standard Weighted Correlation Network Analysis (WCNA), which constructs networks based on a power-law distribution. Our method demonstrated higher clustering accuracy ($p = 1.3e-13$) and purity ($p = 2.22e-16$) compared to WCNA. The improvement was particularly notable at higher signal-to-noise ratios and fewer reference clusters. We further tested our method on human multi-omics cohort data (Arivale Wellness Study), our strategy outperformed the standard WCNA, achieving a higher average silhouette score and improved entropy, leading to better clustering of proteins, metabolites, and laboratory analytes. This approach is powerful for integrating multimodal molecular data in a biologically meaningful way, enabling the discovery of cross-platform relationships that reveal functional connections across biological layers

Abstract citation ID: igaf122.2879

### EXAMINING INSULIN RESISTANCE AND BMI IN THE CONTEXT OF ALPHA-KLOTHO AND FUNCTIONAL DECLINE

Anam Ahmad[1], Alan Rathbun[2], Michelle Shardell[2], Richard Semba[3], Rita Kalyani[4], and Luigi Ferrucci[5], *1. University of Maryland School of Medicine, Cary, North Carolina, United States, 2. University of Maryland School of Medicine, Baltimore, Maryland, United States, 3. Johns Hopkins University, Baltimore, Maryland, United States, 4. Johns Hopkins University School of Medicine, Baltimore, Maryland, United States, 5. NIA, Bethesda, Maryland, United States*

Alpha-klotho, a longevity-associated protein, has been linked to aging-related physical and cognitive decline. Research implies that alpha-klotho is associated with insulin resistance (IR), and with cognitive and physical decline. Body mass index (BMI) is also a risk factor for IR and both functional declines. However, it is unknown whether IR mediates the relationship between alpha-klotho and dual cognitive-physical decline, or if this relationship differs by BMI. Therefore, we assessed mediation with stratification by BMI. Older adults ($n = 461$, age ≥65 years) from the Invecchiare in Chianti study were phenotypically categorized based on cognitive (Mini-Mental State Examination) and physical (4-meter gait speed) using group-based trajectory analysis with data up to 15 years of follow-up. Overall, $n = 149$ (32.3%) participants were categorized as experiencing dual cognitive-physical decline. IR was operationalized using the homeostasis model for insulin resistance (HOMA-IR). Mediation models were implemented to estimate total, natural direct, and natural indirect effects (TE, NDE, NIE, respectively) of alpha-klotho on dual cognitive-physical decline. Adjusted odds ratios (ORs) of dual cognitive-physical decline per doubling of log-transformed alpha-klotho for TE ($OR = 0.45$, 95% Confidence Interval[CI]= 0.11-1.48), NDE ($OR = 0.44$, 95%CI= 0.12-1.38) and NIE ($OR = 1.01$, 95%CI=0.70-1.28) among $n = 225$ overweight (BMI=25-29.9 kg/m2) participants indicated negligible (-0.5%) mediation by HOMA-IR in the relation between alpha-klotho and dual cognitive-physical decline. No significant effects were found overall or among the obese ($n = 68$, BMI > =30 kg/m2) or normal/underweight ($n = 168$, BMI< 25 kg/m2) strata. Further research is needed to better understand potential mechanisms linking alpha-klotho and insulin resistance to cognitive and physical performance over time in older adults.

Abstract citation ID: igaf122.2880

### INNATE IMMUNE CHANGES S IN AGING AND IN OLDER T2DM SUBJECTS WITH OR WITHOUT OBESITY

Tamas Fulop[1], Abdelouahed Khalil[1], Jacek Witkowski[2], and José Morais[3], *1. Université de Sherbrooke, Sherbrooke, Quebec, Canada, 2. University Medical School of Gdansk, Gdansk, Pomorskie, Poland, 3. McGill University, Montréal, Quebec, Canada*

: It could be that in older adults with diabetes mellitus type 2 (T2DM), especially associated with obesity ("diabesity"), their innate immune system changes would be more accentuated than in normal aging. To investigate the monocytes and macrophages phenotypes and functions. Methods: We recruited 48 older subjects (aged from 65 to 80 years), 16 as healthy controls, 16 non obese and 16 obese T2DM patients. Obesity was determined by BMI over 30. We separated the monocytes by adherence and studied their phenotypes by using CD14 and CD16 and diferentiated them in macropages. The monocytes phenotype distribution in older and T2DM non obese subjects resembled that in young subjects, while T2DM obese subjects had a significant increase in the intermediate subpopulation (pro-inflammatory). When monocytes were incubated with M-CSF or autologous sera, they became predominantly M2 in healthy and T2DM non-obese subjects, while GM-CSF induced M1 phenotype in healthy and non-obese T2DM subjects. The M2 had an increased phagocytic capacity but produced less ROS. In contrast the M1 had a great ROS production capacity but much less phagocytic activity. Macrophages of obese T2DM subjects incubated in autologous sera differentiated in M1 in contrast to the healthy and non-obese T2DM subjects, and produced a significantly higher amount of ROS after opsonised E.coli and LPS stimulation. TNFα is increased in both T2DM group compared to healthy subjects. There is an inflammatory status with aging which is not modified by T2DM but much increased when obesity is present concomitantly with T2DM.

Abstract citation ID: igaf122.2881

**INTEGRATIVE MULTI-OMIC SIGNATURES OF LONGEVITY AND HEALTHY AGING**

Lance Pflieger[1], Kengo Watanabe[2], Theinmozhi Arulraj[3], Max Robinson[3], Oliver Fiehn[4], Robert Moritz[3], Jodi Lapidus[5], and Noa Rappaport[6], *1. Phenome Health, Seattle, Washington, United States, 2. Tokushima University Graduate School of Biomedical Sciences, Tokushima, Tokushima, Japan, 3. Institute for Systems Biology, Seattle, Washington, United States, 4. University of California, Davis, Davis, California, United States, 5. Oregon Health & Science University, Portland, Oregon, United States, 6. Institute for Systems Biology, Buck Institute, Phenome Health, Seattle, Washington, United States*

There is a growing interest in serum-based biomarkers that reveal the mechanisms of healthy aging. The Longevity Consortium generated untargeted mass spectrometry-based proteomic and metabolomic datasets from serum samples across four cohorts; the Osteoporotic Fractures in Men (MrOS) Study, the Study of Osteoporotic Fractures (SOF), the Health, Aging, and Body Composition (Health ABC) Study, and the Cardiovascular Health Study (CHS) totaling 3380 total participants with a 1:3 case-cohort design. This dataset offers a unique opportunity to identify a robust prospective signature of Longevity, defined as survival to the 98th percentile based on sex- and birth cohort-specific survival distributions. We applied multiblock sparse partial least squares discriminant analysis and systems biology approaches to integrate and construct multi-omic signatures predictive of longevity. From approximately 5000 metabolites and 500 proteins, we identified biomarkers associated with coagulation and complement and cholesterol transport. Interestingly, the cholesterol transport signature (apolipoproteins) was only enriched in females, suggesting sex-specific mechanisms of longevity. Furthermore, we validate these findings by comparing omics data from human centenarian and mouse longevity studies that also capture molecular signatures induced by life-extending interventions. This study underscores the power of integrative systems biology methods in characterizing the heterogeneity of molecular aging phenotypes, ultimately enabling the development of robust longevity signatures. The identified biomarker signatures have potential implications for personalized interventions aimed at promoting healthy aging and mitigating age-related diseases.

Abstract citation ID: igaf122.2882

**INTERCONNECTIONS OF HYPOXIA AND AGING IN LONG-LIVED SPECIES**

Damla Yalcin[1], Hsin-Yu Fang[2], Erdogan Oguzhan Akyildiz[1], Sudenaz Fatma Oner[3], Anil Sukru Dogan[4], Thomas Park[5], and Perinur Bozaykut Eker[2], *1. Acıbadem Mehmet Ali Aydınlar University, Ataşehir/İstanbul, Istanbul, Turkey, 2. Tufts University, Boston, Massachusetts, United States, 3. Acıbadem Mehmet Ali Aydınlar University, Istanbul, Istanbul, Turkey, 4. Bogazici University, Beşiktaş, Istanbul, Turkey, 5. University of Illinois Chicago, Chicago, Illinois, United States*

Hypoxia is traditionally considered harmful to cells, yet emerging evidence suggests it may promote longevity through unclear mechanisms. Two evolutionarily distant but ecologically similar species, the naked mole-rat (NMR) and blind mole-rat (BMR), exhibit exceptional longevity and hypoxia tolerance, making them ideal models for studying the molecular links between hypoxia and lifespan. Our previous work indicated that hypoxia is associated with several longevity interventions like calorie restriction at the transcriptomic level. This study explored whether hypoxia tolerance pathways overlap with longevity mechanisms, particularly within the nutrient-sensing axis. To investigate, we exposed NMRs, BMRs, and mice to normoxia and severe hypoxia (0% $O_2$ for species-specific survival times) and assessed survival rates. We analyzed AMPK/mTOR signaling, autophagy, mitochondrial function, and inflammation in heart tissue, alongside metabolomics and oxidative phosphorylation (OXPHOS) assays. Our results demonstrated that long-lived species survived hypoxia significantly better than mice. Notably, enhanced AMPK signaling, elevated autophagy-related protein levels, and reduced oxidative stress in these species suggest a strong link between longevity and hypoxia resilience. Moreover, long-lived species preserved mitochondrial health under hypoxia, likely contributing to their extended lifespan. These findings highlight hypoxia's potential role in longevity and suggest new therapeutic avenues for age-related diseases.

Abstract citation ID: igaf122.2883

**MACHINE LEARNING–BASED MOTOR RESERVE INDEX AND NIGRAL FREE WATER IN OLDER ADULTS**

Edward Ofori[1], Alexander Belnavis[1], Roxana Burciu[2], and Sydney Schaefer[3], *1. Arizona State University, Phoenix, Arizona, United States, 2. University of Delaware, Newark, Delaware, United States, 3. Arizona State University, Tempe, Arizona, United States*

Objective To develop and test a machine learning–based, Motor Reserve Index (MRIx), integrating a comorbidity-based frailty measure, gait parameters, balance measures, and sex, and to evaluate its associations with free water in the anterior/posterior substantia nigra and amyloid burden. Methods De-identified data from the Health and Aging Brain Study: Health Disparities (HABS-HD) were collected from 84 older adults (mean age = 60.6 ± 9.5 years; ~60% female). Frailty was determined via sum of chronic conditions (e.g., hypertension, cancer, diabetes, stroke, and kidney disease). Lower extremity motor measures included mean gait speed and inter-trial variability, timed up and go trial variability, Short Physical Performance Battery performance and PET SUVr (florbetaben uptake; PET-FBB) were also computed. An unsupervised dimensionality-reduction method yielded the MRIx from these features. Pearson correlations were used to test MRIx against nigral free-water measures, followed by correlations of anterior and posterior nigral free-water with PET FBB. Significance was set at $p < 0.05$. Results Participants with lower MRIx (suggesting diminished motor reserve) exhibited higher posterior nigral values ($r = -0.29$, $p < 0.05$). Additionally, posterior nigra free-water was positively related to Global FBB SUVR ($r \approx 0.26$, $p \approx 0.02$), indicating that elevated posterior nigral free water corresponded with greater amyloid signal. Anterior nigra showed no significant associations with MRIx or PET FBB SUVRs. Conclusion These preliminary data indicate that a machine learning–derived Motor Reserve Index, reflecting multiple factors including frailty and gait, may offer insights into posterior nigral changes and amyloid pathology in older adults.

Abstract citation ID: igaf122.2884

### SEX AND AGE DIFFERENTIALLY REGULATE THE GUT MICROBIOTA AND GUT HOMEOSTASIS IN MICE

Megan Wong[1], Audrey Pierce[1], Kerry Wellenstein[2], and Jennifer Lee[3], *1. Tufts University School of Medicine, Medford, Massachusetts, United States, 2. JM-USDA HNRCA at Tufts University, Boston, Massachusetts, United States, 3. The JM-USDA Human Nutrition Research Center on Aging at Tufts University, Boston, Massachusetts, United States*

The gut microbiota and gastrointestinal tract serve essentials roles in regulating host metabolism, but our understanding of how sex and age contribute to these processes remains unclear. We metabolically phenotyped young (6-month-old) and old (16-23-month-old) C57BL6 female and male chow-fed mice. We collected terminal blood for serum biochemistry and cecal contents for metabolomics. Small and large intestines were collected for histology, qRT-PCR, and immune cell levels by flow cytometry. Both sexes of old mice had higher body weight, adiposity, and plasma insulin levels versus young controls. However, old female mice had 2-fold higher adiposity versus old male mice, and this was associated with a distinct sex-specific cecal lipidome profile. Old female mice had elevated circulating levels of lipopolysaccharide and cecal short-chain fatty acids versus young female mice, and these were not different within male mice. Within old mice, levels of colonic intraepithelial TCRγδ, a key gut barrier regulator, and CD4+ T-cell cytokines were higher in females versus male mice. By contrast, old male mice had increased expression of intestinal barrier genes versus young male mice, with fewer genes differentially regulated in female mice. Similarly, colon mucus thickness and mucus protein levels were higher in old male mice versus young male controls but not different within female mice. These data identify distinct sex- and age-specific effects on multiple components of the gut microbiome and gut homeostasis that may regulate host metabolism. These differences may lead to sex-specific gut-based strategies to improve host metabolism across the lifespan.

Abstract citation ID: igaf122.2885

### SEX, STERILIZATION AND METABOLIC PROFILES SHAPE AGING TRAJECTORIES AND AGE-RELATED DISEASE RISK IN DOGS

Filippo Artoni[1], Ben Harrison[2], Laura Corlin[3], and Daniel Promislow[1], *1. Jean Mayer USDA Human Nutrition Research Center on Aging, Boston, Massachusetts, United States, 2. University of Washington, Seattle, Washington, United States, 3. Tufts University, Medford, Massachusetts, United States*

The metabolome is a highly conserved network of small molecules that plays a central role in cellular metabolism and signaling, yet its role in aging remains poorly understood. Here, we leverage the companion dog as a powerful translational model to investigate age-related metabolic changes and their relationship with disease. Dogs share a similar environment with humans, exhibit age-related multimorbidity, and have shorter lifespans, making them ideal for studying aging dynamics. Using machine-learning models trained on longitudinal data from the Dog Aging Project (DAP), we constructed a comprehensive model of the aging metabolome. Our dataset included metabolomics, blood chemistry, and complete blood counts from DAP's Precision cohort (n = 976), along with demographic, health, and lifestyle data from DAP's Pack cohort (>50,000). We identified metabolic shifts associated with aging and age-related conditions, including osteoarthritis (OA), where metabolites such as dimethylglycine and betaine were associated with disease risk. Additionally, large, giant, and mixed breeds, as well as sterilized dogs, exhibited an increased risk of OA, whereas no significant sex-based differences were observed. These findings provide new insights into the metabolic underpinnings of age-related diseases and highlight the influence of body size and sterilization status on disease risk.

Abstract citation ID: igaf122.2886

### THE ASSOCIATION BETWEEN FRAILTY AND INFLAMMATORY BIOMARKERS IN THE ELDERLY

Xiaotian Shi, [1]Qing Ma, [1]Wen Tang, [1]Shan Wang, [1] Huayu Yang, [1]Deqiang Zhang, [1]Yuanyuan Li[1], and Ying Sun[1], *1. Beijing Friendship Hospital, Capital Medical University, Beijing, Beijing, China (People's Republic)*

Objectives: This study aims to characterize associations between frailty and systemic inflammation using blood-derived indices and cytokines, and identify causal mechanisms through proteogenomic integration. Methods: We analyzed neutrophil-to-lymphocyte ratio (NLR), derived NLR (dNLR), monocyte-to-lymphocyte ratio (MLR), and hemoglobin-to-red cell distribution width ratio (HRR) from routine blood tests. Serum interleukin-6 (IL-6), IL-1β, IL-18, and tumor necrosis factor-α (TNF-α) were quantified. Mendelian randomization (MR) integrated plasma proteome-wide genome-wide association study (GWAS) data (N = 54,219) with UK Biobank frailty genetics (n = 175,226) for causal inference. Functional annotation of differentially expressed proteins employed Gene Ontology (GO) and Kyoto Encyclopedia of Genes and Genomes (KEGG) pathway analyses. Results: Adjusted analyses identified NLR (OR = 1.57, 95%CI=1.07–2.30), dNLR (OR = 1.59, 1.11–2.27), and HRR (OR = 0.13, 0.03–0.44) as independent frailty predictors. Elevated IL-6 (OR = 1.14, 1.01–1.30) and TNF-α (OR = 1.21, 1.07–1.36) correlated with frailty. MR revealed 26 plasma proteins causally linked to frailty, including five inflammation-associated proteins. Pathway analysis demonstrated JAK-STAT signaling enrichment, connecting chronic inflammation to frailty pathogenesis. Conclusions: NLR, dNLR and HRR serve as accessible frailty biomarkers, while IL-6 and TNF-α are cytokine drivers of frailty. The MR-validated plasma proteome implicates JAK-STAT axis activation as a key mechanism driving inflammation-associated frailty, providing therapeutic targets for this geriatric syndrome.

Abstract citation ID: igaf122.2887

### THE IMPACT OF LIFE HISTORY EVOLUTION ON THE MICROBIOTA OF DROSOPHILA MELANOGASTER

Nourhan Mahmoud, *California State University, Fullerton, Downey, California, United States*

Manipulation of aging components through laboratory evolution of Drosophila melanogaster offers valuable insights into fast adaptive evolution and has implications for agriculture, conservation, and healthcare. Despite the extensive research on phenotypic and genetic divergence in laboratory populations of D. melanogaster, the role of the microbiome in host adaptation has been largely overlooked. This study examines how life history selection of D. melanogaster impacts its microbiome using the Drosophila Experimental Evolution Population (DEEP) resource. We hypothesize that evolutionary changes in the host phenotype and genotype would correlate with distinct, repeatable patterns in microbiome composition. Four selection regimes, based on age-of-first-reproduction (A-type, 10-day; B-type, 14-day; T-type, 21-day; C-type, 28-day), were compared using metagenomic analysis, colony-forming unit (CFU) plating, and gut dissection. Our results revealed that the microbiomes of each population reflected their evolutionary history, with significant differences in microbial abundance and composition. A-type flies exhibited higher bacterial loads, dominated by lactic and acetic acid bacteria, supporting their faster life history strategy. In contrast, C-type flies, which have a slower life history strategy, exhibited higher Wolbachia abundance, which is known to enhance fecundity and viral resistance. These findings suggest that microbiota composition changes alongside host life history evolution, with certain microbes conferring adaptive benefits that align with the host's life history strategy. This study highlights the importance of genetic factors in shaping microbiota diversity and underscores the microbiome's role in host fitness and aging.

### SESSION 7320 (POSTER)

Abstract citation ID: igaf122.2888

### CARDIOVASCULAR DISEASE ASSOCIATION BETWEEN COMORBIDITY AND CARDIOVASCULAR AND CEREBROVASCULAR EVENTS IN OLDER ADULTS WITH NSTE-ACS

Jingwen Shi, Ying Sun, Wen Tang, Yunli Xing, and Qing Ma, *Beijing Friendship Hospital, Capital Medical University, Beijing, Beijing, China (People's Republic)*

Objective: To investigate the association between comorbidity and the occurrence of Major Adverse Cardiovascular and Cerebrovascular Events (MACCE) in older adults with Non-ST-Segment Elevation Acute Coronary Syndrome (NSTE-ACS) during one-year discharge. Materials and Methods: A total of 528 adults aged ≥ 65 years old were enrolled from January 2020 to November 2021 at Beijing Friendship Hospital, Capital Medical University for NSTE-ACS. The age-adjusted Charlson Comorbidity Index (aCCI) was calculated based on age and comorbidities. Participants were stratified into the low aCCI group (aCCI < 5) and the high aCCI group (aCCI ≥5). Multivariate COX regression analysis was employed to assess the association between aCCI and the occurrence of MACCE. Results: The high aCCI group included 251 patients (47.5%). This population exhibited advanced age, higher myocardial injury biomarker levels, and higher prevalence of frailty, polypharmacy, and reduced activities of daily living. When aCCI was analyzed as a continuous variable, the MACCE risk increased by 21.3% (adjusted HR = 1.213, 95%CI:1.043~1.412, P = 0.012) as aCCI increased by one unit. When aCCI was analyzed as a categorical variable, it is exhibited that the MACCE risk increased by 85.8% (adjusted HR = 1.858, 95% CI: 1.119~3.083, P = 0.017) in the high group compared to the low group. Conclusion: Higher comorbidity is associated with increased risk of MACCE during one-year discharge, suggesting that aCCI may serve as a predictor for adverse cerebrocardiovascular outcomes. Early assessment of comorbidity could conduct risk stratification in older adults with NSTE-ACS to implement health management for older adults.

Abstract citation ID: igaf122.2889

### BATHTUB CURVE PATTERNS OF MORTALITY RISK IN CARDIOMETABOLIC DISEASES: A COHORT STUDY USING THE UK BIOBANK

Yuming Chen[1], Shui-kit Cheuk[2], Hui Liu[2], Yanran Deng[1], and Beibei Xu[2], *1. Peking University Health Science Center, Beijing, Beijing, China (People's Republic), 2. Peking University, Beijing, Beijing, China (People's Republic)*

Observational studies suggest nonlinear mortality patterns in cardiometabolic diseases (CMDs): acute events show early risk spikes, while chronic conditions exhibit gradual risk accumulation. These trends superficially align with the bathtub curve—an engineering model describing triphasic failure rates. However, systematic validation of this framework in chronic disease epidemiology remains limited. This study aims to explore and validate the nonlinear temporal dynamics of mortality risk in CMD patients based on bathtub curve theory. Using data from the UK Biobank (n = 502,130), we analyzed 17 CMDs with linked primary care, inpatient and death records among 73,908 patients. A piecewise exponential survival model segmented mortality risk into intervals to capture time-dependent hazard rates. Chronic (e.g., diabetes) and acute diseases (e.g., myocardial infarction) were analyzed separately. Models were adjusted for age, sex, BMI, education, and smoking/alcohol status. Chronic diseases, such as diabetes, exhibited a bathtub-shaped mortality risk: elevated initially, declining in mid-term, and rising again long-term. Each phase showed increased risk with older age at diagnosis. Acute conditions (e.g., heart failure) demonstrated rapidly declining risks over time. Heterogeneity in high-risk time windows was observed across diseases, with hypertension and diabetes consistently following the bathtub curve. Using the piecewise exponential model, we validate the bathtub curve framework could provide a novel nonlinear perspective for understanding mortality risk evolution in CMDs. This approach supports stratified patient management, intensive early intervention, stable-phase monitoring, and late-phase complication control, enhancing precision in public health strategies. The findings underscore the theory's potential in optimizing lifecycle management of CMDs and guiding personalized interventions.

Abstract citation ID: igaf122.2890

### CARDIOMETAGE: A PRACTICAL CLINICAL AGING CLOCK FOR PREDICTING CARDIOMETABOLIC MORTALITY AND MORBIDITY

Yucan Li, Xinming Xu, Kelin Xu, and Xingdong Chen, *Fudan University, Shanghai, Shanghai, China (People's Republic)*

Background: Existing biological age (BA) models primarily focus on systemic changes, overlooking alterations crucial for cardiometabolic diseases (CMDs). Methods: In this study, we developed the CardioMetAge model, a novel aging clock tailored to predict CMD-related outcomes. Trained in the NHANES-III, the model was applied to the continuous NHANES and UK Biobank. We evaluated it on the prediction of cardiometabolic mortality, morbidity, and disease state transitions. Its associations with proteomic pathways, lifestyle factors, and socioeconomic status, as well as the impact of caloric restriction intervention on its change, were also assessed. Results: The final form of CardioMetAge was concise, incorporating chronological age (CA) and 16 common clinical biomarkers. Its age deviation (CardioMetAgeDev) demonstrated stronger associations with CMD-related mortality, morbidity, and multi-morbidity than age deviations of traditional BA models. It also outperformed CA and PhenoAge in predicting 10-year incidence risks for CMDs. Beyond prediction, our findings highlighted the biological determinants of cardiometabolic aging, with proteomic analyses linking CardioMetAgeDev to inflammatory activation and metabolic disorders. Analysis of modifiable factors revealed that lifestyle and socioeconomic status influenced CMD risks, partly through their effect on CardioMetAgeDev, with mediation proportions of 30.3% and 11.1%, respectively. Additionally, two-year caloric restriction slowed the progression of CardioMetAge by 0.92 years (95% CI: 0.27 to 1.57) compared to the ad libitum group. Conclusion: CardioMetAge outperformed existing BA models in simplicity and in predicting CMD outcomes. It provides valuable insights into the mechanisms of cardiometabolic aging and holds potential for clinical monitoring and evaluating the effectiveness of interventions.

Abstract citation ID: igaf122.2891

### ENHANCING DASH DIET ADHERENCE: A CULTURAL AND RESOURCE-TAILORED APPROACH FOR AFRICAN AMERICAN WOMEN

Angela Groves[1], and Yasir Mehmood[2], *1. Western Michigan University, Portage, Michigan, United States, 2. Michigan State University, East Lansing, Michigan, United States*

Hypertension disproportionately affects African American women, placing them at a heightened risk for cardiovascular disease, kidney disease, and stroke. The Dietary Approaches to Stop Hypertension (DASH) diet lowers blood pressure, yet adherence is challenged by cultural food preferences, limited access to healthy foods, and affordability concerns. This study aimed to develop a culturally tailored DASH diet manual by engaging midlife and older African American women with hypertension to identify their food preferences, dietary practices, and available resources. Using a single-category focus group design, we recruited 11 participants from a church congregation in the Southwest United States. Key findings revealed six major themes and four subthemes influencing DASH diet adherence: (1) perspectives on diet, emphasizing sodium reduction and balanced nutrition; (2) awareness and education needs, including subthemes on (a) food label literacy and cooking skills and (b) tailoring materials for different reading levels; (3) community disparities in food access, with subthemes on (a) affordability challenges and (b) thrifty nutrition strategies; (4) challenges of Southern cooking traditions; (5) home appliance diversity in meal preparation; and (6) tailoring the DASH manual to dietary needs, including vegetarian and lactose-intolerant adaptations. Findings underscore the need for culturally sensitive interventions that integrate practical cooking skills, visual aids, and community-based support to improve adherence. Future research should explore culturally tailored DASH education programs, peer support models, and digital tools to enhance long-term adherence and blood pressure control among midlife and older African American women.

Abstract citation ID: igaf122.2892

### PERCEIVED NEIGHBORHOOD ENVIRONMENTS AND CARDIOVASCULAR RISK AMONG OLDER ADULTS: THE ROLE OF COGNITIVE ACTIVITIES

Jeein Law (Jang), *University of Massachusetts Boston, Boston, Massachusetts, United States*

Cardiovascular disease (CVD) is the leading cause of death in the United States, contributing to significant disability and healthcare costs. The neighborhood environment plays a critical role in cardiovascular health, particularly for older adults aging in place. Prior research has shown that higher physical disorder is associated with greater CVD risk, while greater social cohesion is associated with lower CVD risk. However, the role of cognitive activities as a moderator of these relationships, as well as the influence of subjective perceptions of neighborhood characteristics (PNC) on CVD rather than objective indicators, remains underexplored. Using pooled data from the 2016-2018 Health and Retirement Study (n = 6,248), logistic regression examined the likelihood of CVD based on PNC, adjusting for sociodemographic and health-related factors. CVD was defined as a self-reported diagnosis of heart-related diseases (e.g., heart attack, coronary heart disease, angina). Neighborhood cohesion and disorder were assessed based on perceptions of safety, trust, neighbor support, and signs of neglect or disrepair. Cognitive activities included reading, playing word or card games, and using a computer. Results showed that higher social cohesion was associated with a lower likelihood of CVD, while higher physical disorder was linked to greater CVD risk. Additionally, cognitive activity engagement weakened the positive association between physical disorder and CVD risk. These findings highlight the need for policies promoting cognitive engagement among older adults, particularly in disadvantaged neighborhoods. Expanding community-based programs that provide access to cognitive activities, such as libraries, senior centers, and digital literacy initiatives, could serve as protective health interventions.

Abstract citation ID: igaf122.2893

### RESEARCH PARTICIPATION AND HYPERTENSION: EXAMINING THE EFFECTS OF BLOOD PRESSURE MONITORING IN A VALIDATION STUDY

Mitchell Roberts[1], Edward Hammel[2], Erica Sappington[2], Roma Tremblay[2], Alyssa Persad[2], Samantha Hoffman[2], Cyriah Carey[2], and Carla VandeWeerd[2], *1. University of Florida, Gainesville, Florida, United States, 2. UF Health - Precision Health Research Center, The Villages, Florida, United States*

Unmanaged BP and Cardiovascular disease (CVD) are leading health concerns among adults 55+ resulting in increased costs and mortality. This research explored whether participation in an observational BP study influenced CVD management. Participation included validation measurements for The Heart Seat (THS), a novel BP device, alongside a gold-standard BP cuff. Participants received their gold-standard BP readings, potentially increasing awareness of their BP status.491 older adults (M:71.9, SD:6.8, range:50–89 years, 50% female) participated in the validation study at UFHealth PHRC- The Villages, FL (UFIRB202400472) from 5/27/24–10/17/24. All participants completed two study visits, providing demographic, BP, and medical history data. Those with systolic BP < 100mmHg (hypotensive) or ≥ 150mmHg (hypertensive) readings were invited for a third visit (V3) to enhance validation data on high/low BP cases. Of the 491 participants, 45(9.2%) reported CVD-related medical history change between 1st and 2nd visits. Changes included following up to receive new hypertension and/or arrhythmia diagnosis, and/or start of novel medications or medication adjustments. Additionally, among 41 participants with high/low BP invited for V3, 16(39%) reported changes post-visit 2, with 13(81%) initiating BP medications. Though research participation can influence health behaviors, this is often overlooked in the design and follow-up of non-interventional studies. 10% of study participants self-reported changes in CVD management following study participation, with higher rates amongst those at BP extremes. Findings highlight the need to better understand how study participation can positively impact long-term health decisions and clinical care even when non-interventional. Implications for policy and practice will be discussed.

Abstract citation ID: igaf122.2894

### TARGETING MGLL: TERAZOSIN REGULATES TRIGLYCERIDE METABOLISM TO MITIGATE ENDOTHELIAL CELL SENESCENCE

Jie Huang[1], Jinghua Yan[2], Hao Nie[2], and Cuntai Zhang[3], *1. Tongji Hospital, Tongji Medical College, Huazhong University of Science and Technology, Wuhan, Hebei, China (People's Republic), 2. Tongji Hospital, Tongji Medical College, Huazhong University of Science and Technology, Wuhan, Hubei, China (People's Republic), 3. Huazhong University of Science and Technology, Wuhan, Hubei, China (People's Republic)*

Metabolic disorders often arise in senescent endothelial cells, impairing endothelial function, leading to diminished vasodilation and increased vascular stiffness, contributing to the development of cardiovascular disease (CVD). Despite notable advancements, the molecular mechanisms driving endothelial cell senescence and its contribution to vascular aging remain poorly understood. This knowledge gap hinders the development of effective therapeutic strategies. This study investigates the role of terazosin (TZ) in mitigating vascular endothelial cell senescence, with emphasis on its impact on glycerolipid metabolism. Using both in vivo (aged mice) and in vitro (human umbilical vein endothelial cells, HUVECs) models, the research employs techniques such as senescence-associated β-galactosidase (SA-β-gal) staining, lipidomics analysis, and molecular docking simulations to uncover the underlying mechanisms. The results demonstrated that TZ treatment could significantly improve endothelial-dependent vasodilation, reduce vascular stiffness, and attenuate senescence markers in aged mice. Lipidomics analysis revealed that TZ could modulate glycerolipid metabolism, specifically by reducing palmitic acid accumulation in senescent endothelial cells. Further investigation identified monoglyceride lipase (MGLL), which hydrolyzed monoglycerides into palmitic acid and glycerol, as a key player in endothelial cell senescence. TZ reduced palmitic acid accumulation by targeting MGLL, thereby mitigating endothelial senescence. In conclusion, this study provides novel insights into the anti-senescence effects of TZ, highlighting its potential as a therapeutic agent for age-related vascular diseases. Moreover, the identification of MGLL as a critical regulator of glycerolipid metabolism and endothelial cell senescence offers a promising target for future interventions in vascular aging.

Abstract citation ID: igaf122.2895

### THE IMPACT OF THE INTERACTION BETWEEN SARCOPENIA AND FRAILTY ON MORTALITY IN OLDER ADULT WITH CARDIOVASCULAR DISEASE

Alberto Frisoli, Amanda Diniz, and Giovana Menin, *Universidade Federal de Sao Paulo, São Paulo, Sao Paulo, Brazil*

Introduction: Sarcopenia and physical frailty are very prevalent in the elderly with cardiovascular disease (CVD) and increase the risk of mortality. However, it is still unclear whether there is an interaction between them in relation to mortality or whether they act independently. Objective: To assess the predictive value of sarcopenia and frailty in the mortality of older adults with CVD. Methods: Longitudinal analysis of SARCOS, an epidemiological study on sarcopenia and osteoporosis for mortality in the older adults with CVD. Ethic committee - CAE:24412713.6.0000.5505. Sarcopenia was diagnosed by the SDOC recommendation and frailty by the Hopkins criteria at the beginning of the study. Mortality was assessed by telephone call at 6-12-18 months. Survival analyses were performed by Cox regression ($p < 0.08$). Results: In 496 subjects, the mean age was 77.85 (±7.8) and 56.7% were women (p.ns). The mortality rate was 8.1% ($n = 40$), and among those who died, 67.5% ($n = 27$) had frailty, 27.5% ($n = 11$) pre-frailty ($p < 0.001$) and 65% ($n = 26$) sarcopenia ($p = 0.001$). Heart failure, atrial fibrillation, CRF, age, stroke, hospitalizations, falls, and alcohol consumption were also associated with mortality ($p < 0.05$). The interaction between sarcopenia and frailty was significant ($p < 0.001$). In the adjusted multivariate analysis for the variables and the interaction between sarcopenia and frailty, frailty showed HR: 4.16 (1.12-15.42; $p = 0.033$) and sarcopenia HR: 4.13 (0.95-17.95; $p = 0.058$). In the subtraction model of the non-significant variables frailty remained significant with HR 3.80 (1.11-13.01; $p = 0.033$). Conclusions: In older adults with CVDs, frailty and sarcopenia had similar mortality risk values, regardless of the interaction between them

Abstract citation ID: igaf122.2896

### TRENDS IN COMPLEMENTARY AND ALTERNATIVE MEDICINE USE IN ANGINA

Kevin Kozakowski[1], Najah Khan[2], and Audrey Shawley[1], *1. Scripps Health, San Diego, California, United States, 2. Scripps Green Hospital, San Diego, California, United States*

Complementary and alternative medicine (CAM) is a group of diverse medical and health care systems, practices, and products that are not generally considered part of conventional medicine. There is an increasing interest in CAM use in patients with conditions such as angina. This study strives to evaluate CAM use among adults with a history of angina. A cross-sectional analysis using data from the Integrated Public Use Microdata Series - National Health Interview Survey from 2002 was performed to assess CAM use in adults with a history of angina. This is the last year CAM data was known to be collected. Descriptive statistics and Fisher's exact test for any single CAM modality vs demographic variables were performed. Of 1,331 respondents with a history of angina, 18 reported using at least one CAM modality. The mean age was 59-years-old with other demographic variations. The most used modalities were relaxation and herbal therapy. No other associations were identified between demographic variables and CAM use. Though the sample size was limited, this study highlights the potential trends and increased use of CAM in adults with a history of angina. Healthcare providers should be cognizant of CAM use in patients with angina and discuss the benefits and risks with their patients to ensure safe and well-coordinated care. There is a need for more rigorous research to determine the effects and long-term benefits on cardiovascular morbidity and mortality with CAM usage in patients with a history of angina.

Abstract citation ID: igaf122.2897

### TRENDS IN COMPLEMENTARY AND ALTERNATIVE MEDICINE USE IN MYOCARDIAL INFARCTION

Audrey Shawley[1], Najah Khan[2], and Kevin Kozakowski[1], *1. Scripps Health, San Diego, California, United States, 2. Scripps Green Hospital, San Diego, California, United States*

Complementary and alternative medicine (CAM) is a group of diverse medical and health care systems, practices, and products that are not generally considered part of conventional medicine. There is increasing interest in CAM use among patients with conditions such as myocardial infarction (MI). This study aims to evaluate CAM use among adults with a history of MI. A cross-sectional analysis using data from the Integrated Public Use Microdata Series - National Health Interview Survey from 2002 was performed to assess CAM use in adults with a history of MI. This was the last year CAM data was known to have been collected. Descriptive statistics and Fisher's exact test were used to analyze the association between any single CAM modality and demographic variables. Of 1,845 respondents with a history of MI, 14 reported using at least one CAM modality. The mean age was 67 years, with other demographic variations. The most commonly used modalities were relaxation and diet therapy. No other associations were identified between demographic variables and CAM use. Though the sample size was limited, this study highlights potential trends and the increased use of CAM in adults with a history of MI. Healthcare providers should be aware of CAM use in patients with MI and discuss its benefits and risks with their patients to ensure safe and well-coordinated care. More rigorous research is needed to determine the effects and long-term benefits of CAM use on cardiovascular morbidity and mortality in patients with a history of MI.

### SESSION 7325 (POSTER)

### COGNITION III

Abstract citation ID: igaf122.2898

### BIDIRECTIONAL ASSOCIATIONS BETWEEN LONGITUDINAL CHANGE IN COGNITION AND INSTRUMENTAL ACTIVITIES OF DAILY LIVING

Sydney Schaefer[1], Marie Ernsth-Bravell[2], and Deborah Finkel[3], *1. Arizona State University, Tempe, Arizona, United States, 2. Jönköping University, Jönköping, Jonkopings Lan, Sweden, 3. University of Southern California, Los Angeles, California, United States*

Changes in daily function and cognition are two of the primary indicators of the aging process. However, how these two domains decline with respect to each other remains unclear. Recent work has suggested accelerated declines in fine motor function in instrumental activities of daily living (IADLs) precede the onset of dementia in older adults. However, whether early changes in IADLs contribute to subsequent changes in cognition remains unclear. Random intercept cross lag panel models (RI-CLPM) allow us to examine the temporal dynamics of the relationships between two variables that change over time and test hypotheses about direction of influence. This study applied age-based RI-CLPM to longitudinal changes in performance on a timed fine motor IADL test (which included five subtests, e.g., putting coins into a slot) and changes in the Mini-Mental Status Exam (MMSE). Up to 20 years of follow-up were available for 1,462 participants (aged 62-88 years at baseline) from three longitudinal Swedish datasets of older adults (OCTO-Twin, GENDER, SATSA). Model comparisons indicated a unidirectional relationship when participants were in their 60s and 70s: IADL time contributed to subsequent MMSE scores, but MMSE scores did not contribute to subsequent IADL time. For participants in their 80s, the relationship was bi-directional: both variables contributed to subsequent performance on the other variable. These data not only give insights into the temporal dynamics of decline in IADLs and cognition, but also provide proof-of-concept that tests involving motor performance, particularly when evaluated in ecologically-valid contexts, can be used for monitoring progression towards dementia.

Abstract citation ID: igaf122.2899

### CHARACTERIZING OCCUPATIONAL COMPLEXITY: INSIGHTS FROM THE CANADIAN LONGITUDINAL STUDY ON AGING

Catherine Gosselin[1], Annick Parent-Lamarche[2], and Benjamin Boller[3], *1. Université du Québec à Trois-Rivières, Repentigny, Quebec, Canada, 2. Université du Québec à Trois-Rivières, Trois-Rivieres, Quebec, Canada, 3. Université du Québec à Trois-Rivières, Trois-Rivières, Quebec, Canada*

Using data from the Canadian Longitudinal Study on Aging (CLSA), we found that previous research suggests cognitive trajectories following retirement exhibit heterogeneity (Gosselin & Boller, 2022). One potential explanatory factor is the complexity of one's profession, which has been linked to cognitive reserve theory. However, the specific structure of occupational complexity within this cohort remains to be fully characterized. This study aims to identify and define the key dimensions of occupational complexity among participants from the CLSA using a data-driven approach. The sample consisted of 8,243 workers (M = 54.18, SD = 6.09). Occupational information was classified using the Dictionary of Occupational Titles (DOT) to derive complexity scores based on levels of interaction with data, people, and materials. A principal component analysis (PCA) was conducted to extract underlying dimensions of occupational complexity. PCA revealed two distinct dimensions of occupational complexity, explaining 82.5% of the variance: the first, "Coordination and Support" (51.4%), encompasses professions characterized by high levels of social interaction, mentoring, and personnel management (e.g., psychologists). The second, "Autonomous Management" (31.1%), comprises professions requiring elevated autonomy in decision-making and the performance of specialized, often technical, tasks (e.g., engineers). These dimensions highlight distinct patterns of professional task demands within the CLSA cohort. By identifying key occupational complexity profiles, this study enhances our understanding of the professional backgrounds within the CLSA. These findings provide a foundation for future research examining the potential long-term cognitive and health-related implications of occupational characteristics.

Abstract citation ID: igaf122.2900

### CO-EVOLUTION OF COGNITIVE AND HEALTH TRAJECTORIES AMONG U.S. OLDER ADULTS WITH COGNITIVE IMPAIRMENT

Yifan Lou[1], Emma Zang[2], and Yanjun Wu[3], *1. Virginia Commonwealth University, Richmond, Virginia, United States, 2. Yale University, New Haven, Connecticut, United States, 3. Cleveland Clinic, Cleveland, Ohio, United States*

Few studies have explored how multiple health aspects co-evolve over time among persons with cognitive impairment. This study examines the interplays of health status and cognitive trajectories in persons with cognitive impairment. We examine how health status trajectories predict cognitive trajectories, focusing on conditional probabilities to highlight how broader health dynamics shape cognition through shared biological and behavioral pathways. We analyzed data from 2,569 persons with cognitive impairment (PCIs) in the 2011–2021 National Health and Aging Trends Survey. Bayesian dual trajectory models identified distinct health status and cognitive trajectories. We examined their sociodemographic and health correlates. Conditional probabilities of cognitive group membership based on health status group membership were also estimated. We identified five distinct trajectory groups each for health status and cognition, ranging from high-stable to low-declining patterns. The most common health trajectory is "Medium start, moderate decline" (28.8%), followed by "High start, stable". For cognition, the largest group of people fall into the "Medium start, slight decline" (29.7%) category, with "Medium-high start, stable" next (27.6%). Respondents with the most favorable health status trajectories were more likely to have the best cognitive outcomes, while those with sharp declines in health status experienced worse cognitive outcomes, despite starting at higher baseline health levels. To conclude, we find that stable health trajectories are strongly linked to better cognitive outcomes, while sharp health declines predict poorer cognition. These findings emphasize the need for integrated interventions to support physical and cognitive health in older adults.

Abstract citation ID: igaf122.2901

### COGNITIVE INTERVENTIONS FOR CANCER-RELATED COGNITIVE IMPAIRMENT: A META-ANALYSIS OF RCTS

Ah Rim Lee[1], Moonkyoung Park[1], Moira Visovatti[2], Kyeongin Cha[1], and Misook Jung[1], *1. Chungnam National University, Daejeon, Republic of Korea, 2. Eastern Michigan University School of Nursing, Ypsilanti, Michigan, United States*

Cancer-related cognitive impairment (CRCI) is a common challenge among cancer survivors of both older and younger ages, adversely affecting attention, processing speed, memory, and executive function. These deficits often compromise daily activities and occupational performance. Given the limited efficacy of pharmacological treatments, non-pharmacological cognitive interventions have emerged as promising alternatives, although their effectiveness remains variable. This meta-analysis consolidates current evidence on the efficacy of cognitive interventions for enhancing cognitive function in individuals with CRCI. A systematic search of six databases was conducted to identify randomized controlled trials that evaluated cognitive training interventions for CRCI. Studies were included if they assessed cognitive outcomes using standardized neuropsychological tests and/or self-reported measures. The risk of bias was assessed using Cochrane's RoB 2.0, and effect sizes were calculated using Hedges' g with a random-effects model in CMA 4.0. A total of 28 RCTs ($n=1{,}998$) were included, with outcomes from both neuropsychological tests and self-reported measures. The interventions yielded significant improvements in several cognitive domains, including attention ($k=24$, $g=0.24$), processing speed ($k=19$, $g=0.26$), verbal memory ($k=22$, $g=0.31$), visuospatial memory ($k=9$, $g=0.33$), and executive function ($k=23$, $g=0.20$). Additionally, subjective cognitive function showed notable improvement ($k=25$, $g=0.40$). Subgroup analyses revealed that cognitive training ($k=19$, $g=0.31$) and meditation ($k=7$, $g=0.32$) produced significant effects, while exercise-based intervention did not yield significant benefits. This meta-analysis provides robust evidence supporting beneficial effects of cognitive interventions for managing CRCI. Further research is needed to identify the optimal training modalities and to assess long-term benefits, which guide clinical practices.

Abstract citation ID: igaf122.2902

### DIFFERENCES IN ESTIMATED VS MEASURED GFR ASSOCIATIONS WITH COGNITIVE FUNCTION IN AFRICAN AMERICAN OLDER ADULTS

Harshin Sanjanwala[1], Xiaoqian Zhu[2], B. Gwen Windham[2], Srishti Shrestha[2], Michael Griswold[2], Andrew Rule[3], Thomas Mosley[2], and Kevin Sullivan[2], *1. University of Mississippi Medical Center, Jackson, Mississippi, United States, 2. University of Mississippi Medical Center - The MIND Center, Jackson, Mississippi, United States, 3. Mayo Clinic, Rochester, Minnesota, United States*

Impaired kidney function is a risk factor for cognitive decline and dementia, but most studies use serum creatinine-based equations to estimate kidney function as estimated glomerular filtration rate (eGFR); these equations include age, also a strong driver of cognitive impairment, and produce eGFR estimates with substantial error compared to gold-standard objective measures of GFR (mGFR). The purpose of this study was to determine eGFR and mGFR associations with cognitive function in African American adults, a population disproportionately impacted by both chronic kidney disease and dementia. We conducted a cross-temporal analysis of 368 participants (Age: 66.4 ± 7.4y, 75% Female) in the Genetic Epidemiology Network of Arteriopathy study with eGFR (CKD-EPI 2021 Creatinine Race-Free Equation) and mGFR assessed. eGFR was assessed using the CKD-EPI 2021 Creatinine Race-Free Equation and mGFR (urinary clearance of non-radiolabeled iothalamate). Memory (Rey Auditory Verbal Learning Test; RAVLT) and executive function (Digit Symbol Substitution Test; DSST) were assessed. Linear regression models were used to relate eGFR, mGFR, and serum creatinine to cognitive function with and without adjustment for age and sex. In unadjusted models, eGFR was positively associated with DSST scores ($\beta=0.19$, 95%CI:0.11, 0.27) and RAVLT scores ($\beta=0.10$, 95%CI:0.04, 0.16). mGFR was marginally associated with DSST scores ($\beta=0.07$, 95%CI:-0.00, 0.14), but not RAVLT scores ($\beta=0.03$, 95%CI:-0.01, 0.07). Associations of serum creatinine with cognitive function were mostly null to marginal. Age and sex adjustment greatly attenuated all associations. The observed differential renal function associations with cognition suggest age may largely explain prior reported associations.

Abstract citation ID: igaf122.2903

### EDUCATION DID NOT EXPLAIN GENDER DIFFERENCES IN EPISODIC MEMORY IN OLDER ADULTS

Judith Rijnhart, and Ryan Bailey, *University of South Florida, Tampa, Florida, United States*

Previous studies have shown that, on average, women and individuals with more education perform better on verbal episodic memory tests. Historically, women completed fewer education years compared to men and faced structural barriers that prevented them from capitalizing on their education. Therefore, education may suppress gender differences in episodic memory. We aimed to study education as a potential suppressor of gender differences in episodic memory. We used data from 19,623 community-dwelling adults aged 51+ from the United States of America who participated in the 2016 wave of the Health and Retirement Study. Episodic memory was measured using immediate and delayed word recall tests (range 0-20). Education was measured as the number of education years completed (range 0-17). We used the four-way mediation decomposition method to determine whether gender differences in episodic memory were suppressed by fewer education years in women, lower impact of education on episodic memory among women, or both. All models were controlled for confounding by ethnicity, race, parental education, and birth year. Missing data were addressed with multiple imputation and all models were weighted using survey sampling weights. On average, women had a 0.87 higher episodic memory score compared to men (95% confidence interval=0.75;0.99). Estimates of mediated effects and interaction effects from the four-way mediation decomposition were not statistically significant, indicating that education did not suppress gender differences in episodic memory. Future studies are needed that focus on the biological, psychological, and social factors that explain why women on average perform better on episodic memory tests.

Abstract citation ID: igaf122.2904

### ENDURANCE AND GAIT SPEED ASSOCIATIONS WITH INCIDENT DEMENTIA IN OLDER ADULTS: THE ARIC STUDY

Jamesia Kenney[1], Hunter Sylvester[1], Michael Griswold[2], Rebecca Gottesman[3], David Knopman[4], Kelley Pettee Gabriel[5], Kevin Sullivan[2], and B. Gwen Windham[2], *1. University of Mississippi Medical Center, Jackson, Mississippi, United States, 2. University of Mississippi Medical Center - The MIND Center, Jackson, Mississippi, United States, 3. National Institutes of Health, Bethesda, Maryland, United States, 4. Mayo Clinic Department of Neurology, Rochester, Minnesota, United States, 5. The University of Alabama at Birmingham, Birmingham, Alabama, United States*

Faster gait speed and better endurance have been cross-sectionally and jointly associated with a lower likelihood of dementia. However, gait speed associations plateau above 1m/s, suggesting gait speed may be less predictive at near normal speeds. This study examined prospective associations of gait speed, endurance, and moderating effects of endurance on gait speed associations with incident dementia among ARIC participants without dementia at Visit 6 (2016-2017) who completed assessments of endurance ( 2- Minute Walk (2MW), meters) and usual-pace 4-meter gait speed (m/s) (n = 2767, mean age 78+/- 4.3 years, 43% male, 18% Black). Dementia was adjudicated using neuropsychological tests, functional assessments, informant interviews, and medical/vital record surveillance over a median of 3.3 years follow-up. Cox regression models estimated the hazard ratio (HR, 95% CI) for dementia, incorporating gait speed, 2MW, gait speed-by-2MW interaction, demographic, and cardiovascular factors. In separate models, each standard deviation higher 2MW [HR = 0.62, (0.50,0.76)] and faster gait speed [HR = 0.63, (0.50,0.81)] were associated with lower incident dementia risk. However, when jointly adjusting for 2MW and gait speed, higher 2MW was associated with a 32% lower incident dementia risk [HR = 0.68,(0.51, 0.90)], but faster gait speed was not statistically associated with incident dementia [HR = 0.85, (0.62,1.16)]. Moderating effects were not supported (gait speed-x-2MW interaction, p = 0.14). Although both endurance and gait speed predict dementia, endurance may be more informative across the gait speed spectrum, whereas gait speed may be more feasible for clinical settings. Longer follow-up and more dementia events may better inform differential utilities of gait speed and endurance measures.

Abstract citation ID: igaf122.2905

### INFLAMMATION AND COGNITION: THE MODERATING ROLE OF NEIGHBORHOOD DISADVANTAGE

Hye Won Chai[1], Abigail Stephan[1], Vanessa Martinez[1], Alexa Allan[2], Christopher Engeland[2], Alyssa Gamaldo[1], and Lesley Ross[1], *1. Clemson University, Clemson, South Carolina, United States, 2. The Pennsylvania State University, University Park, Pennsylvania, United States*

Extant literature suggests neighborhood disadvantage and inflammation as significant correlates of cognitive function among older adults. However, the joint effects of the contextual and biological factors on cognition have received less attention. The biopsychosocial model of dementia posits that understanding the interplay between biological and socio-contextual determinants of cognition is integral for more effective identification and prevention of Alzheimer's disease and related dementias (ADRD). Therefore, this study examined whether and how the associations between inflammation and cognition vary by neighborhood disadvantage. Participants were drawn from a subsample of the Elucidating the Necessary Active Components of Training (ENACT, NCT05366023) study that assessed a wide range of biological, psychological, and social factors related to cognitive health from older adults aged 55 to 80 years (N = 51; Mean age=64.3). Inflammation was measured using C-reactive protein (CRP) and cognition was measured using Test My Brain (TMB) tasks. Neighborhood disadvantage was measured using the Area Deprivation Index (ADI). Results showed significant interactions between CRP and ADI for TMB tasks measuring processing speed (Digit Symbol Coding; accuracy) and working memory (Trail Making B; time to completion in milliseconds). Specifically, higher inflammation was associated with worse processing speed (B = -0.01; $p < .05$) and working memory (B = 8323.66; $p < .1$) only among those from more disadvantaged neighborhoods. Findings suggest older adults from socioeconomic disadvantaged neighborhoods may be more vulnerable to the cognitive health costs of inflammation, highlighting the need for tailored prevention and intervention efforts that specifically target older adults living in these neighborhoods.

Abstract citation ID: igaf122.2906

### MEDIATING EFFECT OF SOCIAL ENGAGEMENT BETWEEN NEIGHBORHOOD ENVIRONMENT AND COGNITIVE DYSFUNCTION

Min Kyung Park[1], Bada Kang[1], Chang Won Won[2], Dahye Hong[1], Seok-Jae Heo[1], Chang Gi Park[3], Miji Kim[4], and Eleanor McConnell[5], *1. Yonsei University College of Nursing, Seoul, Republic of Korea, 2. Kyung Hee University, Seoul, Republic of Korea, 3. University of Illinois Chicago, Chicago, Illinois, United States, 4. Kyung Hee University College of Medicine, Seoul, Republic of Korea, 5. Duke University, Durham, North Carolina, United States*

Cognitive dysfunction among older adults is a significant health and social issue worldwide; however, previous studies have primarily focused on individual-level risk factors. Therefore, this study examined the longitudinal relationship between older adults' neighborhood environment and cognitive dysfunction and investigated the mediating role of social engagement in this relationship. Using data from the Korean Frailty and Aging Cohort Study (2016–2023), we analyzed 1,262 community-dwelling older adults aged 70–84 years. Cognitive dysfunction was measured based on the number of cognitive tests results in a seven-test battery that were below the cutoff point. The neighborhood environment was assessed using the Perceived Neighborhood Environment Scale, used to evaluate physical and social aspects of a residential area. Social engagement was measured based on participation in eight types of social gatherings or group activities. To examine the longitudinal relationship and mediation effect, we used linear mixed-effects models and Sobel's test. At baseline, the participants had an average age of 76.37 years, 51% were women, and 955 individuals (75.7%) exhibited cognitive dysfunction on at least one cognitive test. Access to destinations had a statistically significant negative relationship with the extent of cognitive dysfunction ($\beta$=-0.056, p=<.001), with social engagement partially mediating this relationship (mediation effect: $\beta$=-0.002, p<.001). Moreover, aesthetic quality also had a negative relationship with the extent of cognitive dysfunction; however, social engagement did not significantly mediate this relationship. These findings highlight the importance of community interventions aiming to improve neighborhood accessibility and encourage social engagement for supporting cognitive well-being in older adults.

Abstract citation ID: igaf122.2907

### PATTERNS OF PHYSICAL ACTIVITY ACROSS THE RETIREMENT TRANSITION IN OLDER ADULTS

Hyesu Yeo, and Michelle Gray, *University of Arkansas, Fayetteville, Arkansas, United States*

Purpose Many studies highlight the health benefits of retirement and its positive relationship with physical activity (PA) and cognition. To better understand this interaction, this study examined PA patterns and their associations with physical health, mental health, and cognition across the retirement transition. Methods Using data from the 2006–2018 RAND Health and Retirement Survey (HRS) and the 2007–2017 HRS Consumption and Activities Mail Survey, this study analyzed 91 retirees aged 60 to 80 (226 non-retirees as a reference group). Graphical analyses compared retirees and non-retirees across eight PA types adapted from the International Classification of Functioning. Bivariate and correlation analyses examined associations between PA, health, cognition, and demographics across pre-retirement and post-retirement. Results After retirement, PA in personal care, caring for housework, social activities, and arts & crafts significantly increased. Most PA declined pre-retirement but showed a rebound at retirement, except for solitary activities, which continued to decline. In post-retirement, PA levels decreased again. PA patterns among non-retirees remained stable. Education($\rho$=0.36), women($\rho \approx$ 2.64-2.89), White race(U $\approx$ 2.43-2.28), urban living (U = -2.44), mental health($\rho$=0.28), and cognition($\rho \approx$ 0.24-0.39) were positively related to specific PA types at retirement, which, in turn, positively influenced mental health($\rho \approx$ 0.27-0.32) and cognition($\rho \approx$ 0.21-0.34) post-retirement. Conclusion This study found a PA decline before retirement, an increase at retirement, and a later decrease, mirroring changes in mental health and cognition. Interventions should aim to prevent PA decline pre-retirement and support sustained PA during the transition into retirement while considering individual differences.

Abstract citation ID: igaf122.2908

### PERCEIVED CONTROL AND COGNITIVE AGING: THE MEDIATING ROLE OF MEDITERRANEAN DIET ADHERENCE

Kelly Parker, and Jeremy Hamm, *North Dakota State University, Fargo, North Dakota, United States*

Consistent evidence has shown that perceived control (PC) buffers against age-related cognitive declines over periods of up to 20 years. However, little is known about how core health behaviors (e.g., diet) and demographic factors (e.g., socioeconomic status (SES)) may mediate and moderate this relationship. Using national data from the Midlife in the United States (MIDUS) study (n=831, age range = 34-83), this study examined the role of a Western Mediterranean Diet (wMD) as a mediator between, and SES as a moderator of, PC and cognitive functioning. Participants provided complete data for age, sex, education, income, race, diet, and health locus of control (PC) at Wave 2, as well as 9-year longitudinal data on episodic memory (EM) at Waves 2 and 3. Hayes' PROCESS Macro was utilized to identify how the relationship between PC and longitudinal EM may be mediated by wMD and moderated by central indicators of SES (income, education). Step 1 mediation models showed that wMD mediated the association between PC and 9-year episodic memory (b = .0084, CIs = .0002-.0211). Step 2 moderated mediation models showed that this wMD mediated pathway was pronounced for only those with less education (b = .0216, CIs = .0045-.0469). Results advance the literature by providing new evidence for (a) the mechanistic health behavior processes (wMD) that account for the relationship between PC and episodic memory and (b) the sociodemographic populations for whom this mediated pathway is most pronounced (those with lower SES).

Abstract citation ID: igaf122.2909

### SELF-REPORTED HEALTH AND FLUID INTELLIGENCE IN OLDER ADULTS

Liu Chen[1], David Salat[2], Steven Arnold[2], and Hiroko Dodge[1], *1. Massachusetts General Hospital, Harvard Medical School, Boston, Massachusetts, United States, 2. Massachusetts General Hospital, Boston, Massachusetts, United States*

Introduction: Stress, arising from physical, social, or emotional factors, can induce lasting neurobiological alterations that may contribute to cognitive impairment, particularly in domains such as fluid intelligence and memory. We hypothesized that poorer self-reported health scores, indicative of higher stress burden, would be associated with lower cognitive performance in older adults. Method: Participants' well-being in physical, mental, and social health domains were quantified using PROMIS® Global Health measures, where higher scores indicate better health within the corresponding domain. General linear regression was then used to examine the association between health domains and cognitive factors (Memory, Crystallized Intelligence [CrystIQ], and Fluid Intelligence [FluidIQ], with higher scores indicating better cognition), while controlling for demographic variables (i.e., age, gender, race, and education). The analysis was conducted on participants aged 60 and older from the Aging Adult Brain Connectome-Human Connnectome Project - Aging (AABC-HCA) baseline visit data. Results: Health scores showed significant positive associations with FluidIQ (Physical health: coefficient=0.026 [95% CI: 0.002-0.047], P=0.030; Mental health: coefficient=0.018 [95% CI: 0.003-0.032], P=0.017; and Social health: 0.226 [95% CI: 0.087-0.365], P=0.001), with the strongest associations shown in social health. No significant associations were observed for health scores and the other two cognitive factors; CrystIQ and Memory. Conclusion: Our findings demonstrate a significant link between self-reported health and FluidIQ in older adults. These findings encourage further investigation into the mechanisms linking psychosocial stress to cognitive trajectories in aging.

Abstract citation ID: igaf122.2910

### SPOUSAL CONCORDANCE IN DEPRESSIVE SYMPTOMS AND THE FOLLOWING COGNITIVE IMPAIRMENT AND MORTALITY

Xuechun Wang, *Peking University, Beijing, Beijing, China (People's Republic)*

Objective: Prior studies have focused on individual depression's health impact, with less attention to spousal depressive symptoms and their health effects. This study examines the association between spousal depressive symptom concordance, mild cognitive impairment (MCI) and mortality risk among middle-aged and older couples. Method: We analyzed five waves of China Health and Retirement Longitudinal Study (2011 to 2020) data. A group-based multi-trajectory model identified trajectories of spousal depressive symptoms. Cox proportional hazards models examined associations between depressive concordance, cognitive decline, and mortality risk. A multi-state survival model estimated transition risks from normal cognition to MCI and to mortality. Linear mixed-effects models evaluated depressive concordance and cognitive functions (mental intactness and episodic memory). Results: Among 11912 respondents, four depressive symptom trajectories were identified: Stable Low-Low (50.29%), Stable High-Mild (21.25%), Stable Mild-High (20.92%) and Stable High-High (7.54%). Compared with baseline neither-depressive-symptoms group, couples in the both-depressive-symptoms group exhibited the highest MCI (HR = 1.33,95% CI: [1.22, 1.45]) and mortality (HR = 1.36, 95% CI: [1.15, 1.62]) risk, with greater transition risks to MCI ($\beta = 0.31$, 95% CI: [0.09, 0.53]) and to mortality ($\beta = 0.19$, 95% CI: [0.04, 1.35]). They also exhibited poorer episodic memory ($\beta$ = -0.30, 95% CI: [-0.37, -0.24]) and mental intactness ($\beta$ = -0.60, 95% CI: [-0.69, -0.51]), with faster decline in episodic memory ($\beta$=-0.01, 95% CI: [-0.03, -0.0002]). Conclusion: Spousal depressive symptom concordance remained stable over time. Couples with high-symptom patterns had increased risks of MCI and mortality, highlighting the need for couple-centered mental health interventions.

Abstract citation ID: igaf122.2911

### STIMULATING NEUROPLASTICITY THROUGH LANGUAGE LEARNING: INNOVATIVE PATHWAYS TO HEALTHY AGING IN OLDER ADULTS

Ladan Ghazi Saidi[1], Yunju Im[2], Yijun Lu[2], Cary Savage[3], and Douglas Schultz[3], *1. University of Nebraska Kearney, Kearney, Nebraska, United States, 2. University of Nebraska Medical Center, Omaha, Nebraska, United States, 3. University of Nebraska-Lincoln, Lincoln, Nebraska, United States*

Healthy aging relies on innovative interventions to sustain cognitive abilities. Language learning is a novel approach to stimulate cognitive function and neuroplasticity. We examine structural brain changes, focusing on cortical thickness variations, in older adults participating in new language acquisition. Healthy monolingual participants (n = 41) aged 60 to 80 residing in a monolingual environment engaged in an online language-learning program. The intervention involved 90-minute daily sessions, five days per week, over four months. Brain cortical thickness was measured before and after the intervention and compared. Behavioral results suggest that all participants successfully learn their language of choice with an average performance score of 96% in the post intervention proficiency test. Neuroimaging analyses reveal a weak trend ($p < 0.01$, uncorrected) indicating structural brain alterations associated with language learning. Changes were observed in the left middle frontal sulcus, the right superior frontal sulcus, and the right transverse temporal sulcus—regions implicated in working memory, attention, cognitive control, executive functions, and language processing. Although these findings did not survive multiple comparisons correction (FWE correction), they suggest structural changes in brain areas crucial for cognitive performance and healthy aging, particularly in executive functions. These results indicate that four months of language learning can induce structural neuroplasticity in older adults, with particularly notable changes in the right hemisphere. While these findings support the potential of language acquisition as an intervention for enhancing brain plasticity in aging, greater effects may be achieved with a higher dosage or longer duration of training, warranting further investigation.

Abstract citation ID: igaf122.2912

### THE FACIAL FEEDBACK EFFECT: CONSISTENT ACROSS AGES, DIVERGENT ACROSS EMOTIONS

Nikolina Kravljaca, and Jennifer Stanley, *The University of Akron, Akron, Ohio, United States*

Emotions are more than just a psychological experience; they are expressed through the body in ways that influence how emotions are felt. This bidirectional relationship between mind and body reflects embodied cognition, which suggests that physical expressions are not merely passive reflections of emotion but active contributors (Costello & Bloesch, 2017). One example is the facial feedback effect, where forming a facial expression, such as a smile, can elicit changes in emotional state (James, 1890). Few studies have examined whether the facial feedback effect operates similarly in older adulthood. This study investigated potential age differences in facial feedback effects by comparing young (n = 69) and older adults' (n = 65) emotional ratings while holding posed expressions. Participants posed facial expressions (happy, angry, or neutral) while completing an emotion-rating questionnaire assessing feelings of happiness and anger. Contrary to our hypotheses, both young and older adults reported increased anger when scowling, but no significant changes in happiness while smiling. Young adults' (but not older adults') baseline anger scores positively correlated with their anger ratings after posing an angry facial expression. Coupled with the relatively low ratings of anger overall, future work should investigate whether individual differences in trait-level emotions moderate facial feedback effects. These findings suggest facial feedback effects are not exclusive to young adults; however, additional emotional context may be necessary to reliably influence the emotional experience. These results contribute to a growing understanding of how physical expression and emotional experience interact across the lifespan.

Abstract citation ID: igaf122.2913

### ULTRA-PROCESSED FOOD INTAKE AND COGNITIVE IMPAIRMENT IN NATIONALLY REPRESENTATIVE OLDER U.S. ADULTS

Heejin Lee[1], Elizabeth Ludwig-Borycz[2], and Cindy Leung[3], *1. Harvard School of Public Health, Boston, Massachusetts, United States, 2. University of Michigan, Ann Arbor, Michigan,*

*United States, 3. Harvard T.H. Chan School of Public Health, Boston, Massachusetts, United States*

Ultra-processed food (UFP) consumption accounts for more than 50% of energy intake in the US, steadily increasing among older adults over the decade. UPF consumption is associated with cognitive decline, but fewer studies have examined the association between UPF consumption and individual cognitive domains. In this study, we examined associations between UPF consumption and impairment in memory, executive function, language, visuospatial, and orientation in the nationally representative Health and Retirement Study (HRS) of adults aged ≥50. Participants without dementia or memory problems at baseline (2012 HRS) were included (n = 1,408). Dietary intake was assessed using the food frequency questionnaire from 2013 Health Care and Nutrition Study (HCNS). UPF was classified using the NOVA categorization. The percentage of energy intake from UPF was grouped into sex-specific quintiles (Q). Cognitive impairment was assessed from the 2016 Harmonized Cognitive Assessment Protocol (HCAP) based on cognitive domain scores, defined as > 1.5 SDs below the mean or a T-score=35. Weighted multivariate-logistic regression was used to estimate the associations between UPF consumption and cognitive outcomes. In the analytic sample, median UPF intake was 42.3% energy/day. After adjustment for baseline sociodemographic and health characteristics, higher UPF consumption was associated with greater impairment in executive function (Q4 vs Q1, OR 2.12, 95% CI 1.17-3.83; Q5 vs Q1, OR 1.77 95% CI 0.98-3.17; P-trend=0.04). UPF consumption was not associated with impairment in other cognitive domains in multivariate models. Our findings highlight the need for support in reducing UPF intake for better cognitive health among older adults.

### SESSION 7330 (POSTER)

### DEMENTIA II

Abstract citation ID: igaf122.2914

### A MULTI-SITE PROSPECTIVE STUDY OF PARADOXICAL LUCIDITY IN MODERATE TO SEVERE DEMENTIA

Maria Tollock, Natalia Leontovich, Anelly Gonzalez, and Sam Parnia, *New York University Langone Health, New York, New York, United States*

Background: Paradoxical episodes of lucidity (paradoxical lucidity [PL]/terminal lucidity) especially near the end of life have been reported in patients with severe dementia. Although little is known about PL, its occurrence challenges current assumptions about dementia and highlights the possibility of a network-level return of cognitive function despite severe dementia. Methods: We conducted the first prospective investigation of PL among severe dementia patients. Among 1,768 patients at NYU Langone, VNS Health, and Bellevue Hospital, 1,405 (79.5%) met inclusion criteria [moderate/severe dementia, life expectancy < 12months]. Of these, 151 (10.7%) were enrolled, 823 (58.6%) declined, 239 (17.0%) were already deceased, and 192 (13.7%) didn't respond to attempted contacts. Primary caregivers completed a log to report instances of perceived mental clarity. Results: Of 151, 93 (61.6%) reported lucidity, with 267 distinct events that were consistent with reality: 1) appropriate orientation to events (67.8%), 2) return of old memories (34.8%), 3) return of functional abilities (27.7%), 4) nonverbal communication (25.1%), 5) terminal lucidity (memories consistent with near-death experiences) (4.1%). A further 9.7% reported hallucinations/delusions, which were not consistent with reality. Potential triggers to PL episodes were identified: music, anniversaries, emotional distress, and medication changes, including immunomodulating agents and antihistamines. Conclusion: Multiple types of sudden paradoxical lucid events occur in end-stage dementia. These can be triggered by multiple factors including medications. PL is common and may provide opportunities for future therapeutic interventions. Research is needed to identify the triggers and underlying mechanisms driving PL.

Abstract citation ID: igaf122.2915

### ADVANCE DIRECTIVES & UNLIMITED TREATMENT PREFERENCE IN DEMENTIA SCENARIO: INSIGHTS OF COMMUNITY-DWELLING ADULTS

Yuchi Young[1], Yichun Liu[2], Yufang Tu[3], Wan-Yu Chiu[4], Ashley Shayya[5], and Thomas O'Grady[6], *1. University at Albany College of Integrated Health Sciences, Albany, New York, United States, 2. New York-Presbyterian Hospital, New York, New York, United States, 3. North Dakota State University, Fargo, North Dakota, United States, 4. National Taiwan University, Zhongzheng District, Taipei, Taiwan (Republic of China), 5. Center for Human Services Research, University at Albany College of Integrated Health Sciences, Albany, New York, United States, 6. University at Albany, Albany, New York, United States*

Dementia leads to progressive cognitive decline, impairing self-care and decision-making. Advance directives (AdvDirs) enable individuals to document healthcare preferences while cognitively capable, ensuring value-aligned care and reducing caregiver burden. This study explores factors influencing preferences for unlimited medical treatment in hypothetical Alzheimer's disease/dementia scenarios among community-dwelling adults. This cross-sectional study surveyed 163 community-dwelling adults (18+), using structured questionnaires to collect sociodemographic, health, and AdvDirs-related data. Key predictors included attitudes toward life-sustaining treatments, comfort discussing death, religious practices, and interest in quality-of-life information related to end-of-life care. The primary outcome was preference for unlimited medical treatment in hypothetical dementia scenarios. Bivariate and multivariate logistic analyses assessed associations, adjusting for covariates. In the dementia scenario, 26.9% of participants preferred unlimited medical treatment. This preference was strongly associated with a pre-existing attitude favoring life-sustaining treatments (OR = 4.24, 95% CI: 1.73 – 10.37, p = 0.002) and religious beliefs (OR = 5.68, 95% CI: 1.51–21.43, p = 0.01). Conversely, an interest in learning about quality of life at the end of life was negatively associated with preferring unlimited treatment (OR = 0.29, 95% CI: 0.09-0.89, p = 0.03). Our findings highlight the need to align advance care planning with individuals' values, beliefs, and religious practices. Raising awareness of quality-of-life considerations in end-of-life care may lead to a shift in preference toward palliative care rather than aggressive treatment. Healthcare providers should discuss treatment trade-offs with cultural and religious sensitivity to support informed decision making.

Abstract citation ID: igaf122.2916

### BALANCING CLOSENESS AND DETACHMENT IN FAMILY DEMENTIA CAREGIVING: A MIXED METHODS REVIEW

Josey Batura[1], Gavin Green[1], Heather Kelly[2], and Elizabeth Fauth[1], *1. Utah State University, Logan, Utah,*

*United States, 2. Institute for Disability Research, Policy & Practice, Logan, Utah, United States*

Background: Caring for a family member with Alzheimer's disease and related dementias (ADRD) involves significant relational shifts, including changes in reciprocity, emotional closeness, and relational strain. While caregiver burden is well-documented, less is known about how caregivers perceive and adapt to changes in relationship closeness and whether closeness is uniformly beneficial. Objectives: This mixed methods systematic review examines (1) how family caregivers experience changes in relationship closeness, (2) the psychosocial factors that predict closening versus detachment, and (3) the impact of perceived closeness on caregiver well-being and caregiving outcomes. Methods: A systematic search was conducted following PRISMA guidelines across PubMed, EBSCOhost, Scopus, and ProQuest. Inclusion criteria required empirical, peer-reviewed studies published since 2015 focusing on relational closeness in spousal ADRD caregiving. A total of 15 studies met the criteria. Results: Three themes emerged: (1) shifting relational roles, (2) emotional adaptation, and (3) the bidirectional impact of closeness on caregiver and care recipient well-being. Some caregivers experienced deepened bonds, particularly when they maintained a sense of partnership despite role transitions. Others experienced detachment, often as a psychological buffer against the emotional distress of declining reciprocity. Closeness was typically linked to lower burden and greater resilience, but also greater anticipatory grief and distress. Conclusion: Closeness is not universally beneficial; while it can foster resilience, it may also intensify grief and distress. Future research should explore longitudinal trajectories of closeness and assess whether interventions (e.g., dyadic counseling, structured grief support) can help caregivers balance emotional connection with adaptive coping strategies.

Abstract citation ID: igaf122.2917

### DEMENTIA KNOWLEDGE ASSESSMENT POINTS TO NEED FOR GREATER UNDERSTANDING OF MODIFIABLE RISK FACTORS IN RURAL AREAS

MC Ehlman[1], Suzanne Leahy[2], Laurel Standiford Reyes[2], and Reagan Lawrence[2], *1. University of Nevada, Reno, Evansville, Nevada, United States, 2. University of Southern Indiana, Evansville, Indiana, United States*

Current lifetime risk of dementia is higher than previously thought (Fang, 2025), reinforcing the importance of public health initiatives focusing on brain health across the lifespan. The aim of this project was to assess baseline knowledge about Alzheimer's disease (AD) in a mostly rural Midwest region to inform educational initiatives among a network of eight Dementia Friendly Communities (DFC). A public university supported the network development as a part of its Geriatric Workforce Enhancement Program funded by the United States Health Resources and Services Administration (HRSA), designed to impact rural communities. The regional assessment surveyed 159 community members and healthcare professionals from 11 regional counties, using Weise's 30-item Basic Knowledge of Alzheimer's Disease, a survey instrument validated for use with rural participants. The assessment was conducted in two stages. Local purposive samples were gathered by network DFCs later supplemented by convenience sampling at regional rural dementia workshops. The sample was 77.4% rural and medically underserved as defined by HRSA. Consistent with previous literature, 64.8% of participants responded that "Persons with a history of diabetes or high blood pressure are at greater risk of AD" (35.2% answered incorrectly). Additional gaps in knowledge were identified where respondents answered false (incorrectly) that "There may be a link between serious head injury and AD" (32.1% answered incorrectly) and "Healthy eating may decrease your AD risk" (26.4% answered incorrectly). Results highlight the need to examine the theoretical underpinnings linking brain health knowledge and positive health behaviors in rural areas to address modifiable risk factors.

Abstract citation ID: igaf122.2918

### DYNAMICS BETWEEN SLEEP PATTERN AND BEHAVIORAL AND PSYCHOLOGICAL SYMPTOMS OF DEMENTIA (BPSD)

Eun Kyo Kim[1], Sinwoo Hwang[1], Minhee Yang[1], Eunhee Cho[1], Jungwon Cho[1], and Chang Gi Park[2], *1. Yonsei University, Seoul, Republic of Korea, 2. University of Illinois Chicago, Chicago, Illinois, United States*

A higher prevalence of behavioral and psychological symptoms of dementia (BPSD) is associated with a higher care burden in caregivers and increased mortality in people living with dementia. However, from the perspective that unmet needs trigger BPSD, there is a lack of time series analysis on whether sleep disturbances can predict the occurrence of BPSD the following day. In this study, older adults with dementia wore an actigraphy device on their wrists continuously for two weeks for sleep data, and caregivers recorded BPSD in a daily symptom diary for two weeks. The panel vector autoregression model analyzed data from 154 older adults living with dementia and their caregivers and found that the previous day's awakenings during sleep had a significant effect on irritability and appetite or eating disorders. Conversely, some symptoms of BPSD, including delusions, had significant effects on the next day's sleep patterns, resulting in increases in total sleep time, number of awakenings, and mean awakening length. In particular, irritability among BPSD was found to have a bidirectional causality with number of awakenings. This study demonstrates that the relationship between BPSD and sleep patterns is dynamic, characterized by a vicious cycle. Consequently, early intervention to alleviate symptoms is imperative, and strategies to enhance sleep quality should be prioritized during the initial stages when sleep patterns are disturbed.

Abstract citation ID: igaf122.2919

### FAMILY AND FRIENDS' SUPPORT TO PEOPLE WITH DEMENTIA IN ACCESSING OLDER AMERICANS ACT SERVICES

Emerson McSparran, Heather Menne, Kate Singer, and Ian Matthew Nelson, *Miami University, Oxford, Ohio, United States*

Some people living with dementia (PLWD) depend on Older Americans Act (OAA) services, including case management, congregate meals, home-delivered meals, homemaker services, and transportation services, to maintain their quality of life and ability to remain independent. Having someone facilitate their access to these services may increase their ability to gain the associated benefits. Using data from the 2023 National Survey of Older Americans Act Participants (NSOAAP), we explored how family and friends are involved in the coordination and use of OAA services for PLWD compared to people without dementia. Descriptive statistics and Fisher's exact tests were conducted to compare the rates at which participants with dementia and participants without dementia rely on family or friends to help arrange their OAA services. Among PLWD, the proportion of family and friends arranging services was significantly higher than those living without dementia (p<.01). Across services, the proportion of friends and family helping PLWD to arrange services were 56.7% for case management, 34.9% for congregate meals, 65.1% for home-delivered meals, 67.6% for homemaker services, and 48.1% for transportation services. Service providers and policymakers should be aware that more PLWD rely on others to help them access OAA services and account for this in service delivery considerations. While PLWD are not the majority of participants, their perspective is important to the OAA mission of helping older adults live independently.

Abstract citation ID: igaf122.2920

### FAMILY SUPPORT AND MODIFIABLE DEMENTIA RISK FACTORS AMONG COGNITIVELY IMPAIRED OLDER ADULTS IN SOUTH KOREA

Minji Shim, and Giyeon Kim, *Chung-Ang University, Seoul, Republic of Korea*

With a rapidly growing aging population, cognitive impairment is a pressing public health issue, and the importance of family support is growing. This study investigates the relationship between family support and modifiable dementia risk factors among older adults with cognitive impairment in South Korea. Family support was categorized by the presence of a spouse, children, and co-residence with children. For risk factor selection, we focused on three key modifiable dementia risk factors identified by the Lancet Commission: smoking, depressive symptoms, and social isolation. We analyzed data from the 2023 Korean National Living Survey for older adults with cognitive impairment(n = 4,009). Binomial logistic regression was conducted to examine the association between these family support categories and three modifiable dementia risk factors. The results indicated significant associations between family support and dementia risk factors. Older adults without a spouse and children were more likely to experience social isolation (OR = 1.51, p<.001) and depressive symptoms (OR = 1.35, p < 0.001) compared to those with a spouse and co-residing children. However, smoking was not significantly associated with family support. These findings highlight the protective role of family support in mitigating modifiable dementia risk factors among cognitively impaired older adults. Given the evolving care culture in Korea due to modernization and nuclear family structures, policies and interventions that strengthen family and social support networks may help reduce these risk factors and improve overall well-being in this population.

Abstract citation ID: igaf122.2921

### HIERARCHICAL CHARACTERISTICS OF DEMENTIA COMMUNITY SUPPORT IN COMMUNITY GENERAL SUPPORT CENTERS

Taeko Nakashima[1], Kei Sugiyama[2], and Etsuyo Kamiyamasaki[3], *1. Nihon Fukushi University, East Greenbush, New York, United States, 2. Osaka Metropolitan University, Osaka, Osaka, Japan, 3. Nihon Fukushi University, MIhama, Aichi, Japan*

Objective: This study aims to evaluate the community work practices of professionals engaged in dementia care at Community General Support Centers using the Latent Rank Theory and to clarify the hierarchical characteristics of their implementation. Background: In Japan, the Basic Act on Dementia to Promote an Inclusive Society, was enacted in 2024. This law aims to foster safe communities where people with dementia can live independently and securely with others. Methods: A questionnaire survey was conducted at 2,908 Community General Support Centers across 32 prefectures, with responses obtained from 876 professionals. After excluding cases with missing data, 515 responses were analyzed. The analysis utilized items related to dementia community work practices and was conducted based on the Latent Rank Theory. Results: Preliminary results suggest that a four-rank model best represents the practice levels. Support initiatives targeting commercial and public facility employees were generally low across all ranks. In Rank 1, items related to engaging with these sectors scored below 2 (“rarely implemented”), except for identifying potential collaborators. On the other hand, initiatives involving multidisciplinary professionals and local residents were recognized to some extent, even in the lower ranks, with a particularly strong presence in higher-ranked groups. Discussion: The importance of engaging with commercial and public facility employees is recognized among dementia community support professionals; however, actual efforts remain insufficient. To enhance these practices, it is essential to establish clear strategies for collaborating with these stakeholders.

Abstract citation ID: igaf122.2922

### KEY FACTORS IN THE QUALITY OF LIFE OF AFRICAN AMERICAN PARENTS LIVING WITH DEMENTIA

Daum Chung, and Kalisha Bonds Johnson, *Emory University, Atlanta, Georgia, United States*

Relationship quality is crucial for the quality of life (QoL) of people living with dementia (PLWD), yet cognitive impairment often limits their participation in decision making. This cross-sectional secondary data analysis aimed to investigate how QoL of African American parents was influenced considering life course. Data were originally collected from 39 African American parent–adult daughter dyads. Parent data with no missing values (N = 33) were included in the analysis. PLWD reported their age, QoL, self-rated health, their perceptions of the dyadic relationship quality, and their perceived level of difficulty in having their opinions heard by family members. PLWD’s dementia severity was assessed by their adult daughters using the Dementia Severity Rating Scale. In the final hierarchical regression model, “good” and “very good” self-rated health, positive interaction of the dyadic relationship, and having “some” difficulty in having opinions heard by family members were identified as significant predictors of QoL. Better self-rated health and positive interaction in the relationship quality were associated with better QoL, while greater difficulty in having their opinions acknowledged by family was associated with worse QoL. Findings suggest that maintaining a positive dyadic relationship alone is not sufficient to enhance African American PLWDs’ QoL. Their perceptions of how difficult it is to have their opinions heard by their family members seem to matter. Therefore, QoL intervention for African American PLWD may benefit from strengthening positive interaction in the dyadic relationship while fostering strategies to support parents’ opinions are heard without difficulty.

Abstract citation ID: igaf122.2923

### PERSONALITY AND INCIDENT DISABLING DEMENTIA AMONG COMMUNITY-DWELLING OLDER ADULTS: A 20-YEAR LONGITUDINAL STUDY

Yukiko Nishita[1], Chikako Tange[1], Shu Zhang[1], Sayaka Kubota[1], Mana Tateishi[1], Fujiko Ando[2], Hiroshi Shimokata[3], and Rei Otsuka[1], *1. National Center for Geriatrics and Gerontology, Obu, Aichi, Japan, 2. Aichi Shukutoku University, Nagakute, Aichi, Japan, 3. Nagoya University of Arts and Sciences, Nisshin, Aichi, Japan*

Objectives: This study examines the effect of personality on incident disabling dementia in community-dwelling older adults. Methods: We analyzed 898 adults (65 – 84 years) from the National Institute for Longevity Sciences-Longitudinal Study of Aging 2nd (2000 – 2002) or 5th (2006 – 2008) waves, with baseline established at their first personality questionnaire response. Participants with baseline cognitive impairment (MMSE ≤ 23), incident disabling dementia before baseline or within five follow-up years were excluded. Personality was assessed using NEO Five-Factor Inventory, measuring neuroticism, extraversion, openness, agreeableness, and conscientiousness. Disabling dementia was defined as ≥ rank II on the "Independence Degree in Daily Living for Older adults with Dementia" based on Doctor's Opinion Paper (from baseline to January 2022). Cox proportional hazards models estimated adjusted hazard ratios (aHR) and 95% confidence intervals (CI) for one standard deviation (SD) increase of personalities, controlling for age, sex, education, hearing and vision impairment, LDL cholesterol, depressive symptoms, physical activity, history of diabetes and hypertension, smoking, alcohol drinking, body mass index, social isolation, APOE ε4, and survey wave. A stratified analysis of APOE ε4 was also conducted. Results: Over a mean (SD) follow-up of 12.3 (5.8) years, 196 individuals (21.8%) developed disabling dementia. Openness was negatively associated with overall incident disabling dementia risk (aHR [95%CI]: 0.82 [0.70–0.96]). Stratified analysis showed that openness reduced dementia risk in ε4 non-carriers (0.79 [0.67–0.94]), while conscientiousness reduced it in ε4 carriers (0.56 [0.37–0.84]). Conclusion: Openness may reduce incident disabling dementia risk, while conscientiousness might suppress disabling dementia onset in ε4 carriers.

Abstract citation ID: igaf122.2924

### SLEEP DISTURBANCE AND DEPRESSION IN OLDER ADULTS: THE ROLE OF DEMENTIA SEVERITY

Hye Rim Ryu[1], Cari Cohen[2], Jerry J. Sweet[2], Christopher Williams[2], Elizabeth K. Geary[2], Julia Thomas[2], and Leslie Guidotti Breting[2], *1. Rosalind Franklin University of Medicine and Science, Glenview, Illinois, United States, 2. Endeavor Health - NorthShore Hospitals, Chicago, Illinois, United States*

Between 40-70% of older adults in the U.S. report sleep disturbance (Crowley, 2022; Smagula et al., 2016). Poor sleep is common in dementia (Wong & Lovier, 2023) and widely associated with adverse outcomes among older adults, including depression (Li et al., 2022). However, less is known about how the severity of dementia influences this association. The present study thus examined whether dementia severity moderates the relationship between sleep disturbance (gathered from clinical interview) and depressive symptoms (measured by the Geriatric Depression Scale). Participants were older adults with a primary dementia diagnosis following outpatient neuropsychological evaluation (N = 196, Mage = 76.60, SDage = 9.71). Dementia severity was categorized as mild (n = 122), moderate (n = 62), and severe (n = 12). Moderated regression analyses demonstrated that increased sleep disturbance predicted higher depressive symptomatology (B = 6.24, p < .01), with dementia severity moderating this association (p = .04). This moderating relationship was significant among older adults with moderate to severe dementia. Results indicated stronger effects in older adults with severe dementia (B = 10.56, p < .01) compared to those with moderate dementia (B = 6.24, p < .01). Overall, these findings demonstrate that that the association between increased sleep disturbance and higher depressive symptoms becomes stronger as dementia progresses. This study provides further insights into the complex intersection between sleep and mood within the context of neurodegenerative disorders. Results underscore the need for targeted interventions addressing sleep disturbance at varying levels of dementia severity to mitigate depressive symptoms.

Abstract citation ID: igaf122.2925

### STAFF CARE APPROACHES ARE ASSOCIATED WITH MEALTIME BEHAVIORS IN OLDER ADULTS WITH DEMENTIA

Kyuri Lee[1], and Wen Liu[2], *1. The University of Iowa, Iowa City, Iowa, United States, 2. Penn State University, University Park, Pennsylvania, United States*

Staff mealtime caregiving is essential to support nursing home residents with dementia, who often experience declines in eating performance and challenging behaviors that interfere with their engagement and food intake during mealtimes. This study examined the associations between staff care approaches and resident behaviors during mealtimes. This study is a secondary analysis of observational data from the OPTIMAL study that tested the impact of a person-centered mealtime care intervention in nursing home dementia care. The sample included 241 full-meal videos, capturing 17 residents with dementia (age 73.9±7.9 years; 88.2% male; 88.2% white, 41.2% married) and 54 direct care staff (age mean ± SD = 44.5±11.6 years; 93.3% female; 80.0% white; 15.8±12.3 years worked as a caregiver). Videos were coded for staff person-centered and task-centered care approaches and resident mealtime behaviors using the refined Cue Utilization and Engagement in Dementia (CUED) mealtime video coding scheme. Resident positive mealtime behaviors were associated with staff person-centered verbal care (β = 0.18, p<.001) and modification of the dining environment (β = 0.41, p=.045), controlling for resident demographic characteristics. Resident challenging mealtime behaviors were associated with staff person-centered verbal care (β = 0.12, p=.019), task-centered nonverbal care (β = 0.98, p<.001), and modification of the dining environment (β = 0.85, p=.004). Staff person-centered care may help promote resident positive behaviors. Minimizing task-centered care may improve resident mealtime challenging behaviors. The association between challenging behaviors, person-centered care, and environmental modifications suggests these strategies may often be reactive rather than proactive. Findings highlight the need for anticipatory care strategies to better support residents who experience mealtime challenges.

Abstract citation ID: igaf122.2926

### THE COMBINED ASSOCIATION OF HEARING LOSS AND SOCIAL ISOLATION ON DEMENTIA

Xi Wang[1], Jason Smith[2], Thomas Cudjoe[3], Jennifer Schrack[2], Nicholas Reed[4], Jennifer Deal[2], and Pablo Martinez-Amezcua[2], *1. Johns Hopkins University Bloomberg School of Public Health, Baltimore, Maryland, United States, 2. Johns Hopkins Bloomberg School of Public Health, Baltimore, Maryland, United States, 3. Johns Hopkins School of Medicine,*

*Baltimore, Maryland, United States, 4. New York University Grossman School of Medicine, New York, New York, United States*

Hearing loss (HL) and social isolation (SI) are considered independent, modifiable risk factors for dementia. However, the modifying role of SI in the HL–dementia relationship remains unclear. We conducted a cross-sectional analysis of community-dwelling adults aged ≥65 in the U.S. from the nationally representative 2022 National Health and Aging Trends Study (NHATS) (n = 4,712). HL was defined as a better-ear pure tone average >25 decibels using a tablet-based auditory assessment. We measured binary SI using a four-domain typology (living arrangement, core discussion network, religious services attendance, and social participation). Dementia was evaluated using the NHATS dementia algorithm (reported dementia diagnosis, AD8, and neurocognitive tests). We used survey-weighted Poisson regressions to test if the additional presence of SI among older adults with hearing loss was associated with a higher risk of dementia. The prevalence of HL, SI, both combined and dementia, were 56.0%, 21.9%, 14.4%, and 9.2%, respectively. Compared to those without HL or SI, the prevalence ratio for dementia was 1.95 (95% CI: 1.38, 2.75) for HL alone, and 1.30 (95% CI: 0.75, 2.25) for SI alone, and 2.42 (95% CI: 1.66, 3.52) for those with both conditions. The super-additive interaction between HL and SI was not statistically significant (Synergy Index = 1.1, 95% CI: 0.4, 1.9). These findings suggest the presence of both HL and SI is associated with a higher prevalence of dementia compared to either factor alone. While no significant interaction was observed, HL management should consider coexisting isolation for dementia prevention.

Abstract citation ID: igaf122.2927

### THE EFFECTS OF HISPANIC-FOCUSED DEMENTIA EDUCATION ON NURSING STUDENTS' AWARENESS, KNOWLEDGE, AND CONFIDENCE

Jessica Sanchez, and Aleatha Rossler, *Texas Woman's University, Dallas, Texas, United States*

Background: Hispanic Americans are 1.5 times more likely to develop dementia than non-Hispanic White Americans, yet experience a greater delay in diagnosis. The cause for delay is multifactorial, including discrimination when seeking healthcare, predisposition to diseases that increase dementia risk, language and financial barriers, and lack of knowledge. Nurses can play a critical role in addressing these barriers, yet nursing curricula lack formal programs to prepare students for this topic. Purpose: To evaluate the effects of a Hispanic-focused dementia educational intervention on nursing students' awareness, knowledge, and confidence in caring for this patient population. Methodology: A single-group, pretest-posttest design was employed, utilizing a convenience sample of undergraduate nursing students. Participants completed assessments to measure their awareness, knowledge, and confidence levels before and after the educational intervention. Results: There was a statistically significant increase in awareness of prevalence of health conditions that increase dementia risk ($p = 0.002$) and prevalence of dementia ($p = 0.02$) within communities of color; increase in confidence in ability to educate Hispanic patients diagnosed with dementia ($p = 0.003$); and increase in the mean knowledge of dementia within the Hispanic population. Conclusions: These findings support the data-driven change of integrating this educational intervention into the nursing curriculum to prepare nurses for delivering culturally competent care. This initiative is an important step to empower future nurses to improve health outcomes and ensure equitable care for vulnerable populations in professional practice.

Abstract citation ID: igaf122.2928

### THE PAINCHEK® PAIN ASSESSMENT TOOL: HARNESSING AI AND REDUCING SUBJECTIVITY TO ASSESS PAIN IN PEOPLE WITH DEMENTIA

Wingyun Mak[1], Orah Burack[2], Kreshnik Hoti[3], Jeff Hughes[3], and Kimberly Bergen-Jackson[4], *1. The New Jewish Home Research Institute on Aging, New York, New York, United States, 2. The New Jewish Home, New York, New York, United States, 3. PainChek, Sydney, New South Wales, Australia, 4. University of Iowa, Iowa City, Iowa, United States*

Pain assessment in people with dementia is challenging and can result in inadequate treatment. Most pain assessment tools require subjective quantification of pain/discomfort. PainChek® is a new assessment tool that harnesses a) artificial intelligence to provide objective pain ratings of facial expression and b) dichotomous ratings of observable non-facial pain indicators. This study aimed to validate PainChek® by examining the agreement, reliability, and predictive validity between PainChek® and the Abbey Pain Scale (APS), a more subjective pain scale commonly used in Australia. Participants (N = 103) were US nursing home residents with moderate-to-severe cognitive impairment. Participants were assessed for pain during rest and post-movement by two blinded raters, each administering one pain assessment tool. Agreement between raters was 92.7% (no pain), 69.1% (mild), 75% (moderate), and 83.3% (severe), suggesting high levels of agreement between PainChek® and APS assessments. Agreement rates, except in the mild condition, exceeded a priori hypotheses and were similar across rest/post-movement conditions. Intraclass correlations show that PainChek® (.73, 95% CI: .62-.81) outperformed APS (.59, 95% CI: .44-.71) on test-retest reliability. Bootstrapping methods yielded predictive values that were 75.5%, 89%, 82.8%, and 38.3% for no pain, mild, moderate, and severe. Low predictive value for severe pain was likely due to few occurrences of severe pain assessments. Results suggest that PainChek® performs on par with the APS, while providing a more objective format that enables greater accessibility for a variety of staff to administer reliably. Decreasing subjectivity from the pain assessment process may facilitate accuracy and lead to more appropriate treatment.

Abstract citation ID: igaf122.2929

### UNTANGLING THE ASSOCIATION BETWEEN AGE, RACE, FAMILY HELP AND SELF-REPORTED STRESS AMONG DEMENTIA CAREGIVERS

Obinna Odo[1], and Tochukwu Ozor[2], *1. Miami University, Oxford, Ohio, United States, 2. Nnamdi Azikiwe University, Nnewi, Anambra, Nigeria*

Studies show that providing care to older adults with Alzheimer's Disease and Other Related Dementias [ADRD] is stressful because the care needs are often challenging and extensive. Self-reported caregiving stress is someone's cognitive appraisal of threats emanating from caregiving stressors such that the demands of the stressors outweigh the individual's available resources. Caregiving stress results in loss of leisure time, emotional exhaustion, and work overload, affecting caregivers' physical and emotional health. This study examined factors that affect self-reported stress among caregivers of older adults with ADRD. Data from the 2017 National Study on Caregiving (NSOC III round 7) and the NSOC Time data subset were merged (N = 1341; Mean age = 58.3) and analyzed using bivariate and multiple linear regression analysis. Among caregivers who identified as Black, dementia caregiving was associated with a decrease in self-reported stress ($\beta$ = -0.095, $p < 0.05$). Age was also negatively associated with self-reported stress, with higher age predicting lower caregiving stress ($\beta$ = -0.007, $p < .001$). Feelings of frustration was strongly associated with caregiving stress ($\beta = 0.492$, $p < .001$). Surprisingly, access to family help was associated with higher caregiving stress ($\beta = 0.12$, $p < 0.05$). These findings underscore the need for tailored caregiving interventions and programs that address the unique needs of dementia caregivers, such as respite care and mental health support services, to mitigate emotional exhaustion, anxiety, and burden. The study also highlights the need for family caregiver training aimed at easing the stress on dementia primary caregivers.

### SESSION 7335 (POSTER)

### DYADIC RESEARCH

Abstract citation ID: igaf122.2930

### BEYOND THE INDIVIDUAL: THE DYADIC ASSOCIATION BETWEEN SPOUSAL PERCEIVED DISCRIMINATION AND DEPRESSIVE SYMPTOMS

Soohyeon Ko, *Harvard University, Boston, Massachusetts, United States*

Perceived discrimination increases psychological distress across the life course. However, little is known about whether one spouse's perceived discrimination similarly affects the depressive symptoms of the other spouse and whether this effect varies by gender or closeness to the spouse. Using data from 6,146 older couples (12,292 individuals aged 60 years or older) in the first wave of the Longitudinal Aging Study in India (2017–2018), we applied multilevel regression models to examine the dyadic associations between perceived discrimination and depressive symptoms (measured by the CES-D) in both oneself and one's partner. We further explored whether this crossover association differs by gender and perceived closeness to one's spouse. Results indicate that after accounting for covariates, perceived discrimination is associated with increased depressive symptoms not only in the individual (Men: $\beta = 0.14$, $p < 0.001$; Women: $\beta = 0.16$, $p < 0.001$) but also in their partner (Men: $\beta = 0.08$, $p < 0.001$; Women: $\beta = 0.09$, $p < 0.001$). Interaction analyses reveal that closeness to one's spouse intensifies the partner effect of perceived discrimination for women ($\beta = 0.07$, $p = 0.03$), whereas no such effect is observed for men. These findings highlight the dyadic implications of perceived discrimination for both partners. Specifically, women who perceive greater closeness to their spouse experience a stronger crossover effect of their partner's perceived discrimination on their own depressive symptoms.

Abstract citation ID: igaf122.2931

### DEPRESSION AS A MEDIATOR BETWEEN HEARING IMPAIRMENT AND COGNITIVE FUNCTION AMONG OLDER COUPLES

Suyeong Bae[1], Chang Dae Lee[2], and Ji-Hyuk Park[1], *1. Yonsei University Mirae Campus, Wonju, Republic of Korea, 2. Indiana University Indianapolis, School of Health & Human Sciences, Indianapolis, Indiana, United States*

Our study investigated how depression mediated the relationship between hearing impairment and cognitive function in older couples, addressing previous limitations by focusing on partner interdependence. This secondary data analysis used the 2023 Korea Elderly Survey. Hearing impairment, measured by a self-reported item regarding hearing inconvenience, was the independent variable. The dependent variable was cognitive function measured by the Korean-Mini Mental State Examination-Second edition, and the mediator was depression, assessed by the Korean version of the short Geriatric Depression Scale. We utilized the actor-partner interdependence mediation model (APIMeM) to examine the association between hearing impairment and cognitive function, as well as the mediating effect of depression in the context of interdependence. Our sample consisted of 2,233 older adult couples. The mean age was 71.65±5.57 years for females and 74.62±6.11 years for males. Based on the APIMeM model, an individual's hearing impairment was significantly associated with their own cognitive function (males: coefficient = 1.93; females: coefficient = 2.02) and their partners' cognitive function (males: coefficient = 0.84; females: coefficient = 1.37). Additionally, an individual's cognitive function was significantly associated with the partner's hearing impairment, and this association was mediated by the individual's own depressive symptoms for both males (coefficient = 0.22) and females (coefficient = 0.25). Our findings highlight the mediating role of depression in the association between hearing impairment and cognitive function in older couples, considering both individual and interdependent effects. These results provided evidence for considering partner interdependence when addressing cognitive health in later life.

Abstract citation ID: igaf122.2932

### DYADIC ASSOCIATIONS BETWEEN DAILY SPOUSAL ARGUMENTS AND AFFECTIVE REACTIONS: THE MODERATING ROLE OF EMOTION WORK

Dakota Witzel[1], Madeline Nichols[2], Kelly Chandler[2], Kelly Cichy[3], and Robert Stawski[4], *1. South Dakota State University, Brookings, South Dakota, United States, 2. Oregon State University, Corvallis, Oregon, United States, 3. Kent State University, Stow, Ohio, United States, 4. Utah State University, Logan, Utah, United States*

Daily stressors, like arguments with spouses, are predictors for changes in same-day affect. Emotion work - defined as activities done to promote another's positive emotional state - has been shown to have mixed associations with health and well-being. Notably, emotion work is more prevalent during stressful experiences. Using the 2015 wave of the Health and Relationships Study, we explored dyadic associations between daily arguments with spouses in gender-diverse marriages and both negative and positive affect and whether emotion work modified associations. Middle-aged couples (Ncouples = 378; 30% different-gender; Mage = 48.22, Agerange = 35-65) in same-gender and different-gender marriages completed 10 days of daily diary measurements on daily stressors, emotion work, and affect. Preliminary results using 3-level multilevel models suggest the odds of reporting an argument did not differ by gender composition (ps<.05), but did differ by the amount of emotion work by the respondent (OR = 3.88; p<.05), and their partner (OR = 2.51, p<.05). Significant differences in negative and positive reactivity emerged when respondents or their partners reported a spousal argument that day compared to non-argument days (ps<.05). On days when a spousal argument was reported, more emotion work from the respondent was related to greater negative and positive affective reactivity (ps<.05). Discussion will focus on the importance of spousal arguments and emotion work for shaping couple wellbeing, and their relevance throughout adulthood. Future work will aim to examine whether associations occur for longer-term affective reactions (e.g., affective residue) and differ by relationship composition (e.g., different-gender, same-gender).

Abstract citation ID: igaf122.2933

### DYADIC CARE TYPES AND QUALITY OF LIFE OF PATIENT-CAREGIVER DYADS IN MULTIPLE CHRONIC CONDITIONS

Maddalena De Maria[1], Jeffrey Stokes[2], Harleah Buck[3], and Elliane Irani[4], *1. Link Campus University, Rome, Lazio, Italy, 2. University of Massachusetts Boston, Boston, Massachusetts, United States, 3. University of Iowa, Iowa City, Iowa, United States, 4. Case Western Reserve University, Cleveland, Ohio, United States*

Purpose: To examine how congruence in the appraisal of dyadic Multiple Chronic Condition (MCC) care type affects the quality of life (QOL) in patient-caregiver dyads for one year. Methods: Patient-caregiver dyads were recruited. Patients aged 65 years and older with a diagnosis of at least two chronic illnesses were enrolled. Those affected by cancer or dementia were excluded. Caregivers were eligible if they were identified by the patient as the primary caregiver. Patient and caregiver appraisal of dyadic care type was assessed using the Dyadic Symptom Management Type instrument. Congruence occurred when patients and caregivers agreed in their appraisal of who is responsible for MCC management. QOL was measured with the Short Form-12. Controlled longitudinal multilevel dyadic models were applied to assess how congruence in dyadic care type influenced both patients' and caregivers' mental and physical QOL over one year. Results: A convenience sample of 2,343 observations from 871 patient-caregiver dyads participated (mean age of 77 and 52 years, respectively). Both patient-oriented and collaborative-oriented congruence in care type were associated with higher levels of mental QOL for patients, while collaborative congruence was linked to higher levels of mental QOL for caregivers. In contrast, caregiver-oriented congruence was associated with lower Mental QOL for patients. For Physical QOL, patient-oriented congruence was associated with better and caregiver-oriented congruence with worse QOL for patients, whereas for caregivers only caregiver-oriented congruence was associated with worse QOL. Conclusion: Congruence itself was not a predictor of QOL, but rather the type of congruence played a key role.

Abstract citation ID: igaf122.2934

### IF THESE WALLS COULD TALK: IN-HOME ACTIVITY AND RELATIONSHIP RATINGS AMONG OLDER ADULT COUPLES AT RISK FOR DEMENTIA

Lyndsey Anderson[1], Joel Steele[2], Wan-Tai Au-Yeung[1], Thomas Riley[1], Victoria Sanchez[1], Zachary Beattie[1], and Jeffrey Kaye[1], *1. Oregon Health & Science University, Portland, Oregon, United States, 2. University of North Dakota, Grand Forks, North Dakota, United States*

Functional decline related to Alzheimer's disease is an interdependent process among older adult couples due to mutual health behaviors, as well as shared stressors and daily activities. The objective of this study was to examine variability in activity in the home among older adult couples that include a partner who is at high-risk of developing AD, and relate levels of variability in activity to perceived spousal support. Data were derived from the study: Remote Sensing of (older adult partners') Engagement in Life and Variability in Everyday Support (RSELVES), which is prospective longitudinal and uses home-based assessment to continuously generate digital evaluations of engagement in life and weekly dyadic support appraisals among couples . Multiple in-home sensors were used to create a whole house activity state array (WHASA), encoding minute level activity over the day, that was subsequently modeled along with weekly self-reported measures of perceived spousal support (Secure Base Characteristics Scale). Activity entropy was aggregated hourly to capture trends in daily activity variability. Results showed that polynomial coefficients of variability were significantly associated with differences between partners' support ratings (encouragement, availability, and intrusiveness) and average support levels. Higher in-home activity variability correlated with greater incongruence in couples' weekly reports of encouragement and availability. Additionally, higher average encouragement levels related to later-day peaks in activity variability. The WHASA approach effectively captured patterns of dyadic home activity and demonstrated relationships with older couples' perceptions of partner support, indicating interdependence in both functional and relational aspects of life engagement.

Abstract citation ID: igaf122.2935

### MARITAL QUALITY AND SMOKING AMONG COUPLES IN MID- AND LATER-LIFE

Angela Curl, Amy Roberts, and Jennifer Bulanda, *Miami University, Oxford, Ohio, United States*

This study investigates the relationship between smoking and marital quality in older married couples, focusing on how marital dynamics influence smoking behaviors in later life. We hypothesized that both own and spouse's ratings of negative marital quality would increase the likelihood of smoking as a coping strategy (Smith & Gibson, 2019). Using pooled data (N = 1,239 individuals) from the 2014-2018 waves of the Health and Retirement Study, we examined smoking status in a sample of older married couples who had reported being smokers at some point in their lives. Actor-Partner Interdependence Models (APIM) were analyzed with HLM 6.08 software. Marital quality was measured using a 3-item positive scale and a 4-item negative scale, controlling for baseline smoking status. Contrary to expectations, higher negative marital quality in husbands was associated with decreased odds of smoking at Wave 2 (OR = 0.33, p<.05), while positive marital quality was not significant for either partner, and wives' negative marital quality was also not significant. The strongest predictor of smoking was baseline smoker status, with husbands showing an odds ratio of 137.28 and wives an odds ratio of 896.11 (p<.01). Our findings of negative marital quality being associated with better health outcomes are consistent with Liu, Shen, and Waite (2016), who found that negative marital quality lowered the risk of developing diabetes and increased diabetes management. These findings highlight the persistent long-term influence of past smoking behaviors over current marital quality in shaping smoking patterns in later life.

Abstract citation ID: igaf122.2936

### OLDER COUPLES' JOINT AND SEPARATE LEISURE TIME AND DAILY MOOD: RESULTS FROM THE KOREAN TIME USE SURVEY

Kyungmin Kim[1], Seung-Eun Cha[2], and Meng Huo[3], *1. Seoul National University, Seoul, Republic of Korea, 2. The University of Suwon, Suwon, Republic of Korea, 3. University of California, Davis, Davis, California, United States*

As demands from work and family decline, older adults typically focus more on leisure activities and time with their spouses. However, less is known about how older married adults engage in leisure activities (i.e., together with or apart from a spouse) and its cross-spousal associations with daily mood within couples. This study utilized couple data from the 2019 Korean Time Use Survey. Both spouses of older couple households (aged 65 – 94; N = 1,174) completed 2-day time diaries, reporting all leisure activities (e.g., social meetings, eating out, exercise) performed in 10-minute intervals each day and indicating the presence of spouse for each time slot. We examined how much time respondents spent on leisure activities together with or separately from their spouses on a daily basis and how their joint and separate leisure time was associated with daily mood. On average, husbands reported more leisure time than wives—together with their spouses (212 vs. 195 mins) and apart from their spouses (305 vs. 214 mins). Results from Actor-Partner Interdependence Models revealed that couples' joint leisure time was positively associated with daily mood only for husbands. Wives' separate leisure time was positively associated with their own daily mood (actor effect) as well as husbands' daily mood (partner effect), whereas husbands' separate leisure time was not significant for either spouse. These findings highlight the role of leisure activities in the daily lives of older adults, suggesting that the implication of leisure activities for well-being in late years should be understood at the couple contexts.

Abstract citation ID: igaf122.2937

### PATIENTS' AND CARE PARTNERS' EXPERIENCES WITH BIOMARKER-INFORMED EVALUATION FOR COGNITIVE IMPAIRMENT

Cristina de Rosa[1], Jeong Eun Kim[1], Joshua Grill[2], and Jennifer Lingler[1], *1. University of Pittsburgh, Pittsburgh, Pennsylvania, United States, 2. University of California, Irvine, Irvine, California, United States*

As biomarker-informed diagnoses for Alzheimer's disease and related dementias move from occurring primarily in controlled research settings to widescale clinical practice, understanding experiences of individuals with cognitive impairment and their care partners will be essential to meeting their unique needs. The Patient And family member Reactions to biomarker-informed ADRD DiagnosEs (PARADE) study conducted focus group discussions (FGDs) with diverse patients and care partners, with primary analysis identifying broad categories of information and support needs. This secondary analysis emphasized an in-depth examination of the broader emotional and interpersonal context in which needs arise. In PARADE, virtual FGDs with trained facilitators following a semi-structured guide asked participants about information and support needs following amyloid PET testing. Secondary thematic analysis of verbatim transcripts included 9 focus group participants (mean age 65.2 years, 55.56% female, 4 patient-care partner dyads and 1 other care partner) and preliminarily identified three themes. Patients and care partners described emotional and personal contexts, like past experiences caring for a family member with dementia, that were integral to their perspectives. Participants shared information within dyads (e.g., attending support groups alone and bringing back information for spouse) and with others with similar experiences. Knowledge of needs and disease trajectory allowed participants to advocate for themselves and anticipate future needs, like considering long-term care. Findings may suggest potential antecedent and contextual factors can contribute to identifying and meeting needs for support and continuing care. Both individuals undergoing biomarker-informed evaluations and family care partners may benefit from inclusion in care and planning discussions.

Abstract citation ID: igaf122.2938

### SPOUSAL WORK EXPECTATIONS AND PSYCHOLOGICAL DISTRESS: INSIGHTS FROM THE UNITED STATES AND SOUTH KOREA

Linh Dang[1], Richard Gonzalez[1], Carlos Mendes de Leon[2], and Briana Mezuk[1], *1. University of Michigan, Ann Arbor, Michigan, United States, 2. Georgetown University, Washington, District of Columbia, United States*

The decision to retire or continue to work past typical retirement age often involves a joint decision-making process between spouses/partners. However, it is unclear what role, if any, spousal decision on employment influences mental health among older couples. Using the Actor-Partner Interdependence Model, this study examined the dyadic associations of work expectations with psychological distress among heterosexual older couples in the United States and South Korea. Data came from the 2016-2018 Health and Retirement Study (n = 2,009 couples) and the Korean Longitudinal Study of Aging Study (n = 385 couples). Perceived expectation of working in the next five years was reported on a probability scale (0-100%). Psychological distress was assessed by the Center for Epidemiologic Studies Depression Scale. The study found moderate to strong correlations between men and women for work expectations and psychological distress among couples. In both countries, the respondents' own work expectations were not significantly associated with their psychological distress (OR(US) =1.01 [0.95, 1.07], OR(Korea) = 0.94 [0.68, 1.30]). There were significant spousal effects of work expectations on psychological distress among US couples (OR(US) = 1.06 [1.00, 1.11]), but no spousal effects were found among Korean couples (OR(Korea) = 0.90 [0.65, 1.24]). Moreover, the association between spousal work expectations and psychological distress did not vary by gender in either country setting. Findings emphasize the value of integrating both individual risk factors as well as contextual characteristics such as spouse/partner, gender, and country to inform mental health efforts supporting older adults during retirement transition.

Abstract citation ID: igaf122.2939

### THE IMPACT OF RESILIENCE ON MENTAL HEALTH OUTCOMES IN DYADS OF INFORMAL CAREGIVERS AND THEIR OLDER CARE RECIPIENTS

Jingyuan Liu, *The Hong Kong Polytechnic University, Hong Kong, China (People's Republic)*

Background: Resilience is widely recognized as a critical protective factor for mental health. However, existing research has predominantly focused on its individual-level effects, overlooking potential dyadic mechanisms in highly interdependent relationships. This gap is particularly salient in caregiver-older care recipient dyads, where mutual psychological interdependence is pronounced. To address this limitation, the present study applies the Actor-Partner Interdependence Model (APIM) to examine how resilience operates within caregiver-recipient dyads. Methods: A sample of 120 dyads of caregiver (Mage = 60.31 years, SDage = 11.10 years) and older care-recipients (Mage = 76.06 years, SDage = 8.46 years) were recruited in China. The APIM approach was used to analyze the dyadic effects of resilience on mental health outcomes, including depression, anxiety, and general well-being. Multilevel modeling (MLM) was employed to estimate the resilience's actor and partner effects on mental health outcomes within the dyads. Results: Results from APIM revealed the actor effect of resilience on both caregivers and care-recipients' own mental health outcomes. A significant partner effect on anxiety was observed ($\beta = -0.133$, $p < 0.05$), indicating that a resilient partner can significantly reduce one's own anxiety levels. Conclusion: The findings reveal that resilience influences not only an individual's mental health but also their partner's anxiety. This underscores the need to consider reciprocal resilience dynamics in resilience building programs. Interventions targeting only one member of the dyad may miss the crucial interdependencies between caregivers and recipients. Therefore, integrated interventions that enhance resilience in both parties should be prioritized in future service design.

Abstract citation ID: igaf122.2940

### THE ROLE OF SYMPTOM APPRAISALS ON QUALITY OF LIFE IN COUPLES DIAGNOSED WITH CANCER

Silvia Cilluffo[1], and Karen Lyons[2], *1. University of Milan, Milan, Lombardia, Italy, 2. Boston College, Chestnut Hill, Massachusetts, United States*

Although survival rates for many cancers are increasing, most cancer survivors continue to experience symptoms that can impact their quality of life and that of their spouse/partner. Guided by the Theory of Dyadic Illness Management, the current study focused on symptoms shared appraisal in survivor- spouse's dyads affects their quality of life. The sample was composed of 49 young-midlife couples within two years of a cancer diagnosis living in urban and rural areas. Actor partner interdependence models examined the association of survivor's symptoms (reported by both the patient and their spouse) on the physical and mental quality of life (QoL) of couples. Significant actor effects were found for survivor's reports of symptoms.; Higher survivor fatigue, pain severity, and pain interference on activities of daily living were significantly associated with worse survivor physical QoL($p = <.001$); higher survivor fatigue and pain severity were significantly associated with worse survivor mental QoL ($p<.05$)., For spouses, a significant actor effect was found for spouse mental QoL; when spouse's perceived survivor pain to be high they reported worse mental QoL ($p<.05$). A significant partner effect was found for pain interference. When survivors rated their pain interference high, their spouses were significantly more likely to report poor physical QoL ($p<.001$). Discussion will focus on the importance of examining appraisals of cancer symptoms within dyads to optimize QoL of both members. Discussion will also focus on the need for further research on the role of symptom appraisal on health behaviors and symptom management in couples living with cancer.

### SESSION 7340 (POSTER)

### EDUCATION: GERONTOLOGY/GERIATRIC EDUCATION

Abstract citation ID: igaf122.2941

### AN EDUCATIONAL BOARD GAME ON AGING THAT INCORPORATES THE 4MS AND SIMULATES AGING EXPERIENCES

Sue Hazelett[1], Lori Kidd[2], Michele Gareri[1], Denise Kropp[3], Margaret Sanders[3], Brandi Chrzanowski[4], and Darcia Simpson[5], *1. Summa Health System, Akron, Ohio, United States, 2. The University of Akron, Akron, Ohio, United States, 3. Northeast Ohio Medical University, Rootstown, Ohio, United States, 4. Direction Home Akron Canton Area Agency on Aging & Disabilities, Green, Ohio, United States, 5. Hospice of the Western Reserve, Cleveland, Ohio, United States*

Innovative approaches to imparting geriatrics knowledge are needed. We will demonstrate a board game developed as part of a HRSA Geriatric Workforce Enhancement Program grant to teach important geriatric issues and simulate common geriatric experiences. The game begins by assigning participants marital status, children, income, health, and chronic conditions by chance. Participants then choose their "life goal" (i.e., either money, family/friends, or health) which guides how they play the game. Progressing through the game simulates real life events and requires decisions that are driven by each player's goals, aligning with a 4Ms approach to Age Friendly care. We received feedback from $n = 13$ participants who all expressed high levels of satisfaction with the game. Effectiveness of the game as an educational tool was rated average 4.25 out of 5, how well it simulated real life challenges was rated average 4.6 out of 5, how well it incorporated "what matters most" was rated 4.5 out of 5. The overall rating was 4.5 and whether they would recommend the game was 4.5. Like popular poverty simulations, this game is a unique way to impart/reinforce important geriatric concepts for a wide range of learners. Participants experience the frustrations of unplanned financial setbacks with fixed incomes (e.g., losing dentures, paying for medications) as well as the importance of specific decisions (e.g., advance care planning, accruing social supports). The impact of random bad luck is also ever present. The game allows them to experience the challenges of aging in a simulated environment.

Abstract citation ID: igaf122.2942

### BUILDING AN AGE-FRIENDLY WORKFORCE BY EMBEDDING THE 4MS FRAMEWORK IN A GERIATRICS CLERKSHIP

Lenny Powell, Jennifer DeGennaro, Christian White, and Kevin Overbeck, *Rowan-Virtua School of Osteopathic Medicine, Stratford, New Jersey, United States*

Rowan-Virtua School of Osteopathic Medicine (RVSOM) Department of Geriatrics and Gerontology embedded the 4Ms framework (What Matters, Medication, Mentation, Mobility) into the required 4-week Geriatrics Clerkship curriculum. Students participate in a full day of in-person didactics delivered by an interdisciplinary team of faculty during the first week of the clerkship designed to teach them to use evidence-based screening tools and employ best practices when caring for older patients. A survey on the 4Ms approach is distributed at the beginning of the rotation and again at the end of the 4 weeks. Pre- and post-clerkship data for 184 students show significant differences on all but 1 of 16 items [e.g., knowledge to perform a Geriatric Assessment: $t(183) = 19.32$, $p = <.001$, $d = 1.42$, identifying inappropriate medications: $t(183) = -18.51$, $p = <.001$, $d = -1.36$, and initiating an Advance Care Planning conversation: $t(182) = -13.57$, $p = <.001$, $d = -1.03$]. In the post survey, students are asked to specify any new practices in their care of patients after the Geriatrics clerkship and over half (54%) reported new care practices. Commonly noted were increased attention to medication lists, functional history, goals of care, and use of evidence-based screening tools. The statement "Knowing what questions to ask geriatrics pts even when I'm in another specialty" illustrates that providing foundational knowledge on aging and training students to assess patients using the 4Ms Framework creates a healthcare workforce able to recognize key issues critical to caring for an aging population.

Abstract citation ID: igaf122.2943

### DEVELOPMENT AND IMPLEMENTATION OF AN INTERPROFESSIONAL, AGE-FRIENDLY LEARNING ACADEMY

Kristin Zimmerman[1], Erika Nixon-Lambert[1], Elvin Price[1], Lana Sargent[2], Antara Sarkar[3], Thomas Walker[3], Glenda Watkins[4], and Sarah Marrs[4], *1. Virginia Commonwealth University School of Pharmacy, Richmond, Virginia, United States, 2. Virginia Commonwealth University School of Nursing, Richmond, Virginia, United States, 3. Virginia Commonwealth University School of Medicine, Richmond, Virginia, United States, 4. Virginia Commonwealth University College of Health Professions, Richmond, Virginia, United States*

There is a shortage of interprofessional geriatrics clinical training and education experiences for health professions students. To address this, we developed an eight-week longitudinal clinical experience for interprofessional learners, including pharmacy, medicine, nursing, social work, physical therapy, and occupational therapy students. The program features co-learning in classroom and clinical spaces, and includes a learner-led design of a quality improvement plan to encourage team-based, age-friendly learning that may translate to collaborative, innovative, person-centered, and continuously improving care. The program was developed by an interprofessional team of faculty and students from the representative disciplines. The development team was guided by the ADDIE (analyze, design, develop, implement, evaluate) model for instructional design. In the analyze phase, the team sought input from faculty and students. In the design phase, the team created learning objectives from key competencies. In the development phase, the four stages of experiential learning from Kolb's experiential learning cycle (concrete experience, reflective observation, abstract conceptualization, and active experimentation) was used to construct the curriculum. The initial implementation featured iterative feedback from the learner cohort; feedback was incorporated with predetermined learning outcomes and evaluation metrics during the evaluation phase. This presentation will discuss program development and share feedback and learning outcomes from the initial implementation cohort. Specifically, we will share quantitative outcomes related to changes in knowledge of age-friendly care, confidence in providing age-friendly care, interprofessional collaborative competency, dementia comfort and knowledge as well as qualitative outcomes related to intent to change practice and commitment to a career caring for older adults.

Abstract citation ID: igaf122.2944

### EXPLORING LIFELONG LEARNING ORGANIZATION WITH ENDOGENOUS AND EXOGENOUS CONCEPT: A CASE STUDY OF JAPAN AND TAIWAN

Yunann Lin[1], Huichuan Wei[1], and Wang Chih-Liang[2], *1. National Chung Cheng University, Minxiong, Chiayi, Taiwan (Republic of China), 2. National Cheng Kung University, Tainan City, Tainan, Taiwan (Republic of China)*

In response to the VUCA (Volatility, Uncertainty, Complexity, Ambiguity) era, lifelong learning organizations must undergo transformative changes as societies emphasize mutual recognition and self-discovery through interaction. This study explores Taiwan's transition by analyzing Japan's multi-layered community-based lifelong learning organizations. The Tamago project in Kashiwa City fosters lifelong learning through community cafés and diverse activities, including educational programs, board game creation, festivals, and holiday events. The Huwei Active Aging Learning Center (AALC), established by Taiwan's Ministry of Education, offers diverse courses focusing on intergenerational interaction, health promotion, and liberal arts. This study employs Bereday's comparative method through interviews, on-site visits, and literature review to analyze the organizational structure, mission, curriculum design, and participant demographics of these two lifelong learning organizations. The analysis reveals distinct differences: Kashiwa operates as an autonomous, resident-managed organization, while Huwei AALC functions as a government-commissioned institution with certified instructors and local managers. Kashiwa emphasizes community problem-solving and collaborative teaching, facilitating intergenerational skill-sharing, whereas Huwei follows a curriculum-based approach targeting older adults over 55. Despite similarities in course content and formation, the two models reflect fundamentally different developmental approaches—endogenous (Kashiwa) versus exogenous (Huwei). Both successfully strengthen localized small-scale social interactions, fostering environments for social connection, self-actualization, and self-identity. Taiwan could benefit from Kashiwa's intergenerational approach to enhance intrinsic motivation among older adults. Future implications include optimizing the learning environment to enhance civic literacy and redefining government roles to view older adults as valuable societal resources

Abstract citation ID: igaf122.2945

### GERONTOLOGY EDUCATION IN SOCIAL WORK: THE ROLE OF POLITICAL CONTEXT AND ALZHEIMER'S AND RELATED DEMENTIA PREVALENCE

Grace Yi, and Madelyn Martinez, *California State University, Fullerton, Fullerton, California, United States*

As the aging population grows, there is an increasing need for professionals trained to address older adults' diverse health and social needs. Social work, a discipline focused on supporting vulnerable populations, has played a key role in aging services. However, a shortage of professionals, including social workers, remains a concern. Limited research exists on the availability and content of gerontology courses in social work education programs. This current study aimed to examine factors influencing the inclusion of gerontology courses in social work programs and explore key themes in course content. A mixed-methods approach was applied. Using the list of CSWE-accredited MSW programs as a sampling framework, the top 150 universities ranked in U.S. News & World Report's Best Schools for Social Work (44.1% of the total) were selected. The dataset included state-level factors (percentage of adults aged 65+, the prevalence of Alzheimer's and related dementias (ADRD), political affiliation, and geographic region) and institutional characteristics. Aging course descriptions on institutions' websites were analyzed qualitatively. Data analysis was conducted using Stata 18 and NVivo 14. While no direct association was found between state-level factors and course availability, a significant interaction effect was observed (B =-1.25, p =.03). Universities in Democratic-led states were more likely to offer courses as ADRD prevalence increased, whereas those in Republican-led states were less likely. Qualitative analysis identified key themes: family, care, clinical practice, and health. Findings highlight the role of the political climate in shaping gerontology education and the need to expand coursework to include macro-level practice.

Abstract citation ID: igaf122.2946

### GROWING PAINS: THE SEARCH FOR AGING STUDIES INTERNSHIPS FOR UNDERGRADUATE MAJORS

Nasreen Sadeq, Debra Dobbs, Lindsay Peterson, Sara Hackett, and Lu Norstrand, *University of South Florida, Tampa, Florida, United States*

The School of Aging Studies at the University of South Florida recently completed a major curriculum revision of our B.S. in Health Care Administration program. Formerly the B.S. in Long-Term Care Administration, this undergraduate program was originally developed with guidance from the Florida Board of Nursing Home Administrators and remains the only program in Florida offering students the opportunity to complete a 9-credit (650 hour) Administrator in Training (AIT) internship. To increase student enrollment in our revised Health Care Administration major, we focused on emphasizing the opportunity to complete an internship as a key recruitment strategy. Our updated curriculum that allows students to choose between the AIT program or a 3-credit part-time internship resulted in 15 interns at 14 different internship sites in 2024, generating 4,850 service hours. We are expecting a two-fold increase in next year in the number of students needing an internship placement. Strategies implemented to improve the overall efficiency of the internship program will be presented, including the development of standard operating procedures, a database for tracking student progress, and online modules that provide students with an orientation to the internship program. Challenges we have experienced including the limited availability of internship sites and the extensive time and effort required to develop new community partnerships and to match students with gerontology-oriented internships aligned with their career goals will also be discussed. With the increasing trend of undergraduates seeking internships and field experiences, we will share our ongoing plans to continue addressing these challenges in the future.

Abstract citation ID: igaf122.2947

### IMPACT OF COLLABORATIVE LEARNING ON INTERNET KNOWLEDGE LEARNING PERFORMANCES AND EXPERIENCES OF OLDER ADULTS

Zhiyu Fan, Yinong An, and Huamao Peng, *Beijing Normal University, Beijing, China (People's Republic)*

Lifelong learning is an important part of active aging. Collaborative learning, characterized by learner-centered autonomy and social interaction, is generally considered more effective than individual learning. However, its applicability to older adults remains unclear due to their distinct learning characteristics. Cognitive interactions in collaboration may deepen learning but could also impose additional cognitive burdens, while socio-emotional interactions may foster engagement and mitigate cognitive aging. This study examined the effect of collaborative learning on older adults' Internet knowledge learning outcomes and experiences, and examined the role of different interactions in the process. Study 1 (n = 167) employed a 3 × 2 mixed design, with learning approach (individual/ dyads collaborative/ triads collaborative) as between-subject variable and test (pre/ post) as within-subject variable. While no significant differences in learning performance were observed across conditions, the dyads reported more positive learning experiences (higher satisfaction and greater willingness for continued learning). Study 2 (n = 76) utilized a 2 × 2 mixed design, with collaborative learning approach (cognitive interactions guided dyads/ socio-emotional interactions guided dyads) as between-subject variable and test (pre/ post) as within-subject variable. Study 2 incorporated data from Study 1's individual and unstructured dyadic conditions. Results replicated Study 1's findings, showing no performance differences. However, dyads without structured guidance (in Study 1) reported more positive learning experiences (higher learning satisfaction). These findings suggest that unstructured dyadic collaboration offers a balanced approach for older adults, maintaining learning performance while enhancing learning experiences. This approach appears particularly suitable for Internet knowledge acquisition in older populations, providing a supportive yet flexible learning environment.

Abstract citation ID: igaf122.2948

### IMPROVING NURSING STUDENTS' ATTITUDE TOWARD AGING THROUGH ACADEMIC AND COMMUNITY PARTNERSHIPS

Angela Braswell, *Wentworth Douglass Hospital, Portsmouth, New Hampshire, United States*

"Maybe the time to learn old age's lessons is in advance. The lessons are out there. Six million teachers are in it now" (Leland, 2018, p. 22). For this 15-week undergraduate nursing course aimed to promote healthy aging and explore nursing issues related to older adults, 94 students engaged in an intergenerational learning opportunity with volunteer "consultants" from a local Continuing Care Retirement Community (CCRC). Using the 4-Ms Framework for Age-Friendly Health Systems and John Leland's "Happiness is a Choice You Make: Lessons From a Year Among the Oldest Old" for inspiration, students and their consultants met on average five times to complete course assignments, culminating in a video presentation that shared the consultant's story, highlighted students' key takeaways, and explained how the experience will inform their future nursing practice. What lessons did they learn? Narrative data and word cloud analysis showed improved student attitudes towards older adults, enhanced empathy, and a deeper appreciation for essential gerontological nursing competencies. The project promoted the growth of transferable essential skills in communication, assessment, and ethical and clinical decision-making. Feedback from consultants highlighted mutual learning, increased connection with younger generation, and satisfaction having contributed to the education of future nurses. Partnerships with local CCRCs offer valuable real-world experiences and a competency-based approach to improve student attitudes toward aging, thereby enhancing health outcomes for older adults. Additionally, these academic and community partnerships can further foster interdisciplinary collaborations, enriching student educational experiences and broadening the program's impact on community health and well-being.

Abstract citation ID: igaf122.2949

### INTERPROFESSIONAL CURRICULUM DESIGN: FROM CONCEPTION TO DELIVERY

Lisa Bodenheimer[1], Anne Rosenfeld[1], and Stephanie Berdugo-Hernandez[2], *1. Rowan University, Glassboro, New Jersey, United States, 2. Rowan Virtua School of Osteopathic Medicine, Stratford, New Jersey, United States*

With funding awarded through the DHHS-HRSA Geriatric Workforce Enhancement Program Grant, the Rowan-Virtua School of Osteopathic Medicine (RV-SOM) has partnered with the Rowan University- Masters in Social Work Program (RMSW) to develop an interprofessionally designed and delivered post-graduate certificate in Geriatrics and Gerontology. RMSW is a new graduate program launched in 2023 and currently has no elective courses in aging. In 2024, initial pre-planning meetings were convened between both departments to conceptualize the components necessary to train the future workforce in geriatric and gerontology best practices with a specific focus on Alzheimer's Disease and Related Dementia. A (5) course elective program was conceived to be offered to master level students university-wide, not just RMSW matriculated students, aiming to recruit a diverse target audience including students in non-clinical programs. Once complete, the (5) course program will be offered as a post-graduate certificate. During AY 24/25, a work group began syllabus development for the first elective course, "Introduction to Geriatrics and Gerontology". The work group has a tripart mission; (1) to obtain stakeholder buy-in from multiple disciplines to co-develop modules from their scope of practice, (2) obtain buy-in from those practitioners to teach in the graduate program, allowing students the opportunity to learn directly from multiple disciplines and (3) create a syllabus which merges theory with practical approaches and interventions. To date, we have successfully engaged high-level faculty in geriatrics, neuropsychology, social work, and occupational therapy. All disciples will be engaged in didactics, small group activities, reading assignments and evaluation.

Abstract citation ID: igaf122.2950

### NEVER TOO "OLD": INSIGHTS FROM THE INTERGENERATIONAL CLASSROOM

Jessica Hsieh[1], Raza Mirza[1], Christopher Klinger[2], Alexis Hart[1], and Florene Shuber[3], *1. University of Toronto, Toronto, Ontario, Canada, 2. McMaster University, Hamilton, Ontario, Canada, 3. Christie Gardens, Toronto, Ontario, Canada*

The topic of aging demands greater attention in education systems, and intergenerational approaches help to combat ageism and improve the lives of older adults, now and in the future. Taking an intergenerational approach to contextualizing the experiences of older adults is a step towards addressing challenges commonly associated with aging. The University of Toronto (UofT) partnered with Christie Gardens, a retirement community, to launch an innovative experiential learning initiative: The Intergenerational Classroom. Half the students were UofT undergraduates; the other half were older adults residing at Christie Gardens. Through interactive seminar-style discussions, collaborative projects and mentorship, the course, which was held at Christie Gardens, and offered in the Fall 2023 and Fall 2024 semesters, provided a semester-long exploration on aging. This study explored the impacts of the Intergenerational Classroom from the perspectives of undergraduate students and older adults who had participated in the program. Guided by a phenomenological qualitative methodology, this study conducted in-person, semi-structured interviews with a sample of undergraduate students (n = 10) and older adults (n = 16). To enhance trustworthiness, two researchers independently analyzed transcript data to identify key transcript statements into themes. Outcomes of program success were identified across domains related to lasting friendships and bonds, increased awareness of aging issues, reduced ageist attitudes, and community and civic engagement. The taxonomy developed provides a comprehensive and conceptually organized range of successful outcomes to serve as infrastructure for the development of meaningful intergenerational programming outcome measures.

Abstract citation ID: igaf122.2951

### ONLINE LEARNING AMONG OLDER ADULTS: LEARNING EFFICACY AS A KEY PREDICTOR FOR MENTAL HEALTH

Junhua Xiao[1], and Huamao Peng[2], *1. Beijing Normal University, Beijing, Beijing, China (People's Republic), 2. Beijing Normal University Institute of Developmental Psychology, Beijing, Beijing, China (People's Republic)*

More than 50% of older adults in China now use the Internet, making online learning a key approach for lifelong learning. With age, older adults tend to prioritize positive emotional experiences, indicating that online learning experiences may have a more direct relationship with mental health than learning performance. This study examines whether learning performance influences mental health through learning experiences, particularly focusing on the effect of online learning efficacy, as it aligns with higher psychological needs of older adults. A total of 998 community-dwelling older adults in China (age = 68.50 ± 5.37 years) completed questionnaires assessing self-rated learning performance, learning experiences (via the Online Learning Efficacy Scale), and mental health (via the Geriatric Depression Scale, Social Participation Scale, Subjective Cognitive Decline Scale). Hierarchical regression and mediation analyses were conducted. The results showed that sixty percent of older adults spent more than 1 hour daily on online learning. Learning performance negatively predicted depression, subjective cognitive decline, social participation with demographic variables controlled. After adding the online learning efficacy into the regression model, the regression model's explanatory power significantly increased. Further analysis showed that online learning efficacy mediated the relationship between learning performance and mental health, with the strongest mediated effect observed on social participation. These findings suggest that for older adults, online learning efficacy is closely linked to their mental health, particularly for social health. Educational programs should prioritize fostering older adults' sense of competence to promote mental health and positive ageing.

Abstract citation ID: igaf122.2952
### ORAL HEALTH IN PHYSICAL THERAPY CURRICULUM: AN ESSENTIAL COMPONENT FOR THE PRACTICE OF PHYSICAL THERAPY
Julie Hartmann, Tara Granada, Victoria Steele, and Zachary Tussey, *Bellarmine University, Louisville, Kentucky, United States*
Literature has revealed a link between oral health and overall systemic health and that a correlation between poor oral health and several chronic diseases, such as diabetes and heart disease exist. Encountering patients with comorbidities that are associated with poor oral health are of high frequency in the practice of physical therapy. It is imperative that physical therapists are equipped with tools necessary to screen for poor oral health and appropriately refer to a dental health professional. The physical therapist should have the knowledge and ability to perform oral health screening as indicated for an older individual in order to provide a comprehensive examination. This reinforces the importance of incorporating oral health content and interprofessional collaboration with dentistry into Doctor of Physical Therapy (DPT) curriculum. Suggestions have been provided for integration into a sample University's DPT curriculum, which have been categorized by body systems, disease processes with delineation of the associated oral health connection. The purpose of this work is threefold. First, it is pertinent to relay the importance that a healthcare professionals' recognition of integrating oral health screening into their practice may elevate the standard of care provided to older adults. Second, a framework has been provided to encourage the incorporation of oral health screening and education into physical therapy practice for older adults. Lastly, it would be beneficial to integrate oral health information into physical therapy curriculum to improve educational opportunities for physical therapy students as well as to promote collaboration with dentistry.

Abstract citation ID: igaf122.2953
### RETHINKING AGING: DOES AN AGING-RELATED COURSE INFLUENCE STUDENT PERCEPTIONS OF OLDER ADULTS?
Lisa Wagner, Suniska Patel, Courtney Muchow, Elizabeth Hough, and Bhumi Jagadish Kotian, *University of San Francisco, San Francisco, California, United States*
Today's college students are living through a societal age transformation: older adults were 12.4% of the population when they were born (Administration on Aging, 2006) and will be 23% of the population when they reach older adulthood themselves (Mather & Kilduff, 2020). Yet, many college students have limited experience with older people and little knowledge about aging. In addition to increasing students' knowledge about aging, aging-related college courses may improve attitudes toward older people. Using a pre-test/post-test design, we assessed attitudes toward older people in college students enrolled in aging-related and non-aging-related courses to determine whether attitudes toward older adults changed following the courses. We hypothesized that attitudes toward older people would improve in Adulthood & Aging and would remain unchanged in non-aging-related courses (Social Psychology, Psychology of Prejudice). We measured attitudes using an allophilia scale (liking for another) modified to measure attitudes toward older adults in five domains: affection, comfort, kinship, engagement, enthusiasm (Wagner & Luger, 2017). Students in Adulthood and Aging had significant increases in comfort, $F(1, 58) = 6.8$, $p = .012$), and enthusiasm, $F(1, 58) = 8.86$, $p = .004$) for older adults compared to students in non-aging-related courses. Students' increase in affection $F(1, 58) = 3.05$, $p = .086$) was marginally significant. Adulthood and Aging student ratings of kinship and engagement with older adults were significantly higher than non-aging-related courses, $F(1, 58) = 9.95$, $p = .003$) and $F(1, 58) = 21.1$, $p < .001$, respectively, but the increase over time was not significantly different.

Abstract citation ID: igaf122.2954
### STUDENT-LED ENVIRONMENT FOR GERIATRIC INTERPROFESSIONAL EDUCATION: A FEASIBILITY AND ACCEPTABILITY STUDY
Kristin Collins[1], Anne Hunt[2], Sylvia Davidson[2], and Dorothy Kessler[3], *1. Queen's University, Stouffville, Ontario, Canada, 2. University of Toronto, Toronto, Ontario, Canada, 3. Queen's University, Kingston, Ontario, Canada*
The field of geriatric medicine is multidisciplinary and requires healthcare professionals to work collaboratively to address the complex needs of older adults. Providing students with clinical training opportunities for interprofessional collaboration can help prepare future clinicians for effective interprofessional practice. This study aimed to explore the acceptability and feasibility of a student-led environment in a Specialized Geriatric Clinic through an implementation science lens. Occupational therapy, pharmacy, and social work students collaborated to deliver brain health education to clients and families. Preliminary data demonstrates that the student-led environment is perceived by both interprofessional staff ($N = 4$) and students ($N = 6$) as acceptable, with an average rating of 4.3/ 5 on the eight domains of the Theoretical Framework of Acceptability. The domains of perceived effectiveness (4.6), intervention coherence (4.5) and overall acceptability (4.8) received the highest ratings. Perceived burden (3.8) received the lowest rating, highlighting the ongoing barriers these environments pose. Client feedback affirmed the environment's acceptability, with a satisfaction survey ($N = 18$) reporting an average rating of 4.3/ 5. Clients found they were treated respectfully (4.9) and had confidence in the students (4.8), however, clients reported that more time could have been spent getting to know them individually (3.5). Operational clinic statistics tracked across the study timeframe found benchmarks for feasibility were met for indicators such as staff workload, number of students placed in the clinic environment, and number of clients who participated. These results will help guide future implementations of student-led environments in this local context and may assist organizations with similar structures.

Abstract citation ID: igaf122.2955
### TASK RELEVANCE OVER EMOTIONAL FACTORS IN OLDER ADULTS' NON-FORMAL LEARNING
Yang Wang, Heng Zhao, and Huamao Peng, *Beijing Normal University Institute of Developmental Psychology, Beijing, Beijing, China (People's Republic)*
Despite the prevalence of non-formal learning among older adults, research in this area remains limited. Therefore, this study aims to analyze older adults' non-formal learning processes, with a particular focus on continuance intention of learning, and to compare the distinct effects of task relevance and emotional factors. Experiment 1 was conducted in a laboratory setting, involving 77 young adults (Mage = 22.49, 18-30 years) and 80 older adults (Mage = 68.41, 61-81 years), randomly assigned to three groups: positive emotion, negative emotion, and control, each receiving different manipulations of learning-related emotion. Participants completed both high-relevance and low-relevance learning tasks. Experiment 2 was conducted in a real-world context, involving 44 older adults (Mage = 69.43, 63-78 years) who were randomly assigned to two groups, with one group completing high-relevance courses and the other group completing low-relevance courses during a six-day intensive tracking study. Both experiments measured participants' continuance intention of learning, learning performance, and subjective mental effort. Additionally, experiment 1 assessed objective mental effort through changes in pupil diameter. The results proved that increased task relevance significantly enhanced older adults' continuance intention of learning, mental effort, and learning performance in non-formal learning contexts, while its impact on young adults was negligible. Meanwhile, positive emotion was a significant predictor of older adults' continuance intention of learning, but only under low-task-relevance conditions. Our study confirms the crucial role of task relevance in non-formal learning for older adults, surpassing the impact of emotional factors, providing clear guidance for curriculum design and educational practices.

Abstract citation ID: igaf122.2956

### TEACHING STRATEGIES FOR INTERDISCIPLINARY COLLABORATION WITHIN GERONTOLOGY EDUCATION

Elaine Jurkowski, *Southern Illinois University, Carbondale, Illinois, United States*

A critical area within gerontological education and medical/health/behavioral professions is to educate and train students for competence within interprofessional collaboration. Based on the experiential learning theory (Kolb & Kolb, 2017) and drawing from the IPCE model (Interprofessional Education Collaborative, 2023), this study has creatively designed a training /education model for students within gerontological fields of practice to enhance their ability to understand the concept of working collaboratively across disciplines. Students were placed in teams utilizing one of at least three models of experiential education and worked across teams with real patient/consumer consultations/case management strategies. The goal was to achieve both patient/consumer outcomes and learn about professional practice with people growing older using an interdisciplinary collaborative approach. Three specific strategies used include "Student Hotspotting", an Interdisciplinary Day (IPE), and a collaborative teaching clinic. The Student Hotspotting approach, modelled after the Camden Coalitions' program was an academic year long experience working with a older person with complex care needs. The IPE day focused on a case study approach and guest presenters via a team-based approach with health-oriented disciplines working as a team to identify a case management plan. The Teaching Clinic brought together medical, social work, nursing, physician assistant and nutrition students together in an outpatient clinic setting to address patients with complex care needs and aging related health conditions. All three strategies examined care through the lens of Social Determinants of Health. Evaluation outcomes presented positive outcomes and benefits to the students' learning outcomes and patient outcomes.

Abstract citation ID: igaf122.2957

### WHAT DRIVES STUDENTS TO PURSUE GERONTOLOGY AND GERIATRICS EDUCATION?

Isaac Asirifi Boateng[1], and Rona Karasik[2], *1. St. Cloud State University, St. Cloud, Minnesota, United States, 2. Saint Cloud State University, Saint Cloud, Minnesota, United States*

Despite the growing need for practitioners, researchers and policymakers knowledgeable about aging and older adults, student interest in pursuing gerontology and geriatrics at all levels (e.g., undergraduate, graduate, post-graduate) and related fields (e.g., human services, medicine, PT/OT, social work) is failing to keep pace with demand. While student recruitment concerns in gerontology are not new, the situation is becoming increasingly dire. Previous studies have shown students are reluctant to enter the field because of ageism/negative attitudes toward aging and older adults (e.g., Jester et al., 2021), lack of awareness of career options (e.g., Dassel et al, 2014) and expectations of lesser prestige and compensation (e.g., Cheung et al., 2023). There is limited understanding, however, of what does draw potential students to gerontology and geriatrics. To develop effective strategies for increasing student interest in gerontological education, this study explored the motivations of individuals who chose the field. Findings from the first wave of data obtained through an email survey of current and recently graduated students taking courses in aging (N = 67) revealed several factors leading to their decisions, including: personal experiences with aging family members (n = 43), desire to improve quality of life for older adults (n = 39), policy advocacy (n = 24), ability to educate others on aging issues (n = 23), positive academic influences (n = 18), and aging-related service experiences (n = 18). Implications for these preliminary findings include opportunities for fostering greater interest in the field, enhancing an age-savvy workforce, and advocating for policies to support the growth of academic gerontology programs.

Abstract citation ID: igaf122.2958

### YOGA AND MINDFULNESS FOR SUCCESSFUL AGING: AN INDIGENOUS LIFE COURSE PERSPECTIVE FROM SOUTH ASIA

Kamala Ramadoss[1], and Ishan Shivanand[2], *1. Syracuse University, Syracuse, New York, United States, 2. Yoga of Immortals (USA) and Indian Institute of Technology (India), Manlius, New York, United States*

Research on yoga and mindfulness-based interventions have focused on the positive impact of yoga and mindfulness-based interventions on health, wellbeing and successful aging. However, some researchers have simultaneously cautioned about weak effects and/or lack of evidence about the effectiveness of yoga and mindfulness-based interventions and the decline in the adoption of yoga and mindfulness as alternative therapies. A major gap in the literature is a lack of critique about the basic concepts and theoretical considerations, as the variables used to measure these concepts need to be grounded in theoretical rigor. In this paper, the authors will examine the history and key concepts of yoga and mindfulness from an indigenous perspective from the Indian sub-continent where these methods originated, along with a discussion on how it contributes to successful aging. Specifically, yoga of immortals from a life course perspective and the concept of "ichha mrutyu" (living a full and healthy life and leaving the body when one has finished living a full and healthy life in a manner and at a time of one's own choosing – not to be confused with euthanasia) versus death (where one has no control) will be discussed. Simultaneously, the authors will examine how these concepts have been adopted in the West and the gaps that arise from this translation of these conceptualizations from East to West. Consequently, the validity and vigor of research, teaching and practice of yoga and mindfulness-based interventions for successful aging will be strengthened.

### SESSION 7345 (POSTER)

Abstract citation ID: igaf122.2959

### EPIDEMIOLOGY A METABOLITE SCORE EXPLAINS RELATIONSHIPS OF UNINTENTIONAL WEIGHT LOSS WITH MORTALITY AND MOBILITY LIMITATION

Shanshan Yao[1], Megan Marron[2], Samaneh Farsijani[2], Iva Miljkovic[2], George Tseng[2], Ravi Shah[3], Venkatesh Murthy[1], and Anne Newman[2], *1. University of Michigan, Ann Arbor, Michigan, United States, 2. University of Pittsburgh, Pittsburgh, Pennsylvania, United States, 3. Vanderbilt University Medical Center, Nashville, Tennessee, United States*

Unintentional weight loss (UWL) is related to mortality and functional decline. Using 77 metabolites associated with UWL risk in the Health, Aging and Body Composition participants, we investigated whether a composite metabolite score explained the relationship of UWL with mortality and mobility limitation. First, we selected 27 metabolites associated with incident UWL (>3% annual loss vs weight stable) using penalized logistic regression. Next, we created an UWL metabolite score, sum of 27 standardized metabolites weighted by their association with incident UWL, with a higher score indicating a greater potential risk for UWL. Among 2,286 participants (mean age 75, 37% Black, 51% women), those with UWL had 60% higher mortality and 25% higher hazard of mobility limitation risk after weight loss compared with weight-stable individuals, adjusting for age, race, sex, and study site. Older adults with one-SD higher UWL metabolite score had significantly higher mortality (HR = 1.44 [1.23, 1.52]) and mobility limitation risk (HR = 1.23 [1.15, 1.32]). The UWL metabolite score attenuated 39% of the relationship UWL and mortality, greater than 11% attenuated by traditional risk factors (i.e. demographics, lifestyle, and health conditions) and 16% by clinical biomarkers (cholesterol, glucose, kidney function, and inflammation biomarkers). Similarly, the UWL metabolite score attenuated 27% of the relationship between UWL and mobility limitation risk, contributing an additional 22% beyond traditional risk factors. This targeted metabolic characterization of UWL captures a substantial portion of the mortality and mobility limitation risks not explained by commonly measured health indicators, highlighting its potential value in risk assessment among older adults.

Abstract citation ID: igaf122.2960

### ASSOCIATION OF URINARY BIOMARKERS AND JOINT LONGITUDINAL COGNITIVE-GAIT TRAJECTORIES: HEALTH ABC FINDINGS

Aman Shrestha[1], Michelle Shardell[2], Chixiang Chen[2], Stephen L. Seliger[2], Charles Ginsberg[3], Lindsay Miller[3], and Peggy Cawthon[4], *1. University of Maryland, Baltimore County, Baltimore, Maryland, United States, 2. University of Maryland School of Medicine, Baltimore, Maryland, United States, 3. University of California, San Diego, San Diego, California, United States, 4. California Pacific Medical Center, San Francisco, California, United States*

Emerging urinary biomarkers are unlocking novel insights into the association between kidney tubular injury and cognitive-physical function. Using data from the Health, Aging and Body Composition Study, we examined associations between baseline urinary markers—uromodulin, alpha-1 microglobulin (α1M), amino-terminal propeptide of type-III procollagen (PIIINP), neutrophil gelatinase-associated lipocalin (NGAL), interlukin-18 (IL-18) and kidney injury molecule-1 (KIM-1)—with simultaneous longitudinal cognition-gait trajectories among initially high-functioning older adults. IL-18 and KIM-1 were analyzed in a larger ancillary study (n = 1902), while uromodulin, α1M, PIIINP and NGAL were assessed in a random sub-cohort (n = 502). Grouped-based trajectory analysis of 20-meter usual gait speed (years 3-6, 8, 10) and modified mini-mental state (years 3, 5, 8, 10) identified three groups: Group 1 with consistently high cognitive-physical performance, Group 2 with stable cognition but declining gait, and Group 3 with rapid declines in both domains. In separate models (Model-1, unadjusted), higher α1M, PIIINP, IL-18 and KIM-1 concentrations were associated with poorer trajectories. After adjusting for sociodemographic factors (model-1A), only α1M ($p = 0.043$) and KIM-1 ($p = 0.005$) remained significant. Further adjustment for estimated glomerular filtration rate (eGFR), urine albumin-to-creatinine ratio (UACR) and c-reactive protein (CRP) (Model-1B) attenuated all associations, leaving only KIM-1 marginally significant ($p = 0.059$). In a fully adjusted model that included all urinary markers (Model-2), only KIM remained individually significant ($p = 0.012$), though the collective effect of six urinary markers was also significant ($p = 0.022$). Of all urinary biomarkers, KIM-1 emerged as the robust predictor of cognitive-gait decline in understanding the interplay between kidney tubular injury and aging-related functional outcomes.

Abstract citation ID: igaf122.2961

### CYTOMEGALOVIRUS, MIDLIFE INFLAMMATION, AND SUBCLINICAL CARDIOVASCULAR DISEASE: THE SWAN STUDY

Aleda Leis[1], Michelle Hood[2], Wade Sanders[1], Laura McLees[1], and Carrie Karvonen-Gutierrez[1], *1. University of Michigan, Ann Arbor, Michigan, United States, 2. University of Michigan School of Public Health, Ann Arbor, Michigan, United States*

Cytomegalovirus (CMV) is a common beta herpesvirus with over half of US adults infected by age 40. Following an often asymptomatic acute infection, the virus remains latent with the potential for reactivation across the lifecourse. We examined associations of CMV seropositivity with the inflammatory marker c-reactive protein and carotid intima media thickness (cIMT) in the Michigan site of the Study of Women's Health Across the Nation (SWAN). Qualitative serum CMV immunoglobulin G (IgG) and M (IgM) assays were conducted in 100 pre- or peri-menopausal women (50 White, 50 Black, mean age 48.3 years) with carotid artery ultrasounds approximately 10 years later. Latent CMV seropositivity was defined as being IgG positive and IgM negative. Overall, 61% of samples were seropositive for latent CMV infection, with higher CMV seropositivity in Black (78%) than White (44%) women. Those with latent CMV seropositivity had almost two times higher C-reactive protein levels those without (8.7mg/L (standard deviation (SD):14.4) vs. 4.6mg/L (SD:4.7)). Latent CMV seropositivity was associated with future subclinical cardiovascular risk. Those with CMV seropositivity had larger (worse) average carotid intima media thickness (cIMT) (mean 0.86mm (SD:0.12) vs. 0.76mm (SD:0.10); $p < 0.001$) and maximal cIMT (mean 1.01mm (SD:0.14) vs. 0.90mm (SD:0.1); $p < 0.001$) compared to those who were not CMV seropositive. Results were similar when stratified by race. Together, the results suggest CMV seropositivity may be associated with greater inflammation and cardiovascular risk as women enter older adulthood.

Abstract citation ID: igaf122.2962

### DIETARY HABITS IN THE OFFSPRING AND GRANDCHILDREN OF LONG-LIVED SIBLINGS

Katrine Kristensen[1], Jacob Pedersen[1], Matthew Keys[1], Jytte Halkjær[2], Anne Tjønneland[2], Mary Wojczynski[3], Kaare Christensen[1], and Christine Dalgård[4], *1. University of Southern Denmark, Odense M, Syddanmark, Denmark, 2. Danish Cancer Society, Copenhagen, Hovedstaden, Denmark, 3. Washington University School of Medicine, St. Louis, MO, Missouri, United States, 4. Clinical Pharmacy, Pharmacology and Environmental Medicine, Odense M, Syddanmark, Denmark*

Descendants of Danish longevity-enriched sibships demonstrate a general health and survival advantage throughout their lives. Diet may be one contributing mechanism. This study evaluates the dietary habits by estimating whether 203 descendants (median age 50, range 18-67, 58% females) of long-lived siblings in the Long Life Family Study adhered more to the official Danish dietary guidelines than 1015 age- and sex-matched population controls. From a comprehensive food frequency questionnaire (366 questions), participants' adherence to dietary guidelines was calculated using a continuous score ranging from 0 (no adherence) to 6 (maximum). Data were collected from 2014 to 2018 (controls) and 2019 to 2023 (descendants). The median adherence score for male descendants was 4.2 (interquartile range 3.7-4.6) compared to 4.6 (4.1-4.9) for male controls, while the median score for female descendants was 4.9 (4.4-5.3), the same as for female controls. The adherence score for male descendants was 0.34 points lower than that for male controls (95% confidence interval -0.51;-0.18), with no significant difference for women (0.03, CI -0.11;0.16). When adjusting for birth year, the adherence score was 0.43 points lower for male descendants (CI -0.85;-0.02) and 0.10 points higher for women (CI -0.23;0.43). Descendents had a higher body mass index (men mean difference 0.7 kg/m2 (-0.2;1.6), women 0.6 kg/m2 (-0.3;1.6)), and fewer were current smokers (male descendants 15.1% vs 17.9% for the controls ($p = 0.641$); women 6.8% vs 13.8%($p = 0.053$). In conclusion, the study found no evidence that descendants of long-lived siblings adhere more to the Danish dietary guidelines than a population-based control group.

Abstract citation ID: igaf122.2963

### DIVERSITY IN SOCIAL NETWORKS AND BRAIN AGING IN COMMUNITY-DWELLING OLDER ADULTS IN JAPAN: THE NEIGE STUDY

Hiroshi Murayama[1], Shiho Amagasa[2], Masaki Machida[3], Shigeru Inoue[3], Takeo Fujiwara[4], and Yugo Shobugawa[5], *1. Tokyo Metropolitan Institute for Geriatrics and Gerontology, Tokyo, Japan, 2. Teikyo University, Tokyo, Tokyo, Japan, 3. Tokyo Medical University, Tokyo Medical University, Tokyo, Japan, 4. Institute of Science Tokyo, Tokyo, Tokyo, Japan, 5. Niigata University, Niigata, Niigata, Japan*

As the global population ages, understanding factors influencing brain health in older adults is crucial due to its impact on cognitive decline. Age-related changes in key regions like the hippocampus, gray matter, and prefrontal cortex are associated with cognitive decline. This study investigated the influence of social network structure on brain aging in older Japanese adults, focusing on regions linked to cognitive decline. We used longitudinal data from the Neuron to Environmental Impact across Generations Study (NEIGE Study), which is a prospective cohort study of randomly-sampled community-dwelling individuals aged 65–84 years living in Tokamachi City, Niigata Prefecture, Japan. The baseline survey was conducted in 2017 and included 527 independent older people. The follow-up survey was completed in 2021. This study analyzed 279 who had magnetic resonance imaging at both baseline and follow-up (47.6% men; mean age: 73.0 ± 5.1 years). Social network homogeneity/heterogeneity was assessed based on similarities or diversity in relationships, and factor analysis identified two network types. Multiple linear regression analyses with inverse probability weighting indicated that homogeneous social networks were not associated with brain volume changes. However, lower heterogeneity in social networks was linked to greater hippocampal volume decline, after adjusting for sociodemographic factors and health behaviors. This association was prominent in right hippocampus. No significant associations were observed for gray matter or prefrontal cortex. More heterogeneous social networks may help mitigate age-related decline in hippocampal volume in older adults, suggesting the cognitive health benefits of social diversity. Further research is needed to explore underlying mechanisms.

Abstract citation ID: igaf122.2964

### HETEROGENEITY IN AGING: EXPLORING GRIP STRENGTH VARIABILITY IN POPULATION-BASED AGE-SEX COHORTS

Richard Fortinsky[1], Katherine Zavez[1], Lisa Barry[1], George Kuchel[2], and Ofer Harel[1], *1. University of Connecticut, Farmington, Connecticut, United States, 2. UCONN HEALTH, Farmington, Connecticut, United States*

Heterogeneity in aging is defined as variability in characteristics associated with chronological age. The concept of increased heterogeneity of health-related characteristics with increasing age has long been recognized as a tenet of geriatric medicine. However, a surprisingly limited amount of research has tested this concept. We used grip strength, an objective measure of physical function and frailty, to explore patterns of grip strength variability in population-based age-sex cohorts cross-sectionally and longitudinally. We constructed a study cohort from the UK Biobank database consisting of adults (52% female) with two measures of grip strength ($n = 73{,}134$). Age groups at initial measurement were: 40-44, 45-49, 50-54, 55-59, 60-64, and 65-69. In females and males separately, we compared: (1) patterns of grip strength variability across successively older age groups at one time point; and (2) changes over time in grip strength variability for individuals within the same age group cohort. Cross-sectional results revealed that, for females, grip strength variability did not differ significantly between pairs of successively older age groups; for males, we found statistically significant decreases in grip strength variability across successively older age groups. Longitudinal results showed that grip strength variability did not change over time within age group cohorts among females. In contrast, grip strength variability increased over time within nearly all age group cohorts among males. We conclude that heterogeneity in grip strength with aging operates differently for females and males, and that patterns of heterogeneity for males are strikingly different depending on whether age is explored cross-sectionally or longitudinally.

Abstract citation ID: igaf122.2965

**METHODS TO ASSESS BIAS AND POOL RESULTS OF STUDIES OF DRUGS ASSOCIATED WITH FALL-RELATED INJURIES**

Jimmie Roberts[1], Andrew Zullo[2], and Sarah Berry[3], *1. Hebrew SeniorLife, Boston, Massachusetts, United States, 2. Brown University, Providence, Rhode Island, United States, 3. Hebrew SeniorLife & Harvard Medical School, Hebrew SeniorLife, Massachusetts, United States*

Studies of older adults could benefit from an index that quantifies the cumulative effects of medications on fall outcomes, but no such evidence-based, composite outcome measure exists. Our objective was to develop an index for the burden of fall-related injury (FRI)-inducing medications while adjusting estimates to account for potential bias in the existing literature. A literature review was conducted to identify full-text studies estimating the effects of medications on FRIs from January 1990 to present. Studies were assessed for bias by a trained epidemiologist using either the Risk of Bias in Non-randomized Studies of Interventions (ROBINS-I) or the Revised Cochrane risk-of-bias (RoB) tool for randomized controlled trials (RCTs). Each effect estimate was then corrected for the degree of potential bias assessed – a correction determined by the research team (e.g., moderate bias=20% reduction). Corrected estimates were standardized, then pooled into a single summary estimate for each medication class. In total, 106 articles were identified: 75 observational studies and 5 RCTs were assessed for RoB. Among observational studies, 67 had serious RoB, 8 had moderate RoB, and none had low or critical RoB. Among RCTs, 4 had some concerns, 1 had low RoB, and none had high RoB. For bias among 5 observational studies of statins with FRIs, statins increased the risk of FRIs by 151% before and 91% after bias correction. Correcting for potential bias ensures our index is robust despite heterogeneous findings. Our approach to developing this index could be extended to assess other critical outcomes like major bleeds.

Abstract citation ID: igaf122.2966

**OLFACTION AND SARCOPENIA IN OLDER ADULTS: A LONGITUDINAL STUDY**

Rui Liu[1], Honglei Chen[2], Chenxi Li[2], Anna Kucharska-Newton[3], Eleanor Simonsick[4], and Yaqun Yuan[2], *1. Sacred Heart University, Fairfield, Connecticut, United States, 2. Michigan State University, East Lansing, Michigan, United States, 3. The University of North Carolina at Chapel Hill, Chapel Hill, North Carolina, United States, 4. National Institute on Aging, Baltimore, Maryland, United States*

Olfactory impairment is associated with greater weight loss in older adults. We examined olfaction in relation to sarcopenia in 2503 participants (aged 71-82, 51.7% women, and 38.4% Black) of the Health, Aging, and Body Composition study. Olfaction was assessed in Year 3 using the 12-item Brief Smell Identification Test, categorized as good (test score: 11-12), moderate (9-10), hyposmia (7-8), and anosmia (0-6). We measured sarcopenia as slow gait speed (20-meter usual walking speed < 0.8 meters/second) or low muscle strength (grip strength < 35.5 kg for men and < 20 kg for women), which were assessed annually/biennially for up to 7 years. We conducted joint model analyses to account for covariates and potential bias due to attrition. Compared with participants with good olfaction, the multivariable adjusted odds ratio (OR) and 95% confidence interval (CI) associated with slow gait speed at baseline was 1.05(0.86, 1.29) for moderate olfaction, 1.13(0.87, 1.46) for hyposmia, and 1.98(1.50, 2.61) for anosmia. Notably, associations strengthened over time. At the end of follow-up, corresponding ORs increased to 1.80(1.17, 2.76) for moderate olfaction, 2.68(1.54, 4.67) for hyposmia and 3.74(2.09, 6.71) for anosmia. For muscle strength, we only found an association with anosmia but not hyposmia or moderate olfaction. At the end of the follow up, compared to those with good olfaction, the ORs for low muscle strength were 1.32(0.80, 2.19) for moderate olfaction, 1.19(0.63, 2.24) for hyposmia, and 3.12(1.48, 6.55) for anosmia. Poor olfaction was associated with markers of sarcopenia in older adults, particularly reduced mobility.

Abstract citation ID: igaf122.2967

**SENMAYO BASED AGING SIGNATURE (SEN-AGE) IN CANCER SURVIVORS: THE HEALTH AND RETIREMENT STUDY (HRS)**

Gokul Seshadri, Shuo Wang, Weihua Guan, Bharat Thyagarajan, and Anna Prizment, *University of Minnesota, Minneapolis, Minnesota, United States*

The SenMayo gene set identifies senescent cells, one of the hallmarks of aging, especially among those with cancer. In the HRS cohort, we constructed a SenMayo based aging signature (Sen-Age) among those without cancer (controls) and examined its association with 4-year mortality in cancer survivors. In the 2016 wave, HRS measured 125 SenMayo genes in blood samples. We applied Lasso regression to participants without cancer in 2016 (N = 3076) and trained SenMayo genes against chronological age; this resulted in a subset of 97 genes with which we created Sen-Age. This Sen-Age signature demonstrated a correlation of 0.46 with chronological age in 609 cancer survivors. We then examined the associations of Sen-Age with 4-year mortality (N = 112) in cancer survivors using Cox Proportional Hazards regression model after adjusting for age, sex, and race. Sen-Age was associated with increased risk of mortality in cancer survivors (HR: 1.47 [1.17–1.85] per 1 year, p = 0.0008). This association remained robust (HR: 1.31 [1.04–1.66], p = 0.02) upon additional adjustment for education level, smoking status, BMI, inflammation and comorbidities. Moreover, cancer survivors have higher Sen-Age score (adj mean: -0.44 ) than non-cancer controls (adj mean: -0.83). Overall, our findings highlight the potential of the Sen-Age signature as a biomarker of aging and mortality in cancer survivors.

Abstract citation ID: igaf122.2968

**THE IMPACT OF T CELL REPERTOIRE ON MORTALITY: FINDINGS FROM THE HEALTH AND RETIREMENT STUDY**

Zach Flaten[1], Ryan Martinez[1], Sithara Vivek[1], Shijia Zhu[1], Jessica Faul[2], Eileen Crimmins[3], Gokul Seshadri[1], and Bharat Thyagarajan[1], *1. University of Minnesota, Minneapolis, Minnesota, United States, 2. University of Michigan, Ann Arbor, Michigan, United States, 3. University of Southern California, Los Angeles, California, United States*

The T and B cell repertoire is the sum of all the T-cell receptors (TCRs) and B cell receptors (BCRs) in an individual and is an indicator of the cumulative exposure of immune challenges over the life course. The biological importance of T/B cell repertoire diversity in a general population of older adults remains unclear. In the Health and Retirement Study, we used MiXCR on bulk RNAseq data to estimate T/B receptor chain numbers (total clones) and T/B receptor chain diversity (number of unique clones) for 3,758 participants. We evaluated the demographic (age, sex, race) and other factors (smoking, CMV seroprevalence) associated with T/B cell repertoire using type-II ANOVA. We used unconditional logistic regression models to evaluate the association between T/B cell repertoire and 4-year mortality. All the models were adjusted for age, sex, race, CMV seropositivity, smoking status, total number of sequencing reads, and total T cell/ B cell counts. The number and diversity of T and B cell receptor chains identified were higher among African Americans as compared to other racial groups. The number and diversity of TCR chains were more common in women, elevated in CMV seropositive individuals. TCR chains were more common in never smokers, but the TCR diversity was higher in current smokers. Moreover, higher numbers of TCR chains (OR: TRA=0.74;p=0.0008, TRB=0.76;p=0.0018) and TCR clonal diversity (OR: TRA=0.66; $p < 0.0001$, TRB=0.7; $p < 0.0001$) were significantly associated with reduced mortality. Further research is needed to understand mechanisms by which TCR impact population health.

Abstract citation ID: igaf122.2969

### WORLD TRADE CENTER DISASTER: A QUALITATIVE STUDY OF OLDER ENROLLEES IN THE WORLD TRADE CENTER HEALTH REGISTRY

Robert Brackbill[1], Alexis Merdjanoff[2], Lydia Leon[1], Janna Metzler[1], Felix Ortega[1], Ashley Rojas[3], and Ndidi Umezinwa[1], *1. New York City Department of Health and Mental Hygiene, Long Island City, New York, United States, 2. New York University, New York, New York, United States, 3. New York City Department of Health and Mental Hygiiene, Long Island City, New York, United States*

The World Trade Center disaster on September 11, 2001 has had long-lasting effects on the health and quality of life of those exposed to the disaster, including lung problems, GERD, cardiovascular diseases, PTSD, depression and other ailments that are likely to worsen with age. The World Trade Center Health Registry, a longitudinal exposure cohort of 71,436 persons who were working or living in the impacted area, has embarked on a mixed model study of older persons in the Registry (55+) that includes a qualitative study followed by survey-based data collection, and in-person cognitive assessment. Recruitment for the qualitative study consisted of a random sample of enrollees 55 years and older stratified by age and sex with a cooperation rate of 67%. This presentation will describe the findings from 50 in-depth interviews with Registry enrollees that explored topics including activities and interpersonal relationships; current health and cognitive issues; stress and well-being; quality of life; the impact of 9/11 on aging; and the use of technology to support health. An overarching theme was that more than twenty years later these respondents continue to grapple with their 9/11 experience. For many, 9/11 was a key turning point in their lives that influenced how they approach growing older; their relationships with others; their own mortality; and, their concerns for their health in the future. This study further supports the need for long-term data collection and analysis of exposed cohorts following a collective trauma like 9/11, especially as the populations grow older.

### SESSION 7350 (POSTER)

Abstract citation ID: igaf122.2970

### FAMILY CAREGIVING III A TELEPHONE & ONLINE INTERVENTION FOR SPANISH-SPEAKING POST-STROKE CAREGIVERS: ENHANCING SELF-EFFICACY

Janet Lopez[1], Melanie Orejuela[2], Nathaniel Eliazar-Macke[3], Arkaprava Roy[4], Tatiana Orozco[2], and I Freytes[2], *1. University of Central Florida, Orlando, Florida, United States, 2. US Department of Veterans Affairs, Gainesville, Florida, United States, 3. Veterans Health Administration, Miami, Florida, United States, 4. University of Florida, Gainesville, Florida, United States*

Caregivers play a crucial role in managing the health and well-being of Veterans with stroke. However, the demands of caregiving can significantly impact their self-efficacy, which is essential for effective caregiving. While there is some evidence suggesting interventions can improve self-efficacy among stroke caregivers, little is known about self-efficacy among Spanish speaking caregivers of Veterans. We report the impact of a problem-solving telephone and online intervention on Spanish-speaking caregivers of Veterans post-stroke's self-efficacy. The study was a two-arm parallel randomized controlled trial with repeated measures and mixed methods that tested the effect of a telephone and online problem-solving intervention for Spanish-speaking stroke caregivers. We utilized the Caregiver Self-Efficacy tool (Spanish Version), a 15-item measure assessing caregivers' confidence in obtaining respite, responding to disruptive patient behaviors, and controlling upsetting thoughts. Self-efficacy was assessed at baseline, post-intervention 1, and post-intervention 2 in 105 participants. Wilcoxon rank sum test for statistical analysis. At baseline, there were no significant differences between the intervention and standard care groups across all domains. Post-intervention 1 showed slight improvements in the intervention group, though not statistically significant. By post-intervention 2 (12 weeks), significant improvements were observed in the intervention group across all domains: obtaining respite care ($p = 0.003$), responding to disruptive behaviors ($p = 0.018$), and controlling thoughts ($p = 0.045$). The intervention significantly enhanced caregiver self-efficacy over time, particularly post intervention. Findings suggest that structured support and training can effectively boost Spanish-speaking caregivers' confidence in managing caregiving tasks, ultimately improving Veterans and caregiver well-being.

Abstract citation ID: igaf122.2971

### ADAPTING WITH AGE: PERSPECTIVES ON CARETAKING IN OLDER ADULT ROMANTIC RELATIONSHIPS

Katelyn Vozza, Jillian Rants, Melinda Heinz, and William Henninger, *University of Northern Iowa, Cedar Falls, Iowa, United States*

Establishing and maintaining quality romantic relationships is an important part of older adulthood. However, older adults experience unique challenges related to health as they explore potential relationships that add meaning to their lives. The growing likelihood of health conditions may change relationship dynamics and introduce a caretaking dynamic into their relationships. This study investigated the differences in how a caretaking role is perceived among single older adults and those in committed relationships, or pursuing new relationships. Interviews were conducted from a sample of 20 older adults aged 65 + (N = 10 single, N = 10 committed relationships) who completed a survey on dating and relationships. Interviews were transcribed verbatim and analyzed using Braun and Clarke thematic analysis approach. Participants' experiences related to past and current relationships often included caretaking responsibilities of a partner. Findings suggested that older adults in long-term committed relationships adapted to the changing health and needs of their partner, stating, "It's something I kind of wish I wouldn't have to do, but there have been times where I've been ill and he's taken care of me, so…fair play." Single/widowed older adults, however, viewed caretaking as a significant concern, "It was so hard to be supportive of somebody with a disability, and I just don't think I could do it again." Women in particular may be hesitant towards new caregiving responsibilities in later life, suggesting that couples may want to proactively discuss these concerns while seeking out romantic partners, and negotiate attitudes of independence and compromise.

Abstract citation ID: igaf122.2972

### AFRICAN AMERICAN RURAL CAREGIVING: ASSESSING NEEDS AND IDENTIFYING SOLUTIONS

Laura Ainsworth, Scott Wilks, and Rhonda Bardales, *Louisiana State University, Baton Rouge, Louisiana, United States*

Uncompensated caregiving for Americans with Alzheimer's disease and related dementias (ADRD) is valued at $339.5 billion annually. With the national rise in older adults reaching 73 million by 2030, the prevalence of individuals with ADRD and their care partners also will rise. Underrepresented care partners (e.g., ethnic minority and non-urban residents) face caregiving burdens unique to cultural identities and geographical contexts. Yet, a dearth of literature exploring their experiences is glaring. The purpose of this study is to redress this gap, to gain a qualitative, meaningful understanding of caregiving perceptions and experiences among rural African American (RAA), ADRD care partners. Three separate, 2-hour focus groups were conducted with RAA ADRD care partners (N = 31) in south Louisiana to identify thematically specific caregiving needs and perceptions of resources to alleviate said needs. Prior to group discussions, participants completed a questionnaire with demographics and measures of anxiety, depression, and social isolation. Constructivist grounded theory methods were used to analyze narrative group data to construct concepts based on care partner perspectives, noting consistent themes. One-quarter of the sample reported symptoms of anxiety and depression;15% reported social isolation. The following themes were identified: lack of household role balance; negativity toward caregiving duties; lack of self-care; hardships exclusive to ethnic and rural cultural differences; and technology needed to lessen strain. This study revealed the daily struggles faced by an underresearched group of ADRD care partners. Highlighting technological innovations to address their struggles faced revealed a potential direction for African American, ADRD care partners in rural communities.

Abstract citation ID: igaf122.2973

### AN INNOVATIVE APPROACH TO DEVELOPING EDUCATIONAL MATERIALS FOR VETERANS' CAREGIVERS USING CO-DESIGN

Jacqueline Boudreau[1], Lynette Kelley[2], Aileen McGrory[1], Marianne Desir[3], and Eileen Dryden[1], *1. Veterans Health Administration, Bedford, Massachusetts, United States, 2. Veterans Health Administration, Aurora, Colorado, United States, 3. Veterans Health Administration, Miami, Florida, United States*

Informal caregivers are invaluable members of many older adults' care teams. Supporting caregivers improves their wellbeing and engagement with patient care teams, improving patient outcomes including longevity of aging in place. The Veterans Health Administration (VA) houses an array of caregiver resources which many caregivers are unaware of. Further, caregiver perspectives have had limited integration into caregiving educational materials. Thus, to increase caregiver engagement and connection to resources, we co-designed caregiver-facing educational materials with caregivers of VA-enrolled Veterans. Caregivers were oriented to project goals and design principles. They contributed to every dimension of development: content, messaging, design, and tailoring by media type. Researchers and caregivers used these dimensions to structure monthly virtual meetings, during and after which researchers summarized and incorporated caregiver feedback, noting any exclusions and rationale (e.g., outside of project's scope). Caregivers noted that meetings were well organized and enjoyed paying their knowledge forward to help peers. Reviewing how their feedback was integrated made caregivers feel valued. Orientation to design and live edits helped caregivers envision their suggestions. Given that caregivers value learning from peers, materials made with caregivers for caregivers are likely to be more appealing, accessible, and useful. Notably, our four caregiver co-designers were White; three were wives and one an adult daughter of VA-enrolled Veterans. Future research should replicate this process with caregivers in different contexts. Co-design with recent caregivers provides them a meaningful experience and leverages their collective wisdom to empower other caregivers to sustain themselves and the older adults they care for.

Abstract citation ID: igaf122.2974

### CAREGIVER VERSION SHARED DECISION-MAKING QUESTIONNAIRE FOR ADULT CHILD AND PARENTS WITH DEMENTIA: A DELPHI STUDY

Liu Huanran, and Vivian Lou, *The University of Hong Kong, Hong Kong, China (People's Republic)*

Adult children constitute the largest group of family caregivers globally and play a pivotal role as primary decision-makers and long-term caregivers for parents with dementia. While shared decision-making (SDM) traditionally involves patients and physicians in clinical settings, dementia care often extends beyond formal healthcare, with decisions about care arrangements, daily care, medication, and finances frequently made by people with dementia and adult child caregivers. Despite the importance of SDM in dementia care, existing research has primarily focused on physician-patient dynamics, leaving a significant gap in understanding patient-caregiver SDM. To address this gap, we adapted the Shared Decision-Making Questionnaire (SDM-Q) into an adult-child caregiver version and evaluated its content validity through a two-round online Delphi study. This adapted tool aims to assess caregivers' perspectives on decision-making with their parents. After translation and a focus group with seven caregivers, nine experts (three researchers, four practitioners, and two caregivers) evaluated the scale's importance, relevance, and clarity using a nine-point Likert scale. Changes, such as replacing "patient" with "my father/mother," were made based on expert feedback to reduce stereotypes. In the first round, item-level content validity indices (I-CVI) ranged from 0.78 to 1 across domains, with Ave-CVI scores of 0.93 (importance), 0.94 (relevance), and 0.89 (clarity). All items achieved moderate to high consensus. In the second round, I-CVI and Ave-CVI improved to 1, indicating excellent content validity and high expert consensus. These results suggest the SDM-Q-Carer is a valid tool for exploring decision-making involvement among adult child caregivers, though further validation is recommended.

Abstract citation ID: igaf122.2975

### CHARACTERIZING KEY DRIVERS OF RESPITE USE AMONG VETERAN CAREGIVERS

Melissa Harris-Gersten[1], Colleen Blue[2], Susan Hastings[1], Zhen Li[1], Jeanie Lo[3], Nadya Majette[1], Jennifer Zervakis[1], and Megan Shepherd-Banigan[4], *1. Durham VA Healthcare System, Durham, North Carolina, United States, 2. Durham VA Health Care System, Durham, North Carolina, United States, 3. Veterans Health Administration, Menlo Park, California, United States, 4. Duke University, Durham, North Carolina, United States*

Respite care has been shown to reduce caregiver burden and may delay long-term care placement, yet it remains underutilized, with only 12-15% of US caregivers reporting use of these services. Understanding drivers of respite use is critical to informing initiatives to improve access. This convergent mixed-methods study aimed to characterize factors influencing respite use among caregivers and Veterans enrolled in the Veterans Health Administration's (VHA) Caregiver Support Program. A retrospective analysis used machine learning (random forests) to analyze caregiver survey data (2018–2021) linked to Veteran electronic health records (N = 1,727), ranking Veteran and caregiver factors associated with respite use. Semi-structured interviews were conducted with a separate sample of caregivers (N = 20) in 2024, with transcripts analyzed using directed content analysis. Data were integrated using Andersen's Behavioral Model for Health Service Use. Top predictors included Veteran's functional limitations, age, health risk, and caregiver's age and caregiver perceived integration of care with the clinical team. Enabling factors included in-home care support and financial resources; need factors encompassed caregiver burden, depression, loneliness, and Veteran's chronic conditions, dementia, and frailty. Qualitative findings reinforced the importance of Veteran and caregiver needs while highlighting how VHA's respite payment model alleviates financial strain. Caregivers described psychosocial and system-level factors, including lack of awareness, concerns about access and quality, and scheduling barriers as reasons not to use respite. Findings provide actionable insights for improving respite uptake by enhancing caregiver-clinician communication, tailoring services, and increasing outreach. Future research should examine how these factors impact respite use in non-VHA settings.

Abstract citation ID: igaf122.2976

### FAMILY COMMITMENT, APPRECIATION, AND THE PURSUIT OF FUTURE GOALS AMONG YOUNG ADULT CAREGIVERS

Maya Brown[1], Sibo Gao[2], and Karen Fingerman[1], *1. The University of Texas at Austin, Austin, Texas, United States, 2. University of Texas at Austin, Austin, Texas, United States*

Up to 10 million young adults are involved in caregiving for older family members with physical or cognitive impairments, but it is not clear whether these young adults feel acknowledged for their efforts. Family commitment may foster feelings of appreciation within the caregiving network. However, this sense of commitment and feeling appreciated could hinder the pursuit of future goals (e.g., education, romantic relationships) due to their greater investment in providing care. African American, Hispanic, Asian American and non-Hispanic White caregivers aged 18 to 29 (N = 141) of family members aged 65+ completed a 30-minute survey regarding caregiving tasks, family commitment, feelings of appreciation from other members of the caregiving network, ability to pursue future goals, and background information. Linear regression models revealed that young adult caregivers with higher family commitment reported greater feelings of appreciation from others in their caregiving network, controlling for caregiving tasks. Contrary to the hypothesis, young adult caregivers reported a greater ability to pursue future goals when they had higher family commitment and felt more appreciated. Findings suggest that young adult caregivers may feel more appreciated when their values (i.e., commitment) are consistent with their behaviors (i.e., providing care for older family member). This sense of appreciation may enhance their pursuit of future goals, likely because they integrate caregiving into their identity rather than viewing it as nonnormative. These issues may be more specific to young adults than midlife and older caregivers and warrant distinct interventions.

Abstract citation ID: igaf122.2977

### IDENTIFYING COMMUNITY-BASED NEEDS OF OLDER FAMILY CAREGIVERS: A SYSTEMATIC REVIEW & META-SYNTHESIS

Arielle Galinsky[1], Clara Sorkin[1], and Elizabeth Marfeo[2], *1. Tufts University Health & Productive Aging Lab, Medford, Massachusetts, United States, 2. Tufts University, Medford, Massachusetts, United States*

The demand for family caregiving in the United States is significant, with millions providing unpaid care for individuals with dementia, often encountering substantial emotional, financial, and physical challenges. Expanding home and community-based support services is essential to reducing family caregiver burden, improving the quality of life for individuals with dementia, facilitating aging in place, and decreasing dependence on long term institutional care. A systematic review and meta-synthesis of qualitative research was conducted to examine unmet needs among older adult family caregivers of individuals with dementia. A comprehensive search strategy was implemented across multiple electronic databases spanning medical, psychological, social, and nursing sciences from 2014 to 2025. A total of 33 studies met inclusion criteria, representing perspectives from 879 family caregivers aggregated across all studies included. Key themes identified include: (1) the influence of cultural and social factors on coping strategies and access to support; (2) the evolving dyadic nature of caregiving as dementia progresses; and (3) the prevalence of unmet emotional and mental health needs. These findings provide a robust evidence base for informing the development of targeted home and community-based support programs, as well as future research initiatives aimed at addressing the diverse needs of older adult family caregivers of individuals with dementia.

Abstract citation ID: igaf122.2978

**IDENTITY RESOLUTION IN THE CONTEXT OF DEMENTIA CAREGIVING: A LONGITUDINAL STUDY**

Lauren Mitchell, Miriam Likhterman, Laura Sanchez Concepcion, Shaye Child, Zena Halwani, and Grace Kavanagh, *Emmanuel College, Boston, Massachusetts, United States*

Becoming a caregiver for a loved one with dementia involves identity adjustment as caregivers' self-concept shifts to accommodate their new role. Most research on caregivers' identities has relied on qualitative methods; there is a need for quantitative, longitudinal research on caregivers' identities over time. The present study used a validated measure rooted in Erikson's (1968) classic theory of identity development to estimate trajectories for caregivers' identity resolution. Caregivers (N = 81) of individuals with mild-to-moderate cognitive impairment completed the identity subscale of the Erikson Psychosocial Stage Inventory (Rosenthal et al., 1981), assessing overall sense of self-continuity and identity coherence, at 0, 3, and 6 months. Latent growth curve models estimated trajectories of change in identity resolution. Measures of caregiving tenure, depressive symptoms, and caregiver burden were included as moderators. Results indicated that caregivers on average experienced a high, stable level of identity resolution (I = 3.95, VarI=.34, S=-.001, VarS=.004). Time spent as a caregiver was not associated with level or change in identity resolution. Caregivers with greater depressive symptoms and higher burden at baseline experienced consistently lower identity resolution across the six-month period. Overall, caregivers' identities seem to be highly resilient. Despite the identity-related challenges documented in qualitative literature (e.g., new responsibilities; changing relationship to the care recipient), caregivers may dynamically adapt and retain a high level of self-continuity and a clear, coherent self-concept. Conversely, caregivers who experience depression and high burden may benefit from support focused directly on identity adjustment.

Abstract citation ID: igaf122.2979

**IMPACT OF CHRONIC PAIN ON CAREGIVING TASKS FOR DEMENTIA FAMILY CAREGIVERS**

Aryn Lee[1], Shelbie Turner[2], Karl Pillemer[3], and M. Carrington Reid[2], *1. Cornell University Medical College, New York, New York, United States, 2. Weill Cornell Medicine, New York, New York, United States, 3. Cornell University, Ithaca, New York, United States*

Recent evidence suggests pain is highly prevalent among dementia family caregivers, but questions about how caregivers' pain impacts care quality remain unanswered. Accordingly, we used a nationwide sample of dementia family caregivers with chronic pain to identify the extent to which caregivers attribute caregiving difficulty to pain. Caregivers (n = 269) reported whether they were responsible for helping care recipients with ten basic and instrumental activities of daily living (BADL & IADL), and whether they experienced difficulty completing each task. If they did report difficulty, they then rated how much of that difficulty they attribute to their pain. We first ran descriptive statistics to determine the prevalence of care task difficulty and mean pain-attributed difficulty and then ran ANOVA models to assess for between-group differences (e.g., racial differences) in pain-attributed difficulty with the care tasks. 205 caregivers helped their care recipient with at least one BADL and reported difficulty with an average of 2.65 BADLs, while 263 helped with at least one IADL and reported difficulty with an average of 2.90 IADLs. When asked about the extent to which pain contributed to the difficulty helping care recipients with BADL/IADLs, caregivers' average response was 6.81 for BADLs and 6.49 for IADLs. Compared to White caregivers, Black caregivers attributed less difficulty with both BADLs and IADLs to pain. Pain appears to be a major contributor to the difficulty dementia family caregivers experience when helping care recipients with daily activities, suggesting that pain is not only highly prevalent but also highly consequential to caregiving outcomes.

Abstract citation ID: igaf122.2980

**LONGITUDINAL IMPACT OF TRANSITION TO CAREGIVING ON COGNITIVE FUNCTIONING: A MATCHED CASE-CONTROL STUDY**

Joanne Elayoubi[1], William Haley[2], Monica Walters[3], Michael Crowe[4], and Gizem Hueluer[5], *1. University of South Florida, Tampa, Florida, United States, 2. University of South Florida, University of South Florida, Florida, United States, 3. University of Michigan, Ann Arbor, Michigan, United States, 4. University of Alabama at Birmingham, Birmingham, Alabama, United States, 5. University of Bamberg, Bamberg, Germany*

Objectives: Chronic stress has a strong theoretical link to poorer cognitive aging outcomes. Stress from caregiving, especially dementia caregiving, is associated with worse cognition; however, most prior studies have serious methodical limitations. We examined the longitudinal impact of transitioning into caregiving (including dementia caregiving) on cognition, compared to carefully matched non-caregiver controls and possible mediating role of depressive symptoms and perceived stress. Methods: Participants in the Caregiving Transition Study (CTS) who transitioned into caregiving (n = 251) were compared to sociodemographically and health-matched non-caregiving controls (n = 251). Data included 14 years of repeated assessments including timepoints before and after transitions on global cognition (six-item screener), episodic memory (word list learning, delayed word recall), and verbal fluency (letter and animal fluency). Results: Compared to non-caregivers, transitioned caregivers performed worse on episodic memory and global cognition after transitions with less decline in word list learning and global cognition post-transitions. All cognitive effects were small (Cohen's d approximately .2 SD units). In adjusted models for subgroup analyses, dementia caregivers performed worse after transitions than non-dementia caregivers in delayed word recall but better on animal fluency. Increases in depressive symptoms mediated the association between caregiving transitions and episodic memory but not global cognition. Perceived stress did not have a mediating role. Discussion: Caregiving stress was associated with worse cognition, but effects were domain-specific for memory and global cognition, and short-lived. Dementia vs. non-dementia caregiving also had differential effects on cognition. Future studies should examine whether these small, temporary declines in caregiver cognition improve with caregiver interventions.

Abstract citation ID: igaf122.2981

**STATE OF THE SCIENCE OF DEMENTIA CARE FOR ASIAN AMERICANS: INTEGRATIVE REVIEW**

Jiyoon Jang, Kalisha Bonds Johnson, Glenna Brewster, and Mi-Kyung Song, *Emory University, Atlanta, Georgia, United States*

Asian Americans account for >8% of persons living with Alzheimer's disease and related dementia (ADRD, PLwD) despite being < 6% of the nation's population. Many are cared for at home by family members or relatives, but the literature is sparse about their specific caregiving experiences. We conducted an integrative review of the existing literature to examine the extent of dementia caregiving research in this population, including study characteristics and empirical evidence of caregiving experiences. We searched PubMed, CINAHL, PsycINFO, Embase, and Web of Science for reports of studies of Asian American caregivers of PLwD conducted in the U.S. published in English between 1999 and 2024. The final sample included 35 articles: of which, 51.4% (n = 18) were conducted in California, and 51.4% (n = 18) were qualitative studies using semi-structured interviews and focus groups. Chinese and Korean caregivers were most frequently studied (14 and 12 articles, respectively) followed by Vietnamese caregivers. Seventeen (48.6%) articles reported a negative impact of caregiving on mental health, e.g., depression, burden, and anxiety. Caregiving challenges included limited access to culturally competent health and community services, distance from home, costs, and stigma. Caregiver intervention studies, (n = 8, 22.9%) including cognitive strategies, self-care, and religious practices demonstrated mixed outcomes in improving caregiver outcomes. Studies predominantly focused on negative outcomes of caregiving. Current evidence suggests that caregiving has a negative impact on the psychological health of Asian American caregivers. However, additional research is needed to examine positive effects of caregiving or more balanced perspectives and experiences of caregiving in this population.

Abstract citation ID: igaf122.2982

### SUPPORTING SPOUSES' CAREGIVING COMMUNICATION SKILLS IN DEMENTIA CARE: A COMMUNICATION PROCESS MODEL

Tyler Nesbit, Emma Bryan, Melissa Armstrong, Carma Bylund, Easton Wollney, and Carla Fisher, *University of Florida, Gainesville, Florida, United States*

Individuals caring for spouses living with Alzheimer's disease and Alzheimer's disease-related dementias (AD/ADRD) report distress tied to caregiver burden. Significant communication skills are required to navigate triadic interactions between caregivers, patients, and clinicians. Caregivers with these skills report less distress, but do not typically receive clinical communication guidance. The evidence-informed cancer-Caregiver Communication Process Model (CCPM) illustrates key communication skills that caregivers enact before, during, and after clinical appointments to attain care-related goals. To adapt CCPM for dementia caregiving, we conducted 15 interviews with AD/ADRD spousal caregivers and thematically analyzed transcripts to confirm and potentially expand the CCPM skills. AD/ADRD caregivers confirmed the importance of the cancer-CCPM skills: they kept lists/notes about what to address before visits, exchanged information or asked questions during visits, and debriefed with their spouse after appointments. Additionally, caregivers identified skills specific to AD/ADRD caregiving, including shift focus/mindset, a communication approach involving multiple skills and implemented before, during, and after appointments to prioritize their spouses' emotional well-being. For example, before appointments, caregivers primed their spouse to help them manage appointment-related anxiety. During appointments, they emphasized emotionally supportive skills, like reassurances when spouses became confused/upset. After appointments, caregivers incorporated a relational activity/ritual (e.g., getting ice cream) to shift their spouse's emotional state. Findings support the value of CCPM for caregiver skill development across disease contexts while demonstrating the need for adaptation to provide targeted guidance to caregivers of individuals with dementia. The dementia-CCPM can be implemented in interventions to enhance AD/ADRD caregivers' clinical communication skills and alleviate distress.

Abstract citation ID: igaf122.2983

### THE "LONG ARM OF CHILDHOOD" INFLUENCES CURRENT CAREGIVERS' HEALTH

Payton Radcliffe, and Julie Hicks-Patrick, *West Virginia University, Morgantown, West Virginia, United States*

Currently, 1 in 5 Americans are providing daily care to friends and family members. Informal caregiving exerts economic, physical, and emotional stress, often affecting health outcomes. We sought to understand whether supportive relationships in childhood influenced adult health and whether such buffers were more important for family caregivers than non-caregivers. We used the 2023 BRFSS (N = 9428; M_age=54.0, SD = 17.8; 52% females; 80.8% white nonHispanic), to test a moderated regression model examining the effects of unmet needs in childhood upon current physical health and whether these were altered as a function of current caregiver status (23.3% current caregivers). The moderated regression model assessing physical health was significant, F(3, 9424)=35.07***, R^2= .011. Direct effects of caregiver status (b = 18.6***) and lower unmet needs (b = 8.2***) were qualified by the interaction between the two (b=-2.6*). Post hoc analyses showed that caregivers whose childhood needs were never met reported more poor health days (M = 17.6) than non-caregivers whose needs were never met (M = 13.8), and more than all others whose basic childhood needs were met. Caregiving is difficult, but taking on that role may be more challenging for those whose childhood needs were not met. More attention is needed to lifespan contributors to caregiving.

Abstract citation ID: igaf122.2984

### THE IMPACT OF DEMENTIA CAREGIVING ON SLEEP AND DEPRESSION: THE ROLE OF SOCIAL SUPPORT AND RURAL-URBAN DIFFERENCES

Heehyul Moon[1], Sunshine Rote[1], Ann Folker[2], and Kathryn Williams Sites[1], *1. University of Louisville, Louisville, Kentucky, United States, 2. University of Massachusetts Amherst, Amherst, Massachusetts, United States*

Dementia caregivers experience greater psychological distress, including sleep disturbances and depression, compared to non-dementia caregivers. Poor sleep is a key predictor of depressive symptoms, yet the role of social support and rural-urban differences in shaping these associations remains unclear. Using data from the National Study of Caregiving (NSOC, N = 1,422), this study applies moderated mediation analysis to examine whether dementia caregiving predicts depressive symptoms via interrupted sleep and whether social support moderates this relationship. In addition, separate analyses were conducted for rural and urban caregivers to assess contextual differences. Results indicate that dementia caregivers report significantly more sleep disturbances than non-dementia caregivers, which in turn contribute to higher levels of depression. Social support moderates the relationship between sleep disturbances and depression, with dementia caregivers who receive greater support exhibiting a stronger indirect effect of caregiving on depression through sleep disruptions compared to non-dementia caregivers. However, separate rural-urban analyses revealed that these effects were present only among urban caregivers. Among rural caregivers, dementia caregiving was not significantly associated with sleep disturbances or depression, suggesting that traditional stress and coping models may not fully capture the experiences of rural caregivers. These findings underscore the need for interventions targeting sleep health and social support among dementia caregivers, particularly in urban settings. Also, alternative frameworks may be necessary to address the unique challenges faced by rural caregivers. Understanding these contextual differences is crucial for developing targeted policies and support systems to improve caregiver mental health and well-being.

### SESSION 7355 (POSTER)

### FRIENDSHIP, SOCIAL NETWORKS, SOCIAL SUPPORT

Abstract citation ID: igaf122.2985

### CONGREGATIONAL INFORMAL SUPPORT NETWORKS FOR OLDER ADULTS WITHOUT ADVOCATES IN BLACK CHURCHES

Miriam Gofine[1], Carolyn Berry[2], Stephanie Zhang[2], and Antoinette Schoenthaler[2], *1. New York University, Brookline, Massachusetts, United States, 2. New York University, New York, New York, United States*

Increasing proportions of older adults (OAs) have unmet care needs due to limited or no familial support ("Older Adults Without Advocates" [OAWA] or "elder orphans"). This prevalence is expected to rise, especially among Black/African American (B/AA) OAs. Both the peer-reviewed and grey literatures suggest that many OAWA receive at least some support from church-based informal (i.e., unpaid) social networks. To our knowledge, no published research exists on B/AA OAWA, but prior work emphasizes the significance of spiritual, emotional, and instrumental support provided by volunteers in congregational informal support networks (CISN) to community-dwelling B/AA OAs more generally. This study synthesizes the work of expert scholars on church-based informal social support networks of B/AA OAs with the Convoy of Care Model and the American Geriatrics Society's identification of "elder orphans" as a distinct clinical population to explore the role of CISN in B/AA OAWA's care networks. Guided by a community-engaged framework, 18 semi-structured interviews with OAWA and CISN members affiliated with 12 predominantly B/AA Baptist and Methodist churches across New York City were thematically analyzed. Preliminary results recognize CISN as a critical element of care networks of OAs and OAWA alike, identifying discordance between clinical and social definitions of OAWA and noting overlap between OAWA and CISN groups. This research enhances understanding of caregiving patterns related to OAWA within urban Black/African American faith communities, highlighting the essential role of CISN in promoting successful aging in place for urban B/AA OAWA.

Abstract citation ID: igaf122.2986

### DEPRESSION, SOCIAL ENGAGEMENT, AND COGNITIVE FUNCTION: A MEDIATION ANALYSIS

Yuchen Gu, *Boston College, Brighton, Massachusetts, United States*

Cognitive abilities are essential for older persons to maintain functional independence. Previous studies have shown that depression is linked to cognitive decline and a higher risk of dementia, while increased social engagement is associated with better cognitive performance. Since depressive disorders are frequently coexisted with severe and extensive social isolation, it is possible that social engagement is a mediator between depression levels and cognitive function. Nevertheless, there is no standardized measurement of social engagement. Whereas some researchers operationalize social engagement as a composite scale of various activities, others examine distinct forms of social engagement based on various theories. I examine the mediating effect of social, physical, and cognitive activities categorized by exploratory factor analysis. Using the KHB decomposition method from waves 14 (2018) and 15 (2020) of the Health and Retirement Study (HRS), I investigate how three different types of social activities can mediate the association between depression levels and cognition among older persons aged 65 and over ($n = 1{,}896$). This study finds that depression is negatively associated with cognitive function, and social, physical, and cognitive activities each partially mediate this relationship. Additionally, social activity has the strongest mediating effect, followed by physical and cognitive activities. These findings suggest that future interventions on promoting older people's social engagement could be a potential strategy to mitigate the negative effects of depression on cognitive function.

Abstract citation ID: igaf122.2987

### DEVELOPING COMMUNITY-CLINICAL LINKAGES FOR BLACK/AFRICAN AMERICAN OLDER ADULTS WITHOUT ADVOCATES

Miriam Gofine[1], Carolyn Berry[2], Stephanie Zhang[2], and Antoinette Schoenthaler[2], *1. New York University, Brookline, Massachusetts, United States, 2. New York University, New York, New York, United States*

The terms Older Adults (OAs) Without Advocates (OAWA) or "elder orphans" refer to OAs lacking surrogates in the event of losing decision-making capacity, typically due to absence of kin support. OAWA can face heightened unmet care needs and healthcare access challenges. Prior research identifies limitations in identifying OAWA across clinical and community settings and disparities in healthcare access among Black/African American (B/AA) OAs. While research on B/AA OAWA is limited, sociologists suggest that B/AA OAs receive substantial informal support from networks of church-based community volunteers. Community-clinical linkage models (CCLM) integrate clinical and community settings, including churches, to improve healthcare access for vulnerable populations. While we are unaware of research on age-friendly CCLM, CCLM that meet the specific needs of aging populations, especially those with unmet healthcare needs, such as B/AA OAs, is essential. Guided by a community-engaged framework, this study aimed to identify barriers and facilitators to connecting community-dwelling B/AA OAWA with church-based CCLM through existing church-based informal support networks. We thematically analyzed interviews and focus groups with 18 OAWA and church community volunteers affiliated with predominantly B/AA churches in New York City. Preliminary analyses reveal CISN as highly trusted care network members and facilitators of healthcare access. CISN recognize OAWA as an OA sub-population but do not report unique needs among OAWA in comparison to OA

in general. Findings advance ongoing development of an age-friendly CCLM by considering accessibility of CCLM to B/AA OAWA, a uniquely high-need population, and introduce a novel approach to identification of community-dwelling OAWA.

Abstract citation ID: igaf122.2988

### EFFECTS OF THE LEVEL AND INSTABILITY OF SELF-ESTEEM ON HELP-SEEKING AND ITS ORIENTATION

Jun Nakahara, *Chukyo University, Toyota, Aichi, Japan*

Some older adults do not seek or even refuse help when needed or when help is available. Therefore, it is important to examine the factors of help-seeking behavior and its orientation among older adults, particularly self-esteem. Previous theoretical assumptions show that individuals with high self-esteem and instability would estimate the ego-threat associated with help-seeking as high and suppress help-seeking; conversely, those with low self-esteem and high instability would be motivated to improve their self-evaluation and tend to productively cope with the task, which would promote help-seeking. This study aims to test these hypotheses. Data were obtained from 138 older adults through a one-week diary survey using the experience sampling method. The seven-day intra-individual standard deviation was used for the self-esteem instability index, and the mean was used for the level index. Results of the multiple regression analysis for the number of help-seeking behaviors in seven days revealed that the interaction term for the level and instability of self-esteem was significant. The simple slope test showed that the slope of self-esteem instability was positive and significant when the level of self-esteem was 1-SD below. Conversely, it was negative and not significant when the level of self-esteem was 1-SD above. There was no relationship between self-esteem indices and help-seeking orientations. This result suggests that some older adults were motivated to improve their self-evaluation and tended to productively cope with tasks, which promoted help-seeking. The lack of a relationship between help-seeking orientations and self-esteem suggests that the psychological process regarding help-seeking is unconscious.

Abstract citation ID: igaf122.2989

### ELIMINATING LONELINESS AND ENHANCING SOCIAL SUPPORT: FINDINGS FROM COMMUNITY FOCUS GROUPS

Vivian Miller[1], Caleb Skinner[1], and Daniel Maitland[2], *1. Bowling Green State University, Bowling Green, Ohio, United States, 2. University of Missouri-Kansas City, Kansas City, Missouri, United States*

Social support and social connection are vital for older adults. Older adults may be at increased risk for loneliness and a lack of social support as this cohort may live alone, experience the loss of friends, or have chronic illness. Failure to have social connections and social support results in significant consequences. Consequences of a lack of connection and support include risk for mental illness, risk for cognitive decline, and risk for physical health concerns (e.g., high blood pressure). To mitigate the consequences of loneliness and to increase support efforts, it is important to explore the lived experiences of older adults with a focus on time periods, activities, and engagement that promoted a sense of support. A total of seventeen (N = 17) participants across two focus groups engaged in this study. Through semi-structured interviews, participants shared that they felt supported by participating in enriching experiences at their local senior center. Through transportation provided by their senior center, community members otherwise transportation disadvantaged were able to engage with others, establish a sense of belonging, and maintain support through programs and services. Additionally, multiple participants explained that their church and pastor provided a sense of connection and were instrumental to their support system after the loss of loved ones. Findings from this study indicate the need to promote senior centers as a community focus. Additionally, findings suggest that senior centers can be a resource through networking of members and attendees. Authors share policy, research, and program implications.

Abstract citation ID: igaf122.2990

### ETHNIC FRIENDSHIP NETWORKS OF KOREAN-AMERICAN OLDER ADULTS IN SUBSIDIZED SENIOR HOUSING

Kyungmin Kim[1], Juyoung Park[2], and Yuri Jang[3], *1. Seoul National University, Seoul, Republic of Korea, 2. Emory University, Georgia, United States, 3. University of Southern California, Los Angeles, California, United States*

Senior housing communities are an important social environment for older adults, offering opportunities for interactions with peers and a sense of community. Older adults from ethnic minorities often find their ethnic peers within housing communities as a primary source of social and emotional support. This study examined ethnic friendship networks that older immigrants experience in senior housing and related individual characteristics. This study analyzed data from Korean-American older adults who reside in subsidized senior housing in the Greater Los Angeles Area (person N = 342; aged 65–102; facility N = 6). Participants named ethnic friends within their housing facilities up to three, using a name-generator approach of social network analysis. Poisson regression models were estimated to examine participants' characteristics associated with degree centrality of ethnic friendship network: (a) out-degree (i.e., self-reported friendship with other residents) and (b) in-degree (i.e., nominated friendship from other residents). Out-degree centrality was positively associated with female gender, participation in community activities, social engagement, and years of residence in the facility. In-degree centrality was positively associated with participation in community activities, chronic medical conditions, and social interactions in the facility. We also found that both indicators of degree centrality substantially differed across six housing facilities, suggesting that each facility may serve as unique social and built environments for ethnic friendship networks of residents. Our findings underscore identifying different levels of factors contributing to ethnic friendship in the senior housing community, given the benefits of being part of a friendship network in later life.

Abstract citation ID: igaf122.2991

### FRIEND NETWORK GROWTH THROUGH SOCIAL ACTIVITIES: A LONGITUDINAL ANALYSIS OF PARTICIPATION IN SPECIFIC ACTIVITIES

Haosen Sun, *University of Nevada, Reno, Reno, Nevada, United States*

Engaging in social activities and maintaining broader, more diverse social networks help to mitigate loneliness and support cognitive health in older adults. However, which activities (volunteering, courses, clubs, organizations, and interactive games) are more effective in fostering new friend connections for intimate discussions—while accounting for changes in participation over time—remains unclear. We use Wave 4 (2011) and Wave 6 (2015) of the Survey of Health, Aging and Retirement in Europe (SHARE), to conduct negative binomial regression with a longitudinal change score framework. Continued monthly participation in social activities is consistently associated with adding more new friends, with clubs and organizations showing the strongest effects. Starting to participate also shows a significant positive effect, particularly for clubs and interactive games where the magnitude exceeds that of continued participation. However, for other activities, starting participation tends to have similar or weaker effects than sustained engagement. Surprisingly, quitting activities is also often positively associated with having more new friends, although the magnitude is generally smaller. Meanwhile, quitting courses and games does not show a significant positive association. Sustained participation in social activities, particularly in clubs and organizations, significantly contributes to bringing more friends into core discussant network. Starting new activities—especially shared-interest-based ones like clubs and games—is also effective. While quitting activities is often accompanied by active adjustments in one's social convoy, more attention is needed for individuals leaving courses or games, as these activities tend to be more structured and based on fixed membership.

Abstract citation ID: igaf122.2992

### FRIENDSHIP RELATIONSHIP QUALITY AND COGNITIVE FUNCTION: A CROSS-LAGGED MODEL OF THE ROLE OF DEPRESSIVE SYMPTOMS

Changmin Peng, and Jeffrey Burr, *University of Massachusetts Boston, Boston, Massachusetts, United States*

Higher social support and lower social strain from friends have been found to be associated with better cognitive performance. Additionally, adequate cognitive function is considered an essential characteristic for maintaining social interactions with friends in late adulthood. However, research on the phycological mechanisms linking friendship quality and cognitive function is limited. Using data from the 2006-2014 Health and Retirement Study, this study examines the bidirectional relationship between positive and negative friendship quality and cognitive function with depressive symptoms as one potential underlying mechanism among older adults aged 51 and older (N = 6,344). A cross-lagged panel model within the structural equation modeling framework is performed. The results showed that older adults who perceived higher friend support tended to experience fewer depressive symptoms, which in turn, was associated with better cognitive function (indirect effect:b=0.001, p = 0.018). However, the reverse direction was not supported-depressive symptoms was not associated with friend support. Further, older adults who reported more strain with friend reported more depressive symptoms, which in turn, was associated with poorer cognitive function (indirect effect:b=-0.002, p = 0.034). Conversely, older adults with better cognitive function were more likely to report fewer depressive symptoms, which in turn, was associated with less strain with friends (indirect effect:b=-0.003, p = 0.047). These results supported hypotheses that depressive symptoms is one explanation for the bidirectional relationship between friend strain and cognitive function, as well as the unidirectional between friend support and cognitive function. The study has implications for intervention programs aiming at helping older adults stay socially connected and maintain cognitive function.

Abstract citation ID: igaf122.2993

### GENDER DIFFERENCES IN OLDER ADULT SOCIAL PARTICIPATION

Eric Vogelsang[1], and Sara Moorman[2], *1. California State University, San Bernardino, San Bernardino, California, United States, 2. Boston College, Chestnut Hill, Massachusetts, United States*

A growing body of research has found that older adult social participation may benefit various aspects of health and well being. While some of this work has found these associations differ by gender, there has been little rigorous investigation into how social participation itself varies between older men and women. Using 10 waves of the Health and Retirement Study, we study how (a) the breadth of social activities, (b) social activity frequency, and (c) the social determinants of social participation differ by gender. Generally, we find that men and women were involved in approximately the same number of social activities and at the same total level of activity frequency. However, gendered differences in population composition (e.g., educational attainment, marital status) suppress greater social participation for women. After controlling for these compositional differences, we find that women had slightly greater participation scores—mostly attributable to volunteering and religious service attendance. We also found that social activity decline begins at about age 65 for men, but age 70 for women. When investigating the social determinants of social participation, our results indicate that men suffer greater "penalties" for being divorced or never married. In addition, we found that increased social participation among those currently working only applied to men. Last, we found that the positive associations between high socioeconomic status and social participation was much stronger in magnitude for women. These results demonstrate how gendered social norms and inequality may impact this important aspect and correlate of "healthy aging."

Abstract citation ID: igaf122.2994

### HEALTH BENEFITS OF A VARIETY IN SOCIAL ACTIVITIES: BIDIRECTIONAL ASSOCIATIONS WITH PHYSICAL AND COGNITIVE HEALTH

Sangha Jeon, *University of Michigan, Ann Arbor, Ann Arbor, Michigan, United States*

Engagement in diverse social activities is linked to better cognitive functioning, fewer depressive symptoms, and greater longevity. Social activity variety may provide unique cognitive, emotional, and physical benefits by fostering purpose in life, stimulating cognitive flexibility, and promoting health behaviors, all of which contribute to overall well-being. Yet, most studies rely on cross-sectional or unidirectional models, leaving the temporal relationship between social activity variety and health outcomes unclear. Drawing on longitudinal data from the Health and Retirement Study (2008, 2012, and 2016) with 2,087 participants aged 50 and above, we examined the dynamic interplay between social activity participation and health outcomes, including self-rated health, chronic conditions, and cognitive functioning. Using a Random Intercept Cross-Lagged Panel Model (RI-CLPM), our findings indicate that greater social activity variety predicts improved physical and cognitive health at subsequent time points. Conversely, individuals with better baseline health are more likely to maintain or increase their engagement in diverse social activities over time. These results suggest a reinforcing cycle in which social activity variety and health mutually support one another. Our findings underscore the importance of interventions that foster diverse social participation as a preventive strategy against cognitive and physical decline in older adults.

Abstract citation ID: igaf122.2995

**OLDER ADULTS AND GENERATIONAL ENGAGEMENT ACROSS SETTINGS**

Janna Heyman[1], Henry Davis[1], and Priscilla Allen[2], *1. Fordham University, New York, New York, United States, 2. Louisiana State University, Baton Rouge, Louisiana, United States*

The aging population in the United States is steadily increasing and new models of intergenerational and multigenerational engagement are developing in diverse settings. This presentation will discuss innovative generational engagement in community centers, senior centers, assisted living, and nursing homes. Detailed insights obtained through research will be discussed. The presentation will present research from two universities' ongoing work with community partners. Highlights include key findings from a collaborative study with a large community center providing generational programming and services to older adults, families, and youth throughout New York City. We will introduce ways of bringing generations together in inpatient settings. These approaches will highlight the evolving role and meaning of older adults in generational programming, illustrating their impact on economic mobility, social connections, and overall well-being. Generational programming focused on areas including mentorship, economic empowerment, and co-housing models, emerged as strategies to improve social connections. A qualitative study, using interviews with older adults, families and staff, underscores the importance of autonomy, social connections, financial issues, and inclusion in intergenerational and multigeneration initiatives. The session will include policy and practice implications for social workers and helping professionals to facilitate generational models to support older adults. Best practices including older adults and their lived experiences with community-based initiatives. Participants will gain practical and research-supported insights to generational programming and foster supportive environments.

Abstract citation ID: igaf122.2996

**RELATIONSHIP BETWEEN RECEIVING SOCIAL SUPPORT AND THE HEALTH OF GRANDPARENT CAREGIVERS**

Courtney Gage[1], and Elizabeth Rickenbach[2], *1. Saint Anselm College, Concord, New Hampshire, United States, 2. Saint Anselm College, Goffstown, New Hampshire, United States*

Raising grandchildren has been linked to increased depression, stress, and worsened physical health. Research has shown that having social support may decrease stress and depression, but less is known about the impact of social support on physical health and chronic conditions among grandparent caregivers. The current study analyzed data from grandparent caregivers (n = 234) who participated in Wave 3 (2013) of the Midlife in the United States longitudinal study (Ryff., et al 2013). Correlational analysis showed that greater support from family correlated significantly with fewer chronic conditions (r=-.257, p = < 0.01), better physical health (r=-.275, p = < 0.01), and better mental/emotional health (r=-3.48, p = < 0.01). Greater support from friends predicted better physical health (r=-.163, p=.019). Independent samples t-tests were conducted to compare grandparents with high (4 or greater) versus low (3 or less) depressive symptoms. Support from family was higher among grandparents with fewer depressive symptoms (M = 3.53, SD =.56) as compared to grandparents with more depressive symptoms (M = 2.94, SD= .88) (t = 2.99, df = 21.87, p=.007). Similarly, support from friends was higher among grandparents with fewer depressive symptoms (M = 3.32, SD=.63) compared to grandparents with more depressive symptoms (M = 2.87, SD=.82) (t = 3.04, df = 22.74, p=.003). The findings show social support is important for grandparent caregivers' physical and emotional health. This study demonstrates that increasing support groups and social assistance is important for grandparent caregivers.

Abstract citation ID: igaf122.2997

**SOCIAL CONNECTEDNESS AS FACILITATORS OF HEALTHCARE ACCESS FOR OLDER ADULTS LIVING ALONE WITH DISABILITIES**

Natalie Turner, Hyun-Jun Kim, Hailey Jung, and Karen Fredriksen-Goldsen, *University of Washington, Seattle, Washington, United States*

Older adults with disabilities experience challenges accessing healthcare, resulting in preventable morbidity and mortality. Of particular concern is the disproportionate prevalence of living alone among people with disabilities – over 30% of people with disabilities live alone. While reflecting autonomy, living alone can contribute to the disablement process, social isolation, and increased healthcare needs. Using the Network Episode Model and data from the Health Equity and Intersectionality Study (n = 260), we examine the role of social connections as facilitators of health information and service navigation as well as individual and structural factors in predicting healthcare access measured by routine checkups, usual source of care, and having a personal doctor. We further estimate the interaction effects to examine if the relationships between these predictors and healthcare access differ by disability status and living arrangements. Logistic regression analyses, controlling for chronic conditions and sociodemographic characteristics, reveal that having more core confidants who provide support for health needs and share health-related information significantly predicts a higher likelihood of receiving routine check-ups, especially for older adults living alone with disabilities. Having a usual source of care is significantly associated with health literacy, with this relationship more pronounced among those living alone with disabilities. Having a personal doctor is positively associated with both health literacy and help-seeking behaviors regardless of disability status and living arrangements. Our findings inform the development of a conceptual model of the social dynamics that either facilitate or impede healthcare access among older adults, particularly those living alone with disabilities.

Abstract citation ID: igaf122.2998

**SOCIAL SUPPORT IN ADULTHOOD BUFFERS THE EFFECTS OF ADVERSE CHILDHOOD EXPERIENCES ON MORTALITY RISK**

Meredith Willard, Ryan Best, and Nicholas Turiano, *West Virginia University, Morgantown, West Virginia, United States*

Exposure to Adverse Childhood Experiences (ACEs) is associated with life-long consequences that can negatively affect aging, such as increased risk for health conditions and premature mortality (Anda et al., 2006; Brown et al., 2009). However, supportive social networks can provide buffering effects for certain individuals, which may be critical for those who experience ACEs. However, more evidence suggests that strainful social networks can amplify the negative consequences of ACEs (Rook, 1990). Thus, we utilized data from the Midlife in the United States (MIDUS) study (N = 6,150), to examine if latent profiles of social support and strain buffered/amplified the effect of ACEs on 26 year mortality risk in adulthood. In 1995, respondents (aged 25-74) answered retrospective reports of childhood adversity and social support and strain questions. A latent profile analysis revealed a 5-class solution was most appropriate (AIC: 52868.923; Entropy: 0.828). Next, interactions were created between ACEs and each of the 5 classes to predict mortality risk via a Cox Proportional Hazards model. Although higher ACEs predicted a significantly increased mortality risk ($p < .05$), being in a class characterized by high support and low strain, 'Optimal network' class, or average levels of support and strain, the 'Average network' class, mitigated this risk. This study provides evidence that ACEs continue impacting health well into older adulthood, and that using a person-centered approach to capture broader aspects of social support/strain is useful in understanding the protective effects these social relationships exert throughout adulthood.

Abstract citation ID: igaf122.2999

### THE DIFFERENTIAL NATURE OF WEAK TIES: RACE DIFFERENCES IN ADULTS' DAILY INTERACTIONS WITH OTHERS

Katherine Fiori[1], Amy Rauer[2], Kira Birditt[3], Angela Turkelson[4], and Oliver Huxhold[5], *1. Adelphi University, Garden City, New York, United States, 2. University of Tennessee, Knoxville, Tennessee, United States, 3. University of Michigan, Ann Arbor, Michigan, United States, 4. University of Michigan Institute for Social Research, Ann Arbor, Michigan, United States, 5. German Centre of Gerontology, Berlin, Berlin, Germany*

Recent research has highlighted the importance of interactions with weak ties for well-being. However, structural, cultural, and individual-level racism may influence the ways in which racially minoritized groups (e.g., Black Americans) interact with others, particularly those individuals outside the network ('weak ties'). Yet most studies on race differences in social ties take a global approach rather than examining daily encounters. Thus, we used ecological momentary assessment and hierarchical network mapping data to examine whether there were race differences in types of daily social interactions (close/in network vs. weak/outside network interactions) and in their valence (positive, negative, ambivalent). Participants from the Stress and Well-being in Everyday Life (SWEL) study (N = 168; M age = 52.96, range = 33-91; 66.1% female; 47.6% Black) completed surveys every three hours for 4-5 days. Results showed that 36.6% of all interactions occurred with people outside of the network. Although White Americans reported having more interactions than Black Americans, the proportions of interactions with close and weak ties did not differ. Interestingly, we found that the proportion of interactions with weak ties was negatively associated with the proportion of positive interactions and positively associated with the proportion of ambivalent interactions for Black Americans only, controlling for age, gender, education, and total number of interactions. Follow-up analyses consider daily experiences of discrimination as a potential mechanism. Findings point to the importance of acknowledging the role of context in shaping the nature and potential effects of daily social interactions with others.

Abstract citation ID: igaf122.3000

### THE IMPACT OF ACTIVE INGREDIENTS OF SOCIAL NETWORKS ON THE TRAJECTORY OF COGNITIVE DECLINE IN OLDER ADULTS

Jingchao Feng, *Tsinghua University, Beijing, Beijing, China (People's Republic)*

Background Social networks, which are closely related to cognitive health and well-being in older adults, undergo significant changes as individuals enter old age. However, there is a lack of longitudinal studies examining the relationship between cognitive decline trajectories and the structural and qualitative aspects of social networks. Our study aims to explore this association and identify the influence of different social network dimensions across relationship types on cognitive trajectory development. Methods The Health and Retirement Study (HRS) is a nationally representative cohort study of older person in the United States. A psychosocial survey assessed the structural and qualitative aspects of social networks, including network size, close relationships, social support provided by the network, and social stress caused by the network. Cognitive function was measured on a 27-point scale every two years. This study employed group-based trajectory modeling to estimate cognitive decline trajectories and used multinomial logistic regression to analyze the association between trajectory categories and components of social networks. Results Among the 10,365 participants aged 50 and above at baseline, trajectory analysis identified three distinct longitudinal cognitive decline trajectories: Persistently low trajectory (n = 1,669, 16.1%), Persistently moderate trajectory (n = 5,058, 48.8%), Persistently high trajectory (n = 3,628, 35.1%). A larger social network and higher social support were protective factors associated with a slower cognitive decline and a higher trajectory. In contrast, social stress increased the risk of a rapid decline in cognitive function but also played a protective role in maintaining a higher cognitive trajectory.

Abstract citation ID: igaf122.3001

### THE ROLE OF SOCIAL RELATIONSHIP QUALITY IN WOMEN DIAGNOSED WITH FIBROMYALGIA

Minju Seong, Hyunjoo An, Inhye Jung, and Hyo Jung Lee, *Yonsei University, Seoul, Republic of Korea*

Fibromyalgia is a syndrome characterized by widespread chronic pain, fatigue, sleep disturbance, and depression. Though its severe pain symptoms disrupt individuals' daily life, the invisible nature of pain and the lack of effective treatment add to patients' suffering. While the exact cause of onset of this syndrome remains unclear, about 90% of fibromyalgia patients are women and research on its contributing factors such as life stressors is still developing. Thus, this study investigates the relationship quality of older women with family and friends and its association with fibromyalgia diagnosis according to biopsychosocial model. Using data from the Health and Retirement Study (HRS, 2016, 2018, 2019), the study sample consisted of women aged 50 and older who completed the 2019 Health Study Survey and reported information on their fibromyalgia diagnosis (n = 1,900). The relationship quality with family (spouse, children, and relatives) and friends was measured using both positive and negative dimensions. We conducted Chi-square tests and logistic regression models. Findings suggest that both positive ($OR=.647$, $p<.05$) and negative ($OR=.619$, $p<.05$) spousal relationship scores were linked to a lower likelihood of fibromyalgia diagnosis. However, negative relationship scores with other family members were associated with a higher risk of being diagnosed with fibromyalgia (OR = 1.502, p≤.01). These results further emphasize the need to consider psychosocial factors when dealing with fibromyalgia patients. Family, as the most intimate social network for individuals, may affect the onset of the condition not only in a genetic context, but also through interpersonal interactions.

### SESSION 7360 (POSTER)

### HOUSING

Abstract citation ID: igaf122.3002

### EVALUATION OF A WELLNESS PROGRAM FOR AFFORDABLE SENIOR HOUSING RESIDENTS

Verena Cimarolli[1], Alisha Sanders[2], Molly Wylie[1], and Robyn Stone[1], *1. LeadingAge, Washington, District of Columbia, United States, 2. Volunteers of America National Services, Washington, District of Columbia, United States*

Volunteers of America National Services' (VOANS) Aging with Options (AWO) program - funded by and developed in partnership with Parker Health Group - is designed to support wellness and improve chronic disease management for residents in affordable senior housing. The intervention, piloted at one housing site, provided in addition to an existing service coordinator, a wellness nurse and a community health worker to help engage residents in healthy behaviors and connect them with resources. The evaluation effort assessed participants' perceptions of the program's impact by implementing a resident survey (N = 69); and determined the program's effect on health outcomes by analyzing service- and health-related data collected regularly by program staff (N = 99). Over 94% of AWO participants reported that the supports received from AWO program staff were helpful. The most frequently mentioned program benefits were an improved sense of health, enhanced well-being and safety, and greater social connection. Residents who developed a wellness plan - for which they identified health and wellness goals and steps to achieve these goals - reported experiencing positive changes such as eating healthier food, greater success controlling health conditions, and exercising more. Regression analyses conducted to identify service-related predictors of health outcomes six months after the program's initiation showed that higher life optimism was associated with receiving more service instances of 'follow-up with service providers.' Further, better self-rated physical health was associated with receiving more service instances of 'exercise/physical fitness', 'socialization', and 'encouragement to attend event/program.'" Evaluation findings underscore the program's benefits for resident health and well-being.

Abstract citation ID: igaf122.3003

### HOUSING CONDITIONS CHANGE AND ITS ASSOCIATION WITH HEALTH AMONG MIDDLE-AGED AND OLDER CHINESE

Peiyi Lu, Tarani Chandola, and Vivian Lou, The University of Hong Kong, Hong Kong, China (People's Republic)

Housing, as an important and modifiable social determinant of health, is closely related to health. Improvement of housing conditions therefore presents an opportunity to promote health. However, prior research mostly focused on one single health outcome and lacked evidence from developing countries. This study investigated the relationship between housing condition changes and various health domains among middle-aged and older Chinese. Longitudinal data from China Health and Retirement Longitudinal Study were used. Participants (aged 45+, N = 8,423) reported if their homes had some amenities (e.g., barrier-free facilities, toilet) in 2011-2013. Participants' mobility limitations, depressive symptoms, and lung function were assessed in 2013-2018. Regression models with interaction terms examined if housing condition changes modified the association between baseline housing and health. Respondents reported improvement in the physical environment and utilities from 2011 to 2013. Individuals living in homes with better physical environment (i.e., barrier-free, bathing/toileting facilities) and more utilities (i.e., gas, water, electricity, telephone and internet) reported better health (IRR< 1 for mobility limitation and depressive symptoms, b > 0 for lung function, Ps < 0.05). Additionally, home improvement in utilities lowered the risk of mobility limitations and improved lung function especially among individuals with no or few utilities at baseline. However, home improvement in physical environment did not modify the relationship between baseline physical environment and health. Our findings suggested many older Chinese resided in homes lacking essential aging-friendly amenities, which was associated with worse health. Enhancing utilities at home has the potential to modify the negative impact of earlier housing conditions on functional and lung health.

Abstract citation ID: igaf122.3004

### HOUSING, SOCIAL SUPPORT, AND TRANSITION IN CONNECTICUT'S MONEY FOLLOWS THE PERSON PROGRAM

Amal Alsamawi, Asmita Aasaavari, Christine Bailey, Kathy Kellett, Tyfany Fabian, Alexandra Sanders, Julie Robison, and Ellis Dillon, *University of Connecticut, Farmington, Connecticut, United States*

Money Follows the Person (MFP) is a Medicaid program that helps older adults move from Long-Term Care institutions to the community. In this study, we examined the role of housing and social support on older adults' ability to move out of institutional settings through the experiences of MFP stakeholders in Connecticut. Preliminary findings from semi-structured interviews with older adults who are eligible or applied to MFP (n = 25), caregivers (n = 16) and MFP professional staff (n = 43) are presented. Qualitative analysis included key word searching, inductive coding, and thematic analysis. Older adults and their caregivers reported that access to housing increased opportunities to leave nursing facilities. However, access to housing was not always sufficient. An older adult with dementia who was reinstitutionalized recounts losing her apartment because she did not have familial support, "I lost everything... My niece never went to the apartment". Housing related assistance from family members included moving in with a family member, support finding a place, and facilitating the move, "My family is the one that went out and found this apartment and they moved all my stuff". An MFP staff described the challenge for older adults, in particular people with dementia to transition if they do not have family support – "It's possible but it is really hard…going home with family is definitely the easier route". The presence of informal support shapes transition and can improve access to housing opportunities. Innovative approaches to housing are imperative for the growing population of older adults with minimal family support.

Abstract citation ID: igaf122.3005
### INFLUENCE OF RESIDENTIAL ENVIRONMENTS ON OLDER ADULTS' SOCIAL AND PHYSICAL ACTIVITIES: A SCOPING REVIEW
Seokyung Park, Wenjin Wang, and Xuemei Zhu, *Texas A&M University, College Station, Texas, United States*

This scoping review examines how older adults' residential environments—homes of community-dwelling older adults and long-term care facilities—impact their social and physical activities, which are key components of active aging. While prior research on active aging has explored broad environmental influences, such as nature, pollution, neighborhood environments, and social environments, relatively few studies have focused on older adults' homes and long-term care communities. A systematic search of four databases (PubMed, PsycINFO, CINAHL, and Web of Science) was conducted, using 14 keywords related to older adults, residential environments, social activity, and physical activity. A total of 3,333 studies were screened following the Preferred Reporting Items for Systematic Reviews and Meta-Analyses (PRISMA) guidelines, with 70 meeting the eligibility criteria for data extraction. Older adults' social and physical activities have been studied across various types of housing, ranging from large-scale multifamily housing to single-family homes. The findings were categorized into four spatial scopes within residential environments: (1) indoor spaces, (2) indoor-outdoor transitional areas, (3) outdoor spaces within the property or site, and (4) immediate surroundings (e.g., sidewalks connected to buildings and nearby houses). This scoping review clarifies the current state of research on how residential environments correlate with older adults' social and physical activity at a more granular spatial scale, highlighting existing knowledge gaps. The findings underscore the need for further investigation to better inform and refine the design of age-friendly residential environments that support older adults' well-being and active lifestyles.

Abstract citation ID: igaf122.3006
### OLDER ADULTS' PARTICIPATION IN SOCIAL ACTIVITIES DIFFER BY WHETHER THEY RENT OR OWN THEIR HOMES
Ebenezer Kyeremeh[1], Justine Sefcik[2], Rose Ann DiMaria-Ghalili[3], Chanee Fabius[4], and Safiyyah Okoye[1], *1. Drexel University, Philadelphia, Pennsylvania, United States, 2. Drexel University College of Nursing and Health Professions, Philadelphia, Pennsylvania, United States, 3. Drexel University, Drexel University, Pennsylvania, United States, 4. Johns Hopkins University, Baltimore, Maryland, United States*

Prior research has substantiated social engagement as a beneficial determinant of psychological and social well-being in later life. International studies find that renting in congregate housing can provide opportunities for social participation among older adults; however, there has been little study of whether social participation varies between older adults who rent vs own their homes in the U.S. Understanding the relationship between housing tenure (i.e., renting vs owning) and social participation can inform programs and policies, tailored to housing tenure status, to support social engagement. We employed a cross-sectional design using nationally representative data from $N=5137$ community-dwelling older adults (≥65 years) from the 2022 National Health and Aging Trends Study (NHATS) to explore the association between owning or renting and social participation (measured as self-report of ≥ 1 of the following in the previous month: visiting family/friends, attending religious services, attending club meetings/group activities, going out for enjoyment, or doing volunteer work). We found a higher proportion of homeowner participants reporting social participation (96.7%) compared to renters (89.2%). In an unadjusted logistic regression model, renters had much lower odds of participating in social activities than owners (OR 0.28, 95% CI: 0.20, 0.40). The association weakened after adjusting for sociodemographic (age, gender, education, race, financial strain, income, living arrangement) and health characteristics (chronic conditions and cognitive, functional, and sensory impairments) (OR for renters 0.71, 95% CI: 0.50, 1.02). Further studies are needed to describe barriers to social participation among older adult renters, focusing on sociodemographic, financial, and health factors.

Abstract citation ID: igaf122.3007
### RELATIONSHIP BETWEEN HOUSING AND PALLIATIVE NEEDS AMONG HOSPITALIZED OLDER ADULTS
Abigail Latimer, and Lynden Bond, *University of Kentucky, Lexington, Kentucky, United States*

In 2024, over 146,000 adults aged 55 and older faced homelessness, many for the first time, with one in four living in poor conditions. Rising housing costs and income loss increase worries over losing housing. Many housing insecure older adults live with chronic health conditions and unmet palliative needs. Our objective is to describe the relationship among homelessness, housing conditions, and palliative needs in a sample of hospitalized older adults. We surveyed 139 hospitalized older adults (≥ 55 years, $M=68 \pm 9$) about housing conditions and health outcomes during a hospital-based point in time count on January 29, 2025. We used the 5-item Integrated Palliative Care Outcome Scale (IPOS) to assess palliative needs. Most were white (86.4%) men (51.8%) living in a home or an apartment ($n=117$), with 16 doubled up and two were experiencing homelessness for the first time. About 15% were worried about their housing; 24.4% reported poor housing conditions with at least one problem. Pain was moderate to overwhelming for 68.9%, 35.8% experienced anxiety most or all of the time, and 46.4% felt at least some peace and most had as much information as they wanted regarding their illness (73.9%). While there were no significant associations between symptoms and housing status, those worried about housing reported greater anxiety over their illness ($t(133)= 2.70$, $p= .008$), felt less peace ($t(134)= 2.83$, $p=.003$), and did not have as much information as they wanted ($t(21.24=3.11$, $p=.005$). Housing worries can heighten health-related anxiety and affect how older adults receive information.

### SESSION 7365 (POSTER)

### LIFE COURSE AND DEVELOPMENTAL CHANGE

Abstract citation ID: igaf122.3008
### AGE DIFFERENCES IN OLDER ADULTS' USAGE OF SELECTION, OPTIMIZATION, AND COMPENSATION: FINDINGS FROM THE SONIC STUDY
Momoho Kakuta[1], Takeshi Nakagawa[2], Yukie Masui[3], Kei Kamide[4], Kazunori Ikebe[4], Takumi Hirata[5], Yasumichi Arai[6], and Yasuyuki Gondo[1], *1. The University of Osaka, Suita, Osaka, Japan, 2. The University of Osaka, Osaka, Osaka,*

*Japan, 3. Tokyo Metropolitan Institute of Gerontology, Itabashi City, Tokyo, Japan, 4. Osaka University, Osaka, Osaka, Japan, 5. Tokyo Metropolitan Institute of Gerontology, Itabashi, Tokyo, Japan, 6. Keio University Graduate School of Health Management, Fujisawa, Kanagawa, Japan*

Selection, Optimization, and Compensation (SOC) were life-management strategies to maintain well-being against function declines. The number of SOC strategies decreased from middle to old age due to fewer resources. However, little is known about the change in SOC strategy from young-old to very old despite a highly significant resource decrease. This study aimed to clarify the age differences in SOC strategy. Data collected from community-dwelling Japanese people participated in the longitudinal cohort study of Septuagenarians, Octogenarians, and Nonagenarians Investigation with Centenarians (SONIC). The samples were divided into 70s (n = 983, 48.2% male, range:68-72), 80s (n = 957, 47.1% male, range:78-82), and 90s (n = 212, 47.2% male, range:88-92). We conducted multiple group factor analysis using polychoric correlation, ANOVA, and correlation analysis between SOC subfactors, subjective well-being, personality traits, and cognitive function. Model fit indices were adequate to compare scores with three generation cohorts (CFI=.960, TLI=.945, RMSEA=.041, SRMR=.078). Only the use of elective selection was significantly higher with aging, but other subfactors were higher from the 70s to 80s and were lower from the 80s to 90s. The only elective selection was negatively correlated (r =-.09$^{4}$-.01), and other subfactors were positively correlated (r =.02$^{4}$.30) with well-being in the 70s and 80s, but the correlation coefficients were weaker in the 90s, respectively (r =-.09$^{4}$.13). We emphasized that the use and the impact of SOC was maximized until the 80s and changed in the 90s. The key factor of well-being in the 90s was not only the SOC strategy.

Abstract citation ID: igaf122.3009

### CHANGE IN WORK-FAMILY SPILLOVER ACROSS 20 YEARS: LONGITUDINAL ANALYSIS OF THE MIDLIFE IN THE UNITED STATES STUDY

Jeongmin Park, Kyoungmin Cho, Soomi Lee, and David Almeida, *The Pennsylvania State University, University Park, Pennsylvania, United States*

Background: Work and family are two of the most central roles in adulthood, yet little is known about how these experiences evolve with aging. This study examined changes in work–family spillover over a 20-year period and explored whether long-term trajectories vary by age and gender. Methods: Data were from the Midlife in the United States (MIDUS) study across three waves (N = 665, employed at all waves; 1995-1996, 2004-2006, 2013-2014). Four types of work–family spillover were measured: negative work-to-family, negative family-to-work, positive work-to-family, and positive family-to-work. Multilevel growth curve models with random slopes and intercepts assessed longitudinal trajectories of work–family spillover controlling for baseline age, gender, race, and education. We also tested whether changes in work-family spillover differed by age and gender. Results: Negative spillover (work-to-family and family-to-work) declined over time, with no quadratic effects. Positive spillovers followed an inverted U-shape, increasing from the first to the second wave and declining by the third. Older adults experienced steeper declines in negative spillovers, but their changes in positive spillovers were similar with those of younger adults. Gender was not associated with changes over time, although females reported higher initial levels of negative family-to-work spillover. These patterns remained consistent after covariate adjustment. Conclusions: Work-family spillover change over time, with negative spillover showing a linear decline and positive spillovers following an increase-decrease pattern. Age-related differences in negative spillover trajectories and gender differences in initial negative spillover levels highlight the need for workplace policies that support work-family balance for different population groups.

Abstract citation ID: igaf122.3010

### EXPLORING THE PHYSIOLOGICAL PATHWAYS CONNECTING ADVERSE CHILDHOOD EXPERIENCES TO LATER LIFE MORTALITY RISK

Taylor Brown, *West Virginia University, Morgantown, West Virginia, United States*

Adverse childhood experiences (ACEs) impact health and development well into adulthood, but less clear are the precise mechanisms. Thus, the current study utilized a sample of 1,198 participants (Mean age = 55;) from the Midlife Development in the U.S. (MIDUS) study to test whether retrospective reports of ACEs led to higher allostatic load (AL) scores in adulthood, resulting in an increased risk of death. An ACEs count score ranging from 0 (no adversity) to 20 (greater adversity) was created (physical and emotional abuse by family; low socioeconomic status). AL score (0 = lowest; 1 = highest) was constructed using 24 biomarkers from 7 different physiological systems measured from 2005-09 (para/sympathetic systems, HPA axis, cardiovascular, lipid/glucose metabolism, inflammation). Death status and survival time was recorded from 1995-96 when ACEs were retrospectively reported, through the censor date of December 2021(deceased = 217). Cox proportional hazards models were estimated in a structural equation modeling framework to assess indirect effects of ACEs on mortality risk via AL (controlling for age, sex, race, education, marital status). Higher ACEs predicted a significantly greater level of AL (B = 0.67; $p < .05$) and higher AL predicted an increased risk of death (HR = 1.46; $p < .05$). A significant mediated effect (IE = 0.03; TOT = 0.30; $p < .05$) suggested that ACEs predicted an increased risk of death due to accumulated physiological damage. Findings elucidate how early life events can lead to a cascade of physiological changes that persist into adulthood and ultimately impact longevity.

Abstract citation ID: igaf122.3011

### HOW SOCIAL PARTICIPATION ENHANCES "GENERATIVITY" AMONG OLDER ADULTS IN JAPAN

Chiharu Miura[1], and Keiko Katagiri[2], *1. Advanced Research Center for Well-being of Kobe University, Ashiya, Hyogo, Japan, 2. Kobe University, Kobe, Hyogo, Japan*

Japan is the world's leading super-aged society. In contemporary Japan, older adults are no longer merely recipients of social support but have emerged as active contributors to society. They are expected to pass on their knowledge and experiences to the next generation, making generativity increasingly essential. One key factor influencing generativity in later life is social participation. Social participation is an accessible and familiar activity for older adults. It provides opportunities to foster new relationships and facilitates learning through various activities. This study examines whether new encounters, new friendships, and learning through acctivities enhance older adults' generativity. A mail survey was conducted targeting residents aged 60-79 in an urban

Japanese city (n = 1045). Out of 456 respondents, data from 299 participants engaged in social participation activities were analyzed. Serial mediation analysis using Hayes' PROCESS macro (Model 6) was applied. The analysis revealed that 'new friends' and 'learning' fully mediated the relationship between 'new encounters' and 'generativity.' Specifically, new encounters positively influenced the acquisition of new friends, which subsequently facilitated learning, ultimately enhancing generativity. These findings suggest that new encounters play a crucial role in fostering generativity among older adults. Social participation, which generates these new encounters, is essential for the well-being and active contribution of contemporary and future older adults in society.

Abstract citation ID: igaf122.3012

### LIFE STRESS AND COMPLEXITY OF RELIGIOUS IDENTITY

Sophia Paleski[1], Alessia Petroni[1], Kyra Hultgren[1], Sarah MacDougall[1], Edward Thompson[2], Christopher Turner[1], Michiko Iwasaki[1], and Andrew Futterman[1], *1. Loyola University Maryland, Baltimore, Maryland, United States, 2. College of the Holy Cross, Worcester, Massachusetts, United States*

This study, using a random sample of 274 older adults and a "snowball" sample of 80 African American older adults (aged 57-100) living in Massachusetts, explores causes of having "straightforward" vs. "complex" orientation to religion in later life. Individual interviews included standard measures of religious affiliation, religious belief and behavior, religious doubts, physical and psychiatric health, life stresses and coping strategies, and social support networks. Personality characteristics were completed two times, 12 months apart, as a part of religious involvement and chronic stress in later life. Fifty-one individuals were identified in both samples with high levels of religious involvement and religious doubt – indicating cognitively complex, reflective religiousness (Batson, 1976). By contrast, 42 older adults were identified having extremely elevated levels of religious involvement with little if any religious doubt – indicating a more "straightforward" form of religiousness. Using a list of potential "causal" factors derived from interview data, discriminant function analyses were conducted to predict membership in either complex or straightforward religious groups. Initial analyses indicated that more education, being female, aged less than 80 years old, fewer functional impairments, onset of less severe, acute social stressors (e.g., breakup of relationships, loss of income) were associated with "complex" religiousness. By contrast, older age (> 80 yrs), increased infirmity, serious chronic stressors, and social isolation were associated with displaying more "straightforward" religiousness. These results are discussed considering our ongoing study of religious development using qualitative interview methods and previous research on cognitive and emotional complexity in later life (Labouvie-Vief, 2016).

Abstract citation ID: igaf122.3013

### LIFE-COURSE SES DISADVANTAGE AND DUAL-POVERTY TRAJECTORIES IN LATER LIFE: EVIDENCE FROM CHINA

Ting Hu[1], and Yu-Chih Chen[2], *1. University of Illinois--Urbana-Champaign, Champaign, Illinois, United States, 2. National Taiwan University, Taipei, Taipei, Taiwan (Republic of China)*

Research on life-course socioeconomic status (SES) and health outcomes in later life has been widely examined, but the application on financial health remains underexplored. Furthermore, financial health measurement tends to be a single dimension focusing on income or assets. To comprehensively understand financial health, this study adopts an asset-based perspective to construct financial health using income and asset poverty as measurements. Guided by the life course models, we examined whether and how four life course models (latency, pathway, cumulative, and mobility) were associated with dual poverty trajectories measured by income and assets. Using 1,976 respondents aged 45 and older from five waves (2011–2018) of the China Health and Retirement Longitudinal Study, sequence analysis and multinomial regression were used to examine how SES risk in childhood and early adulthood was associated with dual-poverty trajectories in later-life. Four types of dual-poverty trajectories were identified through sequence analysis: persistent non-poor, persistent asset-only poverty, transitioning to non-poor from asset poverty, and those switched between income and asset poverty. Regression results showed that the relationship between early-life SES disadvantages and later-life poverty was partially mediated by the SES risk in early adulthood. Furthermore, individuals with persistent disadvantage or downward mobility showed higher risks of all types of poverty. Findings highlight the importance of addressing cumulative SES disadvantages in both early-life and adulthood stages. Policies that support education, improve financial literacy, provide financial assistance, and promote asset-building initiatives should be established to promote financial health and economic security in later life.

Abstract citation ID: igaf122.3014

### MULTIGENERATIONAL SOCIAL MOBILITY AND DEPRESSIVE SYMPTOMS AMONG MIDDLE-AGED AND OLDER CHINESE

Yi Huang[1], Peiyi Lu[1], and Chihua Li[2], *1. The University of Hong Kong, Hong Kong, China (People's Republic), 2. University of Macau, Macao, Macao, China (People's Republic)*

Intergenerational social mobility is associated with mental health in older age. However, most research focused on mobility across two generations and used a single indicator (e.g., education). This study explored the pattern of social mobility across three generations using four socioeconomic indicators and examined their association with depressive symptoms among middle-aged and older Chinese. Respondents (aged ≥ 45) from the China Health and Retirement Longitudinal Study were included (N = 3,314). Information regarding the hukou, education, occupation, and Communist Party membership of the respondents' parents, the respondents themselves, and the respondents' children was retrieved. Latent class analysis identified groups with similar social mobility patterns across three generations. Mixed-effects models examined the association of the class memberships with depressive symptoms trajectory in 2011-2018. Five classes were identified: Class 1 (37.90%) showed a slow rise in social status while remaining rural hukou; Class 2 (26.69%) exhibited a steady rise in social status while remaining rural hukou; Class 3 (24.11%) experienced a rapid rise in social status and hukou converting from rural to urban; Class 4 (1.12%) showed a steady rise in social status while remaining urban hukou; Class 5 (7.18%) maintained a high social status and remained urban hukou. Compared to the

"Slowly rising rural", steadily or rapidly rising or stably high social status was associated with a lower risk of depressive symptoms at baseline. This study underscores the disadvantaged status of individuals with rural hukou and slow multigenerational upward social mobility, calling for more targeted mental health support for them.

Abstract citation ID: igaf122.3015

### PARSING AGE EFFECTS IN FUTURE SELF-CONTINUITY THROUGH GOAL ORIENTATION AND LIFE SATISFACTION

Laken Mooney, and Ryan Best, *West Virginia University, Morgantown, West Virginia, United States*

Future self-continuity (FSC), the feeling of connectedness between the current and the future self, has been shown to predict positive outcomes such as dieting and the likelihood of saving for retirement. Limited additional work has investigated the trajectory of FSC across adulthood, generally finding that FSC increases with age. Possible mechanisms considered in this work such as future time perspective, cognition, personality, and health were not found to account for the observed age effects in multiple regression models. The current study builds on this earlier work by using a parallel mediation model to test if age related differences in goal orientation and life satisfaction may account for age-related increases in FSC. An online sample of American adult participants ($n = 479$) was collected from Prolific. Using a hierarchical regression model, previous findings were replicated showing age as a significant predictor of FSC after controlling for income, education, time perspective, goal orientation, and life satisfaction ($p < .001$). Using a parallel mediation model, we found significant indirect effects for gain-oriented goal orientation ($\beta = .022$) and life satisfaction ($\beta = .016$) but not for maintenance or loss prevention goal orientation. Increased age was associated with a decrease in gain orientation which in turn is associated with a decrease in FSC while age was associated with an increase in life satisfaction which was associated with an increase in FSC. Results are discussed in light of the role of our goals and satisfaction in life in establishing a sense of continuity.

Abstract citation ID: igaf122.3016

### PRECARITY AND SOCIAL EXCLUSION AMONG OLDER WOMEN LIVING ALONE

Sena Odabas, *Newcastle University, Newcastle upon Tyne, England, United Kingdom*

The growing number of older women living alone presents unique challenges and opportunities. Framed by social inclusion, exclusion, and precarity, this paper examines the lived experiences of diverse women, aged 60–92, in Newcastle-upon-Tyne, UK, focusing on how life-course transitions shape socio-economic conditions in later life. Based on 26 in-depth interviews, the paper explores significant life events alongside key features of women's social inclusion/exclusion such as social relationships, financial circumstances, and civic participation. Older women's past experiences, present circumstances, and future expectations are analyzed through the lens of precarity.Interview data were inductively coded and interpreted using social inclusion/exclusion theory, precarity, critical gerontology, and the life-course perspective. Participants highlighted the role of evolving social relationships and life transitions in shaping their current situations and anticipated futures. Many participants associated living alone with independence and autonomy while recognizing the value of seeking advice. For most, living alone did not equate to loneliness, as their experiences were shaped by frequent social interactions and stable routines. Staying engaged and maintaining an active lifestyle were essential for well-being. While some felt financially secure, concerns about future care costs persisted. Changes in neighborhood dynamics were generally viewed positively, with strong emphasis on relationships with neighbors. This paper contributes to understanding social inclusion, exclusion, and the conditions necessary for women to live without uncertainty in later life. Further research should explore intersectional factors shaping aging trajectories to inform inclusive social policies.

Abstract citation ID: igaf122.3017

### THE EFFECT OF PHYSICAL IMPAIRMENT EVENTS ON THE MENTAL HEALTH OF OLDER ADULTS

Bolin Fan, Vivian Lou, and Yuting Huang, *The University of Hong Kong, Hong Kong, China (People's Republic)*

From a life-cycle perspective, the mental health of older adults is closely related to both past and present physical impairments. Understanding the impact of physical impairment events on the mental health of older adults is crucial for developing effective interventions to improve their life quality and well-being. Using data from the 2015-2018 waves of the China Health and Retirement Longitudinal Study, hypotheses about the relationship between physical impairment events at different stages of life and depression levels and life satisfaction in older adults were tested. The study found that physical impairment events occurring before the age of 50 significantly increased depression levels in older adults but had no significant impact on life satisfaction. Physical impairment events in the past three years had negative effects on both depression and life satisfaction, but the moderating effect of past physical impairments was not significant. These results indicate that both past and recent physical impairment events can lead to negative emotions in older adults, but only recent events can affect life satisfaction. Past physical impairments cannot prepare individuals for recent events. This study provides evidence of the time-dependent impact of physical impairment events on the mental health of older adults, drawing on hedonic adaptation and life course theory. It shows that older adults gradually adapt to physical impairments, with emotional responses regarding life satisfaction decreasing over time. This theoretical contribution highlights the long-term effects of both early and recent health events on mental health and offers valuable insights for improving life-cycle health management services.

Abstract citation ID: igaf122.3018

### THE MODERATING EFFECTS OF PERCEIVED CONTROL ON THE RELATIONSHIP BETWEEN CHILDHOOD TRAUMA AND ADULT COGNITIVE HEALTH

Chloe Horowitz[1], Eric Cerino[2], Laura Noll[2], and Nora Dunbar[2], *1. Oregon State University, Corvallis, Oregon, United States, 2. Northern Arizona University, Flagstaff, Arizona, United States*

Trauma exposure has been shown to predict change in both executive function (EF) and episodic memory (EM), such that individuals with greater numbers of trauma exposure experienced greater rates of cognitive decline in both cognitive domains than those with fewer exposures. While it is clear that trauma exposure is a risk factor for cognitive decline in adulthood, it is unclear what factors may act as a buffer in this relationship and protect against the deleterious effects of childhood trauma. Perceived control is an important psychosocial resource for cognitive health that may serve as a buffer against these deleterious effects. This study seeks to examine the extent to which perceived control moderates the relationship between childhood trauma and cognitive health in adulthood. We hypothesized that the detrimental impact of childhood exposure to potentially traumatic events (PTE) on adult cognitive health will be attenuated among those with greater perceived control. We used data from waves 2 and 3 of the Midlife in the United States study. Results from multilevel models revealed that perceived control buffered the detrimental impacts of childhood PTE on cognitive health, but the buffering effect was specific to level and change in EF. Exploratory analyses revealed that higher levels of emotional neglect were associated with worse EF performance only among those with lower perceived control. Perceived control did not moderate effects of childhood PTE on EM outcomes. These findings characterize relationships between childhood PTE and cognitive health later in life by identifying the protective effects of perceived control.

Abstract citation ID: igaf122.3019

### UNRAVELING THE EARLY LIFE IMPACTS ON HEALTHY AGING: INSIGHTS FROM THE CHINA HEALTH AND RETIREMENT LONGITUDINAL STUDY

Liangyi Jin, and Chenkai Wu, *Duke Kunshan University, Kunshan, Jiangsu, China (People's Republic)*

Early-life socioeconomic and environmental factors play a crucial role in influencing the onset of diseases in later life. However, their impact on healthy aging remains understudied. This study examined the association between early-life circumstances and healthy aging among community-dwelling older adults in China, assessed by the Chinese Healthy Aging Index (CHAI). Data were from the China Health and Retirement Longitudinal Study, including 3,069 participants aged ≥60 years who completed the life history survey and had data to calculate the CHAI. The CHAI comprised six components, and each was scored 0 (healthiest), 1, and 2 (unhealthiest) according to sex-specific tertiles or clinically relevant cut-points. The scores were summed to construct the CHAI (0–12). Seven Early life risk factors were included, and we used the logistic regression to determine their associations with CHAI both individually and cumulatively. The average CHAI score was 5.91 (SD = 2.10); 4.82% had a score of 0-2 (healthiest). After multivariable adjustment, personal education attainment and family economic status were individually associated with healthier CHAI scores; the cumulative early-life risk factor score was significantly associated with the CHAI, both in categories and continuously. Each additional score was related to a 34% (95% CI: 20%, 50%) higher odds of being in the healthiest CHAI category. Favorable early-life socioeconomic conditions are associated with healthier aging outcomes among Chinese community-dwelling older adults. Addressing socioeconomic inequalities early in life is pivotal in promoting healthy aging.

Abstract citation ID: igaf122.3020

### WHAT CHARACTERISTICS DEFINE THOSE LIVING ALONE ACROSS STAGES OF THE LIFE COURSE?

Aarohi Deshmane[1], and Ross Andel[2], *1. Arizona State University, Chandler, Arizona, United States, 2. Arizona State University, Arizona State University, Arizona, United States*

As mobile applications become increasingly used to support older adults' mental health, these apps can provide useful insight about factors influencing health and wellbeing across the life course. We set out to investigate correlates of living alone in users of the mobile application Terrapino, launched in December 2022 and developed to help support cognitive health. The entire sample of Terrapino app users aged 18 years and older with complete information on sociodemographic, mental/physical health, and lifestyle factors (n = 8,395) were included and classified into age groups of 18-29 (n = 402), 30-44 (n = 1,128), 45-54 (n = 2,002), 55-64 (1,916), 65-75 (n = 2,078), and 75 + (n = 869). Overall, about equal proportion of individuals lived alone in the youngest (47%) and oldest (44%) age group, with ~20-25% living alone in other age groups. However, men were significantly more likely to live alone early in adulthood (55% vs. 44%; $\chi2[1]=4.57$, p=.033), whereas women were significantly more likely to live alone at ages 55-64, 65-74, and 75 + ($\chi2[1]=20.64$, 127,43, 104.72, respectively, ps<.001). In fully adjusted logistic regression models, those living alone were less educated, engaged in more mental activity but also had poorer sleep, were more likely to smoke and be depressed, but were less likely to drink alcohol in high amounts (ps<.05). The differences, outside of sex differences, higher likelihood of depression and poor sleep in those living alone, started to emerge after age 44. Focusing on treating depression and improving sleep may serve as effective areas for intervention with those living alone at any stage of adulthood.

Abstract citation ID: igaf122.3021

### WHAT MAKES PEOPLE COMMIT TO LEISURE ACTIVITIES? A SELF-DETERMINATION THEORY AND SERIOUS LEISURE APPROACH

Sua Im, Rokbit Lee, Yaeeun Yoo, and Jinmoo Heo, *Yonsei University, Seoul, Republic of Korea*

This qualitative study explores the motivators that facilitate individuals' deep engagement in leisure activities. Park-golf is a form of golf that has catered to the needs and abilities of older adults. Park-golf has recently been popular in Asian countries, and it is known as the fastest growing sport among older adults in Korea. We conducted in-depth interviews with 11 park-golfers who were members of a local club in Korea. The participants included eight males and three females, aged 57 to 74 years (M = 64.5), with experience ranging from 1 to 11 years. Our analysis revealed that (1) pure enjoyment, (2) self-identity, and (3) personal goals played key roles in the participants' intense engagement. Specifically, pure enjoyment was characterized by a sense of refreshment, stress relief, and interest, all of which contributed to a high level of participation. In terms of self-identity, when participants transferred their past experiences to the current leisure activity and perceived the activity as meaningful to their sense of self, it led to increased commitment. Personal goals, such as improving scores, health, or social connections, motivated participants to become more active players. Based on self-determination theory and the serious leisure framework, the results suggest that both self-determined motivators and durable benefits contribute to commitment to leisure activities. Additionally, social contexts positively influence engagement, highlighting the need for supportive environments. Our findings suggest that, in addition to gratification from the activity itself, a sense of alignment with valued desires and personal integrity is essential for sustaining dedication.

### SESSION 7370 (POSTER)

### LONG TERM CARE II

Abstract citation ID: igaf122.3022

### BARRIERS & FACILITATORS OF INNOVATION IN LONG-TERM CARE HOMES DURING COVID-19 IN BRAZIL: VIEWS FROM KEY STAKEHOLDERS

Suzanne Santos[1], Gilciney Rabello[1], Gabriela Nacimben[1], Marisa Domingues[1], Meire Cachioni[1], Patrick Wachholz[2], Charlene Chu[3], and Ruth Melo[1], *1. Universidade de São Paulo, São Paulo, Sao Paulo, Brazil, 2. Universidade Estadual Paulista, Botucatu, Sao Paulo, Brazil, 3. University of Toronto, Toronto, Ontario, Canada*

The COVID-19 pandemic had a significant effect on individuals residing and working in long-term care homes (LTCHs). This scenario highlighted systemic disadvantages and vulnerabilities and called for innovations in the sector around the world. In Brazil, the LTC segment is historically marked by inequalities, underfunding, absence of systematically organized data and research. Therefore, little is known about the perception of key stakeholders about innovation in LTC. This cross-sectional qualitative study, part of the CONNECTED consortium, investigates potential barriers and facilitators in implementing innovations during COVID-19 from the perspective of key stakeholders from four LTCHs. The team conducted 15 semi-structured interviews (residents: n = 5, family members/caregivers: n = 5, staff: n = 5) from March to October 2024. Interviews were transcribed verbatim and the results were analyzed using thematic analysis through Rapid Assessment Procedure (RAP) sheets. Besides financial constraints and workforce shortage, barriers to innovation during COVID-19 included lack of involvement in decision-making for residents, family members, and direct care workers. Residents reported uncertainty in whether their opinions were considered. Communication issues were shared by all groups and influenced their ability to adapt to the new protocols. Facilitators included the willingness to change and the perceived gains of innovations, including enhanced safety measures and the use of technology, which was critical for staying connected. Staff emphasized the need for structured support, while families focused on emotional connections. Such insights underscore the need for inclusive, co-designed practices in Brazilian LTCHs in order to empower the sector and ensure the dignity of such populations.

Abstract citation ID: igaf122.3023

### BARRIERS & FACILITATORS OF INNOVATION IN LONG-TERM CARE HOMES DURING COVID-19: INSIGHTS FROM BRAZILIAN EXPERTS

Gilciney Rabello[1], Suzanne Santos[1], Gabriela Nacimben[1], Marisa Domingues[1], Meire Cachioni[1], Patrick Wachholz[2], Charlene Chu[3], and Ruth Melo[1], *1. Universidade de São Paulo, São Paulo, Sao Paulo, Brazil, 2. Universidade Estadual Paulista, Botucatu, Sao Paulo, Brazil, 3. University of Toronto, Toronto, Ontario, Canada*

The COVID-19 pandemic profoundly disrupted global healthcare systems, with Long-Term Care Homes (LTCHs) facing unprecedented challenges that underscored the critical role of innovation in crisis response. This cross-sectional qualitative study, part of the CONNECTED consortium, examined insights from 12 Brazilian experts (researchers, LTCH managers, industry representatives, and heads of local/national LTCH organizations) to explore barriers and facilitators in implementing innovations during COVID-19. Data were collected through 12 semi-structured interviews (from March to October 2024) and analyzed using thematic analysis. The innovations implemented during COVID-19 were related to: 1) safety and preventive measures, 2) communication and social connections tools, 2) telehealth, 3) remote training programs for LTCH staff, and 4) national voluntary movement to support the LTCHs. Interviewees identified multiple systemic barriers to innovation implementation in LTCHs, including resource constraints (financial and human), digital illiteracy among residents, institutional resistance to change, inadequate staff training, regulatory hurdles, infrastructural limitations, and entrenched cultural stigma surrounding LTCH care. Conversely, experts highlighted key facilitators: targeted governmental and organizational funding, national-driven knowledge sharing, implementation of rapid communication channels, and cross-sector partnerships between public health authorities and LTCHs—all of which proved critical in mitigating pandemic-related disruptions. In Brazil, while the COVID-19 pandemic pressured catalyzed rapid adoption of vital innovations, from telehealth platforms to national solidarity initiatives, systemic barriers like resource scarcity, infrastructural gaps, and cultural stigma persisted as ingrained systematic issues. Therefore, addressing these systemic challenges is imperative to develop the resilience of the Brazilian long-term care sector and ensure preparedness for future health crises.

Abstract citation ID: igaf122.3024

### EVALUATING THE IMPACT OF MANAGED LONG-TERM SERVICES AND SUPPORTS ON HCBS PROVIDERS: FINDINGS FROM A SEVEN WAVE SURVEY

Heather Mentch, Keri Kastner, and Howard Degenholtz, *University of Pittsburgh, Pittsburgh, Pennsylvania, United States*

Community HealthChoices (CHC), Pennsylvania's managed long-term services and supports (MLTSS) program, was designed to improve service coordination, expand access to home and community-based services (HCBS), and ensure provider financial sustainability. This study presents findings from a seven-wave (2018–2024) statewide survey of HCBS providers conducted by the Medicaid Research Center at the University of Pittsburgh. The survey assessed provider experiences with managed care organizations (MCOs), satisfaction with CHC implementation, financial viability, service accessibility, and provider-MCO interactions. Response rates ranged from 19% to 49%, with data analyzed to examine regional trends and provider perspectives. Findings indicate that provider sentiment evolved over time, with initial optimism declining post-implementation before returning to or exceeding pre-implementation levels. While MCO-provider relationships strengthened, there are persistent differences in the rank ordering of perceptions of MCOs. Providers increasingly recognize the importance of upgrading technology. Providers' perceptions of quality and access have generally improved over time, with the notable exception of a slight decrease in perceptions of quality in the Southeast. Providers' expectations regarding financial viability initially declined post-implementation, but have generally improved over time, apart from a recent slight decrease in the Southeast. This multi-year evaluation highlights the complexities of CHC implementation from the provider perspective. While progress has been made in communication, administrative efficiency, and service accessibility, opportunities for improvement related to regulatory burden and financial stability persist. Regional differences suggest that implementation experiences vary, underscoring the need for targeted policy improvements. Future efforts should focus on strengthening provider financial stability and supporting technological improvement.

Abstract citation ID: igaf122.3025
### FACTORS INFLUENCING NURSING PERFORMANCE OF LONG-TERM CARE HOSPITAL NURSES IN KOREA
So Young Shin, and Shin Young Park, *Inje University, Busan, Busan, Republic of Korea*

Purpose: The purpose of this study is to identify the effect of resilience, communication competence, nursing work environment on nursing performance of nurses at long-term care hospitals in Korea. Methods: Among staff nurses at 5 long-term care hospitals located in B metropolitan city in Korea, 137 subjects who have a long-term care experience of more than 1 year participated in this study. Data collection was conducted with the structured self-report questionnaire survey. The collected data were analyzed with descriptive statistics, independent t-test and one-way ANOVA, Scheffé test for post-hoc analysis, Pearson's correlation coefficient and multiple linear regression. Results: The mean age of 137 subjects was 50.96(±10.17) years. Nursing work performance was positively correlated with resilience (r=.70, p<.001), communication competence (r=.59, p<.001), and nursing work environment (r=.23, p=.007). In the multiple linear regression analysis, factors influencing nursing performance of subjects were resilience (β=.54, p<.001) and communication competence (β=.19, p=.037). The explanatory power of resilience and communication competence on nursing performance was 51% (F=24.13, p<.001). Conclusion: The results from this study show that for long-term care hospital nurses, resilience and communication competence have a significant effect on their nursing performance. To enhance nursing performance of nurses at long-term care hospitals, it is necessary to develop effective mediating programs specifically improving resilience and communication competence.

Abstract citation ID: igaf122.3026
### FACTORS RELATED TO RETENTION OF FOREIGN "SPECIFIED SKILLED NURSING CARE WORKERS" IN JAPAN
Noriko Tsukada[1], and Jun Nishimura[2], *1. Nihon University, Tokyo, Tokyo, Japan, 2. Rissho University, Tokyo, Tokyo, Japan*

The Japanese government created a new residence status, "Specified Skilled Workers," to recruit foreign workers in 2019 to cope with serious labor shortages in the 14 industries, including nursing care. There is no restriction on the countries eligible to enter Japan. This study examined factors related to the retention of Specified Skilled nursing care (SSNC) workers. Structured questionnaires were sent to randomly selected 1000 corporations/hospitals nationwide that hired SSNC workers. 9 questionnaires were sent to each address, and SSNC workers were asked to respond to the survey electronically from October 31 to November 30, 2024. Despite the low effective response rate(4%), a total of 340 responses were analyzed in this study. About 93% of the respondents came from 5 countries, including Vietnam(34.1%), Philippines(25.3%), Indonesia(14.1%), Myanmar(10%) and Nepal(9.4%). Preliminary bivariate analyses revealed that "levels of desire to obtain national nursing care certificate" and "job satisfaction" were found to be statistically significantly related to retention of SSNC workers; however, its strength of the relationships varied among countries. For example, as for the levels of desire to obtain a national certificate, while it was the highest(r=.807**) for Philippine SSNC workers, it was the lowest(r=.361*) for Nepal SSNC workers. Moreover, among 37 factors asked, 11 factors were statistically significantly related to retention for Myanmar SSNC workers, while for Indonesians one factor, "increase the number of retakes of national exams," was statistically significant. It is suggested that the government be aware of these differences among countries and develop country-specific-policies to promote retention of SSNC workers.

Abstract citation ID: igaf122.3027
### FOREGROUNDING DIGNITY AND KINDNESS ACROSS CARE FACILITIES: AGING AROUND THE WORLD
Luis Cordero, and Elissa Kozlov, *Rutgers School of Public Health, Piscataway, New Jersey, United States*

As global demographics shift, innovative models of care are emerging to address the evolving needs and preferences of older adults worldwide. Traditional, one-size-fits-all approaches to long-term care are increasingly insufficient, necessitating adaptable and community-centered solutions. This study draws on field observations from ten countries across South America, Central America, Europe, Asia, and Australia between 2022 and 2023. Site visits encompassed both rural and urban care settings, including nursing homes and community-based programs. A key finding across all settings was the central role of community engagement in enhancing well-being, even within institutional environments. For example, Argentina's government-funded initiatives, such as Estaciones Saludables and free day centers, extend opportunities for older adults to remain engaged in community life and delay institutionalization. Similarly, intergenerational exchange programs within care settings foster mutual benefits for residents and the broader community. Additional innovative models include Spain's integration of coworking spaces within nursing homes, Australia's Community Home Model, and Nepal's extensive community event programs for older adults. These approaches highlight the importance of fostering social connections and reimagining traditional care structures to align with cultural contexts and demographic changes. As societies worldwide adapt to aging populations, the future of long-term care lies in models that emphasize community integration, social engagement, and cultural responsiveness.

Abstract citation ID: igaf122.3028
### HEALTH OUTCOMES OF OLDER ADULTS IN LONG-TERM CARE HOMES FOLLOWING HOSPITALIZATION FOR HIP FRACTURE SURGERY
Tiffany Yu[1], Maya Murmann[2], Nicholas Yee[3], Benoît Robert[1], David Kirkwood[4], Richard Perez[4], and Amy Hsu[2], *1. University of Ottawa, Ottawa, Ontario, Canada, 2. Bruyère Health Research Institute, Ottawa, Ontario, Canada, 3. University of Toronto, Toronto, Ontario, Canada, 4. ICES McMaster, Hamilton, Ontario, Canada*

Hip fractures are among the most common and debilitating conditions in the older adult population, particularly for those living in long-term care (LTC) homes. These fractures can reduce quality of life and increase mortality risk. However, surgical management carries potential complications. As a result, older adults and their families often confront the difficult decision of whether to undergo surgery. In this study, we compare outcomes of hospitalized older adults (65+ years old) in Ontario LTC homes that did or did not undergo hip fracture surgery. Data was obtained through the Institute for Clinical Evaluative Sciences (ICES) from 2015 to 2019. Older adults were categorized based on presence of a recorded surgical code. Following this, baseline characteristics and post-discharge outcomes, including mortality, were retrieved from ICES. Outcomes were further stratified by various frailty indices. Among 5,279 older adults meeting inclusion criteria (mean age: 87.01 years, 74.2% female), 4,547 (86.1%) underwent surgery. At baseline, non-surgically managed older adults had higher rates of osteoporosis, hypertension, renal failure, as well as greater functional impairment. Six months post-discharge, older adults who were surgically managed reported fewer deaths in the hospital (4.7% vs 12.8%, $p < 0.0001$), were less likely to experience frequent pain (6.6 vs 7.3%, $p < 0.0001$), and saw improved functional independence (13.8% vs 11.9%, $p < 0.0001$). However, they were also more likely to be physically restrained (11.5% vs 6.3%, $p < 0.0001$). These findings highlight key factors that influence the decision to undergo hip fracture surgery, which offers invaluable insights for clinical decision-making and care planning in LTC.

Abstract citation ID: igaf122.3029

### IMPACT OF ALZHEIMER'S DISEASE ON ORAL HEALTH AND REGIONAL VARIATIONS IN LONG-TERM CARE (2020-2024)

Joohyun Chung, Weidong Wang, and Jing Qian, *University of Massachusetts Amherst, Amherst, Massachusetts, United States*

Around 1.3 million older adults in long-term care (LTC) facilities are at high risk for poor oral health, often neglected, particularly among those with Alzheimer's Disease and Related Dementias (ADRD). This study examines the prevalence of oral health issues, such as tooth loss and gum disease, in LTC residents at various stages of ADRD. It also explores the impact of Alzheimer's on oral health and regional disparities using state-level data from the Minimum Data Set (MDS) from 2020 to 2024. Using a non-experimental, quantitative, longitudinal study design, the research analyzes time trends in the prevalence of oral health problems across various ADRD stages. Descriptive statistics and mixed logistic models were used to examine the relationship between ADRD stages (intact/borderline impairment, moderate, and severe) and oral health outcomes. The study also explores regional differences in the prevalence of tooth loss and gum disease across U.S. The findings show that ADRD significantly affects oral health. Residents with intact/borderline ADRD exhibit poorer oral health, while moderate and severe ADRD significantly increases the prevalence of tooth loss/ gum disease ($p < 0.001$). States like Texas, California, and Florida have seen declines in tooth loss rates, while Kentucky and West Virginia continue to experience high rates. For gum disease, reductions were noted in California, Georgia, and Florida, while Hawaii and Montana saw sharp increases. These results highlight the need for targeted oral health interventions, especially in states with high prevalence rates, to improve care for ADRD residents and address regional disparities in oral health outcomes.

Abstract citation ID: igaf122.3030

### LONGITUDINAL PATTERNS OF CARE TRANSITIONS AMONG FIRST-TIME NURSING HOME RESIDENTS: A FIVE-YEAR STUDY IN MINNESOTA

Dongjuan Xu, Emily Garcia, and Greg Arling, *Purdue University, West Lafayette, Indiana, United States*

The objectives were to: 1) model the 5-year care trajectories of a cohort of first-time admissions to Minnesota nursing homes (NHs), while considering multiple outcomes of discharges to the community, NH readmission, acute hospitalizations, and mortality; and 2) determine the percentage of admissions and resident characteristics related to each trajectory. Using the Minimum Data Set and the Medicaid claims data files, we selected 11,943 residents, age 65+, admitted to Minnesota NHs in 2015 with no prior NH stays. Admissions were followed for 5 years (2015-2019) for survival and care transitions between NH, community, and acute hospitals. Latent class growth analysis was used to analyze longitudinal care trajectories. The optimal number of latent classes was four. Trajectory 1 (37%) had the shortest survival and highest initial (30-day) rate of rehospitalizations. Trajectory 2 (11%) had the second shortest survival and the highest hospitalization rate during years 4–5. These residents were initially discharged to the community but experienced a steadily increasing rate of NH readmission. Trajectory 3 (22%) demonstrated longer survival and the highest hospitalization rate during years 1-3. Trajectory 4 (30%) experienced the most successful transitions to the community and the best long-term outcomes, characterized by the fewest NH readmissions, longest survival, and lowest hospitalization rates. Our findings highlight the heterogeneity of outcomes for the NH population. The trajectories were distinguished by age, dementia, ADL dependency, and comorbid conditions. Understanding this heterogeneity is crucial for developing appropriate care models and policies to meet the needs of an increasingly diverse NH population.

Abstract citation ID: igaf122.3031

### MINNESOTA MEDICAL DIRECTORS: CHALLENGES AND OPPORTUNITIES

Rajean Moone, Teresa McCarthy, and Megan Hakanson, *University of Minnesota, Minneapolis, Minnesota, United States*

Nursing homes must appoint a physician as a Medical Director, yet little is known about their education, challenges, and needs. We conducted an online survey to assess the credentials, responsibilities, and difficulties faced by Minnesota Medical Directors. Since no comprehensive list existed, we called all Minnesota nursing homes to identify their Medical Directors. At 147 facilities (43%), staff were unaware of who held this role. A total of 26 Medical Directors, representing 42% of state nursing homes, responded. Most were male, white, and had served an average of 14 years, spending about six hours on-site monthly. Only 19% completed a geriatrics fellowship, and 46% held a medical director certification. Employment status varied: 69% with a nursing home, 42% through a medical group, and 4% via a stipend. All participated in their facility's quality committee. Qualitative analysis revealed 11 key challenges, including staffing shortages, communication issues, excessive regulations, clinical complexity, leadership struggles, and time constraints. Four main needs emerged: additional education, peer support, regulatory consistency, and more time for patient care. As Medical Directors'

roles evolve, understanding their needs and challenges is crucial to enhancing support and improving nursing home care.

Abstract citation ID: igaf122.3032

**NURSE AIDE TURNOVER IN LONG-TERM AND ACUTE CARE SETTINGS: WORKPLACE DYNAMICS, INCONTINENCE CARE, AND JOB DEMANDS**

Vivian Miller[1], Lauren Maziarz[1], Eric Cooke[1], Julia Bell[2], Jennifer Wagner[1], and Melissa Burek[1], *1. Bowling Green State University, Bowling Green, Ohio, United States, 2. Northeastern University, Boston, Massachusetts, United States*

Turnover rates among Nurse Aides (NAs) within long-term and acute care settings are incredibly high; annual turnover rates within these settings exceed 100%. Nurse Aide (NA) turnover at this rate jeopardizes resident care and places increase burden on remaining staff within these settings. Much research has focused on working conditions and staffing ratios as impacting NA turnover but has yet to examine specific challenges including incontinence care and workplace dynamics. Guided by the Triple Aim, Quadruple Aim, and Quintuple Aim frameworks and the socio-ecological model, this study utilizes a cross-sectional survey to analyze the relationship between perceived workplace challenges, time spent on incontinence care, and turnover intentions. Findings from a sample of 307 NAs ($N=307$) reveal that time spent on incontinence care significantly predicts perceptions of work being challenging but is not directly associated with turnover intentions. Rather, factors such as physical demands, workplace culture, and poor management support were more predictive of turnover. These results underscore the complexity of NA turnover within long-term and acute care settings. Workplace culture, physical demands, management support, and incontinence care are found to be challenging and may impact NAs intention to leave their job. Efforts to reduce turnover among NAs should focus on addressing broad workplace challenges, including incontinence care practices, such as NA training and assistive equipment. Future research may examine incontinence care and workplace considerations over time. Policies may consider initiatives to reduce disparities in NA turnover and improve overall quality of care within and across long-term care settings.

Abstract citation ID: igaf122.3033

**OLDER PERSON'S PARTICIPATION IN LIFE-ENHANCEMENT ACTIVITIES: A MIXED-METHODS OBSERVATIONAL STUDY**

Amarjot Gill[1], Sharon Hewner[2], Jihnhee Yu[1], and Tania Von Visger[1], *1. University at Buffalo, Buffalo, New York, United States, 2. University at Buffalo, New York, United States*

This study examines older persons' experiences and participation in life-enhancement activities in a Canadian long-term care (LTC) facility. Naturalistic observations of 20 life-enhancement activity sessions were conducted in a single LTC facility that includes 111 older persons in September 2024. Data were collected through field notes and guiding questions for systematic observation. NVivo software (version 1.7.1) was used for qualitative thematic analysis, and SPSS software (version 30.0) was used for quantitative analysis. We used Kruskal-Wallis and Mann-Whitney U tests for analysis, with results considered significant if $p<0.05$. There were significant differences in engagement levels: self-initiative ($p<0.001$), assistance-seeking frequency ($p=0.002$), and social interaction frequency ($p<0.001$) across activities. Social interaction frequency also significantly differed by mobility status (wheelchair, walker, independent) ($p=0.014$). Interpersonal conflict incidents ($p<0.001$) and positive emotional expressions ($p<0.001$) varied across activity locations (TV room versus Activity Room). Participants displayed significantly more distractions in the TV Room than in the Activity Room ($p=0.001$). Four themes emerged from thematic analysis: 1) participation barriers, 2) activity contextual factors, 3) facilitator support strategies, and 4) social interactions and emotional well-being. Individual (mobility) and situational (activity location) factors can influence older persons' engagement in life-enhancement activities. For older persons to achieve active participation in life-enhancement activities, we must consider their mobility needs, a non-disruptive environment, and facilitator support. Life enhancement activities benefit from facilitators promoting independence and teamwork, improving participation and social connections.

Abstract citation ID: igaf122.3034

**PERSON-CENTERED CARE PLANNING IN MEDICAID 1915(C) HOME AND COMMUNITY BASED SERVICE (HCBS) WAIVERS FOR OLDER ADULTS**

Natalie Turner, *University of Washington, Seattle, Washington, United States*

Person-centered care planning is an approach to service coordination that prioritizes an individual's goals, needs, preferences, and desires. Over the past decade, the Administration for Community Living and the Centers for Medicare & Medicaid Services have worked to advance person-centered planning within home and community-based services (HCBS). 1915(c) waivers can offer insights into how states apply these principles in care policy. This qualitative study examined how person-centered care planning was described in 63 1915(c) waivers for older adults using the Frameworks Approach, a qualitative method designed for applied policy research. Codes were developed deductively based on waiver application questions and applied to select appendices detailing care planning processes. Data were summarized into matrices and analyzed to identify four key themes: (1) Care planning led and owned by participants; (2) Holistic approaches to care planning; (3) Individualization of services; and (4) Care plans as living, adapting documents. Waivers emphasized participant-driven decision-making, with language such as "Member choice drives all decisions." Care planning was also holistic, considering not only health and functional needs but also social life, community engagement, and other enriching activities voiced by participants. Plans required documentation of individual preferences on service delivery, including schedules and desired activities. Finally, care plans were expected to evolve alongside participants' changing needs. These findings highlight how person-centered care planning is operationalized in care policy and underscore the importance of individual agency in HCBS. Future research should examine how the operationalization of person-centered planning compares to its implementation to assess alignment and effectiveness.

Abstract citation ID: igaf122.3035

**PROMOTING FREEDOM AND AUTONOMY IN RESIDENTIAL DEMENTIA CARE: LESSONS FROM GREEN CARE FARMS**

Gijs Steinmann, Maud Hamers, and Hilde Verbeek, *Maastricht University, Maastricht, Limburg, Netherlands*

Environmental factors play a crucial role in promoting the functional capacities, well-being, and autonomy of older adults, particularly those with dementia. However, by prioritizing safety and control, traditional nursing homes often create restrictive environments. Green Care Farms (GCFs) are increasingly recognized as a promising alternative, integrating physical, social, and organizational aspects that differ radically from traditional settings. This study examines how residential care environments can be (re)designed to structurally promote the freedom and remaining capacities of people with dementia. Specifically, we identify the environmental aspects through which GCFs foster autonomy and explore their potential implementation in other settings. Our findings are based on qualitative research at two Dutch care settings: a GCF and a regular nursing home (RNH). At the GCF, we combined participant observation with semi-structured interviews to immerse ourselves in residents' everyday surroundings and identify aspects that enhanced or constrained freedom and autonomy. At the RNH, we conducted participant observation as active members of a project team in which staff sought to integrate GCF elements into their traditional setting. Our analysis reveals that the GCF environment promotes freedom and autonomy through a consistent alignment of physical and organizational aspects. This generates environmental presses that stimulate physical activity to maintain physical functioning, and it enables the acceptance of risks to enhance freedom. In principle, each of these aspects could be implemented in other settings. However, this would require profound organizational changes in RNHs, as staff would need to adopt values and practices that directly oppose traditional ways of working.

Abstract citation ID: igaf122.3036

### RECRUITMENT OF NURSING HOME RESIDENTS FOR RANDOMIZED CONTROL STUDY: CHALLENGES AND STRATEGIES

Diana Sturdevant[1], Julie Gordon[2], and Barbara Carlson[3], *1. The University of Oklahoma Health Sciences Center, Oklahoma City, Oklahoma, United States, 2. The University of Oklahoma Fran and Earl Ziegler College of Nursing, Oklahoma City, Oklahoma, United States, 3. The University of North Carolina at Wilmington, Wilmington, North Carolina, United States*

Numerous studies attest to the challenges of recruiting and retaining nursing home (NH) residents in clinical trials. In this presentation, we present methods we used to recruit and retain NH residents in a randomized, double-blinded, comparative study that assessed the effects of the Shingrix vaccine on the innate immune system residents (age 65-XXX) during the COVID-19 pandemic. The protocol was quite complex (1) recruitment/consent, (2) Baseline blood draw and 1st injection, and (3) a second injection/blood draw (3 months later). The post-treatment control group had the same number of visits but had two additional visits to receive the vaccine. Of the 217 residents enrolled in the study, 160 residents from 23 NHs completed the study. Our success relied upon working alongside corporate and facility leaders to tailor our protocols to the ever-evolving clinical landscape, which included (1) fluctuating trends in staff/resident outbreaks, (2) weather emergencies, (3) the introduction of new local/federal government regulations (i.e., regular COVID testing, rules governing entry into a facility), and (4) introduction of the COVID vaccine/vaccine hesitancy. While corporate endorsement helped the team to gain entrance, our strategies enabled nursing home staff to become vested in the research. Recognizing the value, both socially and medically, the protocols had for their clients, they worked with us to recruit and retain resident research participants.

Abstract citation ID: igaf122.3037

### REDUCING MINIMUM DIRECT CARE STAFFING REQUIREMENTS IN FLORIDA NURSING HOMES: IMPLICATIONS AND FUTURE DIRECTIONS

Lindsay Peterson[1], and Dylan Jester[2], *1. University of South Florida, Tampa, Florida, United States, 2. VA Palo Alto Healthcare System, Palo Alto, California, United States*

A robust body of research associates higher numbers of direct care staff hours with better quality of care in nursing homes. More RN hours are associated with fewer regulatory deficiencies, pressure ulcers, urinary tract infections, and emergency department visits. More CNA hours are associated with fewer deficiencies, hospitalizations, and pressure ulcers. In 2022, the state of Florida passed legislation to reduce the minimum nursing care requirements for the roughly 700 nursing homes statewide. Prior to the reduction, Florida was a national model with a required 3.6 hours of direct care per resident per day, including 2.5 hours of Certified Nursing Assistant (CNA) care and 1 hour from a licensed nurse. The 2022 legislation reduced the CNA requirement to 2 hours of care per resident per day. Licensed nurses remained at 1 hour and other staff, such as feeding assistants, were authorized to fulfill the remaining 0.6 hours. This study's purpose was to assess the effect of the change using staffing and quality measures data from the Centers for Medicare and Medicaid Services. We found that as of April 2021, Florida nursing home residents received, on average, 3.93 hours of direct nursing care per day. By October 2022, the number had dropped to 3.60. CNA time dropped the most, from 2.55 hours to 2.33 hours per resident per day. Additionally, we found during that same time an increase in resident emergency department visits and hospitalizations. We discuss the implications and nursing home staffing trends across the United States.

Abstract citation ID: igaf122.3038

### THE DEVELOPMENT OF AN UNMET NEEDS APPRAISAL SCALE FOR LONG-TERM CARE SERVICE USERS WITH DISABILITY CERTIFICATES

Peng-Li Wang[1], Shih-Cyuan Wu[1], Hsiao-Wei Yu[1], Shwu-Chong Wu[1], Shwn-Jen Lee[2], Shu-Chen Lu[3], Shiau-Fang Chao[1], and Ya-Mei Chen[4], *1. National Taiwan University, Taipei, Taipei, Taiwan (Republic of China), 2. National Yang Ming Chiao Tung University, Taipei, Taipei, Taiwan (Republic of China), 3. Taiwan Occupational Therapy Association, Taipei, Taipei, Taiwan (Republic of China), 4. National Taiwan University, National Taiwan University, Taipei, Taiwan (Republic of China)*

In 2017, Taiwan launched Long-Term Care4LTC4Plan 2.0, expanding services to include those under 49 with disability certificates. The LTC unmet needs of this group can be different from those general LTC users. By 2021, 182,555 people with disability certificates451% of LTC users4had received LTC services. The Unmet Needs Appraisal Scale (UNAS) for general LTC users, developed by Professor YM Chen's team, is a tool with one domain and 11 items. The purpose of this study was to develop UANS for People with Disability Certificate (UNAS-Disability People) . After expert panel, three items were added to the tool with 14-item version of the

UNAS- Disability People. The revised tool was pilot tested in collaboration with city governments and NGOs associated with various types of disabilities. We examined the tool's properties, including reliability and validity, and included Short-Form 8 as a measure of concurrent validity. From northern and central Taiwan, 620 questionnaires were collected. The UNAS-Disability People initially had 14 items across two domains: unmet needs for mental and physical health (Cronbach's $\alpha = 0.91$). Ultimately, 13 items and two domains were retained, showing good reliability and validity [$\chi^2 = 384.582$, df = 64, p<.0001; CFI=0.906; RMSEA=0.085; SRMR=0.051]. The two domains were moderately correlated (r = 0.69, p<.0001). Both domains showed moderate negative correlations with Physical Component Summary and Mental Component Summary (r = -0.450, p < .0001; r = -0.414, p < .0001), confirming good concurrent validity. The UNAS-Disability People can provide better LTC unmet needs assessment for people with disability certificates with two domains of unmet needs.

Abstract citation ID: igaf122.3039

### THE ROLE OF ENVIRONMENTAL AND SPATIAL FACTORS FOR PERSON-CENTRED CARE IN RESIDENTIAL LONG-TERM CARE FACILITIES

Oscar Ribeiro[1], Miguel Padeiro[2], Flavia Borges-Machado[1], Liliana Sousa[1], and Paula Santana[2], *1. University of Aveiro, Aveiro, Aveiro, Portugal, 2. University of Coimbra, Coimbra, Coimbra, Portugal*

This cross-sectional study investigated variations in person-centred care (PCC) across Portuguese residential long-term care facilities, focusing on environmental and spatial determinants, including facility location, surrounding local environment, and outdoor spaces. Data were collected through a self-administered anonymous online survey distributed nationwide to care home directors between January and June 2024. PCC was assessed using the Person-Centred Care Assessment Tool (P-CAT), and statistical analyses, including bivariate and multivariate models, were conducted using RStudio. A total of 424 directors participated, with the majority representing non-profit cooperative and social sector entities (81.56%). Facilities had an average of 39.63 residents, of whom approximately one-third (31.66%) were people living with dementia (PLwD). P-CAT scores ranged from 34 to 65. Higher PCC levels were positively associated with a perceived sense of security in the local environment, the presence of local commerce, and perceived neighborhood quietness. Concerning outdoor spaces, a sense of security and the presence of an orchard or vegetable garden were also significant predictors of PCC. These findings highlight the relevance of environmental and spatial factors, which are often overlooked in existing research, emphasizing the need to integrate territorial and external contextual variables when assessing PCC variations in long-term care settings. Funding by Fundação para a Ciência e a Tecnologia: SINDIA (2022.04684.PTDC), CEGOT (UIDB/04084/2020), RISE (LA/P/0053/2020).

Abstract citation ID: igaf122.3040

### TRANSITIONS BETWEEN SELF AND PROXY COMPLETION OF NURSING HOME RESIDENT CARE AND ACTIVITY PREFERENCES

Laura Block, and Tonya Roberts, *University of Wisconsin-Madison, Madison, Wisconsin, United States*

Identification of nursing home (NH) resident preferences for care and activities is crucial to informing person-centered care. Preferences are systematically recorded in the Minimum Data Set (MDS) and assessed through self and proxy report. Concerns surrounding accuracy of proxy report exist. Yet, transitions between self and proxy report of preferences, related predictors, and impact on preference patterns are unknown. This retrospective cohort study identified all residents with two preference assessments completed in the 2019 MDS 3.0 (N = 357,321) and examined transitions across preference respondent. Of the residents for whom preference respondent changed (n = 40,799), 31.8% transitioned from self to family report while 14% transitioned from self to staff report. In contrast, 21.8% transitioned from family to resident report and 12% from staff to resident report. Rates of dementia were only somewhat higher among residents who transitioned from resident to family (50.0%) versus family to resident report (46.5%). Cognition worsened in all cases of loss of self-report and improved in all cases of resuming self-report. Preferences reported as important varied across reporter. Findings have implications for understanding the potential impact of proxy report of preference reports and the importance of early preference assessment among individuals with dementia. Future research examining a wider range of factors that predict return of self-report capacity is needed.

### SESSION 7375 (POSTER)

### MENTAL HEALTH II

Abstract citation ID: igaf122.3041

### A NEURODEVELOPMENTAL MODEL OF EATING DISORDER RISK IN MIDLIFE AND OLDER ADULTHOOD

Hannah Heintz-Monette[1], Ann Haynos[1], Kelsey Hagan[2], and Laura Berner[3], *1. Virginia Commonwealth University, Richmond, Virginia, United States, 2. Virginia Commonwealth University School of Medicine, Richmond, Virginia, United States, 3. Icahn School of Medicine at Mount Sinai, New York, New York, United States*

Prior research has identified the interaction of rapid neurocognitive changes with psychosocial stressors as a major precipitant of disordered eating in adolescence. However, there has been little discussion of how similar processes may set the stage for disordered eating in later life. Here, we present a novel framework for the promotion and maintenance of eating pathology in midlife and beyond. First, we review the critical neurobiological alterations that occur during this unique developmental stage and connect these alterations to established risk factors for disordered eating in adolescence. Second, we discuss how unique, aging-related psychosocial stressors interact with brain aging processes to increase vulnerability. We then highlight how our framework sheds light on potential new targets for psychopharmacology and psychotherapy in older adults experiencing disordered eating. Based on this framework, we outline the following future research directions: 1) directly investigate the influence of hormonal, neurocognitive, neurobiological, and environmental aging changes on eating disorder behavior; 2) distinguish pathways from aging to eating pathology that differ as a function of aspects of identity or specific developmental period; 3) establish shared and distinct mechanisms influencing the emergence or worsening of different eating disorder diagnoses and presentations in later life; and 4) identify potential targets for prevention and treatment of eating disorders as vulnerable individuals age. Finally, we advocate for enhanced awareness and screening for eating pathology in midlife and older adults, as well as for a multidisciplinary approach to the treatment of eating disorders in this population.

Abstract citation ID: igaf122.3042

### ABSOLUTE VS RELATIVE INCOME AND LATE-LIFE DEPRESSIVE SYMPTOMS: VARIATIONS BY EMPLOYMENT

Veronica Howell, and Benjamin Shaw, *University of Illinois Chicago, Chicago, Illinois, United States*

Prior research generally finds a positive association between income and well-being. This work builds on absolute and relative deprivation theories. By modeling absolute and relative income, this study examines whether these measures differ in association with late-life depressive symptoms and evaluates the role of employment. Data come from the National Social Life, Health, and Aging Project (NSHAP). Respondents reported baseline absolute income alongside income relative to other Americans and their broader social network. After controlling for covariates and baseline depressive symptoms, the association between income and later-life depressive symptoms was examined using linear regression. Five years after baseline, income relative to other Americans was significantly associated with increases in depressive symptoms ($\beta$ = .432, p = .031), with no evidence of variation by baseline employment. Ten years after baseline, both income relative to other Americans ($\beta$ = .760, p = .003) and relative to one's own network ($\beta$ =.598, p = .027) were significantly associated with increases in depressive symptoms. Interactions with employment were significant, indicating a reversal in the direction of these associations among those employed at baseline. These preliminary results suggest relative income may better predict depressive symptoms over time than absolute income. Further, both relative income measures suggest higher income is linked to reductions in depressive symptoms for those working during baseline. In comparison, higher income is associated with increased symptoms for those not working. Future research will explore how depressive and other mental health trajectories evolve based on income measures, employment transitions, and potential non-linear associations.

Abstract citation ID: igaf122.3043

### ADJUSTMENT DISORDER IN OLDER ADULTS: INSIGHTS FROM A NATIONWIDE SURVEY

Sang Hui Chu[1], Subin Park[2], Hun Kang[1], and Ocksim Kim[2], *1. Yonsei University, Seoul, Republic of Korea, 2. Yonsei University College of Nursing, Seoul, Republic of Korea*

Adjustment disorder (AjD) is a stress-related condition that varies across age groups. Younger adults frequently develop AjD due to work and educational stressors, whereas older adults face challenges such as declining health and social isolation. This study compared the prevalence and risk factors of AjD in adults aged 60 and above versus those aged 59 and younger using the International Adjustment Disorder Questionnaire (IADQ). A total of 916 participants from a nationwide online survey were analyzed, with older adults (>60 years, n = 255) and younger adults (< 60 years, n = 661). The overall prevalence of AjD was 20.5%, with a significantly lower rate in older adults (11.4%) compared to younger adults (24.1%, p<.001). Younger adults were more likely to report work-related (53.3% vs. 28.2%, p<.001) and educational stressors (23.9% vs. 7.1%, p<.001), whereas older adults were significantly more affected by health-related stressors (67.8% vs. 51.3%, p<.001). Logistic regression showed that an increasing number of stressors significantly predicted AjD in younger adults (e.g., OR = 3.900 for five stressors, p<.001). In contrast, residing in the capital region was a significant risk factor for older adults (OR = 4.130, p=.004). Anxiety symptoms were the strongest common predictor of AjD across both groups, with a greater effect in older adults (OR = 21.310, p=.030). These findings highlight the unique vulnerabilities of older adults in developing AjD, particularly to health-related stressors and urban living. Age-specific interventions should prioritize mental health support tailored to older adults, with a focus on mitigating health concerns and enhancing social well-being.

Abstract citation ID: igaf122.3044

### ADVANCING MENTAL HEALTHCARE ACCESS FOR OLDER PEOPLE: RESULTS FROM THE NATIONAL MEDICARE ACCESS INITIATIVE

Jordan Westcott[1], Alexis Isaac[2], and Matthew Fullen[2], *1. University of Tennessee at Knoxville, Knoxville, Tennessee, United States, 2. Virginia Tech, Blacksburg, Virginia, United States*

Historically, older adults have had limited access to mental health services despite having unmet mental health needs (Adepoju et al., 2018). After more than 30 years of advocacy, licensed mental health counselors and marriage and family therapists became eligible for participation in the Medicare program on January 1, 2024 (Consolidated Appropriations Act, 2023), leading more than 60,000 new mental health professionals to enroll in the Medicare program (CMS.gov, n.d.). Therefore, the authors developed the national Medicare ACCESS research initiative to understand initial experiences, motivating factors and concerns, and gerontological counseling competencies with the Medicare program among the newly enrolled mental health provider workforce. Among 127 respondents, 87.4% had provided services to one or more older adult clients since enrolling in the Medicare program. Most highly rated motivating factors for Medicare enrollment included expanding mental healthcare access for older adults (60.8% rated as high importance) and supporting people in one's community (57.6%), and providers were most concerned about Medicare reimbursement rates (68.0%) and compliance with Medicare policies (56.0%) after enrolling. These findings will be contextualized with textual responses illuminating provider perspectives. Additionally, provider age (b = .186, p = .035) and provider attitude toward aging (b = -.319, p < .001) had significant linear relationships with gerontological counseling competencies, R2 = .164, F(2, 114) = 11.16, p < .001 with a moderate effect size (f2 = .196; Cohen, 1988). Implications for provider enrollment efforts, service delivery for older adults seeking mental healthcare, and Medicare advocacy will be discussed.

Abstract citation ID: igaf122.3045

### AGE DIFFERENCES IN AFFECTIVE EXPERIENCES IN SOLITUDE: THE MODERATING ROLE OF NOSTALGIA

Xinyuan Peng[1], Jennifer Lay[2], Yuen Wan Ho[3], Dwight Tse[4], Helene Fung[5], and Da Jiang[1], *1. The Education University of Hong Kong, Hong Kong, China (People's Republic), 2. University of Exeter, Exeter, England, United Kingdom, 3. Lingnan University, Hong Kong, China (People's Republic), 4. University of Strathclyde, Glasgow, Scotland, United Kingdom, 5. The Chinese University of Hong Kong, Hong Kong, China (People's Republic)*

Solitude, defined as the absence of social interaction, is a common experience that increases with age. Research indicates that older adults enjoy solitude better than younger adults, influenced by gender, culture, and social networks. However, the role of nostalgia remains underexplored. Nostalgia, recalling time with close others, is a bittersweet emotion. Older adults experience nostalgia three times more frequently than younger adults. Based on socioemotional selectivity theory, older adults benefit from emotionally-meaningful goals. In accordance with the broaden-and-build theory of positive emotions, nostalgia correlates positively with positive emotions and well-being. This study investigated whether and how dispositional and daily nostalgia moderate associations between solitude, age, and affective experiences. Using the experience sampling method, participants ($N = 188$; Mage = 51.47, SDage = 17.75, range = 19-93, 66.50% women) completed smartphone-based assessments of momentary solitude, daily nostalgia, and positive (PA) and negative affect (NA) five times daily for seven consecutive days. Dispositional nostalgia was measured in a pretest. Results showed that nostalgia moderated associations between solitude, age, and affective experiences. For younger adults, higher dispositional nostalgia intensified the negative link between solitude and PA, while higher daily nostalgia amplified the positive link between solitude and NA. Nostalgia did not moderate the solitude-affect association in older adults. These findings challenge the typical view of nostalgia as a positive contributor to well-being and highlight its negative association with better affective well-being during solitude among younger but not older adults.

Abstract citation ID: igaf122.3046

### BEHAVIORAL ACTIVATION AS A MECHANISM OF CHANGE IN PSYCHOTHERAPY FOR LATE-LIFE DEPRESSION

Lilla Brody[1], Maayra Butt[2], and Brenna Renn[2], *1. University of Nevada, Las Vegas, Henderson, Nevada, United States, 2. University of Nevada, Las Vegas, Las Vegas, Nevada, United States*

Late-life depression is a common condition that, left untreated, exacerbates negative health outcomes. Despite availability of effective psychotherapeutic treatments for depression, few trials have explicitly investigated treatment mechanisms. Engage is a new, brief behavioral psychotherapy for older adults with depression. This secondary data analysis aims to evaluate behavioral activation as a mediator of the relationship between baseline and post-test depression, hypothesizing that behavioral activation would be a mechanism of change for those in the Engage group. Analysis included 92 older adults with depression who participated in the randomized controlled noninferiority trial comparing Engage with Problem Solving Therapy (PST). Depression (measured via the Hamilton Depression Rating Scale; HAM-D) and behavioral activation (measured via the Behavioral Activation for Depression Scale; BADS) were recorded at baseline and 9 week follow up. Participants were an average of 67.5 years old ($SD = 6.19$), mostly female (70.7%), Non-Hispanic (92.4%), and white (85.9%) . Analyses were conducted in R and utilized the bruceR package for mediation. HAM-D scores improved for both groups ($ps < 0.05$). Separate mediation analyses in the Engage and PST groups indicated a direct effect between HAM-D scores across time ($p < 0.01$). Change in BADS was associated with significant decreases in HAM-D across time for both groups. Results suggest that behavioral activation contributes to but does not fully explain change in depressive symptoms during brief psychotherapy for late-life depression. Psychotherapy treatment mechanisms warrant further study in more diverse samples to better understand treatment response.

Abstract citation ID: igaf122.3047

### CHILDHOOD ADVERSITIES AND LATER-LIFE LONELINESS: MEDIATING ROLES OF HEALTH AND INTERGENERATIONAL RELATIONSHIPS

Ying Xu[1], Merril Silverstein[1], Tianqi Zhou[1], and Xiaoyu Fu[2], *1. Syracuse University, Syracuse, New York, United States, 2. Syracuse University, Los Angeles, California, United States*

Social scientists have long recognized the significance of early-life experiences for later-life well-being. However, the implications of these early-life experiences on loneliness in later life have been largely overlooked. To address this gap, this study investigates the association between childhood adversities (CAs: hunger, lack of medical care, parental loss, and poor health) and later-life loneliness among older adults in rural China, with a focus on the mediating roles of self-rated health and emotional closeness with adult children. Using data from the 2021 wave of the Longitudinal Study of Older Adults in Anhui Province ($N = 1{,}554$; males = 810, females = 744), we conducted regression-based mediation analysis for two mediators separately, controlling for respondent age, gender, marital status, educational level, living arrangement, and instrumental activities of daily living. Findings indicate that CAs were associated with greater loneliness ($\beta = 0.473$, $p < 0.001$). Self-rated health partially mediated this relationship ($\beta = 0.4131$, $p < 0.001$), with a significant indirect effect ($\beta = 0.0621$). Similarly, emotional closeness with adult children partially mediated this association ($\beta = 0.4291$, $p < 0.001$), with a significant indirect effect ($\beta = 0.0435$). These findings underscore the long-term consequences of childhood adversities and highlight the importance of promoting health and strengthening intergenerational relationships as potential interventions to mitigate loneliness among older adults in rural China.

Abstract citation ID: igaf122.3048

### DISCRIMINATION AND DEPRESSIVE SYMPTOMS: THE ROLE OF PSYCHOLOGICAL RESILIENCE ACROSS RACIAL AND ETHNIC GROUPS

Yi Wang[1], Yifan Lou[2], Huei-wern Shen[3], and Man Guo[4], *1. University of Iowa School of Social Work, Iowa City, Iowa, United States, 2. Virginia Commonwealth University, Richmond, Virginia, United States, 3. University of North Texas, Denton, Texas, United States, 4. University of Iowa, Iowa City, Iowa, United States*

Despite extensive research on the detrimental effects of discrimination on health, less is known about how multi-dimensional discrimination experiences affect mental health and the potential role of psychological resilience across racial/ethnic groups. To address these gaps, this study analyzed data from 11,833 respondents in the 2016/2018 Health and Retirement Study Psychosocial Leave-Behind Questionnaire (65% non-Hispanic White, 18% non-Hispanic Black, 13% Hispanic, and 4% Other). Respondents reported on six forms of discrimination: disrespect, poor service in restaurants, poor treatment from doctors, perceived as unintelligent, fear from others, and threats. Psychological resilience, defined as the ability to navigate adversity through positive adaption, was assessed using a 12-item index covering domains such as perseverance despite major setbacks, adaptability to change, and recognition of inner strengths. Latent class analysis identified three distinct typologies: (C1) few experiences (70%)—low across all experiences, (C2) microaggressions (25%)—high on disrespect, poor service at restaurant, and perceived as unintelligent, and (C3) intensive discrimination (5%)—high across all experiences. Structural equation modeling revealed that for non-Hispanic Whites and Blacks, C2 and C3 were associated with higher depressive symptoms, both directly and indirectly via lower psychological resilience. Among Hispanics, resilience mediated the effects of microaggressions (C2) on depressive symptoms, while for Other, this mediation effect was observed for intensive discrimination (C3). The findings highlight discrimination as a mental health risk factor for older Americans across racial/ethnic groups and suggest that psychological resilience may mitigate its impact, contingent on experience type and racial/ethnic background. Implications for future research and practice are discussed.

Abstract citation ID: igaf122.3049

### EFFECT OF SOCIAL ROBOTS IN REDUCING LONELINESS AMONG COMMUNITY-DWELLING OLDER ADULTS: A MIXED-METHOD APPROACH

Hiroshi Murayama[1], and Mai Takase[2], *1. Tokyo Metropolitan Institute for Geriatrics and Gerontology, Tokyo, Japan, 2. Tokyo Metropolitan Institute for Geriatrics and Gerontology, Itabashi City, Tokyo, Japan*

Most studies on interventions using social robots to reduce loneliness have been conducted in facilities in Western nations. This study evaluated the effectiveness of social robot interventions in reducing loneliness among community-dwelling older Japanese adults using a randomized controlled trial and qualitative analysis. Individuals aged ≥65 years who lived alone in Tokyo and neighboring areas and experienced loneliness were recruited. Seventy-three eligible participants were randomly assigned to either an intervention or control group. The four-week intervention involved a humanoid social communication robot (BOCCO emo), which facilitated conversations with human operators and/or family members and reminded participants of daily tasks. The primary outcome was loneliness, with psychological well-being, depression, and laughter frequency as secondary outcomes. Participants were evaluated at baseline and follow-up. In the follow-up survey, participants in the intervention group provided open-ended responses regarding their experiences using the social robot. A total of 68 participants completed both the baseline and follow-up surveys (n = 34 in each group; mean age: 82.3 years; 94.1% women). Loneliness decreased more in the intervention group than in the control group. Psychological well-being improved, and the frequency of laughter tended to increase in the intervention group. Content analysis identified four categories: "emotional support and psychological connection," "lifestyle assistance," "enrichment of social interaction," and "cognitive and mental stimulation." Social robots can reduce loneliness among community-dwelling older adults in non-Western societies. Information and communication technology appears to be an effective approach to alleviating loneliness and enhancing well-being among older adults in community settings.

Abstract citation ID: igaf122.3050

### EXPLORING THE RELATIONSHIP BETWEEN MENTAL HEALTH AND EARLY RETIREMENT IN U.S. OLDER ADULTS: A GENDERED PERSPECTIVE

Josephine Boateng[1], Eric Frimpong[2], Seokmin Kim[3], and Jeffrey Burr[2], *1. University of Massachusetts Boston, Dorchester, Massachusetts, United States, 2. University of Massachusetts Boston, Boston, Massachusetts, United States, 3. University of Massachusetts Boston, Quincy, Massachusetts, United States*

Aging populations present significant challenges for workforce retention. However, limited research has investigated how mental health, especially depression, is associated with early retirement decisions. This study examined whether depression is associated with early retirement among older adults aged 51-64. We also explored whether there are gender differences in this relationship using longitudinal data from the 2020 and 2022 Health and Retirement Study (N = 4,915). For a sample of persons engaged in paid work in 2020, employment status in 2022 was measured as retired, engaged in paid work, and "other" (e.g., disabled, not in labor force). Multinomial logistic regression models are employed to investigate these research questions. Depression is measured in 2020 with CESD depressive symptoms scale (range 0-8). Descriptive characteristics showed that 52% of respondents were engaged in paid work in 2022. In 2020, the mean age of the sample was 59 years, 60.2% were females, 65% were married, 40% had a college degree or more, and 73% had self-reported good health. Regression results showed that as depression symptoms increased, the likelihood of engaging in paid work in 2022 decreased as compared to being retired (RRR = 0.9467, p = 0.029), suggesting respondents in 2020 with depressive symptoms were more likely to retire two years later. Finally, the relationship between depression and early retirement was statistically significant for men but there was no corresponding effect for women. Study findings provided insights into how mental health challenges are associated with labor force retention informing policies aimed at supporting older workers.

Abstract citation ID: igaf122.3051

### FINANCIAL STRAIN, FOOD INSECURITY, AND SPIRITUAL BELIEFS ON DEPRESSION IN MIDDLE-AGED AND OLDER INDIGENOUS WOMEN

Soonhee Roh[1], Yeon-Shim Lee[2], Erna Dinata[1], Sukyung Yoon[3], Joel Steele[4], Elizabeth Peffer Talbot[5], and Sasheen T. Stone[6], *1. University of South Dakota, Sioux Falls, South Dakota, United States, 2. San Francisco State University, San Francisco, California, United States, 3. University of North Texas, Denton, Texas, United States, 4. University of North Dakota, Grand Forks, North Dakota, United States, 5. Wright State University, Dayton, Ohio, United States, 6. Yankton Sioux Tribe, Wagner, South Dakota, United States*

Purpose: This study investigates how financial strain and food insecurity contribute to depression among middle-aged and older Indigenous women. It further explores whether religious and spiritual beliefs serve as a buffer against the impact of these stressors, providing insight into their potential protective role in mental health. Method: In collaboration with a Northern Plains tribe, this study included 133 self-identified Indigenous women aged 40 to 70 years. The stress process model (SPM) provided the theoretical framework. Standardized assessments, including the Patient Health Questionnaire (PHQ-9), measures of food insecurity, financial strain, and religious/spiritual beliefs, were administered. Path analysis was used to assess both direct and indirect effects. Results: The study found a significant burden of depression, with 19.5% of participants experiencing mild depression, 11.3% moderate depression, 5.3% moderately severe depression, and 3% severe depression. Results support the SPM, indicating that financial strain and food insecurity are significantly associated with depressive symptoms. Financial strain influenced depression indirectly through its association with food insecurity. However, religious and spiritual beliefs did not act as protective factors against depressive symptoms in this population. Conclusion: This study underscores the complex interplay of financial strain, food insecurity, and depressive symptoms among middle-aged and older Indigenous women. Findings highlight the need for targeted, multidimensional interventions addressing both economic and psychosocial factors to improve mental health outcomes in this population. Future research should refine strategies to enhance well-being and resilience.

Abstract citation ID: igaf122.3052

### MENTAL HEALTH AND FALL RATES AMONG OLDER ADULTS: INFLUENCE OF EXERCISE AND UNMET HEALTH CARE

Idorenyin Udoh[1], Wah Wah Myint[2], and Matthew Smith[3], *1. University of Michigan Flint, Flint, Michigan, United States, 2. Texas A&M University, College Station, College station, Texas, United States, 3. Texas A&M University, College Station, Texas, United States*

Background: Physical activity and unmet health care (UNHC) are important factors influencing poor mental health and increased risk for falls among older adults. This study examines the impact of physical activity on UNHC needs and its subsequent effects on mental health outcomes and fall risk among community-dwelling older adults. Methods: Data were analyzed from 164,955 participants in the 2023 Behavior Risk Factor Surveillance System dataset. Two models were fitted: A multinomial logistic regression model with past-month poor mental health days (PMHD) (a three-categorical variable-i.e., 0 days, 1-13 days, 14-30 days) and a binary logistic regression model with past-year fall (a dummy variable, i.e., a fall or not). Both models adjusted for socio-demographics characteristics, physical activity, and UNHC. Results: Sixty percent of participants had no past-month PMHD, 26% reported 1-13 PMHD, and 14% reported 14-30 PMHD. Nearly 29% reported a fall -year fall. Participants with past-year fall were more likely to report 1-13 (RR = 1.96) and 14-30 (RR = 2.64) PMHD, respectively. Participants without physical activity limitations were less likely to report 1-13 (RR = 0.92) and 14-30 (RR = 0.49) PMHD, respectively. Individuals with UNHC were likely to report 1-13 (RR = 1.86) and 14-30 (RR = 3.33) PMHD, respectively. Participants with PMHD 1-13 (aOR=1.96) and 14-30 (aOR=2.64) were likely to report 1+ falls in the past-year fall, respectively. Those with UNHC were more likely to fall (aOR=1.63), while those who exercised were less likely to fall (aOR=0.69). Conclusions: Findings highlight the importance of physical activity and healthcare utilization in the prevention and interventions for poor mental health status and falls.

Abstract citation ID: igaf122.3053

### MENTAL HEALTH SERVICE USE AMONG OLDER ADULTS AT RISK OF SUICIDE

Afroze Shaikh, *The University of Texas at Austin, Austin, Texas, United States*

Older adults present with historically high rates of suicide (CDC, 2023), yet little research has examined the help-seeking behavior of older adults at elevated risk (Wang et al., 2023). Recent changes in Medicare policy also allow for licensed professional counselors (LPCs) to serve as Medicare providers, potentially increasing access to mental health care for older adults. This study, therefore, sought to examine predictors of proximal suicide risk factors (e.g., thwarted belongingness and perceived burdensomeness; Van Orden et al., 2012) among a national sample of older adults (n = 806) and factors that influence mental health service use among those at elevated risk of suicide using the Andersen Behavioral Model of Health Service Use (Andersen, 1995). Predictors of proximal suicide risk factors included difficulty accessing medical care and lower incomes. We found that 370 participants met clinical cutoff scores for elevated suicide risk (Mitchell et al., 2017) and that only 12.7% (n = 47) reported service use for mental health concerns within the past year, whereas only 2 (0.54%) reported service use for suicide concerns. Among the factors that influenced the need for services, greater levels of suicide behavior (OR = 1.191, p = .009), pain-related emotional burden (OR = 1.078, p = .004), and psychological distress (OR = 1.101, p = .011) increased the likelihood of mental health service use over the past year. Implications to promote belongingness and increase mental health service use among older adults will be discussed.

Abstract citation ID: igaf122.3054

### OLDER ADULT (65+) DEPRESSION PREVALENCE INCREASING IN NEW ENGLAND: FINDINGS FROM THE 2025 HEALTHY AGING DATA REPORTS

Qian Song[1], Taylor Jansen[1], Tiffany Tang[1], Shan Qu[1], Yan-Jhu Su[2], and Elizabeth Dugan[1], *1. University of Massachusetts Boston, Boston, Massachusetts, United States, 2. The Pennsylvania State University, University Park, Pennsylvania, United States*

In 2023, the Surgeon General declared an “epidemic of loneliness” as a poll reported about one in three older adults’ experiences feeling socially isolated. The Healthy Aging Data Reports (HADR) (www.healthyagingdatareports.org) are a rich data source to study rates of older adult rates of depression. The HADR calculates the prevalence of 38 chronic diseases for each community in a state using Medicare Beneficiary Summary File (MBSF), representing 100% of traditional Medicare beneficiaries in a state, aggregating the prevalence to the community and neighborhood-levels using small area estimation techniques. The present descriptive study will compare community and neighborhood-level 65+ “ever-diagnosed” prevalence of depression over three time points (Time 1: 2014-2015; Time 2: 2017-2018; Time 3: 2020-2021) in five New England states: Connecticut (CT), Massachusetts (MA), Maine (ME), New Hampshire (NH), and Rhode Island (RI). Over the three time points, 65+ depression increased for all states. CT (T1:27.75%; T2:29.32%; T3:31.63%), MA (T1:29.52%; T2:31.2%; T3:33.02%), ME (T1:32.50%; T2:33.48%; T3:33.83%), NH (T1:26.84%; T2:27.82%; T3:28.7%), and RI (T1:31.16%; T2:31.7%; T3: 33.37%). While rates increased overtime for all states, CT reported the largest increase from time 1 to time 3 (3.9%). NH had the lowest 65+ depression prevalence in time 3 (28.7%), and Maine reported the highest (33.83%). These trends demonstrate that depression among older adults was highest during the COVID-19 pandemic (Time 3: 2020-2021) and are continuing to increase. The 2025 HADRs can be used to direct policy efforts to treat and address the growing mental health needs of the aging population.

Abstract citation ID: igaf122.3055

**PERCEPTION OF MAJOR LIFE EVENTS AND DEPRESSION IN MID TO LATER LIFE: FINDINGS FROM A SWEDISH LONGITUDINAL STUDY**

Deborah Finkel[1], Lawrence Sacco[2], Shireen Sindi[3], Caroline Hasselgren[4], Martin Hyde[5], and Charlotta Nilsen[6], *1. University of Southern California, Los Angeles, California, United States, 2. Stockholm University, Stockholm, Stockholms Lan, Sweden, 3. Imperial College London, London, England, United Kingdom, 4. University of Gothenburg, Gothenburg, Vastra Gotaland, Sweden, 5. University of Leicester, Leicester, England, United Kingdom, 6. Jönköping University, Jönköping, Jonkopings Lan, Sweden*

Several major life events (MLEs) that can negatively affect mental health are common in mid to later life. While MLEs are generally associated with depression, individuals' subjective perception of such events can further explain this relationship. However, there is a lack of longitudinal studies on perception of MLEs particularly with samples including older adults. This study evaluated how MLEs and their perception are related to depression symptoms in mid to later life. The role of individual factors (e.g. sex and age) in shaping any effect of MLEs on depression was examined. Data from five waves (2010-2018) of the biennial Swedish Longitudinal Occupational Survey of Health (SLOSH) were analyzed using linear random effects regression models. The Symptom Checklist-core depression (SCL-CD6) scale was used to measure levels of depression symptoms. Respondents were asked whether in the previous two years they experienced a series of MLEs (i.e. the illness or accident of a partner; death of a partner; death of a relative or friend; marital separation; partnership formation) as well as their appraisal for each event. Analyses were adjusted for potential confounders. Results indicate that most MLEs were associated to subsequent higher levels of depression to varying extents, with bereavement being the strongest predictor and partnership formation not being associated. Perceiving any MLE as more important increased the strength of the relationship. Significant effects were found also for partnership formation once individual perception was included. Findings underscore the importance of supporting individuals in later life during transitions and key life events.

Abstract citation ID: igaf122.3056

**RISK FACTORS FOR SUICIDE ATTEMPTS AMONG OLDER ADULTS WITH SUICIDAL IDEATION: A CROSS-SECTIONAL STUDY**

Gwang Suk Kim[1], Jae Jun Lee[1], Min Kyung Park[1], Layoung Kim[2], Sooyoung Kwon[3], Eun Ju Park[1], Hayejin Yang[3], and Seungbum Yang[3], *1. Yonsei University College of Nursing, Seoul, Republic of Korea, 2. The University of Suwon, Hwaseong, Republic of Korea, 3. Graduate School of Yonsei University, Seoul, Republic of Korea*

Suicidal ideation is a significant public health issue among older adults; however, not all individuals with suicidal thoughts attempt suicide. Identifying the factors that distinguish people who attempt suicide from those who do not is essential for targeted prevention efforts. The aim of this study was to identify risk factors associated with suicide attempts among older adults with suicidal ideation. This cross-sectional study analyzed data from the 2021–2022 Korean Community Health Survey, a community-based nationwide survey. The study included adults aged 65 years and older expressing suicidal ideation within the past year. Independent variables included demographic, socioeconomic, lifestyle, and health-related factors; the dependent variable was suicide-attempt history within one year. Multiple logistic regression analysis was conducted to identify significant risk factors for suicide attempts. Among 14,626 older adults with suicidal ideation, 352 (2.4%) attempted suicide. Demographic and socioeconomic factors associated with higher suicide-attempt risk included younger age, women, financial difficulty (public welfare recipients), and living alone. Lifestyle and health-related factors, such as smoking, shorter sleep duration, depressive symptoms, higher perceived stress, and lower quality of life, were also significant predictors. These findings underscored the need for suicide-prevention efforts among older adults that prioritize mental-health support, financial and social assistance, sleep intervention, and smoking cessation. Identifying high-risk individuals through routine screening and providing tailored interventions may help prevent suicide attempts in this vulnerable population.

Abstract citation ID: igaf122.3057

**SELF-REPORTED VS OBJECTIVE CEREBROVASCULAR BURDEN: ASSOCIATION WITH DEPRESSION AND COGNITIVE PERFORMANCE**

Emma Churchill[1], Daniel Paulson[1], Nichole Lighthall[1], Apollonia Lysandrou[1], Nicole McClure[1], Madison Maynard[1], Quinn Allen[1], and Nick James[2], *1. University of Central Florida, Orlando, Florida, United States, 2. VA Orlando Health Care, Orlando, Florida, United States*

Introduction: Cerebrovascular burden (CVB) contributes to neurocognitive and behavioral decline in aging. While MRI is the gold standard for assessing CVB, its availability in large community samples is limited. Previous studies have used biomarkers (e.g., A1C) or self-reported comorbidities (e.g., diabetes) to assess CVB. This study evaluates the concordance of these methods, hypothesizing that self-reports serve as a valid alternative to biomarkers in predicting depressive symptoms and cognitive performance. Methods: Data from wave 16 of the Health and Retirement Study (HRS), a nationally representative sample of older adults, were analyzed. Biomarker (A1C, cholesterol, blood pressure, C-reactive protein, cystatin C) and self-reported CVB variables (heart conditions, hypertension, hypercholesterolemia, smoking) were collected. Depression and cognition were assessed using the CES-D 8 and the Harmonized Cognitive Assessment Protocol (HCAP), respectively. Multiple stepwise regression analyses examined relationships among self-reported and objective CVB, depressive symptoms, and cognitive functioning. Results: The sample included 916 (60.2% female) participants with an average age of 75.02 (SD = 7.18). Self-reported CVB predicted depressive symptoms and cognitive performance for Symbol Digit Modalities, Raven's Matrices, and Trail Making Test-B ($p < .05$). Objective CVB also significantly predicted cognitive performance. Discussion: Self-reported CVB may offer comparable (or greater) predictive value than biomarkers alone, especially for measures of mood. These findings support the use of self-reported cerebrovascular morbidities for the study of cognitive aging as they could reduce costs and enhance the utility of large scale studies. This highlights the value of integrating both methods when feasible.

Abstract citation ID: igaf122.3058

**TEST- RETEST RELIABILITY OF THE THAI VERSION OF THE MILD BEHAVIORAL IMPAIRMENT-CHECKLIST (MBI-C)**

Nussaba Sompanich[1], Ross Andel[2], Arunya Tuicomepee[1], and Phot Dhammapeera[1], *1. Chulalongkorn University, Bangkok,*

*Krung Thep, Thailand, 2. Arizona State University, Arizona State University, Arizona, United States*

Mild Behavioral Impairment (MBI) is characterized by persistent behavioral symptoms. Unlike mild cognitive impairment, it focuses on changes in behavior, mood, and personality rather than cognitive deficits. Mild Behavioral Impairment-Checklist (MBI-C) is a screening tool for MBI. Respondents answer yes/no and rate severity from 1 (Mild) to 3 (Severe) for 34 items covering changes in motivation, mood/anxiety, impulsivity, abnormal thoughts, and social appropriateness. This study aims to assess MBI-C in Thailand. We double-translated the MBI-C to Thai and examined its test-retest reliability among 73 Bangkok residents over 50 (mean age= 58.94 ± 5.621, 75.3% were women), administering the test twice over a two-week interval. Spearman's rho correlation yielded an overall test-retest reliability of .423, $p < .001$. In a post-hoc, per-item assessment, we found that 8 of the 24 items correlated perfectly and 10 items yielded correlations <.10. Overall, we observed lower reliability for items capturing momentary emotions like being anxious or worried about mundane things, erratic behaviors like poor judgement during driving, and emotions directed towards significant others like irritability or stubbornness. Items with high reliability included those reflecting changes in mundane behaviors like being more impatient or having more difficulty staying relaxed. Finally, items with zero variance included more extreme behaviors like sexual disinhibition or new bad habits like excessive alcohol drinking or shoplifting. These results offer novel, general and Thai-culture specific insights into the underlying characteristics of items within the MBI-C scale. The presumed role of Thai collectivism among Thai older adults will also be discussed.

Abstract citation ID: igaf122.3059

### THE EFFECTS OF ICT USE ON DEPRESSION IN OLDER ADULTS: THE MEDIATING ROLE OF SOCIAL PARTICIPATION

Mina Hwang, and Yeji Hwang, *Seoul National University, Seoul, Republic of Korea*

As information and communication technologies (ICTs) become integral to daily life, their role in reducing depression among community-dwelling older adults has gained attention. However, the underlying mechanisms remain unclear. As one possible mechanism is via social participation, this study aimed to examine the effects of ICT usage on depression and whether social participation mediates the relationship. This study was a secondary data analysis using the National Health and Aging Trends Study (NHATS) Round 13 (N = 6,722). Participants were categorized into 4 types of ICT usage: Non-use, CT-use, IT-use, and ICT use. Social participation was classified as formal and informal participation. For data analysis, multiple parallel mediation analyses were conducted. Findings revealed that both formal (B=-0.098, 95%CI=-0.148,-0.053) and informal (B=-0.139, 95%CI=-0.188,-0.093) social participation had an indirect effect on the relationship between ICT use and depression. Additionally, ICT use had a direct effect on depression (B=-0.491, 95%CI=-0.701,-0.280). When examining CT use, both formal (B=-0.041, 95%CI=-0.069,-0.019) and informal (B=-0.093, 95%CI=-0.133,-0.058) social participation exhibited an indirect effect on depression. However, there was no direct effect of CT use on depression (B=-.183, 95%CI=-0.428,0.063). For IT use, only informal social participation (B =-0.060, 95%CI=-0.098,-0.029) had an indirect effect on depression, while no direct effect of IT use on depression was observed (B=-0.109, 95%CI=-0.424,0.206). These findings suggest ICT use mitigates depression through social connections, though effects vary by types of ICT use. A comprehensive approach is needed when designing ICT-based interventions. Such intervention should promote social participation to maximize their effectiveness to reduce depression among older adults.

Abstract citation ID: igaf122.3060

### THE RELATIONSHIP BETWEEN GUILT AND HELP-SEEKING IN PARENT-ADULT CHILD DYADS: A DAILY DIARY STUDY

Zewen Huang, and Da Jiang, *The Education University of Hong Kong, Hong Kong, China (People's Republic)*

Help-seeking behaviors play a crucial role in maintaining the quality of parent-adult child relationships. However, little is known about the psychological factors that influence whether parents and adult children seek help from each other. Guilt, an emotional response to perceived wrongdoing or unmet expectations, may discourage help-seeking, as individuals may view it as a burden on family or a sign of dependency. This study examined the within-person association between guilt and help-seeking and whether this relationship differs by family role (i.e., parent vs. adult child) using a 14-day daily diary survey from 103 parent-adult child dyads (Mage (parents) = 57.18, SD = 6.70, 68.93% women; Mage (adult children) = 22.67, SD = 4.94, 84.47% women). Guilt was measured as participants' anticipated guilt when imagining seeking help from their partner on that day, and help-seeking was assessed by asking whether they sought any of six types of support (e.g., companionship, talking about daily events, emotional support, practical support, giving advice, and financial assistance) that day. Multilevel modeling revealed that on days when parents and adult children felt guiltier about seeking help, they were less likely to do so ($\beta = -.04$, $p < .05$). Such relationships did not differ by family role (i.e., as a parent or a child; $\beta = .06$, $p = .096$). These findings highlight that individuals who experience guilt may avoid seeking support even from close family members. Understanding this dynamic provides insights into potential interventions to increase help behavior and promote healthier parent-adult child relationships.

Abstract citation ID: igaf122.3061

### TRACKING THE TRAJECTORY OF RESILIENCE: THE IMPACT OF SIGNIFICANT LIFE EVENTS ON RESILIENCE IN OLDER ADULTS

Jakob Phillips, Jaclyn Bergstrom, Rebecca Daly, Danielle Glorioso, and Anthony Molina, *University of California, San Diego, San Diego, California, United States*

Resilience can change over time and in response to challenging life events, yet the underlying changes in older adulthood and the influence of demographic factors remain unclear. This study examined the 10-year trajectory of resilience in community-dwelling adults and its association with demographic factors and the experience of past-year life events. We analyzed self-report survey data from 1,081 adults in the Successful Aging Evaluation (SAGE) study, a longitudinal study in San Diego County, CA. Resilience was measured using the CD-RISC-10 scale and recent life events with the WHI-LES in all yearly surveys. Linear mixed-effects models assessed associations between recent life events (frequency and perceived impact), demographics, and resilience measured 6 times over 10 years. Results indicated that older age and socioeconomic factors, particularly gender and income, are associated with better resilience trajectories. While resilience showed a small decline with age (b=-0.02, p=0.008), the decline was greater with a higher number of life events (b=-0.25, p<.001). Notably, the effect of life events on resilience trajectory was not as severe for adults over 65 (b=0.003, p=0.017). Household income above $75,000 was associated with greater resilience at baseline (b=0.89, p=0.001), underscoring the protective role of economic resources. Men reported significantly higher resilience trajectories than women (b=0.86, p=0.012). These findings highlight the dynamic nature of resilience in adulthood and the critical role of socioeconomic factors in fostering stability. Future research should explore protective factors that help sustain resilience across the lifespan and implement interventions to support individuals facing cumulative life challenges.

### SESSION 7380 (POSTER)

### PSYCHOSOCIAL WELL-BEING

Abstract citation ID: igaf122.3062

### AFFECTIVE REACTIVITY TO DAILY ROMANTIC RELATIONSHIP TENSION AT MIDLIFE AND OLDER ADULTHOOD

Jeesun Lee, David Almeida, and Steffany Fredman, *The Pennsylvania State University, University Park, Pennsylvania, United States*

Affective reactivity to daily stressors is a marker of psychological vulnerability at midlife and older adulthood. However, little is known about affective reactivity to daily relationship tension specifically and whether this reactivity depends on global romantic relationship quality and gender. Using data from the National Study of Daily Experiences (N=2,804; Mage = 53.31, range = 33 – 83; 51.7% female), we investigated the daily linkages between relationship tension (e.g., argument or disagreement) and same day negative affect (NA) and positive affect (PA) in an 8-day daily diary study, examining whether global relationship strain, support, and gender moderated these associations. Multilevel modeling revealed main effects at the between- and within-person levels. Individuals who experienced greater relationship tension reported higher NA and lower PA, on average, and on days when tension was higher, they reported elevated NA and lower PA. Moderation analyses indicated that those with more strained or less supportive relationships showed stronger NA reactivity to daily relationship tension. In addition, although men and women did not differ in affective reactivity to relationship tension, women with more strained relationships had higher average NA than men, suggesting that the cumulative effects of relationship tension may be worse for women's emotional well-being. These findings highlight the link between daily relationship tension and NA at midlife, particularly among individuals with lower quality romantic relationships. Interventions that reduce global relationship strain and promote relationship support may protect health and well-being in midlife and older adulthood by reducing affectivity reactivity to daily relationship tension.

Abstract citation ID: igaf122.3063

### ASSESSING THE RELATIONSHIP BETWEEN RELIGIOUS BELIEFS AND DEATH ANXIETY: AN EXPLORATIVE STUDY

Tara Kirkpatrick[1], and James Houston[2], *1. Middle Tennessee State University, United States, 2. Middle Tennessee State University, Murfreesboro, Tennessee, United States*

This study aimed to examine the relationship between death anxiety, religiosity, and psychological well-being from an inter-religious perspective, addressing a notable gap in existing literature that primarily focuses on Christian populations. Data were collected through self-report measures from 114 primarily undergraduate college students to assess correlations among death anxiety, psychological well-being, religious coping, private religious practices, religious support, and belief in an afterlife. Contrary to prior research linking higher death anxiety with lower psychological well-being, this study found a significant positive correlation, indicating that higher psychological well-being predicts higher levels of death anxiety. These unexpected findings may suggest that individuals with greater psychological well-being might engage more frequently and openly with thoughts about mortality, potentially interpreting death anxiety as a manageable stressor. The implications of these results highlight a need to revisit theoretical frameworks regarding death anxiety and coping mechanisms. Future research should utilize larger, more diverse samples, and combine objective and self-report methodologies to clarify the complexities identified in this relationship, ultimately informing better clinical practices and educational programs addressing existential concerns.

Abstract citation ID: igaf122.3064

### COHORT DIFFERENCES IN OLDER ADULTS' MARITAL STATUS, TRANSITIONS, AND SOCIAL AND MENTAL HEALTH

Nell Compernolle[1], and Ruoqi Zheng[2], *1. NORC at the University of Chicago, Chicago, Illinois, United States, 2. National Opinion Research Center at The University of Chicago, Chicago, Illinois, United States*

Background and Objectives: Long-examined links between marriage and health emphasize marital status and physical dimensions of health. This study expands current conversations by considering vastly under-studied never-married older adults, marital transitions on social and mental health dimensions, and variation across cohorts. Methods: We analyzed four rounds of data from the National Social Life, Health and Aging Project (NSHAP) spanning 2005 to 2021. A series of linear regression models were used to examine associations between marital status (currently, formerly, never married), transitions (in and out of marriage), and dimensions of health (loneliness, happiness, depression), and moderation by cohort (the Silent Generation, Baby Boomers), respectively. Results: Our findings suggest marriage benefits, but only for certain dimensions: transitions mattered for loneliness and, to some extent depression, whereas status mattered for happiness. Interestingly, Baby Boomers' happiness is more strongly associated both with their marital status and transitions than it is for the Silent Generation. Next, never-married older adults comprise a population with unique experiences from other singles: they do not differ from their married peers in terms of happiness or depression; they are lonelier but not as lonely as those formerly married. Finally, never-married Baby Boomers were less depressed than those never-married in the Silent Generation. Discussion: Explicit consideration of never-married older adults and cohort differences provide novel insights into long-studied marriage-health links. Specifically, the social context—marriage norms—in which older adults entered and experience marriage and any subsequent transitions continue to shape their social and mental health in later life.

Abstract citation ID: igaf122.3065

### DAILY STRESS, CORTISOL DYSREGULATION, AND SCREEN TIME IN MIDLIFE AND OLDER ADULTS: FINDINGS FROM THE MIDUS STUDY

Rinanda Shaleha, David Almeida, and Nelson Roque, *The Pennsylvania State University, University Park, Pennsylvania, United States*

As digital engagement increases, its relationship with daily psychological stress and physiological regulation in midlife and older adults remains underexplored. Guided by the Strength and Vulnerability Integration (SAVI) model, this study investigated whether daily stress and cortisol dysregulation predict screen time behaviors, specifically social media use and TV watching using data from the Midlife in the United States (MIDUS) Refresher 1. 542 participants (Mage = 54.53, SD = 9.53) completed 8-day daily diaries, and a subset provided cortisol data across 4 days. Separate multilevel models tested within-person associations, adjusting for age, sex, self-rated health, chronic conditions, and weekday/weekend variation. Same-day stress was not associated with social media use ($\beta$ = -0.59, p = .705) or TV watching ($\beta$ = -4.75, p = .114). However, higher stress predicted marginally less TV viewing the next day ($\beta$ = -5.64, $p < .05$). A flatter cortisol slope also predicted reduced next-day TV time ($\beta$ = -0.08, $p < .05$), though the effect was small, consistent with subtle physiological influences on behavior. Same-day cortisol effects were not significant. Poor self-rated health was consistently linked to lower screen time. Older adults watched more TV overall (same-day: $\beta$ = 2.17; next-day: $\beta$ = 1.63, $p < .01$), but age did not moderate stress or cortisol effects. Findings suggest that next-day disengagement from TV follows heightened psychological or physiological stress, aligning with SAVI's view that older adults regulate emotions but remain stress-sensitive. This may inform strategies for balanced media use in aging populations.

Abstract citation ID: igaf122.3066

### FINDING SATISFACTION WITH LIFE THROUGH SERIOUS LEISURE: OLDER MEN AND RAILROAD MODELING

Melinda Heinz, and Hillary Wehe, *University of Northern Iowa, Cedar Falls, Iowa, United States*

This study aimed to provide support for railroad modeling as a serious leisure activity for older adult males and explore its relationship with their satisfaction with life. A convenience sample of males (n = 31) attending a regional railroad modeling conference were recruited. Participants completed the serious leisure inventory measure (SLIM) and satisfaction with life scale (SWLS). Hierarchical regression showed that serious engagement in railroad modeling positively predicted increases in life satisfaction, controlling for the physical health of the participants (R2 = .51, adjusted R2 = 0.47, F(2, 28)= 14.0, p <.001). A subsample of participants also completed interviews (n = 9) which were analyzed using Braun and Clarke's reflexive thematic analysis. Theme 1: Adaptability of the Hobby, demonstrated diverse ways men could be involved and benefit from the hobby. Some were drawn to creative aspects while others were focused on socially connecting to other modelers. . Conferences and special interest groups provided opportunities to learn new skills. Theme 2: Lifelong Identity as a Railroad Modeler, reflected its consistent presence in the lives of most men, allowing them to take comfort in turning to the hobby during times of transition or stress. These findings highlight benefits of serious leisure involvement, particularly engagement in outlets that are adaptive . This allows men to participate in different ways with varied ability levels. This suggests that despite health changes, men may be able to remain involved with the hobby by modifying their participation while still maintaining their identity as a railroad modeler.

Abstract citation ID: igaf122.3067

### GEROTRANSCENDENCE INTERVENTION: INSIGHTS FROM A PILOT RCT

Lia Araujo[1], Taiane Abreu[2], Oscar Ribeiro[2], and Laetitia Teixeira[3], *1. RISE-Health, Porto, Porto, Portugal, 2. University of Aveiro, Aveiro, Aveiro, Portugal, 3. ICBAS-UP, Porto, Porto, Portugal*

As the gerotranscendence theory gains visibility in the gerontological field, activities and programs exploring its assumptions with older adults are emerging. Despite the potentialities of the existing interventions, these studies have key limitations, such as inconsistent outcome measurement, lack of randomized controlled trials (RCTs), and insufficient focus on long-term care (LTC) populations. This pilot study aims to present the structure and the benefits of a gerotranscendence program developed with older adults in LTC settings. Forty-four older adults (23 in the experimental group and 21 in the control) have participated in a RCT of a gerotranscendence intervention program, with six weekly sessions, conducted in four different LTC facilities. A protocol with the Gerotranscendence Scale (GST2), Satisfaction with Life Scale (SWLS) and the Geriatric Depression Scale (GDS-15) was used at the beginning and at the end of the intervention. After the post-test, two focus groups were conducted with participants from the experimental group to gather their views on the intervention and how gerotranscendence theory applied to their experiences. Although quantitative analyses revealed no significant changes in the outcome measures (GDS, SWLS, and GST2), qualitative findings provided valuable insights into participants' perceptions and experiences with gerotranscendence, highlighting their redefined views on aging, the benefits of self-reflection, the importance of group dynamics, and the challenges of aging and living in LTC facilities. This study addresses a critical gap in the literature by exploring the structure and impact of a gerotranscendence program designed for older adults in LTC settings, using a mixed-methods approach.

Abstract citation ID: igaf122.3068

### HEDONIC AND EUDAIMONIC WELL-BEING ACROSS THE LIFESPAN: DIFFERENCES BETWEEN WORKING AND NON-WORKING ADULTS

Piaopiao Cai[1], and Silvia Sörensen[2], *1. University of Rochester, Rochester, New York, United States, 2. University of Rochester Warner School of Education and Human Development, Rochester, New York, United States*

The development trajectories of well-being across the lifespan have garnered significant interest in research. Most studies have found evidence of a U-shaped curve trajectory, indicating that midlife adults experience lower well-being compared to younger and older adults across the lifespan. However, the well-being indicators in research have predominantly focused on hedonic aspects, such as hedonic emotions and life satisfaction. The developmental trajectories of eudaimonic well-being, such as six factors of psychological well-being (autonomy, environmental mastery, personal growth, positive relationships, purpose in life, and self-acceptance), have received less attention, particularly concerning the working population. This study utilized a national dataset (n = 2321) to understand the well-being development trajectories between working(n = 1295) and non-working adults (n = 1026) aged 23 to 76. The results of polynomial regressions indicate that only life satisfaction and self-acceptance exhibit a significant quadratic (U-shaped) trend for working and non-working adults. However, the two groups exhibit significantly different ages for minimum life satisfaction (working adults, aged 41.88; non-working adults, aged 37.48) and self-acceptance (working adults, aged 42.98; non-working adults, aged 37.38). Hedonic emotions, autonomy, environmental mastery, and positive relationships, non-working adults exhibit a slightly steeper linear upward trend across the lifespan compared to the linear trajectories of working adults. Additionally, there was a consistently flat linear trajectory for personal growth and purpose in life among both groups. The findings of the present study support Baltes's (1987) theoretical perspective that individual development is lifelong, multidirectional, and malleable. Further investigation may be needed to better understand the differing trajectories between working and non-working groups.

Abstract citation ID: igaf122.3069

### INCREASED DELAYED HEALTHCARE AMONG PEOPLE WITH DEPRESSION AND MULTIMORBIDITY: ANALYSIS OF THE 2019-2023 NHIS DATA

Arum Lim[1], Chitchanok Benjasirisan[2], Soo Hyun Kim[2], Cheryl Himmelfarb[2], Patricia Davidson[3], and Binu Koirala[2], *1. Johns Hopkins University School of Nursing, Baltimore, Maryland, United States, 2. Johns Hopkins University, Baltimore, Maryland, United States, 3. University of New South Wales Sydney, Kensington, New South Wales, Australia*

Depression is common among individuals living with multimorbidity, causing adverse health outcomes. Although timely healthcare access is crucial to this population due to the burden of illness and the complexity of care, it has not been studied extensively how depression hinders healthcare access. This study aimed to examine the prevalence of delayed healthcare and its association with depression and multimorbidity, stratified by age (18-64 vs. ≥65 years). A cross-sectional analysis was performed using 56,727 individuals' data from the 2019-2023 National Health Interview Survey (NHIS). Multimorbidity was defined by a self-reported history of two or more physical chronic conditions (e.g., hypertension, diabetes, stroke, cancer), and a self-reported depression history was assessed additionally. Individuals who experienced delays in healthcare over the past 12 months for any reason, including insurance issues, scheduling conflicts, transportation challenges, or costs, were regarded as having delayed healthcare. Social determinants of health, such as race/ethnicity, education, and socioeconomic status, were adjusted in survey-weighted Poisson regression analyses. The mean (±SE) ages of older and younger adults were 75±0.6 and 51±0.1, respectively, with 54% females in both groups. The adjusted prevalence of delayed healthcare was higher in younger adults (22.0%) than in older adults (7.6%). However, older adults with depression had a 2.19 times higher prevalence of delayed healthcare (95% CI: 1.96-2.43), while occurrence was 1.78 times higher in younger adults (95% CI: 1.67-1.91) than those without depression. Depression screening should be administered early for individuals with multimorbidity to identify the risk for delayed healthcare.

Abstract citation ID: igaf122.3070

### INTERSECTIONAL CHALLENGES OF ADL AND FINANCIAL LIMITATIONS: THE MEDIATING ROLE OF PARTICIPATION IN WELLBEING

Qiwei Li[1], Xiaoli Li[2], and Cheng Yin[3], *1. California State University, Fresno, Fresno, California, United States, 2. Southern Illinois University, Carbondale, Illinois, United States, 3. University of North Texas, Denton, Texas, United States*

Background: Older adults with both ADL limitations and financial constraints face significant challenges in achieving well-being. While community participation was reported as a factor to improved well-being, little is known about its mediating role in this population facing the intersectional difficulties brought by ADL and financial limitations. This study applies Structural Equation Modeling (SEM) to examine the relationships between ADL, financial constraints, participation, and well-being. Methods: Using waves 5 to 9 of NHATS data (n = 4,152), we specified latent constructs for Well-being, Participation, ADL, and Financial Constraints within an SEM model. Mediation effects were assessed by decomposing direct and indirect effects and calculating the mediated proportion. Covariates were included in the model as well. Results: The model demonstrated good fit. ADL had a direct negative association with well-being ($\beta = -0.423$, $p < .001$) and participation ($\beta = -0.475$, $p < .001$). Participation positively predicted well-being ($\beta = 0.255$, $p < .001$), indicating mediation. Financial constraints negatively impacted participation ($\beta = -0.075$, $p < .001$) and well-being ($\beta = -0.083$, $p < .001$). Marital status ($\beta = 0.041$, $p = .008$) and race ($\beta = 0.035$, $p = .027$) were also significant well-being predictors. Participation mediated 22.3% of the ADL to well-being relationship and 18.8% of the financial constraints to well-being relationship. Conclusion: Participation serves as a key mediator between ADL limitations, financial constraints, and well-being. These findings highlight the importance of social engagement interventions to support well-being among older adults facing both functional and financial barriers.

Abstract citation ID: igaf122.3071

### LONGITUDINAL TRAJECTORIES OF HEALTH AS PRECURSORS OF AVERAGE LEVELS AND TEMPORAL PATTERNS OF SELF-CONTINUITY

Yi Lu[1], Urmimala Ghose[2], Denis Gerstorf[3], and Corinna Löckenhoff[1], *1. Cornell University, Ithaca, New York, United States, 2. Humboldt University in Berlin, Humboldt University in Berlin, Berlin, Germany, 3. Humboldt University Berlin, Humboldt University Berlin, Berlin, Germany*

Self-continuity, the sense of being the same person across the past, present, and future, is associated with physical and mental health and sensitive to prior life experiences. However, little is known about the association between self-continuity and longitudinal health trajectories across the adult life span. In this pre-registered study, we used data from 1,655 participants (aged 18-94, M = 52.73, 53% women) in the German Socio-economic Panel Innovation Sample. We examined the implications of longitudinal trajectories of mental and physical health for subsequent self-continuity—both with respect to average self-continuity and with respect to temporal patterns of self-continuity (i.e., relative self-continuity for past vs. future and close vs. distant time points). Self-continuity was assessed across three temporal distances (1/5/10 years) into the past and future. Health-related predictors captured both broad variations in general mental and physical health (i.e., life satisfaction and self-rated health) and more specific aspects (e.g., recent affective states, illness burden). Latent growth models were applied to examine the effects of longitudinal health trajectories on both average levels and temporal variations of past/future self-continuity. Multiple health factors, including illness burden, self-rated health, physician visits, life satisfaction, and affective states, significantly predicted both average levels of self-continuity and decrements in self-continuity for more distant time points. Interactions with age and gender were observed, and, consistent with prior research, self-rated health and negative affect had a stronger impact on past versus future self-continuity. Findings extend our understanding of longitudinal shifts in health as precursors of self-continuity across the life span.

Abstract citation ID: igaf122.3072
**PREDICTING ARTS ENGAGEMENT IN OLDER ADULTS: A PROSPECTIVE ANALYSIS USING THE MIDUS STUDY**
Jacquelyn Stephens[1], and Jennifer Smith[2], *1. Mather Institute, Illinois, United States, 2. Mather Institute, Evanston, Illinois, United States*

Regular participation in the arts shows promise for increasing older adults' well-being (Fancourt et al., 2019): Previous work has found associations between arts engagement and increased well-being, as well as reductions in loneliness, depression, and health problems. However, there has been less attention on what factors predict later arts participation in middle- and older age. The current study draws from two waves of the Mid-life in the United States (MIDUS) study, the Refresher 1 (2011-14) and the Refresher Arts Surveys (2021-22), to examine how facets of subjective well-being predict arts engagement over 9 years. The sample (N = 1545), restricted to ages 55+, had an average age of 68.32 (SD = 7.73), was 54.2% female, and 79% white. Predictive factors included Ryff's six aspects of eudaimonic well-being (WB), life satisfaction (LS), and positive affect (PA). Logistic regression models were used to examine associations between well-being and later monthly arts participation, with covariates of age, gender, education, retirement status, and health. Several aspects of WB predicted greater likelihood of arts participation (purpose in life [OR: 1.03, 95% CI = 1.01, 1.05], personal growth [OR: 1.06, 95% CI = 1.04, 1.08], environmental mastery [OR: 1.02, 95% CI = 1.01, 1.04], and autonomy [OR: 1.03, 95% CI = 1.01, 1.05]), as did LS (OR: 1.19, 95% CI = 1.05, 1.29) and PA (OR: 1.22, 95% CI = 1.02, 1.38). These findings suggest that cultivating well-being may serve as a pathway to arts participation, enhancing quality of life in later years.

Abstract citation ID: igaf122.3073
**RELATIONSHIP BETWEEN ENGAGEMENT WITH LIFE AND AARC IN OLDER ADULTS OVER 80**
Natsue Shiraishi[1], Roman Kaspar[2], Takeshi Nakagawa[3], Yasuhiro Yamamoto[1], Ally Brothers[4], and Takashi Horiuchi[1], *1. Okayama University, Okayama, Okayama, Japan, 2. Charlotte Fresenius Hochschule, Cologne, Nordrhein-Westfalen, Germany, 3. The University of Osaka, Osaka, Osaka, Japan, 4. Colorado State University, Fort Collins, Colorado, United States*

The AARC theoretical model (Diehl et al., 2010) indicates the need to consider Engagement with Life. Kaspar et al. (2022) examined it in older adults aged 80 years and older, but their scope was limited. Therefore, this study examined the relationship between Engagement with Life and subjective aging (AARC). In this context, we investigated both the perceived importance and the actual frequency of activities. A cross-lagged model analysis was conducted using data from the German NRW80+ project, including data from 909 participants aged 80 and older over a two-year period. The results showed that higher importance of "creative activities" at Time1 predicted lower AARC-Losses at Time2 (β = -0.113). Additionally, higher AARC-Gains predicted higher importance of "solitary activities" for having peace and time for yourself (β = 0.137) and "mental activities" to deal with something in greater depth or to study a topic in more detail (β = 0.122). Higher frequency of "creative activities" predicted lower Losses (β = -0.132). Frequent participation in "mental activities" predicted higher Gains (β = 0.116). Higher Gains predicted higher frequency of "physical activity" (β = 0.093) and higher frequency of "mental activity" (β = 0.114). Frequent participation in "social activities" such as spending time with others like relatives or friends predicted lower Gains (β = -0.098). Higher frequency of "mental activities" (lifelong learning) predicted positive subjective aging, and the reverse was also observed. Higher importance and frequency of "creative activities" also predicted lower negative subjective aging.

Abstract citation ID: igaf122.3074
**RELATIONSHIP BETWEEN GARDENING AND STRESS ON OLDER ADULT PHYSICAL AND MENTAL HEALTH**
Laurel Mertz, and Jennifer Smith, *Mather Institute, Evanston, Illinois, United States*

The act of gardening has significant potential to improve successful aging among older adults, including through improved physical health factors such as better flexibility, blood pressure, and cholesterol. Beyond physical health, gardening may also enhance older adults' ability to age well through improved socialization, quality of life, cognition, and mental health. This study aimed to extend upon previous research by evaluating the frequency of gardening among older adults and exploring how gardening may attenuate the relationship between stress and health. A total of 3,293 older adults, ages 55 and better, were recruited via an online research panel (mean age = 65.6 years, 52.9% female, 69.6% White). Participants completed a survey assessing frequency of gardening, stress levels, and physical and mental health. Descriptive and independent t-test analyses revealed that 61.6% of older adults reported gardening at least sometimes, and that older adult women gardened significantly more frequently than older adult men. Multiple regression analyses controlling for gender, income, and age indicated that engaging in more frequent gardening attenuated the negative relationship between stress and mental health, but not physical health. That is, higher levels of stress were related to worsened mental health, but this relationship was weaker for those who gardened more frequently. These findings provide evidence that gardening may be effective in protecting against the negative impact of stress on older adults' mental health. Given the potential protective factor of gardening, future research should explore ways to address barriers to gardening in older adults, especially older adult men.

Abstract citation ID: igaf122.3075
**RELATIONSHIP BETWEEN PHYSICAL HEALTH, DISABILITY, AND GERIATRIC SYNDROMES AND DEPRESSIVE SYMPTOMS IN OLDER ADULTS**
Poshan Dahal, *Case Western Reserve University, Cleveland, Ohio, United States*

Depression in older adults is associated with various negative health consequences. Combination of chronic conditions, functional limitations and geriatric syndromes have the potential to cause detrimental physical and psychological health outcomes. This paper aims to identify the combinations of chronic conditions, functional limitations, and geriatric syndromes that are associated with depressive symptoms among older adults. We used data from 2016-2018 Health and Retirement Study (HRS) in this study. The HRS provides a representative sample of adults aged 50 years and above in the US. We used Classification and Regression Tree Analysis in R program and calculated descriptive statistics in STATA program. Sample size of this study is 15,957. Our outcome variable was depressive symptoms. Key independent variables were chronic conditions, functional limitations, and geriatric syndromes. Other covariates included in the analysis were age, sex, marital status, income, race, and education. Fifteen percent of respondents reported depressive symptoms at 2 years. The top combinations of variables identified through the classification and regression tree analysis for depressive symptoms included having IADL limitations, being troubled with pain, and having persistent dizziness and lightheadedness (>40%). Among older adults, functional limitations and geriatric syndrome were significantly associated with psychological wellbeing than chronic conditions. This finding underscores the clinical and programmatic importance of functional limitations and geriatric syndromes in understanding and addressing the health need of older adults.

Abstract citation ID: igaf122.3076

### SECONDARY CONTROL: A SELF-REGULATORY RESOURCE THAT PROMOTES EMOTIONAL STABILITY IN DAILY LIFE

Matthew Pierce, Laura Klepacz, and Jeremy Hamm, *North Dakota State University, Fargo, North Dakota, United States*

Although secondary control beliefs (e.g., "all clouds have a silver lining") reflect a self-regulatory resource that protects well-being, little is known about how secondary control may help stabilize day-to-day emotional experiences and how aging might affect this regulatory process. Our study examined whether individual differences in secondary control predicted day-to-day variability in positive and negative affect, loneliness, stress, and depressed mood. We further examined how this relationship may differ across the adult lifespan. Using 1 week of daily data from a sample of adults aged 30 to 80 ($n = 217$), we employed multilevel heterogenous variability models to test whether higher levels of secondary control predicted less variability (the Level-1 residual variances) in emotional well-being. All models controlled for study day, age, sex, education, and financial hardship. Results showed that individuals with higher levels of secondary control experienced reduced day-to-day variability in negative affect ($\alpha$=-.16, $p<.005$), loneliness ($\alpha$=-.33, $p<.005$), and depressed mood ($\alpha$=-.44, $p<.005$). Significant Age x Secondary Control interactions were observed for negative affect ($\alpha$=.02, $p<.005$), loneliness ($\alpha$=.02, $p<.005$), and depressed mood ($\alpha$=.01, $p<.005$). These interactions indicated that secondary control was a stronger predictor of reduced day-to-day variability in negative affect, loneliness, and depressed mood for younger (vs. older) adults, who typically experience more volatility in their daily well-being. These findings further inform lifespan theories of development and control by documenting the self-regulatory benefits of secondary control for stabilizing day-to-day emotional well-being, especially among individuals in early adulthood and midlife.

Abstract citation ID: igaf122.3077

### SELF-PERCEPTION OF AGING AND LIFE SATISFACTION OF HISPANIC OLDER ADULTS: THE ROLE OF FAMILY RELATIONSHIP

Dahee Kim, Thuc Luong, Janet Lopez, Ayse Malatyali, Ladda Thiamwong, Abigail Tice, Yingru Li, and Rui Xie, *University of Central Florida, Orlando, Florida, United States*

Individual self-perception of aging (SPA) is shaped by socio-cultural experiences across life stages and may affect emotional well-being in late adulthood. However, the association between SPA and emotional well-being in Hispanic older adults (HOA), as well as the contextual factors affecting this association, has not been explored. We aimed to (1) investigate the relationship between HOAs' SPA and life satisfaction, one of emotional well-being indicators and (2) test the moderating effects of spousal relationships and relationship quality with children. We analyzed data of 143 HOAs from the Health and Retirement Study collected in 2016-2020. SPA was measured using the Attitudes Toward Own Aging (ATOA), and life satisfaction was measured using The Satisfaction with Life scale. Spousal relationship was assessed based on perceived closeness with spouses, and relationship quality with children was evaluated by the frequency of contact with children. The two moderation models were tested using SPSS PROCESS Macro. HOAs with more positive SPA were likely to have higher life satisfaction ($r=.231$, $p<.01$). In HOAs with less frequent contact with children, the more positive SPA, the higher life satisfaction they had. In contrast, in HOAs with more frequent contact with children, the more positive SPA, the lower life satisfaction they had ($B=-.235$, $p<.05$). There was no moderating effect of spousal relationships. This study highlights HOAs' SPA and emotional well-being and showed frequent contact with children may have a negative effect on emotional well-being. Future research is expected to continue exploring the associations among HOA's SPA, health/well-being indicators, and contextual factors.

Abstract citation ID: igaf122.3078

### SOCIAL ENGAGEMENT, LONELINESS AND DEPRESSION AMONG OLDER ADULTS LIVING ALONE: A SYSTEMATIC REVIEW

Genevieve Ebulum[1], and Obinna Ezeihuoma[2], *1. David Umahi Federal University of Health Sciences (DUFUHS) Uburu, Ohaozara LGA, Ebonyi state, Nigeria, Enugu, Enugu, Nigeria, 2. University of Pittsburgh, Pittsburgh, Pennsylvania, United States*

Loneliness and depression among older adults living alone is a critical public health issue. It is associated with physical and mental health consequences, including increased mortality and morbidity. Social engagement has emerged as a potential intervention to address these issues, yet existing research remains fragmented and lacks comprehensive evaluation. A systematic review was conducted on the role of social engagement in mitigating loneliness and depression among older adults living alone. We reviewed articles published between 2018 and 2024 was conducted, following PRISMA guidelines. Databases including PubMed, CINAHL and PsycINFO were searched using predefined inclusion criteria. The Critical Appraisal Skills Programme (CASP) tool was used to assess the quality of the included studies.

Data were extracted and synthesized based on objectives, research design, participants, context, and outcomes. The review included five high-quality studies. Findings revealed that interventions promoting social engagement such as group activities, community programs, and digital platforms significantly reduced loneliness and depression among older adults. However, the effectiveness of these interventions was influenced by factors like cultural norms, resource accessibility, and individual preferences. The review highlights the importance of tailoring social engagement interventions to the specific needs and contexts of older adults. It emphasizes the role of community support, digital inclusion, and policy frameworks in enhancing the reach and effectiveness of these interventions. This systematic review underscores the critical role of social engagement in improving mental well-being among older adults living alone. Evidence-based strategies should prioritize accessibility, inclusivity, and cultural sensitivity to maximize their impact.

Abstract citation ID: igaf122.3079

### THE EFFECT OF DISCORDANCE BETWEEN FELT AGE AND IDEAL AGE ON SUBJECTIVE WELL-BEING

Isu Cho, and Hyung In Park, *Sungkyunkwan University, Seoul, Republic of Korea*

The discrepancy between felt age and ideal age is termed as subjective age discordance (SAD). According to self-discrepancy theory, individuals tend to feel frustrated from experiencing discrepancies between how they perceive themselves and what they desire, which in turn can be associated with decreased well-being. Based on this theory, SAD is proposed to capture the status quo of individual well-being, separate from the discordance between felt age and chronological age that previous literature has been extensively tested. The current study examined the relationship between SAD and indicators of subjective well-being (i.e., life satisfaction, depression, and self-reported physical health) using polynomial regressions and response surface graphs. Data were collected from 318 Korean adults (aged 40 to 79) who responded twice with a 3-month interval. Results showed that a greater felt age against ideal age in Time 1 was linked to decreased life satisfaction in Time 2, whereas a greater ideal age against felt age was associated with enhanced life satisfaction, aligning with self-discrepancy theory. Plus, the greater SAD was in both directions, the higher depression was. However, SAD did not predict physical health. Besides, these patterns were moderated by chronological age such that the effects of SAD on life satisfaction and physical health were more prominent in younger adults (aged 59 or below, $n = 164$) than in older adults (aged 60 or above, $n = 154$). The findings suggest that older adults' better socioemotional abilities (e.g., emotion regulation skills) may alleviate the negative effect of SAD on life satisfaction.

Abstract citation ID: igaf122.3080

### THE IMPACT OF PET OWNERSHIP ON OLDER SINGLE EXPATRIATE FEMALES IN PANAMÁ

Melissa Mansfield, *The Gerontechnology Group, Houston, Texas, United States*

This qualitative hermeneutic phenomenological study explored the lived experience of singlehood and pet companionship among expatriate women aged 55 and older in the Panamá Oeste Province. The research aimed to understand how pet companionship shapes the aging experience and impacts mental health, relationships, and loneliness for this demographic. Semistructured interviews were conducted with 15 single expatriate women who were current or prior pet owners, supplemented by participant observation and photo elicitation techniques. Four themes emerged from the interpretative phenomenological analysis: (a) companionship and emotional bonds, (b) daily structure and routine, (c) personal security and safety, and (d) challenges of pet ownership. Findings suggest pet companionship significantly enhances the aging experience, positively impacting mental health, social connections, daily structure, security, and independence. The study contributes to understanding human–animal bonds in later life, particularly in expatriate contexts. It also supports attachment theory and activity theory of aging as frameworks for understanding how pet companionship influences overall well-being and social engagement in a cross-cultural context. Results highlight the multifaceted role of pets in the lives of older expatriate women living alone in Panamá. Future researchers should investigate the experiences of single expatriate men with close pet relationships across diverse cultural settings to broaden the understanding of this phenomenon.

Abstract citation ID: igaf122.3081

### THE IMPACT OF TECHNOLOGY-ASSISTED INTERVENTIONS ON LONELINESS IN OLDER ADULTS: A META-ANALYSIS

Yu-Ping Chang[1], Ashleigh Holmes[2], Weijun Wang[1], and Jihnhee Yu[1], *1. University at Buffalo, Buffalo, New York, United States, 2. New York University, New York, New York, United States*

Loneliness and social isolation among older adults are critical public health concerns in the United States, affecting a substantial portion of this population and increasing their risk of cognitive decline, as well as severe mental and physical health consequences. Although recent research highlights the potential of technology-assisted interventions in mitigating these issues, offering promising solutions to enhance social connectivity and well-being, a systematic evaluation of their effectiveness in reducing loneliness among older adults is needed. A meta-analysis of randomized controlled trials was conducted, with a comprehensive literature search of studies published up to November 2024 across four major databases: PubMed, PsycINFO, CINAHL, and Web of Science. Two independent reviewers screened and evaluated the records, resulting in 25 studies meeting the inclusion criteria. These studies examined various types of technology-assisted interventions such as robotic assistants, web-based modules, and mobile applications. Statistical analyses were conducted using R (Version 4.0.2). A heterogeneity analysis using Cochran's Q test indicated significant variability among studies ($Q = 157.21$, $df = 24$, $p < 0.001$), with an $I^2$ estimate of 84.7%, suggesting substantial heterogeneity. Given this, a random-effects meta-analysis was employed to pool effect sizes. The overall effect size (Cohen's d) was statistically significant at 0.309 ($p = 0.007$, 95% CI: 0.084–0.534), demonstrating a significant reduction in loneliness among older adults who participated in technology-assisted interventions. These findings underscore the potential of technology as a valuable tool for addressing loneliness. Future research should assess the long-term effectiveness of these interventions and identify best practices for implementation to maximize impact and accessibility.

Abstract citation ID: igaf122.3082

### THE POWER OF PETS: HOW COMPANIONSHIP AND SOCIAL CONNECTIONS SHAPE AGING WELL

Marielena Barbieri, *Mather Institute, United States*

As people age, maintaining strong social connections becomes essential for well-being. While pets are often considered loving companions, their impact on older adults' social and emotional health is not fully understood. This study explores how pet ownership influences loneliness, happiness, and life satisfaction, mediated by social cohesion. Utilizing 4-years of data from the Age Well study (N = 2,863, Mage = 82.31), we examined both the direct and indirect effects of pet ownership on well-being over time. Findings revealed that while pet ownership in 2019 alone had limited direct effects, it significantly enhanced social cohesion during 2020-2021, which in turn reduced loneliness ($b = -.29$, $p < .001$) and increased happiness ($b = .51$, $p < .001$) and life satisfaction ($b = .44$, $p < .001$) by 2022. Differences emerged between pet types: dog owners experienced greater social cohesion ($b = .12$, $p = .001$), leading to improved well-being outcomes, while cat ownership showed no significant indirect effects. These results highlight the power of pets—particularly dogs—in fostering social connections that enhance well-being in later life (e.g.,Hajek & Konig, 2020). As loneliness and isolation remain pressing public health concerns among older adults, promoting pet companionship may serve as a meaningful intervention (Holt-Lunstad, 2017; Krause-Parello et al., 2019). This study highlights the need for aging policies and community programs that recognize and support the social benefits of pet ownership, ensuring that more older adults have access to the companionship and connection that can help them thrive.

Abstract citation ID: igaf122.3083

### TRAJECTORIES OF PSYCHOSOCIAL PHENOTYPES IN OLDER ADULTS WITH PAIN: A LATENT TRANSITION ANALYSIS

Yu-Ping Chang[1], and Ashleigh Holmes[2], *1. University at Buffalo, Buffalo, New York, United States, 2. New York University, New York, New York, United States*

Despite the high prevalence of pain in older adults, research on multivariable contributors to pain outcomes remains limited. Psychosocial phenotyping, which identifies patterns of psychological and social characteristics, requires further longitudinal analysis to better understand its role in older adults experiencing pain. This study explored the stability of psychosocial phenotypes longitudinally in older adults with pain and identified predictors of phenotype transitions over time. Using 2018-2022 annual data from the National Health and Aging Trends Study, latent transition analysis was performed in older adults consistently reporting pain ($n = 813$) to assess psychosocial phenotype transitions longitudinally. Psychosocial phenotypes comprised clusters of similar scores on depression, anxiety, affect, self-realization, resilience, self-efficacy, and social participation. Baseline variables (pain characteristics, biological, cognitive) associated with transitions in phenotypes were determined via logistic regression. Three psychosocial phenotypes (Adverse, Favorable, and Intermediate) were identified. Longitudinally, phenotype membership remained largely stable with a trend towards increased psychosocial adversity. A temporary decline in psychosocial well-being was observed during the COVID-19 pandemic, highlighting the impact of major stressors on these trajectories. Baseline cognition and physical performance predicted transitions to less adverse phenotypes, whereas poorer sleep, diminished physical performance, greater pain-related limitations, and lower self-rated health predicted transitions toward more adverse phenotypes. Older adults with pain exhibit a notable degree of psychosocial stability and resilience, even in the face of significant external stressors. Future research should explore interventions that promote transitions to more favorable phenotypes, support point-of-care clinical decision-making, and advance the integration of precision medicine approaches in pain management.

Abstract citation ID: igaf122.3084

### WISDOM IN OLDER ADULTS AGING WITH A MOBILITY DISABILITY

Hye Soo Lee[1], Olivia Rojas[2], and Wendy Rogers[2], *1. University of Illinois--Urbana-Champaign, Champaign, Illinois, United States, 2. University of Illinois Urbana-Champaign, Champaign, Illinois, United States*

Whereas wisdom is seen as a beneficial concept for navigating life, it lacks a clear definition in academia. Researchers have suggested investigating the contextual factors of wisdom, such as culture, to enhance our understanding of wisdom. We extended this approach by examining a population that shares a specific life context. We used interview data from the Aging Concerns, Challenges, and Everyday Solution Strategies (ACCESS) study. The sample was 60 adults aged 60-79 ($M = 69.4$, $SD = 5.7$) who have lived with a mobility disability for at least 10 years. There were 26 males and 34 females, 86.7% of whom identified as white. We conducted a thematic analysis on the advice participants would offer to a similar other. Our coding scheme included a priori codes based on existing wisdom literature (i.e., self-regulation; cognitive and meta-cognitive realm; social realm; and spirituality and religiosity) as well as additional codes we identified from the data (i.e., physical health; perseverance; attitudes about receiving help; and thinking about the act of giving advice). We found the theme dependence/independence, reflecting participants' discussion of maintaining independence and accepting help. Most of the wisdom mentioned helped participants to be on their own; however, some participants discussed the wisdom of allowing oneself to depend. Wise people are often characterized as advisors, supporters, and givers. This study allowed us to capture the wisdom of dependence, which may benefit all individuals facing declines in functioning. We also discuss the implications of finding wisdom codes that are not widely recognized in the extant wisdom literature.

### SESSION 7385 (POSTER)

### RESEARCH METHODS AND ISSUES: QUANTITATIVE

Abstract citation ID: igaf122.3085

### A SYSTEMATIC APPROACH TO BUILDING HIGH-IMPACT AGING DATASETS FOR RESEARCHERS

Riya Goyal, Yater Henry, Tasnim Raisa, Jessica Fleck, and Duo Wei, *Stockton University, Galloway, New Jersey, United States*

Access to relevant and high-quality secondary datasets is essential for advancing research on the older population across diverse domains such as mental health, health services, and life satisfaction. This study presents a systematic approach to identifying and curating a searchable database of the most significant secondary datasets in these fields. Our method begins with domain experts defining key content domains and their respective subdomains. Using Claude AI, we conduct targeted searches by combining subdomains with data analytics keywords and the term "older adults" to retrieve relevant datasets. To assess the importance of each dataset, we develop a Python algorithm that analyzes the frequency of dataset occurrences across multiple search combinations. For instance, by leveraging Claude AI to generate 100 datasets through a query of "older adults," "Alzheimer's," and "regression," we subsequently matched these datasets against PubMed's repository, revealing 7,561 relevant publications, with 7,030 specifically addressing aging research. This iterative process allows us to identify the most commonly used and impactful datasets for researchers in aging studies. The resulting database will provide a structured, accessible, and efficient resource for scholars and practitioners seeking high-value secondary data to support evidence-based research on aging-related topics.

Abstract citation ID: igaf122.3086

### A THREE-BY-THREE CLUSTER CROSSOVER TRIAL OF POST-ACUTE CARE PATIENTS WITH FRACTURE

Thomas Travison[1], Cathleen Colon-Emeric[2], Mark Toles[3], Andrew Zullo[4], Stephanie Kissam[5], and Sarah Berry[1], *1. Hebrew SeniorLife & Harvard Medical School, Hebrew SeniorLife, Massachusetts, United States, 2. Duke University School of Medicine, Durham, North Carolina, United States, 3. The University of North Carolina at Chapel Hill, Chapel Hill, North Carolina, United States, 4. Brown University, Providence, Rhode Island, United States, 5. American Health Care Association, Washington, District of Columbia, United States*

Later-life fractures are a leading cause of disability. Medication optimization improves outcomes, but no studies have compared models to optimize medications in skilled nursing facilities (SNFs). To address this gap, we designed a pragmatic three-by-three cluster-crossover trial to evaluate three post-acute care models: 1) a Bone Health model providing osteoporosis management recommendations; 2) a Deprescribing model targeting medications that increase fall and fracture risk; and 3) a Combined Care model incorporating both models. In all models, a remote nurse consultant collaborates with an interprofessional team to provide personalized medication recommendations to SNF providers. Forty-two SNFs will be randomized to deliver each model for six months in a randomized sequence such that all SNFs eventually deploy all three models. Approximately 3,780 patients with fracture will receive the intervention while in SNF care. 126 control facilities will be matched to the intervention facilities to compare outcomes. The primary endpoint, injurious falls, will be identified through Medicare claims linked with electronic health record data in the Long-Term Care Data Cooperative, for up to two years post-discharge. Secondary endpoints include fall risk-increasing medication exposure, falls self-efficacy, pain, and sleep quality, measured over 90 days post-discharge. This study is one of few cluster-crossover trials comparing more than two interventions. The presentation will highlight key design features, statistical power computations and considerations, planned statistical analyses, and the trial progress, offering insights for researchers interested in applying this unique cluster-crossover design.

Abstract citation ID: igaf122.3087

### CHALLENGES AND LESSONS FROM A REMOTE INTERVENTION FOR FAMILY CAREGIVERS: A FEASIBILITY STUDY OF THE EMPOWER PROGRAM

Katie Trainum[1], Rosa Schnyer[2], and Bo Xie[2], *1. University of Pennsylvania, Philadelphia, Pennsylvania, United States, 2. The University of Texas at Austin, Austin, Texas, United States*

Remotely delivered interventions offer advantages like broader geographic reach and increased accessibility. However, they also introduce challenges, including scams in the recruitment and implementation as well as technological barriers. This paper reports challenges encountered and lessons learned through a feasibility study of EMPOWER (Engage your Mind and Body to Promote your Own Wellness, Energy and Relaxation), a self-care health intervention incorporating complementary and integrative techniques. In Fall 2024, we conducted four EMPOWER sessions (three via Zoom, one in-person). In the first two Zoom sessions, to ensure participants' privacy and comfort, we did not require verbal or camera engagement, nor did we have a moderator present in the breakout room discussions. After the first two Zoom sessions, we discovered suspicious activity, including similar email formats, inconsistent demographics (i.e., higher-than-expected male and younger participants), and IP address anomalies (survey responses originating from outside the research area and multiple responses from single IP addresses). As a result, data collected from those first two Zoom sessions had to be discarded. Based on these experiences, we recommend several strategies for future virtual caregiving interventions. These include recruiting participants through established in-person groups; implementing rigorous participant vetting processes to verify authenticity; limiting responses to one per IP address; requiring verbal or camera engagement during the intervention sessions; and moderating Zoom breakout room discussions. While remotely delivered interventions present challenges, creative solutions can help maintain research integrity and enhance participant experience. Further work is needed to evaluate the efficacy of these strategies.

Abstract citation ID: igaf122.3088

### IDENTIFICATION OF ADVERSE DRUG WITHDRAWAL EVENTS AFTER OLDER ADULTS DISCONTINUE STATINS

Jonathan Norton[1], Glenn Goodrich[2], Jennifer Barrow[2], Valerie Paolino[2], Ariel Green[1], Orla Sheehan[3], Elizabeth Bayliss[2], and Cynthia Boyd[1], *1. Johns Hopkins University School of Medicine, Baltimore, Maryland, United States, 2. Kaiser Permanente Colorado, Aurora, Colorado, United States, 3. Royal College of Surgeons in Ireland, Dublin, Dublin, Ireland*

Assessing the safety of deprescribing interventions is difficult due to inadequate methods of accurately identifying adverse drug withdrawal events (ADWE) at scale. We explored methods to identify ADWEs after a medication discontinuation using electronic health record (EHR) data, and report on the relationship between statin discontinuations and potential ADWEs. The study population was older adults (65+), taking a statin, and had a confirmed statin discontinuation by chart review. Potential ADWEs were identified by ICD-10 and procedure codes (e.g. stroke, carotid artery disease, ischemic heart disease, coronary artery bypass graft, or percutaneous coronary intervention) within a 12-month time frame following the discontinuation. Two clinicians adjudicated each record to determine if the identified events were related to discontinuations and could constitute

ADWEs. Outcome categories were: doubtful, possible, probable, or definite ADWE. Among 44 potential ADWEs, 48% (21/44) were determined to be doubtful (e.g. stroke during the 12 months following statin discontinuation was more likely related to atrial fibrillation than discontinuation), 48% (21/44) as possible (e.g. myocardial infarction (MI) occurred after discontinuation, but cancer may have also contributed to the MI), 4% (2/44) as probable (e.g. factors other than discontinuation couldn't explain the ADWE), and 0% (0/44) as definite (e.g. same as probable, and confirmed improvement in medical condition after restarting of statin). These exploratory methods of identifying possible ADWEs with ICD-10 and procedure codes after a confirmed discontinuation, while still requiring record review, are new tools that could be enhanced and applied at scale in future deprescribing studies classes to assess safety.

Abstract citation ID: igaf122.3089

### INVESTIGATION OF EXTREME RESPONSE STYLE IN FAMILY CAREGIVING

Mustafa Yildiz[1], Qiyun Liu[2], and Carolyn Pickering[2], *1. Cizik School of Nursing at UTHealth Houston Texas, Houston, Texas, United States, 2. UTHealth Houston, Houston, Texas, United States*

Extreme Response Style (ERS) is a response bias introducing systematic measurement error because individuals differ in their tendency to select extreme end points when responding to surveys with rating categories. Since caregiving studies heavily rely on self-report measures, ERS may pose significant threats to measurement validity in family caregiving. Understanding its predictors is essential for improving validity of inferences in caregiver populations. While prior research links ERS to intelligence, culture, age, and ethnicity among many other variables, determinants of ERS in family caregiving is an unknown phenomenon. This study extends the prior research by examining ERS predictors in family caregiving using a national sample of caregivers (N = 453). ERS was measured with a latent variable through item response tree models by employing a 10-item scale with 5 response categories ranging from Strongly Disagree to Strongly Agree. Predictors of ERS are demographics, caregiving specific variables, and mental health variables. Findings indicated that caregiver ethnicity, gender, education, age, poverty, family issues, anxiety, depression, avoidant/social coping and hours cared do not predict ERS scores. However, increased emotion dysregulation predicts lower scores in ERS. In contrast, increases in positive health status, number of medications used, and problem-based coping predict higher ERS scores. Lastly, being White determines lower ERS levels compared to non-White participants. These results underscore the importance of accounting for ERS in family caregiving research to improve the validity of self-report measures. Understanding ERS predictors can inform survey designs and statistical corrections, ensuring more accurate assessments of caregiver samples.

### SESSION 7390 (POSTER)

### SLEEP

Abstract citation ID: igaf122.3090

### A QUALITATIVE EXPLORATION OF MULTILEVEL FACTORS AFFECTING SLEEP QUALITY AMONG MINORITIZED OLDER ADULTS

Mary Janevic[1], Kimberlydawn Wisdom[2], Martha Quinn[1], Daniel Whibley[3], Philip Cheng[2], and Rebecca Lindsay[1], *1. University of Michigan School of Public Health, Ann Arbor, Michigan, United States, 2. Henry Ford Health, Detroit, Michigan, United States, 3. University of Michigan Medical School, Ann Arbor, Michigan, United States*

Poor sleep is a risk factor for dementia and other chronic conditions, and disproportionately affects African American and economically marginalized older adults. Drivers of sleep inequities exist at the individual, household/neighborhood, and structural levels. We conducted 21 semi-structured interviews with adults aged 50 to 74, 77% African American, who reported persistent "poor" or "very poor" sleep quality over 2+ months. We sought to identify the multilevel factors that impact sleep and elicit feedback on potential components of a behavioral sleep intervention tailored for this community. Using a rapid qualitative analysis approach, we first developed a template reflecting domains of interest (e.g., factors hindering sleep), and then extracted data from transcripts for each domain. We aggregated and reviewed data for main findings. Results revealed that sleep impediments were multilevel. Individual level factors included feeling worried/anxious and being disturbed by pain. Household/neighborhood factors were needing to be "on alert" at night due to security concerns or to care for elderly parents, noise from adult children or pets, and sleeping in a too-warm room. Most participants did not have difficulty falling asleep but woke up repeatedly and had trouble going back to sleep. Appealing features of an intervention included improving sleep without medications; having regular contact with a community health worker; wearing a watch to track sleep; and resources such as videos and podcasts. Findings suggest that a behavioral sleep intervention tailored to the specific needs of older adults in a marginalized community has the potential to be both acceptable and efficacious.

Abstract citation ID: igaf122.3091

### A SCOPING REVIEW OF STRATEGIES AND OUTCOMES OF SLEEP EDUCATION INTERVENTIONS FOR OLDER ADULTS

Soeun Jang[1], Kathy Siepker[2], and Christine Spadola[2], *1. University of Texas at Arlington, The University of Texas at Arlington, Texas, United States, 2. The University of Texas at Arlington, Arlington, Texas, United States*

As sleep disturbances are prevalent among older adults, it is well-established that sleep health issues are negatively impacting their physical, cognitive, and emotional well-being. Although sleep health interventions have been developed and tested as a key non-pharmacological strategy to improve sleep quality in older adults, the scope of their educational components and their effectiveness across different delivery modalities remain unclear. We conducted a scoping review to assess the impact of sleep education interventions on older adults' health and well-being. A search of databases was conducted in September 2024, yielding 1,452 studies published in the past 30 years. After conducting title/abstract and full-text review, 33 studies met our inclusion criteria. The inclusion criteria for included studies were: (1) targeting adults age 60+; (2) including sleep education interventions; and (3) in English; Studies including non-educational or self-guided interventions were excluded. Interventions spanned various countries, with the U.S. (n = 14), followed by Iran, Canada, Japan, and Brazil. Randomized Controlled Trials (RCTs) were the most common (n = 21), followed by quasi-experimental, and pilot studies. Sleep education modalities mostly utilized Cognitive Behavioral Therapy for Insomnia (CBT-I), sleep hygiene education, and relaxation training. CBT-I, interventions that also included physical activity, music, and light therapy showed the greatest improvements in sleep quality and reductions in insomnia severity. Additional benefits from sleep education delivered virtually were also highlighted. Our research highlights that sleep education can be a feasible and effective modality to improve sleep in older adults. Future research should explore tailored interventions for marginalized subgroups of aging populations.

Abstract citation ID: igaf122.3092

### ASSOCIATION BETWEEN SELF-REPORTED SLEEP PROBLEMS AND COGNITIVE DECLINE: A TWO-WAVE PROSPECTIVE STUDY

Fumiko Hamada[1], Monica Walters[2], and Victor Molinari[3], *1. University of South Florida School of Aging, Tampa, Florida, United States, 2. University of Michigan, Ann Arbor, Michigan, United States, 3. University of South Florida, Tampa, Florida, United States*

Sleep is critical to our health, and multiple studies suggest sleep health relates to cognition. However, there are individual differences in sleep problem trends that may influence patterns of cognitive decline. We examined associations between sleep problem trends and cognitive decline among 437 older adults (MageT1=70.3, female=60.0%) from Midlife in the United States (MIDUS) II (Time 1 [T1]:2005-2006) and III (Time 2 [T2]:2013-2017). We created four sleep problem trends from T1-T2 based on self-reported difficulty staying asleep: (1) "no sleep problems" at either time point (n = 259), (2) "developed" sleep problems between T1-T2 (n = 53), (3) "recovered" from T1 sleep problems at T2 (n = 48), and (4) "sustained" sleep problems from T1-T2 (n = 77). Cognition was measured with the Brief Test of Adult Cognition by Telephone. Covariates included age, race/ethnicity, education, and self-reported physical health. Residualized change regressions assessed differences in cognitive decline between groups. Compared to participants with no sleep problems, participants with the other sleep problem trends did not have greater decline in cognition in unadjusted or adjusted models. Regardless of sleep problem trends, all groups experienced significant cognitive decline. While individual differences in sleep problems exist, the findings suggest that sleep problems, whether sustained, developed, or recovered, may not exacerbate cognitive decline. This underscores the importance of considering broader factors influencing cognitive health beyond self-reported sleep difficulties. Further research is needed to explore potential interventions that could help preserve cognitive function in the context of age-related sleep changes.

Abstract citation ID: igaf122.3093

### BIDIRECTIONAL ASSOCIATIONS BETWEEN SLEEP AND ALCOHOL USE IN MIDLIFE AND LATE ADULTHOOD: THE ROLE OF CHILDHOOD TRAUMA

Eunjin Tracy[1], Jichan Kim[2], Pei-Shu Chao[1], Eunjung Kim[1], and Shadi Ansari[1], *1. University of Missouri, Columbia, Missouri, United States, 2. Liberty University, Lynchburg, Virginia, United States*

Childhood trauma, including physical, sexual, and emotional abuse, has lasting effects on sleep in adulthood. It may also increase the risk of alcohol use disorders, as individuals use alcohol to self-medicate distress. Sleep disturbances can contribute to increased alcohol consumption, while alcohol use may further impair sleep quality, reinforcing a cycle of disrupted sleep and excessive drinking. However, bidirectional daily associations between sleep and alcohol use remain understudied, particularly in midlife and late adulthood, and the potential moderating role of childhood trauma is unclear. This study analyzed data from 436 participants (M age = 54.11, range = 34–83) in the Midlife in the United States (MIDUS) Biomarker Project, assessing daily fluctuations in sleep quality, sleep efficiency (SE), and alcohol use over seven days, along with retrospective reports of childhood trauma. Individuals with greater childhood trauma exposure reported poorer sleep quality on average. While sleep quality was not significantly associated with alcohol use, SE and alcohol use demonstrated a bidirectional relationship. Better SE predicted lower alcohol use at both the within- and between-person levels. However, at the within-person level, greater alcohol consumption was linked to higher SE, whereas at the between-person level, individuals who drank more on average had lower SE. These findings suggest a complex interplay between sleep and alcohol use. While alcohol may temporarily aid sleep, it could ultimately be associated with poorer SE over time in midlife and late adulthood. Future research should explore the long-term health implications of these daily interactions and inform clinical interventions.

Abstract citation ID: igaf122.3094

### DAILY SLEEP AND LANGUAGE USE IN LATE LIFE: A LATENT PROFILE ANALYSIS APPROACH

Zexi Zhou, Shiyang Zhang, and Karen Fingerman, *The University of Texas at Austin, Austin, Texas, United States*

Language is crucial for older adults' cognitive activity and social communication, and everyday sleep may play a role in how they talk. This study examines the associations between cognitive functioning, daily sleep, and language use in late life. We used intensive longitudinal data of 266 older adults over 5–6 days from the Daily Experiences and Well-being Study (Mage = 73.74). At baseline, participants completed a battery of standard cognitive tests. Across the study period, Electronically Activated Recorder recorded participants' ambient sound 30 seconds every 7 minutes. Every morning, participants reported their sleep the prior night. Linguistic Inquiry and Word Count software was used to generate linguistic features from transcriptions of recorded speech, including word count, complexity (i.e., words per sentence, words more than 6 letters), emotionality (positivity, negativity), cognitive processes, and informal language. Day-level Latent Profile Analysis identified 2 classes of older adults' daily language: cognitively engaged complex language (i.e., more words, more complex language, higher cognitive processing; 90%) and emotionally expressive informal language (i.e., simpler language, more emotional and informal expressions; 10%). Older adults were more likely to generate cognitively engaged complex language when they had longer-than-usual (versus shorter-than-usual) sleep the prior night, especially for those with better cognitive functioning. Reporting higher-quality prior night's sleep was also linked to a greater likelihood of generating cognitively engaged complex language regardless of cognitive functioning. Findings suggest specific patterns of daily language use in late life, and highlight the importance of sleep in supporting cognitively engaged language use among older adults.

Abstract citation ID: igaf122.3095

### ENVIRONMENTAL INFLUENCES ON SLEEP: NOISE, LIGHTING, & SOCIAL SUPPORT IN SOUTH ASIAN CAREGIVERS FOR OLDER ADULTS

Srujana Chekuri[1], Dinesh Karki[2], Akankshya Chataut[2], and Julie Blaskewicz Boron[2], *1. University of Nebraska Omaha, Elkhorn, Nebraska, United States, 2. University of Nebraska Omaha, Omaha, Nebraska, United States*

Caregivers for older adults often experience disrupted sleep quality due to environmental stressors, affecting their health, well-being, and caregiving capacity. This mixed-methods study examined how noise, lighting, and social support influence self-reported sleep quality among South Asian caregivers who do not reside with care recipients. Quantitative data were collected from 78 South Asian caregivers (62% male; 38% female), aged 19–73 years (M = 26, SD = 7). Nearly half (48.7%, n = 38) reported frequent noise disturbances, significantly associated with lower sleep quality scores (r= –0.47, p < 0.01). Additionally, 47.4% (n = 37) reported high artificial light exposure before bedtime, linked to slightly lower sleep duration (M = 6.54 hours, SD = 1.59) compared to those with lower exposure (M = 6.78 hours, SD = 1.37), though not statistically significant (t= –0.71, p > 0.05). Higher social support was positively correlated with better sleep quality (r = 0.49, p < 0.001), while 33.3% (n = 26) reported social isolation. Qualitative data from five focus groups (n = 21) were analyzed using inductive thematic analysis. Noise was discussed by 16 participants (76%), including external (e.g., traffic, sirens) and internal (e.g., roommates, appliances) sources, contributing to sleep fragmentation. Lighting was discussed by 14 (67%), who reported screen use and limited daylight as disruptors. Community support, emphasized by 15 (71%), buffered stress and enhanced sleep, while isolation increased anxiety and sleep disruption. Findings highlight the need for culturally responsive environmental and social interventions to improve sleep quality among long-distance caregivers, whose unique challenges, such as physical separation and limited direct support, require targeted strategies to enhance caregiving sustainability and overall well-being.

Abstract citation ID: igaf122.3096

### EXPLORING SLEEP BEHAVIORS AND ROUTINES, AND MINDFULNESS PRACTICES AMONG SENIORS RESIDING IN LOW-INCOME HOUSING

Chidera Ejikeme[1], Robert Linscott[2], Stuart Quan[3], Matthew Weaver[3], Charles Czeisler[1], and Rebecca Robbins[1], *1. Brigham and Women's Hospital, Boston, Massachusetts, United States, 2. City of Boston, Boston, Massachusetts, United States, 3. Harvard Medical School, Boston, Massachusetts, United States*

Poor sleep is common among older adults and the general population. Low socioeconomic status is associated with sleep disturbance and short sleep duration. Sleep disturbance is associated with an increased risk for Alzheimer's disease and incident dementia. Evidence indicates that mindfulness can improve sleep duration and quality. We conducted a qualitative study of sleep and mindfulness practices among seniors in a low-income housing facility in the US Northeast. Participants completed the Montreal Cognitive Assessment (MoCA), a brief demographic questionnaire, and an interview about sleep and mindfulness. Interviews were audio-recorded and transcribed verbatim. Qualitative analysis proceeded in accordance with the Constant Comparative Method. Among participants (n = 12), average age was 70.1 (s.d.=6.7); 83.3% of participants were White and 16.7% were Black. Participants were 50% male. Half the sample reported sleeping 7 hours, 25.0% 6 hours, and 25% 8 or more hours. Average score on the MoCA was 23.3 (s.d.=4.03). Common issues related to healthy sleep included taking long naps, nighttime television use, waking from sleep and having trouble falling back asleep, and nighttime checks interrupting sleep conducted by staff members of the residents. Participants reported limited awareness of mindfulness, but in one case, that learning mindfulness skills was 'life changing.' Several reported attempting mindfulness, but being unable to implement a regular practice. Another resident mentioned interest in social support for mindfulness practice. Results from this study highlight sleep struggles, and opportunities for tailoring sleep and mindfulness content in programs for older adults residing in low-income housing.

Abstract citation ID: igaf122.3097

### EXPLORING THE SLEEP-SARCOPENIA RELATIONSHIPS: RESULTS FROM THE LONGITUDINAL STUDY OF AGEING AND HEALTH IN VIET NAM

Thai Le[1], Tuo Yu Chen[2], Cong Nguyen Vu[3], and Yasuhiko Saito[4], *1. Taipei Medical University, Taipei City, Taiwan (Republic of China), 2. Academia Sinica, Taipei City, Taiwan (Republic of China), 3. Institute of Population, Health and Development, Ha Noi, Ha Noi, Vietnam, 4. Nihon University, Tokyo, Tokyo, Japan*

Existing research demonstrates conflicting findings on the relationship between nighttime sleep (sleep quality and quantity) and sarcopenia. Meanwhile, the effects of daytime napping on sarcopenia remain unclear. This study utilized data from the Longitudinal Study of Ageing and Health in Viet Nam to investigate the relationships between sleep with sarcopenia. This cross-sectional study comprised 2,863 adults aged 60 + (mean-age 68.84±7.08 years, 54.7% female). Sarcopenia was assessed using the criteria of the Asian Working Group for Sarcopenia (AWGS) 2019. Self-reported sleep measures included sleep duration (short sleep [< 6 hours], normal sleep [6-9hours], long sleep [>9hours]), sleep satisfaction (yes/no), non-restorative sleep (yes/no), total number of insomnia complaints (scored 1-3, higher score indicating severe insomnia), and daytime napping (none, short nap [< 1hour], long nap [>1hour]). Logistic regression analysis was used to analyze the data, adjusting for sociodemographic and health covariates. A total of 549 participants (19.3%) were diagnosed with sarcopenia, with greater prevalence in males. Short sleep (OR = 1.23,95%CI=1.00-1.50), long sleep (OR = 2.29,95%CI=1.30-4.03), and total number of insomnia complaints (OR = 1.09,95%CI=1.00-1.19) were significantly related to an increased likelihood of sarcopenia. Sleep satisfaction (OR = 0.81,95%CI=0.66-0.99) and daytime napping >1 hour (OR = 0.79,95%CI=0.63-0.99) were related to reduced risks of sarcopenia risk. Non-restorative sleep was not related to sarcopenia. When combining all sleep parameters in the analysis, only long sleep was significantly related to increased risks of sarcopenia (OR = 2.41,95%CI=1.37-4.25). Long sleep was found to raise the risks of sarcopenia among older adults. More research using data from different cohorts is needed to investigate the longitudinal relationships between sleep measures and sarcopenia.

Abstract citation ID: igaf122.3098

### HOME ENVIRONMENTAL FACTORS ASSOCIATED WITH HEALTH OUTCOMES IN COMMUNITY-DWELLING OLDER ADULTS

Youngmin Cho[1], Jing Huang[1], Jiaying Li[1], Claire Wang[1], Russell Calderon[2], Michelle Liu[1], Katherine Ornstein[1], and Junxin Li[1], *1. Johns Hopkins University, Baltimore, Maryland, United States, 2. Johns Hopkins School of Medicine, Baltimore, Maryland, United States*

Older adults spend a substantial proportion of their time indoors, making them particularly vulnerable to home environmental factors. However, existing research has focused on outdoor exposures near residential addresses, leaving a critical gap in understanding the scope of indoor environmental impacts on aging. We investigate the associations between indoor environment and health outcomes (sleep, mental, and cognitive health) in community-dwelling older adults without dementia, using the baseline data from an ongoing RCT. Indoor environment includes self-reported environmental comfort—encompassing light, air quality, temperature, and noise—and continuous 24/7 sensor-based measurements of air pollution ($PM_{2.5}$, $CO_2$, $NO_x$, and TVOCs), humidity, temperature, light, and sound levels. Sleep health was assessed via self-reported measures (Pittsburgh sleep quality index [PSQI], and insomnia severity) and objective measurement devices—ActiGraph and ambulatory EEG. Cognitive function was evaluated using the Everyday cognition scale (ECog) and the NIH Toolbox, while mental health was measured through the Geriatric depression scale, Geriatric anxiety scale, and Perceived stress scale. Our findings (n = 39) showed lower perceived environmental comfort was significantly associated with poorer sleep quality (PSQI, r = 0.36), increased insomnia severity (r = 0.32), worsened mental health (depression, r = 0.34; anxiety, r = 0.36; stress, r = 0.35), and lower cognitive function (ECog, r = 0.32). Additionally, increased evening light exposure correlated with higher stress (r = 0.75), higher $CO_2$ levels were associated with poorer delayed recall (r=-0.64), and higher humidity was linked to increased insomnia severity (r = 0.64). These findings highlight the need for targeted interventions to improve indoor environmental conditions, which may mitigate risks to sleep, mental, and cognitive health in community-dwelling older adults.

Abstract citation ID: igaf122.3099

### INCOME ADEQUACY, PRE-SLEEP AROUSAL, AND MULTIDIMENSIONAL SLEEP HEALTH AMONG OLDER ADULTS

Caitlan Tighe, Brooke Sullivan, Grace Lynch, and Declan Miles, *Providence College, Providence, Rhode Island, United States*

Existing literature indicates income adequacy is associated with general health perceptions among older adults; less is known about its association with sleep health. We investigated if older adults' multidimensional sleep health differs by income adequacy and to evaluate if income adequacy relates to sleep health indirectly through experiences of pre-sleep arousal. We analyzed data from a cross-sectional online survey study of participants recruited from Prolific. Income adequacy was assessed with a self-report item wherein respondents indicated the extent of difficulty they have paying for basic necessities. The Pre-Sleep Arousal Scale assessed cognitive and somatic pre-sleep arousal. The RU-SATED v4.0 questionnaire assessed multidimensional sleep health. Aims were evaluated with an independent samples t-test and regression-based parallel mediation analysis. The sample included 179 adults aged 65-82 years (M = 69.66, SD = 4.20). On average, individuals who reported difficulty paying for basic necessities had worse multidimensional sleep health (M = 12.95, SD = 4.13) than those who did not endorse difficulty (M = 14.89, SD = 3.96), t(177)=3.10, p=.001. Income adequacy was associated with multidimensional sleep health indirectly, B=-.94, 95% CI [-1.62, -.39], through cognitive arousal, B=-.87, SE=.32, 95% CI [-1.59, -.31], but not somatic arousal, B=-.07, 95% CI [-.46, .24]. These findings suggest that, in older adults, feeling unable to pay for basic necessities is associated with more pre-sleep cognitive arousal which is, in turn, associated with worse multidimensional sleep health. Prospective research is needed to clarify this association and determine if sleep health interventions that are cognitively-oriented and consider social determinants of health are beneficial for older adults experiencing income inadequacy.

Abstract citation ID: igaf122.3100

### LATE-LIFE DISABILITY MAY INCREASE WITH MORE FREQUENT INSOMNIA SYMPTOMS AND SLEEP MEDICATIONS USE OVER TIME

Tuo Yu Chen[1], Soomi Lee[2], and Orfeu Buxton[2], *1. Academia Sinica, Taipei City, Taiwan (Republic of China), 2. The Pennsylvania State University, University Park, Pennsylvania, United States*

Study Objectives: This study investigated whether disability status increased with the frequency of insomnia symptoms and sleep medication usage over a 5-year period and whether frequent use of sleep medication modified the longitudinal effects of insomnia symptoms on disability among community-dwelling older adults. Methods: Data were from the National Health and Aging Trends Study (2011-2015; n = 6,722). Disability was assessed with a validated disability index. Insomnia symptoms were assessed by the average frequency of longer sleep onset latency and trouble staying asleep. General sleep medication usage frequency was assessed. Multilevel modeling was used to analyze the data, considering health status, demographic information, and risky health behavior. Results: In the conditional growth model, for every one-unit higher in the frequency of insomnia symptoms and sleep medication usage, disability scores increased by 0.20 (SE = .02, p < .001) and 0.19 (SE = .02, p < .001) every year, respectively, adjusting for covariates. Moreover, the frequency of sleep medication usage influenced the relationship between insomnia symptoms and disability. Specifically, more frequent insomnia symptoms were associated with higher disability scores, and using sleep medications more often led to an even greater increase in disability scores than insomnia symptoms alone. Conclusions: Disability increased with more frequent insomnia symptoms and more frequent sleep medication usage each year. More frequent sleep medication usage has detrimental implications on disability beyond insomnia symptoms. Treating insomnia and managing sleep medication properly may help prevent disability among older adults.

Abstract citation ID: igaf122.3101

### NOVEL APPROACHES FOR EARLY DETECTION OF CHRONIC INSOMNIA AMONG OLDER PRIMARY CARE PATIENTS

Jaime Hughes[1], Corey Obermiller[2], Laura Jakiela[1], Case Peters[1], Lindsay Munn[1], and Deepak Palakshappa[1], *1. Wake Forest University School of Medicine, Winston-Salem, North*

*Carolina, United States, 2. Atrium Health Wake Forest Baptist Medical Center, Winston-Salem, North Carolina, United States*

Sleep disturbance is common among older adults, contributing to social, cognitive, and functional impairments when untreated. Given sleep disturbance is not routinely addressed within primary care, novel methods for its early identification are needed. In partnership with five primary care clinics belonging to a multistate healthcare system, older adults at risk for insomnia, defined by presence of prespecified comorbidities, were identified from the electronic health record and sent a validated sleep screener. Screener domains included sleep schedule, nighttime sleep disturbances, daytime consequences of poor sleep, overall sleep quality, and history of talking with a provider about sleep. Respondents meeting clinical criteria for insomnia were contacted for a telephone interview and invited to participate in additional research activities (interview, co-design, or CBTI treatment). Of 3455 surveys distributed, 446 older adults (mean age: 75(7.2) years, 37% Male, 67% White, 42% pre-frail, 23% poor health) returned a completed survey. One-quarter of respondents screened positive for chronic insomnia (N = 117); yet, only 44 (38%) had an EHR-documented insomnia diagnosis. When asked to describe their sleep in subsequent interviews (N = 94), older adults endorsed a range of symptoms including nighttime sleep complaints (N = 39 (40%), difficulty falling or staying asleep) and negative impacts on daytime function (N = 54 (56%)). These findings indicate insomnia is prevalent yet underdiagnosed and variable in its symptom profile. Patients' preferences for integrating insomnia screening into primary care included completing a screener via the patient portal prior to a scheduled encounter, tips on communicating sleep problems (e.g., nighttime vs. daytime symptoms), and age-friendly sleep recommendations.

Abstract citation ID: igaf122.3102

### RHYTHM AND REST: SEX, CIRCADIAN PREFERENCE AND SLEEP DURING AGING

Natasa Billeci, and Ashley Curtis, *University of South Florida, Tampa, Florida, United States*

Circadian rhythms and sleep health are linked to cognitive decline and Alzheimer's disease (AD) risk. Sex differences exist in sleep and circadian patterns, with men experiencing greater declines in non-rapid eye movement (NREM) sleep and women reporting more sleep disturbances and insomnia. However, the interactive role of sex in circadian preference and polysomnography (PSG)-assessed sleep remains unclear. Understanding these relationships is critical, as women constitute 67% of AD cases. This study examined sex differences in the association between circadian preference and objective sleep parameters in older adults. Cognitively healthy older adults (N = 66; 38 women; Mage=68.05, SD = 5.70) completed overnight PSG and the Morningness-Eveningness Questionnaire. Moderated regressions tested interactions between sex and circadian preference on PSG-measured sleep outcomes, including sleep onset latency, wake time after sleep onset, total sleep time, %N1-N3, and %REM, controlling for age, anxiety, depression, and apnea-hypopnea index. Sex significantly moderated the association between circadian preference and %N3 sleep ($R^2$=0.08, p=.01). In women, greater eveningness preference was associated with lower %N3 (B = 0.80, SE = 0.39, p=.047), while no such association was observed in men (p=.10). The findings suggest that circadian preference and slow-wave sleep relationships may be sex-dependent. Women with an eveningness preference may have a lower %N3, a pattern linked to increased AD risk. Monitoring slow-wave sleep in these individuals could aid in early intervention. Further research should explore sex-specific mechanisms, including postmenopausal hormone changes, circadian gene polymorphisms, and metabolic influences on aging sleep patterns.

Abstract citation ID: igaf122.3103

### SLEEP DISTURBANCE AS A PREDICTOR OF LEARNING AND MEMORY IN OLDER ADULTS: THE MODERATING ROLE OF DEPRESSION

HyeRim Ryu[1], Cari Cohen[2], Jerry J. Sweet[2], Christopher Williams[2], Elizabeth K. Geary[2], Julia Thomas[2], and Leslie Guidotti Breting[2], *1. Rosalind Franklin University of Medicine and Science, Glenview, Illinois, United States, 2. Endeavor Health - NorthShore Hospitals, Chicago, Illinois, United States*

Sleep disturbance, a risk factor for depression (Steiger & Pawlowski, 2019) and memory decline (Crowley, 2011), has become increasingly prevalent among older adults (Reynolds & Adams, 2019). Depressive symptoms are widely associated with lower cognitive functioning in this population (Wei et al., 2019). This study thus investigated whether depressive symptoms moderated the association between sleep disturbance and memory performance. Participants were older adults who received neuropsychological evaluations in an outpatient setting (N = 726, Mage = 76.08, SDage = 6.60). Auditory and visual memory were assessed using the Hopkins Verbal Learning Test (HVLT) and the Brief Visual Memory Test (BVMT), respectively. Depression was assessed using the Geriatric Depression Scale (GDS). The presence or absence of sleep disturbance was assessed during the clinical interview. Results demonstrated that sleep disturbance predicted HVLT immediate recall ($p < .01$), delayed recall (p = .03), percent retained (p = .01), and recognition discrimination scores (p = .04). GDS scores only moderated the relationship between sleep disturbance and immediate recall scores, such that the negative effect of sleep disturbance on immediate recall became stronger as depressive symptoms increased ($p < .01$). Regarding visual memory performances, GDS scores moderated the relationship between sleep disturbance and BVMT immediate recall (p = .04), delayed recall (p = .02), and percent retained (p = .01), with particularly strong moderation for delayed recall scores ($R^2$ Change = .14). These findings further underscore the complex interplay between comorbid sleep disturbance and depressive symptoms impacting learning and memory among older adults.

Abstract citation ID: igaf122.3104

### THE CORRELATION BETWEEN OPTIMISM AND MULTIDIMENSIONAL SLEEP HEALTH IN ADULTS AGED 65 AND OLDER

Brooke Sullivan, and Caitlan Tighe, *Providence College, Providence, Rhode Island, United States*

Optimism has been explored in conjunction with sleep and sleep-related experiences in adults. Fewer studies have examined these associations among older adults specifically and most have investigated associations with single dimensions of sleep or insomnia symptoms. The current study adds to the literature by investigating associations of optimism, positive and negative emotions, and multidimensional sleep health in adults aged 65 years and older. We investigated the study aims with data from a cross-sectional online survey study. The sample included 179

participants ranging in age from 65 to 82 years old (M = 69.7, SD = 4.2). The Comprehensive Inventory of Thriving was used to measure optimism, positive emotions, and negative emotions. The RU_SATED V4.0 Questionnaire was used to measure multidimensional sleep health. Pearson correlations were used to test bivariate associations between multidimensional sleep health and optimism, positive emotions, and negative emotions, respectively. Higher levels of optimism were significantly associated with better multidimensional sleep health ($r = .31$, $p < .001$). Higher levels of positive emotions ($r = .34$, $p = < .001$) and lower levels of negative emotions ($r = -.32$, $p < .001$) were associated with better multidimensional sleep health. Taken together, these findings point to links between optimism, emotional experiences, and self-reported multidimensional sleep health among older adults. Future studies may investigate possible causal links between optimism, emotional experiences, and multidimensional sleep health and, ultimately, attempt to determine if interventions to improve optimism and positive emotional experiences could also improve multidimensional sleep health among older adults.

Abstract citation ID: igaf122.3105

### THE MEDIATING ROLE OF SLEEP QUALITY IN THE ASSOCIATION BETWEEN URINARY INCONTINENCE AND DEPRESSIVE SYMPTOMS

Seo-Yun Choi[1], Yuri Jang[1], and Ju Hyun Kim[2], *1. University of Southern California, Los Angeles, California, United States, 2. Chungnam National University, Daejeon, Republic of Korea*

Urinary incontinence (UI) is a prevalent health issue in the older adult population. Although the negative mental health impact of UI is widely known, the underlying mechanisms remain unclear. Given that UI's physical and emotional discomfort make older adults prone to impaired sleep, we conceptualized sleep quality as a mediator between UI and depressive symptoms. The target group of the present study is older Korean Americans living in subsidized senior housing, who are part of an older immigrant population with socioeconomic disadvantages and health disparities. Using survey data collected from older Korean-American residents of subsidized senior housing in Los Angeles in 2023 ($N = 306$, Mean age = 79.4, $SD = 6.65$), we examined the direct effect of UI on depressive symptoms and the mediating role of sleep quality. UI was indicated by the frequency of experiencing involuntary urine leakage (never/infrequent/frequent), sleep quality was self-rated on a scale from excellent to poor, and depressive symptoms were assessed using the Patient Health Questionnaire (PHQ-9). Multivariate analyses showed the significant direct impact of UI on depressive symptoms. The Hayes PROCESS macro provided supportive evidence for the indirect effect of UI on depressive symptoms through sleep quality (B [SE] = .53 [.15], bias-corrected 95% Confidence Interval = [.24, .85]). The indirect effect accounted for 26.9% of the total effect. Sleep quality was identified as an important pathway through which UI undermines mental health. The study's findings highlight the importance of incorporating sleep-focused strategies into mental health interventions for those affected by UI.

Abstract citation ID: igaf122.3106

### WAKING UP YOUNGER TOGETHER: BETTER SLEEP QUALITY IS LINKED TO A YOUNGER SUBJECTIVE AGE IN OLDER COUPLES

Sierra Birthelmer[1], Juhyeong Lee[1], Elizabeth Zambrano Garza[1], Yoonseok Choi[2], Klaus Rothermund[3], Carsten Wrosch[4], Denis Gerstorf[5], and Christiane Hoppmann[6], *1. The University of British Columbia, Vancouver, British Columbia, Canada, 2. Simon Fraser University, Vancouver, British Columbia, Canada, 3. Friedrich-Schiller University Jena, Institute of Psychology, Jena, Thuringen, Germany, 4. Concordia University, Montreal, Quebec, Canada, 5. Humboldt University Berlin, Humboldt University Berlin, Berlin, Germany, 6. UBC, Vancouver, British Columbia, Canada*

The Contextual Model of Subjective Age proposes that daily experiences, including health and recovery, influence the salience of one's age leading to within-individual variability in subjective age. Daily sleep quality may be one indicator shaping subjective age. Since both sleep quality and subjective age can be shaped by social partners, this study extended past work by examining daily life fluctuations in sleep quality and subjective age among Canadian older couples. We analyzed data from 67 older couples living in Canada (Age: M = 68.4 years, SD = 6.3; 49% female) who provided morning ratings of sleep quality and subjective age over ten consecutive days. We pre-registered the study. After accounting for covariates, findings using the Actor-Partner Interdependence Model indicate that, as hypothesized, increases in sleep quality in both actors and partners were associated with decreases in subjective age. This suggests that when older adults and their partners experience better sleep quality, they report a younger subjective age upon waking, relative to days following poor sleep quality. These findings will be discussed in the context of daily experiences shared by older couples, which may influence variability in their subjective age.

### SESSION 7395 (POSTER)

### SOCIAL ISOLATION AND LONELINESS II

Abstract citation ID: igaf122.3107

### ACTIVITY ENGAGEMENT AND LONELINESS AMONG OLDER ADULTS WHO LIVE ALONE: HETEROGENEITY BY RACE/ETHNICITY

Maki Karakida, Sung Park, Sara Minemoto, and Richard Viskochil, *University of Massachusetts Boston, Boston, Massachusetts, United States*

Social and physical activities play a key role in reducing a feeling of loneliness among older individuals, especially those living alone. Yet, little is known about the associations between activity engagement and loneliness symptoms in older U.S. adults living independently by race/ethnicity. This longitudinal study examines the relationships between activity engagement (social, household, and physical activity; volunteering) in relation to race/ethnicity and loneliness among community-dwelling Americans aged 70+ who live alone. Using the most recent data from the National Health and Aging Trends Study (NHATS), we examined associations between activity engagement and levels and loneliness by race/ethnicity, accounting for sociodemographic, health and other characteristics. Results highlighted that the Hispanic group consistently showed significantly higher loneliness symptoms than their non-Hispanic White peers, even after adjusting for covariates. On the contrary, the non-Hispanic Black group reported significantly lower loneliness scores than non-Hispanic White persons. Activity engagement also signaled a significantly lower likelihood of loneliness across all the models. The model examining activity engagement and race/ethnicity together demonstrated that, compared to the non-Hispanic White group, non-Hispanic Black adults had significantly higher rates of loneliness whereas Hispanic adults indicated significantly lower levels of feeling lonely. These findings suggest that among older U.S. adults living alone, race/ethnicity in conjunction with social and physical activity involvement may lead to differential impacts on their loneliness outcomes and support the development of race/ethnicity-specific interventions.

Abstract citation ID: igaf122.3108

**CHRONIC CONDITIONS AND LONELINESS AMONG NURSING HOME RESIDENTS: AN ANALYSIS OF THE HEALTH & RETIREMENT STUDY WAVE 15**

LaTonya Smith[1], and Laurie Theeke[2], *1. The George Washington University, Westfield, New Jersey, United States, 2. The George Washington University, Washington, District of Columbia, United States*

Approximately 22-42% of nursing home residents report loneliness due to limited social interactions, loss of autonomy, and deteriorating health. This examines the relationships among loneliness and chronic physical and psychological conditions in nursing home residents using data from the Health and Retirement Study (HRS) Wave 15 (2020). Data from 131 nursing home residents were analyzed to explore associations between loneliness and chronic conditions, while considering demographics of age, gender, education, income, and marital status. Descriptive and bivariate statistics were used. The median number of nights in the nursing home was 609. The mean age of participants was 81.7 years (SD = 10.78), 69.5% female, majority reported no current income. 74.4% were high school graduates or higher, and the majority (55%) were widowed. Feeling lonely was prevalent (37.7%), 22.6% felt depressed and 34% rated their memory poor. Functional impairment was evident with 33.3% struggling with instrumental ADLs, reporting difficulty with walking (64.6%), dressing (62%), eating (33.1%), toileting (56.8%) in/out of bed (51.2%). Loneliness correlated with depressive symptoms ($r = .509$, $p < .001$). Psychological problems were related to high blood pressure ($r = .920$, $p < .001$), stroke ($r = .942$, $p < .001$), and diabetes ($r = .940$, $p < .001$), highlighting the interconnectedness of mental and physical health conditions. Residents with lung disease felt more loneliness than without ($X2 = 4.418$, $p=.036$). The findings of this study highlight interplay between loneliness and physical and psychological conditions for those in long-term care, making it critical to address loneliness in this setting.

Abstract citation ID: igaf122.3109

**EFFECTS OF SOCIAL ISOLATION ON MORTALITY IN OLDER ADULTS: DO AGE AND GENDER MATTER?**

Lydia Li, Jay Kayser, and Kyungeun Song, *University of Michigan, Ann Arbor, Michigan, United States*

Social isolation is a recognized risk factor for adverse health outcomes, yet the magnitude of its effect on mortality and differences between genders remain unclear. This study examines whether social isolation predicts all-cause mortality and whether this relationship varies by gender. We analyzed data from the National Health and Aging Trends Study (NHATS), an ongoing nationally representative panel study of Medicare beneficiaries that began in 2011. The analytic sample included 7,186 community-living respondents at baseline, of whom a weighted 28.5% had died by 2023. Despite being a widely used construct, a gold standard to measure social isolation does not exist. Researchers have developed two NHATS-based measures (Pohl and Cudjoe). We used both in weighted Cox proportional-hazard models to test the main effect of social isolation and its interaction with gender, adjusting for sociodemographic, neighborhood, and health characteristics. Both measures of social isolation were significant predictors of mortality risk, though Pohl's measure demonstrated a stronger effect than Cudjoe's. Further, only Pohl's measure had a statistically significant interaction effect with gender ($F [2, 55] = 4.67$, $p =.01$). Specifically, the risk of all-cause mortality was 56% and 2.1 times higher, respectively, for somewhat and very isolated men compared to not-isolated men. Among women, the risk of mortality was 33% higher for the very-isolated relative to the not-isolated. The findings suggest that the operationalization of social isolation matters. Overall, our findings show that social isolation hastens death in older adults, particularly for men. Targeted interventions to reduce social isolation may prevent premature mortality.

Abstract citation ID: igaf122.3110

**FREQUENCY OF CONTACT WITH CONFIDANTS AND COGNITIVE DECLINE OVER TIME IN OLD AGE**

Veronica Howell, Benjamin Shaw, and Uchechi Mitchell, *University of Illinois Chicago, Chicago, Illinois, United States*

Network size and frequency of contact are both positively associated with cognitive function among older adults, however intervention recommendations stemming from these associations remain relatively vague. For example, preventing social isolation is a common goal for reducing dementia risk, but our current conceptualization of the personal network characteristics that constitute social isolation remains imprecise. The purpose of this study is to add precision to our understanding of links between personal network factors and cognitive decline, thus highlighting potential targets for preventing social isolation. Data are from the National Social Life, Health, and Aging Project. Round 1 (2005) network size and frequency of contact measures were combined to create a new set of measures describing: number of confidants with whom the respondent talks frequently (daily or multiple times per week), periodically (weekly or monthly), and seldomly (< monthly). Associations between these measures and cognitive function 5 and 10 years later were estimated while controlling for baseline cognitive function and other covariates. On average, respondents identified 2.5 frequently contacted confidants, 1.5 periodically contacted confidants, and 1 seldomly contacted confidant. Number of periodically contacted confidants was most strongly associated with future cognitive function ($p<.001$). These findings suggest that maintaining contact with confidants outside of one's daily circle may be an important way to prevent social isolation and its associated dementia risk. More research is needed to understand why periodically contacted social ties might bring cognitive benefits, with likely explanations involving regular exposure to diverse information and perspectives while limiting opportunities for interpersonal stress.

Abstract citation ID: igaf122.3111

**IDENTIFICATION OF LATENT PROFILES OF SOCIAL ISOLATION BASED ON STRUCTURAL AND FUNCTIONAL ASPECTS**

Dokyung Yoon[1], Elizabeth Zelinski[2], and Teal Eich[2], *1. University of Southern California Leonard Davis School of Gerontology, Los Angeles, California, United States, 2. University of Southern California, Los Angeles, California, United States*

Social isolation negatively impacts health, and incorporating multidimensional indicators into its measurement may better capture its complexity. This study examined latent profiles of older adults based on social isolation indicators and explored how various factors are associated with profile membership. Participants included 5,466 older adults aged 65 + (Mage = 75.16) from the 2012 and 2014 waves of the Health and Retirement Study. Social isolation was measured through both structural (contact frequency with children/other family members/friends and social participation) and functional (loneliness and social support) aspects. Furthermore, the study incorporated sociodemographic, psychological, environmental, and health-related factors as covariates. Latent Profile Analysis and logistic regression were conducted using Mplus. Results exhibited five social isolation group profiles: (1) 'highly socially engaged' (6%), (2) 'moderately socially engaged' (20%), (3) 'moderately isolated, but not lonely' (46%), (4) 'socially active but unsupported' (12%), and (5) 'most socially isolated' (16%). Structural and functional aspects of social isolation were generally aligned. However, the presence of the 'socially active but unsupported' group suggests that engagement in social activities does not necessarily equate to strong social support. Lower education levels were consistently associated with less social engagement across groups and men tended to belong to socially isolated groups more than women. Compared to the 'highly socially engaged' group, all other groups showed lower levels of extraversion and openness. The 'moderately isolated, but not lonely' and 'most socially isolated' groups exhibited worse cognition, more chronic conditions, and greater depressive symptoms. The findings reveal distinct typologies of social isolation among older adults.

Abstract citation ID: igaf122.3112

### INTERVENTION PROGRAM FOR SOCIAL ISOLATION: THE WAIGAYA PROJECT'S LIFELONG EDUCATION APPROACH

Keiko Katagiri[1], Nahyun Kim[1], Madoka Ogawa[2], and Chiharu Miura[3], *1. Kobe University, Kobe, Hyogo, Japan, 2. Osaka University, Osaka, Osaka, Japan, 3. Advanced Research Center for Well-being of Kobe University, Ashiya, Hyogo, Japan*

In Japan, social isolation and loneliness among older adults have become serious social issues. The purpose of this study is to examines the effects of the Waigaya Project, a lifelong education intervention program launched in 2023 to address these challenges among residents in urban rental apartment complexes, where interpersonal indifference is more pronounced. A mail survey was conducted in January 2025, targeting all residents aged 18 and older in a rental apartment complex in Kobe. A series of Analysis of Covariance (ANCOVA) were performed, with program participation as the independent variable. Dependent variables included the number of weak and strong social ties, sense of belonging, and loneliness. Control variables encompassed sex, education, years of residence, age, parental status, marital status, health and financial status, single-household status, and employment status. The results demonstrated significant positive effects of the intervention of this action research. Participants exhibited increased social ties, a stronger sense of belonging, and lower levels of loneliness than non-participants, with greater effects among those who participated more frequently. The program covered various topics, such as disaster preparedness, financial planning, well-being and generative AI. Lectures were followed by small-group discussions to enhance participant interaction. While most lifelong education programs in Japan rely solely on lectures, our findings emphasize the importance of interactive program design. This action research incorporated diverse data sources, such as real-world data from ICT devices and interviews. Further analysis is required to integrate these data and examine how the intervention reduced loneliness and what factors influenced participation.

Abstract citation ID: igaf122.3113

### LONELINESS AMONG THE SILENT GENERATION AND BABY BOOMERS: A REPLICATED CROSS-SECTIONAL ANALYSIS

Amin Hashemzehi, and Peter Martin, *Iowa State University, Ames, Iowa, United States*

A significant portion of older Americans feels lonely. The relationship between age and loneliness is unclear, and few studies have considered cohort effects. This study investigated whether belonging to the Silent Generation or to Baby Boomers predicts loneliness levels. Analyses were computed on data from waves 9 (2008, $n = 5201$) and 15 (2020, $n = 3278$) of the Health and Retirement Study (HRS). Loneliness was measured using the 11-item UCLA loneliness scale. At wave 9, a t-test indicated significantly higher loneliness in Baby Boomers ($t = -5.99$, $p < .001$). Belonging to the Silent Generation, being female, higher education, African American ethnicity, better subjective health, fewer ADL difficulties, frequent contact with children, friends, and cohabitating with a partner significantly predicted lower loneliness in the final blocked regression model testing four models. The final model explained 17.4% of the overall variance. At wave 15, the loneliness mean was not significantly higher in Baby Boomers ($t = -1.58$, $p = .12$). Nevertheless, belonging to the Silent Generation, fewer difficulties in ADL, frequent contact with children, friends, and cohabitation significantly predicted lower levels of loneliness in the final regression model, explaining 15.4% of the variance. Cohort effects persisted, with Baby Boomers experiencing higher loneliness levels, highlighting the importance of cohort-specific factors. Future studies should explore period effects like social media and COVID-19, and the possible confounding role of age in cohort differences. Developing community support programs for Baby Boomers that emphasize social engagement and raising awareness about intergenerational interactions may be beneficial.

Abstract citation ID: igaf122.3114

### LONELINESS AS A PREDICTOR OF DEPRESSION IN ADULTS 50+: FINDINGS FROM THE HEALTH AND RETIREMENT STUDY

Helena Polansky, Debra Dobbs, and Hongdao Meng, *University of South Florida, Tampa, Florida, United States*

Loneliness is a public health concern that negatively impacts mental well-being among older adults. Understanding its role in depressive symptoms is crucial for developing effective interventions to enhance psychological health and quality of life in aging populations. This study uses a cross-sectional design to examine loneliness as a predictor of depressive symptoms among adults 50 years and older, utilizing a nationally representative sample from Wave 15 (2020) of the Health and Retirement Study (HRS). The analytic sample ($N = 4,212$) self-reported data on the 11-item UCLA Loneliness Scale and the 8-item CES-D Scale. Additional variables included demographic factors (age, gender, education), self-rated health, chronic conditions, and functional limitations (Activities of Daily Living [ADLs], Instrumental Activities of

Daily Living [IADLs]). Spearman correlation and multiple linear regression analyses were employed to assess the relationship between loneliness and depressive symptoms while controlling for covariates. Results indicated a significant positive correlation between loneliness and depressive symptoms ($r = 0.35$, $p < 0.01$). Regression analyses revealed loneliness significantly predicted depressive symptoms ($\beta = 0.11$, $p < 0.01$). Other significant predictors included self-rated health ($\beta = 0.35$, $p < 0.01$), chronic conditions ($\beta = 0.12$, $p < 0.01$), functional limitations (ADLs $\beta = 0.24$, $p < 0.01$; IADLs $\beta = 0.38$, $p < 0.01$), younger age, and female gender. The model explained approximately 29% of the variance in depressive symptoms. The findings underscore loneliness as a critical modifiable risk factor for depression among older adults. Addressing loneliness through targeted interventions could substantially improve mental health outcomes and overall quality of life for older adults. Future research should explore longitudinal designs to establish causality and investigate mechanisms linking loneliness and mental health outcomes.

Abstract citation ID: igaf122.3115

### MACHINE LEARNING-BASED PREDICTION OF LONELINESS IN OLDER ADULTS WITH COGNITIVE DECLINE

Bada Kang, Min Kyung Park, Dahye Hong, Sion Kim, Minji Kang, and Haneul Oh, *Yonsei University College of Nursing, Seoul, Republic of Korea*

Loneliness, a significant public health concern—particularly among older adults—is associated with several adverse health outcomes. Individuals with mild cognitive impairment (MCI) and subjective cognitive decline (SCD) are more likely to experience loneliness owing to reduced social engagement and poor emotional regulation. Because previous research has primarily focused on cross-sectional associations between health-related factors and loneliness levels, the predictors of future loneliness in this population remain largely unexplored. Using a two-year longitudinal dataset, this study aimed to develop machine learning-based predictive models to identify factors associated with loneliness levels one year after baseline assessment in older adults with MCI and SCD. A total of 78 older adults ($n = 56$ SCD and 22 MCI) completed assessments at two time points. In the first year, baseline demographic and health-related data were collected through surveys, while sleep and physical activity data were obtained over two weeks via a 24-hour wrist-worn Actiwatch. Twelve months later, real-time loneliness levels were measured over two weeks employing a mobile Ecological Momentary Assessment approach. Among the tested machine learning models, the Random Forest model demonstrated the highest predictive performance for loneliness one year later (area under the receiver operating characteristic curve [AUC] = 0.970). Sedentary behavior emerged as the strongest predictor, followed by physical movement. These findings highlight the potential of machine learning to predict loneliness in older adults at risk of dementia and emphasize the importance of maintaining adequate physical activity to mitigate loneliness in this vulnerable population.

Abstract citation ID: igaf122.3116

### MARITAL STATUS AND LONELINESS DURING THE PANDEMIC: THE COMPENSATORY ROLE OF ONLINE SOCIAL ACTIVITIES

Ji Hyun Lee[1], and Jeehoon Kim[2], *1. Montana State University, Bozeman, Montana, United States, 2. Idaho State University, Pocatello, Idaho, United States*

An increase in loneliness has been a public concern during the COVID-19 pandemic and heightened loneliness was reported among at-risk groups, including unmarried older adults. When social distancing policies changed the amount and modality of how people connected with others, it is important to examine how online social activities helped alleviate loneliness. The current study examined the effects of online social activities on loneliness for married and unmarried older adults in 2021. Data comes from Round 11 of the National Health and Aging Trends Study ($n = 2,849$, Age M(SD)=78.4, 55% female, 82 % White, 47% married/partnered). Participants reported their loneliness during the last month with one item (dichotomized as never/less than once a week and more than several days a week). Survey-weighted logistic regression models were performed stratified by marital status. Covariates included age, gender, education, race, income, self-rated health and memory, mental health (PHQ-4), physical activity, close social ties, and various social participation. Unmarried older adults were lonelier (35% vs. married 11%) and less likely to be online. When examining the effects of engagement in three online social activities (email/text, SNS, and video chat) during the last month on loneliness, we found that using video chat with family and friends was associated with lower loneliness among unmarried older adults ($OR = 0.65$, $p = .008$). The findings suggest that real-time video-based communication played a compensatory role in reducing loneliness for unmarried older adults. We discuss the value of social technology and its public health implications to reduce the digital divide.

Abstract citation ID: igaf122.3117

### MILES APART- THE NEEDED BRIDGE BETWEEN SOCIAL ISOLATION AND TRANSPORTATION: THE CASE OF CENTRAL MINNESOTA

Claudia Burgos Zuniga[1], Isaac Asirifi Boateng[2], and Phyllis Greenberg[2], *1. Saint Cloud State University, Saint Cloud, Minnesota, United States, 2. St. Cloud State University, St. Cloud, Minnesota, United States*

Social relationships and its connection to transportation play a crucial role in the quality of life of many older adults (Black et al., 2015). While existing literature highlights the relationship between transportation access and social isolation, this poster focuses on the specific transportation challenges faced by older adults in Central Minnesota. Older adults in Central Minnesota expressed that lack of information (14%), availability of transportation (13%) and transportation costs (13%) were their most common barriers to accessing services (e.g., activities, wellness programs and IADL's) (CMCOA, 2024). Additionally, some of the critical factors highlighted by research that can compromise older adult's access to services and activities includes winter conditions, language barriers, technological gaps and safety concerns (Afnan et al., 2022; Dabelko-Schoeny et al.,2021). This poster proposes the following recommendations for consideration: · Promotion of age-friendly rural communities; · Direct (e.g., mobile, in-home) rural health care services; · Door to door transportation that is ADA compliant; · Liaison between school districts / colleges and senior centers in development of intergenerational exchange programs (e.g., skills and culture). By emphasizing localized solutions, this poster contributes to a deeper understanding of social isolation and offers actionable strategies to enhance the quality of life of older adults throughout the United States and internationally.

Abstract citation ID: igaf122.3118

**RETHINKING SOCIAL DISCONNECTION: DOES IT ALWAYS HAVE A NEGATIVE IMPACT?**

Hyojin Choi, Robert Gramling, and Maija Reblin, *University of Vermont, Burlington, Vermont, United States*

Social disconnection, which includes social isolation and loneliness, can negatively impact health and well-being in older adults. However, in reality, these relationships among social isolation, loneliness and well-being are complex. For example, isolated older adults might still feel lonely, but are satisfied with their situation, while those with numerous social connections may still experience loneliness. This study aimed to examine distinct subtypes of social disconnection based on the combination of loneliness and social isolation, and assess their impact on well-being. We analyzed data for all community-dwelling adults (n = 2,542) who participated in both the 2021 and 2022 samples of the National Health and Aging Trends Study. We found that 65% of older adults were neither isolated nor lonely (connected), 14% were only isolated, 15% were lonely but not isolated (lonely in a crowd), and 6% were both isolated and lonely. After controlling for demographic and socio-economic status, regression models show that, compared to the connected group, those who were disconnected or lonely in a crowd were likely to have more depression symptoms and negative emotions in the following wave. However, those who were only isolated did not show a significant difference in depression compared to the connected group. Compared to the connected group, all three other groups were more likely to report fewer positive emotions and more negative attitudes toward life. The findings suggest that feeling lonely is detrimental to well-being while social isolation alone may not be so. It highlights the need for community-based efforts to reduce loneliness.

Abstract citation ID: igaf122.3119

**RISK FACTORS OF LONELINESS IN COMMUNITY-DWELLING SOCIALLY ISOLATED OLDER ADULTS**

Eliza Lai-Yi Wong, Clement Cheuk-Wai Ng, Carol Ka-Po Wong, Shirley Shuk-KuenLui, and Eng-Kiong Yeoh, *The Chinese University of Hong Kong, Hong Kong, China (People's Republic)*

This study aims to identify the risk factors leading to loneliness in community-dwelling socially isolated older adults. The Lubben Social Network Scale-6 determined social isolation, whereas the UCLA 3-item loneliness scale assessed loneliness. A cross-sectional survey was conducted from September 2023 to February 2025, the age group and gender weightings were applied to ensure the population representative. Of the 596 older adults approached in the Hong Kong SAR, China, 269 (45.1%) were considered socially isolated, of which 62 (23.0%) socially isolated older adults experienced loneliness. Results of the logistic regression indicated that socially isolated older adults who attained primary education or below and had chronic disease had the odds ratio of 2.57 (95% CI: 1.31, 5.02) and 3.44 (95% CI: 1.15, 10.33) in undergoing loneliness comparing to those with secondary education or above, and healthy older adults respectively. Socially isolated older adults who reported deaf or severe hearing loss also revealed amplified loneliness risk with an odds ratio of 3.51 (95% CI: 1.04-11.90). Socially isolated older adults with poor quality of life of EQ-5D-5L utility ≤0.801 had enhanced loneliness risk with an odds ratio of 8.75 (95% CI: 3.05, 25.08) in contrast to those with EQ-5D-5L utility =1. Recognizing the leading factors to loneliness in socially isolated older adults provides insights to develop effective community interventions to combat loneliness and social isolation.

Abstract citation ID: igaf122.3120

**RURAL-URBAN DIFFERENCES IN SOCIAL ISOLATION AND DEMENTIA RISK AMONG OLDER ADULTS IN INDIA**

Aarushi Vatsyayan, and Nasim Ferdows, *Northeastern University, Boston, Massachusetts, United States*

Background: Social isolation is a well-documented risk factor for dementia, but its impact varies by demographic and geographic context. Older adults in India face unique structural and social challenges that may exacerbate dementia risk, particularly in rural areas where healthcare access and support networks are weaker. Understanding these disparities is critical for designing targeted interventions that address region-specific needs. Methods: This study utilizes data from the Longitudinal Ageing Study in India (LASI) to examine differences in social isolation between rural and urban older adults (ages 45+). We conducted a descriptive analysis to explore variations in social isolation patterns, marital status, family structure, and community engagement. Key variables include demographics, health indicators (Alzheimer's/dementia history and treatment), family structure, and psychosocial factors (loneliness, depression, and social engagement). Results: Among 71,513 participants, rural adults exhibited higher rates of social isolation than urban counterparts. Rural older adults were more likely to be married (70.16% vs. 66.49%) but reported lower levels of community engagement. Additionally, rural participants experienced loneliness more frequently, with 22.08% reporting feeling lonely sometimes (vs. 20.04% urban) and 3.79% feeling lonely most/all of the time (vs. 3.51% urban). Conclusion: Older adults in rural India experience higher social isolation due to limited social and healthcare resources. This study highlights the need for community-based programs, strengthened family and social networks, and improved access to mental health support in rural settings.

Abstract citation ID: igaf122.3121

**SHOW ME THE MEANING OF BEING LONELY: PATHWAYS FROM COGNITIVE FUNCTIONING TO LONELINESS AND SOCIAL ISOLATION**

Lydia Grenko, Elizabeth Anquillare, Caroline Collins-Pisano, and Andrew Lac, *University of Colorado Colorado Springs, Colorado Springs, Colorado, United States*

Social isolation and loneliness (SI/L) are increasingly prevalent in older adults and connected with worse outcomes in later life. Previous research has focused on the effects of SI/L on cognitive functioning in older adults. However, fewer studies have investigated the pathways from cognitive impairment to SI/L. Mental health and social networks may serve as intermediate constructs that help to account for the link between cognition and SI/L. The present study examined the predictive pathways from cognitive functioning to SI/L through mental health and quality of relationships using data from the National Social Life, Health and Aging Project (NSHAP). Community-dwelling older adults (N = 4,337, Mage=67.63, SD = 10.95) completed a cognitive assessment and mental health and social functioning surveys. Structural equation modeling produced satisfactory fit indices, CFI=.97, TLI=.96, RMSEA=.03. Specifically, higher cognitive functioning predicted higher perceived quality of social relationships (β=.22, p<.001), fewer mental health symptoms (β=-.25, p<.001), greater frequency of social interaction (β=.22, p<.001), and higher self-reported loneliness (β=.18, p<.001). Additionally, higher quality of social relationships predicted less social isolation (β=.22, p<.001) and loneliness (β=-.14, p<.001), whereas greater mental symptoms predicted greater social isolation (β=-.13, p<.001) and loneliness (β=.66, p<.001). Less loneliness in the context of lower cognitive functioning may reflect a sense of subjective closeness with fellow older adults experiencing normative cognitive change. Older adults experiencing cognitive decline may benefit from interventions addressing mental health symptoms, fortifying social supports, and compensatory cognitive strategies to improve social functioning and overall well-being

Abstract citation ID: igaf122.3122

### SOCIAL CONNECTEDNESS AND LONELINESS AMONG U.S. OLDER ADULTS DURING THE COVID-19 PANDEMIC

Qiyu Deng, *Syracuse University, Syracuse, New York, United States*

Using data (N = 4,261) from the Health and Retirement Study (HRS) 2020, this study examines how pandemic-related social connectedness associates with loneliness among U.S. older adults. Pandemic-related social connectedness was measured by a composite indicator integrating social network size, frequency of interactions with children and other family members, and instrumental and emotional support provided and received outside the household. Loneliness was operationalized as a binary outcome using UCLA 3-item scale. Logistic regression incorporating sampling weights was estimated, adjusting for age, gender, race, marital status, living arrangement, functional limitations, self-rated health, depression, education, household size, household income, employment status, and urbanicity. Results showed that greater social connectedness significantly lowered the odds of loneliness (OR = 0.79; p = 0.01). Older adults aged 65 to 79 and those aged 80+ reported lower odds of loneliness compared to adults aged 50 to 64. Moderate and severe functional limitations were significantly associated with higher loneliness odds (OR = 1.49 and OR = 1.68 respectively, p < 0.01). Depression was strongly predictive of loneliness (OR = 1.33–4.58, p < 0.001). Adults living with others, but unmarried or unpartnered, had substantially higher odds of loneliness compared to married or partnered adults (OR = 2.14, p = 0.002). Household size, household income, race, self-rated health, education, employment status, and geographic location were not significantly associated with loneliness. These findings highlight that older adults experiencing functional limitations and depressive symptoms may be particularly vulnerable to loneliness, emphasizing the need for targeted support and interventions to enhance social connectedness during challenging times such as the COVID-19 pandemic.

Abstract citation ID: igaf122.3123

### THE HEAL-HOA DUAL RANDOMIZED CONTROLLED TRIAL: 12-MONTH EFFECTS ON LONELINESS IN OLDER ADULTS

Vivien Tang[1], Da Jiang[1], Jojo Yan Yan Kwok[2], Namkee Choi[3], Dannii Yeung[4], Lisa Warner[5], Rainbow Tin Hung Ho[2], and Kee-Lee Chou[1], *1. The Education University of Hong Kong, Hong Kong, China (People's Republic), 2. The University of Hong Kong, Hong Kong, China (People's Republic), 3. The University of Texas at Austin, Austin, Texas, United States, 4. City University of Hong Kong, Hong Kong, China (People's Republic), 5. MSB Medical School Berlin, Berlin, Berlin, Germany*

Loneliness is a growing public health concern, yet the long-term effectiveness of psychosocial interventions remains uncertain. As part of the Helping Alleviate Loneliness in Hong Kong Older Adults, a dual randomized controlled trial conducted during the COVID-19 pandemic, this study focused on the long-term outcomes of older adults (Mage=76.6, SDage=7.8) who lived alone, felt lonely, faced financial difficulties, and were digitally excluded. The interventions consisted of behavioral activation (Tele-BA), mindfulness (Tele-MF), and befriending (Tele-BF: attention control). Each intervention comprised eight 30-minute telephone-delivered sessions over one month, led by trained laypersons. The lay interventionists were older adult peers who also experienced loneliness. Outcomes were assessed at baseline and 1-, 3-, 6-, and 12-month post-intervention. Compared to Tele-BF, Tele-BA and Tele-MF showed sustained reduction of loneliness at12-month follow-ups. Secondary outcome were also found to improved significantly for sleep quality, life satisfaction, and psychological well-being. Social isolation at 6 months mediated the effects of Tele-BA and Tele-MF at the 12-month follow-up, with a larger increase in social network leading to a more substantial reduction in loneliness. This RCT demonstrated the feasibility of scalable telephone-based interventions beyond the pandemic, with Tele-BA emerging as a promising intervention for alleviating loneliness and improving overall well-being in older adults. Future psychosocial interventions for late-life loneliness should specifically target social isolation, as it mediates the long-term effects of Tele-BA and Tele-MF on loneliness.

Abstract citation ID: igaf122.3124

### THE IMPACT OF SOCIAL ISOLATION AND LONELINESS ON HEALTH OUTCOMES AFTER TRAUMATIC BRAIN INJURY: A SCOPING REVIEW

Wonkyung (Kelly) Jung[1], Tess Chernauskas[1], and Kuan-Ching Wu[2], *1. Boston College, Chestnut Hill, Massachusetts, United States, 2. Emory University, Atlanta, Georgia, United States*

Traumatic brain injury (TBI) often leads to social isolation and loneliness, which can significantly affect an individual's mental, physical, and social well-being, particularly in older adults. Despite these serious consequences, the extent to which social isolation and loneliness impact health outcomes after TBI has not been thoroughly explored. This scoping review aims to map the literature on the effects of social isolation and loneliness on health outcomes following TBI. By examining studies across a range of health domains, we seek to better understand the association between these psychosocial factors and recovery trajectories in TBI populations. A scoping review was conducted following the Preferred Reporting Items for Systematic Reviews and Meta-Analyses (PRISMA) extension for Scoping Reviews. A comprehensive search across PUBMED, CINAHL, EMBASE, PsycINFO, and Web of Science identified studies published between 2014 and 2024. A total of 1,250 studies were screened after duplicates were removed, and eight studies met predefined inclusion criteria. The results indicate that social isolation and loneliness after TBI are associated with worse health outcomes, including higher rates of depression, PTSD, slower recovery, and reduced functional abilities. These psychosocial factors also contribute to increased morbidity, mortality, and reduced quality of life. Social isolation and loneliness pose significant barriers to recovery after TBI. Addressing these factors through targeted interventions may improve both short- and long-term health outcomes. Further research is needed to explore underlying mechanisms and develop effective strategies to mitigate their negative effects in TBI populations.

Abstract citation ID: igaf122.3125

**THE ROLE OF LONELINESS IN PERCEIVED MASTERY AND LIFE SATISFACTION AMONG OLDER ADULTS**

Inhye Jung, and Hyo Jung Lee, *Yonsei University, Seoul, Republic of Korea*

This study examines the impact of loneliness on older adults' quality of life from a relational autonomy perspective. From this perspective, an individual's perceived social connectedness can influence their psychological and behavioral health. Considering loneliness as a negative aspect of perceived social connectedness, this study explores its impact on perceived autonomy and health status. Utilizing data from the 2018 Health and Retirement Study (HRS) (N = 2,279), we conducted multiple mediation analyses using PROCESS Macro Model 6 to assess whether these factors explain the association between loneliness and subjective well-being. The results indicate that higher levels of loneliness are significantly associated with lower perceived mastery ($b = -0.7107$, $p < .001$), which, in turn, is related to poorer self-rated health and lower life satisfaction. Overall, the total indirect effect of loneliness on life satisfaction is significant ($b = -0.2286$, 95% CI [-0.2918, -0.1741]). Among the mediation pathways, the primary pathway (Loneliness → Perceived Mastery → Life Satisfaction) shows a significant indirect effect ($b = -0.19$, 95% CI [-0.2520, -0.1388]). Additionally, a smaller but significant mediation pathway (Loneliness → Perceived Mastery → Self-Rated Health → Life Satisfaction) further contributes to the overall effect ($b = -0.0131$, 95% CI [-0.0208, -0.0071]). These findings underscore the detrimental effects of loneliness on older adults' psychological and physical well-being, highlighting the need for interventions that enhance an individual's sense of control over their lives and foster a more informed perception of their well-being.

### SESSION 7400 (POSTER)

### SPIRITUALITY AND RELIGION

Abstract citation ID: igaf122.3126

**A NEW OLDER ADULT MINISTRY CURRICULUM: INSIGHTS FROM A PILOT COURSE**

Beth Truett, Lisa Peters-Beumer, and Eric Moeller, *Concordia University Chicago, River Forest, Illinois, United States*

Faith based communities which effectively engage older persons can satisfy the desire for meaning, benefit from wisdom and experience and positively impact congregational connectedness. A search for nationally available programs to train leaders among Christian denominations about the dynamics of ministry for, by and with older adults revealed an absence of an educational curriculum. The Center for Gerontology @ Concordia University Chicago developed the Specialist in Aging Ministry course to educate leaders about key gerontological and theological imperatives for effective ministry, including the desire to age in place; need for physical, emotional and financial support; and surviving loss, grief and bereavement. The six-module curriculum has online and in-person options. Congregations (N = 12,227) from four Midwestern states were invited to webinars with the objective of recruiting pilot participants. Twenty-five faith communities representing three denominations committed to and five completed the course. Among sixty-nine participants, fifty-three (77%) completed evaluations rating course content, format, applicability and identifying developmental needs. Evaluators scored the value and time spent on course aspects, rating discussion high or medium (77%) but recommending practice exercises (47%). Modules rated most meaningful addressed ageism, isolation and loneliness, followed by caregiving, grief and loss. Pilots revealed most (83%) will use their training as volunteers. Sixty-two percent cited personal growth, and thirty-two percent will develop a specialized ministry. The findings informed revision objectives: edit less highly rated modules; expand to 90-minute format; create discussion exercises; target marketing to communities desiring a specialized older adult ministry and those interacting with older persons through existing volunteer activities.

Abstract citation ID: igaf122.3127

**AGING MEN'S AND WOMEN'S RELIGIOUS IDENTITIES: STRAIGHTFORWARD OR COMPLEX?**

Kyra Hultgren[1], Sarah MacDougall[1], Sophia Paleski[1], Alessia Petroni[1], Edward Thompson[2], Christopher Turner[1], Michiko Iwasaki[1], and Andrew Futterman[1], *1. Loyola University Maryland, Baltimore, Maryland, United States, 2. College of the Holy Cross, Worcester, Massachusetts, United States*

This study explores the determinants of older adults taking on a "straightforward" or a "complex" orientation to religious belief and behavior. Two hundred and seventy-eight semi-structured interviews of older individuals describing their religious belief and behavior, their religious doubts, and their religious development were used. From these, 40 interviews were selected as either prototypically "straightforward" or "complex" (20 each). Complexity was defined primarily in cognitive and emotional terms as a form of post-formal reasoning and reflective emotionality (Labouvie-Vief, 2000) where religious doubt and faith coexist and individuals describe their religious life as involving a "reflective component" (Labouvie-Vief, 2015). By contrast, religious straightforwardness is defined by individuals who demonstrate either religious faith or doubt, but not both, without extensive reflection. Identification of interviews demonstrating prototypically "straightforward" and "complex" religious belief and behavior was first agreed upon by all researchers, and then the reasons for religious straightforwardness and complexity were assessed. Initial analyses indicated that life stressors, i.e., events beyond one's control (Labouvie-Vief, 2015), were primarily the reason for increased religious straightforwardness. Individuals with "complex" religiousness described little vulnerability and dependence, and an openness to new experience. Other factors described as important reasons for straightforwardness were being above 80-85 years of age (age range 60-101 years), membership in a "conservative" denominational family, and being male. We compare these results to results from our previous quantitative study of religious complexity using a random sample of older adults and discuss our findings in terms of theories of age-related cognitive and emotional complexity.

Abstract citation ID: igaf122.3128

### OLDER WOMEN AGING POSITIVELY IN CUSTODY: IMPACT OF SOCIAL AND RELIGIOUS CONNECTIONS ON DEPRESSIVE SYMPTOMS

Alex Bishop[1], George Randall[2], and Elizabeth Piippo[2], *1. Oklahoma State University, Stillwater, Oklahoma, United States, 2. Sam Houston State University, Huntsville, Texas, United States*

The study's conceptual model focused on a validated outcome measure of depressive symptomatology and was based on the Model of Developmental Adaptation. The model included assessments from 92 women at least 45 years of age who were behind bars in the state of Oklahoma. The hierarchical regression model included distant past assessments (before age 18): adverse childhood experiences, drug use, and alcohol use. Near assessments included social support and religious coping. Covariates include crime type, race/ethnicity, education, age, and marital status. None of the covariates and none of the distal predictors were significantly associated with the outcome. However, in the final model, both religious coping and social support were significant, negatively associating with the outcome. This final model explained 30 percent of the variability in the outcome, depressive symptomatology. Discussion will focus on assessing the hierarchical regression model and the significant role of religious coping and social support being negatively associated with the samples' depressive symptomatology. Findings from this study emphasize that both religious coping and social support independently contribute to the well being of women in custody.

Abstract citation ID: igaf122.3129

### RELIGIOSITY AND SELF-RATED HEALTH AS PREDICTORS OF WELL-BEING AND DEPRESSION IN OLDER ADULTS

Nicholas Prefontaine, and Andrew Revell, *University of Massachusetts Dartmouth, Dartmouth, Massachusetts, United States*

Depression and the well-being of older adults is crucial to public health, as this population faces heightened vulnerability to mental health challenges (Wang et al., 2022). In this present study, the role of religion and self-rated health (SRH) concerning depression and subjective well-being (SWB) were examined. Specifically, the objective of this examination was to identify the predictive nature of religiosity and SRH in a cohort of older adults aged 65 and older (M = 74) as a secondary data analysis. Data were drawn from the Religion, Aging, and Health Survey (RAHS; Krause, 2018), which included 1,500 participants, all Christian (N = 1,500), predominantly White (n = 748) and African American (n = 752), sampled through stratified sampling methods. Measures included Private Religious Practices, Self-Rated Health, and the Center for Epidemiological Studies Depression Inventory (CESD). Life satisfaction, self-esteem, and hope/optimism scales were used to assess SWB. Linear regression analyses indicated religiosity positively predicted SWB variables, such as life satisfaction, $t(1220) = 8.81$, $p = .001$, self-esteem, $t(1313) = 6.07$, $p = .001$, and hope/ optimism, $t(1167) = 11.09$, $p = .001$. Results further indicated that religiosity may offer meaning and purpose, enhancing SWB. Relationships with SRH were also explored. Future research would ideally be longitudinally designed to grasp how religiosity could impact individuals over time and include diverse populations to better understand how religiosity and SRH affect older adults' health.

Abstract citation ID: igaf122.3130

### RELIGIOUS SOCIAL CAPITAL AND PERCEIVED HEALTH AMONG US OLDER ADULTS: A PATH ANALYSIS MODEL

Seoyoon Woo[1], Jeonghwa Lee[2], Yeoun Soo Kim-Godwin[2], and Jeeyae Choi[2], *1. University of North Carolina Wilmington, Wilmington, North Carolina, United States, 2. The University of North Carolina at Wilmington, Wilmington, North Carolina, United States*

Rationale/Objectives--Perceived health and social connectedness are significant indicators of overall health status and well-being among older adults. This study aimed to examine the relationship between religious/spiritual belonging and perceived physical and mental health among older adults in the US. Methods--This secondary cross-sectional study included 2,163 adults aged 50–80(mean=64.5) from the National Poll on Healthy Aging(Wave11), a nationally representative U.S. health survey. Independent variables included religious/spiritual belonging, chronic health conditions, healthy eating habits, BMI, and age, while dependent variables were perceived physical and mental health. Mediators examined were confidence in helping others, feelings of isolation, lack of companionship, and frequency of social contact. Path analyses were conducted using the lavaan package in R. Results--All subjects have some form of religion: 56.4% feel they are part of a community that shares their beliefs. The path model for mediation demonstrated a good fit to both perceived physical health (X^2(17)=85.136, p < 0.001; RMSEA = 0.065, 90% CI[0.051-0.079]; CFI = 0.950; SRMR = 0.050; R^2 = 0.295) and perceived mental health (X^2(13) = 75.253, p < 0.001; RMSEA=0.071, 90% CI[0.056-0.087]; CFI = 0.946; SRMR = 0.055; R^2 = 0.287). Having chronic health conditions ($\beta = 0.19$, $p < 0.001$), healthy eating habits ($\beta$=-0.24, p< 0.001), and BMI ($\beta = 0.02$, $p < 0.001$) have a direct effect on perceived physical health. Religious/spiritual belonging ($\beta = 0.16$,p=0.004), healthy eating habits ($\beta$=-0.15,p=0.004), and age ($\beta$=-0.01,p=0.011) have a direct effect on perceived mental health. Religious/spiritual belonging has an indirect effect on perceived physical and mental health, being mediated by confidence in helping others, feelings of isolation, lack of companionship, and frequency of social contact. Conclusion--Findings enhance the understanding of religious social capital's impact on perceived physical and mental health through mediating factors.

Abstract citation ID: igaf122.3131

### SPIRITUAL AND RELIGIOUS CONNECTIONS TO REDUCING RISK OF COMORBIDITY AMONG MIDDLE AGED WOMEN IN CORRECTIONAL CUSTODY

Serena Gray, and Alex Bishop, *Oklahoma State University, Stillwater, Oklahoma, United States*

Data for this study originated from a sample of N = 220 middle-aged women, aged 40 and older (M = 49.05, SD = 7.23), under state correctional custody in Oklahoma. Logistic regression analyses were computer to examine the relationship of socio-demographic characteristics, adverse childhood experiences, mental health, and religious and spiritual connections to comorbid health impairment. Results indicate that age, (OR = 1.16, CI: 1.03-1.29), attachment to God (OR = 1.11, CI: 1.03-1.18), and religiosity (OR = 1.28, CI: 1.09-1.50) are associated with increased odds of comorbid health impairment; whereas forgiveness was associated with reduced odds (OR = .96, CI: .92-1.00).

Middle-aged women who continue to age while in correctional custody appear to be at a greater risk for experiencing comorbid health problems. Yet, a greater disposition to engage in forgiveness may help off-set this risk. Findings further suggest that a desire to strengthen a relationship and make things right with God, as well as become more actively involved in religious activities may signifying behaviors aimed at resolving increased occurrence of comorbid health impairments. Study results have implications relative to how correctional case managers, forensic social workers, and prison chaplains can better identify and meet the religious, spiritual, and health needs of middle-aged women under correctional custody.

Abstract citation ID: igaf122.3132

### SUSTAINING IDENTITY THROUGH SPIRITUALITY IN END-OF-LIFE DEMENTIA CARE: AN ANALYTIC AUTO-ETHNOGRAPHY

Eunkyung Kim[1], Soobin Park[2], and Sooyoung Park[3], *1. Midwest University, Wentzville, Missouri, United States, 2. Washington University in St. Louis, St. Louis, Missouri, United States, 3. Samsung Medical Center, Gangnam-gu, Seoul, Republic of Korea*

Spirituality, originating from Latin meaning “breath of life,” transcends intellectual capacity, deeply defining who we are. Yet, spirituality remains one of the most neglected dimensions in end-of-life dementia care. Although spiritual support for persons living with dementia (PLWD) is recognized as important, it often remains unaddressed because care providers find it challenging to identify or apply spiritual needs, particularly during advanced dementia. Consequently, spiritual needs frequently go undocumented in care plans and discussions. Additionally, limited empirical research on the spiritual needs of PLWD contributes to uncertainty about integrating spirituality into dementia care. Using analytic auto-ethnography, which enables researchers to utilize personal experiences to generate specific, contextually relevant understanding, this study draws upon my perspectives as an Asian woman, former caregiver to my husband diagnosed with dementia, and current social work scholar. Reflecting on over a decade of caregiving through reflexive approach of archival materials (memoirs, photographs) and concurrent self-observations (diaries, audio-visual recordings), key themes emerged highlighting the nuanced nature of spiritual caregiving. Findings revealed spirituality as highly individualized, extending beyond religious rituals and deeply connected to life history, identity, and meaningful experiences. Assessing spiritual needs involves actively recalling significant past conversations and preferences through dialogue with family and friends. For example, attending annual academic conferences with my husband, a former professor, exemplified spiritual care rooted in personal history. The study implies that recognizing and including individualized spiritual practices restores dignity and personhood, guiding care providers toward spiritually competent and person-centered dementia care, thus addressing key gaps in practice and research.

Abstract citation ID: igaf122.3133

### THE RECIPROCITY OF CHURCH-BASED SUPPORT AND PSYCHOLOGICAL WELL-BEING: RELIGION, AGING AND HEALTH SURVEY (RAH)

Landon Peeples, Lauren Chrzanowski, and Benjamin Mast, *University of Louisville, Louisville, Kentucky, United States*

Church-based support provides both social and emotional benefits, yet little is known about its reciprocation and effects on well-being. This study uses Religion, Aging, and Health (RAH) survey data from older adults aged 65 and over within the United States to investigate (1) how receiving pastoral and congregational support influences the likelihood of providing church-based support and (2) the psychological correlates of providing supporting. Linear regression analysis ($N = 813$) evaluated the effects of receiving support on providing support, controlling for age, gender, race, and denomination. MANOVA ($N = 1118$) assessed the impact of providing support on depression, self-esteem, life satisfaction, and self-rated health. The regression model was statistically significant ($R^2 = 0.225$, $F (9,803) = 25.907$, $p < 0.001$). Receiving congregational support ($B = 0.249$, $p = 0.002$) and age ($B = 0.012$, $p = 0.002$) was positively associated with providing support. Pastoral support, gender, race, denomination, and interaction terms were not significant. MANOVA showed a significant multivariate effect (Pillai’s Trace = .018, $F (4, 1113) = 4.979$, $p < 0.001$). Providing support was associated with lower depression ($F (1, 1116) = 7.410$, $p = 0.007$), higher self-esteem ($F (1, 1116) = 9.997$, $p = 0.002$), and greater life satisfaction ($F (1, 1116) = 6.857$, $p = 0.009$) but did not impact self-rated health ($F (1, 1116) = 1.037$, $p = 0.309$). These findings highlight how providing support can influence psychological well-being within faith communities. Future research should explore how other church-based support contributes to well-being in diverse populations.

Abstract citation ID: igaf122.3134

### WHAT IS ‘SPIRITUAL HEALTH’- A SURVEY OF UK SOCIAL PRESCRIBERS

Ishbel Orla Whitehead, Amy O’Donnell, and Barbara Hanratty, *Newcastle University, Newcastle upon Tyne, England, United Kingdom*

Background: Holistic (whole person) health includes spiritual health. Social prescribing is a UK initiative that aims to provide holistic, or salutogenic (health creating) care to patients, utilising community links, beyond that offered in consultations with health professionals. Support may be directed at many aspects of health and wellbeing, including spiritual health. Spiritual health is associated with many other physical, mental and rehabilitation (restorative care) outcomes. However, how social prescribers define ‘spiritual health’ is unknown. To explore what social prescribers understand by the term “spiritual health.” We designed a custom online survey of social prescribers in the UK and asked ‘what does spiritual health mean to you?’ Free text data were subject to thematic analysis using a priori themes from the literature on definitions of spiritual health. One hundred and seventy one social prescribing link workers responded; 153 gave a definition of spiritual health. Twice as many participants described themselves as spiritual than described themselves as religious. A very small minority found the term ‘spiritual health’ to be meaningless. Definitions of spiritual health were categorised into themes of sense of self, peace, meaning and purpose; Connections to others, the world, or a deity; Spiritual or religious practice. Spiritual health appears to be a term with meaning to UK social prescribers, distinct from religiosity. This ‘working definition’ of spiritual health will allow further research into how spiritual health fits within social prescribing in the UK.

### SESSION 7405 (POSTER)

### ADULT PROTECTION AND ELDER ABUSE (POSTERS)

Abstract citation ID: igaf122.3135

### BARRIERS TO HOUSING FOR APS CLIENTS: PERSPECTIVES FROM APS STAFF AND HOUSING PROVIDERS

Samantha Tuft, Courtney Reynolds, and Mia Canzone, *Benjamin Rose, Cleveland, Ohio, United States*

Oklahoma Adult Protective Services (APS) relies on a patchwork of housing providers, including traditional shelters and nursing homes, to provide clients with emergency housing. To better address its clientele's needs, APS is partnering with Benjamin Rose to develop an innovative, evidence-based shelter program for APS clients in Oklahoma. To develop the program, a needs assessment was conducted through six focus groups—three with APS staff (n = 20) and three with housing providers (n = 21)—via Microsoft Teams. Thematic analysis revealed significant barriers to housing access, including affordability, lack of available resources, and pets. A common theme was the lack of affordable housing, with one participant noting, "As living costs have increased and those fixed incomes have not, people are shuffling… Do I pay my rent or do I pay for food or do I pay for my medication?" A lack of available resources were also highlighted, as one participant stated, "As soon as you have a list of all the resources in your area, it's out of date…" Participants also emphasized pets, with one participant sharing, "That's their best friend, their buddy. Sometimes it's the reason to continue to live…" These findings underscore the need for safe and affordable housing options (including for those with pets), supportive services, and enhanced collaboration between APS and housing providers. The discussion will address these barriers and offer recommendations to improve housing and service coordination for adults experiencing abuse, neglect and/or exp loitation.

Abstract citation ID: igaf122.3136

### BARRIERS TO SCREENING FOR ABUSE IN OLDER ADULTS BY HEALTHCARE PROVIDERS IN RURAL COMMUNITIES

Christopher Gravett[1], Rebekah Perkins[2], Lynn Reinke[2], and Kara Dassel[2], *1. University of Utah College of Nursing, Salt Lake City, Utah, United States, 2. University of Utah, Salt Lake City, Utah, United States*

Older adult abuse is a critical yet underreported issue, leading to severe health outcomes and increased mortality. Despite its importance, screening remains inconsistent in rural healthcare settings, where providers face unique barriers. This study examines screening practices, provider confidence, and barriers in rural Southeastern Utah, a region with limited geriatric care resources. A cross-sectional survey was conducted via Qualtrics with 32 healthcare providers across settings of care. Findings indicate that 42.3% of providers rarely or never screen for abuse, despite 56.5% agreeing that screening is essential. Additionally, 52.2% are unfamiliar with standardized screening instruments, and 30.4% report low confidence in identifying abuse. Providers overwhelmingly cited lack of time, training, and uncertainty in intervention protocols as key barriers. While 72.7% were very likely to report abuse to Adult Protective Services, only 31.8% were likely to engage caregivers or family members, suggesting hesitancy in direct intervention. Qualitative insights revealed strong concerns about patient safety, a desire to improve health outcomes through the early identification and treatment of abuse-related complications; yet, many providers lacked the resources and guidance needed to act effectively. Older adult abuse screening in rural healthcare is limited, providers rarely screen patients, and the majority are unfamiliar with screening tools. Barriers include time constraints, lack of training, and unclear protocols. While providers are willing to report abuse, hesitancy in engaging caregivers highlights the need for better interventions. Improving screening requires training, standardized protocols, and policy reforms. Future research should explore training effectiveness and policy implementation to enhance detection and response.

Abstract citation ID: igaf122.3137

### CHARACTERISTICS OF OLDER INTIMATE PARTNER VIOLENCE CASES IN JAPAN

Asako Katsumata[1], Kiyoko Fukushima[1], and Noriko Tsukada[2], *1. Japan Lutheran College, Mitaka, Tokyo, Japan, 2. Nihon University, Tokyo, Tokyo, Japan*

This study examines factors influencing collaboration between Japan's Community Comprehensive Support Centers (CCSCs) and Domestic Violence (DV) Support Agencies in addressing cases of older Intimate Partner Violence (IPV). Using data from a national survey conducted in November 2022, we analyzed responses from 1,472 Certified Social Workers (CSWs) at randomly selected CCSCs (n = 3,563). Among 406 identified female IPV cases that persisted from younger to older age (excluding missing values), CSWs sought DV Support Agency cooperation in 102 cases (25.8%) but did not in 293 cases (74.2%). Comparative analysis using the $\chi^2$ test revealed that CSWs were significantly more likely to seek cooperation when victims were physically independent ($p < 0.01$), had no dementia ($p < 0.001$), experienced psychological abuse ($p < 0.05$), and wished to separate from their abuser ($p < 0.05$). When seeking cooperation, CSWs were more likely to guide victims toward living apart ($p < 0.01$). Conversely, in cases where cooperation was not sought, CSWs often addressed IPV as a caregiving issue, focusing on reducing caregiver burden when the abuser was also a caregiver ($p < 0.01$) or instructing the abuser to cease abuse ($p < 0.01$). These findings suggest that IPV in later life is often reframed as a caregiving issue, potentially hindering appropriate intervention. To better support older IPV survivors, CSWs require specialized training to distinguish IPV from caregiver burden and collaborate effectively with DV Support Agencies.

Abstract citation ID: igaf122.3138

### DEMOGRAPHIC AND FAMILY NETWORK INFLUENCES ON PSYCHOSOCIAL HEALTH IN AGINCOURT, SOUTH AFRICA: A SEM ANALYSIS

Lawrence Ugwu[1], JohnBosco Chika Chukwuorji[2], and Erhabor S. Idemudia[1], *1. North-West University, Mafikeng, North-West, South Africa, 2. Michigan State University, East Lansing, Michigan, United States*

Demographic determinants, family networks, and daily activity play critical roles in shaping the psychosocial health of older adults. In this study, we examined the predictive effects of age, gender, marital status, and family structure, as indicated by the number of children and grandchildren, including resident grandchildren, on subjective well-being (SWB), depression, and physical activity among older adults in Agincourt, South Africa. Our representative sample, with a mean age of 71.96 years (SD = 8.81) and nearly equal gender distribution (1,330 males and 1,445 females), was analysed using structural equation modelling. Results indicate that higher daily activity levels and robust family engagement, particularly having resident grandchildren, were significantly associated with reduced depression, enhanced physical activity, and improved SWB. Being currently married and maintaining active family interactions further strengthened these associations. These findings emphasise the importance of social integration and family support in mitigating mental health challenges and promoting physical activity among older adults. The study offers valuable insights for developing community-based interventions and policies to enhance the quality of life for ageing populations by reinforcing family networks and encouraging active engagement in daily life.

Abstract citation ID: igaf122.3139

### ELDER ABUSE FROM LIFE COURSE PERSPECTIVE: EFFECTS OF CHILDHOOD VICTIMIZATION AND MEDIATING ROLE OF SOCIAL FACTORS

Louis To, Elsie Yan, and Xue Bai, *The Hong Kong Polytechnic University, Hong Kong, China (People's Republic)*

Developing effective intervention strategies for elder abuse requires a comprehensive understanding of its risk and protective factors. Since aging is a continuous process, elder abuse should be understood from a life course perspective. The present study aims to explore the mediating effects of ageism, family functioning, and satisfaction towards social policies and environments on the relationship between childhood victimization and present elder abuse experience, using a life course approach. A total of 5,007 persons aged 60 or above participated in this cross-sectional quantitative survey in Hong Kong. A significant and positive total effect ($\beta$=0.199, SE = 0.007, t = 28.726, CI [0.186, 0.213]) was found, indicating that experiences of childhood victimization were associated with more frequent elder abuse. The mediation analysis identified significant and positive indirect effects of child victimization through family functioning ($\beta$=0.010, SE = 0.002, CI [0.006, 0.015]), ageism ($\beta$=0.056, SE = 0.005, CI [0.047, 0.065]), and social and policy environments ($\beta$=0.004, SE = 0.002, CI [0.001, 0.008]) on current elder abuse experiences. Childhood victimization is associated with more frequent elder abuse experiences among older adults who experienced high levels of ageism, family dysfunction, and dissatisfaction with social and policy environments. The significant direct effect ($\beta$=0.129, SE = 0.007, t = 17.719, CI [0.115, 0.143]) indicates that these factors serve as partial mediators. Our results confirmed the lifelong detrimental impacts of childhood victimization. Addressing ageism, family function, social and policy environment may provide a possible point of intervention to mitigate the long-term impact of childhood victimization and prevent elder abuse.

Abstract citation ID: igaf122.3140

### FAMILY CAREGIVER PERSPECTIVES ON ELDER MISTREATMENT AND CARING FOR OLDER ADULTS WITH COGNITIVE IMPAIRMENT

Karen Schlag[1], Leila Wood[2], and Monique Pappadis[1], *1. University of Texas Medical Branch at Galveston, Galveston, Texas, United States, 2. University of Texas Health Science Center Houston, Houston, Texas, United States*

Older adults with cognitive impairment are at elevated risk for elder mistreatment (EM), with family caregiving strain recognized as a common risk factor. This qualitative study seeks to better understand how family caregiving for older adults with cognitive impairment and EM risk is influenced by interrelated interpersonal and social factors to provide a more holistic look at caregiving and vulnerability to mistreatment. Specifically, we examined perspectives from family caregivers about their knowledge of EM and their experiences with caregiving, carer strain, social support, and navigating healthcare systems. We conducted semi-structured interviews with 28 family caregivers from a culturally diverse, small urban U.S. community as part of a larger EM screening and support intervention study. Transcribed interviews were analyzed using reflexive thematic analysis. Drawing from a social-ecological framework, we identified three caregiver-focused themes: 1.) Tension between care recipient compliance gaining and elder mistreatment; 2.) Family dynamics and reduced patient care; 3.) Appropriate and desired healthcare interactions around EM. Findings underscore individual-, family-, and institutional-level influences on caregivers that can contribute to EM risk for older adults with cognitive impairment. Results also highlight a need for family-centered EM education and healthcare professionals' proactive engagement with patients and families on caregiving stress management and patient safety. Finally, this research can inform future intervention efforts and policy to address EM of patients with cognitive impairment and their families.

Abstract citation ID: igaf122.3141

### FINANCIAL PROTECTION VS AUTONOMY, PERSPECTIVES FROM FRAUD VICTIMS ON A FINANCIAL ACCOUNT TEMPORARY HOLD POLICY

Louise (Siyu) Gao[1], Marguerite DeLiema[2], and Sohum Bindra[3], *1. University of Minnesota, Minneapolis, Minnesota, United States, 2. University of Minnesota School of Social Work, Minneapolis, Minnesota, United States, 3. University of Minnesota Twin Cities, Minneapolis, Minnesota, United States*

A temporary financial account hold policy, also known as an authorization hold, is a temporary restriction on transferring funds out of a bank or brokerage account. This policy may be utilized as a consumer protection tool to prevent older adult accountholders from elder financial exploitation. Our qualitative study interviewed 12 older fraud victims or their authorized proxies (mean age: 75; mean loss amount: $52,690 ranging from $3500 to $240,000) who experienced a temporary account hold to understand the benefits, costs, and the psychological impact. More than half of the participants showed appreciation and understood the intent of their financial institution(s) to keep them safe from fraud or abuse. However, some perceived the protective temporary hold as infringing on their right to financial self-determination and were upset. A minority of participants faced negative economic impacts, such as credit card companies closing victims' accounts due to risk concerns, difficulty opening new accounts elsewhere, credit score drops, and inability to access their funds for daily activities. Psychosocial outcomes included feeling anxious, depressed, ashamed, and lonely. Participants recommended more transparency, communication, emotional support, and education. Using thematic analysis, this study will offer victims' perspectives on the impact of the temporary account hold policy and facilitate a critical discussion on the importance of self-determination in the consumer protection field. Victims' stories of financial exploitation and their attitudes toward perpetrators will also be discussed.

Abstract citation ID: igaf122.3142

**MAKING THE CALL – CONNECTING APS CLIENTS TO PRIMARY CARE**

Ronan Factora[1], Anna Smith[2], Camille Smith[2], and Kelly Clemings[3], *1. Cleveland Clinic, Cleveland, Ohio, United States, 2. Case Western Reserve University, Cleveland, Ohio, United States, 3. Division Of Senior and Adult Services, Cleveland, Ohio, United States*

Virtual Capacity Evaluations (VCE) are used by the Behavioral Health Unit at Adult Protective Services in Cuyahoga County, Ohio, (APS) to evaluate elder abuse/exploitation/neglect cases where client's decision making is questioned. The evaluation includes confirming if the client has a primary care physician (PCP) which is important as many have chronic illnesses and observed medication non-adherence. Prior pilot data showed many clients were not connected to a PCP before the VCE (PMID: 36123772), leading to implementation of a recommendation to connect the client with their prior OR new PCP upon VCE completion. This quality improvement project followed up with these clients to see if this recommendation was implemented. Phone contact was made to the responsible party for APS clients who completed VCE from 2023-2024 to confirm if a PCP visit was made 3-6 months following VCE completion. Thirty-eight APS clients underwent VCE, of which 17 (44.7%) were already receiving medical care. Successful follow up with 19 clients showed 14 (73.7%) had care with a PCP 3-6 months after VCE. Of the remaining 5, 2 were still without care, but 3 received care more than 6 months after VCE. These results suggest a significant increase in the percentage of persons who receive medical care after completion of a VCE, though greater improvements can be made. An additional protocol connecting APS with a client's guardian or family member soon after VCE may improve these numbers, which is particularly important for clients who have active and unmanaged chronic medical illnesses.

Abstract citation ID: igaf122.3143

**SOCIAL NETWORKS RESHAPE THE IMPACT OF ELDER ABUSE ON SOCIAL WELL-BEING: EXAMINING URBAN-RURAL DISPARITIES IN CHINA**

Xin Sun[1], and Zi Yan[2], *1. Fudan University, Shanghai, Shanghai, China (People's Republic), 2. Waseda University, Tokyo, Tokyo, Japan*

The contextual theory of elder abuse emphasizes that elder abuse arises from the interaction of individual, relational, community, and societal contexts. This study tests this theory using nationally longitudinal data from 7,125 older adults (aged ≥60 years) in the 2018 and 2020 China Longitudinal Ageing Social Survey. We regressed changes in social networks, and social well-being in 2020 on elder abuse experience in 2018 and found that the victims have better social well-being two years later than those who did not report abuse. A novel association was identified between elder abuse, reduced contact, and emotional networks, and improved social well-being among rural victims. Conversely, among urban victims, elder abuse was associated with an expanded instrumental network and improved social well-being. Our findings point to the transforming impact of social networks on elder abuse victims' social well-being. These findings underscore the importance of considering urban-rural disparities in designing culturally adaptive interventions and support systems that leverage social networks to buffer the adverse effects of elder abuse.

Abstract citation ID: igaf122.3144

**STRATEGIES TO ADDRESS OR PREVENT SELF-NEGLECT AMONG OLDER ADULTS: A SYSTEMATIC REVIEW**

Zhiqi Yi[1], Lora Chehab[2], Joy Ernst[3], Fei Sun[2], and Eleanor Smith[4], *1. University of Kansas, Lawrence, Kansas, United States, 2. Michigan State University, East Lansing, Michigan, United States, 3. Wayne State University, Detroit, Michigan, United States, 4. University of Michigan, Ann Arbor, Michigan, United States*

Self-neglect is a growing form of elder maltreatment characterized by one's inability to perform essential self-care tasks due to physical or mental impairment or diminished capacity. Reports of self-neglect to Adult Protective Services far outnumber other types of maltreatment. While research has identified characteristics and risk factors for self-neglect, the best practices to address or prevent elder self-neglect remain unclear. An overview of theoretical models and practical approaches can enhance understanding and how to successfully intervene with older adults who self-neglect. A search of peer-reviewed literature between January 1990 and September 2024 across six databases (i.e., PsycINFO, PubMed, Cochrane Library, EMBASE, WOS, and MedLine) yielded twenty eligible articles with empirical, conceptual, and case research designs. Two reviewers independently screened, read, and rated the quality of all articles. Three authors independently identified themes using a thematic analysis approach and reached a consensus. Various intervention models and approaches with different focal points were identified. Approaches targeting older adults, their caregivers or both, used nutritional intervention, case management, and psychosocial intervention. Approaches targeting service professionals emphasized enhancing service techniques, multidisciplinary collaboration, and community support. Macro-level approaches stressed the need for changes in the community and social systems. These findings emphasized the complexities of self-neglect in older adults, the importance of helping techniques, and the need for an integrated and holistic approach. Although no single best practice is concluded, a holistic, multidisciplinary, and multilevel approach to self-neglect is recommended.

Abstract citation ID: igaf122.3145

**WHO'S AT RISK AND WHY? MULTILEVEL FACTORS OF LATER-LIFE FINANCIAL FRAUD IN CHINA**

Ting Hu[1], Flavia Andrade[2], and Yu-Chih Chen[3], *1. University of Illinois--Urbana-Champaign, Champaign, Illinois, United States, 2. University of Illinois--Urbana-Champaign, Urbana, Illinois, United States, 3. National Taiwan University, Taipei, Taipei, Taiwan (Republic of China)*

Older adults are particularly vulnerable to financial fraud. However, research on risk factors of later-life fraud has focused more on individual attributes without considering contextual factors. Additionally, these studies fail to examine diverse types and amounts of financial loss when measuring financial fraud. We address these gaps using a comprehensive framework to explore the multilevel risk factors of fraud in later life. Using 8,151 participants aged 57+ from the 2015-2018 China Health and Retirement Longitudinal Study, we employed mixed-effects models to examine how factors regarding suitable targets (e.g., previous fraud experience, socioeconomic status, cognition, etc.), capable guardians (e.g., marital status, social participation, etc.), and motivated offenders (e.g., locality, available financial institutions, GDP, etc.) were associated with exposure, types of victimization, and amount of financial loss. Findings showed that prior fraud experience was linked to all three measures of financial fraud. Better episodic memory and greater social activity were associated with higher fraud exposure, while better mental intactness and greater social activity were linked to lower financial loss. Additionally, contextual vulnerabilities—such as urban areas and more developed regions (e.g., more financial institutions and tertiary sectors or Eastern/Central areas)—were associated with increased fraud exposure or financial loss. Our findings highlight the need to target older adults living in urban and Eastern/Central areas, particularly those with prior fraud experiences, better cognitive well-being, higher socioeconomic status, and active social lives. Program and policy efforts related to anti-scam hotlines, educational workshops, and psychological support should be established to combat later-life financial fraud.

### SESSION 7410 (POSTER)

### AGE-FRIENDLY

Abstract citation ID: igaf122.3146

### 4MS INTERVENTIONS IN HOSPITALIZED OLDER ADULTS: WHAT ARE "ACT-ONS" FOR POSITIVE SCREENS?

Justin Lam[1], Susan Kwiatek[2], Shahidul Islam[2], Mark Tursi[2], Christian Nouryan[3], Philip Solomon[2], Maria Carney[4], and Edith Burns[5], *1. Donald and Barbara Zucker School of Medicine at Hofstra/Northwell, Brooklyn, New York, United States, 2. Northwell Health, New Hyde Park, New York, United States, 3. Northwell Health, Great Neck, New York, United States, 4. Zucker School of Medicine at Hofstra/Northwell, New Hyde Park, New York, United States, 5. Zucker School of Medicine Hofstra-Northwell, Manhasset, New York, United States*

The 4Ms framework -What Matters Most (WMM)/Medication/Mentation/Mobility- facilitates addressing issues critical to care of older adults. We identified different interventions, "Act-ons", for positive screens in hospitalized patients assessed for all 4Ms. Unique patients identified by Age-Friendly Health System (AFHS) dashboard aggregating data on adults ≥65y/o admitted to one of 8 hospitals and assessed for all 4Ms. Individual EMR manually reviewed for demographic and clinical data, and for each positive M to determine Act-ons. Admissions reviewed from July-September 2024. A total of 297 patients received all 4Ms (9.6% of 3104 admissions). Median age: 84y/o (IQR:76-90); 57% female, (85%); race: 63% White, 13% Black/AA, 12% Asian, 13% other; 85% English speaking Median LOS: 9 days (IQR:6-15). All 297 had GOC documented but 58.3% did not include WMM. Act-ons: managing team discussed with patient (60%); palliative care consult (40%). For 121 positive Medication assessments, 57.8% had Act-ons: documented reason for no change (73.6%); decrease/deprescribed (26.4%). For 297 Mobility screens, 83.1% had Act-ons: 99% PT consult; 1% RN plan. For 13 positive Mentation screens, 2 (15.4%) had Act-on: electrolytes reviewed/reported; medication side effect treated. This is one of the first reports of Act-ons for 4Ms. Mobility had most Act-ons, then Medication, WMM, lastly Mentation. Act-ons were similar for 3 of the 4Ms across different hospitals within the health system. Delirium was essentially not detected across locations, suggesting need for a root-cause analysis and further education/training. In the future, structured processes for Act-ons may help ensure patient needs are optimally met.

Abstract citation ID: igaf122.3147

### A PILOT PROGRAM OF VIRTUAL HEALTHCARE DELIVERY TO OLDER VETERANS

Sangeeta Rana, Michelle Rossi, Stephanie Sodders, Tammy Knell, and Coleen Cardamone, *VA Pittsburgh Health Care System, Pittsburgh, Pennsylvania, United States*

Telemedicine and ECHO® programs have successfully assisted in delivering specialized medical care as part of outreach efforts to rural and underserved areas. We describe a new pilot, Tele Geriatric Evaluation & Management program (TeleGEM) undertaken by our GRECC (Geriatric Research Education and Clinical Center) using telemedicine to deliver an interdisciplinary team-based model of Comprehensive Geriatric Assessment to Veterans in the network who lacked access to this service. Our team included a geriatrician, registered nurse, pharmacist, and administrative assistant with rehab services and neuropsychology available on a consultative basis. Thirty-three frail Veterans,65y & older (average 77y) were referred to our program for evaluation during a 10-month period starting January 2024. Telemedicine technologies used to provide this service from the tertiary care facility included the use of telemedicine carts available at the local VA medical centers (67%), in Veterans' homes using audiovisual digital devices and/or audio visits by telephone (24%), in addition to E-consults (9%) completed by chart reviews. Veterans were assessed using the 5M framework: what Matters, Mind, Multicomplexity, Medications, and Mobility. Other measures included mileage saved by avoiding travel to the geriatric clinics located at the tertiary care facility. The Tele GEM platform can serve as a novel and valuable healthcare delivery model of care providing comprehensive geriatric assessments which can help improve function and quality of life for frail older Veterans, thus reducing the burden of travel to tertiary care centers.

Abstract citation ID: igaf122.3148

### A STUDY ON THE COMPETENCIES AND CHARACTERISTICS REQUIRED BY INTERGENERATIONAL PROGRAMMING PROFESSIONALS

Stephanie Yu-Ching Chen, *National Chung Cheng University, Minxiong, Chiayi, Taiwan (Republic of China)*

Taiwan faces the challenge of a low birth rate and an aging population, resulting in social problems such as labor shortages and generational conflicts. How to promote cross-generational communication and solidarity has received attention. Intergenerational programs are gradually being promoted in various fields with the support of different government departments. However, there is still a lack of consensus on the professionalism and quality of such projects. This study used face-to-face interviews with 25 scholars and practitioners with experience in intergenerational programming to understand the characteristics and professional abilities required of intergenerational programming professionals. The research results found that intergenerational programming professionals mainly include three roles: leaders, managers, and lecturers. Their main task is to promote interaction, learning, and solidarity across generations. To competently promote intergenerational programs, the promoter should have certain characteristics, including being warm and enthusiastic, easy to interact with others, open and inclusive, flexible and adaptable, and innovative and flexible. Due to the differences in their roles, they need to have the general and the professional capabilities for intergenerational programs. Including professional knowledge such as aging literacy, life course and development, intergenerational learning and intergenerational program; having the attitude of caring and investing in intergenerational issues, respecting multiculturalism, liking both the old adults and children, valuing fairness between generations, and liking teamwork; and having the skills of resource connection and integration, intergenerational communication, promoting intergenerational interaction and connection, and intergenerational program planning and evaluation. These traits and abilities are the key to the success of intergenerational programs.

Abstract citation ID: igaf122.3149

### ACCEPTANCE OF DEPRESCRIBING RECOMMENDATIONS FOR FALL RISK INCREASING MEDICATIONS

Michelle Paulsen[1], Katherine Ritchey[1], Karl Brown[2], and Erica Martinez[1], *1. Department of Veterans Affairs, Seattle, Washington, United States, 2. VA Puget Sound Health Care System, Seattle, Washington, United States*

Fall risk increasing medications (FRIMs) have negative effects on balance and psychomotor functioning which increase the risk of falling in older adults. The Drug Burden Index (DBI) measures total exposure to anticholinergic and sedative medications and has been associated with falling risk in older adults. We evaluated whether reporting DBI score would influence deprescribing of FRIMs in older veterans at risk of falling. From January 2020 to September 2024, a pharmacist conducted a medication review for FRIMs in older veterans at risk of falling. The primary care prescriber (PCP) was alerted to recommendations through the electronic medical record. DBI was reported to PCP in a subset of patients. Changes to FRIMs and DBI score were assessed for all participants three months after initial medication review. Across the 112 study participants, the mean age was 75, 93 (83%) were male, and 89 (80%) were White. Medication-related recommendations were given to 72 (64%) of the 112 participants, with a mean (SD) of 1.9 (1.2) recommendations per patient. Of these, 37 (51%) had a recommendation accepted. The DBI was shared with care providers for 47 (65%) of the 72 who received a recommendation. Recommendation acceptance was higher in the group that did not have their DBI shared (17/25, 68%) compared to the group that did have their DBI shared (20/47, 43%). Though pharmacy intervention led to a reduction in FRIMs, DBI score did not appear to increase the acceptance of FRIM deprescribing recommendations by PCP.

Abstract citation ID: igaf122.3150

### ADVANCING PRIMARY CARE FOR OLDER ADULTS THROUGH THE 4M FRAMEWORK AND RESOURCE EXCHANGE

Victoria O'Connor[1], Christine Ferrone[2], Susanne Campbell[3], Suzanne Herzberg[3], Nijah Venditelli[3], and Jayne Daylor[3], *1. Rhode Island Department of Health, Providence, Rhode Island, United States, 2. University of Rhode Island, Kingston, Rhode Island, United States, 3. Care Transformation Collaborative of Rhode Island, Providence, Rhode Island, United States*

The growing population of adults aged 65+ will strain primary care, increasing demand on a limited workforce. The Age-Friendly 4M framework—Medication, Mentation, Mobility, and What Matters—offers a structured, evidence-based approach to team-based primary care. This project used a resource-exchange strategy to support 4M adoption in primary care. The Rhode Island Department of Health Alzheimer's Disease and Related Disorders "BOLD" Program, the Care Transformation Collaborative of RI, the RI Geriatrics Workforce Enhancement Program, and UnitedHealthcare collaborated to launch two six-month quality improvement initiatives to assist primary care practices with implementing the 4M framework and achieving the Institute for Healthcare Improvement's (IHI) Age-Friendly recognition. Identification of needed resources and support for what matters most to care partners of patients with dementia were key added components. Practices responded to a call for applications and received funding, individualized support from a practice facilitator, and structured workplans to engage at Level 1 (preparing for 4Ms) or Level 2 (expanding 4M processes). Practices met with facilitators monthly and joined three peer learning sessions. Over two years, 11 practices participated. All practices achieved IHI Level 1 designation, and all five that applied advanced to Level 2. Pre- and post-program ratings showed a 22% overall improvement in adherence to 16 distinct 4M guidelines and dementia care partner engagement. By pooling resources, this initiative advanced 4M adoption in primary care, enhancing care for older adults and dementia patients by identifying care partners, addressing their needs, and offering community support. Partner collaboration ensured engagement and sustainability.

Abstract citation ID: igaf122.3151

### AGE FRIENDLY HEALTH SYSTEM 4M COMPETENCY-BASED CURRICULUM FOR INTERNAL MEDICINE RESIDENTS

Tiffany Hu, Cecily McIntyre, Gregory Ouellet, Marcia Mecca, and Jennifer Ouellet, *Yale School of Medicine, New Haven, Connecticut, United States*

The Age Friendly Health System 4Ms is an evidence-based framework to provide high value care to older adults, with which educational content in Geriatrics is increasingly aligned. Focusing on Internal Medicine residents, who provide care to older adults in a variety of settings, we aimed to assess perceived proficiency in core competencies, as identified by AGS/ADGAP and organized using the 4Ms framework. We surveyed Internal Medicine residents in three programs at one academic medical center. The survey asked residents about their self-reported proficiency in AGS/ADGAP competencies on a scale of 1-5 (1 "completely unable to perform this skill", 5 "I am expert and can teach this skill to others"). A total of 35 residents responded to the pre-curriculum survey, including 13 PGY1, 17 PGY2 and 5 PGY3 residents. Most (71%) had no Geriatrics-focused experience in medical school, yet 27 individuals (77%) had >1 Geriatrics rotation during residency. Residents self-reported lower proficiency scores in competencies related to mobility (3.21) and multicomplexity (3.39). Of the competencies, residents scored lowest in their ability to screen for pressure injuries (2.95) and develop a multifaceted plan for fall prevention (3.00). Our findings highlight gaps in Internal Medicine residents' self-reported proficiency in key Geriatrics competencies. These results will inform our development of a QR-generated skills tracker for residents to have skills in Geriatrics evaluated in real time by trained faculty. This will also help align curricular content in didactics and rotations to target highest priority learning needs.

Abstract citation ID: igaf122.3152

### AGE FRIENDLY, BUT NOT FOR ME? RESOLVING COGNITIVE DISSONANCE TO IMPROVE CONTINUITY OF CARE

Joshua Gerlick[1], Betty Napoleon[1], Grace Armstrong[1], Nicholas Schiltz[1], Anne Pohnert[2], and Mary Dolansky[1], *1. Case Western Reserve University, Cleveland, Ohio, United States, 2. CVS Health, Woonsocket, Rhode Island, United States*

A persistent challenge in geriatric healthcare is ensuring that older adults effectively transition from Age-Friendly Health Systems (AFHS) care provided at retail clinics to ongoing follow-up with their primary care providers (PCPs). We conducted three focus groups (N = 20, ages 64–81) to understand older adults' experiences receiving care through the AFHS 4Ms framework at MinuteClinics in select CVSHealth locations, as well as the barriers and facilitators influencing their decisions to follow up with a PCP. Using a constructivist grounded theory approach, we applied team-based initial (open) coding techniques to capture participants' lived experiences during visits to a MinuteClinic. Axial coding then identified deeper relational themes, most notably cognitive dissonance and information uncertainty. Participants frequently expressed contradictory views on the relevance of AFHS, describing it as beneficial in general yet personally irrelevant. One participant captured this tension by stating, "This doesn't apply to me, but it's valuable for others with more serious conditions." We supported these findings with members of the CVS MinuteClinic AFHS Patient Advisory Council (PAC), which highlighted identity-related discomfort with aging. One PAC member remarked, "We don't think of ourselves as old," and this creates a barrier to embracing AFHS recommendations. Focus group and PAC participants reported uncertainty about how to best follow-up and integrate AFHS recommendations with their PCP, suggesting a need for clearer and more personalized messaging. Our findings suggest that providers must explicitly link AFHS 4Ms recommendations with patients' individual health contexts to reduce cognitive dissonance, enhance clarity, and improve the likelihood of follow-up.

Abstract citation ID: igaf122.3153

### AGE-FRIENDLY CARE: WHAT MATTERS MOST TO OLDER ADULTS IN AMBULATORY CARE CLINICS

Sherry Greenberg[1], Nicholas Schiltz[2], Grace Armstrong[2], Anne Pohnert[3], and Mary Dolansky[2], *1. Hunter-Bellevue School of Nursing, New York, New York, United States, 2. Case Western Reserve University, Cleveland, Ohio, United States, 3. CVS Health, Woonsocket, Rhode Island, United States*

Considering patient preferences by asking 'What Matters' most, key to the Age-Friendly Health Systems 4Ms framework, fosters patient-centered care and improves health outcomes. MinuteClinic, comprised of approximately 1,100 convenient care clinics across 35 states and DC, implemented the 4Ms framework in May 2020 as part of routine care for nearly all older adults. This retrospective cross-sectional study aimed to describe What Matters most to older adults at MinuteClinic locations across the US between January 2021 and March 2024. Data were extracted from electronic health records of those aged 65+ who received 4Ms care and responded to the 'What Matters' question, "What matters most to you?" Responses were categorized by providers into predefined categories: social activities/inclusiveness, health, family togetherness, independence, and other. Results demonstrated that 388,046 older adult patients responded to the 'What Matters' question. The most common response was engagement in social activities/inclusiveness (49%), then health (21%), independence (17%), and family togetherness (11%). Over time, responses for "social activities/inclusiveness" increased from 38.6% to 54.9%, while "health" decreased from 26.3% to 19.1%, and "family togetherness" decreased from 16.8% to 6.3%. This is the largest reporting of What Matters most to patients in ambulatory care settings. Approximately 80% of patients identified something other than their own health as what matters most to them, noteworthy given MinuteClinic commonly addresses urgent health needs. This study highlights the significance of understanding "What Matters", revealing that social activities and inclusiveness are priorities, thereby underscoring the need to promote patient-centered care that aligns with values and preferences.

Abstract citation ID: igaf122.3154

### AGE-FRIENDLY PALLIATIVE CARE FOR VETERANS AND CAREGIVERS IN A RURAL VA HEALTHCARE SYSTEM -- THE EASTERN CO EXPERIENCE

Stephanie Hartz[1], Alexander Garbin[2], Courtney McGuire[3], Jill Steagall[4], Jocelyn McCauliff[5], and Kathryn Nearing[6], *1. VA Eastern Colorado Health Care System, Aurora, Colorado, United States, 2. Eastern Colorado VA Health Care System, Aurora, Colorado, United States, 3. Geriatric Research Education and Clinical Center, Aurora, Colorado, United States, 4. Rocky Mountain Regional VA Medical Center, Aurora, Colorado, United States, 5. Veterans Affairs, Aurora, Colorado, United States, 6. University of Colorado Anschutz Medical Campus, Denver, Colorado, United States*

Introduction: Age Friendly Health Systems (AFHS) provide care to older adults aligned with geriatric best practices to improve healthcare quality, minimize harms and support older adults' goals of care. AFHS designation means that providers consistently address the Geriatric 4Ms during clinical visits: Mentation, Mobility, Medication and What Matters Most. Methods: Within the VA Eastern Colorado Health Care System (ECHCS), our rural tele-Palliative Care clinic achieved AFHS Level 2 Committed to Care Excellence recognition in December 2021, becoming the first AFHS-designated telemedicine clinic in the nation. We engaged older Veterans in planning and implementation. Using our existing interprofessional team and clinic workflow, we addressed the Geriatric 4Ms during visits. Results: FY23-24, we conducted 192 AFHS tele-Palliative Care visits, 81% with rural/highly rural Veterans. We served 108 unique patients (FY23: 57; FY24: 51). Compared to Colorado's Veteran population, racially minoritized Veterans and women were underrepresented, while older Veterans were overrepresented. The majority were White (82% and 73%), not Hispanic/Latino (83% and 73%), male (100% and 98%), and ≥65 (90% and 89%). All 4Ms were addressed for 86% (FY23) and 76% (FY24) of unique patients. AFHS tele-Palliative Care saved Veterans/caregivers 23,622 (FY23) and 18,632 (FY24) miles of travel. Conclusion: Congruent with AFHS, Palliative Care focuses on physical, emotional, and psychosocial aspects of serious illness. Our work to achieve AFHS Level 2 designation in a tele-Palliative Care clinic is novel nationally. We demonstrated that evidence-based care can be provided to every older adult, regardless of care modality, without expanding staff or changing clinical workflows.

Abstract citation ID: igaf122.3155

### AN AGE FRIENDLY CROSS-SECTOR PROGRAM TO ADDRESS HEALTH-RELATED SOCIAL NEEDS AND PREVENT AVOIDABLE HOSPITALIZATION

Jose Martinez Escudero[1], and Arielle Basch[2], *1. JASA, New York, New York, United States, 2. JASA, Litchfield, Connecticut, United States*

Older adults face post-hospitalization risks including medication complexity, barriers to follow-up care, and limited understanding of how to manage health conditions at home. These challenges are exacerbated for patients with limited English proficiency, lower health literacy, and complex social needs. JASA Aging Services has partnered with hospitals and health plans in NYC to create an age-friendly hospital and home-based transitional care model to address social needs and reduce readmissions. It includes several innovative elements: age-friendly care across settings driven by "what matters" to the patient; home-based education to build trust and learn about the patient's environment; an in-language, culturally appropriate model led by international medical graduates with extensive clinical knowledge and concordance of (10+) languages and cultures with patients. The program provides education, medication reconciliation, and vital sign monitoring while addressing social needs. The model shows early signs of success. Between February 1, 2024 and January 31, 2025, 1353 patients received JASA's transitional care services of which 83% were connected to essential social services, 56% completed a healthcare proxy form, and 72% received essential support with their medications. We achieved a 38% reduction in readmissions and patients were 21% more likely to engage with a PCP compared to baseline data. Patient satisfaction scores exceed 97%.

Abstract citation ID: igaf122.3156

### ARE WE THERE YET? SHARED DECISION-MAKING NEEDS IN OLDER ADULTS WITH ATRIAL FIBRILLATION AND MULTIMORBIDITY

Hawa Abu[1], Jane Saczynski[2], Michelle Nabi[1], Annie Ferris[1], Jerry Gurwitz[1], David Dosa[1], Alok Kapoor[1], and David McManus[1], *1. University of Massachusetts Chan Medical School, Worcester, Massachusetts, United States, 2. Northeastern University, Boston, Massachusetts, United States*

Background: Older adults with atrial fibrillation (AF) and multimorbidity face complex stroke prevention decisions. Identifying those with unmet Shared Decision-Making (SDM) needs can enhance patient-provider communication. Objective: Examine the prevalence and characteristics of older adults with AF and multimorbidity expressing a need for greater SDM involvement for stroke prevention. Methods: Patients aged 65 years and older with AF were enrolled in a prospective cohort study (2016-2018) from clinics in Massachusetts and Georgia. Participants with one or more chronic conditions were included. At one-year follow-up, participants on anticoagulation were asked about their preference for greater involvement in decision-making regarding the decision to use anticoagulation and the choice of anticoagulant. Affirmative responses indicated a need for greater SDM engagement. Multivariable logistic regression identified patient characteristics associated with preference for more SDM engagement. Results: Participants' (n = 532) mean age was 75 years; 48% were women, and 87% were White. Overall, 41% had 1–4 chronic conditions, 40% had 5–7, and 19% had 8 or more. One-third expressed a need for greater SDM in both anticoagulation initiation and selection of anticoagulant. After multivariable adjustment, participants who were younger (aged 65–74 years), non-White, had lower education level, fewer comorbidities or a higher perceived burden from anticoagulation use, reported a need for greater SDM engagement for stroke prevention. Conclusions: Clinicians need to recognize the specific needs of older patients with AF and multimorbidity that seek greater SDM involvement for stroke prevention. Providing tailored interventions can enhance the decision-making process for stroke prevention in this vulnerable population.

Abstract citation ID: igaf122.3157

### BRIDGING GAPS: THE POWER OF THE GERIATRICS 5M'S IN TRANSFORMING KNOWLEDGE

Ashmeet Bhatia, Keisha Velazquez-Diaz, Kamal Patel, James Meisel, Supriya Parvatini, and Lionel Lim, *VA Bedford Health Care System, Bedford, Massachusetts, United States*

Age-friendly care improves health outcomes for older adults. The Geriatrics 5M's framework—Mind, Mobility, Medications, Multi-complexity, and Matters Most—serve as a guide to ensure that healthcare providers address the multifaceted needs of older patients. However, awareness and perceived application of these principles among hospital staff may be inconsistent. Literature reveals limited studies focusing on increased awareness and application after 5M's workshop. VA Community Living Centers present opportunities for interprofessional education to identify gaps in knowledge and areas for improvement to foster a more age-friendly healthcare environment. This cross-sectional study aims to assess awareness and understanding of the Geriatrics 5M's before and after an educational intervention across health professions, including nurses, physicians, trainees, and allied health professionals (n = 39). Staff were surveyed for knowledge before and after an educational intervention, which was delivered through workshops and learning materials. Results indicate a variable level of awareness of the 5Ms across health professions. Medical care providers showed higher familiarity compared to nurses and allied health professionals. There was improvement in knowledge of the 5M's amongst physician trainees on geriatrics rotation at the VA and after a 5M's workshop. There was increased knowledge across all groups using a Geriatrics 5M's-focused educational curriculum, with notable increases in the "Medications" and "Matters Most" components. Our findings highlight the importance and impact of geriatric education using the Geriatrics 5M's approach in improving interprofessional knowledge of age-friendly care. Further research is needed to explore the long-term impact of these educational efforts on health outcomes and patient care quality.

Abstract citation ID: igaf122.3158

### CASE STUDY ON PROMOTING STRATEGIES AND COPING WITH DIFFICULTIES IN EXCELLENT INTERGENERATIONAL PROGRAMS

Stephanie Yu-Ching Chen, *National Chung Cheng University, Minxiong, Chiayi, Taiwan (Republic of China)*

Eleven units that promote excellent intergenerational programs were selected for the case study to improve the quality of intergenerational programs and effectively construct a training model for intergenerational professionals through the recommendation of the Ministry of Education and the Ministry of Health and Welfare in Taiwan. The research subjects include five organizational types: senior learning centers hosted by primary schools, senior learning centers hosted by community organizations, universities that promote intergenerational learning programs, community care centers, and elderly day care centers. 19 relevant personnel were interviewed, and relevant documents and information were collected. The research results found that (1) the purpose of promoting intergenerational programs is that schools aim to cooperate with the government in promoting senior education policies and reducing age discrimination among students, while community organizations aim to reduce generational gaps and social isolation and increase community inclusiveness. But whether it is a community or a school, they all hope to enhance social cohesion through interaction between different generations. (2) Difficulties in promotion and coping strategies: Difficulties on the school side include low willingness of teachers to participate, insufficient manpower to organize the program, difficulty in balancing the needs of different generations in curriculum design, and parents' concerns about the safety of the elderly entering campus. The difficulties faced by the community are mainly insufficient funding and manpower, generational barriers, and diverse community needs. Schools and communities have different response strategies depending on organizational attributes and resources. Finally, we propose strategies to promote intergenerational programs.

Abstract citation ID: igaf122.3159

### DEVELOPMENT AND VALIDATION OF A MOBILE HEALTH APPLICATION USABILITY SCALE FOR OLDER ADULTS WITH CHRONIC DISEASES

Hongyu Yu[1], Weiyu Qiu[2], Yanfeng Wang[3], Qinyang Wu[3], Ke Hu[3], and Qiaohong Yang[3], *1. University of Washington, Seattle, Washington, United States, 2. The First Affiliated Hospital of Jinan University, Guangzhou, Guangdong, China (People's Republic), 3. Jinan University, Guangzhou, Guangdong, China (People's Republic)*

Background: Chronic diseases are a leading cause of disability and death in older adults. Mobile health (mHealth) applications can improve chronic disease management and reduce healthcare costs, but many are not designed for older users. A reliable tool is needed to assess mHealth usability for this population. Objective: To develop and validate an mHealth usability evaluation scale for older adults with chronic diseases. Methods: The scale was developed (March–September 2022) through literature review, interviews, team discussions, and the Delphi method. A pilot test refined the scale, followed by surveys in Guangzhou, China (October–December 2022), using Item Discrimination Index, correlation analysis, internal consistency tests, and exploratory factor analysis. Reliability and validity evaluations were conducted from October 2022 to February 2023. Results: The final scale included six dimensions and 23 items. Content validity indices ranged from 0.85–1. Fit indices were strong ($\chi^2/df$ = 1.728, fit indices = 0.817–0.928). Average Variance Extraction (>0.4) and Composite Reliability (>0.6) met criteria. Cronbach's $\alpha$ ranged from 0.758–0.911, indicating good internal consistency. Conclusions: The usability scale was rigorously developed and demonstrated strong reliability and validity. It provides a valuable tool for assessing mHealth usability among older adults, supporting age-friendly digital health solutions.

Abstract citation ID: igaf122.3160

### ENHANCING POLST ACCESSIBLY TO ACHIEVE AGE FRIENDLY HEALTH SYSTEM (AFHS) RECOGNITION IN LONG TERM CARE: WHAT MATTERS

Kevin Overbeck, Elyse Perweiler, Jennifer DeGennaro, and Cindy Nolan, *Rowan-Virtua School of Osteopathic Medicine, Stratford, New Jersey, United States*

The NJ POLST document addresses what matters most to older individuals through its "Goals of Care" section ensuring that patient priorities are clearly communicated. As part of the pursuit of Age Friendly Health System (AFHS) recognition, a nursing home utilizing PointClickCare (PCC) as its EMR aimed to address the availability of the NJ POLST forms. While POLST documents are consistently scanned into PCC, they were not readily accessible to acute care teams in the regions three (3) leading hospital systems which use Epic EMR. This gap in accessibility posed a critical barrier to seamless, patient-centered care transitions. An interprofessional team reviewed baseline metrics as well as the current workflows for scanning and storing NJ POLST forms in PCC. Collaborative efforts with hospital system representatives explored technical solutions for integrating POLST documents into Epic. Metrics tracked included the number of residents with completed POLST forms, the proportion of forms successfully transferred to Epic, and the time required to complete this transfer process. 25% of fifty (50) long-term care residents in the nursing home had a completed NJ POLST document accessible in PCC. Prior to implementation 5% had the NJ POLST document electronically accessible in the Epic system of partnering hospitals and 100% had this document accessible upon project completion. This approach can serve as a model for other nursing homes pursuing AFHS recognition. The project demonstrated the feasibility of improving NJ POLST document accessibility across EMR platforms ensuring that patient preferences are available to acute care teams when it matters most.

Abstract citation ID: igaf122.3161

### EVALUATING COGNITIVE SCREENING TOOLS FOR OLDER ADULTS IN AMBULATORY CARE SETTINGS

Shannon Foran[1], Grace Armstrong[2], Sherry Greenberg[3], Nicholas Schiltz[2], Anne Pohnert[4], and Mary Dolansky[2], *1. Case Western Reserve University, Frances Payne Bolton School of Nursing, Cleveland, Ohio, United States, 2. Case Western Reserve University, Cleveland, Ohio, United States, 3. Hunter-Bellevue School of Nursing, New York, New York, United States, 4. CVS Health, Woonsocket, Rhode Island, United States*

Implementation of the Age-Friendly Health Systems 4Ms framework ensures that assessing and acting on What Matters, Medication, Mentation, and Mobility is included for all patients aged 65 and older. In ambulatory settings, patients in this age group are often assessed annually for dementia using brief cognitive screening tools to screen for Alzheimer's Disease/Related Dementias (ADRD). Providers often report that older adults are hesitant to engage in the screening, frequently stating that they do not perceive any memory issues. Additionally, controversy exists on the benefits for screening all older adults. The purpose of this literature review is to present: (a) current instruments used for cognitive screening such as the Mini-Cog, the Eight-item Informant Interview to Differentiate Aging and Dementia (AD8®), Memory Impairment Screen (MIS), and Montreal Cognitive Assessment (MoCA); (b) barriers to dementia screening from both provider's and patients' perspectives; and (c) the ongoing controversy surrounding population screening for dementia in the ambulatory care setting. This review will include a comparison of these instruments in terms of time to complete, acceptability, specificity, and sensitivity. Current utilization and recommendations for these screening tools in ambulatory care settings will be reported. Strategies for implementing these assessments in ambulatory care, where patients often expect short and succinct appointments, will also be presented.

Abstract citation ID: igaf122.3162

### EVALUATION OF AN AGE-FRIENDLY HEALTH SYSTEM: 4MS ASSESSMENTS AND OUTCOMES IN HOSPITALIZED OLDER ADULTS

Justin Lam[1], Susan Kwiatek[2], Shahidul Islam[2], Mark Tursi[2], Christian Nouryan[3], Philip Solomon[2], Maria Carney[4], and Edith Burns[5], *1. Donald and Barbara Zucker School of Medicine at Hofstra/Northwell, Brooklyn, New York, United States, 2. Northwell Health, New Hyde Park, New York, United States, 3. Northwell Health, Great Neck, New York, United States, 4. Zucker School of Medicine at Hofstra/Northwell, New Hyde Park, New York, United States, 5. Zucker School of Medicine Hofstra-Northwell, Manhasset, New York, United States*

The Institute for Healthcare Improvement developed the 4Ms framework (What Matters Most/Medication/Mentation/Mobility) to facilitate Age-Friendly Health Systems (AFHS). Each M has evidence-based screening tools leading to improved outcomes among older adults. We report screening 4Ms vs. 1-3Ms outcomes for hospitalized adults aged ≥65 across hospitals in a large healthcare system. Demographics and clinical data abstracted manually from EMR and from 4Ms AFHS dashboard. Population: Patients ≥65y/o admitted July-September 2024 to eight hospitals committed to care excellence. Outcomes: Length of stay (LOS), 30-day readmission, 30-day ED visits, mortality. Of 3,103 older adult admissions, 298 (9.6%) received all 4Ms and 2,805 received 1-3Ms. Median age: for 4Ms, 84 (IQR:76-90) and for 1-3Ms, 77 (IQR:71-84). Primary language was English for both. Median LOS: for 4Ms, 9 days (IQR:6-15) and for 1-3Ms, 9 days (IQR:5-16). Those receiving 4Ms had a lower AMPAC mobility score (13 vs 18, $p<0.001$) fewer 30-day ED visits (16% vs. 22%, $p=0.02$), higher mortality (11% vs. 5.3% $p<0.001$), and trend towards lower 30-day readmissions (13% vs. 16%, $p=0.2$). In conclusion, a small proportion of hospitalized older adults were screened for all 4Ms. Those with 4Ms were older with lower mobility. They had fewer post-discharge ED visits and a trend towards lower readmissions which may become significant with accrual of more events. Higher mortality may reflect age, greater illness burden. This preliminary assessment provides evidence for 4Ms care as a quality framework associated with key patient outcomes.

Abstract citation ID: igaf122.3163

### GOT CARE! 2.0: A SECONDARY ANALYSIS OF AN INTERPROFESSIONAL GERIATRIC CARE MODEL

Millicent Malcolm[1], Catherine Rees[2], Greg Rhee[3], Anna-Rae Montano[4], and Juliette Shellman[5], *1. University of Connecticut, Storrs, Connecticut, United States, 2. Middlesex Health, Middletown, Connecticut, United States, 3. University of Connecticut School of Medicine, Farmington, Connecticut, United States, 4. Hartford Hospital, Hartford, Connecticut, United States, 5. University of Connecticut, Farmington, Connecticut, United States*

Geriatric Outreach and Training with Care (GOT Care!) was created as a model for interprofessional geriatric education and practice. An interprofessional team of geriatric expert clinical faculty guided students from seven health professions in learning about and caring for chronically ill homebound older adults experiencing high emergency department (ED) use. The GOT Care! project was evaluated using the IHI Quadruple AIM framework and positive outcomes supported development of this model within a health system, however, gaps and need for further inquiry were also identified. GOT Care! 2.0, a secondary analysis of GOT Care! program data, was conducted with the aim of better understanding medical expenditures, healthcare utilization risk factors, and the observed reduction in ED visits for GOT Care! participants. Electronic health records, billing records and medical expenditures were extracted and analyzed for a 6 month pre- and post-program period. Direct costs were reduced significantly in the post-period (mean=$1,264.85) compared to the pre-period (mean=$4,457.44) ($p<0.001$) and at 12 months ($p<0.001$). Multiple factors associated with the likelihood of increased or decreased medical expenditures at 6 and 12 months were identified. With the support of the findings of the secondary analysis, an in-home interprofessional geriatric service pilot was launched. This pilot was based on the GOT Care! model and integrates the IHI Age-Friendly health system criteria focused on improving the IHI Quadruple AIM for a health system's vulnerable older adult population.

Abstract citation ID: igaf122.3164

### HEALTHCARE PROFESSIONALS' PERSPECTIVES ON AGE-FRIENDLY PRIMARY CARE: TECHNOLOGICAL AND CULTURAL CHALLENGES

Lia Ring[1], Paule-Sarah Fraiman[2], Yosefa Birati[3], Reem Nashef-Hamuda[4], Shoshi Keisari[2], and Anna Zisberg[2], *1. Department of Social Work, Ruppin Academic Center, Emek Hefer, Israel, 2. University of Haifa, Haifa, HaZafon, Israel, 3. The Cheryl Spencer Department of Nursing Faculty of Social Welfare and Health Science, Hanaton, HaZafon, Israel, 4. University of Haifa, Tel Aviv University, Haifa, HaZafon, Israel*

Older adults face unique healthcare challenges, necessitating age-friendly primary care approaches to better meet their needs, particularly in highly multi-cultural societies like Israel where language and cultural factors may impact healthcare needs and accessibility. Despite practical efforts to improve primary healthcare for older adults the extent to which primary care clinics meet their unique needs remains unclear. This qualitative study explores how healthcare professionals perceive and implement age-friendly adaptations in primary care, focusing on technological and cultural considerations. Semi-structured interviews were conducted in Israel with 21 Jewish and Arab primary healthcare professionals, aged 31 to 69, examining perspectives on clinic adaptations to older patients' needs. Thematic analysis revealed three main themes regarding the perception of professionals: 1) Age as a Core Identity; 2) Technology, Infrastructure and Administrative Challenges in Primary Care for Older Adults; and 3) Cultural and Linguistic Issues in Older Adult Primary Care.

Each category was further defined into specific themes. Examples from each category include: "Challenges and Special Needs Navigating the Healthcare System and Ensuring Continuity of Care"; "The Shift to Digital and Telephone Interfaces for Healthcare Services and Administration"; and "Differences Between Communities". Although these themes have been discussed, to our knowledge, they haven't been examined together. The findings provide insights into adaptations to improve primary care for older adults, including tailoring technologies and enhancing communication. This study underscores the importance of language, cultural awareness, and healthcare providers' age-related perceptions. By integrating these dimensions, this research expands knowledge and offers new perspectives on age-friendly healthcare practices.

Abstract citation ID: igaf122.3165

### IMPLEMENTING AGE FRIENDLY HEALTH SYSTEMS: ENHANCING CARE FOR OLDER VETERANS

Lana Brown, Hallie Keller, and Olive Phillips, *Central Arkansas Veterans Healthcare System, North Little Rock, Arkansas, United States*

The Age Friendly Health Systems (AFHS) is an initiative of The John A. Hartford Foundation and the Institute for Healthcare Improvement, in partnership with the American Hospital Association and the Catholic Health Association of the United States. In March 2020, the Veterans Affairs' (VA) Office of Geriatrics and Extended Care joined the AFHS movement to deliver safe, reliable, high-quality, evidence-based health care in every setting based on what matters most to older Veterans and their caregivers. AFHS entails providing four evidence-based elements of high-quality care, known as the "4Ms," to older adults: What Matters, Medication, Mentation, and Mobility. One VA Geriatric Patient-Aligned Care Team received AFHS Level II recognition in January 2023. Forty-two Veterans, 6 health care providers (MDs/APRNs), and 7 nurses were surveyed in 2024 to gather opinions regarding Age Friendly care. Four out of 6 providers agreed that AFHS practices were worthwhile to improve care of Veterans, and none expressed difficulty aligning "What Matters" to the Veteran's plan of care. Five out of 7 nurses reported that assisting Veterans with AFHS duties was not burdensome, and none found it difficult to complete screening. Veterans agreed that they felt respected and comfortable during their visit (95%), providers listened to them carefully (98%), providers included what matters most in plans for managing health and wellbeing (95%), and providers talked with them about specific goals for health (92%). Staff and Veteran responses suggest that AFHS practices were successfully adopted, fostering plans of care that align with Veteran goals and preferences.

Abstract citation ID: igaf122.3166

### INTEGRATING GENETICS AND METABOLOMICS REVEALS PUTATIVE MECHANISMS FOR HIPPURIC ACID DELAYING KIDNEY AGING

Yaxin Li[1], Bangwei Chen[1], Li Luo[1], Shengyin Zeng[1], Lei Ruan[4], Shida Zhu[1], Cuntai Zhang[4], and Tao Li[1], *1. BGI Genomics, Shenzhen, Guangdong, China (People's Republic), 3. Tongji Hospital, Tongji Medical College, Huazhong University of Science and Technology, Wuhan, Hubei, China (People's Republic), 4. Huazhong University of Science and Technology, Wuhan, Hubei, China (People's Republic)*

Increased life expectancy has led to a rise in age-related diseases. Hippuric acid (HPA) is a potential biomarker for aging. We aim to examine the associations between plasma HPA and biological aging of different organs, and identify the influence factor by integrating genetic and metabolomic data. In 777 healthy participants, Klemera-Doubal method was used to estimate biological aging and the discrepancy (Δage) between biological age and chronological age. Multiple linear regression was used to assess the relationship between plasma HPA and organ age or Δage (heart, kidney, liver, vascular). Genetic and metabolomic data were integrated to identify factors influencing HPA and kidney aging, and Mendelian randomization (MR) analysis was performed to examine causality. Plasma HPA levels were positively associated with chronological age and liver Δage, but negatively correlated with Δage of heart, kidney (β: -0.794, 95% CI: -1.352, -0.235), and vascular. The MR analysis further support a causal relationship between the HPA and kidney Δage (β: -0.009, 95% CI: -0.015, -0.003). What's more, the INPP4B and STEAP2 gene regions were identified as modulating HPA's effects on kidney aging. Metabolomic analysis showed that specific pathways, including alanine, aspartate, and glutamate metabolism; arginine biosynthesis; and glyoxylate and dicarboxylate metabolism, may play a role in HPA against kidney aging. In conclusion, HPA has distinct associations with different organic aging, especially acting as a protective biomarker for kidney aging. The association is influenced by both genetic and metabolic factors. HPA may provide for early intervention in kidney aging.

Abstract citation ID: igaf122.3167

### PILOTING CURRICULUM ABOUT LIVING TO 100: A NEW APPROACH WITH ELEMENTARY STUDENTS

Hallory Domnick, and Melinda Heinz, *University of Northern Iowa, Cedar Falls, Iowa, United States*

The purpose of this project was to pilot a curriculum for children about aging and centenarians, to promote more positive views of older adults. A total of 125 students in grades Pre-School – 6th grade were reached. The curriculum was inspired by the 100th Day of School Celebration Toolkit (Newsham & Hancock, 2024). The curriculum included reading a book (from the Toolkit) to students about centenarian abilities and accomplishments and watching a clip about an interaction between children and centenarians where both groups learn new things about each other. During the presentation, students are asked to reflect on positive and negative portrayals of older adults and consider that not all older adults are frail or live in a nursing home. Students used common terms to describe a person 100 years old, such as 'old,' 'elder,' 'grandparent,' or 'great-grandparent.' When discussing the appearance of centenarians, students were more likely to identify physical traits, such as being old, having wrinkles, grey or white hair, and possible use of assistive devices. When describing their personalities, students frequently used words like 'happy,' 'kind,' and 'gentle.' To promote more positive views of aging, students colored and designed cards to be given to centenarians in the community. Celebrating the 100th Day of School offers a unique opportunity for children to gain a greater understanding of aging and older adulthood. Future educators planning 100th Day of School Celebration activities could focus on promoting positive events that offer students a more uplifting perspective on aging and centenarians.

Abstract citation ID: igaf122.3168

### PRIMARY CARE PATIENTS' PREFERENCES AND WILLINGNESS TO PAY FOR HOME-BASED HEALTHCARE IN CHINA

Sixian Du[1], and Yaqing Liu[2], *1. Huazhong University of Science and Technology, Wuhan, Hubei, China (People's Republic), 2. Huazhong University of Science and Technology, Hubei, Hubei, China (People's Republic)*

Population aging and the increasing burden of chronic diseases have created urgent demand for high-quality, patient-centered home-based healthcare and support services (HHSS) for older adults. However, evidence on patient preferences for HHSS in developing countries remains limited. This study aimed to identify preferences and willingness to pay (WTP) for HHSS among primary care patients in China, focusing on the aging population. A discrete choice experiment (DCE) was conducted with 312 patients from 13 community health centers in Wuhan and Kunming between January and May 2023. Five attributes were included: scope of services, type of healthcare professional, service institution, insurance reimbursement, and visiting fee. Mixed logit models revealed that the scope of services was the most influential attribute (relative importance 67.33%), with strong preferences for physician-led diagnostic and treatment services over preventive care, mental health support, or daily living assistance. Participants favored services provided by hospitals (19.84% importance) and qualified physicians (12.42% importance), reflecting trust in formal medical institutions. Latent class analysis identified three distinct subgroups: 60.2% prioritized comprehensive medical services; 16.6% preferred services delivered by reputable institutions and professionals; and 23.2% were highly sensitive to cost and reimbursement. WTP analysis indicated patients were willing to pay more for diagnostic and treatment services but less for mental health or care support services. These findings suggest that expanding physician-led, home-based medical care while addressing affordability barriers is critical for advancing geriatric HHSS in China. Policymakers should prioritize developing integrated, multidisciplinary HHSS models to promote healthy aging and support aging in place.

Abstract citation ID: igaf122.3169

### RURAL PRIMARY CARE WORKFORCE DEVELOPMENT IN SERIOUS ILLNESS CARE: THE VA PALLIATIVE SCHOLARS PROGRAM

Sumi Misra[1], and Katharina Echt[2], *1. Vanderbilt University Medical Center, Nashville, Tennessee, United States, 2. Emory University, Atlanta, Georgia, United States*

Evidence demonstrates the quality of advanced and serious illness care is substantively improved by integration of Palliative Medicine practices. The demand for trained palliative care professionals remains unmet with pronounced national workforce gaps in rural communities. We developed and implemented an interdisciplinary primary care team training initiative that leverages the experience of well-established palliative care teams to provide an immersive virtual training experience that blends live case-based learning embedded in clinical encounters with interdisciplinary role appreciation, teamwork, prognostication, breaking bad news, pain, symptom management, team dynamics, and value-based goals of care discussions. Between May and September 2024 six multidisciplinary cohorts of palliative scholars (N = 32) completed the 3-day practicum. In-depth pre-post practicum metrics measured change in knowledge, attitudes, perceived skill, comfort, confidence with recognizing, addressing, and holding end-of-life conversations with patients and families. The scholars post evaluations demonstrated learners' experienced significant gains in knowledge of and comfort with providing essential palliative and supportive care in the primary care setting. Knowledge increased by 85%, 100% indicated having gained valuable communication skills to discuss advance care planning and goals of care, 90% ranked their experience as highly satisfactory, and 100% indicated intention to recommend the practicum to others. Scholars reported feeling empowered to continue learning as champions for Palliative Care principles at their primarily rural settings of care. Initial evaluation of the Palliative Scholars model supports it viability as one effective solution to expanding the primary palliative care competency of our workforce to meet the needs of older adults with life-limiting illness.

Abstract citation ID: igaf122.3170

### STRENGTHENING COMMUNITY-UNIVERSITY ENGAGEMENT AT ILLINOIS TO SUPPORT AGING AND DISABILITY

Carrie Wennerdahl[1], Laura Rice[2], and Wendy Rogers[1], *1. University of Illinois Urbana-Champaign, Champaign, Illinois, United States, 2. University of Illinois College of Applied Health Sciences, Urbana, Illinois, United States*

University and community engagement creates meaningful and mutually beneficial partnerships that enriches learning experiences and provides a foundation for research that is relevant and impactful; addresses community needs; builds trust; and enhances the validity and application of the findings. At the University of Illinois Urbana-Champaign, the College of Applied Health Sciences has two research themes: CHART (Collaboration on Health, Aging, Research, and Technology) and CARD (Collaborations in the Advancement of Research on Disability). Together, they represent consortia of faculty across campus and are proactively developing community connections with local leaders, healthcare providers, community agencies, older adults, people with disabilities, and caregivers. We will demonstrate how CHART and CARD have played a critical role in fostering community and university partnerships with successful programs and events this past year, as part of our Age-Friendly University mission. We hosted an open house and tour of the McKechnie Family LIFE Home during Active Aging Week to showcase research innovations with smart home technologies and robotics for people of all ages and abilities. We conducted a symposium entitled "Community/University Engagement to Support Aging and Disability" with over 100 attendees that brought together community members and researchers to explore strategies for effective research partnerships. Presentations and a panel discussion highlighted the importance of collaboration and addressing the needs of diverse communities. These initiatives not only strengthen university-community connections but also pave the way for continued collaboration and implementation of research initiatives to promote meaningful change and enhance well-being of individuals across all generations and abilities.

Abstract citation ID: igaf122.3171

### THE SOCIAL RESPONSIBILITY OF UNIVERSITIES IN PROMOTING SENIOR UNIVERSITIES – FROM A SOCIOLOGICAL PERSPECTIVE

Weng Pi Ching, *National Chung Cheng University, Minxiong, Chiayi, Taiwan (Republic of China)*

In 2025, Taiwan has officially entered a super-aged society. The social responsibility of universities extends beyond providing formal education to young students; they also play a crucial role in supporting and promoting senior universities. Since 2010, Taiwan's Ministry of Education has sought contractors through a bidding process to implement senior universities. Currently, 83 universities in Taiwan operate senior universities, with 3,500 elderly students enrolled, fulfilling their dreams of attending university. However, more than 10,000 elderly individuals are still eager to join. This study employs in-depth interviews and survey methods. From a sociological perspective, interviews were conducted with the project director and an assistant. From the perspective of resource allocation, interviews were held with personnel responsible for program implementation at eight universities across Taiwan, from north to south. From the standpoint of Symbolic Interactionism, 30 elderly students participating in senior universities were interviewed, followed by a questionnaire survey. The study concludes that the project director aims to uphold social justice and equal opportunity through the implementation of senior universities. Program coordinators utilize university-specific resources to design courses, which account for 40% of the entire curriculum, ensuring that educational funding is used effectively. Elderly students, despite not earning a formal degree, construct their self-identity through interactions, pursuing their studies based on personal interests. They find joy in learning at senior universities, establish positive interpersonal relationships, and ultimately fulfill their dream of attending university. These valuable experiences can serve as a reference for other countries.

### SESSION 7415 (POSTER)

Abstract citation ID: igaf122.3172

### ALZHEIMER'S DISEASE AND RELATED DEMENTIAS IV A DECADE OF PET FOR AMYLOID IMAGING: INSIGHTS FROM REAL-WORLD EVIDENCE SUPPORTING ALZHEIMER'S DIAGNOSIS

Margaret Cadden[1], Samantha Rossano[1], Brittany Watson[1], Marianne Chapleau[1], Santiago Bullich[2], Andrew Stephens[2], Norman Koglin[2], and Aleksandar Jovalekic[2], *1. Life Molecular Imaging, Boston, Massachusetts, United States, 2. Life Molecular Imaging, Berlin, Brandenburg, Germany*

Positron emission tomography (PET) scanning with florbetaben (FBB) was approved in the US in 2014 after validation in histopathological studies for imaging of amyloid plaques in individuals being evaluated for Alzheimer's disease (AD). The current abstract incorporates real-world evidence of FBB's clinical and research use, including results from visual and quantitative assessment of amyloid PET images. The objectives of this work included 1) Evaluating the efficacy of the approved visual assessment method in the routine clinical practice and 2) Presenting results of 15 quantitative pipelines to underscore reliability and added value of quantification. The studies' results confirmed that, both experts and naïve readers accurately assess FBB PET scans and maintain these abilities over time (correct assessments: 96% at baseline and 92% at 6 months follow-up). Quantitative methods, including 510(k) approved software, consistently demonstrated robust and homogeneous performance (accuracy 96.4±1.1%), offering valuable support for visual assessments. Since its approval, visual assessment and quantification of FBB images have undergone additional validation across diverse settings including clinical routine. FBB PET imaging is a validated, reliable, and available tool in the diagnostic pathway of AD, as indicated within the Alzheimer's Association's (AA) Updated Criteria for AD Diagnosis and Staging and the AA/Society for Nuclear Medicine's Appropriate Use Criteria for PET. With the approval of amyloid-targeting therapies, standardized quantification approaches, such as the Centiloid scale, are becoming increasingly important for patient selection and treatment monitoring.

Abstract citation ID: igaf122.3173

### ALZHEIMER'S DISEASE KNOWLEDGE, SOCIAL SUPPORT, AND DEPRESSIVE SYMPTOMS AMONG AMERICAN INDIANS

Yeon-Shim Lee[1], Soonhee Roh[2], Seungjong Cho[3], Soeun Jang[4], Heehyul Moon[5], Donald K. Warne[6], Joel Steele[7], and Sasheen T. Stone[8], *1. San Francisco State University, San Francisco, California, United States, 2. University of South Dakota, Sioux Falls, South Dakota, United States, 3. Texas Tech University, Lubbock, Texas, United States, 4. University of Texas at Arlington, The University of Texas at Arlington, Texas, United States, 5. University of Louisville, Louisville, Kentucky, United States, 6. Johns Hopkins University, Baltimore, Maryland, United States, 7. University of North Dakota, Grand Forks, North Dakota, United States, 8. Yankton Sioux Tribe, Wagner, South Dakota, United States*

Objectives: Alzheimer's disease (AD) is a public health priority, affecting millions of older Americans. American Indian (AI) populations, in particular, experience a disproportionate rate of underdiagnosis and underreporting of AD, despite having a high prevalence of its risk factors. The present study examined the relationship between AD knowledge, social support, and depressive symptoms among American Indians, with a specific focus on how social support might moderate the relationship between AD knowledge and depressive symptoms. Design and Methods: The sample consisted of 227 AI adults aged 18 to 80 residing in the Northern Plains. Multiple-group structural equation modeling assessed the relationships of latent variables to each construct with goodness-of-fit indices. Results: With a 91% response rate, over two-thirds of participants showed signs of cognitive impairment. We found that greater knowledge of AD was associated with lower depressive symptoms and that depressive symptoms was weaker among those with higher social support. However, no significant differences were observed in these relationships between AI individuals with prior exposure to AD and those without. Discussion and Implications: The findings indicated that AD knowledge and social support could be significant factors in improving depressive symptoms within AI populations. Healthcare and social services that reinforce social support systems could be particularly beneficial for addressing AIs' depressive symptoms, especially among those at risk for AD. Health initiatives that incorporate unique cultural and health behavior constructs into AD and mental health promotion are a critical first step toward developing culturally tailored, targeted, community-based interventions for AI populations.

Abstract citation ID: igaf122.3174

### ASSESSING CHANGE IN SLEEP LATENCY AS A POTENTIAL INDICATOR OF ALZHEIMER'S PATHOLOGIES IN ESSENTIAL TREMOR

Hasib Mia[1], Silvia Chapman[2], Sandra Rizer[2], Phyllis Faust[3], Elan Louis[4], and Stephanie Cosentino[2], *1. Columbia*

*Presbyterian Medical Center, New York, New York, United States, 2. Columbia University Irving Medical Center, New York, New York, United States, 3. New York-Presbyterian Hospital, New York, New York, United States, 4. UT Southwestern Medical Center, Dallas, Texas, United States*

Sleep disturbances, including prolonged sleep latency, are frequently reported in Essential tremor (ET) and other neurodegenerative conditions, yet their underlying etiology remains unclear. This study examines whether an increase in sleep latency over time—measured by the Pittsburgh Sleep Quality Index (PSQI)—is associated with tau pathology and amyloid burden in older adults with ET. In a sample of 24 older adults, we assessed changes in sleep latency over time in relation to Alzheimer's disease (AD) neuropathological outcomes, including amyloid plaques and neurofibrillary tangles (tau). Tau pathology was estimated using Braak Stage (AD), and amyloid burden through CERAD scores. Pearson correlations and multiple regression analyses were conducted, adjusting for age, education, and time between sleep data collection and death. Correlational findings indicate an association between increased sleep latency and tau pathology ($r = 0.473$, $p = 0.023$) and amyloid burden ($r = 0.448$, $p = 0.028$). However, when examined in an adjusted regression, the change in sleep latency was not associated with pathological outcomes (tau: $\beta = 0.129$, $p = 0.664$; amyloid: $\beta = 0.257$, $p = 0.324$). Results suggest that while sleep latency may be linked to neurodegenerative pathology, additional factors contribute to this relationship, warranting further research. This study contributes to the growing body of literature on sleep disturbances in dementia by highlighting the influence of confounding factors. Understanding these relationships may help establish sleep latency as a diagnostic tool or biomarker for neurodegenerative conditions, while emphasizing the need for a more nuanced interpretation of its predictive value.

Abstract citation ID: igaf122.3175

### BLACK PEOPLE LIVING WITH MEMORY CHALLENGES & STUDY PARTNERS' PREFERRED ACTIVITY TRACKER FEATURES

Rebecca Lassell[1], Christopher Carey[2], Lola Sample[3], Hanley Elftmann[4], Laura Gitlin[5], Jaroslaw Harezlak[1], Richard Holden[1], and NiCole Keith[3], *1. Indiana University Bloomington, Bloomington, Indiana, United States, 2. Indiana University School of Medicine, Indianapolis, Indiana, United States, 3. Indiana University School of Public Health Bloomington, Bloomington, Indiana, United States, 4. A.T. Still University School of Osteopathic Medicine, Mesa, Arizona, United States, 5. Drexel University, Philadelphia, Pennsylvania, United States*

Activity tracker preferences of Black people living with memory challenges (PLMC) or dementia and study partners are largely unknown, which can impact wear-time adherence. We sought to identify Black PLMC and study partners' preferred activity tracker features. Participants ($n = 10$) were recruited from Eskenazi Health in Indianapolis, Indiana. Data were collected through two 90-minute focus groups with Black PLMC participants ($n = 5$) and Black study partners ($n = 5$), respectively, and a PLMC and study partner interview. The focus group was guided by a semi-structured questionnaire and involved interacting with an Apple Watch SE, Oura Ring Heritage, Fitbit Inspire 3, and Polar Watch Ignite. Participants ranked each device based on comfort (device size, weight, material), convenience (battery life, water resistance, style, emergency button), and features (e.g., activity, steps, time standing, calories, distance/GPS, heart rate, respiratory rate) from 1-5 using checklists and picture cards. Focus groups were audio recorded. Device rankings from each category (comfort, convenience, and features) were summarized with descriptive statistics. Audio recordings were analyzed using the Rapid Identification of Themes from Audio recordings method and transcribed. PLMC rated the Apple Watch SE with mean scores in comfort (4.00), convenience (3.33), and features (4.3),30) emphasizing screen size, viewing progress on the watch, and an emergency button. Study partners rated the Fitbit Inspire 5 most in comfort (5), while the Apple Watch SE had the most rankings for convenience (4.75) and features (5.00), highlighting the emergency button and GPS as key attributes. Findings can guide activity tracker selection in future studies with this population.

Abstract citation ID: igaf122.3176

### COMBINED IMPACT OF PLASMA PHOSPHO-TAU 217, GFAP AND NFL ON LONGITUDINAL DOMAIN-SPECIFIC COGNITIVE DECLINE

Chao-Yi Wu[1], Liu Chen[1], Jennifer Gatchel[2], Sudeshna Das[1], Pia Kivisäkk[2], Steven Arnold[2], and Hiroko Dodge[1], *1. Massachusetts General Hospital, Harvard Medical School, Boston, Massachusetts, United States, 2. Massachusetts General Hospital, Boston, Massachusetts, United States*

Clinical trials are increasingly focused on pre-manifest and early Alzheimer's disease. Accurately predicting clinical progression is important to avoid unnecessary treatment and improve trial efficiency. Plasma p-tau217, an indicator of tau pathology with strong associations to amyloid-beta pathology, NfL, a marker of axonal damage and neurodegeneration and GFAP, a marker of inflammation, are promising diagnostic and prognostic tools. Their combined use could offer more accurate prognostic insights than either biomarker alone. We examined the trajectories of domain-specific cognitive functions by stratifying participants based on plasma p-tau217, NfL and GFAP levels (high/low). Participants were from the Massachusetts Alzheimer's Disease Research Center cohort ($n = 523$). Cognitive functions were assessed using the National Alzheimer's Coordinating Center Uniform Data Set v1-3: global cognition (CDR sum of box; MMSE, MoCA converted in v3), memory (Logical Memory), executive functions (Trail Making Test B), language (Boston Naming), and language-based executive function (Category Fluency Animals). We used linear mixed-effects models with 8 groups combining 3 plasma biomarkers to predict cognitive trajectories over 7 years, controlling for age, sex, and education. High p-tau217 alone was significantly associated with declines in Logical Memory (coefficient=-0.12; $p = 0.03$) and Boston Naming (coefficient=-0.16; $p < 0.01$), but not associated with decline in CDR sum of box and MMSE unless combined with a high burden of NfL and/or GFAP (CDR group*time coefficients=0.17-0.34, $p < 0.01$; MMSE group*time coefficients=-0.39 to -0.69, $p < 0.01$). Neither high GFAP alone nor high NfL alone was associated with significant cognitive declines. The combined use of plasma biomarkers provides a promising approach for predicting domain-specific cognitive decline.

Abstract citation ID: igaf122.3177

### DEVELOPMENT AND SYNTHESIS OF NEW THERAPEUTICS FOR ALZHEIMER'S DISEASE

Lilianna Tchatlian[1], and Stevan Pecic[2], *1. California State University, Fullerton, Glendale, California, United States,*

2. *California State University, Fullerton, Fullerton, California, United States*

Alzheimer's disease (AD) is the sixth leading cause of death among older adults, with 1 in 3 seniors dying with AD or another form of dementia. In 2022, the U.S. had an age-adjusted mortality rate of 28.9 deaths per 100,000 people due to AD. A key therapeutic target for managing AD symptoms is acetylcholinesterase (AChE), an enzyme that breaks down acetylcholine. Donepezil, the most prescribed FDA-approved drug, inhibits AChE but only addresses mild to moderate symptoms in the early stages of AD and is inefficient in stopping the progression of the disease. The long-term goal of our lab is to develop new AChE inhibitors as potential anti-AD therapeutics. In this study we evaluated 25 new compounds, organized in 4 different libraries for their activity in AChE inhibition assay. As part of our drug design process, we first performed several pharmacokinetic predictions. Next, we experimentally determined the solubility, followed by AChE inhibition assay. Finally, using molecular modeling and docking studies, we evaluated the binding modes of the most active analogs. Our structure-activity relationship studies showed that coumarin is better tolerated in the AChE binding site than chromene, yielding low micromolar inhibitors. Modifications at position 3 on the chromene moiety did not significantly affect activity. Pharmacokinetic predictions suggest all new analogs can cross the blood-brain barrier, have moderate oral bioavailability and stability, and are non-toxic at therapeutic doses. The data obtained suggests that these new AChE inhibitors are good drug candidates to be further explored in preclinical studies as AD therapeutics.

Abstract citation ID: igaf122.3178

### DEVELOPMENT OF DIGITAL TWIN CLINICAL DECISION SUPPORT TOOL FOR ALZHEIMER'S PREDICTION AND PREVENTION

Alex Bangs[1], Thomas Paterson[2], Jennifer Rohrs[2], Don Breuner[2], and Cory Funk[2], *1. Fulcrum Neuroscience, Inc., Minnesota, United States, 2. Fulcrum Neuroscience, Inc., Palo Alto, California, United States*

The Fulcrum Brain Health and Neurodegeneration (BHN) model is the product of reconciling over 1,500 peer reviewed papers and accompanying data sets. Our model is coupled with a computational software platform to create a simulated population of millions of virtual individuals representing genetic and environmental variations observed in clinical and observational studies. Using a Bayesian approach, data from individuals in a clinic or trial setting are used to create digital twins. Each digital twin can simulate the predicted risk of mild cognitive impairment (MCI) and dementia with age. Additionally, we have performed preliminary assessments of a set of established interventions around lifestyle interventions, off-label drugs and supplements and how they can be personalized to extend normal cognition. Our digital twins can be further evaluated for these combination therapies, to provide a rank-ordered set of intervention combinations that are predicted to be most effective. We've developed a user interface for reviewing digital twin results, starting with a static, graphical prototype and then a running prototype able to create digital twins based on user-provided data. We refined these prototypes following interviews with gerontologists, neurologists, neuropsychologists, and primary care physicians. Interviews included discovery questions about how cognitive assessments were done, how treatments were selected, and how the clinical workflow operated, in addition to feedback on the presented prototype. Anonymized patient cases were used to create digital twins and discuss how the results could be utilized. This process resulted in a roadmap for development including integration into electronic health record systems and clinical workflows.

Abstract citation ID: igaf122.3179

### HOME-BASED PALLIATIVE CARE AND OUTCOMES AMONG PERSONS LIVING WITH DEMENTIA: A SYSTEMATIC REVIEW

Xuefan Ji, *Columbia University, New York, New York, United States*

Background: Persons living with dementia (PLWD) have high symptom burdens and significantly diminished quality of life due to the progressive nature of the disease. Palliative care is suitable for PLWD as it can provide symptom relief and comfort-based care throughout the disease trajectory. Home-based palliative care (HBPalC) is a model of care that expands palliative care access for PLWD as majority of PLWD live in their own homes. Aim: To systematically review and synthesize features of currently available HBPalC in use for PLWD and evaluate healthcare utilization and cost outcomes. Methods: PubMed, Embase and CINAHL were searched from inception until October 2024 and all U.S. based studies that included PLWD in patient samples and examined the effect of HBPalC on healthcare utilization and cost outcomes were included. Results: A total of seven studies were included, only two provided PLWD-specific HBPalC characteristics and outcomes. HBPalC interventions designed for PLWD involved addressing neurobehavioral symptoms. HBPalC was associated with fewer hospitalization, hospital length of stay, and inpatient and total costs for PLWD. Racial and ethnic PLWD were largely underrepresented in study samples. Discussion and Implications: More studies are needed to exclusively evaluate HBPalC's impact on PLWD. There are gaps in knowledge about HBPalC's impact on racial and ethnic minority PLWD healthcare utilization and cost outcomes. Future studies need to sample racial and ethnic minority PLWD intentionally and use more representative data to evaluate healthcare utilization and cost outcome data.

Abstract citation ID: igaf122.3180

### IDENTIFYING UNMET NEEDS OF DEMENTIA CAREGIVERS MANAGING DYSPHAGIA

Matthew Zuraw[1], Zayn Boustani[2], Rachel Radspinner[1], Himalaya Patel[3], Teresa Thuemling[4], Nicole Rogus-Pulia[5], and Nicole Werner[4], *1. Better Brains, Inc, San Diego, California, United States, 2. Indiana University Bloomington, Chicago, Illinois, United States, 3. Indiana University Bloomington, Bloomington, Indiana, United States, 4. Vanderbilt University Medical Center, Nashville, Tennessee, United States, 5. University of Wisconsin–Madison School of Medicine and Public Health, Madison, Wisconsin, United States*

The estimated prevalence of dysphagia (swallowing dysfunction) is as high as 85.9% for the more than 6 million Americans living with dementia. Dementia caregivers face significant challenges in managing dysphagia, contributing to suboptimal outcomes. Successfully intervention development requires identifying the unmet needs of dementia caregivers managing dysphagia. We conducted a needs assessment using a virtual contextual inquiry method with n = 19 dementia caregivers managing dysphagia to identify unmet needs. Virtual contextual inquiry occurred in three stages: 1) initial virtual interview focused on general dysphagia management tasks, barriers, and strategies; 2) seven days in which we prompted participants twice daily via text to send videos, images, or audio/text descriptions of daily dysphagia management activities and perform an evening reflection; 3) final Zoom interview Zoom to discuss additional context of the daily messages. We analyzed transcripts and messages using team-based analysis to identify categories of unmet needs. We identified 14 categories of unmet needs: the progression of swallowing challenges with dysphagia, how to transition from one stage of dysphagia to another, understanding when to use alternative hydration and nutrition, lack of confidence around negative outcomes, getting trustworthy information from others, ensuring successful mealtimes, what tools to use to support dysphagia, finding trustworthy knowledge, managing costs associated with dysphagia, ensuring quality of life for the person being cared for, getting proper support from professionals, handling emergencies, ensuring adequate nutrition, and managing specific dietary needs. Findings highlight the necessity for targeted resources and interventions to support dementia caregivers in dysphagia management to reduce their burden.

Abstract citation ID: igaf122.3181

### IMPACT OF ACO ENROLLMENT ON PRESCRIPTION DRUG COSTS FOR MEDICARE FFS BENEFICIARIES NEWLY DIAGNOSED WITH ADRD

Seyeon Jang[1], and Jie Chen[2], *1. University of Maryland School of Public Health, College Park, Maryland, United States, 2. University of Maryland College Park, College Park, Maryland, United States*

Medications are a cost-effective treatment for managing chronic conditions and reducing downstream medical costs. Accountable Care Organizations (ACOs) can enhance medication adherence through improved coordination and integration of care, promote the substitution of brand-name drugs with generics, engage pharmacists in direct patient care, and educate patients on therapeutic alternatives. This study examines the effects of ACO enrollment on Part D prescription drug costs among Medicare beneficiaries newly diagnosed with Alzheimer's Disease and Related Dementias (ADRD). We used a 2016–2022 longitudinal cohort dataset integrating the Medicare Beneficiary Summary File, MBSF Cost and Use, and Medicare Shared Savings Program ACO beneficiary-level files. The study included 111,235 beneficiaries newly diagnosed with ADRD in 2017 who remained in Medicare fee-for-service throughout the study period. Generalized estimating equation models were applied, controlling for individual- and neighborhood-level characteristics. Our findings show that prescription drug costs for newly diagnosed ADRD patients increased over time. While ACO enrollees without multiple chronic conditions (MCC) had consistently higher predicted drug costs than non-ACO counterparts, those with MCC encountered progressively growing cost savings, from $55 in 2016 to $93 in 2022, and those with multisystem morbidity (five or more chronic conditions), from $70 to $119. These findings suggest that ACO participation may help lower medication costs for patients with complex health needs while improving care coordination. Further incentives are needed to encourage ACOs to take a more proactive role in managing prescription drug use, given its significant contribution to reducing overall healthcare costs.

Abstract citation ID: igaf122.3182

### INTRODUCING AN EFFECTIVE DEMENTIA TRAINING FOR COMMUNITY HEALTH WORKERS

Kerstin Reinschmidt[1], Zahra Alhay[1], Keith Kleszynski[2], and Lee Jennings[3], *1. The University of Oklahoma Health Sciences Center, Oklahoma City, Oklahoma, United States, 2. University of Oklahoma Health Campus, Oklahoma City, Oklahoma, United States, 3. University of Oklahoma Health Sciences, Oklahoma City, Oklahoma, United States*

Community health workers (CHWs) have been identified as a public health workforce that can help remedy the national dementia crisis by supporting individuals and communities to prevent and recognize dementia. CHWs who completed workforce-appropriate trainings to address dementia are likely to have a positive impact on healthy aging and living with dementia. The Oklahoma Dementia Care Network developed the Dementia Training for Community Health Workers by combining knowledge about CHWs in Oklahoma, national recommendations on the CHW scope of work, and evidence-based dementia care strategies. Utilizing the Kirkpatrick Model, we evaluated the training effectiveness with a focus on knowledge and skill acquisition, CHW training feedback, and application of knowledge and resources shared during the training. We conducted 15 trainings with 307 trainees between June 2020 and March 2024. Data was collected on pre/post knowledge, self-efficacy, and post-training feedback. Analyzing a subsample of trainees (50%; n = 154), we found that both knowledge and some of the self-efficacy items showed statistically significant improvements. Trainees were satisfied with the training content, format, and delivery. Responding to a follow-up survey, they shared their appreciation of the practical value of the training to their jobs. Our training has practical implications for workforce development as it prepares CHWs to address dementia within their broad scope of work. The training can be adapted by different communities using culturally relevant narratives that reflect unique values and experiences.

Abstract citation ID: igaf122.3183

### KETONES AND COGNITION: INSIGHTS FROM A REVERSE-TRANSLATION APPROACH IN ALZHEIMER DISEASE MICE

Karl Fernandes[1], and Paule M'Bra[2], *1. Université de Sherbrooke, Sherbrooke, Quebec, Canada, 2. Université de Montréal, Montreal, Quebec, Canada*

Lifestyle factors are estimated to account for 40% of the risk of developing dementia. Despite this, trials of lifestyle-based interventions, including ketogenic dietary interventions, have shown mixed results in delaying dementia and evidence of both responder- and non-responder participants. In order to understand and eventually optimize ketogenic interventions for dementia, we are using a "reverse-translation" approach in which ketogenic strategies that show promise in humans are modeled in transgenic mouse models of Alzheimer's disease (AD) in order to more clearly understand their mechanisms of action. Behavioral, anatomical and transcriptomic analyses in AD mouse models confirmed that two distinct ketogenic interventions (dietary enrichment with medium chain triglycerides and a high fat/low carb diet) both improved hippocampal-dependent learning and memory and modulated hippocampal neuronal structure and gene expression. Unexpectedly, despite their similar effects on brain function, these two ketogenic interventions showed markedly different effects on circulating ketone levels, suggesting underlying mechanisms that are independent of ketones. Indeed, metabolic and RNA sequencing experiments identified striking, diet-specific effects on multiple peripheral pathophysiological features of AD, including on glucose homeostasis, liver structure-function and the gut microbiome. These findings have important implications for the design of ketogenic and combinatorial lifestyle-based strategies for dementia.

Abstract citation ID: igaf122.3184

### LATENT VARIABLES IN COGNITIVE DECLINE: CAREGIVER BURDEN AND BEHAVIORAL AND PSYCHOLOGICAL SYMPTOMS OF DEMENTIA

Lauren Chrzanowski, Landon Peeples, and Benjamin Mast, *University of Louisville, Louisville, Kentucky, United States*

Latent variable modeling has been used to model dementia by examining the covariance between cognitive decline (g) and daily function (f – decline in ability to perform instrumental activities of daily living) as a common factor (gf). The current study uses confirmatory factor analyses (CFA) to replicate this model and expand it to include behavioral and psychological symptoms (p) and family caregiver burden (b) as a second common factor (pb). Participant data (N = 915; older adults without cognitive impairment, with mild cognitive impairment, or with Alzheimer's disease) was obtained from the Alzheimer's Disease Neuroimaging Initiative (ADNI) wave 4. To replicate the gf factor, scores from the Montreal Cognitive Assessment (MoCA), logical memory, delayed recall, and Functional Activities Questionnaire (FAQ) were fit in the first model. CFA results supported a 4-variable structure of the neurological factor with acceptable global fit. The second model included the pb factor comprised of Neuropsychiatric Inventory (NPI) subscales (caregiver burden/distress, behavioral and psychological symptoms of dementia (BPSD) frequency, and BPSD severity) and Geriatric Depression Scale scores. The global fit of this second model was excellent (CFI = .998, RMSEA = 0.054). The two-factor model had better fit than a single factor model (CFI = .609, RMSEA = .368), suggesting gf and pb are distinct factors reflecting two aspects of dementia: cognitive/functional decline and behavior change/caregiver burden. These results replicate prior literature and highlight a latent interpersonal and distress variable, which may be experienced by individuals and families impacted by dementia.

Abstract citation ID: igaf122.3185

### LIVING ALONE WITH COGNITIVE IMPAIRMENT: A QUALITATIVE SYNTHESIS

Violet Chernoff, and Elyse Couch, *Brown University, Providence, Rhode Island, United States*

Older adults with cognitive impairment, and particularly those with dementia, often rely on informal caregivers like friends or family members. However, the number of dementia patients with limited social or familial support is growing. Research suggests that living alone with cognitive impairment could lead to poorer health and wellbeing outcomes. But, the specific needs of this population are less understood. This qualitative review seeks to summarize the experiences of people living alone with cognitive impairment. We searched PubMed, MEDLINE, PsycINFO, CINAHL, and SocIndex in February 2024. Articles were screened for inclusion by two reviewers. Data from the included studies were synthesized using thematic synthesis. Fifteen studies are included in this review. We identified four themes: (1) coping with uncertainty, (2) loss of connection, (3) decreasing mobility, and (4) a changing sense of self. Participants described the distress, relief, and selective ignoring of receiving an irreversible diagnosis. They reported persistent feelings of loneliness, mainly pertaining to boredom, loss of loved ones, or physical isolation. Perspectives on the future varied, but most participants recognized their growing care needs and dreaded the inevitable move to a nursing home. People with cognitive impairment living alone experience significant isolation, loneliness, and a loss of identity as their condition deteriorates. The lack of in-home support systems exacerbates these challenges, leaving individuals vulnerable to physical harm and inadequate treatment. Further research and health policy should focus on providing social support and individualized care.

Abstract citation ID: igaf122.3186

### MEALTIME BEHAVIORS IN DEMENTIA: A THEORY-BASED UNDERSTANDING

Zih-Ling Wang[1], Hilaire Thompson[2], Oleg Zaslavsky[2], and Basia Belza[2], *1. University of Washington School of Nursing, Seattle, Washington, United States, 2. University of Washington, Seattle, Washington, United States*

Many people living with dementia (PLWD) struggle with mealtimes, and these mealtime behaviors become more difficult as the disease progresses. As their condition advances, they gradually transition from eating independently to needing support from care partners. These mealtime behaviors are influenced by a complex interplay of physical and cognitive deterioration, behavioral and psychological symptoms, social dynamics, and environmental and cultural factors. The purpose of this paper is to systematically examine and compare three theoretical frameworks: (1) the Person-Environment-Occupation (PEO) Model, (2) Environmental Gerontology, and (3) the Social-Ecological Model (SEM), which enhances our understanding of these mealtime behaviors in PLWD. The PEO Model highlights the dynamic interaction between the person's capacities, the environment, and the occupation of eating, identifying barriers and facilitators to mealtime performance. Environmental Gerontology underscores the critical role of the physical and social environment, advocating for modifications to better suit the needs and preferences of PLWD and promote well-being. The SEM provides a multilevel perspective, considering intrapersonal, interpersonal, environmental, and policy-related factors that collectively influence mealtime behaviors in the community. By examining theoretical frameworks and conceptual models, this paper aims to provide a theoretical foundation for understanding the factors influencing mealtime behaviors in PLWD. This analysis contributes to the development of strategies that enhance mealtime enjoyment, satisfaction, and nutritional well-being, thereby supporting the maintenance of eating independence across different stages of dementia.

Abstract citation ID: igaf122.3187

### PERSON-ENVIRONMENT-OCCUPATION MODEL OF MEALTIME PERFORMANCE IN OLDER PEOPLE WITH DEMENTIA: THEORY ANALYSIS

Zih-Ling Wang[1], and Basia Belza[2], *1. University of Washington School of Nursing, Seattle, Washington, United States, 2. University of Washington, Seattle, Washington, United States*

Mealtime challenges are prevalent among people living with dementia (PLWD) and significantly affect their quality of life and nutritional status. Mealtime performance differs from concepts like "eating behaviors" or "feeding difficulties" by focusing on the holistic functional capacity rather than isolated challenges. This paper presents a theory analysis of the Person-Environment-Occupation (PEO) Model as applied to mealtime performance in older people with dementia, using Walker and Avant's systematic approach. The PEO Model provides a holistic framework for understanding the dynamic interactions between individuals, their environments, and their occupations. The analysis examines the model's origins, meaning, logical adequacy, usefulness, generalizability, parsimony, and testability in the context of mealtime performance for PLWD. Findings indicate that the PEO Model effectively conceptualizes how personal factors, environmental factors, and occupational factors interact to influence mealtime performance. The transactions between person-environment, environment-occupation, and person-occupation provide valuable insights into barriers and facilitators of successful mealtime engagement. While the PEO model has been extensively used as a framework in occupational therapy practice, it has yet to be fully applied within nursing science, particularly in dementia care. This model has potential applications in long-term care settings, where environmental modifications and personalized approaches can enhance mealtime participation. This analysis contributes to nursing science by providing a theoretical foundation for developing person-centered interventions for PLWD across different care settings.

Abstract citation ID: igaf122.3188

### PROTOTYPE FEATURES FOR A SLEEP-ENHANCING MUSIC APP IN OLDER ADULTS LIVING WITH DEMENTIA

Osborn Owusu Ansah[1], Justine Sefcik[2], Nancy Hodgson[3], Bei Wu[4], and Darina Petrovsky[1], *1. Duke University, Durham, North Carolina, United States, 2. Drexel University College of Nursing and Health Professions, Philadelphia, Pennsylvania, United States, 3. University of Pennsylvania, Philadelphia, Pennsylvania, United States, 4. NYU Shanghai, Shanghai, China (People's Republic)*

Music interventions show promise for addressing insomnia in persons living with dementia (PLWD), as musical memory often remains intact despite cognitive decline and can be personalized to individual preferences. The study aimed to identify the prototype features of the mobile application, "Calming Music Personalized for Sleep Enhancement in PeRsons living with Dementia" (CoMPoSER) for PLWD and their caregivers. We conducted two rounds of interviews in a collaborative design process. In round one, we held 13 virtual interviews (N = 20), including 6 PLWD and 14 caregivers (PLWD average age 80 years old, SD = 12.1; caregiver average age 61, SD = 16.1). Seven interviews were conducted with both PLWD and their caregivers, and six were conducted with caregivers only. Most were women (n = 14) and 12 out of 20 (60%) identified themselves as White/Caucasian. In round two, we conducted 7 virtual interviews with 10 participants returning from the first round (2 PLWD and 8 caregivers) using the mHealth Usability framework and directed content analysis. We asked participants to select their preferred features and layout options for the CoMPoSER application. When presented with five visual design options, participants recommended labeled symbols, simple layouts, and tutorial options for those with memory problems. Most preferred darker blue color schemes to promote sleep and limited customization options. These insights directly informed the mobile application design, which will be presented.

Abstract citation ID: igaf122.3189

### RELATIONSHIP BETWEEN PEER CONTACT AND QUALITY OF LIFE IN DEMENTIA CAREGIVERS

Hailey Chatterton, Aimee Mooney, Heather Franklin, and Allison Lindauer, *Oregon Health & Science University, Portland, Oregon, United States*

Recent reviews of multi-component dementia caregiving interventions have indicated gaps in mechanism-driven social connection facets. Furthermore, relationships between peer support and quality of life among caregivers are not fully understood. Using data from a family caregiver psychoeducational intervention, Tele-STELLA (Support via Technology: Living and Learning with Advancing Dementia, NIA R01AG067546), we analyzed relationships between peer contact and caregivers' quality of life (QoL), and between perceived contact helpfulness and likelihood of future contact. A merged dataset combined QoL assessments with self-reported peer contact data, totaling 423 observations from 153 participants. During intervention (n = 151) and follow-up (n = 142), participants reported past-month peer contact and future contact intentions. Generalized linear models assessed associations between helpfulness ratings and peer contact. QoL changes were analyzed using descriptive statistics and Cohen's d for peer contact groupwise comparisons. Higher helpfulness ratings at baseline were associated with greater peer contact at follow-up (OR = 1.68, p = 0.03) and stronger intentions for future contact (OR = 5.49, p = 0.02). Among participants with "Good or Excellent" baseline QoL, peer contact had a small negative effect on QoL (d = -0.10); those with "Poor or Fair" QoL showed a moderate positive effect (d = 0.43). These values suggest that while peer contact had minimal association with QoL change for participants starting with higher QoL, it was moderately beneficial for those with lower baseline QoL. Additional research is needed to develop mechanism-informed interventions that promote social connection and improve QoL for dementia caregivers.

Abstract citation ID: igaf122.3190

### SEX DIFFERENCES IN LIFE EXPECTANCY IN DEMENTIA, MILD COGNITIVE IMPAIRMENT, AND SUBJECTIVE COGNITIVE DECLINE

Rachel Amland[1], and Bjorn Heine Strand[2], *1. University of Oslo, Oslo, Oslo, Norway, 2. Norwegian Institute of Public Health, Oslo, Oslo, Norway*

Introduction: It is unclear how dementia affects loss in life expectancy (LE). In this registry-based study, we aimed to study sex differences in LE and loss in LE in dementia, mild cognitive impairment (MCI), and subjective cognitive decline (SCD). Methods: 16,358 patients diagnosed with dementia, MCI or SCD from the Norwegian registry of persons assessed for cognitive symptoms (NorCog) during 2009-2022 were included and followed up for mortality. Sex differences in LE, and loss in LE, were predicted using flexible parametric survival models and sex specific mortality in the general population as reference. Results: Women with dementia had the largest loss in LE; 17 years loss at 60 years, correspondingly men lost 13.5 years. Similar patterns were observed for MCI and dementia subtypes. Discussion: Women with dementia or MCI had larger loss in LE compared to men with these diagnoses.

Abstract citation ID: igaf122.3191

**THE INTERPLAY BETWEEN COMT AND MIR146A SUPPORTS THE ROLE OF IMMUNITY IN ALZHEIMER'S DISEASE**

Anatoliy Yashin, Deqing Wu, Konstantin Arbeev, Olivia Bagley, Hongzhe Duan, Rachel Holmes, and Svetlana Ukraintseva, *Duke University, Durham, North Carolina, United States*

Accumulating evidence points to a major role of compromised immunity in Alzheimer's Disease (AD). The MIR146a and COMT genes are known for their involvement in both immune responses and AD. COMT also regulates the metabolism of catecholamines, which are linked to inflammation and trained immunity. We hypothesize that the interplay between MIR146a and COMT genes influences the risk of AD. To test this hypothesis, the logistic regression model with the interaction term has been applied to the analysis of the Cardiovascular Health Study (CHS) data on males and females combined. We used the binary AD trait with cases (coded as 1) including participants who survived age 85 and did not have AD before or after this age (N = 1,421) and controls (coded as 0) including participants who acquired AD at any age (N = 257). We found that the interaction between SNPs rs2910163 in MIR146a and rs4818 in COMT is significantly associated with AD ($\beta$=-0.48, p = 1.62E-02, Bonferroni=1.67E-02). This result for the same SNPs was confirmed in the Framingham Heart Study (FHS) data analysis, with case and control groups including 903 and 354 participants, respectively ($\beta$=-0.41, p = 3.63E-02). The SNP rs2910163 is in high linkage disequilibrium (LD) with rs2910164, D'=1, R2=0.77. In other studies, SNPs rs4818 and rs2910164 (studied separately) have been associated with multiple health-related traits. The results of this study support the role of immunity in AD and emphasize the importance of studying the roles GxG interactions in AD.

Abstract citation ID: igaf122.3192

**TRENDS AND HEALTHCARE PROFILES OF OLDER ASIAN AMERICAN ADULTS WITH ADRD IN U.S. NURSING HOMES**

Yiyang Yuan[1], Bei Wu[2], and Kate Lapane[1], *1. University of Massachusetts Chan Medical School, Worcester, Massachusetts, United States, 2. NYU Shanghai, China (People's Republic)*

The growing population of older Asian American adults with ADRD, increasing demand for nursing homes, and prevalent preference for non-English languages, which may contribute to social isolation, underscore the need to examine trends and healthcare profiles of non-Hispanic Asian adults with ADRD in U.S. nursing homes. Using Minimum Data Set 3.0, we identified 55,693 non-Hispanic Asian older adults newly admitted for skilled nursing facility (SNF) care and 59,063 for non-SNF care. We report key findings for SNF residents, as results were largely consistent. From 2011 to 2022, the proportion of older non-Hispanic Asian residents with ADRD doubled from 1.2% to 2.4%, with the highest annual growth rate (0.09%, p < 0.0001) among all racial/ethnic groups. About 40% required an interpreter. The top preferred non-English languages were Chinese (17.4%), Korean (12.8%), and Vietnamese (8.1%). Average ages ranged from 82.8 (SD: 7.9, Vietnamese-preferred) to 85.4 years (SD: 7.4, Chinese-preferred). Compared to non-Hispanic White, English-preferred residents, non-Hispanic Asian residents had higher rates of admission from acute hospitals, higher prevalence of severe cognitive impairment, frailty, hypertension, diabetes, and stroke, and higher CHESS and mortality risk scores. Notable within-group variations included frailty proportions from 66.9% (Korean-preferred) to 81.4% (Chinese-preferred), severe cognitive impairment from 12.5% (English-preferred) to 19.3% (Vietnamese-preferred), and average mortality risk scores from 5.2 (SD: 2.6, Korean-preferred) to 6.2 (SD: 3.0, Chinese-preferred). The growing presence and diverse healthcare needs of non-Hispanic Asian residents with ADRD emphasize the necessity of understanding health outcomes by specific subgroups and culturally and linguistically tailored care to address specific challenges.

### SESSION 7420 (POSTER)

### ASSISTED LIVING (POSTERS)

Abstract citation ID: igaf122.3193

**"WHO'S CALLING?" CHALLENGES AND STRATEGIES FOR RECRUITMENT OF ASSISTED LIVING COMMUNITIES IN RESEARCH STUDIES**

Debra Dobbs[1], Jonathan Clapp[1], Hongdao Meng[1], Lindsay Peterson[1], Layla Katharine Santana[1], and William Haley[2], *1. University of South Florida, Tampa, Florida, United States, 2. University of South Florida, University of South Florida, Florida, United States*

The Assisted Living and Residential Care (AL/RC) industry is a complex, state regulatory model of care has been of keen interest to researchers for the last 25 years. Of particular note is the fact there has been a cadre of researchers in the U.S. that have been part of the Center for Excellence in Assisted Living (CEAL, now CEAL@UNC) since the early 2000s invested in addressing the care quality of residents who receive and staff who provide care. Because AL/RC communities vary by state in their regulatory structures and policies, and there is frequent turnover of ownership and administrative staff, recruitment of AL/RCs can be challenging for researchers. This study will detail AL/RC recruitment strategies for a National Institute on Aging funded clinical trial to implement the Palliative Care Education in Assisted Living for Dementia Care Providers (PCEAL-DCP). Effective strategies for recruitment have included: 1) developing a community/university partnership with key current and previous AL owners and operators who can assist with outreach to their networks; 2) building a consortium of AL communities from prior study participation; 3) direct in-person and social media inquiries versus costly and timely traditional recruitment of mail and telephone calls; 4) making the business case for the value of participation in research to corporate AL chains; and 5) coordinating with hospice community partners who provide continuing education incentives and existing relationships with AL community providers. Study design in terms of budgetary and timeline issues to consider in recruitment of ALs will also be discussed.

Abstract citation ID: igaf122.3194
### DIRECT CARE WORKERS RETENTION AND RECRUITMENT TRENDS IN OHIO RESIDENTIAL CARE FACILITIES PRE-POST COVID-19
Oksana Dikhtyar, and Ian Matthew Nelson, *Miami University, Oxford, Ohio, United States*

Retention and recruitment of direct care workers (DCWs) in nursing homes and residential care facilities (RCFs) has been a challenge even before the COVID-19 pandemic, but became even more pronounced during and after. While there are various public data sources about nursing homes, the information about RCFs, most of which are assisted living facilities, is not collected. In Ohio, the Biennial Survey of Long-Term Care Facilities mandated by the state provides comprehensive data on RCFs. Using three waves of the Ohio Biennial Survey of Long-Term Care Facilities, we examined changes in retention and turnover of DCWs, facilities' perceptions of the seriousness of the problem, recruitment approaches, and workplace environment and financial strategies for retention prior to, during, and post COVID-19 pandemic. The average retention rate of DCWs has increased from 64.7% in 2017 to 68.5% in 2023, but the turnover rate stayed the same at 78.3%. Four in ten facilities ranked DCW retention and recruitment as serious problem across all three waves. However, the proportion of facilities that had an insufficient number of qualified applicants fell by 10% during each wave and was 41.4% post COVID. There have been across-the-board increases in the proportion of facilities that implement a variety of workplace environment and financial strategies to retain workers. In terms of recruitment, almost twice as many facilities provided same day pay in 2023 compared to 2021 and 40% stopped requiring drug testing post COVID compared to 17.9% during COVID. Implications for policy and practice are discussed.

Abstract citation ID: igaf122.3195
### FACTORS INFLUENCING RENOVATION DECISIONS BY CARE FACILITY OPERATORS: AN INVESTIGATIVE STUDY IN AUSTRALIA
Si Chen, *Bond University, Southport, Queensland, Australia*

A supportive built environment is essential in residential care facilities for individuals with long-term care needs, both physically and emotionally. However, upgrading existing facilities presents various challenges. Before bridging the gap between theoretical and practical needs in supportive built environment adaptation, it is crucial to first understand the reasons behind renovation decisions from the perspective of care facility operators, a topic that has received limited attention in the past. This study aims to identify the key drivers of renovation decisions and explore the interrelationships between supportive design, renovation project management, and the decision-making process. Interviews were conducted with professionals from three major stakeholder groups: designers, project managers and consultants, and care facility operators. Participants were selected based on their involvement in past or current renovation projects in residential care facilities in Australia. Inductive analysis of interview transcripts identified three main themes: (1) information to guide renovation decisions, (2) decision-making efficiency, and (3) information mismatch. The study found three primary drivers of renovation decisions: budget, regulatory compliance, and operational and workflow efficiency. While some findings did not directly address the research questions, they provide valuable insights into the challenges and decision-making processes in residential care facility renovations. This research contributes to the development of practical tools and frameworks to enhance decision-making and project execution. Additionally, it offers insights from a broader group of stakeholders, informing more effective and strategic renovation processes in residential care facilities.

Abstract citation ID: igaf122.3196
### RESIDENTS' PERCEPTIONS OF LIFE IN ASSISTED LIVING FACILITIES
Katie Trainum[1], Heather Becker[2], Stephanie Morgan[2], and Alexa Stuifbergen[2], *1. University of Pennsylvania, Philadelphia, Pennsylvania, United States, 2. The University of Texas at Austin, Austin, Texas, United States*

Assisted living facilities (ALFs) account for half of all long-term care beds in the United States. Although focused on providing supportive and responsive personal care assistance, few studies have assessed residents' perceptions of their ALFs, particularly as related to facility characteristics. In this study, 364 ALF residents living in 364 ALFs throughout a large southwestern state were interviewed about their perceptions of their ALFs using a closed-ended interview schedule. Their ALFs had an average of 42 beds (ranging from 5 to 177 beds) and occupancy rates from 10% to 100%. The average age of interviewed residents was 84.9 years (SD = 14.2), and most were non-Hispanic White women. Residents' ratings of the availability of certain factors in their ALF were compared with ALF characteristics, such as occupied beds and on-site medical/nursing support. Most residents were satisfied with their ALF and reported they frequently had privacy, access to favorite foods, felt safe, and were respected by staff. Although most ratings had negligible correlations with facility characteristics, they were significantly correlated with concerns about staffing levels ($r = -.23$) and fear of retaliation ($r = -.14$). ALFs must address the delicate balance between resident acuity, safety, and autonomy. This balance becomes even more precarious as the medical complexity of ALF residents increases. Therefore, quality of life measures must take into account all these factors to provide a comprehensive understanding of residents' perceptions of their ALF experience. Future studies should use well-developed measures to assess residents' perceptions in diverse ALF settings.

Abstract citation ID: igaf122.3197
### USING THE LOW-INCOME HOUSING TAX CREDIT TO FUND ASSISTED LIVING: A MIXED-METHODS STUDY
Jordan Kaplan[1], Rebecca Smith[2], Taylor Craig[2], Paula Carder[3], Janiece Taylor[2], Gauri Gadkari[4], Craig Pollack[2], and Kali Thomas[2], *1. Johns Hopkins University School of Medicine, Baltimore, Maryland, United States, 2. Johns Hopkins University, Baltimore, Maryland, United States, 3. Portland State University, Portland, Oregon, United States, 4. Brown University, Providence, Rhode Island, United States*

In the United States, over 31,000 assisted living communities (ALs) serve over 950,000 Americans, providing them with personal care and assistance. At an average monthly cost of $4,500, ALs are often out of reach for middle and low-income older adults. We identified ALs serving low-income populations by focusing on the Low-Income Housing Tax Credit (LIHTC), the country's largest funder of affordable units. We matched an inventory of ALs to HUD's database of LIHTC developments. To identify barriers and opportunities, we conducted semi-structured interviews with 25 stakeholders in roles related to affordable AL development. We identified 197 LIHTC-funded

ALs concentrated in a handful of states including Massachusetts (n = 31), Indiana (n = 26), Iowa (n = 13), Colorado (n = 12), and Maine (n = 12). Thirteen states lacked any LIHTC-ALs. LIHTC-funded ALs were more likely to be in a medically underserved areas compared to other ALs (12% vs 5.8%, $p < 0.001$). Themes identified from interviews include: (1) LIHTC can be paired with other funding streams to build affordable AL in the right regulatory environment; (2) sufficient Medicaid reimbursement rates are critical to a developer's success; and (3) affordable AL development often requires both LIHTC and Medicaid funds, and the complexity of pairing the two often stymies development. In summary, we conducted a novel data match and found LIHTC has been used to fund affordable AL in 37 states. Themes identified from qualitative analysis underscore the extent to which state-level factors affect the viability of LIHTC-AL development.

Abstract citation ID: igaf122.3198

### ANALYZING FALL INCIDENTS AND FALL-RELATED INJURIES IN WISCONSIN ASSISTED LIVING COMMUNITIES

Sohee Kim[1], and James Ford[2], *1. University of Wisconsin - Madison, Madison, Wisconsin, United States, 2. University of Wisconsin–Madison, Madison, Wisconsin, United States*

This study examines trends in fall incidence and fall-related injuries in Wisconsin assisted living communities (ALCs) from 2017 to 2022. It aims to identify differences in fall trends among three ALC types—Adult Family Homes (AFHs), Community-Based Residential Facilities (CBRFs), and Residential Care Apartment Complexes (RCACs)—and to propose targeted interventions for reducing falls and enhancing resident safety. The study analyzes data from the Wisconsin Coalition for Collaborative Excellence in Assisted Living (WCCEAL), incorporating fall reports from ALCs across the state. The dataset includes quarterly records from 2017 to 2022, covering facilities that voluntarily tracked and shared fall and hospitalization data. Falls and fall-related injury data were evaluated using incidence rates per 1,000 resident days, stratified by facility type. Facility characteristics, including occupancy, primary resident population, and location, were analyzed to identify patterns in falls. The study used chi-square tests to compare sample and non-sample cohorts. Overall, the total number of falls in ALCs increased during the study period, whereas fall-related injuries decreased. CBRFs and RCACs showed substantial increases in fall incidence rates, while AFHs showed a declining trend. Findings emphasize the need for customized fall prevention interventions across different ALC types. AFHs may benefit from individualized interventions, CBRFs from staff training and structured fall protocols, and RCACs from self-management strategies like emergency response systems. Standardizing fall reporting practices and enhancing fall prevention policies at both the state and facility levels could improve resident safety and reduce fall-related injuries.

### SESSION 7425 (POSTER)

### CANCER

Abstract citation ID: igaf122.3199

### ADVERSE EVENTS IN SYMPTOM MANAGEMENT INTERVENTIONS USING IMMERSIVE VIRTUAL REALITY IN OLDER ADULTS WITH CANCER

Kailei Yan, and Victoria Loerzel, *University of Central Florida, Orlando, Florida, United States*

Emerging evidence shows that Virtual reality (VR) based interventions can improve motivation and engagement in health-promoting behaviors, offering a promising approach for cancer patients who struggle with symptom burden during and after treatment. While VR-based interventions show therapeutic potential, its effectiveness cannot be fully validated without a critical evaluation of its potential risks. The goal of this report is to describe the adverse events (AE's) reported from our systematic review of randomized controlled trials (RCTs) of symptom management interventions using VR among cancer populations, especially older adults. Among the 29 studies reviewed, 2 studies included adults over age 65. Of these, 11 did not present information on AE's and 5 stated that no AE's were observed. In the remaining studies, difficulty concentrating, mild nausea, tired or aching eyes, moderate fatigue, drowsiness, dizziness and vomiting were reported. Of the 2 studies with older adults, one reported similar AE's as above, the other recorded no AE. One study found that pre-existing symptoms before VR interventions were significantly associated with post-VR AE's. Validated instruments were used to assess AE's in only 5 studies. One study used semi-structured interviews. Overall, this review identified substantial gaps in the reporting of adverse events, especially among older adults. This emphasizes the need for more rigorous documentation and evaluation of the risks associated with VR interventions. Because, current clinical trial reporting primarily focuses on severe AE's, such as impairment on overall health, a more VR specific approach of evaluation of AE's, including vestibular and psychological impacts, is needed.

Abstract citation ID: igaf122.3200

### CHANGING BELIEFS IN MANAGING NAUSEA AMONG OLDER ADULTS WITH CANCER FROM A LONGITUDINAL RANDOMIZED CONTROLLED TRIAL

Kailei Yan[1], Arsham Alamian[2], Michael Owings[1], and Victoria Loerzel[1], *1. University of Central Florida, Orlando, Florida, United States, 2. University of Miami, Coral Gables, Florida, United States*

Chemotherapy-Induced Nausea and Vomiting (CINV) is one of the most feared side effects of chemotherapy, with more severe consequences in older adults, including dehydration, electrolyte imbalance [1], frailty [2], and poor adherence to treatments [3]. A perception gap exists regarding CINV management, with only 38% of patients fully complying with treatment, while clinicians underestimate CINV's impact on patients' daily lives [4]. Understanding beliefs in managing nausea is crucial to improving symptom management among older adults with cancer. This analysis examines the beliefs in managing nausea among older adults enrolled in a longitudinal, multicenter randomized controlled trial (RCT), evaluating Manage At Home (MAH), a novel serious game designed to help older adults self-manage CINV [5, 6]. The sample included in this analysis were 177 participants who completed baseline assessment, and 81 participants completed both baseline and the final assessment. Confirmatory factor analyses (CFA) were conducted to evaluate the construct validity of Symptom Representation Questionnaire (SRQ) [7], adapted to assess beliefs for both time 1 and final assessment. Results from CFA supported a four-factor structure—emotion, cure, time, and cause—at both baseline and final assessments, consistent with original instrument development [7]. However, Item 14 loaded onto multiple factors, suggesting potential ambiguity in wording. Linear mixed models indicated significant within-group improvements over time for all factors ($p < .001$), but no significant between-group differences over time, likely due to limited statistical power. MAH shows promise in improving beliefs related to self-management.

Abstract citation ID: igaf122.3201

**COPING EXPERIENCES OF MIDDLE-AGED FEMALE SURVIVORS OF SEX-SPECIFIC CANCER**

Sunui Shin, Hyun-E Yeom, Misook Jung, and Eunyoung Park, *Chungnam National University, Daejeon, Republic of Korea*

Women diagnosed with sex-specific cancers encounter multifaceted physical and psychological challenges throughout survivorship, particularly concerning their perception of femininity. Alongside aging, middle-aged female survivors must navigate role transitions as mothers and shifts in spousal relationships, leading to broader family and social changes. This study explored their coping experiences, focusing on identity and role transitions. A phenomenological qualitative approach was employed, using purposive sampling to recruit 10 female cancer survivors. The participants had a mean age of 49.1 years, including four diagnosed with breast cancer, five with ovarian cancer, and one with uterine cancer. Data were collected through semi-structured, in-depth interviews and analyzed using Colaizzi's phenomenological method. Four key themes emerged: (1) Threatened Femininity – participants experienced a disrupted sense of femininity due to hair loss, breast changes, and ovarian removal; (2) Concerns and Guilt for Their Children – anxiety about leaving their children behind and guilt over the hereditary nature of cancer; (3) Changes in Marital Relationships – physical changes challenged marital life, yet spousal care and emotional support strengthened relationships; and (4) Shifts in Life Perspectives – despite emotional distress, participants developed a greater appreciation for daily life, leading to renewed purpose. These findings highlight the need to understand the transitions experienced by middle-aged female cancer survivors. They emphasize the importance of a comprehensive approach that considers physical, identity, relational, and life changes to support survivors better.: : Sex-Specific Cancer Survivors, Identity Change, Qualitative research

Abstract citation ID: igaf122.3202

**FATIGUE IN WOMEN WITH GYNECOLOGIC CANCER COMPARED TO PATIENTS WITH OTHER CANCERS RECEIVING CHEMOTHERAPY**

David Asakitogum[1], Arsham Alamian[2], and Victoria Loerzel[1], *1. University of Central Florida, Orlando, Florida, United States, 2. University of Miami, Coral Gables, Florida, United States*

Cancer-related fatigue is reported as the most worrisome symptom in people with cancer. Evidence suggests that chemotherapy is associated with higher levels of fatigue and fatigue variability exists in patients with different cancers. However, there is limited research that compare fatigue levels in patients with gynecologic cancer and other cancers. Therefore, this study aimed to: evaluate and compare fatigue levels in patients with gynecologic, breast, colo-rectal, head and neck cancers; and evaluate demographic and experiential characteristics associated with fatigue severity in these cancer types. Older outpatients (≥50 years) with cancer (n = 99) were recruited before initiation of chemotherapy. Patients completed the Symptom Representation Questionnaire and the EORTC-Core 30 over four cycles of chemotherapy. Parametric and non-parametric tests were used to evaluate differences within and between groups. Logistic regression was used to identify risk factors associated with the severity of fatigue. Patients with gynecologic cancer reported higher mean fatigue scores at Baseline and at Time-4 [3.18(2.63) and 4.81(3.03), respectively] compared to other cancers. Patients with breast cancer reported the highest increased of mean fatigue score from Baseline to Time-4 [2.25(2.54) Vs 4.33(3.38)] (i.e., 94.44%). Patients with gynecologic and breast cancer were more likely to report moderate or severe fatigue at baseline and Time-4 compared to other cancers [(OR = 2.68, 95% CI: 0.873 – 8.206, p = 0.085); OR = 4.90, 95% CI: 0.671 – 35.819, p = 0.117] respectively. Patients with gynecologic cancer experienced higher levels of fatigue compared to other patients with cancer. Clinicians can use this information to identify high risk patients for early fatigue interventions.

Abstract citation ID: igaf122.3203

**GERIATRIC ASSESSMENT IN PATIENTS AGED 70 AND OVER CONSIDERED FOR CAR-T THERAPY: ADESCRIPTIVE STUDY**

Rachel Boisvert, *Université Laval, Quebec, Quebec, Canada*

Geriatric oncology addresses the challenges of treating elderly cancer patients, who are often undertreated due to complex care needs. CAR-T therapy, effective for some hematologic cancers, presents significant side effects that complicate its use in patients over 70. Comprehensive geriatric assessment may help guide treatment decisions in this population. This study aims to describe the profile of elderly patients referred for potential CAR-T therapy, assess the impact of the geriatric evaluation on treatment decisions, examine the occurrence of ICANS and cytokine release syndrome in relation to frailty, and monitor clinical outcomes such as hospitalizations and mortality. This retrospective study will include patients aged 70 and over referred to the Quebec Geriatric Clinic for pre-treatment assessment before May 2025. Data will be collected from medical records, including patient profiles, initial treatment plans, recommendations from geriatric oncology, final therapeutic plans, and clinical outcomes. No complex statistical analysis will be performed due to the limited sample size (approximately 30 patients). Results will be presented descriptively. We expect that the geriatric evaluation will lead to modifications in treatment plans and identify risk factors for severe adverse effects, helping to refine eligibility criteria for CAR-T therapy in elderly patients. The findings could enhance clinical practices by promoting personalized care in geriatric oncology. This study seeks to demonstrate the importance of integrating a complete geriatric evaluation into treatment planning for elderly cancer patients, optimizing care and decision-making for CAR-T therapy.

Abstract citation ID: igaf122.3204

**IMPACT OF FINANCIAL BURDEN ON QUALITY OF LIFE OF CAREGIVERS FOR CANCER: THE MODERATING ROLE OF EMPLOYMENT STATUS**

Khunza Asma, *University of South Florida, Tampa, Florida, United States*

Caregiving for cancer patients imposes substantial financial burdens on caregivers, which can adversely affect their quality of life. This study investigates the relationship between the financial burden of caregiving for cancer patients and the quality of life of caregivers, with a focus on the moderating role of employment status. Using data from the HRS 2020 dataset, we conducted a moderation analysis to explore how employment status influences this relationship. The independent variables in this study are income and wealth, representing the financial burden, while the dependent variable is the quality of life, measured by life satisfaction. Employment status serves as the moderating variable. Our findings reveal that financial burden significantly impacts the quality of life of caregivers. Caregivers with higher financial burdens report lower quality of life. However, employment status plays a crucial moderating role in this relationship. Employed caregivers report higher quality of life compared to their unemployed counterparts, even when experiencing similar levels of financial burden. This suggests that employment provides not only financial resources but also social support and a sense of purpose, which can mitigate the negative effects of financial strain. The results underscore the importance of policies that support employment opportunities for caregivers and provide financial assistance to those who are unemployed. By addressing the financial challenges faced by caregivers, we can improve their overall well-being and ability to provide care. These findings have significant implications for policymakers, healthcare providers, and support organizations aiming to enhance the well-being of caregivers.

Abstract citation ID: igaf122.3205

### MEDIATING ROLE OF CMV INFECTION AND GRIMAGE IN THE ASSOCIATION OF EDUCATION WITH HEALTH OUTCOMES IN CANCER SURVIVORS

Shuo Wang, Weihua Guan, Heather Nelson, Bharat Thyagarajan, Susan Everson-Rose, Anne Blaes, and Anna Prizment, *University of Minnesota, Minneapolis, Minnesota, United States*

Cancer survivors with lower education experience a disproportionate burden of health outcomes. These health disparities may be partially explained by cytomegalovirus (CMV) infection and aging biomarkers. In the Health and Retirement Study (HRS) – a nationally representative cohort of older adults, we examined whether CMV infection and GrimAge, an epigenetic clock, explained the associations of education (< high school vs. ≥high school) with comorbidity index and muscle weakness. HRS measured CMV Immunoglobulin G antibody levels in blood samples from 1,337 cancer survivors in 2016 (mean age=72 years, 54% female, 16% Non-white). A comorbidity index was constructed using five conditions (hypertension, lung disease, cardiac disorders, stroke, diabetes), and muscle weakness was assessed using grip strength in the dominant hand for these survivors. Additionally, GrimAge was calculated for 574 of the survivors. We calculated age acceleration for GrimAge as residuals after regressing GrimAge on chronological age to capture its effect independent of age (GrimAge_r). All analyses were adjusted for survey weights to generate nationally representative estimates. In the structural equation model adjusted for age, sex, race, BMI, and smoking, CMV infection explained 10.2% ($p = 0.03$) of the association between education and comorbidity index (total effect=0.30, $p < 0.01$). GrimAge_r explained 18.9% ($p = 0.01$) of the association between education and comorbidity index (total effect=0.41, $p < 0.01$). Neither CMV infection nor GrimAge_r mediated the association between education and muscle weakness; however, the sample size was limited. Our findings suggest that CMV infection and epigenetic clocks may mediate the association between education and health outcomes in cancer survivors.

Abstract citation ID: igaf122.3206

### PREDICTORS OF COGNITIVE IMPAIRMENT IN CANCER SURVIVORS: USING THE KOREAN LONGITUDINAL STUDY OF AGING (KLOSA)

Ah Rim Lee, Kyeongin Cha, Xirong Cui, Jeongeun Hwang, Sun Suk Bhin, Dahyeon Lee, and Misook Jung, *Chungnam National University, Daejeon, Republic of Korea*

Aging populations and increasing numbers of older cancer survivors demand targeted cognitive health management. Cancer diagnosis and treatment may acccelerate cognitive aging, leading to faster decline than normal aging. Advanced statistical methods are crucial to unravel the complex factors influencing post-treatment cognition. Accordingly, this study employed latent growth mixture models to delineate heterogeneous cognitive trajectories, followed by the development of machine learning model to predict key factors influencing cognitive changes among cancer survivors. Data from 313 cancer survivors, aged from 55 to 95 years, were extracted from a cohort of 6,940 older individuals who participated in the 1st and 7th waves of the Korean Longitudinal Study of Aging. The analysis included demographic information, health-related behaviors, dementia-related comorbidities, cancer-related factors, and cognitive screening scores. Two cognitive trajectories emerged: a maintenance group exhibiting stable, normal scores (84.0%) and a decline group with decreasing scores (16.0%). A broad set of predictors was used in the predictive models to classify membership in the identified cognitive trajectories. The machine learning models-logistic regression, random forest, artificial neural network, support vector machine-achieved balanced accuracies ranging from 0.84 to 0.86 and AUC values between 0.74 and 0.88. Key predictors included age, difficulty with daily activities due to cancer, depression, time since diagnosis, and social networks. These findings underscore the heterogeneous nature of cognitive aging in older cancer survivors and both cancer-related and dementia-related factors play critical roles in predicting cognitive decline. Comprehensive, personalized interventions to mitigate the multifactorial influences on cognition are needed in this vulnerable population.

Abstract citation ID: igaf122.3207

### ROLE OF SOCIAL SUPPORT AND ENGAGEMENT FOR CANCER SURVIVORS WHO BECOME CAREGIVERS

Natalia Babenko[1], William Haley[2], and Judith Rijnhart[1], *1. University of South Florida, Tampa, Florida, United States, 2. University of South Florida, University of South Florida, Florida, United States*

Depressive symptoms significantly affect the quality of life of cancer survivors. Cancer survivors who become caregivers may experience increased caregiver burden, which may exacerbate their psychological distress. Despite these compounding challenges, the intersection of caregiving and cancer survivorship remains understudied. Social support is known to buffer against the negative psychological impact of both cancer and caregiving. This study aimed to examine the role of social engagement and social support in alleviating depressive symptoms among cancer survivors with caregiving responsibilities. A cross-sectional study was conducted with 1,388 participants from the 2021 National Study of Caregiving, focusing on caregivers aged 40 and older. The study explored the moderating and mediating roles of emotional support, service use, instrumental support, and social engagement in the relationship between cancer history and depressive symptoms. While only emotional support moderated the relationship between cancer history and depressive symptoms, the direction of effect estimates across all social support and social engagement domains consistently indicated that cancer survivors who lacked social support or engagement experienced higher depressive symptoms compared to caregivers without a history of cancer. Additionally, receiving financial help and participating in organized activities mediated the relationship between cancer history and depressive symptoms. Thus, caregivers with a history of cancer are more vulnerable to depressive symptoms in the absence of emotional support, monetary assistance, and participating in group activities. Emotional and instrumental support are critical resources for cancer survivors with caregiving responsibilities. Effective mental health strategies should maximize participation in group activities and provide emotional and financial support.

Abstract citation ID: igaf122.3208

### SKELETAL MUSCLE ADIPOSITY IS ASSOCIATED WITH LOWER COGNITIVE FUNCTION IN OLDER CANCER SURVIVORS

Brendan McNeish[1], Iva Miljkovic[2], Anne Newman[2], and Caterina Rosano[2], *1. University of Michigan, Ann Arbor, Michigan, United States, 2. University of Pittsburgh, Pittsburgh, Pennsylvania, United States*

Cancer is associated with accelerated aging and changes in both muscle and cognition. In cancer-free older adults, an emerging association between muscle adiposity and cognition has been observed; however, this relationship has not been investigated in older cancer survivors. This study aimed to evaluate the association between skeletal muscle adiposity and cognitive function in older cancer survivors. This study included 75 cancer survivors who developed cancer within the first five years of the study (age range: 69–79; 65% men; 31% Black; 41% prostate cancer; 21% breast cancer). Thigh intermuscular fat area, a measure of skeletal muscle adiposity, was obtained via computed tomography at Year 6. Cognitive function was assessed using the Digit Symbol Substitution Test (DSST) and the Mini-Mental Status Exam (MMS) at Year 10. Multivariable models adjusted for demographic factors (age, gender, race, education) and thigh muscle area at Year 6. Sensitivity analyses separately adjusted for dementia risk factors, including diabetes, hypertension, APOE4 allele, physical activity, and adiposity measures at Year 6 (BMI, abdominal visceral and subcutaneous adiposity, and total body fat mass). Greater thigh intermuscular fat area was associated with lower DSST scores (B= -0.298, $p < 0.05$) and lower MMS scores (B= -0.195, $p < 0.05$) at Year 10. These associations remained significant and were not attenuated by dementia risk factors or other adiposity measures. Higher skeletal muscle adiposity is independently associated with lower cognitive function in older cancer survivors. Further research is needed to confirm this relationship and explore the underlying biological mechanisms.

Abstract citation ID: igaf122.3209

### SYMPTOM CLUSTERS IN MIDDLE AGE AND OLDER ADULTS PRIOR TO TREATMENT FOR CANCER

Victoria Loerzel, and Kailei Yan, *University of Central Florida, Orlando, Florida, United States*

Health at the beginning of cancer treatment is becoming more complex. While much of the focus on symptoms is centered on during treatment, many adults start treatment for cancer with multiple symptoms related to their diagnosis and other co-morbid illnesses due to age. Identifying symptom clusters (SC) prior to treatment is critical so they can be managed, and we can assist aging adults in maintaining well-being and quality of life. The goal of this study was to identify symptom clusters in middle age and older adults prior to being enrolled in an RCT for symptom management. We used exploratory factor analysis to extract symptom clusters, and model fit indices were evaluated. Then, the same method was applied to analyze subgroup difference between older (aged at least 65) and middle-aged (>50, < 65) participants. 141 adults aged 50 and over were included in analysis. The 14-item Symptom Representation Questionnaire was used to report symptoms. Participants aged 65 and older had 2 distinct SC: Physical and Cognitive/Emotional. Participants aged 64 and younger had 3 SC with unsimilar and difficult to categorize symptoms: Emotional/Neurological, Physical, and Miscellaneous. Each cluster had between 4 and 8 individual symptoms and the Physical clusters were not identical. SC appears to differ by age as does the clarity of the cluster. Assessing SC prior to treatment may lead to early awareness, symptom self-management education, and prevention of worsening symptoms and quality of life in these complex adults.

Abstract citation ID: igaf122.3210

### THE IMPACT OF TRUST IN HEALTH INFORMATION SOURCES ON CANCER BELIEFS AMONG OLDER ADULTS

Jonathan Nutakor, *School of Public Administration, Guizhou University of Finance and Economics, Guiyang, Guizhou, China (People's Republic)*

Beliefs in cancer prevention and causation shape older adults' health behaviors. Trust in sources of health information, i.e., doctors, family, and government, may affect these beliefs. This study explores the association between trust in health information sources and cancer beliefs in older adults. Data was analyzed from older adults (60+) in the Health Information National Trends Survey (HINTS) 5. Logistic regression models were used to test for relationships between trust levels and cancer beliefs, adjusting for demographic factors. Respondents aged 85+ were more likely to believe that everything causes cancer (Coef.=0.775, $p = 0.034$). Women were less likely than men to believe that everything causes cancer (Coef.=-0.306, $p = 0.038$). Those who were living as married were more likely to hold the belief that it is impossible to reduce cancer risk (Coef.=2.080, $p = 0.044$). Trust in doctors was inversely related to beliefs that anything causes cancer (Coef.=-0.242, $p = 0.089$). Belief in government was strongly related to reduced belief in the impossibility of risk reduction (Coef.=-0.436, $p = 0.001$). Trust in the government was negatively associated with willingness for behavioral change (Coef.=-0.588, $p = 0.001$), and trust in charities had a positive trend (Coef.=0.295, $p = 0.072$). In conclusion, trust in health information sources has a profound influence on older adult's cancer-related beliefs and behavior. Lower trust in doctors and government sources is also associated with greater levels of cancer-related myths. Building trust in credible health sources could potentially augment cancer prevention in older adults.

### SESSION 7430 (POSTER)

### CHRONIC DISEASE MANAGEMENT

Abstract citation ID: igaf122.3211

### AGE-RELATED DIFFERENCES IN EMOTIONAL RESPONSES AMONG INDIVIDUALS WITH MULTIPLE SCLEROSIS

Kyungmi Lee, and Matthew Plow, *Case Western Reserve University, Cleveland, Ohio, United States*

Socioemotional theories explain that despite physical decline, psychological changes in aging occur due to adaptation. Aging-related psychological changes may impact emotional responses, such as happiness, stress, and fatigue. However, research on how age affects these factors in individuals with multiple sclerosis (MS) is limited. This study examines how aging affects emotional responses (i.e., happiness, stress, and fatigue) by comparing individuals with MS who are under 55 and over 55, focusing on age-related differences in emotional responses over time. Data from 107 MS participants were collected using ecological momentary assessment, with five daily measurements over 10 days, yielding 6,600 observations. A multilevel model assessed both within-person and between-person differences, controlling for gender and race. In the within-person analysis, previous-day happiness, stress, and fatigue significantly predicted current levels (happiness: $\beta = 0.194$, $p < 0.001$; stress: $\beta = 0.247$, $p < 0.001$; fatigue: $\beta = 0.217$, $p < 0.001$). Weekly patterns showed increasing happiness toward the end of the week ($\beta = 0.106$, $p < 0.01$), decreasing stress ($\beta$ = -0.213, $p < 0.01$), and mid-week fatigue slightly increased ($\beta = 0.075$, $p < 0.05$). Although between-person differences were not statistically significant, younger adults showed greater emotional fluctuations (happiness: $\beta = 0.220$ vs. $\beta = 0.160$; stress: $\beta = 0.260$ vs. $\beta = 0.230$; fatigue: $\beta = 0.230$ vs. $\beta = 0.060$). Aging in MS may be associated with reduced emotional variability due to adaptive mechanisms in aging. Further research is needed to explore the mechanisms and implications for age-specific MS interventions.

Abstract citation ID: igaf122.3212

### AGE-RELATED PHYSICAL ACTIVITY DETERMINANTS IN BREAST CANCER SURVIVORS: A THEORY-BASED MULTIGROUP ANALYSIS

Jeongeun Hwang, Xirong Cui, Ah Rim Lee, Moonkyoung Park, Eunyoung Park, Hyun-E Yeom, and Misook Jung, *Chungnam National University, Daejeon, Republic of Korea*

Background: Physical activity is a proven non-pharmacological intervention that alleviates treatment-related symptoms, enhances cardiovascular health, lowers cancer recurrence and mortality risks, and improve daily functioning and quality of life. However, older breast cancer survivors tend to be less active than younger survivors. This age-related disparity may stem from challenges unique to older survivors-such as reduced self-efficacy due to physical limitations or past negative experiences-which can diminish their motivation to initiate or maintain exercise routines. Yet, it remains unclear whether these motivational factors influence physical activity engagement differently across age groups. This study compared the effects of motivational and volitional factors on physical activity among older and younger cancer survivors. Methods: A total of 218 older and 287 younger breast cancer survivors were recruited. Key constructs from the Health Action Process Approach-including self-efficacy, planning, risk perception, outcome expectancy, intention, and physical activity-were assessed. Structural equation modeling and multi-group analysis were employed to examine the data. Results: Motivational constructs explained 27.8% of the variance in intention among older survivors compared to 3.2% among younger survivors, while volitional constructs explained 42.1% of physical activity variance for older survivors and 88.1% for younger survivors. Significant group differences were observed in the pathways linking risk perception to intention, action planning to physical activity, and recovery self-efficacy to physical activity. Conclusion: These findings indicate that motivational factors exert stronger influences on older survivors, whereas volitional factors are more critical for younger survivors. Tailored interventions addressing these age-specific influences may be key to enhancing physical activity engagement.

Abstract citation ID: igaf122.3213

### EFFECTIVENESS OF DIGITAL THERAPEUTICS ON CHRONIC PAIN IN OLDER PEOPLE: A SYSTEMATIC REVIEW AND META-ANALYSIS

Jeongeun Lee, and Seyeon Park, *Chungnam National University College of Nursing, Daejeon, Republic of Korea*

Background: Chronic pain in older people is a global health issue, reducing quality of life and increasing healthcare costs. Traditional treatments have limited long-term effectiveness due to side effects and dependency risks, driving interest in non-pharmacological approaches like digital therapeutics (DTx), which use technologies such as mobile apps, VR, AI, and digital CBT to modulate pain and promote neuroplasticity. Purpose: This objective of this study is to evaluate the effectiveness of DTx in reducing chronic pain in older people through a systematic review and meta-analysis to assess the effect size. Methods: A systematic review and meta-analysis following PRISMA 2020 guidelines. A comprehensive search of major E-databases was conducted to identify studies examining the effects of digital therapeutics on chronic pain management. A total of 106 studies were initially retrieved, and 6 studies that met the inclusion criteria were selected for analysis. The effect size of Pain reduction was analyzed using Standardized Mean Difference (SMD) and a random-effects model to address heterogeneity. Results: The meta-analysis revealed that digital therapeutics had a statistically significant small effect on reducing chronic pain in older people, with an SMD of -0.31 (95% CI: -0.57, -0.05, $p < 0.05$). Additionally, all 6 included studies were recent (published between 2000-2004), and no studies had a high risk of bias. Conclusion: The findings suggest that DTx can be an effective non-pharmacological strategy for pain management in older adults, but further large-scale and long-term studies are needed to confirm their sustained effectiveness and broader applicability.

Abstract citation ID: igaf122.3214

### EFFECTIVENESS OF INTERVENTIONS TO REDUCE BLOOD PRESSURE IN OLDER ADULTS WITH HYPERTENSION: A NETWORK META-ANALYSIS

Noppawan Piaseu[1], and Phatcharaphon Whaikid[2],
*1. Ramathibodi Hospital, Bangkok, Krung Thep, Thailand,*
*2. Mahidol University, Bangkok, Krung Thep, Thailand*

This study evaluated the effectiveness of interventions in managing blood pressure among older Thai adults. A systematic search was conducted in PubMed, Embase, Scopus, and the

Thai-Journal Citation Index Centre for studies published from 2018 to 2024. Eligible studies included randomized controlled trials and quasi-experimental designs. Two independent reviewers assessed study quality using the Joanna Briggs Institute critical appraisal tool. A network meta-analysis was performed using Stata software. The study protocol was registered on PROSPERO (CRD42025634594). A total of 27 studies, including 1,828 participants over 60, met the inclusion criteria and were included in the analysis of three interventions: Self-Management (SM), Theory-Based Health Behavior Modification (TBHBM), and Exercise. The network meta-analysis revealed that Exercise was the most effective intervention for lowering systolic blood pressure (SBP). Compared to SM, Exercise significantly reduced SBP (MD = -6.75 mmHg, 95% CI: -18.59, -5.09), followed by TBHBM (MD = -0.80 mmHg, 95% CI: -11.54, -9.91). For diastolic blood pressure (DBP), Exercise was also the most effective intervention. Compared to SM, Exercise led to a greater reduction in DBP (MD = -4.98 mmHg, 95% CI: -12.80, 2.84), and compared to TBHBM, the reduction was also greater (MD = -3.14 mmHg, 95% CI: -10.10, 4.12). This provides evidence supporting health behavior modification as an effective strategy for blood pressure management in older Thai adults. Exercise was most effective for SBP reduction, while TBHBM had the most significant impact on DBP. These findings emphasize the importance of tailored interventions to optimize hypertension management in aging populations.

Abstract citation ID: igaf122.3215

### EHEALTH LITERACY IS ASSOCIATED WITH SELF-EFFICACY IN ADULT PATIENTS WITH CHRONIC DISEASE

Onome Osokpo[1], Andrea Jackson-Sagredo[2], Dima Kenj Halabi[2], Vineet Arora[2], David Meltzer[2], and Valerie Press[2], *1. University of Illinois Chicago, Chicago, Illinois, United States, 2. The University of Chicago, Chicago, Illinois, United States*

Self-management self-efficacy—the ability to achieve positive health outcomes by successfully completing self-care tasks—is a strong predictor of effective disease self-management among patients with chronic conditions. However, little is known about how self-management self-efficacy is related to eHealth literacy (eHL), the digital skills needed to seek and use health information from electronic sources. Using a cross-sectional observational design and convenience sampling, we enrolled 1259 English-speaking hospitalized patients ≥18 years of age to test the hypothesis that self-efficacy is related to eHL. Self-efficacy was measured with a single question: "How confident are you that you can manage your health conditions on your own?" Participants responded on a five-point scale ranging from not confident at all (1) to very confident (5) (high self-efficacy: ≥4; low self-efficacy: < 3). eHL was measured with a validated eight-item questionnaire (composite score: 8–40; low eHL: < 24; adequate eHL: ≥24). The sample (54 ± 17.3 years) mostly identified as Black (73%), female (53%), single/widowed/divorced (70%), and was educated with at least some college (54%). Chronic disease was common: 29% had diabetes, 27% had heart disease, 54% had hypertension; 54% were hospitalized in the last 12 months excluding current hospitalization Unadjusted logistic regression revealed a significant association between self-efficacy and eHealth literacy (eHL) (p = 0.003). Patients with lower eHL had significantly lower odds of feeling confident in managing their medical care or illness compared to those with adequate eHL (OR = 3.62, 95% CI: 1.54–8.78, p = 0.003). Interventions promoting eHL may enhance self-efficacy, resulting in effective disease self-management.

Abstract citation ID: igaf122.3216

### HEALTH-INFORMATION TECHNOLOGY USE IN PEOPLE WITH MULTIMORBIDITY: NATIONAL HEALTH INTERVIEW SURVEY 2022-2023

Binu Koirala[1], Arum Lim[2], Chitchanok Benjasirisan[1], Soo Hyun Kim[1], Asma Rayani[1], Cheryl Himmelfarb[1], and Patricia Davidson[3], *1. Johns Hopkins University, Baltimore, Maryland, United States, 2. Johns Hopkins University School of Nursing, Baltimore, Maryland, United States, 3. University of New South Wales Sydney, Kensington, New South Wales, Australia*

Multimorbidity is a key healthcare challenge in the US and highly prevalent among older adults. Health-information technology (HIT) use has been identified as a valuable tool to facilitate multimorbidity management. This study aims to explore the characteristics associated with HIT use among individuals with multimorbidity. A cross-sectional analysis of 2022-2023 National Health Interview Survey (n = 41,194) data was conducted. Presence of multimorbidity was defined as the self-reported history of ≥ 2 chronic conditions: diabetes, hypertension, asthma, stroke, cancer, chronic obstructive pulmonary disease, heart disease (coronary heart disease, heart attack), weak/failing kidneys, and chronic liver conditions. HIT use was defined as any of three aspects of internet use for: health information, communicating with the doctor's office, and test results. Survey-weighted Poisson regression models were developed for statistical analysis. Majority of individuals with multimorbidity [mean (SD) age: 59(0.19) years] reported often use of the internet for all three aspects of HIT use. In an adjusted analysis, characteristics associated with a lower prevalence ratio of HIT use were older age; male; not married; of a racial and ethnic minority group; lower education and income; no insurance; no usual place for medical care; and reporting excellent perceived health status. Compared to adults aged 18-64 years, adults ≥65 years group were 23% less likely to report HIT use (APR: 0.77, 95% CI: 0.74-0.79). Although HIT use is common among adults with multimorbidity, social determinants of health and age affect usage. Careful consideration of these factors is essential when designing a digital health intervention, particularly for older adults.

Abstract citation ID: igaf122.3217

### LOW BACK PAIN AND DISABILITY IN ACTIVITIES OF DAILY LIVING MMONG MIDDLE-AGED AND OLDER ADULTS IN CHINA: A COHORT STUDY

Bo Liang, Xiaoxuan Liang, and Gong Chen, *Peking University, Beijing, Beijing, China (People's Republic)*

Chronic diseases are associated with disability in activities of daily living (ADL), but the relationship between low back pain (LBP) and the risk of ADLs disability remains unclear. This study aimed to estimate the effect of LBP on ADLs disability and explore variations in the risk across gender and age among middle-aged and older adults in China. Participants from the China Health and Retirement Longitudinal Study (CHARLS) 2015 were followed up to assess disabilities of basic ADL (BADL) and instrumental ADL (IADL) in 2018 and 2020. The cohorts comprised 19,223 participants aged ≥45 with intact BADL, and 18,226 with intact IADL, respectively. Cox proportional hazards model was employed to investigate the association between LBP and BADL and IADL disabilities. The incidence rates for BADL and IADL disabilities were 17.81 and 38.32 per 1,000 person-years, respectively. Compared to participants without LBP, those with either back or waist pain had a 64% (HR = 1.64, 95%CI: 1.43-1.87) increased risk of BADL disability, and those with both types of pain showed a 105% (HR = 2.05, 95%CI: 1.79-2.35) increased risk. Similarly, those with only back or waist pain, and pains combined presented a 61% (HR = 1.61, 95%CI: 1.46-1.78) and 122% (HR = 2.22, 95%CI: 2.01-2.45) increased risk of IADL disability, respectively. Subgroup analyses indicated that LBP increased the risk of ADLs disabilities across genders and age groups. Therefore, the increased risk of disabilities in BADL and IADL may be affected by LBP among middle-aged and older Chinese. Early prevention of LBP occurrence can delay or reduce ADLs disabilities risk.

Abstract citation ID: igaf122.3218

### MEDICATION CONFIDENCE AND EMOTIONAL DISTRESS IN OLDER ADULTS WITH TYPE 2 DIABETES: A MODERATED MEDIATION APPROACH

Qiwei Li[1], Elias Mpofu[2], Xiaoli Li[3], Cheng Yin[2], Garikayi Chemhaka[2], Amos Mareverwa[2], and Kaye Brock[4], *1. California State University, Fresno, Fresno, California, United States, 2. University of North Texas, Denton, Texas, United States, 3. Southern Illinois University, Carbondale, Illinois, United States, 4. The University of Sydney, Sydney, New South Wales, Australia*

Background and Aim: Emotional distress is common among individuals with type 2 diabetes (T2D) and would be higher with lower education levels and poor health literacy. High distress is linked to poor self-care and treatment adherence, increasing risks related to fatigue and comorbidities. Confidence in medication adherence and fatigue self-management would relief emotion distress with T2D, but evidence remains unclear. This study examines the relationship between medication confidence and emotional distress management, with fatigue management and comorbidities as mediators and education level as a moderator. Methods: Using a cross-sectional design, we analyzed data from the Diabetes Self-Management Program in Arlington, Texas. The study included 144 older adults (≥60 years; 30% male) with self-reported T2D. Confidence in emotional distress management, medication adherence, fatigue management, comorbidities, and education level were assessed. Multiple linear regression, parallel mediation, and moderated mediation analyses were conducted, controlling age, gender, and race. Results: Medication confidence was significantly associated with emotional distress management ($\beta = 0.71$, $p < 0.01$). Fatigue management confidence strongly mediated this relationship ($\beta = 0.74$, $p < 0.01$, $R^2 = 0.54$), while multimorbidity had no significant mediation effect. Education level moderated the direct effect, with stronger associations in participants with at least a bachelor's degree. The overall model explained 67% of variance in emotional distress confidence ($R^2 = 0.67$). Conclusion and Implications: Interventions for emotional distress in older adults with T2D should enhance medication and fatigue management confidence. Education plays a key moderating role, underscoring the importance of improving health literacy for lower-educated individuals.

Abstract citation ID: igaf122.3219

### MIND-BODY CONNECTION: IMPACT OF SELF-REPORTED MENTAL HEALTH ON CHRONIC DISEASE IN OLDER WOMEN (NSHAP WAVES 2 AND 3)

Annelie Persson[1], and Julie Blaskewicz Boron[1], *University of Nebraska Omaha, Omaha, Nebraska, United States*

Women experience significant hormonal changes during menopause, which can affect both mental health and the risk of chronic diseases. Although the relationship between subjective and objective mental health is not well understood, subjective cognitive and emotional health has been linked with cognitive decline and chronic disease outcomes. This raises the question of whether self-reported mental health (SRMH) might serve as a valuable metric for predicting chronic disease outcomes among post-menopausal women. The current study investigated SRMH as a predictor of chronic disease outcomes, including heart conditions, non-skin cancers, diabetes, and dementia/mild cognitive impairment (MCI). Participants included 578 women (Mean age: 70.5; Range:62-89) from the National Social Life, Health, and Aging Project who participated during waves 2 (2010-2011) and 3 (2015-2016), and were free of chronic disease diagnoses at wave 2. Emotional/mental health was rated on a scale from poor to excellent (Wave2), and modeled as a predictor for chronic disease outcomes (Wave3). Logistic regression analyses showed relationships between SRMH (Wave2) and chronic disease outcomes five years later for dementia/MCI (p=.02, SE: 0.40, CI: -1.78 to -0.18, OR:0.39), heart conditions (p = 0.0054, SE: 0.182, CI: 0.159 to 0.874, OR: 1.66), and a trend for cancers other than skin (p = 0.087, SE:0.23, CI: -0.834 to 0.0582, OR:0.68). Findings suggest that improving mental health may lower cancer risk and reduce dementia/MCI in older women. The unexpected positive relationship with heart conditions warrants further investigation. Future research should explore variables that might mediate/modify these relationships, such as lifestyle factors or social support systems.

Abstract citation ID: igaf122.3220

### RELATIONSHIPS AMONG SYMPTOMS, SELF-CARE, AND QUALITY OF LIFE IN INDIVIDUALS WITH MULTIMORBIDITY

Soo Hyun Kim[1], Arum Lim[2], Chitchanok Benjasirisan[1], Cheryl Himmelfarb[1], Patricia Davidson[3], Rebecca Wright[1], and Binu Koirala[1], *1. Johns Hopkins University, Baltimore, Maryland, United States, 2. Johns Hopkins University School of Nursing, Baltimore, Maryland, United States, 3. University of New South Wales Sydney, Kensington, New South Wales, Australia*

Multimorbidity is a major concern for aging individuals with heart failure (HF), increasing hospitalization, mortality, and healthcare costs. However, relationships among symptoms, self-care, and quality of life (QoL) remain underexplored. This study analyzed baseline survey data from a cohort study (2022–2023) at a university-affiliated hospital. Participants were aged ≥50 years with HF and at least one additional chronic condition. Socio-demographic and clinical data were collected; symptoms were assessed using the Edmonton Symptom Assessment Scale, QoL via the EuroQoL-5D-5L, and self-care using the Self-care of Heart Failure Index. A cross-sectional regression analysis was performed on a sample of 353 participants, with a mean (±SD) age of 70 (±9.5) years. Among them, 277 (78.5%) identified themselves as their primary caregiver. In the unadjusted analysis, all nine symptoms, self-care maintenance, and self-care confidence were significantly associated with QoL. In the adjusted model, after controlling for age, gender, social support and including all symptoms and self-care domains, pain ($\beta = -0.035$, $p<.001$), shortness of breath ($\beta = -0.011$, $p<.05$), well-being ($\beta = -0.018$, $p <.01$), self-care maintenance ($\beta = 0.004$, $p<.001$), and self-care monitoring ($\beta = -0.001$, $p<.05$) remained significant for QoL. These findings highlight the negative association of symptoms on QoL in those with multimorbidity, while self-care maintenance has positive association with QoL. As multimorbidity becomes increasingly prevalent with aging, further research is needed to better understand the complex relationships among symptoms, self-care, and QoL to inform the development and implementation of effective, tailored multimorbidity management strategies.

Abstract citation ID: igaf122.3221

### THE EFFECT OF ASTAXANTHIN ON FATIGUE IN DIABETIC PATIENTS FROM A NUTRITIONAL NURSING PERSPECTIVE

Hongyu Li[1], Rui Zhao[2], Juan Li[1], Guifang Guo[3], and Wei Sang[4], *1. Liaoning Polytechnic Vocational University, Jinzhou, Liaoning, China (People's Republic), 2. Taizhou University, Faculty of Medicine, Taizhou, Zhejiang, China (People's Republic), 3. Peking University, Beijing, Beijing, China (People's Republic), 4. Zhejiang Houde Mingxin Technology Service Co., Taizhou, Zhejiang, China (People's Republic)*

Background Diabetic patients often experience increased fatigue due to inflammation, depression, and poor glycemic control. Astaxanthin, with its anti-inflammatory and antioxidant properties, has shown potential in alleviating diabetic fatigue in animal studies. Objective We aimed to investigate the effects of astaxanthin nutritional care on diabetic fatigue in a type 2 diabetes mouse model over 12 weeks, focusing on gut microbiota-related mechanisms. Methodology A type 2 diabetes mouse model was established using a 60% high-fat diet and low-dose streptozotocin injection. Mouse running exhaustion tests assessed fatigue status. Astaxanthin was added to the diet at 0.01% and 0.02% for 12 weeks. Key fatigue indicators were compared before and after intervention and between groups. Gut microbiota was analyzed via 16S rRNA sequencing. Results The diabetes model group showed significantly higher fasting blood glucose ($p<0.01$), insulin ($p=0.009$), and insulin resistance index ($p=0.002$) than controls. Astaxanthin significantly reduced serum LPS (by 22.70% and 29.88%) and TMAO (by 33.82% and 28.57%). Gut microbiota diversity was modulated, with increased beneficial genera and suppressed harmful genera, influencing fatigue and renal function. Discussion The study demonstrated that increasing beneficial gut genera and inhibiting harmful genera effectively alleviated diabetic fatigue. Future research should explore long-term effects and human applications.: Astaxanthin, Nutrition nursing intervention, Diabetes fatigue, Gut flora

Abstract citation ID: igaf122.3222

### THE INTERSECTION OF CHRONIC DISEASE AND ECONOMIC HARDSHIP IN RURAL AFRICAN AMERICAN COMMUNITIES

Idethia Shevon Harvey, *University of Missouri, Columbia, Missouri, United States*

Introduction Multiple chronic conditions disproportionately affect rural African Americans who face higher prevalence and worse outcomes for arthritis, diabetes, and hypertension. Those living in challenging environments experience higher rates of chronic comorbidities due to the interaction between rural limitations and persistent structural factors. The research investigates using syndemic theory to forge new ground in rural minority health research. Methods Semi-structured interviews and photo-documentation were conducted among African Americans (mean age = 63.2, SD + 9.93 years) with long-term diabetes (mean diagnosis = 14.7, SD + 10.8 years) and hypertension (91.3%). Quantitative analysis included univariate and bivariate statistics describing demographics and self-management behaviors by composite score (0 – 6) counting depression, hypertension, arthritis, smoking, obesity, and poverty. Qualitative methods included semi-structured interviews, photo-documentation, thematic content analysis, and critical visual methodology. Triangulation occurred when photographic themes aligned with interview themes. Results Participants with higher syndemic scores (4.31, SD + 0.48) showed lower days of health eating, physical activity, and glucose testing adherence. Qualitative analysis revealed two themes: "chronicity of poverty" and "contextual factors affecting health management." Limited economic resources not only discouraged positive diabetes management but triggered clustering of health complications, supporting syndemic theory that individuals with diabetes experience magnified health-related and socioenvironmental burdens. Discussion This syndemic approach provides a novel perspective for understanding contributing factors influencing health in rural African American communities. A comprehensive approach addressing biological and social elements is necessary to reduce disease burden, improve patient-centered care, and develop targeted programs supporting self-management practices in these communities.

### SESSION 7435 (POSTER)

### COGNITION IV

Abstract citation ID: igaf122.3223

### ACUTE EFFECTS OF A SHORT BOUT OF WALKING ON DOMAIN-SPECIFIC COGNITION IN ADULTS WITH KNEE REPLACEMENT

Jongwon Lee, Scott Jamieson, Clare Kennerley, Christine Pellegrini, and Chih-Hsiang Yang, *University of South Carolina, Columbia, South Carolina, United States*

The total knee replacement (TKR) is a common elective surgery in older adults, and adults with TKR remain physically inactive and are at high risk for Alzheimer's and related dementia (ADRD). Shorter bouts of walking may be more feasible for this population to reduce the risk of ADRD because prolonged physical activity could be challenging due to lower physical functioning. This study investigated the acute effects of the 6-minute walk on processing speed, visuospatial memory, and visual memory in adults with a history of TKR ≤ 6 months ago. They were randomized into two sequences with two periods: 6 minutes of walking or resting first. Cognition was assessed via brief phone-based tests at baseline, immediately after the first and second periods. Symbol search, grid memory, and color shapes tests were used to measure processing speed, visuospatial memory, and visual memory, respectively. The analysis included 52 participants (female = 40 (77%), age = 65.06 (9.06) yrs, time since surgery = 94.08 (21.66) days). Controlling for demographics and potential learning effects, mixed-effects models revealed that 6 minutes of walking was associated with improved visuospatial memory, as indicated by shorter error distances on the grid memory test ($b = -3.87$, $p=0.04$). The walking effect was not observed for processing speed ($b=178.82$, $p=0.45$) or visual memory ($b=0.01$, $p=0.92$). Although the physiological mechanisms to which exercise benefits domain-specific cognition remain unclear, shorter bouts of walking may immediately improve visuospatial memory in this population.

Abstract citation ID: igaf122.3224

### AGE RELATED CHANGE IN FINANCIAL LITERACY: AN ANALYSIS OF LONGITUDINAL DATA

Ryan Best, and Laken Mooney, *West Virginia University, Morgantown, West Virginia, United States*

Financial literacy is a robust predictor of financial behaviors and outcomes. Aging is not associated with a reduction in the need to make complex financial decisions; for example, individuals must decide when to begin receiving Social Security and how to manage defined-contribution retirement plans. Understanding age differences in financial literacy is an important component of supporting good financial choices and promoting financial wellbeing. Though commonly studied cross-sectionally, longitudinal studies investigating age related change in financial literacy are relatively rare. Existing studies have found moderate declines in older (60+ years old) but not younger (18-59) adults across two measurement points separated by 4 years (Angrisani et al., 2023) and linear declines in octogenarians across 6-10 years (Boyle et al., 2025; Yu et al., 2021). The current study builds on this prior work by investigating declines in the "Big Three" financial literacy questions across seven surveys spanning 17 years. Using data from the RAND American Life Panel ($n = 564$), a representative probability sampled online panel, we evaluated age related change in financial literacy. Controlling for gender, income, and education, a linear mixed effects model found a significant interaction between age and year of measurement, $p = .013$, where financial literacy generally increased across measurement points for younger adults, stabilized in middle and late adulthood, and declined for the oldest old. Results are discussed in regard to financial literacy's trajectory with age suggesting a greater similarity with trends in crystalized, rather than fluid, intelligence and implications for financial wellbeing into old age.

Abstract citation ID: igaf122.3225

### ASSOCIATION OF AEROBIC FITNESS WITH MNEMONIC DISCRIMINATION AND GENERALIZATION IN OLDER ADULTS

Jeongwoon Kim[1], Bryan Montero Herrera[2], Chadsley Wessinger[2], Kylie Kayser[3], Brittany Armstrong[2], Christopher Wahlheim[2], Jennifer Etnier[2], and Kyoung Shin Park[1], *1. Emory University School of Medicine, Atlanta, Georgia, United States, 2. University of North Carolina at Greensboro, Greensboro, North Carolina, United States, 3. The University of North Carolina at Greensboro, Greensboro, North Carolina, United States*

The hippocampus is responsible for learning and memory and vulnerable to age-related decline. Mnemonic discrimination (MD) and mnemonic generalization (MG) are sensitive markers of hippocampal integrity. MD reflects the ability to successfully separate similar information while MG reflects an increasing overlap when encoding similar information. A growing body of research suggests that aerobic fitness (AF) may protect hippocampal integrity in older adults. However, the extent to which MD and MG are associated with AF remains underexplored. In this preliminary study, we examined cross-sectional associations between AF with MG and MD in 60 cognitively unimpaired, ambulatory older adults (mean age 70.6±4.6 years; 86.7% female). We hypothesized negative and positive associations of AF with MG and MD, respectively. AF was assessed through the total distance walked in 6 minutes (6MWD). MG and MD were assessed through the Mnemonic Similarity Task and operationalized as the old (incorrect) and similar (correct) categorization of lure items, respectively. The hypothesis was tested through hierarchical regressions with covariates (age, sex, and education) in step 1 and 6MWD in step 2. Upon adjusting for covariates, 6MWD was inversely associated with MG ($\beta = -0.30$, $p = 0.041$) and had a trend-level positive association with MD ($\beta = 0.25$, $p = 0.060$). These findings indicate that older adults with higher AF may better separate and store information in a non-overlapping manner, implying protective effects of AF on hippocampal-dependent mnemonic discrimination in cognitive aging. Enhancing AF may represent a promising strategy to protect hippocampal integrity among the growing aging population.

Abstract citation ID: igaf122.3226

### ASSOCIATIONS BETWEEN ARTERIAL STIFFNESS AND EXECUTIVE FUNCTION IN OLDER ADULTS: PRELIMINARY ANALYSIS

Jeongwoon Kim, Kaetlin Marsh, and Kyoung Shin Park, *Emory University School of Medicine, Atlanta, Georgia, United States*

Evidence suggests that arterial stiffness is linked to age-related cognitive decline. Majority of research has utilized measures of central tendency (CT) by averaging the outcomes across trials. This approach overlooks intra-individual variability (IIV) which can provide greater sensitivity to subtle cognitive changes. However, the link between arterial stiffness and cognitive IIV remains unexplored. This study utilized preliminary baseline data from ongoing clinical trials to explore how arterial stiffness is associated with IIV and CT scores of executive function. We hypothesized that more arterial stiffness is associated with greater IIV and lower CT scores of executive function. Twelve cognitively unimpaired, female older adults (68.5±2.4 yrs) completed the Flanker and Dimensional Change Card Sort (DCCS) from the NIH Toolbox© V3 to assess cognitive flexibility and inhibitory control, respectively. Arterial stiffness was assessed through carotid-femoral pulse wave velocity (cfPWV) using SphygmoCor XCEL©. IIV outcomes were calculated as the standard deviation of response time (SDRT). CT outcomes utilized the average response time (mRT) across correct trials and the response time corrected accuracy rate (AccRT). Due to limited sample size, the hypothesis was tested using Spearman correlations. For DCCS, cfPWV was associated with greater SDRT ($\rho = 0.81$, $p = 0.001$) with a trend level association for mRT ($\rho = 0.53$, $p = 0.075$) and AccRT ($\rho = -0.55$, $p = 0.063$). cfPWV was not associated with Flanker SDRT, mRT, or AccRT. These findings suggest that IIV offers unique insights into cardiovascular associations with cognitive aging. Furthermore, the selective associations with cognitive flexibility but not inhibitory control suggest that arterial stiffness influences different domains of executive function.

Abstract citation ID: igaf122.3227

### ASSOCIATIONS BETWEEN METABOLIC AND CARDIOVASCULAR BIOMARKERS AND COGNITION IN A NATIONALLY REPRESENTATIVE SAMPLE

Adea Rich[1], Sheina Emrani[2], Kristen George[3], Jennifer Manly[4], Richard Jones[5], and Zachary Kunicki[6], *1. Columbia University, New York, New York, United States, 2. University of Pennsylvania, Philadelphia, Pennsylvania, United States, 3. University of California, Davis, Davis, California, United States, 4. Columbia University Irving Medical Center, New*

*York, New York, United States, 5. Brown University, Brown University, Rhode Island, United States, 6. Warren Alpert Medical of Brown University, Providence, Rhode Island, United States*

Inflammatory, metabolic, and cardiovascular dysregulation contribute to cognitive impairment and Alzheimer's disease and Alzheimer's disease and related dementias (AD/ADRD) in older adults. AD/ADRD blood-based biomarkers are a cost-effective and accessible tool for diagnosis and monitoring, and routine blood biomarkers may offer additional insights. However, their associations with high-quality cognitive assessments remain understudied in nationally representative samples. We analyzed 1,335 older adults (mean age: 74.2; 57% female) from the 2016 Health and Retirement Study (HRS) biomarker data and Harmonized Cognitive Assessment Protocol (HCAP). Biomarkers included total cholesterol, high-density lipoprotein, hemoglobin A1C, c-reactive protein, and cystatin C. Cognitive domains (memory, executive function, language fluency) and diagnostic classifications (normal, mild cognitive impairment [MCI], AD/ADRD) were determined using the Manly-Jones algorithm. Missing data were handled with multiple imputation, and survey weights were incorporated in all analyses. We assessed biomarker levels across diagnoses. Linear regression models examined associations between biomarkers and cognitive domains, and ordinal logistic regression assessed associations between biomarkers and HCAP diagnosis. Models adjusted for age, sex, education, and race/ethnicity. Cystatin C differed across diagnostic groups ($p = 0.004$), being higher in MCI than normal ($p < 0.01$). Higher cystatin C was linked to poorer language fluency ($\beta = -0.08$, $p = 0.02$) and executive functioning ($\beta = -0.07$, $p = 0.02$). Higher A1C was also associated with lower executive functioning ($\beta = -0.07$, $p = 0.02$). Elevated cystatin C and A1C can indicate inflammation and poor cardiovascular and metabolic health, which may contribute to impairments in executive function and language fluency domains.

Abstract citation ID: igaf122.3228

### COMMUNITY BUILT ENVIRONMENTS AND COGNITIVE FUNCTION: EVIDENCE FROM A NINE-YEAR LONGITUDINAL STUDY

Jiaan Zhang, *Fudan University, Shanghai, Shanghai, China (People's Republic)*

In recent years, increasing attention has been given to the role of community built environments in shaping health and well-being among older adults. This study examines the relationship between community built environments and cognitive function among Chinese older adults. Data were drawn from the China Health and Retirement Longitudinal Study (CHARLS) (2011–2020), a nationally representative sample of community-dwelling older adults. The analysis was restricted to individuals aged 60 and older at baseline, who were followed over a nine-year period. Cognitive function was assessed using a modified version of the Telephone Interview for Cognitive Status (mTICS), which evaluates episodic memory, numerical ability, orientation, and visuospatial skills. Community built environments were measured based on the availability of numbers of libraries, theaters, sports facilities, arts venues, and social organizations. Three-level mixed effects generalized linear models that account for clustering of observations within a subject over time and subjects within a community were employed for the study. The results showed that older adults residing in communities with greater availability of intellectually stimulating sites and sports facilities experienced a slower rate of cognitive decline over time, adjusting for individual-level demographic characteristics, socioeconomic status (SES), health characteristics and behaviors, and community-level SES ratings. This study highlights specific community infrastructure that may contribute to the preservation of cognitive function among older adults. The findings suggest the importance of incorporating cognitive health considerations into urban planning and community development initiatives in China.

Abstract citation ID: igaf122.3229

### CONTRAST SENSITIVITY, VISUAL ACUITY, AND COGNITION IN OLDER ADULTS FOR THE HEALTHY AGING INITIATIVE

Molly Quigley[1], John Woolley[1], Talia Gilfix[1], Heather Andrews[2], Yael Koren[1], On-Yee Lo[3], Davide Cappon[4], and Alvaro Pascual-Leone[4], *1. Hebrew SeniorLife, Boston, Massachusetts, United States, 2. Harvard T.H. Chan School of Public Health, Boston, Massachusetts, United States, 3. Harvard Medical School/ Marcus, Hebrew SeniorLife, Boston, Massachusetts, United States, 4. Hebrew SeniorLife & Harvard Medical School, Boston, Massachusetts, United States*

Visual dysfunction in older adults can be associated with cognitive impairment. Cognitive assessments are not regularly utilized by many clinicians as they are time-consuming, susceptible to learning effects, and require complex instructions. Assessing visual function through contrast sensitivity and visual acuity tests may help predict cognitive decline and aid clinicians in its early detection while minimizing implementation barriers. We analyzed data from the first 148 participants of the Healthy Aging Initiative (HAI), a longitudinal observational study designed to track health changes over time in residents living independently in Hebrew SeniorLife Senior Housing Communities. We assessed visual acuity (Rosenbaum Near-Vision Card), contrast sensitivity (Pelli-Robson Contrast Sensitivity Chart), and cognitive function (Mini-Cog, Trail Making Test Part B, and Rey-Auditory Verbal Learning Test [RAVLT]). Cognitive measures were adjusted for demographic norms. We analyzed cross-sectional associations between visual and cognitive measures using Pearson correlations. Contrast sensitivity was significantly associated with both Mini-Cog and RAVLT ($p = 0.019$; $p = 0.006$, respectively). A trend was observed between visual acuity and Mini-Cog scores ($p = 0.092$). No other significant associations were identified. Our results align with existing literature indicating that contrast sensitivity is significantly associated with cognition. The trend between visual acuity and cognition also corresponds with existing literature supporting its value. Annual wellness exams for older adults should include assessments of visual domains, especially in senior housing communities where resident health is closely monitored. Contrast sensitivity, which is associated with memory deficits, may be particularly valuable as a brief and low-cost method for early detection of Alzheimer's disease and related dementias.

Abstract citation ID: igaf122.3230

### EFFECTS OF CHILDHOOD STRESSORS ON MEMORY: MEDIATING ROLE OF PSYCHOLOGICAL RESILIENCE

Guoping Jin, *University of Pittsburgh, Pittsburgh, Pennsylvania, United States*

Memory decline in later life is a growing public concern. While prior research suggests that childhood stressors are associated with lower cognitive functioning in older adults, less is known about their specific impact on memory performance and the potential mechanisms underlying this relationship. Drawing on the theoretical framework of resilience, this study investigated the long-term effects of childhood stressors on memory trajectories in later life, with a particular focus on the mediating role of psychological resilience. Data were from adults aged 65 and over who completed the Leave Behind Questionnaire in the 2006/2008 wave of the Health and Retirement Study (n = 9,069). Path analysis and latent growth curve modeling within a structural equation modeling framework were used to examine the direct and indirect pathways linking childhood stressors to memory trajectories through psychological resilience. The results indicated that respondents who reported a greater number of childhood stressors had higher baseline memory performance ($\beta = 0.12$, $p < .001$). However, childhood stressors were not significantly associated with memory decline over time. Moreover, psychological resilience partially mediated the relationship between childhood stressors and baseline memory (indirect effect = -0.01, $p < .05$). These findings highlighted the complex role of childhood stressors in shaping later-life memory function, suggesting that early-life adversity may foster cognitive adaptations while also undermining psychological resilience. Future research should explore additional mediators and potential moderators to better understand the mechanisms linking childhood experiences to memory function. Other implications for practice and interventions are discussed.

Abstract citation ID: igaf122.3231

### LONGITUDINAL RELATIONSHIPS OF DEPRESSIVE SYMPTOMS AND COGNITION IN OLDER ADULTS GROUPED BY DEPRESSION AGE OF ONSET

Noah Green, Monica Walters, and Laura Zahodne, *University of Michigan, Ann Arbor, Michigan, United States*

Depression is a known risk factor for cognitive impairment in older adults; however, it is unclear how age of onset moderates concurrent and longitudinal relationships between depressive symptoms and cognition in later life. This longitudinal study examines the relationship between depressive symptoms and episodic memory in older adults reporting a history of depression across different life stages. Participants included 373 adults aged 55+ from the Michigan Cognitive Aging Project (MCAP), who reported at least one lifetime depressive episode. Episodic memory was operationalized as the sum of learning trials from the CERAD word list learning task. Depressive symptoms were measured using the ten-item Center for Epidemiological Studies Depression Scale. Age of onset was categorized into four groups according to first depressive episode (childhood [ages < 12; n = 69], adolescence [ages 13-17; n = 73], early adulthood [ages 18-44; n = 155], middle adulthood [ages 45-59; n = 55], late adulthood [ages 60+; n = 21]. Cross-lag panel analyses stratified by age of onset modeled relationships between depressive symptoms and episodic memory across three time points over four years, while controlling for age, gender, race, and education. Longitudinal associations between more depressive symptoms and worse subsequent memory were only identified among adolescent ($\beta$=-0.241, p = 0.017) and early adulthood ($\beta$=-0.139, p = 0.026) onset groups. A longitudinal association between worse memory and more subsequent depressive symptoms was only identified among those with early adulthood onset ($\beta$=-0.270, p = 0.010). Results point to potential critical developmental periods for depression onset with implications for later life prognosis and/or mechanisms involving depressive symptoms and cognitive aging.

Abstract citation ID: igaf122.3232

### PAIN, PHYSICAL ACTIVITY, AND SUBJECTIVE MEMORY COMPLAINTS: EVIDENCE FROM THE HEALTH AND RETIREMENT STUDY

Chang Pu Liang[1], Wuyi Dong[1], and Yan-Jhu Su[2], *1. University of Massachusetts Boston, Boston, Massachusetts, United States, 2. The Pennsylvania State University, University Park, Massachusetts, United States*

This study investigates the intricate relationships between physical activity, pain, and subjective memory complaints (SMCs) among older adults using data from the 2020 Health and Retirement Study. The primary aims were to examine the association between physical activity and SMCs, evaluate the independent contribution of pain to SMCs, and explore the potential moderating role of pain in the relationship between physical activity and SMCs. Results from linear regression models indicated that the associations of pain and physical activity with SMCs were significant in unadjusted analyses but became non-significant after controlling for covariates, suggesting that self-rated health and depression may mediate these relationships. Stratified analyses further confirmed these findings, showing that physical activity did not significantly predict SMCs in either pain-stratified group. Interaction terms between physical activity and pain were not significant, indicating no moderating effect of pain on the physical activity-SMCs relationship. These results emphasize the importance of promoting physical activity and managing pain to support cognitive health in older adults, and the need for further research to elucidate underlying mechanisms and mediating roles of self-rated health and depression.

Abstract citation ID: igaf122.3233

### PHOSPHATIDYLSERINE SUPPLEMENTATION ON PSYCHOMOTOR SPEED AMONG HEALTHY ADULTS WITH SUBJECTIVE COGNITIVE DECLINES

Yin Liu[1], Yangfan Lu[2], and Yunping Tang[3], *1. Utah State University, Logan, Utah, United States, 2. ECA Healthcare Inc., Shanghai, Shanghai, China (People's Republic), 3. Zhejiang Ocean University, Zhoushan, Zhejiang, China (People's Republic)*

About 12% of healthy adults in the U.S. have subjective cognitive decline (SCD). SCD coupled with fine motor impairment can increase risks of developing dementia. Supplementation with phosphatidylserine (PS), a fatty substance that improves blood lipid profiles, is associated with improved cognitive functioning in older adults and those with cognitive impairments. However, its benefits for fine motor functioning and SCD is not clear. We conducted a randomized clinical trial to evaluate the safety and potential efficacy of daily PS supplementation on psychomotor speed. The sample included 70 healthy Japanese who self-reported SCD at baseline (mean age = 50.23, sd = 8.68, range = 35-65). The 12-week trial used a stratified blocked randomized, double-blind, and parallel design. Participants received either placebo (n = 37), or DHAPS® product sourced from herring roe at 300 mg/day (n = 33). Computerized finger tapping test measured psychomotor speed. We conducted growth curve analysis to examine the main-effect on changes in psychomotor speed, and the moderating effect of time-varying omega-6/ omega-3 fatty acid ratio. We controlled time-invariant covariates on overall cognitive functioning and demographics measured at baseline. No adverse events were observed. Compared with that of the placebo group, the standardized motor speed (β = 37.73, se = 17.51, p = .03) was significantly faster in the DHAPS® group especially when the omega-6/omega-3 fatty acid ratio improved from the baseline. Regular daily consumption of DHAPS® at 300 mg/day as a nutritional supplement is safe and can benefit fine motor performance for healthy adults experiencing SCD.

Abstract citation ID: igaf122.3234

### REMOTE BADUANJIN IMPROVES COGNITION AND HIPPOCAMPAL CONNECTIVITY IN SUBJECTIVE COGNITIVE DECLINE

Yuan Feng, Sierra Hodges, Bryn Siegel, Valeria Saccà, Yu Liu, Hana Gross, Bonnie Wong, and Jian Kong, *Massachusetts General Hospital, Boston, Massachusetts, United States*

Subjective Cognitive Decline (SCD) is gaining attention as a risk factor for Alzheimer's Disease (AD), representing an optimal intervention stage before irreversible damage occurs. This randomized controlled study examines the effects of Baduanjin (BDJ), a mind-body exercise, on the modulation of cognitive function and its underlying mechanisms in individuals with SCD. A total of 120 SCD participants were randomly assigned to the BDJ group (n = 60) or the cognitive training (CT) group (n = 60) for six months. All interventions were administered remotely. MRI and cognitive assessments including Preclinical Alzheimer's Cognitive Composite (PACC) and Everyday Cognition scales (ECog) were conducted at baseline, 3 months, and 6 months, with an additional cognitive follow-up at 9 months. Transcriptome-neuroimaging analysis using the Allen Human Brain Atlas explored genetic associations with hippocampal functional connectivity differences. Both groups showed significant PACC improvement at 3 and 6 months (p < 0.001), with no significant group differences. No significant improvement was reported on the ECog in either group. Neuroimaging analysis revealed BDJ training is associated with increased hippocampal connectivity with dorsolateral prefrontal cortex, precentral gyrus, and precuneus from baseline to 6 months compared to CT. Transcriptome-neuroimaging analysis linked group differences in hippocampal functional connectivity to neurotransmitter regulation, synaptic plasticity and neuroinflammation-related pathways. Our findings suggest that on-line BDJ training may modulate hippocampal-cognitive and hippocampal-motor cortical functional connectivity, potentially facilitating cognitive improvement.

Abstract citation ID: igaf122.3235

### SLEEP QUALITY AND COGNITIVE FUNCTIONING AMONG CHINESE OLDER ADULTS LIVING IN THE US

Guoping Jin, and Fengyan Tang, *University of Pittsburgh, Pittsburgh, Pennsylvania, United States*

Racial and ethnic disparities in sleep quality and cognitive health are increasingly recognized, yet little is understood about their associations among Chinese older adults living in the U.S. This study aims to examine the relationships between sleep health and cognitive functioning in this population. This observational study utilized a two-wave panel design as part of the Population Study of Chinese Elderly in Chicago, including 2,228 participants aged 65 years or older who self-identified as Chinese. Participants completed interviews at two time points. Mixed-effects regression models were used to assess the effects of sleep parameters on baseline cognitive functioning and cognitive change over time. Participants had an average age of 77.42 years (±7.57) at baseline, with about 39% reporting fairly bad or very bad sleep quality. Poorer overall sleep quality (B = -0.01, SE = 0.01, p<.01), and more insomnia symptoms (B = -0.01, SE = 0.00, p<.001) were associated with lower baseline global cognition. However, these associations diminished over time (sleep quality: B = 0.01, SE = 0.00, p<.05; insomnia: B = 0.00, SE = 0.00, p<.05). Among sleep quality subdomains, all except sleep efficiency had significantly negative relationships with baseline global cognition. The associations between sleep parameters and the four cognitive domains were less consistent. The findings highlight cross-sectional negative relationships between self-reported sleep parameters and cognition, showing distinct associations between various aspects of sleep quality and cognitive domains. Targeted interventions to improve sleep quality may have the potential to enhance cognitive health outcomes.

Abstract citation ID: igaf122.3236

### SPANISH WORD MEANING IN MOBILE TOOLBOX (MTB): ASSOCIATION WITH LANGUAGE PREFERENCES IN DIFFERENT CONTEXTS

Jiwon Kim, Elizabeth Dworak, Y. Catherine Han, Miriam Novack, Cindy Nowinski, Zahra Hosseinian, and Richard Gershon, *Northwestern University, Evanston, Illinois, United States*

Given that Spanish is the second most spoken language in the US, the lack of cognitive assessments in Spanish for older adults presents a significant gap in clinical care and research for both Spanish-speakers and Spanish-English bilinguals. For bilinguals in particular, the optimal assessment language remains unclear, particularly as their language use and preferences may vary across contexts. The Mobile Toolbox (MTB), available in English and Spanish, provides brief, sensitive, cognitive measures for assessing functioning across the lifespan. In this study, we examined the association between language preference in various contexts and vocabulary scores on the MTB Spanish Word Meaning using regression models with orthogonal contrast codes. The sample included 1,620 Spanish-speakers aged 18-64 (M = 42.79, SD = 14.45), all of whom chose to complete all assessment materials in Spanish. Results revealed that preferring to speak English at home was negatively associated with Spanish vocabulary performance (beta=-.27). Conversely, English preferences in work communication (beta=.37), reading official documents (beta=0.35), media consumption (e.g., TV: beta=.59, news: beta=.36), and smartphone settings (beta=.21) were positively associated with Spanish vocabulary scores. English preferences for reading personal content (books) and community communication did not predict vocabulary performance. In sum, these findings suggest language engagement between home and work settings differentially affect Spanish vocabulary performance in Spanish bilinguals. Future work may examine this effect across different MTB assessments (e.g., executive functioning) as well as in clinical samples with dementia-related diagnoses.

Abstract citation ID: igaf122.3237

### SUBJECTIVE MEMORY COMPLAINTS AND OBJECTIVE MEMORY: THE ROLE OF DEPRESSIVE SYMPTOMS AND HEALTH INSURANCE COVERAGE

Hyun Moon[1], Jordana Breton[1], and Elizabeth Muñoz[2], *1. The University of Texas at Austin, Austin, Texas, United States, 2. University of Texas at Austin, Austin, Texas, United States*

Research indicates that higher subjective memory complaints (SMC) prospectively predict memory performance, with depressive symptoms mediating this process. We examined access to health insurance coverage as a marker of access to and availability of timely healthcare as a moderator of this indirect association. Using two-wave data from the Health and Aging Brain Study – Health Disparities, we expected to replicate the indirect effect reported in the literature from SMC (Time 1) to memory performance (Time 2) via depressive symptoms (Time 2). We expanded on this by testing the hypothesis that this mediation effect would be stronger for those without health insurance coverage compared to those with health insurance. We constructed a variable for memory performance, and fitted a multigroup structural equation model with 1,000 bootstrap samples to test group differences. Covariate adjusted models showed that higher SMC at Time 1 predicted greater depressive symptoms at Time 2, which were associated with lower memory performance at Time 2 ($b = -0.027$, $p = .005$, 95% CI [-0.045, -0.009]). Contrary to our expectation, a significant group difference ($\chi2(2) = 11.112$, $p = .004$) indicated that this indirect effect was observed only among individuals with health insurance coverage ($b = -0.026$, $p = .016$, 95% CI [-0.049, -0.005]). These findings suggest that the role of SMC on memory performance may be stronger among those with health insurance coverage. Future research should explore mechanisms underlying this phenomenon (e.g., ongoing monitoring of memory concerns leading to greater depressive symptoms) to inform practical applications.

Abstract citation ID: igaf122.3238

### THINKING ABOUT FORGETTING: AWARENESS OF AGING AND ITS EFFECT ON MEMORY LAPSES

Ryan Fenstermacher[1], and Taylor Nguyen[2], *1. University of California, Irvine, Carlsbad, California, United States, 2. University of California, Irvine, Irvine, California, United States*

Awareness of aging (AoA) refers to a person's perceptions of age-related changes across adulthood. Whereas some people may focus on perceived age-related gains with age, others may instead focus on age-related losses. These perceptions of aging are important for emotional well-being and physical health. The current study examines how AoA is related to one aspect of daily cognitive function, and how this association is related to daily stressors. We hypothesized that perceived age-related losses would be associated with increased memory lapses, an aspect of cognitive functioning that has been linked to later cognitive decline. Additionally, we explored whether daily stressors moderated this relationship. We used the Daily Diary Project and Daily Inventory of Stressful Events (DISE) from the third wave of the Midlife in the United States (MIDUS) study to examine the relationship between perceived age-related losses and memory lapses ($N = 1{,}236$). Multilevel models indicated that individuals who perceived greater age-related loss were more likely to experience memory lapses over a week, after adjusting for demographic factors and objective cognitive functioning. In addition, on days when they reported experiencing stressors, the positive relationship between perceived age-related loss and memory lapses was more pronounced compared to days without stressors. These findings suggest that one's awareness of age-related loss may inform cognitive trajectories. Future research should examine longitudinal relationships between AoA, daily stresses, and long-term cognitive and physical outcomes.

### SESSION 7440 (POSTER)

### COMMUNITY ENGAGED RESEARCH

Abstract citation ID: igaf122.3239

### A COMMUNITY TO CARE FOR ALL GENERATIONS: A UNIVERSITY, MUNICIPALITY, AND NEIGHBORHOOD COLLABORATION

Yuka Sumikawa[1], Mana Shirouchi[2], Hiroshige Matsumoto[2], Kyoko Yoshioka-Maeda[2], Ayumi Igarashi[2], and Noriko Yamamoto-Mitani[3], *1. University of Tokyo, Bunkyo-City, Tokyo, Japan, 2. The University of Tokyo, Tokyo, Tokyo, Japan, 3. The University of Tokyo, Bunkyo, Tokyo, Japan*

Japan is facing unprecedented challenges to sustain the care delivery system, due to the sharp population decline and the increase of super-aged population. Sustainable care system cannot be actualized without working across research, policy planning, and community design with residents, government agencies, and universities in an interdisciplinary effort. Innovative new perspectives on care and well-being are needed. Our university is implementing a Community Based Participatory Research (CBPR) aimed at extending well-being and happiness expectancy of people in the community. At the heart of this project is a hub within our campus where we aim at various intergenerational community activities and exchange. We conducted a "Community Building Workshop" with community residents, public health representatives, and university members. This collaborative workshop brought together different stakeholders to share perspectives on community needs and explore ideal campus-community relationship. The workshop helped participants understand and identify meaningful roles university campuses could play in communities. Through lectures, campus tours, and World Café sessions, insights were gathered from 40 participants, half of whom were local residents. Attendees expressed various expectations for a community-accessible campus, including spaces for casual health consultations, care education opportunities, disaster prevention functions, and intergenerational interaction venues. Post-workshop survey results showed over 80% of respondents were satisfied with the experience. Through this collaborative workshop, we identified invaluable insights that will guide our efforts to create an effective intergenerational campus-community partnership model contributing to sustainable care systems in Japan with its changing demographic profile. This study was approved by our affiliated university ethics committee (2024314NI).

Abstract citation ID: igaf122.3240

### CO-CREATION AND PRELIMINARY EVALUATION OF INTERGENERATION DIALOGUE BOARD GAME FOR HEALTHY AGING

Eliza Lai-Yi Wong, Clement Cheuk-Wai Ng, Carol Ka-Po Wong, Shirley Shuk-Kuen Lui, and Eng-Kiong Yeoh, *The Chinese University of Hong Kong, Hong Kong, China (People's Republic)*

Social isolation is regarded as a priority global health issue, especially among older adults, while intergenerational communication could be a potential mitigating factor to alleviate social isolation by strengthening social connections and interactions between generations. The board game "Tackling Social Isolation: Dialogue Game on Healthy Growing and Aging", which was developed by an expert panel comprising of a nurse, a social worker and a public health worker, covers 77 dialogue cards under six themes: 'Grow-up Experience', 'Daily Life Encounters',

'Interpersonal Relationship', 'Health Literacy', 'Digital Literacy', and 'Action and Body Movement'. A total of 68 participants were recruited based on their age group as youth (18-39), middle-aged (40-64), and older adults (≥65) to enhance the intergenerational mix for the game dynamics. A pre-post intervention survey was conducted in 19 games to evaluate the board game experience. Results showed that most respondents agreed or strongly agreed that the board game could enhance their intergeneration communication (97.0%) and health knowledge (95.6%) as well as feeling better/much better (80.9%) in 'able to cope with life' post-intervention. The paired t-test results revealed that participants improved significantly on intergeneration communication, communication competence, and health literacy ($p < 0.05$). Focusing on the older adults ($n = 31$), medium effect sizes were observed in intergeneration communication (0.639) and health literacy (0.572), while small effect size was revealed in health literacy (0.402). This gamified intervention contributes to fostering engagement of all generations and improving social connection.

Abstract citation ID: igaf122.3241

### COMMUNITY-BASED PARTICIPATORY RESEARCH ON ART ENGAGEMENT FOR PEOPLE WITH DEMENTIA IN JAPAN

Mika Sugiyama[1], Ayako Edahiro[1], Unno Takako[1], Furuta Akiko[2], and Fumiko Miyamae[1], *1. Tokyo Metropolitan Geriatric Hospital and Institute of Gerontology, Itabashi City, Tokyo, Japan, 2. The Juntendo Tokyo Koto Geriatric Medical Center, Koutou City, Tokyo, Japan*

In Japan, Dementia Friendly Communities promote inclusion and social participation for people with dementia. However, they often face difficulties in engaging in social and cultural activities. This study, part of a community-based participatory research project in Chiyoda City, Tokyo, examined whether participatory art, implemented as a community activity rather than in hospitals or care facilities, could serve as a local resource for post-diagnosis support. The program consisted of an 80-minute pastel and colored pencil participatory art activity, followed by a 40-minute peer support meeting. Six participants with Mild Cognitive Impairment (MCI) or dementia participated, with MMSE-J scores ranging from 14 to 30 (mean 24.7 ± 5.5) and ages from 59 to 97 years. Participants were primarily referred by municipal staff, with some introduced by family. Conducted four times over two months in a community setting, the program had a 91.6% attendance rate. A semi-structured group interview was conducted in the final session, and thematic analysis was performed. Thematic analysis revealed that participatory art activities provided "fun and enjoyment," opportunities to "try new things," and the "joy of self-expression." Many participants noted that they had not engaged with art on their own and expressed a decrease in opportunities to experience art in daily life. Many hoped for continued participation in participatory art activities. These findings suggest that participatory art, when integrated into community activities, can be a valuable resource for people with dementia and MCI, fostering social participation and self-expression. Future research should explore sustainable models for community-based dementia-friendly initiatives.

Abstract citation ID: igaf122.3242

### EMPOWERING HOME HEALTH AIDES: IMPACT OF EDUCATION ON JOB SATISFACTION AND PERCEIVED VALUE IN PATIENT CARE

Sasha Vergez[1], Hannah Mitlak[2], Joanna Bryan[3], Margaret McDonald[4], and Madeline Sterling[3], *1. VNS Health, Bronx, New York, United States, 2. Tufts University, Medford, Massachusetts, United States, 3. Weill Cornell Medicine, New York, New York, United States, 4. VNS Health, New York, New York, United States*

Home health aides (HHAs) play a critical role in patient care, yet their contributions are often overlooked, and high turnover rates warrant the need for intervention. This abstract examines HHAs experiences, attitudes toward their work, and perceptions of their overall value in patient care after receiving educational training on heart failure (HF), as a part of a randomized controlled trial - education only vs. education plus use of chat application with nurse managers. Overall, 102 HHAs participated in the study (mean age: 54 years, 98% women, 73% with >5 years experience). Job satisfaction was measured before and 90 days after the course. While the percentage of participants who agreed they were satisfied with their job increased, the change was not statistically significant. Interviews, conducted 45 days post-course with a sub-set of HHAs (64%), were thematically analyzed. HHAs found the training engaging and informed. Many reported applying their knowledge to improve patient care, educate patients, and support families. Participants valued the training professionally and personally, including enhancing their confidence in patient care. Additionally, HHAs noted improved communications and stronger patient-relationships. Many HHAs expressed a desire for additional learning opportunities. The study highlights the potential of educational programs to improve care for other chronic conditions, while simultaneously supporting and improving engagement of HHAs, enhancing workforce efficacy, job satisfaction and possibly patient outcomes. Continuous learning fosters positive attitudes toward professional development and can help retain HHAs, a profession with historically high turnover rates.

Abstract citation ID: igaf122.3243

### ENHANCING RELAXATION THROUGH CO-DESIGN WITH PEOPLE LIVING WITH DEMENTIA: THE CALM ROBOT

Lillian Hung, Irene Chen, Harleen Hundal, Lynn Jackson, Albin Soni, and Jason Fu, *The University of British Columbia, Vancouver, British Columbia, Canada*

The involvement of individuals living with dementia in the co-design of assistive technologies is crucial to ensuring that these innovations meet their needs, preferences, and lived experiences. This study presents insights from a co-design process involving 4 people living with dementia (PLwD), 6 caregivers, and 2 healthcare professionals to develop a social robot designed to facilitate relaxation and deep-breathing exercises. The research employed a co-design framework, integrating PLwD as active participants in design workshops, prototype evaluations, and reflection meetings. Qualitative data were collected through structured discussions, observation notes, and participant feedback. The iterative design process allowed for continuous refinement of the robot's features, focusing on tactile engagement, intuitive interactions, and non-verbal communication. Findings highlight the importance of structured workshop discussions, the use of visual aids, and the creation of an inclusive environment to facilitate participation by PLwD. Participants reported that the robot provided a calming experience and demonstrated potential for use in care settings. However, some PLwD expressed hesitation in sharing opinions when caregivers were present, suggesting the need for separate workshops to foster uninhibited feedback. This study provides a model for integrating intended users into the design process, offering insights for researchers developing assistive technologies in dementia care.

Abstract citation ID: igaf122.3244

**FOSTERING CIVIL DISCOURSE ACROSS GENERATIONS: INTERGENERATIONAL DIALOGUE ON SOCIAL JUSTICE THROUGH ART**

Kozbi Bayne[1], Rouida Siddiqui[1], Thomas Lu[1], Ruy Tobar-Mosqueira[2], Todd Prusin[1], and Madeleine Hackney[1], *1. Emory School of Medicine, Atlanta, Georgia, United States, 2. Emory Primate Center, Atlanta, Georgia, United States*

Little is known about generational differences and similarities in beliefs about social justice. Science Gallery Atlanta (SGA) is a museum integrating science and art to foster dialogue about challenging topics, including social justice. Previous research suggests intergenerational communication about social and political issues could positively impact older and younger adults. This study investigated perceptions of justice among a diverse sample of older (65+ years) and younger (18–26 years) adults through intergenerational dialogue on justice-focused art offered through SGA's JUSTICE exhibition. This exploratory pilot study recruited ten randomized older-younger dyads who received a mediated tour of JUSTICE. Attitudes about discrimination, equity, and activism were assessed pre- and post-tour through self-report measures, including the Belief in a Just World, Addiction Belief, and Everyday Discrimination Scales, concurrent interviews, and focus groups. Qualitative data were recorded, transcribed, and analyzed thematically using a nodal framework. Pre and post-tour, older adults were more confident than their younger counterparts about the involvement of others in social justice work. Post-tour, young adults rated community involvement in social justice work more favorably and expressed greater support for activism in daily life ($p = 0.05$). Interestingly, younger adults reported experiencing greater discrimination than older adults pre and post-tour ($p < 0.05$). Based on qualitative results, intergenerational bridging may help young adults trust in community activism's efficacy and help older adults understand technology's potential role in social change. Emergent themes illuminate generational differences and similarities in perceptions of social justice, suggesting SGA's interdisciplinary experience effectively promotes discourse across different age groups.

Abstract citation ID: igaf122.3245

**IMPROVING VIETNAMESE IMMIGRANTS' COGNITIVE HEALTH LITERACY: UNIVERSITY-COMMUNITY PARTNERSHIPS**

Christina Miyawaki[1], Nguyen Nguyen[1], Tuong-Vi Ho[2], and Angela McClellan[1], *1. University of Houston, Houston, Texas, United States, 2. Texas Woman's University, Houston, Texas, United States*

After the fall of Saigon in 1975, waves of Vietnamese refugees/immigrants migrated to the United States (U.S.). Currently, 2.3 million Vietnamese live in the U.S. Many came with adverse conditions; however, their health data is scarce. After 50 years, many are aged and may have unmet health needs. To fill this gap, we developed the Vietnamese Aging and Care Survey and collected their health data in Houston, Texas, which revealed a high prevalence of physical, mental, and cognitive disabilities. Using the university-community partnerships, we developed a linguistically and culturally tailored dementia one-pager, formed the Cognitive Health Initiative (CHAIN), and offered Vietnamese refugees/immigrants complimentary memory tests. This study explains how the CHAIN program operates and fosters intergenerational relationships. Using the Cultural Exchange Model, we trained bilingual/bicultural Vietnamese college students (Cohort 1) and conducted cognitive assessments, Vietnamese Montreal Cognitive Assessment (V-MoCA), in 2023. In 2024, when Cohort 2 students joined, we formed mentor (Cohort 1)-mentee (Cohort 2) dyads. Cohort 1 demonstrated the assessment while Cohort 2 observed their mentors perform assessments. After several demonstrations, Cohort 2 tried the assessments supervised by Cohort 1. They repeated this sequence until Cohort 2 felt comfortable conducting the assessments independently. During 2023-2024, we recruited 42 students, attended 24 health fairs, and offered 406 V-MoCA. Mentors (Cohort 1) completed training their mentees (Cohort 2) with tips for conducting the V-MoCA and how to work with older adults. Through the mentor-mentee relationships, the CHAIN promoted teamwork, accountability, ethics, and responsibility to the community, and demonstrated successful university-community partnerships.

Abstract citation ID: igaf122.3246

**LEARNING AND GROWING TOGETHER: MORE FULLY INTEGRATING RESEARCH, PRACTICE, AND ORGANIZATIONAL POLICY**

Lea Efird-Green[1], Jennifer Terray[2], Heidi Gil[2], and Sheryl Zimmerman[3], *1. The University of North Carolina at Chapel Hill, Chapel Hill, North Carolina, United States, 2. LiveWell, Plantsville, Connecticut, United States, 3. University of North Carolina at Chapel Hill, Chapel Hill, North Carolina, United States*

Community-engaged research is important if results are to be relevant to end-users and inform practice and policy; however, the extent to which such research truly partners with community members is highly variable. This project expanded a partnership that developed two "living well with dementia" measures, such that the community partner was fully engaged in actually designing and conducting the evaluation of those measures; in this way, the extent to which the partner functioned as the researcher and informed the research process constitutes a new model for community-engaged research. Process findings indicated full partnership requires that (1) the mission of the partner be fully aligned with and supported by the focus of the research (in this case, LiveWell, a dementia-focused services organization committed to a strengths-based approach to supporting the wellbeing of people living with cognitive change or dementia); (2) the partner be invested in learning about and contributing to the research process (including the development of pragmatic study protocols); (3) the researcher be open to developing protocols that extend beyond traditional research (e.g., being willing to solicit IRB-approval that confidential information may be incorporated into the partner's care plans – notably, to date nearly 80% of consents have included permission for this sharing); and (4) the researcher allow that the protocol may expand to include participants ineligible for study. This presentation will detail these and other novel findings to serve as a model of research that benefits community partners and researchers and has true promise to inform practice and policy.

Abstract citation ID: igaf122.3247

NOURISH TO FLOURISH: A MINDFUL EATING PROGRAM'S IMPACT ON HEALTH AND WELLBEING AMONG OLDER ADULTS

Ajla Basic[1], Teresa Fung[2], and Catherine O'Brien[1], *1. Mather Institute, Evanston, Illinois, United States, 2. Harvard T.H. Chan School of Public Health, Boston, Massachusetts, United States*

Mindful eating is an evidence-based approach that enhances awareness of food choices, eating behaviors, and overall well-being. This study explores outcomes of Nourish to Flourish, a four-week mindful eating program designed for adults 50+ across seven sites in the Chicagoland area from July to October 2024. Facilitators guided participants through a structured curriculum that emphasized eating with intention, recognizing hunger and fullness cues, and fostering a positive relationship with food. Participants completed pre- and post-program surveys assessing mindful eating behaviors, self-reported health, spirituality, and engagement in healthy behaviors. Among 98 participants who attended the first session, 78 (89.6% females) completed the pre and post survey. Findings revealed statistically significant improvements from pre to post in the following measures: mindful eating (M=-.416; SD=.527); [t(77) = -6.97, p <.001], healthy eating identity (M=-.319; SD=.755) [t(73) = -3.64, p < .001] and spirituality (M=-1.08; SD = 2.62); [t(70) = -3.47, p < .001] Statistically improvement in self-reported health was also observed (M=-.197; SD=.748); [t(75) = -2.29, p < .024]. Additionally, participants evaluated the program highly; 93.4% rated their satisfaction with the program highly and 100% claimed they would continue mindful eating practices. In conclusion, a 4-week mindful eating program was successful in improving a number of aspects in dietary and holistic health. Future studies may focus on the long term sustainability of these results.

Abstract citation ID: igaf122.3248

RECALIBRATING THE VILLAGE: NURTURING HOLISTIC WELL-BEING FOR BLACK OLDER ADULTS

Raymond A. Jetson, *Aging While Black, Baton Rouge, Louisiana, United States*

In the face of persistent disparities and social inequities, Black older adults continue to demonstrate remarkable fortitude, often rooted in the strength of their communities – the modern-day "villages" that provide multifaceted support. We explore how these existing community structures can be leveraged and enhanced to promote comprehensive well-being among Black older adults, addressing the complex interplay of factors that contribute to quality of life in later years. Drawing on recent studies and innovative community initiatives, we will examine how traditional support systems like churches, fraternal organizations, and community centers are evolving to meet the multidimensional needs of today's Black older adults. Through case studies of programs that have successfully "recalibrated" traditional community structures to address contemporary challenges holistically, we will describe church-based wellness initiatives that integrate physical, mental, and spiritual health; fraternity-led mentorship programs that foster purpose and social engagement for older men; and community center-based initiatives that address digital empowerment, financial wellness, and creative expression. Our presentation will conclude with a framework for gerontologists, policymakers, and community leaders to assess and enhance the "village" support system in their own communities. This framework emphasizes culturally relevant approaches that honor the strength and determination of Black older adults while addressing their unique experiences and needs. By recalibrating these vital community networks through a holistic lens, we can create more robust, responsive, and effective support systems for Black older adults, ultimately improving their overall well-being, sense of belonging, and quality of life across multiple dimensions.

Abstract citation ID: igaf122.3249

VOICES OF OUR ELDERS: COMMUNITY ENGAGED RESEARCH WITH WABANAKI TRIBES IN MAINE ADDRESSING DEMENTIA

Ralph Cammack[1], and Patrik Johansson[2], *1. Wabanaki Public Health and Wellness, Bangor, Maine, United States, 2. Washington State University, Everett, Washington, United States*

Wabanaki Public Health and Wellness is a Tribally operated public health that serves the four federally recognized Tribes in the state of Maine . Wabanaki Public Health and Wellness is the principal investigator on the Wabanaki Native American Research Center for Health (Wabanaki NARCH), which is supported by the National Institutes of Health. Wabanaki NARCH aims to enhance support systems, promote cultural continuity, and improve the overall well-being of Wabanaki Elders living with dementia. In partnership with two academic institutions, Washington State University and the University of Miami, Wabanaki Public Health and Wellness, through Wabanaki NARCH, is conducting Voices of Our Elders (VOE), an epidemiologic study that seeks to better understand the prevalence of Alzheimer's disease and Alzheimer's disease and related dementia (AD/ADRD) and cognitive decline. VOE also seeks to assess health and lifestyle risk factors for AD/ADRD and cognitive decline among Wabanaki Elders. This presentation will offer the perspectives and lessons learned from a Tribal public health district and its academic partners on: 1. The process of developing, adapting, and Indigenizing study designs and research methodologies of an AD/ADRD study that seeks to better serve the needs and perspectives of Wabanaki Elders; 2. Community engagement strategies employed to reach Wabanaki Elders to participate in VOE; and 3. Ethical aspects and strength-based and capacity building approaches for all research partners to consider when conducting research with Tribal communities in addition to the engagement with academic institutional review boards.

### SESSION 7445 (POSTER)

### CROSS-CULTURAL/CROSS-NATIONAL STUDIES

Abstract citation ID: igaf122.3250

BEYOND INDEPENDENCE: CULTURAL VALUES AND DEPENDENCE AVOIDANCE AMONG OLDER ADULTS IN JAPAN AND SOUTH KOREA

Tomoko Ikeuchi[1], Tzu-Yu Lin[2], and Donghee Han[3], *1. Takachiho University, Tokyo, Tokyo, Japan, 2. Kaohsiung Medical University, Kaohsiung, Kaohsiung, Taiwan (Republic of China), 3. Research Institute of Science for the Better Living of the Elderly, Busan, Busan, Republic of Korea*

Japan and South Korea are experiencing rapid depopulation and population aging, with a growing number of older adults living alone while striving to remain independent. In these East

Asian cultures, maintaining harmony and avoiding burdening others are highly valued. This study examines whether the desire to avoid dependence differs from a preference for independence. Data were collected in February 2025 through online surveys of 800 Japanese and 600 Korean participants (Mage=58.0, SD = 10.2, range=40-81; 50.9% women). Multiple regression analyses revealed that, in both countries, women and older age were associated with greater reluctance to depend on or inconvenience people outside their in-group. However, when considering in-group members (i.e., family, close friends, colleagues), this association was found only for older adults in Japan. Similarly, a preference for independence was linked solely to older age in Japan. These findings suggest that women and older individuals may refrain from seeking support from those outside their in-group, such as community members or government service providers, even when their in-group members are no longer dependable. Furthermore, a reluctance to depend on others may not necessarily equate to a preference for independence. Given that women in these nations have some of the world's longest life expectancies, our findings highlight the need for accessible support systems tailored to older women while respecting their cultural values. This study underscores the importance of culturally sensitive approaches to aging support in East Asian societies, recognizing the distinction between avoiding dependence and preferring independence.

Abstract citation ID: igaf122.3251

### CROSS-NATIONAL COMPARISON OF HEARING STATUS, SOCIAL ACTIVITIES, AND VERBAL MEMORY: UNITED STATES, MEXICO AND CHINA

Chengming Han[1], Brian Downer[2], Rebeca Wong[3], and Zhiwei Hu[4], *1. UTMB Health, The University of Texas Medical Branch, Galveston, Texas, United States, 2. UTMB, Galveston, Texas, United States, 3. The University of Texas Health Science Center at San Antonio, San Antonio, Texas, United States, 4. the University of Texas Medical Branch, Galveston, Texas, United States*

Objective: This study explores the mediating effect of social activities on the association between hearing status and cognitive function across three countries. Method: Data from three waves of the HRS (United States), MHAS (Mexico), and CHARLS (China), collected from 2011 to 2021, were analyzed. Multilevel models were used to examine the association between self-reported hearing status (fair/poor vs. good/excellent) and verbal memory. The mediating effects of social activities were assessed using the Karlson-Holm-Breen (KHB) method. Results: Fair/poor hearing was associated with lower verbal memory in the HRS (b=-0.145, SE = 0.023) and CHARLS (b=-0.056, SE = 0.020), but not in MHAS (b=-0.001, SE = 0.018). Social activities were positively associated with verbal memory in all three datasets. Fair/poor hearing was associated with reduced social activities in the HRS (OR = 0.838, $p < 0.001$), MHAS (OR = 0.847, $p < 0.01$), and CHARLS (OR = 0.779, $p < 0.001$). The association between self-rated hearing and verbal memory remained statistically significant after adjusting for social activities in the HRS (b=-0.144, SE = 0.023) and CHARLS (b=-0.045, SE = 0.021). Conclusion: We did not find universal mediation effects of social activities on the association between hearing status and verbal memory. These findings underscore the need for context-specific approaches to addressing sensory loss and cognitive health.

Abstract citation ID: igaf122.3252

### HEALTH-RELATED QUALITY OF LIFE AMONG OLDER ADULTS IN KAZAKHSTAN DURING THE PANDEMIC

Assel Izekenova[1], Aigulsum Izekenova[2], Yineng Chen[3], Dinara Sukenova[2], and Fei Sun[1], *1. Michigan State University, East Lansing, Michigan, United States, 2. Asfendiyarov Kazakh National Medical University, Almaty, Almaty, Kazakhstan, 3. University at Albany, Albany, New York, United States*

The COVID-19 pandemic has significantly impacted the well-being of older adults, raising concerns about their quality of life (QOL). This study examines the factors influencing QOL among older adults in Kazakhstan during the pandemic, specifically focusing on health-related outcomes. A cross-sectional survey was conducted from June to July 2022 with 445 community-dwelling individuals aged 60 and above. The Older People's Quality of Life (OPQOL) scale was used to assess QOL, while psychological distress and loneliness were measured using the PHQ-4 and UCLA-3 scales, respectively. Univariate and multivariate regression analyses were applied to explore associations between QOL and demographic characteristics, pandemic-related experiences, psychological distress, and loneliness. Results revealed a mean OPQOL total score of 124.1 (SD = 11.3) and a mean health-related sub-score of 13.8 (SD = 2.9). Higher QOL was significantly associated with the absence of chronic diseases ($p < 0.001$), Kazakh ethnicity ($p = 0.003$), and minimal psychological distress ($p < 0.001$). Participants who downplayed concerns about COVID-19 tended to report better overall and health-related QOL ($p \leq 0.005$). Additionally, younger age ($p \leq 0.031$), better self-reported health ($p < 0.001$), and lower concern for family members' infection risk ($p = 0.024$) were linked to higher health-related QOL scores. These findings emphasize the role of health status, psychological resilience, and perceptions of the pandemic in shaping older adults' QOL. Targeted interventions addressing health disparities and pandemic-related stress may improve well-being among older populations in Kazakhstan.

Abstract citation ID: igaf122.3253

### OLDER ADULTS AS EDUCATORS: INSIGHTS ON TEACHING, LEARNING AND WELL-BEING ACROSS TWO CONTINENTS

Giuliana Casanova[1], Margarida Cerqueira[1], and Joyce Weil[2], *1. University of Aveiro, Aveiro, Aveiro, Portugal, 2. Towson University, Towson, Maryland, United States*

Older adults are often perceived as passive recipients of care, overlooking their capacity for meaningful contributions. Older adults also actively engage in lifelong learning, taking on the role of educators, sharing knowledge gained through their professional careers or hobbies with their peers at universities of the third age (U3As) in Portugal and in lifelong learning institutes (LLIs) in the USA. Still understudied in lifelong learning research, this study provides empirical insights into the experiences of older adults as educators. By analysing motivations, rewards, challenges, improvement opportunities and well-being outcomes, this research highlights the individual benefits and informs broader discussions on aging, education and best practices. This multiple case study includes a total of 35 retired older adults, aged 61 to 84. Semi-structured interviews and reflexive thematic analysis were used to explore key themes. Purpose driven engagement and passion for sharing knowledge emerged as primary motivations. Mutual learning, community and social connections as well as student development were the most valued rewards. Challenges related to teaching preparation, classroom dynamics and environmental constraints were identified. Marketing and outreach were noted as strategies for improvement. Emotional and cognitive health were identified as significant well-being outcomes. The Portuguese data reveal some similarities with the USA sample, particularly in relation to mutual learning, growth and classroom dynamics. This cross-cultural comparison provides a deeper understanding of the role of older adults as educators, highlighting the importance of their involvement in their lives and offering insights to enhance their engagement and contributions to education and their community.

Abstract citation ID: igaf122.3254

### TRANSFORMING ANXIETIES OF AGING IN SOUTH-EASTERN EUROPE: A PUBLIC HEALTH HUMANITIES INITIATIVE

Ulla Kriebernegg, *University of Graz, Graz, Steiermark, Austria*

This paper presents the interdisciplinary research project "Transforming Anxieties of Aging in South-Eastern Europe (2023–2027)", funded by the German Volkswagen Foundation. The project investigates demographic nationalism in the region, where rapid population aging is increasingly framed as a societal "burden." South-Eastern Europe has transitioned from high population growth to the fastest aging rates in Europe, with alarmist narratives in media and policy shaping attitudes toward aging and migration. The COVID-19 pandemic further exacerbated these issues, with the region experiencing some of the highest global mortality rates, particularly among older populations. Using an innovative Public Health Humanities approach, this project explores how ageist narratives articulate broader societal anxieties—ranging from concerns over the welfare state and territorial disputes to migration and shifting minority demographics. By examining how fears are transferred across different social domains, the study critically analyzes the metaphors and framings of demographic change. A transnational, interdisciplinary team from nine countries integrates Age Studies and Area Studies, combining qualitative and quantitative methods from social history, anthropology, sociology, demography, political science, cultural studies, and literary studies. The project employs an intersectional, participatory Citizen Science approach to reshape aging narratives—moving beyond alarmist portrayals toward empowering perspectives. Reflexively addressing "age bias" within the research team itself, this study highlights the essential role of Age Studies / Cultural Gerontology in understanding demographic discourse and developing socially just, intergenerationally inclusive policies. The paper will argue that the project can be framed as a Public Health Humanities Initiative that opens new horizons in (Cultural) Gerontology.

### SESSION 7450 (POSTER)

### DISASTERS AND EMERGENCIES

Abstract citation ID: igaf122.3255

### "HOPE DIES LAST": OLDER FSU IMMIGRANTS DISPLACED FROM THE GAZA ENVELOPE DURING THE ISRAEL HAMAS WAR

Ksenya Shulyaev[1], Tova Band-Winterstein[1], Pnina Dolberg[2], and Gabriela Spector-Mersel[3], *1. University of Haifa, Haifa, HaZafon, Israel, 2. The Ruppin Academic Center, Kfar Monash, HaMerkaz, Israel, 3. Sapir College, Sderot, HaDarom, Israel*

Introduction The October 7th Hamas attack and the subsequent war led to the immediate evacuation of tens of thousands of residents from the Gaza Envelope. Among them were older immigrants from the Former Soviet Union (FSU) who had lived in a Continuous Traumatic Situation (CTS) for years. This study explores the unique experiences of these displaced older adults, examining the intersections of aging, migration, and prolonged exposure to conflict. Methods This qualitative-narrative study is based on in-depth interviews with ten FSU immigrants (both men and women), aged 75+, who lived in the Gaza Envelope for at least a decade and were displaced following the October 7th attack. The study employed semi-structured interviews to examine their lived experiences of forced displacement, past immigration, and long-term exposure to trauma. Results Four key themes emerged: (1) From trauma to accumulated trauma—a lifetime of exposure to war-related events, (2) "You're not a child anymore. You're an adult—pull yourself together"—self-discipline and emotional regulation as coping mechanisms, (3) Life in limbo between alienation and belonging—navigating identity as older migrants in a conflict zone, and (4) Social networks as resilience anchors—the vital role of community and structured support in coping with displacement. Conclusions The study highlights the complex interplay between past and present traumas in the lives of older FSU immigrants living near Gaza. Findings underscore the need for targeted interventions that enhance resilience, foster a sense of continuity, and provide tailored support to older displaced immigrants facing prolonged adversity.

Abstract citation ID: igaf122.3256

### AGE, RESILIENCE AND HOPE PREDICT DAILY FUNCTIONING AFTER A FLOOD

Katie Cherry[1], Luke Miller[1], Piper Bordes[1], Matthew Calamia[1], Emily Elliott[1], Laura Sampson[2], and Sandro Galea[3], *1. Louisiana State University, Baton Rouge, Louisiana, United States, 2. Stony Brook University, Stony Brook, New York, United States, 3. Washington University in St. Louis, St. Louis, Missouri, United States*

Prior evidence indicates that multiple flood and hurricane exposures may threaten daily functioning and psychological well-being among younger and older adults. In 2005, coastal residents from south Louisiana lost homes in Hurricane Katrina. Many permanently relocated to the greater Baton Rouge region, an inland community on presumably higher and safer ground. In August of 2016, historic flooding brought widespread destruction across a 22-parish (county) area, creating a second round of catastrophic losses for those who had moved to Baton Rouge after Katrina. The present research is part of a larger study on health and well-being after multiple disaster exposures. Here we examined associations among self-reported daily functioning indexed by the Barkley Functional Impairment Scale (BFIS) and resilience and risk factors within the first two years of the flood. Three flood exposure groups were compared: non-flooded (controls), single disaster (flooded in 2016) and double disaster (flooded in 2005 and again in 2016). Results indicated that the flood exposure groups did not differ in self-reported daily functioning, although symptoms of depression and post-traumatic stress were greater for those whose homes flooded relative to the non-flooded controls. Regression analyses indicted that age, resilience, hope, depression, and PTS symptoms predicted daily functioning after key sociodemographic factors, flood stressors, and prior lifetime trauma were accounted for. Strategies for managing the long-term impacts of multiple disasters for people who live in geographic areas that are prone to severe weather events are discussed.

Abstract citation ID: igaf122.3257

### DIFFERENTIAL IMPACT OF NATURAL DISASTERS ON MENTAL HEALTH ACROSS RACIAL AND ETHNIC GROUPS

SM Foysol Ahmed, and Yeon Jin Choi, *University of Kentucky, Lexington, Kentucky, United States*

Natural disasters are increasing in both frequency and severity, affecting 224.1 million people globally each year. These events have profound consequences for public health and well-being, as exposure to natural disasters has been found to be associated with poor mental health outcomes. The adverse impact of natural disasters may vary across racial and ethnic groups due to their differences in socioeconomic status, trauma exposure, and access to healthcare. However, existing literature primarily focuses either on a single racial and ethnic group or broadly on affected populations, limiting our understanding of how natural disaster exposure affects mental health across diverse racial and ethnic groups. This study examines whether lifetime experience of natural disasters is associated with poor mental health and whether the impact differs across racial and ethnic groups, utilizing data from the 2010/2012 Health and Retirement Study ($N = 13,817$). A series of weighted ordinary least squares regression models were estimated, adjusting for sociodemographic characteristics. The results indicate that natural disaster exposure is associated with higher levels of depression and anxiety, with a greater impact observed among older Blacks (depression: $\beta=.52$, $p<.001$; anxiety: $\beta=.19$, $p<.001$) compared to older Whites (depression: $\beta=.11$, $p<.05$; anxiety: $\beta=.07$, $p<.001$). The association was not significant among older Hispanics (depression: -.20, $p=.45$; anxiety: .02, $p=.82$). Our findings align with previous research indicating greater vulnerability to natural disasters among racial and ethnic minorities. They underscore the need for tailored policy and intervention strategies for these communities, such as culturally informed mental health care and disaster preparedness and response plans.

Abstract citation ID: igaf122.3258

### ENVIRONMENTAL ADAPTATION FOR PERSON-CENTERED CARE: LESSONS FROM AN UNDERGROUND MEDICAL FACILITY

Sharon Ost-Mor[1], Elena Titelman[2], Eduard Zlyesov[2], and Inna Shugaev[2], *1. University of Haifa, Haifa, HaZafon, Israel, 2. Fliman Medical Center, Haifa, HaZafon, Israel*

Environmental factors significantly impact health outcomes as people age, yet little research explores strategies for maintaining quality care when traditional healthcare environments become unavailable. This study examines the successful conversion of an underground parking facility into a functional geriatric rehabilitation center during an extended emergency relocation at Fliman Medical Center. Through a detailed case study, including in depth interviews and diaries, the research analyzes key environmental modifications and operational adaptations necessary to sustain comprehensive carein a non-traditional setting. Specifically, it evaluates environmental control systems, space utilization, therapeutic environment creation, and strategies for preserving patient and staff wellbeing. Findings highlight the effective implementation of critical adaptations, including optimized lighting, ventilation, and temperature control tailored to rehabilitation needs; efficient spatial organization to support rehabilitation services; the creation of therapeutic environments despite spatial constraints; and innovative solutions to maintain patient and staff wellbeing under suboptimal conditions. These results demonstrate that high-quality medical care can be preserved in unconventional settings through strategic environmental modifications. This study offers valuable insights for healthcare facility design, emergency preparedness planning, and the adaptation of non-traditional spaces for care delivery, providing practical guidance for maintaining older adults' quality care during emergencies and facility transitions.

Abstract citation ID: igaf122.3259

### GENDER DIFFERENCES IN THE MENTAL HEALTH IMPACT OF NATURAL DISASTER EXPOSURE AMONG OLDER ADULTS

Yeon Jin Choi, and Karen Lawrence, *University of Kentucky, Lexington, Kentucky, United States*

Natural disasters are becoming more frequent and severe, posing significant risks to human health and well-being. The U.S. is among the most disaster-prone countries, with over 85 million people affected by natural disasters in 2016 alone. Prior research links disaster exposure to poor mental health outcomes, yet less is known about how these effects differ by gender, particularly among older adults. Women are generally more vulnerable to mental health disorders following stressful or traumatic experiences, yet the extent to which these gender differences manifest in disaster-related mental health outcomes remains unclear. We examined whether the association between natural disaster exposure and depressive symptoms and anxiety varies by gender, using data from the 2010/2012 Health and Retirement Study ($N = 13,419$; $M_{age}=65.12$, $SD = 10.3$; female: 54.5%). Among the study sample, 19% of men and 17% of women reported natural disaster exposure. A series of weighted ordinary least squares regression models, stratified by gender, were estimated, adjusting for sociodemographic characteristics. Results indicated that natural disaster exposure was associated with increased depressive and anxiety symptom severity in both men and women. However, associated symptom severity differed by gender: men reported higher depressive symptom severity scores ($\beta=.22$, $p<.01$) than women ($\beta=.14$, $p<.05$), whereas women reported higher anxiety symptom severity scores ($\beta=.08$, $p<.01$) than men ($\beta=.09$, $p<.001$). These findings highlight the differential mental health impact of natural disasters by gender. Disaster response efforts should consider gender-specific vulnerabilities and provide tailored mental health resources to address the unique needs of older men and women.

### SESSION 7455 (POSTER)

### EDUCATION AND TRAINING

Abstract citation ID: igaf122.3260

### ADDRESSING STRUCTURAL AND PERSONAL BARRIERS TO ENHANCE DIVERSITY IN STEM FIELDS

Maeva Laflamme[1], Melanie Horn Mallers[2], Jennifer Piazza[2], and Laura Zettel-Watson[2], *1. California State University, Fullerton, Irvine, California, United States, 2. California State University, Fullerton, Fullerton, California, United States*

As the U.S. population rapidly ages, the need for a diverse and highly trained MSTEM (Medicine, Science, Technology,

Engineering, and Mathematics) workforce with expertise in aging science has never been greater. By 2030, adults over 65 will comprise more than 21% of the population, requiring innovative solutions in healthcare, biotechnology, and engineering (Colby & Ortman, 2014; Vespa et al., 2020). However, despite the increasing diversity of the population, MSTEM fields continue to lack representation from underrepresented minority students, first-generation students, and those from disadvantaged backgrounds (National Science Foundation, 2019). Much of the research on STEM disparities focuses on structural barriers, including financial constraints, access to advanced coursework, and systemic bias in academic institutions. However, personal barriers such as self-efficacy, sense of belonging, and psychological well being are often overlooked, despite their significant role in persistence and success (Matthews et al., 2020). Without a sense of belonging or personal confidence in their abilities, underrepresented MSTEM students may internalize failure, contributing to the "leaky pipeline" in STEM education. This study employs semi-structured interviews with 12 students participating in a two-year program focused on aging research. Students were asked seven open-ended questions about their experiences. The questions explored belonging/identity, and self-efficacy. Interviews were recorded for transcription and coding purposes. Results indicate that the two-year program significantly enhanced students' sense of belonging by fostering a supportive community of individuals with shared goals and aspirations. Mentorship contributed to increased self-efficacy, and students reported feeling supported, attributing the program to improvements in their well-being.

Abstract citation ID: igaf122.3261

### AGING KNOWLEDGE MODERATES HOW AGING ANXIETY RELATES TO EXPECTATIONS OF COUNSELING CLIENTS WITH ALZHEIMER'S DISEASE

Mackenzie Kirby, and Grace Caskie, *Lehigh University, Bethlehem, Pennsylvania, United States*

As the aging population grows, psychologists must be prepared to meet older adults' psychological needs, including those with neurocognitive disorders such as Alzheimer's disease (AD). Because few psychology trainees receive any aging-focused training, their clinical expectations for counseling older adult clients with AD may be negatively influenced by a lack of knowledge about aging and by their aging anxiety, which could compromise quality of care. This study examined how trainees' aging anxiety related to their expectations about counseling an older adult client with AD and whether knowledge about aging moderated this relationship. Doctoral trainees (N = 188; 21-35 years) in clinical and counseling psychology completed measures assessing aging anxiety, knowledge about aging (biological, psychological, social domains), and expectations about counseling an older adult recently diagnosed with AD, described in a case vignette. Regression analyses found that greater aging anxiety (surrounding psychological concerns and fear of old people) predicted trainees' perceptions of this client as less open to therapy, having less motivation to engage in therapy, and having less responsibility for their therapy. Moderation analyses indicated having greater psychological aging knowledge made trainees more resilient to the negative influence of psychological concerns about aging on expectations of the client, however with low biological aging knowledge, less fear of old people was related to more positive clinical expectations of the client. These findings highlight the importance of integrating targeted geropsychology education into psychology training to reduce the influence of aging anxiety on clinical expectations and improve preparedness for counseling older adults with AD.

Abstract citation ID: igaf122.3262

### BRIDGING HRSA PRIORITY GAPS IN GERIATRICS EDUCATION: INSIGHTS FROM GERIATRIC FAST FACTS USAGE DATA

Edmund Duthie[1], Angela Beckert[1], Kathryn Denson[1], Amanda Szymkowski[1], Deborah Simpson[2], and Steven Denson[1], *1. Medical College of Wisconsin, Milwaukee, Wisconsin, United States, 2. Advocate Aurora Health, Milwaukee, Wisconsin, United States*

The Health Resources & Services Administration (HRSA) has identified 11 priority topics for interprofessional training and education to address the primary care needs of older adults. Healthcare professionals and learners increasingly rely on online resources for focused education. The Geriatric Fast Facts (GFFs) website is a globally accessed mobile enabled website for geriatrics education. Analyzing user engagement data from this site can identify gaps between expert-defined priorities and user-identified needs, informing targeted educational dissemination efforts. 107 GFFs were mapped to the 11 HRSA age/dementia-friendly priority topics. Google Analytics usage data from 2/25/2024 – 2/24/2025 was analyzed to assess GFF user engagement, including usage per topic and alignment with HRSA priorities. 42% (45/107) of GFFs were associated with 7 HRSA priorities. Of the top 25 viewed GFFs, 8 (32%) were associated with 3 HRSA priorities: Elder Justice (n = 3), Early detection, diagnosis and treatment of dementia (n = 4), and ADRD/ other mental health conditions (n = 1). Elder Justice-associated GFFs had the highest engagement (3,008 views for 7 GFFs), while Health Inequities, Disparities, and Social Determinants of Health had the lowest (35 views for one GFF). Four HRSA priorities were not associated with any GFF. User engagement with GFFs shows selective alignment with HRSA priorities with highest user interest in Elder Justice. This gap analysis reveals the need to enhance geriatrics education by expanding content related to underrepresented HRSA priorities (e.g., abuse prevention and legal protections) and the need to enhance dissemination strategies for under accessed topics.

Abstract citation ID: igaf122.3263

### BUILDING A 'RAMP' TO AGING RESEARCH: DEVELOPING A TWO-YEAR SUMMER HIGH SCHOOL RESEARCH TRAINING PROGRAM

Morgan Fique[1], Elizabeth Dennis[1], Anna Pudder[1], Linda Horn[2], Cara Felter[2], and Bret Hassel[1], *1. University of Maryland School of Medicine, Baltimore, Maryland, United States, 2. University of Maryland, Baltimore County, Baltimore, Maryland, United States*

Participation in structured research and mentorship programs increases students' likelihood of enrolling in college and graduate school while boosting their confidence in becoming researchers or clinicians. There is also a need to expand the aging research workforce; however, students are unlikely to be exposed to aging research at early educational levels. The Research and Mentoring Program (RAMP) is a summer research training program targeting 11th and 12th grade high school (HS) students from Baltimore City. This two-year program was designed to provide foundational knowledge across the translational research continuum while equipping students with basic lab techniques and clinical measurement skills (Year 1) to prepare them for a mentored summer research experience (Year 2) with faculty conducting aging-focused research from the University of Maryland Claude D. Pepper Older Americans Independence Center, University of Maryland Greenebaum Comprehensive Cancer Center (UMGCCC) and Baltimore Geriatric Research, Education, and Clinical Center (GRECC) within the VA Maryland Health Care System. In turn, RAMP faculty and near peer mentors receive mentoring training to enhance their experience in working with HS trainees. Scholars are compensated both summers through a partnership with Youthworks, a workforce development program funded by Baltimore City. This study tests the feasibility of developing and implementing a scalable training and education program that can be disseminated to other academic institutions providing a path to aging-focused research and clinical career pathways for HS students.

Abstract citation ID: igaf122.3264

**ENHANCING INTERGENERATIONAL PROGRAMS: OLDER ADULTS' PERSPECTIVES ON CHALLENGES AND OPPORTUNITIES**

Zeyu Liu[1], Rana Zadeh[2], and Karl Pillemer[2], *1. Cornell University, Jersey City, New Jersey, United States, 2. Cornell University, Ithaca, New York, United States*

Intergenerational programs (IGPs) are critical interventions for enhancing physical, psychological, social, and cognitive well-being by fostering social connectedness and reducing isolation among older adults. However, a gap in existing research is the limited understanding of the factors influencing the acceptability and feasibility of IGPs. Identifying these factors is essential for designing programs that better meet the needs of older adults. This study examines older adults' perspectives on IGPs through semi-structured interviews with 24 participants (ages 65–104). Thematic analysis identified seven key factors that shape engagement: (1) Culture, (2) Knowledge and Experience Exchange, (3) Interest and Curiosity as Motivation, (4) Emotional Support, (5) Social Interaction, (6) Family and Community Support, and (7) Technology Integration. These factors influence how older and younger participants engage in IGPs, shaping their experiences through shared activities and reciprocal knowledge exchange. The findings emphasize the role of structured engagement in sustaining meaningful intergenerational interactions. While older adults show enthusiasm, younger participants often engage due to academic requirements, limiting intrinsic motivation. Differences in digital literacy create barriers to participation, particularly for older adults unfamiliar with technology. The study also found that varying expectations among older participants, limited institutional support, and the digital divide pose challenges to engagement. These insights enhance our understanding of intergenerational dynamics, guiding policymakers and program developers in designing interventions that promote healthy aging and strengthen intergenerational bonds.

Abstract citation ID: igaf122.3265

**FOSTERING HOLISTIC COLLABORATION: HEALTH PROMOTION WITH PHYSICAL THERAPY AND SPEECH-LANGUAGE PATHOLOGY STUDENTS**

Mary Milidonis, Myrita Wilhite, and Matthew Mino, *Cleveland State University, Cleveland, Ohio, United States*

This qualitative study explores the impact of interprofessional education (IPE) on physical therapy (PT) and speech-language pathology (SLP) students in health promotion clinics for older adults. By engaging in collaborative clinical practice, students from both disciplines were given the opportunity to work together in a real-world setting, promoting holistic care and fostering empathy. Thirty (26 DPT, 4 SLP) graduate students (mean age 24 years, 38.7% male) volunteered to answer open ended questions about an IPE experience for Brain Health Promotion with older adults. Teams screened older adults for cognition and endurance. Older adults were instructed in brain health activities. Three researchers reviewed student surveys with thematic analysis. The study highlights several key findings. First, students reported an enhanced ability to adapt their communication styles to ensure better understanding by older adult clients. Additionally, improved communication and collaboration among team members were noted, emphasizing the importance of interprofessional skills in delivering comprehensive care. Students also expressed increased confidence in clinical settings, attributing this to the hands-on experience and exposure to diverse perspectives. Moreover, the study identified the development of empathy as a crucial component of the learning process. Students recognized the importance of empathetic interactions in enhancing patient outcomes. Overall, the research suggests that IPE in health promotion clinics provides valuable benefits in cultivating essential skills for PT and SLP students, particularly in the context of working with older adults. These findings underscore the importance of incorporating IPE opportunities into educational programs to better prepare future clinicians for collaborative, patient-centered care.

Abstract citation ID: igaf122.3266

**FROM CLASSROOM TO CAREER: THE IMPACT OF GUEST SPEAKERS ON MSW STUDENTS' INTEREST IN AGING**

Mbita Mbao, *Salem State University, Salem, Massachusetts, United States*

The growing demand for geriatric social workers highlights the urgent need to attract more students to aging-related careers. However, many MSW students remain reluctant to enter this field. This study examined whether incorporating guest speakers from the aging services sector into social work curriculum could positively influence students' attitudes and career intentions. Using a cross-sectional pre-posttest design, we surveyed 17 MSW students enrolled in a Social Work with Older Adults course. The Willingness to Work with Older People Scale was used to assess changes in students' perceptions. Findings revealed statistically significant improvements in students' confidence, perceived competence, and willingness to work with older adults. A key result was the increase in students' agreement with the statement, "My professors advise me to consider aging career" ($t = -3.31$, $p < .01$), rising from a pretest mean of 3.59 ($SD = 1.54$) to a posttest mean of 4.88 ($SD = 1.22$). Additionally, Perceived Behavioral Control and Intention to Work with Older Adults measures showed significant gains, reflecting students' increased readiness to enter the aging workforce.These findings suggest that guest speakers serve as powerful role models, bridging the gap between classroom learning and real-world practice. By exposing students to the realities and rewards of geriatric social work, guest speakers may play a critical role in shaping future workforce development.

Abstract citation ID: igaf122.3267

### GERIATRICS EDUCATION MENTOR (GEM) PROGRAM: OUTCOMES FROM PAIRING STUDENTS AND OLDER ADULTS DURING MEDICAL SCHOOL

Hannah Griffin[1], David Wihry[2], and Marilyn Gugliucci[3], *1. University of New England College of Osteopathic Medicine, Biddeford, Maine, United States, 2. University of Maine, Bangor, Maine, United States, 3. University of New England College of Osteopathic Medicine, Kennebunk, Maine, United States*

Currently, only 15% of medical schools have mentor programs that educate students to work with older adults and their health. The University of New England College of Osteopathic Medicine Geriatrics Education Mentor (GEM) program is an experiential education component within the 1st and 2nd year curriculum whereby students and older adults are paired to conduct five "home-visit" and complete geriatrics specific assignments. A pre/post survey design assessed students' competence and confidence with older people (assigned GEMs). The survey was emailed to first-year medical students (avg. 174/class) before they began GEMs and was sent again upon completion of the program at the end of their second year during academic years 2020-2021 through 2023-2024. Data were analyzed in RStudio. Results had a 26% response rate that included overall data (N = 133/Pre & N = 138/post), 24 responses were matched assessments. No significant change in students' interest ($p > 0.4$), perceived readiness and competence ($p > 0.07$), or confidence in working with older adults ($p > 0.5$) was revealed. However, perceived readiness and confidence increased from pre- to post-test, indicating that a larger sample size might reveal significance. Self-reported knowledge of older adult healthcare needs increased, pre- to post-test, with the paired subset showing a significant increase ($p < 0.015$). Although a small sample, these findings provide a foundation for medical schools to consider implementing a mentor type program to augment medical student skill/knowledge in older adult health care to advance age-friendly health care.

Abstract citation ID: igaf122.3268

### IMPROVING HIGH RISK CARE MANAGEMENT EDUCATION IN THE OUTPATIENT SETTING

Fiorella Perez[1], Stephanie Chow[1], Danialle Coyne[2], Jodi Payne[1], Lizette Andrango[2], and Susana Lavayen[2], *1. Icahn School of Medicine at Mount Sinai, New York, New York, United States, 2. Mount Sinai Hospital, New York, New York, United States*

The Aging, Life Innovations, Goals & Needs(ALIGN) Program is an innovative intensive ambulatory model of care that manages older "high risk" patients at risk for acute health decline with a thoughtful curriculum designed to highlight approaches to geriatric medical and psychosocial care. ALIGN curriculum (AC) objectives include learning skills in identifying high risk patients and developing a comprehensive geriatric care plan using the 5Ms of Geriatrics framework, plus "Maintaining Care." Fellows were assigned a small patient panel and participated in team huddles. At rotation end, fellows provided a written sign-out of patients. Following the 2-week AC, fellows completed a post-rotation survey. Six fellows completed the survey between June 2021 and June 2022, rating usefulness (Scale 0-5) of online education, team huddles, shadowing team and confidence in managing high risk patients. Results showed fellows appreciated active 1:1 time with attendings. Subsequently, 13 post-rotation surveys were collected from June 2022-January 2024. Shadowing the team was rated most useful (53.8%) as was seeing patients independently (69.2%). Fellows rated their confidence (scale 0-5) in screening for high-risk older patients as high (average 4.3). Moreover, 61.5 % fellows found 1:1 ALIGN attending education useful, as well as 1:1 educational time with the social worker and care coordinator. For many, ALIGN was their first experience in high-risk patient care management. Likelihood to recommend the ALIGN rotation was 6.9 out of 10. In conclusion, AC is beneficial in promoting leadership and innovation for trainees amidst a perpetually changing landscape in geriatric care.

Abstract citation ID: igaf122.3269

### INTEGRATING CHATGPT AND ESCAPE ROOM WITH A GERIATRIC 5MS CURRICULUM FOR INTERPROFESSIONAL RESIDENTS: A PILOT

Huai Cheng, Erica Allen, Jordan Risher, Kimberly Heckmann, Antonio Valenzuela, Jennifer Schutz, Robin Rabey, and Edward Rattner, *Minneapolis VA Hospital, Minneapolis, Minnesota, United States*

Geriatric 5Ms are core components of age-friendly health system (AFHS). Awareness and knowledge of geriatric 5Ms and AFHS among health professional trainees are low. ChatGPT application to geriatrics education is less studied. With growingly ChatGPT application to medical education, we developed a 2-hour case-based interprofessional geriatric 5M curriculum integrated with ChatGPT as an interaction tool and used the Escape Room (ER) as outcome assessment at a VA medical center. This curriculum aims to demonstrate the application of Geriatrics 5Ms and ChatGPT to the care of older adults for Geri-pharmacy, Nurse practitioners (NP) and Internal medicine (IM) residents (N = 6). After a brief didactic introduction to geriatric 5Ms, AFHS, and interprofessional teamwork approach the case was then unfolded with 7 geriatric 5Ms questions, Responses to 7 questions by the trainees versus and ChatGPT were compared and discussed by the trainees (n = 6), pharmacy, NP and geriatrics faculty (n = 4) and simulation center staff (n = 3). All residents attended a 20-minute orientation to ER, they were locked in ER which was designed to assess whether they can efficiently and effectively apply the geriatric 5Ms knowledge to the case of an older patient ready to be discharged from hospital to home. The residents successfully escaped ER as a team in 22 minutes, which was followed by a 15-minute debriefing. This pilot curriculum was simple to implement. Trainee engagement in geriatrics was enhanced using novel ChatGPT and the Escape Room. Further testing and evaluation of this curriculum is planned.

Abstract citation ID: igaf122.3270

### TRAINING THE PROFESSOR: HEALTH POLICY CURRICULUM IN ADULT DEVELOPMENT AND AGING COURSE

Kirsten Graham[1], Sam Webster[1], and Kelly O'Malley[2], *1. Southern Utah University, Cedar City, Utah, United States, 2. VA Boston Healthcare System, Boston, Massachusetts, United States*

Health policy shapes the lives of older adults but is often underrepresented in undergraduate psychology curricula. This study builds on our prior implementation research integrating health policy content into an undergraduate psychology course. Previous research (student reflections, instructor observations, and course assessments) showed significant increases in students' knowledge, understanding of health policy-making, and critical evaluation skills. Building on these results, we developed a Train the Professor package to help faculty incorporate policy content into similar courses. This package includes a structured curriculum planning framework, consultation with a psychologist experienced in policy work, and ready-to-use instructional materials. We also evaluated the Knowledge and Attitude about Social Policy (KASP) scale, which was developed and revised as part of the curriculum evaluation. Confirmatory factor analyses pre ($\chi 2$ (8) = 12.0 p = .152; CFI = 0.993; TLI = 0.988; RMSEA = 0.051; SRMR = 0.031) and post ($\chi 2$ (8) = 12.0 p = .151; CFI = 0.992; TLI = 0.985; RMSEA = 0.057; SRMR = 0.033) indicated that the KASP was a good fit for the data. Findings support integrating policy education into psychology curricula and refining a scale for future research. By equipping educators with these resources, we aim to foster interdisciplinary learning and better prepare students to engage with policy issues related to aging. Our findings contribute to curriculum development discussions in psychology, emphasizing the value of policy integration in enriching student learning and better serving the community.

Abstract citation ID: igaf122.3271

### WHO CARES: WHAT IS THE FUTURE LANDSCAPE OF GERIATRIC AND GERONTOLOGY EDUCATION

Isaac Asirifi Boateng, and Phyllis Greenberg, *St. Cloud State University, St. Cloud, Minnesota, United States*

The need for advancements in geriatric and gerontology education has become increasingly vital. The current workforce has not kept pace with aging demographics. There is a low percentage of students choosing geriatrics as a specialty, with fewer than 5% of physicians specializing in this field (Kusmaul, 2023). Approximately 25% of BSW and 20% of MSW students have taken a course in gerontology. Adding to the issue is that nearly 50% of MSW students state that they have little or no interest in working with older adults after graduation (Cummings & Galambos, 2002). This poster examined the current landscape and future directions of medical and social service education regarding older adults. It stresses the importance of a critical look at gaps to equip future providers with the necessary skills and knowledge to address the complex needs of an aging population. This poster seeks to identify significant barriers, such as limited exposure to geriatric and gerontology training, underrepresentation of geriatric specialists, and a lack of funding and incentives for professionals working with an aging population. Proposed recommendations include all future practitioners having a baseline education in working with older adults, advocating for policy reforms to prioritize funding incentives (e.g.; scholarships, assistantships, internships) for students in the field. Overall, addressing these issues and implementing the recommended strategies, gerontology and geriatrics education can better prepare professionals not only to deliver high quality services for older adults but rather connect passion with their WHY of ultimately improving the lives of older adults.

### SESSION 7460 (POSTER)

### EMOTIONS

Abstract citation ID: igaf122.3272

### AGE AND FLOURISHING MENTAL HEALTH: THE ROLE OF RUMINATION ON WELL-BEING

Seerat Kang[1], Meghan Elliot[1], Susan Charles[1], and Joseph Mikels[2], *1. University of California, Irvine, Irvine, California, United States, 2. DePaul University, Chicago, Illinois, United States*

A growing number of studies find that older age is often related to higher levels of emotional well-being despite marked declines in cognitive and physical function. Although the positivity effect is not an emotion regulation strategy, an age-related preference to attend to more positively valenced affective experiences may contribute to higher levels of well-being. The current study hypothesized that age is related to higher levels of emotional well-being, and that this association is due to age differences in the tendency to dwell on negative emotions (rumination) and not specific coping strategies. Adults (N = 393) ranging from 18 to 83 years old completed measures of emotional wellbeing (positive & negative affect; flourishing mental health), rumination, and coping strategies (avoidant; emotion-focused, problem-focused, and social support). Age was not significantly associated with higher rates of positive affect. However, older age was related to higher levels of flourishing mental health (B(SE) = .012(.003), p = .0002), even with the addition of coping strategies in the model. With the addition of rumination to the model, rumination accounted for 52% of the association between age and flourishing mental health (B(SE) = .005(.003), p = .05), suggesting higher levels of psychological well-being in older age are related to decreased ruminative tendencies rather than previously posited increases in positive affective experiences.

Abstract citation ID: igaf122.3273

### CORTISOL AND DAILY EMOTIONAL WELL-BEING: WEAKENED ASSOCIATIONS WITH AGE

Rebecca Polk[1], Blake Ebright-Jones[1], Alexandra Freund[2], and Derek Isaacowitz[1], *1. Washington University in St. Louis, St. Louis, Missouri, United States, 2. University of Zurich, Zürich, Switzerland*

Older adults often report greater emotional well-being than younger adults despite declines in physical and cognitive functioning. Cortisol, a key stress hormone, may play an important role in these age-related differences in emotional well-being. As part of a larger study on aging, motivation and emotion in everyday life, we examined the relationship between daily cortisol output and emotional experience in 46 younger (M = 24.9 years, SD = 2.8; 36 women) and 51 older (M = 72.2 years, SD = 5.7; 26 women) adults. Participants provided daily saliva samples for cortisol analysis across six days, concurrent with ecological momentary assessments (EMA) capturing current valence and arousal (5-point scales) and affective choice ("If I could choose to engage with something RIGHT NOW, I would choose something that would make me feel…"). Area under the curve (AUC) was calculated to quantify total daily cortisol output. Multi-level linear models, controlling for sex, sampling day, body mass index, and awakening time, revealed significant main effects of Age Group, Cortisol, and Age Group × Cortisol interactions on daily valence and affective choice responses. Higher daily cortisol was associated with lower average daily valence, particularly in younger compared to older adults. Similar patterns emerged for arousal, with an Age Group × Cortisol interaction indicating a stronger relationship in younger adults. These findings suggest that daily cortisol levels may have a weaker impact on emotional well-being with age.

Abstract citation ID: igaf122.3274

### EMOTION REGULATION FAILURES IN DAILY LIFE IN YOUNGER AND OLDER ADULTS

Blake Ebright-Jones[1], Jacob Gurera[2], and Derek Isaacowitz[1], *1. Washington University in St. Louis, St. Louis, Missouri,*

United States, 2. U.S. Center for SafeSport, Denver, Colorado, United States

Can better emotion regulation abilities explain why older adults report higher levels of emotional well-being? While most research compares age groups on emotion regulation attempts, we considered whether older adults report fewer emotion regulation failures than younger adults. We conducted a daily diary study asking for daily reports every day for 10 days (n = 1036 observations). We asked younger adult participants (n = 79, Mage = 20.6, SDage = 1.92) and older adult participants (n = 75, Mage = 73.2, SDage = 8.47) to identify episodes in which they had attempted to regulate their emotions. For these attempts, participants indicated whether they had succeeded or failed to regulate, and the valence, arousal, and effort associated with their regulation attempt. In predicting emotion regulation failures, we did not find a significant age difference in the likelihood of success vs. failures (OR = 0.70, p = 0.111). Instead, the use of positive-approach regulation tactics (i.e., focusing attention on positive aspects of a situation) was associated with emotion regulation failures for all ages (OR = 2.91, p = 0.001). Among those participants who experienced an emotion regulation failure, older adults were more likely to report positive feelings ($\beta = 0.23$, p = .007), higher arousal ($\beta = 1.33$, p < .001), and greater effort expenditure in emotion regulation ($\beta = 0.27$, p = 0.018). Emotion regulation failures may not differentiate younger and older adults overall but may still provide some clues about how age groups differ in their unfolding emotion regulation behavior.

Abstract citation ID: igaf122.3275

### EXPLORING EMOTION REGULATION TACTICS IN YOUNGER AND OLDER ADULTS USING VIRTUAL REALITY

Jasmine Moon, Blake Ebright-Jones, Samantha Furey, and Derek Isaacowitz, *Washington University in St. Louis, St. Louis, Missouri, United States*

Older adults typically report higher levels of emotional well-being compared to younger adults, and emotion regulation may play a key role. While past research investigating age differences in emotion regulation strategy selection has been inconsistent, recent everyday life studies have identified age differences in emotion regulation tactics. Tactics refer to the ways in which individuals implement strategies and can be operationalized in terms of valence and direction: positive-approaching, negative-approaching, and negative-receding. Most tactic studies have used experience sampling, but Virtual Reality (VR) can be used to investigate age differences in an immersive setting that mimics real-life environments, allowing for emotional responses to more controlled contexts. As the first study to examine age differences in tactics within VR, the current study had participants (N = 70; ages 18-79) first interact with a negatively-valenced virtual environment for three minutes. Then, participants reported whether they had regulated their emotions while in the simulation, and if so, which regulation strategy and tactic they had used to regulate their emotions. Participants were also asked whether they thought they were successful in their regulation attempts. Younger adults were more likely to report regulation success compared to older adults, but no other group differences were significant. This finding contrasts with results from traditional lab tasks and everyday life assessments, suggesting VR offers a promising avenue to further investigate individual differences and environmental influences on real-world emotion regulation across the lifespan.

Abstract citation ID: igaf122.3276

### PERSEVERATIVE THINKING AND EXECUTIVE FUNCTION IN ADULTS AGED 45-70 WITH AND WITHOUT MOOD DISORDERS

Jane Lee[1], Katharine Burns[1], Jennifer Nicoloro-Santabarbara[1], Faith Gunning[2], and Katherine Burdick[1], *1. Brigham and Women's Hospital, Boston, Massachusetts, United States, 2. Weill Cornell Medicine, New York, New York, United States*

Emotion regulation (ER) improves with age, while executive functioning (EF) tends to decline. However, the presence of a mood disorder (MD) negatively impacts both processes. We examined whether MD diagnosis and age influence the relationship between ER and EF in 238 adults aged 45 to 70, including individuals with MD (n = 131) and healthy controls (HC; n = 107). ER was assessed using the Perseverative Thinking Questionnaire (PTQ) and an EF composite score was derived from four cognitive tasks. Participants were grouped into three age groups: 45–53 (n = 72), 54–62 (n = 102), and 63–70 (n = 64). Hierarchical regression analysis revealed a main effect of EF on PTQ ($\beta$=-0.264, p < 0.05), though all interaction terms were non-significant. Follow-up correlational analysis showed that EF was negatively associated with PTQ in middle-aged HCs only (r =-.437, p < 0.05). ANOVA results revealed significant age-related differences in the PTQ within the MD group (F(2, 119), 3.769, p<.05). Tukey post hoc comparisons indicated that younger individuals with MD exhibited higher levels of perseverative thinking (PT) compared to older individuals (p < 0.05). These findings suggest that in middle-aged HCs, EF is protective from PT, a form of maladaptive ER. However, in MD, EF was not significantly correlated with PT across age groups, and younger individuals with MD exhibited higher PT than older individuals. This suggests that factors beyond EF contribute to PT in individuals with MD. Therefore, interventions targeting individuals with MD should address both cognitive and emotional factors contributing to PT.

Abstract citation ID: igaf122.3277

### TESTING THE STRENGTH AND VULNERABILITY INTEGRATION (SAVI) MODEL OF EMOTION IN A SAMPLE OF LATINX ADULTS.

Robert Kennison, and Jaiwei Xiang, *California State University, Los Angeles, Los Angeles, California, United States*

The strength and vulnerability integration (SAVI) model identifies two pathways that affect emotional well-being in aging (Turk Charles, 2010). The strengths pathway theorizes that increases in well-being result from age-related changes in perspective (e.g., expansive time horizon), which enhance strengths like emotion regulation (e.g., cognitive reappraisal). The vulnerabilities pathway posits that age-related threats to well-being include greater physiological reactivity to stress. This vulnerability can be offset by avoiding or de-escalating stressful events. These predicted relationships were examined in survey data that included SAVI-proximate variables collected from a predominately Latinx (74.7%) sample of adults aged 18-89 (n = 573). Latent variable modeling was performed. First, latent measures of expansive time horizon, cognitive reappraisal, and well-being were tested in a measurement model that fit the data. Second, measures of age, number of reported health conditions and concern for the consequences of stress were integrated into the model that replicated the pathways identified by SAVI, producing an acceptable fit (CFI=.94, TLI=.93, RMSEA=.06). For the strengths pathway, advancing age predicted less-expansive time horizons (B=-.02), which predicted greater use of cognitive reappraisal

(B=.27) and greater well-being (B=.18); cognitive reappraisal predicted greater well-being (B=.23). For the vulnerabilities pathway, advancing age predicted number of health conditions (B=.02) and greater well-being (B=.01); health conditions predicted concern for the consequences of stress (B=.33), which predicted poorer well-being (B=-.04). These results are broadly consistent with the SAVI model. Importantly, differences between Latinx and non-Latinx groups were not observed, suggesting that the SAVI model translates to cultures typically understudied in aging research.

### SESSION 7465 (POSTER)

### ENVIRONMENT AND AGING

Abstract citation ID: igaf122.3278

### A META ANALYSIS OF INDOOR TEMPERATURE IMPACTS ON SLEEP DURATION, MORTALITY RISK, AND ECONOMIC COSTS IN OLDER ADULTS

Sam Karsky[1], Carina Gronlund[1], Peter Larson[1], Michaela Marincic[2], Dayna Johnson[3], Philippa Clarke[1], Ketlyne Sol[4], and Konstantinos Papaefthymiou[1], *1. University of Michigan, Ann Arbor, Michigan, United States, 2. 3Cubed, Knoxville, Tennessee, United States, 3. Emory University, Atlanta, Georgia, United States, 4. University of Miami, Boca Raton, Florida, United States*

Of U.S. householders aged 60+, 19% reported difficulty paying energy bills and 11% kept their homes at unhealthy indoor temperatures (IT). Extreme IT may impact sleep health. Healthy sleep is essential for daily functioning and long-term health, yet 33% of adults report short sleep duration (SSD, < 7 hours). Quantifying IT-sleep associations can inform residential weatherization and energy-efficiency programs, including insulation and high-efficiency heating/cooling system installation. We characterized the relationship between IT and SSD and the resulting health costs of temperature-associated sleep loss. We conducted a review and meta-analyses of associations between IT and SSD and also SSD and all-cause mortality. We estimated costs of increased all-cause mortality attributable to SSD in uncomfortably warm homes as the product of 4 terms: prevalence (20%, representing probability) of 6-hour (1 hour less than SSD threshold) sleep duration in older adults, the IT-SSD association, the SSD-mortality association, and a value-of-statistical-life metric, with bootstrapped 90% confidence intervals. Based on results from the literature, we assumed that increases in bedroom IT from comfortable (22 °C) to uncomfortable (30 °C) temperatures were linked to reduced sleep duration by 1 hour in older adults. Additionally, our meta-analysis of 18 studies found SSD is associated with increased all-cause mortality in older adults (pooled RR: 1.08, 95% CI: 1.01, 1.14). Sleep-attributable health costs in uncomfortably hot households were $96,590 (90% CI: $10,240, $362,000). Indoor-environment improvements, including weatherization and energy efficiency upgrades, may offset substantial sleep-related health costs, potentially informing funding mechanisms for these programs.

Abstract citation ID: igaf122.3279

### A PATH ANALYSIS OF HOW ENVIRONMENTAL FRIENDLINESS SHAPES SOCIAL CONNECTEDNESS, ATTITUDES TOWARD AGING, AND QOL

Shiau-Fang Chao[1], Jun-Rong Huang[1], Tzai-Hung Wen[1], and Hsuanlei Shao[2], *1. National Taiwan University, Taipei, Taipei, Taiwan (Republic of China), 2. Taipei Medical University, Taipei City, Taipei, Taiwan (Republic of China)*

Background: The Ecological Model of Aging assumes that an individual's aging experience is embedded within their living environment and influenced by community resources. This theoretical perspective suggests that environmentally friendly communities may improve older adults' social connectedness, reinforce positive attitudes toward aging, and subsequently enhance their quality of life. Methods: This study employed path analysis to analyze data from 1,834 older adults drawn from the 2015 Taiwan Longitudinal Study on Aging. Findings: 1.Direct effects: (1) All four environmental friendliness factors (housing satisfaction, medical treatment accessibility, perceived neighborhood safety, and environmental satisfaction) positively predicted higher social connectedness, as well as positive attitudes toward aging. (2) High social connectedness and positive attitudes toward aging were directly linked to older adults' quality of life, with attitudes toward aging exhibiting the strongest direct effect. 2. Indirect effects: (1) Environmental friendliness factors led to enhanced social connectedness, which fostered positive attitudes toward aging, which ultimately improved quality of life. (2) Environmental friendliness factors also contributed to quality of life through their positive impact on attitudes toward aging, demonstrating the crucial mediating role of aging attitudes. Conclusions and Implications: Our findings highlight the significant role of environmental friendliness, social connectedness, and attitudes toward aging in determining older adults' quality of life. Attitudes toward aging emerged as the strongest predictor of well-being. Environmental friendliness creates opportunities for social interaction, positively shaping aging perceptions and daily experiences. These results inform aging-in-place policies and community interventions that foster age-friendly environments supporting social connectedness and positive aging attitudes.

Abstract citation ID: igaf122.3280

### ANNUAL MEDICAL AND LONG-TERM CARE EXPENDITURES THREE YEARS AFTER HOME ADAPTATIONS FOR OLDER ADULTS WITH CARE NEEDS

Rumiko Tsuchiya-Ito[1], Satomi Kitamura[2], Reina Taguchi[2], and Shota Hamada[2], *1. Dia Foundation for Research on Ageing Societies, Tokyo, Tokyo, Japan, 2. Institute for Health Economics and Policy, Association for Health Economics Research and Social Insurance and Welfare, Tokyo, Tokyo, Japan*

Home adaptation is a strategy for aging in place that does not rely fully on human resources in the current situation of workforce shortages. However, the effect of home adaptation on medical and long-term care (LTC) expenditures remains unclear. This retrospective cohort study examined the association between home adaptation and medical and LTC expenditures using these claims from Hachioji City, Tokyo, Japan. The participants were adults aged ≥ 65 years who had not implemented home adaptation and had certified care needs in the Japanese LTC insurance system between April 2015 and February 2016. Participants (N = 8,963) were categorized into non-implementation (no home adaptation; n = 8,237), sub-maximum (home adaptation < USD 1,350; n = 574), and maximum groups (home adaptation at the maximum costs covered by insurance of USD 1,350; n = 153) according to the implementation of home adaptation between April 2015 and March 2016. A generalized linear model for gamma-distributed data with a log-link function was used to evaluate the associations between home adaptation and annual medical and LTC expenditures in 2019, adjusting for individuals' characteristics. The sum of medical and LTC expenditures was lower in the sub-maximum group (RR; 0.86, 95% confidence interval; 0.78–0.95), but not lower in the maximum group (1.07, 0.88–1.29), compared to the non-implementation group. LTC expenditures were lower in the sub-maximum (0.86, 0.75–0.98) and maximum groups (0.76, 0.59–0.98). Home adaptations could support aging in places for older adults with care needs by reducing medical and LTC expenditures.

Abstract citation ID: igaf122.3281

### ASSOCIATION BETWEEN PM2.5 AIR POLLUTION AND ALL-CAUSE MORTALITY AMONG OLDER MEXICAN AMERICAN ADULTS

Sirena Gutierrez[1], Phillip Cantu[2], Kyriakos Markides[2], and Sadaf Milani[3], *1. University of California, San Francisco, San Francisco, California, United States, 2. UTMB Health, The University of Texas Medical Branch, Galveston, Texas, United States, 3. University of Texas Medical Branch, Galveston, Texas, United States*

Exposure to air pollution, including fine particulate matter (PM2.5), is associated with higher all-cause mortality rates. Racial and ethnic minority groups, including Mexican American adults, are disproportionately exposed to higher levels of air pollution. However, it remains unclear whether this increased exposure translates into a higher mortality risk among older Mexican American adults. This analysis included participants ages 74+ from Wave 5 (2004-2005) of the Hispanic Established Population for the Epidemiological Study of Elderly. Baseline PM2.5 was measured as the annual average at the census tract level, and mortality was established through National Death Index linkages up to December 31, 2018. Cox proportional hazard models were used to estimate the hazard ratio for all-cause mortality, adjusting for sociodemographic characteristics, including age, gender, educational attainment, marital status, nativity, state of residence, and urbanicity. Our sample (n = 1,990) had a mean age of 81.9 years [standard deviation (SD): 5.1] and was 61.6% female. Participants lived in census tracts with average PM2.5 concentration of 10.4 µg/m3 (SD: 3.4, range: 5.1-20.0). We analyzed 13,370 person-years with 1,341 deaths. For every 5-µg/m3 increase in PM2.5 exposure there was a 5.4% increase in all-cause mortality, but the estimate was not statistically significant (95% confidence interval: -6.9% to 19.4%). Future work will include longitudinal exposure data and explore effect heterogeneity to understand the long-term pathways through which air pollution impacts mortality in older Mexican Americans.

Abstract citation ID: igaf122.3282

### CHARACTERISTICS OF HOUSING ADAPTATION RECIPIENTS UNDER JAPAN'S LONG-TERM CARE INSURANCE: A CLUSTER ANALYSIS

Satomi Kitamura[1], Tomoyuki Ota[2], and Rumiko Tsuchiya-Ito[3], *1. Institute for Health Economics and Policy, Association for Health Economics Research and Social Insurance and Welfare, Tokyo, Tokyo, Japan, 2. The Hyogo Institute of Assistive Technology, Hyogo, Hyogo, Japan, 3. Dia Foundation for Research on Ageing Societies, Tokyo, Tokyo, Japan*

Housing adaptation is a key strategy for maintaining the functional ability of older adults with disabilities. This study aimed to characterize older adults aged ≥65 years who received housing adaptation under Japan's long-term care (LTC) insurance in 2015. A k-means cluster analysis was conducted using anonymized medical and LTC claims data, and housing adaptation application data from Hachioji City, Tokyo. Clustering variables included age, sex, household income, LTC needs levels, basic activities of daily living, hospitalization history, comorbidities (10 diseases), housing type (detached house/apartment), and co-residence status (alone/spouse/others). In addition to these clustering variables, housing adaptation details and other LTC services were compared across clusters using Chi-square tests. Among 1,062 participants (median age 81[76–87], 57.1% female), three clusters were identified: 1) older adults with frailty (n = 420, 81[76–85]), 2) those with higher age and mild disabilities (n = 343, 88[84–91]), and 3) those with severe disabilities (n = 299, 75[71–79]). LTC needs levels increased from cluster 1 to 3. Cluster 1 had more joint diseases and fewer hospitalizations. Cluster 2 had more dementia, fractures, and non-spousal co-residing family. Cluster 3 included more males and recent hospitalizations. While housing adaptation details were similar across clusters (e.g., handrail installation: 84%, 82%, 86%; p = 0.44), LTC service utilization varied: cluster 1 used fewer services, cluster 2 relied more on home-visiting care and day-care services, and cluster 3 used more assistive product services (e.g., wheelchairs, electronic beds). These findings highlight the heterogeneity among housing adaptation recipients, suggesting the need for personalized planning.

Abstract citation ID: igaf122.3283

### COGNABILITY ACROSS ADULTHOOD: NEIGHBORHOODS AND COGNITIVE HEALTH BEHAVIORS

Grace Savard[1], Desiree Alvarez-McNelis[2], Mallory Sagehorn[3], Grace Bowman[3], Yue Sun[3], Michael Esposito[2], and Jessica Finlay[3], *1. University of Minnesota Twin Cities, Minneapolis, Minnesota, United States, 2. University of Minnesota, Minneapolis, Minnesota, United States, 3. University of Colorado Boulder, Boulder, Colorado, United States*

While geographic variation in Alzheimer's Disease rates suggests that environmental factors are important in the development of dementia, understanding of specific local contextual features that impact dementia risk across the life course is limited. This paper extends Cognability, a theoretical framework that conceptualizes how supportive a geographical area is for cognitive health through social and behavioral pathways, to a life course perspective. The Neighborhoods and Health at All Ages Study employed seated and mobile interviews (August 2023-March 2024) across the Minneapolis-St. Paul (MN) metropolitan area. Participants were on average 42 years old (range: 23-75). About half (53%) identified as female, 40% male, and 7% non-binary; 22% identified as Hispanic, 22% Asian, 18% non-Hispanic White, 17% Multiracial, 15% Black/African American, 3% American Indian/Alaska Native, and 3% Other. Reflexive thematic analysis identified ten key neighborhood sites that support cognitive health behaviors: parks and paths, recreation centers, eateries, grocery stores and markets, stores, civic/social organizations, religious organizations, arts and cultural amenities, libraries, and educational sites. More broadly, the study captured nuanced perspectives from socioeconomically, racially, ethnically, age, and gender diverse adults who uniquely experience and engage with combinations of amenities and hazards in their own neighborhoods. As the global dementia burden grows and disparities widen, our results can inform upstream community-level interventions to create more equitable neighborhoods that offer opportunities to support lifelong cognitive health and well-being.

Abstract citation ID: igaf122.3284

### EXPLORING INFLUENCE OF WINDOW VIEWS, NATURE AND CONTEXT ON HONG KONG'S CARE HOME RESIDENTS' PSYCHOSOCIAL WELL-BEING

Mohana Das, and Peter Hasdell, *The Hong Kong Polytechnic University, Hong Kong, China (People's Republic)*

This study investigates the psychosocial impact of window views, nature access, and culturally mediated spatial dynamics on older adults' Quality of Life (QOL) in Hong Kong's (HK) hyperdense Long-term Care Facilities (LTCFs). The study evaluates how environmental factors affect residents' sense of belonging, wellbeing, and disengagement in care homes with spatial constraints and window access restrictions. The COVID-19 pandemic's impact on confined spaces underscored the importance of indoor environments to mitigate social isolation. A methodologically rigorous mixed-methods approach integrates cross-sectional surveys (n = 408) assessing nature and view perceptions, semi-structured interviews (n = 33) examining lived experiences, and architectural audits of 6 LTCFs to evaluate space configurations. Although quantitative data supports Western literature on nature's healing properties, qualitative and contextual analyses show that HK's hyper-urbanized milieu, with inadequate green/blue infrastructure and people's cultural acclimation to density, favoured socially dynamic urban panoramas over passive nature scenes. Architectural audits revealed systemic design flaws such as tokenistic nature integrations. The study proposes a socio-ecological gerontological design framework that challenges universalist beliefs about nature's importance in well-being and emphasizes culturally appropriate models for hyperdense Asian cities. It presents context-specific design paradigms that prioritize spatial efficiency, dynamic urban imagery, and micro-nature integrations for high-rise limits by combining architectural analysis with psychosocial insights. These findings foster evidence-based LTCF initiatives and advocate for localized design standards and retrofitting solutions that address HK's socio-spatial constraints. The findings indicate that well-being frameworks tailored to the specific contexts of non-Western, densely populated urban areas should be developed to effectively support their rapidly aging populations.

Abstract citation ID: igaf122.3285

### HOME-BASED MONITORING OF SLEEP AND BEDROOM TEMPERATURE IN OLDER ADULTS

Pei-An Lee[1], Peyton Berning[2], Wanting Yu[2], Brad Manor[3], Lewis Lipsitz[4], and Amir Baniassadi[2], *1. Hebrew SeniorLife & Harvard Medical School, Boston, Massachusetts, United States, 2. Hebrew SeniorLife, Boston, Massachusetts, United States, 3. Hebrew SeniorLife/Harvard Medical School, Boston, Massachusetts, United States, 4. Hebrew Senior Life, Boston, Massachusetts, United States*

More than half of older adults complain about sleep problems, while the underlying reason remains undiagnosed in a substantial portion of them. Observational studies conducted in homes have suggested associations between ambient temperature—varying naturally with weather and seasons—and sleep outcomes. Further, a causal link between ambient temperature and sleep has been established within laboratory settings through artificial temperature manipulations. Yet, with many behavioral and environmental confounders, it is not clear whether and to what extent the causal effect of temperature on sleep translates into older adults' own homes. Fortunately, wearable sleep monitors and cloud-connected thermostats now enable us to remotely monitor sleep while varying bedroom temperature. We piloted this approach in cognitively intact, independent-living older adults aged ≥65 years. Thirteen participants (mean age: 82.6±5 years; 7 females) took part in the study. Over 538 analyzed nights, bedroom temperatures averaged 70.5±2.5 °F (range: 65–76 °F), and overall sleep efficiency (the ratio of time spent asleep to time spent in bed) averaged 89.8±9.6%. Preliminary individual-level analyses revealed a within-person relationship between bedroom temperature and sleep efficiency in 4 of the 11 participants with at least 10 nights of data. These findings demonstrate, for the first time in at-home settings, that older adults may exhibit individual-level sensitivities in sleep efficiency related to bedroom temperature, paving the way for future research aiming to better understand the extent to which sleep in older adults is influenced by environmental factors.

Abstract citation ID: igaf122.3286

### HOMEBOUND OLDER ADULTS: NEIGHBORHOOD SOCIAL COHESION AND PHYSICAL DISORDER AND SOCIAL NETWORK SIZE

Brian Fons[1], Kelly Vences[2], Angelina Gutierrez[2], and Namkee Choi[1], *1. The University of Texas at Austin, Austin, Texas, United States, 2. The University of Texas School of Social Work, Austin, Texas, United States*

Research has shown significant associations between neighborhood characteristics and health in older adults, but few focused on homebound older adults. We used the 2022-2023 U.S. National Health and Aging Trends to examine the associations of (1) homebound state (i.e., never/rarely going outside in the past month) with neighborhood social cohesion (NSC), neighborhood physical disorder (NPD), and social network size (N = 5,593); and (2) changes in the homebound state with the changes in NSC, NPD, and social network size (N = 4,907). Older adults reported their perceived NSC and the number of people in their social network. Interviewers rated NPD based on their observations. Logistic regression models were used for multivariable analyses. The results show that homebound older adults were 4.9% of the Medicare beneficiaries age 65+ years in 2022. The odds of a homebound state were lower among those with higher perceived NSC (aOR=0.83, 95% CI = 0.77-0.90). Of those who were interviewed in both 2022 and 2023, 154 (34.1%) were homebound in 2022 but not in 2023, 169 (33.0%) were not homebound in 2022 but homebound in 2023, and 162 (32.9%) were homebound in both years. Compared to those who continued to be or were newly homebound, those who were no longer homebound in 2023 had increased social network size (aOR=2.21, 95% CI = 1.24-3.94). Social care policies and programs for the increasing number of homebound older adults need to be both individual- and community-focused on improving their social capital, including fostering NSC and social connectedness among community residents.

Abstract citation ID: igaf122.3287
### INDOOR HEAT AND SUBJECTIVE WELL-BEING IN OLDER ADULTS
Amir Baniassadi[1], Wanting Yu[1], Thomas Travison[2], Lewis Lipsitz[3], and Brad Manor[4], *1. Hebrew SeniorLife, Boston, Massachusetts, United States, 2. Hebrew SeniorLife & Harvard Medical School, Hebrew SeniorLife, Massachusetts, United States, 3. Hebrew Senior Life, Boston, Massachusetts, United States, 4. Hebrew SeniorLife/Harvard Medical School, Boston, Massachusetts, United States*

Many older adults frequently experience cognitive difficulties, depressed mood, and discomfort in daily life, yet the impact of their home temperature fluctuations on these important subjective outcomes remains understudied. We conducted a 12-month observational study involving 47 community-dwelling older adults in Boston, MA. Participants' home ambient temperatures were continuously monitored, and they completed twice-daily surveys assessing self-reported experience of cognitive difficulties ("difficulty keeping attention"), thermal discomfort ("feeling hot"), and mood ("feeling down or depressed"). We collected 17,201 Ecological Momentary Assessments, which were time-synced with home temperature sensor data. The mean indoor temperature at the time of assessments was 22.46 ± 2.4 °C. Using Generalized Additive Models (GAMs) controlling for humidity and other relevant covariates, we found that the likelihood of experiencing difficulty keeping attention and the likelihood of feeling hot were each lowest between 20–24 °C. Above this optimal range, every 4 °C increase in ambient temperature corresponded to approximately a 10-fold increase in the likelihood of reporting feeling hot ($P < 0.001$) and doubled the likelihood of experiencing difficulty keeping attention ($P < 0.001$). Although temperature showed a statistically significant relationship with negative mood, the magnitude of this effect was negligible and of limited practical significance. Future studies might benefit from evaluating mood using more sensitive measures. Based on our findings, indoor heat fluctuations appear to impact subjective well-being and cognitive difficulties among older adults. A climate- and location- specific optimization of indoor ambient temperature may therefore protect cognitive functioning and enhance comfort in older populations.

Abstract citation ID: igaf122.3288
### LONGITUDINAL ANALYSIS OF DRIVING BEHAVIOR AND COGNITIVE PERFORMANCE IN OLDER ADULTS
Karla Lynch, Melissa Hatch, Jun Ha Chang, Elizabeth Vlock, and Matthew Rizzo, *University of Nebraska Medical Center, Omaha, Nebraska, United States*

The early detection of cognitive impairment is vital to effective intervention and treatment. Real-world driving behavior may serve as an indicator of cognitive function. This study examines longitudinal changes in driving behaviors and cognitive performance. Twenty-two older adults (mean age = 74.15 years; 10 females) were assessed at baseline and at follow-up (range: 4.75–7.24 years; mean = 5.41, SD = 0.67). Cognitive function was evaluated using the Montreal Cognitive Assessment (MoCA). Driving behavior was monitored via vehicle GPS sensors over a three-month period at both time points. Maximum distance from home was defined as the vehicle's displacement at the end of each drive, regardless of where the drive began. Approximately 97,539 miles were recorded. At baseline, the maximum distance from home averaged 466.27 miles (SD = 231.21), which declined to 160.69 miles (SD = 270.27) at follow-up. MoCA scores decreased from a mean of 26.73 (SD = 2.23) at baseline to 25.59 (SD = 2.38) at follow-up. Paired t-tests revealed a significant reduction in maximum driving distance ($t = 3.854$, $p = 0.001$) and a significant decline in MoCA scores ($t = 2.435$, $p = 0.024$). Pearson correlations showed no significant association between MoCA scores and maximum distance from home at baseline ($r = -0.16$, $p = 0.49$) or follow-up ($r = -0.12$, $p = 0.6$). The study found that both driving distance and cognitive performance declined over time. Overall, real-world driving behavior offers a longitudinal index to complement clinical assessments in patients at risk for cognitive decline and neurodegeneration.

Abstract citation ID: igaf122.3289
### MEASURING LIGHT EXPOSURE IN NURSING HOME RESIDENTS WITH DEMENTIA: FACILITY-LEVEL MEASURES VS WEARABLE SENSOR
Ying-Ling Jao[1], Yo-Jen Liao[2], Diane Berish[1], Julian Wang[1], Margaret Calkins[2], Yun-Han Kuan[3], Light Ndurue[1], and Marie Boltz[1], *1. The Pennsylvania State University, University Park, Pennsylvania, United States, 2. IDEAS Institute, Cleveland Heights, Ohio, United States, 3. Far Eastern Memorial Hospital, New Taipei City, Taipei, Taiwan (Republic of China)*

Adequate lighting exposure has positive impacts on circadian health, mood, and behavioral symptoms in people with dementia. However, nursing home (NH) residents with dementia often experience insufficient circadian lighting exposure. Increasing research has investigated lighting interventions in this population with mixed results. Most intervention studies evaluated outcomes based on facility-level lighting measures without considering the individual's actual light exposure. However, light exposures can vary widely between residents, depending on room location, personal behavior, and daily routine. Wearable light sensors allow us to quantify the dosage each individual receives more precisely. This study examined the differences in light intensity (lux) between facility-level and individual-level measures among NH residents with ADRD. The facility-level measure was conducted twice a week in the bedroom, hallway, and dining room, tailored based on individuals' routines. Individual lighting exposure was measured using a wearable light sensor, LYS button, worn on residents' shoulder/collar 24 hours/day continuously for one week. Descriptive statistics and paired t-tests were performed. A total of 29 residents (average age: 87 years; 72.4% female) were recruited from two NHs. The average facility-level lighting intensities from the two NHs were 110, 114, and 85 lux in the bedroom, hallway, and dining room, respectively. In contrast, residents' individual lighting exposure averaged 268 lux. Paired t-tests revealed significant differences between facility-level manual measures and the individual wearable sensor in the bedroom, hallway, dining room, and the overall facility ($p < 0.001$). Future research should consider using individual light sensors for a more precise design and evaluation of lighting interventions.

Abstract citation ID: igaf122.3290
### NEIGHBORHOOD DISADVANTAGE AND COGNITIVE FUNCTION: THE MODERATING ROLE OF SELF-REPORTED LONELINESS
Regina Wright[1], and Desiree Bygrave[2], *1. University of Delaware, Newark, Delaware, United States, 2. Benedict College, Columbia, South Carolina, United States*

Neighborhood disadvantage has been linked to worse cognitive performance and greater cognitive decline among older adults; however, moderators of these associations have not been well studied. Loneliness is a potential moderator of importance—one that also worsens cognitive functioning. Given the documented influence of neighborhood disadvantage and loneliness on cognitive functioning, it is important to understand whether self-reported loneliness exacerbates this relationship. Therefore, the objective of the study was to examine associations between neighborhood disadvantage and cognitive function, and to examine whether associations are moderated by self-reported loneliness. The analysis included 136 older adults (36% male) with a mean age of 68.04y. Neighborhood disadvantage was assessed with the Area Deprivation Index (ADI), that provided state and national rankings of neighborhood deprivation. Cognitive function was assessed with the Verbal Fluency Test (verbal fluency), the Visual Reproductions Test (short- and long-term nonverbal memory), Logical Memory Test (verbal memory) and Digit Span Forward and Backward (working memory). Self-reported loneliness was measured with the UCLA Loneliness Scale. Multivariable regression analyses were run, adjusted for age, sex, and educational attainment. Results showed that greater national ADI rankings (more disadvantage) were associated with worse short-term nonverbal memory. Similarly, greater state and national ADI rankings were associated with worse long-term nonverbal memory. Additional results showed that loneliness moderated the relationship between national ADI rankings and both types of nonverbal memory. Overall, our findings suggest that greater neighborhood disadvantage may influence select domains of memory, and that one's self-reported loneliness may influence how this relationship manifests.

Abstract citation ID: igaf122.3291

### TRACKING CHANGES IN COMMUNITY LIVEABILITY, INTERPERSONAL CONNECTION AND LONELINESS AMONG OLDER ADULTS IN TAIWAN

Shiau-Fang Chao[1], Jun-Rong Huang[1], Tzai-Hung Wen[1], and Hsuanlei Shao[2], *1. National Taiwan University, Taipei, Taipei, Taiwan (Republic of China), 2. Taipei Medical University, Taipei City, Taipei, Taiwan (Republic of China)*

Background: Loneliness has high prevalence among older adults, yet our understanding of how community liveability affects this condition remains limited. Community liveability represents a modifiable environmental factor that may protect against loneliness. Particularly scarce are longitudinal studies examining how changes in environmental and social factors influence loneliness, especially in Asian contexts like Taiwan where rapid aging and changing family structures make this research critically important. Methods: This study employed multilevel modeling to analyze data from 1,834 older adults (3,668 observations) drawn from the 2015 and 2019 waves of the Taiwan Longitudinal Study on Aging. Findings: 1. At the within-person level, increases in individual housing satisfaction and medical treatment accessibility over time were significantly associated with decreased feelings of loneliness. Additionally, maintaining connections with siblings and relatives over time effectively reduced individuals' experiences of loneliness. 2. At the between-person level, all four community liveability factors, including housing satisfaction, medical treatment accessibility, perceived neighborhood safety, and environmental satisfaction, significantly predicted lower levels of loneliness. 3. Furthermore, at the between-person level, individuals who maintained connections with siblings, relatives, neighbors, or friends reported lower levels of loneliness compared to those lacking interpersonal connections. Conclusions and Implications: Both community liveability factors and interpersonal connections serve as protective factors against loneliness among older adults. The longitudinal findings emphasize that sustained improvements in environmental conditions and maintaining consistent social connections over time are particularly effective in reducing loneliness.

### SESSION 7470 (POSTER)

### FAMILY AND INTERGENERATIONAL RELATIONS

Abstract citation ID: igaf122.3292

### ACCULTURATIVE FAMILY DISTANCING: IMPACT ON LATINX/É GRANDCHILDREN'S CAREGIVING TENDENCIES TOWARD GRANDPARENTS

Elisa Gomez, and Mayra Bámaca, *University of California, Merced, Merced, California, United States*

Affectual solidarity is a vital indicator of relationship quality and caregiving practices from the grandchild towards the grandparent (Mansson, 2022). Caregiving practices toward grandparents capture the activities grandchildren participate in with their grandparents such as helping with chores and going to doctor's appointments (Roberto & Stroes, 1992). Given the importance placed on family interconnectedness among Latinx/é families, grandchildren as caregivers are promising. However, the cultural distance between grandchildren and grandparents (i.e., acculturative family distance [AFD]) may play a role in caregiving tendencies. Existing studies have not explored grandchildren as caregivers nor what predicts a grandchild's caregiving tendencies and none, to our knowledge, are dedicated to the relationship between Latinx/é grandchildren and grandparents. Using regression analysis, we investigated 1) whether perceived affection from grandparents and AFD, defined as communication incongruence, predicted participation with grandparents and 2) whether AFD was a moderator. We collected survey data from 399 young adults (Mage = 20.28; 18% males) in the California Central Valley region. Findings indicated that perceived affection from their grandparent predicted greater grandchild participation with their grandparent ($\beta$=.358). However, this association was weaker for grandchildren with higher levels of AFD ($\beta$=-0.086). AFD can lead to interrupted cultural transmission and retention in the next generation, and less social interaction and intimacy between grandparents and grandchildren (Hwang, 2006; Silverstein & Chen, 1999). Thus, AFD between generations can result in less participation with grandparents and potentially less obligation toward caregiving practices. Future research should explore how AFD can impact grandchildren's role as potential family caregivers.

Abstract citation ID: igaf122.3293

### BEYOND THE ABSENT FATHER MYTH: HOW STRUCTURAL VIOLENCE THROUGH ACES AND APIS IMPACTS THE FATHER-CHILD RELATIONSHIP

Milan Riddick[1], Muriel Taks Calle[1], and Paris Adkins-Jackson[2], *1. Columbia University Mailman School of Public Health, New York, New York, United States, 2. Mailman School of*

*Public Health, Columbia University, New York, New York, United States*

Men racialized as Black are often stereotyped as “absent fathers”, a characterization that overlooks their presence and absolves the role of structural racism in their lives. Adverse Childhood Experiences (ACEs) and Adverse Police Interactions (APIs) likely influence fathers’ relationships with their children. We analyzed a purposive sample of fathers racialized as Black from the Health and Retirement Study (aged 51+, N = 147). ACEs were measured using a sum of 10 self-reported adverse experiences occurring before age 18, while APIs were indicated by having had trouble with the police before age 16, experiencing unfair police encounters, and/or imprisonment for at least three days. Dependent variables included Contact — the frequency of interactions between fathers and children; Positive Relation — measuring the extent to which children understand, support, and connect with their father; and Negative Relation —measuring how frequently children make demands of the father, criticize him, disappoint him, or make him feel unnerved. Regression analyses revealed that APIs are associated with decreased contact between fathers and their children (β = -0.920*; 95% CI [-1.93, 0.09]) and increased negative relation (β = 1.022*; 95% CI [-0.06, 1.13]). ACEs are associated with a decrease in positive relation (β = -0.472***; 95% CI [-0.538, -0.130]) and an increase in negative relation (β = 0.249**; 95% CI [0.04, 0.43]). These findings suggest both ACEs and APIs undermine fathers’ abilities to maintain healthy relationships with their children. There is a need to shift from harmful stereotypes to addressing structural violence that shape these integrational dynamics. *p< 0.1, **p< 0.05, ***p< 0.01

Abstract citation ID: igaf122.3294

### DOES HAVING AN ONLY CHILD AFFECT THE HEALTH AND WELL-BEING OF OLDER ADULTS?

Madhyami Deshmukh, and Peter Martin, *Iowa State University, Ames, Iowa, United States*

Loneliness and depression significantly impact older adults’ health, well-being, and life satisfaction, but the role of gender and number of children remains unclear. This study examines how loneliness and depression affect life satisfaction and self-reported health, considering gender (male vs. female) and number of children (one vs. more than one) as moderators. Data from Wave 14 (2018) of the Health and Retirement Study (HRS) RAND Longitudinal Dataset were analysed, including 5,443 individuals (Mean age = 67.5). Bivariate correlations and blocked regressions assessed relationships, while moderation effects were tested using PROCESS MACRO in SPSS. Loneliness and depression negatively correlated with life satisfaction and self-reported health, indicating that individuals with higher loneliness and depression scores reported lower life satisfaction and poorer health. Blocked regressions confirmed that loneliness and depression significantly predicted both outcomes (β = -34, p < .001; β = -278, p < .001). Having more than one child positively influenced life satisfaction (β = .043, p < .001) after controlling for key variables. Gender moderated the relationship between loneliness and self-reported health, with the effect being stronger for females than males. These findings highlight the importance of family structure in aging and suggest that older adults with more than one child experience greater life satisfaction. This study informs future research on aging in single-child families and can guide policymakers in shaping family planning and aging policies.

Abstract citation ID: igaf122.3295

### FAMILY TIES AND WELL-BEING: THE ROLE OF SUPPORT ACROSS GENERATIONS

Yunann Lin[1], Hui-Ching Weng[2], and Wang Chih-Liang[2], *1. National Chung Cheng University, Minxiong, Chiayi, Taiwan (Republic of China), 2. National Cheng Kung University, Tainan City, Tainan, Taiwan (Republic of China)*

Due to aging impact on traditional family structure, exploring their diverse needs is essential for older adult physical and mental health. Thereby, this study aimed to compare the intergenerational relationship to subjective well-being from both parents and children aspect in the baby boomer and generation X. This longitudinal study analyzed data collected from the 2016 Taiwan Social Change Survey, consisting of demographics, self-rated health, subjective well-being, family harmony, family conflict, financial, household, and emotional support. Participants were categorized into two cohorts: the baby boomer generation (1946-1964), and generation X (1965-1980). Logistic regression is adopted to analysis intergenerational associations. After adjusting gender, marriage, self-rated health, income, providing emotional support (p = 0.028) to parents increase subjective well-being in baby boomer generation for generation X. The baby boomer generation’s subjective well-being was positively associated with providing financial support (p < 0.001) to parents and receiving financial (p = 0.011) and household (p = 0.034) support from children. Additionally, family harmony with parents was positively associated with subjective well-being in baby boomer (p = 0.001) and generation X (p = 0.027); whereas family conflict with parents was negatively associated with subjective well-being in baby boomer (p = 0.04) and generation X (p = 0.004). In conclusion, intergenerational relationship is more significant to subjective well-being in baby boomer than generation X. Baby boomer generation concern providing and receiving support from parent and children, especially in financial aspect; while generation X value parent harmony more than family support.

Abstract citation ID: igaf122.3296

### HOW GENDER SHAPES PARENT-ADULT CHILD CONSISTENCY OF REPORTING PROBLEMS IN CHILDREN’S LIVES

Tamrah Verbanac[1], Destiny Ogle[1], Megan Gilligan[2], and J. Jill Suitor[1], *1. Purdue University, West Lafayette, Indiana, United States, 2. University of Missouri, Columbia, Missouri, United States*

Despite both parents’ and adult children’s declarations that they have an accurate picture of one another’s feelings and behaviors, research that has collected data separately from both members of parent-adult child dyads has shown that there are typically substantial discrepancies in their reports. One area in which such discrepancies may be of particular concern is adult children’s problems. Although parents are often a central source of practical support when adult children face crises, even in midlife, providing adequate support at the most advantageous time may be hindered by a lack of information sharing between the generations. In the present paper, we compare children’s reports of their current and recent problems with parents’ reports of their children’s problems, using data collected from mothers and fathers (aged ~70) and at least one of their adult children (aged ~40) in 85 families as part of the Within-Family Differences Study-I. Using a combination of quantitative and qualitative data, we explore how consistency is reporting is shaped by a combination of the type and timing of the problem, and the gender of both the adult child and the parent. Preliminary findings suggest that consistency is highest between mothers' and daughters' reports, and lowest between fathers and daughters. Consistency is also greater when problem are current and are less likely to potentially "reflect badly" on either the child or the parent (e.g., illnesses and non-culpable injuries). These findings can be helpful to clinicians developing strategies to increase communication across generational ties, particularly in times of crisis.

Abstract citation ID: igaf122.3297

### HUMANITY RESTORATION PROJECT: BRIDGING THE AGE GAP THROUGH HANDS-ON INTERGENERATIONAL LEARNING

Renee Beard[1], Lindsay Riordan[1], Katherine Meszaros[1], and Emma Schultz[2], *1. College of the Holy Cross, Worcester, Massachusetts, United States, 2. St. Mary Health Care Center, Worcester, Massachusetts, United States*

Intergenerational learning is thought to be an effective strategy for disrupting ageist stereotypes youth may hold and addressing the social isolation of participants. For the past 5 years, "The Humanity Restoration Project" has studied the benefits of intergenerational learning between college-aged students and nursing home residents and staff using Anne Basting's improvisational storytelling method TimeSlips™ (TS) and the related "beautiful questions". The central goals of the study include exposing students to intergenerational conversations, countering ageist and ableist social assumptions, and supporting community members living and working in institutional care settings. Both residents' quality of life and staff work satisfaction have been demonstrated to improve during arts-based interventions and intergenerational programming. We collected data through observations, written student reflections, student oral presentations, pre- and post-test surveys and semi-structured individual interviews and focus groups. Students report improved ability to imagine themselves as future old people, a reduction of negativity/stigma about aging, and genuine respect for and identification with older adult participants. Residents were more engaged and felt valued through helping "teach" young people about aging. Like students, the project allowed staff to see residents in a new, more positive and personal light. Intergenerational learning has the dual benefit of disrupting negative views of aging and older people while simultaneously providing meaningful connection and social interactions. It also has the emancipatory potential - for students and staff alike - of restoring the humanity to some of the most disenfranchised among us: older adults living in nursing homes.

Abstract citation ID: igaf122.3298

### INTERGENERATIONAL MENTORING AND SOCIAL SUPPORT: EVIDENCE FROM THE BRIDGE2HEALTH PROGRAM

Jill Juris[1], Shannon Jarrott[2], Hannah D'Angelo[1], and Calyope Ortega[1], *1. Appalachian State University, Boone, North Carolina, United States, 2. The Ohio State University, Columbus, Ohio, United States*

Social connections contribute to healthy aging. Intergenerational strategies provide an innovative approach to creating opportunities for meaningful social connections. BRIDGE2Health is a shared mentoring program designed to improve intergenerational social connections. Two cohorts of adults (n=58, age range = 50-79) participated in monthly intergenerational programming with teens in one of two communities. The Interpersonal Support Evaluation List (Cohen et al., 1985), was utilized to measure functional social support. Paired samples T-Tests results indicated significant differences between pre and post-measures in three domains of functional support: appraisal ($p < .001$), tangible ($p < .001$), and belonging ($p < .001$) for adults in both communities. When comparing new (one year of participation) and returning participants (two years of participation), both new and returning participants experienced similar levels of change in social connection. For returning participants, the effect size was greater in appraisal ($d = -2.17$) compared to overall findings ($d = -2$). For new participants, belonging had the strongest difference of the three (appraisal, belonging, tangible). This study illustrates the potential for enhancing social connections through intergenerational programming.

Abstract citation ID: igaf122.3299

### INTERGENERATIONAL SHARING AS A MODERATOR OF LEARNING AND WELL-BEING: A LATE ADULTHOOD PERSPECTIVE

Rong Ren, and Alei Fan, *Purdue University, West Lafayette, Indiana, United States*

This study investigates the relationships between active learning, personal growth, intergenerational sharing, and well-being among adults aged 55 and above. While lifelong learning has been associated with positive aging outcomes, the underlying mechanisms and potential moderators remain partially explored. Drawing on adult development theories and gerontological frameworks, we examined whether personal growth mediates the relationship between active learning and well-being, and whether intergenerational sharing moderates these relationships. Data were collected from 164 participants through validated measures of active learning, personal growth, intergenerational sharing, and multi-dimensional well-being. Regression analyses using PROCESS revealed that personal growth fully mediates the relationship between active learning and well-being (indirect effect = .376, 95% CI [.223, .532]), with the direct effect becoming non-significant when accounting for personal growth. Contrary to our hypothesis, intergenerational sharing did not moderate the relationship between active learning and well-being, nor the pathways in our mediation model. Instead, intergenerational sharing emerged as an independent predictor of both personal growth ($\beta = .587$, $p < .001$) and well-being, with its effect on well-being also fully mediated by personal growth (indirect effect = .212, 95% CI [.134, .301]). These findings suggest that both active learning and intergenerational sharing contribute to well-being through parallel pathways by fostering personal growth. The results expand our understanding of psychological mechanisms in late adulthood and suggest that interventions promoting active learning and intergenerational sharing should emphasize personal growth as a key outcome for enhancing well-being among older adults.

Abstract citation ID: igaf122.3300

### INTERGENERATIONAL TRANSMISSION OF CORPORAL PUNISHMENT: A SCOPING REVIEW

Ying Xu, Xiafei Wang, and Merril Silverstein, *Syracuse University, Syracuse, New York, United States*

Numerous studies have investigated the impacts of corporal punishment (CP) within families. However, there has been limited research on how these practices are transmitted across generations. This scoping review synthesized 18 peer-reviewed articles published between 2000 and 2023 from four databases, including PsycINFO, PubMed, PsycNet, and ProQuest. The findings revealed that 16 out of 18 studies showed a significant positive correlation in the intergenerational transmission of CP. Among them, nine studies indicated the intergenerational transmission of perpetrating CP, while seven found individuals with childhood CP experience would approve CP. Conversely, only one study suggested a negative association, indicating adults experienced childhood CP were less likely to use it on their own children. Additionally, one study found no significant impact of harsh mothering on later harsh parenting behaviors. These mixed findings highlight the intricate relationship between childhood CP experiences and adult disciplinary attitudes and behaviors. Future studies should prioritize longitudinal research and culturally sensitive approaches to better understand the mechanisms underlying CP transmission and develop effective strategies to break this cycle across generations.

Abstract citation ID: igaf122.3301

### KINLESSNESS IN LATER LIFE: A SCOPING REVIEW

Jianan Li[1], and Claire McDonald[2], *1. University of California, Los Angeles, Los Angeles, California, United States, 2. University of Maryland Baltimore County, Baltimore, Maryland, United States*

Shifting adult family structures—driven by rising childlessness, declining marriage rates, and increased life expectancy—have led to a growing population of kinless older adults, defined as those without a partner and children. This group comprises approximately 7% of the aging population in the United States. Despite the growing evidence on this population, research on their multidimensional well-being remains fragmented across disciplines. This scoping review synthesizes 17 existing articles to examine how definitions of kinlessness vary and to assess the well-being of this population using the PRISMA-ScR guidelines. The different definitions highlight the limitations of framing kinlessness solely in terms of traditional family structures, simple measures of kin presence, and deficit-based perspectives. Empirical evidence highlights a complex pattern of vulnerabilities. Physically, kinless older adults report poorer self-rated health despite often having fewer comorbidities, with greater reliance on formal care services and more unmet needs. Mentally, their unpartnered status contributes to greater loneliness and poorer subjective mental health, though aging itself may mitigate these effects as individuals adapt. Socially, they have less tangible support but maintain distinct—not necessarily fewer—social connections compared to those with close kin. However, well-being varies significantly by age, gender, education, and cultural context. Future research should examine pathways to kinlessness through a life-course perspective, explore resilience mechanisms, and consider intersecting social determinants to better support this diverse population. A nuanced understanding of their experiences is essential for addressing their needs in aging societies.

Abstract citation ID: igaf122.3302

### LIVING ARRANGEMENTS, FAMILY SUPPORT, AND HEALTH OUTCOMES AMONG OLDER ADULTS IN CHINA

Sijiu Wang, and Rui Wu, *Tsinghua University, Beijing, Beijing, China (People's Republic)*

As demographic shifts and urbanization reshape family structures in China, understanding the relationship between living arrangements and health outcomes among older adults is critical. This study examines how different living arrangements influence self-rated health, mental health, and functional status, and whether these effects are mediated by family support. Using three waves (2013, 2015, and 2018) of the China Health and Retirement Longitudinal Study (CHARLS), we categorized living arrangements into four groups: living alone without children in the same city, living alone with children nearby, living with a spouse only, and living with others beyond a spouse. Lagged models addressed potential reverse causality, while mediation analyses explored three key support mechanisms: informal care, frequency of child contact (in-person and technology-based), and financial support. Findings suggest that living alone with children nearby and living with a spouse are associated with better self-rated and mental health compared to living alone without children in the same city. Living with others beyond a spouse is linked to improved mental health. Mediation analyses reveal that informal care receipt supports functional health, while all forms of regular child contact enhance mental and functional health. Financial support is associated with improvements across all health outcomes. These results underscore the significance of both living arrangements and family support in shaping older adults' health. Policymakers should consider strategies to promote intergenerational proximity and alternative support mechanisms for aging populations.

Abstract citation ID: igaf122.3303

### MARITAL SATISFACTION AND PSYCHOLOGICAL WELL-BEING IN OLDER ADULTS LIVING WITH HEALTH DIFFICULTIES

Hyunjoo An[1], Minju Seong[1], Inhye Jung[1], Miseon Kang[2], and Hyo Jung Lee[1], *1. Yonsei University, Seoul, Republic of Korea, 2. Symbiotic Life-TECH Institute, Seoul, Republic of Korea*

Difficulties in performing everyday activities, commonly faced by older individuals, are closely linked to a lower level of psychological well-being, often being observed as an increased number of depressive symptoms. Marital relationships in later life have a significance due to various experiences of loss and long-term togetherness. The health of both spouses, including functional and emotional aspects, plays a critical role in shaping their own psychological well-being. This study explores the effect of spouse's health on older individual's psychological well-being, considering the moderating role of marital satisfaction. Using data from the 2022 Korean Longitudinal Study of Aging, we identified 3,820 older adults who were married, with information on spouse's functional and emotional health. PROCESS Macro Model 1 was employed for the analysis. Compared to both spouses without functional health difficulties, marital satisfaction moderated the effect of a spouse's psychological well-being on the individual's psychological well-being when either spouse experienced such difficulties. Particularly when marital satisfaction was high, it served as a protective factor in this association. These findings indicate that older adults' psychological well-being is affected by his or her spouse's health status, especially when facing functional health difficulties. In addition, their positive spousal relationship satisfaction functions as a moderator to buffer this negative effect. These results contribute to an urgent need for better understanding the experiences of older individuals within marriage considering difficulties in health experienced by their spouses. It also suggests that a high level of satisfaction for their own marital relationship can improve older individuals' psychological well-being.

Abstract citation ID: igaf122.3304

**PREDICTORS OF LATE-LIFE DIVORCE IN SOUTH KOREA: A 21-YEAR PANEL STUDY**

Seoyeon Ahn[1], Minyoung Kwak[2], and Sojung Park[3], *1. Duke-NUS Medical School/National University of Singapore, Singapore, Singapore, 2. Daegu University, Gyongsan, Kyongsang-bukto, Republic of Korea, 3. Washington University in St. Louis, St. Louis, Missouri, United States*

This study examines how socioeconomic and marital factors influence late-life separation and divorce men and women aged over 50 in Korea. Using data from the 21-wave Korea Labor and Income Panel Study (1999–2019), we analyze a sample of 4,922 married couples aged over 50 years old. Discrete-time event history models are applied to investigate the impact of socioeconomic and marital factors on marital instability over the 21-year period. Our findings indicate that home ownership and husbands' pension receipt significantly reduce the risk of marital dissolution, while wives' secondary education is associated with a higher likelihood of marital disruption. Additionally, Husbands' higher levels of relationship satisfaction with family was associated with lower risk of marital disruption. These results highlight the importance of considering both socioeconomic resources and interpersonal factors in explaining marital instability among middle-aged and older adults. The complex relationship between education and divorce risk for older Korean women suggests that both financial independence and cultural norms shape divorce decisions. These findings contribute to our understanding of gray divorce in East Asian societies, where traditional family values coexist with rapid socioeconomic change. Recognizing the predictors of late-life marital dissolution has important implications for developing social services tailored to the unique needs of divorced older adults, particularly in aging societies with evolving family structures.

Abstract citation ID: igaf122.3305

**RECIPROCAL INFLUENCE OF CHRONIC STRESS ON DEPRESSIVE SYMPTOMS AMONG MIDDLE-AGED AND OLDER COUPLES**

Kyuho Lee[1], Jaemin Jeon[1], and Wi Hoon Jung[2], *1. Daegu University, Gyeongsan, Kyongsang-bukto, Republic of Korea, 2. Gachon University, Seongnam, Kyonggi-do, Republic of Korea*

Background: Chronic stress in later life is a known risk factor for depression, potentially magnified within spousal dyads as older adults grapple with financial and social challenges. While many studies examine individual predictors of depressive symptoms, fewer focus on how one partner's stress may spill over onto the other's mental health. This study investigated the reciprocal effects of husbands' and wives' chronic stress on each other's depressive symptoms among older couples. Method: We analyzed data from the 2016 and 2020 waves of the Health and Retirement Study (HRS), encompassing 808 married couples aged 50 or older. A two-wave cross-lagged Actor-Partner Interdependence Model (APIM) tested actor (own T1 stress → own T2 depression) and partner (T1 stress → spouse's T2 depressive symptoms) effects, while controlling for T1 depressive symptoms. This design captures both individual continuity of depression and cross-spousal influences of stress over time. Results: Depressive symptoms remained stable for husbands and wives. Husbands' T1 stress did not predict their own T2 depressive symptoms, whereas wives' T1 stress did. Both husbands' and wives' T1 stress predicted spouses' T2 depressive symptoms, revealing robust cross-spousal "spillover." Wives' stress had a stronger impact on husbands' subsequent depression than vice versa. Discussion: These findings underscore the dyadic nature of chronic stress in later life, with notable gender differences. A couple-based perspective for depression interventions is warranted, recognizing each partner's stress can shape the other's mental health.

Abstract citation ID: igaf122.3306

**REMARRIAGE OF MEN IN LATER LIFE**

Cagri Elmas, *Akdeniz University, Antalya, Antalya, Turkey*

This paper explores the decision-making processes and experiences of men who remarry later in life, focusing on the interplay between remarriage, aging, and masculinity. Unlike first marriages, remarriage in later years involves complex social, economic, and emotional dynamics, making it a critical site for examining how masculine identities and gender roles are renegotiated. Through a explanatory mixed- research methods approach, this research highlights how older men navigate remarriage within the framework of hegemonic masculinity. Findings reveal that while remarriage serves as a means of emotional and practical support, it also functions as a space where men seek to reassert or adapt their masculine identities. The weakening of hegemonic masculine ideals with aging often leads to a renewed emphasis on traditional family structures, reinforcing heteronormative relationships. At the same time, remarried men emphasize the importance of open communication, mutual respect, and emotional connection in fostering fulfilling relationships. By adopting a life course perspective, this study provides a deeper understanding of how gender and masculinity shape marital dynamics in later life. Ultimately, the research contributes to discussions on aging, gender identity, and the evolving nature of masculinity in contemporary society.

Abstract citation ID: igaf122.3307

**SPOUSAL ASSOCIATIONS BETWEEN SOCIAL PARTICIPATION AND CHRONIC DISEASES AMONG CHINESE MIDDLE-AGED AND OLDER ADULTS**

Yunchen Ruan, and Mantang Gan, *Fuzhou University, Fuzhou, Fujian, China (People's Republic)*

Objective: While several previous studies have explored the relationship between a spouse's social participation and the physical health of their partner among Chinese middle-aged and older adults, none have investigated the connection between a spouse's social participation and chronic diseases in their partner, or the potential gender differences in this relationship. Our study seeks to address this gap. Methods: This study utilized data from the China Health and Retirement Longitudinal Study (CHARLS) follow-up surveys conducted in 2013, 2015, and 2018. The sample consisted of 3,072 couples aged 45 and older. Chronic diseases were assessed by counting the total number of chronic conditions reported by the participants. Social participation was measured by the number of different types of social activities in which the participants engaged. The actor-partner interdependence model was employed for analysis. Results: Spousal social participation was significantly associated with the partner's chronic diseases, with notable gender differences. The wife's social participation was positively correlated with the husband's chronic diseases, while the husband's social participation was negatively correlated with the wife's chronic diseases. Notably, depressive symptoms played a significant mediating role in the relationship between spousal social participation and the partner's chronic diseases. Conclusions: Spouses' social participation is associated with chronic diseases in their partners among Chinese middle-aged and older adults, with this association being gender-dependent. The government should prioritize building and strengthening social support networks while implementing gender-sensitive measures to promote social participation and improve health outcomes.

Abstract citation ID: igaf122.3308

### SUBJECTIVE AND OBJECTIVE MEASURES OF FAMILY DYNAMICS WITHIN MULTIGENERATIONAL LATE-LIFE FAMILIES

Duyen Tran[1], Yichen Zhang[1], and Brian Carpenter[2], *1. Washington University in St. Louis, St. Louis, Missouri, United States, 2. Washington University, St. Louis, Missouri, United States*

Late-life families are complex systems that play a significant role in older adults' lives, making it essential to understand intergenerational relationships. Traditional measures of family dynamics have relied on reports from just one person in the family and on self-report scales, which are vulnerable to potential biases. To address these issues, this study compared reports from both older parents and adult children on the Family Adaptability and Cohesion Scale IV and examined associations with performance on a triadic problem-solving task. Participants were 32 families, consisting of an older parent (M age = 75; 93% female) and two adult children (M age = 46; 65% female). Results indicated considerable variability in intergenerational concordance about family features. The mean difference across family members was 2.60 for Cohesion (range = -39.33 to + 37.67, SD = 19.85) and 3.44 for Flexibility (range = -38.17 to + 30.50, SD = 15.33). Across all families, parents and children were similar in their reports of Cohesion and Flexibility ($p = 0.72$, $p = 0.25$, respectively). Associations between the problem-solving task and reports of Cohesion and Flexibility were not significant ($p = 0.37$, $p = 0.58$, respectively). However, higher agreement among family members on family features was significantly associated with better performance on the task (Cohesion: $r = -.76$, $p < .001$; Flexibility: $r = -.48$, $p = .005$). These findings underscore the value of integrating both self-report and behavioral assessments to gain a more comprehensive understanding of family functioning in late life.

Abstract citation ID: igaf122.3309

### THE EFFECT OF FAMILY SUPPORT ON THE DEPRESSION LEVELS AMONG COMMUNITY-DWELLING OLDER ADULTS IN CHINA

Yuchen Gu, *Boston College, Brighton, Massachusetts, United States*

Compared to other nations, China has a severe deficit in mental health resources and support systems, particularly in professional counseling and early intervention. Current measurements used in Western samples of social support lack consideration of the unique aspects of Chinese culture, namely the focus on family support. Additionally, previous studies on family support often measure a single dimension without recognizing the complexity of its financial and emotional aspects, as well as the distinction between receiving and giving support. In this study, I categorize family support as: financial support from children (financial/receiving), living with spouse (emotional/receiving), and taking care of grandchildren (emotional/giving). Based on Chinese social and cultural backgrounds, the goal of this study is to establish how family support in Chinese distinct cultural contexts influences depression levels among community-dwelling older adults. By using the 2018 China Health and Retirement Longitudinal Study (CHARLS), I investigate the association between three different forms of family support on depression levels among Chinese older adults aged 60 and over ($n = 5{,}187$) using the regression analysis. The results show that only living with spouse and taking care of grandchildren is negatively associated with depression levels. While financial support did have a negative impact, it is not statistically significant. These results show that emotional and psychological support are more important in the prevention of mental health deterioration for Chinese older adults. The research findings suggest that family support for Chinese older adults should go beyond financial help and focus more on emotional aspects.

Abstract citation ID: igaf122.3310

### THE EFFECTS OF LIVING ARRANGEMENTS ON DEPRESSION AND LIFE SATISFACTION IN RURAL CHINESE OLDER ADULTS: A 20-YEAR STUDY

Jin Guo[1], Andrew Wister[2], Barbara Mitchell[2], and Shuzhuo Li[1], *1. Xi'an Jiaotong University, Xi'an, Shaanxi, China (People's Republic), 2. Simon Fraser University, Vancouver, British Columbia, Canada*

Objectives: China has experienced rapid socio-economic transformations over the past two decades. Based on individualization and intergenerational solidarity theories, this study explores changes in the associations between the living arrangement on psychological well-being over time and across different chronic conditions. Methods: Eight waves of longitudinal data (2001–2021) collected in Anhui, China were employed, including. 9,765 (person-year) observations. Mixed linear models containing interaction terms for time and chronic conditions were used to examine the effects of living arrangements on life satisfaction and depression. Results: Compared to living with children, the negative correlation with life satisfaction and the positive correlation with depression for those living alone or with spouse only decreased over time, while the negative correlation between living in skipped-generation household and life satisfaction also diminished. As the number of chronic conditions increases, life satisfaction declines further for older adults living alone, with a spouse only, or in a skipped-generation household. Living alone leads to a higher increase in depression, but living with others reduces it. Discussion: This study is the first to identify the changes in dynamic relationships between living arrangements on psychological well-being of older adults across time and chronic health conditions, reflecting shifts in filial piety and family culture. While older adults increasingly accept independent living, coresiding with children remains beneficial during multimorbidity. These findings contribute to the understanding of ageing in the context of economic and cultural transitions in developing regions.

Abstract citation ID: igaf122.3311

### THE GRANDMOTHER EFFECT: A QUALITATIVE STUDY EXAMINING HOW WE PASS DOWN VALUES OF BLACK WOMANHOOD

Tanya Burgess, *Western Kentucky University, Bowling Green, Kentucky, United States*

This qualitative study explores the lived experiences of Black women aged 55 and above residing in Maricopa County, Arizona, with a focus on their perspectives of care, social connections, and intergenerational caregiving norms and values. Through in-depth interviews lasting 60 to 90 minutes, participants shared their narratives on how social engagement and caregiving practices shape their sense of belonging and well-being. Findings revealed that all participants attributed their caregiving approach and social interactions to deeply held cultural values, emphasizing mentorship, service, and intergenerational guidance. These values were largely learned through strong relationships with older Black women, particularly grandmothers. The study highlights that Black women's sense of community is reinforced not solely through mentorship but through structured intergenerational support systems that foster belonging and reduce the pressures associated with the "superwoman" schema. The implications of this study underscore the need for community interventions, services, and policies that move beyond traditional mentorship models. Instead, efforts should be directed toward creating spaces where Black women of all ages can engage in reciprocal care and knowledge-sharing without reinforcing harmful narratives of self-sacrifice. By acknowledging and strengthening intergenerational ties, communities can better support the caregiving trajectories of Black women, ensuring holistic and sustainable well-being across generations.

Abstract citation ID: igaf122.3312

### UNDERSTANDING BARRIERS TO ACCESS SERVICES AMONG GRANDPARENTS RAISING GRANDCHILDREN

Julian Montoro-Rodriguez[1], Bert Hayslip[2], Anita Rogers[3], Jaia Lent[3], Emily Patrick[3], and Ana Beltran[3], *1. The University of North Carolina at Charlotte, Charlotte, North Carolina, United States, 2. University of North Texas, Denton, Texas, United States, 3. Generations United, Washington, District of Columbia, United States*

Grandparent caregivers face numerous barriers in accessing needed services, undermining their well-being. This is key to their adjustment in coping with the demands of caregiving. This study explored predictors of multiple dimensions of barriers to service use among135 grandparent and kinship caregivers (Mage = 56.3) living in Philadelphia. Principal components analysis yielded a 3-factor model of barriers 1) negative interactions with service providers, family stress, understanding and accessing services, 2) grandparent/grandchild physical/mental health difficulties, and 3) limited health insurance, unemployment, inadequate housing, lack of transportation). Predictors were based upon the Anderson (1995) Behavioral and Strengths-Oriented (Ronch & Goldfield, 2003) models. Hierarchical regression analyses found that 1) greater age, poorer grandparent mental health, and less personal resilience uniquely predicted ($p < .02$) more Factor 1 barriers. For Factor 2, greater age, poorer grandparent physical health, greater mental health impediments to caregiving, and less resilience each uniquely ($p < .03$) predicted greater barriers. Less income, greater mental health impediments to caregiving, and less resilience uniquely ($p < .03$) predicted greater Factor 3 barriers. These findings suggest that Anderson predisposing variables as well as enabling and need variables are important in understanding the determinants of barriers to service among grandparent caregivers. They also suggest that the attention to one's physical and mental health and the ability to cope with adversity, i.e., personal resilience (Rutter, 2006), are important in allowing grandparent caregivers' to overcome barriers to service. These findings underscore the importance of adaptive coping skills/empowerment in accessing services among grandparent caregivers.

### SESSION 7475 (POSTER)

### FAMILY CAREGIVING IV

Abstract citation ID: igaf122.3313

### A HIDDEN STRUGGLE: UNDERSTANDING THE EFFECTS OF BEREAVEMENT AFTER CAREGIVING ENDS

C. Cassie Heilman, and Julie Hicks-Patrick, *West Virginia University, Morgantown, West Virginia, United States*

Research shows that up to 20% of caregivers of persons with dementia experience an increase in complicated grief and depressive symptoms following the death of the care recipient (Schulz et al., 2006). We used the 2023 BRFSS, which asked caregivers whether their care recipient had died within the past 30 days. We used independent samples t-test to examine mental and physical health differences between recently bereaved ($n = 77$) and current caregivers ($n = 11233$). Bereaved caregivers reported more poor mental health days per month compared to their non-bereaved counterparts ($M = 9.36$ v. 5.88, $t(11379) = 5.07$, $p < .03$). Bereaved caregivers reported a lower incidence of lifetime depression than non-bereaved caregivers (16% v. 27%; $t(11501) = 28.70$, $p < .001$). However, the two groups did not significantly differ on the number of days of poor physical health ($M = 5.34$ and 4.65). Bereaved caregivers report poorer mental health than those who are continuing to provide care, illustrating that even after the burden of caring for a loved one has passed, the hardships for the caregiver are not over. Post hoc analyses examine potential moderators of these differences. Given the lack of interventions available to assist caregivers with their grief (Arruda & Paun, 2017), expanding programs to assist recently bereaved caregivers could alleviate mental health challenges among this group.

Abstract citation ID: igaf122.3314

### A QUALITATIVE STUDY ON CAREGIVING BURDEN & SLEEP PATTERN: COPING AMONG LONG-DISTANCE SOUTH ASIAN CAREGIVERS

Dinesh Karki[1], Srujana Chekuri[2], Akankshya Chataut[1], and Julie Blaskewicz Boron[1], *1. University of Nebraska Omaha, Omaha, Nebraska, United States, 2. University of Nebraska Omaha, Elkhorn, Nebraska, United States*

Long-distance caregiving (LDC) is a unique experience which mitigates some caregiving disturbances (e.g., nocturnal wandering), but exacerbates others (e.g., care recipient monitoring). LDC may increase stress and disrupt regular sleep, negatively affecting the caregiver's health and quality of life. Sleep disturbances in LDC result from a complex interplay of environmental conditions, personal responsibilities, and coping strategies. This study explored how external factors—including neighborhood, community, and social support—affect sleep among South Asian (SA) long-distance caregivers responsible for companionship, financial assistance, and emotional support. SA Caregivers (N = 78) aged 19-73 (M = 26; SD = 7). were recruited for a larger needs assessment. A subset of participants (n = 21, MAge=26, Male=80.1%) completed one of five focus group sessions via Zoom. Thematic analysis was conducted independently by two reviewers using MAXQDA for inductive coding. Results revealed that caregivers employed diverse coping strategies (e.g., white noise machines, earplugs, adjusted sleep schedules) to mitigate sleep disruptions, however, these only partially addressed long-term concerns. Most (71.4%) reported that strong family and community support systems were critical for reducing caregiving stress and improving sleep quality; neighborhood support was also identified as important by 23.8% of caregivers. In contrast, 19% of caregivers with limited social support reported higher stress and poorer sleep outcomes. These findings highlight the importance of interpersonal networks for fostering resilience and well-being for long-distance caregivers. Addressing contextual factors, implementing effective coping strategies, and strengthening community engagement are essential for enhancing long-distance caregivers' sleep quality and overall health. Future research should examine these relationships in other cultural identity groups.

Abstract citation ID: igaf122.3315

### CAREGIVER BURDEN AND CAREGIVING BENEFITS IN LONG-DISTANCE FAMILY CAREGIVERS

Verena Cimarolli[1], Francesca Falzarano[2], and Kathrin Boerner[3], *1. LeadingAge, Washington, District of Columbia, United States, 2. University of Southern California, Los Angeles, California, United States, 3. Carl von Ossietzky University of Oldenburg, Oldenburg, Niedersachsen, Germany*

A rich empirical knowledge base is available on the impact of caregiving on the well-being of geographically proximate caregivers (CGs) of older adults with chronic illness and on the efficacy of interventions that can support them. These research efforts have largely excluded the about 2 million long-distance family caregivers (LDCs) – CGs living over one hour away from their care recipient (CR). Yet, research comparing LDCs with proximate CGs shows that while LDCs report less physical strain, they experience equal or greater levels of burden and emotional stress. For the development of supportive interventions for LDCs, research identifying factors associated with CG burden and caregiving benefits among this growing group of family CGs is needed. Hence, the purpose of this cross-sectional study (N = 304) was to identify socio-demographic characteristics, primary stressors (e.g., distance from CR, CR cognitive status), and internal resources (i.e., coping, social support, familism) associated with LDCs' levels of CG burden and positive aspects of caregiving. Two regression analyses examined associations between these factors and burden and benefits. Higher CG burden was significantly associated with being younger, being White, less optimal income adequacy, CR's worse cognitive status, spending more time helping the CR, and higher actual social support received by the LDC. Higher levels of positive aspects of caregiving were associated with being female, as well as higher levels of problem-focused coping and familism (filial piety). Study findings provide preliminary insights into possible targets of intervention to alleviate caregiving burden and to increase caregiving benefits among LDCs.

Abstract citation ID: igaf122.3316

### CAREGIVING PROFILES: DEMOGRAPHIC AND ECONOMIC SOCIAL DETERMINANTS OF HEALTH

S. Alison Bolling, and Julie Hicks-Patrick, *West Virginia University, Morgantown, West Virginia, United States*

In 2021-22, approximately 37.1 million Americans provided care to family or friends (U.S. Bureau of Labor, 2024). Caregiver strain may differ across age, gender, race (Young et al., 2019). Given the effects of Social Determinants of Health, such as financial stability, health care, and food resources (Duran & Perez-Stable, 2019), some adults may experience additional threats to health and quality of life before entering the caregiver role. Little is known about those who anticipate becoming a caregiver. Knowing key information about anticipated caregivers would allow better programs and policies for future caregivers. The current study includes 97,914 participants (Mean age = 55.56, 45.8% female, 81.6% white non-Hispanic) who completed the 2022 BRFSS. Approximately 20.1% were current caregivers, 10.47% anticipated becoming a caregiver in the next two years, 5.3% were unsure, and 64.11% did not anticipate becoming a caregiver. A multinomial logistic regression examined the contributions of key demographic characteristics and economic social determinants of health (eSDOH) to predicting group membership, $\chi^2(60675) = 1832.60$, $p < .001$. Compared to current caregivers, anticipated caregivers were more likely to be: younger (OR = .99), male (OR = 1.2), and unemployed (OR = .81). Challenges related to eSDOH were higher for current than for anticipated (OR = .89), unsure (OR = .76), and those not anticipating becoming a caregiver (OR = .73). Additional post hoc analyses compared anticipated caregivers to unsure and not anticipating. More resources should be available for those prior to becoming a caregiver to alleviate future caregiver strain.

Abstract citation ID: igaf122.3317

### COGNITIVE DECLINE IN OLDER ADULTS AND CAREGIVER DISTRESS: STRESS-BURDEN PATHWAYS IN DEMENTIA CARE

Rajib Biswas[1], Melinda Higgins[1], Fayron Epps[2], and Kenneth Hepburn[1], *1. Emory University, Atlanta, Georgia, United States, 2. UT Health San Antonio, San Antonio, Texas, United States*

This secondary analysis of the Tele-Savvy randomized trial examines pathways linking cognitive decline in older adults with dementia to caregiver psychological distress. Using baseline data from 261 caregivers (77 male, 184 female), validated measures (IQCODE, PSS, ZBI, CESD) assessed cognitive decline, stress, burden, and depression. Parallel/serial mediation analyses (PROCESS Models 4/6/85) tested stress and burden as mediators of cognitive decline's impact on depression, with mindful self-care as a moderator. Cognitive decline correlated positively with caregiver stress ($\rho = 0.24$), burden ($\rho = 0.31$), and depression ($\rho = 0.23$; $ps < 0.01$). Stress and burden fully mediated the cognitive decline-depression pathway (total indirect effect: $B = 4.38$, 95% CI[2.60,6.31]), explaining 56.6% of depression variance. Female caregivers reported higher stress ($d = -0.57$), burden ($d = -0.52$), and depression ($r = -0.20$; $ps < 0.01$) than males. Mindful self-care inversely correlated with stress ($\rho = -0.36$), burden ($\rho = -0.22$), and depression (ρ = −0.29; ps < 0.01) but did not moderate mediation pathways (ps > 0.05). These findings align with the parent trial, where Tele-Savvy reduced caregiver stress and depression with moderate-to-large effects. However, burden remained unmitigated, underscoring the need for targeted strategies. Results highlight cognitive decline's indirect effects through sequential stress-burden mechanisms, with marked gender disparities. Implications emphasize integrating gender-sensitive approaches into caregiver support programs, prioritizing stress reduction, and advancing longitudinal research to clarify causality. This work advances understanding of modifiable factors in dementia caregiving and aligns with person-centered aging frameworks, promoting equitable solutions for diverse older populations.

Abstract citation ID: igaf122.3318

### COMPASSION SATISFACTION AND FATIGUE IN BANGLADESHI AMERICANS PROVIDING CARE TO AGING RELATIVES

Mohammad Hossain, Natalie Pope, and Abigail Latimer, *University of Kentucky, Lexington, Kentucky, United States*

Caring for aging parents is deeply rooted in Bangladeshi culture. However, despite the significant growth of the Bangladeshi population in the US, there has been limited research on the well-being of Bangladeshi American family caregivers. This study examined compassion satisfaction (CS) and compassion fatigue (CF) among Bangladeshi Americans caring for aging relatives. We conducted a cross-sectional survey of 12 participants using the Professional Quality of Life (ProQOL) Scale, which includes a measure of CS and two measures of CF (burnout (BO) and secondary traumatic stress (STS)). The ProQOL is validated for use in family caregivers. The sample was equally distributed between both sexes, age (M = 41.44 ± 6.5) reporting moderate levels of CS (M = 38.33 ± 6.53), STS (M = 28.17 ± 9.30) and BO (M = 23.25 ± 5.42). A Pearson's correlation analysis revealed a strong, positive, and significant correlation between caregivers' birth order, r(12)= .74, p < .01) and number of siblings (r(12)= .61, p < .05) and CS. Those caregivers born later in their families and those with a higher number of siblings had more satisfaction with their caregiving experiences. There were no significant relationships found between age, birth order, number of siblings and CS and CF. While this sample is small, it is the first to explore caregiving experiences in Bangladeshi Americans, providing insight into an overlooked population. Future research may want to consider familial structures as protective factors to CF.

Abstract citation ID: igaf122.3319

### DEMENTIA CAREGIVERS' COMMUNICATION WITH HEALTHCARE PROVIDERS

Jinmyoung Cho[1], Alan Stevens[2], Jennifer Thorud[3], Jordan Reese[3], and Marcia Ory[4], *1. Saint Louis University, Saint Louis University, Missouri, United States, 2. Baylor Scott & White Health, Belton, Texas, United States, 3. Baylor Scott & White Research Institute, Temple, Texas, United States, 4. Texas A&M University, College Station, Texas, United States*

The scope of caregiving for people living with dementia (PLWD) includes helping with activities of daily living to managing complex medical and nursing-related tasks. Engaging in multiple tasks leads to negative caregiving experiences. As dementia progresses, interactions with healthcare systems (e.g., scheduling appointments, communication about the patient's medical conditions) are critical to providing care for the PLWD. However, caregivers' interaction with healthcare providers remains underexplored. This study examines caregivers' experiences with dementia patients' healthcare providers. Using reports from 240 caregivers of dementia patients enrolled in a clinical trial, we conducted a descriptive analysis of the frequency of caregivers' communication with PLWD's healthcare workers and its relationship to caregivers' experience with providers. Results showed that frequent communication with healthcare providers is significantly associated with tasks relevant to medical care such as scheduling appointments ($\chi2$=12.89, p<.01) and coordinating care ($\chi2$=30.02, p<.001). Caregivers who frequently communicated with patients' healthcare providers reported providers always or usually listened (98.9%; $\chi2$=14.67, p<.001) and asked about their understanding of patients' treatments (77.8%; $\chi2$=9.69, p<.01). Caregivers with limited or no communication with healthcare professionals reported significantly less communication on whether they needed help managing patients' care treatment (64.5%; $\chi2$=26.21, p<.001). Caregivers who did not communicate at all reported significantly lower burden (F = 4.01, p<.05) while caregivers who did communicate a lot reported higher positive aspects of caregiving compared to their counterparts (F = 7.99, p<.001). These findings underscore the importance of structured engagement between dementia caregivers and health professionals, which could alleviate caregiver burden and enhance care patient outcomes.

Abstract citation ID: igaf122.3320

### MIDDLE-AGED AND OLDER CAREGIVERS' DEPRESSIVE SYMPTOMS AND MULTIPLE SOURCES OF SOCIAL SUPPORT

Seungjong Cho[1], Yeon Jin Choi[2], and Sun Kyung Kim[3], *1. Texas Tech University, Lubbock, Texas, United States, 2. University of Kentucky, Lexington, Kentucky, United States, 3. Case Western Reserve University, Cleveland, Ohio, United States*

Providing care in middle and later life can be physically and emotionally challenging, often contributing to heightened depressive symptoms among caregivers. Since depressive symptoms are associated with physical decline, higher mortality risk, and reduced quality of life, it is essential to identify protective factors that can mitigate this adverse effect. While previous research highlights the benefits of social support, most studies aggregate different sources rather than assessing their distinct effects. This study aims to compare the effects of four different sources of social support (spouse or partner, children, relatives, and friends) on depressive symptoms among middle-aged and older caregivers. We analyzed merged data from the 2020 Health and Retirement Study (N = 1,068). The results from multiple linear regression with multiple imputation indicated that only spousal support was a statistically significant predictor of depressive symptoms among caregivers when all social support variables were entered simultaneously. Specifically, the coefficient for spousal support was -.236 (p=.016), meaning that as spousal support increases by one unit, depressive symptoms decrease by an average of 0.236 units. Among the covariates, higher levels of depressive symptoms were also linked to younger age, being female (compared to male), and lower self-rated health. These findings underscore the critical role of spousal support in mitigating caregivers' depressive symptoms. Given its unique significance, interventions should focus on strengthening spousal relationships through counseling, communication training, and caregiver support programs tailored for couples. Additionally, since younger caregivers exhibited higher depressive symptoms, targeted mental health resources and caregiver support services should prioritize these vulnerable groups.

Abstract citation ID: igaf122.3321

### NEIGHBORHOOD HISTORY-BASED SOCIAL WALKING FOR CAREGIVERS AND PEOPLE LIVING WITH EARLY-STAGE DEMENTIA

Patrice Fuller[1], Charles Fennell[2], Nora Mattek[1], Michael Crespo[1], and Raina Croff[1], *1. Oregon Health & Science University, Portland, Oregon, United States, 2. Wake Forest University School of Medicine, Winston-Salem, North Carolina, United States*

The SHARP Caregiver study engaged seven triads (n = 21) consisting of a healthy or mildly cognitively impaired (MCI) caregiver (age >40), their care partner with MCI or early-stage dementia, (age >40), and a healthy or MCI support person (age >18) in Black history-based neighborhood walking. Triads used the SHARP application to walk 1-mile routes with images to prompt conversational reminiscence 3x/week over 16 weeks in Portland, Oregon's gentrifying historically Black neighborhoods. Mean age overall was 69.8 years. Eight (38%) were cognitively healthy [mean MoCA 26.5 (SD 1.9)] and 13 (4 MCI; 9 early-stage dementia) were cognitively impaired [mean MoCA 18.8 (SD 2.8)]. Caregivers and care partners completed pre/post health assessments. Overall, 29% rated themselves very/fairly active at baseline and 57% at end-study; 36% rated themselves in excellent/very good health at baseline and 43% at end-study. Pre/post mean Geriatric Depression Scale scores decreased for caregivers from 1.86 to 1.14 and increased for care partners from 1.40 to 1.80, indicating normal/no depression and consistency over time. Pre/post mean Zarit Burden Interview (short 12-item) scores for caregivers increased from 13.14 to 14.86 indicating mild to moderate burden and consistency over time. Highly motivational program aspects were recording memories for use in community learning sessions about healthy aging and being accountable to their group. Overall, self-rated activity level improved while depression and caregiver burden was maintained at healthy levels. The SHARP-Caregiver study is feasible for caregivers and care partners, providing structure to weekly activity, group accountability and socializing for mental health and community connection.

Abstract citation ID: igaf122.3322

### PROFESSIONALISM AND CHALLENGES OF INDONESIAN CAREGIVERS FOR OLDER ADULTS IN TAIWAN: A QUALITATIVE STUDY

Fang-Lin Kuo[1], Herry Susanto[2], and Widiyaningsih Widiyaningsih[3], *1. National Health Research Institutes, Zhunan Town, Miaoli, Taiwan (Republic of China), 2. Universitas Islam Sultan Agung, Semarang, Jawa Tengah, Indonesia, 3. Taipei Medical University, Taipei City, Taipei, Taiwan (Republic of China)*

Indonesian caregivers are the largest group of migrant workers in Taiwan and play a critical role in the country's social welfare sector, particularly in caring for older adults. Despite their significant contributions, their professionalism and lived caregiving experiences remain underexplored. This study investigated the motivations, challenges, and essential skills required for effective caregiving among Indonesian migrant workers. A qualitative phenomenological study was conducted using semi-structured interviews with 10 female caregivers who had worked in Taiwan for an average of 6.9 years. Interviews explored participants' perspectives on caregiving responsibilities, communication, conflict resolution, emotional resilience, and professional development. Thematic analysis identified three key themes. First, financial and family-related factors serve as primary motivations for pursuing caregiving work in Taiwan, shaping caregivers' long-term career decisions. Second, caregivers identified essential qualities for professional caregiving, including physical endurance, adaptability, mental resilience, and lifelong learning. Third, participants reported significant challenges in their caregiving roles, including language barriers, conflicts with older adults' families and job expectations exceeding initial agreements. Despite these difficulties, caregivers adopted coping strategies such as engaging in social activities, using technology for self-protection and communication, and relying on religious practices for emotional support. Findings demonstrate the complexity of caregiving among migrant workers and the need for enhanced training in language skills, emergency response, and specialized geriatric care techniques. Addressing these areas can improve caregivers' well-being and the quality of care provided to older adults in Taiwan. These insights contribute to policy recommendations for better support systems and workforce development initiatives tailored to migrant caregivers.

Abstract citation ID: igaf122.3323

### SENSE OF PURPOSE IN CAREGIVING: THE BUFFERING EFFECT ON CAREGIVER BURDEN

Lindsay Wilkinson[1], Bethany Smith[2], and Jong Ko[3], *1. University of Nebraska Omaha, Omaha, Nebraska, United States, 2. KEN Consulting, Inc., College Park, Maryland, United States, 3. Johns Hopkins University, Baltimore, Maryland, United States*

While research has predominately focused on the negative consequences of caregiving for health and well-being, less attention has been paid to the positive outcomes of caregiving. One such benefit may be the sense of purpose individuals obtain from their caregiver role. Drawing on the stress process model, this research asks two main questions: Do caregiver burden and hours spent caregiving contribute to lower perceived quality of life? If yes, does sense of purpose derived from caregiving play a moderating role in these associations? This study utilizes a sample of 53 informal caregivers to patients with chronic illnesses recruited through electronic health records. The average age of respondents was 57 years (range: 23-75), with the majority of respondents providing care to a spouse/partner (58%), followed by an aging parent (32%). Close to one-third of respondents (32%) "nearly always" felt that their life had more of a purpose since they began providing care, compared to 38% of respondents who "never" or "rarely" felt this way. Findings from linear regression models adjusting for demographic characteristics show that higher caregiver burden was significantly associated with lower quality of life ($p < 0.001$); however, the impact of caregiver burden was lessened among respondents who found purpose in caregiving ($p < 0.01$). No association was found for hours spent caregiving. This study demonstrates the importance of sense of purpose as a resource for informal caregivers, particularly those experiencing high levels of perceived burden, and could help lead to targeted interventions aimed at improving caregiver quality of life.

Abstract citation ID: igaf122.3324

### SENSORY LOSS AMONG COGNITIVELY IMPAIRED SPOUSAL CAREGIVERS: FINDINGS FROM THE HEALTH AND RETIREMENT STUDY

Shaoqing Ge[1], and Qingyi Jasmine Zeng[2], *1. University of Texas at Austin School of Nursing, Austin, Texas, United States, 2. The University of Texas School of Nursing, Austin, Texas, United States*

With the global trend of aging, more older adults are assuming caregiving roles for their loved ones. Sensory loss and cognitive decline are prevalent among older adult caregivers, exacerbating the stress and burden of caregiving and leading to increased physical and emotional strain. Previous studies lacked an understanding of the inter-relationship between sensory loss, cognitive impairment, and caregiving. This study addresses this gap by analyzing data from the 2020 Health and Retirement Study (HRS), focusing on cognitively impaired spousal caregivers with self-reported sensory data. Using the Langa-Weir (LW) algorithm, among 926 spousal caregivers, 157 (17%) were categorized as cognitively impaired but not dementia (CIND), and 29 (3%) had dementia. Among cognitively impaired spousal caregivers, 116 rated hearing function as “good and above,” while 70 rated it as “fair/poor.” Additionally, 89 rated their eyesight as “good and above,” and 95 rated it as “fair/poor.” Although ANOVA analysis showed a significant relationship between race and hearing status, being African American or Hispanic/Latino did not significantly increase the odds of having fair or poor hearing in the logistic regression models. Conversely, the odds of having “good and above” eyesight decreased among African American (OR = 0.23, 95% CI = 0.07–0.73, p<.05) and Hispanic caregivers (OR = 0.09, 95% CI = 0.02–0.39, p<.01). Other factors, including age, gender, education, income, working status, and Medicaid status, were non-significant in affecting the odds of better hearing or vision. These findings underscore the need for a comprehensive understanding of the risk factors contributing to sensory loss and associated challenges among cognitively impaired spousal caregivers.

Abstract citation ID: igaf122.3325

### SOCIAL SUPPORT NETWORK, NEIGHBORHOOD SOCIAL COHESION, AND PSYCHOLOGICAL DISTRESS AMONG CAREGIVING SPOUSES

Yeon Jin Choi[1], Seungjong Cho[2], and Jihyeong Jeong[3], *1. University of Kentucky, Lexington, Kentucky, United States, 2. Texas Tech University, Lubbock, Texas, United States, 3. University of Maryland Baltimore, Baltimore, Maryland, United States*

Caregiving burden and stress are well-documented risk factors for poor mental health among caregiving spouses. While social support from family and friends can help alleviate caregiver distress, those without social support network may experience greater burden and psychological distress. For these individuals, neighborhood social cohesion may serve as an alternative protective factor providing an additional layer of social support. Using data from the 2006–2012 Health and Retirement Study (HRS), we examined the association between neighborhood social cohesion and psychological distress among caregiving spouses (N = 1,219), stratified by the availability of a nearby social support network. Approximately 13.5% of caregivers reported having no support network, while 86.5% had children, close friends, or relatives in their neighborhood. Caregivers without a support network exhibited higher levels of hopelessness (M = 2.88, SD = 1.42) and depressive symptoms (M = 3.03, SD = 2.50) compared to those with a support network (hopelessness: M = 2.56, SD = 1.30; depressive symptoms: M = 2.59, SD = 2.30). A series of regression models were estimated, controlling for sociodemographic characteristics, caregiving intensity, and the caregiver-care recipient relationship. Social cohesion was significantly associated with lower psychological distress (hopelessness: b=-0.18, p<.001; depressive symptoms: b=-0.20, p<.01). Notably, the protective effect of social cohesion was stronger among caregivers with no support network (hopelessness: b=-0.20, p<.05; depressive symptoms: b=-0.49, p<.01) compared to those with a support network (hopelessness: b=-0.17, p<.001; depressive symptoms: b=-0.14, p<.05). Findings underscore the importance of fostering social cohesion to mitigate psychological distress, particularly among caregivers with limited social support network. Community-based interventions aimed at strengthening social ties may be beneficial in supporting caregiving spouses.

Abstract citation ID: igaf122.3326

### UNDERSTANDING CHALLENGES AND UNMET NEEDS: EXPERIENCES OF LATINO ADULT-CHILD CAREGIVERS OF PERSONS WITH DEMENTIA

Eunbee Kim[1], Hyun Jung Kim[2], Seunghye Hong[3], and Jung-Ah Lee[1], *1. University of California, Irvine, Irvine, California, United States, 2. Seattle University, Seattle, Washington, United States, 3. University of Hawai‘i at Mānoa, Honolulu, Hawaii, United States*

The Latino/Hispanic community faces unique caregiving challenges, with family caregiving largely falling on adult children rather than spouses. As the prevalence of Alzheimer’s Disease and Related Dementias and the Latino/Hispanic population grow, the burden on these caregivers continues to increase. However, limited research has examined this role’s challenges and long-term impact, making it difficult to develop effective support. This study explores these factors among Latino/Hispanic adult-child caregivers of persons with dementia (PWD), highlighting their experiences and unmet needs. This descriptive qualitative study analyzed home-visit logs from ten adult-child caregivers, including three males, who participated in a randomized controlled trial of a 3-month home-based intervention. Key themes emerged, including struggling with financial strain, coping with persistent distress, navigating a fragmented healthcare system, and balancing competing responsibilities. Financial strain often added to caregivers’ burden, not only affecting their ability to afford care-related expenses but also increasing reliance on family or informal networks for support. Many struggled with balancing work, family obligations, and caregiving demands while facing limited support, leading to physical and emotional exhaustion. Navigating a complex healthcare system created additional barriers to securing appropriate care, with caregivers frequently encountering fragmented services, a lack of culturally competent providers, and challenges related to advanced care planning and legal matters. These challenges contribute to heightened emotional distress, increased caregiver burden, and a greater risk of depressive symptoms. Findings underscore the urgent need for targeted interventions, improved healthcare accessibility, and culturally responsive support programs to better serve Latino/Hispanic adult-child caregivers of PWD.

Abstract citation ID: igaf122.3327

### WHO CARES? EXAMINING MARRIAGE, CULTURE, AND FAMILY APPROVAL ON PERCEIVED OBLIGATIONS TO PROVIDE DEMENTIA CARE

Tim Killian[1], and Allison Martin[2], *1. University of Arkansas, Fayetteville, Arkansas, United States, 2. University of Arkasnas, Fayetteville, Arkansas, United States*

Dementia care presents significant challenges for family caregivers, whose perceived obligations are shaped by relational, cultural, and psychosocial factors. This study examines variations in caregiving expectations based on marital status, marital history, gender, sexual intimacy, and adult children's approval. Additionally, we explore how broader cultural values, including familism and individualism, shape perceived caregiving responsibilities. Using a nationally representative Qualtrics sample of 300 U.S. adults aged 55 and older, participants responded to vignettes depicting caregiving scenarios. Data were first analyzed using descriptive statistics and correlation analysis. Next, mixed-effect multiple regression models were used to assess predictors of perceived caregiving obligations while accounting for individual differences. Results indicate that marital status plays a predicted perceived obligations, with married partners having significantly greater caregiving obligations than those in non-marital relationships ($p < 0.0001$). Familism was a strong predictor of caregiving expectations ($p < 0.0001$), reinforcing caregiving as a cultural obligation. However, findings did not support the assumption that sexual intimacy significantly influences caregiving expectations ($p = 0.30$). Additionally, adult children's approval had only marginal effects on caregiving perceptions ($p = 0.08$), suggesting that while intergenerational relationships may shape caregiving roles, they are not the primary determinant. These findings have implications for policy and intervention strategies aimed at supporting family caregivers, particularly in addressing the needs of diverse family structures and cultural backgrounds. Future research should further explore further the roles of cultural values and personal beliefs in shaping caregiving responsibilities.

Abstract citation ID: igaf122.3328

### WHO MOVES IN MATTERS: MOVE-IN PATTERNS, HOUSEHOLD HEADSHIP, & FINANCIAL STRAIN AMONG LATINO ADULT CHILD CAREGIVERS

Elizabeth Vásquez[1], Jacqueline Angel[2], Sunshine Rote[3], Phillip Cantu[4], and Anna Bokun[5], *1. University at Albany (SUNY), Albany, New York, United States, 2. The University of Texas at Austin, Austin, Texas, United States, 3. University of Louisville, Louisville, Kentucky, United States, 4. UTMB Health, The University of Texas Medical Branch, Galveston, Texas, United States, 5. The University of Texas at Austin, Boston, Massachusetts, United States*

While research shows increasing prevalence of multigenerational households, we know little about how different pathways to co-residence affect caregiver well-being. Using the Hispanic Established Populations for the Epidemiologic Study of the Elderly (2010-11), we examine how move-in scenarios and household structure influence financial strain among 659 Mexican-American adult child caregivers and their older parents (80+). Preliminary findings show that financial strain varies by move-in scenario. Adult children who moved in with their parent to provide care experience the highest rates of financial strain (22.2%), followed by adult children whose parents moved in with them for care (19.4%). Homeownership is a key predictor, with financial strain reaching 35.7% among caregivers who moved into a parent-owned home to provide care. Parents with dementia are more likely to move in with their adult children (64.5% vs. 44.4% when children move in), suggesting cognitive decline drives household restructuring. Among adult children who moved in to provide care, only 14.8% are household heads, compared to 57.1% of those who moved in for general reasons. This housing transition correlates with increased caregiving intensity (8.0-8.1 ADL/IADL hours per day) vs. 2.6-3.4 hours for caregivers who moved in for their own housing needs, and 6.2-6.3 hours for caregivers who co-reside without discerning move-in reasons.These findings highlight the need for policies that consider both direction and motivation for household moves when targeting support for caregiving families. Future research incorporating American Community Survey data will examine broader patterns of caregiving-related mobility and how these patterns compare across other populations.

### SESSION 7480 (POSTER)

### GEROSCIENCE

Abstract citation ID: igaf122.3329

### AGING HALLMARKS AND INNATE IMMUNE RESPONSES IN HUMAN MONOCYTES AND SUBSEQUENT CHRONIC CONDITIONS

Madeline Kelly[1], Da Ma[2], and Jingzhong Ding[2], *1. Wake Forest University School of Medicine, Maple Grove, Minnesota, United States, 2. Wake Forest University School of Medicine, Winston-Salem, North Carolina, United States*

Animal studies suggest that the aging process, underlain by interconnected cellular alterations, promote multiple aging-related chronic conditions simultaneously. Translating these animal findings to humans is needed to realize the therapeutic potentials of geroscience. Transcriptomic profiles were determined in circulating monocytes from 1,264 MESA participants aged 55-94 (51% women, 53% minority). Older age was associated with alterations in 24 of 40 co-expression modules (FDR< 0.05), including interconnected ($r > 0.4$ or $r < -0.4$) hallmarks of the aging process (such as cellular senescence, mitochondrial dysfunction, loss of proteostasis, and epigenetic alterations) and innate immune responses of monocytes (such as inflammatory responses and complement activation). Innate immune responses were further elevated in persons with obesity (FDR< 0.05). Innate immune responses were cross-sectionally associated with shorter 6 minute walk distances (inflammatory responses and complement activation) and lower Cognitive Abilities Screening Instrument scores (inflammatory responses) (FDR< 0.05). During a 6-year follow-up, complement activation predicted new events of aging-related diseases including cardiovascular disease, type-2 diabetes, hypertension, cancer, dementia, chronic kidney disease, chronic obstructive pulmonary disease, and hip fracture (RH:1.16-1.18. FDR=0.03). In conclusion, our transcriptomic analyses identified a coordinated network of aging-related dysfunctions of cellular hallmarks and innate immune responses in human monocytes, and these innate immune responses, exacerbated by obesity, may contribute to poor physical and cognitive performance and aging-related diseases.

Abstract citation ID: igaf122.3330

**BEHAVIORAL AND COGNITIVE DECLINE ARE OBSERVABLE IN THE AGING HOUSE CRICKET (ACHETA DOMESTICUS)**

Gerald Liao, Swastik Singh, Sherwin Dai, Elizabeth Bae, and Warren Ladiges, *University of Washington, Seattle, Washington, United States*

Understanding the mechanisms of aging is critical for developing interventions against age-related diseases. While traditional mammalian models provide valuable insights, they are often limited by ethical and financial constraints. Alternative models, such as invertebrates, offer promising avenues for aging research. Here, we investigate the house cricket (Acheta domesticus) as a novel model organism for studying age-related behavioral and cognitive decline. We hypothesize that crickets, like humans and mice, exhibit progressive deterioration in locomotion, exploratory behavior, and cognition with age. Using adult (6-weeks), mid-age (8-weeks), and geriatric (>10-weeks) cohorts, we conducted a series of behavioral assays. In an open-field test, older crickets demonstrated reduced exploration, slower locomotion, and increased periphery preference. A modified treadmill assay revealed declines in maximum jumping distance, running speed, and endurance. Cognitive function was assessed using an aversive-learning escape paradigm, where crickets learned to associate cinnamon scent with a reward and vanilla scent with a punishment. Geriatric crickets exhibited impaired learning, though no differences were observed in short-term memory. Interestingly, mid-age crickets outperformed other groups in long-term memory. Additionally, adult and geriatric crickets reached decision arms faster than mid-age counterparts, with adults making the quickest choices. A scent preference test revealed that only adults demonstrated a significant preference for vanilla. These findings suggest that age-related phenotypic changes in house crickets are quantifiable through behavioral assays, supporting their potential as a scalable, cost-effective model for aging research. Further exploration of molecular and physiological markers could offer cross-species insights into the biology of aging.

Abstract citation ID: igaf122.3331

**EFFECT OF D-ALLULOSE ON FRUCTOSE-INDUCED SKELETAL MUSCLE INSULIN RESISTANCE**

Teruhiko Koike[1], Kamal Shahriar[2], Takamasa Tsuzuki[3], and Ryoichi Banno[2], *1. Research Center of Health, Physical Fitness, and Sports, Nagoya University, Nagoya, Aichi, Japan, 2. Nagoya University, Nagoya, Aichi, Japan, 3. Meijo University, Nagoya, Aichi, Japan*

Consumption of fructose-sweetened beverages, particularly in combination with a high-fat diet, is a major contributor to obesity, diabetes, and nonalcoholic fatty liver disease. Insulin resistance is a key underlying mechanism in the development of these conditions. In particular, ectopic fat accumulation in skeletal muscle plays a crucial role in inducing insulin resistance. This study aimed to investigate the effects of the rare sugar D-allulose, one of the candidates for caloric restriction mimetics, on fructose-induced insulin resistance. Wistar rats were randomly assigned to one of three dietary groups: fructose-free control diet (CD), high-fat diet (HF), or high-fat/fructose diet (HFF). After four weeks, an intraperitoneal glucose tolerance test (IPGTT) and a two-step hyperinsulinemic-normoglycemic clamp (HE-clamp) test were performed. Blood samples were collected after six weeks, and triglyceride levels in the liver and skeletal muscle were measured. Additionally, Western blotting was conducted on skeletal muscle samples. The HFF group was then supplemented with either 5% D-allulose or 5% cellulose, and the same tests were repeated. The HFF group exhibited increased blood glucose levels in the IPGTT and greater systemic and skeletal muscle insulin resistance in the HE-clamp compared to the CD and HF groups. Furthermore, blood triglycerides (TG), hepatic TG, and muscle TG levels were significantly elevated. However, D-allulose supplementation improved insulin resistance in the HFF group and reduced blood TG, hepatic TG, and muscle TG levels. D-allulose may improve insulin resistance by reducing triglyceride accumulation in skeletal muscle. Notably, this effect appears to be independent of its anti-obesity properties.

Abstract citation ID: igaf122.3332

**THE ROLE OF SENESCENCE-ASSOCIATED INTERFERON IN AGE-RELATED INFLAMMATION**

Mansi Shrivastava[1], Reema Banarjee[2], Bradley Olinger[2], Linna Cui[2], Amit Dey[2], Myriam Gorospe[2], and Nathan Basisty[2], *1. National Institute on Aging, Baltimore, Maryland, United States, 2. National Institute on Aging, Bethesda, Maryland, United States*

Inflammaging, age-related deterioration of immune function, features persistent inflammation alongside an exacerbated immune response to pathogens. Senescent monocytes accumulate with age and release harmful proteins, potentially driving hyperinflammation. This study explores how senescent monocytes contribute to inflammaging and evaluates therapeutic interventions targeting these cells. Using an irradiation-induced senescence model in THP-1 monocytes, we analyzed the proteomic changes via data-independent acquisition (DIA) mass spectrometry. We assessed the impact of senescence on pathogen response by measuring LPS-induced inflammatory cytokines in senescent and non-senescent cells using a Mesoscale kit. Additionally, we tested various compounds for their ability to modulate inflammation and evaluated inflammatory signatures in monocytes. Finally, we examined proteomic profiles of circulating monocytes from 94 individuals aged 22-89 in the Genetic and Epigenetic Signatures of Translational Aging Laboratory Testing (GESTALT) study of aging to identify clinical associations between senescence and IFN signatures and aging phenotypes in vivo. Senescent THP-1 monocytes exhibited significant proteome remodeling, with increased 359 IFN-related proteins, including STAT1, OAS1, OAS2, MX1, and ISG15, while 1355 decreased proteins. Moreover, when stimulated with LPS, senescent monocytes exhibited a highly potentiated inflammatory cytokine response, including interleukins and IFN-ɣ, driving inflammaging and susceptibility. Notably, interventions targeting senescence or IFN signaling reduced these inflammatory responses. Further, the proteomic analysis of circulating monocytes in the GESTALT cohort showed that 27 senescence-associated proteins, correlated with CRP, were identified as potential biomarkers, highlighting systemic inflammation. These findings highlight senescence-associated IFN signaling as a key driver of inflammaging and suggest potential therapeutic strategies to mitigate age-related immune dysregulation.

Abstract citation ID: igaf122.3333

**TOTAL MICRONUTRIENT INTAKE AND EPIGENETIC AGING IN THE WOMEN'S HEALTH INITIATIVE**

Lindsay Reynolds[1], Alexandra Cowan-Pyle[2], Regan Bailey[2], Diane Mitchell[2], Eric Whitsel[3], Mara Vitolins[1], and Janet Tooze[1], *1. Wake Forest University School of Medicine,*

*Winston-Salem, North Carolina, United States, 2. Texas A&M University, College Station, Texas, United States, 3. The University of North Carolina at Chapel Hill Gillings School of Global Public Health, Chapel Hill, North Carolina, United States*

Use of dietary supplements (DS) is common among older adult women and represents an important source of micronutrient intake. To understand whether recommended micronutrient intake is related to slower biological aging, we estimated associations between DS use, total micronutrient intake (from diet and DS use), and an epigenetic biomarker of pace of aging (DunedinPACE). We conducted a cross-sectional study of 4,582 postmenopausal women aged 50–79 years at the Women's Health Initiative (WHI) baseline visit (1993-1998), leveraging food frequency questionnaire, DS use, and leukocyte DNA methylation (DNAm) data. We calculated micronutrient intake using the Total Nutrient Index (TNI, range: 0-100), higher values of which indicate greater adherence to recommended Dietary Reference Intakes for six under-consumed micronutrients (calcium; magnesium; vitamins A, C, D, and E from diet and DS) among the U.S. population. We used linear mixed models adjusted for age, self-identified race and ethnicity, education, smoking, physical activity, ancillary study DNAm data source, and leukocyte proportions to estimate DS use- and TNI-DunedinPACE associations. DS use was common among participants (60%) and associated with a slower DunedinPACE (Beta±SE: -0.100 ±0.026, p = 1.5E-4). A higher TNI score also was associated with a slower DunedinPACE (Beta±SE: -0.040 ±0.013, p = 0.0022). The TNI micronutrient component most strongly associated with DunedinPACE was vitamin C (Beta±SE: -0.0025 ±0.0007, p = 1.19E-04). Other TNI components inversely associated with DunedinPACE were calcium (p = 5.9E-4), magnesium (p = 0.021), and vitamin E (p = 0.012). These findings support that DS use and recommended micronutrient intakes are associated with epigenetic aging and may influence the rate of aging.

### SESSION 7485 (POSTER)

### HEALTH CARE

Abstract citation ID: igaf122.3334

### CHRONIC CONDITIONS, IMMUNOSUPPRESSION, AND DELAYED AND MISSED CARE DURING THE COVID-19 PANDEMIC (2020-2021)

Natalie Turner, and Melissa Martinson, *University of Washington, Seattle, Washington, United States*

The COVID-19 pandemic severely disrupted healthcare provision, leading many to delay or miss medical care. Within the first few months, one-third of adults age 65 and older reported delaying or missing care. Additionally, individuals with chronic conditions faced heightened barriers to accessing healthcare. While some research has examined age-related differences in healthcare access, less is known about how age interacts with specific chronic conditions regarding delayed or missed care. This study examined the role of age in delayed or missed medical care using the National Health Interview Survey 2020–2021 waves. Multivariate logistic regression models assessed the relationship between age, chronic illness, and healthcare disruptions, while exploring disparities by race, ethnicity, income, and insurance status. Adults age 65 and older were less likely to delay or miss care than those age 18–44 and 45-64. These findings remained consistent among individuals with hypertension, cardiovascular disease, and lung disease. However, among immunocompromised individuals and those with diabetes, age was not associated with delayed or missed care. Black and Hispanic individuals with diabetes, and American Indian and Alaska Native individuals with cardiovascular disease experienced greater healthcare disruptions. Immunocompromised individuals with the lowest incomes had increased likelihood of delaying or missing care. These results suggest that while older adults generally experienced fewer disruptions in healthcare, certain chronic conditions may pose unique challenges regardless of age. Findings also highlight how racial disparities in access to care vary across chronic conditions. Future research should examine how outreach efforts, including telehealth, may mitigate barriers to healthcare.

Abstract citation ID: igaf122.3335

### COGNITIVE PERFORMANCE AND DENTAL CARE UTILIZATION AND DENTAL HYGIENE PRACTICE AMONG OLDER ADULTS

Huabin Luo[1], Xiang Qi[2], Ruotong Mona Liu[2], and Bei Wu[3], *1. East Carolina University, Greenville, North Carolina, United States, 2. New York University, New York, New York, United States, 3. NYU Shanghai, Shanghai, China (People's Republic)*

Dental care is crucial for oral health and there is growing evidence that poor oral health contributes to cognitive decline. However, little research has assessed the relationship between different domains of cognitive function and dental utilization and dental self-care. Using National Health and Nutrition Examination Survey (NHANES) 2011-2014, we investigated the associations of cognitive performance with dental utilization and self-care. The sample included 2,495 adults aged 60 years or older and having at least one tooth. Cognitive performance was assessed by the Consortium to Establish a Registry for Alzheimer's Disease: immediate word recall (IWR), delayed recall (DR), Animal Fluency Test (AFT), and Digit Symbol Substitution Test (DSST). We also calculated a low cognitive performance score based on education levels, defined as 1 SD below the mean on a composite z-score of these four tests. The three outcome variables were dental visits, preventive dental visits, and dental flossing. Multivariate regression model results showed that higher levels of cognitive performance in DR and DSST were associated with more frequent dental visits (all P<.05). In addition, low levels of IWR, AFT, and DSST were significantly associated with less dental flossing (all P<.05). Overall low cognitive performance was associated with less dental flossing (AOR=0.48, P=.02). These results suggest that older adults with low cognitive performance are less likely to have regular dental visits or conduct proper dental hygiene practice. Oral health awareness and oral health literacy should be further promoted among older adults and family members.

Abstract citation ID: igaf122.3336

### D-SNP ENROLLEE HEALTHCARE ACCESS AND SATISFACTION: DOES INSURANCE LEVEL OF INTEGRATION MAKE A DIFFERENCE?

Morgan Perry, Ishrat Alam, and Geoffrey Hoffman, *University of Michigan, Ann Arbor, Michigan, United States*

Dual Eligible Special Needs Plans (D-SNPs) range from less integrated, coordination-only plans (CO D-SNPs) with enhanced communication between Medicare Advantage and Medicaid to fully integrated plans (FIDE-SNPs) managed under a single entity. Using restricted Medicare Current Beneficiary Survey data (2015-2020) and CMS health plan data, we assessed the relationship between enrollee satisfaction and access with health plan integration. We identified community-dwelling, fully dually eligible individuals enrolled in a D-SNP, resulting in 3,369 CO D-SNP and 341 FIDE-SNP enrollee-year observations. We estimated survey-weighted logistic regression models adjusting for state and year fixed effects plus individual and area-level factors. We conducted subgroup analyses by age and race/ethnicity. Self-reported satisfaction with access and quality of care were generally high. While FIDE-SNP enrollees were more likely to recommend Medicare Advantage (Adjusted Risk Ratio [ARR]: 1.04, p = 0.006), other outcomes did not vary by level of integration. Comparing FIDE to CO plans, enrollees under 65 were more likely to report having a regular source of care (ARR: 1.07, p = 0.019), those 65 and older were more likely to recommend Medicare Advantage (ARR: 1.05, p = 0.003) and report being satisfied with specialty care (ARR 1.04; p = 0.017), and White enrollees were more likely to report being very satisfied with specialty care (ARR: 1.56, p = 0.003) and plan-related costs (ARR: 1.42, p = 0.002). Mixed sub-group findings call into question whether recent investments into FIDE-SNPs are justified and equitable across populations. State policymakers should consider more tailored investments while awaiting more findings on the effectiveness and equity of these approaches.

Abstract citation ID: igaf122.3337

### ENGAGEMENT IN STROKE PREVENTION SHARED DECISION MAKING IN OLDER ADULTS WITH ATRIAL FIBRILLATION AND MULTIMORBIDITY

Hawa Abu[1], Jane Saczynski[2], Jerry Gurwitz[1], Kathleen Mazor[1], Robert Goldberg[1], David Dosa[1], Alok Kapoor[1], and David McManus[1], *1. University of Massachusetts Chan Medical School, Worcester, Massachusetts, United States, 2. Northeastern University, Boston, Massachusetts, United States*

Background: Older adults with atrial fibrillation (AF) and multimorbidity are posed with stroke prevention decisional conflicts. Understanding the factors that predict engagement in shared decision making (SDM) can enhance patient-centered care. Objective: To examine the extent of engagement in SDM on stroke prevention among older adults with AF and multimorbidity. Methods: A prospective cohort study (2016-2018) enrolled patients aged ≥65 years with AF from clinics in Massachusetts and Georgia. Those with one or more chronic conditions were included in the present study. At one-year follow-up, participants on oral anticoagulation were asked about their involvement in both choosing to be on anticoagulation and selecting an anticoagulant. Engagement in SDM was categorized as fully engaged (affirmative to both questions), partially engaged (affirmative to one), or not engaged (non-affirmative to both). Results: Among 808 participants (mean age 75 years, 48% women, 88% White), 33% were engaged in both aspects of SDM, 28% participated in anticoagulation initiation, 4% in selecting a specific anticoagulant, and 35% did not actively participate in either. Non-engaged patients were more likely to be older, non-White, have lower education levels, cognitive impairment, frailty, poor self-rated health, greater dependency in daily activities, higher comorbidity burden, and lower perceived benefit from anticoagulation. Conclusions: In managing older adults with AF and multimorbidity, healthcare providers should be aware of vulnerable populations that are at risk of being excluded from proper engagement in SDM for stroke prevention to ensure a more tailored approach to providing holistic and patient-centered care for improved outcomes.

Abstract citation ID: igaf122.3338

### EXPLORING THE POTENTIAL FOR ANIMAL ASSISTED INTERVENTIONS (AAI) AS A TREATMENT MODALITY FOR AN AGING POPULATION

Brigitte Belanger[1], and David Hage[2], *1. Florida Gulf Coast University, Cape Coral, Florida, United States, 2. Florida Gulf Coast University, Fort Myers, Florida, United States*

Allied health professionals face significant challenges to providing health care services to aging adults. It is imperative to develop creative strategies to address the complex needs of care recipients and those involved in care provision. Animal Assisted Interventions (AAI) have the potential to positively impact treatment and improve health care outcomes. A recent AAI study with a population experiencing neurocognitive deficits reported benefits for all participants. Six dyads consisting of a care recipient and their care partner attending a SW Florida community dementia support center were invited to participate in 90-minute AAI occupational therapy group sessions over the course of six weeks. Each dyad was placed in one of two experimental groups based on care recipient scores on the Allen Cognitive Level Screen (ACLS-5). Both experimental groups were part of a single-group pre-post-test mixed-method pilot study. As care partners modeled strategies implemented during AAI sessions, such as errorless learning, they began to report improvements in care recipient motivation and activity performance. Additionally, researchers noted increased confidence and patience in caregiving. Researchers hypothesized that AAI could be beneficial to promote health prevention strategies given the interest by participants for future AAI opportunities. The current AAI study explores the effects of human-animal interactions as a strategy to monitor vital signs and assess for early warning signs of atrial fibrillation and atherosclerosis.

Abstract citation ID: igaf122.3339

### FACTORS ASSOCIATED WITH HOSPITAL GERIATRIC SERVICE AVAILABILITY BEYOND URBAN-RURAL DIFFERENCES

Teagan Maguire, Alice Yan, and Jie Chen, *University of Maryland College Park, College Park, Maryland, United States*

The study examined variations in the provision of geriatric care among hospitals based on their urban and rural locations. This cross-sectional study utilized data from the 2023 American Hospital Association Annual Survey and the Area Health Resources File. Descriptive statistics and logistic regression models were employed to compare hospital and geographic variations among hospitals with or without geriatric services. Geriatric services were defined as offering one or more of the following: adult day care, Alzheimer's diagnostic-assessment services, comprehensive geriatric assessment, emergency response systems, geriatric acute care units, and/or geriatric clinics. The study included 2,991 hospitals, categorized as metropolitan (61.82%), micropolitan (16.85%), and rural (21.33%). Descriptive analyses indicate that hospitals in rural areas provide fewer geriatric services. After controlling for hospital size, ownership type, teaching affiliation, and community demographics, hospitals in metropolitan areas were significantly less likely to provide hospital-based geriatric services (OR = 0.68, 95% CI: 0.51–0.91) or offer geriatric psychiatry services (OR = 0.60, 95% CI: 0.39–0.94), compared to rural hospitals. Hospitals with larger bed sizes, teaching hospital status, and locations in areas with more skilled nursing facilities were more likely to provide geriatric services. However, hospitals serving higher proportions of minority populations were significantly less likely to offer geriatric services. A similar pattern was observed among hospitals providing Alzheimer's services and geriatric psychiatric care. Results indicate that differences in geriatric service provision between urban and rural hospitals are largely explained by hospital characteristics, local resources, and population demographics rather than location alone.

Abstract citation ID: igaf122.3340

### GOAL OF CARE CONVERSATIONS IN MOTION: USING SYSTEM DYNAMICS TO DEMONSTRATE A COMMUNICATION PRIMING INTERVENTION

Christi Lero, *Washington University in St. Louis, St. Louis, Missouri, United States*

Health systems are challenged with how to best align treatment with patient values, particularly in the face of older adult patients and those with serious illness. As a result, tools have been developed to endeavor facilitation of goals-of-care conversations (GOCC), which is often seen as a clinical endpoint and research outcome during testing of such tools. However, rehospitalizations are a dynamic and somewhat usual feature of serious illness which remains uncaptured by current methodological approaches for GOCC development and testing. System dynamics is a method used to model systems and visualize interactions among its components via feedback loops, delays, and non-linear relationships. Using a recently published randomized clinical trial featuring a GOCC tool, system dynamics methods are applied using a causal loop diagram, demonstrating interactions of the tool within larger healthcare system and highlighting patterns that surface from its interactions. Three major insights emerged from this reconceptualization. First, there are multiple points at which a GOCC tool may interact with components of the healthcare system. Second, feedback loops reveal that GOCC are less likely to occur if they have already been completed and documented in the patient record. Third, GOCC are not 'one and done.' Rather, they need to be revisited at each change in condition or hospital admission. System dynamics offer insight into understanding complex interactions enabling us to predict how GOCC tools might behave within certain systems.

Abstract citation ID: igaf122.3341

### HEALTHCARE UTILIZATION AMONG HISPANIC MEDICARE ENROLLEES WITH COMPLEX CHRONIC CONDITIONS: PUERTO RICO AND FLORIDA

Matthew Campos[1], and Maricruz Rivera-Hernandez[2], *1. Brown University School of Public Health, Rhode Island, United States, 2. Brown University School of Public Health, Providence, Rhode Island, United States*

Despite higher rates of chronic conditions such as dementia and diabetes, Medicare spending per beneficiary in Puerto Rico (PR) remains significantly lower than on the U.S. mainland (Campos & Rivera-Hernandez, 2024). This study compares the prevalence and demographic profile of Medicare fee-for-service (FFS) beneficiaries (65+) with multiple complex chronic conditions (MCCs) in PR to Hispanic FFS beneficiaries in Florida (FL) and examines whether differences in spending may be driven by variations in service use and access. We analyzed data from the 2022 Medicare Beneficiary Summary File (MBSF), including the Cost and Utilization and 30 CCW Chronic Conditions Segments. Eligible beneficiaries were ≥65, Hispanic, non-dual eligible, and continuously enrolled in Medicare Parts A and B. We stratified by MCCs (2+ complex conditions) and assessed service utilization (dichotomous outcomes (zero vs. any use)) across 17 Medicare spending categories. Demographics were compared using Chi-square and ANOVA tests, and logistic regression models estimated service use differences, adjusting for key covariates. Among FFS beneficiaries, 19.15% (n = 4965/25928) of PR beneficiaries had MCCs, compared to 18.26% (n = 12036/65923) in FL. PR beneficiaries were older and more likely to qualify for Medicare through disability insurance benefits (DIB). Chronic kidney disease was the most common complex condition. PR beneficiaries were significantly less likely than FL beneficiaries to have a claim in 16 of 17 spending categories for those with MCCs and in 14 of 17 categories for those without MCCs (both $p < .0001$). These findings highlight potential access and quality-of-care issues in PR, with implications for Medicare Advantage benchmarks.

Abstract citation ID: igaf122.3342

### IDENTIFYING KNOWLEDGE BARRIERS FOR SMART HOME BLOOD PRESSURE MONITORING DEVICE USAGE AMONG MINORITY OLDER ADULTS

Yanjun Dong[1], Jany Sun[2], Jimmie Boliboun[2], Katherine Koo[3], Valeria Vazquez-Trejo[2], Michael Cui[2], Victoria RIzzo[4], and Jeannine Rowe[5], *1. SUNY at Albany, Albany, New York, United States, 2. Rush Medical College, Chicago, Illinois, United States, 3. Rush University Medical Center, Chicago, Illinois, United States, 4. University at Albany, SUNY, Albany, New York, United States, 5. University of Wisconsin-Whitewater, Whitewater, Wisconsin, United States*

Background Hypertension remains a critical health concern among minority older adults, with disparities in management and outcomes. Smart Home Blood Pressure Monitoring Devices (SHDs) offer a promising tool for improving hypertension management, yet adoption remains limited. This study examines knowledge barriers affecting SHD use and their impact on health outcomes and healthcare engagement. Method This qualitative study employed semi-structured interviews with 20 minority older adults (aged 60+), primarily African American and Latinx, diagnosed with hypertension. Participants were recruited from a health equity study at an academic medical center. Data were analyzed using thematic analysis through ATLAS.ti, guided by Andersen's Expanded Behavioral Model of Health Services Use. Results Knowledge was identified as a key predisposing factor influencing SHD adoption. 60% of participants reported no prior awareness of SHDs before being prescribed the device, with healthcare providers serving as the primary information source. Knowledge gaps, including device functionality and data security uncertainty, contributed to inconsistent use. Among those unfamiliar with SHDs, 42% reported negative experiences, and 33% reported psychological barriers. Despite initial challenges, 90% reported regular use after receiving guidance, with self-monitoring and blood pressure management improvements. Discussion Healthcare providers can bridge knowledge gaps through proactive and continuous patient education. Culturally tailored educational interventions and digital literacy programs can improve self-efficacy and support sustained device usage. Incorporating SHD education into chronic disease management programs and leveraging community networks may enhance adoption. Future research should explore the long-term effects of these interventions on SHD usage and health outcomes across diverse older adults.

Abstract citation ID: igaf122.3343

### LVN-LED TRANSITIONAL CARE COORDINATOR PROGRAM FOR HIP FRACTURES REDUCES MORTALITY

Peter Aldo Giammanco[1], Veranika Mejbaryan[2], Kathleen Breda[3], Michelle Keller[4], and Carol Lin[3], *1. California University of Science and Medicine, Colton, California, United States, 2. Cedars-Sinai, Los Angeles, California, United States, 3. Cedars-Sinai Medical Center, Los Angeles, California, United States, 4. University of Southern California, Los Angeles, California, United States*

Hip fractures pose increased risk of morbidity and mortality in geriatric patients due to complex postoperative. This study analyzed the effects an LVN-led transitional care program had on outcomes of geriatric hip fracture patients in a large urban health system. A cross-sectional study with a non-randomized control group was performed from July 2018 to April 2024. An LVN was trained to be a transitional care coordinator (TCC) for the academic orthopaedic faculty, provided with postoperative protocols for pain, mobilization, DVT prophylaxis, and wound care following hip fracture surgery, and performed phone calls to patients at 7-, 14-, 30-, and 90-days post-discharge. The TCC either answered patient questions directly or escalated questions to the orthopaedist or PCP. The study and control groups included patients contacted by the TCC (TCC) and not contacted by the TCC (control). Bivariate analyses were conducted regarding 90-day ED visits, 90-day readmissions, 90-day mortality, and 1-year mortality. A total of 1569 patients underwent hip fracture care during the study period, 620 (39.5%) of which included TCC. The control and TCC group were similar in age (83.13±8.73 vs. 82.58±8.73 years, $p = 0.182$) and sex (67.0% vs. 71.1% female, $p = 0.086$). No differences were seen in 90-day ED visits between control and TCC (6.9% vs 8.7%, $p = 0.175$) or 90-day readmissions (23.7% vs.19.7%, $p = 0.06$). TCC had lower 90-day (7.8% vs. 3.5%, $p = 0.001$) and 1-year mortality rates (11.6% vs. 7.1%, $p = 0.003$). These findings highlight the benefits and importance of a TCC program as it reduced morality and potentially addressed gaps of post-discharge care.

Abstract citation ID: igaf122.3344

### MEDICARE ADVANTAGE RAPID DISENROLLMENT TRENDS

Em Balkan[1], Jay Shroff[2], and David Meyers[1], *1. Brown University, Providence, Rhode Island, United States, 2. Brown University School of Public Health, Providence, Rhode Island, United States*

In 2024, 54% of Medicare beneficiaries chose to receive their benefits from private plans, known as Medicare Advantage (MA). Medicare beneficiaries can switch their coverage during Medicare's annual Fall Open Enrollment, with new coverage effective January 1 of the following year. All MA enrollees can choose to change their coverage again during the Medicare Advantage Open Enrollment Period from January 1 through March 31. Rapid disenrollment is when a beneficiary who enrolled in a new MA plan during the fall decides to disenroll immediately during the subsequent spring. Rapid disenrollment has been severely understudied. In our analysis of national Medicare data from 2017 to 2022, we found the rate of rapid disenrollment more than tripled, from 4 to 12.2%. The rate of rapid disenrollment was highest for Medicare-Medicaid dual enrollees, with 20% of such beneficiaries rapidly disenrolling by 2022. Additionally, higher proportions of Black, Hispanic, and Native enrollees rapidly disenrolled from their MA plans compared to white enrollees. Plans serving many Medicare-Medicaid dual enrollees tended to have higher rates of rapid disenrollment. It is unclear why rapid disenrollment has increased so dramatically, indicating that beneficiaries quickly decide that their new MA plan is not the best option for them. It is also unclear why rapid disenrollment rates are higher for communities of color and those with lower incomes. For these reasons, and as MA becomes more popular, it is critical to better understand rapid disenrollment in order to improve health care access and quality for Medicare beneficiaries.

Abstract citation ID: igaf122.3345

### ROLES AND PRIORITIES OF PAYERS, HOSPITALS, AND HOME HEALTH AGENCIES IN POST-ACUTE HOME HEALTH CARE

Emily Gadbois[1], Marguerite Daus[2], Jennifer Bunker[3], Jamie Smith[4], Christine Jones[5], and Kali Thomas[3], *1. Brown University School of Public Health, Providence, Rhode Island, United States, 2. University of Colorado Anschutz Medical Campus, Aurora, Colorado, United States, 3. Johns Hopkins University, Baltimore, Maryland, United States, 4. Widener University, Philadelphia, Pennsylvania, United States, 5. University of Colorado School of Medicine, Aurora, Colorado, United States*

Home health care (HHC) provides skilled nursing, therapies, and aide services to help older adults - many of whom are recently discharged from the hospital - regain function, restore health, and maintain independence in the community. The provision of post-acute HHC is influenced by multiple players: Medicare Advantage (MA) plans, post-acute care management companies (CMCs), hospitals, home health agencies (HHAs), and patients and families. To understand the roles and priorities of these players in organizing and delivering post-acute HHC, we conducted in-depth, semi-structured interviews with 44 leaders of MA plans, post-acute CMCs, and HHAs across the country. Analyzed interviews revealed that most MA plans and CMCs agreed that home is the ideal setting and that institutional care should be avoided whenever possible; however, MA plans and CMCs varied in their involvement in post-acute home health referrals, and HHAs tended to view MA and CMC involvement as adding burden to the referral process (Theme 1). MA plan, CMC, and HHA leaders viewed the role of the hospital as to select the appropriate post-acute care setting; however, interview participants reported that these actions can be influenced by relationships with MA plans and HHAs (Theme 2). HHAs were reported to try to balance clinical complexity, staffing, patient location, and payer when accepting referrals, but had relatively limited control in the post-acute referral process (Theme 3). Insights from this work can help policymakers, payers, HHAs, hospitals, and patients understand the varied roles and shared incentives in coordinating post-acute HHC for vulnerable older adults.

Abstract citation ID: igaf122.3346

### THE EFFECTIVENESS OF DIGITAL INTERVENTIONS TO INCREASE PREVENTIVE CARE UPTAKE IN OLDER ADULTS: A SYSTEMATIC REVIEW

Lindsay Burton[1], Kathy Rush[2], Mindy A Smith[1], and Robert Janke[1], *1. The University of British Columbia, Vancouver, British Columbia, Canada, 2. The University of British Columbia - Okanagan Campus, Kelowna, British Columbia, Canada*

The coming decade will witness a substantial increase in older adult populations, contributing to a surge in healthcare utilization and costs driven by rising chronic diseases. Addressing these challenges necessitates strategies to promote healthy aging, reduce chronic diseases, and enhance quality of life among older adults. One proactive approach is to encourage older individuals to follow recommended preventive care and immunization schedules. Despite the increasing number of recommended immunizations for older adults, completion rates remain alarmingly low, particularly for pneumococcal and shingles vaccines. What is unknown is how the use of digital interventions can be appropriately leveraged to address this gap in older adult health services utilization. Following PRISMA guidelines, this systematic review aimed to assess the range and effectiveness of digital interventions to improve preventive uptake among older adults. Twenty-seven studies (23 randomized clinical trials and 4 quasi-experimental) were included. The preventive care services targeted in interventions included colorectal cancer screening (n = 14), breast cancer screening (n = 2), influenza vaccination (n = 5), combined vaccinations (n = 2), pneumococcal vaccination (n = 2), herpes zoster vaccination (n = 1), and COVID-19 vaccination (n = 1). Interventions primarily leveraged automated digital communication, combined digital and printed outreach, tailored telephone communication, and supplemented in-person promotion. Studies consistently demonstrated an increase in preventive care uptake post intervention. However, including digital elements did not consistently show a significant improvement over control groups or standard care. These findings emphasize the importance and effectiveness of a combination of approaches in enhancing preventive care uptake among older adults, thus contributing to improved public health outcomes in aging populations.

Abstract citation ID: igaf122.3347

### THE NEED TO ADDRESS ORAL HEALTH CONCERNS FOR END OF LIFE PATIENTS IN LONG TERM CARE

Kadambari Rawal, *Boston University Henry M. Goldman School of Dental Medicine, Boston, Massachusetts, United States*

To understand dental service utilization by frail older adults in a long-term care (LTC) setting in the last 2 years of their life and the characteristics that may be predictors of ‘higher dental service utilization’, a retrospective cohort study was conducted by chart review of LTC patients at 2 sites who had a dental visit in the 2 year period prior to death. Based on the number of dental appointments attended prior to death, the patients were categorized into five groups. A multivariate logistic regression model was created to identify the factors associated with higher dental service utilization. The study found that 84% of patients who died in the study period, utilized on-site dental services in the last 2 years of their life. Approximately 66% had 3 or more dental appointments. Multivariate analysis suggested that Medicaid beneficiaries were more likely to have a higher utilization of dental services than the others. Diagnostic (including emergency & palliative) and preventive procedures were most often performed and denture related procedures (including fabricating new dentures) were commonly performed too. This study showed that a large percentage of frail older adults utilized dental services even in the last years of their lives when given access to these services. As people are living longer and retaining their teeth longer, there is a rising need and subsequent demand for end-of-life dental services. Certain administrative and policy implementation strategies need to be developed to provide dental services to LTC patients in the last years of their lives.

Abstract citation ID: igaf122.3348

### TRENDS IN HEALTHCARE UTILIZATION AMONG BIPOC AND NON-BIPOC OLDER ADULTS: 1996–2016

Yanjun Dong[1], Kun Wang[2], and Victoria RIzzo[3], *1. SUNY at Albany, Albany, New York, United States, 2. University of Alabama at Birmingham, Birmingham, Alabama, United States, 3. University at Albany, SUNY, Albany, New York, United States*

Background Racial and ethnic disparities in healthcare utilization persist among older adults. Limited access to preventive, treatment, and long-term care services among BIPOC (Black, Indigenous, and People of Color ) older adults has significant implications for health outcomes and aging equity. This study examines how these disparities evolved from 1996 to 2016 and explores the interactions of race, income, and education with time in shaping healthcare access. Methods Data from the 1996–2016 Health and Retirement Study (HRS) (N = 42,233) were analyzed using mixed-effects logistic regression models. Healthcare utilization was categorized into preventive care, treatment care, and long-term care. Covariates included demographics, socioeconomic status, health conditions, and insurance coverage. Results Non-Hispanic Black and Hispanic older adults were less likely than non-Hispanic White older adults to use preventive (OR = 0.52 and OR = 0.59, $p < 0.001$) and treatment care (OR = 0.68 and OR = 0.69, $p < 0.001$). Disparities in preventive care utilization widened post-2008. Long-term care utilization increased for non-Hispanic Black older adults (OR = 1.03, $p < 0.01$), widening disparities, while Hispanic older adults’ long-term care use declined over time. Discussion Findings reveal persistent and widening disparities in preventive and treatment care, emphasizing systemic barriers faced by BIPOC older adults. The increasing reliance on long-term care among non-Hispanic Black older adults may reflect limited access to community-based alternatives. Targeted policies are needed to address inequities, improve healthcare access, and ensure equitable aging experiences.

Abstract citation ID: igaf122.3349

### TRENDS IN OLDER ADULTS BOARDING IN US EDS

Natalia Sifnugel[1], Daniel Cruz[2], Molly Jeffery[3], Rohit Sangal[4], Brendan Carr[5], Scott Dresden[6], Cameron Gettel[7], and Ula Hwang[8], *1. NYU Langone Medical Center, New York, New York, United States, 2. Northwestern Memorial Hospital, Chicago, Illinois, United States, 3. Mayo Clinic, Rochester, Minnesota, United States, 4. Yale University, New Haven,*

*Connecticut, United States, 5. Mayo Clinic Rochester, Rochester, Minnesota, United States, 6. Northwestern University, Evanston, Illinois, United States, 7. Yale School of Medicine, New Haven, Connecticut, United States, 8. NYU Grossman School of Medicine, New York, New York, United States*

Inpatient boarding (holding patients in the emergency department [ED] while awaiting an inpatient bed) is associated with increased risk of delirium, medication safety events, and hospital mortality in older adults. We investigated boarding trends across age strata to determine if older patients had elevated rates compared to younger patients. Administrative data for ED encounters resulting in admission from 3 healthcare systems (2 Midwest, 1 Northeast) from 1/1/19 to 12/31/24 were reviewed. Age was categorized by strata: < 21, 21-64, 65-74, 75-84, and ≥ 85. Based on the CMS Age Friendly Hospital Measures, the proportion of patients boarding for 3+ hours was calculated within each year and age strata. Boarding rates varied substantially by system and age group. At Systems 1 (S1) and 3 (S3), ≥ 85 patients had the highest rates (S1: 54.9%, S3: 70.9% in 2024) and, at System 2 (S2), all adult patients (21+) had similar rates (20.5-22.4% in 2024). Boarding rates increased in all systems over the study period. At S1 and S3, < 21 patients had the highest average yearly increase in boarding (S1: 7.4%, S3: 3.9%). At S2, boarding increased most in ≥ 85 (23.9%). In this multisystem evaluation, approximately one in two older adults experience ED boarding, with the oldest patients (≥ 85) facing the greatest exposure to boarding in recent years. These findings taken alongside known delays and safety risks associated with boarding and patient care complexity amongst the oldest adults may result in disproportionate harm to some of the most vulnerable patients.

### SESSION 7490 (POSTER)

### HUMANITIES AND THE ARTS

Abstract citation ID: igaf122.3350

### CONSIDERATIONS FOR DESIGNING DISABILITY-INCLUSIVE MUSIC PROGRAMS FOR OLDER ADULTS

Jennie Dorris, Lauren Terhorst, and Juleen Rodakowski, *University of Pittsburgh, Pittsburgh, Pennsylvania, United States*

Music is a popular modality that has shown promise to support healthy adults and those living with disabilities. As adults age, they are more likely to report a disability and experience health disparities. Purpose: The objective of our exploratory study was to create an understanding of disability and music participation for adults over 65 years old. Materials and methods: We conducted a secondary data analysis of survey data from the 2022 Current Population Survey, administered to homes in the community in the United States. We included adults over 65 years of age. Disability was measured by a self-reported yes/no response. Music participation was measured by a self-reported yes/no response to either singing or playing a musical instrument in the last 12 months. Results: In a simple logistic regression and a model controlling for demographic characteristics, having a disability was not statistically significantly associated with music participation, $\beta = 0.0351$, $p = 0.735$, (OR = 1.073, 95% CI [0.714, 1.611]). Conclusions: These findings lay the groundwork for future Stage 1 research to design music interventions with universal inclusion of those with and without disabilities to maximize music's potential impacts on older adults.

Abstract citation ID: igaf122.3351

### LITTLE OLD LADY, ME? MODERN CINEMATIC REPRESENTATIONS OF OLDER WOMEN AND CHALLENGING THE NARRATIVE OF DECLINE

Neasa Fitzpatrick, *Trinity College Dublin, Waterville, Kerry, Ireland*

Introduction Ageing discourse is dominated by a 'narrative of decline' that leaks into popular culture. Women are disproportionately affected by this and older women have been under-represented in cinema. However their visibility has increased in the past two decades. We explore the representations of older women in modern cinema and their relationship to the narrative of decline and other ageing stereotypes. Methods Films of the past two decades with female leads over the age of 65 were reviewed. Focus was directed on popular and/or acclaimed films in mainstream and independent cinema. Characterisations of older women were analysed for common themes and patterns. Typical characterisations were identified and analysed in the context of the 'narrative of decline'. Results Two stereotypical portrayals of older women were identified and subsequently described: 1. 'Romantic rejuvenation' where the older woman reclaims youthful attributes through romantic affairs, and 2. 'The passive problem' in which the older woman has a degenerative disability a that poses challenges and burdens to her spouse. Both representations were found to reinforce the narrative of decline. A third representation challenged this narrative: 'The "Old Woman" in her own words' – authentic, engaging depictions of older women from older female filmmakers. Conclusion Rhetoric around ageing women remains entrenched in a narrative of decline, framed in modern cinema as something to avoid or lament. The agency of older women is underestimated, which can have implications for health and social care. When voices are given to older women, we can appreciate their rich inner lives.

Abstract citation ID: igaf122.3352

### NARRATIVE RESEARCH IN AN AGING SOCIETY: A MULTIDISCIPLINARY ANALYSIS FOR OLDER PEOPLE IN TAIWANESE FILM YI YI

Sheng-Chin Hsu, *Ming Chuan University, Taipei, Taipei, Taiwan (Republic of China)*

This study adopts narrative methods to investigate the impact of the grandmother character, who requires long-term care due to an accident in the Taiwanese film Yi Yi, on the overall narrative, other characters, and the development of the narrative. This research analyzes the narrative composition of the film's text and then validates and interprets it theoretically from the perspectives of narrative research and gerontology. Additionally, this paper incorporates relevant discussions of Yang De-chang's creative context, attempting to understand through an interdisciplinary approach how the long-term care older people in Taiwanese films impacts other family members and how family risks are formed in the narrative development. The purpose of this study is to illustrate that the older people who needs long-term care due to an accident, although silent, influences the film's narrative structure and the new decisions among family members. Through interdisciplinary knowledge analysis of narrative theory and gerontology, this study finds that the family in the story is the current majority nuclear family in Taiwan. In a state of fewer people, the family support network is weakened, highlighting the challenges of long-term care for nuclear families in modern society. The knowledge spectrum of gerontology expands the path of narrative analysis for understanding the aging society, which reminds contemporary researchers that understanding the aging society must develop interdisciplinary methods and comprehensive explanations to provide culturally diverse solutions to the problems of the aging society and achieve harmony between generations.

Abstract citation ID: igaf122.3353

**PILOT STUDY OF A HYBRID MUSIC THERAPY SUPPORT GROUP FOR FAMILY CAREGIVERS OF OLDER ADULTS WITH DEMENTIA**

Jennifer Stull, and Sarah Saint Hamilton, *University of Georgia, Athens, Georgia, United States*

Music can effectively lower perceived levels of stress, anxiety, and depression, which are symptoms commonly experienced by family caregivers. Virtual program delivery may increase access to music-based support for caregivers in underserved areas. The purpose of this study was to examine the feasibility of a hybrid music therapy support group for family caregivers of persons with dementia, as well as evaluate the impact of the support group on perceived stress, anxiety, and depression in family caregivers. Participants (n = 9) attended five hybrid music therapy support group sessions. Participants met in-person in a designated community location while a board-certified music therapist facilitated the session via Zoom. Each session included movement to music, guided breathing experiences, music listening, song discussion, songwriting, and verbal discussion of the session topic. Participants rated their perceived levels of stress, anxiety, and depression on a scale from 0 to 10 at the start and end of each session. Results showed a decrease in mean pre/post-test scores of stress and depression in all five sessions. Ratings of perceived anxiety decreased in all but one session in which the ratings remained the same at the start and end of the session. Additionally, pre-test scores of anxiety decreased 56% from session 1 to session 5, and pre-test scores of stress decreased 52% from session 1 to session 5, showing a decrease in baseline stress and depression levels across the study period. Regarding feasibility, program evaluation revealed minimal issues with hybrid delivery and good audiovisual quality utilizing existing facility infrastructure.

Abstract citation ID: igaf122.3354

**THE COST OF LONELINESS: AGEISM AND THE POLITICS OF ASSISTED DYING IN CHIE HAYAKAWA'S PLAN 75**

Ulla Kriebernegg, *University of Graz, Graz, Steiermark, Austria*

This paper examines ageism and medically assisted dying as depicted in the Japanese dystopian film Plan 75 (2022), which envisions large-scale, state-organized gerontocide as a solution to the so-called "problem" of old age. In the film, healthy individuals aged 75 and above who voluntarily enroll in the program receive a financial incentive, enabling them to partake in final experiences such as a domestic journey or a luxurious meal before they are put to death in a clinic. The narrative disturbingly normalizes ageism, presenting voluntary death as a practical response to loneliness, poverty, and depression—while simultaneously alleviating Japan's economic crisis. Using a cultural gerontological framework, this study employs film analysis to interrogate how Plan 75 constructs narratives about aging, autonomy, and societal worth. The paper examines the depiction of older adults as isolated, burdensome figures whose existence must be negotiated through institutionalized solutions rather than through meaningful social interventions and compassionate care. The study also considers how Plan 75 reflects broader social inequalities, particularly in relation to class, gender, and government control over aging populations. Finally, this paper highlights the role of cinema in shaping public perceptions of aging, influencing policy debates on end-of-life care, and challenging ageist narratives in both medical and social contexts.

### SESSION 7495 (POSTER)

### MOBILITY/DISABILITY

Abstract citation ID: igaf122.3355

**AGE-RELATED INCREASES IN ADVERSE SEATING AND MOBILITY EVENTS AMONG NEW MOBILITY DEVICE USERS**

Kevin Pritchard[1], Corey Morrow[2], Mariana Pacheco Busquets[3], Gede Pramana[3], Richard Schein[3], and Mark Schmeler[3], *1. Brigham and Women's Hospital, Boston, Massachusetts, United States, 2. Medical University of South Carolina, Charleston, South Carolina, United States, 3. University of Pittsburgh, Pittsburgh, Pennsylvania, United States*

We described age-related increases in hospitalizations, pressure ulcers, or becoming stranded from a mechanical issue after receiving a new mobility device. This retrospective cohort study included 5,983 individuals who received new mobility devices from 87 U.S. clinics (48 states) using the Functional Mobility Assessment Registry from 2015-2024. Age was self-reported and dichotomized at ≥ 65 years old upon device delivery. Outcomes included self-reported hospitalization for a seating-related issue, pressure ulcer, or becoming stranded due to device malfunction. We estimated age-stratified rates of these outcomes using the Kaplan-Meier method. We used proportional hazards regression to assess the association between age and the number of days until each outcome, after controlling for 16 confounding factors like sociodemographic status, prior device use and experience, health status, physical function, and history of each outcome. Our sample was on average age 60 [SD = 18] years old, 47% were ≥65 years old, 54% women, and 26% were racial or ethnic minorities. The 365-day event rates for seating-related hospitalization, pressure ulcer, and stranding were 53.32% (95% CI: 51.59%-55.07%), 57.01% (55.28%-58.74%), and 56.34% (54.61%-58.08%) in the younger group and 55.33% (53.48%-57.19%), 58.38% (56.54%-60.23%), and 58.09% (56.25%-59.94%) in the older group. After multivariable adjustment, adverse events increased with age. Compared to adults under 65 years old, those 65 and older were more likely to have a seating-related hospitalization (HR: 1.12; 95% CI: 1.05-1.19), pressure ulcer (HR: 1.09; 95% CI: 1.03-1.16), or stranding (HR: 1.10; 95% CI: 1.04-1.17). Older age increased the likelihood of adverse events after receiving new mobility devices.

Abstract citation ID: igaf122.3356

**ALL-CAUSE AND CARDIOVASCULAR MORTALITY IN OLDER ADULTS WITH MULTIMORBIDITY : 1999-2018 NATIONAL SURVEY**

Chitchanok Benjasirisan[1], Arum Lim[2], Asma Rayani[1], Soo Hyun Kim[1], Cheryl Himmelfarb[1], Patricia Davidson[3], and Binu Koirala[1], *1. Johns Hopkins University, Baltimore, Maryland, United States, 2. Johns Hopkins University School of Nursing, Baltimore, Maryland, United States, 3. University of New South Wales Sydney, Kensington, New South Wales, Australia*

Multimorbidity, the presence of two or more chronic conditions, is associated with premature mortality and decreasing life expectancy. Cardiovascular disease (CVD) remains a leading cause of death in the U.S. and is often final pathway to death among people living with multimorbidity. This study examined the association between multimorbidity and mortality, specifically CVD and all-cause mortality, among U.S. adults aged 50 years of age and older. Data from the 1999-2018 National Health Interview Survey linked with the National Death Index were analyzed. Primary outcomes were all-cause and CVD mortality (due to heart disease and cerebrovascular disease) years, measured from the interview to death or December 31, 2019. Cox regression models assessed mortality risk differences, adjusting for age, sex, marital status, poverty-income ratio, and education. Among 264,400 adults (39.02% with 0-1 chronic conditions; 60.98% with ≥2 conditions), the median follow-up was 7 years (IQR: 4-12). All-cause mortality was 30.65 per 1,000 person-years and CVD mortality was 9.91 per 1,000 person-years. Multimorbidity was linked to higher mortality rate (all-cause: 38.66 vs. 19.75 and CVD: 12.79 vs. 5.70, per 1,000 person-years). Fully adjusted models showed a 43% higher all-cause mortality risk (95% CI: 1.40-1.46) and 59% higher CVD mortality risk (95% CI: 1.52-1.65) for those with multimorbidity. Additionally, each additional comorbidity was associated with an increased risk in all-cause (aHR:1.06; 95% CI: 1.04–1.09) and CVD mortality (aHR: 1.14; 95% CI: 1.10–1.18). Integrated models of care considering multidisciplinary management is crucial to improve health outcomes and mitigate mortality risk among older adults with multimorbidity.

Abstract citation ID: igaf122.3357

### ASSOCIATIONS OF GAIT CHARACTERISTICS WITH FALL RISK AND FRAILTY IN OLDER WOMEN

Chitra Banarjee, Patria Marcano Maldonado, Md Sanzid Bin Hossain, Hwan Choi, Rui Xie, and Ladda Thiamwong, *University of Central Florida, Orlando, Florida, United States*

Falls are the leading cause of injury-related death for older adults, aged 60 years and older. Because falls often occur during movement, it is crucial to understand dynamic balance. This preliminary work expands the standard Timed Up and Go (TUG) test of dynamic balance by integrating plantar insole sensors to administer the instrumented TUG (iTUG). We aim to identify the relationship between objective data from the iTUG with subjective measures. Gait characteristics consisted of stride velocity, center of pressure (COP) velocity, and single support phase (SSP) duration, and subjective measures were fall risk and frailty, evaluated by questionnaires. The fall risk was assessed using the Center for Disease Control and Injury Prevention (CDC) Stopping Elderly Accidents, Deaths and Injuries (STEADI) screening instrument, and frailty was assessed using Simple Frailty Questionnaire (FRAIL). The study included 13 older women (Mage=74.66±1.48 years) who first completed the standard TUG followed by three iTUG trials. Spearman's correlations were used to evaluate associations between gait characteristics and subjective measures. A paired t-test was used to confirm the validity of the iTUG by comparing the average duration of the iTUG trials with the experimenter-recorded duration ($p < 0.001$). Greater fall risk was correlated with decreased stride velocity ($p = 0.011$), decreased SSP ($p = 0.011$), and increased COP velocity ($p = 0.002$). Increased frailty was correlated with decreased stride velocity ($p = 0.005$), but did not show correlations with SSP and COP velocity. These preliminary results specify dynamic balance deficits in older women showing that greater perceived fall risk and frailty coincides with cautious and unstable gait.

Abstract citation ID: igaf122.3358

### CHILDHOOD HEALTH CONDITION, MIDLIFE LIFESTYLE, AND LATER-LIFE MOBILITY FUNCTION TRAJECTORY

Liu Huanran, Peiyi Lu, and Vivian Lou, *The University of Hong Kong, Hong Kong, China (People's Republic)*

Identifying individuals at risk of mobility decline early can enable timely interventions. Most studies focus on older age and lower limb mobility, overlooking overall mobility changes from middle age. Furthermore, the differential impact of early-life conditions on mobility trajectories across subgroups remains underexplored. Guided by cumulative advantage/disadvantage theory, this study examines how health-related early-life conditions is associated with mobility trajectories in Chinese middle-aged and older adults from a life course perspective. Data from the 2011- 2018 China Health and Retirement Longitudinal Study and the 2014 Life History Survey included 9,576 participants aged 45+. Mobility was assessed via a 7-item self-reported scale on any difficulty with mobility activities. Latent class trajectory modeling identified four trajectories: "High Mobility and Remain Stable" (50.31%), "Moderate Mobility and Deteriorate Swiftly" (24.96%), "Moderate Mobility and Fluctuate" (15.26%), and "Low Mobility and Decline Rapidly" (9.47%). Multinomial logistics regression model suggested individuals with poorer childhood health and a history of smoking in midlife were more likely to report declining or fluctuating trajectories. These findings highlight health-related early life conditions that can inform targeted interventions to promote mobility in older age, particularly those in disadvantaged childhood health condition and a history of midlife smoking behavior. Our research advances the cumulative advantage/disadvantage theory by illustrating how early-life conditions and midlife lifestyle factors collectively shape late-life mobility function trajectories from a comprehensive life course perspective.

Abstract citation ID: igaf122.3359

### FACTORS INFLUENCING LOSS OF INDEPENDENCE AMONG OLDER ADULTS : RACE/ETHNICITY AS A MODERATOR IN NHATS

Young-Shin Lee, Leticia Camacho, and Hee-Jin Jun, *San Diego State University, San Diego, California, United States*

Disability in later life is a significant contributor to loss of independence (LoI). Despite its complexity, late-life disability remains underexplored. This study investigates factors influencing LoI across ethnic/racial groups, using the International Classification of Functioning, Disability, and Health model. Using 2022 National Health and Aging Trends Study data, LoI in older adults was assessed using the Vulnerable Elders-13 Survey (VES: 0–10), where higher scores denote increased vulnerability. Risk factors included body function, physical performance, and social participation; and gender, ethnicity, health issues, and environmental factors as covariates. Multiple regression analyses examined association with LoI and interaction effect among body function, performance, and social participation. Among 7,106 older adults, 42% were at high risk of LoI. Physical performance and limited social participation significantly interacted with race/

ethnicity. Stratified analyses showed that physical performance was most strongly associated with LoI among White group (β = -0.42), followed by Black (β = -0.34) and Hispanic adults (β = -0.33). Among Black group, health conditions (β = 0.54) had the strongest impact on LoI, compared to White (β = 0.30) or Hispanic (β = 0.34) as well as restricted social participation (β = 0.804) on LoI, followed by Hispanic (β = 0.49) and White (β = 0.46) group. Limited English proficiency was a significant risk factor of LoI only for Hispanics (β = 0.57). Findings highlight that while physical function and performance are consistent predictors of LoI, the influence of health conditions, social participation, and language barriers differs across racial/ethnic groups.

Abstract citation ID: igaf122.3360

### GAIT VARIABILITY AND COGNITIVE FUNCTION IN OLDER ADULTS WITH UNSTEADY GAIT

Juhi Digvijay Salecha[1], Pei-An Lee[1], Brad Manor[2], and On-Yee Lo[3], *1. Hebrew SeniorLife & Harvard Medical School, Boston, Massachusetts, United States, 2. Hebrew SeniorLife/ Harvard Medical School, Boston, Massachusetts, United States, 3. Harvard Medical School/ Marcus, Hebrew SeniorLife, Boston, Massachusetts, United States*

Older adults with unsteady gait are at high risk of falls and cognitive decline. However, the cognitive mechanisms underlying unsteady gait patterns remain poorly understood. While previous research has linked gait and cognition in older adults, few studies specifically examined such relationships in individuals with pre-existing unsteady gait. This study examined the relationship between gait variability, defined by the coefficient of variation in stride time, and cognition in older adults with unsteady gait. Twenty-eight community-dwelling older adults with unsteady gait, defined by gait variability ≥0.025 (79±7 years; 19 females; BMI: 29±5, Fall Efficacy Scale: 27±8) completed walking under two conditions: walking quietly and while completing a verbalized serial subtraction dual task. Participants also completed a cognitive assessment using the NIH Toolbox, measuring processing speed (Pattern Comparison Processing Speed), executive function (Flanker Inhibitory Control and Attention), episodic memory (Picture Sequence Memory), and working memory (List Sorting Working Memory). Multiple linear regression models, adjusted for age, sex, and BMI revealed that processing speed was correlated with gait variability specifically within the dual-task condition (Adjusted $R2=0.41$, $\beta=-0.50$, $p = 0.006$). No other cognitive outcomes were correlated with gait variability in either walking conditions. These results suggest that in older adults with unsteady gait, processing speed may play a critical role in locomotor control, especially when walking is challenged by a cognitive dual task. Future studies are needed to validate these observations and to examine the extent to which processing speed and dual-task performance can be modified in this population.

Abstract citation ID: igaf122.3361

### IMPACT OF A SYSTEMS-BASED INTERVENTION TO OVERCOME BARRIERS TO AMBULATION AND IMPROVE UNIT CULTURE

David Tucker[1], Barbara King[1], and Linsey Steege[2], *1. University of Wisconsin–Madison, Madison, Wisconsin, United States, 2. University of Wisconsin–Madison School of Nursing, Madison, Wisconsin, United States*

Low mobility of older adults during hospitalization results in devastating losses in functional ability by discharge. Nursing staff are identified as the primary healthcare provider responsible for maintaining patient functional status. However, nurses often lack knowledge and self-efficacy for assessing patient mobility and may not see patient ambulation as their role. Mobilizing Older adults Via a systems-based INtervention (MOVIN) is a multi-component intervention designed to target system barriers and change in nurse behavior and ambulation culture on inpatient units. This presentation will report changes in nurse perceptions of barriers to ambulation and changes in unit ambulation culture pre/post MOVIN implementation. A cluster randomized controlled trial design was used to measure changes in nurse behavior and unit ambulation culture. Nursing staff (Registered Nurse (RN) and Certified Nursing Assistants (CNAs) from 2 hospitals and 4 inpatient adult medical units completed Barriers to Patient Ambulation and Ambulation Culture surveys. General Linear Modeling and propensity weights to adjust for covariant differences between pre and post were used as a function to determine group differences (pre/post) by units. Nursing staff completed 194 Pre and 169 Post surveys. RNs had a significant reduction in Barriers to Ambulation (knowledge, attitudes, behaviors) post-intervention. Across nursing staff, positive perceptions of Ambulation Culture increased post-intervention. Implementation of MOVIN was effective in reducing barriers to nurse ambulation and improving ambulation culture on hospital units. Addressing these barriers is critical to achieving sustained changes in nurse-initiated patient ambulation and improving functional outcomes for hospitalized older adults.

Abstract citation ID: igaf122.3362

### NOT ALWAYS A WALK IN THE PARK: EVALUATING TRENDS AND DISPARITIES IN COMMUNITY MOBILITY AMONG OLDER ADULTS, 2015-2022

Jason Falvey[1], Chixiang Chen[1], Michelle Shardell[1], Jay Magaziner[1], and C. Daniel Mullins[2], *1. University of Maryland School of Medicine, Baltimore, Maryland, United States, 2. University of Maryland School of Pharmacy, Baltimore, Maryland, United States*

Maintaining the ability to navigate community distances, or at least three blocks (approximately ¼ mile), is crucial for older adults. Inability to do so is associated with diminished well-being. Our sample included community-dwelling participants aged 70 years and older from the National Health and Aging Trends Study, an ongoing nationally representative annual survey of older Medicare beneficiaries Community mobility was defined as the capacity to walk 3+ blocks without assistance. Repeated cross-sectional trends analysis (SAS-callable SUDAAN) and survey-weighted logistic regression was used to evaluate age, sex, and race and ethnicity–stratified proportions of older adults who could walk at least 3 blocks in their community and changes in this rate over time. Overall, 28% of older adults (or an estimated 7.5 million) could not walk three blocks in 2015; 8.8 million older adults (25%) were unable to walk three blocks in 2022. Consistently, Black and Hispanic participants had significantly lower rates of community mobility than White participants (62% and 63% vs 77% by 2022, respectively; all years $p<.0001$). Female participants also demonstrated persistently lower rates. Hispanic older adults experienced a notable decline in community mobility rates from 2015 (70%) to 2022 (63%), and modest improvements were observed for Black older adults (58% to 62%).

Overall, more than 8 million older adults in the US may have difficulty walking 3 blocks outside, which has only modestly improved over the past 7 years. These findings highlight the need for targeted interventions to address limited and inequitable community mobility in the US.

Abstract citation ID: igaf122.3363

### PHYSICAL FUNCTION IN PEOPLE WITH DISABILITIES AND SOCIAL DETERMINANTS: A GROWTH MIXTURE MODELING STUDY

Seeun Park, and Ivan Molton, *University of Washington, Seattle, Washington, United States*

Preservation of physical functioning is crucial for successful aging among people with disabilities (PWD). While physical function is influenced by social determinants of health (SDOH) in the general population, its relationship with PWD remains unclear. This study aimed to identify trajectories of physical functioning and examine the role of SDOH among PWD. This study utilized two years of longitudinal data from the Rehabilitation Research and Training Center on Healthy Aging and Physical Disability at the University of Washington. Participants were community-dwelling adults with disabilities, including those with spinal cord injury, multiple sclerosis, muscular dystrophy, or post-polio syndrome. Physical function was assessed using the PROMIS Physical Function measure. SDOH factors included health service availability, provider relationships, financial and physical barriers to healthcare, disability benefits, transportation accessibility, community accessibility, employment support, and caregiving availability. Growth Mixture Modeling was used to identify distinct physical function trajectory classes, and multinomial logistic regression examined the associations between SDOH and trajectory membership. A total of 1,573 PWD were included. Four distinct trajectory classes were identified: High Stable, High Decline, Moderate Decline, and Low Function. Those in the Low Function class were more likely to experience disadvantaged SDOH, including lower income, greater physical barriers to healthcare, reduced community accessibility, and lack of disability benefits. Similar trends were observed in the High Decline and Moderate Decline classes. Findings highlight the significant role of SDOH in shaping physical function trajectories among PWD, emphasizing the need to improve healthcare accessibility by addressing financial and physical barriers and fostering social inclusion.

Abstract citation ID: igaf122.3364

### PLASMA BIOMARKERS ASSOCIATED WITH INCIDENT MOBILITY, COGNITIVE, AND DUAL IMPAIRMENT IN OLDER ADULTS: THE ARIC STUDY

Jayanti Shukla[1], Chad Blackshear[2], David Knopman[3], Priya Palta[4], Michael Griswold[2], B. Gwen Windham[2], Thomas Mosley[2], and Kevin Sullivan[2], *1. University of Mississippi School of Medicine, Jackson, Mississippi, United States, 2. University of Mississippi Medical Center - The MIND Center, Jackson, Mississippi, United States, 3. Mayo Clinic, Rochester, Minnesota, United States, 4. UNC School of Medicine, Chapel Hill, North Carolina, United States*

Mobility and cognition impairments are common in older adults, but neurodegenerative pathology contributions are unclear. We examined cross-temporal associations of Alzheimer's Disease (AD)-specific and nonspecific neurodegenerative plasma biomarkers neurofilament light (NfL) with incident mobility and cognitive impairment in the Atherosclerosis Risk in Communities (ARIC) study. We hypothesized NfL would show stronger associations with mobility impairment than p-tau181 in a subset of 1,227 cognitively normal participants with biomarker data and no mobility impairment (mean age=75.5±5.0, 56% female, 23% Black) at visit 5 (2011-13). Participants were classified into five functional groups using data from up to four exams: no impairment (n = 587; referent), mobility impairment only (n = 266; gait speed< 0.8m/s), cognitive impairment only (n = 69; adjudicated dementia), dual impairment (n = 243; both) or decline-free death (n = 262) over a mean 6.3 years (0-11 years) of follow-up. A multinomial regression model estimated association of functional groups with p-tau-181 and NfL jointly, adjusted for cardiovascular and demographic factors, BMI, and kidney function. Relative prevalence ratios (RPR; 95% CI) are presented per one-unit higher log-base-2 transformed biomarker (doubling). For mobility and cognitive functional outcomes, p-tau-181 was more robustly supported [Mobility: RPR=1.36, (1.04,1.77); Cognitive: RPR=1.73, (1.10,2.70); Dual: RPR=1.78, (1.15,2.77)] than NfL [Mobility: RPR=1.14, (0.85,1.53); Cognitive: RPR=1.20, (0.70,2.05); Dual: RPR=1.65, (0.86,3.16)]; higher pTau-181 [RPR=1.60, (1.23,2.09)] and higher NfL [RPR=1.94, (1.39,2.71)] were associated with higher risk of decline-free death. A p-tau-181-x-NfL interaction term was not supported. Findings did not support NfL associations with functional outcomes with consideration of p-tau-181. AD blood biomarkers may help identify individuals at greater risk for multifaceted disability.

Abstract citation ID: igaf122.3365

### PSYCHOLOGICAL RESILIENCE AMONG OLDER ADULTS WITH PHYSICAL DISABILITY: A SCOPING REVIEW

BoRin Kim[1], Sarah Sunghye Kang[2], Meagan Berry[3], Ke Li[4], Soobin Park[5], and Sojung Park[5], *1. University of New Hampshire, University of New Hampshire, New Hampshire, United States, 2. University of California, Los Angeles, Los Angeles, California, United States, 3. University of New Hampshire, Durham, New Hampshire, United States, 4. Ohio University, Athens, Ohio, United States, 5. Washington University in St. Louis, St. Louis, Missouri, United States*

With a growing number of older adults experiencing physical disability, understanding psychological resilience—an essential factor in adapting positively to these challenges—is crucial for promoting well-being in later life. However, limited research has specifically examined resilience among older adults with such disabilities. This scoping review aims to synthesize existing conceptual frameworks, measurement tools, and key contributing factors related to resilience in this population. Following PRISMA-ScR guidelines, a systematic search was conducted in PubMed, CINAHL, Web of Science, and PsycINFO for studies published from 2005 to 2024. Inclusion criteria encompassed older Americans aged ≥65 with physical disability not exclusively related to sensory or cognitive impairments, and an explicit focus on psychological resilience. Twenty-two articles were identified. Conceptual frameworks ranged from viewing resilience as an inherent personality trait to a dynamic process shaped by individual, interpersonal, and macro-level factors. Common measurement instruments included the Connor-Davidson Resilience Scale, Brief Resilience Scale, Ego-Resiliency Scale, and Hardy-Gill Resilience Scale. Factors consistently associated with resilience were optimism, positive affect, self-efficacy, social support, and adaptive coping strategies. Environmental facilitators, such as accessible housing and community resources, also emerged as significant. Overall, the findings underscore resilience as a multifaceted phenomenon, influenced by a dynamic interplay between personal attributes and broader sociocultural contexts. These insights highlight the need for multi-level interventions targeting psychological, social, and structural barriers. Future research should refine measurement tools and investigate how sociocultural contexts shape resilience, ultimately informing strategies to enhance well-being in this vulnerable population.

Abstract citation ID: igaf122.3366

### REDUCED ALPHA POWER ASSOCIATED WITH IMPAIRED DUAL-TASK STANDING PERFORMANCE IN OLDER ADULTS WITH MCI

Helia Osareh[1], Brad Manor[2], and Melike Kahya[1], *1. High Point University, High Point, North Carolina, United States, 2. Hebrew SeniorLife/Harvard Medical School, Boston, Massachusetts, United States*

Older adults, particularly those with mild cognitive impairment (MCI), often struggle with standing balance during dual-tasking. These difficulties arise from limited cognitive processing capacity, with MCI individuals showing greater dual-task cost than healthy older adults. While Electroencephalogram (EEG) studies have found that MCI is associated with reduced alpha power (8-13 Hz), the influence of dual-tasking on brainwave patterns and its relationship to dual-tasking standing performance remain unclear. We hypothesized that older adults with MCI would show reduced alpha power during dual-tasking compared to cognitively-intact controls, and that this reduction would also correlate with worse dual-tasking performance. We recruited 15 cognitive-intact participants (Montreal Cognitive Assessment (MoCA) score = 25-30) and ten participants with MCI (MoCA score = 21-24). Participants completed standing balance assessments while performing verbal subtractions. EEG alpha, beta, gamma, and theta power were recorded throughout the task. APDM Opal sensors were used to measure postural sway metrics synchronously with EEG recording. Older adults with MCI, as compared to cognitively-intact controls, exhibited lower alpha power, particularly in the central right ($p = .003$) and anterior left ($p = .002$) regions of the brain. Beta, theta, and gamma brain waves were not significantly different between groups. Reduced alpha power during dual-tasking was associated with increased postural sway path in older adults with MCI ($r = -0.47$, $p = 0.01$). These results suggest that reduced alpha power is correlated with worse dual-task standing performance. Future research should explore whether reduced alpha power contributes to impaired dual-task standing performance in older adults with MCI.

Abstract citation ID: igaf122.3367

### USE OF AND FACTORS ASSOCIATED WITH THE USE OF MOBILITY ASSISTIVE DEVICES AMONG OLDER ADULTS IN LOW-INCOME HOUSING

Stephanie McCorvey[1], Barbara Resnick[2], Nicole Brandt[3], Jennifer Klinedinst[4], and Sarah Holmes[5], *1. University of Maryland at Baltimore, Baltimore, Maryland, United States, 2. University of Maryland, Baltimore, Maryland, United States, 3. UMB School of Pharmacy, 20 N. Pine St. Baltimore, Maryland, United States, 4. UMB SON, 655 W. Lombard Street, Baltimore, Maryland, United States, 5. University of Maryland School of Nursing, Baltimore, Maryland, United States*

There is a growing number of older adults in low-income housing communities that live with disabilities. Many of these individuals use mobility assistive devices (MADs) including walkers, canes, or wheelchairs, which they obtain from others, purchase or are ordered by a health care provider. This study explored the use of MADs among residents in low-income housing and factors associated with MAD use. It was hypothesized that the majority of individuals would use a MAD; and that perceived health status, cognition, exercise, unsteadiness, fear of falling, a fall in the past year, and fall-related injury, dizziness, and musculoskeletal or neurological disease would be associated with use of an MAD. Data were obtained from older adults ($N = 220$) who participated in an Annual Wellness Visit as part of an interdisciplinary wellness clinic. The mean age of participants was 79.5 ($SD = 8.5$), the majority was female (77%), Black (69%) and non-Hispanic (80%). Over half (53%) used a MAD. Worrying about falling (Wald=5.4, $p = .02$), feeling unsteady (Wald=19.7, $p < .001$), and health status (Wald 4.3, $p = .04$) were all significantly associated with MAD use. Those who worried were 2.9 times more likely to use a MAD, those who felt unsteady were 5.8 times more likely to use an MAD, and those who reported worse health status were 0.44 times less likely to use a MAD. Individuals seem to self-select use of a MAD based on concerns around falls. Future research should focus on the appropriateness of these devices at the individual level for safety and cost effectiveness.

Abstract citation ID: igaf122.3368

### VALIDATION OF A SCALE TO MEASURE BARRIERS TO PROMOTING WALKING IN HOSPITALIZED OLDER ADULTS

Carolanne Bianchi[1], Tobie Olsan[2], and Joyce Smith[2], *1. University of Rochester School of Nursing, Rochester, New York, United States, 2. University of Rochester, Rochester, New York, United States*

This study validated the Barriers to Promoting Walking Scale (BPWS) to measure interprofessional team members' perceptions of barriers to walking hospitalized older adults. Addressing the barriers to closing the knowledge-practice gap between promoting walking and the high prevalence of low mobility during hospitalization is critical to improving the effectiveness of quality improvement interventions and reducing hospital-associated functional decline. To assess the psychometric properties of the BPWS, the instrument was administered to two purposive samples of interprofessional team members from medical, surgical, and combined medical-surgical units in two hospitals resulting in 239/726 completed surveys (33% response rate). Descriptive statistics were used to examine distributional characteristics of the variables. Internal reliability was calculated using Cronbach α. Construct validity was evaluated by measuring the correlation of each domain score with the entire scale score and by known-group validity. Rasch analysis was used to eliminate ambiguous scale items or those with low variability. Scale items—developed from published literature, focus groups, and expert review demonstrated face and content validity (S-CVI/Ave, 0.91). Exploratory factor analysis produced a three-factor solution (Team Members' Confidence in Walking Patients, Practices That Limit Patient Walking, and Patient and Family Engagement) with adequate reliability (Cronbach $\alpha = 0.75$ to 0.91) and construct validity. This study provides strong evidence of the reliability and validity of the BPWS. Engaging the interdisciplinary team and identifying their perceived barriers to walking hospitalized older adults provides opportunities to create shared understandings of older adult care and design novel solutions to promote walking and mitigate hospital acquired functional decline.

### SESSION 7500 (POSTER)

### PAIN MANAGEMENT AND PALLIATIVE CARE

Abstract citation ID: igaf122.3369

### ADVANCING INTERPROFESSIONAL PALLIATIVE CARE EDUCATION: FROM NEEDS ASSESSMENT TO PILOT TESTING

Innessa Manning[1], Christina Matz[2], Anna Kennedy[3], Patricia Tabloski[2], and Karen Bullock[2], *1. Boston College, Lexington, Massachusetts, United States, 2. Boston College, Chestnut Hill, Massachusetts, United States, 3. Dana-Farber Cancer Institute, Boston, Massachusetts, United States*

Interprofessional education is essential in preparing health professionals for effective team-based practice, particularly in palliative care, where interdisciplinary collaboration is fundamental. This poster presents findings from a qualitative needs assessment and pilot testing of a newly developed graduate-level palliative care curriculum to enhance interdisciplinary training for social work, nursing, and theology students. A needs assessment survey (n = 25) conducted with palliative care professionals identified key competencies necessary for effective interprofessional practice, including communication, role clarity, and cultural competence. Structural and systemic challenges such as discipline-specific silos, inconsistent training, and workforce development gaps were also highlighted, emphasizing the need for targeted curriculum interventions that promote team-based care and cultural responsiveness. In response, an interdisciplinary faculty team developed a nine-module asynchronous online course designed to: (1) build discipline-specific expertise within a team context, (2) foster cross-disciplinary collaboration, (3) integrate culturally responsive approaches to patient and family engagement, and (4) promote sustainable practices to mitigate clinician burnout. Pilot testing with a Student Advisory Group—comprising graduate students from social work, nursing, and theology—provided feedback on content effectiveness, interdisciplinary integration, and applicability to real-world palliative care settings. Findings from the needs assessment and pilot testing underscore the importance of interdisciplinary education in developing a workforce capable of delivering compassionate, holistic palliative care. This initiative offers a replicable model for interprofessional training, equipping future clinicians with the knowledge and skills necessary to meet the growing demands of an aging and diverse patient population. Focus areas: interprofessional education, palliative care, interdisciplinary collaboration, curriculum development, cultural responsiveness, workforce training

Abstract citation ID: igaf122.3370

### ASSESSING PALLIATIVE CARE NEEDS IN NURSING HOMES AMONG RESIDENTS WITH COGNITIVE IMPAIRMENT: UPLIFT TRIAL FINDINGS

John Cagle[1], Peiyuan Zhang[2], Todd Becker[3], Alex Floyd[4], Tracie Schwoyer-Morgan[5], Becky Ridder[6], and Kathleen Unroe[7], *1. University of Maryland, Batimore, Maryland, United States, 2. University of Maryland, Baltimore County, Baltimore, Maryland, United States, 3. WashU Medicine, St. Louis, Missouri, United States, 4. Regenstrief Institute, Indianapolis, Indiana, United States, 5. Gilchrist, Baltimore, Maryland, United States, 6. Eskenazi Health, Indianapolis, Indiana, United States, 7. Indiana University School of Medicine, Indianapolis, Indiana, United States*

Palliative care (PC) has been shown to improve quality of care and life for nursing home (NH) residents. The PC needs and acuity of NH residents, particularly those with dementia, remain understudied. Using data from a large, NIA-funded trial evaluating PC in NHs, 655 residents with cognitive impairment were enrolled from 16 NHs across two states. NH staff, typically social workers and nurses, were trained as in-house PC leads. PC leads were asked to complete a PC screening on all enrolled residents to: identify needs; determine whether a PC consult is needed; and, if so, determine triage status for a consult visit. PC screening descriptives are presented and predictors of triage status were modeled. Initial screens were completed for 528 residents (80.6%) of all enrolled residents. Reasons for not being screened were resident discharge (e.g., to hospital) and death. Among six possible PC needs, 15% of residents had no identified needs, 42% had one need, and the remainder (42.8%) had ≥2 needs. The most prevalent needs were polypharmacy (58.1%), a lack of goals-of-care documentation (26.5%), functional decline/medical complexity (24.8%), unmanaged symptoms (19.7%), incongruent care (6.3%), and recent hospitalization (4.5%). Among those screened, a majority (n = 461, 87.3%) indicated a PC consult, with 5.4% of those cases being triaged as high priority. A greater number of needs (B=.42), polypharmacy (B=.33), and unmanaged symptoms (B=.31) significantly predicted higher triage priority ($p<.001$). Understanding residents' PC needs can inform tailored policies and practices to address such gaps in care.

Abstract citation ID: igaf122.3371

### ECOLOGICAL PAIN AND PHYSICAL ACTIVITY VOLUME AND PATTERNS IN OLDER ADULTS

Yurun Cai, Paul Scott, Julia Hooker, Ann Barney, and Sophia Holena, *University of Pittsburgh, Pittsburgh, Pennsylvania, United States*

Chronic pain is associated with lower physical activity (PA) levels in older adults. However, pain characteristics change over time, and the impacts of changes in pain levels on daily PA quantities and patterns remain unknown. The PRIME pilot study recruited 30 older adults (mean age=74.1±6.0y) with multisite pain (≥2 pain sites in shoulder, elbow, hand/wrist, back, hip, knee, or ankle/foot) in Pittsburgh and surrounding areas in Pennsylvania. Pain characteristics (e.g., severity, laterality, interference) were measured using questionnaires during the 2-hour clinic visit. After the clinic visit, ecological pain was assessed using Qualtrics surveys four times a day, along with Actigraph accelerometer assessment on the non-dominant wrist for 14 days. Linear regression models showed that participants with back pain had significantly lower activity counts ($\beta$=-493,271, $p = 0.020$), higher activity fragmentation ($\beta = 5.0\%$, $p = 0.005$), and fewer active minutes ($\beta$=-88.4, $p = 0.017$). Number of pain sites and severity of hand/wrist pain decreased over a day ($\beta$=-0.08, $p = 0.019$; $\beta$=-0.09, $p = 0.042$, respectively) but tended to be stable across 14 days. Linear mixed models showed that every one-unit higher in overall pain severity at one time point was associated with 28,480 fewer activity counts at the subsequent time point ($p = 0.012$), and these associations were weaker at later time points over a day ($p = 0.011$). The severity and distribution of pain vary throughout the day, and the impact of pain severity on daily activity weakens as the day progresses. Individualized pain management programs should be provided to older adults with varying pain characteristics, particularly early in the day.

Abstract citation ID: igaf122.3372

### EVALUATING PAIN MANAGEMENT COMPETENCY IN DEMENTIA VIA VIRTUAL SIMULATIONS: RESEARCH IMPLICATIONS

Yo-Jen Liao[1], Ying-Ling Jao[1], Marie Boltz[1], Nai-Ching Chi[2], Diane Berish[1], and Terrence Murphy[1], *1. The Pennsylvania State University, University Park, Pennsylvania, United States, 2. University of Iowa, Iowa City, Iowa, United States*

Pain under-recognition in dementia highlights the importance of clinicians' pain management competencies in improving care for this population. Virtual simulation presents a potentially more efficient alternative to direct observation for competency evaluation. Simulation allows clinicians to review patient histories, ask questions, perform assessments, and administer treatments on mock cases with interactive responses based on pre-designed scenarios. However, virtual simulation has not been extensively used in dementia pain management research. This study reports the challenges and strategies in using virtual simulations to evaluate nurses' pain management competencies for nursing home residents with dementia. Three scenarios, each representing different dementia stages and pain characteristics, were developed in collaboration with a technology company. The competency evaluation involves two steps. First, after viewing an orientation video, nurses performed pain management on two randomly assigned scenarios. Second, raters reviewed the simulation reports and assessed competencies using the Pain Competency Evaluation in Dementia Scale. Seventeen nurses/nursing students participated, with an average age of 25 years (94% female) and pain competency score of 66%. Challenges included participating nurses improperly exiting the simulation, resulting in unsaved reports. Raters encountered errors in simulation reports. These issues were addressed with the technology company. Scenario development took five months, involving scripting, validation, and refinement. Limitations included difficulties in showing subtle facial expressions and word limits in medical records. Subtle expressions were converted into words, and text was condensed in medical records. Future research should consider the benefits and limitations of virtual simulation while ensuring the design is optimized for research objectives.

Abstract citation ID: igaf122.3373

### EXPERIMENTAL PAIN PHENOTYPES IN OLDER ADULTS WITH KNEE OSTEOARTHRITIS: A NEURAL NETWORK-BASED CLUSTERING APPROACH

Chiyoung Lee[1], Juyoung Park[1], Heewon Kim[2], C. Kent Kwoh[1], Xiaoxiao Sun[1], Chen X. Chen[1], and Hyochol Ahn[1], 1. University of Arizona, Tucson, Arizona, United States, 2. Soongsil University, Seoul, Republic of Korea

Research has emphasized the "phenotyping" of knee osteoarthritis (KOA) pain as a priority to effectively target therapies to individual patients. Following this initiative, this study aimed to characterize pain phenotypes based on experimental pain responses in older adults with symptomatic KOA. We utilized baseline data from a clinical trial that combined non-invasive neuromodulation and meditation in its multimodal approach ($N = 200$). Participants completed demographic and clinical questionnaires, followed by a multimodal quantitative sensory testing (QST) battery. For phenotyping, we implemented a two-layer neural network-based k-means algorithm. Four phenotypes emerged, showing significant differences across QST measures ($p < 0.001$) and were characterized as: (1) high pressure pain thresholds and high conditioned pain modulation (indicating low sensitivity to pain and efficient descending inhibition); (2) average pain responses across most QST modalities; (3) low pressure pain thresholds, high punctate mechanical pain, enhanced temporal summation of pain, and low conditioned pain modulation (indicating full manifestation of peripheral, spreading, and central sensitization along with deficient descending inhibition); and (4) low heat pain thresholds, low heat pain tolerance, and high cold pain (indicating high sensitivity to thermal stimuli). Phenotypes differed by gender, marital status, pain severity (measured by the numeric rating scale), and KOA-related symptoms (measured by the Western Ontario and McMaster Universities Osteoarthritis Index) ($p < 0.05$). Our findings suggest that older adults with symptomatic KOA should be treated according to their phenotypes. Additionally, the identified phenotypes may be useful for selecting and stratifying patients in clinical trials evaluating analgesic compounds and non-pharmacological interventions.

Abstract citation ID: igaf122.3374

### FROM PURE CONTENTMENT TO FRUSTRATION: OLDER PAIN CLINIC PATIENTS' EXPERIENCES OF CLOSE RELATIONSHIPS

Meredith Stensland, *The University of Texas Health Science Center at San Antonio, San Antonio, Texas, United States*

Chronic low back pain (CLBP) is a prevalent condition among older adults, and it has profound physical, psychological, and social consequences. Older adults' subjective experiences of close relationships while living with chronic pain have received limited attention, despite the important implications they have for how they manage their pain. Past research has been focused predominantly on younger and middle-aged adults, heavily quantitative, and reliant on statistical analyses that model support and strain as two siloed phenomena. Thus, to address these limitations, the purpose of the present study was to understand how older pain clinic patients subjectively experience close relationships and to examine co-existing interpersonal support and strain. Using van Manen's phenomenological method, semi-structured, in-depth, one-on-one interviews were conducted with 21 older adults living with CLBP recruited from five outpatient pain clinics (aged 66–83 years). Data were iteratively analyzed via line-by-line thematic coding. Findings illustrate how the relationships of the participants were characterized by the co-occurrence of both positive and negative factors, leading to complex connections with their family and friends. Three core qualitative themes emerged, including: (1) Supported but misunderstood; (2) Self-censoring pain from others; (3) Good intentions, poor outcomes. This study contributes a vivid illustration of older adults' relationship experiences in the context of CLBP. Findings highlight the importance of assessing the social context for individuals with chronic pain and implications for interdisciplinary pain management. Further research is needed to understand how coexisting support and strain impact pain and related psychosocial outcomes throughout the life course.

Abstract citation ID: igaf122.3375

### GOAL ATTAINMENT IN MANAGING PAIN AND DEPRESSIVE SYMPTOMS AMONG MIDDLE-AGED AND OLDER WOMEN

Fatmata Barrie[1], Catherine Clair[2], Shelbie Atkins[3], Sarah Szanton[3], and Janiece Taylor[3], *1. Johns Hopkins University, Burtonsville, Maryland, United States, 2. Johns Hopkins School of Nursing, Baltimore, Maryland, United States, 3. Johns Hopkins University, Baltimore, Maryland, United States*

Goal setting is a key component of pain interventions, fostering self-efficacy and engagement while addressing individualized pain and emotional well-being needs. While goal setting is widely recognized as beneficial, less is known about how goal priorities vary by intervention, population characteristics, and lived context. This study examines goal-setting and attainment in two populations: (1) Middle-aged and older African American women who were frail or prefrail in the DAPPER (Depression and Pain Perseverance through Empowered Recovery) study (N = 34) and (2) Middle-aged and older women of any race/ethnicity with mobility disabilities in the Women in PRIME (Pain Reduction and Improved Mood through Empowerment) study (N = 9). This analysis categorizes and compares 75 total goals set across these interventions to examine factors that influence self-management priorities. A comparative analysis of goals and goal domains revealed distinct priorities between the two groups. Goals were categorized into physical activity, pain management, emotional well-being, stress reduction, and social engagement. DAPPER participants prioritized physical activity goals (27.5%), likely influenced by the COVID-19 pandemic, which limited mobility. PRIME participants set broader goals, emphasizing social engagement, stress reduction, and emotional well-being, aligning with post-pandemic recovery. Differences in living situations and social networks may have also influenced goal-setting behaviors. Findings suggest that participants' lived experiences, including temporality and social supports, shape goal-setting behaviors. These results highlight the need for individualized, patient-directed approaches that consider social determinants of health and cultural influences. Future research with larger samples is needed to confirm these patterns and optimize behavioral interventions for diverse populations.

Abstract citation ID: igaf122.3376

### STAFF-IDENTIFIED PALLIATIVE CARE NEEDS AMONG PROGRAMS OF ALL-INCLUSIVE CARE FOR THE ELDERLY (PACE): A SURVEY STUDY

Molly Nowels[1], Karolina Sadowska[1], Mark Unruh[2], M. Carrington Reid[1], Catherine Riffin[1], Manali Saraiya[3], Evan Plys[4], and Daniel Shalev[1], *1. Weill Cornell Medicine, New York, New York, United States, 2. Joan and Sanford I. Weill College of Medicine, Cornell University, New York, New York, United States, 3. New York-Presbyterian Hospital, New York, New York, United States, 4. Massachusetts General Hospital, Boston, Massachusetts, United States*

The Program of All-Inclusive Care for the Elderly (PACE) is an interdisciplinary, capitated care model that brings nursing home-level care to dual-eligible older adults living in their own communities. Because serious illnesses are prevalent among PACE participants, PACE programs are federally required to include palliative care within their networks. However, the specific palliative care needs of this unique population have not been characterized, limiting the development of palliative care-PACE integration. To understand PACE staff perspectives on palliative care, we conducted a palliative care needs assessment survey among 81 staff (including CNAs, RNs, OT/SLP/PT, and prescribing providers [MD, NP, PA]) at a PACE program with three sites in New York. Providers were asked if they would like to increase access to palliative care service for their patients, about the most common ways a palliative care specialist could help their patients, and the domains of palliative care in which most of their patients could benefit from additional services. More than half (53%) of respondents agreed that they would like to increase access to palliative care services. Palliative care was seen as most helpful in pain management (80%), caregiver and family support (57%), and psychiatric symptom management (46%). In contrast, respondents identified the most prevalent needs in their patients in caregiver support (69% of patients), advance care planning and goals of care conversations (67%), and psychiatric symptom management (63%). These findings suggest high psychosocial palliative care needs among PACE participants, potentially informing the evolution of uniquely adapted and integrated palliative care services.

Abstract citation ID: igaf122.3377

### SUBCUTANEOUS METHYLNALTREXONE FOR OPIOID-INDUCED CONSTIPATION IN OLDER ADULTS WITH ADVANCED ILLNESS

Julie Knight[1], Sri Nalamachu[2], and Adam Laitman[1], *1. Salix Pharmaceuticals, Bridgewater, New Jersey, United States, 2. Analgesic Clinical Research, Blue Springs, Missouri, United States*

Opioid-induced constipation (OIC), affecting up to 81% of patients taking opioid analgesics, is caused by opioids binding to peripheral mu-opioid receptors in the gastrointestinal tract. Methylnaltrexone, a peripherally-acting mu-opioid receptor antagonist, reverses opioid-related constipating effects without affecting centrally-mediated analgesia. This analysis evaluated subcutaneous methylnaltrexone efficacy/safety in older adults (≥65 years) with advanced illness (eg, incurable cancer) and OIC. In 2 randomized, double-blind, placebo-controlled, phase 3 trials, patients received subcutaneous methylnaltrexone 0.15 or 0.30 mg/kg, or placebo. Primary endpoint (both trials) was percentage of patients with rescue medication-free bowel movement (RFBM) within 4 hours after first dose. Data were pooled and analyzed posthoc. Overall, 161 patients (49.1% female) were included. Significantly more patients aged ≥65 years treated with subcutaneous methylnaltrexone 0.15 (37/68 [54.4%]) or 0.30 mg/kg (16/31 [51.6%]) had an RFBM within 4 hours versus placebo (9/62 [14.5%]; both $P < 0.001$ vs placebo). When further stratified by age, an RFBM within 4 hours was reported in significantly more patients 65-74 years treated with methylnaltrexone 0.15 (13/31 [41.9%]) or 0.30 mg/kg (8/15 [53.3%]) versus placebo (3/23 [13.0%]; $P \leq 0.03$ for both); for 75-84 years, methylnaltrexone 0.15 (17/25 [68.0%]) or 0.30 mg/kg (7/12 [58.3%]) versus placebo (3/21 [14.3%]; $P < 0.001$ and $P = 0.02$ versus placebo, respectively). For patients aged ≥85 years, significantly more patients had a RFBM within 4 hours in methylnaltrexone 0.15 mg/kg (7/12 [58.3%]) versus placebo (3/18 [16.7%]; $P = 0.04$) group. Subcutaneous methylnaltrexone was well tolerated. In conclusion, subcutaneous methylnaltrexone for OIC was efficacious, with a rapid onset of action, and well tolerated in older individuals.

Abstract citation ID: igaf122.3378

**THE RELATIONSHIP BETWEEN RACE AND PAIN KNOWLEDGE AND ATTITUDES AMONG RURAL OLD ADULTS IN ALABAMA**

Lewis Lee, Hyunjin Noh, Haelim Jeong, Lauryn Emrick, and Madison Hasnani, *The University of Alabama, Tuscaloosa, Alabama, United States*

The literature highlights persistent racial disparities in pain assessment and diagnosis, suggesting that marginalized racial and ethnic groups may have limited access to pain education due to systemic inequities in healthcare and education systems. However, little research has explored the relationship between race and pain knowledge and attitudes, particularly in rural Alabama. This study aims to address this gap by examining the level of pain knowledge and attitudes among rural residents and assessing the extent to which race is associated with these outcomes, controlling for covariates. Using purposeful sampling, 123 participants were recruited. Eligibility criteria included being 65 years or older, living outside nursing homes, being cognitively intact, having two or more chronic illnesses and chronic pain, and taking medications for these conditions, including pain medications. The dependent variable was a composite score derived from summing items in the Knowledge and Attitudes Survey Regarding Pain (Cronbach's alpha=.70). The main independent variable was binary race, and covariates included socio-demographic and health factors, such as the number of chronic health conditions, and pain scores (Cronbach's alpha=.64). A multiple linear regression model with heteroskedasticity-robust standard errors was used for analysis. Results revealed that BIPOC individuals had significantly lower pain knowledge and attitudes scores compared to their White counterparts ($p<.05$). Additionally, higher levels of education were positively associated with pain knowledge and attitudes scores ($p<.05$). These findings emphasize the need to address racial disparities in pain knowledge and attitudes and highlight education's role in improving perceptions and understanding of pain, particularly among BIPOC populations.

Abstract citation ID: igaf122.3379

**THE ROLE OF PALLIATIVE CARE IN NEW PERIOPERATIVE DNR ORDERS: RISK FACTORS AND TIMING**

Sarah Remer[1], Marcia Russell[2], Ronnie Rosenthal[3], and Clifford Ko[2], *1. The American College of Surgeons, Chicago, Illinois, United States, 2. David Geffen School of Medicine at UCLA, Los Angeles, California, United States, 3. Yale School of Medicine, New Haven, Connecticut, United States*

As the population ages, there is an increased need for comprehensive goals of care (GOC) discussions which often include Do-Not-Resuscitate (DNR) orders. DNR orders are being increasingly studied, but it remains unknown how palliative care consultation (PCC) relates in older adult surgical patients. The American College of Surgeons National Surgical Quality Improvement Program Geriatric Surgery Verification pilot program data was utilized from January 1, 2015 to December 31, 2019. The primary outcome was new DNR order within 30 days of primary operation for patients 65 and older. The association between PCC and DNR was assessed including timing. There were 44,172 patients, 2.7% of which had a new DNR order. 21% were placed preoperatively, 33% the day of surgery, 46% postoperatively. Of the patients who had DNR orders placed, 32% had PCCs versus 0.5% of those who did not have DNR orders ($p<0.0001$). 45% of patients with both new DNR and PCC had them placed on the same day, 10% of PCC were before new DNR, and 45% were after. 57% of PCCs were placed ≤1 day prior to death and 49% of PCCs were placed >7 and ≤30 days postoperatively. After adjustment, PCC was significantly associated with new DNR (OR 9.95; 95% CI 6.42-15.42). Almost half of PCC and new DNR orders occur on the same day. Half of PCCs are on the same day as death signifying need for earlier GOC discussions. Further research is needed to better identify risk factors for earlier PCC to inform GOC.

### SESSION 7505 (POSTER)

### PERSONALITY

Abstract citation ID: igaf122.3380

**A CONJOINT EXPLORATORY FACTOR ANALYSIS OF NORMATIVE AND PATHOLOGICAL PERSONALITY TRAITS IN OLDER ADULTS**

Lisa Stone-Bury[1], and Daniel Segal[2], *1. Bucknell University, Lewisburg, Pennsylvania, United States, 2. University of Colorado Colorado Springs, Colorado Springs, Colorado, United States*

The Alternative Model of Personality Disorders (AMPD) is a dimensional model of personality disorders (PD) that measures PD symptoms according to a five-factor pathological personality trait model. This pathological trait model was designed to align with the Big Five model of normative personality traits: Negative Affect-Neuroticism, Detachment-Extraversion, Antagonism-Agreeableness, Disinhibition-Conscientiousness, and Psychoticism-Openness. Theoretically, lower-order facets of both models should overlap in these proposed directions to produce a five-factor model. This study examined this theoretical conjoint structure among community-dwelling older adults ($n=200$) who were recruited online and completed the Personality Inventory for DSM-5 (PID-5; pathological personality traits) and the Big Five Inventory-2 (BFI-2; normative personality traits). Bivariate correlations indicated strong relationships in expected directions. An exploratory principal factor analysis with oblimin rotation was conducted between the PID-5 and BFI-2 facets. Model fit indices (KMO, Bartlett's test, RMSEA) and commonalities of the scales indicated that the analysis could proceed. A six-factor model emerged: (1) Negative Affect-Neuroticism, (2) Detachment-Extraversion, (3) Antagonism-Agreeableness, (4) Disinhibition-Conscientiousness, (5) Psychoticism-obsessive, and (6) Openness. Essentially, four of the joint domains largely replicated as proposed in the AMPD (with some substantial differences between specific loadings). However, the proposed joint Psychoticism-Openness domain did not emerge, instead loading onto two separate factors (with substantial theoretical differences). Normative fluctuations in Big Five traits that occur across the lifespan may create difficulties when attempting to connect it to the AMPD. The present findings suggest that further revision to the Psychoticism domain is needed for the model to be appropriately applied to older adults.

Abstract citation ID: igaf122.3381

**A PSYCHOMETRIC EVALUATION OF THE INTRAPERSONAL PROBLEMS RATING SCALE AMONG OLDER ADULTS**

Colleen Mock[1], Daniel Segal[2], and Andrew Lac[2], *1. University of Colorado Colorado Springs, Shaker Heights, Ohio, United States, 2. University of Colorado Colorado Springs, Colorado Springs, Colorado, United States*

Introduction: Interpersonal difficulties have long been connected to worse psychological functioning among older adults. However, the understanding of intrapersonal problems in older adults is limited. A psychometric evaluation of intrapersonal problems and their relation to diverse psychological constructs among older adults is needed. Method: Older adults (N = 250; 50.4% women) completed the following self-report measures: Intrapersonal Problems Rating Scale, Levels of Personality Functioning Scale-Brief Form 2.0, Brief Geriatric Suicide Ideation Scale, Geriatric Anxiety Scale, Geriatric Depression Scale-Short Form, Depression Anxiety Stress Score-21, Michigan Alcohol Screening Test-Geriatric Version, Insomnia Severity Index, and the Webexec. The study aimed to further examine intrapersonal functioning and its relations to well established constructs of psychopathology and to provide an initial psychometric evaluation of the IPRS in older adults. Results: Measures of internal consistency were good to excellent for IPRS total and the 7 IPRS domain scores. Bivariate correlations revealed medium to large positive correlations between IPRS total scores with all other measures, ranging from .44 (IPRS with MAST-G) to .80 (IPRS with GAS). Gender differences were also found, with women reporting lower domain scores in externalizing, fantasy proneness, and apathy than men with small effect sizes across domains. Discussion: The internal reliability and convergent validity analyses of the IPRS provide solid psychometric evidence in support of the use of the IPRS as a measure of intrapersonal functioning among older adults. Findings also reveal that deficits in intrapersonal functioning are strongly associated with worse psychological functioning across several important psychopathology and cognitive domains.

Abstract citation ID: igaf122.3382

**PERSONALITY TRAITS PREDICT PROFILES OF PHYSIOLOGICAL DYSREGULATION IN ADULTHOOD**

Nicholas Turiano, Taylor Brown, Sarah Miller, and Meredith Willard, *West Virginia University, Morgantown, West Virginia, United States*

Personality traits are robust predictors of health outcomes across the lifespan, but more evidence is needed how traits specifically impact multiple biological systems. Thus, the current study utilized a sample of 1,239 participants (Mean age = 54.61; range 34-84) from the Midlife Development in the U.S. (MIDUS) study to determine if personality traits would predict extracted latent classes of individuals based on their level of multi-system physiological dysregulation. Personality was assessed with the MIDI Big 5 measure. Twenty-four biomarkers were utilized to create highest quartile proportion scores for each of the 7 different physiological systems measured (para/sympathetic systems, HPA axis, cardiovascular, lipid/glucose metabolism, inflammation). These 7 indicators were used in a latent profile analysis where a 3-class solution provided the best model fit. Multinomial logistic regression analyses revealed that higher levels of conscientiousness significantly predicted (p's < .05) an increased odds in being in the "lowest" physiological risk class (N = 809) versus the two other classes with clearly more physiological dysregulation. Unique to these two classes was one that had elevated parasympathetic and HPA system dysfunction (N = 228), while the other had less parasympathetic but more HPA dysfunction (N = 217). These findings pinpoint parasympathetic and HPA axis dysfunction s two defining features of physiological health in older adulthood, which is related to level of conscientiousness. Exploring how psychological characteristics are associated with the aging process is key to identifying the adults at-risk of poor health and mortality.

Abstract citation ID: igaf122.3383

**THE INFLUENCE OF PERSONALITY TRAITS ON DRIVING BEHAVIORS IN PRECLINICAL ALZHEIMER DISEASE**

David Carr[1], Andrew Aschenbrenner[2], and Ganesh Babulal[3], *1. Washington University in St. Louis, St Louis, Missouri, United States, 2. Washington University in St. Louis, St. Louis, Missouri, United States, 3. Washington University School of Medicine in St. Louis, SAINT LOUIS, Missouri, United States*

Alzheimer disease (AD) has a long preclinical phase in which AD pathology is accumulating without detectable clinical symptoms. It is critical to identify participants in this preclinical phase as early as possible since treatment plans may be more effective in this stage. Monitoring for changes in driving behavior, as measured with GPS sensors, has been explored as a low-burden, easy to administer method for detecting AD risk. However, driving is a complex, multi-faceted process that is likely influenced by other factors that may change in preclinical AD, including personality traits. In this study, we examine the moderating influence of neuroticism and conscientiousness on longitudinal changes in driving behavior in a sample of 203 clinically normal older adults who are at varying risk of developing AD. Our results indicate that neuroticism moderated rates of change in the frequency of speeding as well as the number of trips taken at night. Conscientiousness moderated rates of change in typical driving space. We interpret these findings within established changes in personality traits in preclinical AD and suggest that studies of longitudinal changes in naturalistic driving behavior need to accommodate interpersonal differences, including personality. Strengths and limitations of this study are also noted. Future studies should explicitly establish how much benefit or risk is provided by including personality traits in predictive models of AD progression.

### SESSION 7510 (POSTER)

### PHARMACOLOGY

Abstract citation ID: igaf122.3384

**ANTITHROMBOTIC TREATMENT AFTER LEFT ATRIAL APPENDAGE OCCLUSION IN OLDER ATRIAL FIBRILLATION PATIENTS**

Xiaojuan Liu[1], Sebastian Schneeweiss[1], Daniel Singer[2], Jerry Avorn[1], E. Kevin Heist[2], and Joshua Lin[1], *1. Brigham and Women's Hospital, Boston, Massachusetts, United States, 2. Massachusetts General Hospital, Boston, Massachusetts, United States*

Left atrial appendage occlusion (LAAO) is an alternative to oral anticoagulants (OAC) for stroke prevention in nonvalvular atrial fibrillation (NVAF); however, real-world data on adherence to its postprocedural treatment protocol remain limited. We evaluated post-LAAO antithrombotic treatment patterns in a cohort of 16304 NVAF patients (mean [SD] age = 78 [6]; 47% female; CHA2DS2-VASc score = 5.0 [1.5]) undergoing first-time LAAO using Optum claims data 2015-2024. OAC discontinuation was defined as a ≥60-day gap in supply, and prolonged OAC use was operationally defined as either (1) an OAC refill following a TEE performed 30–90 days post-implantation or (2) OAC refill beyond 90 days post-implantation, regardless of TEE completion. Among LAAO recipients, 9844 (60%) received concomitant OAC and 3499 (10%) received P2Y12 inhibitor at implantation. Transesophageal echocardiography (TEE) was performed in 11237 (69%) patients at 45 (±15) days. Among OAC users (n = 9844), 30% and 85% discontinued OAC by 45 days and 6 months post-implantation, respectively; only 23% patients followed the standard postprocedural protocol. Common deviation from the protocol included no TEE at 45 (±15) days (30%), early OAC discontinued (22%), continued OAC use post-TEE (10%), and P2Y12 inhibitor initiated early or not initiated (10%). Prolonged OAC use was observed in 1970 patients. Factors associated with a higher likelihood of prolonged OAC use included older age, Black race, cardio ablation, and a longer duration of prior OAC use. These findings suggest moderate to low adherence to the LAAO postprocedural protocol, warranting further evaluation of the clinical impact of these adherence patterns.

Abstract citation ID: igaf122.3385

### BEYOND THE RASH: AN ATYPICAL CASE OF VANCOMYCIN-RELATED DELAYED CUTANEOUS HYPERSENSITIVITY

Paul Chung[1], Audrey Shawley[2], Ganeev Singh[3], and Eric Dahms[3], *1. University of California, San Diego School of Medicine, San Diego, California, United States, 2. Scripps Health, San Diego, California, United States, 3. Scripps Mercy Hospital, San Diego, California, United States*

Severe Cutaneous Adverse Reactions (SCARs), including Drug Reaction with Eosinophilia and Systemic Symptoms (DRESS) and Stevens-Johnson Syndrome (SJS)/Toxic Epidermal Necrolysis (TEN), are life-threatening and often linked to commonly used antibiotics. Older adults are particularly vulnerable due to polypharmacy and age-related pharmacokinetic changes. In this case, a 72-year-old male with basal cell carcinoma and global aphasia from a left parietal stroke developed delayed-onset exfoliative erythroderma. He was hospitalized 5 weeks prior for left leg osteomyelitis, undergoing multiple surgical debridements. Discharged to a skilled nursing facility (SNF) on IV vancomycin and piperacillin-tazobactam, he was switched to daptomycin after a week due to an IV antibiotic shortage. Three weeks later, the SNF noted a worsening full-body, erythematous, non-blanching rash. Despite discontinuing daptomycin for doxycycline, his condition progressed. On admission, he had diffuse petechial and maculopapular lesions with scaling involving his entire body, including the face, palms, and soles, but sparing mucosal membranes. Labs showed mild leukocytosis, elevated neutrophils, no eosinophilia, and elevated CRP/ESR. His rash resolved with antibiotic discontinuation, topical corticosteroids, and IV methylprednisolone. This case underscores the diagnostic challenge of SCARs in polypharmacy-prone older adults, particularly in patients who also face communication barriers secondary to their past medical histories. The delayed onset suggested vancomycin hypersensitivity despite lacking classical DRESS features. SJS/TEN were ruled out due to the timeline. Ultimately, Vancomycin was the most likely culprit for this unusual rash. Clinicians must recognize atypical drug reactions in geriatric patients, where polypharmacy and relevant past medical history complicates diagnosis and management.

Abstract citation ID: igaf122.3386

### GASTROINTESTINAL BLEEDING RISK WITH ORAL ANTICOAGULANT AND PROTON PUMP INHIBITOR CO-THERAPY IN NURSING HOMES

Iju Shakya[1], Yoojin Lee[1], Andrew Zullo[1], Jimmie Roberts[2], and Sarah Berry[3], *1. Brown University, Providence, Rhode Island, United States, 2. Hebrew SeniorLife, Boston, Massachusetts, United States, 3. Hebrew SeniorLife & Harvard Medical School, Hebrew SeniorLife, Massachusetts, United States*

Direct oral anticoagulants (DOACs) are essential for preventing and treating thrombosis but increase upper gastrointestinal bleeding (UGIB) risk. Proton pump inhibitors (PPIs) may reduce UGIB risk, but it is unclear whether this protective effect extends to nursing home (NH) residents, who are at high bleeding risk. We aimed to estimate the effect of DOAC and PPI co-therapy, compared to DOAC only, on 1-year UGIB hospitalization risk among NH residents. We conducted a retrospective cohort study emulating a hypothetical target trial. Adults aged ≥ 66 years, enrolled in fee-for-service Medicare, and living in NHs between March 1, 2013, and December 31, 2019, were included. Residents were new DOAC users (no use in prior 60 days) and were classified as exposed if they initiated PPI within 31 days of DOAC dispensing or as unexposed if they did not. UGIB hospitalizations were identified from claims during one-year follow up. A clone-censor-weight approach addressed immortal time bias and discrete-time hazards models estimated UGIB risk in each treatment group. Among 149,686 new DOAC episodes, 10,445 (7.0%) were exposed to PPI co-therapy while 139,241 (93.0%) were unexposed. UGIB incidence was similar in the exposed group (2.8%) and the unexposed group (2.5%). Further analysis with covariate adjustment is needed, but our preliminary results do not suggest a difference in UGIB with DOAC-PPI co-therapy in NH residents.

Abstract citation ID: igaf122.3387

### LEFT ATRIAL APPENDAGE OCCLUSION VERSUS ORAL ANTICOAGULANTS IN OLDER ATRIAL FIBRILLATION PATIENTS

Xiaojuan Liu[1], Sebastian Schneeweiss[1], Daniel Singer[2], Jerry Avorn[1], E. Kevin Heist[2], and Joshua Lin[1], *1. Brigham and Women's Hospital, Boston, Massachusetts, United States, 2. Massachusetts General Hospital, Boston, Massachusetts, United States*

Real-world evidence comparing left atrial appendage occlusion (LAAO) devices to oral anticoagulants (OAC) in nonvalvular atrial fibrillation (NVAF) remain limited. We emulated a target trial and evaluated the effectiveness and safety of LAAO device versus OAC in 35326 NVAF patients (aged ≥65 years, CHA2DS2-VASc score ≥2 [males] or ≥ 3 [females]) using Medicare 2016-2020 and Optum 2016-2024 data. LAAO recipients on OAC (warfarin, apixaban, rivaroxaban, or dabigatran) at implantation (index date) were 1:1 matched to patients who received these medications alone via propensity score matching based on 75 pre-treatment covariates. Outcomes included hospitalization for major bleeding events, ischemic stroke, and all-cause mortality. Follow-up was censored at 2 years or upon treatment deviation (OAC discontinuation or LAAO implantation in OAC users and OAC continuation beyond 90 days in LAAO users). Database-specific rate ratios (RR) were estimated via Poisson regression with inverse probability of censoring weighting and pooled using fixed-effect meta-analysis. Compared to OAC users, LAAO users had a higher bleeding rate over 2 years (RR 1.29; 95% CI 1.11-2.49); however, beyond 6 months post-implantation, LAAO users had a 36% reduction in bleeding rate (0.64; 0.51-0.80). LAAO was also associated with a higher rate of ischemic stroke over 2 years (1.43; 1.10-1.85), though the effect size diminished over time and was no longer statistically significant beyond 6 months (1.16; 0.84-1.62). We found no evidence of association with mortality. These findings suggest that LAAO may reduce long-term bleeding risk but may carry an early increased risk of bleeding and ischemic stroke compared to OAC.

Abstract citation ID: igaf122.3388

### TOWARDS SAFER MEDICATION USE IN OLDER ADULTS: INVESTIGATING BARRIERS AND FACILITATORS TO DEPRESCRIBING

Madhavi Eerike[1], Gomathi Ramaswamy[2], Priyadharsini Rajendran[3], Paul Mathai[4], Veena Nayak[5], and Vijay Kumar Karra[2], *1. All India Institute of Bibinagar Hyderabad, Hyderabad, Telangana, India, 2. All India Institute of Bibinagar Hyderabad, All India Institute of Bibinagar Hyderabad, Telangana, India, 3. Jawaharlal Institute of Postgraduate Medical Education and Research, Pondicherry, Puducherry, India, 4. Malabar Medical College Hospital & Research Centre, Modakkallur, Kerala, India, 5. Kasturba Medical College, Manipal, Manipal, Karnataka, India*

Background: Deprescribing, the systematic reduction or discontinuation of unnecessary medications, is crucial for optimizing pharmacotherapy in older adults. However, its implementation faces challenges, including systemic barriers, knowledge gaps, and patient-specific complexities. Understanding healthcare professionals' perspectives is vital to improving deprescribing practices and enhancing elderly patients' health outcomes. Methods: This qualitative study employed in-depth interviews with healthcare professionals (n = 52) to explore barriers, facilitators, and potential improvements in deprescribing. Thematic analysis was used to identify key insights and patterns from the collected data. Results: Key barriers included limited access to healthcare services, particularly in rural and underserved areas, and knowledge gaps in geriatric pharmacology among resident doctors. Patient-specific challenges, such as advanced age and multiple comorbidities, compounded these difficulties. Chronic use of medications like proton pump inhibitors (PPIs) and benzodiazepines was a prominent obstacle. Systemic issues, such as inefficient workflows and poor interdisciplinary coordination, also hindered deprescribing. Facilitators included patient acceptance and active participation in shared decision-making, supported by effective communication skills. The integration of electronic health records (EHRs) enhanced continuity of care and medication tracking. Increasing acceptance of deprescribing among doctors, especially in government healthcare settings, was also observed. Conclusion: Addressing systemic barriers, enhancing patient education, and implementing structured deprescribing guidelines are essential for improving practices. Strengthening interdisciplinary collaboration and leveraging digital tools, such as EHRs, can support safe and effective medication discontinuation. These findings underscore the need for targeted interventions to optimize deprescribing and improve health outcomes for elderly patients.

### SESSION 7515 (POSTER)

### POLICY

Abstract citation ID: igaf122.3389

### ARTIFICIAL INTELLIGENCE APPLICATIONS IN ADVANCE CARE PLANNING

Girish Hemrajani, and Debra Dobbs, *University of South Florida, Tampa, Florida, United States*

This scoping review examines artificial intelligence (AI) applications in advance care planning (ACP), synthesizing evidence from databases like PubMed, Web of Science, and Scopus (2019-2024), ten articles, using PRISMA guidelines. The review focused on AI tools for ACP documentation, decision-making, and workflow optimization, excluding studies lacking clinical validation or patient-centered outcomes. Findings reveal promising AI applications in ACP: predictive models, like Stanford's EHR-based mortality predictor, improved high-risk patient identification, triggering earlier ACP discussions. Natural Language Processing reduced ACP documentation review time significantly. AI-powered chatbots increased ACP literacy among older adults by 40%, addressing health disparities. Challenges like algorithmic bias from non-diverse datasets exacerbates inequities in ACP access persist. Clinician resistance stems from unclear liability frameworks and poor EHR interoperability. Only 25% of tools address caregiver burden or surrogate decision-maker needs. Research gaps include limited evidence on AI-driven e-planning tools' cost-effectiveness, lack of standardized metrics for evaluating AI's impact on patient autonomy and understudied ethical risks of over-reliance on AI for sensitive end-of-life conversations. Future directions focus on equity-driven design, the integration of e-planning into electronic medical records, and the assessment of AI's capacity to alleviate caregiver burden and enhance preparedness of surrogate decision-makers. This research emphasizes the necessity to transform ACP from a documentation-oriented process to a communication-centered continuum, utilizing AI to democratize access and tackle systemic inequalities. It highlights the potential of AI to transform ACP, while emphasizing the importance of ethical implementation and patient-centered outcomes.: Keywords: Artificial intelligence, advance care planning, health equity, predictive analytics, natural language processing.

Abstract citation ID: igaf122.3390

### CARING FOR THE AGING: A SOCIAL EXCHANGE PERSPECTIVE ON TAIWAN'S SHARED CARE MODEL

Shu-Chuan Jennifer Yeh[1], and Ying-Ying Lo[2], *1. National Sun Yat-sen University, Kaohsiung, Kaohsiung, Taiwan (Republic of China), 2. I-Shou University, Kaohsiung, Kaohsiung, Taiwan (Republic of China)*

The global population aged 60 and above is expected to grow by 34%, reaching 2.1 billion by 2050 (WHO, 2020), bringing significant challenges to families, healthcare systems, and governments. As aging populations face rising rates of chronic diseases such as cardiovascular conditions, diabetes, and dementia, the demand for long-term care increases, straining healthcare infrastructures globally. In Taiwan, approximately 840,000 individuals require long-term care, affecting 11% of families. Care responsibilities often fall on family members or outsourced caregivers, leading to financial and emotional strain, a trend observed in many countries facing similar challenges. To address this, the Taiwanese government has implemented a shared caregiving policy, allowing multiple patients to share one caregiver. This model reduces financial burdens on families and provides caregivers with shift-based work to prevent burnout, rather than continuous 24-hour care. Personal caregivers typically cost NT$2,400 per day, creating financial stress for families, while caregivers often face exhaustion. Using social exchange theory (Cook, 1987; Thibaut & Kelley, 1959), this study evaluates the shared care policy's impact, revealing high satisfaction rates among registered nurses (88%), nursing aides (81.5%), and families (82%). The policy subsidizes caregiving costs, reduces stress, and improves job satisfaction for healthcare professionals. By balancing the costs and rewards for caregivers and families, the policy fosters a fairer exchange of resources and labor. While the policy has addressed caregiver shortages and improved caregiving standards, sustainable government funding remains essential for its long-term success. Future efforts should focus on ensuring financial stability and refining the policy.

Abstract citation ID: igaf122.3391

### CHANGE AND STABILITY IN POLITICAL IDEOLOGY AND ENGAGEMENT OVER THE LIFESPAN: A RETROSPECTIVE STUDY

Kyla Prussman, Elizabeth Rickenbach, Jennifer Lucas, and Kevin Doran, *Saint Anselm College, Goffstown, New Hampshire, United States*

Voters aged 60+ years old are historically important for US elections, due to their persistently high voting rates, the significance of Medicare and Social Security, and population aging (Binstock, 2012; Campbell & Binstock, 2011; Super, 2020). Research has long sought to understand older voter (OV) lifetime experiences with politics and the development of their political ideology across their lifetime. Most research on OV's political ideology is cross-sectional and focuses on general polling data, and research on lifetime political experiences is often framed through a political science or economic lens. The current study incorporated a lifespan and psychological perspective with semi-structured interviews to retrospectively examine perspectives of OVs. In particular, the current study sought to understand whether political preferences vary over a person's life and whether stability or change differs across gender or political party. Participants (n = 88) included New Hampshire registered voters aged 60+ years. The sample included 29 Independent voters (14 women, 15 men), 31 Democratic voters (16 women, 15 men), and 28 Republican voters (15 women, 13 men). Qualitative findings showed change and stability across OVs. Many voters' shared stability across their lifespan and increased intensity towards their political engagement. Others discussed shifts from more conservative values to more liberal values, or from a lack of political engagement to more engagement. OVs discussed how their shifts were influenced by lifetime experiences with work, family, and health, as well as shifting perspectives towards politics. This research contributes to a better understanding of the dynamic nature of OV's political views.

Abstract citation ID: igaf122.3392

### FROM POLICY TO PRACTICE: EVALUATING THE IMPACT OF ALZHEIMER'S STATE PLANS ACROSS THE U.S.

KoriAnne Moslander, Christopher Kelly, and Julie Blaskewicz Boron, *University of Nebraska Omaha, Omaha, Nebraska, United States*

The National Alzheimer's Project Act was signed into law on January 4, 2011, establishing the Advisory Council on Alzheimer's Research, Care, and Services, creating a national plan, and supporting states serving this population. This study utilized a policy analysis framework to examine the implementation of Silver Alert, early detection programs, and dementia training requirements across all 50 states. Further analysis of these policies identified the success rates of the proposed policies compared across each state. It exposed the gaps present in serving the residents of each state living with Alzheimer's disease and other dementias. Results revealed that 64% of states, along with the District of Columbia and Puerto Rico, have implemented a Silver Alerting program or similar, require a certain level of dementia training, and provide early detection programs and campaigns. The leading diagnostic test for early detection of dementia is biomarker testing. Legislation to ensure coverage for this test has passed in 16 states. Illinois was the first state to enact this legislation in August of 2024. Washington state has the greatest number of hours required for dementia training for direct care workers, with 12 hours pertinent to dementia care. California requires fewer but mandates two hours of dementia training within the first 40 hours of employment and five hours of dementia-specific in-service training annually. Florida, California, and Utah lead with the most success stories from their Silver Alert programs. Further discussion will examine the substantial progress made in these state's Alzheimer's state plans.

Abstract citation ID: igaf122.3393

### MEDICARE-MEDICAID DUAL ELIGIBLE SPECIAL NEEDS PLAN ENROLLMENT IN MARYLAND

Christine Gill, Roberto Millar, and Christin Diehl, *University of Maryland, Baltimore County, Baltimore, Maryland, United States*

Nationally, health care spending for Medicare-Medicaid duals is higher than for non-duals in either program. Programs integrating care across these separate programs—one type being dual eligible special needs plans (D-SNPs), a Medicare Advantage (MA) plan—produce mixed results on improving care coordination and reducing costs. This retrospective cohort study examined sociodemographic and geographic factors associated with 2021 D-SNP enrollment among duals in Maryland—a state with low MA enrollment. We report descriptive statistics by Medicare program type (D-SNP or Traditional Medicare [TM]) and logistic regression analyses. Among 109,646 duals, 9% were enrolled in D-SNPs, and 60% were 65 years or older. We found regional differences: Baltimore City enrollees were 1.6 (95% confidence interval (CI) 1.5-1.7) times more likely than those in Central Maryland to select D-SNPs compared with TM, and neighborhoods with the highest quintile of disadvantage had 1.4 (95% CI: 1.3-1.5) times the likelihood of selecting D-SNPs compared with TM. Full duals were less likely to select D-SNPs over TM compared with partial duals (0.8 [95% CI: 0.7-0.8]); those 65 years and older were more likely compared with younger (1.6 [95% CI: 1.5-1.7]); and Black (2.2 [95% CI: 2.1-2.3]) and

Latino (1.6 [95% CI: 1.4-1.8]) were more likely compared with White duals. Given that racial and ethnic minorities and those from more disadvantaged neighborhoods are enrolled in D-SNPs disproportionately in Maryland, additional research should be conducted to examine whether health outcomes and care quality are equitable across programs to inform policy integrating care for this high-needs population.

Abstract citation ID: igaf122.3394

### POLICY COORDINATION OR FRAGMENTATION? A CONTENT ANALYSIS OF DIGITAL AGING POLICIES IN CHINA

Sihan Lu[1], and Lili Shang[2], *1. Institute for Philanthropy Tsinghua University, Beijing, Beijing, China (People's Republic), 2. University of New South Wales, Sydney, Sydney, New South Wales, Australia*

To address population aging, digital technology has become a crucial policy domain for improving aged care services and enhancing the well-being of older adults. The degree of policy coordination would significantly impact the implementation and effectiveness of digital initiatives. By synthesizing key digital aging policy documents from 2020-2024, this study aims to examine the degree of policy coordination, alignment of policy instruments as well as government organization in the Chinese policy landscape. Informed by one policy instrument framework, this study categorizes digital aging policies into supply-side, demand-side and environmental policy instruments. Through iterative coding, this study conducts content and cluster analysis to examine policy content. Findings show that while central government efforts promote coordination, local governments exhibit fragmented policy formation with inconsistent standards, imbalanced resource allocation, and disjointed governance. Three major policy challenges are identified: (1) data interoperability gaps due to a lack of national standards; (2) uneven regional funding distribution, with limited incentives for sustainable industry development; (3) overlapping stakeholders and redundant responsibilities, leading to regulatory inconsistencies. To mitigate fragmentation, we propose establishing an inter-ministerial coordination framework and strengthening digital infrastructure integration for nationwide interoperability in aged care services. As digital technologies are increasingly integrated into aging policies worldwide, ensuring policy coherence is crucial for effective governance. This study contributes to the discourse on technology-driven aging policy design and provides evidence-based insights for policy development.

Abstract citation ID: igaf122.3395

### SMART HOME SAFETY TECHNOLOGY IN MEDICAID LTSS: A POLICY REVIEW OF HCBS WAIVER COVERAGE

Meredith Hughes, Yong Kyung Choi, Jon Pearlman, Pamela Toto, Everette James, and Steven Handler, *University of Pittsburgh, Pittsburgh, Pennsylvania, United States*

Medicaid is the largest payer for long term-services and supports (LTSS) in the United States. Though Medicaid services are subject to federal minimum standards, as well as federal review, states have some flexibility to offer new service categories in Medicaid LTSS and to define the scope of existing service categories, such as assistive technology and home environmental modifications. Smart home safety technology, is a subset of smart home technology specifically designed to enhance home-based safety by detecting and preventing home-based injuries such as falls, improving security, and supporting the aging in place of older adults. Specific examples of smart home safety technology covered in Medicaid waivers included: smart devices to control light switches, smart bulbs, electronic systems that allow individuals to control appliances and security systems, and voice activated door locks. Several states are taking advantage of this flexibility to leverage smart home safety technology to support individuals with disabilities in the home. To better understand the landscape of potential smart home technology coverage within Medicaid, a review of Section 1915(c) home and community-based services waivers covering individuals with age-related and physical disability was conducted. Section 1915(c) waivers allow for the provision of services that help Medicaid LTSS beneficiaries live independently at home, such as personal care, specialized medical equipment, and transportation. This poster will present the results of a Section 1915(c) waiver services policy scan, which looked for language indicating explicit or potential coverage of smart home safety technology within Medicaid HCBS.

Abstract citation ID: igaf122.3396

### THE EXPERIENCE OF SSDI BENEFICIARIES DURING THE MEDICARE WAITING PERIOD

Sunny Kang, *The University of Baltimore, Baltimore, Maryland, United States*

This study examined the experiences of Social Security Disability Insurance (SSDI) beneficiaries during the Medicare waiting period using a mixed-methods approach. It combined semi-structured individual interviews with analyses of the National Health Interview Survey (NHIS) linked to Social Security Administration (SSA) administrative data. The Medicare waiting period represents a critical time for SSDI beneficiaries, given their poorer health and limited financial resources. In 2021, there were 572,000 new SSDI awards, with the number in the Medicare waiting period slightly lower due to deaths, recoveries, and repeat entitlements. Prior research found that before the Affordable Care Act (ACA), 25.8% of these beneficiaries lacked health insurance during this period, with male and Black beneficiaries disproportionately affected. The ACA significantly expanded insurance options, potentially altering their experiences. The quantitative component analyzed NHIS data from 2019-2024, linked to SSA's Master Beneficiary Record. We examined health insurance coverage, healthcare access, and use among SSDI beneficiaries, applying multivariate logistic regression to assess disparities. Interaction terms evaluated moderator effects related to race, ethnicity, and income. The qualitative component involved interviews with 25 SSDI beneficiaries in Baltimore, focusing on their healthcare challenges and coping mechanisms. Thematic analysis identified key barriers to care and policy implications. Findings highlighted persistent disparities in health insurance coverage and access despite ACA-related reforms. Beneficiaries who remained uninsured reported delayed care and worsened health outcomes. This study underscores the need for policy interventions to improve coverage and healthcare access for SSDI beneficiaries during the Medicare waiting period.

Abstract citation ID: igaf122.3397

### THE IMPACT OF THE SUPPORT ACT ON TREATMENT OF OPIOID USE DISORDER

Junjie Gai, and Kanika Arora, *University of Iowa, Iowa City, Iowa, United States*

On January 1, 2020, Medicare Part B began covering opioid use disorder (OUD) treatments, including medications and outpatient services, under the implementation of the Substance Use-Disorder Prevention that Promotes Opioid Recovery and Treatment for Patients and Communities (SUPPORT) Act of 2018. However, little is known about how this coverage expansion affects OUD treatment uptake among Medicare beneficiaries. This study examines whether the passage of the SUPPORT Act influenced the likelihood of medication treatment and treatment completion for OUD patients in Medicare compared to privately insured individuals. We applied a difference-in-differences and event study methodology to examine the causal impact of the SUPPORT Act among Medicare beneficiaries with OUD. We acquired datasets from 2015 to 2022 Treatment Episodes Datasets - Discharge. Our primary outcome is a binary variable (1/0) that indicates whether the use of opioid medications such as methadone or buprenorphine is part of the treatment. Other outcomes include outpatient and inpatient service, and treatment completion rate. All analyses are clustered at the state level and adjusted by covariates. After Medicare's coverage in 2020, Medicare beneficiaries with OUD were 11.9 percentage points (pp) more likely ($p < 0.05$) to receive medication for OUD relative to the privately insured group. Notably, Medicare beneficiaries are more likely to receive non-intensive services, showing a 27.1 pp increase ($p < 0.05$). Our findings indicate a positive significant effect of Medicare's expanded coverage on increasing access to medication treatment and outpatient services among older adults with OUD.

### SESSION 7520 (POSTER)

### PUBLIC HEALTH

Abstract citation ID: igaf122.3398

### DIGITAL DIVIDE OR EDUCATIONAL DIVIDE? IMPACT OF SOCIAL MEDIA ON COVID-19 VACCINATION IN OLDER ADULTS

Mason Dupre[1], Radha Dhingra[2], Truls Østbye[2], Teah Bayless[2], and Hanzhang Xu[3], *1. Cary Academy, Cary, North Carolina, United States, 2. Duke University School of Medicine, Durham, North Carolina, United States, 3. Duke University, Durham, North Carolina, United States*

The spread of disinformation and harmful content on social media is well-known. However, most research focuses on the negative influence of social media on the well-being of adolescents and teenagers. We used data from the 2022 Health and Retirement Study ($n = 4,038$) to examine how the use of social media (Facebook, Twitter [X], and Instagram) was associated with vaccination uptake for COVID-19 among U.S. middle-aged and older adults. Frequency of social media use was measured on a scale ranging from 0=never to 5=daily. In our study (mean (SD) age: 69.4 (9.7) years), approximately 36% of participants used social media daily while 34% never did. Frequent media users were more likely to be younger, women, and more educated compared with less frequent/non-users of social media. Logistic regression models showed that social media use was not directly associated with Covid-19 vaccination. However, we found a significant interaction effect that vaccination uptake for COVID-19 was significantly lower in older adults with low education who frequently used social media. Among adults who used social media daily, those with less than a high school education were significantly less likely to get vaccinated than those with a college degree (odds ratio [95% CI]: 0.35 [0.15-0.82]; P=.016). These results remained after accounting for differences in participants' sociodemographic background, health status, religiosity, and other factors. Furthermore, social media use was not associated with vaccination uptake for influenza, pneumonia, or shingles. Our results highlight the importance of delivering tailored public health messages when promoting vaccine uptake among less-educated older populations.

Abstract citation ID: igaf122.3399

### PATTERNS OF DELAYED DIAGNOSIS OF HYPERTENSION AND DIABETES AMONG MIDDLE-AGED AND OLDER ADULTS IN UK

Yuming Chen[1], Shui-kit Cheuk[2], Yanran Deng[1], and Beibei Xu[2], *1. Peking University Health Science Center, Beijing, Beijing, China (People's Republic), 2. Peking University, Beijing, Beijing, China (People's Republic)*

Delayed diagnosis can adversely impact outcomes, leading to increased complication risks. This study aimed to investigate the influence of sociodemographic factors on diagnostic delay and associations between diagnostic delay and mortality among adults aged 37-73 years. A total of 130,843 hypertension-free individuals with abnormal blood pressure (BP) biomarker and 9,909 diabetes-free individuals with abnormal diabetes-related biomarkers, aged 37-73, from the UK Biobank, were included in two cohorts. Diagnoses were obtained through linked database, coded by ICD-10, and supplemented by self-report diagnoses or medications. Abnormal BP biomarker was defined as $\geq 140/90$ mmHg; abnormal diabetes-related biomarkers included fasting glucose >7.0 mmol/L or HbA1C >48.0 mmol/mol. Logistic regression models were employed to examine the associations. During follow-up, there were 40,055 and 4,603 participants who had hypertension and diabetes diagnosis, respectively. The median diagnostic delay was 6.67 years for hypertension and 4.47 years for diabetes. Compared to individuals without cardiometabolic diseases (CMD) at baseline, those with abnormal blood pressure biomarker who had any disease of diabetes, cerebral vascular disease, and other 10 CMDs, and those with abnormal diabetes-related biomarkers who had any disease of obesity, hypertension and other 3 diseases were more likely to experience diagnostic delay. Additionally, higher levels of abnormal biomarker, older age, male, higher BMI, higher annual income, and former smoking were positively associated with diagnostic delay for both cohorts. Longer diagnostic delay, older age, higher annual income, and smoking were significantly associated with increased mortality. Baseline health conditions, age, income, and lifestyles may contribute to diagnostic delays and mortality.

Abstract citation ID: igaf122.3400

### SUBJECTIVE HEALTH STATUS AND UNMET MEDICAL NEEDS IN OLDER ADULTS WITH HYPERTENSION

Seolah Yoon[1], Min Kyung Park[1], Dahye Hong[1], Sikyeong Park[2], and Bada Kang[1], *1. Yonsei University College of Nursing, Seoul, Republic of Korea, 2. Yonsei University, Seoul, Republic of Korea*

Hypertension is a common chronic disease among older adults that necessitates regular medical care. However, when individuals need medical care but cannot access it, they experience unmet medical needs (UMN). Poor subjective health status and depression may exacerbate this situation by discouraging healthcare-seeking behaviors; however, few studies have explored this relationship. This study examined the association between subjective health status and UMN and the moderating role of depression using data from the Korea Community Health Survey (KCHS). A secondary data analysis was conducted on 44,400 individuals aged 65 and older diagnosed with hypertension. A logistic regression model was used to assess the relationship between subjective health status and UMN, incorporating depression as a moderating factor, while adjusting for covariates such as age, sex, stress level, education, and economic activity. Among older adults with hypertension, 15.6% experienced depression, and 5.0% reported UMN. Individuals with poorer subjective health were 46% more likely to experience UMN than those with better health perceptions (Odds Ratio [OR] = 1.46, 95% CI: 1.36–1.56, $p<0.001$). However, among those with depression, the impact of subjective health deterioration on UMN was 11% lower ($OR=0.89$, 95% CI: 0.78–0.98, $p=0.048$). Future research should further categorize UMN and examine the mechanisms linking subjective health status and depression to UMN. These findings can inform targeted interventions to address UMN in aging populations, emphasizing the importance of addressing psychological barriers to healthcare access.

Abstract citation ID: igaf122.3401

### TEACHING PUBLIC HEALTH AND AGING USING A WORKSHOP MODEL

Kristine Mulhorn, *Drexel University, Philadelphia, Pennsylvania, United States*

Aging populations in low- and middle-income (LMI) countries such as Cameroon face multiple adversities such as poor healthcare access, social isolation, and financial insecurity among others. To tackle these adversities necessitates an interdisciplinary methodology that incorporates varied health approaches. Thus, this paper presents insights from workshops conducted in Cameroon as part of a Fulbright program. The interdisciplinary point of view across multiple health topics was designed to facilitate collaboration among students from nursing, public health, medical laboratory science, nutrition, and mental health disciplines. The workshops provided a platform for students with hands-on experience in community health to engage in cross-disciplinary dialogue, share expertise, and develop innovative solutions tailored to the needs of older persons in Cameroon's rural and urban landscape. One aspect of the workshops explored the implications of fluctuating demographics and public health trends in older populations, particularly through the lens of the International Classification of Disability, Functioning, and Health (ICF). Participants evaluated real-world aging-related barriers, such as limited healthcare access in remote areas, the psychological effects of social isolation, and the financial constraints. Solutions included mobile healthcare clinics to address accessibility gap for older adults in rural regions, community-based social programs to reduce loneliness and enhance mental well-being, and microfinance initiatives aimed at providing financial stability for aging individuals. The workshop model fosters interdisciplinary collaboration, critical thinking, and problem-solving skills among students and the moderating professionals. This model offers a replicable framework that can ultimately contribute to more sustainable and impactful interventions for aging populations in LMI countries.

### SESSION 7525 (POSTER)

### REMINISCENCE/LIFE REVIEW

Abstract citation ID: igaf122.3402

### CAN LIFE STORYBOOK-BASED GROUP REMINISCENCE THERAPY BENEFIT OLDER ADULTS WITH MILD COGNITIVE IMPAIRMENT?

Minhee Yang[1], Eunhee Cho[1], Kyung Hee Lee[1], Bada Kang[2], Chang Gi Park[3], and Jun-Ah Song[4], *1. Yonsei University, Seoul, Republic of Korea, 2. Yonsei University College of Nursing, Seoul, Republic of Korea, 3. University of Illinois Chicago, Chicago, Illinois, United States, 4. Korea University, Seoul, Republic of Korea*

Preventing dementia in older adults with mild cognitive impairment (MCI) is critical; however, existing interventions often overlook psychosocial well-being. Life storybook (LSB)-based group reminiscence therapy (RT) provides a structured and engaging approach to support both cognitive and psychosocial health. This cluster-randomized trial aimed to evaluate the effectiveness of LSB-based group RT on cognitive function, self-esteem, depression, and social interaction among community-dwelling older adults with MCI in South Korea. Thirty-six participants were randomly assigned to either a 6-week LSB-based group RT or a control group with self-directed dementia prevention training materials. Data from 31 participants, after accounting for dropouts, were analyzed using a linear mixed-effects model. The intervention group showed significantly greater self-esteem and cognitive function improvements than the control group. However, no significant changes were observed in depression or social interaction. Qualitative findings suggested that the LSB-based group RT fostered engagement, empathy, and mutual support, potentially enhancing social interaction within the group beyond the scope of quantitative measures. While the small sample size of this pilot study may limit generalizability, these findings highlight the potential of the LSB-based group RT as an effective community-based intervention for promoting cognitive and psychosocial well-being in older adults with MCI. Future research should explore its long-term effects and broader implementation to inform innovative dementia prevention strategies.

Abstract citation ID: igaf122.3403

### INNOVATIONS IN OLDER ADULT MUSIC ANDRAGOGY: A GERONTOLOGICAL FRAMEWORK FOR ADAPTIVE LEARNING

Bei-Yun Wang, *National Chung Cheng University, Chiayi, Taiwan (Republic of China)*

This study employs an andragogical perspective and a gerontological framework to examine innovative approaches in older adult music education. Focusing on a music appreciation course at a community-based learning program for older adults, it investigates how personalized teaching strategies, diverse musical content (from classical works to film scores), and multi-sensory methods address older people's cognitive and social needs. Based on qualitative data from one instructor and eight older learners (aged 63 to 77), the findings underscore the importance of linking life histories with new musical knowledge to enhance motivation, memory retention, and interpersonal engagement. Additionally, incorporating digital platforms and extending learning to public spaces, such as concert halls, fosters a more sustained impact on learners' social interactions and quality of life. The study offers practical insights into designing adaptive andragogical practices, wherein older learners' experiences serve as a vital foundation for curriculum development, thus contributing to broader gerontological discourse and advancing lifelong learning initiatives. Gerontology, Andragogy, Older Adult Music Education, Adaptive Learning, Public Spaces.

Abstract citation ID: igaf122.3404

### LIFELONG MEMORIES THAT HELP PERSONS LIVING WITH DEMENTIA AND DEPRESSION TO ENGAGE AND THRIVE

Christina Miyawaki, and Angela McClellan, *University of Houston, Houston, Texas, United States*

Dementia and depression are common neuropsychiatric disorders in older Americans and 30% experience both conditions. Yet, interventions to address both disorders are scarce. Life review, a structured reminiscence in which people recall their lives chronologically, has demonstrated efficacy in depression. We developed a life review-based intervention, Caregiver-Provided Life Review (C-PLR), for people living with dementia and depression (PwDD) and their family caregivers. We trained caregivers in life review skills, and caregivers conducted life reviews with their PwDD at home. The purposes of this study were to report PwDD's most pleasant and challenging life review sessions and how C-PLR can provide dyads with meaningful activities despite the disorders. Forty-five dyads completed the study. PwDD were 81 years old (mean), widowed, retired, female, and in poor/fair health. Caregivers were 58 years old, married, working, college-educated, female, and in good/excellent health. Dyads enjoyed the early childhood sessions because PwDD articulated their vivid long-term memories. However, older adulthood sessions were challenging because PwDD could not recall recent events highlighting their loss of short-term memories. Observing this progressive decline in memory across the intervention, however, is beneficial because caregivers can determine the extent of their PwDD's decline, adjust their expectations, and plan their lives accordingly. With a lack of meaningful activities as a common unmet need in PwDD's lives, C-PLR can provide PwDD meaningful opportunities to share their life stories from early childhood to the present. Thus, caregivers have a significant role to play for PwDD who wish to leave a legacy in their lives.

Abstract citation ID: igaf122.3405

### PROTOCOL FOR A VIRTUAL LIFE STORY CLUB TO ADDRESS APATHY AND LONELINESS IN OLDER ADULTS

Atul Bhattiprolu[1], Eunah Song[1], Sophie Anfang[1], Samantha Yip[1], Lily Zhou[2], Jennifer Wong[2], Ying Jin[1], and Mirnova Ceïde[1], *1. Albert Einstein College of Medicine, Bronx, New York, United States, 2. Life Story Club, Brooklyn, New York, United States*

Life Story Club (LSC) offers storytelling groups as virtual reminiscence therapy (vRT) to help older adults reduce loneliness and build social connections. Apathy and loneliness are significant modifiable risk factors for dementia, contributing to cognitive decline and emotional distress. This mixed-methods feasibility study evaluates the impact of LSC's vRT on apathy and loneliness in older adults, aged 60+ and without dementia, who are at risk for social isolation. Participants (n = 18) were recruited from community organizations, with inclusion criteria requiring self-reported loneliness. vRT sessions were conducted in English and Spanish to enhance accessibility for Bronx residents. Apathy was measured using the GDS3A, a subset of the Geriatric Depression Scale, and loneliness was assessed with the UCLA Loneliness Scale. Qualitative interviews were also used to assess participant satisfaction and the perceived impact of vRT on social connections. Data were collected at baseline, with follow-up 3 months later. Follow-up data were available for 11 participants regarding apathy, 5 of whom had scores demonstrating apathy at baseline, and 10 participants regarding loneliness. In the initial phase of the pilot, 60% of participants showed stable or improved loneliness scores, whereas 64% showed stable or improved apathy scores, at 3 months compared to baseline. Participants also reported that vRT facilitated meaningful social connections, with one group organizing two in-person events. Further analysis of participants' experiences will refine the LSC protocol, informing future randomized controlled trials and potentially providing a scalable intervention to address cognitive decline, loneliness, and social isolation in the Bronx community.

Abstract citation ID: igaf122.3406

### RECALLING LIFE'S HIGHS AND LOWS IN OLDER AGE: HIERARCHICAL CLUSTERING METHODS TO TOPICS, THEMES, AND STRUCTURES

Hsiao-Wen Liao[1], Nikhil Shanbhogue[1], Hongchen Wu[1], and Thomas Oltmanns[2], *1. Georgia Institute of Technology, Atlanta, Georgia, United States, 2. Washington University in St. Louis, St. Louis, Missouri, United States*

Autobiographical memory becomes richer and more complex, forming a life story, as people develop and grow. This study applies BERTopic—a transformer-based modeling method that leverages BERT-based sentence embeddings and hierarchical clustering—to identify interrelated topics and themes in older adults' life story events of high and low points. Participants, part of the St. Louis Personality and Aging Network study (N = 468), completed a life story interview. Memories were transcribed verbatim for text analysis. Central analyses involved transforming the text into numerical representations by generating embeddings, reducing dimensions following cluster analysis, and extracting keywords for topic representation. Ward's method was used to inform hierarchical dendrograms, through which broader themes and memory structures were inferred. Results indicated that older adults' high points contained more topics and were less semantically similar (k = 8; cosine similarity ranging from 0.63 to 0.87) than their low points (k = 4; cosine similarity ranging from 0.74 to 0.9). Three distantly related (i.e., family joy, career milestones, religious fulfillment) and two tightly clustered themes (i.e., illnesses and loss, marital conflicts) respectively emerged from the topics in high and low points. This study sheds new light on applications of natural language processing techniques to the mental representations of key elements and links in life stories. Findings suggested that older adults' high points encompassed relatively loosely interconnected experiences across multiple facets of life. Experiences in low points were more thematically alike. In discussing these findings, we draw on the life story schema theory and research on autobiographical memory development.

Abstract citation ID: igaf122.3407

**THE LIFE STORY PRACTICE, EDUCATION, AND RESEARCH COLLABORATIVE: PROGRAM NEEDS AND DEVELOPMENT**

Millicent Malcolm[1], and Juliette Shellman[2], *1. University of Connecticut, Storrs, Connecticut, United States, 2. University of Connecticut, Farmington, Connecticut, United States*

Life stories play a vital role in promoting the health and well-being of older individuals, families, and communities. Formal training is essential for delivery of safe and effective life story work. The Life Story Collaborative, an emerging program of The International Center for Life Story and Innovations, aims to foster the growth of knowledge, skills, and interdisciplinary partnerships in Life Story education, research, and practice. A needs assessment conducted at our inaugural meeting identified learning needs, interests, participation goals, program suggestions, and meeting time preferences. Participants (N = 15) were from across the United States, the Netherlands, and Japan representing 5 time zones. Ten of fifteen participants completed the survey. The group consisted of academic researchers and educators, clinicians, ethnographers, and product and service innovators. Participants shared interests for professional development in life story approaches to enhance health outcomes in older adults including end-of-life, safe practices with culturally diverse and underrepresented groups, networking and resource sharing, and disseminating work to advance and draw attention to the field. Suggestions for future meetings included specific guest speakers and topics such as use of artificial intelligence, brain research, practice methods, and funding mechanisms. Positive comments were shared about the meeting and future directions for this group. Results indicate there is a wide variety of interests in the life story field, but few opportunities for formal learning, engagement and collaboration. We have taken the first step to fill this gap with a needs assessment that will guide the development and implementation of The Life Story Collaborative.

### SESSION 7530 (POSTER)

### SENSORY HEALTH (VISION, HEARING)

Abstract citation ID: igaf122.3408

**HEARING AID USE AND VERBAL FLUENCY AS A MEASURE OF COGNITIVE FUNCTION: A CROSS-SECTIONAL STUDY USING SHARE DATA**

Meghana Rajashekara Swamy, and Adam Mecca, *Yale University, New Haven, Connecticut, United States*

Background: Hearing aids may mitigate cognitive decline risk. We analyzed its association with verbal fluency, as a surrogate measure for cognition, using the Survey of Health, Ageing, and Retirement in Europe (SHARE) data. Methods: This cross-sectional study analyzed SHARE Wave 9 (2021–2022) data. Verbal fluency, measured via an animal naming task, was the primary outcome. Self-reported hearing aid use was the primary exposure. Exclusions included dementia, missing data, or age < 50 years. Multivariable linear regression adjusted for demographic, social, and cardiovascular health factors. Analyses used Python 3.9, with $\alpha = 0.05$ for significance. Results: After exclusions, 57,433 participants remained. Hearing aid users were older (mean age 76.9 vs. 68.2 years), more often male (50.9% vs. 42.5%), had smaller households (1.77 vs. 2.01), higher net worth (€410,960 vs. €305,221), were less likely to have a household partner (61.2% vs. 68.0%), and reported poorer hearing (28.7% vs. 16.5%). Hearing aid users exhibited higher verbal fluency scores ($\beta = 0.92$; 95% CI: 0.18–1.67; $p = 0.015$). Better hearing level correlated positively with verbal fluency ($\beta = 0.38$; $p < 0.001$), but its interaction with hearing aid use was nonsignificant ($p = 0.099$). Statistically significant predictors of higher verbal fluency included better social connectedness, education, physical activity, and household net worth, while older age, male gender, stroke, diabetes, hypertension, and larger household size predicted lower performance. Conclusion: Hearing aid use is linked to better verbal fluency, highlighting opportunities for hearing interventions in cognitive rehabilitation and independence. These findings may reflect reduced cognitive load from treatment, reinforcing hearing loss interventions and expanded hearing aid coverage.

Abstract citation ID: igaf122.3409

**HOMEBOUND RISK AND SAFETY HAZARDS IN OLDER ADULTS WITH VISION IMPAIRMENT**

Jonathan Thomas[1], Xindi Chen[2], Louay Almidani[2], Seema Banerjee[2], Zhuochen Yuan[2], Aleksandra Mihailovic[2], and Pradeep Ramulu[2], *1. Texas A&M University Health Science Center, Bryan, Texas, United States, 2. Johns Hopkins University School of Medicine, Baltimore, Maryland, United States*

Homebound status is associated with multiple risk factors and worse health outcomes. However, limited knowledge exists about the association between vision impairment (VI) and becoming homebound. Therefore, this study examined the associations between VI and homebound status, utilization of home-based care, presence of tripping hazards, and likelihood of transitioning to homebound status in United States (US) older adults. Longitudinal data was collected from the 2021-2023 National Health and Aging Trends Study and included Medicare beneficiaries ≥71 years. The main exposure was any VI, defined as impairment in either distance (DVA) or near (NVA) visual acuity (>0.3 logMAR), or contrast sensitivity (CS;< 1.55 logCS). Outcomes included homebound status, presence of safety hazards, and utilization of home-based care. 3,002 participants (mean [SD] age, 78.8 [5.6] years; 55.3% female; 81.8% non-Hispanic White) were included in our study. The prevalence of being homebound was higher among those with any VI (9.2%,95%CI:7.3-11.5%) compared to no VI (4.0%,95%CI:3.0-5.3%). In multivariable logistic regression models, individuals with any VI had a significantly higher risk of becoming homebound or semi-homebound (HR = 1.35,95%CI:1.04-1.74,p=.02) compared to peers without any VI. Likewise, participants with any VI had a greater likelihood of having home hazards (OR = 1.88,95%CI:1.32-2.69, p=.001) and utilizing home-based care (OR = 1.31,95%CI:1.06-1 .62,p=.01) compared to those without any VI. In conclusion, older US adults with VI were more often homebound, more likely to become homebound, had more home hazards, and more often utilized home-based care, highlighting the need to holistically evaluate a patient when determining the need for home care and tailoring interventions to maximize independence.

Abstract citation ID: igaf122.3410

**LANCET COMMISSION ON HEARING LOSS: D/DEAF AND HARD OF HEARING STIGMA MEASUREMENT TOOLS**

Jessica West[1], Rachel Stelmach[2], Melissa Stockton[3], Howard Francis[1], John Kraemer[4], Khalida Saalim[2], Elizabeth Troutman Adams[2], and Laura Nyblade[2], *1. Duke*

*University School of Medicine, Durham, North Carolina, United States, 2. RTI International, Durham, North Carolina, United States, 3. University of Pennsylvania Perelman School of Medicine, Philadelphia, Pennsylvania, United States, 4. Georgetown University, Washington, District of Columbia, United States*

The Lancet Commission on Hearing Loss Measures, Models, and Reduction of Stigma Subgroup aims to advance understanding of d/Deaf or hard of hearing (d/DHH) stigma and actions to address it. We identified a lack of tools to measure the different types of stigma faced by d/DHH people and their close associates. Here, we describe the development and preliminary validation of novel d/DHH stigma measurement tools. We used a multistep exploratory sequential design: survey development, modified Delphi process, cognitive interviewing, and pretesting. Psychometric validation occurred in the U.S. (high-income country) and Ghana (low-middle income country). Stigma measurement tools were developed for 1) d/DHH individuals (experienced, perceived, internalized, anticipated hearing device-related stigma [HDRS]); 2) care partners of d/DHH adults (observed and perceived stigma toward d/DHH people; care partners' internalized, experienced, perceived stigma; perceived HDRS); 3) parents of d/DHH children (parental observations and perceptions of stigma; parental secondary experienced, perceived, internalized stigma; perceived HDRS); and 4) health care providers (HCPs) (perceived and enacted stigma; secondhand patient-experienced stigma; secondhand patient-perceived stigma; perceived HDRS). Additional ageism scales measure d/DHH peoples' experienced ageism and observed ageism among care partners, HCPs, and the general population. These tools, available in Ear and Hearing, fill a critical gap in d/DHH stigma measurement. Ongoing research using these tools will enable monitoring of d/DHH stigma, refinement of interventions, and assessment of societal attitudes toward d/DHH people. Leveraging these tools in clinical and public health settings can help reduce stigma, improve patient experiences, and lead to better overall outcomes in hearing healthcare.

Abstract citation ID: igaf122.3411

### SELF-REPORTED HEARING LOSS AND EMOTIONAL DISTRESS IN OLDER ADULTS: EVIDENCE FROM THE HEALTH AND RETIREMENT STUDY

Hannah Crowder, and Jessica West, *Duke University School of Medicine, Durham, North Carolina, United States*

Hearing loss (HL) is the most prevalent sensory disability in U.S. older adults and is associated with negative markers of emotional distress including depression, loneliness and anxiety. While research typically focuses on these global emotional states, less studied are emotions like anger. Anger tends to be experienced by older adults when they face changes in health status (e.g., HL). Understanding the extent to which HL is linked to anger expression is important, as translational studies have shown anger to be a predictor of negative health outcomes such as cardiovascular disease events and stroke. The current study uses wave-based mixed models to estimate 1) the association between HL and hearing aid use on expression of internalized anger (anger-in) and externalized anger (anger-out) and 2) how this association varies by gender using nationally-representative, longitudinal data from 10,721 adults aged 50 and older in the Health and Retirement Study (HRS) (2006-2012). The sample is predominantly female (68.4%) and participants self-reported normal hearing (85.1%), well-aided HL (1.3%), unaided HL (13.0%), or poorly aided HL (0.6%). Mixed effects models indicate that participants with unaided or poorly-aided HL reported greater anger-in ($\beta$=.06, p<.0001; $\beta$=.13, p<.010, respectively) and anger-out ($\beta$=.05, p<.001; $\beta$ =.12, p≤.001, respectively) compared to those with normal hearing (no difference observed for well-aided HL), net of covariates. Greater on average anger-in ($\beta$=.03, p=.031) and anger-out ($\beta$=.05, p<.001) was experienced among male compared to female respondents. Findings underscore the importance of effective hearing interventions, as poor hearing—especially when untreated—is associated with increased anger expression.

Abstract citation ID: igaf122.3412

### THE ASSOCIATION BETWEEN HEARING AND DRIVING PERFORMANCE AMONG OLDER ADULTS

Richard Marottoli[1], Margaret Doyle[1], and Margaret Wallhagen[2], *1. Yale University School of Medicine, New Haven, Connecticut, United States, 2. University of California, San Francisco, San Francisco, California, United States*

The ability to continue to drive is often essential to an older adult's independence yet may pose a safety risk. Visual and cognitive impairment have been associated with driving performance and crashes, yet less is known about the potential effects of hearing loss. We examined the association between hearing and on-road driving performance scores among 494 community living adults aged >70. Eligibility criteria included cognitive status of > 18 on the Mini-Mental State Examination and visual distance acuity > 20/70. Hearing was assessed by the whisper test (6 numbers/ear, possible range 0-12 correct). Driving performance was rated on a previously validated 35-item scale derived from the Connecticut Department of Motor Vehicles exam (scored 0-70, higher score better). Participants had a mean age of 78.6; 87% were male. In negative binomial regression models the number of errors on the hearing test was significantly associated with poorer driving scores (Rate Ratio (CI)=1.03 (1.02, 1.05)). This association was attenuated in a model adjusted for potential demographic and clinical confounders, including cognitive function. With the addition of an item indicating whether the driver understood oral instruction, per the evaluator, the adjusted association became only marginally significant. Findings suggest that hearing deficits may be associated with poorer driving performance and possibly mediated by the inability to adequately hear test instructions. Further research is needed to explore this relationship to assure older adults are not disadvantaged during driving tests.

Abstract citation ID: igaf122.3413

### UNMET HEARING AID NEED LINKED TO LOWER COMMUNITY ENGAGEMENT SATISFACTION IN VULNERABLE OLDER ADULTS

Dana Urbanski[1], Jack Wolf[2], Romil Parikh[2], Benjamin Langworthy[2], Eric Jutkowitz[3], and Tetyana Shippee[3], *1. Indiana University Bloomington, Bloomington, Indiana, United States, 2. University of Minnesota School of Public Health, Minneapolis, Minnesota, United States, 3. University of Minnesota, Minneapolis, Minnesota, United States*

Hearing loss is associated with dementia, possibly by driving social isolation that contributes to cognitive decline. Hearing aids can improve social engagement among older adults with hearing loss, yet only one-third of those who could benefit from hearing aids use them—often due to barriers such as cost, access, and lack of social support. Consequently, many vulnerable older adults report unmet hearing aid need; however, little research has examined social/community engagement in this population, which may be amenable to treatment but has not received hearing aids. This study examined a single-item measure of community engagement satisfaction among older adults receiving publicly funded home- and community-based services (HCBS; an indicator of social and financial vulnerability) stratified by self-reported hearing aid need. Using data from the 2017-2019 National Core Indicators-Aging and Disability Adult Consumer Surveys, the sample included 10,861 HCBS users aged ≥65 years, 14% of whom reported unmet hearing aid need. Unadjusted satisfaction rates were lower for those who reported unmet need (38.8%) compared to those who reported not needing hearing aids (49.4%) or already using hearing aids (52.3%). Adjusting for covariates, a Poisson model with robust standard errors pooled across multiple imputations revealed that HCBS users reporting unmet need were still 12% (95% CI, 5%-19%) and 17% (95% CI, 11%-24%) less likely to report satisfaction with their community engagement compared to those who reported not needing or already using hearing aids, respectively. Addressing unmet hearing aid need among vulnerable older adults may promote social and community engagement.

Abstract citation ID: igaf122.3414

### URBAN-RURAL DISPARITIES IN SENSORY DISABILITY AND FATAL CAR CRASHES AMONG OLDER ADULTS IN MISSISSIPPI

Wuyi Dong[1], Yan-Jhu Su[2], Taylor Jansen[1], and Elizabeth Dugan[1], *1. University of Massachusetts Boston, Boston, Massachusetts, United States, 2. The Pennsylvania State University, University Park, Massachusetts, United States*

Understanding urban-rural disparities in sensory disabilities and traffic safety is essential for developing targeted interventions that enhance mobility and quality of life among older adults. Using data from the 2023 Mississippi Healthy Aging Data Reports (HADR), this cross-sectional study examines county-level cases of fatal car crashes involving individuals aged 60 and older and the rates of self-reported sensory difficulties (vision and hearing impairments) in Mississippi. Analyzing 82 counties (17 urban, 65 rural), we identify variations in fatal crash (urban: 15.76; rural: 7.88; state average: 9.51) and sensory difficulties (state averages—hearing: 15.5%, vision: 8.7%; urban ranges—hearing: 8.5%–27.7%, vision: 3.1%–15.1%; rural ranges—hearing: 5.4%–35.0%, vision: 0%–20.6%). Bivariate mapping overlays these data to pinpoint counties with both high fatal crash rates and sensory impairment prevalence. This exploratory analysis offers critical insights into the intersection of sensory disabilities and traffic safety, guiding policymakers and service providers in prioritizing transportation interventions and support services for older adults in urban and rural communities.

### SESSION 7535 (POSTER)

### SOCIAL AND HEALTH DISPARITY

Abstract citation ID: igaf122.3415

### AGING ABROAD: DO EASTERN EUROPEANS EXPERIENCE THE HEALTHY IMMIGRANT EFFECT?

Adriana Scanteianu, *University of Pennsylvania, Philadelphia, Pennsylvania, United States*

As the European continent continues to undergo population aging, there is a growing number of migrants who are also aging alongside the native population. Immigrants occupy a unique socioeconomic position and often face health risks and care access issues that are poorly understood, yet critical to informing policy and facilitating healthy aging. In particular, post-Communist Eastern European migration has received little attention in the public health literature despite constituting a sizable immigrant population in Western Europe and Israel. Furthermore, existing literature points to Eastern Europeans as potentially presenting an exception to the "healthy immigrant effect," whereby immigrants usually experience healthier and longer lives than native-born populations despite often having relatively lower socioeconomic status and lacking institutional support. Using data from the Survey of Health, Aging, and Retirement in Europe (SHARE), which provides longitudinal coverage of all EU countries and Israel, this study aims to understand whether Eastern European migrants in Western Europe and Israel experience a health disadvantage compared to other migrant groups and the native-born populations. Findings reveal stark disparities in health outcomes between Eastern European migrants when compared to other migrant groups, as well as the native-born populations in Western Europe and Israel, including differences in mental health, disability, and chronic disease prevalence. These results have strong policy implications, as they imply distinct health and social support needs for the Eastern European migrant population in receiving countries.

Abstract citation ID: igaf122.3416

### ASSOCIATIONS OF DISCRIMINATION AND PAIN AMONG OLDER ADULTS: RESULTS FROM THE EINSTEIN AGING STUDY

Linying Ji[1], Monique Balthazar[2], Yuqi Shen[2], Margeaux Gray[2], Richard Lipton[3], Jennifer Graham-Engeland[2], Carol Derby[3], and Orfeu Buxton[2], *1. Montana State University, Bozeman, Montana, United States, 2. The Pennsylvania State University, University Park, Pennsylvania, United States, 3. Albert Einstein College of Medicine, Bronx, New York, United States*

Chronic pain among older adults varies by race and sex, suggesting the value of examining social determinants (e.g. discrimination) that may drive pain-related outcomes. We examined how discriminatory experiences influence pain reported in daily life and whether those associations vary by race and sex. Participants (N=296, M_age=77.5 years, 69% women; 46% nonhispanic White, 41% nonhispanic Black, 13% Hispanic/other) are from the Einstein Aging Study. Using ambulatory mobile assessments (6 daily, 2 weeks), person-level average scores were estimated for pain intensity ( mean level, peak), pain interference with cognition (PIC), pain interference with function (PIF). A modified Williams' Everyday Discrimination questionnaire assessed the frequency and number of reasons for discrimination. Linear regressions, stratified by race and sex, were used to test associations between pain and discrimination, controlling for age, education, BMI, pain medication use, and chronic diseases. Among women, discrimination frequency was associated with PIF; number of reasons for discrimination was associated with average pain intensity and PIF. For men, discrimination frequency was associated with PIC. Among Blacks, discrimination frequency was associated with peak pain and PIC; number of reasons for discrimination was associated with pain intensity and PIF. For White participants, discrimination frequency (not number of reasons for discrimination) was associated with average pain intensity, PIC, and PIF. Discriminatory experiences are associated with increased pain reports that differ by race and sex. Discriminatory experiences had stronger associations with pain for women than men. Number of reported reasons for discriminatory experiences was more strongly associated with pain outcomes in Black participants.

Abstract citation ID: igaf122.3417

### DEPRESSION DISPARITIES IN OLDER KOREAN AND CHINESE IMMIGRANTS IN THE UNITED STATES

Ruimin Yang[1], Jeongone Seo[2], Shirley Qiu[3], and Gracie Shao[4], *1. Columbia University, Teachers College, New York, New York, United States, 2. Sungkyukwan University, Seoul, Republic of Korea, 3. University of California, Santa Barbara, Santa Barbara, California, United States, 4. The University of British Columbia, Vancouver, British Columbia, Canada*

This meta-analysis compares depression levels among Chinese and Korean immigrants aged 60 and older in the United States. Studies published between 2010 and 2024 were synthesized using standardized mean differences (SMDs) from a random-effects model. Korean immigrants reported higher depressive symptoms than Chinese immigrants when assessed with the Patient Health Questionnaire (PHQ) (SMD = 0.0778, SE = 0.0193, p < .0001, 95% CI [0.0409, 0.1147]), but no significant difference emerged with the Geriatric Depression Scale (GDS) (SMD = -0.0060, SE = 0.0625, p = 0.9233, 95% CI [-0.1284, 0.1164]). Within-group analyses indicated that GDS produced higher depression scores than PHQ for both groups. Among Chinese immigrants, PHQ scores were significantly lower than GDS scores (SMD = -0.2430, SE = 0.0411, p < .0001, 95% CI [-0.3235, -0.1625]), suggesting PHQ may underestimate depression. Likewise, Korean immigrants recorded lower PHQ scores than GDS scores (SMD = -0.1601, SE = 0.0569, p = 0.0049, 95% CI [-0.2715, -0.0486]). Korean immigrants' CES-D scores were also lower than GDS scores (SMD = -0.0998, SE = 0.0417, p = 0.0167, 95% CI [-0.1816, -0.0181]). Potential contributors included acculturation, social support, socioeconomic status, language barriers, healthcare availability, and intergenerational dynamics. These findings underscore the importance of culturally tailored screening and interventions. Research should address measurement disparities, investigate longitudinal patterns, and develop targeted strategies to reduce depression among older Asian immigrants. This could inform policies and optimize mental health outcomes for this vulnerable population. Clinicians and policymakers should prioritize culturally responsive mental health screening, interventions, and policy reforms.

Abstract citation ID: igaf122.3418

### DISPARITIES IN PREVENTIVE HEALTHCARE UTILIZATION & OUTCOMES AMONG SEXUAL MINORITY AND HETEROSEXUAL OLDER ADULTS

Julia Fox, and Jing Li, *University of Washington, Seattle, Washington, United States*

Background: Research on health disparities among older sexual minority adults (SMA) remains limited. Evidence suggests SMA may have lower preventive healthcare utilization and poorer health outcomes than heterosexual peers, partly due to discomfort in medical settings. However, comprehensive studies outside HIV/AIDS and mental health remain scarce. Objective: This study examines differences in health outcomes and healthcare utilization between SMA and heterosexual older adults using Health and Retirement Study (HRS) data. Methods: Data from the 2018–2022 HRS surveys were analyzed to compare self-reported health, chronic conditions, and preventive healthcare utilization (e.g., vaccinations). Descriptive statistics and Pearson's t-tests assessed group differences, with adjusted models planned. Results: The sample includes 463 SMA and 8,755 heterosexual older adults (mean age: 70, 60.6% female). SMA were significantly less likely to receive a shingles vaccine and more likely to have missed a flu shot (p < 0.05), with no differences in COVID-19 or pneumonia vaccinations. SMA had higher rates of poor self-rated health, hypertension, diabetes, and cancer (p < 0.05) but lower arthritis prevalence (p < 0.05). No differences were found in chronic pain, osteoporosis, or glaucoma. Conclusions: SMA experience disparities in some preventive healthcare measures and health outcomes, though patterns vary. These findings highlight the need for targeted interventions and further research to understand underlying factors.

Abstract citation ID: igaf122.3419

### DYNAMIC SURVIVAL PATTERNS IN RURAL-URBAN HEALTH DISPARITIES AMONG CHINESE OLDER PEOPLE

Shibin Yan, *University of Pennsylvania, Philadelphia, Pennsylvania, United States*

China's rapidly aging population faces significant rural-urban health disparities, yet limited research has applied survival analysis to assess their impact on longevity. This study investigates how rural-urban disparities influence life expectancy among Chinese older adults (aged 65+) using data from the Chinese Longitudinal Healthy Longevity Survey (CLHLS, 2005–2010). Survival analysis, including Kaplan-Meier estimates and Cox proportional hazards models, was employed to compare survival experiences and identify predictors of mortality. The sample comprised 7,472 older adults (3,408 urban, 4,064 rural). Results revealed dynamic survival patterns: urban residents exhibited higher survival probabilities between years 10–13, but rural older adults showed marginally better survival after 14 years. Cox regression found urban residence associated with a 10.7% higher mortality hazard (HR = 1.11, p = 0.01), contrary to initial hypotheses (H1: significant rural-urban disparities; H2: urban advantage in longevity). Key predictors of longevity included age, financial sufficiency (HR = 0.86, p < 0.001), female gender (HR = 0.80, p < 0.001), and better self-rated health (HR = 0.94, p = 0.004). Social networks in rural areas may partly explain later-life survival advantages. The study highlights the complex role of geographic disparities and socioeconomic factors in shaping longevity. Findings underscore the need for policies addressing structural inequities in healthcare access while leveraging rural social capital to improve older adults' well-being. This research contributes novel insights by applying survival analysis to rural-urban health disparities in China, emphasizing temporal variations in mortality risks and advocating for targeted interventions to reduce health inequalities.

Abstract citation ID: igaf122.3420

### EARLY-LIFE DISADVANTAGES AND LATE-LIFE CARDIOMETABOLIC HEALTH AMONG OLDER ADULTS IN BRAZIL

Brenda Marques[1], and Mateo Farina[2], *1. The University of Texas at Austin, Austin, Texas, United States, 2. University of Texas at Austin, Austin, Texas, United States*

Exposures to early-life disadvantages, including low parental socioeconomic status (SES), have been linked to long-term health consequences through multiple physiological and social pathways, contributing to higher mortality risk and increased cardiometabolic dysfunction. However, research on these associations remains limited, particularly in low- and middle-income countries. Hence, research on the long-term effects of early-life SES on cardiometabolic risk factors in late adulthood remains scarce and nevertheless needed, with some works calling for attention to the role of socioeconomic-related childhood adversities in cardiometabolic health among Brazilian adults (Andrade et al., 2016). Using data from the 2015 wave of the Brazilian Longitudinal Study of Aging (ELSI-Brazil), this study examines one source of early-life disadvantage and its association with cardiometabolic health among 1408 older Brazilian adults. We have applied generalized linear regression models for continuous metabolic health parameters and logistic regression models for categorical outcomes. Preliminary findings are mixed: on the one hand, having parents with at least some education is protective against stroke risk in comparison to individuals whose parents never went to school and individuals whose parents had bachelor's degree. When it comes to diabetes, individuals whose parents had bachelor's degree or higher education attained have 43.7% higher odds of diabetes compared to individuals whose parents never went to school.

Abstract citation ID: igaf122.3421

### EXAMINING INTERSECTIONAL ROLE OF RACE, ETHNICITY, AND SEXUAL ORIENTATION IN HEALTH DISPARITIES AMONG OLDER ADULTS

Hyun-Jun Kim[1], Hailey Jung[1], Austin Oswald[2], and Karen Fredriksen-Goldsen[1], *1. University of Washington, Seattle, Washington, United States, 2. Dalhousie University, Halifax, Nova Scotia, Canada*

Lesbian, gay, and bisexual (LGB) older adults who are racially/ethnically minoritized may face heightened health disadvantages due to their combined impact inclusive of the risks due to each social position and any excess risks beyond (i.e., synergistic effect), yet empirical evidence remains scarce. To address this gap, we used the 2012-2023 National Health Interview Survey to examine intersectional health disparities among Hispanic, Black, Asian, and other individuals of color within LGB adults age 50+ in the United States. Using non-Hispanic White heterosexuals as the referent and stratifying by gender, we estimated joint health disparities by sexual orientation and race/ethnicity and tested synergistic effects while adjusting for age, education, and income. Among older women, we observed joint disparities, predominately driven by synergistic effects, in cardiovascular disease for other LGB individuals of color (risk difference [RD] 15.5 percentage points [pp], 95% CI 5.5-25.6). Among older men, joint disparities, largely driven by synergistic effects, were observed in asthma (6.1 pp, 95% CI 0.0-12.2) and mental distress (16.4 pp, 5.5-27.3) for Hispanic LGB individuals; cognitive (12.7 pp, 3.0-22.4) and vision impairments (3.4 pp, 3.7-23.1) for Black LGB individuals; and limited physical functioning (15.1 pp, 3.2-26.9), anxiety (28.8 pp, 12.8-44.9), depression (24.5 pp, 8.4-40.5), and poor general health (21.9 pp, 8.1-35.7) for other LGB individuals of color. Overall, health disparity patterns in LGB older adults varied across race/ethnicity, lending some support for the synergistic multiple disadvantage hypothesis. There is a need for future research on intersectionality to identify modifiable mechanisms to eliminate health disparities.

Abstract citation ID: igaf122.3422

### MEDIATORS OF RACIAL DISPARITIES IN HOSPICE USE DURING THE LAST SIX MONTHS OF LIFE

Zhiwei Hu[1], Chengming Han[2], Efstathia Polychronopoulou[2], Mukaila Raji[3], and Yong-Fang Kuo[4], *1. The University of Texas Medical Branch, Galveston, Texas, United States, 2. UTMB Health, The University of Texas Medical Branch, Galveston, Texas, United States, 3. University of Texas Medical Branch at Galveston, Galveston, Texas, United States, 4. University of Texas Medical Branch, Galveston, Galveston, Texas, United States*

Racial disparities remain a concern in hospice care use, especially during the last six months of life. Social determinants of health factors such as low income and suboptimal housing (e.g., inconvenient housing conditions: lacking elevators in multi-level building, non-private bathrooms, or living in multi-level building) impact utilization, but their roles are not fully understood. We analyzed 2015–2020 Medicare Current Beneficiaries Survey data from 1,736 deceased beneficiaries (1,418 white, 318 non-white). Mediation analyses were conducted to test whether income or housing inconvenience explained racial differences in hospice use in the last six months of life. Causal mediation analyses adjusted for confounders, and diagnostic checks ruled out multicollinearity. Overall, 48% of patients used hospice. After adjustment for age, sex, and region, non-white patients were 25% less likely to use hospice. Income partially mediated this disparity (NIE = 1.0687, $p = 0.0576$), accounting for approximately 24% of the total effect. Housing inconvenience exhibited a negligible mediation effect (NIE = 1.0018, $p = 0.8794$). In fully adjusted models, older age (OR = 1.039, 95%CI [1.031, 1.051]) and higher income (OR highest income = 1.423, 95%CI [1.091, 1.856]) were significantly associated with hospice use. Higher housing inconvenience scores were associated with 48.1% lower likelihood of hospice use (OR = 0.519, 95%CI [0.352, 0.764]). Racial disparities in hospice utilization persist during the last six months of life. Income partially mediates these differences, whereas housing inconvenience may directly influence hospice use.

Abstract citation ID: igaf122.3423

### THE AMERICA I KNOW COULD USE A GOOD CRY: IMPACT OF STATE AND CIVILIAN-PERPETRATED RACIAL VIOLENCE ON COGNITIVE HEALTH

Muriel Taks Calle[1], Betselot Wondimu[1], Cailyn Clemons[1], and Paris Adkins-Jackson[2], *1. Columbia University Mailman School of Public Health, New York, New York, United States, 2. Mailman School of Public Health, Columbia University, New York, New York, United States*

From a life course approach and relying on the Weathering Hypothesis as a primary pathway, this study acknowledges that cumulative stressful and fear-related experiences, whether endured directly or indirectly, have a detrimental impact on the individual's health. Prior investigations show that exposure to the American criminal justice system experienced by individuals racialized as Black correlates with adverse cognitive effects. This analysis seeks to highlight not only the role of governmental and institutional bodies in perpetuating structural racism but also the contributions of civilian actors to racial terror which in turn, increases the chances of cognitive impairment among those racialized as Black. We estimate the association between living in a county with a history of structural racism and racially disproportionate incarceration rates (in the decade preceding the passing of the 1994 Crime Bill) and having at least one incident of cognitive impairment (2008-2020), based on cognitive test performance scores from the Health and Retirement Study (HRS). Binomial logistic regression results suggest that participants racialized as Black in counties with high structural racism (lynching history, and disproportionate deaths due to police violence) and disproportionate incarceration have 21.3 times higher odds ($\beta = 3.059$; 95%CI [0.242, 5.875]) of having at least one incident of cognitive impairment compared to those racialized as Black in counties with the same conditions except disproportionate incarceration.

Abstract citation ID: igaf122.3424

### THE NEUROBIOLOGY OF SOCIAL ADVERSITY AND DISPARITY: ANALYZING SOCIAL-STRESS AND HEALTH INEQUITIES

Christina Anurum-Anyanwu[1], and Nasim Ferdows[2],
*1. Institute for Health Equity and Social Justice, Union, New Jersey, United States, 2. Northeastern University, Boston, Massachusetts, United States*

Social stress, stemming from institutional and interpersonal hardships, has been shown to diminish long-term health and increase mortality. These adverse health outcomes are strongly linked to dysregulation in neurochemical stress pathways and can include anxiety, depression, cardiovascular disease, metabolic disorders, and immune deficiency. As exposure to such adversity is often manifested in social inequities, the focus of this analysis, aims to examine how this harm is further exacerbated within historically marginalized populations especially amongst racial and ethnic minorities. This study explores the neurobiological and psychosocial mechanisms underlying the effects of social and environmental inequities on long-term health in disenfranchised communities.

Abstract citation ID: igaf122.3425

### THE ROLE OF LANGUAGE IN THE HEARING-COGNITION ASSOCIATION: HISPANIC COMMUNITY HEALTH STUDY/STUDY OF LATINOS

Pablo Martinez-Amezcua[1], Xi Wang[2], Laura Coco[3], Ariana Stickel[3], Hector Gonzalez[4], Linda Gallo[3], and Daniela Sotres-Alvarez[5], *1. Johns Hopkins Bloomberg School of Public Health, Baltimore, Maryland, United States, 2. Johns Hopkins University Bloomberg School of Public Health, Baltimore, Maryland, United States, 3. San Diego State University, San Diego, California, United States, 4. University of California, San Diego, San Diego, California, United States, 5. The University of North Carolina at Chapel Hill, Chapel Hill, North Carolina, United States*

Background: Hearing loss (HL) is linked to lower cognitive function, yet most research has focused on non-Hispanic/Latino White adults and English speakers. In 2020, 60 million Hispanic/Latino individuals lived in the US, including 16 million with limited English proficiency. Lower English acculturation—a proxy for proficiency—is associated with poorer cognitive performance, but its role in moderating the HL–cognition relationship remains unclear. Methods: We analyzed cross-sectional baseline data from 8,089 Hispanic/Latino adults aged 45 to 74 (mean age = 55; 61% female; 86% Spanish speakers) from the multi-site Hispanic Community Health Study/Study of Latinos cohort. HL was defined as a better-ear pure tone average >25 dB. Language acculturation was assessed using the language domain of the Short Acculturation Scale for Hispanics and categorized into tertiles. We derived global cognitive function scores (z-transformed) from a neurocognitive battery. We used survey-weighted regression models, adjusted for demographics and health factors, to examine the interaction between HL and acculturation tertiles on cognitive function. Results: Among individuals with high acculturation, those with HL (prevalence 14%) had a 0.045-unit lower cognitive score than those without. HL and cognition associations were stronger for those with medium and low acculturation (0.097 and 0.099 units lower cognitive scores, respectively). However, the differences in the HL coefficients across acculturation levels were not significant ($\beta$ HL* medium = –0.052 and $\beta$ HL* low = –0.054, $p > 0.05$ for both). Conclusions: Among Hispanic/Latino adults, HL is associated with lower cognitive function. The heterogeneity in this association across acculturation levels was not statistically significant.

### SESSION 7540 (POSTER)

### SOCIAL SERVICES: POLICY, FINANCING, AND DELIVERY SYSTEMS

Abstract citation ID: igaf122.3426

### AN UPDATED NEEDS ANALYSIS OF MICHIGAN'S AREA AGENCIES ON AGING: SUPPORTING THOSE WHO SUPPORT OTHERS

Natalie Delemeester[1], Joan Ilardo[2], and Angela Zell[2],
*1. Michigan State University, Okemos, Michigan, United States, 2. Michigan State University, East Lansing, Michigan, United States*

Background: As individuals age, they often develop more complex medical needs that may necessitate assistance with activities of daily living. Such assistance has traditionally been provided by children, and in 2019, there were an estimated 11 million Americans caring for both their own children and their parents. The Michigan Area Agencies on Aging (AAA) are an instrumental part of connecting caregivers with resources such as respite care, caregiver training, and care plan monitoring and development. Methods: In 2020 and in 2024, a needs assessment of the 16 AAAs in Michigan was conducted via an online questionnaire sent to each AAA director. In 2020, all 16 responded. In 2024, nine responded. The data from the nine from both points in time were then compared. Results: The responding AAAs prioritized providing respite care and caregiver training in 2020 and 2024. The AAAs reported a 50% decrease of in-person provision of services with a corresponding growth of 125% increase in telephone and use of online service provision. This was in the setting of the elimination of phone and online services from POS contracts. There was also a 228% increase in persons from POS contracts doing caregiver actives who were identified as "other". All nine AAAs predicted future growth over the next three years. Conclusion: Understanding the needs of the AAAs is essential for continued governmental planning and distribution of funds. The AAAs anticipate increased future demand and are expanding how they reach caregivers while also utilizing more diverse contracted workers.

Abstract citation ID: igaf122.3427

### CITIZEN-LED INTERVENTION TO ENHANCE SOCIAL ENGAGEMENT OF OLDER ADULTS IN DEPOPULATED RURAL AREAS IN KOREA

Suhyeon Choi[1], Susan Park[2], Sunyoung Park[1], and Soong-nang Jang[1], *1. Chung-Ang University, Seoul, Republic of Korea, 2. Inha University Medical School, Incheon, Republic of Korea*

Background This study evaluated the effectiveness of a citizen-led intervention designed to enhance social engagement and improve health among older adults in the depopulated areas of Jeongeup City, Korea. These areas face population aging, economic stagnation, and limited infrastructure, increasing healthcare and social service needs. Method The citizen-led intervention included 1) assessing the care needs of older adults, 2) coordinating necessary healthcare and social welfare services accordingly, and 3) implementing a 12-week group frailty management program at community centers. Baseline and follow-up measures were conducted in November 2023 and August 2024, respectively, among older adults aged 65 years and older. Social engagement, subjective health status (SHS), and care fulfillment were measured. The treatment and control groups were conveniently assigned 157 and 2,474 participants, respectively. To ensure group comparability, propensity score matching (1:3) was performed based on demographic and health factors (age, sex, disability, economic status, the number of chronic diseases, etc.), resulting in 267 matched control participants. Results Social engagement significantly increased in the treatment group ($\beta = 0.24$, $p = 0.016$). Care fulfillment was significantly higher in the treatment group ($M = 8.34$, $SD = 1.61$) than in the control group ($M = 6.11$, $SD = 1.81$, $p < 0.01$). SHS declined over time ($OR = 0.54$, $p < 0.01$), with no significant difference between groups. Conclusion Citizen-led interventions improved social engagement and care fulfillment but had limited effects on health outcomes. Trained community members may play a role in addressing care needs by organizing local support networks.

Abstract citation ID: igaf122.3428

### EFFECTS OF MEDICARE ADVANTAGE GROWTH IN NURSING HOMES ON CARE QUALITY AND RESIDENT OUTCOMES: USING SHIFT-SHARE IV

Hyunkyung Yun[1], David Meyers[1], Vincent Mor[2], Cyrus Kosar[1], Peter Hull[1], Hye-Young Jung[3], and Momotazur Rahman[1], *1. Brown University, Providence, Rhode Island, United States, 2. Brown University School of Public Health, Providence, Rhode Island, United States, 3. Joan and Sanford I. Weill College of Medicine, Cornell University, New York, New York, United States*

Medicare Advantage (MA) now covers 54% of all Medicare beneficiaries. MA growth has expanded to nursing home (NH) setting, covering 37% of short-stay patients and 32% of long-stay residents in 2021. The impact of MA growth in NHs remains understudied. Using a shift-share instrumental variable (SSIV), we estimated effects of MA growth in NHs on care quality. We identified MA enrollment of Medicare-enrolled NH residents using the Medicare enrollment file (2015-2019). NH quality measures included CMS five-star overall and staffing ratings and health deficiencies. Population-specific outcomes, derived from claims, included proportion of short-stay residents with 30-day rehospitalization and death, and the quarterly rate of hospitalization and death among long-stay residents. The main explanatory variable was high MA share in NHs (top quartile NH MA share annually). We used a SSIV, leveraging an exogenous shift—the annual national MA contract growth—linked to baseline NH contract share. We estimated a two-stage least squares (2SLS) regression with NH and year fixed effects, adjusting for NH and county characteristics and clustering standard errors for NHs. Among 18,711 NHs (83,215 observations over five years), 31% had high MA share. The first-stage F-statistic was 528.7 (Coef. 0.12, $P < 0.001$), confirming IV strength. In 2SLS results, high MA share in NHs was associated with a 0.6 increase in staffing ratings (95% CI 0.4-0.9; $P < 0.001$), an 18.8% relative increase, but not with overall star-rating, deficiencies, or population-specific outcomes. Although these results suggest MA growth has improved staffing levels at NH, the mechanisms underlying these findings warrant further investigation.

Abstract citation ID: igaf122.3429

### ENGAGING "NO WRONG DOOR" STAKEHOLDERS TO IMPROVE AWARENESS, ACCESS AND EXPERIENCES

Sara Murphy[1], Jennifer Severance[2], Douglas Mains[3], Esme Anaab[3], and R. Andrew Yockey[4], *1. University of North Texas Health Science Center, Fort Worth, Texas, United States, 2. University of North Texas Health Science Center - Fort Worth, TX, Fort Worth, Texas, United States, 3. The University of North Texas Health Science Center at Fort Worth, Fort Worth, Texas, United States, 4. University of Mississippi, Oxford, Mississippi, United States*

Many individuals with chronic comorbidities and functional limitations rely on a patchwork of long term services and supports (LTSS) to access health care, social services, and personal care as they age. However, individuals trying to navigate LTSS face a complex and often fragmented myriad of options that can delay care or lead to inadequate support or unmet needs. In 2012, the No Wrong Door (NWD) Model was implemented by states as a network of local LTSS access points designed to provide person-centered and comprehensive information, assessment, and services. In 2023, the state of Texas issued a survey to determine current awareness, perceptions, and experiences associated with the state's NWD system. In collaboration with multiple state organizations, a survey was designed around the key elements of the NWD framework and distributed to consumers aged 50 and over, caregivers, people with disabilities, veterans and LTSS providers. Of 4,642 survey respondents, 56% identified as aged 50 and over, 22% as LTSS providers, and 20% as caregivers. Responses suggest positive experiences overall with NWD services. Consumers rated confusion (72%), long wait times (73%), not knowing how to access help (69%), and ineligibility (56%) as the most common problematic experiences with the NWD system. Most consumers (75%) and providers (66%) were unaware of the NWD System in their local communities. Recommendations are provided to guide the state in streamlining processes and engaging stakeholders to improve the consumer experience. Findings can inform policies that help states optimize awareness of and coordination between entry points.

Abstract citation ID: igaf122.3430

### EXPANDING THE SOCIAL CARE NETWORK: A REVIEW OF OHIO'S HEALTHY AGING INITIATIVE

Bailee Brekke[1], Robert Applebaum[2], and Jennifer Heston-Mullins[2], *1. Miami University, Waddington, New York, United States, 2. Miami University, Oxford, Ohio, United States*

For older adults with a disability, Medicaid provides the majority of support for long-term services and supports. However, only 10% of individuals age 65 and older are financially eligible for Medicaid. Older Americans Act funding has failed to keep up with the growth in the older population and inflation, creating a service gap for older adults who are not eligible for Medicaid. As a result, states have explored other avenues of funding for social care services. Ohio recently allocated $40 million in county-level funding for older adults in need of social care in their community. Each county was required to spend 20% of authorized funds on housing and food insecurity and 10% towards internet access/digital literacy. The remaining 50% could be spent on services that met the unique needs of each county. Overall, the program served more than 130,000 individuals across Ohio. A review of program reporting showed that counties utilized their discretionary funds in such areas as health and wellness programs (n = 48 counties, mean expenditure=$29,250), transportation services (n = 41 counties, mean expenditure=$45,780) and strategies to address social isolation (n = 36 counties, mean expenditure=$93,690). Qualitative interviews with 27 county officials also provided insights into innovative service strategies and cross-sector collaborative efforts, such as partnerships with law enforcement and local businesses. Findings from this study highlight the importance of offering non-Medicaid funded services to support older adults to remain independent in their local communities.

Abstract citation ID: igaf122.3431

### IMPACTS OF AGING SERVICES: INSIGHTS FROM THE NATIONAL SURVEYS OF AAAS AND TITLE VI PROGRAMS

Heather Menne, Krishnaa Nadig Nair, and Kate Singer,
*Miami University, Oxford, Ohio, United States*

Aging services operate within a shifting landscape shaped by the evolving needs and requirements of consumers and providers, legislative challenges, and external—often unpredictable—factors. The National Survey of Area Agencies on Aging (AAA) and the National Survey of Title VI Programs are conducted regularly to understand how AAAs and Tribal organizations are responding to the evolving needs of older adults and caregivers in communities around the country. This review identifies 26 publications that draw on data from the National Survey of Area Agencies on Aging (AAAs), with some also incorporating findings from the National Survey of Title VI programs. These publications cover six themes: COVID-19 (5 publications), healthcare and healthcare collaborations (6), general AAA operations (5), home and home modifications (3), communities (2), and staffing (1), and point to a system under strain, requiring more adaptable and equitable responses. Research on COVID-19 highlights heightened social isolation of older adults, disrupted healthcare access, and the critical role of AAAs in vaccine outreach and emergency support. Healthcare-related studies explore integrated care models, systemic inequities, and the power of community partnerships in expanding care access. These findings provide critical insights for policymakers, practitioners, and researchers, offering a foundation for informed decision-making and program development. Ensuring this data is used effectively can help strengthen aging services, making them more adaptable, inclusive, and better equipped to support older adults.

Abstract citation ID: igaf122.3432

### RESEARCH RELEVANCE AND READINESS AMONG AGING CARE PROFESSIONALS

Aryn Lee[1], Jerad Moxley[2], Jenna Gladfelter[3], Allison Nickerson[3], Elaine Wethington[4], M. Carrington Reid[2], and Patricia Kim[5], *1. Cornell University Medical College, New York, New York, United States, 2. Weill Cornell Medicine, New York, New York, United States, 3. LiveOn NY, New York, New York, United States, 4. Cornell University, Ithaca, New York, United States, 5. Geriatrics and Palliative Medicine, New York City, New York, United States*

Integration of evidence-based programming is central to aging care policy and practice, yet little is known about aging care professionals' (ACPs) receptivity to research. This study examined research relevance and ACPs' readiness to translate findings into practice. In collaboration with LiveOn NY, an advocacy organization supporting community-based aging care agencies throughout New York state, we surveyed individuals working in member agencies in 2023. We collected information on respondents' agencies, priorities, and assessed perceptions of research relevance and readiness using a 5-point Likert scale (1=strongly disagree, 5=strongly agree). Of 1164 email solicitations, 172 (24.78%) ACPs responded, consisting of executives (15.88%), supervisors (40.5%), social workers (24.71%), and case workers (13.53%), averaging 15.5 years of human/aging service experience (SD = 11.79). A regression model analyzed predictors of research relevance and readiness. ACPs rated research as highly relevant (M = 4.4, SD = 0.5). Relevance positively correlated with ACPs' Hispanic/Latino ethnicity (β=-0.27, p=.003), desire to learn more about research (β = 0.24, p=.01), and research access (β = 0.22, p=.02). However, ACPs reported less readiness to translate research findings into their practices (M = 2.8, SD = 0.68). Readiness was negatively correlated with agencies serving largely multiracial older adults (β=-0.237, p=.01), and positively correlated with desire to learn more about research (β = 0.20, p=.02), lack of support from team members, and ACPs' agencies to implement evidence-based practices (β = 0.306, p<.001). Despite strong recognition of research relevance, ACPs reported feeling less ready to interpret and integrate research into practice. Addressing barriers (e.g., access and professional development) is crucial for enhancing evidence-based aging care in the community setting.

Abstract citation ID: igaf122.3433

### STATE SPENDING ON HOME AND COMMUNITY-BASED SERVICES AND RATES OF LOW-CARE RESIDENTS IN NURSING HOMES

Blythe Chen[1], and Elizabeth White[2], *1. Brown University, Providence, Rhode Island, United States, 2. Brown University School of Public Health, Providence, Rhode Island, United States*

States are increasingly investing in home & community-based services (HCBS) to support older adults "aging in place" and prevent unnecessary nursing home (NH) admission of individuals with low care needs. This observational study examined associations between state HCBS spending (i.e. the percent of state Medicaid long-term care dollars spent on HCBS) and "low care" nursing home use in 2021 using the AARP LTSS State Scorecard and LTCFocus. In linear regression models controlling for state and NH characteristics, we estimated the association of state tertile of HCBS spending with percent of low-care residents in

U.S. NHs (n = 14,105). The final model included an interaction between average state NH cost and HCBS spending. Compared to NHs in lowest-HCBS spend states, NHs in the highest-spend HCBS tertile had 1.08 percentage points (95% CI: -2.15, -0.01) fewer low-care residents. There was a significant interaction between state median NH cost and HCBS spending. In low-cost states, adjusted mean of low-care residents was 11.0% (95% CI: 10.55, 11.46) in lowest HCBS tertile, and 9.92% (95% CI: 9.47, 10.04) in highest HCBS tertile. In high-cost states, adjusted mean of low-care residents was 7.47% (95% CI: 6.94-8.00) in lowest HCBS tertile, and 9.93% (95% CI: 9.01-10.84) in highest HCBS tertile. Higher state HCBS spending is associated with a lower prevalence of low-care NH residents, but this relationship is attenuated in higher-NH cost states. Further research is needed to examine specific HCBS waiver-covered services associated with reducing NH admissions for low-care residents.

Abstract citation ID: igaf122.3434

### THE ACCESSIBILITY AND SITE SELECTION OPTIMIZATION OF ELDERLY CARE INSTITUTIONS IN CHINA: A CASE STUDY

Rong Peng, and Yuanqing Huang, *Guangdong University of Finance and Economics, Guangzhou, Guangdong, China (People's Republic)*

Adjusting and optimizing the spatial layout of elderly care facilities is of great significance to maximize the utilization of elderly care resources for urban residents. Guangzhou is a megacity in southern China and is one of the most economically developed cities in China. The POI data of Guangzhou's elderly care institutions is used to measure the spatial accessibility of elderly care institutions from the township (street) scale based on the Gaussian based 2-step floating catchment area method (Ga2SFCA). This study constructs the index system for the site selection decision of elderly care institutions, and uses the random forest algorithm to select the grid suitable for the layout of elderly care institutions. The results show that the accessibility of elderly care institutions in Guangzhou's central area is relatively high, followed by the main urban area and the suburbs. The accuracy of the simulated location results of elderly care institutions and the real location of elderly care institutions is 90.1%, indicating that the random forest model constructed in this study is reliable. From the perspective of the characteristics of the sites suitable for newly built institutions, it is mainly the areas with dense elderly population and the areas with serious shortage of elderly care institutions. The contribution of this study is to construct a site selection decision index system based on POI data, and use the random forest algorithm to optimize site selection decision of elderly care institutions. This study provides a case study in optimizing the layout of elderly care resources.

### SESSION 7545 (POSTER)

### TECHNOLOGY: RESEARCH APPLICATION/ MEASUREMENT/DEVICES/EDUCATION

Abstract citation ID: igaf122.3435

### ARE OLDER ADULTS READY FOR AUGMENTED REALITY TO BE THE NEW FACETIME?

Mahrukh Tauseef[1], Zalen Ingram[1], Rendong Zhang[1], Cathy Maxwell[2], Lisa Juckett[3], Judith Tate[3], Lorraine Mion[3], and Nilanjan Sarkar[1], *1. Vanderbilt University, Nashville, Tennessee, United States, 2. University of Utah, Salt Lake City, Utah, United States, 3. The Ohio State University, Columbus, Ohio, United States*

Rapid technological advances in augmented reality (AR) expand on existing video technologies (e.g., FaceTime), by offering greater social presence, realism, and immersion. Thus, AR is likely to enhance social connectedness and reduce isolation for older adults. However, older adults are often excluded during product development and/or testing. We conducted a pilot study with 6 pairs of older adults and designated family/friend to determine acceptability of AR telepresence. Each pair engaged in a shared activity through their photorealistic avatars during 8 sessions over 4 weeks. Photorealistic avatars were co-created with each participant, who provided feedback and approval on theirs and their partner's avatars. We developed shared AR activities (checkers and home decoration) in earlier work. Data were collected at baseline, each session, and final session using surveys, semi-structured interviews, and observations. All participants completed all sessions with zero dropouts and no incidences of AR sickness. Four (25%) wanted more than one iteration to create an avatar meeting their approval. All expressed a positive attitude towards the AR experiences, e.g., "I could use this to engage with my friend in Texas" and "I like it better than video call because I can see them here". Three requested a video of their avatars to share with family. Two expressed interest in learning how to use AR to connect with grandchildren. Challenges included a steep learning curve, limited network, and lack of realistic avatar creation tools. Nevertheless, recent improvements in network capabilities and avatar generation technology enhance AR's likelihood as the next FaceTime.

Abstract citation ID: igaf122.3436

### CENTER OF PRESSURE DENSITY MAPS AS POSTURAL ASYMMETRY INDICATORS AND BIOFEEDBACK FOR OLDER ADULTS

Jethro Raphael Suarez[1], Camilla Torres[2], Joon-Hyuk Park[2], and Ladda Thiamwong[2], *1. University of Central Florida, Florida, United States, 2. University of Central Florida, Orlando, Florida, United States*

Nonvisual balance tests enhance proprioceptive and vestibular system use to maintain balance due to the absence of visual input. Visual tools exist that show center of pressure (COP) movement during such tests, but many are difficult to understand visually and do not give focus to the initial starting point. This study aimed to develop density maps from raw force plate data for use as a form of biofeedback for community-dwelling older adults. The Balance Tracking System (BTrackS) is a simple, cost-effective force plate system that records COP movement and was used for balance assessments. R software was utilized to generate density maps using the "stat_density2d" function in the "ggplot2" library from the raw data produced by the BTrackS. We collected force plate data from 21 community-dwelling older adults aged 64 to 83 (mean age = 72.3 ± 5.9 years) who completed a static balance test on the BTrackS. The static balance test involved standing still with feet shoulder-width apart, hands on hips, head forward, and eyes closed for four 20 second trials with 10 seconds rest between. Density maps revealed that the highest COP density areas were not located near the initial starting point for a few older adults, indicating postural asymmetry. The results show that COP density maps could serve as beneficial feedback for older adults by allowing for the recognition of postural sway asymmetry. Future research should determine the feasibility of providing such feedback for older adults, as well as investigate factors that could cause such asymmetry.

Abstract citation ID: igaf122.3437

### COLLECTING LONG-TERM WEARABLE DATA IN OLDER ADULTS: ATHEROSCLEROSIS RISK IN COMMUNITIES NEUROCOGNITIVE STUDY

Erin Spaulding[1], Jie Ding[2], Henry Zhao[2], Pamela Lutsey[3], B. Gwen Windham[4], Katherine Ornstein[1], Josef Coresh[5], and Seth Martin[2], *1. Johns Hopkins University, Baltimore, Maryland, United States, 2. Johns Hopkins University School of Medicine, Baltimore, Maryland, United States, 3. University of Minnesota, Minneapolis, Minnesota, United States, 4. University of Mississippi Medical Center - The MIND Center, Jackson, Mississippi, United States, 5. New York University, New York, New York, United States*

Traditional cohort studies, relying on periodic visits, may introduce bias by capturing data largely when participants feel well. Wearables enable continuous data collection, potentially mitigating this bias, but low adoption among older adults could result in data gaps. Fitbit Charge 6 devices were used to collect long-term health data among Atherosclerosis Risk in Communities Neurocognitive Study (ARIC NCS) participants with a smartphone in 2024-2025. Staff provided Fitbit setup and training, encouraging daily wear and regular syncing, with ongoing technology support. Fitbit wear time, measured by heart rate data, was calculated as a percentage. Mann-Whitney U tests assessed differences in wear time by age, sex, and race. Approximately one-third of eligible participants offered a Fitbit chose to enroll. A total of 309 ARIC NCS participants (84.0±3.2 years [36% ≥85 years], 60% women, 82% White, 45% ≤high school/vocational school) contributed a median of 125 (IQR: 148) days of Fitbit data (~4.2 months). In this sample of older adults aged 79-95 years, the median wear time was 99% (IQR: 3%). A Mann-Whitney U test revealed a significant difference (z=-4.08, $p<0.001$) in median wear time between Black (98%, IQR: 10%) and White (99%, IQR 3%) older adults, indicating greater variability in wear time among Black adults. These findings suggest that with the proper onboarding and ongoing technology support, wearable devices can be used to collect long-term data among adults 80 years and older. Future research could examine potential reasons for greater wear time variability among Black adults, including digital literacy and sensor accuracy.

Abstract citation ID: igaf122.3438

### EVALUATING THE ACCURACY OF AUTOMATIC SPEECH RECOGNITION SYSTEMS IN HOME HEALTHCARE SETTINGS

Dayoung Yu[1], Sasha Vergez[2], Grace Flaherty[1], Maryam Zolnoori[3], Nicole Onorato[1], Julia Hirschberg[3], Maxim Topaz[3], and Margaret McDonald[1], *1. VNS Health, New York, New York, United States, 2. VNS Health, Bronx, New York, United States, 3. Columbia University, New York, New York, United States*

Recent developments in automatic speech recognition (ASR) systems have resulted in increased use of automated transcription services to analyze verbal communications. We aimed to analyze the accuracy of AWS General Transcribe (AWS-GT) on audio recordings of home healthcare patients. These audio recordings included in-person clinician visits and phone calls with research assistants (RAs). Study staff rated the clarity of the audio using a scale of low, medium and high. The audio quality was similar among the two types of recordings. Our results are a part of a larger study aiming to use automated speech processing to identify risk factors for hospitalization and emergency department visits. Overall, 4002 utterances, defined as uninterrupted blocks of speech with four or more words, were analyzed— 3,472 (87%) from clinician recordings and 520 (13%) from RA recordings. Word error rate (WER; range 0(best)-1(worst)) was used to assess the performance of AWS-GT, compared to a gold-standard manual transcription. We found a statistically significant difference in WER for clinician recordings (.26) and RA recordings (.19) ($p<0.05$). We also observed that the WER was poorest for short, four-to-eight-words, utterances (.39), and that this improved as utterances grew longer ($p<0.05$). Clinician recordings (35%) had a greater number of short utterances than RA recordings (25%) ($p<0.05$). This shows how differences in conversation dynamics can impact the accuracy of ASR systems. It is incredibly important that we determine whether existing software is accurate enough for use in clinical documentation and prediction models dependent on ASR systems.

Abstract citation ID: igaf122.3439

### EXPLORING WEARABLE DEVICE USE AMONG DEMENTIA CAREGIVERS: BENEFITS, CHALLENGES, AND IMPROVEMENTS

Jung-Ah Lee[1], Hyun Jung Kim[2], and Kyeung Mi Oh[3], *1. University of California, Irvine, Irvine, California, United States, 2. Seattle University, Seattle, Washington, United States, 3. George Mason University, Fairfax, Virginia, United States*

Caregivers of persons with dementia experience significant challenges and stress due to round-the-clock responsibilities. Wearable devices (WD), such as smartwatches and smart rings, can provide real-time health data, enhancing caregivers' self-care. This study explored caregivers' experiences using WD as part of a three-month in-home educational intervention, which included stress management strategies. Participants ($N=101$) were recruited through community outreach and completed exit interviews. The sample included 14 non-Hispanic White, 13 Hispanic, 35 Korean, and 39 Vietnamese caregivers. Qualitative analysis identified four key themes: enhancing usability, encouraging data engagement, improving wearability and convenience, and expanding health-tracking features. Most caregivers found WD beneficial for increasing health awareness, motivation, and self-monitoring. However, they highlighted the need for additional education on device functions, app navigation, and data interpretation. Non-English-speaking caregivers emphasized the importance of language accessibility. Many preferred larger screens for better readability and reminders to improve adherence. Some struggled with discomfort, forgetting to wear or charge devices, and limited battery life. Caregivers expressed interest in additional health-tracking features, including blood pressure, blood sugar, body fat, and muscle mass monitoring. Addressing these challenges could enhance adherence, particularly among older caregivers. Participants recommended tailored education for caregivers with varying levels of digital literacy, alternative charging methods, and visible battery indicators to improve usability. Findings highlight the need for comprehensive education, frequent reminders, and improved usability to maximize WD benefits. By addressing these factors, WD can better support caregivers in managing their well-being and sustaining their caregiving roles with greater resilience.

Abstract citation ID: igaf122.3440

**NEUROPSYCHOLOGICAL ASSESSMENT: DIGITAL VS. PAPER COMPARABILITY IN OLDER ADULTS**

Claire Snyder[1], Cay Anderson-Hanley[2], Aydana Djoroeva[2], Connor Cavanaugh[2], Shaelyn Kelley[2], Catherine Dacey[2], Samantha Corman[2], and Lily Schwarcz[2], *1. Schenectady, New York, United States, 2. Union College, Schenectady, New York, United States*

The use of digital neuropsychological assessment has increased since the COVID-19 pandemic and continues to be essential (Crivelli, 2024). While digital administration may be more efficient, the accuracy of the results may be questionable (due to potential differences in performance, etc.). This study aimed to evaluate the comparability of brief neuropsychological tests in similar digital and paper forms. Participants completed two cognitive screening tests and two brief tests of executive function: the paper MoCA, and the digital Boston Cognitive Assessment (BoCA), as well as the paper Stroop task ("pStroop"), and an electronic version "eStroop," respectively. A study conducted by Castelli et al. (2021) found that the paper cognitive screener: the Montreal Cognitive Assessment (MoCA), is a sensitive and specific test used to detect cognitive impairment. In this study, it was hypothesized that the performance of digital and paper-and-pencil assessments would be similar, evidenced by positive correlations. Twelve older adults with a mean age of 68.5 years (SD = 8.07) completed a combined lab protocol. Among older adults, the MoCA and the BoCA were significantly moderately correlated, $r(10) = .62$, $p = .03$, while the pStroop and eStroop were significantly strongly correlated, $r(10) = .90$, $p = .002$. This study aimed to determine if digital and paper-and-pencil neuropsychological tests performance would be comparable among brief screening tests. The results showed that for older adults, there was a moderate to strong correlation between paper-and-pencil and digital neuropsychological testing, suggesting that the use of electronic tests may, in some situations, be interchangeable.

Abstract citation ID: igaf122.3441

**READINESS, ACCEPTABILITY AND USE OF REMOTE MONITORING TECHNOLOGIES TO DETECT FRAILTY**

Hema Ramamurthi[1], Greg Kirk[2], Ann Gruber-Baldini[3], and Denise Orwig[4], *1. Johns Hopkins University, Baltimore, Maryland, United States, 2. Bloomberg School of Public Health, Baltimore, Maryland, United States, 3. University of Maryland School of Medicine, Baltimore, Maryland, United States, 4. University of Maryland, Baltimore, Baltimore, Maryland, United States*

Frailty affects a significant portion of older adults and has consistently been shown to lead to adverse health outcomes, especially among People Living with HIV (PLWH). Data from Remote Monitoring Technologies (RMTs) could be used to detect small but significant changes that signal the onset of frailty. Despite the benefits offered by RMTs, there is a paucity of information about essential aspects of RMT use among PLWH. To assess the level of readiness and use of RMTs, we initiated a pilot study to include 30 PLWH aged ≥50 living in the community for a 2-week monitoring period using RMTs (app-based hand grip measurement, motion sensors, accelerometer for gait speed, and digital weighing scale). Surveys included questions about the readiness to use new technology. Of the initial 8 participants, the average age was 61+ 5.3 years, 25% were female, 50% were living alone. Seven participants had access to high-speed internet, and 100% used smartphones. One participant reported using an exercise app, and two reported using a wearable device. The role of technology in providing freedom of mobility was expressed by 50%, and the same respondents somewhat agreed that technology increased productivity. Seven of the 8 participants agreed that people were becoming too dependent on technology and that technological systems are not designed for ordinary people. There was 100% adherence in completing the frailty-related measurements at home. Additional analysis will report on: 1) level of consistency of RMT based measurements to clinic based observations and 2) self-reported acceptability and usability of RMTs.

Abstract citation ID: igaf122.3442

**REFINING THE INTERGENERATIONAL TECHNOLOGY CHECKLIST FOR HIGHER EDUCATION THROUGH A DELPHI PANEL**

Jill Juris[1], Skye Leedahl[2], Shannon Jarrott[3], and Caroline Halliday[1], *1. Appalachian State University, Boone, North Carolina, United States, 2. University of Rhode Island, Kingston, Rhode Island, United States, 3. The Ohio State University, Columbus, Ohio, United States*

The aim of this study was to evaluate the InterGenerational Technology Engagement Checklist for Higher Education (IG TECH) using the Delphi approach that builds expert consensus. We conducted three rounds with the Delphi panel comprised of researchers and practitioners with expertise in intergenerational programs. In each round, participants evaluated the IG TECH using a Criteria Rating Form that included quantitative and qualitative responses related to: 1) Construction, 2) Appropriateness and, 3) Relevance. Responses on a four-point Likert scale ranged from "Poor" to "Excellent". Consensus was pre-defined as 80% of ratings for each item "Good" or "Excellent." The Criteria Rating Form was modified in subsequent rounds to include items with less than 80% approval. Participants were invited from a repository of 283 programs identified through the Generations United database, previous research participation, and gerontology networks. Of those, 47 participants responded to the interest survey, and 39 responded to the Round 1 survey (83% response rate). In Round 2, the Round 1 panelists were invited, and 33 completed the questionnaire (85% response rate). In Round 3, the final round, Round 2 participants were invited and 30 responded (91% response rate). In each round, the research team reviewed quantitative and qualitative data from the Criteria Rating Form and identified specific changes to the IG TECH. Changes were categorized and shared with the Delphi panel. In Round 3, panelists reached 100% consensus on all items. A final version of the IG TECH was developed that includes six sections and a total of 45 items.

Abstract citation ID: igaf122.3443

**USABILITY, ACCEPTABILITY, AND FEASIBILITY OF A REMOTE MONITORING SYSTEM FOR RURAL-DWELLING AFRICAN-AMERICANS**

Otis Owens[1], Zachary Beattie[2], Nicole Sharma[2], Don Campbell[3], Thomas Riley[2], Jon Yeargers[3], Larry Frye[3], and Jeffrey Kaye[2], *1. University of South Carolina, Columbia, South Carolina, United States, 2. Oregon Health & Science University, Portland, Oregon, United States, 3. Life Analytics Incorporated, Woodside, California, United States*

Background and Purpose: This study examined the usability, acceptability, and feasibility of deploying a remote monitoring system in the homes of cognitively healthy rural-dwelling African Americans. Methods: Ten homes in a southeastern state were outfitted with seven sensors/smart devices for up to three months. A 'one-time' survey was distributed online to collect demographic and technology use data. A 'weekly' online survey was also distributed to assess changes in health and home occupancy. Thirty-minute interviews were conducted at the study's conclusion. Descriptive statistics were calculated for quantitative data. Qualitative data was analyzed using thematic analysis. Feasibility was determined by the percentage of days data were collected across all sensors. Results: Participants, on average, were 72 years old, married, had a college or higher education, and owned a smartphone. Themes emerging from interviews related to technology-use satisfaction, ease of technology use, barriers to technology use, openness to remote monitoring, perceived usefulness of remote monitoring for others, and data usefulness. Overall, users were satisfied with the system, especially its passive nature and ease of use. Some, but few, barriers existed to system use (e.g., remembering to weigh). Participants were open to continuous remote monitoring if their health declined. All participants thought receiving sensor data routinely would support their health maintenance. Suggestions for system improvement will be presented. Data across sensors was continuously collected over half of the days installed. Implications: Remote monitoring may be culturally-appropriate for empowering rural-dwelling African Americans to age-in-place, but these systems should be easy-to-use and designed with community input.

### SESSION 7900 (POSTER)

### FLASH POSTER SESSION: FACES OF EQUITY: DIVERSE REALITIES SHAPING AGING IN AMERICA

Abstract citation ID: igaf122.3444

### GENDER DIFFERENCES IN PREDICTORS OF DEPRESSIVE SYMPTOMS AMONG OLDER HISPANIC WOMEN IN THE UNITED STATES

Jaminette Nazario-Acevedo[1], and Takashi Yamashita[2],
*1. University of Maryland, Baltimore & Baltimore County, Martinsburg, West Virginia, United States, 2. University of Maryland, Baltimore County, Baltimore, Maryland, United States*

Older Hispanic women are more likely to experience depressive symptoms than older Hispanic men in the United States. Mental health challenges faced by older Hispanic adults, in general, and their gender differences, in particular, are often overlooked. Previous research highlights that a form of social relationship --- marriage, can serve as a protective factor for mental health. However, gender differences in specific factors associated with depressive symptoms among older Hispanic women and men have not been extensively examined. Given the intersectionality theory, the present study compared the known predictors of depressive symptoms among older Hispanic women and men. Nationally representative samples of Hispanic adults aged 51 years and older ($n = 2564$) are obtained from the 2018 Health and Retirement Study. Binary logistic regression shows that married older Hispanic adults (Odds ratio = 0.69, $p < 0.05$) are less likely to be depressed than their counterparts. No gender difference in the marital status on depressive symptoms is observed. However, there is a significant gender difference in the effect of educational attainment. Older Hispanic women with a college degree or higher (Odds ratio = 0.57, $p < 0.05$) are less likely to be depressed than their counterparts. Yet, educational attainment is not associated with lower depressive symptoms among men (Odd-ratio = 1.02, $p > 0.05$). Findings suggest differential impacts of socioeconomic status on mental health by gender. To improve the mental health of older Hispanic adults in the United States, a more targeted and gendered public health approach may be considered.

Abstract citation ID: igaf122.3445

### ACCELERATED MOLECULAR AGING IN NEIGHBORHOOD POVERTY: A RACIAL/ETHNIC COMPARISON

Jennifer Robinette[1], and Jennifer Smith[2], *1. Chapman University, Orange, California, United States, 2. University of Michigan, Ann Arbor, Michigan, United States*

In the US, racial/ethnic health disparities are undeniable and partially stem from residing in low SES neighborhoods. Associations between neighborhood SES and health may have some underlying molecular mechanisms reflected in the epigenome. Yet, neighborhood characteristics are not always experienced the same way for all residents, and questions remain regarding whether those most exposed to low SES neighborhoods build greater resilience to, or embody greater harmful and cumulative outcomes from, such neighborhoods. The present study tested the hypothesis that greater neighborhood poverty would relate to accelerated epigenetic aging on the Horvath, Hannum, PhenoAge, and GrimAge clocks, and that these associations would differ in strength by race/ethnicity. A national sample of 3,790 older non-Hispanic White, non-Hispanic Black, and Hispanic participants from the 2016 wave of the Health and Retirement Study and census tract poverty rates from the 2012-2016 American Community Survey were used to test these questions. Results of weighted linear regressions suggested that living in higher poverty neighborhoods was associated with accelerated PhenoAge and GrimAge (not Hannum or Horvath), adjusting for age, sex, and educational. After further considering smoking status, however, only the association with GrimAge remained. Investigating interactions with race/ethnicity suggested that poverty was more strongly associated with accelerated GrimAge among non-Hispanic White participants, but this racial/ethnic difference was completely reduced after considering smoking. These results further support the need for economic investment in low SES neighborhoods and encourage more research to determine the social and physical resources needed in such neighborhoods to attenuate accelerated epigenetic aging.

Abstract citation ID: igaf122.3446

### THE RECIPROCITY OF CHURCH-BASED SUPPORT AND PSYCHOLOGICAL WELL-BEING: RELIGION, AGING AND HEALTH SURVEY (RAH)

Landon Peeples, Lauren Chrzanowski, and Benjamin Mast, *University of Louisville, Louisville, Kentucky, United States*

Church-based support provides both social and emotional benefits, yet little is known about its reciprocation and effects on well-being. This study uses Religion, Aging, and Health (RAH) survey data from older adults aged 65 and over within the United States to investigate (1) how receiving pastoral and congregational support influences the likelihood of providing church-based support and (2) the psychological correlates of providing supporting. Linear regression analysis (N = 813) evaluated the effects of receiving support on providing support, controlling for age, gender, race, and denomination. MANOVA (N = 1118) assessed the impact of providing support on depression, self-esteem, life satisfaction, and self-rated health. The regression model was statistically significant ($R^2 = 0.225$, F (9,803) = 25.907, p < 0.001). Receiving congregational support (B = 0.249, p = 0.002) and age (B = 0.012, p = 0.002) was positively associated with providing support. Pastoral support, gender, race, denomination, and interaction terms were not significant. MANOVA showed a significant multivariate effect (Pillai's Trace = .018, F (4, 1113) = 4.979, p < 0.001). Providing support was associated with lower depression (F (1, 1116) = 7.410, p = 0.007), higher self-esteem (F (1, 1116) = 9.997, p = 0.002), and greater life satisfaction (F (1, 1116) = 6.857, p = 0.009) but did not impact self-rated health (F (1, 1116) = 1.037, p = 0.309). These findings highlight how providing support can influence psychological well-being within faith communities. Future research should explore how other church-based support contributes to well-being in diverse populations.

Abstract citation ID: igaf122.3447

### ENHANCING MINDFULNESS IN OLDER ADULTHOOD: MIXED-METHOD FINDINGS FROM A COMMUNITY-BASED GROUP INTERVENTION

Tiana Broen[1], Jonathan Sundby[1], Christopher Griffith[1], and Leilani Feliciano[2], *1. University of Colorado Colorado Springs, Colorado Springs, Colorado, United States, 2. University of Colorado Colorado Springs Department of Psychology, Colorado Springs, Colorado, United States*

Research indicates that mindfulness skills, such as breathing exercises and meditation, effectively reduce psychological symptoms and increase overall well-being. In older adulthood, mindfulness-based interventions are an accessible, cost-effective, and adaptable way to support emotional well-being in everyday life. The current study aimed to examine the efficacy of an 8-week mindfulness skill-building groups on older adults in a community outpatient clinic in the Rocky Mountain Region. Quantitative and qualitative data were gathered across four groups between 2023 and 2024 (N = 22, Mage = 72.13, SD = 5.42, 77.27% female). Participants completed pre- and post-measures of depression, anxiety, stress, well-being, pain, sleep, and trait-mindfulness. To provide a subjective account of specific changes participants had noticed in themselves because of their mindfulness practice, participants were asked to provide narrative feedback, which was recorded and transcribed for thematic analysis. Paired-sample t-tests indicated a significant decrease in perceived stress [t(21) = 3.01, p<.01], depressive symptoms [t(16) = 2.09, p<.05], and anxiety symptoms [t(21) = 3.18, p<.01], as well as an increase in trait mindfulness [t(20) = -4.65, p< .01] and overall well-being [t(21) = -3.24, p<.01]. Thematic analyses were conducted with moderate-good inter-rater reliability (κ=.59-.63), and revealed themes related to the primary outcome variables, in addition to themes of overall effectiveness, interpersonal functioning, group cohesion, mindfulness skills, insight, and positive self-concept. These results indicate that mindfulness-based group interventions can be effective for reducing psychological symptoms, increasing overall well-being, and providing an opportunity for meaningful connection with older adult peers in a group setting.

Abstract citation ID: igaf122.3448

### ASSOCIATION OF FUNCTIONAL LIMITATIONS (ADLS) AND SEXUAL IMPORTANCE IN MIDDLE-AGE AND OLDER U.S. ADULTS.

Lucas Prieto, Claire Leonard, and Gilbert Gimm, *George Mason University, Fairfax, Virginia, United States*

Although prior studies have examined functional limitations (ADLs) in older adults, relatively few U.S. studies examine the association of disability and importance of sex in middle-age and older adults. Sexual health is a vital part of overall well-being in adults, therefore, understanding how older adults prioritize sex is essential. Given this gap, the goal of this study was to analyze a national sample of Wave 3 NSHAP participants and assess the relationship between disability as measured by the count of functional limitations (ADLs) and having any sexual importance among U.S. adults (50 years or older). Using respondent data from 4,371 NSHAP participants, we conducted bivariate and multivariate analyses of the likelihood of reporting sex as important among middle-age (50-64 years) and older U.S. adults (65 years or more). Nearly one-quarter (24.5%) of participants had any ADL limitations and a majority (55.5%) reported sex as important. Compared with middle aged adults (50-64 years), older adults (65+ years) had lower odds (OR = 0.30, p<.001) of reporting sex as important. Also, participants with 3 or more ADLs had lower odds of expressing sexual importance compared to zero ADLs (OR=.64, p=.005). Other factors that were significant with sexual importance included gender, education, race/ethnicity, relationship type, and health. These findings suggest that health care providers should include sexual health questions in their interactions with older adult patients regardless of sociodemographics. Results suggest the need to assess the link between functional limitations and sexual importance among middle-age and older U.S. adults to maintain well-being and healthy aging.

Abstract citation ID: igaf122.3449

### THE EFFECTS OF LIVING ARRANGEMENTS ON DEPRESSION AND LIFE SATISFACTION IN RURAL CHINESE OLDER ADULTS: A 20-YEAR STUDY

Jin Guo[1], Andrew Wister[2], Barbara Mitchell[2], and Shuzhuo Li[1], *1. Xi'an Jiaotong University, Xi'an, Shaanxi, China (People's Republic), 2. Simon Fraser University, Vancouver, British Columbia, Canada*

Objectives: China has experienced rapid socio-economic transformations over the past two decades. Based on individualization and intergenerational solidarity theories, this study explores changes in the associations between the living arrangement on psychological well-being over time and across different chronic conditions. Methods: Eight waves of longitudinal data (2001–2021) collected in Anhui, China were employed, including. 9,765 (person-year) observations. Mixed linear models containing interaction terms for time and chronic conditions were used to examine the effects of living arrangements on life satisfaction and depression. Results: Compared to living with children, the negative correlation with life satisfaction and the positive correlation with depression for those living alone or with spouse only decreased over time, while the negative correlation between living in skipped-generation household and life satisfaction also diminished. As the number of chronic conditions increases, life satisfaction declines further for older adults living alone, with a spouse only, or in a skipped-generation household. Living alone leads to a higher increase in depression, but living with others reduces it. Discussion: This study is the first to identify the changes in dynamic relationships between living arrangements on psychological well-being of older adults across time and chronic health conditions, reflecting shifts in filial piety and family culture. While older adults increasingly accept independent living, coresiding with children remains beneficial during multimorbidity. These findings contribute to the understanding of ageing in the context of economic and cultural transitions in developing regions.

Abstract citation ID: igaf122.3450

### THE AMERICA I KNOW COULD USE A GOOD CRY: IMPACT OF STATE AND CIVILIAN-PERPETRATED RACIAL VIOLENCE ON COGNITIVE HEALTH

Muriel Taks Calle[1], Betselot Wondimu[1], Cailyn Clemons[1], and Paris Adkins-Jackson[2], *1. Columbia University Mailman School of Public Health, New York, New York, United States, 2. Mailman School of Public Health, Columbia University, New York, New York, United States*

From a life course approach and relying on the Weathering Hypothesis as a primary pathway, this study acknowledges that cumulative stressful and fear-related experiences, whether endured directly or indirectly, have a detrimental impact on the individual's health. Prior investigations show that exposure to the American criminal justice system experienced by individuals racialized as Black correlates with adverse cognitive effects. This analysis seeks to highlight not only the role of governmental and institutional bodies in perpetuating structural racism but also the contributions of civilian actors to racial terror which in turn, increases the chances of cognitive impairment among those racialized as Black. We estimate the association between living in a county with a history of structural racism and racially disproportionate incarceration rates (in the decade preceding the passing of the 1994 Crime Bill) and having at least one incident of cognitive impairment (2008-2020), based on cognitive test performance scores from the Health and Retirement Study (HRS). Binomial logistic regression results suggest that participants racialized as Black in counties with high structural racism (lynching history, and disproportionate deaths due to police violence) and disproportionate incarceration have 21.3 times higher odds ($\beta = 3.059$; 95%CI [0.242, 5.875]) of having at least one incident of cognitive impairment compared to those racialized as Black in counties with the same conditions except disproportionate incarceration.

Abstract citation ID: igaf122.3451

### DOCUMENTING GRIEF AND BEREAVEMENT EXPERIENCES AMONG OLDER CHINESE IMMIGRANTS IN LA: A PHOTOVOICE PILOT STUDY

Qianyun Wang, and Cindy Sangalang, *University of California, Los Angeles, Los Angeles, California, United States*

This pilot study explored grief and bereavement experiences among older Chinese immigrants in Los Angeles using a Photovoice approach. The COVID-19 pandemic not only triggered global waves of grief but also intensified anti-Asian racism, disproportionately affecting older Asian immigrants with severe symptoms of depression and anxiety. Older Chinese immigrants faced unique challenges in grief adjustment due to cultural and linguistic barriers, limited social support, and discrimination in healthcare settings. This study employed Photovoice, a participatory action research strategy, to authentically capture and discuss these experiences through visual data. The research, conducted in collaboration with the Chinatown Service Center, addressed the following research questions: 1) What were the lived experiences of grief and bereavement among older Chinese immigrants? 2) How did intersecting factors like age, immigration status, and ethnicity impact their experiences? 3) What challenges and support did they encounter in adapting to grief? A purposive sample of 20-30 participants in total aged 65 or older, born outside the United States, and having lost a loved one over six months ago will be recruited. The methodology included two individual interviews and a focus group discussion, where participants took and discussed photographs representing their grief experiences. Preliminary results were documented and will be presented in a community photographic exhibition. This study aimed to provide insights into the nuanced grief experiences of older Chinese immigrants, contributing to culturally sensitive support frameworks.

Abstract citation ID: igaf122.3452

### GENDER, SOCIAL TIES, AND OLDER ADULT ALCOHOL USE

Eric Vogelsang, and Zackary Zanotelli, *California State University, San Bernardino, San Bernardino, California, United States*

There is little empirical research focusing on how community, family connections, and gender may influence the risk of older adult alcohol use. This is important, given growing concerns over (a) the long-term effects of even moderate alcohol use worldwide, (b) growing alcohol consumption among women, and (c) health impacts of social isolation. Using The Irish Longitudinal Study on Ageing (18,805 observations from 7,705 respondents), we examine how "social ties" (family characteristics and social participation) predict three measures of alcohol use and abuse among adults aged 50-plus. We also test to what extent alcohol use and its correlates may differ by gender. Our results indicate that having three or more close friends (when compared to having no close friends) is associated with regular alcohol consumption, and (for men) greater odds of possible alcohol abuse. We also find that, for women, being a member of a social organization is associated with greater odds of drinking, when compared to those who are not. Unlike similar prior studies, religious participation (which was more common among women) was negatively associated with all three types of alcohol consumption. Last, and contrary to expectations, greater education was positively associated with all three measures of alcohol use and abuse. In total, these findings suggest one potential drawback to close social ties in older ages. They also suggest ways in which a country's history and gendered social norms may influence health behavior in later life.

Abstract citation ID: igaf122.3453

### GENDER DIFFERENCES IN OLDER ADULT SOCIAL PARTICIPATION

Eric Vogelsang[1], and Sara Moorman[2], *1. California State University, San Bernardino, San Bernardino, California, United States, 2. Boston College, Chestnut Hill, Massachusetts, United States*

A growing body of research has found that older adult social participation may benefit various aspects of health and well being. While some of this work has found these associations differ by gender, there has been little rigorous investigation into how social participation itself varies between older men and women. Using 10 waves of the Health and Retirement Study, we study how (a) the breadth of social activities, (b) social activity frequency, and (c) the social determinants of social participation differ by gender. Generally, we find that men and women were involved in approximately the same number of social activities and at the same total level of activity frequency. However, gendered differences in population composition (e.g., educational attainment, marital status) suppress greater social participation for women. After controlling for these compositional differences, we find that women had slightly greater participation scores—mostly attributable to volunteering and religious service attendance. We also found that social activity decline begins at about age 65 for men, but age 70 for women. When investigating the social determinants of social participation, our results indicate that men suffer greater "penalties" for being divorced or never married. In addition, we found that increased social participation among those currently working only applied to men. Last, we found that the positive associations between high socioeconomic status and social participation was much stronger in magnitude for women. These results demonstrate how gendered social norms and inequality may impact this important aspect and correlate of "healthy aging."

### SESSION 7905 (POSTER)

### FLASH POSTER SESSION: FALLS

Abstract citation ID: igaf122.3454

### ASSESSING FALL RISK FACTORS IN ADULTS 65+ LIVING IN RHODE ISLAND, MASSACHUSETTS AND NEW HAMPSHIRE

Nachalie Rodriguez-Cruz[1], Sophia Casale[2], Qinglin Gao[1], Calvin Tran[1], and Elizabeth Dugan[1], *1. University of Massachusetts Boston, Boston, Massachusetts, United States, 2. University of Massachusetts Boston, Red Bank, New Jersey, United States*

Falls are one of the leading causes of injury and death for older adults 65+. A number of risk factors for or consequences of falls include hip fractures, osteoporosis, and difficulty with ambulation. This study aims to describe/contrast disparities in community rates of hip fractures, osteoporosis, and ambulatory difficulty in adults 65+ in Massachusetts, Rhode Island, and New Hampshire. This study utilizes data from the 2025 Healthy Aging Data Reports (healthyagingdatareports.org). The hip fracture and osteoporosis rates were calculated from Medicare fee-for-service data of adults 65+(2020-2021), and ambulation rate difficulties were calculated from American Community Survey (2018-2022). Results showed that state rates for osteoporosis were MA (20.1%) [13.58; 28.6%], RI (18.9%) [13.0; 24.3%], and NH (16.3%) [9.9; 25.7%]. Difficulty with ambulation state rates were MA (18.6%) [6.12; 41.3%], RI (18.7%) [5.77; 27.9%], and NH (16.7%) [0; 42.9%]. Hip fracture state rates were MA (3.20%) [1.53; 4.59%], RI (3.09%) [2.00; 4.07%], and NH (2.79%) [2.04; 3.99%]. A trend was observed across small towns in MA experiencing the greatest rates of osteoporosis (Lenox, 28.6%; Brookline, 27.9%). New Hampshire community and state rates of hip fractures and osteoporosis were lower than MA and RI. The rates of ambulatory difficulty were lower in the southern, wealthier areas of RI. Understanding community rates of the indicators can identify areas underserved and in need of programs or services. Awareness of the disparities across states and within states can help fall prevention, physical activity programming, and hospitals assess the risks individuals within their community experience.

Abstract citation ID: igaf122.3455

### DEVELOPMENT OF A FALL PREDICTION RISK USING MULTIDIMENSIONAL DATA FROM THE CANADIAN LONGITUDINAL STUDY ON AGING

Paulo Roberto Carvalho do Nascimento, Marla Beauchamp, Jinhui Ma, Luciana Macedo, and Lauren Griffith, *McMaster University, Hamilton, Ontario, Canada*

Falls rank first in injury prevention priorities in Canada. Approximately 40% of falls among community-dwelling older adults could be prevented with proper strategies. This study aimed to develop a fall risk index using multidimensional data from the Canadian Longitudinal Study on Aging. We selected 36 potential fall risk factors from systematic reviews and fall guidelines. Predictor variables were extracted from baseline data of older adults (≥ 65 years, n = 12,646), while incident injurious falls were retrieved from follow-up 1 (FUP 1). At FUP1, 8.07% of the participants had an injurious fall. A stepwise multivariable logistic regression model identified 14 predictors associated with injurious falls. Significant ($p < 0.05$) predictors including age, previous falls, previous injurious falls, vision impairment, pain, home dissatisfaction, comorbidities, grip strength, and use of antidepressants were used to create the final model. The dataset was split into training (70%) and test (30%) datasets for model fitting and predict the probabilities, respectively. The model's performance was modest (AUC = 0.63), with a sensitivity of 75.50%, specificity (46.77%), positive predictive value (10.07%), and negative predictive value (96.03%). Although these results limit the model's reliability in confirming high-risk cases, it suggests that the model is more effective at ruling out individuals at low risk of injurious falls. Therefore, while the model may have limited utility for identifying those at high risk, it could be valuable as a screening tool to exclude low-risk individuals and focus preventive strategies on those more likely to experience falls.

Abstract citation ID: igaf122.3456

### COMPARISON OF THE BURDEN OF FALLS AMONG OLDER ADULTS AGED 70 YEARS AND OVER BETWEEN GLOBAL AND CHINA

Xiaodong Chen, and Liping Li, *Shantou University, Shantou, Guangdong, China (People's Republic)*

Objective: To estimate the burden of falls in older adults globally and in China, and project the burden through 2030. Methods: Using data from the Global Burden of Disease Study 2021, we calculated age-standardized incidence rate (ASIR), mortality rate (ASMR), and disability-adjusted life years (ASDR) for falls in people aged ≥70 years from 1990 to 2021. Joinpoint regression was used to analyze the trend of falls disease burden during 1990-2021, and Bayesian age-period-cohort (BAPC) modeling was used to predict the trend of falls disease burden in global and China during 2022-2030. Results: From 1990 to 2021, the global burden of falls increased, with AAPCs of 0.60 for ASIR, 0.69 for ASMR, and 0.30 for ASDR. In China, the increase was more pronounced, with AAPCs of 2.44, 1.47, and 0.89, respectively. Projections for 2022-2030 show global ASIR will rise from 7,672.94 to 8,036.87 per 100,000, while ASMR will decrease from 106.72 to 96.96. In China, ASIR will increase from 7,143.75 to 8,757.76, and ASMR will decrease from 92.52 to 84.95. The global ASDR will decrease from 2,795.73 to

2,711.72, while China's ASDR will rise from 2,293.30 to 2,503.68. Conclusions: The burden of falls disease continues to increase globally and in China in the older adult population aged 70 years and older, with a significant rise in ASIR in China in particular. Compared to global, more attention should be paid to falls ASIR and ASDR in China in the future.

Abstract citation ID: igaf122.3457

### PREDICTING FUNCTIONAL DECLINE AND FALLS IN OLDER ADULTS PARTICIPATING IN GEROFIT

Ben Friedman[1], Jamie Giffuni[2], Megan Kelly[2], Julie Rekant[2], and Odessa Addison[3], *1. University of Maryland, Baltimore County, Baltimore, Maryland, United States, 2. Veterans Affairs, Baltimore, Maryland, United States, 3. University of Maryland School of Medicine, Baltimore, Maryland, United States*

Purpose: Functional limitations contribute to disability and fall risk in older adults. This analysis examined patterns of limitations over 12 months and if baseline function predicts falls in older Veterans participating in GeroFit, a supervised but unstructured VA exercise program. Methods: 199 Veterans (71.9±6.7 years) completed functional assessments at baseline and 12 months (12M), including Gait Speed (GS), Short Physical Performance Battery (SPPB), Four Square Step Test (FSST), and 30-Second Chair Stand (30sCS). Functional limitations were defined by published cut-off scores. Paired t-tests and Wilcoxon signed-rank test assessed changes over time, chi-square tests examined co-occurrence, and logistic regression evaluated predictors of falls at 12M. Results: At baseline, 40% of participants had limitations in at least two measures, and at 12M, 30% remained below cut-off in multiple tests. Overall, Veterans improved significantly ($p < 0.01$) on all functional measures from baseline to 12M (SPPB: 10±2 to 11±2; GS: 1.10±0.26 m/s to 1.14±0.24 m/s; FSST: 13.12±6.90s to 11.63±6.54s; 30sCS 12±5 to 15±6 reps). Persisting below cut-offs was strongly predicted by baseline scores (OR = 2.58, 95% CI: 1.95–3.44, $p < 0.001$). Baseline 30sCS was the only significant predictor of falls at 12M (OR = 0.76, 95% CI: 0.56–0.98, $p = 0.049$), with each additional repetition in the 30sCS at baseline associated with a 24% lower odds of reporting a fall at 12 months. Conclusion: Lower extremity strength (30sCS) may be key for fall risk screening. Functional limitations frequently co-exist, and identification may guide interventions for older Veterans in exercise programs.

Abstract citation ID: igaf122.3458

### ANALYZING FALL INCIDENTS AND FALL-RELATED INJURIES IN WISCONSIN ASSISTED LIVING COMMUNITIES

Sohee Kim[1], and James Ford[2], *1. University of Wisconsin - Madison, Madison, Wisconsin, United States, 2. University of Wisconsin–Madison, Madison, Wisconsin, United States*

This study examines trends in fall incidence and fall-related injuries in Wisconsin assisted living communities (ALCs) from 2017 to 2022. It aims to identify differences in fall trends among three ALC types—Adult Family Homes (AFHs), Community-Based Residential Facilities (CBRFs), and Residential Care Apartment Complexes (RCACs)—and to propose targeted interventions for reducing falls and enhancing resident safety. The study analyzes data from the Wisconsin Coalition for Collaborative Excellence in Assisted Living (WCCEAL), incorporating fall reports from ALCs across the state. The dataset includes quarterly records from 2017 to 2022, covering facilities that voluntarily tracked and shared fall and hospitalization data. Falls and fall-related injury data were evaluated using incidence rates per 1,000 resident days, stratified by facility type. Facility characteristics, including occupancy, primary resident population, and location, were analyzed to identify patterns in falls. The study used chi-square tests to compare sample and non-sample cohorts. Overall, the total number of falls in ALCs increased during the study period, whereas fall-related injuries decreased. CBRFs and RCACs showed substantial increases in fall incidence rates, while AFHs showed a declining trend. Findings emphasize the need for customized fall prevention interventions across different ALC types. AFHs may benefit from individualized interventions, CBRFs from staff training and structured fall protocols, and RCACs from self-management strategies like emergency response systems. Standardizing fall reporting practices and enhancing fall prevention policies at both the state and facility levels could improve resident safety and reduce fall-related injuries.

Abstract citation ID: igaf122.3459

### FALLS AMONG OLDER ADULTS WITH AND WITHOUT ALZHEIMER'S DISEASE AND RELATED DEMENTIAS IN LOS ANGELES COUNTY: 2016-2022

D'Artagnan Robinson[1], Noel Barragan[1], Emiley Chang[1], Dalia Regos-Stewart[1], Mariana Reyes[1], and Tony Kuo[2], *1. Los Angeles County Department of Public Health, Los Angeles, California, United States, 2. County of Los Angeles, Los Angeles, California, United States*

Falls are a leading cause of injury, hospitalization, and mortality for older adults in the United States. Those living with Alzheimer's disease and related dementias (ADRD) are at heightened risk for falls due to cognitive impairment and functional limitations. As part of the Los Angeles County BOLD Initiative, the Los Angeles County (LAC) Department of Public Health conducts surveillance and research on ADRD and their associated risks. Using patient discharge data from the California Department of Health Care Access and Information, this study examines fall-related hospitalizations among LAC adults aged ≥50 years during the period 2016-2022. Falls were identified using external cause of morbidity codes (ICD-10-CM: W00-W19) and ADRD status was determined via principal and secondary diagnosis codes. All falls were analyzed by admission year, age, sex, race/ethnicity and fall type (e.g., falls from same level, falls from bed), with low-frequency categories collapsed into "Other Falls." Chi-square tests were employed to compare fall type by ADRD status. A total of n = 243,136 fall-related hospitalizations occurred during this timeframe, with 20.5% involving ADRD patients. A higher proportion of ADRD patients experienced falls from beds (8.0% vs. 4.3%, $p < 0.0001$) and non-motorized mobility devices (2.4% vs. 1.2%, $p < 0.0001$), whereas a lower proportion fell from stairs and steps (1.9% vs. 4.5%, $p < 0.0001$) compared to non-ADRD patients. Study findings highlight the burden of falls among this population and underscore the need to effectively prevent and manage falls, especially through strategies that address environmental and mobility risks.

Abstract citation ID: igaf122.3460

### SYNERGIC TRIAL SECONDARY OUTCOMES RESULTS ON GAIT AND FALLS

Frederico Faria[1], Surim Son[2], Teresa Liu-Ambrose[3], Quincy Almeida[4], Laura Middleton[5], Louis Bherer[6], and Manuel Montero Odasso[7], *1. Western University Lawson*

*Health Research Institute, London, Ontario, Canada, 2. Western University, London, Ontario, Canada, 3. The University of British Columbia, Vancouver, British Columbia, Canada, 4. Wilfrid Laurier University, Waterloo, Ontario, Canada, 5. University of Waterloo, Waterloo, Ontario, Canada, 6. Université de Montréal, Montreal, Quebec, Canada, 7. University of Western Ontario, London, Ontario, Canada*

Background: Older adults with Mild Cognitive Impairment (MCI) have higher risk of gait impairments and falls, yet the effects of multimodal interventions, including combinations of exercises with cognitive training, on improving their mobility remain unclear. Objectives: To investigate the synergistic effects of aerobic-resistance exercise combined with cognitive training, with or without vitamin D supplementation, on gait performance and fall risk in older adults with Mild Cognitive Impairment (MCI). Methods: The effect of 20 weeks of aerobic-resistance exercise, cognitive training, and vitamin D supplementation (10,000 IU 3x/week) on gait and falls in older adults with MCI was evaluated in the SYNERGIC Trial, using a fractional factorial design. Results: Among 161 participants, exercise-based arms ($n = 4$) improved gait speed (+7.5 cm/s, $p < 0.001$) and reduced injurious falls by 62% (IRR=0.38, $p = 0.04$). Exercise plus cognitive training showed the greatest gains in gait speed and reduced injurious falls (IRR=0.13, $p = 0.05$). Vitamin D did not enhance outcomes and increased gait instability. Conclusion: Aerobic-resistance exercise combined with cognitive training improves gait performance and decreases the risk of injurious falls in older adults with MCI. The addition of vitamin D did not produce benefits.

Abstract citation ID: igaf122.3461

### COST-ANALYSIS OF A GERIATRICIAN-LED FALLS PREVENTION CLINIC AMONG OLDER ADULTS AT HIGH RISK OF FUTURE FALLS

Jennifer C. Davis, Elise Wiley, Larry Dian, Kenneth Madden, *Karim Khan, Naaz Parmar, Katrina Loutet, and Teresa Liu-Ambrose, The University of British Columbia, Vancouver, British Columbia, Canada*

A geriatrician-led Falls Prevention Clinic is a best practice and evidence-based approach for falls prevention that has demonstrated feasibility and acceptability. The economic impacts of this approach, which directly impact its sustainability within the healthcare system, remain unknown. Our primary objective was to determine the costs and potential savings associated with a multi-disciplinary geriatrician-led Falls Prevention Clinic among older adults at high risk of falls. Operating costs of the Falls Prevention Clinic were determined based on operating costs (i.e., personnel, equipment, overhead), medical services plan costs, and personnel costs. Falls Prevention Clinic usage over 12 months was detailed based on monthly frequencies of new and repeat visits. Longitudinal health resource utilization was ascertained over 12 months. Cost savings from falls averted over 12 months were estimated. Main outcome measures included: operating costs of the clinic, estimated annual health resource utilization savings, number of falls over 12 months and health care costs (i.e., health care practitioner, hospital admissions, laboratory tests/investigations). A total of 543 patients were seen over a year, with 240 new and 303 follow-up patients. The total direct health resource utilization costs were 4,892 (7,767) (2024 CAD$) per person over 12 months. The annual estimated cost-saving of the clinic from fall prevention is 956,288 (2024 CAD$). The Fall Prevention Clinic provides a multi-disciplinary approach that is best practice, evidence-based for fall prevention. This approach has demonstrated feasibility and effectiveness and saves health care dollars; thus, it is an effective and economically attractive approach to consider for implementation.

Abstract citation ID: igaf122.3462

### FEAR OF FALLING: 30 YEARS OF RESEARCH

Helen Lach, and Joseph Trinitas, *Saint Louis University, St. Louis, Missouri, United States*

Fear of falling has the potential to reduce activity and quality of life for community-dwelling older people, and an increased risk of falling. This topic has been explored for over 30 years. As a result, the aim of this umbrella review is to explore the state of the science on fear of falling to determine implications for future research. We searched databases including CINAHL, Medline (OVID), Scopus, and Cochrane for reviews (systematic, integrative, metanalyses, metasyntheses) since 1990. Studies with a focus on fear of falling in community-dwelling older adults as the primary topic or outcome in English were included, resulting in 879 articles. After removing duplicates, 606 articles remained for title and abstract screening. Studies of specific conditions such as stroke or specific settings (e.g. nursing home) were excluded. Data was extracted to examine designs, samples, measures, and outcomes of reviews of descriptive and experimental studies for the final 47 articles. Overall, 30 years of evidence confirms that that fear of falling is a significant public health problem for older people, common even among those who have not had a fall. Fear of falling leads to negative outcomes including reduced activity, depression and increased risk of nursing home placement, and is a risk factor for future falls. Further evidence supports exercise and multifactorial interventions to reduce fear of falling and improve quality of life for older people. Future research could help target interventions to improve precision of outcomes.

### SESSION 7910 (POSTER)

### FLASH POSTER SESSION: CAREGIVING

Abstract citation ID: igaf122.3463

### EMPOWER: A FEASIBILITY STUDY OF A REMOTE AND IN-PERSON INTEGRATIVE INTERVENTION FOR CAREGIVER SUPPORT

Katie Trainum[1], Rosa Schnyer[2], and Bo Xie[2], *1. University of Pennsylvania, Philadelphia, Pennsylvania, United States, 2. The University of Texas at Austin, Austin, Texas, United States*

Family caregivers face substantial caregiving-related burdens, needing supportive interventions, particularly those incorporating self-care strategies that can be delivered remotely with broad accessibility. We developed EMPOWER (Engage your Mind and Body to Promote your Own Wellness, Energy and Relaxation), an intervention incorporating complementary and integrative strategies like breathing exercises, Tai Chi, and self-acupressure. In Fall 2024, we conducted four sessions (3 via Zoom; 1 in-person) to evaluate EMPOWER's feasibility. Following recruitment challenges, including scammers in the first two Zoom sessions, the final sample included 8 caregivers (7 female; age range 55-90; mean = 68.38; 6 remote, 2 in-person). Each 90-120-minute session involved instruction and practice led by an experienced clinician. Participants received program handouts and audio recordings for at-home practice. Two weeks later, 75%

(n = 6) completed a post-intervention questionnaire online or over the phone, including 2 from the in-person session and 4 from the third Zoom session. Data were analyzed using content analysis and paired t-tests. T-tests revealed no significant changes in caregiver burden, stress, anxiety, or quality of life from pre- to post-intervention across all groups. Content analysis of qualitative data suggested improved relaxation, stress management, empowerment, and regular practice. Participants also reported challenges such as technological barriers, physical limitations, and time constraints in both session formats. These findings support the feasibility of delivering EMPOWER remotely and in-person for caregivers. Future work should refine the intervention to overcome the identified challenges, incorporate a mobile application for shorter, more accessible sessions, and test with a larger sample.

Abstract citation ID: igaf122.3464

### CHALLENGES AND LESSONS FROM A REMOTE INTERVENTION FOR FAMILY CAREGIVERS: A FEASIBILITY STUDY OF THE EMPOWER PROGRAM

Katie Trainum[1], Rosa Schnyer[2], and Bo Xie[2], *1. University of Pennsylvania, Philadelphia, Pennsylvania, United States, 2. The University of Texas at Austin, Austin, Texas, United States*

Remotely delivered interventions offer advantages like broader geographic reach and increased accessibility. However, they also introduce challenges, including scams in the recruitment and implementation as well as technological barriers. This paper reports challenges encountered and lessons learned through a feasibility study of EMPOWER (Engage your Mind and Body to Promote your Own Wellness, Energy and Relaxation), a self-care health intervention incorporating complementary and integrative techniques. In Fall 2024, we conducted four EMPOWER sessions (three via Zoom, one in-person). In the first two Zoom sessions, to ensure participants' privacy and comfort, we did not require verbal or camera engagement, nor did we have a moderator present in the breakout room discussions. After the first two Zoom sessions, we discovered suspicious activity, including similar email formats, inconsistent demographics (i.e., higher-than-expected male and younger participants), and IP address anomalies (survey responses originating from outside the research area and multiple responses from single IP addresses). As a result, data collected from those first two Zoom sessions had to be discarded. Based on these experiences, we recommend several strategies for future virtual caregiving interventions. These include recruiting participants through established in-person groups; implementing rigorous participant vetting processes to verify authenticity; limiting responses to one per IP address; requiring verbal or camera engagement during the intervention sessions; and moderating Zoom breakout room discussions. While remotely delivered interventions present challenges, creative solutions can help maintain research integrity and enhance participant experience. Further work is needed to evaluate the efficacy of these strategies.

Abstract citation ID: igaf122.3465

### CARE PARTNER VIEWS ON A PROGNOSIS-INFORMED DEMENTIA ROADMAP

Nancy Schoenborn, and Halima Amjad, *Johns Hopkins University School of Medicine, Baltimore, Maryland, United States*

Almost all older adults with Alzheimer's disease and related dementias (ADRD) receive help from care partners. An important unmet need in ADRD care partner support is anticipatory guidance and individualized prognostic information. Given a growing compendium of prognostic tools for older adults, including specifically for people living with dementia (PLWD), the science is ripe for developing a prognosis-informed roadmap in PLWD. However, prognostic tools will have little or negative impact if they are not relevant or acceptable to end-users. We aimed to explore care partners' ADRD prognosis information needs and preferences using qualitative interviews. The study is actively recruiting, with 9 completed interviews thus far. Interviews were analyzed using qualitative thematic analysis. We found that care partners often reported not receiving adequate guidance at the time of ADRD diagnosis. Care partners consider multiple prognostic outcomes important, including life expectancy, likelihood of unsafe behaviors, need for supervision and personal care assistance, and likelihood of mobility challenges. Outcomes that impact living arrangements and safety were the highest priority. Care partners were uniformly accepting of the potential uncertainty inherent in predictions and would nonetheless find the prognostic information useful. Some preferred more personalized predictions while others preferred more general guidance. Care partners reported that the prognosis information would impact healthcare decisions as well as finances, living arrangements, and family members' life plans. Our preliminary findings demonstrate that ADRD care partners strongly desire prognosis information for anticipatory guidance despite potential uncertainties and prioritize predictions about safety challenges and care needs as the most important.

Abstract citation ID: igaf122.3466

### EXPLORING WEARABLE DEVICE USE AMONG DEMENTIA CAREGIVERS: BENEFITS, CHALLENGES, AND IMPROVEMENTS

Jung-Ah Lee[1], Hyun Jung Kim[2], and Kyeung Mi Oh[3], *1. University of California, Irvine, Irvine, California, United States, 2. Seattle University, Seattle, Washington, United States, 3. George Mason University, Fairfax, Virginia, United States*

Caregivers of persons with dementia experience significant challenges and stress due to round-the-clock responsibilities. Wearable devices (WD), such as smartwatches and smart rings, can provide real-time health data, enhancing caregivers' self-care. This study explored caregivers' experiences using WD as part of a three-month in-home educational intervention, which included stress management strategies. Participants (N = 101) were recruited through community outreach and completed exit interviews. The sample included 14 non-Hispanic White, 13 Hispanic, 35 Korean, and 39 Vietnamese caregivers. Qualitative analysis identified four key themes: enhancing usability, encouraging data engagement, improving wearability and convenience, and expanding health-tracking features. Most caregivers found WD beneficial for increasing health awareness, motivation, and self-monitoring. However, they highlighted the need for additional education on device functions, app navigation, and data interpretation. Non-English-speaking caregivers emphasized the importance of language accessibility. Many preferred larger screens for better readability and reminders to improve adherence. Some struggled with discomfort, forgetting to wear or charge devices, and limited battery life. Caregivers expressed interest in additional health-tracking features, including blood pressure, blood sugar, body fat, and muscle mass monitoring. Addressing these challenges could enhance adherence, particularly among older caregivers. Participants recommended tailored education for caregivers with varying levels of digital literacy, alternative charging methods, and visible battery indicators to improve usability. Findings highlight the need for comprehensive education, frequent reminders, and improved usability to maximize WD benefits. By addressing these factors, WD can better support caregivers in managing their well-being and sustaining their caregiving roles with greater resilience.

Abstract citation ID: igaf122.3467

**BALANCING CARE AND CHRONIC ILLNESS: A CONCEPTUAL FRAMEWORK FOR OLDER CAREGIVERS OF ADVANCED ILLNESS PATIENTS.**

Hyeyoung Park, and Joohyun Chung, *University of Massachusetts Amherst, Amherst, Massachusetts, United States*

Aging caregivers are a growing yet under-recognized group. Many older caregivers provide care for individuals with advanced illness while also managing their own chronic conditions. Self-management plays a crucial role in chronic illness, and illness perception influences an individual's self-management. However, the caregiving context—particularly when caring for someone with advanced illness—may alter how caregivers perceive their own illness, subsequently affecting their self-management. Previous research has primarily categorized individuals as either patients with chronic conditions or caregivers, rather than considering these roles simultaneously. To address this gap, this paper develop a conceptual framework that integrates the caregiving context as a moderating or mediating factor between illness perception and self-management, supported by preliminary findings. We conducted an exploratory study (N = 25) assessing illness perception, caregiving strain, anticipatory grief, and self-management among older caregivers of individuals with advanced illness. Descriptive statistics and non-parametric analyses were used to identify patterns. Given the small sample size, results were interpreted as preliminary insights informing theoretical development rather than definitive conclusions. Preliminary findings indicate that while illness perception among caregivers is heightened, this heightened awareness does not necessarily lead to improved or diminished self-management, pointing to the potential influence of other mediating factors. While prior research has suggested that caregiving stress may reduce illness perception due to self-neglect, our data reveal a different pattern, that caregiving stress seems to actually increase illness awareness.

Abstract citation ID: igaf122.3468

**RECRUITMENT OF PEOPLE WITH COGNITIVE IMPAIRMENT AND CAREGIVERS FOR A RANDOMIZED CLINICAL TRIAL**

Pei-Shiun Chang[1], Amy Katz[2], Richard Passey[3], Ziyi Yang[3], Sujuan Gao[3], Liana Apostolova[4], and Yvonne Lu[2], *1. Indiana University Bloomington, Bloomington, Indiana, United States, 2. Indiana University School of Nursing, Indianapolis, Indiana, United States, 3. Indiana University School of Medicine, Indianapolis, Indiana, United States, 4. Indiana University, Indianapolis, Indiana, United States*

Recruiting older people with cognitive impairment (PwCI) and their caregivers (CG) for research studies is an ongoing challenge, which was magnified by the onset of COVID-19. This study describes the unique recruitment of PwCI-CG dyads for a Randomized Control Trial using a tailored Daily Engagement in Meaningful Activity (DEMA) intervention to improve the quality of life for both PwCI and their CGs. Instead of traditional in-person recruitment, telephone-based recruitment was a driving factor for successfully contacting participants. Using a state-wide healthcare system database, we identified 4133 potential participants who met study criteria and could identify a primary caregiver. By mailing study information to potential PwCI-CG dyads, we observed a significant increase in initial contact, returned calls, and general interest in study participating. In-person recruitment, direct referrals from physicians in the clinic setting, and partnered local agencies were among the least successful strategies for establishing initial contact and recruiting dyads. After implementing new recruitment strategies, we recruited 152 dyads in 16 months (mean = 10 dyads/month). We found that establishing a monthly recruitment goal was a beneficial strategy. The healthcare system database, followed by an organizational registry, demonstrated higher rates of making initial contact and reaching eligible dyads (46% and 37%, respectively). Specific dyadic recruitment challenges included finding a suitable CG to participate and the extended follow-up timeframe from initial contact to screening and enrollment. There is a growing need to better understand successful strategies for didactic recruitment, particularly among older PwCI and their CGs.

Abstract citation ID: igaf122.3469

**USABILITY AND ACCEPTABILITY OF A DIGITAL HEALTH ACTIVITY PROGRAM AMONG OLDER HEART FAILURE FAMILY CARE PARTNERS**

Dawon Baik[1], Catherine Jankowski[1], Larry Allen[1], Colleen McIlvennan[1], Heather Coats[1], and Blaine Reeder[2], *1. University of Colorado, Aurora, Colorado, United States, 2. University of Missouri, Columbia, Missouri, United States*

Family care partners of persons with heart failure (HF-FCPs) face significant stress and exhaustion from prolonged caregiving, impacting their health. As they age, their increased burden and limited support raise the risk of poor health outcomes for both themselves and the care recipients. To support older HF-FCPs' health management, we developed a tailored digital health physical activity (PA) program, called TPA4You, that includes videoconferencing with a health coach, wearable sensors, and motivational messaging. We conducted a 1-week, two-phase, iterative field test with 11 participants to evaluate the usability and acceptability of TPA4You. Modifications were made based on Phase 1 participants' feedback (i.e., reducing weekly PA sessions from three to two, replacing the automated messaging system with manually sent motivational messages). All participants in both phases (mean age 68.2±7.1 years, 91% female, 82% spouses) attended 100% of Zoom PA sessions. Key themes from Phases 1 and 2 were similar. Overall, participants found TPA4You acceptable and useful. One key theme was TP4AYou is a digital tool that builds confidence in PA capabilities by increasing awareness and motivation. Another key theme was TPA4You's ability to provide emotional and psychosocial benefits by reducing caregiver stress and facilitating human contact with a health coach. A third theme was TPA4You served as a valuable reminder to take care of themselves. In Phase 2, manual motivational messages led to higher satisfaction, less confusion, and greater PA engagement as compared to Phase 1 automated messaging. These findings will inform further revisions and a pilot RCT of TPA4You.

Abstract citation ID: igaf122.3470

**AGE-FRIENDLY PALLIATIVE CARE FOR VETERANS AND CAREGIVERS IN A RURAL VA HEALTHCARE SYSTEM -- THE EASTERN CO EXPERIENCE**

Stephanie Hartz[1], Alexander Garbin[2], Courtney McGuire[3], Jill Steagall[4], Jocelyn McCauliff[5], and Kathryn Nearing[6], *1. VA Eastern Colorado Health Care System, Aurora, Colorado, United States, 2. Eastern Colorado VA Health Care System, Aurora, Colorado, United States, 3. Geriatric Research Education and Clinical Center, Aurora, Colorado, United States, 4. Rocky Mountain Regional VA Medical Center, Aurora, Colorado, United States, 5. Veterans Affairs, Aurora, Colorado, United States, 6. University of Colorado Anschutz Medical Campus, Denver, Colorado, United States*

Introduction: Age Friendly Health Systems (AFHS) provide care to older adults aligned with geriatric best practices to improve healthcare quality, minimize harms and support older adults' goals of care. AFHS designation means that providers consistently address the Geriatric 4Ms during clinical visits: Mentation, Mobility, Medication and What Matters Most. Methods: Within the VA Eastern Colorado Health Care System (ECHCS), our rural tele-Palliative Care clinic achieved AFHS Level 2 Committed to Care Excellence recognition in December 2021, becoming the first AFHS-designated telemedicine clinic in the nation. We engaged older Veterans in planning and implementation. Using our existing interprofessional team and clinic workflow, we addressed the Geriatric 4Ms during visits. Results: FY23-24, we conducted 192 AFHS tele-Palliative Care visits, 81% with rural/highly rural Veterans. We served 108 unique patients (FY23: 57; FY24: 51). Compared to Colorado's Veteran population, racially minoritized Veterans and women were underrepresented, while older Veterans were overrepresented. The majority were White (82% and 73%), not Hispanic/Latino (83% and 73%), male (100% and 98%), and ≥65 (90% and 89%). All 4Ms were addressed for 86% (FY23) and 76% (FY24) of unique patients. AFHS tele-Palliative Care saved Veterans/caregivers 23,622 (FY23) and 18,632 (FY24) miles of travel. Conclusion: Congruent with AFHS, Palliative Care focuses on physical, emotional, and psychosocial aspects of serious illness. Our work to achieve AFHS Level 2 designation in a tele-Palliative Care clinic is novel nationally. We demonstrated that evidence-based care can be provided to every older adult, regardless of care modality, without expanding staff or changing clinical workflows.

Abstract citation ID: igaf122.3471

**CAREGIVER SPOUSES OF PERSONS WITH DEMENTIA WHO ENGAGE WITH AN EMERGENCY PREPAREDNESS TOOLKIT INTERVENTION**

Anna Satake[1], Rebecca Boxer[1], Andrea Daddato[2], Kris Wain[2], Blythe Dollar[2], and James Lagrotteria[2], *1. University of California, Davis, Davis, California, United States, 2. Kaiser Permanente Colorado, Aurora, Colorado, United States*

Hospitalizations of a spousal caregiver (SC) of a person with dementia (PWD) can significantly disrupt caregiving and negatively impact the SC. This study conducted a low-touch intervention by offering a SC an emergency preparedness toolkit (EPT) to prepare for their own unexpected hospitalization. In January 2023, 1,062 caregivers of PWD who live together and are not institutionalized were invited to request the EPT and use it to prepare for their own healthcare emergency. Of caregivers notified, 489(46%) requested the EPT. Caregivers who didn't request the EPT n = 573(54%) were more likely to be male [261(46%) vs 188(38%);p=.02], have higher Elixhauser comorbidity scores (3.0±2.6 vs 2.5±1.9, p<.01), have more ED visits[132(23%) vs 88(18%)p=.04], and hospitalizations [77(13%) vs. 34(7%);p = <.01] in the prior 12 months compared to caregivers who requested the EPT. Caregiver outcomes by EPT request were measured 6-month after the EPT was sent. Caregivers who didn't request the EPT were no different in number of hospitalizations [52(9% vs 34(7%), p=.2] than those who did; however, they had a higher number of deaths [13(2%) vs 2(.4%), p=.01] at 6 months. For the caregiver, there were no significant differences in outcomes such as ED visits/hospitalizations between the two groups. Findings indicate that the health severity of caregivers of PWD was associated with the caregiver's response to a supportive intervention. Outreach and engagement to caregivers may need to be tailored to a range of caregiver health conditions and severity to be successful. Health of caregivers and their uptake of support and resources warrants further study.

### SESSION 7915 (POSTER)

### FLASH POSTER SESSION: FROM RESEARCH TO REFORM: POLICY PATHWAYS IN AGING AND PUBLIC HEALTH

Abstract citation ID: igaf122.3472

**A HOME AND COMMUNITY-BASED MULTICOMPONENT PHYSICAL ACTIVITY INTERVENTION FOR OLDER ADULTS**

Jing Chen, Nan Hua, and Qin Zhang, *The First Affiliated Hospital, Zhejiang University School of Medicine, Hangzhou, Zhejiang, China (People's Republic)*

Feasible physical activity (PA) interventions to promote multicomponent PA among community-dwelling older adults in China are limited. We aimed to develop and test the implementation feasibility of a multicomponent PA intervention for community-dwelling older adults. The intervention was developed by a multidisciplinary working group based on behavior change theories and techniques. A 12-week feasibility study was conducted in a community in Hangzhou, China. Feasibility was tested by Bowen's framework for assessing acceptability, demand, implementation, practicality, adaptation, integration, expansion, and limited efficacy of the PA intervention program. The PA intervention combined face-to-face group exercise (twice a week) in a community center and home-based exercise assisted by a WeChat applet or exercise manuals (at least once a week) with a weekly goal of engaging in multicomponent exercise at least three days per week. Overall, 19 participants (95%) completed the intervention (mean age of 77 years) with 12 participants using the applet and seven using the exercise manuals for home-based exercises. The intervention satisfied all Bowen's feasibility criteria. In the third month, 93% of the applet users and 79% of the non-applet users adhered to weekly exercise goals. Significant improvements were observed in grip strength (+1.4 kg, 95% CI 0.1 to 2.7) and chair stand test (-3.5 seconds, 95% CI -4.9 to -2.2). The findings suggested that the PA intervention program was feasible in real-world settings, demonstrating its potential for promoting multicomponent PA among community-dwelling older adults. A large-scale randomized controlled trial is needed to test the long-term effectiveness.

Abstract citation ID: igaf122.3473

### REFRAMING AGING AND HIV: INTEGRATING GERIATRIC CARE INTO PRIMARY HIV CLINICS TO CLOSE THE GAP IN ACCESS

Athina Schmidt[1], Jennifer Sergile[1], Irina Vovnoboy[2], and Archana Asundi[2], *1. Boston Medical Center, Boston, Massachusetts, United States, 2. Boston University - Chobanian & Avedisian School of Medicine, Boston, Massachusetts, United States*

The BMC HIV-Endurance (HIVE) Clinic implemented a "pop-up" geriatric care model to address the reluctance of people living with HIV (PLWH) to leave their primary care settings for geriatric services. Patients reported concerns about losing trusted providers. While systemic challenges at our clinic required an increase to the geriatric referral age to 70. The integrated HIVE model introduced specialized geriatric expertise directly into primary care settings to improve access and improve healthcare outcomes. Despite proactive referrals that originated from the patient's primary care provider with specific concerns to address, some patients still required guidance to grasp the rationale behind their referral to a geriatric specialist. Many expressed reluctance, perceiving such care as unnecessary and associating it with an outdated notion of aging, underscoring the need for targeted education on the benefits of specialized geriatric services In this paper presentation session, we describe the implementation of the HIVE Clinic and the strategies used to address barriers experienced by PLWH in accessing geriatric care. These strategies include enhanced communication, improved scheduling, and targeted patient education on aging-related needs to keep them safe and independent at home. Additionally, partnerships with local aging services, including visiting nurse associations (VNA), adult day health programs, and meal services, provided further support to patients. Results demonstrated improved healthcare proxy documentation, addressing polypharmacy concerns, expanded referrals, and strengthened care coordination. The HIVE Clinic's innovative model successfully improved access to geriatric care, emphasizing the value of familiar care settings and targeted patient education to address gaps in HIV-aging care.

Abstract citation ID: igaf122.3474

### DIGITAL MENTAL HEALTH INTERVENTIONS FOR OLDER ADULTS: A META-ANALYSIS OF RANDOMIZED CONTROLLED TRIALS

Yahui Wang, and Dannii Yeung, *City University of Hong Kong, Hong Kong, China (People's Republic)*

With growing mental health concerns among older adults and limited access to traditional services, digital interventions may offer a scalable solution. However, evidence regarding their effectiveness across different mental health outcomes remains unclear. Using multilevel modelling, a meta-analysis of randomized controlled trials examining digital interventions for older adults was conducted. Forty studies comprising 5,148 participants were analysed. Primary outcomes were depression and anxiety, with secondary outcomes including cognitive functioning, loneliness, and quality of life. Compared to control conditions, digital interventions demonstrated significant beneficial effects for depression ($g = -0.28$, 95% CI [-0.43, -0.13], $p < .001$) and anxiety ($g = -0.22$, 95% CI [-0.45, 0.00], $p = .05$). Secondary outcomes showed effects for cognitive functioning ($g = 0.35$, 95% CI [0.06, 0.64], $p < .05$) and loneliness ($g = -0.29$, 95% CI [-0.50, -0.08], $p < .01$), with effects on quality of life approaching significance ($g = 0.22$, 95% CI [-0.02, 0.46], $p = .07$). Moderator analyses for depression revealed larger effects for interventions employing guided human support and for smartphone-delivered interventions compared to unguided or internet-based approaches. Digital interventions appear effective for addressing mental health concerns in older adults, depression and anxiety, improving cognitive functioning and loneliness. The significant heterogeneity suggests that effectiveness varies based on intervention delivery method and the inclusion of human guidance. These findings not only highlight the potential of digital mental health tools for expanding psychotherapy options for older adults, but also suggest that personalized intervention addressing the individual needs can maximize the intervention outcomes.

Abstract citation ID: igaf122.3475

### A SUMMARY OF ORGANIZATIONS PROVIDING EVIDENCE-BASED DEMENTIA CAREGIVING PROGRAMS IN BEST PROGRAMS FOR CAREGIVING

Zoe Fete[1], Megan Huth[2], David Bass[2], Rachel Cannon[2], Sara Powers[1], and Morgan Minyo[2], *1. Benjamin Rose, Cleveland, Ohio, United States, 2. Benjamin Rose Institute on Aging, Cleveland, Ohio, United States*

Best Programs for Caregiving (BPC) is a free online resource of 45 evidence-based dementia caregiving programs. The Public Version of BPC, launched in 2024, enables family/friend caregivers to use a zip code search to find BPC programs available in their communities. Data were analyzed from a structured survey of 206 organizations identified by program developers as delivering their BPC program and used to populate the Public Version. The most common types of delivery organizations were healthcare or single-service community organizations (37; 18.0%), AAAs (37; 18.0%), multi-service community organizations (31; 15.0%), and senior centers/meal programs (28; 13.6%). There were 28 (13.6%) organizations offering a BPC program nationwide to caregivers living anywhere in the US, and 178 offering a program locally or statewide (86.4%). Most organizations were delivering a BPC program partially or fully remote, with only 34 (16.5%) exclusively in-person. An overwhelming majority were delivered exclusively by professionals (160; 77.7%) and were free (175; 85%). Nearly a quarter of programs (49; 23.8%) were adapted for caregivers identifying with diverse communities, most commonly Hispanic and Latino/Latina, Black/African American and LGBTQ. There were 73 (35.8%) programs delivered in languages other than English, with the most frequent offering being Spanish (69). Findings demonstrate that caregivers throughout the US, including those who identify with underserved populations or live in underserved areas, have evidence-based support programs available in their communities, with many being free. Findings also illustrate variation in the characteristics of programs, enabling caregivers to choose options that most closely match their preferences.

Abstract citation ID: igaf122.3476

### UTILIZATION AND PERCEIVED NEED FOR CAREGIVER SUPPORT SERVICES: IMPLICATIONS FOR EMOTIONAL WELLBEING

Tochukwu Okolie[1], and Heather Menne[2], *1. University of Connecticut, Mansfield Center, Connecticut, United States, 2. Miami University, Oxford, Ohio, United States*

The demonstrable efforts of family members providing care to an older person in the United States continue to make research headlines. Nonetheless, family caregivers providing hours of unpaid care are disproportionately at risk of declining emotional wellbeing. One of the protective mechanisms against this decline may be caregiver programs and services including: caregiver education/training, counseling, and support services. Hence, the aim of the study was to explore the differences in emotional wellbeing associated with: (i) attending caregiver support services, and (ii) a perceived need for caregiver support services. Data from the 2023 wave of the National Survey of Older Americans Act Participants (NSOAAP) was used ($n = 1004$). Univariate, bivariate, and regression techniques assessed the relationship between emotional wellbeing and the predictors of interest. The regression results show that compared to those who have attended counseling services, emotional wellbeing was about 0.17 points higher among those who have not attended, controlling for other variables in the model ($\beta = 0.166$, $p = 0.004$). Also, compared to those who perceived the need for counseling services, emotional wellbeing was about 0.3 points higher among those who do not, controlling for other variables in the model ($\beta = 0.312$, $p < .0001$). Also, being a male or a Black/African American were each significantly associated with an increase in emotional wellbeing, while accounting for other factors. The results suggest a need to understand the motivations and barriers to counseling services. There is also a need to sensitize caregivers on the need for these services.

Abstract citation ID: igaf122.3477

### DEVELOPMENT OF THE BAJJAJJA INTERVENTION DESIGNED TO MEET GRANDMOTHER-CAREGIVERS' NEEDS IN UGANDA

Schola Matovu[1], Jacqueline Eaton[2], Lee Ellington[2], and Heather Young[3], *1. University of Utah College of Nursing, Salt Lake City, Utah, United States, 2. University of Utah, Salt Lake City, Utah, United States, 3. Betty Irene Moore School Of Nursing at UC Davis, Sacramento, California, United States*

The financial, social, health and overall well-being of Ugandan grandmothers who care for their grandchildren has been significantly affected by years of ongoing socioeconomic, political, and historical HIV/AIDS-related challenges. We engaged a Community Advisory Board (CAB) of key community partners to co-develop a culturally responsive Building A Joint Action for JaJJAs (BAJJAJJA) intervention to address the most salient financial, social, and health challenges. Using a community-engaged and qualitative research approach, we worked with a CAB ($N = 10$) that consisted of grandmother-caregivers, local council leaders, income generating activity experts, and health providers from local health centers in a peri-urban setting of central Uganda. This participatory approach ensured that intervention development aligned with the Uganda National Council for Science and Technology's priorities for community engagement while also promoting acceptability and sustainability. The developed BAJJAJJA manual details three core intervention components: (1) Income-Generating Activities (IGA)—training in small business skills and cooperative saving practices to enhance financial security; (2) Social Support Groups—peer-led gatherings to strengthen emotional resilience and shared problem-solving; and (3) Health Coaching—education on chronic disease prevention, mental well-being, and self-care strategies with culturally relevant materials tailored for low-literacy populations. This study highlights the effectiveness of a community-engaged approach in designing interventions that address the multidimensional needs of aging grandmother-caregivers. Findings from this study will inform future research that will refine, adapt and test the efficacy of similar interventions in both low-resource and other global settings such as rural areas in the US.

Abstract citation ID: igaf122.3478

### ENHANCING INTERGENERATIONAL PROGRAMS: OLDER ADULTS' PERSPECTIVES ON CHALLENGES AND OPPORTUNITIES

Zeyu Liu[1], Rana Zadeh[2], and Karl Pillemer[2], *1. Cornell University, Jersey City, New Jersey, United States, 2. Cornell University, Ithaca, New York, United States*

Intergenerational programs (IGPs) are critical interventions for enhancing physical, psychological, social, and cognitive well-being by fostering social connectedness and reducing isolation among older adults. However, a gap in existing research is the limited understanding of the factors influencing the acceptability and feasibility of IGPs. Identifying these factors is essential for designing programs that better meet the needs of older adults. This study examines older adults' perspectives on IGPs through semi-structured interviews with 24 participants (ages 65–104). Thematic analysis identified seven key factors that shape engagement: (1) Culture, (2) Knowledge and Experience Exchange, (3) Interest and Curiosity as Motivation, (4) Emotional Support, (5) Social Interaction, (6) Family and Community Support, and (7) Technology Integration. These factors influence how older and younger participants engage in IGPs, shaping their experiences through shared activities and reciprocal knowledge exchange. The findings emphasize the role of structured engagement in sustaining meaningful intergenerational interactions. While older adults show enthusiasm, younger participants often engage due to academic requirements, limiting intrinsic motivation. Differences in digital literacy create barriers to participation, particularly for older adults unfamiliar with technology. The study also found that varying expectations among older participants, limited institutional support, and the digital divide pose challenges to engagement. These insights enhance our understanding of intergenerational dynamics, guiding policymakers and program developers in designing interventions that promote healthy aging and strengthen intergenerational bonds.

Abstract citation ID: igaf122.3479

### LEARNING AND GROWING TOGETHER: MORE FULLY INTEGRATING RESEARCH, PRACTICE, AND ORGANIZATIONAL POLICY

Lea Efird-Green[1], Jennifer Terray[2], Heidi Gil[2], and Sheryl Zimmerman[3], *1. The University of North Carolina at Chapel Hill, Chapel Hill, North Carolina, United States, 2. LiveWell, Plantsville, Connecticut, United States, 3. University of North Carolina at Chapel Hill, Chapel Hill, North Carolina, United States*

Community-engaged research is important if results are to be relevant to end-users and inform practice and policy; however, the extent to which such research truly partners with community members is highly variable. This project expanded a partnership that developed two "living well with dementia" measures, such that the community partner was fully engaged in actually designing and conducting the evaluation of those measures; in this way, the extent to which the partner functioned as the researcher and informed the research process constitutes a new model for community-engaged research. Process findings indicated full partnership requires that (1) the mission of the partner be fully aligned with and supported by the focus of the research (in this case, LiveWell, a dementia-focused services organization committed to a strengths-based approach to supporting the wellbeing of people living with cognitive change or dementia); (2) the partner be invested in learning about and contributing to the research process (including the development of pragmatic study protocols); (3) the researcher be open to developing protocols that extend beyond traditional research (e.g., being willing to solicit IRB-approval that confidential information may be incorporated into the partner's care plans – notably, to date nearly 80% of consents have included permission for this sharing); and (4) the researcher allow that the protocol may expand to include participants ineligible for study. This presentation will detail these and other novel findings to serve as a model of research that benefits community partners and researchers and has true promise to inform practice and policy.

Abstract citation ID: igaf122.3480

### CO-CREATING A VIRTUAL REALITY PROGRAM FOR OLDER ADULTS WITH DEMENTIA IN HOSPITAL

Lillian Hung[1], Lily Haopu Ren[2], W. Ben Mortenson[2], Angelica Lim[3], Jim Mann[2], Lily Wong[2], Christine Wallsworth[2], and Jennifer Boger[4], *1. University of British Columbia, Vancouver, British Columbia, Canada, 2. The University of British Columbia, Vancouver, British Columbia, Canada, 3. Simon Fraser University, Vancouver, British Columbia, Canada, 4. University of Waterloo, Waterloo, Ontario, Canada*

Virtual Reality (VR) is promising in improving the well-being of older adults with dementia in hospitals; however, traditional VR design and VR experience delivery to meet their diverse needs. Involving older adult patient partners, family caregivers, and staff in the co-design of gerontechnology is considered best practice, yet few researchers have adopted this inclusive approach. Appreciative Inquiry offers a collaborative framework to engage relevant users, leveraging their expertise and lived experiences for innovation. Our study aims to understand the contribution of Appreciative Inquiry in engaging multiple partners in co-creating a VR program for Older Adults with Dementia in hospitals. We co-created a VR program guided by Appreciative Inquiry principles, engaging patient partners, families, hospital staff and leaders. We adapted methods to facilitate meaningful participation, including six focus groups and interviews with 56 participants across two hospital units. Participants' insights informed the iterative development and refinement of the VR program. Patient partners, families, staff, and leaders meaningfully contributed to the design process. The collaborative process fostered a sense of ownership among participants, challenged assumptions about dementia care, and promoted positive interactions and experiences within the project team. Our approach demonstrated that using tailored methods is essential for authentic engagement, ensuring the developed technology meets real-world needs and will more likely be adopted in care practice. The study demonstrates the value of Appreciative Inquiry-informed co-design partnerships in co-creating technology. Future studies should further explore co-design methods across diverse cultural and organizational contexts with diverse older adults and other partners.

Abstract citation ID: igaf122.3481

### SELF-COMPASSION AND CAREGIVING OUTCOMES: INSIGHTS FROM A NEW THEORETICAL MODEL

Claire Grant[1], and Katherine Judge[2], *1. VA, Waltham, Massachusetts, United States, 2. Cleveland State University, Cleveland, Ohio, United States*

An expansion of theoretical modeling of the stress process for caregivers of people living with dementia (PLWD) is essential for the advancement of theoretical and applied literatures. The proposed presentation will outline results from a recent study testing an adapted model of self-compassion in caregivers of PLWD (Cha et al., 2023). The model tests parallel lines of mediation by which self-compassion, a way of relating to oneself, influences outcomes including depression, anxiety, and positive aspects of caregiving through mediators including the general resource, emotion regulation, and coping strategies. Caregivers of PLWDs were recruited through CloudResearch and social media posts (N = 226). Participants were majority female (50%), White (70%) and were middle aged (M = 40.5, SD = 12). Findings demonstrated support for self-compassion impacting depression though emotion focused and dysfunctional coping (b=-.05,95%CI [-.0877,-.0139]); b=-.15, 95%CI [-.2150,-.0874]). The general resource (b=-.09,95%CI[-.1777,-.0018]) and dysfunctional coping (b=-.11,95%CI[-.1964,.0583) mediated the relationships between self-compassion and anxiety. Finally, self-compassion indirectly impacted positive aspects of caregiving through problem focused coping (b=.13,95%CI[.0595,.2264]). and the emotion regulation technique of cognitive reappraisal (b=.15, 95%CI[.0143,.2699]). Overall, the results highlight the role of coping as a mechanism of self-compassion to impact a range of caregiver outcomes as well as the important similarities and differences in the significant mediators and relationships. The findings may be extended in future research to further refine theoretical understanding of the caregiving process and may have implications in intervention development. Current applications of these findings as well as future implications will be discussed.

### SESSION 7920 (POSTER)

### FLASH POSTER SESSION: AGING CURRICULUM DESIGN STRATEGIES

Abstract citation ID: igaf122.3482

### INTERPROFESSIONAL CURRICULUM DESIGN: FROM CONCEPTION TO DELIVERY

Lisa Bodenheimer[1], Anne Rosenfeld[1], and Stephanie Berdugo-Hernandez[2], *1. Rowan University, Glassboro, New Jersey, United States, 2. Rowan Virtua School of Osteopathic Medicine, Stratford, New Jersey, United States*

With funding awarded through the DHHS-HRSA Geriatric Workforce Enhancement Program Grant, the Rowan-Virtua School of Osteopathic Medicine (RV-SOM) has partnered with the Rowan University- Masters in Social Work Program (RMSW) to develop an interprofessionally designed and delivered post-graduate certificate in Geriatrics and Gerontology. RMSW is a new graduate program launched in 2023 and currently has no elective courses in aging. In 2024, initial pre-planning meetings were convened between both departments to conceptualize the components necessary to train the future workforce in geriatric and gerontology best practices with a specific focus on Alzheimer's Disease and Related Dementia. A (5) course elective program was conceived to be offered to master level students university-wide, not just RMSW matriculated students, aiming to recruit a diverse target audience including students in non-clinical programs. Once complete, the (5) course program will be offered as a post-graduate certificate. During AY 24/25, a work group began syllabus development for the first elective course, "Introduction to Geriatrics and Gerontology". The work group has a tripart mission; (1) to obtain stakeholder buy-in from multiple disciplines to co-develop modules from their scope of practice, (2) obtain buy-in from those practitioners to teach in the graduate program, allowing students the opportunity to learn directly from multiple disciplines and (3) create a syllabus which merges theory with practical approaches and interventions. To date, we have successfully engaged high-level faculty in geriatrics, neuropsychology, social work, and occupational therapy. All disciples will be engaged in didactics, small group activities, reading assignments and evaluation.

Abstract citation ID: igaf122.3483
### NEVER TOO "OLD": INSIGHTS FROM THE INTERGENERATIONAL CLASSROOM
Jessica Hsieh[1], Raza Mirza[1], Christopher Klinger[2], Alexis Hart[1], and Florene Shuber[3], *1. University of Toronto, Toronto, Ontario, Canada, 2. McMaster University, Hamilton, Ontario, Canada, 3. Christie Gardens, Toronto, Ontario, Canada*

The topic of aging demands greater attention in education systems, and intergenerational approaches help to combat ageism and improve the lives of older adults, now and in the future. Taking an intergenerational approach to contextualizing the experiences of older adults is a step towards addressing challenges commonly associated with aging. The University of Toronto (UofT) partnered with Christie Gardens, a retirement community, to launch an innovative experiential learning initiative: The Intergenerational Classroom. Half the students were UofT undergraduates; the other half were older adults residing at Christie Gardens. Through interactive seminar-style discussions, collaborative projects and mentorship, the course, which was held at Christie Gardens, and offered in the Fall 2023 and Fall 2024 semesters, provided a semester-long exploration on aging. This study explored the impacts of the Intergenerational Classroom from the perspectives of undergraduate students and older adults who had participated in the program. Guided by a phenomenological qualitative methodology, this study conducted in-person, semi-structured interviews with a sample of undergraduate students ($n = 10$) and older adults ($n = 16$). To enhance trustworthiness, two researchers independently analyzed transcript data to identify key transcript statements into themes. Outcomes of program success were identified across domains related to lasting friendships and bonds, increased awareness of aging issues, reduced ageist attitudes, and community and civic engagement. The taxonomy developed provides a comprehensive and conceptually organized range of successful outcomes to serve as infrastructure for the development of meaningful intergenerational programming outcome measures.

Abstract citation ID: igaf122.3484
### ARTIFICIAL INTELLIGENCE AIDS IN THE DEVELOPMENT OF MULTIDISCIPLINARY AGE-FRIENDLY EDUCATION
Suzanne White, Robin McAtee, and Laura Spradley, *University of Arkansas for Medical Sciences, Little Rock, Arkansas, United States*

This work describes a structured approach to leveraging Artificial Intelligence (AI), specifically ChatGPT, a Large Language Model (LLM), to streamline and accelerate the development of educational content while maintaining accuracy and adherence to clinical best practices. Goals were developed to meet the learning objectives of the targeted learners and then AI was introduced. The process began by prompting ChatGPT to generate an e-learning module with specific instructional design elements based on goal-oriented outcomes while integrating age-friendly content into specialized geriatric modules for healthcare professionals. For example, the prompt for "Introducing the 4Ms of Age-Friendly Care" included: "Generate a structured 15-minute e-learning module introducing the 4Ms Framework to licensed and unlicensed healthcare professionals, 8th grade reading level, include an introductory paragraph, key sections with structured content, a conclusion paragraph, key takeaways, and a scenario-based application activity. Use content from the IHI website and the John A. Hartford Foundation only". In seconds, ChatGPT generated the requested e-learning content. The output was reviewed and refined to ensure the desired competencies were appropriately addressed. Subject matter and educational experts conducted a final review to ensure accuracy, adherence to the desired instructional design parameters, and applicability to real-world healthcare settings. After the final review, ChatGPT was prompted to generate knowledge assessments to test key concepts. Key takeaways included: ChatGPT, when coupled with human expertise and oversight for educational content development, was a useful tool to streamline module creation, ensure alignment with instructional design principles, integrate age-friendly care elements, and generate knowledge assessments to evaluate learning.

Abstract citation ID: igaf122.3485
### IT'S NEVER TOO EARLY: HOW COLLEGE STUDENTS VIEW AGING AND PREPARE FOR IT
Kelly O'Sullivan[1], Raven Weaver[1], Jill Juris[2], and Cory Bolkan[3], *1. Washington State University, Pullman, Washington, United States, 2. Appalachian State University, Boone, North Carolina, United States, 3. Washington State University, Vancouver, Washington, United States*

As society ages rapidly, people must prepare for their own healthy aging trajectories. Perceptions of aging and expressions of ageism negatively influence aging experiences, contributing to poorer outcomes (e.g., chronic disease, decreased independence, earlier mortality). In contrast, aging preparedness (e.g., advanced care planning, financial security, retirement planning) leads to better outcomes, including increased autonomy and independence. This study examined how college students ($N = 277$, $M_{age}$=19.83; 66.1% White) perceived their own aging in relation to family, friends, religion, leisure, lifestyle, money, work, and health, and how these perceptions related to their preparedness for aging. We sought to answer two research questions: (1) how do college students perceive aging in these domains? and (2) how do perceptions of aging influence views of aging preparedness? Results indicated that students generally held positive aging perceptions, expressing optimism about anticipated later life experiences. Linear regression revealed significant associations between perceptions of aging preparedness in later life and perceptions of friends ($p = 0.016$), religion ($p < 0.001$), leisure ($p = 0.008$), lifestyle ($p = 0.044$), money ($p = 0.010$), and health ($p < 0.001$). These findings suggest that it is never too early for college students to prepare for aging. Students with positive perceptions of aging felt more prepared for older adulthood; thus, it is important to disrupt negative perceptions of aging early in life, which has the potential to improve health outcomes and foster greater autonomy in later years.

Abstract citation ID: igaf122.3486

**FOSTERING NEXT-GEN GERONTOLOGISTS: AI-POWERED RESEARCH TRAINING FOR UNDERGRADUATES**

Catheryn Koss, *California State University, Sacramento, Sacramento, California, United States*

Artificial intelligence (AI) is increasingly being integrated into academic and professional training as educators explore its potential to enhance student learning. By lowering barriers to entry, AI tools can help students who are intimidated by research gain hands-on experience in the research process, enabling them to produce high-quality, professional-level outputs such as research posters. This presentation will demonstrate how AI can be effectively incorporated into undergraduate curricula, drawing from experience using these tools in a research course focused on aging and the life course. Participants will learn practical strategies for leveraging different AI models to support student engagement in research, from understanding and evaluating academic literature to synthesizing key findings. Additionally, the session will showcase how AI tools, particularly ChatGPT, can assist students in conducting statistical analyses, interpreting results, and visualizing data through graph generation. By integrating AI into coursework, educators can help students develop critical thinking skills, improve their ability to navigate complex research, and gain confidence in quantitative methods. The presentation will include hands-on examples of AI applications in research assignments, discussions on ethical considerations, and best practices for balancing AI assistance with traditional learning methods. Attendees will leave with concrete strategies for integrating AI into their teaching to better prepare students for an evolving technological landscape in gerontology and related fields.

Abstract citation ID: igaf122.3487

**AN EDUCATIONAL BOARD GAME ON AGING THAT INCORPORATES THE 4MS AND SIMULATES AGING EXPERIENCES**

Sue Hazelett[1], Lori Kidd[2], Michele Gareri[1], Denise Kropp[3], Margaret Sanders[3], Brandi Chrzanowski[4], and Darcia Simpson[5], *1. Summa Health System, Akron, Ohio, United States, 2. The University of Akron, Akron, Ohio, United States, 3. Northeast Ohio Medical University, Rootstown, Ohio, United States, 4. Direction Home Akron Canton Area Agency on Aging & Disabilities, Green, Ohio, United States, 5. Hospice of the Western Reserve, Cleveland, Ohio, United States*

Innovative approaches to imparting geriatrics knowledge are needed. We will demonstrate a board game developed as part of a HRSA Geriatric Workforce Enhancement Program grant to teach important geriatric issues and simulate common geriatric experiences. The game begins by assigning participants marital status, children, income, health, and chronic conditions by chance. Participants then choose their "life goal" (i.e., either money, family/friends, or health) which guides how they play the game. Progressing through the game simulates real life events and requires decisions that are driven by each player's goals, aligning with a 4Ms approach to Age Friendly care. We received feedback from n = 13 participants who all expressed high levels of satisfaction with the game. Effectiveness of the game as an educational tool was rated average 4.25 out of 5, how well it simulated real life challenges was rated average 4.6 out of 5, how well it incorporated "what matters most" was rated 4.5 out of 5. The overall rating was 4.5 and whether they would recommend the game was 4.5. Like popular poverty simulations, this game is a unique way to impart/reinforce important geriatric concepts for a wide range of learners. Participants experience the frustrations of unplanned financial setbacks with fixed incomes (e.g., losing dentures, paying for medications) as well as the importance of specific decisions (e.g., advance care planning, accruing social supports). The impact of random bad luck is also ever present. The game allows them to experience the challenges of aging in a simulated environment.

Abstract citation ID: igaf122.3488

**AGE FRIENDLY HEALTH SYSTEM 4M COMPETENCY-BASED CURRICULUM FOR INTERNAL MEDICINE RESIDENTS**

Tiffany Hu, Cecily McIntyre, Gregory Ouellet, Marcia Mecca, and Jennifer Ouellet, *Yale School of Medicine, New Haven, Connecticut, United States*

The Age Friendly Health System 4Ms is an evidence-based framework to provide high value care to older adults, with which educational content in Geriatrics is increasingly aligned. Focusing on Internal Medicine residents, who provide care to older adults in a variety of settings, we aimed to assess perceived proficiency in core competencies, as identified by AGS/ADGAP and organized using the 4Ms framework. We surveyed Internal Medicine residents in three programs at one academic medical center. The survey asked residents about their self-reported proficiency in AGS/ADGAP competencies on a scale of 1-5 (1 "completely unable to perform this skill", 5 "I am expert and can teach this skill to others"). A total of 35 residents responded to the pre-curriculum survey, including 13 PGY1, 17 PGY2 and 5 PGY3 residents. Most (71%) had no Geriatrics-focused experience in medical school, yet 27 individuals (77%) had >1 Geriatrics rotation during residency. Residents self-reported lower proficiency scores in competencies related to mobility (3.21) and multicomplexity (3.39). Of the competencies, residents scored lowest in their ability to screen for pressure injuries (2.95) and develop a multifaceted plan for fall prevention (3.00). Our findings highlight gaps in Internal Medicine residents' self-reported proficiency in key Geriatrics competencies. These results will inform our development of a QR-generated skills tracker for residents to have skills in Geriatrics evaluated in real time by trained faculty. This will also help align curricular content in didactics and rotations to target highest priority learning needs.

Abstract citation ID: igaf122.3489

**WHAT DRIVES STUDENTS TO PURSUE GERONTOLOGY AND GERIATRICS EDUCATION?**

Isaac Asirifi Boateng[1], and Rona Karasik[2], *1. St. Cloud State University, St. Cloud, Minnesota, United States, 2. Saint Cloud State University, Saint Cloud, Minnesota, United States*

Despite the growing need for practitioners, researchers and policymakers knowledgeable about aging and older adults, student interest in pursuing gerontology and geriatrics at all levels (e.g., undergraduate, graduate, post-graduate) and related fields

(e.g., human services, medicine, PT/OT, social work) is failing to keep pace with demand. While student recruitment concerns in gerontology are not new, the situation is becoming increasingly dire. Previous studies have shown students are reluctant to enter the field because of ageism/negative attitudes toward aging and older adults (e.g., Jester et al., 2021), lack of awareness of career options (e.g., Dassel et al, 2014) and expectations of lesser prestige and compensation (e.g., Cheung et al., 2023). There is limited understanding, however, of what does draw potential students to gerontology and geriatrics. To develop effective strategies for increasing student interest in gerontological education, this study explored the motivations of individuals who chose the field. Findings from the first wave of data obtained through an email survey of current and recently graduated students taking courses in aging (N = 67) revealed several factors leading to their decisions, including: personal experiences with aging family members (n = 43), desire to improve quality of life for older adults (n = 39), policy advocacy (n = 24), ability to educate others on aging issues (n = 23), positive academic influences (n = 18), and aging-related service experiences (n = 18). Implications for these preliminary findings include opportunities for fostering greater interest in the field, enhancing an age-savvy workforce, and advocating for policies to support the growth of academic gerontology programs.

Abstract citation ID: igaf122.3490

### TEACHING STRATEGIES FOR INTERDISCIPLINARY COLLABORATION WITHIN GERONTOLOGY EDUCATION

Elaine Jurkowski, *Southern Illinois University, Carbondale, Illinois, United States*

A critical area within gerontological education and medical/health/behavioral professions is to educate and train students for competence within interprofessional collaboration. Based on the experiential learning theory (Kolb & Kolb, 2017) and drawing from the IPCE model (Interprofessional Education Collaborative, 2023), this study has creatively designed a training/education model for students within gerontological fields of practice to enhance their ability to understand the concept of working collaboratively across disciplines. Students were placed in teams utilizing one of at least three models of experiential education and worked across teams with real patient/consumer consultations/case management strategies. The goal was to achieve both patient/consumer outcomes and learn about professional practice with people growing older using an interdisciplinary collaborative approach. Three specific strategies used include "Student Hotspotting", an Interdisciplinary Day (IPE), and a collaborative teaching clinic. The Student Hotspotting approach, modelled after the Camden Coalitions' program was an academic year long experience working with a older person with complex care needs. The IPE day focused on a case study approach and guest presenters via a team-based approach with health-oriented disciplines working as a team to identify a case management plan. The Teaching Clinic brought together medical, social work, nursing, physician assistant and nutrition students together in an outpatient clinic setting to address patients with complex care needs and aging related health conditions. All three strategies examined care through the lens of Social Determinants of Health. Evaluation outcomes presented positive outcomes and benefits to the students' learning outcomes and patient outcomes.

### SESSION 7925 (POSTER)

### FLASH POSTER SESSION: CARING, ENGAGING, AND WELL-BEING IN COMMUNITIES AROUND THE WORLD

Abstract citation ID: igaf122.3491

### PREDICTORS OF LATE-LIFE DIVORCE IN SOUTH KOREA: A 21-YEAR PANEL STUDY

Seoyeon Ahn[1], Minyoung Kwak[2], and Sojung Park[3], *1. Duke-NUS Medical School/National University of Singapore, SINGAPORE, Singapore, 2. Daegu University, Gyongsan, Kyongsang-bukto, Republic of Korea, 3. Washington University in St. Louis, St. Louis, Missouri, United States*

This study examines how socioeconomic and marital factors influence late-life separation and divorce men and women aged over 50 in Korea. Using data from the 21-wave Korea Labor and Income Panel Study (1999–2019), we analyze a sample of 4,922 married couples aged over 50 years old. Discrete-time event history models are applied to investigate the impact of socioeconomic and marital factors on marital instability over the 21-year period. Our findings indicate that home ownership and husbands' pension receipt significantly reduce the risk of marital dissolution, while wives' secondary education is associated with a higher likelihood of marital disruption. Additionally, Husbands' higher levels of relationship satisfaction with family was associated with lower risk of marital disruption. These results highlight the importance of considering both socioeconomic resources and interpersonal factors in explaining marital instability among middle-aged and older adults. The complex relationship between education and divorce risk for older Korean women suggests that both financial independence and cultural norms shape divorce decisions. These findings contribute to our understanding of gray divorce in East Asian societies, where traditional family values coexist with rapid socioeconomic change. Recognizing the predictors of late-life marital dissolution has important implications for developing social services tailored to the unique needs of divorced older adults, particularly in aging societies with evolving family structures.

Abstract citation ID: igaf122.3492

### REVISITING THE HISPANIC PARADOX: NEIGHBORHOOD CHARACTERISTICS AND LIFE SATISFACTION FOR OLDER IMMIGRANTS

Morgan Stangl[1], Lauren Gil Hayes[1], Yi Wang[2], and Man Guo[1], *1. University of Iowa, Iowa City, Iowa, United States, 2. University of Iowa School of Social Work, Iowa City, Iowa, United States*

Nativity status (foreign-born, FB vs. native-born, NB) is a social determinant of well-being, yet its impact on life satisfaction may vary depending on neighborhood characteristics. Current literature discusses the Hispanic Paradox, where FB Hispanics report higher life satisfaction despite immigration challenges, attributed to strong family support. However, neighborhood characteristics remains underexamined, offering new insights. The current study seeks to explore 1) Whether the association between neighborhood characteristics (cohesion/disorder) and life satisfaction is stronger for FB middle-aged/older adults than for NB middle-aged/older adults and 2) if this association varies by race (White, Black, Hispanic). This study utilizes data from the 2018 Health and Retirement Study (HRS) Psychosocial Leave-Behind Questionnaire, a nationally representative survey of middle-aged/older adults, including both NB (N = 4,398) and FB (N = 722). Life satisfaction was regressed on neighborhood cohesion and disorder separately, controlling for demographics and socioeconomic factors. Interaction terms between neighborhood characteristics and nativity status were added to test moderation. These interaction models were then stratified by race/ethnicity. Results show that FB middle-aged/older adults are more sensitive to neighborhood characteristics, with a significant interaction between nativity and neighborhood disorder ($\beta = 0.15$, $p < 0.001$). Neighborhood cohesion shows a strong positive association with life satisfaction for FB Hispanics ($\beta = 0.38$, $p < 0.001$). Neighborhood disorder has a strong negative association for FB Hispanics ($\beta$ = -0.32, $p < 0.001$). These findings suggest that neighborhood characteristics may play a role in shaping life satisfaction, particularly for FB middle-aged/older Hispanic adults. These findings provide another dimension in understanding the Hispanic Paradox.

Abstract citation ID: igaf122.3493

### CONSIDERING COMMUNITY: EXAMINING CIVIC ENGAGEMENT AND WELL-BEING IN LATER LIFE

Lauren Gil Hayes[1], Morgan Stangl[1], Man Guo[1], and Yi Wang[2], *1. University of Iowa, Iowa City, Iowa, United States, 2. University of Iowa School of Social Work, Iowa City, Iowa, United States*

The socio-ecological model (SEM) highlights how well-being is shaped by individual, interpersonal, and community-level factors. While individual behaviors and interpersonal relationships are well-documented influences on well-being, civic engagement—through volunteer activities, adult learning, and special interest group participation—remains an underexamined community-level engagement factor. This study investigates whether civic engagement is associated with loneliness and health in aging adults and whether interpersonal support moderates these associations. Using data from the 2018 Health and Retirement Study (HRS) Leave-Behind Questionnaire (n = 5,530), a nationally representative survey of aging U.S. adults, we employ three survey-weighted Ordinary Least Squares (OLS) regressions with robust standard errors, adjusting for demographic and socioeconomic characteristics. Results indicate that higher civic engagement is significantly associated with lower loneliness ($\beta$ = -0.0436, $p < 0.001$) and better self-rated health ($\beta = 0.1386$, $p < 0.001$). The magnitude of the association between civic engagement and self-rated health is larger than its association with loneliness. Interpersonal support from spouses, children, and friends does not significantly moderate these relationships, inviting further analysis to determine if civic engagement may be beneficial for those with limited support networks. These findings position civic engagement as a potential public health strategy, reinforcing and expanding SEM's multi-dimensional emphasis on structural and social contexts in well-being. Expanding opportunities for volunteerism, lifelong learning, and group participation may enhance both social and health outcomes, reinforcing the importance of community involvement in shaping a holistic understanding of well-being.

Abstract citation ID: igaf122.3494

### HEDONIC AND EUDAIMONIC WELL-BEING ACROSS THE LIFESPAN: DIFFERENCES BETWEEN WORKING AND NON-WORKING ADULTS

Piaopiao Cai[1], and Silvia Sörensen[2], *1. University of Rochester, Rochester, New York, United States, 2. University of Rochester Warner School of Education and Human Development, Rochester, New York, United States*

The development trajectories of well-being across the lifespan have garnered significant interest in research. Most studies have found evidence of a U-shaped curve trajectory, indicating that midlife adults experience lower well-being compared to younger and older adults across the lifespan. However, the well-being indicators in research have predominantly focused on hedonic aspects, such as hedonic emotions and life satisfaction. The developmental trajectories of eudaimonic well-being, such as six factors of psychological well-being (autonomy, environmental mastery, personal growth, positive relationships, purpose in life, and self-acceptance), have received less attention, particularly concerning the working population. This study utilized a national dataset (n = 2321) to understand the well-being development trajectories between working(n = 1295) and non-working adults (n = 1026) aged 23 to 76. The results of polynomial regressions indicate that only life satisfaction and self-acceptance exhibit a significant quadratic (U-shaped) trend for working and non-working adults. However, the two groups exhibit significantly different ages for minimum life satisfaction (working adults, aged 41.88; non-working adults, aged 37.48) and self-acceptance (working adults, aged 42.98; non-working adults, aged 37.38). Hedonic emotions, autonomy, environmental mastery, and positive relationships, non-working adults exhibit a slightly steeper linear upward trend across the lifespan compared to the linear trajectories of working adults. Additionally, there was a consistently flat linear trajectory for personal growth and purpose in life among both groups. The findings of the present study support Baltes's (1987) theoretical perspective that individual development is lifelong, multidirectional, and malleable. Further investigation may be needed to better understand the differing trajectories between working and non-working groups.

Abstract citation ID: igaf122.3495

### SEXUALITY AND WELL-BEING AMONG COMMUNITY-DWELLING OLDER ADULTS IN BOGOTÁ, COLOMBIA

Maria Reyes, Natalie Levy, Maria-Jose Castro, Gabriela Morales, Gabriela Garcia, Maria Ordoñez, and Angie Riveros, *Universidad de los Andes, Bogota, Distrito Capital de Bogota, Colombia*

Sexuality in late life is often overlooked, yet it plays a significant role in the well-being and mental health of older adults. We conducted a mixed methods study to explore the relationship between sexuality, well-being, and psychosocial factors among older adults in Bogotá, Colombia. The quantitative phase included 615 older adults (Mage= 66), 57.3% women, 40.9% men, and 1.4% transwomen. Surveys measured sexual well-being, depression, resilience, life satisfaction, and social support. The qualitative phase involved interviews with 19 participants (7 women, 6 men, and 6 transwomen), exploring experiences of sexuality, intimacy, and interactions with healthcare providers. Results: multiple linear regression analysis showed that overall sexual satisfaction was predicted by age and awareness of the right to a fulfilling sexual life. Significant correlations were found between higher satisfaction with social support and perceived sexual compatibility and overall sexual satisfaction. Low social support was associated with higher distress in sexual functioning. Qualitative findings highlighted stigma surrounding aging and sexuality, particularly among transgender individuals.

Participants highlighted the continued relevance of sexuality, especially for those in active relationships. While some saw no changes with aging, most reported significant shifts. Participants revealed that professionals rarely addressed sexuality. Discussion: social support, awareness of sexual rights, and resilience were positively associated with higher sexual satisfaction. Participants recognized changes in their sexuality as they aged. Cultural and religious beliefs played a crucial role in discussing sexuality, as many participants initially felt barriers to speaking openly. However, they acknowledged that sexuality is an important yet hidden topic.

Abstract citation ID: igaf122.3496

### REWARDING VOLUNTEERING & ENHANCING PSYCHOLOGICAL MEANINGFULNESS IN VOLUNTEER ROLES BOOST LATE-LIFE WELL-BEING

Shiyu Lu, *The University of Hong Kong, Hong Kong, China (People's Republic)*

Although late-life volunteering is considered a health promotion strategy, few studies explored the characteristics of volunteering activities, including the psychological meaningfulness of volunteer roles, the perceived balance between rewards and efforts, and the types of activities that impact the healthy aging process. This study investigates how these dimensions of the volunteering experience influence subjective well-being (SWB) through the lens of belonging and empowerment processes. This cross-sectional study involved 457 volunteers aged 55 and above in Hong Kong from 2023 to 2024. The sense of belonging and general self-efficacy were utilized to capture the belonging and empowerment processes. Results from structural equation models indicated that the perceived balance between efforts and rewards in volunteer roles ($\beta = 0.83$, $p = 0.002$), sense of belonging ($\beta = 0.25$, $p < 0.001$), self-efficacy ($\beta = 0.31$, $p < 0.001$), and engagement in companionship volunteering ($\beta = 1.36$, $p = 0.005$) positively correlated with subjective well-being (SWB) among older adults. The SWB benefits of one-year volunteer hours ($\beta = 0.001$, $p = 0.032$) are mediated by the sense of belonging. Furthermore, the perceived balance between efforts and rewards ($\beta = 0.70$, $p = 0.001$) and the psychological meaningfulness in volunteer roles ($\beta = 1.09$, $p < 0.001$) are jointly mediated by both belonging and empowerment processes. These findings emphasize the significance of achieving a balanced sense of rewards and efforts and psychological meaningfulness in late-life volunteering. This study also highlights specific volunteering activities (e.g., companionship) that can create meaningful volunteering experiences for healthy aging.

Abstract citation ID: igaf122.3497

### NETWORK ANALYSIS ON MULTI-DOMAIN LONELINESS FACTORS AMONG THREE AGING GROUPS WITH KINSHIP VULNERABILITY

Jianan Li, *University of California, Los Angeles, Los Angeles, California, United States*

Close kin (i.e., a partner and children) has been identified as important supporting systems against loneliness in later life, with those with kinship vulnerability—lacking traditional family structures—facing heightened risk. While multi-dimensional factors contributing to loneliness have been studied, their associations across different kinship vulnerability groups remain unexplored. Using the 2016 and 2018 Health and Retirement Study, this study employed network analysis to construct partial correlation networks with 22 factors across six domains (demographics, socioeconomic status, physical, psychological, cognitive, and social well-being) among adults over 50 with kinship vulnerability across three groups: having a partner but no children ("Partner-only," $N = 331$), having children but no partner ("Children-only," $N = 2635$), and having no partner and no children ("kinless," $N = 487$). Centrality measures (betweenness, closeness, strength) showed that psychological well-being factors (depression, hopefulness) were central for Partner-only and Kinless groups, while physical health and demographic factors were prominent for the Children-only group. Ego network analyses revealed that social well-being factors had the strongest direct connections with loneliness: friend support was positively associated with loneliness in Partner-only adults ($r = .296$), while friend strain was the strongest predictor in Children-only ($r = .270$) and Kinless groups ($r = .255$). PageRank analysis identified self-reported health as the most influential bridge across all three networks, especially for Partner-only and Kinless groups. These findings demonstrate distinct loneliness mechanisms across kinship vulnerability groups, informing tailored interventions targeting central factors, factors with the strongest direct and indirect effects, and critical bridges between factors to effectively address loneliness in older adults based on their specific kinship vulnerability.

Abstract citation ID: igaf122.3498

### "WE CAN'T POUR WATER FROM AN EMPTY CUP": CAREGIVERS' EXPERIENCES WITH A DEMENTIA SPECIFIC TRAINING PROGRAM

Brittney Pond[1], Kattia Suarez Vargas[2], Melinda Neri[2], and Jarmin Yeh[2], *1. Sandhills Community College, North Carolina, United States, 2. University of California, San Francisco, San Francisco, California, United States*

The demand for home and community-based services continues to rise in the United States, as does the prevalence of people living with Alzheimer's Disease and Related Dementias (ADRD). However, little is known about the qualitative impacts of dementia specific training programs for California's Medicaid-Funded In-Home Supportive Services (IHSS) caregivers who provide care and services for people living with ADRD. The IHSS + ADRD Training Project implemented and evaluated a 10-week, online, dementia-specific training program offered in English, Spanish, and Chinese (Cantonese and Mandarin) for IHSS caregivers and their care recipients living with ADRD. This poster is based on open-ended qualitative responses ($n = 470$) drawn from a larger quantitative survey of IHSS caregivers who participated in the training program. Caregivers responded to questions about what they learned in the program, its application to everyday work and life, personal impacts, and how the training could be improved. Based on our qualitative analysis using thematic coding, there are three main findings: 1) increased dementia knowledge and skills, 2) the importance of social support from classmates and instructors, and 3) personal development related to self-care and increased confidence. This research has several implications for policy and practice, as it provides evidence to support the ongoing development and implementation of dementia-specific caregiver training programs. This work suggests that establishing policies to support training opportunities are vital for this workforce.

Abstract citation ID: igaf122.3499

**INTERVENTION PROGRAM FOR SOCIAL ISOLATION: THE WAIGAYA PROJECT'S LIFELONG EDUCATION APPROACH**

Keiko Katagiri[1], Nahyun Kim[1], Madoka Ogawa[2], and Chiharu Miura[3], *1. Kobe University, Kobe, Hyogo, Japan, 2. Osaka University, Osaka, Osaka, Japan, 3. Advanced Research Center for Well-being of Kobe University, Ashiya, Hyogo, Japan*

In Japan, social isolation and loneliness among older adults have become serious social issues. The purpose of this study is to examines the effects of the Waigaya Project, a lifelong education intervention program launched in 2023 to address these challenges among residents in urban rental apartment complexes, where interpersonal indifference is more pronounced. A mail survey was conducted in January 2025, targeting all residents aged 18 and older in a rental apartment complex in Kobe. A series of Analysis of Covariance (ANCOVA) were performed, with program participation as the independent variable. Dependent variables included the number of weak and strong social ties, sense of belonging, and loneliness. Control variables encompassed sex, education, years of residence, age, parental status, marital status, health and financial status, single-household status, and employment status. The results demonstrated significant positive effects of the intervention of this action research. Participants exhibited increased social ties, a stronger sense of belonging, and lower levels of loneliness than non-participants, with greater effects among those who participated more frequently. The program covered various topics, such as disaster preparedness, financial planning, well-being and generative AI. Lectures were followed by small-group discussions to enhance participant interaction. While most lifelong education programs in Japan rely solely on lectures, our findings emphasize the importance of interactive program design. This action research incorporated diverse data sources, such as real-world data from ICT devices and interviews. Further analysis is required to integrate these data and examine how the intervention reduced loneliness and what factors influenced participation.

### SESSION 7930 (POSTER)

### FLASH POSTER SESSION: HEALTH CARE AND SERVICES: POLICY AND PROGRAMS ACROSS THE SPECTRUM

Abstract citation ID: igaf122.3500

**ROLES AND PRIORITIES OF PAYERS, HOSPITALS, AND HOME HEALTH AGENCIES IN POST-ACUTE HOME HEALTH CARE**

Emily Gadbois[1], Marguerite Daus[2], Jennifer Bunker[3], Jamie Smith[4], Christine Jones[5], and Kali Thomas[3], *1. Brown University School of Public Health, Providence, Rhode Island, United States, 2. University of Colorado Anschutz Medical Campus, Aurora, Colorado, United States, 3. Johns Hopkins University, Baltimore, Maryland, United States, 4. Widener University, Philadelphia, Pennsylvania, United States, 5. University of Colorado School of Medicine, Aurora, Colorado, United States*

Home health care (HHC) provides skilled nursing, therapies, and aide services to help older adults - many of whom are recently discharged from the hospital - regain function, restore health, and maintain independence in the community. The provision of post-acute HHC is influenced by multiple players: Medicare Advantage (MA) plans, post-acute care management companies (CMCs), hospitals, home health agencies (HHAs), and patients and families. To understand the roles and priorities of these players in organizing and delivering post-acute HHC, we conducted in-depth, semi-structured interviews with 44 leaders of MA plans, post-acute CMCs, and HHAs across the country. Analyzed interviews revealed that most MA plans and CMCs agreed that home is the ideal setting and that institutional care should be avoided whenever possible; however, MA plans and CMCs varied in their involvement in post-acute home health referrals, and HHAs tended to view MA and CMC involvement as adding burden to the referral process (Theme 1). MA plan, CMC, and HHA leaders viewed the role of the hospital as to select the appropriate post-acute care setting; however, interview participants reported that these actions can be influenced by relationships with MA plans and HHAs (Theme 2). HHAs were reported to try to balance clinical complexity, staffing, patient location, and payer when accepting referrals, but had relatively limited control in the post-acute referral process (Theme 3). Insights from this work can help policymakers, payers, HHAs, hospitals, and patients understand the varied roles and shared incentives in coordinating post-acute HHC for vulnerable older adults.

Abstract citation ID: igaf122.3501

**PERSON-CENTERED CARE PLANNING IN MEDICAID 1915(C) HOME AND COMMUNITY BASED SERVICE (HCBS) WAIVERS FOR OLDER ADULTS**

Natalie Turner, *University of Washington, Seattle, Washington, United States*

Person-centered care planning is an approach to service coordination that prioritizes an individual's goals, needs, preferences, and desires. Over the past decade, the Administration for Community Living and the Centers for Medicare & Medicaid Services have worked to advance person-centered planning within home and community-based services (HCBS). 1915(c) waivers can offer insights into how states apply these principles in care policy. This qualitative study examined how person-centered care planning was described in 63 1915(c) waivers for older adults using the Frameworks Approach, a qualitative method designed for applied policy research. Codes were developed deductively based on waiver application questions and applied to select appendices detailing care planning processes. Data were summarized into matrices and analyzed to identify four key themes: (1) Care planning led and owned by participants; (2) Holistic approaches to care planning; (3) Individualization of services; and (4) Care plans as living, adapting documents. Waivers emphasized participant-driven decision-making, with language such as "Member choice drives all decisions." Care planning was also holistic, considering not only health and functional needs but also social life, community engagement, and other enriching activities voiced by participants. Plans required documentation of individual preferences on service delivery, including schedules and desired activities. Finally, care plans were expected to evolve alongside participants' changing needs. These findings highlight how person-centered care planning is operationalized in care policy and underscore the importance of individual agency in HCBS. Future research should examine how the operationalization of person-centered planning compares to its implementation to assess alignment and effectiveness.

Abstract citation ID: igaf122.3502

### MEDICARE ADVANTAGE RAPID DISENROLLMENT TRENDS

Em Balkan[1], Jay Shroff[2], and David Meyers[1], *1. Brown University, Providence, Rhode Island, United States, 2. Brown University School of Public Health, Providence, Rhode Island, United States*

In 2024, 54% of Medicare beneficiaries chose to receive their benefits from private plans, known as Medicare Advantage (MA). Medicare beneficiaries can switch their coverage during Medicare's annual Fall Open Enrollment, with new coverage effective January 1 of the following year. All MA enrollees can choose to change their coverage again during the Medicare Advantage Open Enrollment Period from January 1 through March 31. Rapid disenrollment is when a beneficiary who enrolled in a new MA plan during the fall decides to disenroll immediately during the subsequent spring. Rapid disenrollment has been severely understudied. In our analysis of national Medicare data from 2017 to 2022, we found the rate of rapid disenrollment more than tripled, from 4 to 12.2%. The rate of rapid disenrollment was highest for Medicare-Medicaid dual enrollees, with 20% of such beneficiaries rapidly disenrolling by 2022. Additionally, higher proportions of Black, Hispanic, and Native enrollees rapidly disenrolled from their MA plans compared to white enrollees. Plans serving many Medicare-Medicaid dual enrollees tended to have higher rates of rapid disenrollment. It is unclear why rapid disenrollment has increased so dramatically, indicating that beneficiaries quickly decide that their new MA plan is not the best option for them. It is also unclear why rapid disenrollment rates are higher for communities of color and those with lower incomes. For these reasons, and as MA becomes more popular, it is critical to better understand rapid disenrollment in order to improve health care access and quality for Medicare beneficiaries.

Abstract citation ID: igaf122.3503

### MEDICARE-MEDICAID DUAL ELIGIBLE SPECIAL NEEDS PLAN ENROLLMENT IN MARYLAND

Christine Gill, Roberto Millar, and Christin Diehl, *University of Maryland, Baltimore County, Baltimore, Maryland, United States*

Nationally, health care spending for Medicare-Medicaid duals is higher than for non-duals in either program. Programs integrating care across these separate programs—one type being dual eligible special needs plans (D-SNPs), a Medicare Advantage (MA) plan—produce mixed results on improving care coordination and reducing costs. This retrospective cohort study examined sociodemographic and geographic factors associated with 2021 D-SNP enrollment among duals in Maryland—a state with low MA enrollment. We report descriptive statistics by Medicare program type (D-SNP or Traditional Medicare [TM]) and logistic regression analyses. Among 109,646 duals, 9% were enrolled in D-SNPs, and 60% were 65 years or older. We found regional differences: Baltimore City enrollees were 1.6 (95% confidence interval (CI) 1.5-1.7) times more likely than those in Central Maryland to select D-SNPs compared with TM, and neighborhoods with the highest quintile of disadvantage had 1.4 (95% CI: 1.3-1.5) times the likelihood of selecting D-SNPs compared with TM. Full duals were less likely to select D-SNPs over TM compared with partial duals (0.8 [95% CI: 0.7-0.8]); those 65 years and older were more likely compared with younger (1.6 [95% CI: 1.5-1.7]); and Black (2.2 [95% CI: 2.1-2.3]) and Latino (1.6 [95% CI: 1.4-1.8]) were more likely compared with White duals. Given that racial and ethnic minorities and those from more disadvantaged neighborhoods are enrolled in D-SNPs disproportionately in Maryland, additional research should be conducted to examine whether health outcomes and care quality are equitable across programs to inform policy integrating care for this high-needs population.

Abstract citation ID: igaf122.3504

### CHARACTERIZING KEY DRIVERS OF RESPITE USE AMONG VETERAN CAREGIVERS

Melissa Harris-Gersten[1], Colleen Blue[2], Susan Hastings[1], Zhen Li[1], Jeanie Lo[3], Nadya Majette[1], Jennifer Zervakis[1], and Megan Shepherd-Banigan[4], *1. Durham VA Healthcare System, Durham, North Carolina, United States, 2. Durham VA Health Care System, Durham, North Carolina, United States, 3. Veterans Health Administration, Menlo Park, California, United States, 4. Duke University, Durham, North Carolina, United States*

Respite care has been shown to reduce caregiver burden and may delay long-term care placement, yet it remains underutilized, with only 12-15% of US caregivers reporting use of these services. Understanding drivers of respite use is critical to informing initiatives to improve access. This convergent mixed-methods study aimed to characterize factors influencing respite use among caregivers and Veterans enrolled in the Veterans Health Administration's (VHA) Caregiver Support Program. A retrospective analysis used machine learning (random forests) to analyze caregiver survey data (2018–2021) linked to Veteran electronic health records (N = 1,727), ranking Veteran and caregiver factors associated with respite use. Semi-structured interviews were conducted with a separate sample of caregivers (N = 20) in 2024, with transcripts analyzed using directed content analysis. Data were integrated using Andersen's Behavioral Model for Health Service Use. Top predictors included Veteran's functional limitations, age, health risk, and caregiver's age and caregiver perceived integration of care with the clinical team. Enabling factors included in-home care support and financial resources; need factors encompassed caregiver burden, depression, loneliness, and Veteran's chronic conditions, dementia, and frailty. Qualitative findings reinforced the importance of Veteran and caregiver needs while highlighting how VHA's respite payment model alleviates financial strain. Caregivers described psychosocial and system-level factors, including lack of awareness, concerns about access and quality, and scheduling barriers as reasons not to use respite. Findings provide actionable insights for improving respite uptake by enhancing caregiver-clinician communication, tailoring services, and increasing outreach. Future research should examine how these factors impact respite use in non-VHA settings.

Abstract citation ID: igaf122.3505

### IMPACT OF POLICY REFORMS ON HOSPITAL-AT-HOME SERVICES: AN INTERRUPTED TIME SERIES ANALYSIS OF SHANGHAI

Sixian Du[1], and Yaqing Liu[2], *1. Huazhong University of Science and Technology, Wuhan, Hubei, China (People's Republic), 2. Huazhong University of Science and Technology, Hubei, Hubei, China (People's Republic)*

Background: The rapid growth of aging populations and chronic disease prevalence has increased demand for hospital-at-home (HaH) services, which offer home-based alternatives to traditional inpatient care. However, the policy impact on HaH service development in China remains understudied. Objective: To evaluate the effects of two major policy reforms on HaH services in Shanghai from 2007 to 2022. Methods: A two-stage interrupted time series (ITS) analysis with a control group was conducted. Data from Shanghai Statistical Yearbooks were used to assess changes in institutional capacity, home-based medical services, and HaH beds across hospitals and community health service centers. Eleven outcome indicators were analyzed. Results: Following the first policy in 2010, the number of HaH service providers increased significantly, particularly in community health service centers ($\beta1 + \beta3 + \beta5 + \beta7 = 8.200$, $P < 0.001$). HaH beds per 10,000 population also rose ($\beta1 + \beta3 + \beta5 + \beta7 = 0.406$, $P < 0.001$). However, home-based medical visits declined after both policies ($\beta1 + \beta3 + \beta5 + \beta7 = -90{,}214.350$, $P < 0.01$), likely due to workforce shortages and service delivery risks. After the second policy in 2020, hospital-based HaH beds decreased ($\beta1 + \beta3 + \beta9 = -76.500$, $P < 0.01$). Conclusion: While policy reforms expanded HaH service capacity, challenges remain in sustaining home-based medical visits and ensuring workforce supply. Future policies should prioritize multidisciplinary care teams and address barriers to home-based geriatric healthcare to meet the needs of China's aging population.

Abstract citation ID: igaf122.3506

### MEDICARE PLAN SWITCHING AMONG ASSISTED LIVING RESIDENTS: THE ROLE OF MEMORY CARE

Wanru Liu[1], Gauri Gadkari[1], Jennifer Bunker[2], Kali Thomas[2], and Eric Jutkowitz[3], *1. Brown University, Providence, Rhode Island, United States, 2. Johns Hopkins University, Baltimore, Maryland, United States, 3. University of Minnesota, Minneapolis, Minnesota, United States*

Switching between Medicare Advantage (MA) and Fee-for-Service (FFS) commonly occurs after a health shock or residential relocation. However, little is known about how moving to an assisted living (AL) community influences enrollment decisions. This study examines Medicare plan switching among beneficiaries relocating to assisted living (AL) licensed for memory care versus general AL. Memory care ALs provide specialized dementia services but are approximately 36% more expensive than general ALs, potentially influencing Medicare choices. Using 2021 Medicare Beneficiary Summary Files and a national dataset of ALs, we tracked Medicare enrollment before and after moving to AL ($N = 63{,}395$) across 34 states licensing memory care ALs. A secondary analysis investigated switching among AL residents with dementia. Medicare plan switching varied based on AL licensing: FFS beneficiaries entering memory care-licensed ALs switched to MA less often than those entering general ALs (6.47% vs. 9.16%, $p < 0.05$). MA beneficiaries relocating to memory care compared to general AL were less likely to switch to another MA plan (10.95% vs. 13.08%, $p < 0.05$) or to FFS (4.31% vs. 5.55%, $p < 0.05$). Among individuals with dementia, residents with dementia in memory care ALs exhibited less FFS-to-MA switching compared to those in general ALs (3.40% vs. 4.76%, $p < 0.05$). These findings suggest that moving to an AL influences Medicare enrollment decisions; and, variation in switching exists between the type of AL (general vs memory care) and whether or not an individual has dementia.

Abstract citation ID: igaf122.3507

### ECONOMIC BENEFITS OF CARE COORDINATION FOR OLDER ADULTS

Erblin Shehu[1], and Brian Kaskie[2], *1. State University of New York at Cortland, United States, 2. University of Iowa, Iowa City, Iowa, United States*

In response to rising costs associated with providing healthcare services to Americans over 65 years old, policymakers have called for the expansion of care coordination programs to reduce total spending while improving patient outcomes. This study estimates the financial impact of the Iowa Return to Community (IRTC) initiative, a care coordination program designed to identify at-risk older adults and support their transitions from nursing homes and hospital settings to community living. We apply a Markov chain model with Monte Carlo simulations to capture all possible transitions among hospitals, nursing homes, and community settings. Using de-identified data from the State of Iowa ($N = 523$; 2020–2022) and a statistically matched control group from the National Health and Aging Trends Study (NHATS, 2020–2021), we compare IRTC outcomes with projections of what would occur without the program. Matching criteria include race, ethnicity, gender, income, living status, and 12 measures of instrumental (IADLs) and basic (ADLs) activities of daily living. Findings suggest IRTC participation is associated with reduced healthcare service utilization, resulting in per capita cost savings of $7,920.24 over five years. Subgroup analysis reveals that including informal care partners (ICPs) further increases these savings, as their involvement was linked to lower inpatient hospitalizations and deferred nursing home admissions. Findings call for IRTC expansion as a viable strategy to reduce aggregate healthcare spending while supporting aging in place.

### SESSION 7935 (POSTER)

### FLASH POSTER SESSION: RETHINKING AGING AND AGING EDUCATION

Abstract citation ID: igaf122.3508

### THE SOCIAL RESPONSIBILITY OF UNIVERSITIES IN PROMOTING SENIOR UNIVERSITIES – FROM A SOCIOLOGICAL PERSPECTIVE

Weng Pi Ching, *National Chung Cheng University, Minxiong, Chiayi, Taiwan (Republic of China)*

In 2025, Taiwan has officially entered a super-aged society. The social responsibility of universities extends beyond providing formal education to young students; they also play a crucial role in supporting and promoting senior universities. Since 2010, Taiwan's Ministry of Education has sought contractors through a bidding process to implement senior universities. Currently, 83 universities in Taiwan operate senior universities, with 3,500 elderly students enrolled, fulfilling their dreams of attending university. However, more than 10,000 elderly individuals are still eager to join. This study employs in-depth interviews and survey methods. From a sociological perspective, interviews were conducted with the project director and an assistant. From the perspective of resource allocation, interviews were held with personnel responsible for program implementation at eight universities across Taiwan, from north to south. From the standpoint of Symbolic Interactionism, 30 elderly students participating in senior universities were interviewed, followed by a questionnaire survey. The study concludes that the project director aims to uphold social justice and equal opportunity through the implementation of senior universities. Program coordinators utilize university-specific resources to design courses, which account for 40% of the entire curriculum, ensuring that educational funding is used effectively. Elderly students, despite not earning a formal degree, construct their self-identity through interactions, pursuing their studies based on personal interests. They find joy in learning at senior universities, establish positive interpersonal relationships, and ultimately fulfill their dream of attending university. These valuable experiences can serve as a reference for other countries.

Abstract citation ID: igaf122.3509

### TRAINING THE PROFESSOR: HEALTH POLICY CURRICULUM IN ADULT DEVELOPMENT AND AGING COURSE

Kirsten Graham[1], Sam Webster[1], and Kelly O'Malley[2], *1. Southern Utah University, Cedar City, Utah, United States, 2. VA Boston Healthcare System, Boston, Massachusetts, United States*

Health policy shapes the lives of older adults but is often underrepresented in undergraduate psychology curricula. This study builds on our prior implementation research integrating health policy content into an undergraduate psychology course. Previous research (student reflections, instructor observations, and course assessments) showed significant increases in students' knowledge, understanding of health policy-making, and critical evaluation skills. Building on these results, we developed a Train the Professor package to help faculty incorporate policy content into similar courses. This package includes a structured curriculum planning framework, consultation with a psychologist experienced in policy work, and ready-to-use instructional materials. We also evaluated the Knowledge and Attitude about Social Policy (KASP) scale, which was developed and revised as part of the curriculum evaluation. Confirmatory factor analyses pre ($\chi2$ (8) = 12.0 p = .152; CFI = 0.993; TLI = 0.988; RMSEA = 0.051; SRMR = 0.031) and post ($\chi2$ (8) = 12.0 p = .151; CFI = 0.992; TLI = 0.985; RMSEA = 0.057; SRMR = 0.033) indicated that the KASP was a good fit for the data. Findings support integrating policy education into psychology curricula and refining a scale for future research. By equipping educators with these resources, we aim to foster interdisciplinary learning and better prepare students to engage with policy issues related to aging. Our findings contribute to curriculum development discussions in psychology, emphasizing the value of policy integration in enriching student learning and better serving the community.

Abstract citation ID: igaf122.3510

### GERIATRICS EDUCATION MENTOR (GEM) PROGRAM: OUTCOMES FROM PAIRING STUDENTS AND OLDER ADULTS DURING MEDICAL SCHOOL

Hannah Griffin[1], David Wihry[2], and Marilyn Gugliucci[3], *1. University of New England College of Osteopathic Medicine, Biddeford, Maine, United States, 2. University of Maine, Bangor, Maine, United States, 3. University of New England College of Osteopathic Medicine, Kennebunk, Maine, United States*

Currently, only 15% of medical schools have mentor programs that educate students to work with older adults and their health. The University of New England College of Osteopathic Medicine Geriatrics Education Mentor (GEM) program is an experiential education component within the 1st and 2nd year curriculum whereby students and older adults are paired to conduct five "home-visit" and complete geriatrics specific assignments. A pre/post survey design assessed students' competence and confidence with older people (assigned GEMs). The survey was emailed to first-year medical students (avg. 174/class) before they began GEMs and was sent again upon completion of the program at the end of their second year during academic years 2020-2021 through 2023-2024. Data were analyzed in RStudio. Results had a 26% response rate that included overall data (N = 133/Pre & N = 138/ post), 24 responses were matched assessments. No significant change in students' interest ($p > 0.4$), perceived readiness and competence ($p > 0.07$), or confidence in working with older adults ($p > 0.5$) was revealed. However, perceived readiness and confidence increased from pre- to post-test, indicating that a larger sample size might reveal significance. Self-reported knowledge of older adult healthcare needs increased, pre- to post-test, with the paired subset showing a significant increase ($p < 0.015$). Although a small sample, these findings provide a foundation for medical schools to consider implementing a mentor type program to augment medical student skill/knowledge in older adult health care to advance age-friendly health care.

Abstract citation ID: igaf122.3511

### ADVANCING GERIATRIC CARE: HARNESSING AI AND HUMAN EXPERTISE TO ENHANCE CNA TRAINING

Suzanne White[1], Stephanie Trotter[2], Robin McAtee[2], and Valerie Bearden[3], *1. College of Nursing - University of Arkansas for Medical Sciences, Little Rock, Arkansas, United States, 2. University of Arkansas for Medical Sciences, Little Rock, Arkansas, United States, 3. Oaklawn Center on Aging, Hot Springs, Arkansas, United States*

Staff of the Arkansas Geriatric Education Collaborative (AGEC), a HRSA-funded Geriatric Workforce Enhancement Program, employed a strategic collaboration between human expertise and artificial intelligence (AI), specifically ChatGPT, a Large Language Model (LLM) to develop a robust 40-hour Advanced Geriatric Specialty curriculum for Certified Nursing Assistants (CNAs). Subject matter experts (SMEs) first conducted a thorough review of the Arkansas State CNA curriculum, identifying opportunities to supplement geriatric knowledge and practice. Based on this analysis, they developed a list of learning module topics to include in the specialty curriculum, beginning with an introduction to the 4Ms of Age-Friendly Care – What Matters, Medication, Mentation, and Mobility. ChatGPT was then employed to synthesize information from reputable sources for each topic and accelerate the generation of content for each module, linking content across modules to the 4Ms where relevant. Each module included a scenario-based application activity or a reflection exercise to reinforce learning and encourage critical thinking. Pre/post tests were also generated to assess the learning of key concepts. Subject matter experts reviewed and refined all AI-generated content to ensure accuracy, clinical relevance, and alignment with best practices in geriatric care. This innovative integration of AI-driven content development with expert oversight allowed for the accelerated creation of a high-quality, evidence-informed curriculum to enhance geriatric competency for CNAs.

Abstract citation ID: igaf122.3512
### IN THEIR OWN WORDS: AN INNOVATIVE APPROACH TO INTERGENERATIONAL LEARNING IN AGING COURSES
Carly Chak, Eleni Vodantis, and Nicole Alea, *University of California, Santa Barbara, Santa Barbara, California, United States*

Educational initiatives that encourage younger adults to interact with older adults is one way to reduce negative age-related stereotypes in university students. Incorporating intergenerational interactions into aging-related courses can be challenging when the class size is large. To address this challenge, this project included the development of pre-recorded "Aging Insight Videos". The videos feature five to six different older adults of varying backgrounds and lived experiences. The older adults answer instructor-developed questions that link with course concepts (e.g., social relationships, cognitive aging). These videos provide students the opportunity to learn about course material directly from the life experiences of older adults, in their own words. The current presentation focuses on: how older adult interviewees were chosen, the development of the questions, how the videos were edited, and ideas for implementing the videos into aging courses. Initial evidence, from week one of our adult development and aging course (N = 125), in which the videos were not included, suggests this instructional pedagogy is needed. Students held implicit ageist attitudes: older adult faces were rated significantly lower across five positive traits (warmth, competence, likeability, physical health, memory ability) compared to middle-aged and younger adult faces. We anticipate perceptions to positively change by the end of the course. However, the discussion will focus on how inclusion of the Aging Insight Videos as part of the course curriculum, and having students learn from and interact with older adults through these videos, in a next iteration of the course, should further reduce age-related negative stereotypes.

Abstract citation ID: igaf122.3513
### A STUDY ON THE COMPETENCIES AND CHARACTERISTICS REQUIRED BY INTERGENERATIONAL PROGRAMMING PROFESSIONALS
Stephanie Yu-Ching Chen, *National Chung Cheng University, Minxiong, Chiayi, Taiwan (Republic of China)*

Taiwan faces the challenge of a low birth rate and an aging population, resulting in social problems such as labor shortages and generational conflicts. How to promote cross-generational communication and solidarity has received attention. Intergenerational programs are gradually being promoted in various fields with the support of different government departments. However, there is still a lack of consensus on the professionalism and quality of such projects. This study used face-to-face interviews with 25 scholars and practitioners with experience in intergenerational programming to understand the characteristics and professional abilities required of intergenerational programming professionals. The research results found that intergenerational programming professionals mainly include three roles: leaders, managers, and lecturers. Their main task is to promote interaction, learning, and solidarity across generations. To competently promote intergenerational programs, the promoter should have certain characteristics, including being warm and enthusiastic, easy to interact with others, open and inclusive, flexible and adaptable, and innovative and flexible. Due to the differences in their roles, they need to have the general and the professional capabilities for intergenerational programs. Including professional knowledge such as aging literacy, life course and development, intergenerational learning and intergenerational program; having the attitude of caring and investing in intergenerational issues, respecting multiculturalism, liking both the old adults and children, valuing fairness between generations, and liking teamwork; and having the skills of resource connection and integration, intergenerational communication, promoting intergenerational interaction and connection, and intergenerational program planning and evaluation. These traits and abilities are the key to the success of intergenerational programs.

Abstract citation ID: igaf122.3514
### WHO CARES: WHAT IS THE FUTURE LANDSCAPE OF GERIATRIC AND GERONTOLOGY EDUCATION
Isaac Asirifi Boateng, and Phyllis Greenberg, *St. Cloud State University, St. Cloud, Minnesota, United States*

The need for advancements in geriatric and gerontology education has become increasingly vital. The current workforce has not kept pace with aging demographics. There is a low percentage of students choosing geriatrics as a specialty, with fewer than 5% of physicians specializing in this field (Kusmaul, 2023). Approximately 25% of BSW and 20% of MSW students have taken a course in gerontology. Adding to the issue is that nearly 50% of MSW students state that they have little or no interest in working with older adults after graduation (Cummings & Galambos, 2002). This poster examined the current landscape and future directions of medical and social service education regarding older adults. It stresses the importance of a critical look at gaps to equip future providers with the necessary skills and knowledge to address the complex needs of an aging population. This poster seeks to identify significant barriers, such as limited exposure to geriatric and gerontology training, underrepresentation of geriatric specialists, and a lack of funding and incentives for professionals working with an aging population. Proposed recommendations include all future practitioners having a baseline education in working with older adults, advocating for policy reforms to prioritize funding incentives (e.g.; scholarships, assistantships, internships) for students in the field. Overall, addressing these issues and implementing the recommended strategies, gerontology and geriatrics education can better prepare professionals not only to deliver high quality services for older adults but rather connect passion with their WHY of ultimately improving the lives of older adults.

Abstract citation ID: igaf122.3515
### GROWING PAINS: THE SEARCH FOR AGING STUDIES INTERNSHIPS FOR UNDERGRADUATE MAJORS
Nasreen Sadeq, Debra Dobbs, Lindsay Peterson, Sara Hackett, and Lu Norstrand, *University of South Florida, Tampa, Florida, United States*

The School of Aging Studies at the University of South Florida recently completed a major curriculum revision of our B.S. in Health Care Administration program. Formerly the B.S. in Long-Term Care Administration, this undergraduate program was originally developed with guidance from the Florida Board of Nursing Home Administrators and remains the only program in Florida offering students the opportunity to complete a

9-credit (650 hour) Administrator in Training (AIT) internship. To increase student enrollment in our revised Health Care Administration major, we focused on emphasizing the opportunity to complete an internship as a key recruitment strategy. Our updated curriculum that allows students to choose between the AIT program or a 3-credit part-time internship resulted in 15 interns at 14 different internship sites in 2024, generating 4,850 service hours. We are expecting a two-fold increase in next year in the number of students needing an internship placement. Strategies implemented to improve the overall efficiency of the internship program will be presented, including the development of standard operating procedures, a database for tracking student progress, and online modules that provide students with an orientation to the internship program. Challenges we have experienced including the limited availability of internship sites and the extensive time and effort required to develop new community partnerships and to match students with gerontology-oriented internships aligned with their career goals will also be discussed. With the increasing trend of undergraduates seeking internships and field experiences, we will share our ongoing plans to continue addressing these challenges in the future.

### SESSION 8000 (POSTER)

### LATE BREAKING POSTER SESSION I

Abstract citation ID: igaf122.3516

### A CLASSIFICATION APPROACH TO DETERMINE COGNITIVE IMPAIRMENT STAGE FROM METABOLOMIC DATA

Araya Cserepy, and Joshua Chuah, *Union College, Schenectady, New York, United States*

Alzheimer's Disease (AD) is a neurodegenerative disorder often marked by amyloid beta and tau accumulation, but metabolic changes can precede clinical onset by several years. Metabolites in particular have been implicated as early indicators of brain dysfunction. As such, this study applied machine learning to identify serum-derived metabolites distinguishing early (EMCI) from late mild cognitive impairment (LMCI). Using publicly available metabolite data from the Alzheimer's Disease Neuroimaging Initiative (ADNI), we analyzed baseline profiles from 643 participants (222 EMCI, 421 LMCI) across 104 features. A two-stage feature selection framework combined univariate ANOVA F-testing (top 30 features) with multivariate recursive feature elimination (final 15 features) to find the most important metabolites for diagnosis. This feature selection framework was repeated across different training/testing set splits to ensure the reliability of the features selected. Random Forest classification with balanced weighting addressed dataset imbalance. Across all repetitions, a core panel of five metabolites emerged consistently across validation runs: glyceric acid, hyocholic acid (HCA), hyodeoxycholic acid (HDCA), tauro-muricholic acid (TAMCA), and succinic acid. These metabolites achieved 76% ± 3% accuracy on unseen validation data and 78% ± 6% on development data. Notably, the panel included bile acid metabolites (HCA, HDCA, TAMCA) and energy-related metabolites (glyceric acid, succinic acid), pointing to disruptions in the gut–liver axis and cellular metabolism during disease progression. Overall, this work highlights metabolic alterations that accompany the transition from EMCI to LMCI and demonstrates the potential of machine learning–based metabolomics for early detection and diagnosis in AD.

Abstract citation ID: igaf122.3517

### A DOUBLE-BLIND TRIAL TO TEST IF NAD+ IMPROVES COGNITION

Karina Tavares, and Cindy Tsotsoros, *University of Rhode Island, Providence, Rhode Island, United States*

Pharmaceutical companies market nicotinamide adenine dinucleotide (NAD+) as a supplement that helps support cognitive functioning in aging adults, selling it as a potential shield against age-related decline and Alzheimer's disease. Although clinical trials are emerging, current research on its efficacy remains limited. This double-blind clinical trial aimed to determine if individuals who consumed oral NAD+ would show greater improvement on cognitive test scores than those taking a placebo. Thirty-seven women residing in New England who identified as Latina (17 NAD+, 18 placebo; 10 English speakers, 25 Spanish speakers) completed the NIH Toolbox Cognition Battery at baseline and after 28 days of daily supplementation. Measures included the Flanker Inhibitory Control and Attention, Dimensional Change Card Sort, List Sorting Working Memory, Pattern Comparison Processing Speed, and Picture Sequence Memory tasks. These tests assess executive function, working memory, and processing speed. Fully adjusted T-scores were analyzed. Paired-samples t-tests revealed significant pre-post improvements across all measures ($p < .001$–.079) with medium-to-large effect sizes (NAD+ Cohen's $d = 5.623$–11.986; placebo $d = 5.140$–10.453). ANCOVAs adjusted for baseline revealed no significant between-group differences. English speakers outperformed Spanish speakers, though language did not moderate treatment effects. Findings suggest NAD+ supplementation did not improve cognition more than the placebo. However, medium effect sizes suggest larger studies may reveal treatment effects. This work emphasizes the importance of including women and underrepresented populations in cognitive aging research, and highlights the need for methodologically sound trials to determine whether supplements produce the cognitive benefits they claim.

Abstract citation ID: igaf122.3518

### A ML APPROACH FOR MACES PREDICTION IN PATIENTS WITH UA AND HFPEF

Yijun Wang, and Jinhui Wu, *West ChinaHospital, Sichuan University, Chengdu, Sichuan, China (People's Republic)*

Background Heart failure with preserved ejection fraction (HFpEF) and unstable angina (UA) often coexist in clinical practice, constituting a high-risk cardiovascular phenotype with a markedly increased incidence of major adverse cardiovascular events (MACEs). Objective This study aimed to develop models based on machine learning (ML) algorithms to estimate the risk of MACEs in patients with coexisting UA and HFpEF. Methods This cohort study included 2,638 patients with both HFpEF and UA between January 1, 2015, and December 31, 2021. Patients were divided into the derivation cohort ($n = 1,367$) and validation cohort ($n = 1,271$) based on geographic regions. Clinical, laboratory, and imaging data were extracted from electronic medical records. Key predictors were identified using a hybrid feature selection method combining LASSO and Boruta algorithms. Nine ML models were developed: XGBoost, LGBM, RF, DT, GBDT, KNN, SVM, MLP, and GNB. Model performance was evaluated for the area under the receiver operating characteristic curve, accuracy, sensitivity, specificity, recall, F1-score, precision-recall curve, Brier scores, calibration curves and decision curve analysis.

The best-performing model was deployed as a web-based risk calculator. Results Using a combination of LASSO regression and the Boruta algorithm, seven key predictors were identified: Diabetes Mellitus, TG/HDL-C ratio, SIRI, TyG-BMI index, Creatinine, NT-proBNP, and Gensini score. The XGBoost-based model exhibited superior performance with an AUC of 0.825 in the derivation cohort and an AUC of 0.781 in the validation cohort based on seven features. Conclusion XGBoost-based predictive model constitutes an innovative approach to risk stratification for the complex dual phenotype of HFpEF and UA.

Abstract citation ID: igaf122.3519

### A SENESCENCE ASSOCIATED SECRETORY PHENOTYPE (SASP) IN INDOLENT SYSTEMIC MASTOCYTOSIS COMPARED TO HEALTHY CONTROLS

Jennifer Nicoloro-Santabarbara[1], Rohan Ravindran[1], Matthew Giannetti[1], Dan Dwyer[1], Michael Lam[2], Pui Lee[2], Marianna Castells[1], and Judith Carroll[3], *1. Brigham and Women's Hospital, Boston, Massachusetts, United States, 2. Boston Children's Hospital, Boston, Massachusetts, United States, 3. University of California, Los Angeles, Los Angeles, California, United States*

Indolent systemic mastocytosis (ISM) is a disease characterized by an abnormal proliferation and hyperactivity of neoplastic mast cells in bone marrow and various organ systems, marked by high rates of cognitive dysfunction, musculoskeletal deterioration, and chronic fatigue; clinical manifestations commonly associated with aging. ISM's dual identity as clonal and inflammatory may uniquely accelerate biological aging progression with translational relevance to geroscience. However, no studies have explored cellular senescence and related senescence-associated secretory phenotype (SASP) in ISM. We enrolled 40 adults with ISM and 40 age-and sex-matched healthy controls (HC). Plasma protein levels quantified using the Olink 48 inflammatory panel were used to create a 20-protein SASP index. Welch's t-test revealed ISM patients had 2- to 3-fold greater SASP index scores ($M = 3611.6$) compared to HCs ($M = 1{,}355.2$; $t(39.56) = -7.27$, $p < 0.001$). Within the ISM sample, IL1α, a SASP protein, was positively associated with general fatigue subscale of the Multisystem Fatigue Inventory-Short Form ($p < 0.05$). Patients with CD25+ mast cells, a sign of active disease state, had significantly higher SASP values ($M = 3884$) compared to patients with inactive disease ($M = 2599$; $t(30.99) = -3.1461$, $p < 0.05$). Findings demonstrate a highly active SASP profile among ISM patients compared to controls, and this pro-SASP profile is more pronounced in patients with active ISM disease. These results point to ISM as a driver of accelerated 'aging', informing future clinical and geroscience approaches in this patient population.

Abstract citation ID: igaf122.3520

### ACCEPTABILITY OF GENERATIVE AI-AUTHORED MESSAGES FOR PROMOTING PHYSICAL ACTIVITY FOR MIDDLE-AGED AND OLDER ADULTS

Allyson Tabaczynski[1], Yingjia Liu[1], Saeed Abdullah[2], Lizbeth Benson[1], and David Conroy[1], *1. University of Michigan, Ann Arbor, Michigan, United States, 2. The Pennsylvania State University, University Park, Pennsylvania, United States*

The delivery of digital health interventions for middle-aged and older adults can be streamlined by using generative artificial intelligence (GenAI) to write text message content. Biases in GenAI systems could lead to culturally insensitive or low-quality messages. Evaluating the acceptability of GenAI-authored messages is crucial before use in health interventions. This research examined middle-aged and older adults' perceptions of the cultural sensitivity and quality of GenAI-authored text messages for promoting physical activity, and the person- and message-level factors influencing these perceptions. In a cross-sectional survey, middle-aged and older adults (i.e., ≥ 40 years of age; $N = 630$; Mage=56.8 ±10.1 years) read 80 text messages written by GenAI and identified those that were culturally insensitive or had other problems. Descriptive statistics identified the proportion of GenAI-authored messages labeled as having issues. Separate negative binomial regressions examined the participant (zero-inflated) and message factors associated with message issues. Of 49,859 and 49,894 messages sorted to determine cultural sensitivity and quality, only 4.9% and 6.1% of the messages, respectively, were labeled as having issues. Knowledge of AI-authorship and more favorable attitudes towards AI were associated with identifying more messages as culturally insensitive. Messages generated by prompts that targeted sitting less (compared to moving more) or that described preparing for activity (compared to performing physical activity) received more labels as containing quality issues. Overall, GenAI can be prompted to write high-quality, culturally sensitive text messages for promoting physical activity for middle-aged and older adults, but message content and participants' knowledge of AI use could influence perceptions.

Abstract citation ID: igaf122.3521

### ACCESS AND ENGAGEMENT: DISTINCT ROLES OF DIGITAL TECHNOLOGY IN REDUCING LONELINESS IN LATER LIFE

Jaesung Lee, Liwei Zhang, and Hee Yun Lee, *University of Georgia, Athens, Georgia, United States*

The growing integration of digital technologies in later life raises important issues about how both access and use influence loneliness. Although prior studies have examined technology access and technology use separately, little is known about how they jointly influence loneliness. This study addresses this gap by examining their independent and combined relationship on loneliness among older adults. Using data from 4,285 participants aged 50 and older in the 2020 Health and Retirement Study, weighted multivariate regression models were estimated. In models including each predictor separately, both device ownership ($\beta = -0.153$, $p < 0.001$) and digital activities ($\beta = -0.195$, $p < 0.001$) were associated with lower loneliness. When both predictors were included together, device ownership ($\beta = -0.079$, $p = 0.004$) and digital activities ($\beta = -0.153$, $p < 0.001$) remained significant, with activities showing the stronger association. While device ownership is linked to lower loneliness, the results from the joint model indicate that active engagement through diverse digital activities demonstrates a stronger association. This study supports the view that while ownership provides the foundation for digital inclusion, it is active and diverse engagement with these devices that more strongly enhances well-being in later life.

Abstract citation ID: igaf122.3522

### ACCESS TO CARE AMONG PERSONS WITH END STAGE RENAL DISEASE AFTER ENROLLMENT IN MEDICARE ADVANTAGE

Denise Tyler[1], Emily Gadbois[2], Joan Brazier[3], and Amal Trivedi[3], *1. Miami University, Hancock, Maine, United States, 2. Brown*

*University School of Public Health, Providence, Rhode Island, United States, 3. Brown University, Providence, Rhode Island, United States*

Prior to the 21st Century Cures Act, enrollment in Medicare Advantage (MA) by people with end-stage renal disease (ESRD) was only allowed in limited circumstances. Enrollment in MA by those with ESRD has grown by over 70 percent since the Cures Act, but little is known about the effects of this policy change on those with ESRD and the organizations involved, such as MA plans and dialysis providers. This study, part of a larger mixed methods study on this topic, used qualitative methods to explore access to health care and other services by those with ESRD after enrollment in MA. We conducted 48 interviews with representatives from MA plans, dialysis organizations, and kidney care management companies and qualitatively analyzed these to identify key themes. Participants reported that MA enrollment has impacted patient access to dialysis, health care providers, transplant services, and supplementary benefits. Participants reported that the use of home dialysis modalities had possibly increased, that patients sometimes neglected to consider reduced access to other providers, especially specialists, when selecting MA, and that network restrictions on transplant centers may be affecting patients' ability to receive transplants. Finally, while some touted the benefits of vision and dental coverage for this population, others reported limited coverage and networks resulting in patients being unable to utilize these benefits. These results suggest that older adults with ESRD may need additional information and resources to ensure their insurance decisions don't affect their access to needed health care, services, and preferred providers.

Abstract citation ID: igaf122.3523

### ACOUSTIC FEATURES DIFFERENTIATE SUBTYPES OF COGNITIVE IMPAIRMENT IN A DIGITAL COGNITIVE ASSESSMENT

Tanya Talkar, Daniel Schulman, Connor Higgins, Kan Kawabata, and Sean Tobyne, *Linus Health, Boston, Massachusetts, United States*

The Digital Assessment of Cognition (DAC) is a brief, automated, remote-capable cognitive assessment that includes an animal naming task, a backward digit span test (BDST), and a 6-word Philadelphia Verbal Learning Test (PVLT). Patient speech produced during these tasks is automatically analyzed, yielding a rich set of acoustic and prosodic features that are potentially informative of cognitive impairment. We leverage a recently-developed clustering model that differentiates subtypes of mild cognitive impairment (MCI) and dementia using DAC outputs, and examine between-cluster differences in acoustic features in a dataset (N = 1188) gathered from the Apheleia-001 pre-screener study. Across all tasks, speaking rate, pause duration (mean and std.), pause percentage, and reaction time increase with worsening cognitive impairment, while we see a decrease in voice quality (cepstral peak prominence and harmonic to noise ratio). We particularly see an increase in reaction time during PVLT immediate recall trials with the presence of increased cognitive impairment, but an even more pronounced difference between dementia and MCI subtypes during the delayed recall trial. Similarly, speaking rate showed significant differences between subtypes during the delayed recall task. Our results suggest that response latency related features, and voice quality, can be affected within tasks due to the presence of cognitive impairment, with differences in subtypes likely due to differences in the domains of impairment. We conclude that speech-based features are informative of characterizing both cognitive impairment overall, and of specific subtypes of MCI and dementia. Future work can leverage these features to improve detection of cognitive impairment subtypes.

Abstract citation ID: igaf122.3524

### ADRD CAREGIVERS' HESITANCY TO RECEIVE PREPAREDNESS PROGRAM: CAREGIVING CONTEXT AND RESILIENCE

Emily Killian[1], Sato Ashida[1], Nicholas Ostrem[1], and Aaron Seaman[2], *1. University of Iowa, Iowa City, Iowa, United States, 2. University of Iowa, University of Iowa, Iowa, United States*

Preparedness is paramount for caregivers of persons living with dementia (PLWD) who face unique challenges in disaster situations, yet some are hesitant to participate in programs that help them develop emergency plans. Understanding factors associated with hesitancy can guide recruitment and program adaptation. Caregivers of PLWD randomized into the intervention arm (n = 112) of the Disaster PrepWise-Caregiving trial completed surveys and received a preparedness program. We compared caregivers who expressed hesitancy (n = 16) with those who did not (n = 96) on caregiving context, burden, self-efficacy, and resilience using t-tests and descriptive statistics, and analyzed post-study interviews guided by the PRISM framework and interventionists' notes. Hesitant caregivers reported lower caregiving demands, providing fewer weekly hours (31% < or equal to 8 hrs/week vs. 16%), shorter caregiving duration (38% < 2 years vs. 19%), and were more likely to care for PLWD in mild FAST stages (25% vs. 8%). Hesitant caregivers reported significantly lower Zarit burden scores (M = 2.19) than non-hesitant caregivers (M = 2.95, p = 0.015), but higher self-efficacy to manage dementia symptoms (p = 0.037) and higher CD-RISC perseverance (p = 0.032) and adaptability (p = 0.018) scores. Qualitative data reinforced these patterns: hesitant caregivers describe preparedness as currently unnecessary because care demands were not severe, or they felt equipped to cope through existing supports. Findings highlight that caregivers of individuals in earlier stages may not perceive preparedness as a current priority, indicating need for primary prevention messaging and recruitment strategies that emphasize planning benefits within ADRD caregiving context. Future research should identify strategies that motivate engagement across the spectrum of perceived need.

Abstract citation ID: igaf122.3525

### ADULTHOOD EARNINGS TRAJECTORY AND 10-YEAR MEMORY DECLINE: FINDINGS FROM ADMINISTRATIVE EARNINGS DATA

Calvin Colvin[1], Katrina Kezios[2], Samuel Swift[3], M. Maria Glymour[2], and Adina Zeki Al Hazzouri[4], *1. Columbia University, New York, New York, United States, 2. Boston University, Boston, Massachusetts, United States, 3. University of New Mexico Health Sciences Center, Albuquerque, New Mexico, United States, 4. Columbia University Medical Center, New York, New York, United States*

Financial resources are associated with cognitive functioning in older age, but prior studies do not characterize the dynamic trajectory of lifecourse financial status. Here, we examine the relationship between adulthood earnings trajectories and memory decline in older age using data from Health and Retirement Study participants linked to Social Security Administration earnings records. We included participants with any earnings data when 25 to 59 years old and ≥1 memory assessment on/after age 59 (N = 10,566). Memory was assessed using immediate and delayed 10-word recall or, for those unable to complete these tests, a proxy's interview. We categorized age 25-59 earnings using group-based trajectory modeling, separately for each birth cohort and gender. Using confounder-adjusted linear mixed models, we examined the association of earnings trajectory with 10-year memory decline. The median age at first memory assessment was 60 (IQR: 59-62) years. Across birth cohorts and gender, participants with the highest earnings over adulthood had the slowest decline in memory. For example, among men in the earliest birth cohort (1931-1941), those with high initial earnings that increased over time had an annual rate of memory decline of -0.029 standard units (95% CI: -0.032, -0.026). In comparison, those who maintained low earnings experienced faster declines (βLowxTime = -0.012, 95% CI: -0.016, -0.007), corresponding to an annual rate of decline of -0.041 standard units (95% CI: -0.044, -0.037) Associations generally followed a dose-response pattern. These findings add to the growing literature showing relationships between financial resources and cognition. More work is needed to identify underlying mechanisms.

Abstract citation ID: igaf122.3526

### AFRICAN AMERICAN ADULTS WITH HYPERTENSION MANAGING LONG COVID AND ACCESSING HEALTHCARE: A SYSTEMATIC REVIEW

Raymond Akorlatse, Rae Walker, Raeann LeBlanc, and Michael Lepore, *University of Massachusetts Amherst, Amherst, Massachusetts, United States*

Hypertension (HTN) is a significant risk factor for cardiovascular disease (CVD) and stroke, contributing notably to cardiovascular morbidity and mortality in the United States and globally. The emergence of Long COVID (LC) has expanded the scope of managing hypertension as a cardiovascular and chronic disease in Black and African American adults. This study describes how African American adults exposed to COVID-19 have managed HTN and healthcare access since the pandemic's onset. We applied the Preferred Reporting Items for Systematic Reviews and Meta-Analyses (PRISMA) 2020 guidelines to synthesize existing knowledge. Research articles from 2020-2025 were identified across the Web of Science (Core Collection), MEDLINE (Ovid), and CINAHL databases. English language peer-reviewed studies that reported Social determinants of health (SDOH) factors, COVID-19, HTN, CVD, and healthcare access among Black and African American adults were included. Twenty-three articles were selected. Results revealed common barriers to healthcare, including lack of insurance, transportation challenges, and poor continuity of care. Existing research identified SDOH factors such as employment, redlining, housing insecurity, Medicaid/uninsured, low-income status, low education, rent moratorium, and ethnicity as drivers of health inequities. Though telehealth access and use increased significantly during the pandemic, Black and African American adults continued to face health care access barriers such as lack of access to high-speed internet or broadband, variable levels of digital literacy, and mistrust. Diagnosis of long COVID is a predictor for further health inequities among Black and African American persons managing a CVD diagnosis, geographical location, language, and sociodemographic characteristics (age, sex, gender).

Abstract citation ID: igaf122.3527

### AFRICAN AMERICAN CANCER CAREGIVERS' DECISION ON PALLIATIVE CARE USE: A SOCIAL ECOLOGICAL PERSPECTIVE

Moses Akwobugi[1], and Elisha Oduro[2], *1. University of Maryland Baltimore, Baltimore, Maryland, United States, 2. University of Maryland Baltimore, BALTIMORE, Maryland, United States*

African American (AA) cancer caregivers are less likely to pursue palliative care for their relatives compared to Whites leading to differences in cancer outcomes. However, little is known about the multi-level influences on AA cancer caregivers' palliative care decision-making. Using the Social Ecological Model as a conceptual lens, this study develops a theoretical framework to understand the multi-level influences on African American cancer caregivers' palliative care decision-making. The model highlights five levels of palliative care decision-making influences: intrapersonal, interpersonal, organizational, community, and policy. At the intrapersonal level, health literacy and education level influence how AA cancer caregivers perceive the purpose and benefits of palliative care. Interpersonally, family dynamics and trust in clinicians influence their willingness to engage in shared decision-making about palliative care. Organizationally, the availability of culturally sensitive palliative care services and clinicians' culturally aligned communication influences their palliative care acceptance. Community partnerships with churches and faith leaders validate palliative care as consistent with cultural and spiritual traditions, influencing their palliative care acceptance. At the policy level, strengthening Medicare coverage for palliative care services and affordable community-palliative care programs reduces cost barriers and improves access, influencing AA cancer caregivers' palliative care decisions. This framework underscores the need for coordinated, multi-level palliative care interventions to support AA cancer caregivers to make informed palliative care decisions and reduce disparities in cancer outcomes. Further, it provides a theoretical foundation for future research and practice aimed at improving palliative care use in AA communities.

Abstract citation ID: igaf122.3528

### AGE DIFFERENCES IN AFFECTIVE OUTCOMES OF DAILY EMOTION SHARING

Jane Stephenson[1], Enna Chen[1], Kathrine Whitman[2], and Laura Carstensen[1], *1. Stanford University, Stanford, California, United States, 2. Stanford University, Palo Alto, California, United States*

According to socioemotional selectivity theory (SST), as people age and future time horizons shorten, emotionally meaningful goals are prioritized over exploratory ones. Relatedly, people increasingly engage in selective pruning of social networks that focus on emotionally close relationships. Research has not explored the degree to which older adults discuss emotional experiences with close partners and whether potential benefits of emotional disclosures vary by age. An experience sampling study that sampled emotional experiences at five randomly selected times per day for seven days, generated the data analyzed in the present study. A lifespan sample of 180 participants (aged 18 to 93, M = 57.3) reported whether they shared their emotions with someone and, if so, the person with whom they shared, viz., spouse/partner, other family member, friend, acquaintance, coworker, and stranger. They also reported the degree to which they were currently experiencing 8 positive and 11 negative emotions. Although there were no age differences in the likelihood of sharing emotions with any specific partner, sharing emotions with non-spousal family members was associated with more positive emotional experience and less negative emotional experience in older participants than younger participants; and sharing emotions with spouses or romantic partners was associated with relatively more positive experience in younger and middle-aged adults than in older adults. Gender differences and associations with discrete emotions are also discussed. Findings indicate that emotional disclosures in close relationships play different roles at different ages, which have implications for overall well-being.

Abstract citation ID: igaf122.3529

### AGE DIFFERENCES IN ASSOCIATIONS BETWEEN DAILY EMOTIONAL SUPPORT PROVISION AND MEMORY LAPSES

Faith Shannon, and Eric Cerino, *Northern Arizona University, Flagstaff, Arizona, United States*

Subjective cognitive complaints (e.g., memory lapses) may be an early warning sign of dementia detectable prior to declines in performance-based cognitive function. Identifying modifiable behaviors related to memory lapses is needed to target ways to promote cognitive health in everyday life. The role of emotional support provision, an interpersonal behavior requiring cognitive resources, has not been examined. We address this gap by assessing age differences in associations between emotional support provision and memory lapses. In 1,236 adults from the National Study of Daily Experiences (average age=67.67 years, SD = 10.34, 57% female), participants completed surveys for 8 days with questions regarding whether they gave emotional support and experienced retrospective or prospective memory lapses. We aggregated responses across days to calculate individual differences in proportion of days participants gave emotional support and reported memory lapses. Regression analyses adjusted for age, sex, education, race, caregiving status, and receipt of emotional support. People who gave more emotional support reported more retrospective (Est.=0.08, SE = 0.04, p = 0.02, 95% CI: 0.01, 0.16) and prospective memory lapses (Est.=0.14, SE = 0.03, p<.001, 95% CI: 0.08, 0.20). Age moderated the association between emotional support provision and prospective lapses (Est.=0.01, SE = 0.01, p<.01, 95% CI: 0.002, 0.01) such that the association between emotional support provision and prospective memory lapses was amplified in comparatively older adults (Est.=0.21, SE = 0.04, p<.001, 95% CI: 0.13, 0.30). Results indicate emotional support provision is linked to elevated risk of memory lapses, especially for comparatively older adults that may have less resources to manage demands of emotional support provision and prospective memory.

Abstract citation ID: igaf122.3530

### AGE STEREOTYPES AND STEREOTYPES OF PERSONS LIVING WITH MEMORY LOSS WITH HEALTH IN PARENT-ADULT CHILD DYADS

Yunke Gu[1], Joan Monin[2], Becca Levy[1], Marianne LaFrance[3], and Heidi Gil[4], *1. Yale School of Public Health, New Haven, Connecticut, United States, 2. Yale, Yale, Connecticut, United States, 3. Yale, New Haven, Connecticut, United States, 4. LiveWell, Plantsville, Connecticut, United States*

Negative stereotypes of aging have been associated with poor overall health for the people that hold them. However, research examining stereotypes of persons living with memory loss and how they relate to health remains limited. Little is known about interpersonal influence of these stereotypes within intergenerational caregiving dyads. This study examines the dyadic associations between self-perceptions of aging and persons living with memory loss, and health outcomes in parent-adult child caregiving dyads. This study is a secondary analysis of 126 dyads from the Families Coping Together with Alzheimer's Disease study. Measures included self-reported depressive symptoms and physical and mental health. Positivity scores of age stereotypes and stereotype of persons living with memory loss were derived from participant self-reported words coded on a 5-point scale by independent raters. Actor-Partner Interdependence Models were used to examine intrapersonal and interpersonal associations between the dyads. Our dyadic analyses indicate that positive age stereotypes were significantly associated with better mental health and fewer depressive symptoms for both dyad members (actor effects). Positive stereotypes of people living with memory loss were associated with greater physical health in both dyad members (actor effects). No partner effect emerged. Parent and child stereotypes were not significantly associated. The study underscores the role of self-perceptions of aging and memory loss in shaping individual health outcomes within the context of caregiving dyads. While stereotypes may be more impactful for health intrapersonally than interpersonally, fostering positive perceptions on aging and memory loss may support well-being in caregiving dyads and broader society.

Abstract citation ID: igaf122.3531

### AGE-DEPENDENT DECLINE IN SKELETAL MUSCLE OXYGENATION REVEALED DURING INTERMITTENT HYPOXIA AND RECOVERY

Stefano Donega[1], Kenneth W. Fishbein[2], Rolando J. Hernandez[2], Ross McDevitt[2], Cynthia Chisolm[3], Martina Rossi[4], Mirko Baranzini[2], and Luigi Ferrucci[5], *1. NIA/NIH Biomedical Research Center, Baltimore, Maryland, United States, 2. National Institute on Aging, Baltimore, Maryland, United States, 3. NIA, Baltimore, Maryland, United States, 4. NIA/NIH, Baltimore, Maryland, United States, 5. NIA, Bethesda, Maryland, United States*

Aging and age-associated syndromes such as peripheral artery disease have been previously associated to impaired mitochondrial aerobic metabolism and to ultimately contribute to sarcopenia. The mechanisms underlying this decline are still unclear, partly because existing indirect methods - such as near-infrared spectroscopy - lack the required resolution to accurately assess intramuscular dynamics. In this study, we measured hamstring muscle oxygenation in 34 C57BL/6 mice, clustered as young (~4 months), adult (~11 months), and old (~21 months). Intramuscular oxygen concentration was measured with a fiber-optic probe at a rate of 1 sample per second, alongside continuous monitoring of vital signs under isoflurane anesthesia at three stages during: (I) 1:30 minutes baseline, (II) five 2:30 minutes cycles of acute intermittent hypoxia (21–5% $O_2$), and (III) 1:30 minutes recovery at normoxia (21% oxygen). Baseline oxygenation declined significantly with age and the difference persisted across all five IH cycles. Age-impairments were mostly pronounced during the reoxygenation phase, where older mice showed moderated curve slopes and lower zenith values across all cycles without differences in the kinetics of oxygen depletion during the descending phase, indicating age-preserved hypoxic response. Oxygen levels during recovery remained significantly reduced with age. Our findings reveal a robust age-dependent decline in skeletal muscle oxygenation not only during a IH stress, but also at baseline and recovery, offering new tool and insights to investigate impaired oxygen dynamics in aging and muscle dysfunction.

Abstract citation ID: igaf122.3532

### AGE-RELATED CHANGES IN DEPRESSIVE SYMPTOMS IN GHANAIAN ADULTS

Nutifafa Dey, and Frank Infurna, *Arizona State University, Tempe, Arizona, United States*

Depression is a common mental disorder that is among the leading causes of disability worldwide, linked to poor physical health and decreased quality of life trajectories in adulthood. Current evidence from Western contexts suggests depression follows a U-shaped curve trajectory across the lifespan. However, its patterning is unclear in low- and middle-income countries, representing distinct cultural, economic, and societal environments. We address this gap by examining age-related depression changes in Ghanaian middle-aged and older adults using longitudinal data from ISSER-Northwestern-Yale Long Term Ghana Socioeconomic Panel Survey (GSPS). Nonlinear Growth Curve models were applied through the Multilevel Framework to three waves of data from 5337 (observations=14645) participants aged 30+(baseline: M=48.67, SD=13.89). The average depression at age 60 (centering age) was 18.62 and rose by 0.38 units per decade after 60. Depression shows a curvilinear association with age, accelerating at older ages. Trajectories of depressive symptoms across adulthood in Ghana seem to follow the popular U-Shape curve pattern. Age alone explains little within-person depression variability, and more research is needed to examine the effects of other predictors. Intervention should target depression in later life to address increasing mental health issues and promote quality of life.

Abstract citation ID: igaf122.3533

### AGE-SENSITIVE USABILITY IN CONVERSATIONAL AI AGENTS: A SYSTEMATIC REVIEW

Agnes Jihae Kim, and Moon Choi, *Korea Advanced Institute of Science and Technology, Daejeon, Republic of Korea*

The rapid rise of conversational AI agents, exemplified by ChatGPT, has shaped daily life from health information seeking to emotional support. While offering opportunities and challenges for aging populations, most designs remain youth-centric. This systematic review critically assesses existing knowledge on usability evaluation of conversational AI agents for older adults. Following the PRISMA-ScR guidelines, four major databases (Web of Science, Scopus, ACM Digital Library, and PubMed) were searched using the keywords "older adults," "conversational AI agents," and "usability." After removing duplicates, 873 journal articles were identified. Screening through Covidence software yielded 15 studies that met the inclusion criteria by targeting or separately reporting on older adults. Analysis showed that healthcare (42.9%) and independent living activities (33.3%) were the primary domains of application, while companion support (14.3%) and social care (9.5%) were less frequently studied but emerging. Most studies were published after 2020, with nine conducted in Europe and the U.S. and five in Asia. Regarding measurement, the System Usability Scale (SUS) and satisfaction surveys were most commonly applied, while AI-specific measures (e.g., the Chatbot Usability Scale) were rarely used. Across the seven studies employing SUS, the average score was 70.6, exceeding the benchmark of 68 points and suggesting generally acceptable usability. This review synthesizes current evidence on usability evaluation of conversational AI agents among older adults, who often present distinct cognitive, physical, and behavioral characteristics related to digital technology use. It underscores the need for age-sensitive design and evaluation frameworks to move beyond trial-and-error practices and inform inclusive AI policies.

Abstract citation ID: igaf122.3534

### AGING AND EMOTION: REGULATION TACTICS ALTER SELF-REPORTED AND PHYSIOLOGICAL AROUSAL

Ceresa Munjak-Khoury[1], Rebecca Polk[2], and Derek Isaacowitz[1], *1. Washington University in St. Louis, St. Louis, Missouri, United States, 2. Washington University in St. Louis, St Louis, Missouri, United States*

Older adults consistently report higher emotional well-being despite some physical and mental declines with age. Some theorize this is due to differences in emotion regulation, however no conclusive evidence for age differences in emotion regulation strategies has emerged. Emotion regulation tactics, such as positive-approaching (enhancing the positivity of a situation) and negative-receding (reducing the negativity of a situation), hold promise for uncovering age differences. However, no studies to date have considered whether tactic vary in their physiological profiles. Thus, this study investigated 35 younger (M=19.06 years, SD=3.58; 17 women) and 42 older (M=74.02 years, SD=4.69; 23 women) participants who viewed emotionally-evocative videos and regulated emotions using positive-approaching and negative-receding techniques (with both regulation blocks compared to a neutral video block to control for baseline differences in psychophysiology). Multi-level linear models revealed significant Age x Sex x Tactic interactions, such that positive-approaching tactics (vs negative-receding) were associated with lower arousal ratings for younger women and older men (but not younger men or older women). While there was no tactic difference in self-reported arousal for older women, Respiratory Sinus Arrythmia (a measure of heart activity associated with calm parasympathetic states) was higher during positive-approaching for them. Interestingly, older men had higher RSA during negative-approaching tactics as compared to positive-approaching. These findings suggest emotion regulation tactics have age and sex differential impacts on self-reported and physiological arousal. Future work may disentangle physiological activation and self-reported behavior by also considering the role of interoceptive awareness.

Abstract citation ID: igaf122.3535

### AGING IN GHANA: A SYSTEMATIC REVIEW OF AGING TRENDS, CHALLENGES, AND DIRECTIONS FOR FUTURE POLICY

Deborah Dadson[1], Alfred Boakye[2], Henrietta Bennett[3], Ebenezer Martey[3], Amma Aboagye Kyei[4], Josephine Boateng[5], and Adam Mohammed[6], *1. University of Alabama,*

*Birmingham, Birmingham, Alabama, United States, 2. University of Maryland, Baltimore, Baltimore, Maryland, United States, 3. University of Alabama at Birmingham, Birmingham, Alabama, United States, 4. Central University, Tema, Greater Accra, Ghana, 5. University of Massachusetts Boston, Dorchester, Massachusetts, United States, 6. University of Virginia, Charlottesville, Virginia, United States*

Population aging has received considerable attention across economies presenting significant social, economic and health challenges. Despite this demographic shift, the older adult population in Ghana remains an understudied group with little attention to the context, processes and outcomes of active aging. We conducted a systematic review to compile and report on existing empirical evidence on population trends, age-related health issues, available support systems, and needs of older adults in Ghana to inform age-friendly services and policies. In accordance with PRISMA guidelines, we screened peer-reviewed articles published between 2015 and 2025 using search terms related to aging in Ghana. The review identified 56 articles, and four main themes were developed through thematic synthesis – social support, needs, health outcomes and policy implications. Increased emotional, relational and instrumental support from family and community members were associated with higher levels of well-being, increased healthcare utilization and reduced feelings of isolation. Unmet needs of older adults included food insecurity, financial hardship, lack of assistance with daily activities, and limited access to healthcare. In addition, older adults who reported multiple chronic conditions reported high levels of functional disabilities and had trouble with physical functioning while those who were more physically active had fewer problems with physical functioning. This review emphasizes an immediate, coordinated policy intervention and research action to prepare Ghana for its next demographic phase. Priority action should focus on enhancing healthcare access, fostering social inclusion and promoting community engagement among older adults in Ghana to improve their quality of life.

Abstract citation ID: igaf122.3536

### AGING INITIATIVE: A STUDENT-LED MODEL FOR ADVANCING AGING RESEARCH WITH A TRANSLATIONAL FOCUS

Maks Fedorovskyy[1], Satvik Dasariraju[1], Sean Simonini[2], Eshan Mehra[1], Chancen Law[1], Nina Khera[1], Krishna Rajagopal[1], and Ariela Orkaby[3], *1. Harvard University, Cambridge, Massachusetts, United States, 2. University of Massachusetts Lowell, Lowell, Massachusetts, United States, 3. Brigham & Women's Hospital, Harvard Medical School, Boston, Massachusetts, United States*

Aging research is advancing rapidly but faces a striking shortage of young talent; new educational frameworks are needed to equip future generations with a "full pipeline" viewpoint (spanning research, drug development, and policy) required to bring new science to the clinic. The Aging Initiative is a new undergraduate-led nonprofit, representing a scalable model for student-driven efforts to propel the field. By equipping students and early-career researchers with direct mentorship and hands-on interdisciplinary opportunities in research, biotechnology, and policy, we aim to dismantle barriers that hinder translation. Founded at Harvard in 2023 with seven members, the organization has grown in two years into a Boston-wide community of over 1700 students, researchers, and industry professionals. This rapid growth underscores strong interest and the need for structures that direct it toward lasting impact. Our programs are intentionally designed to integrate disciplines, bridge academia-industry gaps, and confront historically overlooked challenges in therapeutic development for age-related disease. In the preceding months, we hosted a 400-participant muscle aging symposium with world experts on frailty and patient care, biotech founders, and pharmaceutical company executives. In addition to a steady calendar of journal clubs, mixers with hundreds of attendees, and research seminars, we launched collaborations with partners including the Pepper Center, Biolearn, and Nucleate, spanning clinical development, biomarker analysis, and education. With upcoming events poised to bring together thousands, the Aging Initiative's approach provides a replicable blueprint for student-driven engagement that institutions worldwide can adopt to accelerate progress and catalyze the next generation of leaders in aging science.

Abstract citation ID: igaf122.3537

### AI-CONECT: DESIGNING RESPONSIBLE-AI-BASED CONVERSATIONAL CHATBOTS FOR DEMENTIA INTERVENTION

Junyuan Hong[1], Wenqing Zheng[1], Han Meng[2], Siqi Liang[2], Liu Chen[3], Hiroko Dodge[3], Jiayu Zhou[2], and Zhangyang Wang[1], *1. University of Texas at Austin, Austin, Texas, United States, 2. Michigan State University, East Lansing, Michigan, United States, 3. Massachusetts General Hospital, Harvard Medical School, Boston, Massachusetts, United States*

Social isolation is recognized as a major modifiable risk factor for dementia. The I-CONECT clinical trial demonstrated that semi-structured, cognitively stimulating conversations can be an effective strategy for combating social isolation and cognitive decline among older adults with mild cognitive impairment (MCI). However, scaling such interventions is limited by the availability of trained human interviewers. To address this challenge, we developed the AI-CONECT Chatbot, a Responsible-AI system designed to implement the I-CONECT conversational intervention protocol using large language models (LLMs). This approach enables broad, scalable deployment without the human resource limitations of the original intervention. Our approach combines a privacy-preserving learning framework to automatically generate and evaluate protocol-compliant instructional prompts for LLMs with a novel evaluation strategy using "virtual users"—AI-based replicas of participants with MCI and participants with normal cognition (NC) from the I-CONECT clinical trial. In simulated conversations between chatbots and the virtual users, we found that optimized prompts can improve the user's word ratio significantly over ChatGPT (ChatGPT: mean=0.235, std=0.059; ours: mean=0.489, std=0.065, $p = 0.0004$ by Mann-Whitney U test). The high word ratio implies better users' conversational engagement. These findings suggest that LLMs, when carefully prompted, can engage older adults in cognitively stimulating dialogues consistent with established clinical interventions. Our preliminary results demonstrate a pathway for integrating responsible AI into enhancing cognitive functions, offering a practical and scalable alternative to human-facilitated conversational interventions. In our future work, we aim to improve the friendliness and other human-centered attributes of the chatbot.

Abstract citation ID: igaf122.3538

### ALCOHOL CONSUMPTION AND ASSOCIATED FACTORS AMONG ELDERLY PEOPLE IN CENTRAL BRAZIL

Larissa Magalhaes[1], Milara Barp[2], Bruno Vinícius Diniz e Silva[2], Sheila Teles[2], Erika Aparecida Silveira[2], Winny Eveny Moura[2], Pao-Feng Tsai[1], and ´Valeria Pagotto[2], *1. Auburn University, Auburn, Alabama, United States, 2. Federal University of Goias, Goiania, Goias, Brazil*

Background: Alcohol is a legal substance widely consumed across different social strata and age groups. Among older adults, alcohol use poses growing challenges for health management due to age-related physiological vulnerability. This study aimed to estimate alcohol consumption patterns and identify associated risk factors in older adults. Methods: A survey was conducted in Goiânia, Central-West Brazil, from July 2018 to March 2019, using the Alcohol Use Disorders Identification Test (AUDIT). Participants were part of the second wave of a cohort study. A total of 221 older adults were interviewed and included in the analysis. Results: Among the participants, 14.9% reported alcohol use in the past year. Most showed low-risk consumption patterns (97.3%), and 1.8% reported binge drinking. Gender (adjusted odds ratio [aOR] = 2.57), religion (aOR = 0.45), and education level (aOR = 0.18) were significantly associated with alcohol use. Conclusions: Male gender was associated with greater alcohol use, while higher education and religious affiliation appeared protective. Although the prevalence of alcohol consumption was low in older adult population, the findings highlight the need for targeted actions in older men. These results contribute to understanding the health behaviours of older adults and emphasize the importance of tailored health promotion strategies considering the heterogeneity of aging.

Abstract citation ID: igaf122.3539

### AN INTEGRATED PSYCHOSOCIAL THEORY OF LONELINESS: A MULTILEVEL SOCIOLOGICAL AND PSYCHOLOGICAL VIEW

Bianca Suanet[1], Marja Aartsen[2], and Denis Gerstorf[3], *1. Linköping University, Norrköping, Ostergotlands Lan, Sweden, 2. Oslo Metropolitan University, Oslo, Oslo, Norway, 3. Humboldt University Berlin, Humboldt University Berlin, Berlin, Germany*

Loneliness is increasingly recognized as a pressing societal issue, yet theoretical integration across disciplines remains limited. While psychological research often focuses on individual factors, sociological approaches emphasize broader structural and cultural conditions. This article proposes an integrative framework that bridges micro-, meso-, and macro-level perspectives to better understand the complex, multi-layered nature of loneliness across the life course with a focus on later life. We synthesize insights from psychological and sociological perspectives, emphasizing how community, network, and institutional contexts shape individual experiences of loneliness. Building on existing conceptual models and empirical findings, we identify core contextual pathways and mechanisms that operate across levels. The proposed systems model outlines how loneliness emerges through dynamic interactions between societal structures, cultural norms, community settings, and individual factors. We emphasize key intersections between levels, including macro-meso (e.g., welfare policy and community resources), macro-micro (e.g., cultural norms and non-normative life transitions), and meso-micro (e.g., community characteristics and social support). The model supports a context-sensitive understanding of how structural inequalities and cultural norms affect loneliness. Our framework contributes to a more interdisciplinary and multi-level understanding of loneliness by linking structural, community, and individual processes. It provides a foundation for developing context-sensitive, equity-informed intervention elements that address loneliness not only through personal support, but also by transforming the social environments in which loneliness emerges, across different life stages and population groups.

Abstract citation ID: igaf122.3540

### ANTICIPATING DISASTER: EMPOWERING CAREGIVERS AS ESSENTIAL FIRST RESPONDERS

Díana Poncé, Jarmin Yeh, Kattia Suarez Vargas, and Melinda Neri, *University of California, San Francisco, San Francisco, California, United States*

Older adults and adults with disabilities face increased risks during climate disasters, like wildfires, and depend heavily on caregivers for physical and health-related support. Emergency evacuations during these events are particularly challenging due to mobility limitations and cognitive impairments, making caregivers critical in ensuring their care recipients' safety. Yet, many caregivers may be underprepared to respond effectively without prior training. To address this gap, we conducted semi-structured in-depth interviews with 25 Medicaid-funded In-Home Supportive Services caregivers in San Bernardino County, California, who participated in an Emergency and Disaster Readiness Training between June 2024 and March 2025. The interviews explored caregivers' experiences with mitigating risks and preparing for disasters, focusing on how they applied the training skills and concepts in practical scenarios. Through thematic analysis, our findings revealed that caregivers played a proactive role in safeguarding their care recipients by anticipating disaster risks and implementing preparedness measures. Notably, caregivers recognized their role as experts about their care recipients' needs and as essential first responders in preparation for the arrival of official emergency services. They employed three key approaches to mitigate risks: modifying their care recipients' homes to reduce the potential for injury, preparing and rehearsing emergency evacuation plans, and personalizing and supplementing emergency kits to address their care recipients' specific needs. These findings highlight the importance of specialized training programs and policies to empower caregivers as essential first responders. By equipping caregivers with skills and resources to protect vulnerable populations, we can enhance disaster preparedness and improve outcomes for older adults.

Abstract citation ID: igaf122.3541

### ASSESSING THE STABILITY OF COGNITIVE STIMULATION THERAPY'S EFFECTIVENESS ON DEMENTIA ACROSS US AND HONG KONG

Ebow Nketsiah[1], Marla Berg-Weger[2], Max Zubatsky[3], Allison Gibson[4], Jinmyoung Cho[4], and Cara Wallace[5], *1. Florida Atlantic University, Boca Raton, Florida, United States, 2. Saint Louis University - St Louis, MO, St. Louis, Missouri, United States, 3. Saint Lewis University, Saint Louis, Missouri, United States, 4. Saint Louis University, Saint Louis University, Missouri, United States, 5. Saint Louis University, Saint Louis, Missouri, United States*

Cognitive Stimulation Therapy (CST) has demonstrated effectiveness in improving cognition and quality of life for people with mild to moderate dementia and is recognized as a cost-effective psychosocial intervention worldwide. Since its development in the United Kingdom over twenty years ago, CST has been implemented in more than thirty countries across North America, Africa, Asia, Australia, and Europe. Despite this wide adoption, few studies have examined CST's cross-cultural effectiveness. This study explores the stability of CST outcomes across the United States (US) and Hong Kong (HK). Paired sample t-tests and effect size calculations (Cohen's d) were used to assess CST's impact on cognition, quality of life, depression, and physical function among persons with dementia. Findings revealed significant improvements in cognition among CST-US ($t(261) = 7.41$, $d = .458$) and CST-HK participants ($t(204) = -3.13$, $d = -.218$). Quality of life improved significantly in the US group ($t(199) = 8.71$, $d = .616$), while depression significantly decreased in both the US ($t(254) = -6.35$, $d = -.397$) and HK ($t(236) = -3.08$, $d = -.200$) groups. Physical function improved significantly in the US ($t(33) = -2.06$, $d = -.344$) but not in HK ($t(84) = -.89$, $p = .188$). These results suggest that CST is effective across cultural contexts, though the magnitude and significance of outcomes vary. The findings highlight the importance of considering cultural and contextual factors when implementing CST globally and support further investigation into adapting interventions to optimize their effectiveness across diverse settings.

Abstract citation ID: igaf122.3542

### ASSOCIATION BETWEEN NON-OBESE DIABETES VERSUS OBESE DIABETES ON DEMENTIA AND MORTALITY ALL OF US RESEARCH PROGRAM

Junyu Sui[1], Bei Wu[2], Ross Andel[1], and Xiang Qi[3], *1. Arizona State University, Phoenix, Arizona, United States, 2. NYU Shanghai, China (People's Republic), 3. New York University, New York, New York, United States*

Diabetes mellitus represents a significant risk factor for dementia, with both Type 1 and Type 2 diabetes contributing to cognitive decline through distinct mechanisms including insulin resistance, tau hyperphosphorylation, β-amyloid accumulation, chronic inflammation, and oxidative stress. To compare dementia risk between non-obese and obese individuals with type 1 diabetes (T1D) and type 2 diabetes (T2D) among US adults aged over 50 years, this retrospective study utilized data from the All of Us research program and ICD-9/10 codes to identify T1D, T2D, and dementia cases. Participants were classified as non-obese based on BMI (< 30 kg/m² for non-Asian races; < 27.5 kg/m² for Asian Americans) and waist circumference (< 102 cm for males, < 88 cm for females). Statistical analyses employed Cox-proportional hazard models and competing risk models accounting for mortality. Among 268,648 participants with a median follow-up of 10 (interquartile range, 6–14) years, 216 (17.8%) participants developed dementia. After full adjustment, non-obese T1D demonstrated a 4 times higher risk of dementia (95% CI: 2.86-5.60), while non-obese T2D showed a 1.82 times higher risk (95% CI: 1.45-2.28) compared to those without diabetes. These associations persisted when accounting for competing mortality risk and when using waist circumference as an obesity measure. Non-obese diabetes, particularly T1D, is associated with a significantly greater dementia risk compared to both obese diabetes and no diabetes. These findings underscore the importance of early cognitive screening, careful glycemic management, and targeted prevention strategies for non-obese individuals with diabetes.

Abstract citation ID: igaf122.3543

### ASSOCIATION OF PAIN, INSOMNIA, AND DEPRESSIVE SYMPTOM CLUSTERS WITH COGNITIVE DECLINE

Matthew Phan[1], Utpol Das[1], Tyler Bell[2], Mukaila Raji[1], and Sadaf Milani[1], *1. University of Texas Medical Branch, Galveston, Texas, United States, 2. University of California, San Diego, San Diego, California, United States*

Pain-Insomnia-and-Depression syndrome (PIDS) refers to the triad of chronic pain, sleep disturbances, and depressive symptoms disproportionately affecting older adults. Limited data exist on the rates and patterns of these symptom clusters and their association with cognitive function. Our objectives were to characterize symptom clusters of pain, insomnia, and depression and to examine their association with cognitive decline among middle-aged and older Americans. We used data from the Health and Retirement Study (2010-2018), a nationally representative longitudinal study of Americans over age 50 (n = 18,599). Latent class analysis identified clusters based on pain prevalence, insomnia, and depressive symptoms. Mixed-effects linear regression models examined the associations between these clusters and cognitive decline. Five clusters were identified: 1) minimal symptoms (56.90%), 2) moderate pain, low insomnia, low depressive symptoms (7.3%), 3) no pain, high insomnia, moderate depressive symptoms (6.4%), 4) high-impact pain, moderate insomnia, moderate depressive symptoms (11.8%), and 5) high-impact pain, high insomnia, high depressive symptoms (7.5%). Compared to those with minimal symptoms, individuals in the "no pain, high insomnia, and moderate depression" and "high-impact pain, moderate insomnia, and moderate depression" clusters experienced the fastest rates of cognitive decline ($\beta = -0.07$, 95% CI: -0.13, -0.01; $\beta = -0.07$, 95% CI: -0.11, -0.02, respectively). These results suggest a complex interplay among pain, insomnia, and depression, particularly in relation to the development of cognitive decline in older age. Screening for co-occurring symptoms may inform therapeutic clinical decision-making aimed at mitigating cognitive decline.

Abstract citation ID: igaf122.3544

### ASSOCIATIONS AMONG HANDGRIP STRENGTH, BRAIN PATHOLOGY, AND COGNITION IN AUTOSOMAL DOMINANT ALZHEIMER'S DISEASE

Nadeshka Ramirez-Perez[1], Ana Baena[2], Daniel Vasquez[2], Lusiana Martinez[1], Averi Giudicessi[3], Liliana Ramirez Gomez[3], David Aguillon[3], and Yakeel Quiroz[4], *1. Massachusets General Hospital, Boston, Massachusetts, United States, 2. Universidad de Antioquia, Medellin, Antioquia, Colombia, 3. Massachusetts General Hospital, Boston, Massachusetts, United States, 4. Boston University, Boston, Massachusetts, United States*

Objective: Grip strength decline, and cognitive impairment are prevalent in aging and may reflect shared underlying biological processes. This study examined associations between handgrip strength, brain pathology and cognition in individuals with/without a PSEN1 E280A mutation for autosomal dominant Alzheimer's disease (ADAD). We investigated whether handgrip strength differed by mutation status and its association with age and tau pathology. Methods: 52 participants from the Colombia-Boston Biomarker Study (COLBOS) (28 PSEN1-E280A carriers, 24 non-carriers; MAge 34.1 ± 5.5 years; MEducation =9.96 ± 3.94 years; 57.8% female) completed the Mini-Mental State Examination (MMSE), CERAD Word List Memory Test, handgrip test via dynamometer, and MRI and PET imaging for amyloid and tau pathology. Group differences were assessed using Mann–Whitney U tests. Spearman correlations explored associations between handgrip strength, cognition, and PET biomarkers. Results: Handgrip strength did not differ between carriers and non-carriers (Carriers: 74.44 +/- 35.34; non-carriers: 75.53 +/- 26.82; p = .62). Carriers had lower MMSE scores (U = 183.50, p = .004) and greater tau PET burden in the entorhinal cortex (p = .002–.037). Handgrip strength was unrelated to age, cognition or amyloid. Lower handgrip strength was significantly associated with greater tau burden in precuneus in the left hemisphere (ρ = –0.560, p = .019). Conclusion: Although handgrip strength was not linked to carrier status, cognition or amyloid, lower grip strength was associated with greater tau accumulation in the precuneus. These findings highlight handgrip strength as a potential low-cost marker of tau-related neurodegeneration in ADAD. Larger longitudinal studies are needed to extend these results.

Abstract citation ID: igaf122.3545

### ASSOCIATIONS BETWEEN PAIN REACTIVITY TO WORSE SLEEP AND HEALTH OUTCOMES

Christina Mu[1], Brent Small[2], Christina McCrae[3], Lindsay Peterson[3], Ross Andel[4], Katie Stone[5], and Soomi Lee[6], *1. University of California, San Francisco, University of California, San Francisco, California, United States, 2. University of North Carolina at Chapel Hill, Chapel Hill, North Carolina, United States, 3. University of South Florida, Tampa, Florida, United States, 4. Arizona State University, Arizona State University, Arizona, United States, 5. California Pacific Medical Center Research Institute, Castro Valley, California, United States, 6. The Pennsylvania State University, University Park, Pennsylvania, United States*

Objectives: Sleep and pain are co-occurring issues in adulthood. Pain reactivity to worse sleep refers to person-specific changes in daily pain following nights of shorter-than-usual sleep duration or poorer-than-usual sleep quality. We examined the cross-sectional and longitudinal associations of sleep-related pain reactivity with psychological distress and the number of chronic conditions. Methods: Data were obtained from the Work, Family, and Health Study, which collected data from healthcare and information technology workers (n = 311; Mage=41.38 years), who completed 8-days of daily diary. We controlled for sociodemographic and health covariates in a series of multilevel structural equation models and growth curve models in Mplus. Sensitivity analyses adjusted for sleep and pain medications and stratified analyses by age. Results: Those who reported more pain locations and severity following poorer-than-usual sleep quality had more concurrent distress (higher pain location reactivity to worse sleep quality: B = 1.03, p<.001; higher pain severity reactivity to worse sleep quality: B = 0.56, p=.001). In the growth curve models, higher pain location reactivity to poorer sleep quality was associated with higher concurrent distress (Path Est.=1.67, p=.023) and decreases in distress over time (Path Est.=-0.74, p=.032). Higher pain severity reactivity to poorer sleep quality was associated with higher concurrent distress (Path Est.=0.62, p=.030) but not changes in distress over time. Pain reactivity to worse sleep was not associated with concurrent or change in the number of chronic conditions. Discussion: Future directions are to examine the influence of pain reactivity to worse sleep in larger and more diverse samples across longer follow-up periods.

Abstract citation ID: igaf122.3546

### ASSOCIATIONS OF INSTRUMENTAL ACTIVITIES OF DAILY LIVING, FALLS, AND DEPRESSION WITH SUBJECTIVE COGNITIVE DECLINE

Idorenyin Udoh[1], and Matthew Smith[2], *1. University of Michigan Flint, Flint, Michigan, United States, 2. Texas A&M University, College Station, Texas, United States*

Background: Subjective cognitive decline (SCD) has been associated with challenges performing instrumental activities of daily living (IADL), such as managing finances, doing laundry, or taking medication. Such IADL impairments can contribute to an increased risk of falls and a higher likelihood of depression among older adults. The purposes of this study were to assess the prevalence of SCD among older adults and examine the factors associated with SCD (with an emphasis on IADL difficulties, depression, and falls). Methods: Data were analyzed from 1,791 older adults ages 65 years and older from the 2022 National Health and Aging Trends Study. A binary logistic regression model was fit to identify the association of sociodemographics, IADL, self-reported falls, and depression on SCD. Results: About thirteen percent of participants reported SCD, 28.3% reported one or more IADL difficulty, and 39.4% reported depressive symptoms. About 24% reported one or more falls in the past year. Relative to adults ages 65-69 years, those ages 80-84 (aOR=1.74, P = 0.037) and 85-89 (aOR=2.35, P = 0.004) were more likely to report SCD. Participants who reported IADL difficulties (aOR=1.94, P < 0.001), depressive symptoms (aOR=3.33, P < 0.001), and falling in the past year (aOR=1.47, P = 0.017) were more likely to report SCD, respectively. Conclusion: Findings reinforce the role of functional decline, depression, and falling on cognition among older adults. The interrelated nature of these variables highlights the importance of timely cognitive function screening when subjective memory problems are recognized. Mitigation of risk factors through mental health intervention and fall prevention programming may help offset cognitive declines.

Abstract citation ID: igaf122.3547

### BEYOND TECHNOCRACY: HOW CAPITAL DISPARITIES SHAPE GERONTECHNOLOGY EXPECTATIONS ACROSS AGE COHORTS

Yuhan Gao[1], and Yajun Song[2], *1. Peking University, Beijing, Beijing, China (People's Republic), 2. East ChinaUniversity of Science and Technology, Shanghai, Shanghai, China (People's Republic)*

Gerontechnology holds transformative potential for aging-in-place. However, its prevailing designs often reflect biomedical and technocratic biases, overlooking critical disparities in older adults' technological engagement that fundamentally reflect inequities in cultural capital (electronic-health literacy and self-rated health) and social capital (intergenerational support). Grounded in Bourdieu's capital theory, this study investigates how these multidimensional factors differentially shape expectations toward health-focused gerontechnology across age cohorts. We analyzed data from 869 Chinese older adults aged 60-99, categorized into three cohorts (60-64, 65-79, and 80-99). Path analysis was conducted to assess how electronic-health literacy, intergenerational support, self-rated health and other socioeconomic characteristics relate to older people's expectations of two health-focused gerontechnology types: safety-monitoring and health-management. Distinct patterns emerged. The young-old cohort (60–64 years) with higher e-health literacy and poorer health status demonstrated significantly stronger expectations for both gerontechnology, whereas the middle-old cohort (65–79 years) with health declines primarily anticipated safety-monitoring benefits. The oldest-old cohort (80–99 years) revealed divergent dynamics—informational support increased expectations for both gerontechnology, yet reduced instrumental support correlated with health-management interest. These findings underscore that gerontechnology expectations are not merely a function of individual capability but reflect systemic inequities in lifelong capital accumulation. By moving beyond individual-centric adoption models, we argue for structural interventions that address disparities in e-health literacy and social support systems.

Abstract citation ID: igaf122.3548

**BEYOND THE FUNDING: BARRIERS AND FACILITATORS TO VA MEDICAL FOSTER HOME PROGRAM SUSTAINMENT**

Chelsea Manheim[1], Rachel Fix[2], and Leah Haverhals[3], *1. VA, Aurora, Colorado, United States, 2. VA Health Services Research and Development, Aurora, Colorado, United States, 3. VA Eastern Colorado Healthcare System, Aurora, Colorado, United States*

The VA Medical Foster Home (MFH) program provides home-based long-term care for Veterans requiring nursing home level support. VA MFH coordinators recruit community-based non-VA caregivers and match them with Veterans to live in the caregiver's home. Veterans pay caregivers directly and caregivers provide them around the clock care while caring for up to three residents. Since 2017, the VA Office of Rural Health (ORH) has funded new MFH programs in rural areas. The purpose of this abstract is to describe barriers and facilitators to MFH program sustainment identified through six interviews conducted from 2024-2025 with MFH coordinators from six ORH-funded MFH sites across six US states. To analyze data, we applied the REAIM implementation science framework and a team-based inductive and deductive thematic approach. Barriers to expanding reach included lack of timely identification of Veterans appropriate for MFH when space in a MFH became available. This left some caregivers with open rooms in their MFH longer than was ideal. Challenges of recruiting caregivers in rural areas emerged as a barrier to program adoption. Certain state laws acted as barriers to implementation, causing complications for MFH program growth. Veterans quality of life proved very high in MFHs, which facilitated program effectiveness. When MFH coordinators had longer tenure in their role and more experience, this facilitated maintenance and areas to target to expand and sustain programs. As the Veteran population rapidly ages demand for the MFH program will increase. These findings will inform program leadership in strategizing for effective program sustainment.

Abstract citation ID: igaf122.3549

**BIDIRECTIONAL LINKS BETWEEN SLEEP AND MARITAL QUALITY: AN EIGHT-YEAR LONGITUDINAL STUDY OF OLDER COUPLES**

Hanamori Skoblow, Eunjin Tracy, and Megan Gilligan, *University of Missouri, Columbia, Missouri, United States*

The Model of Dynamic Association Between Relationship Functioning and Sleep positions sleep as a fundamentally dyadic process, underscoring the need to study sleep within its relational context and highlighting the value of longitudinal, dyadic research to better understand interconnected pathways between sleep and close relationships. Although sleep problems and the salience of marital ties increase with age, few studies have tested links between partners' sleep and marital quality with older samples. Therefore, we investigated longitudinal and bidirectional within- and between-person associations between sleep and marital quality in couples aged 50 + ($M = 63.12$; $SD = 7.87$). Using dyadic random intercept cross-lagged panel models, we tested associations between multiple domains of sleep (i.e., insomnia and subjective sleep quality) and marital quality (i.e., support and strain) over three waves of data spanning eight years in a nationally representative sample of 2,343 couples from the English Longitudinal Study of Ageing. Women's reports of greater spousal strain predicted increases in their own insomnia symptoms and decreases in their subjective sleep quality over time. Additionally, women's higher-than-average perceptions of spousal support predicted men's later improvements in subjective sleep quality. No significant cross-lagged effects emerged between insomnia and spousal support, nor did we detect partner effects between spousal strain and subjective sleep quality. These findings suggest that women's reports of marital quality are more consequential for both partner's sleep, emphasizing the importance of considering gendered processes and interpersonal dynamics when examining the reciprocal links between sleep and relationship functioning across time.

Abstract citation ID: igaf122.3550

**BOLD IN ACTION: PARTNERING TO PROVIDE COMMUNITY-BASED DEMENTIA EDUCATION IN TEXAS**

Andrew Crocker[1], Rachel Brauner[2], Bailey Young[3], Jenna Perales[4], and Ryan Williams[5], *1. Texas A&M AgriLife Extension Service, Amarillo, Texas, United States, 2. Texas A&M AgriLife Extension Service, College Station, Texas, United States, 3. West Texas A&M University, Canyon, Texas, United States, 4. Alzheimer's Association, Fort Worth, Texas, United States, 5. Texas Tech University, Amarillo, Texas, United States*

Due to dementia's impact on public health, the Building Our Largest Dementia Infrastructure for Alzheimer's Act (BOLD) directs the United States Centers for Disease Control and Prevention to enhance public health infrastructure for dementia risk reduction, early detection, and diagnosis. As a recipient of BOLD funding, the Texas Department of State Health Services (DSHS) embraced the effort in its four-year state plan for Alzheimer's disease to, in part, "leverage existing and new partnerships to provide education to the public and healthcare professionals through community education events and awareness campaigns." In 2024, Texas A&M AgriLife Extension Service, the community-based education and outreach arm of the Texas A&M University System, partnered with DSHS and Alzheimer's Association Chapters across Texas to implement three hour-long programs to help improve dementia literacy and reduce controllable risk factors. In this exploratory research (IRB # 2024-0796), we seek to determine the effectiveness of the educational programming as well as any nuance in the results related to age, residing in a rural area, and residing in a medically underserved area. This study analyzes pre-post evaluation data from three community-based programs: 10 Warning Signs of Alzheimer's

Disease, Healthy Living for Your Brain and Body, and Understanding Alzheimer's and Dementia. Results reveal increase in knowledge with mixed patterns across groups, highlighting both similarities and notable differences based on demographics and residency. Findings from this research help reinforce the need for this type of inter-agency partnership and the resulting community-based education for helping improve dementia literacy and mitigating risk factors.

Abstract citation ID: igaf122.3551

### BRIDGING EDUCATIONAL GAPS IN MID- TO LATE-LIFE COGNITIVE FUNCTION THROUGH ACTIVE COGNITIVE ENGAGEMENT

Thakshila Dasanayake[1], Nikki Hill[1], Diane Berish[1], and Jacqueline Mogle[2], *1. The Pennsylvania State University, University Park, Pennsylvania, United States, 2. RTI Health Solutions, Durham, North Carolina, United States*

Higher education levels may establish a foundation for ongoing intellectual stimulation throughout life, leading to better cognition, including episodic memory (EM) and executive functioning (EF). Additionally, cognitive activity engagement may mitigate declines in cognition as individuals age. However, the relationship between the potential compensatory benefits of education and late-life cognitive engagement is somewhat unclear. This study examined the interaction between level of education and current frequency of cognitive activity on EM and EF in adulthood, using participants from the second wave (2004–2006) of the Midlife in the United States study ($n = 3{,}462$; Mage = 55.99, $SD = 12.21$; range: 32–84 years). Principal component analysis was used to calculate composite cognitive activity scores (intellectual, problem-solving, technological, and social domains) from self-reported frequencies of engagement (never to daily). Multiple linear regressions, controlling for covariates, tested associations of education, cognitive activity, and their interaction, followed by age-stratified analyses (32–64, 65–74, and 75+ years). Significant negative interactions between education and cognitive activity were observed for both EM ($\beta = -0.010$, $p = .042$) and EF ($\beta = -0.009$, $p = .030$), indicating that the cognitive advantage of higher education is smaller at higher cognitive activity levels. Both education and cognitive activity independently predicted better EM and EF. The compensatory effect was stable across age groups for EM, whereas education's benefit for EF was smaller in older adults ($p = .037$), though not moderated by activity. Findings support active cognitive engagement as a strategy to reduce education-related disadvantages in cognition.

Abstract citation ID: igaf122.3552

### BUILDING CONFIDENCE IN AGE-FRIENDLY CARE IN NURSING FACILITIES AND CARE SYSTEMS THROUGH THE KANSAS 4M PARTNERSHIPS

Frances Yang[1], Shawna Wright[2], Barbara MacArthur[3], Tracy Davies[4], Jacqueline Beldeen[2], Colleen Paramesh[2], Kristine Williams[3], and Teri Kennedy[2], *1. University of Kansas, University of Kansas Medical Center, Kansas, United States, 2. University of Kansas Medical Center, Kansas City, Kansas, United States, 3. University of Kansas, Kansas City, Kansas, United States, 4. Washburn University, Topeka, Kansas, United States*

Kansas 4M Geriatric Workforce Education Program is a statewide community-academic partnership to advance evidence-based Age-Friendly Health Systems (4Ms: What Matters, Medication, Mentation, and Mobility) care. In partnership between Kansas 4M, Project Extension for Community Healthcare Outcomes (ECHO), Kansas Nursing Workforce Center, and Advanced Nursing Education Workforce, the Kansas 4M Project ECHO series introduced and applied the 4Ms to the care of nursing home residents, such as communication strategies. This series was designed for Long-Term Services and Support (LTSS) staff, administration, nurses, nurse educators, advanced practice clinicians, physicians, and behavioral health clinicians ($N = 37$). Significant improvement in confidence was found after seven sessions for the statement: "I am confident in my ability to describe effective strategies to incorporate the 4Ms framework into areas of care for nursing facility residents and older adults such as chronic care management, end-of-life support, memory care, and telemedicine." With responses ranging from Strongly Disagree (1 point) to Strongly Agree (5 points). Based on paired t-tests, the average increase was 1.59 points (Standard Deviation (SD)=1.12, $t = 8.68$, $p < 0.001$) in confidence from the beginning (Mean (M)=2.7, $SD = 1.03$) to the latest follow-up ($M = 4.3$, $SD = 0.80$). There was also improvement found for the statement: "I am confident in my ability to explain the importance of implementing age-friendly care in nursing facility and nursing care systems that support older adults" by an average of 1.38 points ($SD = 1.09$, $t = 7.70$, $p < 0.001$). The Kansas 4M model with Project ECHO has shown statistically significant evidence for improvement in confidence among health care workers in LTSS.

Abstract citation ID: igaf122.3553

### CARE TRANSITIONS IN OLDER COLORECTAL SURGERY PATIENTS: SYSTEM, PROVIDER, AND PATIENT INSIGHTS

Mark Iskandar[1], Ferhat Yildiz[2], Christine Ritchie[3], and Sevdenur Cizginer[3], *1. Central Michigan University, Mount Pleasant, Michigan, United States, 2. Harvard Medical School, Boston, Massachusetts, United States, 3. Massachusetts General Hospital, Boston, Massachusetts, United States*

Older adults (age ≥65) undergoing colorectal surgery are at risk of medication errors, complications, and comorbidity exacerbation during care transitions. In this population, approximately 31% experience postoperative complications, with a 1.8-fold increase in functional decline and a 15% chance of readmission within 30 days (May, 2025). Given colorectal surgeries in older adults are projected to increase 50% by 2040, improving post-surgical transitions is important (Xie, 2025). From September 2023 to April 2024, we conducted semi-structured interviews with 42 stakeholders, including older surgical patients, caregivers, and providers from 12 disciplines. Using an inductive–deductive approach, we developed a preliminary codebook organized around system, provider, and patient domains containing codes for challenges, determinants, and solutions. Two coders independently applied and refined the codebook during thematic analysis, resolving discrepancies through discussion. Thematic analysis of transcripts revealed gaps and potential solutions in care transitions. At provider level, challenges included disorganized handoffs, poor medical team continuity, and limited collaboration with primary care. At system level, barriers included inadequate interdisciplinary coordination, insufficient post discharge resources, and difficulty accessing specialty services. At patient level, challenges included medication adherence, understanding discharge instructions, and avoiding readmission. Proposed solutions included stronger provider communication, enhanced education and support for patients and caregivers, and early discharge planning initiated before surgery, extending to virtual follow-ups post-discharge and connection to community resources. Improving transitions requires a multilevel approach that addresses communication gaps, system barriers, and patient vulnerabilities. These stakeholder-identified solutions highlight feasible interventions that could improve care quality and outcomes in this growing population.

Abstract citation ID: igaf122.3554

### CAREBUDDY: A MULTI-AGENT CONVERSATIONAL AI FOR ALZHEIMER'S CARE AND ASSISTANCE

Wordh Ul Hasan[1], Ayanle Aideed[2], Kimia Tuz Zaman[3], and Juan Li[3], *1. University of Minnesota Crookston, Fargo, North Dakota, United States, 2. North Dakota State University, North Dakota State University, North Dakota, United States, 3. North Dakota State University, Fargo, North Dakota, United States*

Managing Alzheimer's disease presents daily challenges for both the individuals living with the condition and their informal caregivers. While conversational AI offers potential support, traditional single-agent systems often lack the specialization and context-awareness required for comprehensive care. This study introduces and evaluates CareBuddy, a modular, multi-agent conversational AI system designed to provide proactive, personalized assistance to both persons with Alzheimer's and their caregivers. CareBuddy features a layered architecture with specialized agents for medical inquiries, appointment scheduling, meal planning, and reminders, coordinated by a central orchestrator. A mixed-methods usability study was conducted with 20 participants—including family caregivers, older adults with and without early-stage memory impairment, and healthcare professionals—to assess effectiveness and usability. Results demonstrated high task completion rates and user satisfaction, with 85% of users rating appointment scheduling 5/5 and 90% rating grocery planning 4 or 5. The system significantly reduced task completion time and cognitive load. The findings indicate that a modular, context-aware multi-agent AI framework can substantially improve daily management and confidence for the entire care dyad, holding promise for integration into broader healthcare platforms.

Abstract citation ID: igaf122.3555

### CENTERING THE VOICES OF DUAL-ROLE CAREGIVERS THROUGH POETIC INQUIRY

Charlotte Weiss, Karen Hirschman, and Mary Naylor, *University of Pennsylvania, Philadelphia, Pennsylvania, United States*

Dual-role caregivers, those who are paid caregivers at work and informal caregivers at home, provide invaluable services to society, and their stories should be heard and valued. In our primary study, we interviewed eight women of color employed at a long-term care (LTC) facility in Pennsylvania, all of whom also served as informal caregivers of family members in their personal lives. These interviews explored the complexities of balancing professional caregiving responsibilities with familial caregiving demands. As a secondary analysis, we used a qualitative, arts-based research method of poetic inquiry, known as I-Poems, to enhance the visibility and representation of dual-role caregivers. Poetic inquiry, as a postmodern and feminist-informed research method, is particularly well-suited to study and represent caregivers' lives. I-Poems were created by lifting the participants' statements representing or speaking of self from an interview text and positioning them into lines of a poem that were then constructed into poetic stanzas. By separating the "I-voice," the participants' sense of agency and self is repositioned to the forefront of the caregiver story. We will present multiple I-Poems to illustrate the contextual intersections of caregiving at work and at home. These poetic representations invite the audience to feel, rather than simply understand, the multidimensional and embodied realities of dual-role caregiving. These participant-voiced research poems contribute to new knowledge and foster humanization of caregivers who navigate care responsibilities across both professional and personal spheres.

Abstract citation ID: igaf122.3556

### CHANGES IN POST-ACUTE TRAJECTORIES FOR PATIENTS WITH SERIOUS MENTAL ILLNESS AFTER PAYMENT REFORMS

Evelyn Hart[1], Harsha Amaravadi[1], Rachel Prusynski[1], Amber Sabbatini[1], Natalie Leland[2], Donald Fogelberg[1], and Tracy Mroz[3], *1. University of Washington, Seattle, Washington, United States, 2. University of Pittsburgh, Pittsburgh, Pennsylvania, United States, 3. University of Washington School of Medicine, Seattle, Washington, United States*

Post-acute care (PAC) services help older adults transition home after hospitalization, yet people with serious mental illness (SMI) may experience barriers to accessing PAC services from skilled nursing facilities (SNFs) and home health agencies (HHAs). We examined whether SMI as a secondary diagnosis was associated with PAC use, and whether PAC use changed following implementation of Medicare payment reforms: the Patient Driven Payment Model (PDPM) in 2019 for SNFs and the Patient Driven Groupings Model (PDGM) in 2020 for HHAs. We analyzed claims for Traditional Medicare beneficiaries aged 18+ hospitalized between 2018–2021 (n = 29,001,357), excluding those with SMI as the primary reason for hospitalization; 18% were admitted to a SNF and 16% to HHA after hospital discharge. Fixed-effects logistic regression with two-way clustered standard errors estimated odds of SNF and HHA admission (in separate models) comparing those with versus without SMI, adjusting for patient, facility, and community characteristics. Interaction terms assessed whether associations between PAC admissions and SMI differed following payment reforms. Secondary SMI was associated with higher odds of SNF admission (OR = 1.48) but lower odds of HHA admission (OR = 0.95). PDPM was linked to slightly reduced SNF use (OR = 0.98), while PDGM was associated with increased HHA use (OR = 1.10). Interaction terms indicated modest attenuation of baseline differences (SNF: OR = 1.06; HHA: OR = 1.02). All findings were statistically significant. Though both PDPM and PDGM aim to compensate facilities fairly for patient care needs, payment reform had differing effects by PAC setting, reflecting underlying divergent pathways to PAC for patients with secondary SMI.

Abstract citation ID: igaf122.3557

### CHILDHOOD ADVERSITY PREDICTS LATER-LIFE MEMORY LAPSES, IRRITATION AND INTERFERENCE

Negar Mokhtari[1], Kat Barrett[1], Heejung Jang[2], Jody Nicholson[1], and Lulu Hottinger[1], *1. Clemson University, Clemson, South Carolina, United States, 2. The Pennsylvania*

*State University, Asian/Pacific Islander, Pennsylvania, United States*

This study investigated relationships between childhood adversity (CA) domains—abuse (physical, sexual, emotional) and neglect (physical, emotional) — daily prospective memory (PM) and retrospective memory (RM) lapse frequency, and memory lapse (ML) related irritation and interference in middle-to-older adults. Participants (N = 1,236; Mage=62.5±10.2) disclosed histories of CA in the 2013-2014 wave of the Midlife in the United States longitudinal study and completed eight nightly reports on MLs in the 2017-2019 wave. In univariate analyses, all five CA domains were significantly associated with PM (p<.001 - .007). Additionally, emotional and physical neglect were univariately significant for RM (p<.001). In the full ML model, no CA domains significantly predicted RM, and only emotional neglect (p=.010) significantly predicted PM. Emotional abuse (p=.039), sexual abuse (p=.032) and physical neglect (p=.007) predicted PM-related irritation, while sexual (p=.044), physical abuse (p=.045) and physical neglect (p=.001) predicted PM-related interference. Physical neglect (p =.035) also remained significant in the full PM model. For RM-related irritation and interference, all CA domains were significant univariately (p<.001). In the full RM model, physical abuse predicted irritation (p =.008), while physical (p=.003) and sexual (p=.017) abuse predicted interference. Overall, every domain of CA predicted daily PM lapses (p = <.001 - .007) and even more strongly predicted RM lapse-related irritation and interference (p = < .001). Findings suggest a history of CA increases the severity of RM lapse-related interference and irritation more than PM lapse frequency in later life. Future person-centered analyses are needed to assess the potential for shared variance between CA domains resulting in suppression effects.

Abstract citation ID: igaf122.3558

### CHRONIC PAIN MANAGEMENT FOR CAREGIVERS OF PERSONS LIVING WITH DEMENTIA

Bianca Shieu[1], M. Carrington Reid[2], Deami Cardenas[3], Glycel Vidaurri[3], Yousef Qan'ir[3], Min Wang[4], and Lixin Song[3], *1. University of Texas Health Science Center at San Antonio, San Antonio, Texas, United States, 2. Weill Cornell Medicine, New York, New York, United States, 3. UT Health San Antonio, San Antonio, Texas, United States, 4. UTSA, San Antonio, Texas, United States*

Chronic pain is highly prevalent yet undertreated in persons living with dementia (PLWD), affecting up to 80% of PLWD and creating significant challenges for their family caregivers. The serious risks associated with pharmacological treatments like opioids necessitate safer, non-pharmacologic options. Auricular Point Acupressure (APA), a non-invasive and low-cost therapy, constitutes a promising alternative to mitigating pain in this expanding patient population. This study explored caregiver perspectives on using APA to inform the development of a caregiver-delivered training program. We conducted a qualitative descriptive study, guided by Bandura's self-efficacy theory, with 22 primary family caregivers who provided care for PLWD between February and July 2025. Following IRB approval, semi-structured interviews were used to understand caregiver needs, interests, and preferred learning formats. Thematic analysis was performed using Atlas.ti V9. Caregivers expressed significant interest in learning APA, viewing it as a potentially valuable tool. They expressed a preference for a multi-faceted training program. Participants further requested a hybrid model that combines instructional videos for clear demonstrations with printed materials for quick reference. In addition, they identified a need for an introductory in-person workshop to become familiar with APA and an AI chatbot that would provide immediate answers to pressing questions. Participants felt that the training materials should focus on managing common conditions like low back, shoulder, and leg pain. These user-centered findings provide a foundational blueprint for developing a tailored APA training program that is designed to enhance family caregiver self-efficacy and provide a practical, safe strategy for pain management among PLWD.

Abstract citation ID: igaf122.3559

### CLASSIFICATION MATTERS: DIVERGENT ESTIMATES OF DEMENTIA RISK FACTORS IN THE HEALTH AND RETIREMENT STUDY

Gina Nam[1], Junxian Liu[2], Jacqueline Torres[3], and Eleanor Hayes-Larson[2], *1. USC Leonard Davis School of Gerontology, Los Angeles, California, United States, 2. University of Southern California, Los Angeles, California, United States, 3. University of California, San Francisco, San Francisco, California, United States*

Clinical dementia diagnosis is costly and time-consuming. Therefore, large population studies often use classification algorithms employing various neurocognitive, functional, and health-related measures to identify participants with dementia. We aimed to determine whether choice of classification algorithm affects conclusions regarding dementia risk factors in the nationally representative Health and Retirement Study (HRS). We compared four commonly used classification algorithms (Langa-Weir, Wu, Expert, Hudomiet). For comparability across algorithms, we restricted to HRS participants age 70+ in 2010 and excluded prevalent dementia cases. We followed participants until first classification of dementia, death, loss to follow-up or administrative censoring (2020), calculated dementia incidence rates (IRs), and estimated hazard ratios (HR) from Cox models for dementia risk factors (e.g., sociodemographics, health) adjusted for baseline age, using sampling weights for representativeness. Dementia IRs over follow-up were broadly similar across three algorithms (Langa-Weir, Wu, Hudomiet), ranging from 26.5 (95% CI 24.6-28.4) to 33.8 (31.9-35.8), and higher for Expert (61.7 [59.1-64.7]). HRs for risk factors and incident dementia varied substantially across algorithms; comparing at least high school versus less than high school, HRs ranged from 0.39 [0.35-0.43] (Langa-Weir) to 0.66 [0.56-0.78] (Hudomiet) and comparing Black vs. White participants, HRs ranged from 1.81 [1.54-2.12] (Expert) to 2.69 [2.30-3.15] (Langa-Weir). Because HRS lacks gold standard longitudinal diagnosis and classification algorithms can yield different conclusions, more work is needed to understand optimal choice of algorithm for a given research question.

Abstract citation ID: igaf122.3560

### CLASSIFYING DEMENTIA DIAGNOSES IN THE BALTIMORE EXPERIENCE CORPS TRIAL USING MEDICARE AND MARYLAND'S HEALTH DATA

Vishaldeep Sekhon[1], Qian-Li Xue[2], Kyle Moored[3], Halima Amjad[1], Emily Richards[3], Eric Slade[4], George Rebok[2], and Michelle Carlson[3], *1. Johns Hopkins University School of Medicine, Baltimore, Maryland, United States, 2. Johns Hopkins University, Baltimore, Maryland, United States,*

*3. Johns Hopkins Bloomberg School of Public Health, Baltimore, Maryland, United States, 4. Johns Hopkins University School of Nursing, Baltimore, Maryland, United States*

Centers for Medicare and Medicaid Services (CMS) administrative data are among the most practical sources for understanding the incidence of dementia over time in older populations, but have yet to be widely used in intervention studies. Through the National Institute of Aging's LINKAGE program, the Baltimore Experience Corps Trial (2006-2022) is one of the first intervention studies to leverage Maryland's state-level Health Information Exchange (HIE) (2013-2022) data, along with CMS administrative data (2008-2022) to sensitively capture participants' healthcare utilization. After evaluating multiple established dementia classification algorithms, we used the Bynum evaluation and management (Bynum-EM) algorithm to identify dementia using International Classification of Diseases (ICD-9,10) codes from CMS Fee-for-Service, Medicare Advantage, and HIE data. We further adjudicated dementia diagnosis by its subtypes (Alzheimer's, mixed/vascular) and incorporated dementia-free mortality. Of 702 participants, 520 were successfully linked with CMS/HIE administrative data. These linked participants were predominantly female (84%), Black (95%), with an average age of 68±6 years and an average of two comorbidities. Over the 14-year follow-up, 26% (n = 135) of the BECT cohort developed dementia, with 8% (n = 32) of them diagnosed with vascular or mixed dementia. About 44% (n = 230) of participants died over time, with 27% having dementia-free mortality. Our study demonstrated the value of both CMS Medicare and state-level HIE data in classifying incident dementia and its subtypes among older adults, providing a useful approach to evaluating long-term intervention effects on dementia risk without requiring in-person follow-up.

Abstract citation ID: igaf122.3561

### CMS GUIDE MODEL: LESSONS LEARNED BY AN ESTABLISHED TRACK PROGRAM AT END OF YEAR ONE

Jessica Mongelli[1], Kaitlin Tangredi[2], Marzena Gieniusz[1], Christian Nouryan[3], Christie Ulbricht[1], Megan Carroll[1], Maria Carney[4], and Edith Burns[5], *1. Northwell Health, New Hyde Park, New York, United States, 2. Northwell Health, New Hyde Park, New York, New York, United States, 3. Northwell Health, Great Neck, New York, United States, 4. Zucker School of Medicine at Hofstra/Northwell, New Hyde Park, New York, United States, 5. Zucker School of Medicine Hofstra-Northwell, Manhasset, New York, United States*

'Guiding an Improved Dementia Care Experience' (GUIDE) is a CMS Innovation Center pilot program implemented January 2024, as an alternate payment model for interprofessional teams to provide comprehensive dementia care for people living with dementia and their caregivers. Program criteria encompass screening and recruitment, comprehensive and home safety assessment, ongoing beneficiary monitoring, data collection/management/reporting, respite, caregiver supports, etc. We report first-year implementation and evolution as an 'Established Track' program in a large integrated health system in a major metropolitan area. To date, the program has enrolled 158 beneficiaries and their caregivers; 19% low, 73% moderate, 8% high complexity. Average age of beneficiaries 79 years, caregiver relationship 53% child, 35% spouse, 10% other. Over 35% of eligible beneficiaries have utilized respite services. Approximately 9% have disenrolled, the majority (64%) expired/hospice. An adaptable team-based approach has been a critical strategy for success. Helpful processes that have evolved include structured recurring meetings, weekly review of data and submissions, care coordination and scheduled monitoring of required care-delivery services. Education and outreach with all stakeholders (e.g. interprofessional healthcare providers, lay community) is essential. Internal challenges have included need for ongoing adjustment of workflows and processes, and system constraints on structuring database/submission/reporting. Additional challenges include changing program requirements, recognizing and addressing the need to supplement internal resources (e.g. home visits, respite care), and identifying and establishing partnerships with external entities. These successes and challenges may significantly impact effectiveness of this pilot model program. Providing additional resources for this vulnerable population requires a true interprofessional, multidisciplinary approach.

Abstract citation ID: igaf122.3562

### CO-DEVELOPING CARED: A PERSONALIZED DEMENTIA OUTCOME MEASURE WITH CAREGIVERS

Sara Masoud[1], Ayse Kuspinar[2], Eunung Na[3], Dale Dauphinee[4], Susan Macaulay[5], and Nancy Mayo[6], *1. UT Health San Antonio, San Antonio, Texas, United States, 2. McMaster University, Hamilton, Ontario, Canada, 3. McMaster, Ontario, Ontario, Canada, 4. McGill University, Montreal, Quebec, Canada, 5. Amazing Women Rock, Amazing Women Rock, Ontario, Canada, 6. McGill, Montreal, Quebec, Canada*

Background: Outcome measures for dementia often fail to reflect the wide variation in symptoms, behaviors, and functional changes experienced by persons living with dementia and their caregivers. Standardized tools may not capture what matters most to patients and families, limiting clinical relevance and applicability. This poster describes the development of the Caregiver-Reported Outcome Measure for Dementia (CARED), a novel tool co-developed by researchers and family caregivers that integrates both standardization and personalization. Methods: CARED was developed through a community–academic partnership in which family caregivers served as co-researchers. Caregivers contributed to defining domains, refining items, and making decisions about measure structure. Surveys, co-created with community partners, collected caregiver responses from the US and Canada to identify CARED domains and items. Results: The final measure consists of 27 total items, four of which are core items to be completed by all respondents, and 23 additional items from which caregivers select the four most relevant to their situation. The personalized structure of CARED ensures the inclusion of universally important outcomes while accommodating individual caregiving circumstances. The co-development process demonstrates the feasibility and value of embedding caregiver partners as partners in measurement design. Implications: CARED represents an innovative approach to dementia outcomes measurement. Through a partnership of researchers and family caregivers, the tool was developed with the lived experiences and perspectives of the population of interest. This framework may guide future efforts to develop outcome measures that are both personalized and standardized, ensuring greater clinical applicability and responsiveness to patient and caregiver needs.

Abstract citation ID: igaf122.3563
### COLORECTAL CANCER SCREENING BARRIERS IN PERSISTENT POVERTY COMMUNITIES: A QUALITATIVE ANALYSIS
Margaret Salisu, *SUNY @ Downstate, Bayshore, New York, United States*

Background: Colorectal cancer (CRC) is the second leading cause of cancer death in the U.S., yet screening uptake is low in communities experiencing persistent poverty. Black and Hispanic adults face disproportionate burden due to intersecting socio-economic and structural inequities. Guided by the Andersen Behavioral Model, this study explored multilevel barriers and facilitators to CRC screening. Methods: Nineteen semi-structured interviews and two focus groups ($n = 25$) were conducted with Black and Hispanic adults aged 45–70, along with provider key informant interviews. Data were analyzed thematically using Andersen's domains of predisposing, enabling, and need factors. Results: Predisposing factors included limited awareness, cultural stigma, fear of diagnosis, and mistrust of the healthcare system. During interviews, several participants reported wanting to get screened after learning more, demonstrating the potential of real-time education. Enabling barriers included cost concerns, transportation challenges, scheduling complexity, and inadequate accommodations for people with disabilities. Need factors reflected divergent perceptions of risk: individuals with family history or symptoms expressed urgency, while others delayed screening due to low perceived vulnerability or fatalistic beliefs. Providers emphasized systemic inefficiencies, while patients highlighted emotional and cognitive barriers. Conclusions/Implications: CRC screening disparities in persistent poverty communities are driven by informational, structural, and psychosocial barriers. Findings highlight the need for culturally tailored, disability-inclusive interventions that build trust, simplify access, and leverage community-based education. Brief, personalized conversations may serve as powerful catalysts for screening uptake. These insights inform equity-focused strategies to reduce CRC disparities in underserved aging populations.

Abstract citation ID: igaf122.3564
### CONTEXTUAL DETAIL, BUT NOT AGE, REDUCES MEMORY ILLUSIONS DURING FREE RECALL
Alana DeLawter, and Sara Lute, *University of North Carolina Asheville, Asheville, North Carolina, United States*

Research exploring reduced memory illusions is helpful for people of all ages and applies to everyday scenarios. However, this research may be most beneficial for older adults, as they are more likely to show recall memory illusions compared to younger adults (Balota et al., 1999; Pierce et al., 2004). Illusions vary depending on presentation and age, with images accompanying word lists being a benefit (Israel and Schacter, 1997; Smith et al., 2015; Smith and Hunt, 1998). Additionally, increased context could be partially effective in reducing memory illusions for older adults (Skinner & Fernandes, 2010). To test this, we employed the Deese–Roediger–McDermott (DRM) paradigm and measured word list recall across four presentation types ($N = 235$). Older participants (60-81 years old) and younger participants (18-35 years old) were randomly assigned to one of four groups: an audio-only group, an audio with text group, an audio with low-context image group, or an audio with high-context image group. Results indicated a main effect for presentation type $F(3, 227) = 2.82$, $p = 0.04$, $\eta^2 = 0.035$, where participants presented with contextually rich images had fewer memory illusions than participants in the text condition. However contrary to our predictions there was no main effect of age and no interaction between age and presentation type. These findings highlight the importance of information presentation and the encoding process regardless of age.

Abstract citation ID: igaf122.3565
### CORTISOL-TO-DHEAS AWAKENING RATIO VARIES WITH DEMENTIA SEVERITY, AGITATION, AGE, AND SEX
Wanrui Wei[1], Töres Theorell[2], Gabriella Engstrom[3], and Azita Emami[1], *1. Yale University, Orange, Connecticut, United States, 2. Karolinska Institutet, Stockholm, Stockholms Lan, Sweden, 3. Florida Atlantic University, Boca Raton, Florida, United States*

The cortisol-to-dehydroepiandrosterone sulfate (DHEAS) ratio in the awakening response has emerged as a promising biomarker of stress-related dysregulation in neurodegenerative conditions. However, little is known about how this ratio varies among people with dementia (PWD) and their family caregivers, particularly across demographic and clinical subgroups. This study aimed to compare awakening cortisol-to-DHEAS ratios between PWD and caregivers and to examine whether age, sex, agitation, and dementia severity predict hormonal imbalance using nonlinear modeling. We analyzed 1,093 saliva samples from 32 PWD and 31 family caregivers. Log-transformed cortisol-to-DHEAS ratios were evaluated through group comparisons, stratified regressions, and generalized additive models. No significant overall group difference emerged ($p = 0.405$). However, subgroup analyses revealed elevated ratios in males under 75 with dementia ($p = 0.048$, Cliff's $\delta = 0.15$). Nonlinear age effects were observed across subgroups, particularly among female caregivers and PWD. Dementia severity was positively associated with ratio elevation, while agitation showed a wave-like nonlinear pattern. In sex-stratified regressions, dementia severity, agitation, and quadratic age terms significantly predicted ratio variation in males ($R^2 = 0.221$) but not in females. These findings suggest that the awakening cortisol-to-DHEAS ratio reflects nonlinear, age- and sex-specific patterns of stress regulation. The ratio may serve as a sex-sensitive biomarker of neuropsychiatric vulnerability in dementia, highlighting the need for tailored approaches in neuroendocrine aging research.

Abstract citation ID: igaf122.3566
### CULTURAL ADAPTATION OF STRESS MANAGEMENT INTERVENTION FOR KOREAN AMERICAN DEMENTIA FAMILY CAREGIVERS
Hyejin Kim[1], Eunbee Liu[1], Olimpia Paun[1], Jaehyuk Song[1], Bruno Kajiyama[2], Seok Gyu Han[1], and Dolores Gallagher-Thompson[3], *1. Rush University, Chicago, Illinois, United States, 2. Photozig, Inc., Moffett Field, California, United States, 3. Stanford University, Stanford, California, United States*

Korean American (KA) family caregivers of individuals with Alzheimer's disease and related dementias (ADRD), particularly those caring for immigrants at home, face unique stress due to culturally specific care needs. We developed the Korean version of iCare (K-iCare), a culturally adapted, cognitive behavioral therapy (CBT)-based stress reduction intervention that has been widely adapted for various ethnic groups, but not for KAs. Guided by the Framework for Reporting Adaptations and

Modifications-Enhanced (FRAME), K-iCare was developed through a five-stage process: (1) initial modification by the research team, (2) feedback from two focus groups, former KA ADRD caregivers (n = 5) and KA community service providers (n = 5), (3) refinement of K-iCare based on focus groups input, (4) validation by focus groups and the original iCare developers, and (5) finalization for implementation. Intervention materials were translated into Korean with careful cultural and linguistic adaptations. Terms without equivalent meaning in Korean were supplemented with both Korean and English wording for clarity. The total number of chapters was consolidated from nine to five, and caregiving scenarios were replaced with culturally relevant examples, such as emphasizing the traditional role of the eldest adult child in caregiving responsibilities. The intervention format was revised from self-paced asynchronous modules to synchronous weekly small-group sessions delivered in a hybrid format. A bilingual and bicultural doctoral-level nurse scientist with expertise in ADRD care will serve as the interventionist. Supporting materials (e.g., fidelity monitoring checklist) were developed to ensure consistent delivery. The next phase will evaluate the feasibility, acceptability, and preliminary efficacy of K-iCare.

Abstract citation ID: igaf122.3567

### DEVELOPMENT AND VALIDATION OF DYNAMIC MULTI-PROTEIN INFLAMMATORY SCORES ASSOCIATED WITH DELIRIUM

Peter Wang[1], Emily Wolfson[2], Yi-Hsiang Hsu[3], Towia Libermann[2], Sharon Inouye[4], Edward Marcantonio[2], Long Ngo[2], and Sarinnapha Vasunilashorn[2], *1. Tufts University School of Medicine, Boston, Massachusetts, United States, 2. Beth Israel Deaconess Medical Center, Boston, Massachusetts, United States, 3. Marcus Institute for Aging Research, Hebrew SeniorLife, Harvard Medical School, Roslindale, Massachusetts, United States, 4. Marcus Institute for Aging Research, Hebrew SeniorLife, Harvard Medical School, Boston, Massachusetts, United States*

Individual inflammatory markers have been associated with delirium. However, previous work to aggregate these markers to more fully capture this joint effect did not consider correlation among the markers, nor did they yield a single inflammatory score. To address these limitations, we aimed to develop and validate preoperative (PREOP) and postoperative day 2 (POD2) inflammatory scores for delirium. We used the Successful Aging after Elective Surgery (SAGES) I study (N = 560, mean age 76.7 ± 5.2 years, 42% male) to develop these scores, and the SAGES II study (N = 420, mean age 73.3 ± 5.6, 35% male) and UK Biobank (N = 32,147, mean age 57.7 ± 8.1, 46% male) for independent validation. 6 inflammatory markers associated with delirium were considered: C-reactive protein (CRP), interleukin-6 (IL-6), soluble tumor necrosis factor alpha receptor-1 (sTNFαR1), chitinase-3 like protein-1 (CHI3L1/YKL-40), interleukin-1 receptor antagonist (IL-1RN), and lipocalin-2 (LCN-2). We used LASSO regression (outcome=delirium) to select markers for inclusion in each score. Beta coefficients from logistic regression were then employed to compute weights attributed to each marker, resulting in the following equations: PREOP score = (1/9)*log(CRP) + (5/9)*log(YKL-40) + (1/3)*log(IL-1RN) [AUC=0.684; SAGES I], validated in SAGES II (AUC=0.617) and UK Biobank (AUC=0.649); POD2 score = (1/8)*log(IL-6) + (1/5)*log(YKL-40) + (1/4)*log(sTNFαR1) + (2/5)*log(IL-1RN) [AUC=0.664; SAGES I], validated in SAGES II (AUC=0.64). In summary, the PREOP and POD2 inflammatory scores both included YKL-40 and IL-1RN. These scores may inform clinical risk assessment or future research on the interrelationship of factors such as genetics, inflammation, and delirium.

Abstract citation ID: igaf122.3568

### DID I DO THE RIGHT THING? MORAL AND ETHICAL DILEMMAS IN ONCOLOGY FAMILY CAREGIVING

Jacquelyn Benson[1], Christi Lero[1], J. Peter Siriprakorn[2], Anaika Bedi[1], and Nashua Haque[3], *1. Washington University in St. Louis, St. Louis, Missouri, United States, 2. Washington University in St. Louis, Saint Louis, Missouri, United States, 3. Georgetown University School of Medicine, Washington D.C., District of Columbia, United States*

Family caregivers are central to cancer care, yet many difficulties extend beyond logistics of caregiving to dilemmas marked by moral uncertainty and ethical tension. Such dilemmas require value trade-offs rather than straightforward problem solving. U.S. research richly documents caregiver challenges but seldom characterizes moral/ethical dilemmas as caregivers themselves experience them. We report preliminary descriptive findings toward a caregiver-informed typology of moral and ethical dilemmas in cancer caregiving. Within an ongoing qualitative study, we conducted inductive thematic analysis of the initial interviews (20% of total sample). Two coders independently reviewed transcripts, wrote reflexive memos, iteratively refined a codebook, and resolved differences by consensus. This analysis was restricted to segments coded as moral or ethical dilemmas; purely practical challenges without explicit value/principle conflict were noted but not analyzed here. Four moral themes emerged: (1) truth-hope stewardship (balancing honesty with preserving hope); (2) rescue vs acceptance near end of life (e.g., whether to call 911 or escalate care); (3) good-caregiver identity vs. self-protection (managing others' judgements while guarding emotional limits); and (4) goal-concordance uncertainty when preferences were unknown. Two ethical tensions were prominent: surrogate autonomy vs. best interest and decisional capacity/understanding at treatment turning points. Hybrid cases (moral + ethical) were common, and moral residue ("what ifs") frequently followed decisions. Preliminary findings indicate caregiver routinely face moral/ethical dilemmas distinct from logistical barriers. A caregiver-informed typology can guide targeted supports (e.g., disclosure coaching, crisis planning, advance-care-planning prompts, post-decision debriefing) and sets up subsequent analyses of how caregivers navigate dilemmas and what impedes coping.

Abstract citation ID: igaf122.3569

### DIFFERENTIAL IMPACT OF LIVING ARRANGEMENT ON DIETARY QUALITY BETWEEN OLDER MEN AND WOMEN

Qun Le, Elizabeth Procter-Gray, Lingming Chen, Danielle LoPilato, Kevin Kane, Sabrina E. Noel, and Wenjun Li, *University of Massachusetts Lowell, Lowell, Massachusetts, United States*

Consuming a healthy diet is essential for healthy aging and longevity. Due to health conditions, living environment, and other factors, older adults are at increased risk of malnutrition. Our study aims to explore association between living arrangement and diet quality, and how gender may modify this association. The Healthy Aging and Neighborhood Study (2021-2025) collected data from 614 community-dwelling people aged 65 years and older living in Central and Northeastern Massachusetts. Living arrangement was measured as living alone vs. living with another adult(s). Diet was evaluated via the Dietary Screening Tool (range 0-100) with a higher score indicating higher diet quality. Generalized linear regression models assessed associations between living arrangement and dietary quality overall, and stratified by gender, adjusting for socio-demographic information including age, race, education, income, and activity-specific balance confidence. Participants were an average age of 74.6 (±6.2) y, 57.7% were women, and 56.0% Non-Hispanic White, 32.9% Asian, and 8.6% Hispanic. The mean diet quality score was 67.3 (±12.5). After adjustment, women had higher dietary quality than men (b (SE) = 3.96 (1.15)). Living alone had a differential impact on dietary quality between men and women. Among men, the 16.9% who lived alone had better diets (b (SE) = 4.81 (1.97)). However, among women, those who lived alone (37.3%) had lower dietary quality (b (SE) = -5.93 (2.29)). In conclusion, living status may differentially impact of dietary quality for men and women. Future studies should explore the underlying mechanism of such gender differences, and provide gender-specific effective interventions.

Abstract citation ID: igaf122.3570

### DIGITAL EMPATHY IN AGING SOCIETIES: CO-CONSTRUCTING THE CONCEPT WITH STUDENTS ACROSS FINLAND AND CHINA

Yaru Li[1], Honglin Chen[1], Yanan Jiang[2], and Ning An[3], *1. University of Eastern Finland, Kuopio, Pohjois-Savo, Finland, 2. University of Wisconsin-Madison, Madison, Wisconsin, United States, 3. Hefei University of Technology, Hefei, Anhui, China (People's Republic)*

As services and education digitize in aging societies—accelerated by rapid advances in AI—cultivating "digital empathy," the cognitive, affective, and communicative expression of empathy via digital technologies, is essential to support older adults' inclusion. We co-constructed this concept with social work students in two contexts (62 master's students in fall 2024 at the University of Eastern Finland, Finland; 52 undergraduate students in spring 2025 at Anhui Institute of Medicine, China) and identified actionable behaviors and design cues. In each setting, students received a brief empathy primer, viewed a short video of older adults encountering digital barriers, then wrote their own definition of "digital empathy." Responses were analyzed using bilingual reflexive thematic analysis with a hybrid codebook and back-translation. Across sites, students converged on three dimensions: (1) communicative presence and empathic signaling online (e.g., appropriate camera use, timely responses, check-ins, supportive reactions); (2) perspective-taking with respectful, clear, plain-language communication; and (3) inclusive support and empathic design for older adults, including non-digital alternatives, patient coaching without shaming, and advocacy for accessibility/usability. Contextual emphases differed: Finnish definitions highlighted communicative presence and leadership-driven psychological safety in hybrid teams, whereas Chinese definitions foregrounded perspective-taking, practical assistance for older adults, and empathic, personalized design (with some invoking AI-enabled support). Findings inform curricula and service norms (e.g., check-in rituals, consent-based communication, plain language) and translate into older-adult-inclusive design prompts (offer access alternatives; prioritize accessibility/usability; normalize supportive reactions).

Abstract citation ID: igaf122.3571

### DISPARITIES IN ADVANCE CARE PLANNING: GAPS IN CLINICAL NOTES ON COMFORT VS. LIFE-SUSTAINING TREATMENT PREFERENCES

Yuwen Ji[1], Alaa Albashayreh[1], and Stephanie Gilbertson-White[2], *1. University of Iowa, Iowa City, Iowa, United States, 2. University of Iowa, University of Iowa, Iowa, United States*

Background: Goal-concordant care is essential, yet disparities in advance care planning (ACP) persist. Electronic health record (EHRs) documentation of specific preferences, such as comfort and life-sustaining treatments (LST) and natural language processing (NLP) to extract free-text data, provides real-world information to explore patterns. Objective: To identify predictors associated with documentation of specific preferences in a cohort of older adults with chronic conditions. Methods: In a dataset of records from 14,729 older adults with heart failure, cancer, or dementia from a large academic medical center, we used a validated NLP model, Priorities-BERT (accuracy= 90.91%), to categorize documented care preferences from 3.6 million EHR notes into four groups: comfort-only, LST-only, mixed, or none. Logistic regression identified factors associated with each preference category (vs. no preference). Results: The mean age of the sample was 78.67 years (SD = 6.05). Female patients (vs. male) (OR = 1.11, 95% CI: 1.04–1.19), heart failure (OR =1.59, 95% CI: 1.34–1.89), dementia (OR = 1.33, 95%CI: 1.25-1.43), were associated with higher odds of having any priorities of care documented. Increasing age was associated with lower odds of LST (OR = 0.94, 95%CI: 0.94-0.95). Patients with heart failure (OR = 1.50, 95%CI: 1.39-1.61) or dementia (OR = 1.56, 95% CI: 1.42-1.71) were more likely to have comfort preferences documented. Conversely, cancer patients were more likely to have LST documented (OR = 1.12, 95%CI: 1.03-1.21). Conclusion: Significant disparities existed across these three diagnoses in documentation ACP preferences. Targeted clinical and health-system interventions are needed to promote equitable and timely ACP conversations for patients likely to benefit from this care.

Abstract citation ID: igaf122.3572

### DIVERGING LIVES AFTER DEATH: DIFFERENCES IN LONELINESS IN WIDOWHOOD ACROSS RACE IN THE UNITED STATES

Micah Tan, *Princeton University, Princeton, New Jersey, United States*

While racial heterogeneity in the widowhood effect on mortality has been explored, heterogeneous effects on social outcomes like loneliness across race in the United States have yet to be investigated. Using a sample of widow(er)s from the Health and Retirement Study (HRS) aged 50 and above (n = 4,835), this study addresses this gap by examining if the impact of widowhood on loneliness varies across black and white widow(er)s. An event study approach with two-way fixed effects models is employed to examine how the likelihood of feeling lonely changes after widowhood for black and white widow(er)s. Results suggest that white respondents experience a stronger widowhood effect on loneliness than black respondents, and that group-level differences across race are driven by differences among widow(er)s living alone with differences in the widowhood effect on loneliness across race being statistically insignificant among those living with others. Differences in the availability of proximate social resources and in the level of stigma experienced after widowhood are argued to explain heterogeneous effects across race, with black widow(er)s having greater access to proximate social resources and experiencing less stigma in widowhood. Preliminary tests find evidence in support of the mediating effects of these mechanisms in explaining racial differences in the widowhood effect on loneliness. These findings shed light on why racial disparities in well-being diminish in later life, with transitions like widowhood making social resources that black Americans have greater access to more important for well-being, thus extending existing life course perspectives on racial disparities in well-being.

Abstract citation ID: igaf122.3573

### DOES PRESENTATION OF LATE-LIFE GENERALIZED ANXIETY DISORDER CHANGE WITH ADVANCING AGE?

Yusuf Lahlou[1], Philippe Landreville[1], Patrick Gosselin[2], Sébastien Grenier[3], and Pierre-Hugues Carmichael[4], *1. Université Laval, Québec, Quebec, Canada, 2. Université de Sherbrooke, Sherbrooke, Quebec, Canada, 3. Université de Montréal, Montréal, Quebec, Canada, 4. Centre d'Excellence sur le Vieillissement de Québec, Québec, Quebec, Canada*

Generalized anxiety disorder (GAD) is one of the most common anxiety disorders in older adults. Given the aging population, it is important to determine if there are age-related variations in the presentation of GAD in older adults. The present study examined the relationship between age and the clinical presentation of GAD in people aged 60 to 87 years (N = 109, M = 68.2). A structured diagnostic interview was conducted and participants completed measures of GAD severity, tendency to worry, behavioral manifestations of GAD, depression, and disability. Most of the analyses performed did not show a significant relationship between age and different indices of GAD presentation. A comparison of the youngest (≤67 years) and oldest (>67 years) participants, based on the median age of the sample, showed that the second group had significantly higher rates of comorbid anxiety disorders (60.8% vs 39.7%; $\chi^2$ = 4.85, p = .03) and death-related worries (54.9% vs 34.5%; $\chi^2$ = 4.44, p = .04). Conversely, the youngest group reported more work or school-related worries (60.5% vs 25.0%; $\chi^2$ = 9.15, p = .002). Overall, the results show that the clinical presentation of GAD does not vary much according to age among older adults. However, the observed differences suggest that clinicians should take into account the presence of comorbid anxiety disorders and the sources of worries according to the age of their client.

Abstract citation ID: igaf122.3574

### DUAL SENSORY LOSS AND RATE OF DEMENTIA SYMPTOM PROGRESSION IN COGNITIVELY NORMAL OLDER ADULTS

Kathryn Hand[1], Lilah Besser[2], Walter Kukull[3], and Willa Brenowitz[4], *1. Oregon Health and Science University, Portland, Oregon, United States, 2. University of Miami, Boca Raton, Florida, United States, 3. University of Washington, Seattle, Washington, United States, 4. Kaiser Permanente, Portland, Oregon, United States*

Loss of both hearing and vision may serve as a stronger predictor of the rate of dementia symptom progression (i.e., clinical progression) than loss of either sense individually. We evaluated the longitudinal associations of hearing, vision, and dual sensory loss with clinical progression among 21,098 participants in the National Alzheimer's Coordinating Center's Uniform Data Set, restricting to those who were cognitively normal at baseline. Participants were 69 ±11 years at baseline, and followed annually for an average of 3.7 years, 66% were female, 78% White, 2.9% visually impaired, 18.6% hearing impaired, and 1.6% dual sensory impaired. Participants were rated by clinicians as vision impaired if they had trouble with vision after correction and as hearing impaired if they had trouble with hearing without any correction. Dementia staging was measured using the Clinical Dementia Rating Sum of Boxes (CDRSUM). Linear mixed-effects models estimated associations, adjusting for age, sex, race, education, cardiovascular disease, diabetes, hypertension, depression, and APOE-ε4 status. At baseline, sensory impairment was not significantly associated with CDRSUM; however, significant interactions with time were observed. Compared to those with no sensory impairments, individuals with dual impairment had the highest annual increase in CDRSUM ($\beta$ = 0.075, 95%CI:0.048–0.102), followed by those with vision impairment alone ($\beta$ = 0.028, 95%CI:0.006–0.049) and hearing impairment alone ($\beta$ = 0.019, 95%CI:0.011–0.028). Dual sensory impairment (versus single sensory impairment) is associated with greater clinical progression of dementia symptoms over time, highlighting the potential importance of early identification and management of dual sensory loss.

Abstract citation ID: igaf122.3575

### E-CHARM HEART AGE TRIAL IN PRIMARY CARE: DESIGN AND BASELINE FINDINGS

Guanzhou Wang[1], Lei Wu[2], Weijing Song[3], Yuzhou Sun[4], You Zuo[2], Yuxuan Huang[2], and Boyuan Li[2], *1. Duke University, Durham, North Carolina, United States, 2. Duke Kunshan University, Kunshan, Jiangsu, China (People's Republic), 3. Duke Kunshan University, Durham, North Carolina, United States, 4. University of St. Andrews, St. Andrews, Scotland, United Kingdom*

Cardiovascular risk awareness among older adults often fails to align with physiological risk, limiting prevention. "Heart Age" Re-frames risk as an intuitive biological age; however, evidence from community primary care, particularly in resource-limited settings, remains scarce. We designed an individually randomized trial in a community health system to test a structured Heart Age risk-education report generated from ASCVD components fully extracted from Chinese government-funded annual physical exams and delivered with a brief physician explanation and tailored lifestyle guidance during routine follow-up, with no extra tests or visits. Older adults with hypertension were allocated to usual care plus the Heart Age report (intervention) or usual care (control). Baseline measures included demographics, a 10-item cardiovascular risk awareness score (0–10), lifestyle indicators (e.g., fruit-intake days/week), medication adherence by the 4-item Morisky scale (MMAS-4; 0–4, higher=better), and routine clinical data. We enrolled 378 participants (174 intervention; 204 control), mean age 66.8±12.0 years. The awareness score averaged 7.16/10; fruit-intake frequency was median 5 days/week (IQR 3–7); medication adherence was mean 2.71/4, median 3 (IQR 2–4). Among intervention participants with complete Heart Age data (n = 174), the Heart Age gap (Heart Age – chronological age) was 8.2±11.7 years. Risk awareness showed near-zero correlation with the Heart Age gap (r≈0.05, 95% CI – 0.10 to 0.20); age-sex adjustment gave similar results. Leveraging routinely collected exams to produce an ASCVD-based Heart Age report with brief lifestyle counseling appears feasible at no added cost; the baseline misalignment highlights room, and opportunity, for improved risk communication. Endline effectiveness evaluation is ongoing.

Abstract citation ID: igaf122.3576

### EFFECTS OF EXERCISE ON INFLAMMATION AND STRESS IN OLDER ADULTS WITH VARYING CHRONIC DISEASE BURDEN

Emma Plank[1], Ethan Ouimet[2], Emily Erlenbach[3], Stephanie Voss[4], Edward McAuley[5], Nicholas Burd[5], and Neha Gothe[1], *1. Northeastern University, Boston, Massachusetts, United States, 2. Northeastern University of Bouve College of Health Sciences, Boston, Massachusetts, United States, 3. EXOS, South San Francisco, California, United States, 4. Mass General Brigham, Boston, Massachusetts, United States, 5. University of Illinois Urbana-Champaign, Urbana, Illinois, United States*

Regular exercise has been shown to lower both inflammatory and physiological stress markers, such as interleukin-6 (IL-6) and cortisol. Individuals living with chronic diseases (CD) often have higher baseline values of these markers than healthy individuals. The purpose of this study was to examine the effects of 6-month exercise interventions on IL-6 and cortisol in older adults with varying CD burden. Sedentary older adults ($n = 142$, 64.2 years) participated in 60-min. sessions, 3x/week as a part of an exercise-based randomized controlled trial. IL-6 and cortisol levels were measured at baseline and following the intervention. Participants were categorized into 1 of 3 categories based on health status: healthy (CD0, $n = 43$), 1 chronic disease (CD1, $n = 43$), or $\geq 2$ chronic diseases (CD2, $n = 59$). No significant differences were noted on baseline BMI and fitness between CD1 and CD2 however CD0 was significantly different from CD2 (p's <.02). Baseline BMI was correlated with IL-6 change (r=-.24, p <.05) but not cortisol while baseline fitness was not correlated with either marker. Repeated measures ANOVA demonstrated significant decrease in both IL-6 and cortisol for CD1 (p's <.01) following the intervention, but not for CD0 and CD2. Participating in three, 60-minute bouts of exercise per week may improve IL-6 and cortisol levels of older adults with 1 CD, thus reducing inflammation and stress associated with CD. Our findings have implications for prevention and CD management, and further research is needed to explore the optimal exercise dosage required to elicit similar reductions for individuals living with a greater CD burden.

Abstract citation ID: igaf122.3577

### EMERGENT UNDERSTANDING: FIRST RESPONDERS' FEEDBACK TO A DEMENTIA EDUCATION TRAINING

Keith Kleszynski[1], Zachary Chandler[2], Jack Stanfield[3], Andrea Golden-Pogue[4], Shirley James[5], Thomas Teasdale[6], and Lee Jennings[6], *1. University of Oklahoma Health Campus, Oklahoma City, Oklahoma, United States, 2. The University of Oklahoma, Oklahoma City, Oklahoma, United States, 3. University of Oklahoma College of Medicine, Oklahoma City, Oklahoma, United States, 4. Oklahoma University Health Sciences Center, Oklahoma City, Oklahoma, United States, 5. Hudson College of Public Health, University of Oklahoma Health Sciences, OKLAHOMA CITY, Oklahoma, United States, 6. University of Oklahoma Health Sciences, Oklahoma City, Oklahoma, United States*

To address the need for increased first-responder (fire, police, emergency medical services) training in dementia awareness and response, the Oklahoma Dementia Care Network developed a novel first-responder training program, consisting of didactic overview and four case-based, role-playing scenarios featuring common experiences from the field. Training content was developed and refined with input from a first-responder focus group. We describe perceptions from the first 34 participants to complete the training. An inductive, thematic analysis examined open-ended responses; questions explored participant experiences, learning outcomes, and recommendations for future content. Two coders coded responses and collaboratively grouped them into themes. Three themes emerged: importance of a practical training focus, profession-specific applications, and increased understanding and empathy. First-responders emphasized actionable strategies over theoretical knowledge, aligning with the time-sensitive, high-stakes environment they operate in. Participants identified specific communication techniques (i.e., "slowing down," using body language/positioning, and de-escalation strategies) as elements missing in their current skill sets. After completing training, participants reported increased confidence interacting with PWD (37 percentage point increase, $p < 0.01$), ability to respond to PWD with understanding (34 percentage point increase, $p < 0.01$), motivation to get to know PWD better (27 percentage point increase, $p = 0.03$), 88% learned about new dementia community resources, and 88% rated the training as very good or excellent. A hands-on training to equip first-responders with tools to better navigate emergency response situations with PWD was well-received and filled a learning gap. These findings highlight the practical value of a dementia-focused training developed with first-responder input that resonates with real-world field experiences.

Abstract citation ID: igaf122.3578

### ENDOTHELIAL-MEDIATED VASCULAR FUNCTION IN FRAIL OLDER ADULTS: A PILOT STUDY

Anna Dietz[1], Sung Gi Noh[2], Laura Mangone[2], Jacob Earp[3], George Kuchel[4], and Oh Sung Kwon[2], *1. University of Connecticut, University of Connecticut, Connecticut, United States, 2. University of Connecticut, Storrs Mansfield, Connecticut, United States, 3. University of Connecticut, Storrs, Connecticut, United States, 4. UCONN HEALTH, Farmington, Connecticut, United States*

Frailty, sarcopenic obesity, endothelial dysfunction, and cardiovascular diseases (CVD) increase with aging. We investigated relationships between frailty, body composition, and endothelial dysfunction using flow-mediated dilation (FMD) and passive limb movement (PLM)-induced hyperemia. 46 participants included Healthy Young (HY, 28 ± 4 yrs, $n = 16$), Healthy Old (HO, 73 ± 6 yrs, $n = 21$), and Frail Old (FO, 78 ± 9 yrs, $n = 9$). Analyses in FO were stratified by body composition. Endothelium-dependent vasodilation was assessed using FMD and PLM. Plasma nitrite and nitrate were measured as NO biomarkers, and oxidative stress was quantified. FMD decreased with age (HY: 10.42 ± 0.8%; HO: 7.45 ± 0.3%, $P = 0.021$) and was lowest in FO (5.31%). PLM peak LBF and AUC declined with age (HY: 692 ± 107 ml/min, 249 ± 104 ml; HO: 289 ± 92 ml/min, 128 ± 45 ml; FO: 212 ± 101 ml/min, 96 ± 38 ml). Within FO, obese individuals had lower FMD and LBF than lean counterparts. Plasma nitrite decreased (HY: 140 ± 6.1 μmol/L; HO: 117 ± 7.3 μmol/L, P = 0.04; FO: 103 ± 6.6 μmol/L). Oxidative stress increased (HY: 0.45 ± 0.3 AU; HO: 2.15 ± 0.5 AU; FO: 2.88 ± 0.5 AU). Endothelial dysfunction increased with aging, frailty, and obesity. Reduced NO bioavailability and oxidative stress likely contribute. FMD and PLM assessments may aid early detection and guide interventions, including exercise, diet, and pharmacological strategies, to preserve vascular function and reduce CVD risk in frailty.

Abstract citation ID: igaf122.3579

### ESTABLISHING INITIAL GUIDELINES FOR HOSPICE CAREGIVER OUTREACH AND RECRUITMENT

Felix Vasquez[1], Elizabeth Luth[2], Veerawat Phongtankuel[3], and Kira Sheldon[4], *1. VNS Health, Brooklyn, New York, United States, 2. Rutgers University, New Brunswick, New Jersey, United States, 3. Weill Cornell Medicine, New York, New York, United States, 4. Rutgers University-New Brunswick, New Brunswick, New Jersey, United States*

Caregiver recruitment is historically challenging in home hospice research, and there are few established best practices recruiting from this population. This analysis aimed to identify trends in home hospice caregiver recruitment to aide investigators in planning studies focused on enrolling hospice caregivers. Recruitment data was compiled from two intervention studies (EDITH-HC and I-HoME) intended to improve the quality of care for home hospice patients and their caregivers and reduce caregiver burden. Caregivers contacted for participation from both studies were aggregated, and 144 of 461 (33%) eligible caregivers enrolled. Recruitment began 14-days after the start of care (SOC) OASIS assessment was completed. We found that thirty-two percent (32%, n = 111 of 342) caregivers who were contacted between 14-90 days from SOC enrolled. Eighty-one percent (81%, n = 90 of 111) of caregivers who enrolled within 14-90 days from SOC also enrolled within the first two recruitment calls. Out of the 1312 calls made, the highest percentage of caregivers answered calls and enrolled during the early afternoon (12pm-3pm: 31% n = 22 of 71), followed by evening (6-8pm: 31%, n = 10 of 32), morning (9am-12pm: 29%, n = 15 of 51), and late afternoon (3pm-6pm: 19%, n = 81 of 436). Eighteen percent (18%, n = 232 of 1312) of calls are missing the time-of-day caregivers were contacted. Recommendations to improve future recruitment practices include calling at least twice, within the first 14-90 days of hospice enrollment, and having the flexibility to call at all times of the day. These findings provide initial guidelines for maximizing recruitment among the hard-to-reach population of hospice caregivers.

Abstract citation ID: igaf122.3580

### EVALUATING RANDOMIZED ASSESSMENT TIMING DESIGN IN A SIMULATION STUDY

Wenshan Yu, Valerie Smith, and Sarah Peskoe,
Duke University, Durham, North Carolina, United States

Longitudinal studies are vital in aging research because they enable researchers to trace trajectories of risk factors and outcomes, such as pain, physical and cognitive function, and biomarkers. In a typical longitudinal design, follow-up time is predetermined and fixed for every individual to ensure that differences in outcomes can be attributed to true temporal changes rather than variability in measurement timing, and to prevent time-related factors from being conflated with treatment or group effects. While this approach maintains internal validity, it can also limit the ability to explore alternative follow-up schedules that might yield richer information or greater efficiency. We propose a randomized follow-up design, in which the timing of some follow-up measurements is randomized across subjects within a longitudinal study. This approach has the potential to better estimate the underlying trajectory of change. Through a series of simulation studies, we demonstrate that the proposed design can substantially reduce bias when estimating between-group differences and when extrapolating to time points not covered in a fixed-time design. It also improves the overall mean squared error in predicting outcomes across all time points, when higher-order terms in the trajectory cannot be estimated due to insufficient data. This design may be valuable for both clinical trials and prospective observational studies in settings where the trajectory pattern is unknown at the design stage. The promising simulation results motivate further application to real data, to assess the utility of the design in reducing participant burden in data collection while increasing statistical power for trajectory estimation.

Abstract citation ID: igaf122.3581

### EVALUATION OF AN OLDER ADULT MEDICAL SCHOOL ALUMNI MENTORSHIP PROGRAM FOR MEDICAL STUDENTS TO LEARN ABOUT AGING

Micayla Flores[1], Emily Harris[2], Louise Aronson[3], Beth MacGillivray[3], and Andrea Wershof Schwartz[4], *1. Massachusetts General Hospital, Boston, Massachusetts, United States, 2. Harvard Medical School, Harvard Medical School, Massachusetts, United States, 3. Harvard Medical School, Boston, Massachusetts, United States, 4. Veteran's Affairs, Jamaica Plain, Massachusetts, United States*

Medical school training must prepare future clinicians to care for our aging population by providing them with skills and knowledge about older adults, including tools for avoiding the negative health impacts of ageism. AGE- PAIRS (Aging and Geriatrics Education: Physician Alumni Intergenerational Relationships with Medical Students) was conducted at Harvard Medical School from Aug 2024- June 2025, matching medical school students with older adult medical school alumni. The program consists of an introductory session, calls focused on 4 themes (medicine as an evolving profession, perceptions of aging, retirement, ageism), and a pre- reading or podcast. The program was evaluated using focus groups and a pre and post program survey that also assessed attitudes towards aging with a pre-validated questionnaire. 148 medical student- mentor dyads participated in the program. Before program initiation, there was no difference between students' and mentors' attitudes towards aging, and there was no change in the mentors' attitudes towards aging following program completion. 78% mentors expressed the experience in the program was moderately, very, or extremely effective, and 81% mentors expressed the experience with their student was very or extremely positive. 92% mentors would like to mentor again. Next steps include post- program student evaluations, including a survey and focus groups. This highly- rated program proved to be a positive experience, providing a balance of career advising and insight into the aging experience, and offering a potentially powerful model for preparing students to care for older adults with empathy and excellence.

Abstract citation ID: igaf122.3582

### EXPECTATIONS MATTER: SOCIAL AND EMOTIONAL LONELINESS AND ATTITUDES TOWARD AGING

Abigail Hoffman[1], and Kimberly Van Orden[2], *1. University of Rochester, Fairport, New York, United States, 2. University of Rochester Medical Center, Rochester, New York, United States*

This study examines relationships between attitudes towards aging (internalized ageism) and loneliness using multidimensional measures. Few studies differentiate between types of loneliness or dimensions of internalized ageism, which is needed to develop targeted interventions. We hypothesized that greater global internalized ageism would be associated with both greater social and emotional loneliness because negative attitudes towards aging could impact an older person's willingness to engage socially (resulting in social loneliness) as well as experiences of feeling connected to others (emotional loneliness). We also hypothesized that all three dimensions of internalized ageism (psychosocial loss, physical change and psychological growth) will be associated with both greater social and emotional loneliness. This is a secondary data analysis from the HOPE clinical trial (NCT03343483) that enrolled participants with clinically significant loneliness (n = 276; ages 60-92; 76.1% female; 90.6% white; 32.2% married) and who completed the 6-item DeJong Gierveld Loneliness Scale and the 24-item Attitudes to Ageing Questionnaire (AAQ). A multiple regression model with both forms of loneliness as simultaneous predictors of global internalized ageism indicated that both social (ß = -0.305, p<.001) and emotional loneliness (ß = -0.352, p<.001) were independently associated with internalized ageism (R2 = 0.321). Three regression models (for each dimension of internalized ageism) also indicated significant independent effects of both social and emotional loneliness on ageism dimensions. Limitations and future directions will be discussed, including planned analyses examining impacts of loneliness interventions provided in the HOPE RCT on dimensions of internalized ageism.

Abstract citation ID: igaf122.3583

### EXPERIENCES OF DEMENTIA CAREGIVING AMONG KOREAN AMERICANS AND KOREAN FAMILY CAREGIVERS IN A CULTURAL CONTEXT

Ji Yeon Lee[1], Kyung Hee Lee[2], Eunhye Hong[2], Jing Wang[3], and Bei Wu[4], *1. Inha University, Incheon, Republic of Korea, 2. Yonsei University, Seoul, Republic of Korea, 3. University of New Hampshire, Durham, New Hampshire, United States, 4. NYU Shanghai, Shanghai, China (People's Republic)*

Many studies have focused on dementia care experiences; however, few have examined these experiences through a cross-cultural lens among individuals sharing the same ethnic background but living in different sociocultural environments. This study aimed to explore how family dementia caregiving experiences were shaped by cultural and societal contexts among Korean-American and Korean caregivers. This international collaborative qualitative study was conducted in the US and South Korea. Participants were family dementia caregivers who provided care to community-dwelling older adults. Researchers interviewed two groups of 11 Korean-Americans in New York and three groups of 14 Koreans in Seoul. Semi-structured interviews explored challenges in dementia care, familial and personal changes due to dementia care, and the impact of culture and social resources on dementia care. Four layers of themes emerged: "Ongoing challenges within the current cultural context," "support and limitations of the social system," "the dynamics of family relationships," and "the impact and life changes." Eleven categories emerged from four themes and some were unique to each group. For example, "differences and inconveniences experienced as Korean-Americans living in the US" was only from Korean-Americans, whereas "feeling disappointed and regretful toward family" emerged exclusively among Korean participants. This study deepens our understanding of both the differences and similarities in the dementia experiences of Korean-American and Korean family caregivers, who share a cultural heritage but live in distinct societal contexts. The findings highlight the need for culturally sensitive, family-centered approaches to dementia care that address the unique challenges faced by caregivers in different settings.

Abstract citation ID: igaf122.3584

### EXPLORING THE CAREGIVING CONTEXT OF MEMORY CHALLENGES IN BOTH CARE RECIPIENTS AND CARE PARTNERS

Sophie Park[1], Jessica Hahne[2], Ella Kohn[3], Kejia Zhang[3], Sara Czaja[1], Megan Shen[4], and Kelly McConnell[5], *1. Weill Cornell Medicine, New York, New York, United States, 2. Washington University in St. Louis, St. Louis, Missouri, United States, 3. Weill Cornell Medicine, Weill Cornell Medicine, New York, United States, 4. Fred Hutchinson Cancer Center, Seattle, Washington, United States, 5. Memorial Sloan Kettering Cancer Center, Memorial Sloan Kettering Cancer Center, New York, United States*

Many care partners (CPs) of care recipients (CRs) with memory challenges also experience cognitive challenges. While the role of "care partner" carries the assumption of intact cognition, there is limited research examining memory challenges among CPs. In a pilot study of an advance care planning intervention for CRs with memory challenges and their CPs, 16 unpaired CRs and CPs and 21 CR-CP dyads were screened for study eligibility with the Montreal Cognitive Assessment-Blind (MoCA-B). Descriptive statistics were used to examine CP scores. Audio recordings of the intervention sessions were coded to quantify explicit references to memory challenges among CPs and CRs serving dual roles as a CR & CP. Five CPs had MoCA-B scores indicating cognitive impairment (MoCA-B< 19; M = 17, SD = 1.22). MoCA-B scores were identical for the CR and CP in three dyads (Range=18-19). In two dyads, the CR had a higher MoCA-B score than the CP (CR Range=18-21, CP Range=17-18). The results also identified two CRs who reported being a CP in another caregiving relationship. Additionally, four of nine CPs reported that they experience memory challenges and described reciprocal caregiving relationships where they received help from their CRs. These findings challenge the prevailing assumption that CPs are fully prepared to meet caregiving demands and suggest that there is significant variability in the cognitive capacity of CPs. Overlooking these differences may underrepresent the challenges CPs face and the support they may require. There is further need to address this issue to accurately understand care dynamics and develop effective care models.

Abstract citation ID: igaf122.3585

### EXPLORING THE IMPACT OF CHORAL SINGING ON OLDER ADULT COMMUNITY HEALTH AND WELL-BEING

Noell Rowan[1], Sandra Quinn[2], Mary Ann East[3], Meg Jordan[2], and Marieh Arnett[2], *1. University of North Carolina Wilmington, Wilmington, North Carolina, United States, 2. University of Maryland, College Park, College Park,*

*Maryland, United States, 3. Encore Creativity for Older Adults, Washington D.C., District of Columbia, United States*

There is increasing interest in community engaged research on the impact of singing on health and well-being of older adults. As its aim, Community-based participatory research (CBPR) blends knowledge with action to create positive and lasting change. Encore Creativity for Older Adults (hereafter Encore) is the largest choral program for older adults in the United States. Community members within Encore were engaged as members of the research team as advisors to the long-term planning process. This cross-sectional survey explored the musical/singing experience, physical/mental health, social connection, loneliness and well-being of singers. Of the (N = 886) participants, over 41% have been with Encore for four years or more, including 15% with more than 10 years. Key findings included 73% reported their health as very good/excellent, with a mean of 4.11 (out of 5) on mental health. Statistically significant differences were noted in life satisfaction with those newest to Encore reporting lower life satisfaction than those with 1 year or more Encore experience. Significant predictors of current health were mental health, health limitations, and life satisfaction. Encore experience became a significant predictor when it interacted with mental health. Singers reported that having a sense of purpose, life-long learning, health and social benefits, social connection, and ease of participation kept them engaged with Encore. There were no significant differences in loneliness by years of Encore experience, suggesting protective benefits across all stages of participation. Implications include practice skills and advocacy to increase music engagement and the potential impact on health/mental health and overall well-being.

Abstract citation ID: igaf122.3586

### EXPLORING THE PHENOMENON OF LONELINESS AMONG OLDER ADULT INFORMAL CAREGIVERS: AN INTEGRATIVE REVIEW

Alyssa Abbott, Jane Flanagan, and Karen Lyons, *Boston College, Chestnut Hill, Massachusetts, United States*

As caregivers age alongside their care recipients, they face unique health challenges, including loneliness. While aging and informal caregiving have been established as risk factors for loneliness, less is known about its manifestation among older adult caregivers. This integrative review, conducted in accordance with the Preferred Reporting Items for Systematic Reviews and Meta Analysis (PRISMA) guidelines, aimed to synthesize current research on loneliness in older adult informal caregivers. Seventeen studies met the inclusion criteria. All but one study were descriptive in nature. One cross-sectional study used a mixed methods approach to explore the impact of an online communication strategy on loneliness. Of the remaining studies, nine were qualitative, four were secondary analysis, one employed a survey design. Per inclusion criteria, all participants were aged 65 or older. Across studies, caregiver characteristics were often underreported; however, when available, most caregivers were female (e.g., 72%) and caring for a spouse. Only one study focused exclusively on male caregivers, and few reported race, ethnicity, or other sociodemographic variables. Findings highlighted that caregiving transitions, particularly involuntary separation due to long-term care placement and the end-of-life period, were associated with heightened loneliness. Notably, loneliness was rarely assessed or addressed by healthcare providers. Loneliness is a salient concern among older adult informal caregivers, especially during caregiving transitions. Recognizing the intersecting stressors faced by this population is essential for informing the design of interventions and guiding policies to better support caregiver well-being.

Abstract citation ID: igaf122.3587

### EXPLORING THE RELATIONSHIP BETWEEN ZINC STATUS AND FRAILTY: OBSERVATIONAL AND GENETIC ANALYSES

Sangyong Choi[1], Kelin Zhong[1], and Chia-Ling Kuo[2], *1. University of Connecticut, Storrs, Connecticut, United States, 2. UCONN HEALTH, Farmington, Connecticut, United States*

Frailty increases the risk of adverse outcomes in older adults, reflecting diminished physiological reserve and vulnerability to stressors. Zinc, an essential mineral, supports immune function and oxidative stress regulation, with circulating levels generally lower in older than younger adults. This study investigates the potential causal relationship between zinc status and frailty using observational analyses and Mendelian Randomization (MR) in the UK Biobank. Dietary zinc intake was assessed by self-reported questionnaire and categorized per UK guidelines. We assessed frailty in older adults using two complementary indices: the Fried frailty phenotype (physical frailty, defined by $\geq 2$ of 5 specific criteria) and a 49-item Frailty Index (FI-49, representing the accumulation of health deficits across multiple systems). Observational analyses revealed divergent associations: excessive zinc intake was linked to higher risk of frailty by FI-49, while zinc deficiency intake was associated with greater risk according to the Fried frailty phenotype, reflecting the conceptual distinctions of each index. MR analyses did not show evidence of a causal effect of genetically predicted blood zinc levels on frailty. These results suggest that both zinc deficiency and excess may contribute to frailty risk, depending on the aspect of frailty measured, while genetically determined zinc levels do not influence status. Despite the possibility of confounding or threshold effects in observational findings, the lack of blood zinc measurements limits conclusions about the relationship between intake, circulating levels, and frailty. Further research should clarify zinc's effects and assess supplementation in those with low intake or at-risk genotypes.

Abstract citation ID: igaf122.3588

### FACILITATORS AND BARRIERS TO SOCIAL ENGAGEMENT AND HEALTHY AGING AMONG OLDER ADULTS

Hin Wing Tse, Emily West, Catherine Sherk, Zoraida Garcia, Frida Ortega-Ruiz, Asma Maredia, Mattea Rodgers, and Elizabeth Muñoz, *The University of Texas at Austin, Austin, Texas, United States,*

Social relationships and community involvement are essential to successful aging, defined as maintaining mental and physical well-being in later life. The COVID-19 pandemic significantly increased social isolation among older adults in the United States and globally. This study utilized the World Café method, a Community-Based Participatory Research (CBPR) approach, to facilitate open and meaningful discussions in the community and foster collaborative learning. It explored the lived experiences of 41 older adults with diverse sociodemographic characteristics, including age (50 years and older), language (English or Spanish), and marital status (married, divorced, widowed, or never married), who were recruited from three Central Texas community centers. Discussions focused on patterns of engagement in social and physical activities, perceived links with mental health, and barriers and facilitators to participation in center activities before, during, and after COVID-19 restrictions. Data were audio-recorded, transcribed verbatim, and thematically analyzed using a deductive approach in Dedoose software. Emerging themes include: Physical activity improved mental health and reduced loneliness; social connection served as a strong motivator for community involvement and engagement in physical activity; lingering impacts of COVID-19, such as fear of illness, reduced program availability, and transportation challenges, continued to limit their participation. These results highlight the need for community-level capacity building to enhance engagement among older adults, particularly those most vulnerable to social disconnection.

Abstract citation ID: igaf122.3589

### FAMILIES' PERSPECTIVES ON PHYSICAL RESTRAINT USE IN OLDER ADULTS: A SCOPING REVIEW

Eun-Hi Kong, *Gachon University, Incheon, Republic of Korea*

Despite many harmful effects, physical restraints have been widely used for older adults in many care settings. Most earlier studies have focused on formal caregivers and little is known about family caregivers of older adults with physical restraints. Therefore, the aim of this review to synthesize research evidence of the literature related to families' perspectives on physical restraint use. Systematic literature search was conducted using electronic database search including MEDLINE, EMBASE, CINAHL, Cochrane Library, KoreaMed, RISS, and Google scholar from the earliest year to July, 2025. The combination of the keywords related to physical restraint, family, and older adults was used. Using EndNote21, search results were imported and duplicates were removed. Grey literature as well as published articles were included in this review. This review followed the JBI guidance and the PRISMA-ScR extension for scoping review. Eleven studies were included in this review. The studies were conducted in many countries on three continents. Six studies were quantitative studies (non-experimental studies) and five studies were qualitative descriptive studies. Family caregivers of older adults receiving home care or long-term care were included. This review found that many family caregivers had mixed perceptions towards physical restraint use on their loved ones. Also, this review pointed out family caregiver's lack of education regarding physical restraint. This review shows that there is a dearth of intervention studies (experimental studies) targeting family caregivers for physical restraint reduction. Future studies are required to develop and evaluate educational programs of restraint-free care and person-centered care for informal caregivers.

Abstract citation ID: igaf122.3590

### FAMILY MATTERS: HOW FAMILY MEMBER INCARCERATION, AND WHO WAS INCARCERATED, IS ASSOCIATED WITH OLDER ADULTS' HEALTH

Louisa Holaday[1], Albert Siu[2], Brie Williams[3], Brita Roy[4], Julia Wright[2], Pranav Gwalani[1], and Emily Wang[5], *1. Icahn School of Medicine at Mount Sinai, New York, New York, United States, 2. Icahn School of Medicine at Mount Sinai, new york, New York, United States, 3. University of California, San Francisco, san francisco, California, United States, 4. NYU Langone Health, brooklyn, New York, United States, 5. Yale University, New Haven, Connecticut, United States*

Intro: Having ever had an incarcerated family member is associated with worse health, but little is known about specifics among older adults, who have lived their entire adult lives in the era of mass incarceration. Methods: In a cross-sectional analysis of FamHIS data (2018), a nationally representative survey designed to understand family member incarceration in the United States, we used logistic regression models to examine associations with self-reported physical and mental health. Our primary exposure was any immediate family member incarceration. Secondary analyses examined relationship of incarcerated family member. Covariates were age, gender, race/ethnicity, education, income, employment, marital status, and personal incarceration history. Results: Among 1,319 subjects, of whom 63% experienced incarceration of an immediate family member, family member incarceration was independently associated with increased odds of reporting "fair" or "poor" physical and mental health (aOR 1.46; 95% Confidence interval: 1.00-2.14); $P = 0.05$ and aOR 2.13; 95% CI: 1.27-3.58 ; $P = 0.004$, respectively). This differed by relationship, with ever parental incarceration independently associated with the highest odds of "fair" or "poor" physical and mental health (aOR 1.99, 95% CI: 1.07-3.68, $P = 0.03$; aOR 2.95, 95% CI: 1.48-5.90 respectively, $P = 0.002$), followed by children and siblings, regardless of when that incarceration occurred. Conclusion: Ever incarceration of a family member was independently associated with poor health among older adults, even for those whose parent was incarcerated in the distant past. Researchers should work to understand the mechanisms behind this, and clinicians and policy makers should intervene at earlier ages to ensure all Americans can age healthfully.

Abstract citation ID: igaf122.3591

### FEASIBILITY OF NEUROSPECTIVE: A MULTI-DOMAIN COGNITIVE SCREENING PLATFORM WITH GAMIFIED TASKS FOR EARLY DETECTION

Lenora Smith, *University of Alabama in Huntsville, Huntsville, Alabama, United States*

Background: Early detection of cognitive decline requires accessible tools that assess multiple domains. Neurospective is a mobile gamified platform to assess a wide range of cognitive functions through short digital games intended for large-scale community-based use. Objectives: To evaluate feasibility and usability of a comprehensive mobile cognitive battery incorporating both traditional neurocognitive tasks and novel gamified paradigms, with plans for ML-based scoring. Methods: The current prototype consists of 24 mobile tasks spanning executive function, attention, memory, psychomotor speed, and visuospatial processing. Stroop serves as the control task. MoCA-aligned tasks include naming animals, a letter-tap inhibition task, serial subtraction, connect-the-dots, and cube drawing. Additional tasks include four versions of memory match, animal and shape counting, naming colors and shapes, digital bingo, motion detection, whack-a-mole, pattern recognition, a block game, and a puzzle game. All tasks were built for compatibility with iOS and Android, and user interactions are logged for duration, touch precision, and error rates. Launch to initial users is planned within the month, with gameplay data sent for machine learning model development. Results: Pre-launch testing shows platform stability and high task engagement. Users report ease of navigation and positive feedback on task enjoyment. Formal usability data collection is forthcoming. Conclusions: Neurospective appears feasible as a scalable, user-friendly screening tool integrating both validated and innovative cognitive tasks. The breadth of domains and planned ML scoring may allow for more nuanced detection of cognitive change, positioning Neurospective as a next-generation tool for early cognitive assessment.

Abstract citation ID: igaf122.3592

**FEW PHARMACOLOGIC INTERVENTIONS PREVENT OR MITIGATE FRAILTY: A SYSTEMATIC REVIEW**

Leah Bolles[1], Michael DeGloria[1], Hailey Cray[1], Rebekah Harris[1], Saadia Qazi[1], and Ariela Orkaby[2], *1. VA Boston Healthcare System, Boston, Massachusetts, United States, 2. Brigham & Women's Hospital, Harvard Medical School, Boston, Massachusetts, United States*

Frailty is a multidimensional clinical syndrome which limits the ability of older adults to cope with both everyday and acute stressors. To date, most interventions for frailty target lifestyle improvements, however, uncertainty remains as to whether pharmacologic interventions may prevent or mitigate this syndrome. Therefore, this systematic review aims to explore the efficacy of existing pharmacologic options for the treatment of frailty. Following PRISMA guidelines, we conducted our search using PubMed, CINAHL, and ClinicalTrials.gov from article inception to 10 June 2025. Only randomized controlled trials evaluating the effect of pharmacologic interventions on validated frailty measures were included. Two authors independently screened articles and extracted data, and disagreements were resolved through a third author. After screening 747 titles and abstracts, 33 full text manuscripts were extracted, and 10 trials were included. Results indicated varying efficacy across unique pharmacologic interventions. L-carnitine, protein supplementation at higher doses, testosterone therapy, and intravenous allogenic mesenchymal stem cells show promise in preventing and/or reducing frailty. Prebiotic administration, aspirin, vitamin D3 with omega-3 fatty acids, canakinumab, and metformin showed no reduction in overall or incident frailty. Notably, the combined supplementation of vitamin D3, omega-3s, and a structured exercise program reduced the risk of becoming pre-frail. Data were insufficient to conduct a meta-analysis. Although some pharmacologic interventions exhibit potential benefits, data remains limited in guiding therapeutic approaches to prevent or mitigate frailty in older adults.

Abstract citation ID: igaf122.3593

**FROM DECADES TO DAYS: LONG-TERM COGNITIVE FUNCTIONING AND MOMENTARY STRESS-CONTROL DYNAMICS IN OLDER ADULTS**

Urmimala Ghose[1], Oliver Schilling[2], Ute Kunzmann[3], NIlam Ram[4], Christiane Hoppmann[5], and Denis Gerstorf[6], *1. Humboldt University in Berlin, Humboldt University in Berlin, Berlin, Germany, 2. Heidelberg University, Germany, Heidelberg, Baden-Wurttemberg, Germany, 3. Universität Leipzig, Leipzig, Sachsen, Germany, 4. Stanford University, Stanford University, California, United States, 5. UBC, Vancouver, British Columbia, Canada, 6. Humboldt University Berlin, Humboldt University Berlin, Berlin, Germany*

Multiple-time scale approaches offer new opportunities to examine how developmental processes unfolding over decades shape moment-to-moment experiences in daily life. Developmental theory suggests that long-term, age-related changes provide a foundation for short-term dynamics of stress and control. Supporting this view, prior work has shown that older adults whose disease burden had increased more across the past two decades experience more daily negative affect and heightened stress reactivity. Extending this line of inquiry, we combined data from 123 old adults (65–69 years, 47% women) and 32 very old adults (85–88 years, 59% women) who provided 24+ year within-person longitudinal data on cognitive (perceptual-motor) functioning and subsequently also completed repeated daily-life assessments of control beliefs and stress six times a day over 7 consecutive days as they were going about their daily-life routines. Using Dynamic Structural Equation Models that integrate long-term growth models with location-scale multilevel models revealed that older adults with steeper cognitive decline reported lower average base levels of perceived control, more reactivity to daily stressors, and more pronounced moment-to-moment fluctuations in perceived control that were independent of stressor exposure. We take our findings to showcase developmental processes evolving over vastly different timescales are closely intertwined and discuss how such new insights will inform developmental theories of control and daily-life functioning.

Abstract citation ID: igaf122.3594

**FROM NEEDS TO ASSETS: DATA-DRIVEN LESSONS FROM THE KENTON COUNTY AGE WELL INITIATIVE**

Ting Hong[1], Laura Allen[2], Michael Marcus[3], and Dana Bradley[4], *1. University of Maryland, Baltimore County, Baltimore, Maryland, United States, 2. University of Maryland Baltimore County, Covington, Kentucky, United States, 3. University of Maryland Baltimore County, Raleigh, North Carolina, United States, 4. University of Maryland Baltimore County, The Erickson School of Aging Studies, Baltimore, Maryland, United States*

This poster presents a case study of the Kenton County Age Well Initiative, a one-year, asset-based community development assessment project focused on leveraging assets to address the challenges of aging in Kenton County, KY. A first suburb of Cincinnati, Ohio, Kenton County is a geographically diverse community with a strong desire to support its older population, which is expected to grow from 16.5% of the population to 19% by 2030. The project began by forming an action committee of 25 local stakeholders to facilitate the team's immersive understanding of individual, institutional, and association-based resources and county-specific challenges related to navigating aging services. Data were collected through community conversations, qualitative surveys, focus groups, and stakeholder interviews across the county with a total of 377 community members and 44 professionals. Four domains were identified as essential for supporting aging in Kenton County: housing, transportation, long-term care, and community vibrancy. Local demographic, organizational, and sector-specific data were then pooled and analyzed in each domain to identify assets and goals to address challenges. The project timeline is presented, highlighting key community engagement events from the project start to finish, including four county-wide workshops for professionals, older residents, and their caregivers. This poster details the lessons learned from the case study, including translating complex data sets to have high impact in community meetings, focusing on collaborative strategies for identifying key data points rather than relying solely on research team expertise, and reframing aging from deficit-based to asset-based in local planning efforts.

Abstract citation ID: igaf122.3595

### FUNCTIONAL STATUS, DAILY FUNCTIONAL CHANGES, AND COGNITIVE FUNCTION IN LOW-INCOME OLDER ADULTS

Renata Komalasari[1], Ni Gusti Eka[2], Martina Pakpahan[3], Vivien Puspitasari[3], Peggy Tahulending[4], Rita Chang[5], Michael Dino[6], and Ladda Thiamwong[7], *1. Akademi Keperawatan Andalusia Jakarta, Jakarta, Jakarta Raya, Indonesia, 2. Universitas Pelita Harapan, Universitas Pelita Harapan, Banten, Indonesia, 3. Universitas Pelita Harapan, Tangerang, Banten, Indonesia, 4. Mayapada Hospital, Mayapada Hospital, Jakarta Raya, Indonesia, 5. Western sydney university, Penrith, New South Wales, Australia, 6. University of Central Florida, University of Central Florida, Florida, United States, 7. University of Central Florida, Orlando, Florida, United States*

Despite consistent evidence on the association between functional status and cognitive function, there is a lack of understanding on daily functional changes in community-dwelling older adults in Indonesia. We examined associations between functional status, healthy brain aging, and cognitive function among 150 adults aged ≥60 (60.7% female, Mage=65.54, SD ± 4.65) in low-income settings, Tangerang. The six-item Rowland Universal Dementia Assessment Scale (RUDAS-Ina) was used to assess cognitive function. Functional status was measured with the eight-item Lawton Instrumental Activities of Daily Living (IADL) Scale. The ten-domain Healthy Brain Aging form assessed functional changes in daily functioning within the past twelve months. Covariates were dementia severity rating scale and sociodemographic. We used generalized linear models with a Poisson distribution and a log link, which is robust to violations of normality in outcome measures. Findings showed functional status was significantly related to cognitive function (e1.079= 0.076, p = < 0.001), but functional changes were not, controlling for dementia severity rating scale and sociodemographic. For every one unit increase in IADL scores, the RUDAS scores increase 7.6%. Mann-Whitney U test was conducted to determine if there were differences in cognitive function. There was a significant difference between higher (RUDAS-Ina scores ≥23) (n = 121) and lower cognitive status group (RUDAS-Ina scores < 23) (n = 29): U = 2774.00, p <.001. Older adults who maintained independent functional performance tended to enjoy healthier cognitive function than their counterparts. Older adults should strive to maintain their independence in performing daily activities to promote healthy cognition, supporting more active aging.

Abstract citation ID: igaf122.3596

### GAIT SPEED AND MORTALITY RISK AMONG COMMUNITY-DWELLING OLDER ADULTS WITH HEART DISEASE: EVIDENCE FROM THE RAND HRS

Melanie Navos[1], and Yong-Fang Kuo[2], *1. University of Texas Medical Branch, Galveston, Texas, United States, 2. University of Texas Medical Branch, Galveston, Galveston, Texas, United States*

We examined the relationship of heart disease and gait speed with all-cause mortality over 11 years of follow-up among community-dwelling older adults. Participants (N = 1153) were from the RAND Health and Retirement Study Longitudinal 2020 (v2) dataset, a nationally-representative longitudinal study that began in 1992. Measures included socio-demographics, multi-morbidity status, depressive symptoms, cognitive function, and all-cause mortality. We used Cox-Proportional Hazards to estimate the hazard ratio (HR) and 95% confidence interval (CI) of all-cause mortality as a function of gait speed. We also examined the nonlinear form of gait speed to obtain the best cut-off points for predicting mortality that are more applicable in the clinical setting than its linear form. Participants were categorized into four groups based on gait speed cut-off (≤0.5 m/s, 0.5-≤0.75 m/s, 0.75-≤1.0 m/s, >1m/s). Older adults with heart disease and slower gait speed of ≤ 0.5m/sec are more at higher risk for mortality (HR = 3.02, 95% CI = 2.18-3.06), while, those with gait speed 0.5-≤0.75 m/s are at moderately elevated risk for mortality (HR = 2.25, 95% CI 1.61-3.16), and those with gait speed 0.75-≤1.0 m/s are at lower risk than the slow walking groups (HR = 1.85, 95% CI 1.38-2.47), compared to those with gait speed >1m/sec after controlling for all covariates. These findings underscore the value of gait speed as a prognostic marker and highlight the need for targeted interventions such as lower-extremity strengthening and endurance training to preserve mobility and reduce mortality risk in this vulnerable population.

Abstract citation ID: igaf122.3597

### GENERATIONAL STRENGTHS HELP BRIDGE POLITICAL DIVIDES: AN ANALYSIS OF CROSS-PARTISAN CONVERSATIONS

Mary Rose Bell[1], Anna Pot[2], and Laura Carstensen[2], *1. Stanford University, Palo Alto, California, United States, 2. Stanford University, Stanford, California, United States*

Older adults' socioemotional strengths, such as emotional stability and positivity, could be a valuable resource for navigating political discussions. When combined with younger adults' curiosity and open-mindedness, these intergenerational strengths have the potential to foster respectful dialogue across political divides. We analyzed conversations from StoryCorps' One Small Step initiative, which pairs individuals with different political views for 50-minute conversations. The sample included 105 conversations with young-young, old-old, and young-old dyads (N = 210 participants; M_young = 29 years, M_old = 71 years). Transcripts were analyzed using LIWC to measure the frequency of prosocial, polite, tentative, and positive words. Results showed that intergenerational (young-old) dyads used more polite words than young-young (b = 0.144, p < 0.001) and old-old dyads (b = 0.090, p = 0.033). Intergenerational dyads also used more prosocial words than young-young dyads (b = 0.146, p = 0.006). Additionally, intergenerational and old-old dyads used more positive words than young-young dyads, while intergenerational and young-young dyads used more tentative words (e.g., maybe, perhaps, wonder) than old-old dyads. These findings suggest that intergenerational dyads foster more positive and courteous political dialogue than same-age dyads. Social norms of deference may explain intergenerational dyads' greater polite and prosocial word use. Moreover, the combined strengths of each generation (e.g., older adults' positive reframing and younger adults' open-mindedness) may underlie intergenerational dyads' greater positive and tentative word use. In a time of marked political polarization in the U.S., intergenerational political discussions may offer a promising path to bridge divisions.

Abstract citation ID: igaf122.3598

### GLOBAL NARRATIVES IN GERONTOLOGY EDUCATION: AN AUTOETHNOGRAPHIC APPROACH FROM EAST ASIA

Takashi Yamashita[1], Yoon Chung Kim[2], Giyeon Kim[3]Tsuann Kuo[4], and Darren Liu[5], *1. University of Maryland, Baltimore County, Baltimore, Maryland, United States, 2. University of Maryland School of Medicine, Baltimore, Maryland, United States, 3. Chung-Ang University, Seoul, Republic of Korea, 4. Chung Shan Medical University, Taichung, Taichung, Taiwan (Republic of China), 5. West Virginia University, Morgantown, West Virginia, United States*

Conventional gerontology courses in higher education often prioritize theory and factual content over the cross-cultural experiences of aging, constraining students' development of cultural competency, such as open-mindedness, empathy, and the ability to challenge age-related stereotypes. While experiential learning, like study abroad, is effective, financial and logistical barriers compromise accessibility for many students. To address this gap, a team of international gerontology educators employed an autoethnographic methodology—a recognized approach for understanding personal and cultural experiences—to conduct immersive fieldwork in Okinawa (Japan), Seoul (South Korea), and Taipei (Taiwan). Through in-depth interactions with local gerontology researchers and community leaders (n = 18), the team collected rich narratives and observations, illuminating unique cultural perspectives on aging. These stories can be integrated into existing gerontology curricula to humanize abstract concepts, highlight cross-cultural differences and commonalities, and challenge age-related stereotypes. For example, a researcher from the Okinawa Centenarian Study shared the historical transition of what it means to be over 100 years old, emphasizing the need to revisit the concept of longevity. Other socioculturally rooted stories, such as a fake bus stop at the care facility in Seoul, and an inclusive support program for diverse older adults with various disabilities, aging stages, and lifestyles at the Red Cross community wellness hub in Taiwan, were illustrated. Such narratives, contextualized with the educators' lived experiences, can convey insider cultural perspectives in class, promoting their learning and cultural competency. Strategies to effectively share specific narratives with a larger gerontology education community and practical applications are evaluated.

Abstract citation ID: igaf122.3599

### HEALTHCARE ACCESS AND UTILIZATION AMONG OLDER U.S. IMMIGRANTS: A POPULATION-BASED ANALYSIS

Irtiza Faruque[1], Aastha Dharia[2], and Mohammad Usama Toseef[3], *1. Oakland University William Beaumont School of Medicine, Auburn Hills, Michigan, United States, 2. Oakland University William Beaumont School of Medicine, Rochester Hills, Michigan, United States, 3. Corewell Health, Farmington Hills, Michigan, United States*

Immigrants comprise a growing segment of the aging U.S. population, with those aged 65 and older now representing approximately 14% of all older adults. Despite their increasing numbers, older immigrants often encounter unique barriers to healthcare, including linguistic, financial, and systemic challenges. Yet, there remains limited research evaluating how these individuals perceive access to and quality of care. Using 2018–2022 data from the Medical Expenditure Panel Survey (MEPS), we conducted a retrospective analysis of self-reported healthcare access among adults aged 65 and older. Immigration status was categorized as U.S.-born, long-term immigrants (≥15 years in the U.S.), and recent immigrants (< 15 years in the U.S.). We examined key aspects of usual source of care (USC), including whether respondents reported having a USC, access to after-hours or weekend care, delays in receiving care or filling prescriptions due to cost, and whether providers communicated in the respondent's preferred language. Survey-weighted logistic regression models revealed that both long-term and recent immigrants were significantly less likely to report having a USC compared to U.S.-born individuals (OR = 0.7 and OR = 0.4, respectively; p = 0.004). Additionally, recent immigrants were more likely to report delayed medical care due to cost (OR = 2.2, p = 0.029). These findings contribute to growing evidence on how immigration status and length of U.S. residency shape access to care among older adults. By identifying disparities rooted in both migration history and broader social determinants, this study informs targeted strategies to promote health equity in aging populations.

Abstract citation ID: igaf122.3600

### HEALTHSPAN-LIFESPAN TRADE-OFF UNDER ACCELERATED BIOLOGICAL AGING: EVIDENCE FROM THE HEALTH AND RETIREMENT STUDY

Heming Pei[1], Arun Balachandran[2], Yifan Shi[3], and Daniel Belsky[4], *1. UNC Gillings School of Global Public Health University of North Carolina at Chapel Hill, Chapel Hill, North Carolina, United States, 2. Columbia University, New York City, New York, United States, 3. New York University, New York, New York, United States, 4. Columbia University, New York, New York, United States*

While life expectancy (LE) has increased globally, the rise in healthy life expectancy (HLE) has not kept pace. This widening gap between HLE and LE—years lived in poor health—could be impacted by the pace of physiological deterioration. This study investigates how accelerated biological aging affects transitions between health states and to assess how it contributes to the compression or expansion of healthspan relative to lifespan, and whether this effect is more pronounced in socially disadvantaged groups. Using longitudinal data from the U.S. Health and Retirement Study, we quantified biological aging with the Pace of Aging metric. Health transitions—including chronic disease, functional decline, cognitive impairment, and death—were modeled using a continuous-time Markov multi-state approach to simulate and calculate healthspan and lifespan across different aging profiles. The analysis revealed that individuals with faster pace of aging had increased risk of transitioning from healthy to unhealthy states, more so than the risk of death once already unhealthy. This imbalance led to an extended duration of life spent in poor health. The HLE–LE gap was consistently wider among those aging faster across all outcomes. And this effect was strongest in individuals with lower educational attainment, minority status, or unhealthy behaviors, suggesting systemic inequities amplify the impact of biological aging. Accelerated biological aging predicts shorter healthy life and exacerbates health inequities. Interventions aimed at slowing biological aging may yield dual benefits—improving overall population health and reducing disparities in how long and how well people live.

Abstract citation ID: igaf122.3601

### HOME HEALTH ACCESS AMONG PERSONS WITH DEMENTIA: TRADITIONAL MEDICARE VS. MEDICARE ADVANTAGE

Chenjuan Ma, *New York University, New York, New York, United States*

Home health care (HHC) is the most used home and community-based service for community-dwelling Americans, particularly persons living with dementia. There are two ways of accessing HHC: entry directly from the community or following institutional care (primarily after hospitalization for acute care). This study compared HHC access (community-entry vs. hospital entry) between persons with and without dementia and examined the role of Medicare Advantage (MA) HHC access. It used 2019 national home health assessment data (i.e., OASIS) linked to the Medicare Beneficiary Summary File. Multivariate logistic regression was used in the analysis, adjusted for patient sociodemographic and clinical conditions, and considering clustering within each agency. Of the 3,174,874 home health episodes in 2019, 42% were community-entry HHC, 22% were from persons with dementia, and 35% were from MA beneficiaries. Persons with dementia were more likely to be traditional Medicare (TM) beneficiaries (31% TM with dementia vs. 6% MA with dementia). Persons with dementia were significantly more likely to enter HHC directly from the community (60% vs. 37%, adjusted OR = 1.52, 95% CI = 1.50-1.53). Among persons with dementia, having MA was less likely to enter HHC from the community (adjusted OR = 0.93, 95% CI = 0.90-0.95), compared to having TM; while among persons without dementia, having MA was more likely to enter HHC from the community (adjusted OR = 1.08, 95% CI = 1.06-1.11). This study indicates the importance of community-entry HHC for high-need populations such as persons with dementia. It highlights the need for continuing monitoring of community-entry HHC accessibility under a rapidly changing healthcare system.

Abstract citation ID: igaf122.3602

### HOSPITALS' AGE-FRIENDLY HEALTH SYSTEMS RECOGNITION AND 30-DAY MORTALITY FOLLOWING HIP FRACTURE SURGERY

Hiroshi Gotanda, Teryl Nuckols, Brandon Truong, Zhaohong Feng, Carol Lin, Kathleen Breda, and Mark Vrahas, *Cedars-Sinai Medical Center, Los Angeles, California, United States*

Hip fractures among older adults present complex clinical challenges that extend far beyond surgical repair. Age-Friendly Health Systems (AFHS) recognition—a geriatric care initiative created to advance evidence-based, interdisciplinary geriatric care, with formal recognitions beginning in 2019—offers a promising pathway to better hip-fracture outcomes. However, the national impact of this initiative on outcomes among older adults with hip fracture remains largely unknown. In this context, we sought to compare 30-day postoperative mortality rates between older adults with hip fracture treated at acute care hospitals with AFHS recognition vs. those treated at hospitals without such recognition. We identified Medicare fee-for-service beneficiaries 65 years and older who underwent hip fracture surgeries based on relevant Current Procedural Terminology codes using 2021 Medicare data. We determined AFHS recognition status using the AFHS database. We fitted a multivariable linear regression model adjusted for patient characteristics. Our study included 27,201 observations (across 2,764 hospitals), 1,964 (7.2%) of which were treated at 141 hospitals with AFHS recognition. We found no evidence that 30-day postoperative mortality rates differ between hospitals with vs. without AFHS recognition (adjusted mortality rate, 6.7% vs. 7.5%; adjusted difference, -0.7 percentage points; 95% confidence interval, -1.7 to + 0.3; P-value = 0.17). While the difference was not statistically significant, the magnitude may present a clinically meaningful difference. Further multi-year, methodologically robust studies are needed to assess the national impact of the AFHS recognition initiative on outcomes among older adults with hip fracture and guide health policy, including CMS's Age-Friendly Hospital Measure beginning in 2025.

Abstract citation ID: igaf122.3603

### HOW DOES FAITH IMPACT END OF LIFE CARE PREFERENCES AMONG OLDER CHINESE IN THE UNITED STATES: A QUALITATIVE PILOT STUDY

Xuehan Zhang, and Amanda Woodward, *Michigan State University, East Lansing, Michigan, United States*

Previous studies have found that religious participation is closely related to older people's overall well-being and life satisfaction. However, the specific impact of religion on older individuals' end-of-life care preferences and death attitudes from racial minority groups remains underexplored. This qualitative pilot study aims to better understand the impact of religion (e.g., Christianity) on beliefs and preferences related to end-of-life care services among older Chinese adults in the United States, particularly those who are not immediately facing death or the need to make these care decisions. 13 semi-structured interviews were conducted in Ann Arbor, Michigan during the summer of 2025. All interviews were in Chinese and then translated into English for coding and analysis. Each interview was coded in Dedoose. Three main findings were identified: 1) Participants in the present study indicated that having no fear of death, not only because of their religious beliefs, but also because of their great confidence in the medical care services in the United States. 2) Participants in the present study were open to talking about euthanasia as a potential option for treatment in the end. 3) Most people who were interviewed in the present study lacked information about end-of-life care options but were willing to go to the "hospital" to receive care in the last stage of life. These findings highlight a critical need for culturally and religiously sensitive end-of-life care services and education among the older Chinese population in the United States.

Abstract citation ID: igaf122.3604

### HOW FAMILY COMMUNICATION ABOUT EARLY-STAGE DEMENTIA IS SILENCED: SELF-CENSORSHIP AND ACCOMMODATION STRATEGIES

Shicheng Xu[1], Vivian Lou[2], Ernest Gonzales[1], and Sehyun Baek[1], *1. New York University, New York, New York, United States, 2. The University of Hong Kong, Hong Kong, China (People's Republic)*

The prevalence of Alzheimer's disease and related dementias in China is significant, with an estimated 15.07 million people in 2020. However, this may be an underestimate as many older adults and family members avoid discussing dementia and undergoing assessment and treatment. This qualitative study explores intergenerational family communications styles on why it is difficult to broach dementia-related issues with family members, and how intergenerational family communication about early-stage dementia is silenced in Chinese families. Participants were recruited from Chinese social media and dementia care centers in Shanghai. Guided by constructivist grounded theory, we conducted three rounds (convenience sampling, theoretical sampling, member checking) of semi-structured interviews with 28 Chinese participants from three generations, including 9 people living with dementia (PLwD), 7 spouses of PLwD, 5 adult children of PLwD, and 6 adult grandchildren of PLwD. "Censoring communicability" emerged as a core category, with two properties: "conducting self-censorship" and "adjusting communication strategies". There appears to be a tension between communication and self-censorship based on a continuum from communicating directly, informally, indirectly, matter-of-factly without emotions, and gradually lowering the level of communicability to silencing. Internalized hierarchy and designated roles within the family are the key barriers that reduced the opportunity for family members to communicate concerns about initial memory problems, help-seeking, and diagnostic disclosure, particularly with the family decision makers, who are usually the spouse or adult child caregivers. Direct and informal communication styles across three generations appear to be ideal pathways to seeking assessment and medical treatment.

Abstract citation ID: igaf122.3605

### HYPOXIA REDUCES TAU AGGREGATION CAUSED BY MITOCHONDRIAL BIO-ENERGETIC DEFICITS

Sushila Thapa[1], Raghbendra Dutta[2], Esther Ojumah[1], Isaiah Smith[3], Matt Kaeberlein[4], and Anthony Grillo[2], *1. University of Cincinnati, Cincinnati, Ohio, United States, 2. University of Cincinnati, University of Cincinnati, Ohio, United States, 3. University of Cincinnati, CIncinnati, Ohio, United States, 4. Optispan, Optispan, Washington, United States*

Tauopathies are marked by abnormal phosphorylation of the tau protein and are often linked to mitochondrial dysfunction. Among mitochondrial complexes, Complex I impairment has been associated with enhanced tau pathology, but the exact underlying mechanism remains unknown. Our study examines whether hypoxia can modulate tau phosphorylation and neuroinflammatory responses in models of mitochondrial and tau pathology. In Ndufs4 knockout (KO) mice, a model of primary Complex I deficiency, abnormally high brain oxygen tensions contribute to disease progression. Chronic exposure to a reduced oxygen environment (hypoxia) normalized these elevated oxygen levels, significantly decreasing astrogliosis and microglial activation. Additionally, hypoxia-treated Ndufs4 KO mice exhibited a reduction in hyperphosphorylated tau, suggesting that oxygen normalization mitigates tau pathology in the context of mitochondrial dysfunction. In contrast, tauopathy P301S mutant mice showed reduced astrogliosis and microglial activation under hypoxia, but tau phosphorylation remained unaltered. We further investigated tau expression in human neuroblastoma cell treated with a Complex I inhibitor, and found increased tau phosphorylation, reinforcing the link between mitochondrial impairment and tau pathology. Although the precise mechanisms remain to be clarified, our findings suggest that hypoxia may activate adaptive cellular pathways that mitigate mitochondrial dysfunction and influence tau phosphorylation. These results highlight hypoxia as a potential therapeutic strategy for tauopathies associated with mitochondrial deficits.

Abstract citation ID: igaf122.3606

### I HEAR YOU, I FEEL YOU: EVALUATING THE IMPACT OF HEARING LOSS AMONG RURAL HOMEBOUND OLDER VETERANS

Nathan Sheets[1], Elizabeth Reilly[2], Sandra Sanchez-Reilly[3], Jose Mendoza[2], and Michael Mader[2], *1. University of Texas Health Science Center at San An, San Antonio, Texas, United States, 2. South Texas Veterans Health Care System, San Antonio, Texas, United States, 3. South Texas Veterans Health Care System and the University of Texas Health Science Center at San Antonio, San Antonio, Texas, United States*

Background: Over 50% older Veterans (OV) live with disabling hearing loss (HL), often undiagnosed and/or untreated. HL consequences include increased depression/dementia risk, PTSD exacerbation, social isolation, caregiver-burden and adverse events when hospitalized. Homebound rural OV consequences of HL remain unknown. Objective: Investigate the relationship between HL, quality-of-life and caregiver-burden among homebound rural OV Methods: Homebound rural OV (Del Rio, TX) were evaluated for HL (audiometer), caregiver-burden (Zarit), hearing-loss-impact-on-quality-of-life measured with Hearing-Handicap-Scale (HHS), and overall quality-of-life (SF-12). Results: N-19. Median Age 77 (74-81), 58% Hispanic, 100% male. Spouses were main caregivers. 58% had three-or-more chronic conditions: 63% mental-health-related, 26% cardiac, 21% pulmonary. 42% reported toxic exposures. 90% OV had HL; 69% found to have moderate-to-severe HL (41-70 dB of loss, 84% bilateral). 57% OV exposed to agent-orange had severe HL ($p = 0.089$). Caregiver-burden scores were higher among OV with severe HL (p=NS) and quality-of-life was not significantly correlated with HL (Physical: $p = 0.93$, Mental: $p = 0.47$, Total: $p = 0.71$). OV with Mild or Normal HL had lower total values of HHS vs. moderate HL ($p = 0.035$) and severe HL ($p = 0.016$). Further, both emotional and social HHS components showed negative significant correlations with severe HL ($p = 0.013$ and $p = 0.021$, respectively). Conclusions: Homebound OV likely suffer from multiple chronic conditions including HL (90%). Severe HL affects quality-of-life and causes caregiver-burden. Self-perceived HL social/emotional impairment significantly correlates to HL severity. In-home interventions such as standardized screening and provision of simpler/cost-effective devices (pocket-talker, alternative to hearing-aids) are needed to avoid further decline among homebound rural populations.

Abstract citation ID: igaf122.3607

### IDENTIFICATION OF COGNITIVE SUPERAGING AND ITS PREDICTORS AMONG OLDER ADULTS: A LATENT PROFILE ANALYSIS

Maham Khan[1], Laura Sands[2], and Benjamin Katz[2], *1. Virginia Tech., Blacksburg, Virginia, United States, 2. Virginia Tech, Blacksburg, Virginia, United States*

Older adults demonstrate substantial variability in cognitive functioning, with some maintaining exceptional performance, called Cognitive SuperAging (CSA). This study identified cognitive superagers and other cognitive profiles among individuals ≥ 75 years in 2020 Health and Retirement Study, and examined demographic, health, and behavioral predictors of CSA. The sample included 5,646 individuals with scores on different cognitive domains, including memory, attention, executive functioning (EF), and fluid intelligence-related tasks. Latent Profile Analysis models with 3-6 profiles were compared using fit indices (AIC, BIC, adjusted-BIC, entropy). A four-profile solution best fit the data, comprising: Class 1 Superagers (11.5%), Class 2 Average Memory, Impaired Attention & EF (39.9%), Class 3 Impaired Memory & EF, High Attention (37.2%), and Class 4 Globally Impaired (11.4%). A conditional model using R3STEP indicated sharpest separation between Globally Impaired and Superagers (Entropy = 0.77). The likelihood of not being Superager increased by 27% with age, 15% with each additional chronic condition, and nearly doubled with higher depressive symptoms. Higher physical activity was associated with more than twice the odds of being in Superaging profile (OR = 2.43, 95%CI [2.25, 2.64]). Biological sex showed no significant association, and sleep disturbance was associated with marginal negative effects (OR = 1.033, 95%CI [1.005 – 1.063]) on superaging. The intermediate profiles also showed similar but less pronounced trends. The results have implications for interventions and policies aimed at promoting resilience in cognitive aging. Findings support targeting physical activity, mental health, and chronic disease management to help maintain high cognitive functioning into advanced age.

Abstract citation ID: igaf122.3608

### IDENTIFYING PREFERRED LEARNING APPROACHES FOR DEMENTIA TRAINING OF DIRECT CARE WORKERS

Troy Andersen[1], Daniel Kaplan[2], Norman Foster[3], Jeanine Stefanucci[1], Andrea Dremann[4], Chad Moffitt[1], and Bonnie Bechek[4], *1. University of Utah, Salt Lake City, Utah, United States, 2. Adelphi University, Garden City, New York, United States, 3. ProActive Memory Services, Inc., Proactive Memory Services, Utah, United States, 4. ProActive Memory Services, Inc., Salt Lake City, Utah, United States*

Training direct care providers to care for individuals with dementia is challenging. Cultural differences, varying education levels, and high staff turnover further complicate consistent training. In our previous studies, direct care providers emphasized the need for training that is relevant, flexible and interactive. Our project aims to improve dementia care competencies through an innovative, flexible web-based platform that presents content in written and audio narration formats. We interviewed and tracked server data to identify preferences of 23 direct care providers (16 PCA, 7 ALC, 95.65% women, mean age 37.5) from personal care agencies (PCA) and assisted living communities (ALC) using training software an average of 1 hour per week for one month. Their use of audio versus written materials was tracked and analyzed to better understand learning preferences. All participants read the materials and 82% listened to the audio. We also captured the time windows of when the training was access to determine if integration of learning occurs within or outside of the work schedule. Highlights from the findings include 61% of participants accessed the training during traditional work hours, while 39% engaged the training outside of a traditional work schedule. This finding speaks to the challenges of integrating learning opportunities into the traditional work schedule. Direct care providers have diverse educational background, experience, and time constraints. To best meet their needs, content should be brief and presented in multiple formats for trainees to select as they believe will best fit their needs.

Abstract citation ID: igaf122.3609

### IMPLEMENTATION OF THE AGE-FRIENDLY HEALTH SYSTEM FRAMEWORK AT FOUR CLINICAL SITES: THE UTHEALTH HOUSTON CASE

Rafael Samper-Ternent[1], Mei Robson[2], Maureen Beck[2], Cindy Woolverton[2], Min Ji Kwak[2], Ezenwa Onyema[2], and Cristina Murdock[2], *1. University of Texas Health Science Center at Houston, Houston, Texas, United States, 2. UTHealth Houston, Houston, Texas, United States*

The Age-Friendly Health Systems (AFHS) framework promotes evidence-based care for older adults through the 4Ms: What Matters, Medication, Mentation, and Mobility. UTHealth Houston joined the Institute for Healthcare Improvement (IHI) System-Wide Spread Collaborative to expand AFHS adoption across four clinical sites: the Center for Healthy Aging (CHA), John S. Dunn Behavioral Health Sciences Center (Dunn), Harris Health System (HHS), and Memorial Hermann Hospital (MHH). We evaluate AFHS implementation using chart reviews from April 2024 to April 2025 to capture patient counts, 4Ms assessments, and corresponding clinical actions. We identify barriers to implementation via review of meeting notes and reports submitted to IHI. Across 9,664 patients, 1,598 charts were reviewed. All sites showed improvement with 67% of CHA patients, 92% of Dunn patients, 80% of MHH patients, and 60% of HHS having complete 4Ms assessments by April 2025. Progress in acting on the 4Ms varied: 66% of CHA, 33% of Dunn, 45% of MHH, and 58% of HHS patients had 4Ms-informed clinical actions documented. Barriers included integrating 4Ms into Epic workflows, capturing meaningful "What Matters" data, and adapting the model to inpatient and outpatient contexts. Despite challenges, all sites advanced toward AFHS goals. Findings highlight the need for site-specific strategies, continuous data use, and sustained support. Strategies to support automatic data collection and reporting are needed. Linking the 4Ms to risk stratification and performance incentives may further enhance adoption and scalability.

Abstract citation ID: igaf122.3610

### INFORMAL DEMENTIA CAREGIVER OUTCOMES IN RELATION TO CARE SETTING AND LONELINESS- AN INTEGRATIVE REVIEW

Maria Weibel, and Judith Tate, *The Ohio State University, Columbus, Ohio, United States*

Background: Globally, loneliness is prevalent and expected to continue increasing. Informal dementia caregivers are lonelier than the general population. Loneliness is associated with greater risk for dementia, cardiovascular disease, depression, and premature mortality. Loneliness has also been associated with greater burden and lower quality of life among caregivers. This review investigates the experience of loneliness among dementia caregivers with a focus on the setting in which the caregiving occurred. The dementia caregiving role does not end when the person with dementia (PWD) transitions to a long-term care (LTC) community. Instead, the caregiver's responsibilities often just shift. Presently, the dementia caregiving experience for caregivers of PWD in LTC is not well studied. Future research is needed to address the dementia caregiver experience of loneliness in both the home/community and LTC settings. Methods: We conducted a literature review across the PubMed, Embase, and CINAHL databases. Results: Caregivers of PWD in the home/community setting experienced loneliness differently than caregivers of PWD in LTC. Some caregivers in the home/ community setting reflected a sense of duty or a cultural norm to provide care that could contribute to loneliness and prolonged caregiving led to increased loneliness. Caregivers whose PWD lived in LTC demonstrated a continuation of the caregiving role and loneliness due to the absence of the PWD. Conclusion: Identifying the differing risk factors, antecedents, and implications associated with the experience of loneliness by dementia care setting is essential to undertaking a comprehensive approach towards improving the caregiving experience for informal dementia caregivers.

Abstract citation ID: igaf122.3611

### INTERFERON-Γ RELEASE ASSAY CONTROL RESULTS ARE ASSOCIATED WITH ALL-CAUSE MORTALITY

Benjamin Seligman[1], David Ganz[2], Matthew Goetz[2], and David Beenhouwer[3], *1. UCLA School of Medicine, Los Angeles, California, United States, 2. VA Greater Los Angeles Healthcare System, Los Angeles, California, United States, 3. University of California, Los Angeles, Los Angeles, California, United States*

Impaired immune responses are a key feature of aging. However, there are no clinical tests of immune function. Interferon-gamma release assays (IGRAs) for tuberculosis screening may provide such a measure. We assess this measure's relationship with all-cause mortality. IGRAs quantify release of interferon-gamma by T-cells and the difference between unstimulated and mitogen-stimulated T-cells is assessed for test validity. This mitogen-nil difference captures T-cell function. We obtained all negative and indeterminate IGRA tests from a large health system along with demographics, frailty, and area deprivation index (ADI) data. We limited our sample to the most recent test given to outpatients not receiving hemodialysis or immunosuppressive medications. We assessed the association of the mitogen-nil difference with mortality at 6 months, 1 year, and 5 years by Cox regression. Among 16,105 unique individuals, reported mitogen-nil results ranged from < 0.01 to > 10 IU/mL. Relative to values from 0-1, values >10 had HRs (95%) for mortality of 0.33 (0.18 - 0.63), 0.41 (0.26 - 0.65), and 0.52 (0.39 - 0.70) at 6 months, 1 year, and 5 years respectively after adjustment for age, sex, and test result (indeterminate versus negative). Including frailty, HRs were 0.36 (0.19 - 0.68), 0.45 (0.29 - 0.70), and 0.57 (0.42 - 0.77). With frailty and ADI, HRs were 0.41 (0.20 - 0.83), 0.50 (0.30 - 0.84), 0.60 (0.43 - 0.83). Greater T-cell response to mitogen stimulation in IGRAs is associated with lower hazard of mortality. This common, commercially available lab test may provide additional information to risk-stratify patients.

Abstract citation ID: igaf122.3612

### INTERGENERATIONAL WISDOM EXCHANGE PROGRAM (WISE): REDUCING SOCIAL ISOLATION FOR BLACK AND HISPANIC OLDER ADULTS

Athena Chan[1], and Rodlescia Sneed[2], *1. Texas Tech University, Lubbock, Texas, United States, 2. Wayne State University, Detroit, Michigan, United States*

Black and Hispanic older adults face disproportionately high rates of social isolation and loneliness, yet culturally tailored interventions remain scarce. The Intergenerational Wisdom Exchange Program (WisE) addresses this gap through a community-engaged initiative that pairs college students with socially isolated Black and Hispanic older adults in an 8-week program focused on reciprocal learning. College students share digital literacy skills, while older adults share their life stories, creating opportunities for meaningful intergenerational exchange. A Community Advisory Board of Black and Hispanic older adults and college students guides the design, refinement, and cultural tailoring of WisE. This collaborative process informs program structure, builds trust, and addresses barriers to participation, ensuring reminiscence and digital literacy remain meaningful and empowering. In this presentation, we describe how community engagement shapes WisE's development, highlighting lessons learned from iterative feedback and co-design with community members. We discuss how the advisory board influences recruitment strategies, session activities, and approaches to fostering intergenerational connection. Through this process, we demonstrate that combining reminiscence with digital literacy supports meaningful intergenerational relationships, reduces social isolation, and promotes psychosocial well-being. WisE illustrates how community-guided design strengthens intervention relevance and sustainability. By centering the voices of Black and Hispanic older adults and students, we create a sustainable model that can be adapted and replicated to address social isolation and loneliness in diverse communities.

Abstract citation ID: igaf122.3613

### INTRODUCING MEDICAL STUDENTS WITH SOCIAL ASPECTS OF OLD AGE

Yehudit Reuveni, *The university of Haifa, Haifa, Hefa, Israel*

During the second year of medical school at the "Ruth and Bruce Rappaport Faculty of Medicine" in the Technion, students participate in workshops that also introduce social aspects of old age. Workshops are conducted and held by a teamwork and leadership expert and usually take place in an assisted-living facility, also can be framed as "protecting housing", in which independent individuals choose to live during their elderhood. Within the workshop, students get to meet the residents, have conversations with them, and then, in small groups, act together as committees that has to propose recommendations to improve health services for the elderly. During this activity, students are exposed to strengths and practical wisdom gained over the years, and are witnessing the bright side of elderhood, through which they get a real opportunity to reframe aging, to reframe and withdraw agist attitudes, and build a more age-friendly professional identity.

Abstract citation ID: igaf122.3614

### INVESTIGATING HOW PHYSICAL ACTIVITY INTENSITY RELATES TO RESTLESS SLEEP: A CASE STUDY USING A SMART RING

Kangeun Lee[1], Jungjoo Lee[2], and Junhyoung Kim[3], *1. Indiana University, College Station, Texas, United States, 2. Texas A&M University, Texas A&M University, Texas, United States, 3. Texas A&M University, College Station, Texas, United States*

Background: Older adults with mild cognitive impairment (MCI) often experience restless sleep. Physical activity is a coping strategy to reduce such disturbances, but self-reported data are limited by recall bias and cognitive difficulties. Aim: This study examined the longitudinal relationship between restless sleep and physical activity intensity in older adults with MCI using continuous monitoring with a smart ring system. Method: Seven older adults with MCI were monitored using Oura smart rings for 14 days. The rings, equipped with photoplethysmography, accelerometer, and gyroscope sensors, tracked changes in movement and heart rate to identify sleep stages and periods of restlessness. Physical activity intensity was computed from heart rate variability and skin temperature, and classified as light, moderate, or vigorous using the Metabolic Equivalent of Task. Generalized estimating equations were applied to account for the small sample size. Results: Vigorous activity was significantly associated with reduced restlessness, with each additional second in activity leading to a 0.18-second decrease in restlessness (B = -0.18, SD = 0.05, p < .01, 95% CI [-0.29, -0.07]). Moderate activity showed a small, non-significant positive association (B = 0.01, SD = 0.01, 95% CI [-0.01, 0.03]). Light activity showed a significant but slight reduction in restlessness with more time in light activity (B = -0.01, SD = 0.01, p < .01, 95% CI [-0.01, -0.01]). Conclusion: Vigorous and light physical activity were significantly linked to reduced restlessness during sleep, whereas moderate activity showed no meaningful effect. Further studies should examine potential confounding factors affecting moderate activity outcomes.

Abstract citation ID: igaf122.3615

### KEEPING OUR EYES ON OLDER ADULTS: TRAINING CHWS IN GERIATRIC CARE

Kevin Espinoza[1], Joycelyn Lawrence[2], Cherell Cottrell-Daniels[3], Katherine Chung-Bridges[3], Diego Shmuels[4], Deborah Gracia[4], and Sweta Tewary[5], *1. Health Choice Network, Inc., Miami, Florida, United States, 2. Jessie Trice Community Health System, Miami, Florida, United States, 3. Health Choice Network, Miami, Florida, United States, 4. Borinquen Healthcare Center, Inc., Miami-Dade, Florida, United States, 5. Jessie Trice Community Health Center, Miami, Florida, United States*

Background: The growing aging population in the U.S. highlights the urgent need for specialized geriatric care and trained professionals. Community Health Workers (CHWs) play a vital frontline role in supporting older adults, yet many lack formal geriatric training. The Geriatrics Workforce Enhancement Program (GWEP), in partnership with a local Area Health Education Center (AHEC), implemented a training based on the 4Ms framework—What Matters, Medication, Mentation, and Mobility—to address this gap and equip CHWs with evidence-based, patient-centered approaches. Methods: Twelve Community Health Workers (CHWs) from Jessie Trice Community Health System and Borinquen Medical Centers participated in a 30-hour geriatric-focused training in October 2024, focused on social determinants of health (SDOH) among older adults (aged ≥65 years). Pre- and post-exams assessed knowledge. Additionally, focus groups explored CHWs' experiences, training relevance, and future needs. Eleven CHWs participated in two separate virtual focus groups, which were audio-recorded and transcribed. Representative quotations will be included in the presentation. Results: Participants showed statistically significant knowledge gains, as measured by a Wilcoxon signed-rank test (z = 3.91, p=.003). Focus group findings revealed seven key themes: (1) enhanced knowledge and confidence; (2) practical skill application to address complex needs like housing and behavioral health; (3) interdisciplinary-CHWs and Health Navigator collaboration; (4) supportive onboarding; (5) electronic health records (EHR) training; (6) regular refresher sessions; and (7) gaps in community resource accuracy. Conclusion: Structured, evidence-informed training significantly enhances CHW capacity in geriatric care and addressing SDOH. Sustained investment in CHW training is essential to build an age-friendly workforce.

Abstract citation ID: igaf122.3616

### LACK OF AWARENESS OF SEPSIS AMONG SEPSIS SURVIVORS IN ACUTE CARE

Katherine Pitcher, Kathryn Bowles, and Michael Stawnychy, *University of Pennsylvania School of Nursing, Philadelphia, Pennsylvania, United States*

Sepsis is a life-threatening disease with high 30-day hospital readmission rates (17% to 35%). Sepsis disproportionately affects older adults (≥60 years), who often experience new functional limitations and cognitive impairment after sepsis. Timely surveillance post-discharge has been shown to reduce readmissions in this population. Prevailing nursing theories—Meleis's Transitions Theory and Riegel's Self-Care Theory—emphasize the importance of patient awareness and reflection to support effective transitions and self-care. However, patients may not be aware of the sepsis diagnosis. Sepsis survivors who were offered home health care (HHC) were surveyed for a study examining illness representation and decision-making around HHC and outpatient follow-up. Quantitative data collection was guided by theories of illness representation, dyadic illness management, and social determinants of health. Among 179 sepsis survivors (mean age 61.8±15.6 years, 48.6% female, 55.9% White), 41.3% of sepsis survivors were unaware that they had been diagnosed with sepsis. Sepsis survivors who were unaware of their diagnosis were more likely to be male and Black, and more likely to have required intensive care, than those who were aware of their diagnosis. Unaware patients also received timely HHC at lower rates and were readmitted within 30 days at higher rates than those who knew their diagnosis. These findings reveal sepsis survivors' lack of awareness of their diagnosis as a critical barrier to post-discharge engagement and challenge assumptions embedded in transitional care models. A high rate of poor diagnostic communication highlights an urgent need for enhanced sepsis communication strategies and targeted education for patients.

Abstract citation ID: igaf122.3617

### LATE-LIFE CHANGES IN HYPOTHALAMIC-PITUITARY-TESTOSTERONE AXIS, HEALTH, AND SURVIVAL IN MEN WITH LONGEVITY

Noah Gold[1], Mary Biggs[2], Michelle Odden[3], Diana He[4], Anne Newman[5], Alvin Matsumoto[6], Sofiya Milman[1], and Sandra Aleksic[1], *1. Albert Einstein College of Medicine, Bronx, New York, United States, 2. University of Washington, Seattle, Washington, United States, 3. Stanford School of Medicine, Palo Alto, California, United States, 4. University of Pennsylvania, Philadelphia, Pennsylvania, United States, 5. University of Pittsburgh, Pittsburgh, Pennsylvania, United States, 6. University of Washington School of Medicine, Seattle, Washington, United States*

While lower testosterone levels in middle-aged men have been associated with poorer metabolic health and mortality, similar associations were not demonstrated in our previous work in nonagenarians and centenarians. As more men reach longevity, it is important to characterize late-life trajectory and etiology of testosterone decline, and their relationship with health and survival. Among 134 men in the Cardiovascular Health Study who had reached 85+ years of age (mean 88±3 years) by the 2005-6 visit, we measured total testosterone (TT; mass spectrometry), luteinizing hormone (LH) and sex hormone-binding globulin (SHBG) (immunoassay) in serum from 2005-6 and another visit 9-13 years earlier. Free testosterone (FT) was calculated. Mean age at death was 93±4 years. Between two visits, TT did not change (463±187 vs. 437±219 ng/dL, $p = 0.17$). However, FT decreased, while LH and SHBG increased (75±26 vs. 62±28 pg/mL, 6.6±6.3 vs. 11.2±10.2 mIU/mL, 48±20 vs. 57±23 nmol/L, respectively; $p < 0.001$), remaining significant after adjusting for baseline age, body mass index (BMI), comorbidities, education, and lifestyle. Men transitioned from biochemical eugonadism (normal FT, LH; 81 vs. 53%) to: compensated (normal FT, high LH; 8 vs. 18%); and overt testicular dysfunction (low FT, high LH; 4 vs. 20%); and rarely hypothalamic-pituitary dysfunction (low FT, non-elevated LH; 6 vs. 8%). At the final visit, TT, FT, and LH were not associated with BMI, diabetes, or glucose levels and did not predict survival. Men with longevity commonly developed biochemical testicular and rarely hypothalamic-pituitary dysfunction with late-life FT decline, which was not associated with metabolic profile or survival.

Abstract citation ID: igaf122.3618

### LESBIAN, GAY, & BISEXUAL HEALTH INEQUITIES AND FINANCIAL BURDEN: HEALTH & RETIREMENT STUDY INSIGHTS

Jennifer May[1], Hanna Smyles[2], Swann Adams[1], and Matthew Lohman[1], *1. University of South Carolina, Columbia, South Carolina, United States, 2. University of South Carolina- College of Nursing, Columbia, South Carolina, United States*

Older lesbian, gay, and bisexual (LGB) adults often face distinct health and social challenges compared to their heterosexual counterparts. This study leverages the nationally representative Health and Retirement Study (HRS) to examine health inequities and financial burden among LGB older adults compared to their non-LGB counterparts in the United States. Using the harmonized RAND dataset from waves 2016, 2018, and 2020, we compared self-identified LGB and non-LGB adults aged 51 and older, including respondents under 50 living in age-eligible households. Weighted descriptive statistics were calculated for prescription drug use, self-rated health, chronic conditions, and hospital nights, with differences by LGB status assessed using chi-square tests. Across all waves, prescription drug use, chronic conditions, and hospital nights did not significantly differ by LGB status. However, consistent and statistically significant differences in self-rated health were observed. LGB adults were more likely to report fair or poor health than non-LGB peers (e.g., in 2020, fair; 27.9% LGB vs 20.2% non-LGB; poor: 6.0% LGB vs 4.6% non-LGB; $p < 0.0001$), with fewer LGB respondents reporting very good or excellent health. These gaps in subjective health status persisted over time despite similar patterns of healthcare utilization and chronic disease burden. Findings highlight the need for clinicians and policymakers to be aware of and address the distinct challenges of older sexual minorities through targeted health promotion, culturally competent care, and inclusive policy initiatives.

Abstract citation ID: igaf122.3619

### LIFE STRESS AND DEMENTIA RISK IN ADULTS WITH DOWN SYNDROME

Alexandra Linker[1], Sigan Hartley[2], Shahid Zaman[3], and Luciana Fonseca[4], *1. Washington State University, Spokane, Washington, United States, 2. University of Wisconsin - Madison, Madison, Wisconsin, United States, 3. University of Cambridge, Cambridge, England, United Kingdom, 4. Krieger Klein Alzheimer's Research Center and the Rutgers Brain Health Institute, Piscataway, New Jersey, United States*

Down syndrome (DS) is the most common genetic cause of intellectual disability, resulting from a triplication of chromosome 21. Individuals with DS are at a higher risk of developing Alzheimer's Disease (AD) compared to the general population. Previously, exposure to stress has been suggested as a risk factor for dementia and AD in the general population. However, the role of life stress in the timing of AD in DS is underinvestigated. Using the Alzheimer Biomarkers Consortium - Down Syndrome (ABC-DS) dataset, we analyzed the association between baseline life stressors, and later cognitive decline and dementia at 16 or 32-month follow-up. We found that a higher baseline sum of stress events was associated with greater risk of conversion from baseline stable cognition to mild cognitive impairment (MCI) or dementia at follow up, even when controlling for age, sex, premorbid level of intellectual disability, site, clinical latency between assessments, and the presence of anxiety or depression (odds ratio = 1.53, $p = 0.021$). Additionally, the death of a loved one at baseline, when rated as having a negative or complex impact, was associated with a decline of more than 6 points on the Block Design test ($p = 0.009$), compared to those without this experience. These results suggest that life stressors may predict cognitive decline and dementia in adults with DS, highlighting a potential target for treatment or intervention. Results also suggest that there may be similar risk factors for AD in adults with DS as those found in the general population.

Abstract citation ID: igaf122.3620

### LINKING FUNCTIONAL LIMITATIONS AND RELATIONSHIP SATISFACTION: THE ROLE OF HELPING AND INTERNET USE

Debarati Kole, and Heather Fuller, *North Dakota State University, Fargo, North Dakota, United States*

As people age, maintaining satisfying relationships is important for overall well-being; however, functional limitations can challenge relationship quality in part due to increased need for caregiving-related help and decreased ability to reciprocate that help. In today's digital age, internet use may help older adults to stay engaged in giving and receiving help despite any functional limitations. The present study sought to understand: 1) Does the frequency of receiving and giving help to friends, family, and neighbors mediate the relationship between functional limitation and relationship satisfaction?, and 2) Does internet use play a moderating role? The data is drawn from the Social Integration and Aging Study (2013), a community-based survey of older adults (60+) in the Upper Midwest ($N = 416$). Bootstrap analyses were conducted with PROCESS macro models 4 and 58. Frequency of receiving help did not mediate the association between functional limitations and relationship satisfaction. However, frequency of giving help meditated the association between functional limitations and relationship satisfaction ($a = -0.05^{***}$, $b = 0.36^{***}$, $c = -0.05^{***}$, $c' = 0.03^{**}$). Furthermore, moderated mediation was evident as frequency of internet use moderated the effect of helping others, indicating that those with greater functional limitations provide more help if they are internet users. Findings highlight the role of giving help to others for older adults to maintain satisfying relationships. The study also indicates that internet use can create opportunities for older adults to offer support. Future research should investigate these effects longitudinally and explore interventions to enhance giving and receiving support among older adults by improving their digital skills.

Abstract citation ID: igaf122.3621
**MACHINE LEARNING PREDICTION FOR FUNCTIONAL IMPAIRMENT, FALLS, AND FRACTURES IN POSTMENOPAUSAL WOMEN**
Junya Uchida, *University of Toronto, Toronto, Ontario, Canada*
Machine learning-based prediction models have gained support for their robustness and flexibility, particularly with complex data common in geriatrics research. They may be well-suited to studying complex aging questions, such as predicting functional status among postmenopausal women. The objective of this study is to develop an interactive prediction model for prevalent, clinically relevant, geriatric health outcomes: functional impairment, falls, and fractures. We simulated a dataset (n = 20,000) to emulate baseline characteristics of the Women's Health Initiative (WHI) study population, a large study of postmenopausal women. The simulated dataset had a mean age of 63.2 years (SD: 7.25), 82.4% non-Hispanic White. Maximum follow-up was 25 years. We examined random survival forest, XGBoost, and Lasso-Cox models. Models were trained and compared across multiple domain-specific feature sets comprising demographic, socioeconomic, behavioral, functional, and health-related features, selected from existing evidence, with time-dependent ROC-AUC, calibration, and Brier score. SHapley Additive exPlanations (SHAP) guided feature selection. The random survival forest outperformed other models in predicting functional impairment, falls, and fractures. Reduced models incorporating 30 features demonstrated comparable performance relative to the comprehensive models with 57 features, while improving computational efficiency. These reduced models demonstrated strong discrimination (ROC-AUCs >0.80) and predictive accuracy (Brier scores < 0.10) across most time points and were subsequently deployed in the interactive application. This study demonstrates the utility of machine learning-based prediction to guide targeted interventions by capturing the complex, heterogeneous nature of geriatric health outcomes. Deploying such advanced models into a user-friendly application may support data-driven decision-making in geriatric clinical practice.

Abstract citation ID: igaf122.3622
**MEDICAID REIMBURSEMENT RATES AND THE QUALITY OF MENTAL HEALTH CARE IN U.S. NURSING HOMES**
Adrita Barooah, and Pamela Nadash, *University of Massachusetts Boston, Boston, Massachusetts, United States*
Quality concerns about U.S. nursing homes are long-standing and have been highlighted by the COVID-19 pandemic. However, mental health quality in these settings remains understudied. Although state Medicaid reimbursement rates have been associated with overall nursing home quality, it is not known whether they affect mental health-specific outcomes. Understanding this relationship is important given facilities' reliance on Medicaid funding and the high prevalence of psychiatric disorders among residents. Using 2019 CASPER data merged with Care Compare, Long-Term Care Focus, and the Area Health Resource File, we conducted a multilevel analysis of nursing homes nested within states to assess the association between higher Medicaid payment rates and the odds of receiving mental-health-related deficiency citations, adjusting for facility- and market-level characteristics. Results indicate that higher Medicaid reimbursement rates (Estimate = 0.0331, p = 0.0344) were significantly associated with lower odds of mental-health-related citations. Additionally, a higher proportion of Medicaid residents (p = 0.0232) and larger facility size (p = 0.0037) were associated with increased odds. Moreover, higher occupancy rates (p = 0.0053) and total staffing hours per resident day (p < 0.0001) were linked to reduced odds of deficiency citations. Study findings suggest that Medicaid payment policy, which has been associated with overall nursing home quality in prior research, may also play a crucial role in shaping mental health-related nursing home quality. The study emphasizes the importance of reexamining Medicaid reimbursement rates, given their broader implications for resident well-being, particularly for individuals with mental illness living in nursing homes.

Abstract citation ID: igaf122.3623
**MEDICARE SAVINGS PROGRAMS, PART D LOW-INCOME SUBSIDY, AND CHARACTERISTICS OF MEDICARE BENEFICIARIES**
Nicha Thiamwong[1], and Boon Peng Ng[2], *1. University of Central Florida, Orlando, Florida, United States, 2. University of Central Florida, Orladno, Florida, United States*
Medicare Savings Programs and Part D Low-Income Subsidy program assist low-income beneficiaries with healthcare and prescription costs. However, information about eligible Medicare beneficiaries' characteristics is limited. This study aimed to examine the socio-demographic characteristics of beneficiaries in these programs. The 2022 Medicare Current Beneficiary Survey Public Use File, a nationally representative data, of Medicare beneficiaries aged ≥65 years (n = 10,186), was used. A three-level categorical dependent variable was created: (1) has Medicare Savings Program and Part D Low-Income Subsidy, (2) has either program, and (3) has neither (reference group). A survey-weighted multinomial regression model was used to examine relationships between socio-demographics and the dependent variable, adjusted for health conditions. Of the study beneficiaries, 12.0% had both programs, 2.1% had either, and 85.9% had neither. About 25% of those with incomes < =100% of the Federal Poverty Level (FPL) did not enroll. Non-Hispanic Blacks (NHB), Hispanics, and Other were more likely than non-Hispanic Whites to have both programs (e.g., for NHB, OR = 2.74, 95% CI [2.01-3.64]). Beneficiaries with less than a high school education were more likely to have both (OR = 2.14, [1.61-2.84]) or have either program (OR = 2.37, [1.33-4.24]) than those with higher education. Beneficiaries with income >200% of the FPL were less likely to have both (OR = 0.01, [0.01-0.02]) or either program (OR = 0.07, [0.04-0.13]) than those < =100% of the FPL. Both programs aided ~15% (representing 7.5 million) of beneficiaries, mainly from lower socio-demographic groups. However, about 1 million beneficiaries had either program, and some eligible individuals were unenrolled, emphasizing policy reforms to improve enrollment processes.

Abstract citation ID: igaf122.3624

**MINDFULNESS TRAINING IMPROVES ATTENTION IN VETERANS WITH MCI AND MILD DEMENTIA: A PILOT STUDY**

Grace Desmond[1], Heera Kamaraj[2], Yasemin Yilmaz[2], Brenna Hagan[2], Kathy Xie[2], and Katherine Turk[2], *1. University of Massachusetts Chan Medical School, Worcester, Massachusetts, United States, 2. VA Boston Healthcare System, Boston, Massachusetts, United States*

Mild cognitive impairment (MCI) due to Alzheimer's disease (AD) is often marked by early and progressive deficits in attention. Mindfulness—defined as focused, nonjudgmental awareness of the present moment—has been shown to improve attention and induce neuroplastic changes in healthy older adults. This pilot study examined whether a 10-week mindfulness intervention could improve attention in individuals with MCI. Participants were recruited from the VA Boston Memory Disorders Clinic and pseudo-randomly assigned to a virtual mindfulness-based stress reduction (MBSR) class ($n = 9$, mean age = 78.4 ± 2.06) or a waitlist control group ($n = 11$, mean age = 78.4 ± 4.76). The MBSR group received weekly group instruction, engaged in focused attention and open monitoring practices, and completed daily meditation via the Happier app. Attention was assessed at baseline and post-intervention using the Digit Span and Symbol Coding subtests from the Repeatable Battery for the Assessment of Neuropsychological Status (RBANS), and the AX-Continuous Performance Task (AX-CPT). Compared to the control group, MBSR participants showed significantly greater improvement in Digit Span ($F(1,18) = 10.71$, $p = 0.004$, $\eta^2 = 0.37$) and AX-CPT D' scores ($F(1,18) = 6.73$, $p = 0.018$, $\eta^2 = 0.27$), suggesting enhanced attention processes. While group differences in Symbol Coding and CPT reaction time and accuracy were not statistically significant, this study provides preliminary evidence that mindfulness may improve attentional functioning in Veterans with MCI. Ongoing work includes examining event-related potential (ERP) components (e.g., N200, P300) to clarify underlying mechanisms of attention change.

Abstract citation ID: igaf122.3625

**MOMENTARY ENVIRONMENTAL CONTEXT AND AGITATION IN PERSONS WITH DEMENTIA AT HOME SETTINGS: A TIME-SERIES ANALYSIS**

Chunhong Xiao[1], Azziza O. Bankole[2], Rita Jablonski[3], Frank Puga[4], Arie Nakhmani[3], Roberto Fernandez-Romero[5], Tami Wyatt[5], and Robert Davis[5], *1. The University of Tennessee at Knoxville, Knoxville, Tennessee, United States, 2. Virginia Tech, Virginia Tech /Carilion School of Medicine, Virginia, United States, 3. UAB, Birmingham/University of Alabama at Birmingham, Alabama, United States, 4. University of Alabama at Birmingham, Birmingham, Alabama, United States, 5. The University of Tennessee, Knoxville/ University of Tennessee, Tennessee, United States*

Dementia-related agitation often occurs in unpredictable episodes throughout the day, contributing to increased caregiver stress and early nursing home placement. There is a lack of knowledge about the dynamics of momentary agitation influenced by home environment triggers. We conducted a secondary time-series analysis of the Behavioral and Environmental Sensing and Intervention (BESI) dataset to examine how home environmental conditions relate to agitation episodes reported by caregivers of PWD at home. The time-series analysis used data from 13 BESI deployments (Phase II: eight 30-day, Phase III: five 60-day). Environmental features, including light level, temperature, humidity, air pressure, and noise/audio features, were collected via room-level sensors. Agitation was captured using wrist-worn accelerometers. Caregivers provided timestamped agitation reports and contextual surveys. Data were harmonized across modalities using UNIX (a standardized digital timestamp format) and relative time formats. Agitation windows (±5 minutes) were labeled, and environmental features were normalized. Generalized linear mixed models were used to estimate agitation likelihood based on environmental fluctuations, controlling household-level clustering. Preliminary modeling revealed that elevated noise levels and abrupt light changes significantly increased agitation odds ($p < 0.05$), especially bedroom and kitchen. Caregiver-targeted interventions in Phase III deployments demonstrated reduced agitation sensitivity to environmental volatility. This suggests that real-time contextual feedback enhanced caregiver responsiveness and behavior management. Momentary environmental context plays a critical role in dementia-related agitation. Time-series data offer a novel lens for understanding agitation triggers in dementia care. Findings support the development of context-aware, just-in-time interventions to empower caregivers and improve dementia care in home settings.

Abstract citation ID: igaf122.3626

**MULTILEVEL PREDICTORS OF DEMENTIA PROGRESSION IN MILD COGNITIVE IMPAIRMENT: A NATIONWIDE CASE-CONTROL STUDY**

Song Hee Hong, and Hayun Lim, *Seoul National University, Seoul, Republic of Korea*

Introduction: Mild cognitive impairment (MCI) represents an early stage of cognitive decline with a high risk of progression to dementia. Since no effective drug treatments exist for dementia, identifying modifiable risk factors is critical. This study examined individual- and regional-level factors associated with dementia diagnosis among patients with MCI. Methods: A nested case-control study was conducted using nationwide health insurance claims data. The cohort included patients first diagnosed with MCI in 2017. Cases were individuals newly diagnosed with dementia in 2022 ($n = 1,344$), while controls were MCI patients without dementia ($n = 5,376$), matched 1:4 by age and sex. Individual-level variables included demographics, comorbidities, lifestyle, and medication use. Regional-level variables encompassed demographic aging indices, healthcare resources, and elderly welfare facilities. Conditional multivariate logistic regression was applied to individual-level factors, and multilevel logistic regression assessed regional-level influences. Results: Significant individual-level predictors of dementia included type 2 diabetes (OR = 1.14, 95%CI=1.00–1.31), depression (OR = 1.64, 95%CI=1.43–1.88), Parkinson's disease (OR = 2.49, 95%CI=1.79–3.47), and hospitalization days (OR = 1.03, 95%CI=1.02–1.05). Protective factors included dyslipidemia (OR = 0.79, 95%CI=0.69–0.90), moderate physical activity (OR = 0.76, 95%CI=0.66–0.87), and adherence to dementia medications (OR = 0.71, 95%CI=0.58–0.87). At the regional level, a higher proportion of older adults increased dementia risk (OR = 1.01, 95%CI=1.00–1.02), while greater availability of elderly residential welfare facilities was protective (OR = 0.96, 95%CI=0.92–0.99). Conclusion: Both individual and regional factors significantly influence dementia risk among MCI patients.

These findings underscore the need for integrated prevention strategies that combine clinical risk management with community-level resource planning to delay dementia onset and reduce its societal burden.

Abstract citation ID: igaf122.3627

### MULTIPARAMETER PROFILING DISTINGUISHES FUNCTIONAL SUBSETS OF PLATELETS IN AGING

Ruth Montgomery[1], Adam Tanguay[1], Subhasis Mohanty[1], Ashish Shelar[1], Mathew Rondina[2], Hailong Meng[1], Steven H. Kleinstein[1], and Albert Shaw[1], *1. Yale School of Medicine, New Haven, Connecticut, United States, 2. Univ. Utah, Salt Lake City, Utah, United States*

Recent studies have revealed important signaling roles in immunity for platelets, which are highly abundant in the blood and largely appreciated for their role in coagulation. Healthy older (age 65-80) and younger (age 21-35) volunteers were enrolled for studies of immune aging, and blood was collected in ACD tubes. Platelet-rich plasma was labeled fresh for profiling by mass cytometry (CyTOF) with metal-conjugated antibodies to surface markers and frozen. Randomized batches of samples were thawed, fixed, and permeabilized for labeling of intracellular markers prior to CyTOF acquisition. FCS files were bead-normalized and manually-gated platelet populations (CD45-, CD41+, CD61+) were analyzed using unsupervised FlowSOM clustering to define clusters based on surface markers. CD107b, with a role in adhesion to endothelium, characterizes distinctions between older and younger participants. The frequency of an activated cluster with low expression of CD107b was higher in platelets from older participants (10.6% vs 6.8%, $p<0.0001$) and, conversely, an activated cluster with higher expression of CD107b had a lower frequency in older participants (2.9% vs 5.4%, $P<5$ x10-7). Platelets from older participants also showed higher expression of CD62p, with a critical role in platelet aggregation, in both activated and resting clusters. Platelets from young participants broadly expressed higher IL-10. Notably, flow cytometry validated elevated expression of CD62p and CD63 in older adults at baseline. However, TLR-induced activation of platelets revealed blunted CD62p and CD63 responses in platelets of older individuals. Differential expression of markers and response to stimulation in platelets may contribute to aberrant immune responses during aging.

Abstract citation ID: igaf122.3628

### NEED SATISFACTION INDIRECTLY ASSOCIATED WITH LOWER CAREGIVER BURDEN & CYTOKINE PRODUCTION IN DEMENTIA CAREGIVERS

Kate Bishop[1], Jensine Paoletti-Hatcher[1], Daniel L. Argueta[2], Kelly N. Brice[2], Bryan T. Denny[2], Charles Green[3], Samantha Henry[4], and Christopher P. Fagundes[2], *1. Portland State University, Portland, Oregon, United States, 2. Rice University, Houston, Texas, United States, 3. UTHealth, Houston, Texas, United States, 4. Baylor College of Medicine, Houston, Texas, United States*

Spousal dementia caregiving is a chronic stressor linked to systemic inflammation and poor health. Self-determination theory (SDT) may provide a useful framework for identifying which caregivers are at the greatest risk. SDT posits that psychological needs satisfaction (PNS) may increase the relative autonomy caregivers perceive within their relationship, contributing to increased well-being overall. We tested a causal association hypothesized in SDT: higher PNS should be indirectly associated with greater well-being through more autonomous motivation. We examined the indirect effect of PNS on self-reported caregiver burden and cytokine production through autonomous motivation for caregiving in cross-sectional analyses of 108 caregivers ($M=72.18$, $SD=7.69$; 72.22% women; 53.70% advanced degree holders). Participants reported demographics and health information, caregiver burden (ZBI), autonomous motivation for caregiving (modified Self-Regulation Questionnaire; $\alpha = .84$), and relationship-oriented PNS (modified Needs Satisfaction Scale; $\alpha = .86$). LPS-stimulated cytokines (IL-6, IL-10, TNF-$\alpha$, and IL-1$\beta$) from whole blood were combined in a linear composite ($\alpha = .85$) before we ran mediation analyses in lavaan. We found that higher PNS was associated with lower cytokine production through higher relative autonomy (indirect effect = -0.15, 95% CI [-0.28, -0.013]). We also found that higher PNS was associated with lower caregiver burden through higher relative autonomy (indirect effect = -0.11, 95% CI [-0.21, -0.023]). Dementia is degenerative and requires caregivers to adapt regularly to meet changing demands; longitudinal or time-lagged research is needed to capture the role of PNS as a possible causal factor in caregiver resilience.

Abstract citation ID: igaf122.3629

### ONLINE TRAINING ON WORKPLACE EDUCATION IN END-OF-LIFE CARE FOR HOME CARE EDUCATORS: A RANDOMIZED CONTROLLED TRIAL

Ryousuke Yamada[1], Hanako Numata[2], Kosuke Kashiwabara[3], Maiko Noguchi-Watanabe[4], Yuka Sumikawa[5], Kayoko Kurita[1], and Noriko Yamamoto-Mitani[4], *1. University of Tokyo, Bunkyo, Tokyo, Japan, 2. University of Tokyo, University of Tokyo, Tokyo, Japan, 3. University of Tokyo Hospital, University of Tokyo Hospital, Tokyo, Japan, 4. The University of Tokyo, Bunkyo, Tokyo, Japan, 5. University of Tokyo, Bunkyo-City, Tokyo, Japan*

No well-established and effective evidence-based training programs exist for home-care nursing educators to provide workplace education in end-of-life (EOL) home care. We developed and evaluated an online training program for educators to improve their on-the-job training (OJT) in EOL home care. We conducted a randomized controlled trial involving 108 educators, assigning them to either an intervention or a waitlist control group. The program included a lecture, two case-study sessions, and a follow-up message. The primary outcome was the change in general EOL educators' attitudes, as measured by the End-of-Life Nursing Education Questionnaire, while the secondary outcomes included changes in OJT-specific knowledge, attitude, and educational practices. We analyzed the data using analysis of covariance, adjusting for the baseline. The intervention group demonstrated significantly higher post-intervention scores in confidence in education (a primary outcome component; $p < .001$), and in the secondary outcomes of OJT attitude ($p = .003$), and practice ($p < .001$). No significant inter-group differences were found regarding educational motivation, perceived impact on learners (primary outcome components), or OJT-related knowledge. These findings suggest that a brief, structured online program can effectively improve educators' confidence in EOL education and OJT-related attitudes, as well as their self-reported practice of OJT for EOL home care. This program offers a scalable solution for improving staff training quality in home-care nursing settings. This study was approved by the Research Ethics Committee of the Graduate School of Medicine, The University of Tokyo (Approval No. 2024359NI-[2]) and registered with the University Hospital Medical Information Network (UMIN000056165).

Abstract citation ID: igaf122.3630

### OPTIMISM AND THE RISK OF DEVELOPING DEMENTIA

Säde Stenlund[1], Hayami Koga[2], Peter James[3], Justin Farmer[1], Colleen McGrath[1], Francine Grodstein[4], and Laura Kubzansky[1], *1. Harvard T. H. Chan School of Public Health, Boston, Massachusetts, United States, 2. Harvard T.H. Chan School of Public Health, Boston, Massachusetts, United States, 3. University of California, Davis, California, United States, 4. Rush University Medical Center, Chicago, Illinois, United States*

Previous studies suggest that higher optimism is associated with better cognitive function and slower cognitive decline in aging. Using data from the Health and Retirement Study, a nationally representative sample of older U.S. adults, we examined whether optimism was associated with lower risk of developing dementia in different population groups and if it was maintained after accounting for initial health status and other potential confounders as well as in critical sensitivity analyses. Optimism was measured in 9,071 cognitively healthy individuals within 2 years before each person's analytic baseline using the validated Life Orientation Test-Revised and dementia onset was identified by an algorithm developed to perform well across major racial and ethnic groups over eight waves of data collection in 2006–2020. Using Cox proportional hazards models, we observed that a 1-standard deviation increase in optimism was associated with a lower hazard of developing dementia (hazard ratio = 0.82, 95% confidence interval 0.79–0.85) over follow-up ranging up to 14 years. Associations did not substantially change when adjusting for age, sex, race and ethnicity, education, depression, major health conditions, health behaviors and depression, or when we removed the first two years of follow-up to address reverse causation or excluded individuals with poorest mental health. When stratifying by race and ethnicity, we observed similar associations in the Non-Hispanic White and Black sub-populations. Findings suggest optimism may be a novel target for interventions seeking to lower the risk of dementia.

Abstract citation ID: igaf122.3631

### ORAL HEALTH ACCESS AMONG ETHNIC MINORITY OLDER ADULTS IN THE UNITED STATES

Xiaochuan Wang, and Jasleen Sodhi, *University of Central Florida, Orlando, Florida, United States*

Oral healthcare is a critical yet often overlooked component of overall health among U.S. adults. Ethnic minority older adults, in particular, are disproportionately affected by poor oral health and related health conditions (e.g., cardiovascular disease and diabetes). These populations also experience significant disparities in access to dental care and preventive services, which can exacerbate existing health inequities. The present study aimed to systematically examine and synthesize existing literature to assess the extent to which oral health disparities exist among ethnic minority older adults in the U.S. and explore the roles of socioeconomic factors on these disparities. Guided by the Preferred Reporting Items for Systematic Reviews and Meta-Analyses (PRISMA) framework, we conducted a systematic review of peer-reviewed articles published in English between 2015 and 2025. Searches were conducted in electronic databases including PubMed, ProQuest, and EBSCOhost. The initial search yielded 464 unduplicated articles. After excluding articles based on title and abstract screening, 75 articles were reviewed in full text. Of these, a total of 59 articles met the inclusion criteria and were included in the qualitative synthesis. Across these studies, three primary barriers to oral healthcare among ethnic minority older adults were identified, including: systemic inequities in oral healthcare, cultural and linguistic barriers between providers and patients, and limited education and access specific to this population. These results underscore the need to improve access to care, implement culturally responsive provider training programs, and develop targeted educational resources to address oral health disparities among this underserved population.

Abstract citation ID: igaf122.3632

### OUTCOMES FOLLOWING CATHETERIZATION FOR ST-SEGMENT ELEVATION MYOCARDIAL INFARCTION IN OLDER ADULTS WITH DEMENTIA

Michael Nanna[1], Leila Agha[2], Ashleigh Erickson[3], Bangyao Sun[2], Raiza Rossi[4], Christopher Leggett[3], Douglas Staiger[5], and Andrew Cohen[6], *1. Yale University, New Haven, Connecticut, United States, 2. Harvard Medical School, Boston, Massachusetts, United States, 3. Geisel School of Medicine, Lebanon, New Hampshire, United States, 4. Yale University, Yale University, Connecticut, United States, 5. Dartmouth College, Hanover, New Hampshire, United States, 6. Yale School of Medicine, New Haven, Connecticut, United States*

ST-segment elevation myocardial infarction (STEMI) is a common cardiac emergency involving complete occlusion of one of the coronary arteries, for which left heart catheterization is the gold standard treatment. Older adults with Alzheimer's disease and related dementias (ADRD) are less likely to receive this treatment, but little is known about the outcomes among those who do. We used a 100% Medicare fee-for-service sample from 2017-2022 to compare outcomes following STEMI among individuals with and without ADRD who underwent catheterization, stratified by pre-event care setting (community versus nursing home). The primary outcome was days alive at home in the year following STEMI; secondary outcomes included one-year mortality, proportion of alive days at home, and long-term care use. Differences between groups were adjusted for age, sex, and comorbidities. Among 117,318 patients, 6.3% had ADRD and 97.8% were admitted from the community. Compared to those without ADRD, community-dwelling patients with ADRD had higher one-year mortality rates (36.6% vs 16.6%; adjusted difference, 10.0%) and fewer home days (225 vs 299; adjusted difference, 37.4), yet over two-thirds spent more than 300 days at home and only 3.9% entered long-term nursing home care. Patients admitted from nursing homes had worse outcomes, regardless of ADRD status, with one-year mortality rates of 57.6% (ADRD) and 40.3% (without ADRD; adjusted difference, 17.3%). Our findings suggest that older adults with ADRD who undergo catheterization for STEMI, particularly if admitted from the community, have health outcomes that many may find acceptable. These results may help guide prognosis-informed, patient-centered decision-making.

Abstract citation ID: igaf122.3633

**PAINFUL LIFE EVENTS ARE ASSOCIATED WITH BRAIN AGING IN THOSE HIGH IN CATASTROPHIZING: AN OBSERVATIONAL STUDY**

Josue Cardoso, and Elizabeth Losin, *Pennsylvania State University, University Park, Pennsylvania, United States*

Chronic pain becomes more common and burdensome with age, and past work has linked brain aging with chronic pain. Nonetheless, it remains unclear whether painful life experiences and pain coping styles are related to brain age in individuals who do not yet have chronic pain. In this secondary analysis, we examined associations between pain coping, painful life experiences, and brain age in a sample of 97 healthy adults between ages 18 and 54. Brain age (brain-PAD) was estimated using a machine learning model trained on T1-weighted MRI data (brainageR). Given the overlap in variables collected for the parent study, Stochastic Search Variable Selection was used to identify important predictors of brain age, as this exploratory approach handles correlated data and model uncertainty more robustly than traditional methods. A moderated mediation model, informed by past literature, was then specified to assess relationships among variables. We found that catastrophizing significantly moderated the link between the number of major painful life events and brain aging. Specifically, as the number of major painful life events increased, individuals who tended to catastrophize more also tended to have higher fear of pain, which, in turn, was related to higher brain age. These findings suggest that maladaptive coping may impact brain aging even in individuals without chronic pain. Pain catastrophizing, particularly, is highlighted as a potential target for intervention. Lastly, the present findings may inform future mechanistic and multilevel longitudinal research aimed at further disentangling the complex relationships between brain aging and acute, subacute and chronic pain.

Abstract citation ID: igaf122.3634

**PARALLEL ASSOCIATIONS BETWEEN COGNITIVE AND NEUROPSYCHIATRIC SYMPTOM DOMAINS IN OLDER ADULTS**

Alyssa De Vito[1], Adea Rich[2], Megan Barker[1], and Zachary Kunicki[3], *1. Brown University, Providence, Rhode Island, United States, 2. Columbia University, New York, New York, United States, 3. Warren Alpert Medical of Brown University, Providence, Rhode Island, United States*

Despite the high prevalence of neuropsychiatric symptoms (NPS) in those with Alzheimer's disease and Alzheimer's disease-related dementias (AD/ADRD), longitudinal research examining the co-occurrence of NPS and cognition has been underexplored, especially during earlier stages of the disease. Our study aimed to investigate the co-occurring trajectories between specific domains of NPS and cognition in those with preclinical or prodromal AD/ADRD. Participants were 1,255 individuals from the Alzheimer's Disease Neuroimaging Initiative (ADNI)-2 and ADNI-3 cohorts who were cognitively normal (CN) or had mild cognitive impairment (MCI) at baseline. This cohort was evenly split by sex and was predominantly White and non-Hispanic. Parallel process models were used to examine how a change in cognition was associated with a change in NPS as measured by the Neuropsychiatric Inventory (NPI) over 48 months. We ran 9 models total to examine relationships between 3 cognitive domains (memory, executive function, and language) and 3 NPS domains (impulse dyscontrol, mood, and somatic disturbance). Cognitive domain and NPS slopes were negatively correlated in each model, such that as cognition decreased, NPS increased. Follow-up testing using Steiger's z-test to compare the correlations across domains suggested no significant differences in the strength of the relationship between mood or somatic disturbance with cognition across domains. However, impulse dyscontrol had a significantly stronger relationship with language than with other cognitive domains. These findings extend prior cross-sectional work that found communication-impulse dyscontrol behavior (e.g., aggression) associations to demonstrate strong co-occurrence longitudinally in those with early-stage AD/ADRD.

Abstract citation ID: igaf122.3635

**PERCEIVED BENEFITS AND IMPLEMENTATION FACTORS OF A MULTICOMPONENT EXERCISE PROGRAM IN CARE FACILITIES IN HONG KONG**

Kin Tung Chan[1], Chi Tat Fung[1], Ziwei Zeng[2], and Yijian Yang[2], *1. Hong Kong Sheng Kung Hui Multidisciplinary Outreaching Support Teams for the Elderly (Kowloon Central Cluster), Hong Kong, China (People's Republic), 2. The Chinese University of Hong Kong, Hong Kong, China (People's Republic)*

Older adults in long-term care (LTC) face accelerated functional decline and high fall risk, traditional prevention programs often overlook upper-limb and core strengthening, which is important for safe mobility. Mobility-Fit, a multicomponent exercise program, was developed to address these gaps in care facilities. This study explored perceived benefits and factors influencing program implementation through semi-structured interviews with 21 LTC residents (13 intervention, 8 control) and 4 care staffs following a cluster randomized controlled trial. Participants reported enhanced health-related quality of life, increased confidence, and enjoyment, with 76.9% describing the program as novel and beneficial. Nearly half (46.2%) highlighted meaningful outcomes such as mutual respect, personalized suitability, and enjoyment from group-based exercise. Additionally, 38.5% of participants in the intervention group reported no fear of falling, compared to 25% in the control group. Key facilitators included tailored exercise adaptations, professional supervision, supportive management by the care professional team. The use of familiar music and culturally resonant exercise incorporating simple equipment such as TheraBands and fitness balls, further enhanced engagement among participants. Challenges included poor vision, limited mobility, and fluctuating health among residents, with difficulties in maintaining engagement and adapting content for diverse functional levels. Nevertheless, staffs emphasized that adaptive strategies and collaborative problem-solving supported program feasibility. Involving family members and integrating culturally relevant elements proved effective. Mobility-Fit was perceived as safe, enjoyable, and feasible when tailored to residents' capacities and cultural context. Our findings support integrating culturally adapted exercise programs in LTC settings to promote physical and psychosocial well-being of older adults.

Abstract citation ID: igaf122.3636

### PERCEIVED COGNITIVE FUNCTION AND MOOD IN SPANISH LANGUAGE HISPANIC AND LATINO FAMILY CAREGIVERS

Sophia Salinas[1], Liliana Ramirez Gomez[1], Paulina Gutierrez-Ramirez[1], Maria Galvez[1], and Felipe Jain[2], *1. Massachusetts General Hospital, Boston, Massachusetts, United States, 2. Massachusetts General Hospital / Harvard Medical School, Boston, Massachusetts, United States*

Spanish-speaking Hispanic and Latino family caregivers of individuals with dementia experience high levels of stress, depression, and emotional strain. These challenges can also impair cognitive functioning. Limited access to culturally and linguistically appropriate support compounds difficulties in Latino communities. We examined correlates of perceived cognitive function in Spanish language caregivers and how improvements in psychological symptoms after a mindfulness intervention correlated with improvements in perceived cognitive function. Data were combined from two pilot, open-label feasibility studies of mentalizing imagery therapy (N = 17), a mindfulness and guided imagery program delivered via telehealth. At baseline and follow-up, participants completed measures of depression, stress, caregiver burden, mindfulness, social support, well-being, and neurological quality of life (Neuro-QoL). Pearson correlation coefficients were calculated for baseline data and changes over time. At baseline, lower depressive symptoms, stress, and caregiver burden were significantly associated with higher Neuro-QoL scores (depression: r=–.447, p=.022; stress: r=-.396, p=.041; caregiver burden: r=–.459, p = 0.016). Improvements in well-being from before to after the trial were linked to better Neuro-QoL outcomes (r=.440, p=.025), but changes in mindfulness or social support were not associated with Neuro-QoL outcomes. These findings suggest that improving mental well-being is closely linked to improved perceptions of cognitive function in Spanish-speaking caregivers. However, targeting trait mindfulness as a means to improving perceived cognitive function in this population was not supported by this data. Culturally tailored interventions addressing multiple aspects of caregiver distress can enhance emotional well-being and cognitive health, offering a promising approach for underserved Spanish-language populations.

Abstract citation ID: igaf122.3637

### PERFORMANCE FATIGABILITY IS ASSOCIATED WITH DIFFERENTIAL GENE EXPRESSION PATTERNS IN HUMAN SKELETAL MUSCLE

Emma Gay[1], Paul Coen[2], Rebecca Deek[1], Brenda Diergaarde[1], Samaneh Farsijani[1], Michael Jurczak[1], Yujia Qiao[1], and Nancy Glynn[1], *1. University of Pittsburgh, Pittsburgh, Pennsylvania, United States, 2. AdventHealth, Orlando, Florida, United States*

Performance fatigability, the degree one is limited by fatigue during a physical task, is a common trait among older adults and is associated with physical and cognitive function. Knowledge of the biological mechanisms underlying performance fatigability is limited; the skeletal muscle transcriptome may reveal important pathways to prevent or mitigate performance fatigability and promote longer healthspan. The accelerometry-based Pittsburgh Performance Fatigability Index (PPFI) quantified percentage of cadence decline (i.e., slowing down) during the usual-paced 400m walk (range 0-100%). We used available cross-sectional skeletal muscle RNAseq data from the Study of Muscle, Mobility and Aging (n = 724, 55.5% Women, 86.9% White) to identify differentially expressed genes in participants with performance fatigability (PPFI>0%, n = 455, 62.8%) and those without performance fatigability (PPFI=0%, n = 269, 37.2%). A total of 1,935 differentially expressed genes were identified (DESeq2; adjusted for age, sex, and batch, false discovery rate adjusted p-value < 0.05). Of these genes, 594 were down-regulated and 1,341 were up-regulated in participants with performance fatigability vs. those without. Gene set enrichment analysis identified up-regulation in those with performance fatigability in four key muscle-related pathways: collagen organization/processing, immune system activation/response, extracellular organization and muscle cell development/function pathways; no pathways were significantly enriched in the under-expressed genes. Upregulation of these four pathways is known to be associated with phenotypic changes in aging muscle that leads to loss of strength and function, all important for maintaining mobility in older age. Pharmaceutical or therapeutic interventions that improve muscle quality may also concomitantly reduce performance fatigability, its sequelae, and extend healthspan.

Abstract citation ID: igaf122.3638

### PERSISTENT GAPS IN DISABILITY-FREE LIFE EXPECTANCY AMONG OLDER ADULTS WITH MENTAL ILLNESS

Yuelin Li[1], Yuelong Xu[2], Zhirui Deng[2], Rezvaneh Manzour[2], Kyeongra Yang[3], Yue Coco Dong[2], and Heeyoung Lee[2], *1. University of PIttsburgh, Pittsburgh, Macau, 2. University of Pittsburgh, Pittsburgh, Pennsylvania, United States, 3. Rutgers University, Newark, New Jersey, United States*

Mental illness affects about 14% of U.S. adults aged 50 and older and may compromise healthy aging, yet its long-term impact on disability-free life expectancy (DFLE) remains under-examined. This study estimated and compared DFLE by mental illness status, assessed temporal trends, and evaluated transitions between healthy and disabled states. Using nationally representative data from the U.S. Health and Retirement Study (1994–2020; N = 14,553), we applied multi-state Markov models to estimate DFLE for adults without mental illness (Neither), with either depressive symptoms or a psychiatric history (Either), or with both (Both). In models adjusted for age and sex, DFLE at baseline was 32.37 years for the Neither group, 26.75 years for the Either group, and 21.76 years for the Both group; by 2020, it declined to 19.87, 15.53, and 13.77 years, respectively. Annual decline rates were similar across groups (Neither = –1.13; Either = –1.02; Both = –0.92 years per wave). Data showed that individuals with mental illness transitioned from a healthy state to disability (transition intensity 0.15 to 0.12/person-year) at approximately twice the rate of those in the general population (transition intensity: 0.06/person-year), while disability-to-health transition rates were similar across groups. The findings indicate the Either and Both groups experience earlier aging-related decline, yet functional recovery is possible even with mental illness. Overall, mental illness was consistently associated with shorter DFLE and longer disabled years, highlighting the need for integrated care for people with mental illness that effectively address both mental and physical health across the care settings.

Abstract citation ID: igaf122.3639

**PERVASIVE DISCRIMINATION IS ASSOCIATED WITH INTERNAL HEALTH LOCUS OF CONTROL IN THE MIDUS STUDY**

Carolina Hamatsu, David Almeida, and Alexis Santos, *Pennsylvania State University, University Park, Pennsylvania, United States*

Objectives: Drawing on Learned Helplessness Theory, the current study explores the association between Internal Health Locus of Control (I-HLOC) and Pervasive Discrimination (PD). We hypothesize that higher levels of PD are associated with a lower I-HLOC, potentially reflecting the impact of cognitive and behavioral processes shaped by repeated experiences of discrimination. Method: I-HLOC and a PD composite score were calculated using data from the second wave of the Midlife in the United States study (MIDUS II), a nationally representative sample of adults in the United States ($n = 2{,}406$). Linear regression models were conducted to study the association between PD and I-HLOC and its dimensions (i.e., everyday, lifetime, and workplace discrimination), while controlling for age, sex, race, marital status, income level, employment status, and educational attainment. Results: We found that individuals in the top tertile on at least one dimension of discrimination reported lower I-HLOC. Separate models for each dimension revealed significant associations for the top tertile of everyday discrimination and workplace discrimination with lower levels of I-HLOC. No association was found for lifetime discrimination. Discussion: Our findings suggest a potential link between I-HLOC and PD. The main contributors are having experienced everyday and workplace discrimination, lending support to our hypothesis. These findings offer a promising explanation for how intra-individual cognitive and behavioral processes may contribute to health disparities by shaping personal beliefs about the level of control people perceive they have when living in the context of repeated or chronic experiences of unfair treatment in the United States.

Abstract citation ID: igaf122.3640

**PET PLAY AND SOCIALIZATION IN AGING COMMUNITIES: EXPLORING ENGAGEMENT, SATISFACTION, AND AGE DIFFERENCES**

Erreannau Zellous[1], and Taylor Pope[2], *1. Miami University, Oxford, Ohio, United States, 2. Purdue University, West Lafayette, Indiana, United States*

Animal companionship among older adults, including therapy and pet ownership, has been associated with improved socialization and well-being. Older adults often face a heightened risk of social isolation and loneliness, with pet interactions potentially facilitating social connection and companionship. While research links the general benefits of pet ownership, fewer studies examine how active pet engagement, such as play, relates to socialization in later life. Guided by Socioemotional Selectivity Theory, pet play may align with emotional goals by fostering satisfying interpersonal exchanges. The Biophilia Hypothesis further supports this connection, by framing pet play as a natural and rewarding form of human connection. Using data from the General Social Survey (GSS, 2018; $N = 239$, aged 55+), an ordinal logistic regression model examined the association between hours spent playing with pets and social satisfaction, controlling for demographic and socioeconomic factors. Pet play was positively associated with higher social satisfaction ($\beta = 0.16$, $p = 0.01$). To explore age differences, a second set of regressions was estimated, stratified by age group (ages 55–64, 65–74, and 75+). This analysis used the same independent, dependent, and control variables within each age subgroup (significant positive association for ages 55–64: $\beta = 0.56$, $p = .005$; nonsignificant for ages 65–74: $\beta = 0.35$, $p = .122$, and ages 75+: $\beta = 0.11$, $p = .736$). Findings suggest that pet play may support socialization among older adults, particularly the youngest-old. Results have implications for aging services and future research exploring pet play's benefits across the lifespan.

Abstract citation ID: igaf122.3641

**PLACE OF DEATH, CARE INTENSITY, SYMPTOM PREVALENCE, AND SYMPTOM MANAGEMENT FOR OLDER ADULTS WITH STROKE AT END OF LIFE**

Hanna Ramsburg[1], Janice Hinkle[2], and Meredith MacKenzie Greenle[2], *1. University of Pennsylvania School of Nursing, Philadelphia, Pennsylvania, United States, 2. Villanova University, Villanova, Pennsylvania, United States*

Each year in the United States, 795,000 people are diagnosed with a new or recurrent stroke, resulting in 165,393 deaths. However, little is known about the quality of end-of-life (EOL) care in older adults diagnosed with stroke. This secondary analysis examined whether place of death and the intensity of care received in the last month of life were correlated with the prevalence and management of EOL symptoms in older adults with stroke, using data from the National Health and Aging Trends Study, a representative sample of Medicare beneficiaries. Logistic regression was used to identify whether place of death and care intensity received in the last month of life were associated with symptom prevalence and symptom management in patients diagnosed with stroke, adjustive for demographic covariates. Of older adults diagnosed with stroke ($n = 257$), 62.6% reported pain, 50.6% reported dyspnea, and 57.6% reported emotional distress in the last month of life. Older adults who died in the acute care setting were half as likely to report pain ($OR = 0.51$, $p = 0.05$), but twice as likely to report dyspnea ($OR = 2.07$, $p = 0.05$) and adequate dyspnea management ($OR = 1.97$, $p = 0.05$). Place of death was not associated with emotional distress ($OR = 1.62$, $p = 0.05$), pain management ($OR = 0.67$, $p = 0.05$), or emotional distress management ($OR = 0.97$, $p = 0.05$). Age ($OR = 0.56$, $p = 0.05$) and race/ethnicity ($OR = 4.80$, $p = 0.05$) were associated with care intensity, but symptom prevalence and management were not associated. Results suggest that older adults with stroke have distressing symptoms at EOL, but future innovative research is needed in the evaluation and management of symptoms at EOL.

Abstract citation ID: igaf122.3642

**PLURALITY OF PRIMARY AND SPECIALTY CARE IN OLDER ADULTS WITH DEMENTIA AND MULTIMORBIDITY**

Sijia Wei[1], Eleanor McConnell[2], Daniela Ladner[3], Blade Robelly[3], and Jeffrey Linder[3], *1. Northwestern University, Chicago, Illinois, United States, 2. Duke University, Durham, North Carolina, United States, 3. Northwestern University Feinberg School of Medicine, Chicago, Illinois, United States*

Older adults with comorbidities often require care across specialties. Those with Alzheimer's Disease and Related Dementias (ADRD) face additional challenges that require coordinated outpatient care. However, the plurality of primary care

(PC) and specialty use among this population remains understudied. This retrospective cohort study used electronic health records from a multi-site academic health system in the Chicago metropolitan area. The cohort included older adults (≥65 years) with ≥2 chronic conditions and regular outpatient visits between 2016-2024, stratified by ADRD diagnosis. Outpatient encounters were classified into PC (internal/family medicine), ADRD specialty (geriatrics, neurology, psychiatry), or other specialty. Patients were categorized based on whether their care was PC-dominant (> mean proportion of PC encounters) and whether they accessed any ADRD specialty. Among 64,279 patients, 16.2% had ADRD. Of 10,687,974 outpatient encounters, only 11.2% were PC. Overall, 18.6% of patients never accessed PC, more often among those without ADRD (21.6%) than those with (16.8%). Among patients with any PC, the average proportion of PC encounters was 16.7%±13.3%. Patients with ADRD had less specialty-dominant care (50.0% vs. 61.1%). Regardless of ADRD status (with ADRD vs without), patients without PC had the lowest ADRD-specialty use (55% vs 26%), while those with specialty-dominant care had the highest (78% vs 42%). Survival was highest among patients with PC-dominant care (57% with ADRD; 81% without ADRD). These findings highlight distinct outpatient care patterns and potential coordination gaps among PC and specialty care that need further study, which may impact outcomes among older adults with ADRD and multimorbidity.

Abstract citation ID: igaf122.3643

### POLYGENIC NATURE OF CORTICAL AGING REVEALS CYTOSKELETAL REGULATION AS A CORE PATHWAY OF RESILIENCE

Ayati Mishra, Nicholas Kim, Nahian Chowdhury, and Andrei Irimia, *University of Southern California, Los Angeles, California, United States*

Brain aging is spatially heterogeneous, with some cortical regions more resilient than others. To investigate the genetic basis of this variation, we developed a deep learning architecture to estimate voxel-wise local brain age (LBA) from T1-weighted MRI scans of 41,708 cognitively normal adults (21,983 females) aged 45 to 83 years in the UK Biobank. LBA provides a spatially resolved phenotype of cortical aging, where higher values indicate older-appearing regions. We conducted the first genome-wide association study of LBA and identified 1,212 SNPs significantly associated with region-specific cortical aging. Genome-wide significant SNPs were enriched in biological processes related to cytoskeletal regulation, highlighting it as a key determinant of resilience. Variants in NUAK1, a serine/threonine kinase regulating tau phosphorylation and microtubule stability, were associated with widespread decreases in LBA, suggesting a protective role by promoting cytoskeletal stability and slowing cortical aging. Prior studies link NUAK1 variations to gray matter differences in regions vulnerable to degeneration, and experimental models indicate that its inhibition can reduce tau pathology and mitigates neurodegeneration. Variants in LPAR1, encoding a receptor regulating actin dynamics, neurogenesis, and white matter integrity, were also linked to decreased LBA in parietal and frontal cortices. This aligns with evidence that LPAR1 supports myelination and plasticity through cytoskeletal regulation and prior studies showing loss of LBAR1 leads to demyelinating disease, underscoring its protective role. Altogether, these findings demonstrate that cytoskeletal regulation provides a genetically encoded protective mechanism that helps preserve cortical structure and slow the aging process.

Abstract citation ID: igaf122.3644

### PREDICTING TRAJECTORIES OF HOSPITALIZATIONS IN U.S. OLDER ADULTS WITH NEWLY DIAGNOSED HEART FAILURE

Hanzhang Xu[1], Radha Dhingra[2], Bradley Hammill[2], Scott Lynch[1], Jessica West[2], Michael Green[2], Lesley Curtis[2], and Matthew Dupre[2], *1. Duke University, Durham, North Carolina, United States, 2. Duke University School of Medicine, Durham, North Carolina, United States*

Heart failure (HF) remains the leading cause of hospitalizations among older adults. Little is known about the longer-term risks of hospitalizations and whether high-risk patients can be identified at the time of HF diagnosis. We analyzed a national sample of Medicare beneficiaries with newly-diagnosed HF between 2010-2015. Patients were then followed for up to 5 years to identify all-cause hospitalizations after their initial diagnosis. Group-based trajectory models were used to identify patients with differing trajectories of hospitalizations and LASSO regression was used to identify patients' characteristics at the time-of-diagnosis to predict their trajectory of hospitalizations. Model calibration was evaluated graphically (observed vs predicted) and discrimination was assessed using c-statistics. Of 84,597 beneficiaries (mean age: 77.4 [± 7.1] years and 58.3% male), we identified four distinct trajectories of hospitalizations over follow-up: Group 1 (n = 19,340; 22.9%) had "low risks" of hospitalization, Group 2 (n = 53,922; 57.9%) had elevated risks of admission shortly after diagnosis ("early risk" group), Group 3 (n = 5,035; 9.8%) had elevated risks at later stages of illness ("late risk" group), and Group 4 (n = 6,300; 9.4%) had consistently "high risks" of hospitalization. Models were well-calibrated predicting patients' hospitalizations and discrimination (c-statistics) were 0.63 (Group 2), 0.67 (Group 3), and 0.77 (Group 4) when comparing to patients with low risks of hospitalization (Group 1). Predicting hospitalizations among beneficiaries improved in those who were diagnosed with HF at younger ages (≤70 years). Patients with high risks of hospitalizations could be identified at the time-of-diagnosis and should be targeted for interventions to improve HF management.

Abstract citation ID: igaf122.3645

### PROFILING MEALTIME VERBAL INTERACTIONS BETWEEN NURSING HOME STAFF AND PERSONS WITH DEMENTIA

Annika Ellis[1], Kyuri Lee[2], and Wen Liu[3], *1. University of Iowa, Eagan, Minnesota, United States, 2. The University of Iowa, Iowa City, Iowa, United States, 3. Penn State University, University Park, Pennsylvania, United States*

Effective communication is crucial to Person-Centered Care (PCC) for Persons with Dementia (PWD) during mealtimes as verbal interactions can influence engagement. This study analyzed 12,959 verbal behaviors in 245 full-mealtime videos in long-term care settings using the CUED coding scheme. Staff produced 9,176 positive verbalizations, including "Orientation/Giving Instruction" (24.80%) and "Giving Choices" (16.46%). Of staffs' 1,781 negative verbalizations, most involved side conversations (42.57%). Residents contributed 2,066 verbalizations in the dyadic conversations (86% positive, 14% negative). Notable resident unintelligible utterances were coded as "Unsure – positive" (30.66%). Additional common verbal resident behaviors included "Expressing personal need or preference" (16.33%) and "Showing approval/agreement" (16.28%). Together, these dyadic interactions indicate an overall positive conversational tone with relatively infrequent negative themes, facilitating a deeper understanding of the context within dementia care communication. Pearson correlations revealed significant associations between staff and resident behaviors. Staff positive behaviors were positively correlated with both resident positive ($r = .288$, $p < .001$) and negative behaviors ($r = .275$, $p < .001$), suggesting that staff engagement prompted a range of resident responses. Staff negative behaviors were associated with resident negative behaviors ($r = .153$, $p = .016$). Additionally, longer durations of staff's negative side conversations were correlated to increased number of total negative staff behaviors ($r = .173$, $p = .007$). These associations indicate that while staff demonstrate many PCC behaviors, negative or off-topic conversations can still shape resident responses in unhelpful ways. Strengthening staff communication strategies to remain focused, positive, and inclusive may enhance mealtime engagement for PWD.

Abstract citation ID: igaf122.3646

### PROGNOSTIC IMPORTANCE OF FRAILTY AND MULTIMORBIDITY ALONGSIDE DISEASE RISK IN OLDER ADULTS WITH MULTIPLE MYELOMA

Samantha Rizzo[1], Hannah Tosi[1], Chunlei Zheng[1], Jennifer La[1], Jane Driver[2], Nikhil Munshi[1], Nathanael Fillmore[1], and Clark DuMontier[1], *1. VA Boston Healthcare System, Boston, Massachusetts, United States, 2. VA Boston Healthcare System, Brockton, Massachusetts, United States*

Older adults with multiple myeloma (MM) often have frailty and multiple conditions (multimorbidity) at the time of diagnosis, representing a more complex population than younger, healthier patients in clinical trials. With data from the national Veterans Affairs (VA) electronic health record and VA Cancer Registry, we used machine learning to understand the degree to which frailty (measured via the electronic VA-Frailty Index) and multimorbidity (chronic conditions from the CMS Chronic Conditions Warehouse) predict clinical outcomes alongside MM stage and other markers of disease-risk. We performed repeated cross-validation to train and test models to predict 3-year mortality, starting by only including MM-specific predictors in the first round of training-testing, followed by adding in frailty and multimorbidity predictors in the second round (holistic model). A total of 4416 patients age ≥ 65 years with newly diagnosed MM who received treatment at the VA between 2004 and 2020 were included. The holistic model showed superior predictive performance (AUROC 0.71) compared to the model that was developed using MM-specific predictors only (AUROC 0.66, t-test $p < 0.001$). The top predictors that contributed to mortality risk were age, hospitalization in the 180 days before starting MM treatment, diuretic use, hemoglobin < 10 g/dL, and frailty. A significant portion of each predictors' impact on mortality was mediated through interactions with other variables. Although disease risk normally drives prognostication in MM, our findings reveal that frailty and multimorbidity significantly improve mortality prediction through both their individual contributions and synergistic interactions.

Abstract citation ID: igaf122.3647

### RECOGNIZING AND RESISTING AGEISM: VIEWS FROM OLDER ADULTS

Aaron Li[1], Natalie Galucia[2], Michele Dinman[1], Brian Carpenter[1], and Nancy Morrow-Howell[1], *1. Washington University, St. Louis, Missouri, United States, 2. Washington University, Saint Louis, Missouri, United States*

This study aims to generate knowledge for the development of anti-ageism interventions for older people. We conducted 12 focus groups (N = 75) to explore experiences with interpersonal and internalized ageism, reactions to ageist incidents, and ideas about resisting. We conducted six-month follow-up survey interviews to gain understanding about how focus group discussions changed awareness and actions regarding ageism. Participants ranged from 60 to 91 years (M = 75); 76% were female, 56% White, 41% Black, and 20% identified as LGBTQIA+. Thematic analysis revealed that not all participants understand the concept of ageism, especially internalized ageism. Among those who did, lack of respect and feeling invisible were identified as hallmarks. Ageism most often appeared among families and in businesses and took the form of disregard and paternalism. Fewer examples were provided in health care and employment, although harmful incidents mentioned. Internalized ageism wasn't well understood; participants often talked about the challenges of aging instead. For participants with minoritized identities, racism, ableism, and homophobia played a more central role than ageism in experiences of discrimination. Reactions to ageism ranged from nonresponse to context-dependent actions shaped by relationships with perpetrators. In ongoing survey follow-ups (n = 52 to date), 75% shared new examples of ageism; 63% described new examples of internalized ageism; 51% reported a conversation about ageism with someone; 38% had addressed an ageist comment directed toward them; and 84% reported increased awareness. These findings suggest that simple discussions about ageism can serve as an intervention, raising awareness and encouraging resistance.

Abstract citation ID: igaf122.3648

### REGIONAL DIFFERENCES IN CHARACTERISTICS, SERVICE USE, AND OUTCOMES IN PATIENTS RECEIVING HOME VISIT CARE IN JAPAN

Jun Komiyama[1], Yu Sun[2], Masao Iwagami[1], Nobuo Sakata[2], Satoru Yoshie[2], Tomoko Ito[1], Kimikazu Kashiwagi[3], and Nanako Tamiya[1], *1. University of Tsukuba, Tsukuba, Ibaraki, Japan, 2. University of Tsukuba, Tsukuba, Ibaraki, Ibaraki, Japan, 3. National College of Nursing, Kiyose, Tokyo, Japan*

Aim: Physician-led home-visit care has become essential in the super-aged society of Japan. To develop region-specific service systems, understanding regional differences in patient profiles is essential. This study aimed to describe regional differences in patient characteristics, service utilization, and outcomes, and to assess the associations between regional healthcare resources and patient outcomes. Methods: This retrospective study used linked data from the National Database of Health Insurance Claims and Specific Health Checkups and the Japanese Long-term Care Database (April 2020–March 2021). Patients aged ≥65 years receiving regular physician-led home visits in October 2020 were included. Analyses were conducted by prefecture and region. Associations between regional healthcare resources and patient outcomes were assessed using Spearman's correlation coefficients

(ρ). Results: Among 337,863 patients, regional variations were observed. Western Japan had higher prevalence of cancer, heart disease, respiratory disease, and orthopedic disorders, whereas eastern Japan showed higher rates of high care-need levels and home nursing utilization. Hospitalization risk was higher in western Japan. In-home deaths were more frequent in eastern Japan. Hospital bed density was positively correlated with hospitalization risk (ρ = 0.62) and negatively with 6-month mortality risk (ρ=-0.44). Utilization of enhanced home care support clinics/hospitals was moderately associated with higher in-home death risks (ρ = 0.49). Conclusions: Substantial regional differences exist in patient characteristics, service utilization, and outcomes among older adults receiving physician-led home-visit care. Healthcare resources were strongly associated with hospitalization and end-of-life care, highlighting the need for region-specific strategies to optimize healthcare delivery and resource allocation for the super-aged society of Japan.

Abstract citation ID: igaf122.3649

### RISK OF DEMENTIA IN IMMUNOCOMPROMISED AND NON-IMMUNOCOMPROMISED PERSONS WITH COVID-19 INFECTION

Winnie Xu[1], Yonah Ziemba[2], James Crawford[2], Michael Diefenbach[2], Shahidul Islam[3], Cristina Sison[2], Christian Nouryan[4], and Edith Burns[5], *1. Hofstra University, Hempstead, New York, United States, 2. Northwell, Manhasset, New York, United States, 3. Northwell Health, New Hyde Park, New York, United States, 4. Northwell Health, Great Neck, New York, United States, 5. Zucker School of Medicine Hofstra-Northwell, Manhasset, New York, United States*

COVID-19 infection is associated with an increased risk of developing dementia, particularly among older adults. We examined the impact of immune status on this risk using the COVID-19 Real World Data Infrastructure (DOI: 10.1093/0fid/ofaf021), a publicly available resource of 5.2 million unique deidentified patient records, linked with the NCI SEER registry. Two cohorts of patients were defined for the study period (12/1/2018-12/31/2024): COVID-19 Positive Persons (CPP) with positive SARS-CoV-2 NAAT and/or COVID-19 ICD-10 code. COVID-19 Negative Persons (CNP) had COVID-related information (including SARS-CoV-2 diagnostic or serologic testing and/or documented vaccination), but no positive NAAT or COVID-19 ICD-10 code. Patients < 65 years, with history of dementia or psychotropic medication use on or before index date (1/1/2020) were excluded. There were 225,852 CPP and 499,295 CNP eligible patients. Incidence of newly-diagnosed dementia among the CPP cohort was 25.6%, vs. 17.3% in CNP (Relative Risk [RR] 1.48; 95% CI: 1.46-1.49). Stratification by immunocompromised status (473,559 immunocompromised; 251,588 non-immunocompromised) showed no significant difference between RR for dementia associated with COVID-19 (RR = 1.48, 95% CI:1.46-1.50 and RR = 1.47, 95% CI: 1.45-1.50 respectively). These unadjusted analyses align with prior findings that COVID-19 infection in patients aged ≥65 years is associated with a greater risk of subsequent dementia diagnosis but suggest immunocompromised individuals have the same increased risk as the general population. Further analysis will examine timing of diagnoses, and relevance of covariates such as vaccination status and anti-viral medication use on dementia risk in CPP patients.

Abstract citation ID: igaf122.3650

### ROOTED IN JOY: EXPLORING SOURCES OF JOY AMONG OLDER AFRICAN AMERICAN WOMEN

Jacquelyn Coats[1], Sheltoria Love[2], and Anais Mahone[3], *1. Duke University School of Medicine, Durham, North Carolina, United States, 2. Washington University in St. Louis, St. Louis, Missouri, United States, 3. University of Alabama at Birmingham, Birmingham, Alabama, United States*

Older African American women demonstrate remarkable resilience despite persistent stressors and disproportionate health outcomes compared to other groups. While joy has been identified as a potential contributor to health and well-being, it remains understudied among this population. This qualitative study addresses that gap by exploring sources of joy in later life for this important group. The analysis is nested within a larger constructivist grounded theory project that used semi-structured interviews to examine stress and coping among college-educated African American women. This study focuses on responses to the final interview question: “What brings you joy?” Participants reflected on joy within the context of their broader life experiences. Sixteen women aged 65 to 91 were recruited through community organizations, libraries, and social networks in the Midwest. Guided by Black Feminist Theory, a collaborative, discussion-based thematic analysis was used to interpret the data. Narratives revealed diverse and multidimensional sources of joy. Four overarching themes emerged: (a) experiencing and preserving Black culture, (b) play and adventure, (c) spirituality and life purpose, and (d) community and connection. Findings suggest that joy is a vital and culturally grounded source of resilience and well-being for older African American women. For aging and gerontology researchers and health practitioners, understanding and integrating elements that foster joy may enhance culturally responsive care and support. This study also underscores the need for focused research on joy as a distinct phenomenon shaped by cultural and structural contexts, and its potential role in promoting health equity in later life.

Abstract citation ID: igaf122.3651

### SELF-PERCEPTIONS OF AGING, RACE-BASED DISCRIMINATION, AND TOTAL COGNITION AMONG DIVERSE OLDER ADULTS

Andrew Steward[1], Cliff Whetung[2], Yura Lee[1], and Tyrone Hamler[3], *1. University of Wisconsin-Milwaukee, Milwaukee, Wisconsin, United States, 2. University of Minnesota Duluth, Duluth, Minnesota, United States, 3. University of Denver, Denver, Colorado, United States*

Research drawing from stereotype embodiment theory suggests that positive self-perceptions of aging (SPA) are associated with enhanced cognitive function among older adults. While everyday discrimination is associated with negative SPA and reduced cognitive function, the relationship between race-based discrimination, SPA, and cognitive function remains unknown. Therefore, we drew from the Health and Retirement Study to examine associations between SPA, race-based discrimination, and cognition among a racially diverse sample of adults 50+ years of age (N = 5,949) between 2014 and 2020. We computed weighted descriptive statistics and estimated mixed-effect regression models, adjusting for theoretically informed covariates. Among respondents who primarily attributed everyday discrimination to race (N = 894), the self-reported racial identity profile was 46.17% Black/African American, 40.73% White, 9% Asian, and 4.53% Indigenous, with 18.19% reporting Hispanic ethnicity. Those who primarily attributed discrimination to race reported the highest standardized everyday discrimination scores (0.72) and lowest standardized SPA scores (-0.19) on average. Overall, more positive SPA was associated with higher cognition (b = 0.49). In mixed-effect models adjusted for upstream predictors of cognition (age, time on study, sex, race, ethnicity, education, income), more frequent experiences of everyday discrimination were associated with lower cognition scores (b=-0.12), but race-based attributions were not associated with differences in cognition. In models stratified by discrimination attributions (no attribution, any race-based attribution, other attributions), SPA was consistently associated with higher cognition scores. These findings may suggest the importance of upstream interventions to promote positive SPA as part of efforts to mitigate the effects of racial discrimination on cognitive health in later life.

Abstract citation ID: igaf122.3652

### SENOLYTICS ATTENUATES INFLUENZA- INDUCED MUSCULAR DECLINE IN AGED MICE

Nagaraju Marka[1], Andreia Cadar[1], Zena Haddad[1], Anchala Anil Rao[1], Rakshit Raj Bisoi[1], Erica Lorenzo[1], Laura Haynes[1], and Jenna Bartley[2], *1. University of Connecticut Health, University of Connecticut Health, Farmington, Connecticut, United States, 2. University of Connecticut School of Medicine, University of Connecticut Health, Connecticut, United States*

Influenza (flu) remains a major burden for older populations with increased morbidity and mortality. In older people, flu infection can lead to loss of physical function. In preclinical murine studies, the Bartley lab previously determined that flu leads to functional decline, muscle-localized inflammation, and muscle degradation and atrophy; and that these effects are exaggerated and prolonged with aging. Cellular senescence accumulate with aging and leads to excessive inflammation via the senescent-associated secretory phenotype (SASP). Treatment with senolytics, dasatinib and quercetin (D+Q), eliminates senescent cells and improves the health span, metabolic functions, reduces muscle inflammation and promotes satellite cell proliferation in aging animal models. Hence, we aimed to determine if pretreatment with D+Q could protect against flu-induced muscle declines in aging. Aged (18-20M) C57BL6/J male and female mice were treated with D+Q prior to sublethal flu infection. Immune responses, physical function, and skeletal muscle parameters were assessed prior to and following flu challenge. D+Q did not impact flu-induced weight loss, lung viral load, and flu-specific CD4 and CD8 T cells responses in the mediastinal lymph node and spleen. However, D+Q treatment mitigated flu-induced functional declines in both male and female with increased grip strength compared to vehicle control. In male D+Q treatment led to reduced gene expression of muscle atrophy markers Atrogin-1 and MuRF-1 compared to vehicle treated mice at 11- days post-infection. In totality, our results suggest senolytics may protect against flu-induced muscular declines without impacting immune responses in aged mice, which may offer insights for potential therapeutic implications.

Abstract citation ID: igaf122.3653

### SMARTWATCH MEASURES, COGNITIVE FUNCTION, AND OLDER ADULTS: ELECTRONIC FRAMINGHAM HEART STUDY (EFHS)

Jie He[1], Yuankai Zhang[1], Xuzhi Wang[1], Nicole Spartano[2], Jian Rong[2], Emelia Benjamin[2], Chunyu Liu[1], and Joanne Murabito[3], *1. Boston University School of Public Health, Boston, Massachusetts, United States, 2. Boston University Chobanian & Avedisian School of Medicine, Boston, Massachusetts, United States, 3. Boston University Chobanian & Avedisian School of Medicine and Boston Medical Center, Boston, Massachusetts, United States*

Smartwatches can continuously monitor heart rate (HR) and step counts. A recent report of wearable-derived daily heart rate per step (DHRPS) observed a stronger association with cardiovascular outcomes than either daily HR or steps alone. However, it remains unclear whether DHRPS is associated with cognitive function. We conducted a cross-sectional study with 249 older adults (mean age 73, cognitively intact, mean Mini-Mental State Examination score of 28.8; 56% women, 14% non-White; median follow-up 662 days [426, 818]) from eFHS with smartwatch data and neuropsychological (NP) assessments within 5 years of eFHS data collection (2021-2024). DHRPS (primary exposure; median 0.02) was calculated as daily average HR (median 74 [69, 78]) divided by daily step counts, averaged across all follow-up days. Secondary exposures included average daily "non-active" HR (median 68 [63, 73]) and average daily step counts (median 4810 [3270, 6831]). Cognitive function was assessed by a composite of NP tests (cNP) spanning memory, executive function, language, and visuospatial domains. Linear regression models were used to examine the association between exposures and cNP, adjusting for demographic, lifestyle, and health-related covariates. Each 1-SD higher DHRPS was associated with a 0.06-SD lower cNP (95% CI: -0.11 to -0.01; $p = 0.011$). Additionally, we found each 1000 more steps per day was associated with a 0.03-SD higher cognitive score (95% CI: 0.004 to 0.05; $p = 0.023$), but daily "non-active" HR was not ($p = 0.77$). Our findings suggest wearable-derived metrics like DHRPS may help identify early cognitive decline. Larger, longitudinal studies are needed to confirm our findings.

Abstract citation ID: igaf122.3654

### SOCIAL DETERMINANTS OF HEALTH IMPACT CARDIOVASCULAR OUTCOMES IN HOSPITALIZED OLDER ADULTS

Vania Remenik-Zarauz[1], Ruth Pius[1], Malay Das[1], and Andrea Reyes-Vega[2], *1. Lincoln Medical and Mental Health Center, Bronx, New York, United States, 2. University of Louisville, University of Louisville, Kentucky, United States*

Social determinants of health (SDoH) have been reported to exert a significant impact on cardiovascular risk. However, there is limited research on the role of SDoH in cardiovascular outcomes in hospitalized older adults. This study aims to examine the impact of socioeconomic status, housing and healthcare access as SDoH on in-hospital outcomes among older adults with acute decompensated heart failure (ADHF), using the National Inpatient Sample (NIS) database from 2016 to 2020. We analyzed 9.2 million ADHF patients stratifying them according to age 65-79 (53%) and ≥80 (47%). The outcomes studied were death, cardiac arrest and cardiogenic shock. A multivariable logistic regression model was used ($p < 0.05$ was considered statistically significant). Within the 65-79 subgroup, all examined SDoH were statistically significant risk factors for cardiogenic shock ($p = < 0.001$) and death (lack of health insurance and homelessness $p = < 0.001$, poor income $p = 0.008$). Interestingly, homelessness was the only SDoH significantly associated with cardiac arrest in this age group ($p = < 0.001$). Within the ≥80 subgroup, lack of health insurance was the only SDoH associated with death ($p = < 0.001$), while cardiogenic shock and cardiac arrest were not significantly associated with any SDoH. These findings reinforce the role of social disparities in adverse outcomes among older adults, highlighting age-related vulnerabilities and the need for interventions addressing both socioeconomic and pathophysiologic mechanisms of disease.

Abstract citation ID: igaf122.3655

### STAFFING INSTABILITY AND FUNCTIONAL DECLINE IN LONG-STAY NURSING HOME RESIDENTS WITH DEMENTIA

Talia Benheim, Christopher Santostefano, and Fangli Geng, *Brown University, Providence, Rhode Island, United States*

Staffing instability in nursing homes may have adverse outcomes for residents with Alzheimer's disease and related dementias (ADRD), yet most prior work has focused on average staffing levels. We linked Payroll Based Journal data to Minimum Data Set assessments from 15,828 facilities from 2018-2022 to examine associations between changes in staffing instability and care outcomes among long-stay residents with ADRD. The primary exposure was the quarterly number of "low outlier days" (LOD), defined as days with total direct care hours per resident at least 20% below the facility's quarterly mean. We used an event study framework leveraging variation in the timing of changes in LOD, with facility and quarter fixed effects, adjusting for time-varying characteristics (overall staffing hours, staffing composition, mean census, resident case mix, resident COVID-19 cases), and weighting by the ADRD resident census. A 10-day increase in LOD was associated with a 0.5 percentage point (pp) increase in the share of residents with ADRD experiencing decline in Activities of Daily Living, a 0.3pp increase in worsened ability to move independently, a 0.2pp increase in significant weight loss, and a 0.1pp increase in pressure ulcers (all $p<.001$), but was not significantly associated with preventable hospitalizations or antipsychotic prescribing. Associations were largest for instability in certified nursing assistant (CNA) staffing, followed by licensed practical nurses and registered nurses, which aligns with CNAs' roles in mobility and feeding assistance. These findings suggest that beyond increasing the quantity of staffing, reducing day-to-day instability should be a target for quality improvement.

Abstract citation ID: igaf122.3656

### STATE SAFETY NET POLICIES AND HEALTH OF ADRD CAREGIVERS: EVIDENCE FROM BRFSS AND SPPD

Talha Ali[1], Siyao Lu[2], Julie Strominger[3], Erin Kent[4], Toni Antonucci[5], and Wassim Tarraf[6], *1. Tufts University, Medford, Massachusetts, United States, 2. Yale University, New Haven, Connecticut, United States, 3. The Ohio State University, Columbus, Ohio, United States, 4. University of North Carolina at Chapel Hill, Chapel Hill, North Carolina, United States, 5. University of Michigan, Ann Arbor, Michigan, United States, 6. Wayne State University, Detroit, Michigan, United States*

Caregivers of individuals with Alzheimer's Disease and Related Dementias (ADRD) experience substantial physical and mental health burdens, often shaped by structural factors such as policy environments. This study examines whether state safety net policies—Medicaid expansion, Medicaid generosity, unemployment benefits, and paid sick leave (PSL)—affect the health of ADRD caregivers, and whether these associations differ by caregiving complexity and sex. We identified ADRD caregivers across 47 states from the U.S. Behavioral Risk Factor Surveillance System (2021–2022; $N = 12{,}025$ representing 7,480,376 nationally) and obtained policy data from the State Policy and Politics Database (2020). Outcomes included caregivers' self-reported poor physical and mental health days in the last month. Negative binomial regression models assessed policy associations with healthy days and tested interactions by caregiving complexity and sex. Northeastern and West Coast states ranked higher in policy generosity, and most Southern and some Midwestern states ranked lower. More generous policies were associated with 0.1-0.5 fewer poor physical and 0.2-0.5 fewer poor mental health days per month among ADRD caregivers, though effects were not statistically significant. Greater Medicaid generosity, unemployment benefits, and PSL were linked to 1–3 and 2–6 fewer days of poor physical and mental health, respectively, among caregivers providing personal care (not significant). However, caregivers providing high care hours without personal care experienced 7–13 more poor mental health days in states with vs. without PSL and Medicaid expansion. No significant sex differences were observed. This suggests targeted state policy efforts are needed to address health risks faced by ADRD caregivers providing high-intensity care.

Abstract citation ID: igaf122.3657

### STRESS EXPOSURES AND COGNITIVE HEALTH TRAJECTORIES IN LATER LIFE: THE MODERATING ROLE OF RELIGIOUS INVOLVEMENT

Xi Zhu, *Oregon State University, Corvallis, Oregon, United States*

A growing body of research has identified stress as a significant factor influencing cognitive health in later life. However, it remains unclear how stress exposures impact the rate of cognitive decline over time. As an important part of life for many older adults, religion provides a unique set of psychosocial resources that may function as a stress buffer. Yet, little research has directly examined the role of religious coping in the stress-cognition link. Using data from the Health and Retirement Study (HRS; 2006-2018), this study examines the effects of four types of stress exposures (lifetime stressful events, cumulative chronic stress, financial strain, and everyday discrimination) on cognitive health trajectories among older adults. It also investigates whether religious involvement moderates the potential adverse effects of stress on cognition. Results from growth curve models show that high levels of chronic stress, financial strain and everyday discrimination were associated with poorer cognitive function at baseline, whereas the total number of lifetime stressful events was positively related to baseline cognition. None of the stress exposures was associated with the rate of cognitive decline over time. In addition, religious attendance and perceived religious importance significantly mitigated the negative impact of financial strain and discrimination on baseline cognitive function. This study contributes to a deeper understanding of the relationship between stress, religion, and cognitive health. It highlights the potential protective role of religious involvement in promoting cognitive resilience against stress in the aging population.

Abstract citation ID: igaf122.3658
**STRUCTURED PHYSICAL ACTIVITY INTERVENTIONS FOR OLDER ADULTS WITH DEMENTIA IN AFRICA**
Ahmed Danquah[1], Jennifer Adomako[2], Frank Adutwum[3], Kadir Abdul Daniyalo[3], and Jennifer Etnier[4], *1. University of Pittsburgh School of Nursing, Pittsburgh, Pennsylvania, United States, 2. University of North Carolina, Greensboro, Greensboro, North Carolina, United States, 3. University of Utah, Salt Lake, Utah, United States, 4. University of North Carolina at Greensboro, Greensboro, North Carolina, United States*

Structured physical activity (PA) interventions and their cognitive benefits for older adults living with dementia in Africa remain underexplored, despite the continent's 2-20% current prevalence rate. This, however, is projected to rise by 350% by the year 2050. Thus, we reviewed the extent to which the cognitive and functional benefits of PA for older adults living with dementia are being explored in Africa. We followed the Joanna Briggs Institute (JBI) methodology for conducting a scoping review and reported our findings using guidelines from the Preferred Reporting Items for Systematic Reviews and Meta-analyses Extension for Scoping Reviews (PRISMA-ScR). We identified articles across African journals and international databases, including PubMed, CINAHL, PsycINFO, and Scopus. Dissertations and other non-peer-reviewed works were also considered for analysis. Search terms included 'physical activity intervention' and ' dementia.' We included only articles published in English that had a structured physical activity component and targeted older adults diagnosed with dementia or cognitive impairment. Across 24 publications, we found that most of the interventions were multicomponent (e.g., aerobics, walking, and dancing), facility-based, and delivered over a period of 6 to 12 weeks by physiotherapists or trained caregivers. We also found improved mood, memory, and attention span in various studies. Few interventions incorporated culturally-grounded practices, and none explicitly addressed the needs of older adults with nonverbal dementia. In sum, structured PA interventions show promise for cognitive improvement for dementia care in Africa, but evidence remains limited. Future research should prioritize culturally-tailored, scalable models to meet the continent's rising dementia burden.

Abstract citation ID: igaf122.3659
**STUDENT PERSPECTIVES ON AGING, DEMENTIA, AND HEALTH CARE: LESSONS LEARNED FROM A COMMUNITY BASED MENTORSHIP PROGRAM**
Rachel Abramson[1], Mengru Wang[1], Marigrace Becker[2], Stephanie Adaniya[1], and Michael Rosenbloom[1], *1. University of Washington, Seattle, Washington, United States, 2. UW Medicine, Seattle, Washington, United States*

Health professional students have limited exposure during their training to the patient and caregiver perspective of aging and living with dementia. Prior studies have shown that community-based service-learning experiences enhance empathy among health professional students. There is limited understanding of how these programs can impact health professional student perspectives on aging, dementia, and the health care system. Ten older adults with mild stage dementia and their caregivers were recruited from a community center that hosts dementia friendly programs. Seven medical students and three social work students at the University of Washington participating in a service-learning program were matched with an older adult mentor and their caregiver based on common interests. Students met with their older adult mentor and caregiver monthly over the course of one year, with activities ranging from walks and attending musical performances, to observing medical appointments. Eight students participated in semi-structured interviews at the conclusion of the program. A thematic analysis was performed by two reviewers using an inductive and deductive approach. Themes identified from the qualitative analysis included appreciating the uniqueness of each person's experience with dementia, the significant stigma surrounding dementia and aging, barriers to accessing care (technology, transportation, prolonged waiting times to seeing providers), and the importance of community for aging and living well with dementia. Next steps in the study include a qualitative analysis of the older adult mentor and caregiver experiences, and assessment of self-reported quality of life among older adults and perceived caregiver burden pre and post program.

Abstract citation ID: igaf122.3660
**SUBSTANCE USE DISORDER DIAGNOSES AMONG OLDER MEDICARE BENEFICIARIES HOSPITALIZED WITH TRAUMATIC BRAIN INJURY**
Yoon Chung Kim, *University of Maryland School of Medicine, Baltimore, Maryland, United States*

Traumatic brain injury (TBI) is a common, fall-related injury among older adults. Studies have reported high rates of pre-existing alcohol and/or substance use disorders (AUD/SUD) among younger adults with TBI, however, less is known about AUD/SUD among older adults with TBI. We estimated the prevalence of pre-existing AUD/SUD among older adults hospitalized with TBI and identified demographic and clinical characteristics associated with AUD/SUD status. We conducted a retrospective cohort study using Medicare administrative claims data, including beneficiaries aged ≥65 years hospitalized with TBI between 2010-2017. Pre-existing AUD/SUD was defined using diagnostic codes during the 6 months pre-TBI and 3 months post-TBI. Among beneficiaries hospitalized with TBI (n = 20,350), prevalence of pre-existing AUD/SUD diagnoses was 8.4% (n = 1,713). AUD diagnoses were most common (7.1%); 1.8% had SUD diagnoses. Among those with any AUD/SUD, 84.8% had AUD and 20.9% had ≥1 SUD diagnoses. Opioid use disorder was the most common SUD (10.4% SUD diagnoses). Compared to Medicare beneficiaries without an AUD/SUD diagnosis, beneficiaries with a pre-existing AUD/SUD diagnosis had lower odds of being older (odds ratio (OR) 0.91; 95% confidence interval (CI): 0.90, 0.92), being female (OR 0.53; 95% CI 0.48, 0.59), higher odds of having chronic obstructive pulmonary disease (OR 2.04; 95% CI 1.82, 2.28) or depression (OR 1.50; 95% CI 1.34, 1.67), and higher odds of dual eligibility for Medicare and Medicaid (OR 1.26; 95% CI 1.12, 1.42). Older Medicaid beneficiaries hospitalized with TBI who have pre-existing AUD/SUD have unique clinical complexities and may benefit from integrated and targeted treatments.

Abstract citation ID: igaf122.3661
**SUPPORTING CAREGIVER WELL-BEING THROUGH A VIRTUAL ART EXHIBIT: PILOT STUDY QUALITATIVE FINDINGS**
Charlotte Weiss[1], Katherine Cotter[1], Morgan Delizia[1], Connie Ulrich[2], and Karen Hirschman[1], *1. University of Pennsylvania, Philadelphia, Pennsylvania, United States,*

2. *University of Pennsylvania School of Nursing - Philadelphia, PA, philadelphia, Pennsylvania, United States*

The growing number of recently hospitalized older adults with multiple chronic conditions (MCCs) increasingly relies on family caregivers (FCGs) for essential support. While FCG involvement is critical, the caregiving role often has a negative impact on their social, emotional, and spiritual well-being. Emerging evidence suggests that engaging with visual art, including museum visits, can enhance well-being across these domains. However, little is known about the use of virtual art exhibits to support FCGs of older adults with MCCs. This exploratory, multi-methods feasibility study aimed to develop and pilot a virtual art museum experience for FCGs of recently hospitalized older adults with MCCs. We first conducted semi-structured interviews with five FCGs to explore their perceptions of virtual art engagement, including potential benefits and barriers. Next, we used the insights from these interviews to inform the curation of a 14-day virtual art exhibit using images from Google Arts & Culture. The exhibit was piloted with a convenience sample of FCGs (n = 15). A daily diary approach was used to explore the associations between daily engagement and perceived stress, social connectedness, emotional well-being, and spirituality. The qualitative findings gathered from the daily diary entries suggest that daily engagement with a virtual art exhibit is both acceptable and accessible for FCGs. The themes identified will be highlighted. Virtual art engagement may offer a scalable, low-cost behavioral intervention to support the holistic well-being of family caregivers managing the complex demands of caregiving for older adults with MCCs.

Abstract citation ID: igaf122.3662

### SYMPTOM BURDEN AND MOBILITY IN OLDER ADULTS: A QUALITATIVE STUDY TO INFORM CLINICAL INNOVATION

Michelle McKay[1], Tasha Martin-Peters[2], Kerry Shields[2], Rebekah Deroo[2], and Christina Whitehouse[2], *1. Villanova University, Norristown, Pennsylvania, United States, 2. Villanova University, Villanova, Pennsylvania, United States*

Approximately 17.6 million adults aged ≥65 years have mobility limitations, which may include the inability to walk a quarter of a mile or climb stairs independently. Mobility limitations are the most common limitation among older adults. Limitations in mobility can lead to disability, decreased quality of life, and poor health outcomes. Older adults report a variety of symptoms that contribute to their mobility limitations, which impact their participation in activities of daily living. There is a lack of data regarding the assessment and management of the symptom burden experienced by older adults. The objective of this study was to explore the perspectives of nurse practitioners (NPs) in identifying and managing the symptom burden associated with mobility limitations in community-dwelling older adults. We conducted 14 semi-structured interviews of NPs in primary care settings. Interviews were professionally transcribed and analyzed using thematic analysis. Six themes were identified: 1) symptom burden over disease labels; 2) functional status and daily living; 3) interprofessional and family involvement; 4) prevention of hospitalization and functional decline; 5) motivation and what matters most; and 6) conservative and holistic treatment approaches. The results of this study provided insight on healthcare provider's assessment and management of symptoms responsible for mobility limitations in community-dwelling older adults. These results will support the development of a symptom burden risk assessment tool and innovative interventions focused on symptom management to improve mobility and decrease the associated negative outcomes.

Abstract citation ID: igaf122.3663

### TEMPORAL ASSOCIATIONS BETWEEN T-CELL SENESCENCE AND ARTERIAL STIFFNESS IN MIDDLE-AGED /OLDER ADULTS

Theodore DeConne, Stephen Kritchevsky, Jamie Justice, and Jingzhong Ding, *Wake Forest University School of Medicine, Winston-Salem, North Carolina, United States*

Age-related structural arterial remodeling (S) and load-dependent (LD) arterial stiffening can be calculated using arterial pulse-wave velocity (PWV) and blood pressure. Senescence-associated T-cells are cross-sectionally associated with both S-PWV and LD-PWV; however, the temporality isn't known. This analysis aimed to determine whether higher T-cell senescence precedes or follows higher S-PWV and LD-PWV leveraging data from the VEGGIE trial. The VEGGIE trial was an 18-week randomized, controlled, caloric restriction weight-loss trial in middle-aged and older adults (age:57.6 ± 5.8 years, n = 58, systolic blood pressure=129±17mmHg at baseline). PWV was estimated (ePWV) using mean arterial pressure and age at each timepoint with previously established reference values. S-ePWV and LD-ePWV were calculated from total ePWV (T-ePWV) using participant-specific exponential models. CD3+ T-cell senescence gene expression (p16INK4a, p21CIP1/WAF1, BCL2, and BAK1) was measured. Multivariable linear regressions tested associations between baseline T-cell senescence and arterial stiffness with 18-week change in PWV or T-cell senescence. Models were adjusted for biologic sex, BMI, race, and treatment-group. Higher baseline p16 ($\beta = 0.546$,P=0.0688) and p21 ($\beta = 0.398$,P=0.0593) were nominally associated with increased T-ePWV. Higher baseline BAK1 was associated with increased S-ePWV ($\beta = 0.0987$,P=0.037). A treatment-group-by-baseline p16 interaction effect was observed with delta T-ePWV (p = 0.0234), where T-ePWV only increased in participants with high p16 at baseline in the caloric restriction group ($\beta = 1.16$,P=0.00405). Interestingly, higher baseline T-ePWV ($\beta$=-0.329,P=0.0556) and S-ePWV ($\beta$=-0.378,P=0.048) were associated with decreased p21. In conclusion, temporal associations between CD3+ senescence and arterial stiffness may be bi-directional and future research is needed directly measuring PWV and T-cell senescence over longer periods.

Abstract citation ID: igaf122.3664

### THE DIFFERENT EFFECTS OF RETIREMENT ON LIFE SATISFACTION BETWEEN THE YOUNG-OLD AND OLD-OLD.

Meeryoung Kim, *Daegu University, Daegu, Republic of Korea*

Korea's retirement age is 60, the lowest among OECD countries. Therefore, older adults must take steps to prepare for 20-30 years after retirement. The effects of retirement can vary depending on whether it is mandatory or voluntary. According to Lowe and Kahn's theory of successful aging and Havigust's activity theory, active lifestyles can contribute to retirees' life satisfaction. As Neugarten defined age differences, this study divided older adults into young-old (ages 60-79) and old-old (age 80 and older) and compared differences in retirement adjustment. This study used the Korean Retirement Income Study data(the ninth and ninth additional panel: 399 young-old and 155 old-old). Hierarchical multiple regressions were used for data analysis. Demographic variables were added in the first step, followed by retirement-related variables, and social variables were included in the third step. The findings showed that the effects of voluntary and mandatory retirement on life satisfaction were statistically significant only for young-old. Furthermore, economic independence was important for young-old. Physical health was important for both young-old and old-old. For young-old, leisure time was a significant factor influencing life satisfaction. However, social relationships were significant indicators for both groups. This implied that leisure time is crucial for the post-retirement adjustment of young-old because they often have limited opportunities to enjoy leisure time due to time constraints before retirement. Furthermore, social relationships that foster a sense of belonging are crucial regardless of age. This suggests that programs and policies that support the continued social relationships of physically frail old-old are particularly desirable.

Abstract citation ID: igaf122.3665

### THE IMOVE TRIAL: BEHAVIORAL AND IMAGING RESULTS OF AN ARTS-BASED INTERVENTION IN PEOPLE WITH AD

Christina Hugenschmidt[1], Jason Fanning[2], Hannah Menaker[1], Santiago Saldana[1], Deepthi Thumuluri[1], Jessie Laurita-Spanglet[2], Edward Ip[1], and Christina Soriano[2], *1. Wake Forest University School of Medicine, Winston-Salem, North Carolina, United States, 2. Wake Forest University, Winston-Salem, North Carolina, United States*

The IMOVE study was a randomized, single-blind trial in dyads (n = 101) of people with MCI or early-stage Alzheimer's disease (PWAD) and their caregivers. IMOVE used a factorial design with four arms designed to test a Movement factor (MOV) and a Social Engagement factor (SOC): the Movement Group (MG) used a dance intervention (MOV+/SOC+); the Movement Alone (MA) arm used the same dance stimulus, but not in a group (MOV+/SOC-); the Social Group (SG) included arts-based social games (MOV-/SOC+); and a Usual Care (UC) control arm (MOV-/SOC-). The primary outcome was quality of life (QOL). Secondary outcomes included physical function, mood, cognition, and structural and functional brain imaging. Behavioral outcomes were assessed using nonparametric methods. Brain imaging outcomes were tested in SPM. Models tested the effects of factors, interactions between factors, and switch to virtual intervention in response to COVID. No significant effects of MOV or SOC were observed for QOL. For secondary outcomes, randomization to a MOV study arm resulted in significant improvements in postural sway on a foam mat ($P < 0.05$) and the Fullerton Advanced Balance Scale ($P < 0.05$). Randomization to a SOC study arm resulted in improved cognitive performance using the RBANS ($P < 0.05$) and depression scores ($P < 0.05$). Diffusion imaging results showed associations with MOV and SOC factors and their interactions in the superior longitudinal fasciculus, posterior limb of the internal capsule, and corticospinal tracts. Overall results suggest that 12 weeks of arts-based movement and social engagement may have separate and beneficial effects in PWAD.

Abstract citation ID: igaf122.3666

### THE IMPACT OF A LIFE STORY INTERVENTION ON DEPRESSIVE SYMPTOMS OF LONG-TERM CARE RESIDENTS WITH DEMENTIA

Lacey DiFranco[1], Silvia Orsulic-Jeras[1], Sara Powers[1], Farida Ejaz[1], and Lisbeth Sanders[2], *1. Benjamin Rose Institute on Aging, Cleveland, Ohio, United States, 2. LifeBio, Marysville, Ohio, United States*

Reminiscence therapies have been shown to positively impact cognition, mood, and other behavioral and psychological symptoms in persons living with dementia (PLWD). LifeBio, a life story intervention, has shown promise in reducing depressive symptoms in nursing home residents with dementia. To address the paucity of scalable life story interventions, a technological adaptation of LifeBio was conducted. LifeBio Memory™, utilizes text-to-speech technology to record resident interviews; machine learning and artificial intelligence enabled production of life story products, such story books, one-page resident bios, and care plans for staff to connect with residents during social or care-based interactions. This paper describes two quasi-experimental, pre–post studies conducted three years apart, testing each version of LifeBio: the first (n = 238) utilized the original, rudimentary protocol and the second (n = 107), used the tablet-based LifeBio Memory™. Both studies demonstrated reductions in depressive symptomology. Study 1, conducted in 16 Ohio nursing homes, showed a significant decrease from baseline (M = 5.59, SD = 5.10) to follow-up (M = 4.71, SD = 5.46); $t(168) = 2.46$, $p = 0.015$, using the Patient Health Questionnaire (PHQ-8). Study 2, conducted in 11 distinct Ohio nursing homes, also showed a significant decrease from baseline (M = 11.23, SD 8.56) to follow-up (M = 9.87, SD = 8.04); $t(106) = 1.99$, $p = 0.024$ using the Center for Epidemiologic Studies Depression Scale (CES-D). Future research plans to evaluate LifeBio Memory™ in randomized trials, identify mechanisms of reducing depression, and assess cost-effectiveness to inform integration into routine dementia care.

Abstract citation ID: igaf122.3667

### THE IMPACT OF STEREOTYPE THREAT ON TECHNOLOGY USAGE IN OLDER ADULTS

Anna Robison[1], and James Houston[2], *1. University of South Florida, Tennessee, United States, 2. Middle Tennessee State University, Murfreesboro, Tennessee, United States*

Negative stereotypes surrounding older adults and technology use, such as incompetency or inability to learn new technologies, continue to permeate American society. These negative stereotypes may serve as a barrier to technology adoption through a concept known as age-based stereotype threat, further contributing to the digital divide and preventing older adults from utilizing technologies that may improve quality of life, independence, and feelings of social isolation. The present study aimed to explore age-based stereotype threat in relation to an objective smartphone use task. The research questions asked if there will be differences between the control and experimental groups on performance on the smartphone task, time to complete the task, and anxiety. We also compared age group differences on the smartphone task. The sample (n = 33) was collected from a community senior center in Tennessee and consisted of older adults aged 55 or older. We divided the sample into those 55-65 (n = 6), 66-75 (n = 13), and 76 + (n = 13) for comparison. We saw no group differences across conditions for the smartphone task, time, or anxiety, but did see a significant difference in the smartphone task by age group, $\chi^2$ (2) = 11.97, p = .003. Post hoc tests revealed that the 76+ age group scored lower on the smartphone task than other age groups, indicating potential cohort effects. Reasons for no differences may include a lack of pressure on the experimental group and stereotype threat construct validity concerns. Future research should increase difficulty on the smartphone task and explore confounds.

Abstract citation ID: igaf122.3668

### THE INTERSECTION OF PHYSICAL DISABILITY AND HOMELESSNESS: A COMMUNITY-ENGAGED RESEARCH INITIATIVE

Natalie Leland[1], Evan Cole[1], Max Hurwitz[2], Julia Lam[3], Carin Wong[4], and Charity Patterson[1], *1. University of Pittsburgh, Pittsburgh, Pennsylvania, United States, 2. UPMC, PIttsburgh, Pennsylvania, United States, 3. Outreach Therapy, Outreach Therapy, Pennsylvania, United States, 4. University of Pittsburgh, University of Pittsburgh, Pennsylvania, United States*

People experiencing homelessness (PEH), compared to their housed peers, experience higher rates of chronic conditions and accelerated disease progression, which can result in a physical disability (e.g., stroke). This project co-designed and executed an examination of homelessness and healthcare service use and outcomes of PEH with a physical disability compared to their peers. This project was a collaboration between the PEH community and academic researchers, which was guided by a co-created shared governance structure and the 10-Step Framework for Continuous Community Engagement. Collaborative prioritization efforts identified outcomes of interest (e.g., duration of homeless shelter use, 30-day readmissions). To examine homelessness service use among PEH, we leveraged Medicaid claims to identify those who had an index stroke, traumatic brain injury (TBI), or amputation as a surrogate for physical disability and linked to the Homelessness Management Information Systems. To examine healthcare utilization and outcomes we used electronic medical records (EMR) to create a cohort of patients with an index physical disability, stratified by housing status (i.e., PEH vs. housed). The PEH cohort (n = 16,024) included those with an index homeless service event between July 2015 and June 2023, of which 5% (n = 746) had a physical disability. Our one-year EMR cohort included patients experiencing an index physical disability—stroke (n = 9,547), TBI (n = 6,708), or amputation (n = 3,494). PEH had more 30-day readmissions (e.g., 46% vs. 32%, P value=0.01, after TBI) than their housed peers. This collaboration highlights the importance of community partnerships to identify populations to target in future quality initiatives.

Abstract citation ID: igaf122.3669

### THE INVISIBLE BURDEN: SELF-CARE AND STRESS IN DUAL CAREGIVERS WORKING IN LONG-TERM CARE

Karen Hirschman, Charlotte Weiss, and Mary Naylor, *University of Pennsylvania, Philadelphia, Pennsylvania, United States*

Long-term care (LTC) employees often juggle caregiving roles at work and home, yet the effects of this dual responsibility are not well understood. This study examined the experiences of LTC employees who also provide unpaid care to family or friends, using surveys and interviews conducted as part of an employer-based support program evaluation. At baseline screening, 107 of 143 employees (75%) reported caregiving outside of work, with nearly half providing over 30 hours per week. Among survey respondents (N = 76), high levels of self-care neglect were reported, with most indicating low to very low engagement in managing, maintaining, or monitoring their own self-care. Stress levels were moderate for 60% and high for 30% of participants. Employees reported high self-care neglect and low to very low management, maintenance, or monitoring of their self-care. Stress levels were moderate for 60% of employees, with an additional 30% reporting high stress. Despite the support program, health self-care neglect worsened over time for some employees (p = 0.01), with no changes in other self-care measures or stress. Employee interview transcripts were coded using a conventional content analysis approach. The key theme identified, "Caregiving is my life, caregiving all around," was supported by subthemes: "I take care of everything," "Work at work, work at home," and "Always something to do, always doing for others." These qualitative findings illustrate the quantitative findings. Suggestions for developing employer-based support programs that align with the lived experiences of LTC employees who also care for family members or friends will be presented.

Abstract citation ID: igaf122.3670

### THE LIFE COURSE PROCESS OF COMING OUT ON THE RURAL NORTHERN GREAT PLAINS

Bryce Van Vleet[1], Heather Fuller[2], and Alyssa Willis[2], *1. Eastern Mennonite University, Harrisonburg, Virginia, United States, 2. North Dakota State University, Fargo, North Dakota, United States*

Identity development for queer individuals involves recognizing non-majority sexual or gendered feelings. Emerging research has identified milestones in queer identity development, indicating coming out can be a developmental process across adulthood. Yet, these studies have excluded rural-dwelling adults who have unique geographical identities. Thus, the current project sought to examine the developmental process of coming out as queer in rural America from retrospective recollections of midlife and older adults (M Age = 61.5). Oral history data was collected beginning in 2017 from regional queer trailblazers in the Northern Great Plains, 28 of whom grew up in a rural area. Abductive thematic analysis was conducted using Elder's life course theory as the guiding deductive framework and resulted in three themes: antecedents to coming out, coming out processes, and consequences of coming out. Participants were likely to feel pressured by traditional gender norms and thus out of place in their rural community of origin. Participants also noted the importance of representational media or people in their rural community that helped them come to terms with their identity. Many participants also indicated using therapy and/or migrating to an urban area to accept their identity. Social networks also helped support or challenge them during their identity exploration and achievement. Findings provide important geographic nuance to the developmental literature and suggest that the availability of cultural resources in rural areas profoundly impacts the ease of queer identity development among rural-dwelling queer people.

Abstract citation ID: igaf122.3671

**THE ROLE OF ATTACHMENT SECURITY IN ADULT CHILDREN'S CAREGIVING FOR PARENTS WITH COGNITIVE IMPAIRMENT OVER TIME**

Angeliki Karachaliou[1], Mary O'Dea[2], and Joan Monin[3], *1. Yale School of Public Health, new haven, Connecticut, United States, 2. Yale School of Public Health, New Haven, Connecticut, United States, 3. Yale, Yale, Connecticut, United States*

Having a parent who is beginning to experience cognitive impairment is a challenging time for adult children both physically and emotionally. Yet, little is known about the potential positive aspects of caregiving including viewing the role of caregiving as rewarding and enriching individuals' life experiences at this stage of a parent and adult child's relationship. One theoretical framework which could explain the variance in caregiver perceptions is attachment theory. Attachment styles of emotional bonding, based on the nature of early emotional connections with parental figures, persist into adulthood impacting the way an individual later receives and provides care. Two main patterns characterize insecure attachment: anxiety and avoidance. People who score low on both are considered securely attached, which is thought to be the most adaptive to stress. Our study consisted of 150 early-stage cognitively impaired parents and one adult child who completed surveys at baseline and one year later, and the aim was to examine whether the adult child's attachment anxiety and avoidance to their parent with cognitive impairment was associated with lower positive views of caregiving. Results from the statistical model showed that only avoidant attachment was associated with lower positive experiences on caregiving at baseline. Attachment dimensions at baseline did not predict year two caregiving positivity controlling for baseline attachment dimensions. As attachment and familial care in later life remains a highly under-researched area, understanding the factors forming the caregivers' experiences early in the caregiving journey is critical to design effective psychosocial interventions and better support parent-child dyads.

Abstract citation ID: igaf122.3672

**THE ROLE OF SOCIAL ISOLATION AND DEPRESSIVE SYMPTOMS IN THE LINK BETWEEN PHYSICAL ACTIVITY AND COGNITION IN OLDER ADULTS**

Daniel Leme[1], Neil Charness[2], Dawn Carr[1], and Zhe He[1], *1. Florida State University, Tallahassee, Florida, United States, 2. Weill Cornell Medicine, New York, New York, United States*

Although the direct relationship between physical activity (PA) and mild cognitive impairment (MCI) in older adults has been widely discussed in the literature, evidence on the indirect association through psychosocial factors is limited. This study aimed to verify both direct and indirect associations between PA and MCI, considering depressive symptoms and social isolation as mediators in a subset of 2933 participants (60 years or older) from the National Health and Nutrition Examination Survey 2011–2014. PA was categorized into four levels (none, low, moderate, high) based on the intensity and duration of activity across occupational, leisure, and transportation domains. Social isolation was determined through a 0–4 ordinal scale, where higher scores indicate greater isolation. One point was assigned for each of the following characteristics: being unmarried, living alone, reporting difficulty leaving home, and difficulty engaging in social activities. Depressive symptoms were measured using the PHQ-9 as follows: minimal, mild, and moderate or severe. MCI was defined as performance ≥1 standard deviation below the mean on at least two of three cognitive tests. The path analysis results showed that higher PA levels were directly associated with lower MCI ($\beta = -0.08$, $p < .001$) and indirectly through reduced depressive symptoms and social isolation ($\beta = -0.001$, $p = .037$). These findings highlight the complex relationship between PA and MCI in older adults, suggesting that PA influences cognition through psychosocial factors in this subgroup that is at greater risk of social isolation and mental and physical health vulnerabilities.

Abstract citation ID: igaf122.3673

**THE VALUE AND THE NEED: PREPARING BSN STUDENTS FOR LONG-TERM CARE EMPLOYMENT**

Kimberly Delgado[1], Donna Roberson[2], and Jean Matthews[3], *1. East Carolina University College of Nursing, Greenville, North Carolina, United States, 2. East Carolina University, Greenville, North Carolina, United States, 3. East Carolina University, Ahoskie, North Carolina, United States*

Long-term care (LTC) settings consistently struggle to meet staffing demands, especially adequate registered nurse (RN) coverage. With an increasing US older adult population, the need for LTC is expected to grow. The Center for Medicare and Medicaid made staffing recommendations to increase RNs employed in LTC. BSN nursing education focuses on general nursing knowledge heavily centered on acute care settings, not specific training in LTC. With support from the Carolina Geriatric Workforce Enhancement Program grant, the team developed an innovative course with tuition support for five students selected from a competitive pool of thirteen applicants. The course provided skills and knowledge focused on LTC settings, care of older adults, and 24 hours of clinical experience in LTC. Qualitative inquiry throughout the course included pre/post course discussion and reflection on the experience. Analysis indicated that the students developed an improved attitude to LTC work, including increased appreciation for the RN role in LTC. The students found the course to be comprehensive, yet reasonable to accomplish with other educational demands of their BSN program. All five students indicated intention to seek LTC employment, if not upon graduation, at some point in their career. One student said that she could "really see the value and need" for BSN RNs in residential care. To meet the demand for BSN RNs in LTC, such programming is essential in BSN programs (eventually nationwide). The course will be repeated with ongoing data collection and tracking of employment sites upon participant graduation.

Abstract citation ID: igaf122.3674

**TIMING OF MENTAL HEALTH DIAGNOSIS IN OLDER IMMIGRANTS AND NON-IMMIGRANTS: EVIDENCE FROM SHARE**

Ana C Teixeira-Santos[1], Joana Carvalheiro[2], and Anja Leist[3], *1. Institute for Research on Socio-Economic Inequality, Department of Social Sciences, University of Luxembourg, Esch-sur-Alzette, Diekirch, Luxembourg, 2. University of Glasgow, School of Psychology and Neuroscience, University of Glasgow, Scotland, United Kingdom, 3. University of Luxembourg, Esch-sur-Alzette, Grevenmacher, Luxembourg*

The risk of mental health problems is high in older adults. Older immigrants may be at even higher risk due to social and socioeconomic disparities. However, little is known about the timing of their first diagnosis compared to native-born peers. This study examined whether older immigrants have a higher risk of earlier mental health diagnosis compared to non-immigrants and the contribution of social determinants. Data came from SHARE waves 5–9, including 48,154 participants (55.1% women) aged 55+ with no prior mental health diagnosis. The outcome was first self-reported diagnosis of affective or emotional disorders. Stepwise hierarchical Cox mixed-effects models with country-level random intercepts sequentially adjusted for sociodemographic, socioeconomic, family, community engagement, and health care satisfaction factors. In the basic model, migrant status was associated with an 18% increased hazard of diagnosis (HR = 1.18, 95%CI: 1.08–1.29). All included covariates were statistically significant ($p < .002$). After full adjustment, the HR decreased to 1.16 (95%CI: 1.05–1.27), suggesting that community engagement and health care satisfaction partially account for the higher risk among migrants. Individuals from low-income countries showed a higher risk (HR > 1.30, $p < .08$) and substantial between-country variation was identified ($p = .02$). These results suggest that older immigrants were diagnosed earlier, with factors such as social engagement and satisfaction with health care partly explaining this difference, highlighting the importance of culturally sensitive interventions that enhance engagement and participation, as well as policy measures to reduce disparities in mental health outcomes among aging immigrant populations across Europe.

Abstract citation ID: igaf122.3675

### TRAINING EXPERIENCE AND FAMILY INTERACTIONS IN CARE SETTINGS: FINDINGS FROM THE NATIONAL DEMENTIA WORKFORCE STUDY

Sunshine Rote, Heehyul Moon, and Kathryn Williams-Sites, *University of Louisville, Louisville, Kentucky, United States*

This study analyzes recently released data from the National Dementia Workforce Study (NDWS). The NDSW is the first large, annual survey of the dementia care workforce in the United States and sponsored by the NIA/NIH. Our analysis examines the relationships between training experiences, family interactions, and worker confidence among direct care workers in nursing home (n = 390) and assisted living facility settings (n = 445). Over 90% of direct care workers in both settings report formal or informal training in dementia, injury prevention, and responding to the needs of residents, while the largest informal and formal training gaps identified were in content related to working with families of people living with dementia. Bivariate analyses show that direct care workers with extensive formal and informal, on-the-job training report significantly greater confidence in the ability to adapt to the changing needs of residents living with dementia compared to those with less training in both nursing homes and assisted living facilities. Direct care workers who report problems with residents' family members are significantly less confident in their ability to remain motivated in providing care. These findings suggest that comprehensive training initiatives combined with family relationship management strategies may be essential for optimizing dementia care quality and worker retention in long-term care settings.

Abstract citation ID: igaf122.3676

### TRAJECTORIES OF SENSE OF PURPOSE LATER IN LIFE: A COORDINATED ANALYSIS OF SIX LONGITUDINAL STUDIES

Olivia Duchow[1], and Gabrielle Pfund[2], *1. Auburn University, Davis, California, United States, 2. Auburn University, Auburn, Alabama, United States*

A sense of purpose is defined as the extent to which individuals perceive their lives as having direction, goals, and meaning. Additionally, having a higher sense of purpose is consistently linked to healthier aging outcomes in older adulthood, including slower cognitive decline, reduced mortality risk, and less allostatic load. However, despite its importance, the trajectory of purpose across the lifespan remains understudied, particularly in later life. In this pre-registered work, we examined age-related trajectories of purpose using coordinated data analysis across six longitudinal studies: Health and Retirement Study (N = 14,000), Midlife in the United States (N = 4,000), Rush Memory and Aging Project (N = 2,300), Rush Minority Aging Research Study (N = 950), Rush Latino Core (N = 250), and Wisconsin Longitudinal Study (N = 10,300). These datasets include diverse populations with at least three measurement occasions of purpose with individuals spanning years to decades. These strengths enable the harmonized examination of purpose development within adults over time. We incorporated demographic and health covariates including race, gender, education, relationship status, and health conditions to examine their impact on purpose trajectories. Using a coordinated analysis approach, this study used multilevel linear and quadratic growth models to characterize how sense of purpose changes as individuals age across diverse groups. Sense of purpose tends to decline later in the lifespan, highlighting the vulnerability of older adults to maintain a sense of purpose. Findings inform the importance of future intervention development aiming to promote purpose into older adulthood.

Abstract citation ID: igaf122.3677

### TRAJECTORIES OF SOCIAL ISOLATION AND LONELINESS AND ADVANCE CARE PLANNING AMONG U.S. OLDER ADULTS

Ke Li[1], Yifan Lou[2], and Jing Wang[3], *1. Ohio University, Athens, Ohio, United States, 2. Virginia Commonwealth University, Richmond, Virginia, United States, 3. University of New Hampshire, Durham, New Hampshire, United States*

Social isolation and loneliness are critical public health concerns that may interfere with end-of-life care preferences. However, their relationships with advance care planning (ACP) remain unclear. Using three-wave data from the Health and Retirement Study, this study identified trajectory patterns of social isolation and loneliness and explored their associations with ACP. Social isolation index was generated using five indicators of marital status, social participation, and frequency of contact. Loneliness was measured by the UCLA loneliness scale. Three dimensions of ACP were considered: living will, Durable Power Attorney for Health Care (DPAHC), and ACP discussions. Group-based trajectory model identified four distinct trajectories for both social isolation (low start-decreasing, low start-increasing, moderate-start stable, high start-stable) and loneliness (low start-stable, low start-increasing, moderate start-decreasing, high start-stable). The results of logistic regression models indicated that both trajectories were significantly related to discussion and DPAHC, but not living will. The high start-stable isolation group had the lowest odds of having discussion across groups and lower odds of having DPAHC than those with moderate start-stable isolation. Compared to low start-stable loneliness group, low start-increasing loneliness group was less likely to have DPAHC. The odds of having discussion were significantly lower among the groups of high start-stable loneliness and low start-increasing loneliness than those with low start-stable loneliness. Gender-specific analyses showed that females were affected by both social isolation and loneliness, whereas males were solely affected by social isolation. The findings highlight the importance of developing targeted interventions to address social isolation and loneliness to increase ACP.

Abstract citation ID: igaf122.3678

### TRANS*FORMING THE LIFE COURSE: NEW DIRECTIONS FOR CRITICAL GERONTOLOGY

Jen Justice, *Miami University, Oxford, Ohio, United States*

The life course perspective proposes life timelines to which individuals must adhere in order to minimize disruptions to their developmental trajectory and age optimally. These normative timelines are constructed and enforced with the ultimate goal of upholding traditional Western values such as reproductive futurity and capitalistic productivity. The framework is predicated on the assumption of a homogenous society; equal opportunity and desire to access each life transition is assumed and thus deviation in their sequence or timing is seen as a personal failure rather than cultural difference. The life course perspective introduces normative constructions of time that lack applicability to individuals of non-normative identities, whose lives often do not follow the linear and teleological progression proposed by the model, instead unfolding in nonlinear, nonconsecutive, and multidirectional paths. By delineating 'on-time' and 'off-time' events and, by extension, 'right' and 'wrong' ways to age, the life course perspective acts as a mechanism of oppression employed by social institutions to pathologize individuals of non-normative identities, particularly in later life. Drawing from critical, cultural, queer, and trans* studies and bringing these disciplines into dialogue with gerontological theory, this paper offers a theoretical trans*formation of time, aging, and the life course.

Abstract citation ID: igaf122.3679

### TRENDS IN ECONOMIC INSECURITY AMONG OLDER ADULTS IN CALIFORNIA, 2015 - 2023: USING THE CALIFORNIA ELDER INDEX

Qianyun Wang[1], Lauren Vuong[2], Natalie Kim[1], Mohammad Khorgamphar[1], Sithara Diunugala[3], Shreya Paul[1], Kathryn Kietzman[4], and D. Imelda Padilla-Frausto[5], *1. University of California, Los Angeles, Los Angeles, California, United States, 2. Stanford University, Stanford, California, United States, 3. UC Irvine Joe C. Wen School of Population & Public Health, Irvine, California, United States, 4. UCLA Fielding School of Public Health, Department of Community Health Sciences, Los Angeles, California, United States, 5. UCLA Center for Health Policy Research, Los Angeles, California, United States*

The California Elder Index (CEI) provides a better measurement of the income older adults need to cover basic living expenses compared to the Federal Poverty Level (FPL) guidelines. The CEI includes costs for housing, healthcare, food, transportation, and miscellaneous, while the FPL includes food costs only. Using the CEI, we document economic insecurity among older Californians from 2015 to 2023. Since the FPL is widely used to determine eligibility for public programs, we provide CEI and FPL comparisons to show that many older adults with income above the FPL, but below the CEI, lack the income needed for day-to-day expenses. This group falls within the "CEI Income Gap" which we disaggregated into two categories: "CEI Low-Income Gap"(below the CEI and 100-199% FPL) and "CEI Middle-Income Gap" (below the CEI and ≥ 200% FPL). Analyses also include those classified as "very-low-income" (below the CEI and ≤99% FPL). Our findings document trends and persistent disparities in economic insecurity: 1) The percentage of older adults with income below the CEI and FPL steadily increased from 2015 to 2023. 2) Economic insecurity among older adults has shifted, with fewer falling into the CEI Low-Income Gap, and more falling into very-low-income or the CEI Middle-Income Gap. 3) Single older adults living alone consistently face higher rates of economic insecurity than older couples, especially single renters, women, Latinos, and Blacks/African Americans. Our findings underscore the value of using the CEI and an income gap framework to better assess needs and allocate resources for California's aging population.

Abstract citation ID: igaf122.3680

### UPSTREAM DRIVERS OF RECEIVING CARE IN MINORITY-SERVING HOSPITALS: A CROSS-SECTIONAL STUDY USING MCBS DATA

Louisa Holaday[1], Alina Kung[1], Yingtong Chen[2], Karen McKendrick[1], Bian Liu[1], and Albert Siu[2], *1. Icahn School of Medicine at Mount Sinai, New York, New York, United States, 2. Icahn School of Medicine at Mount Sinai, new york, New York, United States*

Intro: Racial separation of care contributes to racial disparities in health. Little is known about upstream drivers of receiving care in a minority-serving hospital (MSH) and differences by race/ethnicity. Methods: Cross-sectional analysis of MCBS data (2010-2019) with binary outcome of hospitalization in MSH versus not. Exposures of interest were individual race/ethnicity and insurance; neighborhood disadvantage (SDI); and regional residential racial segregation. We included individual and regional covariates. In fully adjusted models, we tested whether race/ethnicity modified the association between SDI or residential segregation and hospitalization in MSH. Results: Among 8,735 hospitalizations, race/ethnicity (aOR 3.19 for Black vs. White patients; 95% Confidence Interval (CI): 2.60-3.91; $P<0.001$); dual-eligible status (aOR: 1.30, 95% CI: 1.05-1.60; $P<0.05$), neighborhood disadvantage (aOR for most disadvantaged versus least: 2.03, 95% CI: 1.56-2.63, $P<0.001$); and residential segregation (aOR for high segregation versus low: 2.99, 95% CI: 1.98-4.52; $P<0.001$) were independently associated with hospitalization in MSH. In the least disadvantaged or segregated areas, all patients were unlikely to be hospitalized in MSH; however, in the most disadvantaged or segregated areas, White patients had only a small increase in odds of MSH hospitalization while Black patients had a very large increase (P for interaction terms = 0.001 and 0.04, respectively). Conclusion: Our findings on differences by race/ethnicity suggest a need for further research on factors driving site of care, to address the upstream socio-structural determinants of health, and to invest in minority-serving hospitals given the practical challenges of eliminating neighborhood disadvantage and residential segregation.

Abstract citation ID: igaf122.3681

**USING METAPHORICAL NUDGES TO PROMOTE MENTAL HEALTH HELP-SEEKING IN OLDER ADULTS: PRELIMINARY RESULTS OF AN RCT**

Tianyin Liu, and Ho Kan Liu, *The Hong Kong Polytechnic University, Hong Kong, Hong Kong, China (People's Republic)*

Older adults are at an increased risk for mental health issues; however, their intention in seeking help is relatively low. To promote mental health help-seeking, nudging could be a viable strategy, but it has rarely been examined in older adults. This study evaluated the effects of pictorial metaphorical nudges (PMNs) and descriptive metaphorical nudges (DMNs) versus help-seeking information (Control) on promoting mental health help-seeking using a three-arm randomised controlled trial (RCT). The PMNs and DMNs were co-developed by older volunteers (N = 12) with experience in mental health services and delivered daily to participants via WhatsApp for two weeks. The primary outcomes were changes in subjective norms and help-seeking intention; secondary outcomes included perceived behavioral control, help-seeking attitude, perceived barriers to help-seeking, and mental health assessed by PHQ-9, GAD-7 and UCLA-3. In total, 280 participants (average age, 67.9 years; 214 females) were recruited from the community from February 1 to July 31, 2025, and they completed the baseline (T0), post-intervention (T1), and three-month follow-up (T2) questionnaires. Linear mixed models were used for data analysis. Results showed that PMN was more effective in improving subjective norms ($\beta = 0.46$, $p < .05$) and help-seeking attitudes ($\beta = 0.28$, $p < .05$) from T0 to T1 than Control; DMN and Control showed no significant difference. Data collection of more participants and at T2 is ongoing. Preliminary results suggest the feasibility and effects of metaphorical nudges in changing subjective norms and attitudes towards mental health help-seeking. Additionally, visual images may communicate complex information more effectively than text alone.

Abstract citation ID: igaf122.3682

**VARIATIONS IN TRANSPORTER GENES ARE LINKED TO REGION-SPECIFIC PATTERNS OF CORTICAL BRAIN AGING**

Nicholas Kim, Ayati Mishra, Nahian Chowdhury, and Andrei Irimia, *University of Southern California, Los Angeles, California, United States*

Deep learning-derived local brain age (LBA) provides a regional indicator of cortical aging, with higher values reflecting more senesced brain regions. Elucidating the genetic architecture of LBA can reveal mechanisms underlying individual differences in brain aging and susceptibility to neurodegeneration. We conducted the first genome-wide association study of LBA in 41,708 cognitively normal adults (21,983 females) aged 45 to 83 years in the UK Biobank, testing genetic variants for associations with regional cortical aging. Our analysis identified 1,212 significant variants, with the most prominent signals mapping to genes involved in ion and metabolite transport. Two independent variants near KCNK2, which encodes a potassium channel, exhibited the strongest associations with LBA. These alleles were linked to faster aging in the cingulate, precuneus, and parietal cortices, brain regions that undergo early atrophy in Alzheimer's disease (AD). Additional associations implicated variants mapped to SLC25A13, a mitochondrial aspartate-glutamate carrier, and SLC6A20, a transporter of proline and glycine. The SLC25A13 variant was associated with slower aging in frontal and temporal cortices. These brain regions support executive function and episodic memory, both disrupted in AD. In contrast, the SLC6A20 variant was linked to faster aging across parietal and sensorimotor cortices, regions implicated in neurodegenerative motor symptoms such as gait disturbance in Parkinson's disease. Together, these findings suggest a polygenic architecture of cortical aging anchored in transport pathways, highlighting potential targets for early intervention. Future work will test if polygenic risk scores capturing the combined effects of transport-related variants can predict trajectories of regional cortical aging.

Abstract citation ID: igaf122.3683

**VIRTUAL REALITY FOR GERONTOLOGICAL EDUCATION: A PILOT STUDY OF INTERDISCIPLINARY HEALTHCARE TRAINING**

Mingyang Zheng[1], and Sarah Rakes[2], *1. Radford University, Blacksburg, Virginia, United States, 2. Radfford University, Radford, Virginia, United States*

The United States is facing a workforce shortage in caring for its aging population. To address this issue, we developed a training program using Virtual Reality (VR) technology to help healthcare professionals and students better understand and care for older adults, with a specific focus on Alzheimer's disease and social isolation. Participants included nurses, therapists, counselors, students, and other healthcare professionals. We conducted pre- and post-training assessments measuring Alzheimer's knowledge (Carpenter et al., 2009), confidence in working with older adults (Elvish et al., 2018), awareness of social isolation risk factors and resources, and overall training satisfaction. The program trained 107 participants, and among the 48 who completed both pre- and post-training assessments, knowledge about Alzheimer's disease increased significantly, from a mean score of 16.63 to 22.15 ($t = 4.38$, $p < 0.001$). Similarly, confidence in working with older adults improved from 25.38 to 31.29 ($t = 4.671$, $p < 0.001$). Participants also showed increased awareness of social isolation, identifying more risk factors (from 3.33 to 3.81) and available resources (from 3.06 to 3.56) after the training. Nearly all participants (42 out of 44) reported learning something new, and 39 said they could apply what they learned in their work. The vast majority (43 out of 44) felt the training surpassed their initial expectations. This pilot project demonstrates that an immersive, VR-based approach to geriatric education significantly improves both knowledge and confidence among healthcare professionals and students.

Abstract citation ID: igaf122.3684

**WHEN DISABILITY BEGINS MATTERS: LATER-ONSET DISABILITY AND MASTICATORY DISCOMFORT IN OLDER ADULTS**

Chan-Young Park[1], and Nam-Hee Kim[2], *1. Yonsei University, Yonsei University, Kangwon-do, Republic of Korea, 2. Yonsei University, Wonju, Kangwon-do, Republic of Korea*

This study investigates the effect of disability onset timing on masticatory discomfort among older adults, drawing on nationally representative data from the 2023 Korean National Disability

Survey. We classified 6,180 adults aged 50 and older into two groups: Aging with Disability (AWD; disability onset before age 50) and Disability with Aging (DWA; onset at age 50 or later). Masticatory discomfort was measured via self-report, and weighted logistic regression was used to identify predictors, adjusting for age, gender, disability type and severity, oral health behaviors, and healthcare access. Masticatory discomfort was significantly more prevalent in the DWA group (37.1%) compared to the AWD group (22.2%, $p < 0.001$). Even after full adjustment, DWA status remained an independent predictor (odds ratio [OR] 1.23; 95% confidence interval [CI] 1.04–1.45). Other significant predictors included infrequent tooth brushing (once per day or less; OR 1.84, $p < 0.001$), no dental checkups in the past year (OR 1.46, $p < 0.001$), unmet healthcare needs (OR 1.73, $p < 0.001$), older age (age 80 or above; OR 3.23, $p < 0.001$), lower income (OR 2.06, $p < 0.001$), and lower educational attainment (elementary or less; OR 1.39, $p = 0.002$). These findings suggest that later-life disability onset increases vulnerability to oral health problems, likely due to limited adaptation time, weaker coping strategies, and barriers to care. The results highlight the need for life-course–sensitive oral health interventions and suggest that the timing of disability onset should be a key consideration in geriatric care and public health policy.

Abstract citation ID: igaf122.3685

### WHY AGETECH REMAINS INVISIBLE TO OLDER ADULTS: GLOBAL BARRIERS AND INTEGRATION GAPS

Ryan Jo[1], and Chorong Park[2], *1. Elphi, West Lafayette, Indiana, United States, 2. University of Houston, Houston, Texas, United States*

AgeTech has surged—from remote monitoring and smart homes to wearables, safety, and social platforms—yet everyday use by older adults remains modest. We conducted a systematic scan of 300+ companies across North America, Europe, Asia, and Australia and interviewed older adults and caregivers; we triangulated findings with adoption metrics and public reviews to map barriers and enablers. Discoverability is often the first hurdle: products are hard to find until a crisis. Usability and accessibility issues persist (cognitive load, language fit, aesthetics that "signal decline"), alongside cost and reimbursement gaps, limited training or in-life support, and fragmented ecosystems that lack interoperability and electronic health record/personal health record integration. Caregiver-centric marketing sidelines older adults as primary users, reinforcing stigma, privacy and data-governance worries, and low trust. Cultural and linguistic misalignment further depresses sustained use, particularly among independently living older adults and globally diverse communities. Findings point to a practical path forward: integrated, standards-based platforms; co-created, dignity-affirming experiences; plain-language transparency and consent; funded onboarding plus ongoing support; multilingual, culturally tailored outreach; and distribution that meets people where they are (community centers, libraries, faith and cultural organizations). We conclude with procurement criteria for providers, reimbursement recommendations for training/support, and policy levers on interoperability and privacy to enable equitable, scalable AgeTech adoption—and to reframe aging as engaged, capable, and worthy of joyful technology.

Abstract citation ID: igaf122.3686

### WORK PRECARITY, THE GREAT RECESSION, AND DISPARITIES IN HBA1C CHANGES IN OLDER WORKERS

Miriam Mutambudzi, *Syracuse University, Syracuse, New York, United States*

Racial health disparities are exacerbated during economic recessions, when employment disruptions including precarious employment intensify. This study examines whether cumulative precarity, occurring before and during the Great Recession, contributes to worsening diabetes risk, as measured by HbA1c, among older Black and White workers. Data from the Health and Retirement Study (2006–2016) were used to examine changes in HbA1c over 4-year period among 1,573 older Black and White workers. Precarity i.e. experiencing involuntary unemployment, short job tenure, or reduced work hours, and was classified as pre-recession only (2006), Great Recession only (2008–2010), both periods, or neither (referent). Conditional change regression models assessed 4-year follow-up biomarker changes from baseline, adjusting for relevant covariates. Baseline HbA1c strongly predicted follow-up HbA1c ($\beta = 0.75$, $p < 0.001$). While precarity did not show a uniform effect across all participants, race-specific analyses revealed meaningful differences: race*precarity interactions indicated stronger effects among Black participants for pre-recession ($\beta = 0.39$, $p = 0.028$), Great Recession ($\beta = 0.37$, $p = 0.003$), and both periods ($\beta = 0.37$, $p = 0.005$). Stratified analyses confirmed that among Black participants Great Recession only precarity predicted higher follow-up HbA1c ($\beta = 0.30$, $p = 0.031$), with pre-recession only and both-period exposure showing similar marginal trends ($p = 0.056$). Among White participants, no significant associations were observed. Employment precarity during and prior to the Great Recession was associated with higher follow-up HbA1c among older Black workers, but not Whites, highlighting racial disparities in vulnerability to economic shocks.

### SESSION 8005 (POSTER)

### LATE BREAKING POSTER SESSION II

Abstract citation ID: igaf122.3687

### A BODY SHAPE INDEX SHOWS STRONGER ASSOCIATION WITH FALLS THAN BMI IN OLDER ADULTS

Zhenxuan Ma[1], and Indrakshi Roy[2], *1. Indiana University at Bloomington, Bloomington, Indiana, United States, 2. Indiana University, Bloomington, Bloomington, Indiana, United States*

Falls are a major public health concern among older adults, frequently leading to severe injuries, loss of independence, and increased mortality. Identifying individuals at higher risk of falls is therefore a priority in geriatric care and preventive strategies. While Body Mass Index (BMI) has traditionally been used to assess body composition, it fails to account for fat distribution and central obesity, which critically influence postural stability and fall risk. A Body Shape Index (ABSI), a novel anthropometric measure incorporating waist circumference with height and weight, has been proposed as a more accurate indicator of abdominal adiposity. This study aimed to compare the association of ABSI versus BMI in relation to falls among older adults, utilizing data from the National Health and Aging Trends Study (NHATS). This retrospective cohort study analyzed data from 5,343 NHATS participants aged 65 years and older. In adjusted models, ABSI was associated with both any fall (OR 1.15, 95% CI 1.04–1.27, p = 0.009) and multiple falls (OR 1.20, 95% CI 1.03–1.39, p = 0.018), while BMI showed weaker associations (any fall: OR 1.02, 95% CI 1.00–1.03, p = 0.030; multiple falls: OR 1.01, 95% CI 0.995–1.03, p = 0.158). Associations were stronger among adults ≥80 years, those with multiple chronic conditions (MCC), and in subgroups with specific diseases such as arthritis, hypertension, or diabetes. This study suggests ABSI may be a more sensitive measure than BMI for identifying fall risk and may provide added value for geriatric fall risk assessment.

Abstract citation ID: igaf122.3688

### A DEEP LEARNING MODEL FOR PREDICTING PATIENT AGE AND DISEASE STATUS USING PATTERN ERG DATA

Hunter Porter, and Jonathan Wren, *Oklahoma Medical Research Foundation, Oklahoma City, Oklahoma, United States*

The eye is an extension of the central nervous system and can act as a window into neural, vascular, and metabolic health. One measure of retinal function, the pattern electroretinogram (PERG), is used to assess central vision by presenting patients a patterned stimulus (e.g. – an alternating checkerboard). These data have proven useful in understanding a variety of conditions such as diabetic retinopathy, glaucoma, and age-related macular degeneration. However, PERG data currently requires expertise in both performing and interpreting the assay. We hypothesized that an artificially intelligent (AI) model could help with PERG interpretation and potentially provide novel insights outside of ocular dysfunction. We used a publicly available dataset of human PERG traces (PERG-IOBA) to train a deep-learning AutoEncoder (AE) model by tasking it with reproducing real sample data. Then, we used the rich encoder portion of the AE as input for new models tasked with predicting patient age and clinician-annotated disease status which were evaluated on left out test data. These predictor models showed great accuracy in identifying normal from abnormal PERG traces (AUC = 0.834) and were much better age predictors (mean absolute error = 11.7 years) than the naïve model (MAE = 14.8 years). Our next steps involve collecting data from a variety of clinicians to ensure generalizability and to predict non-ocular conditions (e.g. – Alzheimer's and diabetes status). Finally, we want to share these models to enable earlier identification of functional deficits and broader adoption of formerly specialist-only assays such as PERG.

Abstract citation ID: igaf122.3689

### A HIDDEN CRISIS IN NURSING HOMES: EXAMINING DEMENTIA CONFIDENCE AND NEGATIVE JOB EXPERIENCE

Joonyoung Cho[1], and Angela Perone[2], *1. University of Hawai'i at Mānoa, Honolulu, Hawaii, United States, 2. University of California, Berkeley, Berkeley, California, United States*

While dementia care needs in nursing homes are increasing, staff positions are often unfilled. Research suggests that some workers report challenging experiences by residents (e.g., communication problems, disrespectful behaviors). Confidence in providing care might shape their experiences, yet we know little about whether confidence in providing dementia care is associated with negative experiences. To address this gap, we used data from the National Dementia Workforce Study Nursing Home Survey Wave 1, an annual survey of dementia workers in the US. Our final sample size was restricted to respondents who provided information on confidence and negative experiences with residents (N = 374). Confidence in providing dementia care is coded as a continuous variable by summing the following 4 dimensions: (1) use past information to improve communication, (2) adapting working style for tailored care (3) keep motivating, and (4) play an active role in the team. Two separate multivariate logistic regressions revealed that confidence in providing dementia care is associated with lower odds of experiencing communication difficulties with residents (OR = 0.70, p ≤ 0.001). In contrast, confidence in providing dementia care is not associated with experiencing disrespectful behaviors from residents (OR = 0.85, p = 0.176). This study suggests that interventions that build confidence in dementia care (e.g., targeted training), including strategies for navigating communication and behavior challenges, could improve dementia care worker experiences and strengthen this workforce overall.

Abstract citation ID: igaf122.3690

### A NOVEL, MULTI-INSTITUTIONAL GERIATRICS ASSESSMENT IDENTIFIES KNOWLEDGE GAPS IN HEALTH PROFESSIONS TRAINEES

Apoorva Rangan[1], Malvika Varma[2], Rachel Jantea[3], Mengru Wang[4], Yochai Shavit[5], Stephen Pelletier[6], Andrea Schwartz[7], and Deborah Kado[8], *1. Stanford University, Los Altos Hills, California, United States, 2. Beth Israel Deaconess Medical Center, Boston, Massachusetts, United States, 3. UTHealth Houston, Houston, Texas, United States, 4. University of Washington, Seattle, Washington, United States, 5. Stanford University, Stanford, California, United States, 6. Harvard Medical School, Boston, Massachusetts, United States, 7. VA Boston/Harvard Medical School, Boston, Massachusetts, United States, 8. Stanford School of Medicine, Palo Alto, California, United States*

Medical students are expected to care for older people shortly after graduation, yet there are no standardized assessments of geriatrics knowledge. We describe the development and multi-site pilot of the Age-Friendly Knowledge Assessment Tool (AF-KAT), a new assessment evaluating Geriatrics knowledge in clinical trainees. The AF-KAT is a 20-item, case-based multiple-choice questionnaire written by Geriatrics experts and reviewed by general internists and professional editorial staff, including several rounds of peer-review for psychometric and content validity. The AF-KAT questions address the Geriatrics 5M's and were mapped to updated Geriatrics competencies for medical students and residents. In the first at-scale deployment of this tool, we describe findings from the assessment of 275 graduating medical students across four medical schools (University of Washington, UTHealth Houston, Stanford, and Harvard). Participants self-reported their Geriatrics exposure, attitudes towards aging, and perceived sufficiency of training and completed the AF-KAT. Despite high self-reported preparedness, the mean proficiency score was 60%. Only 25% of students reached a "passing" threshold of 70%. Linear modeling revealed that more curricular exposure ($\beta = 0.37^{*}$) and greater understanding of age-friendly systems ($\beta = 0.55^{*}$) significantly predicted performance. Negative attitudes toward older people correlated with worse performance ($r = -0.20^{***}$). This case highlights the feasibility and diagnostic value of using the AF-KAT as an individual-level and program-level assessment tool, with potential uses across health professions trainees. Results underscore the need for a universal and competency-based approach to Geriatrics education, to ensure new physicians are equipped to deliver competent, age-friendly care in an aging society.

Abstract citation ID: igaf122.3691

### A PSYCHOLOGICAL DISTRESS-LINKED PLASMA METABOLITE SCORE PREDICTS COGNITIVE AGING

Yiwen Zhu[1], Tianyi Huang[2], Raji Balasubramanian[3], Clary Clish[4], Timothy Hughes[5], Jerome Rotter[6], Susan Hankinson[3], and Laura Kubzansky[1], *1. Harvard T.H. Chan School of Public Health, Boston, Massachusetts, United States, 2. National Institute on Aging, Baltimore, Maryland, United States, 3. University of Massachusetts Amherst, Amherst, Massachusetts, United States, 4. Broad Institute of MIT and Harvard, Cambridge, Massachusetts, United States, 5. Wake Forest University School of Medicine, Winston-Salem, North Carolina, United States, 6. The Lundquist Institute at Harbor-UCLA Medical Center, Torrance, California, United States*

Chronic psychological distress, such as depression and anxiety, is associated with cognitive decline and increased risk of Alzheimer's disease and related dementias. We previously developed and validated a plasma metabolite-based distress score (MDS), which predicted incident risks of cardiometabolic conditions in independent samples. Components of the MDS reflect metabolic consequences of chronic distress, such as pathways of inflammation and neurotoxicity. In this study, we examined whether baseline MDS was linked to faster cognitive decline and elevated risk of mild cognitive impairment (MCI) or dementia among 3103 participants (mean age 58.2 years at baseline) from the Multi-Ethnic Atherosclerosis Study (MESA) over 24 years of follow-up. Global cognitive function was assessed using a summary score of the Cognitive Abilities Screening Instrument (CASI) at four time points spanning 10-24 years after baseline. Adjudicated cognitive status was obtained 20 years post-baseline based on expert reviews of clinical and informant materials beyond the CASI. Analyses adjusted for ages at baseline and follow-up, study visit, test language, gender, race/ethnicity, socioeconomic status, and a range of biobehavioral and health-related factors. High MDS at baseline was associated with faster declines in global cognitive function measured by repeated CASI assessments (beta coefficient from a linear mixed model for the interaction between MDS and age=-0.03, 95% CI[-0.05, -0.004], $p = 0.02$) and slightly higher odds of MCI or dementia (logistic regression Odds Ratio=1.13[1.00, 1.27], $p = 0.05$). Metabolic alterations linked to psychological distress may contribute to accelerated cognitive aging. Characterizing these pathways could inform strategies to mitigate the impact of distress on cognitive health.

Abstract citation ID: igaf122.3692

### A SEAL THAT HEALS: RESIDENT & STAFF PERSPECTIVES ON PARO IN A LONG-TERM CARE HOME

Yuka Ohno, Janna Zeid, and Lillian Hung, *University of British Columbia, Vancouver, British Columbia, Canada*

In Canada, dementia is highly prevalent in long-term care (LTC), with estimates suggesting that nearly two-thirds of residents live with some form of cognitive impairment. Within LTC, residents often face social isolation, loneliness, and agitation, challenges compounded by limited staffing resources. Socially assistive robots, such as PARO, have been developed to reduce stress and promote well-being. Yet, most research has examined either LTC residents' or staff's perspectives in isolation, limiting understanding of its broader impact. Our study explored the experiences of both residents with dementia and staff when using PARO in an LTC setting. Over four weeks from February to March 2025, residents ($n = 10$) engaged with PARO during group session, while staff ($n = 10$) participated in reflective sessions informed by video excerpts of these interactions. Reflexive thematic analysis revealed three themes from residents' and staff's perspectives respectively. For residents, PARO brought delight, fostered emotional validation, and promoted social connections and shared experiences. Staff feedback corroborated those themes, adding that PARO was a potential resource in the care toolkit to support person-centered care, and that its unique features generated strong engagement, but also presented physical accessibility, feasibility, and sustainability challenges. Findings highlight that PARO can be a valuable tool to enhance dementia care when both resident and staff perspectives are considered. Sustainable adoption requires collaboration among staff, administrators, and researchers to address accessibility, integration, and long-term feasibility. This study contributes to a holistic understanding of PARO's potential role in LTC and informs strategies for advancing socially assistive technologies in dementia care.

Abstract citation ID: igaf122.3693

### A STATE-OF-THE-ART REVIEW OF DEMENTIA CAREGIVERS WITH CARDIOMETABOLIC DISEASES: EVIDENCE AND FUTURE DIRECTIONS

Jin Su[1], Shicheng Xu[1], Jiaming Xiong[2], Xiang Qi[1], Yaguang Zheng[1], Jing Wang[3], Weiyu Mao[4], and Bei Wu[5], *1. New York University, New York, New York, United States, 2. The Hong Kong Polytechnic University, Hong Kong, Hong Kong, China (People's Republic), 3. University of New Hampshire, Durham, New Hampshire, United States, 4. University of Nevada, Reno, Reno, Nevada, United States, 5. NYU Shanghai, Shanghai, China (People's Republic)*

Informal caregivers of people with dementia face an increased risk of cardiometabolic diseases due to demanding caregiving responsibilities. Notably, 80% of these caregivers experience at least one chronic condition, yet they often prioritize caregiving over their health. Despite this vulnerability, evidence on interventions targeting dementia caregivers with cardiometabolic diseases remains limited. This State-of-the-Art review aims to synthesize existing knowledge on dementia caregivers with cardiometabolic diseases, identify research challenges, and highlight future directions. A systematic search of Medline, CINAHL, Embase, and PsycINFO identified 6,657 articles published between Jan 1996 and May 2025. Inclusion criteria focused on studies of dementia caregivers with at least one cardiometabolic or chronic disease, excluding non-English studies, conference abstracts, reviews, and editorials. Five studies remained in the final review: four survey studies (three cross-sectional and one longitudinal) and one intervention proposal study. Three themes emerged from the studies: (1) the synergistic effects of multiple cardiometabolic diseases on stress responses and metabolic risks among dementia caregivers; (2) the need for multicomponent interventions addressing stress and lifestyle in dementia caregivers with cardiometabolic diseases, and (3) the underexplored potential of mobile health technology to support self-management among dementia caregivers with cardiometabolic diseases. The review highlights the scarcity of research on this population, likely due to methodological challenges related to their compounded health risks and complex support needs. Future studies should explore innovative and flexible methods to engage dementia caregivers with cardiometabolic diseases, particularly those from diverse cultural and socioeconomic backgrounds, and develop tailored strategies.

Abstract citation ID: igaf122.3694

### "I MIGHT NEED THIS FOR LATER." "BACK POCKETING" HOSPICE INFORMATION AMONG CAREGIVERS OF BLACK PLWD

Emika Miller[1], Karen Moss[2], Karen Rose[3], Seuli Brill[4], and Mary Beth Happ[5], *1. Ohio State University College of Nursing, Fairborn, Ohio, United States, 2. The Ohio State University, Blacklick, Ohio, United States, 3. The Ohio State University, The Ohio State University, Ohio, United States, 4. College of Medicine, Ohio State University, Ohio, United States, 5. Ohio State University, Columbus, Ohio, United States*

Despite the benefits associated with hospice (e.g., improved quality of life at end-of-life), Black people living with dementia (PLwD) are less likely to utilize hospice services compared to non-Hispanic White persons. Exposure to hospice information may impact end-of-life decision-making for Black PLwD. This qualitative descriptive study examined the sources and characteristics of hospice information among caregivers and its influence on hospice decision-making. We employed semi-structured interviews with family caregivers (N = 25) of deceased Black PLwD. Longo's Expanded Model guided the initial coding process. Thematic narrative analysis was applied to coded data through an iterative process. The study sample was predominantly female (72%), with an average age of 55.5 ± 14.7 years, and served as caregivers for 5.0 ± 4.0 years. Most participants (75%) enrolled their PLwD in hospice. When encountering dementia, hospice or other health-related information, some participants chose to "back pocket" it. "Back pocketing" refers to saving information for later consideration and possible action. Reasons for "back pocketing" information included: 1) deeming the information or source questionable in credibility, or 2) providing a knowledge bank to "pull from" for future decision-making in their caregiving role. One participant found "back pocket" information instrumental in securing a dementia diagnosis for their PLwD. This study refutes the notion that Black people do not receive or retain hospice information. It also emphasizes the importance of providing caregivers with credible hospice information and asking caregivers about "back pocketed" information. These findings may assist clinicians in determining when and through which avenues to provide hospice-related information.

Abstract citation ID: igaf122.3695

### "THE SHOP IS NOT CLOSED": SEX AND SEXUALITY AMONG OLDER ADULT DATERS

Lauren Harris, and Celia Melanson, *University of New Hampshire, Durham, New Hampshire, United States*

There is a common myth that older adults are not sexual people, either from a lack of desire or ability. This study focuses on the experiences of single older adults on the dating market to understand the role of sexual desire and expectation for a population often assumed to be uninterested in or incapable of sexual activity. Previous research has largely focused on younger or partnered individuals, leaving the experiences of single older adults under-explored. Through this study, we seek to understand the desires, preferences, and challenges faced by single older adults who are seeking romantic and sexual relationships. It investigates how factors such as sexual dysfunction, stereotypes about aging bodies, and limited opportunities for finding sexual partners impact their willingness to pursue and their expectations of intimacy. Results indicate that, despite physical and emotional challenges related to aging, sexual activity remains an important aspect of romantic relationships for many single older adults. Both men and women recognize the impact of aging on sexual function but do not view these changes as insurmountable barriers. Rather, they express a desire for intimacy and adjust their expectations to accommodate physical changes. The study challenges agist stereotypes and highlights the need to normalize sexual desire in later life. The findings contribute to a broader understanding of older adult sexuality, particularly for those navigating the dating market, and provide insight into how aging adults perceive and prioritize sexual intimacy in relationships.

Abstract citation ID: igaf122.3696

### ACCULTURATION AND AGE ACCELERATION: DIFFERENCES BETWEEN HISPANIC AND NON-HISPANIC IMMIGRANTS

Antonio Bustillo[1], M. Maria Glymour[2], Kaylie Moropoulos[3], Katrina Kezios[2], and Adina Zeki Al Hazzouri[3], *1. Columbia University Mailman School of Public Health, New York, New York, United States, 2. Boston University, Boston, Massachusetts, United States, 3. Columbia University Medical Center, New York, New York, United States*

Acculturation by immigrants to the United States is tied to dissipating immigrant health advantages, but its relationship to epigenetic age acceleration (AA) has not been studied. Further, acculturation may affect Hispanic and non-Hispanic immigrants differently. We estimated the effects of acculturation on epigenetic AA by Hispanic ethnicity using 2nd & 3rd generation epigenetic age clocks from the Health and Retirement Study (N = 4014). AA scores were defined as standardized residuals from models regressing each clock on chronological age. Acculturation measures included immigration age (0-15 years,16-40, 41-90 vs. non-immigrant); time since immigration (0-39 years, 40+ vs. non-immigrant); immigration year (1923-1964, 1965-2009 vs. non-immigrant); and English spoken at home (yes, no vs. non-immigrant). We predicted mean AA scores per acculturation measure level from confounder-adjusted regression models separately for Hispanics and non-Hispanics; we computed contrasts & 95% confidence intervals (Contrast [95% CI]) between predicted means for each acculturation measure level vs. non-immigrants. Hispanic immigrants showed net age deceleration if they migrated between ages 0-15 (Levine/PhenoAge= -0.28 [-0.56, 0], migrated after 1965 (Levine/PhenoAge = -0.18 [-0.36, -0.01]); GrimAge = -0.17 [-0.32, -0.01]), and regardless of whether they spoke English at home (Levine/PhenoAge = -0.3 [-0.57, -0.04]; GrimAge = -0.16 [-0.31, 0]). Non-Hispanic immigrants showed net age acceleration if they migrated between ages 41-90 (Levine/PhenoAge=0.49 [0.14, 0.84]; DunedinPoAm38 = 0.33 [0.02, 0.65]); immigrated 0-39 years ago (Levine/PhenoAge = 0.26 [0.04, 0.49]); and if they migrated after 1965 (Levine/PhenoAge = 0.2 [0.02, 0.38]). Acculturation may lead to aging advantages for Hispanic immigrants, but disadvantage for non-Hispanics immigrants.

Abstract citation ID: igaf122.3697

**ADAPTING THE 4MS FRAMEWORK FOR AMERICAN INDIAN AND ALASKA NATIVE COMMUNITIES**

Richard Marottoli[1], Bill Benson[2], Breana Dorame[2], Megan Dicken[2], Crystal Gwizdala[3], Barry Wu[3], and Jennifer Ouellet[3], *1. Yale University School of Medicine, New Haven, Connecticut, United States, 2. International Association for Indigenous Aging, Silver Spring, Maryland, United States, 3. Yale School of Medicine, New Haven, Connecticut, United States*

The 4Ms (Matters Most, Medication, Mentation/Mind, Mobility) provide a valuable framework for older individuals, and their caregivers and clinicians, for consideration of factors influencing health, daily function, and well-being. Through an iterative process involving internal discussions and external listening sessions including tribal organizations, brain health advocates and experts, we sought to adapt the language and visual imagery associated with the 4Ms to be more relatable and relevant to American Indian and Alaska Native communities. The final version reflects the individual 4M components and also incorporates cultural elements: a tree at the center, roots representing traditional core values, branches representing the 4Ms, and leaves depicting the daily activities and expressions of the 4Ms. The resulting visual images and terms can be further modified and adapted to reflect local tribal geographic elements, language, and traditions.

Abstract citation ID: igaf122.3698

**ADVANCING COMMUNITY-BASED DEMENTIA SUPPORT: INSIGHTS FROM SOUTH KOREA'S DEMENTIA MANAGEMENT SYSTEM**

Yoon Chung Kim[1], Takashi Yamashita[2], and Giyeon Kim[3], *1. University of Maryland School of Medicine, Baltimore, Maryland, United States, 2. University of Maryland, Baltimore County, Baltimore, Maryland, United States, 3. Chung-Ang University, Seoul, Republic of Korea*

Globally, over 55 million people are living with dementia, with numbers doubling every 20 years. While super-aged societies adopt innovative strategies to support the growing number of people living with dementia (PLWD), gaps remain in systematically sharing practical, scalable models of prevention, diagnosis, and care coordination. This study investigates South Korea's nationally coordinated, community-based dementia management system. Researchers from the United States and South Korea visited Seoul Metropolitan Center for Dementia (SMCD), participated in a leadership forum with administrative directors, conducted in-depth discussions, and reviewed documents from the National Institute of Dementia (NID) and SMCD. Using a descriptive case study approach, we identified an emphasis on community-based support through five core domains of dementia prevention and awareness, early detection, case registration and management, community resource networking, and online-based support and monitoring. South Korea's dementia management system demonstrates integration across central (NID), metropolitan/provincial (e.g., SMCD), and local (Dementia Relief Centers; DRC) levels. Notable initiatives include "Ten Million Citizen Memory Friends," "Memory Café," and "Dementia-Friendly Villages," which promote public awareness through community engagement. Local DRCs offer cognitive screening, counseling, diagnostic referrals, and support for PLWD and their families. Dementia service networks are further strengthened through community resources, such as welfare centers, daycare centers, and primary care physicians, via education/training programs and technical assistance. Additionally, mobile applications such as "Wrist Doctor 9988" facilitate health monitoring and prevention of dementia. Our findings highlight valuable insights for developing and promoting dementia-inclusive, community-based models internationally. Future studies are needed to examine dementia management systems across countries.

Abstract citation ID: igaf122.3699

**AGE BIAS IN EMPLOYMENT-RELATED DESCRIPTIONS: A WORD EMBEDDING ANALYSIS OF PEOPLE'S DAILY**

Chunyan Mai, and Yue Hu, *The Chinese University of Hong Kong, Hong Kong, China (People's Republic)*

Background: The population is aging globally and older adults' labor participation is rising. It's critical to explore factors for post-retirement reemployment. Yet research on the role of aging attitudes is rare. This study investigated age biases in employment-related descriptions in domains of health, personality, skills, employment in People's Daily from year 1950 to 2021 by exploring whether older age groups are framed as more positively or negatively compared to younger cohorts amid China's socioeconomic transitions. Methods: Using natural language processing, word embedding with cosine similarity was applied to analyze associations between age-related words and descriptive words on these people. It was hypothesized that there were periodic fluctuations in age bias across historical stages and stronger associations of younger groups with employment-related words. Results: Key findings included persistent youth-centric bias, especially post-1980s; fluctuating bias (balanced 1950s–1970s, youth-tilted 1980s–2000s, partially corrected post-2010s); and dimensional differences–health tied to medical resources/aging demands, stable personality perceptions, volatile skills due to industrial shifts, and persistent negative employment bias toward older age groups. Discussion: Trends reflect adaptations to economic/industrial changes, with societal reliance on youth vitality as a common thread. Differences across domains stem from alignments with age-based values (experience/vitality), illustrating dynamic workplace value adjustments during China's transitions.

Abstract citation ID: igaf122.3700

**AGING WITH PURPOSE: THE IMPACT OF SUBJECTIVE AGING**

Yiqing Yang[1], and Kelin Li[2], *1. Western Carolina University, Cullowhee, North Carolina, United States, 2. California State University Dominguez Hills, California State University Dominguez Hills, California, United States*

Subjective aging is a well-established predictor of health and well-being in older adults, while a strong sense of purpose in life (PIL) has consistently been linked to positive health outcomes. Despite PIL's role as a core dimension of eudaimonic well-being, its relationship with subjective aging remains underexplored. This study examines the correlates of PIL, measured using Ryff 's 7-item Purpose in Life Subscale, with a focus on two widely used subjective aging measures among 150 community-dwelling older Americans. Spearman correlation indicates that age, gender, race, and partner status are not significantly correlated with PIL. Ordinary least squares regression analyses reveal that felt age and self-perceptions of aging, assessed via an 8-item Attitudes towards Own Aging subscale, are significant predictors of PIL.

In the overall model ($R^2 = 0.50$, $F(12, 137) = 11.62$, $p < 0.0001$), both measures of subjective aging remain significant after adjusting for education, financial strain, religiosity, self-rated health, and depressive symptoms. Additionally, higher educational attainment, greater religiosity, and fewer depressive symptoms are significantly associated with higher PIL, while financial strain and self-rated health are not. These findings suggest that fostering positive subjective aging perceptions may be a promising target for interventions aimed at enhancing PIL, ultimately promoting healthy and successful aging.

Abstract citation ID: igaf122.3701

### AI-DRIVEN HUMAN POSE ESTIMATION REVEALS DISTINCT GAIT PATTERNS IN OLDER ADULTS WITH PHYSIO-COGNITIVE DECLINE

Zilong Zhang, Yiwen Xing, and Lina Ma, *Xuanwu Hospital, Capital Medical University, Beijing, Beijing, China (People's Republic)*

Physio-Cognitive Decline Syndrome (PCDS) is a geriatric condition characterized by coexisting Mobility Impairment No Disability (MIND) and Cognitive Impairment No Dementia (CIND). Gait dysfunction, which commonly occurs in older adults, can serve as an indicator of underlying pathological and functional states. This study employed an AI-based human pose estimation algorithm to analyze gait patterns and quantitatively extract gait parameters in older adults with PCDS. A total of 755 community-dwelling adults aged 65 years or older were included in this study. Participants performed a 4-meter walking test while being recorded via intelligent software; gait information was extracted from 17 key skeletal points using pose estimation technology, and an action recognition algorithm was applied to analyze and predict gait abnormalities. After adjusting for sociodemographic factors, lifestyle, chronic diseases, polypharmacy, nutrition, and psychological conditions, gait dysfunction identified by the pose estimation algorithm was associated with an increased risk of PCDS. Higher connection velocity, joint point acceleration, and joint point angular velocity were protective against PCDS. Stride length showed a positive correlation with immediate and delayed recall memory among PCDS participants, even after adjusting for age and sex. These findings demonstrate that AI-driven pose estimation offers valuable clinical insight into identifying PCDS-related gait impairments, highlighting its potential as a tool for early screening and mobility-cognitive integration assessment in community-dwelling older adults.

Abstract citation ID: igaf122.3702

### ALCOHOL AND PSYCHOLOGICAL PREDICTORS OF DAILY FUNCTIONING BY SEXUAL MINORITY STATUS IN OLDER ADULTS

Carolina L. Costa[1], Carlos Araujo-Menendez[2], and Ariana Stickel[1], *1. San Diego State University, San Diego, California, United States, 2. San Diego State University/ University of California, San Diego, San Diego, California, United States*

Sexual minority (SM; e.g., lesbian, gay, bisexual, or queer+) adults are at increased risk of poor physical and mental health outcomes, likely due to discrimination and related stress. Stress may also drive health behaviors, including increased alcohol use. Therefore, we examined if alcohol use interacts with psychological factors to predict activity of daily living (ADL) limitations among SM and non-SM older adults. Participants included 5,643 adults (SM = 321; M = 72-years, SD = 10-years) from the 2022 Health and Retirement Study. Three-month alcohol use was assessed through five self-reported items (ever drank, ever drank more than 12+ drinks, drinks per week, drinks per day, binges). ADL limitation (yes/no) was defined as 1+ self-reported difficulties across five items. Psychological factors included depressive symptoms (CESD-8) and loneliness (yes/no; 1 item). SM-status-stratified logistic regression models were performed to assess the moderating effect of alcohol in the association between psychological factors and ADL limitations while adjusting for sociodemographic variables. In a model with non-SM adults, higher depression scores were associated with greater odds of ADL limitations, especially for non-drinkers compared to drinkers (OR = 0.90, 95% CI [0.82, 0.97]). In a model with SM adults, endorsing loneliness was associated with more ADL limitations at higher levels of number of drinks per day (OR = 4.45, 95% CI [1.39, 18.55]). These differences may reflect how alcohol use interacts with mental health in distinct ways for SM and non-SM older adults.

Abstract citation ID: igaf122.3703

### AN INTERVENTION-FOCUSED MODEL FOR UNDERSTANDING FULFILLMENT AMONG OLDER ADULTS

Joy Collins[1], Carly Woods[1], Lance Kruse[1], Benton Smith[2], Jeremy Cochran[2], and Lisa D'Ambrosio[3], *1. Humana, Louisville, Kentucky, United States, 2. Burke, Inc., Cincinnati, Ohio, United States, 3. Massachusetts Institute of Technology, Cambridge, Massachusetts, United States*

Older adults experience a range of health issues as they age. Many community and policy initiatives focus primarily on physical health, overlooking the concept of fulfillment — the satisfaction of a life well-lived and needs being met. Prior studies have shown that fulfillment and purpose in life are linked to reduced anxiety and depression, which in turn support better physical health and lower chronic disease risk. Our study aimed to develop and validate a model of fulfillment among American adults aged 62+, using both qualitative (expert interviews, discussions, n = 23) and quantitative methods (survey, n = 1150). The qualitative phase clarified what fulfillment means to older adults, while quantitative analysis using Bayesian Network Analysis identified the key factors and drivers of fulfillment. In the second research phase (n = 5500; completion August, 2025), we will validate the model in a larger sample and analyze fulfillment among key subgroups. Average fit was tested by introducing data noise across 100 iterations. The model demonstrated good reliability (average fit score = 72.1%, SD = 5.8%) and all items within each factor achieved statistical significance ($p < .01$). A Bayesian Network structural model predicted fulfillment agreement ratings with factors as the independent variables: Precision = 70.7%, ROC Index = 89.8%. By identifying the activities and emotions that drive fulfillment, this research offers tools for policymakers and healthcare providers to design more effective, patient-centered interventions. Ultimately, measuring and improving fulfillment can enhance the well-being and health of older adults, guiding targeted support where it is most needed.

Abstract citation ID: igaf122.3704

### ANALYSIS OF A MULTI-SECTOR REGIONAL COALITION TO COMBAT OLDER ADULT ISOLATION IN A RURAL U.S. COUNTY

Michael Castellano[1], Lindsey Skripka[2], Christine Marcos[3], and Lenard Kaye[4], *1. United Way of Lackawanna, Wayne and Pike, Scranton, Pennsylvania, United States, 2. Meals on Wheels of Northeastern Pennsylvania, Scranton, Pennsylvania, United States, 3. Moses Taylor Foundation, Scranton, Pennsylvania, United States, 4. University of Maine, orono, Maine, United States*

Older adults residing in rural communities are at high risk of becoming socially isolated. The traditional scarcity of social and other supportive services in such settings often results in delays in effective community response. With Moses Taylor Foundation funding, a community collaborative of more than 30 health/human service, spiritual, philanthropic, and public agencies have created a formalized multi-sector organizational coalition to address older adult isolation in largely rural Lackawanna County, in northeastern Pennsylvania. Utilizing a collective impact model, a centralized infrastructure with its hub at the United Way of Lackawanna, Wayne, & Pike (UWLWP) has been successfully established. The Age Friendly Lackawanna Collaborative's (AFLC) key components include dedicated staff, volunteer peer navigators, a common goal, shared measurement and data collection protocols, and coordinated, reframed aging communication channels. Key programmatic components that have been established, including screening, navigation, and awareness, ensure identification, guiding, and campaigning functions are performed, respectively. The AFLC has registered more than 167,000 Facebook views and 2,400 hits on the website in the first 6.5 months of 2025. Administering the evidence-based Wilder Collaboration Factors Inventory, confirms that a large majority of organizational partners (71% to 100%) concur that the seven (7) inventory factors aligned with the collective impact model have been successfully implemented. Strongest factor scores confirm achievement of an appropriate cross-section of members (90%) and shared membership stake in both process and outcome (100%). Best practice recommendations for replicating this collective community impact model to combat older adult social isolation in other rural communities are offered.

Abstract citation ID: igaf122.3705

### ANALYSIS OF SEXUAL RISK BEHAVIORS AND PSYCHOSOCIAL DETERMINANTS AMONG OLDER MSM IN MAINLAND CHINA AND HONG KONG

Alex Siu Wing Chan[1], Hok Bun Ku[2], and Elsie Yan[2], *1. Hong Kong Baptist University, Hong Kong, China (People's Republic), 2. The Hong Kong Polytechnic University, Hong Kong, China (People's Republic)*

Older men who have sex with men (OMSM) in Asia face significant sexual health and mental health challenges, yet they are often overlooked in HIV prevention efforts. The psychosocial factors, including substance use, mental health issues, and social stigma, further exacerbate these risks. This study aims to investigate the sexual risk behaviors and psychosocial factors influencing older MSM in Mainland China and Hong Kong, with a focus on identifying key predictors of HIV risk. This cross-sectional comparative study surveyed 302 older MSM (151 from Mainland China and 151 from Hong Kong). Participants, aged 60 and above, were recruited through partnerships with LGBT organizations and NGOs. Data were collected on demographic characteristics, sexual risk behaviors, and psychosocial factors, including mental health and substance use. The study found that only 24.3% of participants aged 50–59 years and 17.1% of those aged 60–69 years reported using condoms at last intercourse. Furthermore, 25% of participants reported having multiple sexual partners. Substance use, particularly methamphetamine, was linked to decreased condom use, with 30% of participants reporting recreational drug use. Mental health issues, including depression and anxiety, were prevalent, with 40% reporting symptoms of depression. Social stigma was a significant barrier to seeking care, further exacerbating these issues. Older MSM in Mainland China and Hong Kong engage in high-risk sexual behaviors influenced by mental health struggles, substance use, and stigma. Tailored, culturally sensitive interventions that address both sexual health and mental health are urgently needed to reduce HIV and STI transmission in this underserved population.

Abstract citation ID: igaf122.3706

### ANGER AND OPTIMISM MODERATE THE RELATIONSHIP BETWEEN NEIGHBORHOOD DISADVANTAGE AND BIOLOGICAL AGING IN MESA

Joyce Woo[1], James Pike[1], Timothy Hughes[2], Jana Hirsch[3], Priya Palta[4], and Ganga Bey[1], *1. The University of North Carolina at Chapel Hill, Chapel Hill, North Carolina, United States, 2. Atrium Health Wake Forest Baptist, Winston-Salem, North Carolina, United States, 3. Drexel University, Philadelphia, Pennsylvania, United States, 4. UNC School of Medicine, Chapel Hill, North Carolina, United States*

We investigated whether individual-level psychosocial risk and resilience differentially moderate the relationship between neighborhood disadvantage and biological aging across ethnoracial groups. Using the Multi-Ethnic Study of Atherosclerosis (MESA), our study included 1,229 participants with DNA methylation (DNAm) data collected in 2010-2012. Neighborhood disadvantage proximal to DNAm assesment was captured using the 2015 Area Deprivation Index (ADI) at the census tract-level. Psychosocial factors included optimism, measured at Exam 2 (2002-2004) using the Life Orientation Test-Revised, and anger reactivity/temperament, measured at Exam 1 (2000-2002) using the State-Trait Anger Expression Inventory. Pace of aging was assessed as the difference between chronological age and GrimAge, a DNAm-based epigenetic clock. Mixed effects models with interaction terms evaluated whether the gender-adjusted relationship between ADI and pace of aging was moderated by optimism and anger. Among participants (mean age: 69.6 years, 51% female, 47% white, 21% Black, 31% Hispanic), greater neighborhood disadvantage was associated with increased age acceleration ($\beta = 0.27$, 95% CI: 0.05, 0.49). Unexpectedly, optimism amplified this association ($\beta = 0.33$, 95% CI: 0.05, 0.60). Stratified models revealed that moderation by optimism was present only for Hispanic participants ($\beta = 1.73$, 95% CI: 0.71, 2.74), whereas anger reactivity was predictive of slower biological aging ($\beta = -1.63$, 95% CI: -3.03, -0.24) among Black participants only. This study highlights the importance of modifiable psychosocial factors in shaping the impact of neighborhood disadvantage on aging. Our findings point to a need for interventions tailored to the unique sociocultural contexts of ethnoracial groups to mitigate disparities in aging.

Abstract citation ID: igaf122.3707

### ANNUAL WELLNESS VISITS AND TIMING OF ADVANCE CARE PLANNING AMONG MEDICARE BENEFICIARIES WITH COGNITIVE IMPAIRMENT

Zhiwei Hu[1], Mukaila Raji[2], Yong Shan[3], Huey-Ming Tzeng[4], Sean O'Mahony[5], and Yong-Fang Kuo[6], *1. the University of Texas Medical Branch, Galveston, Texas, United States, 2. University of Texas Medical Branch at Galveston, Galveston, Texas, United States, 3. UTMB, Galvesto, Texas, United States, 4. School of Nursing, University of Texas Medical Branch, Galveston, Galveston, Texas, United States, 5. University of Texas Medical Branch, Galveston, Texas, United States, 6. University of Texas Medical Branch, Galveston, Galveston, Texas, United States*

Annual wellness visits (AWVs) offer an important opportunity to promote timely advance care planning (ACP) among older adults with mild cognitive impairment or dementia, but the real-world timing and equity of ACP initiation following AWVs remain poorly characterized. We conducted a retrospective cohort study of 959 405 fee-for-service Medicare beneficiaries aged 68 years or older newly diagnosed with cognitive impairment in 2018, using claims data through December 31, 2021, to examine whether AWV receipt was associated with earlier ACP initiation. Propensity-score matching balanced sociodemographic, clinical, and utilization factors, and conditional Fine-Gray models accounting for death as a competing risk estimated hazard ratios. AWV receipt was associated with an 86% higher rate of first ACP use (HR, 1.86; 95% CI, 1.84–1.89). Cumulative incidence analyses showed that 9.0% of AWV recipients initiated ACP on the same day as their AWV, increasing modestly to 9.41% at 30 days, 14.78% at one year, and 27.62% at four years, whereas only 0.01% of non-AWV beneficiaries initiated ACP on their index date, reaching 17.46% by four years. Subgroup analyses showed the strongest effects among rural (HR, 3.34; 95% CI, 3.16–3.52) and Hispanic beneficiaries (HR, 2.78; 95% CI, 2.55–3.03). Sensitivity analyses censoring the switch of AWV between two groups or time-dependent AWVs yielded similar results. These findings indicate that AWVs serve as a key trigger for same-day ACP initiation and may help promote more timely and equitable ACP engagement in cognitively impaired older adults.

Abstract citation ID: igaf122.3708

### APPLYING A DESIGN THINKING × COM-B HYBRID FRAMEWORK TO MOW VOLUNTEER RECRUITMENT & RETENTION

Ziqi Ma[1], and Cecilia Wang[2], *1. University of Minnesota, Twin Cities, Minneapolis, Minnesota, United States, 2. University of Minnesota Twin Cities, St Paul, Minnesota, United States*

This study explores the challenges faced by Meals on Wheels (MOW), including an aging volunteer base, low youth participation, and unstable retention rates, and analyzes how these issues impact service quality and customer satisfaction. To address these challenges, we propose an embedded framework that combines design thinking (empathy, define, ideate, prototype, test) with the COM-B behavior change model (capacity, opportunity, motivation), ensuring that each ideation phase aligns with behavioral drivers. This study employs a mixed-methods approach: quantitative metrics include monthly attendance rates, retention rates at 6 and 12 months, route coverage, and time to fill vacant positions; qualitative data is derived from semi-structured interviews, participant observation, post-intervention surveys, and field notes. Participants included existing volunteers, new volunteers, volunteers who had left within the past year, and project coordinators, ensuring diversity in age, gender, years of service, and route type. Evaluation metrics are used to measure capacity building, changes in motivation, improvements in opportunities, and recurring themes in volunteer feedback. Expected outcomes include increased volunteer numbers, improved recruitment efficiency, improved 6-month and 12-month retention rates, enhanced satisfaction, increased route coverage and reduced last-minute cancellations, and better alignment between volunteer availability and organizational needs. Limitations of the study include geographical constraints, cultural differences, time sensitivity, and self-selection bias. Overall, this framework provides a replicable approach for resource-constrained nonprofit organizations to enhance volunteer recruitment and retention while improving the customer experience.

Abstract citation ID: igaf122.3709

### ASSESSING DRIVERS OF MEDICATION MANAGEMENT HASSLES AMONG CARE PARTNERS OF OLDER ADULTS

Sohee Kim[1], Te-Lien Ku[2], Shu-Ying Wu[3], and Yen-Ming Huang[4], *1. University of Wisconsin - Madison, Madison, Wisconsin, United States, 2. University of Wisconsin-Madison, Madison, Wisconsin, United States, 3. Sijhih Cathay General Hospital, New Taipei City, New Taipei, Taiwan (Republic of China), 4. National Taiwan University, Taipei City, Taipei, Taiwan (Republic of China)*

Care Partners (CPs)—such as family members or friends—play a critical role in assisting older adults with multiple chronic conditions in managing complex medication regimens. However, CPs frequently report feeling burdened by these responsibilities. This study aimed to examine factors associated with medication management hassles reported by CPs. We surveyed 138 CPs in Northern Taiwan who assisted with or were responsible for managing medications for family members aged 65 or older. Data collection included demographics of both CPs and care recipients, care recipient health status, CPs' caregiving experiences, and perceived stressors related to medication management—measured using the Traditional Chinese version of Family Caregiver Medication Administration Hassles Scale (FCMAHS-TC). Data analysis included LASSO (Least Absolute Shrinkage and Selection Operator) regression to select key predicting variables, followed by multivariable linear regression to examine associations with FCMAHS-TC scores. Time spent on medication management ($\beta = 0.18$, $p = 0.002$) and strained (difficult or uneasy) CPs–recipient interactions ($\beta = 7.84$, $p = 0.040$) were significantly associated with higher hassle scores. Housekeeping CPs showed significantly lower hassles than those in full-time working CPs ($\beta = -9.74$, $p = 0.035$). Future research should further explore the contextual and relational mechanisms underlying the associations between identified factors and CPs' perceived medication management hassles, informing the development of interventions that better support CPs in navigating their medication responsibilities.

Abstract citation ID: igaf122.3710

**ASSOCIATED FACTORS OF FALL RISK IN OLDER ADULTS LIVING IN THE COMMUNITY: INSIGHTS FROM THE FRRISQUE ASSESSMENT**

Alvaro Maciel[1], Sabrina Macedo[2], Paulo Barbosa[3], Wesley Sales[1], and Edgar Vieira[4], *1. Universidade Federal do Rio Grande do Norte, NATAL, Rio Grande do Norte, Brazil, 2. Universidade Federal do Rio Grande do Norte, Universidade Federal do Rio Grande do Norte, Rio Grande do Norte, Brazil, 3. universidade Estadual da Paraiba, NATAL, Rio Grande do Norte, Brazil, 4. FLORIDA INTERNATIONAL UNIVERSITY, MIAMI, Florida, United States*

Aging reduces physiological reserves and mobility, increasing vulnerability to falls, which are a major public health concern in older populations. Identifying predictive factors for fall risk is essential for implementing effective prevention strategies. This cross-sectional study, part of the PRO-EVA project, included community-dwelling adults aged 60 years or older residing in Parnamirim, Brazil. Sociodemographic characteristics, lifestyle habits, health conditions, and measures of physical performance and cognitive function were collected using the Older Adults' Health Record Booklet. Fall risk was assessed with the FRRISque questionnaire score. Linear regression analysis was performed to identify associations between potential predictors and fall risk. The study was approved by the Research Ethics Committee of the Federal University of Rio Grande do Norte (protocol no. 2.996.329). The sample comprised 735 participants, with a mean age of 70.1 years (±7.1), predominantly female (60.8%). Regression analysis revealed significant predictors of fall risk: sex ($p = 0.03$), polypharmacy defined as the use of five or more medications ($p = 0.001$), total Short Physical Performance Battery (SPPB) score ($p = 0.01$), presence of comorbidities ($p = 0.05$), and pain ($p = 0.01$). The adjusted model explained 25% of the variance in fall risk ($R^2 = 0.25$). These findings underscore the relevance of clinical and functional factors, such as sex, polypharmacy, physical performance, comorbidities, and pain, in predicting fall risk among older adults. Monitoring these predictors may guide targeted interventions to enhance safety and quality of life in this population.

Abstract citation ID: igaf122.3711

**ASSOCIATION BETWEEN DIETARY PATTERNS AND FRAILTY IN CHINESE LONGEVITY REGIONS: EVIDENCE FROM THE HABCS**

Shi Yin[1], Wuqi Wang[2], Yuebin Lv[3], and Xiaoming Shi[3], *1. The First Affiliated Hospital of USTC, University of Science and Technology of China, Hefei, Anhui, China (People's Republic), 2. School of Public Health, Anhui Medical University, Hefei, Anhui, China (People's Republic), 3. Chinese Center for Disease Control and Prevention, Beijing, Beijing, China (People's Republic)*

Frailty, a geriatric syndrome characterized by reduced physiological reserve and increased vulnerability to stressors, has been associated with dietary habits, though previous studies have focused mainly on Western nations and select Asian regions, leaving a significant gap in systematically examining the distinct dietary patterns and their associations with frailty in Chinese longevity regions. This study leveraged data from 3242 older adults in the Chinese Healthy Ageing and Biomarkers Cohort Study (HABCS) to explore dietary patterns and associations with frailty. Dietary intake was assessed via a validated Food Frequency Questionnaire (FFQ), and frailty was defined using a 39-item index (cutoff >0.25). Mediation analysis examined serum biomarkers (superoxide dismutase [SOD], high-sensitivity C-reactive protein [hsCRP]). Principal Component Analysis revealed four major dietary patterns among older adults in Chinese longevity regions, which were Pattern 1, Wheat-fruits and Vegetables-Dairy and Eggs (15.9% variance), Pattern 2, White Rice-Aquatic products-Red meat and Vegetables (13.3% variance), Pattern 3, Pickled Foods (8.4% variance), and Pattern 4, Grains-Starchy vegetables-Legumes-White Meat (10.0% variance), collectively explaining 47.5% of variance. Pattern 2 was protective against frailty (OR = 0.56; 95%CI: 0.45–0.72), while Pattern 4 increased frailty risk (OR = 1.43; 95%CI: 1.32–1.80). Patterns 1 and 3 showed no significant associations. Mediation analysis revealed that hsCRP mediated 2.7% ($P = 0.028$) of Pattern 2's protective effect; SOD had no significant role. The findings suggest that a diet rich in rice, aquatic products, red meat, and vegetables may reduce frailty risk, potentially due to high-quality protein and anti-inflammatory components. This highlights the importance of region-specific dietary guidance for healthy aging.

Abstract citation ID: igaf122.3712

**ASSOCIATION OF PHYSICAL EXERCISE AND LOCUS COERULEUS-SALIENCE NETWORK FUNCTIONAL CONNECTIVITY**

Nethra Rajeshkumar, Mali Madison, Madeline Stefano, Emily Jon, Sean Lawrence, Elayna Seago, Tae-Ho Lee, and Benjamin Katz, *Virginia Tech, Blacksburg, Virginia, United States*

Physical exercise is a modifiable lifestyle factor that supports brain health and cognition. The locus coeruleus (LC), the brain's primary source of norepinephrine, plays a critical role in attention, and is among one of the first sites affected by Alzheimer's-related pathology. Prior research suggests exercise promotes LC integrity, plasticity, and LC-dependent memory improvement. The salience network (SN), including the dorsal anterior cingulate cortex (dACC) and anterior insula, is crucial for identifying and responding to behaviorally relevant stimuli. LC-SN functional connectivity is thought to support efficient attentional control. Investigating the association between exercise and LC-SN connectivity may provide insight into neural resilience in the aging brain in a region implicated in Alzheimer's pathology. Associations between LC-SN functional connectivity, response time, and exercise were compared in 43 older adults (65% female). fMRI data and response times were collected as participants completed the Place Discrimination Task (PDT), a measure linked to memory and attention. Weekly exercise was assessed via self-report. Linear regression models were used to examine the association between exercise and LC-SN connectivity and exercise and response time while controlling for age, sex, and family history of Alzheimer's disease. The association between exercise and LC-dACC connectivity was marginally significant ($\beta = .34$, $p = 0.0717$). The association between exercise and LC-insula connectivity was not significant ($\beta = -.03$, $p = 0.8481$). However, task response time was significantly associated with exercise ($\beta = -.50$, $p = 0.005$). These findings suggest that while exercise may be linked to cognition during aging, associations with LC-SN functional connectivity are more tenuous.

Abstract citation ID: igaf122.3713

### ASSOCIATIONS BETWEEN HEART RATE VARIABILITY AND INFLAMMATION FROM TWO AGING STUDIES: RACIAL AND AGE DIFFERENCES

Molly Wright[1], Richard Sloan[2], Carol Derby[3], David Almeida[1], and Christopher Engeland[1], *1. The Pennsylvania State University, University Park, Pennsylvania, United States, 2. Columbia University Irving Medical Center, New York, New York, United States, 3. Albert Einstein College of Medicine, Bronx, New York, United States*

The vagus nerve regulates inflammation via a cholinergic anti-inflammatory pathway, supported by findings of a negative association between high-frequency heart rate variability (HF-HRV)—a marker of cardiac vagal activity—and inflammation. This study sought to examine whether there are racial and age differences in this association among mid-life and older adults. In the Einstein Aging Study, 74 adults (Mage=78.3; 45.9% Black) wore ambulatory electrocardiogram (ECG) devices for 7 days to measure HF-HRV and provided blood samples for inflammatory markers, including tumor necrosis factor (TNF)-α. Covariate-adjusted regression models showed HF-HRV was negatively associated with TNF-α ($\beta$=–0.323, $p = 0.005$); this was apparent in Black participants ($\beta$=–0.469, $p = 0.008$) but not White participants ($p = 0.69$). No significant associations with other inflammation markers were seen. To replicate and assess age differences, data (including TNF-α levels and HF-HRV determined from 11-minutes of ECG recordings) from the Midlife in the United States Study (MIDUS) 2 and Refresher Biomarker Projects (N = 1,664, Mage=55.4) were analyzed. Among adults ≥60 years, the negative HF-HRV–TNF-α association was evident for Black/Black Biracial participants (N = 80, $\beta$=–0.280, $p = 0.008$) but not White participants (N = 474, $p = 0.15$) in adjusted regression models. For those < 60(years), this association was evident for White participants (N = 703, $\beta$=–0.081, $p = 0.037$) but not for Black/Black Biracial participants (N = 199, $p = 0.85$). These findings suggest race- and age-specific patterns in the vagal regulation of inflammation. This could have important implications for understanding and addressing cardiovascular health disparities, such as suggesting that interventions targeting HRV may be particularly efficacious among Black individuals in later life.

Abstract citation ID: igaf122.3714

### ASSOCIATIONS BETWEEN NEIGHBORHOOD WALKABILITY, CONVENIENCE STORES, AND COGNITIVE DECLINE AMONG BLACK ADULTS

Ketlyne Sol[1], Melissa Lamar[2], Ana Capuano[3], and Lisa Barnes[2], *1. University of Miami, Boca Raton, Florida, United States, 2. Rush University Medical Center, Chicago, Illinois, United States, 3. University of Chicago, Chicago, Illinois, United States*

Neighborhood walkability facilitates life sustaining activity such as obtaining nutrition, which is vital for cognitive health, but mechanisms are unknown. Convenience stores within the neighborhood may provide a source of nutrition if options are limited. The current study tested whether existence of convenience stores explained the relationship between neighborhood walkability and cognitive decline in Black older adults. Participants (N = 786; M-age=73.4; 79% female) were drawn from two prospective community-based aging studies in Chicago in which participants were administered a battery of cognitive tests assessing five cognitive domains from which a global composite was computed. Participant addresses were geocoded and linked to walkability scores and sum of convenience stores within 0.5 miles of the participant's home. Multilevel regression analyses adjusting for demographics, health factors, and neighborhood SES examined whether the association between neighborhood walkability and cognitive trajectories was explained by convenience stores. More walkability was associated with better baseline visuospatial ability (B=.0015, SE=.0008, p=.0683), but faster decline (B=-.0002, SE=.0001, p=.0139). When adding convenience stores to the fully adjusted models, the effect of walkability on visuospatial ability became slightly stronger for the baseline effect (B=.0022, SE=.0009, p=.0219), while the longitudinal effect was unchanged (B=-.0002, SE=.0001, p=.0183). Walkability was not associated with any other cognitive domains at baseline or longitudinally. Although walkability may have a selective effect on decline in visuospatial abilities, these findings do not support convenience stores as a potential mechanism underlying neighborhood walkability and cognitive trajectories. Future studies are needed to examine other potential mechanisms linking walkability to change in cognition.

Abstract citation ID: igaf122.3715

### ASSOCIATIONS OF SOCIAL DISCONNECTION RISK AND ACTIVITIES OF DAILY LIVING AMONG OLDER ADULTS

Amy Neal, and Matthew Smith, *Texas A&M University, College Station, Texas, United States*

Age-related changes such as sarcopenia, certain chronic conditions, and physical inactivity can cause older adults to become frail. Frailty can make activities of daily living (ADLs) difficult and increase the risk of falling. While mobility impairments may diminish social connection among older adults, there is limited documentation of this relationship. The purpose of this study was to examine the relationship between social connection and difficulty performing three mobility-based ADLs (i.e., walking/climbing stairs, dressing/bathing, doing errands). A sample of 3,602 adults aged 65+ from an internet-delivered survey were analyzed using multinomial logistic regression. Participants were grouped into three groups: no ADL difficulties (71.6%), 1 ADL difficulty (21.3%), and 2+ ADL difficulties (7.1%). The primary predictor variable was social disconnection risk measured using the Upstream Social Interaction Risk Scale (U-SIRS-13). On average, participants were age 70.57(±4.76) and had 3.29(2.52) self-reported chronic conditions. About 16% of the sample reported falling one or more times in the last 12 months. Compared to participants with no ADL difficulties, participants with more social disconnection risk ($p < 0.001$), more self-reported chronic conditions ($p < 0.001$), and who fell one or more times in the past year ($p < 0.001$) had significantly higher odds of reporting 1 ADL difficulty and 2+ ADL difficulties, respectively. Results suggest a that older adults with more mobility impairments may feel more socially disconnected and have more complex disease profiles. Findings support that older adults may benefit from evidence-based fall prevention programming, which can alleviate fall-related risk factors, while improving physical function and promoting social connection with others.

Abstract citation ID: igaf122.3716

### ASYNCHRONOUS PEER SUPPORT CHAT GROUPS FOR FAMILY CAREGIVERS: LESSONS LEARNED

Felipe Jain[1], Maria Galvez[2], Noura Attili[3], and Paulina Gutierrez-Ramirez[2], *1. Massachusetts General Hospital / Harvard Medical School, Boston, Massachusetts, United States,*

*2. Massachusetts General Hospital, Boston, Massachusetts, United States, 3. Massachusetts Institute of Technology, Cambridge, Massachusetts, United States*

Peer support groups serve as a vital resource for family caregivers of people living with dementia. Smartphone apps provide opportunities for convenient, asynchronous interaction, without the need to travel or plan for meetings. However, the value of text-based chat peer support groups for family caregivers is unclear. We aimed to determine the feasibility of a mobile App peer support chatroom embedded within a randomized, controlled trial of caregiver skills with or without mentalizing imagery therapy. Participants received the MGH CareDoc application over 24 weeks, which provided daily learning modules alongside peer support chat. For the first 8 weeks, caregivers could opt to receive weekly telehealth groups that provided psychoeducation on study topics. Caregivers were divided into chatgroups of 4-6 participants based on their telehealth group assignment and on dementia severity of the relatives, to encourage small-group participation and similarity of caregiving experiences. Feasibility and acceptability were determined based on chatroom posts and semi-structured interviews. The trial included 110 participants (mean age 68.8), divided into 29 chat groups. High variability in chatgroup activity was observed, with an average of 104 messages per chatgroup (range 1 to 904). Individual users posted an average of 29 messages (range 1 to 299). Although some participants found the chatgroups to be supportive and helpful, several participants expressed that their chat group did not fully establish cohesion. Findings highlight both potential and challenges of mobile chatrooms, and caregiver feedback on ways to improve the structure of the chatgroups.

Abstract citation ID: igaf122.3717

### AT RISK AND UNDERPREPARED: TRAINING GAPS IN PHYSICAL MOBILITY SUPPORT AMONG AGING INFORMAL CAREGIVERS

Rodney Weir[1], Heather Renter[2], Olivia Brunner[1], Connor Lynch[1], Juliana Roller[1], and Chloe Tomasic[1], *1. Western Michigan University, Kalamazoo, Michigan, United States, 2. Heritage Community of Kalamazoo, Kalamazoo, Michigan, United States*

This qualitative research project explored the educational needs of informal, unpaid caregivers who assist individuals with chronic illnesses in performing physical mobility tasks. Many caregivers find themselves responsible for complex physical tasks which may include transfers, lifting, and the use of adaptive equipment. Previous research has documented that informal caregivers often perform such tasks with little or no training or support. This lack of preparation can lead to caregiver injury, emotional stress, and compromised patient safety. To investigate these challenges, researchers conducted focus group interviews with seven informal caregivers. Using a grounded theory approach, transcripts were analyzed to identify recurring themes related to caregiver education and training. Five key themes emerged: (1) "Making Do," where caregivers improvised with equipment due to insufficient instruction; (2) emotional and psychological stress stemming from low confidence and self-efficacy; (3) physical strain and lack of self-care; (4) limited access to training and essential resources; and (5) difficulty navigating the healthcare system. The findings highlight a critical gap in caregiver education, particularly in the safe and effective use of mobility aids and techniques. Physical therapists, with their expertise in adaptive equipment and movement strategies, are well-positioned to address these gaps by providing targeted education and support. Enhancing caregiver training not only improves safety and care quality but also supports caregivers' well-being and sustainability in their roles. Future research should examine the needs of older caregivers and the potential for physical therapy to extend its educational role in community-based care settings.

Abstract citation ID: igaf122.3718

### BARRIERS AND FACILITATORS TO LONG-TERM SERVICES & SUPPORTS FOR OLDER ADULTS LIVING ALONE WITH COGNITIVE IMPAIRMENT

Elena Portacolone[1], Robyn Stone[2], Matthew Growdon[1], Thi Tran[1], Daisy Feddoes[1], and Andrew Cohen[3], *1. University of California, San Francisco, San Francisco, California, United States, 2. LeadingAge, Washington, District of Columbia, United States, 3. Yale School of Medicine, New Haven, Connecticut, United States*

Background: More than 4 million older Americans with cognitive impairment (CI) live alone. These individuals often face unique challenges due to limited or non-existent support from caregivers. Identifying the barriers and facilitators to accessing long-term services and supports (LTSS) is critical to preventing the adverse outcomes to which this population is particularly vulnerable, including self-neglect, untreated medical conditions, medication mismanagement, falls, and fires. Methods: This qualitative study, conducted between May 2016-February 2024, included adults aged 55+ from diverse racial and ethnic backgrounds who were living alone with CI in California, Louisiana, and Michigan, as well as members of their social circle. Factors influencing LTSS access were identified via in-person, semi-structured interviews conducted in English, Spanish, Cantonese, or Mandarin. Transcripts were analyzed with combined inductive and deductive content analysis drawing from the micro-meso-macro framework. Results: A total of 119 older adults living alone with cognitive impairment (88 [71.5%] women; median age, 78 years [range, 57-103]) and 56 members of their social circle (45 [80.3%] women; median age, 45 years [range, 39-89]) were interviewed multiple times for a total of 558 interviews. Barriers to accessing LTSS included: healthcare providers being unhelpful, distrustful, dismissive, and unaffordable, with rigid protocols and long waiting periods; cognitive impairment itself in the context of living alone; limited resources. Facilitators included: having chronic medical conditions; "hunting" for services; information from attentive healthcare providers; support with transportation. Conclusions: Findings underscore the importance of ensuring that critical LTSS are tailored around the needs of older adults living alone with CI.

Abstract citation ID: igaf122.3719

### BATTLEFIELD ACUPUNCTURE (BFA) FOR MANAGEMENT OF CHRONIC PAIN IN AN OUTPATIENT SETTING

Ana Maria Pasatiempo[1], Rachel Ceria[2], Stephanie Hicks[2], and Jessika Dayrit[2], *1. VA Pacific Islands Health Care System, Department of Veterans Affairs, Alaska, United States, 2. VA Pacific Islands Health Care System, Honolulu, Hawaii, United States*

Background: Battlefield Acupuncture (BFA) is a non-pharmacological auricular acupuncture technique used to manage chronic pain. This study evaluated the short-term and longitudinal effectiveness of BFA among veterans receiving care in the Veteran Health Administration (VHA) community clinic. Methods: Prospective pre-post interventional study was conducted at the VHA Guam Community-Based Outpatient Clinic (2018-2019). Fifty-four veterans with chronic pain received BFA delivered by trained clinicians. Pain intensity was assessed immediately before and after each session using the Defense and Veterans Pain Rating Scale (DVPRS). Final analytic sample included19 participants who completed at least five sessions. Short-term effects were examined by comparing pre-post pain scores for each session; cumulative change was evaluated by comparing pre-session scores from first and fifth visits. Non-parametric tests accounted for the ordinal nature of pain ratings. Results: Significant reductions in pain were observed after each session (mean= 2.68, SD = 1.73). Subsequent treatments also demonstrated significant pain reduction (2nd session, mean=2.05 with SD = 2.09, 3rd session, mean=2.16 with SD = 1.92, 4th session, mean =1.94 with SD = 1.84, 5th session with mean 1.58 with SD = 1.92). However, cumulative improvement across sessions was not statistically significant ($\chi^2(4) = 3.40$, $p = 0.49$). Conclusion: Older Veterans often rely on opioids for chronic pain, which may lead to dependency and side effects. BFA produced short-term effectiveness in reducing chronic pain, with significant relief after individual sessions. However, sustained improvement over multiple treatments was not observed. Limitations include small sample size, lack of a control group, and potential confounders. Larger controlled studies are warranted to assess long-term efficacy.

Abstract citation ID: igaf122.3720

### BEHIND THE SILENCE: TRAINING CHWS TO IDENTIFY AND RESPOND TO SOCIAL ISOLATION

Daniel Moran, *Dartmouth Hitchcock Medical Center, Grantham, New Hampshire, United States*

Social isolation (SI) is a critical social determinant of health that disproportionately affects older adults, increasing the risk of chronic disease, cognitive decline, and mortality. Community health workers (CHWs) are well-positioned to address SI due to their trusted relationships and community-based roles; however, most lack formal training specific to SI. This quality improvement project implemented and evaluated a structured SI training for CHWs at a rural tertiary medical center, guided by Kirkpatrick's Four Levels of Learning Model. Six CHWs completed pre-training, post-training, and one- and two-month follow-up surveys assessing self-reported knowledge, confidence, and application of an SI component within the Aging Wellness Pathway. The two-hour, case-based training covered distinguishing SI from loneliness, identifying risk factors, communication strategies, connecting to community resources, and integrating interventions into electronic health record workflows. Results demonstrated knowledge gains of 18–26% and confidence gains of 12–32% across 11 SI-related domains, with improvements largely sustained at follow-up. CHWs reported using the SI component in an average of 55% of older adult interactions at two months, facilitating connections to senior centers, companion care, transportation, and the Dartmouth Aging Resource Center. Reported barriers included patient resistance, cognitive decline, and workflow integration challenges. Findings indicate that structured SI training can enhance CHW capacity to identify and respond to SI among older adults, supporting early intervention and connection to evidence-based resources. Sustained use may require ongoing reinforcement, workflow alignment, and strategies to address patient-level barriers.

Abstract citation ID: igaf122.3721

### BELONGING AND HEALTH OUTCOMES IN OLDER AMERICANS: A SCOPING REVIEW

Charlotte Moss[1], Zander Vasquez[2], and Raj Shah[1], *1. Rush University Medical Center, Chicago, Illinois, United States, 2. Rush Medical College at Rush University, Chicago, Illinois, United States*

As the number of older Americans grows, there is a movement to reframe aging as being about extending healthspan rather than just life expectancy. A sense of belonging has been theorized to be a positive influence on healthy longevity. This scoping review examined how belonging is associated with mental and physical health outcomes in older adults. We conducted a literature search on PubMed/MEDLINE, Scopus, PsycINFO, CENTRAL, and Google Scholar for studies published between 2015 and 2025. We followed the PRISMA Scoping Review guidelines and registered our protocol. We included original research articles conducted in the United States conceptualizing belonging as a predictor of various health behaviors or outcomes. We excluded studies not available in English, lacking a measure of belonging, and whose populations included individuals under 50 years old. After 1167 titles and abstracts and 88 full manuscripts were triaged, our search yielded 23 studies. Almost half of the articles examined thwarted belongingness and found it was a significant predictor of suicidality. Other outcomes included depression, loneliness, life satisfaction, and physical health. Belonging—or its absence—significantly predicted mental health outcomes, though its effect on physical health were mixed. These findings highlight associations of belonging and health outcomes in older Americans. Few articles studied the direct effects of belonging on chronic health conditions and longevity, highlighting gaps in the literature. More research on this topic is warranted to delineate if sense of belonging is a modifiable resilience factor for increasing healthspan.

Abstract citation ID: igaf122.3722

### BEYOND FINANCIAL ACCESS: SOCIOECONOMIC DETERMINANTS OF HEALTH ENGAGEMENT IN SINGAPORE'S HEALTHIER SG

Seoyeon Ahn[1], Alec Morton[2], Zhi Zhen Lim[2], Shumian Yeo, Cynthia Chen[2], Linda Wei Lin Tan[2], Xueling Sim, and Ian Yi Han Ang[2], *1. Duke-NUS Medical School/National University of Singapore, Singapore, Singapore, 2. National University of Singapore, Singapore, Singapore*

Background: Singapore's Healthier SG, launched in 2023, promotes voluntary primary care enrollment and participation in recommended screenings and vaccinations. Initially targeting adults aged sixty years and above, eligibility expanded to those aged forty years and above by 2025. This study examines how socioeconomic factors influence participation across engagement stages. Methods: We analyzed Singapore Population Health Studies data of adults aged forty years and above (N = 3,801) using multinomial logistic regression. Four Healthier SG engagement stages were examined: (1) unaware and unenrolled (reference), (2) aware but unenrolled, (3) enrolled but unscreened, and (4) enrolled and screened. Results: Older adults aged sixty years and above, those with more chronic conditions, and never-married individuals showed higher engagement rates. Compared to

Chinese participants, Indians showed lower screening completion rates. Notable socioeconomic differences emerged: university education, homeownership, and non-labor force status significantly predicted being enrolled and screened. Marginal effects analysis revealed distinct age-related patterns—being enrolled and screened increased from 10.5% in the 40-49 age group to peak at 25.6% in the 60-69 age group, while program unawareness decreased sharply with age (59% to 45.7% respectively). Conclusions: Despite Singapore's comprehensive preventive healthcare framework, engagement gaps persist across educational, housing, employment, ethnic, and age-related factors. This demonstrates that removing financial barriers alone is insufficient for achieving universal health access. Results underscore the need for targeted interventions addressing age, socioeconomic, and ethnic disparities through differentiated outreach, flexible implementation strategies, and culturally responsive approaches. This research provides actionable insights for designing inclusive health service delivery in diverse aging societies.

Abstract citation ID: igaf122.3723

### BRIDGING GENERATIONS: BRAIN HEALTH EDUCATION WITH OLDER ADULTS AND UNDERGRADUATES IN COMMUNITY SETTINGS

Jian Han, Morgyn Edwards, Madison Edwards, Khalil McKoy, Ashley Sanderlin, and Travonia Hughes, *North Carolina Agricultural and Technical State University, Greensboro, North Carolina, United States*

Intergenerational service learning has the potential to bridge the gap between younger and older populations while promoting mutual educational and social benefits. This project examined the outcomes of a community-based educational intervention in which undergraduate biology students delivered health-related presentations to older adults residing in independent living facilities across North Carolina. Eleven students developed and delivered 10 interactive presentations on brain health, nutrition, physical activity, and healthy aging. Sessions also featured hands-on activities such as pill sorting games, exercise bingo, and MyPlate-based nutrition planning to enhance participant engagement. Quantitative data from pre- and post-program surveys (analyzed using paired t-tests at $p = 0.05$) revealed significant student learning gains in communication, nutrition, and activity planning. The Classroom Undergraduate Research Experience (CURE) survey further supported these findings, with strong gains noted in the "Understanding" domain of Bloom's taxonomy. Older adult participants rated the sessions highly in satisfaction (79%) and engagement (76%), citing both the educational value and interactive components. Findings suggest that intergenerational service learning effectively promotes healthy aging while enhancing students' academic skills, empathy, and commitment to community engagement.

Abstract citation ID: igaf122.3724

### CAPTURING THE PAIN EXPERIENCE: CREATION AND TESTING OF AN AI-DRIVEN PAIN ASSESSMENT APP

Marcia Shade[1], Mrinal Rawool[2], and Stephen Scott[2], *1. University of Nebraska Medical Center, Omaha, Nebraska, United States, 2. University of Nebraska-Lincoln, University of Nebraska Lincoln, Nebraska, United States*

Background: Pain is undertreated and poorly managed in older adults. Pain symptoms may not be reported or identified, and time is limited in clinical practice to address pain among multiple comorbid conditions. Can artificial intelligence assist? The purpose of this project was to create and test a machine learning model (MLM) that captures the remote biopsychosocial pain experience. Methods: Mock data interactions were used to fine-tune a pre-trained model. The model was integrated with a mobile software application's architecture and deployed for testing. Metric data was collected on the MLM's response time, crash, and transcription grammatical accuracy. Results: The MLM generated pain assessment questions, such as, Is the pain worse at certain times of the day, like mornings or evenings? During the initial model creation, synthetic conversation testing yielded ROUGE scores of 0.5–0.6 and BLEU scores of 0.62. During testing, the MLM's average response time was 4 seconds per request. Requests were impacted slightly by the length of the spoken message from the user. One app crash occurrence was documented. The MLM was grammatically accurate; however, the user utterance transcription grammatical error rate was 1.2%. Conclusions: These findings demonstrate the promising metrics from iterative development, deployment, and testing of an MLM designed to engage with older adults with chronic pain. The response time underscores the preliminary efficiency of this integrated system. Continued refinement of the model with performance and user experience testing in real-world settings will help optimize this solution.

Abstract citation ID: igaf122.3725

### CAREGIVER MASTERY AS A MEDIATOR BETWEEN DEMENTIA BEHAVIORAL AND PSYCHOLOGICAL SYMPTOMS AND DEPRESSIVE SYMPTOMS

Kyungmi Lee, Faith Jackson, and Kylie Meyer, *Case Western Reserve University, Cleveland, Ohio, United States*

Self-efficacy is an individual's belief in their ability to handle challenging situations. Caregivers who experience greater bother from behavioral and psychological symptoms (BPSD) of dementia are at high risk of experiencing depressive symptoms. While many studies have explored the impact of BPSD on caregivers' depression, limited research has examined how caregivers' self-efficacy may buffer the negative effects of BPSD. This study investigates whether caregiver self-efficacy, and the related concepts mastery and confidence (i.e., care tasks, decision-making, and knowledge), mediate the relationship between BPSD and caregivers' depression. Mediation analysis was performed using baseline data from an ongoing clinical trial with 130 caregivers, and indirect effects were assessed via bootstrapping with 95% confidence intervals. Results show that mastery significantly mediates the effect of BPSD on depressive symptoms, even after controlling for caregiver age, sex, and caregiving duration. Higher BPSD was associated with lower mastery ($\beta$ = -0.066, SE = 0.019, p = .0009); lower mastery was also associated with higher depressive symptoms ($\beta$ = -0.400, SE = 0.118, p = .0010). The indirect effect of mastery was statistically significant ($\beta$ = 0.027, SE = 0.011, 95% CI [0.009, 0.051]). Among covariates, sex was positively associated with mastery ($\beta$ = 0.216, p = .013), while age and caregiving duration were not significantly related to mastery or depressive symptoms. Other self-efficacy components did not show significant mediation effects. These findings support mastery as an independent mediator between BPSD and caregiver depressive symptoms, highlighting the importance of interventions targeting mastery to reduce depression in caregivers.

Abstract citation ID: igaf122.3726

### CAREGIVING NETWORKS AND FUTURE CARE PLANNING OF OLDER LGBTQ+ ADULTS

Barbara Castro Sanchez, Sophia Tsuker, Natasha Nemmers, and Amanda Leggett, *Wayne State University, Detroit, Michigan, United States*

LGBTQ+ older adults experience significant health disparities, including increased risk for chronic conditions that may necessitate formal or informal care. Many rely on chosen families rather than biological kin for caregiving, which can lead to challenges in recognition by healthcare and legal systems. Past literature has examined LGBTQ+ adults' social networks and primary caregivers; however, little is known about how these networks translate into available or actual caregiving arrangements in later life. In-depth qualitative interviews with a focus on social networks and potential or actual caregiving networks were conducted with 12 community-dwelling LGBTQ+ older adults. Participants were aged 55 to 80 with 50% female- and 50% male-identifying. A thematic analysis identified three themes shaping caregiving networks and planning. (1) Partnership status: single participants anticipated relying on friends and chosen families, while partnered participants were more likely to list family members as potential caregivers. Single participants expressed more concerns over future care planning, listing more deliberate, active future care plans, compared to partnered individuals who reported expectations to rely on their partner. (2) Age and caregiving experience: older participants had more caregiving experience, either as providers or recipients, and more established caregiving networks. (3) The sociopolitical climate: participants expressed significant concern about the broader political climate and its long-term implications often more so than experiencing direct discrimination from individuals or healthcare providers. As aging researchers and practitioners prepare for the needs of an increasingly diverse older population, attention to LGBTQ+ caregiving dynamic and care planning is critical.

Abstract citation ID: igaf122.3727

### CHAMPIONING OLDER ADULTS IN CLIMATE EMPOWERMENT (COALESCE) PHASE 1 - A PARTICIPATORY RAPID REALIST REVIEW

Mei Fang[1], Rebecca White[1], Farnoush Mansourian[1], Sreya Ajay[2], Sharon Sa[1], Dannah DeSouza[1], Claire Dreyfuss[1], and Elise Stone[1], *1. Simon Fraser University, Vancouver, British Columbia, Canada, 2. SFU, Vancouver, British Columbia, Canada*

Background: The COALESCE Project (Phase I) investigates how older adults experience and prioritise the effects of climate breakdown. By engaging them as citizen science co-researchers, the project aims to co-develop resilience strategies tailored to their needs. Partnering older adults with graduate students fostered intergenerational exchange, enhancing research capacity and generating practical interventions to address severe weather impacts on ageing populations. Methods: A participant-led rapid realist review (PRRR) was piloted to explore how best to identify climate change priorities and effective interventions. Five graduate students and seven older adult co-researchers were trained to collaboratively undertake a time-limited PRRR, including literature search, data extraction, and synthesis. Student–older adult pairs conducted targeted searches of academic and grey literature on climate-related impacts and resilience strategies for older people. Guided by older adults' lived experiences, the review process integrated life-course interviews and deliberative dialogues, enabling reflection, critique, and adaptation of the PRRR method. Findings: The pilot showed that effective co-research requires working to the unique strengths of both older adults and students. Rather than duplicating tasks, collaboration was optimised when students applied academic research skills alongside older adults' lived experience and contextual knowledge, ensuring shared responsibility and ownership of the review. Discussion: This intergenerational co-research model deepened understanding of climate impacts on older people, built mutual research capacity, and empowered participants. Critically, the project demonstrated the potential of participatory realist reviews to co-produce knowledge and inform interventions, ensuring older adults define their own climate change contexts and resilience needs.

Abstract citation ID: igaf122.3728

### CHANGES IN AGEISM AND LGBTIA CLINICAL COMPETENCY FOLLOWING AN LGBTIA OLDER ADULT COMPETENCY TRAINING

Jennifer Smith[1], Sharon Bowland[2], Jordan Westcott[3], and Joseph Winberry[4], *1. University of Tennessee, Knoxville, Knoxville, Tennessee, United States, 2. University of Tennessee, Knoxville, Tennessee, United States, 3. University of Tennessee at Knoxville, Knoxville, Tennessee, United States, 4. UNC Chapel Hill, Chapel Hill, North Carolina, United States*

Lesbian, gay, bisexual, transgender, intersex, agender/asexual, and other sexual and gender minority (LGBTIA) older adults face health disparities relative to the general population (Lampe et al., 2023), due in part to unmet health needs through available healthcare and aging services (Gibson et al., 2019). Lack of culturally competent services emerged in a community needs assessment as a concern among LGBTIA older adults (Winberry, 2023). To address this, we conducted an LGBTIA older adult cultural competency training focused on preparing healthcare and other service providers with the knowledge and skills necessary to meet the needs of this population. Using paired sample t-tests, we examined how the training impacted ageist attitudes and LGBTIA clinical competency among participants ($n=43$). The training reduced ageist attitudes among participants, $t(33) = -3.94$, $p < .001$ with a moderate effect size ($d = .47$; Cohen, 1988). We also found that the training improved self-perceived clinical competency for working with LGBTQ+ individuals among participants, $t(37) = 7.64$, $p < .001$ with a moderate effect size ($d = .58$; Cohen, 1988). We found similar results for clinical preparedness, $t(39) = 2.57$, $p = .014$ with a moderate effect size ($d = .53$; Cohen, 1988); attitudes toward LGBTIA individuals, $t(39) = 2.57$, $p = .014$ with a small effect size ($d = .22$; Cohen, 1988); and knowledge about LGBTIA individuals, $t(39) = 2.57$, $p = .014$ with a moderate effect size, ($d = .44$; Cohen, 1988). Implications for interdisciplinary trainings focused on LGBTIA older adults will be discussed.

Abstract citation ID: igaf122.3729

### CHANGING HEALTH BEHAVIORS USING ANNUAL WELLNESS VISITS AMONG SENIOR HOUSING RESIDENTS

Barbara Resnick[1], Nicole Brandt[2], Sarah Holmes[3], Jennifer Klinedinst[4], and Anne Hagan[5], *1. University of Maryland, Baltimore, Maryland, United States, 2. UMB School of Pharmacy, 20 N. Pine St. Baltimore, Maryland, United States,*

*3. University of Maryland School of Nursing, Baltimore, Maryland, United States, 4. UMB SON, 655 W. Lombard Street, Baltimore, Maryland, United States, 5. UMB SON, ELKRIDGE, Maryland, United States*

More than 1.8 million older adults receive rental assistance in federally subsidized low-income housing communities. These individuals are mostly racial and ethnic minorities and experience multiple comorbid medical conditions, functional and cognitive impairment and have poor health behaviors. To help these individuals maintain or improve healthy behaviors we provided monthly interdisciplinary wellness clinics in four senior housing communities. Examples of services provided included blood pressure monitoring, cerumen removal, cognitive evaluations and management, nail and skin care, medication education and management, falls assessments and individualized exercise programs, among others. Annually, during the residents' birthday month, they were invited to have an Annual Medicare Wellness Visit (AWV) which included setting health behavior goals. A sample of 105 residents had two Medicare Annual Wellness Visits. The mean age of the participants was 79(SD = 8), most were female (77%) and Black older adults (75%), with only 21% being Hispanic, 16% married, and 90% had at least some high school education. Changes over time were evaluated using general linear modeling for continuous variables and chi-square for dichotomous variables. There was no change in perceived health status, falls, help with activities of daily living, or alcohol use. There was a decrease in the percentage of residents that worried about falling, were smoking, not exercising, or did not have an advanced directive. Cognition declined over the two years based on the CLOCK test and the individual's subjective memory complaints. Providing AWVs to senior housing residents may be helpful to maintain and improve healthy behaviors over time.

Abstract citation ID: igaf122.3730

### CHANGING PERCEPTIONS OF END-OF-LIFE CARE AMONG OLDER CHINESE AMERICANS: ROLE OF ACCULTURATION

Peiyuan Zhang[1], Jing Wang[2], Fei Sun[3], Xiaoyouxiang Li[4], Yaofeng Chen[5], and Kaipeng Wang[4], *1. University of Alabama, Tuscaloosa, Alabama, United States, 2. University of New Hampshire, Durham, New Hampshire, United States, 3. Michigan State University, East Lansing, Michigan, United States, 4. University of Denver, Denver, Colorado, United States, 5. huazhong university of science and technology, huazhong university of science and technology, Hubei, China (People's Republic)*

Chinese Americans are one of the largest and fastest-growing immigrant groups in the United States, yet end-of-life (EOL) care remains disproportionately underutilized among this population. This study explores perceptions of end-of-life care and how they were shaped by acculturation among older Chinese Americans. Researchers conducted seven focus groups in person or via Zoom with 46 Chinese Americans aged 60 or above living in the United States in 2024. The study included three Mandarin (n = 22; mean years lived in the US: 18), two Cantonese (n = 9; mean years lived in the US: 33), and two English (n = 15; mean years lived in the US: 53) focus groups. All interviews were recorded, transcribed, and thematically analyzed using open coding. Major findings were (1) limited awareness of EOL care before immigrating to the U.S., (2) increased understanding of palliative and hospice care through the experiences of informal social networks post-immigration, (3) adaptation of traditional Chinese cultural views, such as filial piety, which influenced EOL preferences for life-prolonging care, (4) a shift toward greater autonomy and control in EOL decision-making, and (5) growing openness to discuss death and dying. Findings highlight the role of immigration history and acculturation in shaping Chinese American older adults' evolving perceptions of end-of-life care, underscoring the need for clinicians to adopt culturally attuned communication strategies and decision-making support tools tailored to the values and experiences of immigrant older adults.

Abstract citation ID: igaf122.3731

### CHILDHOOD EXPOSURE TO WAR AND DEPRESSIVE SYMPTOMS OR A CURRENT MAJOR DEPRESSIVE EPISODE IN LATE ADULTHOOD

Jin-kyung Lee, and Gwang Suk Kim, *Yonsei University College of Nursing, Yonsei University, Seoul, Republic of Korea*

At the end of World War II, the Korean War occurred from 1950 to 1953. During the war, people experienced unwanted separation from family members and/or leaving their hometown. It is well-known that children exposed to war have a higher risk of post-traumatic stress and depression soon after. However, little is known about its long-term effects, primarily on mental health in late adulthood. This research investigates whether the generation exposed to the war in their childhood is more likely to be vulnerable to depression in late adulthood compared to other generations. Out of 287 participants aged 55 to 89 years old, 124 older adults experienced the Korean War in their childhood. Data collected via self-report surveys and face-to-face interviews with trained researchers were analyzed by multivariate regression and logistic regression. Hamilton depression rating scale and the Mini-International Neuropsychiatric Interview were used to screen for depressive symptoms or a current major depressive episode. The results demonstrate that the older generation exposed to the Korean War in their childhood compared to others had a higher likelihood of experiencing depressive symptoms (B(SE) = -.14(.06), 95% CI = -.26~-.01, p<.05) or a current major depressive episode (OR = .81, 95% CI = .68~.98, p <.05) in low-stress situations. Considering the higher risk of experiencing depressive symptoms and a major depressive episode in late adulthood among the generation exposed to the war, periodic screening and targeted interventions are essential to relieve stress and improve mental health for supporting their healthier aging.

Abstract citation ID: igaf122.3732

### CLINICIAN DELIVERY STRATEGIES FOR EXERCISE-BASED PAIN MANAGEMENT IN DEMENTIA: A QUALITATIVE STUDY

Annalisa Na[1], Amy Kwok[1], Ben Senderling[1], Jenna Lisa[1], Peter Gliebus[2], Julie Fritz[3], Joke Bradt[1], and Laura Gitlin[1], *1. Drexel University, Philadelphia, Pennsylvania, United States, 2. Baptist Health, Boca Rotan, Florida, United States, 3. University of Utah, Salt Lake City, Utah, United States*

Chronic pain affects over 50% of community-dwelling people living with dementia (PLWD), compromising function and quality of life. Despite its prevalence and poor outcomes, evidence-based, non-pharmacological interventions for PLWD remain limited. Guided exercise programs at therapeutic doses targeting underlying pain mechanisms (e.g., osteoarthritis) and associated impairments (e.g., range of motion, weakness) show promise but lack applicability to PLWD. Hence, clinicians rely on trial-and-error, resulting in care variability, resource waste, diminished care quality, and highlighting the need for intervention development. An initial step in developing and testing exercise-based pain interventions is to assess the range of current practices used by clinicians. This qualitative study explores strategies currently used by clinicians to engage PLWD in exercise-based pain management. Sixteen clinicians with at least two years of experience managing pain and working with PLWD in non-institutional settings (e.g., outpatient, home-health) participated in focus groups. We analyzed deidentified transcripts using conventional content analysis and identified an overarching theme that clinicians adopted person-centered approaches. Clinicians explained person-centered approaches include 1) Being flexible with aiding and cueing based on real-time assessments; 2) Employing hands-on strategies to direct attention, support movement recognition, and enhance body awareness. (3) Allowing personalized selection of music through accessible technology to offer rhythmic cueing, foster motivation, support sensory regulation, and create supportive environments. These clinician-described strategies demonstrate that one-size-fits-all models are inadequate and emphasize the need for adaptive, person-centered approaches. These findings support the development of an evidence-informed framework for exercise-based pain management tailored to community-dwelling PLWD.

Abstract citation ID: igaf122.3733

### COGNITIVE RESERVE AND GAIT DECLINE ACROSS THE PARKINSON'S SPECTRUM: EVIDENCE FROM PPMI LONGITUDINAL COHORTS

Han-yun Tseng, *University of Colorado, Aurora, Colorado, United States*

Background: Cognitive reserve has been linked to slower decline in mobility and daily function in Parkinson's disease (PD), but its role across the prodromal-to-diagnosed spectrum, and it shifts over time, remains unclear. Methods: We analyzed longitudinal data from the Parkinson's Progression Markers Initiative (PPMI; baseline N = 3,971; Mage = 62.4 years, 42% female) across four annual visits. Participants included healthy controls (HC; n = 331), prodromal subgroups (hyposmia, RBD, LRRK2 carriers; n = 2,190), and early PD (≤ 2 years from diagnosis; n = 1,450). Mixed-effects models tested between-person (average Montreal Cognitive Assessment [MoCA]) and within-person (time-specific deviations) effects on gait impairment (PIGD), controlling for age, sex, and education. Results: All groups showed significant gait decline over four years. Higher between-person MoCA predicted slower decline, whereas within-person deviations did not. Subgroup analyses revealed the strongest protective effect in LRRK2 carriers, with weaker effects in hyposmia and RBD. In PD, the reserve effect was attenuated but remained present. Growth models showed significant slope-slope coupling between cognition and gait decline ($r = -.24$, $p < .01$), evident when four visits were modeled. Conclusions: Cognitive reserve helps preserve mobility even before PD diagnosis, but once PD manifests, cognitive and motor decline become closely intertwined This stage-specific shift marks the point where resilience gives way to vulnerability, aligning with biopsychosocial and mind-body frameworks that guide intervention in aging and neurodegeneration. Clinically, findings underscore stratified prevention that harnesses reserve before diagnosis and multimodal interventions that address cognitive–motor interdependence once PD manifests.

Abstract citation ID: igaf122.3734

### COMBATTING THE CAREGIVER CRISIS: HEALTH AND SOCIAL INEQUITIES FOR ADRD CAREGIVERS WITH DISABILITIES

Nicholas Mirin, Laurin Bixby, and Joe Caldwell, *Brandeis University, Waltham, Massachusetts, United States*

As the population ages and the care workforce crisis persists, an increasing number of adults are becoming unpaid caregivers for aging parents or other family members with ADRD. While people with disabilities are often assumed to be on the receiving end of care, we estimate 36.4% of disabled adults are caregivers themselves, many of whom provide care to individuals with ADRD. Prior research documents disparities for people with disabilities and caregivers separately, but little attention has been given to the intersection of disability and caregiving. As such, disabled caregivers are largely left out of research and policy discussions related to the impact of ADRD on family caregivers. To address that literature gap, this study uses multivariate logistic regression and data from the 2021–2023 Behavioral Risk Factor Surveillance System (n = 310,722) to estimate health and social determinants of health risks as a function of the interaction between disability and ADRD caregiving status among unpaid caregivers, adjusting for demographic and household characteristics. Results indicate that disabled ADRD caregivers report poorer physical, mental health, and social support outcomes relative to non-disabled caregivers – including elevated risks of housing, food, and job insecurity, as well as significant barriers to transportation. We also find disabled ADRD caregivers disproportionately rely on public assistance programs such as SNAP. Taken together, these findings suggest disabled ADRD caregivers experience compounded risks of adverse health and SDH outcomes. Future policies must address the challenges experienced by family caregivers of adults with ADRD, particularly among those who have disabilities themselves.

Abstract citation ID: igaf122.3735

### COMORBIDITY RISK ASSESSMENT IN MEDICARE BENEFICIARIES WITH NEURODEVELOPMENTAL DISORDERS

Bianca Rodriguez, and Allison Willis, *Perelman School of Medicine, Philadelphia, Pennsylvania, United States*

Neurodevelopmental disorders (NDDs) are lifelong conditions that affect brain development and functioning, with varying levels of impairment on daily life. Advances in technology have extended life expectancy for individuals with NDDs; however, little is known about their health in older adulthood. Using a 20% random sample of Medicare beneficiaries in 2021, we identified adults aged 65 and older with autism spectrum disorder, cerebral palsy, muscular dystrophy, or spina bifida. Demographic characteristics and comorbidities were compared to a control group without NDDs. Compared with controls, older adults with NDDs were younger on entry into Medicare, had distinct sex distributions, and were less racially and ethnically diverse. Neurological conditions—including epilepsy, multiple sclerosis, and migraine—were markedly more prevalent among NDD groups, as were psychiatric disorders such as anxiety, depression, and psychosis. Substance use disorders, particularly alcohol abuse and dependence, occurred at nearly twice the rate of controls. Vascular conditions, including myocardial infarction, atrial fibrillation, heart failure, ischemic heart disease, and stroke, were also elevated, averaging twofold higher in NDD groups.

Neurodegenerative diseases, notably Alzheimer's dementia and Parkinson's disease, were up to three times more common among individuals aging with NDDs, underscoring the dual impact of lifelong neurodevelopmental conditions and aging-related decline. These findings demonstrate that while vascular and substance use disorders contribute to the excess burden of disease, the most pronounced disparities lie in neurological, psychiatric, and neurodegenerative comorbidities. This highlights the urgent need for research on neurodegeneration risk in NDD populations and for healthcare adaptations to better support individuals with lifelong NDDs.

Abstract citation ID: igaf122.3736

**COMPLEX POST-TRAUMATIC STRESS DISORDER IS LINKED WITH ACCELERATED EPIGENETIC AGING IN FEMALE YAZIDI EX-CAPTIVES.**

Yaakov Hoffman[1], Jan Kizilhan[2], Amit Shrira[1], David Cheishvili[3], Moshe Syzf[4], and Ari Zivotofsky[1], *1. Bar-Ilan University, Ramat Gan, HaMerkaz, Israel, 2. State University in Baden-Württemberg, Stuttgart, Baden-Wurttemberg, Germany, 3. McGill University, Montreal, Quebec, Canada, 4. EpiMedTech Global, Singapore, Singapore*

Previous theories have linked exposure to trauma, and particularly trauma symptoms, to greater physical "wear and tear" that manifests as poorer health and metabolic syndrome. Very recent studies have shown that post-traumatic stress disorder (PTSD, which comprises, according to the ICD-11, three symptom clusters; intrusions, avoidance and arousal) longitudinally leads to more accelerated epigenetic aging. This study examines for the first time how complex-PTSD (CPTSD; which includes PTSD in addition to another three symptom clusters related to disturbances in self-organization; affect dysregulation, negative self-concept, and difficulties in forming and maintaining relationships) is linked with epigenetic accelerated aging. The sample comprised 94 female Yazidi women who had been held as sex slaves by ISIS and emigrated to Europe after their release ad who were resettled in Europe after their release from captivity were sampled (Mage 38.41±11.93, range 18-72.81; 63.2% female). Measures included demographic variables, PTSD and CPTD questionnaires along with salvia from which epigenetic aging was derived. Results show older epigenetic aging in those with CPTSD. Their epigenetic age was 4 years greater than ex-captives who did not display trauma symptoms. The data also revealed shorter telomere length for those with CPTSD. Although preliminary, these results demonstrate how complex trauma is associated with accelerated epigenetic mechanisms and address factors that speed up biological aging. The results have implications for normal aging, especially when one ages in the shadow of stress and trauma.

Abstract citation ID: igaf122.3737

**CONCURRENT SEDATIVE DOSING RISKS IN OLDER ADULTS: EVIDENCE FROM A NATIONAL EMS SAMPLE**

David Hancock[1], Rana Barghout[1], Josh Lachs[2], and Tony Rosen[1], *1. Weill Cornell Medicine / NewYork-Presbyterian Hospital, New York, New York, United States, 2. Weill Cornell Medicine, New York, New York, United States*

The rapid control of agitation in older adults often requires sedatives. Despite the heightened vulnerability in this group, little is known about how multiple medications are combined to address agitation in prehospital care. We analyzed national EMS data to characterize both timing and dosing patterns of sedative administration among patients ≥65 years (N = 3,132) who received more than one sedative medication during emergency responses. Ketamine-midazolam was the most common combination (61.7% of cases), followed by haloperidol-midazolam (18.4%). Ketamine-midazolam showed moderate concurrent use (39.5% within five minutes), with ketamine typically administered first when medications were staggered. Most haloperidol-midazolam administrations occurred within five minutes of each other (66.1%), suggesting concurrent use that maximizes sedation and potentially compounds age-related pharmacokinetic changes. By contrast, lorazepam-based combinations more often followed a sequential pattern, with more than five minutes between administrations. These differences are clinically meaningful: concurrent administration of sedatives in older adults presents elevated risk for oversedation, respiratory depression, and delirium, complications that disproportionately affect this vulnerable population and can trigger severe adverse outcomes. Staggered use of lorazepam combinations aligns more closely with geriatric emergency medicine recommendations to "start low and go slow." However, across all combinations, the median doses administered to older adults were consistently above geriatric dosing protocols recommended for emergency settings, instead mirroring prescribing patterns used in younger adults. These findings underscore the need for age-specific prehospital protocols that balance the immediacy of behavioral emergencies with medication safety, ensuring strategies reflect the vulnerability of older adults to adverse sedative effects.

Abstract citation ID: igaf122.3738

**CONNECT POWERED BY BRAIN: HARNESSING AI-DRIVEN BEHAVIOR ANALYSIS TO FOSTER PERSON-CENTERED DEMENTIA PROGRAMMING**

Michael Skrajner[1], Gregg Gorzelle[1], John Zeisel[1], Sucheendra Palaniappan[2], and Vivek Saxena[2], *1. Hopeful Aging, Winchester, Massachusetts, United States, 2. Vikasietum Tecknology LLC, Plano, Texas, United States*

Person-centered activity programming can reduce responsive behaviors and support meaning and purpose for persons living with dementia (PLwD; Chenoweth et al., 2009; Lee et al., 2012). Yet tailoring activities to an individual's cognitive level and interests typically depends on front-line staff, a time-intensive process often infeasible in long-term care. Hopeful Aging, through an NIA-funded SBIR Phase II project, is developing CONNECT powered by BRAIN, an AI-driven platform that personalizes and streamlines activity programming. The system draws from a growing, curated library of over 1,000 dementia-appropriate activities across 12 types. Based on a PLwD's cognitive profile and interests, the platform generates personalized recommendations, each scored for fit (0–100). To minimize decision fatigue, the top seven recommendations appear on the first screen. Recommendations then adapt dynamically through AI-based behavioral engagement analysis derived from session recordings (with consent/assent). Engagement is quantified using behavioral markers—verbal communication, visual attention, pleasure, and hand and head gestures—and compared against the individual's evolving baseline to refine subsequent suggestions. In Phase I, 22 participants (M age=83.7; 82% female; 73% Caucasian) tested an alpha prototype. A subsequent Model Training Study in the ongoing Phase II project with 26 participants (M age=84.5; 76% female; 68% Caucasian) refined behavioral analyses. Post-training F1 values were: Verbal Communication=90%; Visual Attention=87%; Pleasure=86%; Hand Gestures=82%; Head Gestures=76%, with a mean improvement of+9%. Findings demonstrate the feasibility of automated AI engagement analyses for adaptive, person-centered activity programming. A forthcoming cluster-randomized trial will examine impacts on quality of life, responsive behaviors, and depression.

Abstract citation ID: igaf122.3739

### CONNECTION OVER CONVENIENCE: WHY COMMUNICATION MATTERS MORE THAN LOGISTICS IN LTC VISITS

Nicole Praska[1], Caroline Collins-Pisano[1], and Rachel Weiskittle[2], *1. University of Colorado Colorado Springs, Colorado Springs, Colorado, United States, 2. University of Colorado, Colorado Springs, Colorado Springs, Colorado, United States*

Residents of long-term care (LTC) facilities often experience social isolation, even with regular visits from family and friends. Satisfaction with these visits supports relationship maintenance and residents' emotional well-being. Both structural factors and interpersonal dynamics may shape visitor satisfaction, yet their relative contributions remain underexplored. Identifying these predictors can inform programs that foster family engagement and enhance residents' social well-being. This cross-sectional study examined whether LTC facility characteristics (facility type, travel time, length of stay) and resident communication factors (verbal communication ability, recognition of visitor, alertness) were associated with visitor satisfaction. An online national survey was completed by 175 community-dwelling adults (M age = 49.58, SD = 17.86, range 20-82) who had visited a loved one in LTC within the past 12 months. Pearson correlations assessed associations between visitor satisfaction and key variables. No significant associations emerged between visitor satisfaction and facility characteristics (years living in facility: $B = 0.05$, $p = .09$; minutes traveled: $B = 0.001$, $p = 0.82$; independent/assisted living: $B = 0.19$, $p = 0.46$; nursing home/skilled nursing: $B = 0.17$, $p = 0.42$). Verbal communication ability was significantly positively associated with visitor satisfaction ($B = 0.18$, $p = 0.02$). Recognition of visitor and alertness were not significant predictors (recognition: $B = 0.04$, $p = 0.61$; alertness: $B = -0.08$, $p = 0.39$). Findings suggest that residents' verbal communication may play a more critical role in shaping visitor satisfaction than structural or logistical aspects of visits.

Abstract citation ID: igaf122.3740

### CORRELATES OF MENTAL ILLNESS AMONG OLDER ADULTS USING COMMUNITY WELFARE CENTERS IN SOUTH KOREA

Jung eun Yun[1], Sangyong Cho[2], Jihee Kang[3], Sueun Shin[4], Minjin Kim[5], Young Woo Kim[1], Jin-Ah Sim[6], and Dong-Soo Shin[6], *1. Hallym University, Chunceon, Kangwon-do, Republic of Korea, 2. University of Cincinnati, Cincinnat, Ohio, United States, 3. hallym University, Chunceon, Kangwon-do, Republic of Korea, 4. Hallym University, ChunCheon, 5. Cincinnati University, Cincinnati, Ohio, United States, 6. Hallym University, ChunCheon, Kangwon-do, Republic of Korea*

Purpose: This study examined differences by the presence of mental illness among community welfare center users in an urban–rural complex city. As South Korea pursues deinstitutionalization for people with mental illness, welfare centers must adapt their roles to meet community needs. Methods: A cross-sectional survey was conducted with 153 older adults from two community welfare centers. The dependent variable was the presence of mental illness (0 = no, 1 = yes). Independent variables included mental health confidence, age, gender, education, income, living-alone, self-rated health, exercise, diet, marital status, and social capital (trust in neighbors, mutual help). Logistic regression analysis was performed. Results: The model was significant (adjusted $R^2 = 0.346$, $p < 0.001$). Older age was linked to lower likelihood of mental illness ($OR = 0.89$, $p = 0.004$), while women showed higher likelihood than men ($OR = 11.52$, $p = 0.021$). Never-married individuals tended toward higher risk ($OR = 4.65$, $p = 0.107$). Mutual help was positively associated with mental illness ($OR = 8.30$, $p = 0.005$). Conclusion: Age and gender were key predictors, while marital status and social capital warrant further study. Higher prevalence among women likely reflects their greater representation in welfare centers. The positive link between mutual help and mental illness may reflect supportive networks among users with illness, while those without illness may be less engaged due to negative perceptions of welfare centers. Findings underscore the need to tailor programs for users with mental illness and strengthen welfare centers as hubs for aging-in-place and self-management.

Abstract citation ID: igaf122.3741

### COST AND ACCESS CHANGES APPRECIATED WITH TNFA INHIBITOR BIOSIMILARS

Joseph Visingardi[1], and Bryan Sherwood[2], *1. Albany Medical College, Albany, New York, United States, 2. Dartmouth Hitchcock Medical Center, Lebanon, New Hampshire, United States*

Biologic therapies, such as adalimumab, rank among the most costly drugs in the United States. This study aimed to project how pricing and access to adalimumab may shift once it loses patent protection, using the experiences of other biologics that have already gone off-patent as a reference. To evaluate market dynamics, CMS Medicare expenditure data from 2017 through 2021 were examined for several branded biologics and their biosimilars. Analyses focused on changes in unit cost, the number of beneficiaries using the branded product, and shifts in the total population using either branded or biosimilar versions. During this period, branded adalimumab—without biosimilar competition—saw a 72% increase in cost per unit alongside a 23% rise in beneficiary numbers. In contrast, branded infliximab, which faced biosimilar competition, experienced a 53% decline in unit cost, a 26% reduction in branded drug users, and a modest 1% overall decline in total beneficiaries across all formulations. Similar patterns were observed with most other drugs analyzed, showing decreased costs and reduced reliance on branded versions, but with mixed results in overall patient uptake across both branded and biosimilar products. These findings suggest that the loss of exclusivity for biologics, including adalimumab, generally results in lower prices and diminished market share for the brand. However, whether these shifts translate into improved affordability and access remains uncertain, as the anticipated increase in total beneficiaries following the introduction of lower-cost biosimilars was not consistently observed.

Abstract citation ID: igaf122.3742

**CUMULATIVE MTBI BURDEN IN AGING VETERANS: REGIONAL ACCELERATED WHITE MATTER DETERIORATION IDENTIFIED ALONG TRACTS**

Tyler Robinson, Emily Van Etten, Shira Hao, David Rothlein, William Milberg, Catherine Fortier, and David Salat, *Department of Veterans Affairs, Boston, Boston, Massachusetts, United States*

Along-tract analysis of white matter (WM) aging offers a spatially specific approach to identifying effects in WM tissue with greater specificity than established tract-averaged approaches, while maintaining anatomical interpretability. As such, incorporating both whole-tract means and along-tract clusters improves our ability to identify unique and complementary patterns of effects across tracts due to differences in effect distribution and cluster thresholding in both age effects and specific WM insult such as traumatic brain injury (TBI). In this study, we seek to assess the impacts of cumulative mild traumatic brain injury (mTBI) on WM tissue microstructural degeneration in aging in a veteran sample using both whole-tract means and along-tract effect clustering via Freesurfer's TRACULA package. Whole-tract effects were found in the splenium, bilateral ventral cingulate bundle, and bilateral optic radiation. Along-tracts analysis corroborates effects in the ventral cingulate bundle and optic radiation, with further bilateral clusters of effects in the acoustic radiation, and laterally specific clusters in the fornix, middle longitudinal fasciculus, and arcuate fasciculus. In all cases, the higher mTBI burden was associated with greater degrees of WM degeneration in advancing age as reflected by microstructural measures, suggesting mTBI is associated with accelerated age-related declines in the veteran population well after recovery and into later age. Furthermore, these findings specify anatomically founded regions of localized mTBI impacts that are not captured by whole-tract methods and contribute to the aim of improving understanding of anatomical mechanisms of mTBI impact and accuracy of targeting for future diagnosis and intervention.

Abstract citation ID: igaf122.3743

**DAILY STRESSOR EXPOSURE AND SUBJECTIVE MEMORY: AGE MODERATION AND WITHIN-PERSON VARIATION ACROSS DAYS AND YEARS**

Nicole Stuart, and Nancy Sin, *University of British Columbia, Vancouver, British Columbia, Canada*

Growing research indicates that daily stress is associated with poor same-day memory performance. However, it is unclear how these associations may unfold over time, and how age may moderate this relationship. In this pre-registered study, ecological momentary assessment data were collected from adults aged 25-89 ($M = 46.23$, $SD = 17.44$ at baseline) across British Columbia, Canada. Annually for 3 years, participants ($N = 169$; 70% women) reported daily stressor exposure and subjective memory performance for 14 days. Multilevel models evaluated daily stressor exposure and an Age x Yearly Stressor Exposure interaction as predictors of subjective memory, controlling for demographics and depressive symptoms. Subjective memory was rated as relatively worse on days when at least one stressor occurred, compared to stressor-free days (b = -0.126, CI[-0.044,-0.199]). Participants also reported poorer average memory during years with a higher proportion of stressor days, compared to years with fewer stressor days (b = -0.092, CI[-0.064,-0.118]). Baseline age moderated this relationship, showing the strongest deleterious associations in younger (25-39y) adults; however, this relationship remained significant across age groups. Extending prior naturalistic research, the present findings suggest that more stressful periods in one's life are associated with poorer subjective memory, and this is particularly pronounced among younger adults. Future work should evaluate potential mechanisms underlying this relationship, including age-related differences in stressor qualities, emotion regulation, perseverative cognitive processes, and coping.

Abstract citation ID: igaf122.3744

**DELIRIUM DETECTION USING POINT-OF-CARE SINGLE-CHANNEL EEG: A PILOT STUDY**

Rebecca Heinze[1], Tage Runkle[2], Yvonne Larson-Smith[2], Emma Behnken[2], Gen Shinozaki[3], and Allyson Palmer[2], *1. Macalaster College, Rosemount, Minnesota, United States, 2. Mayo Clinic, Rochester, Minnesota, United States, 3. Stanford University, Palo Alto, California, United States*

Delirium is a common yet underdiagnosed medical emergency involving acute deficits in cognition and attention. It affects over 20% of hospitalized patients and up to 80% of ICU patients, with increased prevalence in older adults. Delirium is associated with increased mortality, prolonged hospitalization, long-term cognitive decline, and substantial healthcare costs. However, current screening tools are subjective and difficult to implement consistently. Traditional EEGs show diagnostic utility; however, they are costly and require specialized interpretation. We conducted a prospective pilot study evaluating a point-of-care, single-channel EEG device for delirium detection in hospitalized older adults. EEG spectra were utilized to calculate a 'bispectral EEG' (BSEEG) score reflecting the ratio of slow to fast brain waves. Twenty-two postoperative patients (mean age 75; 59.09% female) were assessed daily for up to seven days using both the BSEEG and the gold standard 3-Minute Diagnostic Interview for Confusion Assessment Method (3D-CAM). ROC analysis was utilized to determine an optimal threshold BSEEG score to indicate delirium positivity. BSEEG scores were significantly higher in the 3D-CAM-positive group than in the 3D-CAM-negative group (1.60 and 1.45, respectively; $p < 0.05$). A BSEEG threshold of 1.5 demonstrated 77.78% sensitivity and 56.10% specificity for delirium. In contrast, route clinical screenings (Delirium Triage Screen and Brief CAM) showed 0.00% sensitivity and 96.30% specificity, and failed to detect all true delirium cases. Although further validation in larger cohorts is needed, these findings suggest that the point-of-care single-channel EEG is a promising, non-invasive screening tool for delirium screening, particularly in fast-paced clinical settings.

Abstract citation ID: igaf122.3745

**DEMENTIA SPECIALISTS' PERSPECTIVES ON IMPROVING RURAL VETERANS' ACCESS TO CARE**

Sarah McDannold[1], Brittany Anderson[2], Tammy Walkner[2], Michelle Hilgeman[3], Hilary Mosher[4], Lauren Moo[1], Guneet Jasuja[5], and Heather Davila[6], *1. VA Bedford Healthcare System, Bedford, Massachusetts, United States, 2. Iowa City VA Health Care System, Iowa City, Iowa, United States, 3. Tuscaloosa VA Medical Center, Tuscaloosa, Alabama, United States, 4. Minneapolis VA Medical Center, Minneapolis, Minnesota, United States, 5. Center for Health Optimization and Implementation Research, VA Bedford, Boston,*

*Massachusetts, United States, 6. Iowa City VA Healthcare System, Iowa City, Iowa, United States*

Outside the Veterans Health Administration (VHA), rurality-based differences in dementia care access have been reported (e.g., fewer physician and home health visits among rural vs. urban residents). However, this has not been examined within the VHA, which prioritizes rural healthcare access. To address this gap, we conducted semi-structured interviews with dementia specialists (N = 20) at five rural VHA medical centers in two regions. Based on Drebig's "pathways to dementia care" framework, interviews focused on understanding gaps and strategies to meet rural Veterans' care needs at three stages: dementia warning signs; evaluation and diagnosis; and aligning treatment and services to optimize patient and caregiver quality of life. We analyzed the thematic content of interviews using rapid qualitative analysis. Common gaps reported were 1) need for earlier recognition of dementia warning signs to access services; 2) lack of dementia specialists, particularly in the community; 3) limited resources to support family caregivers; and 4) limited availability of supportive services such as home care, respite, and residential long-term care. Common strategies to address gaps included 1) education and outreach to primary care clinicians; 2) tele-consult services; and 3) care transitions programs for Veterans with challenging behavioral symptoms. Clinicians consistently described VHA as having more robust dementia specialty care services than what was available in the community. Considerable gaps remain in rural access to dementia care around recognition and evaluation of dementia, and coordination and availability of supportive services. Optimizing access to dementia-related services for rural Veterans requires tailored approaches to leverage VHA and community resources.

Abstract citation ID: igaf122.3746

### DEPRESSION PARTIALLY MEDIATES THE ASSOCIATIONS OF LONELINESS WITH MEMORY, EXECUTIVE FUNCTION AND PROCESSING SPEED

Carmen Jia-Wen Chek[1], Carlos Araujo-Menendez[2], Ariana Stickel[3], and Wassim Tarraf[1], *1. Wayne State University, Detroit, Michigan, United States, 2. San Diego State University/University of California, San Diego, San Diego, California, United States, 3. San Diego State University, San Diego, California, United States*

Loneliness and depression are linked to poorer cognition in older adults. While prior research has often examined loneliness as a mediator between depression and cognition, few studies have investigated depression as a mediator between loneliness and specific domains of cognition (e.g., memory vs. executive). This study addresses that gap by examining whether depression mediates the relationship between loneliness and memory, executive functioning, and attention/processing speed using structural equation modeling (SEM). Cognitive domains were derived using confirmatory factor analysis from the 2016 Harmonized Cognitive Assessment Protocol dataset of the Health and Retirement Study (n = 3,293; Mage = 72.71, SDage = 7.47), with loneliness (UCLA 11-item) and depression (CESD-8) from Waves 2012/2014 were tested in an SEM mediation model. Sociodemographic and physical health covariates were added stepwise. Loneliness significantly predicted increased depressive symptoms ($\beta = 0.39$), and had significant direct effects on all cognitive domains, except for memory in a fully adjusted model ($\beta = -0.04$, $p = .06$). Depression significantly predicted worse cognitive outcomes across all domains. Significant indirect effects on memory ($\beta = -0.05$), executive functioning ($\beta = -0.09$), and attention/ processing speed ($\beta = -0.08$) indicated partial mediation by depression. These indirect effects remained significant after covariate adjustment, with direct effects being slightly attenuated ($p < .01$). Findings highlight that depression only partially accounts for the cognitive risks introduced by loneliness. Loneliness presents a multi-pathway risk for cognitive decline, warranting direct intervention beyond its socioemotional effects.

Abstract citation ID: igaf122.3747

### DO PDGM CLINICAL GROUPS DISADVANTAGE PEOPLE WITH DEMENTIA? A PRE-IMPLEMENTATION BASELINE ANALYSIS

Maxwell Cutty[1], Carmen Pfeil[2], and Sara Knox[1], *1. Medical University of South Carolina, Charleston, South Carolina, United States, 2. University of California, Los Angeles, Los Angeles, California, United States*

In January 2020, Medicare implemented the Patient-Driven Groupings Model (PDGM), linking 30-day home health payments to clinical (case-mix) groupings. How this affects individuals with Alzheimer's Disease and Related Dementias (ADRD) is still not well understood. To establish a pre-PDGM baseline, we retrospectively applied PDGM rules to 2019 Medicare fee-for-service claims to simulate clinical grouping assignment for beneficiaries with and without ADRD (N = 1,432,374; 90% non-ADRD, 10% ADRD). We repeated this in a 1:1 propensity score-matched cohort stratified by ADRD status (129,428 per group), matching on demographics, comorbidity burden, prior health care use, social support, and baseline function/cognition. Before matching, ADRD patients were disproportionately represented in Neuro Rehab, while non-ADRD patients were concentrated in Surgical Aftercare. Notably, Behavioral Health, though small (0.94%), was the only clinical group with a majority of ADRD patients (65% vs. 3.1–17.7% in other groups). The top clinical groups in the stratified pre-match cohort were Musculoskeletal Rehabilitation (non-ADRD 34.0%; ADRD 20.1%) and Cardiac & Circulatory (non-ADRD 16.6%; ADRD 21.1%). After matching, most clinical groups converged toward a 50/50 mix of non-ADRD/ADRD patients. However, Behavioral Health and Neuro Rehab skewed toward ADRD (92.1%, 59.3%), while Infectious Disease, Surgical Aftercare, and Respiratory skewed toward non-ADRD (64.0%, 64.1%, 59.3%). These findings provide the first pre-PDGM characterization of ADRD patient distribution across clinical groupings, highlighting how reimbursement incentives may differentially affect beneficiaries with ADRD. This timely evidence establishes a foundation for evaluating whether the PDGM inadvertently disadvantages patients with ADRD, informing policy, clinical practice, and future research on equitable home health delivery.

Abstract citation ID: igaf122.3748

### DOMAIN-SPECIFIC COGNITION MEDIATES THE ASSOCIATION BETWEEN MOBILITY AND WAYFINDING ABILITY IN OLDER ADULTS

Alexis Chargo, Cheryl Dahle, and Ana Daugherty, *Wayne State University, Detroit, Michigan, United States*

Older adults frequently report difficulties with community wayfinding, which may be further exacerbated by age-related mobility decline; however, the ways in which these processes intersect to support navigation as a fundamental behavior for independent functioning is poorly understood. The connection between mobility and wayfinding may be attributable to a shared set of cognitive processes that are vulnerable to decline. Thus, we examined the contributions of domain-specific cognition as a potential mediator between mobility and wayfinding in a community-based sample of older adults (N = 74, age 53-89 years). Participants underwent a comprehensive assessment of cognitive and mobility function and completed a seated virtual assessment of wayfinding with subsequent recall of environment details. A structural equation model was specified in which a latent mobility construct (indicators: balance, timed forward and backward walking) predicted recall of environment details via three cognitive mediators: processing speed (SDMT), visuospatial working memory (BVMT-R), and declarative memory (VAL-delay). The hypothesized model demonstrated excellent fit and accounted for 16.3% of the variance in environment recall ($p = .04$). While the total effect of mobility on recall of environment details was not significant ($p = .072$), evidence for mediation was supported by a significant cumulative indirect effect ($p = .038$). A comparison of the percentages of unique effects revealed processing speed as the largest contributor, followed by declarative and visuospatial working memory function. The results suggest a role of mobility in the acquisition and shaping of our memory for the environment that is explained in part by a shared cognitive-mobility architecture.

Abstract citation ID: igaf122.3749

### EARLY POLICY IMPACT ON PREVENTIVE HEALTH: REGRESSION DISCONTINUITY STUDY OF SINGAPORE HEALTHIER SG

Seoyeon Ahn[1], Alec Morton[2], Zhi Zhen Lim[2], Shumian Yeo[2], Cynthia Chen[2], Linda Wei Lin Tan[2], Xueling Sim[2], and Ian Yi Han Ang[2], *1. Duke-NUS Medical School/National University of Singapore, Singapore, Singapore, 2. National University of Singapore, Singapore, Singapore*

Background: Singapore's Healthier SG program, launched in 2023, promotes voluntary primary care enrollment and preventive health screenings. Initially targeting adults aged 60 and above, eligibility expanded to those aged 40 and above by January 2024. This study evaluates the causal effect of program enrollment on screening uptake using regression discontinuity design centered at the age-40 eligibility threshold. Methods: We applied fuzzy regression discontinuity design using the age-40 cutoff to estimate local average treatment effects of Healthier SG enrollment on screening behavior. The analytic sample included 522 adults aged 37 to 42 from the Singapore Population Health Studies surveyed from June 2024 to January 2025. We estimated age-based eligibility effects on enrollment (first stage) and used two-stage least squares to assess enrollment impact on screening uptake. Results: Turning 40 increased enrollment probability by 38.6 percentage points ($p < 0.001$). In the second stage, enrollment raised screening uptake by 33.5 percentage points (95% CI: 13.9–53.1; $p < 0.001$). Reduced-form estimates showed that eligibility increased screening rates by 12.9 percentage points ($p < 0.01$). While the policy increased population-level screening modestly, it induced substantial behavioral change among enrollees. Results remained robust across model specifications, bandwidths, and covariate adjustments. Discussion: Healthier SG enrollment substantially increases screening uptake among newly eligible adults at age 40. The age-40 threshold represents a critical juncture for health system engagement to improve aging trajectories. These findings offer robust causal evidence for life-course triggers in preventive health policy and highlight the value of early mid-life interventions.

Abstract citation ID: igaf122.3750

### EARLY USER EXPERIENCES OF CAREMOBI: MHEALTH APPLICATION FOR INFORMAL CAREGIVERS AND CARE COORDINATION

Ashley Wei[1], Laura Peralta[2], and Tina Sadarangani[2], *1. Royal College of Surgeons in Ireland, Edmonton, Alberta, Canada, 2. New York University, New York, New York, United States*

As care of older adults continues to shift toward community and home-based settings, many responsibilities fall to informal caregivers. Despite this, few comprehensive digital tools exist to support their needs. We evaluated early user satisfaction and perceived utility of CareMobi, a mobile application designed to support caregiving for medically complex adults. A cross-sectional post-download survey was distributed to eligible users via the CareMobi newsletter, assessing satisfaction, ease of use, most helpful features, and desired improvements. Users could also provide demographic information, describe how they discovered the app, and share real-world use cases. Informal caregivers most valued features that supported communication and organization. The top 3 features identified were: appointments and calendar tools, health tracking (e.g. vital signs, nutrition, daily updates, etc.), and secure file storage. Overall, 82% of respondents reported being satisfied (37%) or very satisfied (45%) with the app and 76% found CareMobi easy or very easy to use. The most frequently requested feature was a provider contact list ($n = 4$). Open-ended responses highlighted three key benefits associated with app use: enhanced communication with family members and collaboration with healthcare providers, streamlined information management, and improved health tracking and scheduling. Early users reported that CareMobi reduces caregiver burden by centralizing care management. These initial findings underscore the potential of mHealth solutions to support informal caregivers of older adults and inform future development priorities, as well as the need to integrate these into clinical practice.

Abstract citation ID: igaf122.3751

### ECONOMETRIC ANALYSIS OF OPTIMAL SITE OF CARE FOR PREFRAIL AND FRAIL PATIENTS UNDERGOING MASTECTOMY FOR BREAST CANCER

Claire Morton[1], Yu-Jen Chen[2], Kenneth Williams[2], Randall Bloch[3], Ezra Brooks[2], Christina Minami[2], Zara Cooper[2], and Louis Nguyen[2], *1. Center for Surgery and Public Health, Boston, Massachusetts, United States, 2. Brigham and Women's Hospital, Boston, Massachusetts, United States, 3. Boston Medical Center, Boston, Massachusetts, United States*

Patients with breast cancer undergo mastectomy in ambulatory surgery centers (ASCs) or inpatient settings, each considered safe for appropriate patients. Appropriateness remains poorly defined and relies on subjective clinician assessment contributing to variability in practice patterns that may be suboptimal. Utilizing the AHQR HCUP NIS and NASS, we sought to understand the association of frailty, defined by the Hospital Frailty Risk Score, with site of care. Secondary outcomes included transfer to an acute care hospital from an ASC and cost of care. 85.3% of all patients (190,148) and 51.3% (2,603) of prefrail or frail patients underwent surgery in ASCs. On multivariable analysis, frailty or prefrailty (OR 5.856) was associated with increased inpatient care ($p < 0.001$). Rates of transfer were low (0.1%). Odds of transfer were higher among prefrail and frail patients (OR 2.640, $p < 0.05$) but rates remained low (< 0.4%). Given clinician sorting, expected costs of mastectomy for prefrail and frail patients were greater than those for robust patients ($56,036.23, SE $396.05, vs $49,929.62, SE: $142.89). We calculate that rates of transfer that would negate cost savings associated with ambulatory procedures is over 100 times the currently observed rate (38%, SE 4.7%). If all prefrail and frail patients received care at ASCs with transfer rates at currently observed levels expected cost savings would average $8,404 per patient. Our findings suggest that despite slightly higher rates of transfer to an acute care hospital following ASC care, clinicians should consider treating frail and prefrail older adults in ASCs more often.

Abstract citation ID: igaf122.3753

### EFFECT OF LONG-TERM CALORIC RESTRICTION ON EPIGENETIC PROXIES OF INFLAMMAGING: CALERIE™ 2 TRIAL ANALYSIS

Waylon Hastings, *Texas A&M University, College Station, Texas, United States*

Background: Inflammaging, the age-related increase in systemic inflammation, has received particular attention as a target for epigenetic proxies derived using machine learning approaches. Examples include InflammAge, a translational epigenetic biomarker derived from 120+ distinct epi-scores representing various inflammatory biomarkers and/or diseases, and InflClock, a measure developed to predict differences between healthy aging volunteers and patients with chronic inflammatory diseases such as rheumatoid arthritis and multiple sclerosis. Initial reports support the ability of these proxies to distinguish inflammaging phenotypes, but concerns remain about whether such measures capture meaningful change above and beyond age-related redistribution of immune cell populations. Moreover, their responsiveness to gero-protective interventions also remains untested. Methods: To address this gap, we are implementing a panel (n = 130) of inflammatory-related epi-scores in the CALERIE™ clinical trial (n = 594), including three proxies assessing global inflammaging. The overarching goal of this research proposal is to assess longitudinal concordance between epi-scores and serum biomarkers of inflammation (e.g., CRP, IL-6, TNFa), oxidative stress (e.g., isoprostanes), and immune cell distributions. Further, we leverage mixed-effect modeling to test treatment effects of the CALERIE™ intervention on these epigenetic proxies for inflammaging. Results: Preliminary analyses support associations between epigenetic proxies and some, but not all inflammatory biomarkers, and nearly all measures are associated with complete blood count differentials. Although de-noising epigenetic proxies using DNA-methylation deconvolution references increases their measurement sensitivity, intervention effect sizes remained modest to insignificant. Conclusions: Although epigenetic proxies are gaining momentum as surrogate endpoints, additional methodological refinement is necessary to achieve the rigor necessary for clinical translation.

Abstract citation ID: igaf122.3754

### EFFECT OF THE TEACHING NURSING HOME ON NURSING HOME STAFF SATISFACTION

Howard Degenholtz[1], Nancy Zionts[2], Anny Treat[3], Kenna Campbell[3], and Chelsea Dickson[2], *1. University of Pittsburgh, Pittsburgh, Pennsylvania, United States, 2. Jewish Healthcare Foundation, Pittsburgh, Pennsylvania, United States, 3. University of Pittsburgh, School of Public Health, Pittsburgh, Pennsylvania, United States*

The Teaching Nursing Home Collaborative (TNHC) aims to enhance the long-term care workforce and improve care quality in nursing homes through clinical education, and quality improvement organized around the Institute for Healthcare Improvement's Age-Friendly Health System "4Ms" framework: What Matters Most, Mobility, Mentation, and Medication. Nursing homes also participate in an online learning community. Staff satisfaction data were collected using the Centers for Medicare and Medicaid Services (CMS) Nursing Home Employee Satisfaction Survey from two nursing homes between 2022 and 2023, and three in 2024. A total of 726 surveys were available for analysis. In addition, two nursing homes that are part of the TNHC shared staff satisfaction survey data collected by commercial vendors. A subset of five items that also appear on the CMS Survey were analyzed for 2021 – 2024; the effective sample size for these five items was 1,317. The results were mixed. Overall job satisfaction decreased significantly at one nursing home (p=.0000), but did not change significantly at the other two facilities. For all five facilities, there was an increase in satisfaction related to team dynamics (i.e., cross-departmental p=.000 and supervisory engagement p=.0300). However, ratings declined significantly for items related to recommending the facility as a workplace, having adequate resources, and opportunities for professional growth (p < .0001). As the Age-Friendly Nursing Home program is promulgated nationally monitoring staff experience and satisfaction remains critical to sustaining workforce engagement and care quality.

Abstract citation ID: igaf122.3755

### EFFICACY, FEASIBILITY, AND SAFETY OF A GAME-BASED HOME EXERCISE PROGRAM TO IMPROVE BALANCE: A PILOT STUDY

Jordan Kurth[1], Emily Cornish[1], Alexander Wiggins[2], Allisyn Hetherington[3], Robert Creath[2], and Christopher Sciamanna[1], *1. Penn State College of Medicine, Hershey, Pennsylvania, United States, 2. Lebanon Valley College, Annville, Pennsylvania, United States, 3. Penn State College of Medicine Milton S. Hershey Medical Ctr, Hershey, Pennsylvania, United States*

One-third of older adults fall each year, leading to loss of independence and costing billions. While exercise is widely proven to reduce falls, many older adults do not do enough to benefit. Game-based activities are inherently more enjoyable than longer, traditional exercise, but no study has examined the impact of brief, game-based workouts on balance. Our objective was to understand the preliminary efficacy, feasibility, and safety of a 5-minute daily game-based exercise program among older adults at-risk of falling. A total of 16 participants (12 female; mean age 72 years) who had fallen in the past year and performed less than 60 minutes of weekly moderate-to-vigorous exercise completed the 2-week intervention. The intervention involved 3x1-minute sessions (followed by 1-minute rests) of paddling a foam ball against a wall in their home, trying to prevent the ball from hitting the ground. Study staff met virtually with each participant once over the two-week intervention. Timed Up-and-Go scores decreased by an average of 0.76 seconds from baseline (9.35s) to follow-up (8.59s; SMD = -0.5). Average 6-minute walk test distances improved by an average of 30 meters from baseline (341m) to follow-up (371m; SMD 0.48). Zero participants reported a fall. Adherence was also extremely high (98% of days). The average program satisfaction score was 8.5/10. Brief, game-based at-home exercise programs appear to be effective, feasible, and safe for older adults at risk of falling. Future randomized controlled trials should be conducted to test program efficacy on clinically-relevant outcomes over a longer period of time.

Abstract citation ID: igaf122.3756

### ENGAGING OLDER CHINESE-SPEAKING CANCER PATIENTS IN SUPPORT GROUPS: POST-PANDEMIC LESSONS LEARNED

Qi Chen[1], and Xiaoli Li[2], *1. Hunter College - City University of New York, Hunter College - City University of New York, New York, United States, 2. Hunter College - City University of New York, New York, New York, United States*

Older Chinese immigrants with cancer in the U.S. face linguistic, cultural, and psychosocial barriers, posing challenges for support group engagement. The COVID-19 pandemic accelerated the adoption of online group models, creating both challenges and opportunities for care delivery. This qualitative study explored strategies for engaging Chinese-speaking older adults with cancer support groups in the post-pandemic era. Using a community-engaged approach, we recruited Chinese-speaking cancer patients, caregivers, and social workers or community workers who have experience working with older Chinese-speaking cancer patients. Semi-structured interviews were conducted in English or Mandarin, online or in person, and analyzed inductively using thematic analysis. Nineteen participants were interviewed, including patients (n = 7), one caregiver (n = 1), and practitioners (n = 11). Strategies for engaging older patients included: (1) building trust through shared language and cultural familiarity, facilitated by bilingual practitioners who understood norms around emotional restraint and family duty; (2) embedding health education in accessible, culturally grounded language to address misinformation and reduce uncertainty; (3) introducing emotional support gradually, through activities or informational sessions rather than direct questioning about distress; (4) aligning group design with family-centered values, recognizing older adults' prioritization of family needs; and (5) leveraging online formats to reduce barriers of transportation, stigma, and disclosure. In the post-pandemic era, online group-based care presents promising avenues to reduce barriers and foster engagement among Chinese-speaking older adults with cancer. Culturally responsive strategies that build trust, deliver accessible health information, and use flexible virtual formats can enhance equitable access to psychosocial support.

Abstract citation ID: igaf122.3757

### EVALUATING THE LIVED EXPERIENCES OF OLDER ADULTS WITH FALL-MONITORING TECHNOLOGY

Alisha Johnson[1], Chang-Chun Chen[2], K. Melinda Fauss[3], and Wung Shu-Fen[4], *1. University of Missouri, Columbia, Missouri, United States, 2. University of Arizona, Tucson, Arizona, United States, 3. Sinclair School of Nursing, Columbia, Missouri, United States, 4. University of California, Davis, Betty Irene Moore School of Nursing, Sacramento, California, United States*

Every year in the US, 30% of older adults experience falls, resulting in three million visits to emergency departments. Fall-monitoring technologies have proven effective in improving the quality of life for older adults, allowing them to age in place. Though these technologies enhance safety and keep caregivers informed, they also raise concerns regarding autonomy and privacy. However, there is limited understanding of the perspectives of older adults living with these technologies. We used an interpretive phenomenological approach, interviewing eight older adults, with and without dementia, residing in two independent living facilities who had experienced fall-detection technologies for the previous 9-12 months. The interviews focused on understanding how technology influences their self-identity, ability to live independently, safety, and age-in-place. To ensure trustworthiness, field-notations, reflexive journaling, and participant check-backs were used. Several themes emerged reflecting the influence of technologies on lived experiences: the freedom to age in place, the necessity for active intervention, individual approaches to technology based on personality/cognition, and feelings of discomfort and fear. Older adults were more inclined to engage with monitoring technologies if they were open to intervention, had their concerns addressed, and perceived the technology as useful for enabling aging-in-place. Older adults, with and without dementia, are often the target audience for monitoring technologies, yet their perspectives are frequently overlooked. This study significantly enhances the understanding of effective implementation strategies for monitoring technologies. It serves as a valuable resource for individuals and facilities seeking successful integration of fall-detection and prevention technologies to support older adults to age-in-place.

Abstract citation ID: igaf122.3758

### EVERYDAY DISCRIMINATION AND COGNITIVE FUNCTION AMONG OLDER LATINO ADULTS

Mayra Estrella[1], Maude Wagner[1], Lisa Barnes[1], Brittney Lange-Maia[1], David Marquez[2], and Melissa Lamar[1], *1. Rush University Medical Center, Chicago, Illinois, United States, 2. University of Illinois Chicago, Chicago, Illinois, United States*

Greater exposure to everyday discrimination is associated with poorer cognitive health among older Black adults; however, its role in cognitive function among older Latino adults remains largely unexplored. This study examined associations of self-reported experiences of everyday discrimination with cognitive level and change among older Latino adults. We included 351 Latino participants (age=71±7yrs; 79% female) from Rush Alzheimer's Disease Center cohort studies without clinical dementia at baseline and who completed ≥2 cognitive evaluations over time (mean follow-up=7±4yrs). Everyday discrimination was assessed at baseline using the Detroit Area Study Everyday Discrimination Scale (range=0-9). Separate linear mixed-effects models tested associations of everyday discrimination with initial level and annual rates of change in global cognition and five cognitive domains (episodic, working, and semantic memory; perceptual speed and visuospatial ability), adjusting for age at baseline, sex, education, and preferred language (English/Spanish) for testing. Higher levels of everyday discrimination were associated with worse initial global cognition ($\beta$=-0.048, Standard Error [SE]=0.012, $p = 0.0001$), and separate domains including episodic memory ($\beta$=-0.042, SE = 0.015), semantic memory ($\beta$=-0.047, SE = 0.017), working memory ($\beta$=-0.078, SE = 0.017), and perceptual speed ($\beta$=-0.066, SE = 0.018; all $P < 0.01$). There were no associations with cognitive decline. Findings extend previous evidence from cohorts of older Black adults to older Latino adults showing that greater everyday discrimination is associated with worse initial global cognition and most cognitive domains, but not with rates of cognitive decline. As baseline cognitive level may shape dementia risk, this study underscores the importance of addressing social exposures among Latinos to support healthy cognitive aging.

Abstract citation ID: igaf122.3759

### EVERYDAY STRESSORS, DISCRIMINATION, AND HEALTH AMONG LGBTQ+ OLDER ADULT CAREGIVERS OF ADULTS LIVING WITH MCI/ADRD

Nik Lampe[1], Victoria Money[2], Kirsty Clark[3], and Tara Mckay[3], *1. University of South Florida, Tampa, Florida, United States, 2. University of South Carolina, Columbia, South Carolina, United States, 3. Vanderbilt University, Nashville, Tennessee, United States*

Using LGBTQ+ Social Networks, Aging, and Policy Study Wave 3 data (N = 982), this study examines how caregiving for individuals with mild cognitive impairment, Alzheimer's disease, and related dementias (MCI/ADRD) impacts everyday stressors, discrimination, and health outcomes among LGBTQ+ older adults in the Southern United States. Using chi-square tests, Fisher's exact tests, logistic regression, and Poisson regression models, we assessed associations of caregiver role with stress and health outcomes. We then conducted moderation analyses to test interactions between caregiver role and LGBTQ+ minority stress. LGBTQ+ caregivers of individuals with MCI/ADRD were three times more likely to experience proximal minority stress ($p < 0.05$), twice as likely to report suicidal ideation ($p < 0.1$), and more likely to report cognitive problems ($p < 0.05$) and high blood pressure ($p < 0.05$) than non-caregivers. Interaction effects suggest that minority stress may exacerbate mental health problems among MCI/ADRD caregivers. Findings underscore the need for targeted supports and interventions for LGBTQ+ MCI/ADRD caregivers.

Abstract citation ID: igaf122.3760

### EXAMINING CAREGIVER BURDEN IN LOW- AND MIDDLE-INCOME COUNTRIES: A SCOPING REVIEW

Wenjie Chen[1], Aasha Paredes[2], Yiwei Zhang[3], Christian Noval[2], Wai Yan Min Htike[1], Haolin Li[1], Chenkai Wu[1], and Hanzhang Xu[2], *1. Duke Kunshan University, Kunshan, Jiangsu, China (People's Republic), 2. Duke University, Durham, North Carolina, United States, 3. Duke Kunshan University, Suzhou, Jiangsu, China (People's Republic)*

Most research regarding caregiver burden was conducted in high-income countries. However, for low- and middle-income countries (LMICs), structural challenges and limited resources exacerbate caregiver burden. For instance, while government support programs and formal caregivers exist, they are largely inaccessible and unaffordable. Additionally, long-term care systems are underdeveloped overall leaving family members as the primary source of care. This scoping review aims to describe current evidence on caregiver burden in LMICs, including the severity and types of caregiver burden, factors associated with caregiving burden, and existing interventions. We applied PRISMA extension for scoping reviews and searched PubMed for relevant articles up to Aug 2024. We used the Joanna Briggs Institute Manual for Evidence Synthesis and Critical Appraisal Checklist to evaluate quality of evidence. A total of 43 peer-reviewed articles were included, encompassing data from 15,503 caregivers across 12 countries, spanning Asia, Latin America, and Africa. On average, caregivers reported moderate to high levels of perceived burden, particularly in emotional, physical, and social domains. Higher caregiver burden was observed among older, female caregivers characterized by lower educational attainment and limited social support. The most frequently examined care recipient factors included cognitive impairment and depressive symptoms. Common interventions to reduce caregiver burden include structured caregiving activities, discharge planning, spiritual therapy, and social-emotional support. Findings from this scoping review highlight substantial emotional, physical, and social burden faced by caregivers in LMICs, particularly among women. We also found considerable variability in the measures of caregiving burden, study design, and quality of the evidence across studies.

Abstract citation ID: igaf122.3761

**EXAMINING COMORBIDITY PATTERNS AMONG PROSTATE CANCER PATIENT-PARTNER DYADS**

Chunxuan Ma[1], Bianca Shieu[2], and Lixin Song[3], *1. Case Western Reserve University, Cleveland, Ohio, United States, 2. University of Texas Health Science Center at San Antonio, San Antonio, Texas, United States, 3. UT Health San Antonio, San Antonio, Texas, United States*

Background: Prostate cancer (PCa) patients and their partners both experiences reduced quality of life and challenges in maintaining well-being when comorbidities are present. This study aims to describe the pattern of comorbidities for PCa patients and partners. Methods: We defined comorbidity burden as the mean of the Charlson Comorbidity Index (CCI) score, and comorbidity congruence as the absolute difference in the CCI score within each dyad. Using the baseline data from a randomized clinical trial (RCT), we fitted multilevel models to examine how social determinants of health (SDOH) influenced comorbidity congruence. We used network analysis to identify comorbidity co-occurrent patterns. Results: Among 273 dyads in the RCT, 118 dyads lived in high Area Deprivation Index (ADI) ranking areas and 54 patients were Black. Dyads whose partner reported non-white and non-Black (N = 13) exhibited significantly greater discrepancies in comorbidity counts compared to whose partner was White or Black (p = 0.04). Dyads who lived in high ADI areas, who's income >$90,000, or with higher mean age had significantly higher CCI burden (ps < 0.01). We observed co-occurrence of myocardial infarction–heart failure, chronic liver disease–stroke, and stroke–heart failure among patients. We observed myocardial infarction–heart failure and stroke–heart failure among partners. Conclusion: The overall comorbidity characteristics of PCa patient-partner dyads vary by SDOH and neighborhood deprivation. Cardiovascular and stroke comorbidity patterns differ between patients and partners. Understanding comorbidity from a dyadic perspective is essential for designing comprehensive, postoperative well-being interventions for PCa patients and partners, particularly in socioeconomically disadvantaged areas.

Abstract citation ID: igaf122.3762

**EXAMINING PREDICTORS OF PSYCHIATRIC HOSPITALIZATION OUTCOMES IN OLDER ADULTS WITH AND WITHOUT DEMENTIA**

Sophia McInturff[1], Cindy Woolverton[2], Rafael Samper-Ternent[3], Carolyn Pickering[4], Michelle Patriquin[1], and Scott Lane[1], *1. University of Texas HSC at Houston, Houston, Texas, United States, 2. UTHealth Houston, Houston, Texas, United States, 3. University of Texas Health Science Center at Houston, Houston, Texas, United States, 4. UTHealth, Cizik School of Nursing, Houston, Texas, United States*

Older adults with dementia present for acute inpatient psychiatric care to manage dementia-related neuropsychiatric symptoms and co-occurring mental health conditions. Given the complexity of needs, these individuals may be at increased risk for poor outcomes when compared to those without dementia, such as prolonged hospitalization and fragmented discharge planning. This study aimed to examine predictors of health outcomes among this population. Electronic health record data was extracted for patients aged 65+ admitted to the UTHealth Houston Behavioral Sciences Center from 2021-2024. Regression models assessed predictors of length of stay (LOS) and admission type (involuntary vs. voluntary) for individuals with (n = 94) and without (n = 276) a dementia diagnosis. Results indicated that dementia diagnosis, financial status (payer status: indigent, government-assisted, or secure), psychotic disorder diagnoses, and discharge disposition significantly predicted LOS. The length of stay increased by an estimated 3 days with a dementia diagnosis, 3 days with a psychotic disorder, and 20 days with secure payer status. Discharge to assisted living was more common among individuals with dementia (41.7%) than those without (13.9%). The logistic regression predicting admission type was significant ($\chi^2(4) = 32.23$, $p < .001$); dementia diagnosis, psychosis symptoms, and secure payer status increased the likelihood of involuntary admission. These findings suggest that older adults with dementia are at increased risk for longer hospitalizations and involuntary admissions, highlighting the need for tailored discharge planning and improved care transitions in psychiatric settings.

Abstract citation ID: igaf122.3763

**EXPERTISE AT RISK: RECENT POLICY CHANGES AT HHS ADVISORY COMMITTEES**

Apoorva Rangan, *Stanford University, Los Altos Hills, California, United States*

Within the federal government, scientific advisory committees play a critical role in shaping evidence-based research priorities, clinical practice guidelines, and healthcare delivery for older people. There are over 1,000 advisory committees currently advising the federal government, 268 within the Department of Health and Human Services. Committee members are subject-matter experts who participate in committee work on a voluntary basis. As of July 2025, 79 committees have been terminated, and 23 are administratively inactive, representing a significant shift in governance and domain expertise across the Department.1 There have also been significant changes in the membership of several committees.2 This session will use the Advisory Committee on Immunization Practices (ACIP) as a paradigmatic case to describe the direct and indirect roles of scientific committees in guiding healthcare delivery and clinical practice.3, 4 We will also examine recent threats to the ACIP's autonomy and outline potential consequences for clinicians, professional societies, and older patients. Data sources will include administrative notices of meeting postponements, CMS quality measures which incorporate ACIP recommendations, and legislation introduced by current members of Congress.5 We identify other committees related to aging which have faced interruptions, including the National Advisory Committee on Aging and identify the GSA's role in restoring committee meetings. We advocate for HHS leadership and policy makers to preserve the ability of committees to function independently, promote certainty in guideline development, and improve stakeholder confidence. Finally, we address how organizations can advocate for these critical independent scientific committees and describe other routes to communicate science to policymakers.

Abstract citation ID: igaf122.3764

**EXPLORING A LIFE LIVED WITH IRRATIONAL FALL RISK APPRAISAL**

Aleatha Rossler[1], Victoria Loerzel[2], and Ladda Thiamwong[2], *1. university of central florida, willow park, Texas, United States, 2. University of Central Florida, Orlando, Florida, United States*

Fall risk assessment has been focused on the physiological risk factors that can be seen, and interventions are centered on physical strengthening or assistive tools. Fear of falling (FOF) is a psychological risk factor for falls and has been classified as having worry of falling or a lack of balance confidence. Irrational Fall Risk Appraisal (FRA) is when a person has a high level of FOF with a low physical risk; however, little is known about the experience of this group in relation to falls. A qualitative descriptive design was used to learn more about their experience with FOF and any effect it may have on their daily lives. 14 older adults (OAs) with Irrational FRA were interviewed. Participants completed a semi-structured interview that was recorded and transcribed for analysis. Four themes were derived: Experiences with Falls, Acceptance of age-related decrease in ability, Perceptions of Mobility and Fears, and Awareness and Support. 13 of the participants had a prior fall. This study showed that OAs are unaware whether they are experiencing FOF or of the impact that FOF may have on them. FOF is not a common assessment done by healthcare providers and shared with the OAs, and therefore, the older population needs more education on the topic. Education for healthcare providers is needed to raise awareness of how and why to integrate these assessments into their regular practices. Combined interventions to address both psychological and physiological risks, along with community education, may provide added support to maintain independence.

Abstract citation ID: igaf122.3765

### EXPLORING MENTAL DISTRESS AMONG OLDER WORKERS WITH MILD COGNITIVE IMPAIRMENT BY RACE AND ETHNICITY

Ronica Rooks[1], Mason Pearce[2], Joyce Lopis[1], and Paulina Erices-Ocampo[1], *1. University of Colorado Denver, Denver, Colorado, United States, 2. Metropolitan State University of Denver, Denver, Colorado, United States*

Older Black and Latinx adults experience faster cognitive declines and higher dementia prevalence than older White adults. Meaningful activities, like employment, may help slow cognitive declines among older adults with mild cognitive impairment (MCI), but little is known about whether employment can reduce cognitive declines or racial/ethnic disparities in this population. Our pilot study explored mental distress associated with employment among older adults aged 55+ with MCI and gathered insights from community navigators to assess if and how employment may support cognitive functioning across racial/ethnic groups. We conducted three qualitative interviews with older White adults with MCI and two focus groups with Latinx community navigators (n = 12). Data were analyzed using thematic analysis with Atlas.ti. Older White adults with MCI enjoyed working and felt employment supported cognitive functioning through social engagement and mental activity. But, they reported needing family and coworker support. Stress stemmed from complex job tasks and supervisors' performance expectations, but workplace accommodations improved their job satisfaction. Racial/ethnic workplace challenges were not reported. Latinx community navigators described cultural biases in cognitive assessments, stigma around cognitive health, and limited discussion of cognitive health in Latinx families. They noted economic constraints and family dynamics influencing older adult women's caregiving roles and barriers to recognizing and addressing cognitive decline in Latinx communities. These findings highlight the potential of employment to support cognitive functioning in older adults with MCI and cultural and structural barriers for Latinx individuals. Ongoing research will include older African-American and Latinx adults and the nationally-representative Health and Retirement Study.

Abstract citation ID: igaf122.3766

### EXPLORING OLDER ADULTS' ENHANCED ACTIVITIES OF DAILY LIVING

Hye Soo Lee[1], Varitnan Hattakitjamroen[2], Hannah Morrow[3], Michael Bixter[3], Tracy Mitzner[4], Laura Payne[1], and Wendy Rogers[1], *1. University of Illinois-Urbana-Champaign, Champaign, Illinois, United States, 2. Champaign, Illinois, United States, 3. Montclair State University, Montclair, New Jersey, United States, 4. Person in Design, Atlanta, Georgia, United States*

The concept of Enhanced Activities of Daily Living (EADL) recognizes that older adults engage in activities that go beyond the basic and instrumental ADLs. EADLs contribute to health, well-being, and social engagement, which are critical for successful aging. However, they are understudied using the ADL framework, including what older adults consider EADLs. We conducted an online survey wherein participants chose four EADLs from 31 activities (e.g., arts and crafts, working, hiking) developed based on the literature and prior interviews. Then, they rated their satisfaction and challenges for each activity (1 = strongly disagree to 5 = strongly agree). The final sample was 106 participants (age M = 70.6, SD = 6, range=60-92) who were 66% female, 86% with a bachelor's or higher degree, and 87% White. Half had an impairment in vision, hearing, mobility, or cognition (53%). Most participants chose from the activities provided, with only 14 of 424 responses (3%) selecting others. We identified two new activities from the open-ended responses for others (family time, spiritual or religious). The most frequently chosen activities were exercise (n = 35), followed by reading (n = 28), volunteering (n = 28), and music (n = 27). Participants were generally satisfied (M = 4, SD = 0.5) and reported few challenges to engaging in EADLs (M = 1.8, SD = 0.5). In conclusion, despite the diversity in our sample's functional capacity, our activity list was comprehensive, and participants showed high EADL satisfaction. These data provide insights about the range of activities that contribute to the quality of life for older adults and will guide the development of an EADL satisfaction measurement scale.

Abstract citation ID: igaf122.3767

### EXPLORING THE SELECTIVE VULNERABILITY OF VON ECONOMO NEURONS IN NEURODEGENERATIVE DISEASE

Maximilian Berry-Stoelzle[1], Yiming Liu[2], and Georgina Aldridge[2], *1. Cornell University, Ithaca, New York, United States, 2. University of Iowa, Iowa City, Iowa, United States*

Alzheimer's disease, Lewy Body dementia, and frontotemporal dementia are neurodegenerative diseases characterized by cognitive decline and neuronal loss. These diseases are diagnosed using pathological markers of protein aggregation in the brain, including beta-amyloid, tau, alpha-synuclein, and TDP-43. Studies have shown that copathologies, or the presence of more than one pathological protein occur frequently in patients, and may be a contributor to disease severity alongside synapse loss.

The impact of protein copathologies on the synapses, however, remains unclear. Moreover, the selective impact of copathologies on the synapses of different classes of neurons in the human brain is not fully understood. Von Economo neurons (VENs) are a class of neurons found only in highly social animals including humans. VENs have been previously shown to be selectively vulnerable in frontotemporal dementia. We hypothesize that the presence of local copathology leads to a greater loss in dendritic spine density of Von Economo neurons. To test this hypothesis, we collected fresh tissue from the anterior cingulate during brain autopsy of consecutive neurodegenerative disease donors and control autopsies. Using Golgi-Cox staining, we visualize and quantify dendritic spine density and morphology, and neuronal complexity. Strategic dual-immunostaining of adjacent brain sections allows us to assess the impact of co-pathologies on the synapses of VENs. Our preliminary analysis has shown that the anterior cingulate is a locus of copathological build-up within both dementia and control subjects. This study provides a novel evaluation of the impact of copathologies on the synapses of vulnerable neuronal classes in neurodegenerative disease.

Abstract citation ID: igaf122.3768

### EXTRACTING COGNITIVE SLUMS SCORES FROM UNSTRUCTURED NATIONAL VETERANS CLINICAL NOTES WITH AI

Rui Ouyang[1], Christine Rizk[2], Amir Sharafkhaneh[3], Sanam Sharafkhaneh[3], Jose Rios-Monterrosa[3], Dashiell Helmer[3], and Javad Razjouyan[3], *1. Boston Veterans Healthcare Administration, Boston, Massachusetts, United States, 2. Michael E. DeBakey VA Medical Center, Houston, Texas, United States, 3. Baylor College of Medicine, Houston, Texas, United States*

Clinical assessment of cognitive status is critical to evaluating patient health risk and outcomes yet rarely found within the structured data in the electronic health record (EHR). The Saint Louis University Mental Status (SLUMS) Examination is a cognitive screening tool widely used within the Veterans Affairs (VA). Compared to the Mini-Mental State Examination (MMSE), the SLUMS score has greater sensitivity to earlier stages of cognitive impairment. We present a natural language processing (NLP) method for extracting SLUMS scores from unstructured EHR notes. We identified clinical notes from VA patients that contained the word "SLUMS" and a number within a 500 character window. Two researchers independently annotated 1,275 notes. Of these, 899 contained a single SLUMS score and 376 contained missing, multiple, invalid (typo), or qualitative scores. We developed a rule-based system incorporating regular–expression–based pattern matching. The algorithm was developed on the full set of notes and optimized for precision over recall (i.e., only makes predictions when confident). It achieved 83.0% accuracy and 99.6% precision with an F1 score of 78.2%. We further ran the algorithm on 2.96 million unannotated notes. The algorithm throughput exceeded 20 thousand notes per minute when run on a single laptop processor. This work demonstrates how NLP can extract SLUMS scores with high accuracy at scale from millions of unstructured clinical notes. By creating structured cognitive assessment data from information previously buried in free-form clinical text, our work enables cognitive impairment research at the population scale.

Abstract citation ID: igaf122.3769

### FALL RISK FACTORS IN THE MEXICAN AMERICAN POPULATION: AN ANALYSIS USING THE WHO FALLS RISK FACTOR MODEL

Hadi Kooshiar, and Faezeh Babaieasl, *Texas A&M University-Corpus Christi, Texas A&M University-Corpus Christi, Texas, United States*

Background: Falls are a serious cause of injury, disability, and death among older adults. However, there is limited research that shows the World Health Organization (WHO) falls classification model has been applied to very old Mexican American populations. This study examined fall rates and associated risk factors among Mexican Americans aged 89 and above using the WHO model. Methods: This study uses data from Wave 10 (2020–2021) of the Hispanic Established Populations for the Epidemiologic Study of the Elderly (HEPESE). Results: The sample was composed of 66.4% females, with a mean age of 93.84 years (SD = 2.78, range = 89–103 years). 73.2% of respondents had mild cognitive impairment. The fall rate was 39.7%, with 20% of participants experiencing multiple falls, and 16.8% of falls leading to injury. Additionally, 74.3% of respondents reported a fear of falling. According to the WHO falls risk model, in the biological domain, fallers scored lower on Instrumental Activities of Daily Living (IADL) and Activities of Daily Living (ADL), had higher depression rates, and more often had dementia. In the socioeconomic domain, marital status differed significantly between fallers and non-fallers. In the environmental domain, homeownership was significantly associated with fall status. Conclusion: Applying the WHO fall risk factor model revealed multiple areas of vulnerability in very old Mexican Americans. Cognitive and functional impairments, environmental factors, and marital status are significant factors that increase the risk of falls. These findings underscore the importance of culturally tailored, comprehensive fall prevention programs for this aging population.

Abstract citation ID: igaf122.3770

### FAMILY CAREGIVERS' PROVISION OF TEETH CARE TO OLDER ADULTS: THE ROLE OF CAREGIVER STRESS, CARE DEMANDS, AND TRAINING

Yuanchang Zhao[1], and Mengzhao Yan[2], *1. Case Western Reserve University, Cleveland, Ohio, United States, 2. University of Southern California, Los Angeles, California, United States*

Teeth care is essential not only for older adults' dental health but also for their overall quality of life. However, family caregivers often overlook this aspect of care. Limited studies have identified the factors influencing caregivers' actions in providing teeth care, especially using nationally representative data. Guided by the caregiving reluctance framework and the caregiver stress model, this study aims to examine factors associated with family caregivers' provision of teeth care. Using Round 13 of the National Study of Caregiving (NSOC) data (N = 2,557), we employed nested logistic regressions to test the effects of care demands, caregiving training, and stressors on the provision of teeth care, controlling for demographic characteristics. We found that stressor variables (longer caregiving hours, OR = 1.1; caring for someone with a disability, OR = 2.6; caregiver overload, OR = 1.18), demand variables (dementia, OR = 2.58; chewing problems, OR = 1.65), and training (OR = 1.92) were all linked to higher odds of providing teeth care. We also found that caregiver stressors, demands, and training variables were positively correlated. These findings suggest that caregiving stressors, demands, and training are intertwined in influencing the provision of teeth care. Furthermore, the association between caregiving stress and teeth care provision signals potential mental health risks associated with providing such care. This study underscores the importance of training family caregivers to recognize and provide effective teeth care for older adults and offers insights for public policies aimed at improving older adults' dental health and supporting caregivers.

Abstract citation ID: igaf122.3771

### FEAR OF FALLING AND SUBJECTIVE COGNITIVE DECLINE IN RACIALLY DIVERSE ADULTS AGED 60+ IN LOW-INCOME COMMUNITIES

Renata Komalasari[1], Nikki Hill[2], Ayse Malatyali[3], Janet Lopez[3], Sergi Garcia-Retortillo[3], Jian Zou[3], Veronica Decker[3], and Ladda Thiamwong[3], *1. Akademi Keperawatan Andalusia Jakarta, Jakarta, Jakarta Raya, Indonesia, 2. The Pennsylvania State University, University Park, Pennsylvania, United States, 3. University of Central Florida, Orlando, Florida, United States*

Subjective cognitive decline (SCD) and perceived falling or fear of falling (FOF) share similar mental health consequences. Yet, few studies have explored their relationship in racially diverse older adults. This study examined associations between SCD, SCD-related functional difficulties, and FOF, along with the moderating effects of depressive and anxiety symptoms in 91 community-dwelling adults aged 60+(Mean age = 73.26, SD±6.99; 89% female; 96.7% non-White) in Central Florida. FOF was measured using the short Fall-Efficacy Scale International. SCD was assessed using a single question on confusion or memory loss, with a follow-up Likert-scale item for functional difficulties. Depression and anxiety were measured using validated screening tools, including the Patient Health Questionnaire-9 (PHQ-9) and Geriatric Anxiety Inventory-Short Form. We found that SCD, SCD-related functional difficulties, and anxiety symptoms were not significantly associated with FOF, but depressive symptoms were (e.021= 1.021, p=.020). A Poisson regression analysis examined associations between the variables and FOF. Higher depressive symptoms were associated with higher FOF. The interaction of SCD-related functional difficulties with depressive symptoms (e-.002= .998, p=.843) was not associated with FOF, but its interaction with anxiety symptoms was (e.073= 1.075, p=.035). The association between SCD-related functional difficulties and FOF was accentuated as anxiety level increased and attenuated with lower anxiety symptoms. Older adults with more depressive symptoms report greater FOF. Anxiety amplifies the link between FOF and functional difficulties due to subjective cognitive decline. Future research should investigate how FOF and emotional well-being evolve as cognitive deterioration progresses and daily functioning becomes increasingly impaired.

Abstract citation ID: igaf122.3772

### FEASIBILITY OF AN INTERGENERATIONAL PROGRAM SUPPORTING HEALTHY AGING FOR PEOPLE LIVING WITH TRAUMATIC BRAIN INJURY

Pascale Simard[1], Megan Veilleux[1], Valérie Poulin[2], Marie-Christine Ouellet[1], Marie-Eve Lamontagne[1], Krista Best[1], Thierry Belleguic[1], and Samuel Turcotte[1], *1. Université Laval, Québec, Quebec, Canada, 2. Université du Québec à Trois-Rivières, Trois-Rivières, Quebec, Canada*

Introduction: Individuals living with traumatic brain injury (TBI) experience barriers to social participation, limiting healthy aging. While intergenerational programs may offer a solution, their feasibility with the TBI population remains unexplored. Using a community-engaged approach, we co-developed and delivered an intergenerational TBI program in a community organization dedicated to TBI health promotion. Intergenerational dyads (n=8) partnered with professional photographers over seven structured sessions, sharing their thoughts or experiences about aging with disability, while turning words into art. The program culminated in a traveling art exhibition, launched at a national museum. Objectives: (1) To explore perceived program impacts, and (2) to assess feasibility and acceptability. Method: Post-intervention, we conducted semi-structured interviews with 15 TBI participants, a community worker, and the director, using a qualitative descriptive design guided by the Theoretical Framework of Acceptability. Data were analyzed with mixed methods and reported per the COREQ checklist. Results: TBI dyad participants experienced greater social participation, felt more valued and empowered, and perceived increased public awareness of their realities. The program reached TBI members who had not been involved with the organization before, creating opportunities for meaningful social connections. Participants valued and supported the program, but improving its flexibility to diverse participants' abilities would enhance its feasibility. Conclusion: Co-creating and delivering inclusive health promotion initiatives like an art-based intergenerational program for individuals living with TBI is feasible and can promote social connection, participation and healthy aging.

Abstract citation ID: igaf122.3773

### FINANCIAL EXPLOITATION IN PRIVATE HOME CARE: FINDINGS FROM A MULTI-STATE CARE MANAGER SURVEY

Shadi Gholizadeh[1], Megan Harris[2], and Lucas Melville[2], *1. TheKey, coto de caza, California, United States, 2. TheKey, San Diego, California, United States*

Financial exploitation is a critical yet under-recognized risk in private home care, particularly for older adults living with dementia. To better understand its scope and potential safeguards, we conducted a survey of 14 private-duty care managers across 14 states who collectively oversee approximately 700 clients per month. Respondents indicated that they occasionally encounter suspected or confirmed cases of financial exploitation in their professional roles. The most frequently described concerns included theft of valuables, misuse of credit or debit cards, and solicitation of gifts or loans. A number of care managers shared that, across their careers, they had removed caregivers from cases due to concerns about financial boundaries. Despite these observations, relatively few families proactively raised financial concerns, suggesting that many situations may go undetected or unreported. Respondents expressed openness to preventive strategies, including the use of prepaid cards, financial alert systems, and structured caregiver training on boundary-setting and red flags. Most emphasized that reinforcing safeguards at the start of care would be particularly valuable, for example through clear documentation of financial preferences and explicit plans for caregiver involvement in purchases such as groceries. Findings highlight both the potential frequency and complexity of financial exploitation in private home care, as well as opportunities for structured, proactive interventions. These results underscore the shared responsibility of agencies, caregivers, and families in protecting vulnerable clients, and they point to the need for further practice, policy, and research efforts to strengthen safeguards and promote elder justice in the growing private home care sector.

Abstract citation ID: igaf122.3774

**FINANCIAL TOXICITY IN OLDER ADULT SURGICAL PATIENTS: THE IMPACT OF INSURANCE TYPE**

Ezra Brooks[1], Claire Morton[2], Randall Bloch[3], and Louis Nguyen[1], *1. Brigham and Women's Hospital, Boston, Massachusetts, United States, 2. Center for Surgery and Public Health, Boston, Massachusetts, United States, 3. Boston Medical Center, Boston, Massachusetts, United States*

Surgical patients incur significant, harmful medical costs, termed financial toxicity (FT). After 65, most patients elect coverage through subsidized public insurance (Medicare), subsidized private insurance (Medicare Advantage [MA]), or unsubsidized private insurance. We investigated the financial impacts of insurance type in the older adult surgical population. Using the Medical Expenditure Panel Survey (2017-2022), we identified patients ≥65 years covered by Medicare, MA, or private insurance who underwent inpatient surgery. The primary outcome was FT, measured as reported difficulty paying medical bills or delays seeking care or filling prescriptions. Secondary outcomes were out-of-pocket (OOP) costs and total costs (OOP plus premium). Multivariable regression models estimated the risk-adjusted odds of FT, OOP costs, and total costs by insurance type. We identified 4,089 patients, weighted to 6,670,937. Insurance coverage was 24.4% Medicare, 35.3% MA, and 40.4% private. FT was present in 21.9% of patients (24.6% Medicare, 23.2% MA, 19.2% private; P-value< 0.05). Average OOP costs were $2395 ($2451 Medicare, $2291 MA, $2452 private; P-value=0.72). Average total costs were $9243 ($6071 Medicare, $7823 MA, $9975 private; P-value< 0.001). On multivariable analysis, relative to Medicare patients, private and MA patients had similar odds of FT (private OR: 0.89, 95%, CI: 0.64-1.23; MA OR: 1.07, 95% CI: 0.79-1.42), but higher total costs (private: +$3842, 95% CI $2170-$5513; MA: +$1823, 95% CI $114-$3533). Despite the availability of subsidized health insurance, FT remains prevalent in the older adult surgical population. Our data suggest that, relative to Medicare, private and MA plans cost more, but provide no additional benefit to FT.

Abstract citation ID: igaf122.3775

**FREQUENT IN DEMENTIA, DEADLIEST WITHOUT IT: DELIRIUM AND MORTALITY IN HOSPITALIZED OLDER ADULTS**

Mfon Umoh[1], Thiago Junqueira Avelino-Silva[2], Marlon Juliano Romero Aliberti[3], Flavia Barreto Garcez[4], and Esther Oh[5], *1. Johns Hopkins University School of Medicine, Baltimore, Maryland, United States, 2. University of California, San Francisco, University of California, San Francisco, California, United States, 3. University of Sao Paulo Medical School, Baltimore, Maryland, United States, 4. University of Sao Paulo Medical School, Sao Paulo, Sao Paulo, Brazil, 5. Johns Hopkins School of Medicine, Johns Hopkins School of Medicine, Maryland, United States*

Dementia increases vulnerability to delirium and adverse outcomes, yet the prognostic role of delirium across cognitive status is uncertain. Using data from the CHANGE Study, a prospective cohort of adults aged ≥65 years admitted to 43 hospitals in five countries (Brazil-Angola-Chile-Columbia-Portugal), we examined the association between delirium and 90-day mortality and the modifying effect of cognitive status. Delirium (prevalent and incident) was assessed using the Confusion Assessment Method (CAM), and baseline cognitive status with the Clinical Dementia Rating (CDR). Multi-level mixed effects survival models, adjusted for sociodemographic, clinical, and hospital-related factors, were used to examine the association between delirium and mortality, with stratified analyses by CDR to test interaction. Among 2,556 patients (mean age=79±9, 56% female), 37% experienced delirium during hospitalization. However, delirium occurrence increased significantly with worsening baseline cognitive status, affecting 126/765 (16%) patients with normal cognition (CDR=0), 266/976 (27%) with mild cognitive impairment (CDR=0.5), 208/350 (59%) with mild dementia (CDR=1), and 357/465 (77%) with moderate/severe dementia (CDR=2-3), p < 0.001. After adjustment, delirium was associated with increased 90-day mortality (HR = 3.43, 95%CI=2.81-4.18). The largest mortality difference between patients with and without delirium was observed in those with normal cognition (63% vs. 18%; HR = 4.36, 95%CI=3.11-6.11), and the smallest difference in those with moderate/severe dementia (41% vs. 26%; HR = 2.22, 95%CI=1.34-3.66), with a p-value for interaction=0.04. While delirium was more common among patients with dementia, its prognostic impact on mortality was strongest in patients with normal cognition at baseline. Hospital-wide strategies to urgently detect, prevent, and manage delirium are critical for all older patients.

Abstract citation ID: igaf122.3776

**FROM PROTOCOL TO PRACTICE: ENHANCING FIDELITY, SUPPORTING ADAPTATION, AND ADVANCING SCIENCE**

Claire Regan, *University of Pennsylvania, Philadelphia, Pennsylvania, United States*

As the global population ages, ensuring that evidence-based interventions (EBIs) are effectively translated into real-world settings is critical to meeting the complex and evolving needs of older adults. This population often experiences fragmented care and is particularly vulnerable to poor outcomes. Effective translation of EBIs hinges on balancing fidelity, the extent to which an intervention is delivered as originally intended, with the necessary adaptations required for diverse, real-world settings. Rigid adherence to fidelity can undermine local applicability and contextual relevance, but divergence from fidelity may compromise theoretical integrity and patient outcomes. This work explores the importance of and barriers to measuring and reporting both fidelity and adaptation, with a focus on their implications for successful implementation and scalability. Drawing on implementation science frameworks and using the Transitional Care Model (TCM) as a case study, we examine the interplay between fidelity, adaptation, and context. Emphasis is placed on creating practical tools, fostering bidirectional learning, and establishing fidelity standards in intervention protocols. The work concludes with recommendations aimed at enhancing training, reporting, and collaboration to support equitable and effective implementation of EBIs across diverse settings in meaningful ways.

Abstract citation ID: igaf122.3777

**GENDER DIFFERENCES IN YOUNG ADULT ATTITUDES TOWARDS AGING: A QUALITATIVE ANALYSIS OF AGING WELL NARRATIVES**

Saahithi Punnam[1], Patricia Chilton[2], and Matthias Mehl[1], *1. University of Arizona, Tucson, Arizona, United States, 2. St Mary's College, St Mary's College, Maryland, United States*

This study explores young adults' attitudes towards aging, with a focus on understanding their perceptions of older adulthood. While there is existing research that has examined perceptions of aging well among older populations, there is less data on how young adults conceptualize aging well and how these perceptions are formed. Our goal was to qualitatively identify themes in young adults' aging well narratives and analyze gender differences in the prevalence of these themes. The sample consists of 99 students between ages 17 and 26 studying psychology at the University of Arizona. The participants were asked to provide a 4-minute free spoken response to the question, "In your own words, what does aging well mean to you?" in a Zoom interview. Themes and sub-themes were extracted using inductive analysis and represent a broad view of aging perceptions. The themes that emerged were physical health, physical appearance, elders as role models, mental health and fitness, social network, and attitudes of aging, life satisfaction, and well-being. MAXQDA software was used to identify gender differences in these themes. The largest gender differences emerged within the sub-themes of cognitive health, living life to the fullest, and elders as positive role-models. Female participants mentioned cognitive health and positive role models more than male participants, while male participants mentioned living life to the fullest more than females.

Abstract citation ID: igaf122.3778

**GENDERED INCOME TRAJECTORIES AND HEALTH DISPARITIES AT MIDLIFE: EVIDENCE FROM THE NLSY79 COHORT**

Mingyue Ma, *Purdue University, West Lafayette, Indiana, United States*

The body of literature on significant gender inequality in health in the United States, while extensive, has paid relatively little attention to how these disparities originate from longitudinal income trajectories. This study adopts a gender perspective to determine whether women's health disadvantages stem from early experiences in the labor market. Using data from National Longitudinal Survey of Youth 1979 (NLSY79), this study applies Group-Based Trajectory Model (GBTM) to depict distinct types of time-varying income trajectories since their entry into the labor market at ages 25 until their exit at ages 49. I first use Multinomial Logistic regression to analyze gender differences in these trajectories. Further, I use Ordinary Least Square regression to predict later-life health outcomes, including physical and mental health, to investigate how income trajectories contribute to the observed disadvantage of women's health outcomes. Finally, I employ KHB (Karlson–Holm–Breen) method to investigate whether and to what extent the mediators -income trajectories could explain the gender difference in health outcomes. I found that from a temporal perspective, women's income trajectories are more likely to remain in low-status or show a downward trend, while men are vice versa. Compared to men, women are more likely to exhibit worse health outcomes at midlife. Further, gender differences in income trajectories significantly mediate gender inequality in physical health, while partially account for gender inequality in mental health. This evidence highlights the need for policies that secure women's labor rights and equitable pay, as traditional female roles may impose hidden burdens that harm midlife health.

Abstract citation ID: igaf122.3779

**GOAL SETTING FOR HEALTHY BRAIN AGING IN OLDER ADULTS: WHAT MATTERS MOST TO PARTICIPANTS OF THE AGEWISE-AP PROGRAM**

Jaye McLaren[1], Andrew Nguyen[2], Emily Metcalf[3], and Maureen O'Connor[4], *1. VA Bedford Healthcare System, Bedford, Massachusetts, United States, 2. Department of Veterans Affairs, Bedford, Massachusetts, United States, 3. VA Bedford Health Care System, Bedford, Massachusetts, United States, 4. Bedford Veterans Health Care System, Bedford, Massachusetts, United States*

Reducing cognitive decline is described as an important aspect of successful aging. VA supported Age Friendly Healthcare Systems focus on the 4Ms framework (What Matters Most, Medication, Mentation, and Mobility) to improve quality of care and care delivery for older Veterans. The Aging Well through Interaction and Scientific Education– Action Plan program (AgeWISE-AP) emphasizes the What Matters Most element of the 4Ms as we collaborate 1-1 with Veterans to identify individualized lifestyle goals that support positive brain health outcomes. AgeWISE-AP is comprised of two components: an initial 12-week group to educate participants on brain aging and health factors followed by 8 individual sessions supporting participants in the creation and implementation of client-centered goals within the domains of healthy brain aging (e.g. diet, exercise, personal development, socialization etc.) outlined in the Whole Health Circle of Brain Health. Led by a Brain Health Interventionist (BHI), AgeWISE-AP bridges the gap between education and action. Here we report on 36 participants, who completed a total of 282 individual sessions, making 120 lifestyle goals within the 8 domains of healthy brain aging. Using case examples, we will illustrate emerging trends regarding domain selection and goal creation for participants to date. Understanding broadly what matters most to Veterans (their "why") as well as, more specifically, what personal interests, activities, or values drive them to consistently participate in lifestyle behavior changes for healthy brain aging (their "what") is essential to the success of this intervention.

Abstract citation ID: igaf122.3780

**HANDGRIP STRENGTH AND WHITE MATTER INTEGRITY IN MIDLIFE WOMEN AT RISK FOR ALZHEIMER'S DISEASE**

Chadsley Wessinger[1], Jennifer Etnier[1], Samantha DuBois[2], Samuel Kibildis[3], Jarod Vance[4], Brittany Armstrong[1], Laurie Wideman[1], and Alexis Slutsky-Ganesh[5], *1. University of North Carolina at Greensboro, Greensboro, North Carolina, United States, 2. Appalachian State University, Boone, North Carolina, United States, 3. Athens-Clarke County Unified Government, Athens, Georgia, United States, 4. Longwood University, Farmville, Virginia, United States, 5. North Carolina Agricultural and Technical State University, Greensboro, North Carolina, United States*

Poor muscle health is an emerging risk factor for Alzheimer's disease (AD), yet the pathways linking muscle health to AD risk remain unclear. Handgrip strength (HGS), a marker of muscle health, is associated with episodic memory (EM), the hallmark cognitive symptom of AD. White matter microstructural integrity (WMI) is also associated with EM and implicated in AD pathology. While HGS is associated with global WMI, it is unclear if it specifically relates to WMI in tracts critical for EM, like the fornix and hippocampal cingulum (CGH). Given that women are disproportionately affected by age-related muscle loss and AD, investigating these relationships in women may help uncover a mechanistic link between muscle health and AD risk in this population. Participants (N = 18 women, Mage=55.7±7.0, 66% post-menopause) completed HGS using a handgrip dynamometer; operationalized as the sum of the maximum of two trials per hand. Diffusion tensor imaging was used to estimate fornix and CGH fractional anisotropy (FA), mean diffusivity (MD), and axial diffusivity (AxD) as WMI measures. Linear regression models tested the extent to which HGS predicted WMI. The overall model predicting fornix AxD was significant (p=.026). Greater HGS predicted lower fornix AxD (p=.043), whereas no significant associations were observed between HGS and other WMI measures (p's>.08). This suggests that greater muscle strength is predictive of more favorable WMI, (i.e., lower fornix AxD, reflecting less diffusion perpendicular to axonal fibers), suggesting better axonal integrity. Future work should use longitudinal designs to investigate if changes in muscle health predict changes in WMI.

Abstract citation ID: igaf122.3781

### HANDGRIP STRENGTH, DEPRESSIVE SYMPTOMS, AND COGNITIVE FUNCTION IN OLDER MEXICAN AMERICAN ADULTS

Divya Kashyap[1], and Soham AlSnih[2], *1. University of Texas Medical Branch, Galveston, Texas, United States, 2. The University of Texas Medical Branch at Galveston, Texas, Galveston, Texas, United States*

High depressive symptoms (DS) are associated with cognitive decline, while high handgrip strength (HGS) is associated with lower cognitive decline. This study examined nativity differences in the relationship between HGS and DS with cognitive impairment (CI) over a 12-year period among 984 Mexican American adults aged 75 and older who scored ≥21 on the Mini-Mental State Examination (MMSE) at baseline. CI was defined as an MMSE score < 21. HGS was classified as low or high (< 27/≥27 kg for men, < 16/≥16 kg for women), and high DS was defined as a score ≥16 on the Center for Epidemiologic Studies Depression Scale. Covariates included socio-demographics and health characteristics. Participants were categorized into four groups: low HGS-high DS, low HGS-low DS, high HGS-low DS, and high HGS-high DS. Among US-born participants, those in the low HGS-low DS and high HGS-low DS groups had 59% and 60% lower odds of CI over time, respectively, compared to the low HGS-high DS group, controlling for covariates. Foreign-born participants in the low HGS-low DS, high HGS-low DS, and high HGS-high DS groups had 67%, 73%, and 80% lower odds of CI over time, respectively, compared to the low HGS-high DS group, controlling for covariates. Among foreign-born participants with high DS, high HGS was significantly associated with 24% lower odds of CI over time, but this association was not found among US-born participants with high DS, controlling for covariates. These findings suggest that interventions targeting both DS and physical strength may benefit cognitive health in this population.

Abstract citation ID: igaf122.3782

### HARD TO FIND, HARD TO RIDE: FINDINGS FROM DEVELOPING A STATEWIDE TRANSPORTATION DATABASE

Elizabeth Pinyan, Shannen Johnson, Kristel Robison, Daniel Levitt, and Jonathon Weisenfeld, *UNC-Chapel Hill, Chapel Hill, North Carolina, United States*

Transportation access is critical for health, independence, and social connection, especially for older adults. Yet finding alternatives to driving is often difficult, even when services exist. In North Carolina, being unaware of available services is the top barrier to access for older adults. To address this gap, we developed a searchable database of non-fixed-route transportation services across all 100 North Carolina counties. We identified providers (n = 116) and services (n = 298) and gathered details on service area, eligibility, trip purpose, accessibility, transportation type, and scheduling. Reviewing provider websites revealed significant barriers to finding and understanding transportation information. Accurate details were rarely available in one place and often required piecing together multiple sources and contacting providers directly. Common issues included vague eligibility criteria (e.g., "elderly" instead of a specific age), emphasis on administrative details over practical rider information, and frequent use of transportation jargon without explaining what it means for the rider. Transportation services go unused when people do not know they exist, how to use them, or do not see themselves as users. When websites are not user-friendly, potential riders may assume the service itself will be difficult to use. In North Carolina, older adults most often rely on family, friends, or websites for information, highlighting the value of accessible transportation information for older adults and their support systems. This database is an important step forward, but broader improvements are needed. Future directions include formal analysis of provider websites and development of best-practice guidelines for age- and accessibility-friendly transportation communication.

Abstract citation ID: igaf122.3783

### HEARING CARE ACCESS AMIDST DEMENTIA IN PRIMARY CARE PRACTICE: IDENTIFYING OPPORTUNITIES TO IMPROVE INTEGRATION

Danielle Powell[1], Ariella Sapoznick[1], Afia Obeng[1], Christina Koch[2], Stephanie Nothelle[3], Esther Oh[3], Nicholas Reed[4], and Jennifer Wolff[5], *1. University of Maryland, University of Maryland College Park, Maryland, United States, 2. University of Maryland Medical System, University of Maryland Medical System, Maryland, United States, 3. Johns Hopkins School of Medicine, Johns Hopkins School of Medicine, Maryland, United States, 4. New York University Grossman School of Medicine, New York, New York, United States, 5. Johns Hopkins University - School of Public Health, Baltimore, Maryland, United States*

Older adults with hearing loss and dementia face unique communication and care challenges which may inhibit navigation of the hearing care process. Current care models rarely equip primary care providers to initiate hearing care, especially for patients managing dementia. Few interventions have been co-designed with input from persons with dementia, care partners, and providers to understand how hearing needs are addressed outside the audiology clinic. We conducted thematic analysis of semi-structured interviews with primary care providers (n = 6), older adults with hearing loss and self-reported dementia (n = 6), and their care partners (n = 5), recruited from large Mid-Atlantic academic health centers. Most older adults were 76–85, White, male, with ≥5 years of hearing loss, and cared for by a spouse. Providers were predominantly internal medicine physicians with ≥5 years' experience. Interviews explored four domains: 1) hearing loss impact on life, 2) caregiving perspectives, 3) hearing care needs, and 4) system-level implementation. All participants acknowledged hearing's value for health and function but recognized cognitive and other concerns often took precedence during visits. Still, older adults and care partners preferred providers inquire about hearing to support connection and daily function. All desired improved interprofessional communication to ease self-advocacy and education burdens. Providers described hearing health as a "black box," limiting conversation engagement. They expressed interest in accessible educational materials and structured resources to support patient conversations but noted limited time to address hearing during visits. Findings highlight opportunities for practical strategies and interventions to relieve provider burden and improve hearing care integration in primary care.

Abstract citation ID: igaf122.3784

### HEARING LOSS AND COGNITION IN THE MID-TO-LATE LIFE TRANSITION

Willa Brenowitz[1], Pamela Schreiner[2], Kathryn Hand[3], Paige Wartko[4], John Dickerson[5], Kelly Reavis[6], and Kristine Yaffe[7], *1. Kaiser Permanente, Portland, Oregon, United States, 2. University of Minnesota, Minneapolis, Minnesota, United States, 3. Oregon Health and Science University, Portland, Oregon, United States, 4. Kaiser Permanente Washington Health Research Institute, Seattle, Washington, United States, 5. Kaiser Permanente Center for Health Research, Portland, Oregon, United States, 6. VA Portland Health Care System, Portland, Oregon, United States, 7. University of California, San Francisco, San Francisco, California, United States*

Hearing loss is associated with cognitive decline in later life, but few studies have established whether this association emerges earlier in midlife. We evaluated the cross-sectional association between audiometric hearing loss and cognition in individuals spanning ages 53-65 years. We studied 1,486 participants in the Coronary Artery Risk Development in Young Adults (CARDIA) study at a mean age of 61.2 (SD: 3.6), with 58.4% female, 46.4% Black, and 53.6% White. Hearing loss was defined as a pure tone average (PTA) threshold ≥25dB for speech (500-4000 Hz) and high (4000-8000 Hz) frequencies in the better hearing ear. Cognition was measured with the Montreal Cognitive Assessment (MoCA), digit symbol substitution test (DSST), and the Rey Auditory Verbal Learning Test delay recall score (RAVLT). Linear regression models estimated mean differences and 95% confidence intervals (CIs) for the associations between hearing loss in each frequency range and separate cognitive tests, adjusting for age, sex, education, race, health care access, diabetes, prevalent cardiovascular disease, stroke, hypertension, depression, body mass index, and smoking. Hearing loss (11% prevalence) in the speech detection range was associated with lower cognition in all tests (MoCA: -1.31 [95% CI:-1.81,-0.82]; DSST: -2.59 [-5.05,-0.14]; RAVLT: -0.69 [-1.23,-0.15]). High frequency hearing loss (43% prevalence) was also associated with lower cognition in (MoCA: -0.85 [-1.18, -0.52]; DSST: -2.82 [-4.43, -1.23]; and borderline associated with RAVLT: -0.32 [-0.67, 0.03]). Most hearing loss was mild. These findings in a population-based sample suggest that the associations between hearing loss and cognition may be detectable by midlife.

Abstract citation ID: igaf122.3785

### HEARING LOSS INCREASES HOSPITALIZATIONS AMONG U.S. OLDER ADULTS WITH HEART FAILURE

Jessica West[1], Hanzhang Xu[2], Howard Francis[1], Sherri Smith[1], and Matthew Dupre[1], *1. Duke University School of Medicine, Durham, North Carolina, United States, 2. Duke University, Durham, North Carolina, United States*

Effective communication is essential to patient-provider interactions, chronic disease self-management, and ultimately reducing excess healthcare utilization. This study investigated whether hearing loss (HL) was associated with hospitalizations among adults managing heart failure (HF). Nationally-representative prospective cohort data from the 1998-2020 Health and Retirement Study (HRS) were used to examine adults who were diagnosed with HF (n = 3,274). Hearing status was ascertained at each wave by patient-reported hearing and hearing-aid (HA) use (normal hearing, unaided HL, aided HL). Hospitalizations were assessed at each wave from participants' reported number of hospital admissions in the prior two years. Negative binomial mixed models examined numbers of hospitalizations over time by hearing status. Among study participants (mean age 71.46 years [±10.59]), approximately 63.84% reported normal hearing, 28.53% had unaided HL, and 7.64% had aided HL. Adults with unaided HL had significantly more hospitalizations than adults with normal hearing (incidence-rate ratio [IRR]=1.14, 95% CI = 1.07-1.22, P<.001). The association was partly attenuated after adjusting for the sociodemographic and health-related characteristics of adults with HF (IRR=1.07, 95% CI = 1.00-1.14, P=.040). Adults with aided HL had no significant difference in hospitalizations compared to adults with normal hearing. Among HF patients with HL, those who were unaided had significantly more hospitalizations compared to those who wore HAs (IRR=1.26, 95% CI = 1.06-1.49, P=.008). Associations were consistent over the course of the illness and did not vary across demographic groups. Healthcare providers should consider routine hearing assessments in HF patients to identify those who may benefit from HAs to reduce their risk of potentially preventable hospitalizations.

Abstract citation ID: igaf122.3786

### HEART-TO-HEART CAFÉ: AN INNOVATIVE INTERVENTION TO PROMOTE ADVANCE CARE PLANNING AMONG CHINESE AMERICANS

Kaipeng Wang[1], Yaolin Pei[2], Xiaoyouxiang Li[1], Aprille Arena[1], Jina Park[1], Leslie Hasche[1], and Sandy Stokes[3], *1. University of Denver, Denver, Colorado, United States, 2. The University of Texas at Austin, Austin, Texas, United States, 3. Chinese American Coalition for Compassionate Care, Cupertino, California, United States*

Advance care planning (ACP) is associated with improved end-of-life (EOL) care quality, reduced use of aggressive medical treatments, and lower decisional burden for healthcare surrogates. Older Chinese Americans have a significantly lower completion rate of advance directives (ADs) compared to older Americans in general. Thus, a culturally responsive intervention is needed to promote ACP among Chinese Americans. Using a one-group pretest-posttest mixed methods design, this project aims to study the feasibility of Heart-to-Heart (HTH) Café on attitude and readiness regarding ACP among Chinese American adults with a family member aged 60 or older. The program consists of four sessions: (1) HTH Café card games, (2) legal documents lecture, (3) palliative and hospice care lecture, and (4) AD completion workshop. All sessions were virtually offered in Mandarin over a total of five weeks. We evaluated the program's feasibility using a mixed-methods approach, incorporating pre-post surveys, focus groups, and individual interviews. Seventeen participants were successfully enrolled in the program, and 15 (88%) attended at least three sessions and completed the post-test survey. The results of paired t-tests showed statistically significant improvements in knowledge of ACP and palliative care, as well as a decrease in fear of death. While supporting these results, qualitative findings from thematic analysis further revealed the high feasibility of the program, participants' improved knowledge of ACP, and enhanced readiness for discussions about death and ACP. Findings highlight the feasibility of a culturally innovative group approach and underscore the need for continued community outreach in ACP promotion among Chinese Americans.

Abstract citation ID: igaf122.3787

### HEBREW SELF-TRANSCENDENCE: TRANSLATION AND VALIDATION FOR FAMILY CAREGIVERS OF PEOPLE WITH DEMENTIA

Dalit Zaguri-Greener[1], Ruth Lopez[2], Nabel Gara[3], Lola Kolpin[1], and Anna Zisberg[1], *1. University of Haifa, Haifa, Hefa, Israel, 2. MGH Institute of Health Professions, Sharon, Massachusetts, United States, 3. Shoham Medical Center, Shoham Medical Center Pardes Hanna-Karkur, HaMerkaz, Israel*

Background: While family caregivers of individuals with advanced dementia often experience high levels of emotional and physical burden, less attention has been given to their sources of resilience and well-being. Tools like the Self-Transcendence Scale (STS) can help assess these positive dimensions, but culturally adapted versions are needed. Objective: To translate and culturally adapt the STS into Hebrew and evaluate its psychometric properties among family caregivers of nursing home residents with moderate-to-severe dementia. Methods: A sample of 175 Hebrew-speaking caregivers completed the STS. The scale underwent translation and cultural adaptation, followed by exploratory and confirmatory factor analyses. Results: Factor analyses revealed a four-factor structure: Intrapersonal, Interpersonal, Transpersonal, and Temporal, aligned with Reed's theoretical framework. Convergent validity was demonstrated, with higher ST scores significantly associated with lower caregiver burden ($r = -.34$, $p < .001$). Internal consistency was acceptable (Cronbach's alpha = .72). The Temporal dimension emerged as a key contributor, reflecting caregivers' integration of past, present, and future experiences. Conclusions: The Hebrew STS is a valid and reliable tool for assessing self-transcendence among family caregivers. It offers new insights into their psychological adaptation and may support future cross-cultural research and intervention development.

Abstract citation ID: igaf122.3788

### HOME HEALTH INITIATION BY PAYOR TYPE AND ASSOCIATED OUTCOMES AMONG MEDICARE-ENROLLED VETERANS

Roman Ayele[1], Frank DeVone[2], Kate Magid[3], Andrew Zullo[4], Ellen McCreedy[5], Pedro Gozalo[6], and Stefan Gravenstein[2], *1. VA Eastern Colorado Health Care System, Aurora, Colorado, United States, 2. Providence VA Medical Center, Providence, Rhode Island, United States, 3. Eastern Colorado Healthcare System, Veterans Health Administration, Aurora, Colorado, United States, 4. Brown University, Providence, Rhode Island, United States, 5. Brown University, School of Public Health, Providence, Rhode Island, United States, 6. Brown University School of Public Health, Providence, Rhode Island, United States*

Background: Medicare-enrolled Veterans may receive Veterans Health Administration (VA), Traditional Medicare (TM), or Medicare Advantage (MA) funded home health care (HH). Payor type influences rehospitalization and mortality, but does days to HH initiation by payor relate to rehospitalization, nursing home (NH) admission, and death? Methods: Our retrospective cohort study evaluated 72,743 Medicare-enrolled Veterans discharged from VA medical centers (2017–2019) who initiated HH within 14 days for 30- and 90-day rehospitalization, death, and NH admission. We also evaluated timeliness of HH initiation (within vs. >2 days after discharge), relative risks by payor, and for Veterans at lower baseline mortality risk. Results: Among Veterans starting HH within 2 days (n = 35,988), 34.8% received VA-paid HH, 56.3% TM, and 8.8% MA. Rehospitalization within 30 days was 18.5% for VA, 19.5% TM, and 19.7% MA; 90-day rates were 33.3%, 33.8%, and 33.5%, respectively. Thirty-day mortality was higher for VA-paid HH (3.3%) vs TM (2.9%) and MA (3.0%), and at 90 days (9.6% vs. 8.3% and 8.9%). NH admission at 30 days was similar. For HH initiation timeliness, VA-paid HH more often began ≤2 days ($RR_{adj}$=0.95, 95% CI 0.93–0.98 for all Medicare; 0.95, 95% CI 0.92–0.99 for TM; 0.87, 95% CI 0.83–0.91 for MA). Conclusions: Preliminarily, outcomes differ by payor type: VA-paid HH had lower rehospitalization but higher short-term mortality than TM, and faster initiation than Medicare-paid HH. These findings have policy relevance of payor-driven differences in access, quality, and outcomes. This novel study has implications for quality and access monitoring across payors.

Abstract citation ID: igaf122.3789

### HOME REPAIR POLICIES AND THE UNEVEN LANDSCAPE OF AGING IN COMMUNITY FOR LOW-INCOME OLDER ADULT HOMEOWNERS

Leiha Edmonds, *Indiana University Bloomington, Bloomington, Indiana, United States*

Federal, state, and local policies in the United States are increasingly recognizing home repair as a strategy for supporting age-friendly cities and communities. Ensuring access to home maintenance, repairs, and modifications is crucial to support aging in place, enhance the physical and mental health of older adults, and enable low-income homeowners to resist displacement pressures. This article investigates the influence of housing policy on aging-in-place experiences among low-income older adults by examining the work of the Orange County Home Preservation Coalition (OCHPC) in the Triangle Region of North

Carolina. The OCHPC comprises local residents, municipal governments, nonprofit organizations, and home repair providers who collaboratively deliver free home improvements to older adults with limited incomes. Using policy analysis, administrative and interview data from coalition partners, we assess how federal, state, and local policies shape the implementation and reach of these repair programs. Our findings indicate that, despite strong rhetorical commitments to affordable housing stability and climate resilience for older adults, geographic eligibility criteria embedded in policy frameworks result in uneven access to home repair services, particularly disadvantaging those living in peri-urban areas (regions adjacent to metropolitan centers). We further explore how collaborative, cross-sector coalitions can leverage and integrate diverse funding streams and programs to more comprehensively address the home repair needs of low-income older adults.

Abstract citation ID: igaf122.3790

### HOSPITALIZATION AND DEATH AFTER NURSING HOME-TO-NURSING HOME TRANSFER

Ana Montoya, Pil Park, Slim Benloucif, Matthew Davis, and Julie Bynum, *University of Michigan, Ann Arbor, Michigan, United States*

Although some nursing home (NH)-to-NH transfers have been associated with negative outcomes, it is unclear whether they are associated with a higher risk of hospitalization/death among long-term NH residents. Paired cohort analysis was used to evaluate whether long-term residents who experience a transfer are at greater risk for hospitalization or death compared to residents who remain in the same facility. Data: 20% national Minimum Data Set (MDS) assessments linked to Medicare claims. Long-term NH residents who underwent a NH-to-NH transfer in 2019 were matched 1:1 with residents who did not transfer—matching variables included residing in the same NH and month. The primary outcomes were acute care hospitalization and all-cause death within 90 days following the transfer. A total of 1,595 matched resident pairs were analyzed. Hospitalization rates (127.9 vs 126.6 per 1000 residents, $p = 0.92$) and all-cause mortality (9.4 vs 13.2 per 1000 residents, $p = 0.32$) did not differ between the transfer and non-transfer groups. After adjusting for demographics (age, sex, race/ethnicity, marital status), dual eligibility status, activities of daily living (ADL) scores, cognitive function scores (CFS) and comorbidity scores, transfer status was not associated with a higher risk of 90-day hospitalization (RR = 0.99; 95%CI [0.82-1.19]) or death (RR = 1.40; 95%CI [0.72-2.72]). Among long-term NH residents, transfer to another NH was not associated with a higher risk of hospitalization or mortality within 90 days post-transfer, after accounting for resident characteristics and comorbidities. These findings suggest that NH-to-NH transfer when necessary, may not inherently increase acute health risks for this population.

Abstract citation ID: igaf122.3791

### HOW GROUP-BASED PHYSICAL AND SOCIAL ACTIVITIES PRESERVE COGNITIVE FUNCTION IN OLDER ADULTS

Jisu Seo, Seohye Jeong, Eunna Oh, and Rhayun Song, *Chungnam National University, Daejeon, Republic of Korea*

Both structured physical exercise and socially engaging group-based interventions have been shown to support cognitive health in older adults. This study aimed to compare the effects of Tai Chi (a physical activity) and Laughing Therapy (a social activity) to a wait-list control group on cognitive function in community-dwelling frail older adults. A three-arm randomized controlled trial was conducted over 12 weeks. Participants were randomly assigned to one of three groups: Tai Chi ($n = 70$), Laughing Therapy ($n = 46$), or a wait-list control group ($n = 44$). The Tai Chi group attended sessions twice a week for one hour, while the Laughing Therapy group met once a week for one hour. Cognitive function was assessed using the MoCA-K, including subdomain scores. The mean age of participants was 78.83 years (SD = 4.35). Repeated measures ANOVA, controlling for age, was used to examine group differences. After controlling for age, participants in both intervention groups showed trends of improvement across most MoCA-K domains. However, a statistically significant group difference was observed only in the language domain compared to the control group ($F = 4.46$, $p = .013$). The difference in total MoCA-K scores was not statistically significant ($F = 1.20$, $p = .304$). Group-based Tai Chi and Laughing Therapy programs demonstrated potential to enhance language-related cognitive functions among community-dwelling frail older adults. These findings underscore the importance of community-based interventions in maintaining cognitive function and highlight the need for further research to explore the underlying mechanisms and optimal strategies.

Abstract citation ID: igaf122.3792

### IF DIGITAL LITERACY IS AN OVERARCHING DETERMINANT OF HEALTH, HOW CAN WE MEASURE AND CONFIRM E-LITERACY IN OLDER ADULTS?

Michael Splaine[1], Nora Reder[2], and Kate Gordon[1], *1. Splaine Consulting, Columbia, Maryland, United States, 2. University of California, Berkeley, Berkeley, California, United States*

Older adults need to navigate an increasingly digital world-and the heterogeneity of the population's digital skills (and those of their family caregivers) can be a facilitator or a barrier to care and support and best lives. This poster presents the case for viewing digital literacy as a social determinant of health and a summary of the research validated measures of digital literacy that can be put into place in health and social care organizations. The last portion of the paper outlines a process which can be used to adopt in measures and to assist older adults with access to digital competence and tools.

Abstract citation ID: igaf122.3793

### IMPROVING FUNCTION IN OLDER VETERANS WITH LOW VISION USING NEW ACCESSIBLE TECHNOLOGY

Sonia Madera[1], Gabino Lares[2], Tahira Tyrell[3], Mythili Bharadwaj[4], Alyssa Benjamin[5], Nora Carolina Cabrera[5], and Ivana Yero[5], *1. Veterans Administration, Fort Lauderdale, Florida, United States, 2. WPB VA MEDICAL CENTER, West Palm BEach, Florida, United States, 3. WPB Veterans Administration, West Palm BEach, Florida, United States, 4. Veterans Administration, West Palm BEach, Florida, United States, 5. VETERANS ADMINISTRATION, WEST PALM BEACH, Florida, United States*

Introduction: Vision decreases with aging. In the US approximately 18% of people over 65 demonstrate some form of low vision leading to a major impact on ADLs and quality of life with increasing dependence and potential institutionalization. We have demonstrated objectively using FIM scores that accessible technologies such as ORCAM can have a significant impact on improving the ADLs of older adults with low vision. A major drawback is the prohibitive cost of $4500 for the device. Recent advances have adapted the common iPhone to utilize similar programs which are applied on smart devices (Android & iOS) at no cost to the user. Objectives: Comparing the IPHONE and ORCAM in improving functional independence in Veteran older than 60 to regain their desired level of independence using apps on smart devices with training provided. Methodology: comparing data from the Functional Independence Measurement (FIM) scale in comparing the ORCAM and mobile phone app (Seeing AI). Results: Since 2015, 210 Veterans were trained on mobile phones. Technology can capture images and extract text, read aloud, identify people, currency, barcodes, colors, and detect light. The ORCAM and iPhone can increase the overall FIM score average by approximately (3-4.5) once training is provided, including safety awareness (5.10), product identification (4.5), facial recognition and hand-eye coordination with use of camera scores (4.5). The iPhone and ORCAM technologies can improve the quality of life of older Low Vision Veterans and can measurably improve their independence and with the issuance of a smart phone and specialized training provided.

Abstract citation ID: igaf122.3794

### IMPROVING OLDER ASIAN IMMIGRANTS STRUCTURAL CHALLENGES IN ACCESSING HEALTHCARE SERVICES

Sabita Shrestha, and Matthew Sprong, *Governors State University, University Park, Illinois, United States*

Older Asian immigrant population is rapidly growing in the United States. According to the US Census Bureau, this population growth is projected to increase by 6.8 million by 2050. This fast-growing older immigrant population comes with the demand of increased access to improved care and services. However, the system of care and services are often complex and confusing. It is daunting to navigate healthcare services in general. Especially, for older Asian immigrants, the navigation of care and service is even more puzzling who have less familiarity of western system of care. Research has shown that older Asian immigrants often underutilized healthcare services compared to other older population in the United States, thus, presenting poorer health outcomes. One of the reasons that older Asian immigrants underuse healthcare services is due to structural challenges/barriers placed within the healthcare system. Often older Asian immigrants in their later-life restricts opportunities to learn new skills, language, systems, etc., therefore, limiting to understand the complex nature of healthcare services hindering access to needed cares and services that impacts their health and well-being. One way to safeguard older Asian immigrants' health is to focus on improving structural challenges/barriers within the healthcare system that ultimately results into improved access, care, and services. This poster presentation, based on ecological model, will showcase interventions grounded in health education that aimed to improving older Asian immigrants' structural challenges/barriers. Additionally, the application of intervention strategies will lead to increased and improved access to healthcare and services for older Asian immigrants.

Abstract citation ID: igaf122.3795

### INFLUENCE OF FEAR OF FALLING AND BUILT ENVIRONMENT ON PHYSICAL ACTIVITY AMONG DIVERSE OLDER ADULTS WITH CANCER

Kristen Fessele[1], Justin O'Leary[2], Anita Yafeh Mfuh[2], and Jaime Gilliland[2], *1. Memorial Sloan Kettering Cancer Center, Bridgewater, New Jersey, United States, 2. Memorial Sloan Kettering Cancer Center, New York, New York, United States*

Motivation for older adults with cancer (OAC) to engage in physical activity (PA) during and after cancer treatment may be limited by many factors. OAC may be affected by persistent symptoms limiting PA capability such as fatigue, pain and dyspnea, or lack safe home or neighborhood spaces to engage in activity. Fear of falling (FOF) is a major concern among OAC and may decrease PA and life-space mobility. To understand how OAC perceptions of cancer treatment, FOF and their local built environment (BE) influence PA behaviors, we utilized ResearchMatch.org to recruit a sample of 19 people with a history of cancer aged 65+ who were diverse in self-identified race, ethnicity, gender and sexual orientation. We developed an interview guide using Michie's COM-B model (Capability, Opportunity, Motivation – Behavior) and conducted semi-structured interviews ranging from 24 to 60 minutes. Thematic Content Analysis was conducted by two investigators in NVivo v14. Critical themes included 1) regaining past PA levels vs. adjusting to new limitations; 2) high FOF levels may limit PA selection, regardless of fall history; 3) avoiding socialization and PA in public due to post-surgical changes in appearance; 4) home and local neighborhood layout impact opportunity to access safe spaces for PA and 5) desire to receive customized PA and health promotion education near end of treatment instead of while overwhelmed near diagnosis. Factors influencing motivation for PA in OAC are complex; clinicians should assess FOF and local BE to better customize PA recommendations that address these concerns and limitations.

Abstract citation ID: igaf122.3796

### INSIGHTS ON LEVERAGING VIRTUAL REALITY AS A MEDIUM FOR INTERVENTION DELIVERY FOR OLDER ADULTS

George Mois[1], Emre Eraslan[2], Tongxin Sun[3], Qiyuan Cheng[2], Avinash Gupta[2], and Wendy Rogers[2], *1. University of Georgia, Athens, Georgia, United States, 2. University of Illinois Urbana-Champaign, Champaign, Illinois, United States, 3. Tsinghua University, Beijing, Beijing, China (People's Republic)*

Virtual reality (VR) is a growing immersive technology with potential to enhance access and adaptability to sustain participation in social, cognitive, and activity engagements that cannot easily be facilitated through other information communication technologies. Leveraging VR presents a unique opportunity to support resource development and improve access to services and activities that are tailored to meet older adults' needs and preferences. However, there is limited understanding pertaining to the design and adaptation of off-the-shelf VR solutions to support adoption and use by older adults, and how VR can be used to enhance access to interventions, services, and programs. We conducted a mixed methods study in a home simulation environment to explore the application of VR as a vehicle for intervention delivery. We recruited 21 community dwelling older adults aged 61-80 (M = 69, SD = 5.45) to participate in passive VR demonstrations and gathered insights into their experience through questionnaires and a semi structured interview. Participants perceived VR as an immersive medium with the potential of adapting to their own interests and preferred activities. Our findings provide insights into design and implementation considerations in utilizing VR as a medium for intervention delivery for older adults. Based on these findings, we outline opportunities (visual and audio capabilities, tailored experiences) and challenges (disorientation, physical discomfort, usability limitations) in leveraging immersive technologies to support various types of VR engagement activities (e.g., activity, social, cognitive). These considerations can improve effectiveness of VR-based interventions and provide steps to meet the needs and preferences of older adult VR users.

Abstract citation ID: igaf122.3797

### INTERGENERATIONAL INTERVENTIONS TO REDUCE AGEISM: ROLES AND OUTCOMES OF THE OLDER PARTICIPANTS

Brian Carpenter[1], Sizhe Li[2], Claire Wininger[2], Natalie Galucia[2], Aaron Li[1], and Nancy Morrow-Howell[1], *1. Washington University, St. Louis, Missouri, United States, 2. Washington University, Saint Louis, Missouri, United States*

Intergenerational interventions to reduce ageism have focused primarily on the important aim of changing attitudes among younger people. Older adults are included as essential components of interventions, but they are rarely viewed as targets of change or beneficiaries themselves. In the current analysis, we synthesize literature to understand how older adults are included in anti-ageism interventions. We build from a systematic review of interventions by Apriceno and Levy (2023) that summarized intervention effectiveness. We analyzed these articles for the complementary purpose of identifying roles of older adults in interventions. Of 123 articles in the systematic review, 88 included interventions involving personal contact with older persons. In those 88 interventions, older participants were mostly female and Caucasian, with a wide age range, and they were recruited primarily from residential facilities (n = 30 interventions, 34%) and via general community outreach (n = 28, 32%). The most common activities in the interventions were guided conversations/discussions (n = 52, 59%) and creative activities (n = 21, 24%). Only 13 of those 88 programs (15%) evaluated older adults directly and reported outcomes for them. Outcomes focused on attitudes toward younger people, self-esteem, life satisfaction, and generativity and were generally positive, particularly for attitude shifts. However, even when studies did evaluate older adults, they had substantial methodological weaknesses that limit rigorous hypothesis testing and generalizability. In sum, older adults receive cursory attention in intergenerational interventions to combat ageism, despite their central role. Future studies should integrate older adults more systematically into anti-ageism interventions.

Abstract citation ID: igaf122.3798

### INTERVENTIONS FOR IMPROVING SLEEP IN DEMENTIA INFORMAL CAREGIVERS: A SYSTEMATIC REVIEW AND META-ANALYSIS

Jia Yin Ruan[1], Xiang Qi[1], Jin Su[1], Weiyu Mao[2], Eunjung Ko[1], and Bei Wu[3], *1. New York University, New York, New York, United States, 2. University of Nevada, Reno, Reno, Nevada, United States, 3. NYU Shanghai, Shanghai, China (People's Republic)*

Although interventions are available to improve sleep for informal caregivers of people with dementia, summarized evidence is still lacking. This study aimed to systematically review and quantify the effects of non-pharmacological interventions on sleep in this population. A literature search was conducted on eleven databases from inception up to 30 June 2025 to retrieve randomized controlled trials. Meta-analyses were performed to estimate the mean difference (MD) of Pittsburgh Sleep Quality Index (PSQI) (primary outcome) using a random-effects model. Risk of Bias 2 tool was applied to measure methodological quality. Twenty-one RCTs encompassing 1964 adults (74.94 % female) were included. The interventions examined in this study refer to behavioural intervention, psychoeducational intervention and physical intervention. The pooled results showed that non-pharmacological interventions were superior to passive controls (e.g., waitlist, usual care), for improving sleep quality (MD = 1.265, 95% confidence interval [0.194, 2.355], p = 0.021) regarding the PSQI score. All included trials had a high overall risk of bias. The overall evidence evaluated by the Grading of Recommendations, Assessment, Development, and Evaluations framework was very low. Future research is encouraged to generate robust evidence by developing well-designed non-pharmacological sleep interventions that incorporate the 4 P Model of insomnia (Predisposing, Precipitating, Perpetuating, Protective), process evaluation and treatment fidelity.

Abstract citation ID: igaf122.3799

### IS MOTOR FUNCTION IN MCI RELATED TO CAREGIVER RATINGS OF COGNITION AND EVERYDAY FUNCTION?

Kyoung Shin Park, Jeongwoon Kim, Kayci Vickers, Emily Giannotto, Jacquelyn Thelin, Liselotte De Wit, and Amy Rodriguez, *Emory University School of Medicine, Atlanta, Georgia, United States*

Mild cognitive impairment (MCI) often affects daily functioning, which is typically assessed by subjective caregiver ratings. Previous research shows that caregiver ratings may be biased by individual factors, such as caregiver burden and stress. We aimed to extend this work by further examining whether decline in motor function in MCI also influences caregiver perceptions, potentially leading to lower ratings of patients' cognition and everyday function. A cross-sectional study was conducted with 124 MCI patient-caregiver dyads (patients: age=74.7±6.6 years, 49% female; caregivers: age=65.6±13.3 years, 76% female). Caregivers rated MCI patients' cognition and daily function using the Everyday Cognition Scale (ECog-39) and Functional Activities Questionnaire (FAQ) and completed the Zarit Burden Interview Short Form (ZBI-12) and NIH Toolbox Perceived Stress Scale (PSS). Patients completed the Montreal Cognitive Assessment (MoCA) and motor assessments (10-meter walk, handgrip strength, and 30-second chair stands). Multiple linear regressions were fitted while controlling for patient (age, sex, education, MoCA) and caregiver (age, sex) covariates. Consistent with the prior work, higher caregiver burden and stress were associated with lower caregiver ratings on ECog-39 (ZBI-12: B = 0.86, p<.001; PSS: B = 0.94, p<.001) and FAQ ratings (ZBI-12: B = 0.03, p<.001; PSS: B = 0.22, p=.026). In contrast, walking speed, grip strength, and chair stands were not associated with ECog-39 and FAQ ratings (all p>.10). Caregivers' subjective ratings of MCI patients' cognition and daily functioning were more closely tied to their own burden and stress than to patients' motor performance. These findings highlight the need to consider caregiver factors when interpreting subjective functional ratings in MCI.

Abstract citation ID: igaf122.3800

### KEEPING OLDER ADULTS AT HOME: PREDICTION MODELING FOR 30-DAY OUTCOMES AFTER SEPSIS DISCHARGE

Sang Bin You[1], Jiyoun Song[2], Miriam Ryvicker[3], Yolanda Barron[3], and Kathryn Bowles[2], *1. University of Pennsylvania, Philadelphia, Pennsylvania, United States, 2. University of Pennsylvania School of Nursing, Philadelphia, Pennsylvania, United States, 3. VNS Health, New York, New York, United States*

Many older adults prefer to remain in their homes and communities, yet sepsis survivors receiving home healthcare (HHC) often experience readmissions or transition to other facilities. This study aims to identify modifiable risk factors that can alert clinicians and trigger interventions to keep older sepsis survivors at home. We analyzed Medicare fee-for-service beneficiaries (2021-2022) hospitalized for sepsis and discharged with HHC using claims and home care assessment data. The outcome, days-not-at-home, was defined as binary indicator of whether patients spent time in hospitals, skilled nursing, or long-term facilities within 30 days. Logistic regression, random forest, neural network, and XGBoost models were developed, tuned with cross-validated grid search, and evaluated using 10-fold cross-validation. Performance was assessed using the area under the receiver operating characteristic curve (AUC). The cohort included 19,019 older adults [mean age 78.5 years, standard deviation (SD)=8.1] with an average of 5.5 chronic conditions (SD = 2.0), mean hospital stay of 8.1 days (SD = 7.0), and nearly all (96.9%) used ≥5 medications. Within 30 days, 25.5% experienced days-not-at-home. The neural network performed best (AUC=0.6052), followed by logistic regression (AUC=0.6044), XGBoost (AUC=0.6037), and random forest (AUC=0.5955). Top predictors included receiving enteral nutrition therapy, having an ostomy, ≥2 hospitalizations in the past 6 months, positive or missing depression screening, and having pressure ulcer(s). Although overall accuracy was modest, results suggest that addressing depression, enhancing nutrition support and providing close scrutiny of wounds may reduce risk and prevent institutional care among sepsis survivors. Findings may guide risk stratification and tailored interventions to reduce preventable utilization.

Abstract citation ID: igaf122.3801

### KOTT GERONTOLOGY SCHOLARS PROGRAM EVALUATION

Heather Menne[1], Bailee Brekke[2], Jennifer Kinney[1], and Lisa Peters-Beumer[3], *1. Miami University, Oxford, Ohio, United States, 2. Miami University, Waddington, New York, United States, 3. Concordia University Chicago, River Forest, Illinois, United States*

The Kott Gerontology Scholars Program (KGSP) has provided internship scholarships to promising advanced degree graduate students interested in the aging field who are attending Chicagoland area universities for 20 years. The KGSP encourages and fosters interdisciplinary cohorts of graduate students to develop hands-on skills that will engage and prepare them to continue working with and on behalf of older adults after graduation. The Scripps Gerontology Center conducted a mixed-methods approach to evaluate the overall program. Through the use of survey data (n = 74), 3 focus groups (n = 9), and key informant interviews (n = 5) with both alumni scholars and host agencies, the evaluation sought to a) assess the impact of KGSP on scholars' career trajectories and professional development and b) evaluate the engagement and effectiveness of KGSP with partners' agencies. It was evident that many KGSP scholars went on to pursue careers in the field, and 92% of Scholars working in the field of aging are likely or very likely to continue working in the field. Preliminary results from qualitative interviews suggest continued communication and networking opportunities for current and alumni scholars to engage in mentorship. Agency staff also recommended more communication and involvement with all of the current and previous agencies to support program growth. There is a recognized scarcity of gerontological expertise and there are significant gaps in the workforce in the field of aging. The KGSP helps prepare individuals to successfully enter and remain working in the aging network.

Abstract citation ID: igaf122.3802

### LATENT PROFILE ANALYSIS OF OLDER ADULTS BY VOLUNTEER STATUS

Elnaz Abaei[1], and Shannon Jarrott[2], *1. Iowa State University, Ames, Iowa, United States, 2. The Ohio State University, Columbus, Ohio, United States*

Volunteering is generally associated with purposeful activity and social connection, which supports well-being among older adults. Intergenerational volunteering offers additional benefits by supporting principles to reframe aging. Utilizing HRS data, we explored older adult volunteer profiles and outcomes. The sample included 13,135 older adults (M age = 67.26, SD = 10.70), mostly female (58.0%), White/Caucasian (79.0%), and married (59.6%), with a mean12.77 years of education. Using latent profile analysis (LPA), three distinct classes emerged. Class 1 (non-volunteers, 74.6%) showed the least favorable psychological and functional outcomes, with higher loneliness, depressive symptoms, perceived control, and lower mastery and life satisfaction. Class 2 (general volunteers, 20.0%) showed the most favorable profile across indicators. Class 3 (intergenerational volunteers, 5.4%) had a mixed profile, with high mastery and life satisfaction like general volunteers, but elevated loneliness and depressive symptoms like non-volunteers. ANOVA results supported distinctions, showing significant differences across groups in loneliness ($F(2, 12{,}253) = 134.53$), perceived control constraints ($F(2, 12{,}498) = 120.39$), depression ($F(2, 12{,}686) = 126.93$), mastery ($F(2, 12{,}541) = 51.00$), life satisfaction ($F(2, 12{,}705) = 106.54$), and ADL difficulties ($F(2, 13{,}113) = 89.21$), all ps < .001. Small effect sizes (omega-squared = .008–.021) are still meaningful given the large sample, reflecting differences in older adults' lived experiences. Next, growth mixture models will compare trajectories across profiles to examine change over time. Findings highlight broad benefits of volunteering, particularly general volunteering, including the potential psychological value of intergenerational volunteering as a means to positively reframe aging from youth's and older volunteers' perspectives.

Abstract citation ID: igaf122.3803

### LIFESTYLE ACTIVITIES AND INCIDENT DEMENTIA FROM CLAIMS IN THE BALTIMORE EXPERIENCE CORPS STUDY

Kyle Moored[1], Vishaldeep Sekhon[2], George Rebok[3], Kate Gordon[1], Jeanine Parisi[1], Qian-Li Xue[3], and Michelle Carlson[1], *1. Johns Hopkins Bloomberg School of Public Health, Baltimore, Maryland, United States, 2. Johns*

*Hopkins University School of Medicine, Baltimore, Maryland, United States, 3. Johns Hopkins University, Baltimore, Maryland, United States*

Lifestyle activity engagement (e.g., social clubs, classes) in later life has been linked with reduced risk of dementia, but studies have often been limited to short follow-up periods. We examined whether variety and frequency of activity engagement predicted incident dementia risk, leveraging 15 years of linked longitudinal claims data in the Baltimore Experience Corps Study (BECS). Participants were 520 individuals from BECS (2006-2012) with available claims data (mean age=68.2, 84% women, 95% Black/African American). Participants self-reported baseline engagement in 27 activities, and their total number of activities (activity variety) and average frequency of engagement across activities (activity frequency) were calculated. Incident dementia diagnoses (2008-2022) were derived from linked CMS and private claims using the Bynum algorithm. We used Cox proportional hazards models, adjusting for age, sex, education, and intervention group. Participants reported an average baseline variety of 18.8 activities (SD = 3.3) and average activity frequency of 8.8 days per month (SD = 2.6). Hazard rates of incident dementia were 5.2% lower for each additional activity (activity variety: HR=.948, 95% CI: 0.89-1.00), which was slightly attenuated after further adjusting for baseline health conditions and depression (HR=.953, 95% CI: 0.90-1.01). Results were similar in magnitude but not statistically significant for activity frequency (p>.05). In a sample of primarily Black older adults, higher activity variety in later life predicted lower dementia risk over 15 years. Importantly, lifestyle activity engagement was previously shown to be increased by the Experience Corps intervention, suggesting that it may be a potentially modifiable target to promote cognitive resilience with aging.

Abstract citation ID: igaf122.3804

### LINGUISTIC FEATURES OF OLDER ADULTS' DAILY SELF-TALK: A NATURALISTIC OBSERVATIONAL APPROACH

Sibo Gao[1], and Karen Fingerman[2], *1. University of Texas at Austin, Austin, Texas, United States, 2. The University of Texas at Austin, Austin, Texas, United States*

Research supporting socioemotional selectivity theory finds that older adults tend to have better emotion regulation than younger adults, particularly with regard to negative emotion. Verbalized self-talk (i.e., audible speech when alone) may be an important but understudied emotion regulation strategy in late life, promoting psychological distance through second-person pronouns and structured speech, and providing an opportunity to express negative emotions. The current study sought to characterize linguistic features of verbalized self-talk descriptively and in comparison to conversations with social partners. Older adults (N = 333; Mage=73.9) reported whether they had been with social partners every three hours across 5–6 days. Electronically Activated Recorders captured 30-second sound snippets every 7 minutes. Coders transcribed 109,609 snippets; 918 snippets contained verbal utterances when participants were alone (self-talk). Using the Linguistic Inquiry and Word Count, we extracted linguistic features including second-person pronouns, formal and structured language, and emotional tone. Multilevel models revealed no significant within-person association between being alone and second-person pronoun use. However, when participants were alone, they used more structured language and expressed more negative emotion compared with language when with social partners. This study makes an important contribution by capturing naturalistic self-talk as it occurs in daily life and suggests self-talk may help older adults regulate negative emotions. Future research using wearable sensors might capture arousal or allow self-reported experience sampling of unspoken self-talk to tease out when and who uses self-talk to regulate emotions.

Abstract citation ID: igaf122.3805

### LONG LIFE FAMILY STUDY PARTICIPANTS CARRY RARE VARIANTS THAT CORRELATE WITH BETTER COGNITIVE FUNCTION

Hannah Lords[1], Mengze Li[1], Zeyuan Song[2], Anastasia Leshchyk[2], Harold Bae[3], Stacy Andersen[1], Anastasia Gurinovich[2], and Paola Sebastiani[2], *1. Boston University, Boston, Massachusetts, United States, 2. Tufts Medical Center, Boston, Massachusetts, United States, 3. Oregon State University, Corvallis, Oregon, United States*

Cognitive impairment is a growing healthcare and quality-of-life challenge as more individuals reach older ages. Without effective interventions, the prevalence of decline will continue to rise. To identify genetic factors underlying cognitive function, we performed GWAS on nine neuropsychological test scores in the Long Life Family Study (LLFS). We then compared results with UK Biobank GWAS of general cognitive ability and reaction time, conducted quantitative trait locus (QTL) analyses of whole blood transcriptomic and serum metabolomic data for genome-wide significant variants ($p < 5e-8$), and performed gene set enrichment analyses of nominally significant transcripts. We identified 12 genome-wide significant variants across 7 tests in LLFS. Comparison with UK Biobank data revealed test-specific replication patterns, suggesting that loci for general cognitive function and reaction time reflect distinct cognitive domains. QTL analyses identified one SNP–transcript association ($p < 0.05/16{,}300$) but no significant SNP–lipid or SNP–polar metabolite associations. Three rare protective variants—rs190287985, rs75730801, and rs552842447—were linked to semantic fluency (animal), phonemic fluency (FAS), and digit span forward, respectively. Two rare deleterious variants, rs556333682 and rs188304645, associated with performance on the Hopkins Verbal Learning Test (HVLT) total recall, lie in ischemia-related genes SH3TC1 and RPH3A. Another variant, rs180691759, associated with HVLT delayed recall, was proximal to RSPO3 and linked to decreased ECHDC1 expression, implicating ischemic and unexplained ethylmalonic acid pathways. Gene set enrichment identified three pathways (FDR < 0.05), including KEGG JAK-STAT signaling and oxidative phosphorylation. These findings highlight domain-specific mechanisms of cognitive resilience and decline.

Abstract citation ID: igaf122.3806

### LONGITUDINAL ASSOCIATIONS OF REST-ACTIVITY RHYTHM PROFILES WITH PERCEIVED PHYSICAL FATIGABILITY

Namhyun Kim[1], Terri Blackwell[2], Katie Stone[3], Reagan Garcia[1], Peggy Cawthon[4], Melissa Erickson[5], Karyn Esser[6], and Nancy Glynn[1], *1. University of Pittsburgh, Pittsburgh, Pennsylvania, United States, 2. California Pacific Medical Center Research Institute, San Francisco, California, United States, 3. California Pacific Medical Center Research*

*Institute, Castro Valley, California, United States, 4. California Pacific Medical Center, San Francisco, California, United States, 5. AdventHealth, Orlando, Florida, United States, 6. University of Florida College of Medicine, Gainesville, Florida, United States*

Rest-activity rhythms (RARs), 24-hour activity and sleep patterns, are cross-sectionally associated with fatigability, a prognostic marker of functional decline. Participants (N = 821, age=76.4±5.0 years) from the Study of Muscle, Mobility and Aging had perceived physical fatigability measured using the validated 10-item Pittsburgh Fatigability Scale (PFS; 0-50, higher score=greater fatigability) at baseline and up to three follow-up visits over 2.6±0.4 years. RARs were derived from 7-day wrist-worn accelerometry using the extended cosine model (parametric: amplitude, acrophase, alpha, beta) or non-parametric approaches (interdaily stability, intradaily variability, relative amplitude). Three RAR profiles (Synchronized, Concentrated High-Activity, Low-Fragmented) were identified using the k-means clustering algorithm. Compared to Synchronized, the Concentrated High-Activity group had the highest amplitude (5,176 vs. 2,258 counts/min) and earliest acrophase (11:54AM vs. 2:18PM). The Low-Fragmented group vs. Synchronized had the lowest amplitude (1,462 vs. 2,258 counts/min), interdaily stability (0.46 vs. 0.63), and highest intradaily variability (1.10 vs. 0.81). PFS score varied by RAR groups: Synchronized=15.2±8.5, Concentrated High-Activity=14.2±7.9, and Low-Fragmented=17.3±8.7, p = 0.004. Using linear mixed models, the Low-Fragmented group had higher perceived physical fatigability at baseline (β = 1.30, p = 0.046) and a steeper increase in PFS score over time (β = 0.46, p = 0.03) vs. Synchronized, adjusted for demographics, health conditions, lifestyle factors, and sleep efficiency. After further adjustment for cardiorespiratory fitness (VO2peak from treadmill testing) and daily step count, the Low-Fragmented group showed a significantly larger increase in PFS score over time (β = 0.46, p = 0.03; β = 0.49, p = 0.02), although the baseline association was attenuated. Our findings reveal that disrupted RARs are independently associated with persistently worsening perceived physical fatigability.

Abstract citation ID: igaf122.3807

### LOW-INTENSITY PEER-LED EXERCISE IMPROVES FALL RISK APPRAISAL IN OLDER WOMEN

Chitra Banarjee[1], Kworweinski Lafontant[1], Jethro Raphael Suarez[2], Rui Xie[1], and Ladda Thiamwong[1], *1. University of Central Florida, Orlando, Florida, United States, 2. University of Central Florida, Florida, United States*

About 64-79% of older adults aged 60+ have maladaptive fall risk appraisal (FRA), a discrepancy of perceived and physiological fall risks, leading to reduced physical activity and multiple falls. Older adults have been classified into four groups of FRA by integrating fear of falling (FoF) and balance, as measured by the Short Falls Efficacy Scale-International, and BTrackS Balance Test (BBT); a) rational (low FoF and normal balance), b) incongruent (low FoF despite poor balance), c) irrational (high FoF despite normal balance), and d) congruent (high FoF and poor balance). This study examined the effect of an 8-week low-intensity peer-led intervention (PEER) on changes in FRA. We recruited 314 community-dwelling older women (mean age=74.3±6.9years) at four timepoints (baseline and follow-ups at 2, 4, and 6 months) to evaluate longitudinal changes in quantified FRA, or FRA distance, a quantified FRA adapted using the distance formula, where greater values indicate composite deficits in FoF and balance. A linear mixed effects model included fixed effects of intervention, original FRA group, age, and race, with random effects of participant and timepoint. The intervention shows a slight but significant reduction in FRA distance (β=-2.59, p = 0.047), indicating improved outcomes. Compared to the rational group, all other original FRA groups—except irrational—had significantly worse FRA scores (p < 0.001), and advanced age was associated with worse FRA distance (p < 0.001). These results demonstrate the potential of the PEER intervention for shifting FRA, but the limited size of the effect suggests that greater exercise intensity may further improve FRA outcomes.

Abstract citation ID: igaf122.3808

### MAHJONG COMMUNITY: A CASE STUDY ON A SPONTANEOUSLY FORMED ADULT DAY CARE MODEL IN UNDERDEVELOPED REGIONS OF CHINA

Yan Ming[1], Jie Yang[2], and Yuehua Li[3], *1. Capital Normal University, Beijing, Beijing, China (People's Republic), 2. East Carolina University, Greenville, North Carolina, United States, 3. Minzu University of China, Beijing, Beijing, China (People's Republic)*

The underdeveloped areas in southwestern China are characterized by a triple pressure of low economic development, a high density of older adults, and a lack of long-term care (LTC) resources. Social isolation and loneliness are risk factors for mortality of older adults and negatively impact their health and well-being. A new model of home-based community care for older adults has emerged spontaneously, playing a unique role in filling the structural gap in the LTC system. We refer to it as the "Mahjong Community." Through a case study, this article explores the formation and characteristics of this new LTC model and discusses its implications for LTC policies in China. Relying on neighborhood social capital and the mahjong culture, Mahjong Communities transform family space into adult day care settings during the daytime. An average of 15-20 older adults participated daily from 11:30 am to 3:30 pm. The host also accommodated older adults with disabilities who particularly risked social isolation. The activities included mahjong games, socialization, tea and snacks, meals and gift exchanges during holidays. The participants and host formed an emotional bond which created a sense of community. The daily cost of individuals was four yuan (less than one dollar). This low-cost model creates sustainable micro-economic units across the studied geographic area and proves to be scalable across the country due to the similar mahjong culture in different regions. The government should provide support in areas such as improving universal design in infrastructure and subsidies to scale these communities.

Abstract citation ID: igaf122.3809

### MAINTAINING BALANCE:: EXAMINING MENTAL WELLNESS IN BLACK WOMEN DURING THE MENOPAUSAL TRANSITION

Graham McDougall[1], Elyria Kemp[2], Xueyan Liu[2], Candice Sorapuru[3], and Kelly Jones[4], *1. University of Alabama-Tuscaloosa, New Orleans, Louisiana, United States, 2. University of New Orleans, New Orleans, Louisiana, United States, 3. Tulane University, New Orleans, Louisiana, United States, 4. Steady Mindz, Kelly Lyn Health, Indianapolis, Indiana, United States*

Black women begin the menopausal transition at an earlier age than other women and experience more severe menopausal symptoms such as depression and anxiety. The “Strong Black Woman Schema” (SBWS) is a cultural expectation that these women portray strength and caretaker qualities, while suppressing their emotions. This research had two aims (1) what factors influenced how Black women in midlife perceive, seek, and access mental health care and (2) how these factors could enhance mental well-being. Using a mixed-methods approach, the research integrated qualitative interviews (n = 23) and a segmentation analysis (n = 231) to uncover nuanced insights into the lived experiences of Black women and their mental health decision-making. In the qualitative component the participants ranged from 42–60 in age; the mean age was 49. Approximately 60% of the participants sought mental healthcare. Qualitative findings revealed key themes related to emotional suppression, stigma, cultural expectations, structural barriers, and the role of self-care. In the next phase a survey was distributed online to a pre-established national consumer panel of self-identified Black women using a market research firm in the United States. The quantitative segmentation analysis (N = 231), performed using K-means clustering, identified four distinct groups—Reluctant Avoidants, Evolving Discerners, Practical Engagers, and Steady Seekers—each with unique orientations toward mental health care. Based on these profiles, this research proposes targeted engagement strategies for enhancing access to care. These insights into how Black women navigate mental health care may assist in identifying effective strategies for improving access and support to women.

Abstract citation ID: igaf122.3810

### MEDICAID MANAGED LONG-TERM SERVICES AND SUPPORTS IN RELATION TO NURSING HOME STAFFING AND CARE DIVERSION

Alison Trinkoff[1], Jennifer Choi[2], and Michael Lepore[3], *1. University of Maryland School of Nusing, Baltimore, Maryland, United States, 2. Sejong University, Sejong University, Seoul-t'ukpyolsi, Republic of Korea, 3. University of Massachusetts Amherst, Amherst, Massachusetts, United States*

Background: In 2020, Medicaid accounted for approximately 61% of total U.S. spending on long-term services and supports (LTSS), or $200 billion. As the primary LTSS payer, Medicaid faces significant financial pressures from rising demand. In response, US states have implemented Medicaid-Managed LTSS programs, shifting from fee-for-service models to managed care systems, yet few studies have evaluated the impact of these shifts on care. Purpose: This examined two outcomes: care diversion from institutions, and changes in nursing home staffing levels that could mediate the programs' impact on care quality. Methods: The study used data from six states that implemented MLTSS between 2010-2014. We examined the impact on care diversion and staffing levels using a staggered difference-in-differences framework that captures dynamic effects over time. Results: The proportion of Medicaid residents in nursing homes significantly decreased following MLTSS program implementation. Also, the proportion of residents with other payer sources increased, along with a significant rise in total nursing hours per resident day. Cumulative gains across nurse staffing categories contributed to the significant rise in overall staffing, supporting program goals of improving care quality. For diversion, we identified a shift in nursing home resident composition by payer groups, without significant changes in the total number of residents, potentially reflecting diversion of Medicaid beneficiaries to alternative care settings. This suggests that the observed changes reflect a reallocation in resident mix rather than fluctuations in overall nursing home censuses. Conclusions: Findings offer new evidence on the potential beneficial effects of MLTSS implementation across multiple US states.

Abstract citation ID: igaf122.3811

### META-ANALYSIS OF SPATIAL NAVIGATION SKILLS IN MILD COGNITIVE IMPAIRMENT AND ALZHEIMER'S DISEASE

Dorota Kossowska-Kuhn, and Gillian Gouveia, *Florida State University, Tallahassee, Florida, United States*

Dementia affects millions worldwide, with spatial navigation impairments emerging early and becoming more pronounced as the disease progresses. Spatial navigation, the ability to orient oneself and move effectively through the environment, is essential for independent living, yet its trajectory across the dementia spectrum remains insufficiently characterized. Difficulties in spatial navigation manifest in both individuals with Mild Cognitive Impairment (MCI) and Alzheimer's Disease (AD). Two previous meta-analyses demonstrated significant differences ($p < .001$) in navigation skills between cognitively healthy older adults (CHOA) and individuals with MCI, with a large effect size (Hedges' $g = 0.88$), and between CHOA and individuals with AD, with a very large effect size (Hedges' $g = 1.80$). The present meta-analysis compared spatial navigation performance in individuals with MCI and AD by synthesizing evidence from 21 studies, yielding 57 effect sizes. Results indicated that individuals with AD performed significantly worse on spatial navigation tasks than those with MCI, with a small effect size (Hedges' $g = 0.16$, $p < .001$). These findings are based on a diverse range of spatial navigation tasks administered through real-world, virtual reality, computer-based, and paper-and-pencil formats, and encompass various subtypes of navigation including arena, environmental, maze, map drawing, and matrix tasks. This synthesis highlights the need for further research investigating spatial navigation performance differences across the dementia spectrum. Future studies should prioritize longitudinal designs and standardized task protocols to better map trajectories of decline and improve the clinical utility of spatial navigation assessments.

Abstract citation ID: igaf122.3812

### MIDLIFE WORK DISABILITY AND ILLNESS SPELLS: NEW DATA FROM THE HRS 2019 LIFE HISTORY SURVEY

Jessica Kelley[1], John Whesu[1], Yuanchang Zhao[1], and Wenxuan Huang[2], *1. Case Western Reserve University, Cleveland, Ohio, United States, 2. Johns Hopkins University, Baltimore, Maryland, United States*

Midlife is a critically important time in the life course. Adults in midlife are the intergenerational linkage between their (grand) parents and their (grand)children. Spells of ill health or disability can place pressures on the time, money, and energy that midlife individuals, potentially setting off a cascade of adverse circumstances that degrade later-life well-being. Adding the newly released data (2019) from the Health and Retirement Study Life History Survey to the previous two (2015, 2017), we construct joint sequences on 12,909 adults linking their health and work status between the ages of 20 and 63. Health sequences included spells of serious illness, injury, and disablement. Work sequences included work status (FT, PT, retired, not working, education), along with spells of non-work due to medical or disability reasons from age 20 to 63. Combining these sequences, we show that 78% of adults never had a serious illness or had to leave a job for medical reasons, while 17% experienced at least one serious illness that lasted more than a year, or left a job for medical reasons, or both. Another 4.5% were ill their entire lives or never worked due to disability. Median age of first serious illness is 41, and median age of leaving work for medical reasons is 50. We present these joint sequences, along with preliminary associations with later-life outcomes of depressive symptoms, loneliness, and wealth. We further discuss the value of combining work and health sequences in life history data for a fuller picture of midlife health.

Abstract citation ID: igaf122.3813

### MIXED METHODS ANALYSIS OF STATE POLICIES ON CARE PLANNING AND FAMILY ENGAGEMENT IN ASSISTED LIVING

Paula Carder[1], Erh-Chi Hsu[2], Lindsey Smith[3], Cassandra Hua[4], Adam Pennavaria[3], and Eric Jutkowitz[5], *1. Portland State University, Portland, Oregon, United States, 2. Johns Hopkins University, Baltimore, Maryland, United States, 3. Oregon Health & Science University, Portland, Oregon, United States, 4. University of Massachusetts Lowell, Lowell, Massachusetts, United States, 5. University of Minnesota, Minneapolis, Minnesota, United States*

Involving family in care planning is a person-centered care strategy that enhances assisted living (AL) residents, families, and staff well-being. This study analyzes AL care planning and family engagement requirements through a health services regulatory analysis of 256 AL licenses across all 50 states and DC associated with 37190 ALs in 2023. Using a mixed methods approach, we examined license requirements for the presence and qualitative context of family engagement in care planning, staff training on family involvement, and person-centered care. Nearly 80% of ALs were required to have care plans. A larger share of ALs designated for memory care (MC) had family engagement requirements (47%) compared to non-memory care ALs (32.1%). Half of licenses encourage family engagement in care planning, while the remainder used passive language about ensuring family roles or encouraging involvement in activities. Person-centered care policies were less common, appearing more often in MC licenses. Only licenses in 3 states emphasized respecting individuals' cultural and ethnic values in care planning and staff training. Qualitative analysis identified the following categories in family engagement: providing direct care, and involvement in care planning and legal aspects (e.g., lease, payment). Person-centeredness was described in the context of care planning and staff training. The level of specificity and stringency of these categories ranged from passive to detailed and directive. These findings provide the first comprehensive profile of AL care planning requirements, establishing baseline data for future outcome evaluations.

Abstract citation ID: igaf122.3814

### MODERATING ROLE OF POSITIVE EMOTIONS ON EMOTIONAL REACTIVITY TO DAILY EVENTS AND NEGATIVE EMOTIONS IN OLDER ADULTS

Rose Lin, *University of Rochester, Rochester, New York, United States*

Background: Daily stressors accumulate to negatively affect emotional well-being and may accelerate cognitive decline in older adults. Emotional reactivity, or heightened emotional responses to stress, is a key contributor. Positive emotions may buffer these effects, yet it remains unclear whether this protective role is preserved across levels of cognitive functioning and decline. Objective: To test whether positive emotions moderate the association between emotional reactivity to daily events and negative emotions, and whether cognitive ability or cognitive decline influences this effect. Methods: Participants were 2,041 adults aged 50–94 from the MIDUS study who completed cognitive assessments at two waves (2004–2006, 2013–2014). Emotional reactivity was assessed with the Multidimensional Personality Questionnaire, affect with the Positive and Negative Affect Schedule, and cognition with the Brief Test of Adult Cognition by Telephone. Hierarchical regressions examined the moderating role of positive emotions across cognitive ability and cognitive decline subgroups. Results: Higher emotional reactivity was significantly associated with greater negative emotions. Positive emotions moderated this association, such that individuals with higher positive emotions showed weaker links between reactivity and negative emotions. This buffering effect was consistent across levels of cognitive ability. However, when stratified by cognitive decline, moderation was observed only among those with high decline, but not moderate decline. Conclusions: Positive emotions mitigate the impact of stress reactivity on negative mood in later life, even in the presence of cognitive impairment. Findings highlight positive emotion as a resilience factor and support interventions that enhance positive affect to promote emotional well-being.

Abstract citation ID: igaf122.3815

### MORTALITY ACROSS ADULTHOOD: THE IMPACT OF COGNITIVE CHANGE

Olivia Chervenick, Meredith Willard, and Nicholas Turiano, *West Virginia University, Morgantown, West Virginia, United States*

Cognitive performance is a key indicator of health and may also serve as a predictor of mortality. While previous research has linked lower cognition to increased mortality, less is known about whether changes in certain cognitive abilities are stronger predictors of mortality, and the specific ages in adulthood when changes may be most detrimental. Data from 4,152 individuals from the Midlife Development in the U.S. Study (MIDUS) was utilized. Objective cognitive screeners for episodic memory and executive functioning were obtained at two time points (2005 through 2015) through the Brief Test of Adult Cognition via telephone (BTACT). Utilizing Cox proportional hazards modeling (497 deceased over 7 years), we found that those scoring higher in episodic memory ($HR = 0.88$) and executive functioning (0.85) were at a significantly decreased risk of dying (all p's less than .05), controlling for age, gender, race, education level, and marital status. Moreover, those who decreased in episodic memory ($HR = 1.50$) and executive functioning ($HR = 1.14$) had a significantly elevated risk of dying. Age moderation analyses were not significant, suggesting that declines in these cognitive abilities universally predicted an increased risk of death for younger and older adults. Overall, reductions in both episodic memory and executive functioning were linked to greater mortality risk across adulthood, regardless of age. Monitoring cognitive change over time could aid in identifying individuals with higher mortality risk, in hopes to create interventions to potentially increase longevity.

Abstract citation ID: igaf122.3816

### NEUROEXERGAMING FOR MCI: COIN-PLAY IN THE INTERACTIVE PHYSICAL AND COGNITIVE EXERCISE STUDY (IPACES)

Winry Devenport[1], Valerie Needham[2], Paul Arciero[2], and Cay Anderson-Hanley[1], *1. Union College, Schenectady, New York, United States, 2. iPACES LLC, Clifton Park, New York, United States*

Older adults face challenges to maintaining cognitive function and a paramount goal is stemming decline into Alzheimer's Disease and related dementias (ADRDs). Physical exercise is a known protectant and may even slow changes (Kramer et al., 2018). Combining physical activity with mental exercise seems to further enhance benefits, as in a pedal-n-play neuro-exergame: iPACES (an interactive physical and cognitive system; Anderson-Hanley et al., 2018). Research continues to explore how to tailor such interventions to maximize benefits to specific brain functions. Herein, the impact of coin collection on task performance was examined. This gaming feature was added to increase interest in a structured mental exercise program for those with mild cognitive impairment (MCI). As part of a clinical trial, 80 households were enrolled in iPACES; both an MCI patient and a normative caregiver (CG) were followed for a year to examine the impact of pedal-n-play. Households were provided an under-table pedaler and a tablet to play iPACES multiple times per week. Over 3,000 rides were recorded and analyses revealed a significant interaction effect between MCI vs. CG, and coins on vs. off ($p < .001$). Those with MCI performed worse when collecting coins vs. not, while CGs performed better. Thus, coin collection may engage attention for normative persons, while interfering with performance for MCI. Future research could examine possible causality. The implications of this finding for the current refinement of iPACES and other prescriptive digital therapeutics (PDTx) suggests the importance of intentional game design and prescription according to cognitive abilities.

Abstract citation ID: igaf122.3817

### NORADRENERGIC LOCUS COERULEUS HYPERACTIVITY ALTERS ATTENTION IN COGNITIVELY HEALTHY OLDER ADULTS

Joshua Senior[1], Andy Kim[2], Santiago Morales[3], and Mara Mather[2], *1. Massachusetts General Hospital, Harvard Medical School, Boston, Massachusetts, United States, 2. Leonard Davis School of Gerontology, University of Southern California, Los Angeles, California, United States, 3. University of Southern California, Los Angeles, California, United States*

Animal models of Alzheimer's disease have shown that neuropathology in the locus coeruleus (LC) leads to increased neuronal firing, while human studies demonstrate that age-related dysregulation of the LC-noradrenaline (LC-NA) system is associated with cognitive decline. However, due to limitations in directly measuring LC function in vivo, it remains unclear whether age-related alterations in humans reflect tonic LC-NA system hyperactivity. In this study, we tested the hypothesis that cognitively healthy older adults sustain tonic LC hyperactivity, by acquiring electrophysiological, pupillometric, and behavioral measures during a passive and active auditory oddball paradigm. We capitalized on the LC-NA system's role in arousal regulation and manipulated state arousal using the threat of electric shock. We hypothesized that if older adults maintain elevated LC activity compared with young adults, task-evoked noradrenergic responses would be less responsive to arousal in older adults. Consistent with this hypothesis, arousal elicited weaker behavioral responses, pupil dilation responses, and P300 event-related potentials in older adults compared with young adults. Linear mixed models revealed an arousal by modality interaction and revealed that arousal differentially modulated attentional control to salient but task-irrelevant distractors between both age groups. Collectively, these findings support the hypothesis that aging is associated with tonic LC-NA system hyperactivity in humans, with downstream consequences for mechanisms of attentional control. These multimodal findings underscore the potential of non-invasive physiological markers to assess LC-NA system function throughout aging and identify individuals at elevated risk for neurodegenerative progression prior to the emergence of clinical biomarkers.

Abstract citation ID: igaf122.3818

### NOT ONE STORY: A SCOPING REVIEW OF ETHNIC AND CULTURAL FACTORS IN DEMENTIA AMONG ASIAN AMERICANS

Soohyun Park[1], Sylvia Wang[2], Arianna Patel[1], Eilis Reardon[1], Grace Jung[1], Anthony Mark[3], and Ashley Paro[1], *1. Tufts Medical Center, Boston, Massachusetts, United States, 2. Widener University, Chester, Pennsylvania, United States, 3. Tufts University School of Medicine, Boston, Massachusetts, United States*

Asian Americans (AA) represent a rapidly growing and highly diverse population, yet dementia research often aggregates them into a single category. This practice limits culturally competent understanding and care for dementia in AA. This scoping review examines dementia terminology, risk and protective factors, cultural beliefs, caregiving practices, and cognitive screening tools across East AA (Chinese/Taiwanese, Japanese, Korean), South AA (Bangladeshi, Bhutanese, Indian, Maldivian, Nepalese, Pakistani, Sri Lankan), and Southeast AA (Burmese, Cambodian, Filipino, Hmong, Indonesian, Laotian, Malaysian, Thai, Vietnamese). Peer-reviewed articles published in English between 2000 and 2025 were identified through multiple databases, with findings synthesized narratively. Most studies focused on East AA, while South and Southeast AA remain markedly underrepresented. Across AA, terminology for dementia frequently carries negative connotations, though some East AA communities have begun adopting less stigmatizing language. Limited dementia literacy, misconceptions, and stigma are widespread, contributing to underdiagnosis and inadequate treatment. Caregiving is strongly shaped by familial and religious expectations, which often discourage institutional care and may result in social isolation, caregiver burden, and ethical dilemmas. Most validated cognitive screening tools are available for East AA. For South and Southeast Asian groups, only a few tools exist in their countries of origin, and some subgroups have no standardized assessments. This review provides a comprehensive summary of cultural nuances influencing dementia care and highlights critical gaps, particularly for South and Southeast AA. Future research should prioritize ethnically disaggregated data, culturally/linguistically tailored screening instruments, and partnerships with community and faith-based organizations to promote equitable dementia care for AA.

Abstract citation ID: igaf122.3819

### NOVEL VARIANTS AFFECTING PLASMA INTERLEUKIN-6 AMONG A HEALTHY AGING POPULATION: THE LONG LIFE FAMILY STUDY

Mary Wojczynski[1], Lihua Wang[2], Bharat Thyagarajan[3], Kaare Christensen[4], Joseph Zmuda[5], Alexander Kulminski[6], and Michael Province[2], *1. Washington University School of Medicine, St. Louis, MO, Missouri, United States, 2. Washington University School of Medicine, Saint Louis, Missouri, United States, 3. University of Minnesota, Minneapolis, Minnesota, United States, 4. University of Southern Denmark, Odense M, Syddanmark, Denmark, 5. University of Pittsburgh, Pittsburgh, Pennsylvania, United States, 6. DUKE University, Durham, North Carolina, United States*

Aging is associated with chronic low-grade inflammation. Interleukin-6 (IL-6) is a pro-inflammatory cytokine, linked to many clinical conditions, and a marker of chronic inflammation. IL-6 increases with normal aging, and higher levels of IL-6 in older individuals have been associated with physical and cognitive impairment, as well as increased dementia risk. IL-6 may be a pathway to reduce chronic low-grade inflammation. We investigated if there were regions of the genome harboring protective IL-6 variants. We used data from the NIA Long Life Family Study (LLFS, N = 4,084), a multi-national, multigenerational study of healthy aging. Participants underwent a comprehensive in-home examination at baseline, including a blood draw. Plasma IL-6 (pIL-6) concentration and whole genome sequencing (WGS) was performed using blood samples. We completed a linkage analysis for pIL-6 (covariate adjusted) using SOLAR followed by association of WGS variants under the peak. Chromosome 22 at 8cM (21,830,838 base pairs) had a LOD of 2.77. A subset of 20 families, demonstrated a heterogeneity LOD score of 9.54. Association on these 20 families found eight variants with suggestive association ($p < 1E-04$), half protective. Via model selection, 5 variants (3 protective and 2 risk) remained in the model, collectively explaining 20% of the variation in pIL-6, and explaining 41.7% of the linkage. One variant was associated with an RNA transcript for the IGLV3-25 gene ($p = 8.03E-09$), which is a critical gene for the formation of functional antibodies produced by plasma cells. Further analyses of the association of these novel variants with healthy aging and related phenotypes is warranted.

Abstract citation ID: igaf122.3820

### NURSING RESOURCES AS MEDIATORS OF COVID-19 MORTALITY IN HIGH MEDICAID-SERVING HOSPITALS

Sherif Olanrewaju, Ann Kutney-Lee, Daniela Gollinelli, and Margo Brooks Carthon, *University of Pennsylvania, Philadelphia, Pennsylvania, United States*

The COVID-19 pandemic exposed profound disparities within the United States' healthcare system, notably highlighting significant variations in mortality outcomes across different patient groups, particularly among older adults and those from economically disadvantaged populations. Higher rates of mortality among patients hospitalized with COVID-19 were noted in hospitals that cared for larger proportions of Medicaid-insured patients. While some have attributed these differences to hospital and patient characteristics, a potential factor that has been largely underexplored is whether nursing resources, including staffing levels and the quality of the work environment, were associated with the observed disparities in outcomes. Using an innovative hospital benchmarking methodology, this cross-sectional study examined whether nurse staffing and the work environment acted as mediators of the relationship between high Medicaid-serving hospitals and COVID-19 mortality. Using linked nurse survey, administrative hospital, and patient discharge data from 2020, our study included 109,900 patients (mean age=63) hospitalized with COVID-19 in 118 hospitals located across New York. Findings from the hospital-level analysis suggested that nurse staffing and the nurse work environment act as partial mediators of the relationship between high Medicaid-serving status and COVID-19 mortality, with nurse staffing demonstrating the largest mediation effect (28%). Our findings suggest that bolstering nursing resources in hospitals that serve higher proportions of Medicaid-patients may be an important key to reducing disparities and planning for future public health emergencies. Insights gained from this research could significantly inform healthcare policy decisions and resource allocation strategies, particularly in hospitals serving high proportions of older adults insured by Medicaid.

Abstract citation ID: igaf122.3821

### OLDER ADULTS' EXPECTATIONS OF LIVING IN NURSING HOME DECLINED DURING THE PANDEMIC

Soohyun Kim, Yulya Truskinovsky, and Emily Wiemers, *Syracuse University, Syracuse, New York, United States*

The COVID-19 pandemic disproportionately affected nursing homes in the United States. Between March 2020 and January 2022, more than 200,000 nursing home residents died, accounting for roughly one-fourth of all U.S. COVID-19 deaths (Chidambaram, 2022). In addition, residents experienced extreme social isolation and widespread staffing shortages, contributing to physical and psychological decline (Abbassi, 2020), all widely documented in the media. Against this backdrop, we examine how the pandemic shaped community-dwelling older adults' perceptions of nursing homes. Using data from the Health and Retirement Study (HRS), we analyze how older adults' self-reported probability of moving to a nursing home within the next five years changed between 2016 and 2022. Our findings show that expectations of future nursing home use declined after the onset of the pandemic. These results are robust across alternative specifications and placebo tests. This study advances understanding of the pandemic's long-term health implications by highlighting how it shifted expectations about long-term care. Because expectations of nursing home use are predictive of actual utilization, our findings have important implications for long-term care planning. If demand for nursing homes falls faster than anticipated, this may accelerate facility closures while increasing pressure on both unpaid family caregivers and the paid home care workforce.

Abstract citation ID: igaf122.3822

### PATTERNS OF DEMENTIA AND SERIOUS MENTAL ILLNESS CO-OCCURRENCE AMONG NURSING HOME RESIDENTS: A LATENT CLASS ANALYSIS

Laura Block[1], Roger Brown[1], Donovan Maust[2], Megan Zuelsdorff[3], Andrea Gilmore-Bykovskyi[4], and Tonya Roberts[1], *1. University of Wisconsin-Madison, Madison, Wisconsin, United States, 2. University of Michigan Medical*

*School, Ann Arbor, Michigan, United States, 3. University of Wisconsin Madison, Madison, Wisconsin, United States, 4. University of Wisconsin-Madison School of Medicine and Public Health, Madison, Wisconsin, United States*

Over half of all nursing home (NH) residents live with Alzheimer's disease and Alzheimer's disease related dementias (AD/ADRD), and a third with serious mental illness (SMI). AD/ADRD and SMI frequently co-occur among older adults, with research suggesting co-occurrence associates with heightened symptom burden and poor outcomes. Despite high rates of AD/ADRD and SMI among NH residents, patterns of AD/ADRD and SMI co-occurrence among this population and related resident-level characteristics have not been robustly examined. This study conducted a cross-sectional analysis of NH resident quarterly assessment data from the Minimum Data Set (MDS) 3.0 FY2019, utilizing unconditional latent class analysis (LCA) to examine patterns of AD/ADRD and SMI co-occurrence. Among a sample of 227,651 residents with AD/ADRD and/or SMI, a six-class model provided the best-fitting, clinically relevant solution. Classes were labeled according to most probable diagnoses: Predominantly Depression (n = 74,298, 32.6%), Predominantly Dementia (n = 53,667, 23.6%), Anxiety-Depression-Dementia, (n = 68,854, 30.2%) Mixed Diagnoses with Dementia (n = 12,945, 5.7%), Mixed Diagnoses (n = 13,517; 5.9%), and Predominantly Schizophrenia (n = 4,370, 1.9%). Classes differed significantly across clinical characteristics; for example, residents in the Anxiety-Depression-Dementia class had higher rates of pain while residents in the Mixed Diagnoses with Dementia, Mixed Diagnoses, and Predominantly Schizophrenia classes had the highest rates of behavioral symptoms. A more nuanced understanding of AD/ADRD and SMI co-occurrence among NH residents and class differences suggest diagnostic case-mix and resident-level care needs can together inform resource allocation and care delivery. Future work incorporating additional data sources and longitudinal approaches to explore relationships between symptoms, care measures, and outcomes is merited.

Abstract citation ID: igaf122.3823

### PERCEIVED HEALTHCARE DISCRIMINATION AND BARRIERS TO CARE FOR OLDER ADULTS LIVING WITH TRAUMATIC BRAIN INJURY

Yalian Pei, and Haowei Wang, *Syracuse University, Syracuse, New York, United States*

Traumatic brain injury (TBI) is a leading cause of morbidity and mortality in the aging population. Older adults living with TBI have higher risks of hospitalization and often need frequent healthcare. Despite these risks, limited research has examined the quality of healthcare and barriers to healthcare access among older adults living with TBI. Drawing on a large, nationally representative dataset from the All of Us Research Program, this study examined perceived discrimination in healthcare settings and its associations with healthcare utilization and delayed care among older adults with and without TBI. In the sample of N = 70,070 older adults aged 60 and above, 1,713 had a clinical diagnosis of TBI from Electronic Health Records. Respondents were evaluated on a 7-item scale of perceived healthcare discrimination and reported their healthcare utilization and delayed care in the last year. After adjusting for sociodemographic and health conditions, regression results show that compared to those who did not have TBI, older adults living with TBI were more likely to utilize healthcare (OR = 1.29, $p< .05$) but were not more likely to delay care (OR = 1.01, p=.883). Among older adults with TBI, perceived discrimination in healthcare settings was significantly associated with higher risks of delaying care (OR = 1.43, $p<.001$) but was not significantly associated with healthcare utilization (OR = 0.96, p=.227). These findings underscore the need to improve healthcare quality and reduce discriminatory experiences among older adults living with TBI.

Abstract citation ID: igaf122.3824

### PERCEIVED RELATIONSHIP BENEFITS OF CORESIDENT CAREGIVING IN PARENT-ADULT CHILD DEMENTIA CARE DYADS

Courtney Polenick, Kabreah Gunter, Sree Saripalli, Abbie Schoepke, Bhavana Rajesh, and Charity Garner, *University of Michigan, Ann Arbor, Michigan, United States*

The transition to coresident caregiving may be both challenging and rewarding for people living with dementia (PLWD) and the adult child care partners who support them. However, we currently know little about how this transition can impact the parent-adult child relationship from the perspectives of both PLWD and their adult child care partners. In this mixed methods study, we evaluated perceived relationship benefits or improvements following the transition to coresident caregiving among PLWD and their adult child care partners. Participants included 13 people living with dementia (PLWD; M = 84.38 years, SD = 8.30) and their adult child care partners (M = 55.85 years, SD = 5.86) who started coresiding with and supporting the PLWD within the past 5 years. Both care dyad members separately completed recorded phone interviews that included semi-structured questions about how sharing a home and caregiving has benefited or improved the parent-adult child relationship. We conducted qualitative content analysis to determine major themes in the transcribed semi-structured interview data at the dyad level. There were five major themes related to perceived relationship benefits or improvements within care dyads: 1) increased time spent together; 2) mutual exchanges of emotional and practical support; 3) caregiving rewards for both care partners and PLWD; 4) care partner empathy toward PLWD; and 5) resilience in maintaining a close and supportive pre-caregiving relationship. These findings inform targeted interventions to improve well-being among PLWD and their adult child care partners during the critical transition to coresident caregiving.

Abstract citation ID: igaf122.3825

### PERCEPTIONS AND EXPERIENCES OF ORAL HEALTH MANAGEMENT IN DEMENTIA: A QUALITATIVE SYSTEMATIC REVIEW

Jia Yin Ruan[1], Jin Su[1], Xiang Qi[1], Weiyu Mao[2], Yuning Xie[3], Junwen Yu[4], Jing Wang[5], and Bei Wu[6], *1. New York University, New York, New York, United States, 2. University of Nevada, Reno, Reno, Nevada, United States, 3. Renmin University of China, Beijing, China (People's Republic), 4. Fudan University, Shanghai, China (People's Republic), 5. University of New Hampshire, Durham, New Hampshire, United States, 6. NYU Shanghai, Shanghai, China (People's Republic)*

People with dementia often experience oral health problems, and challenges have been identified in managing oral health within this population. Although qualitative studies have explored these challenges from the perspectives of patients, caregivers, and healthcare professionals, there remains a lack of studies that synthesize perceptions and experiences of oral health management from multiple perspectives across diverse contexts. This study aimed to address this gap through a qualitative systematic review. A comprehensive literature search was conducted across eleven databases, covering their inception through July 2025, to identify relevant qualitative studies. Gray literature search was conducted using OpenGrey, Google, and Google Scholar. Hand searches of the reference lists of the included studies were conducted. The Critical Appraisal Skills Programme checklist for qualitative studies was applied to measure the quality of the studies. Data were extracted and analyzed using thematic synthesis. The degree of confidence in the findings was evaluated using the Grading of Recommendations Assessment, Development, and Evaluation Confidence in Evidence approach. A total of 15 studies were included, and three themes were identified: (1) realizing the importance of oral health in dementia as an evolving process, (2) acknowledging the distinct needs of oral health management in dementia, and (3) developing oral health management in dementia across multiple domains. This study reveals stages in dementia oral health management and highlights its unique needs. Future studies should address these unmet oral health needs and explore how caregivers and healthcare professionals can more effectively support oral health management in both clinical practice and research.

Abstract citation ID: igaf122.3826

### PILOTING A VIRTUAL GRADUATE LEVEL COURSE IN TRANSLATIONAL GEROSCIENCE AND PRECISION GERONTOLOGY

Iman Al-Naggar[1], Ellis Dillon[2], Christine Thatcher[1], Richard Fortinsky[2], David Steffens[2], and George Kuchel[3], *1. UConn Health, Farmington, Connecticut, United States, 2. University of Connecticut, Farmington, Connecticut, United States, 3. UCONN HEALTH, Farmington, Connecticut, United States*

Translational Geroscience is a rapidly growing field of research that seeks to prevent, delay, or reverse multiple chronic diseases of aging simultaneously, thereby increasing the human healthspan, or the years of life free of disease and disability. It aims to do so by targeting the biology of aging, which contributes to loss of resilience and disease onset. Research in Translational Geroscience requires a unique set of skills and has its challenges. Engaging in Translational Geroscience also requires knowledge about issues specific to older adults that are not traditionally covered in clinical and translational research educational programs. As interest in Translational Geroscience grows among clinicians and scientists, the need to train these professionals with various backgrounds has become very clear. To fill this gap, the UConn Older Americans Independence "Pepper" Center (P30AG067988) piloted a virtual, foundational, graduate-level course in Translational Geroscience and Precision Gerontology that was attended by approximately 20 trainees at varying training levels engaged or planning to engage in geroscience research. "Scientific Foundations of Translational Geroscience and Precision Gerontology" was a 34-hour course, divided over 2 semesters (Fall 2023 and Spring 2024) and offered in the late afternoons in 2-hour weekly sessions. Each session was presented by a topic expert who lectured for 40 minutes and took questions for 15 minutes. Two papers per topic, including one comprehensive review, were shared with trainees in advance to read. The course was highly rated by trainees who were engaged and interested, despite difficulties related to the virtual platform.

Abstract citation ID: igaf122.3827

### POST TRAUMATIC GROWTH: A NOVEL FRAMEWORK TO STRENGTHEN RESILIENCE AND HEART FAILURE SELF-CARE

Rebecca Meraz[1], and Jocelyn McGee[2], *1. Baylor University, Dallas, Texas, United States, 2. Baylor University, Waco, Texas, United States*

Despite the health benefits of heart failure (HF) self-care, more than 50% of patients are nonadherent to self-care practices. Many individuals struggle due to psychological distress, burden, and diminished resilience. Traditional interventions often neglect enhancing positive psychological growth as a strategy for improving self-care adherence. Using a case study methodology, we examined the experiences of two individuals participating in a resilience-based intervention to improve HF self-care to demonstrate the applicability of the PTG framework to future intervention design. The pilot intervention, StruggleStrong, was a structured 6-week, telehealth, program designed to bolster key aspects of resilience, including personal strengths, optimism, meaning in life, gratitude, and deeper relational connections. Our findings illustrated that participants' experiences aligned seamlessly with the PTG framework. Growth in personal strength empowered participants to view HF self-care challenges as opportunities to apply their own capability. Leaning on faith and personal values helped participants transform the adversity of adhering to stressful self-care tasks into a deepened sense of meaning and purpose. Gratitude and appreciation in life were important to fostering positive psychological growth and resilience. Gratitude was also significant to relational growth, fostering a greater appreciation for the caregivers, doctors, and friends who were resources for navigating burdensome self-care tasks. A greater sense of confidence, purpose, and gratitude led to new possibilities and self-care goals. The PTG framework could be a novel framework for strengthening resilience and improving HF self-care.

Abstract citation ID: igaf122.3828

### PREDICTING RE-INSTITUTIONALIZATION IN A SAMPLE OF OLDER ADULTS WITH SEVERE MENTAL ILLNESS

Deborah Finkel[1], Gavin Green[2], Marie Ernsth-Bravell[3], Pia Bülow[4], and Per Bülow[4], *1. University of Southern California, Los Angeles, California, United States, 2. Utah State University, Logan, Utah, United States, 3. Jönköping University, Jönköping, Jonkopings Lan, Sweden, 4. School of Health and Welfare, Jönköping, Jonkopings Lan, Sweden*

After the Swedish Mental Health Care reform enacted in 1995, many individuals with severe mental illnesses (SMI) were released from long-term care institutions. Many older adults with SMI experienced most of their care during an era of care ideology characterized as that of the total institution, which meant longer periods of institutionalization. Managing new living and support situations challenged many older adults with SMI and as a result, many experienced re-institutionalization. The aim of the current analyses was to identify the older adults with SMI who were re-institutionalized after the 1995 care reforms. We used data from surveys collected in 1996, 2001, 2006, and 2011 from 441 older adults with SMI in a region in southern Sweden (mean age in 1996 = 61.2, SD = 11.4; 54.6% female), and data from national registers. The sample was divided into 3 groups: remained in their own home (N = 251), moved from their home to an institution (N = 37), and remained in an institution (N = 153). Logistic regression indicated that total years of institutionalization and current level of functioning distinguished all 3 groups. Compared with group 1, adults in group 2 were older, were younger at first admission to a mental hospital, had more total years of institutionalization, more needs assessed by the Camberwell Assessment of Needs, and less education. Results reveal the long-term consequences of lengthy institutionalization, including greater risk of developing “institutional syndrome” that can inhibit ability to adjust to living in the community, particularly for adults also experiencing the challenges of aging.

Abstract citation ID: igaf122.3829

### PREDICTIVE MODEL FOR MORTALITY RISK STRATIFICATION USING LABORATORY BIOMARKERS IN SINGAPOREANS

Xinru Lim[1], Jiangfeng Ye[2], Denise Goh[1], Jess Vo[3], Roger Ho[2], Min-Han Tan[3], and Joe Yeong[1], *1. Institute of Molecular and Cell Biology, Singapore, Singapore, 2. National University of Singapore, Singapore, Singapore, 3. Lucence Diagnostics Pte. Ltd., Singapore, Singapore*

Current clinical decision-making tools often rely on aggregate scoring systems or subjective assessments, which may not capture early or subtle physiological changes indicating high mortality risk. While laboratory tests are ubiquitously available and inexpensive, their prognostic potential is underutilized. There is a need for an evidence-based, quantitative tool that can harness the predictive power of routine laboratory data to improve patient outcomes by identifying high-risk individuals earlier and more accurately. With two independent cohorts of older Asians, aged ≥55 years, from the Singapore Longitudinal Ageing Studies, we investigated their basic clinical laboratory biomarkers (i.e. full blood count and biochemistry variables) and its relation to all-cause mortality. Based on risk score derived from a Cox model trained to predict mortality risk, we identified a group of individuals with significantly shorter overall survival ($P < 0.01$), using 17 years of follow-up data. Subsequent Cox Regression analysis demonstrated robust predictive performance for 5-year and 10-year mortality. This result was also validated in the National Health and Nutrition Examination Survey cohort, achieving a concordance index of 0.79 and 0.78, respectively (for aged ≥40 years). Furthermore, we revealed that the biological age was significantly elevated in deceased individuals compared to their chronological age ($P < 0.0001$), while surviving individuals exhibited a younger biological age relative to their chronological age ($P < 0.0001$). Consequently, we developed a predictive model that utilizes routinely analyzed laboratory biomarkers-including components of the immune cell count and other clinical variables-to assess and stratify mortality risk, capable of identifying high-risk individuals earlier and more accurately.

Abstract citation ID: igaf122.3830

### PROFILING COGNITIVE RESERVE AMONG COMMUNITY-DWELLING OLDER ADULTS: A LATENT CLASS ANALYSIS

Wanrui Wei[1], Kairong Wang[2], Shuaifang Wei[3], Huan Zhang[3], Gabriella Engstrom[4], Azita Emami[1], and Zheng Li[3], *1. Yale University, Orange, Connecticut, United States, 2. Peking Union Medical College Hospital, Beijing, Beijing, China (People’s Republic), 3. Chinese Academy of Medical Sciences & Peking Union Medical College, Beijing, Beijing, China (People’s Republic), 4. Florida Atlantic University, Boca Raton, Florida, United States*

Cognitive reserve (CR) is a multidimensional construct shaped by education, occupation, and leisure activities, yet traditional scoring approaches obscure heterogeneity among older adults. This study applied latent class analysis (LCA) to identify distinct CR profiles and examine their clinical correlates. A cross-sectional sample of 426 community-dwelling older adults aged ≥65 years in Beijing, China, was assessed using the Cognitive Reserve Index questionnaire (CRIq). Two CR profiles emerged: Class 1, characterized by low reading and leisure engagement (51.9%), and Class 2, defined by higher educational attainment and cognitively demanding occupations (48.1%). Compared with Class 1, Class 2 participants had higher Montreal Cognitive Assessment scores (26 vs. 25, $p < 0.001$), fewer subjective cognitive decline symptoms (3.5 vs. 4.5, $p < 0.001$), and lower frailty burden (Fried phenotype score: 0 vs. 1, $p = 0.006$). Class 2 membership was also associated with better chewing ability ($p = 0.004$), higher household income ($p < 0.001$), and greater likelihood of being male ($p = 0.007$). Age was inversely associated with Class 2 membership ($\beta = -0.060$, $p = 0.024$). These findings reveal meaningful heterogeneity in CR within aging populations and highlight the importance of stratifying older adults by CR profiles. Identifying subgroups at risk for cognitive decline and frailty provides new opportunities for targeted interventions to promote resilience for aging.

Abstract citation ID: igaf122.3831

### PROGRAM STAFF PERSPECTIVES ON VIRTUAL AND HYBRID VOLUNTEERING IN SENIOR COMPANION AND FOSTER GRANDPARENTS PROGRAMS

Cynthia Cushing[1], Jennifer Crittenden[2], Rachel Coleman[2], and Nathan Tarbox[1], *1. University of Maine, Orono, Maine, United States, 2. University of Maine Center on Aging, Orono, Maine, United States*

Volunteering has been linked to improved health and well-being, increased socialization, and reduction of social isolation among older adults. During the COVID-19 pandemic, virtual volunteering was introduced to existing volunteer programs as a strategy to retain volunteers while staying physically distanced. Semi-structured Zoom-based interviews were conducted with 18 program directors across the U.S. within two predominantly in-person volunteer programs, the Senior Companion Program (SCP) and Foster Grandparent Program (FGP). Interviewees ($N = 18$) included eight SCP, eight FGP, and two representing both programs. Qualitative analysis yielded 16 parent codes within the data. Findings suggest COVID-19 was an impetus for increased VV use in volunteer programs. Post-pandemic, the study found the majority of volunteering has returned to in-person activities, with increased technology adoption for administrative functions with volunteers. Interviewees reported that VV has predominantly occurred on a case-by-case basis for individual volunteers that prefer this modality. Program directors expressed mixed views on VV and technology use with older adult volunteers, with some optimistic and open to future opportunities. Technology access, digital literacy, negative attitudes towards technology, lack of funding, staffing, training capacity, and administrative barriers were found to be challenges to implementing and maintaining VV over the long-term.

Abstract citation ID: igaf122.3832

### PROMOTING SMARTPHONE USAGE AMONG OLDER ADULTS: THE ROLE OF PERCEIVED EMOTIONAL VALUE

Suyi Wang[1], and Minjie Lu[2], *1. Beijing Normal University, Zhuhai, Guangdong, China (People's Republic), 2. Beijing Normal University at Zhuhai, Zhuhai, Guangdong, China (People's Republic)*

As society ages and the digital era rapidly evolves, older adults often face challenges in adapting to new technologies. Socioemotional Selectivity Theory (Carstensen et al., 1999) posited that with age, individuals tend to prioritize emotional motivation over instrumental motivation. According to Perceived Value Theory (Zeithaml, 1988), consumers may weigh the value of a product differently, and this could predict purchase decisions (Sweeney & Soutar, 2001). This study examined whether older adults' prioritization of emotional motivation may make them weigh emotional values (enjoyment or social meanings) of using smartphone over functional values (utilitarian performance). In study 1, 195 participants aged 18 - 80 (108 females, Mage = 45.90, SD = 14.38) reported their social motivations and perceived values of smartphone applications. Results showed that older participants placed greater emphasis on the emotional value of smartphone apps, and this age-related change was mediated by the age-related increase in emotional motivation. Study 2 manipulated the perceived value of smartphone apps through advertising slogans, and 60 younger adults (40 females, Mage = 26.55, SD = 2.87) and 53 older adults (15 females, Mage = 61.20, SD = 2.12) were recruited. Results showed that older adults preferred choosing emotionally appealing apps over functionally appealing apps, but younger adults didn't show this preference. These findings have practical implications that highlighting the emotional values of smartphone may help promote smartphones usage among older adults. (This project was funded by the National Natural Science Foundation of China, Grant No. 72201037).

Abstract citation ID: igaf122.3833

### PROTEOMIC SIGNATURE OF FRAILTY PACE REFLECTS BIOLOGICAL AGING AND PREDICTS DISEASE AND MORTALITY

Jiachen Chen[1], Sandhya Iyer[2], Margaret Doyle[3], Joanne Murabito[4], and Kathryn Lunetta[1], *1. Boston University School of Public Health, Boston, Massachusetts, United States, 2. Boston University School of Medicine, Boston, Massachusetts, United States, 3. University of Vermont, Colchester, Vermont, United States, 4. Boston University Chobanian & Avedisian School of Medicine and Boston Medical Center, Boston, Massachusetts, United States*

Frailty is a clinical manifestation of aging characterized by reduced physiological resilience and increased vulnerability to adverse outcomes. Plasma proteomics offers potential for tracking biological aging, yet few studies have used it to quantify frailty progression over time. We developed a proteomic signature representing the pace of frailty, derived from longitudinal trajectories of weight, grip strength, and gait speed in the Framingham Heart Study (FHS) Offspring cohort (N = 2,078, mean age 67 (SD = 9), 55% female). Using elastic net regression on 2,799 plasma proteins measured by the OLINK platform, we constructed a 112-protein signature spanning pathways related to muscle contraction, extracellular matrix interactions, homeostasis, immune responses and inflammation, metabolism, and neuronal system development. We validated the signature in two independent cohorts with plasma proteins measured by OLINK: the FHS Generation 3 (N = 3,130, mean age 46 (SD = 9), 53% female) and UK Biobank (N = 44,949, mean age 57 (SD = 8), 54% female). The signature was informative across adulthood and significantly associated (FDR ≤ 0.05) with the incidence of 19 major chronic diseases, including cardiometabolic, renal, pulmonary, hepatic, anemia, neurodegenerative, cerebrovascular, musculoskeletal, and oncologic conditions, as well as with all-cause mortality and age-related phenotypes such as telomere length, frailty index, blood pressure, lung function, reaction time and fluid intelligence score in UK Biobank, independent of age and other relevant covariates. Our findings demonstrate that the proteomic pace of frailty captures multiple biological processes underlying physiological decline and may serve as a prognostic biomarker of biological aging.

Abstract citation ID: igaf122.3834

### PSYCHOLOGICAL RESILIENCE AS A MODERATOR OF THE DISABILITY–MENTAL HEALTH ASSOCIATION IN OLDER ADULTS

Sarah Sunghye Kang[1], Sehyun Baek[2], Oejin Shin[3], Soobin Park[4], Sojung Park[4], and BoRin Kim[5], *1. University of California, Los Angeles, Los Angeles, California, United States, 2. New York University, New York, New York, United States, 3. Illinois State University, Normal, Illinois, United States, 4. Washington University in St. Louis, St. Louis, Missouri, United States, 5. University of New Hampshire, University of New Hampshire, New Hampshire, United States*

Later life is often characterized by disruptive events such as the onset of disability, which can diminish one's sense of control and purpose. Declines in functional capacity and mobility introduce new stressors that are closely tied to poorer mental health among older adults. Although recent aging research has highlighted the protective role of psychological resilience in responding to major stressors, its role in mitigating symptoms of depression and anxiety among older adults with disabilities remains understudied. Using data from Round 13 of the National Health and Aging Trends Study (n = 7,373), we examined whether psychological resilience moderates the association between physical disability and symptoms of depression and anxiety. Multiple linear regression analyses revealed that greater disability status was significantly associated with higher levels of both depression and anxiety ($b = 0.880$, $p < .001$). In contrast, higher psychological resilience was strongly associated with lower levels depression and anxiety ($b = -0.131$, $p < .001$). A significant interaction between disability and psychological resilience was identified ($b = -0.037$, $p < .001$), indicating a moderation effect. The protective role of psychological resilience on depression and anxiety became more pronounced with increasing disability status. Identifying psychological resilience as a buffer for mental health issues has the potential to inform interventions in aging populations. Moving beyond a deficit-based perspective, these findings support the development of approaches that enhance adaptive capacities and psychological well-being among older adults living with disabilities.

Abstract citation ID: igaf122.3835

### QUANTIFYING DELIVERY OF EXERCISE: BEYOND ATTENDANCE

Valerie Shuman[1], Jessie VanSwearingen[2], Subashan Perera[2], Janet Freburger[2], Kathleen Mangione[3], and Jennifer Brach[2], *1. University of Pittsburgh, PITTSBURGH, Pennsylvania, United States, 2. University of Pittsburgh, Pittsburgh, Pennsylvania, United States, 3. Arcadia University, Glenside, Pennsylvania, United States*

Most studies assessing the effectiveness of multi-component exercise programs in older adults report attendance as a proxy for dose without accounting for the extent to which intervention components were completed as intended. We examined associations of component-specific doses of a 12-week, 24-session multi-component walking intervention (including endurance treadmill walking and hip extension/abduction strength training) with 12-week change in gait speed. Dose for each component was quantified as total volume, average intensity, and progression. Participants (N = 118) included community-dwelling older adults aged ≥65 years (77.4 ± 6.3) who reported walking difficulty and had a baseline gait speed between 0.6–1.2 m/s (1.1 +/- 0.2). They attended an average of 21.0 ± 3.7 sessions and walked on the treadmill between 1.0 – 3.4 mph on the treadmill. Strength load progressed by an average of 45.3 ± 38.5 and 29.1 ± 47.0 per session for hip extension and abduction, respectively. Attendance was not associated with improvement. However, higher average treadmill speed, faster progression in treadmill speed, and greater progression of hip strength training loads were each associated with greater gains in gait speed. For example, increasing the treadmill speed by 0.1 mph every 5 sessions was associated with a 0.2 m/s increase in gait speed. The findings illustrate the fallacy of using only attendance as a proxy for dose. A detailed assessment of dose received for each component of a multicomponent exercise program offers valuable insight into which components drive functional improvement. Such findings can inform better care delivery.

Abstract citation ID: igaf122.3836

### RATES OF NEW ADRD DIAGNOSIS WITHIN 1 YEAR OF ENTERING ASSISTED LIVING FACILITIES: GENERAL CARE VERSUS MEMORY CARE

Gauri Gadkari[1], Cassandra Hua[2], Jennifer Bunker[3], Kali Thomas[3], and Eric Jutkowitz[4], *1. Brown University, Providence, Rhode Island, United States, 2. University of Massachusetts Lowell, Lowell, Massachusetts, United States, 3. Johns Hopkins University, Baltimore, Maryland, United States, 4. University of Minnesota, Minneapolis, Minnesota, United States*

Assisted living (AL) is a growing option for long-term residential care among older adults. Some ALs hold specialized licenses to provide memory care (MC) and are better equipped to support residents experiencing cognitive decline, making them more likely to attract individuals anticipating such needs, regardless of their diagnosis status. Using Medicare claims, we identified beneficiaries who newly moved to ALs during 2020 and 2021 across 34 states with available MC licensure data. We examined rates of new Alzheimer's disease and related dementias (ADRD) diagnoses within a year from entry into AL. Residents with an existing ADRD diagnosis (no MC: 24%, MC: 27%) were excluded, resulting in a final cohort of 95,207 beneficiaries. We fit logistic regression models to examine whether having MC licensure was associated with higher probability of new ADRD diagnoses. Risks were derived from predictive margins after adjusting for demographics, co-morbidities, and prior health utilization. The adjusted risks showed on average 10.9% (95% CI: 10.6-11.1) of beneficiaries receive an ADRD diagnosis within a year of moving to an AL without MC compared to 11.7% (95% CI: 11.3-12.0) among those who move to AL with MC. The small difference was statistically significant ($p < 0.001$). These findings suggest that while individuals in ALs with MC are more likely to receive an ADRD diagnosis, the difference in rate of new diagnosis is small. Many ALs providing MC also offer general care and thus may have a sufficient population not at risk for ADRD.

Abstract citation ID: igaf122.3837

### RECRUITMENT OF UNDERREPRESENTED OLDER ADULTS IN MENTAL HEALTH RESEARCH: A SCOPING REVIEW

Maayra Butt, Vanessa Flores, Kimberly Cobos, and Brenna Renn, *University of Nevada, Las Vegas, Las Vegas, Nevada, United States*

Older adults, especially those identifying as multiracial or as racial or ethnic minorities, are drastically underrepresented in mental health research. Underrepresentation limits generalizability of study findings and hinders equitable mental health. This scoping review: 1) summarizes strategies to successfully recruit and retain diverse participants and 2) identifies recruitment barriers and proposed solutions. PubMed, PsycInfo, and Embase were searched from inception to March 2025. Eligible studies included Census-defined racial and ethnic minority middle-aged and older adults; focused on recruitment and retention; and addressed late-life affective disorders (depression, anxiety, and PTSD). Two independent raters assessed studies at each phase, with consensus adjudicated by the senior author. A total of 1091 papers were screened at the title/abstract stage and 325 papers were screened at the full text stage, yielding 35 papers for inclusion. Recruitment and retention strategies focused on partnering with community groups (i.e. faith based organizations), alleviating structural barriers (i.e. lack of transportation), and providing education regarding study purpose. Recruitment was sometimes hindered by mental health stigma, mistrust, eligibility requirements of a formal mental health diagnosis, and general difficulty enrolling racial and ethnic minority men. Papers underscored the importance of developing pre-recruitment community partnerships, involving community members on study design, and presenting information through discussion groups and at organizations serving minority populations. Effective recruitment of racially and ethnically diverse older adults into mental health research requires strategies that advance equity, accessibility, and community engagement. Future work should assess older adult perspectives and factors they consider when deciding to participate in research.

Abstract citation ID: igaf122.3838

### REMOTE PATIENT MONITORING: A CASE OF LOWER UTILIZATION, ENHANCED SELF-MANAGEMENT, AND IMPROVED QUALITY OF LIFE

David Picella[1], Valerie Smith[1], Ji Yoo[2], Sanggon Nam[1], Diane Chau[1], and Ashraf Memon[3], *1. Azusa Pacific University, Azusa, California, United States, 2. Kirk Kerkorian School of Medicine at UNLV, Las Vegas, Nevada, United States, 3. Hifinite Health, San Diego, California, United States*

Older adults with complex chronic cardiac conditions often face frequent hospitalizations, escalating costs, and diminished well-being. Remote patient monitoring (RPM) serves as a proactive tool to reduce costs in geriatric populations by enabling early intervention and self-management, though its adoption is limited by reimbursement and implementation barriers. This case study illustrates RPM's multifaceted impacts, such as cost savings, empowerment, and the patient perspective. An older adult with a history of cardiac valve disorder, arrhythmia, oxygen dependency, and extended hospice care underwent a transcatheter valve procedure at a tertiary care center. RPM was initiated post-procedure using a home-based system to track daily weight, blood pressure, and wellness ratings. Medication adjustments were guided by thresholds, with remote oversight prompting targeted interventions. A semi-structured interview was analyzed using Grok AI to thematically capture RPM's effects. Prior to RPM, the patient experienced multiple emergency room visits monthly due to symptom exacerbations, leading to high costs and significant life disruption. Post-implementation, no hospitalizations occurred for more than 8 months, as monitoring supported early detection and self-management skills. This fostered independence, reduced symptom burden, and improved emotional outlook, with the patient reporting greater control and optimism. As a proactive cost-reduction strategy, RPM benefited this older adult by minimizing utilization, building self-efficacy, and enhancing quality of life, aligning with innovative gerontological approaches. The case supports expanded access, simplified technologies, and policy reforms to broaden RPM's application in diverse geriatric populations. Future research should assess these outcomes in larger cohorts for equitable integration.

Abstract citation ID: igaf122.3839

**RESIDENTIAL SEGREGATION EXACERBATES THE EFFECTS OF CARDIOVASCULAR DISEASE ON COGNITION IN BLACK OLDER ADULTS**

Alexandra Clark[1], Ashley Chikkala[1], Abbey Hamlin[1], Adam Bush[1], Wassim Tarraf[2], Alexandra Weigand[3], Michael Marsiske[4], and Kelsey Thomas[5], *1. University of Texas at Austin, Austin, Texas, United States, 2. Wayne State University, Detroit, Michigan, United States, 3. University of California, San Francisco, San Francisco, California, United States, 4. University of Florida, Gainesville, Florida, United States, 5. University of California, San Diego, La Jolla, California, United States*

Black older adults experience higher rates of cardiovascular disease (CVD) compared to non-Hispanic Whites, and CVD is a known risk factor for dementia. Black older adults are also more likely to reside in racially segregated neighborhoods with limited resources and access to healthcare, which may exacerbate the negative effects of CVD on cognition. The present study examined the interactive effects of CVD and segregation on memory trajectories and 10-year incidence of mild cognitive impairment (MCI) among 662 Black older adults enrolled in the Advanced Cognitive Training for Independent and Vital Elderly study. Baseline residential segregation was assessed at tract and county levels using dissimilarity and interaction indices. Blood pressure measurements (systolic ¬– diastolic) were used to calculate pulse pressure as a proxy for CVD. Outcomes included memory composites for baseline, post-intervention, 1-, 2-, 3-, 5-, and 10-year follow-ups as well as 10-year incidence of MCI. Latent growth curve models, adjusted for demographic and health factors, revealed significant segregation x CVD interactions on average memory levels (Bs≥-.48, ps≤.02), but not rates of decline. In highly segregated counties, Black older adults with elevated CVD demonstrated worse memory than those with lower CVD; in less segregated counties, memory levels did not differ by CVD. Logistic regressions revealed a similar interactive effect on 10-year incidence of MCI (OR = 1.22, p=.04); in highly segregated counties, individuals with high CVD demonstrated higher odds of MCI compared to those with low CVD. Multi-domain interventions addressing CVD risk and structural inequality may reduce dementia disparities in Black older adults.

Abstract citation ID: igaf122.3840

**REVEALING WITHIN-AGENCY VARIATION IN MEDICARE HOME HEALTH QUALITY RATINGS**

Shekinah Fashaw-Walters[1], Olga Jarrín[2], and Anum Zafar[2], *1. University of Pennsylvania, Philadelphia, Pennsylvania, United States, 2. Rutgers, The State University of New Jersey, New Brunswick, New Jersey, United States*

The Centers for Medicare & Medicaid Services (CMS) publicly reports Home Health Agency (HHA) quality ratings using a composite 5-star system based on seven assessment- and claims-based quality indicators. These ratings are intended to guide consumer decision-making and encourage performance improvement among providers. However, because the ratings reflect agency-level averages, they may mask important variation in the quality of care delivered to different patient subgroups. This study examines within-agency variation in reported performance using national Medicare administrative, assessment, and claims data. We applied CMS's published star rating methodology to simulate overall and group-specific quality ratings and assess the reliability and informativeness of disaggregated quality ratings. Our findings show that agencies with average to high overall ratings (3–5 stars) can exhibit substantial variation in care outcomes across patient subgroups. This variability is obscured in the reported rating, limiting its utility for some consumers and stakeholders, typically those from marginalized groups. By identifying patterns of intra-agency variation, this analysis highlights opportunities to enhance the information provided by publicly reported quality measures to increase usability for all patient groups. These refinements could better equip consumers to make informed choices and support data-driven improvement efforts among providers. This work demonstrates the feasibility of generating more detailed quality information using existing administrative data sources and methods. These findings provide timely insights into how quality measurement tools can evolve to capture meaningful variation across different patient populations—enabling a clearer understanding of what works best, for whom, and under what conditions.

Abstract citation ID: igaf122.3841

**RISK FACTORS FOR FALLS IN ELDERLY PATIENTS WITH MODERATE-TO-SEVERE DEMENTIA.**

Huixian Li, Tianyi Luo, and Yanyan Wang, *West China Hospital, Sichuan University, Chengdu, Sichuan, China (People's Republic)*

Dementia is a progressive neurodegenerative disease that affects millions of older adults worldwide, leading to cognitive and functional decline and significantly increases the risk of falls. While physiological risk factors are well documented, evidence on environmental and behavioral contributors remains limited.

Using data from the Chengdu Long-term Care Insurance (LTCI) database (2018–2022), we conducted a cross-sectional study to evaluate factors influencing fall risk among older adults with moderate-to-severe dementia. We applied univariate and multivariate logistic regression analyses to examine the associations of residential environment, physical function, and neuropsychiatric symptoms with fall risk. A total of 5,806 participants were included, with a median age of 82.0 years (IQR, 76.0–87.0); 62.2% were female. Multivariate logistic regression analyses showed that participants living at home, compared with those in skilled nursing facilities, had a significantly higher risk of falls (adjusted odds ratio [aOR] = 2.15, 95% CI: 1.60–2.89). Other independent risk factors included aphasia (aOR = 2.11 [1.67–2.66]), dysphagia (aOR = 1.69 [1.32–2.17]), hallucinations/delusions (aOR = 1.30 [1.04–1.62]), genitourinary disorders (aOR = 1.36 [1.08–1.71]), and rheumatic autoimmune diseases (aOR = 1.31 [1.02–1.68]). Notably, participants with mental illness had a lower risk of falls (aOR = 0.53 [0.43–0.66]), which may reflect more intensive medical care and management in this population. Our findings indicate that fall risk assessment and prevention for individuals with moderate-to-severe dementia should systematically incorporate residential environment, neuropsychiatric symptoms, and functional impairments. Addressing these factors is essential for improving patient safety, ensuring high-quality medical services, and reducing caregiver burden.

Abstract citation ID: igaf122.3842

### SAFETY AND DIGNITY IN OLDER ADULT MENTAL HEALTHCARE: EMBEDDING GERONTOLOGICAL INNOVATION IN PRACTICE

Kristine Lee[1], and Katarina Friberg-Felsted[2], *1. University of Utah College of Nursing, Cedar Hills, Utah, United States, 2. University of Utah, Salt Lake City, Utah, United States*

The growing demographic of older adults calls for urgent considerations for age-friendly outpatient and community mental health services on their behalf. Rates of depression and anxiety are high in later life, yet few clinicians receive gerontological training. This lack of expertise undermines quality and safety. Although cognitive behavioral therapy (CBT) and reminiscence therapy are effective in older populations, they remain underutilized. Workforce shortages intensify these gaps: fewer than 4% of psychologists and 2% of social workers pursue advanced training in gerontology. The expansion of ketamine for treatment-resistant depression and anxiety illustrates the risks of innovation outpacing evidence. While FDA-approved for adults with depression under strict REMS monitoring, the scientific literature regarding ketamine use in older adults is minimal. Published data consist primarily of case reports and one small pilot study in mild cognitive impairment; there are no randomized controlled trials in Alzheimer's disease, dementia syndromes, or residential care populations. Known adverse effects—dissociation, hypertension, sedation, increased fall risk, and delirium are particularly concerning. In the absence of gerontological expertise, off-label use in outpatient or assisted-living settings risks compromising patient safety. This project proposes an evidence-based framework for outpatient mental health care in aging populations. Drawing on Erikson's psychosocial theory, Butler's life review model, and Pearlin's stress process model, the Gerontological Mental Health Framework integrates CBT, reminiscence therapy, psychoeducation, and caregiver support groups. Interdisciplinary staffing, including geropsychiatrists, gerontologists, social workers, and caregiver peer facilitators, ensures developmentally informed, team-based care. This scalable framework addresses geropsychology, offering strategies to strengthen outpatient mental health services.

Abstract citation ID: igaf122.3843

### SEX DIFFERENCES IN ARTHRITIS, PAIN, AND FRAILTY AMONG MEXICAN AMERICAN OLDER ADULTS

Jamil Okada[1], Soham Al Snih[2], and Marielle Rominger[3], *1. UTMB, Galveston, Texas, United States, 2. The University of Texas Medical Branch at Galveston, Texas, Galveston, Texas, United States, 3. Dell Medical School, Austin, Texas, United States*

Frailty is a significant public health concern in older adults because of its associated increased risk of falls, disability, cognitive decline, decreased quality of life, and mortality. This study investigated sex differences in the relationship between arthritis and pain with frailty over 21-years of follow-up among non-frail at baseline Mexican American older adults. Participants (N = 1632) were from the Hispanic Established Population for the Epidemiologic Study of the Elderly. Measures included socio-demographics and health characteristics. Independent variables were self-reported physician diagnosed arthritis and pain on weight bearing. Frailty (outcome variable) was defined as ≥ 3 criteria: unintentional weight loss of > 10 pounds, weakness, exhaustion, low physical activity, and slowness. Participants were divided into four groups: no arthritis-no pain, pain-no arthritis, arthritis-no pain, and arthritis-pain. Generalized estimating equation models were used to estimate the odds ratio (OR) and 95% confidence interval (95% CI) of frailty as a function of arthritis and pain over time by sex, controlling for covariates. Among male participants, those with pain only or arthritis and pain had greater (OR = 2.53, 95% CI = 1.50-4.25 and OR = 2.33, 95% CI = 1.06-5.12, respectively) odds of frailty over time than those without pain and without arthritis after controlling for covariates. Among female participants, those with pain only had greater (OR = 1.82, 95% CI = 1.15-2.88) odds of frailty over time than those without arthritis and without pain after controlling for covariates. Pain was a significantly associated with frailty over time in both males and females. However, arthritis and pain were significantly associated with frailty over time in male participants only.

Abstract citation ID: igaf122.3844

### SHARING FUTURE CONCERNS ABOUT DEMENTIA: DIFFERENCES IN CARE AND LOGISTICAL PLANNING CONCERNS ACROSS DYAD MEMBERS

Amanda Piechota[1], Emily Mroz[2], Yifan Lou[3], Thi Vu[4], Dustin Gad[5], and Joan Monin[6], *1. University of Alabama, Tuscaloosa, Alabama, United States, 2. Emory University, Atlanta, Georgia, United States, 3. Virginia Commonwealth University, Richmond, Virginia, United States, 4. Yale University, New Haven, Connecticut, United States, 5. Cornell University, Ithaca, New York, United States, 6. Yale, Yale, Connecticut, United States*

The progressive and degenerative symptoms of dementia cause families to develop disease-related problems or worries, or "future concerns." Adult children represent more than half of the primary caregivers to a person living with dementia (PLWD), but may have different future concerns than their parents, causing communication and planning disconnects as they become increasingly involved in care. In this study, we aimed to 1) compare the frequency of nominating concerns related to "care and logistical planning" across adult children and parents living with memory concerns or dementia, and 2) explore which sub-types of care and logistical planning concerns may be drivers of differences across dyad members. We analyzed videos of discussions about future concerns between 122 adult children and 108 parents who self-reported memory concerns and scored 12 or higher on the Mini Mental Status Examination. Participants were asked to share three future concerns with one another. Four coders established reliability (ICC = .92, kappa = .71-.83) to quantitatively content-analyze videos for future concerns. Paired samples t-test demonstrated adult children (M= .93/2.54) shared more care and logistical planning concerns than PLWDs (M=.47/2.17, p<.001). Results were driven by adult children's (M=.37/2.54), as compared to parents' (M=.10/2.17) frequency of sharing concerns related to planning for PLWDs' safety (p<.001), and adult children's (M=.25/2.54) as compared to parents' (M=.02/2.17) frequency of sharing concerns related to challenges with disease co-management (p<.001). Results inform development of resources to help parent- adult child dyads understand each other's concerns, make decisions, and plan for the future.

Abstract citation ID: igaf122.3845

### SOCIAL ISOLATION AND FRAILTY AMONG CHINESE OLDER ADULTS: ROLE OF DIETARY PATTERNS

Zhongfei Pei[1], and Ming Xu[2], *1. Peking University, Beijing, Beijing, China (People's Republic), 2. Peking University School of Public Health, Beijing, Beijing, China (People's Republic)*

Background: Frailty is a health problem that is extremely prone to arise with aging and is more prevalent in socially isolated older adults. The dietary pattern often serves as an important determinant of health in later life that cannot be ignored. Identifying the role that a specific dietary pattern plays in social isolation and frailty can provide insights to improve the quality of life of older adults. Methods: This cross-sectional study used data from the eighth wave of the Chinese Longitudinal Healthy Longevity Survey (CLHLS) conducted in 2018. A total of 8889 older adults (aged 60+) were included in our study. Frailty was assessed using FRAIL Scale. Social isolation was defined by marital status, living arrangement, frequent visit by family members, and social participation. Dietary patterns were explored by principal component analysis. The logistic regression was used to carry out the moderating effect analysis. Results: Four dietary patterns were identified, namely "vegetable-nut-mushroom or alga", "egg-milk", "meat-fish", and "pickle". Subjects with higher levels of social isolation were associated with a higher risk of frailty (OR = 2.31, 95%CI=2.17-2.46). We observed that the egg-milk pattern as a moderator alleviated this relationship (OR = 0.91, 95%CI=0.86-0.97). Conclusions: Our study suggested that social isolation was positively correlated with frailty and the egg-milk pattern is a moderator in this relationship. Improving the social isolation of older people was beneficial to prevent frailty. It is necessary to advocate the dietary pattern characterized by eggs and milk in socially isolated older adults to reduce the risk of frailty.

Abstract citation ID: igaf122.3846

### STRENGTHENING COMMUNITY BELONGING: SURVEY EVIDENCE FROM A CO-GENERATIONAL PILOT PROGRAM

Sofia Holmes[1]BoRin Kim[2], Kelley Petralia[3], Laura Cerniglia[3], and Tracy LaCroix[3], *1. University of New Hampshire, Durham, New Hampshire, United States, 2. University of New Hampshire, University of New Hampshire, New Hampshire, United States, 3. Westborough Connects, Westborough, Massachusetts, United States*

Intergenerational programs play a vital role in building inclusive communities by reducing isolation, challenging ageism, and fostering belonging across generations. Developed in collaboration with a local community organization, this study contributes to the limited quantitative evidence on their impacts. We examined pre- and post-program changes in ageism, perceived social cohesion, and social connection among participants in a community-based co-generational program. Eleven dyads of older adults (65+) and adolescents (14–17) completed structured activities weekly over six weeks, with pre- and post-program surveys administered. Paired-sample t-tests assessed within-group changes, and independent t-tests examined generational differences. The adolescent group (n = 11) was 73% female, 64% non-white, and over half had lived locally their entire lives. The older adult group (n = 11) was 64% female, all white, mean age 70.5; two-thirds had recently moved to town, while one-third had lived there 25+ years, and over half were single. Perceived social cohesion improved after the program (t(21)=1.743, p = 0.096), with a stronger effect among older adults. Pre-program, adolescents scored higher on the ageism scale than older adults (t(20)=-1.863, p=.077), but this gap narrowed and was no longer significant post-program (t(20)=0.746, p=.464). No significant changes were observed in social connection, though small positive trends emerged. Findings suggest that co-generational programs can enhance social cohesion and help reduce intergenerational biases. While limited by small sample size, this community–academic partnership provides preliminary evidence that can inform the development of future intergenerational initiatives, with particular potential to benefit vulnerable older adults at high risk of isolation.

Abstract citation ID: igaf122.3847

### SUBJECTIVE AND OBJECTIVE APPRAISALS OF SELF-RATED HEALTH

Abolade Oladimeji[1], Kristen Berg[2], Douglas Einstadter[2], and Adam Perzynski[2], *1. Samford University, Birmingham, Alabama, United States, 2. Case Western Reserve University, Cleveland, Ohio, United States*

Introduction: Positive self-perceptions of aging are linked to better self-rated health, we examined the impact of clinical diagnoses (e.g., sleep disorders and comorbidities) and subjective factors (e.g., sleep appraisal, social and physical limitations) on self-rated health. Methods: Our cohort consisted of 1,523 older adults (65+) with at least two outpatient primary care visits and one Medicare Annual Wellness Visit. We used an ordered logistic regression model to predict self-rated health, which was rated on a five-point scale from "poor" to "excellent." Results: The regression analysis showed that older adults with sleep difficulties (based on the PHQ-9 sleep item) had poorer self-rated health than those without any sleep difficulties. However, there was no significant difference between those with and without a diagnosed sleep disorder. This suggests that subjective sleep appraisal is associated with self-rated health, while sleep disorder diagnosis is not. We also found that higher social limitations were linked to poorer health, while increased physical activity was associated with better self-rated health. Higher comorbidity scores were also associated with poorer health outcomes. Also, African Americans reported lower self-rated health compared to Whites and others. Discussion: Our findings indicate that self-rated health is influenced by both subjective and objective factors, including perceived sleep difficulties, social and physical limitations, and physical health status (e.g., comorbidities). This study highlights the significance of physical functioning and social engagement in shaping perceived health among older adults

Abstract citation ID: igaf122.3848

### SYNERGISTIC MODULATION OF GTPASE HYDROLYSIS AND MTORC1 ACTIVITY IN LRRK2-G2019S PARKINSON'S DISEASE

Zainab Cheema, *"Bergen County Academies", Maywood, New Jersey, United States*

The LRRK2-G2019S mutation is implicated in the dysregulation of GTPase hydrolysis activity, which affects the mechanistic target of rapamycin complex 1 (mTORC1) signaling pathway in Parkinson's disease (PD). This study aims to investigate how this mutation influences GTPase activity and its downstream effects on cellular processes in an in-vitro model of Parkinson's Disease. It is hypothesized that treatment with Rapamycin, an mTORC1 inhibitor, in combination with Methyl 2-Hydroxybenzoate (Methyl Salicylate), a small molecule targeting GTPases, will restore GTPase hydrolysis activity, mitigate autophagic fluidity, regulate mTORC1 signaling, and reduce neurodegenerative features. Additionally, the treatment is expected to decrease reactive oxygen species (ROS) accumulation and apoptosis markers, promoting cell survival by preventing oxidative damage. Experimental techniques include MTS-based cell viability, Fluorescence-based GTPase activity, ROS-Glo, Caspase-Glo 9 and enzyme-linked immunosorbent assays (ELISA) analysis to assess changes in cell health, autophagic flux, and GTPase cycling. Genetic knockdown approaches combined with fluorescence microscopy will be used to visualize mTORC1 activity responses. Preliminary findings suggest that targeting GTPase dysfunction in mTORC1 signaling could offer novel therapeutic strategies for PD and broader neurodegenerative disease management, but further data analysis is required to validate these results.

Abstract citation ID: igaf122.3849

### SYSTEMS MAPPING OF THE COMPLEX EXPERIENCES OF FAMILY CAREGIVERS AND CARE RECIPIENTS WITH COGNITIVE IMPAIRMENT

Doyoung Kim[1], Eleanor Hummel[1], Samuel Baxter[1], Anna Kahkoska[1], Leah Frerichs[1], Erin Kent[1], Katherine Ornstein[2], and Kristen Hassmiller Lich[1], *1. University of North Carolina at Chapel Hill, Chapel Hill, North Carolina, United States, 2. Johns Hopkins University, Baltimore, Maryland, United States*

Emotional well-being of family caregivers and older adult care recipients often fluctuates over the illness trajectory. Both parties influence each other's emotional states within a complex web of interrelated factors, yet few studies have mapped the interconnected pathways underlying these dynamics. This study applied a participatory systems science approach to address this gap. Family caregivers of older adults (≥65 years) with dementia or other cognitive impairment residing in North Carolina participated in a four-hour group model building session involving drawing and explaining trend lines of emotional states and structured group discussions. Focus group data were analyzed using qualitative causal loop diagramming to depict factors, pathways, and feedback loops (i.e., a closed chain of causal connections) influencing emotional well-being over time. Between 12/2024 and 05/2025, ten sessions (n = 2–5 participants) were held with 29 unique caregivers (mean age 63.7 ± 11.9 years; 86.2% female; 72.4% White; 48.3% spousal; 62.1% caring for individuals at moderate-to-severe stages [FAST 5+]) of Alzheimer's disease and other forms of cognitive impairment. Multiple complex system dynamics themes emerged, describing pathways and feedback loops that both influence and are influenced by changes in the emotional well-being of caregivers and care recipients. These themes relate to: caregiver and care recipient characteristics; interpersonal and family dynamics; social and formal support systems; healthcare and long-term care settings; legal and employment contexts; and technology use. Findings provide a systems-level foundation for designing interventions that holistically address interconnected drivers of well-being.

Abstract citation ID: igaf122.3850

### TARGETING VASCULAR AGING IN PROGERIA BY MTOR INHIBITORS FROM MULTI-OMICS SCREENING

Hsin-Yu Fang[1], Helin Sera Şan[2], Fatma Akar[2], Sudenaz Fatma Oner[2], Ceren Şinikçi[2], Alexander Tyshkovskiy[3], Vadim Gladyshev[3], and Perinur Bozaykut Eker[1], *1. Tufts University, Boston, Massachusetts, United States, 2. Acıbadem Mehmet Ali Aydınlar University, Istanbul, Istanbul, Turkey, 3. Harvard Medical School, Boston, Massachusetts, United States*

Aging is a predominant risk factor for many chronic diseases, yet shared mechanisms underlying effective longevity interventions remain poorly understood. To address this, we established a cross-species, multi-omics discovery platform that integrates conserved gene expression signatures to identify novel compounds with lifespan and healthspan extending potential. Here, we investigated whether top-ranked mTOR inhibitors could mitigate accelerated vascular aging in progeria, a premature aging disorder that manifests with early-onset vascular pathology. Induced pluripotent stem cell-derived vascular smooth muscle cells (iPSC-VSMCs) from progeria patients were treated with selected mTOR inhibitors and comprehensively assessed for cellular and molecular biomarkers of vascular aging. Cellular assays demonstrated a significant change of senescence phenotype through SA-β-gal and EdU incorporation, together with decreased inflammatory signaling, without induction of apoptosis. Drug treatment also lowered intracellular reactive oxygen species (ROS), in both replicatively senescent cells and cells subjected to biomechanical stress using a progeria-on-a-chip vascular model, underscoring their robustness across distinct aging-related stress conditions. Furthermore, drug treatment reduced both epigenetic and transcriptomic age, with transcriptomic improvements most prominently reflected in extracellular matrix organization and inflammation-related modules. These findings highlight the therapeutic potential of novel mTOR inhibitors identified through multi-omics screening in attenuating vascular aging in progeria and suggest broader strategies for preventing aging-associated vascular disease.

Abstract citation ID: igaf122.3851

**TAU-INDUCED ACTIVATION OF THE DROSOPHILA RETROTRANSPOSON COPIA CAUSALLY MEDIATES NEURODEGENERATION**

Cynthia Schulz[1], Joanna Sohn[1], Peter M'Angale[2], Travis Thomson[2], and Bess Frost[1], *1. Brown University, Providence, Rhode Island, United States, 2. University of Massachusetts Chan Medical School, Worcester, Massachusetts, United States*

Tauopathies are a group of neurodegenerative disorders characterized by the deposition of hyperphosphorylated tau protein. Evidence across model systems of tauopathy and postmortem human brain tissue identify heterochromatin decondensation as a mechanistic driver of retrotransposon activation from dark regions of the genome, ultimately driving neuronal death. Since retrotransposons are genetic remnants of ancestral viral infections integrated into the genome, many retrotransposons can encode the same viral proteins that form mature virions. Recent findings suggests that the Drosophila retrotransposon Copia, which encodes retroviral-like proteins that can form capsids, mediates synaptic plasticity at the neuromuscular junction through transsynatptic capsid transfer. While we know that copia transcripts are elevated in tau transgenic Drosophila, it is unknown how Copia protein expression is disrupted or whether Copia dysregulation mediates tau-induced neurotoxicity. We find that despite the fact that copia transcripts are elevated in tau transgenic Drosophila brains, full length Copia polyprotein (Copia Full) and the Copia Gag protein are not significantly changed. Although Copia Full and Copia Gag expression levels are unchanged, Copia Full relocalizes to the nucleus whereas Copia Gag relocalizes into distinct puncta in tau transgenic fly brains. Surprisingly, we find that knockdown of full length copia and copia gag rescue tau-induced neurotoxicity. Taken together, our findings present further evidence of retrotransposon activation in tauopathy and suggest a unique mechanism of toxicity.

Abstract citation ID: igaf122.3852

**TECHNOLOGY USE AND HOSPITALIZATION RISK IN OLDER ADULTS WITH AND WITHOUT DIABETES**

Mohammed Alqurashi[1], Martha Coates[2], and Rose Ann DiMaria-Ghalili[3], *1. Drexel University, Alexandria, Virginia, United States, 2. Drexel University, Philadelphia, Pennsylvania, United States, 3. Drexel University, Drexel University, Pennsylvania, United States*

Hospitalizations are a significant concern among older adults with diabetes. Technology-related health, including online prescription refills, telehealth visits, and internet health information seeking, are becoming more prevalent, it is important to examine their impact on hospitalization risk. This study investigated the association between digital health technology use and overnight hospitalization in the past year among community-dwelling older adults aged 65 and older, with and without diabetes. We utilized the National Health and Aging Trends Study Round 13 (2023) dataset and the sample included 7,049 community-dwelling older adults aged 65 and older categorized by diabetes status ($n = 2{,}159$ with diabetes, $n = 4{,}890$ without diabetes). Overnight hospitalization in the last year was reported by 508 (23.6%) of diabetic participants and 801 (16.4%) of non-diabetic participants ($p < 0.001$). Logistic regression analysis indicated that diabetes was significantly associated with increased odds of hospitalization (OR = 1.57, 95% CI: 1.36–1.81, $p < 0.001$). Compared to those without diabetes, participants with diabetes were significantly less likely to use computers or tablets ($p < 0.001$) and telehealth services ($p = 0.02$), but more likely to refill prescriptions online ($p < 0.001$). No significant difference was found between groups in using the internet to search for health information ($p = 0.34$). These findings show that older adults with diabetes are more likely to be hospitalized and less likely to use some digital health tools. Health technology use did not affect hospitalization odds, but further research is needed to explore its impact on outcomes in older adults with diabetes.

Abstract citation ID: igaf122.3853

**TECHNOLOGY-BASED PROGRAM EFFECTS ON HEALTH OUTCOMES AND CAREGIVING EXPERIENCE IN ASIAN AMERICAN CAREGIVERS**

Jiwon Baek[1], Dongmi Kim[1], Seulgi Ryu[2], Yu-Shan Cheng[1], Wonshik Chee[1], and Eun-Ok Im[1], *1. University of Texas at Austin, Austin, Texas, United States, 2. The University of Texas at Austin, Austin, Texas, United States*

Background: Midlife women caring for people living with Alzheimer disease face substantial burdens that jeopardize health. Such burdens are often greater among ethnic minority women due to cultural norms surrounding illness and caregiving. We hypothesized that a culturally tailored and technology-based program would improve (i) health outcomes of caregivers and care recipients and (ii) caregiving experience. Methods: This study developed a 3-month intervention program based on Bandura's Self-Efficacy Theory of Behavior Change and delivered a coaching/support intervention program via a project platform and the Alzheimer's Association website. Although the study used random assignment, analyses were restricted to the intervention group due to an underpowered control group. This preliminary analysis included 30 caregivers with repeated measures of caregivers' and care recipients' health outcomes (stress and physical and psychological symptoms of caregivers; cognitive, mood, and quality-of-life status of care recipients) and caregiving experiences (caregiving activity, caregiving competence, and coping and behavioral skills). Linear mixed-effects panel models were estimated with adjusting for sociodemographic and health/disease-related covariates. Results: Caregivers demonstrated statistically significant improvements following the technology-based interventions. Perceived stress and acculturation stress of caregivers decreased. Although behavioral and psychological symptoms of care recipients showed no significant change, caregivers' reaction to their symptoms decreased. Both care recipients' mood and caregivers' caregiving skills showed significant improvement. Conclusions: A 3-month information and coaching/support intervention was associated with meaningful short-term improvements in caregiver's and care recipients' health and caregiving experience. Findings support the potential of culturally aligned interventions to reduce health disparities among ethnic minority women caregivers.

Abstract citation ID: igaf122.3854

**TESTING A STRUCTURAL EQUATION MODEL OF NURSING HOME ORGANIZATIONAL CHARACTERISTICS**

Sorah Levy[1], Sarah Holmes[2], Barbara Resnick[1], Kelly Doran[3], Elizabeth Galik[2], and Tara McMullen[4], *1. University of Maryland, Baltimore, Maryland, United States, 2. University of Maryland School of Nursing, Baltimore, Maryland, United*

*States, 3. University of Maryland, School of Nursing, Baltimore, Maryland, United States, 4. Centers for Medicare & Medicaid Services, Centers for Medicare and Medicaid Services, Maryland, United States*

Nursing home (NH) organizational characteristics such as staffing, profit status, and community size are important contributors to the quality of care delivered. These organizational characteristics are linked to resident outcomes such as the prevalence of pressure injuries, rehospitalization, morbidity, and mortality. Guided by the Social Ecological Model and Donabedian's Structure-Process-Outcome framework, this study tested a comprehensive measurement model of NH organizational factors. The hypothesized measurement model specified two latent factors: Operational (profit status, size, percent Medicaid) and Care Delivery (minutes per resident per day RN, LPN, CNA). This cross-sectional secondary data analysis included 55 NH communities in Maryland and Pennsylvania using data from the Evidence Integration Triangle for Behavioral and Psychological Symptoms of Dementia (EIT-4-BPSD) combined with data from LTCFocus. Structural equation modeling using Mplus was used to test the model. Fit statistics include $\chi^2$ /df, Tucker Lewis Index (TLI), and root mean square error of approximation (RMSEA). The model demonstrated excellent fit ($\chi^2$ /df = 0.798, TLI = 1.00, RMSEA = 0.000). CNA staffing was a significant indicator for the Care Delivery factor ($\beta = 0.565$, $p = 0.008$), and percent Medicaid ($\beta = 0.950$, $p < 0.001$) and profit status ($\beta = 0.485$, $p < 0.001$) were significant indicators of the Operational factor. This study provides empirical support for a theoretically based measurement model of NH organizational factors. Future research should explore the impact of NH organizational factors on resident outcomes. Findings may also inform policy and operating decisions aimed at strengthening infrastructure to support quality care.

Abstract citation ID: igaf122.3855

### THE DEVIL IS IN THE DETAILS: FACETS OF IMPULSIVITY AS CORRELATES OF SOCIAL CIRCLE CHANGE OVER TIME

Sydney Demchak[1], Mary Cox[2], Michael Boudreaux[3], Thomas Oltmanns[2], and Patrick Hill[4], *1. Washington University in St. Louis, Pittsburgh, Pennsylvania, United States, 2. Washington University in St. Louis, St. Louis, Missouri, United States, 3. Hogan Assessments, Tulsa, Oklahoma, United States, 4. Washington University in St. Louis, Saint Louis, Missouri, United States*

Impulsivity is a multifaceted trait that influences life outcomes such as financial stability, purposefulness, and connection. The current study examined the relationship between impulsivity and changes in social network during older adulthood. We employed data gathered over the span of three years from participants who were part of the St. Louis Personality and Aging Network. Impulsivity-related traits, such as self-discipline, deliberation, and excitement-seeking, were also assessed to determine more specific associations with the size and stability of older adults' inner circle, middle circle, and outer circle social ties across two time points. Social circle information was collected from participants ($n = 815$, mean age: 62.5 years) across two waves, and the NEO Personality Inventory served as the baseline for the level of impulsivity and impulsivity-related traits. Results indicated that global impulsivity showed no significant associations with inner or middle circle size, or with overall social changes over time. However, the facet of self-discipline was positively related to the number of inner circle members at Wave 1 ($r = .08$) and Wave 2 ($r = .07$), and to middle circle members at both waves ($r = .09$ each). Additionally, deliberation was associated with fewer individuals dropping out of the inner circle over time ($r = -.08$). Excitement-seeking though showed no significant correlations. These findings suggest that viewing impulsivity as a singular variable would be insufficient, as it could obscure potentially relevant trait-level associations. Moreover, self-discipline and deliberation could prove important for maintaining and growing social networks in older adulthood.

Abstract citation ID: igaf122.3856

### THE EDUCATION IN AGING AND GEROSCIENCE RESEARCH (EAGR) PROGRAM FOR UNDERGRADUATE STUDENTS

Kenneth Campellone[1], Rosie Mirabella[1], Iman Al-Naggar[2], and Lisa Nigro[3], *1. University of Connecticut, Storrs, Connecticut, United States, 2. UConn Health, Farmington, Connecticut, United States, 3. Central Connecticut State University, New Britain, Connecticut, United States*

Aging is the greatest risk factor for most chronic illnesses including cardiovascular diseases, cancer, diabetes, and neurodegenerative disorders. As our population ages, the personal, professional, and economic burdens that accompany age-associated conditions continue to grow. The interdisciplinary field of geroscience aims to understand and manipulate the mechanisms of biological aging that impact the development of chronic diseases with the goal of improving human healthspan. While this concept is gaining traction in some graduate programs and medical schools, geroscience is not well recognized at the undergraduate level. To address the need for growing the geroscience workforce, the UConn Education in Aging and Geroscience Research (EAGR) Program provides teaching and training to the most foundational population for strengthening the geroscientist pipeline – undergraduate students. Our EAGR Program (R25 AG083251) provides a holistic multi-campus experience in which students are: (1) instructed in the breadth and depth of geroscience concepts through a customizable literature-based coursework track consisting of content, writing, and seminar classes; (2) trained in modern experimental approaches from basic to translational geroscience during a 2-week methods workshop with the themes of 'Molecules-to-Cells' and 'Flies-to-Humans'; (3) immersed in geroscience research through individualized laboratory experiences in the summer and academic year; and (4) enabled to hone their communication skills and preparedness for advanced graduate, medical, or professional training. The structure, content, and outcomes from the first two years of the UConn EAGR program can help guide the development of similar geroscience training programs across other universities and medical school campuses.

Abstract citation ID: igaf122.3857

### THE EFFECT OF PRE-RETIREMENT PREPARATION ON POST-RETIREMENT WELL-BEING: THE MEDIATING ROLE OF SENSE OF CONTROL

Gloria Lin, Dannii Yeung, and Edwin KaHung Chung, *City University of Hong Kong, Hong Kong, China (People's Republic)*

Previous research has established the linkage between pre-retirement preparation and post-retirement well-being

(Yeung, 2018), but the underlying mechanisms have not been fully explored. Drawing on Social Cognitive Theory (Bandura, 1978), this study examined the mediating effect of sense of control on this relationship in 943 Hong Kong Chinese retirees (Mage = 71.76 years, SD = 5.28, range = 65 – 95). Using a cross-sectional design, retirees' retirement preparation, sense of control (i.e., perceived constraints [PC] and personal mastery [PM]), life satisfaction, positive and negative affect, and depressive symptoms were assessed. Results demonstrated a significant main effect of retirement preparation on all well-being measures. Both PC and PM mediated the effect of retirement preparation on life satisfaction, negative affect, and depressive symptoms, while only PM mediated the effect on positive affect. Specifically, retirees with more retirement preparation exhibited greater PM and fewer PC, resulting in higher life satisfaction ($\beta = .02$, 95%CI = .00, .03 for PC; $\beta = .03$, 95%CI = .01, .06 for PM), more positive emotions ($\beta = .02$, 95%CI = .01, .04 for PM), and fewer negative emotions ($\beta = -.03$, 95%CI = –.06, –.00 for PC; $\beta = -.01$, 95%CI = –.02, –.00 for PM) and depressive symptoms ($\beta = -.03$, 95%CI = –.05, –.01 for PC; $\beta = -.02$, 95%CI = –.03, –.01 for PM). The results remained significant even after controlling age, gender, and socioeconomic status. Our findings underscored the protective role of retirement preparation in sustaining sense of control and post-retirement psychological well-being.

Abstract citation ID: igaf122.3858

### THE INFLUENCE OF SELF-REPORTED EXERCISE AND SLEEP DURATION AND QUALITY ON NEXT-DAY CORTISOL RHYTHMS IN OLDER ADULTS

Taylor Fein, Orfeu Buxton, and Soomi Lee, *The Pennsylvania State University, University Park, Pennsylvania, United States*

Cortisol shows high day-to-day variability and reflects physiological stress and functioning of the hypothalamic-pituitary-adrenal (HPA) axis. This study examined how sleep and exercise interact with cortisol rhythms, specifically the cortisol awakening response (CAR) and diurnal cortisol slope (DCS). Data from the National Study of Daily Experiences (NSDE 3; 2016-2020), with 699 CAR observations from 279 participants (Mage=63, SDage=10) and 546 DCS observations from 227 participants. For four consecutive days, participants provided four saliva cortisol samples daily and reported time engaging in moderate-to-vigorous exercise, and sleep duration and quality. Multilevel models adjusted for trait (e.g., age, sex, race, education, BMI, smoking, drinking, medication) and state-like (e.g., wake time, prior day CAR/DCS, weekend/weekday) covariates examined independent and interactive effects of sleep and exercise on cortisol. There were no independent associations, however interactive associations were found. Cross-level interactions emerged between within-person prior-day exercise hours and between-person sleep hours ($B = 0.06$, $SE = 0.03$, $p = 0.035$). Simple slope analyses showed that among adults who slept longer than others on average (≥8.2 hours), controlling for within-person sleep, exercising more than usual on the prior day was associated with a flatter DCS the following day. This effect was driven by higher than usual bedtime cortisol, suggesting residual physiological stress and fatigue. Second, a within-person interaction between prior-day exercise and previous-night sleep quality was observed ($B = -0.18$, $SE = 0.08$, $p = 0.021$). On days following more exercise and better sleep quality, DCS had a steeper decline than usual. These findings suggest that daily exercise may influence cortisol rhythm depending on both habitual and nightly sleep.

Abstract citation ID: igaf122.3859

### THE INTERSECTION OF CHRONIC DISEASE AND ECONOMIC HARDSHIP IN RURAL AFRICAN AMERICAN COMMUNITIES

Idethia Shevon Harvey, *University of Missouri, Columbia, Missouri, United States*

Introduction Multiple chronic conditions disproportionately affect rural African Americans, who face higher prevalence and worse outcomes for arthritis, diabetes, and hypertension. Those living in challenging environments experience higher rates of chronic comorbidities due to the interactions between rural limitations and persistent structural factors. This research investigates using syndemic theory to forge new ground in rural minority health research. Methods Semi-structured interviews and photo-documentation were conducted among African Americans (mean age = 63.2, SD + 9.93 years) with long-term diabetes (mean diagnosis = 14.7, SD + 10.8 years) and hypertension (91.3%). Quantitative analysis included univariate and bivariate statistics describing demographics and self-management behaviors by composite score (0 – 6) counting depression, hypertension, arthritis, smoking, obesity, and poverty. Qualitative methods included semi-structured interviews, photo-documentation, thematic content analysis, and critical visual methodology. Triangulation occurred when photographic themes aligned with interview themes. Results Participants with higher syndemic scores (4.31, SD + 0.48) showed fewer days of healthy eating, physical activity, and glucose testing adherence. Qualitative analysis revealed two themes: "chronicity of poverty" and "contextual factors affecting health management." Limited economic resources not only discouraged positive diabetes management but also triggered clustering of health complications, supporting syndemic theory that individuals with diabetes experience magnified health-related and socioenvironmental burdens. Discussion This syndemic approach provides a novel perspective for understanding contributing factors influencing health in rural African American communities. A comprehensive approach addressing biological and social elements is necessary to reduce disease burden, improve patient-centered care, and develop targeted programs supporting self-management practices in these communities.

Abstract citation ID: igaf122.3860

### THE LIVED EXPERIENCES OF BEREAVED DEMENTIA CAREGIVERS ON THE ALZCONNECTED PEER SUPPORT FORUM

Mary Gemma O'Donnell, Zachary Baker, and Sabrina Garcia-Arias, *Arizona State University, Phoenix, Arizona, United States*

Bereaved dementia caregivers face a 6–26% risk of complicated or prolonged grief, yet little is known about how they adapt after loss in the expanding landscape of online peer support. Digital forums provide spaces where caregivers share narratives, preserve bonds with the deceased, and seek meaning beyond the caregiving role, but these expressions remain largely undocumented in dementia bereavement research. This qualitative study examined how bereaved dementia caregivers express continuing bonds (CBs) that support adaptive grief in an online peer support context. Guided by Continuing Bonds Theory and a constructivist lens, the study employed a qualitative netnographic approach with reflexive thematic analysis to analyze posts from the ALZConnected caregiver forum. Data included 1,771 threads (2,012,195 words) from the "Supporting Those Who Have Lost

Someone" discussion board, posted between 2011 and March 2023. Purposive sampling identified relevant narratives. Coding was conducted both deductively, using existing CB typologies, and inductively to capture novel caregiver-generated expressions. Six interpretive themes were identified: Living for Them Now, Love That Outlives Loss, Seeing Them in a New Light, Touchstones and Traces That Bond, Honoring Bonds in Shared Spaces, and Still Teaching, Still Giving. These themes illustrate how caregivers sustain ongoing connections that foster personal growth, meaning-making, and integration of loss. Findings offer practical relevance for clinicians, grief counselors, and support providers by identifying adaptive pathways that can be encouraged in bereavement interventions. This study advances theoretical understanding of CBs in dementia bereavement and underscores the role of online peer support in promoting adaptive grief.

Abstract citation ID: igaf122.3861

### THE MELODY PROJECT: A MUSIC-BASED INTERVENTION TO REDUCE LONELINESS AND FOSTER BELONGING IN ASSISTED LIVING

Gemma Wang[1], Lena Thompson[2], and Jordan Lewis[2], *1. The Chapin School, New York, NY, New York, United States, 2. University of Alaska Fairbanks, Fairbanks, Alaska, United States*

Despite living in communal environments, approximately 55% of older adults in assisted living facilities experience loneliness, which is linked to a 29% higher risk of premature death. The Melody Project, developed by Gemma Wang, strives to address this issue. Moved by the isolation of assisted living residents living near her school, Wang drew on her eleven years of musical experience to lead music-based sessions at nearby facilities. After recognizing the residents' profound joy, she established The Melody Project to continue fostering intergenerational engagement. This initiative hosts sessions connecting music and interaction through icebreaker activities, collaborative art inspired by music, and group discussions. The Melody Project has reached three nursing homes in New York City and has been received with positive feedback by residents and staff. The activities leave older adults with personalized keepsakes to remember their time with the students, and they remain deeply touched by the stories shared and relationships forged. With the support of her mentors, Wang plans to implement pre-post evaluation surveys to provide quantitative support for The Melody Project in the coming school year. She has also begun making connections in different states to establish state chapters and is researching aging and neurodegeneration at the University of California, Santa Barbara, this summer. By bridging generational divides through creative expression, The Melody Project reduces loneliness while fostering empathy, purpose, and belonging across generations, promoting meaningful relationships that help rediscover community and camaraderie. The Melody Project is thus a scalable, community-based, and evidence-informed approach to aging well together.

Abstract citation ID: igaf122.3862

### THE QUALITY-OF-LIFE IMPLICATIONS OF HELPLESSNESS IN MIDDLE-AGE AND OLDER NIGERIANS LIVING WITH HIV

Victor Elisha[1], Tochukwu Okolie[2], Anthony Briggs[3], Chinelo Nduka[4], and Candidus Nwakasi[5], *1. University of Connecticut, Farmington, Farmington, Connecticut, United States, 2. University of Connecticut, Mansfield Center, Connecticut, United States, 3. NYU Grossman School of Medicine, New York, New York, United States, 4. Nnamdi Azikiwe Teaching Hospital, Nnewi, Anambra, Nigeria, 5. University of Connecticut, Storrs, Connecticut, United States*

Background: In 2023, the estimated HIV prevalence in Nigeria was 2.1%, ~ 2 million people living with HIV (PLWH). While interventions have improved physical health and reduced mortality among PLWH in Nigeria, limited research has examined the impact of perceived helplessness on their overall wellbeing. This study investigated the effect of helplessness on wellbeing by focusing on health-related quality-of-life (HRQoL), subjective health status (SHS), and subjective cognitive decline (SCD). Methods: We recruited 150 PLWH (mean age = 55.2 years) to complete a questionnaire assessing social, psychosocial, and health factors. We modeled whether perceived helplessness was associated with physical-HRQoL (P-HRQoL) and mental-HRQoL (M-HRQoL), SHS, and SCD. Results: Higher perceived helplessness was associated with lower physical health-related quality of life (P-HRQoL; $\beta$ = -0.275, $p < 0.001$) and mental health-related quality of life (M-HRQoL; $\beta$ = -0.180, $p < 0.001$). It was also associated with subjective health ($\beta = 0.010$, $p < 0.001$) and higher report of SCD ($\beta = 0.443$, $p < 0.001$. Increased age was associated with lower P-HRQoL, M-HRQoL, SHS, and higher SCD. Additionally, more years since HIV diagnosis was associated with lower physical and mental health-related quality of life. Discussion: While medical advancements and public health efforts improve longevity among PLWH, many PLWH in Nigeria face persistent stressors from HIV-related stigma, and social disadvantages. These may contribute to perceived helplessness, and a diminished sense of control, thus impacting their wellbeing. Our findings underscore the importance of developing psychosocial interventions for middle-age and older PLWH in Nigeria

Abstract citation ID: igaf122.3863

### TOKTOGETHER: HARNESSING TIKTOK'S POTENTIAL TO ADDRESS AGEISM IN HEALTH EDUCATION

Kathryn Coccia, *Saint Louis University, Indianapolis, Indiana, United States*

Background: Experts predict that the geriatric provider workforce needs to expand by over 100% by 2030 to meet projected population needs. Prior research has called for novel interventions to increase interest in geriatric specialization among health students. Methods: Surveying a sample of 110 interprofessional health students at Saint Louis University, this study tested the preliminary efficacy, feasibility, and acceptability of a novel intervention called TokTogether. Utilizing an explanatory mixed-methods design with an experimental group ($n = 78$) and a control group ($n = 32$), this study examined whether engaging with TikTok content created by and featuring older adults could effectively reduce participants' ageism and age anxiety and increase interest in working with older adults (OAs) Results: Exposure to the TokTogether intervention significantly reduced ageism ($t = 3.40$, $p = 0.001$) and age anxiety ($t = 4.80$, $p < 0.001$) within the experimental group. It did not significantly increase interest in working with OAs ($t = 0.79$, $p = 0.43$). Participants in the experimental group showed a significantly greater reduction in ageism scores compared to those in the control group ($t = 2.14$, $p = 0.03$). Mixed-methods results supported the feasibility and acceptability of the intervention, with qualitative themes revealing that participants experienced increased feelings of connection with OAs, gained insight into the aging process, and reflected on and reevaluated their beliefs about aging and OAs. Conclusion: Engaging with TikTok content produced by and featuring older adults may be a scalable and effective way to reduce ageism and age anxiety among health students. Further investigation is needed about interventions to increase student interest in working with OAs.

Abstract citation ID: igaf122.3864

### TRANSPORTATION USE AND ACCESSIBILITY AMONG PERSONS WITH DEMENTIA WHO RESIDE ALONE IN THE COMMUNITY

Laura Girling, Yat Tung Hung, and Mary Nemec, *Towson University, Towson, Maryland, United States*

Transportation access is a critical component of independent living and community engagement, particularly for older adults with cognitive impairment. Despite this importance, limited research has examined how community-dwelling person with dementia who live alone navigate their transportation needs. To address this gap, an exploratory descriptive study was conducted using data from a National Institutes of Health-funded protocol. In-depth semi-structured interviews were conducted with live-alone persons with dementia and their collaterals (N = 96). Inductive content analysis revealed that most participants had stopped driving, and alternative transportation options such as public transit, paratransit, ride-sharing services, and informal support networks were inconsistently available or underutilized. Key barriers included cognitive and physical limitations, lack of dementia-sensitive services, and geographic constraints. Participants expressed a desire to remain engaged in their communities but faced significant obstacles in accessing essential services, social activities, and healthcare. Findings underscore the need for dementia-sensitive transportation planning, including training for transit staff, flexible scheduling, and integration with community-based support services. Improving transportation access for this vulnerable and growing population can foster autonomy, reduce isolation, and support aging in place for longer durations.

Abstract citation ID: igaf122.3865

### UNCOVERING MECHANISMS OF NRTI INDUCED CELLULAR SENESCENCE

Niharika Kura[1], Chisaka Kuehnemann[1], Gailan Constantino[1], and Christopher Wiley[2], *1. Tufts University, Boston, Massachusetts, United States, 2. Jean Mayer HNRCA at Tufts University, Boston, Massachusetts, United States*

People living with HIV (PLWHIV) currently have extended lifespans, but experience increased age-related diseases and younger ages compared to their HIV negative counterparts. Nucleotide reverse transcriptase inhibitors (NRTIs) are breakthrough medications at the center of HIV therapy. However, these drugs have been known to cause side effects associated with premature aging, Early generation NRTIs, such as thymidine analogs AZT and d4T, caused lipodystrophy and myopathies, while modern NRTIs, such as the adenosine analogs TDF and TAF, cause renal toxicity and dyslipidemia. All of these drugs induce senescence in vitro; however, their mechanism of senescence induction is distinct. In this study, we show that early generation NRTIs cause senescence via mitochondrial dysfunction, whereas modern NRTIs cause senescence via genotoxic stress. This study demonstrates potential cellular mechanisms associated with accelerated aging seen in PLWHIV, as well as potentially linking methods of senescence induction to clinical presentations of aging pathologies.

Abstract citation ID: igaf122.3866

### UNDERSTANDING THE RESPITE FOR ALL MODEL: EXPERIENCES OF PEOPLE LIVING WITH DEMENTIA, CAREGIVERS, AND VOLUNTEERS

Eleanor Hummel, Camille Murray, Siyue Fang, Karl Umble, and Erin Kent, *University of North Carolina at Chapel Hill, Chapel Hill, North Carolina, United States*

Respite care is a long-term service and support (LTSS) that provides instrumental support to family caregivers. The volunteer-run Respite for All (RFA) model has sought to increase access to LTSS. There are over fifty iterations of the model across fifteen states. Our study aimed to qualitatively evaluate the RFA model. During the first phase, the RFA model was characterized through semi-structured Key Informant Interviews (KIIs) with RFA leaders. After characterizing the RFA model, semi-structured in-depth interviews (IDIs) were conducted to understand the experiences of caregivers, PLWD, and volunteers with one iteration of the model. Thematic analysis was performed. KIIs were conducted with RFA leaders (n = 7), and IDIs were conducted with PLWD (n = 3), caregivers (n = 5), and volunteers (n = 5). A logic model was used to organize program inputs, activities, outputs, and impacts. Program leaders reported distinctive characteristics about the RFA model, including high replicability, engagement, and the ability to deliver person-centered care. Program experiences of PLWD, caregivers, and volunteers were reported to be overwhelmingly positive and included increased feelings of well-being, acceptance, purpose, social support, engagement, and community. Facilitators to accessing programs included: word-of-mouth, diverse volunteers, geographic proximity, feedback, inclusivity, other program supports, strong connections with caregivers, and honoring and remembering participants. Barriers to access were also described: stigma and cultural beliefs around respite care and dementia, program capacity and awareness, distrust, geographic limitations, lack of diversity, dementia progression, and cost. This study provides a foundation for future research on the RFA model, an innovative approach to respite care.

Abstract citation ID: igaf122.3867

### VALIDATION OF COMPUTER VISION FOR SEGMENTING TIMED UP AND GO SUBTASKS FROM CONVENTIONAL VIDEO RECORDINGS

Chitra Banarjee, Sarah Reynolds, Zengyan Wang, Chen Chen, Rui Xie, and Ladda Thiamwong, *University of Central Florida, Orlando, Florida, United States*

The Timed Up and Go (TUG) test is a standardized clinical tool used to assess the dynamic balance of older adults for fall prevention. It is typically assessed using a stopwatch recording the total duration to complete the test. Recently, studies have focused on the relevance of the subtasks of the TUG: sit-to-stand, 3-meter forward-walk, turn, back-walk, stand-to-sit. Studies using wearable devices or depth cameras have introduced new metrics for assessing fall risk in clinical settings. These metrics have been shown to be associated with lower limb strength, motor impairments, and executive function. We aimed to utilize an affordable video camera and computer vision (CV) to detect the durations of TUG components and validate it through comparison with manual coding. The sample included 17 older adults

(70.6 + 7.3 years, 70.6% female), who completed four trials of the TUG. The trials were recorded on a GoPro Hero 12 video camera, placed 110 inches away from the turning point with a frontal view of the participant. The videos were segmented into subtasks by trained research assistants and analyzed using AlphaPose and MotionBERT for 2D and 3D human pose estimation, respectively. Subtasks were extracted using the head and shoulder coordinates from the CV output. Spearman correlations were utilized to compare the annotated and CV-extracted durations of the subtasks, revealing comparable durations ($\rho = 0.479$, $p < 0.001$). These preliminary results indicate the potential of CV in primary care settings as an affordable method of expanding clinical evaluation of dynamic balance in older adults by medical professionals.

Abstract citation ID: igaf122.3868

### WEIGHTED VESTS PRESERVE RESTING ENERGY EXPENDITURE DURING INTENTIONAL WEIGHT LOSS IN OLDER ADULTS

Cassidy Guida[1], Lauren Chandler-Holtz[2], Daniel Beavers[2], Philip Kramer[1], Sarah Wherry[3], Barbara Nicklas[4], and Kristen Beavers[1], *1. Wake Forest University School of Medicine, Winston-Salem, North Carolina, United States, 2. Wake Forest University, Winston Salem, North Carolina, United States, 3. University of Colorado Anschutz Medical Campus, Aurora, Colorado, United States, 4. Wake Forest University School of Medicine, Winston Salem, North Carolina, United States*

Weight regain following intentional weight loss in older adults is a near expected occurrence. The gravitostat theory suggests that gravitational loading – in the form of a weighted vest, for instance – might dampen the body's perception of weight loss, thus mitigating signals driving weight regain, such as reduced resting energy expenditure (REE). The 12-month INVEST in Bone Health Trial (NCT04076618), compared WL alone (targeting 10% WL), WL plus weighted vest use (WL+VEST; 8 hours/day, weight replacement up to 10% total WL), or WL plus resistance training (WL+RT; 3 supervised sessions/week) on bone health in 150 older adults, is an ideal platform to examine this concept. A subset of participants ($n = 21$) with baseline characteristics similar to the original cohort (66.9±5.4 years; 71% women; 67% white; body mass index [BMI] 33.3±3.5 kg/m$^2$) returned for a 24-month follow up visit (12 months post-intervention) to collect REE and body weight. At intervention end (12-months), REE was better preserved in WL+VEST [+146.9 kcal/day (95% CI: 32.4, 222.4)] compared to WL alone [-52.5 kcal/day (95% CI: -137.5, 32.5)] and WL+RT [-89.7 kcal/day (95% CI: -160.5, -18.8)]; $p = 0.02$. From 12 to 24 months, WL+VEST regained the least weight [+0.74 kg (95% CI:(-3.75, 5.23)], compared to WL+RT [+4.74 kg (95% CI: 0.51, 8.96)] and WL alone [+4.40 kg (95% CI (2.03, 6.77)]; $p = 0.084$. Findings suggest that weighted vest use may attenuate long-term weight regain in older adults by preserving REE. Future work will examine other measures of energy balance, including physical activity energy expenditure and appetite hormones.

Abstract citation ID: igaf122.3869

### WHAT DO OLDER ADULTS WANT FROM SOCIAL ROBOTS AND ARTIFICIAL INTELLIGENCE? A PRODUCT-ORIENTED PERSPECTIVE

Nicole Long-ki Fung[1], Yueyuan Zheng[2], Bertram Shi[2], Janet Hui-wen Hsiao[2], and Jean Woo[1], *1. The Chinese University of Hong Kong, Hong Kong, Hong Kong, China (People's Republic), 2. Hong Kong University of Science and Technology, Hong Kong, Hong Kong, China (People's Republic)*

The global aging population is placing increasing pressure on healthcare and social resources. Many have turned to technology to offset the impact of manpower shortage in eldercare. Social robots and artificial intelligence hold promising potential in enhancing older adults' well-being and quality of life. While older adults generally express positive attitudes towards these technologies, adoption rates remain relatively low, with older adults citing concerns about design, usability, and relevance. Existing technological acceptance models emphasize the effects of individual factors or subjective evaluations (e.g., perceived usefulness) on product acceptance, offering limited guidance on the product attributes that foster positive perceptions among older adults. This study adopted a product-oriented perspective to understand older adults' preferences for social robots and artificial intelligence. We searched through five databases, including PsycINFO, Web of Science, IEEE Xplore Digital Library, Scopus, and ACM Digital Library, and identified 243 relevant articles published until January 2025. We extracted 22 product features deemed desirable or undesirable by older adults and organized them according to self-determination theory and socioemotional selectivity theory. Older adults valued features that support their autonomy (e.g., customization), relatedness (e.g., facilitating social connections), and competence (e.g., providing living assistance), as well as features serving emotional regulation (e.g., providing entertainment), pro-closeness (e.g., grandchildren-like voices), and self-relevance goals (e.g., offering culturally specific features like dialects). Our synthesis highlights the psychological needs and motivational priorities underlying older adults' technology preferences. These findings provide actionable insights for designing human-centered technologies that are more likely to be accepted and adopted by older adults.

Abstract citation ID: igaf122.3870

### WHO LISTENS, HOW WE SHARE: SOCIAL CONTEXT EFFECTS ON MOOD IN BRIEF REMINISCENCE AMONG OLDER ADULTS IN CHINA

Keyi Shi, *Chinese University Hong Kong, St. Louis, Missouri, United States*

Older adults face heightened risks of depression and affective dysregulation, particularly in low-resource contexts. Reminiscence, involving the structured recall of autobiographical memories, has been shown to improve emotional well-being, but most evidence comes from multi-session interventions in Western populations. The immediate effects of single-session reminiscence and the influence of social context, including listener familiarity and sharing modality, remain underexplored. I conducted a quasi-randomized, mixed-design study with 91 community-dwelling older adults (mean age = 67.7 years, 54% female) in Jiujiang, China. Participants were assigned to online writing, offline writing, oral sharing with a relative, or oral sharing with an unfamiliar young adult listener. Mood was assessed before and after the session using the Brief Introspection Scale (16 items; alpha = .84) and a global affect rating. A two-by-four mixed ANOVA tested main and interaction effects, followed by item-level analyses. No significant time by group interaction was observed ($p > .05$). Item-level analyses revealed significant pre-post increases in positive affective states including lively, content, loving, and active (all $ps < .05$), with trends favoring oral sharing over writing. Brief reminiscence improved specific positive affect and proved feasible in a low-resource, non-Western context. These findings highlight its potential as an accessible, person-centered strategy for enhancing emotional well-being and underscore the importance of social context in optimizing intervention benefits.

### SESSION 8010 (POSTER)

### LATE BREAKING POSTER SESSION III

Abstract citation ID: igaf122.3871

### A HELPFUL TOOL OR ANOTHER BARRIER? EXPLORING OLDER ADULTS' PERSPECTIVES ON PATIENT PORTALS

Chung Hyeon Jeong[1], BoRin Kim[2], and Amy Tietjen[3],
*1. University of New Hampshire - eHealth readiness, Durham, New Hampshire, United States, 2. University of New Hampshire, University of New Hampshire, New Hampshire, United States, 3. University of New Hampshire, Durham, Massachusetts, United States*

While patient portal use among older adults increased during the COVID-19 pandemic, their perceptions and experiences with these tools remain underexplored. The Unified Theory of Acceptance and Use of Technology (UTAUT) highlights perceived usefulness, effort expectancy, and social influence as predictors of technology adoption. This qualitative study examined how these constructs are reflected in older adults' perceptions of patient portals in the post-pandemic context. In spring 2025, we conducted semi-structured Zoom interviews with 19 community-dwelling adults aged 65 and older who had visited a healthcare provider in the past six months. Eligible participants lived independently, had internet access, and no cognitive impairment. Thematic analysis generated three themes that both reflect and extend the UTAUT framework: (1) "Not for Us, But for Them"—participants reported low perceived usefulness, viewing portals as serving providers' efficiency more than patient needs; (2) "Technology with Warmth"—older adults valued human connection in care and emphasized the importance of tailoring portals to their needs and readiness; and (3) "We Don't Talk About It"—participants described limited social influence, noting that portals were rarely discussed among peers, challenging assumptions that peer endorsement strongly predicts adoption. These findings suggest that older adults' portal use is shaped not only by functional barriers but also by emotional trust and limited social diffusion. To foster meaningful adoption, strategies should enhance perceived usefulness, incorporate relational and culturally sensitive design, and provide education that improves both relevance and usability.

Abstract citation ID: igaf122.3872

### A REVIEW OF TECHNOLOGY-ENHANCED RECREATIONAL THERAPY IN MILD COGNITIVE IMPAIRMENT

Hayun Back[1], Yongseop Kim[2], and Rebecca Westenskow[1],
*1. University of Utah, Salt Lake City, Utah, United States, 2. University of Utah, University of Utah, Utah, United States*

As the prevalence of mild cognitive impairment (MCI) and dementia continues to rise, there is growing interest in non-pharmacological interventions that promote cognitive and psychosocial well-being. Technology-enhanced recreational therapy (TERT) represents a promising approach that aligns with recreational therapy's emphasis on activity-based cognitive rehabilitation. However, the scope, effectiveness, and limitations of TERT in supporting individuals with MCI and dementia remain insufficiently understood. This scoping review mapped current evidence on TERT interventions for older adults with MCI and dementia, identifying technologies, therapeutic goals, outcomes, and methodological gaps. According to Arksey and O'Malley's framework and PRISMA guidelines, five databases were searched for peer-reviewed studies (2000–2024). Forty-three studies met inclusion criteria and were charted by technology type, population, goals, outcomes, and design. Immersive and mixed/unspecified VR dominated (n = 36), with few studies using mobile apps (n = 2), robots (n = 1), or multimodel platforms (n = 1). Populations included MCI (n = 11), dementia (n = 8), and broader aging groups (n = 16), often in long-term care (n = 16). Therapeutic aims clustered around cognition (n = 15), affect (n = 13), social engagement (n = 11), and reminiscence (n = 5). Most studies (31/43) reported positive outcomes in cognition; mixed findings (n = 12) reflected small samples and short interventions. Study designs included RCTs (n = 11) and reviews (n = 11), though many were preliminary. Common barriers included usability, cybersickness, and equipment costs. Evidence indicates TERT can improve cognitive stimulation, affect, and social connection for older adults with cognitive decline. However, methodological limitations and inequities in access restrict implementation. Future work should pursue rigorous, longitudinal trials and culturally responsive designs to ensure equitable translation into recreational therapy practice.

Abstract citation ID: igaf122.3873

### A SCOPING REVIEW OF COGNITIVE SCREENING AMONG OLDER ADULTS WITH HEART DISEASE

Christopher Griffith[1], Lindsey Nelson[1], and Helen Graham[2],
*1. University of Colorado Colorado Springs, Colorado Springs, Colorado, United States, 2. UCCS, University of Colorado Colorado Springs, Colorado, United States*

Strong associations exist between cognitive impairment (CI) and heart disease in older adults, and such conditions are often comorbid. Early identification of CI can improve health outcomes. However, standard screening protocols are lacking, and it is uncertain whether healthcare providers routinely conduct formal or informal assessments for CI. The purpose of this review was to better understand the extent to which healthcare providers screen for CI in older adults with heart disease within clinical settings and to identify which screening methods are used. A scoping review was conducted using Arksey and O'Malley's systematic framework. Articles were included if they featured information about frequency, application, or other contextual factors about CI screening for adults with heart disease in clinical settings. Of 876 potential sources identified, 9 were included for the full review. The articles represented international screening practices, including the United States, Australia, the Netherlands, China, and Uganda. Most articles (n = 6) assessed healthcare providers' practices and perspectives on cognitive screening. Results included a range of providers (e.g., nurses, psychologists, rehabilitation specialists, cardiologists) who routinely screen, 3% to 39%, and various screening measures (e.g., Montreal Cognitive Assessment, Mini-Mental Status Exam, Mini-Cog, and more). Barriers to regular screening were feasibility concerns and provider-specific attitudes and beliefs, among others. Recommendations included implementation of standardized screening protocols, increasing providers' awareness of age-related considerations, and greater interdisciplinary collaboration, among others. Findings demonstrated that some providers recognize the importance of routine cognitive screening, but many lack adequate knowledge, training, and logistical support.

Abstract citation ID: igaf122.3874

### "COFFEE WITH ALEXA" – FACILITATORS AND BARRIERS TO ADOPTION OF VOICE-ACTIVATED VIRTUAL ASSISTANTS

Patricia Oh[1], Susan Wehry[2], Jennifer Jain[1], Ruth Dufresne[3], Katie Keough[2], Rachel Coleman[4], and Lenard Kaye[4], *1. University of Maine, Bangor, Maine, United States, 2. University of New England, Portland, Maine, United States, 3. Center for Excellence in Public Health, University of New England, Portland, Maine, United States, 4. University of Maine Center on Aging, Orono, Maine, United States*

Background: Virtual home assistants (VHAs) have been promoted to support healthy aging and assist people living with early-stage dementia by facilitating daily tasks, providing reminders, and fostering social connections. Adoption is impeded by ageist stereotypes often direct responsibility for installing and monitoring the devices to family care partners. Additional barriers include limited access to affordable internet and insufficient training to use the VHA comfortably. Project: To evaluate a community-based VHA training and installation program across rural and urban Maine communities that replicated an initiative in an age-friendly suburban community. Twenty-nine volunteers were trained to install VHA in 27 rural communities, each receiving equipment to install VHA in up to 15 homes. Four urban areas participated, each receiving supplies for 25 homes. Methods: Data collection included volunteer surveys (n = 29), focus groups (n = 24), recipient surveys (n = 201, 68% response rate), and semi-structured interviews (n = 19). Results: Most volunteers were very comfortable with installation (67%) and had prior VHA experience (59%). Rural best practices included (1) 1:1 custom installation, (2) follow-up, and (3) word-of-mouth recruitment. Urban success depended on (1) collaboration with congregate housing and with an organizational training partner. Among recipients, 72% expressed confidence using their device and 27% customized their VHA routine to meet additional needs after they were installed. Benefits included (1) social connection; (2) independence; (3) fun; and (4) confidence. Conclusion: These findings challenge ageist assumptions about technology adoption by older adults living with cognitive changes and highlight the potential for community-specific programs with geographically-tailored approaches to distributing VHA.

Abstract citation ID: igaf122.3875

### "I'M NOT ALONE ON THIS JOURNEY—THERE ARE GOOD PEOPLE OUT THERE": SOCIAL CONNECTEDNESS AMONG PEOPLE WITH LTSS NEEDS

Sarah Sunghye Kang[1], Jianan Li[1], Lené Levy-Storms[2], and Kathryn Kietzman[3], *1. University of California, Los Angeles, Los Angeles, California, United States, 2. UCLA Luskin School of Public Affairs/Social Welfare, Los Angeles, California, United States, 3. UCLA Fielding School of Public Health, Los Angeles, California, United States*

The demand for Long-Term Services and Supports (LTSS) in California continues to rise, with an estimated 4.1 million adults living with disabilities or chronic conditions. People with LTSS needs often experience physical isolation, which reduces opportunities to build and maintain social relationships and stay engaged in society. At the same time, social connections support well-being; yet, the lived experiences of social connectedness among this population remain understudied. The California LTSS Study includes a qualitative component that explores the experiences of older adults and adults who have difficulties with activities of daily living. We conducted open-ended, in-person interviews with a purposive sample of 90 participants. Preliminary analyses with a subgroup of 12 participants revealed insights about their experiences of social isolation and connectedness. Using analytical approaches guided by constructivist grounded theory, we identified three categories describing the lived experiences of social connectedness among people with LTSS needs: 1) the sources of social connections (e.g., family and friends, cohabitants, caregivers, community involvement, and employment), 2) perceived changes in social connections (e.g., COVID-19, worsening health conditions, and loss or gain of loved ones or caregivers), and 3) barriers and bridges to social connections (e.g., technology, mobility, accessibility, social perceptions of disability, and internal resources). These preliminary findings characterize aspects of social connectedness using the voices of people with LTSS needs and suggest the need for more in-depth, qualitative analyses that examine both opportunities and challenges related to the social well-being of this vulnerable population.

Abstract citation ID: igaf122.3876

### ACCESS AND USE OF DIGITAL DEVICES ARE ASSOCIATED WITH LESS LONELINESS OVER TIME AMONG OLDER ADULTS

Deborah Finkel[1], and Anna Dahl Aslan[2], *1. University of Southern California, Los Angeles, California, United States, 2. University of Skövde, Skövde, Vastra Gotaland, Sweden*

Background: Loneliness, common among older adults, is a public health problem. The roots of loneliness are multifactorial, ranging from individual to structural factors. The digitalization of the society has the potential to influence older adults' level of loneliness through individual factors, e.g., older adults' access and use of digital devices, as the structure for how we interact and communicate has changed. Aim: to study the association between access and use of digital devices and loneliness over time. Methods: At baseline in 2004, (n = 785, mean age=70.1, SD = 11.2; 57.9% female; 100% European ancestry) and consecutively 4 more times over 10 years, participants in the longitudinal population-based Swedish Adoption/Twin Study of Aging responded to question about their access and use of computer and cellphone, and whether they are bothered by loneliness. Results: At baseline, access to cellphone and computer was 71% and 42% and daily use was 17% and 19%. By 2014, access increased to 86% and 64% and daily use increased to 46% and 37%. Repeated measures ANOVA indicated higher digital access (p = 0.001) and digital use (p = 0.054) as time-varying covariates were associated with less loneliness, while controlling for age, sex, and education. Conclusion: Among early adopters of digital devices both access and use are associated with less loneliness. The findings agree with a growing body of literature indicating that higher access and use of digital devices in old age potentially buffer against loneliness. Future studies need to explore the causal relation and control for a broader range of risk factors of loneliness.

Abstract citation ID: igaf122.3877

**ADDRESSING BARRIERS TO ENROLLING PAID CAREGIVERS IN CALIFORNIA: SYSTEMATIC REVIEW AND FIELD PERSPECTIVES**

Aaron Luo, *Yorba Linda High School, Yorba Linda, California, United States*

Home care systems face persistent workforce shortages, with significant barriers to enrolling paid caregivers. This study examines barriers to the enrollment of paid home care workers, focusing on California's regulatory and market contexts. A systematic literature review was conducted, supplemented by the perspective of practice from a field practitioner. Commonly identified barriers to enrolling paid home care workers include low wages, administrative burden, and limited career mobility. In California, state-specific enrollment requirements such as Home Care Aide (HCA) registration and Livescan background checks present additional challenges. These upfront expenses, often $70–$100 or more, reduced recruitment effectiveness by discouraging applicants and diminishing trust. Solutions identified vary based on who eventually pays for the costs, mainly including: (1) Agencies covering or paying back the upfront fees; (2) government help through subsidies or vouchers for those who qualify; (3) using workforce development funds to cover credentialing costs, and (4) making the application and background check process simpler and faster. Each has its own advantages and disadvantages. Pros and cons of each recommendation were discussed from the perspective of field practice. General recommendations include: implementing state-funded cost-offset programs for HCA registration and Live Scan; piloting expedited or mobile Live Scan services; broadening awareness campaigns in underserved communities; and creating public–private partnerships to cover credentialing costs. Addressing these barriers is critical to strengthening California's paid home care workforce and providing essential services to older adults and those who rely on the services.

Abstract citation ID: igaf122.3878

**AGE-FRIENDLY HEALTH CARE IN RURAL WASHINGTON: ASSESSING SYSTEM READINESS AND ADVANCING RECOGNITION**

Anita Souza[1], Katherine Bennett[2], Aimee Verrall[3], Leigh Ann Mike[1], Barbara Cochrane[1], and Elizabeth Phelan[4], *1. University of Washington, Seattle, Washington, United States, 2. University of Washington School of Medicine, Seattle, Washington, United States, 3. University of Washington / School of Medicine, Seattle, Washington, United States, 4. University of Washington, School of Medicine, Seattle, Washington, United States*

Aging demographic trends in Washington State reflect national patterns, with rural communities in particular experiencing significant growth in adults over age 65 along with an overwhelmed healthcare workforce and accessibility challenges. As this population expands, health systems are called upon to collaborate with older adults, address their unique health needs, and apply the 4Ms of Age-Friendly Health Systems across the continuum of care. To support this mandate, this project engaged rural health systems to formalize Age-Friendly care by implementing structured assessments and evidence-based improvements for Institute for Healthcare Improvement (IHI) recognition. An Age-Friendly Outreach Coordinator provided targeted support and strategic collaboration with three health systems in rural counties. Each system reviewed its current care and then implemented education and evaluation to align with Age-Friendly care across its sites. Within six months, all three participating health systems – including five primary care clinics, one critical access hospital, and one nursing home – achieved IHI's Age-Friendly Level 1 recognition. This consistent attainment of Level 1 highlights the feasibility and adaptability of Age-Friendly interventions in different rural sites and demonstrates the readiness of rural providers to support older adults through integrated, person-centered models of care. These findings suggest that with targeted support and strategic collaboration, Age-Friendly practice can be promoted in rural healthcare organizations, with the goal of improving outcomes and care experiences for older adults. Future efforts will focus on sustaining these changes, technical assistance to track Age-Friendly care processes and outcomes and expanding the evaluation/dissemination of outreach coordinator support.

Abstract citation ID: igaf122.3879

**AGEISM AND KNOWLEDGE OF AGING AMONG FUTURE HEALTHCARE PROFESSIONALS**

Frances Hawes[1], ShuangShuang Wang[2], and Inessah Cernohous[1], *1. University of Wisconsin-Eau Claire, Eau Claire, Wisconsin, United States, 2. Southwest Jiaotong University, Chengdu, Sichuan, China (People's Republic)*

As the global population ages, addressing ageist attitudes among future healthcare professionals is essential. This study surveyed 148 undergraduate students pursuing health-related careers at a large Midwestern university to assess their knowledge and attitudes about aging using the Ageism Attitude Scale (AAS) and Palmore's Facts on Aging Quiz (FAQ II). Students demonstrated limited knowledge of aging but generally positive attitudes, with low negative ageism, moderate positive ageism, and some belief that aging restricts life. Regression analyses showed that attitudes varied by academic major, experience, and knowledge. Nursing majors reported higher positive ageism than pre-med students, and frequent contact with older adults was linked to lower restrictive views. Negative ageism was lowest among healthcare administration majors and students with formal training in aging. Aging knowledge was positively associated with more favorable attitudes and varied by school year, with sophomores and juniors scoring lower than freshmen.

Abstract citation ID: igaf122.3880

**AGE-RELATED STEREOTYPE THREAT AND ITS IMPACT ON WORKING MEMORY IN OLDER ADULTS: A SYSTEMATIC REVIEW OF THE LITERATURE**

Julie Pham, Thomas Ritz, and Holly Bowen, *Southern Methodist University, Dallas, Texas, United States*

Stereotype threat related to aging represents a growing area of concern, with evidence suggesting it may impair functions like working memory that are vulnerable to age-related decline. This systematic review examines how age-related stereotype threat impacts working memory in older adults. The current review synthesizes 20 empirical studies (across 22 samples) that used experimental stereotype threat manipulations and assessed working memory outcomes in participants aged 59 and older. Studies were coded by working memory component and function, type of stereotype threat (blatant versus subtle), and sample characteristics. Findings indicate that subtle stereotype cues may impair working memory performance more consistently than blatant threats. Specifically, five of eight samples (62.5%) exposed to subtle stereotype threat showed significant impairments, particularly on tasks involving storage, manipulation, and updating. In contrast, only three of 14 samples (21.4%) exposed to blatant stereotype threat showed significant effects. When analyzing specific working memory components, five of the 13 samples assessing the central executive component showed poorer performance under threat, including two under subtle threat. Of the six samples examining the phonological loop, two found significant effects under subtle threat, as did both studies on visuospatial working memory. One study also reported that subtle threat impaired overall working memory performance. No consistent link was found between participant demographics and stereotype threat effects. Overall, the review suggests that stereotype threat, especially in subtle forms, may compound age-related working memory decline, and highlights the need for more rigorous studies to clarify these effects and guide interventions to support cognitive aging.

Abstract citation ID: igaf122.3881

### AGE-SPECIFIC LONG-TERM EFFECTS OF SLEEP ON MEMORY AND EXECUTIVE FUNCTION: MIDUS 2 AND 3

Sunbok Park, Jeongok Logan, and Ha Do Byon, *University of Virginia School of Nursing, Charlottesville, Virginia, United States*

Maintaining cognitive health during aging is critical, and sleep quality has emerged as a modifiable factor in cognitive decline. Yet, age-specific associations between sleep and long-term cognitive changes, independent of mental and neurological conditions, remain underexplored in nationally representative samples. Using data from the Midlife in the United States (MIDUS) study, sleep quality was assessed at MIDUS 2 with the Pittsburgh Sleep Quality Index (PSQI), and cognitive function was reassessed approximately 9.2 years later at MIDUS 3. Cognitive domains included episodic memory, executive functioning, and a composite score, standardized to MIDUS 2 means and standard deviations. Partial correlations examined associations between baseline sleep and cognitive change, controlling for age, sex, education level, psychiatric visits in the past 12 months, and neurological disorders in the past year from MIDUS 2. Analyses were stratified by age: older adults (≥65 years, n = 92) and young–middle-aged adults (< 65 years, n = 537). Among older adults, episodic memory decline was linked to poorer global sleep (r=-0.286, p = 0.005), longer sleep latency (r=-0.244, p = 0.018), and lower habitual sleep efficiency (r=-0.306, p = 0.003), with no associations for executive functioning. In contrast, among young–middle-aged adults, executive functioning decline was associated with poor subjective sleep quality (r=-0.116, p = 0.007) and shorter sleep duration (r=-0.113, p = 0.009). These findings highlight distinct age-specific vulnerabilities: episodic memory in older adults linked to structural sleep elements, and executive functioning in younger adults linked to subjective quality and duration. Tailored sleep interventions, emphasizing latency reduction and efficiency in older adults and duration/quality in younger adults, may help preserve long-term cognitive health.

Abstract citation ID: igaf122.3882

### AGING ATTITUDE AND POST-RETIREMENT RE-EMPLOYMENT AMONG RETIRED AND UNRETIRED HONG KONG OLDER ADULTS

Yue Hu, Helene Fung, and Chunyan Mai, *The Chinese University of Hong Kong, Hong Kong, China (People's Republic)*

Literature about views on aging and preparation for late life indicates the pivotal role of attitude towards aging in planning for post-retirement reemployment. However, it still remains unclear whether negative age stereotypes impede post-retirement re-employment among retired and unretired older adults. Using a cross-sectional design, 317 Hong Kong older adults (50 – 80 yrs, mean age 60.99 ± 6.17, 54% females, 113 unretired) were recruited by using a convenience sampling method. We found that unretired older adults' negative attitudes toward older population in personality and mental health predicted a lower likelihood of post-retirement employment planning at a marginally significant level (p = .07), even after controlling socioeconomic status. Retired older adults' negative attitudes toward older adult population in their societal participation were significantly associated with a lower likelihood of actual post-retirement employment (p = .03 < .05). Meanwhile, negative attitudes toward the older adult population in their physical aspects were significantly associated with a higher likelihood of actual post-retirement employment (p = 0.002 < .01). Findings suggest that societal expectations about older adults' vulnerability in personality, mental health and social participation may hamper their productivity in late life. Future studies are warranted to further examine how self-perceptions about aging in different domains shape post-retirement employment planning in a larger sample.

Abstract citation ID: igaf122.3883

### AGING, WEALTH, AND PROSOCIALITY: INSIGHTS FROM LARGE NATIONALLY REPRESENTATIVE SURVEYS

Jack Kirwan[1], Enna Chen[2], and Laura Carstensen[2], *1. Stanford Center on Longevity, Stanford, California, United States, 2. Stanford University, Stanford, California, United States*

Socioemotional selectivity theory (SST) posits that as future time horizons shorten, older adults increasingly prioritize emotional meaning. Reflecting this shift, prosocial behavior such as charitable giving tends to rise with age. Yet this pattern has not been tested in large, nationally representative surveys. This gap is important given that older adults hold a majority of national wealth and their giving decisions carry broad societal implications. Using data from the Federal Reserve's Survey of Consumer Finances, we found that in 2022, adults aged 55 and over held 73% of the national wealth (ages 40-54: 20%; under 40: 7%), up from 53% in 1989, a one-third surge across three decades. Prosocial behavior was examined in the Panel Study on Income Dynamics, a nationally representative sample collected in 2023 (N = 7,694; ages 18–98, M = 48, SD = 16). Consistent with prior findings, older adults were more likely to donate (β = 0.01, p<.001, $R^2$=.081) and donated larger amounts (β = 38.47, p<.001, $R^2$=.013) than younger adults. Similarly, older adults were more likely to volunteer (β = 0.002, p<.001, $R^2$=.005) and volunteered more hours (β = 0.01, p<.001, $R^2$=.004) than younger adults. These effects held after controlling for wealth, race, and marital status. Taken together, the growing concentration of wealth among older adults and their heightened prosociality suggest they may represent a strategic focus for philanthropy. Encouraging charitable giving in later life could help redirect intergenerational wealth transfers to promote equity.

Abstract citation ID: igaf122.3884

### ANALYSIS OF GREEN HOUSE HOME PERFORMANCE ACROSS DOMAINS OF THE CMS NURSING HOME FIVE-STAR QUALITY RATING SYSTEM

Dan Andersen[1], Marla DeVries[2], Margaret Calkins[3], Christina Schilstra[4], Patrick Miller[4], and Susan Ryan[5], *1. Explore Digits, Sykesville, Maryland, United States, 2. AgingIN, Linthicum Heights, Maryland, United States, 3. IDEAS Institute, Cleveland Heights, Ohio, United States, 4. Explore Digits, Rockville, Maryland, United States, 5. Center for Innovation, Linthicum, Maryland, United States*

The Green House (GH) model of nursing home care, characterized by fewer beds per unit, empowered staff, and a resident-directed philosophy, has long been associated with culture change innovation. To evaluate GH performance on national metrics of quality, we linked 53 sites that include GH homes to CMS data sources including the Five-Star Quality Rating System. We compared sites with GH homes to all other nursing homes across multiple domains: overall star rating, health inspections ratings, staffing ratings, quality measure ratings, and individual indicators such as case-mix adjusted staff time and quality measures. Sites with GH homes outperformed the national average across most metrics. For example, 78% of sites with GH homes received a 4- or 5-star rating for staffing compared to 31% nationally; sites with GH homes provided 56% more adjusted nurse aide time than the national average; and 68% of sites with GH homes earned a 4- or 5-star rating for quality measures compared to 49% of non-GH sites. Sites with GH homes also demonstrated fewer low-star ratings across every domain. Weekend staffing levels, a common area of decline in traditional homes, remained significantly higher in sites with GH homes. These differences are even more pronounced in a sub-analysis of GH-only campuses (i.e., sites with no traditional nursing home beds), though this subgroup was not reported separately due to small sample size. These findings suggest that adopting the GH model supports consistently higher quality outcomes, reinforcing its potential as a scalable strategy for improving nursing home care nationwide.

Abstract citation ID: igaf122.3885

### ASSOCIATION OF ORAL HEALTH AND GENETIC RISK WITH INCIDENCE OF DEMENTIA

Yunrui Liu[1], Xiang Qi[1], Ruotong Mona Liu[1], Sophie Zhijing Xu[1], Huabin Luo[2], and Bei Wu[3], *1. New York University, New York, New York, United States, 2. East Carolina University, Greenville, North Carolina, United States, 3. NYU Shanghai, Shanghai, China (People's Republic)*

Genetic predisposition and oral health problems both contribute to the risk of dementia, yet it remains unclear whether poor oral health confers similar risk across different levels of genetic susceptibility. We investigated the joint effect of oral health problems (painful gums, bleeding gums, loose teeth, toothache, and dentures) and genetic risk on incident dementia in the UK Biobank cohort. Participants (N = 364,557) free of dementia at baseline (2006–2010) were followed until 2022. Genetic risk was defined by polygenic risk score (PRS; categorized as low, intermediate, and high) and APOE ε4 carrier status. Cox proportional hazards and competing risk regression models were used to estimate associations with all-cause dementia (ACD) and its major subtypes, Alzheimer's disease (AD) and vascular dementia (VaD), adjusting for demographic, socioeconomic and health-related factors. During follow-up, 1,915 cases of ACD, 3,278 AD, and 1,581 VaD were identified. Oral health problems, particularly painful gums and dentures, were associated with increased risk of ACD, AD, and VaD. Higher genetic risk, assessed by both PRS and APOE ε4, was also strongly associated with incident dementia. Analyses of joint effects indicated that painful gums and dentures demonstrated additive interactions with genetic risk, although no multiplicative interaction was observed. These findings suggest that poor oral health is independently associated with elevated dementia risk and may exacerbate the burden among genetically susceptible individuals. Improving oral health care could represent a modifiable strategy for dementia prevention.

Abstract citation ID: igaf122.3886

### ASSOCIATIONS BETWEEN RETINAL NERVE FIBER LAYER CHANGES IN GLAUCOMA AND FUNCTIONAL GAIT METRICS

Niranjani Nagarajan[1], Neil Alexander[1], Mark Redfern[2], Emma Baillargeon[3], Lauro Ojeda[1], Srinath Soundararajan[1], Rakie Cham[2], and Amanda Bicket[1], *1. University of Michigan, Ann Arbor, Michigan, United States, 2. University of Pittsburg, Pittsburg, Pennsylvania, United States, 3. Northwestern University, Chicago, Illinois, United States*

Glaucoma affects mobility measured in clinic. This work investigated associations of structural measures of glaucoma with at-home mobility, an indicator of real-world function. We recruited 38 participants with glaucoma and captured spatiotemporal gait characteristics in clinic and at home using inertial measurement units. Recent optical coherence tomography retinal nerve fiber layer (RNFL OCT) measurements – a structural severity indicator – were obtained from participants' medical records. We performed two sets of regression analyses: In the first, each gait metric was a dependent variable and RNFL OCT an independent variable; in the second, within-subject differences between in-clinic and at-home gait were dependent variables. Thinner superior RNFL (more advanced glaucoma) in the better eye was associated with reduced step regularity ($p = 0.04$) and increased step duration variability ($p = 0.03$) in clinic. Thinner inferior RNFL in the worse eye was associated with slower gait ($p = 0.05$) and shorter steps ($p = 0.03$) at home. Additionally, thinner inferior RNFL in the worse eye was associated with slower gait ($p = 0.01$), shorter step length ($p = 0.02$), and longer step duration ($p < 0.01$) at home vs in-clinic. Our findings expand the common perceptions that a person's better eye drives function in glaucoma and that mobility is most impacted by superior RNFL thinning. We find new associations of inferior RNFL thinning in the worse eye with slower gait and shorter steps at home and with greater differences between at-home and in-clinic gait. The finding that more glaucomatous worse eye may make someone susceptible to gait change in real-world environments has not been reported previously.

Abstract citation ID: igaf122.3887

**BARRIERS TO TREATMENT INITIATION AMONG ELDER ABUSE VICTIMS: AN ANALYSIS OF REFERRALS TO THE PROTECT PROGRAM**

Clare Culver[1], Rebecca Kiflom[2], Isabel Rollandi[3], Tobi Abramson[4], and Jo Anne Sirey[1], *1. Weill Cornell Medical College, New York City, New York, United States, 2. Weill Cornell Medical College, NEW YORK, New York, United States, 3. Weill Cornell Medicine, New York City, New York, United States, 4. NYC Department for the Aging, New York, New York, United States*

Elder abuse (EA) is a growing public health concern, affecting 1 in 6 older adults globally. Among victims, one-third experience significant depression, yet most remain untreated due to barriers to mental health care access. We analyzed factors influencing therapy initiation among EA victims referred to the PROTECT program, an evidence-based, short-term depression treatment provided alongside abuse amelioration services. We assessed demographic and abuse-specific barriers to treatment initiation (defined as attending an initial evaluation visit), as well as reasons for exclusion or attrition. Of 501 eligible referrals received between May 2020-December 2024, 78.2% initiated treatment, 8.4% were unreachable, and 13.4% refused services prior to initiating treatment. EA victims were racially diverse (57.6%) and mostly female (81.6%). No significant differences were found between initiators and refusers in gender, race, abuse status, legal action, time to assignment, or depression severity. Initiators were significantly younger than those who refused, p =. 045. Clients speaking English or Spanish were significantly more likely to start treatment compared to speakers of other languages (p = 0.010; OR = 4.40). Findings suggest that demographic and legal factors may not significantly influence treatment engagement in this population while language and age may continue to act as barriers for EA victims. PROTECT was designed to help reduce these barriers by addressing socioeconomic limitations and by proactively reaching out to participants rather than requiring them to seek services. Continuing to address these factors is essential for designing targeted strategies to enhance mental health service access and initiation in this vulnerable population.

Abstract citation ID: igaf122.3888

**BASELINE COGNITIVE CONTROL PREDICTS WEIGHT LOSS AFTER INTERVENTION IN MIDDLE AND OLDER ADULTS**

Marrium Mansoor[1], Kristen Howard[1], Jenna Warnock[2], Theresa Libera[1], Jyoti Savla[3], Kevin Davy[1], Brenda Davy[1], and Benjamin Katz[1], *1. Virginia Tech, Blacksburg, Virginia, United States, 2. Virginia Polytechnic Institute and State University, Blacksburg, Virginia, United States, 3. Dept. of Human Development and Family Science, Blacksburg, Virginia, United States*

Cognitive processes, including attention and executive function (EF), are linked to multiple health behaviors including weight maintenance. In a 12-week clinical weight loss trial, 109 middle aged and older adults (50-70 years old) who had a BMI above 25 were randomized to control or lifestyle intervention (diet and physical activity) groups. Participants (76.4% female, 80% White) completed a set of cognitive tasks including the Attention Network Task (ANT) as well as physiological measurements at baseline and week 12. A mean weight change of -4.5 kgs (SD = 3.3) was observed across 12 weeks. A linear regression model was created with BMI change as the outcome with age, sex, race, education, intervention group, baseline BMI, and ANT performance as covariates. While experimental group was not associated with differences in BMI, reaction time (RT) on correct incongruent trials of the ANT significantly predicted BMI change (B= -.23, p=.033) suggesting that faster RTs on incongruent trials was linked to a larger decrease in BMI. Incongruent trials of the ANT are thought to reflect executive attention, the cognitive process that confers the ability to ignore irrelevant stimuli while focusing on the target stimulus or goal. This finding is consistent with other studies on EFs and weight loss and add to the literature by suggesting that executive attention may be an important cognitive process to consider in interventions aimed at helping individuals make lifestyle changes for weight loss health more generally. However, further research is needed to fully explore these links between EFs and weight maintenance.

Abstract citation ID: igaf122.3889

**BEER PIMS AND GEMS-RX PRESCRIBING IN TREAT-AND-RELEASE ED VISITS AMONG OLDER VETERANS: A PROOF-OF-CONCEPT STUDY**

Michael Zanchelli[1], Kimberly Judon[2], Orna Intrator[3], William Hung[4], and Ula Hwang[5], *1. James J. Peters VAMC, Bronx, New York, United States, 2. James J. Peters VA Medical Center, Bronx, New York, United States, 3. James J Peters VA Medical Center, New York, New York, United States, 4. James J. Peters Department of Veterans Affairs Medical Center, New York, New York, United States, 5. NYU Grossman School of Medicine, New York, New York, United States*

Nearly half of older Veterans (≥65 years) visiting emergency departments (EDs) receive new medications. Potentially inappropriate medication (PIMs) lists, such as Beers Criteria, primarily address long-term use, yet consideration of indications, dosing, and shorter therapies with ED prescribing may not apply. The Geriatrics Emergency Medicine Safety Recommendations (GEMS-Rx) is a Beers subset of high-risk ED medications that include first-generation antihistamines, first-generation antipsychotics, benzodiazepines, barbiturates, hypnotics ("Z-Drugs"), metoclopramide, skeletal muscle relaxants, and long-acting sulfonylureas. This proof-of-concept study used a convenience sample of Veterans to provide initial understanding of GEMS-Rx use in VA EDs. We conducted retrospective analysis used VA's Corporate Data Warehouse (CDW) for Veterans who received care in 12 VA Hospital-In-Home (HIH) programs between 10/2021 and 3/2024 and among them any presentation to the ED during this period. Data included patient demographics, ED visit details, and medications prescribed from the ED. Among 20,034 ED visits by 3,684 unique Veterans, mean age was 76 years ± SD 7.7, 60% were non-Hispanic White, 12% were non-Hispanic Black, 5% were female. Of these, 7,112 (35%) of the ED visits had at least one ED medication prescribed. 2,450 (34%) of these ED prescribed medications were categorized as Beers PIMs. 1,674 (68%) of the Beers PIMS were considered high-risk ED medications according to the GEMS-Rx criteria. Many ED medications prescribed to older Veterans are considered potentially inappropriate or even high-risk, underscoring gaps in age-specific medication safety. Incorporating ED-focused guidance such as GEMS-Rx could promote safer medication selection and potentially reduce adverse events.

Abstract citation ID: igaf122.3890

### BRAIN NETWORK PREDICTION OF OUTCOMES FOR FAMILY CAREGIVER DEPRESSION

Belise Swartwood[1], Felipe Jain[2], and Benjamin Wade[3], *1. The General Hospital Corporation, Boston, Massachusetts, United States, 2. Massachusetts General Hospital / Harvard Medical School, Boston, Massachusetts, United States, 3. Massachusetts General Physicians Organization, Inc., Boston, Massachusetts, United States*

Alzheimer's disease and related dementias affect over seven million Americans. Family members who provide care and assistance to their relatives often experience psychological burden and emotional distress, including elevated rates of depression. Caregiver response to depression treatment is highly variable, resulting in delays and inefficacious interventions for many, but there are no validated biomarkers to guide therapy. Overall brain function and depression treatment response is thought to be determined in part by the strength of large-scale brain connectivity networks. Resting-state functional magnetic resonance imaging (fMRI) can estimate these connectivity strengths. In a pilot study, we examined whether baseline connectivity could predict depressive symptom improvement following Mentalizing Imagery Therapy (MIT) or control conditions in family caregivers of people living with dementia ($N=43$). Participants underwent resting-state fMRI and completed a depression measure before and after treatment. Independent Component Analysis extracted 45 functional connectivity components per participant. Least Absolute Shrinkage and Selection Operator (LASSO) regression with 10-fold cross-validation identified features associated with changes in QIDS scores. SHAP values were obtained to determine feature strength. Permutation testing (1,000 iterations) assessed statistical significance. Connectivity patterns within several networks—including the dorsal and ventral default mode networks, a somatosensory network, and central executive network—were significantly associated with symptom change in combination with baseline depression and treatment group ($R2=0.71$; $p<0.05$). These findings suggest that pre-treatment brain network connectivity may serve as a biomarker for identifying caregivers most likely to benefit from intervention. Such neurobiologically informed predictions might help personalize depression treatment for family caregivers.

Abstract citation ID: igaf122.3891

### CARDIOVASCULAR DISEASE AS A RISK FACTOR FOR DEPRESSION IN KOREAN OLDER PERSONS

Heashoon Lee, *Hannam University, Daejeon, Republic of Korea*

As populations live longer, supporting emotional well-being alongside physical health is essential to healthy aging. This study explores how specific cardiovascular diseases (CVDs) are associated with depressive symptoms among Korean older persons. While earlier studies often examined depression as a risk factor for CVD, this research reverses that approach—investigating CVD as a predictor of late-life depression using newly available national data. Data were drawn from 1,648 Korean people aged 65 years and older, using the 2023 Korea National Health and Nutrition Examination Survey (KNHANES), which became publicly available after the general GSA abstract deadline. Analyses used a complex sampling design and included t-tests, Rao–Scott chi-square tests, and multiple logistic regression adjusted for relevant covariates. Results showed that older persons diagnosed with angina pectoris were 3.62 times more likely to report depressive symptoms, those with dyslipidemia were 1.91 times more likely, and those with stroke were 2.32 times more likely, compared to those without these conditions. In contrast, hypertension, systolic/diastolic blood pressure, and myocardial infarction were not significantly associated with depression. Additionally, women were 3.03 times more likely than men to experience depressive symptoms. These findings underscore the value of integrating cardiovascular and mental health services in age-inclusive care models. Tailored interventions for those living with angina pectoris, dyslipidemia, or stroke may enhance both emotional well-being and quality of life for older people in diverse aging societies.

Abstract citation ID: igaf122.3892

### CAREGIVER IDENTITY AND RESILIENCE ACROSS AND BEYOND DEMENTIA CARE: A QUALITATIVE STUDY

Ida Ghaemmaghamfarahani, Chizobam Nweke, and R. Amanda Cooper, *University of Connecticut, Storrs, Connecticut, United States*

With increasing dementia prevalence and reliance on spousal care partners, understanding how caregiving identity evolves and how resilience is fostered across the care trajectory is crucial. While dementia research often emphasizes caregiver burden, fewer studies explore how identity and resilience develop in response to shifting roles and changing relationship dynamics. Guided by Caregiver Identity Theory, we conducted a thematic analysis of 18 in-depth interviews with long-term spousal caregivers of people living with dementia to examine how they experience identity transitions and cultivate resilience as care dynamics evolve. Five interrelated themes emerged: disruption of familiar roles and relational structures, highlighting loss of shared routines and partner reciprocity; internalizing the caregiver identity, reflecting deepened commitment and sense of duty; navigating uncertainty and strain, encompassing emotional, physical, and existential stressors, including ambiguous loss and caregiving self-doubt; sustaining identity through adaptation and engagement, illustrating how caregivers reframed challenges, sought support, and retained purpose; and meaning-making through connection and shared history, capturing how long-term bonds, lucid moments, and partner appreciation contributed to resilience and meaning. Findings highlight caregiving identity as a dynamic, evolving process. Caregivers developed resilience by adapting to changing roles, remaining grounded in personal values, and making meaning of their experiences over time. For some, caregiver identity continued beyond active care, offering insight into how long-term roles and meaning-making evolve. These insights can inform research and guide efforts to better understand and support caregivers' evolving identities, needs, and processes through which they construct resilience and meaning across and beyond the care journey.

Abstract citation ID: igaf122.3893

### CARING FOR CAREGIVERS OF OLDER ADULTS: A QUALITY IMPROVEMENT INNOVATION APPROACH

Diane Mariani, and Alison Wagner, *Rush University Medical Center, Chicago, Illinois, United States*

Caregivers of older adults in the United States are crucial partners in the health and well-being of older adult care recipients. Despite their key role in caring for the nation's older adults, which is in existence due to the nation's lack of a plan for care for those who are not in need of residential care, caregivers are rarely asked whether or not they are ready, willing or even able to provide care, and neither are their needs identified or addressed within health systems. Caring for Caregivers (C4C) was developed at Rush University Medical Center to create system change by addressing how caregivers are identified within an Age-Friendly Health Systems, and to provide services and support for family caregivers that focus on their physical and emotional health and well-being. The first model to formally integrate caregivers into Age-Friendly Health Systems, C4C provides a comprehensive assessment of needs, strengths, and the 4Ms namely What Matters, Medication, Mentation, Mentation, and Mobility and then develops an individualized intervention plan for each caregiver. Follow up assessments for burden depression, and anxiety are completed at both one-month and three-month post intervention. Late-breaking data demonstrating statistically significant reductions in burden, depression, and anxiety symptoms for caregivers will be shared. Caregivers who completed an initial assessment and at least one follow-up assessment after completing the intervention, demonstrated a decrease in symptoms of depression via PHQ-9 ($n=47$; $z= -134.5$, $p=0.03$), anxiety via GAD-7 ($n=46$; $z=203.5$, $p=0.002$), and caregiver burden via the Burden Scale for Caregivers ($n=45$; $z=-193.5$, $p=0.01$).

Abstract citation ID: igaf122.3894

### CHARACTERISTICS OF THE DEMENTIA CARE WORKFORCE IN NURSING HOMES AND ASSISTED LIVING COMMUNITIES IN THE U.S.

Lei Chen[1], Donovan Maust[2], and Joanne Spetz[1], *1. University of California, San Francisco, San Francisco, California, United States, 2. University of Michigan, Ann Arbor, Michigan, United States*

The professional dementia care workforce directly supports people with Alzheimer's disease and related dementias across multiple care settings. Although much research has focused on family and unpaid caregivers, less is known about those providing care in a professional capacity and the extent to which their characteristics influence outcomes for people with dementia. This study analyzes the first wave of the National Dementia Workforce Study (NDWS) Nursing Home ($N=394$) and Assisted Living ($N=447$) Staff Surveys' Public Use Files. Weighted descriptive statistics and chi-squared tests were used to examine the overlap and distinct attributes of workers across these two settings. Findings indicate that nursing home staff were more likely to hold licenses (e.g., licensed practical/vocational nurse; certified nursing assistant), have higher education attainment, and earn higher hourly wages compared to assisted living workers. They were also more likely to report holding multiple jobs for pay and to get health insurance and benefits from their current employer. However, nursing home staff reported greater challenges in communication and more disrespectful behavior from residents' family members. Significant differences were also observed between the two settings by country of origin and race/ethnicity. This study provides a population-level portrait of the dementia care workforce in the U.S., highlighting both commonalities and unique challenges across nursing homes and assisted living facilities. Findings can inform policy and program development to strengthen the recruitment, retention, and support of a diverse professional dementia care workforce and to improve care quality for people living with dementia.

Abstract citation ID: igaf122.3895

### CHARACTERIZATION OF DELIRIUM AND ITS SEVERITY ACROSS THREE DISTINCT CLINICAL SETTINGS

Jason Albaum[1], Tamara Fong[2], Richard Jones[3], and Sharon Inouye[4], *1. Marcus Institute for Aging Research, Hebrew SeniorLife, Boston, Massachusetts, United States, 2. Beth Israel Deaconess Medical Center, Harvard Medical School, Boston, Massachusetts, United States, 3. Brown University, Brown University, Rhode Island, United States, 4. Marcus Institute for Aging Research, Hebrew SeniorLife, Harvard Medical School, Boston, Massachusetts, United States*

Delirium and its severity (i.e., the intensity or degree of delirium symptoms) can be difficult to measure in people with dementia due to substantial overlap in symptoms and clinical features, complicating both diagnosis and research. In this descriptive study, we used data from the Better Assessment of Illness II (BASIL II) study of adults aged ≥70 years recruited from medical inpatient (Site 1), inpatient elective surgery (Site 2), or skilled nursing facility (Site 3) settings, to characterize delirium and its severity in settings with varying dementia prevalence. Trained staff conducted patient and proxy interviews, chart review, and delirium assessments at baseline and 1-2 days after acute illness or surgery using the Confusion Assessment Method (CAM; delirium present/absent) and CAM-Severity (CAM-S; scored 0-19, 19 = worst). Among 488 participants (mean [SD] age 79 [6] years; 58% female; 75% white), dementia prevalence was 23% (Site 1), 11% (Site 2), and 83% (Site 3). Following acute illness or surgery, the proportion meeting criteria for delirium and median (IQR) CAM-S scores among those with delirium were: Site 1, 50%, score 6 (4-6); Site 2, 1%, score 3 (3-3); Site 3, 24%, score 8 (6-10). Delirium rates and severity showed different patterns of variation across settings. Dementia prevalence may be associated with greater delirium severity; however, it is possible that chronic cognitive symptoms may also elevate delirium severity scores. These findings underscore the pressing need for studies validating measures of delirium severity in populations with dementia to advance research and patient care.

Abstract citation ID: igaf122.3896

### CLINICIAN PERSPECTIVES ON WORKFLOW, EHR INTEGRATION, AND DEMENTIA CARE MANAGEMENT FOR HOSPICE TRANSITIONS

Daniella Torres[1], and Komal Murali[2], *1. NYU College of Global Public Health, New York, New York, United States, 2. New York University Rory Meyers College of Nursing, New York, New York, United States*

This study explores healthcare professional perspectives on workflow, electronic health record (EHR) use, and care management during hospice transitions for persons with dementia and their care partners. Eighteen nurses, nurse practitioners, and physicians (with experience in acute care and home healthcare) participated across three semi-structured virtual focus groups. Inductive coding was applied to identify key themes. The thematic analysis revealed three interconnected domains of facilitating optimal hospice transitions: structured workflows, EHR integration, and optimizing care manager roles. Participants emphasized the importance of structured, multi-step communication to clarify responsibilities, improve care partner support, and ensure continuity of care. Workflow integration was described as requiring clinical flexibility to balance thoroughness with feasibility, including proper timing, patient privacy, follow-up, and interdisciplinary collaboration. EHR use emerged as central to supporting seamless workflow and documentation. Clinicians preferred direct messaging over automated flags to reduce alert fatigue and favored embedding hospice transitions checklists and advanced care planning templates within the EHR to facilitate information sharing and linking documentation to actionable outcomes. Care managers were identified as facilitators rather than decision-makers who provide targeted guidance and have defined responsibilities in hospice transitions. Telephonic, virtual, and in-person communication were highlighted as context-dependent strategies, complemented by follow-up hospice resources for caregivers. Findings suggest that hospice transitions care relies on structured workflows, EHR optimization, and care manager roles. Interventions that balance standardized tools with personalized communication and provider workflow and caregiver experience may improve the quality and consistency of hospice care delivery for persons with dementia and their care partners.

Abstract citation ID: igaf122.3897

### COGNITIVE FUNCTION AND 30-DAY READMISSION RISK AFTER SKILLED NURSING FACILITY-TO-HOME TRANSITIONS

Indrakshi Roy[1], Jennifer Carnahan[2], James Slaven[3], and Emily Fox Ludden[4], *1. Indiana University, Bloomington, Bloomington, Indiana, United States, 2. Indiana University Center for Aging Research, Indianapolis, Indiana, United States, 3. Indiana University, Indianapolis, Indiana, United States, 4. Regenstrief Institute, Inc., Indianapolis, Indiana, United States*

Transitions from skilled nursing facilities (SNFs) to home are common among Medicare beneficiaries, yet they remain one of the least studied and most vulnerable care transitions. Unlike patients discharged directly home from the hospital, those who first receive SNF care are typically more medically complex, experience multiple transitions, and face a heightened risk of adverse events such as hospital readmissions. Because most SNF patients ultimately return home, understanding this second transition is essential for ensuring quality of care, continuity, and patient and caregiver well-being. We used Health and Retirement Study data linked to Medicare claims and Minimum Data Set (MDS) assessments from 2000–2019 to examine the association between cognitive function and 30-day hospital readmission after SNF-to-home discharge. Cognition was measured using the MDS-based Cognitive Function Scale (CFS). Among 4,527 beneficiaries, we applied generalized estimating equations to generate odds of readmission among those with moderate to severe impairment. After adjusting for demographic and clinical characteristics, CFS was not significantly associated with 30-day readmission (OR = 1.17; 95% CI = 0.91–1.50). However, patients with moderate or severe cognitive impairment had modestly higher unadjusted readmission rates (18.0%) compared with those with intact or mild cognition (15.3%), suggesting unmet needs in discharge readiness or community support. Our findings indicate that cognition alone may not independently predict short-term rehospitalization, but it remains clinically relevant in transitional care planning. By focusing on SNF-to-home discharges, this study highlights a population where targeted interventions—such as enhanced caregiver engagement and community-based services—may reduce preventable readmissions and improve outcomes for older adults.

Abstract citation ID: igaf122.3898

### COGNITIVE RESERVE MODERATES THE IMPACT OF DEPRESSION ON COGNITIVE PERFORMANCE IN AGING

Rabia Khalaila, *Zefat Academic College, Zefat, HaZafon, Israel*

depression and cognitive performance in older adults and explores how cognitive reserve (CR)—measured through a composite score of education, occupational complexity, and cognitive activities, as well as each individual component—moderates this relationship over a four-year period. Methods: Data from 32,325 participants aged 50+ in the Survey of Health, Aging, and Retirement in Europe (SHARE) were analyzed. Cognitive performance was measured using memory, numeracy, and verbal fluency tests at baseline and after four years. Depressive symptoms were assessed with the EURO-D scale, and CR was evaluated through education, occupational complexity, and cognitive activity engagement. Moderation effects were tested using bootstrapping with resampling strategies. Results: Lower and average CR levels were linked to a stronger negative association between depression and cognitive performance, while higher CR showed no adverse effects. Education and cognitive activities significantly reduced depression's impact on cognitive function, whereas occupational complexity had no significant effect. Conclusions: Cognitive reserve, particularly through education and cognitive activities, moderates the impact of depression on cognitive performance in older adults. Public health strategies should promote CR-enhancing activities to protect cognitive health in later life.

Abstract citation ID: igaf122.3899

### COLLABORATING WITH PATIENTS AND COMMUNITIES TO IDENTIFY HEALTHY REENTRY NEEDS OF OLDER WOMEN AFTER INCARCERATION

Amanda Emerson[1], Jill Peltzer[1], Amanda Thimmesch[2], Alexandria Thompson[1], Bernard Schuster[1], Sharla Smith[1], and Janet Severine[1], *1. University of Kansas Medical Center, Kansas City, Kansas, United States, 2. University of Kansas Medical Center, University of Kansas Medical Center, Kansas, United States*

Background: Older women's health needs after incarceration have not been a focus in research, resulting in a lack of evidence to guide the design of programs to support health in reentering older women. In Older Women Leading Healthy Aging Research Together (OWLHART), our Kansas City-based team worked with a patient-based community group to answer two questions: "What are priority health needs for older women after incarceration?" and "What are opportunities and obstacles to supporting older women in healthy aging after incarceration?" Method: With the 12-member OWLHART Network, we planned listening sessions and developed interview guides. Group and individual interviews took place on Zoom with three participant groups in February 2025: older women with lived experience of incarceration (LEI), community and peer advocates, and healthy aging researchers. OWLHART then joined us over several months in thematically analyzing deidentified data using the framework method and comparing themes across groups. Results: Five individual or group interviews were conducted with seven women (age 48-68) with LEI, four community advocates, and two researchers. Theme clusters included "priority health-related needs of older women during reentry"; "solutions that do or could work"; and "what's research got to do with it." Convergence was observed across the three clusters with notable divergences in priority needs and research. Conclusion: Women, advocates, and researchers mostly agree about priority needs and what works in supporting older women post-incarceration. Future intervention and health promotion design would benefit from leveraging older women's desire to be involved in developing and implementing solutions.

Abstract citation ID: igaf122.3900

### COMMUNITY HEALTH WORKER DELIVERED PAIN SELF MANAGEMENT IMPROVES FUNCTION IN OLDER ADULTS WITH COGNITIVE IMPAIRMENT

Jennifer Blackwood[1], Donovan Maust[2], Rachel Davis[3], Elizabeth Brines[3], Charity Hoffman[4], Amanda Leggett[5], Joan Ilardo[6], and Mary Janevic[7], *1. University of Michigan-Flint, Flint, Michigan, United States, 2. University of Michigan Medical School, Ann Arbor, Michigan, United States, 3. University of Michigan, Ann Arbor, Michigan, United States, 4. University of Michigan Health System, Ann Arbor, Michigan, United States, 5. Wayne State University, Detroit, Michigan, United States, 6. Michigan State University, East Lansing, Michigan, United States, 7. University of Michigan School of Public Health, Ann Arbor, Michigan, United States*

Older adults with chronic pain and mild cognitive impairment or Alzheimer's disease–related dementias (MCI/ADRD) face dual challenges that undermine function and quality of life, yet few interventions address both conditions. We developed and tested STEPS-CI, a telephone-based pain self-management program delivered by community health workers (CHWs) and adapted for cognitive impairment. Fifty-two participants (mean age 67.1; 83% women; 46% African American; 27% rural, 44% Detroit-based) were randomized to intervention (n = 23) or control (n = 29). Participants in the intervention arm received seven weekly CHW-led sessions, supported by tailored materials co-designed with an advisory council from the National Council of Dementia Minds. At post-program follow-up, Global Impression of Change ratings showed significant improvements in function, pain, and quality of life for intervention vs. control participants (all p <.01) as well as in social participation (PROMIS 4-item scale, p=.03). These gains were accompanied by favorable trends in reduced pain interference and decreased self-reported pain medication use. The program was feasible and well-accepted, with 82% retention, >90% CHW fidelity to session protocols, and strong qualitative feedback highlighting relevance and accessibility. Findings suggest that CHWs can successfully deliver adapted behavioral pain management strategies to older adults with MCI/ADRD, producing functional benefits that matter to patients and families. This pilot study provides new data supporting advancement to a fully powered Behavioral Intervention Stage 3 trial to evaluate the efficacy of STEPS-CI for improving pain-related outcomes in diverse older adults living with both pain and dementia.

Abstract citation ID: igaf122.3901

### COMMUNITY PERCEPTIONS ON DEMENTIA-FRIENDLY INFRASTRUCTURE NEEDS: A MIXED-METHOD STUDY

MD Sarafat Hossain, Sudikshya Sahoo, Nicole Ruggiano, and Amie Brunson, *University of Alabama, Tuscaloosa, Alabama, United States*

Developing dementia-friendly communities with necessary infrastructural support can largely contribute to ensuring equitable access to care and public life among individuals with dementia. As part of the Building Our Largest Dementia Infrastructure (BOLD) initiative, this study investigated the perspectives of stakeholders (e.g., caregivers, providers, educators, NGO workers, Govt. employees) about the current infrastructural preparedness in Alabama and assesses the remaining needs to support dementia patients and their caregivers. Using a convergent mixed methods design, a statewide survey and series of focus groups were conducted to identify public priorities for supporting people with dementia, their families, and their providers. Quantitative data underwent uni- and multi-variate analyses and qualitative data underwent a multi-step coding process to identify themes. Data were also triangulated to enhance the trustworthiness of findings. A total of 29 people participated in four focus group discussion sessions and 397 people completed the online survey. The analysis identified four themes related to physical and social infrastructure that community members identified as priorities for improving dementia care: (1) infrastructural barriers to accessing information and care; (2) insufficient interventions to educate people about dementia; (3) unmet dementia caregivers' needs for their social determinants of health; and (4) multi-system factors to improve the quality of dementia care. Survey data confirmed qualitative thematic findings. These findings have significant implications for how investments in practice, policy, and research may help close gaps in dementia care and promote dementia-friendly communities. Theoretically it reinforces the ecological system theory drawing attention to built and service environments for dementia.

Abstract citation ID: igaf122.3902

### COMMUNITY SERVICE UTILIZATION AND AGING ATTITUDES IN OLDER CHINESE ADULTS: THE ROLE OF CHRONIC CONDITIONS

Zhijing Xu[1], Yaguang Zheng[1], and Bei Wu[2], *1. New York University, New York, New York, United States, 2. NYU Shanghai, Shanghai, China (People's Republic)*

Abstract As China faces an aging population and rising chronic disease prevalence, understanding factors shaping aging attitudes is essential. This study investigates how community service utilization was associated with aging attitudes among older Chinese adults and whether having chronic conditions made a difference. Data from the 2020 China Longitudinal Aging Social Survey (CLASS) were analyzed using multiple regression. Among 7,111 participants aged 60 and older, 5,574 (78.38%) had chronic conditions. Among individuals with chronic conditions, home visits were significantly associated with more negative attitudes toward aging (b = -1.241, p < 0.001), while services such as accompaniment to medical appointments (b = 1.185, p = 0.026), household assistance (b = 0.628, p = 0.033), and participation in daycare center activities (b = 1.279, p = 0.006) were associated with more positive attitudes toward aging. Among older adults without chronic diseases, elderly meal delivery was the only service significantly associated with more negative attitudes toward aging (b = -3.065, p = 0.048). In conclusion, community service utilization plays a significant role in shaping aging attitudes, with varying effects depending on the presence of chronic conditions. Services that promote independence, such as accompaniment and household assistance, are more likely to foster positive aging perceptions, whereas services like home visits and meal delivery may reinforce negative views, especially among those with chronic diseases.: Community Service Utilization, Aging Attitudes, Chronic Conditions

Abstract citation ID: igaf122.3903

### COMPARING DXA AND BIA FOR ASSESSING CHANGES IN MUSCLE MASS IN OLDER ADULTS FOLLOWING STRENGTH TRAINING

Daniel Lee, Afeo Ikhide, Noberto Quiles, Bridget McFadden, and Anoop Balachandran, *Queens College, Flushing, New York, United States*

Dual-energy X-ray absorptiometry (DXA) is frequently used to assess sarcopenia and muscle mass in older adults. However, DXA is expensive, requires a licensed X-ray technician, and involves radiation exposure. In contrast, bioelectrical impedance analysis (BIA) is quick, cheaper, requires no expertise, and does not involve radiation. Despite these practical advantages, comparative studies investigating change in fat-free mass (FFM) in response to resistance training in healthy older adults are limited. We compared DXA vs. BIA for FFM assessment in response to a strength training program in older adults. Community-dwelling older adults (n = 11, M = 68 years [SD 3.3], 75% female) underwent both DXA and BIA measurements under standardized conditions at baseline and after 20 weeks. Full-body strength training regimen of eight exercises was performed twice a week for 20 weeks using plate-loaded machines. The intraclass correlation coefficient (ICC) was used to assess absolute agreement. Bland–Altman (BA) plots were used to evaluate measurement error and limits of agreement. Absolute agreement for change in FFM between DXA and BIA was good, with an ICC of 0.86 (95% CI [.56, .96]), $F(10,10) = 12.38$, $p < .001$. The BA analysis showed a mean difference of 0.04 kg (95% CI [−0.35, 0.43]), with limits of agreement ranging from −1.09 to 1.17 kg. Our findings demonstrate good agreement between DXA and BIA for change in FFM in response to strength training in older adults. However, a larger sample size is needed to draw stronger conclusions about the utility of BIA.

Abstract citation ID: igaf122.3904

### COMPARING LIVING EXPERIENCES IN SUBDIVIDED UNIT AND PUBLIC HOUSING IN HONG KONG: A QUALITATIVE STUDY

Peiyi Lu, *University of Hong Kong, Hong Kong, Hong Kong, China (People's Republic)*

Crowded housing conditions have been linked to poorer health, with these effects potentially intensifying in older age. Yet, existing research has largely focused on general adult population without comparing aging experiences across housing types. This study explores how housing environments shape aging by comparing middle-aged and older adults (aged 45+) living in subdivided units (SDUs) and public housing in Hong Kong. Thirty participants—15 from SDUs and 15 from public housing—were purposefully selected. Face-to-face interviews using open-ended questions explored how individuals perceived their housing conditions to affect their daily lives, health, and social relationships. Thematic analysis was conducted to identify key patterns. Most participants had low incomes and received government aid. SDU residents, mainly single men in bedspace apartments, reported strained family ties and poorer living conditions, including noise, unsanitary environments, air pollution, and lack of elevators. As they aged, they expressed a need for age-friendly housing with more space, cleanliness, privacy, and accessibility. In contrast, public housing residents were generally satisfied with their living environments. The findings suggest that public housing offers a more supportive environment for aging than SDUs. The inadequate conditions in SDUs pose risks to physical and mental health as individuals grow older. To promote healthy aging, it is recommended that the government strengthen and enforce building regulations for subdivided units to meet aging-related health standards.

Abstract citation ID: igaf122.3905

### COMPLEX CARE IN DEMENTIA: OLDER CAREGIVERS PROVIDE MORE, LONGER-HOUR CAREGIVERS PROVIDE LESS

Sutthinee Thorngthip, and Kylie Meyer, *Case Western Reserve University, Cleveland, Ohio, United States*

As the backbone of dementia care, family caregivers play an essential role in supporting individuals living with dementia as they provide a variety of care, from helping with daily activities (e.g., dressing) to assisting with complex care tasks (e.g., wound care, managing medication). Family caregivers often provide complex care without sufficient training, which can negatively affect their mental health and increase burden. However, few studies focus on characterizing complex care provided by family caregivers to inform such intervention. This study identified complex care tasks performed by caregivers and explored associations between the number of tasks provided and caregiver age, weekly hours of caregiving, and confidence with caregiving. A total of 131 caregivers completed surveys. Complex care questions were based on 17 items from the 2021 national AARP Home Alone report. Findings revealed that caregivers performed an average of 5.11 complex care tasks (SD = 2.54). The most frequent tasks were managing medications (93.9%, n = 123), preparing special diet (57.3%, n = 75), using telehealth equipment (56.5%, n = 74). Furthermore, older caregivers provided more complex care tasks ($r = .215$; $p = .014$), while caregivers providing a higher number of caregiving hours performed fewer tasks ($r = .302$; $p < .001$), potentially due to the integration of formal care support. Caregivers performing more complex care tasks reported lower confidence in taking care of different aspects of dementia care ($r = .234$; $p = .007$). The findings highlight the need for tailored caregiver training programs and accessible resources to equip family caregivers with skills and confidence for complex care tasks.

Abstract citation ID: igaf122.3906

### CONNECTION AND PROTECTION: A LIFESPAN ANALYSIS OF TECHNOLOGY USE IN LGBTQ+ ADULTS

Emily Briggs, and Jess Francis-Levin , *University of Michigan, Ann Arbor, Michigan, United States*

Finding community and forming social ties looks a lot different today than it did 50 years ago. For members of the LGBTQ+ community, formation of a safe and affirming community adds a layer of complexity to the ever evolving process of developing meaningful social connections. This exploratory study investigates responses to an open-ended prompt in a large multi-measure survey inquiring about technology use in the LGBTQ+ community (N = 338). Participant responses (N = 89) about their technology use provided insight into how social technology is uniquely harnessed by members of the LGBTQ+ community in a sample of young (Age, 18-34, n = 45), midlife (Age, 35-64, n = 31), and older adults (Age, 65+, n = 13). Themes generated from our thematic analysis highlight the different expectations held across the lifespan for community using social technology. While young adults sought out niche spaces tailored to their specific interests, midlife adults discussed using social media to form in person connections, and older adults disclosed details about their in-person community. Furthermore, while older adults expressed the most caution for interacting via online platforms, the theme of using social technology to maintain boundaries with toxic relations was present across all age groups. Taken together, these responses highlight the importance of online spaces as a place for the intentional formation of safe social communities inclusive of the evolving social community needs for adults across the lifespan.

Abstract citation ID: igaf122.3907

### COVID-19 EXPOSURE, TREATMENT PATTERNS, AND HEALTHCARE UTILIZATION IN US LONG-TERM CARE: A RETROSPECTIVE EHR STUDY

Sohini Ganguli[1], Brittin Wagner[2], Florin Draica[1], Daniel Verdi[1], Kate Mathers[3], Ralph Valeriote[3], and Stefan Gravenstein[4], *1. Shionogi Inc, Florham Park, New Jersey, United States, 2. PointClickCare, Bloomington, Minnesota, United States, 3. PointClickCare, Mississauga, Ontario, Canada, 4. Alpert Medical School of Brown University, Providence, Rhode Island, United States*

Long-term care (LTC) residents remain disproportionately affected by COVID-19 due to age-related vulnerabilities and complex comorbidities. Here we characterize COVID-19 exposure, treatment, and healthcare resource utilization (HCRU) in U.S. LTC facilities using a large-scale electronic health record (EHR) database. We conducted a retrospective observational study using PointClickCare's Life Sciences EHR database (2022–2025). COVID-19 exposure, diagnosis, and treatment events were identified using ICD-10 codes and medication orders. Outcomes included demographic and clinical characteristics, antiviral treatment patterns, contraindicated medication use, and HCRU metrics. Among 1.4 million COVID-19-related events documented in the EHR, we found 143,943 exposures, 932,821 diagnoses, and 350,421 treatments. Residents with documented exposures to COVID-19 were younger (mean age 72) than those diagnosed (77) or treated (79). Just under 20% of medication orders for COVID-19 antiviral treatment were associated with EHR documented diagnosis within 14 days. Paxlovid was the most prescribed antiviral, though ~43% of treatments occurred without an EHR-documented diagnosis or exposure. Medication adjustments to reduce interactions between chronic disease medications and antiviral treatments were made for approximately one-third of patients on a contraindicated medication (35% on Paxlovid and 28% on molnupiravir). COVID diagnosis was associated with increased MACE, hospitalizations, and oxygen use, especially in the first week following diagnosis. This study highlights critical gaps in exposure documentation and antiviral treatment uptake in LTC settings. Findings underscore the need for improved coding practices, prescriber education, and understanding timely post-exposure prophylaxis strategies to reduce clinical burden and improve outcomes for LTC residents.

Abstract citation ID: igaf122.3908

### CROWDSOURCED DRUG DISCOVERY APPROACH IDENTIFIES BET INHIBITORS AS NOVEL GEROPROTECTORS PROMOTING HEALTHY AGING

Byungju Kim, and Samuel Beck, *Boston University, Boston, Massachusetts, United States*

The significant growth of the elderly population, driven by advances in biomedical science and public health, poses a critical socio-economic challenge. Identification and targeting of reliable biomarkers are essential for developing effective strategies to extend healthspan and lifespan. One promising target is age-associated misexpression of genes lacking CpG islands (CGI-genes), which is commonly observed across a broad range of aged tissues and contributes to chronic inflammation and loss of cell identity. Notably, established anti-aging interventions, such as calorie restriction, fasting, and a methionine-restriction diet, inhibits this dysregulation, suggesting that pharmacological suppression of CGI- gene misexpression would provide an effective strategy to promote healthy aging. To streamline this process, we developed a novel in silico drug discovery approach leveraging crowdsourced transcriptomic data and machine learning. Our approach significantly outperformed existing resources such as Connectivity Map, which exhibit crucial weaknesses in capturing pathological gene expression patterns in vivo. We analyzed CGI-gene expression in drug-treated vs. control groups using over 100,000 GEO samples and identified BET-inhibitors as potential geroprotectors suppressing CGI- gene misexpressions. Our experimental validation demonstrates that long-term (14-week) treatment with BET inhibitor effectively suppresses age-associated decline in muscle strength, body balance, and memory function, delays epigenetic aging, and promotes better survival in aged mice. Taken together, our study demonstrates that a pharmacological targeting of CGI- gene misexpression using a BET inhibitor provides an effective strategy for promoting healthy aging. In addition, our novel crowdsourced drug discovery approach offers an effective and practical solution for combating aging and associated diseases.

Abstract citation ID: igaf122.3909

### CULTIVATING CULTURAL SENSITIVITY FOR PROSPECTIVE GERIATRIC PROFESSIONALS THROUGH STUDY ABROAD IN JAPAN

Aya Yoshikawa[1], Kenshi Nishino[2], and George King[3], *1. Texas Woman's University, Texas, United States, 2. Furate Medical and Welfare Corporations, Kitakyushu, Fukuoka, Japan, 3. Texas Woman's University, Denton, Texas, United States*

Studying abroad provides a unique experiential learning opportunity to develop students' cultural sensitivity, critical thinking, problem-solving, and collaboration skills, which are vital for improving the health and well-being of increasingly diverse older adults. In May 2025, 15 undergraduate students from nursing and social work took part in a three-week study abroad course in Japan, one of the fastest-aging societies in the world. During this course, students visited a geriatric care organization in Kitakyushu that provides clinical care and community support for older adults. This learning environment allowed students to investigate various determinants of health and techniques for promoting health among older adults. Students gained greater understanding of aging issues by interacting with older adults, clinicians, the city mayor, city officials, and representatives of aging agencies. Students also engaged in hands-on therapeutic sessions with older adults and an intergenerational service-learning event with a university student organization. Through reflection journal exercises, students recognized similarities and differences in population aging between Japan and the United States and discussed how culture influences healthcare, as well as how to apply their course experience and knowledge to their future professions. Toward the end of the course, students collaborated to create group presentations on risk factors for pre-assigned chronic illnesses (i.e., dementia, cardiovascular disease, age-related joint and bone disorders) and recommended prevention/management strategies from both Japanese and American perspectives. The challenges of course design and delivery, as well as changes in students' perspectives on public health and aging issues and healthcare over the course, will be highlighted.

Abstract citation ID: igaf122.3910

### CULTIVATING EARLY INTEREST IN AGING-RELATED PROFESSIONS: EVALUATION OF A HIGH SCHOOL–BASED "SILVER AGE COURSE"

Xinxin Cai, Xinyu Yi, Xiang Li, and Xue Bai, *The Hong Kong Polytechnic University, Hong Kong, China (People's Republic)*

Background: The rising demand for care professionals highlights the need to cultivate early career interest. As high school students increasingly align studies with career goals, introducing aging-related careers presents a strategic opportunity. This study evaluated whether a weekly "Silver Age Course," exposing students to aging, older adults and care professionals, could foster orientation toward care careers. Methods: A mixed-methods sequential explanatory design was adopted. Phase 1 involved a quasi-experiment with enrolled students ($n = 28$) and a control group ($n = 31$). Career orientation, willingness and confidence in communicating with older adults, understanding and application of knowledge and skills, impression of older adults, and compassion were measured before and after the course. Repeated-measures MANCOVA was used for analysis. Phase 2 included four rounds of reflective diaries (T1 to T4) from enrolled students ($n = 28$), analyzed using thematic trajectory analysis. Results: Significant time*group interactions were found for career orientation and understanding of knowledge and skills after controlling for social relationships ($p < 0.05$). Thematic trajectory analysis revealed that the course fostered care and aging-related career orientation, shifting students from non-care to care-related interests, vague to specific career goals, and unrelated aspirations to incorporating a caring mindset. These changes stemmed from students' deeper understanding of aging, enhanced compassion, and maturity. Growth extended into school and family life, reinforcing motivation to care careers. Conclusions: High school students' interest in aging-related professions can be fostered through experiential and project-based learning. Such programs support holistic growth of adolescents and help cultivate future care professionals.

Abstract citation ID: igaf122.3911

### CURRICULUM DEVELOPMENT OF A CLINICAL COURSE ON AGE-FRIENDLY HEALTH SYSTEM 4MS CARE IN NURSING HOMES

Megan Kazakoff, Yurun Cai, Beth Schwartz, Jacqueline Dunbar-Jacob, and Elizabeth Schlenk, *University of Pittsburgh, Pittsburgh, Pennsylvania, United States*

As the U.S. population ages, there is an urgent need to prepare nurses specialized in geriatric care, particularly in long-term care. Clinical curricula in nursing homes (NHs) are needed to enhance geriatric training for prelicensure students to improve patient outcomes. The purpose of this project was to design a practicum course with input from both NH staff and experienced instructors focused on principals of geriatric nursing care. University of Pittsburgh School of Nursing developed and implemented an 8-week, 1-credit elective clinical course based on the Age-Friendly Health Systems (AFHS) 4Ms framework through an academic-practice partnership with The Willows NH. Undergraduate nursing students engaged in weekly learning activities and developed a case study applying the 4Ms. Evaluation included pre- and post-course surveys of student and faculty competency ratings. Post-course data showed positive trends in student competency, especially in clinical assessment skills and person-centered care. Qualitative feedback highlighted improved understanding of geriatric care and nurse-resident relationships. The AFHS 4Ms-based course demonstrates that NHs can be effective clinical learning environments. Structured and competency-based curricula can enhance student preparedness for geriatric nursing. By leveraging structured orientation, strong academic-practice collaboration, and reflective learning, nursing programs can prepare students to meet the complex needs of aging populations in a way that also challenges misperceptions about NH practice.

Abstract citation ID: igaf122.3912

### DEVELOPING AND IMPLEMENTING AGE FRIENDLY CARE: INTERPROFESSIONAL COURSE FOR UNDERSERVED POPULATIONS

Diana Woods[1], David Picella[2], Adria Navarro[3], Adam Solomon[4], Diane Chau[2], Nghia Pham[4], Amit Khurana[5], and Traci Frees[6], *1. Azusa Pacific University, Azusa Pacific University, California, United States, 2. Azusa Pacific University, Azusa, California, United States, 3. University of Southern California, Los Angeles, California, United States, 4. Care Connect, Costa Mesa, California, United States, 5. Medica, Minnetonka, Minnesota, United States, 6. Azusa PAcific University, Azusa, California, United States*

Background: To enhance the geriatric workforce capacity in underserved settings, Azusa Pacific University (APU) Geriatric Workforce Enhancement Program (GWEP) established an Interprofessional Education (IPE) council. Representing academia and practice in medicine, nursing, pharmacy, social work, physical therapy, and spiritual care ministry, the council developed a training program to promote interprofessional (IP) team-based care for minority and underserved older adults using the Age-Friendly Health Systems' 4Ms framework (What Matters, Medication, Mentation, Mobility). Description: The council met regularly over three months to create a 12-week, 20-hour-per-week hybrid course, delivered using Canvas with instructional design support. The 12-week curriculum, focused on the 4Ms, includes modules about the fundamentals of geriatrics, normal aging, polypharmacy, dementia, falls, and ethics. Knowledge is integrated during the last 2 weeks of the course. Diverse students apply knowledge in IP teams during weekly faculty-led online case study discussions. Comments include "We are learning so much from each other"; This course is extremely helpful". Baseline RedCap surveys gathered demographics, consent, attitudes on aging, age-friendly care, and IPE; post assessment will mimic baseline surveys plus, competencies, and knowledge implementation. Students earn an APU Interprofessional Geriatric Program certificate upon completion. Evaluation: Fourteen students applied; 10 were accepted from an assisted living facility and hospice programs. Discussions focus on real-world underserved cases. We are compiling pre-post data on attitudes and impact. Implications: This model advances geriatric workforce development through interprofessional collaboration, and has a potential for improved competencies, practices, and advocacy. Future cohorts will include degree-seeking IP students.

Abstract citation ID: igaf122.3913

### DEVELOPING AND VALIDATING NOVEL EHR-BASED MULTIMORBIDITY-WEIGHTED INDEX ACROSS COMORBIDITY MEASURES

Melissa Wei[1], Ashley Kang[2], Alexandra Klomhaus[2], Lucia Chen[2], and Chi-Hong Tseng[2], *1. UCLA and VA, Los Angeles, California, United States, 2. University of California, Los Angeles, Los Angeles, California, United States*

Multimorbidity and physical functioning are important to measure for older adults, but tools capturing both are lacking in electronic health record (EHR) data. The multimorbidity-weighted index is weighted to physical functioning and has been validated for claims and EHR data but is not designed specifically for clinical data available in the EHR (medications, labs). Herein, we develop an EHR-based multimorbidity-weighted index (eMWI) for use in EHR systems using the Observational Medical Outcomes Partnership (OMOP) Common Data Model and validate its performance against prior comorbidity measures. To develop our 76-condition eMWI, we applied our previously validated physical functioning-based disease weights to 1) published condition algorithms when available, and 2) chart review-validated ICD-code-based definitions for remaining conditions. Using a University of California Health Data Warehouse dataset of adults ≥18 years with ≥2 outpatient encounters, 2012-2024, we compared multimorbidity values and distributions for eMWI, Charlson, Elixhauser, and simple disease count. Our final cohort included 4,672,662 adults, 55.7% female, with mean±SD age 46.3±16.8. Patients had higher multimorbidity values (mean±SD) using eMWI (5.66±7.85) vs Charlson (1.11±1.92), Elixhauser (1.89±2.51), and disease count (3±3.55). eMWI demonstrated superior performance in quantifying unique values of multimorbidity burden, exhibiting the widest range of scores (0-91) with only 26% having a score of 0. By comparison, Charlson scores ranged from 0-25 and 59% had scores of 0. We demonstrate that our newly developed eMWI can be used in EHR data for quantifying multimorbidity burden in comorbidity adjustment and risk stratification tools for adults across the lifespan, with advantages over prior measures.

Abstract citation ID: igaf122.3914

### DEVELOPMENT OF A 6-YEAR SARCOPENIA RISK PREDICTION MODEL BASED ON AN ELDERLY POPULATION: A PROSPECTIVE COHORT STUDY

Yu Wang, Maolin Zhou, Xiaochu Wu, and Yanyan Wang, *West China Hospital, Sichuan University, Chengdu, Sichuan, China (People's Republic)*

Sarcopenia, a progressive age-related condition that is difficult to diagnose, increases the risk of adverse outcomes, including falls, frailty, and Alzheimer's disease in older adults.Existing prediction models are limited by small sample sizes, difficult-to-collect predictors, and short prediction horizons. This study aimed to develop and validate a 6-year sarcopenia risk prediction model using routinely available clinical data.We employed a prospective cohort design, analyzing data from the West China Health and Aging Trend (WCHAT) study (baseline in 2018, 6-year follow-up in 2024), The baseline sample comprised 1,184 participants without sarcopenia, and data were collected on demographics, physical examinations, questionnaires, and laboratory tests. A support vector machine (SVM) model was developed, and sarcopenia was diagnosed according to the 2019 Asian Working Group for Sarcopenia (AWGS) criteria.During the 6-year follow-up, 78 participants (6.6%) developed sarcopenia.The final model included 19 predictors: age, weight, height, BMI, tooth count, toothbrushing frequency, waist-to-hip ratio, skeletal muscle mass, usual gait speed over 3 meters, mid-upper arm circumference, height-to-arm-circumference ratio, calf circumference, height-to-calf-circumference ratio, waist circumference, hip circumference, mobile phone use, fasting insulin, and HDL.Model validation showed a sensitivity of 0.71 (95% CI: 0.49–0.83), specificity of 0.78 (95% CI: 0.77–0.86), and AUC of 0.84 (95% CI: 0.77–0.90), respectively.This model, based on easily accessible clinical variables, showed good predictive performance for 6-year sarcopenia risk, indicating its potential clinical utility for identifying high-risk individuals who may benefit from closer monitoring or early intervention.

Abstract citation ID: igaf122.3915

### DISCRETE EMOTIONS, DEPRESSIVE SYMPTOMS, AND CAREGIVER BURDEN IN ADULT CHILDREN OF PARENTS WITH COGNITIVE CHANGE

Dustin Gad[1], Jenna Wells[1], and Joan Monin[2], *1. Cornell University, Ithaca, New York, United States, 2. Yale University, New Haven, Connecticut, United States*

Family dementia caregivers often struggle with caregiver burden and higher rates of depression than similarly-aged non-caregivers. Specific negative emotions including guilt and anger have been implicated in family dementia caregivers' mental health cross-sectionally. However, few studies have investigated discrete positive emotions and how discrete emotions influence caregivers' mental health longitudinally. Our study addresses these gaps in N = 145 adult children of parents with cognitive change, who completed questionnaires at baseline (T1) and one year later (T2) about their emotions (PANAS), depressive symptoms (CES-D), and caregiver burden (ZBI-12). For cross-sectional associations (T1), we conducted Pearson's correlations between each emotion and outcome. For longitudinal associations, we conducted linear regressions with each emotion at T1 predicting changes in each outcome from T1 to T2, controlling for T1 levels of the outcome. All emotions that were significantly correlated with outcome variables ($p < .0025$ with Bonferroni correction) were then included in a regression predicting the respective outcome variable. Cross-sectionally, greater distress, upset, shame, and fear were associated with greater depressive symptoms; greater guilt was associated with greater caregiver burden. Longitudinally, greater guilt was associated with increased depressive symptoms (B = .345); greater determination was associated with decreased caregiver burden (B = -.281). Findings suggest alleviating guilt (i.e., feeling that one has upset or harmed another person) through cognitive restructuring may help protect caregivers' mental health; and promoting determination through caregiver skills trainings, support groups, and strength recognition techniques (e.g., benefit-finding), may mitigate caregivers' long-term feelings of burden.

Abstract citation ID: igaf122.3916

### DO ADVERSE CHILDHOOD EXPERIENCES (ACES) INFLUENCE GETTING RECOMMENDED VACCINES FOR FLORIDA RESIDENTS AGES 50+?

Stephanie Clervil[1], Roseline Ukenenye[2], Sandro Louis[1], Priscilla Ryder[3], and Aymara Diaz[4], *1. Miami Dade College, Miami, Florida, United States, 2. Howard University, Washington, District of Columbia, United States, 3. Larkin University College of Pharmacy, Miami, Florida, United States, 4. Larkin University, Miami, Florida, United States*

Adverse childhood experiences (ACEs) are traumatic events that occur before the age of 18, including abuse, neglect, and family dysfunction. ACEs are associated with many adult physical and mental health outcomes (e.g., cardiovascular disease, diabetes, cancer, arthritis, depression and anxiety, premature death). ACEs are also associated with negative health behaviors (e.g., tobacco use, binge drinking). Little is known about the effects of ACEs on other health behaviors, such as getting recommended immunizations. This investigation used data from the 2023 Behavioral Risk Factor Surveillance System (BRFSS) from Florida for respondents ages 50 and older. ACEs were summed to give a score of 0 (no adverse experiences) to 8 (maximum number). The typical respondent was a White, retired, female, high school graduate who received Medicare. 3679 (44.6%) and 4597 (55.0%) reported getting shingles and pneumonia vaccines, respectively. The median number of ACEs was 1, with 0 being the most common value. In bivariate analysis, ACEs were highly associated with being vaccinated (Chi-square (8) = 26.557, $p < 0.001$)—the higher the number of ACEs, the smaller the percentage vaccinated. ACEs lost significance in binary logistic models for receiving the vaccines. Factors that increased the odds of being vaccinated were having a personal health care provider and being retired, while being Black or Hispanic (both vaccines), being Asian (pneumonia vaccine only), and reporting poor mental health decreased the odds of vaccination, adjusting for education and home ownership. The relationship between ACEs and immunization may be mediated by socioeconomic factors.

Abstract citation ID: igaf122.3917

### DO HIGH VOLUME ANTI DEMENTIA DRUG PRESCRIBING DOCTORS BILL MEDICARE FOR CARE PLANS?

Michael Splaine, and Kate Gordon, *Splaine Consulting, Columbia, Maryland, United States*

Medical providers can bill the Centers for Medicare and Medicaid Services (CMS) for dementia care plans under code 99483. Dementia care plans provide written information to help provide education and support to the patient and their care provider. The purpose of this data analysis was to determine the relationship between high-volume dementia medication prescribers to providers of dementia care plans. Identifying the relationship between these two factors may help shine a light on a way to improve care for individuals with dementia. The billing codes analyzed were for dementia care plans by high-volume dementia prescription providers across five U.S. markets states from the 2021 Centers for Medicare and Medicaid Services (CMS) database. The main finding across all five was that there was a significantly low use (5% or less) of the dementia care plan billing code amongst the high-volume dementia medication prescribers.

Abstract citation ID: igaf122.3918

### DOES FRAILTY MODIFY THE ASSOCIATIONS OF LATE-LIFE VASCULAR RISK FACTORS WITH INCIDENT DEMENTIA?

Jason Smith[1], A. Richey Sharrett[2], Priya Palta[1], B. Gwen Windham[3], Pamela Lutsey[4], Alden Gross[5], and Jennifer Deal[5], *1. UNC School of Medicine, Chapel Hill, North Carolina, United States, 2. Johns Hopkins University, Baltimore, Maryland, United States, 3. University of Mississippi Medical Center - The MIND Center, Jackson, Mississippi, United States, 4. University of Minnesota, Minneapolis, Minnesota, United States, 5. Johns Hopkins Bloomberg School of Public Health, Baltimore, Maryland, United States*

The contribution of late-life vascular risk factors—hypertension in particular—to dementia remains controversial. Given the association of frailty with lower blood pressure (BP) specifically, we hypothesized that hypertension, but not diabetes or smoking, is more strongly associated with dementia risk in robust than in pre-frail/frail older adults. We used Cox proportional hazards models, adjusted for demographic and clinical/lifestyle characteristics, with a multiplicative interaction term between vascular risk factors and physical frailty (robust versus pre-frail/frail) to estimate cause-specific hazard ratios (HRs) of 11-year incident dementia associated with elevated BP, hypertension, diabetes and current smoking in community-living White and Black participants aged 67-89 years without dementia in the Atherosclerosis Risk in Communities Neurocognitive Study. We then stratified HRs of dementia by frailty. Overall, there were 377 (15.8%) dementia cases in robust participants ($n = 2383$; mean age, 74.2 years; 55.4% female) and 812 (30.0%) in pre-frail/frail participants ($n = 2710$; mean age 76.4 years; 61.3% female). There was an interaction between BP and frailty on dementia risk (P=.04). For robust participants, HRs were 1.06 (95% CI: 0.63-1.77) for elevated BP and 1.34 (95% CI: 0.92-1.94) for hypertension relative to normal BP. For pre-frail/frail participants, HRs were 0.59 (95% CI: 0.40-0.86) for elevated BP and 0.83 (95% CI: 0.67-1.04) for hypertension relative to normal BP. Diabetes and smoking were associated with increased dementia risk in both robust and pre-frail/frail individuals. Our findings suggest frailty status could be useful for clinical management of BP in older adults to support brain health.

Abstract citation ID: igaf122.3919

### DULOXETINE PLUS EXERCISE FOR KNEE OSTEOARTHRITIS: TARGETING PAIN AND DEPRESSION USING COMBINED TREATMENT

Alan Rathbun[1], Yu Dong[1], Brock Beamer[2], Mark Luborsky[3], Michelle Shardell[1], Claudia Campbell[4], Grace Lo[5], and Alice Ryan[1], *1. University of Maryland School of Medicine, Baltimore, Maryland, United States, 2. University of Maryland, Baltimore, Maryland, United States, 3. Wayne State University, Detroit, Michigan, United States, 4. Johns Hopkins University School of Medicine, Baltimore, Maryland, United States, 5. Baylor College of Medicine, Houston, Texas, United States*

Pain and depressive symptoms represent the most common knee osteoarthritis (KOA) phenotype, and their co-occurrence causes bidirectional effects that complicate treatment. Intervening on one condition does not necessarily improve the other. This pilot study tested a 12-week hybrid aerobic exercise program plus duloxetine to treat pain and depressive symptoms in KOA.

Between May 2023 and March 2025, participants with symptomatic KOA and mild to severe depressive symptoms were recruited from the University of Maryland, the VA Maryland Health Care System, and the Baltimore area. The intervention combined a hybrid walking program (two home-based and one supervised session per week) with duloxetine, initiated at 30 mg and titrated to 60 mg. Outcomes were collected at baseline and 12 weeks: Knee injury and Osteoarthritis Outcome Score (KOOS) pain subscale, Hamilton Depression Rating Scale (HAM-D), 6-Minute Walk Distance (6MWD), and Chair Stands. Acceptability was evaluated through recruitment, adherence, and dropout. Recruitment exceeded targets (N = 20), and 34 participants enrolled; 21 initiated treatment and 17 completed the protocol, with >80% adherence. KOOS pain scores increased, indicating less pain (+14.2, 95% CI [6.5, 21.9], d = 0.95), while HAM-D scores decreased, implying fewer depressive symptoms (−5.5, 95% CI [−9.0, −2.0], d = 0.81). Physical function also improved (6MWD = +130 ft, 95% CI [56.7, 204.1], d = 0.91; Chair Stands = +1.8 repetitions, 95% CI [0.7, 2.8], d = 0.88). This study demonstrates acceptability, high adherence, and large efficacy signals for a combined exercise–pharmacologic protocol and underscores the need for rigorous evaluation in clinical trials.

Abstract citation ID: igaf122.3920

### DYNAMIC ASSOCIATIONS BETWEEN INTRINSIC CAPACITY AND DISABILITY BEFORE DEATH AMONG OLDER ADULTS

Olivia Malkowski[1], Kendra Plourde[2], Max Western[3], and Thomas Gill[1], *1. Yale University, New Haven, Connecticut, United States, 2. Yale School of Public Health, New Haven, Connecticut, United States, 3. University of Bath, Bath, England, United Kingdom*

Intrinsic capacity is negatively associated with disability; however, little is known about how the magnitude of this association varies leading up to death. Using time-varying effect modeling (TVEM), a statistical tool for capturing dynamic patterns of change, we evaluated the association between intrinsic capacity and disability as a function of time before death. Data were analyzed from the 746 (of 754 enrolled) participants aged 70+ years of the Precipitating Events Project cohort study who died through November 2024. Intrinsic capacity was assessed at baseline and subsequently at 18-month intervals using 25 self-reported and physical performance measures. Intrinsic capacity scores (range: 0–100) were derived using Bayesian Multilevel Item Response Theory. Disability was assessed monthly and defined as dependence in instrumental activities of daily living. TVEM was applied to evaluate the association between intrinsic capacity and disability, anchored to participants' time of death. Among the 746 decedents (64.2% female), the mean age at death was 88.4 years. Trajectories using a backwards timescale spanning 20 years showed a gradual decline in average intrinsic capacity from 80 to 47 points; the change in disability prevalence was non-linear. The inverse association between intrinsic capacity and disability strengthened as individuals approached death. In the last four years of life, a ten-point increase in intrinsic capacity was associated with 47–48% lower odds of disability. The heightened association between intrinsic capacity and disability at the end of life underscores the importance of monitoring changes in a person's intrinsic capacity, which might offer opportunities for preventive action.

Abstract citation ID: igaf122.3921

### EFFECTIVENESS OF NON-PHARMACOLOGICAL INTERVENTIONS FOR OLDER ADULTS WITH DEMENTIA: AN UMBRELLA REVIEW

Taeko Saito[1], Natsumi Shimizu[2], and Li Yao[3], *1. Nippon Medical School, Inzai, Chiba, Japan, 2. Musashino University, Tokyo, Tokyo, Japan, 3. Chiba University, Chiba, Chiba, Japan*

Dementia is a major global public health concern, with older adults with dementia particularly vulnerable to complications such as delirium and psychological symptoms of dementia (BPSD). Non-pharmacological interventions (NPIs) have been found to be effective for these symptoms, and several meta-analyses have shown NPIs are effective in reducing BPSD delirium. However, evidence on NPIs remains fragmented across existing reviews and there has been no comprehensive evaluation of the strength and validity of NPIs. Therefore, this study aimed to identify assess the previous evidence using umbrella review consolidates data from systematic reviews and meta-analyses. A comprehensive literature search was conducted across PubMed, Medline, Web of Science, CHINALE, PsycINFO from inception to March 2025, 15systematic reviews that met the inclusion criteria were identified. Two reviewers independently screened selected studies, extracted data, and assessed methodological quality using the AMSTAR 2 tool. NPIs included interventions using ICT, exercise, music, light therapy, and various combinations of these approaches. In addition, BPSD and delirium symptoms such as agitation, depression, anxiety, and cognitive function were also addressed. A meta-analysis of non-pharmacological interventions (NPI) for these symptoms was conducted. As a result, NPI was suggested to be effective for several symptoms. Providing integrated knowledge on the current initiatives, this umbrella review study is the first step towards better crafted interventions that support NPI to elderly people with dementia and designing appropriate interventions for BPSD and delirium.

Abstract citation ID: igaf122.3922

### EMPATHY-BASED TRAINING IN A GERIATRIC WORKFORCE ENHANCEMENT PROGRAM (GWEP) FOR DEMENTIA CARE

Guillermina Solis, Emre Umucu, Andrew Vernon, Beatrice Lee, Cecilia Fierro, and Hyejin Jung, *The University of Texas at El Paso, El Paso, Texas, United States*

In the southwest border region, formal dementia training has traditionally been limited, and educational resources and community support for care have been scarce. As a first-year Geriatric Workforce Enhancement Program (GWEP) recipient of a HRSA grant, the goal is to enhance training and educational resources for the formal and informal healthcare workforce in the region. Programs, such as Dementia Live®, that align with provision of content within age-friendly and dementia friendly care are selected to address the critical gap of knowledge as an entry point into dementia care education for diverse disciplines and caregivers. The initiative implemented Dementia Live® training, which provides an immersive, empathy-based experience to 119 Hispanic/Latino participants, who held various care roles in a variety of health care settings. The effectiveness of the training was evaluated with a pre and post training survey and statistical comparison with paired t-test was conducted to examine changes in dementia knowledge. The results showed a statistically significant increase in knowledge scores from pre-training (M = 3.29)

to post-training (M = 3.81), t(118)=5.92, p<.001. Additionally, a moderate effect size (d = 0.54) was observed indicating a meaningful practical impact. This study supports that an empathy-focused educational approach is highly effective in boosting dementia knowledge among multidisciplinary care teams. By reaching a broad, underserved population, this project demonstrates a replicable model for GWEP initiatives to build geriatric care readiness and establish essential educational and community support infrastructure in areas lacking resources. A key strength of this initiative was its ability to engage a diverse workforce.

Abstract citation ID: igaf122.3923

### EMPOWERING HEALTHY HABITS THROUGH COMMUNITY BASED HYPERTENSION SCREENING AND COUNSELING BY HEALTHCARE STUDENTS

Don'aa Williams[1], Taqwa Naas[2], Anna Azuz[2], Maria Sobhie[2], Jennifer Mendez[1], Brittany Stewart[2], and Rima Charara[1], *1. Wayne State University School of Medicine, Detroit, Michigan, United States, 2. Wayne State University, Detroit, Michigan, United States*

Hypertension is highly prevalent in the community, yet it often goes undetected, especially among older adults with limited access to care. Although blood pressure is routinely checked during doctor visits, individuals who do not seek regular care may dismiss their symptoms. As a chronic, multifactorial illness, hypertension can lead to poor health outcomes and is known as a "silent killer." To address this, Wayne State healthcare students provide free blood pressure screenings and health counseling to the community every week from June to August at the Detroit Riverwalk. Supervised by physicians and pharmacists, students gain hands-on experience conducting holistic assessments and providing medication and lifestyle counseling, with particular attention to hypertension among older adults. In the summer of 2025 alone, the program conducted approximately 470 screenings. A total of 82 students participated, including 66 medical students and 16 pharmacy students, all of whom completed the AMA blood pressure training modules prior to volunteering. These in-person screenings not only strengthened students' skills in accurate blood pressure measurement but also provided valuable experience in patient-centered counseling and interprofessional collaboration. Riverwalk participants who agreed to be screened received health resources, information on free clinics and primary care providers, as well as nutrition guidance. Survey data revealed that participants left screenings with greater confidence in managing their blood pressure and a strong intention to continue engaging in future health checks. This initiative demonstrates a replicable model of community-centered, interprofessional education that enhances student learning while building trust and promoting autonomy among older adults.

Abstract citation ID: igaf122.3924

### EMPOWERING RURAL DEMENTIA CAREGIVERS THROUGH A TECHNOLOGY-BASED PLATFORM: A CASE STUDY FROM MICHIGAN

Lora Chehab, Rhea Shenoy, Fei Sun, and Daniel Velez Ortiz, *Michigan State University, East Lansing, Michigan, United States*

Providing care for relatives with dementia poses additional challenges for families in rural communities where access to information, education, and supportive services is often limited. This case study examines the implementation of a technology-based platform, Trualta, to support dementia caregivers in 12 selected rural counties in Michigan by providing education, skills training, peer connection, and local resources (e.g., adult day centers, county specific resource guides). Once logged in, caregivers have on demand access to the platform based upon their needs. Data from 172 caregivers enrolled between July 2023 to July 2025 were analyzed to assess usage patterns, impact, and lessons learned. On average, caregivers completed 13.5 learning modules (SD = 32.37), which are dementia care education and skills training; 41 participants attended at least one synchronous seminar. Participants viewed local resources an average of 4.2 times (SD = 5.0). Trualta's peer support forum provides caregivers with space to share questions, reflections, and experiences. On average, participants contributed 2.74 entries to the forum (SD = 10.40), reflecting meaningful engagement. A thematic analysis of posts revealed that the two most prominent categories were daily care techniques and emotional support, which accounted for 33.0% and 17.4% of all entries, respectively. Interaction analysis found posts were notably detailed and insightful, with an average length of 633 characters and 58.0% using structured titles. Findings indicate technology-based interventions can empower rural dementia caregivers by addressing their needs for education, emotional support, and community resources. However, future research needs to evaluate the program's long-term impact on caregiver stress and well-being.

Abstract citation ID: igaf122.3925

### ENHANCING ACTIVITY ENGAGEMENT TO OPTIMIZE MEDICARE HOME HEALTH PATIENT OUTCOMES: A PILOT RCT

Chiung-ju Liu[1], Dorian Rose[2], Peihua Qiu[2], Zibo Tian[2], Alexandra Armstrong[3], and Yvonne Lu[4], *1. University of Florida College of Public Health and Health Professions, Gainesville, Florida, United States, 2. University of Florida, Gainesville, Florida, United States, 3. Duke University, Durham, North Carolina, United States, 4. Indiana University School of Nursing, Indianapolis, Indiana, United States*

Reducing task demands early in rehabilitation and gradually increasing them may enhance functional outcomes for patients receiving home health therapy. This pilot randomized controlled trial, completed in March 2025, examined the feasibility and preliminary effects of the Home-based Activity Reactivation Program, which integrates compensatory and restorative strategies to calibrate task demands as an adjuvant treatment to usual therapy. Forty-seven patients (mean age 82) were randomized to receive the Home-based Activity Reactivation Program plus usual therapy (n = 24) or usual therapy alone (n = 23). The Home-based Activity Reactivation Program was delivered in six weekly home visits, separate from usual therapy sessions. The primary outcome was Motor Skills from the Assessment of Motor and Process Skills; secondary outcomes included multiple measures of physical function. The Home-based Activity Reactivation Program demonstrated high acceptability, with 95% of completers reporting satisfaction and no severe or serious adverse events. Unexpectedly, the control group showed greater improvement in Motor Skills at postintervention (estimated difference -0.29 logits, p = 0.02), though this was not sustained at one-month follow-up. No significant between-group differences were found over time for secondary outcomes. These findings suggest that the Home-based Activity Reactivation Program did not yield superior outcomes compared to usual therapy alone. Potential contributing factors include limited intervention intensity, scheduling challenges, and practitioner bias. The study highlights the complexities of integrating and evaluating adjuvant interventions within routine home health care and underscores the need for further refinement and testing of the Home-based Activity Reactivation Program to optimize its effectiveness.

Abstract citation ID: igaf122.3926

### ENHANCING UNDERGRADUATE INTEREST AND PREPARATION FOR RESEARCH IN AGING AND NEUROLOGIC DISEASES

Natalie Galucia, Brian Carpenter, Claire Wininger, and Michele Dinman, *Washington University in St. Louis, Saint Louis, Missouri, United States,*

Many students pursuing careers in research, policy, or practice in the social and health sciences overlook gerontology and geriatrics due to limited exposure to aging. Additionally, negative stereotypes about older adults and aging discourage students from pursuing careers in these fields. Our NINDS-sponsored summer research program aims to meet this workforce need by recruiting undergraduate trainees with varied academic backgrounds and interests to provide rigorous didactics, structured mentoring, and hands-on research experience. The goal of the program is to foster interest, attitudes, and skills in the aging field and research pertaining to neurologic disorders among older adults. Students spend eight weeks working in research labs under the guidance of faculty mentors and attending professional development seminars. Seven cohorts of participants (n = 88, 68.8% female, 29.1% male, 46.5% White, 19.8% Black, 25.6% Asian) were surveyed before and after the program. Students' self-reported knowledge of research methods increased 34.5%, and skills in data visualization increased 36.3%. The age they consider someone to be "old" increased from 66 to 73 years, suggesting a reframing of how they view aging. Students also considered aging more relevant to their personal lives. A significant proportion of alumni have enrolled in research-oriented graduate school or medical school (36.8%) and pursued public health- or research-related positions (72.3%). Overall, the program has had a positive impact, increasing knowledge and confidence in conducting research, and encouraging students to consider aging in their personal and professional lives.

Abstract citation ID: igaf122.3927

### EVALUATING A CO-GENERATIONAL PROGRAM: BUILDING SOCIAL COHESION ACROSS GENERATIONS

BoRin Kim[1], Sofia Holmes[2], Kelley Petralia[3], Laura Cerniglia[3], Tracy LaCroix[3], Chung Hyeon Jeong[4], and Alexa Johnson[2], *1. University of New Hampshire, University of New Hampshire, New Hampshire, United States, 2. University of New Hampshire, Durham, New Hampshire, United States, 3. Westborough Connects, Westborough, Massachusetts, United States, 4. University of New Hampshire - eHealth readiness, Durham, New Hampshire, United States*

Meaningful intergenerational interactions support both older adults' well-being and adolescent development, yet declining opportunities for engagement contribute to widening social divides in communities. Intergenerational solidarity theory offers a framework for understanding how structured cross-generational contact fosters affective, associational, and functional ties that strengthen cohesion and belonging. Guided by this framework and developed in collaboration with a local community organization, this study evaluated a dyadic co-generational program designed to promote reciprocal connection and civic engagement. We examined how participation influenced social cohesion, perceived connections, and community belonging among older adults and adolescents. Eleven intergenerational pairs of older adults (65+) and adolescents (14–17) participated in a six-week program in which dyads selected and completed weekly community-based activities of their choice. Data were collected through semi-structured post-program group interviews and weekly activity logs, enabling triangulation across self-reported experiences and observed practices. Thematic analysis identified mechanisms of connection, perceived benefits, and barriers to sustained engagement. Shared activities emerged as the primary mechanism fostering connection and reciprocity. Reported benefits included reduced social isolation, stronger community belonging, and more positive perceptions of both age groups. Older adults emphasized the program's role in countering isolation in age-segregated housing, while adolescents valued opportunities to engage with the broader community beyond peers. Barriers included transportation, communication differences, and informal power dynamics. Findings highlight the importance of designing programs collaboratively with community partners to enhance feasibility, cultural fit, and long-term impact. Insights from this program advance understanding of intergenerational solidarity while offering practice-based strategies to foster more inclusive, connected communities.

Abstract citation ID: igaf122.3928

### EVALUATING THE EFFECTIVENESS OF HOME IV THERAPY ADMINISTRATION MODALITIES IN HOMEBOUND OLDER ADULTS

Kimberly Oakman[1], Tammy Sanders[2], Laura Medlin[2], Sandra Sanchez-Reilly[3], Hanh Trinh[4], Valerie Escamilla[5], and Michael Mader[2], *1. Veterans Administration, San Antonio, Texas, United States, 2. South Texas Veterans Health Care System, San Antonio, Texas, United States, 3. South Texas Veterans Health Care System and the University of Texas Health Science Center at San Antonio, San Antonio, Texas, United States, 4. Veterans Health Administration, Veterans Health Administration, Texas, United States, 5. South Texas Veterans Health Care System and UT Health San Antonio, San Antonio, Texas, United States*

Background: Over 80% of older adults (OA) suffer from one or more chronic conditions which affect their ability to cope with injuries or disease exacerbations. When acutely ill, OA usually require intravenous medication administration (IVMA), which traditionally has been done when hospitalized, exposing them to increased adverse events. Home-care-programs have increased their scope-of-practice to allow home-IVMA with >85% goal that medication-enters-the-body (METB). Administration methodology comparisons between home infusions (HI) and intravenous-push-administration (IVP-100% METB) have not been studied. Objective: To compare the IVMA effectiveness using HI vs. IVP in a population of OA receiving IV-treatment at home. Methods: Using three infusion companies and guaranteeing nurses-led visits, first-part-of-study collected one-year detailed data on IVMA methodology/complications via HI. Subsequently, IVP was encouraged (vs. HI) whenever recommended by pharmacy. One-year data on recommendation follow-up/complications/satisfaction was analyzed. Results: Overall, 67% were>65, 95%male, age=43-87. First-part-of-study: N = 15, 60% had>10 IVMA episodes using PICC/midline/porta-cath. Only 53% participants met >85% METB goal with standard HI. Second-part-of-study: N = 154, 28% IVMA were recommended to IVP, those subjects receiving 100% METB. When comparing the pre-and post-groups, complication rate was 10% for both, High satisfaction rates were found among the IVP group. Conclusions: On this preliminary study and when recommended by pharmacy, IVP was shown to be an effective IVMA method as demonstrated by 100%METB without higher complications compared to HI and indicated high patient satisfaction. Further research is needed to evaluate effectiveness of IVP vs. HI among OA, considering nurse manpower, cost-effectiveness (tubing/supplies/pump, infusion preparation, etc.), time-to-completion, METB rates, complications and patient-satisfaction.

Abstract citation ID: igaf122.3929

### EVALUATION OF A POSITIVE PSYCHOEDUCATIONAL INTERVENTION FOR INFORMAL CAREGIVERS: RANDOMIZED CONTROLLED TRIAL

Yoko Moriyama[1], Akemi Matsuzawa[2], Tomoko Wakui[3], Nanako Tamiya[4], and Hideto Takahashi[5], *1. National Institute of Public Health, Wako, Saitama, Saitama, Japan, 2. Hokkaido University, Sapporo, Hokkaido, Japan, 3. Tokyo Metropolitan Institute for Geriatrics and Gerontology, Tokyo, Tokyo, Japan, 4. University of Tsukuba, Tsukuba, Ibaraki, Japan, 5. Teikyo Heisei University, Nakano, Tokyo, Japan*

The purpose of this study is to evaluate the impact of a positive psychology-based online program on the well-being of working-age informal caregivers. We have developed a positive psychoeducational program to enhance the well-being of working-age informal caregivers because we have confirmed it is low. We incorporated three themes in positive psychology—strengths, self-compassion, and best possible selves into the program and, set a two-month implementation period. Five hundred caregivers of older family aged 30 to 59, recruited from a web-based research company, were randomly assigned to an intervention group of 350 and a control group of 150. Surveys on well-being were conducted at four points: baseline, mid-program, post-program, and one month later: follow-up. The dependent variable was the change in Subjective Happiness Scale: SHS scores from baseline to 1) post-program and 2) follow-up, with program participation as the independent variable. Multiple regression analysis was performed, adjusting for baseline SHS, caregiver age, cohabitation status, older family age, care level, dementia, primary caregiver or not, and caregiving frequency. One hundred eighty intervention participants worked on all sessions and 118 control participants responded to the end point survey, and 167 and 104 of whom responded to the follow up survey. At the end point, a significant positive correlation was observed among change in SHS and intervention (coefficient;0.187, P-value;0.036), however at the follow up, it was not observed (-0.04, 0.717). The effect of intervention was observed immediately after implementation, but it was not sustained, indicating further efforts are needed to maintain the effects.

Abstract citation ID: igaf122.3930

### EXAMINING THE ASSOCIATION BETWEEN NURSING HOME LEADERSHIP TURNOVER AND RESIDENT SATISFACTION

Jenny Kwon[1], and John Bowblis[2], *1. University of California, San Francisco, San Francisco, California, United States, 2. Miami University, Oxford, Ohio, United States*

Leadership stability in nursing homes (NHs) plays a critical role in shaping residents' care experience. However, the impact of nursing home administrator (NHA) and director of nursing (DON) turnover on resident satisfaction has not been thoroughly examined. This study investigates the relationship between NHA/DON turnover and resident satisfaction scores. An aggregate dataset of Ohio NHs in 2017 was constructed (N = 752) by merging five data sources: The Ohio Nursing Home Resident Satisfaction Survey, the Ohio Biennial Survey of Long-Term Care Facilities, the Certification and Survey Provider Enhanced Reports, Medicaid Cost Report, and LTCFocus. The outcome variables were eight resident satisfaction scores: overall satisfaction score and seven individual domain scores. The scores were continuous variables, with higher scores indicating greater resident satisfaction (range: 0-100). Leadership turnover was categorized into four predictors: one NHA turnover, two or more NHA turnover, one DON turnover, and two or more DON turnover over the past three years. Multiple linear regressions, including control variables, were conducted via SAS. Results show that two or more DON turnover is associated with a lower overall resident satisfaction score by 1.14% points ($p < .05$) and lower scores of Environment and Caregivers domains. However, NHA turnover is not statistically associated with overall resident satisfaction score, while positively associated with two domain scores. The findings of this study highlight the critical role of DON stability in improving resident satisfaction and suggest that having one leadership turnover may not compromise NH quality; however, frequent turnover, particularly among DONs, could be detrimental.

Abstract citation ID: igaf122.3931

### EXPLORING SOCIAL MEDIA ABUSE IN NURSING HOMES: A CONVERT FORM OF ELDER ABUSE

Wei-Lin Xue[1], Eilon Caspi[2], Layana Aoun[3], Christy Aoun[3], and Pi-Ju Liu[3], *1. Case Western Reserve University, Cleveland, Ohio, United States, 2. University of Minnesota, Minneapolis, Minnesota, United States, 3. Purdue University, West Lafayette, Indiana, United States*

Elder abuse in nursing homes manifests in various forms, including physical harm by staff and neglect of resident care. A newly emerging form of abuse and privacy violation, social media abuse, has become increasingly prevalent but remains largely unrecognized. Social media abuse involves the unauthorized taking and posting of photos and/or videos of residents on platforms such as Snapchat, Facebook, and TikTok, often accompanied by demeaning or abusive contents. Social media abuse in nursing homes has not been systematically examined, with little understanding of characteristics and impact of this type of abuse on residents. This exploratory qualitative study utilizes de-identified data (state investigation reports) from 100 nursing homes across 31 states, accessed through the Nursing Home Inspect (ProPublica) website, to understand social media abuse in nursing homes. Preliminary analysis of investigation reports reveals evidence of privacy violations and abuse affecting 152 nursing home residents. Approximately 85% of these victims exhibited some degree of cognitive impairment, with at least 50 residents suffering from moderate-to-severe cognitive deficits. The findings aim to inform care practices and policy changes to protect residents' privacy, dignity and safety.

Abstract citation ID: igaf122.3932

### FAITH-PLACED SERVICES AND SUPPORTS FOR ALZHEIMER'S DISEASE AND RELATED DEMENTIAS: A SCOPING REVIEW

Noelle Fields[1], Ling Xu[2], Mary Stringfellow[3], Katherine Kitchens[4], and Soeun Jang[5], *1. The University of Texas at Arlington, Arlington, Texas, United States, 2. University of Texas at Arlington, Arlington, Texas, United States, 3. UT Southwestern Medical School, Dallas, Texas, United States, 4. UT Southwestern Medical Center, Dallas, Texas, United States, 5. University of Texas at Arlington, The University of Texas at Arlington, Texas, United States*

Faith-based communities, including churches, temples, and other places of worship, have been identified as important sources of support for Alzheimer's disease and related dementias (AD/ADRD) family caregivers. The role of faith communities as important sources of support has been deeply rooted in cultural, religious, and historical contexts. However, the extent and nature of services and supports provided in faith-placed settings remain unclear. This scoping review focused on the existing literature on faith-placed interventions and services that support AD/ADRD family caregivers. Following Arksey and O'Malley's (2005) scoping review framework, we searched seven databases, including Academic Search Complete, CINAHL Complete, and PsycINFO, for peer-reviewed studies published through August 2024. Studies were eligible if they examined services, interventions, or supports for AD/ADRD family caregivers in places of worship. Two independent reviewers screened articles, and data were charted and thematically analyzed. Eleven studies met inclusion criteria. Faith-placed supports varied widely, including dementia education, caregiver support groups, respite care, and dementia-friendly worship services. These interventions were particularly impactful in underserved and rural communities. Common themes included increased dementia knowledge, emotional and social support, and dementia-friendly modifications. Barriers to implementation included limited clergy training, resource constraints, and cultural stigma around dementia. Faith-placed settings play a critical role in supporting AD/ADRD caregivers.The geographical context of the studies may play an important role in shaping the design and effectiveness of faith-placed interventions for AD/ADRD. Barriers to faith-placed interventions remain, highlighting the need for continued refinement and expansion of such programs.

Abstract citation ID: igaf122.3933

### FEASIBILITY OF A PEER SUPPORT INTERVENTION FOR FAMILY CAREGIVERS OF BLACK OLDER ADULTS LIVING WITH DEMENTIA

Karen Moss[1], Abraham Brody[2], Karen Bullock[3], Alai Tan[4], Kathy Wright[5], and Mary Beth Happ[6], *1. The Ohio State University, Blacklick, Ohio, United States, 2. NYU Rory Meyers College of Nursing, New York, New York, United States, 3. Boston College, Chestnut Hill, Massachusetts, United States, 4. The Ohio State University, Columbus, Ohio, United States, 5. The Ohio State University, Powell, Ohio, United States, 6. Ohio State University, Columbus, Ohio, United States*

Black family caregivers of older adults living with dementia are at high risk for negative impacts of caregiving, including poor overall health. Culturally responsive evidenced-based caregiving support interventions are lacking and may help to meet the unmet needs of Black family caregivers of people living with dementia. The purpose of this study was to test the feasibility and acceptability of the Peer Support for Family Caregivers of Black Older Adults Living with Dementia (Pair 2 Care) Intervention. Pair 2 Care is a 6-month, flexible, virtual peer support intervention co-designed with Black current family caregivers, former family caregivers (care recipient is deceased), community leaders, and healthcare providers. This intervention leverages the experiences of trained former caregivers as mentors to current caregiver mentees. A total of 15 current caregiver mentees were mentored by 11 trained former caregivers; all were female and mostly daughters who were caring or cared for a parent living with dementia. Pair 2 Care was found to be feasible and acceptable. All participants were enrolled, mentors were trained, and mentees and mentors were paired within 10 weeks. Former caregiver mentors were retained at 90% and mentees at 93%. Both mentors and mentees rated their overall Pair 2 Care experience as very high (9/10). Qualitative feedback was overwhelmingly positive, with several participants requesting to continue the program. Pair 2 Care may provide an innovative approach to improving family caregiver health outcomes. This award-winning intervention is poised to become part of existing innovations in aging that promotes health equity.

Abstract citation ID: igaf122.3934

### FEASIBILITY OF MOTIVATIONALLY ENHANCED COMPENSATORY COGNITIVE TRAINING FOR OLDER VETERANS WITH MCI

Kate Shirley[1], Laura Campbell[2], Jacqueline Maye[3], Emily Sano[4], Delaney Pickell[3], Amy Jak[3], Maya O'Neil[4], and Elizabeth Twamley[3], *1. Brown University, Providence, Rhode Island, United States, 2. University of California, San Diego, San Diego, California, United States, 3. VA San Diego Health Care System, San Diego, California, United States, 4. VA Portland Health Care System, Portland, Oregon, United States*

Mild Cognitive Impairment (MCI) increases risk for functional decline and dementia, yet effective interventions for older adults remain limited. Motivationally Enhanced Compensatory Cognitive Training (ME-CCT) is a manualized, 8-week cognitive rehabilitation group intervention delivered in-person and via telehealth that integrates compensatory cognitive strategies, motivational interviewing, and lifestyle education. In this multisite randomized controlled trial, 156 older Veterans (Mean age = 71.5, SD = 7.7) with MCI were randomized to ME-CCT (n = 84) or a Goal-Focused Supportive Contact control condition (SC; n = 72). Among participants who attended at least one session, mean attendance was high in both groups (ME-CCT: 7.7 ± 1.3 of 8 sessions; SC: 7.5 ± 1.7), with few never attending (ME-CCT: 5; SC: 6). Dropout among those who started was low and comparable across groups (ME-CCT: 5.1%; SC: 7.6%). Session attendance for ME-CCT was consistent across delivery formats, with in-person participants (n = 63) attending 7.7 ± 1.2 sessions on average and telehealth participants (n = 21) attending 7.5 ± 1.7 sessions. Dropout rates were also comparable across delivery formats (In-person: 8.3%; Virtual: 10.5%). ME-CCT was well received: 96.9% rated quality as good to excellent, 92.2% reported improved problem management, and 95.2% were satisfied with services. Findings indicate ME-CCT is feasible and acceptable for older Veterans with MCI across delivery formats, with high attendance, low dropout, and strong satisfaction. Future research should prioritize optimizing telehealth delivery and evaluating whether sustained engagement yields cognitive and functional benefits.

Abstract citation ID: igaf122.3935

### FINANCIAL HARDSHIP IN DEMENTIA: COMMUNITY FOCUS GROUP FINDINGS ON MEASURE DEVELOPMENT

Jacqueline Telonidis[1], Nicole Fleming[1], Kara Dassel[1], Jace Flatt[2], Hanna Hedges[1], Jia-Wen Guo[1], Eli Iacob[1], and Sara Bybee[1], *1. University of Utah, Salt Lake City, Utah, United States, 2. University of Nevada Las Vegas, Nevada, United States*

By 2060, nearly 14 million Americans will live with dementia. As one of the most expensive diseases, dementia costs primarily burden persons living with dementia (PLWD) and their care partners. Given the association between financial hardship and worsening health, this study aimed to develop and validate an inclusive dementia-focused measure of financial hardship for LGBTQ+ and non-LGBTQ+ PLWD and their care partners. We conducted two 90-minute focus groups with aging and/or LGBTQ+ health professionals (n = 7)—including social workers, researchers, and nurses—and non-LGBTQ+ PLWD and their care partners (n = 8). The LGBTQ+ PLWD focus group remains to be conducted. Using the Financial Hardship Typology framework, we examined material costs, emotional responses, and coping behaviors. Participants reviewed 27 potential items adapted from the COmprehensive Score for financial Toxicity (COST) used in cancer care, discussing each item's relevance to financial hardship and quality of life using a 4-point Likert scale. Focus groups were audio recorded, transcribed verbatim, and analyzed using qualitative content analysis. Ratings were summarized with descriptive statistics. The most relevant items included: using savings for medical care, worrying about affording care, worrying if income covers care costs, and distress about future financial security. Both groups recommended separate measures for care partners and PLWD based on disease severity. Dyads emphasized psychological distress from dementia-related financial hardships. These findings will inform development of a more feasible, accessible, and useable financial hardship measure for PLWD and their care partners.

Abstract citation ID: igaf122.3936

### FINANCIAL STATUS, BURDEN, AND FAMILY CAREGIVER WELL-BEING: A CROSS-SECTIONAL ANALYSIS

Megan Thomas Hebdon[1], Amy Patten[2], Sandi Alshahwan[3], and Carolyn Phillips[2], *1. The University of Utah, Salt Lake City, Utah, United States, 2. University of Texas at Austin, Austin, Texas, United States, 3. University of Utah, Salt Lake City, Utah, United States*

Family caregivers are fundamental to the care and support of individuals with chronic illnesses and disabilities. Both caregiver burden and financial challenges may impact the health and well-being of family caregivers. The purpose of this study was to explore the impact of financial status and caregiver burden on caregiver well-being. Family caregivers (N = 60) of individuals with chronic illnesses and/or disabilities were recruited using online recruitment methods. A rigorous fraud protocol was used to screen potential participants. After screening and consent procedures, participants completed an online cross-sectional survey with demographic questions, including income and perceived financial adequacy, and measures for caregiver burden and holistic well-being. Data were analyzed using descriptive statistics, correlation analysis, and hierarchical linear regression. Caregivers were primarily women (n = 48; 80%) and White (n = 40; 67%). Half reported having an adequate income (n = 30; 50%; average income $58,873.25, SD = 41,187.05). Annual income was significantly associated with financial adequacy (r=.42, p<.01) and meaning and purpose (r=.28, p < 01), but no other well-being domains. Annual income and perceived financial adequacy were not significantly related to caregiver burden. Perceived financial adequacy was significantly positively associated with all well-being domains (r=.30-.47, p<.01,). Caregiver burden was significantly negatively associated with all well-being domains, except financial and material stability (r=-.23, p=ns). When controlling for income, the relationships among financial adequacy, caregiver burden, and well-being domains remained. Our findings suggest that caregivers' perceptions of their financial situation have more impact on well-being than income. In addition, burden and perceived financial adequacy may be key independent factors related to caregiver well-being.

Abstract citation ID: igaf122.3937

### FOUR-DIMENSIONAL CONTINUITY GAPS IN VOLUNTEER-INTEGRATED SERVICES FOR OLDER ADULTS: MIXED-METHODS STUDY IN CHINA

Mingzhu Yang[1], Shuang Cai[2], and Jinsong Pan[3], *1. Shanghai General Hospital Shanghai Jiao Tong University School of Medicine, Baltimore, Maryland, United States, 2. Shanghai General Hospital Shanghai Jiao Tong University School of Medicine, Shanghai General Hospital, Shanghai Jiao Tong University School of Medicine, Shanghai, China (People's Republic), 3. Shanghai General Hospital, Shanghai Jiao Tong University School of Medicine, Shanghai, China (People's Republic)*

Rapid urban population ageing in China is increasing demand for community-based services for older adults. Volunteer-supported programs, offering companionship, emotional support, and assistance with daily tasks, are promoted as complements to formal care, yet their integration into resilient systems with functional, relational, informational, and managerial continuity remains unclear. This study aimed to identify barriers, examine service discontinuities, and integrate findings within a four-dimensional continuity framework to inform strategies for cohesive volunteer engagement. An explanatory sequential mixed-methods design was applied in districts with the highest proportion of older adults, Hongkou and Huangpu, Shanghai. A cross-sectional survey of 880 older adults (response rate 92.9%) measured willingness, utilization, and predictors of underuse or dissatisfaction. Multivariable logistic regression, adjusted for demographic and health factors, identified predictors. In-depth interviews with 21 older adults, volunteers, and Red Cross staff explored mechanisms. Findings were integrated via the Pillar Integration Process, mapping to four continuity domains. Although 96.3% expressed willingness, only 46.5% used services in the past year. Digital illiteracy, particularly among those aged > =80 years (89.3%), and high volunteer turnover ( > =3 changes in 6 months) significantly predicted underuse and dissatisfaction (adjusted odds ratio=2.31). Qualitative results revealed policy–service dissonance shifting unmet needs to volunteers, relational instability from turnover, digital–informational gaps causing fragmented handovers, and governance fragmentation marked by absent procedures and siloed coordination. Bridging this gap requires expanding Long-Term Care Insurance to moderately disabled adults, embedding volunteers in stable interdisciplinary teams, introducing care navigation for unmet needs, and improving digital literacy via hybrid record systems.

Abstract citation ID: igaf122.3938

### FRAILTY SCREENING IN WORLD TRADE CENTER RESPONDERS: VALIDATION OF THE WTC CLINICAL FRAILTY INDEX

Chinmayi Venkatram[1], Ghalib Bello[1], William Hung[2], Olivia Alpizar[3], Hannah Thompson[1], Elena Colicino[1], Katherine Ornstein[4], and Fred Ko[2], *1. Icahn School of Medicine at Mount Sinai, New York, New York, United States, 2. James J. Peters Department of Veterans Affairs Medical Center, New York, New York, United States, 3. University of Massachusetts Amherst, Amherst, Massachusetts, United States, 4. Johns Hopkins University, Baltimore, Maryland, United States*

World Trade Center (WTC) responders are a unique cohort whose environmental exposures place them at increased risk for earlier onset of aging-related conditions, including frailty. Detecting frailty in this group is critical for guiding interventions to support healthy aging. The WTC Clinical Frailty Index (WTC FI-Clinical) is a continuous measure constructed from 30 health items routinely collected during responder monitoring visits. We aimed to use the frailty phenotype to validate and establish a meaningful cutoff for identifying frailty using the WTC FI-Clinical. We enrolled WTC responders aged 50 and older who received annual health monitoring at Mount Sinai's WTC Health Program (WTCHP). Frailty was measured using both the frailty phenotype and WTC FI-Clinical. ROC (Receiver Operation Characteristic) analysis, with the frailty phenotype as reference, was used to assess discrimination and identify an optimal cutoff for the WTC FI-Clinical. Among 390 responders (median age 62, IQR 57–67), the frailty phenotype classified 54.1% robust, 40.8% pre-frail, and 5.1% frail. The WTC FI-Clinical demonstrated good discrimination for frailty (AUC 0.907, 95% CI: 0.858–0.952), and ROC analysis identified an optimal cutoff of 0.237, corresponding to 85% sensitivity and 76% specificity. Using this cutoff, 27.2% were classified as frail by the WTC FI-Clinical (versus 5.1% by the phenotype). These findings support its use as a practical screening tool to identify at-risk responders and guide healthy aging interventions within the WTCHP. It also extends frailty detection beyond traditional older cohorts, offering insight into how frailty can emerge earlier in populations with unique environmental exposures.

Abstract citation ID: igaf122.3939

### FROM "CAN I HANDLE THIS?" TO "IS IT WORTH IT?": THE INTERPLAY OF EXPERIENCE & PSYCHOLOGICAL BARRIERS IN TECH ADOPTION

Joonhyeog Park[1], Jinbo Niu[2], and Tamara Cadet[1], *1. University of Pennsylvania, Philadelphia, Pennsylvania, United States, 2. University of Southern California, Los Angeles, California, United States*

Assistive smart home technologies (SHTs), such as motion sensors and voice assistants, may enhance independence for older adults living at home. However, the decision to use these devices involves a complex trade-off between the benefits they offer and legitimate concerns about privacy and personal autonomy. Guided by the Unified Theory of Acceptance and Use of Technology to assess behavioral intention and its key predictors, such as usefulness and social influence, we aimed to understand how psychological barriers influence the intention to use SHTs and whether this behavior changes based on a user's prior experience with smart devices. We analyzed online survey data from 421 U.S. adults aged 55-80 (Mage=64.5), recruited via the CloudResearch Survey Platform. The analysis employed hierarchical multivariate regression models with robust standard errors. Findings suggested for inexperienced users, the importance of both ease of use and social influence grew in decision-making as their privacy concerns increased ($p<.10$). Furthermore, the need for an easy-to-use system became more critical as their fear of dependency grew ($p<.10$). For experienced users, technology's usefulness became the key factor in overcoming their fear of dependency ($p<.05$), while social influence was most critical in mitigating privacy concerns ($p<.05$). These findings suggest the need for tailored support in decision-making about SHTs. Inexperienced users should receive clear demonstrations of ease of use to alleviate dependency concerns, while also leveraging social proof to address their anxieties. For experienced users, support should include evidence of the technology's benefits and endorsements from trusted sources to help navigate privacy implications.

Abstract citation ID: igaf122.3940

### FROM BARRIERS TO ACTION: OPERATIONALIZING IMPLEMENTATION STRATEGIES FOR THE GOC VIDEO IN NURSING HOMES

Latarsha Chisholm[1], Susan Hickman[2], and Kathleen Unroe[3], *1. University of Central Florida, Florida, United States, 2. Indiana University, Indianapolis, Indiana, United States, 3. Indiana University School of Medicine, Indianapolis, Indiana, United States*

Implementation challenges have limited the widespread adoption of evidence-based strategies to improve advance care planning for nursing home residents and their families. Implementation strategies are critical for influencing outcomes and enhancing the adoption of evidence-based interventions. This study explored the use of implementation strategies to support the Goals of Care (GOC) video—a 20-minute decision aid designed to help family caregivers of persons living with dementia make healthcare decisions aligned with their values. The GOC video is part of a broader intervention that includes staff training and structured care plan meetings. Semi-structured group interviews were conducted with 12 staff members from six Florida nursing homes. Content analysis identified key barriers to implementation, and the CFIR-ERIC matching tool was used to align appropriate strategies to address them. Common barriers included the video's length, limited access to understandable health education, caregiver readiness, and cultural or family background differences. Recommended implementation strategies included promoting adaptation, distributing diverse educational materials, assessing readiness, identifying barriers and facilitators, and fostering learning collaboratives. Action staff can take include adapting the video to local contexts while preserving core components and integrating it into Quality Assurance & Performance Improvement (QAPI) programs to promote advance care planning. Action the research team can take includes supporting implementation by co-developing culturally appropriate materials, conducting pre-implementation readiness assessments, identifying enablers and barriers, and sharing best practices across sites. This study highlights key barriers and actionable strategies for implementing the GOC video in nursing home settings.

Abstract citation ID: igaf122.3941

### FROM DIAGNOSIS FORWARD: HOW EARLY INFORMATION SHAPES THE FAMILY CAREGIVING EXPERIENCE IN DEMENTIA

Tomoko Wakui[1], Tsuyoshi Okamura[2], Kae Ito[2], Takumi Hirata[3], and Tomoyuki Yabuki[4], *1. Tokyo Metropolitan Institute for Geriatrics and Gerontology, Tokyo, Tokyo, Japan, 2. Tokyo Metropolitan Institute for Geriatrics and Gerontology, Itabashi, Tokyo, Japan, 3. Tokyo Metropolitan Institute of Gerontology, Itabashi, Tokyo, Japan, 4. University of Kochi, Kochi, Kochi, Japan*

Beyond helping individuals and their families come to terms with a dementia diagnosis, appropriate informational support at the time of diagnosis may influence how they manage life with dementia in the years that follow. This study aimed to examine the impact of informational support provided at the time of diagnosis on the current caregiving burden experienced by family members. A self-administered survey was conducted in January 2025 with 164 family caregivers of individuals diagnosed at dementia medical centers and by certified dementia support physicians across Japan. The primary outcome was caregiver burden, assessed using the short version of the Japanese Zarit Caregiving Inventory (J-ZBI_8). Regression analyses controlled for care recipient characteristics, caregiver demographics, and caregiving context, while examining associations between nine types of informational support received at diagnosis and current caregiver burden. Caregivers most frequently reported receiving information about the disease itself (72.0%), long-term care services and Community General Support Centers (58.5%), and local medical institutions (37.2%). Practical information on legal, financial, or employment-related matters was less commonly provided. Among all types of support, only dementia-related information ($B = -5.07$, $p < .001$) and information about local medical institutions ($B = -2.93$, $p = .02$) were significantly associated with lower caregiver burden. These findings indicate that early, focused informational support—particularly regarding the disease and access to local dementia-specialized clinics—may enhance caregivers' preparedness and ease anxiety about managing future care needs.

Abstract citation ID: igaf122.3942

### FROM ISOLATION TO INTEGRATION: HOW SENIOR CENTERS ARE POISED TO TREAT THE LONELINESS EPIDEMIC AMONG OLDER ADULTS

Lauren Roberson[1], Kimberly Parker[2], Enid Borden[3], Sarah Anderson[1], Susan Zies[1], Misty Harmon[1], Sudha Sankar[4], and Abby Pound[5], *1. The Ohio State University, Columbus, Ohio, United States, 2. University of Kentucky, Lexington, Kentucky, United States, 3. National Foundation to End Senior Hunger, Alexandria, Virginia, United States, 4. North Carolina State University, Raleigh, North Carolina, United States, 5. Mid Ohio Food Collective, Columbus, Ohio, United States*

As the aging population grows, there is a rise in mental health challenges, including loneliness. Loneliness contributes to decreased quality of life and increased morbidity and mortality. One in three older adults in the U.S. are lonely. As physical places where older adults engage in activities promoting social connection, physical activity, and nutrition, senior centers are an underutilized resource for addressing loneliness. To understand senior center utilization in Ohio, we conducted a cross-sectional survey among older adults aged 60+ who regularly attend senior centers and those who have not within the last three months. Reasons for attending include social support, companionship, recreation, and receiving a nutritious meal. According to both groups, the purpose of a senior center is to provide a place for fellowship, camaraderie, laughter, and life enrichment to keep the golden years active. Among non-users, primary reasons for not attending include a lack of need, interest, and salient programming. Non-users believe senior centers are for "old" people who are clinging to life. This perception could not be further from the truth. ANOVA was used to detect differences in perceptions of senior centers between users and non-users. There were significant differences in perceived importance, with users more likely to agree that senior centers are important. Findings provide insight on the need for tailored outreach to combat the stigma associated with senior centers, their purpose, and the population they serve. This would enable more older adults to benefit from socialization and nutritious meals, improving mental and physical health.

Abstract citation ID: igaf122.3943

### FULL LONGITUDINAL MEDIATION MODELING ANALYSIS OF FRAILTY, DEPRESSION, AND COGNITIVE IMPAIRMENT

Namhee Kim[1], Wongyeong Lee[2], Doukyoung Chon[3], Miji Kim[4], and Chang Won Won[4], *1. Yonsei University, Wonju, Republic of Korea, 2. Yonsei University College of Nursing, Seoul, Republic of Korea, 3. Yonsei University, Industry-Academic Cooperation Foundation of Future Medicine, Seoul, Republic of Korea, 4. Kyung Hee University College of Medicine, Seoul, Republic of Korea*

Frailty, depression, and cognitive impairment (CI) are interrelated geriatric syndromes that significantly impact the functioning and quality of life of older adults. In the context of a super-aging society, a clear understanding of their long-term interactions is essential for timely intervention and evidence-based policymaking. This study aimed to identify the directionality, mediating pathways, and critical time points for intervention by analyzing the dynamic interactions among them over time using the four-wave Korean Frailty and Aging Cohort Study ($N = 1{,}377$). Data were collected from a nationally representative sample of older adults in Korea from 2016 to 2023. Descriptive statistics characterized baseline levels and wave-specific changes. Pearson's correlation coefficients assessed cross-sectional associations, while autoregressive and cross-lagged panel models evaluated temporal stability and causal directionality. Full Longitudinal Mediation Modeling (FLMM) explored potential mediation effects. All three variables showed significant temporal stability ($\beta = .60$–$.75$, $p < .001$). Depression in the initial stage significantly predicted increased frailty ($\beta = .18$, $p < .01$) and CI ($\beta = -.12$, $p < .05$) in subsequent waves, while the reverse effects were weak or insignificant. FLMM showed that depression mediated the effect of baseline cognitive function on future frailty, explaining approximately 35% of the total effect. Depression was identified as a significant mediator and longitudinal predictor of late-life physical and CI. Early screening and intervention for depressive symptoms can mitigate the progression to frailty and CI. These findings support integrated and timely strategies in community-based geriatric care and provide practical guidance for the effective allocation of preventive resources.

Abstract citation ID: igaf122.3944

### GENDER DIFFERENCES IN LONELINESS – FOUR DECADES OF EVIDENCE FROM THE FRAMINGHAM HEART STUDY

Mitzi Gonzales[1], Matthew Scott[2], Alexa Beiser[2], Allison Mays[1], and Joel Salinas[3], *1. Cedars-Sinai Medical Center, Los Angeles, California, United States, 2. Boston University, Boston, Massachusetts, United States, 3. New York University Grossman School of Medicine, New York, New York, United States*

Technological developments over the past four decades have altered social communication, potentially shaping experiences of loneliness. We evaluated loneliness prevalence between ages 25–94 years in men and women in the Framingham Heart Study from 1980-2019. Loneliness was assessed with the Center for Epidemiologic Studies-Depression scale. Analyses included 14,261 responses from men and 17,625 from women. Women were more likely than men to report loneliness at least one (23.7% vs. 17.5%) and ≥3 days (8.8% vs. 5.6%) per week. Using the average prevalence across four decades, 6.7% of men ages 25-34 reported loneliness ≥3 days weekly, which declined to 4.6% at ages 65–74, then rose to 7.3% at ages 75-84, and rose sharply to 12.9% at ages 85–94. Among women, prevalence remained stable from ages 25–34 (6.3%) through 54–64 (6.5%), but increased at ages 65-74 (8.7%), 75–84 (14.5%) and 85–94 (17.4%). At ages 75–84, loneliness prevalence (≥3 days/week) was twice as high in women than men (14.5% vs. 7.3%). Loneliness ≥3 times weekly in men peaked in 1980–1989 (8.6%) and then remained stable from 1990-1999 through 2010-2019 at 4.9–5.2%. In women, prevalence declined from 15.6% in 1980–1989 to a low of 5.5% in 2010–2019, a level comparable to men in this period (4.9%). In summary, women reported higher prevalence of loneliness than men and may be particularly vulnerable earlier in late life. Encouragingly, loneliness declined over the past four decades with the gap between men and women narrowing.

Abstract citation ID: igaf122.3945

### GERIATRIC INTERPROFESSIONAL EDUCATION IMMERSION: A STUDENT PERSPECTIVE APPLYING THE 4MS

Grace Mulligan[1], Jezebel Ortiz Villaman[2], Nelson Thomas[3], Maud Formson[4], Emilia Sanchez[2], and Emily Balog[3], *1. Rowan University, Stratford, New Jersey, United States, 2. Rutgers University, Camden, New Jersey, United States, 3. Rowan University School of Osteopathic Medicine, Stratford, New Jersey, United States, 4. Thomas Jefferson University, Philadelphia, Pennsylvania, United States*

The U.S. healthcare system is not prepared to address the complex needs of the older adult population nearing 78 million by 2050. This requires a proactive shift toward age-friendly healthcare systems. The Age-Friendly Health Systems initiative developed the 4Ms framework: What Matters, Mentation, Medication, and Mobility which seeks to address the geriatric workforce needs. This poster presents how an innovative geriatric education and training program influenced interdisciplinary students' understanding and application of person-centered care. Over eight weeks, medical, nursing, social work, and occupational therapy students participated in a 160-hour immersion that combined didactic sessions, community site visits, and application at a Fair Share Housing community in Camden, NJ (i.e., the interprofessional supersite). Students integrated knowledge through hands-on experience, developing interventions responsive to resident needs at the interprofessional supersite. Key themes included the importance of identifying patient values (What Matters), understanding cognitive and social engagement needs (Mentation), addressing polypharmacy and food-as-medicine (Medication), and assessing fall risks, transportation, and accessibility challenges (Mobility). Students also gained insights into how social determinants and systemic disparities influence health equity across communities. Interprofessional collaboration and cultural humility emerged as essential elements for personal and professional growth necessary for delivering effective, compassionate care. This educational model demonstrated that early exposure to age-friendly care fosters critical thinking, enhances empathy, and prepares students to deliver patient-centered, interdisciplinary care for a growing aging population. The program's outcomes support embedding the 4Ms into clinical training to create a workforce prepared to meet the evolving demands of geriatric medicine.

Abstract citation ID: igaf122.3946

### GIS ANALYSIS OF ALABAMA'S AT-RISK HOSPITALS: CONSIDERING NEW MEDICAID CHANGES AND POTENTIAL SOLUTIONS

Avani Shah[1], and Joseph Swain[2], *1. University of Alabama, Tuscaloosa, Alabama, United States, 2. Arkansas Tech University, Russellville, Arkansas, United States*

With recent passage of the One Big Beautiful Bill Act (OBBBA), Medicaid changes are likely to impact older adults. One concern is that hospitals that depend on Medicaid revenue from higher Medicaid patient volumes may be at greater risk of closure. Alabama serves as an example of a state that did not expand Medicaid and provided limited aid to struggling hospitals. Even before the passage of the bill, a majority of Alabama's hospitals were struggling partially due to a Medicare wage index calculation that underpaid hospitals (AHA, 2014). To better understand potential hospital access gaps in Alabama, we 1) coded media reports of at-risk hospitals in Alabama and hospitals who had reduced services/ converting to a rural emergency hospital (REH) 2) used the percent of Medicaid enrollment/eligibility by county in 2024. We then conducted GIS analyses to map the hospitals that may be at risk of closing or reducing services while examining Medicaid eligibility rates by county in Alabama. GIS analyses results revealed more at-risk hospitals in areas with higher Medicaid enrollment. Health access gaps were most pronounced in the lower half of the state, westernmost side of Alabama, and a pocket of 3 counties in Northeast Alabama. One hospital converted to an REH losing its geriatric psychiatry unit. While conversion to a REH may result in saving a hospital from closure and access to a nearby ER, the result is hardship for older adults depending on nearby inpatient services. Potential solutions to offer quality health care options are discussed.

Abstract citation ID: igaf122.3947

### GOLDEN TEA PROGRAM: EXAMINING THE FEASIBILITY OF A CULTURALLY-BASED, MINDFULNESS INTERVENTION FOR OLDER CHINESE

Keith Chan[1], Wenting Zhang[2], Isabel Ching[3], Rosemary Li[4], and Soko Setoguchi[5], *1. Hunter College, New York, New York, United States, 2. Tea Arts & Culture, Brooklyn, New York, United States, 3. Hamilton-Madison House, New York, New York, United States, 4. Rutgers University, New Brunswick, New Jersey, United States, 5. Rutgers University, Rutgers University, New Jersey, United States*

The Golden Tea Program is a 7-week culturally-based mindfulness intervention that aims to improve overall mental health, reduce loneliness, delay cognitive decline, and strengthen a sense of cultural connectedness, conducted with a community-based sample of 32 Chinese older adults in New York City. The intervention is rooted in the study and appreciation of tea by creating the space and time to slow down with intention and experience the beauty of everyday interactions. Drinking tea is a sensory experience that can be appreciated through a multicultural and multigenerational lens. The overall goal is to: 1) Determine the feasibility of this culturally-based mindfulness intervention rooted in tea culture with Chinese older adults, 2) Test the influence of this program to reduce social isolation and loneliness, increase social connections, strengthen cultural identity, delay cognitive decline and improve overall mental health, and 3) Conduct a careful mixed methods evaluation of the proposed intervention using community-based intervention framework. Preliminary findings indicated acceptability and feasibility of the Golden Tea program, with participants reporting improvements in cognition, social well-being and mental health. This pilot study can serve as a demonstration of a feasible, low-risk and low-cost community-based program for Chinese older adults.

Abstract citation ID: igaf122.3948

### GRIEF AND ITS IMPACT IN OLDER ADULTS: FINDINGS FROM THE NATIONAL SOCIAL LIFE, HEALTH, AND AGING PROJECT, WAVE 2

Swasati Handique, *University of Texas at Arlington, Arlington, Texas, United States*

This study examined how feelings of being stunned and angry after the death of someone close have an impact on the physical and mental health of older adults. Data for this study was drawn from a nationally representative sample of 3196 individuals (aged 60-plus) from the NSHAP, Wave 2. The primary outcomes were self-rated physical health and mental health, measured on a 5-point Likert scale, with independent variables as responses to death of feeling stunned and feeling angry, both measured on a 5-point scale. Multiple linear regression analyses was conducted using Stata 17.0. Results: Descriptives revealed a mean age of 73.2 years, with the sample being 53% female. Self-rated physical health was statistically significant, $F(6, 1935) = 18.05$, $p < .001$. Feeling stunned was negatively associated with physical health ($\beta = -0.10$, $p < .001$), suggesting that those who reported being more emotionally overwhelmed by a death also rated their physical health lower. Self-rated mental health was also statistically significant, $F(6, 1938) = 20.11$, $p < .001$. Feeling stunned ($\beta = -0.10$, $p < .001$) and feeling angry ($\beta = -0.11$, $p < .001$) were significant negative predictors of mental health. Findings from this study highlight that prolonging grief has the potential of awakening the memories of past losses compounding the grief and impacting the physical and mental health of older adults. Therefore, grief support and community-based groups can be serve as essential resources to foster grief literacy and provide support to older adults navigating a loss.

Abstract citation ID: igaf122.3949

### GRIP STRENGTH AS A MEDIATOR LINKING MOTORIC COGNITIVE RISK SYNDROME TO INSTRUMENTAL ACTIVITIES OF DAILY LIVING

Wan-Yun Chou, and Su-I Hou, *University of Central Florida, Orlando, Florida, United States,*

Motoric cognitive risk syndrome (MCR) represents a preclinical condition characterized by the simultaneous presence of gait impairment and cognitive complaints, typically manifesting before dementia onset. This study explores the complex mediating relationships between MCR, instrumental activities of daily living (IADL), and grip strength among Taiwanese older adults. The cross-sectional study analyzed 204 community-dwelling adults aged 65 and older who participated in annual health screenings between March 2021 and November 2022. Participants were categorized as either robust or having MCR, with MCR diagnosed when individuals scored below 1.5 standard deviations of age- and sex-matched norms on timed-up-and-go speed assessments while experiencing subjective cognitive complaints. Bootstrap analysis, with 5000 resamples, was employed to investigate how grip strength mediates the relationship between MCR and daily functioning. The prevalence of MCR reached 25% among participants. Results demonstrated a significant negative correlation between IADL score and MCR presence, with grip strength serving as a crucial mediating factor ($\beta$=-1.1055, $p = 0.003$). Bootstrap analysis confirmed the partial mediating effect of grip strength, explaining 33% of the total effect of MCR presence on IADL score. These findings underscore the critical importance of monitoring grip strength alongside gait speed and cognitive health to preserve daily activity function in aging populations. Understanding MCR's underlying mechanisms could facilitate early dementia detection and prevention strategies. The research highlights the necessity of monitoring both grip strength and MCR in older adults. However, the convenience sampling methodology may limit generalizability, necessitating further investigation into grip strength's impact on MCR-IADL relationships for broader clinical applications.

Abstract citation ID: igaf122.3950

### HEAD-MOUNTED DISPLAY VIRTUAL REALITY FOR COGNITIVE AND PHYSICAL FUNCTION IN OLDER ADULTS: A SCOPING REVIEW

Ferdinand Delgado, and Sanghee Moon, *University of New Hampshire, Durham, New Hampshire, United States*

Head-mounted display virtual reality (HMD-VR) shows promise for supporting cognitive and physical function in older adults, yet researchers face challenges identifying appropriate applications for interventions. This scoping review systematically mapped HMD-VR applications used in research with older adults, creating a comprehensive catalog to guide future interventions, support new development, and help clinicians identify appropriate interventions. Following PRISMA-ScR guidelines, we searched PubMed, CINAHL, and Web of Science using terms related to VR technology, older adults, and cognitive/physical function. Twelve studies published 2009-2024 were included, encompassing 809 participants aged 55+ years (average age range: 67.9 to 85.9 years). We identified 32 distinct HMD-VR applications across platforms, including Oculus Quest (n = 6 studies), HTC VIVE (n = 3), and others (n = 3). Custom-developed applications dominated the field (87.5%, n = 28 applications), with only three commercially available applications used: Wander, YouTube VR, and Maze Walk, with one unknown. From the identified applications, attention was the most frequently targeted cognitive domain (n = 24), followed by executive function (n = 17), visuospatial function (n = 12), and memory (n = 9). Upper limb movement was the primary physical target (n = 15), followed by lower limb movement (n = 12), and balance training (n = 9). Eleven out of twelve studies reported positive cognitive outcomes, while six out of eight studies showed improved physical outcomes. This mapping reveals that researchers predominantly create specialized VR, suggesting the need for more accessible, evidence-based applications. Our scoping review provides researchers and clinicians with potential application options for designing HMD-VR interventions targeting cognitive and physical function in older adult populations.

Abstract citation ID: igaf122.3951
### HEALTH DISPARITIES AMONG ASIAN IMMIGRANTS IN THE UNITED STATES: THE IMPACT OF ARRIVAL COHORTS AND COUNTRIES OF ORIGIN

Yingzhi Xu, *Duke University, Durham, North Carolina, United States*

Asian Americans are the fastest-growing racial or ethnic group in the United States, having experienced an 81% increase in the population since 2000. Seventy-one percent of adult Asian Americans are immigrants (Pew Research Center, 2022). Much previous research focused on the optimistic social standing of Asian Americans as Asian immigrants' successful incorporation into American society. However, their health assimilation process and the health disparities among Asian immigrants have been largely underexplored. Using data from the 2008 to 2023 American Community Survey (ACS), this study indicates that the health (cognitive difficulty, physical disability, and independent difficulty, etc.) of Asian immigrants differs by both the arrival cohorts and the sending countries.

Abstract citation ID: igaf122.3952
### HEALTHCARE DEBT AMONG OLDER ADULTS WITH LOW-INCOME: EVIDENCE FROM A MIXED-METHODS

Anam Bhatti, Ivan Avila, Hannah Beesley, and Jeffrey Greger, *AARP Foundation, Washington, District of Columbia, United States*

Although Medicare and Medicaid extend broad coverage, some older adults with low-income (OALI) still experience healthcare debt, underscoring ongoing vulnerabilities in financial and health security. The AARP Foundation conducted 12 focus groups (n = 81), 13 expert interviews, and a nationally representative survey (n = 2,066) of adults aged 50+ with low-income. Qualitative data analysis primarily followed a grounded theory approach. Nationally weighted survey data were examined via cross-tabulations to assess debt prevalence, drivers, and consequences by income, insurance type, age, and comorbidity status. Using a broader definition of healthcare debt than benchmark studies (e.g., SIPP and KFF), this study finds that coverage gaps and provider misperceptions about financial aid create lasting financial burdens for OALI. Survey findings show that 33% of Medicare and 39% of Medicaid beneficiaries carry healthcare debt, most often linked to dental, and vision services. Qualitative themes reinforce survey findings: charity care screening targets uninsured OALI yet falls short for those on high-deductible plans, contributing to low awareness (26%) and use (16%). Debt forces tradeoffs: 44% of OALI cut back on basic needs and 45% of those owing over $1,000 delay or forgo treatment altogether. The study findings underscore the need to boost assistance awareness and tailor interventions by income and coverage type. The findings offer insights for stakeholders to design financial assistance programs that prevent debt accumulation and safeguard both the health and financial security of older adults.

Abstract citation ID: igaf122.3953
### HEALTHCARE STUDENTS' EXPOSURE AND KNOWLEDGE OF THE 4MS FRAMEWORK FOR AN AGE-FRIENDLY HEALTH SYSTEM

Laura Finn[1], Isabel Coulter-Daly[2], and Leah Peterman[3], *1. Philadelphia College of Pharmacy / St Joseph's University, Philadelphia, Pennsylvania, United States, 2. Philadelphia College of Pharmacy, LANGHORNE, Pennsylvania, United States, 3. Philadelphia College of Pharmacy at Saint Josephs University, Philadelphia, Pennsylvania, United States*

Pharmacists are essential healthcare professionals directly involved in the care of older individuals. To effectively and holistically fulfill the needs of these patients, pharmacists must possess a strong knowledge of the foundations of geriatric care; this is represented by the 4Ms Framework for an Age-Friendly Health System. The framework is one that all professionals should be exposed to, particularly those in the early stages of their careers as to adopt this approach for their future practice. The aim of this study at St. Joseph's University was to evaluate pharmacy and physical therapy students' familiarity with the 4Ms framework through an 18-question online survey that included multiple choice and open-ended responses. The results demonstrated that 88% percent of the surveyed healthcare students either never heard of the 4Ms framework or had heard of the approach but were unclear of its meaning. Findings highlight the lack of awareness future professionals have on how to properly care for an older individual. Almost 70% of responding students reported that their program either did not have a required geriatrics course or they were unsure if it did, and only 4% of participants took a geriatrics elective. These results emphasize that training and exposure to caring for this patient population should be incorporated into curricula, experiential programs, and extracurricular activities. Doing so will be beneficial to both students by increasing their preparedness and to older adult patients by improving the quality of their care.

Abstract citation ID: igaf122.3954
### HIGHER POSITIVE AFFECT IS ASSOCIATED WITH LOWER SYSTEMIC INFLAMMATION IN WOMEN BUT NOT IN MEN: FINDINGS FROM MIDUS

Rui Wang, Derek Spangler, Christopher Engeland, and Harold Lee, *The Pennsylvania State University, University Park, Pennsylvania, United States*

Higher positive affect is associated with healthy aging, with inflammation proposed as a mediating mechanism. However, most studies assessed inflammation primarily via CRP and/or IL-6, which may insufficiently capture the breadth of systemic inflammation. We examined the association between positive affect and a cytokine composite consisting of four inflammatory cytokines. We hypothesized that higher positive affect would be associated with lower cytokine composite levels, and this association would vary based on sex given women's higher inflammation. We used data from Midlife in the United States (N = 1023, mean age 46 yrs, female 54%). Positive affect was measured by Positive Affect and Negative Affect Schedule (range: 1-5, mean score: 3.67) between 2004 and 2006. The cytokine composite was assessed using IL-6, IL-8, IL-10, and TNF-α, and was collected between 2004 and 2009. The cytokine composite was regressed on positive affect, with covariates: time difference between positive affect and blood draw for cytokines, sociodemographic factors (e.g., age, sex), negative affect, and health behaviors and conditions (e.g., smoking, BMI). Positive affect was not associated with the cytokine composite in the total sample (r2=.14, b=-0.04, p = 0.06, 95% CI [-0.08, 0.01]). However, sex moderated the association (p = 0.03): among women, higher positive affect was associated with lower cytokine composite (r2=.13, b=-0.07, p = 0.02, 95% CI [-0.13, -0.01]), but no association was observed in men (r2=.15, b=-0.01, p = 0.70, 95% CI [-0.08, 0.05]). In women, higher positive affect was prospectively associated with lower systemic inflammation, which leads to healthy aging. Future studies should investigate mechanisms underlying this sex-specific association.

Abstract citation ID: igaf122.3955

### HOSPITALIZATIONS FOR PEOPLE AGING WITH SCHIZOPHRENIA

Tracie Harrison[1], Cherisse Madlock-Brown[1], You Wang[1], Karen Dunn Lopez[2], Ryan Carnahan[3], and Veronica Garcia Walker[4], *1. University of Iowa College of Nursing, Iowa City, Iowa, United States, 2. University of Iowa, Iowa City, Iowa, United States, 3. The University of Iowa College of Public Health, Iowa City, Iowa, United States, 4. The University of Texas at Austin, Austin, Texas, United States*

Aging with schizophrenia or schizoaffective disorder is associated with high mortality. Quality of acute care in general hospital systems for older adults with long-term psychiatric conditions like schizophrenia and schizoaffective disorders is not well studied. After IRB approval, we extracted real-world data from 9,868 persons with a diagnosis of schizophrenia/schizoaffective disorder (62% female and 38% male) who received care in a midwestern, rural-designated hospital in the past 5 years. The majority were single and dismissed to self-care/home at an average age of 49 years. The length of stay for those over 65 (length of stay mean = 11.2 days) compared to under 65 (length of stay mean = 10.16) years of age was significantly (p = 0.008) higher. The length of hospitalization for men (11.97) was also higher than for women (9.35; p < 0.001). There were 17% with a 30-day readmission; the top readmission diagnoses included: shortness of breath, chest pain of unspecified type, and suicidal ideation, the same top three reason for initial admission. Chief complaints were non-specified in 42% of admissions. Over 93% of 30-, 60-, and 90-day readmissions were for reasons other than schizophrenia. Further explorations of quality care delivery, especially sex by age interactions for those with a diagnosis of schizophrenia, are needed.

Abstract citation ID: igaf122.3956

### HOW FINANCIAL CHALLENGES AFFECT HEALTH AND LIVED EXPERIENCES OF ADULTS WITH LONG-TERM SERVICES AND SUPPORTS NEEDS

Lei Chen[1], Qianyun Wang[2], and Kathryn Kietzman[3], *1. University of California, San Francisco, San Francisco, California, United States, 2. University of California, Los Angeles, Los Angeles, California, United States, 3. UCLA Fielding School of Public Health, Los Angeles, California, United States*

Older adults and people with disabilities living at home with chronic care needs may require Long-Term Services and Supports (LTSS). However, little is known about the diverse financial challenges they face due to these care needs, and how these challenges may impact their health and daily lives. This study draws on analyses of population-level quantitative survey data (2019-2020, n = 2030) and a subgroup of 18 in-depth qualitative interviews (2023-2025, n = 90) collected as part of the California Long-Term Services and Supports study. Employing a sequential mixed-method approach, we used descriptive statistics and grounded theory to identify financial challenges experienced by adults with LTSS needs and examine how these challenges affected their health and well-being. Survey findings show that 61% of California's adults with LTSS needs experienced financial worries that were associated with their self-rated health and serious psychological distress. We also found an association between lower incomes and poorer health outcomes. Qualitative interview participants identified key financial challenges including: barriers to accessing public benefits (e.g., age, employment, or education restrictions); inadequacies in caregiving and LTSS services (e.g., insufficient caregiving hours); services, supports, and equipment not covered by insurance; high housing cost and poor housing quality; and heightened vulnerability to financial insecurity. These results highlight the urgent need to refine LTSS eligibility criteria and integrate appropriate age, housing, employment, and income considerations into policy development. Findings also underscore the importance of developing inclusive programs that reduce financial disparities and strengthen the quality of care provided to people with LTSS needs.

Abstract citation ID: igaf122.3957

### HOW PSYCHOLOGICAL RESILIENCE MEDIATES THE EFFECT OF CAREGIVER FRUSTRATION ON THE HEALTH OF CAREGIVERS

Victor Elisha[1], Emil Coman[2], and Richard Fortinsky[3], *1. University of Connecticut, Farmington, Connecticut, United States, 2. UConn Health Disparities Institute, Farmington, Connecticut, United States, 3. University of Connecticut, Farmington, Connecticut, United States*

Family caregivers are critically important in supporting older adults living with dementia. Caregiving responsibilities often lead to negative emotional responses including frustration, which could negatively affect caregivers' health and well-being. However, adverse effects of caregiver frustration might be mitigated by psychological resilience, which is an established mediator in the manifestation of negative health outcomes in other populations. This cross-sectional study used path analysis to examine relationships between caregiver frustration, psychological resilience, and self-reported health in 291 caregivers of older adults living with dementia. The model hypothesized direct paths connecting caregiver frustration, psychological resilience and self-reported health and used maximum likelihood estimation to obtain path coefficients. Results revealed that increased caregiver frustration was associated with lower psychological resilience (β = -0.41: p < 0.0001, 95% CI [-0.59, -0.22]) and psychological resilience was positively associated with self-reported health (β = 0.32: p < 0.0001, 95% CI [0.17, 0.48]. The indirect effect of caregiver frustration on self-reported health via resilience was estimated as -0.13 (p = 0.003, 95% CI [-0.22, -0.05]), while the direct effect of caregiver frustration on self-reported health was not statistically significant (β = -0.083: p = 0.52, 95% CI [-0.34, -0.17]). We conclude from this pattern of results that the compromising effect of caregiver frustration on health outcomes among caregivers of people living with dementia occurs through diminished psychological resilience, highlighting the potential benefit of psychological resilience-building interventions in caregiver support programs.

Abstract citation ID: igaf122.3958

### HYBRID VALUE-AWARE TRANSFORMER IDENTIFIES NOVEL AND SUSPECTED DRUGS FOR ALZHEIMER'S DISEASE

Qing Zeng[1], Yijun Shao[2], Ying Yin[3], Edward Zamrini[4], Ali Ahmed[5], and Cheng Yan[6], *1. The George Washington University, Washington, District of Columbia, United States, 2. George Washington University, Washington, DC, Washington, District of Columbia, United States, 3. George Washington University, George Washington University, District of Columbia, United States, 4. Irvine Clinical Research, Irvine, California, United States, 5. Washington DC VA Medical Center, Washington, District of Columbia, United States, 6. George Washington University, Washington, District of Columbia, United States*

Drug discovery for Alzheimer's disease and related dementias (ADRD) faces many challenges including the complex and multifactorial nature of ADRD. Our research team developed the Hybrid Value-Aware Transformer (HVAT), a novel deep learning architecture designed to model multimodal health data. HVAT extends Transformer models, a powerful AI technology originally developed for natural language processing and generation, with "clinical tokens" that represent both longitudinal and non-longitudinal data, while also incorporating numerical values such as laboratory results and cumulative drug doses. We used this hybrid, value-aware model to identify dose-dependent associations between drug exposures and ADRD risk. Specifically, we trained an HVAT model on a case-control cohort of approximately one million U.S. Veterans aged 65 years and older, with up to 10 years of medical history. HVAT achieved fair predictive performance (area under the curve [AUC] = 0.775) in distinguishing ADRD cases from controls. We then derived population-level drug impact scores, a novel explainable AI measure similar to odds ratio, to quantify the association between normalized cumulative dose and ADRD risk. Our analyses yielded both suspected candidates, such as varenicline, which has supportive preclinical and early clinical evidence in ADRD, and novel candidates, such as metolazone, a thiazide-like diuretic not previously linked to dementia. These findings demonstrate that HVAT can recover known signals while also uncovering new drug repurposing opportunities. By combining deep learning with explainable AI analysis, this project represents a generalizable AI framework for accelerating drug discovery in aging research.

Abstract citation ID: igaf122.3959

### IDENTIFYING AND VALIDATING CLUSTERS OF MENOPAUSAL SYMPTOMS ACROSS REPRODUCTIVE STAGES

Alan Rathbun[1], Rebecca Brotman[1], Joanna Borgogna[2], Sarah Brown[1], Jacques Ravel[1], and Michelle Shardell[1], *1. University of Maryland School of Medicine, Baltimore, Maryland, United States, 2. McLaughlin Research Institute, Great Falls, Montana, United States*

Biological changes during the menopausal transition produce symptoms across multiple domains, yet clinical profiles remain poorly understood, hindering screening and targeted care. This study aimed to identify and validate menopausal symptom clusters and examine their variation across reproductive stages. Women aged 35–60 years (n = 741) enrolled in the Human Papillomavirus in Perimenopause study were followed for two years and completed four semiannual follow-up visits assessing genitourinary, vasomotor, other physical, and mood-related menopausal symptoms. Latent class models were fit using all available data (2,360 person-visits) to identify symptom clusters. Models with two to six classes were compared via fit statistics, posterior probabilities, item-response probabilities, and interpretability. Participants were assigned to the symptom cluster for which they had the highest posterior probability. Four classes emerged: the multi-domain cluster (21.0%) had high probabilities for all symptoms; the asymptomatic cluster (30.6%) had uniformly low symptom burden; the mood/somatic cluster (27.0%) showed elevated psychological and non-specific physical symptoms; and the genitourinary/vasomotor cluster (21.4%) was marked by vaginal dryness, decreased sexual interest, and vasomotor symptoms. Age and the proportion of postmenopausal women were significantly higher in the multi-domain (49.8 years; 47.1%) and genitourinary/vasomotor (50.8 years; 46.3%) clusters than in the mood/somatic and asymptomatic clusters. These participants were also more likely to exhibit clinical signs of genitourinary syndrome of menopause, have lower current hormone use and estradiol levels, and were less likely to have Lactobacillus-dominant vaginal microbial communities. Menopausal symptoms cluster differently across reproductive stages, which may inform screening and targeted care.

Abstract citation ID: igaf122.3960

### IMPACT OF HEART FAILURE ON RECOVERY AFTER TRAUMATIC BRAIN INJURY IN ADULTS 65 AND OLDER

Sofio Dumbadze[1], Jennifer Albrecht[2], and Jason Falvey[3], *1. University of Maryland, Baltimore, Baltimore, Maryland, United States, 2. University of Maryland, Baltimore, Maryland, United States, 3. University of Maryland School of Medicine, Baltimore, Maryland, United States*

Heart failure (HF) is common among older adults with traumatic brain injury (TBI). Understanding how HF affects days spent at home, a meaningful measure of recovery following an acute event, can inform care management and improve outcomes in this vulnerable population. We examined the impact of HF on days at home following TBI among adults aged 65 and older, hypothesizing that people with HF would spend fewer days at home after TBI compared to those without HF. We conducted a retrospective cohort study among adults aged 65 and older who were hospitalized with TBI using Medicare administrative claims data from 2010 to 2018. TBI and HF were identified using disease classification codes. We measured days at home over 1-year post-TBI by subtracting the number of days spent in inpatient care, skilled nursing facilities, nursing homes, emergency or outpatient observation settings, and the number of days spent deceased from 365. We modeled days at home as a function of HF using a negative binomial model. Among 20,206 older adults with TBI, 9,056 (44.8%) had HF. After TBI, older adults with HF spent fewer days at home than without HF (adjusted rate ratio 0.96; 95% confidence interval 0.94-0.99). This translates to an adjusted difference of 9 fewer days at home over the year following TBI among older adults with HF. Integration of chronic disease management into rehabilitation pathways for TBI survivors with HF may be warranted.

Abstract citation ID: igaf122.3961

### IMPACT OF THE TEACHING NURSING HOME ON NURSING STUDENT'S WILLINGNESS TO WORK IN LONG-TERM CARE

Howard Degenholtz[1], Nancy Zionts[2], Anny Treat[3], Elizabeth Schlenk[1], Erin Kitt-Lewis[4], Lori Ingleton Gold[5], Kenna Campbell[3], and Chelsea Dickson[2], *1. University of Pittsburgh, Pittsburgh, Pennsylvania, United States, 2. Jewish Healthcare Foundation, Pittsburgh, Pennsylvania, United States, 3. University of Pittsburgh, School of Public Health, Pittsburgh, Pennsylvania, United States, 4. Penn State, State College, Pennsylvania, United States, 5. University of Pennsylvania, Philadelphia, Pennsylvania, United States*

The Teaching Nursing Home Collaborative (TNHC) aims to strengthen the long-term care workforce and improve quality of care. The initiative integrates several key components: clinical rotations for nursing students in partnered nursing homes, implementation of the Institute for Healthcare Improvement's Age-Friendly Health System "4Ms" framework (What Matters, Mobility, Mentation, Medication), and an online learning collaborative. This study reports on the experience of nursing students in select courses from three schools of nursing who participated in clinical rotations at four nursing homes from 2022 to 2024. Students reported their interest in working with older adults and in long-term care settings and rated their competencies at the beginning and end of the semester: interdisciplinary collaboration, conducting resident assessments, eliciting resident values, health promotion, resident interviewing, medication review, assessing what matters most, and conducting an evidence-based project. School of nursing faculty instructors evaluated student competencies at the end of the term. Analysis included 438 student responses at baseline and 521 at the end of the semester, along with 290 faculty assessments. Student self-reported competence significantly improved across all eight domains ($p < .001$). Overall, faculty rated students significantly lower than the students' self- ratings. Over time, faculty ratings of student competence increased ($p < .001$). Students reported relatively weak preferences for working with older adults or in long-term care post-graduation. This preference did not change over time. These findings suggest that while the TNH program is meeting pedagogical goals, additional strategies are needed to influence student attitudes toward careers in long-term care.

Abstract citation ID: igaf122.3962

### IMPACT OF UNMET CARE NEEDS ON RESILIENCE IN MULTIMORBID OLDER ADULTS

Boah Kim[1], Lun Li[2], and Andrew Wister[3], *1. Gerontology Research Centre, Simon Fraser University, Vancouver, British Columbia, Canada, 2. MacEwan University, Edmonton, Alberta, Canada, 3. Simon Fraser University, Vancouver, British Columbia, Canada*

Older adults with multimorbidity (i.e., two or more chronic conditions) are more likely to face an enduring risk of unmet (care) needs in accessing healthcare services due to their unique and complex form of health adversity. Multimorbidity resilience (MR), the ability to bounce back from multiple chronic illness-related challenges, is an important indicator of positive adaptation to multimorbidity. However, despite the importance of addressing unmet needs among multimorbid older adults, there is sparse knowledge on the impact of unmet needs on resilience. This study aims to fill these gaps through an investigation of the key factors affecting resilience among multimorbid older adults. A social determinant of health model is used to frame the study. Using the Canadian Longitudinal Study on Aging (CLSA), a total of 12,677 multimorbid older adults were analyzed. Hierarchical linear regression was conducted to identify the relationship between resilience and four sequentially ordered blocks of predictors: 1) unmet needs, 2) sociodemographic characteristics, 3) health context, and 4) social/environmental supports. It was found that the level of resilience was lower among multimorbid older adults who reported having unmet needs compared to those without unmet needs. Several significant covariates across multiple domains were also identified (e.g., sex, urban/rural status, household income, perceived healthy aging, community friendliness). This study extends our understanding of potential protective and/or risk factors of MR among older adults. To address the challenges of accessing healthcare services among multimorbid older adults, there is a need to implement health policies/initiatives to fill their unmet care needs.

Abstract citation ID: igaf122.3963

### IMPLEMENTING AN INTERVENTION FOR BPSD AT INPATIENT MENTAL HEALTH: FEASIBILITY & ACCEPTABILITY

Melissa Meynadasy[1], Kelly O'Malley[2], Holly Marston[3], Amanda Smith[3], and Kelly Klein[3], *1. VA Boston Healthcare System, West Roxbury, Massachusetts, United States, 2. VA Boston Healthcare System, Boston, Massachusetts, United States, 3. VA Boston Healthcare System, Brockton, Massachusetts, United States*

Given population aging and increased dementia risk in substance use and serious mental illness populations, inpatient mental health (MH) will likely admit more older adults with comorbid dementia. Behavioral and psychiatric symptoms of dementia (BPSD) also result in admissions. Inpatient MH staff do not always receive training in BPSD. STAR-VA is an evidence-based intervention for BPSD developed for VA nursing homes, with demonstrated feasibility and efficacy in acute medical settings. Using the Institute for Healthcare Improvement model, this quality improvement project assessed feasibility and acceptability of STAR-VA in inpatient MH. Needs assessment examined interest and utility of dementia education; post-implementation assessment focused on acceptability and ongoing needs. Implemented changes focused on: education, staff support, collaboration, milieu. All respondents (N = 14; 78.6% nursing) reported caring for patients with dementia. Staff shortages (71.4%), BPSD (50%), lack of resources (50%), placement difficulty (42.9%), and burnout (42.9%) were most endorsed challenges. Only 28.6% of respondents agreed they had tools to improve the milieu. Educational needs included brief in-services (85.7%), dementia workshops (57.1%), and informal consultation (42.9%). Over 7 months, 17 patients were enrolled, 3 formal behavior plans were created, and nursing attendance at educational rounds ranged from 5-13 (M = 9). Follow-up questionnaires (N = 9; 77.8% nursing) revealed managing BPSD (66.7%) and burnout (55.6%) remained most challenging. Lack of resources decreased to 33.3% endorsed. Respondents agreed they had tools needed to improve the milieu (66.7%). Majority (88.9%) reported educational efforts were helpful for managing BPSD. It appears it is feasible and acceptable to adapt STAR-VA to inpatient MH.

Abstract citation ID: igaf122.3964

### IMPROVING BLOOD PRESSURE CONTROL THROUGH A TECHNOLOGY-BASED HYPERTENSION SELF-MANAGEMENT INTERVENTION

Carolyn Still[1], Parishma Guttoo[1], Rania Aljohani[1], Siobhan Aaron[1], and Carla Harwell[2], *1. Case Western Reserve University, Cleveland, Ohio, United States, 2. University Hospitals Cleveland Medical Center, University Hospitals Cleveland Medical Center, Ohio, United States*

Hypertension affects 48% of U.S. adults and remains the leading cause of cardiovascular disease (CVD), with smaller demographic groups experiencing the highest rates and adverse health burden. Evidence suggests that the integration of digital health and technology-based interventions may offer strategies to self-management engagement, promote adherence to treatment regimens, and sustain lifestyle modifications, especially for populations experiencing despair associated with hypertension. However, evidence on the effectiveness of such technologies in African Americans (AA) remains sparse. This study investigates the effectiveness of a refined, multi-component technology-based intervention—OPTIMA-BP (OPtimizing Technology to Improve Medication Adherence and BP Control; n = 63) vs a Wait-list control group (n = 56) using a prospective, randomized controlled trial. The intervention integrates technology with tailored education and clinical support to enhance hypertension self-management. The sample comprised AA adults (mean age 63), diagnosed with hypertension, taking at least two antihypertensive medications, and owning a smartphone. Data was collected to assess demographic characteristics, health behaviors, medication adherence, technology adoption, and use. The primary outcome was systolic BP < 130 mmHg after 6 months. At baseline, the mean systolic BP for the intervention group was 137.89 mmHg (SD = 14.64), compared to 136.70 mmHg (SD = 13.73) for the Wait-list group. At 6 months, a greater reduction of systolic BP was noted in the intervention group, a decrease of 7.07 mmHg vs 1.93 mmHg for the Wait-list group. Preliminary findings suggest that technology-based interventions could be effective in improving hypertension self-management and BP control in the AA population.

Abstract citation ID: igaf122.3965

### IMPROVING NURSING HOME READINESS: PPE ACCESS AND USE DURING COVID-19

Melissa Taylor, Anna Jones, Steven Miller, Linda Anderson, Lori Popejoy, and Amy Vogelsmeier, *University of Missouri - Columbia, Columbia, Missouri, United States*

The COVID-19 pandemic created unprecedented challenges for nursing homes (NHs), particularly challenges related to personal protective equipment (PPE). This analysis explored PPE access and use by NH staff, residents, and visitors during the pandemic. As part of a larger study aimed at developing recommendations to improve NHs' preparedness for infectious disease outbreaks, 116 interviews were conducted with leaders (n = 43), staff (n = 140), infection preventionists (n = 16) and residents/families (n = 63) from 24 Missouri NHs. Data were analyzed using directed content analysis to examine patterns of PPE access, usage, and challenges. To further verify challenges related to PPE access and use during the pandemic, a systematic review of 31 articles published between 2020 and 2023 was conducted. Interview findings revealed that all 24 NHs experienced PPE shortages due to supply chain disruptions and had to rely on corporate, state, and community support. Despite federal mandates, PPE use was inconsistent due to supply shortages, staff resistance, and communication difficulties with residents with dementia and hearing impairments. Training staff on PPE use and adapting care during shortages were key concerns. The systematic review findings echoed the interview findings, revealing a lack of streamlined PPE acquisition systems for NHs, significantly affecting supply and use. Few innovative solutions for maintaining resident care during PPE shortages were documented. Recommendations include ensuring NHs have reliable access to PPE, awareness of supply resources, and inclusive implementation processes. Special consideration for PPE use should be given to residents with memory and/or hearing impairment challenges.

Abstract citation ID: igaf122.3966

### INCENTIVES FOR ACCESS: EXAMINING INCREASED ADMISSIONS FOR CANCER PATIENTS AFTER THE PATIENT DRIVEN PAYMENT MODEL

Harsha Amaravadi[1], Rachel Prusynski[1], Paul Fishman[1], Natalie Leland[2], and Tracy Mroz[3], *1. University of Washington, Seattle, Washington, United States, 2. University of Pittsburgh, Pittsburgh, Pennsylvania, United States, 3. University of Washington School of Medicine, Seattle, Washington, United States*

Metastatic cancer prevalence is increasing as the population ages, highlighting the need for adequate cancer care in skilled nursing facilities (SNFs) which primarily serve older adults. However, cancer care in SNFs has typically been suboptimal. In October 2019, Medicare implemented the Patient Driven Payment Model (PDPM) which aligned SNF payment with patient medical complexity, rather than service volume . While PDPM was not cancer-specific, we hypothesize that PDPM's incentives impacted SNF patient selection, resulting in increased SNF admissions and altered SNF length-of-stay (LOS) for cancer patients. We used adjusted interrupted time series analysis, leveraging 100% Traditional Medicare claims from 2018-February 2020 to examine SNF admissions pre/post PDPM. Analyses account for patient complexity, discharge-relevant hospital factors, seasonality, and other post-acute care policies. We identified 1,096,484,782 hospital stays for metastatic cancer patients, 13% of which resulted in a SNF stay. We observed opposing patterns for the effect of PDPM on SNF admissions and LOS. For admissions, PDPM was associated with an immediate relative decline of 5.6% in October 2019 followed by a consistent monthly increase of 3.2%. The converse occurred for LOS; we observed an immediate increase (3.8 days, 18%), followed by a consistent monthly reduction of .73 days (-3.6%). Though both total effects (i.e., immediate + monthly trend through February 2020) were positive, the contrasting patterns may suggest changing severity among cancer patients entering SNFs. Findings may reflect PDPM's financial incentive to admit complex patients, underscoring the need for commensurate care delivery to improve patient outcomes in addition to access.

Abstract citation ID: igaf122.3967

### INHIBITING KMO BLOCKS KYNURENINE METABOLITES' INDUCTION OF SENESCENCE IN HUMAN NEURONS, MICROGLIA, AND 5XFAD MICE

William Hill, Dmitry Kondrikov, Steve Dixon, and Gavin Wang, *Medical University of South Carolina, Charleston, South Carolina, United States*

There is a growing interest in the involvement Kynurenine (KYN) Pathway (KP) metabolites in neurodegenerative disorders like Alzheimer's and Parkinson's disease. Research in this area is still limited but shows potential for providing novel mechanisms of action to target Alzheimer's disease therapeutically. Neuroinflammatory factors like INF-g, TNF-a, and IL-6 upregulate IDO-1 conversion of the essential amino acid tryptophan into KYN. In turn kynurenine monooxygenase (KMO) modifies KYN into 3-hydroxykynurenine (3-HK). Importantly, we have demonstrated that downstream KYN metabolites 3-HK, 3-Hydroxyanthranilic Acid (3-HAA), and quinolinic acid (QA) act in part via the Aryl hydrocarbon receptor (AhR), signaling system to induce senescence, which can be blocked by AhR inhibition (siRNA and DFM). Our preliminary data demonstrated that 3-HK,3HAA, and QA induced senescence in human neurons (SH-SY5Y cells) and microglial cells (HMC3), by multiple assays (including p21, SAb-Gal, H3K9Me3 methylation nuclear staining, and DNA damage), in a dose dependent fashion, as well as generating reactive oxygen species (ROS).These metabolites also increased tau phosphorylation in SH-SY5Y cells. Inhibiting KMO with siRNA, JM6, R0-61-8048 or GSK366 reduces generation of sub-KYN metabolites and decreases their ability to induce senescence or generate inflammatory SASPs and ROS. We treated 3-month-old 5xFAD Alzheimer's disease model mice with 3-HK or with the KMO inhibitor JM6 for 2 months. We saw an increase in senescence biomarkers with 3-HK (PAI-1, p21, IL-6, TIMP2) relative to a decrease with JM6 KMO inhibition. Our studies support exploring the targeting of KMO to potentially reduce central nervous system senescence.

Abstract citation ID: igaf122.3968

### INTEGRATING OLDER ADULTS' VOICES ON CLIMATE CHANGE INTO ONE HEALTH FRAMEWORK TO PROMOTE HEALTHY AGING

Hui Zhao[1], Anne Stewart[2], Francesca Ezeokonkwo[3], Joey Oi Yee Wong[4], Kayoung Lee[4], Karen Lok Yi Wong[4], Kai Zhang[5], and Lillian Hung[4], *1. James Madison University, Harrisonburg, Virginia, United States, 2. James Madison University, JMU Dept of Graduate Psychology, Virginia, United States, 3. Holy Family University, Philadelphia, Pennsylvania, United States, 4. University of British Columbia, Vancouver, British Columbia, Canada, 5. UNT Health, Fort Worth, Texas, United States*

Climate change poses threats to population health, affecting not only physical health, but also mental, social, and environmental well-being. Older adults are especially vulnerable, yet their perspectives are often overlooked in climate and health research. This qualitative pilot study foregrounds the voices of older adults, applying the One Health Framework to examine their perspectives on how climate change affects overall health. Employing an autophotography process and semi-structured interviews, the study also explores older adults' knowledge and beliefs about climate-related issues. Seventeen community-based older adults were recruited and 14 of them completed the study. Participants are white, aged 67-85, and mostly with graduate degrees. Constant comparison was used to analyze data. Key themes included 1) Vulnerability—participants highlighted challenges such as self-care and inadequate infrastructure during climate change and linked these challenges to their impacts on participants' care for plants and wildlife; 2) Recognizing Ecosystem Disruption—participants noted disruption of local weather patterns, and food systems and changing patterns in plant and wildlife interactions; and 3) Nurturing Resilience—participants described adapting strategies, including engaging in energy saving, plant care and sustaining strong bonds with wildlife as sources of well-being. Participants emphasized the need for cross-sector collaboration and intergenerational knowledge sharing to strengthen resilience. Findings indicate that acknowledging older adults' perspectives is critical for achieving holistic health goals. Within the One Health Framework, these insights highlight the interconnectedness of human, wildlife, and environmental health, and confirm that older adults' health outcomes cannot be addressed in isolation from ecological and social systems.

Abstract citation ID: igaf122.3969

### INTEGRATING SMART TECHNOLOGY INTO HEALTH PROMOTION FOR OLDER ADULTS IN TAIWAN: MANAGING CARDIOVASCULAR RISKS

Jen-Yu Wang[1], and Li-Hui Lin[2], *1. National Chung Cheng University, Hsinchu, Taiwan (Republic of China), 2. National Chung Cheng University, Chiayi, Minxiong, Taiwan (Republic of China)*

As Taiwan rapidly transitions into a super-aged society, cardiovascular disease has become a critical health concern for older adults. This study investigates the integration of smart technology into an active aging health promotion program and its impact on cardiovascular risk management and learning motivation. Grounded in achievement motivation theory, the research employs a quasi-experimental design with qualitative inquiry to evaluate both physiological outcomes and participants' learning experiences. A total of 30 older adults were recruited and assigned to either an experimental group, which received a 8-week program integrating smart technology (e.g., heart spectrum analyzer) with YSD Therapy massage, or a control group, which participated in traditional exercise sessions. Cardiovascular indicators—including blood pressure, heart rate, and heart spectrum indices (I1, I2, I3)—were measured before and after the intervention. Qualitative data from questionnaires and semi-structured interviews were analyzed using Braun and Clarke's six-phase thematic analysis to identify themes related to motivation, participation, and health self-management. Results showed that the experimental group demonstrated greater improvements in cardiovascular stability, especially in reducing arrhythmia and coronary risk, compared to the control group. Qualitative findings indicated that smart technology enhanced health data visualization, strengthened participants' sense of control, and fostered stronger motivation to sustain health behaviors. This study demonstrates the feasibility and value of integrating smart technology with traditional health promotion practices. It provides empirical evidence for education-based interventions that improve cardiovascular outcomes while empowering older adults' motivation, agency, and long-term participation.

Abstract citation ID: igaf122.3970

### INTENSIVE CARE OUTCOMES IN OLDER ADULTS WITH AND WITHOUT DEMENTIA: A SYSTEMATIC REVIEW AND META-ANALYSIS

Camila Badell[1], Maria Jose Lizarazo[2], Dustin M. Solorzano-Salazar[3], Luis A. Figueroa[4], Katherine K. Fung[5], Eric T. Y. Chyn[5], Eloy F. Ruiz[5], and Oscar J. Ponce-Ponte[6], *1. Rutgers New Jersey Medical School, Lima, Lima, Peru, 2. Mayo Clinic, Minnesota, United States, 3. Knowledge and Evaluation Research Unit, Mayo Clinic, Rochester, Minnesota, United States, 3. Universidad Peruana de Ciencias Aplicadas (UPC), Lima, Lima, Peru, 4. Universidad San Francisco de Quito, Quito, Pichincha, Ecuador, 5. Rutgers New Jersey Medical School, Newark, New Jersey, United States, 6. Derriford Hospital, University Hospital Plymouth NHS Trust, Plymouth, England, United Kingdom*

Older adults with dementia account over 15% of intensive care unit (ICU) admissions and face higher risks of functional decline and complications. We systematically evaluated the difference in ICU-related outcomes in patients with and without dementia, (e.g., mortality, resuscitation and code status decisions, complications, duration of life support and hospital stay). Searches were performed in MEDLINE, EMBASE, SCOPUS, and Web of Science between 2003 and 2024. A random-effects meta-analysis was done of odds ratios (OR) and mean differences (MD) and their corresponding 95% confidence intervals (CI) were calculated. Fifteen studies, including 1,231,037 patients (12% with dementia), met the inclusion criteria. Adjusted analysis showed that patients with dementia had a higher risk of ICU mortality (OR = 1.05, 95% CI 1.00–1.11), 30-day mortality (OR = 1.54, 95% CI 1.47–1.62) and 12-month mortality (OR = 1.95, 95% CI 1.88–2.02). They were less likely to receive mechanical ventilation (OR = 0.73, 95% CI 0.62–0.85, I2 0.0%) and more likely to develop delirium (OR = 4.80, 95% CI 1.81–12.72). In patients with dementia, ICU stays were slightly shorter (MD = –1 day, 95% CI –1.7 to –0.4), and discharge home was less common (OR = 0.40, 95% CI 0.39–0.42). Older adults with dementia experienced greater risk of mortality and delirium, alongside less aggressive intervention use and healthcare utilization (i.e., shorter ICU stays). These findings underscore the need for prospective studies to optimize ICU decision-making and post-discharge care for this vulnerable population.

Abstract citation ID: igaf122.3971

### IPA IMPROVES MUSCLE FUNCTION AND REDUCES BONE LOSS IN AGED MICE AND MODULATE LIFESPAN IN DROSOPHILA MELANOGASTER

Sagar Vyavahare[1], Ford Berger[2], Shelton Swint[3], Roger Zhong[2], Marion Cooley[4], Carlos Isales[5], Jessica Hoffman[2], and Sadanand Fulzele[2], *1. Augusta University, Martinez, Georgia, United States, 2. Augusta University, Augusta, Georgia, United States, 3. College of Science and Mathematics, Augusta, Georgia, United States, 4. Dental College of Georgia, Augusta, Georgia, United States, 5. Augusta Universoty, Augusta, Georgia, United States*

Aging is associated with alterations in endogenous tryptophan (TRP) metabolism that contribute to musculoskeletal decline. In this study, we investigated the effects of the microbiota-derived tryptophan (TRP) metabolite, indole-3-propionic acid (IPA), on musculoskeletal health in aged mice. Additionally, we assessed the impact of IPA on the lifespan of the fruit fly, Drosophila melanogaster. Our findings revealed that IPA-treated aged mice exhibited enhanced muscle function (grip strength and hang time). Histological and bone microCT analyses revealed no changes in muscle fiber size but enhanced bone microarchitecture. Furthermore, molecular studies have elucidated that IPA treatment prevents oxidative stress and reduces senescence, indicating improved cellular survival. Our Drosophila melanogaster longevity analysis revealed a significant extension of lifespan, but lifespan effects were genotype- and sex-specific. Collectively, our findings identify IPA as a promising microbiota-derived metabolite that improves musculoskeletal health and promotes longevity, highlighting its potential as a therapeutic intervention for age-related decline.

Abstract citation ID: igaf122.3972

### JUST SAY IT SIMPLY: HOW PROMPTING SHAPES AI RESPONSES FOR OLDER ADULTS

Litzy Puma[1], Kalyani Nair[2], and Walter Boot[3], *1. Weill Cornell Medicine, Jamaica, New York, United States, 2. Weill Cornell Medicine, New York City, New York, United States, 3. Weill Cornell Medicine, New York, New York, United States*

Large language models (LLMs) have strong potential as decision aids for older adults seeking information related to their health, well-being, and independence. Langston et al. (2025) found that off-the-shelf LLMs, such as Bard (now Gemini) and ChatGPT, provide quick and generally accurate responses to questions relevant to older adults. However, these responses are often long and complex, which may create difficulties due to age-related changes in information processing, particularly for those with mild cognitive impairment (MCI). We tested whether simple prompt modifications, such as stating that the user is an older adult, has MCI, or is seeking a simple explanation, could improve the readability of responses. Three independent raters evaluated a subset responses by ChatGPT4o to questions from Langston et al. (2025), scoring responses on readability metrics. Prompts indicating the user had MCI resulted in slightly simpler language (1.75 vs. 1.80 syllables per word, $p < .05$), but had little effect on other measures. In contrast, prompts that explicitly requested simple answers led to significantly improved readability, including shorter responses (209 vs. 279 words, $p < .05$) and fewer syllables per word (1.59 vs. 1.80, $p < .001$). Sentence length increased (10.87 vs. 9.15 words, $p < .001$), perhaps because responses contained fewer short filler sentences, more information-dense phrasing, and greater context. These results suggest that prompt engineering can improve LLM usability for older adults, but requests must be direct. LLMs do not reliably adapt based only on descriptions of the user.

Abstract citation ID: igaf122.3973

### LIMITED ACCESS TO POST-ACUTE REHABILITATION CARE AT SKILLED NURSING FACILITIES WITH MEDICAID

Lilly Estenson, Erin Duffy, Jonathan Cloughesy, and Mireille Jacobson, *University of Southern California, Los Angeles, California, United States*

Medicaid beneficiaries often have trouble finding healthcare providers that accept their insurance due to Medicaid's relatively low reimbursement rates. We conducted an audit study to examine Medicaid beneficiaries' access to post-acute rehabilitation care at skilled nursing facilities (SNFs) in California, comparing SNF bed availability for patients with Medicare and Medicaid ("Medi-Medi") to patients with Medicaid alone. We made calls between June and August 2025 to 968 SNFs listed as accepting Medicare and Medicaid in CMS Nursing Home Compare to inquire about bed availability for a fictitious grandparent being discharged from the hospital after hip fracture surgery. Patient insurance coverage was randomly assigned as Medi-Medi for calls to 483 SNFs and Medicaid alone for calls to 485 SNFs. Patient gender was randomized within each health insurance group. Callers did not specify patient age or additional health insurance details. Admission coordinators were reachable after up to five calls and able to provide information on bed availability at 72% of the SNFs. Among these SNFs, approximately 85% signaled bed availability and encouraged hospital transfer initiation for Medi-Medi patients. Coordinators were about 35 percentage points less likely to signal bed availability when patients had Medicaid alone. Bed availability for Medicaid-only patients was sometimes contingent on enrollment in a specific managed Medicaid plan. Our results indicate that Californians with Medicaid alone may experience difficulty accessing post-acute SNF care following a hospitalization relative to Californians dually eligible for Medicare and Medicaid. Recent Medicaid budget cuts may lower reimbursement rates and further limit post-acute care access.

Abstract citation ID: igaf122.3974

### LONGITUDINAL MEASUREMENT INVARIANCE OF THE HARMONIZED COGNITIVE ASSESSMENT PROTOCOL IN MEX-COG

Sangha Jeon[1], Zachary Kunicki[2], Lily Kamalyan[3], Sarah Prieto[4], Rebeca Wong[5], Miguel Rentería[3], and Emily Briceño[6], *1. University of Michigan, Ann Arbor, Ann Arbor, Michigan, United States, 2. Warren Alpert Medical of Brown University, Providence, Rhode Island, United States, 3. Taub Institute for Research on Alzheimer's Disease and the Aging Brain, Columbia University College of Physicians and Surgeons, New York, New York, United States, 4. Brown University, Providence, Rhode Island, United States, 5. University of Texas Health San Antonio, San Antonio, Texas, United States, 6. University of Michigan Medical School, Ann Arbor, Michigan, United States*

With the projected increase of older adults with Alzheimer's disease and Alzheimer's disease-related dementias (AD/ADRD) in Mexico, there is a growing need for consistent measurement of cognitive functioning over time. Ensuring measurement invariance is critical so that observed changes reflect true cognitive decline or improvement, rather than differences in how the test functions across time. This study evaluated the longitudinal measurement invariance (MI) of the Harmonized Cognitive Assessment Protocol (HCAP) from the Mexican Cognitive Aging Ancillary Study (Mex-Cog). Participants were 1,372 adults aged 55 and older who completed both Wave 1 (2016) and Wave 2 (2021) assessments. We evaluated longitudinal invariance using unidimensional models for five domains (orientation, language, memory, executive functioning, and visuospatial functioning), and a separate higher-order model representing general cognitive performance (GCP). All domain-specific models showed good fit (comparative fit index [CFI] > .95), and the higher-order model fit was acceptable (CFI = .943), with good to acceptable RMSEA (.000-.077) and SRMR (.00-.080). Longitudinal invariance analyses yielded mixed results across cognitive domains: language met only configural invariance, memory showed metric invariance, orientation and executive function showed partial metric invariance, and visuospatial functioning achieved scalar invariance. Results suggest that the factor structure of cognitive domains in Mex-Cog remains stable over 5 years. However, some items functioned differently over time, possibly due to practice effects, indicating that observed changes in these items may not solely reflect true cognitive change. This highlights the importance of examining the longitudinal consistency of cognitive scores over time in population-based cognitive aging studies.

Abstract citation ID: igaf122.3975

### MARKETING TO THE 'RIGHT' OLDER ADULT: WHOSE AGING IS REPRESENTED IN RECRUITMENT MATERIALS?

Regan Metz[1], Kyra Corbisiero[2], Abigail LeGars[1], and Kaitlyn Langendoerfer[1], *1. Wilkes University, Wilkes-Barre, Pennsylvania, United States, 2. College of the Holy Cross, Worcester, Massachusetts, United States*

There are nearly 11,000 older adult centers in the US. While well attended by previous generations, as more baby boomers aged, most centers experienced a declining clientele. These centers have begun to reconceptualize their role to service the interests of the new cohort. They now include cafes, wellness programing, and lifelong learning. This begs the question, who are older adult centers recruiting and what are the potential impacts? This study was designed to examine older adult center promotional materials using content analysis of the images featured within brochures, newsletters, and social media pages from 140 centers located in Pennsylvania. Three raters independently coded the images on a range of variables including observed demographics, health status, physical limitation, appearance (e.g. wrinkles/gray hair), and overall portrayal of aging. Our findings demonstrated that promotional materials reflected the demographics of Pennsylvania's aging population in terms of race and gender. However, the images overwhelmingly portrayed older adults as healthy (93%), happy (71%), and without any significant signs of decline; emphasizing a general image of "successful aging." Notably, only 40% of images were coded as representing "inclusive aging," meaning that most promotional materials do not accurately reflect the broader realities and diversity of the overall aging population. Failing to promote inclusivity, marketing materials currently frame the centers as recreation-style clubs, rather than spaces supportive of underrepresented groups. Discussed are the impacts on public funding of older adult centers and on marginalized community-dwelling older adults more generally.

Abstract citation ID: igaf122.3976

### NAVIGATING CONFLICTING EXPECTATIONS AND DEVALUATION: A QUALITATIVE STUDY OF HOME HEALTH AIDE EXPERIENCES

Chenjuan Ma, Sora Aikawa, Hillary Dutton, Soo Yeon Kim, and Ching Chen, *New York University, New York, New York, United States*

Home health aides (HHAs) are crucial in supporting individuals aging in place, offering companionship and assistance with daily activities. Despite their vital role, a lack of understanding regarding their work experiences has impeded efforts to address the HHA workforce shortage. This qualitative study explored HHAs' experiences, their views on roles and responsibilities, and the unique challenges they face. Data were gathered through five focus groups with a total of 47 HHAs and analyzed using a qualitative thematic approach. The analysis identified two main themes and five sub-themes. Theme 1, "Facing Conflicting Expectations Regarding Job Responsibilities," addresses the inconsistencies HHAs encounter in role expectations from patients, family members, and the interdisciplinary care team. This theme is supported by three sub-themes: 1) Experiencing a discrepancy in role interpretation, 2) Struggling with ambiguous care plans, and 3) Navigating conflicting demands. Theme 2, "Struggling to be Recognized and Respected as Part of the Care Team," highlights a key dilemma for HHAs: though often seen as the "eyes and ears" of home care patients, they are not always acknowledged as essential members of the care team. This disconnect can have serious implications for patient care. The sub-themes for this section are: 1) Feeling devalued and unappreciated by clients, colleagues, and employers, and 2) Impacting care quality and patient safety. The findings suggest that clearly defined HHA roles and improved communication between HHAs and other care team members are essential for reducing conflict, fostering collaboration, and ensuring quality care and patient safety.

Abstract citation ID: igaf122.3977

### NEIGHBORHOOD POLLUTING INFRASTRUCTURE AND COGNITIVE HEALTH IN RACIALLY DIVERSE OLDER ADULTS

Vivian Ku[1], Ketlyne Sol[2], Monica Walters[3], Noah Green[3], and Laura Zahodne[3], *1. Boston University, Boston, Massachusetts, United States, 2. University of Miami, Boca Raton, Florida, United States, 3. University of Michigan, Ann Arbor, Michigan, United States*

Pollution exposure relates to worse cognitive health. Modifiable sources of pollution may serve as an intervention target, but less is known about how polluting infrastructure in the built environment relate to distinct cognitive domains and racial disparities. 772 adults ages 55+ from the Michigan Cognitive Aging Project completed a neuropsychological battery measuring five cognitive domains (episodic memory, executive function, processing speed, language, visuospatial function). Participants' addresses were geocoded and linked to data from the National Neighborhood Data Archive. Polluting infrastructure was operationalized at the census-tract level as the presence of Toxic Release Inventory (TRI) sites and total highway length. Generalized estimating equations accounting for geographic clustering modeled relationships between polluting infrastructure and cognition, controlling for individual-level and neighborhood-level covariates. The presence of TRI sites related to worse language ($\beta$=-.140, p=.031), while greater total highway length related to worse executive function ($\beta$=-.007, p=.036) and language ($\beta$=-.011, p=.016). Race modified the relationship between total highway length and visuospatial function ($\beta$=-.024, p=.008). Greater total highway length related to better visuospatial function only among non-Hispanic Black participants ($\beta$=.027, p=.038). Highways and TRI sites may represent modifiable polluting infrastructure that influences older adults' cognition through distinct pathways. The unexpected finding between highways and visuospatial function among Black older adults may reflect unmeasured effects of highway accessibility (e.g., access to resources, social networks). Future research should explore how older adults interact with their neighborhood to inform interventions that promote cognitive health in older adults.

Abstract citation ID: igaf122.3978

### NEIGHBOURHOOD COHESION AND CHRONIC LONELINESS IN THE UK: ANALYSIS OF THE UK HOUSEHOLD LONGITUDINAL STUDY

Christina Victor, and Isla Rippon, *Brunel University London, Guildford, England, United Kingdom*

Loneliness has been identified as a major global public health challenge. There is substantial evidence reporting prevalence and risk factors for loneliness in older adults. Longitudinal evidence about loneliness is less well established and there is no consensus on the typology characterising longitudinal loneliness trajectories. Our evidence examining neighbourhood level factors is limited mostly focussing upon walkability or access to blue-green spaces. We investigate the role of neighbourhood cohesion as a 'protective' factor against experiencing chronic loneliness and focuses upon two under-researched groups of older adults, minoritised ethnic groups and sexual minorities. We use data for 10,916 adults aged 50+ who participated in waves 9-11- and 13 of the United Kingdom Household Longitudinal Survey (UKHLS). Loneliness was measured using the three-item UCLA scale with a score of 6+ defining loneliness and neighbourhood cohesion by the Buckner scale. We define four loneliness trajectories: not lonely, transient (lonely once), fluctuating (two or three occasions of loneliness) and chronic (lonely at every wave). Chronic loneliness is higher among black, Indian, white Irish and mixed ethnicity populations compared to white British respondents. Sexual minority participants had higher levels of chronic loneliness than heterosexual participants. Neighbourhood cohesion is highest for the white Irish group and heterosexual groups and lowest for mixed race and sexual minority participants. High neighbourhood cohesion is protective against chronic loneliness for white British, Irish and for both sexual minority and majority groups. Adjustment age, sex, living alone, material resources and mental well being attenuated these relationships.

Abstract citation ID: igaf122.3979

### NETWORK ANALYSIS OF HOW DIFFERENT PHYSICAL ACTIVITY DOMAINS RELATE TO COGNITION IN OLDER ADULTS

Daniel Leme, and Zhe He, *Florida State University, Tallahassee, Florida, United States*

Cognitive performance in older adults is shaped by a complex interplay of clinical and psychosocial factors. While previous studies have demonstrated bivariate associations between physical activity (PA) and cognitive decline, less is known about how distinct PA domains are simultaneously associated with mild cognitive impairment (MCI), particularly when accounting for potential confounding variables. This study examined the simultaneous associations between specific PA domains and MCI, while controlling for a broad range of clinical, psychosocial, and demographic factors, using network analysis. Data from 3,172 adults aged 60 years and older who participated in the 2011–2014 National Health and Nutrition Examination Survey (NHANES). PA was assessed using the Global Physical Activity Questionnaire (GPAQ), which classifies activity into three domains: occupational PA (OPA), including household tasks and food gathering; transportation PA (TPA), such as walking and cycling; and leisure-time PA (LTPA), encompassing sports, exercise, and recreational activities. Among participants, 10.3% reported moderate to high levels of OPA, 3.7% engaged in TPA, and 4.4% in LTPA. The final network model revealed significant negative associations between both OPA (edge weight = –0.22) and LTPA (edge weight = –0.20) with MCI. No meaningful association was found between TPA and MCI. Additionally, depressive symptoms, social isolation, and polypharmacy were positively associated with MCI (edge weights ≤ 0.22). Our findings indicate that the association between PA and MCI in older adults is domain-specific and emphasize the need for a multidimensional approach to healthy aging that integrates PA promotion with clinical and psychosocial strategies to support cognitive health.

Abstract citation ID: igaf122.3980

### NIH RESILIENCE MODEL: LONGITUDINAL EFFECTS OF SURGERY ON GENETICS, PHYSICAL CAPACITY & CHRONIC CONDITIONS

Kenneth Seldeen[1], and Frances Yang[2], *1. University of Kansas Medical Center, Kansas City, Kansas, United States, 2. University of Kansas, University of Kansas Medical Center, Kansas, United States*

The National Institutes of Health (NIH) Resilience Research Working Group has put forth the resilience model that over time, a system's dynamic response to a challenge depends on the severity of the stressor, the length of time exposed, and innate biological factors. Measuring resilience therefore creates opportunities to evaluate predictive potential for future health outcomes, relationship to underlying biology, and capacity to be enhanced through intervention. This study hypothesized that older adults (aged 65+), who are resilient will have higher physical capacity and fewer chronic health conditions over time (response) after experiencing one or more surgeries within a 12-month period (challenge) due to a better polygenics score (system). Therefore, this study examined resilience in the 2016 through 2020 waves of the National Health and Aging Trends Study (NHATS), which consists of a nationally representative sample of older adults. There were 2,827 older adults with polygenics score for BMI. Using latent variable modeling, we found that with each increase in the number of hip, knee, heart, cataract, or back surgeries during 2017 there was and associated decline in physical capacity (walking 3 or more blocks, b=-0.14, p<.001) and polygenic BMI scores (b=-0.002, p<.001 - with a response of increasing physical capacity from 2016 to 2019 with overall declines in the number of chronic conditions in 2020. These data provide new insights into the predictive potential of resilience measurement and support the goal of NIH to establish fundamental knowledge about resilience to enhance health, lengthen life, and reduce illness and disability.

Abstract citation ID: igaf122.3981

### NON-TOXIC TAU OLIGOMER POLYMORPHS ISOLATED FROM NON-DEMENTED INDIVIDUALS WITH ALZHEIMER'S NEUROPATHOLOGY

Thai Nguyen[1], Jutatip Guptarak[2], Batbayar Tumurbaatar[2], Michela Marcatti[2], Wen-Ru Zhang[2], Anna Fracassi[2], and Giulio Taglialatela[2], *1. John Sealy School of Medicine, Galveston, Texas, United States, 2. The University of Texas Medical Branch, Galveston, Texas, United States*

Background: Alzheimer's disease (AD) is a neurodegenerative disorder that is characterized by the presence of extracellular Aβ plaques and intracellular tau neurofibrillary tangles. A subset of individuals classified as A+T+N–, termed Non-Demented with Alzheimer's Neuropathology (NDAN), maintain normal cognition despite harboring full AD pathology. This suggests that tau oligomers (TauO) may exist as distinct polymorphs with divergent functional consequences as well as the existence of innate protective mechanisms opposing the events that normally lead to cognitive impairment. Methods: We investigated whether NDAN brain-derived tau oligomers (NDAN-BDTOs) differ from AD-BDTOs in toxicity and microglial responses. Wild-type mice received intracerebroventricular injections of AD- or NDAN-BDTOs. Brain tissue was analyzed by immunofluorescence and western blotting to characterize microglial phenotypes (Iba1, CD68, TREM2) and assess pathways associated with phagocytosis, autophagy (Atg5, Atg7, LC3A/B, p62), and oxidative stress (SOD1/2, catalase, 8-oxo-dG). Results: AD- and NDAN-BDTOs were identified as structurally distinct polymorphs with divergent biological effects. AD-BDTOs induced neuronal apoptosis and pro-inflammatory microglial activation, while NDAN-BDTOs enhanced autophagy, preserved antioxidant defenses, and reduced oxidative DNA damage. Furthermore, NDAN-BDTOs elicited a specialized microglial phenotype distinct from that induced by AD-BDTOs, suggesting differential engagement of neuroinflammatory pathways. Conclusion: Our findings support the existence of a non-toxic, propagating tau oligomer in NDAN brains that promotes resilience by avoiding neurodegeneration. These results highlight the therapeutic potential of strategies that stabilize or redirect tau oligomers into non-toxic conformations, offering a novel avenue for disease-modifying interventions in AD.

Abstract citation ID: igaf122.3982

### NUTRITIONAL DENSITY AND ITS IMPACT ON BIOLOGICAL AGE: INSIGHTS FROM LARGE COHORT STUDIES

Eleonora Porcu, Charlotte Debras, Shu Wang, Li-Tang Tsai, Alberto Conde Freniche, Philipp Gut, and Sébastien Herzig, *Nestle Institute of Health Science, Lausanne, Vaud, Switzerland*

Relationships between nutrition and aging represent a critical area of research, as dietary choices can influence health and disease risk. In this study, we used Clinical PhenoAge (PA) as an indicator of health status reflecting biological processes of aging better than chronological age. We calculated biological age advancement (BAA) in two populations (UKBiobank, N = 103,344 and NHANES 2015-2018, N = 4,264) as the difference between PA and chronological age and explored the association with specific diet quality scores. To this end we used nutrition density (measured using NRF9.3 score, a method evaluating adequate intakes of 12 nutrients) as well as individual nutrient intake. After adjusting for relevant anthropometric, sociodemographic and health-related covariates, the linear regression analyses revealed that a 50-point increase of NRF9.3 is associated with lower BAA in UKBiobank (β=-0.093[95% CI: -0.103,-0.083], P<0.001) and in NHANES (β=-0.152[-0.192,-0.112], P< 0.001). Given that BMI is often influenced by dietary choices and could be associated with PA, we aimed to investigate whether the association between NRF9.3 and BAA could be mediated by BMI. Mediation analysis indicated that a modest part of the association was mediated by BMI (19%[16%-22%] in UKBiobank, and 12%[6%-19%] in NHANES, P<0.001). Furthermore, we identified specific nutrients associated with lower BAA, including fiber (β_UKBiobank=-0.11[-0.13,-0.09], β_NHANES=-0.44 [-0.59,-0.30], P< 0.001) and magnesium (β_UKBiobank=-0.11[-0.13,-0.09], β_NHANES=-0.46[-0.60,-0.31],P< 0.001). Conversely, nutrients associated with higher BAA included added sugars (β_UKBiobank=0.15[0.13,0.17], β_NHANES=0.35[0.21,0.50], P< 0.001). Overall, these findings suggest the relevant role of diet in biological aging, highlighting the potential for targeted nutritional interventions promoting healthier aging, ultimately leading to healthy longevity.

Abstract citation ID: igaf122.3983

### OBESITY CARE IN PRIMARY CARE: IMPLEMENTING THE GSA KAER TOOLKIT FOR OLDER ADULTS

Anna Pendrey[1], Triccia Aparicio[2], Miguel Paz[2], Mariel Zelaya[2], Samir Cabrera[3], Javier Sevilla[1], Paola Rodriguez[1], and Omar Suazo[3], *1. Indiana University, Zionsville, Indiana, United States, 2. Universidad Nacional Autonoma de Honduras, Zionsville, Indiana, United States, 3. UNITEC, Zionsville, Indiana, United States*

Background Obesity affects nearly 40% of US adults aged ≥65 years and contributes to cardiovascular disease, diabetes, functional decline, and reduced quality of life. Age-related physiological changes, multimorbidity, and polypharmacy complicate obesity management. The Gerontological Society of America's KAER (Kickstart, Assess, Evaluate, Refer) Toolkit offers structured, age-appropriate guidance for primary care providers. Methods This quality improvement project was conducted in a geriatric primary care clinic from September 2023 to June 2025. The KAER Toolkit was used in routine visits for patients aged ≥65 years. Providers were trained to discuss weight without stigma, assess body composition and comorbidities, evaluate contributing factors, and refer patients. Data on screening, provider engagement, and referrals were analyzed. Results Among 41 older adult patients, 34% were identified with obesity, predominantly females aged 66–70 years. Most had class I obesity. Patients were diverse, with Hispanic (43%) and African American (36%) representation. Functional assessments showed that while most remained independent in basic activities of daily living, 71% required assistance with instrumental activities. High rates of depression (86%) and cognitive impairment (64%) were observed, along with prevalent comorbidities such as hypertension (93%), chronic kidney disease (86%), and hyperlipidemia (71%). Nutritional deficiencies were prevalent, with 64% deficient in vitamin D. Conclusions The GSA KAER Toolkit facilitated systematic, age-sensitive obesity management in primary care. Its user-friendly design supports early identification, targeted intervention, and coordinated care for obesity in older adults.

Abstract citation ID: igaf122.3984

### OLDER ADULTS' PROPENSITY FOR COMMUNITY: AGE DIFFERENCES IN THE EFFECTS OF CLOSENESS ON SHARED POSITIVITY

Sydni Adams, and Joseph Mikels, *DePaul University, Chicago, Illinois, United States*

Positivity resonance describes a connection involving shared positive affect and biobehavioral synchronization (Fredrickson, 2013), which improves one's health (Major et al., 2018; Wells et al., 2021). Positivity resonance may also increase communal strength (e.g., going out of one's way to meet others' needs), which improves relationships (Mattingly et al., 2011). Though older adults' social networks are comprised largely of close relationships (Moore et al., 2016), older relative to younger adults may be more likely to engage more distant others with positivity resonance, which may explain why weak ties are predictive of healthy aging (Huxhold et al., 2020). We explored the relationship between positivity resonance and communal strength for older and younger adults across strong, weak, no tie conditions. 250 participants (125 younger adults; 125 older adults) reported their feelings of positivity resonance and communal strength after recalling recent interactions across each level of tie strength. Using multilevel regression, we found an interaction of age and tie strength on positivity resonance, $F(1, 468) = 1.38$, $p < .05$, such that older adults experienced greater positivity resonance than younger adults for weak and no tie interactions. There was also an interaction between tie strength and positivity resonance on communal strength, $F(1, 600) = 8.85$, $p < .01$. The more positivity resonance participants experienced, the more communal strength they reported. This research advances our understanding of the quality of network diversity among younger and older adults. Our findings suggest that older adults have improved relationships with acquaintances and strangers compared to younger adults.

Abstract citation ID: igaf122.3985

### OLDER ADULTS' MOTIVATION AND VOLITION IN SUSTAINING EXERCISE AFTER A STRUCTURED PROGRAM

Bora Jin[1], and Elizabeth Roumell[2], *1. Winston Salem State University, Kernersville, North Carolina, United States, 2. Texas A&M University, Texas A&M University, Texas, United States*

Sustaining exercise habits is important for supporting health and independence in later life (World Health Organization, 2024). While exercise adherence has been widely studied, less is known about how older adults continue activity once structured programs conclude. Drawing on Self-Determination Theory (Ryan & Deci, 2017) and Volitional Competences (Kuhl, 2000; Elsborg et al., 2019), we explored how motivation and volition influence exercise continuation among older adults who completed an eight-week structured exercise program. Using a critical realist approach (Fletcher, 2017), we conducted 29 semi-structured interviews, analyzed through abductive and retroductive reasoning, and coded with Atlas.ti. We identified motivational factors that explained why participants continued exercising (e.g., perceived physical improvements, accountability, intergenerational influence, and social connection and enjoyment). We also found volitional factors that described how participants sustained routines despite challenges, including embedding exercise into daily life, using self-regulation strategies, adapting routines to prevent injury, and drawing on a lifelong exerciser identity. Participants also described barriers such as health limitations, emotional and cognitive challenges, hesitancy to act once programs ended, and financial or logistical constraints. Our findings suggest that older adults build on motivation to engage and volition to persist once a structured program ends, balancing supportive daily practices with barriers such as health concerns, emotional challenges, and financial constraints. We consider how autonomy-supportive practices, accountability structures, and program designs that acknowledge these barriers may help sustain participation. We conclude by outlining implications for fitness instructors, health educators, and practitioners seeking to promote meaningful exercise engagement in older adults.

Abstract citation ID: igaf122.3986

**OLDER DRIVERS: ATTENTION, HABITS AND MEMORY**

Laura Hemmy, Katelyn Schwieters, Marshall Mabry, William Kessler, Brian Davis, and Nichole Morris, *University of Minnesota, Minneapolis, Minnesota, United States*

Older drivers are at increased risk for negative outcomes and generating knowledge to promote safe driving and when to consider retirement from driving are of the utmost importance. This study evaluated how demographics and cognitive state are related to driver habits and attention. A sample of community dwelling older adults (n = 111) without global cognitive impairment on the Minnesota Cognitive Acuity Screen completed baseline assessments before the initiation of a driver safety intervention. Participants reported as 61% female, mean age 69.1 (SD 6.2), 15.9 mean years education (SD 2.4), and 66% retired. 19% reported living in an urban setting, 47% suburban, and 34% rural. Self-reported measures included the Driving and Riding Avoidance Scale (DRAS), Cognitive Failures Questionnaire - Driving (CFQ), Attention-Related Driving Errors Scale (ARDES), Mindfulness Attention Awareness Scale (MAAS), and Aggressive Driving Behavior Questionnaire (ADBQ). No measure was significantly related to participant demographics. Brief performance measures of cognition administered by telephone, including the Montreal Cognitive Assessment (MoCA) Blind and oral Trails A and B, were mostly uncorrelated with the self-reported driving and attention measures. One exception was the MoCA word recall and DRAS, r(106) = -.224, p=.02, in which poorer memory was associated with greater driver avoidance. These findings are noteworthy for 1) a lack of correlation between self-reported measures of driver attention and either age or cognition, 2) no gender differences found in measures of driver behavior, and 3) in a sample pre-screened for cognitive impairment, the word recall was significantly related to older adults' self-reported driver avoidance behaviors.

Abstract citation ID: igaf122.3987

**OMITTED MEDICATIONS AMONG HOSPITALIZED OLDER ADULTS AND PERSONS WITH DEMENTIA USING MIMIC-IV**

Ellen Munsterman[1], Jiyoun Song[1], Marie Boltz[2], and Pamela Cacchione[3], *1. University of Pennsylvania School of Nursing, Philadelphia, Pennsylvania, United States, 2. Pennsylvania State University, University Park, Pennsylvania, United States, 3. University of Pennsylvania, Philadelphia, Pennsylvania, United States*

Omitted medication occurs when a prescribed drug is not administered. Hospitalized persons with dementia (PWD) may experience omitted medications due to delirium, lack of routine/familiarity, and/or lack of dementia-specific training among clinicians. The impact of omitted medications ranges from negligible to fatal, but little research has been done on omitted medications focused on hospitalized persons with dementia. This study aimed to examine the association between having a dementia diagnosis and omitted medications and explore factors that may influence this relationship. We utilized the MIMIC-IV-Hosp dataset, de-identified EHR data from adults admitted to Beth Israel Deaconess Medical Center from 2008-2019. Our sample included admission-level data for adults aged >60 with hospital stays >48 hours. PWD were identified using ICD diagnosis codes. Omitted medications were identified based on event documentation in the medication record. Preliminary findings: Among 95,421 admissions, 10,921 (11.4%) were PWD. In unadjusted comparisons, PWD had a 2.65% higher proportion of omitted medications ($p < 0.001$). After adjusting for age and length of stay (LOS), PWD had a 1.95% higher proportion of omitted medications ($p < 0.001$). While statistically significant, the effect size was small, and the model explained only 2.3% of the variance. Among PWD, delirium was independently associated with a higher proportion of omitted medications ($\beta = 2.61$, $p < 0.001$), adjusting for age and LOS. This model explained 3.9% of the variance in omitted medications among PWD. Further research is warranted to identify types of medications most affected by omissions among PWD and to clarify the underlying mechanisms.

Abstract citation ID: igaf122.3988

**ORGANIZATIONAL FACTORS INFLUENCING PROGRESS IN TAIWAN'S DISABILITY PREVENTION PROGRAMS**

Wan-Yu Chiu[1], Hui-Fen Mao[2], Ling-Hui Chang[3], and Ya-Mei Chen[4], *1. National Taiwan University, Zhongzheng District, Taipei, Taiwan (Republic of China), 2. National Taiwan University, Taipei City, Taipei, Taiwan (Republic of China), 3. National Cheng Kung University, 701, Tainan, Taiwan (Republic of China), 4. National Taiwan University, National Taiwan University, Taipei, Taiwan (Republic of China)*

Taiwan's nationwide Prevention and Delay of Disability (PDD) program plays a key role in promoting older adults' function. However, health progress varies by frailty level and may be shaped by organizational characteristics. This study investigated how such characteristics influence different types of health progress among older adults. The cross-sectional study (2019–2020) involved 392 administrators from PDD service centers and 13,213 participants, categorized as frail (563), pre-frail (2,397), or healthy (10,253). Over a 12-week period, multiple centers implemented a multi-domain intervention composed of similar elements, such as physical exercise, nutrition, cognitive training, and social engagement. Organizational-level data were collected from administrators using six dimensions: efficiency, responsiveness, professionalism, cooperation, environment, and sustainability. Health progress among participants was classified into three distinct types for each frailty level: stable, multidimensional, and unique, resulting in a total of 9 progress types. Using multilevel analysis, higher organizational responsiveness was associated with greater progress among frail and pre-frail participants. Frail individuals were more likely to exhibit depressive-social progress (β = 0.746, p = 0.013), while pre-frail participants showed a tendency toward multidimensional progress (β = 0.312, p = 0.012). Organizations managing a greater variety and volume of programs were more likely to facilitate multidimensional progress in both frail (β = 0.148, p = 0.004) and pre-frail groups (β = 0.034, p = 0.008), while organizational factors appeared to have limited influence on healthy participants. These findings highlight the role of organizational factors in influencing health outcomes among older adults.

Abstract citation ID: igaf122.3989

### PARENTAL AGE AND STRESS IN CAREGIVERS OF INDIVIDUALS WITH ANGELMAN SYNDROME: INSIGHTS FROM THE LADDER COHORT

Sean Halpin[1], Sarah Potter[2], Wen-Hann Tan[3], Anjali Sadhwani[3], Batsheva Friedman[3], and Anne Wheeler[4], *1. RTI International, Decatur, Georgia, United States, 2. RTI International, Research Triangle, North Carolina, United States, 3. Harvard Medical School, Boston, Massachusetts, United States, 4. RTI International, Research Triangle Park, North Carolina, United States*

Angelman syndrome (AS) is a rare neurogenetic disorder characterized by severe developmental delay including minimal speech and motor impairments. As individuals with AS grow older, many parents provide intensive support into their own later life. Using data from the Angelman Syndrome Natural History Study, via the Linking Angelman and Dup15q Data for Expanded Research (LADDER) Database, we examined how maternal age relates to stress domains. Our sample included 268 participants (591 observations), with maternal ages of 23–64 years (baseline mean = 36, SD = 7.1). Mixed-effects REML regression models assessed associations between child and maternal age and 16 stress domains from the Parenting Stress Index. Maternal age was significantly linked to increased stress in child adaptability (β = 0.574, p = .014), reinforcement (β = 0.566, p = .008), demandingness (β = 0.692, p < .001), mood (β = 0.864, p < .001), isolation (β = 0.653, p = .005), and total stress (β = 0.548, p = .012). These effects held after adjusting for child age, which also correlated with stress in adaptability (β = 1.007, p < .001) and mood (β = 1.269, p < .001). Older caregivers face compounding stress from aging children's behavioral challenges and their own age-related vulnerabilities. Age-responsive interventions are needed to support long-term caregivers and guide future research into late-life caregiving in rare neurodevelopmental disorders.

Abstract citation ID: igaf122.3990

### PATH ANALYSIS OF FACTORS THAT INFLUENCE FAMILY CAREGIVER BURDEN IN DEMENTIA CARE

Jiyoung Kim, EunKyo Kim, Minhee Yang, Eunhee Cho, Sinwoo Hwang, and Jungwon Cho, *Yonsei University, Seoul, Republic of Korea*

Dementia, affecting over 55 million people worldwide, demands urgent attention to caregiver burden, but the interplay of contributing factors remains unclear. This study aimed to identify factors from both people living with dementia (PLWD) and their family caregivers that influence caregiver burden, and to examine the interrelationships among these factors. Data were drawn from 178 dyads of community-dwelling PLWD and their family caregivers in Korea. Family caregiver burden was assessed through caregivers' subjective health and perceived stress related to their family member's behavioral and psychological symptoms of dementia (BPSD). The analysis was grounded in the Stress Process Model, which provided a theoretical framework for examining caregiver burden. We modified the model by repositioning BPSD as the primary stressors of caregiver burden and additionally incorporating PLWD factors as antecedents to BPSD. Path analysis was conducted using JASP 0.19.3.0, and model fit was evaluated using multiple fit indices. Agitation and depression—key BPSD of PLWD—were negatively associated with both caregivers' subjective health and stress levels. These associations were also indirectly influenced by PLWD's age. Furthermore, caregivers' sex, age, and economic status were directly associated with their subjective health. Neither caregiving hours nor caregiving duration significantly influenced caregiver burden. These findings underscore the significant impact of agitation and depression on caregiver burden and indicate that when BPSD manifest, the burden experienced by caregivers should also be addressed. Thus, tailored dementia interventions addressing these factors need to be developed to mitigate caregiver burden and improve outcomes for both PLWD and their families.

Abstract citation ID: igaf122.3991

### PERCEIVED HEALTH STATUS AMONG OLDER ADULTS WITH INTELLECTUAL AND DEVELOPMENTAL DISABILITIES

Jihyeong Jeong[1], Changhyun Kim[2], Sehyun Baek[3], and Amanda Lehning[1], *1. University of Maryland Baltimore, Baltimore, Maryland, United States, 2. Busan National University, Busan, Republic of Korea, 3. New York University, New York, New York, United States*

With growing interest in the age-friendly community (AFC) movement, empirical research has increasingly explored the associations between AFC characteristics and various health outcomes. However, prior studies have primarily focused on the general older population, with limited attention given to more vulnerable subgroups, particularly older adults with intellectual and developmental disabilities (IDD). Using the National Core Indicators® - Intellectual and Developmental Disabilities in-person survey 2022-2023 data, we analyzed the association between AFC indicators and self-rated health among older adults with IDD aged 55 or older who were living in the community (N = 688). The AFC features analyzed are: (a) housing, (b) outdoor spaces and buildings, (c) transportation, (d) social participation and inclusion, (e) health and wellness, and (f) volunteering and civic engagement. Perceived health status was measured using a single item: "Overall, how would you describe your health?" We performed bivariate and logistic regression analyses controlling for covariates. Our study demonstrated significant associations between better self-rated health and housing satisfaction (OR = 3.13, p = .012), access to adequate transportation (OR = 1.94, p = .019), and social participation (OR = 1.15, p = .001). Based on the findings, we suggest that housing satisfaction for older adults with IDD should ensure cognitively accessible residential environments. Transportation services should address additional challenges that they encounter (e.g., understanding complex routes, coping with unexpected changes, and communication barriers). Promoting social participation should entail cognitive accessibility, trained support staff, and creating inclusive community environments that understand and accommodate communication difficulties.

Abstract citation ID: igaf122.3992

**PERCEPTIONS OF VALUE AND ADOPTION OF ADRD MODIFYING DRUGS AMONG PHYSICIANS AROUND THE U.S.**

Dinithi Perera[1], Michelle Keller[1], Zaldy Tan[2], Chanelle Mizrahi[1], Nabeel Qureshi[3], Golnaz Yadollahikhales[2], and Sarah Kremen[2], *1. University of Southern California, Los Angeles, California, United States, 2. Cedars-Sinai Medical Center, Los Angeles, California, United States, 3. Cedars-Sinai Medical Centers, Los Angeles, California, United States*

Following recent FDA approvals in the U.S. of two anti-amyloid medications for Alzheimer's Disease, lecanemab and donanemab, we aimed to examine clinicians' perceptions of the value of and real-world adoption experiences with prescribing these medications. While these monoclonal antibody treatments are the first medications to clear amyloid in the brain, they come with significant treatment burdens and risks. Our objective was to better understand the risk-benefit evaluations physicians make when selecting candidates for treatment; the practice-level, insurance, and health system obstacles they've had to navigate when implementing these complex medications into their practices; and how they communicate the risks and benefits to patients and their families. Using purposive and snowball sampling, we interviewed 10 physicians including geriatricians, neurologists and psychiatrists to understand the drivers of and barriers to adoption of these medications by physicians. We identified 5 themes from our qualitative interviews: 1) a broad continuum of wariness to avid proponent of the medications; 2) differences in a propensity to prescribe these medications and approaches to care between different clinical specialties, with strong support from neurologists and a more cautious approach from geriatricians; 3) varied communication styles in how physicians present the risks and benefits with patients and their families, 4) significant variation in practice and health-system implementation, from a planned approach to a less organized approach, and 5) clinicians facing shifting roles of balancing hope with limited data, managing ethical complexity, and bearing the emotional weight of guiding patients through novel therapies.

Abstract citation ID: igaf122.3993

**PERCIEVED MEMORY DECLINE AND ADVANCE CARE PLANNING ENGAGEMENT: COMPARING LGBTQ+ AND NON-LGBTQ+ ADULTS**

Olivia (YuHsuan) Wang[1], and Mireille Jacobson[2], *1. USC, Los Angeles, California, United States, 2. University of Southern California, Los Angeles, California, United States*

Background: Advance care planning (ACP) allows individuals to discuss future medical decisions while cognitively capable. LGBTQ+ adults may benefit disproportionately, given weaker family support networks. They also report higher rates of perceived memory decline, but how cognitive concerns impact ACP and healthcare communication among LGBTQ+ adults remains understudied. Methods: We surveyed 2,398 adults (510 LGBTQ+) in the Understanding America Study, a national panel of U.S. adults aged 18+, to examine ACP preparedness and discussions in medical settings, and health care communication. Logistic regression models tested differences by LGBTQ+ status and interactions with perceived memory decline, controlling for demographics. Results: Compared to non-LGBTQ+ adults, LGBTQ+ adults were 3% more likely to have thought about ACP ($p<.05$) and 5% more likely to report ACP discussions in medical settings ($p<.05$). Among LGBTQ+ adults, perceived memory decline was associated with a higher likelihood of ACP discussions in medical settings compared with non-LGBTQ+ adults, and also with a greater likelihood of never having considered ACP. Perceived memory decline also reduced comfort with health care communication among LGBTQ+ adults. Conclusions: LGBTQ+ adults show greater ACP engagement overall, but perceived memory decline is linked to both higher likelihood of ACP discussions and higher likelihood of never having considered ACP. This suggests that ACP engagement among LGBTQ+ adults with perceived memory decline may be concentrated at different points along the planning spectrum. Providers should assess each patient's starting point, validate perceived decline, and offer stage-appropriate interventions to foster communication and move patients toward timely ACP participation.

Abstract citation ID: igaf122.3994

**PHASE 1 EVALUATION OF MF-300: AN INVESTIGATIONAL FIRST-IN-CLASS ORAL CANDIDATE FOR SARCOPENIA**

Leigh MacConell[1], Amrik Shah[2], Diane Mould[3], Siva Lavu[4], and Micah Webster[5], *1. Alchemeos Clinical Consulting, Encinitas, California, United States, 2. Karma Statistics LLC, Skillman, New Jersey, United States, 3. Projections Research Inc, Phoenixville, Pennsylvania, United States, 4. RedOwlet LLC, Wellesley, Massachusetts, United States, 5. Epirium Bio, San Diego, California, United States*

Age-related sarcopenia, a progressive chronic disease involving loss of skeletal muscle mass and function, contributes to disability and loss of independence, affecting 1/3 of Americans over 60 years. Despite being associated with increased risk of falls, hospitalization, and all-cause mortality, no pharmacologic treatment is approved for sarcopenia, representing an important unmet medical need. MF-300 is an investigational, first-in-class, orally administered, 15-hydroxyprostaglandin dehydrogenase inhibitor that improves aged muscle force in preclinical studies and is in development for sarcopenia. Safety, tolerability, pharmacokinetics (PK) and pharmacodynamics (PD) of MF-300 were evaluated in healthy adults in a Phase 1 double-blind Single Ascending Dose (SAD) and Multiple Ascending Dose (MAD) study. In the SAD, MF-300 or placebo were administered to 40 subjects at 5 doses (75mg to 800mg). In the MAD, dose escalation included QD doses of 75mg, 125mg, and 200mg for 5 days to 30 subjects. Urinary prostaglandin E2 and its metabolites were investigated as MF-300 target engagement biomarkers. All participants completed the study. MF-300 was observed to be safe and well-tolerated; all but one adverse event was mild with no dose relationship. There were no serious adverse events. The PK profile showed rapid absorption (Tmax 1.17h-2.58h) and a T1/2 supporting QD dosing (12.2h-15.9h). Exposure metrics (Cmax, AUC) increased slightly greater than dose-proportionally and were consistent with anticipated therapeutic activity based on preclinical data. Dose-related changes in PD biomarkers were consistent with MF-300 target engagement. These findings support further Phase 2 evaluation of MF-300 in age-related sarcopenia.

Abstract citation ID: igaf122.3995

**POOR MENTAL HEALTH IN MIDDLE-AGED AND OLDER ADULTS WITH COGNITIVE DIFFICULTIES**

Zoe Fagan, and Amy Fiske, *West Virginia University, Morgantown, West Virginia, United States*

Cognitive difficulties and depressive symptoms are interrelated in older adults. There is some evidence that depression may be a prodromal symptom of dementia (Brommelhoff et al., 2009). In addition, depression may arise in the early stages of Alzheimer's Disease as an emotional reaction to the awareness of loss of cognitive abilities (Rosanna et al., 2022). The primary goal of this analysis was to examine the association between cognitive difficulties and mental health in older adults in a large national dataset, and to test whether the relation can be explained by prior depressive disorder diagnosis. An additional aim was to test possible predictors of poor mental health among older adults who endorsed thinking or memory problems. The study involved analyzing data (N = 133,857 ) from the 2023 wave of the Behavior Risk Factor Surveillance System (BRFSS). Participants included adults ages 45-80 + (mean age= 55.8). Hypotheses were tested using a series of hierarchical linear regression models. Demographics included age, sex, and education. Independent variables included self-reported cognitive difficulties and life-time history of depressive disorder. Cognitive difficulties were significantly associated with the number of days of poor mental health, controlling for demographics as well as history of depression, $F(5, 130{,}674) = 8749.54$, $p < 0.01$, change in $R2 = .034$. These findings suggest a unique role for cognitive difficulties in explaining mental health status. Future studies should focus on understanding mental health outcomes in older adults with cognitive difficulties, and developing interventions to address these issues.

Abstract citation ID: igaf122.3996

### POSTURAL SWAY MODULATION AND EXECUTIVE FUNCTION PERFORMANCE DURING THE N-BACK TASK IN YOUNGER AND OLDER ADULTS

Azizah Jor'dan[1], Anastasiia Shmidt[1], Olga Gjika[1], Ikechukwu Iloputaife[2], Wanting Yu[2], Jonathan Bean[3], and Brad Manor[4], *1. University of Massachusetts Boston, Boston, Massachusetts, United States, 2. Hebrew SeniorLife, Boston, Massachusetts, United States, 3. VA Boston Healthcare System, Boston, Massachusetts, United States, 4. Hebrew SeniorLife/Harvard Medical School, Boston, Massachusetts, United States*

Maintaining postural control during cognitively demanding "dual tasks" is critical for daily functioning, especially in older adults. Prior work suggests that postural sway is modulated based on the demands of the secondary task to facilitate visual performance. However, it is unclear whether postural sway adapts to facilitate performance on the n-back task of executive function, or whether age influences this relationship. Twenty-three healthy young (26±3 years) and 24 older (76±6 years) adults performed the less demanding identify X (IdX) and more demanding 2-back tasks while standing. Postural sway acceleration (elliptical area, range, and path length) was measured with a wearable motion sensor. N-back task efficiency was reported indexed with the Balance Integration Score (BIS; composite of reaction time and accuracy; lower = better efficiency). During the IdX task, greater sway magnitude was linked to poorer efficiency across participants ($p = 0.02$), though this attenuated after adjusting for age, sex, and BMI ($p = 0.08$). In young adults, greater sway magnitude and longer range showed non-significant trends toward worse efficiency ($p = 0.07$-$0.08$), trends which were absent in older adults ($p > 0.18$). In the 2-back task, shorter path length showed non-significant trends toward poorer efficiency both across groups ($p = 0.07$) and within the older group only ($p = 0.08$). These results indicate that the association between postural sway and task performance is dependent upon age group and task difficulty. Older adults may not modulate sway to support cognitive efficiency under low demands but may show unique cognitive-motor interactions during more complex tasks.

Abstract citation ID: igaf122.3997

### PREDICTION OF DELIRIUM IN ICU PATIENTS AND DEVELOPMENT OF A CDSS MODEL USING CLINICAL DATA WAREHOUSE

Sun Ju Kim, *Chungnam National University Se-Jong Hospital, Se-Jong, Ch'ungch'ong-namdo, Republic of Korea*

Delirium in intensive care unit (ICU) patients is a critical condition associated with prolonged hospital stay, increased mortality, and higher healthcare costs, underscoring the need for early prediction and prevention. This study aimed to identify risk factors for ICU delirium using a Clinical Data Warehouse (CDW) and to develop a Clinical Decision Support System (CDSS) integrating machine learning models. A retrospective cohort study was conducted on adult patients (≥18 years) admitted to the ICU of a tertiary hospital in Daejeon, South Korea, from May 2015 to May 2024. Patients were classified into two groups: those who developed delirium within five days of ICU admission and those who did not. Predictive models were constructed using logistic regression, decision tree, and XGBoost. Variables included demographics, biometric indicators, vital signs such as Glasgow Coma Scale (GCS), blood pressure, oxygen saturation, and past medical history. The decision tree model demonstrated high sensitivity, while the XGBoost model achieved the highest accuracy (0.66) and specificity. Logistic regression offered interpretability but showed limited sensitivity (0.22). Key predictive variables were GCS-Verbal, GCS-Eye, age, blood pressure, and oxygen saturation. Based on these findings, a CDSS was designed to proactively assess the risk of delirium in ICU patients. The results suggest that machine learning-based models, particularly XGBoost, have potential clinical utility for early detection and intervention strategies. Future research should focus on optimizing predictive models and evaluating real-time implementation to support clinical decision-making and improve outcomes for critically ill patients.

Abstract citation ID: igaf122.3998

### PREDICTORS OF MOBILITY DISABILITY IN OLDER ADULTS WITH PHYSICAL FRAILTY AND SARCOPENIA

Riccardo Calvani[1], Matteo Tosato[1], Stefano Cacciatore[2], Emanuele Marzetti[1], and Francesco Landi[1], *1. Università Cattolica del Sacro Cuore, Rome, Lazio, Italy, 2. Università Cattolica del Sacro Cuore, Rom, Lazio, Italy*

Older adults with physical frailty and sarcopenia (PF&S) face a high risk of mobility disability. Early identification of predictors is essential to develop targeted preventive strategies. We performed a secondary analysis of the SPRINTT trial (NCT02582138) including community-dwelling older adults with PF&S and preserved mobility at baseline. To avoid intervention-related confounding, only participants in the control group were analyzed. Incident mobility disability was defined as inability to complete a 400-meter walk test within 15 minutes. Eighty-eight baseline variables (demographic, clinical, functional, social, and laboratory) were tested as candidate predictors using multivariate classification methods and machine learning (Partial Least-Squares Discriminant Analysis [PLS-DA], Random Forest, Gradient Boosting). Cox proportional hazards models were applied to confirm associations. Among 759 participants (71.3% women; median age 78 years), 354 (47%) developed mobility disability over 26 months. Model accuracy was similar across approaches (best PLS-DA: 67.6%). Baseline 400 m walk time was the strongest and most consistent predictor. In multivariate Cox regression, longer walking time, older age, lower SPPB score, and higher CES-D and SARC-F scores were independently associated with disability risk. Notably, depressive symptoms modified the impact of walking time (interaction $p < 0.001$): participants with both slow gait (≥480s) and CES-D ≥9 had the highest risk (HR = 2.69; 95% CI: 1.89–3.84). Kaplan–Meier curves confirmed the additive effect of gait speed and depression. Slower gait and depressive symptoms independently and synergistically predict mobility disability in older adults with PF&S. Simple cutoffs (400 m walk ≥480s, CES-D ≥9) may help identify high-risk individuals and inform preventive interventions.

Abstract citation ID: igaf122.3999

### PREDICTORS OF RECEIVING HELP WITH BANKING AMONG OLDER ADULTS WITH PROBABLE DEMENTIA

Alina Kung, and Claire Ankuda, *Icahn School of Medicine at Mount Sinai, New York, New York, United States*

Background: Receiving help with financial matters has the potential to protect older adults with cognitive impairment from adverse economic consequences. Little is known on who receives help with banking among these older adults. Methods: We used data from the National Health and Aging Trends Study to identify a cohort of older adults (aged 65 years and older) with probable dementia (n = 4048) (hereafter, OAPD). We used the first survey where the participant reported probable dementia, and excluded observations with incomplete data (n = 851). We used multivariate logistic regression to identify sociodemographic, household, health, and functional predictors associated with receiving help with banking in the last month. Results: Only about half of OAPD received help with banking (n = 1656, 52.7%). In adjusted analyses, being older (aOR 2.42 for age group >90 versus age group 65-69), female (aOR 1.33), not living with a spouse (aOR 1.69), on Medicaid (aOR 1.33), in poorer self-reported health (aOR 2.29 for poor health versus excellent health) or having a proxy answer the interview (aOR 2.92) were associated with receiving help with banking in the last month. Conclusion: About half of OAPD do not receive help with banking, indicating a potential risk and unmet need. OAPD who are younger, male, living with a spouse, and not on Medicaid are more likely to not receive help with banking. Future work should explore which OAPD are at greatest risk of adverse economic and health consequences as a result of not having received helped with banking.

Abstract citation ID: igaf122.4000

### PSYCHOEDUCATION PROGRAMMING FOR THE NEW CAREGIVER: MASTERING THE FIRST 100 DAYS

Carolyn Clevenger[1], Karah Alexander[1], Molly Perkins[2], Fayron Epps[3], and Kenneth Hepburn[1], *1. Emory University, Atlanta, Georgia, United States, 2. Emory University School of Medicine, Atlanta, Georgia, United States, 3. UT Health San Antonio, San Antonio, Texas, United States*

A dementia diagnosis marks a defining transition for individuals who must quickly assume the role of "caregiver." While many programs exist for caregivers of persons living with dementia (PLWD), most are designed for those in the later stages of their conditions, when substantial cognitive losses have occurred and behavioral and psychological signs of dementia are manifesting themselves. This leaves a gap in support for caregivers at the time of initial diagnosis. As part of this two-year, two-phase project, we engaged a diverse group of experienced family caregivers to co-develop and prototype-test the Caregiver Bootcamp program, Caregiver Bootcamp is a synchronous online, interactive psychoeducation program aimed at building caregiving competence and confidence (mastery) among caregivers for persons recently diagnosed with dementia. We report on prototype testing outcomes and subsequent course revisions prior to the Stage Ib single-group clinical trial of the finalized program. Twenty-two caregivers participated in five live or recorded two-hour sessions over six-weeks. The course covered four key areas: processing the diagnosis and its implications (including self-care), understanding the basics of dementia conditions, taking the first legal and financial logistical steps, and building a support network. Participants were predominantly Black or African American (60.9%), female (87%), and were currently caring for a parent (55.6%). Based on feedback, program changes included shortening weekly sessions to 90 minutes, integrating video narratives from seasoned caregivers to illustrate real-world implementation of course recommendations, simplifying "academic presentation" of theoretical models, increasing interactivity, and expanding resource offerings to address various learning styles.

Abstract citation ID: igaf122.4001

### REFRAMING THE RIGHTS TO CARE THROUGH THE ETHICS OF CARE

Aeji Jang, *Dongguk University, Gyeongju-si, Kyongsang-bukto, Republic of Korea*

The purpose of this study is to clarify the rights to care for older people and caregivers from the perspective of the public ethics of care. Despite increased attention and efforts toward caring for older people in welfare states, concerns about their care persist. By assuming the ideal of independent citizens, welfare states have overlooked the fact that everyone experiences dependency and lives within caring relationships. Furthermore, attempts to manage care within the private sphere in a cost-effective way have led to neglecting the intrinsic value of care work. This suggests the need for a fundamental shift in our approach to designing care policies. As public ethics of care does not exclude individuals who live with unavoidable dependency, such as older adults, it offers a normative framework that considers care as a right. It distinguishes between the unavoidable dependency of care receivers and the derived dependency of caregivers, emphasizing the state's responsibility to support both. For conceptualizing 'the rights to care', we conducted a narrative literature review. An in-depth review of studies and books about public ethics of care was carried out, with a focus on the lived realities of care-receiving and caregiving. Finally, this study distinguishes the rights to care as 'rights to receive' for older people and 'rights to provide' for caregivers, with four sub-principles: Access, Individuality, Compensation, and Security. This study not only contributes to the development of care ethics but also provides a practical and policy foundation for an aging society.

Abstract citation ID: igaf122.4002

**RETHINKING HEALTHSPAN: BEYOND THE ABSENCE OF DISEASE**

Nicolai Wohns, and Sarah McKiddy, *University of Washington, Seattle, Washington, United States*

Healthspan is a central concept in aging studies and geroscience, typically equating health with the absence of disease or disability. We argue, however, that 'healthspan' rests on a narrow and often unexamined set of assumptions about what it means to be healthy and thereby marginalizes subjective and relational aspects of aging, especially the experiences of those living with chronic conditions, disabilities, or conditions outside normative biomedical models. This orientation risks pathologizing the experience of aging that may, in fact, be meaningful in important and underappreciated ways. The conventional definition of healthspan frames aging-related decline as categorically negative, which sidelines the potential for meaning, growth, or transformation within the experience of aging. It relegates illness and frailty to a deficit narrative rather than as conditions that can prompt reflection, deepen relationships, or cultivate virtues such as patience, humility, or resilience. In light of these limitations, we suggest a more comprehensive approach that deemphasizes the normalizing effect of healthspan, instead allowing what we value in life (e.g., flourishing, relational connection, or moral development) to take pride of place in shaping our metrics and priorities in geroscience.

Abstract citation ID: igaf122.4003

**RETROTRANSPOSON-DERIVED DOUBLE-STRANDED RNA AS A DRIVER OF REACTIVE ASTROGLIOSIS IN TAUOPATHY**

Alyssa Cavalier, Paulino Ramirez, and Bess Frost, *Brown University, Providence, Rhode Island, United States*

Nearly half of the human genome is composed of retrotransposons, virus-like sequences thought to be genetic fossils from ancient viral infections. Due to their likely viral origins, retrotransposons are typically highly suppressed; however, retrotransposons become activated in neurodegenerative diseases including Alzheimer's disease and other tauopathies. Pathological forms of tau cause heterochromatin decondensation, leading to increased transcription of previously silenced retrotransposons. While much of the work on retrotransposons focuses on their potential to propagate throughout the genome, retrotransposon-derived products such as double-stranded RNA (dsRNA) can drive toxicity through innate immune activation. Prior work in our lab found elevated levels of astrocytic dsRNA and MDA5 (a dsRNA sensor) in human tauopathy and tau transgenic mice; additionally, pan-neuronal overexpression of Dicer-2 reduced total dsRNA burden in tau transgenic Drosophila. These data suggest neuronal origin of dsRNA that accumulates in astrocytes in tauopathy. Using iPSC-derived neurons carrying the IVS10 + 16 MAPT mutation, we observed increased retrotransposon transcripts via RNA-seq when compared to isogenic controls; however, we do not know if neurons expressing pathogenic tau have elevated dsRNA levels in vitro. RNA-seq analyses also show a broad dysregulation of pathways related to innate immune activity. Our preliminary data suggest that dsRNA burden is elevated in tau mutant iPSC-derived neurons, as well as increased expression of antiviral dsRNA sensors such as TLR-3. Current analyses are focused on downstream immune signaling pathways and potential interaction with dsRNA in tau mutant neurons. Future research aims to characterize potential modes of neuronal dsRNA release and detection by cultured astrocytes.

Abstract citation ID: igaf122.4004

**ROLE OF APPETITE LOSS AND INFLAMMATION ON INCIDENT DISABILITY AMONG COMMUNITY-DWELLING OLDER ADULTS**

Germine Soliman[1], Anne Newman[2], Shanshan Yao[3], Xiaomin Lu[1], George Kuchel[4], and Lisa Barry[1], *1. University of Connecticut, Farmington, Connecticut, United States, 2. University of Pittsburgh, Pittsburgh, Pennsylvania, United States, 3. University of Michigan, Ann Arbor, Michigan, United States, 4. UCONN HEALTH, Farmington, Connecticut, United States*

Aging is associated with reduced appetite and increased pro-inflammatory status, both of which may contribute to disability. We evaluated whether appetite loss and inflammatory markers were associated with incident disability in community-dwelling older adults. Health, Aging, and Body Composition Study participants ($n = 3,071$) were asked about their appetite in the past month, with responses dichotomized as good versus impaired. Participants were followed up to 6 years to determine if they experienced incident Persistent Lower Extremity Limitation (PLL), defined as two consecutive self-reports of difficulty walking a quarter mile and/or climbing 10 steps without resting. Biomarkers (IL6, TNF-α, CRP) were assessed at baseline. 388 (12.6%) individuals reported poor appetite, mainly female (59%), Black (54%), with a mean age of 73.9 (±3) years. A total of 64% reporting poor appetite and 47% reporting good appetite developed PLL ($p < 0.001$). Levels of IL6, TNF-α, and CRP were higher among those with poor appetite ($p < 0.001$ for each comparison). A Fine-Gray proportional hazards model adjusted for demographics, chronic diseases, anxiety, depression, prescribed medications, and sensory factors, indicated that poor appetite (hazard ratio[HR]= 1.56; 95% CI 1.29, 1.87) and inflammatory markers ((HR IL6=1.05; 95% CI 1.02, 1.09); (HR TNF-α =1.05; 95% CI 1.02, 1.08); (HR CRP=1.02; 95% CI 1.00, 1.04)) were each associated with developing PLL. Our findings show that reducing appetite loss and inflammation may be promising targets for minimizing disability in older adults. Additional research will assess the potential mediating role of inflammation in the relationship between appetite loss and disability.

Abstract citation ID: igaf122.4005

**SELF-RATED HEALTH, PHYSICAL ACTIVITY, MEASURES OF PHYSICAL FUNCTIONING AND MORTALITY AMONG OLDER U.S. ADULTS**

Peter Hart, *Kinesmetrics, Tallahassee, Florida, United States*

The objective of this study was to determine if different measures of physical functioning (PF) can predict mortality independent of self-rated health (SRH) and physical activity (PA). A baseline sample of 6,173 adults 65+ years of age were included from the 2001-2018 NHANES. A SRH variable was created with categories of excellent/very good, good, fair, and poor. PA status was based on participants reporting either no (inactive) or at least some (active) recreational PA. Seven different PF measures were used and included a 19-item total PF score (PFT), activities of daily living (ADL), instrumental activities of daily living (IADL), leisure and social activities (LSA), general physical activities (GPA), lower extremity mobility (LEM), and an IRT-derived total PF score (PFIRT). All PF measures were scored so larger values represented greater PF limitation. Seven Cox regression models were employed each with a different PF measure and adjusted for age, sex, race, income, SRH, PA, BMI, BSI, smoking, alcohol consumption, and comorbidities. A total of 2,103 deaths occurred during a median follow-up of 10.3 years. Risk of death decreased for 1st (HR = 0.70, 0.60-0.82), 2nd (HR = 0.77, 0.64-0.93), and 3rd (HR = 0.82, 0.72-0.94) PFIRT quartiles (reference: 4th), increased for poor (HR = 2.11, 1.51-2.97), fair (HR = 1.82, 1.54-2.15), and good (HR = 1.20, 1.07-1.35) SRH (reference: excellent/very good), and increased for inactive (HR = 1.27, 1.12-1.43) PA status (reference: active). All PF models saw similar results less LSA where LSA lost its predictive ability. These findings indicate that SRH, PA, and PF are robust independent predictors of all-cause mortality in older adults.

Abstract citation ID: igaf122.4006

### SENSE OF CONTROL AND COGNITIVE AGING: THE MEDIATING ROLE OF RISK AND PROTECTIVE FACTORS

Nathan Merin[1], Kylie Schiloski[2], and Margie Lachman[2], *1. Brandeis University, Burlingame, California, United States, 2. Brandeis University, Waltham, Massachusetts, United States*

Sense of control is a psychosocial factor that is known to be associated with engagement in protective health behaviors and cognitive and physical health outcomes in middle age and later life. The present study tested whether sense of control was related to risk and protective factors contributing to brain health measured by the McCance Brain Care Score (BCS), an index of modifiable physical (blood pressure, blood glucose, cholesterol, Body Mass Index), lifestyle (nutrition, exercise, smoking status, sleep, alcohol consumption), and psychosocial factors (stress, relationships, purpose in life). We hypothesized that the BCS would mediate the relationship between sense of control and 9-year changes in cognitive and physical health. Data were collected from the second (M2) and third (M3) waves of the Midlife in the United States (MIDUS) Study and biomarker sub-study. Results showed that the relationships between sense of control and 9-year residual change in episodic memory, functional health, and chronic conditions were all mediated by the BCS, with each model controlling for age, sex, race, and level of education. The BCS also mediated the relationship between sense of control and level of performance 9-years later for executive functioning. These findings demonstrate that those with a higher sense of control were more likely to have a higher brain care score, which in turn was related to a greater maintenance of specific cognitive and physical health outcomes. This work suggests that promoting a sense of control shows potential for enhancing brain health and maintaining favorable cognitive and physical health.

Abstract citation ID: igaf122.4007

### SERVICE-USE SUBTYPES AND CAREGIVER BURDEN IN HOME-LIVING ADULTS WITH SEVERE CARE NEEDS IN JAPAN

Yu Sun, Masao Iwagami, Nobuo Sakata, Satoru Yoshie, Yoko Hamasaki, Shoichi Masumoto, Gen Nakayama, and Ryohei Goto, *University of Tsukuba, Tsukuba, Ibaraki, Ibaraki, Japan*

Objectives: As Japan transitions from hospital- to community-centered care, the burden on family caregivers is a growing concern. We aimed to identify subgroups of community-dwelling adults with severe care needs based on patterns of medical and long-term care (LTC) service use and to examine associations with caregiver burden. Methods: We conducted a cross-sectional survey in four municipalities of Ibaraki Prefecture in Japan, targeting dyads of care managers and primary caregivers of adults with care-need level ≥3 living at home. Latent class analysis (LCA) was applied to service-use indicators from care manager questionnaires. Then, caregiver questionnaires were linked to assess high caregiver burden, defined by the Burden Index of Caregivers-11 (BIC-11) and dichotomized at the median. Additional analyses examined five BIC-11 domains (time-dependent, emotional, existential, physical, service-related). Adjusted prevalence ratios (aPRs) were estimated using modified Poisson regression with robust variance, adjusting for patient and caregiver covariates. Results: Of 320 care manager respondents, LCA identified three subtypes: (1) outpatient/day services–centered, (2) multi-component home-visit services (mainly physician and nurse home visits), and (3) outpatient/day rehabilitation–centered. After linkage and exclusion of missing data, 243 dyads were analyzed. Compared with the outpatient/day services subtype, the multi-component home-visit subtype was associated with a lower prevalence of high caregiver burden (aPR 0.66; 95% CI 0.46–0.95). Domain-specific analyses showed significantly lower emotional and existential burden in this group. Conclusions: Among adults with severe care needs at home, a multi-component home-visit service pattern was associated with lower caregiver burden, suggesting that appropriate service combinations may help mitigate caregiver strain.

Abstract citation ID: igaf122.4008

### SEX DIFFERENCES IN BLOOD PRESSURE AND COGNITIVE AGING: NATIONAL ALZHEIMER'S COORDINATING CENTER (NACC_

Carlos Araujo Menendez[1], Alonzo Mendoza[2], Carolina L. Costa[2], and Ariana Stickel[2], *1. San Diego State University, La jolla, California, United States, 2. San Diego State University, San Diego, California, United States*

Introduction: Elevated blood pressure (BP) is a known risk factor for cognitive decline that fluctuates over time, yet most studies rely on single or few BP measures and do not examine potential sex differences. Therefore, we investigated the associations between longitudinal BP and cognitive aging trajectories in Latino adults. Methods: Participants included 800 Latino adults without dementia from the NACC (2015–2022, 28 sites) with an average of 7-years of repeated BP assessments. Mixed-effects regression models evaluated the effects of time-varying systolic (SYS), diastolic (DIAS), and pulse pressure (PP) on trajectories of global cognition, executive functioning, episodic memory, and language. Models sequentially adjusted for demographics (age, education, language, heritage, cognitive status) and health variables (time-varying BMI, baseline hypertension). Results: A significant DIAS × Age × Sex interaction emerged for executive functioning ($\beta = 0.41$, SE = 0.18, $p = 0.02$). Among females, higher diastolic BP predicted steeper age-related decline (slope=-0.06, $p = 0.01$), whereas in males the effect was attenuated and slightly protective (slope = +0.02, $p = 0.04$). For global cognition, higher PP predicted greater decline across both sexes ($\beta$=-0.36, SE = 0.13, $p = 0.01$). No other significant effects were observed. Conclusion: Over 7-years of follow-up, diastolic BP had sex-specific associations with executive functioning trajectories, while PP broadly predicted global cognitive decline regardless of sex. These findings highlight that ongoing BP monitoring and consideration of sex-specific risks are essential for identifying Latino individuals who are at risk of cognitive decline.

Abstract citation ID: igaf122.4009

**SHINING A SPOTLIGHT ON GERONTOLOGY: ENHANCING VISIBILITY AND IMPACT IN AGING PROGRAMS**

Naomi Adjei[1], Julie Masters[2], Elsa Wilcox[3], Mark Staley[3], and Hailey Baca[4], *1. Dartmouth College, The Dartmouth Institute for Health Policy & Clinical Practice, New Hampshire, United States, 2. UNO, Omaha, Nebraska, United States, 3. University of Nebraska-Lincoln, University of Nebraska-Lincoln, Nebraska, United States, 4. University of Nebraska-Lincoln, Lincoln, Nebraska, United States*

Despite the rapid aging of the U.S. population and the growing demand for professionals trained to support older adults, Gerontology programs across the country have experienced a troubling decline in visibility, enrollment, and institutional support. Over the past decade, several undergraduate and graduate Gerontology programs have been downsized, merged with other disciplines, or eliminated entirely. This decline persists even as the societal need for aging-focused expertise intensifies. This study investigates the visibility and enrollment dynamics of Gerontology programs across the United States by analyzing institutional size, program-level enrollment, recruitment strategies, and disciplinary intersections. The sample included 74 colleges with Gerontology programs. Results indicate that a majority of institutions offering Gerontology programs are small to mid-sized, with 0–5,000 and 5,001–10,000 student enrollments comprising the largest segments highlighting the predominance of smaller educational settings in the field. Analysis of recruitment methods shows that college advisors (over 70%), in-class presentations (50%), and printed materials (approximately 47%) were perceived as the most successful strategies for attracting students from other disciplines. Less effective approaches included community presentations and partnerships with high schools. These findings indicate the importance of direct academic engagement of program awareness. In addition, data suggests strong interdisciplinary overlap between Gerontology and human services or behavioral health fields, emphasizing the need for cross-departmental collaboration in curriculum design and student recruitment. Together, the data highlight the opportunities and challenges facing Gerontology programs. Improving visibility through targeted advising and strategic partnerships, along with exploring interdisciplinary collaborations, may enhance program growth and sustainability.

Abstract citation ID: igaf122.4010

**SOCIAL ISOLATION, LONELINESS, AND PACE OF AGING AMONG OLDER ADULTS IN THE ENGLISH LONGITUDINAL STUDY OF AGEING**

Yifan Shi[1], Arun Balachandran[2], Heming Pei[3], and Daniel Belsky[4], *1. New York University, New York, New York, United States, 2. Columbia University, New York City, New York, United States, 3. UNC Gillings School of Global Public Health University of North Carolina at Chapel Hill, Chapel Hill, North Carolina, United States, 4. Columbia University, New York, New York, United States*

Loneliness and social isolation are prevalent among older adults and have been linked to increased morbidity and mortality. Emerging evidence suggests that social disconnection may also accelerate biological aging, yet few studies have examined the bidirectional relationship between psychosocial factors and aging processes using longitudinal data and validated biological aging measures. Using data from the English Longitudinal Study of Ageing, we investigated reciprocal associations between loneliness, social isolation, and the Pace of Aging (PoA), a biomarker-based measure of multisystem physiological decline. The analytic sample included 5,659 adults aged ≥50 with repeated blood biomarker, physical, and functional assessments across three timepoints. Loneliness and social isolation were measured at baseline and follow-up. Linear regression models were fitted, stratified by sex and age group, and adjusted for demographic, socioeconomic, health, and behavioral covariates. We found that baseline loneliness, but not social isolation, predicted faster PoA ($\beta = 0.03$), with stronger effects among men and adults aged younger than 70. In turn, faster PoA prospectively predicted higher levels of both loneliness ($\beta = 0.12$) and social isolation ($\beta = 0.11$) at follow-up, independent of baseline levels. These bidirectional associations persisted after adjustment, suggesting a reinforcing cycle between social and biological vulnerability. Findings highlight loneliness as a distinct and modifiable risk factor for accelerated aging and demonstrate the value of PoA as a responsive endpoint in social and behavioral intervention research. Addressing loneliness, particularly among men and the younger-old adults, may be critical for promoting healthy longevity and reducing disparities in aging outcomes.

Abstract citation ID: igaf122.4011

**SPATIAL HETEROGENEITY OF SOCIAL VULNERABILITIES ASSOCIATED WITH COLORECTAL CANCER SCREENING RATES IN CALIFORNIA**

Jinbo Niu[1], Joonhyeog Park[2], and Tamara Cadet[2], *1. University of Southern California, Los Angeles, California, United States, 2. University of Pennsylvania, Philadelphia, Pennsylvania, United States*

Colorectal cancer (CRC) is the second leading cause of cancer-related deaths in the United States, and the American Cancer Society recommends CRC screening beginning at age 45. Despite its economic prosperity, California in 2022 reported the lowest CRC screening rates among adults aged 45 to 75 in the U.S. This counterintuitive phenomenon raises concerns about which social vulnerability factors are associated with the low rate in California. However, prior research on social vulnerability has largely focused on national-level patterns, with limited attention to California-specific disparities and the spatial heterogeneity of social vulnerability factors associated with CRC screening rates. This study aimed to explore the spatial heterogeneity of social vulnerabilities influencing CRC screening rates in California by analyzing the California tract-level CRC screening rate from the CDC PLACES data and the total 16 social vulnerability variables from the CDC Social Vulnerability Index (SVI) data in 2022. The most associated SVI factors were selected through stepwise regression, with their spatially varying effects examined via Geographically Weighted Regression. Among the top five associated SVI factors selected by stepwise regression, educational attainment and limited English proficiency showed the greatest spatial heterogeneity, with effects ranging from strongly negative to positive across regions. The percentage of adults over 65 maintained an overall positive association with screening rates but with little spatial variation. These findings highlight the need for spatially adaptive language and education interventions that address local vulnerabilities. Meanwhile, prioritizing CRC screening among adults aged 45–65 is essential to narrowing screening gaps in California.

Abstract citation ID: igaf122.4012

**SPIRITUAL AND RELIGIOUS INFLUENCES ON HEALTHCARE PROFESSIONALS' CAREER MEANING AND PERCEPTIONS OF AGING**

Heidi Ewen, Anne Fish, James Fish, and Asiya Patel, *SUNY Binghamton University, Binghamton, New York, United States*

Religious and spiritual beliefs play a significant role in shaping healthcare professionals' attitudes, behaviors, and perceptions of patient care. This study explores how personal spirituality and religiosity contribute to professionals' sense of meaning in their careers and influence their views on aging. Participants reported their levels of spirituality and religiosity using a four-point scale and completed two reliable instruments: the Spiritual Meaning of Healthcare Career scale ($\alpha = .84$) and the Aging as Punishment for Sin subscale ($\alpha = .88$). ANOVA results revealed that those identifying as "very" or "moderately" religious or spiritual scored significantly higher on the Spiritual Meaning scale. While most participants disagreed with the notion that aging is a punishment for sin, individuals who were "very religious" scored higher on this belief subscale. Regression analyses revealed that spiritual intensity was a strong predictor of the perception of aging as punishment for sin, while both spiritual and religious intensity were significant predictors of the spiritual meaning of a healthcare career. Notably, the Aging as Punishment for Sin subscale demonstrated strong psychometric validity and holds promise for future research, particularly in understanding and addressing ageist beliefs in clinical settings. Interestingly, even among those endorsing this belief, highly religious participants expressed less overall negativity toward aging. These findings underscore the complex interplay between faith and professional identity and highlight the need for nuanced consideration of spirituality in healthcare ethics and education.

Abstract citation ID: igaf122.4013

**STATIC BALANCE FROM THE SHORT PHYSICAL PERFORMANCE BATTERY COMPARED WITH BTRACKS AMONG OLDER ADULTS**

Jethro Raphael Suarez[1], Kworweinski Lafontant[1], Daniel Goble[2], Joon-Hyuk Park[3], Michael Dino[1], and Ladda Thiamwong[1], *1. University of Central Florida, Florida, United States, 2. Oakland University, Rochester, Michigan, United States, 3. Daegu Gyeongbuk Institute of Science and Technology, Daegu, Republic of Korea*

The static balance portion of the Short Physical Performance Battery (SPPB) has rarely been compared directly with gold standard force plate balance assessments. This study aimed to compare static balance metrics from a cost-efficient force plate between community-dwelling older adults with low and high SPPB balance scores. The Balance Tracking System (BTrackS), a stationary and simple balance assessment instrument, was used for measuring static balance metrics during a non-visual static balance test. The static balance portion of the SPPB ranges from a total score of 0 to 4 and involves unsupported standing for 10 seconds in a side-by-side, a semi-tandem, and a tandem stance. We cross-sectionally compared center of pressure (COP) path length and sway area from 204 community-dwelling older adults aged 61 to 96 (176 women, mean age = 75 ± 7 years) who completed a BTrackS and SPPB assessment. A total of 146 older adults exhibited a high SPPB balance score (= 4), while 58 exhibited a low SPPB balance score (≤ 3). The Low SPPB Balance group had significantly greater COP path length ($p = 0.006$) and sway area ($p < 0.001$) compared to the High SPPB Balance group. These results suggest that the balance score from the SPPB is a valid indicator of static balance performance when compared to a gold standard force plate assessment and therefore, provide evidence for clinicians with limited technological resources to use the SPPB in a similar population and infer postural sway metrics based on high or low SPPB balance scores.

Abstract citation ID: igaf122.4014

**STRESSORS & WELL-BEING: PSYCHOMETRIC & CORRELATIONAL FINDINGS FROM INTERVIEWS WITH PERSONS LIVING WITH DEMENTIA**

Emma Jackson[1], and Katherine Judge[2], *1. Cleveland State University, Westlake, Ohio, United States, 2. Cleveland State University, Cleveland, Ohio, United States*

The poster will present psychometric and correlational results from measures not extensively used with self-reported data of persons living with dementia (PLWD). The pilot study, guided by the Stress Process Model for Individuals with Dementia, used the following scales: Mini-Mental State Examination (MMSE), Perceived Memory Difficulty Scale (PMDS), BRIA Behavioral Frequency and Distress Scale, Difficulties in Emotion Regulation Scale (DERS-16), Hospital Anxiety and Depression Scale (HADS), and Quality of Life-Alzheimer's Disease Scale (QOL-AD). Thirty-eight participants were interviewed, of which 66% were women, 63% White, and had an average MMSE score of 20.57. Cronbach alpha scores demonstrated good reliability for all scales, ranging from .71 to .89. MMSE scores only correlated with behavioral frequency scores, such that those with more cognitive impairment also experienced more behavioral symptoms (-.363, $p = .025$). In contrast, higher perceived memory difficulty scores were associated with more behavioral symptom frequency (.431, $p = .007$) and difficulties with emotion regulation (.567, $p < .001$). Perceived memory difficulty scores also correlated with all well-being variables, with higher scores being associated with more depression (.385, $p = .017$), anxiety (.623, $p < .001$), and lower quality of life (-.417, $p = .009$). Similarly, more difficulties with emotion regulation, a newer construct to PLWD research, correlated with more behavioral symptom frequency (.580, $p < .001$). Finally, higher DERS scores correlated with more depression and anxiety (.451, $p = .004$; .685, $p < .001$, respectively). Implications focusing on the novelty of these relationships will be discussed further.

Abstract citation ID: igaf122.4015

**SUICIDE IDEATION IN HISPANIC AND LATINO FAMILY CAREGIVERS: DAILY PREVALENCE AND RISK FACTORS**

Felipe Jain[1], Natashia Bibriescas[2], Loreli Alvarez[3], and Frank Puga[2], *1. Massachusetts General Hospital / Harvard Medical School, Boston, Massachusetts, United States, 2. University of Alabama at Birmingham, Birmingham, Alabama, United States, 3. The University of Alabama at Birmingham, Birmingham, Alabama, United States*

Family caregivers face elevated risk of suicide ideation (SI). Hispanic and Latino (H&L) family caregivers of people living with dementia experience high burden yet remain understudied regarding SI. In H&L caregivers, we examined SI prevalence and associated risk factors using data from the Nuestros Días (Our Days) Study. 178 H&L family caregivers of people living with dementia (age: M = 56.3 (SD = 10.1), sex: 89.3% female) completed daily diary surveys (N = 3,237 observations) assessing SI over 21 days. Predictors, including depressive symptoms, coping strategies, emotion regulation, perceived stress, alcohol use, global health, and social isolation were assessed at baseline. Caregivers completed four questions daily to assess passive or active SI for 21 days (mean completion 18.2 (SD = 3.61) days). A stepwise zero-inflated Poisson regression, controlling for differences in the number of days observed with an offset, was used to predict total days of reported SI. 27.0% of participants (N = 48) reported passive or active SI on at least 1 day. Of these participants, the median number of days with SI was 2 (interquartile range 1 to 10.25). The final predictive model for days of SI included depression, emotional suppression, dysfunctional coping, emotion-focused coping, and social isolation (R2=.77, p<.001). Emotion-focused coping (sIRR=.637, 95% CI [0.534, 0.760], p<.0001) was significantly associated with fewer days of reported SI while social isolation (sIRR=2.337, 95% CI [1.680, 3.251], p<.0001) was significantly associated with more days of SI. In conclusion, SI is common among H&L family caregivers. Therapies that target improving emotion-based coping strategies and reducing social isolation might reduce SI.

Abstract citation ID: igaf122.4016

**T CELL ACTIVATION TRIGGERS CALCIUM-DEPENDENT EXPRESSION OF THE AGING MARKER P16INK4A**

Noah Lepola, Xiangnan Guan, and Christin Burd, *Ohio State University, Columbus, Ohio, United States*

p16INK4a is a senescence-associated cell cycle inhibitor and aging biomarker. Gerontogenic factors, including chemotherapy, smoking, and physical inactivity lead to durable increases in human peripheral blood T cell (PBTL) p16INK4a levels. However, the mechanisms that regulate p16INK4a in T cells remain uncertain. Here, we show that T cell activation signals induce p16INK4a mRNA and protein in mouse and human T cells. This induction is primarily driven by CD3 signaling, and is further enhanced by IL-2, but not by CD28 co-stimulation. Using luciferase reporter assays in Jurkat cells, we identify a predicted NFAT/Sp1 binding site in the p16INK4a promoter that is required for reporter activation following T cell stimulation. Furthermore, cyclosporine A-mediated inhibition of calcium signaling and NFAT activation limits endogenous p16INK4a expression during T cell stimulation. These findings suggest that p16INK4a is primarily regulated by calcium-dependent NFAT signaling downstream of CD3 engagement in T cells.

Abstract citation ID: igaf122.4017

**TACKLING BARRIERS TO INCREASE EARLY PALLIATIVE CARE ACCESS FOR OLDER ADULTS**

Natalie Martinez[1], Vanessa Gonzalez[1], Gabriela Zaragoza[2], Melida Juarez-Briggs[1], Anjuli Vasquez[1], Michael Mader[1], Sandra Sanchez-Reilly[3], and Jennifer Barker[4], *1. South Texas Veterans Health Care System, San Antonio, Texas, United States, 2. South Texas Health Care System, San Antonio, Texas, United States, 3. University of Texas Health San Antonio, San Antonio, Texas, United States, 4. University of Texas HSC at San Antonio, San Antonio, Texas, United States*

Background: Early palliative care (EPC) has been shown to provide a myriad of benefits including improved quality of life for older adults. Despite this, there is continued underutilization of palliative care (PC) in the geriatric population, and many barriers to obtaining timely consultations. Methods: This Quality Improvement (QI) project utilized the IPAL-ICU screening tool with modifications (PCST) to prioritize PC consultations for seriously ill older adults. Building upon our original project "Increasing Palliative Care in the Progressive Care Unit"1, we expanded use of the PCST tool to two additional units within our veteran's hospital from August 2023 -July 2024. Multiple QI strategies were used: dedicated RN-led QI project, RN unit champions, physician education campaigns, flyers, brochures, feedback gathering, and regular audits of progress. Results: N = 621 PCST screens completed. 494 unique veterans, 96% male, 398 new and 223 established. The median age was 73 with an interquartile range (IQR) of 64 to 78. Of the new patients, 122 were seen inpatient and 146 were transferred to outpatient consultations. The PCST contributed to an increase in 40% of inpatient consultations and 30% increase in outpatient consultations, allowing for earlier PC access for seriously ill older adults. Conclusion: PCST is an effective means of prioritizing PC consultations for seriously ill older adults. Implications for Research, Policy, or Practice: Our QI process has led to optimizing a standardization tool for screening which can be disseminated on a national level to successfully move PC consultations earlier for seriously ill older adults.

Abstract citation ID: igaf122.4018

**TELOMERE LENGTH MEASUREMENT USING CRISPR-CAS12A**

Waylon Hastings, and Anna Tillinghast, *Texas A&M University, College Station, Texas, United States*

Background: Mixed results of meta-analyses have led to questions about the validity of telomere length (TL) as a biomarker of aging. Conflicting findings are in part attributable limitations in TL measurement methodology, which for epidemiological studies is often quantitative-PCR (qPCR). Although high throughput and minimal DNA input make qPCR extremely cost-effective, this comes at the cost of precision and accuracy, which is challenged by the need for non-linear transformations to estimate final TL. Methods: We outline a pipeline to validate a novel methodology to measure TL using CRISPR-Cas12a that includes quality of control of key reagents, optimization of assay conditions, and experimental design to generate comparative data using qPCR. Results: Preliminary quality control using gel electrophoresis confirmed length of synthesized oligomer standards. Validation assays further supported methodological design, such that measurements of dsDNA standards were proportional to total telomere content (R2>0.999) and not influenced by oligomer length (p > 0.05) when assessed using either Cas12a or qPCR. Signals from a 2-foldly diluted standard series were each significantly distinct when assessed using Cas12a (p < 0.05) but not qPCR, suggesting Cas12a provides enhanced precision. By contrast, standards with the lowest total telomeric content were not distinguishable from negative controls when assessed by Cas12a (p > 0.05), but were well above background when assessed using qPCR, suggesting the need to further refine the Cas12 assay to establish an acceptable lower limit of detection. Conclusion: Additional methodological validation is ongoing, including generating measurements on a set of 100+ samples with paired data generated using qPCR, hybridization, and sequencing-based approaches.

Abstract citation ID: igaf122.4019

**TEMPORAL TRENDS IN LONELINESS ACROSS FOUR DECADES IN THE FRAMINGHAM HEART STUDY**

Allison Mays[1], Matthew Scott[2], Alexa Beiser[2], Mitzi Gonzales[1], and Joel Salinas[3], *1. Cedars Sinai Medical Center, Beverly Hills, California, United States, 2. Boston University, Boston, Massachusetts, United States, 3. New York University Grossman School of Medicine, New York, New York, United States*

Using the Framingham Heart Study, we aimed to describe the prevalence of loneliness within age groups across four decades. Participants completed the Center for Epidemiologic Studies Depression Scale (CES-D Scale) and responded how often they felt lonely in the past week. We included in our analysis 30,186 observations longitudinally collected between 1980-2019. Loneliness reported at least once weekly was more prevalent in younger age groups, decreased in mid-life and then increased in older age in a U shaped curve as in prior literature; this trajectory persisted across decades with total prevalences of: 26.6%, 22.5%, 20.6%, 18.3%, 16.5%, 23.1%, and 33.7% in age groups 25-34, 35-44, 45-54, 55-64, 65-74, 75-84 and 85-94 respectively. Loneliness three or more times per week decreased from the 1980s for all ages with continued declines in younger (25-34; 35-44) and older (75-84; 85-94) age groups. Despite these decreases, loneliness prevalence remained highest in the oldest age groups (75-84; 85-94) which reported rates of 11.6% and 15.9% respectively, compared to 5.7-6.9% in younger participants. Loneliness prevalence in ages 75-84 and 85-94 declined across decades with rates respectively of 22.4% and 36.0% from 1980-1989; 11.4% and 15.3% from 1990-1999; 9.7% and 13.9% from 2000-2009; and 7.0% and 9.8% from 2010-2019. However, prevalence rates across ages still maintained a J shaped curve. Loneliness has decreased from the 1980s in the Framingham Heart Study, though older adults ages 75-84 and 85-94 maintained the highest prevalence of loneliness. Further research into factors contributing to declining rates of loneliness is merited.

Abstract citation ID: igaf122.4020

**TESTING ASSOCIATIONS BETWEEN KEY PRECARITIES AND ADVERSE OUTCOMES IN OLDER ADULTS WITHOUT CARE PARTNERS**

Brittany Jones-Cobb, *University of Washington, Seattle, Washington, United States*

Many older adults in the U.S. rely on care partners such as a spouse, child, kin, or other social network member to assist with daily needs, yet demographic trends are leaving more individuals without such vital support. This study builds on a scoping review that identified social isolation, future care planning (e.g., advance directives), self-efficacy, and engagement in preventive services as key precarities for older adults without care partners, and hospitalization and functional decline as potential adverse outcomes. Using longitudinal data from the Health and Retirement Study (2006–2020), hybrid mixed-effects logistic regression models with random intercepts estimated within- and between-person effects of these precarities on hospitalization and incident activities of daily living (ADL) dependency among respondents without care partners, controlling for age, sex, race, Hispanic ethnicity, and poverty status. For hospitalization, higher perceived constraints (OR = 1.27, 95% CI = 1.11–1.46) and lower average future care planning (OR = 0.77, 95% CI = 0.68–0.88) were associated with greater odds. For ADL dependency, higher perceived constraints (OR = 1.49, 95% CI = 1.14–1.94), greater social isolation (OR = 1.32, 95% CI = 1.04–1.68), and lower perceived mastery (OR = 0.63, 95% CI = 0.49–0.82) were associated with greater odds, with heightened risks for Hispanic respondents (OR = 3.27, 95% CI = 1.43–7.51) and those experiencing poverty (OR = 2.52, 95% CI = 1.45–4.37). Findings suggest interventions targeting self-efficacy, social isolation, and care planning may reduce risks of hospitalization and ADL dependency among older adults aging without care partners.

Abstract citation ID: igaf122.4021

**TESTOSTERONE THERAPY EFFECTS ADIPOSE DISTRIBUTION IN OLDER FEMALES POST HIP-FRACTURE: THE STEP-HI STUDY**

Jacob Earp[1], Shangshu Zhao[1], Furong Xu[2], Richard Fortinsky[1], Jenna Bartley[3], Chia-Ling Kuo[1], and George Kuchel[1], *1. University of Connecticut, Farmington, Connecticut, United States, 2. University of Rhode Island, University of Rhode Island, Rhode Island, United States, 3. University of Connecticut School of Medicine, University of Connecticut Health, Connecticut, United States*

With aging and injury females experience ectopic redistribution of appendicular adipose tissue (AAT) into the viscera, where visceral adipose tissue (VAT) becomes highly inflammatory and metabolically stressful. This adipose redistribution increases the risk of both reinjury and obesity-related illnesses. Strategies that can disrupt this unhealthy adipose redistribution after hip fracture injury (HF) are of great interest. We aimed to determine how testosterone therapy affects the amount and distribution of total adipose tissue (TAT) in older women following HF. This was a secondary analysis of the STEP-HI study, a multi-site randomized clinical trial that randomized older females recovering from HF to a 6-month resistance exercise intervention while taking either testosterone (EX+T, n = 35, age=79±9 years) or a placebo (EX+P, n = 31, age=76±7 years). Changes in TAT, AAT and VAT mass and percentage of TAT in each region (AAT% and VAT%) were measured using dual x-ray absorptiometry and compared between groups. No significant differences were observed between groups at baseline ($p > 0.05$). Over the intervention period, mean change between baseline and six months were not significantly different between groups for TAT (EX+P: 298±2002 g, EX+T: 419±2086 g, $p = 0.827$), AAT (EX+P: 52±1007 g, EX+T: 39±1078 g, $p = 0.890$) AAT% (EX+P: -0.42±1.40, EX+T: -0.52±1.67%, $p = 0.718$) and VAT (EX+P: 45±232 g, EX+T: -44±151 g, $p = 0.091$), while VAT% improved in the EX+T (-0.26±0.51%) compared to EX+P (+0.12±0.52%, $p = 0.003$). These results demonstrate that while testosterone treatment was unable to decrease overall adipose stores compared to exercise alone in older women recovering from HF, it may help prevent unhealthy adipose redistribution.

Abstract citation ID: igaf122.4022

**THE ASSOCIATION BETWEEN SUBJECTIVE HEARING DIFFICULTY AND COGNITIVE IMPAIRMENT: A STRATIFIED ANALYSIS**

Dukyoo Jung, Leeho Yoo, Soo Gyung Shin, Sukyung Byeon, and Hyein Seo, *Ewha Womans University, Seoul-t'ukpyolsi, Republic of Korea*

Age-related hearing loss (ARHL) affects 50% of older adults, and has become a global concern due to its adverse impact on cognitive function. However, the effects of ARHL within specific subgroups remain unclear. This study aimed to examine the association between ARHL and cognitive impairment (CI) across diverse subgroups of community-dwelling older adults. This is a cross-sectional study using data from the 2023 National Survey of Older Koreans. Hearing-related characteristics included ARHL diagnosis, subjective hearing difficulty, and hearing-aid (HA) use. Complex sample logistic regression was done to examine the relationship between hearing-related characteristics and CI. A total of 7,419 participants were included in the analysis. Among all participants, subjective hearing difficulty (OR = 3.139, p<.001) and HA use (OR = 0.76, p=.044) were both significantly associated with CI in the unadjusted model, while diagnosis of ARHL was not (OR = 1.337, p=.116). These associations remained significant after adjusting for covariates but were attenuated. After stratifying participants by HA use, frailty, sex, age, depression, and social participation, subjective hearing difficulty was significantly associated with CI in the unadjusted model. In the adjusted model, this association remained consistent in all subgroups except for the HA user group. Our findings suggest that subjective hearing difficulty is significantly associated with CI in older adults, even after controlling for covariates. The absence of an association between ARHL diagnosis and CI may reflect limited awareness of ARHL among older adults. Also, stratified analysis suggests the potential preventive role of hearing aids in cognitive decline.

Abstract citation ID: igaf122.4023

### THE ASSOCIATION OF DIVORCE AND LATE-LIFE BRAIN HEALTH IN A RACIALLY AND ETHNICALLY DIVERSE COHORT OF OLDER ADULTS

Suhani Amin[1], Junxian Liu[1], Paola Gilsanz[2], Rachel Whitmer[3], Charles DeCarli[3], and Eleanor Hayes-Larson[1], *1. University of Southern California, Los Angeles, California, United States, 2. Kaiser Permanente Northern California, Pleasanton, California, United States, 3. University of California, Davis, Sacramento, California, United States*

Divorce is a common life stressor that could impact brain health in older adults. Current literature evaluating its relationship with neuroimaging measures, including structural MRI and amyloid PET, is limited and contains mixed findings. Using the Kaiser Healthy Aging and Different Life Experiences (KHANDLE) and Study of Healthy Aging in African Americans (STAR) datasets, we analyzed the impact of history of divorce on MRI outcomes including volumetric measures and white matter hyperintensities (n = 649) using linear regression models and on PET outcomes (n = 374), including standardized uptake value ratio using linear regression and amyloid positivity using relative risk regression. All models adjusted for age at brain imaging, gender, race/ethnicity, southern birth, participant's education, parental separation or divorce, parental education, and childhood socioeconomic status. The sample had a mean age at baseline of 72.4 years (SD = 7.8), with 60.7% of participants female and 42.5% of the sample reporting a history of divorce. Those with a history of divorce had smaller hippocampus volume, greater total white matter hyperintensity volume, and increased amyloid burden, but effect estimates were small, imprecise, and crossed the null. Results were similar when stratified by sex and restricted to individuals age 65+. Thus, a history of divorce was not strongly associated with late-life brain health in our sample, and differences may reflect social and economic contexts surrounding partnership dissolution. Further research should investigate possible sources of bias and heterogeneity, such as differences in the subjective experience of divorce as a stressor, in understanding this relationship.

Abstract citation ID: igaf122.4024

### THE ASSOCIATION OF PHYSICAL ACTIVITY TO COGNITIVE PERFORMANCE IN AFRICAN AMERICAN OLDER ADULTS

Rio Tate[1], Brent Small[1], and William Haley[2], *1. University of South Florida, Tampa, Florida, United States, 2. University of North Carolina at Chapel Hill, Chapel Hill, North Carolina, United States*

The number of older adults in the United States is expected to increase in the years to come. As we age, cognitive performance is noted to decline. African American older adults are more likely than non-Hispanic Whites to experience cognitive impairment and dementia. It is known that physical activity is a modifiable risk factor for dementia. It is not known the extent to which physical activity engagement is related to cognitive performance in African American older adults, and which variables, if any, moderate this association. To answer this question, the authors examined the association between three intensities of physical activity (mild, moderate, and vigorous) and four domains of cognitive performance (episodic memory, working memory, total mental status, and total cognition). Data were analyzed at five time points using ten years of data from the Health and Retirement Study. Data was also analyzed at the between-person and within-person level. Results indicated that greater engagement in physical activity was associated with greater cognitive performance across a variety of domains. Younger age, higher years of education, and female gender moderated this association. Implications from this study indicate the effectiveness of engaging in a modifiable risk factor of dementia with regards to higher cognitive performance in minority older adults. Downstream cross-racial comparisons examining the effectiveness of physical activity on cognitive performance are therefore based in more comprehensive data analysis.

Abstract citation ID: igaf122.4025

### THE EFFECTS OF AGING ON FEAR LEARNING IN MALE MICE

Chad Smies, Jiyeon Baek, and Janine Kwapis, *The Pennsylvania State University, University Park, Pennsylvania, United States*

Cognitive decline is prevalent in the aging population, in both human and rodent models. Mice show age-related impairments in fear learning and dysregulated gene expression following a learning event. While many studies investigate physical decline in aging, little is known about how maladaptive learning observed in post-traumatic stress disorder (PTSD) manifests in the aging population. The current study aims to characterize and identify intervention points to support appropriate fear learning with age. Fear conditioning is commonly used to model PTSD in rodents, and memory is measured as freezing behavior. Contextual fear conditioning (CFC) pairs a shock with a specific context. In contrast, delay (DFC) and trace fear (TFC) conditioning pair a shock that either co-terminates with a cue or is presented after a trace interval, respectively. Extinction is commonly used as a treatment model for PTSD, where the cue or context is repeatedly presented in the absence of a shock. Recent work has revealed age-associated deficits in CFC extinction and TFC using an auditory cue. The current study characterizes fear responses in TFC and DFC and extinction in aged mice using a novel light cue that can be presented outside the conditioning chamber. We found subtle, but reliable, deficits in freezing behavior in aged mice. Importantly, the successful pairing of the shock with the light cue allows future studies to examine brain activity while presenting a shock-associated light cue with functional magnetic resonance imaging (fMRI) to determine activity changes that occur in PTSD with aging, providing insight for future treatments.

Abstract citation ID: igaf122.4026

**THE IMPACT OF GENOMIC BURDEN ON DEMENTIA CAREGIVING: A SCOPING REVIEW**

Wenxin Bian[1], Jing Wang[2], Jamie Conklin[1], Xiangyu Zhang[2], Chloe Mitchell[2], Aubrie Woodward[2], and Rose Mary Xavier[3], *1. University of North Carolina at Chapel Hill, Chapel Hill, North Carolina, United States, 2. University of New Hampshire, Durham, New Hampshire, United States, 3. UTHealth Houston, Houston, Texas, United States*

Background: Although genomic technologies are increasingly used in dementia risk assessment, diagnosis and treatment, the impact of genomic burden in persons living with dementia (PWLD) on caregiving remains largely unexamined. Purpose: We investigated the impact of genomic burden on dementia caregiving and identified gaps to inform future practice and policy on dementia care. Methods: We searched PubMed, APA PsycInfo, and CINAHL through April 2025 for peer-reviewed studies examining caregiver outcomes related to genomic burden in PLWD. Two reviewers independently screened articles, resolving discrepancies by consensus. Data were extracted and thematically analyzed. Results: Of 708 studies identified, 82 underwent full-text screening. 5 studies that met inclusion criteria were conducted in the UK (n = 1) and Brazil (n = 4). Investigated genomic factors included APOE (n = 4), LDLD (n = 1), ACE (n = 1), and MAO-A (n = 1) variants. Care recipients, mainly with late-onset Alzheimer's disease, represented a range of dementia severity from probable to severe. Caregivers were primarily family members. Caregiving outcomes included caregiving burden and caregiving distress. Conclusion: Available evidence to determine the impact of genomic burden in PWLD on caregiving outcomes is limited, but they point to the mediating role of neuropsychiatric symptoms. Longitudinal studies are needed to assess how genomic burden influences caregiving burden. Intervention studies integrating genomic information and/or genetic counseling in dementia care models could clarify clinical utility as well as the psychosocial effects of genomic risk disclosure on the caregiving process.

Abstract citation ID: igaf122.4027

**THE LIVED EXPERIENCE OF LONELINESS IN TRANSGENDER OLDER ADULTS: A QUALITATIVE DESCRIPTIVE STUDY**

Katherine Bowers[1], and Joan Carpenter[2], *1. University of Maryland, Baltimore, Maryland, United States, 2. University of Maryland School of Nursing, Baltimore, Maryland, United States*

Loneliness affects more than a third of community dwelling older adults and is associated with poor physical and mental health, cognitive decline, and increased mortality. Transgender individuals experience health and socioeconomic disparities that place them at high risk for loneliness. The purpose of this study is to examine loneliness in transgender adults aged 50 and over and fill a critical knowledge gap within LGBTQIA+ aging research. Guided by the Health Equity Promotion Model, we conducted a qualitative descriptive study. Participants were sampled through gender affirming care centers in Maryland and local and national transgender support groups. Through semi-structured interviews and journaling, participants described their experiences with loneliness. Data were coded using conventional content analysis. Participants (N = 9) were 56-76 years old and had diverse gender identities. Three themes emerged regarding the lived experience of loneliness in transgender older adults: (1) loneliness on the inside; (2) longing for connection within relationships; (3) loneliness imposed by the outside world. The intersection of age and transgender identity heightens marginalization and elevates loneliness due to discrimination and social exclusion. Transitioning later in life, related to only recent access to gender affirming care, leads to complicated social challenges and partner strain increasing loneliness. Importantly, the current political climate has increased fear of persecution and discrimination; participants reported its tremendous impact on their isolation and loneliness. This study reveals that loneliness is prominent in transgender older adults. Given the unique vulnerabilities this population experiences, it is imperative that loneliness is addressed at both individual and societal levels.

Abstract citation ID: igaf122.4028

**THE MEANING OF A POSITIVE LIVING ENVIRONMENT IN NURSING HOMES: A SCOPING REVIEW AND CONCEPT ANALYSIS**

Lien Janssens, Lisa Geyskens, Ellen Vlaeyen, Bernadette Dierckx de Casterlé, and Mieke Deschodt, *KU Leuven, Balen, Vlaams-Brabant, Belgium*

Many studies explore elements of nursing home living environments that contribute to concepts such as a sense of home, quality of life, or thriving. Yet, the overarching understanding of a 'positive living environment' remains unclear. We aimed to clarify what constitutes a positive nursing home environment and identify its key elements. We combined scoping review with concept analysis methodology to determine the core characteristics of such an environment (defining attributes), the required conditions (antecedents), and outcomes (consequences). Five databases were searched for original qualitative studies published in the past 20 years exploring resident, staff, and informal caregivers' perspectives. In total, 146 studies met inclusion criteria and were categorized based on relevance. We analyzed the 19 highly relevant studies reflecting residents' perspectives. Our findings show that a positive living environment is a flexible concept and is co-created through dynamic interaction between residents, staff, and the broader context. The environment is perceived as positive when residents can continue being themselves. This includes experiencing a balance between support and independence, building and maintaining relationships, engaging in meaningful activities, receiving quality care, staying connected with the outside community and being involved in nursing home life. Achieving this requires organizational conditions such as quality caregivers, a supportive physical environment, as well as resident-related factors including a positive mindset and acceptance. The consequences include dignity, belonging, peace of mind, feeling at home and overall well-being. These elements are closely interconnected. Further research should examine these interrelationships to identify changes most likely to improve the overall environment.

Abstract citation ID: igaf122.4029

### THE MULTI-DOMAIN LONG TERM CARE NEEDS AND UNMET NEEDS OF VETERANS WITH A STROKE DIAGNOSIS

Roshni Singh[1], Diana Ruiz[2], Richard Munoz[3], Marianne Desir[2], and Sandra Garcia-Davis[4], *1. University of Miami, Miami, Florida, United States, 2. Miami Veterans Affairs Hospital, Miami, Florida, United States, 3. South Florida Veteran Affairs Foundation for Research and Education, Miami, Florida, United States, 4. Nova Southeastern University, Fort Lauderdale, Florida, United States*

Stroke survivors frequently experience several unmet needs, which can significantly impact quality of life and ability to age in the community. We used data from the HERO CARE survey, fielded in 2021 to 20,000 community-dwelling Veterans across five VA sites (respondent N = 8,056). Veterans with a history of stroke diagnosis were defined by Hierarchical Condition Category 100 (n = 576). We sought to describe the long-term care needs of stroke survivors across five domains: activities of daily living (ADLs), instrumental ADLs (IADLs), nursing, health care coordination, and social (legal and housing). We classified respondents as having an unmet need if they responded that they needed a little or a lot more help for a specific need. Veteran respondents were mostly men (97.7%), average age 80.4 (SD 8.7), married (61.3%), Non-Hispanic White (74.1%), residing in urban areas (81.9%), low health literacy (61.8%), and with a caregiver (70.4%). Veterans reported difficulties with ADLs (69.3%), IADLs (79.0%), nursing tasks (70.6%), health care coordination (72.2%), and social (48.5%). Overall, 54.2% reported having an unmet need, with ADLs (29.5%), IADLs (38.6%), nursing (29.4%), health care coordination (30.3%), and social (20.5%). Veterans also reported psychological and social concerns: dementia (29%), possible depression (29%), possible anxiety (18.8%), food insecurity (19.1%), and transportation problems (17.1%). Stroke survivors describe complex unmet needs. They require proactive, comprehensive support to address their multi-domain long-term care needs to empower them to live in the community. These data can aid in developing programs and policies to support stroke survivors and their caregivers.

Abstract citation ID: igaf122.4030

### THE RELATIONSHIP OF LANGUAGE BARRIERS TO CAREGIVING RELATIONAL QUALITY AMONG LATINO CAREGIVERS

Michelle Johns[1], Alan Tiburcio[1], Gustavo Arruda Franco[1], Selena Zhong[1], I'deyah Ricketts[1], Melissa Howe[1], and Lissette Piedra[2], *1. NORC at the University of Chicago, Chicago, Illinois, United States, 2. University of Illinois at Urbana-Champaign, Urbana, Illinois, United States*

Sixty-eight million people speak a language other than English at home, with the majority of those speaking Spanish. Language proficiency plays a strong role in shaping individuals' social networks, and language barriers may affect the quality of an individual's relationships, such as that between older adults and informal caregivers. Among Latino caregivers of recipients experiencing cognitive decline and coinciding loss of English as a second language, language barriers may intensify deleterious consequences for health and hinder informal caregivers from connecting care recipients to needed healthcare and other formal services. However, to date, few have examined how language differences between caregivers and care recipients shape relationship quality and care dynamics. Using data from a 2025 nationally representative survey of Latino caregivers (n = 625), we assess the prevalence of language barriers as an obstacle to healthcare seeking and the degree to which these barriers affect the relational quality between informal caregivers and older adults. Results indicate high proportions of the Latino caregiving population experiencing language barriers in supporting older adults in accessing healthcare (e.g., 39% reported barriers talking with doctors and other health providers; 31% reported barriers handling health insurance). The presence of these barriers is highly correlated with greater financial, emotional, and physical strain within the caregiving relationship. We discuss results in relation to the growing literature on social diversity in caregiving experiences and interventions (e.g., staff translators on site at medical facilities) that can improve relationship quality between Latino caregivers and health of the older adults in their care.

Abstract citation ID: igaf122.4031

### THE SENSORY & SOCIAL NEGLECT INDEX: A NOVEL MULTIDIMENSIONAL RISK TOOL FOR METABOLIC AND COGNITIVE DECLINE

Arjun/Aarti Sahai, Ruizhi Huang, and Jinmyoung Cho, *Saint Louis University, Saint Louis, Missouri, United States*

Older adults experience multisensory impairment and low social engagement; their combined impact on metabolic and cognitive decline is rarely examined. Our objective was to develop a Sensory/Social Neglect Index (SNI), integrating four domains and assessing its association with diabetes and cognitive decline in older adults. Using cross-sectional data and multinomial logistic regression, we examined the relationship between SNI levels and diabetes/cognitive decline (alone/combined), adjusting for demographic/health covariates. Data was extracted from the 2020 National Health Interview Survey. The SNI was created by summing 4 domains from NHIS variables: dental neglect, hearing impairment, vision impairment, and social neglect, yielding a cumulative level from 0-4. Respondents (4,573 U.S. adults aged ≥65) were categorized into neither condition, diabetes only, cognitive decline only, or both. SNI levels were right-skewed, with 36.1% of participants at level 0, 35.5% at level 1, 19.5% at level 2, 7.4% at level 3, and 1.5% at level 4. Higher SNI levels were more common in older adults, racial/ethnic minorities, and nonmetropolitan residents. The risk of cognitive decline only was significantly higher at SNI=3 (ARRR=3.65, 95% CI:1.33–10.01) and remained elevated at level 4 (ARRR=4.26, 95% CI:1.25–14.51). The risk of both conditions rose from level 2 (ARRR=18.10, 95% CI:2.07–157.93) to level 4 (ARRR=91.33, 95% CI:6.03–1382.52), though wide confidence intervals reflect small sample sizes. Final analyses will refine model estimates/explore category consolidation. Results suggest that sensory/social neglect is associated with cognitive decline and diabetes. Incorporating SNI-based screening into primary care could identify at-risk individuals and foster preventive care.

Abstract citation ID: igaf122.4032

### TRAIT MINDFULNESS ACROSS ADULTHOOD: CAN MINDFULNESS BUFFER PHYSIOLOGICAL REACTIVITY TO SPOUSAL DISTRESS?

Skylar Weiskittel, and Stephanie Wilson, *The University of Alabama at Birmingham, Birmingham, Alabama, United States*

Older adults tend to have higher trait mindfulness—the capacity to sustain attention to present experiences without judgement—compared to younger adults. This may reflect age-related improvements in emotion regulation due to life experience and a shift in perceived time horizons. Higher trait mindfulness has been linked to reduced physiological reactivity to psychosocial stressors, promoting both physical and mental well-being. However, it remains unclear whether trait mindfulness protects older adults from blood pressure reactivity during exposure to a spouse's emotional distress, an important but understudied marital stressor. To examine this, 204 community adults ages 25–90 (N = 102 couples) reported their trait mindfulness and had their blood pressure measured before and after an emotional disclosure task. First, age was positively correlated with trait mindfulness ($r = 0.40$, $p < 0.001$), replicating prior work. Controlling for the fixed effects of sex, BMI, tobacco, comorbidities, and education, multilevel models indicated that trait mindfulness did not alter blood pressure reactivity (ps > .361). Older adults also did not differentially benefit from mindfulness in terms of systolic or diastolic blood pressure reactivity (ps > .230). However, older adults did show diminished diastolic reactivity compared to younger adults (B = -0.088, SE = 0.029, p=.003), apart from their mindfulness levels. Thus, although older adults reported greater mindfulness and experienced dampened diastolic reactivity to their partner's emotional distress, mindfulness did not account for or amplify this physiological advantage. Future research should examine distinct facets of trait mindfulness and explore other psychosocial factors that may confer benefits to older adults under stress.

Abstract citation ID: igaf122.4033

### TRAJECTORIES OF FUNCTIONAL HEALTH IN LATER LIFE: THE ROLE OF HEARING PROBLEMS AND SOCIAL NETWORK

Markus Wettstein[1], Bettina Williger[2], Lisa Incerti[3], Rosa-Linde Fischer[4], Julia Wolff[5], and Susanne Wurm[1], *1. Institute for Community Medicine, University Medicine Greifswald, Greifswald, Mecklenburg-Vorpommern, Germany, 2. Engineering Psychology / Interdisciplinary Studies, Landshut, Bayern, Germany, 3. Landshut University of Applied Sciences, Landshut, Bayern, Germany, 4. ORCA Labs Europe, Erlangen, Bayern, Germany, 5. IGES Institute, Berlin, Berlin, Germany*

Problems with hearing in midlife and old age are associated with poorer functional health. At the same time, the impact of hearing impairment on health outcomes varies considerably across individuals. Little is known about psychosocial factors that moderate the association between hearing impairment and functional health. We used data from the German Ageing Survey (2008-2023; n = 12,156; mean age = 62.35 years, age range 40-93 years) and investigated whether social network size is such a moderating factor, as a large social network might, for instance, provide support and motivation to maintain one's functional health, particularly in older adults. Based on longitudinal multilevel regression models, controlling for age, gender, education, hearing aid use, self-rated health and region of residence, we found that functional health declined over time and was poorer at baseline in individuals with more self-reported hearing problems. A larger social network size was associated with better functional health at baseline, and among chronologically older adults, this association was stronger. In addition, this association was stronger in older adults who reported more hearing problems. A larger network size was also associated with a less steep functional health decline over time, but only among chronologically older adults. Our findings suggest that older adults, particularly those affected by hearing problems, may benefit from a larger social network which may help to counteract the detrimental effect of poor hearing on functional health. Interventions to increase social embeddedness in older adults, with and without impaired hearing, could thus contribute to promoting healthy aging.

Abstract citation ID: igaf122.4034

### TRANSFORMATIVE LEARNING: VISITING A MULTIGENERATIONAL LIVING COMMUNITY IN SWITZERLAND

Jennifer Leszczynski, and T. Caitlin Vasquez-O'Brien, *Eastern Connecticut State University, Willimantic, Connecticut, United States*

Global studies are transformative for students (Jean-Francois, Sharma, & Obi, 2023) and intergenerational experiences help students to view aging through a cross-cultural lens and better understand the importance of intergenerational connections (Montepare & Leedahl, 2020) and aging in place. In May 2025, 26 undergraduates and two faculty members visited The Foundry (Giesserei) – a grassroots community designed specifically for multigenerational living in Winterthur, Switzerland. Including 155 apartments and housing individuals across the lifespan, the community contains a library, preschool, restaurant and art gallery. As the first Americans to tour the facility, we will highlight our perspectives of this unique opportunity.

Abstract citation ID: igaf122.4035

### TREATMENT DURABILITY AND CONCEPTUAL VALIDITY OF AN INNOVATIVE PT PROGRAM TARGETING MOBILITY AMONG OLDER VETERANS

Jonathan Bean[1], Rebekah Harris[2], Elisa Ogawa[2], Rachel Ward[2], Mary Kate Palleschi[3], Mariana Wingood[4], Thomas Travison[5], and Jennifer Brach[6], *1. Harvard Medical School, Boston, Massachusetts, United States, 2. VA Boston Healthcare System, Boston, Massachusetts, United States, 3. Albany Medical College, Albany, New York, United States, 4. Wake Forest University School of Medicine, Winston-Salem, North Carolina, United States, 5. Hebrew SeniorLife & Harvard Medical School, Hebrew SeniorLife, Massachusetts, United States, 6. University of Pittsburgh, Pittsburgh, Pennsylvania, United States*

We investigated the treatment durability and conceptual validity of an innovative physical therapy program known as Live Long Walk Strong (LLWS). LLWS is designed to enhance mobility by targeting leg power, trunk muscle endurance, gait variability and self-efficacy for exercise, which are all attributes linked to mobility decline. This was a longitudinal study of LLWS participants derived from a randomized controlled trial (RCT). The RCT participants in an 8-week waitlist control group crossed over to LLWS treatment. The combined cohort was then evaluated after treatment and over 16 weeks of subsequent follow up. This study was conducted at an outpatient VA tertiary care center among community-dwelling middle aged and older Veterans with slow walking speed. LLWS is an 8-week 10 (1-hour/session) session outpatient physical therapy program. Mobility measures included walking speed and the Short Physical Performance Battery (SPPB). We also evaluated the attributes LLWS targets. Within robust multivariable linear mixed effect models, we observed significant improvements (p<.05) in walking speed by ≥.09m/s after treatment and this magnitude of difference persisted 8 and 16 weeks after treatment ended. This pattern of improvement was also observed for the SPPB and all four attributes. Within stratified analyses, the initial treatment response on mobility surpassed a clinically meaningful threshold after adjusting for health factors commonly mitigating rehabilitative gains. Our study demonstrates that LLWS treatment effects are robust and sustained up to 4 months after treatment. As conceptually designed, LLWS produces large and prolonged improvements in measures of mobility and the attributes it targets.

Abstract citation ID: igaf122.4036

### TRENDS AND CHARACTERISTICS OF MEDICATION USE AMONG U.S. OLDER ADULTS BY FRAILTY: A 20-YEAR NHANES ANALYSIS

Maryanne Kim, Michael Steinman, and Matthew Growdon, *University of California, San Francisco, San Francisco, California, United States*

Frailty, a syndrome of reduced physiologic reserve, is associated with higher medication burden and vulnerability to adverse drug events. Reducing this burden requires understanding which therapeutic classes predominate and how their distribution varies by frailty status; however, long-term, population-based data remain limited. We analyzed data from the U.S. National Health and Nutrition Examination Survey (NHANES), 1999–March 2020, including 12,766 adults aged ≥65 years with ≥80% of frailty index (FI) components available, categorizing participants as robust, frail or severe frailty. Trends in therapeutic class use were examined across survey years. Mean medication counts rose from 2.60 to 3.52 in robust, 4.23 to 5.66 in frail, and 6.08 to 7.87 in severe frailty. Cardiovascular and metabolic agents predominated in all frailty groups, with higher counts in more frail groups. By 2017–2020, older adults with severe frailty used more than twice as many drugs from cardiovascular (2.59 vs 1.02) and metabolic (1.88 vs 0.81) classes as robust individuals. Over the 21-year period, the use of psychotherapeutic agents rose in all groups, nearly quadrupling in people with severe frailty (0.09 to 0.38), while central nervous system agents use increased minimally in robust and frail groups but meaningfully in severe frailty group (0.66 to 0.90). Medication burden increased across all frailty strata, with severe frailty showing higher use of cardio-metabolic agents and larger increase in psychotherapeutic and central nervous system agents. Medication optimization strategies for older adults should be tailored to frailty status, with attention to therapeutic classes most likely to drive polypharmacy.

Abstract citation ID: igaf122.4037

### TRENDS IN EARLY AND LATE MIDLIFE SUBSTANCE USE THROUGH 2024 FROM THE US MONITORING THE FUTURE PANEL STUDY

Megan Patrick, Yvonne Terry-McElrath, and Joy Bohyun Jang, *University of Michigan, Ann Arbor, Michigan, United States*

Longitudinal data collected across the life course provide unique opportunities to understand how risk factors for health in older adulthood evolve over time. The Monitoring the Future (MTF) Panel study surveys national US samples of ~20,000 adults each year, including a new nationally-representative cohort of 12th grade students every year since 1976 (Patrick et al., 2025). The current study reports on changes over time in substance use among those in early midlife (ages 35-50) and late midlife (ages 55-65) through 2024, focusing on cannabis and nicotine use. Cannabis use overall remains at/near all-time high levels in these age groups, as prevalence levels have doubled or nearly doubled over the past 5 and 10 years (e.g., early midlife past 12-month use increased from 4.4% in 2014 to 26.6% in 2024). The prevalence of vaping cannabis in the past 12 months reached historic highs in both age groups, rising from 8.7% in 2023 to 10.9% in 2024 among early midlife adults and from 3.5% to 5.1% among late midlife adults. Regarding nicotine, the prevalence of nicotine pouch use increased from 3.3% in 2023 to 4.9% in 2024 in early midlife, and from 2.2% to 4.3% for late midlife. Among late midlife adults there were significant increases in the use of hookah (from 2.0% to 3.4%) and smokeless tobacco (from 3.3% to 5.7%) in the past 12 months from 2023 to 2024. These shifts illustrate how substance use is evolving among adults, with implications for health and wellbeing in midlife and beyond.

Abstract citation ID: igaf122.4038

### UNDERSTANDING DEMENTIA CAREGIVER NEEDS: A QUALITATIVE ANALYSIS OF NATIONAL HELPLINE CALLS

Nancy Hodgson[1], Sonia Talwar[1], Kerry Finegan[2], Liming Huang[1], and Todd Myers[1], *1. University of Pennsylvania, Philadelphia, Pennsylvania, United States, 2. Alzheimer's Association, Chicago, Illinois, United States*

Over 11 million Americans provide unpaid care to persons living with dementia (PLWD), leading to social isolation, financial hardship, and increased risk for depression and stress. Despite available resources, dementia caregivers frequently lack access to coordinated support. Telephone support programs offer accessible interventions, but little is known about specific needs caregivers express when seeking help. This study examined challenges and needs reported by callers to the Alzheimer's Association National Helpline (AANH). We completed a qualitative content analysis of 1,000 call logs from the AANH (July 2021 to July 2022). Three researchers independently coded call logs using open coding followed by development of a refined 21-code framework. Caregiver-presenting challenges and needs were identified, categorized, and ranked by frequency. The average Kappa value among coders was 0.47, with 72% agreement. The ten most frequently reported needs were: home and community-based services (19%), communication techniques with PLWD (16%), caregiver self-care and emotional support (15%), managing neuropsychiatric symptoms and behaviors (10%), medical management (9%), relevant educational resources (7%), legal resources (5%), insurance and healthcare financing (5%), home safety resources (5%), and transition to long-term care advice (5%). Most caregivers were women, White, caring for spouses, and over 65 years old. This newly completed analysis reveals that dementia caregivers most frequently sought guidance for concerns routinely not discussed during healthcare encounters. These fresh findings highlight critical gaps in current care delivery, offering opportunities to better support this vulnerable population through proactive assessment by healthcare providers, the development of tailored interventions, and policy efforts.

Abstract citation ID: igaf122.4039

### UNDERSTANDING THE MESOTHELIOMA JOURNEY FROM DIAGNOSIS THROUGH BEREAVEMENT

Amanda Woodward[1], Jenavry Hirsch[1], Xuehan Zhang[1], Maria Towe[2], Claudine Benmar[2], Julie White[2], Brian Lapacek[2], and Melissa Culligan[3], *1. Michigan State University, East Lansing, Michigan, United States, 2. Mesothelioma Applied Research Foundation, Washington, District of Columbia, United States, 3. Temple University Hospital, Philadelphia, Pennsylvania, United States*

The Michigan State University School of Social Work (MSUSSW) and the Mesothelioma Applied Research Foundation (Meso Foundation) are collaborating on a federally funded four-year project to develop and test an online support intervention for those caring for a loved one with mesothelioma. This poster will present results from the first phase of this work within the context of a community-engaged research framework. Twenty-seven semi-structured interviews (5 patients, 12 caregivers, 10 bereaved) were conducted via Zoom between December 2024 and May 2025. Interview topics included the diagnosis, the treatment journey, the experience of providing care, decision making, planning for end of life, and caregiving and bereavement support. Interviews were recorded and transcribed. A codebook was created through an iterative process and each interview was coded in Dedoose by two team members with discrepancies and questions resolved by the full team. Initial thematic analysis identified three main findings. First the uniqueness of mesothelioma compared to other cancer experiences permeated all aspects of caregiving. Second, caregivers and patients experienced a lack of control. For example, limited treatment options meant there were really no treatment decisions to make. Third the impact of health-related social factors was heightened because of the unique aspects of mesothelioma (for example, the need to travel to receive care from experts). Based on these findings, the next phase of this research will include mapping the trajectory from diagnosis through bereavement to identify key time points for intervention and confirming these findings through focus group discussions.

Abstract citation ID: igaf122.4040

### UNMET CARE NEEDS AMONG VETERANS: INTERSECTION OF HOMEBOUND STATUS AND RURALITY

Sandra Garcia-Davis[1], Way Way Hlaing[2], Denise Vidot[3], Daniel Feaster[4], Erin Bouldin[5], Ranak Trivedi[6], Luci Leykum[7], and Stuti Dang[8], *1. Nova Southeastern University, Fort Lauderdale, Florida, United States, 2. University of Miami Division of Epidemiology & Population Health Sciences, Miami, Florida, United States, 3. School of Nursing and Health Studies, Coral Gables, Florida, United States, 4. University of Miami Miller School of Medicine, Miami, Florida, United States, 5. University of Utah, Salt Lake City, Utah, United States, 6. VA Palo Alto Health Care System, Palo Alto, California, United States, 7. South Texas Veterans Healthcare System, San Antonio, Texas, United States, 8. Miami VA Healthcare System, Miami VA Healthcare System, Florida, United States*

Most older adults prefer aging in place, even when homebound. Equitable access to long-term services, including home and community-based services (HCBS), is a Veterans Affairs (VA) priority, yet rural Veterans often face access challenges. Here, we describe the prevalence of activities of daily living (ADL) and instrumental ADL (IADL) unmet needs by homebound status and rurality and assess the association between homebound status and unmet needs, stratified by rurality. We analyzed Veterans aged 65+ with ADL or IADL problems from the HERO CARE survey. Veterans reporting difficulty with at least one ADL or IADL were categorized as having a problem. Unmet need was defined as requiring "a little or a lot more help." Weighted multivariable modified Poisson regressions with robust standard errors assessed the association between homebound status and unmet need, stratified by rurality. Among Veterans with ADL or IADL problems ($N = 5,045$; Weighted $N = 190,224$), 15% were homebound and 21% rural. About 13% of rural and 15% of urban Veterans were homebound. ADL unmet needs were reported by 10% of rural and 15% of urban Veterans ($p<.05$). Rural homebound Veterans had 2.01 (1.14–3.43) times higher prevalence of ADL unmet needs than rural non-homebound Veterans. Urban homebound Veterans had 1.45 (1.18–1.85) times higher prevalence than urban non-homebound Veterans. No significant IADL associations were found. Unmet ADL needs vary by homebound status and rurality. There is a critical need to understand factors driving unmet needs and access disparities among homebound rural Veterans, so equity-focused solutions can be developed.

Abstract citation ID: igaf122.4041

### UNPACKING VALUE TENSIONS IN OLDER ADULTS' ENGAGEMENT WITH GENERATIVE AI

Debaleena Chattopadhyay, and Tasneem Mubashshira, *University of Illinois Chicago, Chicago, Illinois, United States*

Generative artificial intelligence (GAI) refers to AI systems that create new content—such as text, images, audio, or video—based on patterns learned from large datasets. Unlike traditional digital technologies that are task-specific and predictable, GAI systems support flexible, open-ended interaction. However, this flexibility introduces several challenges, including unpredictable outputs (e.g., hallucinations), ambiguous authorship, and humanlike interactions, especially in conversational tools. We used a value sensitive design approach and reflexive thematic analysis to examine how community-dwelling older adults engage with GAI. Semi-structured interviews with twelve U.S.-based older adults (median age = 64; median CFI = 2.5) explored their perceptions, experiences, and concerns. Participants ($n = 8$) who used tools like Copilot or ChatGPT described using them for small, routine tasks such as finding information and editing text ("for the little things"; "find out things that I've forgotten") and appreciated their ease of use ("I could tell it more precisely what I wanted"; "answers me back in detail"; "pretty simple to use"). However, both users and non-users expressed value tensions, particularly around trust ("It's functional, but I think part of it is I don't trust it"), lack of accountability ("I mean who's giving you those answers?"), absence of informed consent ("an excuse to legitimize thievery"), algorithmic bias ("there's so much misinformation out there"), and broader concerns about societal harm ("it scares me about what human nature will do with AI"). Notably, even frequent users demonstrated critical engagement rather than overtrust, pointing to the need for design practices that engage with—not undermine—users' healthy skepticism.

Abstract citation ID: igaf122.4042

### UNRAVELING HEALTHY LONGEVITY: CHARACTERIZING PARTICIPANTS AGED 80+ IN THE MILLION VETERAN PROGRAM

Xuan-Mai Nguyen[1], Yanping Li[1], April Williams[1], Jane Driver[2], Kelly Cho[1], J. Michael Gaziano[1], Stacey Whitbourne[1], and Ariela Orkaby[3], *1. VA Boston Healthcare System, Boston,*

*Massachusetts, United States, 2. VA Boston Healthcare System, Brockton, Massachusetts, United States, 3. Brigham & Women's Hospital, Harvard Medical School, Boston, Massachusetts, United States*

US Veterans aged 80 and older represent one of the fastest growing segment of the aging population, however the functional status and lifestyle behaviors of this population is poorly understood. We extracted survey and health record data from participants in the Department of Veterans Affairs (VA) Million Veteran Program who were 80 years and older upon enrollment. Descriptive statistics were reported for demographics, morbidities and functional status (basic Activities of Daily Living (ADL), Instrumental Activities of Daily Living (IADL), gait speed, requiring the assistance of another person, and the VA Frailty Index (VA-FI)). Adherence to healthy lifestyle behaviors (healthy diet, adequate physical activity, social support, avoiding opioids, not smoking, restful sleep, no heavy drinking, good stress management) was also reported. There were 84,152 participants aged 80 and older. Median age was 84 years (range: 80-109; IQR: 81, 87), 98% were male and 96% were non-Hispanic White. Few (10%) required assistance for 1+ ADLs and 24% required assistance for 1+ IADLs. Almost half (47%) reported a gait speed of normal (2-2.9 mph) or faster, 9% required the assistance of another person, and 36% were classified as robust using the VA-FI. Participants reported adhering to a median of 5 (IQR 4, 6) healthy lifestyle behaviors. This MVP cohort is among the largest group of adults over age 80 and captures a highly functional group with good adherence to healthy lifestyle factors. Future work will better characterize modifiable risk factors and genetic information to guide healthy aging for all.

Abstract citation ID: igaf122.4043

### VALIDATION OF STANDARDIZED NURSING LANGUAGES FOR PEOPLE LIVING WITH HIV OVER 50

Juh Shin[1], Sue Moorhead[2], Elizabeth Swanson[3], Mary Clarke[4], Cheryl Wagner[5], Karen Dunn Lopez[2], Nidhi Naidu[6], and Hritik Majgaonkar[7], *1. George Washington University, Ashburn, Virginia, United States, 2. University of Iowa, Iowa City, Iowa, United States, 3. University of Iowa Center of Nursing Classification and Clinical Effectiveness, Iowa City, Iowa, Iowa, United States, 4. Healthlinx, DAVENPORT, Iowa, United States, 5. University of Iowa College of Nursing, Iowa City, Iowa, United States, 6. Geroge Washington University, Arlington, Virginia, United States, 7. George Washington University, Washington, DC, District of Columbia, United States*

Despite improvements in HIV treatment, nursing care for older adults living with HIV remains insufficiently tailored to their unique needs. This study aimed to identify and validate nursing diagnoses, interventions, and outcomes relevant to this population. A content validation survey was conducted with 27 HIV/AIDS nursing experts who evaluated 45 nursing diagnoses, 79 outcomes, and 82 interventions on their relevance using a five-point scale. Relevance ratios classified each item as critical, supplemental, or unnecessary. Experts identified critical nursing diagnoses such as risk for loneliness, impaired memory, and risk for infection. Key interventions included Medication Management, education on prescribed medications, and Emotional Support. Important nursing outcomes identified were Social Support, Infection Severity, and Immunization Behavior. These validated elements provide an evidence-based framework for developing holistic, individualized care plans addressing both HIV-specific and aging-related health concerns. Incorporating these findings into clinical nursing practice can improve assessment, planning, and care delivery for older adults living with HIV. Nurses are encouraged to use these validated care components to deliver comprehensive, person-centered care that attends to physical, cognitive, and psychosocial needs, thereby enhancing health outcomes and quality of life for this growing population.

Abstract citation ID: igaf122.4044

### VARIATION IN PATIENT OUTCOMES ACROSS NURSE PRACTITIONER SCOPE OF PRACTICE LEVELS

Chih-Ying Li[1], Md Ibrahim Tahashilder[1], Letitia Graves[1], Peter Cram[2], and Yong-Fang Kuo[3], *1. University of Texas Medical Branch, Galveston, Texas, United States, 2. Wright State University, Fairborn, Ohio, United States, 3. University of Texas Medical Branch, Galveston, Galveston, Texas, United States*

This study investigates whether varying degrees of authorized nursing practice patterns (restricted, reduced, full authority with transition, and full authority without transition) across the nation influenced patient outcomes (30-day hospital readmission and in-hospital mortality). The four practice patterns are defined based on the published annual legislative update for advanced practice registered nurses. Utilizing 2019 CMS Medicare administrative claims, we analyzed six medical conditions due to their high prevalence and burdens commonly targeted for quality improvement and policy: acute myocardial infarction (n = 153,870), heart failure (n = 449,232), pneumonia (n = 288,831), stroke (n = 250,291), total hip/knee arthroplasty (n = 538,283), and hip/femur fractures (n = 147,694). We conducted multivariable logistic regression, adjusting for age, sex, race/ethnicity, residential rurality, dual eligibility, Medicare original entitlement, Elixhauser comorbidity, hospital lengths of stay, hospital ownership, and median household income (at the zip-code level). Relative to the restricted practice group, patients in the reduced practice group were more likely to be readmitted (OR = 1.06, 95% CI = 1.04-1.08), whereas patients in both full authority groups demonstrated a reduced likelihood of readmission (with transition: OR = 0.91, 95% CI = 0.90-0.93; without transition: OR = 0.89, 95% CI = 0.88-0.91). Relative to the restricted practice group, patients in the reduced and full authority with transition groups had lower mortality risk (OR = 0.95, 95% CI = 0.93-0.98; OR = 0.99, 95% CI = 0.97-1.02, respectively), whereas patients in full authority without transition group demonstrated an increased mortality risk (OR = 1.11, 95% CI = 1.09-1.13). Our results suggest that the full nursing authority with transition produced relatively balanced and optimal results on both outcomes among Medicare beneficiaries.

Abstract citation ID: igaf122.4045

### VOLUNTEERING TRANSITIONS AND PSYCHOLOGICAL WELL-BEING IN FAMILY CAREGIVERS OF OLDER ADULTS

Sol Baik[1], Jennifer Crittenden[2], and Kyeongmo Kim[3], *1. University of Virginia, Charlottesville, Virginia, United States, 2. University of Maine Center on Aging, Orono, Maine, United States, 3. Virginia Commonwealth University, Richmond, Virginia, United States*

Despite their commitment to caregiving, approximately 25% of family and other unpaid caregivers of older adults in the U.S. participated in formal volunteer activities in 2017. Formal volunteering has been associated with improved psychological well-being, as it offers an additional social role that can enhance self-esteem and expand social networks. However, little is known about how discontinuing volunteering affects caregiver well-being. We hypothesized that ceasing volunteer activity may result in role loss or identity disruption, potentially diminishing psychological well-being, with particular attention to gender differences. Using data from Rounds 5 and 7 of the National Study of Caregiving, we examined the longitudinal effects of starting and stopping formal volunteering on the well-being of 1,148 family and unpaid caregivers of Medicare beneficiaries living in the community. A multilevel mixed-effects generalized linear model was used to assess these associations. Results showed that caregivers who consistently volunteered over time reported significantly better psychological outcomes compared to those who never volunteered. Transitions into or out of volunteering (i.e., starting or stopping) were not significantly associated with changes in well-being overall. However, sensitivity analyses revealed that the positive effects of sustained volunteering were consistent across both women and men. Notably, ceasing formal volunteering was associated with improved psychological well-being among male caregivers. While continued volunteering may generally support caregiver well-being, its psychological impact may vary by gender. For men, discontinuation may offer relief or reprioritization that enhances well-being. These findings highlight the need for gender-responsive strategies to support caregivers through volunteer engagement.

Abstract citation ID: igaf122.4046

### WHEN SLEEP MEETS STRESS: HOW DISCRIMINATION AND SLOW WAVE SLEEP IMPACT MEMORY IN AGING BLACK ADULTS

Osswaah Fariduddin[1], Stirling Crawford[2], Maria Clark[1], Elizabeth Luth[3], and Darlingtina Esiaka[4], *1. University of Kentucky, Lexington, Kentucky, United States, 2. University of Kentucky, Frankfort, Kentucky, United States, 3. Rutgers University, New Brunswick, New Jersey, United States, 4. University of Kentucky - College of Medicine, Lexington, Kentucky, United States*

Background: Black Americans disproportionately experience everyday discrimination—unjust treatment based on race, limiting opportunities—which has been associated with risk of impaired visuospatial memory, an early indicator of cognitive decline. Slow wave sleep (SWS) is critical for memory consolidation but naturally declines with age. The decline is further exacerbated by psychosocial stressors such as discrimination. The purpose of this study is to examine whether SWS moderates the association between visuospatial memory and discrimination among aging Black Americans. Methods: As part of an ongoing study, participants ($N = 59$, Mage=52.76) completed three nights of at-home objective sleep monitoring using a wearable device (Sleep profiler) and measures assessing demographics and discrimination. Visuospatial memory was measured using the Benson Complex Figure Task. PROCESS Macro (Model 1) was used to test the moderating role of SWS on the association between discrimination and visuospatial memory, while adjusting for age, sex, and marital status. Results: SWS significantly moderated the relationship between discrimination and visuospatial memory ($B=-0.0093$, $p = 0.047$). At lower levels of discrimination, SWS had a positive association with visuospatial memory ($B = 0.13$, $p = 0.089$). However, at higher levels of discrimination, the association was negative ($B=-0.089$, $p = 0.32$). No significant main effects were found for age, sex, or marital status. Conclusion: Findings suggest that while SWS may be beneficial for cognitive health, the benefit is eroded by the presence of discrimination in aging Black Americans. Our study highlights how the combined effects of psychological and biological factors can provide crucial insight into cognitive aging among populations at high-risk of developing cognitive decline.

Abstract citation ID: igaf122.4047

### WHEN SOCIAL INTERACTIONS FEEL LESS MEANINGFUL: AGE DIFFERENCES IN SOLITUDE PREFERENCE IN DAILY CONTEXT

Yoonseok Choi[1], Hansol Kim[2], and Christiane Hoppmann[3], *1. Simon Fraser University, Vancouver, British Columbia, Canada, 2. Sogang University, Seoul, Republic of Korea, 3. University of British Columbia, Vancouver, British Columbia, Canada*

Solitude and social interactions have often been studied separately, each linked to distinct personality profiles (e.g., low vs. high extraversion). However, in daily life, these experiences may be interconnected as individuals transition between time alone and time with others. This study investigated whether lower-quality social interactions during the day are associated with a greater preference for solitude at the end of the day. Age moderation in this association was also examined. As younger individuals are more likely to engage in social interactions due to obligatory demands (e.g., work, childcare), we hypothesized that the proposed association is stronger among younger adults compared to older adults. Using daily life assessments up to 10 consecutive days, we assessed perceived meaningfulness of daily social interactions and preference for solitude at the end of the day in 128 individuals from South Korea and Canada (Age: $M = 48.19$ years, Range: 19-93; 67% female; 56% Asian, 32% White). Results based on multilevel modeling with ordinal outcomes showed that individuals who, on average, reported lower meaningfulness in social interactions exhibited a greater preference for solitude versus interacting or staying with others (between-person effect). The within-person association was moderated by age, such that younger individuals showed a stronger preference for solitude on days when their social interactions felt less meaningful. These findings underscore the role of social context and age in shaping motives for seeking solitude in daily life.

Abstract citation ID: igaf122.4048

### WHERE CARE FALLS SHORT: MAPPING THE SUPPLY–DEMAND GAP IN ADRD CARE ACROSS AN AGING UNITED STATES

Yung Chun[1], Oejin Shin[2], Soobin Park[1], and Sojung Park[1], *1. Washington University in St. Louis, St. Louis, Missouri, United States, 2. Illinois State University, Normal, Illinois, United States*

The growing prevalence of Alzheimer's disease and related dementias (ADRD) poses urgent challenges for equitable access to long-term care across the United States. While demand for ADRD care is driven by an aging population and rising cognitive health needs, the availability of facilities and specialized services remains uneven, raising concerns about geographic disparities. This study integrates multiple data sources to evaluate the spatial balance of ADRD demand and care infrastructure at the county level. Demand is estimated using American Community Survey data on older adults with cognitive difficulties, while supply is drawn from National Care Planning Council records covering nine facility types across 31 states. Explanatory Spatial Data Analysis (ESDA) is applied to map demand, supply, and their balance, with results further disaggregated by facility type. Preliminary findings reveal sharp disparities: demand is concentrated in large metropolitan counties, the Sunbelt, and parts of the Midwest and Appalachia. Facilities cluster along the East Coast, California, Florida, and selected Midwestern metros, while deficits are greatest in the rural South, Great Plains, and Mountain West. Specialized services such as adult day programs and Alzheimer's centers are heavily urban, whereas generalized services—nursing homes, assisted living, and retirement facilities—are more dispersed but still unevenly distributed. These findings highlight a persistent spatial mismatch between population needs and care infrastructure. The concentration of specialized services in metropolitan centers risks leaving rural and micropolitan communities underserved. Addressing these inequities is critical to promoting equitable aging, reducing caregiver burdens, and ensuring timely access to ADRD care.

Abstract citation ID: igaf122.4049

### WORKING MEMORY DECLINE AFTER AGE 60 NEGATIVELY IMPACTS MEDICATION ADHERENCE

Candace Ryan[1], Cheryl Dahle[1], Debra Schutte[1], and Ana Daugherty[2], *1. Wayne State University, Detroit, Michigan, United States, 2. Institute of Gerontology, Wayne State University, Detroit, Michigan, United States*

Medication adherence is critical for chronic disease management. Working memory is a putative cognitive process necessary for successful adherence. Working memory is known to decline with age. Working memory processes are hypothesized to support successful adherence, which is vulnerable to age-related decline. Poor understanding of cognitive correlates to adherence among community-dwelling older adults impedes effective strategies to reduce late-life health disparities. A longitudinal analysis tested the association of working memory decline with future medication adherence in 117 community-dwelling participants of the Detroit Brain Aging Study (73.3% female, 84.3% White), mean age = 70.49 (SD = 12.48). Working memory was assessed on three measurement occasions (2-year delays), and medication adherence was reported at a follow-up 14–22 years after baseline. In binomial logistic regression, the interaction age × change in working memory predicted the likelihood of medication adherence ($b = 0.72$, $p = 0.02$). In the oldest age group >80 years, greater working memory declines predicted lesser likelihood of reporting medication adherence in the future, this was attenuated at younger ages, with a negligible effect at 60 years. Concurrent age, delay since baseline, and change in processing speed were not significant (all p's > 0.7), nor was the cumulative model ($p = .25$; $R2 = 0.11$). In this sample, working memory declines had a modest association with future medication adherence behavior, with the effect most prominent among adults over age 60 years. Future studies are needed to determine additional factors, beyond cognitive decline, that contribute to medication adherence.

Abstract citation ID: igaf122.4050

### YOUNG ADULT SUBSTANCE USE AS A PREDICTOR OF POOR MEMORY DECADES LATER IN MIDLIFE

Yuk Pang, Yvonne Terry-McElrath, Joy Bohyun Jang, and Megan Patrick, *University of Michigan, Ann Arbor, Michigan, United States*

Subjective memory decline is an early sign of cognitive decline and dementia. Understanding risk factors associated with midlife poor memory is crucial for effective prevention. This study prospectively examines associations between young adult (YA) heavy substance use and late midlife poor memory, and the mediating role of early midlife problematic substance use. Data on number of waves of YA (age 18-30) heavy alcohol, binge drinking, cannabis and cigarette use, early midlife (age 35) substance use disorder symptoms and heavy cigarette use, and late midlife poor self-rated memory (age 50-65) were from the Monitoring the Future (MTF) Panel study ($N = 14{,}659$). Mediation analyses assessed whether early midlife problematic substance use mediated YA heavy substance use associations with late midlife poor memory. YA heavy alcohol use and binge drinking were associated with higher odds of early midlife disordered (vs. non-disordered) alcohol use (AOR=1.69 and 1.66, both $p<.001$), and with late midlife poor memory (AOR=1.34 and 1.39, both $p<.001$). Partial mediation for alcohol (AOR=1.17, $p<.001$) and full mediation for binge drinking (AOR=1.18, $p<.001$) were observed. YA heavy cannabis use was associated with early midlife disordered use (vs. non-disordered; AOR=1.42, $p<.001$), and with late midlife poor memory (AOR 1.41, $p = 0.008$); full mediation (AOR=1.13, $p = 0.010$) was observed. YA daily cigarette use was directly associated with late midlife poor memory (AOR=1.05, $p = 0.047$), with no evidence of mediation. These findings underscore long-term risks of sustained YA substance use for cognition, and indicate that long-term associations may partially or completely operate through early midlife disordered substance use.

### SESSION 8015 (POSTER)

### LATE BREAKING POSTER SESSION IV

Abstract citation ID: igaf122.4051

### 48 HOUR HOSPICE HOME IMMERSIONS - AY 2018-19: STUDENT LEARNING AND SKILLS TO AUGMENT THEIR CAREER

Tyler Dentry[1], Kevin Reale[1], and Marilyn Gugliucci[2], *1. University of New England College of Osteopathic Medicine, Biddeford, Maine, United States, 2. University of New England College of Osteopathic Medicine, Kennebunk, Maine, United States*

Medical student end-of-life care (EOL) and palliative medicine (PM) training has been cited as inadequate; creating gaps in student preparation and confidence in end-of-life care. To address this, UNECOM developed the Learning by Living: 48-hour Hospice Immersion in 2014, to create a unique immersive learning experience in EOL/PM care. The purpose of this analysis was to determine the changes in students' perspectives before and after the immersion and what learning/skills they plan to apply as a physician. UNECOM student volunteers were immersed for 48 hours in a hospice house to conduct ethnographic research through three phases: (1) Pre-Field Work; (2) Field Work; and (3) Post-Field Work. Twelve student journals from the 2018-2019 academic year were analyzed in five steps: (1) selected student journals read to identify themes; (2) journals re-read notating and selecting quotes; (3) code book created with agreed upon theme definitions (inter-rater reliability); (4) data coded into identified themes; and (5) final analysis conducted using NVivo Qualitative Software. Data analyses resulted in three key themes that reflected knowledge, skills, and attitudes to augment students' success in EOL: (1) preconceived notions of the hospice experience, (2) growth and development as a professional, and (3) person-centered care in the hospice environment. Quotes extracted from student journals highlighted impactful and unique experiences for developing medical students' confidence to work with terminal patients. Impactful immersive education such as this 48-Hour Hospice Immersion provides EOL and PM education in a clinical setting that benefits students and their future patients.

Abstract citation ID: igaf122.4052

**A MODEL FOR INTEGRATED BEHAVIORAL HEALTH TEAMS IN PRIMARY CARE TO SUPPORT COGNITIVE SCREENING FOR OLDER ADULTS**

Louisa Thompson[1], Stephanie Czech[2], Molly Lawrence[1], and Charles Eaton[1], *1. Alpert Medical School at Brown University, Providence, Rhode Island, United States, 2. Alpert Medical School at Brown University, Pawtucket, Rhode Island, United States*

Earlier detection of cognitive impairment among older adults is a growing clinical and public health priority, particularly as new interventions (both medical and behavioral) become available to manage mild cognitive impairment (MCI) due to AD. However, cognitive screening tools remain under-utilized in primary care due to barriers such as time, training deficits, and insufficient supporting research. Integrated Behavioral Health (IBH) teams employed in primary care settings are uniquely positioned to support care for older adult patients with MCI or dementia and their families. However, these teams often receive inadequate and/or inconsistent training in cognitive screening, brain health education, and caregiver support for this population. Further, Community Health Workers on IBH teams are often not equipped to screen or engage patients in care. We will present findings from a pilot study examining the feasibility and acceptability of an IBH-integrated tablet-based cognitive screening protocol. First, we will describe the approach to promoting awareness, training, and workflows for PCPs and IBH clinicians implementing the protocol. Second, we will share feasibility and acceptability findings from protocol implementation. During a 3-month period, 4 participating PCPs attempted 11 warm handoffs to the IBH team for cognitive screening. Thus far handoffs have been most successful during Medicare Annual Wellness Visits (83.3% completion rate) as compared to other routine follow-ups (40% completion rate). Finally, we will share lessons learned from iterative protocol development, examples of training materials, and case studies of successful and unsuccessful warm handoffs.

Abstract citation ID: igaf122.4053

**A QUALITATIVE EXPLORATION OF COMMUNITY PERCEPTION ON DEMENTIA AND DEMENTIA CARE IN NEPAL**

Srijana Shrestha[1], Grace DiBacco[2], Jene Shrestha[3], Rekha Khatri[4], Bibhav Acharya[5], and Sabitri Sapkota[3], *1. Wheaton College, Norton, Massachusetts, United States, 2. University at Albany (SUNY), Albany, New York, United States, 3. Possible Nepal, Kathmandu, Bagmati, Nepal, 4. University of Sydney, Sydney, New South Wales, Australia, 5. University of California, San Francisco, San Francisco, California, United States*

Around 2/3rd of those with dementia live in low- and middle-income countries (LMICs), which face the greatest burden due to limited financial resources and underdeveloped healthcare systems and weak social care networks. However, research on supporting people with dementia (PwD) in these settings is sparse. Our exploratory qualitative study examined dementia care through the perspectives of family caregivers, community health workers (CHWs), primary healthcare providers, and a key informant from the government in Nepal. We conducted focus groups with CHWs and individual in-depth interviews with other study participants. Interviews lasted 25-90 minutes and all audio tapes were transcribed and analyzed using thematic analysis. Study findings showed that the family caregivers and CHWs often attributed memory loss to normal aging, and weren't aware of dementia as a health condition with available resources to help people with dementia and their caregivers but were eager to learn. All interviewees highlighted caregiver burden and the need for caregiver support, especially within the cultural context that expects family members to care for their seniors. Finally, study participants noted the need for institutional support noting the lack of dementia care training for health care providers, need for dementia care capacity building, and an urgent need for government programs and additional support for PwD and their caregivers. Finally, a unique perspective on cultural and community-based care was emphasized. Our study underscores several key challenges and necessary actions to improve dementia care in Nepal.

Abstract citation ID: igaf122.4054

**A REDOX-SENSITIVE JNK-CHOP SIGNALING AXIS DRIVES RUNNING STRESS-INDUCED MTUPR ACTIVATION IN AGED SKELETAL MUSCLE**

Grant Laskin, Baylah Mazonson, Yuhoung Kim, Laura Verdi, and Ladora Thompson, *Boston University, Boston, Massachusetts, United States*

Mitochondrial dysfunction and impaired proteostasis are aging hallmarks. The mitochondrial unfolded protein response (mtUPR) is a transcriptional program that restores mitochondrial integrity following proteotoxic stress, yet its regulation during physiological stress in aged muscle remains poorly defined. Here, we combine in vivo and in vitro approaches to investigate mtUPR regulation in aged skeletal muscle. Young (4-mo) and older (22-24-mo) C57BL/6 mice of both sexes either remained sedentary or performed three consecutive sessions of treadmill running to exhaustion as a physiological mitochondrial stressor. Running induced the putative mtUPR chaperones mtHsp70 in skeletal muscle of older mice and Hsp60 only in older males. mtUPR activation coincided with age-emergent indices of impaired mitochondrial proteostasis, most pronounced in males. Aged muscle exhibited modest carbonylation localized at intermyofibrillar mitochondria and running stress elevated 8-OHdG levels in males, suggesting greater oxidative burden in older males. To assess the role of mitochondrial reactive oxygen species (mtROS) in mtUPR activation, we induced mitochondrial matrix protein misfolding in C2C12 myotubes with or without an mtROS scavenger or stimulator. mtROS modulation amplified (stimulator) or suppressed (scavenger) the mtUPR during proteotoxic stress, but altering mtROS alone was insufficient to adjust mtUPR activation. Pathway screening revealed redox-sensitive activation of JNK-CHOP signaling, consistent with in vivo observations. Adenoviral-mediated shRNA CHOP knockdown and pharmacological JNK inhibition confirmed this axis as an mtROS-sensitive driver of the mtUPR. These findings support a model in which proteostatic and oxidative burdens in aged muscle mitochondria, especially in males, potentiate physiological stress-induced mtUPR activation via a JNK-CHOP-dependent mechanism.

Abstract citation ID: igaf122.4055

### "IT SEEMS TO HAVE DRAWN US CLOSER": PARTICIPANT REFLECTIONS ON DIGITAL ADVANCE CARE PLANNING

Sharon Bigger[1], Jordana Clayton[2], Sara Bybee[2], Nancy Aruscavage[3], Eli Iacob[2], Rebecca Utz[2], and Kara Dassel[2], *1. University of Utah, Asheville, North Carolina, United States, 2. University of Utah, Salt Lake City, Utah, United States, 3. University of Utah College of Nursing, Salt Lake City, Utah, United States*

Persons living with dementia (PLWD) and their care partners (CP) experience improved outcomes when engaging in advance care planning (ACP). We explored participant reflections on a web-based intervention for ACP in dementia. A thematic analysis was conducted on open-text feedback data from N = 47 dyads (PLWD and CP). Two coders analyzed data independently, resolved discrepancies, and collaborated with the research team to determine themes. The sample was mostly female (54%), white (86%), and aged 26 to 93. Among care recipients, 67% had mild cognitive impairment or dementia; the remainder reported early warning signs of Alzheimer's disease. Three themes included: (1) Dynamic nature of ACP in dementia, (2) Cognitive issues, and (3) Emotional issues. Findings revealed that dyads (PLWD and CP) used the web-based intervention for ongoing, dynamic ACP despite cognitive issues and emotional issues. For example, despite PLWD "needing questions clarified" and having "frustration" and "overwhelm," CPs stated, "It seems to have drawn us closer," "These are important and necessary plans to make, but it is sad to go through this process," and "Even though (we) have already completed our directives…this…helped to have additional considerations for our end-of-life plan…" These findings support a Delphi expert panel definition of ACP in dementia as a continued process with family over time that is adapted to the PLWD's capacity. We add that ACP remains beneficial even in the face of cognitive and emotional challenges. Researchers and clinicians should acknowledge cognitive and emotional issues for persons engaging in dynamic ACP in dementia.

Abstract citation ID: igaf122.4056

### ACADEMIC RETIREMENT: A SYSTEMATIC REVIEW OF THE LITERATURE ON RETIREMENT PLANNING AMONG UNIVERSITY FACULTY

Michelle Silver, *University of Toronto, Toronto, Ontario, Canada*

The overarching goal of this study is to inform institutional policy, support systems, and future research focused on enhancing retirement transitions within academia. In this scoping review we: (1) explore key factors influencing the retirement decisions of higher education faculty, including common pathways and barriers faced by faculty; (2) synthesize prominent study designs and findings in this area; and (3) evaluate the current state of the literature. A scoping review was conducted to address the outlined objectives. Relevant studies were identified through comprehensive searches across multiple academic databases using terms related to retirement decisions among university faculty. Four independent reviewers screened the literature and extracted key data using a standardized process to ensure rigor and consistency in study selection and synthesis. A total of 74 studies met the inclusion criteria. Most utilized cross-sectional survey designs, with qualitative interviews also commonly employed. Frequently examined variables included chronological age, partner or spousal retirement timing, health status, institutional policies, and professional identity. Geographical focus and academic subfields varied across the literature, revealing gaps in cross-contextual understanding. A concentration on individual and organizational factors was noted, with limited longitudinal research capturing transitions over time. This review builds on prior work by highlighting the varied nature of retirement among academic professionals, who often work in environments characterized by high autonomy. Understanding these retirement processes is critical for designing policies and practices that support career satisfaction and institutional knowledge retention. The findings have implications for retirement education and the development of inclusive retirement policies.

Abstract citation ID: igaf122.4057

### ACCELERATED PHENOTYPIC AGING IN MIDLIFE IS ASSOCIATED WITH LONG-TERM RISK OF NEURODEGENERATION

Huan Li[1], Mary Ryan Baumann[1], Jessica Malta[1], Lindsay Clark[1], Sterling Johnson[1], James Pankow[2], Art Walaszek[1], and Natascha Merten[1], *1. University of Wisconsin-Madison, Madison, Wisconsin, United States, 2. University of Minnesota, Minneapolis, Minnesota, United States*

PhenoAge is a multi-system aging measure based on easily-obtained clinical blood tests. It has been associated with increased risk of disability, age-related morbidities and mortality. Accelerated PhenoAge (PhenoAgeAccel) measures whether an individual is biologically/physiologically aging faster or slower than expected by their chronological age. It is unclear whether PhenoAgeAccel could also be a marker for increased risk of neurodegeneration. Neurofilament light chain (NfL) is a biomarker for general neurodegeneration, measurable in blood. In this study, we aimed to determine whether PhenoAgeAccel is associated with long-term changes in blood-based NfL levels from mid to later life. This longitudinal study is based on N = 1,508 (mean age=49 years, 54% women) Beaver Dam Offspring Study participants. We measured blood-based clinical markers necessary for calculating PhenoAgeAccel at baseline and serum NfL levels at baseline, 5-year and 10-year follow-up. We used linear mixed-effects models with PhenoAgeAccel as predictor and log NfL levels as outcome, adjusting for random intercept, study wave, sex, education, body mass index and kidney diseases. PhenoAgeAccel was not significantly associated with baseline NfL. With every additional year older in PhenoAge compared to chronological age (PhenoAgeAccel) at baseline, participants had faster NfL increases over 10 years [overall: 0.80% faster, 95% Confidence Interval(0.36%,1.25%); women:0.45%(-0.09%,1.00%); men:1.32%(0.57%,2.07%)]. Accelerated PhenoAge in midlife was associated with 10-year increases in NfL, overall and in men. Longer follow-up will be needed to determine whether PhenoAge is predictive of neurodegeneration later in life. PhenoAge could become a potential cost-effective measure to help identify individuals at risk of neurodegeneration and inform early intervention.

Abstract citation ID: igaf122.4058

### ADAPTING ENHANCE®FITNESS FOR PEOPLE LIVING WITH DEMENTIA AND THEIR CARE PARTNERS THROUGH A RE-AIM LENS

Sarah McKiddy, Wenting Peng, and Basia Belza, *University of Washington, Seattle, Washington, United States*

Multidomain lifestyle trials demonstrate that combined behavior change can support cognitive health. The FINGER demonstrated that a two-year multidomain lifestyle intervention helped maintain or improve cognition compared to general health advice in older adults who had elevated dementia risk factors. Preliminary findings from the U.S. POINTER trial similarly suggests that a multidomain approach led to greater cognitive benefits than self-guided health promotion among adults at elevated dementia risk. Despite these promising studies, widely disseminated community-based exercise programs have rarely been adapted for, or rigorously tested with, people living with dementia (PLWD) and their care partners. Enhance®Fitness (EF) – a long-standing, evidence-based, low-cost group exercise and falls prevention program with broad adoption across community and health system settings in the U.S. – improves physical performance and demonstrates strong participant retention. Remotely delivered EF has proven feasible for older adults with arthritis, and a pragmatic trial is comparing in-person and tele-EF. Yet many EF studies have excluded participants with cognitive impairment and no EF study examined the effectiveness of improving cognitive function, limiting relevance to PLWD. This presentation explores considerations for tailoring EF to support PLWD and their care partners. Embedding EF in dementia prevention trials would allow evaluation of outcomes meaningful to PLWD and families, including social engagement and caregiver support. The RE-AIM framework guides our evaluation to identify facilitators and barriers specific to PLWD and families. EF's national infrastructure and interdisciplinary research base offers a robust pathway to extend dementia-friendly lifestyle interventions to community-based health promotion programs.

Abstract citation ID: igaf122.4059

### ADAPTIVE FAMILY RESPONSES TO DEMENTIA CARE: A FOUR-YEAR LONGITUDINAL STUDY IN HONG KONG

Jacky C. P. Choy[1], Gloria Hoi Yan Wong[2], and Cheng Shi[3], *1. The University of Hong Kong, Hong Kong, Hong Kong, China (People's Republic), 2. University of Reading, Reading, United Kingdom, 3. Lingnan University, Hong Kong, Hong Kong, China (People's Republic)*

Multiple informal caregivers are often involved in dementia caregiving, yet little is known about how families collectively respond to care-related changes over time. Guided by family stress theories, this longitudinal study aimed to 1) examine changes in care demands and use of resources, and 2) identify predictors of adaptive family responses. Participants were 90 informal caregivers (22% spouses; mean age = 55.8 ± 12.4 years) of community-dwelling PLwD in Hong Kong. Data were collected via telephone interviews in 2021 and 2025, and analyzed using repeated measures and multiple regression. Over four years, PLwD showed significant declines in ADL, IADL, and health-related quality of life (QoL) (all p<.001). While the number of informal caregivers remained stable, there were increases in the use of foreign domestic helpers (38% to 52%), formal care services (39% to 62%), and informal care time (512 to 729 hours/month). Caregivers reported increased care burden (p<.001) and reduced health-related QoL (p=.005). After adjusting for functional decline, predictors of involving more informal caregivers were fewer members participating in care-related decision making (B=-0.716, p<.001) and higher care burden (B = 0.043, p=.007). Predictors of increased or sustained use of social services included fewer decision-making caregivers (OR = 0.341, p=.036), better relationship quality with PLwD (OR = 2.13, p=.048), and higher expectations for future caregiving needs (OR = 1.16, p=.028). Our findings suggest that families with enmeshed dynamics and misaligned care expectations may be at higher risk for maladaptive functioning, highlighting the need for early identification and targeted support programs in shaping adaptive responses to dementia care over time.

Abstract citation ID: igaf122.4060

### ADOPTING OR AVOIDING? OLDER ADULTS' RIDE-HAILING PERCEPTIONS AND EXPERIENCES

Paula Carder, and Minju Song, *Portland State University, Portland, Oregon, United States*

This qualitative study explored how older adults perceive ride-hailing services and what factors influence their willingness to use them. Informed by the "accessibility framework," we explored how individual, transport, land-use, temporal, and social-context factors influence older adults' perceptions of and decisions about adopting services like Lyft and Uber. We conducted semi-structured interviews with 18 older adults (ages 66-83) in the Portland metropolitan region. Participants included 6 men and 12 women; 8 had never used ride-hailing, while 10 had done so at least once. Most identified as non-Hispanic White, with two Korean immigrants and one Chinese American. Interview questions included ride-hailing experiences and thoughts in terms of geographical, physical/psychological, and technological accessibility, as well as their transportation needs and the relationship between ride-hailing and public transit. All interviews were audio-recorded, transcribed, loaded into Atlas.ti software, and coded to identify common themes and positive and negative perspectives regarding ride-hailing. Participants' perceptions and prior experiences informed decisions about ride-hailing use. Primary themes included concerns about safety at different times of the day, relative affordability, relative affordability compared to public transit or owning a vehicle, and difficulties with ride-hailing app usability and smartphone access. Participants had suggestions for making ride-hailing services more accessible to older adults and people with disabilities, and viewed ride-hailing as a complement or substitute to other transportation modes. Findings suggest that even when ride-hailing services are available, older adults may not adopt services if they do not perceive them as safe, affordable, easy to use, or physically accommodating.

Abstract citation ID: igaf122.4061

### ADVANCING DEMENTIA RESEARCH IN CANADA: EARLY INSIGHTS FROM A SCOPING REVIEW OF REVIEWS

Juanita-Dawne Bacsu[1], Kiana Mero[1], Alixe Menard[2], Megan E. O'Connell[3], Jennifer Bethell[4], Sarah Fraser[2], Sheila Blackstock[1], and Wendy Hulko[1], *1. Thompson Rivers University, Kamloops, British Columbia, Canada, 2. University of Ottawa, Ottawa, Ontario, Canada, 3. University of*

*Saskatchewan, Saskatoon, Saskatchewan, Canada, 4. University Health Network, Toronto, Ontario, Canada*

Recently, a national dementia strategy was developed to advance the field of brain health and dementia in Canada. However, there is a paucity of information on the current research landscape and evidence available to advance this strategy. Drawing on findings from our recent scoping review of reviews, this late breaking presentation aims to: i) identify the landscape of Canadian-led reviews on brain health and dementia research; and 2) recognize knowledge gaps and areas of innovation to strengthen brain health and dementia research, policy, and practice. Drawing on Arksey and O'Malley's framework, a comprehensive scoping review protocol was developed which guided our search of five databases (CINAHL, PubMed, PsycINFO, Scopus, and Web of Science). The literature was independently screened by two reviewers, extracted into a standardized table, and thematically analyzed. We found a total of 7860 initial articles, with 275 reviews included in our analysis. Findings were identified in three areas: 1) risk reduction strategies related to modifiable risks, lifestyle choices, and pharmacological factors; 2) enhancing therapies and finding a cure ranging from neuroimaging techniques to biomedical research; and 3) enhancing the quality of life of people living with dementia and care partners using a range of strategies to improve mental, social, and physical aspects. Although extensive reviews exist, significant knowledge gaps remain driven by factors such as limited participant diversity, inconsistent measures, and lack of targeted coordination, among other challenges. Findings from this study reveal vital gaps and research priorities that are essential to advancing Canada's dementia strategy forward.

Abstract citation ID: igaf122.4062

### ADVERSE CHILDHOOD EXPERIENCES ARE ASSOCIATED WITH DEVELOPMENT OF PSYCHOLOGICAL RESILIENCE AMONG OLDER ADULTS

Chenguang Du[1], Laura Sands[2], Yan Yuan[1], Yuewen Zhang[1], Caiwen Li[1], and Benjamin Katz[2], *1. Northwest Normal University, Lanzhou, Gansu, China (People's Republic), 2. Virginia Tech, Blacksburg, Virginia, United States*

Background: A growing body of research suggests that adverse childhood experiences (ACE; e.g. physical abuse) are associated with mental and physical health in young and middle adulthood. However, less is known about how ACEs are linked to psychological resilience in older adults. This study explored the long-term impact of childhood adversity on psychological resilience (PR) trajectories among older adults, drawing on the framework of Cumulative Advantage and Disadvantage (CAD) theory. Method: Data include 9,069 respondents aged 65 and above from 2006 to 2020 of the Health and Retirement Study (HRS). We first employed the group-based trajectory modeling to classify heterogeneity in PR's development over time. Next, a multinomial logistic regression was conducted using four different ACE items including: school retention, physical abuse, parental substance abuse, and trouble with police as the main predictors, with covariates (e.g. age, gender, marital status, and education) controlled in the model. Result: The analyses revealed 3 distinct trajectory groups: one group with stable resilience, and two groups with either "sharp" decline or "gradual" decline. In addition, after adjusting for covariates, we found that of the four ACE items, only experiences of physical abuse and parental substance abuse were significantly associated with the reduced likelihood of maintaining a stable trajectory on PR. Conclusion: These findings suggest that early adverse experiences may influence trajectories of psychological resilience well into old age. The results underscore the importance of adopting trauma-informed approaches in resilience-based intervention, emphasizing early identification and intervention for individuals with histories of childhood maltreatment.

Abstract citation ID: igaf122.4063

### AGE AND SEX DIFFERENCES IN DEHYDROEPIANDROSTERONE SULFATE AMONG PEOPLE WITH DEMENTIA AND CAREGIVERS

Wanrui Wei[1], Töres Theorell[2], Gabriella Engstrom[3], and Azita Emami[1], *1. Yale University, Orange, Connecticut, United States, 2. Karolinska Institutet, Stockholm, Stockholms Lan, Sweden, 3. Florida Atlantic University, Boca Raton, Florida, United States*

Dehydroepiandrosterone sulfate (DHEAS) is a key adrenal hormone with neuroprotective and anti-glucocorticoid properties, yet little is known about its role in dementia and caregiving contexts. This study compared basal DHEAS levels and awakening responses between people with dementia (PWD) and their family caregivers, while examining age- and sex-specific patterns. Salivary samples were collected at awakening and 30 minutes post-awakening. Analyses included independent group comparisons, sex- and age-stratified analyses (< 65, 65–75, >75 years), and interaction modeling. Results showed that among females, PWD exhibited significantly higher basal DHEAS than caregivers after age 65, while in males, a crossover pattern emerged: PWD demonstrated higher levels before age 75, whereas caregivers showed slightly higher levels thereafter. Awakening response differences were most pronounced in younger males, with PWD displaying markedly lower responses relative to caregivers. These findings highlight distinct age- and sex-specific trajectories of DHEAS regulation in dementia. In conclusion, salivary DHEAS profiles differ between PWD and caregivers, with sex- and age-related variations. DHEAS may serve as a noninvasive biomarker of neuroendocrine function, providing insights into stress regulation and potential intervention targets for dementia care.

Abstract citation ID: igaf122.4064

### AGENCY AND COMMUNION IN NARRATIVES OF OLDER ADULTS WITH AND WITHOUT MILD COGNITIVE IMPAIRMENT

Xiangyu (Annie) Pei[1], Emily Tso[1], Gelila Ambellu[1], Da Eun Kim[2], Hsiao-Wen Liao[2], and Emily Mroz[1], *1. Emory University, Atlanta, Georgia, United States, 2. Georgia Institute of Technology, Atlanta, Georgia, United States*

Older adults with mild cognitive impairment (MCI) tend to worry about losing their memories and sense of identity, putting them at risk of reduced wellbeing. The retrieval of autobiographical memories (AM) about positive life events predicts greater life satisfaction and positive self-conceptions of future selves in adults. Therefore, autobiographical memory interventions for people with cognitive change have grown in popularity. However, much existing research about AM has focused on dementia rather than MCI, and technology-assisted memory sharing remains empirically underexplored. This study examined agency and communion – two features linked to wellbeing in late life – in AM elicited from adults with MCI ($n = 17$) and those without MCI ($n = 18$). We examined whether technology-enhanced memory sharing (i.e., photo memory viewing in virtual reality (VR) versus positive memory outside VR) influences narration of agency and communion. Transcripts were coded by two coders (kappa = 1.0). Results demonstrated that communion and agency appeared across both groups and conditions. Agency did not differ significantly between both groups and conditions. Communion in non-VR assisted positive memories was higher among adults without MCI (M = 1.00) than those with MCI (M = .29; p = .034). However, both groups included similar amounts of communion in VR-assisted photo memories. During this presentation, we will discuss our protocols and preliminary takeaways, including that the VR-assisted photo memory task may support people with MCI in sharing more communal narratives. Further research can explore how VR environments promote rich memory sharing in different older adult populations.

Abstract citation ID: igaf122.4065

### AGE-RELATED STRESS IN INDIVIDUALS WITH A SUBSTANCE USE HISTORY

Suruchi Ghaiye[1], Ge Wang[2], Daniel Li[3], Runze Ma[1], and Wei Li[4], *1. University of Alabama at Birmingham, Birmingham, Alabama, United States, 2. Huazhong University of Science and Technology, WuHan, Hubei, China (People's Republic), 3. SUNY, New York, New York, United States, 4. UAB, Birmingham, Alabama, United States*

Substance use recovery is a multifaceted process influenced by psychological, physiological, and social factors. Stress remains a critical barrier to sustained recovery, particularly among elderly individuals who may face compounded challenges due to age-related vulnerabilities. Understanding age-related stress in recovery populations can inform targeted interventions and improve program efficacy. Demographic information and vital signs were collected from participants aged from 20 to 80 years old. Stress was assessed using a standardized questionnaire. Correlation analyses was done between demographic factors or vital signs and stress scores using the SPSS. $p < 0.05$ was considered significant. Age was positively correlated with systolic blood pressure ($r = 0.235$, $p = 0.03$) and diastolic blood pressure ($r = 0.258$, $p = 0.017$), indicating higher blood pressure levels in older participants. Conversely, heart rate showed a negative correlation with age ($r = -0.216$, $p = 0.046$). Psychological stress negatively correlated with age and older individuals reported lower levels of perceived stress as indicated by the following negative correlations: 1. feeling nervous or stressed (r=-0.369, $p < 0.001$); 2. feeling unable to control important things (r=-0.256, $p = 0.017$); 3. perceived helplessness (r=-0.286, $p = 0.008$); Total stress score (r=-0.25, $p = 0.02$). In conclusion, age is a significant factor influencing stress in individual enrolled in a local substance abuse recovery program. These findings underscore the need for age-sensitive approaches in substance use recovery services and highlight the importance of continuous psycho-social support.

Abstract citation ID: igaf122.4066

### AIR POLLUTION AND CARDIOVASCULAR RISK FACTORS IN OLDER ADULTS: EVIDENCE FROM THE LOOKUP 8+ PROJECT

Matteo Tosato[1], Riccardo Calvani[2], Stefano Cacciatore[3], Emanuele Marzetti[2], and Francesco Landi[2], *1. Fondazione Policlinico Universitario Agostino Gemelli IRCCS, Rome, Lazio, Italy, 2. Università Cattolica del Sacro Cuore, Rome, Lazio, Italy, 3. Fondazione Policlinico Universitario Gemelli IRCCS, Rome, Lazio, Italy*

Air pollution is increasingly recognized as an important determinant of cardiovascular health, yet evidence in older adults remains limited. We analyzed data from 6077 community-dwelling participants of the Longevity Check-up (Lookup) 8+ cohort across 22 Italian cities spanning mainland Italy and major islands to examine the association between PM2.5 exposure and clinical risk factors. Annual mean PM2.5 concentrations were estimated by integrating ground monitoring station and satellite-based data. Exposure was calculated at the municipality level and restricted to participants residing in urban areas. Participants were stratified by exposure above or below the regulatory threshold of 25 μg/m³. The study sample had a mean age of 70.4 years (53.6% women, BMI 26.0 kg/m²). Of these, 1538 (25.3%) were exposed to PM2.5 ≥25 μg/m³. Compared with those below threshold, highly exposed participants showed significantly higher systolic blood pressure (134.7 vs 128.1 mmHg; +6.5 mmHg, $p < 0.001$), diastolic pressure (77.6 vs 75.6 mmHg; +2 mmHg; $p < 0.001$), and blood cholesterol (213.7 vs 199.8 mg/dL; +13.9 mg/dL; $p < 0.001$). Linear regression analysis confirmed a quantitative dose-response with each 1 μg/m³ increase in PM2.5 exposure being associated with +0.45 mmHg systolic blood pressure and +1.2 mg/dL cholesterol. Associations remained statistically significant after adjustment for major confounders including age, sex, BMI, smoking status, diabetes, and physical activity. These findings provide evidence linking PM2.5 exposure to adverse cardiovascular profiles in older adults and highlight the importance of considering environmental determinants in geriatric healthcare.

Abstract citation ID: igaf122.4067

### ALCOHOL USE TRAJECTORIES ACROSS 24 YEARS AND FUTURE DEMENTIA RISK: EVIDENCE FROM THE HEALTH AND RETIREMENT STUDY

Alonzo Mendoza[1], Carlos Araujo-Menendez[2], Armando Lemus[1], and Ariana Stickel[1], *1. San Diego State University, San Diego, California, United States, 2. San Diego State University/University of California, San Diego, San Diego, California, United States*

We aimed to determine alcohol use trajectories and their relation to future dementia risk. Participants included 3,488 adults from the Health and Retirement Study (HRS; waves 1992-2016) who underwent a dementia evaluation (cognitively intact, mild cognitive impairment, and dementia) as part of the 2016 Harmonized Cognitive Assessment Protocol (HCAP) and self-reported alcohol consumption during at least two study visits. Alcohol consumption composite scores (number of days drinking, drinks per day, and number of binges in the past 3 months) were used to model trajectories using latent class mixed models (LCMM). The number of latent classes was determined based on fit indices (AIC, BIC, log-likelihood, entropy) and the interpretability of trajectories. Multinomial logistic regression evaluated the associations between LCMM-derived alcohol trajectories and future dementia diagnosis, while controlling for demographic characteristics (age at diagnosis, race, ethnicity, and education). The 3-class model fit best. Classes were distinguished by initial alcohol use, and all classes decreased alcohol consumption over time but to varying degrees ("high initial use with declines that then plateau", "very high initial use with later decreased use", and "average users who decreased more with time”). Significant alcohol trajectories by age interactions emerged: Compared to average users, the “high initial users” (OR = 1.03, 95% CI [1.01 - 1.04) and “very high initial users” (OR = 1.39, 95% CI [1.36 - 1.42]) groups showed increased dementia risk with increasing age. No other results were significant. Our findings suggest that early alcohol interventions may be most beneficial in reducing future dementia risk.

Abstract citation ID: igaf122.4068

### ARTIFICIAL INTELLIGENCE IN HOSPICE DEMENTIA CARE: PERSPECTIVES OF HOSPICE STAFF AND FAMILY CAREGIVERS

Oonjee Oh, and George Demiris, *University of Pennsylvania, Philadelphia, Pennsylvania, United States*

Artificial intelligence (AI) has gained attention as a tool to support dementia care through efficient and personalized interventions. However, individuals situated at the intersection of dementia and end-of-life care are particularly vulnerable due to severe conditions and lack of decision-making capacities. Yet, there is limited research on AI acceptability in this specific context. Hence, we aimed to examine the perceptions of family caregivers and hospice staff regarding AI integration in end-of-life care settings for persons with dementia. We adopted a qualitative descriptive design with 19 participants (10 dementia caregivers in hospice and 9 hospice staff members). Dementia caregivers were interviewed individually and staff, including nurses, social workers, bereavement counselors, and a music therapist, participated in 3 focus group interviews. Transcripts were analyzed using a directed content analysis approach; the Technology Acceptance Model served as the guiding framework. We explored themes of “perceived usefulness,” “perceived ease of use,” and the inductive theme of “guiding the new wave of the future.” Caregivers and staff identified strengths and concerns of using AI in hospice dementia care. Example strengths included providing comprehensible informational support to the families and assisting with monitoring symptoms at home. Concerns included the perceived absence of human touch and warmth in AI, which may create resistance among families to adopt AI, and its potential lack of nuanced understanding of dementia-related behaviors. Staff also highlighted critical considerations when designing and deploying AI for this population, such as recognizing the heterogeneity in dementia and integrating the voices of those providing direct care.

Abstract citation ID: igaf122.4069

### ASSESSING THE EFFICACY OF DELIVERING HYBRID DEMENTIA CARE IN A RESOURCE SCARCE RURAL COMMUNITY

Vi Belanger[1], Chris Beaulieu[2], Lenard Kaye[3], and Rachel Coleman[4], *1. Aroostook Area Agency on Aging, Presque Isle, Maine, United States, 2. Aroostook Area Agency on Aging, Presque Isle, Me 04769, Maine, United States, 3. University of Maine, orono, Maine, United States, 4. University of Maine Center on Aging, Orono, Maine, United States*

Older adult cognitive health services, especially dementia care, has been an especially scarce resource in rural communities. The Aroostook Memory Care Center (AMCC), in northern Maine, is testing the efficacy of delivering a hybrid combination of on-site and remote dementia care based at an Area Agency of Aging. AMCC is located in Aroostook County, the largest county east of the Mississippi River (6,700 sq. mi.) with all 24 census tracts designated rural, and the closest dementia specialty practice 150+ miles away. A 3-year ACL grant has enabled delivery of services for persons with dementia (PWD), support for caregivers, and community education. A process protocol for referrals and intake, care coordination and planning, interdisciplinary case reviews, remote specialty geriatrician neuropsychology assessments, and connections to AAA and other community services has now been established and successfully implemented. A Memory Center flow pathway protocol has been constructed and has been adhered to over the life of the program. Over a 15-month period, 282 referrals, 51 on-line referrals to specialty dementia care were made and 206 PWDs and 149 caregivers received services. Pre- and post-measures of the result of on-site and remote training and clinical interventions (changes in levels of understanding Alzheimer’s and dementia; levels of patient and caregiver preparedness because of expert consultation; and increases in caregiver quality of life) confirm significant participant gains. Strategies for sustaining a hybrid dementia care initiative in rural communities are offered focused on creating operational efficiencies, developing a reimbursement model, and tapping sources of philanthropy.

Abstract citation ID: igaf122.4070

### ASSESSING THE FEASIBILITY OF MUSIC THERAPY DURING EXERCISE IN CARDIAC REHABILITATION: A MIXED-METHODS ANALYSIS

Ryan Conard[1], and Wen-Chih Wu[2], *1. The Warren Alpert Medical School of Brown University, Providence, Rhode Island, United States, 2. Brown University Health, Providence, Rhode Island, United States*

Cardiac rehabilitation (CR) is an evidence-based secondary prevention strategy following cardiovascular events. However, appointment adherence is an ongoing challenge, often limited by psychosocial barriers. Music therapy has shown promise in improving emotional well-being, and enhancing exercise endurance while lowering perceived exertion, which could improve experience of CR sessions. This quality improvement pilot project evaluated the feasibility of offering personalized music listening during exercise sessions as a novel and underexplored approach to increasing participation in CR. Over an eight-week period, all patients in a 12-week CR program were offered a personalized music intervention during supervised exercise three times a week. Participants could opt in or out at each session. Playlists were generated using participant preferences and offered via MP3 players or personal devices. Uptake, adherence, and qualitative feedback were tracked across sessions. 160 people participated (65.6% male, mean age 67.7±12.1 years, 80.6% white). 58.4% agreed to listen to music (uptake). Of these, 97.5% continued to adhere to the music intervention across their remaining sessions. An average of 61.5% of CR participants used music daily. Qualitatively, people reported highly enjoying the intervention, characterizing the music as energizing, motivating, and relaxing. Many reported that music motivated them to stay longer each session or return more often. The high uptake and adherence of participants, positive qualitative feedback, minimal disruption to existing clinic operations, and low cost ($21 per MP3 player) illustrate the strong feasibility and suggest that personalized music during exercise may be a scalable strategy to enhance CR engagement and warrant further study.

Abstract citation ID: igaf122.4071

### ASSESSMENT OF CAREGIVER STRESS ACROSS DEMENTIA AND NON-DEMENTIA GROUPS

Urooj Hudda[1], Ethan Bradford[1], Gohar Azhar[2], Regina Gibson[1], Madeline Wood[1], Debbie Brady[1], and Jeanne Wei[2], *1. University of Arkansas for Medical Sciences, Little Rock, Arkansas, United States, 2. University of Arkansas for Medical Sciences, Little Rock, AR, LITTLE ROCK, Arkansas, United States*

As the U.S population ages, informal caregivers have become essential to supporting older adults. This study aims to explore the emotional, financial, and health-related stressors and challenges experienced by caregivers attending to patients living with dementia compared to those without dementia. An anonymous, cross-sectional, convenience sample survey exploring caregiver burden was administered to 114 primary caregivers. A factor analysis of 10 items related to caregiver stress with polychoric correlations was conducted and factor scores were calculated to serve as an index of caregiver stress. Missing data were handled using multiple imputation. Linear regression was conducted using factor scores to examine the relationship between caregiver burden and being a dementia caregiver as well as the number of caregivers. 37 participants were dementia caregivers and 77 were non-dementia caregivers. Most participants were over the age of 60 (59.5%). 37.2% of dementia caregivers responded that they had a stress level of "severe" or higher compared to 21.4% of non-dementia caregivers. 13.9% of dementia caregivers "often" or "always" had trouble paying their bills in the last year, compared to 5.2% of non-dementia caregivers. A linear regression concluded that dementia caregivers had significantly higher burden than those without ($p = 0.040$). Receiving help from other caregivers significantly reduced caregiver burden ($p = 0.016$). Caregivers of patients who are living with dementia experience significantly greater stress, which might be mitigated through shared responsibilities. Addressing stressors of caregivers is critical not only for their wellbeing but can also impact the health outcomes of patients and potentially delay or prevent institutionalization.

Abstract citation ID: igaf122.4072

### ASSOCIATION OF PAIN, INSOMNIA, AND DEPRESSION CLUSTERS WITH COGNITION IN OLDER MEXICAN AMERICANS

Utpol Das[1], Matthew Phan[1], Tyler Bell[2], Mukaila Raji[1], and Sadaf Milani[1], *1. University of Texas Medical Branch, Galveston, Texas, United States, 2. University of California, San Diego, San Diego, California, United States*

Older Mexican Americans—a rapidly growing segment of the U.S. population—experience high rates of co-occurring pain, depression, and insomnia, but data on how these symptom clusters affect their cognition remain limited. We examined patterns of pain-insomnia-depressive symptom clusters and their association with cognitive decline using data from the Hispanic Established Populations for the Epidemiologic Study of the Elderly (2010-2016), a study of older Mexican Americans in the Southwestern United States. Latent class analysis identified clusters based on pain on weight-bearing, depressive symptoms, and insomnia symptoms. Linear mixed-effects models examined the association between different patterns of symptom clusters and cognitive decline, measured by the Mini-Mental State Examination. Three latent classes were identified ($n = 935$): pain only (66.5%), pain and elevated depressive symptoms, (21.0%), and pain, elevated depressive symptoms, and insomnia (12.5%). The pain and elevated depressive symptoms cluster ($\beta$=-3.72, $p < 0.001$) and the pain, depressive symptoms, and insomnia cluster ($\beta$=-2.33, $p < 0.001$) were associated with worse baseline cognition compared to the pain only cluster. Education was a significant effect modifier: in stratified analyses, similar findings were observed for those with 0 and 1-6 years of education. Among individuals with ≥7 years of education, those in the pain and elevated depressive symptoms cluster had greater cognitive decline over time compared to the pain only cluster ($\beta$=-0.65, $p = 0.039$). Our findings highlight the need for targeted interventions addressing co-occurring conditions to mitigate cognitive decline among older Mexican Americans. Education appears to modify this association, suggesting a role in cognitive reserve and warranting consideration in intervention design.

Abstract citation ID: igaf122.4073

### ASSOCIATIONS BETWEEN OBJECTIVE SLEEP MEASURES AND PROTEOMIC AGING: THE ARIC STUDY

Chunyu Liu[1], Sanaz Sedaghat[2], Anna Prizment[2], Shuo Wang[2], Keenan Walker[3], Pamela Lutsey[2], Brion Maher[1], and Adam Spira[1], *1. Johns Hopkins Bloomberg School of Public Health, Baltimore, Maryland, United States, 2. University of Minnesota, Minneapolis, Minnesota, United States, 3. National Institute on Aging, Baltimore, Maryland, United States*

Increasing evidence links poor sleep to accelerated biological aging; however, associations between sleep and proteomic age acceleration (PAC) remain understudied. We examined these associations among 1,554 participants (mean age 60.5±5.5 years, 99.0% White) in the Atherosclerosis Risk in Communities study (ARIC; waves 3 [1993-1995], 5 [2011-2013]) and the Sleep Heart Health Study (SHHS; waves 1 [1996]) who had polysomnography and proteomic data. Predictors were sleep-disordered breathing (SDB) and hypoxia, and sleep architecture, fragmentation, and duration (SHHS w1). Outcomes were proteomic age, derived from plasma (ARIC w3/w5; SomaScan v.4) using four published clocks (Wang, Sathyan, Tanaka, Lehallier). Linear regression assessed cross-sectional associations between sleep and PAC (ARIC w3); linear mixed models assessed associations between sleep and PAC longitudinal changes over time (ARIC w3 and w5). All models adjusted for age, race, education, study site, smoking, alcohol use, BMI, and sleep medication. Cross-sectionally, SDB (Wang: B = 0.59 [0.04, 1.14]; Sathyan: 0.91 [0.06, 1.76]), less REM sleep (Tanaka: -0.04 [-0.07, -0.01]; Lehallier: -0.03 [-0.06, -0.00] ), and lower sleep efficiency (Tanaka: -0.02 [-0.05, -0.00]) were associated with higher PAC. Longitudinally, lower REM oxyhemoglobin saturation (Sathyan: −0.27 [−0.45, −0.08]; Tanaka: −0.17 [−0.31, −0.03]), less slow-wave sleep (Tanaka: −0.03 [−0.05, −0.01]), higher arousal index (Tanaka: 0.03 [0.00, 0.05]), lower sleep efficiency (Wang: −0.05 [−0.09, −0.00]), and sleep > 8 h (Wang: 0.83 [0.05, 1.60]; Sathyan: 1.33 [0.35, 2.31]) were associated with increasing PAC (all $p < 0.05$). Findings suggest poor sleep accelerates proteomic aging, emphasizing sleep health as a target for aging-related disease prevention.

Abstract citation ID: igaf122.4074
### ASSOCIATIONS OF ADVERSE CHILDHOOD EXPERIENCES, SOCIAL FACTORS, AND MENTAL HEALTH IN ALL-CAUSE CAREGIVERS

Maizonne Fields, and Olivio Clay, *The University of Alabama at Birmingham, Birmingham, Alabama, United States*

The number of individuals needing assistance with personal care and household tasks from an informal caregiver is rising. Previous studies have also identified relationships between adverse childhood experiences (ACEs)—traumatic events related to violence, abuse, and/or exposure to mental health or substance use problems in others in the home— and mental health status. Using a life course perspective on aging, it is possible that experiencing could worsen mental health those that eventually served as a caregiver. This study uses data from the 2023 main wave of the Behavioral Risk Factor Surveillance System and includes all-cause caregivers. The study investigates the impact of living with someone that was depressed, mentally ill, or suicidal and being physically harmed (outside of spanking) more than once on the frequency of poor mental health days. Final analyses included 1180 participants that resided in either Oregon or Tennessee, 58.7% were females, and 37.4% being aged 65 and older. Linear multiple regression models revealed that living with someone that was depressed, mentally ill, or suicidal prior to age 18 (B=.174, p<.001) served as a better predictor of mental health than being physically harmed (outside of spanking) more than once (B=.094, p<.001). These models controlled for a series of demographic and social factors. Results suggest experiencing ACEs has the potential to worsen mental health in all-cause caregivers. These findings have implications for mental health providers and policymakers. Finally, results can inform researchers attempting to develop interventions for all-cause caregivers.

Abstract citation ID: igaf122.4075
### AVERAGE LIFETIME WORK EXPOSURES AND IMMUNE FUNCTION AMONG OLDER ADULTS

Rebekah Carpenter[1], Dawn Carr[1], and Grace Noppert[2], *1. Florida State University, Tallahassee, Florida, United States, 2. University of Michigan, Ann Arbor, Michigan, United States*

Overall immune health and functioning plays a critical role in the development of aging-related chronic conditions and diseases over the life course. Recent research shows that differences in immune function in later life are socially patterned and shaped by lifetime exposures. However, limited attention has been given to the potential impact of work-related factors on later-life immune health. Given that the average adult spends approximately one-third of their life working, occupational conditions may have long-term influences on immune function as people age over time. We use a new dataset that leverages occupation-level work exposures from the Occupational Information Network (O*NET), individual-level work history data, and individual immune function data from the Health and Retirement Study (HRS). Ordinary Least Square (OLS) regression models were assessed to evaluate associations between three domains of lifetime work exposures and eight indicators (e.g., Cytomegalovirus IgG antibody levels) of immune function after age 65. We find that we find that multiple stress- (e.g., job demands), physical- (e.g. frequency of bending and twisting the body), and environmental-related (e.g., exposure to contaminants) average lifetime work exposures are associated with later life immune health. We also find that the association between some work exposures (e.g., decision freedom) and immune function are dependent on sex, suggesting that for some occupational exposures, the same level of exposure influences immune function outcomes differently for men and women. Based on these findings, we determine that work exposures may be an important social determinant of immune function as people age over the life course

Abstract citation ID: igaf122.4076
### BENCHMARK STUDY OF 2D AND 3D GAIT ANALYSIS FOR AI-POWERED MOBILE FALL RISK SCREENERS

Zengyan Wang, Chen Chen, Chitra Banarjee, Rui Xie, and Ladda Thiamwong, *University of Central Florida, Orlando, Florida, United States*

Mobile health clinics are commonly used to assess health but rarely equipped to assess fall risk indicators such as gait impairment. To address this gap, we propose AI-driven mobile units integrating video-based gait analysis and inertial measurement units (IMUs) for rapid, low-cost fall risk screening. This study benchmarked eight 2D and 3D gait extraction models (OpenPose, AlphaPose, HRNet, MediaPipe, ViTPose, videopose, MotionBERT, MotionAGFormer) using publicly available videos of older adults completing the Timed Up and Go test (N = 28), which includes one front-facing camera view and another with a higher, top-down viewing angle. Models were compared in their computational, temporal, and spatial gait metrics (including gait speed, cadence, stride time and length). Results show that 2D methods, particularly HRNet, consistently outperformed other models in both successful recognition rate (HRNet 99% vs 88.3% averaged across all other models). HRNet delivered the most reliable ankle tracking and the fastest inference time, key advantages for resource-constrained environments like mobile units. By contrast, 3D models required more computational resources without improving gait recognition. Temporal metrics from the videos were comparable to IMU-derived metrics. However, Spatial alignment with IMU-derived pelvis trajectories failed across all models. While the 2-camera setup provides stable perspectives for gait analyses, it limits visibility of certain side-plane movements, reducing confidence in spatial parameters such as ankle flexion and knee angle. The superior performance of 2D models confirms the feasibility of deploying lightweight, AI-powered mobile systems for fall risk screening, especially in underserved areas with limited access to specialized geriatric care.

Abstract citation ID: igaf122.4077
### BENCHMARKING FOUNDATION MODELS FOR ALZHEIMER'S DISEASE AND RELATED DEMENTIA DETECTION FROM SPONTANEOUS SPEECH

Jingyu Li[1], Lingchao Mao[1], Hairong Wang[2], Zhendong Wang[3], Xi Mao[4], and Xuelei Ni[5], *1. Georgia Institute of Technology, Atlanta, Georgia, United States, 2. The University of Texas at Austin, Austin, Texas, United States, 3. Shandong Mental Health Center, Jinan, Shandong, China (People's Republic), 4. University of Texas Rio Grande Valley, Brownsville, Texas, United States, 5. Kennesaw State University, Kennesaw, Georgia, United States*

Alzheimer's disease and related dementias (ADRD) are progressive neurodegenerative conditions where early detection is critical for timely intervention and care planning. Acoustic biomarkers—such as changes in prosody, fluency, and pause patterns—can be extracted from spontaneous speech and offer a non-invasive avenue for early diagnosis. Foundational speech and language models, which are pre-trained deep learning models, can generate high-dimensional embeddings that capture rich contextual and acoustic information from raw audio or text. Using data from the PREPARE Phase 2 Challenge, which includes recordings from over 1,600 individuals, we examined the potential of foundation models for ADRD detection. Specifically, we benchmarked a range of open-source speech and language models on their ability to classify participants into different stages of cognitive decline. Among speech-based approaches, the Whisper-medium model achieved the highest performance (accuracy = 0.731; AUC = 0.802). Overall, state-of-the-art automatic speech recognition (ASR) models outperformed other models. Incorporating prosodic features such as pauses also improved classification in text-based approaches. Our work presents a comprehensive benchmarking framework built on state-of-the-art speech models and validated on a large, clinically relevant dataset, and demonstrates that foundation models can provide a scalable and cost-effective framework for early ADRD detection. Our findings highlight the promise of speech-derived embeddings as powerful non-invasive biomarkers for cognitive impairment. Future ADRD screening approaches should integrate both semantic and non-semantic features to improve generalizability and support broader clinical application.

Abstract citation ID: igaf122.4078

### BREAST CANCER SCREENING IN OLDER ADULTS LIVING WITH DEMENTIA: A SCOPING REVIEW

Eun-Hi Kong, *Gachon University, Incheon, Republic of Korea*

The utilization of breast cancer screening in older adults with dementia is debatable, challenging, and ethical issue. There has been a lack of evidence on breast cancer screening among older adults living with dementia. The purpose of this review to synthesize evidence of the literature regarding breast cancer screening in older adults living with dementia. Systematic literature search was conducted using electronic databases including MEDLINE, CINAHL, PsycINFO, EMBASE, and Google scholar from the earliest year to July, 2025. Search keywords included dementia, Alzheimer, cognitive impairment, cognitive impaired, cognitive dysfunction, breast cancer screening, mammography, and mammogram. Search results were uploaded and duplicates were removed using EndNote21. Both published studies and grey literature were included in this review. This study followed the PRISMA-ScR and the JBI guidance and for scoping review. Twelve studies were included in this review. Most studies were conducted in USA and used quantitative research methods. Many studies targeted older adults living with dementia and some studies targeted caregivers. The included studies had various focuses such as utilization rate, associated factor, comparison with elders without dementia, decision, benefit, harm, and cost effectiveness of breast cancer screening among older adults with dementia. Further studies are required to explore benefits, harms, decision-making, ethical issues, barriers, and facilitators of breast cancer screening among older adults living with dementia. More qualitative research and mixed methods research are needed. Future studies are needed to develop and evaluate clear and detailed guidelines on breast cancer screening in older adults living with dementia.

Abstract citation ID: igaf122.4079

### BRIDGING AGING AND DISABILITY: A REPLICABLE FRAMEWORK FOR INCLUSIVE POLICY DESIGN

Sonya Durham, Annie Rhodes, and Leland Waters, *Virginia Commonwealth University, Richmond, Virginia, United States*

States are now confronted with a critical policy gap, addressing the growing population of adults with intellectual and developmental disabilities (IDD). Aging services rarely account for the unique needs of these adults who face compounded health, economic, and social inequities. This project tackles this gap by adapting Missouri's Master Plan on Aging (MPA) for Virginia with an emphasis on the IDD population. This approach will be used to reshape aging policy to be more inclusive, evidence-based, and replicable across diverse state contexts. Purpose: The Virginia Adaptation Report evaluates which components of Missouri's MPA Needs Assessment can be transferred and which require modification, considering Virginia's demographics, service systems, and policy landscape. This work includes disability within aging policy, applying innovative aging theories and model-building approaches. Methods: An environmental scan and gap analysis identified existing aging and disability resources and policies across Virginia. A concept map was developed to visualize service silos, theoretical omissions, and potential integration points. Innovation theory, equity-based models, and systems-thinking guided decisions on what to retain, adapt, or eliminate from the Missouri framework. Findings and Recommendations: The adaptation revealed structural and theoretical gaps in Virginia's aging policy, particularly for aging individuals with IDD. Recommendations include implementing cross-sector data-sharing, integrating IDD-specific indicators into statewide aging surveys, expanding stakeholder engagement (including individuals with lived experience), and investing in inclusive training for service providers. By centering inclusive design and theory innovation, the resulting framework bridges traditional aging approaches with disability-inclusive strategies and provides a replicable, evidence-informed roadmap for other states.

Abstract citation ID: igaf122.4080

### BRIDGING THE GAP IN POST-FRACTURE CARE USING THE RE-AIM FRAMEWORK: INSIGHTS FROM THE OPTIONS STUDY

Gretchen Tucker[1], Veysel Baris[2], Min Jeoung Kang[3], Nancy Latham[4], Kumiko Schnock[4], Pamela Garabedian[5], Denise Orwig[6], and Patricia Dykes[7], *1. University of Maryland, Baltimore School of Medicine, Baltimore, Maryland, United States, 2. Dokuz Eylul University, Izmir, Izmir, Turkey, 3. Brigham and Womens Hospital, Boston, Massachusetts, United States, 4. Brigham and Women's Hospital, Boston, Massachusetts, United States, 5. Mass General Brigham, Inc., Somerville, Massachusetts, United States, 6. University of Maryland, Baltimore, Baltimore, Maryland, United States, 7. BWH/Harvard Medical School, Boston, Massachusetts, United States*

An estimated 10 million older Americans have osteoporosis, a leading risk factor for fragility fractures that often result in disability, diminished quality of life, and increased mortality. Despite availability of effective treatments, evidence-based post-fracture care remains underutilized. The OsteoPorotic fracTure preventION System (OPTIONS) is an integrated, multi-modal intervention designed to improve care for community-dwelling older adults following lower limb fractures who transition to skilled nursing facilities and back home. OPTIONS includes clinical decision support tools for providers, patients, and care partners to strengthen fracture prevention efforts across care transitions. In the pre-implementation phase, we used the RE-AIM (Reach, Effectiveness, Adoption, Implementation Maintenance) implementation science framework to explore current fracture care practices in skilled nursing facilities (e.g., exercise, nutrition, and bone health medications) and explored facilitators and barriers to intervention delivery and uptake. We conducted seven interdisciplinary focus groups with physical and occupational therapists, nurses, and dieticians using a semi-structured interview guide. Transcripts were coded and data were analyzed using content analysis. Barriers identified were patients' complex health conditions, staff turnover, and osteoporosis medication costs. Facilitators included strong communication among staff, access to resources, sufficient training, and training offered in multiple formats. Findings were used to inform development of a practical OPTIONS implementation toolkit, including training materials, workflow guides, and care team resources. The toolkit is currently being tested during the study's pilot phase to assess feasibility and inform broader implementation. This work aims to improve the delivery of fracture prevention interventions and reduce secondary fracture risk among vulnerable older adults.

Abstract citation ID: igaf122.4081

### CARDIOMETABOLIC HEALTH, NATIVITY STATUS AND COGNITION AMONG LATINOS IN THE HEALTH AND RETIREMENT STUDY

Kenya Luna, and Lauren Brown, *University of Southern California, Los Angeles, California, United States*

Cardiometabolic disease increases dementia risk for older adults. Latinos face a higher burden of cardiometabolic risk factors than their white counterparts. It is unclear how nativity or location of birth impacts the relationship between cardiometabolic disease and cognition among Latinos. Importantly, these are potentially modifiable factors that can reduce the risk of developing dementia later in life. This study examines cardiometabolic biomarkers among foreign-born and U.S. born Latinos and determines whether these biomarkers are associated with increased risk of cognitive decline. Using the 2016 Health and Retirement Study with venous blood biomarkers ($n = 1027$), descriptive statistics show that U.S. born Latinos had higher history of cardiometabolic diseases [1.29(0.08)], lower fasting glucose [121.52(4.10)], and higher hemoglobin [13.90(0.13)] than foreign born Latinos. OLS models show history of cardiometabolic risk ($\beta$=-0.62; $p < 0.01$) predict worse cognitive function while higher hemoglobin levels ($\beta$= 0.30; $p < 0.01$) predicts better cognitive function among both US and foreign-born Latino populations. However, nativity stratified OLS models show that cardiometabolic history ($\beta$= -0.85; $p < 0.01$) predict worse cognitive function while glucose ($\beta$= 0.01; $p < 0.05$) and hemoglobin ($\beta$= 0.46; $p < 0.01$) predict better cognitive function among foreign-born Latinos. Cardiometabolic history ($\beta$= -0.26; $p = 0.36$), glucose ($\beta$= -0.01; $p = 0.14$) and hemoglobin ($\beta$= 0.13; $p = 0.42$) were not significant predictors of cognitive function among U.S. Born Latinos. Findings highlight early risk factors of cognitive decline for Latinos vary by nativity. These differences may guide culturally and contextually relevant treatments among older Latino populations.

Abstract citation ID: igaf122.4082

### CARE PARTNER AND CARE-RECEIVER KINSHIP INFLUENCES DESIRE TO SEEK LONG-TERM CARE AFTER HOSPITALIZATION

Ahmed-Rufai Yahaya[1], Azza AL Harrasi[1], Samiya AL-Hadhrami[1], Ashley Kuzmik[2], Liza Behrens[1], Diane Berish[1], and Marie Boltz[1], *1. The Pennsylvania State University, University Park, Pennsylvania, United States, 2. Alzheimer's Association, Chicago, Illinois, United States*

Dementia affects 57 million people worldwide, and over 7 million Americans live with Alzheimer's disease. Care partners play vital roles in providing care, but they also express several stressors, including behavioral symptoms, relationship stressors, and preparedness. There is evidence that the kinship (type of relationship) and quality of the care partner/care-receiver relationship are related to long-term care placement and quality of life. However, limited research has examined how kinship influences the desire to seek long-term care, post-acute hospitalization. This secondary analysis of a cluster randomized clinical trial (Family-centered Function-focused Care) examined the association between kinship and desire to seek long-term care for person with dementia at discharge, 2 months, and 6 months post-acute-hospitalization by care partners of hospitalized patients with dementia ($N = 158$). Care partner kinship type was classified as spouse/partner ($n = 47$), adult child ($n = 86$), or other relatives/friends ($n = 25$). Findings showed significant main effects of kinship type with a desire to seek long-term care [discharge covariates: $F(2,158)=4.80$, $p=.010$, partial-$\eta^2=.061$; 2-month: $F(2,145)=4.54$, $p=.012$, partial-$\eta^2=.059$; 6-month: $F(2,145)=4.21$, $p=.017$, partial-$\eta^2=.055$] . Spouse/partner care partners showed lower desire to seek long-term care compared to adult children across timepoints (discharge: $M = 0.96$ vs. 1.34, $p=.019$; 2-month: $M = 0.91$ vs. 1.57, $p=.013$; 6-month: $M = 0.66$ vs. 1.55, $p=.013$) and others at 6-months ($p=.002$). Significantly older care partner age across all timepoints ($p=.012$, $p=.004$, $p=.009$), higher neuropsychiatric symptoms ($p=.024$), and lower care partner preparedness ($p=.030$) were associated with desire to seek long-term care. Our findings support interventions targeted at the care partner-recipient kinship type, aiming at preparedness and behavioral symptom management.

Abstract citation ID: igaf122.4083

### CARE TRANSITIONS PROGRAM FOR SURGICAL PATIENTS WITH OLDER AGE, COGNITIVE DECLINE AND/OR FRAILTY

Marianne Desir[1], Lisa Hue[2], Sofia Economidou[3], and Naixin Kang[4], *1. Veterans Health Administration, Miami, Florida, United States, 2. Hackensack Meridian Health, North Bergen, New Jersey, United States, 3. University of Miami/Jackson Health System Fellowship in Geriatric Medicine, Miami, Florida, United States, 4. Miami Veterans Affairs Healthcare System, Miami, Florida, United States*

C-TraC (Coordinated Transitional Care) is a protocol-driven hospital care transition program that has been offered primarily to patients admitted to medical wards. We adapted C-TraC with a target population of surgical patients with frailty, older age and/or cognitive impairment. A nurse case manager collaborated with a geriatrician and the surgery service to provide pre-discharge visits and weekly post-discharge telehealth visits for up to four weeks. In these visits the nurse: 1) reinforced discharge education; 2) ensured awareness of follow-up appointments; 3) discussed home medications and medication adherence; 4) ensured availability of sufficient functional supports; and 5) ensured awareness of contacts for future questions. After initial piloting in 2023, 51 patients participated in the program during the revised implementation in 2024. Average age was 75 years. Most common surgery types were orthopedic (n = 13), general surgery/surgical oncology (n = 9), vascular (n = 6) and urology (n = 5). 30 patients completed the full C-TraC program. Among historical controls, the readmission rate was 13.3%. Among the 51 patients completing at least the C-TraC visit at hospital discharge, readmission rate was 9.8%. Among the 30 patients who completed the full C-TraC program, the readmission rate was 10.0%. The 30 patients who completed the full program were reached by phone for program evaluation; all described that the program was either moderately or very much a positive aspect of their experience with VA healthcare. Our experience supports that C-TraC can be successfully adapted and implemented for the target population of surgical patients with frailty, older age and/or cognitive impairment.

Abstract citation ID: igaf122.4084

### CAREGIVER DEPRESSION MEDIATES CAREGIVER SELF EFFICACY AND NEGATIVE INTERACTIONS WITH CARE RECIPIENTS

Ayala Tzadikario[1], Au Myat May[2], Maya Sakharov[2], Julian Ortega[2], Abigail Overstreet[2], Bárbara Gómez[2], Hannah Shui-Wah Leung[2], and Rowena Gomez[2], *1. Palo Alto University, Palo Alto, California, United States, 2. Palo Alto Universiety, Palo Alto, California, United States*

Previous research has demonstrated that caregiver self-efficacy, the perceived ability to perform according to caregiving demands and make caregiving decisions, may significantly affect caregiver-care recipient relational dynamics (Leung et al., 2020). Additionally, studies have indicated that caregiver depression significantly predicted negative interpersonal outcomes during transitions to facility-based care (Nunez, 2021). The present study examined how caregiver depression may mediate the predictive relationship between caregiver self-efficacy and negative interactions with care recipients in familial caregivers during facility admission transitions for relatives with dementia. As part of a larger randomized controlled trial of the Residential Care Transition Module (RCTM), participants (N = 234; 40 men; 194 women) responded to several questions and measures including: the Caregiver Self-Efficacy Scale, the Center for Epidemiological Studies-Depression Scale, and the Number of Positive and Negative Interactions scale split into six summed categories: Relative Positive, Relative Negative, Staff Positive, Staff Negative, Other Family Members Positive, and Other Family Members Negative. Using Hayes' PROCESS macro for mediation analysis, the data analyses indicated that caregiver depression fully mediated the relationship between caregiver self-efficacy and negative interactions with care recipients (ps<.05). These results suggest that caregivers with lower self-efficacy may experience increased negative interactions with care recipients because of elevated depression symptoms. These findings may help clinicians better understand the mechanisms contributing to strained caregiver-care recipient relationships during long-term care facility transitions. Furthermore, the findings suggest that interventions targeting depression symptoms may improve interpersonal dynamics between familial caregivers and care recipients during this critical transition period.

Abstract citation ID: igaf122.4085

### CARING FOR OLDER ADULT PARENTS: AN URBAN MALAYSIAN PERSPECTIVE

Karuna Thomas, *Washington University in St. Louis, St. Louis, Missouri, United States*

This study set out to understand the experience of family caregivers in Malaysia who chose to coreside with their older adult parent in order to provide care. With an increasing aging population in Malaysia, along with the lack of policy supporting caregivers, this raised the question of how family caregivers manage the multiple roles in adulthood, along with the responsibilities that come with caring, and cultural expectations of filial piety as discussed in the literature. Twelve qualitative interviews were conducted to explore this experience among urban Malaysians who were coresiding with their parent in order to provide care. Participants were aged between 42 and 59 years old, and answered questions about their decision to coreside and how they manage responsibilities. Thematic analysis revealed that family caregivers are influenced by an ingroup decision making perspective with care of older adults being considered an important part of their cultural identity. Malaysian caregivers expressed a blindness to the burden associated with caregiving in which the responsibilities were viewed as a blessing and not a burden. Caregivers adjust to managing lifestyle changes by employing action-focused, emotion-focused, and spiritual-focused coping strategies. These findings provide further insight into how Malaysian caregivers perceive their experience and how policy can best support caregivers in the country.

Abstract citation ID: igaf122.4086

### CERAMIDE-LOWERING THERAPY IMPROVES METABOLIC HEALTH IN AGED UM-HET3 MICE FED A WESTERN DIET

Hamid Koroni[1], Adolfo Garcia[2], Jiyuan Yin[2], Erik Marchant[2], Vivian Diaz[2], Blake Rasmussen[2], Shangang Zhao[2], and Juan Palavicini[2], *1. University of Texas Medical Branch at Galveston, League City, Texas, United States, 2. University of Texas Health Science Center at San Antonio, San Antonio, Texas, United States*

Aging is the leading risk factor for virtually all chronic diseases, and when combined with obesity from high-fat, high-carbohydrate Western diets, accelerates metabolic decline. Ceramide, lipids whose synthesis is regulated by the longevity-assurance-gene (LAG1), accumulate with age and in metabolic syndrome, impairing glucose and fatty acid metabolism and contributing to insulin resistance, mitochondrial dysfunction, inflammation, and cellular senescence. This study tested whether blocking ceramide synthesis could improve metabolic function in aged, genetically diverse UM-HET3 mice fed a Western diet. Aged mice received myriocin, a serine palmitoyl transferase inhibitor, intermittently (one week on, two weeks off) alongside a Western diet. Comparison groups included Western diet alone, a control diet without added sucrose/cholesterol but similar in calories, and standard chow. Metabolic activity was assessed with the Promethion Metabolic System, analyzing three full days of data. Myriocin increased fatty acid utilization compared to control diet and chow, and showed an increasing trend relative to Western diet, with no differences in energy expenditure. Oral Glucose tolerance tests (OGTT), blood lactate and ketones, indicated that myriocin restored glucose tolerance to control diet levels, without affecting lactate or ketones. Body composition by qMRI showed reduced body fat percentage with myriocin. Liver mitochondrial function assessed by high-resolution respirometry was negatively impacted by Western diet and partially rescued by myriocin. ELISA demonstrated normalized insulin levels without changes in leptin. By modeling the human-like Western diet in a genetically diverse animal model, these findings provide proof-of-concept that targeting ceramide synthesis can extend healthspan in aged, metabolically unhealthy mice.

Abstract citation ID: igaf122.4087

### CHATROOM SENTIMENT PREDICTS MOOD IMPROVEMENT IN FAMILY CAREGIVER MOBILE PEER SUPPORT GROUPS

Noura Attili[1], Paulina Gutierrez-Ramirez[2], Maria Galvez[2], Liliana Ramirez Gomez[2], and Felipe Jain[3], *1. Massachusetts Institute of Technology, Cambridge, Massachusetts, United States, 2. Massachusetts General Hospital, Boston, Massachusetts, United States, 3. Massachusetts General Hospital / Harvard Medical School, Boston, Massachusetts, United States*

A large proportion of family caregivers of people living with dementia experience negative impacts of caregiving on their mental health, including increased anxiety, depression, and social isolation. Online peer support groups provide an important forum for social interaction and connection for these caregivers that may help alleviate these symptoms. However, although many individuals experience peer support groups as beneficial, some find peer support groups to be unhelpful. There is little quantitative research into mechanisms that determine why some groups are effective at alleviating caregiver distress, and others are not. We aimed to study the impact of chat post sentiment within mobile application text-based chatrooms on the mental health of older adult family caregivers of dementia patients (mean age 68.5). We analyzed 3,021 chat messages using natural language processing across 29 chatrooms, which were provided as part of a randomized, controlled trial of caregiver skills with or without mentalizing imagery therapy. Outcomes included assessments of perceived stress, depressive symptoms, mindfulness, and self-compassion. Results showed that overall average positive sentiment within chatrooms, but not the sentiment of individual user posts, was significantly correlated with decreased depressive symptoms ($p = 0.01$), decreased perceived stress ($p = 0.03$), increased self-compassion ($p = 0.05$), and increased mindfulness ($p = 0.05$). These findings suggest that overall positivity within peer support chatrooms might be a mechanism by which caregivers experience clinical improvements. Increasing positivity within chatrooms might be a therapeutic target to improve caregiver mood and enhance positive psychological traits.

Abstract citation ID: igaf122.4088

### COMPASS: A SOFTWARE TOOL FOR CARE PREFERENCE ASSESSMENT OF SATISFACTION IN SKILLED NURSING

Anthony Sterns[1], Katherine Abbott[2], Kimberly VanHaitsma[3], Charles De Vilmorin[4], and Jeffry Moore[4], *1. Miami University, Twinsburg, Ohio, United States, 2. Miami University, Oxford, Ohio, United States, 3. The Pennsylvania State University, University Park, Pennsylvania, United States, 4. LifeLoop, LifeLoop, District of Columbia, United States*

The results of the Compass project demonstrate both the feasibility and significant usefulness of digital resident preference assessments for enhancing person-centered care in nursing home communities. Using the scientifically validated Preferences for Everyday Living Inventory (PELI), Compass-21 was created, consisting of 21 targeted questions—16 aligned with MDS Section F and five additional high-priority preferences chosen by staff after reviewing the remaining 56 PELI questions. Focus groups and formative research with administrators and frontline staff revealed that existing paper-based and EHR systems were not dynamic enough and were poorly updated for sharing preference information, which hindered truly individualized care. Compass-21 enabled systematic, real-time documentation and sharing of residents' preferences and satisfaction. In four nursing home communities, 72 eligible residents (with a Brief Inventory for Mental Status score of 12 or higher) completed assessments of their most important preferences and provided a satisfaction rating for how well each preference was met. The tool produced reports used in care planning meetings, facilitating targeted discussion and improvement efforts. A survey of the reports given to care staff (nursing, social work, activities), family, and residents showed that the reports were helpful (3.9/5.0), understandable (4.44/5.0), and useful (4.16/5.0). Statistical analyses examined the change in preference-congruent care by comparing baseline and 30-day follow-up using repeated measures ANOVA. These results showed positive increases in the number of important preferences that were met, providing a strong basis for expansion and wider adoption across communities.

Abstract citation ID: igaf122.4089

### CONCURRENT PREDICTION OF SUCCESSFUL AGING BY OBJECTIVE MARKERS OF AGING AND PSYCHOLOGICAL MARKERS OF AGING

Fabio Selovin[1], M. Clara de Paula Couto[2], Markus Scholz[3], and Klaus Rothermund[4], *1. Friedrich-Schiller Universität Jena, Institut für Psychologie, Jena, Thuringen, Germany, 2. Friedrich-Schiller-University Jena, Jena, Thuringen, Germany, 3. University of Leipzig, Leipzig, Sachsen, Germany, 4. Friedrich-Schiller University Jena, Institute of Psychology, Jena, Thuringen, Germany*

Objective indicators of cognitive decline, immune function and inflammation are used as objective markers of aging (OMAs) to predict an individuals aging success. Individuals beliefs and evaluations of their own age and aging, like subjective age have been found to significantly impact longevity and health in old age, making them psychological markers of aging (PMAs). However, studies rarely consider PMAs alongside OMAs when predicting successful aging related outcomes. Our study aims to concurrently predict autonomous daily functioning (IADL), health-related quality of life (hQoL) and life satisfaction (LS) using OMAs of cognitive aging (trail making test), inflammation (interleukin-6, c-reactive protein), and immune aging (neutrophil-to-lymphocyte ratio) as well as PMAs, specifically subjective age and future self-views. To this end, we used cross-sectional data from 456 older adults (Md age = 74 years, 60-86 years) from the LIFE Adult Study and predicted the respective outcome by hierarchical linear regression, while controlling for socio-demographic factors. The results show that OMAs of cognitive function predicted IADL (ß = -0.15, $p < .05$) and OMAs of inflammation predicted hQoL (ß = -0.10, $p < .05$) but no OMA significantly predicted LS. Subjective age (ß = -0.17, $p < .05$) and future self-views (ß = 0.15, $p < .05$) predicted LS, but did not predict IADL. Future self-views (ß = 0.27, $p < .001$) but not subjective age was associated with hQoL. Our results suggest that fundamental biological aging pathways and psychological constructions of age are selectively associated with specific form of success in old age.

Abstract citation ID: igaf122.4090

**CONCURRENT VALIDITY OF BRAINCHECK COMPARED TO SLUMS IN OLDER ADULTS USING REAL-WORLD CLINICAL DATA**

Duong Huynh[1], Mary Patterson[1], and Bin Huang[2], *1. BrainCheck Inc., Austin, Texas, United States, 2. BrainCheck Inc, Austin, Texas, United States*

This retrospective study evaluated the psychometric and diagnostic properties of BrainCheck Assess (BC-Assess), a digital cognitive testing tool, compared with the Saint Louis University Mental Status (SLUMS) exam, a widely used paper-based screener. Real-world clinical data collected across 22 clinics from 2,038 individuals aged 50 years or older who completed both instruments on the same day were analyzed. Participants were classified into Normal (N = 354), Mild Cognitive Impairment (MCI; N = 759), and Dementia (N = 925) groups based on SLUMS criteria. The sample was 56% female, 94% had at least a high school education, and the mean (SD) age was 73.6 (9.3) years. Overall scores on BC-Assess and SLUMS were strongly correlated (r = 0.75), indicating substantial convergence in measuring global cognitive function. Subscore-level analyses revealed moderate correlations between conceptually matched tasks. Canonical correlation analysis identified two major shared cognitive dimensions: the first reflected the role of effective encoding, maintenance, and retrieval of information, and the second captured the coordinated engagement of working memory, attentional control, and executive functioning essential for adaptive cognitive regulation. ROC analyses using SLUMS diagnostic categories as reference showed good discriminative ability for BC-Assess, with AUC values of 0.80 (Normal vs. MCI+Dementia) and 0.84 (Normal+MCI vs. Dementia). Optimal BrainCheck cutoff scores demonstrated balanced positive and negative percent agreement (0.71–0.79) across cutoff determination and validation sets, highlighting strong generalizability. These results support BC-Assess as a valid digital screening tool aligned with SLUMS, providing enhanced domain-specific cognitive insights for clinical and research use.

Abstract citation ID: igaf122.4091

**COST-EFFECTIVENESS OF PAPILLARY THYROID MICROCARCINOMA TREATMENT PATHWAYS IN OLDER ADULTS**

Rebecca Kowalski[1], Seth Rabinowitz[1], Julia Slejko[2], C. Daniel Mullins[2], and Yinin Hu[3], *1. University of Maryland, Baltimore, Maryland, United States, 2. University of Maryland School of Pharmacy, Baltimore, Maryland, United States, 3. University of Maryland School of Medicine, Baltimore, Maryland, United States*

Papillary thyroid microcarcinomas (PTMCs) are sub-centimeter cancers with an excellent prognosis. Cancer outcomes are similar across four common treatment pathways, making cost-effectiveness an important factor for treatment selection. In this decision analysis study, we hypothesized that compared to alternative strategies, active surveillance (AS) would be cost-effective for PTMC treatment among older adults. A Markov model was constructed to evaluate the incremental cost-effectiveness of three PTMC treatment pathways – radiofrequency ablation (RFA), partial thyroidectomy (PT), and total thyroidectomy (TT) – compared to AS. The base case was a 70-year-old patient with PTMC without high-risk features. Cost-effectiveness was estimated with a 10-year horizon and a willingness-to-pay threshold of $50,000/quality-adjusted life-year (QALY). Costs and outcome probabilities were derived from the literature. QALY weights for common PTMC-related health states were derived using time trade-off. Probabilistic sensitivity analysis (PSA) accounted for parameter uncertainty for QALY weights, AS & RFA disease progression probabilities, and treatment selection following disease progression. In this model, TT was strongly dominated by PT, and PT by RFA. RFA was cost-effective compared to AS, with an incremental cost-effectiveness ratio of $23,821/QALY. A 1-way sensitivity analysis demonstrated that RFA remained more cost-effective than AS for post-RFA disease progression rates up to 6.3 times that of AS. The PSA demonstrated RFA and AS were optimal in 58% and 36% of model iterations, respectively. In conclusion, for older adults with PTMC, RFA and AS are cost-effective relative to surgery; RFA is more cost-effective than AS, independent of its degree of disease progression risk reduction.

Abstract citation ID: igaf122.4092

**CULTURAL ADAPTATION OF AN EVIDENCE-BASED DYADIC POST DIAGNOSIS SUPPORT INTERVENTION FOR AFRICAN AMERICAN DYADS**

Silvia Orsulic-Jeras[1], Lauren Parker[2], Kalisha Bonds Johnson[3], Donna Salaam[1], and Zoe Fete[1], *1. Benjamin Rose, Cleveland, Ohio, United States, 2. Johns Hopkins Bloomberg School of Public Health, Baltimore, Maryland, United States, 3. Emory University, Atlanta, Georgia, United States*

African American older adults are twice as likely to be affected by dementia than non-Hispanic whites. They report unique cultural, structural, and social challenges that can limit access to and engagement with supportive care interventions. SHARE for Dementia is an evidence-based, early-stage dyadic intervention designed to support persons living with dementia (PLWDs) and their care partners in making informed care planning decisions. This presentation describes the first cultural adaptation of SHARE for African American care dyads using a two-phase, community-engagement approach. Phase 1 consisted of a review of the original SHARE protocol by an Advisory Committee (AC; n = 8) comprised of dementia caregiving and subject matter experts. The AC reviewed existing SHARE materials, identified cultural considerations and priorities needed to improve relevance, accessibility, and acceptability. Using a similar methodology, the second phase convened focus groups of African American caregivers and early-stage PWLDs (n = 11). A thematic analysis revealed the following themes: medical mistrust, stigma, lack of dementia education, importance of faith-based support, complex family dynamics, and familial support with future care. Based on these findings, Version 1 of SHARE was developed. Using an iterative approach, both groups reconvened to evaluate V1, yielding refinements that guided development of Version 2, a testable, culturally tailored SHARE. Intervention modifications were systematically documented using the Framework for Reporting Adaptations and Modifications (FRAME) Model. This presentation addresses critical barriers to care planning engagement in African American dyads and lays the groundwork for future testing to determine preliminary efficacy of the culturally adapted SHARE.

Abstract citation ID: igaf122.4093

**DECOUPLING HEALTH AND NEGATIVE AFFECT IN LATE ADULTHOOD: SELF-PERCEPTIONS OF AGING AS A PSYCHOLOGICAL BUFFER**

Han-yun Tseng[1], Manfred Diehl[2], Stephen Aichele[2], Hans-Werner Wahl[3], and Oliver Schilling[4], *1. University of Colorado, Aurora, Colorado, United States, 2. Colorado State University, Fort Collins, Colorado, United States, 3. Heidelberg University, Heidelberg, Baden-Wurttemberg, Germany, 4. Heidelberg University, Germany, Heidelberg, Baden-Wurttemberg, Germany*

Objectives: This study revisited the paradox of well-being in a 20-year longitudinal dataset, testing whether self-perceptions of aging (SPA), a modifiable psychological resource, buffer the impact of declining health on emotional well-being in midlife and later adulthood. Methods: Data were drawn from the Interdisciplinary Longitudinal Study of Adult Development and Aging (ILSE), which followed participants from age 60 to 80 (C30 cohort) and age 40 to 60 years (C50 cohort). Negative affect was measured using four longitudinally invariant items from the Zung Depression Scale; health was objectively assessed by physicians; SPA was measured by the Attitudes Toward Own Aging scale (ATOA). Latent growth curve models tested longitudinal associations and SPA moderation. Results: Health declined significantly in both cohorts, whereas negative affect remained stable, consistent with the paradox of well-being. SPA significantly moderated the health-affect relationship, but only in the older cohort (C30 cohort). Specifically, in older individuals with more positive SPA, the association between health decline and negative affect was significantly weaker compared to those with more negative SPA. No moderation effect of SPA on health-affect associations emerged in the younger cohort (C50 cohort). The findings held after controlling for a range of socio-demographic and personality characteristics. Conclusions: These findings support the proposition that SPA influence emotional resilience in later life. Additionally, the paradox of well-being may be contingent on SPA, such that positive views of aging help preserve emotional well-being despite physical decline. This finding points to the potential of SPA-focused psychological interventions to promote well-being in aging populations.

Abstract citation ID: igaf122.4094

**DEMENTIA-CAPABLE WORKFORCE: OUTCOMES OF A SCALABLE IPE DEMENTIA CURRICULUM FOR HEALTH PROFESSION STUDENTS**

Jung Kwak[1], Veronica Young[2], Lauren El-Assad[2], Kwaku Duah Oppong[2], Sydney Silverman[2], and Alyssa Aguirre[3], *1. University of Texas-Austin, Austin, Texas, United States, 2. The University of Texas at Austin, Austin, Texas, United States, 3. The University of Texas at Austin Dell Medical School, Austin, Texas, United States*

Health professional students require enhanced training in Alzheimer's Disease and Related Dementias (ADRD) and interprofessional collaboration to address workforce gaps in dementia-capable care. We developed and evaluated an online interprofessional education (IPE) curriculum for students in nursing, medicine, pharmacy, social work, and speech-language pathology at a university in Texas. The curriculum included two asynchronous modules (brain health, early detection, and IPE principles) and a virtual IPE Day with case-based, team-oriented learning. Pre- and post-surveys assessed ADRD knowledge, attitudes, and IPE competencies using validated scales. Qualitative feedback was collected through open-ended responses and analyzed thematically. Of 58 students enrolled, 42 completed both modules and 30 participated in the IPE Day. Statistically significant improvements were observed in ADRD knowledge (mean increase = 1.43, $p < .001$) and preparedness to support families (1.49, $p < .001$). Significant gains were also found across five IPE competency domains: communication (mean increase = 0.48, $p = .003$), collaboration (0.53, $p < .001$), roles and responsibilities (0.43, $p = .005$), patient-centered care (0.50, $p = .002$), and team functioning and conflict management (0.39, $p = .001$). Over 90 percent of participants agreed the curriculum supported their learning and professional development. Qualitative analysis revealed five themes: students valued active learning strategies, clinical relevance, and self-paced flexibility, while identifying opportunities for greater interprofessional integration and extended simulation time. These findings support the feasibility and impact of brief, scalable virtual IPE curricula to enhance dementia care competencies and provide implications for interprofessional curriculum design in ADRD education and training.

Abstract citation ID: igaf122.4095

**DEPRESSIVE SYMPTOMS AND QUALITY OF LIFE IN MULTIMORBIDITY: A SCOPING REVIEW OF COMMUNITY-BASED INTERVENTIONS**

Benita Bamgbade[1], Irina Mindlis[2], Atami De Main[3], Hanzhang Xu[4], Wenhui Zhang[5], Lindsey Randall[6], Abigail Sallings[7], and Isabella Thoene[7], *1. Northeastern University, Boston, Massachusetts, United States, 2. Rutgers University, New York, New York, United States, 3. Weill Cornell Medicine, New York, New York, United States, 4. Duke University, Durham, North Carolina, United States, 5. Emory University, Atlanta, Georgia, United States, 6. Mayo Clinic, Rochester, Minnesota, United States, 7. University of Florida, Gainesville, Florida, United States*

Depressive symptoms and poor quality of life (QoL) are common among over 65% of older adults living with multimorbidity. Community-based interventions have the potential for scalability and may be more accessible for older adults with complex needs compared to interventions delivered in healthcare settings. This scoping review aims to describe the literature on community-based interventions targeting depressive symptoms and QoL for adults with multimorbidity. Six databases were searched for studies published in 2015 or later, focusing on interventions in community-based settings (e.g., community-based organizations, senior centers, retirement communities). Studies focusing on a single condition or an index condition with comorbidities were excluded. Of 7,829 screened studies, 173 articles were included in full-text review. About half of the studies were conducted in high-income English-speaking countries and 11% in low and middle-income countries (LMIC). Study samples consisted mostly of older adults with varied multimorbidity profiles that included diabetes, cardiovascular disease, mental health disorders, and frailty. Nearly 40% of studies reported physical activity and patient education interventions, while peer-led/community health worker interventions were the least reported intervention. This review highlights a gap in studies conducted in LMIC where the burden of multimorbidity is high and resources are limited, and a gap in interventions that are community member-led. More research is needed to understand how these scalable, cost-effective community-based interventions may address the needs of multimorbidity patients in LMIC and how leveraging community members may strengthen interventions in addressing depressive symptoms and QoL in patients with multimorbidity.

Abstract citation ID: igaf122.4096

### DEPRESSIVE SYMPTOMS ATTENUATE THE COGNITIVE BENEFITS OF SLOW WAVE SLEEP IN OLDER BLACK AMERICANS

Stirling Crawford[1], Osswaah Fariduddin[2], Maria Clark[2], Elizabeth Luth[3], and Darlingtina Esiaka[4], *1. University of Kentucky, Frankfort, Kentucky, United States, 2. University of Kentucky, Lexington, Kentucky, United States, 3. Rutgers University, New Brunswick, New Jersey, United States, 4. University of Kentucky - College of Medicine, Lexington, Kentucky, United States*

Background: Slow wave sleep (SWS), critical for memory consolidation, has been linked to better visual-spatial working memory (VSWM)—a sensitive early marker of cognitive decline. Depressive symptoms have been shown to modulate memory performance, but their interactive effects with SWS remain underexplored in Black American populations disproportionately impacted by sleep disturbances. This study investigated the interaction between objectively measured SWS and depressive symptoms on VSWM among older Black Americans. Methods: As part of a larger observational study examining longitudinal benefits of sleep in older Black Americans, participants (N = 59; mean age 52.76) completed three consecutive nights of at-home sleep monitoring using the Sleep Profiler, a validated wearable device. VSWM performance was assessed using standardized memory tasks, while depression was assessed using the Geriatric Depression Scale. Moderation analysis was used to examine whether depression moderated the relationship between SWS and VSWM, while controlling for age, gender and marital status. Results: SWS (B = 0.665, p = 0.006) and depressive symptoms (B = 2.177, p = 0.008) had significant independent effects on VSWM. The moderation analysis revealed a significant interaction (B=-0.1106, p = 0.007) between SWS and depressive symptoms. Further examination of the conditional effects revealed a positive association between SWS and VSWM at low depressive symptom levels (t = 2.8847, p = 0.0057) and a negative association at higher depressive symptom levels (t=-2.7760, p = 0.0076). Conclusions: These findings suggest that while SWS supports VSWM at low levels of depressive symptoms, the benefit diminishes under high depressive symptoms. Thus, emphasizing the need to investigate sleep–mood interactions when assessing the benefits of sleep among aging Black Americans.

Abstract citation ID: igaf122.4097

### DEVELOPING AN ASSISTIVE TECHNOLOGY FOR PERSONS WITH MILD COGNITIVE IMPAIRMENT: A USER-CENTERED APPROACH

Lauren Robinet[1], Liujing Xie[2], Xin Yao Lin[3], Raksha Mudar[4], Walter Boot[3], Sara Czaja[3], Neil Charness[3], and Wendy Rogers[1], *1. University of Illinois Urbana-Champaign, Champaign, Illinois, United States, 2. University of Illinois, Urbana-Champaign, Champaign, Illinois, United States, 3. Weill Cornell Medicine, New York, New York, United States, 4. University of Illinois Urbana-Champaign, University of Illinois Urbana-Champaign, Illinois, United States*

Assistive technology (AT) holds great potential to increase independence for older adults, specifically persons with mild cognitive impairment (PwMCI). MCI affects approximately 20% of individuals aged 50 and above. Despite this prevalence, the existing literature does not include robust information on their attitudes, perceived needs, or desires regarding AT. We conducted six focus groups across two sites (University of Illinois Urbana-Champaign and Weill Cornell Medicine). There were 20 total participants: 6 male, 14 female, aged 60-85, with a TICS-M score between 22-37, indicative of some level of cognitive impairment. The goal of the group discussions was to develop recommendations for an AT system, called SPARC (Supporting Personal Activities and Reinforcing Cognition). The SPARC system offers features designed to aid PwMCI in completing complex activities of daily living, support their cognitive health, increase social engagement, and provide educational opportunities. Discussions focused on content, ease of use, relevance, and integration with other tools. Participants responded favorably to the "all-in-one" nature of the system. They showed some hesitation about potential effort and determination needed to adopt the system. The activity support and education features were well-received. Participants discussed potential barriers to adoption for the social and reminiscence features, most common being relevance to them, ease of use, privacy concerns, and integration with current approaches. These findings underscore the importance of assessing PwMCI's attitudes and opinions during the early stages of the design process, which will impact the adoption and success of the SPARC or other systems intended to support their needs.

Abstract citation ID: igaf122.4098

### DEVELOPMENT AND VALIDATION OF AN INTERACTIVE GAME-BASED DIGITAL COGNITIVE ASSESSMENT TOOL

Ruike Sun, Huixian Li, and Yanyan Wang, *West China Hospital, Sichuan University, Chengdu, Sichuan, China (People's Republic)*

Early screening and intervention are essential for mitigating cognitive decline, particularly for the prevention of Alzheimer's disease (AD) through timely detection of mild cognitive impairment (MCI). Despite advances in digital assessments, barriers still limit their adoption among older Chinese adults. This study developed a digital cognitive assessment tool integrating gaming scenarios and Chinese cultural elements for screening MCI in China (IGD-CAT) and evaluated its concurrent and discriminative validity. A theoretical framework was established through literature review, expert panel, and Delphi survey, and IGD-CAT was created via interdisciplinary collaboration. A total of 218 participants from communities and hospitals completed traditional assessments followed by IGD-CAT and were classified as normal cognition (NC) or MCI. Concurrent validity was examined through correlations with Montreal Cognitive Assessment (MoCA) scores, and discriminatory validity was assessed using a random forest model. IGD-CAT comprises 14 tasks across seven domains: time orientation, attention, memory, language, calculation, visuospatial ability, and executive function. Among 196 participants (mean age 63.6 ± 5.81 years), 93 were classified as MCI and 103 as NC. Task scores correlated positively with MoCA (r = 0.140–0.387), while completion times correlated negatively (r=-0.476 to -0.168). The total IGD-CAT score correlated with MoCA (r = 0.529, P<.001), whereas total completion time correlated negatively (r=-0.549, P<.001). In the test set, the random forest model achieved 89.65% sensitivity, 96.55% specificity, and an AUC of 0.96. IGD-CAT covers multiple cognitive domains, is time-efficient, and age-friendly. With high sensitivity and specificity, it is suitable for application in primary care, community, hospital, and home settings.

Abstract citation ID: igaf122.4099

### DEVELOPMENT OF AN AGE FRIENDLY CURRICULUM FOR LONGITUDINAL MEDICAL STUDENT CLERKSHIPS IN RURAL COMMUNITY SETTINGS

Alpha Scheel[1], Mengru Wang[1], Katherine Bennett[2], Aimee Verrall[3], and Elizabeth Phelan[4], *1. University of Washington, Seattle, Washington, United States, 2. University of Washington School of Medicine, Seattle, Washington, United States, 3. University of Washington / School of Medicine, Seattle, Washington, United States, 4. University of Washington, School of Medicine, Seattle, Washington, United States*

Most medical schools in the U.S. have minimal geriatrics training requirements, and few provide rural healthcare training. The University of Washington School of Medicine has a unique multi-state program that offers medical students opportunities to train across the WWAMI (WA, WY, AK, MT, ID) region, which is predominantly rural and medically underserved. Students can opt for longitudinal training at a rural and/or underserved site through the Targeted Rural and Underserved Track (TRUST) or the WWAMI Rural Integrated Training Experience (WRITE) program. Many students who train in these programs ultimately pursue careers in primary care and return to practice in a rural/underserved area; however, they lack focused training in geriatrics. The goal of our curriculum was to address this training gap. In the pilot phase, a needs assessment was performed via focus groups with 18 medical students in the WRITE program. Results identified a need for 1) templates for evaluating geriatric syndromes, 2) printable resources for patients with limited access to the internet, and 3) audio resources for students. These findings informed the development of four modules on the 4Ms of Age-Friendly Care: Mentation, Mobility, Medications, and What Matters. A multi-modal educational strategy was then implemented with short videos, podcasts, one-page clinical guides, and relevant standardized test practice questions. The refined curriculum was rolled out to one cohort of 18 medical students in August 2025. Next steps include evaluation of self-reported competence in the 4Ms and professional interest in caring for older adults in rural settings pre and post course.

Abstract citation ID: igaf122.4100

### DIET QUALITY OF INDEPENDENTLY LIVING OLDER ADULTS IN MASSACHUSETTS (2021-2025)

Qun Le, Lingming Chen, Danielle LoPilato, Stephanie Chavarria, Jie Cheng, Meng Zhang, Sabrina E. Noel, and Wenjun Li, *University of Massachusetts Lowell, Lowell, Massachusetts, United States*

A healthy diet is key to healthy aging. Access to nutritious food is one of the major social determinants of health in Healthy People 2030. In this analysis, associations between dietary quality and factors such as age, race, and other personal and environmental characteristics were quantified. The Healthy Aging and Neighborhood Study (2021-2025) collected sociodemographic and dietary data from 614 people aged 65 y and older living in communities in Central and Northeastern Massachusetts. Dietary quality was evaluated by the Dietary Screening Tool (range 0-100) with higher scores indicating higher diet quality. Generalized linear regression models were used and regression coefficients (B(SE)) were presented. The cohort was on average aged 74.6(±6.2) y, 57.7% female, 56.0% Non-Hispanic White, 32.9% Asian, and 8.6% Hispanic. Without covariate adjustment, higher diet quality was significantly associated with older age (B(SE) =0.2(0.1)), Asian race (9.7(1.0)), having any difficulty in IADL (2.8(1.0)), being married (2.4(1.0)), and living in suburban areas (5.2(1.5)), and urban areas (5.0(1.3)). Similarly, lower diet quality was related to higher educational attainment (-0.4(0.2)), household income over $50,000/year (-4.8(1.1)), and better self-rated general health (-1.4(0.4)). However, after covariate adjustments, higher diet quality was associated only with female gender (2.3(1.0)) and Asian race (13.0(1.8)). The changes in directions and strengths of the associations indicate the complicated relationships between sociodemographic factors and diet quality in older age. Future research should explore the mechanisms of sociodemographic differences in dietary quality.

Abstract citation ID: igaf122.4101

### DIET-INFORMED METAGENOMIC CLOCKS FOR AGING IN THE INTEGRATIVE LONGEVITY OMICS STUDY

Tanya Karagiannis[1], Anastasia Leshchyk[1], Meghan Short[2], Ye Chen[1], Sarah Bald[3], Stacy Andersen[3], Daniel Segrè[3], and Paola Sebastiani[1], *1. Tufts Medical Center, Boston, Massachusetts, United States, 2. Tufts University School of Medicine, Medford, Massachusetts, United States, 3. Boston University, Boston, Massachusetts, United States*

Aging is a heterogeneous process that unfolds uniquely across individuals and biological systems. Aging clocks, computational models trained on molecular data to estimate biological age, offer a promising framework for investigating aging biology at the individual level. While most clocks rely on epigenetic, transcriptomic, or proteomic data, the human microbiome remains an underutilized source of aging biomarkers despite its central role in host metabolism, immune regulation, and inflammation, all of which are influenced by diet and linked to aging. To explore microbiome-mediated aging, we developed aging clocks using shotgun metagenomics sequencing data from 267 individuals in the Integrative Longevity Omics cohort, including centenarians and their offspring. These clocks estimate biological age based on taxonomic composition across multiple taxonomic levels. To account for dietary influences, we incorporated total caloric intake and a novel metric developed by our group, the Nutrient Variety Index (NVI), which summarizes the distribution of nutrient groups within a diet. Adjusting for caloric intake and NVIs significantly improved clock performance, suggesting that nutrient composition modulates microbiome aging. This integrative approach enhances biological age estimation and has the potential to uncover microbial signatures of healthy aging. Our findings underscore the importance of diet in shaping aging trajectories and highlight the potential of microbiome-based clocks to uncover nutrition-linked aging pathways.

Abstract citation ID: igaf122.4102

### DIFFERENTIATING MEMORY CARE: REGULATORY DIFFERENCES FOR MEMORY CARE IN ASSISTED LIVING

Lindsey Smith[1], Erh-Chi Hsu[2], Adam Pennavaria[1], Kelsey MacKenzie[1], Cassandra Hua[3], Jamie Cho[2], and Paula Carder[4], *1. Oregon Health & Science University,*

*Portland, Oregon, United States, 2. Johns Hopkins University, Baltimore, Maryland, United States, 3. University of Massachusetts Lowell, Lowell, Massachusetts, United States, 4. Portland State University, Portland, Oregon, United States*

While prior research has categorized regulatory oversight of AL, we lack a clear understanding of what regulations differ for dementia-specific compared to general AL. Using a health services regulatory analysis of 2023 AL regulations, we examine how dementia care is regulated across the 45 states with dementia-specific AL policies. States most often add memory care-specific rules beyond general AL, including allowing provision of dementia-specific services (18 states), advertising such services (14), requiring controlled egress (13), addressing dementia-related behaviors (13), limiting admission to people living with dementia (12), requiring a dementia-specific activity program (12), mandating dementia-specific staff training where not required in general AL (10), applying certification to only part of a campus (9), requiring a secured outdoor space (9), involving family in care (8), having defined methods for identifying dementia (8), requiring staffing ratios (7), and cognitive impairment screening at admission (5). These requirements highlight substantial variation in how states define and regulate dementia care in AL. Understanding these regulatory differences is important for assessing how they impact care quality, resident safety, and access to memory care—and can inform decision-making for residents, families, and policymakers.

Abstract citation ID: igaf122.4103

### DOES GRANDPARENTING WORSEN LONELINESS? THE CONDITIONAL ROLE OF CARE AND ACTIVITY

In Jeong Hwang[1], and Joseph Svec[2], *1. Iowa State University, Ames, Iowa, United States, 2. St. Joseph's University, Patchogue, New York, United States*

Research on grandparenting has found seemingly contradictory patterns: grandparental childcare is often linked to poorer well-being, but grandparenting is also associated with reduced loneliness. These inconsistencies highlight that grandparenting is not a monolithic experience. However, little attention has been paid to the different profiles of grandparent–grandchild interaction, as prior work has typically treated it as either caregiving or leisure. This study identifies profiles of grandparenting—care only, activity only, and care combined with activity—and examines how these relate to grandparents' loneliness. Using the last four waves of the Health and Retirement Study (2016, 2018, 2020, 2022; N = 7,586), we estimate random-effects panel models with clustered standard errors. We find that providing care in the absence of activities was associated with significantly greater loneliness, suggesting that purely instrumental caregiving can be burdensome. In contrast, engaging in activities with grandchildren was consistently linked to reduced loneliness, with the strongest protective effect at moderate activity levels. At higher activity levels, caregiving embedded in shared interactions buffers the negative effect of caregiving alone, and in some cases caregivers report less loneliness compared to non-caregiver grandparents. These findings suggest that the well-being consequences of grandparenting are conditional on the balance between caregiving and activity. This study underscores the importance of supporting shared, meaningful activities to strengthen intergenerational ties and reduce loneliness among older adults.

Abstract citation ID: igaf122.4104

### EDUCATION DOMINATES MODIFIABLE PREDICTORS OF LATE-LIFE COGNITION: AN XGBOOST STUDY

Jose Cabrero Castro[1], Eric Klopack[2], Emma Aguila[2], Brian Downer[3], and Jennifer Ailshire[2], *1. University of Texas at El Paso, El Paso, Texas, United States, 2. University of Southern California, Los Angeles, California, United States, 3. University of Texas Medical Branch, Galveston, Texas, United States*

Education, cognitively demanding work, and mentally stimulating leisure activities are theorized to bolster cognitive reserve, but their relative contributions across the life course remain unclear in low and middle income settings. We quantified the effects of education, occupational cognitive complexity, and leisure activities on late life cognition in Mexico, and ranked predictors using Extreme Gradient Boosting (XGBoost). We analyzed cross sectional data from the 2012 Mexican Health and Aging Study including 13,698 adults aged 50 years or older. Global cognition was composite of seven neuropsychological tests. Exposures included years of schooling; occupational cognitive complexity derived by linking longest held occupation to O*NET descriptors; frequency of leisure activities; and current work status. We fit XGBoost with 10 fold cross validation, reported model R squared, and computed gain based feature importance. A secondary model residualized age to isolate modifiable predictors. The full model explained 55 percent of the variance in global cognition (R squared = 0.55). Relative contributions were education 65.4 percent, age 21.1 percent, leisure 9.8 percent, occupational complexity 3.2 percent, and work status 0.5 percent. With age residualized, leisure rose to 21.3 percent and occupational complexity to 10.4 percent. Within leisure, reading contributed the largest gain (11.9 percent), followed by puzzles (3.8 percent) and technology use to communicate with friends (1.7 percent). Schooling was the dominant determinant of late life cognition, but modifiable leisure and occupational characteristics accounted for meaningful additional variation, highlighting opportunities to support cognitive aging in middle income contexts.

Abstract citation ID: igaf122.4105

### EFFECTIVE COMMUNICATION DURING THE COVID-19 PANDEMIC: SOCIAL VERSUS TRADITIONAL MEDIA CHANNELS

Reilly Burns[1], Michele Wood[2], Pimbucha Rusmevichientong[2], and Jennifer Piazza[2], *1. California State University, Fullerton, Moreno Valley, California, United States, 2. California State University, Fullerton, Fullerton, California, United States*

During a public health crisis, it is vital to understand how individuals respond to information regarding potential health risks. The COVID-19 pandemic was an example of such a crisis, where information often conflicted and trust in different sources varied. Understanding which sources improve adherence to established medical guidelines is important, particularly for older adults who are vulnerable to infection. The current study tested whether information communicated through social media (e.g., Facebook, Tik-Tok) versus traditional media sources (e.g., newspapers, television) was differentially associated with actions taken to prevent COVID-19 infection. Participants included 425 Orange County, California residents (Mage: 47.6; range: 18-88), who completed an online survey in July of 2020. Results revealed that people were more likely to receive information on how to prevent COVID-19 infection (e.g., maintaining physical distance, wearing a face covering) from social media sources (b = .127, p = .03) compared to traditional media sources (b = .071, p = .30). Information communicated through social media, however, was unrelated to actions taken to prevent infection (b = -.011, p = .746) or perceived effectiveness of those actions (b = -.018, p = .632). In contrast, information communicated through traditional media sources was related to more actions taken to prevent COVID-19 infection (b = .129, p = .002) and greater perceived effectiveness of those actions (b = .115, p = .008). Findings indicate that although social media is a primary source of health information, it may not be as effective at eliciting behavioral change as traditional media sources.

Abstract citation ID: igaf122.4106

### EFFECTS OF FERMENTED BLACK SOYBEAN SUPPLEMENTATION ON FRAILTY: A DOUBLE-BLIND RANDOMIZED CONTROLLED TRIAL

Chiung-Jung Wen[1], Yen-Chun Koh[2], Yen-Chen Tung[3], Shu-Chen Hsieh[2], Yi-Chen Lo[2], Jaw-Shiun Tsai[1], and Min-Hsiung Pan[2], *1. National Taiwan University Hospital, Taipei, Taipei, Taiwan (Republic of China), 2. National Taiwan University, Taipei, Taipei, Taiwan (Republic of China), 3. National Ilan University, Yilan, Yilan, Taiwan (Republic of China)*

Background In our previous study, fermented black beans and adlay were found to reduce aging biomarkers and improve aging-related gut microbial dysbiosis in aging mice. Building on the potential of these samples, this study evaluated the effects of fermented black beans and adlay on frailty in older adults. Methods This randomized double-blind controlled trial was designed to evaluate the efficacy of a 12-week consumption of a fermented black soybean and adlay supplement in older adults. A total of 116 eligible older adults were randomly assigned to either the experimental or placebo group. Results Both groups showed improvements in physical function (e.g., 5-meter walk, grip strength, and timed up and go test); however, only the experimental group exhibited significant improvements in the Mini Nutritional Assessment and frailty scores. Regarding serum biomarkers, both total cholesterol and HDL levels decreased in the placebo group. Gut microbiota analysis revealed that the intervention increased the abundance of Bacillus spp. Furthermore, functional predictions indicated that gut microbial genes related to cardiomyopathy and isoflavonoid biosynthesis pathways significantly changed in the experimental group but not in the placebo group, suggesting that fermentation may contribute to these changes. Conclusion These results suggest that fermented black beans and adlay may help improve frailty in older adults by modulating gut microbiota. However, further investigation is needed to determine whether this intervention can prevent cardiovascular disease in older individuals and, in turn, improve frailty status. Future research should explore this potential link to better understand its impact on frailty in this population.

Abstract citation ID: igaf122.4107

### EFFECTS OF GERIATRIC EMERGENCY DEPARTMENT IMPLEMENTATION ON HOSPITAL ADMISSIONS: EVIDENCE FROM HCUP

Jasmine Su[1], Cameron Gettel[2], Inessa Cohen[3], Xi Chen[4], Yuting Qian[5], Craig Rothenberg[6], Ula Hwang[7], and Elyssa Grogan[8], *1. New York University School of Medicine, New York, New York, United States, 2. Yale School of Medicine, New Haven, Connecticut, United States, 3. Yale University, Yale University, Connecticut, United States, 4. Yale University, New Haven, Connecticut, United States, 5. Yale School of Public Health, New Haven, Connecticut, United States, 6. Yale University School of Medicine, New Haven, Connecticut, United States, 7. NYU Grossman School of Medicine, New York, New York, United States, 8. NYU Langone Health, New York, New York, United States*

Since 2018, over 500 emergency departments (EDs) have been accredited as geriatric emergency departments (GEDs) in recognition of the age-friendly care they deliver to older adults. However, the impact of GED implementation on healthcare utilization remains unclear. This retrospective study examined the association between GED status and hospital admission using encounter-level data from the Healthcare Cost and Utilization Project (HCUP) from 2017-2022. 350 EDs, encompassing 20 million encounters, were matched 1:1 by GED status using hospital urbanicity, teaching status, state, encounter volume, and geriatric encounter volume. A difference-in-differences event-study design assessed the effect of GED status on hospital admissions over time in seven states. Analyses controlled for patient age, gender, ethnicity, and all matching variables above, with state, year, and ED fixed effects included. Event study estimates indicated a statistically significant decline in admissions at GED sites compared with matched non-GED sites. Relative to non-GEDs, GED implementation led to a 0.5-percentage-point decrease after one year (95% CI: –0.03, 0.02), a 4.6-point decrease (95% CI: –0.08, –0.01) after two years, a 7.5-point decrease (95% CI: –0.12, –0.03) after three years, and an 11.3-point decrease (95% CI: –0.17, –0.06) after four years, from a baseline mean of 51.6% in GED admissions. We saw no indication of pre-trends in the hospital admission outcome, indicating comparability between GEDs and non-GEDs prior to accreditation. These findings suggest that GED implementation may produce meaningful reductions in hospital admissions over time, supporting its potential to improve care for older adults in emergency settings.

Abstract citation ID: igaf122.4108

### EMOTIONAL SUPPORT AND SUBJECTIVE COGNITIVE DECLINE: THE MODERATING ROLE OF ADVERSE CHILDHOOD EXPERIENCES

Boram Lee[1], and Jeehoon Kim[2], *1. Ewha Womans University, Seoul, Republic of Korea, 2. Idaho State University, Pocatello, Idaho, United States*

Emotional support is well-known to protect against older adults' cognitive decline; however, it remains unclear whether this protective effect differs depending on exposure to adverse childhood experiences (ACEs), particularly given the unanticipated stress during the COVID-19 pandemic. We examined the associations between emotional support, ACEs, and subjective cognitive decline (SCD) in American older adults. We analyzed the 2022-2023 Behavioral Risk Factor Surveillance System data for White, Black, and Hispanic adults aged 65 + (n = 53,261). Logistic regressions assessed associations among emotional support, ACEs and SCD, including an interaction of emotional support and ACEs, after adjusting for sociodemographic characteristics, depression, self-reported health status, health insurance, and routine medical care utilization. Overall, 15.3% of the sample reported SCD in the past 12 months. In adjusted models, high levels of emotional support were associated with lower odds of SCD (Adjusted OR = 0.51, 95% CI = 0.45-0.59), whereas ACE exposure was associated with higher odds of SCD (Adjusted OR = 1.27, 95%

CI = 1.13-1.45). The protective association of emotional support was stronger among older adults without ACEs than among those with a history of ACEs (Adjusted OR = 1.39, 95% CI = 1.21-1.59). These findings suggest that emotional support provides a buffer against SCD in later life, but this protective effect is diminished for older adults with a history of ACEs. The reduced emotional support availability among ACE-exposed individuals may reflect smaller social networks and compromised social relationship quality established earlier in life. This underscores the need for targeted interventions to address both cognitive and social deficits in older adults exposed to ACEs.

Abstract citation ID: igaf122.4109

### EVALUATING LARGE LANGUAGE MODELS TO SUPPORT DEMENTIA CAREGIVERS: IDENTIFYING OPPORTUNITIES FOR IMPROVEMENT

Kyeung Mi Oh[1], Sungsoo Hong[1], Ziwei Zhu[2], Huayu Zhou[2], and Jung Ah Lee[3], *1. George Mason University, Fairfax, Virginia, United States, 2. George Mason University, George Mason University, Virginia, United States, 3. University of California, Irvine, University of California, Irvine, California, United States*

Awareness and access to the dementia caregiving resources is crucial for informal caregivers of people with early-stage dementia. Large language models (LLMs) offer easy access to caregiving information, but the risks, challenges, and ways to improve LLM-generated responses remain understudied. This mixed methods study evaluated LLMs, including the baseline ChatGPT-4o model and an enhanced version refined through prompt engineering grounded in health science and gerontology literature, to support informal dementia caregivers. This study aimed to assess key factors influencing preferred responses from LLMs and to identify related risks and challenges, thereby informing opportunities for improvement. Surveys and interviews with 12 stakeholders, including 10 healthcare professionals and 2 caregivers, were conducted to assess model responses to questions commonly asked by caregivers. The responses were assessed using validated multidimensional measures based on a human evaluation framework for LLMs in healthcare. Survey results showed the enhanced ChatGPT-4o model scored significantly higher in actionability, relevance, and satisfaction than the baseline ChatGPT-4o version. However, no significant differences were observed between the models in accuracy, understanding, intelligibility, trust, safety, or potential harm. Key themes from interview data influencing preferred responses included wordiness, in-depth content, empathy, actionability, accuracy, relevance, and bias. Overall, while both models' responses were perceived as overly verbose, the enhanced model provided more comprehensive and caregiver-centered information than the baseline ChatGPT-4o. These findings suggest that LLMs can deliver more practical and satisfying guidance for dementia caregiving. Incorporating domain-specific frameworks into model design may enable scalable, evidence-based support for caregivers in real-world settings.

Abstract citation ID: igaf122.4110

### EVALUATING THE PRESIDENTIALLY MANDATED ACCELERATED EXPANSION OF THE VA VETERAN-DIRECTED CARE PROGRAM

Krysttel Stryczek[1], Wendy Hathaway[2], Stuti Dang[3], and Leah Haverhals[1], *1. VA Eastern Colorado Healthcare System, Aurora, Colorado, United States, 2. Transformative Health Systems Research to Improve Veteran Equity and Independence (THRIVE) Center of Innovation, VA Providence Healthcare System, Providence, Rhode Island, United States, 3. Miami VA Healthcare System, Miami VA Healthcare System, Florida, United States*

The 2023 United Sates Presidential Executive Order 14095 mandated the Veterans Health Administration's (VA) expansion of the Veteran-Directed Care (VDC) program from 71 to all 171 Veterans Affairs Medical Centers (VAMCs) by the end of fiscal year 2024. VDC enables nursing-home eligible Veterans to remain at home and work with Aging and Disability Network Agencies (ADNAs) to coordinate personalized care services and supports. Enrolled Veterans receive an annual budget to hire caregiver employees, often family and friends, and acquire other needed services. Between October 2024 to June 2025, we conducted 55 phone interviews with VA and ADNA program coordinators and staff, Veterans, and caregivers across 10 VDC programs nationwide, including six expansion sites and four established programs. Interviews were recorded, transcribed, and analyzed to identify barriers and facilitators to VDC implementation. Staff data were analyzed using the Consolidated Framework for Implementation Research (CFIR) 2.0, and thematic analysis was applied to Veteran and caregiver data. We found that VDC is highly recipient-centered, focused on Veterans' needs and promotes self-management and aging in place. Facilitators included making VDC a VA performance metric, staff motivation, clear communication and shared missions between VAMCs and ADNAs, and flexible tailoring to each site's context. Program complexity emerged as a barrier due to varying VDC employer models, multi-state and regional coverage requiring different processes, inadequate staffing, and limited rural coverage. Challenges for Veterans included administrative management for employing caregivers. The evaluation identified factors impacting VDC implementation that can inform VA-community partnerships to improve Veteran-centered care delivery.

Abstract citation ID: igaf122.4111

### EXCEPTION TO THE RULES?: INVESTIGATING PREDICTORS OF LATE-LIFE VOLUNTEERISM IN NORTH DAKOTA

Bryce Van Vleet[1], and Emily Kinkade[2], *1. Eastern Mennonite University, Harrisonburg, Virginia, United States, 2. NDSU Extension, Fargo, North Dakota, United States*

Volunteering in late life is predicted by younger age, higher education, and better health among other factors. Despite North Dakota being an aging, rural state with health outcomes and educational attainment lower than the national average, late-life volunteerism outpaces the US national average. The current project investigates predictors of volunteerism in North Dakotan older adults and compares those predictors to control states with similarly high rates of volunteerism, and Nevada, the state with the lowest rate of volunteerism. An older adult (65+) subset of the 2023 Current Population Survey, Volunteering and Civic Life Supplement was utilized. A logistic regression found gender, higher education, and contact with others to be significant predictors of volunteerism in North Dakota, Nebraska, and Nevada. Next, t-tests identified differences in volunteerism predictors between North Dakota and Nevada. Older North Dakotans had less contact with others, were less racially diverse, and were more rural. Last, an ANOVA was conducted to investigate differences in predictors between North Dakota and Nevada, and two regional control states: Utah and Nebraska. North Dakotans were more rural than all other comparison states, had lower education and less contact with others than Nevada only, but had greater contact with others than Utah. These findings indicate that factors other than established demographic predictors of volunteerism, such as contact with others, may be important predictors of volunteerism among older adults. Due to the exploratory nature of this study, future research is needed to investigate these implications further.

Abstract citation ID: igaf122.4112

### EXCEPTIONAL AGING: COGNITIVE AND BRAIN HEALTH IN SUPER MOVERS

Oshadi Jayakody[1], Joe Verghese[2], Helena Blumen[2], Sofiya Milman[1], Nir Barzilai[3], Ying Jin[1], Cuiling Wang[1], and Erica Weiss[1], *1. Albert Einstein College of Medicine, Bronx, New York, United States, 2. Stony Brook University, Stony Brook, New York, United States, 3. Institute for Aging Research, Bronx, New York, United States*

Super movers, individuals aged 80 and older with gait speeds ≥1.5 SD above age- and sex-adjusted means, represent an exceptional aging phenotype and may offer insights into resilience against cognitive decline. We examined their risk of incident cognitive impairment, cognitive trajectories and brain health using data from 1) five Health and Retirement Study International Network of Studies (HRS-INS) 2) the LonGenity study and 3) the RUSH Memory Aging Project (RUSH MAP). In HRS-INS we assessed incident cognitive impairment (>1.5 SD below age-adjusted cognitive test means plus impaired Instrumental Activities of Daily Living) of super movers and conduct a meta-analysis using age- and sex-adjusted hazard ratios (HR) from Cox models of individual studies. LonGenity study data were used to model longitudinal cognitive decline using linear mixed-effects models (adjusted for age, sex, education, and parental longevity) and to compare cortical thickness and hippocampal subfield volumes between super versus non-super movers. RUSH MAP data assessed dementia-related pathology in super movers. In pooled HRS-INS data (n = 358/3,989 super movers; baseline age 83.6–84.4 years; follow-up 3.8–6.1 years), super movers had a lower risk of cognitive impairment (HR 0.50, 95% CI 0.29–0.71). In LonGenity (n = 197; mean age 84.6, SD 3.3), super movers exhibited slower decline in memory and non-memory domains and preserved hippocampal subfield volumes. In RUSH MAP, they had better late-life cognition, despite no differences in dementia-related pathology. Understanding the behavioral and biological traits of super movers may reveal protective mechanisms against cognitive decline and dementia to inform future interventions.

Abstract citation ID: igaf122.4113

### EXPERIENCE WITH CONTINUOUS GLUCOSE MONITOR IN REDUCING CARE BURDEN FOR OLDER CHINESE AMERICANS WITH MCI AND DIABETES

Eric Chen[1], Bei Wu[2], Richard Lipton[3], Katharine Lawrence[4], Susan Zweig[4], Linda Siminerio[5], Jessica Zwerling[3], and Yaguang Zheng[1], *1. New York University, New York, New York, United States, 2. NYU Shanghai, Shanghai, China (People's Republic), 3. Albert Einstein College of Medicine, Bronx, New York, United States, 4. NYU Grossman School of Medicine, New York, New York, United States, 5. University of Pittsburgh, Pittsburgh, Pennsylvania, United States*

Mild cognitive impairment (MCI) can heighten the challenges of managing type 2 diabetes (T2D), particularly among older Chinese Americans due to the interplays of cultural, financial, and health-related burdens. The impact of continuous glucose monitoring (CGM) in this population remains understudied. We conducted a qualitative study to explore their CGM experience. Participants (N = 11) were recruited from NYC community, who utilized CGM for 10 days and shared CGM data with their carepartners. In-depth interviews were conducted, transcribed, and then thematically analyzed using ATLAS.ti software. Participants had a mean age of 74.5 ± 5.2 years, and 63.6% were women. Participants reported CGM helped them assess the influence of various foods on glucose levels (n = 7), choose appropriate foods (n = 10) and portions (n = 5), and enhance carepartner involvement (n = 5) and communication (n = 4). CGM helped them make better dietary decisions, promoting a positive outlook (n = 9) in diabetes self-management. Carepartners echoed similar sentiments, but added that the device enhanced diabetes management as it was more persuasive in motivating behavioral changes (n = 5), and increased carepartner involvement by allowing them to check in more often (n = 6) and review data with the patient (n = 4). For future studies, patients suggested that providers offer more direct feedback on the real-time data (e.g., provide nutritional guidance that reflects culturally specific diets). Our findings indicate that older Chinese Americans with MCI and T2D found that CGM was feasible in easing their management burden. Future studies should couple CGM with education and behavioral approaches to improve diabetes control in older Chinese Americans.

Abstract citation ID: igaf122.4114

### EXPLORING CO-RESIDENTIAL GRANDPARENTS' TIME INVESTMENTS IN GRANDCHILDREN IN THE UNITED STATES

Abigail Stephan[1], Sarah Flood[2], and Jill Juris[3], *1. Clemson University, Clemson, South Carolina, United States, 2. University of Minnesota, Minneapolis, Minnesota, United States, 3. Appalachian State University, Boone, North Carolina, United States*

More than six million households across the United States contain both grandparents and grandchildren. Despite the prevalence of co-residential grandparents in skipped-generation grandfamilies (containing grandchildren and grandparents but no parent generation) or multigenerational households (containing children, parents, and grandparents), detailed knowledge of grandparents' time-based contributions to grandchildren's development is currently limited. Mirroring the vast parental time investment literature, we use nationally representative time diary data from the American Time Use Survey (2003-2022) to test three hypotheses–linked lives, gender, and grandchild needs–around the amount of time grandparents (N = 5,557) spend with grandchildren under age 18. Bivariate results show that (1) co-residential grandparents spend an average of 3.25 hours per day with their grandchildren, (2) co-residential grandmothers spend more time caring for grandchildren than co-residential grandfathers, (3) grandparents in grandfamilies spend more time with grandchildren than grandparents in multigenerational households, and (4) grandparent time investments decline with grandchild age. Analyzing the composition of co-residential grandparents' time with grandchildren and the intersections between gender, family structure, and grandchild needs reveals a more complex and nuanced picture of grandparent investments in grandchildren. Results highlight the need for policies and support systems tailored to co-residential grandparents with varying circumstances who invest in their grandchildren's care and development.

Abstract citation ID: igaf122.4115

### EXPLORING HEALTHY AGING CHALLENGES AMONG VETERANS AND NON-VETERANS IN PERMANENT SUPPORTIVE HOUSING

Jun Heo[1], Janelle Cardenas[2], Teena Ellison[2], April Aiken[2], and Anita Souza[3], *1. University of Washington School of Nursing, Kent, Washington, United States, 2. Compass Housing Alliance, Seattle, Washington, United States, 3. University of Washington, Seattle, Washington, United States*

As individuals experiencing homelessness age, their healthcare needs become increasingly complex. Adults over 50 represent the fastest-growing group among the homeless population, yet the population is highly heterogeneous, and the factors influencing their ability to age well are underexplored. This study examined barriers to healthcare and housing stability among older adults with lived experience of homelessness, focusing on differences between veterans and non-veterans. We used a qualitative design and developed a semi-structured interview guide in collaboration with Compass Housing Alliance. We recruited 20 participants over age 50 who had recently exited homelessness and were residing in Permanent Supportive Housing (PSH). We conducted 45-minute interviews with veterans (n = 10) and non-veterans (n = 10), asking about daily life, healthcare access, social connection, use of support services, and perceptions of aging well. Participants also completed a brief demographic survey. Interviews were recorded, transcribed, and coded to identify key themes. While both groups reported similar overall needs and behaviors, one notable barrier across participants was untreated dental issues, which significantly impacted well-being. Veterans more frequently described challenges with social adjustment and reintegration, whereas non-veterans more often reported difficulty accessing consistent healthcare. These subgroup-specific differences reflect unique structural vulnerabilities shaped by service systems and lived histories. By drawing on lived experience, this study highlights important distinctions within the aging homeless population. Findings can inform policies and services that support healthy aging in Permanent Supportive Housing, helping healthcare providers and housing organizations tailor interventions to the needs of diverse older adults who have experienced homelessness.

Abstract citation ID: igaf122.4116

### FACTORS ASSOCIATED WITH DISSATISFACTION WITH OUT-OF-POCKET MEDICAL COSTS AMONG MEDICARE BENEFICIARIES

Andrew Chern[1], and Boon Peng Ng[2], *1. University of Central Florida, Orlando, Florida, United States, 2. University of Central Florida, Orladno, Florida, United States*

Out-of-pocket medical expenses remain a substantial concern for many Medicare beneficiaries, particularly those with high care needs and limited income. The objective of this study was to examine factors (i.e., sociodemographic characteristics, health status, insurance coverage and benefits) associated with dissatisfaction with out-of-pocket medical costs among Medicare beneficiaries. We conducted a cross-sectional analysis of 9,960 adults aged 65 years and older from the 2022 Medicare Current Beneficiary Survey Public Use File (weighted N = 51.7million). The dependent variable was self-reported satisfaction with out-of-pocket costs, dichotomized as satisfied versus dissatisfied. A survey-weighted logistic regression model identified factors associated with dissatisfaction. Overall, 12.4% (6.4million) reported dissatisfaction. Higher odds of dissatisfaction were associated with age 65–74 years versus ≥75 (OR = 1.41,P< 0.001), non-Hispanic Black (OR = 1.44,P=0.011) or other race/ethnicity (vs non-Hispanic White), income ≤120% federal poverty level (FPL) (OR = 1.46,P=0.023) or > 120% to ≤ 200% FPL (vs > 200% FPL), 1–2 limitations in activities of daily living (ADLs) (OR = 1.60,P< 0.001) or ≥ 3 limitations (vs no functional limitations), reporting good general health (OR = 1.31,P=0.001) or fair/poor health (vs excellent/very good), having a Part D plan (vs no) (OR = 1.29,P=0.033), and self-purchased private insurance (vs no) (OR = 1.30,P=0.013). Full-year Medicare Advantage enrollment (vs. full-year fee-for-service) (OR = 0.62,P< 0.001) and dual Medicare–Medicaid eligibility (vs no dual) (OR = 0.34,P< 0.001) were protective factors against dissatisfaction. These findings highlight inequities in out-of-pocket costs satisfaction among socioeconomically and medically vulnerable older adults. Policy reforms aimed at expanding supplemental coverage and providing targeted financial assistance could improve out-of-pocket cost-related satisfaction and advance health equity in this population.

Abstract citation ID: igaf122.4117

### FAMILY CAREGIVER STRATEGIES TO MANAGE MEDICATION REFUSALS: RESULTS FROM A CROSS-SECTIONAL SURVEY

Reed Bratches[1], Macy Stockdill[1], Alejandra Martinez[2], Elisha Underwood[1], Paul Barr[2], Frank Puga[1], J. Nicholas Odom[1], and Rita Jablonski[1], *1. University of Alabama at Birmingham, Birmingham, Alabama, United States, 2. Dartmouth College, Hanover, New Hampshire, United States*

Family caregivers of persons living with dementia commonly navigate care-resistant behaviors when administering medications. Nonpharmacologic strategies are recommended to manage care-resistant behaviors, but the specific strategies used by family caregivers and their association with caregiver factors including burden, mental health, and perceived medication hassles is unknown. We conducted a survey of 63 family caregivers of persons living with dementia to determine the nonpharmacologic strategies used to manage medication-related care-resistant behaviors and their associations with caregiver factors like burden and mental health. We analyzed strategies descriptively and using univariate statistical tests (t-test and chi-square as appropriate) and used logistic regression to determine strength and direction of statistically significant associations. We found that common strategies included waiting and trying again later (79.4%), covertly administering medications (46.0%), and doing nothing/skipping the medication dose (25.4%). In multivariable regression modeling controlling for demographic factors, we found that higher caregiver mental health scores according to the PROMIS-10 was associated with a decreased likelihood (OR 0.64; 95%CI 0.44, 0.88; p = 0.01) of covertly administering medications. The lower likelihood of covert administration in higher mental health scores indicates the potential for caregiver mental health interventions to affect responses to care-resistant behaviors. Given the cross-sectional nature of our study, additional work is needed to determine the causal pathways between the identified care-resistant behaviors, the strategies used to manage them, and caregiver outcomes, and to test interventions to support caregiver management of medication-related care-resistant behaviors.

Abstract citation ID: igaf122.4118

**FAMILY STRENGTH, DISABILITY ACCEPTANCE, AND LIFE SATISFACTION IN OLDER ADULTS WITH HEARING LOSS**

Seohyeon Kim, and Sunghee Tak, *Seoul National University, Seoul, Republic of Korea*

Family strength is considered a key contributor to life satisfaction among individuals with disabilities. However, little is known about its longitudinal influence on the older adults with hearing loss. This study aimed to examine the long-term effects of family strength on life satisfaction and to investigate the moderating role of disability acceptance. Data were drawn from the Disability and Life Dynamics Panel in South Korea, spanning six waves from 2018 to 2023. A total of 437 participants aged 50 years and above with registered hearing disabilities were included in the analysis. Generalized Estimating Equations (GEE) were employed to analyze the overall associations between main variables across repeated measures, and Group-Based Trajectory Modeling (GBTM) was applied to capture distinct subgroups with different trajectories of disability acceptance over time. At the result, three distinct trajectories emerged: “delayed improvement” (23.3%), “stable moderate” (61.7%), and “initial high then declining” (15.0%). Significant differences across groups were observed in levels of disability acceptance, family strength, and life satisfaction. GEE analysis revealed that higher family strength, educational attainment, adequate sleep duration, and employment were positively associated with life satisfaction. However, the moderating effect of disability was not statistically significant. These findings highlight the importance of the family-based determinants in life satisfaction in individuals with sensory impairments. Therefore, a tailored approach that fosters family support and acknowledges varying trajectories of disability acceptance may be beneficial in promoting well-being among the older adults with hearing impairments.

Abstract citation ID: igaf122.4119

**FAMILY STRENGTHS, SELF-ESTEEM, AND DEPRESSION IN OLDER ADULTS WITH KIDNEY/LIVER DISABILITY: A DYADIC ANALYSIS**

JaeWon Hyun[1], Bomgyeol Kim[2], Hun Kang[1], and JiYeon Choi[1], *1. Yonsei University, Seoul, Republic of Korea, 2. Yonsei University College of Nursing, Seoul, Republic of Korea*

Chronic kidney or liver disease often leads to disability status. As populations age, these challenges increase emotional, physical, and social strain on individuals and family caregivers. While family strengths reduce depressive symptoms, few studies have examined the underlying mechanisms within patient-caregiver dyads affected by these conditions. Using the data from the 2023 Disability and Life Dynamics Panel (DLDP), we examined how self-esteem mediate the relationship between family strengths and depressive symptoms in 161 chronic kidney disease dyads (adults aged ≥50 years undergoing dialysis or post-kidney transplant) and 76 chronic liver disease dyads (adults aged ≥50 years with chronic liver dysfunction). The Actor–Partner Interdependence Mediation Model showed good fit for both groups. In kidney disease dyads, patients’ and caregivers’ family strengths positively influenced their self-esteem ($\beta=0.28$, p=.004; $\beta=0.38$, p<.001), which in turn reduced their own depressive symptoms ($\beta=-0.48$, p<.001; $\beta=-0.28$, p=.002). Additionally, patients’ self-esteem was negatively associated with caregivers’ depressive symptoms ($\beta=-0.18$, p=.026). In liver disease dyads, patients’ family strengths increased self-esteem ($\beta=0.42$, p=.010), decreasing their depressive symptoms ($\beta=-0.58$, p<.001; indirect effect $\beta=-0.24$, p=.041), while caregivers’ family strengths directly reduced their depressive symptoms ($\beta=-0.25$, p=.042). No significant partner effects were observed in liver dyads. Our findings highlight the importance of family strengths and self-esteem in mitigating depressive symptoms among older adults with chronic kidney or liver disease and their caregivers. These results suggest that enhancing family strengths and supporting self-esteem may be effective strategies to promote psychosocial well-being in this population.

Abstract citation ID: igaf122.4120

**FEASIBILITY OF CARE COORDINATION TO REDUCE UNNECESSARY HOSPITALIZATION FOR ASSISTED LIVING RESIDENTS**

Ann Reddy[1], Laura Dionne[1], Grace Wittenberg[1], Peter Serina[2], Nate Hunkins[3], Martha Etzell[3], Aimee VanDenberghe[3], and Ellen McCreedy[4], *1. Brown University, Providence, Rhode Island, United States, 2. Salem Hospital, Salem, Massachusetts, United States, 3. Bluestone Physician Services, Stillwater, Minnesota, United States, 4. Brown University, School of Public Health, Providence, Rhode Island, United States*

Almost half of Assisted Living Community (ALC) residents visit the emergency department (ED) yearly, experiencing more visits and longer stays compared to community-dwelling older adults. People living with Alzheimer’s disease and related dementias (ADRD) are at greater risk for delirium, falls, and accelerated decline associated with increased ED visits. Each transition provides an opportunity for care coordination and avoidance of unnecessary hospital admission. Bluestone Accountable Care Organization developed ED Early Response, a care coordination program. Care managers provide timely, structured information to ED providers via phone and fax within 120 minutes of ED registration. We assessed the feasibility of, and adherence to, the program. Between November 2023 and June 2024, we enrolled 1,376 patients with 1,989 eligible ED visits (mean: 1.4 visits per patient), 1,237 ED visits for patients with ADRD and 752 ED visits for patients without ADRD. Qualifying visits occurred during working hours (8 a.m. - 4 p.m.), with 82.5% of visits ($n=1,641$) identified via electronic admission, discharge, and transfer notifications, and 17.5% of visits ($n=348$) identified through direct communication between ALCs and care managers. Care managers successfully provided real-time information to ED providers for 44% of the eligible ED visits (547 of 1,237) for patients with ADRD, and for 40% of visits (304 of 752) for patients without ADRD. Care managers self-reported a hospital avoidance rate of 11%. While adherence was lower than anticipated, early structured communication could reduce unnecessary hospital admissions for ALC residents.

Abstract citation ID: igaf122.4121

**FEASIBILITY PILOT OF PERSONALIZED DIETARY INTERVENTION TO IMPROVE METABOLITE PROFILING AND DIABETES OUTCOMES**

Jisook Ko[1], Jane Chung[2], and Chun-Liang Chen[3], *1. University of Utah, Salt Lake City, Utah, United States, 2. Emory*

*University, Atlanta, Georgia, United States, 3. UT Health San Antonio, San Antonio, Texas, United States*

Effective dietary modification is crucial for managing type 2 diabetes in older adults, as it helps regulate blood glucose and prevent further severe complications. However, varying metabolic responses, cultural food choices, and low adherence present challenges, especially for ethnic minority populations. We conducted a NIH-funded 4-week feasibility pilot of Personalized Behavioral Nutrition (PBN) in 12 older Korean Americans with type 2 diabetes. The PBN program began with a 7-day assessment of diet and fasting blood glucose prior to the intervention, followed by individualized recommendations targeting three specific nutrients delivered by an interventionist. Participants were involved in digital self-monitoring of food intake and fasting glucose using mobile apps and wearable devices during the intervention period. Measures included serum metabolite profiling to assess PBN adherence, lipids panel, fasting glucose, and HbA1C which were collected at baseline and 4 weeks. Analysis of serum samples identified 28 metabolites consistently present at both time points out of 203 measured. Following the intervention, increases in ornithine, D-ribulose-5-phosphate, L-arginine, urea, and 3-methylhistidine were found, suggesting changes in protein metabolism and nitrogen processing, possibly linked to higher B vitamin intake. Increased propionate indicated greater gut microbial fermentation, likely due to higher fiber consumption. Clinically, participants demonstrated a reduction in HbA1C and fasting glucose levels. These findings show that a personalized, culturally informed dietary approach is feasible and may improve metabolic outcomes in older adults with type 2 diabetes. Metabolite profiling can further help identify biomarkers and inform precision nutrition strategies for the geriatric population.

Abstract citation ID: igaf122.4122

### FORMER APS CLIENTS' PERSPECTIVES ON BARRIERS TO SAFE AND STABLE HOUSING

Mia Canzone, Samantha Tuft, and Courtney Reynolds, *Benjamin Rose, Cleveland, Ohio, United States*

Oklahoma Adult Protective Services (APS) partnered with Benjamin Rose to conduct a needs assessment to better understand/serve APS clients needing safe housing. Ten former APS clients participated in semi structured interviews to educate Benjamin Rose and APS about their housing searches. Fifty percent of participants identified as male (n = 5). Average age recorded was 59 years old (Range = 30-76). Racial backgrounds included White (n = 6), American Indian/Alaska Native (n = 4), and Black/African American (n = 3); 40% identified as multiracial. Participants resided in apartment complexes/ homes (n = 7), an assisted living facility (n = 1), or were unhoused (n = 2). Most described homelessness as a series of unfortunate incidents. Preliminary results included themes of inaccessible housing ("I have a disability of course, and that requires me to use a walker. . . I can't just go pick a house or an apartment that I want that's within my budget . . ."), insufficient emergency housing infrastructure (after living in a mental hospital, hospital, rehab, and assisted living facility, one participant stated, ". . . it was better than being on the street in a tent"), and perceived lack of APS involvement ("I feel like the whole APS is a liar because, they will not find me a place to live"). These findings are informing the development of an innovative elder shelter program for APS clients in need of safe housing. A major implication includes the need for a systemic approach to supporting unhoused APS clients.

Abstract citation ID: igaf122.4123

### FOSTERING EMPATHY IN PATIENT-MEDICAL STUDENT RELATIONSHIPS DURING A 48-HOUR HOSPICE HOME IMMERSION

Brandon Asika Etuka[1], Sophia Moschella[2], Jhanvi Puri[3], and Marilyn Gugliucci[4], *1. University of New England College of Osteopathic Medicine, Middletown, Connecticut, United States, 2. University of New England, Biddeford, Maine, United States, 3. University of New England College of Osteopathic Medicine, Portland, Maine, United States, 4. University of New England College of Osteopathic Medicine, Kennebunk, Maine, United States*

While studies show that empathy declines throughout a medical student's clinical education, fewer than one-third of medical schools have dedicated course content focusing on providing compassionate end-of-life care. Our analysis through the University of New England College of Osteopathic Medicine's "Learning-by-Living: 48-Hour Hospice Home Immersion Project" strived to investigate how second-year medical students' empathy is impacted during this unique hospice patient care immersion. Phenomenologic qualitative analysis was applied to identify themes from accounts expressed in ten student immersion journals written during AY 2020-2021. Manual content data analysis was conducted for each journal, which was the foundation for creating a codebook from which inter-rater reliability was based. Thematic coding of the journal data was conducted manually and then fine-tuned using NVivo-15 software to categorize quotes within each theme. Through analyses, 43 themes were identified with significant quotes stored within each. The top four themes addressing medical students' empathy were: Impact of a Patient's Story on Empathy, Preservation of Patients' Dignity and Humanity, Building an Emotional Toolbox, and Empathy and Appreciation for Colleagues and Caregivers. Students learned from the personal stories and experiences of patients and their families along with working with the interprofessional hospice team. Student reflections demonstrated significant understanding of the services hospice provides. They also gained self-awareness and identified skills applicable to their careers as future physicians, including navigating open discussions surrounding end-of-life care with patients/families. This project creates impactful learning, skill development, and end-of-life experiences that augment students' ability to be empathetic providers.

Abstract citation ID: igaf122.4124

### FOUR TYPES OF HARDSHIP AND THE MODERATING EFFECT OF SOCIAL SUPPORT ON DEPRESSIVE SYMPTOMS IN OLDER KOREAN ADULTS

Meeryoung Kim, *Daegu University, Daegu, Republic of Korea*

Older adults face challenges such as physical frailty, functional impairment, and loneliness. They also face a decline in formal roles after retirement. These challenges can affect their mental health. Pearlin et al.'s (1981) stress theory explains the effects of social support. Long-standing research has argued that social support has both direct and moderating effects depending on the type of challenges older adults face and the availability and use of social support. This study examined the main and moderating effects of social support. This study used panel data from the ninth(2021), ninth additional(2022), and 10th(2023) of the Korea Retirement Income Study(n = 560, 60+). Hierarchical multiple regressions were used for data analysis. In the first step, depressive symptoms (2021), demographic variables, and four hardships (IADL, economic dependence, loneliness, and absence of role) were included. The likelihood of borrowing when financial hardship occurs was used as a moderator variable. The findings showed that gender had different effects on depressive symptoms. Economic dependence had a different effect on depressive symptoms compared to economic independence. Social participation (leisure activities, class reunions, and community activities) and exercise directly reduced depressive symptoms. Instrumental social support had a moderating effect when experiencing economic hardship. These results suggest that not all difficulties experienced by older adults are stressful. Structural social support had a main effect on reducing depressive symptoms in older adults, while functional social support had a moderating effect. Regardless of the difficulties faced by older adults, social support proved to be an important factor in reducing depressive symptoms.

Abstract citation ID: igaf122.4125

### FROM COMMUNITY TO CARE: CHWS ADVANCING AGE-FRIENDLY HEALTH IN FQHCS

Cherell Cottrell-Daniels[1], Joycelyn Lawrence[2], Kevin Espinoza[3], Katherine Chung-Bridges[1], Diego Shmuels[4], Deborah Gracia[4], and Sweta Tewary[5], *1. Health Choice Network, Miami, Florida, United States, 2. Jessie Trice Community Health System, Miami, Florida, United States, 3. Health Choice Network, Inc., Miami, Florida, United States, 4. Borinquen Healthcare Center, Inc., Miami-Dade, Florida, United States, 5. Jessie Trice Community Health Center, Miami, Florida, United States*

Background: The growing older adult population in the U.S. has increased demand for health care professionals with specialized training in geriatrics. Geriatric Workforce Enhancement Programs (GWEPs) across the country are tasked with training Community Health Workers (CHWs) to meet this need. This project integrated CHWs into Age-Friendly Health Systems (AFHS) using 4Ms framework (What matters, Mentation, Medication, Mobility). Goals included assessing changes in CHWs knowledge, intent to use training, and baseline geriatric screenings. Methods: Twelve CHWs completed a 30-hour in-person geriatric care training covering syndromes, community resources, patient-centered care, and the 4Ms of AFHS. Pre/post surveys captured demographics, satisfaction, and self-rated knowledge. The Wilcoxon Signed-Rank Test assessed changes in knowledge. CHWs documented 4Ms discussion with older adults (65+) using the electronic health record (EHR) unstructured notes section and spreadsheets. Data from 55 chart reviews (Feb- April 2025) were assessed for screening rates and documentation consistency. Results: CHWs were 73% female, 45% White, with an average age of 40 years. Pre-training, 36% rated their knowledge as moderate; post-training, 91% reported high knowledge and high likelihood of applying information. Knowledge gains were statistically significant ($z = -2.23$, $p = .026$). CHWs documentation for Medication, Mobility, and What Matters was 100%; Mentation screening was 87% for depression, 31% for dementia, with full completion of all 4Ms in 31% of encounters. Conclusion: Findings suggest strong adoption of the 4Ms components, ease of documentation, with opportunities for improvement in dementia screenings and overall 4Ms completion. Strengthening EHR infrastructure is needed to support age-friendly care.

Abstract citation ID: igaf122.4126

### FROM FEAR TO CONFIDENCE: A QUALITATIVE STUDY OF INTERGENERATIONAL TECHNOLOGY SUPPORT AMONG LOW-INCOME OLDER ADULTS

Andrew Kim[1], John Kim[1], Carissa Liu[1], Ian Lee[2], Mengzhao Yan[1], and Min-Kyoung Rhee[1], *1. University of Southern California, Los Angeles, California, United States, 2. University of California, Los Angeles, Los Angeles, California, United States*

Despite the growing reliance on technology for daily tasks, health management, and social connection, older adults, particularly those of advanced age and low income, continue to have lower rates of technology adoption. However, research shows that when factors like device access and learning opportunities are present, age and income are no longer significant barriers. Training and support also improve confidence in technology use among older adults. This study conducted a qualitative investigation into how intergenerational technical support can enhance technology use among low-income older adults. Participants aged 60 and older, living in affordable senior housing in Los Angeles, received weekly one-hour, one-on-one sessions with university student "tech buddies" over five weeks. They learned to use the devices and applications they were most interested in. Twenty-five participants who completed at least three sessions were included in the final sample, and semi-structured interviews were conducted post-intervention. Using grounded theory and constant comparison methods, we identified five major barriers to technology use: limited infrastructure, restricted access to support, age-related physical and cognitive changes, lack of knowledge and experience, and emotional resistance. Intergenerational support helped mitigate these challenges and led to seven positive outcomes, organized into four main themes: gratitude and satisfaction, improved tech knowledge and skills, interpersonal connection, and empowerment. Findings underscore the potential of personalized, intergenerational support to bridge the digital divide among low-income older adults. The study offers insights for community organizations and local government aiming to promote digital inclusion through targeted support and training for this underserved population.

Abstract citation ID: igaf122.4127

### FRONTOTEMPORAL DEMENTIA AND SUICIDE: A SCOPING REVIEW

Samantha Tinker[1], Noelle Fields[1], Kathy Siepker[1], Jaclyn Kirsch[1], and Soeun Jang[2], *1. The University of Texas at Arlington, Arlington, Texas, United States, 2. University of Texas at Arlington, The University of Texas at Arlington, Texas, United States*

Persons living with frontotemporal dementia (FTD) have the highest suicide risk among persons living with dementia, yet their experiences are often overlooked in research and practice. This scoping review examined the literature on frontotemporal dementia and suicide, identified factors contributing to suicidal behavior, and explored potential treatment and prevention options. This encompassed the full spectrum of suicidality including suicidal ideation, attempts, and completed suicide. After formulating the research question, the researchers proceeded by following the PRISM-ScR guidelines. Five databases were systematically searched using Arksey and O'Malley's framework. Studies were selected if they included FTD and suicide. Nineteen studies met the inclusion criteria. Key findings highlighted subtypes of FTD and suicide, risk factors in FTD and suicide, mental health conditions commonly occurring in FTD, and prevention and treatment methods. Instances of depression and other affective disorders were noted, alongside the characteristics and risk factors of suicide for the different subtypes of frontotemporal dementia. Findings underscore the need for prevention and treatment, family caregiver interventions, and the need for a tailored behavioral assessment for suicide in this population.

Abstract citation ID: igaf122.4128

### GAIT SPEED IS ASSOCIATED WITH EXECUTIVE FUNCTION IN LATIN AMERICAN OLDER ADULTS FROM LATAMFINGERS

Myriam Gutiérrez[1], Alejandra Marroig[2], Carolina Delgado[3], Andrea Slachevsky[4], Ana Charamelo[5], David Aguillon[6], and Lucía Crivelli[7], *1. Hospital Clínico Universidad de Chile, Santiago, Region Metropolitana, Chile, 2. Universidad de la Republica Uruguay, Montevideo, Montevideo, Uruguay, 3. Hospital Clínico Universidad de Chile, Santiago de Chile, Region Metropolitana, Chile, 4. Hospital del Salvador, Santiago de Chile, Region Metropolitana, Chile, 5. Hospital Británico, Montevideo, Montevideo, Uruguay, 6. Universidad de Antioquia, Medellin, Antioquia, Colombia, 7. FLENI, Buenos Aires, Buenos Aires, Argentina*

Dementia prevalence is increasing with aging worldwide, a leading cause of disability in Latin American Countries (LAC). Interestingly, physical performance has been reported as a predictor of cognitive decline, and gait speed has emerged as a potential clinical marker of executive dysfunction. However, evidence of the association between physical and cognitive measures remains scarce, especially in LAC older adults. This study examined the association between gait speed and executive function in older adults from 12 LAC countries. A cross-sectional study including 1,243 participants aged 60 to 77 years from the LatAm-FINGERS randomized multicenter cohort at baseline. Sociodemographic, lifestyle, and health data were collected. Physical performance was assessed using the Short Physical Performance Battery (SPPB), with gait speed measured by a standardized 4-meter walk test. Executive and processing speed were evaluated with the Trail Making Test A and B, Stroop interference index (SII), semantic and phonological fluency, and Concept Shifting Test motor speed (CST-MS). Linear regressions and composite scores were computed. The preliminary results indicate that participants' mean age was 67.5 ± 4.7 years (67.5% female). Faster gait speed was significantly associated with better Stroop C ($p<0.00001$), Stroop P ($p=0.001$), semantic fluency ($p=0.02$), and higher Mini-Mental State Examination (MMSE) scores. Associations with CST-MS and TMT tests were not statistically significant. This study is the first effort in LAC to associate gait speed with executive tests, contributing to the understanding of a useful functional measure as gait speed, and its potential future preventive applications in LAC.

Abstract citation ID: igaf122.4129

### HEALTH AND PSYCHOSOCIAL RISKS OF UNENGAGED RESIDENTS IN LOW-INCOME INDEPENDENT LIVING COMMUNITIES

Jeremy Johnson[1], Khalid Yusuf[2], Emily Campbell[1], Autumn Hart[1], and Victor Ronis-Tobin[1], *1. Xavier University, Cincinnati, Ohio, United States, 2. University of Cincinnati, Cincinnati, Ohio, United States*

Social engagement is a critical determinant of health in older adults, yet many residents of independent living communities remain unengaged despite available opportunities. We report baseline data from a longitudinal wellness program study of residents in low-income independent communities in Southwest Ohio. Assessments measured general health, quality of life, and psychosocial function. Participants ($N=119$) were older adults ($M=73.5$, $SD=8.6$), primarily female (83.2%), African American (60.5%), and with annual incomes below $25,000 (86%). Quartile splits of the Social Engagement and Activities Questionnaire (SEAQ) were used to define engagement, with most participants (69.9%) classified as engaged. Compared to engaged peers, unengaged residents (31.1%) scored significantly lower on domains of physical functioning ($M = -12.27$, $p = .020$), vitality (−9.86, $p = .016$), and emotional well-being (−10.26, $p = .013$), on the SF–36 quality of life instrument, and had higher fall risk scores ($M=1.37$, $p = .035$) on a fall risk assessment. Other SF-36 domains trended in the expected direction but were nonsignificant. Unengaged residents also showed nonsignificant trends toward greater loneliness scores (UCLA-3 Item) and healthcare utilization (annual hospital visits). Examining social isolations using the Lubben Social Network Scale (LSNS-6), unengaged residents were nearly three times more likely to report social isolation than engaged residents (32.4% vs. 12.2%; $OR=3.46$, 95% CI [1.33–8.98]). Findings highlight the need to improve engagement in wellness programming of older low-income adults living independently.

Abstract citation ID: igaf122.4130

### HISPANIC/LATINO(A) AGING WITH HIV: INSIGHTS FROM THE MWCCS COHORT

Mirza Ishrat Noor[1], Mark Kuniholm[1], Allison Appleton[1], Jessica Islam[2], Marlene Camacho-Rivera[3], Gypsyamber D'Souza[4], Deborah Gustafson[3], and Elizabeth Vásquez[1], *1. University at Albany (SUNY), Albany, New York, United States, 2. H. Lee Moffitt Cancer Center and Research Institute, Tampa, Florida, United States, 3. SUNY Downstate Health Sciences University, Brooklyn, New York, United States, 4. Johns Hopkins University, Baltimore, Maryland, United States*

Hispanic/Latino (H/L) individuals living with HIV are an understudied and diverse population with significant health disparities compared to non-H/L individuals. We examined frailty and cardiovascular disease (CVD) risk among H/L and non-H/L White adults aged ≥40 years in the MACS/WIHS Combined Cohort Study (MWCCS). The sample included 621 H/L and 1,293 non-H/L White participants. Frailty was defined using the Fried Frailty Phenotype (FFP), with a score ≥3/5 indicating frailty. Ten-year CVD risk was categorized as low (< 5%), borderline (5–7.4%), intermediate (7.5–19.9%), or high (≥20%). Adjusted odds ratios (aOR) and 95% confidence intervals (CI) assessed associations between ethnicity and outcomes, with models stratified by sex and adjusted for HIV status, age, education, BMI, depression, diabetes, and hypertension. H/L participants were more likely to be women (53.6% vs. 15.9%), frail (22.5% vs. 11.8%), and living with HIV (73.3% vs. 50.7%), but less likely to have hypertension (46.9% vs. 66.3%) or high CVD risk (7.5% vs. 18.4%). Among men, H/L participants had significantly lower odds of intermediate (aOR: 0.32, CI: 0.18–0.57) and high (aOR: 0.20, CI: 0.09–0.45) CVD risk but higher odds of frailty (aOR: 3.54, CI: 1.65–7.58) than non-H/L Whites. Among women, no significant differences were observed in frailty or CVD risk by ethnicity. Despite lower CVD risk, H/L men had higher frailty, suggesting potential sex-specific disparities that require further investigation.

Abstract citation ID: igaf122.4131

### HOSPITALIZATION AND CARE TRAJECTORIES NEAR END OF LIFE AMONG OLDER ADULTS – A NATIONWIDE COHORT STUDY

Naemi Herzog[1], Rahel Laager[2], Philipp Schütz[3], Beat Müller[3], and Alexander Kutz[3], *1. University of Basel / Cantonal Hospital Aarau, Zurich, Switzerland, 2. Children's Hospital Lucern, Lucern, Luzern, Switzerland, 3. Cantonal Hospital Aarau, Aarau, Aargau, Switzerland*

The final year of life accounts for a disproportionate share of health-care utilization. Characterizing hospitalization patterns, causes, and interventions during this period, and examining how these trajectories differ by frailty, is crucial for anticipating needs and improving end-of-life care. In this retrospective cohort study using Swiss national administrative claims of adults aged ≥65 years (2013-2022), we assessed temporal trends in hospitalizations within 1 year of death. The main outcome included causes of hospitalizations and number of invasive care interventions, stratified by frailty. Among 186,349 older adults, causes of hospitalization and interventions remained largely stable over time, with cardiac disease, cancer and injury-related conditions predominating. In frailer individuals, psychiatric and neurological conditions were more frequent in addition to injuries and cardiac diseases. Overall, 51.1% of individuals were hospitalized within the final three months of life. Among less frail individuals, cancer-related hospitalizations increased substantially (8.7% at 12 months before death to 26.6% in the last month of life, standardized mean difference [SMD] −0.482, $p < 0.001$), whereas in frailer individuals, the absolute increase was smaller (1.4% to 6.1%, SMD -0.252, $p < 0.001$), alongside increases observed for cardiac (10.1% to 18.3%, SMD -0.238 $p < 0.001$) and infectious diseases (9.5% to 15.7%, SMD -0.189 $p < 0.001$). Intervention rates increased modestly near death (39.4% to 42.0%, SMD −0.053, $p = 0.005$). In conclusion, hospitalization trajectories in the last year of life differ by frailty, with cancer dominating in the less frail and non-cancer causes in the frail. Tailored approaches are needed to improve alignment of end-of-life care.

Abstract citation ID: igaf122.4132

### HOUSING AND EPIGENETIC AGE ACCELERATION IN THE UNITED STATES: RESULTS FROM A NATIONALLY REPRESENTATIVE SAMPLE

Tongyu Gu, and Jennifer Ailshire, *University of Southern California, Los Angeles, California, United States*

Housing tenure is associated with a number of health outcomes at older ages, with homeowners typically demonstrating better health than renters. The underlying biological mechanisms linking housing to aging, especially in community-dwelling older adults, have yet to be explored. Using data on DNA methylation from the 2016 Health and Retirement Study Venous Blood Subsample, we examine the associations between housing tenure and epigenetic age acceleration in a nationally representative sample of US community-dwelling adults aged 56+ ($n = 3,449$). Survey-weighted linear regression models were used to predict epigenetic age acceleration measured with the following clocks: PhenoAge, GrimAge, PCGrimAge, and DunedinPACE. Compared to homeowners, renters showed age acceleration as measured by GrimAge ($\beta = 0.56$, $p < 0.05$), PCGrimAge ($\beta = 0.49$, $p < 0.05$), and DunedinPACE ($\beta = 0.02$, $p < 0.05$), though not by PhenoAge ($\beta = 0.55$, $p = 0.26$), after adjusting for sociodemographic factors (e.g., chronological age, gender, race/ethnicity, education, non-housing wealth, and number of household members) and health behaviors (e.g., smoking, alcohol use, obesity, and physical activity). Additional analyses did not find differences between homeowners with or without mortgages, or between homeowners and those who lived rent-free with others. These findings suggest renters experience earlier biological aging, which may increase aging-related morbidity and mortality risks. Homeownership is an important dimension of health equity among U.S. older adults.

Abstract citation ID: igaf122.4133

### HYDROGEL-BASED SCAFFOLD DEVELOPMENT FOR ISCHEMIC STROKE IN VITRO PLATFORM

Lidadi Agbomi[1], Richard Goodwin[2], and Bruce Gao[3], *1. Clemson University, Simpsonville, South Carolina, United States, 2. University of South Carolina, Columbia, South Carolina, United States, 3. Clemson University, Clemson, South Carolina, United States*

Background: Age-related neurological disorders, especially cerebrovascular disorders, pose a threat to the ever-increasing geriatric community. Improving tools for clinical studies can aid in the overall outcome of cerebrovascular incidents. In particular, ischemic strokes, a type of cerebrovascular disorder, which is the 2nd leading cause of both death and disability worldwide, can benefit from in vitro platforms that support the development of novel interventions. Methods: While the microfluidic chip was designed in CAD software, parallel efforts focused on creating the hydrogel scaffold. The first step was choosing the correct material. Material selection was based on desired properties for the scaffold; alginate was selected for its mechanical strength. Considering the tests being done, gelatin was added to help with cell adhesion properties. Following material selection, optimal concentrations were determined. The last test was deciding whether a room-temperature or frozen hydrogel would work best at maintaining the structural integrity of the hydrogel. Results: A composition of 1% alginate and 5% gelatin, in a ratio of 40/60, was the most suitable hydrogel. Further testing showed that freezing the hydrogel for 15 hours before dropping it into a calcium chloride bath significantly improved structural integrity. Conclusion: A working hydrogel formulation has been identified, and the next step is to create microchannels within the scaffold for cell culture. Further testing will be done to optimize the materials and techniques to create the channels.

Abstract citation ID: igaf122.4134

### IDENTIFYING THE DIVERGENT RURAL TRAINING NEEDS EXPRESSED BY OLDER ADULTS AND GERONTOLOGICAL PROVIDERS

Rachel Coleman[1], Lenard Kaye[2], Susan Wehry[3], Mary DeSilva[4], Lynette Sirois[4], Ruth Dufresne[5], Katie Keough[4], and Patricia Oh[6], *1. University of Maine Center on Aging, Orono, Maine, United States, 2. University of Maine, orono, Maine, United States, 3. University of New England College of Osteopathic Medicine, Portland, Maine, United States, 4. University of New England, Portland, Maine, United States, 5. Center for*

*Excellence in Public Health, University of New England, Portland, Maine, United States, 6. University of Maine, Bangor, Maine, United States*

Needs assessments of aging-related training and education commonly rely on the perspectives of those with specialized gerontological and geriatrics expertise and less so on the views of older adults themselves. Maine's Geriatrics Workforce Enhancement Program (AgingME2), now in its second operational cycle, conducts an annual assessment of preferred training content and delivery formats identified by both older adults and gerontological professionals. The 2025 assessment (n=254; 53.9% older adults/community members and 85.4% gerontological professionals/caregivers; multiple roles could be selected) is representative of all 16 counties in the state, the most rural in the nation. Substantial proportions of both groups (approximately one-third) indicated awareness of both the 4Ms of age-friendly health care and the age positivity movement and 32.3% and 33.6% of older adults and health care providers, respectively, have discussed 4M's related questions during their doctor/patient interactions. Significantly larger proportions of gerontological professionals and providers prefer in-person training compared to older adults (67.4% vs. 51.8%) as well as asynchronous webinars and other brief video formats. Providers emphasize the value of technology for reducing isolation/loneliness while older adults value it for lifelong learning and locating health resources. Exercise, remaining in the community, and driving safely are prioritized by older adults while providers emphasize end-of-life, cognitive decline and polypharmacy training. There is consensus that webinars of 30-60 minutes are preferable when offered. The implications of these differing perspectives for planning gerontological education and training learning sessions for older adults compared to professional providers are considered.

Abstract citation ID: igaf122.4135

### IMMERSIVE SIMULATION TRAINING: REMOTE PATIENT MONITORING OF COMPLEX OLDER ADULTS

David Picella, Sanggon Nam, and Lowell Renold, *Azusa Pacific University, Azusa, California, United States*

In value-based geriatric care, building practical skills in managing chronic conditions is vital. This pilot study assesses an immersive training program using Remote Patient Monitoring (RPM) to enhance nurse practitioner (NP) students' perceptions and skills. Eight NP students participated in a 10-day immersion simulation, embodying the patient experience by using RPM devices (blood pressure monitors, glucose meters, weight scales, pulse oximeters) to report daily vitals via a secure portal. They also simulated medication adherence to experience challenges of managing complex regimens, through hands-on patient role immersion. Pre- and post-perceptions were measured on a 5-point Likert scale, analyzed via Wilcoxon Signed-Rank and Paired Samples T-Tests. Qualitative responses were thematically analyzed for discoveries, perspective changes, and therapeutic challenges. Quantitative findings showed a significant decrease in perceived RPM cost burden (Wilcoxon $p=0.046$; T-Test $p=0.033$; Pre-Mean=3.38, Post-Mean=2.88), with no significant shifts in RPM's importance, liability, time consumption, feasibility, or medication ease ($p>0.05$). Qualitatively, themes emerged around heightened understanding of older adults' daily struggles with technology, adherence, and complex regimens; appreciation for RPM in averting hospitalizations; and calls for interprofessional collaboration. This pilot demonstrates that RPM simulation training enhances NP students' understanding of geriatric care complexities, critical for value-based care. Interprofessional educators and clinicians can leverage such training to improve chronic disease management, emphasizing simplified regimens and team-based approaches to enhance patient outcomes.

Abstract citation ID: igaf122.4136

### IMPACT OF INTERGENERATIONAL SERVICE LEARNING TO SUPPORT GOAL ATTAINMENT FOR HOMEBOUND OLDER ADULTS

Yeonjae Lee[1], Meti Negassa[2], Maggie Ratnayake[3], and Keith Chan[4], *1. Johns Hopkins University, Baltimore, Maryland, United States, 2. N/A, Pasadena, Maryland, United States, 3. Lori's Hands, Newark, Delaware, United States, 4. Hunter College, New York, New York, United States*

More than two million of older adults are homebound and five million need help leaving their homes. They often experience social isolation, food insecurity, and lack of connection to community resources. To date, home-based services for those aging in place are lacking. Using newly available data, this study examined the benefits of an intergenerational home-based service learning program on goal attainment in 1) social support, 2) home safety and cleanliness, 3) access to care resources, 4) food access, and 5) improving physicality for a community-based sample of 164 homebound older adults. Findings indicated that 75% reported positive changes in at least one of the five target service areas. The majority of participants reported 1) social support and 2) home safety and cleanliness as their most important goals. Results from chi square tests indicated statistically significant differences in goal attainment for 1) social support (Chi-square = 59.28, $p<0.001$, Cramer's V: 0.30) and 2) home safety and cleanliness (Chi-square: 29.50, $p<0.05$, Cramer's V: 0.21). Results from this study suggest that intergenerational in-home support services can improve social support and home safety and cleanliness for homebound older adults, thereby supporting aging in place. Policies and practice can support a pipeline of geriatric health professionals through innovative service learning models to benefit older adults, caregivers, and students.

Abstract citation ID: igaf122.4137

### IMPLEMENTATION OF THE CAPABLE PROGRAM PLUS CRITICAL HOME REPAIRS AMONG OLDER VETERANS

Connor Mitchell[1], Laura Ellen Ashcraft[2], Amanda Peeples[3], Octavia Goodman[4], Robert Burke[5], Allyson Evelyn-Gustave[6], Sarah Szanton[7], and Rebecca Brown[2], *1. Department of Veterans Affairs, Philadelphia, Pennsylvania, United States, 2. University of Pennsylvania, Philadelphia, Pennsylvania, United States, 3. Veterans Health Administration, Philadelphia, Pennsylvania, United States, 4. Corporal Michael J. Crescenz VAMC | U.S. Department of Veterans Affairs, Philadelphia, Pennsylvania, United States, 5. Center for Healthcare Evaluation, Research, and Promotion, Philadelphia, Pennsylvania, United States, 6. Johns Hopkins School of Nursing, Johns Hopkins School of Nursing, Maryland, United States, 7. Johns Hopkins University, Baltimore, Maryland, United States*

Effective programs are needed to facilitate aging in place, particularly among the growing number of older adults with functional decline. The Community Aging in Place-Advancing Better Living for Elders (CAPABLE) intervention, delivered by an interdisciplinary team, helps low-income, community-dwelling older adults to address causes of disability at home. We tested the implementation of CAPABLE among older Veterans in The Safer Aging Though Geriatrics-Informed Evidence-Based Practices Quality Enhancement Research Initiative hybrid type III effectiveness-implementation trial. We adapted CAPABLE to fit the Veterans Affairs (VA) context (CAPABLE-VA) and provided one year of implementation facilitation (e.g., training, implementation team meetings) to sites. Implementation outcomes were measured using the RE-AIM framework, effectiveness outcomes (general health status, ADLs, IADLs, falls self-efficacy, depressive symptoms, pain) using pre- and post-intervention assessments, and qualitative outcomes through post-intervention interviews using the PRISM framework. Four sites enrolled 30 Veterans: 53% (n = 16) received the full clinical dose of CAPABLE-VA; 90% (n = 27) received home modifications (e.g., grab bars, walk-in showers, entryway ramps); and 73% (n = 22) received critical home repairs (e.g. repairing or replacing plumbing, flooring, staircases). Veterans reported significant improvements in overall health status ($p < 0.001$), ADL performance ($p = 0.002$), depressive symptoms ($p = 0.03$), and pain ($p = 0.01$). Interviews with Veterans and staff indicated that CAPABLE-VA made homes safer and aging in place easier, though the program's administrative burden was noted. These findings underscore the need to pair CAPABLE with critical home repairs for low-income older Veterans, while highlighting the challenges of reaching Veterans at scale with a complex, multi-component intervention within the VA.

Abstract citation ID: igaf122.4138

### IMPLEMENTING PALLIATIVE CARE CONSULTATION PROGRAMS IN NURSING HOMES: KEY STAKEHOLDERS' PERSPECTIVES

Elisha Oduro[1], Alexandra Mora[2], Ruth Lopez[3], Kathleen Unroe[4], Moses Akwobugi[2], and Joan Carpenter[5], *1. University of Maryland Baltimore, Baltimore, Maryland, United States, 2. University of Maryland Baltimore, Baltimore, Maryland, United States, 3. MGH Institute of Health Professions, Sharon, Massachusetts, United States, 4. Indiana University School of Medicine, Indiana, Indiana, United States, 5. University of Maryland School of Nursing, Baltimore, Maryland, United States*

Palliative care (PC) consultation is associated with reduced acute care use and burdensome transitions for nursing home (NH) residents. However, little is known about the steps for implementing PC consultations into NHs. This study identified the processes of implementing PC consultations into NH workflows from the perspective of key stakeholders. Using a qualitative descriptive approach, we conducted semi-structured interviews with stakeholders in fourteen NHs in the Eastern United States. We analyzed transcribed interviews using conventional content analysis. Participants (N = 33) were NH direct care workers (e.g., nurses, social workers), PC consultants (e.g., PC nurse practitioners), and leaders (e.g., PC agency and NH directors), predominantly female (93.9%), and White (93.9%). One-third had 20 years of experience working in NHs. We identified a six-step PC consultation implementation process: (1) NH and PC agency establishing partnerships, (2) NH staff identifying and determining the need for PC referral, (3) NH staff initiating PC referrals, (4) PC agency processing referrals, (5) PC provider conducting consult, and (6) PC and NH follow-up to ensure PC care is implemented. Within each step, NH residents and their families, PC agency and NH leaders, and primary care providers significantly shape the process by undertaking actions that either move or stall the PC consultation process. Strategies that support the process include strong NH and PC agency relationships, PC consultant provider availability, PC consultant access to resident information, and NH staff investment in PC consultation services. Our findings provide an actionable framework to guide the implementation of effective NH PC consultations.

Abstract citation ID: igaf122.4139

### IMPLEMENTING THE GSA KAER TOOLKIT TO ENHANCE DETECTION AND MANAGEMENT OF COGNITIVE IMPAIRMENT IN OLDER ADULTS

Anna Pendrey[1], Triccia Aparicio[2], Miguel Paz[2], Mariel Zelaya[2], Javier Sevilla[1], Samir Cabrera[3], Paola Rodriguez[1], and Omar Suazo[3], *1. Indiana University, Zionsville, Indiana, United States, 2. Universidad Nacional Autonoma de Honduras, Zionsville, Indiana, United States, 3. UNITEC, Zionsville, Indiana, United States*

Background Cognitive impairment and dementia affect older adults in the US but often go undiagnosed in primary care due to time constraints, stigma, and lack of standardized protocols. The Gerontological Society of America's KAER (Kickstart, Assess, Evaluate, Refer) Toolkit offers a structured approach to improve early identification and management. Methods This quality improvement project was conducted in a primary care brain clinic from September 2023 to June 2025 for patients aged ≥65 years. Cognitive screening was integrated into routine visits. Patients with concerns identified in the Kickstart phase underwent detailed history-taking, functional assessment, and standardized testing, including the Montreal Cognitive Assessment and Patient Health Questionnaire-9. Reversible causes were investigated, and individualized care plans were developed with referrals to subspecialty care and community resources. Data collected included prevalence, comorbidities, functional status, and referral patterns. Results Among 41 patients screened, 80% had cognitive impairment, most aged 66–75 years. Women were more likely to have dementia; men more often had mild cognitive impairment. Patients comprised 42% African American, 27% Hispanic, and 27% White. Depression (83%) and anxiety (67%) were common, along with comorbidities such as hypertension (23%) and chronic kidney disease (23%). Two patients had positive ApoE genotypes with MRI-confirmed pathology. Conclusions KAER Toolkit implementation identified high rates of cognitive impairment and comorbidities, supporting its user-friendly design to improve early detection, targeted interventions, and coordinated care in older adults.

Abstract citation ID: igaf122.4140

### IMPLICATIONS OF CHANGES TO THE FEDERAL FUNDING LANDSCAPE FOR GERIATRICS TRAINING PROGRAMS

Apoorva Rangan, *Stanford University, Los Altos Hills, California, United States*

This poster session will describe the history of federal Geriatrics workforce development programs and the implications of proposed cuts to the Geriatrics Workforce Enhancement Programs (GWEPs) and Geriatrics Academic Career Awards

(GACAs) on the nation's capacity to train clinicians in age-friendly care. The 2008 Institute of Medicine report Retooling for an Aging America identified clinical complexity, ageist stereotypes, lack of mentors, and financial disincentives as barriers to geriatrics workforce growth[1]. In response, GWEPs (launched in 2015) and GACAs (re-established in 2019 after elimination in 2015) were established to embed interprofessional geriatrics curricula, support early-career educators and researchers, and fortify training sites in underserved settings.[2] These programs received top-line funding of $42 million in fiscal year 2024, faced severe cuts with top-line funding of $28 million in fiscal year 2025, and have uncertain funding this year.3, 4 Drawing on recent National Center for Health Workforce Analysis workforce projections, American Board of Medical Specialties Board Certification Report, American Geriatrics Society policy materials, and HHS' Tracking Accountability in Government Grants System (TAGGS), this session will explore how elimination of these federal investments may: 1) interrupt crucial interprofessional training partnerships; 2) erode a critical pipeline of clinician–educators by withdrawing protected time and mentorship; and 3) exacerbate health disparities by constricting age-friendly care capacity for low-income and rural older people. Finally, this session will engage participants in policy recommendations to preserve and expand these programs' funding, ensuring a sustainable geriatrics workforce equipped to meet the complex needs of all of us as we age.

Abstract citation ID: igaf122.4141

### IMPROVING HEALTH CARE QUALITY FOR OLDER ADULTS IN SUB-SAHARAN AFRICA: LESSONS FOR GLOBAL HEALTH SYSTEMS

Judith Ani, *Walter Sisulu University, Mthatha, Eastern Cape, South Africa*

This study examined how older adults in a Sub-Saharan African community experience healthcare interactions and navigate health and disability challenges, with an emphasis on identifying system-level gaps and opportunities for quality improvement in healthcare delivery. Using a qualitative design, 36 semi-structured interviews were conducted with adults aged 65 years and older, recruited purposively to capture diverse household and social contexts. Data were thematically analyzed to explore lived experiences of care access, quality, and coping strategies. Participants highlighted three recurring challenges in the quality of care: inadequate provider responsiveness to age-related conditions, structural inequities such as long travel distances and out-of-pocket costs, and lack of culturally sensitive communication in clinical encounters. In response, older adults adopted adaptive coping strategies (community caregiving networks, spirituality, and alternative medicine use) and maladaptive strategies (delaying or avoiding care). While these strategies mitigated immediate hardship, they often undermined long-term health outcomes. The findings underscore the urgent need for quality improvement innovations tailored to older populations in low-resource settings. Strengthening provider training in geriatric competencies, integrating community health workers into formal systems, and implementing financial protection measures can enhance care equity. These insights from Sub-Saharan Africa offer globally relevant lessons for advancing age-friendly, patient-centered care in diverse health systems.

Abstract citation ID: igaf122.4142

### IMPROVING QUALITY AND SAFETY IN AGED CARE THROUGH DIGITAL INNOVATION AND DATA-DRIVEN INTERVENTIONS

Francois Xavier Duchateau[1], Aileen Waton[2], Mel White[3], Ana Isabel Fernandez Castello[4], Siobhan Mackintosh[5], and Anne Lepetit[5], *1. BUPA, London, England, United Kingdom, 2. Bupa UK, London, England, United Kingdom, 3. Bupa Australia, Melbourne, Victoria, Australia, 4. Sanitas, Madrid, Castilla y Leon, Spain, 5. Bupa, London, England, United Kingdom*

Introduction Improving the quality of care while minimizing risks remains a core objective for healthcare organizations. Aged care (AC) presents unique challenges associated with the older population, particularly polypharmacy, nutritional deficits, and reduced mobility. This study illustrates how digitalization and data-driven approaches can support quality improvement in AC, through the evaluation of 3 recent initiatives. Methods We are a non-profit international healthcare provider that delivers AC. In the UK, an Electronic Medication Administration Record (eMAR) system was gradually implemented across 120 care homes (CH) between June 2023 and June 2024. The effectiveness of this intervention was assessed through reported medication error rates. In the Australia, 57 CH are supported by 13 Wellness Hubs staffed by 24/7 Nurse Practitioners, who coordinate multidisciplinary teams including dietitians, physiotherapists, wound care specialists... As a quality indicator, we measured the time to healing for pressure ulcers. In Spain, 45 CH adopted a digital care model including voice assistants (Alexa) in each room to provide real-time care instructions, based on individualized care plans. The incidence of falls per 1,000 bed-days was reported. All data were consolidated using Power BI dashboards. Results Following the implementation of eMAR, minor medication errors decreased by 7%, and moderate to major errors declined by 50%. In APAC, the healing time for pressure ulcers was reduced by 42 days. In Spain, the rate of falls decreased to below 5 per 1,000 bed-days. Conclusion These findings suggest that the implementation of tailored, technology-enabled initiatives can significantly enhance quality and safety in AC.

Abstract citation ID: igaf122.4143

### INCLUSION OF OLDER ADULTS IN CONSUMER-DIRECTED HEAT SAFETY MATERIALS: A CONTENT ANALYSIS OF U.S. STATE WEBSITES

Christopher Liu[1], Pierre Jolin[1], Emily Gadbois[2], Elizabeth Fussell[3], Allan Just[3], and Theresa Shireman[2], *1. Brown University, Providence, Rhode Island, United States, 2. Brown University School of Public Health, Providence, Rhode Island, United States, 3. Institute at Brown for Environment and Society, Providence, Rhode Island, United States*

The increasing frequency and duration of extreme heat events places our growing older adult population at heightened risk for adverse, heat-related outcomes. While states provide the public with important heat safety messaging through websites, little is known about the inclusion of targeted information relevant to older adults. We conducted a qualitative content analysis of consumer-directed heat safety information published on state websites to 1) characterize information relevant to older adults and their caregivers and 2) assess information depth and content gaps. Our content analysis revealed five major themes regarding heat-specific risks: 1) acknowledgment of risk in older adults and definitions of older age; 2) chronic conditions or disabilities; 3) use of particular prescription medications or known toxic substances; 4) restricted fluid and salt intake; 5) social isolation. Most websites acknowledged that older adults are at increased risk of heat illness, but only half included a definition for older age. A majority discussed risk in those with chronic conditions—with cardiovascular conditions and obesity included most frequently and cancer and cognitive impairment cited the least. Only half discussed prescription medications, two-thirds discussed toxic substances, and half discussed social isolation as an intersectional concern. Most discussed the importance of consuming fluids and electrolytes, however, few provided recommendations for those with restricted fluid and salt intake. Our results highlight the lack of granular, age-inclusive information in most heat safety material published by state websites. Identified content gaps should inform relevant additions to these materials to promote self-sufficiency in mitigating heat risk among older adults.

Abstract citation ID: igaf122.4144

### INCORPORATING FORCED MIGRATION AND ALTERNATIVE ONTOLOGIES INTO LIFE COURSE THEORY: INSIGHTS FROM HMONG REFUGEES

Michal Engelman, and Maichou Lor, *University of Wisconsin-Madison, Madison, Wisconsin, United States*

Hmong refugees were resettled in Wisconsin after the Secret War in Laos. Their life experiences and unique ontology offer new insights into the intersection of forced migration and life course studies. Hmoob Lub Neej (Hmong People's Lives) is a community-engaged study of aging in Wisconsin's Hmong refugee community. We conducted semi-structured in-depth life history interviews with 55 older Hmong men and women. Interviews in two Hmong dialects were transcribed and translated into English. Using the five principles of life course theory (history and geography; trajectories and transitions; timing; agency; linked lives), we analyze the disruption and reconstitution of Hmong lives and communities. Participants describe the intersection of history, geography, and biography as they experienced violence and forced relocations. They also address the reconstruction of their livelihoods and communities, highlighting agency and linked lives. The narratives tie early and midlife hardships with later-life outcomes, and showcase social ties – and their absence – as sources of both tension and resilience. In addition, Hmong ontology offers a set of culturally and historically-specific ideas of time, place, spirits, health and life that together generate important new considerations for understanding disruptions to individual and collective life courses. Longing for a spiritually meaningful yet unreachable home and rapid social change in the diaspora define this Hmong cohort's experience of aging. Their story connects the universal experience of aging and intergenerational change to themes of displacement and inequality, and to diverse ontologies of the life course.

Abstract citation ID: igaf122.4145

### INFORMAL SOCIAL ACTIVITIES AND COGNITIVE FUNCTION IN LATER LIFE: THE ROLE OF GENDER AND MARITAL STATUS

Haejin Jang[1], and Sae Hwang Han[2], *1. University of Massachusetts Boston, Boston, Massachusetts, United States, 2. University of Texas at Austin, Austin, Texas, United States*

Social engagement with friends and neighbors in informal domains—e.g., having social visits or providing informal helping—is increasingly recognized as a lifestyle factor protective of cognitive function in later life. However, little is known about whether these benefits vary across sociodemographic characteristics, such as gender and marital status. Using eight waves of nationally representative data from the U.S. Health and Retirement Study (person N = 29,777; 131,449 person-wave observations), this study examined how gender and marital status moderated the cognitive outcomes associated with social visits and informal helping. Cognitive function was assessed with the modified Telephone Interview for Cognitive Status (m-TICS), and social visits and informal helping were evaluated as both status and level of involvement (i.e., dose effects). Within-between random effects models were employed to identify within-person effects unbiased by stable person-level characteristics. Findings showed that both forms of social activities were linked to better cognitive function among women, whereas only informal helping predicted better cognitive outcomes among men. Moderate levels of informal helping yielded the greatest benefit for both women and men, while high levels showed diminishing returns; by contrast, only high-frequency social visits were associated with significant cognitive benefits among women. When considering marital status, significant differences appeared only among men, where social visits were linked to better cognition during when men were married, but not when they were unmarried. These findings highlight the importance of tailoring social engagement interventions to reflect differences in gender and marital status, particularly among populations at risk of cognitive decline.

Abstract citation ID: igaf122.4146

### INTERPRETABLE MACHINE LEARNING FOR COGNITIVE AGING: HANDLING MISSING DATA AND UNCOVERING SOCIAL DETERMINANTS

Xi Mao[1], Hairong Wang[2], Lingchao Mao[3], Jingyu Li[3], Zhendong Wang[4], and Xuelei Ni[5], *1. University of Texas Rio Grande Valley, Brownsville, Texas, United States, 2. The University of Texas at Austin, Austin, Texas, United States, 3. Georgia Institute of Technology, Atlanta, Georgia, United States, 4. Shandong Mental Health Center, Jinan, Shandong, China (People's Republic), 5. Kennesaw State University, Kennesaw, Georgia, United States*

Early detection of Alzheimer's disease (AD) is critical, as neuropathologic change and modifiable social behavioral risks accumulate years before diagnosis. Identifying higher-risk individuals earlier enables prevention, timely care, and more equitable resource allocation. We study prediction of cognitive performance from social determinants of health (SDOH) using the NIH NIA supported PREPARE Challenge Phase 2 dataset, derived from the nationally representative Mex-Cog cohort within the 2003 and 2012 Mexican Health and Aging Study (MHAS). The target is a validated composite cognitive score covering orientation, immediate and delayed memory, attention, language, constructional praxis, and executive function, which derived from 2021 and 2016 MHAS. We curated features across demographic, socioeconomic, health, lifestyle, psychosocial, and healthcare access domains to capture multidimensional social and behavioral influences on cognitive aging. Substantial missingness was addressed with a singular value decomposition (SVD)-based imputation pipeline, treating continuous and categorical variables separately. This leverages latent feature correlations to recover missing values while balancing reliability and scalability. To better understand the relationship between input features and the composite cognitive score, we conducted a thorough post hoc analysis of the top contributing features, examining the mechanism of these features are associated with cognitive scores. The study further stratified the analysis by age group to explore whether the most predictive features differ across life stages. The proposed framework demonstrates robustness, interpretability, and computational efficiency, underscoring its potential as a practical modeling strategy for MHAS data in AD and MCI detection with substantial missingness across both continuous and categorical features.

Abstract citation ID: igaf122.4147

### INTERSECTING INEQUITIES IN ISCHEMIC HEART DISEASE AMONG U.S. OLDER ADULTS: TRENDS BY NATIVITY, SEX & RACE, 2005–19

Stuti Das, *Boston University, Boston, Massachusetts, United States*

Ischemic heart disease (IHD) remains a leading cause of morbidity and mortality in later life, with prevalence rising steeply after midlife. While disparities by sex and race/ethnicity are well documented, less is known about how these inequities evolve at the intersection of nativity, sex, and race/ethnicity among U.S. adults aged 45 and older. Using harmonized data from the Integrated Public Use Microdata Series of the National Health Interview Survey (IPUMS NHIS, 2005–2019), I examine trends in self-reported IHD diagnoses. Joinpoint regression identifies inflection points in age-standardized prevalence across subgroups defined by nativity, sex, and race/ethnicity, followed by moderation analyses to assess how these identities jointly shape disease risk. Findings reveal that foreign-born adults consistently report lower IHD prevalence than their U.S.-born peers across sex and racial/ethnic groups. However, this immigrant health advantage is not uniform. Native-born White women are the only group to experience a sustained decline in IHD over time, while the nativity gap is widest among non-Hispanic Black and Asian men. Some immigrant subgroups, such as Asian immigrant women, show slight increases, though still below native-born levels. These findings underscore the importance of an intersectional, equity-oriented lens for understanding disparities in cardiovascular aging and for informing more inclusive public health strategies. As the U.S. population grows older and more diverse, it is critical to examine how migration, racialization, gender, and aging interact to shape chronic disease risk and resilience.

Abstract citation ID: igaf122.4148

### INTERVENTIONS TO ADDRESS FINANCIAL VULNERABILITY IN DEMENTIA: A SCOPING REVIEW

Shadi Gholizadeh[1], Megan Harris[2], and Lucas Melville[2], *1. The Key, Coto de Caza, California, United States, 2. TheKey, San Diego, California, United States*

Older adults living with dementia and other cognitive changes are at elevated risk for financial exploitation, mismanagement of funds, and financial abuse. Despite this, relatively few interventions have been developed to address financial vulnerability in home and community settings, especially when care is also being provided. This scoping review aimed to identify and summarize peer-reviewed studies describing interventions targeting financial harm among people with dementia. PubMed and PsycINFO were searched using terms related to cognitive impairment, financial exploitation, and intervention. Of 39 articles retrieved, 7 were reviewed in full, and 5 met inclusion criteria. Interventions included: a home photo-assessment tool to identify environmental triggers for delusions of theft; a telehealth dementia care coordination model using unlicensed navigators to address financial and legal concerns; a policy-focused interview study with safeguarding professionals on risks tied to personal budget misuse; a multidisciplinary forensic center model that increased referrals for conservatorship; and a behavioral-technology study identifying commonly lost objects to inform assistive robotics. Across studies, interventions varied in scope and rigor. Most were small-scale, lacked formal evaluation, and none were randomized controlled trials. However, they highlighted promising strategies to reduce financial harm through environmental, relational, technological, and legal approaches. Most targeted early to moderate dementia and were caregiver-inclusive. This review underscores the urgent need for scalable, rigorously evaluated interventions that promote financial safety as part of dementia care planning, while preserving autonomy, reducing caregiver strain, and enabling interdisciplinary collaboration across care, legal, and community settings.

Abstract citation ID: igaf122.4149

### LATENT VIRUSES IN OLDER ADULTS: ASSOCIATIONS WITH INFLAMMATION AND IMPLICATIONS FOR HEALTH

Tanvi Tomar[1], Brett Messman[1], Jennifer Graham-Engeland[1], Erik Knight[2], and Christopher Engeland[1], *1. The Pennsylvania State University, University Park, Pennsylvania, United States, 2. University of Colorado Boulder, Boulder, Colorado, United States*

Latent viruses, such as Epstein-Barr Virus (EBV), Cytomegalovirus (CMV), and Human Herpesvirus-6 (HHV-6), are persistent viral infections that can reactivate over time and are highly prevalent in older adults (60+), with prevalence rates ranging from 60% to>95%. The pathogenic burden exerted over the lifespan by such persistent viral infections is increasingly recognized as a risk factor for poorer immunity and health outcomes in older adults. In this context, the current study explored associations between 1) EBV, CMV, and HHV-6 antibody seropositivity or 2) pathogenic burden (i.e., number of seropositive viruses) with C-reactive protein (CRP) and basal and lipopolysaccharide (LPS)-stimulated inflammatory cytokines [interleukin-6 (IL-6), interleukin-8 (IL-8), interleukin-10 (IL-10), tumor necrosis factor-alpha (TNF-a), interferon-gamma (IFN-γ)], assessing basal and reactive immune measures, respectively. Participants were healthy older adults (N=31; ages 60-81; 68% female; 94% White) recruited from central Pennsylvania. All participants were IgG seropositive for at least one latent virus (19% for 1 virus; 58% for 2 viruses; 22% for 3 viruses). Greater pathogenic burden was negatively correlated with LPS-stimulated cytokines: IL-6 (r=-.09, p=.004); IL-8 (r=-.12, p=.038); IL-10 (r=-.04, p=.015); IFN-γ (r=-.09, p=.044); TNF-α (r=-.12, p=.013). Additionally, among individuals with high IgG levels (4th quantile) the strength of the negative correlations was considerably greater for stimulated IL-6 (r=-.26, p<.001), IL-10 (r=-.16, p<.001), and TNF-α (r=-.27, p<.001). These preliminary findings suggest that greater pathogenic burden of latent viruses may impair the ability to mount an appropriate inflammatory response. These results warrant further investigation between cumulative latent viral burden and inflammation among older adults.

Abstract citation ID: igaf122.4150

### LEARNING AND DEVELOPMENT AMONG CARE WORKERS FOR OLDER ADULTS IN FINLAND

Younho Lee, and Yongho Chon, *Incheon National University, Incheon, Republic of Korea*

Expanding learning perspectives can give insight into the mechanisms of developing care workers for older adults and organizations. Care workers learn from lecture-based education and training regarding health and social care while learning from hands-on experiences by observing, asking questions, and interacting with skilled colleagues (Dewing, 2010; Raymond et al., 2014). That is, they experience various types of learning because of the influence of context, relationship, and interaction at the workplace (Marsick & Watkins, 1990; Eraut, 2004; Anvik et al., 2020). However, most interventions in learning are education and training, and these methods are difficult to assess direct effectiveness (Surr et al., 2017, 2019; Latham et al., 2024). Therefore, this study aims to examine the experiences and perspectives of care workers' learning in Siun Sote, Finland. A qualitative study was conducted at the Siun sote center. Eight nurses, seven care assistants, two social workers, three registered nurses, and three managers participated in the interviews. The main finding is as follows. First, care workers participated in organization-led training and obtained knowledge and skills in health and social care. Second, care workers experienced observation, supervision, and delegation, and achieved person-centered care, problem-solving, and situational judgment competencies. Care workers participated in teamwork and multidisciplinary collaboration. Siun sote runs knowledge management systems, organizational training programs, and task guidelines. The organization can strengthen its capacities to provide diverse services for its clients and manage knowledge and skills. This study shed light on various types of learning activities and outcomes for care workers and the organization itself.

Abstract citation ID: igaf122.4151

### LENGTH OF REPRODUCTIVE LIFESPAN AND LATE-LIFE COGNITIVE FUNCTION AMONG U.S. WOMEN: EVIDENCE FROM NHANES 2011-2014

Julia Unsworth, Hannah Jones, and Sahra Sheikhnur, *OHSU-PSU School of Public Health, Portland, Oregon, United States*

Women experience disproportionately higher rates of dementia and cognitive decline than men, and lifetime exposure to sex hormones is hypothesized to contribute to this disparity. Reproductive lifespan, defined as the interval between age at first period (menarche) and menopause, may influence late-life cognitive function through cumulative estrogen exposure, but this relationship remains underresearched in nationally representative U.S. samples. Using cross-sectional data from 1,435 women 60 years and older in the 2011-2014 National Health and Nutrition Examination Survey (NHANES), we examined the relationship between reproductive lifespan and cognitive function. Reproductive lifespan was defined as the duration between age at menarche and age at last menstrual period, categorized into weighted tertiles (≤30, 31-38 years, >38 years). Cognitive function was measured using a composite score derived from the Consortium to Establish a Registry for Alzheimer's Disease Word Learning and Recall, Animal Fluency Test, and Digit Symbol Substitution Test. Survey-weighted multivariable linear regression models estimated associations, adjusting for age, race/ethnicity, nativity, parity, education, and alcohol use. Women with longer reproductive lifespans (>38 years) reported significantly higher cognitive scores compared to those in the shortest tertile (adjusted $\beta = 0.15$; 95% CI: 0.03, 0.26). The association was strongest among college graduates ($\beta = 0.23$; 95% CI: 0.03, 0.42), compared to women with lower education with smaller and not statistically significant results. A formal test for interaction confirmed effect modification by education ($p = 0.025$). These findings suggest that a longer reproductive lifespan is associated with better late-life cognitive function, particularly among women with higher educational attainment.

Abstract citation ID: igaf122.4152

### LESSONS FROM MAPPING THE STRUCTURAL AND SOCIAL DETERMINANTS OF HEALTH FOR OLDER ADULTS LIVING IN NYC CHINATOWNS

Isadora Li[1], Dalilah Mora[2], and Paris Adkins-Jackson[3], *1. Barnard College Department of Political Science, New York, New York, United States, 2. Columbia Mailman School of Public Health, Bronx, New York, United States, 3. Mailman School of Public Health, Columbia University, New York, New York, United States*

Older adults racialized as Asian are an underrepresented group in health disparities research. Neighborhoods commonly referred to as Chinatowns in NYC have older adult populations in which the majority are racialized as Asian and who face unique challenges to healthy aging due to structural determinants of health. This study aimed to compile a comprehensive directory of health resources in three New York City Chinatowns (Manhattan Chinatown, Flushing, and Sunset Park) in order to understand the landscape of health access for older adults in these neighborhoods. The study used a modified approach to community asset mapping, which involved teams of researchers conducting walking surveys within the target neighborhoods. Community asset mapping is a proven method of identifying community resources, but challenges arise when older adults are engaged in this approach due to mobility limitations. The protocol yielded a novel dataset of 1,154 resources (313 in Sunset Park, 364in Manhattan, 477 in Flushing) among four broad categories: healthcare, older adults, social services, and health promotion. The study also produced three neighborhood-specific Google Maps albums of determinants. The team then collaborated with a local organization to further refine a portion of the dataset to align with their efforts to support older adults living in an affordable senior housing building. This project's approach to asset mapping has implications for generating methods of investigation involving older adults which accommodate their needs, such as accommodations due to mobility limitations. The novel dataset and collection of maps will also drive continued work with community organizations.

Abstract citation ID: igaf122.4153

### LEVERAGING ARTIFICIAL INTELLIGENCE IN ADVANCE CARE PLANNING: A SCOPING REVIEW

Minghui Tan[1], Jinfeng Ding[2], and Siyuan Tang[2], *1. Central South University, union city, New Jersey, United States, 2. Central South University, Changsha, Hunan, China (People's Republic)*

Background Advance care planning (ACP) is a process that enables individuals to discuss future health care decisions before they become seriously ill or unable to communicate. Artificial intelligence (AI) has demonstrated promising outcomes in facilitating healthcare, offering the potential to facilitate ACP. However, the current status of using AI to facilitate ACP is unclear. This study aimed to investigate how AI has been leveraged to facilitate ACP, with a particular focus on the intended purposes, AI algorithms used, data sources, and the performance of AI in achieving the intended purposes. Methods The methodology employed in this study adhered to the Scoping Review Methodological Framework. PubMed, EMBASE, Web of Science, CINAHL, Cochrane Library, and IEEE Xplore databases were searched from their inception to July 2025. Descriptive analysis and narrative synthesis were used to summarize findings from the included studies. Results A total of 42 eligible studies was analyzed. The studies were primarily used to detect ACP conversations and documents, identify patients needing ACP, promote ACP education, and explore linguistic features in ACP conversations. Rule-based natural language processing emerged as the most commonly used AI algorithm, with textual data being the primary modality employed. The included studies exhibited significant variation in performance evaluation. Finding The current use of AI in ACP remains limited in scope, primarily focused on extracting ACP documentation from electronic health records and identifying patients who may benefit from ACP. The use of advanced technologies such as generative AI is limited, and performance evaluation primarily relied on discrimination metrics.

Abstract citation ID: igaf122.4154

### LINKAGES OF NANDA INTERNATIONAL, NIC AND NOC FOR PEOPLE LIVING WITH HIV OVER 50

Juh Shin[1], Cheryl Wagner[2], Elizabeth Swanson[3], Mary Clarke[4], Sue Moorhead[5], Karen Dunn Lopez[5], Hritik Majgaonkar[6], and Nidhi Naidu[7], *1. George Washington University, Ashburn, Virginia, United States, 2. University of Iowa College of Nursing, Iowa City, Iowa, United States, 3. University of Iowa Center of Nursing Classification and Clinical Effectiveness, Iowa City, Iowa, Iowa, United States, 4. Healthlinx, Davenport, Iowa, United States, 5. University of Iowa, Iowa City, Iowa, United States, 6. George Washington University, Washington, DC, District of Columbia, United States, 7. Geroge Washington University, Arlington, Virginia, United States*

Despite advancements in HIV treatment, nursing care for older adults living with HIV (OPLWH) remains insufficiently structured to address their complex, age-related needs. The purpose was to develop linkages between NANDA International, Nursing Outcomes Classification, and Nursing Interventions Classification for older adults living with HIV aged over 50. Data were collected from 16 experienced nurses specializing HIV/AIDS care, who evaluated the relevance of 45 NANDA International, 79 Nursing Outcomes Classification, and 82 Nursing Interventions Classification specific to older adults living with HIV. A table was developed to present frequently used 16 NANDA-I diagnoses in the as critical (relevance ratio ≥0.80) or supplemental (0.60–0.79). The Nursing Outcomes Classifications were linked according to the core components of the selected NANDA-I, followed by Nursing Interventions Classification. Finally, we achieved consensus among experts based on clinical judgment, experience, and established NOC/NIC knowledge. Sixteen core NANDA International were validated as critical, including (i) Risk for Excessive Loneliness linked with Loneliness Severity and Emotional Support; (ii) Risk for Infection linked with Infection Severity and Infection Control; (iii) Inadequate Nutritional Intake linked with Nutritional Status: Nutrient Intake and Nutrition Management; (iv) Impaired Tissue Integrity linked with Tissue Integrity: Skin and Mucous Membranes and Wound Care; and (v) Impaired Memory linked with Memory and Cognitive Stimulation. The validated linkages provide a structured, evidence-based framework for nursing care planning for older adults living with human immunodeficiency virus, emphasizing the integration of psychosocial and physiological health needs. This linkage system supports holistic, individualized nursing care.

Abstract citation ID: igaf122.4155

### LIVING ARRANGEMENTS AS DYNAMIC CONTEXTS FOR COGNITIVE AGING: THE PROTECTIVE ROLE OF SOCIAL PARTICIPATION

Hyunjoo Lee[1], Sojung Park[2], Eunsun Kwon[3], Takashi Amano[4], and Minyoung Kwak[5], *1. Daegu University, Kyungsansi, Kyongsang-bukto, Republic of Korea, 2. Washington University in St. Louis, St. Louis, Missouri, United States, 3. Fairleigh Dickinson University, Florham Park, New Jersey, United States, 4. Rutgers University Newark, Newark, New Jersey, United States, 5. Daegu University, Gyongsan, Kyongsang-bukto, Republic of Korea*

Living arrangements are a central part of older adults' daily lives, shaping opportunities for social interaction, support, and cognitive stimulation. Prior studies show that co-residence with a spouse is often linked to better cognitive outcomes, while living alone or with non-spouse others may carry greater risks. Yet most research has focused on cross-sectional differences. Less is known about how changes in living arrangements over time affect cognition and whether these effects vary across subgroups. Using longitudinal data and fixed-effects models, this study examines the association between shifts in living arrangements and cognitive functioning, and the moderating role of social participation. We also examine whether the protective role of social participation differs by gender and education. Data come from 12 waves (2010–2022) of the National Health and Aging Trends Study (NHATS) in the U.S., including 39,676 observations from 6,295 adults aged 65 and older. Fixed-effects models assessed within-person changes in living arrangements, with sociodemographic and health characteristics controlled. Results indicated that transitioning from living with a spouse to living alone was associated with a 0.556-point decline in cognitive scores ($p < 0.01$). Each unit increase in social participation reduced this disadvantage by 0.108 points ($p < 0.01$). Social participation was also independently associated with higher cognitive scores ($\beta = 0.110$, $p < 0.001$). Subgroup analyses revealed stronger protective effects among men and those with higher education. These findings suggest that living arrangements are dynamic contexts shaping cognitive aging. Social participation represents a modifiable pathway to resilience, underscoring the importance of fostering engagement opportunities for older adults navigating household transitions.

Abstract citation ID: igaf122.4156

### LIVING TOGETHER, FEELING APART: LONELINESS AND LIVING ARRANGEMENTS AMONG OLDER US IMMIGRANTS

Mara Sheftel, *Rutgers University, New Brunswick, New Jersey, United States*

Loneliness tends to increase with age after 50 [3–5] and is a key modifiable risk factor for chronic health conditions in older adulthood [8–11]. Immigrants may be especially vulnerable to loneliness due to disrupted social networks and geographic separation from origin-country ties [12–14]. While older immigrants are more likely than their US-born counterparts to coreside with family [15,16], such arrangements may confer either risk [18] or resilience [17] for mental health, depending on context. This presents an empirical puzzle. This paper investigates how patterns of loneliness and living arrangements vary by country of origin, drawing on Health and Retirement Study (HRS) data for foreign-born Mexican older adults and new data from the New Jersey Population Health Cohort Study (NJHealth) for foreign-born Korean and Indian older adults. Findings reveal distinct patterns compared to US-born counterparts: Indian immigrants are less likely to live alone and show no significant differences in loneliness, Korean immigrants report higher loneliness despite similar living arrangements, and Mexican immigrants are both more likely to report loneliness and less likely to live alone. These results suggest that the relationship between living arrangements and loneliness is not uniform across immigrant groups and may reflect differences in cultural norms, migration histories, and socioeconomic contexts. As the US older immigrant population becomes increasingly diverse [19], understanding group-specific vulnerabilities is critical. This study offers early insights into which older immigrant populations may be at heightened risk of loneliness and how living arrangements intersect with mental health outcomes in later life.

Abstract citation ID: igaf122.4157

### LONELINESS, SOCIAL SUPPORT, & PERCEIVED ECONOMIC HARDSHIP IN SELF-MANAGEMENT OF OLDER LIVER TRANSPLANT RECIPIENTS

JiYeon Choi[1], JaeWon Hyun[1], Bomgyeol Kim[2], Hun Kang[1], and Jin-kyung Lee[2], *1. Yonsei University, Seoul, Republic of Korea, 2. Yonsei University College of Nursing, Seoul, Republic of Korea*

Liver transplantation is a highly selective and resource-intensive procedure that challenges both individuals and health systems. With more older adults receiving liver transplantation to treat end-stage liver disease, it is important to recognize that outcomes are shaped not only by aging-related physical and cognitive challenges but also by psychosocial factors, including loneliness, social support, and perceived economic hardship. We examined relationships among loneliness, perceived social support, perceived economic hardship, and self-management in older liver transplant recipients (LTRs) (age≥60), focusing on the mediating role of perceived social support and the moderating role of perceived economic hardship. A cross-sectional analysis was conducted using data from 131 LTRs (Age 65.24±3.91 years, 55.8% men, 27.78±17.00 months post-transplant) recruited from a tertiary academic medical center in South Korea. Measures included Loneliness scale (3-item), Multidimensional Scale of Perceived Social Support (12-item), Modified Transplant Self-management Scale (16-item), and economic hardship (one-item). Perceived economic hardship was classified into three categories: difficult, moderate, and none. Moderated mediation analysis was conducted using Hayes' PROCESS macro (Model 15), controlling for gender, income, education, marital status, living arrangement, return to work status, cognitive status, and frailty. Loneliness was associated with lower perceived social support ($\beta=-0.27$, $p=.002$), and higher social support was associated with better self-management ($\beta = 5.91$, $p<.001$). The indirect effect of loneliness on self-management via social support was significant across perceived economic hardship levels (index=0.503, 95% CI 0.03-1.04). Findings suggest that financial constraints may diminish the protective role of social support. Interventions to enhance self-management should address psychosocial barriers in aging LTRs.

Abstract citation ID: igaf122.4158

### MAPPING DEMENTIA CARE IN U.S. PRISONS: A STATE-LEVEL CHARACTERIZATION OF POLICIES AND PROGRAMS

Elizabeth Breen[1], Indrakshi Roy[2], and Ricky Camplain[3], *1. Indiana University School of Medicine, Indianapolis, Indiana, United States, 2. Indiana University, Bloomington, Bloomington, Indiana, United States, 3. Indiana University Bloomington, Bloomington, Indiana, United States*

The aging of the U.S. prison population has led to a marked increase in incarcerated individuals with Alzheimer's disease and related dementias (ADRD). Despite the clinical complexity and behavioral management needs associated with dementia, carceral health systems remain underprepared to deliver appropriate care. Research on ADRD in correctional settings is sparse, and national policy guidelines are lacking. Because healthcare in prisons is administered at the state level, understanding the landscape of dementia care requires granular, jurisdiction-specific analysis. This project presents a comprehensive, state-by-state review of dementia-related services in U.S. carceral systems. Public records, correctional health documents, and secondary sources were used to categorize each state based on (1) presence of a dementia-specific program, (2) availability of geriatric or hospice care units, and (3) use of compassionate or geriatric release policies. Our analysis reveals wide variability: only 34% of states currently operate dementia-specific prison programs, while others rely on general end-of-life services or ambiguous early release frameworks. Many states have no identifiable infrastructure for supporting cognitive decline among incarcerated individuals. These findings highlight critical gaps in both the delivery and documentation of dementia care in prisons. As the incarcerated population continues to age, developing scalable, evidence-informed strategies for the management of ADRD in carceral settings will be essential for ensuring continuity of care, safety, and resource stewardship across the health and justice systems.

Abstract citation ID: igaf122.4159

### MAPPING HUMAN HEALTH OUTCOMES IN RELATION TO HARMFUL ALGAL BLOOMS USING A TRANSLATIONAL RESEARCH FRAMEWORK

Rebecca Koszalinski[1], Malcolm McFarland[2], John Reif[3], Adam Schaefer[4], Michael Parsons[5], Ann Cary[6], Alex Rockenstyre[1], and Rachael Shinbeckler[6], *1. University of Central Florida, Orlando, Florida, United States, 2. Florida Atlantic University, Fort Pierce, Florida, United States, 3. Colorado State University, Fort Collins, Colorado, United States, 4. Self, Richmond Hill, Georgia, United States, 5. Florida Gulf Coast University, Ft Myers, Florida, United States, 6. Florida Gulf Coast University, Ft. Myers, Florida, United States*

Aim: To evaluate Human Health Outcomes (HHO) after exposure to Harmful Algal Blooms (HABs) in aging adults through the National Institute of Environmental Science,

Research Translational Framework (NIEHS-TRF). Our program of research required a cohesive framework for continued multidisciplinary collaboration (environmental sciences, epidemiology, & nursing) and research dissemination in healthcare literature. The initial research (2018) during a bloom detected cyanobacterial microcystins in the nares of 95% of aging adults (n = 125) while a different analysis (2023) indicated that residential and recreational exposures were significantly associated with increased risks of respiratory (74%), gastrointestinal (35%) and ocular symptoms (62%). A recent study (2025) affirmed that frequencies of reporting for these symptom groups were significantly higher during bloom periods than non-bloom periods. It became important to diagram our research trajectory. The process of cartography consisted of three steps on the NIEHS-TRF: 1) identification of research categories and activities (depicted visually by rings/nodes) that linked to research program outcomes, 2) within each category (visual ring), linked specific works and program outcomes to activities (visual nodes), and 3) coherently depicted visually as an overall map. The outcomes of the cartography process yielded a cohesive research trajectory, informed grant proposals, guided manuscripts and abstracts all focused-on understanding and addressing potential HHO of exposure to HABs in the aging adult population. Cartography using the NIEHS-TRF framework is recommended for nurse scientists and clinical researchers to support purposeful multidisciplinary collaboration and to strengthen planning and evaluation with clear visualization(s).

Abstract citation ID: igaf122.4160

### MAPPING INTERINDIVIDUAL VARIATION IN THE AGING CONNECTOME STUDY RESULTS: EXAMINING FITNESS AND EXECUTIVE CONTROL

Stan Colcombe[1], and Anna MacKay-Brandt[2], *1. Nathan Kline Institute, Orangeburg, New York, United States, 2. The Nathan S. Kline Institute for Psychiatric Research, Orangeburg, New York, United States*

Translational geroscience seeks to identify modifiable factors that can slow or prevent age-related functional decline. Cardiovascular fitness (CF) is one such factor, with intervention studies showing that fitness improvements enhance brain plasticity and executive control. However, little is known about normative variation in CF across mid- to late adulthood or how it predicts cognitive change under naturalistic conditions. We examined data from Mapping Interindividual Variation in the Aging Connectome, a community-ascertained sample aged 40–74 years. Participants completed annual assessments of CF ($VO_2$submax) and executive control (Attention Network Task [ANT] conflict effect) over three years. Among individuals with three assessments (n = 155) fitness was highly stable, ICC(2,1) = 0.83. Such high absolute agreement over time was contrary to our expectation of intraindividual variation, and possibly indicative of stable VO2 set points. However, ANT conflict effect was less stable, ICC(2,1) = 0.57, and decreased over time, consistent with practice effects. Notably, higher $VO_2$max at baseline predicted greater practice effects at follow-up ($\beta = -1.06$, $p = 0.030$, $R^2 = 0.022$). Although intensive programs can improve CF and executive control, our findings suggest that under naturalistic conditions CF is a stable physiological trait in later adulthood. Remarkably, the plasticity of the system at baseline, indexed by CF, appears to result in greater cognitive gains 1 to 2 years later - underscoring the utility of VO2 as a biomarker of brain plasticity. The stability of VO2 around individual set points suggests caution in interpreting exercise studies that lack gold standard VO2 assessment.

Abstract citation ID: igaf122.4161

### MAPPING LIFECOURSE REPRODUCTIVE TRAJECTORIES IN A NATIONALLY REPRESENTATIVE COHORT OF U.S. WOMEN

Brianah McCoy, and Lauren Gaydosh, *University of North Carolina at Chapel Hill, Chapel Hill, North Carolina, United States*

Reproductive timing and experiences vary substantially among U.S. women, yet few studies have systematically classified reproductive life course trajectories in nationally representative, longitudinal cohorts. Using data from Waves I–V of the National Longitudinal Study of Adolescent to Adult Health (Add Health), we constructed reproductive trajectories based on age at menarche, timing of first birth, total parity, reproductive lifespan, and pregnancy-related complications. Variables were harmonized and temporally aligned to accommodate changes in survey structure across waves, and latent class analysis identified distinct reproductive patterns. These trajectories capture meaningful variation in reproductive timing and intensity that is hypothesized to be biologically relevant for chronic disease risk in midlife. This work is novel in its application to a large, nationally representative sample with rich longitudinal reproductive histories. The resulting classifications provide a foundation for ongoing research linking reproductive life course patterns to molecular indicators of aging and long-term health outcomes in women.

Abstract citation ID: igaf122.4162

### METABOLIC REGULATION OF INFLAMMATION IN AGING

Michaella Niceforo[1], Gabrielle Chase[1], Lydia Gugliuzza[1], Ava Lankowski[2], and Leena Bharath[1], *1. Merrimack college, North Andover, Massachusetts, United States, 2. Merrimack college, Merrimack College, Massachusetts, United States*

Age-related cellular changes negatively impact the function of CD4+ T cells. Our prior work showed that mitochondrial complex II (Succinate dehydrogenase (SDH)) expression was upregulated in T cells from older (O) adults (60-80 years old). T cells from O adults also produced higher amounts of cytokines that are generally considered proinflammatory, such as Th17 cytokines, IL-17A/F, IL-21, and Th-17 supportive cytokine IL-6, compared to T cells from young (Y) adults (25-40 years old). The objective of our study is to evaluate whether hyperactivation of SDH is required for the induction of proinflammatory cytokines and the mechanistic link between SDH and Th17 cytokine production. CD4+ T cells were isolated from lean normoglycemic younger (Y; avg: 31.58 yrs; BMI 21.14 kg/m2) and older (O; avg: 64.81 yrs; BMI 21.95 kg/m2) adults. SDH was pharmacologically and genetically modulated, and mitochondrial structure, function, metabolites, and cytokine production were quantified. SDH activation in T cells from O adults induced heightened oxidation of succinate, disrupted the ratio of fumarate to succinate, stabilized HIF-1α, and promoted Th17 cytokines. Genetic and pharmacological inhibition of SDH in T cells from O adults lowered proinflammatory cytokine production, whereas exogenous addition of cell-permeable succinate induced SDH protein in T cells from Y adults, recapitulating the proinflammatory Th17 profile observed in T cells from O adults. These data establish a mechanistic link between SDH and Th17 inflammation.

Abstract citation ID: igaf122.4163

### MIDDLE ADULTHOOD RELATIONSHIPS AND LATER COGNITIVE FUNCTION: AN 18-YEAR STUDY OF MEXICAN-ORIGIN ADULTS

Ariana Guenther[1], Olivia Atherton[2], Angelina R. Sutin[3], Richard Robins[1], and Tomiko Yoneda[2], *1. University of California, Davis, Davis, California, United States, 2. University of California, Riverside, Riverside, California, United States, 3. Florida State University College of Medicine, Tallahassee, Florida, United States*

Extensive evidence shows that close relationships matter for cognitive health, serving as both risk and protective factors. However, most existing literature focuses on older White adults, relies on self-reports, and centers on spousal relationships. Less is known about whether midlife spousal and child relationship experiences affect cognitive function over time, particularly in underrepresented communities. Our study addresses these gaps by focusing on middle adulthood, a period when people balance caregiving, work, and evolving family roles. Specifically, we ask whether relationship quality with partner and child (assessed via parent and child reports) shapes initial levels and long-term change in cognitive function. Drawing on data from an 18-year longitudinal study of Mexican-origin adults (N = 1,111; Mage = 38.3 at baseline), we used latent growth curve modeling to examine several relationship factors (aggregated across Years 1-10) as predictors of level and change in cognitive function assessed at Years 12, 16, and 18. Findings showed that higher relationship stability, satisfaction, warmth, and social support were associated with higher baseline cognitive function. However, higher satisfaction, warmth, and social support were also associated with steeper cognitive declines over time. In contrast, relationship instability, hostility, and conflict were unrelated to cognition, except for relationship instability predicting faster decline. Higher parent–child conflict also predicted higher baseline cognition but steeper decline. Together, these findings emphasize complex links between relationship factors and cognitive aging, suggesting supportive relationships are protective for cognition early in middle adulthood, but may also be associated with more decline later in midlife.

Abstract citation ID: igaf122.4164

### MODULATION OF SERINE METABOLISM IMPROVES HEALTH AND LONGEVITY IN DROSOPHILA MELANOGASTER

Shengshuai Shan[1], Ryan Armant[1], Shelton Swint[2], Hanna Patel[1], Kelsey Patterson[1], Eric Foreman[1], Jessica Alvarez[1], and Jessica Hoffman[1], *1. Augusta University, Augusta, Georgia, United States, 2. College of Science and Mathematics, Augusta, Georgia, United States*

Non-essential amino acids, including L-serine, though often overlooked, are crucial in metabolism and may influence aging. Serine is metabolized through diverse pathways that produce metabolites with different effects on lifespan. Metabolites such as glutathione, spermidine, and glycine are linked to increased longevity, whereas ceramides and sphingolipids are associated with reduced lifespan. These divergent metabolic outcomes may underlie conflicting results in laboratory organisms for previous studies, and the specific metabolites and genes mediating its effects remain largely unidentified. This study aimed to evaluate the effects of serine modulation, via dietary L-serine supplementation and knockdown of key genes involved in L-serine metabolism, on healthspan and lifespan across genetically diverse strains and both sexes of Drosophila melanogaster. Preliminary results indicate that L-serine supplementation significantly extended lifespan and improved healthspan in both sexes. Moreover, knockdown of lace, the first step in sphingolipid biosynthesis and the Drosophila ortholog of serine palmitoyltransferase (SPT), markedly increased lifespan in both sexes, with a more pronounced effect in females. Additionally, knockdown of serine hydroxymethyltransferase (SHMT), which bidirectionally converts glycine to serine, significantly increased lifespan in both sexes. Genetic manipulations increased longevity to a greater degree than serine supplementation. These findings suggest that metabolic reprogramming, achieved by targeting specific branches of the serine metabolic pathway, may be a key mediator of health outcomes. Future directions will analyze metabolomic profiles of serine supplemented and knockdown flies for insights into the molecular mechanism that drive their effects. In conclusion, our results highlight serine metabolism as a potential pro-longevity target for intervention studies.

Abstract citation ID: igaf122.4165

### NOVEL CLUSTERING ANALYSIS UNVEILS ASSOCIATIONS BETWEEN FOOD CHOICES AND VASCULAR AGING

Chen Chen[1], Bangwei Chen[2], Li Luo[1], Shengyin Zeng[1], Jianguo Zhang[1], and Tao Li[1], *1. BGI Genomics, Shenzhen, Guangdong, China (People's Republic), 2. Tongji Hospital, Tongji Medical College, Huazhong University of Science and Technology, Wuhan, Hubei, China (People's Republic)*

Objective Vascular aging, marked by arterial stiffness, is a key risk factor for cardiovascular diseases (CVD) and a target for dietary interventions. However, dietary features influencing vascular aging are poorly understood. This study aimed to identify food choices linked to vascular aging in Chinese adults without CVD. Methods In this cross-sectional study of 1,155 adults, we collected dietary data (yes/no responses to 16 food items), sociodemographic characteristics, and body composition. Brachial-ankle pulse wave velocity (baPWV) was measured as the arterial stiffness indicator. Multiple correspondence analysis with hierarchical clustering on principal components were used to derive dietary clusters. Cramer's V was calculated to measure the strength of association between variables and clusters. Generalized linear models were used to compare baPWV across clusters. Results Three distinct dietary clusters were identified, characterized by whole grain, root vegetables, smoked and salty foods (Cramer's V > 0.5). Chinese-adapted Western diet (High smoked or salty foods intake) showed significantly higher baPWV compared to both Chinese-adapted Mediterranean diet (High whole grains or root vegetables intake, β: 30.52, 95% CI: 10.04-50.98) and conventional Chinese urban diet (balanced diet without favoritism, β: 22.91, 95% CI: 5.75-40.19). In contrast, there was no significant difference between Chinese-adapted Mediterranean diet and conventional Chinese urban diet. Conclusions We identified dietary groups based on simple food choice responses. The group characterized by preference for smoked or salty foods and low intake of whole grains or root vegetables, is associated with vascular aging. Keywords food choice; dietary pattern; vascular aging; arterial stiffness; multiple correspondence analysis

Abstract citation ID: igaf122.4166

### OLDER ADULTS CARE DURING A TIME OF CRISIS: ISRAEL'S EXPERIENCE DURING WARTIME

Yakir Rottenberg[1], and Shelley A. Sternberg[2], *1. Faculty of Medicine, Hebrew University of Jerusalem, Israel, Jerusalem, Yerushalayim, Israel, 2. Maccabi Healthcare Services, Tel Aviv Israel, Tel Aviv, Tel Aviv, Israel*

In recent decades, global crises like the COVID-19 pandemic and natural disasters have significantly challenged the ability to provide care for older adults, highlighting a critical research priority. Israel, with its advanced system of care for older adults and strong community support, performed well during COVID-19. This report describes efforts to maintain care during the June 2025 Israel-Iran war, a period of physical threat, disrupted medical services, psychological distress, and weakened social supports due to displacement and infrastructure damage. We highlight innovative strategies ensuring continuity of medical, mental health, and essential social services for older adults. Israel's approach to emergency care for older adults rested on three pillars: (i) Leveraging past experience: Protocols from previous emergencies, including COVID-19, were quickly activated. This ensured continuity of care for older adults, especially those evacuated or in unsafe apartments, balancing safety with independence. (ii) Coordinated communication: Ongoing national and municipal collaboration and communication about at-risk older adults, enabled effective and timely, targeted interventions. (iii) Volunteerism and Civil Initiatives: A massive surge of community-led volunteer efforts provided transport, medications, and evacuee assistance, critically addressing urgent needs beyond government capacity. In conclusion, these three pillars – rapid response based on prior experience, a strong national and municipal system of communication and collaboration, and robust volunteerism – enabled continuous, high-quality care for older adults during this major crisis., These lessons can inform other systems aiming to enhance their resilience and capacity to provide care to older adults during emergency situations.

Abstract citation ID: igaf122.4167

### OSTEOCLAST-DERIVED SMALL EXTRACELLULAR VESICLE AS NOVEL LIQUID BIOPSY FOR ASSESSING BONE HEALTH IN OLDER ADULTS

Shalini Mishra, Yixin Su, Ashish Kumar, Sangeeta Singh, Kristen Beavers, and Gagan Deep, *Wake Forest University School of Medicine, Winston-Salem, North Carolina, United States*

Age-related bone loss is a highly prevalent condition that contributes substantially to morbidity, mortality, and healthcare costs worldwide. Dysregulated osteoclast activity characterizes aging bone; however, current diagnostic measurements lack specificity, sensitivity, and early detection ability. Small extracellular vesicles (sEV) derived from specific cell types offer a promising non-invasive liquid biopsy approach for assessing hard-to-access tissues, like bone. This proof-of-concept study aimed to isolate osteoclast-specific sEV (sEV-Osteoclast) from blood, laying the foundation for a sensitive, specific, and repeatable method to evaluate osteoclast physiology and molecular status of bone in clinical studies. Four osteoclast-specific surface markers (tartrate-resistant acid phosphatase (TRAP), receptor activator of nuclear factor kappa-B (RANK), integrin alpha V (CD51) and dendritic cell-specific transmembrane protein (DCStamp)) were identified through extensive literature review and validated using the Human Protein Atlas and UniProt databases. Next, total sEV were isolated from human plasma samples ($n = 11$; age: 70.0±2.76 years) and characterized for size (99.65±2.49nm) and concentration (3.58E+11±3.13E+10particle/ml) by nanoparticle tracking analysis. Next, the percentage of sEVOsteoclast markers in the total sEV was ascertained by flow cytometry, revealing that 6.70±0.80% of plasma-total sEV were TRAP positive (+), 2.54±0.27% were RANK+, 7.36±0.64% were CD51+, and 3.63±0.27% were DCStamp+. As any specific sEV population can be reliably isolated from plasma if the percentage positivity is ≥2% in total sEV, these preliminary findings demonstrate feasibility of sEV-Osteoclast isolation from plasma. Future work aims to measure sEV-Osteoclast in a NIA-funded clinical trial of weight loss in older adults (NCT05764733), as a biomarker of early risk assessment and real-time intervention monitoring.

Abstract citation ID: igaf122.4168

### PARENTAL DEPRESSION IN CHILDHOOD AND DEPRESSIVE SYMPTOMS IN LATER LIFE

Wenxing Wei, and Aloen Townsend, *Case Western Reserve University, Cleveland, Ohio, United States*

There is limited research examining parental depression in childhood as a separate risk factor, rather than combining it with other adverse childhood experiences (ACEs). Based on life course theory, this study investigates the association between parental depression during respondents' childhood (neither parent, father only, mother only, or both parents depressed) and later-life depressive symptoms and whether this association differs by respondents' gender, while controlling for other ACEs, sociodemographic characteristics, and health. The study was based on a nationally representative sample of 4,635 males and 5,457 females aged 45 and older from the China Health and Retirement Longitudinal Study (CHARLS). ACE variables in the 2014 Life History Survey were merged with the harmonized 2018 CHARLS dataset. Multiple imputation addressed missing data. One-way ANOVA and hierarchical OLS multiple regression were conducted. The regression model explained significant variance ($R2 = .229$, $p < .001$). Both mother only depressed and both parents depressed were significantly associated with higher later-life depressive symptoms, compared to having neither parent depressed. Respondents with both parents depressed reported the highest depressive symptoms ($b = 1.642$, $p < .001$). Gender did not significantly moderate these relationships. The findings suggest that maternal depression and having both parents depressed have particularly negative long-term effects on respondents' mental health, which should be considered in the development of protective strategies and policies. Even in middle and older age, the study findings highlight the importance of assessing family history of depression.

Abstract citation ID: igaf122.4169

### PATIENT CARE ASSISTANTS MAKING MEANING OF THEIR WORK IN NURSING HOMES

Justina Knowles, *University of The Bahamas, Nassau, New Providence, Bahamas, The*

The purpose of this study was to describe the meaning that patient care assistants (PCAs) ascribe to their work in nursing homes. This study was needed to close existing knowledge gaps, such as the existence of obsolete findings that cannot guide modern practice, lack of generalizability of the outcomes of previous studies, and inadequate empirical evidence to support policies and programs. Guided by the meaning of work level model, the purpose of this qualitative case study was to evaluate what meanings PCAs attach to their work in a profession that is undervalued and underpaid. Data were collected from in-depth interviews with 10 PCAs and four RNs to understand how the benefits of meaning making help PCAs adopt better work culture and institutional practices, and what characteristics RNs perceive as nurturing a positive meaning-making experience. Thematic analysis revealed that a positive workplace culture, strong organizational practices, effective collaboration, caring relationships, opportunities for professional development, and resilience and adaptation contributed to the meaning-making experience for PCAs. The perspectives of PCAs and RNs regarding their work may facilitate the creation of protocols, policies, and programs for empowering PCAs to create positive meaning of their work in nursing homes. The findings may also promote improved care outcomes among older adults in nursing homes. Positive social change may occur when health care teams understand the significant role and value of care that PCAs deliver in nursing homes.

Abstract citation ID: igaf122.4170

### PATTERNS OF MEDICATION REGIMENS AND DRUG-DRUG INTERACTIONS AMONG OLDER ADULTS REQUIRING LONG-TERM CARE

Shotaro Hagiwara[1], Jun Komiyama[2], Masao Iwagami[2], Shota Hamada[3], Hiroyuki Kobayashi[1], and Nanako Tamiya[2], *1. University of Tsukuba, Mito, Ibaraki, Japan, 2. University of Tsukuba, Tsukuba, Ibaraki, Japan, 3. Tokyo University of Pharmacology and Life Sciences, Hachioji, Tokyo, Japan*

Purpose: Although older patients requiring long-term care (LTC) are highly susceptible to adverse drug events, they often experience polypharmacy, leading to potential drug-drug interactions (DDIs). This study examined the patterns of medication regimens and the risks of DDIs among older patients requiring LTC. Methods: We analyzed the medication regimens among 67,531 individuals aged 65 and older who utilized long-term care insurance services in Ibaraki Prefecture, Japan, based on public medical and LTC insurance claims data in 2018. We examined DDIs using package inserts and DrugBank Online (version 5.1.13). An association analysis was conducted using R (version 4.3.3) to calculate Support, Confidence, and Lift values. Medication classification followed the WHO's ATC classification. Results: The median number of drugs was seven (IQR, 5-9). The most frequent two-drug combination was gastric acid-related disorder drugs and calcium channel blockers (CCBs) (Support 0.33, Confidence 0.73, Lift 1.02). For four-drug combinations, it was antithrombotic drugs, CCBs, renin-angiotensin system agents, and gastric acid-related disorder drugs (0.07, 0.78, 1.09). For six-drug combinations, it was antithrombotic drugs, CCBs, renin-angiotensin system agents, lipid-modifying drugs, psychotropic drugs, and gastric acid-related disorder drugs (0.01, 0.84, 1.17). Among the top 30 drugs, co-prescribed medications with Support>0.03 were extracted. The likelihood of being prescribed at least one drug with a moderate or major interaction was highest for clopidogrel (90.1%), zolpidem (88.1%), rosuvastatin (85.9%), celecoxib (84.2%), and spironolactone (83.4%). Conclusion: We identified the most common medication regimens among older patients requiring LTC and high-risk drugs for DDIs. Establishing a prescription check system is needed.

Abstract citation ID: igaf122.4171

### PERCEIVED CONFIDENCE IN OBTAINING AN ALZHEIMER'S DISEASE DIAGNOSIS: EXPLORING RACE AND RURALITY

Hannah Huff[1], Anita Reina[2], Caleb Snead[3], Lydia Burton[1], Don Scott[1], Stephen Correia[1], Lisa Renzi-Hammond[1], and Jenay Beer[1], *1. University of Georgia, Athens, Georgia, United States, 2. Kennesaw State University, Kennesaw, Georgia, United States, 3. Northwestern University, Evanston, Illinois, United States*

Alzheimer's disease (AD) disproportionately affects African Americans, who are twice as likely as Caucasians to develop AD. Research shows; however, that despite higher likelihood of developing AD, African Americans are 35% less likely to get an AD diagnosis. Past research has identified structural barriers (e.g., medical underservice, out-of-pocket costs) that disproportionately affect African American communities, but the intersection of rurality and race on obtaining an AD diagnosis has not been well-studied. In this study, we investigate confidence in obtaining a dementia diagnosis among African American residents of both rural and urban communities. As part of a larger dementia needs assessment, a cross-sectional survey was administered to Georgians ($N = 531$), assessing confidence in getting a dementia-related diagnosis if they started having memory concerns. Surveys were administered at health fairs and community-focused education/programming. Self-reported confidence in obtaining a dementia diagnosis for rural and urban communities combined was greater for African Americans (mdn=4.00; $n = 131$) compared to Caucasians (mdn=3.00; $n = 400$), $U = 31420.50$, $p < 0.001$, $r = 0.15$. Among rural-dwelling participants, confidence in obtaining a dementia diagnosis was greater for African Americans (mdn=4.00; $n = 101$) compared to Caucasians (mdn=3.00; $n = 267$), $U = 16949.50$, $p < 0.001$, $r = 0.20$. Additionally, rural African Americans (mdn=4.00, $n = 101$) expressed greater confidence than urban African Americans (mdn=3.00, $n = 30$), $U = 1068.00$, $p = 0.011$, $r = 0.22$. Although this study indicates rural-dwelling African Americans are confident in obtaining an AD diagnosis, previous studies suggest they lack trust in healthcare providers. Future research on the intersection of race and rurality should attempt to understand why rural African Americans are more confident than Caucasian counterparts.

Abstract citation ID: igaf122.4172

### PERSPECTIVES OF INDEPENDENT PROVIDERS: MOTIVATIONS, CHALLENGES, AND RECOMMENDATIONS FOR LTSS

Rejina Akter[1], Sanjeela Gandhari[2], Heather Reece[1], Leah M. Janssen[1], and Jennifer Heston-Mullins[1], *1. Miami University, Oxford, Ohio, United States, 2. Texas A & M University, College Station, Texas, United States*

In the context of long-term services and supports, independent providers are self-employed individuals who deliver home care services to older adults and people living with disabilities. They play a vital, but often unrecognized role in a system plagued by workforce shortages by supporting individuals living in their homes and communities. Despite their important role in providing long-term care, little is known about the motivations and experiences of independent providers. This study aimed to (1) explore independent providers' motivations for entering the role, (2) examine perceived role benefits and challenges, and (3) identify suggestions for improving recruitment, retention, and recognition of independent providers. To explore their perspectives, in-depth interviews were conducted with 20 independent providers certified to deliver personal care, homemaking, and/or transportation services across 19 counties in a midwestern state. Qualitative descriptive thematic analysis identified that most providers entered the role while caring for loved ones informally, often after negative experiences with agency-based care. Providers valued the flexibility, autonomy, and deeper client relationships that being independent afforded. They also reported burdensome administrative requirements, inconsistent payment schedules, unclear guidance, and limited communication with payors. Suggested improvements included streamlined onboarding, fair and timely compensation, increased community awareness of their role, and digital tools to support care coordination. These findings highlight independent providers as a crucial yet neglected segment of the long-term care workforce. Policy recognition in reducing administrative burdens and improving independent provider retention and recognition is essential to sustaining home care options for older adults and people living with disabilities.

Abstract citation ID: igaf122.4173

### PHYSICAL ACTIVITY, SLEEP, AND HYPERTENSION AMONG U.S. MIDDLE AND OLDER ADULTS: NHANES INSIGHTS

Dennis Miezah[1], Thomas Hinneh[2], Rockson Ansong[3], and Qian Song[1], *1. University of Massachusetts Boston, Boston, Massachusetts, United States, 2. Johns Hopkins University, Baltimore, Maryland, United States, 3. Louisiana State University, Baton Rouge, Louisiana, United States*

Hypertension is a primary risk factor for cardiovascular disease. Physical activity and sleep are critical determinants of blood pressure. This study investigated associations between physical activity, sleep duration, and hypertension in middle-aged and older adults. We analyzed cross-sectional data from the National Health and Nutrition Examination Survey of adults aged 40-80 years (N = 9,133). Hypertension was defined as systolic blood pressure ≥130 mmHg, diastolic blood pressure ≥80 mmHg, or current antihypertensive medication use. Physical activity was categorized as not meeting guidelines (< 500 MET-min/wk), meeting guidelines (≥500-< 1000 MET-min/wk), and exceeding guidelines (≥1000 MET-min/wk). Sleep duration was classified as short (< 7 hours), recommended (7–9 hours), or long (>9 hours). Descriptive statistical analyses were performed. Logistic regression was used to estimate the associations between lifestyle behaviors and hypertension, with adjustments made for sociodemographic and clinical factors. Overall, 61.2% of participants had hypertension. Middle-aged adults (40–64 years) comprised 69% of the sample; older adults (65–80 years) comprised 31%. Meeting physical activity guidelines was associated with lower odds of hypertension (adjusted OR = 0.78, 95% CI: 0.65–0.94, p < 0.01). Short sleep was associated with higher odds of hypertension (adjusted OR = 1.32, 95% CI: 1.12–1.55, p < 0.01). No significant association was observed for long sleep. Stratified analyses suggested stronger associations among older adults and lower-income groups. Physical activity and sleep are independently associated with hypertension in U.S. middle and older adults. Integrated lifestyle interventions may reduce hypertension burden, particularly among high-risk populations.

Abstract citation ID: igaf122.4174

### PILOT VALIDATION OF AI-DERIVED FEATURES FOR PROGNOSTIC MODELS IN GERIATRIC ONCOLOGY

Alaa Albashayreh[1], W. Nick Street[1], and Stephanie Gilbertson-White[2], *1. University of Iowa, Iowa City, Iowa, United States, 2. University of Iowa, University of Iowa, Iowa, United States*

Accurate survival prediction for older adults with lung cancer is critical for guiding supportive care, yet standard models often underutilize valuable information in clinical notes. The objective of this study was to determine if a prognostic model integrating features from unstructured clinical notes could more accurately predict survival in older adults with lung cancer than a model using structured data alone. We studied 1,391 patients (≥60 years at time of diagnosis) with lung cancer, analyzing 19,377 clinical encounters. We compared a baseline survival model using structured electronic health record (EHR) data to an enriched model that integrated previously validated natural language processing (NLP)-derived features, including functional status. Models were evaluated for 12-month mortality using AUC and C-Statistic. Thirty-eight percent of patients presented with metastatic disease. The baseline prediction model achieved good performance (AUC: 0.740; C-Statistic: 0.697). The enriched model demonstrated statistically significant improvement (AUC: 0.768 vs. 0.740; C-Statistic: 0.717 vs. 0.697; DeLong's test p<.0001) with well-calibrated predictions and effective risk stratification into distinct prognostic groups. Feature analysis confirmed NLP-derived functional status and longitudinal chemotherapy timing were key predictors, with functional status ranking among the top three most important features. Clinical notes contain crucial prognostic information not captured in structured data. Leveraging NLP to extract this narrative data offers a powerful pathway to more accurate survival prediction, which could enable better identification of high-risk patients to improve supportive care interventions.

Abstract citation ID: igaf122.4175

### POLYPHARMACY AND MEDICATION SELF-EFFICACY IN OLDER EMERGENCY DEPARTMENT PATIENTS

Inessa Cohen[1], Natalia Sifnugel[2], Ling Han[3], Daniella Meeker[4], Christopher Carpenter[5], William Hung[6], Debra Tomasino[7], and Ula Hwang[8], *1. Yale University, Yale University, Connecticut, United States, 2. NYU Langone Medical Center, New York, New York, United States, 3. Yale School of Medicine, New Haven, Connecticut, United States, 4. Yale School of Medicine, Yale University, Connecticut, United States, 5. Mayo Clinic, Rochester, Minnesota, United States, 6. James J. Peters Department of Veterans Affairs Medical Center, New York, New York, United States, 7. New York University, New York, New York, United States, 8. NYU Grossman School of Medicine, New York, New York, United States*

Over 50% of older adults in the US take ≥5 medications, with prevalence reaching 76.8% in the emergency department (ED) setting. However, little is known about their medication self-efficacy (the ability to understand and follow medication regimens), which may influence adherence and adverse drug events when new prescriptions are added during ED visits. Our objective was to determine whether polypharmacy and potentially inappropriate medication (PIM) are associated with self-efficacy. We performed a logistic regression analysis using 5-site multicenter ED survey data linked to electronic health records from patients aged ≥65 years with baseline ED visits during 2019-2021. The predictors were polypharmacy (≥5 medications) and PIM use (≥1 high-risk medication, GEMRx criteria) of prescriptions taken within 1 year of the ED visit. The outcome was low medication self-efficacy (< 11 points MUSE-taking score) assessed at the ED visit. Covariates included insurance, education, marital status, race/ethnicity, caregiver support, dementia status, and frailty. Among 921 patients, 249 (27.0%) had polypharmacy and 66 (7.2%) had PIM use. Low medication self-efficacy was reported by 76 (37.8%) with polypharmacy and 26 (12.9%) with PIM use versus 125 (62.2%) and 40 (5.6%) without. Both polypharmacy (aOR 6.12, 95% CI 2.05-18.22, $p = 0.001$) and PIM use (aOR 33.65, 95% CI 5.81-214.35, $p < 0.001$) were associated with greater adjusted odds of low medication self-efficacy. Frailty modified this association, with weaker effects in more frail patients (OR 5.51, 95% CI 2.57-11.8, $p = 0.003$). These findings suggest that the ED is an important setting to identify older adults who may struggle with medication use.

Abstract citation ID: igaf122.4176

### PREDICTING PSYCHOLOGICAL RESILIENCE IN OLDER ADULTS DURING THE COVID-19 PANDEMIC: A MACHINE LEARNING APPROACH

Josephine Abrials[1], Xuan Lu[2], Xiwen Guan[1], and Xiaoling Xiang[3], *1. University of Michigan, Ann Arbor, Michigan, United States, 2. University of Arizona, Tucson, Arizona, United States, 3. University of Michigan-Ann Arbor, Ann Arbor, Michigan, United States*

Research has demonstrated that resilience is the norm after disasters, yet not all individuals and communities adapt equally well. Studies on resilience have predominantly been cross-sectional and relied on traditional statistical methods. Adopting a longitudinal design, this study aimed to predict psychological resilience among older adults during the COVID-19 pandemic using machine learning methods. The study sample consisted of 3,364 individuals who completed the 2016 and 2020 Leave-Behind Questionnaire from the Health and Retirement Study. A comprehensive, theory-informed set of predictors at the individual, interpersonal, and community levels was measured in 2016, and resilience was measured using a 6-item scale in 2020 (Cronbach's $\alpha = .81$). Three machine learning algorithms (LASSO, Ridge, and Random Forest) were trained with five-fold cross-validation, with LASSO being the best fit to the data (RMSE = 0.873; $R^2 = 0.195$). SHAP values were used to interpret feature importance. Twenty-four important predictors emerged, including psychological traits (e.g., optimism, openness), pre-pandemic social participation, social support, and neighborhood cohesion, as well as indicators of technology adaptation (e.g., learning a new device) during the pandemic. Non-linear and interaction effects were also identified. Study findings highlight the complex and multifaceted nature of resilience, demonstrating the value of a theory-informed data science approach. Addressing digital inequities and fostering social support and participation are potential targets for resilience promotion. Future studies could build upon our findings and employ causal inference methods to better understand the causes and mechanisms that promote psychological resilience in older adults.

Abstract citation ID: igaf122.4177

### PREDICTION OF SARCOPENIA ONSET IN OLDER ADULTS WITH DIABETES USING URINARY TITIN LEVELS: A JAPANESE COHORT STUDY

Michio Shimabukuro[1], Hayato Tanabe[2], Kiriko Watanabe[1], Rie Tsutsumi[3], Yuna Izumi-Mishima[3], Kazuhiro Nomura[3], Masafumi Matsuo[4], and Hiroshi Sakaue[3], *1. Fukushima Medical University School of Medicine, Fukushima, Fukushima, Japan, 2. Department of Diabetes, Endocrinology, and Metabolism, Fukushima, Fukushima, Japan, 3. Institute of Biomedical Sciences, Tokushima University, Tokushima, Tokushima, Japan, 4. Kobe University, Kobe, Hyogo, Japan*

Standardized biomarkers for incident sarcopenia are still elusive. Recently, we reported that urinary titin serves as a novel, noninvasive biomarker to identify individuals with diabetes at a high risk of muscle damage and sarcopenia (Tanabe, et al. Diabetes Care in press). However, the predictive power of urinary titin for sarcopenia has not yet been clarified in older adults. In Japanese older adults with type 2 diabetes, baseline urinary titin levels were measured, and sarcopenia was evaluated annually using the Asian Working Group for Sarcopenia (AWGS) 2019 criteria. Kaplan-Meier curves, Cox models, and time-dependent receiver operating characteristic analyses were employed to assess incident sarcopenia. Among the 287 participants (median follow-up: 824 days), 39 developed sarcopenia. The high titin tertile was associated with an elevated sarcopenia risk (log-rank $P = 0.008$) and low muscle strength ($P = 0.004$). Cox models associated titin with sarcopenia (adjusted hazard ratio per SD: 1.38, 95% CI: 1.07–1.78, $P = 0.013$) and low muscle strength (HR per SD: 1.37, 95% CI: 1.10–1.70, $P = 0.005$). Risk estimates were consistent across subgroups, including males, individuals with BMI< 25 kg/m2, HbA1c ≥7%, and eGFR ≥60 mL/min/1.73 m2 (P for interaction >0.05). Elevated urinary titin levels predict sarcopenia and low muscle strength in older adults with type 2 diabetes, supporting its use as a noninvasive biomarker in this population.

Abstract citation ID: igaf122.4178

### PREDICTIVE VALUE OF GAIT SPEED AND GRIP STRENGTH FOR MORTALITY IN OLDER ADULTS WITH CARDIOMETABOLIC MULTIMORBIDITY

Hyeon Hong[1], Shweta Gore[2], Lin-Na Chou[3], Julie Keysor[4], Amol Karmarkar[5], and Amit Kumar[1], *1. University of Utah, Salt Lake City, Utah, United States, 2. MGH Institute of Health Professions, Boston, Massachusetts, United States, 3. University of Utah, Saratoga Springs, Utah, United States, 4. University of Massachusetts Lowell, Lowell, Massachusetts, United States, 5. Virginia Commonwealth University, Richmond, Virginia, United States*

This study aims to establish cut-off points for gait speed and hand grip strength based on one-year hospitalization risks and to examine their predictive value for three-year mortality in older adults with cardiometabolic multimorbidity (CMM). This retrospective cohort study utilized data from the National Health and Aging Trends Study (2015-2018) linked to Medicare claims for community-dwelling adults aged 66 years and older. Our primary predictors were gait speed (m/s) and BMI-normalized hand-grip strength. First, we established optimal cut-off values for gait speed and grip strength based on 1-year hospitalization risk, using sensitivity and specificity. Next, we employed Cox proportional hazards regression to estimate the probability of 3-year mortality, with gait speed and grip strength as predictors, stratified by sex and disease group. Among 4,157 older adults,11.3% had CMM (6.5% male, 4.7% female). Among older males, those with CMM had the lowest cut-off values for grip strength at 0.82kg/BMI (sensitivity 0.59; specificity 0.85) and for gait speed at 0.58m/sec (sensitivity 0.62; specificity 0.92) to predict 1-year hospitalization. In females with CMM, the cut-off was 0.52kg/BMI (sensitivity 0.61; specificity 0.84) for grip strength and 0.68m/sec (sensitivity 0.69; specificity0.71) for gait speed. Among males with CMM, those with below the cut-off gait speed had a significantly higher 3-year mortality compared to those with above the cut-off (HR = 0.47, 95% CI = 0.23-0.97, p < 0.05). These validated cut-off points for gait speed and grip strength can assist in risk stratification and mortality prediction in older adults with CMM, serving as a screening tool for identifying high-risk individuals.

Abstract citation ID: igaf122.4179

### PREPARING GERIATRICS-TRAINED PHYSICIANS TO DISCUSS MEDICAL CANNABIS WITH THEIR OLDER ADULT PATIENTS

Alison Moore[1], Ali Punsalan[2], Krish Jagasia[2], Julie Bobitt[3], Jeremy Hirst[2], Michelle Sexton[4], Kathryn Winters[2], and Annie Nguyen[2], *1. University of California San Diego, San Diego, California, United States, 2. UC San Diego, La Jolla, California, United States, 3. University of Illinois at Chicago, Chicago, Illinois, United States, 4. UC San DIego, La Jolla, California, United States*

Older adults are increasingly using cannabis for health conditions (e.g., pain, sleep disorders), often without consulting a healthcare provider. Many providers also feel unprepared to discuss cannabis use with patients. This educational intervention aimed to raise geriatrics-trained physician awareness and promote compassionate dialogue about cannabis use with older adults. Thirty-one geriatrics and geriatric psychiatry physicians and fellows attended a synchronous, online, didactic series on cannabis divided into four, 30-minute modules on: 1) epidemiology of use among older adults, 2) history of legalization and stigma associated with use, 3) medical evidence and patient advising, and 4) having compassionate dialogue related to use. Pre- and post-intervention data were collected (n = 22 pre-test, n = 13 post-test). Wilcoxon signed-rank tests were performed to assess changes. Statistically significant (p-value's<.05) increases were seen in the percentage of physicians who reported being: moderately to extremely knowledgeable about medical cannabis (18.2% to 71.4%), somewhat to very comfortable initiating conversations about cannabis (22.7% to 92.9%), very to extremely confident assessing the risks (4.5% to 28.6%), benefits (0% to 14.3%), and safety of cannabis (0% to 21.4%), and very to extremely confident providing advice about cannabis (0% to 14.2%). This brief training series was effective in raising physicians' knowledge about and comfort with having conversations about medical cannabis with their older adult patients. Similar training can be easily implemented in pre- and post-doctoral settings. As more older adults turn to cannabis as a therapeutic alternative, physicians will need access to formal training to strengthen confidence providing advice.

Abstract citation ID: igaf122.4180

### RACE, RELIGIOSITY, AND NEUROTICISM: INFLUENCES ON END-OF-LIFE READINESS IN LATER LIFE

Satvika Boyina[1], Mary Cox[2], and Patrick Hill[3], *1. St. Louis University, St. Louis, Missouri, United States, 2. Washington University in St. Louis, St. Louis, Missouri, United States, 3. Washington University in St. Louis, Saint Louis, Missouri, United States*

End of life (EOL) care planning, or preparing for future medical decisions and preferences, is essential for promoting autonomy and quality care in later life. Unfortunately, many individuals delay or avoid this process. This study examined how psychological factors specifically race, racial centrality, religiosity, and neuroticism relate to both EOL planning mindsets and behaviors among older American adults (n = 467; ages 60-88). Data was collected online via Prolific in summer 2025. Participants completed surveys assessing psychological readiness (mindset), planning actions (behavior), personality traits, religious reliance, and racial identity. EOL mindset refers to how psychologically ready or comfortable individuals feel discussing death, while behaviors reflect concrete actions like creating a will or naming a decision-maker. Correlational analyses and t-tests revealed that Black participants reported greater psychological readiness for EOL planning than White participants. Among Black individuals, higher racial centrality was associated with stronger EOL mindsets, suggesting that cultural identity may serve as a motivating or protective factor. Religiosity was also positively associated with the EOL mindset, indicating that spiritual frameworks may enhance emotional comfort with end-of-life discussions. In contrast, neuroticism was linked to both lower EOL readiness and fewer completed planning behaviors, highlighting how emotional vulnerability may act as a barrier to both mindset and action. These findings suggest that sociocultural and psychological factors shape how older adults approach end-of-life planning. For healthcare institutions, these results emphasize the importance of developing culturally responsive and emotionally attuned interventions that foster both readiness in EOL care planning across aging populations.

Abstract citation ID: igaf122.4181

### RANDOM FOREST AND SHAP ANALYSIS ON SOCIAL VULNERABILITY AND EDENTULISM AMONG US ADULTS 65 AND OLDER

Jinbo Niu[1], Joonhyeog Park[2], and Tamara Cadet[2], *1. University of Southern California, Los Angeles, California, United States, 2. University of Pennsylvania, Philadelphia, Pennsylvania, United States*

Approximately 17.3% of adults aged 65 and older in the U.S. face complete edentulism. Identifying the social factors that contribute to edentulism and their interactions is crucial for creating predictive models and effective interventions. However, most studies ignored the interactions among social factors on the edentulism prevalence. Using the concept of social vulnerability, we integrated the latest CDC Places data (2022) and Social Vulnerability Index (SVI) data (2022) to analyze the prevalence of edentulism among adults aged 65 and older across over 3,000 U.S. counties. Random Forest (RF) and SHapley Additive exPlanations (SHAP) were applied to identify the main driving factors of edentulism and characterize their interaction effects. Among the factors considered, the poverty rate emerged as the most influential in predicting edentulism prevalence, followed by the no-high-school-diploma rate and the internet inaccessibility rate. The interaction between poverty rate and no-high-school-diploma rate had the strongest effect on edentulism prevalence. On average, the interactions between social vulnerability factors accounted for 123.08% of the direct influence on edentulism prevalence. The analysis also revealed critical thresholds where the combined impact of these factors shifts to either exacerbate or moderate edentulism prevalence. Social vulnerabilities do not operate in isolation, and their interactions can exacerbate or moderate edentulism among older adults. Accounting for interaction effects and thresholds can refine prediction modeling and guide integrated interventions addressing poverty, education, and internet access to better target high-risk communities.

Abstract citation ID: igaf122.4182

### REDESIGNING NURSE–PATIENT ROUNDS TO ADVANCE PATIENT-CENTERED CARE IN COMPREHENSIVE NURSING SERVICE WARDS

Sooyoung Park[1], Youngmi Lim[2], Heekyung Kim[2], Jungha Lee[2], Soojin Roh[2], Jiwon Kim[2], Youngjun Yoo[2], and Dojin Jeon[2], *1. Samsung Medical Center, Gangnam-gu, Seoul, Republic of Korea, 2. Samsung Medical Center, Gangnam-gu, Seoul-t'ukpyolsi, Republic of Korea*

Comprehensive Nursing Service wards in Korea provide 24-hour clinical and daily care by nurses and nurse assistants without family presence, aiming to reduce caregiver burden and ensure equitable care. However, among older patients—many postoperative with high treatment needs—patient experience surveys revealed gaps, including difficulty voicing concerns, limited participation in decisions, and insufficient emotional support. Although a "Happy Rounding" program—routine nurse–patient rounds to proactively identify needs—was in place, nurse surveys indicated inconsistent practice due to lack of standardized criteria, varied interpretations of rounding procedures, and uncertainty about its role in care. This study aimed to develop and evaluate a ward-specific Happy Rounding protocol to enhance proactive nursing and improve patient experience. Through team discussions among nurses, a ward-specific protocol was co-designed into the "Double Happy Rounding Checklist." To enhance nurse engagement, we introduced ward-wide visual reminders and a patient experience process including bedtime pain checks, clear explanations of procedures and medications, and consistent use of empathic language and active listening. A two-week pilot test with patients and nurses confirmed feasibility, guided refinements, and led to ward-wide adoption. Implementation improved nurse involvement and patient satisfaction. Patients reported that nurses listened more attentively (89.9→96.5), responded more promptly (88.9→97.2), explained procedures more clearly (87.0→97.9), and created a safer environment for raising concerns (80.1→94.3). In addition, 81.2% of nurses endorsed the necessity of Happy Rounding, reflecting strong acceptance. This innovation demonstrates that proactive, standardized rounding can strengthen trust, safeguard patient rights, and advance patient-centered care, with future work needed to sustain and scale its impact.

Abstract citation ID: igaf122.4183

### REFRAMING THE TECHNOLOGY NARRATIVE: SUBJECTIVE AGING AND INCLUSIVE CO-DESIGN

Pallabi Bhowmick[1], and Erik Stolterman Bergqvist[2], *1. University of Illinois Urbana-Champaign, Champaign, Illinois, United States, 2. Indiana University Bloomington, Bloomington, Indiana, United States*

Older adults' technology acceptance is shaped not only by functional needs but also by subjective perceptions of aging and ageist stereotypes. While older adults acknowledge the value of assistive technologies, they often view these tools as more suitable for 'others', those older or in poorer health, rather than for themselves. This reluctance to see themselves as potential beneficiaries, coupled with stigma around needing assistance, poses barriers to adoption. For designing a technology to support social connection, we organized a co-design workshop with 15 older adults (ages 70–90, M = 78.1, SD = 6.4) and 6 student designers, deliberately structuring it to create a non-stigmatizing environment that fostered effective engagement. First, a humorous video of a Generation Z teenager struggling with a rotary phone reframed difficulties with new technologies as a universal experience. Second, a "technology timeline" activity highlighted older adults' history of technological adaptability, countering stereotypes of technological ineptitude. Third, the "Someone Who Is Not Me" (SWIM) technique shifted the focus from personal need to a hypothetical 'other', reducing self-stereotyping while still enabling reflection on their own potential use. Interestingly, during ideation, participants transitioned from designing for imagined personas to actively situating themselves as end-users, expressing interest in integrating the technology into their lives. When deficit-based framings were avoided, older adults were receptive to adopting later-life technologies. This work underscores the importance of framing technology for older adults as supporting independence rather than signaling decline, and highlights how strategies that reduce stigma during co-design challenge stereotypes and empower older adults as producers of innovation.

Abstract citation ID: igaf122.4184

### REGIONAL DISPARITIES IN RURAL PENSION PROVISION: EXAMINING EQUITY-EFFICIENCY DYNAMICS IN CHINA'S URBANIZATION

Huijing Kuang[1], Guoyong Ma[1], and Hong Mi[2], *1. Northeast Forestry University, Harbin, Heilongjiang, China (People's Republic), 2. Zhejiang University, Hangzhou, Zhejiang, China (People's Republic)*

Within the context of China's delayed retirement policies, regional variations in demographic structures and social security provision have become increasingly pronounced. The regional equity-efficiency of the basic endowment insurance for urban and rural residents have emerged as pivotal issues in social security system reform. This study employs Suzhou (a high-tech industrial hub) and Daqing (a resource-dependent city) as comparative case studies. Utilizing demographic and social security data alongside policy document analysis, it investigates urban heterogeneity in social security implementation and reveals systemic issues concerning regional equity and efficiency. Key findings reveal: (1)Enrollment Scale Disparity: Daqing's enrollment exceeds Suzhou's by over 15-fold. Beyond rural residents, enrollees primarily comprise land-expropriated farmers generated during urban expansion and flexible workers transitioning from basic endowment insurance for employees;(2)Benefit Equity Gap: Substantial inter-city disparity exists in pension benefits. By 2025, Suzhou's basic pension standard reached RMB 705/month, starkly contrasting with Daqing's minimum standard of RMB 163/month—a gap exceeding fourfold;(3)Subsidy Efficiency Variation: Suzhou's municipal subsidy for the highest contribution tier is 15%, fostering a virtuous "more contributions, higher returns" cycle, while constrained by local fiscal capacity, Daqing's highest-tier subsidy ratio is only 3.5%, severely diminishing enrollment incentives;(4)Pension replacement rates exhibit polarization: For minimum-tier contributions, Suzhou's rate represents 23.4% of rural residents' per capita disposable income, compared to merely 9.8% in Daqing. The study proposes a scheme of "implementing the integration of basic pensions and adopting graded contribution subsidies" to narrow regional pension disparities and addressing issues of systemic equity and efficiency.

Abstract citation ID: igaf122.4185

### REST-ACTIVITY RHYTHMS WITH COGNITIVE IMPAIRMENT AND ALZHEIMER'S DISEASE (AD)-RELATED PLASMA BIOMARKERS

Ahmed Danquah[1], Ann Cohen[2], Beth Snitz[2], and Yurun Cai[2], *1. University of Pittsburgh School of Nursing, Pittsburgh, Pennsylvania, United States, 2. University of Pittsburgh, Pittsburgh, Pennsylvania, United States*

Rest-activity rhythms (RAR) may serve as early indicators of cognitive decline, yet their associations with cognitive impairment and Alzheimer's disease (AD) biomarkers remain unclear. Thus, our study aims to examine the associations between objectively measured rest-activity rhythms, diagnosis of mild cognitive impairment (MCI) and AD, and AD-related plasma biomarkers in older adults in the Connectomics in Brain Aging and Dementia study. A total of 180 participants (mean age=65.0y, 64.4%women, 55.56% Black) had valid 7-day wrist-worn accelerometer assessment and consensus cognitive diagnosis in 2016-2021. RAR measures were computed using nparACT R package including interdaily stability (IS), intradaily variability (IV), relative amplitude (RA), M10 (activity levels at the most active 10 hours), and L5 (activity levels at the least active 5 hours). Plasma biomarkers (Aβ42, Aβ40, phosphorylated tau [p-tau]181, p-tau217, p-tau231, glial fibrillary acidic protein [GFAP], and neurofilament light chain [NfL]) were measured. Logistic regression models showed that higher IS and higher M10 were associated with lower odds of MCI or dementia diagnosis (odds ratio [OR]=0.15, 95% confidence interval[CI]=0.03-0.82, p = 0.03; OR = 0.94, 95% CI = 0.90-0.98, p = 0.003, respectively) after adjusting for sociodemographic factors. No significant associations were found between RAR and Aβ or p-tau biomarkers. However, linear regression models showed that higher RA was significantly associated with lower GFAP levels (β=-164.3, p = 0.02). Findings suggest that more stable (IS) and robust (RA) rest-activity rhythms may serve as non-invasive markers of cognitive health in older adults. Circadian disruptions may be linked to neuroinflammatory processes more than amyloid or tau pathology, but warrant further investigations in longitudinal studies.

Abstract citation ID: igaf122.4186

### REVISITING THE SOCIAL FRAILTY INDEX: A REPLICATION WITH IMPLICATIONS FOR AN EMERGING CONSTRUCT

Charles Collinge, and Monica Luciana, *University of Minnesota, Minneapolis, Minnesota, United States*

Frailty is a manifestation of the aging process associated with adverse outcomes. Methods to quantify it typically focus on physical decline. However, formulations that include social behavior are gaining interest. Recently, Shah and colleagues (2023) developed a 10-item Social Frailty Index (SFI-10) in data from the Health and Retirement Study (HRS) that used 8 social items plus age and sex to successfully predict mortality. This study evaluates the construct and criterion validity of the SFI-10. Utilizing Shah (2023)'s HRS cohorts (n = 8264, ages 66 to 101), and a sample from the Midlife in the United States (MIDUS) study (n = 5619, ages 40 to 75), we constructed the SFI-10 in both samples alongside an 8-item index (SFI-8) that reflects only social behavior. Logistic regression was used to predict mortality from the SFI-10, SFI-8, and models that included only age and sex from midlife to old age. Within MIDUS, we also evaluated the SFI-8, SFI-10, and age against criteria relevant to social frailty. Findings indicate that the SFI-10 relies heavily on age, with marginal contribution from its social items, to predict mortality in the HRS. Although social items had heightened importance for mortality prediction in MIDUS, the reliance on age persisted. While associations were observed between the SFI-10 and external criteria relevant for social frailty, the SFI-8 exhibited even stronger associations with these criteria. These findings raise questions about how social frailty should be quantified. A measure that exclusively indexes social behavior may best reflect the social frailty construct.

Abstract citation ID: igaf122.4187

### ROADMAPS OF RESISTANCE: MOTORIST GREENBOOK SAFE SPACES AND MODERN-DAY SUBJECTIVE COGNITIVE DECLINE

Jamila Rodriguez[1], Sydney Moseley[2], Pedro Pinheiro-Chagas[2], Muriel Taks Calle[3], Paris Adkins-Jackson[3], and Tanisha Hill-Jarrett[4], *1. Boston University, Boston, Massachusetts, United States, 2. University of California, San Francisco, San Francisco, California, United States, 3. Columbia University Mailman School of Public Health, New York, New York, United States, 4. University of California Memory and Aging Center, San Francisco, California, United States*

Introduction: The Greenbook is a historic list of safe spaces for individuals racialized as Black living in the U.S. under Jim Crow. The literature on structural racism and poorer cognitive aging across generations suggests the presence of safe spaces (i.e., structural resilience) may mitigate contemporary disparities in cognitive aging, like subjective cognitive decline (SCD). This study examines the state-level association between Greenbook sites and SCD. Methods: The state-level sum of 1948 Greenbook sites was transformed into quartiles representing sites per square mile (Q1=fewer sites/sq/mi; Q4 = most sites/sq/mi). These data were linked with state-level data from a single-item reporting SCD from 2015/2016 CDC Behavioral Risk Factor Surveillance System (BRFSS) for a sample of older adults (N = 21,378, aged>45). We compared participants across racialized groups and gender living in states with fewer Greenbook sites (Q1,Q2,Q3) to those living in states with more sites (Q4) on modern-day SCD (adjusting for income, degree attainment, and age). Results: Accounting for covariates, women racialized as Black had the highest odds of reporting SCD (OR: 1.26, 95% C.I. 1.11-1.44) compared to men racialized as White. Across all participants except men racialized as Black, living in states with the fewest Greenbook sites per square mile compared to those in states with the highest associated with modern-day SCD. Discussion: Living near fewer Greenbook sites is associated with present-day SCD among older adults. This suggests historic safe spaces served as protective factors against SCD. The Greenbook is a refuge from historic structural harms that shape health inequities today.

Abstract citation ID: igaf122.4188

### SELF-RATED HEALTH IN MONGOLIAN OLDER ADULTS: INSIGHTS FROM CHILDHOOD AND LIFE-COURSE FACTORS

Sugarmaa Myagmarjav[1], Zoljargalan Gantumur[2], Ariunsanaa Bagaajav[3], and Saranchuluun Otgon[1], *1. Mongolian National University of Medical Sciences, Ulaanbaatar, Ulaanbaatar, Mongolia, 2. Virginia Commonwealth University, Richmond, Virginia, United States, 3. City University of New York, Astoria, New York, United States*

An upper-middle-income country in Asia, Mongolia, is experiencing rapid population aging, with the population aged 65 and above projected to triple by 2060. Healthy aging reflects socioeconomic, and health conditions accumulated across the life course rather than being determined only in old age. Recently, life-course factors have increasingly been emphasized in aging studies to understanding healthy aging better. A cross-sectional survey among 149 older adults aged 60 and above in Ulaanbaatar was carried out from January to March, 2025 to examine whether self-rated health across childhood, midlife, and later life is associated with socioeconomic conditions. A single-item question was used to measure self-rated health both in childhood and midlife. Life-course socioeconomic indicators included childhood (family financial status, parental income adequacy), midlife (education, occupation, salary satisfaction), and older age (pension, household income adequacy). Binary logistic regression examined associations between life-course factors and current health. Participants' mean age was 68.8 ± 7.1 years, 40.3% were male, 61.8% married, and 57.7% urban residents. Overall, 43% reported poor health, with women more likely to report poor self-rated health than men. Childhood self-rated health was the only significant predictor of current health. Rural residence was also linked to poorer health, while education, marital status, and income showed no significant effects. Childhood health was a key predictor of current self-rated health. This study underscores the need to conduct future research with larger samples to validate the current results and identify additional life-course determinants of healthy aging.

Abstract citation ID: igaf122.4189

### SENSORIMOTOR MEASURES PREDICT LONGITUDINAL COGNITIVE DECLINE AND CORRELATE WITH CSF AND PLASMA BIOMARKERS

Semere Bekena[1], Ramkrishna Kumar Singh[2], Yiqi Zhu[3], David Carr[4], and Ganesh Babulal[5], *1. Washington University School of Medicine in St. Louis, St louis, Missouri, United States, 2. Washington University School of Medicine in St. Louis, St. Louis, Missouri, United States, 3. Washington University School of Medicine in St. Louis, Washington Universury, Missouri, United States, 4. Washington University in St. Louis, St Louis, Missouri, United States, 5. Washington University School of Medicine in St. Louis, SAINT LOUIS, Missouri, United States*

Identifying early markers of cognitive decline in cognitively normal older adults is critical for dementia prevention. Sensorimotor measures such as gait speed, grip strength, and reaction time may serve as accessible, sensitive indicators of current and future cognitive impairment. This study examined associations between baseline sensorimotor function and cognitive performance and decline, assessed by the Preclinical Alzheimer Cognitive Composite (PACC), in cognitively normal older adults. In this prospective cohort study, 246 cognitively normal older adults from the DRIVES Project completed baseline assessments of grip strength, gait speed, simple reaction time, and PACC scores. Linear mixed-effects models adjusted for demographic, genetic, and neighborhood depravity level. Cross-sectional analyses examined associations between sensorimotor measures and cerebrospinal fluid (CSF) biomarkers of amyloid and tau pathology, and plasma neurofilament light chain (NfL), where available. Participants (mean age: 74.9 ± 5.17 years; 48.8% female) followed on average for 4 years had a mean baseline PACC score of 1.06 ± 0.50. Cross-sectionally, slower gait speed was associated with higher CSF tau/Aβ42 ratio ($p = 0.027$), CSF tTau/Aβ42 ratio ($p = 0.009$), and plasma NfL ($p = 0.024$). Slower reaction time predicted lower baseline PACC scores ($p = 0.028$). Longitudinally, low grip strength ($p = 0.002$) and slow gait speed ($p < 0.001$) predicted lower cognitive performance, with slow gait speed predicting faster decline ($p = 0.015$). In conclusion, sensory-motor function measures are associated with current and future cognitive performance, supporting their role in early identification of older adults (65 and above) at risk for cognitive decline.

Abstract citation ID: igaf122.4190

### SENSORY IMPAIRMENT AND LABOR FORCE PARTICIPATION: EVIDENCE FROM PRE-RETIREMENT AGE ADULTS IN CHINA

Yuetong Toria Liu, and Zhang Zhang, *Johns Hopkins University, Bloomberg School of Public Health, Baltimore, Maryland, United States*

Hearing loss (HL), vision impairment (VI), and dual sensory impairment (DSI) pose challenges to labor force participation (LFP) in midlife. This study examines the impact of HL, VI, and DSI on LFP among adults aged 50-64 and explores differences by hukou-residency status in China. The data were drawn from the China Health and Retirement Longitudinal Study (2011–2018). We conducted analyses using ordinary least squares, random-effects, and fixed-effects (FE) models. FE models were applied for subgroup analyses and interpretation. Among 32,408 person-year observations, 15.4% reported HL, 6.8% reported VI, and 4.6% reported DSI. The weighted prevalence of hearing aid use among people with HL was 1.9%. No significant difference was found in the probability of employment between those with and without sensory impairment, nor across hukou-residency groups. However, HL, VI, and DSI were associated with 12.9% ($p < .01$), 5.5% ($p > .05$), and 21.3% ($p < .01$) fewer annual work hours, respectively, compared to those without sensory impairment. Among rural residents, HL, VI, and DSI corresponded to 16.9% (p < .01), 6.6% (p > .05), and 22.2% (p < .01) reductions in annual work hours, respectively. For rural-to-urban migrant workers, reductions were 0.9%, 6.0%, and 23.8%, respectively, although not statistically significant. Our study suggests that while sensory impairment was not associated with the probability of employment, HL and DSI significantly reduce annual work hours, particularly among rural residents. These findings point to the need for more inclusive health and labor policies to support pre-retirement adults with sensory impairments in China.

Abstract citation ID: igaf122.4191

### SHARED DECISION-MAKING FOR PROSTATE CANCER SCREENING AMONG MINORITY MEN IN THE UNITED STATES: A SYSTEMATIC REVIEW

Hyesong Joung[1], Hyeyeon Shin[2], and Randy Jones[1], *1. The University of Virginia, Charlottesville, Virginia, United States, 2. The Ohio State University, Columbus, Ohio, United States*

Prostate cancer screening (PCS) remains controversial in its efficacy, making shared decision-making (SDM) essential to ensure informed choices. However, racial and ethnic minority men in the United States experience persistent disparities in PCS participation and outcomes. Despite these disparities, how SDM interventions have been designed, implemented, and evaluated for these populations remains unclear. To address this gap, this systematic review examined SDM interventions for PCS among minority men, with a specific focus on the extent to which interventions incorporated key SDM features (information exchange, deliberation, and decision implementation) based on the SDM framework. Six databases (PubMed, Embase, CINAHL, PsycINFO, Web of Science, and Scopus) were searched for U.S.-based, English-language studies published in the past ten years that included minority participants comprising more than 30% of the sample. Eighteen studies met the inclusion criteria. Most targeted African American men, and the majority of interventions were delivered as single-session programs. Participant age criteria were inconsistent across studies. While all studies supported information exchange, few incorporated the full spectrum of SDM components (deliberation and decision implementation). Knowledge improvement was consistently reported, but effects on decision quality and screening behavior remain unclear. These findings highlight challenges in both the design and evaluation of SDM interventions, including limited application of core SDM features and underrepresentation of diverse minority populations. This review calls for future studies to develop and test consistent, comprehensive SDM interventions. Such efforts are needed to advance research on informed, preference-sensitive screening decisions and ultimately contribute to more equitable, patient-centered prostate cancer care.

Abstract citation ID: igaf122.4192

### SIX-MONTH EFFECTS OF THE CAREGIVER SUPPORT MODEL ON REDUCING FAMILY CAREGIVERS' NEEDS AND ENHANCING THEIR RESOURCES

Ka Hung Edwin Chung, Kin-Kit Li, and Dannii Yeung, *City University of Hong Kong, Hong Kong, China (People's Republic)*

Caring for frail older adults imposes significant stress and burden on family caregivers. To address these challenges and enhance their resources, the Caregiver Support Model (CSM) was developed (Leung et al., under review). The model incorporates systematic assessment, personalized intervention plans, and sustained support for caregivers. This longitudinal study examined the long-term effects of the CSM on 162 family caregivers from nine elderly centers (Mage=69.4, SD = 11.8; 79.6% female) between July 2024 and July 2025. Participants were initially assessed for their caregiving needs and resources using the Caregiver Needs and Resources Assessment (CNRA) (Li et al., 2023), followed by the implementation of personalized intervention plans over six months. Data were collected at baseline, three months, and six months. Controlling for demographic factors of both caregivers and care recipients, repeated measures MANCOVA revealed that the intervention program significantly reduced caregiver needs, including physical needs, role conflict, care recipient needs, psychological, and social support needs [F(10, 880) = 6.00, p <.001]. Additionally, caregiver resources — include spirituality, self-efficacy, responsibility, community resources, family support, understanding of care recipients, and a healthy lifestyle — showed significant improvement over the three timepoints [F(14, 876) = 6.85, p <.001]. Improvements were more pronounced at six-month compared to three-month, indicating a lasting effect and consolidation of gains. These findings underscore the importance of personalized interventions that take caregivers' evolving needs into consideration through systematic assessment. The CSM presents a promising approach to enhancing caregiver well-being and managing the complex demands of caregiving, particularly in an aging population.

Abstract citation ID: igaf122.4193

### SLEEP LATENCY AND PHYSICAL ACTIVITY IN OLDER ADULTS WITH MILD COGNITIVE IMPAIRMENT: OURA RING LONGITUDINAL STUDY

Jungjoo Lee[1], Junhyoung Kim[2], Kangeun Lee[3], and Aubrey Carter[2], *1. Texas A&M University, Texas A&M University, Texas, United States, 2. Texas A&M University, College Station, Texas, United States, 3. Indiana University, College Station, Texas, United States*

Background: Older adults with mild cognitive impairment (MCI) experience delayed sleep latency due to neuropsychological imbalance. While physical activity may enhance sleep quality, reliance on self-reported measures can reduce reliability because of recall bias. Aim: To investigate the longitudinal relationship between sleep latency and physical activity intensity in older adults with MCI through continuous monitoring using a smart ring system. Method: Seven older adults with MCI wore Oura smart rings for 14 days. Equipped with photoplethysmography, accelerometer, and gyroscope sensors, the rings calculated sleep latency as the interval from attempting sleep to the first light sleep episode, determined by reduced movement and thermoregulatory shifts. Physical activity intensity was estimated from heart rate variability and skin temperature, and classified as light, moderate, or vigorous based on the Metabolic Equivalent of Task. Data were analyzed using generalized estimating equations. Results: Vigorous activity significantly reduced sleep latency. Each additional second of vigorous activity corresponded to a 0.60 second decrease in latency (B = -0.60, SD = 0.21, p < .01, CI [-1.02, -0.18]). Moderate activity showed no significant association (B = 0.04, SD = 0.11, 95% CI [-0.17, 0.26]). Light activity showed a near significant reduction of 0.02 seconds per second (B = -0.02, SD = 0.01, 95% CI [-0.05, 0.01]). Conclusion: Only vigorous physical activity was significantly associated with reduced sleep latency. Light activity showed a trend toward improvement, while moderate activity had no clear effect. These results suggest that intensity specific interventions may be required to improve sleep in this population.

Abstract citation ID: igaf122.4194

### SLEEP QUALITY AS A MODIFIER OF PLASMA PTAU217 AND GFAP ASSOCIATIONS WITH COGNITIVE FUNCTION

Ramkrishna Kumar Singh[1], Semere Bekena[2], Yiqi Zhu[3], Paris Adkins-Jackson[4], Beau M. Ances[1], and Ganesh Babulal[5], *1. Washington University School of Medicine in St. Louis, St. Louis, Missouri, United States, 2. Washington University School of Medicine in St. Louis, St louis, Missouri, United States, 3. Washington University School of Medicine in St. Louis, Washington Universury, Missouri, United States, 4. Mailman School of Public Health, Columbia University, New York, New York, United States, 5. Washington University School of Medicine in St. Louis, SAINT LOUIS, Missouri, United States*

Background: Plasma biomarkers, such as neurofilament light chain (NfL), glial fibrillary acidic protein (GFAP), phosphorylated tau (pTau217), and total tau (tTau), are associated with cognitive decline. However, the role of sleep quality in modifying these associations remains unclear. This study examines whether subjective sleep quality, as measured by the Pittsburgh Sleep Quality Index (PSQI), modifies the associations between plasma biomarkers and cognitive performance. Methods: We analyzed cross-sectional data from 491 adults aged 36 years or older in the Aging Adult Brain Connectome study. Plasma levels of NfL, GFAP, pTau217, and total tTau were measured. Cognitive performance was assessed using the Montreal Cognitive Assessment (MoCA) and the Preclinical Alzheimer's Cognitive Composite (PACC). Sleep quality was measured using the Pittsburgh Sleep Quality Index (PSQI). Generalized linear models were used to test main and interaction effects while adjusting for demographics. Sensitivity analyses included APOE ε4 status and body mass index. Results: Higher plasma levels of NfL, GFAP, and pT217 were associated with lower cognitive performance on both MoCA and PACC (all $P < 0.05$). Poorer sleep quality was independently associated with worse PACC outcomes. Critically, significant interaction effects were observed: PSQI moderated the negative associations between GFAP and both PACC ($\beta = 0.0003$, $P = 0.039$) and MoCA ($\beta = 0.0019$, $P = 0.021$), and between pT217 and MoCA ($\beta = 0.0299$, $P = 0.004$), indicating a synergistic relationship between sleep quality and glial/amyloid-related pathology in cognitive aging. Conclusion: Sleep quality modifies biomarker-cognition associations, highlighting its potential as a behavioral target to support brain health.

Abstract citation ID: igaf122.4195

### SLEEP QUALITY MODERATES AGE-RELATED DECLINE IN HIPPOCAMPAL-DEPENDENT MEMORY IN MIDLIFE ADULTS

Kyoung Shin Park[1], Samantha DuBois[2], Samuel Kibildis[3], Hadassah Som-Pimpong[4], Jarod Vance[5], Brittany Armstrong[6], Christopher Wahlheim[6], and Jennifer Etnier[6], *1. Emory University School of Medicine, Atlanta, Georgia, United States, 2. Appalachian State University, Boone, North Carolina, United States, 3. Athens-Clarke County Unified Government, Athens, Georgia, United States, 4. Meharry Medical College, Nashiville, Tennessee, United States, 5. Longwood University, Farmville, Virginia, United States, 6. University of North Carolina at Greensboro, Greensboro, North Carolina, United States*

The hippocampus, critical for learning and memory, is vulnerable to age-related decline. Evidence suggests that sleep is associated with hippocampal integrity and cognitive function in older adults. However, few studies have examined whether sleep quality moderates age-related decline in hippocampal-dependent memory in midlife—a critical time point for detecting preclinical cognitive changes. In this cross-sectional study, we tested the hypothesis that poor sleep quality would be linked to steeper age-related decline in hippocampal-dependent memory performance in midlife. We analyzed data from cognitively normal midlife adults with a family history of Alzheimer's disease ($N = 180$; Age= 40–65 years [$M = 56.13 \pm 6.35$ years]; 89% females, MoCA=$27.9 \pm 1.7$) enrolled in a clinical trial (NCT03876314). Participants completed the Pittsburg Sleep Quality Index (PSQI) and Mnemonic Similarity Task (MST)—a sensitive test of hippocampal-dependent memory function. Multiple linear regression was fitted while controlling for potential covariates (e.g., sex, education, APOE-ε4 carriage, and pulse pressure). There was a significant Age × PSQI interaction ($b = -0.0017$, $p = .037$) with lure discrimination index (LDI) as the outcome, such that higher PSQI scores (poorer sleep quality) were associated with steeper age-related decline in hippocampal-dependent memory performance. Simple slopes analysis showed that age predicted lower LDI in participants with average ($b = -0.01$, $p = .01$) and higher (+1 SD) PSQI ($b = -0.01$, $p < .01$), but not those with lower (–1 SD) PSQI ($p = .81$). These findings suggest the potential role of sleep quality in protecting hippocampal-dependent memory function against age-related decline in midlife and support the utility of the MST for detecting subtle cognitive changes during this critical life stage.

Abstract citation ID: igaf122.4196

### SOCIAL CONNECTION AND BRAIN AGE IN FEMALES AND MALES

Helena Blumen[1], Chava Pollak[2], Sanish Sathyan[1], Sofiya Milman[3], and Joe Verghese[1], *1. Stony Brook University, Stony Brook, New York, United States, 2. Stony Brook University, Sto, New York, United States, 3. Albert Einstein College of Medicine, Albert Einstein College of Medicine, New York, United States*

Poor social connection is associated with negative health outcomes in aging – including dementia. Structural aspects of social connection include marital status, living arrangements, employment/volunteering, and social networks. Sex-related differences in the structure of social connection have been observed in aging but how they relate to brainageR – or other machine learning estimates of the biological age of a brain – is currently unknown. This study of 171 Ashkenazi Jewish older adults without dementia from the LonGenity study (M Age = 72.59 years; 59% female) aimed to determine if sex modified associations between structural social connection measures (marital status, living arrangements, employment/volunteering, social networks) and brainageR acceleration (AgeAccel). Brain AgeAccel was defined as the residual from regressing machine learning-predicted brainageR

estimates on chronological age. Separate linear regression models adjusted for potential confounders were applied to each social connection measure, and the interaction term between sex and social connection was the effect of interest in each model. Living arrangements (alone/with someone) and marital status (married/not married) – but not employment/volunteering (yes/no) or social networks (social network index) – were differentially associated with brain AgeAccel in males and females. Males who lived with someone or were married had lower brain AgeAccel (better) than males who lived alone or were unmarried. Females who lived with someone or were married had higher brain AgeAccel (worse) than females who lived alone or were unmarried. These novel sex-specific associations are interesting but need the be confirmed in larger and more diverse samples of older adults.

Abstract citation ID: igaf122.4197

**SOCIAL ISOLATION AND TECHNOLOGY USAGE AMONG MEXICAN AMERICAN FAMILY CAREGIVERS FOR ALZHEIMER'S DISEASE PATIENTS**

Leyi Zhou[1], and Lin Jiang[2], *1. University of California, Berkeley, Berkeley, California, United States, 2. University of Texas Rio Grande Valley, Edinburg, Texas, United States*

The number of Latinos affected by Alzheimer's disease and related dementias (ADRD) is increasing rapidly, with Mexican American family caregivers facing substantial burdens. While information and communication technology (ICT) reduces caregivers' social isolation, research has predominantly focused on White American populations. This study examines how ICT use affects social isolation and loneliness among Mexican American caregivers, and how cultural and religious factors influence this relationship. In-depth interviews were conducted with 24 Mexican American family caregivers of ADRD patients along the South Texas-Mexico border. Thematic analysis revealed that caregivers experienced social isolation and loneliness that intensified during COVID-19. Cultural factors, particularly familism—the obligation to care for family members—both motivated caregiving but also contributed to isolation when family understanding was limited. ICT use significantly reduced social isolation by maintaining social connections, accessing support groups, and facilitating healthcare communication. ICT effectiveness was influenced by cultural preferences for family-centered communication and varying technology comfort levels. Religion shaped ICT use, with online religious participation serving as both a coping strategy and means of maintaining spiritual community connections. Findings suggest future interventions should prioritize culturally tailored ICT solutions addressing Mexican American caregivers' specific needs. Understanding the intersection of cultural values, technology use, and social isolation is essential for designing effective support systems that improve quality of life for both caregivers and patients in Latino communities affected by ADRD.

Abstract citation ID: igaf122.4198

**SOCIAL NETWORKS IN MID-TO-LATE-LIFE ADULTS AND COGNITIVE DECLINE: THE FRAMINGHAM HEART STUDY**

Phillip Hwang, *Boston University School of Public Health, Boston, Massachusetts, United States*

Studies examining the relationship between social networks and cognitive decline mainly include older adults; less is known about the relationship when including adults earlier in life, such as middle age. We investigated the association between social network size among mid-to-late-life adults and cognitive decline in memory, language, and executive functioning. The study sample included 1,297 participants age 40-90 years from the Framingham Heart Study Offspring cohort. Social network size was determined by the Berkman-Syme Social Network Index (SNI), which was measured between 2005-2009, and categorized into four groups (e.g., low, low/medium, medium/high, and high). Cognitive factor scores for memory, language, and executive functioning were previously derived from neuropsychological tests. Linear mixed models were used to estimate mean annual change in each factor score associated with social network size, with the low SNI group as the reference. Models were adjusted for age, sex, education, body mass index, smoking, physical activity, and depressive symptoms. High SNI was significantly associated with declines in memory ($\beta$ = -0.011; 95% CI = -0.02, -0.003) and executive functioning ($\beta$ = -0.011; 95% CI = -0.02, -0.001). Medium/high SNI was also significantly associated with decline in memory ($\beta$ = -0.013; 95% CI = -0.02, -0.005). Individuals with higher SNI, as compared to lower SNI, had greater proportions of prevalent dementia and incident dementia during the follow up period. These findings suggest that reverse causation impacts the association between social networks and cognitive decline and that seeking greater social support may be a sign of worsening cognitive function.

Abstract citation ID: igaf122.4199

**SOCIAL PARTICIPATION AND HRQOL WITH NEURODEVELOPMENTAL DISABILITIES: MEDIATION BY EVERYDAY LIVING CAPABILITIES**

Rongfang Zhan[1], Kayi Ntinda Ntinda[2], and Elias Mpofu[1], *1. University of North Texas, Denton, Texas, United States, 2. UT health Rio Grande valley, Edinburg, Texas, United States*

Background and aims: Social participation is a key determinant of health-related quality of life (HRQoL) of adults with neurodevelopmental disabilities (NDDs), yet their social participation for HRQoL in everyday living remains unknown. This study investigated and social participation HRQoL relationship, with everyday living capabilities as a mediator. Method: The of 304 adults with NDDs (females = 83.3%; age range 18 to 69 years old). completed standardized social participation, Health related quality of life (HRQoL) and everyday capabilities measures. Multi-group path analysis was employed to examine self-perceived everyday living capabilities meditation of the association between social participation and health-related quality of life (HRQoL) across the two groups. Results: For adult autistics, both the indirect effect ($\beta$ = .170, 95% CI [.071, .179]) and the direct effect of social participation on HRQoL ($\beta$ = .177, 95% CI [.040, .221]) were significant, indicating partial mediation of capabilities. For adults with intellectual disability, the direct effect of social participation on HRQoL was nonsignificant ($\beta$ = .174, 95% CI [–.012, .224]), indicating full mediation. Conclusion and implications: These results underscore the importance of personal factors in the HRQoL of adults with NDDs. By implication, policy and program development initiatives should improve everyday living needs for the improved quality of life for this population. Keywords: Social Participation, neurodevelopmental disabilities, health-related quality of life, mediation analysis.

Abstract citation ID: igaf122.4200

### SOCIAL SUPPORT AND MENTAL HEALTH OF OLDER ADULTS IN SUB-SAHARAN AFRICA: AN EXPLORATORY CHAIN MEDIATION ANALYSIS

Wisdom Obioha[1], Nkechi Chukwuemeka[2], Ikenna Okechi[2], and Ifeoma Eze[2], *1. Miami University, Oxford, Ohio, United States, 2. University of Nigeria, University of Nigeria, Enugu, Nigeria*

Social support has regularly been identified as both protective and promotive factor for the mental health of older adults in various contexts and cultures, yet these benefits of social support may require other important factors to be transmitted to the mental health of older adults, especially in Sub-Saharan Africa. Also, given that social support is a multidimensional construct, different types of social support may have varying levels of benefits for older adults, especially when interacting with other factors. We employed survey data from 350 older adults aged between 60 and 93 years old from a rural community in Nigeria to explore the potential sequential mediation effect of self-compassion and life satisfaction on older adults' mental health. Data was derived from the Multidimensional Scale of Perceived Social Support, Self-Compassion Scale-Short Form, Satisfaction with Life Scale, and Psychological Well-being Scale. After controlling for age, marital status, and education; family support, self-compassion and life satisfaction were positive predictors of mental health. Life satisfaction was a significant mediator between social support and mental health, but self-compassion was not. Support from friends and significant others associated positively with mental health only through the mediating effect of life satisfaction. Self-compassion and life satisfaction also did not exert sequential mediation effects on this relationship. The study concludes that self-compassionate older adults who perceive a sufficient amount of support from important sources and maintain high levels of life satisfaction may witness significant improvements in their mental health. Concerted efforts should be tailored towards improving self-compassion among older adults.

Abstract citation ID: igaf122.4201

### SOCIODEMOGRAPHIC AND HEALTH CHARACTERIZATION OF THE SOUTH TEXAS ALZHEIMER'S DISEASE RESEARCH CENTER COHORT

Silvia Mejia-Arango[1], Jeremy A Tanner[2], Campbell Sullivan[2], Ashley LaRoche[2], Jennifer Del Bosque[3], Rosa Pirela[1], Gladys Maestre[4], and Sudha Seshadri[2], *1. University of Texas Rio Grande Valley, Harlingen, Texas, United States, 2. University of Texas Health Sciences Center at San Antonio, San Antonio, Texas, United States, 3. UT Health San Antonio, San Antonio, Texas, United States, 4. University of Texas Rio Grande Valley, Edinburg, Texas, United States*

The South Texas ADRC (STAC) is the only Texas-based ADRC with three sites—San Antonio, Laredo, and the Rio Grande Valley—and leads in Hispanic participant data (about 60%) among the 36 active ADRCs. To better understand the characteristics of our cohort, we analyzed differences in sociodemographic and health conditions by ethnicity, as well as their role as predictors of cognitive status. A total of 773 participants enrolled in STAC who completed a standardized clinical evaluation protocol used in the ADRCs, were included in the analysis. Research participants were aged 71 ± 9.6 years; 60% were female; the mean years of education were 14.6 ± 3.6 years; and 59% were Hispanic. Rates of dementia were significantly higher among non-Hispanics (30% vs. 17.4%), while MCI rates were not significantly different (24.9% vs. 28.3%). Additionally, there were no differences in cognitive impairment not classified as MCI (1% vs. 3.5%). We found a significant trend towards worse conditions among Hispanic participants compared with non-Hispanics. Hispanics had significantly fewer years of education; higher socioeconomic disadvantage at the state level based on the adversity index (ADI), and higher rates of diabetes (32%), hypertension (72.9%), and hypercholesterolemia (64.3%). Predictors of dementia among Hispanics were older age, lower years of education, and being male. Among non-Hispanics, older age and being male were the only significant predictors of dementia. Different recruitment and enrollment strategies across the three STAC sites, leading to high heterogeneity among subsamples, are discussed as possible drivers of our results.

Abstract citation ID: igaf122.4202

### SOCIODEMOGRAPHIC FACTORS AND ACCESS TO DIGITAL RESOURCES AMONG MEDICARE BENEFICIARIES IN NONMETROPOLITAN AREAS

Brian Nguyen, and Boon Peng Ng, *University of Central Florida, Orlando, Florida, United States*

The COVID-19 pandemic accelerated telehealth adoption, but disparities in digital access may hinder its potential, especially for older adults in rural areas. This cross-sectional study examined sociodemographic factors of digital access using 2022 Medicare Current Beneficiary Survey Public Use File. The sample included 1,732 Medicare beneficiaries aged ≥65 living in nonmetropolitan areas. The dependent variable of digital access was categorized as: (1) access to both a computer/tablet and the internet, (2) access to either, and (3) access to neither (reference group). A survey-weighted multinomial logit model was conducted to assess associations between sociodemographic factors and digital access. Overall, 71.7% of nonmetro beneficiaries had both computer and internet access, 14.4% had one or the other, and 13.9% had neither. Beneficiaries aged ≥75 (vs 65–74) had significantly lower odds of having both computer and internet access ($OR = 0.30$, $p < 0.001$) and access to either ($OR = 0.39$, $p = 0.003$). Males had lower odds of having both access ($OR = 0.64$, $p = 0.023$). Non-Hispanic Blacks (NHB) and Other minorities had lower odds of having both access (for NHB, $OR = 0.45$, $p < 0.001$). Beneficiaries with lower education (vs more than a high school education) had lower odds of having both access (for less than a high school education, $OR = 0.13$, $p < 0.001$). Income under $25,000 was associated with lower odds of having both access ($OR = 0.36$, $p < 0.001$). Older age, male sex, minority race/ethnicity, lower education, and lower income are key factors of reduced digital access among nonmetro Medicare beneficiaries, underscoring the need for targeted interventions and policies to expand telehealth access for these at-risk populations.

Abstract citation ID: igaf122.4203

### SUBJECTIVE NEARNESS TO DEATH AND EMOTIONAL-COGNITIVE COMPLEXITY: AN INTEGRATIVE MODEL

Sara Cohen-Fridel, Amit Shrira, Ehud Bodner, and Yaacov Yablon, *Bar-Ilan University, Ramat Gan, HaMerkaz, Israel*

Lifespan developmental psychology emphasizes the role of emotional-cognitive complexity (i.e., the ability to hold conflicting emotions and thoughts) in late-life adjustment and functioning. The present study examines an integrative model that explores the relationship between subjective nearness to death (SNtD) and emotional-cognitive complexity, while considering the mediating role of the hostile world scenario (HWS). SNtD refers to how close one perceives oneself to death and is associated with negative psychological experiences. The HWS refers to an individual's perception of actual or potential threats to their physical and psychological integrity. Partially supporting our hypotheses, our study (N = 1,073, ages 60-86) showed that some dimensions of the HWS mediated the relationship between SNtD and emotional-cognitive complexity. These findings highlight the theoretical contribution of the HWS in revealing the psychological mechanisms linking SNtD perception with emotional-cognitive adaptation in later life.

Abstract citation ID: igaf122.4204

### SYSTEM-SPECIFIC METABOLOMIC CLOCKS REVEAL AGING HETEROGENEITY AND LINKS TO DIET, COGNITION, MORTALITY

Anastasia Leshchyk[1], Nicole Roth[2], Stefano Monti[2], Stacy Andersen[2], Thomas Perls[2], and Paola Sebastiani[1], *1. Tufts Medical Center, Boston, Massachusetts, United States, 2. Boston University, Boston, Massachusetts, United States*

Aging is a heterogeneous process that unfolds differently across individuals and biological systems. While single biological clocks provide valuable insights, they often fail to capture the complex and multidimensional nature of aging. In this study, we developed system-specific aging clocks using metabolomics data from the Integrative Longevity Omics study to better understand the heterogeneity of aging trajectories. Each clock was designed to estimate biological age within a distinct metabolic system, under the assumption that variability in system-specific function reflects unique aspects of the aging process. Our analyses revealed striking inter-individual variability: some participants consistently exhibited age acceleration across systems, others showed age deceleration, and many demonstrated mixed patterns depending on the system measured. To further explore this heterogeneity, we clustered participants into subgroups based on their system-specific aging profiles. We then examined associations between these subgroups and (1) the Nutrient Variety Index (NVI), a comprehensive metric summarizing dietary diversity across 19 nutrient groups, (2) cognitive performance, and (3) mortality risk. We found that several subgroups displayed significant associations with multiple NVIs, particularly those reflecting balanced intake of carbohydrates. These same subgroups also showed more favorable cognitive outcomes and reduced mortality risk, suggesting that consistent patterns of healthy aging may be linked to dietary diversity and nutritional balance. Conversely, other subgroups displayed discordant patterns of aging acceleration and were associated with poorer outcomes. These findings highlight that aging is not uniform but system-specific, and that metabolomic aging clocks offer a promising framework for uncovering distinct pathways shaping healthy aging.

Abstract citation ID: igaf122.4205

### TARGETING ILLNESS PERCEPTION THROUGH TIP: IMPROVING DEPRESSION TREATMENT ENGAGEMENT IN LATE LIFE

Rebecca Kiflom[1], Clare Culver[2], Jialin Wu[3], Isabel Rollandi[4], Helen Kales[5], and Jo Anne Sirey[2], *1. Weill Cornell Medical College, NEW YORK, New York, United States, 2. Weill Cornell Medical College, New York City, New York, United States, 3. Weill Cornell Medical College, New York, New York, United States, 4. Weill Cornell Medicine, New York City, New York, United States, 5. UC Davis Health, Sacramento, California, United States*

Depression remains one of the most prevalent psychiatric disorders among older adults, contributing significantly to disability, mortality, and reduced quality of life. Despite the established effectiveness of antidepressants, adherence to antidepressant treatment remains a challenge. Illness perception, an individuals' beliefs about the cause, timeline, and controllability of their illness, has been linked to treatment engagement across chronic health conditions but is understudied in late-life depression. This study examines whether the Treatment Initiation and Participation (TIP) intervention improved illness perception among 231 adults (>60) with major depression who were newly prescribed antidepressants at primary care clinics in Michigan and New York City. Participants were recruited within 10 days of receiving a new antidepressant prescription and randomized to either TIP or treatment as usual (control). The TIP intervention included three structured, 30-minute sessions over six weeks, during which participants explored barriers to care, discussed misconceptions about depression, addressed stigma, and developed personalized adherence strategies. Illness perception was assessed using the Brief Illness Perception Questionnaire (BIP) at baseline and again at 24 weeks. Antidepressant adherence was tracked at 6 and 12 weeks using self-report and chart verification. A mixed-effects model demonstrated TIP participants had a significantly greater average reduction in BIP scores from baseline to 24 weeks (-17) compared to the control group (-11), p<.05. These findings suggest that targeting illness perception early during antidepressant treatment may enhance understanding of depression and support sustained engagement with care. Interventions like TIP can play a role in correcting misbeliefs about depression and improving long-term adherence.

Abstract citation ID: igaf122.4206

### TASK-SPECIFIC VOCAL ACOUSTIC MARKERS REVEAL AMYLOID BURDEN

Hyunsun Ham[1], Jiwon Son[1], Keun You Kim[2], So Yeon Jeon[3], and Jun-Young Lee[3], *1. Seoul National University, Republic of Korea, 2. Yonsei University College of Medicine, Seoul, Republic of Korea, 3. Seoul Metropolitan Government-Seoul National University (SMG-SNU) Boramae Medical Center, Seoul, Republic of Korea*

This study investigates whether task-specific vocal acoustic features can reliably predict amyloid PET positivity in Alzheimer's disease. While prior works often relied on a single task such as picture description, we systematically designed five distinct speech tasks, each designed at a different linguistic level—from acoustic-phonetic to discourse-level—to examine how the underlying pathology manifests in speech production at varying linguistic levels. Seventy-five participants completed five speech tasks, including 46 amyloid-negative and 29 amyloid-positive individuals, as determined by PET imaging. From each task, we extracted 162 hand-crafted acoustic features using an optimized clinical speech analysis pipeline. These features included spectral features(MFCCs), prosodic features (pitch, intensity, speech rate), and voice quality measures (jitter, shimmer, HNR). Recursive feature elimination and XGBoost were employed for feature selection, followed by task-specific classification using multiple binary classifiers. Among the tasks, the Sequential Motion Rate task yielded the highest accuracy(79%). Pitch-related features consistently ranked among the most important across all tasks, suggesting their robustness regardless of linguistic level. Prosodic features demonstrated increased discriminative power in higher-order tasks, whereas voice quality features were particularly salient in tasks emphasizing phonation and articulation. These findings highlight that vocal acoustic markers from specific speech tasks can effectively reveal amyloid burden, with distinct acoustic patterns depending on the linguistic complexity of the task. These task-dependent speech markers may reflect early neurodegenerative changes, supporting a link between amyloid pathology and speech production.

Abstract citation ID: igaf122.4207

### TECHNOLOGICAL CHALLENGES DURING VIDEO VISITS WITH VETERANS FOR MEDICATION MANAGEMENT

Michael Zanchelli[1], Kristal Xie[2], Chelsea Hawley[3], Sheikh Hossain[4], Akanksha Samant[5], and William Hung[6], *1. James J. Peters VAMC, Bronx, New York, United States, 2. University of Hawaii at Manoa, Honolulu, Hawaii, United States, 3. VA Bedford Healthcare System, Bedford, Massachusetts, United States, 4. James J Peter VA Medical Center, New York, New York, United States, 5. James J Peters VA Medical Center, Bronx, NY, New York, United States, 6. James J. Peters Department of Veterans Affairs Medical Center, New York, New York, United States*

There is limited understanding of technological barriers that occurs leading up to and during video visits for older adults. Successful implementation of video visit to deliver care requires recognizing the specific barriers faced by older adults and developing targeted strategies to address these factors. We share the barriers faced and how we overcame them in 129 video visits with older Veterans. As part of a randomized controlled trial at Bronx and Bedford VAs, 129 Veterans attempted a video consultation with a clinical pharmacist from their home, to assess their medications and answer medication-related questions. Participants were aged ≥65 years, taking ≥5 daily VA medications, having ≥2 chronic conditions, ≥ 1 VA primary care visit within past year with no history or diagnosis of dementia (MoCA score > 24/30 or telephone MoCA [t-MoCA] score ≥ 18/22). Of 129 Veterans, 120 (93%) successfully completed a video visit, with 95% using their own device and 64% using smartphones. Most participants (79%) connected on their first attempt, although 58% required assistance. Average assistance last 17 minutes (range 1-33), primarily provided by study coordinators (74%), and pharmacists (11%). The most common challenges included connection/bandwidth problems (25%), technology literacy (20%), and hearing difficulties (13%). The most common methods for overcoming these challenges were proactive assessment of health literacy, cognitive status, technology comfort, device ownership, and prior video visit experience, as well as the opportunity to practice a video visit before the actual visit to work out technology kinks.

Abstract citation ID: igaf122.4208

### TEMPORAL ASSOCIATIONS BETWEEN DAILY AFFECT, SLEEP, AND PHYSICAL ACTIVITY IN FAMILY CAREGIVERS OF OLDER ADULTS

Soojung Ahn, and Dabin Hwang, *Boston College William F. Connell School of Nursing, Chestnut Hill, Massachusetts, United States*

Informal caregiving can be psychologically taxing and may disrupt caregivers' ability to maintain health-promoting behaviors. Psychological distress is linked to sleep problems and reduced physical activity, yet their temporal relationships remain underexplored in caregiving contexts. This study examined within- and between-person associations among psychological distress, sleep, and physical activity in caregivers of older adults (≥65 years) with chronic illness. Thirty caregivers (mean age, 57 years [SD = 10]; female, 93.3%; white race: 76.7%) completed daily surveys for 7 days, reporting negative affect (e.g., depressed, bored, irritated, dissatisfied with self, worried, and nervous), perceived stress each evening, and sleep quality each morning. Concurrently, they wore an accelerometer (ActiGraph GT9X-Link) to capture sleep and physical activity. Multilevel models accounted for person-level covariates (age, general health status, depression, sleep disorder) and day-level covariates (weekday/weekend, lagged variables). Within-person analyses showed that on the days with higher levels of negative affect and stress, caregivers were more likely to experience shorter sleep duration (B [95% CI] = -1.08 [-2.10, -0.06]; -0.95 [-1.47, -0.43]) and to spend longer time being inactive the following day (3.08 [1.10, 5.06]; 1.41 [0.38, 2.44]). On the days following shorter or poorer-quality sleep, caregivers were more likely to spend longer time being inactive (-0.37 [-0.65, -0.08]; 44.20 [20.97, 67.44]). No statistically significant between-person differences were observed. These findings highlight the daily interdependence of psychological distress, sleep, and physical activity. Interventions should incorporate real-time strategies that address daily stressors, promote sleep hygiene, and encourage physical activity to support sustainable caregiver health and prevent long-term decline.

Abstract citation ID: igaf122.4209

### THE ASSOCIATION BETWEEN VOLUNTEERING AND MEMORY: DOES WEALTH MATTER?

Alanna Frost, *University of Massachusetts Boston, Windham, New Hampshire, United States*

Objective: The benefits of volunteering and cognitive function have been explored in recent years. Although several studies have studied this relationship, no studies have examined the effect of an essential element of socioeconomic status (SES), wealth, for the relationship between volunteering and elements of cognitive function, specifically episodic memory. Therefore, the aims of this study were to investigate (1) whether volunteering is associated with memory, (2) whether wealth is related to memory, and (3) whether wealth moderates the relationship between volunteering and memory. Method: The present study utilized a nationally representative sample of 8,075 respondents aged 60 years and older, who completed both the 2020 and 2022 waves of the Health and Retirement Study. Results: Results from linear regression models revealed that older adults who volunteer have higher episodic memory scores than those who do not volunteer. Further, results indicate that higher levels of wealth are also associated with higher episodic memory scores. Results did not show a significant moderation effect of wealth for the link between volunteering and episodic memory. Conclusion: The results of this study suggest that volunteering offers cognitive benefits among older adults. Respondents who volunteer report higher levels of episodic memory compared to non-volunteers, and individuals who were wealthier also reported higher memory scores. Despite findings from the current literature suggesting that volunteering may have a greater impact on cognitive reserve among individuals of lower SES, wealth did not moderate the relationship between volunteer status and memory.

Abstract citation ID: igaf122.4210

**THE ASSOCIATION OF THE NUMBER OF INFORMAL CAREGIVING ROLES WITH DEPRESSION: MODERATION OF SELF-ESTEEM**

Sehyun Baek, Ernest Gonzales, and Shicheng Xu, *New York University, New York, New York, United States*

Demographic changes, a decline in the ratio of potential caregivers to the number of adults who need care, highlight the growing prevalence of multiple caregiving roles (e.g., double-decker sandwich caregiving). However, little is known about the extent to which the number of caregiving roles is associated with depression. We examined the association between the number of caregiving roles (no role, 1 caregiving role, and 2+ caregiving roles), derived from caregiving for grandchildren, parents with Activities of Daily Livings (ADLs), parents with Instrumental Activities of Daily Livings (IADLs), spouse with ADLs, and spouse with IADLs) and depression, and whether self-esteem moderates this association. We utilized the data from the 2022 Health and Retirement Study (HRS) and RAND HRS (n = 2,259), guided by the Stress Process Model that posits self-esteem as a resource in the association of caregiving on depression. Results indicated that having 1 caregiving role ($b = 1.50$, $p < .01$) and 2+ caregiving roles ($b = 2.37$, $p < .01$) were significantly associated with a higher level of depression compared to non-caregivers. Self-esteem moderated the associations: higher self-esteem buffered the impact of caregiving roles on depression (1 role × self-esteem: $b = -0.043$, $p < .01$; 2+ roles × self-esteem: $b = -0.065$, $p < .01$). Findings underscore the importance of considering the heterogeneity of caregiving roles. For instance, caregiver training and support groups to enhance self-esteem, the National Family Caregiver Support Program should be further expanded, with attention to older adults who shoulder multiple caregiving responsibilities.

Abstract citation ID: igaf122.4211

**THE DEMENTIA DIAGNOSTIC PROCESS: THE CAREGIVER'S ROLE AND THE CHALLENGES FACED**

Lily Gorman, and R. Amanda Cooper, *University of Connecticut, Storrs, Connecticut, United States*

The objective of this study was to explore the role of the caregiver when seeking a dementia diagnosis for their spouse, as well as identify the challenges they faced. The process of seeking a dementia diagnosis can be a long and complex task, with numerous barriers along the way. Familial caregivers are deeply involved in this process, yet the role and challenges faced by caregivers are not often the focus of this type of research. Thematic analysis was used to explore caregivers' experiences of seeking a dementia diagnosis for their spouse. This qualitative study included interviews from 18 caregivers. A number of challenges emerged consisting of both doctor-related barriers: (1) Doctors dismissing or ignoring concerns, (2) Unclear disclosure or miscommunication from doctors, (3) Long, complicated, many step process, (4) Misdiagnosis or mistreatment, (5) Lengthy and frustrating evaluations; and personal or disease-related barriers: (6) Misattribution of symptoms, (7) Fear of hurting their spouse's feelings, and (8) Denial or hesitancy from their spouse. Seven fluid and dynamic caregiver roles were also identified: Fierce Advocate, The Bad Guy, Determined Investigator, Team Player, Secret Agent, Fearful Fighter, and Pragmatic Realist. These findings provide insight into the complex role caregivers play when seeking a dementia diagnosis for their spouse, and may provide both healthcare professionals and fellow caregivers with an understanding of what this process entails. This study highlights the urgent need to streamline the diagnostic process for people with dementia and their caregivers.

Abstract citation ID: igaf122.4212

**THE DUAL CRISIS: EXAMINING OLDER ADULT OPIOID USE AND NURSING COMPETENCY**

Noell Rowan[1], and Tamatha Arms[2], *1. University of North Carolina Wilmington, Wilmington, North Carolina, United States, 2. UNCW, Wilmington, North Carolina, United States*

The opioid crisis represents a significant public health emergency in the United States, profoundly affecting individuals, families, and healthcare systems alike. Ortiz and colleagues (2022) in a comprehensive meta-analysis revealed that the average percentage of total pain knowledge among nurses was only 52.9% (95% CI: 47.2–58.6). Moreover, Kramarow & Tejada-Vera (2022) found a 320% increase in opioid overdose deaths among older adults and a 500% increase in older adult opioid related hospitalizations. Kaiser (2020) found that 75% of nurse participants felt unprepared to handle an opioid related case. These results underscore the urgent need for improved nurse education and training. The intersection of these challenges—inadequate pain management training, an aging population with complex needs, and the ongoing opioid crisis—creates a perfect storm that demands immediate attention and innovative solutions. This cross-sectional study involved nurse participants recruited through emails that were sent to all LPNs, RNs, and advanced practice nurses in one southeastern state of the United States. Participants completed a Knowledge and Attitudes Survey Regarding Pain (KARSP) tool and a second knowledge survey on opioids. A total of 264 nurses responded, with the majority being nurse practitioners. The mean score was 75% (SD ± 11). Only 45% of the participants understood respiratory depression associated with opioids and 36% were able to identify physical withdrawal symptoms. Implications include improvements and innovative strategies for practitioner education and advocacy for older adults and health care needs.

Abstract citation ID: igaf122.4213

**THE IMPACT OF BRAIN AGE VERSUS CHRONOLOGICAL AGE ON COGNITIVE FATIGUE: NOVEL METRICS AND NEW INSIGHTS**

Cristina Roman[1], Glenn Wylie[2], Jay Buckey[3], Nancy Chiaravalloti[4], James Ford[5], Michael Falvo[6], Helen Genova[4], and John DeLuca[2], *1. Keck School of Medicine of USC, Los Angeles, California, United States, 2. Kessler Foundation, West Orange, New Jersey, United States, 3. Dartmouth Geisel School of Medicine, Hanover, New Hampshire, United States, 4. Kessler Foundation, East Hanover, New Jersey, United States, 5. Dartmouth College, Hanover, New Hampshire, United States, 6. Rutgers University, East Orange, New Jersey, United States*

Fatigue impacts 22% of the general population. Yet, our understanding of chronological age and fatigue is mixed, suggesting other more novel age- and fatigue-related metrics, such as brain predicted age difference (brain-PAD; i.e., a measure of accelerated/decelerated brain aging) and Signal Detection Theory

(SDT; i.e., objective behavioral fatigue measures), are needed to better understand the cognitive fatigue (CF)-age relationship. The current study investigates the relationship between chronological age versus brain-PAD and CF in a non-medical lifespan sample ($n = 85$; mean age=46.9 years). Participants completed fatigue inducing tasks during brain scans, capturing real time "state" CF using a visual analog scale. Slope and SDT (Criterion) metrics were calculated for CF. Brain-PAD (i.e., chronological age minus predicted brain age) was estimated using brainageR. Linear mixed effects analyses showed a main effect of Chronological Age ($F(1, 97.1)=7.33$, $p = 0.008$): for every year of increased age, participants reported 0.51 less CF. CF slope showed a significant effect of Brain-PAD ($F(1,79)=4.00$, $p = 0.04$), such that participants with increased brain-PAD fatigued at a faster rate. There was a three-way interaction between CF, Age and Task ($F(1,403.8)=5.52$, $p = 0.02$), resulting from a positive relationship between SDT Criterion and CF when participants were young that became increasingly flat as age increased; slope became negative for older participants. Our results show a critical difference between younger and older participants in relation to CF. In addition, increased brain age, rather than chronological age, predicted faster accrual of CF. These results suggest that CF may be an effective indicator of brain age across the lifespan.

Abstract citation ID: igaf122.4214

### THE IMPACT OF MASCULINITY BELIEFS AND NEGATIVE PERCEPTIONS OF AGING ON OLDER ADULT MEN'S DEPRESSION

Darby Mackenstadt[1], David Buys[2], Mary Dozier[2], and Carolyn Adams-Price[2], *1. Mississippi State University, Durham, North Carolina, United States, 2. Mississippi State University, Starkville, Mississippi, United States*

Stereotype embodiment theory suggests how older adults that hold negative aging stereotypes may come to internalize them and begin to think negatively about their aging. Those who hold negative aging stereotypes also have more negative outcomes in physical and mental health. This project aimed to evaluate the relationship between masculinity standards, negative perceptions of aging, and depression in men aged 55 and older ($N = 438$) living in the southern United States. Results indicated that viewing aging as associated with psychosocial loss was associated with increased depression symptoms ($\beta = .28$, $p < .001$). Stereotypical masculine behaviors were not significantly associated with depression symptoms ($\beta = -.08$, $p = .59$); however, engaging in stereotypical masculine behaviors significantly moderated the effect of viewing aging as psychosocial loss on depression symptoms ($\beta = .31$, $p = .04$). Results suggest that participants who reported a high adherence to masculinity beliefs in combination with a negative perception of aging had higher symptoms of depression. Older adult men who have more negative perspectives of aging as well as participate more in traditional masculine behaviors may be at a higher risk for depression. Mental healthcare providers may benefit from recognizing the relationship between masculinity and negative perceptions of aging, and discussing thoughts or experiences related to aging with their clients who identify as men. Individuals who have a rigid adherence to hegemonic masculinity standards and who perceive aging negatively may benefit from discussing these views, and how to acknowledge the positive aspects of growing older.

Abstract citation ID: igaf122.4215

### THE PARADOX OF INCLUSION: OLDER ADULTS WITH LEARNING DISABILITIES IN POLICY AND PRACTICE

Anastasiia Brus[1], Judith Sixsmith[2], Joe Tai[2], Darren Chadwick[3], and Mei Lan Fang[4], *1. Ukrainian Catholic University, Lviv, L'vivs'ka Oblast', Ukraine, 2. University of Dundee, Dundee, Scotland, United Kingdom, 3. Liverpool John Moores University, Liverpool, England, United Kingdom, 4. Simon Fraser University, Vancouver, British Columbia, Canada*

As part of the UK IncludeAge Study, we explore how policy, service structures, and organisational practices shape opportunities for the inclusion of older adults with learning disabilities. Despite policy frameworks promoting integration, significant barriers persist that paradoxically reinforce exclusion. The aim was to examine these tensions and identify pathways for systemic change. Our approach involved conducting ten semi-structured interviews with representatives from local Older Adult Social Care departments ($n = 5$) and voluntary or charitable organisations ($n = 5$) across England and Scotland. Data were analysed using Reflexive Thematic Analysis to identify recurring themes and contradictions in organisational approaches to inclusion. Substantial and intersecting barriers were identified. The accessibility of information remains a major challenge, with complex language, bureaucratic procedures, and poor dissemination limiting access to services. Tools intended to foster inclusion often perpetuated exclusion. Staff reluctance to address sensitive issues created silences around important areas of life, while paternalistic attitudes framed older adults with learning disabilities as "child-like." Though intended as protection, such practices restricted autonomy and increased dependency. Policy programmes were frequently designed without the involvement of older adults with learning disabilities, resulting in fragmented provision and the need for retrospective "inclusive" fixes. Limited facilitators, such as staff training, voluntary sector initiatives, and community collaborations, were inconsistent and resource-dependent. Findings reveal a paradox of inclusion: older adults with learning disabilities are selectively recognised, experiencing conditional rather than full citizenship. Meaningful change requires embedding accessibility, dignity, and identity at the policy-making stage to ensure genuine participation in community life.

Abstract citation ID: igaf122.4216

### THE ROLE OF ANXIETY AND DEPRESSIVE SYMPTOMS ON NURSE-PATIENT MUTUALITY IN OLDER ADULTS WITH CHRONIC ILLNESSES

Silvia Cilluffo[1], Karen Lyons[2], Bassola Barbara[3], Rosario Caruso[4], Stefano Terzoni[5], and Maura Lusignani[1], *1. University of Milan, Milan, Lombardia, Italy, 2. Boston College, Chestnut Hill, Massachusetts, United States, 3. ASST GRANDE OSPEDALE METROPOLITANO NIGUARDA, MILAN, Lombardia, Italy, 4. University of Milan, Milano, Lombardia, Italy, 5. Università degli Studi di Milano, Milan, Lombardia, Italy*

The interactive process (mutuality) between older patients with multiple chronic illnesses and their nurses has received little investigation, despite the therapeutic nature of the relationship. Even less is known about the role of the older patient's mental health on the perception of this therapeutic relationship. This paper reports novel findings from recently available data from a multi-center study. The sample of 145 patients (Mage= 67.5, ±15.4) were recruited from hospitals across Italy. Patients had to be older adults with 2 or more chronic illnesses. Nurse-patient mutuality was assessed using a validated measure developed by this team consisting of three dimensions: developing rapport/going beyond, becoming a point of reference, and shared decision-making/care. Findings from multiple regression analyses found that both anxiety and depressive symptoms were significantly associated with the patient's interaction with their nurse. Higher levels of anxiety were significantly positively associated with the developing rapport/going beyond dimension (e.g., trusting the nurse, empathic interactions) ($p < .01$), and point of reference dimension (e.g., asking the nurse for advice) ($p < .001$). In contrast, higher levels of depressive symptoms were negatively associated with the developing rapport/going beyond dimension ($p< .01$) and point of reference dimension ($p < .01$). Neither anxiety or depressive symptoms were significantly associated with the shared decision-making/care Discussion will focus on the role of mental health when older patients with multiple chronic illnesses interact with their nurse and implications for optimizing the nurse-patient relationship to support better health outcomes.

Abstract citation ID: igaf122.4217

### THE ROLE OF SOCIAL ISOLATION IN SHAPING HEALTH OUTCOMES IN OLDER ADULTS: A REGIONAL ANALYSIS

Vince Falco[1], Lynn Wiles[1], Jonathan Romero[1], and Leorey Saligan[2], *1. Old Dominion University, Virginia Beach, Virginia, United States, 2. Rutgers University, Newark, New Jersey, United States*

Social isolation is a critical public health concern in older adults and is linked to a range of adverse health outcomes across diverse sociodemographic groups. This study investigated the associations between social isolation with physical and mental health of older adults residing in Hampton Roads, a racially diverse region in Southeastern Virginia, home to a large population of senior military veterans. Secondarily, the study explored how social determinants of health (SDoH) and comorbid medical conditions influenced the relationship of social isolation with physical and mental health of these seniors. Physical and mental health were measured using the Patient-Reported Outcomes Measurement Information System Global Health short form. Social isolation was assessed using the Social Connection and Isolation portion of the National Institutes of Health Social Determinants of Health form. The SDoH included in this analysis as covariates were individual-level sociodemographic data, such as age, sex, race, marital status, highest level of education completed, current employment status and income, all collected via self-report. Comorbidities were assessed using the Charlson Comorbidity Index. Stepwise regression analysis of 174 participants with complete data showed that each one-unit increase in social isolation score (less socially isolated) was associated with a 1.16-point improvement in mental health scores, after adjusting for all covariates. Social isolation accounted for 2.2% of the variance in mental health outcomes. No significant association was found between social isolation and physical health using the same analytical approach. Interventions to reduce social isolation may be a potential strategy to improve mental well-being of older adults.

Abstract citation ID: igaf122.4218

### TRAINING DEMENTIA CARE PARTNERS IN RURAL CHINA: A MIXED-METHODS RANDOMIZED CONTROLLED FEASIBILITY TRIAL

Dexia Kong, *The Chinese University of Hong Kong, Hong Kong, Hong Kong, China (People's Republic)*

China has the world's largest dementia population. Despite a higher prevalence of dementia in rural areas, few dementia family caregiver training intervention studies have been conducted in rural China. To address this gap, we conducted a mixed-methods RCT feasibility trial. In Phase 1, we conducted in-depth qualitative interviews with 25 local family caregivers regarding their caregiving experiences and needs, alongside focus group discussions with 30 local healthcare providers exploring their caregiving experiences, to inform the cultural adaptation of the World Health Organization's iSupport program to fit the rural Chinese context. In Phase 2, we implemented a two-arm randomized controlled feasibility trial ($n=64$) to evaluate the preliminary effectiveness and feasibility of the adapted program delivered via face-to-face group sessions (Arm 1) versus a wait-list control group (Arm 2). Treatment outcomes, including dementia knowledge and caregiving self-efficacy, and caregiver burden, were measured at baseline (T1), 2 months after baseline (T2), and 4 months after baseline (T3), using validated instruments. Feasibility indicators included retention rate and treatment usability. A subset of participants ($n=25$) participated in post-treatment qualitative interviews to understand their program experiences, perceived impacts, and barriers. Intention-to-treat analysis indicates promising preliminary effects on knowledge boost and burden reduction. The training program had high retention rate (81%) and usability scores (mean = 2.8, range: 0-4). Results from both phases suggest the program's potential scalability. The findings on the initial effectiveness of the culturally-adapted caregiver training program can inform key ingredients for a future full-scale pragmatic trial in low-resource settings.

Abstract citation ID: igaf122.4219

### TRAJECTORIES OF DEPRESSIVE SYMPTOMS IN OLDER ADULTS: ANALYSIS OF THE KOREAN RETIREMENT AND INCOME STUDY

Jin-kyung Lee[1], JaeWon Hyun[2], Bomgyeol Kim[1], Hun Kang[2], Hanna Lee[2], Seongmi Choi[3], and JiYeon Choi[2], *1. Yonsei University College of Nursing, Yonsei University, Seoul, Republic of Korea, 2. Yonsei University, Seoul, Republic of Korea, 3. Daegu Catholic University, Daegu, Republic of Korea*

Depressive symptoms are prevalent among older adults. It poses mental health challenges and increases the risks of frailty, disability, and even early mortality. With longer life expectancy, understanding how these symptoms change over time in later life is increasingly important. This analysis aimed to explore characteristics of depressive symptom trajectories among community-dwelling older adults using national survey data, the Korean Retirement and Income Study (KReIS). We analyzed 15,103 data reported by 2,717 older adults (72.77±5.07 years old on average, female: 60.43%) over six waves of the KReIS (2011-2021). As analytic methods, we used a latent growth mixture model (GMM) and longitudinal multilevel models (MLM). The four-trajectory model is chosen because of its best model fit: Constantly low (71.3%), constantly high (1.8%), increasing (16.3%), and decreasing (10.7%) depressive symptoms.

Longitudinal MLM results demonstrate various effects of relevant factors by trajectory group. For example, in the group of reducing depressive symptoms (n = 244), the number of family members living together (β=-.66, p<.05), health satisfaction (β=-2.66, p<.001), hobby satisfaction (β=-.81, p<.05), and relationship satisfaction (β=-1.67, p<.01) were negatively associated with depressive symptoms. On the other hand, in the group of increasing depressive symptoms (n = 411), health satisfaction (β=-2.34, p<.001) and hobby satisfaction (β=-1.15, p<.001) were negatively associated, whereas the amount of medical expenditure (β=.49, p<.01) was positively related to depression. These results suggest that understanding heterogeneity in longitudinal depressive symptom patterns in older adults can guide efficient interventions. Personal and environmental factors may differ in significance, but relationship satisfaction appears central across all trajectories.

Abstract citation ID: igaf122.4220

**TRANSFORMATIVE LEARNING OF RETIRED HIGH-TECH OLDER MALE VOLUNTEERS IN TAIWAN: MOTIVATION, CONTRIBUTION, CHANGE**

Jen-Yu Wang[1], and Li-Hui Lin[2], *1. National Chung Cheng University, Hsinchu County, Hsinchu, Taiwan (Republic of China), 2. National Chung Cheng University, Chiayi, Chiayi, Taiwan (Republic of China)*

As Taiwan rapidly enters a super-aged society, retired older adults are increasingly viewed as important social resources. Among them, men from the high-tech sector—who often experienced intense workloads and occupational stress—are seeking new ways to restore health and pursue meaningful engagement in later life. Guided by Mezirow's Transformative Learning Theory, this study explores the motivations, contributions, and changes of retired high-tech older male volunteers in community-based health promotion programs. Using a qualitative design, purposive sampling recruited six retirees aged 65–75 with high-tech industry backgrounds and over three years of consistent volunteer service. Data were collected through in-depth interviews, participant observation, and document analysis. To ensure rigor, qualitative data were analyzed using Braun and Clarke's six-step thematic analysis, allowing for systematic coding, theme development, and interpretation across multiple data sources. Three major themes emerged. First, Motivation: participants were driven by a desire for self-care, social contribution, and extending professional values. Second, Contribution: they actively supported course instruction, event planning, and organizational management by applying technical and managerial expertise. Third, Change: through reflection and peer support, volunteers overcame initial anxiety, developed a renewed identity, and shifted from health service recipients to active health promoters. Findings demonstrate that retired high-tech professionals possess strong transformative learning potential and can effectively contribute to community health initiatives. The study suggests that nonprofit and adult education organizations design practice-oriented and supportive mechanisms to sustain their participation, thereby fostering active aging and intergenerational inclusion.

Abstract citation ID: igaf122.4221

**TREATMENT OF OBSTRUCTIVE SLEEP APNEA MAY SLOW COGNITIVE DECLINE IN OLDER ADULTS**

Christopher Kaufmann[1], Jennifer Albrecht[2], Vishaldeep Sekhon[3], Halima Amjad[3], Emerson M. Wickwire[2], Alden Gross[4], Atul Malhotra[5], and Adam Spira[4], *1. University of Florida, Gainesville, Florida, United States, 2. University of Maryland, Baltimore, Maryland, United States, 3. Johns Hopkins University School of Medicine, Baltimore, Maryland, United States, 4. Johns Hopkins Bloomberg School of Public Health, Baltimore, Maryland, United States, 5. University of California, San Diego School of Medicine, La Jolla, Florida, United States*

Research links obstructive sleep apnea (OSA) to negative cognitive outcomes, but studies linking OSA treatment to cognitive decline have yielded inconsistent results. We investigated whether continuous positive airway pressure (CPAP) treatment was associated with cognitive performance over 10 years (2011-2021) among older adults with OSA. Data came from the National Health and Aging Trends Study (NHATS) linked with Medicare claims through the NIA LINKAGE Enclave. We identified N = 777 participants in the 2011 NHATS enrollment cohort who had >1 inpatient or outpatient claim with an OSA diagnosis (ICD-9=780.51, 327.23, 780.53, 327.23; ICD-10=G47.33, G47.30) and were free of cognitive impairment at baseline. CPAP treatment was defined as ≥ 1 claim for CPAP (HCPCS Code=E0601) at any time during study period. Cognitive performance was assessed using a factor-based total score derived from NHATS cognitive measures of executive function, orientation, and recall, modeled as a continuous outcome across study years. We fit generalized linear mixed models adjusting for age, BMI, education, and marital status, with interaction term for CPAP use by year. Cognitive scores declined over follow-up. In adjusted models, the annual decline among CPAP-treated participants was -0.03 factor score units per year (95% CI: -0.04, -0.02; p < 0.001). The CPAP*time interaction was -0.02 (95% CI: -0.04, -0.001; p = 0.040), indicating that participants without CPAP declined an additional 0.02 units per year, for an estimated decline of -0.05 units per year. CPAP treatment was associated with slower cognitive decline among older adults with OSA, and may thus represent a modifiable target to promote healthy brain aging.

Abstract citation ID: igaf122.4222

**TRENDS IN UTILIZATION OF GERIATRIC REHABILITATION SERVICES IN SWITZERLAND (2015-2022) – A NATIONWIDE COHORT STUDY**

Rick Veer[1], Naemi Herzog[2], Philipp Schütz[1], Beat Müller[1], and Alexander Kutz[1], *1. Cantonal Hospital Aarau, Aarau, Aargau, Switzerland, 2. University of Basel / Cantonal Hospital Aarau, Zurich, Switzerland*

Population aging increases functional decline, underscoring the role of geriatric rehabilitation in restoring functional capacity after hospitalization or community referral. Yet, patient trajectories in geriatric rehabilitation remain poorly characterized. This cohort study analyzed Swiss national administrative claims (2015–2022) of adults aged ≥65 years comparing temporal trends in geriatric rehabilitation and acute-care hospitalizations. Main outcomes included causes of hospitalizations, length of stay, DRG-based cost weight, in-hospital mortality, and 30-day rehospitalization. Among 4,030,691 hospitalizations, 125,990 (3.1%) involved geriatric rehabilitation, mostly in acute-care hospitals (76.3%), followed by geriatric (20.5%) and other facilities (3.2%). Geriatric rehabilitation admissions increased by 8.3% annually (95% confidence interval [CI]: 5.2–11.5) versus 1.2% (95% CI: 0.3–2.2) in acute-care. Circulatory diseases were the leading cause of acute-care admissions (19.3%), while injuries predominated in geriatric rehabilitation (25.1%) and showed the steepest increase (+9.7% annually; 95% CI: 6.3–13.1), followed by circulatory, musculoskeletal, and mental disorders. Length of stay declined more in geriatric rehabilitation (−4.3% per year; 95% CI: −4.9-−3.7) than in acute care (−2.0%; 95% CI: −2.2-−1.9). DRG-based cost weight decreased annually by -3.09% (95% CI: −4.60%-−1.57%) but remained stable in acute care. Discharge home after geriatric rehabilitation declined by − 1.2% per year (95% CI: −1.6-−0.7), with increasing transfers to other rehabilitation and nursing homes. 30-day rehospitalization and in-hospital mortality were stable in both groups. In conclusion, the proportion of hospitalizations involving geriatric rehabilitation is rising, reflecting growing demand in an aging population. Its effectiveness for patient outcomes and health system performance warrants further evaluation.

Abstract citation ID: igaf122.4223

### TRUST MATTERS: A QUALITATIVE ANALYSIS OF MEDICAL MISTRUST AMONG OLDER BLACK ADULTS

Channing Tate[1], Denae Gerasta[1], and Tsion Shiferaw[2], *1. University of Colorado Anschutz, Aurora, Colorado, United States, 2. St Joseph's Hospital Denver, Aurora, Colorado, United States*

Medical mistrust is a major social driver of health and a persistent barrier to equitable healthcare for Black Americans. Historically, Black communities have been excluded from quality care through systemic racism and unethical practices like the Tuskegee Syphilis Study. These experiences have created deep-rooted mistrust in healthcare systems and providers. This study explores how older Black adults experience and conceptualize trust in healthcare. We focused on how both past and current experiences influence healthcare-seeking behaviors and identified preferred provider characteristics that promote trust. A purposive subsample of 12 Black adults aged 65 and older was drawn from a larger mixed-methods. Semi-structured interviews explored personal past and present experiences with the healthcare system, perceptions of trust and mistrust, and factors shaping care engagement. Thematic analysis identified multilevel determinants—individual, organizational, and systemic—that influence trust in both patient-clinician interactions and broader healthcare contexts. Participants described trust as multidimensional and evolving, shaped by past and ongoing exposure to systemic racism, discrimination, and structural barriers. Mistrust was reinforced by experiences such as microaggressions, dismissive treatment, and a perceived lack of respect. Conversely, trust was strengthened when providers showed competence, cultural humility, honesty, and respect for autonomy. Participants emphasized their own agency, often engaging in self-advocacy, information-seeking, and shared decision-making. These findings highlight the importance of addressing both structural inequities and individual needs. While systemic racism has fueled mistrust, consistent, respectful, and culturally responsive care can help rebuild trust and improve healthcare engagement among older Black adults.

Abstract citation ID: igaf122.4224

### UNDERSTANDING CONVERSATION BETWEEN OLDER ADULTS: AN OBSERVATIONAL STUDY

Fuxi Ouyang, and W. Quin Yow, *Singapore University of Technology and Design, Singapore, Singapore*

Previous studies have shown older adults' conversations featuring reminiscence, elaboration, multimodal expression, and reduced cohesion. However, most research focuses on monolingual populations, with limited attention to multilingual environments. This gap is important because multilingualism shapes cognitive, social, and communicative processes in distinct ways. As part of a larger research project, we conducted an observation study to understand the characteristics of bilingual older adults' conversations in Singapore. Twenty older adults (mean-age=75.6; 19F) were randomly paired and asked to engage in an unstructured conversation with their partner for 20 to 30 minutes. This approach, while situated in a study setting, was intended to elicit spontaneous conversational behavior that approximates naturalistic communication. Conversations were audio-recorded, and two coders applied global topic coding to the transcripts. These conversations were analysed for the frequency and commonality of topics and vocabulary in the conversations. Six categories were found to be frequently referenced (decreasing-order): Community Centre, Recreation, Shopping, Food, Family, and Travel. Analyses also revealed that a single topic typically dominated the discussion, accounting for more than 50% of all segments. In addition, based on the five-person-frame-reference, Self (mean=37.5%), None–Inanimate (mean=20.7%), and Other Speaker (mean=19.5%) were the most frequently referenced. References to Relatives (mean=7.6%) and Service Providers (mean=4.0%) were relatively rare. For time frame references, we found that most conversations were focused on the Present (mean=56.2%) and the Past (mean=33.9%), followed by Future-oriented content (mean=9.9%). These results demonstrated conversational characteristics among older adults in multilingual contexts, contributing to a deeper understanding of communicative patterns in diverse societies.

Abstract citation ID: igaf122.4225

### UNDERSTANDING STAFFING DISPARITIES IN NURSING HOMES: INSIGHTS FROM NEIGHBORHOOD DEPRIVATION

Jenny Kwon[1], Xiao Qiu[2], and Samuel Van Vleet[3], *1. University of California, San Francisco, San Francisco, California, United States, 2. Miami University, Miami University, Ohio, United States, 3. Massachusetts General Hospital | Harvard Medical School, Boston, Massachusetts, United States*

Low staffing levels in U.S. nursing homes (NHs) remain an ongoing concern for the quality of care. Socioeconomic deprivation in the areas surrounding NH may influence its ability to maintain optimal staffing levels, but this relationship has been understudied. This study was to examine the association between neighborhood deprivation and NH staffing levels. An aggregate dataset of Ohio NHs in 2022 was constructed (N = 610) by merging three public data sources: the Area Deprivation Index (ADI) from the Neighborhood Atlas, Nursing Home Archived Data from the Centers for Medicare & Medicaid Services, and LTCFocus from Brown University. Staffing levels for three types of nursing staff, including registered nurses (RNs), licensed practical nurses (LPNs), and certified nursing assistants (CNAs) were measured by hours per resident day (continuous). Neighborhood socioeconomic disadvantage was assessed using the national ADI. The score was a continuous variable, with a higher score indicating greater deprivation within a neighborhood (range: 0-100). Multiple linear regressions, including control variables, were conducted via SAS. Results show that a higher ADI score is significantly associated with a lower CNA staffing level ($p < .05$). Negative associations between ADI and RN/LPN staffing levels approach significance ($p < 0.1$). Given that CNAs provide most hands-on care and closely interact with residents, these findings highlight the need for policy interventions in deprived areas to ensure adequate CNA staffing. Targeted workforce recruitment, improved working conditions, expanded career development and training opportunities, and increased staffing support could help reduce staffing disparities in NHs located in disadvantaged neighborhoods.

Abstract citation ID: igaf122.4226

### UNMET CARE NEEDS AMONG OLDER ADULTS IN THE AMERICAS.

Nekehia Quashie[1], Flavia Andrade[2], Ting Hu[3], and Hayley Berg[4], *1. University of Rhode Island, Kingston, Rhode Island, United States, 2. University of Illinois—Urbana-Champaign, Urbana, Illinois, United States, 3. University of Illinois—Urbana-Champaign, Champaign, Illinois, United States, 4. University of Rhode Island, University of Rhode Island, Rhode Island, United States*

The global region of the Americas is rapidly aging, albeit with significant differences in socioeconomic development and cultural values surrounding care for older adults. As populations age, there is also an increasing risk of health vulnerability that may lead older adults to require assistance with basic and instrumental activities of daily living. Although some studies have examined the prevalence of unmet needs in the region, no studies have examined cross-national variation in care needs based on both activities of daily living and instrumental activities of daily living, as well as the care received. Using harmonized cross-national data from the international family of Health and Retirement Studies, United States (HRS), Mexico (MHAS), Costa Rica (CRELES), and Brazil (ELSI), we examine cross-national trends in care needs (having 1 or more ADL and IADL limitations) and care received among adults 60 years and older. We also examine variation in the prevalence of social care needs and care received by demographic (age, gender), social (living arrangements), and economic (education) characteristics. Our findings suggest that adults 70 and older have higher care needs and receipt, women have higher care needs but lower care receipt than men in some countries, an educational gradient in care need and receipt, and a higher prevalence of care needs among older adults living alone, but higher care receipt among those living with others. We discuss these findings considering the socioeconomic conditions and the options for long-term care and support systems for older adults in the region.

Abstract citation ID: igaf122.4227

### UNMET NEEDS FOR AGING CARE ASSISTANCE AMONG COMMUNITY-DWELLING OLDER ADULTS: A LIFE COURSE INVESTIGATION

Millicent Ngwu, *Department of Sociology and Criminology University at Buffalo, Buffalo, New York, United States*

Unmet care needs are a growing public health and policy issue across developed countries. Older adults are faced with the challenge of maintaining quality care. The unmet need for aging care assistance is shaped by social identities, including gender and race. Although early life disadvantages shape later life outcomes, little is known about how childhood experiences matter for unmet care needs among community-dwelling older adults. Guided by the life course perspective, this study examined how early life experiences, such as childhood financial background and health status, matter for unmet care needs. The data for this study were drawn from Round 12 of the National Health and Aging Trends Study (2022) to statistically analyze how early life experiences matter for unmet care needs among community-dwelling older adults. The study's analysis revealed that early life experiences, such as fair or poor childhood health status, remained a significant predictor of unmet care needs in later life, with fair or poor health status significantly linked to increased odds of older adults having unmet needs for aging care assistance. Sociodemographic factors such as age, education, adulthood health status and income significantly predict unmet needs. Further, as older adults advance in age, they continue to face increased odds of experiencing unmet needs. The policy implications include prioritizing health programs through targeted policies for children and adults who have poor health status.

Abstract citation ID: igaf122.4228

### UNTOLD STORIES OF GRIEF AND JOY IN AGING: AN INTERGENERATIONAL ART-BASED PROJECT

Jeannette Sager[1], Anna Gibbons[1], Gwendolyn Hoeffgen[1], Domenic Toliver[1], Adela Cardona Puerta[1], Max Goldman[1], Cal Storrs[1], and Serena Hasworth[2], *1. Portland State University, Portland, Oregon, United States, 2. Portland State University, West Linn, Oregon, United States*

Narratives around aging are often reduced to cliches, limiting opportunities to recognize complexity, diversity, and dignity in aging. To address this gap, Portland State University's GSA Student Chapter organized Untold Stories of Grief and Joy, an arts-based, intergenerational project designed to foster reflection, dialogue, and connection across the lifespan. The project was hosted in an affordable residential community, where eight older adult participants engaged in guided reflection, relationship building, and artistic creation. An interdisciplinary group of student facilitators supported participants in four weekly workshops that featured a new prompt around joy and grief and an art medium (e.g., photography, poetry). Each week, residents shared their artwork and the stories behind them. The project culminated in a community-wide art exhibition where participants displayed their art and shared reflections. The project was evaluated using a focus group with participants, interviews with facilitators, and survey feedback from exhibition attendees. Findings from the evaluation highlighted the project's impact on community building and intergenerational engagement, with bonds between residents, students, and the broader community. Participants shared how they reframed perspectives on aging, embracing the possibility of new beginnings at every age. The workshops fostered self-exploration, empowerment, vulnerability, healing, and for some, a newfound identity as an artist. Engagement with art, grief and joy, and the aging process provided participants with opportunities to express complex emotions and experiences through creative processes. This project created replicable tools for other GSA Student Chapters to bring this work to long-term care communities, community centers, and other settings.

Abstract citation ID: igaf122.4229

### UNUSUAL PERCEPTUAL EXPERIENCES AT THE END-OF-LIFE: CAREGIVER RESPONSES AND IMPLICATIONS FOR SYMPTOM MANAGEMENT

Kelly Melekis[1], Elizabeth Cepeda[1], Carol Weisse[2], and Meredith Killian[2], *1. University of Vermont, Burlington,*

*Vermont, United States, 2. Union College, Schenectady, New York, United States*

Unusual perceptual experiences (UPEs) occurring near death are a universal human phenomenon, with accounts recorded across time and cultures (Kellehear, 2016). For those on hospice, characterizations of UPEs and levels of both patient and caregiver distress may influence decisions about symptom management. The predominance of the routine home care setting rather than an institutional or hospitalized setting highlights the importance of understanding how informal caregivers manage UPEs in these settings, especially in light of research showing that medication administration for managing EOL symptoms varies by setting (Arevalo et al., 2018). This study utilized a mixed-method retrospective record review of 138 deceased hospice patients to better understand the presence and characterization of UPEs at EOL, caregiver perceptions of symptoms, and symptom management in home care settings. 36% of patients were identified as having > 1 UPE. While the majority (51%) experienced a single UPE, there were a total of 137 instances among 49 patients. Distribution of PRN psychotropic medication following a UPE incident was indicated only 14% of the time. The vast majority never received an antipsychotic PRN (88%) or a benzodiazepine PRN (80%) following a UPE incident, and there was little to no documentation of behavioral interventions for symptom management. The variability with which PRN medication was administered suggests caregivers may be uncertain about when to administer medications. Study results point to a need for enhanced caregiver training on strategies for assessing and documenting UPEs, and decision-making for symptom management, including both pharmacologic and behavioral interventions.

Abstract citation ID: igaf122.4230

### USER-CENTERED LLM CHATBOT TO BRIDGE INFORMATION GAPS IN RETIREMENT COMMUNITY

Amy Deng, Kaylee Zhang, Ethan Liu, Ryker Hou, George Choi, Xingyu Li, and Feng Chen, *University of Washington, Seattle, Washington, United States*

Lack of technology and eHealth literacy limits many retirement-community residents from retrieving essential daily information and engaging with online resources. Existing intranet portals often use small text, complex menus, and keyboard input that clash with age-related changes in vision, dexterity, and search self-confidence. To tackle these barriers, we partnered with older adults to co-design a large-language-model (LLM) powered conversational assistant specially designed for retirement community residents. Iterative interviews, observation sessions, and sketch workshops produced three personas capturing diverse goals and backgrounds. These personas guided the inclusion of large print and high contrast themes, adjustable font and color palettes, voice-to-text input, and one-tap example prompts. In parallel, we tested multiple LLMs (GPT-3.5-Turbo, GPT-4.1, Gemini 2.0, Llama 3.3-70B) with elder-centered prompts and selected GPT-4.1 for deployment based on superior completeness and clarity. Qualitative usability sessions revealed that participants valued immediate formatting controls and hands-free interaction. They conclude that the developed chatbot is friendly and easy to navigate, but also point out the need for transparent source links for reassurance. Participants reported greater confidence when seeking technology tips or health information after short use, suggesting early gains in digital self-efficacy. Collectively, the findings show that an LLM assistant grounded in persona-driven, user-centered design can enhance perceived accessibility, confidence, and efficiency for older adults while illuminating refinements, such as audio read-back and family-mediated configuration, needed for sustained adoption. Future work will examine longitudinal engagement and community-wide rollout to verify the impact of the chatbot.

Abstract citation ID: igaf122.4231

### USING SYNTHETIC DATASETS TO AGGREGATE DATA IN AGING AND LONGEVITY STUDIES: A RANDOMIZED RESAMPLING APPROACH

Sarah Peskoe[1], and Zachary Kunicki[2], *1. Duke University, Durham, North Carolina, United States, 2. Warren Alpert Medical of Brown University, Providence, Rhode Island, United States*

The scarcity of harmonized datasets capturing clinical and cognitive outcomes—such as pain, function, biological, and behavioral measures—in older adults presents a major barrier to reproducible aging research. While federated data models offer one solution, they are often constrained by institutional privacy policies and logistical complexity. This study explores an alternative: pooled synthetic datasets generated using the synthpop package in R, which employs Classification and Regression Trees (CART) to model conditional distributions across mixed data types. We evaluate the utility of this approach using data from the Health and Retirement Study (HRS), focusing on how the choice of synthesis order affects bias and inference. Our findings show that fixed synthesis chains can introduce bias in downstream variables, while varying the chain improves accuracy for key outcomes. We propose a randomized resampling strategy to mitigate these effects, demonstrating improved precision and reduced bias in simulation. This work highlights the potential of synthetic data to support scalable, privacy-preserving, and methodologically rigorous research in aging and longevity, and proposes guidance and strategies to improve the statistical performance and integrity of synthetic datasets.

Abstract citation ID: igaf122.4232

### VALIDATION OF A PHYSICAL ACTIVITY SCALE FOR OLDER ADULTS PARTICIPATING IN THE HEALTH AND RETIREMENT STUDY

Peter Hart, *Kinesmetrics, Tallahassee, Florida, United States*

The aim of this research was to validate a new scale measuring physical activity (PA) using items contained in a large national survey of older adults. Data from 15,335 adults 50+ years of age participating in the 2022 Health and Retirement Study were used. The assessment strategy involved six steps: 1) defining the PA scale (PAS) items and categories, 2) factor analysis, 3) internal consistency reliability, 4) item response theory (IRT) analysis, 5) convergent validity correlations, and 6) modeling PAS scores with a general health (GH) outcome. Polychoric correlations between items were used for the classical analyses. A graded response model (GRM) for polytomous items was employed for the IRT analysis. Multinomial logistic regression was used to model GH categories with PAS scores. The PAS included 3 items of vigorous, moderate, and light PA, each with 3 categories of inactive, low/moderately active, and highly active. Factor analysis retained a single factor with 70% explained variance, whilst the reliability coefficient for items was 0.79. IRT calibration showed category thresholds ranging from -1.90 to 1.05 and item discrimination parameters between 1.39 and 5.00. IRT theta scores correlated with the PAS sum score ($r = 0.97$), age ($r = -0.21$), GH ($r = 0.38$), and timed walk performance ($r = 0.35$). Modeling showed that for each point increase in PAS score, odds of poor (OR = 0.31, 0.27-0.34), fair (OR = 0.47, 0.43-0.52), good (OR = 0.60, 0.56-0.65), and very good (OR = 0.79, 0.73-0.85) GH, as compared to excellent (reference), continually decreased. These results support the use of a simple 3-item PAS to measure PA in older adults.

Abstract citation ID: igaf122.4233

### WITHIN-PERSON LINKS BETWEEN STRESS AND REPETITIVE THINKING INTENSIFY WITH AGE

Julie Kircher[1], Christopher Engeland[1], Dakota Witzel[2], Sarah Lipitz[1], and Jennifer Graham-Engeland[1], *1. The Pennsylvania State University, University Park, Pennsylvania, United States, 2. South Dakota State University, Brookings, South Dakota, United States*

Perceived stress and unconstructive repetitive thinking (URT) – persistent, negative thought patterns – are bidirectionally linked, often following a pattern in which stressors trigger URT that heightens perceived stress. These associations may be particularly strong in midlife, when exposure to and diversity of stressors are greater than in older adulthood. This suggests that older age may be a source of resilience; however, the moderating effect of age on associations between daily stress and URT remains largely untested. Using ecological momentary assessment, we examined how momentary perceived stress relates to URT and whether age affects this relationship. We hypothesized that older adults would report lower URT during moments when they also reported higher than usual perceived stress (compared to their own averages). Data came from a longitudinal measurement burst study, in which participants completed five assessments every day for 14 days. The sample, recruited via systematic probability from the Bronx NY, included 235 community-dwelling adults (Mage=45.99, Range=25–65). Multilevel models revealed that during moments of higher than usual perceived stress, momentary reports of URT were also higher ($\gamma = 0.0344$, SE = 0.0010, t(213)=34.18, p<.001). Age significantly modified the momentary perceived stress and URT association ($\gamma = 0.0002$, SE = 0.0001, t(213)=2.24, p=.025), such that the strength of this association increased with age. Simple slopes showed significant positive associations (p<.0001) across all age groups: (-1 SD/~35 years) b = 0.032, (0 SD/~46years) b = 0.034, (+1 SD/~57years) b = 0.036. These findings highlight the importance of examining real-time links between URT and perceived stress to inform future work and, ultimately perhaps, personalized interventions for older adults.

Abstract citation ID: igaf122.4234

### WORKLOAD ACCOMMODATIONS FOR LATE CAREER PHYSICIANS: ASSOCIATIONS BETWEEN CALL SCHEDULE AND DISRUPTIVE BEHAVIOR

Miranda McDaniel[1], Taylor Evans[1], Michael Williams[2], and Betsy Williams[1], *1. Professional Renewal Center, Lawrence, Kansas, United States, 2. Professional Renewal Center/Wales Behavioral Assessment, Lawrence, Kansas, United States*

The recent Federation of State Medical Board census data revealed a trend of increasing physician attrition1. To maximize retention of late career physicians, it will be important for healthcare systems to consider accommodations that will reduce physician stress and burnout and support maximal performance. Factors such as lack of sleep and workload contribute to feelings of burnout and can be associated with behavioral difficulties and interpersonal problems. 2. Especially for late career physicians, workload is recognized as a potential risk factor and impediment to staying in the workforce4. In a study examining senior emergency physicians, senior physicians noted increasing difficulties with their workload including difficulty recovering from overnight shifts3. Our study explores associations between physicians' call schedules and disruptive workplace behavior across the career span. This study utilizes a sample of physicians between 23 and 84 years of age (N = 89, mean 56) referred for a comprehensive fitness for duty assessment. The findings of this study indicate late career physicians who were referred for a fitness for duty assessment secondary to disruptive behavior had greater call schedule workloads, there is a significant Age X Call Schedule interaction as well. This research has implications for how to retain and optimize the functioning of late career physicians. Additionally, this study has implications for increasing methods of age inclusivity in the workplace.

### SESSION 9000 (PAPER)

### LATE BREAKING: ALZHEIMER'S DISEASE, INCLUDING ALZHEIMER'S DISEASE—RELATED DEMENTIAS I

Abstract citation ID: igaf122.4235

### DEMENTIA CAREGIVERS' EMOTIONAL LANGUAGE IN RELATIONSHIP NARRATIVES AND ASSOCIATIONS WITH WELL-BEING

Jenna Wells[1], Dustin Gad[1], and Emily Mroz[2], *1. Cornell University, Ithaca, New York, United States, 2. Emory University, Atlanta, Georgia, United States*

Dementia drastically alters caregivers' relationships with their care recipients, and these changes can erode caregivers' well-being. Although relationship narratives offer a rich window into individuals' subjective impressions of their relationships, a narrative sharing approach to data collection is rarely applied in the context of dementia caregiving. The present study asked 67 current caregivers of people with dementia to verbally narrate two defining memories from their relationship with their care recipient from: (1) during caregiving and (2) before caregiving began, and to complete self-report questionnaires of their current well-being. We used Linguistic Inquiry and Word Count (LIWC-22) to quantify caregivers' use of positive, negative, anger, sadness, and anxiety language in their relationship-defining narratives. Pearson correlations indicated that greater anger language in the "before caregiving" narratives was associated with higher depressive symptoms; greater sadness language in the "during caregiving" narratives was associated with higher anxiety symptoms and higher premorbid (pre-caregiving) relationship closeness; and greater positive language in the "before caregiving" narratives was associated with higher premorbid relationship closeness (rs = .25-.30, ps < .05). No associations were found with anxiety language in either narrative type. Caregivers who express anger about their pre-caregiving relationship may feel conflicted about their current caregiving role, contributing to increased depressive symptoms. Additionally, caregivers who felt more connected to the care recipient prior to dementia may express more sadness as their relationship changes, which in turn may fuel anxiety symptoms. Future research should evaluate emotional language as a potential target for narrative-based interventions to improve dementia caregivers' well-being.

Abstract citation ID: igaf122.4236

**ENHANCING DEMENTIA CARE VIA GUIDE: FOCUS ON OLDER ADULTS LIVING ALONE WITH LIMITED SUPPORTS**

Elena Portacolone[1], Krista Harrison[1], Jarmin Yeh[1], Sarah Dulaney[1], Katherine Possin[1], Matthew Growdon[1], Andrew Cohen[2], and Robyn Stone[3], *1. University of California, San Francisco, San Francisco, California, United States, 2. Yale School of Medicine, New Haven, Connecticut, United States, 3. LeadingAge, Washington, District of Columbia, United States*

Background: Approximately one-fourth of older adults with dementia live alone in the United States (about 1.7 million people), often with limited or no caregiver support, making them a particularly challenging population to care for healthcare providers. These individuals often have limited support from caregivers, and it is unclear whether they will benefit from the Guiding an Improved Dementia Experience (GUIDE) national demonstration model, launched by the Centers for Medicare and Medicaid Services in July 2024, which emphasizes support for patient/caregiver dyads. To address this knowledge gap, we elicited the perspectives of national dementia experts on the GUIDE model's ability to meet the needs of older adults living alone with limited caregiver support. Methods: From October-November 2024, teleconference interviews were conducted with 10 national dementia policy experts working in research, consulting, and advocacy institutions. Interview transcripts were analyzed using a combined inductive and deductive content analysis approach. Results: All experts concurred that obstacles may prevent older adults living alone with dementia from benefitting fully from GUIDE, for 5 reasons: : 1) no benefit exists equivalent to what is provided for caregivers' respite ($2,500/year); 2) monthly reimbursements of $215 may be insufficient to support this high-needs population; 3) GUIDE-related medication plans may be impractical; 4) navigators are mostly available via telephone rather than in-home; 5) the GUIDE evaluation ignores living arrangements and includes only interviews with caregiver/patient dyads. Conclusions: Findings underscore the importance of developing comprehensive care interventions to effectively support older adults with dementia who live alone with limited caregiver support.

Abstract citation ID: igaf122.4237

**FACTORS INFLUENCING THE PARTICIPATION OF BLACK OLDER ADULTS IN CLINICAL TRIALS ON DEMENTIA**

Elena Portacolone[1], Julio Rojas-Martinez[1], Kim Scott[2], Thi Tran[1], and Catherine Waters[1], *1. University of California, San Francisco, San Francisco, California, United States, 2. Bay Area Black Nurses Association, San Francisco, California, United States*

Background: Black communities are twice as likely as Whites to have dementia and represent 13% of the US population, constituting only 5% of participants in clinical trials on dementia. The goal of this study was to identify barriers and facilitators to participating in the a clinical trial on dementia from the perspective of Black older adults who agreed to be referred to the trialists who were testing a drug to treat Alzheimer's disease. Between 5/1/2023-1/31/2024, we raised awareness of Black communities about the option of being referred by our team to the trial via events endorsed by the National Black Nursing Association and the Alzheimer's Association. Methods: Interviews of Black older adults who agreed to be referred to the clinical trial identified barriers/facilitators to enroll in the trial after allowing our team to forward their details to the trialists. Interview transcripts were analyzed in ATLAS-ti with inductive/deductive content analysis. Results: In April-May 2024, a Black researcher contacted the 43 older adults who agreed to be referred to the trial. She interviewed 17 study participants who self-reported as Black/African American (average age 69, 70% new to research). Facilitators included: wanting medications, understanding the importance of participating in clinical trials, interacting with race-concordant researchers, personal exposure to dementia. Barriers included: limited connections with clinical trialists, logistical constraints, limited trust and understanding of participating in clinical trials. Conclusions: Findings underscore the importance of strong connections between clinical trialists and Black communities, as well as the salience of interdisciplinary partnerships to achieve social justice.

Abstract citation ID: igaf122.4238

**PERFORMANCE OF CLAIMS-BASED DEMENTIA ALGORITHMS IN MEDICARE FEE-FOR-SERVICE AND MEDICARE ADVANTAGE POPULATIONS**

You Wu[1], Chan Mi Park[2], Lan Luo[3], Xiecheng Chen[4], Eunji G. Kim[3], Sandra Shi[5], Dae Hyun Kim[5], and Ellen McCarthy[5], *1. Harvard T.H. Chan School of Public Health, Boston, Massachusetts, United States, 2. Hebrew SeniorLife, Harvard Medical school, Boston, Massachusetts, United States, 3. Marcus Institute for Aging Research, Boston, Massachusetts, United States, 4. Hebrew Rehabilitation Center, Roslindale, Massachusetts, United States, 5. Hebrew SeniorLife, Boston, Massachusetts, United States*

Claims-based dementia algorithms have not been evaluated in the Medicare Advantage (MA) population, a critical gap given the rapid growth of MA and concerns about data completeness. Using community-dwelling participants from the National Health and Aging Trends Study (NHATS) Round 7 (2017; n = 5,058), Round 9 (2019; n = 4,048), and Round 11 (2021-2022; n = 3,109), we validated the Bynum Standard and Chronic Condition Warehouse (CCW) dementia algorithms against NHATS classification for probable dementia. The prevalence of probable dementia was similar between MA (8.2%-9.1%) and FFS (8.1%-8.5%) across rounds. Overall, both algorithms had comparable performance in MA vs FFS populations, demonstrating high specificity (>97.9%) and negative predictive value [NPV] (>92.9%). The Bynum algorithm had similar sensitivity between MA and FFS in Round 7 (30.2% vs. 29.1%) and 11 (25.6% vs. 23.9%), but statistically significant and clinically meaningful lower sensitivity in MA for Round 9 (25.0% vs. 36.4%). However, positive predictive value [PPV] was similar between MA (62.7%-70.6%) and FFS (62.1%-70.4%) in all rounds. Comparatively, the CCW algorithm had statistically significant and clinically meaningful lower sensitivity in MA than FFS for Round 9 (30.8% vs. 46.9%) and 11 (35.0% vs. 49.3%), yet similar sensitivity for Round 7 (40.4% vs. 42.2%). The PPV in MA and FFS varied across rounds, from Round 7 (76.3% vs. 71.2%) to 11 (68.5% vs. 76.4%). Claims-based dementia algorithms have lower or comparable sensitivity in MA vs. FFS, with similarly moderate PPV and consistently high specificity and NPV. Researchers should consider these trade-offs when selecting algorithms for MA and FFS.

Abstract citation ID: igaf122.4239
**REVIEW OF INTERVENTIONS FOR APATHY AMONG PERSONS LIVING WITH DEMENTIA: INTERNATIONAL COMPARISON**

Mariya Kovaleva[1], Peace Johnson[2], and Cindy Schmidt[3], *1. University of Nebraska Medical center, University of Nebraska Medical Center College of Nursing, Nebraska, United States, 2. University of Nebraska Medical Center, University of Nebraska Medical Center College of Nursing, Nebraska, United States, 3. University of Nebraska Medical Center, Leon S. McCoogan Health Sciences Library, Nebraska, United States*

Apathy is the most prevalent neuropsychiatric symptom among persons living with dementia (PLWD). Caregivers frequently struggle with providing appropriate and engaging activities for their PLWD. Lack of appropriate activities also characterizes hospitals and rehabilitation facilities, undermining PLWDs' quality of life, dignity, and personhood. Apathy differs from depression. Despite its prevalence, little is known about efficacious interventions for apathy. We aimed to conduct a scoping review of the literature on evidence-based interventions for apathy developed in the U.S. and internationally in 2000-2025 and to compare these interventions. Specifically, we aimed to describe the intervention details, populations for whom they are offered (e.g., diagnosis, residence types); and outcomes for PLWD, caregivers, and healthcare systems. Guideline-directed (Arksey & O'Malley, 2005) scoping review of the literature. Librarian conducted the literature search using six databases: Medline, PsycINFO, CINAHL, Scopus, EMBASE, and Cochrane Library. Six hundred and thirty-four U.S. articles and 1375 non-U.S. articles were retrieved. Peer-reviewed articles describing intervention testing are selected. Full results with international comparison are currently pending. Preliminarily, we identified 18 relevant articles on the U.S. interventions. Results encompassing interventions developed internationally will be ready by the time of the presentation, if selected. Although dementia is incurable, neuropsychiatric symptoms are modifiable. Non-pharmacological approaches are the mainstay in addressing neuropsychiatric symptoms due to little to no adverse effects, as opposed to pharmacologic management of symptoms. Healthcare organizations and community agencies must prioritize interventions for apathy to uphold PLWDs' dignity and potentially decrease caregiver burden. Such interventions may be feasible for many organizations.

Abstract citation ID: igaf122.4240
**THROUGH THE BURDEN: A MODEL OF SELF-TRANSCENDENCE IN IN FAMILY CAREGIVERS OF NH RESIDENTS**

Dalit Zaguri-Greener[1], Ruth Lopez[2], and Anna Zisberg[1], *1. University of Haifa, Haifa, Hefa, Israel, 2. MGH Institute of Health Professions, Sharon, Massachusetts, United States*

Background: Family caregivers of nursing home (NH) residents with advanced dementia often experience high emotional and logistical burden. While most models emphasize stress and coping, fewer address meaning-making and personal growth. This study applies Reed's Self-Transcendence (ST) Theory to explore how caregiving may foster adaptation and meaning after placement. Objective: To examine whether perceptions of family-centered care predict caregiver burden and ST. Methods: Using a cross-sectional survey of 232 family caregivers of NH residents with advanced dementia, we examined the relationships between "Perception of Family-Centered Care" (basic care, symptom management, communication, trust, shared decision-making, and recognition of personhood), in NH staff, burden, and ST. Results: Structural equation modeling revealed that Perception of Family-Centered Care significantly predicted lower caregiver burden ($\beta = -.611$, $p < .001$). In turn, lower burden was associated with higher ST ($\beta = -.52$, $p = .002$). The direct path from Perception of Family-Centered Care to ST was nonsignificant ($\beta = -.154$, $p = .218$). However, the indirect effect from Perception of Family-Centered Care to ST through caregiver burden was significant ($\beta = .67$, 95% CI [.214 – 2.253], $p = .000$), indicating full mediation. Conclusions: Findings highlight the transformative nature of family caregiving in the NH setting. Recognizing family caregiving as a dynamic, transformative process can guide interventions that help family caregivers move through the burden-transcend toward meaning and connection. NH policies should consider integrating structured communication, family engagement, and decision-making support to reduce burden while fostering resilience, meaning-making, and role adaptation.

### SESSION 9005 (PAPER)

### LATE BREAKING: ALZHEIMER'S DISEASE, INCLUDING ALZHEIMER'S DISEASE—RELATED DEMENTIAS II

Abstract citation ID: igaf122.4241
**CAREGIVER NETWORK ROLES AND HEALTH AND WELL-BEING AMONG LATINO OLDER ADULTS**

Nell Compernolle[1], Ellen Bloss[1], Emily Bray[1], Alan Tiburcio[2], Melissa Howe[1], and Lissette Piedra[3], *1. NORC at the University of Chicago, Chicago, Illinois, United States, 2. NORC, Chicago, Illinois, United States, 3. University of Illinois at Urbana-Champaign, Urbana, Illinois, United States*

Prior research links caregiving to adverse health outcomes, especially when caregivers support individuals with Alzheimer's disease or related dementias. Importantly, studies show that diverse caregiving networks that include kin and non-kin are associated with lower caregiver burden and improved health. Using data from a new, nationally representative survey of ($n = 733$) Latino caregivers, we explore how specific network roles within caregiver networks impact caregiver health and wellbeing. We examine the social contexts of informal caregiving in the United States, focusing on the wellbeing of Latino caregivers of older adults. We define distinct roles within caregiver networks: care helpers (individuals who assist the caregiver in providing care), care disruptors (who hinder/complicate caregiving), and personal helpers (who support the caregiver personally, but not with caregiving). Preliminary findings indicate that kin comprise most roles - 80% of care helpers, 84% of care disruptors, and 87% of personal helpers. Caregivers with care helpers in their network report decreased feelings of loneliness ($OR = 0.84$, $SE = 0.08$, $p < 0.10$), while care disruptors significantly increase loneliness ($OR = 1.62$, $SE = 0.44$, $p < 0.10$). As the proportion of kin in care disruptor roles increases, caregivers experience even greater loneliness ($OR = 1.68$, $SE = 0.45$, $p < 0.10$) and depression ($OR = 1.68$, $SE = 0.44$, $p < 0.05$). Caregivers with personal helpers report slightly increased loneliness, depression and lower self-rated health, but results were not significant. Caregivers of individuals with ADRD reported higher loneliness ($OR = 1.32$, $SE = 0.22$, $p < 0.10$, depression ($OR = 1.40$, $SE = 0.23$, $p < 0.05$), and poorer health (OR = 1.91, SE = 0.31, p < 0.001). This study builds on frameworks situating caregiving within broader social networks by examining specific roles within Latino caregiver networks.

Abstract citation ID: igaf122.4242

### EVALUATING THE IMPACT OF CARE COMMUNITY COACHING INTERVENTION: A CLUSTER RANDOMIZED CONTROLLED TRIAL

Boeun Kim[1], Basia Belza[2], Shawn Johnson[3], Emily Waddington[4], Walter Moczygemba[5], and Lorna Prophater[4], *1. University of Iowa, Iowa City, Iowa, United States, 2. University of Washington, Seattle, Washington, United States, 3. The Alzheimer's Association, Chicago, Illinois, United States, 4. Alzheimer's Association, Chicago, Illinois, United States, 5. University of North Carolina at Chapel Hill, Chapel Hill, North Carolina, United States*

Within care communities, including nursing homes and assisted living settings, person-centered dementia care, highlighted by the 2018 Alzheimer's Association Dementia Care Practice Recommendations (DCPR), is the foundation of quality care and improving staff outcomes. A cluster randomized controlled trial was conducted from November 2022 to April 2025 to evaluate a six-month Care Community Coaching intervention, delivered primarily in person, to enhance person-centered dementia care in alignment with the DCPR. A total of 77 care communities were randomized, with 434 staff members participating—227 from 38 intervention communities and 207 from 39 control communities. Outcomes included DCPR adoption, employee satisfaction, person-centered care practices, and dementia care confidence, measured post-intervention and at three-month follow-up. Generalized Estimating Equations were used to estimate intervention effects. Findings indicate that care coaching increased communities' adoption of the DCPR by an average of 20.0%. Additionally, communities participating in care coaching showed statistically significant improvements in employee satisfaction, and staff perceptions of the delivery of individualized care and workplace practices. No statistically significant impacts on staff perceptions of caregiver-resident relationships or dementia care confidence were noted. Findings provide direction for future research and intervention development, including examining the impact of coaching on resident quality outcomes, and incorporating skills training into future models. Collectively, findings provide evidence of the effectiveness of a Care Community Coaching Program in DCPR adoption and improving staff outcomes and person-centered practices, offering a practical path towards improving the lived experience of residents and staff in care communities across the United States.

Abstract citation ID: igaf122.4243

### FAMILY PERCEPTIONS OF PALLIATIVE CARE CONSULTATIONS FOR NURSING HOME RESIDENTS

Alfred Boakye[1], Kathleen Unroe[2], Mary Ersek[3], Peiyuan Zhang[4], Gretchen Tucker[5], Alex Floyd[6], and John Cagle[7], *1. University of Maryland, Baltimore, Baltimore, Maryland, United States, 2. Indiana University School of Medicine, Indianapolis, Indiana, United States, 3. University of Pennsylvania, Philadelphia, Pennsylvania, United States, 4. University of Alabama, Tuscaloosa, Alabama, United States, 5. University of Maryland, Baltimore School of Medicine, Baltimore, Maryland, United States, 6. Indiana University School of Medicine, Indianapolis, Indiana, United States, 7. University of Maryland, Batimore, Maryland, United States*

People who receive care in nursing homes have a significant burden of serious medical illness, functional impairment, and cognitive impairment, and are known to have unmet palliative care needs. External specialist providers often conduct palliative care consultations (PCCs). PCCs usually include comprehensive physical and psychosocial assessments with accompanying treatment recommendations, goals of care conversations, and support for family members. However, the current understanding of specific interactions that can help improve the quality of PCC visits is limited. This makes the involvement and perceptions of families, who are key stakeholders in care decision-making, critical. Using a descriptive qualitative research design, our study sought to bridge this knowledge gap. As part of an NIH-funded clinical trial (R01 AG066922), we conducted 22 semi-structured interviews with family members of residents with moderate-to-severe cognitive impairment who received PCCs. Residents resided in 1 of 5 nursing homes in either Maryland or Indiana. Family members were recruited within 1 month of the occurrence of a PCC for their associated resident. Data were analyzed using thematic content analysis. According to family members, PCCs promoted physical comfort and family support, attended to residents' social needs, and provided next steps/care planning. Family also reported PCCs were helpful for reasons including that they improved residents' quality of life through offering recommendations/referrals, keeping family informed, and supporting family. Findings provide insights and demonstrate the value of PCCs for family members of residents with dementia.

Abstract citation ID: igaf122.4244

### PERSONAL CARE AIDE STAFFING IN U.S. RESIDENTIAL CARE COMMUNITIES

Erh-Chi Hsu[1], Katherine Kennedy[2], and David Mohr[3], *1. Johns Hopkins University, Baltimore, Maryland, United States, 2. Providence VA Healthcare System, Providence, Rhode Island, United States, 3. Veterans Health Administration, Mason, Ohio, United States*

Residential care communities (RCCs) employ approximately 660,000 personal care aides—nearly three-quarters of the direct care workforce in these settings—yet these workers receive minimal training and are not licensed healthcare professionals. Despite their essential role, national patterns of aide staffing and their relationship to RCC characteristics remain unknown. This study examines how training requirements and organizational factors are associated with aide hours per resident day (HPRD) using data from the 2022 National Post-acute and Long-term Care Study. A weighted sample of 518 RCCs was analyzed using regression models. Higher aide staffing levels were significantly associated with RCCs that reimbursed aides for initial training ($\beta = 0.84$, SE = 0.36) and with facilities operating at greater than 85% occupancy ($\beta = 0.82$, SE = 0.34). In contrast, lower staffing levels were observed in RCCs requiring more than 60 hours of initial training ($\beta$=-1.36, SE = 0.58), serving 1–25% ($\beta$=-1.19, SE = 0.57) or ≥ 25% ($\beta$=-2.01, SE = 0.51) Medicaid-supported residents, and those with more than 50 beds ($\beta$=-2.14, SE = 0.30). Neither continuing education requirements nor ownership type were significantly associated with staffing. Findings underscore the importance of supporting aide training reimbursement in association with higher staffing ratios. However, more extensive initial training requirements may reflect an expectation of greater responsibility per aide, potentially reducing staffing needs. Further research is needed to explore the trade-offs between training hours and staffing levels and to better understand the influence of Medicaid participation on workforce capacity in RCCs.

Abstract citation ID: igaf122.4245

### RACE AND PALLIATIVE CARE ACCESS IN HOSPITALIZED OLDER ADULTS WITH TBI

Jennifer Albrecht[1], Chih Chun Tung[2], and Raya Kheirbek[3], *1. University of Maryland, Baltimore, Maryland, United States, 2. University of Maryland, University of Maryland, Maryland, United States, 3. University of Maryland School of Medicine, Baltimore, Maryland, United States*

Background: Enhanced understanding of the use of palliative care among older adults with traumatic brain injury (TBI) could help guide development of policy and educational interventions. Our objective was to assess racial and ethnic disparities in the receipt of palliative care among older adults with TBI. Methods: We conducted a cross-sectional study using data from the Premier Database from May 2022-May 2023. We included adults aged 65 and older with an admission diagnosis of TBI who died during hospitalization. We compared characteristics and palliative care receipt across racial/ethnic groups. Logistic regression models were used to estimate the unadjusted and adjusted odds of receiving palliative care as a function of race/ethnicity. The primary outcome was receipt of a palliative care consultation. Results: Of 1,119 included patients,76.4% were Non-Hispanic White, 5.1% were Non-Hispanic Black, 5.5% were Hispanic, 4.4% were Asian, and 8.7% were classified as Other/Unknown. The majority (81.7%) received palliative care. In adjusted models, Non-Hispanic Black patients had the lowest odds of receiving a palliative care consultation compared to Non-Hispanic White patients (odds ratio (OR) 0.42; 95% confidence interval, 0.23-0.76). Conclusions: In a cohort of older adults hospitalized with TBI who died in-hospital, Non-Hispanic Black patients were markedly less likely to receive palliative care compared to their White counterparts. This study underscores the need for future work to identify the extent to which historical mistrust, communication barriers, provider bias, and socioeconomic factors contribute to differences in palliative care access among older TBI patients. Keywords: palliative care; racial disparities; traumatic brain injury.

Abstract citation ID: igaf122.4246

### THE ROLE OF STROKE ON VERBAL FLUENCY AND RECALL TRAJECTORIES: A LONGITUDINAL MIXED-EFFECTS ANALYSIS USING ELSA

Franklin Massa[1], Alejandra Marroig[2], Florencia Santiñaque[1], and Graciela Muniz-Terrera[3], *1. UDELAR, Montevideo, Montevideo, Uruguay, 2. Universidad de la Republica Uruguay, Montevideo, Montevideo, Uruguay, 3. Ohio University, Athens, Ohio, United States*

Understanding cognitive decline is a challenge in aging populations, and stroke has emerged as a key event triggering both immediate and long-term impairments. Previous studies have shown that having a stroke is associated with acute impairment in cognitive domains. Furthermore, it may increase the risk of dementia and mortality. However, the evidence on how strokes model the trajectory of different cognitive domains is limited and inconsistent. We evaluated the association between having a stroke and trajectories of cognitive domains using 9 waves from the English Longitudinal Study of Aging (ELSA). We used the Verbal Fluency (VF) test to assess the executive function and Total Recall (TR) test for memory function. We adjusted linear mixed effects models to capture both within and between person variability using the time-to-stroke to model differences by age in cognitive trajectories. The results showed that having a stroke was associated with a decrease of 1.14 words ($p < 0.001$) in the VF mean performance at 70 years old. However, it was not associated with the rate of change of VF ($p = 0.1206$). For the TR, stroke corresponded to an average decrease of 0.66 words ($p < 0.001$) and a pronounced acceleration in the decline of 0.14 words on average per year ($p < 0.001$). These findings showed that stroke is associated with a significant immediate decrease in both cognitive domains. Additionally, memory function exhibits a significantly accelerated decline after stroke. These insights underscore the need for early cognitive monitoring and tailored interventions targeting memory-related functions following stroke.

### SESSION 9010 (PAPER)

### LATE BREAKING: ALZHEIMER'S DISEASE, INCLUDING ALZHEIMER'S DISEASE—RELATED DEMENTIAS III

Abstract citation ID: igaf122.4247

### ENHANCING BRAIN HEALTH PROMOTION AND DEMENTIA DETECTION IN PRIMARY CARE WITH THE KAER FRAMEWORK

Lisa McGuire[1], and Jennifer Pettis[2], *1. Gerontological Society of America, Atlanta, Georgia, United States, 2. Gerontological Society of America, Washington, District of Columbia, United States*

The Gerontological Society of America's (GSA) KAER Toolkit for Brain Health is intended to support primary care teams in implementing a comprehensive approach to initiating conversations about brain health, detecting and diagnosing dementia, and providing individuals with community-based supports. Based on the four steps of the KAER framework—Kickstart, Assess, Evaluate, and Refer (KAER)— the Toolkit includes practical approaches, educational resources, and validated clinical tools that teams can immediately integrate into their workflow. Recently, GSA launched the third version of the publicly available GSA KAER Toolkit that was revised based on expert review and evaluation of the latest scientific evidence for its clinical applicability. This online version allows for frequent updates aligning with the ever-evolving brain health scientific-landscape, including significant advances in brain health promotion, dementia risk reduction, advances in biomarkers, and recent guideline updates on use of screening and detection tools. Attendees at this session will learn about the flexible KAER framework and how it can be integrated into existing workflows within their practice setting through the entire diagnostic process of K to A to E and to R improve care and outcomes of older adults living with dementia and their caregivers. This session will highlight newly added resources to the KAER Toolkit including brain health promotion, dementia risk reduction, common behavioral and psychological symptoms association with dementia, and updated screening and diagnostic tools. Additionally, practice pearls about how aspects of the KAER toolkit have been integrated into various clinical practices will be discussed.

Abstract citation ID: igaf122.4248

### HOW DO HEALTH CARE PROVIDERS WANT TO LEARN? SHAPING BRAIN HEALTH AND DEMENTIA PROFESSIONAL EDUCATION

Jennifer Pettis[1], Nancy Keach[2], Brooks Kenny[3], and Lisa McGuire[4], *1. Gerontological Society of America, Washington, District of Columbia, United States, 2. BrightFocus Foundation, Clarksburg, Maryland, United States, 3. BrightFocus Foundation, BrightFocus Foundation, Maryland, United States, 4. Gerontological Society of America, Atlanta, Georgia, United States*

The Gerontological Society of America (GSA) KAER Toolkit for Brain Health serves as a timely and impactful resource for educating health care professionals across disciplines about brain health and dementia. BrightFocus Foundation's (BFF) Zoom in on Dementia & Alzheimer's is a monthly information program that provides expert insights on key topics within the Alzheimer's landscape. Despite the abundance of education content developed for healthcare professionals, little is known about professionals' needs and preferences about clinical education. To ensure that educational content meets the needs of diverse healthcare professionals, GSA and BFF convened three virtual focus groups during July and August 2025. Thirty-three GSA members recruited from GSA's Health Sciences Section participated. Focus groups included diverse interdisciplinary providers (e.g., doctors, nurses, social workers, physician associates, pharmacists, physical therapists). One-hour groups were conducted by a trained facilitator, and each participant was provided with a small incentive for their time. Each group was queried about their needs and preferences regarding delivery, length, and format for clinical education and content distribution channels or sources where providers obtain their clinical education. Participants were also asked to provide input about topics related to dementia. The focus groups yielded valuable insights about members' needs and preferences around educational programming for dementia including priority areas aligned in each step in the GSA KAER—Kickstart, Assess, Evaluate, Refer—framework and the Zoom In educational series. This presentation will highlight findings from the focus groups of healthcare professionals and describe educational strategies resulting from the findings.

Abstract citation ID: igaf122.4249

### THE DEVELOPMENT OF A MULTICOMPONENT GROUP MUSIC INTERVENTION FOR PERSONS WITH DEMENTIA LIVING IN RESIDENTIAL CARE

Silvia Orsulic-Jeras[1], Gregg Gorzelle[2], Lacey DiFranco[1], Michael Skrajner[3], Sara Powers[1], Sarah Nicolay[1], and Emerson McSparran[4], *1. Benjamin Rose, Cleveland, Ohio, United States, 2. Hopeful Aging, WInchester, Massachusetts, United States, 3. Hopeful Aging, Winchester, Massachusetts, United States, 4. Miami University, Oxford, Ohio, United States*

Engagement-focused, nonpharmacological interventions are essential for enhancing quality of life among persons with dementia (PWD) in long-term care (LTC). Making Connections Thru Music (MCTM), a group-based intervention designed to be delivered by trained older adult volunteers, is grounded in the Comprehensive Process of Group Engagement (Cohen-Mansfield et al., 2017). Two pilot studies demonstrated strong acceptability, feasibility, and high levels of engagement and positive affect, supporting further development. This presentation illustrates how the theoretical underpinnings of MCTM inform its expansion into a comprehensive, technology-supported program designed for scalability. The enhanced intervention consists of two novel components: (1) an online volunteer training course providing dementia education, communication strategies, and facilitation skills; and (2) a tablet-based software application delivering a structured, step-by-step session protocol. Intervention design explicitly considers external elements (e.g., noise, lighting), personal attributes (e.g., communication and cognitive strengths), and stimulus characteristics (e.g., music selection, utilization of props). Evidence-based engagement techniques (e.g., external aids, discussion prompts, tactile materials) are incorporated to increase reminiscence, affect, and social interaction. Implications of intervention design choices and training content for volunteer preparedness and resident outcomes will be discussed. Next steps include a randomized controlled trial (RCT) across 12 assisted living and nursing home communities in Ohio and Massachusetts. The RCT will evaluate feasibility, fidelity, and preliminary outcomes on resident engagement and affect. If successful, MCTM can be the first scalable, volunteer-delivered group music intervention available for PWD living in residential care settings.

Abstract citation ID: igaf122.4250

### THE UNDOING OF A DIAGNOSIS - UNDERREPORTING OF DEMENTIA AMONG OLDER ADULTS

Yuting Qian[1], and Xi Chen[2], *1. Yale School of Public Health, New Haven, Connecticut, United States, 2. Yale University, New Haven, Connecticut, United States*

Accurate recognition of dementia is critical for clinical management, care planning, and decision-making. However, stigma, limited awareness, and inadequate disclosure may hinder recognition, and little evidence exists on the extent to which individuals with a documented diagnosis acknowledge it. We evaluated dementia under-reporting, associated factors, and consequences. We used 1998–2020 Health and Retirement Study (HRS)-Medicare linked data of adults aged ≥65 years, weighted for national estimates. Dementia under-reporting was defined as individuals with a dementia diagnosis in claims who did not report dementia in the matching HRS interview. Generalized Estimating Equation (GEE) models with a logit link estimated adjusted under-reporting rates and assessed patient- and provider-level factors, as well as subsequent health care utilization and financial planning. Among 6,134 persons with probable dementia (PWPD), 41% with a dementia diagnosis did not self-report dementia. Under-reporting was highest among self-respondents (66%) compared with 52% for depression, 39% for diabetes, 23% for hypertension, and 17% for arthritis. Rates exceeded 80% before probable symptom onset and remained about 40% afterward. Under-reporting varied significantly by education, living arrangement, dual eligibility, plan type, and diagnosis setting. PWPD who under-reported were less likely to have problem-based visits (OR, 0.71; 95% CI, 0.54-0.95), influenza vaccination (OR, 0.68; 95% CI, 0.55-0.84), and a witnessed will or trust (OR, 0.62; 95% CI, 0.47-0.81). This is the first national longitudinal study to demonstrate the existence and consequences of dementia under-reporting. Beyond improving detection, policies should ensure that diagnosed individuals, especially in preclinical or prodromal stages, are adequately informed.

Abstract citation ID: igaf122.4251

**UNCOVERING DYNAMICS OF END-OF-LIFE DECISION MAKING AMONG INDIVIDUALS AT RISK OF ADRD: INSIGHTS FROM CLINICAL DATA**

Shaoqing Ge[1], Qingyi Jasmine Zeng[2], Katherine Britt[3], and Bin Huang[4], *1. University of Texas at Austin School of Nursing, Austin, Texas, United States, 2. The University of Texas School of Nursing, Austin, Texas, United States, 3. University of Iowa, Iowa City, Iowa, United States, 4. BrainCheck Inc, Austin, Texas, United States*

This study examined how personal and relational factors from both patients and their caregivers influence advance care planning (ACP) decisions in real-world clinical settings. We focused on four outcomes central to end-of-life decision-making: discussion of end-of-life wishes, designation of financial and health care powers of attorney, and recognition of palliative or hospice care appropriateness. We analyzed data from individuals aged 40 and older who completed computerized cognitive care planning following cognitive assessment failures (n = 522). We applied logistic regression models to identify predictors of ACP engagement, incorporating variables from both patients and caregivers. Among patient-related factors, older age and higher education were consistently associated with increased ACP engagement. In contrast, patients identifying as Non-Hispanic Black or Hispanic/Latino were significantly less likely to engage in end-of-life discussions or designate proxies. Lower functional status also predicted greater planning activity. Caregiver-related predictors revealed that moderate stress and awareness of support resources were positively associated with proxy designation and hospice recognition. However, frequent weekly contact with the patient and perceived ability to provide care were negatively associated with ACP outcomes. Higher caregiver depression scores were linked to a reduced likelihood of health care proxy designation. These findings highlight the importance of dyadic and contextual influences in shaping ACP decisions. Emotional readiness, relational dynamics, and caregiving burden play critical roles in whether and how planning occurs. Our results underscore the need for interventions that recognize the shared nature of end-of-life decision-making and support both parties in navigating the emotional and interpersonal challenges of caregiving.

Abstract citation ID: igaf122.4252

**HEALTH CARE PROVIDERS' AWARENESS, KNOWLEDGE, AND READINESS TO IMPLEMENT DIGITAL COGNITIVE ASSESSMENTS IN PRIMARY CARE SETTINGS**

Jennifer Pettis[1], Lisa McGuire[2], Ben Tiede[3], and Emily Scholler[3], *1. Gerontological Society of America, Washington, District of Columbia, United States, 2. Gerontological Society of America, Atlanta, Georgia, United States, 3. Global CEO Initiative on Alzheimer's Disease, Wayne, Pennsylvania, United States*

The growing number of people with undiagnosed dementia underscores the urgent need to improve routine screening procedures for cognitive impairment in older adults. While health care continues to advance technologically, most traditional cognitive screening tools remain paper based. Emerging research suggests that the use of digital cognitive assessments (DCAs) could improve the detection of cognitive impairment while easing the burden on primary care teams who are already stretched thin. To effectively prepare primary care teams to implement DCAs, it is important to understand their current awareness, knowledge, and readiness to integrate DCAs into their clinical workflows. The Global CEO Initiative on Alzheimer's Disease (CEOi) is leading a global effort to advance the adoption of DCAs in clinical care, aiming to provide patients with a simpler, timelier, and more accurate diagnostic experience. As part of this effort, CEOi commissioned the Gerontological Society of America (GSA) and the American Academy of Family Physicians (AAFP) to survey their members in August and September 2025. The survey explored health care providers' awareness, knowledge, and readiness to use DCAs in primary care settings. Findings from this survey offer key insights to guide recommendations on the information, training, and resources needed to equip the primary care workforce for early detection of cognitive impairment through the utilization of DCAs. Attendees of this session will gain an understanding of the survey results and learn what supports health care providers identified as necessary to effectively integrate DCAs into their primary care practices.

### SESSION 9015 (PAPER)

### LATE BREAKING: ALZHEIMER'S DISEASE, INCLUDING ALZHEIMER'S DISEASE—RELATED DEMENTIAS IV

Abstract citation ID: igaf122.4253

**GETTING THROUGH THE DOOR: LESSONS LEARNED FROM RECRUITING ASSISTED LIVING FACILITIES FOR GROUP INTERVENTIONS**

Chirantana Dayanandaswamy, *University of South Florida GEC, Tampa, Florida, United States*

Engaging assisted living facilities (ALFs) is critical for implementing non-pharmacological social interventions that support residents with dementia. This study reports recruitment outcomes and lessons learned from an 8-week group music intervention conducted in Hillsborough County, Florida, from August 2024 to July 2025. A total of 166 licensed ALFs were identified, with 54 (32.5%) meeting eligibility criteria based on a minimum bed capacity of 20. Facility contact information was collected manually from their websites, and outreach was prioritized by facility size. Recruitment methods included phone calls, email follow-ups, in-person meetings, attendance at facility events, flyer distribution, and professional referrals. For facilities expressing interest by phone, our team visited the sites to conduct informational sessions and review study details. Of the 12 facilities visited, 9 (75%) agreed to start the study; six formally enrolled, and three are in the enrollment phase. Four enrolled facilities completed the intervention, while two withdrew due to staffing limitations. Across enrolled sites, 66 residents and 10 staff participated. Direct phone outreach followed by in-person visits was the most effective recruitment strategy, helping to build trust and address logistical questions. In contrast, email outreach proved ineffective, generating little to no response. Consistent, professional communication throughout the process was essential to maintaining engagement and supporting successful site participation. Our findings emphasize that constant communication, direct engagement through phone and in-person visits, and consistent professional interaction are crucial for improving recruitment and retention. These lessons can inform future efforts to implement and expand group social interventions across diverse care settings.

Abstract citation ID: igaf122.4254

### MOMENTARY AND LONGITUDINAL EFFECTS OF LONELINESS ON DEMENTIA CAREGIVER WELL-BEING

Christopher Fagundes[1], Pauline Goodson[1], Vincent D. Lai[1], Daniel Argueta[2], Paula Lanternier[1], Valentina Maza Santibañez[1], Kelly N. Brice[1], and Brenda Zarazua-Osorio[1], *1. Rice University, Houston, Texas, United States, 2. Rice Univdrsity, Houston, Texas, United States*

Spousal caregivers of individuals with Alzheimer's disease and Alzheimer's disease-related dementias (AD/ADRD) often experience profound loneliness, which contributes to persistent psychological distress and elevated risk for morbidity and mortality. Yet the mechanisms through which loneliness shapes their emotional well-being and caregiving outcomes remain unclear. Guided by stress process and attachment frameworks, we tested both momentary and longitudinal effects of loneliness in AD/ADRD spousal caregivers. Participants (N = 69; mean age = 71.5 years; 64% female; 62% White) completed ecological momentary assessments of loneliness and affect, along with baseline and follow-up surveys of caregiver burden, depressive symptoms, and anticipatory grief. Multilevel models showed that within-person increases in loneliness predicted greater depressed affect (b = 0.72, p < .001), controlling for demographics. Longitudinally, higher baseline loneliness predicted increases in caregiver burden (b = 0.21, p = .02) and anticipatory grief (b = 0.01, p = .01), but not depressive symptoms, beyond prior levels. Moderation analyses indicated that attachment anxiety heightened vulnerability to depressed affect, whereas attachment avoidance buffered the within-person daily link between loneliness and depressed affect. Findings demonstrate that loneliness functions as both a dynamic daily process and a long-term vulnerability factor in AD/ADRD spousal caregiving. By identifying attachment insecurity as a risk amplifier, this work provides mechanistic insight into when and for whom loneliness is most detrimental and highlights loneliness as a promising intervention target to reduce burden, mitigate anticipatory grief, and promote resilience among AD/ADRD spousal caregivers.

Abstract citation ID: igaf122.4255

### QUALITY OF FAMILY RELATIONSHIPS AND DAILY STRESS AMONG MIDDLE-AGED AND OLDER ADULTS

Heejung Jang, and David Almeida, *The Pennsylvania State University, University Park, Pennsylvania, United States*

Relationships with close family members are among an individual's most important social bonds. Although researchers regard stress as a risk factor for eroding relationship quality, more work is needed to understand how family relationships influence exposure and affective response to daily stress. This study explores how the underlying quality of family relationships impacts exposure to daily stressors and the moderating influence of family relationship quality on the link between these stressors and negative affective responsivity. We used data from 1,236 middle-aged and older adults (Mage= 62.48 years, SD = 10.21, range 43-91; 57% female) drawn from the third waves of the Midlife in the United States and National Studies of Daily Experiences. Latent profile analysis (LPA) models identified four family relationship types: pleasant, ambivalent, neutral, and unpleasant. Multilevel mixed effects models examined the associations between these relationship types and daily stressors and tested whether family relationship types moderated the association between each type of daily stressor and negative affect. Results showed that ambivalent and neutral relationships are associated with arguments (OR = 1.86, p<.001; OR = 1.86, p<.01) and avoided arguments (OR = 1.54, p<.001; OR = 1.59, p<.01). Among participants with ambivalent relationships, avoided arguments were associated with blunted responsivity (b= -.04, p<.05). For neutral relationships, network stressors were associated with exacerbated responsivity (b= .13, p<.01). This study contributes to the literature by revealing that family relationships impact an individual's affective responsivity to the stressors encountered in daily life.

Abstract citation ID: igaf122.4256

### RURAL AND URBAN DIFFERENCES IN DEMENTIA DIAGNOSIS IN THE WESTERN U.S.

Lauren Cater[1], Andrew Nute[2], Kara Bensley[2], Maja Pederson[1], Brianna Beck[2], Jessica Fleser[2], Curtis Noonan[1], and Rachel Peterson[3], *1. University of Montana, Missoula, Montana, United States, 2. Providence Health & Services, Renton, Washington, United States, 3. University of Montana, University of Montana, Montana, United States*

Prior research suggests diagnostic prevalence of Alzheimer's disease and related dementias (ADRD) is lower in rural U.S., though noted geographic variations have been observed in studies of the southern and Midwest regions. We compared rural and urban diagnosed prevalence of ADRD and cognitive impairment in eight Western states: Alaska, California, Idaho, Montana, New Mexico, Oregon, Texas, Washington. Diagnostic codes for ADRD and cognitive impairment from Providence Health system's electronic medical records were pulled from Jan. 1, 2018-June 30, 2025 for all active patients. Of 6,911,104 patients (min=Idaho: 45,160; max=California: 2.46 million), we observed a substantial difference in overall diagnosed prevalence across states of residence: Oregon and Montana had the highest diagnosed prevalence (6.6% and 6.5%, respectively); New Mexico had the lowest (2.3%). Overall diagnosed prevalence among those residing in urban and high commuting areas (RUCA 1-3, 4.1, 5.1, 8.1, 10.1) was 5.4% versus 4.7% among those residing in rural and low commuting areas (p < 0.001). Within-state urban versus rural differences in diagnosed prevalence was estimated for Alaska (4.8% vs. 3.5%; p < 0.001), California (4.8% vs. 4.2%; p < 0.001), Montana (7.3% urban vs. 5.1% rural; p < 0.001), New Mexico (3.2% vs. 2.2%; p = 0.01), Oregon (6.7% vs. 5.9%; p < 0.001), Texas (3.9% vs. 3.3%; p < 0.001) and Washington (6.3% vs. 5.3%; p < 0.001), but not for Idaho (3.5% vs. 3.4%; p = 0.57). Our findings within a single health system indicate lower ADRD diagnosed prevalence among patients residing in rural areas in 7 of the 8 states examined, suggesting a pattern of lower health system access and diagnosis.

Abstract citation ID: igaf122.4257

### SBAV: A SEX-STRATIFIED PRECISION FRAMEWORK FOR PREDICTING SLEEP APNEA–DRIVEN NEURODEGENERATION IN AGING WOMEN

Hanan Sheikh Ibrahim, *Cleveland Clinic Abu Dhabi, Abu Dhabi, Abu Dhabi, United Arab Emirates*

Background Obstructive sleep apnea (OSA) is a known risk factor for cognitive impairment, yet remains underrecognized in aging women. Traditional tools such as STOP-BANG and apnea–hypopnea index (AHI) thresholds were validated in male cohorts, failing to account for hormonal decline, altered sleep architecture, and sex-specific neuroinflammation post-menopause. Objective To develop and present the SBAV (Sleep–Brain–Aging Vulnerability) framework—a multi-modal, sex-stratified precision model integrating genomics, electrophysiology, hormonal markers, and AI-supported analytics to improve early detection of OSA-associated cognitive risk in women. Methods A structured scoping review (2018–2024) was conducted using PubMed, Scopus, and Web of Science. Key domains included: - Genomics (APOE ε4, CRY1, CLOCK) - Proteomics (tau, amyloid-β, IL-6, BDNF) - Endocrine markers (estrogen, cortisol) - EEG sleep microarchitecture (spindle loss, REM/NREM fragmentation) - Neuroimaging features (hippocampal atrophy) Findings were synthesized into a sex-stratified dashboard linking EEG and biomarker data with cognitive profiles. Results Women with OSA demonstrate a distinct EEG phenotype: reduced spindle density, altered slow-wave activity, and delayed cortical arousal, correlated with elevated tau and memory decline. Hormonal depletion and APOE ε4 synergistically amplify neurodegenerative vulnerability under intermittent hypoxia. Standard diagnostics miss over 40% of female cases. SBAV integrates omics, EEG, and hormonal data to outperform AHI-based stratification by 35–42%, enabling earlier, sex-specific risk identification. Conclusion SBAV redefines OSA in women as a sex-dependent neurodegenerative amplifier, proposing a paradigm shift in sleep and dementia screening. By leveraging AI-assisted, multi-omic stratification, this model lays the groundwork for tailored women's cognitive prevention in midlife.

Abstract citation ID: igaf122.4258

### SENSORIMOTOR MEASURES PREDICT LONGITUDINAL COGNITIVE DECLINE AND CORRELATE WITH CSF AND PLASMA BIOMARKERS.

Semere Bekena[1], Ramkrishna Kumar Singh[2], Yiqi Zhu[3], David Carr[4], and Ganesh Babulal[5], *1. Washington University School of Medicine in St. Louis, St louis, Missouri, United States, 2. Washington University School of Medicine in St. Louis, St. Louis, Missouri, United States, 3. Washington University School of Medicine in St. Louis, Washington Universury, Missouri, United States, 4. Washington University in St. Louis, St Louis, Missouri, United States, 5. Washington University School of Medicine in St. Louis, SAINT LOUIS, Missouri, United States*

Identifying early markers of cognitive decline in cognitively normal older adults is critical for dementia prevention. Sensorimotor measures such as gait speed, grip strength, and reaction time may serve as accessible, sensitive indicators of current and future cognitive impairment. This study examined associations between baseline sensorimotor function and cognitive performance and decline, assessed by the Preclinical Alzheimer Cognitive Composite (PACC), in cognitively normal older adults. In this prospective cohort study, 246 cognitively normal older adults from the DRIVES Project completed baseline assessments of grip strength, gait speed, simple reaction time, and PACC scores. Linear mixed-effects models adjusted for demographic, genetic, and neighborhood depravity level. Cross-sectional analyses examined associations between sensorimotor measures and cerebrospinal fluid (CSF) biomarkers of amyloid and tau pathology, and plasma neurofilament light chain (NfL), where available. Participants (mean age: 74.9 ± 5.17 years; 48.8% female) followed on average for 4 years had a mean baseline PACC score of 1.06 ± 0.50. Cross-sectionally, slower gait speed was associated with higher CSF tau/Aβ42 ratio ($p = 0.027$), CSF tTau/Aβ42 ratio ($p = 0.009$), and plasma NfL ($p = 0.024$). Slower reaction time predicted lower baseline PACC scores ($p = 0.028$). Longitudinally, low grip strength ($p = 0.002$) and slow gait speed ($p < 0.001$) predicted lower cognitive performance, with slow gait speed predicting faster decline ($p = 0.015$). Simple sensory-motor function measures are associated with current and future cognitive performance, supporting their role in early identification of older adults at risk for cognitive decline.

### SESSION 9020 (PAPER)

### LATE BREAKING: ALZHEIMER'S DISEASE, INCLUDING ALZHEIMER'S DISEASE—RELATED DEMENTIAS V

Abstract citation ID: igaf122.4259

### COMPREHENSIVE DEMENTIA SCREENING IDENTIFIES PATIENTS WITH POOR HEALTH OUTCOMES AND HIGHER HEALTHCARE UTILIZATION

Nabeel Qureshi[1], Pamela Roberts[1], Nancy Sicotte[1], Drew Hirsch[2], Mitzi Gonzales[2], Sarah Kremen[2], and Zaldy Tan[2], *1. Cedars-Sinai Medical Centers, Los Angeles, California, United States, 2. Cedars-Sinai Medical Center, Los Angeles, California, United States*

There are 6.9 million persons living with dementia in the United States, and over 40% of hospitalized patients age 70+ have dementia. Detection rates in hospitals are less than 50%, likely underestimating the true burden. We implemented a comprehensive program to identify cognitive impairment at hospital admission, using a tripartite approach (EHR alerts for dementia, and administration of the 4AT and AD8) in inpatient medical/surgical units. Patients were grouped by screening results and delirium status. We conducted Poisson, logistic, and generalized linear regressions to assess differences in length of stay (LOS), readmission rates, prior hospitalizations, and costs between those with and without cognitive impairment. Over the first 16 months, we identified 11,180 patient encounters. Diagnosed dementia patients had lower LOS (7.5 vs. 8.3 days, $p < 0.001$), while those with undiagnosed cognitive impairment had higher LOS (9.6 vs. 8.3 days, $p < 0.001$). Diagnosed dementia patients had a higher probability of 90-day readmission (17.5% vs. 15.1%, $p < 0.05$), and more hospitalizations in the prior 6 months (6.5 vs. 4.5, $p < 0.001$). Patients with potential cognitive impairment but no delirium also had more prior hospitalizations (5.1 vs. 4.5, $p < 0.01$). Patients with any cognitive impairment incurred lower costs (502K vs. 573K, $p < 0.001$), with differences observed primarily among those discharged home. Implementing systematic screening for cognitive impairment at hospital admission reveals significant differences in health outcomes by dementia status. Early recognition and tailored care strategies for cognitively impaired patients are crucial to improving hospital outcomes.

Abstract citation ID: igaf122.4260

### FALLS PREVENTION BEHAVIORS BY FAMILY CAREGIVER-PERSON LIVING WITH DEMENTIA DYADS AND FALLS

Victoria Panzer[1], Veronica Smith[1], Joanne Maher[1], Bayard Kohlhepp[1], and Richard Fortinsky[2], *1. Brookside Research & Development, Coupeville, Washington, United States, 2. University of Connecticut, Farmington, Connecticut, United States*

We previously reported the successful use of the FallScape-D computer-tailored multimedia falls-prevention intervention (FallScape-D) for family caregivers of persons living with dementia (PLWD). We enumerated caregiver-reported falls prevention behaviors (FPBs) during the 4-6 week intervention. This study enrolled a small intentionally-varied PLWD-caregiver dyad cohort, delivered Fallscape-D, and followed dyads for up to one year. Methods: Eleven family caregivers (6 female; mean(SD) age=65.6(12.8)) delivered FallScape-D, consisting of daily caregiver presentation of brief (< 90s.) individually-relevant stories to the PLWD (5female; age=78.2(5.3); MMSE12-29). Caregivers utilized an on-line daily log to report falls, injury falls, near falls (NFs), FPBs, and other events/issues encountered. A case series approach explored patterns of caregiver-reported FPBs and PLWD falls. Results: Caregivers logged 371 weeks (13-52) of daily reporting, 435 FPBs (12-106) and 14 PLWD falls. The first four weeks, 154 FPBs were reported (average=14(8)). Months 2 + 3 (9 weeks) yielded 148 FPBs (average=13(13). Months 3-6, 6-9, 9-12 (13 weeks each) yielded 81 FPBs (average=12(8), 29 FPBs (average=6(7) and 23 FPBs (average=5(5) respectively. Most falls or NFs, stimulated 1-4 FPBs, reducing or eliminating subsequent NFs. Six falls were caused indirectly (e.g. extraneous party created obstacle-3, caregiver extended absence-2, unrelated hospitalization-1). Eight falls related to health issues or dementia progression occurred 3+ months later. Conclusions: Family caregivers generated falls prevention ideas and encouraged PLWD falls prevention measures. A booster session may have prevented falls that occurred months after FallScape-D by inspiring FPBs matching changed circumstances. We conclude that stimulating FPBs is a viable strategy to prevent falls in PLWD.

Abstract citation ID: igaf122.4261

### ICARE.IT: A LONGITUDINAL STUDY USING WEARABLE SENSORS TO MONITOR INFORMAL CAREGIVERS OF PEOPLE WITH DEMENTIA

Francesca Zanasi, Angela Berardi, Paola Di Florio, Ylenia Druda, Marcello Sicbaldi, Marco Albertini, Marco Domenicali, and Alessandro Silvani, *University of Bologna, Bologna, Emilia-Romagna, Italy*

Informal caregivers play a vital yet often invisible role in supporting individuals with dementia, facing significant psychological and physical burdens. The ICare.IT study is a novel longitudinal investigation that combines subjective assessments (questionnaires) and objective physiological data (wearable sensors) to evaluate caregiver well-being over time. Critically, this is one of the first studies to apply multiple wearable sensor technology—tracking heart rate, sleep, and physical activity—to a large number of informal caregivers in their daily environment. Participants complete validated online questionnaires and wear sensors for one week, with data collected at baseline (T0), six months (T1), and twelve months (T2). This abstract presents preliminary findings from the first 68 caregivers enrolled in Ravenna (Italy). Most were adult children or partners, with high levels of care intensity (median 42.5 hours/week) and moderate-to-severe burden scores (ZBI median = 39). Notably, 84% reported low social support, and 49% experienced reduced friendship interactions. Anxiety and depression scores were in the borderline range and strongly correlated with perceived burden. Poor subjective sleep quality was also prevalent and associated with increased anxiety, depression, and perceived burden of care. However, heart rate and objective sleep duration were not significantly associated with burden. These early results highlight how wearable technology can reveal new dimensions of caregiver stress, opening pathways for targeted, data-informed interventions. Ongoing follow-up will enrich this analysis and improve understanding of caregivers' evolving needs.

Abstract citation ID: igaf122.4262

### IMPACT OF FINANCIAL INCENTIVE FOR DEMENTIA CARE IN ACUTE CARE HOSPITALS: A DIFFERENCE-IN-DIFFERENCES ANALYSIS

Jinyan Wu[1], Kojiro Morita[1], Ayumi Igarashi[2], Hideo Yasunaga[3], Yuya Kimura[1], Hiroki Matsui[4], Fushimi Kiyohide[5], and Noriko Yamamoto-Mitani[6], *1. Graduate School of Medicine, The University of Tokyo, Tokyo, Tokyo, Japan, 2. Graduate School of Nursing, School of Nursing, Chiba University, Chiba, Chiba, Chiba, Japan, 3. School of Public Health, The University of Tokyo, Tokyo, Tokyo, Japan, 4. School of Public Health, The University of Tokyo, Tokyo, Tokyo, Tokyo, Japan, 5. Graduate School of Medical and Dental Sciences, Institute of Science Tokyo, Tokyo, Tokyo, Japan, 6. The University of Tokyo, Bunkyo, Tokyo, Japan*

In 2016, Japan introduced a financial incentive motivating the establishment of multidisciplinary dementia care teams, the Dementia Care Add-on 1 (DCA1), to enhance dementia care in acute hospitals. While studies have assessed short-term effects, few have examined long-term outcomes, care process indicators, or patient-centered outcomes. To assess the long-term impact of DCA1 on older inpatients with dementia, we used data from a national inpatient database in Japan, the Diagnosis Procedure Combination, from 2014 to 2020. Difference-in-differences analysis was used to compare the outcomes between DCA1-implementing hospitals and matched controls. The primary outcome was length of stay (LOS); secondary outcomes included ADL, discharge destination, in-hospital fractures, and potentially inappropriate medication prescriptions. Among 235 matched hospital pairs (309,791 patients), DCA1 implementation alone was not significantly associated with outcomes. Sensitivity analysis showed patients who received care billed under DCA1 (41.5% of eligible cases), were more likely to have reduced LOS (difference, −1.57 days; 95% confidence interval (CI), −2.09 to − 1.06), maintained ADL (odds ratio, 1.96; 95%CI, 1.23 to 3.12) and to have been discharged home (odds ratio, 2.03; 95%CI, 1.28 to 3.20). Another sensitivity analysis excluding hospitals certified for DCA2 (a less stringent version of the incentive) showed modest LOS reduction (difference, −0.12 days; 95%CI, −0.22 to − 0.03). Over 60% of DCA1 hospitals billed fewer than 20% of eligible patients, indicating limited implementation. These findings suggest that the policy itself may have a limited overall impact; however, its effectiveness depends on actual care delivery. Future policies should prioritize implementation quality and contextual support.

Abstract citation ID: igaf122.4263

### NEUROVASCULAR COUPLING, GAIT PARAMETERS, AND COGNITIVE PERFORMANCE AS BIOMARKERS OF MILD COGNITIVE IMPAIRMENT

Yihui Cai[1], Wayne Chan[1], Tongyu Ma[1], Kaixin Zhou[2], Yarou Yuan[1], Shuning Li[1], and Marco Pang[1], *1. The Hong Kong Polytechnic University, Hong Kong, China (People's Republic), 2. The Second Clinical School of Clinical Medicine*

*of Guangzhou, University of Chinese Medicine, Guangzhou, Guangdong, China (People's Republic)*

Neurovascular coupling (NVC) dysfunction and gait disturbances are common in individuals with mild cognitive impairment (MCI), particularly under dual-task conditions requiring simultaneous cognitive and motor performance. This cross-sectional study compared gait parameters, NVC activation, and cognitive performance between 66 MCI participants and 47 healthy controls (mean age = 70.88 ± 4.66 years, 56% female) during three tasks: simple walking, the auditory clock test while walking, and the auditory Stroop test while walking. Gait parameters were recorded using APDM Mobility Lab sensors, cognitive performance (accuracy, reaction time, omission rate) was assessed via PsychoPy software, and NVC activation was measured using functional near-infrared spectroscopy. Feature selection and modeling were performed using Random Forest and Least Absolute Shrinkage and Selection Operator (LASSO) regression. In the simple walking task, MCI participants showed reduced turn counts and asymmetry in the terminal double support phase. During the auditory clock dual-task test, they exhibited reduced cognitive accuracy, incomplete turning, prolonged gait cycle duration, cadence asymmetry, terminal double support phase asymmetry, and increased activation in the left subcentral area. The auditory Stroop test revealed reduced cognitive accuracy, cadence, turn counts, elevated omission rates, stance and swing phase asymmetries, and increased activation in the left subcentral area, right dorsolateral prefrontal cortex, and right frontal pole area. These findings highlight the potential of combining gait parameters, NVC activation, and cognitive performance during auditory dual-task walking tests, particularly the auditory Stroop Test, as promising biomarkers for early detection of MCI. However, a larger sample is needed to enhance these findings' robustness.

Abstract citation ID: igaf122.4264

### PREVALENCE OF POTENTIALLY MODIFIABLE RISK FACTORS IN PERSONS AT-RISK FOR ALZHEIMER'S DISEASE

Zaldy Tan[1], Nabeel Qureshi[2], Drew Hirsch[1], Mitzi Gonzales[1], Sarah Kremen[1], Stephanie Bray[1], Anna Czarny[1], and Nancy Sicotte[2], *1. Cedars-Sinai Medical Center, Los Angeles, California, United States, 2. Cedars-Sinai Medical Centers, Los Angeles, California, United States*

Addressing modifiable medical and lifestyle risk factors may reduce the incidence of Alzheimer's disease and related dementias (ADRD). The Cedars-Sinai Memory & Health Aging Program (MHAP) promotes brain health via personalized risk profiling and risk reduction among asymptomatic adults aged 40 years and older at risk for ADRD. MHAP was launched with an email invitation to Cedars-Sinai Medical Center patients. Eligibility required age 40 or older, no cognitive or neurological diagnosis, and presence of two or more ADRD risk factors. Participants completed a 90-minute in-person visit assessing ADRD risk factors and cognitive performance. Of 123 enrolled patients, 84 (68.2%) had a family history of dementia or carried at least one ApoE4 allele. The mean age was 59.5 years; 63.1% were female. Most were White (77.4%) or Asian (9.5%), non-Hispanic (95.2%), and highly educated (91.7% college graduate or higher). Common risk factors included family history of ADRD in a first-degree relative (92.9%), weak social networks (52.4%), sleep disorder (51.2%), elevated LDL cholesterol (50%), and obstructive sleep apnea (50%). Among those completing the Montreal Cognitive Assessment (MoCA), the average score was 26.0, with frequent difficulties in delayed recall (91.9%), visuospatial/executive function (52.7%), language (36.5%), and attention (27.0%). Personalized risk profiling revealed high rates of modifiable ADRD risk factors, including sleep disorders, elevated cholesterol, and low physical and social activity. Targeted interventions addressing these factors may reduce future ADRD risk in at-risk populations.

Abstract citation ID: igaf122.4265

### METOLAZONE AND ALZHEIMER'S DISEASE RELATED DEMENTIA (ADRD): ARTIFICIAL INTELLIGENCE APPROACH TO DRUG REPURPOSING

Ali Ahmed[1], Sijian Zhang[2], Yijun Shao[3], Andrew Zullo[4], Mo-Kyung Sin[5], Charles Faselis[2], Gregg Fonarow[6], and Qing Zeng-Trietler[2], *1. Washington DC VA Medical Center, Washington, District of Columbia, United States, 2. Veterans Affairs Medical Center, Washington, DC, Washington, District of Columbia, United States, 3. George Washington University, Washington, DC, Washington, District of Columbia, United States, 4. Brown University, Providence, Rhode Island, United States, 5. Seattle University, Seattle, Washington, United States, 6. UCAL, Los Angeles, California, United States*

Currently, there is no drug approved for ADRD prevention. Developing new drugs is costly and time-consuming, and drug repurposing is rare and often serendipitous. In our NIA-funded R01 study, we used a Medication-Wide Association Study (MWAS) Plus approach, where "MWAS", similar to GWAS, identifies medications linked to ADRD from VA's national pharmacy data using deep learning, and the "Plus" examines these links in propensity score (PS) matched cohorts. Metolazone, a diuretic used in heart failure (HF), emerged at the top of the AI-generated list. To avoid confounding by indication bias, we examined this association in 648,552 HF patients in VA (2000–2017) without baseline ADRD and metolazone use, of whom 5068 were initiated on metolazone. We assembled a PS-matched cohort of 5068 pairs of patients balanced on 65 baseline characteristics and followed them until July 31, 2025. HR (95% CI) for ADRD associated with metolazone was 0.94 (0.83–1.06) and for death was 1.40 (1.35–1.46). To assess the impact of competing risk of death, we compared the median (IQR) time-to-death, which was 5.5 (2.1–9.5) years for non-metolazone and 4.7 (1.3–7.3) years for metolazone. When we examined the association between metolazone and incident ADRD in 9151 (90.3%) patients who died (thus, eliminating the competing risk of death), new ADRD occurred in 12.7% and 9.0% of patients in non-metolazone and metolazone groups, respectively (HR associated with metolazone, 0.86; 95% CI,0.76–0.98). These hypothesis-generating findings need to be replicated and confirmed in clinical trials. These findings also support future use of AI in efficient drug repurposing.

### SESSION 9025 (PAPER)

### LATE BREAKING: ALZHEIMER'S DISEASE, INCLUDING ALZHEIMER'S DISEASE—RELATED DEMENTIAS VI

Abstract citation ID: igaf122.4266

### 24-HOUR MOVEMENT BEHAVIOR PREDICT WORKING MEMORY ACROSS TIME IN OLDER ADULTS: NATIONAL HEALTH AND AGING TRENDS STUDY

John Oginni[1], Rebecca Bergee[2], Jennifer Schrack[3], and Zan Gao[2], *1. University Of Tennessee-knoxville, Knoxville, Tennessee, United States, 2. University of Tennessee-Knoxville,*

*Knoxville, Tennessee, United States, 3. Johns Hopkins Bloomberg School of Public Health, Baltimore, Maryland, United States*

Cross-sectional studies have identified associations between 24-hour movement behaviors — moderate-to-vigorous physical activity (MVPA), light physical activity (LPA), sedentary time (SED), and sleep — and cognitive outcomes. However, it remains unclear how these behaviors, measured at a single time point, predict older adults' cognitive outcomes over time. This study investigated the longitudinal relationship between 24-hour movement behaviors and subsequent working memory in older adults, using three waves of follow-up data (2021–2023) from 492 U.S. Medicare beneficiaries aged 65 and older in the National Health and Aging Trends Study (NHATS). Structural equation modeling with an autoregressive cross-lagged panel design revealed that a 60-minute increase in SED at Time 1(T1) and Time 2(T2) predicted declines in working memory at T2 ($B = -0.02$, $p < 0.001$) and Time 3 (T3) ($B = -0.013$, $p = 0.004$), respectively. Conversely, a 15-minute increase in MVPA at T1 was associated with improved working memory ($B = 0.013$, $p = 0.01$) at T2, and increases in MVPA at T2 predicted better working memory at T3 ($B = 0.019$, $p < 0.001$). Similarly, a 60-minute increase in LPA at T1 and T2 predicted better working memory at T2 ($B = 0.02$, $p < 0.01$) and T3 ($B = 0.03$, $p = 0.001$), respectively. Notably, sleep duration at T1 was positively associated with working memory at T2 ($B = 0.016$, $p = 0.008$). These findings suggest that MVPA, LPA, and adequate sleep may support cognitive function over time, while prolonged sedentary behavior may be detrimental.

Abstract citation ID: igaf122.4267

### DEVELOPMENT AND EVALUATION OF A WEB-BASED PLATFORM FOR DEMENTIA CAREGIVERS: A TWO-PHASE MIXED-METHODS STUDY

Qiping Fan[1], Minh-Nguyet Hoang[2], Logan DuBose[2], Laura Kim[3], Louis Fisher[1], Shinduk Lee[4], Marcia Ory[2], and Tokunbo Falohun[2], *1. Clemson University, Clemson, South Carolina, United States, 2. Texas A&M University, College Station, Texas, United States, 3. Texas A&M University, Texas A&M University, Texas, United States, 4. University of Utah, Clemson, Utah, United States*

Dementia caregivers face significant challenges, including managing financial, legal, and daily care responsibilities, yet existing resources often lack the personalization required to meet their complex needs. This study developed and evaluated Olera.care, a web-based platform designed to provide tailored educational and professional resources for dementia caregivers, using a two-phase mixed-methods design. Phase I included 30 family caregivers from Texas and employed a mixed-methods approach to identify caregiving challenges through qualitative interviews, develop and refine platform features, and pilot-test usability using the Mobile Application Rating Scale (MARS). Caregivers, predominantly aged ≥50 years (83%), women (77%), and White (83%), rated the platform highly for overall satisfaction (mean 4.57/5, SD 0.44), functionality (mean 4.46, SD 0.44), aesthetics (mean 4.58, SD 0.53), and personalized information quality (mean 4.76, SD 0.44). Phase II evaluated the platform usability and functionality among 65 caregivers from across the U.S. using the Technology Acceptance Survey (TAS), achieving an average score of 5.83/7 (SD 0.85), demonstrating strong usability and acceptance. Caregiver participants represented diverse demographics, with 69% White and 31% identifying as Black, Asian, Hispanic, or multiracial/ethnic. Care recipients exhibited varying levels of functional impairment (FAST levels 2–7d). Across both phases, caregivers emphasized the value of the platform in addressing caregiving challenges, particularly functional, legal, and financial needs. With its user-friendly design and personalized resources, Olera.care is a scalable, effective tool that supports dementia caregivers and addresses their growing needs. The study received funding support from the Small Business Innovative Research Program of National Institute on Aging.

Abstract citation ID: igaf122.4268

### MULTIDISCIPLINARY CLINICIANS' PERSPECTIVES ON ELDER MISTREATMENT: CHALLENGES AND OPPORTUNITIES IN PRIMARY CARE

Monique Pappadis[1], Monique Brown[2], Karen Schlag[1], and Leila Wood[3], *1. University of Texas Medical Branch at Galveston, Galveston, Texas, United States, 2. University of South Carolina, Columbia, South Carolina, United States, 3. University of Texas Health Science Center Houston, Houston, Texas, United States*

Elder mistreatment (EM) is an underrecognized public health concern, especially among older adults with mild cognitive impairment or Alzheimer's Disease and Related Dementias (MCI/ADRD). However, little is known about the perspectives of healthcare professionals on identifying EM in outpatient primary care settings. This qualitative study aimed to explore the perspectives of primary care providers on the identification of EM in older adults with cognitive impairments and cultural considerations in screening. A multidisciplinary sample of 24 outpatient care providers were recruited and interviewed via Zoom. The interview included questions on identification/recognition of EM, cultural considerations in screening, and considerations of addressing EM with patients with MCI/ADRD. Thematic analysis was used to identify recurring themes. Notable themes included: easily detectable visible signs/symptoms, challenges with distinguishing elder mistreatment from common symptoms of dementia, unmet social needs as risk factors, financial strain/dependency on the older adults' finances, caregivers' need for respite care to reduce caregiving strain, family neglecting to provide basic needs and cleanliness, and family dynamics and silence during clinical encounters. Cultural considerations included the differences in how individuals define EM, hesitancy for seeking assistance, perceptions of caregiver burden, perceptions of respect towards older adults and the provision of care, and screening in one's primary language. Primary care clinicians recognize that EM screening and clinician training are needed to address EM in primary care. Building trust between the clinician, older adult and family are essential to identify EM early and address unmet needs of patients with MCI/ADRD and their families.

Abstract citation ID: igaf122.4269

### PLASMA AB42/GFAP PREDICTS COGNITIVE HEALTH IN YOUNGER ADULTS FROM DIVERSE LATIN AMERICAN COUNTRIES

Erica Diminich, *California State University, Dominguez Hills, Dominguez Hills, California, United States*

Latino adults face nearly twice the risk of Alzheimer's disease (AD) compared to non-Latino Whites, yet remain underrepresented in plasma biomarker research. This study addresses a critical gap by examining blood-based biomarkers of AD-related neuropathology and brain health with cognitive performance in younger adults using community placed research to advance health equity in AD research. Objectives were to determine associations between plasma AD biomarkers with cardiometabolic factors and validate these biomarkers with NIH Toolbox cognitive assessments in community-dwelling Latino adults at early midlife. We enrolled 164 foreign-born Latino adults (mean age 48.43) in a community-placed research study. Plasma biomarkers were quantified using Simoa technology. The NIH Toolbox assessed six cognitive domains. Spearman correlations and multivariable linear regression models examined biomarker-cognition associations, controlling for demographics and cardiometabolic factors. Community engagement successfully recruited 98% monolingual Spanish speakers and 47% Central American participants—populations typically excluded from traditional studies. Blood biomarkers demonstrated significant age-related increases and cognitive associations: GFAP inversely correlated with executive function ($\rho$=-0.37, $p<0.001$) and global cognition ($\rho$=-0.27, $p=0.006$). Novel Aβ42/GFAP ratio positively predicted executive function ($\rho=0.33$, $p<0.001$), episodic memory ($\rho=0.25$, $p=0.01$), and processing speed ($\rho=0.22$, $p=0.03$). This work establishes the feasibility and rigor of community placed AD biomarker research in at-risk populations. Findings support the development of culturally responsive recruitment protocols, inform early intervention studies, and provide evidence for health equity approaches that can guide policy for inclusive clinical trial design and community placed early detection and prevention programs.

Abstract citation ID: igaf122.4270

### THE ROLE OF NEIGHBORHOOD SOCIAL CONTEXT IN THE LINK BETWEEN CUMULATIVE SOCIAL ISOLATION AND COGNITIVE DECLINE

Anna Chupak[1], and Yanping Jiang[2], *1. University of South Carolina, Columbia, South Carolina, United States, 2. Rutgers, The State University of New Jersey, New Brunswick, New Jersey, United States*

Social isolation is prevalent among U.S. adults aged 50–80 and is associated with poor cognition and elevated dementia risk. Few studies have examined how variations in type and repeated exposure to social isolation, including differences by neighborhood context, influence cognitive trajectories. This study investigates how cumulative, subjective and objective social isolation impact adults' cognitive function over time, including variations by neighborhood social cohesion and physical disorder. We used data from the Health and Retirement Study (2006–2016) on American adults aged 50–104 ($N=7{,}410$). Objective and subjective social isolation were measured by the 5-item Steptoe Index and the 3-item UCLA Loneliness Scale, respectively. Cognitive function was assessed using combined memory and executive function scores. We applied weighted mixed-effects regressions to examine associations between each type of cumulative social isolation and cognitive decline, adjusting for sociodemographic and health covariates. Then, we stratified each association by perceived neighborhood social cohesion (e.g., trust) and physical disorder (e.g., crime). Compared to no social isolation, individuals with persistent objective social isolation experienced the steepest cognitive decline over 8 years ($p=0.02$, $SE=0.02$); this association was non-significant among those living in neighborhoods with low levels of physical disorder. Individuals with persistent subjective social isolation had worse baseline cognition ($p<0.01$, $SE=0.18$), but showed more gradual cognitive decline over time ($p<0.01$, $SE=0.02$). These findings highlight the need for community-level strategies alongside individual interventions to reduce loneliness and promote social connectedness and cognitive resilience in older adults. Improving neighborhood conditions may buffer the cognitive impact of social isolation.

Abstract citation ID: igaf122.4271

### THE SIGNIFICANCE AND POWER OF COMMUNITY: BLACK DEMENTIA CAREGIVERS NAVIGATING CARE SYSTEMS

Janelle Gore[1], Mayra Sainz[2], Larry Strickland[3], Stephanie Bennett[2], Carolyn Clevenger[2], and Fayron Epps[3], *1. The University of Alabama, Tuscaloosa, Alabama, United States, 2. Emory University, Atlanta, Georgia, United States, 3. UT Health San Antonio, San Antonio, Texas, United States*

As the prevalence of Alzheimer's Disease and related dementia continues to disproportionately affect the Black community, the need to support caregivers is also expected to increase. A challenge many Black dementia caregivers face is navigating care systems due to the complexities of healthcare in the US. These difficulties can increase levels of isolation, in turn affecting the quality of care provided to their family member living with dementia. Thus, a psychoeducation course, Caregiving While Black LIVE, was developed to foster a community of Black health professionals and caregivers to equip and empower Black dementia caregivers with the sense of mastery needed to navigate care systems while acknowledging the social and cultural realities. Caregivers participated in the 6-week course, led by facilitators, meeting weekly with a group of 6 to 15 Black dementia caregivers either via Zoom or in person. Individual qualitative interviews were conducted with 17 of the 40 study participants to explore their experiences in the course. Most caregivers were women, with an average age of 57. Analysis revealed that participating in a culturally tailored psychoeducational program influences Black dementia caregivers' experiences navigating care systems by 1. Enhancing understanding, trust, and emotional comfort, 2. Empowering ability to make informed decisions, 3. Redefining the caregiving role, and 4. Sharing knowledge within the community. Culturally tailored programming remains a critical need for Black dementia caregivers. Researchers should work to increase the development of spaces that promote communal learning to boost confidence and improve health outcomes for families affected by dementia.

Abstract citation ID: igaf122.4272

### WHITE MATTER, AGE, APOE-E4, AND EPISODIC MEMORY IN MIDLIFE ADULTS AT HEIGHTENED RISK FOR ALZHEIMER'S DISEASE

Chadsley Wessinger[1], Jennifer Etnier[1], Kyoung Shin Park[2], Samantha DuBois[3], Samuel Kibildis[4], Jarod Vance[5], Brittany Armstrong[1], and Alexis Slutsky-Ganesh[6], *1. University of North Carolina at Greensboro, Greensboro, North Carolina, United States, 2. Emory University School of Medicine, Atlanta, Georgia, United States, 3. Appalachian State University, Boone, North Carolina, United States, 4. Athens-Clarke County Unified Government, Athens, Georgia, United States, 5. Longwood University, Farmville, Virginia, United States, 6. North Carolina Agricultural and Technical State University, Greensboro, North Carolina, United States*

Episodic memory (EM) decline is an early cognitive symptom of Alzheimer's disease (AD). White matter microstructural integrity (WMI) supports EM, but it is unclear if AD risk factors of age and apolipoprotein epsilon-4 genotype (APOE-e4) influence the relationship between WMI and EM at midlife prior to AD symptom onset. We investigated if age and APOE-e4 carriage independently moderated the relationship between WMI and EM in cognitively normal middle-aged adults with a family history of AD (FH+). Participants (N = 109[84% female]; Mage=56.2±6.0 years) provided saliva samples for APOE-e4 genotyping (e4 non-carriers/carriers=61/48) and completed diffusion tensor imaging (DTI) and cognitive testing. DTI scalars—mean diffusivity (MD), axial diffusivity (AxD), and radial diffusivity (RD)—were estimated in the fornix and hippocampal cingulum (CGH), tracts critical for EM. EM was assessed with Auditory Verbal Learning Task Immediate Recall (AVLT-IR) and Delayed Recall (AVLT-DR). Moderated regression models were fitted; significant ($p<.05$) and near-significant ($p<.10$) interactions were followed by simple slope analyses. No WMI*APOE-e4 carriage interactions were significant (p's>.10). Age moderated the WMI–AVLT-IR associations for CGH AxD (left: p=.027, right: p=.011), MD (left: p=.011, right: p=.005), and RD (right: p=.044); and the WMI–AVLT-DR associations for CGH AxD (left: p=.009, right: p=.010) and MD (left: p=.014, right: p=.011). In the oldest of participants (Mage=62.7±1.9 years), bilateral CGH AxD and MD negatively predicted AVLT-IR (p's=.011-.023) and AVLT-DR (p's=.012-.062) performance. In cognitively normal middle-aged adults with FH+, negative relationships between CGH WMI and EM were observed, suggesting the possibility of pre-clinical associations between brain health and cognitive behavior.

### SESSION 9030 (PAPER)

### LATE BREAKING: ALZHEIMER'S DISEASE, INCLUDING ALZHEIMER'S DISEASE—RELATED DEMENTIAS VII

Abstract citation ID: igaf122.4273

### ADVANCING PUBLIC HEALTH INFRASTRUCTURE FOR DEMENTIA THROUGH THE PUBLIC HEALTH CENTERS OF EXCELLENCE

Shelby Roberts[1], Joseph Gaugler[2], Joshua Chodosh[3], Soo Borson[4], and Mickal Lewis[5], *1. Alzheimer's Association, Chicago, Illinois, United States, 2. University of Minnesota, Minneapolis, Minnesota, United States, 3. NYU Langone, New York City, New York, United States, 4. University of Southern California, Los Angeles, California, United States, 5. Alzheimers Association, Chicago, Illinois, United States*

Over the past five years, the three Public Health Centers of Excellence (PHCOEs) have advanced the national public health response to Alzheimer's and related dementias by translating research into practice, building capacity, fostering innovation across diverse jurisdictions, and providing critical support to both BOLD program awardees and health departments operating without federal funding. The PHCOE on Dementia Risk Reduction, housed at the Alzheimer's Association, has synthesized scientific evidence on modifiable risk factors for dementia into short research briefs for health department officials and trained 64 local health department officials in 38 states and the District of Columbia. The PHCOE on Early Detection, housed at New York University's Grossman School of Medicine, has addressed the systemic underdetection of dementia, collaborating with over 75 organizations. It has developed toolkits and resources to promote early and equitable detection, delivered 61 presentations, and catalyzed national dialogue on making early detection routine practice. The PHCOE on Dementia Caregiving, housed at the University of Minnesota, has increased awareness of caregiving as a public health priority through the dissemination of toolkits, hosting webinars and virtual panels, and participation in 37 national conferences. Collectively, the Centers have reached over 14,000 professionals, hosted three national conferences, and laid the groundwork for sustained public health action on dementia. These efforts have catalyzed the integration of dementia-related strategies into public health practice, fostering innovation, collaboration, and evidence-based action.

Abstract citation ID: igaf122.4274

### UNDERSTANDING PUBLIC PERCEPTIONS OF NORMAL AGING AND DEMENTIA IN THE UNITED STATES

Lucas Hamilton[1], Max Coleman[2], Anne Sprecher[1], and Lauren Rutter[3], *1. Augustana University, Sioux Falls, South Dakota, United States, 2. The University of Utah, Salt Lake City, Utah, United States, 3. Indiana University Bloomington, Bloomington, Indiana, United States*

The rising prevalence of Alzheimer's disease and related dementia (ADRD) presents major public health concerns. Unfortunately, public knowledge about ADRD significantly diverges from scientific consensus. Misinformation and ageism have led to heightened stigma against people with ADRD. Characteristics of the stigmatized (i.e., people with ADRD) and of the general public both influence stigma; however, this evidence mainly comes from international samples. Building on this evidence, this study addresses ADRD stigma in the United States using stratified quota sampling of 1,115 adults to match national demographics. Using the well-established vignette approach, a 2 (Gender: Male, Female) × 3 (Cognitive Status: Normal, MCI, ADRD) between-subjects design was employed. Symptoms of the vignette character mirror the cognitive domains affected by ADRD and were reviewed by geriatric neuropsychologists to ensure accuracy. Stigma was shaped by individual differences more than vignette factors. Using multiple regression analyses, higher stigma was associated with less knowledge, younger age, being male, non-White race, identifying as a Republican, and living in areas with higher ADRD prevalence. Surprisingly, personal contact was not associated with less stigma. Only a third of participants (35.8%) correctly identified normal cognitive aging, and a sixth (16.8%) mislabeled impairment as normal. Scores on the Dementia Knowledge Assessment scale were roughly 50% with only 77 respondents (6.9%) correctly answering 20 items or more, which is the baseline seen in some international samples. Therefore, efforts to reduce ADRD stigma need to be tailored to address informational deficits and narratives that perpetuate negative perceptions in certain groups.

Abstract citation ID: igaf122.4275

### SOCIAL ENGAGEMENT OF CAREGIVERS: A CONCEPT ANALYSIS

Chih Yu Wang[1], Hyeyeon Shin[1], and Kathy Wright[2], *1. The Ohio State University, Columbus, Ohio, United States, 2. The Ohio State University, Powell, Ohio, United States*

Social engagement is critical for caregivers, as isolation increases their risk for depressive symptoms and anxiety. Social engagement is not well-defined and is used interchangeably with other terms. We aim to define social engagement by examining its attributes, antecedents, consequences, and empirical referents in caregivers. Following Walker and Avant's concept analysis, five databases—PubMed, CINAHL, PsycINFO, Scopus, and Web of Science—were utilized (with no year limit). The Stress Process Model was applied, and the Social-Ecological Model mapped the multilevel contextual influences on caregiver social engagement. Two authors independently screened the abstracts and full texts. Quality was assessed using the Joanna Briggs Institute tool. Twenty-seven studies define social engagement as actively interacting with others in safe, supportive environments where caregivers build meaningful relationships over time." Four attributes were identified: engagement behaviors in social activities, shared and supportive contexts for interaction, meaningful relationships, and level of interaction. Four antecedent categories appeared at all socioecological levels, except for the macrosystem, and consequences were grouped into benefits and risks. None of the studies used a validated instrument or captured all four attributes. Five studies assessed activity types, nine captured shared contexts, two addressed meaningful relationships, two measured engagement levels, six measured contact frequency, and three measured network size. By providing a more precise conceptual definition of social engagement, this review lays the groundwork for future research endeavors. Moving forward, developing validated measures and exploring contextual variations in caregiver social engagement are critical.

Abstract citation ID: igaf122.4276

### SOCIALLY ASSISTIVE ROBOTS FOR COMMUNITY-DWELLING INDIVIDUALS WITH MILD COGNITIVE IMPAIRMENT AND DEMENTIA

Light Ndurue, Ying-Ling Jao, and John Akudugu, *Pennsylvania State University, University Park, Pennsylvania, United States*

Mild cognitive impairment (MCI) and dementia are prevalent conditions that significantly impact the quality of life for community-dwelling older adults. Increasing research has focused on the development of socially assistive robots to support this population; yet the evidence has not been systematically synthesized. This integrative review examines research evidence on the effects of socially assistive robots (SARs) for community-dwelling individuals with MCI and dementia. Databases searched included PubMed, CINAHL, and PsycINFO using terms related to MCI, dementia, robotics/assistive technology, and community. Original research that evaluates SARs on community-dwelling individuals with MCI or dementia with reported outcomes were selected, 15 eligible articles identified. Studies reviewed included randomized control trials, quasi-experimental studies, and or pre–post intervention design; with sample size ranging from 30 to 66. The SARs reviewed included zoomorphic and humanoid robots, used either one-to-one interactions or in small group settings. The SARs were designed to provide companionship, prompt conversation, cue reminiscence or music, structure routines, and encourage gentle physical activities. Findings revealed that SARs had positive effects on engagement, pleasure, and social connectedness and demonstrated good usability and acceptability by users. Yet, SARs did not show significant improvement on global cognition or memory. Results on agitation, apathy, and quality of life were mixed across studies. In conclusion, SARs show promising benefits on engagement, positive affect, and social connection for community-dwelling individuals with MCI and dementia. Further research with larger sample sizes and longitudinal designs is needed to establish effective approaches to integrating SARs in clinical practice to support this population.

Abstract citation ID: igaf122.4277

### STATE-LEVEL STRUCTURAL RACISM AND SUBJECTIVE COGNITIVE DECLINE: REGIONAL VARIATION IN U.S. ADULTS

Kun Wang[1], Joonhyeog Park[2], Suk-Young Kang[3], Yanjun Dong[4], Victoria RIzzo[5], and Danan Gu[6], *1. University of Alabama at Birmingham, Birmingham, Alabama, United States, 2. University of Pennsylvania, Philadelphia, Pennsylvania, United States, 3. Binghamton University, Binghamton, New York, United States, 4. SUNY at Albany, Albany, New York, United States, 5. University at Albany, SUNY, Albany, New York, United States, 6. Jiangsu China, Suzhou, Jiangsu, China (People's Republic)*

Structural racism—geographically patterned across U.S. regions—shapes population health, yet links to cognition remain underexplored. Using 2020–2023 BRFSS data, we evaluated associations between state-level structural racism (SLSR) and subjective cognitive decline (SCD) among non-Hispanic (NH) Black and White adults aged ≥45 (N = 180,790) and assessed regional heterogeneity. SLSR (2020), a six-indicator index of socioeconomic status, healthcare access, and residential segregation, with higher values indicating larger Black–White disparities disadvantaging Black individuals, was grouped into national quartiles (Q1–Q4) for pooled analyses. Primary analyses used survey-weighted logistic regressions; sensitivity analyses used multilevel logistic models; both adjusted for individual- and state-level covariates. In pooled analyses, associations were non-monotonic: versus Q1, Q2 had higher odds of SCD (aOR=1.15; 95%CI 1.07–1.24) and Q4 had lower odds (aOR=0.86; 95%CI 0.79–0.94). Given marked geography (Q1: South, West; Q4: Midwest, New England, Mountain West), we conducted region-stratified analyses using within-region low/medium/high SLSR categories to avoid sparse cells. Compared with low SLSR, medium and high SLSR were associated with higher odds of SCD in the South; high SLSR with higher odds in the Midwest but lower odds in the Northeast and West. These patterns were evident among NH White adults but not NH Black adults (except the South). Results were robust in adults ≥65. Region-dependent findings—especially inverse associations among NH White adults in the Northeast and West—imply stratified advantage in higher-racism settings; largely null associations for NH Black adults may reflect SCD under-detection or distinct pathways. Longitudinal, fine-grained studies using objective cognitive outcomes are needed to clarify mechanisms and guide region-tailored policy.

Abstract citation ID: igaf122.4278

### SEX DIFFERENCES IN ALZHEIMER'S DISEASE RISK FACTORS IN THE SOUTHERN COMMUNITY COHORT STUDY

Kelsie Full[1], Xijing Han[2], Hui Shi[2], Danxia Yu[2], Logan Dumitrescu[2], Atul Malhotra[3], and Loren Lipworth[2], *1. Vanderbilt University Medical Center, Nashville, Tennessee, United States, 2. Vanderbilt University Medical, Nashville, Tennessee, United States, 3. University of California, San Diego, School of Medicine, La Jolla, Florida, United States*

In the United States, aging women bear a disproportionate Alzheimer's disease (AD) and related dementias (ADRD) burden, accounting for nearly 2/3 of cases. This striking sex disparity remains poorly understood and may be driven by differences in ADRD risk factors. In the Southern Community Cohort Study (SCCS), we investigated the sex-stratified prevalence of the 2024

Lancet Commission on Dementia identified ADRD risk factors and associations with incident ADRD. This analysis includes 28,250 SCCS participants (mean age: 60 years; 62% females; 59% Black) who reached ≥65 years prior to end of follow-up and were continuously enrolled in Medicare. ADRD risk factors included education, low physical activity, high sedentary time, smoking, alcohol use, obesity, social isolation, sleep duration, and physician diagnosis of depression, hypertension, and hypocholesterolemia. Incident ADRD was ascertained via Centers for Medicare and Medicaid Services linkage through December 2020. We ran Cox proportional hazard models adjusting for age, race, education, employment and income. Compared to males, females were significantly more likely to have depression, hypercholesterolemia, diabetes, hypertension, obesity, social isolation and short sleep duration (all p-values < 0.001). Over 4.5 years of follow-up, 1,994 ADRD cases occurred among females and 1,095 cases among males. Associations with incident ADRD were stronger among females compared to males for higher education (Hazard Ratios (HRs): 1.33; 1.19), hypercholesterolemia (HRs:1.10; 0.98), depression (HRs: 1.60; 1.39), higher sedentary time (HRs: 1.31; 1.18), hypertension (HRs:1.24; 1.12), and social isolation (HRs: 1.25; 1.19). ADRD risk factors that are more prevalent and impactful in females may inform novel prevention strategies.

### SESSION 9035 (PAPER)

### LATE BREAKING: ALZHEIMER'S DISEASE, INCLUDING ALZHEIMER'S DISEASE—RELATED DEMENTIAS VIII

Abstract citation ID: igaf122.4279

### A NUDGE-BASED CAMPAIGN FOR DEMENTIA PREVENTION OUTCOMES: A PRAGMATIC CLUSTER-RANDOMIZED TRIAL

Jose Aravena[1], Hugo Castro[2], Ronald Poblete[2], Maria Aravena[2], Marilu Budinich[2], Patricio Fuentes[3], Cecilia Albala[4], and Becca Levy[5], *1. Yale University, New Haven, Connecticut, United States, 2. CULTIVAMENTE, Santiago, Region Metropolitana, Chile, 3. Hospital Clinico Universidad de Chile, Santiago, Region Metropolitana, Chile, 4. INTA, University of Chile, Santiago, Region Metropolitana, Chile, 5. Yale School of Public Health, New Haven, Connecticut, United States*

Although dementia prevention is a global priority, few interventions have been successfully translated into public health and community settings. To address this gap, we evaluated the effectiveness of a nudge-based Alzheimer's disease (AD) prevention campaign on dementia prevention outcomes in older adults and healthcare providers. A pragmatic cluster-randomized trial was conducted in seven senior centers in Chile with adults aged ≥60 years who had cognitive impairment but no dementia. Centers were randomized to intervention (n = 3) or control (n = 4). All participants received usual care, including twice-weekly group activities (physical, cognitive, and social) and healthcare provider dementia training. Intervention centers additionally implemented CULTIVAMENTE, a nudge-based awareness campaign delivered through posters, brochures, and web content. We hypothesized that the intervention would improve cognitive healthy behaviors, cognitive performance (memory, executive function, and prevalence of mild-to-moderate cognitive impairment), and provider practices (AD prevention discussions and referrals). Intention-to-treat analyses using linear mixed models and generalized estimating equations confirmed these predictions. After six months, intervention participants (n = 101) demonstrated a 95.4% greater increase in cognitive healthy behaviors than controls (n = 110), significantly higher memory and executive function scores, and a lower prevalence of mild-to-moderate cognitive impairment. They were also more likely to report greater knowledge of AD prevention, to have engaged in discussions with providers about dementia risk reduction, and to have received referrals for managing risk factors. Embedding nudge-based messaging into senior centers improved dementia prevention behaviors, cognitive outcomes, and provider practices. These findings highlight the promise of low-cost, scalable strategies for advancing dementia prevention in real-world settings.

Abstract citation ID: igaf122.4280

### AUGMENTING STANDARD ALGORITHMS TO IMPROVE DEMENTIA DETECTION AND PREDICTIVE ACCURACY IN MEDICARE BENEFICIARIES

Sara Knox[1], Janet Horn[2], Kit Simpson[2], and Bethany Wolf[2], *1. Medical University of South Carolina, Charleston, South Carolina, United States, 2. Medical University of South Carolina, Medical University of South Carolina, South Carolina, United States*

Accurate identification of Alzheimer's Disease and Related Dementias (ADRD) in Medicare populations is critical for research and care planning. This study evaluated whether incorporating medication use and clinical diagnostic criteria into a digital phenotype could improve ADRD ascertainment over the standard CMS Chronic Conditions Warehouse (CCW) algorithm, which relies solely on diagnosis codes. Using data from 205,856 Medicare beneficiaries receiving home health services, we developed two predictive models: one based on the CCW phenotype and a novel ADRD (novel) phenotype based on clinical diagnostic criteria that categorizes individuals as ADRD-positive, ADRD-negative, or uncertain. Individuals with uncertain ADRD status were excluded during model training but analyzed later. Both models incorporated demographic variables, healthcare utilization, psychiatric symptoms, cognitive function, and prescription patterns. Data imputation and stacked elastic net regression were used to develop parsimonious models. Model performance was evaluated using AUC, sensitivity, specificity, and predictive values on an independent test set. The CCW model achieved an AUC of 0.863 for predicting its phenotype, while the novel model achieved an AUC of 0.906 for its respective phenotype. When applied to individuals with uncertain ADRD status, model predictions differed, with the novel model classifying more individuals as ADRD-negative than the CCW model. Overall, our findings suggest that augmenting standard claims-based algorithms with medication and clinical diagnostic criteria improves ADRD classification, particularly in cases where diagnosis is ambiguous. The novel model may offer a more conservative and specific approach to dementia identification, enhancing population-level research and targeted interventions.

Abstract citation ID: igaf122.4281

### CULTURALLY TAILORED COGNITIVE TRAINING FOR OLDER CHINESE AMERICANS: PRELIMINARY RESULTS FROM A PILOT RCT

Tingzhong (Michelle) Xue[1], Aybey Amy Wei[2], Camilla Sanders[2], Runqi Wangqin[2], Bei Wu[3], Eleanor McConnell[2], and Hanzhang Xu[2], *1. University of Massachusetts Amherst, Amherst, Massachusetts, United States, 2. Duke University,*

*Durham, North Carolina, United States, 3. NYU Shanghai, Shanghai, China (People's Republic)*

Older Chinese Americans are at increased risk of developing dementia but have limited access to culturally relevant prevention programs due to linguistic and transportation barriers. To address these issues, we have worked with older Chinese Americans and their adult children to co-design a mobile-based cognitive training intervention. In this study, we evaluated the feasibility and acceptability of this cognitive training intervention through a pilot randomized controlled trial with baseline, 8-, and 12-week follow-ups. Older Chinese Americans aged 60+ with consent capacity were randomized into the intervention (n = 20) or the wait-list control group (n = 10). The Intervention group completes 10 - 15 minutes training daily for 12 weeks from 21 training exercises covering cognitive domains including attention, processing speed, memory and visual spatial ability etc. Feasibility and acceptability were measured by retention rate and training completion rate, as well as a satisfaction survey. We enrolled our participants from January-July 2025. Of 30 participants (mean age 71.2±8.9 years; 60% female), 46.6% had less than college education and 96.7% had limited English proficiency. To date, 22 out of 30 participants have completed the study. The retention rate was 100% in the intervention group and 70% in the control group. Participants in the intervention group rated satisfaction as 9.7 out of 10. We are scheduled to complete all follow-ups and secondary outcomes, including cognition and psychological well-being by mid Oct 2025. Our preliminary evidence suggests that this culturally and linguistically tailored cognitive training intervention is feasible and acceptable among older Chinese Americans.

Abstract citation ID: igaf122.4282

### MEASURING WHAT MATTERS: MULTIDIMENSIONAL DEMENTIA CARE SUPPORT NEEDS IN HOME HEALTH

Sara Knox[1], Janet Horn[2], Kit Simpson[2], and Julie Bynum[3], *1. Medical University of South Carolina, Charleston, South Carolina, United States, 2. Medical University of South Carolina, Medical University of South Carolina, South Carolina, United States, 3. University of Michigan, Ann Arbor, Michigan, United States*

Home health clinicians need scalable tools to assess the complex care needs of people living with dementia (PLWD). Existing approaches are multidimensional, burdensome, or not integrated into routine workflows. The objective of this study was to develop and validate a care needs scale for PLWD using items from the Outcomes Assessment Information Set (OASIS), establishing a foundation for future integration into documentation systems. We analyzed 2019 Research Identifiable Files (OASIS, HHA, MBSF, MedPAR) including 1,376,556 PLWD in the development cohort (60%) and 917,704 in the validation cohort (40%). Factor 1 (functional/ADL support) was analyzed separately due to its dominant variance (eigenvalue 12.18, ~81% variance explained). Four additional factors were derived using exploratory factor analysis with orthogonal rotation. Factor loadings were high (0.70–0.95), communalities exceeded 0.70, and weighted beta scores produced individual-level summary scores. The five care support domains are: (1) Functional & ADL Support, (2) Behavioral/Psychiatric Support, (3) Memory & Decision-Making Assistance, (4) Hospitalization Risk, and (5) Challenging Behaviors & Aggression Management. Functional/ADL items showed broad variation (means 1.72–3.83, SD 1.63–2.30), demonstrating meaningful differentiation across patients. This validated, multidimensional scale leverages routinely collected clinical data to characterize PLWD care needs. Future work will evaluate integration into documentation systems and examine relationships with outcomes, offering a scalable tool to support research, clinical decision-making, and policy innovation in dementia care.

Abstract citation ID: igaf122.4283

### SUICIDE RISK AMONG PERSONS WITH DEMENTIA AND THEIR FAMILIES: INSIGHTS FROM POPULATION MORTALITY REGISTRY

Viktoryia Kalesnikava, *Institute for Social Research, Ann Arbor, Michigan, United States*

The onset and progression of Alzheimer's and related dementias (ADRD) can impact the psychosocial well-being of persons with dementia and their families, increasing the risk of suicidal behavior, particularly among those diagnosed before age 65. While ADRD disproportionately affects racial and ethnic minorities and individuals from disadvantaged backgrounds, research on late-life suicide in persons with ADRD has been limited, especially among underrepresented groups. This study examined individual and contextual suicide correlates among persons with ADRD and their relatives, applying large language models to structured and textual data from the National Violent Death Reporting System (2003-2022). Among suicide decedents aged 45 yo or older, 7654 deaths were linked to ADRD: 51% had confirmed ADRD, 34% had MCI/suspected ADRD, and 14% were relatives of persons with ADRD. Decedents with confirmed ADRD had higher odds of traumatic brain injury (TBI), schizophrenia diagnosis and were twice as likely to die in a long-term care facility compared to age-, sex-, and US State-matched non-ADRD decedents. Decedents with MCI/suspected ADRD more often had college education or beyond and higher odds of depressed mood, anxiety, PTSD, and TBI. Decedent relatives of persons with ADRD were more often married, had higher odds of experiencing family or relationship stress and housing instability, but were less likely to be from racial or ethnic minority groups or have lower education. Our study describes variations in potentially modifiable late-life suicide risk factors across ADRD-related groups, highlighting the need for tailored psychosocial support for both persons with ADRD diagnosis and their families.

### SESSION 9040 (PAPER)

### LATE BREAKING: ALZHEIMER'S DISEASE, INCLUDING ALZHEIMER'S DISEASE—RELATED DEMENTIAS X

Abstract citation ID: igaf122.4284

### COMPRESSION OF COGNITIVE DECLINE AND COGNITIVE RESILIENCE IN EXTREME LONGEVITY

Wenxin Zhang[1], Wenjie Cai[2], Albert Hofman[1], Anand Viswanathan[3], Susanne van Veluw[3], Deborah Blacker[3], Sudeshna Das[4], and Yuan Ma[1], *1. Harvard School of Public Health, Boston, Massachusetts, United States, 2. Johns Hopkins University School of Public Health, Baltimore, Maryland, United States, 3. Massachusetts General Hospital, Boston, Massachusetts, United States, 4. Massachusetts General Hospital, Harvard Medical School, Boston, Massachusetts, United States*

Compressing the duration of cognitive impairment is critical to preserve quality of life until the end. To what extent cognitive decline is compressed and cognitive resilience increases with extreme longevity is not well understood. We used data of 13,999 deceased participants from the National Alzheimer's Coordinating Center cohort, including 8,146 with neuropathological data. Cognitive function was assessed annually (median follow-up: 4.9 years). We evaluated cognitive trajectories before death and cognitive resilience (defined as high neuropathological burden without dementia) across lifespan groups (ages 50-100+). Participants with longer lifespans, particularly centenarians, exhibited slower cognitive decline and shorter periods of cognitive impairment before death, although distinct cognitive trajectories existed among centenarians. Cognitive resilience also increased with longer lifespans, but associated factors varied. APOE ε2 was associated with higher cognitive resilience only in centenarians. Our findings support a general compression of cognitive decline and increased cognitive resilience in extreme longevity.

Abstract citation ID: igaf122.4285

### DEMENTIA CAREGIVER PREPAREDNESS FOR SURROGATE DECISION-MAKING: SPECIFICITY AND CONFIDENCE IN CARE PLANNING

Rachel Bloom[1], Elizabeth Rojas[2], Emily LeRolland[3], and Francesca Falzarano[2], *1. Yale University, New York, New York, United States, 2. University of Southern California, Los Angeles, California, United States, 3. Fordham University, Bronx, New York, United States*

Dementia family caregivers commonly find themselves serving as medical surrogate decision-makers as the person with dementia nears their end of life. Engagement in advance care planning (ACP) conversations has been found to alleviate decision-making burden among surrogates, but these conversations rarely take place before the later stages of dementia. This study examines dementia caregivers' (N = 34) surrogate decision-making preparedness, using a semi-structured interview to ask about care planning conversations with their family member and their role as potential surrogates. We developed and applied a novel content coding system to assess caregivers' confidence and specificity in their planning approach and understanding of preferences. Survey data on self-efficacy, preparedness, mutuality, burden, and ACP engagement were also collected. We found that the presence of confidence was positively correlated with ACP engagement ($r = .42$, $p = .01$), while lack of confidence was negatively correlated with ACP engagement ($r = -.46$, $p < .01$). The presence of specificity was negatively correlated with self-efficacy in obtaining respite ($r = -.38$, $p = .03$), and lack of specificity was negatively correlated with burden ($r = -.45$, $p < .01$) and positively correlated with mutuality ($r = .35$, $p < .05$). The association between a lack of specificity and mutuality may reflect an assumption that specific planning is unnecessary when relationship quality is high. This work helps to elucidate the relationships between different aspects of surrogate decision-making for people with dementia and provides touchpoints for future decision-making preparedness research and interventions involving family caregivers.

Abstract citation ID: igaf122.4286

### ENTRAINING THE BRAIN WITH MUSIC AND SYNCHRONIZED LIGHT TO IMPROVE MEMORY AND PRESERVE COGNITIVE FUNCTION

Edward Large[1], Ji Chul Kim[2], and Psyche Loui[3], *1. Oscillo Biosciences, Storrs, Connecticut, United States, 2. Oscillo Biosciences, Willington, Connecticut, United States, 3. Northeastern University, Boston, Massachusetts, United States*

Worldwide at least 57 million individuals are currently living with Alzheimer's disease (AD), and by 2050 nearly 13 million Americans are projected to have AD. Pharmacological interventions have not been successful in producing significant effects on memory, cognition and behavior, and all pharmaceuticals carry some risk of potentially dangerous side effects. Recent research suggests that rehabilitation of neural oscillations supporting memory and cognition may provide effective treatments for neurological disorders such as AD. However, there are significant challenges in developing non-invasive, feasible, effective and scalable methods for human intervention. We have developed an intervention to rehabilitate theta (4-8Hz) and gamma (30-50Hz) neural oscillations using self-selected music and synchronized light pulses. We are currently running a Phase I Clinical Trial (NCT05984524) in individuals with Mild Cognitive impairment (MCI) and early-stage AD. Our gamma-enhanced music-based intervention (gMBI) is administered in the patient's home 30 minutes/day for 8 weeks; the control intervention includes listening to a health education podcast (the health-education intervention, HEI). Before and after the therapy patients undergo a battery of tests, including electroencephalography (EEG), structural magnetic resonance imaging (sMRI), diffusion tensor imaging (DTI), functional MRI (fMRI), blood draws, and tests of cognition and memory. Patient usage data in both conditions is monitored. We find that patients comply better with the treatment protocol than with the control protocol. After 8 weeks, neural oscillations are strengthened, hippocampal activation increases, memory improves, and cognitive function is preserved in the treatment group relative to controls. Patient recruitment is ongoing.

Abstract citation ID: igaf122.4287

### OLDER AFRICAN AMERICAN WOMEN'S MENTAL HEALTH AND SUBJECTIVE MEMORY: THE ROLE OF SOCIAL DETERMINANTS OF HEALTH

Gail Wallace[1], Ian McDonough[2], Roland Thorpe[3], and George Rebok[1], *1. Johns Hopkins University, Baltimore, Maryland, United States, 2. Binghamton University, Binghamton, New York, United States, 3. Johns Hopkins Bloomberg School of Public Health, Baltimore, Maryland, United States*

This study examined the role of psychosocial coping ability on depression and subjective memory outcomes moderated by social determinants of health in older African American women in the Advanced Cognitive Training for Independent and Vital Elderly (ACTIVE) study. Path analysis modeling was done using an autoregressive cross-lagged modeling approach on longitudinal data with 607 older African American women (Mean Age = 72.17, 65-91) residing in U.S. cities: Birmingham, Boston, Indianapolis, Baltimore, State College, PA, and Detroit as part of the ACTIVE study. Bidirectional cross-lagged effects modeled from depressive symptoms to subsequent subjective memory and vice versa, at each lagged time interval from baseline to 10 years following baseline was implemented. The social determinants of health (SDH) of study participants were divided into high and low groups via a median split and submitted to a multigroup comparison. Covariates included age, training group, booster status, and baseline physical and general health. African American women with higher levels of personal control reported higher levels of subjective memory ($\beta = .20$, $SE = .038$, $p < .001$) and fewer depressive symptoms (β = -.24, SE = .037, p < .001). Longitudinally, higher levels of personal control also were positively associated with improved subjective memory at Year 2 (β = .14, p = .004). These effects were moderated by several SDH factors. The present findings offer a window into how different types of SDHs shape dementia risk differentially through mental and cognitive health, especially in understudied older minority population demographics consisting of AA women.

Abstract citation ID: igaf122.4288

### PREDICTORS OF COGNITIVE RESILIENCE IN THE MULTI-ETHNIC STUDY OF ATHEROSCLEROSIS

Leidys Gutierrez-Martinez[1], Maude Wagner[2], Hodan Mohamed[1], Timothy Hughes[3], Timothy Hohman[4], Rachel F. Buckley[5], M. Maria Glymour[1], and Marcia Pescador Jimenez[1], *1. Boston University, Boston, Massachusetts, United States, 2. Rush University Medical Center, Chicago, Illinois, United States, 3. Wake Forest University School of Medicine, Winston-Salem, North Carolina, United States, 4. Vanderbilt University Medical Center, Nashville, Tennessee, United States, 5. Massachusetts General Hospital, Boston, Massachusetts, United States*

Modifiable predictors of cognitive resilience (CR)—maintaining better-than-expected cognition despite neuropathology—may inform dementia prevention strategies. We quantified CR and identified main predictors in a U.S.-based multi-ethnic cohort. We used data from 1,335 individuals enrolled in 2000-02 (Exam-1; age=45-84) in the Multi-Ethnic Study of Atherosclerosis (MESA) with repeated global cognition scores (z-scores of Cognitive Abilities Screening Instrument, Digit Symbol Coding, Digit Span) administered at Exams 5 (2010-12), 6 (2016-18), and 7 (2022-24). Neuroimaging biomarkers assessed at Exam 6, included white matter fractional anisotropy, white matter hyperintensities, and total brain volume, adjusted for intracranial volume. First, using linear mixed-effects models adjusted for age, sex, and race/ethnicity, we estimated CR as random slopes of global cognition, capturing individual deviations from expected cognitive decline given a specific neuroimaging profile. CR estimates were z-scored; positive values indicated better CR. Second, using general linear models, we assessed whether modifiable social and health-related risk factors at Exam 1 predicted CR a decade later. Each predictor's contribution to CR was assessed by percentage change in R-square by comparing fully-adjusted vs. reduced-models. At first neurocognitive assessment, study participants averaged 66 years old (SD = 8) and 46% were male. In fully-adjusted models, educational attainment (β = 0.19;95% CI=0.11,0.27) and family income (β = 0.12;95%CI=0.07,0.18) were independently associated with higher CR. More depressive symptoms and higher waist girth were independently associated with lower CR (β=-0.01;95% CI=-0.02,-0.01; β=-0.01;95% CI=-0.02,-0.01, respectively). Education and income were main contributors to CR (%R-squared Change=39% and 22%, respectively). Early favorable social (education, income) and health factors (depressive symptoms, waist girth) may promote CR decades later.

Abstract citation ID: igaf122.4289

### TRAJECTORIES OF MID-LIFE JOB INSECURITY AND EMPLOYMENT: ASSOCIATIONS WITH COGNITIVE FUNCTION IN THE HRS-HCAP

Erika Beidelman[1], Xuexin Yu[1], Rachel Donnelly[2], Mateo Farina[3], Constance Beaufils[4], Amanda Sonnega[5], and Yuan Zhang[6], *1. Columbia University Mailman School of Public Health, New York, New York, United States, 2. Vanderbilt University, Vanderbilt University, Tennessee, United States, 3. University of Texas at Austin, Austin, Texas, United States, 4. King's College London, King's College London, England, United Kingdom, 5. University of Michigan, Ann Arbor, Michigan, United States, 6. Columbia University, New York, New York, United States*

Continued workforce participation may preserve cognitive health in older age. However, many Americans face job insecurity and instability in later-life which may undermine these benefits. We examined the association of employment and perceived job insecurity trajectories (1998-2014) with domain-specific cognitive functioning (2016) among Health and Retirement Study Harmonized Cognitive Assessment Protocol participants aged < 67 years in 1998 (n = 2,618). Gender-stratified sequence analyses identified clusters of employment trajectories and adjusted linear regression models—weighted for differential retention and survival—estimated associations with standardized cognitive scores. We observed six trajectory clusters for men and women: predominantly job secure employment (reference), job secure to retirement transition, predominantly job insecure employment, predominantly self-employed, predominantly retirement, not in labor force (women only), and inconsistent employment (men only). Among women, retirement, not in the labor force, and secure-to-retirement paths were linked to lower memory (β range=-2.52 to -3.94) and executive function (β range=-2.65 to -4.00) scores compared to predominantly secure employment. For men, inconsistent employment was linked to underperformance on memory (β=-4.50, 95% CI=-7.12, -1.89), executive function (β=-5.33, 95% CI=-8.09, -2.57), language (β=-3.60, 95% CI=-6.20, -1.00), and orientation (β=-0.43, 95% CI=-0.69, -0.16) while retirement predicted lower memory (β=-3.64, 95% CI=-6.08, -1.19) scores and job insecurity predicted lower language scores (β=-3.01, 95% CI=-5.80, -0.23). Cognitive deficits were concentrated among clusters defined by unstable trajectories, including early labor force exits and inconsistent employment. As interest in extending labor force participation at older ages increases, our findings underscore the role of employment stability and security on cognitive functioning.

### SESSION 9045 (PAPER)

### LATE BREAKING: ALZHEIMER'S DISEASE, INCLUDING ALZHEIMER'S DISEASE—RELATED DEMENTIAS IX

Abstract citation ID: igaf122.4290

### ASSOCIATION BETWEEN COGNITIVE AND PLACE-OF-CARE TRAJECTORIES IN THE LAST THREE YEARS OF LIFE

Haiqun Lin[1], Olga Jarrín[2], Ayana April-Sanders[3], Weiyi Xia[3], and Anum Zafar[2], *1. Rutgers, The State University of New Jersey, Newark, New Jersey, United States, 2. Rutgers, The State University of New Jersey, New Brunswick, New Jersey, United States, 3. Rutgers, The State University of New Jersey, piscataway, New Jersey, United States*

We examined how patterns of place-of-care align with distinct cognitive function trajectories in the last three years of life. We used a 10% random sample of 181,463 Medicare beneficiaries aged 50 or older who died in 2019. Cognitive function was classified, based on assessment and claims Data: no impairment, memory loss, mild impairment, or dementia. Place-of-care was categorized quarterly for the last three years of life as: (1) home without skilled care; (2) home with skilled care (e.g., home health care or home hospice); or (3) facility-based care, including hospitals or skilled nursing facilities. Group-based trajectory models identified 10 distinct cognitive function trajectories and 13 place-of-care trajectories. One-third of decedents (36%) followed a no-impairment trajectory, spending most of their final three years at home. Two trajectories (7.5%) showed a gradual decline from memory loss to impairment near death, while one (12.1%) exhibited persistent severe impairment and dementia. Beneficiaries in these three declining and impaired trajectories (19.6%) were more likely to use sustained facility-based or skilled home care. These groups also had the highest proportions of females, dual Medicare–Medicaid enrollees, and individuals with depression, psychosis, or stroke. Findings suggest that distinct cognitive decline patterns are associated with predictable place-of-care trajectories and socio-clinical profiles. Targeting care models, such as proactive home-based services and enhanced caregiver support, could reduce reliance on facility-based care and better align end-of-life care with patient and family preferences for high-risk cognitive-trajectory groups. These findings can inform emerging dementia care models and national policy initiatives at improving end-of-life care.

Abstract citation ID: igaf122.4291

### ASSOCIATIONS BETWEEN SOCIAL ISOLATION, EDUCATION, AND RURALITY AND COGNITIVE HEALTH IN THE LASI STUDY

Raaga Likhitha Musunuri[1], Kavya Vytla[1], Kajal Dhansingh Bhauryal[1], Aarushi Vatsyayan[1], Amit Kumar[2], and Nasim Ferdows[1], *1. Northeastern University, Boston, Massachusetts, United States, 2. University of Utah, Salt Lake City, Utah, United States*

Social isolation is increasingly recognized as a risk factor for cognitive decline, yet its measurement and implications remain underexamined in low- and middle-income countries. This study examines various indicators of the multidimensional Social Isolation Index (SII) and its association with cognitive functioning among older adults in India. We used nationally representative data from the Longitudinal Aging Study in India (LASI) Wave 1 (2017–2019), focusing on individuals aged ≥60 years (N = 31,300) across 34 states and union territories. The SII combined six indicators (marital status, household structure, child contact, social and religious participation, and loneliness) into a weighted index (0-1). Cognitive function was measured using the composite cognitive index score (0-43). Linear regression models were used to assess the association between SII and cognition after sequential adjustment for socio-demographic, health, and behavioral covariates. Moderation was tested through interaction terms with rurality and education. A higher SII score was associated with lower cognitive functioning, with a 1.04-point difference between the most and least isolated individuals (95% CI: –1.13, –0.96). This association remained robust after full adjustment (–0.67, 95% CI: –0.74, –0.59). No rural-urban differences were observed. Educational attainment moderated the association, particularly in urban settings, where the association was weaker among those with higher education. SSI is consistently associated with lower cognitive functioning in later life, with education appearing to modify this association, particularly in urban settings. Understanding the effects of both objective and subjective dimensions of isolation is crucial for developing strategies to support healthier aging and improve cognitive health.

Abstract citation ID: igaf122.4292

### DELIRIUM INCREASES RISK OF MILD COGNITIVE IMPAIRMENT (MCI) AND DEMENTIA IN THE MAYO CLINIC STUDY OF AGING

Allyson Palmer, Aidan Mullan, Heling Jia, Sunghwan Sohn, and Fernanda Bellolio, *Mayo Clinic, Rochester, Minnesota, United States*

Delirium is a state of acute confusion characterized by deficits in attention that occurs commonly in hospitalized older adults and is associated with adverse outcomes. Delirium may have significant implications for cognitive health, however the relationship of delirium to mild cognitive impairment (MCI) and dementia is not fully understood. To evaluate the impact of delirium on cognitive health trajectories, we leveraged the Mayo Clinic Study of Aging (MCSA), a population-based cohort study of cognitive health and aging in residents of Olmsted County, Minnesota, who are between the ages of 30 and 89 and without a previous diagnosis of dementia at enrollment. Emergency Department and hospital encounters were identified for all patients enrolled in the MCSA, and clinical notes following each encounter up to 30 days were analyzed using a previously validated natural language processing (NLP) algorithm for delirium detection. The association between delirium within 30 days of hospitalization and the development of MCI or dementia was assessed using Cox proportional hazards regression, adjusted for patient age, sex, and level of education. Initial MCSA assessment indicated normal cognition for all individuals in this cohort. We found that delirium was significantly associated with development of MCI and dementia, with 42 individuals experiencing delirium within 30 days of hospitalization; hazard ratio of 10.07 (95% CI 4.14, 24.49) for developing MCI and 19.10 (95% CI 8.40, 43.47) for dementia compared to those without delirium. Our results suggest that hospitalization with an episode of delirium can significantly impact cognitive trajectories in older adults.

Abstract citation ID: igaf122.4293

### INITIATING DISCUSSION OF CARE PARTNER MENTAL HEALTH DURING CLINICAL ENCOUNTERS FOR PERSONS WITH DEMENTIA

Lisa Mistler[1], Kady Sternberg[2], Alejandra Martinez[3], Joshua Chodosh[4], and Paul Barr[3], *1. Geisel School of Medicine, Lebanon, New Hampshire, United States, 2. Dartmouth-Hitchcock Medical Center, Lebanon, New Hampshire, United States, 3. The Dartmouth Institute for Health Policy and Clinical Practice, Lebanon, New Hampshire, United States, 4. NYU Langone, New York City, New York, United States*

Objectives: Care partners (CP) of persons with dementia (PwD) experience considerably higher depression rates than non-CP peers. Current guidelines provide no strategies for implementing recommended mental health assessments for CP, whose interaction with clinicians is primarily through visits accompanying PwD. The objective of this study is to assess CP mental health support by clinicians of PwD during routine office visits for PwD. Methods: Data comprise transcripts of 200 initial clinical visits of PwD accompanied by CP. Two IRB-approved study team members independently analyzed 10 randomly-selected transcripts from urban clinics and 10 from rural clinics. Quantitative analysis included number of visits, number of times CP mental health was mentioned, who initiated the topic and the response to broaching the topic. Qualitative content analysis identified themes and patterns of clinician, CP, and PwD responses and mental health topics discussed. Results: In our preliminary analysis of 20 transcripts, CP mental health was mentioned in 10% of urban and 30% of rural visits. Four patterns were identified: 1) clinician inquired, unprompted, about CP mental health and CP responded; 2) CP reported mental health concerns and clinician did not respond; 3) CP reported mental health concerns and clinician responded; 4) CP mental health not discussed. Implications: Preliminary data indicate a difference in frequency of and approaches to assessing CP mental health during PwD clinician visits in rural versus urban clinics. Knowing factors associated with clinicians asking about CP mental health will help to develop tailored interventions for screening and referring CP for mental health care.

Abstract citation ID: igaf122.4294

### SYSTEMIC INFLAMMATION AND ITS INTERACTION WITH ALZHEIMER'S DISEASE PATHOLOGY IN ACCELERATING COGNITIVE DECLINE

Diefei Chen[1], Keenan Walker[2], Marilyn Albert[3], Jeremy Walston[3], Jacqueline Langdon[3], and Alden Gross[1], *1. Johns Hopkins Bloomberg School of Public Health, Baltimore, Maryland, United States, 2. National Institute on Aging, Baltimore, Maryland, United States, 3. Johns Hopkins School of Medicine, Baltimore, Maryland, United States*

Systemic inflammation may accelerate cognitive decline related to Alzheimer's disease (AD), yet underlying mechanisms remain unclear. We tested whether systemic inflammatory markers are associated with cognitive decline and whether they interact with AD pathology (amyloid, tau) and neuroinflammation (GFAP) to accelerate cognitive decline. We analyzed 161 participants from the BIOCARD study, who were cognitively unimpaired at baseline, with the first biofluid collection at mean age of 59.3 years (SD = 6.2), median of 3 samples (range 2–5) over 19.5 years on average. Blood-based systemic inflammatory markers were: IL1β, IL6, IL8, TNFα, TNFR1, CRP. Baseline Aβ42/40, ptau181, and GFAP were assayed in both CSF and blood. Linear mixedeffects models related (1) baseline systemic inflammatory levels, (2) withinperson change in each systemic inflammatory marker, and (3) interactions between baseline systemic inflammation and Aβ, ptau181, or GFAP to change in a general cognitive factor score, adjusting for age, sex, education, and vascular risk. Systemic inflammatory markers alone were not associated with faster cognitive decline. However, significant interactions indicated steeper cognitive decline when systemic inflammation cooccurred with AD pathology or neuroinflammation. Using CSF markers, faster decline was seen for CRP×Aβ42/40, TNFR1×ptau181, and IL1β×GFAP. Using blood-based markers, similar patterns emerged for CRP×Aβ42/40; IL8, TNFα, TNFR1×ptau181; IL8, TNFR1×GFAP. Systemic inflammation did not predict cognitive decline on its own but amplified the adverse effects of amyloid, tau, and GFAP. These findings support a synergistic model in which systemic inflammation interacts with AD pathophysiology to accelerate cognitive decline and may aid in identifying individuals at elevated risk for targeted intervention.

### SESSION 9050 (PAPER)

### LATE BREAKING: APPLYING PRINCIPLES TO REFRAME AGING IN YOUR WORK I

Abstract citation ID: igaf122.4295

### AGE IS JUST A NUMBER: HEALTH DIAGNOSES AS AN IMPORTANT PREDICTIVE FACTOR OF COGNITIVE FUNCTIONING IN PHYSICIANS

Miranda McDaniel[1], Johanna Carrasco[1], Taylor Evans[1], Michael Williams[2], and Betsy Williams[1], *1. Professional Renewal Center, Lawrence, Kansas, United States, 2. Professional Renewal Center/Wales Behavioral Assessment, Lawrence, Kansas, United States*

The physician workforce is aging. The Federation of State Medical Boards 2024 census indicated that 31% of licensed physicians are 60 years and older1. Late career physicians are an invaluable community asset that comprise an increasing proportion of the physician workforce3. While it is true that aging is associated with changes in physical and cognitive functioning that can affect skills germane to clinical work, late career physicians have knowledge, skills, resilience, and wisdom equitable with, and in many cases greater than, younger colleagues4. To address any concerns that may arise as part of the normal aging process, some health systems are requiring late career physicians to complete a screening process once they reach a target age; for example, the Society of Surgical Chairs recommended mandatory cognitive and psychomotor testing beginning at age 653. Additional factors are associated with cognitive functioning such as medical health2. This study explores the contribution of several health diagnoses as predictive of overall cognitive functioning. This study utilizes a sample of physicians aged 23 - 84 years (N = 189, mean 56) referred for a comprehensive fitness for duty assessment. As part of the evaluation process, participants undergo a full neuropsychological battery including the Wechsler Adult Intelligence Scale (WAIS) 4th edition. Analyses indicate an association among age, physical health, and WAIS-4 Full Scale IQ. This research has implications for the implementation of additional screening measures for health and cognitive impairment across the health professional career span and reducing age related bias in the workplace.

Abstract citation ID: igaf122.4296

### COMPARING MOTIVATIONAL INTERVIEWING WITH EDUCATION FOR LATER LIFE PLANNING: A TWO-ARM RANDOMISED CONTROLLED TRIAL

Youjuan Zhang[1], and Xue Bai[2], *1. The Polytechnic University of Hong Kong, Hong Kong, Hong Kong, China (People's Republic), 2. The Hong Kong Polytechnic University, Hong Kong, Hong Kong, China (People's Republic)*

The prevalent view of ageing as a period of decline often obscures opportunities for growth, agency, and continued contribution. Reframing ageing requires moving beyond simply providing knowledge and instead empower individuals to take proactive action in planning for a thriving later life. This study developed and evaluated a theory-informed intervention designed to reframe later-life planning as an empowering process across health, financial, social participation, family life, and long-term care domains through a randomised controlled trial. Community-dwelling adults (aged 50-75) in Hong Kong (63.0±3.9 years, 72.9% female, 74.6% retired) were randomised to a 4-session strength-based motivational interviewing (MI, n = 30) intervention resolving ambivalence, or a structured education intervention (n = 29) for knowledge/skill-building. Outcomes included attitudes, preparedness, stage of change and confidence in multidimensional later-life planning and psychosocial well-being, assessed at baseline (T0), immediately post-intervention (T1), and 1- (T2) and 3-month (T3) follow-ups. Generalised estimating equations examined differential changes in outcomes over time and groups. Overall, MI demonstrated sustained improvements in planning stages ($p < 0.001$, $d = 0.77$) and preparedness ($p = 0.049$, $d = 0.33$) at T3, outperforming Education. Such effects were particularly significant among social participation (planning stage: $p = 0.02$, $d = 0.52$) and health (preparedness: $p = 0.03$, $d = 0.41$) planning. Financial, family life, or long-term care planning, often requiring household-level consensus, were less responsive, warranting future family-based interventions. Both groups improved psychosocial outcomes (e.g., attitudes toward ageing, life satisfaction, self-efficacy; $p < 0.05$). This strengths-based model effectively empowers adults to engage with their later-life. By reframing planning from a burden to an opportunity for self-determination, it provides a tool for practitioners to advance equitable ageing.

Abstract citation ID: igaf122.4297

### FROM AN INTERSECTIONAL PERSPECTIVE REFRAMING AGING AND CARE EXPERIENCES OF TAIWANESE WOMEN CAREGIVERS

Yen-Chin Lee, *National Chung Cheng University, Taipei, Taipei, Taiwan (Republic of China)*

This study applies an intersectionality framework to fundamentally reframe our understanding of aging and caregiving in Taiwanese society. By meticulously examining the complex experiences of women caring for sick parents-in-law on behalf of their husbands, it aims to expose the systemic social inequalities often obscured by homogeneous views of aging, advocating for more socially just and inclusive gerontology education and long-term care policies. Utilizing a qualitative research approach, the study conducted semi-structured, in-depth interviews with five Taiwanese female caregivers. Reflexive thematic analysis, guided by intersectionality theory, was employed to reveal how deeply ingrained traditional concepts of "male superiority" profoundly influence caregiving roles, familial power dynamics, and well-being, providing a nuanced empirical basis for reframing. The research highlights that caregivers' experiences are shaped by intersecting power relations, key insights include: 1) the "taken-for-granted" role of daughters-in-law due to gendered labor, economic disparities and unpaid labor, 2) the multiplex burden of "double aging care" and 3) the profound impact of urban-rural and ethnic differences. Despite these pressures, caregivers demonstrated remarkable resilience and a desire for "women-empowerment" and "life-awakening". This study calls for structural reforms in family care support, e.g., exploring "community of reciprocity and aging together", social mutual aid mechanisms like a "collecting points to redeem a free service", and expanding gerontology education to all age groups to foster genuine gender equality and social justice. Ultimately, it appeals for the voices of vulnerable Taiwanese women to be heard and seen to build a more equitable and supportive super-aged society.

Abstract citation ID: igaf122.4298

### LONGITUDINAL PHYSICAL FUNCTION TRAJECTORIES IN OLDER ADULTS WITH MENTAL ILLNESS

Yuelin Li[1], Yuelong Xu[2], Zhirui Deng[2], Rezvaneh Manzour[2], Kyeongra Yang[3], Yue Coco Dong[2], and Heeyoung Lee[2], *1. University of PIttsburgh, Pittsburgh, Macau, 2. University of Pittsburgh, Pittsburgh, Pennsylvania, United States, 3. Rutgers University, Newark, New Jersey, United States*

Mental illness is recognized as a risk factor for accelerated physical aging and earlier onset of morbidity and mortality, yet its impact on functional outcomes in aging remains underexplored. This study examined longitudinal changes in physical functioning by mental illness using data from 13,419 adults aged ≥50 in the U.S. Health and Retirement Study (2006–2018). Mental illness was defined by the presence of depressive symptoms and/or psychiatric history, categorized as General (neither), Depression-only, Either, or Both. Physical functioning was assessed with grip strength, balance, and walking speed. Generalized estimating equations modeled longitudinal trajectories. Of the sample, 50.7% were in the General group, 20.9% in Depression-only, 23.7% in Either, and 4.7% in Both. At baseline, adults in the Both group exhibited poorer physical functioning than the General group, including lower grip strength (31.7 vs. 34.7 kg), slower walking speed (3.39 vs. 3.12 seconds), and lower balance test completion (28.5 vs. 35.1; $ps < .001$). Over time, all groups experienced functional decline, but walking speed declined more rapidly in the Both group (from 3.39 to 3.58 seconds) compared with the General group ($p < .05$). Baseline differences in grip strength and balance persisted over time with comparable rates of decline. Findings provide evidence that mental illness is a determinant of functional aging, with physical functioning serving as a measurable phenotype. Results underscore the need to integrate mental and physical health approaches in research and practice to reduce disparities and promote healthy aging among people with mental illness.

### SESSION 9055 (PAPER)

### LATE BREAKING: APPLYING PRINCIPLES TO REFRAME AGING IN YOUR WORK II

Abstract citation ID: igaf122.4299

### "TO KEEP US, HELP US": AGE INCLUSIVITY AT LEHMAN COLLEGE, A CITY UNIVERSITY OF NEW YORK SENIOR CAMPUS IN THE BRONX.

Theresa Lundy[1], Jesus Estrada[1], and Justine McGovern[2], *1. Lehman College, Bronx, New York, United States, 2. CUNY—Lehman College, Bronx, New York, United States*

This paper shares findings from an age inclusivity study conducted at Lehman College, February-April, 2025. Located in the Bronx, Lehman is a commuter campus offering degrees to over 12,000 students. It is a federally designated Hispanic-serving institution. Faculty number over 900 and staff, including leadership, exceed 1,400. Facing mounting pressure to increase enrolment, promote retention, and meet the needs of changing student demographics in the context of political, financial and social change, the timing of this project is urgent. Research aims included developing new knowledge about the experiences of older members of the Lehman community; providing data to promote age inclusive policies and practices; and improving the Lehman experience for older students, staff and faculty.

With student, faculty and staff ages exceeding national norms, Lehman presents an opportunity for leadership in age inclusivity in higher education. Moreover, with student populations trending older and retirement ages extending later, improving conditions for older community members seeking to develop skills, knowledge and networks and participate meaningfully in society with gainful employment, is essential. The Age Inclusivity Survey was sent by email to the Lehman community. Participation was anonymous and voluntary. Key findings include lack of awareness about factors that affect older community members and existing policies and practices designed to enhance the Lehman experience for older members. Qualitative comments revealed disappointment and frustration with, and some appreciation for, campus resources. Implications include raising awareness of diverse experiences at Lehman and extending the reach of inclusivity to improve the Lehman experience for all.

Abstract citation ID: igaf122.4300

### AGING AND RESILIENCE DURING URBANIZATION: A CASE STUDY IN A VILLAGERS' RESETTLEMENT COMMUNITY

Lingyi Shen, *Betty Irene Moore School of Nursing, University of California, Davis, Sacramento, California, United States*

Kunshan is a county-level city near Shanghai, China. Once a poor region reliant on agriculture, this area has undergone rapid industrialization and urbanization since the 1980s. This resulted in many farmlands being replaced and the emergence of various villagers' resettlement communities. Local villagers who are Chinese Baby Boomers faced the dual challenge of population aging and urban transformation. This study explores the aging experience of older adults in one of those villagers' resettlement communities, twenty years after the relocation, investigating their resilience and vulnerabilities emerged in the process. Over a four-month period, ethnographic research was conducted with participant observations, in-depth interviews, as well as oral history interviews focusing on older villagers' life trajectories and their evolving relationships with different spaces and social organizations during urbanization. Data was analyzed using the grounded theory approach. Three factors are identified that contribute to older villagers' resilience: 1) adaptive capacities derived from cumulative adversity in their life course; 2) financial and administrative support from village as a collective community of interests; 3) social and religious networks retained from previous rural livelihoods. However, limited education constrains their capacity to articulate their needs despite the presence of third-party services offered within the community. Disparities on social welfare still exist. Despite older villagers' relatively high satisfaction towards their experience, long-term sustainability of this way of aging is of concern when younger generations are gradually losing the resilience acquired by their parents and gra ndparents.

Abstract citation ID: igaf122.4301

### INTERPROFESSIONAL COLLABORATION AND CARE FOR OLDER ADULTS THROUGH THE 5M'S: WORKSHOP DEVELOPMENT/IMPLEMENTATION

Philip Solomon[1], Marie Dobrayel[2], Michele Pisano[3], Kaitlin Tangredi[2], Barbara Vogel[2], Christian Nouryan[4], and Edith Burns[5], *1. Northwell Health, New Hyde Park, New York, United States, 2. Northwell Health, New Hyde Park, New York, New York, United States, 3. St. John's University, Queens, New York, United States, 4. Northwell Health, Great Neck, New York, United States, 5. Zucker School of Medicine Hofstra-Northwell, Manhasset, New York, United States*

The field of Geriatrics is inherently based on interprofessional collaboration, requiring a variety of health professionals to address the principles and complexity of caring for older adults. A novel workshop curriculum was developed and implemented across a large health system weaving three threads: the 5M's framework, the Interprofessional Education Collaborative (IPEC) core competencies, and health system navigation for patients and caregivers. Stakeholders in numerous health professions were included in content creation and learner recruitment. The interactive, half-day, in-person sessions reframed each "M" in the context of health system navigation: 1) Medications: How do older adults pay for/access them? 2) Mobility: What is the proper venue to care for older adults with debility? 3) Mind: How do we provide support and coverage for custodial care for older adults with dementia? 4) What Matters Most: How can a health system align care and support patients' goals? 5) Multicomplexity: How can we work collaboratively to care for older adults and help them navigate complex health systems? This paper reports results of > 200 learners from nine health professions who have taken the course. Curriculum evaluation was guided by the Kirkpatrick model, assessing learners' attitudes, knowledge, and behavior. Over 93% felt knowledge would help both clinical practice and professional development. There were statistically significant increases in knowledge of IPEC competencies, ability to define roles of other health professions, intent to apply 5M's in clinical practice, and interest in pursuing formal Geriatrics training. Next steps include further internal/external dissemination and adaptation (e.g., asynchronous learning modules).

Abstract citation ID: igaf122.4302

### VALIDATING AGE-EQUITABLE MIXED EMOTION FILM STIMULI FOR LIFESPAN RESEARCH

Ryan Muskin[1], Lucas Hamilton[2], Michelle Moore[1], and Eric Allard[1], *1. Cleveland State University, Cleveland, Ohio, United States, 2. Augustana University, Sioux Falls, South Dakota, United States*

Mixed emotions are increasingly recognized as important for well-being and adaptive functioning in later life. However, research on age differences in mixed emotions is limited by methodological challenges, particularly the lack of age-equitable emotion elicitors. Theoretical frameworks suggest that older adults may experience less intense affective blends than younger adults, raising concerns that commonly used stimuli (e.g., film clips) may not evoke comparable responses across the lifespan . This study tested whether specific film genres can elicit more age-equitable emotional responses, thereby reframing how mixed emotions are studied across adulthood. Participants Participants were younger and older adults recruited via Prolific who viewed one of three clips: a dark comedy (Barry, $N = 258$), a biographical drama (King Richard, $N = 185$), or a fantasy-action film (Avengers: Endgame, $N = 185$). Age (Younger, Older) × Time (Pre, Post) ANOVAs revealed significant age × time interactions for Barry and Avengers ($p < .01$; $\eta p^2 = .03–.05$), with older adults reporting more diminished positive affect. However, King Richard produced no significant age differences, and item-level analyses showed age equivalence in high-intensity positive and negative affect across King Richard and Avengers. Across clips, younger adults reported higher mixed emotions overall, but findings suggest genre and intensity modulate age-related differences. These results support theoretical predictions about mixed emotion intensity while offering a novel approach to designing emotionally evocative stimuli that work equitably across age groups. By identifying affective blends that transcend age-related response biases, this study advances methodological practices that more accurately represent emotional experience across the lifespan.

### SESSION 9060 (PAPER)

### LATE BREAKING: ARTIFICIAL INTELLIGENCE AND EMERGING TECHNOLOGIES I

Abstract citation ID: igaf122.4303

### DEVELOPMENT AND VALIDATION OF AN APP-BASED MINDFULNESS PROGRAM FOR OLDER ADULTS: A RANDOMIZED CONTROLLED TRIAL

Shuting Dai, Yahui Wang, and Dannii Yeung, *City University of Hong Kong, Hong Kong, China (People's Republic)*

Objectives Despite the growing popularity of digital mindfulness-based interventions (MBIs), existing programs often vary in duration and format, and few are tailored for older adults. This study aimed to develop and validate a 4-week, app-based mindfulness program tailored for older adults. Methods Using a randomized controlled trial design, a total of 240 older Chinese adults were recruited and randomized into either a 4-week app-based mindfulness program (MF; n = 120) or a digital health education program (HE; n = 120). Outcome variables include depressive symptoms and state mindfulness level. Results Preliminary analyses included participants who completed baseline (T0) and post-intervention (T1) assessments (MF: n = 46; HE: n = 54), with follow-up (T2) data available for a smaller subset (MF: n = 11; HE: n = 13). At T0, the two groups did not differ significantly on depressive symptoms (MF: M = 7.07, SD = 1.97; HE: M = 6.78, SD = 1.74) and state mindfulness (MF: M = 59.35, SD = 5.13; HE: M = 60.31, SD = 5.64). Mixed-effects models from T0 to T2 showed significant group (2) × time (3) interactions for depressive symptoms (F (2, 134.52) =3.95, p=.019, $\eta p^2$=.055) and state mindfulness (F (2, 128.69) =3.42, p=.032, $\eta p^2$=.050). Conclusions Preliminary findings indicate that the 4-week, app-based mindfulness program is feasible and can reduce depressive symptoms and improve state mindfulness level in older adults. These results suggest that digital mindfulness intervention is an accessible approach to support mental health in late adulthood.

Abstract citation ID: igaf122.4304

### DIGITAL CONFIDENCE MEDIATES THE IMPACT OF VISUAL IMPAIRMENT ON DAILY INTERNET USE IN OLDER ADULTS

Haruno Suzuki, Thomas Hoffmann, Heather Leutwyler, and Margaret Wallhagen, *University of California, San Francisco, San Francisco, California, United States*

Older adults with visual impairment (VI) often experience barriers to engaging with digital technologies, yet remain underrepresented in digital health research. Confidence in using technology may mediate the association between VI and digital behaviors. This study aimed to 1) explore factors associated with digital behaviors among older adults with VI and 2) examine whether confidence in using technology mediates the relationship between VI and digital behaviors in older adults. The study used cross-sectional, nationally representative data from HINTS 2024. The sample included respondents aged 60 and older (N = 3,149). VI was defined using a self-reported item on visual difficulty. Outcomes included daily internet, smartphone, computer, and wearable device use in the past 12 months. Confidence in using applications/programs (e.g., Zoom) without help was the mediator. Multivariable logistic regression and mediation analyses with 1,000 bootstrap replications were adjusted for age, sex, race/ethnicity, education, marital status, self-rated health, and mobility impairment. Among older adults, 7.1% (N = 223) reported VI. Older age and lower educational attainment were significantly associated with lower odds of daily internet and smartphone use (P < 0.05). Statistically significant indirect (IE), direct (DE), and total effects (TEs) were observed for daily internet use (IE: OR = 0.92, 95% CI: 0.86–0.99, P = 0.017; DE: OR = 0.70, 95% CI: 0.53–0.93, P = 0.012; TE: OR = 0.65, 95% CI: 0.49–0.87, P = 0.003; proportion mediated: 20.3%, 95% CI: 1.9–38.7, P = 0.031), suggesting that VI reduces internet use partially through reduced confidence. Interventions targeting digital confidence, including personalized coaching, accessible interface, and peer support, may help bridge the digital divide in this population.

Abstract citation ID: igaf122.4305

### UNIVERSAL SINGLE-CELL TRANSCRIPTOMIC AGING CLOCK POWERED BY LLMS REVEALS TARGETS TO SLOW CELLULAR AGING

Raghav Sehgal, *Yale University, New Haven, Connecticut, United States*

Large language models such as GPT have shown impressive performance in capturing structure and meaning from natural language. We adapt this capability to biology by representing single-cell transcriptomes as ordered sequences of gene names, analogous to sentences in text. Each cell's expression profile is converted into a "cell sentence," enabling pretrained language models to be fine-tuned directly on single-cell data for age prediction. Trained on millions of cells spanning diverse tissues and life stages, the model learns a universal latent representation of aging. It captures both cell-type–specific trajectories and conserved molecular signatures, generalizing across tissues and species. Unlike other foundational models that must be trained from scratch on omics data, our framework leverages already well-validated LLMs, taking advantage of their robust priors while avoiding the cost and instability of building new architectures. The resulting clock achieves state-of-the-art accuracy in predicting biological age at single-cell resolution. Embeddings highlight aging-associated processes such as immune activation, proteostasis decline, and metabolic shifts, aligning with known hallmarks of aging. Importantly, the model can also identify genes whose modulation decreases predicted transcriptional age, nominating candidate targets for therapeutic intervention. These features make the approach valuable both for measuring aging and for prioritizing interventions that may slow or reverse it. This work establishes the first universal, multi-tissue aging clock at single-cell resolution, demonstrating that pretrained language models can be directly adapted to biological data to map, measure, and potentially modify cellular aging.

Abstract citation ID: igaf122.4306
### LATENT PROFILES AND PREDICTORS OF TRANSFER-ASSISTIVE ROBOT ACCEPTANCE AMONG KOREAN CARE WORKERS

Hee Jeong Yoon[1], and Young Sun Kim[2], *1. Kyung Hee University, Yongin-si, Kyonggi-do, Republic of Korea, 2. Kyung Hee University, kyonggi-do, Kyonggi-do, Republic of Korea*

In South Korea, with population aging and a growing shortage of care workers, the adoption of AgeTech in care settings has been steadily increasing, and transfer-assistive robots are increasingly considered to reduce care workers' workload. This study aimed to identify technology acceptance typologies for transfer-assistive robots among Korean care workers and to examine factors influencing these profiles. In 2023, survey data were collected from 421 care workers engaged in transfer tasks. Latent profile analysis (LPA) across six domains—self-efficacy, anxiety, attitude, ease of use, usefulness, and intention—identified three distinct groups: (a) high-acceptance (proactive) group (53%), (b) anxiety-dominant but moderate-acceptance group (42%), and (c) low-acceptance (prospective) group (5%). Personal characteristics (gender, age, education), health characteristics (depression), job-related burden factors (job burnout, job stress), and technology-related attitude (technology enthusiasm) significantly predicted group membership. The low-acceptance group (c) was more likely to be female, have lower education, higher depression, and lower technology enthusiasm than groups (a) and (b). The anxiety-dominant group (b) was older, had higher job stress, and lower technology enthusiasm compared to the high-acceptance group (a). These findings indicate that technology acceptance patterns among care workers are heterogeneous and that personal, health, job-related burden, and technology-related factors should be considered comprehensively when examining transfer-assistive robot adoption. Understanding these patterns and their predictors can guide tailored implementation and training strategies, supporting the effective adoption of transfer-assistive robots in care settings and informing the development of customized interventions to enhance robot acceptance among older adult care workers.

Abstract citation ID: igaf122.4307
### REAL-TIME ASSOCIATIONS BETWEEN DIGITAL INDICATORS AND DAILY PAIN AND DEPRESSIVE SYMPTOMS AMONG KOREAN OLDER ADULTS

Sunmi Song, Seo-Yeon Hwang, Hae-Young Kim, and Junesun Kim, *Korea University, Seoul, Republic of Korea*

Despite the importance of personalized digital healthcare services for addressing pain and emotional health in older adults, the development of digital healthcare interventions has been limited by a lack of evidence on real-time predictors of pain and emotional health among older adults. This study examined whether digital sensing data on heart rate variability, sleep quality, and physical activity could predict same-day or next-day pain and depressive symptoms among socially vulnerable older adults. As part of a larger project evaluating the efficacy of a digital healthcare platform integrated with a public community-based care service, older adult care recipients (n = 35; Mean age = 78.03, SD = 4.10; 80% women) and their community caregivers (n = 16) participated in a 6-week trial. Depressive symptoms were assessed daily using the 9-item Patient Health Questionnaire via scripted chatbot interviews, while pain was measured using a Visual Analogue Scale. Digital biomarkers—including heart rate variability, sleep, and physical activity—were collected via a continuously worn wearable sensor (Fitbit). Multilevel modeling revealed that within-person fluctuations in heart rate variability, mean of normal-to-normal intervals (Mean NNI; p = .016), and moderate physical activity (p = .001) were associated with same-day pain. Changes in sleep duration (p = .031) and efficiency (p = .053) were linked to same-day depressive symptoms. Lagged models further showed that Mean NNI (p = .031) and sleep fragmentation (p = .027) predicted next-day pain. These findings highlight the potential of real-time wearable data to inform personalized pain and mental health management for older adults.

### SESSION 9065 (PAPER)

### LATE BREAKING: ARTIFICIAL INTELLIGENCE AND EMERGING TECHNOLOGIES II

Abstract citation ID: igaf122.4308
### ACCEPTANCE OF DIGITAL LEISURE FOR OLDER ADULTS: FOCUSING ON THE SENIOR TECHNOLOGY ACCEPTANCE MODEL

Hyunjoo Lee, Hyeri Shin, Yeomin Han, Donghyun Lee, and Young Sun Kim, *Kyunghee University, Seoul, Republic of Korea*

Rapid digitalization is reshaping leisure in later life, creating new opportunities for engagement, learning, and social connection (Nimrod & Adoni, 2012). In Korea, smartphone-based leisure use among adults aged 60+ has risen markedly over the past decade (Ministry of Culture, Sports and Tourism, 2018, 2022). Participation in digital leisure—covering activities such as online communication, streaming, gaming, and virtual learning—has been associated with enhanced quality of life, subjective well-being, and social inclusion (Regalado et al., 2023; Gallistl & Nimrod, 2019). Understanding the factors that shape acceptance is therefore essential for policies and interventions that promote active and healthy aging. This study tested a structural model linking gerontechnology anxiety, multidimensional health, control beliefs, attitudinal beliefs, and intention to participate in digital leisure. Data from 300 community-dwelling adults aged 60+ were analyzed using AMOS. Health positively predicted control beliefs ($\beta$=.36, p<.001), attitudinal beliefs ($\beta$=.42, p<.001), and intention ($\beta$=.18, p<.01). Control beliefs ($\beta$=.28, p<.001) and attitudinal beliefs ($\beta$=.51, p<.001) significantly predicted intention, with attitudinal beliefs showing the strongest effect. Technology-related anxiety negatively predicted control beliefs ($\beta$ = −.15, p<.05) but had a small positive association with attitudinal beliefs ($\beta$=.11, p<.05), indicating that mild anxiety may coexist with curiosity and perceived benefits. This dual role suggests that while anxiety can reduce confidence, it may also reflect cautious interest. Interventions that reduce functional barriers, build self-efficacy, and harness the motivational aspects of mild anxiety through supportive, hands-on learning could increase older adults' digital leisure participation, supporting quality of life, psychosocial well-being, and social integration among older adults.

Abstract citation ID: igaf122.4309
### BENEFITS OF SELF-QUANTIFICATION FOR FAMILY CAREGIVERS AND FUTURE MACHINE LEARNING SUPPORT

Tomoko Wakui, *Tokyo Metropolitan Institute for Geriatrics and Gerontology, Tokyo, Tokyo, Japan*

Family caregivers of community-dwelling older adults requiring long-term care often face significant psychological and physical burdens, particularly under the increasingly diverse care arrangements in Japan, where existing support systems are reaching their limits. Self-quantification—systematic daily documentation of caregiving activities and well-being, along with caregivers' sleep, activity, and health—offers a promising approach to capturing fluctuations in caregiver stress and identifying early risks. The purpose of this study was to comprehensively evaluate the impacts of self-quantification on family caregivers. Specifically, we aimed to elucidate the mechanisms underlying these effects by integrating quantitative findings with qualitative insights from caregivers' narratives, and examine the potential of applying machine learning to daily caregiving data for predictive risk detection and future digital health support. We developed the Caregiving Visualization Program (Care-VIP), enabling family caregivers to record daily caregiving activities, personal routines, and emotional well-being. An initial online survey of 3,256 caregivers mapped caregiving situations nationwide, followed by a 60-day self-quantification trial with 201 participants. A follow-up survey assessed the program's perceived benefits and challenges. Program participation was more likely among male caregivers (OR = 1.44), those employed full-time (OR = 1.80), caregivers with longer caregiving duration (OR = 1.17), and those with higher caregiving burden (OR = 1.01). Qualitative feedback indicated that daily documentation fostered reflection, recognition of personal health, and positive behavioral changes, such as the use of respite services and improved health habits. Machine learning analysis of daily sleep/activity data further demonstrated feasibility in detecting patterns predictive of caregiver stress and depressive symptoms.

Abstract citation ID: igaf122.4310

### MACHINE LEARNING ALGORITHM–BASED PREDICTIVE MODEL OF LATE-LIFE LONELINESS LEVEL IN LONG-TERM CARE RESIDENTS

Mo Yi[1], Zhiwen Wang[1], and Anna Beeber[2], *1. Peking University, Beijing, Beijing, China (People's Republic), 2. Johns Hopkins University, Baltimore, Maryland, United States*

Background and Purpose: Late-life loneliness affects nearly one-third of older adults in long-term care facilities (LCFs) and is strongly associated with adverse health outcomes, posing a serious threat to global healthy aging. This study aimed to construct and validate predictive models using five advanced machine learning algorithms to identify residents at risk of loneliness and to rank key contributing factors. Methods: Data were collected from 583 residents across six LCFs in China who had stayed for at least three months. Loneliness was defined as a UCLA-8 score ≥16. Sixteen predictors covering demographic, physical, psychological, and social domains were selected via LASSO approach to train five machine learning algorithms with cross-validation. Model performance was evaluated by the area under the curve (AUC), accuracy, recall, specificity, precision, and Cohen's kappa. Results: The ElasticNet algorithm model achieved the best overall performance, with an AUC of 0.737. At the 0.5 probability threshold, accuracy was 70.6%, specificity 95.3%, precision 70.8%, recall 71.5%, and kappa 0.404. The confusion matrix showed strong ability to identify residents without loneliness but limited sensitivity for detecting loneliness. Feature ranking revealed the top eight predictors as depression, anxiety, instrumental activities of daily living, vision status, multimorbidity, cognitive orientation deficit, language ability, and mobility. Psychological symptoms and functional impairments consistently contributed the greatest weight. Conclusion: Findings suggest its potential use for routine screening and may assist care staff in early identification and targeted interventions for loneliness and the promising use of machine learning for care of older adults in LCFs.

Abstract citation ID: igaf122.4311

### REIMAGINING ENGAGEMENT AND CONNECTION: USABILITY INSIGHTS FROM VIRTUAL REALITY USE AMONG OLDER ADULTS

Pallabi Bhowmick[1], Noah Olivero[1], Tracy Mitzner[2], Avinash Gupta[1], and Wendy Rogers[1], *1. University of Illinois Urbana-Champaign, Champaign, Illinois, United States, 2. Person in Design, Atlanta, Georgia, United States*

Social isolation affects nearly one in four older adults, and virtual reality (VR) has emerged as a promising tool for fostering social connection and engagement. To better understand its potential, we have conducted a needs assessment study with older adults to explore the usability, acceptance, and perceived benefits of VR applications across multiple sites. Analysis of the data from one site (n = 16, aged 65–79, M = 71.56, SD = 4.18) showed that majority of the participants (88%) rated enjoyability, engagement, and appeal between 7 and 10 on a 10-point scale, indicating strong enthusiasm for VR as a novel technology offering a fun alternative to support cognitive engagement and leisure activities. Additionally, participants emphasized the social potential of VR and its ability to facilitate meaningful connection with distant family members, particularly grandchildren, through gaming and collaborative activities. They expressed excitement about experiences that transcend physical limitations, such as virtual travel, noting that immersive VR offers a compelling alternative for exploring new places with friends and family as travel becomes difficult with age. Participants also preferred interactive over passive experiences, highlighting a desire for activities that enabled active engagement within the VR environment. Although some participants initially found the VR applications challenging due to unfamiliarity, they emphasized the need for practice rather than additional training. Many expressed a willingness to adapt and highlighted the importance of home access to support continued use. These findings challenge ageist assumptions about older adults and technology, underscoring the need for inclusive VR design that enhances engagement and quality of life.

Abstract citation ID: igaf122.4312

### FACILITATING RESIDENT INTERACTION THROUGH ROBOT-DELIVERED TRIVIA IN ASSISTED LIVING SETTINGS

Shannon Power[1], Anne Adams[2], Lydia Burton[1], Kasey Smith[2], and Jenay Beer[1], *1. University of Georgia, Athens, Georgia, United States, 2. SimpleC LLC, Marietta, Georgia, United States*

With high dementia prevalence and few professional caregivers, socially assistive robots (SAR) offer a promising alternative to support social and cognitive engagement among older adults in residential care. This study included observation of SAR-facilitated trivia for older residents in assisted living settings. The SAR was a 4-ft humanoid Softbank Robotics Pepper robot with a chest-mounted tablet. Trivia interactions were designed to support cognitive engagement through curated content, timed sequences, and natural language paired with expressive animations. Each session included an introduction, trivia Q&A, discussion prompts, and closing. A total of 82 customized trivia items were created, with tailored speech (pitch, speed, emotion), 70+ matching gestures, expressions, jokes, laughter, and music. Seven observation sessions were conducted across two facilities. The naturalistic setting allowed residents (sample range: 2-16) and staff (sample range: 0-5) to join/leave freely. 289 observational segments were documented by six researchers via a standardized template. Segments were coded using the CREATE Model of Aging and Technology, across four non-exclusive categories: user, technology(robot), environment, and task(trivia). Observations revealed active engagement, prompting frequent interactions between Resident+Task (e.g., answer questions, freq=51), Resident+Resident (e.g., laughing, side conversation, freq=39), and Resident+Robot (e.g., asking robot questions, freq=28). Staff+Task interactions were least frequent (e.g., prompting, repeating questions, freq=16). Data suggest that the trivia tasks were engaging, the robot facilitated much of the social interaction, and the staff were able to monitor and supplement the interaction. Future design recommendations include AI-optimization of speech clarity, volume, and pacing to accommodate differences in resident cognitive abilities.

Abstract citation ID: igaf122.4313

### UTILIZING HUMAN-IN-THE-LOOP AI PREDICTIONS TO PRESCRIBE PERSONALIZED FALLS PREVENTION PROGRAMS

Mahederemariam Dagne, Nathan Green, Diane Murphy, and Patricia Heyn, *Marymount University, Arlington, Virginia, United States*

Falls are the leading cause of fatal and nonfatal injuries in older adults, with over 14 million falls reported annually (CDC). In response, the Administration for Community Living (ACL) launched seventeen Evidence-Based Falls Prevention Programs (EBFPPs) nationwide. From 2013 to 2024, demographic, pre-, and post-program data on 203,838 adult participants were collected. Given the robustness of this dataset, we believe AI could be used to evaluate EBFPPs, identify patterns to predict falls risk, and determine which programs work best for different individuals. This enables a more personalized, precise, and effective fall prevention strategy. Therefore, we analyzed ten key outcomes from the surveys, such as falls reduction, balance, perceived strength, and fear of falling (FoF). Changes in falls, strength, and balance were statistically significant ($p < 0.05$ in most pairwise tests) and showed the highest improvement in participants enrolled in FallScape (39.9%, N = 160), CAPABLE (36.2%, N = 71), Matter of Balance (34.38%, N = 41,515), and Stepping On (32.7%, N = 12,362). These results may be due to the programs' focus on combining behavioral and exercise training rather than relying on a single modality. However, SAIL and EnhanceFitness ranked highest in reducing FoF. Based on these findings, we developed an AI-based algorithm to identify key falls-related factors, such as health status, strength, and FoF, and provide customized prevention recommendations that incorporate participant-identified priorities. Rather than adopting a one-size-fits-all approach, our method emphasizes tailoring fall interventions through human-in-the-loop AI predictions. This personalization aims to better address multiple risk factors and their combined effects on falls prevention.

### SESSION 9070 (PAPER)

### LATE BREAKING: ARTIFICIAL INTELLIGENCE AND EMERGING TECHNOLOGIES III

Abstract citation ID: igaf122.4314

### ARTIFICIAL INTELLIGENCE USE AND VIEWS: KEY FINDINGS FROM THE NATIONAL POLL ON HEALTHY AGING

Erica Solway[1], Robin Brewer[1], J. Scott Roberts[2], Dianne Singer[1], Sydney Strunk[1], Nicholas Box[1], and Matthias Kirch[1], *1. University of Michigan, Ann Arbor, Michigan, United States, 2. University of Michigan School of Public Health, Ann Arbor, Michigan, United States*

The spread of artificial intelligence (AI) technologies offers opportunities to improve home safety, provide health information, and support aging in place, but it also raises concerns about risks to health and well-being. Little is known about older adults' use and views on AI related to healthy aging. In February 2025, the University of Michigan National Poll on Healthy Aging surveyed a diverse sample of 2,883 adults age 50 and older about their experiences with and views on AI technologies. Overall, 55% of adults age 50 and older said they had ever used AI technologies that one speaks or types to, with 14% using AI for health information. Many reported benefits of using AI-powered devices, including voice assistants and home security devices and systems, for aging in place. Overall, 35% of adults age 50 and older reported interest in using AI in their day-to-day lives. At the same time, 92% of adults age 50 and older agreed they want to know if the information they receive is from a person or from AI, and 81% wanted to learn more about the risks of AI. Nearly half (46%) had very little or no trust in AI-generated health information. This presentation will describe notable differences in AI use and perspectives by demographic subgroups and will conclude with a discussion of how these findings can be used for the development of programs to assist older adults in understanding the risks of AI and in safely and effectively using AI to support healthy aging.

Abstract citation ID: igaf122.4315

### DETECTING PRE-FRAILTY OR FRAILTY USING ACTIVITY MONITORING FROM WEARABLE SENSOR

Andrew Song[1], Kailin Xu[1], Kuan-Yuan Wang[2], Lingsong Kong[3], and Dae Hyun Kim[4], *1. Hebrew Seniorlife, Roslindale, Massachusetts, United States, 2. Hebrew SeniorLife, Roslindale, Massachusetts, United States, 3. University of Massachusetts, Amherst, Massachusetts, United States, 4. Hebrew SeniorLife, Boston, Massachusetts, United States*

Early identification of pre-frailty or frailty in community-dwelling older adults can enable targeted interventions, yet clinical assessments are time-consuming and rarely used in routine care. We evaluated whether activPAL, a thigh-worn accelerometer, can identify pre-frail-or-frail status in older adults. In a cross-sectional study, we enrolled 44 participants aged 63–97 years from independent living or assisted living facilities within Hebrew SeniorLife. They completed the Fried Frailty Phenotype (FFP) and Comprehensive Geriatric Assessment-based Frailty Index (CGA-FI) assessments, then wore activPAL for consecutive 10 days. We derived 38 features across activity volume, gait, sedentary behavior, and diurnal patterns from the activPAL data. Lasso and ridge regression, random forest, and a simple neural network were trained to classify robust versus pre-frail-or-frail against each frailty measure. Performance was evaluated using area under the ROC curve, balanced accuracy, and F1 score with nested cross validation. 36 participants were pre-frail-or-frail by FFP (≥1 point) and 28 by CGA-FI (≥0.15). The pre-frail-or-frail group showed less walking time, lower cadence, smaller activity amplitude, and lower variability in total sedentary time. Ridge regression performed better than other models for predicting pre-frail-or-frail by FFP (AUC, 0.81; balanced accuracy, 0.65; F1, 0.76) and by CGA-FI (AUC, 0.78; balanced accuracy, 0.71; F1, 0.79). Our findings demonstrate that thigh-worn activity monitoring can be useful in detecting pre-frail and frail status, highlighting its potential as a scalable, sensor-based screening tool to guide early preventive interventions in older adults. External validation in a larger, diverse sample is warranted.

Abstract citation ID: igaf122.4316

### ENSEMBLEAGE CLOCK: A RELIABLE, ROBUST AND CONVENIENT EPIGENETIC AGE PREDICTION SERVICE FOR PROMOTING HEALTHY AGING

Hayan Lee[1], Akshay Anand[2], Yash Agarwal[3], Tanisha Gupta[4], Jason Lin[5], Mirna Ghemrawi[6], and Glenn Gerhard[7], *1. Fox Chase Cancer Center, Philadelphia, Pennsylvania, United States, 2. University of California, Berkeley, California, United States, 3. University of California, Los Angeles, Los Angeles, California, United States, 4. Georgia Institute of Technology, Atlanta, Georgia, United States, 5. Brown University, Providence, Rhode Island, United States, 6. The Center for Forensic Science Research and Education, Horsham, Pennsylvania, United States, 7. Temple University Health System, Philadelphia, Pennsylvania, United States*

Age is a major risk factor for various diseases, such as cancer, cardiovascular conditions, and neurodegenerative diseases. However, chronological age, the simple number of years one has lived and an unmodifiable risk factor, does not capture individual health differences, prompting the development of methods to accurately estimate biological age, modifiable by healthy habits, instead of relying on chronological age. One of the major molecular approaches exploits DNA methylation (DNAm), an essential epigenetic modifier for regulating gene expression, cell differentiation, and aging. DNAm-based aging clocks have been developed to predict biological age, but the prediction is highly dependent on training data, including assay technologies. To address these clocks' high variance, we present EnsembleAge Clocks, leveraging previously developed DNAm clocks, harnessing the strengths of each methylation age clock, smoothing out individual variances, and providing a more robust estimation of biological age. We validated our EnsembleAge Clock models using DNA methylation data from nine organs in the GTEx dataset. Our EnsembleNaive clock model achieved the lowest median absolute error (MeAE) of 4.04 years in whole blood. The EnsembleLR model demonstrated the lowest MeAE of 6.35 years across multiple tissues, including breast, lung, muscle, ovary, prostate, testis, and colon. Our EnsembleAge clock models are also implemented as a web service (https://ensemble.epiclock.app), allowing users to conveniently upload their DNA methylation data and receive predictions of their biological age, empowering individuals to track their epigenetic age over time and promoting healthy aging with a beneficial lifestyle.

Abstract citation ID: igaf122.4317

### SMALL LANGUAGE MODELS FOR EARLY DETECTION OF ADRD: OPEN-SOURCE LANGUAGE MODELS IN CLINICAL SPEECH CLASSIFICATION

Venkatanand ram Addepalli[1], Praveen Rao[2], Erich Kummerfeld[3], Andrew Kiselica[4], and Knoo Lee[5], *1. Institute of Data Science and informatics, University of Missouri, Columbia, Missouri, United States, 2. University of Missouri, Columbia, Missouri, United States, 3. University of Minnesota - Twin Cities, Twin Cities, Minnesota, United States, 4. University of Georgia, Athens, Georgia, United States, 5. University of Missouri - Columbia, Columbia, Missouri, United States*

Large language models (LLMs) have shown promise in biomedical and clinical applications, including aging research, patient care, and translational geriatrics. However, their use is limited by costs, privacy risks, and HIPAA concerns. These challenges point to an alternative: Small Language Models (SLM; < 2 billion parameters) that run entirely on local hardware, reducing latency and dependence on external services. Recent open-source releases (e.g., DeepSeek R1) highlight the growing capability and efficiency of open-source models. These advances draw attention to SLMs, which are inherently efficient and easier to deploy in local clinical settings. This underscores the need to evaluate smaller, open-source language models on similar tasks. In this study, we evaluated four SLMs (DeepSeek Coder, GPT-2, Microsoft Phi-3, SmolLM2) on 398 transcripts from the Pitt Corpus, comprising dementia patients and control participants evaluated with the Cookie Theft picture description task. To our knowledge, this is the first on-premise deployment of an SLM directly on linguistic features of transcripts on those with ADRD, demonstrating comparable dementia classification accuracy to LLMs under HIPAA-compliant, low-latency conditions. Models were trained and tested on a subset of the Pitt corpus. The best-performing model (GPT-2) reached 78.3% accuracy with inference times under three seconds. This balance of accuracy with efficiency demonstrates the feasibility of locally-hosted SLMs as privacy-preserving tools for dementia detection. Our findings suggest that SLMs may offer practical pathways for deploying AI in aging research and clinical care. Future work will assess real-time speech analysis with integration of multimodal data for comprehensive geriatric assessment.

Abstract citation ID: igaf122.4318

### TECHNOLOGY ADOPTION IN LATER LIFE: CROSS-CULTURAL INSIGHTS FROM HONG KONG, CZECHIA, AND GERMANY

Nia Yuqing Wang[1], Yiwen Wu[1], Josefína Králová[2], Merle Scheipers[3], Jaroslava Hasmanová Marhánková[2], Klaus Rothermund[3], and Helene Fung[1], *1. The Chinese University of Hong Kong, Hong Kong, China (People's Republic), 2. Charles University, Prague, Hlavni mesto Praha, Czech Republic, 3. Friedrich Schiller University Jena, Jena, Thuringen, Germany*

Background: Despite technology's potential to enhance late-life well-being, older adults exhibit low adoption rates. While the Unified Theory of Acceptance and Use of Technology (UTAUT) identifies predictors of technology adoption among older adults, cross-cultural comparisons remain scarce. This study aims to fill in this gap. Methods: Semi-structured interviews with 78 adults (aged 70–95 years) across Hong Kong (n = 30),

Germany (n = 29), and Czech Republic (n = 19) were conducted. Thematic analysis was done. Independent coders also rated attitudes, intention and actual usage. Results: Eight themes emerged in explaining technology adoption among older adults: functionality, readiness to adopt, social influence, effort, support, cost, psychological factors, and physical limitations. While Germans and Czechs emphasized readiness to adopt technology, Hong Kongese prioritized functionality and social influence. Smartphones were generally viewed positively across all cultures, with Czechs showing the most favorable attitudes, Germans the least, and Hong Kongese in between. In contrast, technologies that support independent living had low usage across all cultures. Despite this, Czechs showed the highest willingness to adopt, while Germans and Hong Kongese were more hesitant—Germans being the most negative. Finally, common motivators and barriers emerged across all cultures: the primary motivator for use is functionality (e.g., "It's useful"), while the primary barrier to use is low readiness (e.g., "I don't want to"). Discussion and Conclusion: Findings suggest that similar factors motivate technology adaption among older adults across cultures, but cultures shape their priority.

### SESSION 9075 (PAPER)

### LATE BREAKING: CLIMATE AND AGING

Abstract citation ID: igaf122.4319

### AGE-FRIENDLY AND CLIMATE READY? THE IMPORTANCE OF CLIMATE CHANGE TO AGE-FRIENDLY TRANSFORMATIONS

Erica Husser[1], Erin Kitt-Lewis[1], Grace Behr[2], Michael McShane[1], and Marie Boltz[1], *1. The Pennsylvania State University, University Park, Pennsylvania, United States, 2. Maastricht University, Maastricht, Limburg, Netherlands*

Significant investments in Age-Friendly initiatives demonstrate a growing social commitment to reducing ageism and enhancing quality of life for older adults. Exposure to the health impacts of climate change place older adults living alone at high risk for suffering and mortality. The purpose of this qualitative study was to explore perceptions of the natural environment (importance and concern) and preparation for extreme weather events across the age-spectrum. Researchers in a college of nursing partnered with a mobile clinic and attended eight events in five rural communities. We conducted 41 in-person face-to-face semi-structured interviews with randomly selected participants. Iterative thematic analysis resulted in six major themes: Importance of the Natural Environment, Concerns about the Natural Environment, Experiences with Extreme Weather, Preparing for Weather Emergencies, Sharing and Acts of Care, and Causes and Control. Findings illustrate 1) continuity in the value placed on the natural environment across the age-spectrum, 2) a common perception that "others don't care enough", and 3) a desire to share knowledge. Findings can be applied to Age-Friendly initiatives to promote shared benefits of healthy environments, address the misperception that no one cares to help normalize conversations about climate change and preparedness, and provide opportunities for age-integration through program activities, civic events, and the built environment. Integrating climate change into Age-Friendly movements is necessary to keep older adults safe, healthy, and thriving, but also to promote age-integration as a powerful mechanism for building strong communities capable of caring for one another during climate-related disasters.

Abstract citation ID: igaf122.4320

### AIR POLLUTION AND POST-DISCHARGE RECOVERY AMONG OLDER ADULTS HOSPITALIZED FOR HEART FAILURE

Tong Wen, Jingwen Hu, Michelle Shardell, Rozalina McCoy, Shuo Chen, Kathleen Ryan, Jason Falvey, and Chixiang Chen, *University of Maryland School of Medicine, Baltimore, Maryland, United States*

While exposure to fine particulate matter (PM2.5) has been associated with an increased risk of hospitalization for heart failure among older adults, its impact on post-discharge recovery in older adults already hospitalized for heart failure remains unclear. We evaluated associations between PM2.5 exposure during the month of hospital admission and days spent at home (DAH) as well as mortality in a nationwide representative sample of US adults aged 65 and older. Data from 66 854 Medicare Fee-for-service beneficiaries with heart failure hospitalization (2017-2019) were linked with validated, model-derived mean PM2.5 concentrations at Zip Code Tabulation Areas level. Post-discharge six-month DAH was defined as days alive minus days spent in inpatient hospitals, hospital observation units, nursing facilities, or emergency departments. All-cause mortality was assessed as time from hospital discharge to death within six-month. Quantile regression and Cox proportional regression models, adjusted for covariates, were used to quantify associations. Compared with those exposed to the lowest PM2.5 category (< =5.9 μg/m3), those in the highest quartile PM2.5 level (>8.6 μg/m3) was associated with 4.4 fewer days (95% CI: 0.9-8.0; P = 0.014) at the 20th percentile of DAH. Exposure to the highest quartile PM2.5 level was also associated with increased six-month all-cause mortality, as compared to the lowest PM2.5 category (hazard ratio = 1.066 (95%CI: 1.022-1.113, P = 0.003). These findings suggest that air pollution may negatively impact recovery more strongly at the lower tail of recovery than at the median or higher tail, highlighting the need for targeted intervention strategies to protect the most vulnerable patients.

Abstract citation ID: igaf122.4321

### ENVIRONMENTAL STRESSORS AND SLEEP HEALTH AMONG LOW-INCOME OLDER ADULTS: A MIXED-METHODS STUDY

Shuxian Hua[1], Youngmin Cho[2], Sofia Liu[2], Hannah Fu[2], Michelle Liu[2], Claire Wang[2], Russell Calderon[3], and Junxin Li[2], *1. University of North Carolina at Chapel Hill, Chapel Hill, North Carolina, United States, 2. Johns Hopkins University, Baltimore, Maryland, United States, 3. Johns Hopkins School of Medicine, Baltimore, Maryland, United States*

Environmental factors are increasingly recognized as critical determinants of sleep health, particularly among older adults in low-income communities. This convergent mixed-methods study aimed to: (1) explore sleep environment stressors (SES) reported by community-dwelling, low-income older adults; and (2) compare subjective sleep health between those affected by SES and those who were not. Twenty-four older adults participated. SES were measured with Assessment of Sleep Environment (ASE), a 4-point Likert scale from "Strongly Agree" to "Strongly Disagree". Participants were classified as Environmentally Impacted Sleepers (EIS, n = 14) if they endorsed any AES items, or as Environmentally Unimpacted Sleepers (EUS, n = 10) if they did not. Sleep health was assessed with the Pittsburgh Sleep Quality Index (PSQI), with group comparisons analyzed using Welch's t-tests and effect sizes (Cohen's d with Hedges'

correction). Semi-structured interviews further explored perceived environmental stressors and sleep experiences, which were inductively coded and thematically analyzed. The most frequently self-reported stressors were: "too soft mattress" (n = 7), "uncomfortable smell" (n = 6), "too noisy" (n = 5), "too humid" (n = 5), "too cool" (n = 5), "too much light" (n = 5). Qualitative findings revealed that outdoor noise and thermal conditions as major stressors, and participants noted that adjusting their environment (e.g., lighting, sound, or fans) improved sleep; most participants described a cool, dimly lit room with some background noise as ideal. EIS group had worse sleep health (p<.001, d = 1.87). Both groups perceived poor sleep quality, frequent night awakenings, and medical aid use; yet EIS group more commonly described challenges returning to sleep. Addressing environmental stressors is essential for healthy aging sleep.

Abstract citation ID: igaf122.4322

### EXPOSURE TO HEATWAVES AND HIGH DEPRESSIVE SYMPTOMS AMONG OLDER ADULTS IN MEXICO

Brian Downer[1], Joseph Saenz[2], Rebeca Wong[3], Emma Aguila[4], and Jorge Peniche[4], *1. University of Texas Medical Branch, Galveston, Texas, United States, 2. Arizona State University, Phoenix, Arizona, United States, 3. University of Texas Health San Antonio, San Antonio, Texas, United States, 4. University of Southern California, Los Angeles, California, United States*

Heatwaves can increase the risk of depressive symptoms among older adults. However, it is unknown whether this association is modified by the structural and physical characteristics of a person's home. We used data from 8,952 participants in the Mexican Health and Aging Study who were aged 60 and older in 2018. Participants were linked to municipality-level data for daily maximum temperatures from May 1st to August 31st of each year from 1981-2018. A heatwave was defined as > 2 consecutive days with a maximum daily temperature >95th percentile of municipality-specific temperatures from 1981-2017. We created a variable for the total number of heatwaves (0, 1, >2) in summer 2018. Latent class analysis was used to define home types based on the materials for the roof, walls, floor, and the number of levels and rooms. Logistic regression models that adjusted for demographic characteristics were used. Participants who experienced >2 heatwaves had 1.15 (95% CI = 1.01-1.31) higher odds for high depressive symptoms compared to those who experienced no heatwaves. The interaction term was not significant, but this association was significant for participants living in a single-story home with mixed floor materials, concrete roof, and some rooms (OR = 1.27, 95% CI = 1.05-1.55), but not in a multi-story home with wood floors, concreate roof, and many rooms (OR = 1.16, 95% CI = 0.91-1.48) or a single-story home with concrete floors, laminate roof, and a few rooms (OR = 0.92, 95% CI = 0.71-1.19). Heatwaves are a risk factor for high depressive symptoms, the effects of which may differ based on a home's characteristics.

Abstract citation ID: igaf122.4323

### URBAN SHRINKAGE AND DEPRESSIVE SYMPTOMS AMONG OLDER ADULTS IN CHINA: RURAL BURDEN AND THRESHOLD EFFECTS

Xinyue Wen[1], and Fernando DePaolis[2], *1. Middlebury College, Monterey, California, United States, 2. Middlebury Institute of International Studies at Monterey, Monterey, California, United States*

Urban shrinkage is rising globally, yet its mental-health implications for older adults—and how they differ by urban–rural context—remain unclear. Using four waves of the China Health and Retirement Longitudinal Study (CHARLS; unbalanced panel of 19,126 adults; 38,366 person-waves) linked to city-level population change, we examined depressive symptoms in later life. City-level data were defined at the prefecture level, encompassing both urban districts and rural counties; "urban/rural" denotes respondents' residence. Individual fixed-effects models with time-varying covariates (age, gender, income, education, marital status) and wave controls revealed a robust gradient: shrinking rural > non-shrinking rural > shrinking urban > non-shrinking urban, indicating that the depressive-symptom burden is concentrated in shrinking rural contexts rather than uniformly across "shrinking cities." To probe nonlinearity, we estimated segmented (piecewise linear) regressions of depressive symptoms on the continuous population-change rate, overall and stratified by residence, with the same covariate adjustment. Breakpoint analysis identified an adjusted overall threshold at − 12.0% population change (95% CI: −15.7%, −8.3%), beyond which depressive symptoms rose sharply. In rural areas, the threshold was −10.7% (95% CI: −13.6%, −7.8%); in urban areas, the estimated breakpoint was −5.7% (95% CI: −18.1%, 6.6%) and not statistically distinct. Findings highlight urban shrinkage as a place-based determinant of mental-health inequality in aging, with severe demographic decline disproportionately elevating depressive symptoms among rural older adults. As shrinkage becomes a global reality, strategies for mental health and aging should move beyond growth-oriented models toward targeted, place-sensitive interventions that bolster services, social support, and infrastructure in shrinking rural prefectures.

Abstract citation ID: igaf122.4324

### WHEN DISASTER STRIKES, AREA AGENCIES ON AGING HELP OLDER ADULTS PREPARE AND RESPOND

Traci Wilson, *USAging, Washington, District of Columbia, United States*

Global climate change has increased the frequency and intensity of natural disasters, which can have a disproportionate impact on older adults. According to data from a new module in the 2025 National Survey of Area Agencies on Aging (72% response rate) 65% of AAAs reported that a community within their service area has experienced a significant emergency or disaster within the past five years. This presentation will share nationally-representative data about the ways in which AAAs support older adults to prepare for emergencies, help the community prepare to respond to consumers, and ensure consumer safety. AAAs support individual emergency preparedness through providing educational resources (67%), addressing emergency preparedness as a standard part of the care planning process (45%), and maintaining a prioritized consumer registry (44%). AAAs also helps the community prepare to respond to consumer needs during disasters: 63% are involved in emergency planning collaboratives, 47% require providers to have emergency response plans in place, 45% are in regular two-way communication with EMS. The data will be supported by case examples describing how AAA emergency preparedness and response plays out before, during and after a natural disaster, including lessons learned.

### SESSION 9080 (PAPER)

### LATE BREAKING: CLINICAL PRACTICE INNOVATIONS I

Abstract citation ID: igaf122.4325

### ADAPTIVE RIDING: AN INTERVENTION TO IMPROVE FALLS IN INDIVIDUALS WITH PARKINSON'S DISEASE

Betsy Kemeny, Whitney Angelini, Chase Decker, Emma Lemire, and Alyssa Snyder, *Slippery Rock University, Slippery Rock, Pennsylvania, United States*

More than 60% of individuals living with Parkinson's disease fall each year and experience postural instability, gait changes, and fear of falling. Due to both the human-animal bond and activation of the neuromuscular system, equine-assisted services (EAS) are an innovative approach for falls prevention. Both simulated horseback movement and groundwork have shown improved balance in individuals with Parkinson's disease. No known research focuses on mounted EAS for falls prevention. This pilot waitlist control study measured the effectiveness of six sessions of horseback riding on fall prevention in 12 participants over 60 with Parkinson's disease using: 1) Tinetti Balance and Gait, 2) Posture Screen App, 3) Adverse Events Questionnaire, 4) Anchored Rating Scale, 5) Activities-Specific Balance Confidence Scale. Findings suggest improvements, trending toward significance, in these specific variables: 1) confidence walking on different surfaces (e.g. ramp, parking lot, mall ($p$=.06); 2) overall Tinetti score ($\bar{x}$=22.6, to $\bar{x}$=24.2), 3) gait [path($\bar{x}$=1.44, to $\bar{x}$ =1.78), trunk ($\bar{x}$=1.11 to $\bar{x}$=1.44), walking time ($\bar{x}$=.89 to $\bar{x}$=1)], 4) balance [chair stand ($\bar{x}$=1.56,to $\bar{x}$=1.67), nudged($\bar{x}$=1.67, to $\bar{x}$=1.89), step continuity($\bar{x}$=.67 to $\bar{x}$=.78), and steadiness($\bar{x}$=.67 to $\bar{x}$=1)]. When comparing first to last session, there was significant improvement in maintaining ankle dorsiflexion in saddle ($p$<.05) and body control ($p$<.05). Pre to post posture screen comparison suggests improved alignment of the shoulders, hips, and knees. While further research on larger samples with randomized controls are needed, this research demonstrated improvements and feasibility for mounted EAS for older adults living with Parkinson's disease.

Abstract citation ID: igaf122.4326

### CLAIMS-BASED FRAILTY AMONG NEWLY ADMITTED LONG-STAY NURSING HOME RESIDENTS

Kailin Xu[1], Chan Mi Park[2], Brianne Olivieri-Mui[3], Sarah Berry[2], Dae Hyun Kim[1], and Sandra Shi[1], *1. Hebrew SeniorLife, Boston, Massachusetts, United States, 2. Hebrew SeniorLife & Harvard Medical school, Boston, Massachusetts, United States, 3. Northeastern University, Boston, Massachusetts, United States*

Admission to a long-stay nursing home (LNH, defined as ≥ 30 accumulative days within an episode) is a sentinel event for older adults, often signaling limited life expectancy or increased risk of adverse health outcomes. However, the prevalence of frailty and its prognostic value at LNH admission remain poorly characterized. In a 7% random sample of fee-for-service Medicare beneficiaries from 2015 to 2019, we identified 66,407 new LNH residents. Frailty was measured using the claims-based frailty index (CFI) on claims in the 12 months prior to LNH admission and categorized as non-frail (CFI < 0.25), mild frailty (0.25-0.34), moderate frailty (0.35-0.44) or severe frailty (≥0.45). We examined the association of frailty category with mortality, hospitalization, ED visits, injurious falls, and pressure ulcers over one-year follow-up, using Cox proportional hazards or Fine–Gray competing-risk models adjusted for age, sex, and dual eligibility. Frailty was present in 77.1% at LNH admission, with 46.7% mild frailty, 25.6% moderate frailty, and 4.8% severe frailty. One-year risk of mortality increased with frailty: 20.4% in non-frailty, 26.3% in mild frailty, 30.6% in moderate frailty, and 33.5% in severe frailty. Differences in mortality persisted when stratified by age and sex. Compared with non-frail beneficiaries, those with severe frailty had higher rates of mortality (HR [95% CI], 2.0 [1.8, 2.1]), hospitalization (2.8 [2.6–2.9]), ED visits (1.8 [1.6–1.9]), injurious falls (1.2 [0.9–1.5), and pressure ulcers (3.9 [3.4–4.4]). These results highlight that a claims-based frailty assessment can be useful for prognostication and risk stratification among newly admitted LNH residents.

Abstract citation ID: igaf122.4327

### CHOOSE HOME: EFFECTIVENESS AND ACCEPTABILITY OF A NOVEL GERIATRIC HOME-BASED PROGRAM

Caroline Madrigal[1], Rebecca Howe[2], James Rudolph[3], Meghan Makela[4], Alexandra Nothern[1], Wendy Abrams[1], and Jane Driver[5], *1. VA Boston Healthcare System, Boston, Massachusetts, United States, 2. VA Providence Healthcare System, Providence, Rhode Island, United States, 3. VA Providence, Providence, Rhode Island, United States, 4. VA Boston Healthcare System, West Roxbury, Massachusetts, United States, 5. VA Boston Healthcare System, Brockton, Massachusetts, United States*

Older Veterans prefer to 'age in place' in their homes/communities. However, complex, chronic medical conditions and limited resources put Veterans at risk for avoidable acute care use and long-term institutionalization. VA Boston Healthcare System developed Choose Home, an interdisciplinary, home-based program that provides intensive, short-term support via telephone and in-home medical and psychosocial case management. The program is designed to stabilize high-risk Veterans in their homes, promoting independence and connecting them to needed services. To evaluate the program's impact from Veterans' and their caregivers' perspectives, we collected anonymous surveys at program discharge (n = 68) and conducted in-depth, semi-structured interviews with 12 Veterans (including 8 caregivers). 84% (n = 57) of Veterans/caregivers felt they were doing better at home after program participation, 90% (n = 61) felt better able to manage their care independently, and 88% (n = 60) felt satisfied with the program. We used thematic analysis to identify key programmatic outcomes from Veterans' viewpoints, including: 1) independence/aging in place, via providing supports that allow Veterans to maintain autonomy and dignity; (2) holistic care during health crises, by offering coordinated support to help Veterans and families navigate overwhelming medical events; (3) caregiver empowerment, through education, respite, and practical assistance to mitigate burnout. Veterans' perceptions of how Choose Home impacted their trajectory provide important insights into the program's mechanisms of success which are essential for sustainment and scalability. Findings reflect the perceived effectiveness and acceptability of the program and highlight the value of integrated, community-based care models in enhancing the well-being and autonomy of Veterans and their caregivers.

Abstract citation ID: igaf122.4328

### IDENTIFYING PERIMENOPAUSE PHENOTYPES AMONG WOMEN LIVING WITH AND WITHOUT HIV: A LATENT CLASS ANALYSIS

Maeve McNamara, Lauren Collins, C. Christina Mehta, Anna Rubtsova, Camille Vaughan, and Alexis Bender, *Emory University, Atlanta, Georgia, United States*

There is increasing appreciation for the multidimensional impact that perimenopause symptoms have on women in mid-life. We sought to identify distinct classes of menopause symptomatology and whether these classes differ by HIV status and sociodemographic and clinical characteristics. We conducted a latent class analysis (LCA) of women with and without HIV in late perimenopause enrolled in the Women's Interagency HIV Study who attended at least two study visits between 2007-2019. Late perimenopause was defined using STRAW +10 criteria. We used LCA to identify unique groups of participants based on the frequency of nine perimenopause symptoms (hot flashes, cold sweats, night sweats, musculoskeletal stiffness/soreness, irritability, nervousness, and three types of sleep disturbances). We assessed differences in class membership by HIV serostatus; sociodemographic and clinical characteristics, including non-AIDS comorbidity (NACM) burden, a measure of multimorbidity. Among 818 women (590 with HIV, 228 without HIV) aged 35-58 years (Median age 48), 67% identified as Black, and 9.3% reported hormonal birth control use. Age and symptom frequency did not differ by HIV serostatus. LCA identified five symptom classes: (1) sore, tired, and on edge, (2) hot, sore, and tired, (3) tired, (4) hot, sore, and irritable, and (5) overall low symptoms. Class membership significantly differed by depressive symptoms, age, hormonal birth control use, NACM burden, alcohol use, and income, but not by HIV serostatus, race, tobacco use, or BMI. We identified heterogeneity in perimenopause symptomology regardless of HIV. These data may inform provider education to improve patient validation and agency in seeking perimenopausal care.

Abstract citation ID: igaf122.4329

### OLDER ADULTS' VIEWS OF SMARTPHONE-BASED MONITORING FOR DEMENTIA RISK

Katherine Hackett[1], Heather Wurtz[2], Frandys Berroa[1], Hillary Ramos Espinoza[1], Maria Loizos[1], Carolyn Zhu[1], Alex Federman[1], and Mary Sano[1], *1. Icahn School of Medicine at Mount Sinai, New York, New York, United States, 2. University of Southern California, Los Angeles, California, United States*

Clinical assessment of function in dementia risk evaluations is challenging and may benefit from passive smartphone monitoring, which enables low-burden, continuous tracking of everyday behaviors outside the clinic. However, acceptability of passive monitoring in older adults is required for such technologies to be useful. To assess acceptability, 17 cognitively normal older adults (mean age=76.9, SD = 8.7; mean education=15.8, SD = 3.4; 59% White, 41% Hispanic) from the Mount Sinai Alzheimer's Disease Research Center completed a 1-hour semi-structured interview in English or Spanish assessing attitudes toward smartphone monitoring, followed by a 5-point acceptability rating (likelihood of future study participation). Our interview guide drew from the Technology Acceptance Model and preliminary passive smartphone studies to explore a priori themes. Transcripts were analyzed using a hybrid inductive-deductive approach and content analysis. Emergent themes included perceived utility, privacy/ethical concerns, personal characteristics, logistical considerations, and overall comfort level. Key barriers to acceptability included privacy concerns (e.g., unease with monitoring, risk of data breach) which varied by data type (GPS versus call/text meta-data). Some participants questioned the clinical utility and identified potential confounding variables. Factors contributing to acceptability included recognized scope (i.e., perceived value in tracking behaviors not routinely assessed in clinic), altruistic attitudes toward research, clear understanding of privacy protections, and interest in early detection of dementia. Post-interview, 76% were likely to participate in a future digital monitoring study and 94% agreed to recruitment contact. These findings offer insights to enhance acceptability in future clinical implementation, including transparent review of privacy safeguards, existing evidence, and clinical integration logistics.

Abstract citation ID: igaf122.4330

### OLDER MALES EXHIBIT GREATER DUAL-TASK GAIT VARIABILITY AND POSTURAL AMPLITUDE THAN OLDER FEMALES

Abeera Maham[1], Katherine Hsieh[2], Ashwini Pandey[1], and Deborah Jehu[1], *1. Augusta University, Augusta, Georgia, United States, 2. Georgia State University, Atlanta, Georgia, United States*

Greater dual-task cost (poorer cognitive and/or motor performance) heightens fall risk in older adults. There may be sex differences in dual-task cost that may inform tailored fall prevention strategies, but further research is needed. The purpose of this study was to examine whether sex differences influence dual-task cost during posture and gait in older males and females with poor mobility. A cross-sectional study was conducted in 85 older adults with self-reported poor mobility with no significant cognitive impairment (aged ≥65 years, 50 females; Montreal Cognitive Assessment scores>18). Using APDM wearable inertial sensors, participants stood with their feet apart and eyes open while completing no cognitive task, and while counting backwards by 3's. Participants also walked at a comfortable walking speed while completing no cognitive task, and while naming words starting with "F", "A", or "S". Two gait (step time coefficient of variability (CV), stride length CV, gait speed, double-support time, elevation CV) and postural (velocity, root mean square error (RMS), jerk, frequency) trials were completed. Males exhibited a greater step time CV during dual-task gait (F = 5.23, p=.009, males: 68.6±89.4%; females: 26.6±59.5%) and RMS during dual-task posture (F = 1.33, p=.009, males: 2884.5±1701.9m/s2; females: 2020.39±1458.19m/s2) than females in analyses of covariance, controlling for age and height. These findings suggest that greater variability in step time during gait and amplitude of postural sway in males relative to females serve as sensitive markers of dual-task cost in older adults. These results improve understanding of sex-specific differences in dual-task performance, guiding targeted interventions to enhance mobility.

### SESSION 9085 (PAPER)

### LATE BREAKING: CLINICAL PRACTICE INNOVATIONS II

Abstract citation ID: igaf122.4331

### CONTINUATION OF GLP-1 RECEPTOR AGONISTS DURING POST-ACUTE SKILLED NURSING FACILITY STAYS AND 30-DAY OUTCOMES

Chan Mi Park[1], Eunji G. Kim[2], Lan Luo[2], Xiecheng Chen[3], Dae Hyun Kim[4], and Ellen McCarthy[4], *1. Hebrew SeniorLife, Harvard Medical school, Boston, Massachusetts, United States, 2. Marcus Institute for Aging Research, Boston, Massachusetts, United States, 3. Hebrew Rehabilitation Center, Roslindale, Massachusetts, United States, 4. Hebrew SeniorLife, Boston, Massachusetts, United States*

In Medicare Part A– skilled-nursing facility (SNF) care—where facilities receive a predetermined, fixed amount per day —continuity of GLP-1 receptor agonists (GLP-1RAs) is unknown. Using the Long-Term Care Data Cooperative, a national provider-led EHR data linked to Medicare claims (>2,500 SNFs), we assembled a retrospective cohort of 7,037 older adults who received a GLP-1RA for ≥90 days before SNF admission. Exposure was percent days covered during the SNF stay: discontinuers (0%), partial continuers (>0–< 90%), and complete continuers (≥90%). Propensity scores with overlap weighting balanced demographics, frailty, primary diagnosis of hospitalization, healthcare utilization, facility ownership, glucose and kidney functions, cognition/ADLs, BMI, chronic conditions, and concomitant medications across three exposures (SMD < 0.10). After weighting, sample sizes were: discontinuers n = 612.6, partial n = 615.2, complete n = 612.6. Weighted baseline characteristics were similar: mean age 74.8 years; 38% male; race/ethnicity 79% non-Hispanic White; diabetes treatment profile ~44% insulin, ~33–34% metformin, and ~37–38% other second-line therapy at baseline. The primary endpoint was a 30-day composite of death or cardiovascular hospitalization (heart failure, acute myocardial infarction, stroke); the safety endpoint was 30-day hospitalization for diabetic ketoacidosis or hypo/hyperglycemia. 30-day cumulative incidence for the composite was 10.66% (discontinuers) vs 8.26% (partial) vs 8.59% (complete). The hazard ratio for the composite was 0.77 (95% CI, 0.63–0.93) for partial and 0.80 (95% CI, 0.63–1.00) for complete compared to the discontinuers. Safety events were lower with continuation (1.19% vs 0.69% vs 0.63%). GLP-1RA continuation—partial or complete—was associated with lower 30-day mortality/CV risk than discontinuation, arguing against stopping during short SNF stays.

Abstract citation ID: igaf122.4332

### EFFECTIVENESS OF A DIGITAL FALLS PREVENTION (KOKU) TO IMPROVE FUNCTION AND REDUCE FALLS RISK IN OLDER ADULTS.

Emma Stanmore[1], Amelia Parchment[2], Bolanle Odebiyi[2], Peter Bower[2], Chloe French[2], Chunhu Shi[2], Dawn Skelton[3], and Chris Todd[2], *1. University of Manchester, Stoke-on-Trent, England, United Kingdom, 2. University of Manchester, Manchester, England, United Kingdom, 3. Glasgow Caledonian University, Glasgow, Scotland, United Kingdom*

Functional decline and falls are common, challenging problems for older adults. Access and uptake to evidence-based exercise programmes are often sub-optimal. This study investigated the effectiveness of the KOKU digital strength and balance program for improving physical function and reducing falls risk among community-dwelling older adults. This two-arm, parallel group randomized controlled trial with an intention-to-treat data analysis enrolled eligible community-dwelling older individuals adults (60 years and older). Participants were randomized to either the intervention group comprising the digital strength and balance program (KOKU) alongside standard care (strength and balance exercise advice and falls prevention leaflet) or to the control group, receiving standard care only. The primary outcome measure was balance function (Berg Balance Score). Secondary trial outcomes included: lower limb strength; healthcare utilisation and health-related quality of life; concerns about falling; physical activity; falls risk, pain, mood, fatigue, self-reported falls, acceptability and usability of the KOKU program. 202 participants were recruited with a mean (SD) age of 76 (8.5) years, majority women (73%). At 3-month follow-up,181 participants (90%) had completed the trial. There was a statistically significant Berg Balance Score (mean difference 6.35 at 12-weeks, 95% CI, 4.48, 8.22), in favour of KOKU. Participants receiving the KOKU intervention had improved lower-limb strength as measured by the 5X Sit to Stand Chair test at 12-weeks compared to participants in the control group (mean difference: -6.85; 95% CI: -10.47, -3.24). The KOKU digital program could be considered as an effective self-management option to improve functional outcomes for older adults.

Abstract citation ID: igaf122.4333

### EXPLORING COMPLEX DISCOURSE TO INDEX EXECUTIVE FUNCTIONING IN OLDER ADULTS WITH COGNITIVE IMPAIRMENT

Eliza Baby[1], Lizzy Lydon[2], Natalia Rzepa[3], Sara Czaja[4], Walter Boot[4], Neil Charness[4], Wendy Rogers[5], and Raksha Mudar[6], *1. University of Illinois at Urbana-Champaign, Champaign, Illinois, United States, 2. Miami University, Oxford, Ohio, United States, 3. University of Illinois Urbana-Champaign, Urbana-Champaign, Illinois, United States, 4. Weill Cornell Medicine, New York, New York, United States, 5. University of Illinois Urbana-Champaign, Champaign, Illinois, United States, 6. University of Illinois Urbana-Champaign, University of Illinois Urbana-Champaign, Illinois, United States*

Discourse production engages multiple cognitive domains including executive functions and is known to be disrupted in individuals with cognitive impairments. Studying discourse reveals how cognitive impairments affect daily functioning by highlighting challenges in communication. We evaluated utility of a complex discourse production task in characterizing differences across older adults (n = 90) with mild cognitive impairment (MCI), traumatic brain injury (TBI), post-stroke cognitive impairment (PSCI), multiple etiology cognitive impairment, and cognitively normal older adult controls as part of the Everyday Needs Assessment for Cognitive Tasks (ENACT) study. Using a mixed-methods, cross-sectional design (n = 18 per group; mean age=71.81 ± 3.36 years) participants completed a discourse production task requiring executive processes (e.g., abstraction, planning) along with standard cognitive assessments. Discourse was analyzed using manual and automated methods. Manual coding identified critical elements (travel, activity, packing, and stay) and elaborations within categories. Automated analysis via CLAN software extracted linguistic metrics such as mean length of utterance (MLU) and semantic density. Group differences were analyzed using ANCOVA with age as a covariate. Significant group differences ($p < 0.05$), controlling for multiple comparisons, emerged across several discourse measures. The MCI and multiple etiology groups produced fewer critical elements compared to controls, whereas all other clinical groups showed reduced elaborations and semantic density. Additionally, participants in the TBI and PSCI groups had shorter utterances reflected in the lower MLU scores. Findings suggest that the discourse task distinguishes group-level differences and offers clinically relevant insights to guide assessment of functional challenges and inform targeted cognitive rehabilitation strategies including technology support.

Abstract citation ID: igaf122.4334

### INTEGRATED DIGIT-IN-NOISE TEST: A RAPID SCREENING TOOL FOR HEARING AND COGNITIVE FUNCTION

Lena Wong, June Tung, and Shangqiguo Wang, *The University of Hong Kong, Hong Kong, China (People's Republic)*

A rapid and easy-to-administer screening tool is essential for community-based detection of hearing loss and cognitive decline. The Integrated Digit-in-Noise Test (iDIN) extends the traditional Digit-in-Noise Test (DIN) by incorporating 2- to 5-digit sequences, as the only test to simultaneous assess and differentiate hearing and cognition. Speech Reception Thresholds (SRTs) are measured as the signal-to-noise ratio (SNR) at which 50% of digits are correctly identified. Specifically, 3-digit SRTs are used for hearing screening, while the difference between backward and forward 3-digit SRTs (SRT3b-3) serves as an indicator of cognitive function. In this study, 601 community-dwelling participants with potential but undiagnosed hearing loss were recruited (mean age 76.0 ± 8.8 years; education 6.4 ± 4.4 years; MoCA 21.5 ± 6.4). In terms of hearing screening, the average 3-digit SRT was -4.3 ± 7.0 dB SNR, with a cutoff of -7.7 dB SNR for hearing loss detection (35 dB HL in better ear) (sensitivity 0.85, specificity 0.73). For cognitive screening, the mean SRT3b-3 was 5.03 ± 7.32 dB SNR. Using MoCA thresholds of 21/22 for MCI, the optimal SRT3b-3 cutoff was 3.3 dB (sensitivity 0.74, specificity 0.76); for dementia (MoCA 15), the cutoff was 5.5 dB (sensitivity 0.83, specificity 0.77). No significant correlation was found between SRT3b-3 and better ear hearing levels, indicating that iDIN can effectively be used for cognitive screening in older adults with hearing impairment. In conclusion, iDIN shows promise as a quick, dual-purpose screening tool for early detection of hearing and cognitive issues in community settings.

Abstract citation ID: igaf122.4335

### THRIVING BETWEEN WORK AND CARE IN HONG KONG: ASSOCIATIONS OF WORK-FAMILY ENRICHMENT WITH DEPRESSION

Bobo Lau[1], Crystal Ng[1], Crystal Ying Chan[2], Steve Fu-Fai Fong[1], Florence Fong[3], Doris S. F. Yu[2], and Rebecca Choy-Yung[4], *1. Hong Kong Shue Yan University, Hong Kong, China (People's Republic), 2. The Chinese University of Hong Kong, Hong Kong, China (People's Republic), 3. Lingnan University, Hong Kong, China (People's Republic), 4. Golden Age Foundation, Hong Kong, China (People's Republic)*

Six out of ten American family caregivers are employed. In Hong Kong, up to one-third of the over 1 million family caregivers for older adults and adults with disabilities are in full-time employment. Work-family enrichment postulates that people gain happiness, knowledge, and tangible resources from one role (e.g., caregiving) and may be transferred to benefit another role (e.g., work), challenging the notion that roles compete for resources and are often conflictual. Between May and July 2025, this online survey collected data from 353 Hong Kong Chinese adults providing care to an adult relative and working full- or part-time (72.0% female; mean age = 46.1 (SD = 11.4)). It explored the impact of work-family enrichment domains on caregiving burden, gains and depression. Based on the Patient-Health Questionnaire-9, 21.8% and 14.7% of the sample showed moderate and severe depressive symptoms respectively. Path analysis tested the mediating roles of burden and gains between six enrichment domains and depression. Two indirect effects were significant: (i) work-to-family development enhances gain and suppresses depression (95% C.I. = -0.78 to -0.17); (ii) transfer of positive mood from family to work reduces burden and depression (95% C. I. = -1.43 to -0.13). The model explained 45% of depression's variance. Work-family enrichment works bidirectionally (family to work and work to family) and both affectively and cognitively. With an example from a family-friendly social enterprise, we will discuss how employers may facilitate social capital and benefit-finding in employees' dual role, besides adjustable work hours and shifts, for effective support.

Abstract citation ID: igaf122.4336

### TIME TO LOOK BEYOND AGE? FRAILTY AS THE KEY DETERMINANT OF EVT PROGNOSIS IN ACUTE ISCHEMIC STROKE PATIENTS ≥75 YEARS

Wenhua Yu, and Lina Ma, *Xuanwu Hospital, Capital Medical University, Beijing, Beijing, China (People's Republic)*

Background: Stroke is a major public health concern worldwide and the primary cause of adult mortality in China. Endovascular treatment (EVT) has been increasingly utilized in older acute ischemic stroke (AIS) patients; however, there is limited data regarding the association between frailty and prognosis after EVT in individuals aged ≥75 years. Objective: To evaluate frailty assessment as a prognostic indicator following EVT in patients with AIS aged ≥75 years. Methods: We included 193 patients with AIS aged ≥75 years who underwent EVT at Xuanwu Hospital of Capital Medical University from May 2013 to July 2023. Frailty was assessed using the Clinical Frailty Scale. The primary outcome was defined as a favorable modified Rankin Scale score of 0–1 at 3 months after EVT. We used multivariable logistic regression to evaluate the impact of frailty on clinical outcomes. Results: The mean age of participants was 80.34±4.39 years. Frailty, older age, female sex, consumed less alcohol, stroke history, coronary heart disease, more chronic diseases, lower Barthel Index, higher admitted National Institutes of Health Stroke Scale scores, higher fasting blood glucose levels, higher neutrophil percentage, higher D-Dimer levels and lower hemoglobin levels were associated with unfavorable outcomes. Logistic regression analysis revealed that frailty and stroke history were associated with poor prognosis after EVT in older adults with AIS. Conclusions: Frailty is an indicator of poor prognosis following EVT in patients with AIS aged ≥75 years. Frailty assessment provides valuable insights for clinical decision-making, helping guide more informed EVT decisions in this age group.

### SESSION 9090 (PAPER)

### LATE BREAKING: CLINICAL PRACTICE INNOVATIONS III

Abstract citation ID: igaf122.4337

### A SYSTEMATIC REVIEW OF RISK AND PROTECTIVE FACTORS FOR DEPRESSIVE SYMPTOMS IN OLDER ADULTS WITH VISUAL IMPAIRMENT

Haruno Suzuki, Heather Leutwyler, Jacqueline Torres, Kenneth Covinsky, and Margaret Wallhagen, *University of California, San Francisco, San Francisco, California, United States*

Depressive symptoms are prevalent among older adults with visual impairment (VI), yet there is no synthesis of modifiable factors to support prevention and early treatment programs. This systematic review aimed to identify risk and protective factors associated with depressive symptoms in older adults with VI. The protocol was registered with PROSPERO (CRD42024602520). Eligible studies were quantitative research that examined factors associated with depressive symptoms in adults aged 60 or older with VI. Five electronic databases (PubMed, Web of Science, Embase, PsycINFO, and CINAHL) were searched in November 2024 in collaboration with a medical librarian. Three reviewers independently screened titles, abstracts, and full-texts and assessed methodological quality using the National Heart, Lung, and Blood Institute's Quality Assessment Tool. Out of 13,761 records identified, 22 studies met the eligibility criteria, including 17 cross-sectional and 5 longitudinal designs. The majority of studies were assessed as "Fair" quality due to a lack of sample size justification and the inability to establish temporality. Common risk factors for depressive symptoms included older age, greater self-reported visual difficulty, poor self-rated health, and greater ADL/IADL limitations. Protective factors included greater social support and use of rehabilitation services. This review suggests that functional limitation may be a key modifiable risk factor associated with depressive symptoms. Our analysis also highlights the critical need for more robust longitudinal studies, standardized definitions of VI, and assessment of cognitive function in future research. Findings may guide clinicians, researchers, and policymakers in developing novel strategies for clinical screening and targeted interventions.

Abstract citation ID: igaf122.4338

### ADDRESSING COMPLEX SYMPTOM BURDEN IN AGING AND LONG COVID THROUGH A REMOTE YOGA AND SELF-MANAGEMENT

Jennifer Portz[1], Christine Fruhauf[2], Stacey Schepens[3], and Arlene Schmid[2], *1. University of Colorado School of Medicine, Aurora, Colorado, United States, 2. Colorado State University, Fort Collins, Colorado, United States, 3. University of Southern California, Los Angeles, California, United States*

Older adults with multiple chronic conditions (MCC) represent a population at particular risk for the long-term consequences of COVID-19. MCC are frequently accompanied by overlapping symptoms such as fatigue, pain, and depression, which long COVID can both mimic and intensify, accelerating functional decline and reducing overall quality of life. Older adults with MCC are not only more likely to develop long COVID but also experience greater difficulty recovering due to the complexity of managing multiple conditions simultaneously. Despite emerging guidelines, effective MCC/Long-COVID treatment strategies remain limited. This research evaluated the feasibility, usability, and preliminary impact of MY-Skills Mobile, a novel, 8-week, fully remote intervention that integrates therapeutic yoga with digital self-management education to improve physical, emotional, and social well-being across populations with high symptom burden. Two single-arm pilot studies tested MY-Skills Mobile: Study 1 enrolled 24 older adults (mean age 70±5) with MCC experiencing depressive symptoms, along with 9 care partners, and Study 2 involved patients (mean age 55.8±10.9) with long COVID (N=9). Both studies met feasibility benchmarks for recruitment, retention, and attendance. Participants rated the intervention positively, noting benefits such as relaxation, increased energy, and emotional relief. Improvements were observed in depressive symptoms, fatigue, pain interference, and self-efficacy, with care partners reporting stronger social support. No adverse events occurred, though suicide risk screening identified five participants needing follow-up. MY-Skills Mobile represents an innovative, scalable model for delivering support to populations with complex, overlapping health concerns. Future research will evaluate efficacy through fully powered trials.

Abstract citation ID: igaf122.4339

### EVALUATION OF THE CHOOSE HOME INTERVENTION TO REDUCE HEALTH SERVICES USE AND PROMOTE AGING IN PLACE

Daniel Harris[1], James Rudolph[2], Christopher Halladay[3], Malisa Barber[4], Alexandra Nothern[5], and Jane Driver[6], *1. University of Delaware, Newark, Delaware, United States, 2. VA Providence, Providence, Rhode Island, United States, 3. Providence VAMC THRIVE-COIN, Providence, Rhode Island, United States, 4. Department of Veteran Affairs, Providence, Rhode Island, United States, 5. VA Boston Healthcare System, Boston, Massachusetts, United States, 6. VA Boston Healthcare System, Brockton, Massachusetts, United States*

Nearly all older Veterans have expressed the desire to remain in their homes and to avoid the use of acute health services, yet complex comorbidity and limited resources at home represent key barriers towards achieving "aging in place." To address gaps in the care continuum for older Veterans at-risk for acute health services use and to promote aging in place, VA Boston Healthcare System developed Choose Home (CH), an interdisciplinary team providing short-term, in-home medical and social care to stabilize veterans at risk of losing independence. Between 2023 and 2025, a total of 219 Veterans (mean age = 80.7 years; 85.8% White; 38.5% diagnosed with dementia) were admitted to CH. We used electronic health records to measure emergency department (ED) visits, inpatient admissions, and hospital length of stay before and after CH admission. Poisson regression with robust standard errors estimated incidence rate ratios (IRRs) and 95% confidence intervals (CIs) comparing health services in the 61 to 120 days before admission (reference) to 60-day periods following admission. Compared to the reference period, ED visits decreased by 40% (IRR=0.60, 95%CI=0.34-1.02) and 63% (IRR=0.37, 95%CI=0.20-0.69) in the 61-120 days and 121-180 days after admission, respectively. Hospital length of stay (121-180 days IRR=0.45, 95%CI=0.26-0.76) and inpatient admissions (121-180 days IRR=0.52, 95% CI=0.34-0.79) decreased by 55% and 48%, respectively. The CH program is a scalable intervention that appears to be effective at reducing acute health services use among older Veterans at increased risk of health-related complications.

Abstract citation ID: igaf122.4340

### PATIENT AND CARE PARTNER REACTIONS TO A PRIMARY CARE BRAIN HEALTH VISIT FOR COGNITIVE ASSESSMENT

Clarissa Hsu[1], Mikael Anne Greenwood-Hickman[1], Alana Elop[2], Leah Karliner[2], Tyler Barrett[1], Leonardo Coleman[1], Sascha Dublin[1], and Deborah Barnes[3], *1. Kaiser Permanente Washington, Seattle, Washington, United States, 2. UCSF, San Francisco, California, United States, 3. University of California, San Francisco, San Francisco, California, United States*

The eRADAR study is a randomized controlled trial in two United States healthcare systems testing targeted dementia screening in primary care. High-risk individuals ≥ age 65 were identified using a predictive algorithm—eRADAR (electronic health record Risk of Alzheimer's and Dementia Assessment Rule)—and invited for a "brain health" screening visit (BHV). Patients and their primary care providers (PCPs) were given visit findings (possible dementia, possible mild cognitive impairment [MCI], or normal cognition) and follow-up recommendations. A subset of participants and their care partners (CPs) were invited to complete in-depth telephone interviews based on a purposeful sampling that prioritized participants who screened positive for possible dementia or MCI. We conducted interviews representing 40 BHVs (38 patients and 19 CPs). Forty-eight percent of patients screened positive for possible dementia, 38% for MCI, and 15% were cognitively normal. Patient reactions to the BHV were positive overall. Very few participants reported that the BHV caused distress or anxiety, but they also did not feel the BHV provided them with new information. Patients and CPs often downplayed results suggestive of cognitive impairment or attributed them to other causes, and many were reluctant to follow up with their PCP. Interview feedback indicates the BHV did not consistently motivate people to seek further evaluation or care for cognitive issues. Similar screening programs should consider more direct options to motivate follow-up for positive findings (e.g., directly scheduling a follow-up with the PCP, direct referral to specialty care, screening immediately before a PCP appointment).

Abstract citation ID: igaf122.4341

### REIT INVESTMENT IN US SKILLED NURSING FACILITIES AND THE QUALITY AND COST OF CARE FOR RESIDENTS

Mark Unruh[1], Jiebing Wen[2], David Stevenson[3], Hye-Young Jung[1], and Robert Braun[1], *1. Joan and Sanford I. Weill College of Medicine, Cornell University, New York, New York, United States, 2. Weill Cornell Medicine, New York, New York, United States, 3. Vanderbilt University, Nashville, Tennessee, United States*

Real estate investment trusts (REITs) are publicly-traded or privately-owned companies that invest in the real estate of income producing properties. REIT investments in skilled nursing facilities (SNFs) have been associated with reductions in registered nurse staffing and worse health deficiency scores. However, the relationship between REIT investment in SNFs and resident outcomes is not known. We examined the association of REIT investment in SNFs with 4 measures of quality (30-day rehospitalization, discharge to the community, functional improvement, 180-day mortality), in addition to length-of-stay and Medicare costs for residents receiving post-acute care. A national database of REIT investments in SNFs was constructed and merged with Medicare claims and Minimum Data Set assessments for the period 2014 to 2021. In facility-level analyses, changes in outcome measures for SNFs with REIT investment were compared to those of for-profit SNFs without these investments using a difference-in-differences approach, adjusting for resident and facility characteristics. Among 9,936 SNFs in our sample, 1,174 (11.8%) had REIT investment. Following REIT investment, rates of discharge to the community worsened by 4.8 percentage points (95% CI: -8.2, -1.4), an 11.3% decrease compared to the baseline mean, relative to other SNFs. Estimates for other quality measures, SNF length-of-stay, and Medicare costs were not statistically significant. REIT investments may negatively impact SNF quality through lower rates of discharge to the community but are not associated with other quality outcomes or with Medicare costs for residents receiving post-acute care.

Abstract citation ID: igaf122.4342

### USE OF CLINICAL EMR SYSTEMS FOR TARGETED RECRUITMENT OF OLDER ADULTS FOR RESEARCH: FACILITATORS AND BARRIERS

Lisa Kenyon-Pesce[1], Roshanak Sharafieh[1], Karina Berg[1], Tina Ferrarotti[1], George Kuchel[2], Richard Fortinsky[3], Iman Al-Naggar[1], and Julie Robison[3], *1. UConn Health, Farmington, Connecticut, United States, 2. UCONN HEALTH, Farmington, Connecticut, United States, 3. University of Connecticut, Farmington, Connecticut, United States*

Identifying participants with specific diagnoses through the Electronic Medical Record (EMR) represents one of the most promising strategies for targeted research recruitment. Given the remarkable heterogeneity of the aging population in terms of functional and cognitive status, clusters of co-morbidities, medication use, decline of physical function and increased risk of frailty, EMR-based approaches should be especially helpful for recruitment in observational and interventional studies involving older adults. However, the implementation of this approach faces real-world obstacles that prevent expensive institutional investments in EMRs from meeting their full potential. These barriers include the absence of functional and accurate data in EMRs, provider burden and burnout resulting from EMR alert fatigue, compliance and regulatory issues, as well as occasional misperceptions and pushback among institutional leaders. This presentation aims to discuss facilitators and barriers of utilizing EMR alerts as a recruitment method for identifying patients for research studies. A case study highlights the challenges and benefits of using Electronic Medical Records (EMR) systems to recruit older adults with early-stage lower extremity wounds. In addition, we discuss potential solutions, such as better stratifying frailty, function, and care preferences, while overcoming regulatory obstacles in the effective implementation of these important tools.

### SESSION 9095 (PAPER)

### LATE BREAKING: CLINICAL PRACTICE INNOVATIONS IV

Abstract citation ID: igaf122.4343

### PSYCHOLOGICAL WHIPLASH: FACTORS LINKED TO PERITRAUMATIC DISTRESS IN OLDER ADULTS FIVE DAYS AFTER MISSILE ATTACKS

Boaz Ben-David[1], Efrat Arieli Zaks[1], and Yuval Palgi[2], *1. Reichman University, Herzliya, Tel Aviv, Israel, 2. University of Haifa, Haifa, Hefa, Israel*

The rise of civilian-targeted warfare, including ballistic missile and drone (BMD) attacks, has globalized the need for rapid, trauma-informed mental health care for older adults. This study contributes to system-level innovation by identifying, in the immediate days following exposure, clinical predictors of peritraumatic distress (PD), a key risk factor for long-term psychopathology, in aging populations. Amid the Iran-Israel-US armed conflict, a 12-day conflict in June 2025 with daily BMD attacks affected tens of thousands of Israelis (infrastructure damage, evacuations, injuries and deaths), underscoring the importance of mental health preparedness. Yet, the psychological toll of BMD attacks on civilians has mostly been explored only months after exposure, and even less is known about their immediate impact on older adults. Two to five days after the cease-fire, we surveyed a national sample of 710 Israeli adults aged 60–85 (M = 71.86). More than half (56%) exceeded the clinical threshold of PD (PD-Inventory score ≥14). Logistic regressions (Nagelkerke-$r^2$=.53), adjusting for sociodemographic and situational factors, revealed five significant predictors: (A) Exposure to the BMD trauma (OR = 2.3); (B) Pre-existing probable-PTSD from the October-7 attack (OR = 4.9); (C) Sleep difficulties (OR = 1.1); (D) Intensive media engagement (OR = 1.3); and (E) Decreased national identity (OR = 0.7). Findings illustrate the severe immediate psychological impact of BMD attacks on older adults. They identify scalable, clinically actionable indicators (behavioral reactions, pre-existing conditions and emotional factors) of acute stress within days of large-scale national trauma. Health systems globally can strengthen age-friendly mental health care by integrating such early screening tools into post-crisis response.

Abstract citation ID: igaf122.4344

### EFFICACY OF AN INNOVATIVE PT PROGRAM TO ENHANCE MOBILITY IN OLDER VETERANS WITH SLOW WALKING SPEED

Jonathan Bean[1], Rebekah Harris[2], Elisa Ogawa[2], Elizabeth Rathje[3], Jennifer Brach[4], Paige Burns[3], Addie Middleton[5], and Thomas Travison[6], *1. Harvard Medical School, Boston, Massachusetts, United States, 2. VA Boston Healthcare System, Boston, Massachusetts, United States, 3. VA Boston Healthcare System, VA Boston Healthcare System, Massachusetts, United States, 4. University of Pittsburgh, Pittsburgh, Pennsylvania, United States, 5. Univ of Colorado, Anschutz Medical Campus, University of Colorado School of Medicine, Colorado, United States, 6. Hebrew SeniorLife & Harvard Medical School, Hebrew SeniorLife, Massachusetts, United States*

We investigated the efficacy of an innovative physical therapy program targeting mobility known as Live Long Walk Strong (LLWS). This was a single-blind randomized controlled trial conducted at an outpatient VA tertiary care center among community-dwelling middle-aged and older Veterans with slow walking speed. LLWS is an 8-week 10 (1-hour/session) session outpatient physical therapy program. It was designed to enhance mobility by targeting impairments linked to mobility decline and fostering long-term engagement in physical activity through coaching and behavior change strategies. LLWS treatment was compared to participants within a waitlist control group. Walking speed was the primary outcome and secondary outcomes included measures of mobility. We randomized 150 participants and experienced a 25% dropout rate, which was in part due to the COVID-19 pandemic shutdowns of human research (LLWS n = 56, control n = 58). We observed significantly faster (p<.05) walking speed (mean change 0.08±0.02 m/s) after the 8-week program, as well as significantly greater improvements in the Short Physical Performance Battery (mean change 0.95±0.32) and the Activity Measure for Post Acute Care (mean change 2.1±0.8) withing the LLWS group (all p < 0.05). These observed increases were consistent with large clinically meaningful differences. Our study demonstrates that LLWS enabled statistically significant and clinically meaningful gains in mobility among middle aged and older adult Veterans with slow walking speed, justifying its efficacy as a treatment for mobility limitations. Separate analyses of this clinical trial will evaluate long term outcomes and be better powered to evaluate changes in the attributes LLWS targets.

Abstract citation ID: igaf122.4345

### MULTIMORBIDITY PATTERNS AMONG OLDER WOMEN PARTICIPANTS OF THE ALL OF US RESEARCH PROGRAM

Mayra Tisminetzky[1], Mara Epstein[2], and Yanhua Zhou[2], *1. University of Massachusetts Chan Medical School, shrewsbury, Massachusetts, United States, 2. univ of Massachusetts medical school, worcester, Massachusetts, United States*

Despite the fact that older women typically outlive men in industrialized countries and experience a higher prevalence of multimorbidity (the presence of two or more chronic conditions) with advancing age, limited knowledge exists regarding the specific patterns of chronic conditions that affect the lives of older women. We aimed to identify the prevalence and patterns of multimorbidity in older women (aged ≥65 years). Our study sample included 56,220 women aged 65 or older at study enrollment, who completed the three baseline surveys for the All of Us Research Program (AoURP). Overall mean age of this study population was 71 years; 71% were white and 9.7% were Hispanic. Overall, the five most common chronic conditions among AoURP participants were hyperlipidemia, hypertension, osteoarthritis, obesity and chronic pain (65%,62%, 61%, 47% and 42% prevalence, respectively). The top five pairs of co-occurring chronic conditions were: hyperlipidemia + hypertension; osteoarthritis + hyperlipidemia; osteoarthritis +hypertension; chronic pain + osteoarthritis; obesity + hypertension (51%,49%, 46%, 36% and 35% prevalence, respectively). More than 80% of study participants presented with two or more chronic conditions. Our findings also suggested that 25% of the older women participants of the AoURP reported having issues to complete daily activities and 1 out of 5 women reported having poor health. Given the high prevalence of chronic conditions and growing health-care needs within this aging population, understanding the distribution of specific multimorbidity combinations is of particular importance to optimize care delivery, reduce disparities, and address unmet healthcare needs.

Abstract citation ID: igaf122.4346

### CULTURAL ADAPTATION OF ADVANCED-COMFORT FOR CHINESE AMERICAN FAMILY CAREGIVERS OF NURSING HOME RESIDENTS

Andrea Wei[1], Benjamin Semple[2], and Ruth Lopez[3], *1. MGH Institute of Health Professions, Boston, Massachusetts, United States, 2. Wake Forest University, Charlotte, North Carolina, United States, 3. MGH Institute of Health Professions, Sharon, Massachusetts, United States*

Family caregivers of nursing home (NH) residents with advanced dementia often prioritize comfort-focused care.

However, culturally tailored tools to support this preference are lacking. This study aimed to culturally adapt the web-based ADVANCED-Comfort intervention and 6M Care Planning Tool to align with the values and caregiving practices of Chinese American families. We used a community-engaged, qualitative approach guided by principles of cultural tailoring and intervention mapping. A Chinese American Community Advisory Board (CAB) participated in a series of virtual meetings that included a concept mapping activity to define comfort and provide feedback on the intervention. Data were analyzed using qualitative description. CAB members identified five core themes of comfort: familiarity, respect, trust and emotional safety, connection to culture and family, and minimizing distress. These were richly illustrated with personal narratives, such as the importance of continuity in caregiving and culturally appropriate forms of address. A content mapping exercise showed strong alignment between these themes and the 6Ms of the intervention. Cultural adaptations included aesthetic refinements, incorporation of culturally specific imagery and symbolism, and greater emphasis on family naming and rituals. The 6M Care Planning Tool, co-completed by families and staff, generates a person-centered care plan that reflects shared values and is posted in the resident's room. This cultural adaptation process demonstrated how participatory methods can enhance the relevance and acceptability of web-based interventions for diverse populations. The adapted ADVANCED-Comfort intervention is now better positioned to support culturally congruent, comfort-focused care for Chinese American families in NH settings.

Abstract citation ID: igaf122.4347

### VA NURSING HOME TO HOME PROGRAM: MIXED METHODS EVALUATION OF AN INNOVATIVE TWO-YEAR PILOT

Rachel Fix[1], Chelsea Manheim[2], Orna Intrator[3], Winifred Scott[4], and Leah Haverhals[5], *1. VA Health Services Research and Development, Aurora, Colorado, United States, 2. VA, Aurora, Colorado, United States, 3. James J Peters VA Medical Center, New York, New York, United States, 4. VA, Menlo Park, California, United States, 5. VA Eastern Colorado Healthcare System, Aurora, Colorado, United States*

As the United States Veteran population ages, the Department of Veterans Affairs (VA) is expanding initiatives to ensure Veterans age at home. In 2023, the VA developed the Nursing Home to Home (NHTH) pilot program to assist Veterans living in VA-paid community nursing homes (CNH) to return home. This abstract describes cost savings and Veteran and caregiver satisfaction with NHTH. Cost analyses were conducted through analyzing 24 NHTH Veterans discharged from CNHs across three VA Medical Centers. Analyses were based on long-term services and supports (LTSS), assuming other costs did not change before/after NHTH discharge, during four, 30-day intervals before and after discharge. Average savings of LTSS was $5,496 per Veteran per month (equivalent to $65,952 per Veteran per year). Additionally, 67 satisfaction surveys were completed by Veterans and caregivers, at two points in time, post-CNH discharge. Qualitative interviews were conducted with 21 Veterans or their caregivers. We applied thematic analysis to analyze Veterans' and caregivers' satisfaction surveys and interview data. Results showed Veterans and caregivers having high satisfaction with NHTH. The NHTH dyadic teams of one nurse and one social worker acted as liaisons to Veterans receiving VA LTSS. LTSS received post-discharge facilitated Veterans' safety once home. Evaluation results suggest NHTH to be a promising pilot program with a high likelihood of future success in ensuring Veterans placed in CNHs can transition safely back to the community. This is especially critical as VA costs for CNH care are projected to double by 2037.

### SESSION 9100 (PAPER)

### LATE BREAKING: COMMUNITY-ENGAGED RESEARCH APPROACHES I

Abstract citation ID: igaf122.4348

### LATE BREAKING: COMMUNITY-ENGAGED RESEARCH APPROACHES I COPING WITH LATE LIFE LOSS AND BEREAVEMENT: THE HEALING POWER OF TRANSCENDENCE AND CREATIVITY

Jasmin Tahmaseb McCon, *West Chester University, West Chester University of PA, Pennsylvania, United States*

Losses associated with late life can lead to meaninglessness, loneliness, isolation, and despair. When facing grief and mourning, life review is an effective psychological process that can be healing. Focusing on past strengths and contributions can be helpful when faced with adversity. This qualitative study examined the coping strategies of a diverse group of adults who experienced a significant loss. Utilizing a life review process, we explored how memories of the past could help manage current realities. We identified activities that were healing and fostered meaning, purpose, and ego integrity. Interviews were conducted with 23 men and women over 70. Participants were a part of a nine-year "Life History Project" (2016 – 2025), a part of the WHO and AARP' Livable Communities Project. All participants (16 women and 7 men) experienced a significant loss during the time of the study. Interviews explored the ways they coped with changing personal and social realities, mourned their losses, and managed their grief. Results of thematic analysis indicated that the primary challenges were 1) personal losses (health and death of loved ones), 2) cultural bereavement (loss of social trust), and 3) stress and concern about the future of the planet (climate anxiety). Effective coping included three interrelated strategies: transcendence (religious/spiritual and natural connections), creative activities (music, painting, writing), and personal and cultural rituals. Transcendence, in particular, represented a shift from egocentricity to a broader awareness of self and the world and resulted in increased peace, acceptance, and coherence of past experiences with present realities.

Abstract citation ID: igaf122.4349

### CREATING CONNECTION BEYOND ACCESS: A STRUCTURED DIGITAL ENGAGEMENT PLATFORM FOR OLDER ADULTS

Soohyoung Rain Lee[1], Andrea Maxi[1], Sharon Turnage[1], Cathy Sasscer[1], Sarah Kaufman[1], Hang Liu[2], Ana Semedo[2], and Grace Yi[3], *1. Yeshiva University, New York, New York, United States, 2. Selfhelp, New York, New York, United States, 3. California State University, Fullerton, Fullerton, California, United States*

Loneliness and depressive symptoms are persistent concerns in later life, particularly for those with functional or sensory limitations. Digital programs may help, yet evidence on feasibility and mental health correlates in diverse samples is limited. This study evaluated the Virtual Senior Center (VSC), a facilitator-led, routine-based online program, examining loneliness, depressive symptoms, digital confidence, and perceived benefits, supplemented with focus group interviews. Twenty-three racially diverse older adults completed baseline surveys after enrollment and participation in at least one session. Measures included the UCLA Loneliness Scale, Geriatric Depression Scale-10, digital confidence, functional limitations, and participation. Descriptive statistics, Mann-Whitney U, and Spearman's ρ were used. Focus groups were analyzed using rapid content analysis. Participants reported moderate loneliness (M = 41.4, SD = 15.0) and mild depressive symptoms (M = 2.3, SD = 2.2). Digital confidence was negatively associated with loneliness (ρ = -.49, p = .02) and depressive symptoms (ρ = -.54, p = .01). Functional limitations were associated with higher depressive symptoms (ρ = .53, p = .01), but not loneliness, and did not reduce program use. Focus group themes emphasized the value of structured routines and consistent opportunities for peer connection, making VSC a meaningful part of weekly life. Findings suggest that digital confidence supports emotional well-being and that structured, inclusive programs can sustain engagement even among older adults with impairments. By fostering routine, familiarity, and continuity, areas where many digital interventions failed to provide, VSC demonstrates potential to reduce isolation and support aging well in the community.

Abstract citation ID: igaf122.4350

### DIALOGUE: UNDERSTANDING TECHNOLOGY USE IN SOCIAL CARE DELIVERY

Hannah Marston[1], Katie Brittain[2], Jennifer Lynch[3], Matthew Lariviere[4], Raj Mehta[5], Joanna Thorn[6], and Grant Gibson[7], *1. The Open University, Milton Keynes, England, United Kingdom, 2. Newcastle University, Newcastle, England, United Kingdom, 3. University of Hertfordshire, Hertfordshire, England, United Kingdom, 4. Northumbria University, Newcastle, England, United Kingdom, 5. n/a, n/a, England, United Kingdom, 6. University of Bristol, Bristol, England, United Kingdom, 7. University of Stirling, Stirling, Scotland, United Kingdom*

UK social care services for older people, typically provided by local authorities, are struggling to meet demands placed upon them leading to a sense of crisis in the sector. Capitalizing on digital transformation, social care providers are exploring how widely available household smart technologies (e.g., virtual assistants, medication boxes) can be utilized within existing social care services. Underpinned by life course theory, this study explored older adults experiences of, and opinions regarding the continuing and expanding use of technology in social care. Eighteen older (65+) users of social care services were recruited to take part in four co-production workshops in three sites in England (North East, South West and Central England). Workshops employed vignettes of exemplar cases of technology use in social care to generate discussion amongst participants. Descriptive insights were provided offering responses to challenging social care situations encountered by the participants. Findings identified four themes, 1. Current (or everyday) technology use, 2. Benefits of technology use, 3. Concerns with technology use, 4. Key priorities for technology enabled care. Across the four themes, participants noted areas for improvement including clear signposting of information/services provided, in-person care, the want and need to maintain independent living, digital literacy/accessibility concerns, trust in technologies, and training and support. Findings highlight priorities, challenges and concern the ongoing digital transformation of social care in the context of increasing demand and reducing financial resources, for services and their clients. Findings inform educators in gerontology and social work regarding the impacts of technology on social care delivery.

Abstract citation ID: igaf122.4351

### FALL PREVENTION FOR RURAL SPANISH-SPEAKING COMMUNITIES: PILOT STUDY

Janet Lopez, Ladda Thiamwong, Rui Xie, and Victoria Loerzel, *University of Central Florida, Orlando, Florida, United States*

This community-engaged research focuses on the information gathering phase for culturally adapting the Physio-feedback Exercise Program (PEER) for middle-aged and older Spanish-speaking adults in rural Central Florida communities. PEER is a technology-based intervention combining real-time balance feedback using the BTrackS Balance System, cognitive reframing through a fall risk appraisal matrix, and peer-led balance and strength training exercises. While PEER has demonstrated effectiveness in urban community-dwelling older populations, rural communities face distinct barriers including limited technology access, different cultural contexts, and unique health challenges related to falls and frailty. Using the Heuristic Framework and Ecological Validity Model, this study presents the first phase of a four-step cultural adaptation process: information gathering. Through participatory methods involving community partners, we collected comprehensive data about rural Spanish-speaking middle-aged and older adults (N = 30) to inform subsequent PEER adaptation. We evaluated community needs for fall prevention and conducted a rapid analysis. Findings revealed that most participants were female (n = 22) and had a lower than high school education (n = 24). Some indicated that they had never heard of fall prevention programs or did not understand their purpose. None were aware of such programs in their communities. Many expressed interest in participating in fall prevention programming. They emphasized the importance of providing these programs in Spanish, ensuring scheduling flexibility (including evenings and weekends), and offering free programming. This gathering of information focuses on adapting evidence-based interventions for underserved rural populations. These findings will directly inform the cultural adaptation of PEER for middle-aged and older Hispanic adults in rural communities.

Abstract citation ID: igaf122.4352

### FROM JOY TO GENERATIVITY: 2-YEAR SHIFT IN HIP-HOP DANCE TEAMS AGED 65+ AT A NATIONAL COMPETITION

Atsuko Miyazaki, *The University of Tokyo, Tokyo, Tokyo, Japan*

Community-based hip-hop dance teams may do more than keep older adults moving—they may shift motives from enjoyment to contribution in later life. We examined two-year within-person change among older adults engaged in community hip-hop dance teams in Japan. At baseline (Year 1), 79 participants across seven municipalities were surveyed; longitudinal analyses included 62 participants (mean age 75.2±6.1; 96.8% women) from six municipalities with data at both Year 1 and Year 2. All teams practiced hip-hop and competed annually in a national competition. By Year 2, the mean participation duration was 2.73±0.99 years. A 40-item questionnaire spanned seven psychosocial domains (Helping Others, Belongingness, Relationships, Status, Social Identity, Enjoyment, Information).

Change scores (Year 2 minus Year 1) were tested against zero using one-sample t-tests with Cohen's d and 95% confidence intervals; participation averaged 2.16 sessions/week for 49.35 minutes/session. Prosocial orientation increased modestly: Helping Others Δ = +0.76 (95% CI 0.15–1.37; d≈0.32). Other domains showed small, directionally positive but non-significant changes—Status Δ = +0.63 (−0.29–1.55), Belongingness Δ = +1.47 (−0.40–3.34), Relationships Δ = +0.31 (−0.52–1.14), Information Δ = +0.19 (−0.49–0.87), Social Identity Δ = −0.02 (−1.12–1.08)—while Enjoyment declined slightly, Δ = −0.55 (−1.59–0.49). Participants frequently noted "more friends," "moving more every day," and "greater purpose," echoing the quantitative pattern. Over two years, community hip-hop dance teams were associated with a small yet consistent shift from initial enjoyment toward contribution and connectedness. Although most effects were small and CIs included zero, the Helping Others CI did not, suggesting practical salience; implications include peer-mentor roles, structured helping opportunities, and intergenerational sessions.

Abstract citation ID: igaf122.4353

### PREVENTING SEXUAL VIOLENCE IN LATER LIFE: EXAMINING PRE- AND POST- BYSTANDER TRAINING RESULTS

Michelle Hand, *George Mason University, Fairfax, Virginia, United States*

Pressing needs persist for practitioner trainings on sexual violence (SV) in later life, which bystander programs have been recommended to address. Yet before this research, no known bystander training on SV in later life existed. This study aimed (a) to explore training needs that may exist for practitioners who serve older adults or survivors and their recommendations for refining a bystander training to prevent SV in later life, then (b) to pilot and evaluate the effectiveness of this training. An interview and focus groups were conducted with 16 practitioners who serve older adults or survivors, and with people older than 50, whose recommendations were used to refine the training, which was piloted with 51 practitioners who serve older adults and survivors. Before the training, participants were asked the extent to which they believed SV in later life is a problem, their intentions to help prevent and address it, and a scenario involving SV in later life was presented, after which participants were asked the extent to which they felt obligated to prevent SV in later life, whether they knew where to report, and if their help was required. These questions were repeated post-training. Although modest improvements yet no significant changes were found in beliefs of SV in later life requiring one's help or knowing where to report, paired samples t-test results demonstrated significant positive changes across all other measures, reflecting increased awareness of SV in later life, intention and obligation to help prevent and address it. Implications will be discussed.

### SESSION 9105 (PAPER)

### LATE BREAKING: COMMUNITY-ENGAGED RESEARCH APPROACHES II

Abstract citation ID: igaf122.4354

### AGING LANDSCAPES: CLUSTERING NEIGHBORHOOD RESOURCES FOR OLDER ADULTS

Eric Delmelle, *Lehigh University, Bethlehem, Pennsylvania, United States*

The neighborhoods where older adults live and interact strongly shape opportunities for physical activity, social engagement, and aging in place, yet research often examines these features in isolation rather than as holistic neighborhood types. We introduce a typology of "aging landscapes" that captures how combinations of environmental and social resources evolve over time and may support or hinder healthy aging. Drawing on a longitudinal dataset (1990–2019) at the U.S. census tract level, we compiled fine-scale measures of neighborhood resources reflecting three domains: (A) physical function (e.g., recreational facilities, greenness, walkability), (B) cognitive function (e.g., cultural and civic organizations, learning locations, hobby spaces), and (C) aging in place (e.g., health and social services, food access, third places). Variables were rescaled and reduced using Principal Component Analysis within each domain, and k-means clustering was applied to identify tract-level neighborhood types across three decades. This approach allows neighborhoods to change cluster membership over time, reflecting dynamic improvements or losses in resources. We also conduct exploratory PCA across all variables to evaluate whether data-driven structures align with theoretically defined domains. The resulting typology provides new insights into the spatial distribution and temporal evolution of resource-rich and resource-poor environments for older adults. This work provides a scalable framework for identifying and monitoring aging-supportive communities, offering new directions for urban planning and public health practice aimed at promoting environments that enable healthy aging.

Abstract citation ID: igaf122.4355

### COMMUNITY LANDSCAPE OF AGING AND HOMELESSNESS IN DENVER: A QUALITATIVE STUDY

Pilar Ingle[1], Riley Coyne[1], Myra Nagy[2], John Olander[2], Ingrid Backes[1], Johnna Flood[2], Liane Morrison[2], and Asia Cutforth[3], *1. University of Denver, Denver, Colorado, United States, 2. Elevated Denver, Denver, Colorado, United States, 3. University of Denver, University of Denver, Colorado, United States*

Older adults (50+) represent the largest age cohort of single adults experiencing homelessness in the U.S. However, they are largely unrecognized by policy makers, and many homeless service organizations are unequipped to appropriately serve their aging clients. This community-engaged qualitative project centers the perspectives of unhoused older adults to identify strengths and gaps in services to inform opportunities for meaningful change. Twenty individuals (ages 61-77, 11 females, 9 males) currently or recently unhoused were recruited to participate in semi-structured interviews from two Denver, CO nonprofits dedicated to serving older unhoused clients. An inductive thematic analysis approach was used to code interviews and identify themes. Interviews lasted an average of 43 minutes. The following themes were identified: 1) unaffordable housing costs contributed to loss of housing, despite most participants having worked most of their lives; 2) overnight shelters, while providing necessary services, feel unsafe and often do not help individuals move forward; 3) aging-specific homeless service organizations were supportive for enrolling in benefits and finding housing, yet needed healthcare services were difficult to access for many; and 4) community and sense of belonging are critical, along with a willingness to ask for help. Meaningful inclusion of older adults who have experienced homelessness is essential for research and program/policy development to ensure relevant, appropriate services and reforms. Our findings further validate systemic failures in supporting our elders' stability and opportunities to thrive in later life, and underscore the need for homelessness and healthcare services dedicated to unhoused older adults.

Abstract citation ID: igaf122.4356

### CROSSING A CENTURY: BLACK MALE RESILIENCE, SPIRITUALITY, AND NEW HORIZONS

Stephanie Boddie, Jocelyn McGee, and Rebecca Meraz, *Baylor University, Waco, Texas, United States*

Background and Objective The U.S. centenarian population has nearly tripled over the last three decades (Schaeffer, 2024). Centenarians are disproportionately white women, with only 8% identifying as Black (Schaeffer, 2024). This study explores the lived experiences of two Black men who achieved extreme longevity, highlighting the role of spirituality as a protective factor that fosters resilience and provides insights into navigating stressful life events. Research Method Data were drawn from oral history collections of Black elders. The analysis employed Elder's (1998) Life Course Theory and Dannefer's (2003) Cumulative Advantage/Disadvantage (CAD) Theory to conduct thematic analysis of two oral histories. Findings Charles Wiggins (d. 103) and Louis Tucker (d. 105) were both raised in the Black church and sustained strong religious and spiritual practices throughout their lives. Over the course of a century, they endured wars, illness, discrimination, and domestic terrorism. Their spirituality, coupled with resilience cultivated in early life, served as a buffer against stress in later years (Jones et al., 2022). Both men also maintained social engagement, independence, and mobility, including driving, well into advanced age. Discussion and Implications This study underscores the importance of spirituality as a dimension of resilience in extreme longevity, particularly among Black men. Findings suggest that attention to spiritual practices can enhance understanding of health, social support, and family dynamics across the life course. Implications extend to caregiving practices and gerontological research, pointing to the need for greater consideration of cultural and spiritual factors in supporting centenarians.

Abstract citation ID: igaf122.4357

### IMPACT OF SOCIAL DETERMINANTS OF HEALTH ON UTILIZATION OF TCM AND CCM SERVICES: DIFFERENCES BY DISABILITY STATUS

Eunji G. Kim[1], Ellen McCarthy[2], Dae Hyun Kim[2], Lan Luo[1], and Chan Mi Park[3], *1. Marcus Institute for Aging Research, Boston, Massachusetts, United States, 2. Hebrew SeniorLife, Boston, Massachusetts, United States, 3. Hebrew SeniorLife, Harvard Medical school, Boston, Massachusetts, United States*

Transitional Care Management (TCM) and Chronic Care Management (CCM) services aim to improve post-hospital and chronic condition care, but utilization varies across populations. Social determinants of health (SDOH) can contribute to disparities in access to these services, especially among beneficiaries with disabilities. We examined how SDOH factors relate to TCM and CCM utilization, stratified by beneficiaries' disability status. Using 2016-2020 Medicare Current Beneficiary Survey data linked to fee-for-service claims, we conducted matched case-control studies (1:2 ratio) of TCM-eligible (n = 4,762) and CCM-eligible (n = 25,380) beneficiaries. Eight SDOH domains, derived from 26 survey items, were standardized and analyzed via conditional logistic regression, adjusting for demographics and comorbidities. For TCM, lack of healthcare access (adjusted OR [95% CI]: 0.85 [0.76-0.95]) and poor neighborhood environment (0.83 [0.75-0.93]) were associated with lower TCM utilization. The associations were stronger among those without disability than those with disability for healthcare access (0.80 [0.64-0.99] vs. 0.86 [0.74-0.99]), but stronger among those with disability than those without for neighborhood environment (0.81 [0.70-0.93] vs. 0.89 [0.74-1.07]). For CCM, economic instability (1.16 [1.01-1.34]) and lack of transportation (1.21 [1.05-1.40]) were associated with greater utilization of CCM. However, stratified by disability status, these associations were attenuated and not statistical significant. SDOH impacts TCM and CCM utilization differently, with distinct patterns by disability. TCM under-serves those facing environmental and access barriers, especially individuals with disabilities, while CCM reaches people who are socioeconomically disadvantaged and less affected by disability. Integrating SDOH screening into care planning and aligning TCM and CCM delivery may improve equitable care.

Abstract citation ID: igaf122.4358

### RETHINKING ELDERCARE IN A DIGITAL AGE: INTERNET USE AND SHIFTING ATTITUDES IN CHINA

Mengting Li[1], Zhiyong Lin[2], and Yibo Li[1], *1. Renmin University of China, Beijing, Beijing, China (People's Republic), 2. University of Texas at San Antonio, San Antonio, Texas, United States*

This study examines how internet use is associated with older adults' attitudes and preferences regarding eldercare in China. Specifically, it explores whether internet access and usage patterns are linked to views on who should be primarily responsible for caring for older adults and to preferences for informal, formal, or mixed care arrangements. Data come from the 2020 wave of the China Longitudinal Aging Social Survey (CLASS), a nationally representative survey of community-dwelling adults aged 60 and above. Multinomial logistic regression models were used to assess the associations between four indicators of internet use (access, frequency, number of online activities, and digital proficiency) and two outcome measures: (1) perceived responsibility for eldercare (family, government/society, or shared), and (2) personal care preferences (family-based caregiving, community-based services or institutional care). Internet access was associated with lower support for formal care but greater endorsement of shared responsibility, compared to family-only care. Greater online activities and digital proficiency were positively associated with support for shared responsibility. In terms of care preferences, internet access was negatively associated with preference for community-based services and institutional care, whereas online activity and proficiency were positively associated with such preferences. Digitally engaged individuals were more likely to consider formal care than non-users, though family care remained the most preferred option. Internet use is linked to more nuanced and flexible views on eldercare among older Chinese adults. Digital engagement may play a role in reshaping traditional expectations and expanding consideration of formal care in aging societies.

Abstract citation ID: igaf122.4359

### SUPPORTING OLDER ADULTS' UNDERSTANDING OF HEALTH RESEARCH: INSIGHTS FROM COMMUNITY ENGAGEMENT STUDIOS

Samuel Van Vleet[1], Nicholas Cloney[2], Ruochen Yang[3], Alice Xie[4], Kristina Brackpool[5], Hugo Aparicio[6], Evan Plys[3], and Jin hui Joo[3], *1. Massachusetts General Hospital | Harvard Medical School, Boston, Massachusetts, United States, 2. Brigham & Women's Hospital, Boston, Massachusetts, United States, 3. Massachusetts General Hospital, Boston, Massachusetts, United States, 4. University of Nevada, Las Vegas, Las Vegas, Nevada, United States, 5. Mass General Brigham, Santa Clarita, California, United States, 6. Boston University, Boston, Massachusetts, United States*

Dementia research studies often struggle to recruit and retain participants from ethnic minority groups. Community Engagement (CE) studios have been used to facilitate discussion within a population of interest to obtain their perspectives. Presentations by experts on relevant topics also draw listeners, so combining the two may increase participation in the studios. To conduct a public engagement program using CE studios to transfer health and research knowledge and to understand the community's views on research and research participation, eleven modified CE studios were conducted in older adult housing authorities. These studios included presentations on relevant health-related topics to encourage turnout. In addition, study staff interviewed eight resource coordinators and followed up several months later to confirm initial analyses and gather additional suggestions. Through the CE studies, information on health topics such as dementia, loneliness and mental health were provided. Attendees of the CES and the resource coordinators shared their perceptions of health research, described barriers that currently limit research participation and provided a number of recommendations for improving community partnerships. By implementing these modified CE studios in communities of low-income older adults, researchers could obtain new insights on how research is viewed and what can be done to increase participation within these groups. Modified Community Engagement Studios may be a valuable tool for improving participation compared to traditional CE Studios.

### SESSION 9110 (PAPER)

### LATE BREAKING: COMMUNITY-ENGAGED RESEARCH APPROACHES III

Abstract citation ID: igaf122.4360

### BALANCING OBLIGATION AND AUTONOMY: A CHATBOT FOR FAMILY CAREGIVERS FACING LIFE-SUSTAINING TREATMENT DILEMMAS

Seonghyun Ellin Jeong, *University of Toronto at St. George Campus, Toronto, Ontario, Canada*

Family caregivers often expend excessive energy balancing care, personal life, and emotional stability, especially when illness limits the patient's autonomy and intensifies ethical dilemmas surrounding the caregiver's decision making. These dilemmas intensify in the absence of caregiver centered communication and support programs. This project seeks to fill the gap by proposing a self-reflective guiding AI chatbot designed to offer early stage emotional, ethical reflection and informative data before treatment decisions are made. This project builds on an ethical analysis framework that combines principal bioethics to address the moral and emotional costs of choosing life-sustaining treatment. Recognizing the need for early-stage emotional support before a decision is made, we propose a self-reflective AI chatbot prototype to help caregivers articulate their values, emotional reasoning, and potential areas of internal conflict. The chatbot includes a DASS-21-based emotional check-in, CBT-informed narrative prompts, and live-updating local welfare resource information when financial strain is detected. All interactions are anonymous and non-directive. The methodology includes a psychology-based assessment to measure stress levels, experiences of ethical conflict, and financial pressures. Qualitative research will be applied to explore family caregiver's ethical and emotional pattern, aiming to reveal social and structural foundations in their decision-making process. This study offers a roadmap through an ethical lens for examining real world treatment decisions on behalf of patients. By addressing these systemic challenges and suggesting an intervention model, the research seeks to reduce the risk of unethical decisions among family caregivers, ultimately safeguarding patient rights.

Abstract citation ID: igaf122.4361

### BEYOND INDIVIDUAL RISK: NEIGHBORHOOD EFFECTS ON LONELINESS IN PREDOMINATELY BLACK ADULTS IN PITTSBURGH

Moka Yoo-Jeong[1], Greta Jianjia Cheng[2], Tamara Dubowitz[3], Wendy Troxel[4], Gerald Hunter[5], Bonnie Dastidar[6], Tanisha Hill-Jarrett[7], and Andrea Rosso[2], *1. Northeastern University, Boston, Massachusetts, United States, 2. University of Pittsburgh, Pittsburgh, Pennsylvania, United States, 3. University of Pittsburgh School of Public Health, Pittsburgh, Pennsylvania, United States, 4. RAND, Pittsburgh, Pennsylvania, United States, 5. Rand Corporation, Pittsburgh, Pennsylvania, United States, 6. RAND, Santa Monia, California, United States, 7. University of California Memory and Aging Center, San Francisco, California, United States*

While individual correlates (age, sex, physical function) of loneliness have been well-documented, less is known about whether neighborhood characteristics modify these associations. Guided by the Ecological Theory of Aging, this study examined whether subjective and objective neighborhood factors moderate the associations between individual correlates and loneliness among adults in two predominantly Black neighborhoods. Data were drawn from the Think PHRESH study (N = 753), an ancillary study to a longitudinal cohort study of the Pittsburgh Hill/Homewood Research on Neighborhood Change and Health (PHRESH). Loneliness was measured using the UCLA 3-item scale. Subjective neighborhood factors included perceived safety and neighborhood satisfaction. Objective factors included socioeconomic deprivation and residential instability. Multivariable linear regressions with interaction terms tested moderation effects. Among those perceiving their neighborhoods as less safe, middle-aged adults (50-65 years) and older adults (≥65 years) reported significantly higher loneliness compared to younger adults (31-49 years). These age-related differences were not observed among those reporting high safety. A similar pattern emerged for neighborhood satisfaction: greater loneliness was reported among middle-aged and older adults with low neighborhood satisfaction, but not among those with high satisfaction. Functional limitations were also linked to greater loneliness among those with low perceived safety or neighborhood satisfaction. Objective neighborhood measures did not moderate associations. Findings demonstrate that perceptions of neighborhood safety and satisfaction interact with individual vulnerabilities to shape loneliness. Supportive neighborhood conditions may buffer loneliness among high-risk groups. These findings highlight the need for community-based interventions that enhance subjective neighborhood experiences to buffer against loneliness in historically marginalized communities.

Abstract citation ID: igaf122.4362

**COGCARE: TAILORING COGNITIVE REHABILITATION FOR BLACK/AFRICAN AMERICAN OLDER ADULTS**

Dumichel Harley[1], Yoojee Kim[2], and Ryan Mace[2], *1. Massachusettes General Hospital BHX-1, Cambridge, Massachusetts, United States, 2. Massachusetts General Hospital, Boston, Massachusetts, United States*

Cognitive rehabilitation (CR) programs provide cognitive strategies for individuals with cognitive decline and improve cognitive/functional outcomes. African American (AA) individuals are disproportionately affected by dementia and have cultural preferences not addressed in CR programs currently. Resources are needed to aid this underserved population. This concurrent mixed methods study guided development of CogCare, which would provide tailored CR strategies to improve stress/mood and cognitive/daily functioning for older AA adults with mild/moderate dementia and their caregivers. Older adults and dementia caregivers ($N = 16$ older adults; $N = 3$ caregivers) first completed self-report measures of emotional/cognitive/daily functioning, and were administered a cognitive screener (MCQ, CRS, PROMIS-CF). They then participated in one-time focus groups ($N = 5$). Select members participated in one-on-one interviews to understand their experiences with dementia/dementia caregiving. Qualitative analyses identified needs/knowledge/cultural preferences of this population. Quantitative analysis explored correlations between treatment targets. We triangulated the mixed methods results to ensure that tailoring of CogCare is empirically/culturally supported. Thematic analyses highlighted the following themes: (1) CR strategies, (2) cultural preferences, (3) caregiver considerations, (4) treatment barriers, and (5) awareness/recognition of cognitive decline. Quantitative analyses showed that (a) greater compensation strategy use correlated with better self-perceptions of cognitive functioning ($p < 0.001$) and (b) caregiver burden and competence correlated with a loss of sense of self and personal growth, respectively ($p = .004$; $p = .000$). Participant feedback underscores the demand for accessible, community-informed CR. These findings will inform an open pilot to evaluate feasibility and refine CogCare prior to conducting a randomized trial.

Abstract citation ID: igaf122.4363

**HYBRID EFFECTIVENESS-IMPLEMENTATION TRIAL OF A PILOT RENTAL SUBSIDY PROGRAM AT AN AREA AGENCY ON AGING.**

Anthony Traver[1], Katie Calhoun[2], and Marisa Sheldon[2], *1. University of Kentucky, Lexington, Kentucky, United States, 2. The Ohio State University, Columbus, Ohio, United States*

The prevalence and severity of housing insecurity among older adults in the U.S. are rising and leading to unmet needs, premature nursing admissions, and homelessness. Across the country, area agencies of aging (AAA) are developing new programs and partnerships to address housing insecurity in later life. In 2024, our research team partnered with an AAA to develop a shallow rental subsidy program that provided $330 of rental assistance a month for 12 months to housing-insecure home and community-based (HCBS) clients and evaluated the program through a hybrid effectiveness-implementation trial. We assessed program effectiveness through a 12-month mixed-methods randomized controlled trial with 40 HCBS clients that focused on housing, health, and service utilization outcomes. Treatment effects were estimated using longitudinal analysis of covariance and mixed with qualitative data using the primary data analysis integration procedure. Implementation facilitators and barriers were assessed through surveys and focus groups with AAA staff and interpreted using the Consolidated Framework for Implementation Research (CFIR). Mixed-methods results demonstrated positive program impacts on well-being by alleviating financial distress associated with rising rental costs. Qualitative results that reflected positive impacts on housing security diverged from quantitative results of a null effect. Implementation results revealed a strong tension for change within the AAA that facilitated implementation, but a lack of program compatibility and limited information dissemination were barriers. Our results demonstrate that shallow rental subsidies can be a meaningful complement to HCBS for housing-insecure AAA clients and identify key agency-level features that can promote implementation within the AAA context.

Abstract citation ID: igaf122.4364

**INTERGENERATIONAL PARTNERED DANCE FOR PD AND AD: EVALUATING THE FEASIBILITY OF THE APPROACH PROTOCOL**

Madeleine Hackney[1], Kozbi Bayne[1], Jill Bishop[2], and Marcel Foster[3], *1. Emory School of Medicine, Atlanta, Georgia, United States, 2. Emory University School of Medicine, Atlanta, Georgia, United States, 3. Performance Hypothesis, Atlanta, Georgia, United States*

Dance, often conducted in an intergenerational context, may provide unique learning strategies that promote neuroplasticity to mitigate Parkinson's Disease (PD). Dance's capacity to influence creativity is understudied. The Adaptango Performance Process for Older Adult Community Rehabilitation (APPROACH) protocol explores community building between People with PD and young adults. Partnering young adults with older adults with PD may lead to an intergenerational increase in creativity and understanding between generations, increasing social support and decreasing depression. We studied the intergenerational interactions occurring during a three-month choreographic process, using adapted tango dance. We define the impact of the instructor/older adult/young adult co-experiences and reveal the relationship between creativity and psychosocial status in older adults and younger adults. Six dyads of People with PD (71+/-9 y) and young adults (age= 21+/-2 y) participated in a three-month choreographic workshop involving classes twice per week. At baseline, participants completed cognitive and neuropsychological tests of creativity, and self-reports measuring social support and quality of life. Participants were qualitatively interviewed during the tenth choreographic class and at study end provided qualitative feedback about the extent to which APPROACH classes impacted their well-being and attitudes toward intergenerational relationships. All participants performed in the final performance. Initial analyses show correlations between creativity, social support, and depression in younger adults and older adults with PD. The intergenerational approach may be effective for delivering therapy. Further implementation of the APPROACH protocol could lead to increased creativity and social support for young adults and older adults with PD.

Abstract citation ID: igaf122.4365

**LEVERAGING DESIGN AND MANAGEMENT OF COMMON SPACE OF SENIOR HOUSING TO PROMOTE SOCIAL CONNECTIONS AMONG OLDER ADULTS**

Astrid Jing Weinberg, *Massachusetts Institute of Technology, Massachusetts Institute of Technology, Massachusetts, United States*

The development of quality independent-living senior housing is a critical component for cities and towns to support residents to 'age well'. This can contribute to a physical and social environment for older adults that reduces potential social isolation and loneliness and promotes healthy ageing. This study investigates the use of common areas within independent-living senior housing sites and its potential implications for residents' social interactions and overall well-being. It draws upon a case study performed at three residential buildings in the '2Life Communities' network in urban, suburban, and urban periphery settings within the Boston Metropolitan area, USA. Data was collected from residents (N = 153, CI = 95%) and staff members (N = 8), applying a random sampling strategy and a paper-and-pencil researcher-administered questionnaire for residents and the Delphi technique with a two-round online questionnaire for staff members. Additional site observations of the residents' use of common spaces and related social interactions and behaviors were conducted. The study found similarities in the types of activities residents preferred to do when using common spaces as well as in their evaluations of community programs they most actively participated in. It also found that the design and availability of common spaces and activities varied across the three sites. Most participants stated satisfaction with their living environment and engagement in their community, though some noted infrequent use of common spaces or curated activities. It concludes with discussions on the value of common space and potential factors that could encourage active use of common areas by residents for enhancing social connectedness.

Abstract citation ID: igaf122.4366

**SUBJECT MATTER EXPERT INSIGHTS FOR ENGAGING UNDERREPRESENTED OLDER ADULTS WITH DISABILITIES IN RESEARCH**

Elena Remillard[1], Maurita Harris[2], Kyra Miller[1], and Wendy Rogers[3], *1. Georgia Institute of Technology, Atlanta, Georgia, United States, 2. Wilfrid Laurier University, Toronto, Ontario, Canada, 3. University of Illinois Urbana-Champaign, Champaign, Illinois, United States*

The population of older Americans is increasingly diverse. To keep pace with this demographic shift and advance gerontological research, it is critical that research samples reflect this diversity, so findings are generalizable and representative. However, there are barriers to engaging individuals from underrepresented racial/ethnic groups, such as a mistrust of government institutions, historical precedence of unethical research, language barriers, and logistical challenges that often impede participation. There are some general recommendations for expanding diversity of research samples and overcoming these barriers, but there is a need to understand more about best practices for older adults with disabilities, which is a population that can be especially challenging to reach. We interviewed Subject Matter Experts (SMEs) who had direct experience (professional or personal) engaging older adults and/or people with disabilities from underrepresented racial/ethnic groups (e.g., Black/African American, Asian American, Hispanic/Latino American, and Native American). Participants (N = 13) included researchers, clinicians, and advocacy group leaders. The SMEs shared insights on effective strategies for recruitment, outreach, study design, and knowledge dissemination. Their recommendations had key themes including establishing reciprocal community partnerships, involving community members on the research team, offering flexible ways to participate, and carefully considering terminology when referring to disability, race, and ethnicity. These interviews with SMEs provided important insights and actionable solutions to improve the diversity and inclusivity of research samples. Ensuring that participant samples reflect the range of persons who will use a product, technology, intervention, or community program will contribute to successful implementation, ultimately improving quality of life for older adults.

### SESSION 9115 (PAPER)

### LATE BREAKING: COMMUNITY-ENGAGED RESEARCH APPROACHES IV

Abstract citation ID: igaf122.4367

**APPLICATION OF COMMUNITY-BASED PARTICIPATORY RESEARCH TO SUPPORT CHINESE AMERICAN DEMENTIA CAREGIVERS**

Jinyu Liu[1], Ada Yuk-Sim Mui[2], Ethan Siu Leung Cheung[3], Yifan Lou[4], and Qin Sun[1], *1. Baylor University, Waco, Texas, United States, 2. Columbia University, New York, New York, United States, 3. The University of Hong Kong, Hong Kong, 4. Virginia Commonwealth University, Richmond, Virginia, United States*

Objectives: This study aims to apply community-based participatory research (CBPR) to develop and evaluate a culturally sensitive intervention to reduce the stress of Chinese American dementia caregivers, an understudied population in implementation science. Methods: Guided by CBPR principles, we used multiple strategies for partnership formation, community assessment, intervention development, feasibility evaluation, and intervention improvement. Results: We formed a CBPR team including administrators, social workers, and caregivers from the community and investigators and research assistants. Using mixed methods, we assessed the needs and strengths of Chinese ADRD caregivers through 30 interviews and a survey (N = 165). Based on assessment results, the CBPR team developed a peer mentoring program engaging former caregivers to support current ones. A pilot test showed high feasibility and potential to reduce loneliness and depression while enhancing mentors' sense of purpose. The team has refined the intervention for further evaluation. Discussions: CBPR was useful to facilitate trust-building, cultural tailoring, and shared decision-making for intervention development grounded in caregivers' experiences and strengths. This study contributes to the growing evidence supporting CBPR as a powerful framework for advancing racial minority's engagement in health studies and intervention development. Through deep community engagement, the project addressed both the unmet needs and unique strengths of an underserved population. Our findings emphasize that CBPR not only enhances research participation and intervention relevance, but also fosters mutual learning, community empowerment, and sustainable support systems. research should expand on this work by applying and tailoring CBPR to other social and cultural contexts to support diverse caregiving communities.

Abstract citation ID: igaf122.4368

### CO-DEVELOPING SKILL TRAINING WITH OLDER VOLUNTEER RESOURCE NAVIGATORS

Patricia Oh[1], and Elizabeth Gattine[2], *1. University of Maine, Bangor, Maine, United States, 2. State of Maine Governor's Office, Augusta, Maine, United States*

Background: Older rural dwellers face significant barriers to essential resources, healthcare, and social connections. To increase access, the Governor's Cabinet on Aging, UMaine Center on Aging, and five Area Agencies on Aging, launched the Community Connectors project, a volunteer neighbor-to-neighbor resource navigator model, in 12 age-friendly communities. Connectors were trusted community members with varying backgrounds and professional skills. Project: The research team and subject matter experts collaborated with the Community Connectors to co-develop skills training that supports age-friendly community volunteers and the resource navigation role. Method: Fourteen Connectors (age range 45 – 78) participated in this rapid realist evaluation. Semi-structured interviews (n = 14) and 2 focus groups with connectors and age-friendly leaders identified 15 skill chapter topics, structure, and components. Subject matter experts collaborated with Connectors to develop chapters tailored to adult learning styles, while plain language experts ensured accessibility for diverse educational backgrounds and non-native English speakers. Connectors reviewed skill chapters before distribution. Surveys (n = 67), semi-structured interviews (n = 23), and 2 focus groups (n = 13) collected data about the skill chapter experience. Results: During the initial 9 months, 156 age-friendly leaders and community volunteers completed skill chapters. Despite initial reluctance, 71% of Connectors (10/14) completed 10 skill chapters, earning Certificates of Age-Friendly Community Development. Themes identified included (1) microlearning; (2) applied reflection; (3) peer learning; and (4) connection to experts. Conclusion: This collaborative approach developed accessible training materials for older community volunteers serving as resource navigators. Replication may provide guidance to organizations training older adults for community-based roles in geographically diverse locales.

Abstract citation ID: igaf122.4369

### COMMUNITY-ENGAGED LANGUAGE AND CULTURAL ADAPTATION OF A MOBILE HEALTH BEHAVIORAL INCONTINENCE MANAGEMENT PROGRAM

Alison Huang[1], Celia Kaplan[1], Alayne Markland[2], Claudia Vila Manes[1], Jenny Chen[1], Joyce Cheng[3], Joana Mattero[4], and Leah Karliner[1], *1. University of California, San Francisco, San Francisco, California, United States, 2. University of Utah School of Medicine, Salt Lake City, Utah, United States, 3. Chinese Community Health Resource Center, San Francisco, California, United States, 4. Mission Neighborhood Centers, San Francisco, California, United States*

Language and cultural barriers prevent many midlife and older ethnic minority women from accessing first-line behavioral management interventions for urinary incontinence, despite these techniques' proven effectiveness. This interdisciplinary, community-engaged project adapted an interactive smartphone program for behavioral incontinence management (MyHealtheBladder) to enable its use by midlife and older Latina and Chinese American women with limited English proficiency. Using Barrera and Castro's cultural adaptation framework, we engaged geriatrician and incontinence experts, health communication scientists, and community advocates, in addition to Latina and Chinese American women with incontinence across the aging spectrum. The adaptation process involved systematic review and translation of program content, iterative discussion and refinement by community partners, and content pilot testing in Spanish- and Chinese-speaking women aged 46-77 years with weekly incontinence. Community engagement revealed universal challenges in discussing clinical and anatomical concepts related to incontinence, while identifying culture-specific considerations including varying comfort levels with bladder/bowel discussions, preferences for medical versus colloquial terminology, and differing expectations regarding patient self-management versus clinician consultation. The adaptation process resulted in multiple changes to the language or terminology used to describe toileting activities, pelvic anatomy, and patient-clinician interactions, as well as patient case examples used to reinforce behavioral management techniques. Results demonstrate how meaningful community partnership can identify both shared and distinct cultural needs in health intervention adaptation. The project's community-engaged approach addressed language barriers while honoring cultural values and communication preferences, offering a model for adapting digital health interventions for diverse aging populations facing similar barriers for sensitive health conditions.

Abstract citation ID: igaf122.4370

### EXPLORING NEW WAYS OF USING SIMULATIONS FOR TEACHING ACCESSIBILITY WITH GERONTOLOGY AND URBAN DESIGN STUDENTS

Tamara Smith, and Alina Gross, *Westfield State University, Westfield, Massachusetts, United States*

At Westfield State University two departments regularly teach undergraduate students about physical and cognitive accessibility in urban/interior design. One is the Health Sciences, offering courses such as GERO 101: Introduction to Gerontology and GERO 0102: Health and Physical Aging, and the other is Geography, Planning, and Sustainability, offering courses that cover these issues such as GPS 216: Social Justice and the City. Teaching undergraduate-level students about accessibility across a range of disabilities has a complicated history, particularly around the usage of so-called "simulations", which ask students to do activities such as wear a blindfold to understand visual impairment, wear ear coverings to understand auditory impairment, or use a wheelchair to learn about physical disabilities. While there may be benefits to versions of these exercises and facilitators are likely well-meaning, disability simulations also risk of being offensive, oversimplified, insensitive, and ineffective in their purpose. As faculty from the above departments, our conference session will present ways that we have undertaken simulation-like activities while also addressing the concerns around such activities by reworking and reframing them in a variety of ways to address potential pitfalls. We will share how we have achieved some of the positive objectives of disability simulations, while also making sure students understand context and critiques of these activities appropriately. We will also share ways that we have students focus on the experience of these activities rather than emphasizing such activities will provide specific insights into a particular disability, and have students reflect on these activities in new ways.

Abstract citation ID: igaf122.4371

### EMOTIONALLY RESPONSIVE TEACHING IN AGING EDUCATION: A FRAMEWORK FOR TRANSFORMATIVE PEDAGOGY

Juanjuan "June" He, *Drexel University, Philadelphia, Pennsylvania, United States*

As aging education increasingly intersects with design, community practice, and care, the emotional dimension of teaching remains under-recognized. This presentation introduces a framework for emotionally responsive teaching—a pedagogical approach that centers emotional presence, vulnerability, and cultural humility as catalysts for transformative learning. Grounded in a multi-year Aging & Design course series, this framework emerged from immersive collaborations between university students and older Asian immigrants in Philadelphia. Students engaged in community-based co-design not only developed inclusive design responses, but also confronted personal assumptions, ageism, and deep cultural difference. Through structured reflection and experiential learning, students showed significant shifts in empathy, cultural awareness, and their understanding of aging as a lived and social experience. The presentation shares practical strategies for cultivating emotionally attuned classroom environments, drawing from student reflections, pedagogical tools, and qualitative and quantitative insights. It proposes that emotionally responsive teaching is not ancillary, but foundational: enabling students to engage more fully, listen more deeply, and reimagine aging not just as a demographic issue, but a human one. This session is designed for educators in gerontology, design, social work, and health professions seeking to bridge knowledge and care, and to reshape the classroom into a space of intergenerational connection, inclusion, and ethical growth.

Abstract citation ID: igaf122.4372

### IDENTIFYING CONTRIBUTORS TO UNMET COMMUNITY MOBILITY NEEDS IN OLDER ADULTS AFTER HOME PHYSICAL REHABILITATION

Lindsey Mathis[1], Morgan Fique[1], Jason Falvey[1], and Jasmine Cooper[2], *1. University of Maryland School of Medicine, Baltimore, Maryland, United States, 2. University of Maryland School of Pharmacy, Baltimore, Maryland, United States*

Community mobility impairments after major injuries can persist even after completing home physical rehabilitation programs. Neighborhood-level barriers are potential contributors, but their specific impacts on long-term outcomes are unclear. Identifying these relationships can inform care to address mobility deficits in relation to complex neighborhoods. As such, this study explores therapy experiences and neighborhood conditions to identify contributors to community mobility deficits after rehabilitation. Semi-structured interviews were conducted between July 2024 and March 2025 via Zoom or telephone. Interviews were recorded and transcribed. De-identified transcripts were analyzed using inductive thematic analysis. Data were collected until thematic saturation was reached and no new findings arose. Reliability was tested between two independent coders using Cohen's Kappa (0.74). Five major themes emerged: 1) Restrictive Impacts of Unmet Needs on Community Mobility; 2) Benefits of Targeting Everyday Challenges along with Primary Rehab Diagnoses; 3) Social Networks Enhancing Recovery; 4) Fears of Violence influencing Voluntary Outdoor Mobility, Mitigating Influences of Social Cohesion; 5) Impacts of Comorbidities on Community Mobility. Ultimately, these findings suggest that integrating higher-level social and environmental support systems could further improve outcomes for rehabilitation recipients. Home therapy providers might consider integrating environmental needs into rehabilitation programs—such as facilitating preferred outdoor mobility routes—both during and after home care to better support sustained mobility beyond the home.

### SESSION 9120 (PAPER)

### LATE BREAKING: EFFECTIVE SCIENCE COMMUNICATION

Abstract citation ID: igaf122.4373

### DEMONSTRATING THE EFFECTIVENESS AND ECONOMIC VALUE OF EBPS: FALL PREVENTION AND CDSME PROGRAMS

Brian Ezeonu, Reena Sethi, and Kathryn Zahm, *National Council on Aging, Arlington, Virginia, United States*

Two growing challenges, falls and chronic diseases, are among the costliest and most debilitating for older adults. Falls are the leading cause of fatal and non-fatal injuries among adults aged 65 and older and 60-75% of older adults are living with multiple chronic conditions in the United States, both risk factors combined lead to the highest health care expenditures for adults aged 65 years and older. With one in five Americans projected to be aged 65 years or older by 2030 the demand for cost-effective strategies to prevent falls and manage chronic diseases is more urgent than ever. This study uses pre-post data drawn from NCOA's Healthy Aging Programs Integrated Database (HAPID), covering over 800,000 participants that enrolled in ACL-funded fall prevention and chronic disease self-management education (CDSME) programs to analyze evidence-based fall prevention and CDSME program effectiveness and economic value. Fixed effects and random effects regression models demonstrate that both programs significantly improve health outcomes such as reduced falls, enhanced self-rated health, lower loneliness, and improved self-efficacy. The ROI analysis for fall prevention using avoided healthcare utilization incidents per participant and literature-based healthcare cost estimates shows economic returns ranging from $8.36 to $38.04 per dollar invested. CDSME results showed a 6% increase in self-rated health, 4% improvement in self-efficacy, and up to $153 million in mental health cost savings. Findings from both studies show that evidence-based falls prevention and CDSME programs are not only effective, but fiscally sound.

Abstract citation ID: igaf122.4374

### EXPLORING SPANISH-SPEAKING PATIENT PERSPECTIVES ON INPATIENT SERIOUS ILLNESS COMMUNICATION EXPERIENCES

Carine Davila, Heily Chavez Granados, Ana Paola Garza Naveda, and Christine Ritchie, *Massachusetts General Hospital, Boston, Massachusetts, United States*

Nearly 3.5 million Americans aged 65 years and older who identify as Hispanic/Latino speak Spanish at home. Compared to non-Latino White older adults, Latino older adults are more likely to have a serious illness, but less likely to engage in advance care planning and serious illness conversations (SIC). It remains unclear what Spanish-speaking older adults view as barriers and factors that impact their SIC experience. To address this, we conducted a qualitative study interviewing 12 Spanish–speaking older adults or their medical decision-makers (mean age 69 years, range 55-95 years, 67% female) who had a documented SIC with a palliative care clinician during a recent hospitalization at an academic medical center. We explored their experiences with SIC with clinicians (not limited to palliative care) and elicited recommendations for improvement then used thematic coding to identify key themes. Participants reported that interpreter interactions with patients and clinicians influence patients' active engagement in language discordant SIC. Triadic patient-interpreter-clinician interactions can be overwhelming for patients as they simultaneously process emotionally laden medical information and wonder if interpreters are accurately relaying information bidirectionally. In addition, patients and care partners emphasized that faith, spirituality, and cultural background impact their illness experience and care planning (e.g., information-sharing preferences, decision-making preferences, and considerations around potential travel to their home country during illness). These findings indicate that clinicians and interpreters need culturally tailored interventions and personalized approaches to SIC, guided by patient and care partner recommendations, to effectively meet the needs of Latino Spanish-speaking patients.

Abstract citation ID: igaf122.4375

### LAYOFF EXPERIENCE IN ADULTHOOD AND ALL-CAUSE MORTALITY AMONG US ADULTS, 1979-2022

Xuexin Yu[1], Katrina Kezios[2], Samuel Swift[3], Kaylie Moropoulos[4], and Adina Zeki Al Hazzouri[4], *1. Columbia University Mailman School of Public Health, New York, New York, United States, 2. Boston University, Boston, Massachusetts, United States, 3. University of New Mexico Health Sciences Center, Albuquerque, New Mexico, United States, 4. Columbia University Medical Center, New York, New York, United States*

Involuntary job loss due to layoffs is increasingly common in the US, while its association with all-cause mortality remains inconclusive. We investigated the association between cumulative layoff experience over 33 peak-working years in adulthood and mortality during the subsequent 10 years. Data were from 7,234 working-age adults (aged 16+) in National Longitudinal Survey of Youth 1979. Cumulative layoff experience was operationalized as the number of jobs ending in a layoff from 1979-2012: never (n = 5,183), one (n = 1,514), and ≥two layoffs (n = 537). Compared to never being laid-off (n = 5,183), layoff experience contributed to 18.71 (95% CI: 1.09-36.23) and 32.61 (95% CI: 2.66-62.56) excess deaths per 10,000 person-years among participants with one and ≥two layoffs. These estimates correspond to 0.27 and 0.39 excess years of potential life lost per person for one and ≥two layoff groups. In the fully adjusted Cox proportional hazards model, compared to those with no layoffs, individuals with one and ≥two layoffs had 1.23 (95% CI: 1.00 to 1.51) and 1.30 times (95% CI: 0.96 to 1.77) higher hazards of death from 2012-2022. The observed association did not statistically differ by timing of layoffs, sex/gender, and race/ethnicity. Findings suggest job insecurity may be an important determinant of life expectancy.

Abstract citation ID: igaf122.4376

### THE TAPE OF LIFE: A MUSICAL ACTIVITY ENGAGING THE PUBLIC WITH AGING RESEARCH

Jessica Foley, *Tufts University, Medford, Massachusetts, United States*

Effective science communication can come in many different forms. In particular, the use of artistic approaches can help to make complex concepts more appealing and accessible to new audiences. Concerns about the globally aging population, along with growing media coverage of the anti-aging industry, make public engagement with aging research of particular importance. Considering the current emphasis on technology-enhanced learning, and the proliferation of accessible tools for music production, we devised an interactive music-based science engagement activity designed to introduce new audiences to the diversity of animal aging, and to explore attitudes to longevity research. Participants recorded a snippet of themselves singing into our "lifespan-emulator" (a musical synthesizer ) and listened as their "tape of life" sped up, decayed, and echoed to mirror the lifespan, aging rate, and reproductive curve of several different animal species. We then used this musical activity as a launching point for more in-depth discussions of the biology of aging, and to explore public perception of work aiming to extend the human lifespan. We presented this at five UK-based science festivals, introducing several hundred participants from diverse demographic backgrounds to aging research. Participants' attitudes were evaluated using non-interventionist methods such as poll questions and drawing activities. We found that broadly, our audiences were not interested in substantial extensions to human longevity, with the average "ideal" human lifespan approximately that of the current maximum. Our work demonstrates the efficacy of creative approaches to science communication, and the value of public engagement as a tool to inform research.

### SESSION 9125 (PAPER)

### LATE BREAKING: MOTIVATING PATIENTS FOR HEALTH BEHAVIOR CHANGE I

Abstract citation ID: igaf122.4377

### CAUSAL THINKING AND TRAUMA DETERMINE DEMENTIA PREVENTION BEHAVIORS IN INDIGENOUS AND NON-INDIGENOUS OLDER ADULTS

Jose Aravena[1], Moises Sandoval[2], Javiera Provis[3], Carolina Herrera[4], Karen Wang[1], Cecilia Albala[5], and Becca Levy[6], *1. Yale University, New Haven, Connecticut, United States, 2. INTA, Universidad de Chile, Santiago, Region Metropolitana, Chile, 3. ComunidadMujer, Santiago, Region Metropolitana, Chile, 4. Hospital Hernan Henriquez Aravena, Temuco, Araucania, Chile, 5. INTA, University of Chile, Santiago, Region Metropolitana, Chile, 6. Yale School of Public Health, New Haven, Connecticut, United States*

Indigenous groups in high-income countries experience a higher risk of dementia than non-Indigenous populations. Internal causal thinking (attributing chronic diseases to factors within the individual) has been linked to healthier behaviors, but its role in cognitive health, and how trauma perceptions shape preventive behaviors, remains unclear. We investigated whether causal thinking about cognitive problems and the identification of traumatic experiences as a cause influence engagement in dementia prevention behaviors among Indigenous and non-Indigenous older adults in Chile. In a sequential mixed-method study, Mapuche (n = 167) and non-Indigenous (n = 245) adults aged ≥60 years completed open-ended questions to classify internal versus external causal thinking toward memory problems and Alzheimer's disease (AD), and whether trauma was perceived as a cause. Dementia prevention behaviors were measured by engagement in healthy lifestyles and lifestyle-related dementia risk factors. Fully adjusted multivariate regressions showed that internal causal thinking toward memory problems and AD was associated with better prevention behaviors in the total sample (memory loss: B = –0.028, SE = 0.011, p = 0.015; AD: B = –0.043, SE = 0.014, p = 0.002). Among Mapuche participants, this association was present only for memory problems (B = –0.046, SE = 0.019, p = 0.017), while among non-Indigenous participants it was present only for AD (B = –0.052, SE = 0.018, p = 0.004). Notably, Mapuche participants were more likely to identify trauma as a cause of cognitive problems, and this was linked to more dementia risk factors (B = 0.536, SE = 0.222, p = 0.017). These findings suggest that trauma-related causal beliefs may be an important, culturally specific barrier to dementia prevention, underscoring the need to address trauma narratives alongside health education in prevention strategies

Abstract citation ID: igaf122.4378

### EFFECTS OF TECHNOLOGY-BASED EXERCISE PROGRAMS IN OLDER ADULTS: A SYSTEMATIC REVIEW AND META-ANALYSIS

Sunghee Tak, Hyein Choi, and Sunjung Kim, *Seoul National University, Seoul National Univerisity, Seoul-t'ukpyolsi, Republic of Korea*

Background: Exergame and virtual reality–based interventions have emerged as innovative strategies to prevent falls in older adults. However, evidence regarding their effects on key outcomes related to fall prevention remains limited. Methods: A systematic review and meta-analysis was conducted following PRISMA guidelines. Six electronic databases were searched to identify randomized controlled trials and quasi-experimental studies involving participants aged ≥60 years that reported at least one relevant outcome (mobility, balance, or fear of falling). Data were synthesized using a random-effects model, and standardized mean differences (SMDs) with 95% confidence intervals (CIs) were calculated. Risk of publication bias was assessed using funnel plots. Results: Nine studies met the inclusion criteria. Meta-analysis showed significant improvements in mobility (SMD = –1.05, 95% CI: –1.58 to –0.51) and dynamic balance measured by the Berg Balance Scale (SMD = 0.46, 95% CI: 0.10 to 0.83). No significant effect was found for static balance assessed by the One-Leg Stance test (SMD = –0.28, 95% CI: –1.00 to 0.43). Fear of falling was significantly reduced (SMD = –0.35, 95% CI: –0.61 to –0.08), but the effect became non-significant after excluding a study with median-based statistics (SMD = –0.021, 95% CI: –0.58 to 0.16). Funnel plots indicated possible publication bias for mobility and fear of falling. Conclusions: Technology-based interventions may improve mobility and dynamic balance in older adults, but their effects on static balance and fear of falling are less certain. High-quality, methodologically rigorous trials are needed to confirm these findings and guide their integration into geriatric fall-prevention programs.

Abstract citation ID: igaf122.4379

### ENGAGING CAREGIVERS, OLDER ADULTS AND COMMUNITY MEMBERS IN ORAL HEALTH EDUCATION: THE MOTIVATE AT HOME PILOT PROGRAM

Jennifer Crittenden[1], Denise O'Connell[2], Tara Wilson[2], and Labrini Nelligan[2], *1. University of Maine Center on Aging, Orono, Maine, United States, 2. Massachusetts General Hospital, Bangor, Maine, United States*

The MOTIVATE at Home (MAH) program brings education to caregivers so they are empowered to provide and/or support oral health care for older adults living at home, a growing population that disproportionately faces oral health disparities and health risks. The MAH pilot included statewide online and in-person educational sessions, learning stations at community-based organizations, and online "Ask the Dental Expert" sessions. Surveys were collected from 121 learners across MAH offerings with six additional qualitative interviews conducted with partnering community-based host sites. The audience ranged in age from 23-94 years old (M = 68 years) with 28% reporting a current caregiving role. The majority of participants (86%) learned something new that they could use to improve their own oral health and 83% of all participants, regardless of current self-reported care partner status, reported learning something new that they could use to improve the oral health of someone for who they provide care. Key findings demonstrate the value of a community-based accessible approach to education. Partners also reported that the MAH program was a valuable addition to their current offerings and collaborations. Findings also underscore the importance of using a brief and engaging format for online learning, integration of staff roles and support into in-person learning stations to encourage participation, and inclusion of "gamification" and "fun" to enhance learning. Participants identified additional ongoing education needs focused on specialty dental topics, the connection between health/mental health and oral health, along with an ongoing need for access to affordable dental care for older adults.

Abstract citation ID: igaf122.4380

### IMPROVING CARE & OUTCOMES FOR PEOPLE WITH THE CHRONIC DISEASE OF OBESITY USING THE KAER FRAMEWORK

Elana Kieffer Blass[1], Jennifer Pettis[1], Lisa McGuire[2], and Ophira Bansal[3], *1. Gerontological Society of America, Washington, District of Columbia, United States, 2. Gerontological Society of America, Atlanta, Georgia, United States, 3. Gerontology Society of America, Washington, District of Columbia, United States*

Obesity prevalence for adults aged 65 years old and older has nearly doubled from 22% in 1988 to 42% in 2020. This increase has brought much needed attention to the chronic disease of obesity in older adults and their unique needs when it comes to discussions about and treatment options for overweight and obesity. The Gerontological Society of America (GSA) KAER Toolkit for the Management of Obesity in Older Adults provides a framework for primary care providers to help older people with obesity address this chronic health condition. The newly updated web-based toolkit utilizes the KAER framework to help providers of older adults with recognize and care for obesity in their patients. The four steps of the KAER framework—Kickstart,

Assess, Evaluate, and Refer (KAER)—were initially formulated to improve health-related outcomes for people with dementia. Building on the success of this initial work, the KAER framework has been leveraged to help primary care providers improve outcomes for other conditions, including obesity. This presentation will demonstrate how providers can help older adults with obesity improve their health, function, and quality of life with practical approaches, educational resources, practice pearls, and validated clinical tools, that teams can immediately integrate into their workflow.

Abstract citation ID: igaf122.4381

### THE ASSOCIATION OF RESILIENCE FACTORS WITH MENTAL HEALTH AND SUBSTANCE USE OUTCOMES AMONG PEOPLE WITH AND WITHOUT HIV

Jeremy Delgadillo, Andres Azuero, Olivio Clay, Michael Crowe, David Vance, and Pariya Fazeli, *The University of Alabama at Birmingham, Birmingham, Alabama, United States*

Intrapersonal resilience factors may reduce the prevalence and severity of mental health and substance use outcomes among people living with HIV (PLWH). Cross-sectional data on intrapersonal resilience factors [i.e., resilience, self-rated successful aging (SRSA), self-perceived age], mental health [i.e., depressive symptoms, clinically relevant depressive symptoms, mental health related quality of life (MHQoL)] and substance use (i.e., illicit substance use, alcoholic drinks per week) outcomes among 174 PLWH (Mage = 51.30) and 105 people living without HIV (PLWoH; Mage = 60.10) was examined. Resilience was measured with the Connor-Davidson Resilience Scale, SRSA was a rating on a scale of 1-10, self-perceived age was subjective age minus chronological age, depression outcomes were measured with the Center of Epidemiologic Studies Depression Scale, MHRQoL was measured with the Medical Outcome Study survey, and substance use outcomes were measured with urine toxicology test and self-report. Analyses included general linear models and logistic regressions. Higher levels of resilience and SRSA were associated with fewer depressive symptoms (Resilience: B=-0.37, $p<0.01$; SRSA: B=-1.24, $p<0.01$), not having clinically relevant depressive symptoms (Resilience: B=-0.07, $p<0.01$; SRSA: B=-.32, $p<0.01$), and higher MHQoL (Resilience: B = 0.48, $p<0.01$; SRSA: B = 1.10, $p<0.01$), only higher resilience was associated with testing positive for THC (B = 0.06, $p<0.05$). The association between SRSA and clinically relevant depressive symptoms was weaker among PLWH than PLWoH (B = 0.59, $p<0.05$). Intrapersonal resilience factors may reduce the severity of depression and promote better MHQoL, and SRSA is associated with lower incidence of depression for PLWH and PLWoH alike.

### SESSION 9130 (PAPER)

### LATE BREAKING: MOTIVATING PATIENTS FOR HEALTH BEHAVIOR CHANGE II

Abstract citation ID: igaf122.4382

### EMPOWERING BLACK CAREGIVERS WITH CULTURALLY TAILORED BLOOD PRESSURE SELF-MANAGEMENT EDUCATION

Fayron Epps[1], Shanae Rhodes[2], Derrick Ndukwe[3], Leonarda Daudi[3], Solee Wirba[3], and Ellen Mikus[3], *1. UT Health San Antonio, San Antonio, Texas, United States, 2. The University of Texas Health Science Center at San Antonio, San Antonio, Texas, United States, 3. UT Health San Antonio, University of Texas Health Science Center San Antonio, Texas, United States*

Hypertension is highly prevalent among Black caregivers, reflecting broader trends in the Black community, where nearly 56% of Black adults have hypertension. Black caregivers, who often experience chronic stress, financial strain, and limited access to healthcare, face an even greater risk. Despite these risks, many Black caregivers do not regularly monitor their blood pressure, highlighting the need for targeted interventions and self-management resources to reduce health risks in this vulnerable population. Therefore, this project aimed to enhance hypertension awareness among the Black community, especially caregivers. We evaluated the feasibility and acceptability of delivering culturally tailored blood pressure self-management education to Black caregivers at the 2025 Alter Dementia Summit. After the session, attendees completed pledge cards outlining their commitments and plans toward blood pressure self-management, with 118 responses collected. A content analysis identified eight key categories that reflect the ways in which the education influenced attendees' intentions for blood pressure management: regularly monitoring blood pressure, engaging in physical activities, gaining and sharing knowledge about blood pressure management, improving nutritional practices, prioritizing overall health, enhancing medication management, reducing stress, and achieving proper rest and sleep. Overall, the pledges reflect a meaningful shift toward proactive, empowered, and holistic health behaviors inspired by the education. Educating and providing resources to populations disproportionately affected by cardiovascular and dementia-related disorders is crucial for disease prevention. This feasibility and acceptability study serves as a foundational step in developing an educational packet that can be integrated into existing forums to promote healthy blood pressure and dementia risk reduction.

Abstract citation ID: igaf122.4383

### HEALTHY AGING EYES & EARS: SENSORY HEALTH NEEDS AND BARRIERS FOR ADULTS AGING WITH HIV

Alison Abraham[1], Erin Burk-Leaver[2], Hannah Kisselburgh[3], and Haley Sanner[2], *1. University of Colorado--Denver, Aurora, Colorado, United States, 2. Colorado Health Network, Denver, Colorado, United States, 3. Colorado School of Public Health, Aurora, Colorado, United States*

Sensory loss is common among aging adults and has been linked to poor health outcomes, including depression, frailty, cognitive decline, falls, and poor quality of life. Vision and hearing loss are more prevalent among adults aging with HIV (AAWH) and are associated with decreased healthcare engagement. This qualitative study used a community-based participatory research approach to explore sensory health experiences, needs, and program preferences of AAWH receiving services at Colorado Health Network, a statewide AIDS Service Organization in Denver. Following purposive sampling, we conducted and audio-recorded ten individual interviews and two focus groups with 23 participants (ages 51-73), who shared their lived experiences with sensory loss and perspectives on sensory care. Audio recordings were transcribed, and data were analyzed through deductive and inductive thematic analysis and team-based triangulation. Six themes were identified: interplay of HIV and aging in sensory decline perceptions; sensory aid stigma and self-perceptions of accelerated aging; navigating affordability of sensory aids; creative behavioral, environmental, and technological adaptations to sensory loss; long-term survivorship as a driver of self-management of care and self-advocacy; integrating ideal sensory care for AAWH. Using the Social Ecological Model, we identified facilitators and barriers to sensory care at the individual, interpersonal/provider, organizational/community, and system levels. Facilitators included motivation to maintain independence, peer modeling, community-based benefits, and care navigation. Barriers included discomfort with assistive devices, stigma, provider bias, limited awareness of HIV-specific health needs, and gaps in insurance coverage. Findings offer guidance for designing community-based sensory health programs tailored to the lived experience of AAWH.

Abstract citation ID: igaf122.4384

### PATHWAYS FROM DEPRESSION TO ELEVATED CARDIOVASCULAR RISK IN OLDER ADULTS THROUGH LIFE'S ESSENTIAL 8 MODEL

Navin Kaushal[1], Lakshmi Naliyat[2], Parvati Naliyatthaliyazchayil[2], and Donya Nemati[3], *1. Indiana University, Indianapolis, Indiana, United States, 2. Indiana University-Indianapolis, Indianapolis, Indiana, United States, 3. Ohio State University, Columbus, Ohio, United States*

Introduction: Depression is the leading cause of disability globally and increases the risk for cardiovascular disease, which is the leading cause of mortality. While it's known that depression negatively affects preventive behaviors, there's limited research on its direct and indirect impact on lifestyle factors in a comprehensive model. The Life Essential 8 (LE8) model emphasizes four healthy behaviors: physical activity, nutrition, sleep, and not smoking, which influence four biomarkers: LDL cholesterol, BMI, fasting glucose, and systolic blood pressure. Guided by LE8, this study aimed to investigate how depression affects health behaviors and biological factors among older adults. Methods: The analysis used NHANES data (2021-2023) on community-dwelling older adults in the U.S. ($n = 887$). A multi-mediation path analysis model was developed where biological factors (LDL cholesterol, BMI and glucose levels) mediate the relationship between systolic blood pressure and health behaviors (nutrition, physical activity, sleep and smoking), predicted by depression assessed by Patient Health Questionnaire-9 (PHQ-9). Results: Higher scores in depression was found to predict less physical activity ($\beta$=-.11, p=.04), worse sleep ($\beta$=-.10, p=.04), worse nutrition ($\beta$=-.10, p=.04), and greater smoking ($\beta$=-.08, p=.04). Accounting for total effects found depression to predict higher BMI ($\beta$=.07, p=.022), LDL cholesterol ($\beta$=.09, p=.012), and blood glucose levels ($\beta$=.11, p=.009). Conclusion: Depression functioned as barrier to all four preventive health behaviors of the LE8 model and additionally contributed to worsening three biological factors. These findings bring attention to the importance of incorporating intervention components to potentially alleviate depressive symptoms to increase likelihood of successful health behavior adoption.

Abstract citation ID: igaf122.4385

### PERSONALIZED, MUSIC-BASED RHYTHMIC AUDITORY STIMULATION FOR EXERCISE PROMOTION OF OLDER ADULTS

Kyoung Shin Park[1], Bryan Montero Herrera[2], Brittany Armstrong[2], Jiyeong Hong[2], Jeongwoon Kim[1], Jasmin Hutchinson[3], David Williams[4], and Jennifer Etnier[2], *1. Emory University School of Medicine, Atlanta, Georgia, United States, 2. University of North Carolina at Greensboro, Greensboro, North Carolina, United States, 3. Springfield College, Springfield, Massachusetts, United States, 4. Brown University School of Public Health, Providence, Rhode Island, United States*

Older adults' low rate of moderate-to-vigorous physical activity (MVPA) poses a public health crisis. This clinical trial (NCT06496425, NCT06364189) aimed to test the effects of personalized, music-based rhythmic auditory stimulation (RAS) on MVPA over 6 months in older adults. Community-dwelling, low-active older adults ($N = 59$, Age=70.4±4.4 years, 81% female) were randomized into exercise only (EX) or exercise with RAS (MEX) groups. Both groups received an identical exercise program with aerobic and strength training over 6 months with bimonthly decreases in supervised group sessions from 3 to 1, then 0 days/week. The MEX group was trained to exercise in sync with the tempo of RAS. Weekly MVPA volume was assessed using a waist-worn triaxial accelerometer for 7 days at baseline and on a monthly basis. The data were fitted to a nonlinear mixed-effects model while controlling for covariates (age, sex, race, body mass index). Results indicate that MVPA followed a nonlinear trajectory over time with a significant quadratic effect ($p<.001$), indicating MVPA increased in the initial months with supervision and decreased during the unsupervised months in both groups. The MEX group had higher MVPA in month 1 (p=.004) and month 2 ($p<.017$) before exhibiting a steeper decline in the following months (B=–0.127, p=.028) with a distinct cubic pattern of change ($B = 0.15$, p=.031) compared to the EX group. Random effects indicated substantial individual variability in both baseline MVPA and time slopes. These findings suggest that RAS can successfully increase MVPA through supervised training, but independent adherence to MVPA may require additional strategies.

### SESSION 9135 (PAPER)

### LATE BREAKING: QUALITY IMPROVEMENT INNOVATIONS IN HEALTH CARE I

Abstract citation ID: igaf122.4386

### IMPROVING CARE TRANSITIONS FOR SEPSIS SURVIVORS TO HOME HEALTH CARE: THE I-TRANSFER EXPERIENCE

Kathryn Bowles[1], Sang Bin You[2], Michael Stawnychy[1], Nancy Hodgson[2], Elaine Sang[1], Katherine Pitcher[1], Sungho Oh[1], and Karen Hirschman[2], *1. University of Pennsylvania School of Nursing, Philadelphia, Pennsylvania, United States, 2. University of Pennsylvania, Philadelphia, Pennsylvania, United States*

Older adult sepsis survivors are at high risk for readmission, with a median time-to-readmission of 11 days, making timely attention post-discharge critical. However, providing timely post-acute care is difficult partly due to care transition challenges —such as barriers in identifying sepsis, sharing critical information, and activating timely post-acute follow-up. I-TRANSFER evaluates the implementation and impact of a protocol for timely home health (HH) and outpatient visits within one-week post-discharge for Medicare sepsis survivors. Herein, we describe ERIC implementation strategies used to overcome barriers to timely post-acute care during the I-TRANSFER implementation. Five health systems (16 hospitals) in four states partnered on implementation with five HH agencies. Barriers and facilitators identified via needs assessment interviews were coded using thematic analysis. Findings were shared and strategies developed with implementation teams over 18 months. Strategies were mapped by the research team to the Expert Recommendations for Implementing Change (ERIC) taxonomy. Barriers and strategies were discussed in 36 planning and 145 implementation meetings. To identify sepsis patients, sites modified EHRs (alerts/reports), educated providers, and improved documentation. For information transfer, strategies included referral flags, reminders, notes, staff education, and securing a new sepsis aftercare ICD-10 code. For timely outpatient care, appointments were achieved using centralized support, revised roles, and telemedicine. Implementation strategies addressed barriers from diagnosis through outpatient follow-up, offering actionable guidance and scalable solutions to enhance hospital-to-home and outpatient transitions for older adults and prevent poor discharge outcomes. Mapping to the ERIC taxonomy provides a standardized way to describe the strategies across implementations.

Abstract citation ID: igaf122.4387

### LEVERAGING THE ELDER MISTREATMENT EMERGENCY DEPARTMENT TOOLKIT TO MEET CMS'S AGE-FRIENDLY HOSPITAL MEASURES

Ruthann Froberg[1], Scott Bane[2], and Kristin Lees Haggerty[1], *1. Education Development Center, Waltham, Massachusetts, United States, 2. The John A. Hartford Foundation, New York CIty, New York, United States*

This year, the Centers for Medicare & Medicaid Services' (CMS) introduced an Age-Friendly Health Systems (AFHS) Hospital Measure to assess hospital commitment to providing care to older adult patients. The free, publicly available Elder Mistreatment Emergency Department (EMED) Toolkit—developed by the National Collaboratory to Address Elder Mistreatment—provides a system intervention that aligns with two of this Measure's domains: social vulnerability and age-friendly care leadership. Utilizing the EMED Toolkit to address this new measure is an opportunity to integrate routine identification of elder mistreatment, robust tracking of referrals for positive screens, and strategic leadership to support these efforts. Beyond its validated screening instrument, the Toolkit integrates decision-support prompts, staff workflows, and community-referral pathways into standard hospital processes, ensuring that social-vulnerability assessments trigger evaluation and service linkage. This presentation will provide a detailed crosswalk of the Toolkit that demonstrates functions corresponding to CMS's social vulnerability and age-friendly care leadership attestations. The presentation will also include examples demonstrating the EMED Toolkit's adaptability to different healthcare settings, offering health systems a scalable solution. This free resource provides a roadmap to assist health systems in meeting requirements of the AFHS measure and provides a framework to develop a multidisciplinary response team, ultimately elevating the care provided to older adults.

Abstract citation ID: igaf122.4388

### MEDICAID EXPANSION AND STATE-LEVEL DIFFERENCES IN LONGEVITY-BASED AGING THRESHOLDS

Arun Balachandran[1], Danny Alex[2], and Daniel Belsky[3], *1. Columbia University, New York City, New York, United States, 2. Florida State University, New York, New York, United States, 3. Columbia University, New York, New York, United States*

The Affordable Care Act (ACA), implemented in 2014, allowed U.S. states to expand Medicaid eligibility to previously uninsured populations. The timing and extent of expansion varied across states. While prior research has examined ACA-related mortality outcomes among younger adults, its long-term effects on older populations remain underexplored. This study examines how Medicaid expansion influenced longevity and wellbeing among older adults using two novel demographic indicators: the Prospective Old Age Threshold (POAT) and the Comparative Prospective Old Age Threshold (CPOAT). POAT represents the age at which remaining life expectancy equals 15 years, while CPOAT adjusts this measure to reflect state-level variation in the rarity of reaching POAT. Both measures capture shifts in the "old-age threshold," with higher thresholds indicating greater gains in life expectancy. We analyzed state-level demographic and mortality data from 2010–2022 linking national datasets, including CDC WONDER, the Behavioral Risk Factor Surveillance System, the Health Resources and Services Administration, the U.S. Census Bureau, and the Bureau of Labor Statistics. States were classified as Medicaid expansion or non-expansion based on Kaiser Family Foundation records. Descriptive analyses and multivariate difference-in-differences models were used to assess annual state-level changes in aging thresholds. Preliminary findings indicate that Medicaid expansion was associated with larger increases in POAT and CPOAT (~1.8 to 2.4 years), with stronger effects in states that expanded earlier. Although the magnitude varied across measures and covariates, the overall trend suggests Medicaid expansion improved longevity among older adults. These findings underscore Medicaid expansion's role in promoting equitable health gains across aging populations.

Abstract citation ID: igaf122.4389

### PERFORMANCE OF CLAIMS-BASED FRAILTY MEASURES IN MEDICARE FFS AND MA: FINDINGS FROM NHATS

Lan Luo[1], Xiecheng Chen[2], Chan Mi Park[3], Sandra Shi[1], Kevin Pritchard[4], Ellen McCarthy[1], and Dae Hyun Kim[1], *1. Hebrew SeniorLife, Boston, Massachusetts, United States, 2. Hebrew Rehabilitation Center, Roslindale, Massachusetts, United States, 3. Hebrew SeniorLife, Harvard Medical school, Boston, Massachusetts, United States, 4. Hebrew SeniorLife, Roslindale, Massachusetts, United States*

Frailty is a state of vulnerability in older adults which predicts adverse health outcomes. Although clinical measures, such as a Comprehensive Geriatric Assessment–Frailty Index (CGA-FI) and the Fried phenotype (FP), are reference standards, claims-based measures are often used for research. Given the rapidly growing Medicare Advantage (MA) population and concerns regarding MA data completeness, we assessed performance of claims-based frailty models in detecting frailty by CGA-FI and FP among community-dwelling MA and fee-for-service (FFS) beneficiaries. We measured the Kim's Claims-based Frailty Index (CFI) and Gilbert's Hospital Frailty Risk Score (HFRS) in the National Health and Aging Trends Study linked to Medicare claims. In 2019 (FFS n = 2,153, MA n = 1,672), CFI had high specificity (FFS 0.97, MA 0.99) but low sensitivity (FFS 0.31, MA 0.24) in detecting frailty by CGA-FI. The CFI's PPV was higher for CGA-FI than FP (FFS: 0.84 vs 0.44, MA: 0.90 vs 0.47). In comparison, HFRS had high sensitivity (FFS 0.91, MA 0.91) but low specificity (FFS 0.38, MA 0.43) for CGA-FI frailty, with similar results for FP. Model discrimination was good (C-statistics: CFI 0.81, HFRS 0.79), with both models performing better when benchmarked against CGA-FI. 2021 analyses were similar. In sensitivity analysis using only inpatient claims, HFRS sensitivity decreased while specificity increased. These results suggest that claims-based frailty measures may perform comparably in MA and FFS beneficiaries. However, performance characteristics between models vary – highlighting the importance of model selection for a given population or purpose.

Abstract citation ID: igaf122.4390

### THE IMPACT OF RELATIONAL COORDINATION ON STAFF OUTCOMES IN LONG-TERM CARE

Lisa Cranley, and Daniyal Zuberi, *University of Toronto, Toronto, Ontario, Canada*

Relational coordination (RC) emphasizes the importance of communication and relationships among staff to effectively coordinate care. This study investigated the impact of RC on staff outcomes: work engagement, job satisfaction, and burnout in seven long-term care (LTC) homes in Ontario, Canada. In phase 1 of this mixed-methods study, an online survey was distributed to staff (nurses, allied health providers, personal support workers) working in LTC homes. Staff were asked about RC, work engagement, job satisfaction, work-related burnout (professional efficacy, emotional exhaustion, cynicism), and demographic information. The impact of RC on staff outcomes was assessed using random-effect modeling to account for the multi-level (staff/ LTC) data, with staff as the unit of analysis and LTC as the random effect. We received 153 completed surveys. RC had a significant positive association with staff work engagement, job satisfaction, and professional efficacy in both unadjusted and adjusted models for staff demographics and LTC size. In contrast, RC had a significant negative association with emotional exhaustion and a non-significant negative association with cynicism. Age had a significant positive association with work engagement and job satisfaction, while the association was inverse with cynicism. Holding a bachelor's degree or higher had a significant positive association with work engagement and job satisfaction; however, the association was inverse with emotional exhaustion. None of the demographics were significantly associated with professional efficacy. Better communication and relationships between staff can enhance their work engagement and well-being, which could improve overall work efficiency and support quality improvement innovations for integrated care.

### SESSION 9140 (PAPER)

### LATE BREAKING: QUALITY IMPROVEMENT INNOVATIONS IN HEALTH CARE II

Abstract citation ID: igaf122.4391

### ASSOCIATION OF GERIATRIC EMERGENCY DEPARTMENT CARE WITH PATIENT OUTCOMES IN OLDER ADULTS

Yuting Qian[1], Cameron Gettel[2], Jasmine Su[3], Elyssa Grogan[4], Inessa Cohen[5], Craig Rothenberg[6], Xi Chen[7], and Ula Hwang[8], *1. Yale School of Public Health, New Haven, Connecticut, United States, 2. Yale School of Medicine, New Haven, Connecticut, United States, 3. New York University School of Medicine, New York, New York, United States, 4. NYU Langone Health, New York, New York, United States, 5. Yale University, Yale University, Connecticut, United States, 6. Yale University School of Medicine, New Haven, Connecticut, United States, 7. Yale University, New Haven, Connecticut, United States, 8. NYU Grossman School of Medicine, New York, New York, United States*

Since 2018, the Geriatric Emergency Department (GED) accreditation program has recognized EDs that provide high-quality care tailored to older adults. Despite its considerable expansion in recent years, no studies have evaluated the impact of GED care on patient-centered outcomes using national data. Our objective was to determine whether GED care is associated with improved outcomes among older adults. We used the 2018-2021 Health and Retirement Study-Medicare linked data of adults aged≥65 years, weighted for national estimates. Receipt of GED care was defined as having an ED visit at a GED (versus not). Patient-level analyses were conducted using each individual's most recent ED visit. Multivariable logistic regression models were used to estimate associations between receipt of GED care and outcomes of hospital admission and 30-day mortality, adjusting for patient demographics, socioeconomic status, health conditions, and hospital-level characteristics. Among 4,570 older adults – representing 25,317,444 adults nationally – with an ED visit, 270 (5.9%) received GED care. Compared with those treated in non-GEDs, patients treated in GEDs had significantly lower odds of hospital admission (OR, 0.710; 95% CI, 0.505-0.998), primarily among adults aged 65-80 years (OR, 0.565; 95% CI, 0.336-0.952) and non-Hispanic White individuals (OR, 0.614; 95% CI, 0.410-0.920). Among non-Hispanic White individuals, GED care was also associated with a significant reduction in 30-day mortality (OR, 0.554; 95% CI, 0.324-0.945). This is the first study using national data to demonstrate that GED care significantly improves outcomes for older adult subgroups. Broader implementation can enhance reach and impact across programs and diverse populations.

Abstract citation ID: igaf122.4392

### CAREGIVER SUPPORT ACCESS AND THE INFORMATION LANDSCAPE: A STUDY OF INFORMATION SOURCES AND FORMAL SUPPORT USE

Joseph Svec[1], Jun Chu[2], Eunbea Kim[3], and In Jeong Hwang[4], *1. St. Joseph's University, Patchogue, New York, United States, 2. University of Maryland, Baltimore County, Baltimore, Maryland, United States, 3. Auburn University, Auburn, Alabama, United States, 4. Iowa State University, Ames, Iowa, United States*

The gap between the need for respite services and caregiver abilities to access them is well documented. (AARP, 2024). One reason for this gap is that many caregivers lack information regarding the cost and availability of respite services (Castro et al., 2023). Previous studies found that getting information on respite services can be cumbersome and the accessed information may be incomplete. (Leocadie et al., 2018). Drawing on institutional models of organizational behavior, we distinguish among three major types of information contexts: 1) Individualized, 2) Community-Level, and 3) Public-Level. The objective of our study is to examine the impact of information gathering on utilization of respite care among caregivers. Using data from the National Study of Caregivers (NSOC), we found that caregivers who seek information in the individual sphere have 16% higher odds (OR = 1.16) of using respite services whereas those who received information from community (OR = 0.82) or public (OR = 0.86) spheres have between 18% and 14% lower odds of using respite services. Rather, caregivers relying on community and public sphere for information had higher odds of receiving caregiving training and utilizing support group services. The results suggest that while individuals are more likely to seek functional, and costly, support via respite, public information sources are more likely to direct caregivers toward individualized self-help approaches via training to enhance caregiving activities or support group coping. These findings suggest that caregiver support policies should account for how informational disparities can create qualitative differences in formal support use.

Abstract citation ID: igaf122.4393

### CASE COMPARISON OF NURSING HOMES DURING COVID-19: LESSONS FOR FUTURE INFECTIOUS DISEASE OUTBREAKS

Amy Vogelsmeier[1], Lori Popejoy[1], Steven Miller[2], Melissa Taylor[1], and Linda Anderson[1], *1. University of Missouri - Columbia, Columbia, Missouri, United States, 2. University of Missouri Sinclair School of Nursing, Columbia, Missouri, United States*

U.S. nursing homes (NH) were affected by the COVID-19 pandemic with over 2.2 million confirmed cases and over 150,000 resident deaths. This analysis explored differences in NHs' response during COVID-19 including activities that may have influenced resident outcomes and was part of a larger mixed-methods study to understand NHs response to COVID-19. Interviews with leaders, infection preventionists, staff, residents/families from 24 Midwestern NHs were analyzed using directed content analysis and summarized into individual NH cases. We selected 10 NHs for case-comparison based on their 2020 Nursing Home Safety Net all-cause mortality Data: five high mortality NHs (HMNH) and five low mortality NHs (LMNH). Most HMNHs were non-corporate/standalone (n = 4, 80%) and urban (n = 3, 60%). Most LMNHs were corporate owned (n = 3, 60%) and rural (n = 3, 60%). Bed size (61-120) was similar (HMNH n = 3, 60%; LMNH n = 4, 80%). No differences were noted for leaders or medical director involvement. LMNHs had more RN infection preventionists compared to HMNH (n = 4, 80%, n = 2; 40%, respectively). Implementation of regulatory-based guidelines e.g. cohort, isolation, quarantine, PPE use, testing/reporting, visitor restrictions were similar, however, HMNHs experienced greater challenges with PPE access and longer test result turnaround times. Both groups used agencies for staffing shortages, however, LMNHs had more consistent processes for managing agency staff. Both groups promoted vaccinations except one HMNH encouraged medical/religious exemptions with the goal of turnover reduction. Both groups shared concerns about the impact of COVID-19 on staff/residents' health/well-being. Differences in how NHs responded to COVID-19 and implications for future infectious disease outbreaks will be described.

Abstract citation ID: igaf122.4394

### EXPANDING GERIATRIC EDUCATION ACROSS THE UNIVERSITY AND COUNTRY: SCHAALMAN SENIOR VOICES PROGRAM

Erin Emery-Tiburcio, Grisel Rodriguez-Morales, Laurin Mack, Franco Sambataro, and Robyn Golden, *Rush University Medical Center, Chicago, Illinois, United States*

The Schaalman Senior Voices (SSV) Program aims to expand awareness of the 4Ms of an Age-Friendly Health System across Rush University, and around the country. This aim was achieved by offering: student fellowships, in which health professions students complete a mentored project focusing on the 4Ms; faculty fellowships, in which health professions faculty develop and integrate the 4Ms into existing curriculum; a six-session, twelve-hour Summer Academy on Aging for Rush University students and staff; and a Thought Leader Lectureship in which a leader in the aging network presents to a national audience. In the 2024-2025 academic year, three student fellows, three faculty fellows, 19 Summer Academy students, and one thought leader with 269 attendees participated in Schaalman activities. Outcomes of student fellowships included significant increases in awareness of audiology services among primary care staff, development of a training module for direct care workers on bathing and toileting within the 4Ms framework, and increased knowledge about delirium among nursing staff. Faculty fellows created unique, interactive 4Ms curriculum within an anesthesiology clerkship, an undergraduate pathophysiology course, and occupational therapy assessment training. Summer Academy learners demonstrated an average of 21.27% increase in knowledge (range 4.41%-65.7% increase). Outcomes of the Thought Leader Lectures included (n = 241 responses) 98% satisfaction with content and 96% found the content directly applicable to practice. Implementation of the SSV project has significantly increased access to aging education and taken great strides in creating the 4Ms as foundational learning across Rush University colleges. Implications for policy and dissemination will be discussed.

Abstract citation ID: igaf122.4395

### INFORMATION DIVERSITY AND HEALTH CARE QUALITY: THE ROLE OF TRUST IN HEALTH CARE SYSTEM AND SELF-CARE CONFIDENCE

Jeong Eun Lee[1], In Jeong Hwang[1], Ansuk Jeong[2], and Yulri Kim[3], *1. Iowa State University, Ames, Iowa, United States, 2. DePaul University, Chicago, Illinois, United States, 3. Iowa State University, Iowa State University, Iowa, United States*

With health information now accessible through diverse channels, older adults are increasingly seeking it from multiple sources using online portals, social media and wearable devices. Whether these emerging health-seeking behaviors complement or conflict with traditional practices such as visiting doctors warrants an empirical investigation. This study first examines the relationship between information-seeking behavior and perceived quality of professional healthcare services and then explores whether this relationship is moderated by trust in the healthcare system and confidence in self-care. Based on 2,203 older adults over 65 (M = 73.3, SD = 6.7) from wave 6 of HINTS (Health Information National Trends Survey), we create health information diversity scores based on standardized measures of health information frequency and variety—derived from patient engagement with online portals, social media, and wearable devices. We then analyze how these diversity scores relate to perceived care quality from health professionals, with healthcare system trust and self-care confidence as moderators. We found that diverse information was in general negatively associated with perceived care quality. However, both healthcare trust and self-care confidence moderated this relationship. In particular, the negative relationship weakened with high trust and hjgher self-care confidence compared to those with lower levels. Health information source diversity may undermine care satisfaction from health care professionals, especially when patients lack trust in healthcare system or confidence in self-management. Healthcare interventions should prioritize building both institutional trust and patient self-efficacy to help patients effectively navigate diverse health information while maintaining positive care experiences.

Abstract citation ID: igaf122.4396

### LEVERAGING ELECTRONIC HEALTH RECORD DATA FROM A NOVEL STANDARDIZED NOTE TEMPLATE TO STUDY ELDER ABUSE IN VETERANS

Lihan Kao[1], Maria Mor[2], Hongwei Zhang[2], Tony Rosen[3], and Lena Makaroun[4], *1. University of Pittsburgh School of Medicine, Pittsburgh, Pennsylvania, United States, 2. VA Pittsburgh Healthcare System, Pittsburgh, Pennsylvania, United States, 3. Weill Cornell Medicine / NewYork-Presbyterian Hospital, New York, New York, United States, 4. VA Pittsburgh Healthcare System/University of Pittsburgh, VA Pittsburgh Healthcare System, Pennsylvania, United States*

Though healthcare systems play an important role in identifying and responding to elder abuse (EA), inadequate electronic health record (EHR) data have hindered EA surveillance and research efforts. In 2025, the Veterans Health Administration (VHA) implemented a national standardized EHR note template to document reports of suspected abuse/neglect. In this retrospective descriptive study, we examined VHA EHR data from the 10-site pilot and initial four months of national implementation of the new note template (3/2024 – 6/2025). To focus on EA, we restricted analyses to veterans aged ≥60 years and excluded reports to Child Protective Services. The final analytic sample consisted of 1,982 reports for 1,684 unique veterans. Reported veterans had a median age of 76 years and were 92.8% male, 20.2% non-Hispanic Black, 3.9% Hispanic, and 30.3% married. While most (86.4%) had one report filed over the study period, 11.0% had two, and 2.7% had ≥3. EA occurred most often in the veteran's home (78.5%). The most frequent abuse types were self-neglect (36.3%), multiple abuse types (24.2%), and financial exploitation (17.6%). Among poly-victimization reports, the most frequent combination was financial exploitation and self-neglect. Among non-self-neglect cases (N = 1,253), the most commonly reported suspected harmers were multiple individuals (23.7%) or adult children (19.2%). This first system-wide description of EA in a national healthcare system, enabled by standardized documentation, highlights prevalent self-neglect, poly-victimization, and multiple suspected harmers, underscoring the complexity of EA identified in healthcare settings. Future efforts using data from structured EHR tools may improve EA detection and prevention efforts.

### SESSION 9145 (PAPER)

### LATE BREAKING: QUALITY IMPROVEMENT INNOVATIONS IN HEALTH CARE III

Abstract citation ID: igaf122.4397

### ACCURACY OF THE CMS CHRONIC CONDITIONS DATA WAREHOUSE ALGORITHMS IN MEDICARE ADVANTAGE AND FEE-FOR-SERVICE DATA

Xiecheng Chen[1], Chan Mi Park[2], Lan Luo[3], Eunji G. Kim[3], Ellen McCarthy[4], and Dae Hyun Kim[4], *1. Hebrew Rehabilitation Center, Roslindale, Massachusetts, United States, 2. Hebrew SeniorLife, Harvard Medical school, Boston, Massachusetts, United States, 3. Marcus Institute for Aging Research, Boston, Massachusetts, United States, 4. Hebrew SeniorLife, Boston, Massachusetts, United States*

Since over half of Medicare beneficiaries now enroll in Medicare Advantage (MA), and Chronic Condition Warehouse (CCW) algorithms are commonly used to identify chronic conditions from Medicare claims data, we evaluated whether their performance extends from fee-for-service (FFS) claims to MA encounter data using NHATS Round 9 (2019) as the reference. NHATS respondents with at least 24 months of continuous MA or FFS enrollment before the survey date were linked to Medicare claims and encounter files. CCW definitions with a two-year look-back were applied for conditions such as arthritis, chronic lung disease (COPD plus asthma), diabetes, heart disease (heart failure plus ischemic heart disease), hypertension, and osteoporosis. Self-reported, physician-diagnosed conditions served as the reference, with estimates weighted to the national survey. The differences between programs were tested using survey-weighted Wald tests. The sample included 1,708 MA and 2,239 FFS beneficiaries. High sensitivity was observed for diabetes and hypertension across both programs, with high specificity for diabetes (0.95 in MA vs. 0.92 in FFS) and moderate specificity for hypertension (0.70 in MA vs. 0.68 in FFS). Chronic lung disease demonstrated moderate sensitivity (0.64–0.66) and high specificity (0.90–0.91), with minimal differences between MA and FFS. Heart disease showed moderate sensitivity and specificity for both programs. Arthritis and osteoporosis had lower sensitivity (0.50 in MA vs. 0.54 in FFS) for arthritis, but were highly specific (0.85 in MA vs. 0.85 in FFS). Overall, CCW algorithms for these common conditions performed comparably in MA and FFS, and the observed differences between programs were small.

Abstract citation ID: igaf122.4398

### ENHANCING QUALITY THROUGH EQUITY AND ACCESS IN AN AGE-FRIENDLY ECOSYSTEM

Robyn Golden, Elizabeth Davis, Walter Rosenberg, Diane Mariani, Alison Wagner, Padraic Stanley, Erin Emery-Tiburcio, and Molly Jenkins, *Rush University Medical Center, Chicago, Illinois, United States*

Ensuring timely, quality, and culturally responsive access to medical and social care is essential to equity in meeting the diverse needs of older adults. Access influences every aspect of care delivery, from early intervention to ongoing management of chronic conditions, and plays a key role in improving health and wellbeing. This symposium will highlight how programs at Rush University Medical Center are breaking down barriers to care through the 4M's of an Age-Friendly Health System Framework and expanding opportunities for older adults, caregivers, and providers. Presentations will feature four initiatives that are built on the foundation of the 4Ms of an Age-Friendly Health System: Schaalman Senior Voices expands access to geriatric education for healthcare providers and students to enhance their ability to care for older adults; Falls Prevention initiatives expand community access to education on risks and strategies by addressing social determinants of health and offering English- and Spanish-language sessions; Rush@Home delivers team-based primary and social care directly to patients' homes; and Caring for Caregivers (C4C) connects caregivers with supportive services that strengthen their capacity to provide care. Speakers will share emerging data and recent program developments, followed by a discussion of practical strategies to expand access to education, caregiver support, coordinated home-based services, and clinical care. The session will conclude with an interactive dialogue offering actionable strategies for clinicians, educators, policymakers, and advocates to take a multi-pronged, holistic approach to ensuring older adults receive quality healthcare.

Abstract citation ID: igaf122.4399

**IMPLEMENTING "GET CONNECTED" TRAINING IN LONG-TERM CARE SETTINGS DURING COVID**

Lené Levy-Storms[1], Eunice Kim[2], and Susan Kohler[3], *1. UCLA Luskin School of Public Affairs/Social Welfare, Los Angeles, California, United States, 2. California State University, Los Angeles, California, United States, 3. University of Southern California, Los Angeles, California, United States*

A lack of emotionally attuned care occurs in long-term care (LTC) settings threatening residents' quality of life, particularly those with cognitive deficits. This study offered "Get Connected (GC)," an evidence-based communication training intervention that teaches nine techniques for attaining emotional connections between direct care workers and residents using the EPIS framework (Exploration, Preparation, Implementation, Sustainment) with a train-the-trainer strategy. In the Exploration phase, this study recruited four of approximately 100 LTC facilities invited. In the Preparation phase, only 4 of these 5 sites ultimately participated. In the Implementation phase, two LTC facilities——each with one assisted living and one nursing home——offered GC with a total of 37 direct care workers over an eight-month period after respective accommodations. Entrenched organizational-level challenges, magnified by the lingering effects of the COVID-19 pandemic, posed sustained difficulties throughout implementation processes. Despite these issues, data analyses on post-training surveys with these 37 direct care workers indicated a high acceptability of GC strategies (95%). Additional results from 6 separate focus groups with three stakeholder types (total N = 34: 11 administrators, 11 staff, 12 residents) reaffirmed GC training as a valuable refresher and emphasized the importance of reinforcement. Additional results from both survey and focus group respondents supporting wider uptake in LTC settings (99%) suggest promise for the Sustainment phase. Implications will require concerted efforts among LTC stakeholders at multiple levels, including policy makers, administration, and the care workforce, to transform LTC training infrastructure to prioritize humanity during care.

Abstract citation ID: igaf122.4400

**REFINING A NOVEL ADVERSE EVENT REPORTING DECISION ALGORITHM IN RESEARCH INVOLVING SERIOUSLY ILL AND OLDER ADULTS**

Abigail Baim-Lance[1], Sara Lubetsky[2], Gabrielle Schiller[2], Brynn Bowman[2], Rachael Heitner[2], Dalane Kitzman[3], and Christopher Woodrell[2], *1. Veterans Health Administration, New York, New York, United States, 2. Icahn school of Medicine at Mount Sinai, New York, New York, United States, 3. Wake Forest School of Medicine, Winston-Salem, North Carolina, United States*

Studies enrolling older adults and people with serious illness are critical to advancing the science of aging. However, current serious and non-serious adverse event [(S)AE] reporting has been excessive in terms of expedited and unrelated (S)AEs reporting, resulting in an administrative burden that may inhibit innovation. In response, an expert panel—comprising representatives from federal agencies, researchers in geriatrics and palliative care, national clinical and scientific organizations, and medical ethicists—convened between 2021-2025 to develop guidance that, in line with existing regulations, improves (S)AE assessment and reporting. The resulting Decision Algorithm and Reporting Toolkit (DART) consists of decision-making support tools accounting for study types, prioritizing event relatedness, and assessing alignment of events with documented patient goals. To improve DART usability and uptake, our next step was to seek feedback on the tools. We presented DART in a structured 90-minute interactive multi-stakeholder meeting. Topics included project background, Toolkit overview, and feedback captured by note-taking synthesized into next steps. There were 31 attendees, many expressing DART's value for the field. Feedback was two-pronged: 1) Toolkit modifications, including nuanced issues that should be addressed via user testing; and 2) dissemination input. The group recommended pilot testing leveraging investigator and sponsor networks and research collaboratives. Involving IRB and regulatory monitors was also suggested. Concurrently, the group endorsed scientific meeting presentations to socialize the approach. Next steps for DART will be ongoing refinement, pilot testing, and stakeholder engagement to improve uptake and use, ultimately with the goal of improving (S)AE reporting.

Abstract citation ID: igaf122.4401

**REIMAGINING CARE: TECHNOLOGY-ENABLED STRATEGIES TO ADDRESS THE DIRECT CARE WORKFORCE SHORTAGE**

Sharmila Suresh, and Jara Pallas-Brink, *Michigan State University, East Lansing, Michigan, United States*

Direct Care Workers (DCWs) provide long-term care and personal assistance services to older adults and those living with disabilities or chronic conditions, ensuring a high quality of life and independence. The shortage of Direct Care Workers in the United States has become a critical challenge for aging and disability services. Contributing factors are high turnover rates, lack of benefits, low wages, racism, lack of respect, and inadequate training. To address the DCW shortage, IMPART Alliance at Michigan State University launched a program that supports employers in testing innovative retention and recruitment strategies. Within this context, this paper examines several organizations that are piloting grassroots innovations that integrate technology into care models. One promising example is to expand residential supported housing offerings by introducing a smart home alternative to traditional residential support models which rely on 24-hour direct support. This initiative aims not to replace DCWs but to utilize technology as a tool to elevate person-centered care, expanding opportunities for communication and enhancing individualized support. Furthermore, these programs emphasize training and the development of new skills, fostering a collaborative relationship between caregivers, technology, and clients. By examining three organizational initiatives that focus on various innovations in care, this paper explores the potential for grassroots, technology-enabled solutions to both strengthen the direct care workforce and inform broader strategies for addressing the shortages.

### SESSION 9150 (PAPER)

### LATE BREAKING: TRANSLATIONAL GEROSCIENCE I

Abstract citation ID: igaf122.4402

### A BODY COMPOSITION AGING CLOCK AND ITS MULTIOMIC PROFILES

Liming Zhang[1], Xueqing Jia[2], Maoyao Xia[3], Shuli Jia[4], Jesse Poganik[5], Xia Jiang[3], Meiling Ge[3], and Zuyun Liu[1], *1. Zhejiang University, Hangzhou, Zhejiang, China (People's Republic), 2. Zhejiang university, Hangzhou, Zhejiang, China (People's Republic), 3. Sichuan University, Chengdu, Sichuan, China (People's Republic), 4. Sichuan University, Chengdu, Zhejiang, China (People's Republic), 5. Harvard Medical School, Boston, Massachusetts, United States*

Body composition represent a fundamental and physiologically relevant dimension of aging. Leveraging deep body composition phenotypic data from a Chinese cohort, we developed BodycomBA, a novel aging clock, and BodycomBAA for assessing aging acceleration. BodycomBA showed robust correlations with chronological age (CA), and BodycomBAA exhibited strong efficacy in capturing a wide spectrum of physical health conditions. These findings were validated in two large independent cohorts (n=9,256 and n=3,403). Additionally, BodycomBAA was effective in discriminating early-onset cases of age-related diseases, and incorporating BodycomBA into models of conventional risk factors (e.g., CA, smoking, and drinking) enhanced the diagnostic power for these diseases. By integrating multiomic data, we identified 350 DNAm sites, 68 proteins, 41 metabolites, and 257 gut microbiota significantly associated with BodycomBAA and elucidated their related biological pathways. Calculating the similarity network between these pathways further identified 2 connected (integrin binding-focal adhesion pathways act as bridges between the 2 modules) and 8 independent functional modules for proteome and DNA methylome, respectively. Subsequent cross-omics analysis revealed interconnected networks of molecular pairs and core molecules (e.g., PLOD1 protein) underlying BodycomBAA. Interestingly, the omic-inferred BodycomBA models demonstrated enhanced capability in elucidating the heterogeneity of physical health conditions (e.g., functions of liver, kidney, and metabolism) compared to the actual BodycomBA. Collectively, this study presents a highly adaptable and valid aging clock derived from body composition data. Its multiomic profiles provide insights into aging biology and a valuable framework for precisely stratifying individuals at risk of accelerated aging, paving the way for personalized anti-aging interventions.

Abstract citation ID: igaf122.4403

### CULTIVATING THE PHYSICIAN-SCIENTIST PIPELINE IN TRANSLATIONAL AGING: EARLY OUTCOMES FROM NIA STARR

Alison Huang[1], Louis Nguyen[2], Heather Whitson[3], Ashley Price[4], Hannah Puttre[5], Zara Cooper[2], Anthony Viera[4], and Christine Ritchie[5], *1. University of California, San Francisco, San Francisco, California, United States, 2. Brigham and Women's Hospital, Boston, Massachusetts, United States, 3. Duke University, Durham, North Carolina, United States, 4. Duke University School of Medicine, Durham, North Carolina, United States, 5. Massachusetts General Hospital, Boston, Massachusetts, United States*

Despite efforts to increase the clinician-scientist pipeline, fewer physicians or surgeons are pursuing scientific careers, particularly in aging, threatening future advances in care. One critical challenge for early-stage physician/surgeon scientists is the demanding and inflexible nature of medical training, particularly residency training that offers few opportunities for sustained research engagement. The National Institutes of Health's Stimulating Access to Research in Residency (StARR) R38 initiative represents a recent effort to directly integrate intensive, 1-2 year-long research experiences into residency. We present early insights from all StARR programs funded by the National Institute on Aging--the University of California San Francisco, Duke University, Brigham and Women's Hospital, and Massachusetts General Hospital--which have collectively engaged 27 physicians/surgeons-in-training in basic, clinical, or translational aging science since 2021. Of the 15 NIA StARR graduates who have already completed residency, including 5 from traditionally underrepresented backgrounds, all have so far continued in academic training or careers, and 9 remain engaged in aging science. Mixed-methods evaluation data from graduating scholars demonstrate StARR's meaningful impact on career trajectories and research commitment. However, program implementation has revealed key educational challenges: establishing effective interdisciplinary mentorship teams; balancing rigorous methodological training with productivity pressures; identifying sustainable funding pathways; and defining aging research focus across diverse specialties. These findings inform broader efforts to restructure medical education for research integration; while StARR's early success suggests that systematic, extended research training during residency can effectively cultivate physician-scientists in aging, programmatic innovations are needed to address identified training barriers and ensure scalability across institutions.

Abstract citation ID: igaf122.4404

### MULTIOMICS MAPPING REVEALS BIOLOGICAL INSIGHTS INTO THE MUSCLE-TO-MENTAL AXIS DURING AGING

Kaili Sun[1], Xucheng Wu[1], Hongwei Chen[1], Xueqing Jia[2], Weijing Gao[2], Xiaoyan Jiang[3], Xiaofeng Wang[4], and Zuyun Liu[1], *1. Zhejiang University, Hangzhou, Zhejiang, China (People's Republic), 2. Zhejiang university, Hangzhou, Zhejiang, China (People's Republic), 3. Tongji University, Shanghai, Shanghai, China (People's Republic), 4. Fudan University, Shanghai, Shanghai, China (People's Republic)*

Peripheral organs, including the skeletal muscle, play an important role in mental health. However, how skeletal muscle degeneration impacts mental disorders and the underlying mechanisms have not been fully understood. This study dissected the muscle-to-mental axis utilizing a multidimensional dataset of samples from the UK Biobank and the Rugao Ageing Study. Longitudinal analysis observed that skeletal muscle degeneration was associated with an increased risk of mental disorders, including depression and anxiety. This study mapped the multiomics signatures linked to muscle and mental disorders, pinpointing >2,000 statistically significant associations and 742 biomarkers, comprising 31 gut microbial species, 71 metabolites and 640 proteins. Notably, deleterious molecules (e.g., glycoprotein acetyls, growth differentiation factor 15, and urokinase plasminogen activator surface receptor) are known for their roles in inflammation, apoptosis and mitochondrial dysfunction. Conversely, protective molecules [e.g., valine (Val), leucine-rich repeat neuronal protein 1, and integrin alpha-11] are implicated in cellular homeostasis, neuroprotection and immune regulation. Further analysis revealed that the impact of skeletal muscle degeneration on mental disorders may occur through immunoinflammatory processes, and alterations in gut microbiome, metabolome and proteome. The multiomics integrative analysis identified an interaction network of key molecules (e.g., Val, T-cell surface glycoprotein CD4, and Paenibacillus thermotolerans) regulating muscle and mental disorders. Taken together, the multiomics mapping deepens our understanding of the muscle-to-mental axis during aging and reveals critical molecules as geroprotectors of multiple systems.

Abstract citation ID: igaf122.4405

### STATE- AND COUNTY-LEVEL SOCIAL DETERMINANTS OF HEALTH-ADJUSTED LIFE EXPECTANCY

Elizabeth Davis[1], Nasim Ferdows[1], and Daniel Kim[2],
*1. Northeastern University, Boston, Massachusetts, United States,*
*2. School of Community Health and Behavioral Sciences, Bouvé College of Health Sciences, Northeastern University, Boston, Massachusetts, United States*

In the United States, health-adjusted life expectancy (HALE) declined by 0.3 years between 2009 and 2019, which may reflect increased deaths related to drug overdose, suicide, and alcohol. To combat the rise in such "deaths of despair," it is critical to identify their root causes including social and economic determinants and address them through corresponding policies. We obtained county-level HALE from 2009 and 2019 through the Global Burden of Diseases, Injuries, and Risk Factors Study. Using state fixed effects models and these panel data, we explored multiple social and economic determinants of HALE simultaneously including the state-level percentage in poverty, gender pay gap ratio, violent crime rate, tax burden, earned income tax credit rate, income inequality, welfare spending, and education spending; and county-level social capital, debt-to-income ratio, and racial segregation. We found several of these factors to be positively associated with HALE including a higher state tax burden and earned income tax credit rate as well as county-level median household income, social capital, and debt-to-income ratio. Moreover, we determined other factors to be inversely linked to HALE including state-level median income, the percentage in poverty, percentage unemployed, and income inequality level; as well as the county-level percentage unemployed. Overall, our findings suggest a variety of specific social and economic determinants of HALE that may help to inform future interventions and policies in order to promote healthy aging and advance healthy longevity among Americans.

Abstract citation ID: igaf122.4406

### TRANSLAGE: A UNIFIED PLATFORM TO BUILD AND TRANSLATE AGING BIOMARKERS INTO CLINICAL SURROGATE ENDPOINTS

Raghav Sehgal, *Yale University, New Haven, Connecticut, United States*

Evaluating the effectiveness of lifespan- and healthspan-extending interventions in human clinical trials requires robust surrogate endpoints. Omics-based aging biomarkers hold promise but face limitations: narrow validation efforts, lack of standardization, and population biases. To address these, we developed TranslAGE, a knowledgebase and clinical trial platform that benchmarks aging biomarkers using three key metrics—Responsiveness, Prognostication, and Reliability (RPR). Responsiveness TranslAGE evaluates biomarkers across 51 interventions, including pharmacological agents, supplements, lifestyle modifications, and medical procedures. This breadth enables identification of biomarkers that consistently capture changes in biological age across distinct mechanistic pathways, guiding trials from metabolic health to cellular senescence. Prognostication The platform integrates data from 50,000+ individuals across diverse populations to assess biomarkers' ability to predict 40+ outcomes—frailty, cognitive decline, cardiovascular disease, and mortality. Unlike prior Western-centric studies, TranslAGE employs standardized metrics to enable cross-population and cross-outcome comparisons, ensuring global relevance. Reliability Utility depends on reproducibility. TranslAGE assesses: Technical reliability using DNA methylation datasets across Illumina 450K, Epic v1, and Epic v2 arrays. Biological reliability by quantifying variability from real-world factors such as circadian timing, stress, and diet—critical for clinical deployment. By uniting these dimensions in a standardized framework, TranslAGE enables shortlisting of biomarkers that are biologically informative, clinically practical, and globally generalizable. This positions the platform as a foundation for advancing the next generation of geroscience clinical trials.

Abstract citation ID: igaf122.4407

### WORK-FAMILY LIFE COURSE TRAJECTORIES AND MENTAL WELL-BEING: EVIDENCE FROM CHARLS, SHARE, ELSA, HRS, NCDS AND BCS70

Jiajia Li[1], Fabrice Kämpfen[2], and Heming Pei[3], *1. Institute of Population Research, Peking University, Beijing, Beijing, China (People's Republic), 2. School of Economics, University College Dublin, Dublin, Hofuoborgarsvaoio, Iceland, 3. UNC Gillings School of Global Public Health University of North Carolina at Chapel Hill, Chapel Hill, North Carolina, United States*

Later-life mental well-being is shaped by cumulative work, partnership, and parenting histories, yet few studies compare these life-course patterns across diverse regions using harmonized designs. We analyzed 61,132 adults aged ≥50 from four HRS-sister studies—the China Health and Retirement Longitudinal Study (CHARLS;n=9,276), Survey of Health, Ageing and Retirement in Europe (SHARE;n=35,087), English Longitudinal Study of Ageing (ELSA;n=5,283), and Health and Retirement Study (HRS;n=11,486)—and supplemented with two prospective British birth cohorts for robustness. Using multi-channel sequence analysis and hidden Markov mixture models, we identified 5–6 work–family trajectory clusters from ages 15 to 50. Stable full-time employment, sustained partnerships, and one to two children were consistently linked to better outcomes. In CHARLS, the agricultural, early marriage, ≥5 children cluster had depressive symptoms 0.271 SD higher (95% CI:0.233–0.310) and self-rated health 0.108 SD lower (95%CI:–0.138 to – 0.077) than the stable non-agricultural, later marriage,1–2 children group. In SHARE, the Home/family+Early Stable+2-Kids cluster had depressive symptoms 0.076 SD higher (95%CI:0.059–0.094) and self-rated health 0.045 SD lower (95% CI:–0.061to –0.029) than the full-time, early stable, two-child cluster. In HRS, the Home/family focus+Early

Diverse + ≥3 children cluster had depressive symptoms 0.227 SD higher (95%CI:0.188–0.267) and self-rated health 0.219 SD lower (95%CI:–0.257 to – 0.181) than the stable reference. Gender disparities were largest in China and smallest in the U.S. Results were robust to early-life adjustment, attrition weighting, and exclusion of adult mediators. This cross-national design highlights the mental-health benefits of stable, balanced work–family patterns and supports policies promoting employment stability, gender equity, and caregiving support across the life course.

Abstract citation ID: igaf122.4408

### OPTIMIZING TAXONOMIC ASSIGNMENTS AND COMMUNITY STATE TYPES FOR THE VAGINAL MICROBIOTA IN OLDER WOMEN

Sarah Brown[1], Michael France[1], L. Elaine Waetjen[2], Michelle Shardell[1], Rebecca Brotman[1], Jacques Ravel[1], and Johanna Holm[1], *1. University of Maryland School of Medicine, Baltimore, Maryland, United States, 2. University of California Davis Health, Sacramento, California, United States*

The vaginal microbiome plays a critical role in protecting urogenital health across the lifespan, yet bioinformatic tools and community state type (CST) definitions derived from 16S rRNA gene amplicon sequencing data have been developed using data from reproductive-age women. As a result, current classification tools may not accurately describe the microbial compositions of peri- and postmenopausal women, whose vaginal microbiota shift significantly with declining estrogen levels. To address this gap, we refined two previously published key tools, vSpeciateDB and VALENCIA, using sequencing data from N = 1924 peri/postmenopausal women aged 45-72 who were enrolled in the Study of Women Across the Nation (SWAN) and the Human Papillomavirus in Perimenopause (HIP) study. We expanded the three region-specific vSpeciateDB databases with 3,092 high-confidence sequences, adding 826, 710, and 572 new species to the V1V3, V3V4, and V4 databases, respectively. This improved species-level classification rates for older women from 95% to 98%. Hierarchical clustering of 2281 peri/postmenopausal samples revealed three novel sub-CSTs of a CST previously described in reproductive-age women as low in Lactobacillus spp, moderate Prevotella abundance and high evenness of other species. These sub-CSTs differed in relative abundances of key taxa, including Prevotella, Finegoldia, Corynebacterium, Faecalibacterium, Fenollaria, and Phocaeicola, and accounted for 24.3% of peri/postmenopausal samples, compared to 1.4% in reproductive-aged women. Including these sub-CSTs improved CST assignment quality for both reproductive-age and peri/postmenopausal women. Ongoing analyses will explore whether these sub-CSTs are differentially associated with health outcomes in older women including genitourinary syndrome of menopause and recurrent urinary tract infections.

### SESSION 9155 (PAPER)

### LATE BREAKING: TRANSLATIONAL GEROSCIENCE II

Abstract citation ID: igaf122.4409

### COMBINED IMPACT OF MULTIPLE NUTRIENTS ON LONGEVITY: FINDINGS FROM THE INTEGRATIVE LONGEVITY OMICS STUDY

Paola Sebastiani[1], Andres Ardisson Korat[2], Anastasia Leshchyk[1], Nicole Roth[3], Toshiko Tanaka[4], Stacy Andersen[3], and Thomas Perls[3], *1. Tufts Medical Center, Boston, Massachusetts, United States, 2. Tufts University, Boston, Massachusetts, United States, 3. Boston University, Boston, Massachusetts, United States, 4. National Institute on Aging, Baltimore, Maryland, United States*

Understanding the effect of nutrition on aging is essential for identifying dietary habits that promote healthy longevity. Many studies have investigated the effect of nutrient patterns on aging trajectories by analyzing nutrients in isolation or aggregated into dietary indices. To consider the joint effects of multiple nutrient groups simultaneously while accounting for their interactions, we developed the Nutrient Variety Index (NVI) that summarizes the distribution of groups of nutrients within a diet. We generated NVIs of 19 nutrient groups from dietary data of 235 centenarians and 469 centenarians' offspring and controls enrolled in the Integrative Longevity Omics study, 8035 Health and Retirement Study participants, and 1500 Baltimore Longitudinal Study of Aging participants. We analyzed NVIs in relation to chronological and biological age, cognitive function, mortality risk, and exceptional longevity. Our analyses showed substantial changes of the NVIs of multiple nutrient groups with older ages. For example, the NVI of proportion of carbohydrates, fats, proteins and alcohol in the diet was negatively correlated with older age and mortality risk and positively correlated with younger biological age based on various metabolomic clocks. The decreasing NVI of these macronutrients with older age captures the shift in balance between carbohydrates that become the dominant nutrients in the diet of individuals aged 60 years and older, compared to decreased consumption of fats and proteins. Individuals selected for familial extreme longevity had a significantly higher NVI of these and other nutrients groups, thus suggesting that they maintain a better balance of many nutrients as they age.

Abstract citation ID: igaf122.4410

### GUT MICROBIAL SIGNATURES OF FRAILTY AND FUNCTIONAL INDEPENDENCE IN CENTENARIANS AND OLDER ADULTS

Meghan Short[1], Tanya Karagiannis[2], Ye Chen[2], Daniel Segrè[3], Sarah Bald[3], Stacy Andersen[3], Thomas Perls[3], and Paola Sebastiani[2], *1. Tufts University School of Medicine, Medford, Massachusetts, United States, 2. Tufts Medical Center, Boston, Massachusetts, United States, 3. Boston University, Boston, Massachusetts, United States*

Frailty and functional limitations decrease quality of life for older adults and are associated with myriad health risks. The gut microbiome is a potential therapeutic avenue for promoting functioning among older adults, but a clearer understanding of mechanisms by which commensal gut bacteria may affect frailty and functional independence is needed. In this study, we analyzed data from an ongoing cohort study, Integrative Longevity Omics (ILO), which has 418 shotgun metagenomics samples of the gut microbiome from centenarians and their offspring. We measured associations of microbial alpha/beta diversity and species with function measures (activities of daily living [ADLs] and instrumental activities of daily living [IADLs]) and Fried frailty phenotype domains (physical activity, fatigability, grip strength, weight loss). Species-level alpha diversity decreased with increasing Pittsburgh fatigability score($\beta$=-0.0051, 95% CI:-0.0098 to -0.0003, $p = 0.038$), adjusting for age, sex, and education. Beta diversity (Bray-Curtis dissimilarity) was associated with ADLs($R2=0.005$, $p = 0.002$), IADLs($R2=0.005$, $p = 0.006$), and fatigability($R2=0.010$, $p \leq 0.001$), adjusting for age, sex, and education using PERMANOVA. Finally, we identified several species associated with multiple frailty and functioning scores, including Anareostipes hadrus, which was associated with improved ADLs, IADLs, fatigability, and lower likelihood of substantial weight loss, and is a known anti-inflammatory butyrate producer. Worse ADLs/IADLs and greater fatigability was associated with Clostridium innocuum, a commensal gut bacteria with documented potential for virulence. These results identify microbial species for further analysis in association with other 'omics layers and for future functional studies to understand their potential mechanistic effects on frailty and functioning outcomes in older adults.

Abstract citation ID: igaf122.4411

### IMPROVING THE EVIDENCE ON LONG-TERM FRAILTY TRAJECTORIES: DATA FROM THE NURSES' HEALTH STUDY

Anna Siefkas[1], Ariela Orkaby[2], Marian Hannan[3], Sebastien Haneuse[1], A. Heather Eliassen[1], and Yuan Ma[1], *1. Harvard. T.H. Chan School of Public Health, Boston, Massachusetts, United States, 2. Brigham & Women's Hospital, Harvard Medical School, Boston, Massachusetts, United States, 3. Hebrew SeniorLife, Boston, Massachusetts, United States*

Evidence on long-term frailty trajectories is crucial for monitoring the dynamic changes in health status in older adults and may better inform prognosis than single-time measurements. However, such evidence is limited due to the scarcity of frailty data over decades as well as methodological challenges including missing data, attrition (study dropout), and truncation due to mortality. Leveraging decades-long data from the Nurses' Health Study (1992–2016) and appropriate methods to address these challenges, we characterized long-term frailty trajectories of 89,312 women (aged 46–71 in 1992). A frailty index (FI) was calculated every four years, using two-stage multiple imputation with generalized linear mixed models to impute FI item non-response in years with available data (stage 1) and complete FI values in years of unit non-response (stage 2). We addressed attrition using inverse probability of censoring weighting. Models for frailty over time were estimated partly conditional on death and operationalized using GEE models with an independence working correlation structure to avoid implicit imputation of trajectories past the point of mortality. Mean FI values increased linearly by 0.0079 annually in the population (95% CI: 0.0078 – 0.0080), adjusting for age. Trajectories differed by baseline frailty status and by age, however, greater variation in trajectories was seen by frailty status than by age. The group that was prefrail at baseline demonstrated the most rapid increase in FI over time. Our results build upon prior findings of increasing FI values over time and provide a framework for rigorous longitudinal studies of aging.

Abstract citation ID: igaf122.4412

### INTEGRATING UNKNOWN METABOLITES TO OPTIMIZE A METABOLOMIC DISTRESS SCORE FOR PSYCHOLOGICAL DISTRESS IN OLDER WOMEN

Yiwen Zhu[1], Raji Balasubramanian[2], Julián Ávila-Pacheco[3], Clary Clish[3], Jie Yao[4], Jerome Rotter[4], Laura Kubzansky[1], and Susan Hankinson[2], *1. Harvard T.H. Chan School of Public Health, Boston, Massachusetts, United States, 2. University of Massachusetts Amherst, Amherst, Massachusetts, United States, 3. Broad Institute of MIT and Harvard, Cambridge, Massachusetts, United States, 4. The Lundquist Institute at Harbor-UCLA Medical Center, Torrance, California, United States*

Chronic distress, including depression and anxiety, contributes significantly to cardiometabolic risk in aging populations, with metabolic alterations acting as a key pathway. We previously developed a metabolomic distress score (MDS) using 20 identified metabolites in older White women, which predicted cardiometabolic risk in aging cohorts. Here, we expand this approach by identifying metabolomic features not yet characterized that are associated with distress and derive a revised score incorporating these features (rMDS). Assays were conducted at the Broad Institute using nontargeted liquid chromatography-mass spectrometry methods. We performed agnostic analyses assessing associations between distress and up to 5539 unannotated features among older White women from three cohorts: Nurses' Health Study (N = 540, mean age=64), Women's Health Initiative Observational Cohort (N = 1107; mean age=67), and Multi-Ethnic Study of Atherosclerosis (N = 770; mean age=63). We selected unknown metabolites and derived an rMDS via the least absolute shrinkage and selection operator (LASSO). We identified 14 features robustly associated with distress across all cohorts ($p < 0.1$), plus 65 features in the two most recently assayed cohorts. Ongoing biochemical identification has led to 26 new metabolite annotations among the features identified, including cholesteryl esters and zeaxanthin. The revised distress score (rMDS) integrated novel signals from 67 LASSO-selected known and unknown metabolites. In NHS, rMDS was more strongly associated with distress than the MDS (OR = 5.15, 95%CI [3.85-7.06] vs. OR = 3.30, 95%CI [2.59-4.27]). Validation of rMDS in independent samples is ongoing. Incorporating previously unidentified metabolomic features greatly enhanced the performance of the metabolomic score, revealing novel pathways linking psychological distress to health and aging.

Abstract citation ID: igaf122.4413

### NEIGHBORHOOD DEPRIVATION, FINANCIAL HARDSHIP, AND DECREMENTS IN KIDNEY FUNCTION ACROSS A DECADE

Agus Surachman, *Drexel University, Philadelphia, Pennsylvania, United States*

Neighborhood factors are critical determinants of accelerated renal aging, an important risk for chronic kidney disease (CKD), a pressing public health problem. Little is known about whether financial hardship plays an important role as an individual-level pathway in the association between neighborhood factors and accelerated renal aging. This analysis utilized data from 653 (M age = 54, SD = 9.5; 55% female; 79% NH white) participants who completed the biomarker data collections in both wave 2 and 3 of the Midlife in the United States (MIDUS) study. The neighborhood factor is based on the national level Area Deprivation Index (ADI), a validated neighborhood disadvantage score based on neighborhood socioeconomic factors (1=least disadvantage, 100=most disadvantage). Financial hardship includes material (e.g., lower income to poverty ratio), psychological (e.g., perceived difficulty paying bills), and behavioral (e.g., sold possessions to meet needs) domains. The main outcome was absolute change in creatine-based estimated glomerular filtration rate (eGFR) across a decade. Analyses were adjusted for age, sex, education, and health-related factors (smoking, obesity, hypertension, and diabetes status). Participants living in a highly deprived neighborhood (third percentile or higher) showed faster decrements in eGFR (Est=3.05, SE = 1.42, p<.05). Financial hardship was also associated with faster decrements in eGFR (Est=3.74, SE = 1.19, p<.01). Indirect effect analysis indicated that higher financial hardship was significant pathway through which neighborhood deprivation was linked to faster decrements in eGFR (Est=0.92, SE = 0.39, p<.05). Neighborhood deprivation is a critical risk factor for accelerated renal aging, highly likely due to the higher experience of financial hardship.

Abstract citation ID: igaf122.4414

**THE ROLES OF SOCIAL SECURITY AND SUPPLEMENTAL SECURITY INCOME AS DETERMINANTS OF COGNITIVE FUNCTION**

Jiaxing Bao, and Daniel Kim, *School of Community Health and Behavioral Sciences, Bouvé College of Health Sciences, Northeastern University, Boston, Massachusetts, United States*

Alzheimer's disease and related dementias (ADRD) pose a growing public health challenge in the U.S. and understanding protective factors against cognitive decline has become urgent, with over 6 million older Americans currently affected and projected 2025 costs of $781 billion. Financial security has been identified as a potential determinant of cognitive health, yet the longitudinal effects of social security (SS) and supplemental security income (SSI) on cognitive function remain underexplored. Using nationally-representative individual-level panel data from the Health and Retirement Study (2000-2018) and controlling for state and time fixed effects, we examined the associations between SS and SSI benefits with cognitive function among older adults. The SS analysis included 6,649 individuals aged 67 and older with at least 10 years' work history; the SSI sub-analysis comprised 971 eligible individuals. Cognitive function was measured using the 27-point Telephone Interview for Cognitive Status (TICS) screening instrument. All models controlled for individual- and state-level covariates and time trends. Each additional $1,000 in annual SS benefits was associated with a 0.014-point increase in the TICS score (P < 0.01). Meanwhile, SSI benefits showed inverse and non-significant associations with cognition. Covariates such as age and comorbidities predicted lower cognitive scores, and health insurance coverage showed strong positive associations with cognition. Overall, SS benefits appear to confer modest and significant benefits to cognitive function among older Americans, suggesting protective effects of financial security on cognitive health. These findings support the importance of public sources of retirement income to help maintain cognitive function in late life for Americans.

### Participant Index

Arabic numbers refer to session numbers.
Duplicate numbers indicate the participant is listed as an author on multiple abstracts within a session.

**A**
Aaron, Siobhan: 8010
Aartsen, Marja: 8000
Aasaavari, Asmita: 1315, 7360
Abadir, Peter: 3390
Abaei, Elnaz: 1065, 4600, 8005
Abbott, Alyssa: 8000
Abbott, Katherine M: 1300, 2490, 7305, 8015
Abdel Magid, Hoda Samir: 2005, 2400
Abdelfattah, Lindsey: 4065
Abdelgawad, Norhan: 4007
Abdi, Lovina: 7240
Abdullah, Saeed: 8000
Abi Nassif, Alexandra: 1495
Ablah, Elizabeth: 7180
Aboagye, Shelly: 2175, 4190
Aboagye Kyei, Amma S: 8000
Abraham, Alison G: 9130
Abrahams, Loes: 7035
Abrahamyan, Lusine: 7090
Abrams, Grace: 2275
Abrams, Wendy: 9080
Abramson, Rachel H: 8000
Abramson, Tobi: 8010
Abreu, Taiane: 7380
Abrials, Josephine M: 8015
Abshire Saylor, Martha: 4450
Abu, Hawa O: 2500, 7410, 7485
Abul, Zeynep: 7255
Acharya, Bibhav: 8015
Acharya, Sharmila: 7100
Acierno, Ron: 4415
Ackerman, Michelle L: 7175
Ackley, Sarah: 4280
Adamek, Margaret E: 4070
Adams, Anne E: 9065
Adams, Swann: 7220, 8000
Adams, Sydni N: 8010
Adams-Campbell, Lucile: 7190
Adams-Price, Carolyn: 8015
Adandom, Isreal: 7300
Adaniya, Stephanie: 8000
Adar, Sara D: 1040
Addepalli, Venkatanand ram: 9070
Addison, Odessa: 7140, 7905
Adebusoye, Lawrence A: 4565
Adegoke, Elikanah Olusayo: 7315
Adeleke, Bisola C: 4420
Adelman, Ronald D: 1170
Adelnia, Fatemeh: 3260
Adeniji, Dolapo O: 4070
Adepoju, Omolola E: 3305, 4395
Aderonmu, Aderonke: 4190
Adfares, Hadil: 7295
Adjei, Mathias: 7065, 7240
Adjei, Naomi: 4150, 8010
Adjei Gyimah, Akwasi: 7065, 7240
Adkins-Jackson, Paris B: 2005, 3345, 4125, 7470, 7535, 7900, 8015
Adomako, Jennifer Asaa: 8000
Adutwum, Frank: 8000
Aelick, Katelynn: 7075
Afonso, Tara McBride: 1050
Agarwal, Yash: 9070
Agbawodikeizu, Patricia U: 1365
Agbomi, Lidadi: 8015
Aggarwal, Neelum: 4570
Agha, Leila: 8000
Aghjayan, Sarah: 4585
Agmon, Maayan: 1110, 2105, 2415, 7040, 7210
Agrawal, Shubham: 3340
Agrawal, Sunil K: 7025
Aguila, Emma: 2060, 8015, 9075
Aguilar, Vanessa: 4475
Aguillon, David: 8000, 8015
Aguilo, Maria Elisa: 1390
Aguirre, Alyssa: 4015, 8015
Aguzzoli Peres, Fernando: 7010
Agyekum, Boadi: 4470
Ahmad, Anam: 7315
Ahmad, Fyzeen A: 2330
Ahmad, Waqar: 4550
Ahmed, Ali: 2460, 8010, 9020
Ahmed, S M Foysol: 7450
Ahmed, Sohel: 1375
Ahn, Bumsoo: 4275
Ahn, Hyochol: 7010, 7500
Ahn, SangNam: 7205
Ahn, Sangwoo: 4580
Ahn, Seoyeon: 7275, 7470, 7925, 8005
Ahn, Soojung: 2190, 2255, 8015
Ahn, Sumyeong: 3090
Aichele, Stephen: 8015
Aideed, Ayanle: 8000
Aikawa, Sora: 2440, 8010
Aiken, April: 8015
Ailshire, Jennifer: 1415, 2250, 2270, 2275, 2345, 2400, 8015
Ainsworth, Laura A: 7350
Ajay, Sreya: 8005
Ajayi, Oluwaseun K: 4565
Ajrouch, Kristine: 3230, 4285
Akar, Fatma: 8005
Akash, Moniruzzaman: 4005
Akenine, Ulrika: 4370
Akiko, Furuta: 7440
Akincigil, Ayse: 2185, 4210
Akmal, Allie: 2400
Akman, Jasmine: 4210
Akorlatse, Raymond: 8000
Akter, Nahida: 1005, 7305
Akter, Rejina: 8015
Akter, Salma: 1005
Akudugu, John: 9030
Akushevich, Igor: 1235, 4010, 4460, 7185
Akwobugi, Moses: 1145, 8000, 8015
Akyildiz, Erdogan Oguzhan: 7315
AL Harrasi, Azza M: 8015
Al Juboori, Ruaa: 4430, 7220
Al Snih, Soham: 7225, 7230, 7255, 8005
Alabi, Aderonke A: 4565
Alagappan, Cecilia M: 7135
Alam, Ishrat: 7485
Alam, Marzan: 4005
Alamian, Arsham: 7425
Albala, Cecilia: 9035, 9125
Albashayreh, Alaa: 8000, 8015
Albaum, Jason A: 8010
Albert, Marilyn S: 9045
Albert, Steven M: 4305
Alberth, Andrew: 7275, 7285
Albertini, Marco: 4575, 7210, 9020
Albrecht, Jennifer S: 2035, 8010, 8015, 9005
Aldridge, Georgina: 8005
Aldridge, Melissa D: 2185
Aldwin, Carolyn M: 3330
Alea, Nicole: 7005, 7935
Alejo, Cherrylyn: 1050
Alejo, Prince M: 4350
Aleksic, Sandra: 8000
Alemi, Farrokh: 2125
Alex, Danny: 9135
Alexander, G Caleb: 7255
Alexander, Gregory L: 7125
Alexander, Karah L: 8010
Alexander, Neil B: 4060, 7225, 8010
Alexander, Rachael: 1025
Al-Hadhrami, Samiya: 8015
Alhay, Zahra: 7415
Ali, Farwa: 7025
Ali, Talha: 8000
Ali, Waad: 4470, 7040
Alio, Paula A: 3165
Aljohani, Rania: 8010
Allan, Alexa C: 3060, 7325
Allard, Eric: 9055
Allebach, Phoebe: 4130
Allen, Elizabeth M.: 4590
Allen, Erica: 7455
Allen, Julie Ober: 1095
Allen, Larry A: 7085, 7910
Allen, Laura D: 8000
Allen, Priscilla D: 7355
Allen, Quinn: 7375
Allen, Rebecca S: 7310
Aller, Ty: 7215, 7305
Allison, David B: 2560
Allison, Theresa A: 2515
Alloy, Linda: 1130
Allsopp, Richard: 7240
Alm, Kylie H: 1155
Almeida, David M: 1175, 1375, 1480, 2095, 2280, 3330, 4000, 4120, 4520, 7365, 7380, 8000, 8005, 9015
Almeida, Quincy: 7225, 7905
Almidani, Louay: 7530

© The Author(s) 2025. Published by Oxford University Press on behalf of the Gerontological Society of America.
This is an Open Access article distributed under the terms of the Creative Commons Attribution License (https://creativecommons.org/licenses/by/4.0/), which permits unrestricted reuse, distribution, and reproduction in any medium, provided the original work is properly cited.

Almroth, Melody: 1425
Al-Naggar, Iman: 1387, 8005, 9090
Alnajar, Malek: 1025, 2190
Alosco, Mike: 1510
Alpizar, Olivia: 8010
Alquist, Jessica: 4590
Alqurashi, Mohammed: 8005
Alsamawi, Amal: 1315, 7360
Alshahwan, Sandi: 8010
Alston, Daniel: 2330
Altizer, Jasmine Travers: 1435, 2440
Altona, Janissa: 3035
Alvarez, Jessica: 8015
Alvarez, Loreli: 2030, 4140, 8010
Alvarez-Alvarado, Stacey: 1285
Alvarez-McNelis, Desiree: 7465
Alving, Loren: 7135
Alzahrani, Mallak: 1220, 2510
Amadae, Sonja M: 7275
Amagasa, Shiho: 7345
Amano, Takashi: 2025, 8015
Amar, Shimon: 7135
Amaravadi, Harsha: 8000, 8010
Ambadar, Zara: 2375
Ambellu, Gelila: 8015
Ambrosio, Fabrisia: 4473
Amer, Hana A: 7125
Ames, Jesse: 1455
Amin, Iftekhar: 7145
Amin, Suhani: 8010
Amini, Reza: 7050
Amir-Koren, Uri: 4130, 4210
Amjad, Halima: 2035, 7010, 7910, 8000, 8015
Amland, Rachel: 7415
An, Hyunjoo: 7355, 7470
An, Ning: 8000
An, Ruopeng: 7015
An, Yang: 4495
An, Yinong: 7340
Anaab, Esme: 7540
Anand, Akshay: 9070
Ances, Beau M.: 8015
Andel, Ross: 3370, 7295, 7365, 7375, 8000
Andersen, Dan: 8010
Andersen, Stacy: 1520, 4440, 7310, 8005, 8015, 9155
Andersen, Troy C: 8000
Anderson, Brittany: 8005
Anderson, Emma: 1350
Anderson, Joel G: 1000, 1420, 2010
Anderson, Keith A: 2455, 4145
Anderson, Linda K: 8010, 9140
Anderson, Loretta: 4070
Anderson, Lyndsey M: 7335
Anderson, Rose: 1240
Anderson, Rozalyn: 3267
Anderson, Ruth A: 2150, 2255, 2475, 4095
Anderson, Sarah: 8010
Anderson, Shannon: 2255
Anderson, Timothy S: 1485
Anderson-Hanley, Cay: 7545, 8005
Ando, Fujiko: 7330
Andonian, Brian J: 2380, 4525
Andrade, Flavia D: 2060, 3265, 7405, 8015
Andrango, Lizette: 7455
Andreoletti, Carrie L: 4007
Andrews, Heather: 7435
Andrews, Ryan: 2000, 4540
Andrews, Sophie C: 4580
Aneja, Ritu: 3095
Anetzberger, Georgia J: 3350
Anfang, Sophie: 7525
Ang, Ian Yi Han: 8005
Ang, Shannon: 1205, 3325
Angel, Jacqueline L: 2060, 3265, 3395, 7475
Angelini, Whitney: 9080
Angell, Sarah C: 3315
Angrisani, Marco: 3010, 4490
Ani, Judith I: 8015
Aniemeke, Chinenye C: 1430
Anil Rao, Anchala: 8000
Ankuda, Claire: 2185, 4430, 8010
Anquillare, Elizabeth: 7200, 7395
Ansah, John Pastor: 2435
Ansari, Shadi: 4335, 7390
Ansong, Rockson: 8015
Anstey, Kaarin J.: 4470
Antic, Mina: 3330
Antonios, Vera: 2395
Antonio-Villa, Neftali Eduardo: 1155
Antonucci, Toni: 3230, 4285, 4490, 8000
Antunes, Kristen: 7230
Anurum-Anyanwu, Christina: 7535
Aoun, Christy: 8010
Aoun, Layana: 8010
Aoun-Barakat, Lydia: 7245
Aparicio, Hugo: 9105
Aparicio, Triccia: 8010, 8015
Apostolou, Hannah L: 7310
Apostolova, Liana: 7010, 7910
Appelhans, Bradley M: 1110
Applebaum, Allison J: 2175
Applebaum, Robert: 4260, 7250, 7540
Applegate, Julia M: 1000
Appleton, Allison: 8015
April-Sanders, Ayana: 9045
Arai, Yasumichi: 7365
Aranda, María P: 1055, 2060, 3265, 3370, 3395
Araujo, Lia: 2120, 7380
Araujo Menendez, Carlos: 8010
Araujo-Menendez, Carlos: 8005, 8015
Aravena, Jose M: 9035, 9125
Aravena, Maria: 9035
Arbeev, Konstantin: 7415
Arciero, Paul: 8005
Ardekani, Azam: 7050
Ardisson Korat, Andres: 4440, 9155
Arena, Aprille: 1515, 8005
Arfanakis, Konstantinos: 4570
Arguelles-Borge, Soledad: 7005, 7110
Argueta, Daniel: 9015
Argueta, Daniel L.: 8000
Arias, Dalmina: 3265
Arieli Zaks, Efrat: 9095
Aris, Izzuddin: 1225
Arling, Greg: 7370
Arlyck, Ralph: 4115
Armant, Ryan: 8015
Armbruster, Bill: 4130
Arms, Tamatha E: 8015
Armstrong, Alexandra: 8010
Armstrong, Brittany D: 7435, 8005, 8015, 9025, 9130
Armstrong, Grace Q: 1485, 7410
Armstrong, Melissa: 7350
Arnett, Marieh: 8000
Arnette, Shannon: 7005
Arnold, Michelle L: 4065
Arnold, Sophie: 7030
Arnold, Steven E: 7325, 7415
Arntsen, Bjørnulf: 7205
Arogundade, Odunayo T: 7150
Aron, Sophia: 1280
Aronson, Louise: 8000
Arora, Arushi: 1270
Arora, Kanika: 1500, 7300, 7515
Arora, Vineet: 7430
Arpawong, Thalida: 3127
Arroyo, Edmond M: 4350
Arruda Franco, Gustavo: 8010
Arteaga, Ignacia: 7135
Arthanat, Sajay: 2410, 4005
Artoni, Filippo: 7315
Arulraj, Theinmozhi: 7315
Aruscavage, Nancy: 8015
Arya, Richa: 7170
Asakitogum, David: 7425
Asante, Samuel: 7065, 7240
Aschenbrenner, Andrew: 7505
Aschwanden, Damaris: 1480
Ashcraft, Alyce S: 7125
Ashcraft, Laura Ellen: 8015
Ashe, Maureen C: 4490
Ashebir, Sophia: 3250, 4410, 7005
Asher, Merav: 2415
Ashida, Sato: 2135, 4350, 8000
Ashiqueali, Sarah: 4347
Ashirifi, Gifty D: 4070
Ashish, Dev: 7010
Ashton, Abbie: 4450
Ask, Levi: 1255
Aslam, Hamza Saghir: 2315
Asma, Khunza: 7425
Asoni, Gesuina: 7265
Asraf, Kfir: 1110
Assakul, Rarinsri P: 1275
Asthana, Sanjay: 4585
Astone, Kristina: 4535
Asundi, Archana: 7245, 7915
Aswani, Truphosa (Posa): 2010, 3155
Atherton, Olivia E: 4110, 8015
Atkins, Peter: 7300
Atkins, Shelbie: 7500
Atshan, Samer: 1040, 1330
Attili, Noura: 8005, 8015
Au, Rhoda: 1510
Austad, Steven N: 2560, 4347
Austin, Nadine C: 1315
Au-Yeung, Wan-Tai M: 7335
Avalos, Maria: 2285
Avelino-Silva, Thiago Junqueira: 4425, 8005
Avent, Elizabeth S: 2295, 3300

Averbukh, Maxim: 4275
Aviña, Sarah: 4260
Avila, Ivan: 8010
Avila, Jaqueline: 7200
Ávila-Funes, José A: 7145
Ávila-Pacheco, Julián: 9155
Avila-Rieger, Justina F: 3380, 7040
Avorn, Jerry: 7510
Awad, Rabab: 7060
Awata, Shuji: 7300
Ayalon, Liat: 2250, 2370, 3080, 3305, 4395
Ayaz, Hasan: 4525
Aycock, Dawn: 3040
Ayele, Roman A: 8005
Azar, Ariel: 3195
Azhar, Esha: 1330, 7035
Azhar, Gohar: 7005, 7010, 7190, 8015
Azuero, Andres: 2030, 4140, 9125
Azuz, Anna: 8010

B
Babaieasl, Faezeh: 8005
Babar, Huma: 2175
Babenko, Natalia: 7175, 7425
Babulal, Ganesh M: 1355, 7505, 8015, 9015
Baby, Bincy: 7230
Baby, Eliza: 9085
Baca, Hailey: 8010
Bachmeier, James: 4290
Bachmeier, Natalie: 4075
Bachner, Yaacov G.: 2370, 7110
Back, Hayun: 8010
Backes, Ingrid: 1215, 9105
Bäckman, Malin: 3145
Backus, Deborah: 1230, 7225
Bacsu, Juanita-Dawne: 2070, 7205, 7220, 8015
Badell, Camila S: 8010
Bae, Elizabeth: 1520, 7480
Bae, Harold: 8005
Bae, Jungyeon: 7295
Bae, Myeongjin: 7265
Bae, Suyeong: 7335
Baehr, Laura: 2380, 4525
Baek, Jihye: 7290
Baek, Jimin: 3235
Baek, Jiwon: 8005
Baek, Jiyeon: 7190, 8010
Baek, Jonggyu: 7255
Baek, Sehyun: 8000, 8005, 8010, 8015
Baek, Seoyoung: 7245
Baena, Ana: 8000
Bagaajav, Ariunsanaa: 8015
Bagley, Olivia: 7415
Bagott, Darcie: 4565
Bahrami, Raheleh: 7305
Bai, Chen: 7145
Bai, Jing: 4575
Bai, Xue: 1515, 2315, 7405, 8010, 9050
Baier, Rosa R: 2055, 4475
Baig, Farah: 3000
Baik, Dawon: 7085, 7910
Baik, Sol: 7260, 7295, 8010
Bailey, Christine: 1315, 7360
Bailey, Regan L: 7480
Bailey, Ryan: 7325
Baillargeon, Emma: 8010
Baim-Lance, Abigail: 4425, 9145
Baker, Elizabeth: 3095
Baker, Jillian S: 1110, 4460
Baker, Jordan: 7265
Baker, Kristin: 1385, 2185
Baker, Tamara A.: 3395, 4095
Baker, Zachary G: 1440, 3375, 8005
Bakerjian, Debra: 2410
Bakitas, Marie: 4445
Bakker, Arnold: 1155
Balachandran, Anoop T: 8010
Balachandran, Arun: 2105, 8000, 8010, 9135
Balasubramanian, Raji: 8005, 9155
Balbale, Salva N: 7185
Bald, Sarah: 8015, 9155
Baldus, Clara M: 3355
Baldyga, Kathryn: 7130
Balk, Deborah: 3385
Balkan, Em: 1465, 7485, 7930
Ball, Karlene: 4030
Ball, Sarah: 1485
Ballew, Shoshana H: 3105, 4450
Ballhausen, Nicola: 1240
Balmuth, Alexa: 3250, 4410
Balog, Emily: 8010
Balser, Sarah: 3245
Balthazar, Monique: 7535
Balz, Magdalen A: 7040
Bámaca, Mayra: 7470
Bambury, Elizabeth: 7180
Bamgbade, Benita A: 8015
Bamonti, Patricia M: 2240, 7265
Banarjee, Chitra: 3030, 7225, 7495, 8005, 8015
Banarjee, Reema: 7315, 7480
Bandeen-Roche, Karen: 2100, 2510, 3345, 4025
Band-Winterstein, Tova: 7450
Bandyopadhyay, Attrayee: 1025
Bane, Scott: 9135
Banerjee, Samprit: 4225
Banerjee, Seema: 7530
Bangerter, Lauren R: 1035, 3340, 4015
Bangs, Alex: 7415
Baniassadi, Amir: 3235, 7295, 7465
Bankole, Azziza O.: 8000
Bankovska, Maryna: 4470, 7210
Banno, Ryoichi: 7480
Bansal, Ophira: 9125
Bao, Jiaxing: 9155
Bao, Qintong: 7310
Bao, Shaorui: 7285
Bapna, Priya: 1470
Barajas-Gonzalez, R. Gabriela: 3155
Baral, Suvab R: 4200
Baranes, Guy: 2245
Baranzini, Mirko: 8000
Barbabella, Francesco: 7070
Barbakoff, Daniel: 7300
Barbara, Bassola: 8015
Barber, Malisa: 9090
Barber, Sian: 1365
Barbera, Mariagnese: 1290
Barbieri, Marielena: 7380
Barbosa, Paulo: 8005
Barbosa Suffredini, Ivana CC: 7300
Barczi, Steve: 3005, 3115, 4350
Bardales, Rhonda: 7350
Barghout, Rana: 8005
Barha, Cindy: 4230
Baris, Veysel: 8015
Barker, Jennifer M: 8010
Barker, Megan: 7290, 8000
Barnes, Deborah E: 2245, 9090
Barnes, Lisa L: 1415, 2025, 2200, 3130, 3395, 4570, 8005
Barnett, Michael: 3165, 4240
Barney, Ann C: 7500
Barooah, Adrita: 8000
Barouch, Stav: 4355
Barp, Milara: 8000
Barr, Paul J: 2045
Barr, Paul: 8015
Barr, Paul J: 9045
Barr, Robin: 2215
Barragan, Noel: 7225, 7905
Barreto Garcez, Flavia: 8005
Barrett, Anne E: 4245
Barrett, Austin: 4020
Barrett, Donna: 4600
Barrett, Kat: 1285, 8000
Barrett, Tyler: 9090
Barrie, Fatmata M: 7500
Barrios, Veronica: 2325
Barron, Yolanda: 8005
Barrow, Jennifer: 7385
Barry, Lisa C: 1110, 1315, 2185, 7205, 7345, 8010
Bartels, Stephen: 1160
Bartlett, Paige: 7100
Bartley, Jenna: 8000, 8010
Bartman, Sydney: 1520
Barton, Kelli: 7205
Barzilai, Nir: 4495, 8015
Barzilay, Shira: 4185
Basch, Arielle: 7410
Bashian, Hannah: 1050, 2240
Basic, Ajla: 7440
Basisty, Nathan: 7315, 7480
Basnet, Lila: 1500
Basrai, Zavera: 7075
Bass, David: 2285, 7010, 7915
Basu, Rashmita: 3310, 7010
Basu, Shayna: 7265
Batchek, Dakota: 1220
Bates, Timothy: 4260
Batista-Malat, Eleanor: 1215, 7230
Batsch, Nicole: 4160
Batsis, John A: 1220, 1235
Battle, Kimberly: 1260, 7095
Batura, Josey: 4085, 7330
Bauer, Julia A: 2040
Baughman, Kristin: 7225
Bauldry, Shawn: 1195, 4315
Baumgart, Matthew: 4160
Baxter, Samuel: 8005
Bayless, Teah: 7520
Bayliss, Elizabeth: 4090, 7385
Bayne, Alycia: 4435
Bayne, Kozbi L: 7440, 9110
Bea, Megan: 3285
Beaird, Genevieve: 1260, 7095
Beamer, Brock: 2125, 8010
Bean, Jonathan F: 7040, 7300, 8010
Bean, Jonathan F: 8010
Bean, Jonathan F: 9095
Beard, Renee L: 7470
Bearden, Valerie: 7015, 7935
Beason, Asia L: 7210
Beattie, Zachary: 7335, 7545

Beatty Moody, Danielle L: 2400
Beauchamp, Jennifer ES: 4415
Beauchamp, Marla: 7225, 7905
Beaufils, Constance: 3225, 9040
Beaulieu, Chris: 8015
Beavers, Daniel P: 7165, 8005
Beavers, Kristen: 7165, 8005, 8015
Bechara, John: 7035
Bechek, Bonnie: 8000
Becher, Robert D: 1375
Beck, Brianna: 9015
Beck, Emorie D: 1265, 4120
Beck, Maureen: 8000
Beck, Mike: 2220
Beck, Samuel: 8010
Becker, Heather: 7420
Becker, Marigrace: 2420, 8000
Becker, Todd D: 2320, 4430, 7260, 7500
Beckert, Angela: 7455
Beckett, Cameron G: 1315
Bedi, Anaika: 8000
Bedjeti, Katy: 4120
Beebe, Timothy: 2160
Beeber, Anna Song: 1270, 2150, 4070, 4095, 9065
Beech, Bettina M: 3180
Beem, Daniel K: 4405
Beenhouwer, David: 8000
Beer, Jenay M: 8015, 9065
Beesley, Hannah: 8010
Begum, Momotaz: 4005
Behnken, Emma: 8005
Behr, Grace: 9075
Behrens, Liza L: 1005, 2170, 3155, 4480, 8015
Bei, Eva: 4575
Beidelman, Erika T: 1495, 9040
Beiser, Alexa: 8010
Beisheim-Ryan, Emma H: 7140
Bekelman, David: 4430
Bekena, Semere: 8015, 9015
Belanger, Brigitte: 7485
Belanger, Emmanuelle: 2160, 4330, 7185, 7220
Belanger, Vi: 8015
Beldeen, Jacqueline: 8000
Belin de Chantemele, Eric: 7245
Belinson, Natella: 1515
Bell, Bethany A: 4125
Bell, Janice F: 2450, 4145
Bell, Julia: 7370
Bell, Mallory: 1080
Bell, Mary Rose E: 8000
Bell, Sarah Beth: 1510
Bell, Tyler R: 3370, 4030, 4560, 8000, 8015
Belleguic, Thierry: 8005
Belli, Hayley M: 2380
Belli, Robert: 2400
Bello, Ghalib: 8010
Bello-Chavolla, Omar Yaxmehen: 1155
Bellolio, Fernanda: 9045
Bellury, Lanell: 2255
Belnavis, Alexander: 7315
Belsky, Daniel: 8000, 8010, 9135
Beltran, Ana: 7470
Beltran, Susanny J: 2265
Belyea, Loni: 7030
Belza, Basia: 3205, 4535, 7100, 7415, 8015, 9005
Ben Harush, Odelia: 7145
Benavides, Megan: 1090
Ben-David, Boaz M: 4235, 9095
Bender, Alexis A: 2160, 4135
Bender, Alexis A: 9080
Benedict, Claire: 4035
Benge, Jared: 4015
Benheim, Talia S: 7050, 8000
Benjamin, Alyssa: 8005
Benjamin, Andrea Z: 7000
Benjamin, Emelia J: 7120, 8000
Benjasirisan, Chitchanok: 7380, 7430, 7495
Benloucif, Slim: 8005
Benmar, Claudine: 8010
Bennett, David A: 2460
Bennett, Henrietta A: 4085, 8000
Bennett, Jill: 4470
Bennett, Katherine: 2205, 8010, 8015
Bennett, Stephanie: 7015, 9025
Bensley, Kara: 9015
Benson, Bill: 8005
Benson, Clark: 7000
Benson, Jacquelyn: 7260, 8000
Benson, Lizbeth: 8000
Benton, Donna: 2390, 3300, 7085
Benyamine, Mohamed: 4430
Benzel, Heather: 1305
Benzo, Roberto: 2220
Berardi, Angela: 9020
Berdugo-Hernandez, Stephanie: 7340, 7920
Beresford, Lea: 7005
Berg, Anne Ingeborg: 4010, 7255
Berg, Hayley: 8015
Berg, Karina M: 1110, 7305, 9090
Berg, Kristen: 8005
Bergee, Rebecca E: 9025
Bergen-Jackson, Kimberly M: 7250, 7330
Berger, Ford: 8010
Bergman, Yoav S: 1100, 1330, 2105, 2390
Bergstrom, Jaclyn: 7050, 7190, 7375
Berg-Wedger, Marla: 3255, 2110, 2335, 8000
Berish, Diane: 3035, 3220, 4380, 7465, 7500, 8000, 8015
Berkley, Amy S: 1150, 1515
Berkowsky, Ronald W: 1135
Berlinger, Nancy S: 2420
Bernacki, Gwen: 4445
Bernard, Kimberly P: 1465
Berner, Kennedy J: 7250
Berner, Laura A: 7375
Bernhardt, Paul: 4560
Berning, Peyton: 7295, 7465
Berridge, Clara: 4200
Berroa, Frandys: 9080
Berry, Carolyn: 1505, 7355
Berry, Meagan: 7495
Berry, Sarah D: 1435, 7345, 7385, 7510, 9080
Berry-Stoelzle, Maresi: 1305
Berry-Stoelzle, Maximilian V: 8005
Berta, Whitney: 3040
Bessen, Sarah: 1075
Besser, Lilah: 2400, 4045, 7040, 8000
Bessette, Lily G: 1485
Best, Krista: 8005
Best, Ryan: 1330, 4420, 7355, 7365, 7435
Best, Thomas: 4560
Bethell, Jennifer: 8015
Bettger, Janet: 4525
Bey, Ganga S: 3185, 7145, 8005
Beydoun, May: 7130
Bhagianadh, Divya: 2185, 4390
Bhananker, Annika: 1090
Bharadwaj, Mythili: 8005
Bharath, Leena: 8015
Bhargava, Mahak: 3095
Bhat, Aarti C: 1375
Bhatia, Ashmeet: 7410
Bhatt, Tanvi: 7225
Bhatta, Tirth: 2500
Bhattacharya, Shelley R: 7225
Bhattacharyya, Kallol Kumar: 4030, 7100
Bhatti, Anam: 8010
Bhattiprolu, Atul K: 7525
Bhauryal, Kajal Dhansingh: 9045
Bherer, Louis: 7225, 7905
Bhin, Sun Suk: 7425
Bholat, Michelle: 2555
Bhowmick, Pallabi: 4080, 4150, 8015, 9065
Bhurtel, Shikha: 2450, 4145
Bian, Jinwei: 4585
Bian, Wenxin: 8010
Bianchi, Carolanne: 7495
Bibbins-Domingo, Kirsten: 7260
Bibbo, Jessica: 1215, 1395, 2485
Bibriescas, Natashia: 2030, 4140, 8010
Bicket, Amanda K: 8010
Biderman, Aya: 7135
Bielak, Allison A M: 4050
Bietsch, Breana A: 4165
Bigger, Sharon: 8015
Biggs, Mary L: 8000
Biglieri, Samantha: 2515
Bigman, Galya: 7130, 7265
Bilau, Ibrahim: 7160
Bilbrey, Ann: 7230, 7305
Billeci, Natasa: 1475, 4230, 7390
Bimman, Rennie: 4575
Bin Hossain, Md Sanzid: 7495
Binaco, Jenna N: 7110
Bindra, Sohum: 7405
Birati, Yosefa: 1485, 7410
Bird, Tess: 2090
Birditt, Kira S: 4195, 4490, 7355
Birkeland, Robyn: 7300
Birthelmer, Sierra: 7390
Bisesti, Erin M: 3020
Bishop, Alex J: 7400
Bishop, Christine E: 3310
Bishop, Jill: 9110
Bishop, Kate: 8000
Bishop, Nicholas J: 1055, 4470
Bishop, Rachel A L: 7145
Bishop, Teddy K: 1060, 4240
Bisoi, Rakshit Raj: 8000
Biswas, Rajib Kumar: 7475
Bivona-Maldonado, Emily: 4225
Bixby, Laurin: 8005
Bixter, Michael T: 8005

Black, Kathy: 4130, 7175
Black, Olivia: 2150
Blacker, Deborah: 7205, 9040
Blackshear, Chad: 7025, 7495
Blackstock, Sheila: 8015
Blackwell, Terri L: 8005
Blackwood, Jennifer: 7160, 8010
Blaes, Anne H: 7425
Blair, Elizabeth: 7170
Blancato, Bob: 3350
Blanchflower, Morgan: 2445
Blanco, Julie: 2110
Blanco, Luisa: 3290
Blank, Sabrina: 2390
Blasco, Drew: 7145
Blaskewicz Boron, Julie: 7095, 7150, 7160, 7390, 7430, 7475, 7515
Blind, Jenna: 2440
Blind, Melissa: 1160, 2170
Blinka, Marcela D: 2035
Bloch, Randall: 8005
Block, Laura: 1025, 2135, 7000, 7370, 8005
Blocker, Erin: 1020
Blodgett, Nicole: 4075
Bloem, Max: 7260
Bloemen, Elizabeth M: 1085, 3300
Blom, Jeanet W.: 7010
Bloom, Rachel F: 3200, 9040
Bloomberg, Mikaela: 4095
Bloss, Ellen: 1115, 1445, 9005
Blue, Colleen M: 7350, 7930
Blumen, Helena M: 8015
Boakye, Alfred: 4550, 8000, 9005
Boamah, Sheila: 2515
Boateng, Isaac Asirifi: 7340, 7395, 7455, 7920, 7935
Boateng, Josephine A: 1075, 7375, 8000
Boateng, Maame-Owusua: 7210
Bober, Timothy: 4305
Bobitt, Julie: 1315, 1500, 3045, 4390, 7185, 8015
Bock, Payton: 1520
Boddie, Stephanie C: 9105
Bodenheimer, Lisa: 7340, 7920
Bodner, Ehud: 3360, 8015
Boerner, Kathrin: 3080, 7200, 7475
Boeve, Angelica R: 1050, 2240
Bogaerts, Stefan: 7035
Bogdan, Ryan: 4040
Boger, Jennifer: 7205, 7915
Bogumil, Elizabeth: 3000
Bohall, Jeriel: 7205
Boisvert, Rachel: 7425
Bokun, Anna: 2060, 3265, 7475
Bolduc, Jessica J: 7225
Boliboun, Jimmie: 7485
Bolkan, Cory: 3295, 7175, 7920
Bollan, Josh: 1085
Bollens-Lund, Evan: 4430
Boller, Benjamin: 7200, 7325
Bolles, Leah: 8000
Bollin, Salli: 2485
Bolling, S. Alison: 7475
Boltz, Marie: 1310, 2180, 3035, 3220, 4380, 4480, 7465, 7500, 8010, 8015, 9075
Bolz, Fabio: 7040
Bonar, Erin E: 4390
Bond, Lynden: 7360
Bonds Johnson, Kalisha: 3375, 4160, 4485, 7330, 7350, 8015
Bonner, Alice: 1435, 4405
Bonnet, Kemberlee: 2255
Boockvar, Kenneth: 4445
Boon, Jeffrey T: 2045
Boot, Walter R: 1285, 3025, 3215, 3340, 4080, 4200, 4320, 4420, 7295, 8010, 8015, 9085
Booth, Richard: 1430
Booth, Sarah: 4295
Borden, Enid: 8010
Bordes, Piper J: 4395, 7450
Borges-Machado, Flavia: 7370
Borgogna, Joanna: 8010
Borgogna, Joanna Lynn: 3260
Borodovsky, Jacob: 4390
Borrero, Lisa: 2330, 2455
Borson, Soo: 7185, 9030
Boscardin, John: 7010, 7140, 7255
Bouchard, L.B.: 4035
Bouchard-Liporto, Madison: 1100
Boucher, Nathan A: 2090, 4210, 4565, 7075
Boudreau, Jacqueline: 2540, 7280, 7350
Boudreaux, Michael: 8005
Bouldin, Erin D: 4265, 8010
Bouranis, Nicole G: 7000
Bourassa, Kyle J: 2025
Boustani, Malaz: 2285, 2555, 3010, 7010
Boustani, Zayn: 7010, 7415
Bowblis, John R: 1150, 3280, 4260, 4365, 4440, 7250, 8010
Bowen, Holly J: 8010
Bowen, Lauren M: 1130, 2145
Bowen, Pamela G: 7245
Bower, Peter: 9085
Bowers, Katherine E: 8010
Bowland, Sharon E: 7110, 8005
Bowles, Kathryn H: 8000, 8005, 9135
Bowman, Brynn: 9145
Bowman, Grace: 7465
Box, Nicholas: 9070
Boxer, Rebecca S: 7085, 7910
Boyd, Cynthia: 7385
Boyina, Satvika: 8015
Boyle, Julia T: 2240
Bozaykut Eker, Perinur: 7315, 8005
Brach, Jennifer: 4525, 8005, 8010, 9095
Brackbill, Robert M: 7345
Brackpool, Kristina: 9105
Bradford, Ethan: 8015
Bradley, Dana B: 8000
Bradt, Joke: 8005
Brady, Debbie: 8015
Brady, McKayla: 1005
Brady, Samantha: 1405, 2050, 3250
Brandas, Benedetta: 7265
Brandis, Leah M: 4400
Brandon, Amanda: 4347
Brandt, Mark J: 3270
Brandt, Martina: 2345
Brandt, Nicole: 7290, 7495, 8005
Brangman, Sharon A: 4455
Branson, Brianna: 7185
Braswell, Angela M: 7340
Bratches, Reed: 8015
Brathwaite, Justin S: 1220
Braun, Robert T: 9090
Brauner, Rachel A: 8000
Braunstein, Joel B: 7010
Bray, Emily: 9005
Bray, Stephanie: 9020
Brazg, Noa: 2205
Brazier, Joan F: 7050, 8000
Brecht, Mary-Lynn: 1295
Breda, Kathleen M: 7485, 8000
Breder, Kelseanne: 3240, 4155
Breen, Elizabeth: 8015
Brehmer, Yvonne: 1240, 1480
Brekke, Bailee: 3125, 7540, 8005
Brennan, Caitlin: 7220
Brennan, Taylor: 3250, 7005
Brennan Ing, Mark: 1490, 2145, 2395, 7265
Brenowitz, Willa: 3050, 8000, 8005
Breton, Jordana: 2345, 7145, 7435
Breuner, Don: 7415
Brewer, Judson: 1160
Brewer, Robin N: 9070
Brewster, Glenna S: 4215, 4335, 7350
Brice, Kelly N.: 8000, 9015
Briceño, Emily: 1500, 2000, 3380, 4540, 8010
Bridgforth, James: 2515
Briggs, Anthony: 8005
Briggs, Emily: 3230, 8010
Brill, Seuli: 8005
Brines, Elizabeth: 8010
Bristol, Alycia: 1035, 1300
Britt, Katherine Carroll: 3025, 3260, 4225, 9010
Brittain, Katie: 9100
Britton, Karysa: 1045
Brock, Kaye: 7430
Brocksmith, Hannah: 7185
Brodaty, Henry: 4470
Brody, Abraham: 1270, 2380, 2440, 3205, 3375, 4015, 7185, 8010
Brody, Lilla: 7375
Broen, Tiana: 7020, 7195, 7900
Brohard, Cheryl: 4265
Bronas, Ulf G: 7020
Bronstein, Laura: 4185
Brooks, Ezra S: 8005
Brooks, Jada L: 1020
Brooks, Ron: 3170
Brooks, Sonya: 4250
Brooks Carthon, Margo A: 8005
Brothers, Ally: 2170, 7380
Brotman, Rebecca M: 8010, 9150
Brotman, Shari: 7065
Brousard, Jordan: 4415
Brown, Amy: 4230
Brown, Cameron: 4440
Brown, Candace: 4007, 4230
Brown, Casey K: 1275, 2025, 4515, 7290
Brown, Diane K: 4600
Brown, Karl J: 4445, 7410
Brown, Lana M: 7220, 7410
Brown, Lauren L: 4440, 8015

Brown, Mallory M: 7030
Brown, Maria T: 2405, 4455
Brown, Maya P: 7350
Brown, Monique J: 9025
Brown, Rebecca: 1220, 2245, 3025, 8015
Brown, Roger L: 8005
Brown, Sarah E: 8010, 9150
Brown, Susan L: 2295
Brown, Susan H: 7120, 7310
Brown, Sylvia: 4200
Brown, Taylor J: 7365, 7505
Browning, Wesley R: 4465
Bruce, Marino A: 3180
Bruni, Pietra T: 1050
Brunner, Olivia: 8005
Brunson, Amie: 8010
Brunswick, Chad A: 7190
Brunt, Christopher S: 4260
Brus, Anastasiia: 8015
Bryan, Emma G: 7350
Bryan, Joanna: 7440
Bryan, William E: 3065
Bryant, Ashley Leak: 4095
Bryant, Natasha: 7075, 7300
Bryer, Chase M: 1225
Buchanich, Jeanine M: 2200
Buchinger, Laura: 4490
Buchman, Aron: 7200
Buck, Harleah: 2070, 3355, 7225, 7335
Buckey, Jay C: 8015
Buckley, Nina: 2265
Buckley, Rachel F.: 9040
Buckwalter, Kathleen: 7005, 7305
Bucy, Taylor: 1010, 1485
Budhathoki, Chakra: 4385
Budinich, Marilu: 9035
Budney, Alan: 4390
Buedo, Paola: 7145
Buffalari, Deanne: 3150
Buffenstein, Shelley: 3267
Buitron, Daphne: 1055, 7255
Bulanda, Jennifer R: 7335
Bullich, Santiago: 7415
Bullock, Karen: 7290, 7500, 8010
Bülow, Per: 8005
Bülow, Pia: 8005
Bulut, Eyuphan: 4200
Bunker, Jennifer: 3280, 7185, 7485, 7930, 8005
Bunn, Melanie Gayle: 2475
Burack, Orah R: 7250, 7330
Burciu, Roxana: 7315
Burd, Christin E: 8010
Burd, Nicholas A: 8000
Burdick, Jennifer: 2355
Burdick, Katherine E: 7460
Burek, Melissa: 7370
Burgdorf, Julia G: 1270
Burgess, Elisabeth O: 4550
Burgess, Tanya L: 7470
Burghardt, Andrew: 1460
Burgos Zuniga, Claudia C: 7395
Burke, Robert E: 8015
Burk-Leaver, Erin Y: 1000, 9130
Burkley, Julie: 4545
Burman, Bonnie K: 2485
Burnes, David: 1085, 1215, 7180
Burnet, Evie N: 7200
Burnett, Jason: 1085, 4415
Burnett-Bowie, Sherri-Ann: 4460
Burnette, Denise: 7260
Burns, Adam: 1460
Burns, Edith: 2175, 7215, 7410, 8000, 9055
Burns, Hannah: 2085
Burns, Jeff: 7265
Burns, Katharine: 7460
Burns, Paige: 9095
Burns, Reilly M: 8015
Burns, Shane D: 4290
Burr, Jeffrey A: 3335, 7080, 7170, 7355, 7375
Burton, Lindsay: 7485
Burton, Lydia M: 8015, 9065
Bush, Adam: 8005
Bustillo, Antonio J: 8005
Buta, Brian: 2100, 3105
Butera, Katie A: 7140
Butt, Maayra I: 7375, 8005
Buxbaum, Channa: 7185
Buxton, Orfeu M: 2230, 3060, 7390, 7535, 8005
Buys, David: 8015
Bybee, Sara G: 4240, 8010, 8015
Byeon, Sukyung: 8010
Byers, Amy L: 7205, 7255
Bygrave, Desiree: 7465
Bylund, Carma: 7350
Bynum, Julie: 7120, 7250, 8005, 9035
Byon, Ha Do: 8010
Byrd, DeAnnah R: 1450
Byrd, Elizabeth: 7245, 7280

**C**

Cabrera, Nora Carolina: 8005
Cabrera, Ryan: 1305
Cabrera, Samir: 8010, 8015
Cabrero Castro, Jose E: 8015
Cacchione, Pamela Z: 3390, 8010
Cacciatore, Stefano: 8010
Cacciatore, Stefano: 8015
Caceres, Maria: 1160, 3355, 7015
Cachioni, Meire: 7370
Cacia, Ava Marie: 7150
Cadar, Andreia: 8000
Cadden, Margaret: 7415
Cadet, Tamara: 8010, 8015
Cadmus, Eniola O: 4565
Cagle, John: 1145, 7220, 7500, 9005
Cahill, Kevin E: 3310
Cai, Piaopiao: 4175, 7380, 7925
Cai, Shuang: 8010
Cai, Wenjie: 4060, 4540, 9040
Cai, Xinxin: 8010
Cai, Xiuting: 2305
Cai, Yihui: 9020
Cai, Yurun: 1255, 7500, 8010, 8015
Cal, Halvorsen: 1195
Calamia, Matthew R: 4395, 7450
Caldera, Selena: 2050, 3070
Calderon, Russell J: 4010, 7390, 9075
Caldwell, Clover: 7305
Caldwell, Joe: 3190, 8005
Calfee, Kris: 1475
Calhoun, Katie: 9110
Caljouw, Monique AA: 1010
Calkins, Maggie: 2080
Calkins, Margaret: 2080, 3035, 7465, 8010
Callahan, Kathryn E: 1110, 1255, 3160
Callaway, Teagan: 7170, 7220
Callow, Daniel D: 1510, 4375
Calloway, Haley: 4065
Calvani, Riccardo: 8010, 8015
Calver, Jen: 2465
Calvo, Esteban: 1425, 4325
Camacho, Cinthia: 4470, 7210
Camacho, David: 1055, 3265, 4150
Camacho, Leticia: 7495
Camacho, Paul B: 1255
Camacho-Rivera, Marlene: 8015
Cameron, Vanessa K: 7245
Cammack, Ralph: 7440
Cammer, Allison: 7205
Campbell, Claudia: 8010
Campbell, Don: 7545
Campbell, Emily: 8015
Campbell, Kenna: 8005, 8010
Campbell, Laura M: 8010
Campbell, Susanne: 7410
Campellone, Kenneth: 8005
Campitelli, Anthony: 7120
Camplain, Ricky: 8015
Campos, Blanca: 2555
Campos, Matthew: 7485
Can, Reyyan: 3360, 4270
Canell, Anastasia E: 7300
Canetto, Silvia Sara: 7255
Canham, Sarah L: 2515
Cannell, Michael B: 1085
Cannon, Melissa L: 7005
Cannon, Rachel: 2285, 7010, 7915
Cantrell, Randall: 4210
Cantu, Phillip A: 2060, 2345, 3265, 4540, 7465, 7475
Canzone, Mia R: 7405, 8015
Cao, Jiawei: 7010
Cao, Jing: 7040
Cao, Katy: 4245
Cao, Rui: 1405
Cao, Ying (Jessica): 4320
Cao, Yunhua: 4255
Capilupi, Michael: 7000
Cappon, Davide Balos: 7025, 7435
Caprio, Thomas V: 2205
Capuano, Ana: 8005
Carasso, Ella: 4185
Carbajal, Berta: 7305
Cardamone, Coleen: 7410
Cardenas, Deami: 8000
Cardenas, Janelle: 8015
Cardenas, Luisa: 3155
Carder, Paula: 3280, 4400, 7420, 8005, 8015
Cardona Puerta, Adela: 8015
Cardoso, Josue: 8000
Cardwell, Christina: 7300
Carey, Christopher: 3085, 7415
Carey, Cyriah: 7320
Carlile, Robert M: 7010
Carlin, Kevin: 2055
Carlozzi, Noelle: 4015
Carlson, Andrea C: 4460
Carlson, Barbara: 7370
Carlson, Bryant: 1490
Carlson, Michelle C: 2400, 8000, 8005
Carlson, Rose L: 4135
Carmichael, Pierre-Hugues: 8000

Carmiol-Rodriguez, Priscilla: 7100
Carnahan, Jennifer L: 1435, 4240, 8010
Carnahan, Ryan: 8010
Carneiro Marques, Fátima Cristina CC: 7300
Carney, Gemma M: 1365, 3140
Carney, Maria: 7410, 8000
Carniol, Renie R: 3000
Carpenter, Brian D: 1390, 7470, 8000, 8005, 8010
Carpenter, Christopher R: 8015
Carpenter, Joan G: 1145, 1300, 1435, 8010, 8015
Carpenter, Molly: 4405
Carpenter, Rebekah: 8015
Carpenter-Song, Elizabeth A: 2045
Carr, Alex: 7090
Carr, Brendan M: 7485
Carr, David B: 1355, 7505, 8015, 9015
Carr, Dawn Celeste: 1285, 2025, 2105, 3175, 3285, 4245, 4560, 8000, 8015
Carr, Deborah: 1140, 2020, 3195, 4000, 7055, 7290
Carrasco, Johanna: 9050
Carrillo, Anthony: 7245
Carrington, Crystal: 4075
Carroll, Judith: 1475, 8000
Carroll, Megan: 8000
Carroll-Sauer, Cathleen: 7225
Carstensen, Laura L: 1195, 1330, 3275, 4040, 8000, 8010
Carswell, Andrew: 4210
Carter, Aubrey: 8015
Carter, Chelsey R: 7210
Caruso, Rosario: 8015
Carvalheiro, Joana: 8000
Carvalho do Nascimento, Paulo Roberto: 7225, 7905
Carwile, Hope: 4475
Cary, Ann H: 8015
Cary Jr., Michael P: 3135, 3355
Casale, Sophia: 4130, 7200, 7225, 7260, 7905
Casanova, Giuliana: 7445
Case, Donna: 7295
Casey, James: 4515
Casey, Kamryn M: 7290
Caskie, Grace I L: 7005, 7455
Caspi, Avshalom: 7025, 7115
caspi, Eilon: 8010
Cassales Tosi, Fabiana: 7140
Cassario, Abigail: 3270
Cassidy, Jessica L: 1020
Cassidy, Weitman: 7050
Castañeda-Avila, Maira: 2555, 7255
Castellano, Michael: 8005
Castellanos, Stacy: 3290
Castells, Marianna: 8000
Castillo-Collazo, Delmarielinnette: 7200
Castle, John E: 1175
Castro, Hugo: 9035
Castro, Maria-Jose: 7285, 7925
Castro Sanchez, Barbara A: 8005
Catalano, Hannah: 7150
Cateau, Deke G: 1005, 4550
Cater, Lauren: 9015
Cauble, Christina: 3255
Caulfield, Maria C: 2335
Cavailles, Clemence: 4335
Cavalier, Alyssa: 8010
Cavanaugh, Connor: 7545
Cavanaugh, Sheamus: 2170
Cawthon, Peggy M: 1460, 7345, 8005
Ceïde, Mirnova E: 7525
Cellarius, Karen: 4400
Cenko, Erta: 1390
Cenzer, Irena K: 2245, 3050, 4070
Cepeda, Elizabeth: 8015
Ceria, Rachel: 8005
Cerino, Eric S: 1175, 2095, 2170, 2280, 4120, 7115, 7365, 8000
Cerino, Lauren: 3250, 4410
Cerniglia, Laura: 8005, 8010
Cernohous, Inessah: 8010
Cerqueira, Margarida: 7445
Cha, Kyeongin: 7325, 7425
Cha, Seung-Eun: 7335
Chaaya, Monique: 1495
Chachlani, Preeti: 7305
Chacon, Elyana: 7185
Chadwick, Darren: 8015
Chai, Hye Won: 1285, 2280, 7325
Chai, Shujun: 1235
Chak, Carly: 7005, 7935
Chakraborty, Shayok: 1285
Cham, Rakie: 8010
Chamberlin, Elizabeth S: 1305, 2365, 7230, 7280
Chamberlin, Keran W: 4375
Chan, Alex Chi-keung: 4180
Chan, Alex Kin-shing: 4180
Chan, Alex Siu Wing: 8005
Chan, Angelique Wei-Ming: 1205, 2435
Chan, Athena: 7185, 8000
Chan, Celine: 3155
Chan, Crystal Ying: 9085
Chan, Keith T: 8010, 8015
Chan, Kin Tung: 8000
Chan, Lily Man Lee: 7085
Chan, Wai Chi: 1235, 4455, 7255
Chan, Wai Sze: 1030
Chan, Wayne LS: 9020
Chan, Ya-Ning: 2255, 2475
Chandler, Blythe E: 1470
Chandler, Kelly D: 7335
Chandler, Rasheeta: 7015
Chandler, Zachary W: 8000
Chandler-Holtz, Lauren: 8005
Chandola, Tarani: 1030, 7360
Chang, Emiley: 7225, 7905
Chang, E-Shien: 2325, 3025
Chang, Jun Ha: 7465
Chang, Ling-Hui: 8010
Chang, Pei-Shiun: 7010, 7910
Chang, Rita: 8000
Chang, Timothy: 2555
Chang, Ya-Han: 1290, 4175, 7145
Chang, Yu-Ling: 4030
Chang, Yu-Ping: 7245, 7255, 7380
Chang-Lee, Shu-Nu: 7205
Chanti-Ketterl, Marianne: 1020
Chao, Pei-Shu: 4335, 7390
Chao, Shiau-Fang: 7165, 7370, 7465
Chao, Ying-Yu: 7255
Chapleau, Marianne: 7415
Chaplow, Zachary: 2220
Chapman, Brian G: 4220
Chapman, Elizabeth N: 2195
Chapman, Silvia: 1100, 7415
Chapman, Susan A: 1370, 3315
Chappell, Richard J: 1200
Charaf, Amani: 1135
Charamelo, Ana: 8015
Charankevich, Hanna: 2480
Charara, Rima: 8010
Chargo, Alexis N: 8005
Charles, Doreek: 4135
Charles, Susan T: 2095, 2280, 4120, 7460
Charlwood, Catherine: 2335
Charness, Neil: 4080, 4420, 8000, 8015, 9085
Chary, Anita: 1090
Chase, Cara: 2220
Chase, Gabrielle: 8015
Chasteen, Alison L. L: 1210, 7205
Chataut, Akankshya: 7390, 7475
Chatterjee, Sudeshna A: 4525
Chatterton, Hailey A: 7415
Chattopadhyay, Anurima: 1190
Chattopadhyay, Debaleena: 2040, 4590, 8010
Chau, Diane: 8005, 8010
Chau, Pui Hing: 4585
Chaudhary, Indra: 1500
Chaudhury, Habib: 2325, 3035
Chauhan, Shekhar: 2105, 3020, 4470, 4560
Chavarria, Stephanie: 8015
Chaverri Carvajal, Alexander: 7250
Chavez, Lorely A: 3355
Chavez Granados, Heily: 7205, 9120
Chee, Wonshik: 4065, 7305, 8005
Cheema, Zainab K: 8005
Chehab, Lora: 7405, 8010
Cheishvili, David: 8005
Chek, Carmen Jia-Wen: 8005
Chekuri, Srujana: 7160, 7390, 7475
Chel, Victor G: 4105
Chemalapatti, Kavya: 7195
Chemhaka, Garikayi: 7430
Chen, Amanda: 2465
Chen, Bangwei: 7410
Chen, Bangwei: 8015
Chen, Bin: 3090
Chen, Blythe: 7540
Chen, Chang-Chun: 8005
Chen, Chen: 2305
Chen, Chen: 8005, 8015
Chen, Chen: 8015
Chen, Chen X.: 7500
Chen, Chen-Fen: 7250
Chen, Ching: 8010
Chen, Chixiang: 3260, 4330, 7345, 7495, 9075
Chen, Chun-Liang: 8015
Chen, Cynthia: 4370, 7070
Chen, Cynthia: 8005
Chen, Diefei: 3380, 9045
Chen, Duan-Rung: 7220
Chen, Enna Y: 1195, 3275, 4085, 8000, 8010
Chen, Eric: 8015
Chen, Feng: 8015

Chen, Gong: 4255, 7300, 7430
Chen, Haiyi: 1235
Chen, Haiying: 1075, 1455, 2470
Chen, Hao-Min: 2300
Chen, Honglei: 1500, 7345
Chen, Honglin: 8000
Chen, Hongwei: 9150
Chen, Irene: 7440
Chen, Jen-Hao: 1490, 7245
Chen, Jenny: 9115
Chen, Jiachen: 8005
Chen, Jiawei: 7025
Chen, Jiayu: 7015
Chen, Jie: 7010
Chen, Jie: 7415, 7485
Chen, Jing: 1220, 1245, 4055, 7095, 7130, 7915
Chen, Jingsha: 1155
Chen, Kay: 1400
Chen, Keer: 4170
Chen, Kevin: 7255
Chen, Kuan-Hua: 4085, 4515, 7290
Chen, Lei: 2260, 4260, 8010
Chen, Li: 4335
Chen, Li-Mei: 7300
Chen, Lin: 7090
Chen, Lin Yee: 4375
Chen, Lingjun: 4060, 7225
Chen, Lingming: 8000, 8015
Chen, Liu: 2360, 3090, 7325, 7415, 8000
Chen, Liying: 7150
Chen, Lucia: 8010
Chen, Natalie: 7185
Chen, Qi: 2540, 8005
Chen, Qiaoxi: 1340
Chen, Ruijia: 3050, 4070, 4280, 7010
Chen, Shiyu: 4105
Chen, Shuo: 3260, 9075
Chen, Shyh-Huei: 2470
Chen, Si: 7420
Chen, Sinan: 7140
Chen, Sinuo: 4255
Chen, Stephanie Yu-Ching: 7410, 7935
Chen, Stephen: 1090
Chen, Tuo Yu: 2230, 7390
Chen, Wei-Ti: 1490, 7245
Chen, Wenjie: 8005
Chen, Xi: 1135
Chen, Xi: 1160, 1180, 2105
Chen, Xi: 3125
Chen, Xi: 3200, 7240, 8015, 9010, 9140
Chen, Xiaodong: 7225, 7905
Chen, Xiaowen: 2100
Chen, Xiayu S: 4565, 7210, 7295
Chen, Xiecheng: 9000, 9085, 9135, 9145
Chen, Xindi: 7530
Chen, Xingdong: 7320
Chen, Xinyi: 1240
Chen, Xuewei: 2290
Chen, Xuhui: 7190
Chen, Ya-Mei: 7140, 7370, 8010
Chen, Yanjun: 1200
Chen, Yaofeng: 8005
Chen, Yasheng: 1355
Chen, Ye: 8015, 9155
CHEN, Yilin: 4455
Chen, Yi-Ling: 7020
Chen, Yineng: 7445
Chen, Yingtong: 8000
Chen, Yu-Chih: 7070, 7365, 7405
Chen, Yu-Jen: 8005
Chen, Yuming: 2545, 7320, 7520
Chen, Yuxuan: 1275
Chen, Zhaojing: 7305
Chen, Zhe: 7050
Chen, Zhirui: 3305, 4530
Chen, Zhuolin: 2455
Chen, Zi: 4585
Chen, Zihan: 7050
Chen-Edinboro, Lenis P: 4070, 7150
Cheng, Bei: 4370, 7310
Cheng, Greta Jianjia: 2200, 9110
Cheng, Hongtao: 1245
Cheng, Huai Y: 2195, 3065
Cheng, Huai: 3065
Cheng, Huai Y: 7455
Cheng, Jie: 8015
Cheng, Joyce: 9115
Cheng, Kent Jason: 3195
Cheng, Kimberly: 7185
Cheng, Mengling: 2120
Cheng, Philip: 7390
Cheng, Qiqi: 1205
Cheng, Qiyuan: 8005
Cheng, Wan-Ju: 7020
Cheng, Yu-Shan: 8005
Chereches, Flavia: 1240
Chern, Andrew: 8015
Chernauskas, Tess M: 7395
Chernoff, Violet: 7415
Cherry, Barbara: 7145
Cherry, Katie E: 4395, 7450
Chervenick, Olivia N: 8005
Chess, Eric: 1215
Cheuk, Shui-kit: 7320, 7520
Cheung, David: 1385
Cheung, Ethan Siu Leung: 7260, 9115
Cheung, Monit: 7075
Chew, Alex: 1115
Chew, Justin: 7120
Chew, Keng Hao: 7235
Chi, Chih-Lin: 7305
Chi, Hua-Chin: 7305
Chi, Nai-Ching: 3220, 7265, 7305, 7500
Chia, Clement: 4370
chia, Hui Xiang: 3325
Chia, Jia Qian: 7120
Chiang, Hao-Ping: 7205
Chiaravalloti, Nancy: 8015
Chidebe, Runcie CW: 2220
Chie, Wei-Chu: 1365
Chih-Liang, Wang: 7340, 7470
Chikkala, Ashley: 4570, 8005
Child, Shaye: 7350
Chilton, Patricia: 8005
Chin, Janecca A: 3050, 4085
Chin, Meejung: 7290
Ching, Isabel: 8010
Chiriboga, David A: 2385
Chiriboga, David A.: 7210, 7305
Chisholm, Latarsha: 2265, 8010
Chisolm, Cynthia: 8000
Chiu, Wan-Yu: 7220, 7330, 8010
Chiu, Yu-Wen: 2300
Cho, Allison: 7000
Cho, Donghyuck: 7305
Cho, Emma: 7290
Cho, Euna: 4345
Cho, Eunhee: 7185, 7295, 7330, 7525, 8010
Cho, Haa-young: 3045
Cho, Hannah: 7295
Cho, Hyeonji: 4315
Cho, Hyunkeun: 2135
Cho, Isu: 7200, 7380
Cho, Jamie: 8015
Cho, Jinmyoung: 3255, 7475, 8000, 8010
Cho, Ji-Young: 1345
Cho, Joonyoung: 8005
Cho, Jungwon: 7185, 7295, 7330, 8010
Cho, Kelly: 8010
Cho, Kyoungmin: 2230, 7365
Cho, KyungAh: 7220
Cho, Sangyong: 8005
Cho, Seungjong: 3245, 4580, 7415, 7475
Cho, Sung Eun: 2275
Cho, Sung-il: 7090
Cho, Tsai-Chin: 1495, 2050, 4060, 4540
Cho, Yein: 3150
Cho, Yesang: 7295
Cho, Youngmin: 2255, 4385, 7390, 9075
Choate, Ashley: 1470
Chodos, Anna H: 4160
Chodosh, Joshua: 2045, 9030, 9045
Choi, Abigail: 7085
Choi, Bokyoung: 7005, 7135
Choi, Byeong Yeob: 3355
Choi, Edmond Pui Hang: 4585, 7085
Choi, Eun Young: 2005, 2250, 2275, 2345
Choi, Eunhee: 7005
Choi, George: 8015
Choi, Ha Young: 3230, 4320, 7295
Choi, HeeSun: 4590
Choi, HwaJung: 1375, 2050, 3010, 3230
Choi, Hwan: 7495
Choi, Hyein: 9125
Choi, Hyojin: 3135, 7395
Choi, Ian: 7085
Choi, Jean: 7200
Choi, Jeeyae: 7400
Choi, Jennifer L: 8005
Choi, Jeongeun: 7060
Choi, Jihee: 7005, 7135
Choi, JiYeon: 2210, 7055, 7300, 8015
Choi, Moon: 3025, 3075, 3340, 4420, 8000
Choi, Namkee G: 2310, 4300, 4415, 4530, 7020, 7155, 7255, 7395, 7465
Choi, Narae: 4180
Choi, Sangyong: 8000
Choi, Seongmi: 2210, 7055, 7300, 8015
Choi, Seo-Yun: 7210, 7390
Choi, Seung: 4015
Choi, Seung-won E: 3020
Choi, Seung-won Emily: 3020
Choi, Shinae L: 1140, 1365
Choi, Suhyeon: 7540
Choi, Yeon Jin: 7035, 7450, 7475
Choi, Yong Kyung: 2375, 3205, 3215, 4305, 7225, 7515
Choi, Yoonseok: 4270, 4490, 7390, 8010
Chon, Doukyoung: 8010
Chon, Yongho: 8015
Chopik, William J: 3270
Choquette, Elise: 1020
Chou, Kee-Lee: 4300, 7395

Chou, Lin-Na: 2190, 4580, 8015
Chou, Wan-Yun: 1290, 8010
Choudhry, Niteesh: 3390
Choula, Rita: 3070, 4100
Chow, Christine: 3340
Chow, Julian Chun-Chung: 2540
Chow, Stephanie W: 4425, 7455
Chowdhury, Nahian: 8000
Choy, Jacky C. P.: 2135, 8015
Choy-Yung, Rebecca: 9085
Christensen, Kaare: 7345, 8005
Christensen, Leah: 2365
Christofi, Sofia: 7115
Chrzanowski, Brandi: 7225, 7340, 7920
Chrzanowski, Lauren: 7305, 7400, 7415, 7900
Chu, Charlene: 7370
Chu, Chenyu: 4275
Chu, Jun: 2305, 7095, 9140
Chu, Ruotong: 4330
Chu, Sang Hui: 7375
Chua, Grace: 1205
Chua, Shiyun: 7120
Chuah, Joshua: 4060, 8000
Chudasama, Darshan: 2190
Chui, Cheryl Hiu-Kwan: 1135
Chukwu, Ngozi E: 1365
Chukwuemeka, Nkechi A: 8015
Chukwuorji, JohnBosco Chika: 2220, 7405
Chulak, Jamy: 1390
Chun, Gloria: 2350
Chun, Yung: 8010
Chung, Daum: 4335, 7330
Chung, Edwin Ka Hung: 2350, 8005
Chung, Hyeran: 1250, 2060
Chung, Jane: 3260, 4200, 8015
Chung, Jayer: 7300
Chung, Ji Won: 4475
Chung, Joohyun: 2465, 4135, 7220, 7370, 7910
Chung, Ka Hung Edwin: 8015
Chung, Paul J: 7510
Chung, Sandrine Man-chi: 1385
Chung, Soondool: 7210, 7290, 7305
Chung, Sulki: 3020
Chunga, Richard E: 7305
Chung-Bridges, Katherine: 8000, 8015
Chupak, Anna L: 9025
Churchill, Emma G: 7375
Chwa, Amir: 4370
Chyn, Eric T. Y.: 8010
Cicero, Ethan: 1000, 2145, 7110
Cichy, Kelly E: 7115, 7335
Cidav, Tom: 1055, 4450
Cieslik, Katarzyna A: 2125
Cifuentes, Christina: 1275
Cigliana, Jill: 7205, 7300, 7910
Cilluffo, Silvia: 7335, 8015
Cimarolli, Verena: 7075, 7280, 7360, 7475
Ciolfi, Mary Lou: 1115, 4480, 7170
Cipolli, Gabriela C: 7090
Cipriano, Andrea: 4107
Cisek, Edward: 1160, 1225
Cizginer, Sevdenur: 8000
Cizik, Amy M: 1025
Clair, Catherine A: 2030, 7500
Clancy, Erin T: 4050
Clancy, Margaret: 1070, 7120
Clapp, Jonathan D: 7420
Clare, Linda: 2335
Clark, Alexandra L: 4570, 8005
Clark, David J: 4525
Clark, Jeff: 7300
Clark, Kirsty A: 8005
Clark, Lindsay R: 1200, 8015
Clark, Maria F: 7200
Clark, Maria: 8010, 8015
Clark, Melissa: 1465, 2160, 7220
Clark, Phillip G: 2205
Clarke, Mary F: 8010, 8015
Clarke, Philippa: 1075, 1370, 1375, 4440, 7465
Clauw, Daniel: 4450
Clay, Olivio J: 3370, 8015, 9125
Clayton, Jordana L: 8015
Clayton, Tanya: 7220
Clayton-Jones, Mary (Kate) C: 1020
Clem, Sarah: 7085
Clements, Nia: 1040
Clemings, Kelly A: 7405
Clemons, Cailyn: 2005, 7535, 7900
Clervil, Stephanie: 8010
Clevenger, Carolyn K: 1275, 2160, 7185, 8010, 9025
Clish, Clary: 8005, 9155
Cloney, Nicholas D: 9105
Cloughesy, Jonathan N: 8010
Clouse, Kate: 3355
Cloyes, Kristin: 2030, 2255
Clutton, Jonathan: 7265
Coates, Martha: 8005
Coats, Heather: 7085, 7910
Coats, Jacquelyn: 8000
Cobb, Ryon J: 4125
Cobos, kimberly: 8005
Coccia, Kathryn W: 8005
Cochran, Jeremy: 8005
Cochrane, Barbara: 8010
Coco, Laura: 7535
Coe, Antoinette B: 7205
Coen, Paul M: 4275, 8000
Cohen, Andrew B: 8000, 8005, 9000
Cohen, Ann: 1255, 2200, 8015
Cohen, Cari: 7330, 7390
Cohen, Deborah: 4470, 7210
Cohen, Inessa: 7090, 8015, 9140
Cohen, Jessica: 7215
Cohen, Marc: 1130, 1150, 3155, 7085, 7250, 7255
Cohen, Olivia: 1160
Cohen, Steven A: 2255
Cohen, Yafit: 4195
Cohen, Yarin: 1090, 2540, 4315
Cohen-Fridel, Sara: 8015
Cohen-Mansfield, Jiska: 3130
Cohn-Schwartz, Ella: 2370, 2390, 7110
Coker, Karen: 7005
Colón-Emeric, Cathleen: 3135
Colón-Semenza, Cristina: 2380
Colcombe, Stan J: 8015
Cole, Connie: 1300
Cole, Evan: 8000
Cole, Marilyn B: 7275
Cole, Steven: 7115
Coleman, Carissa: 1150, 1515, 2225, 7010, 7250
Coleman, Leonardo: 9090
Coleman, Max: 9030
Coleman, Rachel L: 1115, 7170, 8005, 8010, 8015
Coley, Rebekah L: 3305, 4530
Colicino, Elena: 8010
Collie, Angel: 1285
Collinge, Charles W: 8015
Collins, Amber N: 1060
Collins, Joy: 8005
Collins, Kristin: 7340
Collins, Lauren F: 9080
Collins-Pisano, Caroline: 4575, 7395, 8005
Colman, Allison: 4020
Colon-Emeric, Cathleen: 1435, 3065, 7385
Colon-Vargas, Monica: 3370
Colson-Fearon, Brionna: 4530
Colvin, Calvin: 8000
Coman, Emil: 8010
Combs, Collean: 1165
Comer, Russ: 7280
Compernolle, Nell: 1115, 1445, 4085, 7380, 9005
Conant, Rachel: 4160
Conard, Ryan: 8015
Conde Freniche, Alberto: 8010
Condon, Ashely: 4105
Cong, Zhen: 1400, 2275
Conklin, Jamie: 8010
Connell, Elizabeth H: 2310
Connell, James: 1335
Connelly, Denise: 1430
Connolly, Marie-Therese: 1085
CONNOR, KATIE M: 1050
Connor Eruchalu, Jade: 7205
Conroy, David E: 8000
Constantino, Gailan: 8005
Contento, Angela: 3165
Convoy, Dana: 4075
Cook, Diane: 4045
Cooke, Alicia K: 3345
Cooke, Eric: 7370
Cooley, Marion: 8010
Coon, David W: 1110, 7305
Cooper, Jasmine: 9115
Cooper, Lujira: 2525
Cooper, R. Amanda: 7185, 8010, 8015
Cooper, Shelby J: 7250
Cooper, Zara: 8005, 9150
Coppola, Andrea: 1295
Coppotelli, Giuseppe: 1520
Corazzini, Kirsten: 1395, 1400, 2150
Corbisiero, Kyra: 8010
Cordero, Luis J: 7370
Cordova, Lourdes: 7305
Coresh, Josef: 1155, 1200, 2535, 3105, 4385, 7545
Corley, Robin P: 4110
Corley, Samantha S: 7255
Corlin, Laura: 7315
Corman, Samantha C: 7545
Cornelison, Laci: 4405
Cornelius, Kala: 3005, 4350
Cornish, Emily J: 8005
Cornman, Jennifer C: 2375

Coromac-Medrano, Juliamaria: 7200
Correia, Stephen: 8015
Cortes, Constanza: 3397
Cortes, Tara A: 2015
Cosentino, Stephanie: 1100, 7095, 7415
Costa, Amy N: 1475, 4230
Costa, Carolina L.: 8005, 8010
Costanza, Katelyn: 3295
Costello, Erica: 3350
Costello, Janina: 7150
Cotten, Shelia R: 3340
Cotter, Katherine N: 8000
Cotton, Quinton D: 3375
Cotton, Sam: 1060, 2465
Cottrell-Daniels, Cherell: 8000, 8015
Couch, Elyse: 4330, 7185, 7415
Coughlin, Joseph F: 1405, 3250, 4410
Coulourides Kogan, Alexis: 1215
Coulter-Daly, Isabel: 8010
Covarubbias, Anthony J: 2277
Covinsky, Kenneth: 2245, 9090
Cowan, Ronald L: 2045
Cowan-Pyle, Alexandra E: 7480
Cowley, Martha: 1115
Cox, Brian S: 4475
Cox, Carole: 3055
Cox, Mary A: 8005, 8015
Cox, Sarah: 1085, 3300
Coyle, Caitlin: 1410, 2185, 3000, 3080, 3170, 4130, 4545, 7030
Coyne, Danialle: 4425, 7455
Coyne, Riley: 1215, 9105
Craig, Taylor: 7420
Crainiceanu, Ciprian: 4375
Cram, Peter: 7305
Cram, Peter: 8010
Cramer, Candace L: 4545
Cramm, Jane: 7260
Crandall, Jill: 1455
Crane, Paul K: 3380
Crank, Peter J: 3235
Cranley, Lisa: 3040, 9135
Crawford, James M: 8000
Crawford, Kiersten: 2140
Crawford, Stirling: 8010, 8015
Cray, Hailey V: 8000
Creath, Robert: 8005
Crespo, Michael: 7475
Crews, Deidra C: 3345
Crimmins, Eileen: 2137, 2250, 3127, 7345
Crittenden, Jennifer: 1115, 7170, 8005, 8010, 9125
Crivelli, Lucía: 8015
Crocker, Andrew B: 8000
Crocker, Jillian: 7005, 7110
Croff, Raina L: 1280, 7475
Cross, Dori: 1010
Crowder, Hannah: 7530
Crowder, Victoria: 2255
Crowe, Michael: 2030, 3370, 4030, 4140, 4560, 4570, 7350, 9125
Cruikshank, Kirsten: 4045
Cruise, Cathy: 1305, 7280
Crum, Michelle: 2045
Cruz, Daniel S: 7485
Cruz-Carrillo, Yesenia: 4290
Crystal, Stephen: 2020
Cserepy, Araya: 8000
Cubbin, Catherine: 4530
Cudjoe, Thomas: 1360, 2190, 7330
Cuevas, Jadira: 7095
Cuff, Patricia A: 7075
Cui, Linna: 7480
Cui, Michael: 7485
cui, xianmei: 3075
Cui, Xirong: 7425, 7430
Cullen, Katie: 7275
Culligan, Melissa J: 8010
Culver, Clare: 8010, 8015
Cummings, Steve: 3170
Cummings, Steven R: 1460, 4275, 4495
Cuozzo, Juliana: 3070
Curl, Angela L: 7335
Curran, Sean P: 1520, 2137, 3120, 4227
Curriero, Samantha: 4445
Curtis, Ashley F: 1475, 4230, 7390
Curtis, David S.: 7260
Curtis, Lesley H: 8000
Curyto, Kimberly: 1060
Cushing, Cynthia: 8005
Cutforth, Asia: 1215
Cutforth, Asia: 9105
Cutrona, Carolyn: 4195
Cutty, Maxwell: 8005
Czaja, Sara: 1170, 2325, 3025, 4080, 4525, 8000, 8015, 9085
Czarny, Anna: 9020
Czech, Stephanie J: 8015
Czeisler, Charles A: 7390

**D**

Da, Wendi: 4070, 4505
da Rosa, Grace: 1065
Dabelea, Dana: 1455
Dabelko-Schoeny, Holly I: 3125, 3305, 4130
Dacey, Catherine: 7545
Dacks, Penny A: 7185
Daddato, Andrea E: 4090, 7085, 7910
Dadgostar, Porooshat: 3115
Dadson, Deborah A: 8000
Dagan Yarkoni, Ayelet: 1515
Dagen, Nicole: 7220
Dagne, Mahederemariam: 4190, 9065
Dahal, Arati: 1270
Dahal, Poshan: 7380
Dahal, Roshani: 3095
Dahl Aslan, Anna: 8010
Dahle, Cheryl L: 8005, 8010
Dahms, Eric: 7510
Dai, Sherwin: 1520, 7480
Dai, Shuting: 9060
Daks, Jennifer: 2085
Dalal, Taykhoom: 3090
D'Alesio, Mark: 4435
Dalgård, Christine: 7345
Daly, Rebecca: 7050, 7375
D'Ambrosio, Conchita: 1210
D'Ambrosio, Joseph: 2110
D'Ambrosio, Lisa: 3250, 4410, 7005, 8005
Damman, Marleen: 2350
Damoiseaux, Jessica S: 1255
Damul, Benmun: 7055
Danaei, Goodarz: 7010
Daneshvar, Nina: 1235
Dang, Kristina Van: 2250
Dang, Linh: 7335
Dang, Stuti: 8010, 8015
D'Angelo, Hannah: 7470
Daniel, Jacquelin: 1260
Daniyalo, Kadir Abdul: 8000
Dannefer, Dale: 2020
Danquah, Ahmed: 4465
Danquah, Ahmed: 8000, 8015
D'Antonio, Patricia M: 2215, 2355, 3210, 4555
Dark-Freudeman, Alissa: 1390
Das, Malay: 8000
Das, Mohana: 7465
Das, Sayani: 3305
Das, Stuti: 8015
Das, Sudeshna: 7415, 9040
Das, Utpol: 8000, 8015
Das Gupta, Debasree: 1355, 4030, 4440, 4560
Dasanayake, Thakshila: 8000
Dasariraju, Satvik: 8000
Dasgupta, Abhirupa: 4150
Dash, Chiranjeev: 7190
Dassel, Kara B: 1380, 4555, 7405, 8010, 8015
Dastgheyb, Raha: 1155, 7245
Dastidar, Bonnie: 2200, 9110
Daudi, Leonarda: 9130
Daugherty, Ana M: 8005
Daugherty, Ana: 8010
Dauphinee, Dale: 8000
Daus, Marguerite: 7485, 7930
Davey, Adam: 2080
David, Daniel: 2440, 4155, 7250
Davidson, H Edward: 7305
Davidson, Patricia M: 7380, 7430, 7495
Davidson, Sylvia: 7340
Davie, Laura: 1395, 2150
Davies, Casey: 2030
Davies, Tracy: 8000
Davies-Teye, Bernard: 2125
Davila, Ana Luisa: 3370
Davila, Carine: 9120
Davila, Heather: 1305, 2085, 8005
Davila-Roman, Ana Luisa: 3370
Davis, Brian: 8010
Davis, Carter H: 7215
Davis, Elizabeth: 9145
Davis, Elizabeth: 9150
Davis, Henry J: 7355
Davis, Isabelle: 2265
Davis, Jennifer C.: 7225, 7905
Davis, Jonathan: 1500
Davis, Matthew: 8005
Davis, Rachel: 2400
Davis, Rachel: 8010
Davis, Robert: 8000
Davis, Scott: 7225
Davis-Hearn, Chelsea N: 7300
Davitt, Joan: 1145, 7085, 7220
Davlyatov, Ganisher: 3365
Davoudi, Anis: 2100, 2480, 4375
Davy, Brenda: 8010
Davy, Kevin P: 8010
Dawe, Connor: 4500
Dawes, Connor: 4500
Dawson, Walter D: 4400, 7010
Dayanandaswamy, Chirantana: 9015
Daylor, Jayne: 7410

Dayrit, Jessika: 8005
De Anda-Duran, Ileana: 3380
de Boer, Bram: 3035
De Fries, Carson M: 1215, 1240, 4040
De Jong, Marla: 4555
De Jonge, Karl E: 3340
de Klerk-Rubin, Vicki: 7215
De Main, Atami: 1055, 1490, 7255, 8015
De Maria, Maddalena: 7335
de Paula Couto, M. Clara: 1210, 2370, 8015
de Rosa, Cristina: 7335
De Sanctis, Pierfilippo: 7025
De Vilmorin, Charles: 8015
De Vito, Alyssa: 2000, 7290, 7305, 8000
De Wit, Liselotte: 8005
de Wit, Sanne: 4580
Deal, Jennifer: 1075, 1200, 2535, 7330, 8010
Deardorff, W. James: 7140
DeBlois, Jacob P: 2085
Debras, Charlotte: 8010
DeCarli, Charles: 2040, 8010
Decker, Autumn: 3295, 4565
Decker, Chase: 9080
Decker, Veronica B: 1055, 7225, 8005
Declercq, Anja: 1060
DeConne, Theodore: 8000
Deek, Rebecca A: 8000
Deep, Gagan: 8015
Defillo Draiby, Julio: 7000
DeForge, Christine: 7305
Degenholtz, Howard B: 1385, 4100, 4260, 7370, 8005, 8010
DeGennaro, Athena P: 1360
DeGennaro, Jennifer: 7340, 7410
DeGloria, Michael: 8000
Dehghanian, Marjan: 2555
Deichert, Jerome A: 4550
DeJohn, Amber: 4245
Del Bene, Victor A: 4570
Del Bosque, Jennifer: 8015
Del Carpio, Karina: 2275
DeLany, James P: 2470
DeLawter, Alana: 8000
Delemeester, Natalie K: 7540
Delgadillo, Jeremy D: 9125
Delgado, Carolina: 8015
Delgado, Ferdinand: 8010
Delgado, Kimberly: 8000
DeLiema, Marguerite: 1010, 2115, 7405
Deliu, Zane: 7145
Delizia, Morgan R: 8000
Delmelle, Eric: 9105
DeLuca, John: 8015
Demchak, Sydney M: 8005
Demers, Camille: 7065
Demesi, Joy C: 1430
Demeter, Naor: 4195
Demetres, Michelle: 1505, 7105
Demir, Gokhan: 3065
Demir, Murat M: 4350
Demiris, George: 3390, 4200, 7205, 7220, 7260, 7290, 7295, 8015
DeMott, Andrew: 3045
Demuth, Ilja: 4050, 4490
Deng, Amy: 8015
Deng, Qiyu: 7395
Deng, Yanran: 2545, 7320, 7520
Deng, Zhirui: 8000, 9050
Denis Oliva, Francyess: 7170, 7220
Dennis, Elizabeth A: 2395, 7130, 7455
Dennis, Paul: 4430
Denny, Bryan T.: 8000
Denny, Sharon S: 7185
Denson, Kathryn: 7455
Denson, Steven: 7455
Dent, Kallisse R: 7255
Dentry, Tyler: 8015
DePaolis, Fernando: 9075
Depp, Colin: 7050
Derby, Carol A: 1110, 4310, 7535, 8005
Derivaux, Mattie: 2535
Deroo, Rebekah: 8000
Derricks, Veronica: 3085
Dertinger, Melinda: 4045
Deschodt, Mieke: 1060, 8010
Deshaies, Marie-Hélène: 7065
Deshmane, Aarohi: 7365
Deshmukh, Madhyami: 7470
DeSilva, Mary: 8015
Desir, Marianne: 2540, 7280, 7350, 8010, 8015
Desjardins, Michael R: 2400
Desmond, Grace: 8000
DeSouza, Dannah D: 8005
Destrampe, Melissa A: 1130, 3155
Deutsch, Judith: 7140
Devenport, Winry F: 8005
Devlin, Kathryn: 7290
DeVone, Frank: 8005
DeVries, Marla: 8010
Dewey, Erica: 3150
DeWitt, Avery: 7210
Dey, Amit: 7315, 7480
Dey, Nutifafa E Y: 8000
Deyoung, Audrey: 4485
Dhakal, Samitinjaya: 7130
Dhakal, Usha: 1500, 3380
Dhammapeera, Phot: 7375
Dhamne, Sayali: 7120
Dhana, Klodian: 4570
Dhanavanthri Muralidhara, Sneha: 4230
Dharia, Aastha: 1370, 8000
Dhingra, Lara: 7305
Dhingra, Radha: 4095, 7520, 8000
di, liu: 7190
Di Florio, Paola: 9020
Di Gessa, Giorgio: 3145
Di Rosa, Mirko: 7070
Diallo, Ana F: 1260, 7095
Dian, Larry: 7225, 7905
Diaz, Aymara: 8010
Diaz, Vivian: 8015
Diaz Nelson, Michele: 7000
Diaz-Asper, Catherine: 4190
Diaz-Santos, Mirella: 2555
Diaz-Valdes, Antonia: 1425, 4325
DiBacco, Grace M: 8015
DiCarlo, Nicholas: 1420
DiCesare, Elyse: 1340
Dicianno, Brad: 2375
Dicken, Megan: 8005
Dickerson, John: 8005
Dickson, Chelsea: 8005, 8010
Dickson, Daniel: 7065
Dictus, Cassandra: 2255, 4095, 4425, 4565
Dieciuc, Michael A: 1285
Diefenbach, Michael A: 2175, 8000
Diehl, Christin: 7515, 7930
Diehl, Manfred: 8015
Dieppa Colo, Emily: 1005
Dierckx de Casterlé, Bernadette: 8010
Diergaarde, Brenda: 8000
Dietz, Anna: 8000
DiFiglia, Stephanie: 2030, 4140
DiFranco, Lacey: 8000, 9010
Dikhtyar, Oksana: 7420
Dikken, Jeroen: 3080
DiLalla, Linda: 1050
Dill, Janette: 2260, 3075
Dillon, Ellis: 1315, 2185, 4135, 7360, 8005
Dillon, Kayla: 1035
Dillon, Simon T: 2505
DiMaria-Ghalili, Rose Ann: 7360, 8005
Diminich, Erica D: 9025
Dinata, Erna: 7375
Dindo, Lilian: 7030
Ding, Dan: 4305
Ding, Jie: 4385, 7545
Ding, Jinfeng: 8015
Ding, Jingzhong: 7480
Ding, Jingzhong: 8000
Ding, Kedong: 1140, 3245
Ding, Linlin: 4230
Ding, Margaret: 1305, 3315, 4205, 7270
Ding, Ruoxi: 4055
Ding, Weiyang: 3205
Diniz, Amanda: 7320
Diniz, Breno S: 4495
Diniz e Silva, Bruno Vinícius: 8000
Dinman, Michele: 8000, 8010
Dino, Michael: 3030, 4450, 7160, 7225, 8000, 8010
Dinov, Ivo: 1510
Dionne, Laura: 1465, 8015
Diunugala, Sithara D: 8000
Dixon, Steve: 8010
Djoroeva, Aydana: 7545
Do, Hien: 2275
Do, Jonghwa: 7055
Doan, Lan N: 3155
Dobbs, Debra: 2490, 3335, 7040, 7220, 7340, 7395, 7420, 7515, 7935
Dobrayel, Marie: 9055
Dodge, Hiroko H: 1185, 2360, 3090, 7325, 7415, 8000
Dodge, Shana G: 1015, 7185
Doering, Sabrina: 4010
Dogan, Anil Sukru: 7315
Dogari, bolu: 3230
Dohan, Daniel: 7185
Doherty, Fiona C: 3125
Dolan, Hanne R: 1110
Dolansky, Mary A: 1485, 7410
Dolberg, Pnina: 7450
Dold, Anna: 7265
Dolenc Nott, Brooke: 7005
Dollar, Blythe: 7085, 7910
Dolma, Rinchen: 2220
Domenicali, Marco: 9020
Domingorena, Sofia: 7145
Domingues, Marisa: 7370
Dominguez, Ahria J: 7290
Domnick, Hallory A: 7410
Donaher, Brooke: 4175
Donahue, Marissa: 7305
Donega, Stefano: 8000
Donelle, Lorie: 1430
Dong, Fanghong: 7290
Dong, Wuyi: 3320, 7200, 7435, 7530

Dong, Yan: 7145
Dong, Yanjun: 1105, 4395, 7295, 7485, 9030
Dong, Yu: 8010
Dong, Yue Coco: 8000, 9050
Dongmei, Zuo: 3285
Donnelly, Rachel: 9040
Donofry, Shannon D: 4585
Donohe, Krista: 1260
Donohoe, Maria: 2135, 4350
Donorfio, Laura KM: 4220, 7295
Donovan, Heidi: 2375
Donovan, Nancy J: 2310
Dorame, Ashley: 3330
Dorame, Breana: 8005
Doran, Kelly: 8005
Doran, Kevin: 7515
Dore, Emily: 1080
Doron, Malka: 4355, 7175
Dorris, Jennie L: 7490
Dos Santos, Luis Miguel: 1385
Dosa, David: 2500, 7255, 7410, 7485
Dougherty, Maura: 7125
Dougherty, Ryan J: 4375
Douglas, Natalie F: 1165, 1300
Douglas, Sara: 7220
Dove, Abigail: 4370
Dow, Patience M: 2465, 4010
Dowd, James J: 3140
Dowden, Lauren: 1015
Dowhower, Dan P: 7005
Downer, Brian: 1250, 2060, 3045, 7240, 7445, 8015, 9075
Doyle, Cassidy: 3185
Doyle, Margaret: 7530
Doyle, Margaret F: 8005
Dozier, Mary: 8015
Dozier, Stephen: 7190
Draica, Florin: 8010
Dremann, Andrea: 8000
Dresden, Scott M: 7485
Drewelies, Johanna: 4050
Drewelies, Johanna: 4490
Dreyfuss, Claire: 8005
Driver, Jane: 1470, 7220, 8000, 8010, 9080, 9090
Drolet, Marie-Josee: 2235
Drost, Jennifer: 4600, 7225
Druda, Ylenia: 9020
Dryden, Eileen: 2540, 3115, 7280, 7350
D'Souza, Deepak Cyril: 4390
D'Souza, Gypsyamber: 8015
Du, Chenguang: 8015
Du, Sixian: 7300, 7410, 7930
Du, Yao: 1235
Duan, Hongzhe: 7415
Duan, Liping: 4275
Duan, Yinfei: 2150, 7300
Duberstein, Paul R: 2320, 3205
Dublin, Sascha: 9090
Dubois, Lindsay: 3190
DuBois, Samantha L: 8005, 8015, 9025
DuBose, Logan: 9025
Dubowitz, Tamara: 2200, 9110
Duchateau, Francois Xavier: 8015
Duchesneau, Emilie D: 1255, 4065
Duchow, Olivia: 8000
Duchowny, Kate: 4440
Dueck, Kimberly: 3025
Duezel, Sandra: 4050
Duff, Kevin: 1185
Duffy, Erin L: 8010
Dufour, Alyssa: 7130
Dufour, Isabelle: 1080
Dufresne, Ruth: 8010, 8015
Dugan, Beth: 4220
Dugan, Elizabeth: 1075, 1335, 7130, 7170, 7185, 7200, 7210, 7225, 7245, 7255, 7260, 7300, 7305, 7375, 7530, 7905
Dulaney, Sarah OCS: 9000
Dumbadze, Sofio: 8010
Dumitrescu, Logan: 9030
DuMontier, Clark: 8000
Dumuid, Dorothea: 3235
Dunbar, Nora: 7365
Dunbar-Jacob, Jacqueline: 8010
Dunk, Michelle: 4370
Dunlap, Shawn: 2085
Dunn Lopez, Karen: 8010, 8015
Dupre, Mason L: 7520
Dupre, Matthew E: 4095, 8000, 8005
Duque, Gustavo: 1520, 3117, 4473
Durant, Raegan W: 4445
Durette, Kailey: 4500
Durham, Sonya: 8015
Duthie, Edmund H: 7455
Dutta, Chhanda: 4008
Dutta, Naibedya: 4275
Dutta, Raghbendra K: 8000
Dutton, Hillary: 2440, 8010
Dworak, Elizabeth M: 7435
Dwyer, Dan: 8000
Dykes, Patricia: 8015
Dys, Sarah: 3155, 3280

**E**

Eagle, Angelica: 4490
Earls, John C: 7015, 7190
Earp, Jacob E: 8000, 8010
East, Mary Ann: 8000
Eaton, Charles B: 8015
Eaton, Jacqueline: 2135, 2455, 4240, 4355, 4555, 7085, 7915
Eaton, William: 1510
Ebright-Jones, Blake D: 7460
Ebstein, Madison: 7290
Ebulum, Genevieve C: 7380
Echt, Katharina V: 2195
Echt, Katharina V: 7410
Economidou, Sofia: 8015
Edahiro, Ayako: 7440
Edelman, Linda: 2255
Edelstein, Offer E: 7185
Edlund, John E: 3150
Edmonds, Andrew: 2395
Edmonds, Leiha: 8005
Edwards, Madison: 8005
Edwards, Morgyn: 8005
Edwards, Robert: 7120
Edwards, Samuel: 7135
Edwin Sam Devahi, Adharsha Sam: 3090
Eerike, Madhavi: 7510
Efird-Green, Lea: 1435, 7440, 7915
Egbert, Anna R: 7020, 7265
Egge, Robert: 4160
Eglseer, Doris: 1310
Ehlman, MC: 7330
Ehrlich, Joshua R: 1075, 4065
Eich, Teal: 7395
Eichorn, Ann: 7215
Eilat, Shvat: 3080
Einstadter, Douglas: 8005
Eisenstein, Amy R: 2495, 4020
Ejaz, Farida: 1215, 8000
Ejem, Deborah: 4445
Ejikeme, Chidera A: 7390
Eka, Ni Gusti Ayu: 8000
Ekoh, Prince C: 1365
Ekwebelem, Maureen: 4225
El-Abbadi, Naglaa: 4440, 4460
El-Assad, Lauren M: 8015
Elayoubi, Joanne: 7350
Elbejjani, Martine: 1495
ELBOIM-GABYZON, MICHAL: 4355, 7175
Elder, Sadie Leder: 3150
Elfenbein, Pamela: 4355
Elftmann, Hanley: 7415
Elgayar, Sara: 3115
Eliassen, A. Heather: 9155
Eliazar-Macke, Nathaniel: 7350
Eliopoulos, Elaine M: 7270
Elisha, Victor: 8005, 8010
Eliyahou, Ayala: 1090
Elk, Ronit: 3165, 4240
Elkarif, Talia: 1330, 4235
El-Khatib, Lana: 2265
Ellington, Lee: 2135, 2255, 7085, 7915
Elliot, Amy: 4345
Elliot, Meghan R: 7050, 7460
Elliott, Annaliese: 7160
Elliott, Christian: 7305
Elliott, Emily M: 4395, 7450
Elliott, Maxwell: 7025
Elliott, Susan J: 3255
Ellis, Annika M: 8000
Ellis, Katrina: 3245
Ellis, Rebecca: 4390
Ellison, Teena: 8015
El-Makarim Aboueissa, Abou: 4105
Elman, Alyssa: 3300
Elmas, Cagri: 7470
Elmasri, Jeannine: 7015
Elmore, Catherine E: 2190
Elop, Alana: 9090
Elrod, Cathy S: 4190
Emami, Azita: 8000, 8005, 8015
Emans, Sinclair: 2417
Embon Magal, Shiri: 1110, 7040
Emerson, Amanda: 8010
Emery-Tiburcio, Erin E: 2110, 2240, 9140, 9145
Emma, Amanda: 2085
Emrani, Sheina: 4570, 7435
Emrick, Lauryn: 7500
Eng, Chloe: 3380
Engeland, Christopher G: 1445, 3060, 7325, 8005, 8010, 8015
Engelman, Michal: 3265, 4520, 4585, 7260, 7290, 8015
Engelmeier, Joey: 4305
English, Dan: 3255
English, Tammy: 1045, 3270, 4515
Engstrom, Gabriella: 8000, 8005, 8015
Enguidanos, Susan: 2265, 2390, 3300, 7220
Enriquez Hesles, Elisa: 7190
Enroth, Linda: 1080

Ensrud, Kristine: 1455, 1460
Epps, Fayron: 1135, 2420, 3375, 4335, 7015, 7475, 8010, 9025, 9130
Epstein, Mara: 9095
Epstein-Lubow, Gary: 2285
Eraslan, Emre: 8005
Ercan, Semra: 7265
Erices-Ocampo, Paulina: 8005
Erickson, Ashleigh: 8000
Erickson, Kirk: 4585, 7265
Erickson, Melissa L: 8005
Ericsson, Malin: 4030
Erkens, Petra: 7250
Erlenbach, Emily: 7265, 8000
Ermer, Ashley: 4360
Ernst, Jerome: 2395
Ernst, Joy: 7405
Ernsth-Bravell, Marie: 7325, 8005
Erodula, Nandini: 7265
Ersek, Mary: 1300
Ersek, Mary: 9005
Erving, Christy L: 1105, 4250
Escamilla, Valerie: 8010
Escareno, Juana: 3355
Eshraghi, Karen: 7305
Esiaka, Darlingtina: 2220, 2420, 3180, 7200, 8010, 8015
Espejo, Edie: 7010
Espeland, Mark A: 1455, 2470
Espinosa, Cisco: 1170
Espinoza, Kevin Enrique: 8000, 8015
Espinoza, Sara E: 1387, 2557, 4090
Esposito, Michael: 2005, 7465
Essen, Emma von: 1095
Esser, Karyn A: 8005
Estabrooks, Carole A: 2150
Estenson, Lilly: 1250, 8010
Estes, Kayley D: 7050
Estlein, Roi: 1125, 4185
Estrada, Jesus: 9055
Estrella, Mayra L: 8005
Etnier, Jennifer L: 7435, 8000, 8005, 8015, 9025, 9130
Ettman, Catherine: 3315
Etuka, Brandon Asika: 8015
Etzell, Martha: 2055, 8015
Etzkorn, Lacey H: 2100, 3105, 4375
Evangelista, Melanie: 2500
Evans, Abigail: 1145
Evans, Annalise: 7005
Evans, Daniel: 1460, 4495
Evans, Joni K: 1455
Evans, Katie: 4160
Evans, Michele: 7130
Evans, Taylor: 8015, 9050
Evans, Tracy L: 1035
Evans, William J: 1460
Evelyn-Gustave, Allyson: 8015
Everson-Rose, Susan A: 7425
Ewen, Heidi H: 8010
Eze, Ifeoma E: 8015
Ezeihuoma, Obinna: 7380
Ezeokonkwo, Francesca C: 8010
Ezeonu, Brian I: 9120
Ezzi, Zahra: 1310

F

Fabian, Tyfany: 7360
Fabisiak, James: 4305
Fabius, Chanee D: 1010, 2260, 3075, 7360
Facchetti, Teresa: 7095
Factora, Ronan: 7405
Fagan, Zoe: 8010
Fagundes, Christopher: 9015
Fagundes, Christopher P.: 8000
Fahey, Margaret C: 7180
Faieta, Julie: 4305
Falco, Vince: 8015
Falk, Derek: 2060
Falkstedt, Daniel: 1425
Falls, Katherine H: 1260, 7095
Falohun, Tokunbo: 9025
Falvey, Jason R: 7000, 7495, 8010, 9075, 9115
Falvo, Michael J: 8015
Falzarano, Francesca: 2390, 3025, 7040, 7475, 9040
Famula, Jessica: 2450, 4145
Fan, Alei: 7470
Fan, Bolin: 7365
Fan, Erica K: 2200
Fan, Mingming: 7295
Fan, Ning: 7220
Fan, Qiping: 9025
Fan, Xuehong: 4180
Fan, Zhiyu: 7340
Fanelli Kuczmarski, Marie: 7130
Fang, Hsin-Yu: 7315, 8005
Fang, James R: 4060, 7225
Fang, Mei L: 8005
Fang, Mei Lan: 1135, 1325, 8015
Fang, Siyue: 8005
Fang, Ya: 1235, 7010
Fanning, Jason: 2470, 8000
Fanzosa, Emily: 3115
Faran, Yifat: 2105
Faria, Frederico: 7225, 7905
Fariduddin, Osswaah: 8010, 8015
Farina, Mateo P: 1105, 2345, 3380, 7535, 9040
Farmand, Aida: 3310
Farmer, Heather R: 1415, 2345, 4095, 4125, 4360
Farmer, Justin: 8000
Farnham, Trish: 7075
Farrell, David: 4475
Farrell, Timothy W: 7030
Farsijani, Samaneh: 4295, 7345, 8000
Faruque, Irtiza S: 8000
Faselis, Charles: 9020
Fashaw-Walters, Shekinah: 1270, 8005
Fasolino, Tracy: 2075
Fassi, Janelle E: 4115, 7255
Fassnacht, Daniel: 4580
Fastame, Maria Chiara: 7265
Fastbom, Johan: 4370
Faul, Anna: 1060, 2110, 2465
Faul, Jessica: 7345
Faurot, Keturah R: 4535
Fauss, K. Melinda: 8005
Faust, Phyllis: 7415
Fauth, Elizabeth: 4085, 7215, 7305, 7330
Favero, Michelle: 3245
Fazeli, Pariya L: 4570, 7245, 9125
Fazio, Elena: 4008
Fdil, Mariam: 2235
Feaster, Daniel: 8010
Featherston, Kyle G: 7125
Feddoes, Daisy: 8005
Federman, Alex: 9080
Fedorov, Alex: 7015
Fedorovskyy, Maks: 8000
Fedus, Donna B: 2135
Feehan, Jack: 4473
Fein, Taylor: 8005
Feliciano, Leilani: 7020, 7195, 7900
Felix, Cynthia: 4030
Felson, David: 1070
Felt, John M: 1445
Felter, Cara: 7455
Felts, Adam: 3250, 4410
Fender, Chloe: 4347
Fenelon, Andrew: 1375
FENG, GUANG-GANG: 7020
Feng, jingchao: 7355
Feng, Yuan: 7435
Feng, Zhaohong: 8000
Fennell, Charles: 7475
Fennell, Gillian: 1070, 7120
Fenstermacher, Ryan: 7435
Ferdows, Nasim: 1020, 2260, 7210, 7395, 7535, 9045, 9150
Fergen, Joshua T: 4045
Ferguson, Dee: 1405
Ferguson, Erin: 4280
Ferguson, Giselle: 1175
Fermin-Martinez, Carlos A: 1155
Fernandes, Cecilia: 2500
Fernandes, Karl JL: 7415
Fernandes, Marta: 7015
Fernandes Junior, Ezemir Dantas: 4425
Fernandez Cajavilca, Moroni D: 2135, 4260, 7185
Fernandez Castello, Ana Isabel: 8015
Fernandez-Romero, Roberto: 8000
Ferrante, Lauren: 7000
Ferraro, Kenneth F: 1080
Ferrarotti, Tina: 9090
Ferreira Leitao Azevedo, Renato: 1505, 4320
Ferrie, Joseph P: 3330
Ferris, Annie: 7410
Ferrone, Christine F: 7410
Ferrucci, Luigi: 4295, 4495, 7315, 8000
Feryn, Alicia: 1460
Fessele, Kristen L: 8005
Fete, Zoe: 2285, 7010, 7185, 7915, 8015
Fick, Donna M: 3205, 4015, 7060
Fiehn, Oliver: 4495, 7315
Fielding, Roger: 3160
Fields, Beth: 1035, 2445, 4435
Fields, Bronwyn: 2450
Fields, Erica Barnes: 4115
Fields, Maizonne: 8015
Fields, Noelle L: 8010, 8015
Fierro, Cecilia: 8010
Figueiro, Vittoria: 2265
Figueroa, Luis A.: 8010
Fillmore, Nathanael: 8000
Finamore, Victoria: 3355
Finch, Brian: 4030
Findley, Kimberly: 2290, 2445
Finegan, Kerry: 8010
Fingerman, Karen: 3185, 7350, 7390, 8005
Finik, Jackie: 2475, 4505

Fink, Howard A: 3065
Finkel, Deborah G: 4030, 7325, 7375, 8005, 8010
Finkelstein, Ruth: 3385
Finlay, Jessica: 2005, 2400, 4110, 4195, 4505, 7465
Finn, Laura: 8010
Finn, Myrna: 1130, 3155
Fiori, Katherine L: 2085, 4360, 7355
Fique, Morgan T: 7455, 9115
Firman, James Paul: 1515, 3215
Fischer, Mya: 2235
Fischer, Rosa-Linde: 8010
Fischer, Stacy M: 2320
Fish, Anne: 8010
Fish, James: 8010
Fishbein, Kenneth W.: 8000
Fisher, Carla: 7350
Fisher, Dr. Anna: 4405
Fisher, Koren L: 7310
Fisher, Lauren: 1015
Fisher, Louis: 9025
Fishman, Paul: 8010
Fisk, Calley: 4440
Fiske, Amy: 1440, 2240, 3100, 7255, 8010
Fitzgerald Wolff, Sharon: 7180
Fitzgibbons, Joanna: 7170, 7290
Fitzpatrick, Neasa: 7490
Fix, Rachel M: 8000, 9095
Flaherty, Grace K: 7545
Flanagan, Jane M: 8000
Flatt, Jace D: 1000, 1420, 2010, 2145, 2550, 7110, 7145, 8010
Fleck, Jessica: 7015, 7150, 7385
Fleet, Alexa: 3155, 7020
Fleming, Jessica: 7060
Fleming, Nicole: 4240, 8010
Fleser, Jessica: 9015
Fletcher, Aisha D: 4250
Flood, Johnna: 9105
Flood, Sarah: 8015
Flores, Belinda: 3355
Flores, Elena: 7190
Flores, Emily V: 7310
Flores, Micayla: 8000
Flores, Vanessa: 8005
Flores Romero, Karla Renata: 4070
Floyd, Alex: 1145, 7500
Floyd, Alex C: 9005
Fogelberg, Donald: 8000
Fogle, Stephen J: 2500, 3140
Foley, Jessica: 1520, 9120
Folker, Ann: 7350
Folorunsho, Sunkanmi: 7200
Fonarow, Gregg: 9020
Fong, Daniel Yee Tak: 4585, 7085
Fong, Florence: 9085
Fong, Steve Fu-Fai: 9085
Fong, Tamara G: 2505, 8010
Fons, Brian R: 4415, 7155, 7255, 7465
Fonseca, Luciana: 8000
Fonseca Valencia, Carolina: 7310
For, Wing-Yin: 1135
Foran, Shannon: 7410
Ford, Cassandra: 4045
Ford, James H: 2135, 7420, 7905
Ford, James C: 8015
Forden, Jessica: 3310
Foreman, Eric R: 8015
Formson, Maud: 8010
Forrester, Sarah N: 1415
Fortier, Catherine: 8005
Fortinsky, Richard: 1110, 1315, 1385, 2185, 4495, 7305, 7345, 8005, 8010, 9020, 9090
Fosnight, Susan: 4600, 7225
Foster, Charlie E M: 2120
Foster, Marcel: 9110
Foster, Norman: 8000
Fowler, Mackenzie E: 3095
Fowler, Nicole: 1435
Fox, Aimee: 1390
Fox, Julia: 7535
Fox Ludden, Emily: 8010
Fracassi, Anna: 8010
Fraiman, Paule-Sarah: 1485, 7410
Fram, Maryah S: 1090
France, Michael T: 9150
Francis, Dania V: 3310
Francis, Hannah: 1365
Francis, Howard: 7530, 8005
Francis-Levin, Jess: 3230, 8010
Frankel, Emily L: 7130
Franklin, Heather: 1165, 7415
Franzosa, Emily: 1305, 3315, 4205, 7270
Frase, Robert T: 2405, 4040, 4185, 7085
Fraser, Sarah: 7205, 8015
Freburger, Janet: 8005
Freddolino, Paul P: 3215, 4320
Fredman, Steffany J: 7380
Fredrickson, Barbara L: 1275
Fredriksen-Goldsen, Karen: 2155, 2525, 7355, 7535
Free, Peyton: 7305
Freeborn, Todd: 4475
Freedman, Vicki A: 2375
Frees, Traci: 8010
Freitag, Simone: 7125
French, Chloe: 9085
Frerichs, Leah: 8005
Freund, Alexandra: 7460
Freund, Katherine: 3170, 7155
Frey, Scott: 2215
Freytes, I Magaly: 7350
Friberg-Felsted, Katarina: 2455, 4355, 4555, 8005
Fried, Jack J: 4305
Fried, Terri R: 3290
Fried, Terri: 7030
Friedberg, Daniel: 4007
Friedland, Hannah: 7005, 7110
Friedman, Batsheva: 8010
Friedman, Ben: 7140, 7905
Friedman, M. Isabel: 7215
Friedman, Mackey R: 2140
Frimpong, Eric: 1075, 7065, 7240, 7375
Frisoli, Alberto: 7320
Fritz, Julie: 8005
Froberg, Ruthann: 9135
Frost, Alanna F: 8015
Frost, Bess: 8005, 8010
Fruhauf, Christine A: 4075, 9090
Frydenlund, Markus: 7205
Frye, Larry: 7545
Fu, Hannah: 9075
Fu, Jason: 7440
Fu, Lillian X: 2095, 4515
Fu, Peipei: 1180
Fu, Rong: 3125, 7145
Fu, Xiaoyu: 1225, 7085, 7375
Fuccione, James: 1335
Fuentes, Carlos G: 3305
Fuentes, Patricio: 9035
Fugate-Whitlock, Elizabeth: 1390, 2330
Fuglestad, Paul: 4095
Fujiwara, Takeo: 7345
Fujiwara, Yoshinori: 7265
Fukushima, Kiyoko: 7160, 7405
Full, Kelsie M: 9030
Fullen, Matthew: 7255, 7375
Fuller, Heather: 2290, 4045, 4165, 8000
Fuller, Patrice M: 1280, 7475
Fuller, Shannon: 4020
Fulop, Tamas: 7315
Fulp-Cooke, Rachael S: 2450
Fulzele, Sadanand: 7245, 8010
Fung, Chi Tat: 8000
Fung, Helene: 1330, 1350, 2250, 2455, 3360, 4300, 7005, 7375, 8010, 9070
Fung, Katherine K.: 8010
Fung, Long Ki: 2455
Fung, Nicole Long-ki: 1330, 8005
Fung, Teresa: 7440
Fung, Victor: 2165
Funk, Cory: 7415
Funkhouser, Dillon: 4435
Furey, Samantha: 7460
Furht, Borko: 2045
Furihata, Ryuji: 2230
Furman, David: 4107
Fussell, Elizabeth: 8015
Futterman, Andrew: 7365, 7400

### G

Gabriel, Kelley Pettee: 2535
Gad, Dustin: 2095, 8005, 8010, 9000
Gadbois, Emily A: 1465, 2055, 7050, 7220, 7485, 7930, 8000, 8015
Gadkari, Gauri: 3280, 7185, 7420, 7930, 8005
Gage, Courtney A: 7355
Gai, Junjie: 7515
Gale, Seth: 7185
Galea, Sandro: 3315, 7450
Galecki, Andrzej: 4450
Galik, Elizabeth: 2090, 2350, 3220, 4460, 8005
Galinsky, Arielle: 7350
Gallagher, Virginia: 4485
Gallagher Thompson, Dolores: 2240, 7230, 7305, 8000
Gallo, Joe: 4150
Gallo, Linda: 7535
Galperin, Irina: 7200
Galucia, Natalie: 8000, 8005, 8010
Galvez, Maria: 3025, 8000, 8005, 8015
Galvin, James E: 4045
Gamaldo, Alyssa A: 2280, 3060, 7325
Gamble, Laura D: 2335
Gampetro, Pamela J: 1505
Gan, Mantang: 7470
Gan, Siqi: 7010, 7140

Gan, Wenqi: 4135
Gandhari, Sanjeela: 8015
Ganguli, Sohini: 8010
Gans, Hannah: 2370
Gantumur, Zoljargalan: 4325, 8015
Ganz, David A: 8000
Gao, Alvin: 2395
Gao, Bruce: 8015
Gao, Lan: 4335
Gao, Louise (Siyu): 1010, 7405
Gao, Mingui: 7255
Gao, Qi: 4310
Gao, Qinglin: 7130, 7225, 7255, 7300, 7905
Gao, Shangbang: 7190
Gao, Sibo: 7350, 8005
Gao, Sujuan: 7010, 7910
Gao, Sunan: 2100, 3105, 4375
Gao, Weijing: 9150
Gao, Xiang: 2150
Gao, Ying: 4585
Gao, Yuhan: 8000
Gao, Zan: 9025
Gao, Zhengrong: 2245
Gara, Nabel: 8005
Garabedian, Pamela: 8015
Garbe, Renee: 3300
Garbin, Alexander J: 7410, 7910
García-Lara, Juan M A: 7145
Garcia, Adolfo J: 8015
Garcia, Catherine: 1225, 4290, 4315
Garcia, Cheyenne M: 4285
Garcia, Emily: 7370
Garcia, Gabriela: 7285, 7925
Garcia, Gilberto: 4275
Garcia, Kandyce: 2170
Garcia, Lorena: 2450
Garcia, Marc A: 4290
Garcia, Patricia: 3085
Garcia, Reagan E: 8005
Garcia, Zoraida: 7145, 8000
Garcia De La Garza, Angel: 4310
Garcia Morales, Emmanuel E: 4065
Garcia Walker, Veronica: 8010
Garcia-Arias, Sabrina: 8005
Garcia-Chanes, Rosa: 2060
Garcia-Davis, Sandra: 8010
Garcia-Jaramillo, Manuel: 4347
Garcia-Morales, Emmanuel: 1075
Garcia-Retortillo, Sergi: 8005
Gardiner, Lea: 7000
Gardner, Bailey: 2555, 3010, 7010
Gardner, Raquel C.: 7000
Gardner, Sue E: 7265
Gareri, Michele: 7225, 7340, 7920
Garner, Charity: 8005
Garnett, Anna: 1430
Garrido, Melissa: 1190
Gary-Webb, Tiffany L: 2200
Garza Naveda, Ana Paola: 9120
Gaspar, Lauren: 1520
Gasser, Brent: 7160
Gassoumis, Zachary: 1215, 3300
Gatchel, Jennifer: 7415
Gately, Megan: 2365, 7295, 7310
Gatt, Justine M.: 4470
Gattine, Elizabeth: 9115
Gatz, Margaret: 4030
Gau, Wen-Bing: 7080
Gaugler, Joseph E: 1280, 2010, 2305, 3117, 3155, 3375, 4100, 7300, 9030
Gavett, Brandon: 4540
Gay, Emma L: 8000
Gay, Whitney: 1060
Gaydosh, Lauren: 8015
Gaylord, Susan: 4535
Gazaway, Shena: 3165
Gaziano, J.Michael: 8010
Gazit, Eran: 7200
Ge, Jing: 4370, 7310
Ge, Linghan: 4315
Ge, Meiling: 9150
Ge, Shaoqing: 4065, 4225, 7475, 9010
Geary, Elizabeth K.: 7330, 7390
Geisser, Sophia R: 2240, 4475
Geisser, Zoe R: 2240
Gell, Nancy M: 7265
Geller, Aaron M: 4515
Gelmon, Sherril B: 7010
Gems, David: 4473
Généreux, Mélissa: 4150
Geng, Fangli: 7125, 7185, 8000
Genova, Helen: 8015
Gensoli, Lindsey: 2275
George, Claudene: 4200
George, Kristen: 7435
George, Megan: 4525
George, Miriam: 2465
Geraets, Anouk: 1105
Gerasta, Denae: 2320, 8015
Gerhard, Glenn: 9070
Gerlick, Joshua A: 7410
Gerosa, Laura: 4473
Gershon, Richard C: 7435
Gerstorf, Denis: 1350, 2120, 4050, 4490, 4515, 7380, 7390, 8000
Gesel, Paul: 4005
Getahun, Darios: 7110
Gettel, Cameron: 7485, 8015, 9140
Getz, Marjorie A: 7175
Gevaert, Jessie: 1425
Geyskens, Lisa: 1060, 8010
Ghaemmaghamfarahani, Ida: 8010
Ghaiye, Suruchi: 8015
Ghanem, Karyman: 7230
Ghanta, Manohar: 7015
Ghattas, Ola: 4005
Ghazi Saidi, Ladan: 4045, 7325
Gheller, Mary: 7120
Ghemrawi, Mirna: 9070
Ghiasi, Akbar: 3365
Ghimire, Dirgha J: 1500
Ghimire, Saruna: 4465
Ghodsi Jafari, Aveen: 7305
Gholizadeh, Shadi: 8005, 8015
Ghose, Urmimala: 1350, 7265, 7380, 8000
Ghosh, Indrajit: 4465
Giammanco, Peter Aldo: 7485
Gianaros, Peter J: 2200
Giandrea, Michael D: 3310
Giannakopoulou, Parthenia: 3030
Giannetti, Matthew: 8000
Giannotto, Emily L: 8005
Giard, David: 1305
Giatras, Ava: 2445
Gibbons, Anna: 8015
Gibson, Allison: 2110, 2335, 8000
Gibson, Grant: 9100
Gibson, Regina: 8015
Gider, Nathalie: 3370, 4030
Gieniusz, Marzena: 2175, 8000
Giffuni, Jamie: 7140, 7905
Gil, Efrat: 7210
Gil, Heidi: 7440, 7915, 8000
Gil Hayes, Lauren: 7260, 7290, 7925
Gilbert, Lauren R: 3305
Gilbertson-White, Stephanie: 8000, 8015
Gilfix, Talia: 7435
Gilhuly, Devin: 7290
Gill, Amarjot S: 7370
Gill, Christine: 7515, 7930
Gill, Thomas M: 1375, 8010
Gillespie, Kate: 7185
Gillett, Alexis: 7265
Gilligan, Megan: 2405, 3185, 4040, 4185, 4315, 4485, 7085, 7470, 8000
Gilliland, Jaime: 2220, 8005
Gillis, Elizabeth: 4500
Gills, Joshua L: 7120
Gilmartin, Mattia J: 1485
Gilmore-Bykovskyi, Andrea: 7000, 8005
Gilsanz, Paola: 8010
Gimm, Gilbert: 2190, 2375, 7065, 7900
Ginggeaw, Sangduan: 4135
Ginsberg, Charles: 7345
Ginsberg, Terri: 4570
Giordani, Bruno: 1510
Giordano, Stephanie J: 1010, 2260, 3020, 3190
Giorgio, Luciana: 3245
Giraldo-Santiago, Natalia: 2310
Girling, Laura: 8005
Giroski, Lexi B: 7200
Gitelman, Darren R: 7010
Gitlin, Laura N: 1320, 3085, 4210, 7300, 7415, 8005
Gittleson, Alondra: 2275
Giudicessi, Averi: 8000
Gjika, Olga: 8010
Gladfelter, Jenna: 7540
Gladyshev, Vadim: 8005
Glaser, Karen F: 3225
Glavin, Ye-Fan Wang: 2305
Gleason, Carey: 1160, 2170, 7270
Gleason, Kathy S: 4090
Gleason, Kelly T: 1360
Gleason, Lauren J: 2205
Gleason, Shayna R: 3170
Gleason, Shayna R: 4130
Gleason, Shayna R: 7155
Glei, Dana A: 3285
Glenn, Jordan: 7120
Glennie, Kristen S: 7300
Glesby, Marshall J.: 1490
Gliebus, Peter: 8005
Glinka, Allison: 7305
Glorioso, Danielle: 7050, 7375
Glose, Susan: 7005
Glover, Crystal M: 2550
Glymour, M. Maria: 4280, 7260, 8000, 8005, 9040
Glynn, Nancy W: 8000, 8005
Goble, Daniel J: 8010
Goetz, Matthew B: 8000
Gofine, Miriam: 1505, 7355

Goh, Denise: 8005
Goh, Jing Wen: 2435
Goh, Joshua O S: 4030
Goh, Sarah: 4370
Golant, Stephen: 4340
Gold, Noah: 8000
Goldberg, Emily: 2277
Goldberg, Robert: 2500, 7485
Goldblatt, Hadass: 1240
Golden, Robyn L: 9140, 9145
Golden-Pogue, Andrea L: 8000
Goldman, Alyssa: 1105, 3050, 3285
Goldman, Annika: 2085
Goldman, Max: 8015
Golland, Yulia: 4235
Gollinelli, Daniela A: 8005
Golzarri-Arroyo, Lilian: 2555, 3010, 7010
Gomes Goncalves, Natalia: 4540
Gómez, Bárbara: 8015
Gomez, Elisa: 7470
Gomez, Rowena: 8015
Gómez-Martínez, Héctor M: 7200
Gomez-Lopez, Iris: 1370
Goncalves, Marcus D: 3105
Gondo, Yasuyuki: 7365
Gong, Caixia: 1220
Gong, Jessica: 1040
Gong, Jiaowei: 4185
Gonyea, Judith G: 4575
González-Rivera, Christian: 3385
Gonzales, Alexander: 3300
Gonzales, Ernest: 2530, 3045, 7005, 8000, 8015
Gonzales, Mitzi: 1387, 8010, 9020
Gonzalez, Alexa S: 3380, 4540
Gonzalez, Anelly: 7330
Gonzalez, Evelyn: 2005, 2400
Gonzalez, Hector M: 2040, 7535
Gonzalez, Jeffrey S: 4310
Gonzalez, Karen: 2140
Gonzalez, Richard: 2375, 7230, 7335
Gonzalez, Rodriguez: 7010
Gonzalez, Vanessa: 8010
Gonzalez Pyles, Maria S: 7305
Good, Mary K: 1305
Goodman, Michael: 7110
Goodman, Octavia K: 8015
Goodrich, Glenn: 7385
Goodson, Pauline: 9015
Goodwin, James S: 4365
Goodwin, Richard: 8015
Gooldin, Sigal: 7110
Gordon, Barbara: 1060, 2465
Gordon, Julie A: 7370
Gordon, Kate: 1395, 8005
Gordon, Kate: 8005
Gordon, Kate: 8010
Gore, Janelle E: 9025
Gore, Shweta: 8015
Gorman, Emily F: 4465
Gorman, Lily G: 8015
Gorospe, Myriam: 7480
Gorzelle, Gregg: 8005, 9010
Gosselin, Catherine: 7325
Gosselin, Patrick: 8000
Gotanda, Hiroshi: 8000
Gothe, Neha P: 1255, 7265, 8000
Goto, Ryohei: 8010
Gottesman, Elaine: 3300
Gottesman, Rebecca: 1200, 2535, 7325
Gotwals, Amy E: 2355
Gourley, Amy N: 1210
Gouveia, Gillian: 8005
Goyal, Riya: 7015, 7385
Gozalo, Pedro L: 2160, 7125
Gozalo, Pedro: 8005
Grabowski, David: 2465
Gracia, Deborah: 8000, 8015
Grady, James: 1385
Graf, Allyson S: 3150
Graham, Eileen K: 1265, 4120
Graham, Helen L: 8010
Graham, Kirsten L: 7455, 7935
Grahama-Engeland, Jennifer E: 1445, 7535, 8015
Gramling, Robert: 7395
Grams, Morgan E: 1155, 3105
Granada, Tara: 7340
Granbom, Marianne: 4215
Grande, Giulia: 4370
Grant, Claire: 2030, 7230, 7915
Grant, Haley: 4295
Gras, Laura: 7140
Grasso, Anna: 2380
Gravely, Amy A: 3065
Gravenstein, Stefan: 1435, 7305
Gravenstein, Stefan: 8005
Gravenstein, Stefan: 8010
Graves, Letitia: 8010
Gravett, Christopher J: 7405
Gray, Jullie M: 7170
Gray, Margeaux: 7535
Gray, Michelle: 7120, 7325
Gray, Serena: 7400
Gray, Tamryn: 3355
Greaney, Mary A: 2255
Greco, Carol: 4535
Green, Ariel R: 4090, 7385
Green, Brian: 1050
Green, Charles: 8000
Green, Gavin: 4085, 7215, 7330, 8005
Green, Gizelle: 3040
Green, Michael D: 4095, 8000
Green, Nathan: 9065
Green, Noah: 7435, 8010
Greenberg, Jonathan: 2310
Greenberg, Phyllis A: 7395, 7455, 7935
Greenberg, Sherry A: 7410
Greenberg, Steven M: 2460
Greenfield, Emily: 2170, 2185, 3000, 4130
Greenfields, Margaret: 1325
Greenwood-Hickman, Mikael Anne: 9090
Greger, Jeffrey S: 8010
Gregg, Jay: 2085
Grenier, Sébastien: 8000
Grenko, Lydia K: 7395
Grenz, Adele: 3080
Grey-Coker, Sierra: 3345
Grier, Kimberlee: 7305
Griffin, Hannah: 7455, 7935
Griffith, Christopher X: 7195, 7900, 8010
Griffith, Lauren E: 2545, 7225, 7905
Grill, Joshua D: 3375, 7135, 7335
Grillo, Anthony: 8000
Grindrod, Kelly: 7230
Grippo, Angela: 7040
Griswold, Michael: 2535, 7025, 7325, 7495
Groden, Sheryl R: 7295
Grodsky, Eric: 2120, 7010, 7040
Grodstein, Francine: 8000
Grogan, Elyssa FL: 8015, 9140
Grol-Prokopczyk, Hanna: 1070, 4590
Gronlund, Carina: 7465
Gross, Alden L: 1495, 2000, 2035, 3380, 4060, 4280, 4540, 7040, 8010, 8015, 9045
Gross, Alina T: 9115
Gross, Danae: 1220
Gross, Hana: 7435
Groves, Angela: 7320
Groves, Patricia S: 7225
Growdon, Matthew: 7255, 8005, 8010, 9000
Growney, Claire: 1045, 1195, 1330, 3270, 4040
Gruber-Baldini, Ann: 2395, 7545
Gu, Danan: 9030
Gu, Tongyu: 8015
Gu, Yuchen: 7355, 7470
Gu, Yunke: 8000
Guan, Jean: 1345
Guan, Weihua: 7345, 7425
Guan, Xiangnan: 8010
Guan, Xiwen: 8015
Guay, Sandryne: 7200
Guengerich, Terri: 3070
Guenther, Ariana G: 8015
Guerrero, Omar: 7210
Guest, M. Aaron: 2030, 2455, 4355
Gugliucci, Marilyn R: 3130, 7455, 7935, 8015
Gugliuzza, Lydia: 8015
Guida, Cassidy A: 8005
Guidotti Breting, Leslie: 7330, 7390
Guion, Kent: 7005
Gunning, Faith: 7460
Gunter, Kabreah: 8005
Guo, guifang: 7430
Guo, Jia-Wen: 2030, 8010
Guo, Jin: 7470, 7900
Guo, Man: 1250, 7260, 7290, 7375, 7925
Guo, Muqi: 1495
Guo, Sunan: 2100
Guo, Tangmeng: 4370, 7310
Guo, Tianming: 4505
Guo, Xueying: 7070
Guo, Yinning: 2545, 7090
Guo, Zheng: 3275
Gupta, Aditi: 7090
Gupta, Aditya: 7015
Gupta, Avinash: 8005, 9065
Gupta, Ritom: 7225
Gupta, Santosh S: 2500
Gupta, Tanisha: 9070
Guptarak, Jutatip: 8010
Gurera, Jacob W: 7460
Gurevitch, Jacqueline: 2240
Gurinovich, Anastasia: 8005
Gurlu, Merve: 2350
Gurung, Adina: 1500
Gurwitz, Jerry H: 1435, 2500, 7410, 7485
Gur-Yaish, Nurit: 7000
Gusen, Nanle Joseph: 7305

Gusmano, Michael K: 3335
Gustafson, Deborah: 2395
Gustafson, Deborah R: 8015
Gut, Philipp: 8010
Gutchess, Angela: 4030, 7200
Gutiérrez, Myriam: 8015
Gutierrez, Angela: 3265, 4110, 4125, 4250
Gutierrez, Angelina: 4415, 7155, 7255, 7465
Gutierrez, Carmen: 3235
Gutierrez, Sirena: 3380, 7465
Gutierrez-Martinez, Leidys: 9040
Gutierrez-Ramirez, Paulina: 3025, 8000, 8005, 8015
Gutman, Itai: 2245
Guttoo, Parishma: 8010
Guzman, Benedict: 7185
Gwalani, Pranav: 8000
Gwizdala, Crystal: 8005
Gwon, Nayeong: 7035

**H**

Ha, Jung-Hwa: 3045
Haack, Linda: 1035
Haardoerfer, Regine: 2160
Haas, Ashley: 1395
Haas, Steven: 4470
Haase, Claudia M: 2095, 4515, 7265
Habadi, Assma: 7015
Hacker, Eileen: 7265
Hackett, Katherine: 9080
Hackett, Sara: 7340, 7935
Hackney, Madeleine E: 7225, 7440, 9110
Haddad, Zena: 8000
Hadson, Kimberly: 4155
Hagan, Anne: 7290, 8005
Hagan, Brenna: 8000
Hagan, Kelsey E: 7375
Hage, David: 7485
Hagedorn, Aaron: 7300
Hagemann, Lauren: 7230
Haggar, Katherine: 3050
Haggerty, Kristin Lees: 2285
Hagiwara, Shotaro: 8015
Hahn, Samantha L: 7145
Hahne, Jessica: 8000
Haig, Sarah: 4305
Haigh, Sylvia V: 2225, 3040, 4240, 4550
Hajjar, Ihab: 2360
Hakanson, Megan: 7370
Halasz, Ildiko: 7040
Halevi Hochwald, Inbal: 8005
Haley, William E: 2490, 7350, 7420, 7425, 8010
Halkjær, Jytte: 7345
Hall, Charles B: 2400, 4310
Hall, Katherine S: 1470
Hall, LaToya: 2115, 7310
Hall, Lauren M: 1305, 3315, 4205, 7270
Halladay, Christopher: 9090
Haller, Nico: 7125
Halliday, Caroline: 7545
Halloway, Shannon: 4570
Halpin, Sean N: 2220, 8010
Halvorsen, Cal: 1195, 1425, 2170
Halwani, Zena: 7350
Ham, Hyunsun: 8015
Hamada, Fumiko: 7290, 7390
Hamada, Shota: 7250, 7465
Hamada, Shota: 8015
Hamasaki, Yoko: 8010
Hamatsu, Carolina E: 8000
Hamers, Jan: 1310
Hamers, Maud: 3035, 7370
Hamill, Randi: 2260
Hamilton, Lucas J: 1095, 2360, 9030, 9055
Hamler, Tyrone C: 3245, 3395, 8000
Hamlin, Abbey M: 4570, 8005
Hamm, Jeremy: 1175, 7325, 7380
Hamm, Sara: 2275
Hammel, Edward: 7320
Hammel, Iriana S: 2195, 3065
Hammill, Bradley G: 8000
Hampton, Marsha: 3060
Hamrick, Mark: 7245
Han, Bing: 4470, 7210
Han, Chengming: 1180, 3045, 7240, 7445, 7535
Han, Donghee: 7445
Han, Duke: 1100, 3117, 3185
Han, Gaeun: 7080
Han, Gyounghae: 7230
Han, Hae-Ra: 1345
Han, Jian: 8005
Han, Ling: 8015
Han, Lisa F: 7305
Han, Mo: 4590
Han, Sae Hwang: 1195, 2025, 8015
Han, Seok Gyu: 8000
Han, Song: 1110
Han, Soojeong: 7125
Han, Xiaowen: 1180
Han, Xijing: 9030
Han, Y. Catherine: 7435
Han, Yeomin: 9065
Han, Ying: 1235
Han, Yunji: 7155
Hanchate, Amresh D: 1255, 4065
Hancock, Cynthia: 2330
Hancock, David: 8005
Hand, Kathryn: 8000, 8005
Hand, Michelle D: 2165, 9100
Handa, Satoru: 7225
Handique, Swasati: 8010
Handler, Steven M: 4305
Handler, Steven: 4305
Handler, Steven M: 7225, 7515
Haneuse, Sebastien: 9155
Haney, TaFarra: 7220
Hang, Ling: 7090
Hang, Qiqi: 4505
Hankes, Michael: 2245
Hankinson, Susan: 8005, 9155
Hanley, Meredith: 2495
Hannan, Marian: 9155
Hanratty, Barbara: 7400
Hansmann, KJ: 4435
Hanson, Laura: 3015, 3290
Hao, Shira: 8005
Haozous, Emily A: 3345
Happ, Mary Beth: 8005, 8010
Happel, Christine M: 1000
Haque, Nashua: 8000
Harada, Ann SM: 1455
Harada, Ko: 4425
Harden, J Taylor T: 4190
Hardt, Madeleine M: 2220
Harel, Dovrat: 4235
Harel, Ofer: 7345
Harezlak, Jaroslaw: 3085, 7415
Hargand, Sari: 7280
Hariri, Ahmad: 7025
Harley, Dumichel: 9110
Harmon, Misty: 8010
Haro-Ramos, Alein Y: 4095
Harrington, Karra: 2360
Harrington, Samantha: 2365
Harris, Daniel A: 7305, 9090
Harris, Emily: 8000
Harris, Lauren: 8005
Harris, Maurita T: 9110
Harris, Megan: 8005, 8015
Harris, Rebekah: 1470, 7040, 8000, 8010, 9095
Harris-Gersten, Melissa: 7350, 7930
Harrison, Ben: 7045, 7315
Harrison, Krista L: 2185, 9000
Harrison, Tracie C: 7165, 8010
Hart, Alexis: 7340, 7920
Hart, Autumn: 8015
Hart, Evelyn Grace: 8000
Hart, Peter D: 8010, 8015
Hart, Sara E: 4555
Hartley, Gregory W: 7075, 7140
Hartley, Sigan: 8000
Hartlieb, Kristen: 7020
Hartmann, Christine W: 1300, 4475
Hartmann, Julie A: 7340
Hartung, Heike: 2140
Hartz, Stephanie M: 7410, 7910
Harvey, Idethia Shevon: 7430, 8005
Harvey, Jessica: 2120
Harwell, Carla: 8010
Hasan, Sayed: 7190
Hasan, Wordh Ul: 3340, 7015
Hasan, Wordh Ul: 8000
Hasche, Leslie: 1515, 8005
Hasdell, Peter: 7465
Hasegawa, Nanako: 7250
Hashemzehi, Amin: 1065, 7395
Hasin, Deborah: 4390
Hasnani, Madison E: 7500
Hassan, Malaz: 4190
Hassel, Bret: 7455
Hasselgren, Caroline: 7375
Hassmiller Lich, Kristen: 8005
Hastings, Susan: 1470, 4430, 4565, 7350, 7930
Hastings, Waylon J: 8005, 8010
Hasworth, Serena B: 8015
Hatch, Melissa A: 7465
Hathaway, Wendy: 8015
Hattakitjamroen, Varitnan: 4080, 8005
Haub, Mark: 7130
Haurin, Donald: 3285
Hausdorff, Jeffrey M: 7200
Haverhals, Leah M: 1300, 8000, 8015, 9095
Hawes, Courtney: 7050
Hawes, Frances: 4007, 8010
Hawkley, Louise: 1445
Hawks, Zoë: 1265, 1265, 4120
Hawley, Chelsea: 8015
Hayashi, Atsuko: 7140
Hayden, Mary: 3235
Hayek, Roee: 1220, 2245
Hayes, Cellas: 1340
Hayes, Courtney: 4205

Hayes, Jessica M: 1255
Hayes, Kaleen N: 7305
Hayes-Larson, Eleanor: 2390, 4280, 8000, 8010
Hayman, Laura L: 7200
Haynes, Laura: 8000
Haynos, Ann F: 7375
Hays, Ron D: 3290
Hayslip Jr., Bert: 4085, 7470
Hazelett, Sue: 4600, 7150, 7340, 7920
Hazlett, Susan: 2485
Hazuda, Helen P: 1455
He, Caihong: 7130
He, Diana: 8000
He, Jasmin: 1215
He, Jianghua: 7225
He, Jie: 8000
He, Juanjuan "June": 9115
He, Junjie: 1160, 2105
He, Ping: 4370, 7310
He, Ranran: 2405, 4040, 4185, 7085
He, Rendong: 2480, 4070
He, Wenqian: 2275
He, Yilin: 7015
He, Zhe: 1285
He, Zhe: 8000
He, Zhe: 8010
Heath, Diana M: 1275
Heaton, Karen: 4140
Hebdon, Megan: 2190
Heber-Brown, Jennifer: 2330
Heck, Andrew: 2240
Heck, Jennifer: 7005
Heckbert, Susan R: 2400
Heckmann, Kimberly: 7455
Heckman-Stoddard, Brandy: 1455
Hed, Sara: 4010, 7255
Hedges, Hanna: 8010
Heeren, Amanda: 1305
Heffernan, Kevin S: 2085
Heffner, Kathi L: 2310
Heflin, Colleen M: 3285
Heid, Allison R.: 1300, 7305
Heiland, Frank: 3385
Heilman, C. Cassie: 7475
Hein, Maria L: 1150, 1515, 2225, 3220, 4015, 7250
Heinemann, Laura L: 3140
Heintz-Monette, Hannah L: 7375
Heinz, Melinda: 4035, 7015, 7150, 7220, 7275, 7285, 7350, 7380, 7410
Heinze, Rebecca G: 8005
Heist, E. Kevin: 7510
Heitner, Rachael: 9145
Heller, Sloane: 7010
Heller, Tamar: 2430
Helm, Faith: 2205
Helmer, Dashiell: 8005
Helmly, Victoria: 2485
Helsabeck, Nathan P: 2135
Hemmy, Laura: 8010
Hemrajani, Girish: 7515
Hendricks, Justin: 2405
Hendricks-Ferguson, Verna L: 4430, 7220
Hendrix, Cristina: 3355
Henke, Maria: 7300
Henkens, Kene: 2350
Henninger, William: 7285, 7350
Henning-Smith, Carrie: 1010
Henricks, Evan: 3240
Henry, Kevin: 2400
Henry, Maureen: 2075
Henry, Samantha: 8000
Henry, Yater: 7385
Heo, Jinmoo: 7065, 7365
Heo, Jun: 8015
Heo, Seok-Jae: 7325
Hepburn, Kenneth: 1275, 2160, 3375, 4485, 7015, 7475, 8010
Hepple, Russell: 4275
Herbeck, Brandon J: 2485
Herd, Pamela: 4585
Hermens, Daniel: 4580
Hernández, Diana: 7040
Hernández-Ruiz, Virgilio A: 7145
Hernandez, Adriana: 7030, 7050
Hernandez, Mario A: 4080
Hernandez, Rolando J.: 8000
Hernandez-Chilatra, Jessica A: 7015
Hernandez-Tejada, Melba: 4415
Herrera, Carolina: 9125
Herrick, Ashton: 7160
Herrick, Tamara H: 7225
Herring, Laura: 1220
Herzberg, Suzanne: 7410
Herzig, Sébastien: 8010
Herzog, Naemi: 8015
Heselton, Hyeon Jung: 7150
Hess, Moritz: 3080
Heston-Mullins, Jennifer L: 4365, 7540, 8015
Hetherington, Allisyn: 8005
Hetherington-Rauth, Megan: 4473
Hewner, Sharon: 7370
Heyman, Janna C: 7355
Heyn, Patricia C: 2070, 2175, 4190, 9065
Hicken, Bret L: 1305, 2365
Hickey, Johanna: 1435
Hickman, Louis: 3270
Hickman, Susan: 1145, 1435, 8010
Hickner, Andy: 3025
Hicks, Daniel: 4275
Hicks, Stephanie: 8005
Hicks-Patrick, Julie: 3335, 7005, 7350, 7475
Hiersteiner, Dorothy: 3190
Higareda-Garza-Ramos, Andrea: 7145
Higgins, Connor: 8000
Higgins, Maureen: 7225
Higgins, Melinda: 7475
Highberg, Nels: 4220
Higuchi-Sanabria, Ryo: 4275
Hilderbrand, Zoey: 4350
Hilgeman, Michelle M: 1060, 2085, 4240, 7105, 8005
Hill, Carl V: 3180, 4125, 4160
Hill, Cristal: 2417, 4227
Hill, Jordan R: 2555, 3010, 7010
Hill, Nikki L: 8000, 8005
Hill, Patrick L: 4040, 8005, 8015
Hill, William D: 8010
Hill-Jarrett, Tanisha G: 2200, 4280, 8015, 9110
Hillman, Charles: 7265
Himmelfarb, Cheryl R: 7380, 7430, 7495
Himmelmann, Laura: 4450, 7200
Hinkle, Janice: 8000
Hinneh, Thomas: 8015
Hinrichs-Kinney, Lauren A: 7140
Hinyard, Leslie: 4430, 7220
Hirata, Takumi: 7365, 8010
Hiratsuka, Vanessa: 1120
Hirsch, Drew: 9020
Hirsch, Jana A: 2400, 7040, 8005
Hirsch, Jenavry R: 8010
Hirschberg, Julia: 7545
Hirschman, Karen B: 1015, 8000, 9135
Hirsch-Matsioulas, Orit: 1130
Hirsch-Matsioulas1, Orit: 4355
Hirst, Jeremy: 8015
Hiss, Jacob: 7090
Hittner, Emily F: 7265
Hladek, Melissa D: 1220, 3345, 4010, 4445
Hlaing, WayWay M: 8010
Ho, Jessica Y: 1375
Ho, Ken Tsz-Kin: 1135
Ho, Rainbow Tin Hung: 4300, 7395
Ho, Roger: 8005
Ho, Tuong-Vi: 7440
Ho, Yuen Wan: 1350, 7375
Hoang, Bao: 3090
Hoang, Jason N: 7010
Hoang, Minh-Nguyet: 9025
Hobday, Amy: 7300
Hobday, John V: 2010
Hoben, Matthias: 4095
Hock, Robert: 2540
Hodes, Richard: 3033, 4008
Hodges, Sierra: 7435
Hodgson, Nancy A: 7415, 8010, 9135
Hoedjes, Meeke: 7035
Hoedl, Manuela: 1310
Hoeffgen, Gwendolyn: 8015
Hoel, Sydney: 1035, 7305
Hoenig, Helen: 7310
Hoeppner, Bettina: 1160
Hoffman, Abigail: 8000
Hoffman, Charity M: 8010
Hoffman, Elise: 4535
Hoffman, Geoffrey: 7485
Hoffman, Jessica M: 2417, 7045, 8010, 8015
Hoffman, Rashelle: 1020
Hoffman, Samantha: 7320
Hoffman, Yaakov SG: 1100, 8005
Hoffmann, Thomas: 9060
Hofman, Albert: 9040
Hogan, David B: 2545
Hogans, Beth B: 2195, 4600
Hohman, Timothy J: 7145, 9040
Holaday, Louisa W: 8000
Holden, Richard J: 2555, 3010, 3085, 7010, 7415
Holena, Sophia: 7500
Holland, Alexandra: 2005
Holley, Lyn M: 7295
Hollingshaus, Michael: 1025
Hollingsworth, David W: 4240
Hollis, Linda: 1450
Hollister, Brooke A: 7205
Hollman, Nicholas: 1510, 2245
Holm, Johanna B: 9150
Holman, Alison: 7050
Holmes, Ashleigh: 7380
Holmes, Rachel: 7415
Holmes, Sarah: 1395, 2150, 2350, 3220, 4460, 7290, 7495, 8005
Holmes, Sofia: 8005, 8010

Holt, Janet K: 1020, 2170, 4045
Homayouni, Ramin: 1370
Hon, Kristin C: 3025
Honda, Ayumi: 7070, 7235
Honda, Sumihisa: 7235
Honea, Robyn: 4585
Hong, Chenlu: 4455
Hong, Dahye: 7325, 7395, 7520
Hong, Dongfang: 7185, 7200, 7255, 7260
Hong, Eunhye: 8000
Hong, Hyeon: 8015
Hong, Ickpyo: 4525
Hong, Jiyeong: 9130
Hong, Junyuan: 8000
Hong, Kyung-Zoon: 7295
Hong, Michin: 7290
Hong, Seunghye: 7475
Hong, Song Hee: 8000
Hong, Sungjae: 4320
Hong, Sungsoo: 8015
Hong, Ting: 8000
Hong, Wan: 3340, 4420
Hong, YinHui: 2300
Hood, Michelle M: 1110, 4460, 7345
Hoogendijk, Emiel O.: 2510, 7240
Hoogendoorn, Claire J: 4310
Hooker, Julia: 7500
Hooper, Chloe: 7255
Hoppmann, Christiane: 1350, 4270, 4490, 4515, 7390, 8000, 8010
Hoque, Asef Raiyan: 7145
Hord, Norman: 4347
Horiuchi, Takashi: 7380
Horn, Amanda J: 2515
Horn, Janet: 9035
Horn, Linda B: 7455
Horn Mallers, Melanie C: 7455
Horowitz, Chloe S: 7365
Horsfall, Laura: 2250
Horstman, Molly J: 1035
Hort, Jakub: 7295
Horton, Madison: 7185
Hosgood, Dean: 2400
Hossain, MD Sarafat: 8010
Hossain, Mohammad Sajjad: 7475
Hossain, Sheikh: 8015
Hosseinian, Zahra: 7435
Hoti, Kreshnik: 7250, 7330
Hottinger, Lulu: 8000
Hotz, V. Joseph: 3050
Hou, Chien-Chou: 7165
Hou, Ryker: 8015
Hou, Su-I: 1290, 2300, 3030, 4175, 8010
Hou, Yifei: 4040
Hough, Elizabeth A: 7340
Hough, Katrina A: 4020
Houston, Denise K: 1455, 2470
Houston, James R: 7380, 8000
Houston, Leah N: 7310
Houts, Renate: 7115
Hovland, Cynthia: 4600
Howard, Alexandra: 7310
Howard, Kristen R: 8010
Howard, Marjorie: 7165
Howd, Beth: 3220
Howe, Melissa J: 4085, 8010, 9005
Howe, Rebecca: 9080
Howell, Britteny M: 1120, 3295
Howell, Cynthia: 2515
Howell, Veronica: 7375, 7395
Howland, Chelsea: 4230
Howren, Bryant: 1020
Hshieh, Tammy: 2505
Hsiao, Janet Hui-wen: 8005
Hsieh, Evelyn: 7245
Hsieh, Jessica: 7340, 7920
Hsieh, Katherine L: 1230, 9080
Hsieh, Shu-Chen: 8015
Hsu, Amy T: 7370
Hsu, Benjumin: 2125
Hsu, Clarissa W: 9090
Hsu, Erh-Chi: 7200, 8005, 8015, 9005
Hsu, Fang-Chi: 2470
Hsu, Hui-chuan: 7205
Hsu, Kelly: 7215
Hsu, Sheng-Chin: 7490
Hsu, Wan-Ling: 7205
Hsu, Yi-Hsiang: 8000
Hsu, Yu-Chi: 7090
Htoo, Zaw Wai: 7130
Hu, Elise: 1160, 3355, 7015
Hu, Haomin: 2375, 4305, 7225
Hu, Jingwen: 9075
Hu, Jinyu: 4505
Hu, Ke: 7410
Hu, Linlin: 2305, 7145
Hu, Mengyao: 1075
Hu, Rita X: 4285
Hu, Tiantian: 7015
Hu, Tiffany Y: 7410, 7920
Hu, Ting: 7365, 7405, 8015
Hu, Tingfei: 2300, 7100
Hu, Yan: 7015
HU, Yanqiu: 7305
Hu, Yaomin: 3160
Hu, Yinin: 8015
Hu, Yue: 7240
Hu, Yue: 8005, 8010
Hu, Zhiwei: 7240, 7445, 7535, 8005
Hua, Cassandra L: 3280, 8005, 8015
Hua, Nan: 4055, 7095, 7915
Hua, Shuxian: 9075
Hua, Yijing: 7030
Huang, Alison R: 1075
Huang, Alison J: 9115, 9150
Huang, Bin: 3025, 4225, 8015, 9010
Huang, Chih Yun: 7235
Huang, Chiyue: 7045
Huang, Codiez Z. D.: 1225
Huang, Jie: 7320
Huang, Jing: 3015, 4010, 4385, 7390
Huang, Jun-Rong: 7465
Huang, Liming: 8010
Huang, Qiongrong: 7190
Huang, Rui: 1070, 1370, 4590
Huang, Rui: 7025, 7195
Huang, Ruizhi: 8010
Huang, Sean: 3280
Huang, Shuo J: 4125
Huang, Siyuan: 7180
Huang, Tianyi: 8005
Huang, Wenxuan: 1180, 1505, 8005
Huang, Yan: 1125
Huang, Yen-Ming: 1365, 2135, 8005
Huang, Yi: 7365
Huang, Yuanqing: 7540
Huang, Yuncheng: 7030
Huang, Yuting: 7365
Huang, Yuxuan: 8000
Huang, Zewen: 7375
Huang, Zongan: 7015
Huanran, Liu: 7350, 7495
Hubbard, Erin: 7130, 7290
Huberty, Jennifer: 4590
Hubner, Sarah: 7095, 7280
Huckfeldt, Peter: 1455
Hudda, Urooj: 8015
Hudson, Darrell: 7260
Hue, Lisa: 8015
Hueluer, Gizem: 1350, 2120, 7350
Huemer, Marie-Theres: 1245
Huff, Hannah N: 8015
Hugenschmidt, Christina E: 8000
Hughes, Jaime M: 4205, 4525, 7390
Hughes, Jeff: 7250, 7330
Hughes, MacKenzie L: 1120
Hughes, Meredith: 4305, 7515
Hughes, Michelle: 7225
Hughes, Susan: 3045
Hughes, Timothy: 2400, 7040, 8005
Hughes, Timothy: 8005
Hughes, Timothy: 9040
Hughes, Travonia: 8005
Huisingh-Scheetz, Megan: 1220, 2100, 4200
Hulen, Elizabeth: 7135
Hulko, Wendy: 8015
Hull, Peter: 7540
Hulme, Claire T: 2335
Hultgren, Kyra: 7365, 7400
Hummel, Eleanor: 8005
Hundal, Harleen: 7440
Hung, Lillian: 2090, 2165, 3260, 7205, 7235, 7295, 7440, 7915, 8005, 8010
Hung, William: 3115, 4425
Hung, William W: 8010, 8015
Hung, Yat Tung: 8005
Hunkins, Nate: 8015
Hunt, Anne: 7340
Hunt, Lauren: 1025, 2185, 2560, 7010
Hunter, Gerald: 2200, 9110
Hunter, Heather: 7165
Hunter, Sandra K: 7120
Huo, Meng: 1275, 2025, 3185, 4485, 7185, 7335
Hurlburt, Michael: 7085
Hurley, Kelley: 2055
Hurwitz, Max: 8000
Hussain, Saad: 7185
Hussaini, Fareeha: 4230
Hussein, Mostafa: 4005
Husser, Erica K: 7060, 9075
Hutchinson, Jasmin: 9130
Huth, Megan: 1395, 2285, 7010, 7185, 7915
Hutto, Lashawn D: 7255
Huxhold, Oliver: 4490, 7355
Huynh, Duong: 8015
Huynh, James: 4250
Hwang, Dabin: 3205, 8015
Hwang, In Jeong: 8015, 9140
Hwang, Jeongeun: 7425, 7430
Hwang, Jinseon: 7020
Hwang, Mina: 7375
Hwang, Min Jun: 7265, 7305
Hwang, Misun: 7295
Hwang, Phillip: 1510, 8015
Hwang, Seo-Yeon: 9060

Hwang, Sinwoo: 7185, 7295, 7330, 8010
Hwang, Ula: 7090, 7485, 8010, 8015, 9140
Hwang, Woosang: 2405
Hwang, Yeji: 7155, 7290, 7375
Hyde, Martin: 7375
Hyer, Madison: 3285
Hyson, Rosemary T: 3385
Hyun, JaeWon: 2210, 7055, 7300, 8015
Hyun, Jinshil: 2400, 4310

I
Iacob, Eli: 1025, 8010, 8015
Ibrahim, Stephanie: 1295
Idemudia, Erhabor S. S: 7405
Idler, Ellen L: 1080
Igarashi, Ayumi: 2210, 7440
Igarashi, Ayumi: 9020
Ignacio, Daniel: 7145
Igwe, Ivuoma: 3065
Ihara, Emily S: 2165, 4600, 7005
Ijima, Momoka: 7140
Ikebe, Kazunori: 7365
Ikeuchi, Tomoko: 7445
Ikhide, Afeo: 8010
Iktilat, Khalil: 2105, 2415, 7090
Ilardo, Joan L: 4075, 7540, 8010
Iloputaife, Ikechukwu: 8010
Ilugbo, Oluwakayode J: 4565
Im, Eun-Ok: 7305, 8005
Im, Sua: 7065, 7365
Im, Yunju: 7325
in der Schmitten, Jürgen: 7220
Incerti, Lisa: 8010
Infurna, Frank J: 4290, 8000
Ingle, Pilar: 1215, 3245, 9105
Ingleton Gold, Lori: 8010
Ingram, Zalen: 7545
Ingvalson, Stephanie: 7300
Inker, Jenny: 7010
Inoue, Megumi: 2265
Inoue, Shigeru: 7345
Inouye, Sharon K: 2505, 8000, 8010
Intraligi, Valerio: 7070
Intrator, Orna: 3115, 8010, 9095
Ionescu, Elena: 7075
Ip, Edward: 8000
Ipiankama, Sampson C.: 2220
Irani, Elliane: 3300, 4215, 4335, 7335
Irbık, Dilare Ecenur: 4325
Irimia, Andrei: 8000
Irshaid, Hisham: 3230
Irving, Kate E: 7010
Isaac, Alexis: 7255, 7375
Isaac, Gregory: 7000
Isaacowitz, Derek M: 1045, 7460, 8000
Isaacson, michal: 3200
Isales, Carlos M: 8010
Ishikawa, Tomoki: 7250
Ishioka, Yoshiko: 7260
Iskandar, Mark H: 8000
Islam, Jessica: 8015
Islam, Shahidul: 7410, 8000
Islas huertas, Gabriela: 2555
Issac, Gregory: 3220
Isvan, Nilufer: 3190
Ito, Kae: 7300
Ito, Kae: 8010
Ito, Keitaro: 7140
Ito, Tomoko: 8000
Itoh, Sakiko: 4590
Iveniuk, James D: 1105, 4285
Ivenuik, James: 2295
Iwagami, Masao: 8000, 8010, 8015
Iwami, Taku: 2230
Iwasaki, Michiko: 7365, 7400
Iyer, Sandhya: 8005
Izekenova, Aigulsum: 7445
Izekenova, Assel: 7445
Izumi-Mishima, Yuna: 8015

J
Jaber, Sausan: 7265
Jablonski, Rita A: 7280
Jablonski, Rita: 8000
Jablonski, Rita A: 8015
Jacelon, Cynthia: 7250
Jack, Clifford R: 1200
Jacklin, Kristen: 1160, 2170, 4045
Jackson, Dylan B: 3235
Jackson, Emma: 2030, 8010
Jackson, Eva M J: 7010
Jackson, Faith M: 8005
Jackson, Kathryn: 1265, 4120
Jackson, Kelley L: 2045
Jackson, Lynn: 7440
Jackson, Phronie: 7050
Jackson, Phyllis D: 3165
Jackson, Yolanda: 7200
Jackson-Sagredo, Andrea: 7430
Jacob, Alexandra E: 4570
Jacob, Christine: 4155
Jacobs, Jeremy M: 1155
Jacobs, Karen: 4345
Jacobs, Leslie: 7010
Jacobs, Lindsey: 7310
Jacobsen, Juliet: 4575
Jacobson, Mireille: 1250, 8010
Jacobus, Claire: 7150
Jaen, Joce: 4540
Jagasia, Krish: 8015
Jahn, Jacob: 4560
Jain, Felipe A: 3025, 8000, 8005, 8010, 8015
Jain, Jennifer: 8010
Jain, Shalini: 7190
Jak, Amy: 8010
Jakiela, Laura W: 4205, 7390
Jamali, Ali Akbar: 7205
James, Bryan D: 1200
James, Dara L: 1440
James, Everette: 7515
James, Nick: 7375
James, Peter: 2400, 8000
James, Sarah-Naomi: 1105
James, Shirley A: 8000
Jamieson, Scott: 7435
Jamil, Md Shafayat: 7190
Janevic, Mary: 7390, 8010
Jang, Aeji: 8010
Jang, Haejin: 8015
Jang, Heejung: 8000, 9015
Jang, Jeong H: 7305
Jang, Jinwoo: 2045
Jang, Jiyoon: 7350
Jang, Joy Bohyun: 1105, 8010
Jang, Seyeon: 7415
Jang, Soeun: 7390, 7415, 8010, 8015
Jang, Soong-nang: 7005, 7135, 7540
Jang, Sua: 7300
Jang, Yunseo: 7295
Jang, Yuri: 3325, 4465, 7210, 7305, 7355, 7390
Janicki, Matthew P: 1395, 4265
Janke, Robert: 7485
Jankowski, Catherine: 7085, 7910
Jansen, Taylor: 1075, 1320, 3170, 7170, 7210, 7245, 7305, 7375, 7530
Janssen, Leah M.: 8015
Janssens, Lien: 8010
Jantea, Rachel: 8005
Jao, Ying-Ling: 3035, 3220, 7465, 7500, 9030
Jaros, Maria: 1150
Jarrín, Olga F: 3205, 8005, 9045
Jarrott, Shannon E: 1240, 1505, 7105, 7470, 7545, 8005
Jasuja, Guneet K: 2085, 8005
Jayakody, Oshadi: 8015
Jazwinski, S. Michal: 4330
Jeamjitvibool, Thanakrit: 4225
Jeffery, Molly M: 7485
Jehu, Deborah A: 9080
Jen, Sarah: 2525, 7110, 7220
Jenkins, Emerald: 4265
Jenkins, Molly: 9145
Jennette, Kyle J: 3355
Jennings, Lee A: 1510, 2445, 7415, 8000
Jennings, Stephen: 1470
Jensen, Christine J: 7010
Jeon, Dojin: 8015
Jeon, Jaemin: 7470
Jeon, Pureum: 4525
Jeon, Sangha: 7355, 8010
Jeon, So Yeon: 3235, 8015
Jeong, Ansuk: 9140
Jeong, Chung Hyeon: 8010
Jeong, Daeun: 7295
Jeong, Haelim: 7500
Jeong, Jihyeong: 7090, 7475, 8010
Jeong, Seohye: 8005
Jeong, Seonghyun Ellin: 9110
Jesdale, William: 7255
Jesmin, Syeda S: 7145
Jespersen, Brooke V: 7135
Jessee, Lisa: 3195, 4360
Jester, Dylan: 7370
Jetson, Raymond A.: 7440
Jezak, Samantha T: 4347
Jha, Sonya: 1435
Jhaveri, Kavya: 7095
Ji, Linying: 7535
Ji, Minghui: 4505
Ji, Molin: 2160
Ji, Xuefan: 4135, 7415
Ji, Yuwen: 8000
Jia, Heling: 9045
Jia, Shuli: 9150
Jia, Xueqing: 7240, 9150
Jiakponnah, Nwanyieze Ngozi: 4330
Jian, Bo: 4315
Jiang, Da: 1350, 4300, 7375, 7395
Jiang, Jing: 7265
Jiang, Lin: 8015
Jiang, Nan: 7015, 7095
Jiang, Nisi: 4347

Jiang, Xia: 9150
Jiang, xiangxiang: 4265
Jiang, Xiaoqian: 7015
Jiang, Xiaoyan: 9150
Jiang, Yanan: 8000
Jiang, Yanping: 4070, 4505, 7090, 9025
Jiang, Yingyue: 1115
Jiang, Yuling: 7225
Jiang, Yun: 7295
Jiao, Hongmei: 7090
Jimenez, Andreina: 2440
Jimenez, Daniel: 1055, 7035, 7205
Jimoh, Itopa I: 1150
Jin, Bora: 8010
Jin, Guoping: 4530, 7090, 7260, 7435
Jin, Liangyi: 7365
Jin, Yichen: 4295
Jin, Ying: 7525
Jin, Ying: 8015
Jin, Yuanyuan: 1125
Jing, Bocheng: 7140
Jing, Mingrui: 7015
Jing Weinberg, Astrid: 9110
Jingyuan, Xu: 7010
Jo, Ryan: 8000
Johansson, Patrik: 7440
Johns, Lily: 3100
Johns, Michelle: 8010
Johnson, Abree: 2125
Johnson, Alexa: 8010
Johnson, Alisha H: 8005
Johnson, Benjamin: 7005
Johnson, Charles: 1105
Johnson, Chloe: 7245
Johnson, Daniel: 7150
Johnson, Dayna: 7465
Johnson, Elma: 7010
Johnson, Florence: 1510, 2375
Johnson, Ian M: 1420, 4165
Johnson, Jack: 4265
Johnson, January: 2170
Johnson, Jeremy: 8015
Johnson, Julene K: 2135, 7260
Johnson, Kim G: 7010
Johnson, Kimson E: 3175, 4580, 7200
Johnson, LaShaune P: 3140
Johnson, laurinda: 2260
Johnson, Madelyn: 2285
Johnson, Peace: 9000
Johnson, Rhea: 1505
Johnson, Sallie: 1135
Johnson, Shannen: 8005
Johnson, Shawn M: 9005
Johnson, Sterling C: 1200, 8015
Johnson, Ted: 1080, 7225
Johnston, Carrie: 1490
Jolin, James: 1465
Jolin, Pierre: 1465, 8015
Jon, Emily: 8005
Jones, Anna: 8010
Jones, Brian D: 7225
Jones, Christine D: 7485, 7930
Jones, Emily: 7005
Jones, Hannah: 8015
Jones, Katie: 4090
Jones, Kayleigh: 7010
Jones, Kelly: 8005
Jones, London B: 7225
Jones, Mary: 1275
Jones, Megan D: 7120
Jones, Randy A: 8015
Jones, Raymond: 2245, 3180, 7245
Jones, Richard N: 1225, 2000, 2505, 3380, 4540, 7435, 8010
Jones, Samia: 4190
Jones, Tessa: 4430
Jones-Cobb, Brittany R: 8010
Jones-Locklear, Jennifer: 1020
Joo, Jin hui: 1345, 2310, 9105
Jordan, Jacqueline: 3040
Jordan, Meg: 8000
Jor'dan, Azizah J: 8010
Joseph, Joshua: 3285
Joseph, Preetha: 7240
Joseph, Sarah: 2445
Joshi, Amol: 4065
Joung, Hyesong: 8015
Jovalekic, Aleksandar: 7415
Joyce, Jillian: 7095
Ju, Catherine H: 2240, 3100
Juang, Yu-Chen: 7220
Juarez-Briggs, Melida A: 8010
Juckett, Lisa: 7545
Judd, Suzanne E: 7260
Judge, Katherine: 2030, 7230, 7915, 8010
Judon, Kimberly: 8010
Jun, Hee-Jin: 7495
Jun, Jiweon: 7230
June, Andrea: 3150
Jung, Dukyoo: 8010
Jung, Grace: 8005
Jung, Hailey: 2525, 7355, 7535
Jung, Hyejin: 8010
Jung, Hye-Young: 1385, 7250, 7540, 9090
Jung, Inhye: 7145, 7355, 7395, 7470
Jung, Misook: 7220, 7325, 7425, 7430
Jung, Wi Hoon: 7470
Jung, Wonkyung (Kelly): 3205, 7395
Jungo, Katja: 7250
Junker, Tom: 7035
Jurczak, Michael J: 8000
Jurgens, David: 3100
Juris, Jill: 3260, 4600, 7105, 7175, 7215, 7470, 7545, 7920, 8015
Jurkowski, Elaine T: 7340, 7920
Just, Allan: 8015
Just, Danielle: 3040
Justice, Jamie: 8000
Justice, Jen R: 8000
Justin, Moore: 4525
Jutkowitz, Eric: 1010, 1430, 2260, 3020, 3075, 3190, 7185, 7200, 7300, 7530, 7930, 8005
Jylha, Marja: 1080

K

Kabalan, Mayssan: 1495
Kabel, Allison M: 7065
Kabua, Philmar M: 7005
Kachal, Josefa: 7135
Kadiu, Teuta: 4105
Kado, Deborah M: 3065
Kado, Deborah: 8005
Kaeberlein, Matt: 8000
Kahkoska, Anna: 8005
Kahsay, Eskira: 2105
Kahya, Melike: 1255, 7495
Kaiser, Ronald: 3215
Kaiser-Grolimund, Andrea: 7250
Kaizi-Lutu, Marc A: 1510
Kajiyama, Bruno: 7230, 7305, 8000
Kajiyama, Claudia: 7305
Kakuta, Momoho: 4195, 7365
Kalender-Rich, Jessica L: 1485
Kales, Helen C: 8015
Kalesnikava, Viktoryia A: 3100, 9035
Kalir, Daphna M: 4205
Kalousova, Lucie: 1140, 3175, 7055
Kalter, Steve: 7300
Kalu, Michael: 7300
Kalyani, Rita: 7315
Kamalyan, Lily: 8010
Kamaraj, Heera: 8000
Kamath, Vidyulata: 2535
Kamer, Angela: 3110
Kamide, Kei: 7365
Kamis, Christina: 7260
Kamiyamasaki, Etsuyo: 7330
Kämpfen, Fabrice: 9150
Kan, Ge Lin: 7295
Kan, Junpeng: 7090
Kane, Alice: 7090
Kane, Esther: 1015, 7185
Kane, Kevin: 8000
Kang, Ashley J: 8010
Kang, Bada: 2210, 7325, 7395, 7520, 7525
Kang, Chaeryon: 4585
Kang, Donghun: 3025, 4420
Kang, Hun: 2210, 7055, 7300, 7375, 8015
Kang, Jaewon: 2445
Kang, Jee eun: 1445, 2360
Kang, Ji Young: 7275
Kang, Jihee: 8005
Kang, Jinkyung: 4450
Kang, Lin: 3160
Kang, Min Jeoung: 8015
Kang, Minji: 7395
Kang, Miseon: 3325, 7470
Kang, Naixin: 8015
Kang, Ning: 4255
Kang, Sarah Sunghye: 3020, 7495, 8005, 8010
Kang, Seerat K: 7460
Kang, Seokjo: 2277
Kang, Suk-Young: 9030
Kang, Sung Hye: 1405
Kang, Sunny: 7515
Kang, Yu Jin: 3040
Kannan, Lakshmi: 7225
Kanter, Jeremy: 4360
Kao, Hsueh-Fen S: 2175
Kao, Lihan: 9140
Kaplan, Celia: 9115
Kaplan, Daniel: 8000
Kaplan, Jordan: 7420
Kapoor, Alok: 2500, 7410, 7485
Kappler, Caitlin B: 1470
Karabukayeva, Aizhan: 1150
Karachaliou, Angeliki: 8000
Karagiannis, Tanya T: 8015, 9155
Karakida, Maki: 7395
Karakose, Selin: 1120, 1265
Karakullukçu Erdem, Sima: 3310
Karasik, David: 1520
Karasik, Rona J: 2145, 3120, 4035, 7340, 7920
Karayanidis, Frini: 3235
Karel, Daniel: 1190
Karg, Patricia: 4305
Karimi, Zahra: 7060
Karki, Dinesh: 7390, 7475

Karlin, Nancy J: 7280
Karliner, Leah: 9090, 9115
Karlsen, John E: 4200
Karlsson, Ida: 4030
Karmacharya, Isha: 4465
Karmarkar, Amol: 8015
Karmarkar, Shriya: 7290
Karr, Daniel W: 1305
Karra, Vijay Kumar M: 7510
Karsky, Sam: 7465
Karvonen-Gutierrez, Carrie A: 1110, 4460, 7345
Kashiwabara, Kosuke: 8000
Kashiwagi, Kimikazu: 8000
Kashyap, Divya: 8005
Kaskie, Brian: 3150, 4390, 7070, 7185, 7930
Kasl-Godley, Julia: 2240
Kaslow, Angela "Jackie" A: 1370
Kaspar, Roman: 7380
Kastner, Keri: 1385, 7370
Katagiri, Keiko: 7365, 7395, 7925
Katsumata, Asako: 7160, 7405
Katz, Amy: 7010, 7910
Katz, Benjamin: 4007, 7020, 7095, 7200, 8000, 8005, 8010, 8015
Katz, Mindy j: 2400, 4310
Kauffman, Timothy L: 4255
Kaufman, Brystana: 4430
Kaufman, Jennifer E: 2395, 3385
Kaufman, Joel: 2400, 7040
Kaufman, Sarah: 9100
Kaufmann, Christopher N: 1295, 1475, 2035, 3235, 4385, 8015
Kaup, Migette L: 2080
Kaushal, Navin: 4560, 9130
Kavanagh, Grace: 7350
Kawabata, Kan: 8000
Kawachi, Ichiro: 1225
Kawai, Hisashi: 7225
Kaye, Jeffrey: 1280, 4200, 7335, 7545
Kaye, Lenard W: 7170, 8005, 8010, 8015
Kayser, Jay: 3175, 7395
Kayser, Kylie: 7435
Kazakoff, Megan: 8010
Ke, Jin: 7035
Keach, Nancy: 9010
Keage, Hannah: 3235
Keefe, Bronwyn: 2490, 4075, 4345
Keefe, Francie: 4185
Keefe, Francis: 7205
Keisari, Shoshi: 1330, 4205, 4235, 7410
Keiser, Brennan: 4535
Keith, Amy: 2330
Keith, NiCole: 3085, 7415
Keith, Verna: 4140
Keleher, Jennie: 4475
Kelekar, Uma: 1355, 4030, 4190, 4440, 4560
Kelle, Nadja: 4490
Keller, Hallie: 7220, 7410
Keller, Michelle: 7485, 8010
Kellett, Kathy: 1315, 7360
Kelley, Heather: 4085, 7215, 7305
Kelley, Jessica A: 1405, 2020, 3117, 3285, 8005
Kelley, Lynette: 2540, 3115, 7280, 7350
Kelley, Shaelyn R: 7545
Kelly, Christopher M: 4550, 7130, 7515
Kelly, Heather: 7330
Kelly, Kathleen: 2450
Kelly, Madeline: 7480
Kelly, Megan: 7140
Kelly, Megan M: 7300
Kelly, Megan: 7905
Kelson, Mark J: 2120
Kemeny, Betsy: 9080
Kemnitz, Joseph W: 7045
Kemp, Candace L: 1275, 2160, 4550
Kemp, Elizabeth: 4205
Kemp, Elyria: 8005
Kendzor, Darla E: 1510
Kenj Halabi, Dima: 7430
Kennedy, Anna: 7500
Kennedy, Katherine A: 2225, 3040, 4010, 4550, 9005
Kennedy, Meaghan A: 3115, 7300
Kennedy, Richard: 1060
Kennedy, Teri: 3255, 8000
Kennerley, Clare L: 7435
Kenney, Jamesia: 7325
Kennison, Robert F: 7460
Kenny, Anne: 1160
Kenny, Brooks: 9010
Kenowsky, Sunnie: 7010
Kent, Erin E: 4450, 8000, 8005
Kenyon-Pesce, Lisa: 9090
Keough, Katie: 8010, 8015
Kermel-Schiffman, Ile: 1090
Kern, Kristopher C: 7220
Kern-Lyons, Calista: 3295
Kerssens, Chantal: 7225
Kessler, Dorothy: 7340
Kessler, William: 8010
Ketcher, Dana: 1160, 4045
Ketel, Christian: 3355
Key, Jamie: 4240
Key, Kent: 2515
Key, Mickeal N: 7265
Keys, Matthew T: 7345
Keysor, Julie: 8015
Kezios, Katrina: 2390, 8000, 8005, 9120
Khalaila, Rabia: 8010
Khaleghzadegan, Salar: 2045
Khalidi, Sarah: 4140
Khalil, Abdelouahed: 7315
Khan, Karim: 7225, 7905
Khan, Maham: 8000
Khan, Najah A: 7320
Khan, Nasrin: 1060
Khanpour, Sahar: 4590
Khasawinah, Sarah: 7185
Khatri, Rekha: 8015
Khattar, Jayati: 2545
Khatun, Asifa: 1005
Kheirbek, Raya Elfadel: 1190, 2125, 2485, 3235, 4600, 9005
Kheirbek, Raymond: 2125
Khera, Nina: 8000
Khobragade, Pranali Y: 4540
Kholodovsky, Eric: 4560
Khoo, Eliza: 4370
Khor, Ern Chern: 4420
Khorgamphar, Mohammad: 8000
Khorob, Kvitoslava: 3360, 4270
Khosla, Sundeep: 2137
Khurana, Amit: 8010
Kibildis, Samuel W: 8005, 8015, 9025
Kidd, Lori: 4600, 7340, 7920
Kiefer, Lea: 1470, 7030
Kieffer Blass, Elana: 9125
Kiel, Douglas P: 7130
Kietzman, Kathryn: 8000, 8010
Kiflom, Rebecca: 8010, 8015
Kilaberia, Tina R: 7015, 7250
Kilcrease, Amanda K: 4015
Kilduff, Courtney: 2310
Killian, Emily: 2135, 8000
Killian, Meredith: 8015
Killian, Tim S: 7475
Kim, Agnes Jihae: 3075, 8000
Kim, Andrew: 8015
Kim, Andy Jeesu: 8005
Kim, Bo: 7270
Kim, Boah: 8010
Kim, Boeun: 3345, 4125, 9005
Kim, Bo-Kyung Elizabeth: 4250
Kim, Bo-Kyung Elizabeth: 4250
Kim, Bomgyeol: 2210, 7055, 7300, 8015
Kim, BoRin: 4070, 4185, 7495, 8005, 8010
kim, byungju: 8010
Kim, Changhyun: 8010
Kim, Chang-O: 7005
Kim, Chang-O: 7135
Kim, Da Eun: 8015
Kim, Dae Hyun: 1340, 4090, 9000, 9070, 9080, 9085, 9105, 9135, 9145
Kim, Dahee: 1055, 3030, 7090, 7160, 7225, 7380
Kim, Daniel: 9150, 9155
Kim, Dongmi: 7305
Kim, Dongmi: 8005
Kim, Dongwook: 7295
Kim, Doyoung: 8005
Kim, Elissa W: 1415
Kim, Eric S: 7115
Kim, Eunbea: 4195, 4315, 7040, 9140
Kim, Eunbee: 7475
Kim, Eunice: 9145
Kim, Eunji G.: 9000, 9085, 9105, 9145
Kim, Eunjung: 4335, 7390
Kim, EunKyo: 7185, 7295, 7330, 8010
Kim, Eunkyung: 7400
Kim, Gee: 1485
Kim, Giyeon: 3325, 7035, 7080, 7090, 7115, 7165, 7330, 8000, 8005
Kim, Gwang Suk: 7090, 7145, 7225, 7245, 7265, 7300, 7375, 8005
Kim, Haeun: 7035
Kim, Hae-Young: 9060
Kim, Ha-Neul: 3215
Kim, Hansol: 8010
Kim, Heekyung: 8015
Kim, Heewon: 7500
Kim, Hyejin: 8000
Kim, Hyejoong: 7080
Kim, Hyun: 1345
Kim, Hyun Jung: 7475, 7545, 7910
Kim, Hyun-Jun: 7355, 7535
Kim, Jaehyun: 7265
Kim, Jaeyoon: 7290
Kim, Jeehoon: 7295, 7395, 8015
Kim, Jennifer L: 7295
Kim, Jeong Eun: 7335
Kim, Jeong Lan: 3235
Kim, Jeonghun: 7255
Kim, Jeongwoon: 7435, 8005, 9130
Kim, Jeung Hyun: 3370
Kim, Ji Chul: 9040

Kim, Jichan: 4335, 7390
Kim, JinShil: 7220
Kim, Jiwon: 7435
Kim, Jiwon: 8015
Kim, Jiyeon: 3315
Kim, Jiyoung: 8010
Kim, John: 8015
Kim, Joohyun: 7230
Kim, Ju Hyun: 7390
Kim, Jueon Aaron: 7085
Kim, Junesun: 9060
Kim, Jung-Nim: 7220
Kim, Junhyoung: 7265, 7305, 8000, 8015
Kim, Keun You: 8015
Kim, Kyeongmo: 3325, 7260, 8010
Kim, Kyeongwon: 4250
Kim, Kyung Soo: 3355
Kim, Kyungmin: 2025, 3185, 3335, 4485, 7115, 7185, 7230, 7335, 7355
Kim, Laura: 9025
Kim, Layoung: 7090, 7145, 7225, 7245, 7265, 7300, 7375
Kim, Manjun: 7295
Kim, Maryanne: 8010
Kim, Meeryoung: 8000, 8015
Kim, Miji: 7325, 8010
Kim, Min Hee: 2020, 3050
Kim, Minjin: 8005
Kim, Minzae: 1510
Kim, Nahyun: 7395, 7925
Kim, Namhee: 7090, 7225, 8010
Kim, Nam-Hee: 8000
Kim, Namhyun: 8005
Kim, Natalie: 8000
Kim, Nicholas J: 8000
Kim, Ocksim: 7375
Kim, Patricia: 7540
Kim, Sangkyu: 4330
Kim, Sara: 1345
Kim, Sein: 7230
Kim, Seohyeon: 8015
Kim, Seokmin: 1075, 7070, 7375
Kim, Seon: 7260
Kim, Seoyoun: 1195
Kim, Seulki: 3020
Kim, Sewon: 7300
Kim, Sion: 7395
Kim, Sohee: 7420, 7905, 8005
Kim, Sohyun: 3260, 4320
Kim, Soo Hyun: 7380, 7430, 7495
Kim, Soo Yeon: 8010
Kim, Soohyun: 8005
Kim, Steffi: 3165, 4350
Kim, Suhyun: 7100, 7295
Kim, Sun Ju: 7060, 8010
Kim, Sun Kyung: 7475
Kim, Sunjung: 9125
Kim, Suyoung: 3265, 4195
Kim, Tae: 2200
Kim, Taekyung: 3025, 3075
Kim, Tina: 1345
Kim, Yeoeun Kate: 7085
Kim, Yeonwoo: 4580
Kim, Yijung: 3340
Kim, Yongseop: 4580, 7265, 8010
Kim, Yoojee: 2310
Kim, Yoojee: 9110
Kim, Yoon Chung: 1395, 2150, 8000, 8005
Kim, Young Sun: 9060, 9065
Kim, Young Woo: 8005
Kim, Yuhoung: 8015
Kim, Yulri: 1120, 4315, 9140
Kimball, Patricia: 1085
Kimerling, Rachel: 2085
Kim-Godwin, Yeoun Soo: 7005, 7400
Kim-Knauss, Yaeji: 1330
Kimpel, Christine: 3355
Kimron Greenblatt, Lee: 3360
Kimura, Yuya: 9020
Kinariwala, Kush: 2065
King, Angelique: 3295
King, Barbara J: 4425, 7495
King, Caroline: 4500
King, DJ: 7195
King, George A: 8010
King, Jessica: 2225
King, Kelsey: 1160
Kinkade, Emily: 8015
Kinney, Jennifer: 8005
Kinzig, Elizabeth: 2485
Kiosses, Dimitris: 4010
Kirby, Mackenzie: 7455
Kirch, Matthias: 4360, 4390, 7250
Kirch, Matthias: 9070
Kircher, Julie A: 8015
Kirk, Greg: 7545
Kirkegaard, Allison: 3010
Kirkpatrick, Tara: 7380
Kirkwood, David: 7370
Kirsch, Jaclyn: 8015
Kirsch, Julie A: 4215
Kirwan, Jack B: 8010
Kiselica, Andrew: 9070
Kishimoto, Kyoko: 2145
Kisley, Michael A: 2095
Kissam, Stephanie: 7385
Kisselburgh, Hannah: 9130
Kitamura, Koji: 7225
Kitamura, Satomi: 7250, 7465
Kitchens, Katherine: 8010
Kittle, Krystal R: 4140
Kitt-Lewis, Erin: 1310, 2485, 4480, 8010, 9075
Kitzman, Dalane: 2245
Kitzman, Dalane: 9145
Kivipelto, Miia: 1290, 3030
Kivisäkk, Pia: 7415
Kivowitz, barbara: 2500
Kiwak, eliza: 7030
Kiyohide, Fushimi: 9020
Kizilhan, Jan: 8005
Kizony, Rachel: 7210
Klaiber, Patrick: 1480, 7035
Klatt, Maryanna D: 2135
Klayn, Rami: 7060
Kleckner, Amber: 7130
Klein, Kelly: 8010
Klein Druyan, Adar: 1515
Kleinstein, Steven H.: 8000
Klenczar-Castro, Brittany: 2010
Klepacz, Laura: 1175, 7380
Klepin, Heidi D: 1255
Kleszynski, Keith: 7415, 8000
Klinedinst, Jennifer: 7290, 7495, 8005
Klinedinst, Tara: 1510, 2245, 2445
Kling, Mitchel A: 4570
Klinger, Christopher: 7340, 7920
Klomhaus, Alexandra: 8010
Klonover, Eyal: 2105
Klopack, Eric: 8015
Klug, Jenna: 1520
Kluger, Benzi M: 3165
Knebl, Janice: 2065
Knell, Tammy A: 7410
Knibbeler, Marjolein: 7250
Knight, Christy: 4425
Knight, Cynthia M: 1050
Knight, Erik L: 8015
Knight, Joseph: 2010
Knight, Julie: 7500
Knodt, Annchen: 7025
Knopman, David: 2535, 7325, 7495
Knowles, Justina E: 8015
Knox, Sara: 8005, 9035
Ko, Clifford: 7500
Ko, Eunjung: 1400, 2475, 4170, 8005
Ko, Fred: 4425, 8010
Ko, Jisook: 8015
Ko, Jong: 7475
Ko, Kwangman: 7085
Ko, Soohyeon: 7335
Kobayashi, Erika: 2120, 7265
Kobayashi, Hiroyuki: 8015
Kobayashi, Lindsay: 1495, 2000, 3380, 4060, 4455, 4540
Koch, Christina: 8005
Koestner, Sophie A: 7065
Koffer, Rachel E: 4050
Koffski, Maya J: 2330
Koga, Hayami K: 8000
Koglin, Norman: 7415
Koh, Vanessa Jean Wen: 2435
Koh, Yen-Chun: 8015
Koh, YoungBin: 7005, 7080
Kohlbrenner, Briana: 1215
Kohler, Susan: 9145
Kohler, Thibault: 4580
Kohlhepp, Bayard: 9020
Kohli-Hailovski, Tal: 4355, 7175
Kohn, Ella: 8000
Kohon, Jacklyn: 3280
Koike, Teruhiko: 7480
Koirala, Binu: 7380, 7430, 7495
Kojima, Misao: 7265
Kolachalama, Vijaya B: 3127
Kolanowski, Ann: 3220
Kolawole, Francis: 7300
Kole, Debarati: 2290, 8000
Kolluri, Surya: 2115
Kolpin, Lola: 8005
Komalasari, Renata: 8000, 8005
Komiyama, Jun: 8000, 8015
Kondrikov, Dmitry: 8010
Kong, Chunyan: 3020
Kong, Dexia: 7305, 8015
Kong, Eun-Hi: 8000, 8015
Kong, Jian: 7435
Kong, Jooyoung: 4520, 7290
Kong, Lingsong: 9070
Konkle-Parker, Deborah: 2140
Kononowech, Jennifer: 1300
Konopka, Adam: 1387
Koo, Katherine: 7485
Kooij, Dorien: 1480
Kooshiar, Hadi: 8005
Kopera-Frye, Karen: 2290
Koren, Chaya: 1130, 4195
Koren, Omry: 2105
Koren, Yael: 7435
Korenman, Sanders: 3385
Korfage, Ida J.: 7220
Korlat, Selma: 3145
Kornadt, Anna E: 1210, 1480, 3360, 4270
Koroni, Hamid R: 8015
Koroukian, Siran: 7220

Kosar, Cyrus M: 2000, 7540
Kositsawat, Jatupol: 4025
Koss, Catheryn: 1215, 7015, 7920
Kossowska-Kuhn, Dorota: 4420, 8005
Koszalinski, Rebecca S: 8015
Kota, Veda: 2320
Kotal, Ashwin: 4280
Kotian, Bhumi Jagadish: 7340
Kotterman, Amy: 1300
Kottorp, Anders: 4215
Kotwal, Ashwin A: 2190, 3050, 4020, 4070
Kovaleva, Mariya: 7125, 9000
Kowalchick, Kalei: 4480
Kowalski, Rebecca: 8015
Kozakowski, Kevin M: 7320
Koziel, Michele: 1300
Kozlov, Elissa: 2320, 7370
Králová, Josefína: 9070
Kraal, A Zarina: 3380
Kraemer, John D: 7530
Kraft, Malissa: 3005, 7195
Kramer, Arthur: 7265
kramer, Benjamin: 4200
Kramer, Philip A: 2245, 2470, 8005
Kramer-Kostecka, Eydie: 4020
Kraschnewski, Jennifer L: 4480
Kratz, Anna: 4195
Kravchenko, Julia: 1235, 4010, 4460, 7185
Kravljaca, Nikolina: 7325
Kreider, Amanda: 4260
Kreider, Consuelo M: 2290, 2445
Krein, Sarah: 4450
Kremen, Sarah: 8010, 9020
Kremer, Ian N: 4160
Krendl, Anne: 1095, 2360
Kreuzinger, Isa-Marie: 7020, 7265
Krick, Katelynn E: 2460
Kriebernegg, Ulla: 2140, 7445, 7490
Kristensen, Katrine L: 7345
Kritchevsky, Stephen: 1075, 2470, 4275, 4295, 8000
Krok-Schoen, Jessica L: 2220
Kropp, Denise J: 7340, 7920
Krueger, Katie: 3255
Krumm, Isabella: 3150
Kruse, Lance: 8005
Ku, Hok Bun: 8005
Ku, Nai-Wen: 7090
Ku, Te-Lien: 1035, 1365, 2135, 2445, 8005
Ku, Vivian: 8010
Kuan, Yun-Han: 3035, 7465
Kuang, Huijing: 8015
Kube, Erin R: 2240
Kubiak, Jenna: 3170
Kubota, Sayaka: 7330
Kubzansky, Laura D: 8000, 8005, 9155
Kucharska-Newton, Anna: 4450, 7345
Kuchel, George A: 4025, 4473, 4495, 7345, 8000, 8005, 8010, 9090
Kuehnemann, Chisaka: 8005
Kuffel, Randall: 7255
Kuglin Jones, Ann: 2255
Kukull, Walter: 8000
Kullgren, Jeffrey T: 4360, 7250
Kulminski, Alexander: 8005
Kumapayi, Peace: 7300
Kumar, Amit: 8015, 9045
Kumar, Ashish: 8015
Kumar, Manish: 7040
Kumar, Sanjana: 2320
Kumar, Vivek: 7190
Kumfor, Fiona: 4470
Kummerfeld, Erich: 9070
Kung, Alina: 8000, 8010
Kung, Po-Jen: 2480
Kunicki, Zachary J: 2000, 2505, 3380, 4015, 4540, 7290, 7435, 8000, 8010, 8015
Kuniholm, Mark: 8015
Kunik, Mark E: 1035, 4265
Kunkel, Suzanne: 7170
Kunzmann, Ute: 8000
Kuo, Chia-Ling: 4025, 4495, 8000, 8010
Kuo, Fang-Lin: 7475
Kuo, Jasmin: 7130
Kuo, Tony: 7225, 7905
Kuo, tsuann: 7150, 8000
Kuo, Yong-Fang: 1070, 4365, 7305, 7535, 8000, 8005, 8010
Kupferschmidt, Anthony: 1325
Kura, Niharika: 8005
Kuribayashi, Shihomi: 7225
Kurita, Kayoko: 8000
Kurth, Jordan D: 8005
Kurth, Maria: 2280
Kurtz, Chava: 7145
Kurzhiparambil, Leya T: 3280
Kusmaul, Nancy: 1145, 1395, 2150, 4395, 7220
Kuspinar, Ayse: 8000
Kusters, Cynthia DJ: 1475
Kutner, Jean S: 3375
Kutney-Lee, Ann: 8005
Kutz, Alexander: 8015
Kuzmik, Ashley: 2180, 4380, 8015
Kwait, Jennafer: 2140
Kwak, Jung: 2130, 7305, 8015
Kwak, Min Ji: 8000
Kwak, Minyoung: 7470, 7925, 8015
Kwan, Crystal: 1115
Kwan, Ngai: 3205
Kwan, Rick Yiu-cho: 4180
Kwapis, Janine: 2137, 7190, 8010
Kwiatek, Susan: 7410
Kwoh, C. Kent: 7500
Kwok, Alex Pak-ki: 4180
Kwok, Amy: 8005
Kwok, Eric Lun Yiu: 7255
Kwok, Ian B: 3340
Kwok, Jennifer H: 2040
Kwok, Jojo Yan Yan: 4300, 7085, 7395
Kwok, Yu Hon Kenneth: 1030
Kwon, Eunsun: 7275, 8015
Kwon, Jenny: 4345, 8010, 8015
Kwon, Oh Sung: 8000
Kwon, Simona C: 1345
Kwon, Soonhyung: 3325, 7305
Kwon, Sooyoung: 7090, 7245, 7265, 7300, 7375
Kwon, Suyoung: 4125
Kwong, Katherine: 4035
Kyaw, Kaela: 3295
Kyeremeh, Ebenezer: 7360
Kyriacou, Corinne: 3040

L
La, Jennifer: 8000
Létourneau, Dominic: 2235
Lévesque, Marie-Hélène: 2235, 4150
Löckenhoff, Corinna: 3275, 4300, 7380
López-Lorenzo, Karla Daniela: 3355
Laager, Rahel: 8015
Lac, Andrew: 7395, 7505
Lach, Helen W: 7225, 7905
Lachman, Margie E: 4000, 7115, 7200, 8010
Lachs, Josh: 8005
Lachs, Mark: 1170, 1215, 2325, 3275
Lackmeyer, Abbe: 7170
LaCroix, Tracy: 8005, 8010
Ladda, Shawn: 7305
Ladiges, Warren: 1520, 7480
Ladner, Daniela Patricia: 7185, 8000
Laflamme, Maeva: 7455
Lafontant, Kworweinski: 3030, 8005, 8010
Lafountain, Olivia: 2525, 7110
LaFrance, Marianne: 8000
Lagisetty, Pooja: 4450
LaGorio, Kara: 7265
Lagrotteria, James: 7085, 7910
Lahlou, Yusuf: 8000
LaHue, Sara C: 4107
Lai, Chao-Qiang: 4460
Lai, Daniel W. L.: 1215, 1225, 2260, 2315, 2325
Lai, Jin-Shei: 4015
Lai, Jocelyn: 1045, 3270
Lai, Patrick Ho Lam: 1425, 2530
Lai, Vincent D.: 9015
Lai, Wenhua Zoey: 2050, 3010, 7230
Lai, Wen-Hua Zoey: 1060
Laird, Lance: 4535
Laitman, Adam: 7500
Lakomy, Martin: 2405
Lam, Gigi: 4180
Lam, Julia: 8000
Lam, Justin W: 7295, 7410
Lam, Michael: 8000
LAM, Tommy L H: 4455
Lamar, Melissa: 8005
Lambrou, Nickolas: 1160
Lamontagne, Marie-Eve: 8005
Lamp, Amanda H: 3295
Lampe, Nik M: 1315, 4220, 8005
Lamura, Giovanni: 7070
Lan, Yue: 7265
Landi, Francesco: 8010, 8015
Landreville, Philippe: 8000
Lane, Sandi J: 2090, 7075
Lane, Scott: 8005
Lanford, Tiera: 4430
Lang, Frieder R: 1330
Lang, Lang: 7245
Langa, Kenneth M: 4540
Langdon, Jacqueline M: 9045
Lange-Maia, Brittney: 8005
Langendoerfer, Kaitlyn: 8010
Langston, Emily M: 4080
Langworthy, Benjamin: 1430, 3020, 3075, 7300, 7530

Lankowski, Ava: 8015
Lanternier, Paula: 9015
Lanza, Dalila V: 3155
Lanza, Stephanie: 7180
Lapacek, Brian T: 8010
Lapane, Kate: 2555, 7255, 7415
Lapidus, Jodi: 1460, 7315
Lares, Gabino: 8005
Large, Edward W: 9040
Lariviere, Matthew: 9100
Larkin, Jennie: 4008
Larkin, Marni: 7140
Larkina, Marina: 7305
LaRoche, Ashley: 8015
LaRoche, Dain: 4005
Larouche, Julie: 4105
Larson, Kelsie: 4045
Larson, Peter: 7465
Larson, Scott: 7305
Larson-Smith, Yvonne: 8005
Laskin, Grant: 8015
Laskow, Thomas: 2510
Lasseigne, Brittany N: 3397
Lassell, Rebecca KF: 2380, 3085, 4525, 7415
Latham, Nancy: 2395, 8015
Latham-Mintus, Kenzie: 1190, 1415, 2485
Lathrop, Laura: 1440
Latimer, Abigail: 7360, 7475
Lau, Bobo: 9085
Lau, Bobo Hi Po: 1385, 4180, 7205
Lau, Fung Yee: 4585
LAU, Gary K. K.: 1225
Lau, Matthew Zhi Yuan: 3215
Laurienti, Paul J: 1075
Laurita-Spanglet, Jessie: 8000
LaValley, Michael: 1070
Lavayen, Susana: 7455
Lavretsky, Helen: 4535
Lavu, Siva: 8010
Law, Chancen: 8000
Law, Makenna E: 1160, 2310, 7295
Law, Stephanie: 1385
Law (Jang), Jeein: 4030, 4440, 4560, 7320
Lawrence, Carly W: 2085
Lawrence, Jennifer: 2115
Lawrence, Joycelyn: 8000, 8015
Lawrence, Karen A: 7035, 7450
Lawrence, Katharine: 4170, 8015
Lawrence, Molly: 7305, 8015
Lawrence, Reagan W: 7330
Lawrence, Sean: 8005
Lawson, Denise: 7185
Lay, Jennifer C: 1350, 7375
Lazarus, Rachel: 7025
Le, Qun: 8000, 8015
Le, Thai Minh: 7390
Leach, Susan: 7140
Leahy, Suzanne: 7330
Leal, Kelly: 7265
Leary, Natalie: 7205
LeBlanc, Raeann G: 4135, 7110, 8000
LeBrasseur, Nathan: 4107, 7190
LeCouteur, David: 7240
LeCroy, Madison N: 3155
Lee, Ah Rim: 7325, 7425, 7430
Lee, Angella: 7310
Lee, Ann: 2440
Lee, Aryn: 7350, 7540
Lee, Beatrice: 8010
Lee, Boram: 8015
Lee, Chae Man: 1385, 2185, 4135
Lee, Chaiwoo: 3250, 4410
Lee, Chanam: 1135, 3125
Lee, Chang Dae: 7335
Lee, Chioun: 3285
Lee, Chiyoung: 7010, 7500
Lee, Christopher S: 2255
Lee, Dahyeon: 7295
Lee, Dahyeon: 7425
Lee, Daniel: 8010
Lee, David R: 3290
Lee, Dayoung: 2120
lee, Donghyun: 9065
Lee, Ellen: 1295
Lee, Eunju: 4485, 7230
Lee, Eunseo: 7295
Lee, Gina: 1065, 4520, 7290
Lee, Gyeonghee: 7235
Lee, Haerim: 7220
Lee, Hanna: 8015
Lee, Harold H: 8010
Lee, Hayan T: 9070
Lee, Hazy H.Y.: 2315
Lee, Heashoon: 8010
Lee, Hee: 1365, 7295
Lee, Hee Yun: 8000
Lee, Heejin: 7325
Lee, Heeyoung: 8000, 9050
Lee, Hochang Benjamin: 1345
Lee, Hyangkyu: 7060
Lee, Hye Soo: 4080, 7380, 8005
Lee, Hyo Jung: 3325, 7145, 7355, 7395, 7470
Lee, Hyunjoo: 8015
Lee, Hyunjoo: 9065
Lee, Ian: 8015
Lee, Jae Jun: 7145, 7225, 7300, 7375
Lee, Jaesung: 8000
Lee, Jane Y: 2530
Lee, Jane H: 7460
Lee, Jean Mui Hua: 2150, 3135
Lee, Jeesun: 7380
Lee, Jennifer: 4227, 7315
Lee, Jeong Eun: 1120, 4315, 7040, 9140
Lee, Jeongeun: 7430
Lee, Jeonghwa: 7005, 7400
Lee, Jeongmin: 7125
Lee, Jessica: 4415
Lee, Ji Hyun: 4285, 7295, 7395
Lee, Ji Yeon: 8000
Lee, Jiha: 7120
Lee, Jihwan: 7005, 7135
Lee, Jinkook: 2000, 3010, 3380, 4280
Lee, Jin-kyung: 8005, 8015
Lee, Jin-Moo: 1355
Lee, Jongwon: 7435
Lee, Joohyun: 4060
Lee, Joy: 4240
Lee, Juhyeong: 4270, 7090, 7165, 7390
Lee, Jung Ah: 8015
Lee, Jung-Ah: 7220, 7475, 7545, 7910
Lee, Jungha: 8015
Lee, Jungjoo: 7305, 8000, 8015
Lee, Jun-Young: 8015
Lee, Kangeun: 8000, 8015
Lee, Kathy: 1020
Lee, Kayoung: 7125
Lee, Kayoung: 8010
Lee, Knoo: 9070
Lee, Kristine H: 8005
Lee, Kyuho: 7470
Lee, Kyung Hee: 2210, 7525, 8000
Lee, Kyungmi: 7430, 8005
Lee, Kyungun: 7115
Lee, Kyuri: 7330, 8000
Lee, Lewina O: 3330
Lee, Lewis H: 7500
Lee, Linda: 7230
Lee, Mi Jung: 2445
Lee, Mina: 7085
Lee, Miu Yee: 1385
Lee, Othelia O: 4180
Lee, Pei-An: 1110, 7295, 7465, 7495
Lee, Pin-Shiuan: 7020
Lee, Pui: 8000
Lee, Rebekka: 2310
Lee, Rokbit Sanghee: 7065, 7365
Lee, SangA: 7185, 7295
Lee, Sei: 1025, 7140
Lee, Shinduk: 1300
Lee, Shinduk: 9025
Lee, Shwn-Jen: 7370
Lee, Soohyoung Rain: 9100
Lee, Soomi: 1475, 2230, 2385, 4050, 4215, 7365, 7390, 8000, 8005
Lee, Su-In: 2137
Lee, Sun Ah: 2280
Lee, Sung-Jin: 4210
Lee, Sun-Kyung: 7185
Lee, Sunmin: 7210
Lee, Tae-Ho: 8005
Lee, Traci: 1215
Lee, Vincent W. P.: 1225
Lee, Wai Tong: 2455
Lee, Wongyeong: 8010
Lee, Yen-Chin: 9050
Lee, Yen-Han: 1235
Lee, Yeonjae: 8015
Lee, Yeonjung: 2140
Lee, Yeonjung: 3020, 4210, 7005, 7035, 7080
Lee, Yeonjung (Jane): 1065
Lee, Yeon-Shim: 7160, 7375, 7415
Lee, Yoojin: 3370, 7510
Lee, Yookyong: 7300
Lee, Youjung: 2340, 4185
Lee, Young-Shin: 7495
Lee, Younho: 8015
Lee, Yura: 8000
Leedahl, Skye N: 4600, 7105, 7215, 7545
Leeman, Jennifer: 7105
Lees Haggerty, Kristin: 1085, 9135
Leff, Bruce: 1270, 2480
LeGars, Abigail K: 8010
Leggett, Amanda N: 1060, 3010, 4015, 7230, 8005, 8010
Leggett, Christopher: 8000
Lehmberg, Lisa J: 2165
Lehning, Amanda J: 4150, 7090, 8010
Lehr, Marcus: 4460
Lehtisalo, Jenni: 1290
Lei, Lianlian: 4390
Lei, Tingyu: 4055
Leis, Aleda: 2000, 4460, 7345
Leiser, Scott F: 2417
Leist, Anja: 1105, 8000
Lekhak, Nirmala: 2500
Leland, Natalie E: 1270, 8000, 8010
Leme, Daniel: 8000, 8010
Lemire, Emma: 9080
Lemus, Armando: 8015

Lenchik, Leon: 7165
Leng, Iris: 2245
Leng, Sean X: 3160
Lennon, Danielle B: 3005, 4350
Lent, Jaia: 7470
Lentz, Jeffrey G: 4135
Leo, Hubert: 3105
Leon, Lydia: 7345
Leonard, Claire: 7065, 7900
Leong, I tek: 1400
Leontovich, Natalia: 7330
Lepetit, Anne: 8015
Lepinteur, Anthony: 1210
Lepola, Noah: 8010
Lepore, Michael J: 1395, 2150, 2335, 2465, 3117, 4135, 4160, 8000, 8005
Lerma, Sofia: 7190
Lerner, Edie: 7290
Lerner, Emily: 4360, 7290
Lero, Christi M: 7485, 8000
LeRolland, Emily: 9040
Leshchyk, Anastasia: 8005, 8015, 9155
Lesser, Janna: 3355, 7255
Leszczynski, Jennifer: 8010
Letts, Elisa: 4445
Leung, Cindy: 7325
Leung, Daniel Dick-man: 4180
Leung, Dara Kiu Yi: 1235, 4455, 7255
Leung, Hannah Shui-Wah: 8015
Leung, Jasen Kin-Fung: 7145
Leung, Rachel Hoi Laam: 2250
Leung, Vania: 3240
Leutwyler, Heather C: 7130, 7290, 9060, 9090
Levan, Darius: 7290
Levasseur, Mélanie: 2235, 2545, 4150
Levasseur, Melanie: 2235
Leveille, Suzanne: 4560
Levenson, Robert W: 1275, 4085, 4515, 7290
Levin, Gali: 2105
Levin, Jason: 7265
Levin, Michael: 7215
Levitan, Eric: 1230
Levitt, Daniel: 8005
Levkoff, Sue: 1090
Levy, Becca R: 2370, 8000, 9035, 9125
Levy, Cari: 1130, 1145, 1300, 1435
Levy, Natalie: 7285, 7925
Levy, Sorah: 2350, 4460, 8005
Levy-Storms, Lené: 3020, 8010, 9145
Lewinson, Terri: 4150
Lewis, Charity T: 7290
Lewis, Gabrielle: 7265
Lewis, Jordan P: 2420, 3165, 4350
Lewis, Jordan P: 8005
Lewis, Joshua Christian Julian: 2155
Lewis, Lauren: 2135
Lewis, Mickal D: 7010, 9030
Lewis, Nathan: 4490, 4515
Lewis, Stuart: 1085
Leykum, Luci: 8010
Li, Aaron: 8000, 8005
Li, Ai-Tzu: 7080
Li, Boyuan: 8000
Li, Caiwen: 8015
Li, Chenxi: 7345
Li, Chien-Ching P: 2300, 7100
Li, Chihua: 4060
Li, Chihua: 4540, 7365
Li, Chih-Ying: 8010
Li, Daniel: 8015
Li, Guoyan: 7265
Li, Hao: 1460
Li, Haolin: 8005
Li, Haoru: 7025
Li, Hongyu: 7430
Li, Huan: 8015
Li, Huixian: 8005, 8015
Li, Isadora: 8015
LI, Jessica J.: 1225
Li, Jiajia: 9150
Li, Jianan: 3020, 7290, 7470, 7925, 8010
Li, Jiaxin: 7090
Li, Jiaying: 4385, 7390
Li, Jie: 1385
Li, Jing: 2185, 7535
Li, Jingyi: 4180
Li, Jingyu: 8015
Li, Juan: 3340
Li, Juan: 4330
Li, Juan: 7015
Li, Juan: 7030
Li, Juan: 7430
Li, Juan: 8000
Li, Junxin: 2480, 4010, 4070, 4335, 4385, 7390, 9075
Li, Ke: 4070, 4185, 7495, 8000
Li, Kelin: 8005
LI, Kin-Kit: 8015
Li, Lihua: 2185
Li, Ling: 4370
Li, Liping: 7225, 7905
LI, Lun: 2140, 2315, 8010
Li, Lydia W: 7395
Li, Meiyi: 7260
Li, Meng-Hao: 2265
Li, Mengting: 9105
Li, Mengze: 8005
Li, Niying: 2190, 4580
Li, Qian: 7040
Li, Qiwei: 7380, 7430
Li, Rosemary: 8010
Li, Ruoxuan Rosalyn: 2230
Li, Shan: 7240
Li, Shaojie: 7225
Li, Shuang: 4365
Li, Shuang: 7300
Li, Shumin: 1245
Li, Shuning: 9020
Li, Shuzhuo: 7470, 7900
Li, Sizhe: 8005
Li, Tao: 7410, 8015
Li, Tianyuan: 3275
Li, Tianzi: 1245
Li, Wanling: 3075
Li, Wei: 8015
Li, Wenhan: 4370, 7310
Li, Wenjing: 1060, 7230
Li, Wenjun: 8000, 8015
Li, Xiang: 8010
Li, Xiaoang: 4275
Li, Xiaoli: 7250, 7380, 7430
Li, Xiaoli: 8005
Li, Xiaoyouxiang: 8005
Li, Xingyu: 8015
Li, Ya-Hsin: 2300
Li, Yanping: 8010
Li, Yaru: 8000
Li, Yawen: 2305, 7305
Li, Yaxin: 7410
Li, Yezhen: 2000, 3010
Li, Yiang: 2295
Li, Yibo: 9105
Li, Yingru: 7380
Li, Yixia: 7205, 7255
Li, Yuanyuan: 7315
Li, Yucan: 7320
Li, Yue: 4345
Li, Yuehua: 8005
Li, Yuekang: 7145
Li, Yuelin: 7150, 8000, 9050
Li, Yuhang: 1070
Li, Yun: 7125
Li, Zhen: 7350, 7930
Li, Zheng: 8005
Lian, Zheng (Noah): 1140, 7055
Liang, Bo: 7300, 7430
Liang, ChangPu: 7085, 7435
Liang, Jersey: 2120
Liang, Jiaming: 2540, 4565
Liang, Jie: 7030
Liang, Siqi: 3090
Liang, Siqi: 8000
Liang, Xiaoxuan: 7300, 7430
Liang, Yanjing: 4180, 7100
Liang, Zhen: 3160
Liao, Gerald Y: 1520, 7480
Liao, Hsiao-Wen: 2350, 7525, 8015
Liao, Shuyao: 3275
Liao, Yanru: 3370
Liao, Yo-Jen K: 3035, 3220, 7465, 7500
Libby, Julia B: 7145
Libera, Theresa L: 8010
Libermann, Towia A: 2505, 8000
Libon, David J: 4570
Lichtenberg, Peter: 2115, 3130, 7190, 7310
Lieber, Sarah B: 4525
Liebermann, Erica: 7105, 7215
Lifshitz, Rinat: 1090
Lighthall, Nichole: 7375
Likhterman, Mir-iam M: 7350
Lim, Angelica: 7205, 7915
Lim, Arum: 7380, 7430, 7495
Lim, Donovan: 4370
Lim, Hayun: 8000
Lim, Junyub: 3370
Lim, Kimberly: 4475
Lim, Lionel: 7410
Lim, Wee Shiong: 7120
Lim, Wen Huey: 2435
Lim, Xinru: 8005
Lim, Ye-Ji: 7100
Lim, Youngmi: 8015
Lim, Zhi Zhen: 8005
Lima, Julie: 1435
Limani, Merita: 1070
Lim-Soh, Jeremy: 1205, 3325
Lin, Asenath: 2350
Lin, Carol: 7485, 8000
Lin, Chia Jou: 7060
Lin, Feng-Chang: 1235
Lin, Frank: 1075, 1200
Lin, Gloria: 8005
Lin, Haiqun: 3205, 9045
Lin, Hongmei: 1330, 3360, 4300
Lin, Hui-Yi: 2300
Lin, I-Fen: 1280, 3050, 4085
Lin, Jason: 9070
Lin, Joshua: 1340, 4090, 7510
Lin, Ju-Ping: 7165
Lin, Li-Hui: 8010, 8015
Lin, Molly Zhixuan: 1330, 2250, 4300
Lin, Natong: 1110

Lin, Nelson: 3155
Lin, Rose: 8005
Lin, Sabrina: 1185
Lin, Shih-Yin: 1435, 2015, 2380, 3205, 4015, 7185
Lin, Shiqi: 2275
Lin, Tzu-Yu: 7445
Lin, Xin Yao: 3025, 4080, 8015
Lin, Yan: 7305
Lin, Yi: 7190
Lin, Yunann: 7340, 7470
Lin, Yuqian: 7010, 7125
Lin, Zhiyong: 2040, 9105
Lin, Zhuoer: 1160, 2040, 2105, 3200
Lind, Lisa: 2240
Lindauer, Allison: 1015, 1165, 7415
Lindberg, Brian W: 1450, 2215, 2355, 3210
Lindberg, Daniel: 1085, 3300
Linden, Wolfgang: 4490
Lindenberger, Ulman: 4050
Linder, Jeffrey: 8000
Lindsay, Rebecca: 7390
Lingard, Ayaka: 3215
Lingler, Jennifer: 4045, 7335
Link, Jessica: 1315
Linker, Alexandra: 8000
Linker, Maddison: 7200
Linscott, Robert: 7390
Linsky, Amy: 4090
Lipitz, Sarah: 8015
Lipsitz, Lewis A: 1255, 3117, 3235, 7025, 7130, 7295, 7465
Lipton, Richard: 2400, 4310, 7535, 8015
Lipworth, Loren: 9030
Lisa, Jenna: 8005
Littleton, Tenesha: 2340
Liu, Aihong: 4370, 7310
Liu, Amy: 4520
Liu, Bian: 1270, 8000
Liu, Carissa: 8015
Liu, Chiung-ju: 2290, 7265, 8010
Liu, Christopher: 1465, 8015
Liu, Chunyu: 7040
Liu, Chunyu: 8000
Liu, Chunyu: 8015
liu, Dan: 7240
Liu, Darren: 2090, 2300, 8000
Liu, Ethan: 8015
Liu, Eunbee: 8000
Liu, Fangyu: 1155, 4495
Liu, Hang: 9100
Liu, Hao "Howe": 4255
Liu, Haoyuan: 7015
Liu, Ho Kan: 8000
Liu, Hui: 1125, 2295, 3050, 7255
Liu, Hui: 7320
Liu, Jianfang: 7125, 7185
Liu, Jiaru: 3225
Liu, Jingwen: 1240
Liu, Jingyuan: 7335
Liu, Jinyu: 9115
Liu, Julianna: 2505
Liu, Jun: 1340
Liu, Junxian: 8000, 8010
LIU, Karen P. Y.: 4455
Liu, Keyao: 2125
Liu, LiFan: 1290
Liu, Longjian: 7015
Liu, Marian: 1085
Liu, Mengjing: 4200
Liu, Mengshi: 7130, 7185, 7200, 7210, 7255, 7260, 7300, 7305
Liu, Mengting: 2305
Liu, Mengyu: 1515
Liu, Michelle: 7390, 9075
Liu, Peiran: 4495
Liu, Pi-Ju: 8010
Liu, Qiyun: 7385
Liu, Rui: 7345
Liu, Ruotong: 4505
Liu, Ruotong Mona: 3110, 4505, 7485, 8010
Liu, Shao-Hsien: 7255
Liu, Sofia: 4070, 4385, 9075
Liu, Tianyin: 8000
Liu, Wanru: 7010, 7185, 7930
Liu, Wen: 3220, 4015, 4125, 7330, 8000
Liu, Xiaodong: 4030
Liu, Xiaojuan: 1340, 4090, 7510
Liu, Xueyan: 8005
Liu, Yaqing: 7300, 7410, 7930
Liu, Yi: 7160, 7225
Liu, Yichun: 7330
Liu, Yifan: 2480
Liu, Yiming: 8005
Liu, Yin: 4520, 7435
Liu, Ying: 2120, 4435
Liu, Yingjia: 8000
Liu, Yiwen: 1105
Liu, Yu: 7435
Liu, Yuanhao: 7015
Liu, Yuetong Toria T: 8015
Liu, Yujun: 1505, 4030, 7040
Liu, Yunrui: 8010
Liu, Zeyu: 7455, 7915
Liu, Zuyun: 7240, 9150
Liu-Ambrose, Teresa: 7225, 7905
Livingston, Melvin: 1085
Livne, Ofir: 1500, 4390
Lizarazo, Maria Jose: 8010
Llaneza, Danielle H: 7220
Lo, Grace: 8010
Lo, Jeanie: 7350, 7930
Lo, On-Yee: 1110, 1255, 7435, 7495
Lo, Yi-Chen: 8015
Lo, Ying-Ying: 7515
Lober, William B: 4200
Locascio, Joseph: 2310
Locher, Julie L: 1465
Loeb, Susan J: 2485
Loerzel, Victoria: 7425, 8005, 9100
Logan, Jeongok: 8010
Logue, Caroline: 4450
Logue Cook, Rachel N: 7120, 7310
Lohman, Matthew: 8000
Lohmar, Sabine: 1440, 7255
Loibl, Cäzilia: 3285
Loizos, Maria: 9080
Lok, Kris Yuet Wan: 7085
London, Andrew S: 2085
Londono, Valeria: 3340
Long, Armari: 7225
Long, Monica: 2205
Long, Yanan: 4200
Longo, Valter: 3267
Lopez, Bruna C: 1425
Lopez, Janet: 1055, 7090, 7160, 7225, 7350, 7380, 8005, 9100
Lopez, Ruth Palan: 8005, 8015, 9000, 9095
Lopez, Ruth Palan: 1145
Lopez Blanco, Jose David: 7210
Lopez-Ortega, Mariana: 2060
LoPilato, Danielle: 8000, 8015
Lopis, Joyce A: 8005
Lor, Maichou: 8015
Lord, Justin: 3365
Lord, Marie-Michele: 2235
Lord, Sébastien: 4150
Lords, Hannah: 8005
Lorenzo, Erica: 8000
Losin, Elizabeth: 8000
Lou, Vivian Weiqun: 1030, 2350, 7350, 7360, 7365, 7495, 8000
Lou, Yifan: 1140, 2265, 4325, 7005, 7325, 7375, 8000, 8005, 9115
Loui, Psyche: 9040
Louie, Patricia: 4440
Louis, Elan: 7415
Louis, Sandro: 8010
Louis-Charles, Willencia: 4080
Loutet, Katrina: 7225, 7905
Louzoun, Yoram: 2105
Lovasi, Gina S: 2400
Love, Sheltoria: 8000
Lovence, Keisha: 2245
Lowenthal, Julie: 2075
Lu, Hongzhou: 4055
Lu, Jinxia: 7300
Lu, Kevin: 4265
LU, Lucy Xi: 4455
Lu, Minjie: 8005
Lu, Peiyi: 7360, 7365, 7495, 8010
Lu, Shiyu: 4370, 7150, 7220, 7925
Lu, Shu-Chen: 7370
Lu, Sihan: 7515
Lu, Siyao: 8000
Lu, Thomas: 7440
Lu, Xiaomin: 8010
Lu, Xuan: 8015
Lu, Yangfan: 7435
Lu, Yi: 3275, 4300, 7380
Lu, Yifei: 1200
Lu, Yijun: 7325
Lu, Yvonne: 7010, 7265, 7910, 8010
Lubetsky, Sara: 9145
Luborsky, Mark: 8010
Lucantoni, Davide: 7070
Lucas, Jennifer: 7515
Lucas-Wright, Anna: 4250
Lucca, Kaitlyn: 1015
Lucci, Katherine: 7230
Lucey, Brendan: 4385
Luchetti, Martina: 1120, 1265, 1445, 1480, 4360
Luciana, Monica: 8015
Ludwig-Borycz, Elizabeth: 7325
Lui, Fan: 4180
Lui, Shirley Shuk-Kuen: 7395, 7440
Luk, Sau Man Angela: 1030
Lukkahatai, Nada: 4010
Lum, Terry Yat Sang: 1030, 1135, 1235, 4455, 7255
Luna, Kenya: 4440, 8015
Lunak, Azylen M: 2330
Lundebjerg, Nancy: 2215
Lundquist, Tessa: 1440
Lundy, Theresa L: 9055
Lunetta, Kathryn L: 8005
Luo, Aaron: 8010
Luo, Huabin: 3110, 7485, 8010
Luo, Jing: 1265
Luo, Juan: 7045

Luo, Jun: 2380
Luo, Junyuan: 3270
Luo, Lan: 9000, 9085, 9105
Luo, Lan: 9135
Luo, Lan: 9145
Luo, Li: 7410, 8015
Luo, Minxia: 4270
Luo, Tianyi: 8005
Luo, Xiao: 3025
Luo, Yan: 2545
Luo, Yanan: 4055, 4455, 7010
Luo, Yuan: 2460
Luo, Zhehui: 1500
Luong, Gloria: 2095, 3270
Luong, Thuc: 7380
Lusardi, Michelle M: 7140
Lusignani, Maura: 8015
Lussier-Therrien, Marika: 2235
Lustbader, Wendy: 3055
Lustria, Mia Liza A: 1285
Lute, Sara M: 8000
Luth, Elizabeth: 2320, 7220, 8000
Luth, Elizabeth: 8010, 8015
Luther, Brenda: 1300
Lutsey, Pamela L: 4385, 7545, 8010, 8015
Lv, Gang: 4265
Lv, Yuebin: 8005
Lwi, Sandy J: 4085
Ly, Ka: 7000
Ly, Timothy K: 3185
Lydon, Lizzy: 1165, 9085
Lynch, Colleen: 7265
Lynch, Connor: 8005
Lynch, David: 1220, 1235
Lynch, Delanie: 7165
Lynch, Grace: 7390
Lynch, Jennifer: 9100
Lynch, Karla: 7465
Lynch, Kathleen: 1090
Lynch, Scott M: 8000
Lynn, Freda: 2135
Lynn, Rachel: 2255
Lyons, Heidi A: 2295
Lyons, Karen S: 3375, 4485, 7335, 8000, 8015
Lyons, Kathleen: 7300
Lysandrou, Apollonia E: 7375
Lyu, Wangyizhuang: 7305

**M**

Ma, Carol Hok Ka: 7235
Ma, Chenjuan: 2440, 8000, 8010
Ma, Chunxuan: 8005
Ma, Da: 7480
Ma, Gu: 3275
Ma, Guoyong: 2305
Ma, Guoyong: 8015
Ma, Jessica: 4430
Ma, Jinhui: 7225, 7905
Ma, Kaiting: 7015
MA, Lina: 8005, 9085
Ma, Maggie S. L.: 2135
Ma, Mingyue: 8005
Ma, Qing: 7090, 7315, 7320
Ma, Runze: 8015
Ma, Stefan: 2230
Ma, Tongyu: 9020
Ma, Winkie: 1265
Ma, Yuan: 1245, 7090, 9040, 9155
Ma, Yuxuan: 2305
Ma, Zhenxuan: 8005
Ma, Ziqi: 8005
Müller, Beat: 8015
Mabry, Marshall: 8010
MacArthur, Barbara: 8000
Macaulay, Susan: 8000
MacConell, Leigh: 8010
MacConnachie, Lauren: 1110, 4460
MacDonald, Catherine: 1005
Macdonald, Karen C: 7275
MacDonald, Stuart: 7115
MacDougall, Sarah: 7365, 7400
Mace, Ryan A: 1160, 2310, 7295, 9110
Macedo, Luciana: 7225, 7905
Macedo, Sabrina: 8005
MacGillivray, Beth: 8000
Machiano, Joseph: 7225
Machida, Masaki: 7345
Maciel, Alvaro: 8005
Mack, Laurin J: 9140
MacKay-Brandt, Anna: 8015
Mackenstadt, Darby: 8015
MacKenzie, Kelsey: 8015
MacKenzie Greenle, Meredith: 8000
Mackintosh, Siobhan: 8015
MacNeil, Andie: 7180
MacPherson, Andrew: 2355, 2495
Madden, Ken: 4490
Madden, Kenneth: 7225, 7905
Maddow, Charles: 1085
Mader, Michael J: 8000, 8010
Madera, Sonia: 8005
Madison, Mali A: 8005
Madlock-Brown, Cherisse: 8010
Madrigal, Caroline: 1470, 4480, 9080
Maeda, Akari: 2210
Maestre, Gladys Elena: 8015
Magalhaes, Larissa: 8000
Magaziner, Jay: 7495
Magdalena Giovani, Élcio CC: 7300
Mage, Susanna M: 2390, 3300, 7085
Maggs, Jennifer: 7180
Magid, Kate: 1300, 8005
Magnusson, Dawn: 7140
Magruder, Kathryn M: 2085
Maguire, Teagan: 7485
Maham, Abeera: 9080
Maher, Brion S: 1510, 7040, 8015
Maher, Joanne: 9020
Mahfuza Haque, Mahi: 7290
Mahmood, Atiya: 1325, 2515, 3305
Mahmoud, Kafayat: 3195, 4580
Mahmoud, Nourhan: 7315
Mahmoudi, Elham: 1160, 3355, 7000
Mahone, Anais: 8000
Mahoney, Jeannette R: 2535, 4200
Mai, Chunyan: 8005, 8010
Mainieri, Tina: 3230
Mains, Douglas: 7540
Mair, Christina F: 2200
Mair, Christine A: 1240
Maitland, Daniel W M: 7355
Majette, Nadya: 7350, 7930
Majgaonkar, Hritik N: 8010, 8015
Majon-Valpuesta, Dolores: 2235
Mak, Felix: 2350
Mak, Jonathan Ka-Long: 2510
Mak, Wingyun: 7250, 7330
Makaroun, Lena K: 1085, 9140
Makela, Meghan J: 9080
Makita, Meiko: 1135
Makris, Una: 7120
Malani, Preeti: 4390
Malatyali, Ayse: 1055, 4450, 7160, 7380, 8005
Malcolm, Millicent M: 7410, 7525
Malecki, Kristen: 7260
Malette, Holly: 2240
Malhotra, Atul: 2035
Malhotra, Atul: 8015, 9030
Malhotra, Rahul: 1205, 2230, 2435, 3325
Malkowski, Olivia S: 2120, 8010
Maloney, Gabrielle: 4325
Malta, Jessica S: 8015
Manalel, Jasmine A: 2395, 3385
Mandelblatt, Jeanne: 1475, 7190
Mandl, Lisa A: 4525
M'Angale, Peter: 8005
Mangialasche, Francesca: 1290, 3030
Mangiardi, Rosemarie: 7075
Mangila, Jessica: 2275
Mangione, Kathleen: 8005
Mangone, Laura: 8000
Manheim, Chelsea: 8000, 9095
Mani, Sneha Sarah: 1495, 2000, 4060
Manini, Todd M: 1295, 1390
Mankowski, Robert T: 7190
Manly, Jennifer J: 3380, 7040, 7435
Mann, Ajit Singh: 4300
Mann, Frank D: 7255
Mann, Jim: 7205, 7915
Mannan, Masuma: 2300
Mannheim, Ittay: 2370
Manning, Innessa A: 7500
Manning, Kenneth: 1470
Manning, Lydia K: 1135, 3150
Manocha, Samiya: 7290
Manor, Brad: 1110, 1255, 7025, 7295, 7465, 7495, 8010
Mansfield, Kelly: 2030
Mansfield, Melissa: 7380
Mansfield, Tyler: 1460, 4275
Mansion, Natalie: 1260, 7095
Mansoor, Marrium: 7020, 7200, 7230, 8010
Mansourian, Farnoush: 8005
Manzo, Alma A: 7305
Manzour, Rezvaneh: 8000, 9050
Mao, Hui-Fen: 8010
Mao, Jieyu: 7195
Mao, Lingchao: 8015
Mao, Weiyu: 1400, 2475, 4170, 8005
Mao, Xi: 8015
Mao, Ziling: 2245, 4295
Mar, Amanda B: 1185
Marabini, Camilla: 2350
Marcano Maldonado, Patria: 7495

Marcantonio, Edward R: 2505, 7060, 8000
Marcatti, Michela: 8010
Marchant, Erik D: 8015
Marcos, Christine: 8005
Marcus, Cara: 3170
Marcus, Michael: 4595, 8000
Marcus, Zeve: 7155
Maredia, Asma: 8000
Mareverwa, Amos: 7430
Marfeo, Elizabeth: 1305, 2365, 7230, 7280, 7350
Margolis, Rachel: 1405
Marhánková, Jaroslava Hasmanová: 9070
Mariani, Diane: 8010, 9145
Marie, Boltz: 2180
Marincic, Michaela: 7465
Marini, Christina M: 2085
Marino, Francesca R: 1510
Marino, Victoria: 3330
Marinov, Aleksandar: 1325
Mark, Anthony: 8005
Marka, Nagaraju: 8000
Markham Risica, Patricia: 1465
Markides, Kyriakos S: 4290, 7465
Markland, Alayne D: 9115
Marks, Jane: 2205
Marnfeldt, Kelly: 1215, 3300
Marottoli, Richard: 7245, 7530, 8005
Marques, Brenda: 7535
Marquez, David X: 8005
Marr, Calum: 4205
Marroig, Alejandra: 3265, 4110, 7145, 8015, 9005
Marron, Megan M: 2000, 2245, 7345
Marrs, Sarah: 7340
Marsh, Kaetlin F: 7435
Marshall, Gillian: 3180
Marsiske, Michael: 4570, 8005
Marston, Hannah R: 3080, 9100
Marston, Holly: 8010
Martey, Ebenezer: 3370, 8000
Marti, C. Nathan: 7020
Martin, Allison: 7475
Martin, Cindy: 2170
Martin, Corby: 3267
Martin, Diane: 4345
Martin, F. Michael: 7150
Martin, Jennifer L: 1295
Martin, Peter: 1065, 4600, 7040, 7395, 7470
Martin, Seth S: 4385, 7545
Martin, Jr., Wesley T: 3005, 4350
Martindale, Jonathan: 7120
Martine, Shareck: 4150
Martinez, Alejandra: 2045
Martinez, Alejandra: 8015
Martinez, Alejandra: 9045
Martinez, Erica: 4445, 7410
Martinez, Iveris L: 2490
Martinez, Lusiana: 8000
Martinez, Madelyn: 7340
Martinez, Natalie A: 8010
Martinez, Pauline D: 2450, 3000
Martinez, Ryan: 7345
Martinez, Vanessa: 7325
Martinez Escudero, Jose: 7410
Martinez-Amezcua, Pablo: 1075, 4450, 7330, 7535
Martin-Peters, Tasha: 8000
Martinson, Melissa: 7485
Martinson, Wendy: 7305
Martire, Lynn: 1125
Martyr, Anthony: 2335
Marzetti, Emanuele: 8010, 8015
Marziliano, Allison: 2175
Masaki, Harue: 7215
Masaki, Kamal: 1065
Mason, Amara: 2340
Mason, Hannah Ç: 2325, 4080
Mason, Tyara R: 1470
Masoud, Sara S: 1135, 3355, 8000
Massa, Fernando: 7145
Massa, Franklin: 9005
Massihzadegan, Setareh: 7030
Massimo, Lauren M: 1015, 7205
Massingale, Shermetria: 4135
Massizhadegan, Setareh: 1415
Mast, Benjamin T: 7305, 7400, 7415, 7900
Masters, Julie L: 8010
Mastrostefano, Aglaé: 7065
Masui, Yukie: 7365
Masumoto, Shoichi: 8010
Masurkar, Arjun V: 7010
Matchar, David Bruce: 2435
Mathai, Paul: 7510
Mather, Mara: 8005
Mathers, Kate: 8010
Mathew, Jason R: 4200
Mathis, Lindsey M: 9115
Matlock, Daniel D: 2320, 2500
Matos-Moreno, Amílcar: 7200
Matovu, Schola N: 7085, 7915
Matsui, Hiroki: 9020
Matsumoto, Alvin M: 8000
Matsumoto, Hiroshige: 7440
Matsuo, Masafumi: 8015
Matsuzawa, Akemi: 8010
Mattek, Nora: 1280, 7475
Mattero, Joana: 9115
Matteson, Tyler J: 1330
Matthews, Alicia K.: 7100
Matthews, Jean M: 8000
Mattke, Soeren: 4435
Matz, Christina: 1425, 2050, 2530, 7500
Mauck, Erin: 7085
Mauer, Jess: 1335
Maurer, Jürgen: 4070
Mausbach, Brent T: 1295
Maust, Donovan T: 1010, 8005, 8010
Maxfield, Molly: 1440, 7010
Maxi, Andrea: 9100
Maxwell, Cathy A: 7075, 7295, 7545
May, Au Myat G: 8015
May, Jennifer T: 8000
May, Melissa: 4020
Maye, Jacqueline E: 8010
Mayeda, Elizabeth Rose: 4280
Mayer, Jamie: 4030, 7040
Maynard, Madison: 7375
Mayo, Nancy: 8000
Mays, Allison M: 8010
Maza Santibañez, Valentina: 9015
Maziarz, Lauren: 7370
Mazonson, Baylah: 8015
Mazor, Kathleen: 7485
Mazumder, Nikhilesh R: 7185
Mazzarella, Diana: 4070
Mazzola, Paolo: 1220
Mbakile-Mahlanza, Lingani: 7010
Mbam, Kingsley C: 7070, 7080
Mbanefo, Onyinye: 4565
Mbao, Mbita: 7455
M’Bra, Paule: 7415
McAlinden, Michael: 4205
McAllister, Denise: 4210
McArdle, Patrick: 2125
McAtee, Robin: 7015, 7920, 7935
McAuley, Edward: 1255, 7265, 8000
McBee-Black, Kerri: 7065
McBride Afonso, Tara: 1050
McCall, Stephen: 1495
McCall, Stephen: 3315
McCampbel, Jody: 1085
McCarthy, Ellen P: 9000, 9085, 9105, 9135, 9145
McCarthy, Michael J: 2170
McCarthy, Teresa: 2010, 7370
McCartney, Jenn: 4305
McCauliff, Jocelyn: 7410, 7910
McClellan, Angela: 4265, 7440, 7525
McClelland, Robyn: 7040
McClure, Leslie A: 2335
McClure, MaKenna: 2260
McClure, Nicole: 7375
McConeghy, Kevin: 7305
McConnell, Eleanor S: 2210
McConnell, Eleanor: 3040
McConnell, Eleanor S: 3135, 4075
McConnell, Eleanor: 4425
McConnell, Eleanor S: 7325, 8000, 9035
McConnell, Kelly M: 2220, 8000
McConnell, William R: 3050
McCormick, Mark: 2417
McCorvey, Stephanie A: 2350, 7085, 7495
McCoy, Brianah M: 8015
McCoy, Megan C: 1000
McCoy, Rozalina: 9075
McCracken, Courtney: 7110
McCrackin, Sarah: 1315
McCrae, Christina S: 4230, 8000
McCreedy, Ellen: 2055, 4475, 8005, 8015
McCullough, Megan B: 1300
McCune, Teagan: 7050
McDaniel, Miranda: 8015, 9050
McDannold, Sarah: 8005
McDermott, Cara L: 3065
McDermott, Kate: 2310
McDevitt, Ross A: 8000
Mcdonald, Anthony: 4435
McDonald, Claire: 7470
McDonald, Jerry: 1295
McDonald, Margaret: 1170, 7440, 7545
McDonough, Ian: 9040
McDougall, Graham J: 8005
Mcelfish, Pearl A: 7005
McFadden, Bridget: 8010
McFarland, Malcolm N: 8015
McFeeley, Brittany M: 7050, 7075

McGaffigan, Erin A: 1130, 3155
McGee, Jocelyn Shealy: 8005, 9105
McGee Lawrence, Meghan: 7245
McGovern, Justine: 2165, 4115, 9055
McGrath, Colleen B: 8000
McGready, John: 4450
McGregor, Casey: 7105, 7215
McGrory, Aileen: 2540, 7280, 7350
McGuinness, Bernadette: 4205
McGuire, Catherine: 4475
McGuire, Courtney: 7410, 7910
McGuire, Lisa C: 1395, 9010, 9125
McGuire, William: 7020
McIlvennan, Colleen K: 7085, 7910
Mcintosh, Nathalie: 2225, 3040, 4550
McInturff, Sophia: 8005
McIntyre, Cecily: 7410, 7920
McIntyre, Madeline: 7005
McKay, Michelle A: 4560, 8000
Mckay, Tara: 8005
McKendrick, Karen: 2185, 8000
McKenna, Kevin: 4565
Mckenzie, Barbara: 7000
McKibbon, Kristy: 7075
McKiddy, Sarah: 8010, 8015
McKinney, Lana P: 4210
McKinnon, Annette: 7230
McKoy, Khalil: 8005
McLaren, Jaye E: 2365, 3005, 7300, 7310, 8005
McLaughlin, Sara: 2260
McLees, Laura K: 7345
McLennan, Stuart: 7145
McLin, Maia: 2340
McManus, Caoimhe A: 2480
McManus, David: 2500, 7410, 7485
McManus, Regan: 7280
McMillan, Colleen: 7230
McMullen, Tara: 4160, 8005
McNamara, Maeve S: 9080
McNeish, Brendan L: 7425
McNichols, Christine C: 7300
McNicoll, Lynn: 7255
McPeek, Ashley: 2205
McPherson, Rachel: 2180, 3220, 4460
McPhillips, Miranda V: 4215, 4335
McQuown, Colleen: 1085
McShane, Michael: 9075
McSparran, Emerson: 2335, 4160, 7330, 9010
McTigue, Kathleen: 4535
Meadows, Jeff: 3060
Meah, Noah: 4015
Mecca, Adam P: 7530
Mecca, Marcia: 7030, 7410, 7920
Mechaber, Amanda: 7310
Medina, Jose T: 1425, 4325
Medina, Morgan N: 7270
Medina-Junge, Meira: 4235
Medlin, Laura M: 8010
Medya, Sourav: 4590
Meehan, Amy: 7050
Meeker, Daniella: 8015
Mehegan, Laura L: 7025
Mehl, Matthias: 8005
Mehmood, Yasir: 7320
Mehra, Eshan: 8000
Mehrotra, Sanjay: 7185
Mehta, C. Christina: 2395, 9080
Mehta, Raj: 9100
Mei, Haokun: 4505
Mei, Sharon: 2300, 7100
Meier, Madeline H: 4390
Meier, Tabea: 4515
Meier, Tabea: 4515
Meijer, Erik: 3380, 4280
Meisel, James: 7410
Meister, Melanie: 4230
Mejbaryan, Veranika: 7485
Mejia, Shannon: 3230, 4320, 4420, 7225, 7295
Mejia-Arango, Silvia: 8015
Melamud, Eugene: 7045
Melanson, Celia: 8005
Melekis, Kelly A: 4510, 8015
Mellow, Maddison L: 3235
Melms, Tobias: 7125
Melo, Ruth C: 7090, 7140, 7370
Melson, Jacob: 7280
Meltzer, David: 7430
Meltzer, Gabriella Y: 1090, 7040
Melville, Lucas: 8005, 8015
Memmer, Nicole: 3215, 7295
Memon, Ashraf: 8005
Mena, Emily: 1310, 3035
Menaker, Hannah: 8000
Menard, Alixe: 8015
Mendes de Leon, Carlos F: 1495, 1500, 7335
Mendez, Jennifer: 8010
Mendez, Xenia: 4260
Mendez Campos, Barbara: 2050, 7110, 7290
Mendez-Chacon, Ericka: 7090
Mendoza, Alonzo: 8010, 8015
Mendoza, Jose: 8000
Mendoza, Katie C: 1050
Mendoza, Nancy: 2340, 3055
Mendoza Ruvalcaba, Neyda: 7040
Menen, Tetla: 4240
Meng, Hailong: 8000
Meng, Han: 8000
Meng, Hongdao: 7040, 7190, 7305, 7395, 7420
Menin, Giovana: 7320
Menne, Heather L: 1395, 2260, 2335, 4160, 7085, 7330, 7540, 7915, 8005
Mentch, Heather: 7370
Meraiyebu, Ajibola Bart: 1430
Meraz, Rebecca: 8005, 9105
Merdjanoff, Alexis: 1090, 2250, 7345
Merin, Nathan H: 8010
Mero, Kiana: 8015
Merrick, Charlie: 4010
Merrilees, Jennifer J: 4085, 4515, 7290
Merten, Natascha: 1200, 2535, 8015
Mertz, Laurel: 1490, 7295, 7380
Mese, Patrick O: 7250
Messman, Brett: 8015
Meszaros, Katherine: 7470
Metcalf, Emily E: 7295, 8005
Metz, Regan: 8010
Metzler, Janna: 7345
Meuser, Aaron M: 3170
Meyer, Kylie: 3300, 7085, 8005, 8010
Meyers, David: 4430, 7485, 7540, 7930
Meynadasy, Melissa: 1050, 8010
Meynet, Stéphanie: 4150
Mez, Jesse: 1510
Mezuk, Briana: 2105, 3100, 4580, 7335
Mi, Hong: 1090, 2305, 7305, 8015
Mia, Hasib: 7415
Miao, Huali: 3075
Miao, Mary: 2545
Michaud, Francois: 2235
Michaud, Kaleb: 7120
Middleton, Addie: 9095
Middleton, Laura: 7225, 7905
Middleton, Lefkos: 1290
Miezah, Dennis: 8015
Migliaccio, John: 4595
Migneault, Deborah: 1385, 2185
Mihailovic, Aleksandra: 7530
Mike, Leigh Ann: 8010
Mikels, Joseph A: 2095, 7265, 7460, 8010
Mikus, Ellen: 9130
Milad, Elizabeth: 1265
Milani, Sadaf Arefi: 1070, 2005, 2345, 3370, 4560, 7465, 8000, 8015
Milberg, William: 7040, 8005
Mildrum Chana, Sofia: 4140
Miles, Declan: 7390
Milidonis, Mary: 7455
Milisen, Koen: 1060
Miljkovic, Iva: 7345, 7425
Millar, Courtney: 7130
Millar, Roberto J: 7515, 7930
Millenbah, Ashley: 1440
Miller, Amanda: 1265
Miller, Benjamin F: 2555
Miller, Christopher: 4090
Miller, Edward A: 1150, 2130, 2150, 3335, 4365, 7250
Miller, Eleanor: 7305
Miller, Emika: 8005
Miller, Jacqueline: 4260
Miller, Julie: 2530
Miller, Katherine: 1360, 2375, 2520, 3315, 4265
Miller, Kenneth L: 7140
Miller, Kyra A: 9110
Miller, Lindsay: 7345
Miller, Luke R: 4395, 7450
Miller, Matthew: 2245
Miller, Michael E: 1075, 2470
Miller, Patrick: 8010
Miller, Sara: 7180
Miller, Sarah J: 7180, 7505
Miller, Steven J: 8010
Miller, Steven: 9140
Miller, Vivian J: 7355, 7370
Milliard, Carrie: 7185
Milliken, Laurie: 3205
Mills, Corina I: 4250
Mills, Whitney L: 1060, 2225, 3040, 4240, 4550

Milman, Sofiya: 2557, 4495, 8000, 8015
Mimbs, Hope: 4245
Min, Deborah: 1345
Min Htike, Wai Yan: 8005
Minahan Zucchetto, Jillian: 7040
Minami, Christina: 8005
Minami, Kota: 7195
Mindlis, Irina: 1055, 4010, 4225, 7255, 8015
Minemoto, Sara: 7395
Miner, Brienne: 4335
Miner, Jennifer A: 4015
Ming, Yan: 8005
Mingo, Chivon A: 2550
Mino, Matthew: 7455
Minor, Bryan J: 4045
Minyo, Morgan J: 2285, 7010, 7915
Mion, Lorraine: 7545
Mirabella, Rosie: 8005
Mirin, Nicholas L: 8005
Mironi, Nava: 2390
Mirza, Mansha: 3265
Mirza, Raza M: 7340, 7920
Mishra, Ayati: 8000
Mishra, Shalini: 8015
Mishra, Swarup: 7190
Misra, Shalini: 4007
Misra, Sumi K: 2195, 7410
Missell-Gray, Rachel: 7145
Mistler, Lisa A: 2045
Mistler, Lisa A: 9045
Mital, Avani: 7045
Mitchell, Barbara: 7470, 7900
Mitchell, Chloe: 1400, 8010
Mitchell, Connor: 8015
Mitchell, Diane C: 7480
Mitchell, Jamie: 2515, 7190
Mitchell, Lauren: 4195, 7350
Mitchell, Uchechi: 2040, 7395
Mitlak, Hannah: 7440
Mitsova, Diana M: 4045
Mittelman, Mary S: 1400, 4170
Mittman, Brian: 4535
Mitzner, Tracy L: 1230, 2430, 4080, 8005, 9065
Miura, Chiharu: 7365, 7395, 7925
Miwa, Hiroyasu: 4590
Miyamae, Fumiko: 7440
Miyano, Yurina: 2210
Miyashita, Satoshi: 7195
Miyawaki, Christina E: 4265, 7440, 7525
Miyazaki, Atsuko: 9100
Mizrahi, Chanelle: 8010
Mizukami, Taeko: 2210
Mlecko, Maura R: 7200
Mlinac, Michelle E: 1050, 2240
Moberg, Ylva: 1095
Mock, Colleen: 7505
Moczygemba, Walter: 9005
Moeller, Eric: 7400
Moeller, Kelly: 1010
Moen, Phyllis: 1115, 1180
Moffitt, Chad: 8000
Moffitt, Terrie: 7025, 7115
Mogle, Jacqueline: 1175, 1480, 4050
Mogle, Jacqueline: 4310
Mogle, Jacqueline: 8000
Mogos, Mulubrhan F: 2255
Mohamed, Hodan: 9040
Mohamed, Rania E: 7170, 7220
Mohammed, Adam: 8000
Mohanty, Subhasis: 8000
Mohr, David: 2225, 3040, 4550, 9005
Mois, George: 4080, 4320, 8005
Mojtabai, Ramin: 7255
Mokhtari, Negar: 8000
Mola, Giada: 1330
Molina, Anthony: 4107, 7050, 7190, 7375
Molinari, Victor: 7390
Molinsky, Jennifer H: 7300
Moll-Jongerius, Annemarie: 4105
Molton, Ivan: 7495
Momin, Anmol: 7300
Monaghan-Geernaert, Pamela: 2290
Monane, Mark B: 7010
Mondol, Lucy: 1005
Money, Victoria L: 8005
Mongelli, Jessica: 2175, 8000
Monin, Joan K: 2095, 2135, 4490, 7210, 8000, 8005, 8010
Monroe, Todd B: 2045
Monroe-Lord, Lillie: 7050
Montalva, Nicolas: 1425
Montano, Anna-Rae: 7410
Montemuro Rode, Lauren: 7055
Montepare, Joann M: 1130, 2145, 3150
Montero Herrera, Bryan: 7435, 9130
Montero Odasso, Manuel: 7225, 7905
Montesinos, alexandra: 7315
Montgomery, Lynnette: 4525
Montgomery, Ruth R: 8000
Montgomery, Stephen: 1520
Monti, Stefano: 1520, 8015
Montoro-Rodriguez, Julian: 4085, 7230, 7305, 7470
Montoya, Ana: 7250, 8005
Montoya, Yadira: 3070
Moo, Lauren: 1305, 2085, 2365, 3005, 3115, 7230, 7280, 7295, 7310, 8005
Moon, Chooza: 1475, 4230, 7265
Moon, Heehyul E: 7160, 7350, 7415, 8000
Moon, Hyun: 7435
Moon, Jasmine: 7460
Moon, Jennifer: 2045
Moon, Saemi: 3020
Moon, Sanghee: 8010
Moone, Rajean P: 2010, 3000, 4220, 7370
Mooney, Aimee: 1015, 1165, 7415
Mooney, Graham: 2400
Mooney, Laken A: 7365, 7435
Moore, Alison A: 8015
Moore, Jeffry: 8015
Moore, Justin B: 4205
Moore, Kari A: 2400
Moore, Karlin: 4210
Moore, Laura: 1050
Moore, Michelle: 9055
Moore, Miranda: 2160, 3255
Moore, Raeanne C: 1295
Moored, Kyle D: 2400, 8000, 8005
Moorehead, Jeanmarie: 2440
Moorhead, Sue: 8010, 8015
Moorman, Sara M: 2295, 3050, 4520, 7355, 7900
Mor, Maria K: 9140
Mor, Vincent: 7540
Mora, Alexandra: 1145, 8015
Mora, Dalilah: 8015
Morag, Ido: 3200
Moraga Franco, Cristina: 4460
Morais, José: 7315
Morales, Gabriela: 7285, 7925
Morales, Santiago: 8005
Moran, Daniel S: 8005
Morgan, Amanda C: 7005
Morgan, Brianna: 2045, 2555, 4155
Morgan, Jasmine: 7025
Morgan, Jennifer Craft: 1005, 1275, 2225, 3040, 4550
Morgan, Robert O: 4475
Morgan, Sarah S: 3165
Morgan, Stephanie: 7420
Morhardt, Darby: 1015
Mori, Hiroki: 7265
Morin, Ruth: 7205
Morishita-Suzuki, Kumi: 7230
Morita, Kojiro: 9020
Moritz, Robert L: 7315
Moriyama, Yoko: 8010
Morone, Natalia: 4535
Moropoulos, Kaylie: 8005, 9120
Morris, Nichole: 8010
Morrison, Liane: 9105
Morrison, Sean: 4430, 7010
Morrison, Wanda: 7220
Morrow, Corey: 7495
Morrow, Hannah: 8005
Morrow-Howell, Nancy: 1195, 1425, 8000, 8005
Mortby, Moyra E.: 4470
Mortenson, W.Ben: 7205, 7915
Morton, Alec: 8005
Morton, Claire: 8005
Moschella, Sophia: 8015
Moseley, Sydney: 8015
Moses-Eisenstein, Michelle: 7020
Mosher, Hilary: 2085, 8005
Mosing, Miriam: 4030
Moslander, KoriAnne C: 7515
Mosley, Thomas: 1200, 7325, 7495
Mosqueda, Laura: 1215, 3300, 3350, 7230
Moss, Charlotte: 8005
Moss, Karen O: 2135, 8005, 8010
Mott, Catharine: 2475
Mould, Diane R: 8010
Moulton, Stephanie: 3285
Moura, Winny Eveny: 8000
Mowbray, Orion: 2340
Moxley, Jerad: 7540
Moy, Marilyn L: 7265
Moye, Jennifer: 2085, 7265
Moyer, Caleb R: 7200
Moyosoreoluwa, Jacobs: 2375
Mpofu, Elias: 7250, 7430, 8015
Mroczek, Daniel K: 1265, 3330, 4000, 4120
Mroz, Emily L: 1210, 1275, 2135, 3375, 8005, 8015, 9000
Mroz, Tracy M: 1270, 4450, 8000, 8010

Mu, Christina X: 8000
Muñoz, Elizabeth: 2005, 2345, 3060, 3395, 4110, 7145, 7200, 7435, 8000
Mubashshira, Tasneem: 2040, 8010
Muchow, Courtney R: 7340
Mudar, Raksha A: 1165, 4080, 8015, 9085
Mudhur, Jasmine: 7090
Mudrazija, Stipica: 2060
Mueller, Christine: 4340
Mueller, J. Tom: 7180
Mui, Ada Yuk-Sim: 9115
Mukherjee, Namrata: 7110
Mukherjee, Shalini: 4270
Mulhorn, Kristine A: 7520
Mullan, Aidan F: 9045
Mullen, Rebecca: 7290
Muller, Chandra: 2120, 7010, 7040
Muller, Emma: 4230
Mulligan, Grace: 8010
Mullins, C. Daniel: 7495, 8015
Mumma, Kara T: 1230
Munell, Alicia: 4100
Mungas, Dan: 4540
Muniz-Terrera, Graciela: 2510, 2545, 3265, 4110, 4120, 7145, 9005
Munjak-Khoury, Ceresa: 8000
Munly, Kelly: 4035
Munn, Lindsay Thompson: 7390
Munoz, Elizabeth: 1175
Munoz, Richard: 8010
Munro, Cynthia: 1510
Munshi, Nikhil: 8000
Munsterman, Ellen: 8010
Murabito, Joanne M.: 3127
Murabito, Joanne M: 8000, 8005
Murali, Komal Patel: 8010
Muramatsu, Naoko: 1160, 3355, 7015
Murayama, Hiroshi: 7345, 7375
Murdaugh, Kimberly: 3340
Murdock, Cristina: 8000
Murmann, Maya: 7370
Muroff, Jordana: 4075, 4345
Murphy, Blake: 1020
Murphy, Diane: 9065
Murphy, Erin C: 4035
Murphy, Rachel A: 4490
Murphy, Sara C: 2065, 7540
murphy, Terrence: 3220, 4480, 7500
Murray, Andrew: 1300
Murray, Briana: 1395
Murray, Camille: 8005
Murray, Catherine-Anne: 4500
Murray, Deven: 3245
Murray, Dorothea: 7135
Murray, Sarah: 1305
Murthy, Venkatesh: 2000, 2245, 7345
Musema, Jane: 3085
Musi, Nicolas: 2557, 4090
Musinguzi, Nicholas: 3020
Muskin, Ryan: 9055
Musty, Allison: 1255
Musunuri, Raaga Likhitha: 9045
Mutambudzi, Miriam: 8000
Mutz, Julian: 7090
Myagmarjav, Sugarmaa: 8015
Myatt, Glen D: 2535
Myers, Clarice: 2000
Myers, Keith: 2445
Myers, Todd: 8010
Myint, Wah Wah: 7375

**N**

Na, Annalisa: 8005
Na, Eunung: 8000
Na, Hunhui: 4420
Naas, Taqwa: 8010
Naavaal, Shillpa: 4440
Nabi, Michelle: 7410
Nabors, Emily: 4180
Nacimben, Gabriela: 7370
Nadarajah, Nandhini: 4425
Nadarajan, Gayathri Devi D/O: 2150, 3135
Nadash, Pamela: 3335, 7085, 7255, 8000
Nadig Nair, Krishnaa: 7540
Nadorff, Danielle K: 2340, 3335
Nagarajan, Niranjani: 8010
Nagel, Corey: 7165
Nagib, Ahsan: 4347
Nagy, Myra: 9105
Nagykaldi, Zsolt: 1510
Nah, Suyoung: 7230
Naher, Samsun: 2075
Naidu, Nidhi: 8010, 8015
Naik, Aanand D: 1035, 4445, 7030
Naim, Sigal: 1130, 4355
Nair, Anil: 7025, 7195
Nair, Kalyani B: 8010
Nair, Malini: 7025, 7195
Nair, Sridevi Unni: 7190
Najafi, Bijan: 7300
Najar, Mohsen: 3230
Nakagawa, Takeshi: 2120, 7365, 7380
Nakahara, Jun: 7355
Nakamura, Jeanne: 4300
Nakamura, Masahide: 7140
Nakanishi, Miharu: 7220
Nakashima, Taeko: 7330
Nakayama, Gen: 8010
Nakayama, Takeo: 2230
Nakhmani, Arie: 8000
Nalamachu, Sri: 7500
Naliyat, Lakshmi: 9130
Naliyatthaliyazchayil, Parvati: 9130
Nam, Gina E: 8000
Nam, Hee-kyoung: 7090
Nam, Sanggon: 8005, 8015
Nam, Youngwon: 7115
Namkung, Eun Ha: 1405, 7235
Nanna, Michael: 8000
Nanson, Kate: 7205
Napoleon, Betty J: 7410
Narine, Donnette: 7115
Naseem, Rahat: 3230
Nash, Paul: 1450, 2395
Nashef-Hamuda, Reem: 1125, 1240, 4205, 7410
Nathan, Rima: 4245
Navarro, Adria: 8010
Navarro-Millan, Iris: 4525
Navos, Melanie L: 8000
Nayak, Veena: 7510
Naylor, Mary: 8000
Nazario-Acevedo, Jaminette M: 7145, 7900
Ndebele, Hanani: 7010
Ndjaboue, Ruth: 4150
Nduka, Chinelo: 7245, 8005
Ndukwe, Derrick: 9130
Ndurue, Light O: 3035, 7465, 9030
Neal, Amy: 8005
Nearing, Kathryn A: 1130, 2195, 7170, 7410, 7910
Needham, Valerie: 8005
Negassa, Meti: 8015
Nehrkorn-Bailey, Abigail: 7265
Neiberg, Rebecca H: 2470
Neilands, Torsten: 7260
Neises, Kristen: 3240
Neller, Sarah A: 2135
Nelligan, Labrini: 9125
Nelson, Elizabeth: 1190
Nelson, Heather H: 7425
Nelson, Ian Matthew M: 2335, 7330, 7420
Nelson, Lindsey C: 8010
Nelson, Matt: 2335, 4160, 7250
Nemati, Donya: 2135, 4560, 9130
Nemec, Mary: 8005
Nemeth, Samuel: 1195, 2320
Nemia, Gianna: 2275
Nemmers, Natasha L: 1060, 1120, 3010, 4485, 7230, 8005
Neogi, Tuhina: 1070, 7120
Neri, Melinda: 7215, 7925, 8000
Nesbit, Tyler S: 7350
Netta Bentur, Netta Bentur: 7090
Nettles, Brenda: 7030
Neumann, Lycia: 4160
Neupert, Shevaun D: 1120, 1480, 3095, 3360, 4270
Neville, Charlotte: 4205
Nevriana, Alicia: 1425
Newell, Bradley J: 3255
Newman, Anne B: 2000, 2245, 2470, 4295, 7345, 7425, 8000, 8010
Newman, David: 2045
Newsham, Tina: 1380, 1390, 2330, 3120, 3295, 7150
Newton, Wendy: 7045
Ng, Angus Jun Jie: 2150
Ng, Boon Peng: 2435, 8000
Ng, Boon Peng: 8015
Ng, Boon Peng: 8015
Ng, Christine Tsz Ying: 7085
Ng, Clement Cheuk-Wai: 7395, 7440
Ng, Crystal: 4490
Ng, Crystal: 9085
Ng, Wee Qin: 3275
Ng, Xuan Min: 4425
Ng, Yee To (Crystal): 4195, 4360
Ngai, Joe Tsz Kin: 1385
Ngan, Kerry: 1340
Ngandu, Tiia: 1290
Ngo, Duyen: 7280
Ngo, Long H: 2505, 8000
Ngo, Victoria: 1305, 2365, 7230, 7280
Nguyen, Andrew H: 2365, 8005
Nguyen, Anh Thy: 7185
Nguyen, Ann W: 1415, 3245
Nguyen, Annie: 8015
Nguyen, Brian: 8015
Nguyen, Christopher P: 2135
Nguyen, Dinh: 1355
Nguyen, Duy: 7300
Nguyen, Hoang Van: 4347

Nguyen, Louis L: 8005, 9150
Nguyen, Minh: 2275
Nguyen, Nguyen TK: 7440
Nguyen, Tam: 3155
Nguyen, Taylor: 7435
Nguyen, Thai: 8010
Nguyen, Tra: 4535
Nguyen, Xuan-Mai T: 8010
Ngwa, Che Henry: 1495
Ngwu, Millicent O: 8015
Ni, Xuelei S: 8015
Niceforo, Michaella: 8015
Nicholls, Karen: 4500
Nichols, Emma: 1040, 2000, 3380, 4280, 4540
Nichols, Madeline: 7335
Nicholson, Jody S: 1285, 8000
Nickels, Shirley: 7225
Nickerson, Allison: 7540
Nickerson, Brett: 2220
Nicklas, Barbara J: 2470, 4205, 7165
Nicklas, Barbara: 8005
Nicklett, Emily J: 3245
Nicolay, Sarah: 2485, 9010
Nicoloro-Santabarbara, Jennifer: 7460, 8000
Nie, Hao: 7320
Nieboer, Anna P: 7260
Nielsen, Natalia: 7255
Nigro, Lisa: 8005
Nikitin, Jana: 1210, 1350, 3145, 4270
Nikpay, Sayeh: 3095
Nikzad-Terhune, Katherina A: 2330, 3150
Nilsen, Charlotta: 7375
Nimrod, Galit: 1375
Nishar, Shivani: 4010
Nishida, Yoshifumi: 7225
Nishimura, Jun: 7370
Nishino, Kenshi: 8010
Nishita, Yukiko: 7330
Nissen, Adam T: 1265
Niu, Jinbo: 8010, 8015
Nix Parker, Tamara: 4445
Nixon-Lambert, Erika: 7340
Niznik, Joshua: 1235
Nizza, Megan: 4075, 4345, 4530
Nketsiah, Ebow T: 8000
Nkimbeng, Manka: 2010, 3155, 3375
Nnadi, Laura: 2395
Noble, Liz: 4180
Noble, Molly E: 1300, 2260
Nocera, Joseph (Joe): 7225
Noel, Lailea: 4530
Noel, Sabrina E.: 8000, 8015
Nogal, Bartek: 7015
Noguchi-Watanabe, Maiko: 8000
Noh, Heeju: 7090, 7315
Noh, Hyunjin: 1365, 7310, 7500
Noh, Sung Gi: 8000
Nolan, Cindy: 7410
Nolan, Raena N: 7295
Noll, Laura: 7365
Nolte, Julia: 3275
Nomura, Ayano: 7225
Nomura, Kazuhiro: 8015
Noonan, Curtis: 9015
Noonan, Madeline: 2310
Noor, Mirza Ishrat: 8015
Noppert, Grace A: 4440, 8015
Nordyke, Alexandra: 7010
Noriega, Xavier: 2525, 7110
Noriega-Makarskyy, Daisy T: 1100
Norihiro, Nishioka: 2230
Norris, Emily: 7185
Norstrand, Lu: 7340, 7935
Northcutt, Caitlin A: 3370, 4030, 4560
Norton, Jonathan D: 7385
Nothelle, Stephanie: 8005
Nothern, Alexandra: 9080, 9090
Noun, Margaret: 1160
Noureldin, Marwa: 2455
Nouryan, Christian: 7215, 7410, 8000, 9055
Novack, Miriam A: 7435
Noval, Christian: 8005
Novisky, Meghan: 3235
Novruz, Ayla: 2400
Nowakowski, Alexandra “Xan” CH: 4220
Nowels, Molly: 4135, 7500
Nowinski, Cindy J: 7435
Nsor, Neke: 2025
Ntinda, Kayi Ntinda: 8015
Nuckols, Teryl: 8000
Numata, Hanako: 2210, 8000
Nunez, Johanna G: 3265
Nunez, Matthew: 4415
Nurse, Ashley: 7210
Nusetor, Frederick: 4460
Nutakor, Jonathan A: 7425
Nute, Andrew: 9015
Nwakasi, Candidus: 7200, 7245, 8005
Nweke, Chizobam: 7185, 8010
Nyblade, Laura: 7530
Nygaard, Marianne: 4030
Nyhuis, Casandra: 1510

O

Oakes, Connor: 7190
Oakman, Kimberly: 8010
Obando, Viviana: 7025, 7195
Obeid, Carla: 7145
Obeng, Afia: 8005
Obermiller, Corey: 7390
Obi, Perpetua C: 7300
Obioha, Wisdom C: 8015
Oblon, Nicole: 7090
O’Brien, Amanda: 7265
O’Brien, Catherine: 7440
O’Brien, Erin: 2350
O’Brien, Jennifer L: 1285
Ocoko, Faith: 7270
O’Connell, Denise: 9125
O’Connell, Megan E.: 2545, 8015
O’Connor, Maureen K: 2365, 3005, 7195, 8005
O’Connor, Melissa: 4560
O’Connor, Victoria: 7410
Odabas, Sena: 7365
Odden, Michelle: 8000
O’Dea, Mary: 8000
Odebiyi, Bolanle: 9085
Oderberg, Jane: 2065
Odo, Obinna C: 1365, 7200, 7330
Odom, Hannah: 7265
Odom, J. Nicholas: 4445, 8015
O’Donnell, Amy J: 7400
O’Donnell, Arden: 4575
O’Donnell, Mary Gemma: 2170
O’Donnell, Mary Gemma: 8005
Oduro, Elisha B: 1145, 8000, 8015
Ofili, Divine-Favour: 1080
Ofir, Ruty: 1515
Ofori, Edward: 7315
Ofrey, Ebi: 1430
O’Gara-Standiford, Shannon: 1215
Ogawa, Elisa F: 7040, 8010, 9095
Ogawa, Madoka: 7395, 7925
Ogbuniro, Joseph: 7300
Ogden, Lydia P: 1410
Oginni, John: 9025
Ogland-Hand, Suzann: 2240
Ogle, Destiny M: 2405, 4040, 4185, 4315, 4485, 7085, 7470
Ogletree, Aaron M: 1505, 4007, 7105
O’Grady, Thomas: 7330
Ogugu, Everlyne G: 2450
Ogunjesa, Babatope: 3205
Oh, Esther S: 8005
Oh, Eunna: 7150, 8005
Oh, Hamilton Se-Hwee: 1155
Oh, Haneul: 7395
Oh, Hans: 4125, 4250
Oh, Jeewon: 2405
Oh, Kyeung Mi: 7545, 7910, 8015
Oh, Oonjee: 7205, 7220, 8015
Oh, Patricia: 8010, 8015, 9115
Oh, Sungho: 9135
Oh, Yun Taek: 1115, 3175, 7070
Ohana, Irit: 8005
O’Hara, Leeanne: 4205
Ohno, Yuka: 8005
Ohwobete, Jemima: 4305
Ojaimi, Nadim: 2125
Ojeda, Lauro: 8010
Ojumah, Esther O: 8000
Okada, Akira: 7250
Okada, Hiroshi: 2230
Okada, Jamil: 8005
Okamura, Tsuyoshi: 8010
Okechi, Ikenna D: 8015
Okereke, Olivia I: 1160
Okine, Joana: 2340
Okolie, Tochukwu J: 1365, 7085, 7915, 8005
Okoli-Umeweni, Adaora: 4570
Okoye, Safiyyah M: 4210, 7360
Okun, Sarit: 2370
Oladimeji, Abolade: 8005
Olander, John: 9105
Olanrewaju, Sherif A: 8005
Olaru, Gabriel: 1240, 3270
Olayeni, Bolanle: 1505
Olds, Timothy: 3235
O’Leary, Bryant J: 7265
O’Leary, Justin M: 8005
O’Lenick, Cassandra R: 3235
Olinger, Bradley: 7480
Oliveira Bueno de Souza, Roberta: 7140
Oliven, Ron: 7060
Oliver, Brant: 1485
Oliver, Debra: 7220, 7260
Oliver, Emily: 1095
Olivero, Noah: 9065
Olivier, Michael: 3397
Olivieri-Mui, Brianne: 9080
Olsan, Tobie: 7495
Olsen, Bonnie J: 1215, 7230
Oltmanns, Thomas F: 2350, 4040, 7525, 8005

O'Mahony, Sean: 8005
O'Malley, Kelly A: 2110, 7455, 7935, 8010
O'Mara, Megan: 7145
Ommering, Belinda W.C.: 4105
O'Neil, Maya E: 8010
O'Neill, Desmond: 4115
Oner, Sudenaz Fatma: 7315, 8005
Ong, Anthony D: 1265, 4120
Ong, Chong Yau: 2150
Ong, Liane: 7185
Ong, Lydia Q: 1480
Ong, Marcus EH: 2150, 3135
Ono, Mayo: 7235
Onorato, Nicole: 7545
Onsando, Wambui Moraa: 2045, 4150
Onuh, Samuel C: 1365
Onyedibe, Maria Chidi C.: 2220
Onyema, Ezenwa: 8000
Opazo Breton, Magdalena: 1245
Opitz, Alexander: 7025
Oppong, Beatrice: 4325
Oppong, Kwaku Duah: 4445, 8015
Ordoñez, Maria J: 7285, 7925
O'Reilly, Maria: 7155
O'Reilly-Jacob, Monica: 7185
Orejuela, Melanie: 7350
Orewa, Gregory N: 1150, 1430, 3365
Orkaby, Ariela: 8000, 8010, 9155
Orkibi, Hod: 1330, 4205, 4235
Ornelas Van Horne, Yoshira: 2005
Ornstein, Katherine: 1025, 1270, 2190, 2375, 2480, 4070, 4385, 4450, 7390, 7545, 8005, 8010
O'Rourke, Hannah: 3040
Orozco, Tatiana: 7350
Orr, Miranda: 2557
Orsega-Smith, Elizabeth: 7130
Orsulic-Jeras, Silvia: 7185, 8000, 8015, 9010
Ortega, Calyope: 7470
Ortega, Felix: 7345
Ortega, Julian: 8015
Ortega-Ruiz, Frida: 8000
Ortiz, Gabi Celia: 7110
Ortiz Villaman, Jezebel: 8010
Ortiz-Ortiz, Karen: 2555
Orwig, Denise: 2395, 4070, 7545, 8015
Orwoll, Eric: 1460
Ory, Marcia: 7475, 9025
Osal, Ryan: 7225
Osareh, Helia F: 7495
Oseroff, Benjamin H: 2480
Osevala, Nicole M: 4480
Osime, Esther: 7085
Osokpo, Onome H: 4070, 7430
Ospina, Natalia: 4020
Ost-Mor, Sharon: 1090, 1365, 3040, 7450
Østbye, Truls: 7520
Ostrem, Nicholas: 8000
O'Sullivan, Kelly S: 7175, 7920
O'Sullivan, Roger: 1185
Oswald, Austin: 2525, 7110, 7535
Ota, Tomoyuki: 7465
Otgon, Saranchuluun: 8015
Otsuka, Rei: 7330
Otto, Amy: 4045
Otu, Hasan H: 2505
Ou, Alison Y. T.: 1225
Ouellet, Gregory M: 7410, 7920
Ouellet, Jennifer A: 7410, 7920, 8005
Ouellet, Marie-Christine: 8005
Ouimet, Ethan: 7265, 8000
Oullet, Jennifer: 7030
OUYANG, FUXI: 3215, 8015
Ouyang, Meishuo: 1235, 4010
Ouyang, Rui: 8005
Overbeck, Kevin: 4570, 7340, 7410
Overstreet, Abigail: 8015
Owe, Brenda: 7310
Owen, Donna C: 7125
Owens, Otis L: 7545
Owens, Victoria: 1370
Owings, Michael J: 7425
Owoaje, Eme T: 4565
Owusu Ansah, Osborn: 7415
Oyebode, Jan R: 2335
Oyedeji, Charity I: 7170, 7220
Ozawa, Tabasa: 4435
Ozor, Tochukwu: 7330

**P**

Pace, Thomas: 4580
Pacheco, Kelly: 1055
Pacheco, Laura: 7065
Pacheco Busquets, Mariana: 7495
Pack, Lindsay: 1300
Packer-Porat, Tammi: 3040
Padeiro, Miguel: 7370
Padilla, Paola: 3030
Padilla-Frausto, D. Imelda: 8000
Pagan, Michelangelo J: 4200
Pagotto, ´Valeria: 8000
Painter, Mairead: 4405
Pajewski, Nicholas M: 1110, 1255
Pakpahan, Martina: 8000
Paladino, Joanna: 7185, 7205
Palakshappa, Deepak: 7390
Palaniappan, Sucheendra K: 8005
Palavicini, Juan P: 8015
Paleski, Sophia L: 7365, 7400
palgi, Yuval: 1125, 2105, 3200, 3360, 4270, 9095
Palladino, Dianne: 4020
Pallas-Brink, Jara: 9145
Palleschi, Mary Kate: 8010
Pallis, Maryssa: 4565
Palm, Matthew: 4245
Palmer, Allyson K: 8005, 9045
Palmer, Jennifer: 4210
Palta, Priya: 1200, 2535, 3185, 4375, 7495, 8005, 8010
Pan, Honghui: 1290
pan, Jinsong: 8010
Pan, Kuan-Yu: 1425
Pan, Min-Hsiung: 8015
Pan, Qianhui: 7015, 7095
Pan, Wei: 3135
Pan, Xi: 7040
Pandare, Siddhi V: 2350
Pandey, Ashwini: 9080
Pang, Marco YC: 9020
Pang, Yijiang: 3090
Pang, Yuk C: 8010
Pankow, James S: 1200, 8015
Pantzer, Christina: 7210
Panzer, Victoria: 9020
Paoletti-Hatcher, Jensine: 8000
Paolino, Valerie: 7385
Papadopoulos, Airia: 3155
Papaefthymiou, Konstantinos: 7465
Pappa, Sara T: 4190
Pappadis, Monique R: 7230, 7405, 9025
Paramesh, Colleen: 8000
Parchment, Amelia: 9085
Paredes, Aasha: 8005
Paredes, Lucero G: 1375
Parent-Lamarche, Annick: 7325
Parihar, Madhur: 1355
Parikh, Romil R: 1010, 1430, 2160, 2260, 3020, 3075, 3190, 7300, 7530
Parisi, Jeanine M: 8005
Park, Chan Mi: 9000, 9080, 9085, 9105, 9135, 9145
Park, Chang Gi: 7325, 7330, 7525
Park, Chan-Young: 8000
Park, Chorong: 7015
Park, Chorong: 8000
Park, Daniel: 7085
Park, Do-Hyung: 4180
Park, Eun Ju: 7145, 7300, 7375
Park, Eunyoung: 7220, 7425, 7430
Park, Geunhye: 7255
Park, Hyejin: 7265
Park, Hyeyoung K: 3040, 7220, 7910
Park, Hyung In: 7380
Park, Isac: 4200
Park, Jae: 3030
Park, Jeongmin: 7365
Park, Jihoon: 1385
Park, Ji-Hyuk: 7335
Park, Jina: 1515, 8005
Park, Jiwon: 3025, 3075, 4420
Park, Joonhyeog: 7295, 8010, 8015, 9030
Park, Joon-Hyuk: 7545
Park, Joon-Hyuk: 8010
Park, Jung In: 7210
Park, Junmin: 4210
Park, Juyoung: 1020, 4045
Park, Juyoung: 4465
Park, Juyoung: 7010
Park, Juyoung: 7210, 7355
Park, Juyoung: 7500
Park, Kyoung Shin: 7435, 8005, 8015, 9025, 9130
Park, Kyoungjae: 3020
Park, Min Kyung: 7225, 7300, 7325, 7375, 7395, 7520
Park, Moonkyoung: 7325, 7430
Park, Nan Sook: 3325, 7210, 7305
Park, Pil: 8005
Park, SaeMi: 4560
Park, Seeun: 7495
Park, Seokyung: 7360
Park, Seyeon: 7430

Park, Shin Young: 7370
Park, Sikyeong: 7520
Park, Sojung: 7275, 7290, 7470, 7495, 7925, 8005, 8010, 8015
Park, Soobin: 7400, 7495
Park, Soobin: 8005, 8010
Park, Soohyun: 8005
Park, Soojin: 3285
Park, Sooyoung: 7400, 8015
Park, Sophie: 8000
Park, Subin: 7375
Park, Sunbok: 8010
Park, Sung S: 7395
Park, Sunyoung: 7540
Park, Susan: 7540
Park, Thomas: 7315
Parker, Andrew M: 1330
Parker, Daniel C: 7010
Parker, Kelly: 1175, 7325
Parker, Kimberly: 8010
Parker, Lauren J: 8015
Parker, Tassy: 2170
Parkman, Suzanne: 4105
Parks, Anna: 7185
Parks, Christine G: 1500
Parmanto, Bambang: 2375
Parmar, Naaz: 7225, 7905
Parnia, Sam: 7330
Paro, Ashley: 8005
Parrish, Elise: 2375
Parry, Jayne: 7310
Parson, Britney: 2110
Parsons, Helen M: 3095
Parsons, Michael: 8015
Parvatini, Supriya: 7410
Pasarariu, Alexandru L: 2045
Pasatiempo, Ana Maria G: 8005
Pascual-Leone, Alvaro: 7025, 7435
Passey, Richard: 7010, 7910
Passmore, Susan: 7270
Pate, Melannie: 7005
Patel, Arianna: 8005
Patel, Asiya: 8010
Patel, Hanna: 8015
Patel, Himalaya: 7415
Patel, Jaahnvi: 7225
Patel, Jahanvi: 7050
Patel, Kamal: 7410
Patel, Kushang V: 4535
Patel, Nidhi: 1505
Patel, Prisha: 4415
Patel, Priti: 2075
Patel, Ratna: 4470
Patel, Reeya: 4250
Patel, Snehal: 7305
Patel, Suniska: 7340
Patel, Tejal: 7230
Paterson, Thomas: 7415
Pathak, Anangsha: 4580
Patil, Amruta: 4210
Patil, Shivrajo: 7010
Patrick, Emily: 7470
Patrick, Megan E: 1105, 8010
Patriquin, Michelle: 8005
Patskanick, Taylor: 1405
Patten, Amy B: 8010
Patterson, Charity: 8000
Patterson, Joanne G: 1000
Patterson, Kelsey: 8015
Patterson, Logan: 3295
Patterson, Mary: 8015
Patterson, Meagan M: 3150
Patterson, Sarah E: 2375, 3050, 4360, 4485
Patyal, Pankaj: 7010, 7190
Pau, Massimiliano: 7265
Paudel, Anju: 3220, 4460
Paul, Constanca: 7135
Paul, Shreya: 8000
Paul, Todd: 1050
Paulsen, Michelle R: 7410
Paulson, Daniel: 7375
Paulson, Sally: 7120
Pauly, Theresa: 1480, 3305, 4515
Paun, Olimpia: 2300, 8000
Pavlik, Jan: 7295
Pavon, Juliessa M: 3065
Payne, Jodi: 7455
Payne, Laura: 8005
Paz, Miguel: 8010, 8015
Peacock, Brandi: 7270
Pearce, Mason: 8005
Pearl Naim, Sigal: 7175
Pearlman, Jon: 4305, 7515
Pearlstein, Jennifer G: 2155
Pearman, Ann: 1120
Pearson, Megan: 1470
Pecic, Stevan: 7415
Peckham, Allie: 1440, 2030
Pedersen, Jacob K: 7345
Pederson, Maja: 9015
Pederson, Tabitha: 3245
Pedro, Sofia: 7120
Peduzzi, Nora: 7250
Peek, Gina: 4210
Peeler, David: 1230
Peeples, Amanda D: 8015
Peeples, Landon B: 7305, 7400, 7415, 7900
Peereboom, Danielle: 2520
Peerman, Carey: 1135
Pei, Heming: 8000, 8010, 9150
Pei, Xiangyu (Annie): 8015
Pei, Yalian: 8005
Pei, Yaolin: 1400, 2475, 3015, 3290, 4065, 4170, 8005
Pei, Zhongfei: 8005
Peiffer, Elizabeth: 7225
Pekaar, Keri A: 1480
Peleg, Shira: 2390
Pellegrini, Christine: 7435
Pelletier, Stephen: 8005
Peltz, Carrie: 4335
Peltzer, Jill: 8010
Pendergast, Jacquelyn: 4090
Pendrey, Anna: 8010, 8015
Peng, Changmin: 3335, 7355
Peng, Huamao: 7340
Peng, Rong: 7540
Peng, Siyun: 2360
Peng, Wenting: 7100, 8015
Peng, Xinyuan: 7375
Penhale, Bridget L: 3350
Peniche, Jorge: 9075
Pennavaria, Adam: 8005, 8015
Pennella, Jenna: 4095
Pepin, Marc J: 3065
Pepin, Renee: 2395, 4020, 7300
Perales, Jenna: 8000
Perales-Puchalt, Jaime: 4230
Peralta, Laura S: 8005
Perepezko, Kate M: 2445, 3135, 4160
Perera, Dinithi: 1290
Perera, Dinithi: 8010
Perera, Subashan: 8005
Perez, Fiorella: 7455
Perez, Richard: 7370
Perez Montes, Viviana: 4008
Perezalonso Espinosa, Jeronimo: 1155
Perissinotto, Carla A: 3050, 4020, 4070
Perkhounkova, Yelena: 1150, 1515, 2225, 3220, 4015, 7250
Perkins, Molly M: 1275, 2160, 4135, 8010
Perkins, Rebekah: 4555, 7405
Perls, Thomas: 1520, 4440, 7310, 8015, 9155
Peron, Emily P: 1260
Perone, Angela K: 1380, 1450, 2070, 4165, 7235, 8005
Perre, Taylor: 7220
Perrin, Nancy: 1345
Perron, Rebecca: 2530
Perry, Andrea: 7305
Perry, Audrey J: 2455
Perry, Brea L: 1095, 2360
Perry, Dafna: 1520
Perry, Morgan: 7485
Perry, Tam E: 2215, 2515, 3305
Perryman, Erica M: 7005
Persad, Alyssa: 7320
Persky, Victoria: 2040
Persson, Annelie M: 7430
Perweiler, Elyse: 7410
Perzynski, Adam: 8005
Pesavento, Matthew: 3285
Pescador Jimenez, Marcia I: 2400, 9040
Peskoe, Sarah: 8000, 8015
Peterfreund, Ilana: 1485
Peterman, Leah M: 8010
Peters, Case: 4205, 7390
Peters-Beumer, Lisa: 4355, 7400, 8005
Peterson, Colleen M: 7155
Peterson, Elizabeth W: 7225
Peterson, Lindsay J: 7175, 7220, 7290, 7340, 7370, 7420, 7935, 8000
Peterson, Rachel: 9015
Peterson, Tina: 3055
Petralia, Kelley: 8005, 8010
Petroni, Alessia G: 7365, 7400
Petrovsky, Darina: 7415
Petrowitz, Bradley: 7310
Pettee Gabriel, Kelley: 1110, 7325
Pettis, Jennifer: 1395, 9010, 9125
Pfeil, Carmen: 8005
Pflieger, Lance: 7090, 7190, 7315
Pfund, Gabrielle N: 1045
Pfund, Gabrielle: 8000
Pham, Julie Q: 8010
Pham, Nghia: 8010
Pham, Tony V: 2310
Phan, Matthew: 8000, 8015
Phaowiriya, Hathaichanok: 7245
Phelan, Elizabeth A: 4015, 8010, 8015
Phelps, Brenna: 7225
Phibbs, Ciaran: 2225, 4550
Philip, Celene: 7290
Philips, Kira C: 2320
Phillips, Carolyn: 8010
Phillips, Christy: 1285
Phillips, Jakob: 7375
Phillips, Joseph R: 7095
Phillips, Meredith: 2200
Phillips, Olive: 7410
Phinney, Alison: 2420
Phipps, Stephen M: 7190
Phongtankuel, Veerawat: 1170, 8000
Phuah, Chia-Ling: 1355
Pi Ching, Weng: 7410, 7935

Pianosi, Birgit: 7075
Piaseu, Noppawan: 7130, 7430
Piazolo, Felix: 3215
Piazza, Jennifer R: 2095, 2280, 4120, 7310, 7455, 8015
Picella, David V: 8005, 8010, 8015
Pickell, Delaney: 8010
Pickering, Carolyn E: 1085, 4085, 7015, 7385, 8005
Pickett, Andrew C: 3375, 7305
Piechota, Amanda: 4475, 8005
Piedad, Mary: 7090
Piedra, Lissette M: 4085, 8010, 9005
Pierce, Audrey: 4227, 7315
Pierce, Matthew J: 1175, 7380
Piippo, Elizabeth: 7400
Pike, James Russell: 1200, 2535, 3185, 4065, 4450
Pike, James: 8005
Pillemer, Karl: 1215, 2325, 2385, 3145, 7350, 7455, 7915
Pimentel, Camilla B: 3115
Pincus, Harold: 7020
Pinheiro-Chagas, Pedro: 8015
Pinto, Jayant: 1500
Pinyan, Elizabeth C: 8005
Piol, Silvia: 1330, 4235
Pirela, Rosa V: 8015
Pisano, Michele: 9055
Pitcher, Katherine: 8000, 9135
Pittard, Colleen: 4020
Pitts, Jessica: 7225
Pituch, Keenan A: 1110, 2030
Pitzer, Kyle: 7260
Pius, Ruth: 8000
Piyasinchai, Bell: 7035
Plank, Emma: 8000
Plankey, Michael: 2140
Plankey, Michael: 2395
Plasencia, Rosa: 3190
Plassman, Brenda L: 1500, 2475
Platts, Loretta G: 3310
Pless Kaiser, Anica: 2085
Pliskin, Neil H: 3355
Plourde, Kendra: 8010
Plow, Matthew: 7430
Plumb, Jennifer: 4525
Plys, Evan: 2310, 4135, 4590, 7500, 9105
Poblete, Ronald: 9035
Pock, Eva Maria Lissa: 1310
Poe, Abigail: 4140
Poganik, Jesse R: 9150
Poghosyan, Lusine: 7125, 7185
Pogorzelska-Maziarz, Monika: 3040
Pohnert, Anne M: 1485, 7410
Polansky, Helena: 7395
Polenick, Courtney A: 8005
Polk, Rebecca: 7460, 8000
Pollack, Craig: 4210, 7420
Pollack, Kathryn I: 3375
Pollak, Chava: 8015
Polsky, Daniel: 1360, 2520
Polychronopoulou, Efstathia: 7535
Pomeroy, Mary Lou: 2480
Pomeroy, Mary Louise: 2190, 2375, 2480
Pomier Maren, Yisel: 7075
Pompeu, Jose Eduardo: 7140
Poncé, Díana: 1420, 8000
Ponce-Ponte, Oscar J.: 8010
Pond, Brittney L: 1420, 7215, 7925
Pontiff, Mattie: 7140
Ponto, Kevin: 2445
Poole, Patricia: 2475
Poole, Tamara S: 7170, 7220
Poon, Leonard W: 1065
Poot, Charlotte C: 1010
Pope, Natalie E: 2170, 3000, 4130
Pope, Natalie S: 7475
Pope, Taylor A: 2295, 8000
Pope, Zachary C: 1510
Popejoy, Lori: 8010, 9140
Porat-Dahlerbruch, Josh: 7125
Porcu, Eleonora: 8010
Portacolone, Elena: 8005, 9000
Porter, Gillian: 1175
Porter, Hunter L: 8005
Porter, Kristen E: 4220
Porter, Laura: 2110
Porter, Laura S: 7205
Portz, Jennifer D: 4090, 9090
Possin, Katherine: 2245, 9000
Pot, Anna: 4040, 8000
Potter, Claire: 4205
Potter, Sarah Nelson: 8010
Poulin, Valérie: 2235, 8005
Pound, Abby: 8010
Powell, Danielle S: 2520, 8005
Powell, Darcey N: 3150
Powell, Lenny: 4570, 7340
Powell, Victoria D: 4450
Powell, Wizdom: 3375, 4485
Power, Shannon R: 9065
Powers, Becky B: 2195
Powers, James: 3140, 4115
Powers, Sara: 2285, 7010, 7185, 7915, 8000, 9010
Powers, Timothy: 4175
Pradeep Kumar, Danya: 7090
Pradhan, Rohit: 1150, 3365
Pramana, Gede: 7495
Prasad, Anyah: 1095, 2390, 2525
Praska, Nicole A: 8005
Prefontaine, Nicholas M: 7400
Press, Valerie G: 7430
Presser, Kelly: 1300
Presti, Peter: 1230
Pretzer-Aboff, Ingrid: 4200
Preuss, Benedikt: 1310
Previtali, Federica: 3145
Prevratil, Michael J: 4420
Price, Ashley: 9150
Price, Elvin: 1260, 7340
Price, Nathan: 7015, 7190
Prieto, Lucas R: 7065, 7900
Prieto, Sarah: 7290, 7305, 8010
Prina, Matthew D: 2335, 3145
Principi, Andrea: 7070
Pritchard, Kevin T: 1340, 4090, 4525, 7495
Pritchard, Kevin T: 9135
Prizment, Anna: 7345, 7425, 8015
Procter-Gray, Elizabeth: 8000
Promislow, Daniel: 3267, 7045, 7315
Prophater, Lorna: 9005
Proulx, Christine: 4485
Province, Michael A: 8005
Provis, Javiera: 9125
Prusin, Todd: 7440
Prussman, Kyla: 7515
Prusynski, Rachel A: 1270, 8000, 8010
Psyllos, Marco: 3125
Puca, Amy: 1160, 1225
Puccio, Ava M.: 7000
Pudder, Anna: 7455
Puga, Frank: 2030, 3370, 4140, 7245, 8000, 8010, 8015
Pugh, Kendra: 2365
Pullen, Carly E: 7005
Pulpitel, Tamara: 4347
Puma, Litzy: 8010
Punnam, Saahithi: 8005
Punsalan, Ali: 8015
Punshon, Sarah: 3005, 4350
Puri, Jhanvi: 8015
Purol, Mariah: 3270
Purvis, Martha: 1260
Puspitasari, Vivien: 8000
Putnam, Michelle: 1410, 2430, 3117, 3320
Puts, Martine: 7090
Puttre, Hannah: 9150
Putzu, Valeria: 7265
Puxty, John: 1280
Pynoos, Jon: 7300

Q

Qan'ir, Yousef: 8000
Qazi, Saadia: 8000
Qi, Xiang: 1400, 2475, 3015, 3110, 4055, 4170, 4505, 7485, 8000, 8005, 8010
Qian, James: 4030
Qian, Jing: 7370
Qian, Yiqing: 1465, 2190, 2375
Qian, Yuting: 3200, 7250, 8015, 9010, 9140
Qiao, Yujia: 8000
Qin, Chengfan: 4055
Qin, Lu: 1375, 1510, 3010
Qin, Weidi: 1415, 3245, 4520
Qin, Ying: 2305
Qin, Yue: 2120
Qiu, Chen: 2545, 4065
Qiu, Peihua: 8010
Qiu, Shirley S: 7535
Qiu, Weiyu: 7410
Qiu, Xiao: 8015
Qiu, Xichenhui: 4505
Qiu, Yiming: 4385
Qu, Shan: 7170, 7210, 7305, 7375
Qu, Zhijing: 2260
Quach, Emma: 1305, 7295
Quach, Ha-Linh: 1205
Quach, Lien: 7245
Quan, Jianchao: 1030
Quan, Stuart F: 7390
Quashie, Nekehia T: 3195, 8015
Query, Sharon: 2290
Quesnel-Vallée, Amélie: 1080
Quezada, Alejandro: 7020
Quiñones, Ana: 4470
Quiñones-Cordero, Maria M: 3375, 7230, 7305
Quiben, Myla: 7140
Quigley, Denise: 3010
Quigley, Molly: 7435

Quiles, Noberto: 8010
Quiles, Rosalphie: 4090
Quillen, Crystal A: 3150
Quinn, Joseph: 2255
Quinn, Joseph F: 3310
Quinn, Martha: 7390
Quinn, Sandra: 8000
Quinto, Marcelo: 3165
Quiroz, Yakeel T: 8000
Qureshi, Nabeel S: 8010, 9020

**R**

Rabalais, Jennifer: 1335
Rabello, Gilciney A: 7370
Rabey, Robin: 7455
Rabin, Laura A: 4310
Rabinowitz, Jill: 4375
Rabinowitz, Seth: 8015
Racoosin Kornmeier, Jillian: 2495, 4020
Radcliffe, Payton: 7350
Rademacher, Emma: 3305
Radpour, Siavash: 3310, 3385
Radspinner, Rachel: 7415
Raftery, Daniel: 7045
Ragot, Samuel: 7065
Ragsdale, Luna: 1085
Ragsdale, Vickie S: 7125
Rahat, Nazm: 7025
Rahemi, Zahra: 7205, 7220
Rahman, Momotazur: 1270, 2465, 3280, 7540
Raimondi, Alessandra: 3070
Raina, Parminder: 2545
Rainier, Riva: 7050
Rainville, G.: 7025
Raisa, Tasnim: 7015, 7385
Raj, Minakshi: 1165, 4565
Rajagopal, Krishna: 8000
Rajashekara Swamy, Meghana: 7530
Rajendran, Priyadharsini: 7510
Rajesh, Bhavana: 8005
Rajeshkumar, Nethra: 8005
Raji, Mukaila: 1070, 7305, 7535, 8000, 8005, 8015
Rakes, Sarah: 8000
Ram, NIlam: 1350, 4515, 8000
Ramadoss, Kamala: 7340
Ramamurthi, Hema: 7545
Raman, Rema: 3375
Ramaswamy, Gomathi: 7510
Ramirez, Paulino: 8010
Ramirez Gomez, Liliana: 3025, 7185, 8000, 8015
Ramirez-Garcia, Daniel: 1155
Ramirez-Perez, Nadeshka J: 8000
Rammal, Hassan: 1520
Ramos, Katherine: 7205
Ramos Espinoza, Hillary M: 9080
Ramprasad, Nidhi: 4135
Ramsburg, Hanna: 8000
Ramsey, Jennifer: 7230, 7305
Ramsey-Tobienne, Alexis E: 4355
Ramulu, Pradeep: 7530
Rana, Sangeeta: 7410
Rand, Debbie: 7090
Randall, George Kevin: 7400
Randall, Lindsey: 8015
Randolph, Madisin: 7270
Rangan, Apoorva: 8005, 8015
Rani, Neha: 1390
Rants, Jillian: 7350
Rao, Praveen: 9070
Rao, Smitha: 3125, 3305
Rappaport, Noa: 7090, 7190, 7315
Rapport, Lisa J: 7310
Rasmussen, Blake B: 8015
Rataj, Alison C: 2150, 4220, 7275
Ratangee, Brina: 7055
Rathbun, Alan M: 2000, 4330, 7315, 8010
Rathje, Elizabeth: 9095
Ratnayake, Maggie: 8015
Rattner, Edward: 7455
Rauer, Amy: 4490, 7355
Ravel, Jacques: 8010, 9150
Ravindran, Rohan: 8000
Rawal, Kadambari: 7485
Rawool, Mrinal: 8005
Rayan-Gharra, Nosaiba: 8005
Rayani, Asma: 7430, 7495
Rayner, Daniel: 7300
Raza, Sakeena: 7255
Razavian, Narges: 7185
Razjouyan, Javad: 8005
Reale, Kevin: 8015
Reardon, Eilis: 8005
Reaves, Alvin L: 3165
Reavis, Kelly: 8005
Rebadulla, Hannah M: 4350
Reblin, Maija: 2030, 7395
Rebok, George: 4030, 8000, 8005, 9040
Recker, Amy: 4475
Reckrey, Jennifer: 1270, 2480, 3315
Recto, Pamela L: 7255
Reczek, Rin: 1405
Reddy, Ann: 2055, 4475, 8015
Reder, Nora: 8005
Redfern, Mark: 8010
Redman, Leanne: 3267
Redmond, Leslie: 1120
Redondo-Sáenz, Diego: 7020
Reece, Heather: 8015
Reed, Alexandra M: 7185
Reed, Eric: 1520
Reed, Grace: 7220
Reed, Nicholas: 1075, 1200, 2000, 2535, 4065, 7330, 8005
Reeder, Blaine: 7085, 7910
Rees, Catherine: 7410
Reese, Jordan: 7475
Reeve, Charlie: 7230
Reeves, Alexis: 1110, 4460
Regal, Rachel: 1260, 2110, 7095
Regan, Claire: 8005
Regos-Stewart, Dalia: 7225, 7905
Reichman, Rocky: 4535
Reid, M. Carrington: 1055, 4010, 4135, 4525, 4535, 7350, 7500, 7540, 8000
Reider, Lisa: 4450
Reif, John S: 8015
Reiff, Jenni: 4450
Reiffer, Norina: 7250
Reilly, Elizabeth: 8000
Reina, Anita M: 8015
Reinecke, Robert: 4070
Reinke, Lynn: 7405
Reinschmidt, Kerstin M: 7415
Reisberg, Barry: 7010
Rejeski, W. Jack Jack: 2470
Rekant, Julie: 7140, 7905
Remenik-Zarauz, Vania: 8000
Remer, Sarah: 7500
Remillard, Elena T: 1230, 2430, 9110
Ren, Lily Haopu: 7205
Ren, Lily Haopu: 7235
Ren, Lily Haopu: 7915
Ren, Rong: 7470
Renn, Brenna N: 7375, 8005
Renold, Lowell: 8015
Renter, Heather M: 8005
Rentería, Miguel: 3380, 8010, 4540
Renzi-Hammond, Lisa M: 8015
Resendes, Natasha: 3065
Resnick, Barbara: 1260, 1435, 2180, 2350, 3160, 3220, 4460, 7290, 7495, 8005
Resnick, Susan: 4495
Reuben, David B: 2285, 3290
Reuveni, Yehudit: 8000
Revell, Andrew J: 7400
Reyes, Giselle: 2030
Reyes, Jimmy A: 2300
Reyes, Laurent: 1405
Reyes, Maria F: 7285, 7925
Reyes, Mariana: 7225, 7905
Reyes-Vega, Andrea: 8000
Reynolds, Addam S: 4455
Reynolds, Chandra A: 1175, 4030, 4110
Reynolds, Courtney: 1215, 7405, 8015
Reynolds, Lindsay M: 7480
Reynolds, Sarah R: 8005
Reza, Abigail: 2540
Rhee, Greg: 7410
Rhee, Min-Kyoung: 8015
Rhee, Soo: 4110
Rhodes, Alec: 3285
Rhodes, Annie: 1005, 7300, 8015
Rhodes, Ramona: 7220
Rhodes, Shanae: 7255, 9130
Ribeiro, Oscar: 2120, 7370, 7380
Rice, Laura A: 1230, 2430, 7225, 7410
Rich, Adea: 2000, 7435, 8000
Rich, Josiah: 1090
Rich, Scott: 3170
Richards, Emily: 8000
Richards, Kathy C: 4475
Richards, Marcus: 1105
Richards, Nicole E: 1175, 3185
Richards Adams, Ingrid K.: 2135
Richardson, Arlan: 4347
Richardson, James K: 7200
Richman, Katherine M: 4545
Richmond-Rakerd, Leah: 7255
Rickenbach, Elizabeth: 7355, 7515
Ricketts, I'deyah: 4285, 8010
Ridder, Becky: 7500
Riddick, Milan: 7470
Riddle, Nicole: 3397
Riegel, Barbara: 1015
Riffin, Catherine: 4135, 7500
Rijnhart, Judith J M: 7325, 7425
Riklon, Sheldon: 7005
Riley, Jessica: 3115

Riley, Thomas: 7335
Riley, Thomas: 7545
Rilling, James K: 1275
Ring, Lia: 1365, 7410
Ringel, Joanna: 1170
Riordan, Lindsay: 7470
Rios Casas, Francisco: 1105
Riosmena, Fernando: 4470
Rios-Monterrosa, Jose: 8005
Ripperger, Hayley S: 4585
Rippon, Isla: 4360, 8010
Risacher, Shannon L: 3380
Riser, Tiffany J: 1465, 2480
Risher, Jordan: 7455
Ristl, Christina: 1350
Ritchey, Katherine C: 1470
Ritchey, Katherine C: 4445
Ritchey, Katherine C: 7410
Ritchie, Christine S: 1160, 1270, 1400, 2190, 2285, 2310, 2480, 3375, 7185, 7205, 8000, 9120, 9150
Ritter, Ashley Z: 2465, 4010
Ritz, Thomas: 8010
Rivadeneira, Natalie A: 7145
Rivas, Daniel: 1520
Rivera-Hernandez, Maricruz: 3370, 7485
riveros, Angie F: 7285, 7925
Rizer, Sandra: 1100, 7095, 7415
Rizk, Christine: 8005
Rizk, Ryan C: 4560
Rizvi, Anita: 4190
Rizzo, Matthew: 7465
Rizzo, Samantha: 8000
RIzzo, Victoria M: 7485, 9030
Roan, Carol: 4585
Robbins, Rebecca: 7390
Robelly, Blade: 8000
Roberson, Donna: 7010, 8000
Roberson, Lauren: 8010
Robert, Benoît: 7370
Robert, Stephanie: 4520
Roberto, Karen A: 4520, 7230
Roberts, Adam: 7020
Roberts, Amy R: 2325, 7335
Roberts, Emily: 2290
Roberts, Eric T: 1250
Roberts, J. Scott: 4360, 4390, 7250, 9070
Roberts, Jimmie E: 7345, 7510
Roberts, Mitchell: 7320
Roberts, Pamela: 9020
Roberts, Patrick: 4007
Roberts, Shelby: 9030
Roberts, TJ: 1325
Roberts, Tonya: 7370, 8005
Robertson, Mariah: 3240
Robinet, Lauren J: 8015
Robinette, Jennifer W: 7315, 7900
Robins, Diana L: 4525
Robins, Richard: 4110, 8015
Robinson, Anthony: 7310
Robinson, D'Artagnan M: 7225, 7905
Robinson, Kat: 4355
Robinson, Marc: 1090
Robinson, Max: 7090, 7315
Robinson, Millicent: 4250
Robinson, Stephanie A: 7265
Robinson, Tyler D: 8005
Robinson-Lane, Sheria G: 1510, 2375
Robison, Anna: 8000
Robison, Julie: 1315, 1385, 2185, 4135, 4160, 7305, 7360, 9090
Robison, Kristel: 8005
Robson, Mei W: 8000
Roche-Dean, Maria: 1150, 1515, 2225, 2375, 7250
Rockenstyre, Alex: 8015
Rockwood, Kenneth: 7090
Rodakowski, Juleen: 1510, 7490
Rodgers, Mattea: 8000
Rodin, Rebecca: 7010
Rodney, Tamar: 4265
Rodríguez-Díaz, Melina: 7040
Rodrigo, Hansapani: 7190
Rodriguez, Amy: 8005
Rodriguez, Bianca: 8005
Rodriguez, Jamila: 8015
Rodriguez, Miriam Jocelyn: 2555, 3010, 3085, 7010
Rodriguez, Paola: 8010, 8015
Rodriguez, Racheal: 7125
Rodriguez Colmenares, Nury Alejandra: 4140
Rodriguez Cruz, Nachalie: 7300
Rodriguez-Cruz, Nachalie: 7130, 7185, 7225, 7905
Rodriguez-Morales, Grisel: 9140
Roe, Laura: 1135
Roecke, Christina: 4270
Rogers, Anita: 7470
Rogers, Geoff: 1085
Rogers, Wendy A: 1165, 1320, 1505, 2430, 4080, 7380, 7410, 8005, 8015, 9065, 9085, 9110
Rogus-Pulia, Nicole: 7415
Roh, Daniel: 4275
Roh, Soojin: 8015
Roh, Soonhee: 7160, 7375, 7415
Rohlfsen, Leah: 4510
Rohrs, Jennifer: 7415
Rojas, Ashley: 7345
Rojas, Elizabeth: 9040
Rojas, Olivia: 7380
Rojas-Alvarez, Alfonso: 1250, 2060
Rojas-Martinez, Julio: 9000
Rojas-Saunero, L Paloma: 4280
Rollandi, Isabel: 8010, 8015
Roller, Juliana: 8005
Rollings, Kimberly: 1375
Rom, Yifat: 3200
Roma, Corinne: 2055, 4010, 4475
Romañach-Álvarez, Lourdes S: 2345, 7145
Roman, Anna: 3295
Roman, Cristina AF: 8015
Roman Jones, Joanne: 4480
Roman-Woo, Carly: 1240
Romero, Jonathan: 8015
Romero, Sergio: 2445
Romero Aliberti, Marlon Juliano: 4425, 8005
Rominger, Marielle: 8005
Rompilla, David B: 2095, 7265
Rondina, Mathew: 8000
Rong, Jian: 8000
Ronis-Tobin, Victor: 8015
Rook, Erin: 1000
Rooks, Ronica N: 8005
Roque, Nelson A: 2400, 4310, 7380
Rorai, Vanessa O: 2515
Rosano, Caterina: 7425
Rosario, Andre A: 3205
Rose, Dorian: 8010
Rose, Grace: 7265
Rose, Johnie: 7220
Rose, Karen: 4170, 8005
Roseen, Eric: 4535
Rosen, Allyson: 7135
Rosen, Amy: 4090
Rosen, Howie: 7135
Rosen, Jennifer: 4160
Rosen, Tony: 1085, 2325, 3300, 8005, 9140
Rosenberg, Mara: 2190
Rosenberg, Walter: 9145
Rosenbloom, Michael: 8000
Rosenfeld, Anne: 7340, 7920
Rosen-Lang, Yael: 7000
Rosenstein, Benjamin: 3240
Rosenthal, Ronnie: 7500
Rosko, Ashley: 2220
Ross, Clifford: 1140, 7055
Ross, Jaime M: 1520
Ross, Jenifer: 4095
Ross, Lesley A: 1285, 2280, 3060, 4310, 7325
Ross, Sarah: 2065
Rossano, Samantha: 7415
Rosselli, Monica: 2045
Rosser, Mary: 7095
Rossi, Martina: 8000
Rossi, Michelle I: 3115, 7410
Rossi, Raiza: 8000
Rossler, Aleatha: 7330
Rossler, Aleatha M: 8005
Rosso, Andrea L: 2200, 9110
Rostamvand, Maryam: 7245
Rote, Sunshine: 2060, 3265, 7350, 7475, 8000
Roth, Nicole: 8015, 9155
Rothenberg, Craig: 8015, 9140
Rothermund, Klaus: 1210, 2370, 4270, 7390, 8015, 9070
Rothgang, Heinz: 1310
Rothlein, David: 8005
Rottenberg, Yakir: 8015
Rotter, Jerome: 8005, 9155
Roumell, Elizabeth: 8010
Rouse, Lorna: 3080
Routh, Brianna: 7265
Rovianne, Rovianne: 4015
Rovine, Michael J.: 7305
Rowan, Noell L: 8000, 8015
Rowe, Cathy: 3000
Rowe, Jeannine M: 7485
Roy, Arkaprava: 7350
Roy, Brita: 8000
Roy, Deepa: 7190
Roy, Indrakshi: 2260, 8005, 8010, 8015
Roy, Upal: 7190
Royse, Sarah K: 2200
Rozani, Violetta: 8005
Rozenberg, Aliza: 1155
Ruan, Jia Yin: 8005
Ruan, Lei: 7410
Ruan, Yunchen: 2275, 7470
Rubi, Lisbeth: 2220
Rubin, Leah: 1155, 7245
Rubio, Olivia C: 4445
Rubtsova, Anna A: 2395, 9080
Rudolph, James L: 9080, 9090
Rueppel, Elizabeth: 7290
Ruggiano, Nicole: 8010
Ruggles, Sydney C: 7300
Ruiz, Diana: 8010

Ruiz, Eloy F.: 8010
Ruiz, Jessica J: 7005
Rule, Andrew: 7325
Rule, Payton D: 2155
Runkle, Tage: 8005
Runyan, Amanda: 7010
Rupprecht, Fiona S: 1210
Rush, Jonathan: 1175, 2095, 2280, 4120
Rush, Kathy: 7485
Rusmevichientong, Pimbucha: 8015
Russell, Daniel: 4195
Russell, Marcia M: 7500
Russell, Wynfred: 2010, 3155
Rustici, Craig M: 3040
Ruth, Terrance: 7290
Rutt, Joshua L: 4460
Rutter, Lauren A: 1095, 9030
Ryan, Alice: 7130, 7265, 8010
Ryan, Candace EM: 8010
Ryan, Gery W: 3290
Ryan, Kathleen: 9075
Ryan, Lindsay: 1105
Ryan, Susan: 4480, 8010
Ryan Baumann, Mary: 1200, 8015
Rydberg, Michael: 1235
Ryder, Priscilla T: 8010
Ryff, Carol D: 4000, 7055
Ryou, Yeon Ji: 1065, 4195
Ryskina, Kira L: 7270
Ryu, Byeongju: 1195, 7290
Ryu, HyeRim: 7330, 7390
Ryu, Jungsu: 7265
Ryu, Seulgi: 7305, 8005
Ryvicker, Miriam: 7220, 8005
Rzepa, Natalia: 9085
Rzhetsky, Andrey: 4200

S
Sa, Sharon: 8005
Saad, Odelyah: 8005
Saalim, Khalida: 7530
Saar-Ashkenazy, Rotem: 2105
Saavedra, Joey M: 3045
Sabatini, Serena: 1210, 2390
Sabbatini, Amber K: 8000
Sabol, Valerie: 4075
Saccà, Valeria: 7435
Sacco, Lawrence B: 7375
Sacco, Paul: 1145, 7220
Sacks, Frank: 4570
Saczynski, Jane: 2505, 7410, 7485
Sadarangani, Tina: 2440, 4145, 8005
Sadeq, Nasreen: 7340, 7935
Sadhwani, Anjali: 8010
Sadler, Richard C: 2400
Sadow, Sage: 2030
Sadowska, Karolina: 7500
Sadowski, Martin: 7010
Saenz, Joseph L: 1495, 4290, 9075
Safford, Monika M: 1170, 7260
Sagehorn, Mallory A P: 7465
Sager, Jeannette: 8015
Sahai, Arjun/Aarti: 8010
Sahli, MIchelle: 7295
Sahoo, Nirakar: 7190
Sahoo, Sudikshya: 8010
Sailor, David J: 3235
Saini, Geetanjali: 3095
Saint Hamilton, Sarah: 7490
Sainz, Mayra: 1250, 3375, 9025
Saito, Taeko: 8010
Saito, Yasuhiko: 7235, 7390
Sakakibara, Sakura: 4370
Sakata, Nobuo: 8000, 8010
Sakaue, Hiroshi: 8015
Sakharov, Maya: 8015
Saksena, Mallika: 7295
Salaam, Donna: 7185, 8015
Salas, Sarah S: 3265
Salat, David: 7325
Salat, David: 8005
Salazar, Delany T: 4280
Saldana, Santiago: 8000
Salecha, Juhi Digvijay: 1110, 7495
Sales, Wesley: 8005
Saliba, Debra: 1270, 1435
Saligan, Leorey: 8015
Salinas, Joel: 8010
Salinas, Sophia E: 8000
Salisbury, Amy L: 1260
Salisu, Margaret: 8000
Salive, Marcel: 1455
Sallings, Abigail: 8015
Salomon, Amit: 7200
Salter, Allison: 7305
Samaniego, Catherine H: 4225
Samant, Akanksha: 8015
Sambataro, Franco E: 9140
Sames, Carol: 4525
Samper-Ternent, Rafael: 4565, 8000, 8005
Sample, Lola: 3085, 7415
Sampson, Laura: 7450
Samuel, Laura J: 1465, 3345, 4125
Şan, Helin Sera: 8005
San Roman, Irene: 1305
Sanchez, Emilia: 8010
Sanchez, Jessica: 7330
Sanchez, Victoria A: 4065
Sanchez, Victoria: 7335
Sanchez Concepcion, Laura: 7350
Sanchez-Reilly, Sandra: 8000, 8010
Sanchez-Reilly, Sandra: 8010
Sanderlin, Ashley: 8005
Sanders, Alexandra: 7360
Sanders, Alisha: 7360
Sanders, Camilla: 9035
Sanders, Edie C: 3025, 3215, 4420
Sanders, Lisbeth: 8000
Sanders, Margaret: 4600, 7225, 7340, 7920
Sanders, Natalie: 7030
Sanders, Tammy: 8010
Sanders, Wade M: 7345
Sandiford, Dione: 3205
Sandler, Dale: 1500
Sandoval, Moises: 9125
Sandridge, Desirae R: 7200
Sands, Laura P: 7095, 7170, 8000, 8015
Sanford, Jon A: 1230, 1320, 2430
Sang, Dongxia: 2325
Sang, Elaine H: 9135
Sang, Wei: 7430
Sangal, Rohit: 7485
Sangalang, Cindy: 7055, 7900
Sangdahl, Caitlin: 1135
Sanjanwala, Harshin: 7325
Sankar, Sudha: 8010
Sanner, Haley: 1000, 9130
Sano, Emily: 8010
Sano, Mary: 9080
Santamaria-Ulloa, Carolina: 4150, 7090
Santana, Layla Katharine: 7040, 7420
Santana, Paula: 7370
Santiñaque, Florencia: 9005
Santilli, Josie: 7105, 7215
Santomauro, Damian: 7185
Santora, Kenneth: 3033, 4008
Santos, Alexis: 4290, 8000
Santos, Giselle L A B: 7090
Santos, Suzanne T: 7370
Santostefano, Christopher M: 8000
Saper, Robert: 4535
Sapkota, Hardik: 1500
Sapkota, Krishna Prasad: 4465
Sapkota, Sabitri: 8015
Sapoznick, Ariella: 8005
Sappington, Erica: 7320
Sapra, Mamta: 7230
Saptono, Andi: 2375
SAQUING-NWODO, SHALLEE: 1050
Sarabia, Josie: 3305
Saraiya, Manali: 4135, 7500
Sarasty, Oscar: 7130
Saravanan, Anitha: 1505
Sardina, Angie L: 3060
Sargent, Lana: 1260, 7340
Sarig, Jonathan: 3040
Saripalli, Sree N: 8005
Sarkar, Antara: 7340
Sarkar, Nilanjan: 7545
Sarter, Katharina: 1005
Saruhanlioglu, Zeynep N: 1480
Sasscer, Cathy: 9100
Sasser, Janet: 1130
Satake, Anna: 7085, 7910
Sathyan, Sanish: 4495, 8015
Sato, Ashida: 1360
Saunders, Pamela A: 2070
Savage, Cary: 7325
Savard, Grace M: 2330, 7300, 7465
Savela, Roosa-Maria: 3030
Savla, Jyoti: 7230, 8010
Savoye, Louise: 4135
Saxena, Vivek: 8005
Sayer, James: 4435
Scaccabarrozzi, Luis: 2395
Scales, Kezia: 1005, 2225, 7075
Scanteianu, Adriana: 7535
Scerpella, Danny L: 2520
Schütz, Philipp: 8015
Schüz, Benjamin: 3035
Schaefer, Adam M: 8015
Schaefer, Sydney Y: 7185, 7315, 7325
Schafer, Donna E: 7075
Schafer, Markus H: 1105
Scheel, Alpha T: 8015
Scheffer, Julian A: 4085, 7290
Scheffers-Barnhoorn, Maaike N.: 4105
Schein, Richard M: 7495
Scheipers, Merle: 9070
Schell, Brent R: 7265
Schellhorn, Abby: 7220
Schenker, Rony: 1515
Schepens, Stacey: 9090
Scher, Clara J: 2185, 4145, 4530
Scherf, Erick: 3245
Scheunemann, Leslie Page: 2445
Schickedanz, Heather: 4160
Schiller, Gabrielle: 9145
Schilling, Birgit: 4473

Schilling, Oliver K: 8000, 8015
Schiloski, Kylie A: 7115
Schiloski, Kylie: 8010
Schilstra, Christina: 8010
Schiltz, Nicholas: 1485, 7220, 7410
Schindel Martin, Lori: 7075
Schlag, Karen E: 7230, 7405, 9025
Schlenk, Elizabeth A: 8010
Schlomann, Anna: 3215, 7295
Schluter, Emma: 4440
Schmeler, Mark R: 7495
Schmid, Arlene: 9090
Schmidt, Athina L: 7245, 7915
Schmidt, Cindy: 9000
Schmitt, Eva M: 2505
Schneeweiss, Sebastian: 7510
Schneider, AnnMarie: 3215
Schneider, Stefan: 2120
Schneider, Sue: 4075
Schnock, Kumiko: 8015
Schnyer, Rosa N: 7230, 7385, 7910
Schoberer, Daniela: 1310
Schoen, Gena: 0
Schoenberg, Nancy E: 4045
Schoenborn, Nancy: 1360, 2520, 7010, 7910
Schoenfeld, Elinor R: 4200
Schoenfield, Elinor: 3155, 4200
Schoenthaler, Antoinette: 1505, 7355
Schoepke, Abbie: 8005
Scholes, Shaun: 1040, 2250
Scholler, Emily: 9010
Scholz, Markus: 8015
Schork, Nicholas J: 4495
Schrack, Jennifer A: 1075, 2100, 2480, 3105, 3260, 4375, 4450, 7330, 9025
Schreiner, Pamela: 8005
Schrimpf, M. Brooke: 7125
Schroepfer, Tracy A: 2265
Schuler, Emily: 7055
Schulman, Daniel: 8000
Schulman-Green, Dena: 3015, 3290
Schultheis, Maria: 7290
Schultz, Amy: 7260
Schultz, Douglas: 7325
Schultz, Emma: 7470
Schultz, Sydney M: 2100
Schulz, Cynthia L: 8005
Schumacher, John G: 4007
Schurch, Thomas: 1390
Schuster, Amy M: 3340
Schuster, Bernard G: 8010
Schutte, Debra: 8010
Schuttner, Linnaea: 4445
Schutz, Jennifer: 7455
Schwarcz, Lily: 7545
Schwartz, Andrea Wershof: 2195, 8005
Schwartz, Andrea Wershof: 7090
Schwartz, Beth: 8010
Schwartz, Heather M: 7305
Schweikhard, April: 2445
Schwendeman, Hannah: 1190
Schwieters, Katelyn: 8010
Schwoyer-Morgan, Tracie: 7500
Sciamanna, Christopher N: 8005
Scott, Don: 8015
Scott, Kim: 9000
Scott, Matthew: 8010
Scott, Paul W: 7500
Scott, Stacey B: 1175, 4473, 7255
Scott, Stephen: 8005
Scott, Winifred: 9095
Seabury, Joshua: 4555
Seago, Elayna: 7095, 7200, 8005
Seah, Betsy: 4425
Seale Reiff, Jenni: 1360
Seaman, Aaron: 1305, 8000
Sebastiani, Paola: 1520, 4440, 7310, 8005, 8015, 9155
Sedaghat, Sanaz: 8015
Seegmiller, Jesse: 7010
Sefcik, Justine S: 7295, 7360, 7415
Segal, Daniel L: 7505
Segal-Gidan, Freddi: 4160, 7135
Segel-Karpas, Dikla: 1125, 3040, 4185
Segev-Rosenberg, Galia: 1090
Segrè, Daniel: 8015, 9155
Segundo, Joahana: 1440
Sehgal, Raghav: 9060, 9150
Seidel, Elizabeth: 2015
Seidenfeld, Justine: 2475, 4565
Seino, Satoshi: 7265
Sekhon, Vishaldeep: 8000, 8005, 8015
Seldeen, Kenneth L: 8010
Seliger, Stephen L.: 7345
Seligman, Benjamin: 8000
Sellers, Ashley J: 2255
Selovin, Fabio: 8015
Seltzer, Judith A: 3050
Selvin, Elizabeth: 2100
Semba, Richard: 7315
Semedo, Ana: 9100
Semelka, Charles T: 1455
Semple, Benjamin J: 9095
Semple, Paige: 1385
Senderling, Ben: 8005
Senior, Joshua: 8005
Senyk, Oksana: 3360, 4270
Seo, Hyein: 8010
Seo, Jeongone: 7295, 7535
Seo, Jin Young: 7255
Seo, Jisu: 7150, 8005
Seong, Minju: 7355, 7470
Seow, Lek Wei: 7190
Sergile, Jennifer: 7245, 7915
Serier, Kelsey: 2085
Serina, Peter: 2055, 8015
Serpas, Dylan G: 7145
Service, Kathryn: 1395
Seshadri, Gokul: 2345, 7345
Seshadri, Sandhya: 3165
Seshadri, Sudha: 3127, 8015
Sethi, Reena: 9120
Setiawan, I Made Agus: 2375
Setoguchi, Soko: 8010
Severance, Jennifer: 2065, 7540
Severine, Janet: 8010
Sevi, Barış: 3265, 4110
Sevilla, Javier: 8010, 8015
Seward, Morgan: 4590
Sexton, Michelle: 8015
Shaaban, C. Elizabeth: 2200, 3380
Shabbir, Alibhai: 7090
Shachaf, Hadas: 7060
Shade, Marcia: 8005
Shadid, Jamileh: 7185
Shadmi, Efrat: 7000, 7145
Shaffer, Tasha: 7265
Shafiekhani, Parsa: 7295
Shah, Amil: 3185
Shah, Amrik: 8010
Shah, Avani A: 8010
Shah, Raj C: 8005
Shah, Ravi: 2245, 7345
Shah, Sachin: 4280, 7185
Shah, Samantha: 2555
Shah, Yogi: 7185
Shaham, Galia: 7200
SHAHAR, Danit R: 7135
Shahar, Orit: 1515
Shahriar, Kamal Md: 7480
Shaikh, Afroze N: 7375
Shakir, Madiha: 7010
Shakya, Iju: 7510
Shaleha, Rinanda RA: 7380
Shalev, Daniel: 4135, 4225, 7020, 7500
Shalev, Idan: 1375
Shamsi, Masooma: 1505
Shan, Liang: 4140
Shan, Shengshuai: 8015
Shan, Yong: 1070, 7305, 8005
Shanahan, Angela: 7225
Shanbhag, Prajakta: 7290
Shanbhogue, Nikhil M: 2350, 7525
Shane, Jacob: 1175
Shang, Lili: 7515
Shannon, Faith: 1175, 8000
Shao, Gracie Wan Ting: 7535
Shao, Hsuanlei: 7465
Shao, Lu: 1245, 4055
Shao, Sijing: 4120
Shao, Yijun: 8010, 9020
Shao, Yongzhao: 7010
Shapira, Bettina: 7175
Sharafieh, Roshanak: 9090
Sharafkhaneh, Amir: 8005
Sharafkhaneh, Sanam: 8005
Sharbaugh, Michael B: 1385
Shardell, Michelle D: 2000, 3260, 4330, 7315, 7345, 7495, 8010, 9075, 9150
Sharifi, Hasti: 4590
Sharit, Joseph: 4080
Sharma, Anajali: 2395
Sharma, Nicole: 7545
Sharma, Shakshi: 7005
Sharma, Tanya: 7150
Sharma, Uttam: 1500
Sharp, Meaghan: 1015
Sharrett, A. Richey: 8010
Shaughnessy, Marianne: 1470, 2195
Shavit, Yochai Z: 3275, 8005
Shaw, Albert C: 8000
Shaw, Benjamin A: 7375, 7395
Shaw, Clarissa: 1150, 1515, 2225, 3220, 7000, 7250
Shaw, Susan: 7110
Shawley, Audrey: 7320, 7510
Shayya, Ashley: 7220, 7330
Shdo, Suzanne M: 1275
Shea, Kyla: 4295, 4440, 4460
Shealy, Carlisle: 7170
Shean, John: 7010
Sheehan, Cailee: 3150
Sheehan, Orla C: 2480, 7385
Sheets, Nathan: 8000
Shefer-Hilel, Galia: 7135
Sheftel, Mara G: 1405, 4290, 8015
Shehu, Erblin: 7070, 7300, 7930

Sheikh Ibrahim, Hanan: 9015
Sheikhnur, Sahra: 8015
Shelar, Ashish: 8000
Shelawala, Nicole: 4465
Sheldon, Kira G: 7220, 8000
Sheldon, Marisa: 3305, 9110
Shellman, Juliette: 7410, 7525
Shelton, Amy: 7000
Shen, Huei-wern: 7005, 7100, 7375
Shen, Jie: 1245, 7090
Shen, Karen: 3315
Shen, Lingyi: 9055
Shen, Megan: 8000
Shen, Yuqi: 7535
Shenoy, Rhea: 8010
Shepherd, Alexis: 7305
Shepherd-Banigan, Megan: 4565, 7350, 7930
Sheps, Janna: 1485
Sherk, Catherine: 8000
Sherman, Carey Wexler: 2010
Sherman-Wilkins, Kyler J: 2345
Sherry, Laura: 4065
Sherwood, Bryan: 8005
Shetty, Nihar N: 1470
Shewokis, Patricia A: 4525
Shi, Bertram E: 8005
Shi, Cheng: 8015
Shi, Chunhu: 9085
Shi, Hui: 9030
Shi, Huidong: 7245
Shi, Jingwen: 7320
Shi, Keyi: 8005
Shi, Sandra: 9000, 9080, 9135
SHI, Xiaoming: 8005
Shi, Xiaotian: 7315
Shi, Yifan: 8000, 8010
Shiba, Koichiro: 1195
Shields, Kerry: 8000
Shieu, Bianca: 2255, 8000, 8005
Shiferaw, Tsion: 8015
Shih, Ludy C: 1510
Shih, Regina A: 2160, 2375, 4160
Shih, Yao-Chi: 7250
Shillingsburg, Laura: 2340
Shim, Minji: 7330
Shimabukuro, Michio: 8015
Shimamoto, Tomonari: 2230
Shimizu, Natsumi: 8010
Shimokata, Hiroshi: 7330
Shin, Dong-Soo: 8005
Shin, hyeri: 9065
Shin, Hyeyeon: 8015, 9030
Shin, Jiyoung: 2210
Shin, Juh: 7245, 8010, 8015
Shin, Jung-hye: 2445
Shin, Oejin: 8005, 8010
Shin, So Young: 7370
Shin, Soo Gyung: 8010
Shin, Sueun: 8005
Shin, Sunui: 7425
Shin, Yeonsoo: 7090, 7165
Shin, Yoonkyung W: 1415
Shinbeckler, Rachael: 8015
Shinozaki, Gen: 8005
Shiovitz-Ezra, Sharon: 4315
Shippee, Tetyana P: 1010, 1430, 2010, 2160, 2260, 3020, 3075, 3155, 3190, 7270, 7300, 7530
Shiraishi, Natsue: 7380
Shireman, Theresa: 8015
Shirk, Steve: 7230
Shirk, Steven D: 1305, 2365, 7280, 7295, 7310
Shirley, Kate: 8010
Shirouchi, Mana: 7440
Shiu, Cheng-Shi: 1490, 7245
Shivanand, Ishan: 7340
Shivers, Stephani: 1160, 1225
Shmidt, Anastasiia J: 8010
Shmuels, Diego I: 8000, 8015
Shobugawa, Yugo: 7345
Shochat, Tamar: 4335, 7255
Shomee, Homaira Huda: 4590
Shorer, Eran: 1155
Short, Meghan I: 8015, 9155
Shovali, Tamar: 2330, 4355
Shrestha, Aman: 4465, 7345
Shrestha, Jene: 8015
Shrestha, Priyanka: 7060
Shrestha, Sabita: 8005
Shrestha, Srijana: 8015
Shrestha, Srishti: 2535, 7325
Shrikanth, Shalini: 1505
Shrira, Amit: 3360, 4270, 8005, 8015
Shrivastava, Jyotsna: 7190
Shrivastava, Mansi: 7480
Shroff, Jay: 7485, 7930
Shuber, Florene: 7340, 7920
Shu-Fen, Wung: 8005
Shugaev, Inna: 1090, 7450
Shukla, Jayanti: 7495
Shukla, Rohit: 7190
Shulyaev, Ksenya: 7000, 7450
Shuman, Valerie: 8005
Shvedova, Maria: 4275
Si, Wanwan: 1090
Siamdoust, Shahrzad: 2475, 4145
Sibai, Abla Mehio: 1495
Sicbaldi, Marcello: 9020
Sicinski, Kamil: 4585
Siconolfi, Daniel: 4160
Sicotte, Nancy: 9020
Siddiqui, Aziza: 7160
Siddiqui, Rouida: 7440
Sideman, Alissa: 7135
Siebecker, Megan: 3080
Sieber, Frederick: 2510
Siedlecki, Karen: 3200, 7040
Siefkas, Anna: 9155
Siegel, Bryn: 7435
Siegfried, Alexa L: 4435
Siegler, Eugenia L: 1490
Siepker, Kathy L: 7390
Siepker, Kathy: 8015
Sifnugel, Natalia: 7090, 7485, 8015
Silberbogen, Amy: 7265
Silbert, Lisa C: 1185, 2360
Silvani, Alessandro: 9020
Silveira, Erika Aparecida: 8000
Silveira, Maria: 4450
Silver, Michelle: 8015
Silver, Roxane Cohen: 7050
Silverberg, Michael: 7110
Silverblatt, Dana: 2110
Silverman, Sydney: 8015
Silverstein, Merril: 1225, 2405, 4040, 4315, 7085, 7375, 7470
Silverstein, Nina M: 1130, 2145, 3080, 3170, 4555, 7170, 7305
Sim, Jin-Ah: 8005
Sim, Xueling: 8005
Simard, Pascale: 8005
Simbrig, Ines: 3215
Siminerio, Linda M: 8015
Simon, Lena: 3060
Simonds, Wendy: 2225
Simonian, Melissa: 2055, 4475
Simonini, Sean: 8000
Simonsick, Eleanor: 2000, 2100, 2245, 4008, 7345
Simpson, Darcia: 7340, 7920
Simpson, Deborah: 7455
Simpson, Elizabeth: 1150, 3335, 7250, 7255
Simpson, Gaynell M: 3055
Simpson, Kit: 9035
Simpson, Michelle: 4205
Simpson, Stephen: 4347
Sims, Kendra D: 4540, 7260
Sin, Mo-Kyung: 2460, 3065, 9020
Sin, Nancy L: 1480, 8005
Sin, Wilfred Wing Fung: 7085
Sindi, Shireen: 7375
Singer, Daniel E: 7510
Singer, Dianne C: 4390, 7250, 9070
Singer, Jonathan: 4590
Singer, Kate: 2335, 3135, 4160, 7330, 7540
Singer, Neta: 4205
Singh, Ganeev: 7510
Singh, Nandini: 4020
Singh, Navrag: 2435
Singh, Portia: 4305
Singh, Ramkrishna Kumar: 8015, 9015
Singh, Roshni: 8010
Singh, Sangeeta: 8015
Singh, Shifali: 1295
Singh, Swastik: 1520, 7480
Singh, Yashaswini: 3280
Singh Wadhwa, Satpal: 2555
Singleton, Mekiayla (Meki): 2140, 7220
Şinikçi, Ceren: 8005
Sinkewicz, Marilyn: 1370
Sinvani, Liron: 2175
Siren, Anu: 2350
Sirey, Jo Anne: 2220, 8010, 8015
Siriprakorn, J. Peter: 8000
Sirois, Lynette: 8015
Sisco, Shannon M: 4570
Sison, Cristina: 8000
Sista, Anushka: 4210
Siu, Albert: 8000
Sixsmith, Andrew: 1325
Sixsmith, Judith A: 1135, 1325, 8015
Sjöberg, Maja: 4010
Skelton, Dawn: 9085
Skinner, Caleb: 7355
Skinstad, Anne Helene: 4350
Skipper, Antonius D: 2225, 4550
Skoblow, Hanamori F: 4485, 7085, 8000
Skrajner, Michael J: 8005, 9010
Skripka, Lindsey: 8005
Slachevsky, Andrea: 8015
Slade, Eric: 8000
Slade, Martin R: 2370
Slater, Craig: 4345
Slattum, Patricia: 1260, 2110
Slaven, James E: 8010
Slejko, Julia F: 8015
Sliwinski, Martin J: 1175, 1295, 1445, 4050, 4215
Sloan, Caroline E: 2075
Sloan, Richard P: 8005
Sloane, Philip: 7225

Slutsky-Ganesh, Alexis B: 8005, 9025
Small, Brent J: 8000, 8010
Small, Sarah: 2190
Smallwood, Stacy: 4240
Smerker, Nick: 4035
Smichenko, Juliana: 4335, 7000, 7060, 7255
Smies, Chad W: 7190, 8010
Smiley, Adrianne: 7280
Smith, Alex: 7140
Smith, Alexander k: 2190, 7010
Smith, Amanda: 8010
Smith, Anna P: 7405
Smith, Ashleigh: 3235
Smith, Ashley: 4475, 7105
Smith, Benton: 8005
Smith, Bethany: 7475
Smith, Brian N: 2085
Smith, Camille E: 7405
Smith, Eleanor: 7405
Smith, Emily L: 1120
Smith, Isaiah: 8000
Smith, Jacqui: 2050, 3175, 7305
Smith, Jamie M: 1270, 7485, 7930
Smith, Jason: 7330
Smith, Jason: 8010
Smith, Jeffrey S: 7190
Smith, Jennifer L: 1490
Smith, Jennifer L: 7110
Smith, Jennifer L: 7295
Smith, Jennifer: 7315
Smith, Jennifer L: 7380
Smith, Jennifer: 7900
Smith, Jennifer L: 8005
Smith, JoAnn: 2515
Smith, Joyce: 7495
Smith, Kasey N: 9065
Smith, Kimberley J: 4360
Smith, LaTonya: 7255, 7395
Smith, Lenora W: 8000
Smith, Lindsey: 3280, 4400, 8005, 8015
Smith, Matthew Lee: 1185, 2065, 4180, 7085, 7375, 8000, 8005
Smith, Mindy A: 7485
Smith, Rebecca: 7420
Smith, Sharla: 8010
Smith, Sherri L: 8005
Smith, Tamara: 9115
Smith, Terry E: 2170
Smith, Thomas J: 4030
Smith, Valerie: 8000
Smith, Valerie: 8005
Smith, Veronica: 9020
Smith, Wanda: 1015
Smith-Ray, Renae L: 1490
Smyles, Hanna: 8000
Snead, Caleb A: 8015
Sneed, Rodlescia S: 2390, 8000
Snitz, Beth: 1255, 8015
Snow, A. Lynn: 1060, 1300, 4475, 7105, 7310
Snyder, Alyssa: 9080
Snyder, Claire: 7545
Sobhie, Maria: 8010
Soble, Jason R: 3355
Socci, Marco: 7070
Sodders, Stephanie: 7410
Sodhi, Jasleen: 8000
Soh, Yenee: 1225
Sohmer, Dana: 2205
Sohn, Joanna: 8005
Sohn, Sunghwan: 9045
Soiron, Séverine: 7250
Sojib, Noushad: 4005
Sol, Ketlyne: 7465, 8005, 8010
Soliman, Germine: 4135, 8010
Solis, Guillermina R: 8010
Solomon, Adam: 8010
Solomon, Alina: 1290, 3030
Solomon, Philip: 7410, 9055
Solorzano-Salazar, Dustin M.: 8010
Solway, Erica: 4360, 4390, 7250, 9070
Somayaji, Darryl: 2255
Somerville, Ceara: 2185
Sompanich, Nussaba: 7375
Som-Pimpong, Hadassah: 8015
Son, Bo Ran: 3235
Son, Jiwon: 8015
Son, Surim: 7225, 7905
Song, Andrew J: 9070
Song, Eunah: 7525
Song, Jaehyuk: 8000
Song, Jieun: 7055
Song, Jiyoun: 8005, 8010
Song, Jun-Ah: 7525
Song, Jungwoo: 4420
Song, Kyungeun: 7395
Song, Lixin: 8000, 8005
Song, Mi-Kyung: 4135, 7350
Song, Minju: 8015
Song, Qian: 3320, 7170, 7210, 7300, 7305, 7375, 8015
Song, Rhayun: 7150, 8005
Song, Shixing: 7145
Song, Si Young: 1095
Song, Sunmi: 9060
Song, Weijing: 8000
Song, Woojin: 1160, 3355, 7015
Song, Xu: 7225
Song, Yajun: 8000
Song, Yeonsu: 1295
Song, Yuting: 4065
Song, Zachary: 7220
Song, Zeyuan: 8005
Soni, Albin: 7440
Sonnega, Amanda: 9040
Sorapuru, Candice: 8005
Sörensen, Silvia: 1290, 4175, 7145, 7380, 7925
Soriano, Christina: 8000
Sorkin, Clara: 7350
Sorkin, John: 7130
Soske, Jon D: 4010
Sosnoff, Jacob J: 4060, 7225
Sosnowski, David W: 7040
Sotres-Alvarez, Daniela: 7535
Sou, Ka Lon: 3090, 3215
Soulie, Brianna: 4245
Soundararajan, Srinath: 8010
Sousa, Liliana: 7370
Sousa, Susana: 7135
Southerland, Jodi L: 7085
Souza, Anita M: 8010, 8015
Sowan, Azizeh: 1135
Spadola, Christine: 7390
Spangler, Derek P: 8010
Spangler, Hillary B: 1220, 1235
Spartano, Nicole L: 8000
Spaulding, Erin M: 4385, 7545
Speckmann, Elisa-Marie: 7225
Spector-Mersel, Gabriela: 4315, 7450
Spees, Colleen: 2220
Spencer, Chelsea M: 7150
Sperber, Nina: 4430
Spetz, Joanne: 2260, 3315, 4260, 8010
Spira, Adam P: 1510, 2035, 3235, 4375, 7040, 8015
Spiro, Avron: 2085, 3330
Spiteri, Raymond J.: 7205
Splaine, Michael J: 8005, 8010
Split, Molly: 7290
Spooner, Kiara: 3180
Spradley, Laura: 7015, 7920
Sprecher, Anne: 9030
Springer, Shmuel: 1220, 2245
Springstein, Tabea: 1045
Sprong, Matthew: 8005
Spychalla, A.J.: 7025
Squires, PhD, RN, FAAN, Allison P.: 1485
Srivastava, Shobhit: 7040
St. Louis, Renee M: 3170, 4435, 7155
Stadler, Susi: 2515
Staehli, Alexandra: 7250
Staiger, Douglas: 8000
Staker, Erin: 4190
Staley, Mark: 8010
Stam, Elisabeth J: 1355, 7275
Standiford Reyes, Laurel: 7330
Stanfield, Jack E: 8000
Stanford, Ty: 3235
Stangl, Morgan: 7260, 7290, 7925
Stanley, Jennifer T: 7325
Stanley, Padraic: 9145
Stanmore, Emma: 9085
Stansbury, Kim: 7290
Stark, Susan: 1320, 1410, 2430
Starwalt, Susan: 3170
Staton, Ashley: 1260
Staton, Elizabeth W: 7140
Statz, Tamara: 7155
Staudacher, Sandra: 7250
Stavig, Mariana: 2555, 3010, 7010
Stavrinos, Despina L: 4570
Stawnychy, Michael A: 8000
Stawnychy, Michael: 9135
Stawski, Robert S: 1175, 2280, 7115, 7335
Stayer, Sarah: 7305
Steagall, Jill: 7410, 7910
Stearns, Melanie A: 4230
Stechuchak, Karen M: 1470
Steege, Linsey M: 4425, 7495
Steel, Ryan: 1190
Steele, Joel: 7335, 7375, 7415
Steele, Victoria: 7340
Stefano, Madeline: 8005
Stefanucci, Jeanine: 8000
Steffen, Ann M: 2240
Steffens, David C: 1385, 8005
Steinman, Bernard Alex: 4210, 7070, 7300
Steinman, Kenneth J: 3300
Steinman, Michael: 7255, 8010
Steinmann, Gijs: 3035, 7370
Steinmetz, Jaimie: 7185
Stelmach, Rachel D: 7530
Stenlund, Säde: 8000
Stensland, Meredith L: 7500
Stephan, Abigail: 1285, 1390, 3060, 7325, 8015
Stephan, Blossom: 1210
Stephan, Yannick: 1120

Stephen, Kritchevsky: 4525
Stephens, Andrew: 7415
Stephens, Caroline E: 1025
Stephens, Jacquelyn: 7265, 7380
Stephenson, Jane M: 8000
Steptoe, Andrew: 4095, 4120
Sterling, Madeline: 1170, 7440
Sternberg, Kady F: 9045
Sternberg, Shelley A.: 8015
Sterner, Elizabeth A: 1505
Sterns, Anthony: 8015
Sterns, Harvey: 7215
Stessman, Jochanan: 1155
Stessman-Lande, Irit: 1155
Stetten, Nichole A: 1465, 2055
Stevens, Alan B: 1035, 1225, 7475
Stevens-Lapsley, Jennifer E: 1435, 7140
Stevenson, David: 9090
Steward, Andrew: 8000
Stewart, Anne: 8010
Stewart, Brittany: 8010
Stewart, Wynnett C: 7245
Stickel, Ariana: 7535, 8005, 8010, 8015
Stigar, Lorraine: 4240
Still, Carolyn Harmon: 8010
Still, Michael: 4090
Stockdill, Macy: 8015
Stockton, Melissa A: 7530
Stoddard, Gregory J: 2190
Stodola, Allyson R: 4400
Stoeckle, Rebecca J: 2285
Stokes, Andrew: 4280
Stokes, Jeffrey: 1355, 1415, 2345, 3335, 4360, 7050, 7200, 7275, 7335
Stokes, Sandy Chen: 8005
Stokey, Max: 3305
Stolle-Wahl, Claudia: 1310
Stolterman Bergqvist, Erik: 4150, 8015
Stolzmann, Kelly: 4090
Stone, Elise: 1325, 8005
Stone, John: 2470
Stone, Katie L: 4385, 8000, 8005
Stone, Patricia: 7305
Stone, Robyn I: 4405, 4545, 7075, 7280, 7300, 7360, 8005, 9000
Stone, Samantha: 0
Stone, Sasheen T.: 7375, 7415
Stone-Bury, Lisa E: 7505
Stoop, Annerieke: 1010
Storms, Aaron D: 7220
Storrs, Cal: 8015
Stoumbos, Julia A: 3000
Stout, Michael: 4347
Stovrag, Selma: 1300
Stowe, Cynthia L: 2470
Strand, Bjorn Heine: 7415
Strand, Jennifer: 4010, 7255
Strangmann, Iris: 3380
Stratton, Lauren: 2030, 4160
Strauss, John: 2120
Street, W. Nick: 8015
Strickland, Larry C: 9025
Stringfellow, Mary: 8010
Stripling, Ashley: 7005, 7110
Strominger, Julie: 8000
Strommen, Jane: 2290
Strotmeyer, Elsa: 2245
Strough, JoNell: 1330, 3275, 7035
Strouse, John J: 7170, 7220
Strouse, Madison: 2085
Strunk, Sydney N: 4360, 4390, 7250, 9070
Strupeit, Steve: 7125
Stryczek, Krysttel: 8015
Stuart, Nicole S: 8005
Stuckenschneider, Tim: 4450, 7200, 7225
Stuifbergen, Alexa: 7420
Stull, Jennifer Denk: 7490
Stump, Timothy: 1145
Sturdevant, Diana L: 7005, 7370
Stusser, Aaron: 2085
Stutts, Whitney: 1220
Su, Chen-Chang: 7070
Su, Chin-Yi: 2050, 2530
Su, Jasmine IS: 7090, 8015, 9140
Su, Jin: 8005
Su, Linzhi: 7300
Su, Shaoyong: 2475
Su, Yan: 7185
Su, Yan-Jhu: 1075, 7080, 7200, 7210, 7275, 7305, 7375, 7435, 7530
Su, Yixin: 8015
Suamchaiyaphum, Krisada: 4225
Suandi, Matthew: 4245
Suanet, Bianca A: 8000
Suarez, Jethro Raphael M: 3030, 7225, 7545, 8005, 8010
Suarez Vargas, Kattia L: 2515, 7215, 7925, 8000
Suazo, Omar: 8010, 8015
Suberry, Assaf: 2370, 4395
Subramanyan, Sanjana: 1510
Sudore, Rebecca L.: 7220
Suero Samboy, Yudi Pamela: 7240
Sugar, Judith A: 7075
Sugawara, Ikuko: 7260
Sugiyama, Kei: 7330
Sugiyama, Mika: 7440
Suh, JungYeon: 3230, 7295
Suh, Michelle: 1090
Sui, Junyu: 4170, 8000
Suitor, J. Jill: 2405, 4040, 4185, 4315, 7085, 7470
Sukenova, Dinara: 7445
Sulick, Brenda A: 4400
Sullivan, Brooke: 7390
Sullivan, Campbell: 8015
Sullivan, Kevin: 1200, 2535, 7025, 7325, 7495
Sullivan, Shannon M: 7010
Sultana, Shabiha: 7245
Sumikawa, Yuka: 2210, 7440, 8000
Sun, Bangyao: 8000
Sun, Changqing: 2305
Sun, Dongmei: 4525
Sun, Fei: 1145, 2150, 2305, 3215, 7035, 7405, 7445, 8005, 8010
Sun, Haoqi: 7015
Sun, Haosen: 1490, 7355
Sun, Jany: 7485
Sun, Kaili: 9150
Sun, Kang: 7210
sun, lingxue: 7020
Sun, Nina: 7190
Sun, Peter C: 7100
Sun, Qin: 9115
Sun, Ruike: 8015
Sun, Tongxin: 8005
Sun, Tyler: 7300
Sun, Winnie: 2465
Sun, Xiaoxiao: 7500
Sun, Xin: 1205, 4055, 7405
Sun, Ying: 7090, 7315, 7320
Sun, Yu: 8000, 8010
Sun, Yue: 7465
Sun, Yue Yang: 3275
Sun, Yuzhou: 8000
Sundby, Jonathan M: 7020, 7195, 7900
Sunderaraman, Preeti: 1100
Sung, Pildoo: 3325
Sunil, Surya: 7025, 7195
Suo, Shujing: 7125
Supiano, Katherine P: 4240
Surachman, Agus: 9155
Surapaneni, Aditya: 1155, 3105
Suresh, Sharmila: 9145
Suriaga, Armiel A: 2275, 7020
Surood, Shireen: 1225
Susanto, Herry: 7475
Sutin, Angelina R.: 1120, 1265, 1445, 1480, 4110, 4360, 8015
Sutphin, George L: 4275
Sutton, Brad: 1255
Suwanno, Jom: 2435
Suzuki, Haruno: 9060, 9090
Suzuki, Rie: 7145, 7160
Svec, Joseph: 1120, 2305, 4315, 8015, 9140
Swaim, Kathryn E: 3095
Swain, Joseph: 8010
Swanger, Nancy: 4565
Swanson, Elizabeth A: 8010, 8015
Swanson, Helena L: 3150
Swartwood, Belise: 8010
Sweeney, Nicholas: 4240
Sweet, Jerry J.: 7330, 7390
Swenson, Rod: 4570
Swift, Samuel: 8000, 9120
Swint, Shelton G: 8010, 8015
Syed, Alisha: 7025
Sylvester, Hunter C: 2535, 7325
Syzf, Moshe: 8005
Szabo-Reed, Amanda: 7265
Szanton, Sarah: 1320, 2550, 3345, 4125, 4445, 7500, 8015
Szymkowski, Amanda: 7455

### T

T, Muhammad: 2105
Tabaczynski, Allyson: 8000
Tabatabaei, Abbas: 7225
Tabb, Loni P: 7260
Tabloski, Patricia A: 7500
Taei, Afsaneh: 4215
Taglialatela, Giulio: 8010
Taguchi, Reina: 7250, 7465
Tahashilder, Md Ibrahim: 8010
Tahmaseb McCon, Jasmin B: 9100
Tahulending, Peggy: 8000
Tai, Albert: 1520
Tai, Joe: 8015
Tak, Sunghee H: 8015, 9125
Takahashi, Hideto: 8010
Takako, Unno: 7440
Takaoka, Manami: 2210
Takase, Mai: 7375
Takayama, Midori: 7260
Takeya, Yasushi: 7195
Taks Calle, Muriel: 2005, 7470, 7535, 7900, 8015
Talarczyk, Jarett: 4335
Talbot, Elizabeth Peffer: 7375

Talegawkar, Sameera: 4295
Talkar, Tanya: 8000
Talley, Kristine: 2010
Talwar, Sonia: 8010
TAM, Wai San Wilson X: 7040
Tamiya, Nanako: 8000, 8010, 8015
Tan, Alai: 8010
TAN, Davynn G. H.: 4455
Tan, Jing Xuan: 4370
Tan, Kok Yang: 2435
Tan, Linda Wei Lin: 8005
Tan, Micah: 8000
Tan, Minghui: 8015
Tan, Min-Han: 8005
Tan, Nasya S: 1075
Tan, Poh Choo: 7040
Tan, Wen-Hann: 8010
Tan, Zaldy S: 8010, 9020
Tanabe, Hayato: 8015
Tanaka, Keiko: 3010
Tanaka, Toshiko: 4295, 4495, 9155
Tang, Charlotte: 7160
Tang, Fengyan: 4070, 4505, 7090, 7260, 7435
Tang, Mandi: 7295
Tang, Siyuan: 8015
Tang, Tiffany: 7375
Tang, Vera Mun Yu: 1030
Tang, Vivien: 7395
Tang, Wen: 7090, 7315, 7320
Tang, Yongfang: 4105
Tang, Yunping: 7435
Tange, Chikako: 7330
Tanglai, Wirampa: 4225
Tangonan, Andrea A: 1230
Tangpricha, Vin: 7110
Tangredi, Kaitlin: 8000, 9055
Tanguay, Adam: 8000
Tanide, Atsuko: 7265
Tanner, Gwen: 7075
Tanner, Jeremy A: 8015
Tappen, Ruth M: 2045
Tarabochia, Dawn: 7265
Tarbox, Nathan: 8005
Tarraf, Wassim: 1370, 2040, 4570, 4580, 8000, 8005
Tasnova, Sabrina: 7125
Tatar, Raquel G.: 4590
Tate, Channing E: 8015
Tate, Judith A: 7545, 8000
Tate, Rio S: 8010
Tateishi, Mana: 7330
Tateyama, Yukiko: 2230
Tauseef, Mahrukh: 7545
Tavares, Karina: 8000
Tay, Djin: 1025
Taylor, Catherine: 7170
Taylor, Harry O: 2360
Taylor, Janelle S: 2420
Taylor, Janiece L: 2030, 3205, 7420, 7500
Taylor, Melissa L: 8010, 9140
Taylor, Morgan K: 7200
Taylor, Nathan: 7285
Taylor, Olivia: 2055
Taylor, Philip: 1005, 3335, 4550
Taylor, William: 2435
Taylor-McPhail, Tobie E: 4090
Tchatlian, Lilianna: 7415
Teasdale, Thomas: 8000
Teaster, Pamela: 3350, 4565, 7170
Teerawichitchainan, Bussarawan Puk: 2275, 7150
Teixeira, Laetitia: 2120, 7135, 7380
Teixeira, Samantha: 3305, 4530
Teixeira-Santos, Ana C: 8000
Teles, Sheila: 8000
Tell, Eileen J: 4545, 7085
Telonidis, Jacqueline S: 4240, 8010
Teng, Chiao-Hsin: 2255
Teno, Joan: 2160, 7220
Tepper, Angeles: 1255, 7265
Terashima, Rika: 7195
Terhorst, Lauren: 7490
Terhune, Elizabeth: 4190
Terracciano, Antonio: 1120, 1265, 1445, 1480
Terray, Jennifer: 7440, 7915
Terry-McElrath, Yvonne: 8010
Terzoni, Stefano: 8015
Tesar, Sarah: 2330
Testa, Alexander: 2540, 3235
Testoni, Ines: 1330
Teves, Christoph: 3035
Tewary, Sweta: 8000, 8015
Thacker, Ayush: 7185
Thackeray, Anne: 4580
Thalil, Muhammad: 4215, 7040
Than, Myint Myint: 4425
Thangwaritorn, Pilar: 4230
Thapa, Sushila: 8000
Thatcher, Christine: 8005
Thayer, Erin: 1215
Thayer, Rachel E: 7200
Theeke, Laurie: 7255, 7395
Thelin, Jacquelyn: 8005
Theorell, Töres: 8000, 8015
Thiamwong, Ladda: 1055, 2435, 3030, 7090, 7160, 7225, 7380, 7495, 7545, 8000, 8005, 8010, 8015, 9100
Thiamwong, Nicha: 8000
Thielking, Em: 7075
Thierry, Amy D: 2345
Thimmesch, Amanda: 8010
Thoene, Isabella M: 8015
Thoma, Mary: 4070, 4280
Thomas, Amy: 4445
Thomas, Cassandra: 2170
Thomas, Jonathan: 4065, 7530
Thomas, Josha: 7265
Thomas, Julia: 7330, 7390
Thomas, Kali S: 1465, 2055, 2160, 3280, 4095, 7185, 7220, 7420, 7485, 7930, 8005
Thomas, Karuna S: 8015
Thomas, Kelsey R: 4570, 8005
Thomas, Lauren: 4330
Thomas, Nelson: 8010
Thomas, Patricia A: 2295
Thomas, Robert J: 1510, 7015
Thomas Hebdon, Megan: 8010
Thomas Tobin, Courtney S: 3265, 4110, 4125, 4250
Thomeer, Mieke B: 1405
Thompson, Alexandria: 8010
Thompson, Atalie C: 1075, 4065
Thompson, Edward H: 7365, 7400
Thompson, Emily: 2445
Thompson, Hannah: 8010
Thompson, Hilaire J: 7415
Thompson, Katherine: 2205
Thompson, Ladora: 1450, 8015
Thompson, Lena: 2135, 3165, 4350, 8005
Thompson, Louisa I: 7290, 8015
Thomson, Travis: 8005
Thorn, Joanna: 9100
Thorngthip, Sutthinee: 8010
Thornton, Howard: 2515
Thorpe, Joshua: 4430
Thorpe, Lorna: 2380
Thorpe, Roland J: 1505, 3180, 3205, 3235, 3345, 3395, 4095, 4125, 4250, 9040, 3060, 3265
Thorud, Jennifer: 7475
Thuemling, Teresa: 1035, 7415
Thumuluri, Deepthi: 8000
Thyagarajan, Bharat: 1200, 2040, 2345, 7010, 7345, 7425, 8005
Tian, Qu (Teresa): 4495
Tian, Zibo: 8010
Tiburcio, Alan r: 8010, 9005
Tice, Abigail: 3030, 4450, 7090, 7160, 7380
Tice, Maria C: 7005
Tiede, Ben: 9010
Tiemeier, Henning: 1225
Tietjen, Amy: 8010
Tietz, Sarah: 1085, 7290
Tietz, MD, Sarah: 3300
Tighe, Caitlan: 7390
Tijerina, Brenda: 3305
Tilley, J. Lucas: 1095
Tilley, Sarah: 7120
Tillinghast, Anna C: 8010
Timmins, Alexandra M: 4350
Timmons-Crear, ZaKiyah L: 7015
Timonen, Virpi: 3145
Tinetti, Mary: 4445, 7030
Tinker, Samantha: 8015
Tinney, Emma: 7265
Tipaldo, Jenna F: 3385
Tipiani, Dante: 1225
Tischler, Victoria: 1210
Tisminetzky, Mayra: 9095
Titelman, Elena: 1090, 7450
Tjønneland, Anne: 7345
Tjaden, Ashley H: 1455
To, Louis: 7405
Tobar-Mosqueira, Ruy: 7440
Tobias-Wallingford, Hannah: 1520
Tobin, Jonathan N: 1170
Tobin, Karin E: 2030
Tobyne, Sean: 8000
Todd, Chris: 9085
Toh, Wei Xing: 3275
Tolea, magdalena I: 2170
Toles, Mark: 7105, 7385
Toliver, Domenic: 8015
Tollock, Maria: 7330
Tolson, Michele: 3310
Tom, Sarah: 7095
Tomar, Tanvi: 8015
Tomasic, Chloe: 8005
Tomasino, Debra: 7090, 8015
Tommet, Douglas: 2505, 4540
Tompkins, Cathy: 4600, 7005
Tong, Sebastian: 4535
Tong, Wing H.: 4105

Tong, Yuying: 4315
Tongsiri, Sirinart: 2435
Tonkikh, Orly: 2450, 4145
Tooze, Janet A: 7480
Topaz, Maxim: 7545
Topping, Michael: 7040
Torres, Camilla: 7545
Torres, Daniella: 8010
Torres, Jacqueline M: 3050, 4070, 4280, 8000, 9090
Torres, Sandra: 3335
Torres Ruiz, Ilse: 7300
Tortora, Carla: 7135
Tosato, Matteo: 8010
Tosato, Matteo: 8015
Toseef, Mohammad Usama: 1370, 2260, 8000
Tosi, Dominique M: 3065
Tosi, Hannah: 8000
Toto, Pamela E: 2445, 4305, 7515
Touhiduzzaman, MD: 4200
Towe, Maria: 8010
Towns, Juell M: 1280
Townsend, Aloen L: 8015
Townsend, Nick P: 2120
Towsley, Gail L: 1300, 1435, 4555
Toyama, Masahiro: 7200, 7255
Tracy, Eunjin L: 4335, 7390, 8000
Trainum, Katie: 4065, 7230, 7385, 7420, 7910
Tramujas Vasconcellos Neumann, Lycia: 4380
Tran, Calvin K: 7130, 7200, 7225, 7255, 7300, 7905
Tran, Diep: 1355
Tran, Duyen: 7470
Tran, Dylan: 4280
Tran, Karenna: 2500
Tran, Melissa K: 1435
Tran, Rose: 1275
Tran, Thi: 8005, 9000
Tranah, Gregory J: 4275
Trane, Ralph: 4585
Traver, Anthony C: 3305
Traver, Anthony C: 9110
Travis, Levi M: 4560
Travison, Thomas G: 1255, 2505, 4460, 7385, 7465, 8010, 9095
Traywick, LaVona S: 1450, 4115, 7265
Treat, Anny: 8005, 8010
Tremblay, Roma: 7320
Trinh, Hanh: 8010
Trinitas, Joseph: 4430, 7225, 7905
Trinkoff, Alison: 8005
Trinquart, Ludovic: 4535
TRIPATHI, ALOK: 7245
Trittschuh, Emily H: 3005
Trivedi, Amal N: 8000
Trivedi, Ranak: 8010
Troeung, Lakkhina: 2435
Troost, Jonathan: 4015
Tros, Willemijn: 7010
Trotter, Stephanie: 7015, 7935
Troutman Adams, Elizabeth: 7530
Troutman-Jordan, Meredith S: 4007
Troxel, Wendy M: 2200, 9110
Truebig, Janet: 7000
Truett, Beth J: 7400
Trujillo, Janet: 7145
Truong, Brandon: 8000
Truskinovsky, Yulya: 8005
Tsai, Chu-Lin: 7090
Tsai, Jack: 3235
Tsai, Jaw-Shiun: 8015
Tsai, Li-Tang: 8010
Tsai, Pao-Feng: 8000
Tsakiridi, Anastasia: 1095
Tse, Dwight: 1350, 3360, 4300, 7375
Tse, Hin Wing: 8000
Tseng, Chi-Hong: 8010
Tseng, George: 7345
Tseng, Han-yun: 8005, 8015
Tseng, Tung Sung: 2300
Tsimpida, Dialechti: 1095
Tso, Emily: 8015
Tsoi, Tom Chun Wai: 1030
Tsotsoros, Cindy: 8000
Tsuchiya-Ito, Rumiko: 7250, 7465
Tsuda, Shuji: 7300
Tsukada, Noriko: 7160, 7370, 7405
Tsuker, Sophia: 4015, 8005
Tsutsui, Junya: 3010
Tsutsumi, Rie: 8015
Tsuzuki, Takamasa: 7480
Tu, Wanzhu: 1145
Tu, Yufang: 7220, 7330
Tucker, David W: 4425, 7495
Tucker, Gretchen G: 8015, 9005
Tucker, Katherine L: 4295
Tuepker, Anaïs: 7135
Tuft, Samantha E: 1215, 7405, 8015
Tuicomepee, Arunya: 7375
Tuladhar, Lirisha: 1395, 2325, 4365
Tullett, Alexa: 7310
Tumurbaatar, Batbayar: 8010
Tung, Chih Chun: 9005
Tung, June H C: 9085
Tung, Yen-Chen: 8015
Tungaturthy, Neha: 4155
Tuominen, Katariina: 1080
Tupler, Larry: 1235, 4010
Turan, Janet: 2140
Turcotte, Samuel: 8005
Turiano, Nicholas A: 7180, 7355, 7505, 8005
Turjeman, Sondra: 2105
Turk, Katherine: 8000
Turkelson, Angela: 4195, 4490, 7355
Turley, Niels: 7015
Turnage, Sharon: 9100
Turner, Christopher O: 7365, 7400
Turner, Natalie: 4200, 7355, 7370, 7485, 7930
Turner, Shelbie: 1210, 1240, 1505, 2280, 7105, 7350
Turner, Sidney: 4440
Tursi, Mark: 7215, 7410
Tur-Sinai, Aviad: 7090
Turyk, Mary E: 2040
Tussey, Zachary: 7340
Twamley, Elizabeth W: 8010
Twum, Emmanuella Asabea: 2540
Tyler, Denise A: 8000
Tyrell, Tahira: 8005
Tyshkovskiy, Alexander: 8005
Tzadikario, Ayala: 8015
Tzemah-Shahar, Roy: 2105, 2415, 7000, 7210
Tzeng, Huey-Ming: 7305, 8005
Tziarli, Dimitra: 2110, 2335

U
Ucar, Duygu: 2277
Uchida, Junya: 8000
Uddin, Jalal: 1000
Udeh, kingsley C: 1365, 2260
Udo, Charles: 3180
Udoh, Idorenyin I: 7145, 7375, 8000
Ueda, Takuya: 7265
Ughra, Ravjyot: 7300
Ugoh, Munachimso: 4330
Ugwu, Benita A: 7300
Ugwu, Emmanuel O: 1365
Ugwu, Lawrence E: 7405
Ukenenye, Roseline: 8010
Ukonu, Nene: 1070
Ukraintseva, Svetlana: 7185, 7415
Ulbricht, Christie: 8000
Ulloa, Gabriella: 7265
Ulmer, Cameron: 7105
Ulrich, Connie: 7205, 8000
Umans, Jason G: 7190
Umble, Karl: 8005
Umezinwa, Ndidi M: 7345
Umoh, Mfon E: 8005
Umucu, Emre: 8010
Underwood, Elisha: 4140
Underwood, Elisha: 8015
Unroe, Kathleen: 1145, 1300, 1435, 3085, 7220, 7500, 8010, 8015, 9005
Unruh, Mark A: 4135, 7250, 7500, 9090
Unsworth, Julia R: 4400, 8015
Unsworth, Mackenzie: 7150
Urbanski, Dana: 1010, 1430, 2260, 3075, 3375, 7530
Urbina, Ray G: 7120
Urlings, Judith: 1310
Utz, Rebecca L: 1025, 1030, 8015

V
Vader, Daniel: 4210
Vain, Megan: 4200
Vale, Michael T: 7110
Valenti, Korijna: 4240
Valentin, Yves: 4590
Valenzuela, Antonio: 7455
Valeriote, Ralph: 8010
Välimäki, Tarja: 3030
Vallabha, Shree: 3270
Van Bogart, Karina: 1265, 1445, 2360, 4120
van Bruggen, Sytske: 4105
Van den block, Lieve: 7220
van den Heuvel, Edwin: 2545
Van Denend, Toni: 7225
van der Steen, Jenny T: 4105, 7010, 7220
van Ede, Anna FTM: 4105
Van Etten, Emily: 8005
Van Haitsma, Kimberly L: 1300, 4480
van Hoof, Joost: 3080
Van Hook, Jennifer: 4290
Van Houtven, Courtney: 4430, 4565, 7205
Van Orden, Kimberly A: 1185, 3100, 8000
van Rossum, Erik: 7250
van Schooten, Kimberley S: 4060
van Veluw, Susanne: 9040
Van Vleet, Bryce A: 2290, 4165, 8000, 8015
Van Vleet, Samuel: 1190, 3125, 4440, 4580

Van Vleet, Samuel C: 8015, 9105
Van Zutphen, Addison: 4405
van Zyl, Carin: 7220
Vance, David E: 2395, 4570, 7245, 7280, 9125
Vance, Jarod C: 8005, 8015, 9025
VanDenberghe, Aimee: 8015
Vander Meulen, Maria: 1285
Vanderheide, Clara: 2310
VanderWerf, Jessica A: 1315, 7175
VandeWeerd, Carla: 7320
VanDusen, Amy: 7145
VanHaitsma, Kimberly S: 1005, 7305, 8015
VanHeiden, Sarah: 3085
Vanhoutte, Bram: 4325, 4580
VanSwearingen, Jessie: 8005
Varadhan, Ravi: 2510
Varma, Malvika: 8005
Varma, Sashank: 2350
Varma, Vijay R: 2400
Varzino, Michael: 1505
Vashishtha, Rakhi: 1205
Vasireddy, Sindhu: 4440
Vasquez, Alexa: 7305
Vasquez, Anjuli: 8010
Vasquez, Daniel: 8000
Vásquez, Elizabeth: 1190, 2060, 3265, 3390, 7475, 8015
Vasquez, Felix A: 8000
Vasquez, Zander: 8005
Vasquez-O'Brien, T. Caitlin: 8010
Vasunilashorn, Sarinnapha M: 2505, 8000
Vatsyayan, Aarushi: 7395, 9045
Vaughan, Camille P: 9080
Vazquez, Christian E: 2060
Vazquez-Nuñez, Karla Patricia: 7040
Vazquez-Trejo, Valeria: 7485
Veer, Rick: 8015
Vega, Nicole M: 4227
Vega, Pamela Tella: 4150
Veilleux, Megan: 8005
Velasquez, Roberto: 7230, 7305
Velazquez-Diaz, Keisha: 7410
Velez Ortiz, Daniel: 8010
Veliz, Philip: 7295
Vemuri, Prashanthi: 7025
Vemuri, Ramesh: 2555
Vences, Kelly: 4415, 7155, 7255, 7465
Venditelli, Nijah: 7410
Venditti, Elizabeth M: 1455
Venegas, Maria: 1305, 2365, 7280
Venkatram, Chinmayi: 8010
Vera Murillo, Lizbeth C: 1285
Verbanac, Tamrah L: 7470
Verbanac, Viridian: 7085
Verbeek, Hilde: 1310, 3035, 7370
Verdery, Ashton M: 1405, 4290
Verdi, Daniel: 8010
Verdi, Laura: 8015
Verdile, Erin: 4075
Vergez, Sasha M: 1170, 7440, 7545
Verghese, Joe: 4200, 4495, 7025, 8015
Verma, Ambika: 7010, 7190
Vernon, Andrew A: 8010
Verrall, Aimee: 8010, 8015
Versey, H. Shellae: 2420, 4530
Verstaen, Alice: 4515
Vetter, Michael: 7070
Vetter, Valentin M: 4490
Viale, Daniela: 7265
Vickers, Kayci L: 8005
Victor, Christina R: 1185, 4360, 8010
Vidaurri, Glycel: 8000
Vidoni, Eric: 1020
Vidot, Denise: 8010
VIEIRA, EDGAR RAMOS: 8005
Viera, Anthony: 9150
Vila Manes, Claudia: 9115
Villar, Michelle J: 2045
Villa-Sánchez, Bernardo: 7185
Vincenzo, Jennifer L: 1280
Vinge, Jill: 3065
Virata, Michael: 7245
Visaria, Abhijit: 1205, 2230, 2435
Visingardi, Joseph: 8005
Viskochil, Richard: 7395
Visovatti, Moira: 7325
Viswanathan, Anand: 9040
Vitolins, Mara Z: 1255, 7480
Vivarakanon, Phatchanun: 7035
Vivek, Sithara: 7010, 7345
Vivek-Ananth, R.P.: 7190
Vlaeyen, Ellen: 8010
Vlock, Elizabeth: 7465
Vluggen, Stan: 7250
Vo, Jess: 8005
Vo, Quynh: 2450, 4145
Vodantis, Eleni: 7005, 7935
Vogel, Barbara: 9055
Vogel, Selina: 4470
Vögele, Claus: 1210
Vogelsang, Eric M: 7180, 7355, 7900
Vogelsmeier, Amy: 8010, 9140
Volker, Michael: 1325
Von Kay, Karin: 2170
Von Visger, Tania T: 7370
Vonk, Jet MJ: 3380
Voss, Stephanie: 1255, 7265, 8000
Vovnoboy, Irina: 7245, 7915
Vozza, Katelyn: 7350
Vozzolo, Tristan R: 4200
Vrahas, Mark S: 8000
Vranceanu, Ana-Maria: 1160, 2310, 3375, 4590, 7205
Vu, Cong Nguyen: 7390
Vu, Long: 7220
Vu, Milan: 7045
Vu, Thi: 2135, 8005
Vuong, Lauren: 8000
Vupputuri, Suma: 7110
Vyavahare, Sagar: 8010
Vytla, Kavya: 9045

**W**

Wachana, Anne: 1280
Wachholz, Patrick: 7370
Waddington, Emily: 9005
Wade, Benjamin: 8010
Wade, Gregory: 1155
Wadsworth, Sally J: 1175, 4110
Waern, Margda: 4010, 7255
Waetjen, L. Elaine: 9150
Wagenknecht, Lynne E: 1455, 3105
Wagner, Alison: 8010, 9145
Wagner, Brittin: 8010
Wagner, Cheryl M: 8010, 8015
Wagner, Fernando A: 4070, 4150
Wagner, Jennifer: 7370
Wagner, Lisa: 7340
Wagner, Maude: 8005, 9040
Wahl, Hans-Werner: 3215, 7295, 8015
Wahlheim, Christopher: 7435, 8015
Wahrendorf, Morten: 3195
Wahring, Iris: 1350
Waights, Verina: 3080
Wain, Kris: 7085, 7910
Waite, Linda J: 2295
Wakui, Tomoko: 4590, 8010, 9065
Walaszek, Art: 1200, 8015
Waldinger, Robert J: 3330
Waldron, Danielle: 1355
Walker, Keenan A: 1155, 1200, 4495, 7040, 8015, 9045
Walker, Nalani: 7000
Walker, Rae K: 8000
Walker, Thomas: 7340
Walkner, Tammy J: 1305, 2085
Walkner, Tammy: 8005
Walkosz, Barbara J: 2485
Walkup, Michael P: 2470
Wallace, Cara L: 4430, 7220, 8000
Wallace, Gail: 9040
Wallace, Louise Wallace: 3080
Wallace, Meredith: 2230
Waller, Dylan E: 7295
Waller, Lianna: 1085
Wallhagen, Margaret I: 7530, 9060, 9090
Wallsworth, Christine: 7205, 7915
Walsh, Patrick: 1345
Walston, Jeremy D: 2510, 9045
Walters, Monica E: 2005, 7350, 7390, 7435, 8010
Walton, Quenette L: 3305
Wan, Meijun: 3320
Wan, Qiaoqin: 2125, 7265
Wan, Shaowei: 2500
Wang, Ange: 1220
Wang, Anne: 1470, 7030
Wang, Arthur: 3380
Wang, Bei-Yun: 7525
Wang, Bingwen: 7015
Wang, Boxiang: 7265
Wang, Cecilia Xi: 8005
Wang, Chenchen: 4535
Wang, Chen-pin: 4090
Wang, Chih Yu: 9030
Wang, Claire L: 1465, 2480, 4070, 4385, 7390, 9075
Wang, Cuiling: 4310, 8015
Wang, Dahua: 7185
Wang, Di: 2405, 4185, 7085
Wang, Dingyue: 3355
Wang, Dongmin: 4255
Wang, Emily A: 8000
Wang, Ethan B: 7295
Wang, Fei: 3090
Wang, Gavin: 8010
Wang, Ge: 8015
Wang, Gemma: 8005
Wang, Guanzhou: 7240, 8000
Wang, Hairong: 8015

Wang, Haowei: 2345, 3020, 8005
Wang, Hongmei: 3320
Wang, Hongwu: 2290
Wang, Jen-Yu: 8010, 8015
Wang, Jiankun: 3090
Wang, Jiaqing: 7015
Wang, Jing: 1395, 1400, 2150, 2255, 2475, 3015, 4005, 4170, 4330, 7030, 8000, 8005, 8010
Wang, Jingxuan: 4280
Wang, Julian: 3035, 7465
Wang, Juwen: 1125
Wang, Kaipeng: 1145, 2150, 4040, 8005
Wang, Kairong: 8005
Wang, Karen: 9125
Wang, Kuan-Yuan: 9070
Wang, Kun: 1105, 7085, 7295, 7485, 9030
Wang, Leping: 3195
Wang, Lihua: 8005
wang, linying: 3075
Wang, Maggie: 2395
Wang, Mengru: 8000, 8005, 8015
Wang, Min: 8000
Wang, Nia Yuqing: 9070
Wang, Nili: 7220
Wang, Olivia (YuHsuan): 8010
Wang, Panpan: 2305
Wang, Peng-Li: 7370
Wang, Peter: 8000
Wang, Qianyun: 7055, 7900, 8000, 8010
Wang, Qingwei: 7010, 7015, 7255
Wang, Rui: 8010
Wang, Senhu: 7050
Wang, Shan: 7315
Wang, Shangqiguo: 9085
Wang, Shu: 8010
Wang, Shuai: 7015
Wang, Shuaijie: 7225
Wang, ShuangShuang: 1335, 8010
Wang, Shuo: 7345, 7425, 8015
Wang, Sijiu: 1245, 2185, 4055, 7470
Wang, Sophia: 3085
Wang, Suyi: 8005
Wang, Sylvia: 8005
Wang, Tingmei: 7015
Wang, Weidong: 7370
Wang, Weijun: 7380
Wang, Wei-Lin: 2005, 2400
Wang, Weiming: 7255
Wang, Wendy: 4375
Wang, Wenjin: 1135, 3125, 7360
Wang, Wuqi: 8005
Wang, Xi: 7330, 7535
Wang, Xiafei: 7470
Wang, Xiao (Joyce): 1270, 2160
Wang, Xiaochuan: 8000
Wang, Xiaofeng: 9150
Wang, Xiaoling: 2475
Wang, Xingzhi: 1180
Wang, Xinyi: 1235
Wang, Xuechun: 7325
Wang, Xuzhi: 8000
Wang, Yahui: 7295, 7915, 9060
Wang, Yanfeng: 7410
Wang, Yang: 1160, 2105
Wang, Yang: 7340
Wang, Yanhong: 7125
Wang, Yanxin: 2510
Wang, Yanyan: 8005, 8010, 8015
Wang, Yi: 1180
Wang, Yi: 1250
Wang, Yi: 1375, 3200
Wang, Yi: 7005, 7100, 7260, 7290, 7375, 7925
Wang, Yihan: 4425
Wang, Yijun: 8000
Wang, Yilin: 4530
Wang, Yiwen: 1125
Wang, You: 8010
Wang, Yu: 8010
Wang, Yueh-Tzu: 7165
Wang, Zengyan: 8005, 8015
Wang, Zhangyang: 8000
Wang, Zhendong: 8015
Wang, Zhiwen: 9065
Wang, Zih-Ling: 3205, 7185, 7415
Wangi, Karolus Yosef Woitilia: 4480
Wangqin, Runqi: 9035
Wanigatunga, Amal A: 2100, 3105, 4375
Ward, Caitlin: 3220, 7000
Ward, Michelle K: 7185, 7255, 7260
Ward, Nicola Ann: 4205
Ward, Rachel: 8010
Wardlow, Liane: 7020
Ware, Deanna: 2140
Warne, Donald K.: 7415
Warner, David F: 2295, 7220
Warner, Lisa M: 4300, 7395
Warner, Mildred E: 4130
Warnock, Jenna: 8010
Warren, Aaron: 7265
Warren, John Robert: 2120, 7010, 7040
Warren, Kathryn: 4410
Warry, Wayne: 4045
Wartko, Paige: 8005
Washington, Ashley: 7280
Washington, Karla T: 2320, 7220, 7260
Wasserman, Bruce A: 4375
Watanabe, Kengo: 7090, 7190, 7315
Watanabe, Kentaro: 4590
Watanabe, Kiriko: 8015
Waterhouse, Jodi L: 1130, 2070, 4355, 7290
Waters, Catherine: 9000
Waters, Leland: 1005, 1260, 2110, 2205, 7095, 8015
Watkins, Glenda: 1260, 7340
Waton, Aileen: 8015
Watson, Brittany: 7415
Watson, Gwendolyn Paige: 3340
Watson, Jacob C: 4545
Watts, Amber: 4230, 4585, 7265
Wawrzynski, Sarah E: 2030
Wayne, Peter: 4255
Wayt, Cordelia: 1390
Weaver, Ashley: 7165
Weaver, Destiny J: 7310
Weaver, Matthew D: 7390
Weaver, Raven: 3295, 4565, 7175, 7920
Webber, Madeline: 3125
Webber, Sophia: 1130, 3155
Webster, Amy: 1470
Webster, Micah: 8010
Webster, Noah: 2325, 4285
Webster, Sam: 7455, 7935
Webster-Dekker, Katelyn E: 7265
Wec, Aleksandra: 1360, 2520
Weech-Maldonado, Robert: 1150, 3365
Weems, Jacy: 1270, 7220
Wehe, Hillary: 7380
Wehry, Susan: 8010
Wehry, susan: 8015
Wei, Andrea: 9095
Wei, Ashley Z: 8005
Wei, Aybey Amy: 9035
Wei, Cindy: 3305
Wei, Duo Helen: 7015, 7385
Wei, Feng: 7015
Wei, Huichuan: 7340
Wei, Jeanne: 7005, 7010, 7190, 8015
Wei, Linjiang: 1235
Wei, Melissa Y: 8010
Wei, Shuaifang: 8005
Wei, Sijia: 7185, 8000
Wei, Wanrui: 8000, 8005, 8015
Wei, Wenxing: 1105, 3245, 3300, 8015
Weibel, Maria: 8000
Weibel, Robert: 4270
Weidmann, Rebekka: 3270
Weigand, Alexandra: 4570, 8005
Weigler, Elizabeth: 1160
Weil, Joyce: 2515, 7445
Weimar, Lisa: 3220, 7000
Weinberg, Janice: 4535
Weiner, Deborah: 7205
Weinhardt, Michael: 3080
Weinkove, David: 7190
Weir, David R: 4205
Weir, Rodney: 8005
Weisenfeld, Dana: 4460
Weisenfeld, Jonathon: 8005
Weiskittel, Skylar E: 8010
Weiskittle, Rachel: 4575, 8005
Weiss, Charlotte R: 8000
Weiss, David: 1210
Weiss, Erica: 8015
Weiss, Jordan: 3105
Weissberger, Gali: 1100, 2390
Weisse, Carol S: 4510, 8015
Weisskohl, Jack: 1260
Weldrick, Rachel: 2515
Wellenstein, Kerry: 4227, 7315
Weller, Christian E: 3310
Wells, Ashley: 1075
Wells, Jenna L: 1275, 2095, 4085, 7290, 8010, 9000
Wen, Chiung-Jung: 8015
Wen, Frances: 7005
Wen, Jiebing: 9090
Wen, Ming: 4315
Wen, Tong: 9075
Wen, Tzai-Hung: 7465
Wen, Wan: 7295
Wen, Xinyue: 9075
Wendte, Jered: 1260
Weng, Hui-Ching: 7470
Wennerdahl, Carrie L: 7410
Werfelli, Howaida: 1135
Werner, Nicole E: 1035, 2270, 2520, 7305, 7415
Werner, Shoshi: 8005
Wershof Schwartz, Andrea: 8000
Wessinger, Chadsley M: 7435, 8005, 9025
West, Brady: 7205
West, Emily: 8000
West, Jessica S: 7530, 8000, 8005
West, Tim: 7010

Westanmo, Anders D: 3065
Westcott, Jordan B: 7255, 7375, 8005
Westenskow, Rebecca: 8010
Westergard, Angela: 4475
Western, Elizabeth C: 4240
Western, Max J: 2120, 8010
Westlund, Erik: 3345
Westover, M. Brandon: 7015
Wethington, Elaine: 1055, 4285, 7540
Wettstein, Markus: 1210
Wettstein, Markus: 8010
Weuve, Jennifer: 2000
Whaikid, Phatcharaphon: 7130, 7430
Whang, Priscilla H: 4110
Wharton, Whitney L: 1420
Wheeler, Anne C: 8010
Wherry, Sarah J: 7165, 8005
Whesu, John: 8005
Whetung, Cliff: 2530, 3045, 8000
Whibley, Daniel: 7390
Whitaker, Kara M: 7265
Whitbourne, Stacey B: 8010
Whitbourne, Susan K: 1130, 2145
White, Carole: 3355
White, Christian: 7340
White, Corinne: 2310
White, Elizabeth: 7125, 7540
White, Julie: 8010
White, Kina: 1335
White, Mathew: 3145
White, Mel: 8015
White, Rebecca E: 1325, 8005
White, Suzanne M: 7015, 7920, 7935
White Makinde, Keisha: 7260
White Whilby, Kellee: 4125
Whitehead, Ishbel Orla: 7400
Whitehouse, Christina: 8000
Whitfield, Keith E: 3175
Whitlatch, Carol J: 3375
Whitley, Deborah: 2340
Whitman, Ethan: 7025
Whitman, Kathrine: 8000
Whitmer, Rachel: 8010
Whitmore, Celina: 3005, 4350
Whitmore, Corrie: 3295
Whitsel, Eric A: 7480
Whitson, Heather: 1020, 2510, 7205, 9150
Whittier, Jordan M: 4510
Whyman, Jeremy D: 7310
Wickersham, Claire E: 3000
Wickliffe, Jeffrey: 2275
Wickwire, Emerson M.: 2035, 8015
Wideman, Laurie: 8005
Widiyaningsih, Widiyaningsih: 7475
Wiederholt, Senta: 7135
Wiemers, Emily E: 3050, 8005
Wiendrick, Jack: 1460
Wiese, Lisa A: 1020, 2170, 4045, 4450
Wiggins, Alexander: 8005
Wihry, David: 7455, 7935
Wiktorsson, Stefan: 4010, 7255
Wilber, Kathleen H: 2385, 2390, 3300, 7085, 7230
Wilcock, Donna M: 2460
Wilcox, Elsa: 8010
Wild, Tess: 3275
Wiles, Lynn: 8015
Wiley, Christopher D: 4347, 8005
Wiley, Elise: 7225, 7905
Wilhelm, Myia: 3035
Wilhelmi, Morgan: 2255
Wilhelmi, Olga: 3235
Wilhite, Myrita: 7455
Wilkins, James M: 1340, 4090
Wilkins, Jessica E: 4240
Wilkinson, Katherine: 7150
Wilkinson, Lindsay R: 7130, 7475
Wilks, Scott: 7350
Willard, Meredith A: 7180, 7355, 7505, 8005
Willcox, Bradley J: 1065, 7240
Williams, Alisha: 7300
Williams, April R: 8010
Williams, Betsy: 8015, 9050
Williams, Brie: 8000
Williams, Christine L: 1020, 4045
Williams, Christopher: 7330, 7390
Williams, David M: 9130
Williams, Don'aa: 8010
Williams, Ginger: 4075
Williams, Ishan C: 4045
Williams, Johnathan: 1260, 7095
Williams, Kenneth: 8005
Williams, Kristine N: 1150, 1515, 2225, 7250, 8000
Williams, Michael: 8015, 9050
Williams, Michael P.: 4590
Williams, Rhiannon: 3255
Williams, Rua: 7015
Williams, Ryan Blake: 8000
Williams, Victoria J: 4585
Williams Sites, Kathryn: 7350
Williamson, Jeff: 2410
Williams-Sites, Kathryn: 8000
Williger, Bettina: 8010
Williman, Marty: 2485
Willis, Allison: 8005
Willis, Alyssa: 8000
Willroth, Emily C: 1045, 2155, 4110
Wilmoth, Janet M: 2085
Wilson, Annalee: 4075
Wilson, Charlton: 2170
Wilson, DaiLene: 7280
Wilson, Linnea M: 1485
Wilson, Nikki-Anne: 1280, 4470
Wilson, Skylar: 1095
Wilson, Stephanie J: 8010
Wilson, Tara: 9125
Wilson, Tracey: 1155
Wilson, Traci L: 9075
Winberry, Joseph: 7110
Winberry, Joseph: 8005
Windham, B. Gwen: 2535, 3105, 7025, 7325, 7495, 7545, 8010
Windsor, Winrose: 7225
Wingood, Gina: 2395
Wingood, Mariana: 1110, 4525, 8010
Wininger, Claire: 8005, 8010
Winogora, Victoria M: 7305
Winstead, Vicki: 4085
Winters, Kathryn: 8015
Wirba, Solee: 9130
Wirth, Maria: 4270
Wisdom, Kimberly-dawn: 7390
Wisniewski, Thomas: 7010
Wister, Andrew: 7470, 7900, 8010
Witkowski, Jacek: 7315
Wittenberg, Grace: 2055, 8015
Witt-Swanson, Lindsey: 4435
Witzel, Dakota D: 1175, 2280, 7115, 7335, 8015
Wladkowski, Stephanie P.: 2265, 4430, 7220
Woestman, Jenna: 3005, 4350
Wohns, Nicolai: 8010
Wojczynski, Mary K: 7345, 8005
Wolf, Bethany: 9035
Wolf, Jack M: 1430, 2260, 3020, 3075, 3190, 7300, 7530
Wolf, Michael S: 4525
Wolfe, Alex: 7130
Wolfe, Hannah: 1045
Wolfe, Joseph D: 1405
Wolfe, Megan: 3000
Wolff, Allycia: 3375
Wolff, Jennifer: 1360, 2375, 2520, 3315, 4450, 8005
Wolff, Julia: 8010
Wolf-Ostermann, Karin: 1310, 3035
Wolfson, Emily A: 8000
Wollney, Easton: 7350
Womack, Julie A: 7245
Won, Chang Won: 7325
Won, Chang Won: 8010
Wondimu, Betselot: 7535, 7900
Wong, Alex: 1300
Wong, Alice C.: 2315
Wong, Bonnie: 7435
Wong, Carin: 8000
Wong, Carol Ka-Po: 7395, 7440
Wong, Cindy: 7205
Wong, David W: 4030
Wong, Edwin Lok Yan: 7255
Wong, Eliza Lai-Yi: 7145, 7395, 7440
Wong, Gloria Hoi Yan: 1235, 4455, 7255, 8015
Wong, Jennifer A: 2380
Wong, Jennifer: 7525
Wong, Joey Oi Yee: 2090, 7235, 8010
Wong, Karen Lok Yi: 7235
Wong, Karen Lok Yi: 8010
Wong, Lena L N: 9085
Wong, Lily: 7205, 7915
Wong, Megan: 4227, 7315
Wong, Rebeca: 4470, 7445
Wong, Rebeca: 8010, 9075
Wong, Roger: 2140, 7185
Wong, Stella Lai-Kuen: 1135
Wong, Stephanie Ming Yin: 1235, 4455, 7255
Woo, Jean: 2455, 8005
Woo, Joyce: 8005
Woo, Seoyoon: 7005, 7400
Wood, Kristie: 2095
Wood, Leila: 7405, 9025
Wood, Madeline: 8015
Wood, Michele: 8015
Woodhouse, Mark: 1455, 2160
Woodrell, Christopher: 9145
Woods, Carly: 8005
Woods, Diana L: 8010
Woodward, Amanda T: 8000, 8010
Woodward, Aubrie: 1400, 8010
Woolley, John: 7435
Woolrych, Ryan: 1325

Woolson, Sandra: 4430
Woolverton, Cindy B: 8000, 8005
Wormwood, Jolie: 4090
Worthington, Amber: 1120
Wren, Jonathan D: 3127, 8005
Wright, Bernadette: 1370, 1465
Wright, Eric R: 4550
Wright, Julia: 8000
Wright, Kathy D: 2135, 8010, 9030
Wright, Lauri: 4095
Wright, Molly A: 8005
Wright, Morgan M: 2010
Wright, Rebecca: 7430
Wright, Regina S: 7465
Wright, Shawna: 8000
Wroolie, Tonita Emily: 2240
Wrosch, Carsten: 7390
Wrublowsky, Robert: 2080
Wu, Barry J: 8005
Wu, Bei: 1400, 1450, 2475, 3015, 3110, 3290, 4055, 4170, 4330, 4505, 7030, 7415, 7485, 8000, 8005, 8010, 8015, 9035
Wu, Chao-Yi: 2360, 3090, 7415
Wu, Chenkai: 2510, 7365, 8005
Wu, Deqing: 7415
Wu, Fei: 7010
Wu, Heng: 7090
Wu, Hongchen: 2350, 7525
Wu, Huijun: 1245
Wu, Jialin: 8015
Wu, Jing: 7225
Wu, Jinhui: 8000
Wu, Jinyan: 9020
Wu, Katherine: 2140
Wu, Kuan-Ching: 7205, 7395
Wu, Lei: 8000
Wu, Li: 1220
Wu, Lihua: 3075
Wu, Mingche MJ: 2520
Wu, Niels: 3250, 4410, 7005
Wu, Qinyang: 7410
Wu, Rui: 7470
Wu, Samantha: 2300, 7100
Wu, Shih-Cyuan: 7370
Wu, Shu-Ying: 1365, 2135, 8005
Wu, Shwu-Chong: 7370
WU, Vivien Xi: 7040
Wu, Wen-Chih: 7000
Wu, Wen-Chih: 8015
Wu, Xiaochu: 8010
Wu, Xucheng: 9150
Wu, Yanjun: 7325
Wu, Yifei: 7225
Wu, Yingyan: 4540
Wu, Yiwen: 3360, 7005, 9070
Wu, Yiyi: 2395
Wu, Yiyuan: 3300
Wu, You: 1245, 9000
Wu, Yuewen: 7255
Wu, Yuhan: 7010
Wu, Zhen: 2305, 7145
Wurm, Susanne: 8010
Wurtz, Heather: 9080
Wyatt, Tami H: 8000
Wylie, Glenn R: 8015
Wylie, Molly J: 7280, 7360
Wyman, Mary F: 3005, 4350
Wyman, Michelle: 7305
Wyrostek, Lyndi: 2485

**X**

Xavier, Rose Mary: 8010
Xi, Wanyu: 2370
XIA, Keting: 7045
Xia, Maoyao: 9150
Xia, Weiyi: 9045
Xiang, Jaiwei F: 7460
Xiang, Xiaoling: 3175, 8015
Xiao, Chunhong: 8000
Xiao, Jinyi: 7275
Xiao, Junhua: 7340
Xiao, Lulu: 7145
Xiao, Qian: 3185
Xiao, Wendong: 2125
Xiao, Xaviera: 4225
Xiao, Xiaohua: 2275
Xiao, Yexuan: 7015, 7095
Xie, Alice: 9105
Xie, Bo: 3340, 4065, 7015, 7230, 7385, 7910
Xie, Fei: 7125
Xie, Hui “Jimmy”: 4470, 7210
Xie, J. Kathy: 7115
Xie, Kathy: 8000
Xie, Kristal: 8015
Xie, Liujing: 8015
Xie, Rui: 2435, 3030, 4450, 7090, 7160, 7380, 7495, 8005, 8015, 9100
Xie, Serena Jinchen: 4180
Xie, Yuning: 7145, 8005
Xing, Weijie: 7015
XING, YANQIU: 7190
Xing, Yiwen: 8005
Xing, Yunli: 7090, 7320
Xiong, Jiaming: 8005
Xiong, Xuechen: 1030
Xu, Beibei: 2545, 7320, 7520
Xu, Cai: 1235
Xu, Chuanzheng: 1430
Xu, Chun: 7190
Xu, Dongjuan: 7370
Xu, Furong: 8010
Xu, Hanzhang: 2150, 3135, 4095, 4170, 4505, 7520, 8000, 8005, 8015, 9035
Xu, Huiwen: 4365
Xu, Jiahui: 4330
Xu, Jiajia: 7240
Xu, Jianhong: 2510
Xu, Jiayun: 2255
Xu, Kailin: 9070, 9080
Xu, Kelin: 7320
Xu, Ling: 8010
Xu, Mengya: 2005, 2400
Xu, Ming: 8005
Xu, Qin: 2545, 4505, 7090
Xu, Shicheng: 8000, 8005, 8015
Xu, Shu: 1075, 4065, 7275
Xu, Shuo: 3320, 7220
Xu, Sophie Zhijing: 2475, 4505, 8010
Xu, Ting: 4055, 4505, 7090
Xu, Wei: 2350
Xu, Wei: 7260
Xu, Weili: 4370
Xu, Wenqian: 3020
Xu, Winnie: 8000
Xu, Xinming: 7320
Xu, Xinyi: 2545, 7090
Xu, Yanxun: 7245
Xu, Ying: 1225, 7085, 7375, 7470
Xu, Yingzhi: 8010
Xu, Yuelong: 8000, 9050
Xu, Zhijing: 3110, 8010
XUE, Dandan: 7305
Xue, Lingshu: 1385
Xue, Qian-Li: 1220, 2100, 2400, 2510, 8000, 8005
Xue, Tingzhong (Michelle): 2465, 3135, 9035
Xue, Wei-Lin: 8010

**Y**

Yablon, Yaacov: 8015
Yabuki, Tomoyuki: 8010
Yadav, Hariom: 7190
Yadollahikhales, Golnaz: 8010
Yafeh Mfuh, Anita: 8005
Yaffe, Kristine: 4335, 7000, 8005
Yahaya, Ahmed-Rufai: 8015
Yaisikana, Mo’e: 4325
Yajima, Masanao: 1100
Yalcin, Damla: 7315
Yamada, Ryousuke: 8000
Yamada, Yuji: 7195
Yamakawa, Miyae: 7195
Yamamoto, Yasuhiro: 7380
Yamamoto-Mitani, Noriko: 2210, 7440, 8000, 9020
Yamashita, Takashi: 7035, 7115, 7145, 7900, 8000, 8005
Yan, Alice: 7485
Yan, Cheng: 8010
Yan, Elsie: 1215, 2325, 7405, 8005
Yan, Haijuan: 2085
Yan, Jinghua: 7320
Yan, Kailei: 7425
Yan, Mengzhao: 1320, 7300, 8005, 8015
Yan, Shibin: 1205, 7300, 7535
Yan, Xumeng: 4125, 4250
YAN, Zi: 1205, 4055, 7300, 7405
Yanez, Romel: 1135
Yang, Betsy: 3065
Yang, Biying: 7150
Yang, Chih-Hsiang: 7435
Yang, Chun-Ting: 1340, 4090
Yang, Eunhwa: 7160
Yang, Frances M: 1150, 1515, 2225, 3255, 7250, 8000, 8010
Yang, Hayejin: 7300, 7375
Yang, Hee Won: 3235
Yang, Hongyan: 7080
Yang, Huayu: 7315
Yang, Hyewon: 3270
Yang, Janet: 2240
Yang, Jie: 1340
Yang, Jie: 8005
Yang, KwangSoo: 2045
Yang, Kyeongra: 8000, 9050
Yang, Mingzhu: 8010
Yang, Minhee: 7185, 7295, 7330, 7525, 8010
Yang, Qian: 2275
Yang, Qiaohong: 7410
Yang, Qifan: 7010
Yang, Qing: 3355, 4095
Yang, Ruimin: 7535
Yang, Ruochen: 9105
Yang, Seungbum: 7225, 7300, 7375
Yang, Shuqi: 7015
Yang, Siying: 2185
Yang, Wei: 3225, 7240
Yang, Xinyue: 7185
Yang, Yang (Claire): 1495, 3195
Yang, Yijian: 4060, 7090, 7225, 8000
Yang, Yiqing: 8005
Yang, Yong: 1355
Yang, Yulin: 1370, 4070
Yang, Yunjia: 7265

Yang, Yuting: 1285
Yang, Zhou: 2545
Yang, Ziyi: 7010, 7910
Yankeelov, Pamela: 1060, 2465
Yankson, Spendilove: 7240
Yao, Jie: 2475
Yao, Jie: 9155
Yao, Jingxin: 4565
Yao, Li: 7215, 8010
Yao, Shanshan: 2000, 2245, 7345, 8010
Yao, Yao: 7225
Yap, Xin Yi X: 7040
Yarbrough, Courtney: 1080
Yarnall, Jessica: 4560
Yarrow, Linda: 7130
Yashin, Anatoliy I: 7185, 7415
Yashkin, Arseniy: 1235, 4010, 4460, 7185
Yasumoto, Saori: 3010
Yasunaga, Hideo: 9020
Yau, Jessie Ho-Yin: 1135
Yauk, Jessica: 7220
Yazdani, Neshat: 7040
Ye, Fan: 4200
Ye, Jiangfeng: 8005
Ye, Jing: 1430, 7080
Ye, Lichuan: 4265
Yeargers, Jon: 7545
Yee, Claire I: 1275, 4085
Yee, Nicholas J: 7370
Yeh, An-Yun: 7255
Yeh, Jarmin C: 2515, 4160, 7215, 7925, 8000, 9000
Yeh, Shu-Chuan Jennifer: 7515
Yeh, Yen-Po: 7140
Yellon, Tamar: 8005
Yeo, Hyesu: 7325
Yeo, Shumian: 8005
Yeoh, Eng-Kiong: 7395, 7440
Yeom, Hyun-E: 7220, 7425, 7430
Yeong, Joe: 8005
Yero, Ivana: 8005
Yeung, Dannii: 2350, 4300, 7295, 7395, 7915, 8005, 8015, 9060
Yeung, Wei-jun Jean: 1240
Yew, Pui Ying: 7305
Ygrubay, Pamela K: 2245
Yi, Dahyun: 3380
Yi, Grace: 3325, 7340, 9100
Yi, Mo: 4335, 9065
Yi, Stella S: 2380, 3155
Yi, Xingnan: 1030
Yi, Xinyu: 8010
Yi, Yang: 2125
Yi, Zhiqi: 2525, 3320, 7110, 7220, 7405
Yildiz, Ferhat: 8000
YILDIZ, MUSTAFA: 7385
Yilmaz, Yasemin: 8000
Yin, Cheng: 7250, 7380, 7430
Yin, Diana: 3215
Yin, Jiyuan: 8015
Yin, Lijuan: 1160, 3355, 7015
Yin, Na: 3385
Yin, Qingqing: 7090, 7260
YIN, SHI: 8005
Yin, Ying: 8010
Ying, Dire: 2125
Ying, Gelan: 3380, 4540
Yip, Samantha: 7525
Ylitalo, Kelly: 4460
Yockey, R. Andrew: 7540
Yoder, Grant: 2320
Yokoyama, Yuri: 7265
Yolken, Robert H: 7040
Yonashiro-Cho, Jeanine: 3300
Yoneda, Tomiko: 1265, 4120, 4515, 8015
Yoo, Eun-Young: 4525
Yoo, Haeun: 7115, 7230
Yoo, Jaeon: 7080
Yoo, Ji: 8005
Yoo, Leeho: 8010
Yoo, Yaeeun: 7065, 7365
Yoo, Youngjun: 8015
Yoo-Jeong, Moka: 1155, 9110
Yoon, Caroline: 7075
Yoon, Dokyung: 7395
Yoon, Hee Jeong: 9060
Yoon, Seolah: 7520
Yoon, Sukyung: 7375
Yoon, Sumin: 1090
Yoon, Young Ji: 7295
York, Amy M: 2355
Yoshie, Satoru: 8000, 8010
Yoshikawa, Aya: 8010
Yoshimitsu, Takahashi: 2230
Yoshioka-Maeda, Kyoko: 7440
Yost, Govinda: 1455
You, Sang Bin: 8005, 9135
Young, Bailey A: 8000
Young, Heather M: 2450, 4145, 7085, 7915
Young, Kate: 7090
Young, Meghan: 4345
Young, Sarah R: 4525
Young, Veronica: 8015
Young, Yuchi: 7220, 7330
Yow, W. Quin: 3090, 3215, 4560, 8015
Yu, Chang: 4185, 4590, 7040
Yu, Chen-Wei: 2095
Yu, Danxia: 9030
Yu, Dayoung: 7545
Yu, Doris S. F.: 1225, 2545, 4065, 9085
Yu, Gongda: 4590
Yu, Heshuo: 7055
Yu, Hongyu: 7410
Yu, Hsiao-Wei: 7140, 7370
Yu, Jiani: 3300
Yu, Jiao: 1180, 1505, 4580
Yu, Jie: 7305
Yu, Jiening: 7240
Yu, Jihnhee: 7370, 7380
Yu, Junwen: 1245, 4055
Yu, Junwen: 8005
Yu, Kexin: 1185, 2360
Yu, Mingzhi: 7225
Yu, Pei-Ying: 7080
Yu, Tiffany: 7370
Yu, Wanting: 7025, 7295, 7465, 8010
Yu, Wenhua: 9085
Yu, Wenshan: 8000
Yu, Xuexin: 4455, 9040, 9120
Yu, Zhenhan: 7225
Yu, Zilong: 4225
Yuan, changzheng: 7090
Yuan, Jiameng: 2150, 3135, 4505
Yuan, Yan: 8015
Yuan, Yaqi: 1370
Yuan, Yaqun: 1500, 7345
Yuan, Yarou: 9020
Yuan, Yiyang: 7255, 7415
Yuan, Zhuochen: 7530
Yue, Yiwei: 7040
Yuh, Esther L.: 7000
Yun, Hyunkyung: 1385, 7250, 7540
Yun, Ji-Young: 1345
Yun, Jung eun: 8005
Yung-Choy, Rebecca: 7205
Yunru, Chen: 4370
Yurkiv, Halyna: 1430
Yusuf, Khalid: 8015
Yuwen, Weichao: 4180

Z

Zadeh, Rana: 7455, 7915
Zafar, Anum: 3205, 8005, 9045
Zafar, Sahar: 7015
Zagorski, William: 4145, 4160
Zaguri-Greener, Dalit: 8005, 9000
Zahm, Kathryn: 9120
Zahodne, Laura: 3380, 7435, 8010
Zahradka, Nicole: 7300
Zajacova, Anna: 1070
Zakrajsek, Jennifer S: 4435
Zaman, Kimia Tuz: 3340, 7015, 8000
Zaman, Shahid: 8000
Zambrano Garza, Elizabeth: 7390
Zamrini, Edward: 2460, 8010
Zanasi, Francesca: 9020
Zanchelli, Michael C: 8010, 8015
Zang, Emma: 4185, 7325
Zang, Shenghao: 4080
Zanier, Nicole: 4435
Zaninotto, Paola: 1040, 2250, 3145, 7295
Zanjani, Faika: 1260
Zank, Susanne: 4470
Zannas, Anthony: 3185
Zanotelli, Zackary: 7180, 7900
Zanotto, Tobia: 4060, 7090, 7225
Zaragoza, Gabriela: 8010
Zarazua-Osorio, Brenda: 9015
Zarit, Steven H: 2385, 3185
Zaslavsky, Oleg: 7185, 7205, 7415
Zauszniewski, Jaclene A: 3300
Zavala, Daisy V: 1265
Zavez, Katherine: 7345
Žefran, Miloš: 7015
Zegarra Valdivia, Jonathan Adrian: 7010
Zeglin, Lydia: 7130
Zeid, Janna: 8005
Zeisel, John: 8005
Zeki Al Hazzouri, Adina: 8000, 8005, 9120
Zelaya, Mariel: 8010, 8015
Zelinski, Elizabeth: 7395
Zell, Angela: 4075, 7540
Zellous, Erreannau L: 3125, 8000
Zeng, Junyan: 7145
Zeng, Qing: 8010
zeng, Qingyi Jasmine: 7475, 9010
Zeng, Shengyin: 7410, 8015
Zeng, Weicheng: 7015
Zeng, Xiaoyi: 3015, 4015, 4530, 7260
Zeng, Xueling: 7245
Zeng, Ziwei: 4060, 7090, 8000
Zeng-Trietler, Qing: 9020

Zertuche, Juan-Pablo: 1070
Zervakis, Jennifer: 7350, 7930
Zettel-Watson, Laura: 7455
Zhai, Shumenghui: 4180
Zhai, Wanting: 1475
zhai, yanping: 7125
Zhan, Rongfang: 8015
Zhang, Boya: 1040
Zhang, Caifang: 7300
Zhang, Chenxin: 4310
Zhang, Cuntai: 3160, 7190, 7320, 7410
Zhang, Deqiang: 7315
Zhang, Guolei: 7300
Zhang, Hongwei: 9140
Zhang, Huan: 8005
Zhang, Jiaan: 7435
Zhang, Jianguo: 8015
Zhang, Jingyun: 7240
Zhang, Jun-e: 1245
Zhang, Kai: 2475, 8010
Zhang, Kaylee: 8015
Zhang, Kejia: 8000
Zhang, Kelvin Pengyuan: 2000, 4060, 4540
Zhang, kemeng: 4370, 7310
Zhang, Kevin: 7305
Zhang, Le: 7190
Zhang, Liangwen: 1235, 7010
Zhang, Liming: 7240, 9150
Zhang, Liwei: 8000
Zhang, Meina: 1475, 4230, 7265
Zhang, Meng: 8015
Zhang, Minqing: 7240
Zhang, Ning: 4265
Zhang, Peiyuan: 1145, 1365, 3015, 7220, 7500
Zhang, Peiyuan: 8005, 9005
Zhang, Qian: 3075
Zhang, Qiao: 7010
Zhang, Qin: 1220, 1245, 4055, 7095, 7130, 7915
Zhang, Rendong: 7545
Zhang, Rong: 4060, 7125
Zhang, Rui: 7305
Zhang, Ruoying: 3025
Zhang, Shenghao R: 1285
Zhang, Shiyang: 2025, 7390
Zhang, Shu: 7330
Zhang, Sijian: 9020
Zhang, Stephanie: 7355
Zhang, Suyanpeng: 7185
Zhang, Wei: 3320
Zhang, Weiguo: 2315
Zhang, Wen: 1235
Zhang, Wencheng: 1125, 2405
Zhang, Wenhan: 4565
Zhang, Wenhui: 8015
Zhang, Wen-Ru: 8010
Zhang, Wenting: 8010
Zhang, Wenxin: 9040
Zhang, Wuyang: 2000, 4450
Zhang, Xiangyu: 8010
Zhang, Xiao: 4470, 7210
Zhang, Xiaodan: 3090
Zhang, Xiaomin: 7010, 7190
Zhang, Xin: 3275
Zhang, Xiuhong: 7300
Zhang, Xiyuan: 4295
Zhang, Xue: 4130
Zhang, Xuehan: 8000, 8010
Zhang, Yalu: 3320
Zhang, Yan: 1160, 2105
Zhang, Yan: 4255
Zhang, Yan: 7220
Zhang, Yi: 7310
Zhang, Yichen: 7470
Zhang, Yichi: 1340
Zhang, Yiwei: 8005
Zhang, Yongkang: 3300, 7220, 7250
Zhang, Youjuan: 1515, 2315, 9050
Zhang, Yu: 1125
Zhang, Yuan S: 1495, 9040
Zhang, Yuankai: 8000
Zhang, Yuchen: 4485
Zhang, Yuewen: 8015
Zhang, Yuhe: 7015
Zhang, Zhang: 1360, 2520, 3110, 8015
Zhang, Zhe: 1140
Zhang, Zilong: 8005
Zhang, Ziqi: 7300
Zhang, Ziying: 3035, 3125
Zhao, Bochao: 2125
Zhao, Erfei: 4440
Zhao, Ershuo: 7145
Zhao, Fang: 3020
Zhao, Haijun: 7145
Zhao, Heng: 7340
Zhao, Henry: 4385, 7545
Zhao, Hui: 8010
Zhao, Jing: 3320
Zhao, Lihui: 7185
Zhao, Rui: 7430
Zhao, Shangang: 8015
Zhao, Shangshu: 4495
Zhao, Shangshu: 8010
Zhao, Xiaoyan: 7265
Zhao, Xintong: 3320
Zhao, Yang: 7310
Zhao, Yong: 7235, 7295
Zhao, Yuanchang: 8005
Zhao, Yuxiang (Chris): 2370
Zheng, Anqing: 1175
Zheng, Chunlei: 8000
Zheng, Jiashun: 1460
Zheng, Jiayu: 7015
Zheng, Judy: 7025
Zheng, Mingyang: 8000
Zheng, Ruoqi: 7380
Zheng, Shimin: 7085
Zheng, Wenqing: 8000
Zheng, Xiaoping: 4060
Zheng, Yaguang: 4170, 4505, 8005, 8010, 8015
Zheng, Yexin: 2275
Zheng, Yueyuan: 8005
Zhong, Angela: 7265
Zhong, Chuwen: 1185, 4455
Zhong, Kelin: 8000
Zhong, Roger: 8010
Zhong, Selena: 4085, 8010
Zhong, Tangsheng: 4335
Zhou, Huayu: 8015
Zhou, Jiajia: 2260
Zhou, Jiayu: 3090
Zhou, Jiayu: 8000
Zhou, Junhong: 7025
Zhou, Kaixin: 9020
Zhou, Leyi: 2540, 8015
Zhou, Lily: 7525
Zhou, Maolin: 8010
Zhou, Nan: 2020
Zhou, Peter: 2090
Zhou, Sasha: 4565
Zhou, Shuai: 1515, 2315
Zhou, Tianqi: 1225, 2405, 7085, 7375
Zhou, Timothy: 1035
Zhou, Wenjian: 7225
Zhou, Xinkai: 4375
Zhou, Yanhua: 9095
Zhou, Yanjiao: 4227
Zhou, Yatian: 3275
Zhou, Yixuan: 4280
Zhou, Yuanjin: 4015, 4530
Zhou, Yue: 7255
Zhou, Zexi: 1105, 3015, 7295, 7390
Zhou, Zhiyong: 4270
Zhu, Carolyn: 9080
Zhu, Dantong: 7090
Zhu, Haojing: 3145
Zhu, Jiakang: 7010
Zhu, Leticia: 3305
Zhu, Mengya: 7095
Zhu, Peijia: 7010
Zhu, Shida: 7410
Zhu, Shijia: 7345
Zhu, Shijun: 2350
Zhu, Wenjuan: 3075
Zhu, Xi: 3290
Zhu, Xi: 8000
Zhu, Xiaoqian: 2535, 7325
Zhu, Xuemei: 7360
Zhu, Yiqi: 8015, 9015
Zhu, Yiwen: 8005, 9155
Zhu, Yujun: 2390, 7085
Zhu, Zheng: 4055, 7015
Zhu, Ziwei: 8015
Ziemba, Yonah C: 8000
Zies, Susan: 8010
Zieschang, Tania: 4450, 7200, 7225
Zimbroff, Robert M: 2480
Zimmer, Vicki: 1160, 1225
Zimmer, Zachary: 2275
Zimmerman, Kristin M: 7340
Zimmerman, Sheryl: 1435, 3260, 3280, 7105, 7225, 7440, 7915
Zing, Xing: 4520
Zionts, Nancy: 8005, 8010
Zipunnikov, Vadim: 4375
Zisberg, Anna: 1130, 1485, 1515, 4335, 4355, 7000, 7060, 7145, 7175, 7255, 7410, 8005, 9000
Zisk Rony, Rachel Yaffa: 8005
Zissimopoulos, Julie: 2005, 2400
Zivin, Kara: 3100
Zivotofsky, Ari: 8005
Zlyesov, Eduard: 1090, 7450
Zmuda, Joseph M: 8005
Zocchi, Mark: 2085
Zoh, Roger: 3010, 7010
Zolnoori, Maryam: 7545
Zonderman, Alan: 7130
Zonker, Christina R: 1165
Zou, Baiming: 4095
Zou, Jian: 8005
Zubatsky, Max: 2110, 2335, 8000
Zuberi, Daniyal: 9135
Zuelsdorff, Megan: 1160, 8005
Zullo, Andrew R: 1435, 2465, 4010, 7345, 7385, 7510, 8005, 9020
Zuniga, Franziska: 7250
Zuo, Lihan: 3090
Zuo, You: 8000
Zuraw, Matthew: 7305, 7415
Zurlo, Karen: 4210
Zwakhalen, Sandra: 7250
Zweig, Richard: 7005
Zweig, Susan: 4170, 8015
Zwerling, Jessica: 8015
